# Age-related transcriptional drift and physiological adaptation in long-living Ames dwarf skeletal muscle

**DOI:** 10.1093/narmme/ugag018

**Published:** 2026-03-23

**Authors:** Kingsley Mokube Ekumi, Matthew J Johnston, Michelle Pauley Murphy, Sharlene Rakoczy, Taylor R Painter, Holly M Brown-Borg

**Affiliations:** Department of Biomedical Sciences, University of North Dakota School of Medicine & Health Sciences, Grand Forks, ND 58203, United States; Department of Biomedical Sciences, University of North Dakota School of Medicine & Health Sciences, Grand Forks, ND 58203, United States; Department of Biomedical Sciences, University of North Dakota School of Medicine & Health Sciences, Grand Forks, ND 58203, United States; Department of Biomedical Sciences, University of North Dakota School of Medicine & Health Sciences, Grand Forks, ND 58203, United States; Department of Biomedical Sciences, University of North Dakota School of Medicine & Health Sciences, Grand Forks, ND 58203, United States; Department of Biomedical Sciences, University of North Dakota School of Medicine & Health Sciences, Grand Forks, ND 58203, United States

## Abstract

Skeletal muscle aging is accompanied by deterioration in metabolic flexibility, neuromuscular connectivity, and structural integrity, all of which contribute to frailty and the loss of functional independence. Ames dwarf mice exhibit postnatal growth hormone deficiency and an exceptionally long lifespan, providing a unique model for revealing transcriptional programs that support healthy aging. Here, we present the first comprehensive transcriptomic and functional profile of hindlimb skeletal muscle in middle-aged and old-aged Ames dwarf mice. We show that Ames dwarf muscle maintains a transcriptional profile enriched for vascular remodeling, synaptic communication, extracellular matrix organization, and structural resilience while suppressing lipid metabolic pathways and age-associated transcriptional drift. Advanced age in Ames mice is marked by a substantial shift in transcription factors associated with downregulated genes and a temporally coordinated activation of senescence-associated and inflammatory-response signatures that appear to support, rather than impair, tissue maintenance. Functionally, Ames dwarf mice maintain neuromuscular coordination, grip strength, and endurance with age. Collectively, these findings indicate a distinct transcriptional drift in Ames dwarf skeletal muscle that integrates vascular, neuronal, and senescence-related signals to preserve structural and functional resilience. This work implicates molecular mediators, including Apelin, Klotho, and Notch1 that may underlie exceptional healthspan and modulate resistance to frailty.

## Graphical Abstract

**Graphical Abstract:**
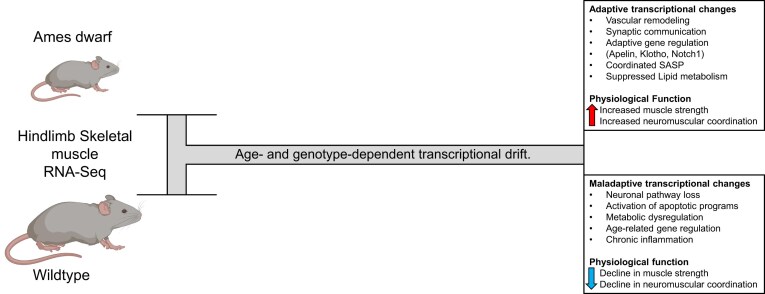

## Introduction

Aging is the strongest risk factor for chronic disease and functional decline, with skeletal muscle deterioration playing a central role in frailty, disability, and loss of independence [1, 2]. Sarcopenia, the progressive reduction in muscle mass and contractile strength, is one of the most debilitating features of aging, contributing to falls, fractures, and impaired metabolic health [1, 2]. At the cellular and molecular levels, skeletal muscle aging is driven by interconnected processes including mitochondrial dysfunction, impaired proteostasis, metabolic reprogramming, and dysregulation of stress-response pathways [3–6]. Another critical hallmark of aging is cellular senescence, a state of essentially permanent cell cycle arrest accompanied by profound transcriptional, epigenetic, and functional changes [7, 8]. Senescent cells accumulate progressively with chronological age and under conditions of stress such as DNA damage, telomere attrition, or mitochondrial dysfunction [7, 8]. A defining feature of these cells is the acquisition of the senescence-associated secretory phenotype (SASP), a pro-inflammatory and tissue-remodeling secretome consisting of cytokines, chemokines, growth factors, and proteases [9, 10]. Persistent SASP activity drives chronic inflammation, fibrosis, and disruption of tissue architecture.

In skeletal muscle, excessive senescence burden and SASP signaling can impair myogenic progenitor cell function, contribute to extracellular matrix (ECM) remodeling, and accelerate the decline in muscle strength with age [11, 12]. Moreover, because senescent cells accumulate across multiple tissues in temporal and spatial synchrony with age-associated functional decline in both animals and humans [9, 13, 14], they have been hypothesized to drive the deterioration underlying numerous chronic diseases [10]. The SASP can also evoke a local inflammatory response with complex effects, including the elimination of senescent cells by phagocytosis, thus leading to tissue remodeling and damage resolution [15–17]. During embryonic development, senescence programs contribute to tissue patterning by eliminating transient structures [18]. In adult tissues, senescence plays critical roles in wound healing and regeneration by coordinating immune surveillance and promoting tissue remodeling [19–22]. Senescence also acts as a potent tumor suppressor mechanism, halting the proliferation of cells at risk of malignant transformation [23]. Thus, cellular senescence represents a double-edged sword; acutely beneficial in transient contexts but deleterious when chronic and unresolved [11, 12, 15, 20, 21, 23].

Reflecting on exceptional human longevity, supercentenarians, individuals who live beyond 110 years [24], demonstrate a striking paradox. Despite their longevous chronological age, they maintain functional independence until very late in life and are resilient against many age-related pathologies [25, 26]. Biological analyses of supercentenarians have revealed evidence of senescence, but importantly, a dampened state of chronic low-grade inflammation, often referred to as ‘inflammaging’ [24, 27–32]. In contrast, in the general older adult population, inflammaging is characterized by persistent activation of innate immune pathways, increased circulating pro-inflammatory cytokines, and a progressive decline in immune regulation, which is strongly linked to frailty, cardiovascular disease, neurodegeneration, and sarcopenia [33–44]. The ability to tolerate or regulate senescence while suppressing chronic inflammation appears to be a key factor underlying their exceptional healthspan.

The Ames dwarf mouse provides complementary insights into the biology of aging and longevity. Ames dwarf mice harbor a mutation in the Prop1 gene that disrupts anterior pituitary development, and thus are deficient in growth hormone (GH), prolactin, and thyroid-stimulating hormone [45–49]. Postnatal GH deficiency leads to markedly reduced insulin-like growth factor 1 (IGF-1) signaling and is associated with a 49%–68% extension of lifespan relative to wildtype controls [48–50]. Importantly, Ames dwarf mice exhibit delayed onset of age-related pathologies, enhanced metabolic efficiency, and preserved organ function well into old age [51–54]. At the molecular level, these mice display reduced oxidative damage [55], improved stress resistance [56, 57], and altered inflammatory signaling [58, 59]. Given that the molecular characteristics of Ames dwarf mice parallel those of human supercentenarians—both demonstrating remarkable resilience to age-related disease and functional decline—we propose that age-associated transcriptional programs in Ames dwarf mice provide a valuable framework for elucidating mechanisms underlying resilience to age-related deterioration in tissue function.

In this study, we focused on the hindlimb skeletal muscle aging process and differences in the genotypes of Ames dwarf and wildtype mice. Skeletal muscle is the primary site of age-related frailty, metabolic regulation, and sarcopenia, but also a source of systemic signals that influence whole-body physiology [1, 33–36, 44, 60]. Loss of both muscle mass and strength leads to sarcopenia [43], which contributes to the risk of falls and fractures in older adults and is the second-leading cause of injury and death [61, 62]. Klotho (Kl), a longevity-promoting gene [63], improves skeletal muscle structure and function through the modulation of genes associated with aging [64]. Apelin (Apln) is an exerkine that regulates skeletal muscle adaptation to exercise through apelin signaling and mitochondrial biogenesis [65–68], and reversal of age-associated sarcopenia [69]. GH deficiency in Ames dwarf mice increases the number of oxidative fibers in the tibialis anterior muscle [54], suggestive of a programmable effect of GH deficiency on oxidative function [55, 57, 70] and exercise endurance [54, 71]. Notch1 improves muscle regeneration and exercise performance [72]. Ames dwarf mice show delayed decline in age-related neuromuscular frailty and bone mineralization [71, 73]. Although a correlation between GH deficiency in Ames dwarf mice and delayed-frailty has been reported [54, 71], it remains unclear how the pro-longevity transcriptional adaptations that delay skeletal muscle frailty in Ames dwarf mice exert their function. Here, we implemented bulk RNA sequencing transcriptomic analysis to meticulously dissect transcriptional drifts in middle- and old-aged Ames dwarf mice. We reveal an unanticipated interaction between altered metabolic states and the preservation of muscle integrity and vascular remodeling in middle-aged and old-aged Ames dwarf mice. Furthermore, we find that cytokines and neurodegenerative disease pathways were associated with the altered transcriptional drift. Transcriptional response of the aging process between old-aged and middle-aged dwarf Ames mice highlights the central role of progressive transcriptional adaptation in maintaining the balance between SASP activation and inflammatory signaling, providing new insight into mechanisms that promote healthy skeletal muscle aging. We further reveal the physiological relevance of the transcriptional signatures in preserving strength and neural coordination, the most functionally relevant traits for avoiding frailty.

## Materials and methods

### Mice

All mice were maintained in group-housed conditions with a 12 h/12 h light/dark cycle at 22 ± 1°C, with free access to food and water at the Center for Biomedical Research at the University of North Dakota. Mice were fed *ad libitum* (Teklad 2016). All mice used for *in vivo* studies were drug and test-naive. Whenever possible, age- and sex-matched littermates were used for the studies. All animal care was executed according to the guidelines of NIH for the care and use of laboratory animals. Animal experiments were approved by the UND Institutional Animal Care and Use Committee (IACUC), and health status checks were performed daily. In general, breeding of Ames dwarf and wildtype mice was performed using homozygous (df/df) or heterozygous (df/+) males with heterozygous (df/+) wildtype females.

### Physical assessment testing

The physical assessment testing was done in the following order: grip strength and latency-to-fall testing on the same day, and endurance exercise the following day.

**Grip strength measurements** were performed using the Maze Engineers Grip Strength Instrument (Skokie, IL, USA) and accompanying software. On testing days, animals were first weighed, and each mouse was assessed with all four paws on the grid for five trials, with approximately 10 min between each measurement. The maximum and minimum forces were excluded, and the remaining three measurements were averaged for downstream analysis. Measurements for each cohort were conducted at the same time of day by the same investigator. The order in which mice were tested was randomized. Grip strength was normalized to body weight to account for size differences between genotypes.

**Latency-to-fall activity** was recorded using the Rotarod apparatus (Maze Engineers, Skokie, IL, USA) and software. The instrument was fitted with 50% smaller rotating dowels in half of the lanes to accommodate the smaller body size of the dwarf mice. The activity involved a maximum latency of 5 min, with an initial rotation speed of 4 rpm, accelerating at 7.2 rpm² to a maximum of 40 rpm. The apparatus motion sensor automatically recorded latency-to-fall. Each mouse completed three trials per session, with 10–15-min intervals between trials. Final scores represent the average latency across three trials.

**Endurance exercise activity** was performed using the Maze Engineers Treadmill Instrument (Skokie, IL, USA) and software. Mice were first allowed to acclimate to the treadmill at a low speed. The speed was set to accelerate at 1 m/min^2^ and monitored. Mice that did not engage in running were given a slight nudge with the use of a spatula. The trial was considered complete when mice consistently resisted engaging in the activity and were considered exhausted. Data are recorded in absolute time in seconds.

### RNA extraction and quantitative polymerase chain reaction

Total RNA was extracted from frozen mixed muscle tissues using Allprep DNA/RNA/Protein mini kit (#80004 Qiagen), and complementary DNA (cDNA) was synthesized using the First-strand cDNA synthesis master mix 4× (MB6008, ScienCell) according to the manufacturer’s instructions. All quantitative polymerase chain reactions (qPCRs) were performed with the Powerup SYBR Green Master Mix (A25778, Applied Biosystems). Each sample was loaded in eight biological replicates, and fluorescence acquisition was conducted on a CFX Connect™ Real-Time PCR Detection System (Bio-Rad). Gene expression differences were calculated using the threshold cycle method, and *B2M* expression levels were used as a reference gene for normalization. Values are plotted as log_2_(foldchange) ± standard error of the mean (SEM). Primer sequences are listed in Supplementary Table primers.

### RNA sequencing

Total RNA isolated was submitted to the UND Genomic Core for library preparation and sequencing. The library was established using NEBNext ultra II RNA Library Prep Kit (nondirectional) with NEBNext ribosomal RNA Depletion Kit to prepare nondirectional ribo-depleted RNA-seq libraries, which were quantified and normalized. Sequencing was performed using the Illumina HiSeq X platform, with 150-bp paired-end reads. Raw RNA sequencing FASTQ files were first evaluated for quality using FASTQC v0.119. Adapter trimming and quality filtering were performed using Trimmomatic v0.39 in paired-end mode. Only read pairs in which both mates passed quality trimming were used for alignment. Alignment to GRCm38/mm10 (mouse reference genome) was performed using Bowtie2 v2.4.2. SAM files generated by Bowtie2 were converted to BAM format, sorted by genomic coordinates, and indexed using SAMtools v1.10. Only properly paired and uniquely mapped fragments were retained for quantification. Gene-level read quantification was performed using featureCounts v1.4.6-p4 (Subread package), assigning fragments to genes based on overlap with annotated exons in the reference GTF. Reads were counted in paired-end mode with both mates required to align, and strand specificity was not applied. Multimapping and chimeric fragments were excluded to ensure high-confidence assignments. The resulting count matrix contained uniquely aligned fragment counts for all annotated genes and served as the input for downstream differential expression analyses.

### Differential gene expression and gene enrichment analysis

Sequence count normalization and differential gene expression analysis were conducted using the R DESeq2 package (version 1.44.0) using the Wald test. Genes with a *P*-adjusted value < 0.05 and |-fold change > 0.58 were considered differentially expressed genes. Enrichment analysis of differentially expressed genes was performed using enrichR (v3.4) with MSigDB for Gene Ontology (GO) Biological process enrichment (*P*-adjusted value < 0.05 and |log_2_-fold change| ≥ 0.58) and Kyoto Encyclopedia of Genes and Genomes (KEGG) pathway enrichment (*P*-adjusted value < 0.05). In specified cases, differentially expressed genes were further analyzed using Metascape (http://metascape.org) with default parameters for tissue-specific gene signature enrichment. In cases of aging data sets, the nominal *P*-value was equal to the *P*-adjusted value since a single data set enrichment was used to compare between two age groups.

### Quantification and statistical analysis

Statistical analyses were performed using GraphPad Prism and R software—indicated in each figure legend. Nested *t*-tests and chi-square tests were used. Results are shown as mean ± SEM. Two-tailed *P*-values <.05 were considered statistically significant. For all experiments, mice were assigned to experimental groups based on their genotypes. Sample sizes were determined based on the means and variation of previous pilot or published experiments. No data were excluded for statistical analysis.

## Results

### Mapping the differential transcriptional profile of middle- and old-aged dwarf and wildtype mouse skeletal muscle

**Figure 1.**
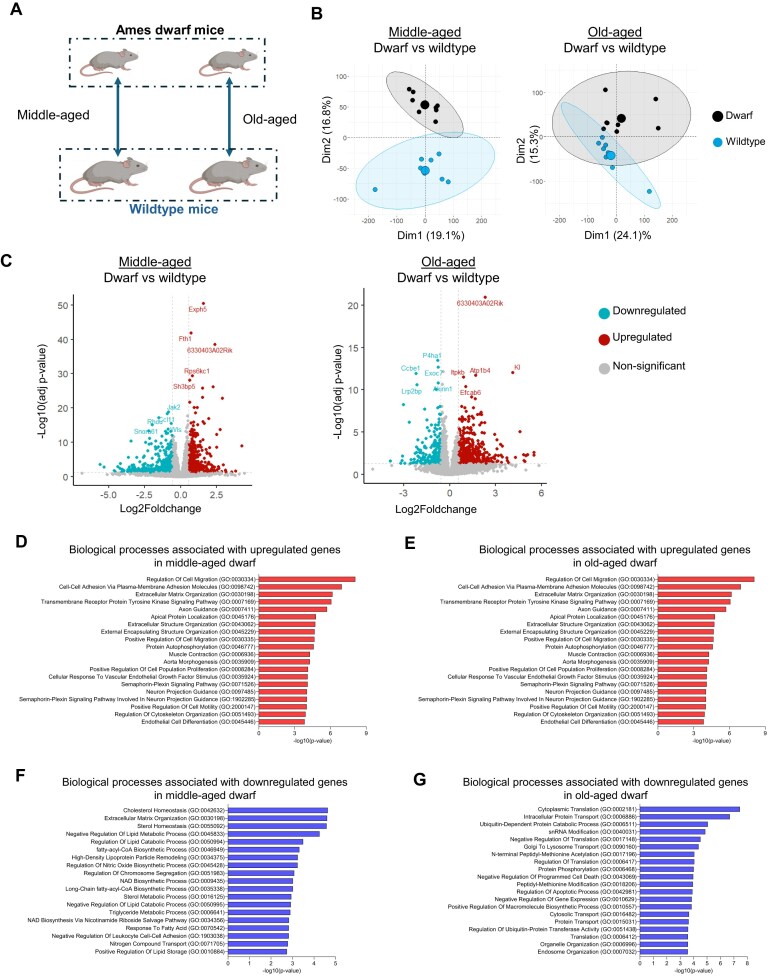
Transcriptional differences between Ames dwarf and wildtype mice across age-matched groups. (**A**) Schematic of the design for analysis of RNA-sequencing experiment for analysis of age-matched transcriptional changes between genotypes. Mouse icons were created in BioRender. Ekumi, K. (2026) https://BioRender.com/zwr9215. (**B**) Middle-aged dwarf versus middle-aged wildtype: principal component analysis (PCA) showing sample separation along Dim1 (19.1% variance) and Dim2 (16.8% variance), indicating distinct transcriptomic profiles between genotypes at middle age. Old-aged dwarf versus old-aged wildtype: PCA plot showing separation along Dim1 (24.1% variance) and Dim2 (15.3% variance), highlighting increased divergence in gene expression with advanced age. (**C**) Middle-aged dwarf versus middle-aged wildtype: Volcano plot displaying differentially expressed genes, with log_2_-fold change on the *x*-axis and log_10_(*P*-adjusted value) on the *y*-axis. Significantly upregulated genes are shown in red, and downregulated genes in blue. Old-aged dwarf versus old-aged wildtype: Volcano plot illustrating the differential expression landscape for aged animals, highlighting shifts in gene regulation with aging in the Ames dwarf model. (**D–G**) GO Biological Process enrichment of differentially expressed genes for the same genotype comparisons. Biological processes associated with the upregulated genes are shown in red, indicating key biological processes altered in (**D**) middle-aged dwarf versus wildtype and (**E**) old-aged dwarf versus wildtype animals. Biological processes associated with the downregulated genes are shown in blue, indicating key biological processes altered in (**F**) middle-aged dwarf versus wildtype and (**G**) old-aged dwarf versus wildtype animals. *n* = 8/group.

To generate the first transcriptional profile of skeletal muscle in middle-aged and old-aged dwarf and wildtype mice, we collected hindlimb muscles from 32 sedentary male and female mice: 16 middle-aged (12–13.5 months old) and 16 old-aged (20–24 months old) dwarf and wildtype mice (Supplementary Dataset 1). Healthy hindlimb muscles were harvested from anatomically equivalent regions (Tibialis anterior, Extensor digitorum longus, Soleus, Gastrocnemius, and Quadriceps) across each mouse group and processed via standardized RNA-sequencing protocols. After quality control to remove low-quality read-counts, the RNA-Seq data quality (Supplementary Fig. 1), indicated by the number of counts and genes with at least 10 counts across samples, was highly correlated (Supplementary Fig. 2). To determine the transcriptional signatures of Ames dwarf hindlimb muscles, the mice were divided into four groups: middle-aged wildtype, middle-aged dwarf, old-aged wildtype, and old-aged dwarf (Fig. 1A). Using DESeq2 normalization, we compared the transcriptomic signatures of these four groups. Principal component analysis revealed significant clustering of middle-aged dwarf mice relative to middle-aged wildtype, with the first and second principal components accounting for 19.1% and 16.8% of the variance, respectively (Fig. 1B). A similar separation was observed between old-aged dwarf and wildtype mice, with the first and second principal components accounting for 24.1% and 15.3% of the variances (Fig. 1B). Overall, principal component analysis of all four groups, plotted on the same scale, demonstrates significant clustering, with the first and second principal components accounting for 23.9% and 14.2% of the total variance, respectively (Supplementary Fig. 3).

By differential expression analysis (*P*-adjusted value < 0.05 and |log_2_-fold change| > 0.58), we identified 1087/16 474 differentially expressed genes between middle-aged dwarf versus middle-aged wildtype, and 788/15 684 differentially expressed genes between old-aged dwarf versus old-aged wildtype. The top 10 differentially expressed genes in the middle-aged dwarf group included Exph5, Fth1, 633040403A02Rik (Stum), Rps6kc1, Sh3bp5, Jak2, Ccl11, Rhou, Wls, and Snora61, whereas those in the old-aged dwarf group included 633040403A02Rik (Stum), Kl, Atp1b4, Itpkb, Efcab6, P4ha1, Ccbe1, Exoc7, Lrp2bp, and Akirin1 (Fig. 1C). These genes were confirmed by RT-qPCR (Supplementary Fig. 4A and 4B). However, the messenger RNA (mRNA) level of 633040403A02Rik (Stum) gene, which is evolutionarily conserved and required for locomotion and mechanical sensing [74], was very low or undetectable, possibly due to its typical localization in neurons and joint-associated tissues. Interestingly, these top differentially expressed genes included both well-characterized and muscle-associated genes. For example, Jak2 and Rps6kc1, components of the PDGF signaling pathway that are known to be upregulated in skeletal muscle following exercise during the active phase, were differentially expressed [75]. Fth1 deficiency leads to muscle atrophy and impaired endurance via altered ferroptosis [76], whereas the Ccl11 gene is considered a pro-aging factor [77, 78]. Rhou is shown to be upregulated in activin-induced muscle wasting, weakness, and fibrosis [79]. Kl is a longevity-promoting gene that improves muscle fitness [63]. Itpkb is expressed in whole muscle but not in myofibers [80]. Atp1b4 and Exoc7 are involved in regulating glucose metabolism in the muscles [81, 82]. Akirin1 plays a role in the regulation of skeletal muscle fiber-type switching [83]. Amongst upregulated genes, 372 (40.5%) are unique to the old-aged dwarf, while 367 (40.0%) are unique to the middle-aged dwarf. Amongst downregulated genes, 130 (19.4%) are unique to the old-aged dwarf, and 434 (64.7%) are unique to the middle-aged dwarf. The overlap of upregulated and downregulated genes between the middle-aged and old-aged dwarf is limited to 179 (19.5%) and 107 (15.9%) genes, respectively. These results indicate that the transcriptional profile of Ames dwarf mouse skeletal muscle differs markedly from that of wildtype mice, with key genes involved in longevity, pro-aging processes, muscle function, and structural stability playing key roles in the observed transcriptomic differences.

To understand the biological significance of these alterations, we performed GO enrichment analysis of biological processes among differentially expressed genes in Ames dwarf mice compared with wildtype controls, focusing separately on the middle-aged and old-aged groups (Fig. 1D–G). In middle-aged dwarf mice, the upregulated genes were significantly enriched for biological processes associated with vascular development and neuronal communication (Fig. 1D). These include blood vessel morphogenesis, vascular endothelial cell migration, vasculogenesis, and blood vessel endothelial cell migration. Additionally, pathways related to synaptic and neuronal functions, such as synapse organization, chemical synaptic transmission, axon guidance, and atrial cardiac muscle cell action potential, were also prominent. In old-aged dwarf mice, upregulated genes were enriched mainly in processes related to tissue structure, cellular maintenance, and neuronal regulation (Fig. 1E). The key GO biological processes included cell–cell adhesion via plasma membrane adhesion molecules, ECM organization, cellular response to vascular endothelial growth factor stimulus, axon guidance, and semaphorin-plexin signaling involved in neuron projection guidance. Notably, axon guidance and transmembrane receptor protein tyrosine kinase signaling pathways were commonly enriched in both age groups (Fig. 1D and E), suggesting a shared mechanism of neurovascular remodeling across the aging stages in Ames dwarf mice. However, unique to old-aged dwarf mice were biological processes involved in muscle contraction and protein autophosphorylation, highlighting age-specific regulatory responses in muscle physiology (Fig. 1E). Together, these findings underscore dynamic and age-dependent transcriptomic shifts in Ames dwarf skeletal muscle, particularly in pathways tied to vascular remodeling, synaptic function, and muscle integrity.

Next, we examined the biological processes associated with the downregulated genes for middle-aged and old-aged dwarf mice (Fig. 1F and G). Indeed, the biological processes were highly divergent from each other among the top 20 biological processes. In middle-aged dwarf mice, the downregulated genes were mostly associated with lipid and energy metabolism processes that included cholesterol homeostasis, regulation of lipid catabolic processes, triglyceride metabolic processes, nitric oxide biosynthetic process, NAD biosynthetic process, and NAD biosynthesis via nicotinamide riboside salvage pathway (Fig. 1F). Of note, lifelong depletion of adult skeletal muscle NAD does not compromise muscle function or accelerate aging [84]. Additionally, extracellular matrix (ECM) organization and regulation of chromosome segregation were uniquely altered pathways not associated with lipid and energy metabolism. However, ECM organization was among the top 20 biological processes observed to be enriched among the upregulated genes in old-aged dwarfs. We identified biological processes involved in protein transport and modification to be enriched in the old-aged dwarf that included intracellular protein transport, Golgi to lysosome transport, ubiquitin-dependent protein catabolic process, protein phosphorylation, and N-terminal peptidyl-methionine acetylation (Fig. 1G). We also observed negative regulation of programmed cell death, and regulation of apoptotic process were associated with the downregulated genes in old-aged dwarfs—biological processes that are usually associated with upregulated genes with aging and muscle loss [85–88].

Deconvolution of the differentially expressed genes to a reference skeletal muscle single-cell RNA sequencing dataset (Supplementary Fig. 5) indicates that downregulated gene sets demonstrate moderate association with tendon, fibroblast, immune-associated, and select nonmyofiber stromal cell signatures, while showing minimal enrichment for dominant myofiber subtype-specific programs. The difference in myofiber types is consistent with research indicating Ames dwarf mice express a higher percentage of type IIa muscle fiber in the tibialis anterior muscle compared to wildtype mice [54]. The upregulated gene sets are predominantly associated with endothelial, pericyte, smooth muscle cell, B cell, and Schwann cell signatures. Together, these results demonstrate that transcriptional differences between Ames dwarf and wildtype muscle are not explained by large compositional shifts in a specific myofiber cell type. Instead, they reflect genotype-specific regulation of muscle fiber integrity and coordinated muscle tissue nonmyofiber cell support with age.

### Cytokine signaling and neurodegenerative disease pathways are altered in Ames dwarf mice

**Figure 2.**
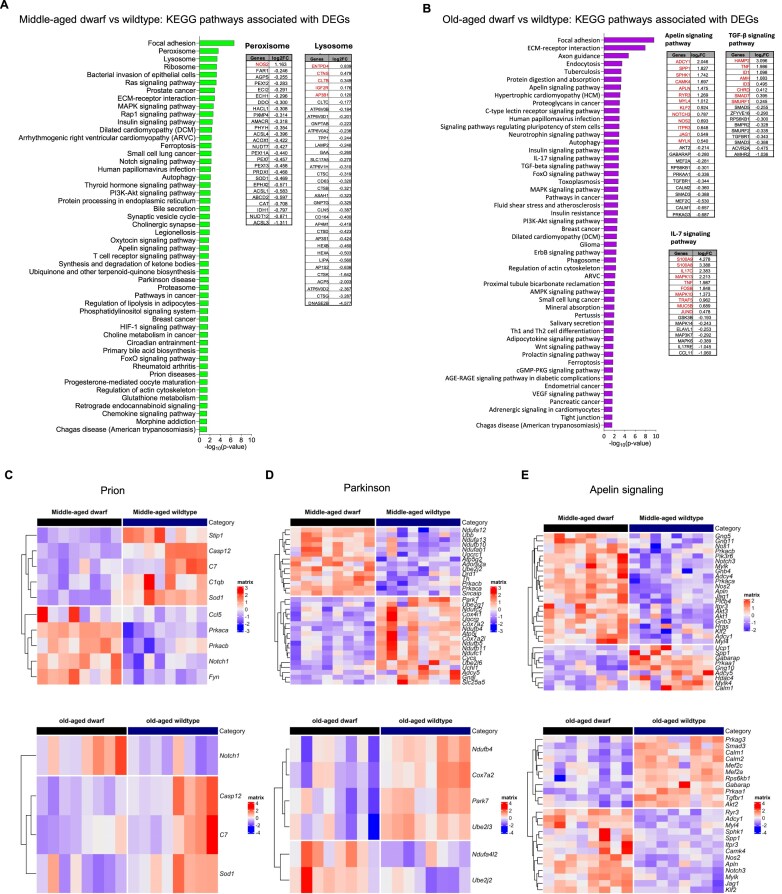
KEGG pathway enrichment analysis of differentially expressed genes in Ames dwarf versus wildtype mice across age-matched groups. (**A**) Middle-aged dwarf versus middle-aged wildtype: Key enriched KEGG pathways include peroxisome and lysosome, revealing metabolic and cellular component remodeling at middle age in the dwarf genotype. (**B**) Old-aged dwarf versus old-aged wildtype: Enriched KEGG pathways indicate significant alterations in cytokine-related and signaling networks, including the TGF-β signaling pathway, Apelin signaling pathway, and IL-7 signaling pathway, reflecting age-associated modulation of immune and cellular communication processes in Ames dwarf mice. (**C**) Prion disease KEGG pathway enrichment, shown for middle-aged dwarf versus wildtype (top) and old-aged dwarf versus wildtype (bottom), showing differential expression patterns associated with proteostasis and neurodegenerative processes. (**D**) Parkinson’s disease KEGG pathway enrichment for middle-aged dwarf versus wildtype (top) and old-aged dwarf versus wildtype (bottom), indicating transcriptional changes linked to mitochondrial function and neuronal maintenance. (**E**) Apelin signaling KEGG pathway enrichment for middle-aged dwarf versus wildtype (top) and old-aged dwarf versus wildtype (bottom), illustrating age-dependent modulation of metabolic and intercellular communication pathways.

Homeostatic maintenance of skeletal muscle during aging and in response to physiological stressors requires the anticipatory production of enzymes, antioxidants, and other protective factors that modulate cellular stress pathways. These pathways are triggered by mechanical loading, accumulation of metabolic byproducts, oxidative stress, and chronic low-grade inflammation [89–91]. To gain mechanistic insights into the transcriptional changes observed in the skeletal muscles of middle-aged and old-aged dwarf mice, we performed KEGG pathway analysis using all significantly upregulated and downregulated genes (*P*-adjusted value < 0.05). In accordance with these differentially expressed genes, the results revealed substantial alterations in canonical pathways when comparing dwarf mice to their age-matched wildtype counterparts, highlighting differences in pathways involved in cellular structure, cytokine signaling, metabolic homeostasis, and the maintenance of cellular integrity (Fig. 2 and Supplementary Dataset 3). These findings suggest that key biological pathways underlying muscle function and adaptability are differentially regulated in Ames dwarf mice across both age groups.

In middle-aged dwarf mice, KEGG pathways such as ECM–receptor interaction (Col2a1, Spp1), focal adhesion (Lamc2, Chad), peroxisome (Acsl3, Nudt12), lysosome (Ctsg, Dnase2b), and ribosome (Rpl22l1, Rps18) pathways were enriched compared to wildtype controls (Fig. 2A). This suggests delayed ECM remodeling, cellular adhesion, and organelle function at this age. In contrast, old-aged dwarf mice exhibited enrichment in several pathways, including focal adhesion (Col2a1, Spp1), ECM–receptor interaction (Col1a1, Sv2a), apelin signaling (Apln, Nos2), interleukin-7 signaling (Il7c, Jund), and TGF-β signaling (Tnf, Chrd) (Fig. 2B). These enriched pathways point to a sustained or compensatory activation of the inflammatory response and tissue remodeling in aged skeletal muscle.

Interestingly, we observed enrichment of age-associated disease pathways in the skeletal muscle of both middle-aged and old-aged dwarf mice. Skeletal muscle from middle-aged dwarf mice was more enriched in genes of neurodegenerative pathways, such as Parkinson’s and prion diseases, than in old-aged dwarf mice when compared to age-matched wildtype counterparts (Fig. 2C and D, respectively). These pathways are often linked not only to neuronal decline but also to impaired muscle function, mitochondrial dysfunction, and homeostatic imbalance [92–98]. Notably, such signatures are commonly suppressed across multiple genetic and lifespan-extending interventions, including caloric restriction or mTOR inhibition [99], suggesting that dwarf mice may exhibit protective transcriptional reprogramming at the expense of anabolic signaling that instead supports muscle integrity and delays age-related functional decline. In the case of prion disease pathways, 10 genes were significantly co-regulated in middle-aged dwarf mice, whereas only 4 of these genes were significantly co-regulated in old-aged dwarf mice (Fig. 2C). These genes included Stip1, Casp12, C7, C1qb, and Sod1 in downregulated genes and Ccl5, Prkaca, Prkacb, Notch1, and Fyn in upregulated genes. Casp12 is a caspase gene activated by ER-stress [100, 101], and reduced Casp12 expression preserves muscle function and compensatory hypertrophy in the mdx-mouse model [102]. The complement gene (C7 and C1qb) activation pathway drives synaptic loss in Alzheimer’s disease-mouse models [103, 104]. Stip1 and Sod1 are stress-responsive genes within the downregulated prion disease-associated pathway. Fyn plays a pivotal role in muscle immune responses following denervation [105, 106]. Interestingly, the expression of Notch1, which improves muscle regeneration and exercise performance in middle-aged wildtype mice and the Duchenne muscular dystrophy mouse (Mdx) model [72], was significantly upregulated in both middle-aged and old-aged dwarf (Fig. 2C), but more so in the middle-aged than in the old-aged dwarf—possibly due to its reduced expression with age [72]. Thus, the contraction from ten to four co-regulated genes with age may indicate that long-lived Ames dwarfs maintain prion-related proteostasis with a smaller, more stable gene set, which is consistent with their delayed aging and highly efficient stress-response pathways [45]. We also examined genes that were altered in Parkinson’s disease pathways. Interestingly, we found a list of genes that are part of the mitochondrial electron transport chain complex I (Ndufa12, Ndufa13, Ndufb10, Ndufab1, Ndufc2, Ndufb4, Ndufb5, Ndufb11, and Ndufc1) in middle-aged dwarfs (Fig. 2D). In contrast, these genes did not co-regulate with the mitochondrial electron transport chain complex-I genes (Ndufb4 and Ndufa4l2) in old-aged dwarf mice (Fig. 2D). Ndufb10 expression is reduced in the skeletal muscle of certain Parkinson’s disease patients [107]. We did observe a downregulation of Ndufb10 in the skeletal muscle of middle-aged dwarf mice; however, this alteration was not present in old-aged dwarfs, which may reflect the inconsistent and variable reports of Ndufb10 involvement in Parkinson’s disease [108]. Th is a gene that codes for tyrosine hydroxylase, which marks the network of sympathetic neurons that contact and maintain homeostasis of the neuromuscular junction and muscle [109–111] , and was upregulated in middle-aged dwarf mice. Importantly, the Park7 gene, which prevents the formation of glycerate-modified metabolites and protein damage by these metabolites [112], is downregulated in both middle-aged and old-aged dwarf mice. The alteration in Park7 may reflect aging in the wildtype, as these mice do not have Parkinson’s disease. These data confirm previous findings that Ames dwarf mice have enhanced antioxidant defenses and reduced oxidative damage to DNA and proteins with age [55].

Finally, we assessed genes altered in the apelin signaling pathway in middle-aged and old-aged dwarf mice. The genes upregulated in both age groups included Apln, Myl4, Itpr3, Nos2, Notch3, Mulk, Jag1, and Klf2 (Fig. 2E). Apelin is an exerkine and pharmacological target that has been shown to reverse age-associated sarcopenia by increasing muscle functional capacity, modulating energy metabolism, activating Apelin-AMPK signaling, reversing muscle degeneration, promoting mitochondriogenesis and autophagy, and reducing inflammation in myofibers [65–69, 113–118]. We also observed changes in genes involved in vascular homeostasis and immune regulation, including Prkaa1, Nos2, and Gabarap (Fig. 2E). Prkaa1 influences endothelial inflammation and regulates glycolytic activity in part through the HIF1a pathway [119], and HIF1a itself promotes angiogenesis and cellular energy homeostasis under hypoxic conditions [120]. Notably, the HIF1a pathway was enriched in middle-aged dwarf mice (Supplementary Dataset 2). Nos2 (nitric oxide synthase 2) contributes to the damping of excessive inflammatory response [121]. Together, these data suggest that Ames dwarf mice exhibit distinct, age-specific alterations in skeletal muscle pathways that may reflect adaptive or pathological responses to aging and altered endocrine signaling.

### Aging is associated with a pronounced increase in the transcription factor signature of downregulated genes in Ames dwarf skeletal muscle

**Figure 3.**
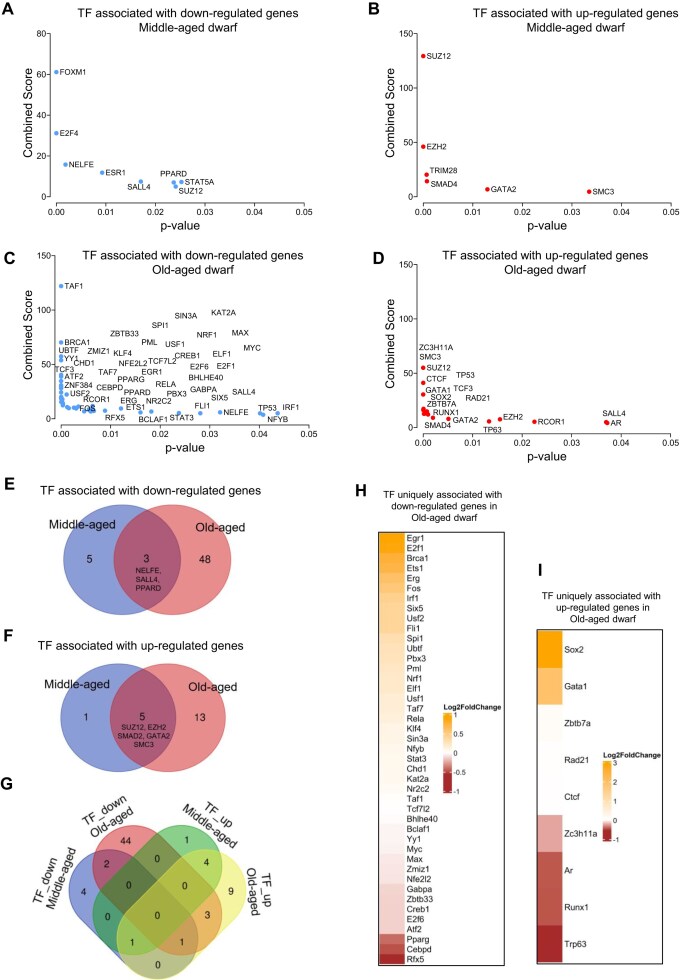
TFs enrichment analysis of differential gene expressions. (**A–D**) TF enrichment analysis of differentially expressed genes using Enrichr gene set library ENCODE_and_ChEA_Consensus_TFs_from_ChIP-X. Plots show the *P*-value (*x*-axis) and combined score (*y*-axis) for TFs associated with middle-aged dwarf downregulated genes (**A**) and upregulated genes (**B**), and old-aged dwarf downregulated genes (**C**) and upregulated genes (**D**). Blue points indicate TFs enriched among downregulated gene sets; red points indicate TFs enriched among upregulated gene sets. (**E, F**) Venn diagrams comparing enriched TFs between m- and o-derived gene sets. (**E**) Overlap of TFs enriched in downregulated genes, highlighting shared regulators NELFE, SALL4, and PPARD. (**F**) Overlap of TFs enriched in upregulated genes, showing shared regulators SUZ12, EZH2, SMAD4, GATA2, and SMC3. (**G**) Four-way Venn diagram illustrating shared and distinct TFs across all categories in panels (E) and (F). (**H, I**) Heatmaps displaying log_2_-fold change values for enriched TFs distinctively associated with old-aged dwarf downregulated (**H**) and upregulated (**I**) gene sets. Color intensity corresponds to the magnitude and direction of expression change.

To investigate potential upstream regulators driving the observed transcriptomic changes in dwarf mice, we performed transcription factor (TF) enrichment analysis using the Enrichr gene set library ENCODE_and_ChEA_Consensus_TFs_from_ChIP-X. This analysis was conducted separately on both upregulated and downregulated genes in middle-aged and old-aged dwarf mice using the differentially expressed gene data set generated in Fig. 1 above (Fig. 3A–D and Supplementary Dataset 2). Focusing on the pairwise comparison strategy and applying a *P*-value threshold of 0.05, we identified eight TFs associated with downregulated genes (Fig. 3A) and six TFs associated with upregulated genes (Fig. 3B) in middle-aged dwarf mice. In old-aged dwarf mice, the analysis revealed 51 TFs associated with downregulated genes (Fig. 3C) and 18 TFs associated with upregulated genes (Fig. 3D). Among these TFs, three overlapping TFs, Nelfe (Negative Elongation Factor E), Sall4 (Sal-like protein 4), and PparD (Peroxisome proliferator-activated receptor delta), were consistently associated with downregulated genes across both age groups (Fig. 3E). Similarly, we identified five TFs, Suz12, Ezh2, Smad4, Gata2, and Smc3, that were shared among the TFs associated with the upregulated gene sets in both middle-aged and old-aged dwarf mice (Fig. 3F). Interestingly, these TFs are functionally linked to critical biological processes, including transcriptional regulation, cytokine signaling, vascular development, and metabolism. For example, Nelfe, Suz12, and Ezh2 are key epigenetic regulators of gene expression; Smad4 and Gata2 are key mediators of cytokine and vascular developmental signaling; and PparD plays a critical role in lipid metabolism and energy homeostasis. Sall4 is a well-known stemness-associated TF implicated in developmental regulations. The enrichment of these TFs aligns with the results from the biological and functional pathway analyses (Fig. 3E and F), suggesting that some conserved and shared regulatory mechanisms are associated with the overall transcriptional phenotype of dwarf mouse skeletal muscle.

Notably, 32.7% (5/14) of the TFs were uniquely associated with the differentially expressed genes observed in middle-aged dwarf group (Fig. 3G). In contrast, 76.8% (53/69) of the TFs were uniquely associated with gene alterations in old-aged dwarf mice (Fig. 3G). Among these TFs, 80.02% (44 out of 53) were linked to downregulated genes, whereas 16.98% (9 out of 53) were associated with upregulated genes in the old-aged dwarf mice (Fig. 3G). These findings indicate that, with advancing age, Ames dwarf skeletal muscle displays a pronounced shift in TF associations, particularly toward downregulated gene networks. Suppression of age-associated transcriptional changes at gene regulatory regions has been reported in liver tissues of Ames dwarf mice [122], which is consistent with our observations. Thus, we further studied the expression of the TFs uniquely associated with changes in old-aged dwarf mice (Fig. 3H and I, and Supplementary Dataset 4). The majority of the TFs associated with downregulated genes in old-aged dwarf mice showed positive expression changes (Fig. 3H). Interestingly, several of these TFs, such as Irf1, Atf2, Rfx5, Cebpd, Zbtb33, and Ubtf, are implicated in immune and inflammatory responses as well as ribosomal protein synthesis (Fig. 3H). In contrast, Ets1 and Erg are involved in vascular homeostasis, whereas Six5 is involved in regulating muscle satellite cell proliferation and differentiation (Fig. 3H). Zc3h11a, Trp63, and Ar are interesting TFs associated with the upregulated genes in old-aged Ames dwarf mice and showed negative expression changes (Fig. 3I) and are involved in inflammatory response suppression during stress-induced mRNA export, regulation of gene expression during muscle atrophy (Trp63), and exercise-induced muscle hypertrophy, respectively. These results are consistent with our biological process and pathway enrichment analyses (Figs. 1 and 2, respectively). The markedly divergent TF profiles between the middle-aged and old-aged groups suggest that TFs play an increasingly selective role in regulating gene expression changes with age. In particular, the higher proportion of TFs associated with downregulated genes in the old-aged group may reflect a broader decline in transcriptional activation or a shift toward repressive or compensatory regulatory programs. Such transcriptional reorganization is likely to influence key physiological, metabolic, and pathological processes associated with advanced aging. In Ames dwarf mice, these regulatory shifts may represent adaptive mechanisms that contribute to their markedly extended lifespan and sustained healthspan, potentially by promoting a balanced inflammatory microenvironment, metabolic efficiency, stabilized muscle integrity, and delayed onset of age-related pathologies in skeletal muscle.

### Aging transcriptomic datasets identify molecular response of healthspan and skeletal muscle microenvironmental regulation in Ames dwarf mice

**Figure 4.**
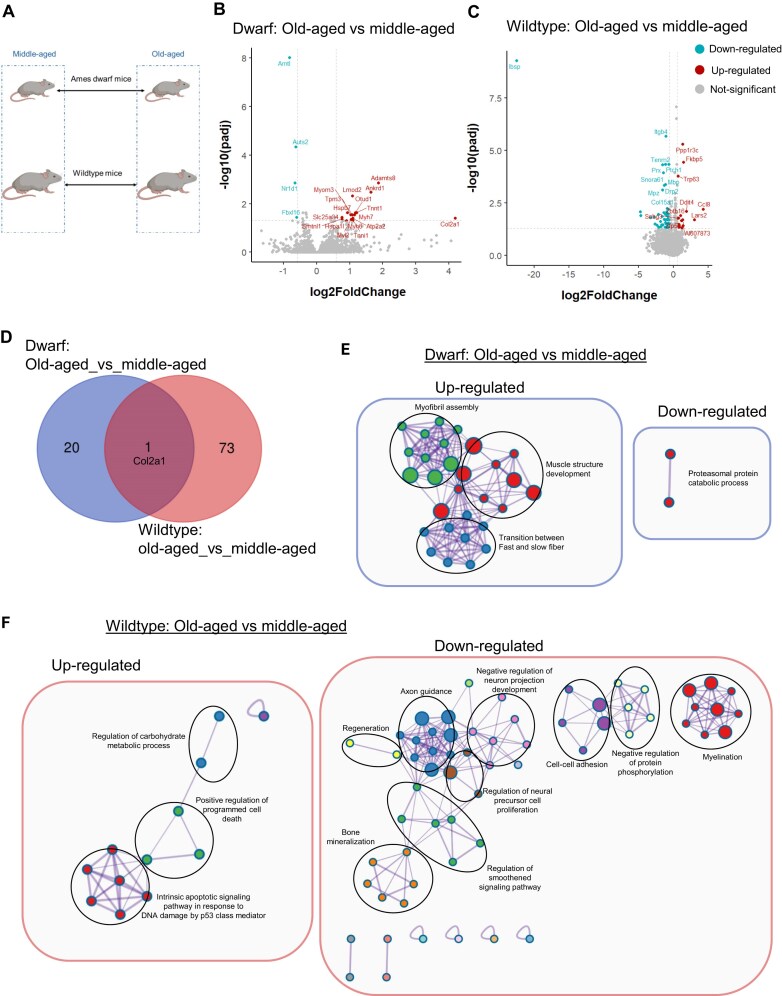
Aging-associated transcriptional and functional changes in Ames dwarf and wildtype skeletal muscle. (**A**) Schematic of the design for analysis of RNA-sequencing experiment of the aging process in each genotype. Mouse icons were created in BioRender. Ekumi, K. (2026) https://BioRender.com/zwr9215. (**B**) Volcano plot of differential gene expression analysis comparing old-aged versus middle-aged Ames dwarf mice identified 21 significantly altered genes, including 17 upregulated and 4 downregulated transcripts. (**C**) Volcano plot of differential gene expression analysis between dwarf mice, old-aged versus middle-aged comparison revealed 74 significantly altered genes (20 upregulated, 54 downregulated), representing a substantially greater aging-associated transcriptional shift than in dwarf mice. (**D**) Venn diagram shows limited overlap of transcriptional change in the aging process between genotypes, with *Col2a1* as the only shared aging-associated gene. (E and F) Metascape enrichment network visualization for results from dwarf and wildtype aging transcriptome, showing the intra-cluster and inter-cluster similarities of enriched terms. Cluster annotations are shown in color code. The color code represents the identities of gene lists. (**E**) GO biological process enrichment of differentially expressed genes in aging dwarf mice shows upregulation of pathways associated with muscle structural maintenance, including myofibril assembly, muscle structure development, and fast-to-slow fiber transition. In contrast, the sole downregulated pathway reflected proteasomal protein catabolism. (**F**) GO biological process enrichment for the wildtype aging transcriptome reveals upregulation of apoptotic processes (e.g. programmed cell death, intrinsic apoptotic signaling) and minor enrichment of carbohydrate metabolic regulation, with downregulated pathways dominated by neuronal and regenerative functions (axon guidance, myelination, neural precursor proliferation, muscle cell regeneration, smoothened signaling), as well as bone mineralization. *n* = 8.

To compare the aging process between Ames dwarf and wildtype mice, we generated differentially expressed genes between old-aged and middle-aged mice within each genotype (Fig. 4 and Supplementary Dataset 5). Comparing old-aged versus middle-aged dwarf mice (Fig. 4A), these data identify 21 differentially expressed genes out of 12 596 tested (*P*-adjusted value < 0.05 and |log_2_-fold change| ≥ 0.58) of which 17 genes were upregulated, and 4 genes were downregulated (Fig. 4B). The majority of upregulated genes were associated with muscle contractile and structural functions, including Tnni1, Myh6, Myh7, Tnnt1, Myl2, Tpm3, and Lmod2, suggesting preserved or reinforced sarcomere integrity in aged dwarf mice. Additional upregulated genes, such as Atp2a2 (involved in calcium handling), Hspb7 and Hspa1l (stress response), and Col2a1 and Adamts8 (ECM remodeling), are indicative of enhanced protective mechanisms and adaptive tissue remodeling that may contribute to sustained muscle homeostasis with age. In contrast, the small subset of downregulated genes, including the circadian regulators Nr1d1 and Arntl, indicates a potential reorganization of circadian and metabolic regulatory processes in old-aged dwarf skeletal muscle.

Next, comparing old-aged versus middle-aged wildtype mice (Fig. 4A), we identified 74 differentially expressed genes out of 14 802 tested (*P*-adjusted value < 0.05 and |log_2_-fold change| ≥ 0.58) of which 54 genes were downregulated, and 20 genes were upregulated (Fig. 4C). This represents a greater percentage of differentially expressed genes (∼70% greater) in the aging process of the wildtype mice than in the dwarf mice, in which only Col2a1 gene was shared between the aging processes of both genotypes (Fig. 4D). The top upregulated genes included Fkbp5, which is notable because its expression increases with age in bone marrow–derived mesenchymal stem cells, where it impairs osteogenic differentiation and enhances cellular senescence [123]. The upregulation of the chemokine gene Ccl8 may delay the resolution of inflammatory responses and suppress muscle regeneration in aging wildtype mice [124]. Senescence-associated genes, including Cdkn1a and the TF Trp63 were among the upregulated genes, which is consistent with the hallmarks of skeletal muscle aging. Genes involved in stress response (Ddit4, Foxo1), mitochondrial regulation and metabolic reprogramming (Ppp1r3c, Nfil3, Foxo1, Lars2, Slc7a8, and Tead4) were also upregulated. Among the top downregulated genes, Tenm2 [125], Prx [126], Ptch1, Mbp [127], and Mpz [127], are essential for maintaining the myelin sheath in peripheral nerves and are associated with disorders such as Charcot-Marie-Tooth disease and Dejerine-Sottas disease. Ibsp [128] is involved in bone mineralization, and Itgb4 and Col15a1 [129] play roles in stabilizing tissue integrity.

Pathway-enrichment analysis of the aging process in dwarf mice revealed upregulation of major muscle-stabilizing processes, including myofibril assembly, muscle structure development, and transition between fast and slow fibers (Fig. 4E, upregulated). The proteasomal protein catabolic process was the only enriched pathway associated with the downregulated genes, possibly due to the small number of genes (Fig. 4E, downregulated). Conversely, pathway-enrichment analysis of the aging process in wildtype mice shows upregulation of apoptotic processes, such as positive regulation of programmed cell death and intrinsic apoptotic signaling pathway in response to DNA damage by p53 class mediators (Fig. 4F, upregulated). Minor terms associated with the upregulated genes were related to the regulation of carbohydrate metabolic processes (Fig. 4F, upregulated), possibly activated to support metabolic adaptation resulting from aging-associated muscle fiber-type composition changes [130]. The pathways associated with genes downregulated during the aging process in wildtype mice were predominantly related to neuronal processes that included axon guidance, myelination, cell–cell adhesion, regulation of neural precursor cell proliferation, and negative regulation of neuron projection development (Fig. 4F, downregulated). Other pathways are involved in the regeneration of muscle cells [131, 132], including regeneration, regulation of the smoothened signaling pathway and negative regulation of protein phosphorylation (Fig. 4F, downregulated). Interestingly, bone mineralization was another pathway included (Fig. 4F, downregulated), given the close interplay between bone and muscle in maintaining musculoskeletal integrity.

**Figure 5.**
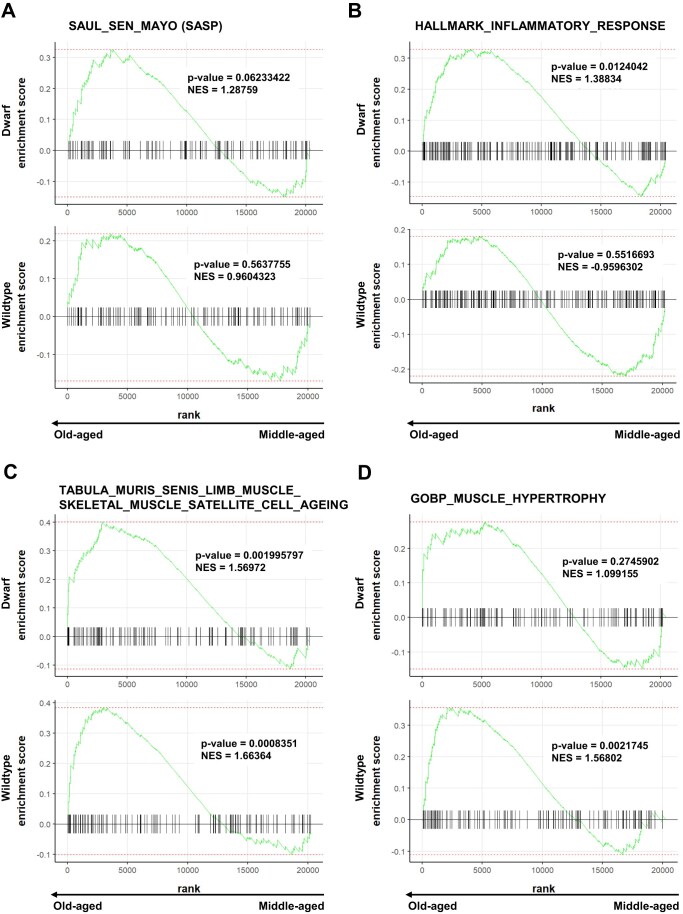
Transcriptional response of aging dataset in the aging process of Ames dwarf and wildtype mice. (**A**) GSEA using the SAUL_SEN_MAYO SASP aging dataset demonstrates significant enrichment of SASP genes in old-aged dwarf skeletal muscle (NES = 1.29, *P*-adjusted value < 0.1), but not in wildtype mice (NES = 0.96, *P*-adjusted value > 0.1). (**B**) GSEA of inflammatory response genes shows that dwarf mice exhibit significant enrichment of HALLMARK_INFLAMMATORY_RESPONSE genes (NES = 1.39, *P*-adjusted value < 0.1) and the wildtype mice show nonsignificant enrichment (NES = 0.96, *P*-adjusted value > 0.1). (**C**) GSEA of TABULA_MURIS_SENIS_LIMB_MUSCLE_SKELETAL_MUSCLE_SATELLITE_CELL_AGEING aging dataset indicating that dwarf and wildtype mice show significant positive enrichment in the aging process (NES = 1.57, *P*-adjusted value = 0.002 and NES = 1.66, *P*-adjusted value = 0.0008, respectively). (**D**) GSEA of muscle hypertrophy genes shows that dwarf mice exhibit nonsignificant enrichment of GOBP_MUSCLE_HYPERTROPHY genes dataset (NES = 1.10, *P*-adjusted value > 0.1) and the wildtype mice show a significant enrichment (NES = 1.57, *P*-adjusted value = 0.0021). The *P*-adjusted value was identical to the binomial *P*-value because the comparison was between two groups and a single reference data set.

Next, we assessed the transcriptional profile of the SASP, a well-established hallmark of the involvement of cellular senescence in aging. We hypothesized that GH deficiency in dwarf mice may contribute to skeletal muscle maintenance and stability in a cell-intrinsic manner through the generation of senescence-controlled tissue microenvironment. To explore this, we used the SAUL_SEN_MAYO gene dataset [133], which includes SASP genes. The SASP genes included cytokine, inflammatory, and metalloproteinase genes. Using a ranked gene list of the differentially expressed genes, gene set enrichment analysis (GSEA) revealed significant positive enrichment of SASP genes in old-aged dwarfs compared with middle-aged dwarfs (Fig. 5A, *P*-adjusted value < 0.1, Normalized enrichment score (NES) = 1.29). Compared with middle-aged wildtype mice, old-aged wildtype mice showed positive enrichment of the SASP genes, but this enrichment did not reach statistical significance (Fig. 5A, *P*-adjusted value > 0.1, NES = 0.96). These results suggest that (i) delayed accumulation of senescent cells in dwarf mice with age as the SAUL_SEN_MAYO gene dataset predicts aging robustly when chronological aging is included, a younger age group, dwarf_Old_vs_Middle compared to wildtype_Old_vs_Middle, and (ii) the old-aged and middle-aged wildtype mice are phenotypically and transcriptionally similar, given the limited number of differentially expressed genes. We further assessed two additional characteristic hallmarks of aging: the inflammatory response, using HALLMARK_INFLAMMATORY_RESPONSE gene dataset, and physiological hypertrophy, using the GOBP_MUSCLE_HYPERTROPHY gene dataset. GSEA revealed similar positive and significant enrichment of hallmark inflammatory response genes as the SASP enrichment for the dwarf mice (Fig. 5B, *P*-adjusted value < 0.1, NES = 1.39) and nonsignificant negative enrichment for the wildtype (Fig. 5B, *P*-adjusted value > 0.1, NES = −0.96). In contrast, old-aged dwarf mice showed positive enrichment of genes involved in muscle hypertrophy, which did not reach statistical significance (Fig. 5C, *P*-adjusted value > 0.1, NES = 1.12), whereas old-aged wildtype mice showed significant positive enrichment (Fig. 5C, *P*-adjusted value < 0.1, NES = 1.57). Thus, Ames dwarf mice maintained balanced anabolic processes in muscle during aging. In contrast, wildtype mice show increased anabolic muscle processes to compensate for the effects of aging, possibly through anabolic hormonal stimulation [134], given that the mice were sedentary and the only load that could induce hypertrophy was their body mass. Lastly, we tested the aging dataset of skeletal muscle satellite cells using TABULA_MURIS_SENIS_LIMB_MUSCLE_SKELETAL_MUSCLE_SATELLITE_CELL_AGEING gene dataset [135], since the mice used in this study had not undergone any injury, which can otherwise activate satellite cells and confound gene-expression signatures [136, 137]. GSEA revealed similar enrichment of genes relating to skeletal muscle satellite cells aging amongst dwarf and wildtype mice aging process (Fig. 5D). Thus, the response to senescence, coupled with inflammatory response, may facilitate tissue repair [138, 139], which is consistent with the enrichment of muscle maintenance and structural organization genes observed (Fig. 4D, upregulated) in the dwarf mice aging process. Collectively, the relatively limited number of differentially expressed genes and divergence of the aging transcriptome, together with the enrichment of SASP and inflammatory responses genes, suggest that dwarf mice maintain a functionally resilient muscle transcriptome that remodels skeletal muscle with increasing age, which is consistent with their delayed onset of age-related sarcopenia [54].

Projection of the SAUL_SEN_MAYO (SASP) and HALLMARK_INFLAMMATORY_RESPONSE gene sets onto a publicly available single-cell skeletal muscle (mouse hindlimb single-cell and single-nuclei) reference atlas [62] revealed that enrichment of these signatures is predominantly localized to nonmyofiber populations (Supplementary Fig. 6). Both gene sets were primarily enriched within fibroblast, endothelial, perivascular, immune, and satellite cell clusters, with comparatively lower expression across mature myofiber populations rather than restricted expansion within immune clusters. These findings indicate that the senescence and inflammatory signatures detected in bulk RNA-seq data likely reflect coordinated microenvironmental remodeling rather than intrinsic senescence of post-mitotic myofibers or pathological immune infiltration.

### Phenotypic physical assessment of Ames dwarf mice at advanced age shows preservation of skeletal muscle function

**Figure 6.**
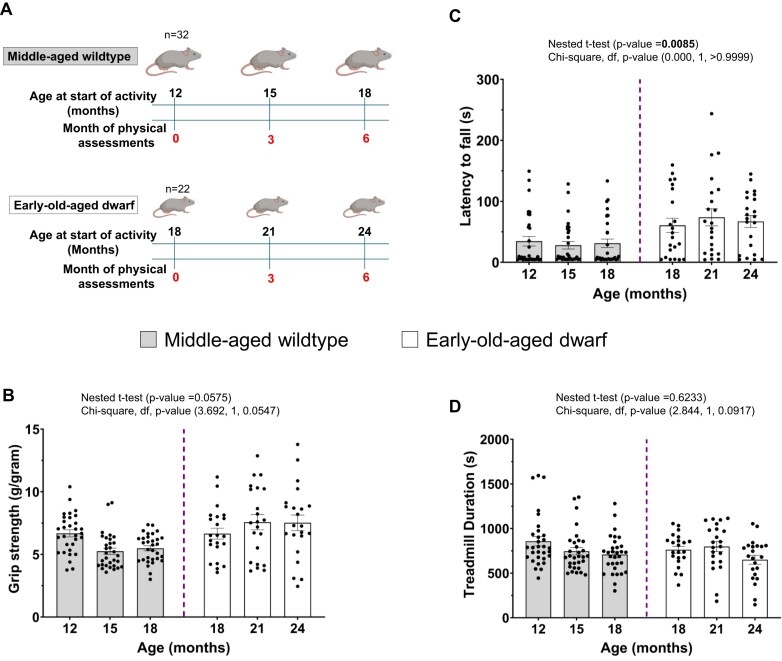
Age- and genotype-dependent differences in physical performance of Ames Dwarf and Wildtype Mice. (**A**) Design of age group and physical assessment study. Mouse icons were created in BioRender. Ekumi, K. (2026) https://BioRender.com/zwr9215. (**B**) Normalized maximal Grip strength measurements showed a significant effect by nested *t*-test (*P* = 0.0085), while the chi-square test was not significant (χ² = 0.000, df = 1, *P* > 0.9999). Data were normalized to body weight. (**C**) Rotarod performance across age groups and genotypes. The nested *t*-test results approached significance (*P* = 0.0575), and the chi-square test similarly trended toward significance (χ² = 3.692, df = 1, *P* = 0.0547). (**D**) Treadmill endurance testing revealed no significant differences by nested *t*-test (*P* = 0.6233) or chi-square test (χ² = 2.844, df = 1, *P* = 0.0917). Data are represented as mean ± standard error of the mean (SEM). Nested *t*-test was used to compare the Mean of the genotypes (nested *P*-value for genotype/main column effect) and age (chi-square test for age/sub column effect).

Transcriptional comparison of age-matched genotypes and the aging process within genotypes indicates that middle- and old-aged wildtype mice are not transcriptionally far apart. In contrast, analysis of the aging process within dwarf mice, together with their comparison to age-matched wildtype controls, suggests that age-associated transcriptional changes may follow a distinct or attenuated trajectory in this genotype. Based on this interpretation, we hypothesized that Ames dwarf mice may preserve transcriptional programs related to muscle stability and maintenance at ages when wildtype mice begin to exhibit age-associated transcriptional changes. To test whether these molecular findings translate into functional outcomes, we conducted a series of physical assessments, including latency-to-fall, grip strength, and treadmill endurance, which collectively provide insight into neuromuscular coordination, muscle strength, and systemic endurance capacity. We initiated physical assessments using 12-month-old wildtype mice (representing middle-aged) and 18-month-old dwarf mice (representing early-old-aged), which are approximately six months older. Mice were evaluated every three months over six months (0, 3, and 6 months), enabling both cross-sectional comparisons and longitudinal evaluation of performance stability (Fig. 6A). This design was chosen to determine whether transcriptional features consistent with preserved muscle maintenance in dwarf mice translate into measurable functional advantages at ages when wildtype mice begin to exhibit functional decline.

The physical assessment revealed several essential trends. Latency to fall, which reflects neuromuscular coordination and gait speed, was significantly higher in dwarf mice compared to wildtype controls (*P*-value = 0.0085; Fig. 6B). Importantly, no significant age-related differences were observed within either genotype over the 6 months (chi-square = 0.000, df = 1, *P*-value > 0.9999), indicating that both groups maintained stable motor function during the experimental window, with dwarf mice consistently outperforming wildtype mice. These results suggest that the functional advantages of dwarf mice persist over time and translate into enhanced motor performance relative to wildtype mice. Grip strength was evaluated as force normalized to bodyweight and was higher in dwarf mice compared to wildtype mice (*P*-value = 0.0575; Fig. 6C). Although this difference did not reach statistical significance, the trend is biologically meaningful as it suggests that dwarf mice maintain superior muscle strength relative to their body size. Additionally, there was a near-significant effect of age within genotype (chi-square = 3.692, df = 1, *P*-value = 0.0547), with maximal grip strength observed at 6 months in early-old-aged dwarf mice. This pattern indicates that muscle function in dwarf mice is preserved mainly with age and may remain stable or improve slightly at later time points. Finally, treadmill endurance testing at accelerated-speeds revealed no significant difference between early-old-aged dwarf and middle-aged wildtype mice (*P*-value = 0.6233; Fig. 6D). There was a modest trend toward age-related differences within genotype (chi-square = 2.844, df = 1, *P*-value = 0.0917), suggesting subtle variability in endurance performance with aging. The maximal running duration was observed in the middle-aged wildtype. Considering Ames dwarf mice have shorter stride length and smaller body weight compared to wildtype mice [54], the short stride length of dwarf mice may be compensated for by high oxygen intake [140, 141]. This finding does not contradict our previous report indicating that 24-month-old male dwarf mice exhibit greater endurance capacity than age-matched male wildtype mice [54].

Together, these results demonstrate that early-old-aged dwarf mice showed improved neuromuscular coordination, muscle strength, and gait speed while maintaining systemic aerobic endurance capacity compared with middle-aged wildtype mice. The preservation of skeletal muscle function overall underscores the functional relevance of transcriptional programs that favor muscle stability and maintenance with increasing age in dwarf mice.

## Discussion

Aging is associated with alterations in inflammatory and metabolic pathways that can coordinate tissue structural and functional changes [35, 36, 38–40, 43, 130]. In skeletal muscle, conditions associated with muscle aging include a decline in mobility and onset of sarcopenia, both of which have been linked to alterations in muscle structural integrity and disruption of mitochondrial processes [1, 3, 5, 42]. Several studies on lifespan-extending interventions have underscored the importance of elucidating the biological adaptations of aging to promote healthier aging trajectories and extend healthspan. Our study provides a comprehensive characterization of transcriptional adaptations and associated functional relevance in hindlimb skeletal muscle of Ames dwarf mice, a well-established longevity model. We found that dwarf mice maintained transcriptional programs related to muscle structural stability, vascular remodeling, and neuronal communication at ages when wildtype mice exhibit age-related transcriptional drift. Additionally, the increase in the number of TFs associated with the downregulated genes in the Ames dwarf mice captures the synergistic suppression of lipid metabolic and proteostasis pathways that can accelerate the aging process and increase chronic inflammation. Functionally, these molecular adaptations translate into preserved grip strength and neuromuscular coordination, highlighting the biological relevance of the observed transcriptional profiles. These findings suggest that the GH-deficient milieu of Ames dwarf mice orchestrates a unique transcriptional network that maintains skeletal muscle integrity and delays frailty [45–54].

Our differential gene expression analysis revealed that middle-aged dwarf mice display upregulation of genes involved in vasculogenesis, endothelial migration, and synaptic signaling, whereas old-aged dwarf mice exhibit upregulation of genes involved in protein transport, autophosphorylation, and ECM organization. Together, these age-dependent shifts suggest a transition from tissue development and remodeling programs in middle-aged Ames dwarf mice to tissue maintenance and repair pathways in old-aged Ames dwarf mice. This temporal switch is consistent with the concept of “adaptive remodeling” during aging, where early activation of vascular and neuronal programs sets the stage for sustained tissue function, whereas later prioritization of ECM and protein homeostasis supports structural stability and repair capacity during advanced age [142]. The observed downregulation of biological processes linked to lipid metabolism, cholesterol homeostasis, and nitric oxide biosynthesis in middle-aged dwarfs may reflect metabolic reprogramming that prioritizes oxidative efficiency and stress resistance [55–59, 70, 84, 143]. These alterations align with previous studies showing enhanced mitochondrial quality control and reduced oxidative damage in these mice [55–57]. This transcriptional reorganization likely contributes to the remarkable ability of dwarf mice to resist sarcopenia and sustain muscle function into advanced age [54, 71]. The downregulation of genes involved in lipid metabolism suggests that Ames dwarf mice maintain efficient lipid metabolism, as has been previously reported [140]. This is potentially mediated by reduced circulating triglyceride, cholesterol, and nonesterified fatty acids level, a trait further corroborated by the UK Biobank publications linking favorable lipid profiles to extended longevity [144]. Similarly, studies on human supercentenarians indicate that preserved lipid homeostasis alongside enhanced metabolic flexibility is a hallmark of exceptional longevity [145]. At the molecular level, highly unsaturated phosphatidylcholines, triacylglycerols, and plasmalogens with higher orders of carbon and double bonds are metabolites that correlate positively with longevity in plasma metabolic profiles [146]. Given that unsaturated lipids are more susceptible to peroxidation than saturated lipids [147], the enhanced antioxidant defense of Ames dwarf mice [55–57] drives the lipid metabolic process toward longevity and healthy lipid metabolic transcriptional changes in skeletal muscle.

A key insight from our work is the enrichment of SASP and inflammatory response signatures in the aging process, specifically in old-aged dwarf mice. While chronic SASP activity is classically associated with tissue dysfunction, stem cell exhaustion, and propagation of pro-inflammatory tissue microenvironment that accelerates skeletal muscle frailty and multimorbidity [11, 12, 14, 21, 42, 148], our data suggest a more nuanced role for senescence in this context. The enrichment of SASP genes during the aging process in Ames dwarf mice coincided with upregulation of pathways linked to ECM remodeling, cytokine/immune signaling, and tissue regeneration, potentially facilitating tissue maintenance rather than degeneration [9–17, 133]. Thus, skeletal muscle in Ames dwarf mice, within a GH-deficient environment, may remodel the composition and temporal dynamics of the SASP, thereby influencing its functional consequences. Importantly, the spatial distribution of SASP and inflammatory response signatures was not concentrated within mature myofibers, which are post-mitotic, nor was it driven by overt immune-cell expansion. Instead, the pattern is consistent with controlled paracrine signaling within the muscle microenvironment. This observation is consistent with the concept of “beneficial senescence”, in which transient senescent cell accumulation supports repair and regeneration [18–22, 138]. Notably, wildtype mice showed weaker or nonsignificant enrichment of SASP genes with age, suggesting that the timing and magnitude of senescence-associated programs may be critical determinants of healthy muscle aging outcomes. Thus, the attenuated enrichment of SASP and inflammatory response genes observed during aging in wildtype mice may reflect dysregulated signaling cascades, characterized either by insufficient activation of regenerative pathways or by abrogation of the reparative phase, leading to a premature transition to dysregulated inflammatory and senescence-associated responses. Testing this hypothesis will require future studies to dissect the composition of the SASP in Ames dwarf versus wildtype mice, including the duration of activation and its impact on immune and muscle cell population and types within skeletal muscle.

Our results from the physical assessments corroborated the molecular findings. Early-old-aged dwarf mice consistently outperformed middle-aged wildtype mice in latency-to-fall testing, reflecting superior neuromuscular coordination. Early-old-aged dwarf mice also exhibited a strong trend toward increased grip strength with age. These results suggest that the transcriptional programs favoring structural stability and neuronal communication are not merely molecular markers but actively contribute to improved physical performance [54, 71, 73]. Keeping in mind that the functional testing was performed on early-old-aged dwarf versus middle-aged wildtype mice that are not chronological age-matched mice, the treadmill endurance did not differ significantly between genotypes. However, our previous findings indicated superior endurance capacity at 24 months when comparing chronologically age-matched male dwarf and male wildtype mice [54]. This may imply that the benefits of the dwarf transcriptome are most pronounced in strength and neural coordination earlier, rather than systemic aerobic capacity [140, 141]. Thus, selective biological processes that provide molecular functional advantage may be sufficient to mitigate age-related frailty and falls, which are major contributors to morbidity in aging populations [61, 62]. The observed gain in strength appears to be primarily due to neural adaptation in Ames dwarf mice, given that old-aged wildtype mice show significant enrichment for muscle hypertrophy genes, which may compensate for the decline in muscle function with age. This distinction is relevant because even modest preservation of strength and coordination is associated with lower disability risk and reduced all-cause mortality in human cohort studies [149, 150]. The preservation of physical function likely reflects tissue- and pathway-specific aging trajectories. Whereas systemic endurance depends heavily on cardiopulmonary function and oxidative muscle metabolism, the preservation of physical fitness with age in Ames dwarf mice may arise from maintained neuromuscular junction integrity, ECM organization, and motor unit synchronization, factors essential for short-duration strength, balance, and fall prevention, but less critical for prolonged aerobic performance [151–153]. This pattern may reflect an adaptive prioritization strategy in Ames dwarf mice, whereby transcriptional drifts in gene expressions are invested in preserving strength and coordination, the most functionally relevant traits for avoiding frailty, rather than enhancing endurance, which contributes less directly to survival and longevity. Taken together, these findings reinforce the notion that Ames dwarf mice provide a valuable model for disentangling the differential contributions of muscle endurance versus neuromuscular strength to healthy aging. Thus, our results support the hypothesis that interventions targeting molecular pathways uncovered in Ames dwarf mice, such as synaptic signaling and ECM remodeling, may preserve neuromuscular coordination and muscle structural integrity more effectively than strategies focused solely on aerobic endurance, offering potential translational opportunities to prevent age-related frailty in humans.

Our results have broad implications for understanding the biology of healthy aging. By mapping transcriptional changes longitudinally, we identified candidate genes, such as Apelin, Klotho, and Notch1 that may serve as molecular drivers of skeletal muscle resilience [63–69, 72, 113–118]. Each of these factors has been independently implicated in muscle maintenance, but their simultaneous enrichment in Ames dwarf mice suggests a coordinated network of longevity-associated regulators. The enrichment of apelin signaling pathways is particularly intriguing, as apelin has been shown to reverse age-associated sarcopenia, restore neuromuscular junction integrity, and increase mitochondrial biogenesis [68, 69, 116]. In addition to its role in muscle metabolism, Apelin also exerts vasoprotective and anti-inflammatory effects, supporting tissue perfusion and resilience against stressors [65, 116–118]. Therefore, targeting apelin or its downstream effectors could represent a promising therapeutic strategy for promoting muscle health in aging humans. Systemic klotho deficiency promotes skeletal muscle weakness, wasting, and impaired contractility through neuromuscular junction remodeling, myofiber denervation, and functional motor unit loss [154]. A previous study reported that therapeutic administration of klotho in old-aged mice inhibited pathways related to cholesterol metabolism and sphingolipid signaling but activated pathways related to cytokine–cytokine receptor interaction, vascular smooth muscle contraction, and cellular senescence—processes that are altered in the opposite direction in aged geriatric mice [63, 64]. This finding implies that the timing of transcriptional drift is critical for early intervention, which may harness beneficial biological processes, whereas delayed intervention could limit efficacy or even exacerbate decline. Notch1 gene expression in middle-aged dwarf mice further strengthens this framework by linking regenerative myogenesis to aging resilience. Notch1 is essential for maintaining satellite cell quiescence and regenerative capacity, thereby delaying stem cell exhaustion and promoting efficient muscle repair [155–157]. Notch1 activation in myotubes improves muscle regeneration and exercise performance in middle-aged wildtype and dystrophic mdx mice [72]. The greater expression of Notch1 in middle-aged dwarf mice compared with both old-aged dwarf and wildtype mice underscores its role as a temporally sensitive mediator of muscle health. This pattern is consistent with the well-documented decline in Notch1 expression with age [72] and suggests that Ames dwarf mice may prolong Notch1 expression, thereby enhancing regenerative and functional capacity at midlife. Taken together, these findings suggest that healthy aging is not the result of a single protective gene or pathway, but rather an orchestrated transcriptional program that integrates regenerative, vascular, and metabolic signals. Therapeutic strategies targeting Apelin, Klotho, or Notch1 may therefore have synergistic potential, especially if they are delivered with attention to the temporal context of aging. Future studies employing time-resolved interventions in preclinical models will be essential for defining the therapeutic window during which these pathways can be most effectively leveraged to prevent sarcopenia and extend healthspan.

Translating these findings to humans will require careful consideration of endocrine and metabolic differences between species. Nevertheless, the parallels between Ames dwarf mice and human supercentenarians, both of which exhibit preserved tissue function, dampened inflammaging, metabolic alterations, and extended healthspan, underscore the relevance of our observations [24–32]. Importantly, interventions that mimic the endocrine and transcriptional states of Ames dwarf mice, such as partial GH/IGF-1 axis inhibition or pharmacologic induction of beneficial SASP responses could potentially delay muscle aging in humans [45–50, 58, 59]. Likewise, pharmacologic or cellular strategies designed to induce beneficial SASP responses may enhance tissue repair and remodeling without promoting chronic inflammation. Targeting specific molecular pathways enriched in Ames dwarf muscle, such as Apelin signaling, Klotho, and Notch1 activity, may synergize with metabolic interventions to preserve both muscle structure and function. In addition, longitudinal studies that integrate muscle transcriptomics with functional phenotyping will be essential for validating these candidate pathways. Our findings demonstrate that Ames dwarf mice maintain a transcriptional and functional muscle phenotype that resists age-related decline through coordinated regulation of vascular remodeling, neuronal communication, senescence-associated signaling, and lipid metabolism. These transcriptional adaptations preserve muscle strength and coordination, potentially contributing to the extended healthspan and longevity of Ames dwarf mice. The longevity of Ames dwarf mice and their biological processes make them a promising model for studying age-dependent therapeutic targets for strategies aimed at ameliorating health problems related to sarcopenia, potentially serving as a model for supercentenarian studies.

## Conclusions

These findings suggest that the GH-deficient milieu of Ames dwarf mice orchestrates a unique transcriptional network that maintains skeletal muscle integrity and delays frailty. The data implicate ‘beneficial senescence’ as one potential mechanism that skeletal muscle of dwarf mice may utilize to support repair and regeneration via remodeling of the SASP. Overall, it appears that an orchestrated transcriptional program integrates many signals that result in healthy aging; where Apelin, Klotho, and Notch1 could serve as potential mediators in a therapeutic approach to prevent sarcopenia and extend healthspan.

## Supplementary Material

ugag018_Supplemental_Files

## Data Availability

The data underlying this article will be shared on reasonable request to the corresponding author. The RNA-seq datasets generated during this study are available in the NCBI SRA repository under BioProject accession PRJNA1404110.
