# Supplementary material for: Age-related transcriptional drift and physiological adaptation in long-living Ames dwarf skeletal muscle
Source: NAR Mol Med. 2026 Mar 23;3(2):ugag018. doi: 10.1093/narmme/ugag018 (PMC13111926; doi:10.1093/narmme/ugag018)
Supplement: ugag018_Supplemental_Files [file ugag018_supplemental_files.zip › Revised_Supplementary figures NARMM.docx]

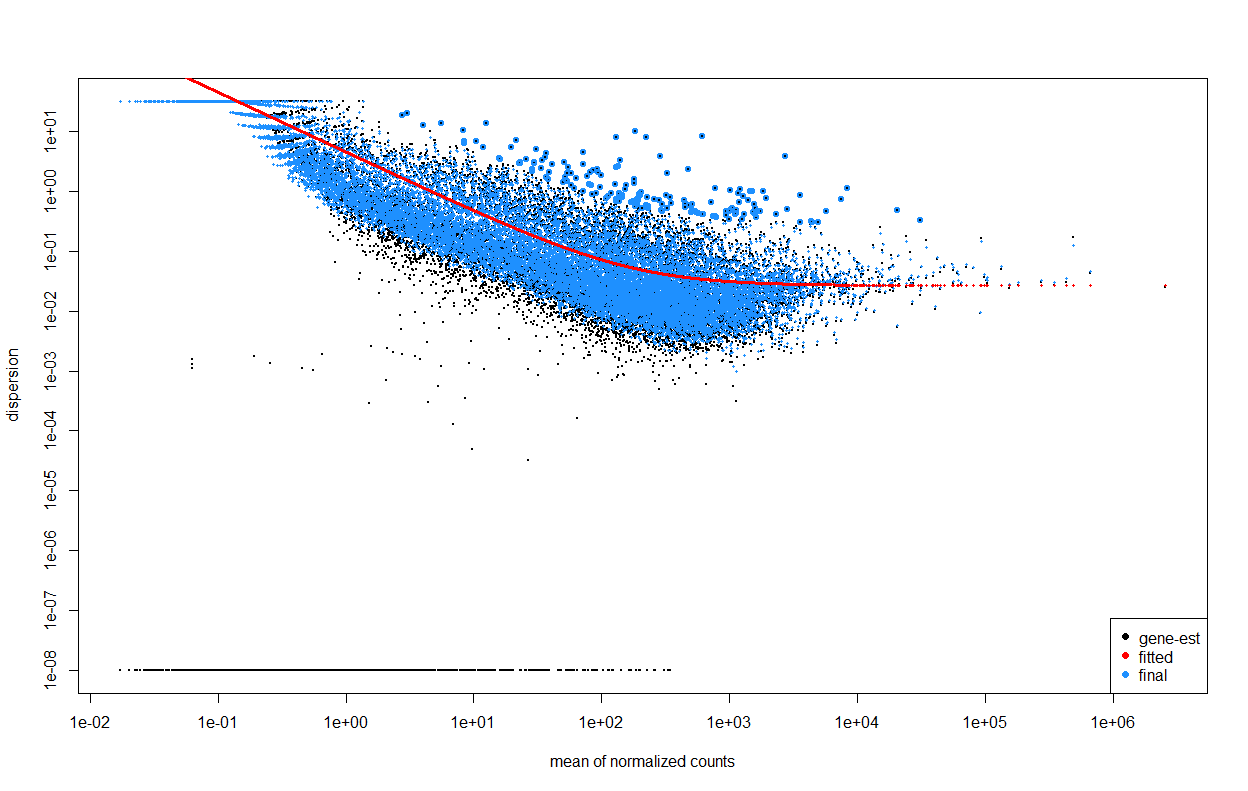


**Supplementary Fig. 1. Dispersion estimation and model fit for RNA-sequencing data from dwarf and wildtype skeletal muscle.** Dispersion estimates were generated using DESeq2 to evaluate variance across genes in RNA-sequencing datasets collected from dwarf and wildtype mice, including both male and female mice at middle-aged and old-aged time points. Each black point represents the gene-wise dispersion estimate prior to shrinkage, reflecting raw variability for individual transcripts across genotypes, ages, and sexes. The red curve shows the fitted mean–dispersion trend estimated by DESeq2, modeling the expected decline in dispersion with increasing gene abundance. Blue points denote the final dispersion estimates after empirical Bayesian shrinkage toward the fitted trend, which stabilizes variance estimates, particularly for low- and moderate-expression genes. The overall pattern demonstrates appropriate dispersion behavior across all sample cohorts and supports the robustness of downstream differential expression analyses comparing age, and genotype effects in the skeletal muscle tissue.


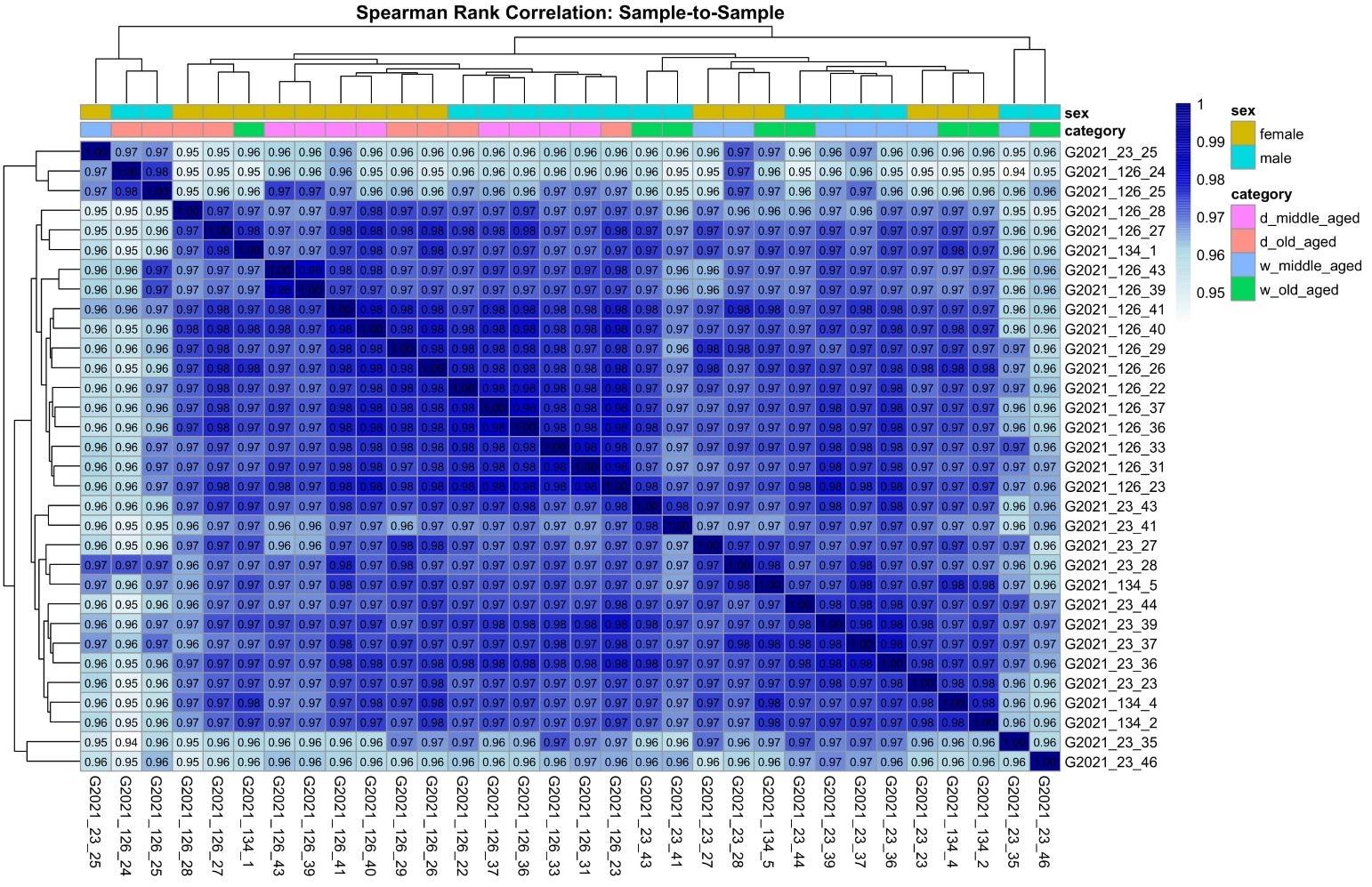


**Supplementary Fig. 2.** **Similarity of sample-to-sample association pattern**. Heatmap displaying the spearman rank correlation between Deseq2 normalized count expression profiles for all samples. The intensity of the blue color indicates higher similarity with the darker blue in the diagonal represents highest similarity within each sample. Samples are clustered based on the pair-wise correlation matrix using hierarchical clustering. Both rows and columns are ordered by hierarchical clustering, allowing visualization of sample grouping patterns. The dendrograms show clustering relationships among samples, highlighting similarities within the G2021_23 (wildtype) and G2021_126 (dwarf) groups as well as overall sample structure. The color bar at the top of the heatmap represents the sexes and genotypes. The spearman rank correlation coefficient is indicated in each square, with values ranging from 0.95 to 1 indicating the strong correlation across all samples which is an indication that the sample are all from the same tissue type (hindlimb muscle).

**Supplementary Fig. 3. Principal component analysis of skeletal muscle transcriptomes across genotypes and age groups.** Principal component analysis (PCA) of normalized RNA-sequencing data from skeletal muscle of middle-aged and old-aged Ames dwarf (d) and wildtype (w) mice. All four groups are plotted on the same axis scale to enable direct comparison of clustering patterns. The first principal component (Dim1) accounts for 23.9% of the total variance, while the second principal component (Dim2) explains 14.2% of the variance. Samples are color-coded by genotype and age: d_middle_aged (green), d_old_aged (red), w_middle_aged (brown), and w_old_aged (yellow). Ellipses represent 95% confidence intervals for each group. Separation along Dim1 primarily reflects genotype-dependent transcriptional differences, whereas Dim2 captures age-associated variation within genotypes. The distinct clustering of Ames dwarf and wildtype samples indicates genotype-specific transcriptional organization, with age-related divergence more pronounced in wildtype muscle.

(B)

(A)

**Supplementary Fig. 4. RT-qPCR validation of the top and selected differentially expressed genes and their fold-change directionality.** (A) Fold-change expression of the selected top differentially expressed genes in middle-aged dwarf versus wildtype skeletal muscle. (B) Fold-change expression of selected top differentially expressed genes in old-aged dwarf versus wildtype skeletal muscle. *Fold-change expression of the top and selected differentially expressed genes identified by RNA-sequencing were validated by RT-qPCR in skeletal muscle from dwarf and wildtype mice (n = 8 per group). Red points represent the upregulated gene, and blue points represent the downregulated gene across middle-aged and old-aged cohorts. Data are presented as Mean ± S.E.M., highlighting fold-change magnitude and directionality. Expression values were normalized to housekeeping genes (B2M) and plotted relative to the **wildtype age-matched** reference group. RT-qPCR analysis confirms the differential gene expression derived directionality of change, with consistent upregulation and downregulation patterns across age and genotype.

**Supplementary Fig .5. Transcriptomic deconvolution of bulk RNA-seq datasets using single-cell skeletal muscle (hindlimb muscles) reference signatures.**

Bulk RNA-sequencing datasets comparing Ames dwarf and wildtype skeletal muscle were deconvoluted using publicly available single-cell skeletal muscle (hindlimb muscles) reference datasets to estimate relative enrichment of cell-type–specific transcriptional signatures. (A) UMAP representation of publicly available single-cell RNA-sequencing data from mouse skeletal muscle, annotated by major cell populations including type I, IIa, IIb, and IIx myofibers, satellite cells, fibroblasts, fibro-adipogenic progenitors, endothelial subtypes (arterial, venous, capillary, lymphatic), pericytes, smooth muscle cells, Schwann cells (myelinating and non-myelinating), immune populations (macrophages, monocytes, neutrophils, B cells), adipocytes, erythrocytes, and tendon cells. (B-C). Differentially expressed genes (DEGs) were analyzed separately for (B) middle-aged dwarf vs wildtype downregulated genes, and middle-aged dwarf vs wildtype upregulated genes, (C) old-aged dwarf vs wildtype upregulated genes, and old-aged dwarf vs wildtype downregulated genes.

**Color intensity represents relative module score (gene set mean expression) of the gene set transcripts within each cell.*

**Supplementary Fig .6. Single-cell projection of SASP (SAUL_SEN_MAYO) and HALLMARK_INFLAMMATORY_RESPONSE gene signatures onto reference skeletal muscle (hindlimb muscle) cell populations.** (A) UMAP representation of publicly available single-cell RNA-sequencing data from mouse skeletal muscle, annotated by major cell populations including type I, IIa, IIb, and IIx myofibers, satellite cells, fibroblasts, fibro-adipogenic progenitors, endothelial subtypes (arterial, venous, capillary, lymphatic), pericytes, smooth muscle cells, Schwann cells (myelinating and non-myelinating), immune populations (macrophages, monocytes, neutrophils, B cells), adipocytes, erythrocytes, and tendon cells. (B) Projection of the SAUL_SEN_MAYO (SASP) gene set onto the UMAP embedding. SASP enrichment is predominantly localized to non-myofiber populations, including fibroblasts, endothelial cells, immune subsets, and satellite cells, with comparatively lower expression across mature myofiber clusters. (C) Projection of the HALLMARK_INFLAMMATORY_RESPONSE gene set onto the UMAP embedding. Inflammatory response genes show a similar distribution pattern, enriched primarily in stromal and immune-associated populations rather than in differentiated myofibers.

* *Color intensity represents relative module score (gene set mean expression) of the gene set transcripts within each cell.*

**Supplementary Method**

Projection of bulk RNA-sequencing data was done using the interactive view of mouse skeletal muscle (hindlimb muscle) single-cell and single-nuclei data. Gene data sets for the downregulated and upregulated genes, and SAUL_SEN_MAYO (SASP) and HALLMARK_INFLAMMATORY_RESPONSE were projected on the UMAP of the clustered cell types.

Interactive link: <https://cellxgene.cziscience.com/e/bedee313-cbf4-44a5-a0cd-2ca5bb2068dc.cxg/>

Reference

Kedlian, V.R., Wang, Y., Liu, T. *et al.* Human skeletal muscle aging atlas. *Nat Aging* **4**, 727–744 (2024). https://doi.org/10.1038/s43587-024-00613-3
