# Supplementary material for: Age-related transcriptional drift and physiological adaptation in long-living Ames dwarf skeletal muscle
Source: NAR Mol Med. 2026 Mar 23;3(2):ugag018. doi: 10.1093/narmme/ugag018 (PMC13111926; doi:10.1093/narmme/ugag018)
Supplement: ugag018_Supplemental_Files [file ugag018_supplemental_files.zip › Suppl. Dataset 1.pdf]

Supplementary Dataset 1

| Column1 | sample | genotype | sex | category | AGE |
|---|---|---|---|---|---|
| G2021_126_29 | G2021_126_29 | dwarf | female | d_old_aged | 23.3 |
| G2021_126_28 | G2021_126_28 | dwarf | female | d_old_aged | 23.3 |
| G2021_126_27 | G2021_126_27 | dwarf | female | d_old_aged | 23.3 |
| G2021_126_26 | G2021_126_26 | dwarf | female | d_old_aged | 23.3 |
| G2021_126_22 | G2021_126_22 | dwarf | male | d_old_aged | 20.13 |
| G2021_126_24 | G2021_126_24 | dwarf | male | d_old_aged | 21.57 |
| G2021_126_23 | G2021_126_23 | dwarf | male | d_old_aged | 21.57 |
| G2021_126_25 | G2021_126_25 | dwarf | male | d_old_aged | 22.3 |
| G2021_134_5 | G2021_134_5 | wildtype | female | w_old_aged | 22.33 |
| G2021_134_4 | G2021_134_4 | wildtype | female | w_old_aged | 22.33 |
| G2021_134_2 | G2021_134_2 | wildtype | female | w_old_aged | 22.33 |
| G2021_134_1 | G2021_134_1 | wildtype | female | w_old_aged | 22.33 |
| G2021_23_46 | G2021_23_46 | wildtype | male | w_old_aged | 22.37 |
| G2021_23_44 | G2021_23_44 | wildtype | male | w_old_aged | 23.23 |
| G2021_23_43 | G2021_23_43 | wildtype | male | w_old_aged | 23.23 |
| G2021_23_41 | G2021_23_41 | wildtype | male | w_old_aged | 23.83 |

| Column1 | sample | genotype | sex | category | AGE |
|---|---|---|---|---|---|
| G2021_126_43 | G2021_126_43 | dwarf | female | d_middle_aged | 12.53 |
| G2021_126_41 | G2021_126_41 | dwarf | female | d_middle_aged | 13.23 |
| G2021_126_40 | G2021_126_40 | dwarf | female | d_middle_aged | 13.23 |
| G2021_126_39 | G2021_126_39 | dwarf | female | d_middle_aged | 13.23 |
| G2021_126_33 | G2021_126_33 | dwarf | male | d_middle_aged | 12.53 |
| G2021_126_37 | G2021_126_37 | dwarf | male | d_middle_aged | 12.63 |
| G2021_126_36 | G2021_126_36 | dwarf | male | d_middle_aged | 12.63 |
| G2021_126_31 | G2021_126_31 | dwarf | male | d_middle_aged | 13.23 |
| G2021_23_28 | G2021_23_28 | wildtype | female | w_middle_aged | 12.2 |
| G2021_23_27 | G2021_23_27 | wildtype | female | w_middle_aged | 12.2 |
| G2021_23_25 | G2021_23_25 | wildtype | female | w_middle_aged | 12.2 |
| G2021_23_23 | G2021_23_23 | wildtype | female | w_middle_aged | 13 |
| G2021_23_39 | G2021_23_39 | wildtype | male | w_middle_aged | 12.2 |
| G2021_23_37 | G2021_23_37 | wildtype | male | w_middle_aged | 12.2 |
| G2021_23_36 | G2021_23_36 | wildtype | male | w_middle_aged | 12.2 |
| G2021_23_35 | G2021_23_35 | wildtype | male | w_middle_aged | 12.2 |

| Column1 | sample | genotype | sex | category | AGE |
|---|---|---|---|---|---|
| G2021_126_29 | G2021_126_29 | dwarf | female | d_old_aged | 23.3 |
| G2021_126_28 | G2021_126_28 | dwarf | female | d_old_aged | 23.3 |
| G2021_126_27 | G2021_126_27 | dwarf | female | d_old_aged | 23.3 |
| G2021_126_26 | G2021_126_26 | dwarf | female | d_old_aged | 23.3 |
| G2021_126_22 | G2021_126_22 | dwarf | male | d_old_aged | 20.13 |
| G2021_126_24 | G2021_126_24 | dwarf | male | d_old_aged | 21.57 |
| G2021_126_23 | G2021_126_23 | dwarf | male | d_old_aged | 21.57 |
| G2021_126_25 | G2021_126_25 | dwarf | male | d_old_aged | 22.3 |
| G2021_126_43 | G2021_126_43 | dwarf | female | d_middle_aged | 12.53 |
| G2021_126_41 | G2021_126_41 | dwarf | female | d_middle_aged | 13.23 |
| G2021_126_40 | G2021_126_40 | dwarf | female | d_middle_aged | 13.23 |
| G2021_126_39 | G2021_126_39 | dwarf | female | d_middle_aged | 13.23 |
| G2021_126_33 | G2021_126_33 | dwarf | male | d_middle_aged | 12.53 |
| G2021_126_37 | G2021_126_37 | dwarf | male | d_middle_aged | 12.63 |
| G2021_126_36 | G2021_126_36 | dwarf | male | d_middle_aged | 12.63 |
| G2021_126_31 | G2021_126_31 | dwarf | male | d_middle_aged | 13.23 |

| Column1 | sample | genotype | sex | category | AGE |
|---|---|---|---|---|---|
| G2021_13 | G2021_13 | wildtype | female | w_old_age | 22.33 |
| G2021_13 | G2021_13 | wildtype | female | w_old_age | 22.33 |
| G2021_13 | G2021_13 | wildtype | female | w_old_age | 22.33 |
| G2021_13 | G2021_13 | wildtype | female | w_old_age | 22.33 |
| G2021_23_ | G2021_23_ | wildtype | male | w_old_age | 22.37 |
| G2021_23_ | G2021_23_ | wildtype | male | w_old_age | 23.23 |
| G2021_23_ | G2021_23_ | wildtype | male | w_old_age | 23.23 |
| G2021_23_ | G2021_23_ | wildtype | male | w_old_age | 23.83 |
| G2021_23_ | G2021_23_ | wildtype | female | w_middle_ | 12.2 |
| G2021_23_ | G2021_23_ | wildtype | female | w_middle_ | 12.2 |
| G2021_23_ | G2021_23_ | wildtype | female | w_middle_ | 12.2 |
| G2021_23_ | G2021_23_ | wildtype | female | w_middle_ | 13 |
| G2021_23_ | G2021_23_ | wildtype | male | w_middle_ | 12.2 |
| G2021_23_ | G2021_23_ | wildtype | male | w_middle_ | 12.2 |
| G2021_23_ | G2021_23_ | wildtype | male | w_middle_ | 12.2 |
| G2021_23_ | G2021_23_ | wildtype | male | w_middle_ | 12.2 |
