# Supplementary material for: Age-related transcriptional drift and physiological adaptation in long-living Ames dwarf skeletal muscle
Source: NAR Mol Med. 2026 Mar 23;3(2):ugag018. doi: 10.1093/narmme/ugag018 (PMC13111926; doi:10.1093/narmme/ugag018)
Supplement: ugag018_Supplemental_Files [file ugag018_supplemental_files.zip › Suppl. Dataset 3.pdf]

baseMean	log2FoldChange	lfcSE	stat	pvalue	padj
14.96166832	0.162731618	0.425438186	0.382503554	0.702087893	0.840383343
18.12156129	-0.139962636	0.457038399	-0.306238241	0.759423258	0.87432656
134.26655	0.872768834	0.165419528	5.276093128	1.31967E-07	5.73621E-06
780.1041582	-0.203852787	0.079697614	-2.557827969	0.010532818	0.054411305
870.6007955	-0.097772747	0.094662946	-1.03285131	0.301673487	0.516769162
331.6815757	-0.126917249	0.076220042	-1.665142738	0.095884282	0.249447772
261.5564175	-0.310113583	0.094483748	-3.282189674	0.001030043	0.009859618
1582.863131	-0.297135746	0.121744433	-2.440651596	0.014660791	0.069097227
1085.671776	-0.321693765	0.081863692	-3.929626868	8.50778E-05	0.00138222
228.9516523	-0.079296162	0.103092776	-0.76917283	0.441790718	0.647633371
504.873057	-0.066537639	0.10891211	-0.610929671	0.541246141	0.728624641
25.5670892	-0.092456616	0.200468574	-0.461202545	0.644653296	0.802783158
13.25120698	-0.038320762	0.309770558	-0.123706922	0.90154733	0.950812936
13.15757853	-0.111958281	0.376538803	-0.297335308	0.766210528	0.87815168
555.2326585	-0.211959042	0.062532908	-3.389559984	0.000700049	0.007294501
114.9327871	-0.319165521	0.124031616	-2.573259389	0.010074567	0.052788937
1.90466419	0.068225523	0.80471847	0.084781853	0.932434821	0.96810558
30.39422747	-0.176227368	0.205790937	-0.856341732	0.391808784	0.604967515
3.037784918	-0.652873221	0.581702982	-1.122348073	0.261714474	0.473092773
1062.307114	0.010787885	0.102112399	0.105647163	0.915862304	0.958430263
101.1390629	0.295748302	0.103998888	2.843764083	0.004458405	0.028939227
2478.048069	-0.261605011	0.103662877	-2.523613269	0.011615563	0.058608242
644.9639961	0.029228762	0.102354681	0.28556351	0.775212455	0.883490141
4.896743936	-3.583855954	0.917858855	-3.90458286	9.4388E-05	0.001491162
273.1516341	-0.012302737	0.061823698	-0.198997113	0.842265003	0.921757179
993.3385605	-0.220360747	0.117383402	-1.877273467	0.060480623	0.184750191
830.381917	0.131941907	0.044936035	2.936216023	0.003322429	0.023410475
341.6254031	-0.074394287	0.099225091	-0.749752767	0.453403621	0.658047187
281.1195227	-0.084303089	0.107845958	-0.781699105	0.434391428	0.641118471
350.3595146	0.047825714	0.097560681	0.490215053	0.62398173	0.78896884
23.04369521	0.838055392	0.230545053	3.635104644	0.000277868	0.003551276
2.353316506	1.811531384	0.779282116	2.324615627	0.020092522	0.086221468
66.5176763	0.132251437	0.129393116	1.022090208	0.306738207	0.521863599
22.33638629	0.037186827	0.286049508	0.130001366	0.896565346	0.947889713
2578.82297	-0.176163738	0.065257033	-2.699536418	0.006943616	0.040108388
84.71588208	0.086637896	0.106510188	0.813423558	0.415975257	0.625481598
488.16205	-0.848167374	0.150211384	-5.646491989	1.63755E-08	9.43251E-07
190.0906586	-0.370060781	0.099111751	-3.733772999	0.000188633	0.002602623
458.3338448	0.026633598	0.119882633	0.222163939	0.824186265	0.911434821
636.49416	-0.029349087	0.101629583	-0.288784878	0.772746005	0.881532974
4.748686111	-0.570818873	0.492654948	-1.158658562	0.246595388	0.45517974
8317.5022	0.115679785	0.116116206	0.996241515	0.319132813	0.534070902
137.0122411	-0.410544662	0.232629955	-1.764797065	0.077597878	0.217813502
53.4866862	0.489920781	0.18617503	2.63150639	0.008500727	0.046914899
2.842254406	0.298513407	0.913843477	0.326657042	0.743927295	0.865559591
54.17669987	0.107544836	0.402257639	0.267353124	0.789197288	0.891087275
7.004862023	-0.707909141	0.767348082	-0.922539794	0.356247083	0.5707853

11.83271096	0.638851899	0.455357792	1.402966877	0.160626716	0.34882211
75.583691	-0.217224935	0.321449525	-0.675766856	0.499188681	0.695150831
26.38319427	0.183478655	0.175647512	1.044584419	0.296215084	0.510925274
8.953871341	-0.026809597	0.332349427	-0.080666896	0.935706864	0.969704969
57.83714105	0.279411553	0.190143513	1.469477183	0.141703406	0.321103427
18.88392232	0.015643494	0.220236596	0.071030402	0.943373559	0.972658406
29.36497582	0.264831573	0.358346798	0.739037084	0.459884479	0.662623194
659.6160471	-0.108964496	0.15631694	-0.697074134	0.485756401	0.684588637
6.560239856	0.382494577	0.490224795	0.780243229	0.435247722	0.641976357
32.98476791	0.568976071	0.480745705	1.183528142	0.2365999	0.443985278
2.473390464	-0.165731992	0.640017249	-0.258949258	0.795674391	0.894773586
9.187549617	-0.046436681	0.345625284	-0.134355566	0.893121416	0.946108145
160.872766	-0.600200028	0.276244323	-2.172714433	0.029801817	0.113489397
12.44699752	-0.26506816	0.318495464	-0.832250973	0.405267298	0.615958434
185.7210239	-0.005907842	0.093033788	-0.063502115	0.949366675	0.976027621
150.1912893	-0.342329886	0.149441272	-2.290731886	0.021978925	0.091837656
458.852656	0.22292575	0.08872912	2.512430542	0.011990271	0.059875028
18.5903419	-0.320148153	0.38219166	-0.837663892	0.402219498	0.613761024
105.0348603	0.905178514	0.210985873	4.290232809	1.78486E-05	0.000387451
751.2153651	-0.202019038	0.084228511	-2.398463837	0.016464002	0.074924854
6.584045004	1.97609428	0.442957872	4.461133675	8.15272E-06	0.000201361
872.1468259	0.016390896	0.058313556	0.281082091	0.778647437	0.884792834
99.91663144	-0.78540287	0.174910159	-4.490321624	7.11157E-06	0.000179138
33.44048828	-0.242090171	0.293015204	-0.826203445	0.408688694	0.619247143
1.951059948	-0.643226729	0.753437191	-0.853723093	0.393258436	0.606491244
92.93094456	0.136743479	0.149533429	0.914467616	0.360471187	0.574702635
162.0791849	-0.049692638	0.10257834	-0.484435968	0.628076498	0.791231339
265.6678748	0.036353064	0.066255828	0.548677232	0.583226974	0.759227189
5.194739767	-0.843776009	0.71498766	-1.180126673	0.237949837	0.445503537
26995.52164	-0.203241812	0.083008237	-2.44845356	0.014347092	0.068074309
112.2277661	-0.325107332	0.17203737	-1.889748332	0.058791627	0.180948038
135.7842789	-0.10111138	0.121200022	-0.83425216	0.404138904	0.614990985
44.14648812	0.111750099	0.30786121	0.362988567	0.716613425	0.84963465
30.38994934	0.416722556	0.17774111	2.344547954	0.019050161	0.083002472
796.0812264	0.271272061	0.047054258	5.765090616	8.1614E-09	5.21127E-07
1113.390324	-0.159350705	0.081922123	-1.945148636	0.051757092	0.165691086
572.1074369	0.21739859	0.067579386	3.216936436	0.001295673	0.011740879
1465.354163	-0.220352006	0.07298177	-3.019274608	0.002533808	0.019280344
31.44076742	0.120482408	0.32024856	0.376215302	0.706756841	0.843214962
157.6140623	0.479510212	0.137682687	3.482719751	0.000496348	0.005539859
969.8937375	-0.138439661	0.060428453	-2.290968159	0.021965255	0.091818223
60.78559288	-0.241268239	0.14886453	-1.62072348	0.105076956	0.265415174
217.8174639	-0.340297288	0.108324818	-3.14145267	0.00168112	0.014199103
249.0018269	0.152899715	0.143101461	1.06847068	0.285308249	0.499698926
390.7305605	0.151681634	0.073671755	2.058884501	0.039505304	0.137475788
25264.51756	-0.135852465	0.126976597	-1.069901602	0.284663602	0.498846463
134.388355	0.468280123	0.133949199	3.495953152	0.000472372	0.005341009
208.2330823	0.392839898	0.131302283	2.99187408	0.002772706	0.020594306

13.87642919	-0.372443256	0.32329503	-1.152022829	0.249311692	0.458337331
2480.703817	0.064847187	0.088238347	0.734909356	0.4623947	0.664876519
1182.577365	0.016446221	0.059667777	0.275629866	0.782832368	0.887263876
3.919210836	0.427730016	0.543532404	0.786944831	0.431314162	0.63834961
8.251935743	1.716214721	0.658982825	2.604339074	0.009205164	0.049573674
1147.818103	0.262369243	0.045559888	5.758777211	8.47254E-09	5.38906E-07
14.19705452	-0.867281708	0.336764542	-2.575335583	0.010014287	0.052556664
670.1863722	-0.084106049	0.091372231	-0.920477128	0.35732348	0.571842531
2038.675818	-0.099466929	0.075339986	-1.320240871	0.18675461	0.385345121
162.8527124	-0.355134262	0.090615855	-3.919118389	8.88735E-05	0.001426999
208.3064329	0.282759816	0.105634848	2.67676644	0.007433643	0.042286548
29.07765016	0.743177889	0.227279788	3.269881128	0.001075927	0.010192536
16.10131241	-1.233906326	0.454404906	-2.715433547	0.006618906	0.038873389
72.50755264	-0.384815748	0.178388551	-2.157177382	0.030991846	0.11645423
72.63869697	-0.257143901	0.141107005	-1.822332644	0.068404528	0.200815147
21.21242172	-0.047951868	0.238439569	-0.201107007	0.840614903	0.921008906
763.5681653	-0.057149615	0.071625368	-0.797896288	0.424930678	0.632424608
294.4403801	-0.168582065	0.073813493	-2.283892274	0.022377863	0.09290648
1217.225941	0.005267931	0.088468297	0.05954597	0.952517252	0.977124952
397.2313063	-0.024392073	0.099870452	-0.244237137	0.807047166	0.901693069
149.35077	-0.309164244	0.248855687	-1.242343494	0.214109854	0.418813315
19.3296122	0.476916493	0.227246594	2.098673893	0.035845658	0.128597858
13.69012333	0.072358865	0.453215594	0.15965661	0.873151582	0.936680519
348.8199138	0.177530354	0.080362472	2.209120114	0.027166287	0.106607294
15.12085	1.350402565	0.302564506	4.463188968	8.07488E-06	0.000200945
1295.729199	0.01468108	0.083569346	0.175675421	0.860548946	0.931104966
375.2416045	0.085922012	0.077188061	1.113151586	0.265643332	0.477355509
395.0428948	-0.023719072	0.074787378	-0.317153408	0.751127201	0.870013027
6.237126026	-1.128510488	0.395503536	-2.853351195	0.004326078	0.028337103
133.7807654	-0.160242629	0.106966773	-1.498059859	0.134117699	0.309882015
4.309472664	0.657194222	0.652248202	1.007583033	0.313654678	0.528554333
1.940292917	0.716623236	0.738553491	0.970306476	0.33189375	0.547363864
3.121916235	0.355066862	0.68430024	0.51887584	0.603847326	0.77420662
791.6443432	0.288606736	0.166822719	1.730020573	0.0836266	0.228885962
12.44120154	-0.232664367	0.359385085	-0.647395723	0.517375863	0.709915873
10.32201591	-0.212013438	0.366988716	-0.577711055	0.563459214	0.74593596
155.249684	0.020809137	0.139654653	0.14900425	0.881550279	0.940708595
32.83730174	0.204106639	0.279475828	0.73031947	0.46519493	0.667272205
44.28755326	-0.370261675	0.203462032	-1.819807217	0.068788368	0.201282341
13.09949432	1.031628214	0.741450312	1.39136527	0.16411469	0.353623737
9.60535425	-0.560925174	0.738907312	-0.759127924	0.447776036	0.65285976
24.73827383	-0.242590257	0.309618496	-0.783513455	0.433325656	0.640328374
525.5199744	-0.094656285	0.085798765	-1.103235983	0.269924699	0.481874674
20.26835938	-0.035444793	0.24599178	-0.144089341	0.885429922	0.942772268
1584.41576	-0.160485172	0.082652222	-1.941692168	0.052174377	0.166315924
298.4804428	-0.051801656	0.089977196	-0.575719829	0.564804571	0.747206097
459.5457425	0.109393456	0.082157287	1.331512518	0.183020429	0.380301606
315.72144	-0.148719815	0.097393769	-1.526995171	0.126762212	0.29809959

32.43452118	-0.484637144	0.289821537	-1.672191615	0.094486543	0.2469964
281.3423321	0.070865074	0.094540479	0.749573885	0.453511384	0.658076856
9.222100481	-0.649112441	0.604029156	-1.074637598	0.282537012	0.496216923
129.8800016	-0.231618071	0.103445996	-2.239024036	0.025154352	0.101071415
819.7639971	-0.217231086	0.089198915	-2.435355711	0.014877157	0.06984505
348.7500393	-0.234306124	0.303475654	-0.772075523	0.440069699	0.645853739
26.0879991	-0.079747911	0.196376756	-0.406096485	0.684671708	0.829651922
68.64459373	0.049838668	0.140171204	0.355555679	0.72217334	0.852432094
127.2943007	-0.064662442	0.113048034	-0.571990854	0.567328171	0.748471553
252.3584223	0.06470278	0.074081593	0.873398875	0.382445704	0.595164418
456.9471907	0.147698093	0.256616302	0.575560057	0.564912586	0.747206097
69.33958736	-0.028879391	0.155635748	-0.18555757	0.85279173	0.927255626
3.94792729	0.434601001	0.463887276	0.936867692	0.34882662	0.56340775
5157.338886	-0.139261484	0.193268346	-0.72056023	0.47118013	0.671530536
687.2268176	-0.575637148	0.209269622	-2.750696166	0.005946877	0.035807331
191.2228628	-0.019304407	0.108357242	-0.178155204	0.858601089	0.929770219
591.765007	0.204346082	0.121669432	1.679518666	0.093051004	0.244836646
12.5507305	0.208248652	0.290534184	0.716778487	0.473510788	0.673691415
160.6006807	-0.209052238	0.104623101	-1.998146072	0.045700826	0.152775043
68.47875786	0.088972958	0.197663569	0.450123199	0.65262161	0.808003036
1046.200575	0.072017388	0.145707186	0.494261062	0.62112182	0.78656014
169.9118412	-0.277943423	0.142491865	-1.950591513	0.051105658	0.164355462
382.8907534	0.600571159	0.123884634	4.847826098	1.24822E-06	4.1795E-05
7.252895792	0.754338508	0.876544229	0.860582368	0.3894681	0.602903353
381.8495514	0.249816536	0.22419922	1.114261398	0.265167062	0.476739298
1294.847262	-0.089091251	0.095688895	-0.931051096	0.351827134	0.566901428
277.3623341	-0.096915412	0.07802741	-1.242068815	0.214211173	0.418912021
25.15093012	0.346572976	0.340295718	1.018446479	0.308465824	0.523559239
146.5438941	-0.148856671	0.117161889	-1.270521259	0.203899015	0.406859542
92.85534064	0.206794702	0.111954315	1.847134716	0.064727618	0.193349552
377.6589116	-0.258922232	0.10009105	-2.586866978	0.009685297	0.051502923
14.89098367	-0.134290778	0.253896683	-0.528918994	0.596861645	0.768780199
700.5927265	-0.120684621	0.221719713	-0.544311642	0.586227037	0.760977281
64.44291062	-0.104091442	0.137427899	-0.757425847	0.448794776	0.653766482
103.2145968	-0.202896046	0.113008483	-1.795405448	0.072589125	0.208884779
317.7830798	-0.184485069	0.12651992	-1.45815038	0.144799097	0.325565761
1153.321898	-0.018926773	0.083854984	-0.22570839	0.821428232	0.910446344
146.4157511	-0.013307648	0.25007997	-0.053213569	0.957561744	0.979090857
194.1887919	0.656291267	0.138679316	4.732438028	2.21839E-06	6.76773E-05
6.425839096	-0.05980406	0.426333684	-0.140275241	0.888442526	0.944210191
227.1471019	-0.035803884	0.119870917	-0.298686996	0.76517888	0.877720432
163.5257555	0.125079798	0.125310685	0.998157486	0.318202998	0.533219021
227.2502039	0.580546844	0.225355683	2.576135803	0.00999114	0.052451891
1826.676297	-0.146016072	0.069764607	-2.092982074	0.036350755	0.129957104
227.5686644	-0.21668549	0.116728389	-1.856322108	0.06340766	0.190651175
1505.596247	-0.245030325	0.104113	-2.353503658	0.018597425	0.081439122
355.6311968	-0.083963927	0.115402573	-0.727574127	0.466874323	0.668633191
398.1613017	-0.271066017	0.127049886	-2.133540036	0.032880451	0.121115816

49.75069261	0.411280297	0.143798682	2.860111723	0.004234918	0.027906415
136.944627	-0.076245437	0.116986498	-0.651745613	0.514565292	0.707828041
596.2288661	-0.026522621	0.093043815	-0.285055179	0.775601868	0.883629572
10.41455358	-0.88779687	0.470531212	-1.88679698	0.059187638	0.181845793
21.89123499	-1.630670401	0.56269965	-2.897941027	0.003756212	0.025506941
10.52759139	-0.811626514	0.451941882	-1.795864792	0.072516023	0.208851218
36.31731312	-0.324648123	0.160878652	-2.01796894	0.043594496	0.147711997
31.84536383	-0.036139288	0.165692245	-0.218110918	0.827342691	0.913759955
167.5070951	0.07935469	0.099208926	0.799874496	0.423783516	0.631486019
56.84993981	0.280345785	0.132760165	2.111670952	0.034714682	0.125717668
71.64355882	-0.008611674	0.132875579	-0.064810057	0.948325234	0.975443925
17.37222912	-0.428228501	0.385845621	-1.109844139	0.267066195	0.478749089
606.9492452	-0.255522969	0.126139176	-2.025722518	0.042793228	0.145571604
35.64598889	-0.445283746	0.367663237	-1.211118492	0.225849995	0.431435274
3.234330627	-0.173238069	0.64432052	-0.268869396	0.788030188	0.890459519
819.4135461	-0.198947275	0.060156441	-3.307164981	0.000942454	0.009197857
713.1512089	-0.024556239	0.095494716	-0.257147625	0.797064814	0.895203555
103.9552064	-0.052049671	0.106917643	-0.486820226	0.626385736	0.790677612
108.155473	-0.074632273	0.107760393	-0.692576105	0.48857561	0.686504952
221.0866429	-0.196399701	0.075023009	-2.617859536	0.008848322	0.048251326
388.4489021	-0.294422186	0.109473801	-2.689430562	0.007157404	0.041026815
642.3471327	0.043936395	0.114807303	0.382696869	0.701944536	0.840272801
11.40327431	-0.51928524	0.50300842	-1.032358941	0.301903999	0.516898552
7.789341869	-0.119347508	0.492792852	-0.242185956	0.808636077	0.902844509
741.2888989	0.088373272	0.04853742	1.820724522	0.068648743	0.201052336
77.08954558	-0.120885685	0.217055236	-0.556935123	0.577571756	0.755670053
2153.583394	-0.144024381	0.076845083	-1.87421725	0.060900488	0.185673903
870.7621555	-0.015209393	0.102567068	-0.148287295	0.882116041	0.940885702
46.96095907	-0.32376426	0.17237644	-1.878239631	0.060348391	0.184483094
5.727850438	-0.019728756	0.403447662	-0.048900409	0.960998663	0.98086139
514.4139343	-0.03944927	0.060013526	-0.657339647	0.510962549	0.705147867
3.357444535	0.123503826	0.632514013	0.19525864	0.845190474	0.92332015
1848.914733	0.164055831	0.114164774	1.437009209	0.150715377	0.334305254
24.20303084	-0.270799002	0.245048332	-1.105084044	0.269123174	0.480860647
277.5179429	-0.08261121	0.123497352	-0.668931019	0.503539482	0.699200053
155.0415041	-0.175893441	0.078227378	-2.248489542	0.024544991	0.099276746
1.928953282	0.326602409	0.68678305	0.475553974	0.634392172	0.794992898
2938.767883	-0.112909743	0.104981237	-1.075523071	0.282140609	0.495967668
296.9064035	0.09530348	0.088548426	1.076286558	0.281799119	0.495832968
10.33751383	0.529165217	0.325044694	1.627976787	0.103529808	0.263161558
182.9434366	-0.302535007	0.089970183	-3.362614122	0.000772082	0.00788548
112.4196453	-0.262028296	0.144215993	-1.816915656	0.069230029	0.202287247
1558.942061	0.073281342	0.084652404	0.865673487	0.386669243	0.599697714
95.63668364	-0.12422464	0.148288802	-0.837720973	0.402187431	0.613761024
335.388622	0.502945436	0.194246852	2.589207657	0.009619706	0.051319639
747.5783969	0.20348374	0.106432094	1.911864468	0.05589358	0.173931024
64.07903859	-2.611499629	0.474910562	-5.498929352	3.82104E-08	1.92501E-06
3.843353023	1.414725569	0.959458425	1.474504295	0.140345862	0.319168654

6.000027658	1.776726868	0.7813919	2.273797397	0.022978171	0.094564673
349.8941039	0.098134289	0.067309626	1.457953263	0.144853426	0.325643469
448.4518859	0.193006804	0.14394014	1.340882429	0.179958628	0.375936905
14.53442958	0.759874764	0.276337578	2.749806121	0.005963054	0.035878504
1005.452028	0.215337065	0.051474927	4.183338885	2.87259E-05	0.000575006
49.22642366	0.588287595	0.223033251	2.637667669	0.008347835	0.046241502
4621.870258	-0.09992287	0.127159471	-0.785807534	0.431980252	0.639050648
1040.575525	-0.135240258	0.102951948	-1.313625051	0.188972414	0.388316272
2.741939914	-1.022286823	0.625957559	-1.633156766	0.10243603	0.261349104
12.7358566	-0.096632558	0.325401979	-0.296963645	0.766494265	0.878311336
98.4817407	2.254064574	0.196879078	11.44897972	2.37929E-30	6.53275E-27
70.30033293	0.504162758	0.328441299	1.535016332	0.124779816	0.295593801
282.1612089	-0.10050188	0.124989634	-0.804081724	0.421349779	0.629615592
5.501937144	-0.183030649	0.424712936	-0.430951433	0.666503684	0.818089403
3.859102805	-0.438955551	0.540463368	-0.812183725	0.416686217	0.626095433
105.6934948	0.305241009	0.105044711	2.905819883	0.003662922	0.025121974
429.1121416	0.005386479	0.086561117	0.062227471	0.950381686	0.976400867
111.2632808	-0.058847349	0.109303563	-0.538384541	0.590311597	0.763627267
265.4565595	-0.349420294	0.134158767	-2.60452821	0.009200085	0.049562523
137.753374	0.089565946	0.118977951	0.752794492	0.451573417	0.656469531
303.2600313	-0.011103605	0.075446037	-0.147172803	0.882995627	0.941585065
496.9448316	-0.796645112	0.165969664	-4.799944119	1.5871E-06	5.10662E-05
379.6547553	-0.097520759	0.086700556	-1.124799696	0.260673923	0.471750216
51.55331648	-0.414132616	0.290513267	-1.425520491	0.154006801	0.338642291
12.79444231	1.033503966	0.512838002	2.015264005	0.043876994	0.148333594
155.7608844	-0.312655064	0.145492484	-2.14894307	0.031638913	0.118003046
554.0169291	-0.023736278	0.091481278	-0.259465963	0.795275741	0.894535883
2758.33482	-0.205323577	0.125975065	-1.629874747	0.103127972	0.26241665
64230.0357	-0.110558616	0.14394778	-0.768046691	0.442459446	0.648148401
436.5964778	-0.166564372	0.064902219	-2.566389457	0.010276337	0.053421389
19.53158852	-1.692292242	1.169842413	-1.446598467	0.148009416	0.330349155
35.17367828	0.265816133	0.258408113	1.02866791	0.303635754	0.518513052
428.0059639	-0.132614139	0.104803098	-1.265364689	0.205740627	0.409245483
12.72114227	-0.936124432	0.612592621	-1.528135338	0.126478939	0.297871199
387.7152377	-0.222019084	0.070218601	-3.161827214	0.001567826	0.013579579
3148.751643	-0.579540503	0.230640842	-2.512740142	0.011979754	0.059840651
1006.626996	0.211326283	0.109632149	1.92759409	0.053905635	0.169960081
159.1718893	0.26514378	0.105065225	2.523611215	0.011615631	0.058608242
398.8204161	0.000182861	0.091765428	0.001992695	0.99841006	0.998773824
9.359749667	-0.008875398	0.389194999	-0.022804501	0.981806217	0.990645901
379.8808555	-0.088623739	0.058383105	-1.517968913	0.129022236	0.301576661
1837.55372	-0.187494556	0.063408679	-2.956922622	0.003107261	0.022343525
3.352497936	-0.47855853	0.745656373	-0.641794998	0.521006298	0.712937765
10002.71431	-0.227849083	0.182906355	-1.245714415	0.21286926	0.417576588
2.129681065	-1.785051031	0.819588336	-2.177984914	0.02940716	0.112502731
11315.46692	0.325512862	0.118806854	2.739849193	0.006146738	0.036745993
6.784468388	-0.90759833	0.523704923	-1.733033793	0.083089663	0.228098501
8.433012606	-0.798939338	0.834937282	-0.956885452	0.338625075	0.553808149

799.9524908	-0.10404668	0.045910134	-2.266311855	0.023432296	0.095835066
876.8521326	0.099253627	0.052972343	1.8736877	0.060973482	0.18584221
312.4681586	0.417978755	0.05909408	7.073107033	1.51503E-12	2.83321E-10
603.4096943	-0.034038287	0.091734289	-0.371053048	0.710598021	0.84564098
2.842996796	1.087930587	0.682528282	1.593971437	0.110942432	0.275345405
50.232736	-0.605692074	0.241381001	-2.509278159	0.012097818	0.060302406
1234.455785	0.20672491	0.053359986	3.874156032	0.000106995	0.001650406
2.110710947	-0.01319171	0.64421221	-0.020477274	0.983662641	0.991966796
4.285703515	0.433485662	0.601093438	0.72116186	0.470809935	0.671362327
295.7609037	-0.043459218	0.073775493	-0.589073913	0.555811692	0.740455696
274.6921143	-0.002976378	0.068300162	-0.043577903	0.965240866	0.98260939
1298.107772	-0.277724409	0.162672483	-1.707261145	0.087773525	0.236117088
314.891461	0.143373839	0.077554965	1.8486739	0.064504915	0.193034326
182.8836263	-0.264514201	0.120512215	-2.194916091	0.028169617	0.109140705
136.9034248	-0.014142362	0.103459475	-0.136694701	0.891272118	0.945526539
11.094854	-0.331418598	0.298313953	-1.110972498	0.266580187	0.478288173
245.8632349	-0.297319687	0.078863722	-3.770043808	0.000163219	0.00231999
669.7921186	-0.402059011	0.13581039	-2.960443685	0.003071963	0.022167111
2191.508006	-0.144293551	0.209492787	-0.68877575	0.490964398	0.688704658
20.7966298	0.049968675	0.277753033	0.179903257	0.857228518	0.92936531
5.372390998	0.252168849	0.496665227	0.507723986	0.611646925	0.780039684
1.982592155	-1.282863026	0.957794537	-1.339392716	0.180442859	0.376614173
32.0800642	-0.820138531	0.232020869	-3.534761912	0.000408143	0.004782179
2.747465304	-0.560772491	0.594762509	-0.942851108	0.345757066	0.560133927
257.0479363	0.067228533	0.086561305	0.776658026	0.437360557	0.64337964
248.2405378	-0.04006483	0.138465764	-0.289348274	0.772314874	0.881285256
108.5351533	-0.085332148	0.106716669	-0.799614043	0.423934449	0.631512443
411.2548041	0.00391848	0.063115661	0.062084119	0.950495844	0.976457254
1920.050173	0.105109211	0.071612544	1.467748607	0.142172522	0.321679732
114.1367596	0.245547157	0.118918807	2.064830306	0.038939059	0.136166857
44.75665892	-0.170863927	0.202935879	-0.841960169	0.399810249	0.612021197
208.537065	-0.13850456	0.077472081	-1.787799655	0.073808337	0.211206972
7399.136564	0.029970523	0.096920579	0.309227648	0.757148363	0.873294276
1.703192417	1.931178437	0.800854801	2.411396466	0.015891563	0.073107403
635.8762429	-0.108197923	0.078214894	-1.383341682	0.166560123	0.357466319
7.991249057	0.649015636	0.558504585	1.162059638	0.245211241	0.453481138
412.1619021	-0.131281727	0.066084271	-1.986580532	0.0469689	0.155499531
38425.96393	0.132162315	0.088330841	1.496219376	0.134596497	0.310726274
111.9568197	-0.401664522	0.178437986	-2.251003452	0.024385319	0.098873675
12791.73143	-0.015361675	0.094619395	-0.162352281	0.871028446	0.935876058
270.306137	0.111875768	0.084058018	1.330935124	0.183210359	0.38041435
1.874906964	0.554461038	0.738178085	0.751120968	0.452579858	0.657130317
124.4941347	-0.209748326	0.170746432	-1.228419964	0.219289357	0.423903045
9.925315593	0.425699387	0.313380508	1.358410546	0.17433345	0.368295621
1467.923552	0.651391362	0.122579131	5.314047798	1.07216E-07	4.76087E-06
42.18862167	0.883679549	0.203137577	4.350153048	1.36043E-05	0.000308276
234.3909902	-0.1157507	0.086067857	-1.344877213	0.17866489	0.374375386
143.6749459	0.498507591	0.13081341	3.810829428	0.000138501	0.00201553

88.75151186	0.516211345	0.167958631	3.07344339	0.002116038	0.016848533
6.726059019	0.144907041	0.865377824	0.167449451	0.867016418	0.934032728
12.7447157	0.436969137	0.307416653	1.421423114	0.155193791	0.340479759
523.4154658	-0.191656134	0.086143821	-2.224839026	0.026092047	0.103691513
14.92254644	-1.125912106	0.527176067	-2.135742073	0.032700443	0.120726961
120.3423528	-1.310885894	0.167576613	-7.822606477	5.17407E-15	1.4954E-12
59.69532539	-0.344682605	0.275550687	-1.250886394	0.210975929	0.415366175
17.50142418	0.213682816	0.234863072	0.909818706	0.362918128	0.576876249
4.508349305	1.585300194	0.837986772	1.891796205	0.058518139	0.180360678
369.3004596	-0.041316097	0.089613822	-0.461046036	0.644765577	0.802801611
278.0861873	0.009356445	0.103686448	0.09023788	0.928098183	0.965637301
956.1107439	-0.314436549	0.056076283	-5.607300168	2.05507E-08	1.13608E-06
12.15261219	0.119819363	0.341968921	0.350380854	0.726052893	0.854845295
3086.325096	-0.110095155	0.108199188	-1.017522937	0.308904727	0.523980279
230.1375519	0.397913492	0.297630756	1.336936738	0.181243288	0.377567264
2446.823616	-0.214712405	0.165419223	-1.297989438	0.194290964	0.39515424
174.0683365	0.091717591	0.092301808	0.99367058	0.32038327	0.535424686
94.5988942	0.482327241	0.271419152	1.777056765	0.075558901	0.214280828
100.3113576	0.842456983	0.201420631	4.182575445	2.88225E-05	0.000576241
1087.045672	0.018863208	0.091873227	0.205317795	0.837323842	0.918686178
5.457419793	0.161742309	0.49270224	0.328275977	0.742703006	0.86500393
478.8628012	-0.081026616	0.058145666	-1.393510831	0.163465372	0.353078345
3.386694128	0.685781543	0.553560326	1.238856021	0.215398823	0.419942337
8.944660398	-0.362073758	0.485314985	-0.746059301	0.455631594	0.659816708
131.3567887	0.00773859	0.175586134	0.044072898	0.964846296	0.98249956
2.694208616	-0.03554381	1.138512346	-0.031219521	0.975094472	0.987719156
3630.89806	-0.088334138	0.060203724	-1.467253719	0.142307048	0.321803316
18.0045075	-0.226993496	0.272160832	-0.834041747	0.404257459	0.614990985
1.818616796	-0.806782754	0.801101747	-1.007091493	0.313890809	0.528654159
4.876552148	0.609256427	0.566120617	1.07619544	0.281839859	0.495833718
3.307072231	0.614825666	0.573581949	1.071905537	0.283762463	0.497886499
5.260290396	-1.129235	0.725786896	-1.55587681	0.119737389	0.288693031
41.17531152	0.497082615	0.395713671	1.256167405	0.209055282	0.413045899
40.88841585	-0.096677274	0.344060435	-0.280989223	0.778718666	0.884792834
168.2600732	0.335645306	0.207847091	1.614866514	0.106339605	0.267537975
15.32087142	0.562004692	0.50610051	1.110460632	0.266800583	0.478475159
2586.44199	-0.168705113	0.098350824	-1.715340113	0.086282888	0.233278573
542.7892098	-0.323118281	0.115727693	-2.792056714	0.005237418	0.032694663
3.974612925	0.238570345	0.524069219	0.455226783	0.648946094	0.805510696
5.579261706	-0.801032017	0.870395107	-0.920308502	0.357411567	0.571927942
2138.926918	0.074450863	0.078020567	0.954246632	0.339958815	0.554777763
49.14339693	0.381635424	0.167033686	2.284781197	0.022325661	0.09273626
59.43176439	-0.004684448	0.157177709	-0.029803514	0.976223756	0.98803896
1979.041638	-0.276968137	0.059470384	-4.657244806	3.20469E-06	9.13393E-05
2.83082183	-0.078781841	0.756794304	-0.104099411	0.917090458	0.95910745
4.089616201	-0.255211552	0.591477376	-0.431481511	0.666118293	0.81795116
1.756581814	-0.561569049	0.94874843	-0.591905116	0.553914128	0.738940914
21.28286122	0.083480474	0.242084453	0.344840293	0.73021444	0.857477559

537.025235	-0.108385897	0.214131652	-0.506164764	0.612740988	0.780808713
213.7746853	0.11667702	0.087222657	1.337691656	0.180996972	0.377244955
146.9888545	-0.020604778	0.118653994	-0.173654315	0.862137143	0.931452496
8.232907178	1.061027778	0.467620889	2.268991402	0.023268849	0.095284866
281.1475531	0.146292462	0.088646132	1.650297192	0.098882166	0.254727883
32.26791003	0.643865448	0.217065806	2.966222365	0.003014824	0.021869755
1.90085446	0.08776297	0.853600679	0.102815019	0.918109785	0.959333553
637.5676407	0.075357713	0.064233277	1.173188038	0.240720408	0.448115254
76.92668435	-0.419621883	0.165079762	-2.541934147	0.011024094	0.056278564
769.29627	-0.03532398	0.064003522	-0.551906812	0.581012201	0.757846001
29.81117211	0.281438616	0.220087545	1.278757574	0.200982438	0.403630951
10.06231633	-2.074645466	0.726486631	-2.855724218	0.004293879	0.02819345
110.49489	-0.005926092	0.190309349	-0.031139257	0.975158483	0.987719156
135.1163142	0.257089651	0.26988718	0.952581929	0.340801937	0.555328498
371.249527	-0.060078449	0.054089791	-1.110716968	0.266690196	0.478381347
388.0813445	0.051693656	0.158099186	0.326969777	0.743690745	0.865506187
4.823294384	-0.012880879	0.550549266	-0.023396416	0.981334064	0.990412141
1132.10496	-0.084229181	0.082552902	-1.02030551	0.307583591	0.522492481
417.8375894	0.101259203	0.079261536	1.277532692	0.201414246	0.404153263
12.55016941	0.046204042	0.445311008	0.103756792	0.917362356	0.959166505
11.49448207	-0.108314753	0.516649535	-0.20964841	0.833942093	0.916929991
9.217552979	0.130565217	0.418061216	0.31231124	0.754803999	0.871827159
3.676236303	-1.127518525	0.762643062	-1.478435432	0.139291268	0.317779302
2.074272674	-0.946570485	0.85161511	-1.111500341	0.266353043	0.478141008
3.423449776	0.318710104	0.724169748	0.44010414	0.659861683	0.812630737
2.179270705	-1.208320457	0.839283209	-1.439705267	0.149950803	0.333185752
32.70868236	0.234662282	0.213228739	1.100519016	0.271106049	0.483145938
47.74421684	-0.040153212	0.171575298	-0.234026763	0.814964201	0.906836897
39.13363381	0.794603162	0.456567453	1.740385032	0.081791432	0.225738218
106.5372706	0.381928315	0.176735206	2.161020002	0.030693794	0.115815293
772.5017198	0.258098571	0.060897262	4.23826229	2.25257E-05	0.000470326
39.75927795	0.291351148	0.18321777	1.590190453	0.111791882	0.276650062
4.453023946	-0.206044608	0.495521694	-0.415813497	0.677546482	0.825889807
311.5521019	0.024071371	0.114592114	0.210061319	0.833619815	0.916902912
273.9480498	-0.400501365	0.108176561	-3.702293368	0.000213659	0.002885102
766.9787238	-0.147875104	0.067409268	-2.193691005	0.028257631	0.109404515
2025.707947	0.026911076	0.144087376	0.186769144	0.851841641	0.926915291
12.83053075	-0.229168341	0.298243271	-0.768394003	0.442253142	0.648039774
1.906363845	-2.29333551	1.228675062	-1.866510994	0.061969907	0.187560583
2169.294239	0.247057842	0.088019307	2.80685966	0.005002703	0.031491988
231.8717717	0.110806264	0.182772783	0.606251443	0.544347797	0.731331399
320.2015979	0.027056811	0.0895939	0.301993893	0.762656718	0.8761511
139.5871952	-0.217369031	0.100149873	-2.170437417	0.029973725	0.113828295
2.841917273	2.076312464	0.73290912	2.832973978	0.004611714	0.0296771
1015.968955	0.284142882	0.069113635	4.111242041	3.93536E-05	0.000737556
41.3025253	1.308648294	0.224569806	5.827356382	5.63123E-09	3.9143E-07
308.1644166	0.017000699	0.056591059	0.300413157	0.763862029	0.876985369
75.13973925	0.020460601	0.115634354	0.176942238	0.859553761	0.930434895

507.3370317	0.007907396	0.127923001	0.061813712	0.950711184	0.976564063
94.63089828	0.12903102	0.107635726	1.19877502	0.230615438	0.436936024
345.7664352	-0.205138197	0.072802409	-2.81773913	0.004836309	0.030655386
18.7258176	-0.023501986	0.271213224	-0.086655015	0.930945734	0.967169075
862.9304096	-0.190223914	0.067555051	-2.815835549	0.004865056	0.030778394
2410.900268	-0.011061635	0.090634472	-0.122046668	0.902862059	0.951311133
462.4816833	-0.279217362	0.063148821	-4.421576808	9.79832E-06	0.000234278
5749.931246	0.168969009	0.102625309	1.646465285	0.099668002	0.255872006
250.6909718	0.010164403	0.083536305	0.121676468	0.903155251	0.951456563
1064.200967	0.104634289	0.065221946	1.604280392	0.108652261	0.271490574
3.779747922	-0.450611675	0.540191853	-0.834169699	0.404185363	0.614990985
89.69056793	-0.062260874	0.119224318	-0.52221623	0.601519787	0.772480361
7.747940276	0.128904735	0.484155399	0.266246612	0.790049287	0.891518046
74.97077339	0.078594503	0.350240812	0.224401328	0.822445037	0.910976907
276.4981652	0.059384876	0.076909783	0.772136838	0.440033386	0.645853739
145.9843061	-0.075532078	0.094370007	-0.800382248	0.423489363	0.631300049
11.73119204	-0.911270577	0.504561319	-1.806065078	0.070908175	0.205513947
279.2697519	-0.127873542	0.075938696	-1.683904892	0.092200051	0.243153154
6726.712252	-0.020667195	0.057727147	-0.358015185	0.72033195	0.851639769
1051.345305	-0.182208343	0.052854672	-3.44734602	0.000566123	0.006164118
243.5063987	0.032357562	0.071226572	0.454290593	0.649619686	0.80577146
735.8191136	0.048652961	0.059821791	0.813298295	0.416047055	0.625532461
34.25271174	0.418049101	0.188707467	2.215328878	0.026737499	0.105623544
397.5849993	0.081919861	0.077753086	1.053589841	0.292070742	0.507295575
266.5352959	0.024248404	0.074116825	0.327164637	0.743543366	0.865479645
120.8175687	-0.185717077	0.137522261	-1.350451018	0.176871355	0.372035075
144.7179519	-0.159643548	0.10905948	-1.463820915	0.143242886	0.323254058
190.8242751	-0.096415584	0.073098538	-1.318981015	0.187175459	0.385875174
4.081032638	0.200988665	0.560526301	0.358571337	0.719915793	0.851514415
596.9222189	-0.077609436	0.06347244	-1.222726532	0.221433002	0.426155056
121.3405492	0.26757377	0.198389816	1.348727348	0.177424558	0.372722796
297.2578816	0.020428035	0.143368839	0.142485876	0.886696235	0.943388903
133.9267805	0.013636654	0.110689008	0.123197909	0.901950382	0.950958758
44.33529489	-0.004817873	0.157960814	-0.030500429	0.975667951	0.987719156
962.9735322	-0.144886908	0.130331426	-1.111680526	0.266275536	0.478053964
8.299762233	0.275439632	0.450881638	0.610891215	0.541271601	0.728624641
2.155158063	-0.580011457	0.83637641	-0.693481368	0.488007515	0.686192881
42.17410731	0.333100713	0.263662552	1.263359968	0.206459844	0.409884246
249.450057	-0.128878564	0.073289616	-1.758483291	0.078665312	0.219761294
317.0458228	0.022525335	0.064285369	0.350395985	0.726041539	0.854845295
62.49220576	-0.316000009	0.239034717	-1.321983736	0.186173567	0.384483307
369.1689293	0.225840317	0.081344691	2.776337515	0.005497511	0.033881777
475.5780424	0.146179289	0.067624777	2.161623243	0.030647228	0.115692585
159.7638994	0.031478258	0.184613158	0.170509288	0.864609631	0.932688731
235.3202844	-0.100450655	0.130681569	-0.768667349	0.442090813	0.647896455
305.3041813	0.128231834	0.071333037	1.797650002	0.072232489	0.208325985
3.788929683	-0.777903428	0.880250629	-0.883729477	0.376842264	0.589841864
28.59950132	-0.724340231	0.29033324	-2.494858088	0.012600751	0.062095357

20.92438227	0.45689489	0.396063669	1.1535895	0.248668505	0.457410111
40.52031582	-0.056220157	0.278552633	-0.201829567	0.840049966	0.920573614
114.350136	-0.213305096	0.126235837	-1.689734871	0.091078689	0.241693028
509.38149	-0.207496812	0.063966849	-3.243817908	0.001179391	0.010970802
175.2492831	-0.375448157	0.089937596	-4.174540701	2.98588E-05	0.000594074
1794.451494	0.202168982	0.061774089	3.272714926	0.001065199	0.01012
13.67718576	-0.763698624	0.29168856	-2.618198756	0.00883953	0.048219344
633.6045764	0.198099589	0.38366238	0.516338321	0.605618136	0.77567875
62.74424992	-0.048447323	0.166650787	-0.290711638	0.77127187	0.880583047
151.4669988	0.202354759	0.366258583	0.552491514	0.580611647	0.757609579
167.5337852	-0.000709371	0.122137439	-0.005807971	0.995365936	0.997576362
88.54168509	-0.218489152	0.126235	-1.730812793	0.083485159	0.228744704
99.20833911	-0.243088387	0.107613633	-2.258899556	0.023889634	0.097367102
613.2175619	-0.004135587	0.092495746	-0.044711102	0.964337584	0.982345867
134.35358	-0.0924534	0.081064433	-1.140492766	0.254081065	0.463389137
68.42733363	-0.853078109	0.203115041	-4.199975067	2.66944E-05	0.00054359
300.2756907	-0.572352717	0.215839188	-2.651755331	0.008007454	0.044716882
861.3430643	-0.182655118	0.208409206	-0.876425382	0.380798825	0.593610885
21.88228572	-0.087940694	0.233504907	-0.37661176	0.706462147	0.843064305
499.252904	-0.206255308	0.090519898	-2.278563187	0.022693044	0.093930958
2.898482865	3.785286708	2.981120565	1.269752976	0.204172636	0.407201766
50.95261027	-0.897812306	0.309675314	-2.899205281	0.003741099	0.025446268
685.1585536	-0.249778806	0.059237654	-4.216554672	2.48063E-05	0.000510694
301.5724662	0.037472662	0.060992979	0.614376661	0.538966455	0.72670923
2.66282844	0.359296996	0.560765393	0.640726051	0.521700686	0.713349603
228.5535364	-0.102113517	0.079264072	-1.288269875	0.197652036	0.399083176
3.431973573	-0.805929083	0.999052446	-0.806693469	0.419843108	0.628733943
614.625571	-0.046091657	0.100763813	-0.457422717	0.647367239	0.80444124
256.4984871	-0.385835736	0.121625707	-3.172320595	0.00151226	0.013241222
33.47491761	0.314485432	0.190975148	1.646734853	0.099612557	0.255837733
58.82837149	-0.113832688	0.275690952	-0.412899614	0.679680164	0.826777746
2.322560049	0.839525703	1.049032296	0.80028585	0.4235452	0.631300049
5.313856219	-0.602457688	0.442868434	-1.360353645	0.17371804	0.367372399
3.500936734	-0.357413548	0.547876311	-0.652361747	0.514167835	0.707591786
579.1389182	-0.016858595	0.075812133	-0.222373315	0.824023283	0.911434821
7.352124774	0.634523723	0.400809392	1.583105924	0.113397324	0.279042177
600.4543299	-0.043178549	0.05979126	-0.72215486	0.470199272	0.670973518
141.8291967	-0.39326593	0.112332309	-3.500915576	0.000463663	0.005253356
1187.109331	0.039216148	0.108079385	0.362845773	0.716720097	0.84963465
758.9148902	-0.152945857	0.079099074	-1.933598591	0.053162501	0.168596518
37.67645853	-1.31259354	0.614599931	-2.135687744	0.032704874	0.120726961
577.0396044	0.079054786	0.08909403	0.887318554	0.374907419	0.588340599
62.86855865	0.684526516	0.293642769	2.331153999	0.019745241	0.085219569
14.81321428	0.292327133	0.277686193	1.052724769	0.292467153	0.507703254
880.7479444	0.122651301	0.140323421	0.874061512	0.382084756	0.594875424
2.674996797	-1.075680964	0.92527108	-1.162557641	0.245009026	0.453310724
1.937050747	-0.98932554	0.711512212	-1.390454757	0.164390829	0.354102316
406.9800244	-0.08418512	0.082187533	-1.024305226	0.30569113	0.520512215

101.9787948	0.056330206	0.099100672	0.568413968	0.569753908	0.750099284
8.152343882	-0.497109834	0.372381092	-1.334949183	0.181892979	0.378537764
6.665291716	1.065892284	0.543658619	1.960591162	0.049926734	0.162087954
6.658950533	-2.027983892	0.916666609	-2.212346204	0.026942753	0.106033185
9.857956845	1.526776854	0.939242589	1.625540485	0.104047445	0.263790105
3529.594879	0.235187437	0.060457625	3.890120321	0.000100195	0.001564554
2.88201227	-2.268085185	1.042783603	-2.175029582	0.0296279	0.113097656
321.9350968	-0.03113528	0.07203407	-0.43222992	0.665574315	0.8175592
57.33799745	0.420523219	0.354657479	1.185716485	0.235734282	0.442966416
36.94298998	0.335005293	0.418873409	0.79977694	0.423840046	0.631486019
421.0449782	0.234707402	0.128900224	1.820845575	0.068630334	0.201034162
4.657251074	-0.026106173	0.48428602	-0.053906517	0.957009644	0.978754462
54.51383032	-1.216817396	0.795759026	-1.529127986	0.126232719	0.297545978
63.66914548	-0.175204356	0.164940183	-1.06222967	0.288131433	0.502825978
39.47938922	-0.922487804	0.341104677	-2.70441265	0.006842526	0.039803591
1162.801526	0.338304401	0.1196199	2.828161554	0.004681617	0.029904989
53.95756619	-0.19077509	0.175546837	-1.08674752	0.277148407	0.490103495
2122.052387	-0.043002812	0.058032616	-0.741011091	0.458686716	0.661797596
93.70354111	-0.185373464	0.216340995	-0.856857775	0.391523491	0.604778059
1068.369677	0.326613379	0.056576444	5.772956967	7.78923E-09	5.05196E-07
752.209566	0.015484969	0.080485532	0.192394443	0.847433245	0.924458083
108.6345131	0.031132321	0.121277378	0.256703451	0.797407708	0.895283485
6.045073359	0.601532025	0.470136126	1.279484795	0.20072639	0.403297314
26.74721622	0.217450485	0.288756474	0.753058388	0.451414828	0.656419939
836.1707272	-0.172861113	0.083890211	-2.06056358	0.039344694	0.13698194
452.2299406	0.052346222	0.061808962	0.84690343	0.397048951	0.609588078
304.4023675	-0.050387141	0.067693489	-0.744342503	0.456669294	0.660390621
27.99326773	0.230266993	0.197487295	1.165983829	0.243620984	0.451249391
17.20398832	1.359716373	0.318135223	4.274020204	1.9198E-05	0.000410737
44.60784605	0.26342331	0.3212324	0.820039666	0.412193495	0.62200931
18.65674942	0.618957832	0.261375435	2.368079589	0.017880689	0.079248445
949.0938701	0.062710794	0.068562846	0.914646894	0.360377032	0.57466323
961.7833942	0.472140366	0.074836757	6.308936762	2.80959E-10	2.72266E-08
672.1697245	-0.207391066	0.077290261	-2.683275518	0.00729049	0.041667753
467.7761541	-0.051026378	0.125740793	-0.405806076	0.684885094	0.829696916
25.92493854	0.549388616	0.206793892	2.656696529	0.007891044	0.044156611
8.642271127	0.293412377	0.425511548	0.68955209	0.490475905	0.688371107
202.130347	0.624000839	0.179007618	3.48588986	0.000490503	0.005489501
121.9810603	-0.442369278	0.116315845	-3.803172977	0.000142855	0.002071642
1671.114363	-0.173767101	0.068851592	-2.523792051	0.011609658	0.058608242
1450.576441	-0.024304988	0.083785364	-0.290086327	0.771750196	0.880884967
11.61186772	1.662895877	1.10010966	1.511572835	0.130642567	0.304328143
761.4955957	-0.012684923	0.194358016	-0.065265757	0.947962406	0.975143522
1.754240081	-0.302578711	0.886725059	-0.341231714	0.732929151	0.859215921
1369.672999	-0.287687147	0.387829631	-0.741787435	0.458216135	0.661594047
721.5402639	-0.791441934	0.451661961	-1.75228822	0.079724261	0.221629678
271.1085965	0.094618499	0.155623795	0.607995064	0.543190748	0.730491786
13.99449232	-0.01768927	0.502987188	-0.03516843	0.971945436	0.985970658

2485.175085	0.190619638	0.253752324	0.751203515	0.452530185	0.65711611
205.649024	-0.218090492	0.278434189	-0.783274831	0.43346574	0.640328374
69.84592923	0.660791887	0.194088158	3.404596616	0.000662619	0.006973833
41.50724187	0.670292081	0.184520675	3.632612334	0.000280566	0.003574672
226.0581613	-0.021577909	0.06554861	-0.329189415	0.742012523	0.864835046
646.4086953	-0.010805393	0.083596069	-0.12925719	0.897154145	0.948086303
3499.733405	0.071700025	0.080347136	0.892378113	0.372190298	0.585510216
362.1568624	-0.096465795	0.086820972	-1.111088638	0.266530198	0.478259224
123.5005351	-0.147985939	0.087605966	-1.689222166	0.091176863	0.241797752
503.7030807	0.029973218	0.057217279	0.523849068	0.600383522	0.771567059
2.114909659	0.318794879	0.747360504	0.426561047	0.669699075	0.820241125
3141.534791	0.001562103	0.088000191	0.017751132	0.985837389	0.992949691
32.89128881	-0.138413246	0.237259612	-0.583383091	0.559635438	0.743622698
7.996062771	-1.055310966	0.461620234	-2.286102058	0.022248289	0.092531761
5.861182789	-1.067885326	0.817089037	-1.306938751	0.191233514	0.391440645
21.584284	0.117068922	0.635850344	0.184113955	0.853924059	0.928183225
243.8793141	0.332979956	0.094030958	3.541173717	0.000398351	0.004680769
9.799483929	0.044279255	0.315414903	0.140384157	0.888356475	0.944210191
9.179598543	-0.264807866	0.439804165	-0.602104042	0.547104884	0.73377887
250.5845465	-0.287143565	0.096585649	-2.972942342	0.002949599	0.021519148
999.8489744	-0.081359358	0.070647917	-1.151617229	0.249478398	0.45849031
141.3993406	-0.155030189	0.130904707	-1.184298048	0.236295101	0.443666001
1270.099231	-0.159781221	0.097553309	-1.637886222	0.101445431	0.259464683
813.7311053	-0.0135272	0.052668514	-0.256836554	0.797304951	0.89525023
831.3169371	0.428162977	0.130933354	3.270083326	0.001075158	0.010191113
373.6791991	0.006076879	0.161708459	0.037579227	0.97002317	0.985321835
1983.871597	-0.258250181	0.125000053	-2.066000577	0.038828425	0.13592424
875.7847495	0.017226092	0.604159758	0.028512479	0.977253415	0.988263026
5.226118927	-0.718155234	0.64740914	-1.109275711	0.26731126	0.478933218
19.54258527	0.59194422	0.313582446	1.887682896	0.059068534	0.181513715
11.04639543	-1.497871935	0.493785674	-3.033445509	0.002417784	0.018550012
1547.085529	0.95123365	0.480081512	1.981400297	0.0475464	0.156667869
155.8511082	0.174426682	0.183731433	0.949356781	0.342439184	0.556619
22082.26129	0.001251231	0.094176104	0.013286081	0.989399553	0.994530438
136.2461817	0.727499338	0.534008377	1.36233694	0.173091575	0.366518073
488.1240418	0.031102374	0.068375937	0.454873092	0.649200542	0.805627137
4.484990493	-0.304285187	0.470313291	-0.646984027	0.517642281	0.710176534
115.3387669	0.164922365	0.134836549	1.223128053	0.221281335	0.42601247
11.85465434	-1.631654948	0.823640915	-1.981027069	0.047588238	0.156731034
276.2865933	-0.005036255	0.08498975	-0.059257208	0.952747244	0.977124952
2.943330223	-0.362843045	0.656436076	-0.55274696	0.580436692	0.757539944
10.33193664	0.507503334	0.503979435	1.006992148	0.313938548	0.528654159
199.5412769	0.906734003	0.65375676	1.386959277	0.165454181	0.355927419
2196.536955	0.037713248	0.113648109	0.33184228	0.740008357	0.863255748
78.81576064	0.439810809	0.154058968	2.854821204	0.004306106	0.028251213
837.7337632	0.085729067	0.110728291	0.77422911	0.438795317	0.644424326
11.40493577	0.648742045	0.307216075	2.111680012	0.034713905	0.125717668
471.6758657	-0.075332461	0.097842443	-0.769936429	0.441337603	0.647195627

28.15804635	-2.239875827	0.783639127	-2.858300142	0.004259173	0.028038321
417.6477988	-0.926945384	0.139679058	-6.636251694	3.2176E-11	3.84107E-09
22.42092927	-0.739203138	0.319710554	-2.312101146	0.02077211	0.088355213
520.6334149	-0.071718058	0.071457264	-1.003649652	0.315547513	0.530279479
267.3042886	-0.288316213	0.225700446	-1.277428637	0.20145096	0.404177702
287.1790889	-5.77476E-05	0.117827904	-0.000490101	0.999608956	0.999734918
250.5392393	-0.218145798	0.095481476	-2.284692343	0.022330874	0.09273626
178.9400574	0.023320915	0.122711579	0.190046571	0.849272637	0.925628813
116.2254089	-0.049813268	0.222420496	-0.223959882	0.82278852	0.911237589
2.735222952	-1.848718995	0.734731635	-2.516182652	0.011863368	0.059439517
15.04050123	-1.000513152	0.439718082	-2.275351394	0.022884859	0.09440045
1.967078036	-1.206856659	1.070933811	-1.126919933	0.259776335	0.470694604
40.38237803	-0.350898405	0.186082504	-1.885714115	0.05933349	0.182056232
7.892775249	-1.515139006	0.455215509	-3.328399357	0.000873466	0.008664779
336.4557362	-0.270835275	0.105382592	-2.570019102	0.010169291	0.05315003
2395.559517	0.053259161	0.07369236	0.72272296	0.469850107	0.670853758
148.5031168	-1.373469408	0.55949844	-2.454822587	0.014095413	0.067345661
250.8341664	0.53518622	0.115595928	4.629801649	3.66016E-06	0.000102547
2184.331937	-0.405231051	0.079024675	-5.127905305	2.92984E-07	1.17722E-05
132.5182425	0.176124693	0.123905587	1.421442703	0.155188099	0.340479759
203.2056716	-0.223747546	0.097176234	-2.302492455	0.021307416	0.089985486
202.9486678	-0.165329811	0.117810342	-1.403355664	0.160510807	0.348617539
264.4856249	0.277424734	0.117727665	2.356495685	0.018448282	0.081001332
776.5479367	-0.239157222	0.067034364	-3.567680931	0.000360155	0.004333958
1666.209932	0.017658128	0.118010195	0.149632226	0.881054781	0.940606342
87.54610649	-0.05702524	0.135390196	-0.421191796	0.673615034	0.823222819
96.14485365	0.071438421	0.155751461	0.458669347	0.646471629	0.80392948
393.8619022	0.13819214	0.140087775	0.986468231	0.323903397	0.539260694
1.77619562	0.99997986	0.93212886	1.072791437	0.283364704	0.497352453
280.6832224	-0.307193221	0.173836728	-1.767136461	0.077205378	0.217034491
49.91545884	-0.240212905	0.3179061	-0.755609613	0.449883291	0.654773154
1109.537965	0.023343742	0.06661222	0.350442338	0.726006757	0.854845295
13.12059785	-0.263335287	0.331946351	-0.793306768	0.427599115	0.634847496
40.37792462	-1.076522442	0.250234975	-4.302046275	1.69228E-05	0.00037122
3071.132373	-0.06367687	0.102708203	-0.619978428	0.535271989	0.723897438
1.873659251	1.007656386	1.084571668	0.929082342	0.352846415	0.56787728
697.0751946	-0.051740891	0.06043172	-0.856187624	0.391894007	0.605010015
55.89178661	0.060691555	0.163170198	0.37195245	0.709928253	0.845284623
11.77532686	-0.35744463	0.330470457	-1.081623553	0.279419835	0.492716073
74.02847339	-0.453556404	0.302321407	-1.500245744	0.133550758	0.308874798
4.180837568	0.318192657	0.551653403	0.576798141	0.564075826	0.746512303
1.754203544	0.689849675	0.761326627	0.90611526	0.364874849	0.578253801
4.209232454	1.29416172	0.51069909	2.534098345	0.011273709	0.057233614
3.272654697	1.212920579	0.573237913	2.115911303	0.034352352	0.124675463
4148.591676	0.009975725	0.134411715	0.074217675	0.940837182	0.971928999
13.94407401	0.776975952	0.548548671	1.416421172	0.156652229	0.342629954
161.5695089	-0.040493013	0.131025186	-0.309047556	0.757285352	0.873391129
225.943718	-0.023936963	0.098509565	-0.242991259	0.808012169	0.902331377

181.1315555	0.209602233	0.115445376	1.815596605	0.069432275	0.202626625
1827.501989	-0.605966383	0.137829961	-4.396477957	1.10022E-05	0.000258558
22.08829158	-1.727791532	0.480374504	-3.596759441	0.000322206	0.003946487
304.1438723	0.078731538	0.062281023	1.264133666	0.206182055	0.409622371
250.4529266	-0.137236812	0.158465983	-0.866033259	0.386471923	0.599504563
2059.78713	0.042566778	0.065533114	0.649546085	0.515985469	0.708985891
2130.870206	-0.036747971	0.089322951	-0.411405694	0.680775079	0.827468213
962.9848558	-0.070487145	0.06989509	-1.008470619	0.313228586	0.527998334
1.729662564	0.057169373	0.726650442	0.078675206	0.937290967	0.970445469
4.075228638	-0.072430893	0.586583559	-0.123479242	0.901727611	0.950906456
77.99180425	-0.376257025	0.194947937	-1.930038503	0.053602067	0.169457006
896.0968123	-0.018314564	0.059690952	-0.306823112	0.758978015	0.874200334
134.1571809	0.002799155	0.09001613	0.031096148	0.975192861	0.987719156
733.9417068	-0.216065738	0.07425139	-2.909921815	0.003615192	0.024919109
77.02013694	0.592832808	0.309509867	1.915392277	0.055442495	0.17295203
116.8248748	-0.489975541	0.131310307	-3.731432456	0.000190394	0.002622535
380.669798	0.098418016	0.083555388	1.177877558	0.238845423	0.446360434
40.55188411	0.47026547	0.166051878	2.832039446	0.004625214	0.029714089
29.47802552	-0.083348153	0.335048528	-0.24876442	0.803543015	0.899315102
81.60154852	-0.149434799	0.241781201	-0.618057973	0.536537125	0.724877003
6.585793627	-0.123677696	0.631460201	-0.195859844	0.844719869	0.922989661
4.95656201	0.223576281	0.850890332	0.262755695	0.792738886	0.893093493
14.58449161	-0.198095809	0.394189046	-0.502540116	0.615287641	0.782406945
797.2620073	-0.174707698	0.083098035	-2.102428748	0.035515735	0.127775982
2.375579225	0.622293988	0.791919191	0.785804909	0.43198179	0.639050648
89.80672967	0.341493597	0.228008676	1.497721942	0.134205509	0.309953953
6.736805511	-0.985529698	0.792898748	-1.242945205	0.213888026	0.418507745
305.0422691	-0.405710475	0.103307668	-3.92720581	8.59384E-05	0.001387837
419.4974155	-0.158662302	0.113836954	-1.393767977	0.163387681	0.352956814
307.4366224	0.050878169	0.13573723	0.374828406	0.707788087	0.844017441
11.22688559	0.311073929	0.315646039	0.985515074	0.324371131	0.539548668
1288.362354	-0.030192133	0.094061645	-0.320982401	0.748223728	0.868038735
8.196947774	-1.197015636	0.548063982	-2.184080099	0.028956363	0.111298908
136.0617702	0.126143296	0.097297799	1.296466081	0.194814958	0.395829011
114.3986525	-0.465033862	0.130417113	-3.565742645	0.000362827	0.004350231
2.839349869	-0.82007782	0.661102442	-1.240470112	0.214801562	0.419270253
256.7132853	-0.248079137	0.082903854	-2.99237159	0.002768191	0.020588342
11.32405667	-0.48957839	0.464563432	-1.053846163	0.291953355	0.507238933
219.821678	-0.011237311	0.075172374	-0.149487246	0.881169173	0.940606554
1084.324689	-0.272799799	0.071465046	-3.81724793	0.000134949	0.001977885
321.9989871	0.053710017	0.076746827	0.699833712	0.484031159	0.683225885
162.3490046	0.043195752	0.084321171	0.512276476	0.608457526	0.777636097
193.6885643	0.660826735	0.127387606	5.187527689	2.13104E-07	8.7987E-06
10.95226268	0.328091376	0.312009684	1.051542285	0.293009599	0.508189707
99.36392726	0.459881961	0.125244743	3.671866388	0.000240786	0.003165763
169.1029794	0.174814522	0.089645165	1.950071953	0.051167544	0.164394587
217.1672268	-0.010911087	0.084034412	-0.129840704	0.896692458	0.947904653
556.7753117	-0.099857756	0.199789665	-0.499814421	0.617205755	0.783631924

1977.477737	0.228207176	0.048315634	4.723257421	2.32097E-06	7.01571E-05
4.096178614	0.552645906	0.593494659	0.931172501	0.35176434	0.566893044
388.3271769	0.168400962	0.159897374	1.053181535	0.2922578	0.507500262
371.5988404	0.051401553	0.082433079	0.623554936	0.532919914	0.722221344
1.935255573	-2.777949228	1.308945598	-2.122280126	0.033814221	0.123433522
1194.25202	-0.241879011	0.109671485	-2.205486783	0.027419954	0.107219636
58.03110899	-0.060277299	0.154253924	-0.390766714	0.695969681	0.836829758
247.5100328	0.067768584	0.153055338	0.442771781	0.657930817	0.811680353
548.5077174	-0.09242663	0.107767394	-0.857649299	0.391086144	0.604272476
153.6590887	0.248339111	0.098752453	2.514763978	0.011911209	0.059570511
9.171651075	0.281420022	0.437076399	0.643869177	0.51966027	0.712042194
28.23793764	-0.143015865	0.194905193	-0.733771443	0.463088048	0.665466896
2.985593205	-1.446451927	0.79719614	-1.814424147	0.069612451	0.202972658
25.88347609	1.426480955	1.069908016	1.333274388	0.182441776	0.379344416
35.13665489	-0.121410421	0.228592211	-0.5311223	0.595334032	0.767361863
38.28029123	-2.669917293	0.742993176	-3.593461392	0.000326314	0.003987906
120.8211968	0.350662916	0.176239901	1.989690836	0.046625003	0.154827715
96.8600078	-0.315335356	0.135949615	-2.319501654	0.020367852	0.087175886
303.2082871	-0.16499694	0.108364102	-1.52261622	0.127854749	0.29952775
31.74257385	-0.30543401	0.171940768	-1.776390863	0.075668516	0.214444028
2565.696696	0.177726063	0.184467017	0.963457131	0.335318168	0.550421632
745.6700717	-0.443366363	0.188123859	-2.356779014	0.018434213	0.080961138
38.16858125	-0.451682299	0.168844011	-2.67514551	0.007469682	0.042476888
664.5616358	-0.140505706	0.062247686	-2.257203677	0.023995351	0.097749606
232.612641	-0.1949762	0.082491875	-2.36358066	0.018099286	0.079959142
28.8216717	0.096089938	0.223552206	0.429832205	0.667317704	0.818667728
3084.519279	0.190237323	0.095102202	2.000346149	0.045462899	0.152164932
2061.124371	-0.228408968	0.101907524	-2.241335671	0.02500434	0.100689684
5.718912093	-0.33472506	0.508334226	-0.658474371	0.51023336	0.704622797
100.6400011	0.559668064	0.123173208	4.543748393	5.52626E-06	0.000144737
1649.112046	0.130102375	0.076275843	1.705682561	0.088067198	0.236636605
592.3306969	-0.380006339	0.068408845	-5.554929909	2.77723E-08	1.47113E-06
36.50746312	1.703693148	0.354698841	4.803210356	1.56142E-06	5.05359E-05
425.7493649	0.222672604	0.071877389	3.097950672	0.001948639	0.015907766
1402.154181	0.539475205	0.098995303	5.449503038	5.05108E-08	2.46188E-06
127.8330413	-0.607200122	0.124351975	-4.882914986	1.04529E-06	3.57263E-05
102.0654321	0.033540257	0.102482781	0.327277	0.743458387	0.865441878
468.3177889	0.144035172	0.060726955	2.371849059	0.01769932	0.078766881
44.45127336	-0.379685277	0.210889894	-1.800395787	0.071798166	0.207400137
122.0128743	-0.186027303	0.13455728	-1.382513848	0.166813981	0.357871276
246.1213669	-0.122622836	0.07376968	-1.662238966	0.09646487	0.250202831
185.9780511	-0.560509107	0.170850652	-3.280696322	0.001035512	0.009895023
1023.750549	-0.210377094	0.089505833	-2.350428869	0.018751791	0.081940852
26.07350102	-0.598295967	0.4593916	-1.302365928	0.192791324	0.39336689
854.8486802	0.061323018	0.128372238	0.477696887	0.632865961	0.794224511
6.769771	0.242766371	0.413624946	0.586923911	0.55725481	0.741574435
638.5600798	0.036576841	0.058440586	0.62588081	0.531393122	0.720863825
5.216710129	-1.665227114	0.752023754	-2.214327812	0.026806237	0.105707454

1278.435624	0.091293756	0.129584341	0.704512251	0.481113823	0.680098603
101.1812056	0.03019391	0.180299984	0.167464851	0.867004302	0.934032728
214.725731	0.429299094	0.21977189	1.953384914	0.050774002	0.163689025
2.22376852	-0.216226728	0.619746519	-0.348895429	0.727167817	0.85562812
784.5867936	-0.420480062	0.062084074	-6.772752462	1.26355E-11	1.77912E-09
568.6409275	-0.423199525	0.060766464	-6.964359889	3.299E-12	5.60286E-10
227.6589115	-0.183622433	0.089725133	-2.046499416	0.04070726	0.140530469
22.02228992	-0.398108223	0.386310359	-1.030539861	0.30275665	0.517785171
2.206473498	-0.41870493	0.749410178	-0.558712629	0.576357859	0.754823068
31.31806058	-0.663212823	0.287983712	-2.302952548	0.021281513	0.089941418
94.576828	-0.621496403	0.252333261	-2.462998344	0.013778055	0.06627144
577.9345382	-0.202517384	0.047834193	-4.23373683	2.2984E-05	0.000478683
72.87131735	0.122552615	0.128806507	0.951447387	0.341377315	0.555826239
6.29078615	2.029048988	1.311665309	1.546925861	0.121881124	0.291793937
40.07064627	-0.137881545	0.242075006	-0.569581916	0.568961297	0.749725517
32.24266856	-0.628603658	0.209580033	-2.999348978	0.002705572	0.020232227
8.578745015	-0.103510979	0.377822951	-0.273966891	0.784110066	0.887950686
2926.357017	0.078356543	0.100035832	0.783284764	0.433459908	0.640328374
506.4988687	0.652406982	0.362301999	1.800726972	0.071745924	0.207285576
274.1622316	-0.796922385	0.343277476	-2.321510851	0.020259289	0.086824019
11.56064274	-1.352666756	0.652016643	-2.074589308	0.038024614	0.134199325
304.3932895	-0.131606446	0.110217917	-1.194056733	0.232455777	0.439160145
20.39596827	-1.70924799	0.824674487	-2.072633527	0.038206398	0.134633627
25.57782278	-0.183143466	0.297851119	-0.614882586	0.538632264	0.726541399
5561.757753	-0.11936255	0.092211365	-1.294445109	0.195511718	0.396559965
8.458635518	0.252662724	0.407434975	0.62013018	0.535172084	0.72384441
116.0547744	0.13805081	0.106679348	1.294072499	0.19564038	0.396674414
134.7852553	-0.148387361	0.094557378	-1.5692838	0.116581825	0.283939827
211.1679023	0.152104134	0.098769938	1.539984105	0.123564228	0.293804883
476.5965008	-0.23688381	0.074012233	-3.200603463	0.001371401	0.012258526
424.7246905	0.103800369	0.077385725	1.341337419	0.179810926	0.375771304
200.3527859	-0.212829429	0.080912835	-2.630354359	0.008529591	0.047042678
187.9795897	0.381704078	0.082284345	4.638842046	3.50367E-06	9.88346E-05
47.69588895	-0.033827567	0.178704192	-0.189293643	0.849862678	0.925926208
7.043816276	-1.317465865	0.670003827	-1.966355729	0.049257533	0.160391836
91.08327977	0.522597294	0.504389881	1.036097895	0.300156474	0.51497373
210.7796028	0.13727628	0.089582905	1.532393701	0.125425311	0.296404118
4.910375728	-0.443233309	0.580696729	-0.763278468	0.445297341	0.650570095
140.2761203	0.424514036	0.137548278	3.086291185	0.002026702	0.016350586
15.5287414	0.063754949	0.307938283	0.207038073	0.835980125	0.918006704
6.120113171	0.393996287	0.54606497	0.721519067	0.470590214	0.671243524
16.66505625	0.706643193	0.624503654	1.131527716	0.257833045	0.468260636
7.010920134	1.168317621	1.009903996	1.156860083	0.247329529	0.455812358
18.69536029	0.004710109	0.415503836	0.011335898	0.990955456	0.995184112
5.704876229	-0.817057495	0.942643587	-0.866772454	0.386066698	0.599289396
45.93486237	-0.165887968	0.513438756	-0.323092026	0.746625549	0.867077573
4.55889796	1.738672538	1.377082583	1.262576812	0.206741305	0.410201451
14.67803948	0.05971432	0.346948337	0.172113002	0.863348691	0.931920109

420.2235314	0.075069147	0.074522305	1.007337956	0.313772396	0.528590495
1783.49924	-0.021925111	0.033113681	-0.662116376	0.507896642	0.70264438
1196.718329	0.027999868	0.058477272	0.478816256	0.632069351	0.793934606
3844.390588	0.122945164	0.084679589	1.451886627	0.146533131	0.328031908
616.7352602	0.091142892	0.112563983	0.809698535	0.41811346	0.627379647
19831.64394	0.166276543	0.117869726	1.41068066	0.158338796	0.345447401
44.91096136	0.443292382	0.290250633	1.527274472	0.126692775	0.2980278
18865.77026	-0.113109497	0.099594575	-1.135699375	0.256082402	0.466272991
126.2878001	-0.034916377	0.150181846	-0.232493994	0.816154351	0.907793315
6.246832378	-0.45612123	0.612506799	-0.744679456	0.456465522	0.660285961
216.0190142	0.121906654	0.071686149	1.700560787	0.089025493	0.238186982
19.44937326	-0.341851852	0.329268322	-1.038216642	0.299169206	0.514082977
6.188512181	-0.531784469	0.679210735	-0.782944736	0.433659565	0.640497371
252.8084183	-0.015434285	0.173289244	-0.089066605	0.929028978	0.965637301
3.131734447	0.081983657	0.731366781	0.112096501	0.910746892	0.955037829
242.367977	0.116212305	0.269317869	0.431506105	0.666100415	0.81795116
3.13499963	-2.063212224	0.9273617	-2.224819317	0.02609337	0.103691513
4.69702381	-0.842158422	0.589649974	-1.428234476	0.153224381	0.337733268
37.24975468	-0.139503006	0.175081531	-0.796788815	0.425573692	0.632924168
229.8963211	-0.464614869	0.115146439	-4.034991222	5.46043E-05	0.00096415
14.22629245	0.500876666	0.543200193	0.922084845	0.356484321	0.571054328
5.034661921	0.216124201	0.698523514	0.309401469	0.757016153	0.873264064
5.312817491	0.905821049	0.589107096	1.537616938	0.124142304	0.294728391
79.19163452	-0.38916724	0.171478147	-2.269485919	0.023238793	0.095256499
43.74814581	-0.723675888	0.18856156	-3.837876001	0.000124103	0.00185524
11.3921246	0.45244276	0.466178824	0.970534775	0.33178	0.547285844
25.33400371	-0.244040148	0.28712221	-0.849952179	0.395351674	0.608239025
138.4764827	0.720414528	0.158138474	4.555593022	5.2238E-06	0.000138355
95.27489894	-0.214040611	0.210352011	-1.017535367	0.308898817	0.523980279
29.50066319	-0.424909161	0.333953967	-1.272358477	0.20324578	0.406097147
126.5463355	-2.316557995	0.750467508	-3.086819841	0.002023102	0.016332601
17.39872329	0.810664274	0.348180285	2.328288842	0.019896771	0.08564918
291.7410174	0.048516825	0.234442238	0.206945752	0.836052225	0.918024685
10.47042637	-2.474280598	0.546318662	-4.529006186	5.92618E-06	0.000153262
2868.113711	-0.165584525	0.114401407	-1.447399377	0.1477851	0.329937897
12.79895727	-0.134281032	0.719251589	-0.186695496	0.851899388	0.926915291
31.5318782	-0.425900283	0.221150174	-1.925841952	0.054124111	0.170322944
70.26991005	-0.116496398	0.131603039	-0.885210553	0.376043082	0.589208078
5.560520175	-2.076836903	0.846717698	-2.452809133	0.014174553	0.067567009
115.11162	-0.369013839	0.196797742	-1.875091838	0.06078009	0.185360106
394.1777705	-0.067486768	0.105958504	-0.636916962	0.524178934	0.715318403
138.8348693	0.002449029	0.134854884	0.018160478	0.985510831	0.992802876
228.5139567	-0.176728691	0.342124443	-0.516562598	0.605461531	0.775551921
250.9097787	0.450194837	0.094662079	4.755809738	1.97653E-06	6.20215E-05
1559.931063	-0.044232426	0.092500445	-0.478186088	0.632517763	0.794214758
2.341051906	-0.096035194	0.68857926	-0.139468613	0.889079857	0.944605583
234.7525751	-0.127800485	0.066303569	-1.927505375	0.053916679	0.169962374
802.1030773	0.012457286	0.099652712	0.125006993	0.900518014	0.95030003

182.0946075	-0.003422294	0.12734755	-0.026873654	0.978560507	0.988823497
2240.937497	0.205098963	0.043163514	4.75167436	2.01739E-06	6.29441E-05
120.4646999	-0.189235226	0.137309689	-1.378163679	0.168152758	0.359542826
91.59954753	0.164899322	0.162846514	1.012605783	0.311248496	0.526264641
109.0195026	0.454819918	0.11364152	4.002233681	6.27473E-05	0.001081275
413.8711795	-0.372558707	0.106115491	-3.510879541	0.000446627	0.00511664
1300.282275	-0.029164301	0.063764401	-0.457375917	0.647400871	0.80444124
364.228939	-0.065750423	0.078415223	-0.838490541	0.401755258	0.613336681
1034.76262	-0.085684951	0.064376702	-1.330993163	0.18319126	0.38041435
876.2150259	0.190105409	0.071363904	2.663887473	0.00772434	0.043430297
9857.291649	-0.106115831	0.16303974	-0.650858687	0.515137712	0.708319729
170.4552001	0.019976262	0.152051784	0.131378019	0.895476277	0.947407114
6.198632547	0.660604871	0.407433324	1.621381541	0.104935837	0.26530279
787.6935705	0.948335257	0.093818422	10.10819881	5.08098E-24	6.97534E-21
137.6851433	2.385595402	0.173576557	13.74376498	5.55123E-43	3.04836E-39
1029.75656	-0.00338126	0.054803239	-0.061698175	0.950803193	0.976590299
53.9128259	-0.381633364	0.156619943	-2.43668435	0.014822612	0.069648519
312.9144655	0.060349952	0.071705578	0.841635393	0.399992072	0.612119776
1193.659905	-0.266351989	0.066927574	-3.979704849	6.90009E-05	0.001174298
186.43588	0.260528356	0.081583598	3.193391352	0.001406122	0.012514565
143.812535	-0.226931524	0.102071044	-2.223270318	0.026197578	0.10401998
13.38004575	-0.342722833	0.286404082	-1.196640882	0.231446559	0.438057285
417.4050476	0.11489609	0.095019191	1.209188262	0.226590526	0.432232145
328.2813802	-0.085842376	0.109239128	-0.785820772	0.431972496	0.639050648
288.1920664	0.105728218	0.0810661	1.30422233	0.192157788	0.392803996
338.1730162	-0.010123541	0.07721822	-0.131103004	0.895693825	0.947455865
2729.601334	-0.012707198	0.060525464	-0.209947959	0.83370829	0.916902912
2.287883979	0.695553357	0.668126153	1.041050935	0.297851909	0.512567884
541.2871358	-0.062270501	0.108642854	-0.573167017	0.566531614	0.748319581
840.4271488	0.075591683	0.137280301	0.550637509	0.58188219	0.758210783
396.1482883	0.04071878	0.073059953	0.557333785	0.577299397	0.755395573
294.533759	-0.170807392	0.138682116	-1.231646856	0.21808103	0.422815921
395.3061969	-0.205624409	0.05298628	-3.88071039	0.000104152	0.001618676
269.5559317	0.171785226	0.087942086	1.9533904	0.050773353	0.163689025
1177.66016	-0.187061534	0.077573146	-2.411421275	0.015890482	0.073107403
5.472105493	1.54870268	0.460617521	3.36223137	0.000773153	0.007886641
33.20684356	0.184948033	0.243568597	0.759326264	0.44765741	0.652824097
101.5175165	0.20586012	0.109948819	1.872326799	0.061161405	0.186095483
371.0648417	-0.037756443	0.066704277	-0.566027316	0.571375211	0.751118187
300.9451965	-0.019495506	0.072933735	-0.267304372	0.789234821	0.891087275
1970.009352	0.063020629	0.079364117	0.794069552	0.427154942	0.634373047
81.32817521	-0.053178021	0.10935032	-0.486308783	0.626748253	0.790677612
1.887352578	-0.351970562	0.855603121	-0.411371292	0.680800301	0.827468213
8.759375281	-0.274938882	0.401888288	-0.684117678	0.49390086	0.690823804
466.4227308	0.203910005	0.095713829	2.130413206	0.033137514	0.12182714
142.1883252	0.225141602	0.104402476	2.15647761	0.03104639	0.116505292
57.31337271	-1.431320747	0.474471684	-3.016662099	0.002555745	0.01941337
625.28031	-0.143458097	0.082904256	-1.730406898	0.083557602	0.2288492

515.1423284	0.532914529	0.247282848	2.155080843	0.031155509	0.116749117
89.21290321	1.084867942	0.193773885	5.598628227	2.16055E-08	1.18249E-06
696.1368741	-0.064937208	0.051190243	-1.268546583	0.204602827	0.407720692
1103.998554	0.005609509	0.051297753	0.109351945	0.912923347	0.956586161
3.153267961	0.63384242	0.552788112	1.146628167	0.25153533	0.46062617
4.845744678	1.118766766	0.446930741	2.503221782	0.01230684	0.061067132
184.8264975	-0.221594053	0.088790196	-2.495704065	0.012570743	0.062003118
3445.831261	-0.148207989	0.068064421	-2.177466399	0.029445786	0.112575976
6.446802106	-0.110898885	0.422267742	-0.262626939	0.792838134	0.893098455
42.8239936	-0.113671127	0.284951581	-0.398913832	0.689956697	0.83271258
501.2721031	0.308767122	0.11840925	2.607626698	0.009117232	0.049261163
88.8225485	0.042151595	0.114999844	0.366536103	0.713965078	0.847585632
267.1590319	-0.025452739	0.065596791	-0.388018057	0.698002671	0.838111954
309.3666551	-0.345207609	0.22587189	-1.528333647	0.12642972	0.297806867
12.72428021	-0.303745592	0.357458957	-0.849735574	0.395472116	0.608310704
1.69595938	-1.805909529	0.839778459	-2.150459456	0.03151889	0.11768862
314.6659186	0.051818308	0.081087716	0.639040167	0.522796802	0.714142166
73.26840674	-0.536134158	0.219206223	-2.445798077	0.014453192	0.068400427
37.1529308	-2.153362477	0.766386708	-2.809759685	0.00495785	0.031293344
1065.833594	0.285830316	0.045404587	6.295185974	3.07032E-10	2.89031E-08
2489.850765	0.237441393	0.13711023	1.731755478	0.083317108	0.228380373
61.1189288	0.374903309	0.253842859	1.476910994	0.139699498	0.318331729
656.3278049	0.808340467	0.066762918	12.10762644	9.62389E-34	3.9636E-30
437.5740031	-0.12385431	0.051749686	-2.393334501	0.016696012	0.075687974
40.9695918	0.474969007	0.235462198	2.017177329	0.043677011	0.147895396
257.6854796	-0.234808555	0.181190565	-1.295920431	0.195002899	0.395966691
4.107165666	0.765851605	0.563508341	1.359077673	0.174121977	0.367943234
95.65663078	-0.082990206	0.111585728	-0.743734949	0.457036841	0.660585609
9.658085357	-0.720619995	0.539327067	-1.336146541	0.18150138	0.37796154
3.25213338	0.666756765	0.819123388	0.813988191	0.415651716	0.625357946
34.79652513	-0.844785061	0.341292208	-2.475254462	0.013314128	0.064694681
341.599848	-0.172238932	0.069453453	-2.479918919	0.013141226	0.064030926
57.75799487	0.033607039	0.160519459	0.209364271	0.834163882	0.916961029
1482.243928	-0.217012256	0.141300187	-1.535824265	0.124581487	0.295345433
16.40002348	-1.975918664	0.722771577	-2.733807923	0.006260655	0.037260851
143.1511617	-0.266618855	0.105575007	-2.525397461	0.01155675	0.058404198
1848.291183	0.042204531	0.260462286	0.162037013	0.871276707	0.935876058
12.94903869	-1.866945994	0.52674985	-3.544274375	0.000393695	0.00463598
4.258733881	-0.571692515	0.493376937	-1.158733762	0.246564724	0.45517974
225.934463	0.029848442	0.080876512	0.369061936	0.71208156	0.846212223
2.965141858	-0.685550992	0.92178403	-0.743721924	0.457044723	0.660585609
54.45509333	0.337248842	0.239841903	1.40612978	0.159685591	0.347190237
490.8048619	-0.013373047	0.092772475	-0.14414887	0.885382915	0.942772268
54.3530319	0.062489547	0.155117712	0.402852426	0.687056791	0.830726992
9.401219691	-0.923713739	0.716914399	-1.288457506	0.197586752	0.399010295
32.93734441	0.230854878	0.184827316	1.249030087	0.21165407	0.41593572
58.95615086	0.407615166	0.230540444	1.768085278	0.077046648	0.216781036
4.501258877	0.714538357	0.511073238	1.398113428	0.162079008	0.351188949

54.25442639	-0.025838022	0.26063538	-0.099134746	0.921031285	0.961050759
187.4593119	0.022200468	0.08665866	0.256182913	0.797809602	0.895595805
26.68521868	-0.468597275	0.362865691	-1.291379389	0.196572154	0.397926968
79.87658986	0.11503658	0.118945831	0.967134196	0.333476953	0.548794652
29.11386273	1.684891403	0.661959242	2.545309885	0.01091808	0.055815905
123.6980733	-1.125032428	0.317090895	-3.547980864	0.000388196	0.004581051
357.5910344	-0.56019614	0.190312321	-2.943562117	0.003244587	0.023059244
26.34128921	-1.312767354	0.388003569	-3.383389894	0.000715969	0.007413498
2.662694101	-0.343779368	0.754492825	-0.455642992	0.648646723	0.805287423
657.8044866	0.565603568	0.102032238	5.543380964	2.96686E-08	1.54671E-06
679.0977063	-0.329499478	0.07250757	-4.544345889	5.51061E-06	0.000144557
50.53519795	0.396724442	0.151920383	2.611397074	0.009017313	0.048881609
572.8170686	-0.511807097	0.115496179	-4.431376864	9.36333E-06	0.000225844
37.89796065	0.534959011	0.194167269	2.755145155	0.00586661	0.035469549
349.3127654	-0.101768141	0.062375618	-1.631537194	0.102777018	0.26200694
23.75877413	1.538284	0.713782973	2.155114449	0.031152879	0.116749117
622.8755246	-0.430842191	0.147694781	-2.91711181	0.003532891	0.024554356
119.8562923	0.853888558	0.154077805	5.541930962	2.99154E-08	1.55466E-06
56.68028298	-0.116549045	0.265597706	-0.438817965	0.660793442	0.813527725
575.5083828	0.432672574	0.094189452	4.593641465	4.35577E-06	0.000118803
121.5804479	0.422333085	0.088487281	4.772811186	1.81672E-06	5.74446E-05
2.885927488	0.202527415	0.616481435	0.328521515	0.74251738	0.86500393
3.513958791	0.476951668	0.55952711	0.85241923	0.393981451	0.606905157
176.5575578	-0.240168335	0.099765228	-2.407335105	0.016069417	0.073658202
12.06091421	-0.257537905	0.298335979	-0.863247893	0.388001186	0.601197474
928.1472049	-0.162384045	0.139312069	-1.165613617	0.2437707	0.451393647
2631.685077	0.139682402	0.122770954	1.137747953	0.255225744	0.465160848
72.3209001	0.417124657	0.115451815	3.612976179	0.000302703	0.003754048
18.02038092	-0.877853971	0.522664016	-1.679576064	0.093039828	0.244836646
87.65315527	-0.094025871	0.124968964	-0.752393784	0.451814284	0.656539518
3.828650498	-0.00838111	0.750254809	-0.011171018	0.991087003	0.995255549
16.07408402	1.118521596	1.035984611	1.079670088	0.280289118	0.493887448
139.3748261	0.448555081	0.093518161	4.796448885	1.61503E-06	5.17627E-05
7.546696855	-0.248193786	0.558297302	-0.444554873	0.656641465	0.81089405
64.74013058	-0.278153754	0.152814012	-1.820211052	0.068726871	0.201173857
1098.778872	0.136509927	0.077310488	1.765736186	0.077440119	0.217481848
12.45292972	-0.266201511	0.273592304	-0.972986107	0.330560207	0.545820272
49.10249692	-0.031801891	0.13503853	-0.235502348	0.813818857	0.906237868
66.17458882	0.475282619	0.137094554	3.466823474	0.000526648	0.005818909
596.6227782	0.100111674	0.059863436	1.672334231	0.094458433	0.24696348
158.120033	-0.299143316	0.08892655	-3.363937033	0.000768391	0.007862403
852.6003962	0.044444734	0.096224456	0.461886046	0.644163041	0.802475948
211.3384892	0.129137237	0.110145112	1.17242822	0.241025175	0.448508837
15.48247237	1.071000355	0.664021212	1.612900815	0.106766058	0.268109014
2225.267348	-0.05390658	0.091976735	-0.5860893	0.557815507	0.741982451
91.98762568	0.062822178	0.192170166	0.326909108	0.743736632	0.865506187
715.4959542	0.33344398	0.146868281	2.270360752	0.023185704	0.09506254
32.86429567	0.305237097	0.220985705	1.381252683	0.167201281	0.358468754

444.6679423	-0.09511784	0.065483907	-1.452537647	0.146352169	0.327758906
6.322418383	-0.939881108	0.498707291	-1.884634784	0.059479163	0.182333406
48.89734512	-0.20222198	0.292004616	-0.692530081	0.488604502	0.686504952
517.214539	0.038832025	0.068900215	0.563598024	0.573027732	0.752170338
363.9864279	-0.119755896	0.10689021	-1.120363556	0.262558869	0.473911998
498.9431201	-0.012574748	0.10938096	-0.114962856	0.908474564	0.953990946
2482.005448	0.252017696	0.118793598	2.121475392	0.033881817	0.123516054
4.684514189	-0.653001599	0.474694848	-1.375623946	0.168938089	0.360643525
14.62611901	-0.458068584	0.500212361	-0.915748229	0.359798963	0.573951606
219.9773171	-0.184045595	0.073979694	-2.487785307	0.01285413	0.063000705
124.843575	0.762940561	0.227143208	3.358852621	0.000782668	0.007963973
8.212379705	0.69368477	0.452630536	1.532562909	0.125383586	0.296351392
208.418749	-0.114784661	0.085517121	-1.342241881	0.179517581	0.375301095
786.5943198	0.292823125	0.076457736	3.829869166	0.000128211	0.001901129
149.1417718	-0.161292259	0.104446853	-1.544251979	0.122527301	0.292501519
124.9105567	-0.22329017	0.114151219	-1.956090977	0.050454438	0.162977727
53.66502189	0.010578259	0.176028139	0.060094138	0.952080659	0.977124952
43.39776314	-0.151797405	0.176701084	-0.859063233	0.390305631	0.603916123
2182.413204	-0.195392215	0.107234358	-1.822104584	0.068439118	0.200815147
72.44954055	0.155872583	0.181369864	0.859418313	0.39010977	0.603669768
188.7097057	-0.129793061	0.091989696	-1.410952166	0.158258719	0.345364172
2.697124713	1.24897309	0.708454442	1.762954702	0.07790813	0.218450565
8.998134865	-0.478245981	0.33536519	-1.426045386	0.153855242	0.33850411
3.210691579	-1.27217356	0.659794964	-1.928134691	0.053838375	0.169878067
701.7566673	0.066308766	0.132161062	0.501726942	0.61585961	0.782777813
6923.082951	-0.193526111	0.114469031	-1.690641647	0.090905265	0.241427268
555.888569	0.043313441	0.063339439	0.683830512	0.494082197	0.691018772
22.14625159	-0.181286011	0.307156134	-0.59020801	0.555051203	0.739858687
320.7703782	0.005141381	0.109286697	0.047044894	0.962477447	0.981463149
60.76678036	-0.639111544	0.135742502	-4.708264079	2.49835E-06	7.44265E-05
249.0996864	-0.011400445	0.142462766	-0.080024034	0.936218141	0.970016204
10.68747297	-0.833918528	0.346869868	-2.404125018	0.016211228	0.074081491
9.779900095	0.127626579	0.511894158	0.249322202	0.803111559	0.899097368
133.7517367	-1.497160305	0.391196176	-3.827134308	0.000129644	0.001912043
159.6914818	0.252327218	0.171051138	1.475156616	0.140170442	0.3189579
452.1773338	0.092556056	0.111898314	0.82714433	0.408155261	0.618920265
2.272417645	3.828293374	1.281518479	2.987310317	0.002814439	0.020819518
1.939142259	-1.531704084	1.620294005	-0.945324787	0.344493088	0.558800623
256.5909606	-0.456722707	0.105876506	-4.313730439	1.60523E-05	0.000355437
358.846808	-0.025830806	0.120183085	-0.214928793	0.829822836	0.915210644
121.6507143	-0.110176687	0.21310643	-0.517003111	0.605153987	0.775218257
3.355082621	-0.471789137	0.776140436	-0.607865684	0.543276561	0.730508517
6.580560499	0.46111225	0.362862688	1.27076237	0.2038132	0.406737571
1030.782389	0.132152659	0.079733862	1.657422034	0.09743418	0.252141168
3.768108187	-1.406358594	0.920355952	-1.528059433	0.126497782	0.297871199
704.3627689	0.026920818	0.126142928	0.213415201	0.831003125	0.915614595
54.50107187	-0.044077804	0.209409579	-0.210486091	0.833288307	0.916833892
22.9946945	-0.994756978	0.608801057	-1.633960662	0.102267111	0.261039415

692.0957181	-0.21200106	0.074319717	-2.852554715	0.004336935	0.028385642
8.723113512	-0.15833109	0.318849448	-0.496570059	0.619492271	0.785160461
1.893186168	-1.268418016	1.23499667	-1.027061891	0.304391322	0.519050061
13.79933406	-1.72058546	0.447342666	-3.846235981	0.000119946	0.001799631
2326.355202	0.026132722	0.157272754	0.166161788	0.86802963	0.934495951
6.614809848	-0.167755466	0.487854697	-0.343863587	0.730948877	0.857789699
397.0997373	0.148336853	0.117488118	1.262568982	0.206744121	0.410201451
9.137333438	0.480689402	0.489418013	0.982165326	0.326018407	0.540977763
8.630398742	0.050250023	0.440514314	0.114071259	0.909181305	0.954307284
231.7042085	0.017076849	0.134269918	0.127182988	0.89879557	0.948877064
212.9887689	0.076488264	0.105849331	0.722614524	0.469916743	0.670859016
3.000997977	-1.354877046	0.648515182	-2.089198653	0.036689845	0.130970423
557.439901	-0.16624341	0.180230095	-0.922395398	0.356322369	0.570850405
1001.775153	-0.216765016	0.082267062	-2.634894349	0.008416348	0.046558398
5.168339983	-0.999893304	0.582932301	-1.715282035	0.086293531	0.233278573
1.96259726	0.642452959	0.66604393	0.964580459	0.334754991	0.550047536
109.1037177	-0.270514854	0.147351968	-1.83584148	0.066381105	0.196736818
6.178348175	-2.609733015	0.684115174	-3.814756804	0.000136317	0.00198952
10.51863689	-1.619318631	0.515894278	-3.138857517	0.001696079	0.014270278
262.5282228	0.165206533	0.072273317	2.285857893	0.022262574	0.092567803
17.63053601	-0.909611635	0.372150245	-2.444205389	0.014517159	0.068643996
267.6060728	-0.144175512	0.277270518	-0.519981401	0.603076538	0.773279575
86.05559192	-0.599477737	0.271408399	-2.208766344	0.027190897	0.106644495
71.24927222	0.165696781	0.138914661	1.192795489	0.232949477	0.439639098
266.609253	0.103339015	0.087997291	1.17434314	0.240257611	0.447687352
11.42547341	-0.540639123	0.507704245	-1.064870205	0.28693467	0.501479298
164.4877151	-0.004316277	0.099582299	-0.043343815	0.965427464	0.982726894
1089.157357	0.08582598	0.176083959	0.487415099	0.625964196	0.790627823
1.992582243	-0.270114435	0.707924791	-0.381558095	0.702789172	0.840673019
125.2853404	0.229888688	0.101175685	2.272173287	0.023076046	0.094848997
206.3030852	0.051237201	0.122997802	0.416570058	0.676992914	0.825613125
1800.236329	-0.262217543	0.108735067	-2.411526938	0.015885878	0.073107403
9.069079143	0.458127433	0.324726686	1.410809314	0.158300847	0.345410352
182.658866	-0.032591588	0.15799491	-0.206282516	0.836570235	0.918328674
3.271816313	1.988355232	1.428887532	1.391540752	0.164061511	0.353617493
15.61436604	-2.452849983	0.339982284	-7.214640587	5.40766E-13	1.09167E-10
1.893476286	-0.087652907	1.064812528	-0.082317689	0.934394088	0.9689185
724.9459195	-0.204916627	0.152470885	-1.343972179	0.178957384	0.374510155
82.40307985	-0.294442643	0.173575288	-1.696339647	0.089821576	0.239547618
512.9259204	-0.066405553	0.063971115	-1.038055276	0.299244322	0.514104803
15.66837796	0.06342354	0.376353801	0.168521056	0.866173381	0.933787619
7.996835074	-0.526460021	0.340354721	-1.546798054	0.12191195	0.291793937
56.87473749	-0.115841317	0.150985873	-0.767232816	0.442943105	0.648528056
433.76998	-0.336057664	0.161169557	-2.085118747	0.037058523	0.131886171
317.450803	-0.138196462	0.121777027	-1.134831961	0.256445732	0.466764666
80.00833576	-0.044804387	0.205863663	-0.217641066	0.827708785	0.913863048
11.81558218	0.338738696	0.373564568	0.906774157	0.364526238	0.578201931
71.94492765	-0.531933792	0.135411797	-3.928267723	8.55599E-05	0.001387164

136.7202472	-0.313209263	0.189653389	-1.651482548	0.098640081	0.254414161
3.470571054	-0.176858802	0.560799433	-0.31536908	0.75248144	0.870899202
385.1001089	0.254451815	0.104926313	2.425052463	0.015306175	0.071339235
10.35219389	0.333691519	0.34883114	0.956598998	0.338769695	0.553934685
11186.57379	-0.248491688	0.169939453	-1.462236602	0.143676385	0.323704153
146.9123187	0.255430326	0.157564511	1.621115851	0.104992795	0.265344941
2.045014266	-0.149182404	0.70274002	-0.212286763	0.831883322	0.91596382
106.946181	0.108313968	0.110104271	0.983739935	0.325243399	0.54023591
143.7906766	-0.077621665	0.141753108	-0.547583518	0.583977909	0.759563671
496.4758171	-0.138127275	0.089463615	-1.543949187	0.122600643	0.292501519
19.6580338	-0.716820199	0.251062946	-2.855141352	0.004301768	0.028233993
3.709931631	0.25673493	0.755898203	0.339642202	0.734125993	0.859864316
202.0945573	-0.512349418	0.168906421	-3.033332979	0.002418686	0.018550012
1142.209794	0.067894736	0.064746066	1.048631069	0.294347951	0.509144071
68.42524792	0.016944603	0.175915844	0.096322209	0.923264673	0.962127781
119.869986	0.047919998	0.105874209	0.452612567	0.650827748	0.806509427
61.94968841	0.364709735	0.141995567	2.568458594	0.010215192	0.053288494
23.54821487	0.040242799	0.225717985	0.178287959	0.858496834	0.929770219
153.0463611	-0.067192733	0.132693966	-0.506373689	0.612594342	0.780789807
615.5351218	-0.014891727	0.065612406	-0.22696511	0.820450873	0.909820137
904.5824396	0.106317066	0.116453729	0.912955452	0.361265971	0.575339666
1029.138772	-0.009423885	0.14272443	-0.066028537	0.947355106	0.974872135
14.94141547	-0.458751934	0.257371184	-1.782452591	0.074675456	0.212874798
17.98618579	-0.33476397	0.27177402	-1.231773258	0.218033796	0.422815921
2.91096562	-1.017323714	0.633973965	-1.604677431	0.108564811	0.271313213
221.6960146	0.498681041	0.100946627	4.940046597	7.81039E-07	2.82788E-05
133.19009	0.19835436	0.133193322	1.489221506	0.136429049	0.313507066
56.56804082	0.233480384	0.135585809	1.722011955	0.085067353	0.231382743
1371.678769	0.166111599	0.097905862	1.696646095	0.08976359	0.239437819
28.53075358	0.26752505	0.184929018	1.44663641	0.147998783	0.330349155
20.30425005	-0.944655652	0.283821299	-3.328346586	0.000873631	0.008664779
14.14187837	-0.131224283	0.269683472	-0.4865863	0.626551534	0.790677612
49.82904779	-0.545545331	0.220814288	-2.470607025	0.013488395	0.06525536
971.7831967	-0.046587363	0.120869195	-0.385436196	0.69991427	0.839012442
99.46348814	-0.146955256	0.114670093	-1.281548233	0.20000117	0.402370007
122.3075877	0.143498987	0.118899468	1.20689343	0.227473188	0.433254357
433.1303059	-0.933470505	0.281612886	-3.314729377	0.000917319	0.009010731
319.6429054	-0.299702105	0.096846744	-3.09460175	0.001970773	0.016048699
18.68243556	-0.018347909	0.315061779	-0.058235907	0.953560719	0.977472422
175.3613489	-0.042382245	0.103694804	-0.40872101	0.682744414	0.828547438
8.234330105	-0.076450336	0.436189062	-0.175268807	0.860868421	0.931184922
211.3630919	-0.082753623	0.075667842	-1.093643222	0.274111485	0.486764321
105.4234128	-0.059329133	0.097982043	-0.605510259	0.54484001	0.731813642
137.937479	0.471045476	0.144210737	3.266368961	0.001089362	0.010303736
112.0107788	-0.129208099	0.103565587	-1.247596845	0.212178734	0.41657975
2.307924013	-0.519444346	0.653350887	-0.795046516	0.426586444	0.633800964
1729.647168	-0.400021404	0.08541993	-4.682998485	2.82709E-06	8.30186E-05
6.957040891	-0.955831088	0.443322423	-2.156063032	0.031078744	0.116547057

40.07929949	0.024210233	0.207016903	0.1169481	0.906901182	0.953303217
435.5783238	-0.086080122	0.187278015	-0.459638158	0.645775965	0.803568408
4.759696136	-0.610108995	0.462448154	-1.319302476	0.18706801	0.385798498
495.9695309	0.121039487	0.108962192	1.110839322	0.266637517	0.478338937
170.2100198	0.016483993	0.102558959	0.160726989	0.872308431	0.936384156
933.7195415	-0.170473209	0.077640432	-2.195675677	0.028115165	0.109057978
429.6608213	-0.567299545	0.105774344	-5.363300039	8.17151E-08	3.75777E-06
601.0512898	0.0620515	0.053539074	1.158994643	0.246458369	0.455072311
21.27768841	0.861005209	0.610034185	1.411404852	0.158125273	0.345118674
916.6133655	0.075969997	0.127714649	0.594841682	0.551949302	0.737753575
29.31633789	0.462298929	0.221389713	2.088168065	0.036782677	0.131136708
246.4188161	0.014705121	0.113662604	0.129375187	0.89706078	0.948048454
186.6349607	-0.066357561	0.089094942	-0.744796053	0.456395021	0.660283795
287.2532083	-0.03358198	0.095893474	-0.350200885	0.726187943	0.854934372
2182.880556	-0.321670171	0.092265117	-3.486368207	0.000489627	0.005483418
4.285069472	-0.976634349	0.744767691	-1.311327492	0.189747144	0.389476547
18.80972914	0.371163421	0.265337206	1.398836699	0.161861958	0.350949448
196.8746187	0.062480314	0.119234974	0.524009961	0.600271611	0.771483424
390.6139135	-0.355342704	0.120227866	-2.955576904	0.003120849	0.022402119
134.0672815	0.384001481	0.196292106	1.95627572	0.050432683	0.162939403
172.3896814	-0.272708576	0.089715835	-3.039692794	0.002368196	0.018339695
28.60914984	0.375404843	0.259003995	1.44941719	0.147221111	0.329035488
400.8639847	-0.062246078	0.063154342	-0.985618352	0.324320429	0.539548668
384.6280168	-0.314279876	0.108416227	-2.89882692	0.003745616	0.025455973
556.8247568	0.128764252	0.106781601	1.205865527	0.227869345	0.433500443
103.8191735	-0.049403216	0.153668713	-0.321491703	0.747837799	0.86771555
10.60691437	-0.049329936	0.30336859	-0.162607262	0.870827669	0.935813113
490.8726542	-0.331502207	0.088530786	-3.744485093	0.000180764	0.002519381
22.52342695	0.169587283	0.413328233	0.410296876	0.681588184	0.827937158
27.90763183	-0.360527295	0.36345828	-0.991935843	0.321228823	0.53627475
28.87816732	-0.636806302	0.30795382	-2.067862974	0.03865291	0.135514844
472.9863545	-0.241635582	0.240161372	-1.006138415	0.314348992	0.529183046
7.292572459	1.173498571	0.571275856	2.054171479	0.039959098	0.138679749
39.91986506	1.026403376	0.186632895	5.499584498	3.80687E-08	1.92376E-06
269.7302239	0.206795751	0.068882741	3.002141717	0.002680873	0.020093134
3.286116445	-0.286930716	0.508962275	-0.563756354	0.572919959	0.752170338
449.2813784	-0.057442609	0.088121025	-0.65186043	0.514491214	0.707791263
3.543187757	-0.216092346	0.490807488	-0.440279237	0.659734877	0.812578183
25.64234011	0.636657115	0.262331887	2.426914706	0.015227835	0.071110984
1181.105666	0.284217601	0.123728055	2.297115233	0.021612197	0.090827655
30.33716001	-0.345638611	0.249592711	-1.384810516	0.166110414	0.356873105
229.924153	0.41652731	0.19155391	2.174465191	0.029670217	0.113216304
14.68227855	0.088862979	0.435499976	0.204048184	0.838315846	0.919223592
49.50523076	0.001084116	0.187129494	0.005793399	0.995377562	0.997576362
211.0189161	-0.03531794	0.076773088	-0.460030206	0.64549454	0.803284261
15.91217894	-0.013697715	0.291201701	-0.047038583	0.962482477	0.981463149
517.7667387	0.049585638	0.143969528	0.344417589	0.730532263	0.85760625
1.874690518	-0.230833833	0.775725843	-0.29757141	0.766030298	0.878006201

124.9662428	0.063304168	0.119273207	0.530749275	0.595592535	0.767505587
22.81342676	0.027893041	0.193348141	0.1442633	0.885292558	0.942747873
338.3110313	-0.132571743	0.158570424	-0.836043317	0.403130535	0.614411364
303.179783	-0.04701639	0.120499954	-0.390177658	0.696405181	0.837047932
2171.103859	-0.230364202	0.073339372	-3.141071392	0.00168331	0.014199103
4.296128783	0.319128082	0.712677075	0.447787775	0.654306367	0.80905525
719.1905332	-0.280426269	0.086562478	-3.239582278	0.001197049	0.011072538
500.9028248	-0.20320488	0.110206654	-1.843853093	0.065204553	0.194400896
795.6095934	-0.234860768	0.125588037	-1.870088701	0.061471501	0.18660061
2776.640044	-0.131197953	0.090189854	-1.454686401	0.145756099	0.32678089
6.306187489	-0.544234028	0.393066418	-1.384585411	0.166179275	0.35695643
6.499804473	-1.66084848	0.554804394	-2.993574848	0.002757299	0.020535144
28.50611624	-0.039382182	0.263956247	-0.149199659	0.881396089	0.940691803
100.5248885	0.0597881	0.100249828	0.59639105	0.550914018	0.736907887
119.5072175	-0.527172436	0.32607231	-1.616734756	0.105935548	0.266918867
467.3946485	-0.781169276	0.117261669	-6.661761549	2.70565E-11	3.27741E-09
1.869531839	-1.838025206	1.077661495	-1.705568227	0.088088499	0.236655241
592.4062662	0.183642763	0.082260053	2.23246589	0.025584187	0.102349174
720.7003857	-0.037447191	0.071235594	-0.525680903	0.599109934	0.770050485
678.4597518	-0.046844764	0.077896109	-0.601374895	0.547590316	0.734310367
171.3069117	0.596098166	0.204501403	2.914885463	0.003558191	0.024670723
333.3065478	-0.135002747	0.081509074	-1.656291008	0.097662901	0.252534708
149.2515024	-0.099312584	0.108660325	-0.913972818	0.360731129	0.575005769
154.951586	0.995618434	0.176891205	5.628422478	1.81865E-08	1.02957E-06
43.23458542	0.916360365	0.450679348	2.033286792	0.042023557	0.143868677
9973.449528	-0.104579284	0.054557186	-1.916874589	0.055253866	0.172625107
254.4913381	0.966066462	0.122335681	7.896849522	2.86041E-15	8.56772E-13
380.6433666	0.097871458	0.207584439	0.471477817	0.637299557	0.797302013
1.885698754	1.278377852	0.709057465	1.802925596	0.071399901	0.206575687
107.3560713	-0.069592082	0.183959393	-0.378301323	0.705206763	0.842341663
172.523502	0.789437694	0.150581518	5.242593535	1.58335E-07	6.81048E-06
9.993148654	-1.334537067	0.754223417	-1.769418766	0.076824016	0.21648971
283.8398233	-0.15213455	0.158058265	-0.962521955	0.33578748	0.550901568
33.57773458	-0.227768561	0.235229639	-0.968281729	0.332903687	0.548476551
4.287606013	0.749211537	0.783688738	0.956006512	0.339068945	0.554005032
273.4872525	0.433036863	0.113524321	3.814485374	0.000136467	0.00198952
3.971902092	-0.348285689	0.490114839	-0.710620576	0.477319389	0.677000397
1536.182571	0.153468018	0.118224434	1.298107447	0.194250416	0.395120552
2076.933462	-0.165151353	0.112105735	-1.473174874	0.140703888	0.319762154
370.96494	-0.306206819	0.066288699	-4.619291427	3.85053E-06	0.000106791
1604.226924	-0.14641983	0.048477067	-3.020393759	0.002524463	0.019218113
305.4458917	-0.031554941	0.088633999	-0.356013966	0.721830106	0.852371097
371.5401279	-0.268432623	0.189792793	-1.414345715	0.157260421	0.343595248
17.30960694	0.037906575	0.325193747	0.116566126	0.907203882	0.953303217
42.54761474	0.923877851	0.249652871	3.70064982	0.000215048	0.002901476
18.556053	-0.175459908	0.280439642	-0.625660147	0.531537879	0.720941463
176.8175425	0.347040468	0.17908614	1.937841024	0.052642621	0.167452122
221.6137112	0.083450491	0.165924974	0.502941112	0.615005675	0.782363204

220.632826	-0.087528268	0.095895299	-0.912748269	0.361374951	0.575363965
4.590018135	0.022365904	0.531576089	0.0420747	0.966439149	0.98333139
508.2194284	-0.194882413	0.078338317	-2.487702302	0.012857131	0.063000705
106.3802996	-0.207461254	0.197490411	-1.050487734	0.293493927	0.508521136
426.5877768	-0.214980668	0.060843505	-3.53333799	0.000410348	0.004800425
740.0755061	-0.026863789	0.056600848	-0.474618137	0.635059176	0.795526186
555.8594696	-0.048125516	0.077644166	-0.619821407	0.535375373	0.723941057
6.327828874	1.055827593	0.397389468	2.656908847	0.007886076	0.044143806
430.4000847	0.071727433	0.084666141	0.847179659	0.396894989	0.609578167
113.3290805	-0.003380167	0.187763452	-0.018002262	0.985637049	0.992869313
447.1416069	-0.409953104	0.065588782	-6.250353988	4.09523E-10	3.72734E-08
23.36065725	-0.466216121	0.366294977	-1.272788736	0.203093019	0.405939626
220.1527027	0.015295378	0.07653679	0.199843469	0.841603003	0.921462698
60.80397932	0.605991822	0.126249419	4.799957317	1.58699E-06	5.10662E-05
17.20811982	-0.790436489	0.344063941	-2.297353472	0.021598614	0.090824396
18.49610547	-0.839224186	0.445921891	-1.881998176	0.059836263	0.18329167
3.663516487	1.21006843	0.613639064	1.971954689	0.048614779	0.159030952
7771.354538	0.153152711	0.089478336	1.711617778	0.08696713	0.234506606
439.7456588	-0.129380027	0.076064331	-1.700929008	0.088956319	0.238171511
211.8109036	0.008088105	0.109474629	0.07388109	0.941105003	0.971961872
148.604083	0.168533257	0.117515807	1.434132652	0.151534411	0.335677626
500.2637159	0.403544045	0.1515791	2.662267057	0.007761627	0.043625058
105.2044795	0.567741954	0.19142054	2.965940609	0.003017588	0.021880166
10.99008533	0.050358469	0.301839158	0.166838755	0.867496924	0.934367069
390.6422923	-0.072636741	0.06800799	-1.068061882	0.285492599	0.499915505
1038.337214	-0.124162252	0.060192594	-2.062749639	0.039136421	0.136509295
145.2395048	-0.007926689	0.099789695	-0.079433939	0.936687474	0.970184161
21.78923714	0.313182429	0.252271155	1.241451601	0.214438967	0.419063612
725.5899215	0.096757564	0.073134139	1.323015012	0.185830386	0.384015275
15.08403612	0.471957255	0.328361732	1.437308946	0.150630228	0.334161376
14.72902423	-0.251683362	0.414711119	-0.606888387	0.543924985	0.730983554
351.7815219	0.299290944	0.088051373	3.399049149	0.000676206	0.007099944
19.86245541	-0.14462315	0.409785672	-0.352923881	0.724145507	0.853557222
540.5777807	-1.021365226	0.213918009	-4.774563996	1.80097E-06	5.70561E-05
14.45532337	-2.355863156	0.736931553	-3.19685478	0.001389349	0.012392056
2783.042874	0.23126118	0.094289884	2.452661634	0.014180366	0.067575164
2484.507821	-0.021844691	0.100124731	-0.218174781	0.827292934	0.913759955
274.7887266	0.083313986	0.154348745	0.539777541	0.589350457	0.76286316
103.1624537	-0.381379464	0.196462711	-1.941230795	0.052230289	0.166429746
112.4666271	0.421509809	0.162592356	2.592433126	0.009529971	0.050939889
3.074782502	-0.006355175	0.589246895	-0.01078525	0.991394782	0.995470966
213.6865847	-0.132825678	0.111495318	-1.191311705	0.23353124	0.440333484
532.4623554	-0.083218686	0.122443232	-0.679651169	0.496725362	0.693461961
1659.695283	0.05429975	0.14150876	0.383720059	0.701185944	0.839689505
1539.002555	-0.171224764	0.09277407	-1.845610137	0.064948834	0.193799508
438.1227927	-0.108768498	0.080533647	-1.350596954	0.176824577	0.371984176
490.3314089	-0.173272373	0.059007373	-2.936452928	0.003319892	0.023410475
6.219319359	3.140341058	1.138934138	2.757263088	0.005828743	0.035328447

14.49337082	-0.187573579	0.314379845	-0.596646325	0.550743536	0.736778211
9.527436517	-0.110866878	0.340102878	-0.325980417	0.744439172	0.865910536
283.2986517	-0.09815932	0.135102282	-0.7265556	0.467498235	0.66923583
1328.624451	-0.058065948	0.044208895	-1.313444892	0.189033079	0.388316967
39.61493679	0.899634919	0.24010102	3.746901691	0.000179032	0.002503715
3.216461456	-0.500266279	0.696804293	-0.717943739	0.472791978	0.673187125
865.0280268	0.309418378	0.1001861	3.088436211	0.002012129	0.01628885
387.5632487	0.570555192	0.125929954	4.530734548	5.8779E-06	0.000152974
8.94722866	0.043679466	0.43393385	0.100659273	0.919820943	0.960396135
3.875063423	-1.381020667	1.415431656	-0.975688696	0.329218755	0.544751886
127.0753501	0.38264576	0.121096152	3.159850697	0.0015785	0.013636188
65.57636218	0.113486313	0.15449822	0.734547707	0.462614996	0.665049916
485.3194716	0.093101148	0.106327207	0.875609836	0.381242176	0.593923904
129.6081617	-0.442853598	0.139051075	-3.184826847	0.001448406	0.012790822
64.36537398	-0.046297161	0.211334764	-0.219070255	0.826595321	0.913363158
144.4255708	-0.224424813	0.182158684	-1.232029169	0.217938188	0.422787766
97.06824968	0.048784379	0.188883963	0.258276978	0.796193152	0.894773586
320.9562976	0.110939603	0.076163687	1.456594442	0.145228363	0.32610914
9.021570812	0.175103791	0.356823188	0.490729853	0.623617529	0.788568865
209.7284667	0.111554432	0.095430828	1.168955922	0.242421391	0.45003944
58.21881058	0.054622682	0.18714955	0.29186649	0.770388705	0.880245771
5031.60324	-0.190570565	0.09116659	-2.090355299	0.036585895	0.130627663
11.1413195	-0.323454276	0.371013644	-0.871812348	0.383310757	0.596229007
275.3525266	-0.024060817	0.137541653	-0.174934764	0.861130894	0.931215742
71.6171662	0.015030966	0.138519558	0.108511511	0.91358995	0.956888548
37.87714103	-0.613668034	0.194069693	-3.16210133	0.00156635	0.013573939
316.1330667	0.415080024	0.09113552	4.554536178	5.25013E-06	0.000138829
2259.926495	0.217394755	0.076092017	2.856998202	0.004276683	0.028102939
9.693668878	0.429024934	0.328990152	1.304066192	0.192211015	0.392828754
2.919073692	-0.629521795	0.588873793	-1.069026679	0.285057648	0.499366195
98.50300892	0.048718171	0.112184527	0.434268183	0.664093706	0.816438784
631.7266231	0.011719958	0.052710422	0.222346127	0.824044446	0.911434821
133.8345715	-0.233598458	0.169918486	-1.374767773	0.169203452	0.361116424
1.707458998	-0.702048082	0.888802399	-0.789880948	0.429597299	0.636609328
2.096700535	0.269812743	0.690206347	0.390916056	0.695859286	0.836819174
1156.616318	0.045724178	0.155788111	0.293502356	0.769138199	0.879610044
1250.75786	0.267697271	0.117726302	2.273895185	0.022972289	0.094564673
52.96097864	0.394747888	0.198897037	1.984684606	0.047179573	0.155946284
98.99105777	-0.126604087	0.110815315	-1.142478245	0.253255284	0.462388069
481.9135273	0.138425801	0.297355119	0.465523518	0.641556599	0.800560779
282.3972536	0.066261296	0.083110908	0.797263532	0.425297995	0.632742632
297.8851036	0.378552588	0.130812541	2.893855467	0.003805433	0.025735099
10.47131678	-0.070057829	0.385805144	-0.181588634	0.855905569	0.929355941
125.7159521	-0.048161261	0.096028306	-0.501531923	0.615996817	0.782777813
110.8078669	-0.278167281	0.377553409	-0.736762732	0.461266651	0.66377753
509.1477695	0.016332559	0.117880778	0.1385515	0.889804571	0.945108995
484.5789665	0.181504558	0.057353274	3.164676505	0.001552555	0.013482754
780.0806589	0.011643589	0.079925106	0.145681243	0.88417303	0.942262895

1.768013441	-0.18766925	1.149775478	-0.163222519	0.870343236	0.935658736
27.45039733	0.21118362	0.354469452	0.595773823	0.551326333	0.737218344
15.30409721	-0.14791103	0.649317091	-0.227794758	0.819805802	0.909423741
72.83706719	-0.239111325	0.1374875	-1.739149561	0.082008459	0.226147866
294.0804647	-0.421318698	0.226511128	-1.860035322	0.062880529	0.189515886
72.58984214	0.630637563	0.353256175	1.785213129	0.074226753	0.211815612
2075.920387	0.052266242	0.091347814	0.57216741	0.567208564	0.748471553
269.3739078	-0.131166431	0.081309648	-1.613171792	0.10670719	0.268053408
1170.111109	-0.030228158	0.040816421	-0.740588159	0.45894319	0.661935747
1465.86021	0.051242468	0.058272312	0.879362182	0.37920493	0.592190921
688.6890703	0.026392227	0.04711133	0.560209777	0.575336359	0.753972704
68.04106734	-0.408444579	0.191087626	-2.137472674	0.032559566	0.120400963
337.5194165	-0.384525599	0.104202033	-3.690192873	0.000224084	0.002998831
121.061149	0.082873347	0.114751907	0.722195814	0.470174096	0.670973518
183.0631895	0.38476274	0.138381723	2.780444791	0.005428449	0.033525412
1.91421533	1.052842266	0.842775587	1.249255771	0.211571539	0.415922379
339.6688595	0.060188784	0.109980565	0.547267457	0.584194998	0.759621118
27.34906622	-0.476920603	0.383134602	-1.244786036	0.21321041	0.417828132
251.9400533	0.13264991	0.089621798	1.480107661	0.138844517	0.317261564
7.288075773	-0.708061104	0.555407019	-1.274850838	0.202362043	0.405117546
95.2345295	-1.013567687	0.309105156	-3.279038433	0.001041614	0.009947569
101.8450739	-0.778137351	0.60191473	-1.292770074	0.196090593	0.397245012
188.7509788	-0.494358903	0.078510018	-6.296762063	3.03927E-10	2.87753E-08
2219.940765	-0.170358534	0.091005779	-1.871952932	0.061213116	0.186159288
56.06428876	0.177566564	0.174787148	1.015901718	0.30967619	0.524856539
5364.939854	0.031772151	0.077301872	0.411013998	0.681062271	0.827664491
38.11633587	0.07743823	0.269456025	0.287387266	0.77381581	0.882447851
566.0414418	0.122672691	0.068690437	1.785877269	0.074119132	0.211719093
2.003659867	0.360210763	0.673339768	0.534961367	0.592676572	0.765244443
546.693865	-0.084398053	0.113774314	-0.741802351	0.458207096	0.661594047
18.95864242	-0.146609932	0.332039904	-0.44154311	0.658819859	0.812363514
2.619585982	-1.502725561	0.822160297	-1.827776853	0.067583048	0.198992518
340.7632743	0.035556293	0.150377406	0.236447044	0.813085795	0.906097599
74.04186069	0.010063132	0.143043117	0.070350337	0.943914818	0.972895359
352.1076618	0.028896434	0.167079266	0.172950448	0.862690377	0.931737465
60.08562746	0.190652202	0.232271067	0.820817694	0.411750118	0.621681922
25.32819704	-2.435028451	1.039402131	-2.342720279	0.01914373	0.0833
14.67960554	-1.611376494	0.950444759	-1.695392056	0.090001072	0.239837861
3197.760303	-0.761939808	0.462757701	-1.646519996	0.099656747	0.255872006
515.9180552	0.26676353	0.057410459	4.646601594	3.37448E-06	9.58469E-05
1276.56082	0.353300118	0.055222595	6.397745644	1.57688E-10	1.64414E-08
11.95852988	0.574678578	0.343902779	1.67104953	0.094711895	0.247154088
94.36836272	0.232148238	0.140911141	1.647479664	0.099459493	0.255770928
384.9915275	-0.233273676	0.061171277	-3.813451124	0.00013704	0.001996102
44.61661287	-0.027739614	0.183899712	-0.150840984	0.880101156	0.939892807
1060.549857	0.213852027	0.060093643	3.558646406	0.000372771	0.004446799
143.1516292	0.097753472	0.323139145	0.302512008	0.762261781	0.875917923
468.6930419	0.115932818	0.084409349	1.373459444	0.169609561	0.361655178

189.6151247	0.558639045	0.586760712	0.952073024	0.341059949	0.555580386
302.4559017	-0.190033761	0.079909616	-2.378108797	0.017401691	0.077964496
1878.833172	-0.349487067	0.050411861	-6.932635704	4.13071E-12	6.6715E-10
271.0100079	-0.001559974	0.173702368	-0.008980732	0.992834509	0.995978303
1605.47854	-0.122643981	0.05064185	-2.421791088	0.015444226	0.071730529
37.00450997	0.121060242	0.208892962	0.579532413	0.562229983	0.745233095
3260.993651	0.131967445	0.080507998	1.639184284	0.101174886	0.25893352
562.8836498	0.373758108	0.062784671	5.9530153	2.63247E-09	1.98932E-07
420.5353297	0.633427632	0.134198762	4.720070616	2.35763E-06	7.10047E-05
67.78329745	0.574608808	0.21113763	2.721489327	0.006498848	0.03831855
12.99074267	0.360983362	0.292669928	1.233414599	0.217421121	0.422248697
369.4193814	-0.008119031	0.078714223	-0.103145665	0.917847363	0.959308302
3681.833302	0.051624886	0.08717454	0.592201424	0.553715716	0.738855719
163.3565823	-0.178735093	0.144957887	-1.23301392	0.217570571	0.422273513
438.5570594	0.246949609	0.078315385	3.153270687	0.00161452	0.013855297
1753.734451	0.332080107	0.060610198	5.47894773	4.27863E-08	2.1167E-06
58.23844484	0.598659635	0.178740683	3.349319385	0.000810104	0.008162474
106.8032205	-0.00806459	0.136519194	-0.059072938	0.952894014	0.977165889
30.30570143	-0.047092716	0.194270669	-0.24240775	0.80846423	0.902713821
75.24545499	-0.570026702	0.25430161	-2.241537921	0.024991252	0.100661586
578.5695328	0.214911644	0.108165923	1.986870161	0.046936787	0.155424448
10.69493567	-0.733750354	0.435523161	-1.684756218	0.092035615	0.242940991
128.6793822	0.440552787	0.104879742	4.200551799	2.66265E-05	0.000542878
832.7908069	-0.125373922	0.052059079	-2.408300798	0.01602697	0.07356598
1569.520049	-0.224050578	0.090689517	-2.470523441	0.013491548	0.06525536
345.4532903	0.292934793	0.127454179	2.298353767	0.021541663	0.090715069
391.6254655	0.335619238	0.064065941	5.238653028	1.61753E-07	6.92134E-06
67.22660324	0.453334443	0.20405245	2.22165646	0.026306531	0.104377118
800.5884316	0.034299104	0.086297481	0.397451971	0.691034198	0.833511303
17.25167658	0.048999699	0.28941385	0.169306683	0.86555542	0.933396663
187.048177	-0.170006645	0.089283764	-1.904116025	0.056895075	0.176613806
1314.114805	0.239957679	0.044902592	5.343960551	9.09374E-08	4.11178E-06
598.67175	-0.018163975	0.057983858	-0.313259167	0.75408377	0.871653489
3862.090957	-0.164495474	0.066005891	-2.492133216	0.01269784	0.062424417
106.2451056	0.101690774	0.24238272	0.419546302	0.674816923	0.824025943
35.74311203	-0.03015672	0.232264112	-0.129838052	0.896694556	0.947904653
870.6443995	-0.010183593	0.06526168	-0.156042456	0.875999556	0.937672708
749.9457074	0.1096132	0.078393253	1.398247875	0.162038645	0.351188949
160.9418747	0.042438201	0.096589114	0.439368364	0.660394645	0.81313543
292.5332571	0.038404469	0.08770377	0.437888467	0.661467136	0.814060183
9.433220339	0.960644329	0.342629979	2.803736936	0.00505141	0.031722851
2946.640135	0.053253342	0.081261816	0.655330449	0.51225502	0.706301407
205.8105047	-0.054288471	0.141558596	-0.383505296	0.701345144	0.839737026
55.39841086	-0.21598959	0.165121795	-1.308062269	0.19085219	0.390833335
254.0430055	0.026133834	0.083885248	0.311542667	0.755388112	0.87230224
9.150769153	0.512943141	0.457268866	1.121753914	0.261967086	0.473242344
514.2618248	-0.071309729	0.078209832	-0.911774476	0.361887449	0.575846019
81.17545567	0.038688163	0.142569971	0.271362637	0.786112135	0.889390242

247.3484358	-0.010986545	0.126223819	-0.087040192	0.930639565	0.967033947
59.55935408	-0.169336793	0.131353824	-1.289165311	0.197340622	0.39875922
3.613469022	0.262187772	0.548609031	0.477913699	0.63271163	0.794224511
335.3103636	0.061677835	0.073788796	0.83586992	0.403228086	0.61450319
1116.939461	0.029933022	0.063623294	0.470472685	0.638017345	0.797775919
328.8576767	-0.01257459	0.07460274	-0.168553995	0.86614747	0.933787619
5.326399874	-1.869951309	0.945521541	-1.977692973	0.047963353	0.157714228
1854.651878	-0.024282265	0.078065123	-0.311051386	0.755761557	0.872365037
840.3721604	0.104265154	0.049095632	2.123715502	0.03369394	0.123156539
243.4471278	-0.057750486	0.088914085	-0.649508862	0.51600952	0.708985891
20740.40673	-0.204355408	0.066994113	-3.05034875	0.002285758	0.017829343
4.368464769	-0.442863077	0.53628197	-0.82580266	0.408916045	0.619446706
312.0569977	-0.12642088	0.092101876	-1.372620036	0.1698705	0.361883695
253.7424624	0.664866672	0.192371172	3.456165827	0.000547918	0.006013593
1532.167594	-0.040507864	0.143374142	-0.282532568	0.777535178	0.884312122
371.9878209	0.912613806	0.162507314	5.615832195	1.95618E-08	1.09613E-06
64.74234463	-0.090159216	0.145381425	-0.620156361	0.535154849	0.72384441
18.79243943	0.430226415	0.247405411	1.738953133	0.082043007	0.226205272
275.4757913	-0.275478636	0.084450881	-3.261998382	0.001106298	0.010432255
752.8975641	-0.160086708	0.063468892	-2.522286169	0.011659481	0.058775488
8.965984267	-0.075028144	0.350925477	-0.213800791	0.830702408	0.915614595
635.5086725	0.350006199	0.087122628	4.017397157	5.88445E-05	0.001022578
203.333706	0.16966217	0.070493015	2.406794056	0.016093242	0.073685401
14.54430316	0.405186922	0.330620379	1.225535231	0.220373624	0.424860747
11.8169009	1.09911508	0.32157122	3.417952265	0.000630942	0.006718897
185.4210857	0.021270079	0.118205815	0.179941054	0.857198844	0.92936531
78.25175852	-0.417641717	0.258295518	-1.616914304	0.10589678	0.266913029
53.88809533	-0.477282602	0.266795614	-1.788944706	0.073623721	0.210898484
315.6222959	-0.13925492	0.100394744	-1.387073811	0.165419257	0.355898764
430.2720865	-0.031477555	0.066166358	-0.475733531	0.634264229	0.794893032
11.07114324	0.279434685	0.351292162	0.795448106	0.426352887	0.633682556
6871.923966	0.138823071	0.070974341	1.955961391	0.050469702	0.162995075
8.157678775	-0.207921028	0.376332154	-0.552493392	0.580610361	0.757609579
7.020138813	0.321514868	0.576499985	0.557701432	0.577048279	0.755186952
645.3056781	0.017617957	0.063398587	0.277891943	0.781095309	0.886011848
33.34549951	1.168260368	0.404383228	2.888993127	0.003864775	0.026033877
15.57873761	-1.059818982	0.442892287	-2.392949736	0.016713531	0.075718733
657.7456538	0.076088991	0.060465997	1.258376515	0.208255621	0.412009499
7486.857233	0.141638105	0.069609089	2.034764523	0.041874572	0.143567472
120.8819806	-0.029576146	0.110868304	-0.266768273	0.789647584	0.891207713
41.05297875	0.050924144	0.163692087	0.311097163	0.755726758	0.872365037
40.34545467	-0.132670475	0.206689012	-0.641884509	0.520948173	0.712937765
81.93058549	0.153500733	0.120283837	1.276154278	0.201900988	0.404784821
111.5333102	0.004511518	0.119809464	0.037655772	0.96996214	0.985321835
7.96505486	-0.132236195	0.365153111	-0.362139034	0.717248136	0.850068042
20.34755566	-0.395093217	0.286156866	-1.380687531	0.167375057	0.358607971
3.958337616	-0.556978022	0.480072899	-1.160194677	0.245969554	0.454526352
31.08964984	-0.073595327	0.219966821	-0.334574673	0.737945953	0.861826289

27.41013672	0.097909667	0.244098059	0.401107929	0.688340663	0.831540961
84.03292501	0.194235167	0.132481881	1.466126281	0.142613887	0.322235794
54.92387828	-0.053705945	0.17966576	-0.298921422	0.765000001	0.877720432
40.33954602	-0.103231866	0.18066963	-0.57138472	0.567738884	0.748773548
301.4146703	-0.057986823	0.068732703	-0.843656956	0.398861127	0.611199292
517.421525	0.076740677	0.117288511	0.654289806	0.512925112	0.706456676
1426.795959	-0.202360959	0.086034218	-2.352098539	0.018667829	0.08163892
48.13136715	0.621826382	0.189360369	3.283825352	0.001024084	0.009823784
73.73764699	-0.768549777	0.308762908	-2.489125984	0.012805758	0.062823721
40.89537432	-0.436639057	0.281336223	-1.552018623	0.120657768	0.290099801
153.1761053	-0.045678605	0.076924891	-0.593807858	0.552640633	0.738078782
77.74666812	-0.124000713	0.207239907	-0.5983438	0.549610557	0.736001001
379.0849333	-0.138134791	0.060110629	-2.298009424	0.021561253	0.090757485
366.9193597	0.114860481	0.068906235	1.666909845	0.095532333	0.24882208
7.126750097	0.401311857	0.393257632	1.020480785	0.307500498	0.522484231
555.3217736	0.239884474	0.091989225	2.607745353	0.009114073	0.049260248
129.3064532	-0.252738847	0.205764078	-1.228294318	0.219336503	0.423903045
449.6374747	0.031438993	0.101850039	0.30867924	0.757565537	0.8735899
293.3337326	-0.245153581	0.087210146	-2.811067213	0.004937747	0.031190354
761.5904962	-0.032085833	0.072743294	-0.441083033	0.659152886	0.812416008
4.363461477	0.083775053	0.47216424	0.177427781	0.859172387	0.930236192
234.0986742	-0.133245121	0.075829206	-1.757174165	0.078888127	0.220159751
109.7385418	-0.2372597	0.169661294	-1.398431515	0.161983525	0.351120604
215.7281686	0.118888899	0.108615694	1.094583063	0.273699339	0.486358831
32.38287781	0.629137881	0.260068691	2.419121958	0.015558023	0.07205591
281.275356	-0.00700687	0.0717579	-0.097645976	0.922213415	0.961560774
332.4113737	-0.197081128	0.076288972	-2.583350156	0.009784596	0.051780095
469.948277	0.061132193	0.06274182	0.974345229	0.329885154	0.545336384
160.4941132	-0.10589096	0.09578286	-1.105531404	0.268929395	0.480722965
75.11396785	0.075617569	0.16108189	0.469435572	0.638758327	0.798267972
2494.455521	0.101162267	0.088018404	1.149330854	0.25041958	0.459400018
63.85185612	0.690738609	0.25081903	2.753932221	0.005888396	0.035585267
1554.870734	-0.20098602	0.057144097	-3.51717901	0.00043616	0.005017662
39.31642329	-0.394384278	0.200903505	-1.963053248	0.049639987	0.161359342
2058.109512	0.246277447	0.123639843	1.991893884	0.046382702	0.154365381
299.162028	0.653572184	0.190067377	3.438634211	0.000584657	0.006332435
21.24530398	0.257250433	0.265599314	0.968565879	0.332761835	0.548337036
3.697335261	-1.58231062	1.265119298	-1.250720483	0.211036475	0.415366175
536.5950803	-0.728774646	0.183859957	-3.963748603	7.3782E-05	0.001233994
6.792557542	-0.524880657	0.7677984	-0.683617805	0.494216538	0.691089318
54.43406032	-0.194977967	0.164002253	-1.188873708	0.234489368	0.441533643
126.3940145	-0.561222189	0.088542546	-6.338446476	2.32093E-10	2.31728E-08
202.3363297	-0.750849773	0.336070521	-2.234203017	0.025469718	0.102015107
508.4519391	-0.22273897	0.078347157	-2.842974503	0.004469465	0.028988173
61.87430861	-0.909143483	0.17265267	-5.265736606	1.39628E-07	6.05326E-06
1288.046388	-0.484189097	0.093901407	-5.156356156	2.51802E-07	1.01423E-05
23021.10516	-0.34844828	0.217217901	-1.604141644	0.108682834	0.271525783
4.456411481	0.764805624	0.696032485	1.09880737	0.271852097	0.48389967

5.37662647	0.43162187	0.398862668	1.082131534	0.279194086	0.492445757
504.3927354	-0.151138523	0.072818546	-2.075549862	0.037935603	0.133965942
478.6027767	-0.088395774	0.081027424	-1.090936501	0.275300825	0.488011752
2.525934908	-3.972460448	1.41552866	-2.806344061	0.005010716	0.031513341
6.943815332	0.007100053	0.398936731	0.017797442	0.985800445	0.992949691
64.45660159	-0.236256497	0.237072246	-0.996559071	0.318978581	0.534029791
4.553504905	-2.758335925	0.885183163	-3.116118833	0.001832484	0.015162402
3.025422048	0.345131662	0.557430452	0.619147484	0.535819204	0.724279063
244.6772841	-0.159103985	0.08723777	-1.823797032	0.068182763	0.200328667
9.337090325	0.285380846	0.480240036	0.594246262	0.552347415	0.737806804
53.39663337	-0.411573521	0.189514573	-2.171724917	0.029876418	0.113668385
556.3072875	-0.622513044	0.088028949	-7.071685556	1.53063E-12	2.83321E-10
6.854940002	0.208540182	0.460173001	0.453177785	0.650420728	0.806308305
110.9011968	-0.141147346	0.153683309	-0.918429901	0.358393842	0.5727765
117.2846599	-0.058303964	0.107881079	-0.540446618	0.588889066	0.76241798
8678.650572	0.148659049	0.093875757	1.583572305	0.11329108	0.278948733
1014.843535	0.135126741	0.07235852	1.867461384	0.061837185	0.187330963
154.5533004	-0.159307775	0.085071757	-1.872628249	0.061119738	0.186047036
179.5956576	0.298709081	0.098380812	3.036253478	0.002395379	0.018459493
2.76846552	0.271405918	0.850180072	0.31923345	0.749549493	0.868971031
271.6400221	0.637182831	0.105918485	6.015784979	1.79017E-09	1.41107E-07
4.822192502	0.895994373	0.450677362	1.98810601	0.046799967	0.155189745
246.5907225	-0.143686617	0.088974752	-1.614914487	0.106329215	0.267537975
119.4906968	-0.732341907	0.249197845	-2.938797107	0.003294887	0.023286129
6.595538577	-2.098523934	1.003090991	-2.092057402	0.036433381	0.130139532
3.028250228	-0.538931525	0.597406603	-0.902118461	0.366993943	0.580606763
7.645389016	0.192844665	0.472797027	0.407880452	0.683361445	0.828701792
217.0260114	-0.216616026	0.128212071	-1.689513509	0.091121065	0.241766537
5.03970061	0.74826573	0.498065159	1.502345058	0.133008018	0.308052029
4.896698674	0.024465712	0.465387565	0.052570619	0.958074028	0.979356635
1867.120176	-0.002441205	0.101303848	-0.024097847	0.980774561	0.990108149
543.3254857	-0.028259394	0.082985711	-0.340533255	0.733454984	0.859506146
157.0412707	-0.076725655	0.09422985	-0.814239384	0.415507827	0.625357946
181.431849	-0.075562881	0.093118822	-0.81146732	0.417097353	0.626482658
1059.65878	0.432863213	0.118546677	3.651415827	0.000260799	0.00337767
698.4005029	-0.023512546	0.079164843	-0.297007417	0.766460846	0.878311336
3.009871958	0.422203248	0.556296685	0.758953376	0.447880447	0.652954203
66.46048653	0.135195108	0.124910223	1.082338216	0.279102271	0.492336525
224.9959829	-0.174655487	0.09679079	-1.804463913	0.071158611	0.206094755
68.01777339	-0.024902507	0.344414498	-0.072303888	0.94236007	0.972429231
75.32940719	-0.95689144	0.213526091	-4.481379476	7.41621E-06	0.000186242
4.332811508	-3.385588069	0.96093172	-3.523234793	0.000426314	0.004931944
119.9279046	-2.193948582	0.310722781	-7.060790893	1.65558E-12	3.03044E-10
2.977228861	-2.364488126	0.67694723	-3.492869192	0.000477861	0.005380252
1116.610221	-0.014099497	0.095237864	-0.14804508	0.882307191	0.940967739
428.3226853	-0.091107191	0.098911838	-0.921094913	0.357000876	0.571548341
5775.116903	0.305966584	0.098281512	3.113165203	0.001850924	0.01528427
13.60699626	0.28990875	0.287225031	1.009343612	0.312809871	0.527591731

794.6522681	-0.075885645	0.080440456	-0.943376613	0.345488301	0.559895217
381.3627842	0.455714626	0.229519843	1.985512979	0.047087428	0.155735451
3976.053948	-0.244970994	0.089547228	-2.735662494	0.006225484	0.03710962
443.6725327	-0.701079415	0.192267485	-3.646375328	0.000265965	0.003417718
34.75496807	-1.034757403	0.52884184	-1.956648143	0.050388851	0.162861669
1599.424029	0.042817662	0.063976406	0.669272694	0.503321542	0.699015266
77.01899031	-0.195179283	0.133424419	-1.462845286	0.143509718	0.323621034
93.52954256	0.031611303	0.12894717	0.245149259	0.806340863	0.901254115
234.0115862	0.127247397	0.157292518	0.808985694	0.418523375	0.627708857
5.038948555	0.29657616	0.447808985	0.662282737	0.507790038	0.702614907
491.4758209	0.278639687	0.068720999	4.054651281	5.02091E-05	0.000896415
222.8420518	-0.101205803	0.090747856	-1.115241814	0.264746812	0.476087652
149.4005902	-0.023843809	0.135101477	-0.176488143	0.859910463	0.930759853
66.52086065	-0.385022202	0.123591201	-3.115288143	0.001837653	0.015197538
35.56243956	-0.137334958	0.20485551	-0.670399138	0.502603384	0.698266548
3.14029641	-0.23699188	0.55600643	-0.426239459	0.669933367	0.820434307
63.22305016	-0.503592205	0.19275761	-2.612567174	0.008986503	0.048762731
5.580031735	0.083675986	0.561081708	0.149133335	0.881448422	0.940691803
61.9620531	0.171895656	0.145960674	1.177684721	0.23892232	0.446360434
87.90974244	0.265001903	0.114178243	2.320949223	0.020289584	0.086908636
14.83385495	-0.202042928	0.356868643	-0.566154891	0.571288491	0.751109865
106.3649926	-0.156101495	0.184010151	-0.848330891	0.396253719	0.609075814
35.09849678	0.434552817	0.247005095	1.759286858	0.078528798	0.219491587
16.32908137	0.680515856	0.3096267	2.197859084	0.027959149	0.108785788
646.5269489	-0.214387542	0.118271174	-1.812677888	0.069881517	0.203505057
26.24545364	-0.508335775	0.28545016	-1.780821474	0.07494162	0.213239062
722.7892584	0.292861388	0.050666324	5.780198047	7.46127E-09	4.87766E-07
30.99771301	-0.317822522	0.304134385	-1.045006871	0.296019794	0.510695369
7.819693411	0.576800184	0.887820588	0.649681019	0.515898287	0.708985891
145.9441746	-0.241790613	0.139225669	-1.736681277	0.082443444	0.226950609
16.74582358	-0.309935019	0.472617659	-0.65578383	0.511963222	0.706072364
33.23901381	0.136079919	0.219799942	0.619108076	0.535845164	0.724279063
389.0501795	0.0550678	0.092861738	0.593008497	0.553175466	0.73855358
603.4514503	-0.245256495	0.05854178	-4.189426708	2.7966E-05	0.000561844
31.34065916	-0.269027135	0.17592505	-1.529214484	0.126211281	0.297539302
758.632286	-0.119806342	0.092345917	-1.297364801	0.194505698	0.39534693
3.455877301	-0.369723905	0.487268526	-0.758768288	0.447991179	0.652990786
68.5246315	-0.212412258	0.295676254	-0.718394715	0.472513945	0.672849401
302.280871	-0.141498816	0.081932693	-1.727012884	0.08416535	0.229787864
5.185921307	-0.204267914	0.662824242	-0.308178097	0.757946818	0.873603573
24.18792317	-0.323029356	0.24290626	-1.32985192	0.183567065	0.380866982
377.6572874	0.103070009	0.083909468	1.228347779	0.219316442	0.423903045
39.81293724	0.054090713	0.182324085	0.296673437	0.766715838	0.878445228
13.61646806	-3.393830566	0.972332463	-3.490401376	0.000482296	0.005412355
441.1521263	-0.127800952	0.086432808	-1.478616216	0.139242916	0.317745772
817.4740178	-0.103378055	0.084002772	-1.230650522	0.218453601	0.423029367
284.040338	-0.298802882	0.089309964	-3.345683614	0.0008208	0.008250066
100.9276023	-0.100353847	0.124230508	-0.807803565	0.419203673	0.628262028

624.5287698	0.108417009	0.074196127	1.461221937	0.143954545	0.324153522
3.621720876	-0.693803989	0.539113268	-1.286935474	0.198116779	0.399727596
853.2670979	-0.520672157	0.164540807	-3.164395297	0.001554056	0.013488679
7.475247961	0.397788943	0.391948801	1.014900267	0.310153371	0.525137224
2225.146811	0.687221133	0.105650002	6.504695882	7.78507E-11	8.43758E-09
374.1873766	0.240396456	0.0746643	3.219697445	0.00128326	0.011668465
272.7177349	-0.232446783	0.117316321	-1.981367825	0.047550039	0.156667869
5.894056012	-0.087516381	0.364027966	-0.240411147	0.810011538	0.903767699
319.2113709	-0.390609202	0.209860872	-1.861276945	0.062705077	0.18913727
1.707862586	0.519706796	0.753947216	0.689314563	0.490625335	0.688433148
1020.980863	-0.154851362	0.055911775	-2.769566187	0.0056131	0.034413921
457.6189215	0.219943611	0.068133144	3.228144172	0.001245962	0.011435082
106.411901	-0.190593711	0.110229874	-1.729056777	0.083798934	0.229143952
740.790446	-0.74969621	0.230266371	-3.255778106	0.001130821	0.010627007
29.70906097	0.250278393	0.43571791	0.574404648	0.565694008	0.748001651
658.5067136	0.170926765	0.070593273	2.421289702	0.015465547	0.071768849
81.12603562	0.932332791	0.111131196	8.389478636	4.88303E-17	2.36597E-14
651.3132106	-0.316827367	0.091599651	-3.458827235	0.000542533	0.005970398
84.66623033	-0.015680598	0.103690816	-0.151224558	0.879798579	0.939813369
132.1367278	0.011061712	0.099779504	0.110861564	0.911726125	0.955699681
441.5230113	-0.079260087	0.080738084	-0.98169394	0.326250652	0.541145111
34.65410917	0.190017812	0.222802485	0.852853197	0.393740719	0.606740045
698.1428697	0.055255307	0.096377401	0.573322236	0.566426532	0.748300778
532.1619384	-0.081960575	0.065516504	-1.250991286	0.210937657	0.415366175
2.956363191	0.398368615	0.593050597	0.671727871	0.501756955	0.697505543
151.966892	0.058448031	0.096771965	0.603976893	0.545859005	0.732704412
111.6713861	-0.188143364	0.106416204	-1.767995449	0.077061664	0.216781036
497.9052541	-0.052823267	0.065822337	-0.802512794	0.42225639	0.630552191
95.67534491	0.188253889	0.118436198	1.589496215	0.111948408	0.276954208
5.539913015	-0.177171997	0.477164447	-0.371301755	0.710412791	0.845617075
20.25125032	0.328597405	0.256188686	1.28263824	0.199618846	0.401872281
2966.844963	-0.159826221	0.139505166	-1.145665254	0.251933686	0.461142645
206.63593	0.399403512	0.15435635	2.587541829	0.009666346	0.051464856
373.0978396	0.30138443	0.062905487	4.791067458	1.65896E-06	5.29647E-05
14.8886605	-0.607086899	0.723013044	-0.839662444	0.401097677	0.612787084
16.76671074	0.592497198	0.287439372	2.061294504	0.039274952	0.136847819
924.4970877	-0.133335049	0.066681057	-1.999594117	0.04554411	0.152343891
418.457738	0.158701253	0.140132163	1.132511263	0.257419552	0.467957282
231.6529739	0.172421272	0.067609267	2.550260918	0.010764233	0.055225776
628.9706128	-0.076500389	0.062743668	-1.219252744	0.222748278	0.427806473
42.17126977	0.048244254	0.179020636	0.269489903	0.787552711	0.890225289
67.43945319	-0.300392319	0.267919794	-1.121202412	0.262201713	0.473474847
6.548033255	-0.221269178	0.366191081	-0.604245133	0.545680678	0.732598025
316.3887132	0.089226037	0.07975233	1.118789095	0.263230125	0.474603599
16.80559008	0.465053636	0.298841264	1.556189493	0.119663039	0.288624815
88.68083294	0.248795123	0.233739044	1.06441405	0.287141172	0.501682433
1.923468568	1.200044637	0.71851395	1.670175835	0.09488458	0.247487106
1426.669455	0.071009585	0.047771566	1.486440403	0.13716267	0.314567529

2620.388285	-0.080380569	0.089515028	-0.897956147	0.369208929	0.582823677
56.2374547	-0.664128723	0.302185294	-2.197753288	0.027966692	0.108789441
130.0861006	0.168202794	0.084674037	1.986474238	0.046980691	0.155507313
27.69903554	0.022316908	0.184671343	0.120846622	0.903812522	0.951582251
2523.072569	0.297310722	0.052252845	5.689847582	1.27153E-08	7.50794E-07
3.34413726	0.670835074	0.585629995	1.145493025	0.252004983	0.461142645
101.6592501	0.315133441	0.206719392	1.524450311	0.12739626	0.298780921
185.7400365	0.070390136	0.094812353	0.742415241	0.457835788	0.661206871
80.19153219	0.1301316	0.15341299	0.848243685	0.396302273	0.609075814
2.201879817	0.30984241	0.676230641	0.458190432	0.646815633	0.804199301
29.34842536	1.057394295	0.317053781	3.335062881	0.000852801	0.008509416
274.6275608	-0.176081969	0.108915887	-1.61667847	0.105947704	0.266918867
247.1773617	-0.263431127	0.087164284	-3.022237032	0.00250914	0.019110296
4.825304375	-0.025551115	0.471111793	-0.054235779	0.956747315	0.978702547
87.3611372	0.668287131	0.196747186	3.396679484	0.000682088	0.007157146
427.5578381	0.006479966	0.129095239	0.050195239	0.959966806	0.9804928
70.09425823	0.742061465	0.779632171	0.951809702	0.341193501	0.555636786
41.38517848	0.356965261	0.225329947	1.584189168	0.113150677	0.278798125
169.9026939	0.256706196	0.100822389	2.546122926	0.010892682	0.055728586
266.1361637	0.113132935	0.083819931	1.349714004	0.177107739	0.372398541
14.35204804	0.293991906	0.24147774	1.217470008	0.223425441	0.428534816
227.5255595	-0.060707466	0.089574859	-0.677728849	0.497943631	0.694532502
40.83651401	0.543502875	0.266704196	2.037848982	0.041565038	0.142714139
890.6773311	0.073354916	0.04670541	1.570587142	0.116278579	0.283452694
349.838183	0.28851748	0.082639237	3.491289242	0.000480696	0.005398077
130.8900754	-0.109984444	0.163387771	-0.673149792	0.500851999	0.696642675
987.2661504	-0.04428423	0.080650535	-0.549087856	0.582945159	0.758985106
42.30076486	0.246797748	0.549293731	0.449300136	0.65321516	0.808434118
36.36374729	-0.11836217	0.184663631	-0.640960914	0.521548078	0.71331773
1885.314385	0.082148249	0.055542172	1.479024799	0.139133687	0.317621792
1788.606934	-0.01180365	0.096695172	-0.122070723	0.902843008	0.951311133
59.35530189	0.491280972	0.191458476	2.565992289	0.010288112	0.053465725
284.9410393	-0.011625256	0.074694087	-0.155638235	0.876318185	0.937737303
2.799085475	2.520571971	0.746473121	3.3766413	0.000733767	0.007556959
6690.794916	-0.241243458	0.1361017	-1.772523477	0.076307702	0.215587906
449.8825848	0.035004473	0.115297544	0.3036012	0.761431737	0.875476441
94.21019862	0.17788288	0.123133436	1.444635069	0.148560417	0.331175143
5.98004733	0.985250829	0.50942403	1.934048594	0.053107153	0.16853925
69.08555935	0.195770432	0.14389405	1.360517912	0.173666089	0.367309686
267.5119512	0.147171435	0.080006803	1.839486521	0.065843659	0.195654479
26.43399511	0.268467743	0.209879704	1.279150572	0.200844038	0.403451369
5.769974079	0.534206327	0.402113656	1.328495857	0.184014351	0.381458717
21.72636503	0.040853059	0.346683976	0.117839479	0.906194848	0.952959157
3.409390749	0.203993043	0.620032626	0.329003725	0.742152873	0.864878344
2.161591951	-2.019595668	1.203966037	-1.677452357	0.093454058	0.245505048
17.92494814	-0.336172215	0.330343195	-1.017645346	0.308846529	0.523980279
7513.446135	0.249400678	0.107303104	2.324263412	0.02011138	0.086279917
671.5230572	-0.289877216	0.074093007	-3.912342439	9.14052E-05	0.001460532

31673.03781	0.021298898	0.081669644	0.260793325	0.794251892	0.893917451
6.467593596	1.390104832	0.466344208	2.98085579	0.002874441	0.021149417
967.0718866	0.174882495	0.055669932	3.141417461	0.001681322	0.014199103
1149.490583	-0.059060576	0.046729066	-1.263893782	0.206268154	0.409701178
7.46963691	-0.107179864	0.439589564	-0.243818036	0.807371751	0.901799595
267.1924665	-0.117510299	0.109911567	-1.069134969	0.285008857	0.499344317
41.37378377	0.099032342	0.295379511	0.335271534	0.737420265	0.861788314
2.248970098	-2.041817542	1.110352323	-1.838891585	0.065931134	0.195773161
6.877056256	-3.694887811	1.295398075	-2.852318436	0.00434016	0.02839547
147.4302108	0.406850349	0.221788748	1.834404822	0.066593924	0.197119235
233.3927755	0.334683703	0.105090559	3.184717118	0.001448956	0.012790822
228.4287154	-0.273810996	0.093607305	-2.92510285	0.003443424	0.02407766
9642.246963	0.010149852	0.070087434	0.144816997	0.884855362	0.942468039
2910.394585	-0.128718234	0.126119023	-1.020609184	0.307439636	0.522484231
1512.759041	0.141612875	0.083992903	1.686010015	0.09179387	0.242536042
13.85321589	0.389303172	0.336361881	1.157393849	0.247111486	0.455777809
317.8633737	0.033754225	0.087388852	0.386253218	0.699309144	0.838702594
363.860984	0.118616211	0.052485252	2.259991249	0.023821794	0.097138672
1366.527291	0.202873844	0.065199622	3.111580039	0.00186089	0.015345564
771.4235806	-0.020449756	0.108044848	-0.189270992	0.849880429	0.925926208
91.40405004	0.070637564	0.201276875	0.350947244	0.725627927	0.854589253
317.0914295	-0.103725337	0.104006759	-0.997294193	0.318621731	0.53371211
979.1355561	-0.126777128	0.181634149	-0.69798069	0.48518927	0.684040054
91.95334791	0.149469418	0.106545981	1.402863029	0.160657688	0.348843383
2880.030885	-0.25530439	0.078421741	-3.255530739	0.001131806	0.010630205
421.0478546	-0.096215546	0.051337682	-1.874170047	0.060906991	0.185673903
103.9966606	-0.520149308	0.236195745	-2.202195925	0.027651472	0.107843358
1097.422331	-0.179419043	0.083442483	-2.15021217	0.031538436	0.117734918
7.466447104	0.539138494	0.448082646	1.203212173	0.228894225	0.434925429
512.1247225	-0.20270107	0.06559245	-3.090311012	0.00199947	0.01621027
1474.61524	0.085291741	0.047796785	1.784466084	0.074347961	0.21208802
72.49090144	-0.044557206	0.179589065	-0.248106451	0.804052044	0.899535041
8.373058716	0.937110346	0.433120151	2.163626756	0.030493006	0.11529634
225.051656	0.483303761	0.231704632	2.085861453	0.036991175	0.131760565
115.9673706	0.411749034	0.107665322	3.824342198	0.000131122	0.001930383
67.10218027	-0.410616996	0.300960176	-1.364356579	0.172455367	0.365405751
49.95817715	-0.235997729	0.159022642	-1.484051115	0.137795361	0.315546396
387.5406573	0.011445246	0.067940778	0.168459159	0.866222071	0.933787619
17.55027818	-0.29225876	0.327400245	-0.892665061	0.372036566	0.585492013
103.3794062	-0.246172411	0.149752741	-1.643859133	0.100205303	0.256851123
47.66750714	-0.823282867	0.223206419	-3.68843724	0.000225636	0.003017144
872.6511843	-0.320277208	0.081316617	-3.938644017	8.19434E-05	0.001345898
30.91893775	0.192442091	0.190225531	1.011652277	0.311704341	0.52645368
416.1511816	-0.304103705	0.093810462	-3.241682196	0.001188265	0.011028201
1518.863063	0.34725839	0.092307113	3.761989515	0.000168567	0.002383669
8.06505046	-0.478656322	0.402504211	-1.18919581	0.234362623	0.441345433
2.781876171	-0.467811122	0.639220329	-0.731846441	0.4642623	0.666514782
10.56232679	2.216914638	0.83511976	2.654606854	0.007940089	0.044370768

491.7971471	-0.08939097	0.141555125	-0.631492289	0.52771869	0.717652113
1467.011871	0.074909369	0.130812042	0.572648882	0.566882455	0.748471553
556.5367515	0.093517375	0.05994595	1.560028236	0.118753209	0.287190305
376.0984419	-0.196970284	0.069575252	-2.831039471	0.004639699	0.029719986
302.6746619	0.287322094	0.084959215	3.381882634	0.000719909	0.00743094
32.26929057	0.056656638	0.22728208	0.249278949	0.803145014	0.899097368
576.7727446	-0.302524405	0.09462068	-3.197233487	0.001387526	0.012382501
337.0890797	-0.050591867	0.063142099	-0.801238293	0.422993707	0.63111002
280.137821	0.075816944	0.060673829	1.249582328	0.211452162	0.415737309
35.50528417	0.371835529	0.212382103	1.750785609	0.079982846	0.222198552
2.223743463	0.08751699	0.633484192	0.138151814	0.890120437	0.945139805
38.84201782	-1.1259352	0.307980935	-3.655860062	0.000256321	0.003330154
94.05732117	-0.088388609	0.118018499	-0.748938598	0.453894215	0.658168585
1186.400048	0.243775764	0.082934041	2.939393305	0.003288555	0.023251352
430.4229717	-0.036897989	0.090258669	-0.40880272	0.682684444	0.828547438
561.4692515	0.040703434	0.072676047	0.560066698	0.575433945	0.753972704
33.68091218	-0.229205786	0.23119504	-0.991395776	0.321492362	0.536568571
3.349665671	-0.115110303	0.554091194	-0.207746134	0.835427194	0.917766577
175.5826249	-0.88313354	0.30076981	-2.936243966	0.003322129	0.023410475
380.1141005	-0.072655511	0.112911866	-0.643471002	0.519918525	0.712159123
281.3906895	-0.248321352	0.135874081	-1.827584406	0.067611948	0.199042035
426.506041	-0.232261473	0.062119182	-3.738965422	0.000184779	0.002565279
7.342637848	0.602290649	0.492768927	1.222257769	0.221610164	0.426346589
5.016032989	-1.358450701	0.862693516	-1.574662005	0.115334482	0.282048078
449.8513626	-0.285812566	0.135935117	-2.102566078	0.035503718	0.127760648
23.20950173	-1.091472638	0.469085905	-2.326807579	0.019975509	0.085865459
95.72570098	0.143488383	0.113489601	1.264330673	0.206111365	0.409537887
2.637336257	1.555136298	0.717662195	2.166947497	0.030238858	0.11454471
145.2383109	0.163769949	0.090368494	1.812246087	0.069948181	0.203576974
414.5315279	0.056447106	0.057951339	0.974043169	0.330035106	0.545419232
77.27873462	-0.070711171	0.115987091	-0.609646902	0.542095735	0.729614799
44.89025744	0.284035503	0.187144781	1.517731362	0.129082134	0.301589735
9.730104789	-0.629389253	0.394187055	-1.596676616	0.110337811	0.274412001
1781.762649	0.0137542	0.045224707	0.304130211	0.761028691	0.875424502
234.6294371	-0.013185617	0.079874117	-0.165079973	0.868881036	0.934607931
2271.110981	-0.083514769	0.089807068	-0.929935376	0.352404545	0.567443308
75.75842489	-0.278708311	0.125606642	-2.218897873	0.026493673	0.104932917
2.075052696	-0.093101932	0.759820508	-0.122531481	0.902478115	0.951164319
1610.370615	-0.054412895	0.043205527	-1.259396616	0.207887111	0.411538253
30.65568182	-0.446419976	0.374696889	-1.191416285	0.233490203	0.440306502
216.6713241	-0.09138391	0.071693951	-1.274639054	0.202437028	0.405216559
3.304657032	0.227546776	0.567212146	0.401166966	0.6882972	0.831540961
5.33348652	0.999920425	0.46870213	2.13338144	0.032893448	0.121115816
918.9103674	0.034184702	0.042325139	0.807668979	0.419281166	0.628262028
153.0237763	-0.025109871	0.185305821	-0.135505033	0.892212585	0.94585057
4.833803443	-0.077000777	0.554248608	-0.138928228	0.889506865	0.944882455
1188.757065	0.315818337	0.07855449	4.020372833	5.81061E-05	0.001011882
279.745651	0.101132966	0.093983924	1.076066648	0.281897451	0.495833718

53.92077839	0.867336966	0.155381614	5.581979371	2.37797E-08	1.28864E-06
1462.29647	0.409351411	0.075063646	5.453390972	4.94183E-08	2.41578E-06
280.7134979	0.161539037	0.07158738	2.256529537	0.024037488	0.097821364
765.6510815	-0.248166622	0.108586085	-2.28543668	0.022287235	0.092646963
441.4342915	-0.146543068	0.111432471	-1.315084069	0.18848165	0.387888408
523.7026842	-0.319124327	0.076342286	-4.18017775	2.91281E-05	0.00058094
987.8957577	-0.023746759	0.072119169	-0.329271117	0.741950773	0.864835046
416.1268807	0.096831839	0.108397146	0.89330616	0.37169324	0.5851316
239.3321789	0.041463627	0.099551577	0.416503971	0.677041262	0.825613125
15293.98075	0.032338257	0.102194814	0.316437357	0.751670565	0.870449943
59.42189337	0.217148911	0.158586449	1.36927784	0.170912442	0.363086692
785.2937244	-0.402565142	0.164504587	-2.44713627	0.014399638	0.068284296
287.0589939	-0.002029661	0.083103709	-0.024423236	0.980515015	0.990070754
63.26171755	-0.509284916	0.272346926	-1.869985914	0.061485774	0.186609551
563.3531107	-0.20020297	0.058874105	-3.400526776	0.000672561	0.007066185
2614.937505	0.177341247	0.082215869	2.157019662	0.031004132	0.11645423
1537.954568	0.20561116	0.054458916	3.775527956	0.000159669	0.002275423
10.70375395	0.30327792	0.325032556	0.933069364	0.350784156	0.565832638
22.03333038	0.487361886	0.252025164	1.933782636	0.053139859	0.168596518
310.0716085	0.106733175	0.078795256	1.354563451	0.175556685	0.369973687
583.8649791	0.045754245	0.058394961	0.7835307	0.433315533	0.640328374
14226.23644	0.0034646	0.087270685	0.039699472	0.968332722	0.984103224
899.5193323	0.253910462	0.081011463	3.134253506	0.001722919	0.01445182
676.3649727	-0.471458935	0.111265557	-4.237240607	2.26284E-05	0.000471873
374.4912036	-0.10218172	0.083288029	-1.226847612	0.219879872	0.42430608
1150.956274	0.038984298	0.063651566	0.612464078	0.540230757	0.727818245
58.52998206	0.577609143	0.459352226	1.257442785	0.208593345	0.412578552
12.67771667	0.165764445	0.260135185	0.637224239	0.523978791	0.715163762
264.0741349	0.276035285	0.077514704	3.561069962	0.000369347	0.004409142
49.3624619	0.627367713	0.154449948	4.061948356	4.86648E-05	0.000872366
643.9403957	0.036142633	0.073290293	0.493143517	0.621911185	0.787067216
29.85247143	-0.592176839	0.208177437	-2.844577426	0.004447038	0.028899605
4.29419386	0.304047545	0.436617754	0.69637009	0.486197089	0.684991948
116.2615557	0.911054226	0.259570663	3.509850512	0.000448359	0.005125788
286.2566502	1.219168583	0.176406485	6.91113244	4.808E-12	7.64825E-10
554.61254	0.02587867	0.129597035	0.19968566	0.841726429	0.921491308
164.1821841	-0.12291358	0.084490284	-1.454765848	0.145734096	0.326776031
5.885296834	-0.273419869	0.444310048	-0.615380792	0.538303274	0.726231114
23.14327498	-2.715325282	1.133032417	-2.396511557	0.016551972	0.075242047
2.440755679	0.231762506	0.820767656	0.282372855	0.777657627	0.884312122
14.14110784	1.024748537	0.318465247	3.217771948	0.001291905	0.011713176
113.721959	0.483840374	0.641004459	0.754815925	0.450359437	0.655292472
11.64558597	-1.490869169	0.591576828	-2.520161539	0.011730099	0.058981994
5.820921864	1.229287522	0.537252193	2.288101451	0.022131614	0.092179298
840.757827	0.309824992	0.174594959	1.774535725	0.075974578	0.214907011
638.941194	0.178965724	0.064897697	2.757659087	0.005821688	0.035304727
3.118606388	0.37058299	0.654201875	0.566465802	0.571077174	0.751109865
275.4630021	-0.197365934	0.106786128	-1.848235687	0.064568255	0.193118633

461.0525468	-0.007036075	0.173662575	-0.040515783	0.967681924	0.98386242
3.791456571	-0.427439288	0.518013904	-0.825150223	0.409286309	0.619722671
94.04052155	-0.754298576	0.338879611	-2.225860017	0.026023559	0.103503649
30.84022949	0.243431357	0.194200254	1.253506894	0.210021292	0.414168188
1922.298999	-0.008070877	0.111265447	-0.072537136	0.942174453	0.972418112
342.7709769	-0.124554365	0.11641823	-1.069887117	0.284670123	0.498846463
99.04458844	-0.101356732	0.127644643	-0.794053942	0.427164029	0.634373047
281.4875452	-0.286908143	0.098948571	-2.899568322	0.003736769	0.025427316
65.79702075	-0.043625361	0.155882945	-0.279859743	0.779585114	0.885082046
130.3475322	0.435076159	0.114530428	3.798782265	0.000145409	0.002104977
172.1145136	0.485181131	0.11814646	4.106607422	4.01513E-05	0.00074656
914.7740663	-0.336443611	0.080974108	-4.154952985	3.25354E-05	0.000638843
1246.085406	-0.039132957	0.065022822	-0.601834189	0.547284515	0.733960037
950.5193738	0.073223808	0.075724569	0.966975569	0.333556247	0.548794652
28.75414507	-0.067105788	0.262385837	-0.255752324	0.798142089	0.895680707
373.4635568	0.168772485	0.07350545	2.296054041	0.021672792	0.090941816
444.4008799	-0.17624769	0.070926186	-2.48494525	0.012957136	0.063340018
1174.270136	-0.161109921	0.125922415	-1.279437982	0.200742866	0.403297314
237.1711809	0.000749674	0.089925641	0.008336601	0.993348432	0.996251191
476.7660334	-0.019892334	0.086671459	-0.229514235	0.818469258	0.908774216
6585.104253	-0.07745782	0.177556631	-0.436242902	0.662660504	0.815346116
13.58021667	0.539349619	0.322472202	1.672546088	0.094416688	0.24693134
1.75012695	-0.851370768	0.865373244	-0.983819149	0.325204443	0.540225672
4838.659554	-0.562604156	0.156059639	-3.605058678	0.000312082	0.003839616
46.80317518	-0.376234582	0.140733448	-2.673384235	0.007509019	0.042623462
443.1344505	0.210393473	0.084356632	2.494095214	0.012627867	0.062210369
34.56092733	-0.338290404	0.17651587	-1.916487195	0.055303112	0.17272797
85.81891514	0.096162539	0.185993513	0.517020928	0.60514155	0.775218257
310.9138978	-0.308253817	0.08467249	-3.640542738	0.000272064	0.003491068
325.7267407	-0.240177551	0.08803598	-2.728174888	0.006368583	0.037726013
1033.31674	-0.176623546	0.07923147	-2.22920949	0.025799969	0.102962378
60.96377014	0.089374532	0.127981211	0.698341041	0.484963939	0.683839419
636.8667097	-0.218518151	0.090237966	-2.421576637	0.015453342	0.071732838
2369.362962	0.037034359	0.066377384	0.55793641	0.576887808	0.755156913
135.698909	-0.306602752	0.105121202	-2.916659486	0.003538018	0.024559206
2.534496348	1.014544129	0.653374372	1.552776131	0.120476627	0.289953536
344.171616	-0.104071351	0.083207024	-1.250751993	0.211024975	0.415366175
422.4908461	-0.458222393	0.1451202	-3.15753695	0.001591081	0.013723279
250.0774647	-0.052049708	0.093818737	-0.554790116	0.579038229	0.756889295
77.80067984	0.469867711	0.150606536	3.119836112	0.001809517	0.015002507
124.76063	0.009712315	0.101531838	0.095657825	0.923792338	0.962529566
16.68200388	0.936233004	1.046550478	0.894589438	0.3710066	0.584485295
39.94958154	0.032545112	0.144525918	0.225185298	0.821835127	0.910668721
61.77010475	-0.274172493	0.15740179	-1.741863884	0.081532265	0.225173937
93.7441086	0.435607507	0.182660109	2.384798249	0.01708849	0.076853886
475.3275127	0.164494611	0.131222831	1.253551761	0.210004974	0.414168188
215.2056371	-0.380997992	0.121108878	-3.145912986	0.001655693	0.014066983
217.6894679	-0.06938066	0.09716883	-0.714021775	0.475213715	0.675059993

534.3928878	0.099962782	0.067176549	1.488060688	0.13673489	0.313907638
105.1963554	-0.063836028	0.124942053	-0.510925075	0.609403522	0.778422394
147.7975802	0.003812574	0.159357125	0.023924719	0.980912657	0.990108149
118.1065598	-0.116280138	0.132171391	-0.879767827	0.378985097	0.592072118
340.9456078	-0.220451553	0.084465285	-2.609966352	0.009055113	0.049021995
752.8791708	0.007318004	0.144782823	0.050544697	0.959688332	0.980337668
7094.074797	-0.217060975	0.083912327	-2.586759096	0.00968833	0.051502923
60.08624826	0.685261938	0.163272732	4.197038481	2.70428E-05	0.000548339
16.75991329	0.859492521	0.239760939	3.584789609	0.00033735	0.004098455
7564.338735	0.235175787	0.13705882	1.715874891	0.086184943	0.233138055
541.7728549	-0.311926015	0.136807248	-2.280040122	0.022605309	0.093614847
5971.575527	-0.353932777	0.13393502	-2.642570835	0.008227925	0.045700216
10.67555229	-0.33927154	0.291067067	-1.16561294	0.243770974	0.451393647
401.1258683	0.011686424	0.073164077	0.159728982	0.87309457	0.936680519
139.2888054	0.053946995	0.103457811	0.521439556	0.602060602	0.772878233
117.7111595	-0.078028685	0.145628383	-0.535806846	0.59209205	0.764669523
79.40409364	0.127983996	0.289335285	0.442338016	0.658244625	0.811853108
1058.86634	-0.052392407	0.064130155	-0.816969911	0.413945623	0.623739156
17.43496827	0.397900297	0.320661402	1.240873688	0.214652414	0.419227489
3209.740839	0.036353072	0.101513346	0.358111258	0.720260054	0.851622172
5.170820481	0.776999639	0.813212134	0.955469806	0.339340168	0.55415245
2.203993149	1.62877127	1.255057685	1.297766063	0.194367735	0.395212795
66.25516184	0.145690407	0.163511999	0.891007437	0.37292518	0.586386315
415.1036382	0.121483231	0.105089499	1.155997806	0.247682055	0.456253929
56.7022712	-0.236902726	0.147629369	-1.604712719	0.108557041	0.271313213
237.315378	0.025781804	0.06956801	0.370598552	0.710936561	0.84576363
2.20888814	-2.569034294	1.163571676	-2.207886585	0.02725218	0.106704405
198.179297	-0.057933681	0.228099485	-0.253984267	0.799507712	0.896484956
160.5251337	0.217355419	0.087380029	2.487472507	0.01286544	0.06300394
9.414249937	0.222324292	0.321449631	0.691630261	0.489169553	0.686965008
218.7258415	0.480809977	0.220666596	2.178897875	0.029339256	0.112351208
913.8380313	0.057798047	0.04980505	1.160485655	0.245851131	0.454358485
16.00826072	0.082050767	0.266325173	0.308084909	0.758017724	0.873624177
778.1696004	-0.234667612	0.088274457	-2.658386339	0.007851583	0.044002853
9.638349506	0.028819123	0.337048885	0.085504283	0.931860491	0.967753245
8.649914211	0.8676206	0.358927573	2.417258145	0.015637922	0.072324293
36.86554784	0.609131152	0.508089274	1.198866387	0.230579904	0.436936024
180.9717632	0.490146193	0.521531137	0.939821533	0.347309112	0.5619851
33.44934385	0.642707406	0.435810726	1.474739761	0.140282522	0.319112713
4.919116272	-0.843535682	0.787269454	-1.071470102	0.283958107	0.498022555
661.9533267	0.22331183	0.197771705	1.129139429	0.25883902	0.469564368
1174.554937	-0.618730592	0.181255529	-3.413581885	0.000641149	0.006796838
425.916178	0.020637388	0.061929996	0.333237363	0.738955117	0.862699071
12.27953661	-3.388667221	0.80062461	-4.232529425	2.31078E-05	0.000480653
41.97862045	-0.167356696	0.175525747	-0.953459525	0.340357294	0.555263029
809.550527	-0.106854149	0.087014013	-1.228010808	0.219442911	0.423921325
8.503389432	-2.115645441	0.817427502	-2.588175019	0.009648594	0.051407161
109.2315083	-0.336040934	0.104414523	-3.2183352	0.00128937	0.011699322

10822.46836	-0.214674827	0.139382849	-1.54018108	0.12351622	0.293792408
5.459094748	-0.466300608	0.525030734	-0.888139641	0.374465642	0.58802278
5.406246281	0.037264674	0.558401288	0.066734577	0.946793007	0.974828397
2.0029942	-2.782607727	1.453313035	-1.914665085	0.055535229	0.173110192
12.67229428	-0.707018491	0.389056724	-1.817263258	0.069176813	0.202203481
433.3959551	-0.049989213	0.09140352	-0.546906868	0.584442717	0.75979398
25.43999096	-0.231591369	0.223580283	-1.035830913	0.300281033	0.515122671
3.96782336	0.967909728	0.621938504	1.556278832	0.119641803	0.288618987
280.7681841	0.027974989	0.072021169	0.388427302	0.697699843	0.838003561
482.9394393	-0.142977252	0.112064077	-1.275852674	0.202007603	0.404850761
14.64908501	-0.638443767	0.402809908	-1.584975331	0.112971939	0.278517
131.6394206	-0.291163623	0.104769697	-2.779082409	0.005451269	0.0336355
336.9144538	0.145684548	0.17464037	0.834197429	0.40416974	0.614990985
493.0250961	0.049667744	0.14835091	0.334799049	0.737776678	0.861800757
8.162385916	-1.028481591	0.366493886	-2.806272167	0.005011834	0.031513341
806.5267648	-0.011042126	0.048510056	-0.227625499	0.819937395	0.90948348
85.32632939	0.12337996	0.104533128	1.180295304	0.237882785	0.445469394
49.65986322	-0.636015662	0.203842474	-3.120133155	0.001807693	0.014994932
212.1232991	0.482228577	0.121479985	3.969613416	7.19893E-05	0.001210155
2.397922561	0.660377118	0.873170691	0.756297852	0.449470635	0.654461662
313.0242772	-0.675657629	0.473880012	-1.425798961	0.153926381	0.338555834
9.519516428	0.117463291	0.598615162	0.196225052	0.844434021	0.922856413
12.60365819	1.811905602	0.769472817	2.354736337	0.018535853	0.081212669
120.9041588	-0.132685283	0.106955635	-1.240563741	0.214766954	0.419252375
3.604945813	-0.370893799	0.789013168	-0.470073015	0.638302853	0.798011778
35.63050406	0.222758281	0.44852335	0.496648125	0.61943721	0.78515108
774.0724718	-0.353710311	0.065426093	-5.406257613	6.43552E-08	3.08194E-06
2929.756205	0.089120868	0.055520932	1.605176014	0.108455075	0.271162377
7.881066322	0.12557817	0.392193478	0.320194438	0.748820939	0.86855426
3.383480351	-0.176347044	0.575108735	-0.306632526	0.759123094	0.874286482
359.7227588	0.167199107	0.060841968	2.748088427	0.005994384	0.036014399
283.7691986	-0.26516085	0.094604591	-2.802832774	0.005065593	0.031790695
195.4940572	0.02959599	0.16386123	0.180616181	0.856668854	0.92936531
7.952554106	-0.686449053	0.420729745	-1.631567679	0.102770592	0.26200694
14.90029072	-1.746496496	0.484294233	-3.606271509	0.000310628	0.00382744
66.46366765	-0.018769356	0.159071831	-0.11799296	0.906073236	0.952959157
26.93201865	0.501146188	0.296346734	1.691080517	0.090821426	0.241282401
6.520892223	0.081586011	0.575959933	0.141652234	0.887354706	0.943568846
20.00368651	1.788745497	0.720706949	2.481931801	0.013067228	0.063726915
19.91261882	1.931205993	0.66385159	2.909092971	0.00362479	0.024952982
16.7683677	1.295603625	0.514694099	2.517230384	0.011828146	0.059335224
57.33170211	0.166641665	0.278976387	0.597332509	0.550285404	0.736546229
318.102539	0.021875361	0.120764625	0.181140472	0.856257318	0.92936531
581.2585225	0.114286597	0.062093873	1.840545486	0.065688193	0.195359761
181.4996498	0.781280885	0.10592806	7.375580057	1.63631E-13	3.7967E-11
2.748723586	-0.17007651	0.602356477	-0.282351924	0.777673675	0.884312122
348.0627977	0.064965162	0.080511891	0.80690146	0.419723258	0.628649963
201.0071948	-0.557523844	0.151290064	-3.685131914	0.000228584	0.00304668

950.1726627	0.039998457	0.077671172	0.514971721	0.606572782	0.776311374
10.44461385	0.835663109	0.389450817	2.145747477	0.031893131	0.118583529
651.6035423	0.062898116	0.058301468	1.078842759	0.28065783	0.494308965
852.4361025	-0.038789942	0.053291852	-0.727877529	0.46668856	0.668563317
68.07904548	-0.564392499	0.160951349	-3.506603094	0.000453866	0.005163661
5125.513694	0.034578893	0.104314816	0.331485922	0.740277473	0.863447401
603.8861802	0.071541558	0.094156019	0.759819267	0.447362624	0.652487992
49.65104224	-0.902642336	0.420502889	-2.146578201	0.031826877	0.118471335
304.587164	0.320358935	0.074464492	4.302170422	1.69133E-05	0.00037122
1692.195195	-0.098224426	0.064040119	-1.533795188	0.125080047	0.295973671
1018.542878	-0.052526673	0.061481994	-0.854342371	0.392915317	0.606245849
363.4226863	0.106449199	0.059604537	1.785924437	0.074111494	0.211719093
486.6914729	-0.31157222	0.164192562	-1.897602524	0.057748462	0.178522829
98.86926836	0.165131453	0.157932105	1.045585081	0.29575264	0.510408791
47.83816795	-0.126394971	0.1746971	-0.72350927	0.469367061	0.670512744
26.34657394	-0.386863212	0.247182637	-1.565090563	0.117561676	0.285344488
89.8907533	-0.080132804	0.108361116	-0.739497768	0.459604795	0.662483978
29.3714513	0.026931814	0.195448435	0.137794982	0.89040245	0.945256474
61.95027563	-0.11471917	0.220622164	-0.519980262	0.603077332	0.773279575
457.5900134	-0.19344758	0.10311416	-1.876052511	0.06064807	0.185158692
92.51141537	1.170496292	0.173035786	6.764475255	1.33793E-11	1.86789E-09
268.0160869	0.268563764	0.078245549	3.432319996	0.000598441	0.00644782
1185.565834	0.086977671	0.045700834	1.903196607	0.057014896	0.176819163
160.4491358	-0.376876077	0.097448652	-3.867432429	0.000109987	0.001690234
168.9333403	-0.205647372	0.072389889	-2.840830067	0.004499628	0.029137923
83.54839111	0.799914026	0.211088942	3.789464376	0.000150972	0.00216837
24.88648636	0.691009744	0.225124279	3.069459003	0.002144468	0.016984602
181.4086572	-0.333893162	0.098480007	-3.390466483	0.000697738	0.007284242
961.9506719	0.205646863	0.09701278	2.119791452	0.034023636	0.123841226
3914.314593	-0.393452773	0.126527331	-3.10962675	0.001873239	0.015422158
315.6110256	0.463967989	0.1202865	3.857190866	0.000114698	0.001749563
300.5641426	0.07359027	0.096706213	0.760967347	0.446676571	0.651775892
3796.427345	-0.371192344	0.125585079	-2.955704193	0.003119561	0.022402119
46.60120006	-0.070775966	0.137478928	-0.514813195	0.606683564	0.776392841
1361.951773	-0.059026623	0.090684281	-0.650902483	0.515109438	0.708319729
6.070693938	0.329759744	0.438394634	0.752198404	0.451931753	0.656598889
5.917629907	0.132912963	0.440113503	0.301997012	0.76265434	0.8761511
1863.525662	-0.272291488	0.093727763	-2.905131634	0.003670987	0.025156338
27.80768818	1.498450434	0.520087227	2.881152153	0.003962243	0.026492884
15457.77449	-0.116878519	0.086203099	-1.355850549	0.175146724	0.369350631
792.6857791	0.098785596	0.056063207	1.762039716	0.078062587	0.218596475
1549.704541	-0.412929341	0.065810267	-6.274542894	3.50663E-10	3.22727E-08
48.93016206	0.103829614	0.143180644	0.725165156	0.468350716	0.670018398
2101.833453	-0.183185124	0.062918609	-2.911461762	0.003597419	0.02484606
6.064647184	-0.200793961	0.36861006	-0.544732721	0.585937357	0.760836448
2832.125402	-0.362938419	0.114969751	-3.156816602	0.001595016	0.013742834
3.631082561	-0.736314218	0.561506332	-1.311319526	0.189749834	0.389476547
17.10366802	-1.021889612	0.45106954	-2.265481307	0.02348316	0.096019254

10.84320164	-1.357013874	0.640979143	-2.11709521	0.034251768	0.124448658
361.4633453	0.129570421	0.119327142	1.085841984	0.277548908	0.490556716
124.4170019	0.054245844	0.105787166	0.512782841	0.608103234	0.777484879
210.2884347	-0.055888019	0.096501394	-0.579142095	0.562493298	0.745278458
1.94683356	0.727444877	0.85796232	0.847875087	0.396507541	0.609334442
219.5860391	0.167782416	0.181683418	0.923487776	0.355753068	0.57032659
19.37227124	0.24674635	0.236319674	1.044121064	0.296429382	0.511207508
224.5903011	-0.046974045	0.094034634	-0.49953983	0.617399134	0.783755788
23.25972859	0.510312396	0.211334517	2.414713904	0.015747573	0.072708948
72.97277022	-0.756638198	0.165940115	-4.559706366	5.12252E-06	0.00013611
64.4706908	0.020619446	0.161077836	0.128009206	0.898141688	0.948475137
281.4477952	-0.198371974	0.090480757	-2.192421687	0.028349072	0.109624542
795.7594912	0.162891129	0.133868082	1.216803338	0.22367905	0.428769484
1634.337748	-0.170497037	0.120539917	-1.414444623	0.157231396	0.3435774
427.2867657	0.22750831	0.141614714	1.606530161	0.108157474	0.270732921
368.9869846	-0.107935742	0.085016833	-1.269580829	0.204233982	0.407201766
81.73254284	-0.246991046	0.14626459	-1.688659195	0.09128476	0.24183615
731.6384917	0.108799575	0.06396577	1.700903071	0.08896119	0.238171511
2340.323041	-0.401139468	0.119981601	-3.343341522	0.00082776	0.008309879
415.5703882	0.06708524	0.092177103	0.727786373	0.466744367	0.668563317
3121.352671	-0.086560331	0.077177648	-1.121572544	0.262044231	0.473242344
52.33706756	-0.29082433	0.205479904	-1.415341961	0.156968258	0.343230034
379.4412943	-0.30749471	0.167174804	-1.839360379	0.065862198	0.195674273
138.573061	-0.954150969	0.348417661	-2.738526418	0.00617152	0.036850172
173.0603633	-0.237019219	0.146527225	-1.617578026	0.105753568	0.266633651
12.11273058	-1.402985238	0.925043782	-1.516669011	0.129350272	0.302000621
4026.112983	-0.533786865	0.256422659	-2.081668085	0.037372798	0.132575252
163.1066342	-0.286202682	0.108199893	-2.645129059	0.008165977	0.045432724
1424.403587	-0.205495319	0.066042957	-3.111540246	0.001861141	0.015345564
12.98727454	-0.39886184	0.27887189	-1.430269075	0.152639807	0.337030985
4.923983528	1.80029031	0.591474003	3.043735314	0.002336606	0.018131536
3.705053249	0.219727268	1.15329904	0.190520637	0.848901173	0.925471374
613.758975	-0.009363769	0.088809681	-0.105436359	0.916029566	0.958442114
250.8394619	0.081955982	0.066738198	1.228022111	0.219438668	0.423921325
126.0137806	-0.068778848	0.260318459	-0.264210415	0.791617786	0.892371111
13.5423717	-0.184144484	0.268484966	-0.685865158	0.492798143	0.690017157
452.7824913	0.114910008	0.056851453	2.021232571	0.043255696	0.146835839
3220.886594	-0.035613751	0.072383716	-0.492013298	0.622709943	0.787723535
369.1895012	-0.208576882	0.106225813	-1.963523507	0.049585376	0.161213633
359.5955833	-0.089087617	0.057837137	-1.540318579	0.123482717	0.293792408
145.0452297	-0.151904219	0.102574604	-1.480914507	0.138629357	0.316927564
7.729490561	1.365016168	0.782871839	1.74360106	0.081228678	0.224657382
112.5178772	-0.101119341	0.215851033	-0.468468182	0.63944982	0.798613269
225.0221871	-0.187982555	0.26539429	-0.70831424	0.478750136	0.678095584
105.5997827	0.27165382	0.141634978	1.917985403	0.055112862	0.172282597
370.5954772	-0.050206519	0.070168073	-0.715517991	0.474289028	0.674262616
428.0954811	0.348896933	0.076340972	4.570244861	4.87155E-06	0.000130494
6.190038815	-2.222432854	1.09591403	-2.027926272	0.042567773	0.14524886

39.75743499	-0.179724934	0.189234314	-0.949748118	0.342240254	0.556467099
98.27221908	-0.487168978	0.129479861	-3.762507723	0.000168218	0.002380867
5.401250483	-3.096107737	1.675671216	-1.847682116	0.064648343	0.193183378
1261.433197	-0.100961112	0.044466205	-2.270513337	0.023176455	0.095048275
8.922835034	-1.693714047	0.783751067	-2.161035714	0.030692581	0.115815293
8.983831343	-0.379478528	0.40837142	-0.929248495	0.35276032	0.567794188
192.2296524	-0.846427009	0.232907284	-3.634180059	0.000278866	0.003561271
16.51270699	-0.196955859	0.24248766	-0.81223044	0.416659416	0.626095433
215.7823383	0.207273418	0.068265159	3.036298774	0.002395019	0.018459493
104243.3436	-0.004015632	0.08857744	-0.045334699	0.96384053	0.982021701
141.5482837	-0.281770819	0.138977104	-2.027462158	0.042615171	0.145351534
7.467280939	1.287448225	0.471232233	2.732088628	0.006293421	0.037347916
269.2865807	-0.261044397	0.104463281	-2.498910584	0.012457574	0.061629451
206.5094423	-0.291919175	0.079026475	-3.693941501	0.000220805	0.002964576
316.044956	0.013915238	0.107309257	0.12967416	0.896824227	0.947955013
53.49405548	-0.008340995	0.177835308	-0.046902918	0.962590602	0.981463149
2501.078579	-0.058204474	0.047149648	-1.234462537	0.217030598	0.422021019
220.6164153	-0.097740306	0.091847622	-1.064157175	0.287257503	0.501827718
361.6238679	0.113835401	0.09001681	1.264601584	0.206014185	0.409517791
290.622192	0.084766123	0.082493195	1.027552925	0.304160178	0.518817019
132.3320212	0.076431476	0.134997446	0.566169797	0.57127836	0.751109865
383.5376234	0.107920572	0.068092136	1.584919755	0.112984567	0.278517
23.79926681	0.16499759	0.206538503	0.798870855	0.4243653	0.631868578
6.744387158	-0.380626931	0.441227343	-0.862654904	0.388327234	0.601532943
2116.358862	0.272316638	0.052397996	5.19708114	2.02442E-07	8.46454E-06
21.85634747	0.536893808	0.2285805	2.348817196	0.01883315	0.082230936
6.470984567	0.57450214	0.381824598	1.504623176	0.132420984	0.306908173
961.3339748	0.096206075	0.094379742	1.019350902	0.308036406	0.523045945
15.55242615	-0.244894065	0.301719286	-0.811661951	0.416985633	0.626371962
545.5132916	-0.264084142	0.111749898	-2.363171223	0.018119296	0.079987271
167.4727133	0.173252675	0.094430342	1.83471404	0.066548071	0.197072249
2.4701656	0.366840404	0.591125767	0.620579282	0.534876475	0.72384441
225.5673722	0.040191462	0.083871032	0.47920553	0.631792421	0.79384855
115.348593	-1.055468694	0.392553063	-2.688728717	0.007172468	0.041098866
26.00346221	-0.49093447	0.314239231	-1.562295285	0.118218442	0.286233189
775.8876683	0.361735364	0.065174646	5.550246678	2.85267E-08	1.49665E-06
1197.424874	-0.601992124	0.202191818	-2.977331776	0.002907692	0.021308413
2176.20016	0.223857712	0.074845028	2.990949688	0.002781113	0.020628571
37.20128159	0.40149124	0.182465733	2.200365153	0.027780998	0.108220422
309.9548867	-0.187002794	0.059343129	-3.151212252	0.001625943	0.013905748
1449.2447	-0.122350969	0.099373756	-1.231220127	0.218240546	0.422975853
256.6632336	-0.005160972	0.087050064	-0.059287399	0.952723198	0.977124952
5.423579178	0.339608821	0.40262273	0.843491427	0.398953658	0.611199292
4375.473809	-0.063005805	0.07188951	-0.876425565	0.380798725	0.593610885
352.4074253	0.19232795	0.063061516	3.049846587	0.002289583	0.017847565
1620.663886	0.058609125	0.048873608	1.199197842	0.230451026	0.436936024
1245.884207	0.114905779	0.092829925	1.237809666	0.215786644	0.420332559
2.476603078	0.012667804	0.593762599	0.021334796	0.982978587	0.991585894

128.9495393	-0.039633563	0.097586288	-0.406138649	0.684640729	0.829651922
792.555424	-0.027638672	0.049224712	-0.561479602	0.574470632	0.753369622
149.1244816	0.051544956	0.206118412	0.250074488	0.802529745	0.899069005
268.826859	-0.450606277	0.078276154	-5.756622593	8.58135E-09	5.39816E-07
419.7158422	-0.027909749	0.084078767	-0.331947643	0.739928794	0.863255748
173.8556122	0.022273565	0.091185462	0.244266629	0.807024327	0.901693069
51.46068353	0.164950532	0.13568252	1.215709519	0.224095597	0.429184035
1512.267437	0.130450714	0.074270482	1.756427447	0.07901545	0.220365755
1478.613283	0.377042148	0.102573964	3.675807525	0.000237098	0.003127267
3.224910975	0.203118413	0.919237945	0.220963912	0.825120532	0.912223049
511.966529	-0.112651861	0.053423599	-2.10865352	0.0349745	0.126297653
4.79741904	0.155333886	0.442218401	0.351260565	0.725392877	0.854556797
242.3125789	-0.308659712	0.130053134	-2.373335439	0.017628246	0.07857352
251.2439111	0.096358416	0.164123287	0.587109956	0.557129862	0.741574435
2.773253876	0.195914504	0.93348578	0.209874117	0.833765924	0.916902912
32.77079069	0.190571976	0.310234989	0.614282667	0.539028554	0.726733481
83.12175343	0.547214126	0.135527703	4.037655127	5.39881E-05	0.000957374
29.95372341	-0.054916649	0.179162836	-0.306518084	0.759210213	0.874325693
641.5689936	0.078936896	0.063149122	1.250007818	0.211296691	0.415530822
61.39628625	0.781114352	0.249104907	3.13568432	0.001714536	0.014396164
2.803789524	1.644176256	0.818996229	2.007550461	0.044691083	0.150222588
22.57678435	-0.585463349	0.238831256	-2.451368214	0.014231429	0.067700999
96.02418101	-0.06368198	0.098153031	-0.648802988	0.516465726	0.709316912
2013.410378	-0.454393907	0.085715622	-5.301179626	1.15057E-07	5.06804E-06
300.4543398	-0.071356058	0.057993415	-1.230416553	0.218541158	0.423060756
98.06139388	-0.690145237	0.317608674	-2.17294203	0.029784681	0.113450365
16.10059646	0.01772207	0.240580008	0.073663936	0.941277795	0.972079388
218.2497633	-0.277566699	0.106127212	-2.615414965	0.008911911	0.048469733
711.2231359	0.003676491	0.089002697	0.041307636	0.967050646	0.983639473
3485.590971	-0.022571273	0.036630719	-0.616184287	0.537772898	0.725807075
69.29525977	-0.015554928	0.120708292	-0.128863791	0.897465428	0.948210116
97.41421332	-0.14559402	0.173855367	-0.837443346	0.402343409	0.613826573
601.9788317	0.496556754	0.107116851	4.635654899	3.55809E-06	9.98569E-05
47.605738	-0.04093448	0.200444192	-0.204218838	0.838182491	0.919138545
42.75641424	0.200973839	0.259477212	0.774533676	0.438615262	0.644328698
65.66147839	-0.42732405	0.131601583	-3.247104182	0.001165857	0.010869456
3.117622366	-0.416278604	0.565134188	-0.736601346	0.461364816	0.6638592
4.280259098	-0.817095378	0.54279034	-1.505360942	0.132231305	0.306770669
61.89979016	0.219979743	0.156314198	1.40729214	0.159340779	0.346773473
27.6683118	-0.387678389	0.247697291	-1.565129706	0.117552499	0.285344488
38.36493073	0.343217371	0.176941973	1.9397171	0.05241408	0.166874627
12.79337068	-0.024585257	0.293458811	-0.083777539	0.933233307	0.96844633
671.4076302	-0.070529407	0.111572702	-0.632138555	0.527296344	0.717492357
11.55685416	0.966568488	0.393867342	2.454045777	0.0141259	0.067432649
79.42508208	-0.429103979	0.133968673	-3.203017307	0.001359958	0.01218648
575.5311432	-0.078779101	0.171416834	-0.459576225	0.645820427	0.803568408
23.9230297	0.42218233	0.465757676	0.906442022	0.364701939	0.578202266
532.7926596	0.223813305	0.094018393	2.380526816	0.017287903	0.077606021

12.80071109	0.956980076	0.33127557	2.888773462	0.003867476	0.026033877
16.69734215	0.40390873	0.294163507	1.373075586	0.16972885	0.36172226
9.553395777	0.765958333	0.408134082	1.876732098	0.060554821	0.184942551
369.1399346	-0.108994148	0.080969807	-1.346108541	0.178267515	0.373921446
250.7778301	-0.717153934	0.174452093	-4.110893264	3.94131E-05	0.000737832
15.25992885	0.821243543	0.414333681	1.982082514	0.047470006	0.156651803
2.544026006	0.728429321	0.759057825	0.959649315	0.337231741	0.552571682
1046.580062	0.007568238	0.112086398	0.067521467	0.946166573	0.974440367
5.27501177	0.767278448	0.4809537	1.595327051	0.110639119	0.275036797
48.1991038	0.21814738	0.15936055	1.368891984	0.171033042	0.363279826
3.628337332	-0.162775259	0.463350959	-0.351300146	0.725363185	0.854556797
5.021551774	-0.37555925	0.570393854	-0.658420926	0.510267692	0.704622797
25.09648295	-0.03900212	0.231947186	-0.168150866	0.866464594	0.933868226
65.89503153	-0.199084801	0.145714285	-1.366268244	0.171854784	0.364414431
119.4708001	-0.138457386	0.110409905	-1.25403048	0.209830927	0.414034162
151.2141022	-0.674046694	0.279585679	-2.410877038	0.015914213	0.073152594
1481.245866	0.113461528	0.188793819	0.600981158	0.547852536	0.734560988
31821.91495	-1.044980635	0.419430928	-2.491424845	0.012723188	0.062511721
82585.18062	0.137217266	0.230248226	0.595953629	0.551206205	0.737179008
703115.6394	0.102704596	0.133214301	0.770972753	0.440723082	0.646478459
771.9192208	-0.263265422	0.147853147	-1.780587204	0.074979912	0.21326296
38.8286677	0.715165315	0.386065719	1.852444494	0.063962021	0.191758023
418.1443722	-0.237708275	0.156132735	-1.522475573	0.127889961	0.299567642
4.602784405	1.286606551	0.614837583	2.092595811	0.036385251	0.130027973
2152.985827	0.425914151	0.150376648	2.832315771	0.004621219	0.029703456
10.86987451	0.409760593	0.372602971	1.099724439	0.271452203	0.483344531
2.789002778	-0.391726773	0.686418237	-0.570682351	0.568214985	0.74897935
175.1690883	-0.02798969	0.08932618	-0.313342514	0.754020454	0.871653489
408.3505169	-0.095564315	0.126598696	-0.754860184	0.450332878	0.655292472
31.21760245	0.259009191	0.534327302	0.48473883	0.627861619	0.791096258
69.42235213	0.530253579	0.152277847	3.482145232	0.000497414	0.005548
6.277646345	0.169426839	0.426901159	0.396876034	0.691458878	0.833840389
616.3910156	-0.01041009	0.205769742	-0.050590966	0.959651462	0.980337668
780.7090705	0.064693006	0.087528472	0.73910814	0.459841334	0.662623194
6.009034861	2.121274985	0.62291297	3.405411493	0.000660644	0.00695873
258.6434373	0.873463322	0.1114562	7.836830248	4.62062E-15	1.35929E-12
74.59376616	-0.154650082	0.157488796	-0.981975136	0.326112098	0.54102424
134.6800714	-0.207186037	0.195713073	-1.058621348	0.289772261	0.504673668
364.6987223	0.031362022	0.204289178	0.153517782	0.877989968	0.938489926
218.8219205	-0.013797031	0.076583835	-0.180155911	0.85703017	0.92936531
10.71989895	-1.763038412	0.733085609	-2.404955697	0.016174426	0.073956537
252.4794596	0.319933732	0.089744749	3.564929824	0.000363954	0.004357392
17.22354937	-0.952328597	0.291086905	-3.271629817	0.001069295	0.010147214
490.6361672	0.198042629	0.05576506	3.55137479	0.000383224	0.004541897
1505.910466	0.588890221	0.115472949	5.099811026	3.39993E-07	1.33358E-05
50.86799722	-0.045485058	0.213097608	-0.21344706	0.830978278	0.915614595
164.9052241	-0.441667518	0.10649292	-4.147388567	3.36289E-05	0.000654076
3.9727685	-0.131371232	0.60358575	-0.217651314	0.8277008	0.913863048

4.362884468	-0.574862771	0.556496393	-1.033003588	0.301602219	0.516736917
347.5951365	0.316825446	0.159313104	1.988696715	0.046734689	0.15512941
66.82381532	0.154266196	0.136240926	1.132304368	0.257506494	0.467957282
119.2433996	-0.417125271	0.132185683	-3.155600973	0.001601678	0.013785813
78.96653096	0.638404211	0.139844559	4.565098673	4.99259E-06	0.000133087
1118.187508	0.031566015	0.096074214	0.328558656	0.742489302	0.86500393
9.194418472	0.158936366	0.468240684	0.339433056	0.73428352	0.859926545
16.91077283	0.423292441	0.30649879	1.381057463	0.167261293	0.358524052
67.53495601	0.021582846	0.119576944	0.180493376	0.856765254	0.92936531
206.3360805	0.134575313	0.091417364	1.472097938	0.140994432	0.320201582
2614.957882	-0.342026302	0.145843684	-2.345156766	0.019019081	0.082899546
36.05152141	-0.127642714	0.180376077	-0.707647689	0.47916407	0.678327657
1726.999885	0.349113915	0.090531497	3.85627022	0.00011513	0.001752916
3.46557237	0.613103238	0.646173815	0.948820928	0.342711696	0.556843129
101.2059318	-0.205383323	0.167487218	-1.226262672	0.220099844	0.424631911
67.46567316	-0.686516007	0.2843362	-2.414451646	0.015758915	0.072720549
928.0227287	-0.210454694	0.060559685	-3.475161638	0.000510546	0.005673753
418.5305944	0.04879324	0.052445852	0.930354612	0.35218751	0.567204717
5.331715467	0.691900335	0.404324433	1.711250366	0.087034905	0.234572086
38.79015011	0.413704109	0.196167089	2.108937389	0.034949986	0.126236807
1634.084405	-0.038089875	0.046598426	-0.817406904	0.413695931	0.623476971
1156.222426	0.426427152	0.06783397	6.286336371	3.25045E-10	3.0425E-08
374.5270122	0.733720506	0.1353436	5.421168829	5.92106E-08	2.85215E-06
85.23902907	1.063569284	0.216380974	4.915262475	8.86636E-07	3.10775E-05
144.8629714	0.737411038	0.115340192	6.393357133	1.62283E-10	1.68141E-08
228.5013673	0.088081311	0.078709715	1.119065299	0.263112283	0.474443054
124.1406288	0.27797881	0.09044215	3.07355377	0.002115256	0.016848533
5.993391902	-0.092848661	0.447115306	-0.207661559	0.835493235	0.917771165
47.65020073	0.049997434	0.165441329	0.302206432	0.762494701	0.87608716
42.77827182	1.408868741	0.636059676	2.214994589	0.026760436	0.105643763
8.354081549	0.09061179	0.369451275	0.245260462	0.806254763	0.901224113
763.7688948	0.104770634	0.05460953	1.9185412	0.055042423	0.172184398
307.8356136	0.064191025	0.088487192	0.725427295	0.468189933	0.66992173
2923.644804	0.014876859	0.046868459	0.317417295	0.750926986	0.870013027
176.9743282	0.337939124	0.18138123	1.863142747	0.062442185	0.18860883
6011.651962	0.159115674	0.072566227	2.192695976	0.028329291	0.109587755
2.123534909	0.862503349	0.72502455	1.189619509	0.234195974	0.441189294
223.0747077	-0.163877797	0.071482387	-2.292561903	0.02187324	0.09152648
1576.930029	0.09036281	0.073422558	1.230722705	0.218426593	0.423029367
1000.23759	-0.326883175	0.056143991	-5.82222909	5.80679E-09	3.98588E-07
204.2716513	0.068769216	0.067388356	1.020491077	0.307495619	0.522484231
130.2404208	0.320146823	0.118753638	2.695890648	0.007020071	0.040394218
4011.365526	-0.186432117	0.123881193	-1.504926711	0.13234292	0.30681677
3.14063672	0.270677561	0.607805758	0.445335632	0.656077221	0.810697281
107.9214373	0.014103201	0.115114794	0.122514235	0.902491773	0.951164319
2.487517871	-0.283728698	0.708912833	-0.400230727	0.688986585	0.832138197
76.53892044	-0.403279649	0.195405389	-2.06381027	0.039035709	0.136275993
83.13991723	-0.452826469	0.247690169	-1.828197181	0.067519964	0.198877863

14.86615773	1.071245953	0.34066472	3.144575564	0.00166328	0.014109612
25.13420125	0.731696819	0.22915593	3.193008448	0.001407988	0.012524405
173.2042306	0.99902125	0.213701375	4.674847094	2.94173E-06	8.54709E-05
164.6143454	0.267897337	0.076234386	3.514127275	0.000441201	0.005065053
382.8438681	-0.016923099	0.079157461	-0.213790321	0.830710574	0.915614595
64.18326541	-0.197526914	0.318524629	-0.620130742	0.535171714	0.72384441
2.408926553	0.449247998	0.700362583	0.641450599	0.521229968	0.713138885
449.201353	0.117297557	0.064701006	1.812917041	0.069844618	0.203433563
113.958493	-0.181197412	0.190068287	-0.953327961	0.340423929	0.555270316
40.08393763	-0.352647984	0.170178703	-2.072221597	0.03824478	0.134740058
27.11411421	0.339799767	0.287988293	1.179908264	0.238036703	0.445615527
8.655311812	1.463358244	0.522542674	2.800456915	0.005103032	0.03197693
1.743575186	-0.101170459	0.955573654	-0.105874057	0.915682278	0.958322206
1147.01326	0.033939489	0.062005744	0.547360404	0.584131153	0.759621118
246.4882279	0.211433761	0.078370177	2.69788545	0.006978145	0.040209149
695.8891017	0.215041526	0.090658118	2.372005185	0.017691842	0.078766881
219.9291489	0.677564848	0.239425136	2.829965387	0.004655304	0.029783098
30.74508334	0.762586221	0.357333821	2.134100319	0.03283457	0.121037526
93.61116978	-0.194252486	0.163196878	-1.190295355	0.233930326	0.440833698
3258.041264	0.043851576	0.12559177	0.349159632	0.72696947	0.855557584
387.0401368	0.082299759	0.068064637	1.209141236	0.226608589	0.432232145
1828.145657	0.028039868	0.057803506	0.485089407	0.627612926	0.790978857
59154.54848	0.335272574	0.118721252	2.824031658	0.00474237	0.030187714
472.791865	-0.043959614	0.070612482	-0.622547355	0.533582015	0.722790508
956.4750636	-0.088180694	0.073688445	-1.196669216	0.23143551	0.438057285
43.33638515	0.11010651	0.158059565	0.696614026	0.486044376	0.684893939
15196.84718	0.271560461	0.069434861	3.911010348	9.19108E-05	0.001465769
184.3300662	0.433362053	0.094783894	4.57210643	4.82846E-06	0.00012955
888.565554	-0.174903865	0.084160742	-2.078212019	0.037689836	0.133347821
470.3719231	0.035415576	0.059761231	0.592617917	0.553436885	0.738722999
58.13912257	-0.149130372	0.143981062	-1.035763806	0.300312347	0.515122671
627.5320618	0.043864469	0.077477542	0.566157215	0.571286912	0.751109865
250.2838986	0.076782774	0.071706507	1.070792286	0.284262836	0.498344787
178.1821976	-0.142797968	0.069569095	-2.052606361	0.04011077	0.139015646
80.74436971	-0.248645885	0.129063745	-1.926535483	0.054037546	0.170131231
5.612212816	1.082324494	0.524220897	2.064634393	0.038957607	0.136202803
6.263663908	-0.708624047	0.477819063	-1.483038459	0.138064193	0.315986318
2.035039082	-2.160390014	1.204065996	-1.794245516	0.07277399	0.209264918
50.42022306	0.722660611	0.150083988	4.815041365	1.47169E-06	4.82002E-05
5.946503753	-0.624068217	0.633441012	-0.985203365	0.324524188	0.539655353
163.8778325	0.966423529	0.196106948	4.928043276	8.30572E-07	2.95526E-05
64.35135037	0.130738868	0.143419032	0.911586606	0.361986376	0.575947803
189.1219138	-0.176620287	0.098330551	-1.796189331	0.072464411	0.208775569
55.72082911	-0.305372611	0.135848609	-2.247889124	0.024583261	0.099407128
10.75177536	0.181623218	0.3137565	0.578866791	0.56267906	0.745322411
3.560492869	-4.122292843	1.525058224	-2.703039647	0.006870855	0.039883885
36.01444273	0.332327465	0.193712781	1.715568084	0.086241124	0.233251027
21.08864807	-0.453406227	0.415096326	-1.092291593	0.274704954	0.487397891

11.00524366	-0.525368043	0.522629066	-1.005240766	0.31478093	0.529679101
4650.702401	-0.246420905	0.159702114	-1.543003395	0.122829952	0.292878945
6.244278071	0.267837425	0.525667216	0.509518983	0.610388496	0.779257601
1417.603435	0.152793067	0.049278843	3.10058145	0.001931411	0.015790602
935.1258951	0.426083261	0.256116623	1.663629856	0.096186422	0.249828924
17.05418414	0.856306573	0.303778121	2.818855323	0.004819524	0.030589661
1590.103164	-0.316515879	0.117033574	-2.704487858	0.006840977	0.039803591
472.9525598	-0.060654119	0.081169121	-0.747256075	0.454909001	0.659117932
174.1384952	-0.486280488	0.164722314	-2.952122727	0.003155975	0.022595188
1076.438881	0.103265651	0.037913537	2.723714493	0.006455228	0.038156954
415.5574751	0.328903409	0.240380772	1.368260056	0.171230689	0.363512162
15.4040738	-0.210697755	0.382313121	-0.551113063	0.581556171	0.758146246
18.11943453	0.061340624	0.282365401	0.217238456	0.828022517	0.914087062
199.1928637	0.022741365	0.086456911	0.263036983	0.792522074	0.893084934
17.5493869	0.751985161	0.683545158	1.100125065	0.271277634	0.483274606
7.344935434	-0.110162956	0.550753781	-0.20002215	0.841463258	0.921448133
112.0498885	-0.218662726	0.151358845	-1.44466434	0.148552191	0.331175143
573.8024082	-0.152361258	0.082178862	-1.854020045	0.063736292	0.191277385
54.77376567	-0.04368211	0.196519013	-0.222279307	0.82409646	0.911434821
112.2242123	0.479243564	0.100791991	4.75477823	1.98664E-06	6.22007E-05
43.6341127	-0.415056253	0.225729349	-1.838734106	0.065954304	0.19580667
15.02378465	0.145320766	0.322503044	0.450602774	0.652275867	0.807757095
4.029549416	-0.768676351	0.502712582	-1.529057315	0.126250236	0.297545978
73.95439793	-0.965951671	0.22081665	-4.374451247	1.21738E-05	0.000281702
1.720617788	-2.134490866	1.109553162	-1.923739158	0.054387287	0.17069574
367.0079162	0.847929105	0.178582079	4.748119792	2.05316E-06	6.36984E-05
10.2324109	-1.084994003	0.614708295	-1.765055087	0.077554508	0.217728862
3933.253767	0.148959222	0.059335176	2.510470721	0.012057032	0.060135497
3035.235664	-0.169848752	0.086056232	-1.973694973	0.048416437	0.158661514
166.7047917	-0.319954324	0.081475581	-3.926996543	8.60132E-05	0.001387837
324.9387366	0.386446606	0.078599472	4.916656509	8.80348E-07	3.0989E-05
121.8894898	0.02657203	0.108419881	0.245084481	0.806391019	0.901254115
287.9640855	-0.326712123	0.10129914	-3.2252211	0.001258754	0.011506219
3.303834992	-0.544937175	0.565814705	-0.963101825	0.335496426	0.550604515
124.5448955	-0.309853609	0.119836282	-2.585641048	0.00971981	0.051586386
628.4872299	-0.003845734	0.047417545	-0.081103598	0.935359565	0.969553481
241.4675087	0.354645558	0.132286877	2.68088238	0.007342832	0.041879287
59.63024134	-0.13314098	0.144613041	-0.920670635	0.357222412	0.571736327
75.77275179	0.019251159	0.132621484	0.145158677	0.884585591	0.942458827
110.7033605	-0.31800473	0.324312175	-0.980551315	0.326814052	0.541951237
868.8891233	0.079997103	0.059503391	1.344412508	0.178815031	0.374375724
116.3866754	-0.027563808	0.118210325	-0.233175973	0.815624763	0.907449338
540.4924769	-0.333203909	0.168720165	-1.974890848	0.048280536	0.158377848
3.400920627	-1.276906904	1.353941169	-0.943103684	0.345627872	0.560034775
333.0198448	-0.125049146	0.088980287	-1.405357863	0.15991489	0.347551174
294.7650303	-0.341747953	0.158466075	-2.156600097	0.031036837	0.116495979
28.16864782	-0.421962485	0.209985921	-2.009479894	0.044486265	0.149748003
3844.218953	-0.068767876	0.023854936	-2.882752533	0.00394217	0.026388992

395.8003895	-0.144291009	0.084868236	-1.700176824	0.08909767	0.238239737
127.8916854	0.58030732	0.096872747	5.990408441	2.09315E-09	1.60384E-07
426.0003595	-0.275079771	0.164142764	-1.67585682	0.093766241	0.246050503
1887.640346	0.331403315	0.098584003	3.361633799	0.000774828	0.007898835
259.6156922	0.21821648	0.115369797	1.89145241	0.058563978	0.180467204
399.6396047	0.105668936	0.062990911	1.677526708	0.093439531	0.245505048
1468.020318	0.125229868	0.138409117	0.904780485	0.365581692	0.579095462
1164.412809	-0.203210716	0.063074701	-3.221746771	0.001274117	0.011615828
4591.918756	0.097707972	0.079319647	1.231825605	0.218014236	0.422815921
18.45449374	0.128571785	0.269755925	0.476622652	0.63363085	0.794703816
141.9977665	0.104720465	0.088012378	1.189837927	0.234110099	0.441122015
248.1979637	0.246974736	0.171387291	1.441032965	0.14957537	0.332560838
361.3092624	0.095938442	0.060430811	1.587574939	0.112382489	0.277652838
12.36530008	0.107431743	0.282053216	0.380891751	0.703283574	0.840959105
91.44108054	0.056785045	0.139024079	0.408454748	0.682939846	0.828594957
273.119221	-0.105048449	0.067579299	-1.554447157	0.120077792	0.289331805
136.029758	-0.109120672	0.15476406	-0.705077601	0.480761945	0.679756282
600.2251063	0.021074975	0.100623946	0.209442936	0.834102478	0.916961029
274.6539666	-0.12903527	0.085041258	-1.517325501	0.129184523	0.301699153
278.7656357	0.514141712	0.109868212	4.679622079	2.87404E-06	8.3983E-05
550.0134691	-0.838381873	0.491713677	-1.705020445	0.088190611	0.23685232
371.4090602	-0.072158928	0.061292783	-1.177282623	0.23908272	0.446593139
1928.800741	-0.430570522	0.071586085	-6.014723672	1.80194E-09	1.41358E-07
3.43296845	0.324131652	0.636446709	0.509283256	0.610553693	0.77930251
300.5538626	-0.045239032	0.08424973	-0.536963518	0.591292814	0.764264772
26.27300962	-0.297514678	0.293008482	-1.015379065	0.309925168	0.525116448
119.127127	0.208534914	0.110883604	1.880665012	0.060017502	0.183641963
12.20777004	0.569845423	0.288505362	1.975164061	0.048249533	0.1583213
1906.208409	-0.011730846	0.076728932	-0.152886871	0.878487489	0.93889989
1428.063703	-0.361836658	0.124906633	-2.89685703	0.003769215	0.025553105
256.7717133	0.354202121	0.094889161	3.732798524	0.000189364	0.00261053
808.3650241	0.200540975	0.046739161	4.290641347	1.78158E-05	0.000387451
87.98485829	0.163084304	0.117919263	1.383016648	0.166659761	0.357632536
86.04319105	-0.67212908	0.343468739	-1.956885746	0.050360903	0.162838644
3035.084119	-0.102665435	0.067801538	-1.514205107	0.129973827	0.303069897
1189.149595	-0.07856568	0.079816688	-0.984326476	0.324955015	0.539974675
7.856043699	0.356397662	0.351838831	1.012957156	0.311080626	0.526046215
4360.140793	-0.000682383	0.070251103	-0.009713478	0.992249888	0.995850406
2.851472334	-1.108192101	0.68148884	-1.626133894	0.103921175	0.263653388
11.4008289	1.008398042	0.411201047	2.452323623	0.014193694	0.067580035
475.8667174	0.236051733	0.060035224	3.931887252	8.42816E-05	0.001371352
25.25613772	0.193354763	0.262702538	0.736021677	0.461717506	0.66425065
1181.98622	0.359027019	0.052958758	6.779370062	1.20701E-11	1.71416E-09
2.212943162	-2.03659903	1.143774986	-1.780594134	0.074978779	0.21326296
300.0507305	0.070856233	0.069687344	1.016773326	0.309261274	0.524531064
177.713057	0.132128334	0.106896811	1.236036265	0.216445084	0.421180759
93.90683173	0.14851248	0.175222915	0.847563116	0.396681323	0.609544643
26.80842449	0.056174453	0.239563384	0.234486809	0.814607073	0.906623221

49.37376202	-0.743287754	0.271859057	-2.734092302	0.006255251	0.037260851
1194.2783	0.224802297	0.058308649	3.855385108	0.000115548	0.001757646
3.275920611	-0.024447819	0.5017981	-0.04872043	0.961142094	0.98086139
1.907353382	-1.33029978	0.715920701	-1.858166383	0.06314539	0.190001308
130.4015963	-0.522766633	0.247340574	-2.113549846	0.034553733	0.125217379
31.63744513	-0.020035858	0.258789643	-0.077421406	0.938288313	0.970668417
1294.434862	-0.048523518	0.050126581	-0.968019691	0.333034536	0.548476551
331.3324676	-0.154211006	0.079879337	-1.930549403	0.0535388	0.169289481
8768.211215	-0.067606537	0.066142587	-1.022133249	0.306717838	0.521863599
16.10735539	-0.075172382	0.329828904	-0.227913264	0.819713671	0.909423741
39.29938422	-1.645559268	0.346397149	-4.750498871	2.02915E-06	6.30722E-05
12.75765631	-0.675166592	0.683243633	-0.988178389	0.32306529	0.538191687
233.7937898	-0.278540948	0.104500547	-2.665449658	0.007688544	0.043273344
71.93138954	-0.068352121	0.287173577	-0.238016748	0.811868104	0.905164804
16.68770635	-0.794336078	0.252013771	-3.151955051	0.001621812	0.013898241
11.31364593	0.220276955	0.280703142	0.784732771	0.432610265	0.63969316
214.9284317	0.168748936	0.298136974	0.566011431	0.57138601	0.751118187
1.789875754	0.929072759	0.77838843	1.193585006	0.232640342	0.439370196
102.0290531	0.143991502	0.09873471	1.458367609	0.144739244	0.325475608
1129.577857	0.156843567	0.060360693	2.598438782	0.009364874	0.050220357
1071.979925	0.18725033	0.079296836	2.36138463	0.018206836	0.080219154
105.4337695	-0.262672706	0.125275554	-2.096759491	0.036014871	0.129081438
32.98213174	-0.071519873	0.251429252	-0.284453273	0.776063039	0.883652841
242.1504093	-0.177915172	0.091430013	-1.945916513	0.051664769	0.16549201
122.3556808	-0.006215619	0.107741023	-0.057690362	0.953995271	0.977641944
229.0549624	0.126667735	0.105244214	1.203560085	0.228759655	0.434802906
77.47536889	1.162540596	0.199565921	5.825346277	5.69943E-09	3.94506E-07
127.4429434	0.037418736	0.191817095	0.195075086	0.845334167	0.923392672
2288.697512	0.323514238	0.107830689	3.000205622	0.002697974	0.020193741
120.4239884	0.486017326	0.120586585	4.030442754	5.56719E-05	0.000979216
174.1705997	0.239365708	0.121261909	1.973956291	0.048386713	0.158599824
1247.198082	0.137092107	0.06191237	2.21429267	0.026808653	0.105707454
17.04658938	0.167512107	0.288735281	0.580158084	0.561808017	0.744947302
25.03401131	0.224409126	0.213690834	1.050157938	0.293645504	0.508620235
43.91540498	-0.469417889	0.158770662	-2.956578274	0.003110733	0.02235873
43.12387635	0.172564984	0.437563028	0.394377434	0.693302402	0.834976033
1.775629125	0.776844823	0.829286199	0.936763236	0.34888036	0.563420748
5.199594267	-0.019515417	0.47685619	-0.040925163	0.967355557	0.983717003
15.13857913	0.287975231	0.265834319	1.08328839	0.278680437	0.491940641
603.9171199	-0.318172614	0.06679835	-4.763180752	1.90565E-06	6.00262E-05
408.4769024	-0.001682634	0.08280806	-0.02031969	0.983788348	0.991966796
120.72044	-0.235796178	0.236791952	-0.995794731	0.319349891	0.534379899
65.55959212	-0.15899408	0.205288015	-0.774492751	0.438639453	0.644328698
143.5307939	-0.103913004	0.109129415	-0.95219977	0.340995678	0.555534296
90.69209802	-0.158305466	0.280783298	-0.56379944	0.572890633	0.752170338
37.62715659	0.283633981	0.175402147	1.617049657	0.105867563	0.266880218
770.0153578	-0.12839546	0.085795907	-1.496521965	0.134517688	0.310587862
161.6584704	0.296226065	0.208147054	1.423157618	0.154690469	0.339828081

1016.537698	-0.079974469	0.122127039	-0.654846543	0.512566561	0.706319325
832.0922743	-0.098963013	0.074369782	-1.33068849	0.183291532	0.380430009
1065.668476	-0.044109479	0.079713931	-0.55334718	0.5800257	0.757293715
16.09997894	0.370422772	0.314523592	1.177726509	0.238905655	0.446360434
224.7385688	0.297568796	0.177687028	1.67467935	0.093997161	0.246225033
5.727076956	1.17250111	0.55417838	2.115746755	0.034366352	0.124675463
33.06415315	-0.356945929	0.226370294	-1.57682319	0.114836214	0.28135214
4.408547625	0.947682227	0.631825119	1.499912234	0.133637138	0.309031193
10.35187383	-0.290367807	0.565018539	-0.51390846	0.607315993	0.776900424
8.538788849	-0.366355473	0.620205783	-0.590699866	0.554721538	0.739598787
181.8441009	-0.943994289	0.097452447	-9.686717186	3.43389E-22	3.77132E-19
2.399236425	0.229577582	0.607488718	0.377912503	0.705495594	0.842625565
16.57389677	0.454677672	0.505596842	0.899288989	0.368498751	0.582134026
3.423162104	-0.067807722	0.504040069	-0.134528436	0.892984727	0.946108145
7.768849843	-0.529398359	0.342753656	-1.544544749	0.122456419	0.29249631
150.8458837	-0.078676805	0.094082844	-0.836250287	0.403014114	0.614325414
9.686548558	-0.017783369	0.356517252	-0.049880808	0.960217371	0.980574075
524.1325082	0.075714084	0.069557671	1.088508037	0.27637089	0.489036955
214.0958767	-0.130833828	0.103403765	-1.265271412	0.205774051	0.409262552
159.4001057	-0.185803771	0.147191426	-1.262327405	0.206830999	0.410275001
5.314673226	0.520185136	0.452039548	1.150751383	0.249834531	0.458821811
84.25307999	0.004172026	0.127821883	0.03263937	0.973962174	0.987205615
3229.801022	-0.352237999	0.100424673	-3.507484635	0.000452364	0.00515014
5.938296258	-0.437093143	0.435389313	-1.003913349	0.315420382	0.530228099
600.3510382	-0.093877499	0.084591936	-1.109768879	0.267098633	0.478749089
14.14755748	-0.591443653	0.517205985	-1.14353598	0.252816125	0.461985981
42.20500001	0.08443317	0.421679433	0.200230704	0.841300155	0.921330769
7.155013553	0.177352015	0.631868866	0.280678515	0.778956989	0.884794608
45.83136311	0.513253909	0.360612086	1.423285378	0.154653444	0.339792056
7.416257358	2.097403378	0.786521076	2.666684267	0.00766036	0.043144195
20.86793098	-2.347847995	0.790492228	-2.970108891	0.002976942	0.021660227
18.25863308	0.318490415	0.306906129	1.037745372	0.299388616	0.514191832
18.20793978	-0.497748322	0.56969949	-0.873703296	0.382279855	0.595018739
9.834923155	-4.064073314	1.16994525	-3.473729488	0.000513278	0.005689309
10.96229668	0.231108442	0.334520026	0.690865789	0.489649887	0.687446075
11.63236181	-0.393327106	0.27022989	-1.455527759	0.145523211	0.32655035
126.6824788	-0.282781996	0.104188861	-2.714128882	0.006645031	0.038985127
943.0497327	-0.090902002	0.0491051	-1.851172339	0.064144764	0.192270895
6.667450889	0.45136893	0.438004324	1.030512498	0.302769489	0.517785171
30.43605315	0.034886817	0.308109427	0.113228657	0.909849277	0.95476508
83.79253393	0.119601896	0.252416117	0.473828285	0.635622363	0.795984758
237.8966453	-0.138360766	0.096498391	-1.43381423	0.151625283	0.335826151
4.047521362	-0.335811161	0.498420614	-0.673750547	0.50046992	0.6962876
5.872921617	2.543129131	0.93870693	2.709183292	0.006744907	0.039430657
62.37064837	-0.721471697	0.27021043	-2.67003645	0.007584301	0.042877069
92.39941963	-0.602820131	0.27251657	-2.212049456	0.026963248	0.106067875
10.06571807	-0.108241647	0.399599059	-0.270875631	0.786486688	0.889541352
11.02686215	-0.710905674	0.410158711	-1.733245339	0.083052072	0.228033305

342.8071982	-0.112824078	0.072835776	-1.549020056	0.121376899	0.291158774
229.5072119	0.042480904	0.099064231	0.428821818	0.6680529	0.819105647
1314.317202	-0.005457139	0.060706675	-0.089893565	0.928371795	0.965637301
53.21571761	-0.719546974	0.288689944	-2.492455965	0.012686306	0.062404958
191.9748045	-0.289925695	0.148008919	-1.958839355	0.050131603	0.162412592
1.965304587	-0.676789213	0.787396366	-0.859527986	0.390049286	0.603632874
956.3806528	-0.703589568	0.38573233	-1.824035771	0.068146664	0.200258322
140.729675	-0.033928805	0.080586335	-0.4210243	0.673737338	0.823222819
104.8657621	0.000495705	0.11432552	0.004335904	0.99654046	0.998004635
343.5357401	0.210000968	0.109630948	1.915526329	0.055425415	0.172931493
8.092982918	0.235008378	0.479692028	0.489915122	0.624193962	0.789055504
667.4764374	0.071278465	0.064916623	1.098000194	0.272204404	0.48431746
19.02609451	0.36404524	0.246384184	1.477551172	0.139527952	0.318054999
62.61767763	0.037343529	0.137024603	0.272531562	0.785213326	0.888692051
81.19015589	-0.047730289	0.11572701	-0.412438625	0.680017958	0.826932461
97.05646988	-0.5183651	0.116235787	-4.459599873	8.21128E-06	0.000202504
69.61655701	0.526380438	0.207795645	2.533163959	0.011303807	0.05735326
499.8344936	-0.260352976	0.065345974	-3.98422369	6.77011E-05	0.001159363
210.5120903	0.193327539	0.095411889	2.0262416	0.042740033	0.145562263
5.078812036	-0.441204688	0.439038439	-1.004934075	0.314928595	0.529706901
135.7075179	0.211361139	0.08501597	2.486134527	0.012913914	0.063203748
560.1493097	-0.049461592	0.074924741	-0.660150321	0.509157369	0.703620375
46.84468405	0.302278287	0.143105788	2.112271562	0.034663164	0.125586311
2.118101955	-0.43150679	0.641627944	-0.672518699	0.50125354	0.697106957
6.755661286	-0.044902628	0.413113334	-0.108693244	0.913445801	0.956832589
15.10832652	0.566474466	0.320793231	1.765855424	0.077420107	0.217462719
88.7876544	0.755552307	0.130159534	5.804817223	6.44363E-09	4.33275E-07
18.03217493	-0.230960655	0.258787958	-0.89247064	0.372140723	0.585510216
7.981524993	-0.15441254	0.515625591	-0.299466401	0.764584205	0.877631005
137.4996171	-0.170913387	0.165509787	-1.032648217	0.301768555	0.516838611
145.2252152	-0.246420315	0.111601465	-2.208038347	0.0272416	0.106704405
1830.808141	-0.079105256	0.065113293	-1.214886433	0.224409409	0.42942509
2.210956373	-0.240539647	0.630765026	-0.381345886	0.702946609	0.840739251
363.9294672	-0.129421538	0.096619908	-1.339491433	0.180410742	0.37659485
625.7787642	-0.012254718	0.08554625	-0.14325255	0.886090729	0.943111133
105.3219201	-0.15117098	0.123096962	-1.228064264	0.219422845	0.423921325
704.0343035	-0.078116735	0.084286012	-0.926805437	0.354027561	0.568833517
4.200544692	0.646538445	0.539488622	1.198428326	0.230750311	0.43704077
160.6291733	0.126699206	0.104371718	1.213922775	0.224777215	0.429829349
3658.284469	-0.177323361	0.041655977	-4.25685277	2.07325E-05	0.000439571
277.3063575	-0.194106984	0.075011207	-2.587706445	0.009661728	0.051460494
226.0248673	-0.160970305	0.131117694	-1.227677975	0.219567878	0.424016647
69.33802649	-0.034833793	0.156281331	-0.222891583	0.823619887	0.91138289
185.8570979	0.255866417	0.098373666	2.600964529	0.009296207	0.049949676
11.22304895	-0.424909824	0.488169561	-0.870414417	0.384073971	0.597190617
37.15403523	-0.514659984	0.159331336	-3.230124067	0.001237365	0.011368853
180.2668767	-0.293634405	0.097425349	-3.013942548	0.002578766	0.019523252
37.95768181	0.500876601	0.192124118	2.607046984	0.009132683	0.049312296

4.7663361	-0.295652706	0.494760218	-0.597567661	0.550128448	0.736514917
2.470405782	0.718250132	0.669420266	1.072943513	0.283296461	0.497338651
159.3870769	0.392217687	0.134277116	2.920957039	0.003489579	0.024323588
410.8278029	-0.1534095	0.066361497	-2.311724531	0.020792869	0.088420681
2.942779861	0.347961835	0.599067481	0.58083913	0.56134888	0.74475811
32.43999281	-0.380606904	0.257337918	-1.479016022	0.139136033	0.317621792
3.132065507	-0.184922255	0.59728458	-0.309604937	0.756861401	0.873190736
190.4829549	-0.445832254	0.107205511	-4.158669188	3.20107E-05	0.000629289
2.95348337	2.140146022	1.055580504	2.027458838	0.04261551	0.145351534
10.09375766	-0.530329798	0.836712518	-0.633825581	0.526194657	0.716822193
48.23803602	-3.556450376	1.118983747	-3.178285998	0.001481485	0.013030427
45.53306473	0.100318918	0.164216998	0.610892409	0.54127081	0.728624641
1.736834112	-1.626546254	1.256137441	-1.294879207	0.195361902	0.39640251
2679.01475	-0.154688034	0.071336298	-2.16843372	0.030125702	0.114241774
1125.556342	-0.020126805	0.079189144	-0.254161167	0.79937105	0.896484956
2297.146784	0.006834171	0.107835493	0.063375897	0.94946718	0.976070036
37.06630435	0.222650886	0.203822257	1.092377685	0.274667127	0.487383267
2544.012868	-0.022974787	0.147082505	-0.156203399	0.875872698	0.937672708
562.8203665	-0.013079766	0.088131305	-0.148412254	0.88201743	0.940841436
2940.024679	0.230875147	0.089560896	2.577856598	0.009941525	0.052291403
13.1294296	0.260077723	0.283573657	0.917143454	0.359067473	0.57346365
20.99678688	0.205964992	0.243796483	0.844823476	0.398209405	0.610697469
21.05837989	0.016356293	0.228701466	0.071518094	0.942985424	0.972623458
1119.969559	-0.13565968	0.096399003	-1.407272644	0.159346558	0.346773473
13.65974829	0.035170607	0.258277374	0.136173784	0.891683899	0.945641854
148.0560781	-0.296399232	0.111503067	-2.658215961	0.007855554	0.044002853
77.11836283	0.244787863	0.112317283	2.179431832	0.029299604	0.112277662
262.4570829	0.326400165	0.106959781	3.051615877	0.002276132	0.017781276
166.4497972	0.209118289	0.107967889	1.936856315	0.05276291	0.167737587
82.78900658	0.307340162	0.192435882	1.597104236	0.110242474	0.274256032
4.482446705	-0.486392122	0.625390768	-0.777741129	0.43672164	0.642902204
6.475129495	-0.042373205	0.573247959	-0.07391776	0.941075824	0.971961872
75.64222614	-0.026609903	0.133703364	-0.199021942	0.84224558	0.921757179
214.573348	0.042055318	0.355264389	0.118377522	0.905768535	0.952959157
1101.350117	-0.597128994	0.381274715	-1.566138459	0.117316206	0.285057385
381.7001063	-0.126726913	0.15589938	-0.812876311	0.416288978	0.625839078
659.3109252	-0.211974717	0.159303839	-1.330631563	0.183310272	0.380430009
243.6205009	0.274384625	0.081756741	3.356110087	0.000790471	0.008023548
169.1628035	-0.347859635	0.141949075	-2.45059459	0.014262048	0.067790907
152.1241922	-0.23992923	0.179758158	-1.334733474	0.181963594	0.378613187
224.3386502	0.018492952	0.085354916	0.216659485	0.828473724	0.914385171
2114.59015	0.032956739	0.069984502	0.47091482	0.637701565	0.797573383
99.96949256	-0.174289062	0.146091141	-1.193015956	0.232863124	0.439576843
5008.770282	-0.043828115	0.094820193	-0.462223433	0.643921101	0.802235212
3265.281011	0.102193264	0.119862343	0.852588572	0.393887502	0.606836502
172.4916864	0.0804502	0.130198838	0.617902595	0.536639549	0.724877003
541.9392646	0.165104358	0.106525695	1.549901719	0.121165107	0.291015304
2832.447168	0.013250031	0.079670084	0.166311241	0.86791202	0.934495951

48.52866418	-1.438528947	0.413030428	-3.482864338	0.00049608	0.005539859
59.89321826	0.503406395	0.261808314	1.922805227	0.054504515	0.170932303
3.873880079	-1.247136496	0.906191789	-1.376239017	0.168747646	0.360377071
121.0266171	-0.125327418	0.130792978	-0.958212128	0.337955803	0.553262834
85.30338122	-0.282436683	0.245374122	-1.151045111	0.249713677	0.458709382
104.1436875	-1.768880169	0.672719109	-2.629448376	0.008552352	0.047117361
568.4309252	-0.073439388	0.110358805	-0.665460159	0.505756218	0.700742467
4.586389027	-0.708768392	0.64381073	-1.100895587	0.270942103	0.482906005
167.7019691	-0.114605455	0.112743777	-1.016512464	0.309385415	0.52466996
157.7494188	0.140982182	0.113357948	1.243690318	0.213613561	0.418140423
5481.526789	-0.917737589	0.493676738	-1.858984875	0.063029282	0.18968659
1659.399839	0.022722143	0.090521026	0.251015081	0.801802449	0.898503064
29.71784372	-0.132730059	0.245072088	-0.541595983	0.58809686	0.761898999
960.4803225	0.191324071	0.167348857	1.143264877	0.252928633	0.462047716
81.6656647	0.042595703	0.208181363	0.204608629	0.837877913	0.918926885
6501.238103	0.430024718	0.086318685	4.981826554	6.29869E-07	2.34232E-05
219.5052858	0.127830858	0.154915005	0.825167696	0.409276391	0.619722671
1.865737884	-2.304484176	1.692270143	-1.361770865	0.173270209	0.366754905
7.175004993	0.722132501	0.444428846	1.624855154	0.104193426	0.264033609
521.215182	0.12170094	0.054164791	2.246864372	0.024648696	0.099588119
98.77752746	-0.33647562	0.241506578	-1.393235843	0.163548484	0.353157197
374.8794684	-0.127080981	0.080960649	-1.569663563	0.116493402	0.283808386
2419.45066	-0.038723492	0.082535921	-0.469171377	0.638947143	0.798267972
7.949839025	0.991263654	0.403828535	2.454664708	0.014101605	0.067355708
13.82038736	0.320303092	0.417186144	0.767770206	0.442623718	0.648331389
1243.549733	-0.180116708	0.091782671	-1.962426083	0.049712899	0.161503179
18.44545612	0.375286439	0.243343083	1.542211244	0.123022269	0.293122879
31.66499312	-0.040296176	0.222281211	-0.181284668	0.85614414	0.92936531
1348.865935	0.013509242	0.090950474	0.148534045	0.88192132	0.940841436
214.1919546	0.456997862	0.145689462	3.136794224	0.00170806	0.014349094
65.99289792	0.817044779	0.180812958	4.518729112	6.22119E-06	0.000160137
16.15410382	0.478735676	0.353535062	1.354139172	0.175691981	0.370084565
5.961116191	0.379768751	0.398028082	0.954125521	0.340020109	0.554822829
125.536209	0.924212486	0.352955296	2.618497292	0.008831799	0.048193131
172.0882578	0.082049795	0.076298562	1.075378002	0.282205526	0.495967668
443.6724996	0.064887175	0.069860324	0.928812974	0.35298602	0.56793551
3662.907585	0.08938399	0.113956876	0.784366798	0.432824915	0.639905007
6.992945126	0.720491762	0.419693123	1.716710906	0.086032006	0.232886268
5.234142357	0.865725857	0.651427053	1.328968229	0.183858452	0.381231484
6.656065564	0.933463036	0.395826135	2.358265296	0.018360566	0.080680706
7.885467641	0.080327496	0.372136162	0.21585512	0.829100682	0.914559484
4.761234221	1.755195793	0.72904738	2.407519513	0.016061304	0.073641503
13.46038975	0.978773112	0.348819971	2.805954912	0.005016771	0.03153235
13.13597669	0.782891441	0.311673458	2.511896413	0.012008433	0.059947554
18.42941786	0.703759661	0.240899414	2.921383866	0.003484802	0.02430509
24.34026927	0.609432974	0.276031988	2.207834599	0.027255805	0.106704405
10.97993058	0.714854445	0.389893823	1.833459271	0.0667343	0.197308853
8.659965394	3.11099678	0.971297908	3.202927499	0.001360382	0.01218648

168.4533263	0.240483759	0.074707016	3.219025116	0.001286272	0.011681394
227.8162755	-0.01691846	0.090683345	-0.186566342	0.852000659	0.926915291
24.8059495	0.018106259	0.18613236	0.097276255	0.922507012	0.961560774
7495.64111	-0.074782814	0.091880861	-0.813910684	0.415696119	0.625357946
210.2701007	-0.034153952	0.082187022	-0.415563816	0.677729209	0.825950823
311.978025	0.23195234	0.077920744	2.97677266	0.002912999	0.021328333
6.261861856	-0.092808995	0.518343226	-0.179049305	0.857898987	0.929613001
135.4949295	0.174902325	0.166023676	1.053478213	0.292121874	0.507317706
36.2824994	0.014224267	0.220036118	0.064645148	0.948456537	0.975518074
207.5052179	0.402697242	0.096809421	4.159690621	3.18679E-05	0.000628242
8.074958694	1.408799332	0.508446531	2.770791511	0.005592022	0.034310231
131.5305601	-0.07048747	0.121318534	-0.581011557	0.561232665	0.74475811
1.925735942	0.857116117	0.88284266	0.970859424	0.331618286	0.547183458
454.089882	-0.216288532	0.069656284	-3.105082851	0.001902257	0.015607133
114.8458863	0.574230299	0.108665406	5.284389197	1.26125E-07	5.49677E-06
1.746640296	1.676086175	0.774734632	2.163432619	0.030507921	0.115310435
22.53452533	0.953671529	0.309169159	3.084626975	0.002038076	0.016426254
206.2123634	0.88512907	0.285419619	3.101150066	0.001927706	0.015775968
1723.653523	-0.085652523	0.041405277	-2.068637852	0.038580083	0.135336351
1206.985067	-0.1878023	0.092487301	-2.030573899	0.042298238	0.144568707
521.0624686	-0.123796683	0.089861615	-1.377636972	0.1683154	0.359716154
13.8752079	0.030885052	0.298854164	0.103344893	0.917689247	0.959308302
1634.127591	0.003936211	0.072708991	0.05413651	0.956826404	0.978702547
366.8515547	0.482865085	0.1699521	2.841183398	0.004494646	0.029128557
8.098256234	0.863008373	0.365270193	2.36265753	0.018144428	0.080051235
110.8258819	0.029989666	0.256729501	0.116814259	0.907007244	0.953303217
13.9948747	0.356431861	0.371407519	0.959678636	0.337216979	0.552571682
2021.636663	0.256802414	0.059511059	4.315204866	1.59455E-05	0.000353998
149.9260808	-0.047461979	0.087584229	-0.541900981	0.587886721	0.761885532
67.84475547	0.464412967	0.178534414	2.601251811	0.009288425	0.049924146
639.9575318	-0.168148839	0.058208853	-2.888715912	0.003868184	0.026033877
1568.583595	0.165028029	0.061347528	2.69005181	0.007144093	0.040964769
161.6591626	0.257650292	0.099930105	2.578305034	0.009928631	0.052240264
3.382817158	-0.312027074	0.552318271	-0.564940706	0.572114095	0.751595502
1059.972597	0.270481667	0.074777562	3.617150105	0.000297865	0.003723081
38.95706541	-0.364015963	0.204965324	-1.775988035	0.075734889	0.214558309
512.0988917	0.154191093	0.224501343	0.686815904	0.492198746	0.6896766
3.165063525	-0.724745776	0.564485837	-1.283904269	0.199175453	0.401273868
6.343933236	-0.301516165	0.369268783	-0.816522217	0.414201522	0.623896487
66.99782589	0.612496496	0.172140148	3.558126928	0.000373509	0.004452377
10.91626077	0.908916064	0.573479718	1.584914053	0.112985863	0.278517
2083.664737	0.103045974	0.09440957	1.091478059	0.275062581	0.487737632
1408.474864	0.034742059	0.068325639	0.508477632	0.611118422	0.779755644
7.059306185	-1.022026127	0.747214283	-1.367781841	0.171380373	0.363595656
727.1173654	0.270203592	0.072600884	3.721767238	0.000197833	0.002695704
817.266853	-0.017466582	0.06062259	-0.288120027	0.773254863	0.882051749
584.2529591	0.210604297	0.056945378	3.69835632	0.000217	0.002920637
5.293460578	0.445641033	0.428925556	1.038970579	0.298818419	0.513735145

5.420470598	-0.493322273	0.758209269	-0.650641311	0.515278057	0.708394451
211.8483968	0.06363324	0.072595361	0.876546929	0.380732776	0.593610885
17566.39649	-0.329465417	0.110579391	-2.979446827	0.002887693	0.021209033
7.473053732	-0.953696484	0.566127465	-1.684596743	0.0920664	0.242983319
39.04249783	0.147196604	0.182738991	0.805501895	0.420530113	0.629066856
123.1515506	0.122213189	0.1533773	0.796814061	0.425559027	0.632924168
129.4019048	0.044610325	0.102484235	0.435289631	0.66335221	0.815802003
9.657159011	-1.2485541	0.654383482	-1.907985355	0.056393109	0.175286808
19.58822054	0.938700836	0.796303937	1.178822297	0.238468944	0.446120529
570.8047287	-0.200539285	0.177084289	-1.132451031	0.257444861	0.467957282
1398.512383	0.315732719	0.062741433	5.032284154	4.8467E-07	1.83974E-05
678.0815766	0.128909934	0.064658638	1.993700104	0.046184838	0.153831181
1084.385006	0.249597018	0.053148879	4.696185956	2.65064E-06	7.83962E-05
2348.977624	0.046947511	0.088565744	0.530086563	0.596051915	0.767938112
815.2696368	0.145194424	0.059994118	2.420144326	0.015514348	0.071914286
13.50294783	-0.117762626	0.259124353	-0.454463755	0.649495074	0.805709937
406.1176464	0.151028431	0.061125244	2.470802903	0.01348101	0.06525536
8.904847795	0.910607612	0.356678255	2.553022503	0.01067926	0.054909527
309.4369836	0.217432084	0.075218752	2.890663267	0.003844298	0.025932706
16.15854456	-1.920601743	0.732831552	-2.62079565	0.008772483	0.047953902
300.3795158	0.445727021	0.090348391	4.933425103	8.08E-07	2.89688E-05
78.5012453	-2.288151023	0.877787048	-2.606726799	0.009141226	0.049326093
1399.858927	0.009980168	0.208752241	0.047808676	0.961868722	0.981287177
4.778721913	-1.035380079	0.655155294	-1.580358259	0.114024845	0.280114121
3.185898634	-0.03458129	0.550236978	-0.062847993	0.949887548	0.976186239
305.8361271	0.10053124	0.06594516	1.524467288	0.127392022	0.298780921
90.65247206	-0.141959338	0.215127979	-0.659883192	0.509328791	0.703680183
21.18270834	-0.193746632	0.208857839	-0.92764836	0.353590003	0.568540551
4.927585358	-0.446834738	0.468958761	-0.952823094	0.340679711	0.555270316
2.157593496	-2.876745727	1.943926757	-1.479863229	0.138909751	0.317331149
13.82632051	-0.349674213	0.290708009	-1.202836532	0.229039585	0.435101259
5.27439791	-2.7135223	0.8882117	-3.055040032	0.002250305	0.017669934
4072.051371	-0.007604819	0.104361257	-0.07287014	0.941909455	0.972365984
8.102292316	0.321434521	0.447777437	0.71784439	0.472853239	0.673216167
1243.960454	0.15733173	0.064925384	2.423269924	0.015381493	0.071560214
121.0119703	-0.110413043	0.15189657	-0.726896225	0.46728953	0.669053339
75.59132439	-0.103084593	0.200814543	-0.513332309	0.607718887	0.777295104
89.99253194	0.232605904	0.110874056	2.097929048	0.035911414	0.12880571
14.12740678	0.094019286	0.302231143	0.311084043	0.755736732	0.872365037
125.641246	0.209004921	0.097450857	2.144721227	0.031975143	0.118799842
739.9152858	-0.793144188	0.461884325	-1.717192258	0.08594405	0.232886268
5.061206223	-0.864872192	0.57377258	-1.507343192	0.131722712	0.305978561
173.6548894	0.249699166	0.129738622	1.924632475	0.054275354	0.170538275
898.8402217	0.262065686	0.093131486	2.813932191	0.004893955	0.030949334
91.70270627	0.497893572	0.125380678	3.971055025	7.1555E-05	0.001204958
10.32985187	1.14831014	0.380677369	3.016491739	0.002557182	0.01941337
19.77417847	0.036361102	0.302016247	0.120394522	0.904170631	0.951715991
214.2566879	0.002693729	0.08733174	0.030844792	0.975393318	0.987719156

3.997069751	0.35716717	0.573128022	0.623189159	0.533160225	0.722368743
554.2357698	-0.017644148	0.102591672	-0.171984216	0.863449938	0.931920109
2.233471405	1.665991067	0.832365863	2.001512965	0.045337138	0.151867428
77.51739621	-0.199539114	0.107877834	-1.849676682	0.064360163	0.192776242
3773.869148	-0.087747035	0.090090641	-0.973986138	0.330063422	0.545419232
346.7034225	-0.189170835	0.141444256	-1.337423236	0.181084524	0.377331957
936.2270623	0.062339328	0.085611398	0.728166223	0.466511839	0.668520881
4593.850661	0.332657659	0.185200331	1.796204446	0.072462008	0.208775569
1050.178421	-0.014737653	0.071372367	-0.206489614	0.836408476	0.918232256
133.069106	-0.076360262	0.114603119	-0.666301775	0.505218231	0.70040942
2.914438188	0.064631396	0.617252425	0.104708209	0.916607348	0.958837688
156.3477985	-0.143712974	0.14314609	-1.003960179	0.315397808	0.530228099
116.9445906	-0.070635363	0.095409117	-0.74034186	0.459092587	0.662074214
16.58490746	-0.237318881	0.328512303	-0.722404849	0.470045606	0.670923708
603.9663096	-0.103268899	0.068385897	-1.510090576	0.131020314	0.304948947
313.2846529	-0.116517944	0.079866828	-1.458902862	0.144591845	0.325277353
27.36014145	0.577368135	0.196182933	2.943008989	0.00325039	0.023070625
738.9192412	0.268518779	0.080643423	3.329704621	0.000869382	0.008643447
15.14256755	0.410861721	0.284147359	1.445945943	0.148192364	0.330578336
69.4412335	0.347262028	0.186316862	1.863825013	0.062346282	0.188526551
683.6517495	0.160091438	0.057974726	2.761400496	0.005755405	0.035022326
156.691903	-0.029687966	0.141235465	-0.210201921	0.833510081	0.916902912
292.0998813	-0.021673642	0.073193815	-0.296113023	0.767143769	0.878716103
19.02801655	0.358293253	0.306030562	1.170776051	0.241688807	0.449287001
300.2706233	-0.315576677	0.089922003	-3.509448937	0.000449036	0.005129975
14.43298754	0.039344212	0.263187499	0.149491189	0.881166061	0.940606554
565.7034173	0.027310647	0.050257341	0.543416079	0.586843357	0.761532688
729.6115812	-0.127346191	0.05751678	-2.214070249	0.026823947	0.10574245
14.64406313	-0.180580254	0.331756735	-0.54431526	0.586224547	0.760977281
1608.013982	-0.216033549	0.421019952	-0.513119504	0.607867729	0.77736477
233.6612982	0.09189346	0.082791998	1.109931672	0.267028471	0.478749089
188.5040361	-0.276834638	0.136279992	-2.031366697	0.04221781	0.144353716
137.0212381	-0.200432303	0.087271946	-2.296640706	0.021639275	0.090857623
768.0565328	-0.02155686	0.070915196	-0.303980825	0.7611425	0.875424502
56.73778837	0.001100739	0.190776167	0.005769793	0.995396397	0.997576362
442.3858985	0.07127134	0.148585353	0.479665988	0.631464916	0.793618632
24.88431465	0.162161159	0.270601965	0.599260836	0.54899896	0.735421115
16.5745192	-1.939189277	0.69583135	-2.786866787	0.005322035	0.033010244
1581.28174	-0.171107861	0.048049487	-3.561075714	0.000369339	0.004409142
68.58178368	-0.376145738	0.200786499	-1.87336171	0.061018454	0.185910488
251.7639137	-0.441011056	0.064452941	-6.8423729	7.7892E-12	1.14571E-09
63.38391054	-0.157315175	0.173589792	-0.906246695	0.364805293	0.578253801
351.1801415	0.036421051	0.07194233	0.506253422	0.612678756	0.780789807
32.84731954	0.283454546	0.206285826	1.37408639	0.169414864	0.361380354
37.54695571	0.435302853	0.216565916	2.010024762	0.044428568	0.14961531
2.019653278	-1.355953558	1.918416181	-0.706808862	0.479685267	0.678836447
2467.181535	-0.03980553	0.128358499	-0.310112148	0.756475674	0.872946221
241.7862068	0.448707918	0.161077275	2.785668673	0.005341744	0.033107559

434.0361756	0.705172641	0.159848875	4.411495814	1.02659E-05	0.000243689
63.62896466	0.080944993	0.197460621	0.409929802	0.681857443	0.828142105
114.0395365	0.039362424	0.121601601	0.323699886	0.746165257	0.866956703
696.1240041	-0.107016818	0.089290732	-1.198521009	0.230714249	0.437038457
443.0805442	-0.137577075	0.070454971	-1.95269507	0.050855738	0.163888387
1333.184278	0.029490611	0.061922938	0.476246964	0.633898445	0.794736908
2.069364056	-1.875466993	1.303498448	-1.438794957	0.150208626	0.333495539
1059.276149	0.201167936	0.10928341	1.84079117	0.065652167	0.195296823
196.9991272	0.107292505	0.081287759	1.319909755	0.18686515	0.385482837
319.7026272	0.146311531	0.080883867	1.808908696	0.070465189	0.204698537
891.5070664	0.262711635	0.051987931	5.053319675	4.34196E-07	1.66348E-05
305.5806274	-2.751215216	0.837042899	-3.28682702	0.001013231	0.009755675
16.30310392	0.424834034	0.291617285	1.456820482	0.145165941	0.326034588
522.8715688	-0.276015584	0.098145951	-2.812297219	0.004918903	0.031095167
578.4332436	-0.266342287	0.186772815	-1.426022773	0.153861769	0.33850411
42.64586905	0.290445969	0.167564043	1.733343046	0.083034714	0.228033305
61.50954008	-1.039653261	0.475052522	-2.188501719	0.028633076	0.11041697
966.8905492	0.197171378	0.092936935	2.121561018	0.033874619	0.123516054
1.713143232	0.220563628	0.740866068	0.297710528	0.765924108	0.878006201
88.47202124	-0.334625133	0.156688623	-2.135605798	0.032711558	0.120726961
2.897611652	-0.897474639	0.698789391	-1.284327797	0.199027285	0.401073454
4.112084406	0.228541801	0.529984248	0.431223761	0.666305678	0.818067386
481.5623444	-0.270085928	0.141345932	-1.910815008	0.056028358	0.174317502
31.22543598	0.886206095	0.374019551	2.369411152	0.017816435	0.079079856
90.96450122	0.75664543	0.171671774	4.407512144	1.04565E-05	0.000247145
18.2185385	0.587639181	0.34117254	1.722410545	0.084995175	0.23136327
451.0087817	0.024387934	0.057060258	0.427406649	0.669083165	0.819834748
4.969191658	-0.208494095	0.685349289	-0.304215818	0.760963474	0.875424502
10.44982788	-2.052782049	0.847447577	-2.422311544	0.015422123	0.071667959
99.88504544	-0.228304213	0.12887727	-1.771485484	0.076480005	0.21592658
586.4766693	-0.266501988	0.053636871	-4.968634129	6.74262E-07	2.4684E-05
171.4739571	0.219935562	0.131148007	1.677002696	0.093541955	0.245618451
372.4336299	-0.110619371	0.156679467	-0.706023408	0.480173581	0.679294081
544.980699	0.22012645	0.073711871	2.986309354	0.002823668	0.02086905
48.30173108	-0.301622168	0.174106585	-1.732399536	0.083202451	0.228255982
187.4082358	0.625655806	0.261469992	2.392839807	0.016718539	0.075718733
139.9558643	0.388267994	0.127136213	3.053952797	0.002258476	0.017708775
133.2858931	0.975755412	0.146133724	6.677140537	2.43649E-11	3.04082E-09
20.52090992	1.072848548	0.300218584	3.573558081	0.000352163	0.004251804
564.2403361	-0.140069432	0.1044794	-1.340641617	0.180036838	0.376052603
17.2848363	-0.538008083	0.369813813	-1.454807972	0.145722431	0.326776031
813.2216961	-0.020929786	0.0586754	-0.356704612	0.721312952	0.852102807
66.4445978	0.184730227	0.161889521	1.141088229	0.253833209	0.463186562
2.840625201	0.966525027	0.624959282	1.546540797	0.121974015	0.291809458
313.7745465	-0.039526241	0.068515481	-0.576895033	0.564010366	0.746485641
232.9423868	-0.116521791	0.078586038	-1.482728919	0.138146448	0.316130655
835.9266141	0.00741359	0.100749918	0.073584082	0.941341337	0.972084071
942.1690344	0.18717391	0.04434898	4.220478368	2.43784E-05	0.000503782

639.9801348	-0.176564863	0.085791774	-2.058062863	0.039584099	0.137662751
108.013961	1.141270112	0.268089962	4.257041559	2.0715E-05	0.000439571
5.524172884	-0.786104356	0.46967578	-1.673717038	0.094186224	0.246563459
134.0433829	-1.212853111	0.52349222	-2.316850305	0.020511888	0.087528478
90.42594103	-0.149284292	0.107677329	-1.38640411	0.165623542	0.356105747
759.8315524	0.244051884	0.059725496	4.08622614	4.38446E-05	0.000804339
79.62670684	-0.148804151	0.148385649	-1.002820369	0.315947539	0.530681052
418.8515557	-0.488193354	0.204255071	-2.390116204	0.016843043	0.076141297
412.3706096	0.343025774	0.106057169	3.234347813	0.001219209	0.011245938
63.91606049	-0.367746359	0.216514317	-1.698485184	0.089416227	0.238932628
828.7171064	-0.069232611	0.183873585	-0.376522874	0.706528214	0.843064305
875.6661726	0.087908081	0.04800859	1.831090685	0.067087009	0.197974384
548.254317	-0.36012932	0.109202098	-3.297824186	0.000974371	0.009453352
537.9638226	0.007541679	0.065562002	0.115031253	0.90842035	0.953990946
144.4934571	0.893054948	0.346902068	2.574371933	0.010042226	0.052686505
181.1312122	0.175132379	0.095644125	1.83108349	0.067088082	0.197974384
1185.167817	-0.266106961	0.089116316	-2.986063308	0.002825941	0.020876483
660.2554692	0.022616547	0.050377123	0.4489448	0.653471477	0.808447666
386.3386959	-0.045240579	0.067112512	-0.674100513	0.500247413	0.696036815
959.1442023	0.023370497	0.057460238	0.40672468	0.684210212	0.829348762
272.498751	0.10005106	0.158816565	0.629978746	0.52870849	0.718760824
2.333813544	-0.205523358	0.613013728	-0.335267138	0.737423581	0.861788314
25.89950586	1.267757711	0.873474251	1.451396775	0.146669407	0.328247768
11806.4726	0.041449259	0.105081664	0.394448063	0.693250265	0.834976033
54.9443858	0.754839611	0.169744459	4.446917544	8.71113E-06	0.000212919
69.13420651	0.77014261	0.21765842	3.538308382	0.000402699	0.004725122
324.810043	0.167285416	0.100497518	1.664572605	0.095998055	0.249601002
15.79343107	-0.417795532	0.342991232	-1.218093911	0.223188287	0.428282335
1312.872973	-0.238280053	0.0969519	-2.457714125	0.013982443	0.066902923
935.0947441	0.530375373	0.093060301	5.699265637	1.20325E-08	7.26091E-07
12.45419764	0.159923184	0.319957589	0.499826194	0.617197465	0.783631924
52.01352153	0.117078477	0.155904474	0.750962904	0.452674981	0.657152594
4096.598089	0.093625116	0.081411909	1.150017453	0.250136683	0.45908553
55.85898992	-0.434476071	0.193125731	-2.249705766	0.024467629	0.099085478
97.52512344	0.085278422	0.120981028	0.704890864	0.480878156	0.6798238
590.7712176	-0.285654513	0.059519809	-4.799318373	1.59207E-06	5.11261E-05
130.6781724	-0.550925272	0.089874912	-6.129911694	8.79279E-10	7.31578E-08
18.18247724	0.422865842	0.227724244	1.856920613	0.063322449	0.190455244
1320.825235	0.252113388	0.061576199	4.094331801	4.23388E-05	0.000782816
219.6275814	-0.074243789	0.080095512	-0.926940689	0.35395733	0.568831631
1401.745527	0.256801377	0.07527864	3.411344519	0.000646434	0.006844053
859.0868468	-0.048512067	0.082379993	-0.588881658	0.555940663	0.740545496
687.437434	0.002771265	0.050030175	0.055391875	0.955826269	0.978293782
2384.148985	-0.24436751	0.153262014	-1.594442771	0.110836899	0.275320729
1.823609427	-0.349683597	0.745743803	-0.468905803	0.639136968	0.798327577
1357.679274	0.056704079	0.090203304	0.628625299	0.529594394	0.719352326
12.03587822	-1.182015514	0.611988728	-1.931433473	0.053429469	0.169091234
39.16839964	0.053218142	0.184065716	0.28912577	0.772485134	0.881357441

249.1772982	0.309850055	0.07763904	3.990905308	6.58216E-05	0.001130703
8.374071882	-0.037122248	0.543500485	-0.068302143	0.945545118	0.974141328
16.24277101	0.395712509	0.23780422	1.664026441	0.096107146	0.249766385
820.3909374	0.107241842	0.078096931	1.373188947	0.169693615	0.36172226
640.68628	-0.057765924	0.087315223	-0.661579061	0.508241031	0.702907521
2.437680304	0.463362107	0.667642444	0.694027337	0.487665065	0.685967982
86.45887258	0.006869257	0.154722778	0.044397195	0.964587798	0.982392126
56.37910407	0.116629746	0.127456004	0.91505886	0.360160731	0.574374432
538.0541772	0.066113602	0.096734793	0.68345215	0.494321176	0.69117697
2306.759626	0.299709798	0.084904456	3.529965478	0.000415614	0.004845593
53.26314246	0.021125582	0.195633379	0.107985569	0.91400714	0.957177142
1052.351276	-0.419748346	0.08531413	-4.920033104	8.65296E-07	3.06557E-05
994.1633847	-0.011400737	0.120325064	-0.094749481	0.924513814	0.963037659
677.5651813	-0.303806023	0.076158119	-3.989148181	6.6311E-05	0.00113767
216.7114659	-0.165575216	0.135640861	-1.220688328	0.222204049	0.427040305
513.6338997	-0.109328955	0.155874736	-0.701389831	0.483059766	0.682205451
449.6199729	-0.077282975	0.137793062	-0.560862604	0.574891205	0.753826883
3.64241209	-1.016928245	1.22368891	-0.831034944	0.405953889	0.616774358
1524.083058	-0.166178011	0.143760226	-1.155938715	0.247706226	0.456253929
35.34627889	-0.523846235	0.312461011	-1.676517123	0.093636945	0.245789521
5.668900094	0.269476611	0.479233314	0.562307759	0.573906353	0.752765709
56.84954557	0.003290695	0.202315951	0.016265127	0.987022878	0.9933542
343.7936478	0.048211858	0.069230756	0.696393632	0.48618235	0.684991948
494.654625	0.081815458	0.073938159	1.106539017	0.268493286	0.480491998
59.55121191	-0.086949384	0.146151615	-0.594925923	0.551892989	0.737738161
7.93789219	0.130429468	0.323099505	0.403682042	0.686446547	0.830470765
265.1415497	0.396899686	0.17490092	2.269283012	0.023251121	0.095283325
21.80056049	-0.472061847	0.480287435	-0.982873614	0.325669646	0.540725836
2.947374238	0.743533841	0.635604672	1.1698055	0.24207925	0.449771264
728.7579892	0.303294336	0.064842836	4.677376152	2.90569E-06	8.4573E-05
428.4465094	0.029587709	0.155825905	0.189876701	0.84940575	0.925653921
3.367403063	0.636179035	0.509822113	1.247845119	0.212087782	0.41657975
24.72418714	0.018228009	0.25050109	0.072766185	0.94199218	0.972366504
3.101618527	0.658303347	0.578893131	1.137175953	0.255464738	0.465528635
648.6933211	-0.267794793	0.144219086	-1.85686098	0.063330935	0.190455244
40.92656562	-0.33466024	0.219883922	-1.521985952	0.128012601	0.299727058
19.5228104	-0.678980048	0.381585806	-1.779364006	0.075180104	0.213501791
15.82976295	-1.047640262	0.431605429	-2.427310207	0.015211243	0.071109541
7.338209734	-0.845686182	0.428392931	-1.974089954	0.048371515	0.158581561
5.091526544	-0.696169066	0.552017724	-1.26113535	0.207260086	0.41080496
12.51942027	0.703763968	0.493277455	1.426710182	0.153663449	0.338354197
6.220935335	-0.922226417	0.771702087	-1.195054972	0.232065553	0.438724801
68.06950119	-0.24869501	0.340291919	-0.730828433	0.464883954	0.667000371
96.98687382	-0.115161818	0.131804907	-0.873729371	0.382265652	0.595018739
187.6435362	-0.015807363	0.10776809	-0.146679435	0.883385052	0.941812409
6.092441615	-0.140448845	0.597134999	-0.235204511	0.814050005	0.906248127
64.43813791	-0.096872116	0.144969422	-0.668224479	0.503990312	0.699472317
68.01937204	-0.528914855	0.153222391	-3.451942307	0.000556567	0.006095692

8.454641575	-0.827736146	0.423676373	-1.953699094	0.050736813	0.163634792
55.89614032	0.317669501	0.183715743	1.729135977	0.083784762	0.229143952
65.84321729	0.302511911	0.211086134	1.433120713	0.151823343	0.336084084
295.7662954	-0.068340803	0.083957566	-0.81399219	0.415649425	0.625357946
2467.075368	-0.214035994	0.107862497	-1.984341174	0.047217819	0.155981164
242.8772791	0.169352388	0.088157822	1.921013737	0.054729976	0.171458081
5.775723561	-0.689862233	0.721945349	-0.955560189	0.339294483	0.554132776
27.49859512	0.274251296	0.257531292	1.064924164	0.286910249	0.501479298
54.20906318	0.055481109	0.23826814	0.232851561	0.815876673	0.907607017
353.4397869	-0.069562162	0.093842884	-0.741261984	0.458534607	0.661694036
330.941231	-0.101424057	0.070769346	-1.433163686	0.151811065	0.336084084
270.5690683	0.050779945	0.075134625	0.675852783	0.499134118	0.69513361
277.0184033	0.120243768	0.062473188	1.924725973	0.054263649	0.170534025
614.3461025	-0.080067672	0.0578039	-1.385160386	0.166003431	0.356697074
362.2493736	-0.092510505	0.059107146	-1.565132322	0.117551886	0.285344488
409.7058097	-0.218037665	0.083105103	-2.623637488	0.008699633	0.047645527
678.0959224	0.047881186	0.048756272	0.982051836	0.326074312	0.541016035
2179.043611	0.308650378	0.099545909	3.100583252	0.001931399	0.015790602
534.1418756	0.45707254	0.09131102	5.005666799	5.5669E-07	2.08905E-05
108.3133862	0.017235314	0.121349461	0.142030411	0.887055985	0.943527912
205.3332204	0.058066731	0.095735748	0.606531334	0.544161982	0.731141382
176.2555806	0.027936868	0.095486653	0.292573539	0.769848142	0.880039215
15.34719344	-0.295037428	0.264906518	-1.113741669	0.265390028	0.477036041
11.52073461	0.218171767	0.30174217	0.72304036	0.469655089	0.670633434
156.8458949	0.03089252	0.073836066	0.418393361	0.67565954	0.824504834
471.5262715	-0.316636115	0.162510225	-1.948407337	0.051366243	0.164856318
67.60667124	-0.000538699	0.207210004	-0.002599775	0.997925682	0.998559673
702.7102238	0.054437331	0.111590326	0.487831986	0.625668853	0.790434715
214.2066611	0.235643986	0.07549185	3.121449346	0.001799632	0.01495065
28.91065396	-0.117011445	0.547407264	-0.21375574	0.830737542	0.915614595
835.3053601	0.070708158	0.067814128	1.042675911	0.297098418	0.511766627
175.5844243	0.206678618	0.131559996	1.570983762	0.116186421	0.283353826
93.73409509	-0.14798778	0.132522267	-1.11670124	0.264122088	0.475535221
482.2733941	-0.072232922	0.075397086	-0.958033338	0.338045948	0.55335542
193.837824	0.273794835	0.073096696	3.745652689	0.000179925	0.002511122
2353.443402	-0.421647368	0.101788687	-4.142379464	3.43721E-05	0.000664608
71.77954859	-0.090609437	0.112985998	-0.8019528	0.422580262	0.630807107
2.590033678	0.462946677	0.788915671	0.586813894	0.557328705	0.741574435
12.63030584	-0.225712576	0.474276475	-0.47590928	0.63413901	0.794857038
1138.076634	-0.027236743	0.069256995	-0.39327064	0.6941196	0.835387176
9.139800858	-0.705738151	0.389183511	-1.813381427	0.069773012	0.203260938
662.1436475	-0.613613677	0.099492533	-6.167434488	6.94069E-10	5.89386E-08
289.5837733	0.125233156	0.145468983	0.8608925	0.389297253	0.602752156
4.336645226	-0.282948412	0.603200832	-0.469078286	0.639013679	0.798267972
176.3110038	0.38808893	0.105283233	3.68614184	0.000227679	0.003037078
17.97957222	-0.298550545	0.46926809	-0.636204659	0.524643037	0.715773863
952.3039215	0.048030075	0.06860089	0.700137789	0.483841258	0.683016357
10.21823249	0.397879186	0.383282998	1.038082013	0.299231874	0.514104803

391.9946938	0.364700184	0.081249165	4.488663767	7.16713E-06	0.000180262
358.2027484	0.073315981	0.133401062	0.549590684	0.58260015	0.758895775
4.319199072	-0.109232007	0.520769228	-0.209751269	0.833861808	0.916902912
43.05192489	-0.215521374	0.150058456	-1.436249441	0.150931375	0.334694235
70.78396824	0.312622329	0.141341741	2.211818868	0.026979183	0.106100515
89.67740056	-0.1432525	0.137972243	-1.038270431	0.299144171	0.514082977
259.7719316	0.132058659	0.073676372	1.792415319	0.073066463	0.209828068
252.3805931	-0.181590749	0.099649583	-1.822293111	0.068410523	0.200815147
1161.484727	0.062619564	0.067664926	0.925436084	0.354739117	0.569365959
248.4008504	-0.161377546	0.083689582	-1.928287142	0.05381942	0.169850791
50.90371436	0.368535787	0.172432645	2.137273872	0.032575723	0.120433675
121.3703609	0.500341209	0.209516129	2.388079676	0.016936669	0.076400518
1129.349465	0.40606553	0.084854145	4.785453057	1.70602E-06	5.42567E-05
297.1434019	0.423132589	0.182648869	2.316645003	0.020523078	0.087544584
5.782922349	-0.176854792	0.45671817	-0.387229596	0.698586242	0.838453107
1129.275907	0.474387728	0.054429409	8.715650885	2.89093E-18	1.64225E-15
108.3019353	0.130213464	0.24269368	0.536534217	0.591589394	0.764439852
34.14960972	0.652963428	0.325114963	2.008407799	0.044599975	0.149977543
2.667536488	-0.125944524	0.818292371	-0.1539114	0.877679596	0.938489926
1650.619177	0.083776156	0.083700704	1.00090146	0.316874451	0.53185835
102.2627288	-0.077942629	0.124872665	-0.624176868	0.532511437	0.721964728
55.45458663	0.270013703	0.217732643	1.240115858	0.214932544	0.419426526
13.30120766	-2.188816492	0.889763121	-2.459999117	0.013893736	0.06666545
72.1275558	-0.171591091	0.116737817	-1.469884356	0.141593077	0.320941711
61.55866733	0.187536162	0.19361129	0.968622035	0.332733805	0.548337036
308.7862761	-0.000909804	0.070929553	-0.012826862	0.989765925	0.994530438
43.18592677	0.496357925	0.180158491	2.755118128	0.005867095	0.035469549
743.7282352	0.27727561	0.050199408	5.523483629	3.32343E-08	1.70561E-06
504.0785298	0.141635235	0.062184197	2.277672501	0.022746097	0.094008833
1835.209849	-0.361237279	0.088987131	-4.059432802	4.91921E-05	0.000879902
39.2044734	0.273031446	0.240856218	1.133586869	0.256967883	0.467560074
296.2377797	-0.525630595	0.232989488	-2.256027084	0.024068935	0.097855783
285.0114103	0.158889272	0.089863703	1.768114008	0.077041846	0.216781036
232.1028163	-0.341722006	0.18333164	-1.863955426	0.062327964	0.188505761
2.744414628	-0.264969715	0.570202035	-0.464694439	0.642150291	0.801058905
140.5335589	-0.310000331	0.13078415	-2.370320336	0.017772679	0.079007019
2.157384364	0.455303938	1.139628056	0.39951977	0.68951026	0.832526533
27.88506597	0.419864108	0.243646082	1.723254095	0.084842585	0.231062448
30.3522129	0.124591286	0.181340961	0.687055401	0.492047818	0.689578541
105.8942868	0.158648589	0.268755504	0.590308243	0.554984014	0.739858687
1075.65822	0.214868326	0.126036435	1.704811197	0.088229643	0.23689486
2.822894597	-0.673979134	0.740619919	-0.910020264	0.362811823	0.576876249
3128.476391	-0.259562397	0.093113187	-2.787600815	0.005309993	0.032989168
347.3217682	-0.077745462	0.065984572	-1.178236964	0.23870215	0.446360434
14.31397527	0.35618466	0.31692834	1.123864973	0.261070313	0.472311919
104.2371402	0.124761247	0.146290148	0.852834239	0.393751234	0.606740045
71.90788658	0.156794404	0.134390159	1.16671046	0.243327317	0.450998082
5.074032167	0.925526397	0.525411984	1.761525098	0.078149569	0.218765675

118.558156	0.082149158	0.098582355	0.833304884	0.404672802	0.61545234
4.813305476	1.158980159	1.337774124	0.866349661	0.38629844	0.599452311
1066.795261	0.125189567	0.043811057	2.857487923	0.004270089	0.028082013
17.91151165	0.316288708	0.235478068	1.343176932	0.179214689	0.37485815
11.03428421	0.187367389	0.287965933	0.650658177	0.515267167	0.708394451
266.6166334	0.217900451	0.069352745	3.141915326	0.001678466	0.014199103
91.72012727	-0.272157222	0.23164126	-1.174908228	0.240031434	0.447417723
207.6831631	0.530548082	0.151823201	3.494512555	0.000474928	0.005366233
155.16652	0.235186291	0.088224801	2.665761658	0.007681413	0.043247983
88.24706662	0.271811104	0.140328475	1.936963287	0.052749831	0.167728377
127.1803247	-0.296148409	0.096510726	-3.068554364	0.002150972	0.017019746
1001.105365	-0.243100493	0.115944019	-2.096705773	0.036019629	0.129081438
53.74511908	0.49206181	0.129619205	3.796210667	0.000146925	0.002121281
494.847135	0.275064202	0.114368811	2.40506305	0.016169676	0.073956537
1324.471506	0.082523094	0.075913407	1.087068767	0.27700642	0.490003625
11.89606143	-0.205775251	0.361318126	-0.569512673	0.569008273	0.749727449
202.8990889	0.360568799	0.1303404	2.766362536	0.005668548	0.034685169
25.92920268	0.706432045	0.387961193	1.82088327	0.068624603	0.201034162
310.6047069	0.034055793	0.117059043	0.290928339	0.771106127	0.880499181
1520.651349	0.198470249	0.103001856	1.926860906	0.053996967	0.17011781
2.306163415	0.168202445	0.678073241	0.248059406	0.804088444	0.899535041
6.683375115	0.510906521	0.358258741	1.426082502	0.153844529	0.33850411
92.71452901	-0.147138928	0.148066723	-0.993733942	0.320352414	0.535424686
74.15304956	0.064293942	0.124771484	0.515293557	0.6063479	0.776144158
43.37213274	0.226449783	0.149950553	1.51016304	0.131001827	0.304948947
413.3449714	-0.058409263	0.055086909	-1.060311134	0.289003075	0.503706799
981.1509357	-0.067400505	0.080714645	-0.835046789	0.403691365	0.614855381
394.819633	-0.934675841	0.44829407	-2.084961418	0.037072803	0.131886171
219.0548088	0.107211969	0.084550005	1.268030306	0.204787128	0.4078906
588.2275793	-0.185577831	0.164012401	-1.131486581	0.257850348	0.468260636
2253.545097	-0.116259187	0.039928094	-2.911713924	0.003594517	0.024838955
1021.89727	0.120835909	0.102063267	1.183931424	0.23644021	0.443858271
288.6314709	-0.17325903	0.090744739	-1.909301095	0.056223261	0.174791848
281.0624822	-0.210477959	0.083300709	-2.526724695	0.011513171	0.058216079
2.549400261	-0.295090352	0.642445699	-0.459323415	0.646001936	0.803672851
346.7091413	-0.07913228	0.103145638	-0.767189785	0.442968685	0.648528056
95.2414166	-0.00232321	0.147468739	-0.015753912	0.987430717	0.993528977
398.7189548	-0.161994111	0.074088686	-2.18648918	0.028779835	0.11077547
34.44104523	0.003672681	0.186078614	0.019737252	0.984252974	0.992142415
1026.288676	-0.070269618	0.077672796	-0.904687626	0.365630898	0.579117721
26.67298244	0.576223225	0.267237835	2.156218725	0.03106659	0.116547057
292.5345459	-0.06559404	0.061671403	-1.063605449	0.287507473	0.502056191
132.6622392	-0.191076788	0.104416591	-1.829946629	0.06725792	0.198283281
104.8761858	0.252078007	0.142190667	1.772816819	0.076259065	0.215487452
16.40506786	0.285915941	0.279120977	1.024344154	0.305672749	0.520512215
102.0110003	-0.02788566	0.145606767	-0.191513489	0.848123313	0.924868171
298.4687657	-0.840340188	0.279138826	-3.010474038	0.002608402	0.019693319
202.2513509	0.305369666	0.105908394	2.883337703	0.003934854	0.026372163

489.4995231	-0.132733415	0.055058653	-2.410763935	0.015919148	0.073152594
352.1458926	-0.010649871	0.083297008	-0.127854185	0.898264369	0.948529403
6767.490199	-0.775129631	0.558064096	-1.388961656	0.16484441	0.354800995
133.681078	0.489343935	0.140081626	3.493277086	0.000477131	0.005380054
3624.929351	0.3931599	0.135702644	2.897216217	0.003764902	0.025543356
1012.702533	0.069846476	0.04570149	1.528319454	0.126433242	0.297806867
2.068686933	1.988286676	1.940607611	1.024569143	0.305566531	0.520512215
22.0009733	0.287085759	0.235222282	1.220487092	0.22228028	0.42713698
93.57351569	-0.235355093	0.101519722	-2.318318923	0.020431995	0.087359637
93.32510878	-0.114391725	0.121381526	-0.942414621	0.345980404	0.560385526
72.7925716	-0.212309389	0.167807707	-1.265194504	0.205801612	0.409267957
2032.47481	0.008232475	0.106941644	0.076981004	0.938638657	0.970696688
818.5770542	0.070627208	0.05937041	1.189602841	0.234202529	0.441189294
263.7304382	-0.157299281	0.074091986	-2.123026937	0.033751595	0.123286867
190.1462054	-0.675301938	0.121360589	-5.564425357	2.63018E-08	1.4068E-06
111.7049994	-0.261650067	0.134942139	-1.938979686	0.052503812	0.167075101
169.5306803	0.095285805	0.132108285	0.721270477	0.470743118	0.671362327
343.9574315	-0.040666127	0.087391734	-0.465331508	0.641694074	0.800671679
24.36539932	-0.229334173	0.256275757	-0.894872677	0.370855154	0.584428073
71.0426842	-0.169977207	0.138685757	-1.225628433	0.220338533	0.424860747
86.63933327	-0.292849727	0.175205122	-1.671467843	0.094629305	0.247020171
82.14343011	0.155775214	0.114490756	1.360592057	0.173642643	0.367307255
4693.268262	-0.000181205	0.098269412	-0.001843966	0.998528729	0.998831883
748.4991578	-0.116944069	0.124264994	-0.941086191	0.346660692	0.561266657
34.35058704	-0.010035537	0.169090215	-0.059350191	0.952673185	0.977124952
763.6605404	0.044572548	0.058815185	0.757840812	0.448546287	0.653462286
45.18072797	0.021273091	0.271767463	0.078276812	0.937607862	0.970479513
1817.227417	0.100275092	0.054946847	1.824947161	0.068009003	0.200032194
19.44082899	0.272542962	0.246932021	1.103716567	0.269716107	0.481711095
4.169247159	0.728638124	0.550705791	1.323098715	0.185802552	0.384015275
228.0116914	0.856687576	0.250025581	3.426399696	0.00061164	0.006534469
1039.863871	0.030182707	0.114583741	0.263411775	0.792233216	0.892820495
104.917451	0.041762542	0.248027349	0.168378778	0.866285302	0.933794678
44.87004482	-0.112432071	0.20082264	-0.559857552	0.575576604	0.754099648
50.43419723	-0.43976789	0.164777415	-2.668860242	0.007610911	0.042968523
940.649384	0.024297916	0.077724151	0.312617325	0.754571414	0.871752087
576.7417475	0.080866272	0.067859344	1.191674825	0.233388773	0.440216011
6.983576965	0.349878042	0.375396835	0.932021821	0.351325249	0.566504355
17.10872614	-0.35368309	0.326062525	-1.084709416	0.278050376	0.49116469
7.486783388	-0.545135714	0.447418396	-1.218402548	0.223071037	0.42812953
3.120461384	-0.051629207	0.718033614	-0.071903607	0.94267862	0.972623458
127.1871977	-0.100699039	0.088255925	-1.140988996	0.253874502	0.463210605
7.051603292	0.033017065	0.440233111	0.074999049	0.940215469	0.971553467
79.69680798	-0.218554625	0.12027303	-1.817154059	0.069193527	0.202216457
33.6123885	-0.232020091	0.482843683	-0.480528377	0.63085173	0.79315053
2080.219726	0.076129242	0.112479648	0.676826816	0.498515842	0.694742406
496.3913696	0.008541047	0.063797885	0.133876648	0.893500116	0.946123642
188.2988454	0.096752617	0.113586839	0.851794253	0.394328297	0.607070016

6.988841823	-0.153185884	0.447136544	-0.342593076	0.731904612	0.858466987
42.015219	-3.014270484	0.884870836	-3.406452514	0.00065813	0.006941122
178.0353233	-0.241023322	0.098874559	-2.43766774	0.014782354	0.069518842
3.974130508	0.239561566	0.500711553	0.478442257	0.632335463	0.794046377
117.2051826	-0.308433505	0.137117723	-2.24940656	0.024486642	0.099113743
696.1089569	0.135809651	0.14303006	0.949518242	0.342357099	0.556594335
40.22608614	0.168767663	0.302460368	0.557982734	0.576856174	0.755156913
2046.491631	-0.169012402	0.068632294	-2.462578362	0.013794203	0.066308971
93.72841835	-0.384342327	0.264080486	-1.455398437	0.145558989	0.326561185
2903.783233	-0.089080108	0.050522255	-1.763185492	0.07786921	0.218389064
2.176782552	-1.483309613	1.728637085	-0.858080407	0.390848066	0.604048588
224.0612054	-0.017824144	0.098596031	-0.180779525	0.856540635	0.92936531
97.04512287	-0.011472506	0.153025859	-0.074971027	0.940237764	0.971553467
58.57483038	0.019002245	0.159016382	0.119498661	0.9048803	0.952300557
420.2263726	-0.198940141	0.089770982	-2.21608516	0.02668567	0.105488056
54.34751747	0.112996759	0.398801924	0.283340557	0.776915791	0.884146915
31.95499053	0.52785403	0.265626336	1.987205177	0.046899664	0.155332744
25.74624018	-0.011209082	0.20466819	-0.054767095	0.956324015	0.978600026
637.8546986	0.053604258	0.083605126	0.641159947	0.521418769	0.713205978
2.902774362	-0.836807537	0.759699481	-1.101498102	0.27067993	0.482543141
126.5997735	0.436945228	0.169524436	2.577476366	0.009952469	0.052315561
5.974891182	0.437647922	0.440134373	0.994350701	0.32005216	0.535174512
19.14900588	0.14469056	0.52654057	0.274794704	0.783473967	0.887685704
1436.669679	-0.127245612	0.077389147	-1.644230719	0.100128554	0.256734287
799.7102852	-0.134754019	0.076791412	-1.754805854	0.079292522	0.220727443
10.38221658	-0.250177446	0.402972344	-0.620830311	0.534711277	0.723766845
395.0846517	0.301453268	0.090223903	3.341168551	0.000834266	0.00835991
4.337368135	0.987093138	0.468005039	2.109150663	0.034931579	0.126197989
5156.463397	0.243083193	0.07068187	3.439116614	0.000583616	0.00632532
26.34688557	0.347453256	0.313379346	1.108730555	0.267546436	0.479134688
28.17745645	-2.111671529	0.503941307	-4.190312446	2.78571E-05	0.000560338
1453.710659	-0.247091157	0.105595002	-2.339989132	0.019284301	0.083756809
3415.494136	-0.100865713	0.099255877	-1.016219049	0.309525087	0.524816415
90.80019195	-0.048119148	0.174287904	-0.276090001	0.782478941	0.887032618
37.70097531	-0.451043897	0.423005159	-1.066284623	0.286294999	0.50074726
539.2598751	-0.267254593	0.10354346	-2.581086174	0.009848999	0.052003979
6.055475226	-1.744097166	0.689501716	-2.529503734	0.011422396	0.057881436
173.9098528	-0.007622018	0.091460128	-0.083337056	0.933583536	0.968634829
530.4677802	-0.544250042	0.169639449	-3.208275235	0.001335336	0.012013626
74.65413366	0.134998872	0.180171358	0.749280425	0.453688203	0.658108374
1.762149666	-0.310878805	0.820516261	-0.378881955	0.704775525	0.842009718
16.89271565	0.332815352	0.362093248	0.919142661	0.358020958	0.572450705
254.1733858	0.265654982	0.114558434	2.318947404	0.020397888	0.08728177
324.8145413	-0.193773662	0.061877539	-3.131567056	0.001738761	0.014547661
231.9306083	-0.031759196	0.100597813	-0.315704637	0.752226707	0.870852794
46.61299382	-0.627058708	0.272485845	-2.301252413	0.021377368	0.090184573
325.7442849	-0.22980908	0.084858832	-2.7081339	0.006766272	0.039541526
230.1904382	-0.02636663	0.166269387	-0.158577779	0.874001537	0.936840479

86.95288799	-0.14624738	0.134788807	-1.085011316	0.277916643	0.491086429
23.21774774	-0.440334602	0.382353162	-1.151643676	0.249467526	0.45849031
179.0491566	-0.000757198	0.179403964	-0.00422063	0.996632434	0.998020302
41.17044002	-0.428356105	0.186409002	-2.297936799	0.021565387	0.090757485
38.5674472	-1.509485304	0.607492045	-2.484782011	0.012963079	0.063350269
20.9422698	-0.550533837	0.287650751	-1.913896745	0.05563335	0.173284896
1349.025574	-0.13925987	0.045265077	-3.076541092	0.002094174	0.016745151
40.99785875	0.061793753	0.285790936	0.216220129	0.828816164	0.914385171
183.0495646	0.415486152	0.217566592	1.909696463	0.056172307	0.174666399
6.137016696	0.816809168	0.419813489	1.945647742	0.051697068	0.165563277
2.792787941	0.886197636	0.783212521	1.13149064	0.257848641	0.468260636
84.06535822	-0.934011071	0.378269545	-2.469168039	0.013542761	0.065445421
54.02708687	-0.252208408	0.251267843	-1.003743278	0.315502371	0.530279479
6.756862754	-0.12321001	0.401366869	-0.306976036	0.758861613	0.874200334
7.859933852	-0.114640551	0.402650403	-0.284714854	0.775862609	0.883652841
1033.544882	0.03703745	0.080688617	0.459017042	0.646221928	0.803825132
61.31608912	0.049303146	0.120678642	0.408549059	0.682870621	0.828578523
655.7301103	-0.0689284	0.144307258	-0.477650264	0.632899149	0.794224511
411.9366612	0.006130341	0.071507743	0.085729756	0.931681247	0.967628097
64.86269995	0.088124606	0.11861718	0.742932904	0.457522304	0.660985919
2.871186256	-1.227292087	0.85863485	-1.429352755	0.15290287	0.337385734
626.3553061	0.334342741	0.16995643	1.967226187	0.04915714	0.160201822
6.70375362	-0.096463444	0.474571936	-0.203264114	0.838928603	0.919711839
21.45119814	0.487970027	0.264411436	1.845495164	0.064965541	0.193814257
134.1314982	0.011262845	0.103955056	0.108343407	0.913723291	0.956940718
10.58879977	0.505614565	0.314282163	1.608791792	0.107661876	0.269752358
204.0211065	-0.215712126	0.077579937	-2.780514327	0.005427286	0.033525412
15.30845657	-0.376631346	0.309458916	-1.217064127	0.223579818	0.42871024
8.179683304	-0.398962697	0.599405749	-0.665597049	0.505668693	0.700680129
1006.346058	0.156108451	0.114052881	1.368737466	0.171081354	0.363288764
2613.585764	-0.193219791	0.107931655	-1.790205019	0.073420959	0.210537314
192.4743755	0.040612728	0.111699048	0.363590636	0.716163721	0.849440394
296.8800472	0.071174045	0.129142907	0.551126243	0.581547137	0.758146246
38.4687527	-0.391961707	0.171271598	-2.288538863	0.02210616	0.092105817
235.3696571	-0.09181005	0.077405514	-1.186091858	0.235586026	0.442880916
25.20276234	0.517767855	0.237533756	2.179765367	0.029274858	0.112208938
1070.830597	-0.213537803	0.095464852	-2.236821178	0.02529803	0.101500181
560.2880253	-0.698392158	0.433673572	-1.61040977	0.107308429	0.268992116
69.58014345	-0.389541737	0.313175824	-1.243843575	0.213557139	0.418106369
196.9273952	-0.350205578	0.089037877	-3.933220222	8.38154E-05	0.001365751
1484.793598	-0.226121279	0.115642057	-1.955355048	0.050541178	0.16312997
150.3440362	0.126811083	0.101420181	1.250353549	0.211170425	0.415431286
13.3411721	0.690484237	0.272457119	2.534285907	0.011267676	0.057220619
1882.80901	-0.231615373	0.120226274	-1.926495481	0.054042536	0.170131231
13.97112433	1.838703541	0.301718601	6.094100704	1.10054E-09	9.06518E-08
8.593024147	0.288658556	0.402719649	0.716772962	0.473514197	0.673691415
257.7672857	0.002033017	0.065422729	0.031075092	0.975209654	0.987719156
12.50968329	0.173331349	0.285221016	0.607708897	0.543380561	0.730508517

145.3164312	-0.016626796	0.114577275	-0.145114253	0.884620665	0.942458827
44.54579064	-0.26271506	0.231328505	-1.13567958	0.25609069	0.466272991
318.4742163	-0.090335294	0.084115132	-1.073948198	0.2828459	0.496600591
1091.132917	-0.20316973	0.105301365	-1.929412116	0.053679721	0.169637391
112.7404143	0.467889126	0.129432511	3.614927362	0.000300432	0.003738153
279.3391896	0.612637739	0.098857044	6.197208769	5.74732E-10	5.02611E-08
8.979493665	0.771765795	0.373021519	2.068957838	0.038550043	0.135327387
1047.308943	0.05938249	0.075249398	0.789142386	0.430028791	0.637008662
116.2084676	-0.258135737	0.125804593	-2.051878478	0.040181474	0.139152744
6.488722964	0.364329224	0.417265174	0.873135951	0.382588981	0.595331148
52.3788321	-0.053692923	0.143840031	-0.373282202	0.708938422	0.844935557
230.7008548	0.29140505	0.101814381	2.862120725	0.004208166	0.027774569
82.75511847	-0.185375821	0.169621546	-1.092878977	0.27444694	0.487131087
21.49057986	0.123454487	0.254719554	0.484668274	0.627911675	0.791096258
54.46760858	-0.231486045	0.174176186	-1.329033835	0.183836807	0.381231484
398.1699844	-0.254574538	0.108187947	-2.353076707	0.018618793	0.081476082
69.17236381	-0.428610507	0.132224849	-3.241527684	0.001188909	0.011028201
304.7592523	0.019793517	0.120976789	0.163614171	0.870034888	0.935388289
502.7085702	0.011828514	0.095322313	0.124089667	0.901244279	0.950648941
11.58374424	0.538005562	0.304158367	1.768833672	0.076921636	0.216653621
4.758503361	-0.75328318	0.463801612	-1.624149552	0.104343895	0.264231535
107.2541486	-0.339830099	0.102634022	-3.311086259	0.000929346	0.009095037
769.2577329	0.291934543	0.075913871	3.845602107	0.000120257	0.001802648
72.76626985	-0.407521488	0.161629427	-2.521332266	0.01169114	0.058881027
41.60262754	-0.274046239	0.157194956	-1.743352621	0.081272039	0.224666226
335.2608453	-0.356987696	0.114155289	-3.127211173	0.001764731	0.014712643
2.980478009	0.720643952	0.562746648	1.280583286	0.200340074	0.402794708
1867.427893	-0.369893891	0.110826765	-3.3375863	0.000845095	0.008447871
18.24081815	-0.126479703	0.281945796	-0.44859581	0.653723257	0.80858327
132.7088618	-0.052526311	0.116513831	-0.4508161	0.652122096	0.80762738
169.81214	-0.237963184	0.139621636	-1.704343183	0.088316992	0.237022358
155.4496579	0.682777369	0.214326938	3.185681538	0.001444135	0.012763237
106.146806	-0.148675043	0.191836123	-0.775010674	0.438333352	0.644127981
227.5401053	-0.181194577	0.087939115	-2.060454871	0.039355076	0.13698194
19.08533597	-0.205463961	0.26652704	-0.770893492	0.440770065	0.646478459
2566.801736	-0.218478011	0.101792847	-2.146300234	0.031849033	0.118518403
476.0178347	-0.091724166	0.079445237	-1.154558404	0.248271309	0.457077732
4990.433573	-0.092300653	0.074787403	-1.234173786	0.217138153	0.422114485
929.8017675	-0.11800267	0.074262598	-1.588991947	0.112062211	0.27702774
95.2290444	0.132795059	0.110801083	1.198499647	0.23072256	0.437038457
94.79525149	-0.255622698	0.130532277	-1.958310261	0.050193617	0.162581529
1174.441857	-0.126431713	0.107545846	-1.17560759	0.239751721	0.447181861
2357.957336	-0.16937843	0.104535146	-1.620301279	0.105167574	0.265568479
3.821936927	-0.075967166	0.744568591	-0.102028432	0.918734107	0.959711208
458.963069	-0.598871527	0.258355009	-2.318017869	0.02044835	0.08740688
191.2585344	-0.02120839	0.079969075	-0.265207389	0.790849703	0.892054639
143.3178475	-0.016734018	0.096503273	-0.173403629	0.862334173	0.931532154
304.7373486	-0.133024421	0.064994807	-2.046693072	0.04068823	0.140523894

2882.141354	-0.071021591	0.128352942	-0.553330448	0.580037155	0.757293715
128.3250533	0.210602384	0.220345742	0.9557815	0.339182637	0.554005032
67.92062916	0.066447797	0.164058284	0.40502555	0.68545873	0.829778815
11.26209221	0.080235341	0.278812276	0.287775497	0.773518595	0.882169978
25.25519902	-0.372212774	0.515113191	-0.722584435	0.469935234	0.670859016
157.2936712	-0.260462862	0.119059358	-2.187672321	0.02869348	0.110624008
119.4554789	-0.174648078	0.12669552	-1.378486613	0.168053098	0.359452966
875.8757097	-0.174028946	0.09299893	-1.871300523	0.061303438	0.186365167
330.2099382	0.35229556	0.091475034	3.851275515	0.000117504	0.001771056
3.183505726	1.699223739	0.653682552	2.599463202	0.009336969	0.050119656
1417.797157	-0.165612975	0.069753025	-2.374276613	0.017583372	0.07842991
201.1791895	-0.023115735	0.106878219	-0.216281064	0.828768669	0.914385171
77.29699697	0.034404923	0.122076119	0.281831724	0.778072544	0.884485723
2.943116441	-0.235434899	0.597298904	-0.394165966	0.693458511	0.83500768
4.566711694	-2.663609567	1.026874375	-2.593900121	0.009489405	0.050739519
455.9916866	-0.272858251	0.115206019	-2.368437458	0.0178634	0.079214443
39.79734045	-0.337404765	0.173320395	-1.94671126	0.051569361	0.165282812
1.843383151	0.915004373	0.783377776	1.168024421	0.242796915	0.450533495
147.042977	0.630006895	0.130296471	4.835180017	1.33025E-06	4.41826E-05
1553.229181	-0.248609844	0.119555464	-2.079451968	0.037575828	0.13309486
51.46462263	-0.367597154	0.168159402	-2.18600417	0.0288153	0.110886068
295.6118668	0.208313327	0.081384529	2.559618248	0.01047872	0.054233878
351.9105206	0.262283484	0.114189141	2.29692142	0.021623253	0.090849647
846.2533321	0.025861005	0.070779615	0.365373631	0.714832522	0.848364741
73.17978258	0.005736356	0.148893759	0.038526501	0.969267902	0.984810622
6.339886525	-0.049520677	0.368230389	-0.134482863	0.893020762	0.946108145
23.72343492	-0.059023783	0.181545009	-0.325119283	0.745090795	0.866362631
171.9757176	-0.119699918	0.097406258	-1.228872981	0.219119433	0.423732075
542.1664144	0.005159327	0.089404835	0.057707472	0.953981642	0.977641944
328.9411318	0.053620093	0.108416329	0.494575801	0.620899587	0.786339161
602.6066702	-0.307082462	0.14552439	-2.110178662	0.03484297	0.125960738
88.20176864	-0.045606031	0.106922754	-0.426532517	0.669719859	0.820241125
7.870813863	-0.988539789	0.71642775	-1.379817838	0.167642739	0.359007538
4.375672682	1.030639847	0.44833366	2.298823264	0.021514977	0.090625859
1624.407243	-0.076221417	0.076474384	-0.996692131	0.31891397	0.534029791
474.2745611	0.028261844	0.09441179	0.299346557	0.764675635	0.8776748
194.7855827	-0.202191708	0.10296512	-1.963691279	0.049565905	0.161182138
35.94578104	0.058911976	0.223622165	0.263444262	0.792208179	0.892820495
34.17292019	-0.1360106	0.235219172	-0.578229227	0.563109369	0.74565258
9.681245848	-0.310490166	0.432726981	-0.717519775	0.473053439	0.673383433
250.4029464	-0.412011773	0.113450894	-3.631630898	0.000281636	0.003582755
1412.325053	-0.26214422	0.084735099	-3.09369107	0.001976832	0.01608214
463.2610983	-0.074260099	0.077245639	-0.961350057	0.336376187	0.551498935
449.0311511	0.011111798	0.113968012	0.097499273	0.922329911	0.961560774
58.30304717	0.490591254	0.130440081	3.761046846	0.000169204	0.002388571
1368.996454	-0.034898943	0.096406657	-0.361997234	0.717354097	0.850132465
644.9247096	-0.121569754	0.130743855	-0.929831497	0.352458336	0.567474455
100.5271194	-0.011195771	0.156355414	-0.071604624	0.94291656	0.972623458

11.95390853	-0.58571622	0.484803876	-1.20815086	0.22698924	0.432703163
16.51803428	0.314852336	0.274750622	1.145956774	0.251813038	0.461032228
622.9551804	-0.041206337	0.073562431	-0.560154636	0.575373966	0.753972704
33.48757523	0.40572092	0.191712743	2.116296042	0.034319637	0.12461576
45.11223942	0.10880863	0.164046577	0.663278883	0.507151958	0.70201787
9.113697104	-0.466483468	0.426048063	-1.094908083	0.273556907	0.48625124
640.7115073	-0.319896912	0.091602471	-3.492230158	0.000479005	0.005382765
1033.795976	0.130625576	0.089968472	1.451903912	0.146528325	0.328031908
304.2869598	-1.559272525	0.448145632	-3.47938798	0.00050256	0.005594041
2.676707257	-0.563419804	1.01543338	-0.554856492	0.578992823	0.756889295
235.9686137	-0.326982379	0.094107342	-3.474568202	0.000511676	0.005680158
535.7526705	0.04552273	0.079271901	0.574260604	0.565791464	0.748001651
499.9760931	-0.101722321	0.073825742	-1.377870627	0.168243235	0.359626191
881.0800919	-0.352755133	0.087833967	-4.016158482	5.91545E-05	0.001026882
39.78080803	0.070563256	0.207006554	0.3408745	0.733198062	0.859266193
336.2720706	0.287161769	0.067715446	4.240712959	2.22811E-05	0.000466402
115.6835893	0.274937627	0.178549888	1.539836454	0.123600224	0.293804883
166.5216728	0.120760928	0.115007402	1.050027448	0.293705493	0.508620235
129.8457426	0.096279357	0.119073775	0.808568949	0.418763131	0.62783981
1.868745137	-0.398511893	0.692076088	-0.575820925	0.564736229	0.747206097
374.9964814	0.086026212	0.075173047	1.144375743	0.252467843	0.461564227
6.084513977	-0.449796626	0.497632348	-0.903873367	0.366062553	0.579522823
43.58382008	-0.777326761	0.270810891	-2.870367429	0.004099951	0.027245901
104.5378974	-0.341817327	0.112613227	-3.0353213	0.002402796	0.018488396
64.50992447	0.225511772	0.165575472	1.361987789	0.173201739	0.366687491
4.841993018	-0.024169591	0.543255534	-0.04449028	0.9645136	0.982392126
133.5703785	-0.356017194	0.105095311	-3.387564979	0.00070516	0.007329216
201.0489632	-0.382216999	0.162974924	-2.345250353	0.019014307	0.082899546
2592.445093	-0.203593783	0.068900355	-2.95490181	0.003127686	0.022431648
2.926147748	-0.771319356	0.594316635	-1.29782562	0.194347264	0.395212795
1159.696489	-0.187325379	0.089887243	-2.084004058	0.037159798	0.132018657
573.7615988	-0.198178082	0.094502645	-2.097063873	0.035987921	0.129023943
230.8337815	-0.103305716	0.085578077	-1.20715163	0.227373755	0.433185525
79.36432176	-0.315297507	0.154170268	-2.045125245	0.040842512	0.140967849
61.31363897	-0.331965518	0.190484007	-1.742747451	0.081377739	0.224822551
6.350735559	0.044809795	0.428119761	0.104666496	0.916640448	0.958837688
12.26481523	-1.567043536	0.673252301	-2.327572493	0.019934815	0.085745731
2.326792533	-0.355737082	0.651979466	-0.545626205	0.585322908	0.760458169
202.6815198	0.757504651	0.125390116	6.041183117	1.52988E-09	1.21755E-07
518.1743519	-0.157567219	0.085676421	-1.839096652	0.065900971	0.195754166
65.58384272	0.030042269	0.134303566	0.223689287	0.822999082	0.911237589
208.591903	0.373003387	0.099245004	3.758409722	0.000170997	0.002407692
754.7478741	-0.120527456	0.053892697	-2.23643393	0.025323361	0.101577074
20.68612859	-0.364141789	0.258882276	-1.406592194	0.159548349	0.347120906
347.5824105	-0.329871853	0.069811163	-4.725202113	2.29887E-06	6.97688E-05
1540.582315	0.251059216	0.123690632	2.029735085	0.042383475	0.144769927
25.84988387	2.508613787	0.313700301	7.996848529	1.27644E-15	4.29145E-13
659.9165812	-0.039503638	0.078089096	-0.505879051	0.61294156	0.780915252

38.63958857	0.038322032	0.159909453	0.239648323	0.810602899	0.904012269
83.78921652	0.225836479	0.126800861	1.781032699	0.074907109	0.213239062
322.9235497	-0.107257323	0.058276658	-1.84048515	0.065697042	0.195359761
94.63951913	-0.259779988	0.174559322	-1.488204612	0.136696941	0.313907638
267.4264464	0.235722948	0.149322498	1.578616428	0.114424064	0.280801733
10.59101749	0.530949973	0.327721642	1.620124841	0.105205462	0.265606064
10643.59955	0.082086789	0.079176789	1.036753193	0.299850895	0.514717479
283.260902	-0.35520578	0.115796401	-3.067502762	0.002158555	0.017071546
53.0820057	-0.65351186	0.376263405	-1.736846719	0.08241423	0.226950609
202.9101429	-0.028792412	0.071574854	-0.402269945	0.687485371	0.831033101
302.4653538	0.146012489	0.088353808	1.652588534	0.098414633	0.253960318
443.8643265	-0.248628128	0.082661976	-3.007768986	0.002631731	0.01980591
655.524893	-0.163126753	0.074570692	-2.187545117	0.028702753	0.11063387
695.774983	-0.040079574	0.140145828	-0.285984781	0.774889778	0.883244599
614.8437939	-0.283534034	0.087249332	-3.249698626	0.001155274	0.010789104
364.5304294	-0.236731841	0.093265808	-2.538248958	0.01114087	0.056769159
4.810762591	-0.341096936	0.527634835	-0.646464019	0.517978892	0.710447445
69.64362443	0.341996038	0.218318728	1.566498853	0.117231877	0.284975349
22.91182836	-0.022075927	0.284499069	-0.077595781	0.938149599	0.970668417
13.4499306	-0.363864123	0.319064635	-1.140408819	0.254116021	0.463394656
5.418184915	-0.397670976	0.602571153	-0.659956876	0.509281503	0.703673865
461.0569494	-0.039910736	0.073527288	-0.542801686	0.587266353	0.761715809
43.52521042	-0.252327139	0.165170717	-1.52767478	0.126593305	0.297885745
6.782942349	-0.079582549	0.462996264	-0.171885941	0.863527199	0.931920109
536.4219395	0.00380537	0.050630106	0.075160218	0.940087236	0.9715198
9.020371515	-0.593796449	0.373161758	-1.591257507	0.111551636	0.276263025
551.2788883	0.377752402	0.115050126	3.283372358	0.001025731	0.009824855
331.3546074	0.631613648	0.13902198	4.543264669	5.53896E-06	0.000144839
292.0614102	0.051533082	0.157696156	0.326787179	0.743828857	0.865506187
1132.994275	-0.123270856	0.084300599	-1.462277344	0.143665224	0.323704153
529.6916038	-0.274561647	0.111776629	-2.456342159	0.014035945	0.067119928
2.552222856	0.292145782	0.60829636	0.480268832	0.631036249	0.793230487
128.6728625	0.066464649	0.185846744	0.357631497	0.720619104	0.851795876
159.5073258	-0.253610069	0.090340544	-2.807267473	0.004996374	0.031476199
252.4726528	-0.048026112	0.066387764	-0.723418118	0.469423043	0.670534572
5.203698491	-0.670445985	0.874298993	-0.766838335	0.443177641	0.648623708
1231.516661	-0.258123732	0.065264636	-3.955032099	7.65244E-05	0.001269549
1271.607608	0.038064789	0.088134554	0.431894048	0.665818421	0.817765799
50.37087268	-0.812620875	0.270462276	-3.004562733	0.002659629	0.019988471
227.6991697	-1.069668693	0.348853741	-3.066238274	0.002167705	0.017127468
1622.032409	0.232973537	0.114285845	2.038516117	0.041498345	0.142574292
89.41291637	-0.26165668	0.126595983	-2.066864001	0.038746971	0.135754486
40.12455563	0.211755133	0.187719759	1.128038589	0.259303622	0.470200096
54.91803867	-0.032922158	0.175016256	-0.188109145	0.850791092	0.926363298
656.7992725	-0.2481784	0.112951314	-2.197215698	0.028005045	0.108835837
4.709807528	-0.466934278	0.482109802	-0.968522681	0.332783397	0.548337036
73.27267015	-0.13792301	0.137479794	-1.003223858	0.315752864	0.530496785
58.22860622	-0.491263567	0.326057976	-1.506675508	0.131893852	0.306246557

40.17270261	0.508322343	0.336817928	1.50919028	0.131250163	0.305182102
2466.66584	-0.058741447	0.092914188	-0.632211815	0.527248478	0.717486488
616.730657	0.301255741	0.099938713	3.014404835	0.00257484	0.019511458
14.06006614	0.299182389	0.241900674	1.236798491	0.216161902	0.420878286
227.7306302	0.217401554	0.106553992	2.040294793	0.041320973	0.142127824
273.2114511	-0.057818119	0.075616229	-0.764625783	0.444494411	0.649789656
589.5439809	0.321894509	0.065849391	4.888344536	1.01687E-06	3.48274E-05
705.8363161	0.172624913	0.086742824	1.990077174	0.046582435	0.154741465
385.0806073	-0.16813516	0.058023409	-2.89771254	0.003758949	0.025515012
80.99957445	-0.224137958	0.167442301	-1.338598172	0.180701522	0.376867562
287.4296278	-0.03145285	0.078862721	-0.398830399	0.690018176	0.83271258
67.00325694	0.040080539	0.138456302	0.289481506	0.77221293	0.881274949
285.7604656	-0.483417798	0.162203036	-2.980325213	0.002879425	0.021167179
13.3001211	-0.764115901	0.317876136	-2.403816499	0.016224915	0.074123476
11.85561649	-0.17945436	0.439415252	-0.408393562	0.682984759	0.828594957
1.965860737	-4.041086688	1.737496061	-2.325810562	0.020028659	0.086059501
42.86510355	-0.296085673	0.185239463	-1.598394141	0.109955288	0.273816978
147.9859012	-0.224728463	0.109032223	-2.061119696	0.039291622	0.136847819
8.714523494	0.040070802	0.359491835	0.111465125	0.91124752	0.955384448
543.9876339	0.289552747	0.068829463	4.206813988	2.58996E-05	0.000528712
915.8544058	-0.31492805	0.094729656	-3.324492698	0.000885796	0.008759061
226.5852919	0.037353026	0.077712283	0.480657945	0.630759625	0.79310033
26.56569472	-0.117465811	0.184718362	-0.635918428	0.524829591	0.715791603
229.3799689	-0.162250515	0.094281995	-1.720906679	0.085267759	0.231764168
325.0921007	-0.120467027	0.092591098	-1.301064891	0.193236244	0.393882135
33.4583878	0.193235145	0.16932192	1.141229349	0.253774494	0.463130721
95.35244026	-0.065462638	0.126372287	-0.518014195	0.604448366	0.774579939
2212.409845	-0.26216873	0.088077982	-2.976552417	0.002915092	0.021334177
134.6394575	0.030486702	0.114284953	0.266760418	0.789653632	0.891207713
101.6445594	-0.03965361	0.121245027	-0.327053495	0.743627425	0.865506187
526.150871	-0.227847536	0.187147871	-1.217473298	0.22342419	0.428534816
233.517412	-0.353233995	0.088811433	-3.977348225	6.96881E-05	0.001182329
83.25002678	-1.26965281	0.644586308	-1.969717312	0.048870778	0.159556541
694.0315938	-0.123180887	0.054150905	-2.274770616	0.022919694	0.094465608
94.86706003	-0.130960856	0.097397423	-1.344602879	0.178753513	0.374375724
713.7381867	0.095786634	0.042980912	2.228585431	0.025841501	0.103059577
46.83281623	0.149473432	0.163857446	0.912216294	0.361654868	0.575601075
3574.94019	-0.055645765	0.083896981	-0.663263013	0.507162121	0.70201787
19.30674891	0.475057471	0.259269782	1.832290158	0.066908203	0.197641336
19.64389024	-0.286701572	0.301812193	-0.949933697	0.342145944	0.556467099
363.8202086	0.136007029	0.073555389	1.849042334	0.0644517	0.192980245
400.2705961	-0.014268593	0.09265261	-0.154000978	0.877608964	0.938489926
100.8622508	0.013662345	0.161363877	0.08466793	0.932525393	0.968136056
46.64173807	-0.204313738	0.144956307	-1.409484984	0.15869181	0.345805408
3126.448624	-0.261527096	0.072352258	-3.614636281	0.00030077	0.003739531
1114.571356	0.038726744	0.062668973	0.617957214	0.536603543	0.724877003
93.99810083	-1.184767476	0.323696631	-3.6601168	0.0002521	0.003286634
78.41342343	-0.068384727	0.1452378	-0.470846621	0.637750269	0.797573383

2.301552105	1.024526615	0.907093818	1.129460475	0.258703634	0.469370448
18.43413835	0.117898172	0.309692219	0.380694654	0.703429836	0.841042248
46.28919186	-0.034423355	0.191482655	-0.179772704	0.857331014	0.92936531
174.8854292	0.174844932	0.074927737	2.333514116	0.019621178	0.084906565
576.5158213	-0.317559917	0.109997389	-2.886976864	0.003889628	0.026143507
60.46290799	-0.413193304	0.169746386	-2.434180272	0.01492556	0.069992504
428.7851607	0.129669917	0.074229095	1.746888023	0.080656764	0.223565708
90.56162815	-0.883500372	0.316370781	-2.792610519	0.00522846	0.03265112
177.3697895	0.492677513	0.15435897	3.191764716	0.001414065	0.012564888
139.531247	-0.110696746	0.103967663	-1.064722844	0.287001369	0.501491362
780.4488135	-0.142788306	0.054349897	-2.627204706	0.008608953	0.047347995
732.6943745	-0.180011488	0.101205756	-1.778668469	0.075294132	0.213750736
2.37814981	0.953093995	0.69072787	1.379840074	0.167635891	0.359007538
2.497263789	0.209204711	0.735121405	0.284585253	0.77596191	0.883652841
168.686676	0.019973074	0.091288087	0.218791681	0.826812328	0.913540506
686.1283736	-0.427635226	0.188064377	-2.273876817	0.022973394	0.094564673
153.8173053	0.108609645	0.120771447	0.899299034	0.368493402	0.582134026
13.83065939	0.08455719	0.286715357	0.294916851	0.768057399	0.879106343
179.7213738	0.014038038	0.090190622	0.155648538	0.876310064	0.937737303
734.7758973	-0.243283067	0.084204687	-2.889186765	0.003862396	0.026033877
2.911297213	0.650650521	0.538112364	1.209135051	0.226610965	0.432232145
160.6129922	-0.040573753	0.100236177	-0.404781534	0.685638104	0.829778815
60.49857427	0.053932904	0.176754732	0.305128485	0.760268297	0.87505484
2.916626363	0.255884137	0.627799482	0.407588958	0.683575472	0.828823311
8.628059956	0.630073022	0.361424302	1.743305633	0.081280242	0.224666226
155.8653554	-0.242397103	0.114040312	-2.125538757	0.033541684	0.122792377
560.4247766	-0.113329668	0.068721538	-1.649114258	0.099124229	0.255075442
310.3876551	-0.1674019	0.093037697	-1.799291088	0.071972647	0.207802772
24.62995264	-0.221685664	0.238953904	-0.92773401	0.353545562	0.568540551
5.309979587	1.286024936	0.813389144	1.581069707	0.1138621	0.279797768
48.17110852	-0.484196919	0.293589173	-1.649232888	0.099099933	0.255075442
54.99432275	-0.219800291	0.309799411	-0.709492281	0.47801904	0.677604968
1.713849731	0.842525956	0.91806664	0.917717646	0.358766707	0.573157878
135.8197763	-0.223481406	0.399760083	-0.559038822	0.576135226	0.754595452
487.1042642	-0.316455027	0.060762874	-5.208032599	1.90853E-07	8.0003E-06
2294.361429	-0.167238259	0.100029348	-1.671891917	0.094545637	0.247020171
1244.077819	-0.040979589	0.058768509	-0.697305232	0.485611794	0.684460019
213.2953342	-0.487853069	0.127392931	-3.829514451	0.000128396	0.00190216
107.0458353	-0.249636549	0.102039759	-2.44646353	0.014426539	0.068348924
6.337019569	0.543832715	0.634894812	0.856571364	0.391681817	0.604965897
54.94141996	-0.11409634	0.130544273	-0.874004946	0.38211556	0.594875424
1123.826212	0.12776717	0.067364596	1.896651633	0.057873931	0.178810042
589.5198696	-0.013472415	0.067675441	-0.199073915	0.842204926	0.921757179
242.7533958	0.105751957	0.074746053	1.414816595	0.157122277	0.34352122
68.51409778	0.117789925	0.128639624	0.915658185	0.359846203	0.573951606
1977.12916	0.076030351	0.06845881	1.110599953	0.266740583	0.478419637
60.08176197	0.086111711	0.132199555	0.651376712	0.51480334	0.708037254
11.78868058	-0.926351658	0.46020999	-2.012889066	0.044126301	0.148931916

997.7814253	-0.031376294	0.099763332	-0.31450728	0.753135788	0.871171356
268.1312911	0.027724567	0.080666699	0.343692842	0.731077295	0.85787929
2.419408543	0.47599547	0.624680504	0.761982273	0.446070584	0.651180045
7342.779575	-0.324353151	0.119497061	-2.714319053	0.006641217	0.038976633
4.99970356	0.056393952	0.41001191	0.137542228	0.890602216	0.945339324
1667.663871	0.096071381	0.100153378	0.959242541	0.337436574	0.552742381
197.1597882	-0.171630508	0.110653026	-1.551069269	0.120885085	0.29055455
183.8166237	-0.248046326	0.110575859	-2.243223137	0.024882429	0.100272293
28.25142491	0.399110599	0.342009447	1.166957821	0.243227403	0.450925978
1228.75539	0.404460273	0.104365523	3.875420379	0.000106441	0.001643808
2.813813919	1.03630778	0.667721287	1.552006503	0.120660668	0.290099801
349.5511773	0.077871525	0.110899764	0.702179353	0.48256732	0.681685305
20.88561079	0.096176248	0.228313845	0.421245798	0.673575605	0.823222819
8.696871923	0.236044131	0.368887489	0.639881097	0.522249904	0.713631795
329.0788994	0.051770098	0.102834207	0.503432655	0.614660117	0.78222563
1114.616467	0.033445358	0.0794567	0.420925579	0.673809426	0.823222819
202.5825705	-0.014762147	0.088618213	-0.16658141	0.867699422	0.934470438
358.5422514	-0.024870418	0.073535885	-0.338207914	0.735206513	0.860688745
168.3720125	0.764551459	0.170070054	4.495508999	6.94037E-06	0.000175361
431.6071158	-0.195586066	0.104417553	-1.873114814	0.061052532	0.185912182
439.246093	0.015142753	0.074733903	0.202622267	0.839430284	0.920078144
165.477752	-0.067046136	0.125979856	-0.532197274	0.594589371	0.766872723
56.02258438	-0.074388991	0.136962416	-0.543134338	0.587037312	0.761579192
31.57948851	-0.13678753	0.187639051	-0.72899287	0.466006024	0.668144756
321.6302664	-0.184745867	0.074645683	-2.474970565	0.013324716	0.064714436
472.088943	-0.084151372	0.056664867	-1.485071363	0.137524921	0.3152777
17.52227592	-0.899574597	0.711447774	-1.264428156	0.206076392	0.409517791
78.70207697	0.324920135	0.268286469	1.211094007	0.225859378	0.431435274
4.714094538	-0.263137032	0.66362803	-0.396512836	0.691726741	0.834041303
7839.490523	0.023605616	0.109096596	0.216373537	0.828696592	0.914385171
191.2664188	-0.197130637	0.172009573	-1.146044567	0.251776712	0.461016956
1113.17074	0.061498751	0.048867991	1.258466949	0.208222933	0.412009499
569.8584723	-1.402873431	0.410856744	-3.414507493	0.000638974	0.006786888
445.2048365	-0.491597149	0.22240369	-2.210382161	0.027078651	0.106364734
12.89350873	0.372913457	0.401093153	0.929742765	0.352504286	0.567492975
2.794067857	1.160415693	1.436228592	0.807960306	0.419113433	0.628199717
3.788425571	-1.48929164	1.240299666	-1.200751464	0.229847624	0.436032907
84.13306115	-2.428446716	0.646666384	-3.755331614	0.000173112	0.002431244
5.790652151	-2.934918731	0.807137906	-3.636204804	0.000276684	0.003538896
24.69433131	-4.190923367	0.920736797	-4.551706179	5.32126E-06	0.00014026
3.095000529	-2.607889742	1.033564077	-2.523200835	0.011629196	0.05865872
2.201941884	-4.224304582	1.168113713	-3.616347053	0.00029879	0.003726517
2.548482488	1.078995582	0.645540255	1.671461345	0.094630588	0.247020171
120.2353186	-0.379468185	0.266691122	-1.422875208	0.154772334	0.339907082
12.04409224	1.286202445	0.650927772	1.975952632	0.048160142	0.158184329
3.260793399	-0.111885233	0.818626293	-0.13667437	0.891288189	0.945526539
7.562992266	-0.237215844	0.674651887	-0.351612214	0.725129104	0.854429358
6.448549629	-2.81957998	1.022909743	-2.756430857	0.005843597	0.035379096

282.5859769	-0.512679695	0.453500901	-1.130493223	0.258268453	0.468787405
2.69470698	-0.702759089	0.816583049	-0.860609451	0.389453179	0.602903353
1318.686667	-0.140801715	0.053950052	-2.609853186	0.009058109	0.049022104
219.6725052	-0.104916357	0.24756422	-0.423794509	0.671715683	0.821580637
311.1013148	-0.022313475	0.151620274	-0.147166827	0.883000344	0.941585065
10.67112473	0.354850606	0.37031832	0.958231301	0.337946137	0.553262834
154.1089202	-0.893721191	0.172343097	-5.18570924	2.15194E-07	8.86278E-06
628.8064753	-0.251997864	0.07594242	-3.318275394	0.000905751	0.008934939
43.76485739	-0.700662238	0.43177153	-1.622761549	0.104640388	0.26483613
7.184640908	1.294926458	0.397943887	3.254042844	0.001137751	0.010673869
932.4122738	0.040588571	0.051831665	0.783084457	0.433577518	0.640433608
115.6931881	-0.273094583	0.108440424	-2.518383568	0.011789485	0.059177326
868.881463	-0.053332758	0.048208535	-1.106292857	0.268599783	0.480491998
106.0919928	0.070063479	0.128988216	0.54317736	0.587007693	0.761579192
6.83169332	-1.177899275	0.674371686	-1.746661819	0.080696017	0.223614161
1698.250274	0.002104628	0.073343728	0.028695411	0.977107517	0.988204876
1586.912545	-0.068411606	0.159258548	-0.429563169	0.667513434	0.818748888
453.0868821	0.188757408	0.098406068	1.918148062	0.05509224	0.172250817
46.16888223	0.330862425	0.147432288	2.2441653	0.024821768	0.100199412
6.189900536	-0.960103017	0.415574666	-2.310302083	0.020871436	0.088686106
1426.036444	0.028713962	0.096185538	0.298526809	0.765301118	0.877781147
14.55191365	0.298783652	0.25958866	1.150988845	0.249736825	0.458709382
69.74072602	0.959136613	0.244591847	3.921376054	8.80447E-05	0.001417839
16.37702082	0.484383094	0.387309646	1.250635245	0.211067586	0.415370705
448.6992654	0.168488928	0.067230786	2.506127578	0.012206157	0.060708845
165.0622148	0.065628061	0.145692986	0.450454496	0.652382758	0.807789472
2.883226379	0.401639675	0.657442558	0.610912194	0.541257712	0.728624641
21.19661147	-0.331848683	0.377602991	-0.878829592	0.379493679	0.592361082
519.0223326	0.255321292	0.116235448	2.19658715	0.028049945	0.108933237
50.76051477	-0.235369195	0.141228501	-1.66658425	0.095597103	0.248912071
10.87303555	-0.001574865	0.36429333	-0.004323069	0.996550701	0.998004635
60.90556663	2.888533169	0.269472374	10.71921817	8.26994E-27	1.51377E-23
356.9910893	1.519357636	0.140670351	10.80083777	3.41077E-27	7.02363E-24
1.910056097	3.124811752	1.014764311	3.079347311	0.002074547	0.016622609
2.133378626	1.885443024	0.918689287	2.052318504	0.040138719	0.139063145
18.2162261	2.001716411	0.300309816	6.665504446	2.63758E-11	3.26059E-09
170.0755707	1.504318871	0.236857196	6.351163878	2.13692E-10	2.18656E-08
2.57480881	2.183395703	0.962242045	2.26907119	0.023263997	0.095284866
2.016531124	2.478361827	0.869147842	2.851484761	0.004351557	0.028436719
3.283847181	2.327227822	0.721068184	3.227472622	0.00124889	0.0114492
8.421193678	1.888722505	0.422023241	4.475399276	7.62686E-06	0.00019066
5.386799356	-0.683480749	0.511704226	-1.335694948	0.181649001	0.378221139
1.895679707	-0.670111587	0.70302393	-0.953184605	0.340496545	0.555270316
1275.564459	-0.018406412	0.076586027	-0.24033643	0.810069456	0.90377111
10.57527255	0.329356418	0.316006641	1.04224524	0.297297994	0.51192666
6840.03217	-0.153760273	0.048499247	-3.170364163	0.00152248	0.013298693
27.36027639	0.58539857	0.250058602	2.341045521	0.019229822	0.083586304
407.7815739	-0.440618036	0.08245662	-5.343634432	9.11012E-08	4.11178E-06

172.3464799	0.237952654	0.102036401	2.332036922	0.019698749	0.085130427
11.2436056	-0.061438931	0.315838144	-0.194526632	0.845763544	0.923706045
206.4178851	0.011726417	0.09568035	0.122558255	0.902456913	0.951164319
170.872983	0.05345363	0.080744069	0.662013084	0.507962836	0.702676947
580.7820607	0.058231649	0.106932901	0.544562513	0.586054443	0.760928506
1586.788271	0.33552448	0.10902921	3.077381553	0.002088278	0.016708253
162.1160011	-0.206467099	0.08461332	-2.440125262	0.01468217	0.069166162
4.139405033	0.498300705	0.563861068	0.883729581	0.376842208	0.589841864
421.6390189	-0.025896824	0.096553748	-0.268211488	0.788536533	0.890703684
1021.437491	0.368357327	0.061056003	6.03310583	1.60838E-09	1.27387E-07
24.65725459	-0.141667443	0.304673588	-0.464981045	0.641945031	0.800863494
552.5357433	-0.389494206	0.107978236	-3.60715474	0.000309573	0.003820155
3.655838798	-0.29813108	0.480413417	-0.620571927	0.534881315	0.72384441
2404.302869	-0.004798405	0.093385063	-0.051383005	0.959020327	0.980080698
32.58677614	-0.357165566	0.222774283	-1.603262103	0.1088768	0.271845468
2.894797034	0.305707043	0.594630956	0.514112224	0.607173532	0.776778501
919.1898521	-0.075545082	0.055547263	-1.360014463	0.173825347	0.367476343
151.5491555	0.086714377	0.229216234	0.378308181	0.705201669	0.842341663
49.41652586	0.047420511	0.181700862	0.260981211	0.794106997	0.893917451
204.7158891	0.157520042	0.075435167	2.088151299	0.036784189	0.131136708
219.7883181	0.017511965	0.076690415	0.228346202	0.819377109	0.909351826
936.465493	0.19780796	0.043979753	4.497705139	6.86909E-06	0.000173911
115.9184613	0.2952099	0.101038234	2.921764266	0.003480549	0.024285709
167.5526189	0.226928041	0.129197757	1.756439473	0.079013398	0.220365755
249.1935738	0.24162349	0.095242362	2.536932975	0.011182836	0.05689501
1202.58227	-0.257585531	0.10914026	-2.360133013	0.018268384	0.080404315
3.20074742	-0.837249656	0.758841522	-1.103326098	0.269885577	0.481874674
12.38870842	-1.102770763	0.587350584	-1.87753412	0.060444925	0.184709646
252.5394413	0.827440953	0.144648533	5.72035495	1.06302E-08	6.53438E-07
166.401146	-0.157362283	0.34590166	-0.454933587	0.649157019	0.805627137
41.50004572	-0.121790474	0.386401067	-0.315191866	0.752615981	0.870932542
401.4789154	0.529977249	0.147058314	3.60385777	0.000313529	0.003854531
9735.666961	0.109101387	0.086981004	1.254312809	0.20972833	0.413928898
124.6523766	0.211587444	0.093008761	2.274919512	0.022910759	0.094452412
622.0187467	0.256829291	0.068712413	3.73774226	0.00018568	0.0025705
24.23643962	0.770496963	0.245100492	3.143596146	0.001668856	0.01414963
395.9761648	0.194537371	0.091000506	2.137761421	0.032536112	0.120341244
96.35868418	-0.211644894	0.228206793	-0.927425916	0.35370544	0.568593229
9.673469992	-0.03925869	0.356852887	-0.110013653	0.912398547	0.956222003
60.55639498	0.063165598	0.200468803	0.315089416	0.752693764	0.870961373
5.799141867	1.285499497	0.812980522	1.581218076	0.113828184	0.279797768
807.1669823	0.485160468	0.082499579	5.880762954	4.0838E-09	2.93784E-07
2.353592775	0.624956393	0.697507292	0.895985461	0.370260528	0.583756526
62.56675048	0.242175267	0.161948517	1.495384285	0.13481418	0.311006321
393.3475856	0.182602384	0.073122751	2.497203417	0.012517713	0.061815587
222.3111968	0.110837771	0.080609473	1.374996846	0.169132422	0.3610116
1041.012854	0.034132077	0.080511967	0.42393794	0.671611074	0.821572795
2.562356394	-0.073580279	0.726310513	-0.101306917	0.919306825	0.960102741

714.9232568	0.11493341	0.070512082	1.629981797	0.103105344	0.262406525
301.0576496	0.482502046	0.102869527	4.690427368	2.72635E-06	8.03469E-05
274.4751636	-0.187302088	0.092978662	-2.01446314	0.043960931	0.148495464
6.938244485	-0.895961431	0.683517854	-1.310809112	0.189922263	0.389684813
407.0294836	-0.216504157	0.106984437	-2.023697687	0.043001267	0.146213182
162.7906091	0.183354447	0.189773931	0.966172995	0.333957623	0.549173277
219.4535539	-0.241043108	0.087596881	-2.751731619	0.005928108	0.035707369
999.3461558	-0.047937511	0.044525738	-1.076624736	0.281647949	0.495712426
17.04261023	-0.124930323	0.258293104	-0.483676571	0.628615423	0.791789164
70.12461436	-0.601382471	0.292395613	-2.056742455	0.039711006	0.137929394
4.398899657	0.755501575	0.722434588	1.045771601	0.295666496	0.510408791
112.8377326	0.690350995	0.140367955	4.91815241	8.73649E-07	3.08852E-05
13.92002748	-0.755354799	0.624414039	-1.209701819	0.226393332	0.432121
1.905978577	1.358498634	0.870794282	1.56006839	0.11874372	0.287190305
332.492438	-0.026301867	0.133091599	-0.197622293	0.843340592	0.922094173
34.64673832	-0.599488118	0.275837034	-2.173341665	0.029754612	0.113414502
1479.480291	-0.21740639	0.056099669	-3.875359593	0.000106467	0.001643808
474.3003524	-0.027979496	0.096671033	-0.289429991	0.772252347	0.881274949
225.1871035	-0.046187671	0.128597389	-0.359164918	0.71947172	0.851125005
2.003612257	-1.780983422	0.956875278	-1.861249281	0.062708982	0.18913727
634.4731436	-0.580532868	0.118101071	-4.915559719	8.85292E-07	3.10775E-05
33.13242295	0.147167156	0.401321261	0.366706602	0.713837882	0.847585632
2.092315079	1.595590195	0.964487436	1.654340052	0.09805844	0.253261618
9.667628335	-0.775767617	0.516935643	-1.500704445	0.133432023	0.308686862
4.14288085	-0.505601063	0.854032191	-0.5920164	0.553839606	0.738901334
1.952985964	-1.397359623	1.177241062	-1.186978324	0.235236171	0.442535192
2.908634309	-3.412603101	1.493685691	-2.28468621	0.022331234	0.09273626
48.60992142	-0.818989324	0.204145377	-4.011794625	6.02589E-05	0.001043854
791.1022734	0.464722459	0.090984478	5.107711452	3.26084E-07	1.29296E-05
521.3116457	-0.05740046	0.064224724	-0.893743983	0.371458887	0.584924703
154.9897153	-0.275098605	0.252493022	-1.089529537	0.275920437	0.488528712
2.78005478	-0.021747313	0.664269557	-0.032738686	0.973882973	0.987186076
611.7293229	-0.054437424	0.071623909	-0.76004542	0.447227436	0.652406338
51.22793185	-0.503954525	0.150198494	-3.355256846	0.000792913	0.008036109
3.214412765	-1.453648195	0.56214841	-2.585879759	0.009713081	0.051579197
442.0710673	-0.090006424	0.075515483	-1.191893633	0.233302955	0.440154933
1554.527139	-0.09344444	0.06571189	-1.422032444	0.155016833	0.340182137
823.2030922	0.180033812	0.068811622	2.616328559	0.008888099	0.048397147
1381.436604	0.029571524	0.064882474	0.455770601	0.648554947	0.805267878
15.32176237	0.274643141	0.339648599	0.808609669	0.418739701	0.62783981
37.86895128	-0.489685516	0.461715078	-1.060579435	0.288881072	0.503654015
187.3764384	-0.119404172	0.124166623	-0.961644678	0.336228121	0.551329232
557.5758492	-0.147439866	0.062222238	-2.369568692	0.017808847	0.079078959
12504.96065	-0.000987739	0.222860775	-0.004432088	0.996463717	0.998004635
337.8901253	-0.104002826	0.087155315	-1.193304458	0.232750157	0.43946353
399.8334884	-0.092215527	0.056871173	-1.621480996	0.104914522	0.265289615
5.164205107	0.574164329	0.726076704	0.790776409	0.429074479	0.636006205
51.012567	-0.068776617	0.203794612	-0.337480055	0.735755045	0.861152614

73.6725863	0.218491955	0.166137395	1.315128092	0.188466857	0.387888408
54.95095277	0.156682463	0.197697867	0.792534916	0.428048841	0.635000145
2890.608751	-0.057389616	0.152267511	-0.376899939	0.706247967	0.843035215
547.6548962	-0.020653634	0.155001961	-0.133247566	0.893997594	0.946388043
4.321144173	-0.752053242	0.580955651	-1.294510588	0.195489114	0.396559965
1258.561266	-0.140864053	0.122255825	-1.152207294	0.249235901	0.458249133
189.2440748	-0.167726327	0.071191819	-2.35597754	0.018474035	0.081092792
125.4858433	-0.138933074	0.114443019	-1.213993432	0.224750232	0.429829349
401.1711044	-0.03185396	0.074994152	-0.424752591	0.671017041	0.821214982
2.138323844	-0.029898174	0.85752676	-0.034865587	0.972186921	0.986031074
164.7243699	-0.188367139	0.126268121	-1.491802814	0.135750846	0.312297086
595.5772901	-0.229269584	0.068222605	-3.36061021	0.000777705	0.007918363
154.5765766	0.064940437	0.114929844	0.565044156	0.572043731	0.751595502
90.21904558	0.013457804	0.136919826	0.098289669	0.921702282	0.961385551
15.103817	-0.409845836	0.385536628	-1.063052913	0.287757957	0.502387089
15.53480508	-0.619765751	0.394124021	-1.572514534	0.115831271	0.282613205
86.12386004	0.047114532	0.11377146	0.41411556	0.678789478	0.826487646
816.6697359	0.106583162	0.052915718	2.014206106	0.043987899	0.148556098
2.200914245	-0.748850977	0.733505473	-1.020920776	0.307291975	0.52240814
388.8841618	0.112875715	0.063601469	1.7747344	0.075941752	0.21488568
144.134781	-0.107244251	0.101286532	-1.058820452	0.289681558	0.50456904
635.7894407	-0.059990028	0.069193234	-0.866992694	0.385946013	0.599253027
13.12469145	0.206469404	0.32007376	0.645068199	0.518882991	0.711272956
83.60722949	-0.144130452	0.13747147	-1.048439008	0.294436389	0.509190119
1576.030382	0.092533472	0.120163807	0.77006109	0.441263656	0.647144793
120.0725951	-1.158078935	0.261113944	-4.435147804	9.20091E-06	0.000222578
6.369611418	0.258431275	0.646991083	0.399435605	0.689572263	0.832540378
4.866074499	-0.922030775	0.7476397	-1.233255503	0.217480453	0.422248697
152.0167632	0.251465218	0.173386194	1.450318575	0.146969701	0.328621371
12.11280591	-0.55340904	0.383141955	-1.444396869	0.148627371	0.331274013
253.8075989	-0.020899508	0.076497233	-0.273206067	0.784694817	0.888396839
111.8221069	0.024165049	0.148476116	0.16275378	0.870712301	0.935813113
253.5663341	-0.102760201	0.077294434	-1.329464439	0.183694791	0.381066783
17.62711181	0.059523027	0.299606438	0.198670722	0.842520328	0.921757179
385.9164565	-0.052774883	0.10669596	-0.494628691	0.620862245	0.786339161
6.207963604	-0.216027603	0.450774173	-0.479236869	0.631770128	0.79384855
14.95448444	-0.253083513	0.339729063	-0.744956911	0.456297769	0.660259065
343.9732945	-0.129240851	0.083211449	-1.553161874	0.120384467	0.289927645
28.8543322	-1.858065364	0.863120938	-2.152728873	0.031339993	0.117126825
15.16434825	-2.481406948	0.878822892	-2.823557478	0.004749391	0.030198811
16.81232299	-1.814314243	0.899671338	-2.016641152	0.043732975	0.147998569
23.19355754	-2.095043439	0.832759368	-2.515784894	0.011876765	0.059481626
18.55260769	-1.002022091	0.631880783	-1.585777125	0.112789877	0.278283724
43.55620093	-1.771967474	0.71788684	-2.468310289	0.013575259	0.065525583
57.83403948	-2.202445123	0.834952007	-2.637810443	0.008344321	0.046237587
39.4560442	-2.111855205	0.840228748	-2.513428885	0.011956388	0.059760175
75.97997029	-2.235407555	0.870368233	-2.568346901	0.010218484	0.053288795
45.24873747	-0.825932022	0.468596862	-1.762564135	0.077974031	0.218534226

4.312872797		-2.257464671	1.214022291	-1.859491945	0.06295744	0.189592266
4.722719541		-2.681302521	1.02006236	-2.628567258	0.00857454	0.047195779
28.96841992		-1.404973078	0.467832526	-3.00315391	0.002671973	0.020072083
5.717828591		0.104714864	0.493464917	0.212203261	0.831948463	0.91596382
5.502274609		-2.406881806	1.080110217	-2.228366855	0.025856061	0.103061398
16.74410876		-2.429224142	0.876373554	-2.771904893	0.005572932	0.03421859
91.69628695		-1.047851545	0.181600177	-5.770101994	7.92236E-09	5.09816E-07
18.58909303		-2.064085204	0.867306358	-2.37988017	0.017318269	0.077696394
29.34965189		-1.991279505	0.623455613	-3.193939494	0.001403455	0.012504339
168.09878		-0.088793686	0.127792886	-0.694824957	0.487165006	0.685605662
124.402417		-0.048339023	0.096993134	-0.498375726	0.618219243	0.784271046
3.538779669		-0.282907591	0.598432551	-0.472747665	0.636393216	0.796591584
1.9405621		-4.393872814	1.94733825	-2.256348025	0.024048844	0.097822384
502.9631528		0.041090118	0.145210664	0.282969012	0.777200591	0.884207847
29.12367439		-2.392182097	0.788553851	-3.033631876	0.002416291	0.018550012
18.46093305		-1.607638028	0.657681573	-2.44440181	0.014509257	0.068626328
29.06140636		-1.509233138	0.635783584	-2.373815831	0.017605329	0.07849261
94.68495109		-0.785974745	0.450404046	-1.745043704	0.080977262	0.224129605
122.3765587		-1.422811105	0.632107316	-2.25090118	0.024391797	0.098875606
160.4656742		-1.124870328	0.499763342	-2.250805997	0.024397828	0.098875722
177.2421827		-0.295375822	0.32426814	-0.910899918	0.362348109	0.576356353
16.5666046		-1.854608183	0.68624124	-2.702560082	0.006880775	0.039916478
26.16571314		-2.043989368	0.672805459	-3.038009487	0.002381465	0.018410252
16.20035277		-1.956437123	0.723720711	-2.703304042	0.006865392	0.039866218
4.372638507		-2.977324142	1.025240791	-2.90402427	0.003683996	0.025193088
31.09796681		-0.898203176	0.424295816	-2.11692678	0.034266062	0.124448658
380.2080814		-0.258618431	0.255941749	-1.01045817	0.312275831	0.527039446
158.4681224		-0.509548557	0.158643695	-3.211905509	0.001318577	0.011915657
526.9164781		-0.280365675	0.208016559	-1.3478046	0.177721238	0.373060611
11.23450921		-0.355476987	0.525108022	-0.676959734	0.498431503	0.694742406
307.1312357		-0.43002949	0.097269171	-4.421025567	9.82335E-06	0.000234536
355.141376		0.284763738	0.139021221	2.048347267	0.040525985	0.140139603
196.2328831		-0.058149382	0.215894957	-0.269341086	0.787667218	0.890293636
1724.713419		-0.190348766	0.139193426	-1.367512613	0.171464687	0.363727691
53.01296423		-2.544830233	0.787480446	-3.231610699	0.001230946	0.011328832
33.70335744		-1.710475555	0.616084006	-2.776367409	0.005497005	0.033881777
16.79109049		-2.157528022	0.961696062	-2.24346143	0.024867074	0.100252501
9.654492156		-1.771977411	1.000253361	-1.771528574	0.076472845	0.21592658
18.93594386		-2.475392316	0.673343089	-3.676271959	0.000236667	0.003126588
89.49754466		-0.626034798	0.478017639	-1.309647904	0.190314975	0.390237882
20.47842882		-1.935126916	0.74977419	-2.580946293	0.009852991	0.052004548
69.33455261		-2.441087595	0.83983653	-2.906622311	0.00365354	0.025082115
66.10945632		-0.21695708	0.260425153	-0.833088038	0.404795079	0.615467848
112.3628111		-0.020038703	0.361438278	-0.055441563	0.955786684	0.978293782
159.2599156		-0.056998903	0.378989192	-0.150397174	0.880451271	0.940144817
3.806287752		1.419200638	0.859139345	1.651886446	0.098557702	0.254289676
30.69516475		-1.179521773	0.232375164	-5.075937336	3.8559E-07	1.49816E-05
1141.951991		-0.002781382	0.051379149	-0.05413446	0.956828037	0.978702547

498.670251	0.097412277	0.106042652	0.918614119	0.358297444	0.572733563
106.876559	-0.072235653	0.143205	-0.504421304	0.613965351	0.781643369
2.807662576	-0.608796537	0.640109251	-0.951082234	0.341562633	0.556073012
217.4956168	0.068228647	0.098775068	0.690747651	0.489724138	0.68749173
7.211143324	0.439798931	0.407122986	1.080260625	0.280026138	0.493490651
382.2129023	0.05197056	0.096907576	0.536289964	0.591758166	0.764478044
454.7481347	-0.141323803	0.074206801	-1.904458914	0.056850443	0.176601263
2.200760297	2.491427551	0.877064129	2.840644679	0.004502245	0.029142828
22.15687299	-0.045987971	0.240924261	-0.190881447	0.848618476	0.925285643
102.0004593	0.183697761	0.283100062	0.648879268	0.516416416	0.709308324
461.5234828	-0.119452802	0.093281221	-1.280566453	0.20034599	0.402794708
293.4995311	-0.160297481	0.081611712	-1.964148006	0.04951293	0.161073463
40.26454468	-0.081372253	0.19325276	-0.421066448	0.673706561	0.823222819
70.28669004	0.322495491	0.280748219	1.148700042	0.250679689	0.45957013
4550.680561	-0.056332387	0.119920265	-0.469748688	0.638534578	0.79822463
470.2923279	-0.214314332	0.089238125	-2.401600584	0.016323521	0.074489983
59.33177557	-0.093374793	0.308299412	-0.302870486	0.761988564	0.875874332
409.4362916	-0.343191285	0.059006285	-5.816181841	6.0207E-09	4.09855E-07
7.288012703	0.429419661	0.353558348	1.214565187	0.224531973	0.429609724
17.7223924	0.59456794	0.543497152	1.093966984	0.273969459	0.486656041
3.27452261	-0.507979888	0.615459296	-0.825367155	0.409163176	0.619650135
75.60377827	0.107164702	0.246003388	0.43562287	0.663110375	0.815611876
34.05092303	-0.017654346	0.228443203	-0.077281117	0.938399912	0.970668417
20368.46237	-0.524225591	0.18352296	-2.856457803	0.004283969	0.028139598
159.0808412	-0.086470854	0.07566705	-1.142780824	0.253129602	0.462209828
70.84531643	-0.632123616	0.262063885	-2.412097403	0.015861044	0.073035402
52.81094099	0.695104024	0.288531916	2.40910619	0.015991644	0.073424289
17.27756772	-0.083296898	0.325300343	-0.256061512	0.79790334	0.895595805
67.83228599	0.174655215	0.158375011	1.10279528	0.270116078	0.482059611
6.088756495	-0.818895937	0.453894473	-1.804154897	0.071207028	0.206179469
2074.519527	-0.521559445	0.227278246	-2.294805837	0.021744254	0.091172013
12.45889007	-0.907006836	0.312956291	-2.898190136	0.00375323	0.025497199
335.9594373	-0.450705419	0.107464023	-4.194012139	2.74063E-05	0.000553299
129.0272027	-0.043964523	0.148727794	-0.295603944	0.767532561	0.878871997
222.6395537	-0.276492993	0.094540874	-2.924586801	0.003449139	0.024097163
95.11991109	0.200089866	0.17150918	1.166642313	0.243354848	0.450998082
22.31458503	0.352719786	0.236378674	1.492181083	0.13565168	0.312156136
48.09595566	0.434019239	0.132134691	3.28467289	0.001021008	0.009807634
374.606398	-0.147280149	0.094942917	-1.551249456	0.120841914	0.29049317
123.8495489	0.56836437	0.18901156	3.007034975	0.002638094	0.019844731
4.318224725	1.034798731	0.520388129	1.988513331	0.046754946	0.155165387
571.4842399	0.156603696	0.077085096	2.031569071	0.0421973	0.144313541
3.161303423	-0.974196682	0.554913319	-1.755583528	0.079159547	0.220604755
6.346451629	-0.718353895	0.65132076	-1.102918776	0.270062439	0.482016103
645.3651709	-0.290782878	0.092390838	-3.147312915	0.001647785	0.014021494
174.6585941	0.011576857	0.102468797	0.112979344	0.910046931	0.954911665
55.0605752	-0.181931085	0.179404474	-1.014083325	0.310542994	0.52547639
68.76592677	-0.166100693	0.129107498	-1.286530183	0.198258092	0.399914756

324.376024	-0.07096432	0.085549706	-0.829509804	0.406815991	0.617614597
22.50338769	-0.925420618	0.420030104	-2.203224507	0.027578929	0.107590346
552.7827999	-0.587662641	0.176640335	-3.326888167	0.000878216	0.008705013
17.74406997	-0.695784314	0.455194872	-1.528541633	0.126378115	0.297762166
1267.244502	0.103697675	0.073786139	1.405381505	0.159907863	0.347551174
896.614447	0.091612224	0.051248056	1.787623405	0.073836787	0.211251689
8.304989439	0.329895398	0.361648245	0.912199637	0.361663635	0.575601075
130.0498841	0.220736715	0.103810266	2.126347663	0.033474322	0.122627526
5.071127514	-1.473433032	0.820674284	-1.795393204	0.072591075	0.208884779
88.10863337	-0.036466959	0.124462796	-0.292994855	0.769526084	0.879931472
212.850555	0.624146166	0.110764818	5.63487737	1.75183E-08	9.9516E-07
3.617325989	0.688261156	0.583405795	1.17972972	0.238107729	0.445647209
757.786663	0.081201267	0.095362151	0.85150415	0.394489359	0.607225165
138.4222115	-0.110811716	0.080912009	-1.369533613	0.170832535	0.363041175
105.2106584	-0.400744823	0.137564613	-2.913138877	0.003578154	0.024765056
55.56036949	0.233462377	0.147284275	1.585114076	0.112940418	0.278517
171.6960234	-0.492279524	0.189938292	-2.591786628	0.009547897	0.051002611
290.0707814	0.000791055	0.076644623	0.010321078	0.991765117	0.995693738
341.3911212	-0.154106945	0.099624735	-1.546874326	0.121893553	0.291793937
2.010681082	0.625814089	0.698172295	0.896360531	0.37006024	0.583496592
1.696747371	-0.29774049	1.123407642	-0.265033349	0.790983771	0.892098821
1080.728853	-0.125589056	0.11054577	-1.136081976	0.255922258	0.46612087
255.8103079	-0.035552363	0.270438365	-0.131461979	0.895409862	0.947397692
982.2484445	-0.168972612	0.098579503	-1.714074494	0.086515045	0.233839025
4.829050112	-3.145355516	0.935348845	-3.362761959	0.000771669	0.00788548
2.245194695	0.835550603	0.763815429	1.093916896	0.273991428	0.486656041
657.5000074	0.203231594	0.052616535	3.862504362	0.000112231	0.001719894
19.76584473	-0.246300938	0.243086468	-1.013223566	0.310953386	0.525939023
65.59319964	0.452169025	0.208716219	2.166429744	0.030278363	0.114641635
106.9629722	-0.291742053	0.121211631	-2.406881665	0.016089382	0.073685401
190.2797572	0.384320723	0.174946696	2.19678754	0.028035623	0.108903291
157.4906481	-0.035139059	0.151373695	-0.232134511	0.816433541	0.907813535
448.0828027	0.148222112	0.085342568	1.736789927	0.082424258	0.226950609
596.2563699	-0.090654236	0.077789279	-1.165382125	0.243864349	0.451497111
641.1264791	-0.21957077	0.052852343	-4.154418814	3.26115E-05	0.000639574
26.00836534	0.122822123	0.440039718	0.279115992	0.780155811	0.885448627
769.1087522	-0.077459418	0.076699588	-1.00990657	0.312540055	0.527269315
429.8330333	0.020657849	0.066644434	0.309971111	0.756582925	0.87294769
362.0747548	-0.02251932	0.119726139	-0.188090257	0.850805899	0.926363298
1727.587062	0.153339111	0.107888385	1.421275435	0.155236702	0.340483215
2057.783554	-0.111362583	0.080040445	-1.391328884	0.164125719	0.353623737
2436.59096	0.072763299	0.111962327	0.649890918	0.515762685	0.708942384
3391.316975	-0.405500428	0.120845502	-3.355527678	0.000792137	0.008035511
500.3001372	-0.060039004	0.086782515	-0.691832956	0.489042238	0.686944221
1102.39457	-0.065712836	0.066410368	-0.989496639	0.322420213	0.537439698
2270.09452	-0.123017938	0.079665926	-1.544172574	0.122546531	0.292501519
137.1260727	-0.121785251	0.1258472	-0.96772317	0.333182643	0.548610781
46.76910727	-0.562006003	0.17523656	-3.207127566	0.001340675	0.012049255

260.39921	-0.270883878	0.077107136	-3.513084408	0.000442937	0.005081434
346.6135768	0.101747662	0.121677184	0.836209864	0.40303685	0.614325414
492.6372145	0.110924459	0.115333492	0.961771447	0.336164424	0.551316348
13.3879944	-0.300765047	0.271910987	-1.10611583	0.268676388	0.480491998
16.23348246	0.044489226	0.496797991	0.089551945	0.928643273	0.965637301
83.98131321	0.072420061	0.14318377	0.505784007	0.613008287	0.780915252
8.84732892	0.138567508	0.430143131	0.322142788	0.747344525	0.867452526
6.264699557	-0.607716199	0.468413539	-1.297392473	0.194496181	0.39534693
3.885886889	-0.070230533	0.538141763	-0.130505635	0.896166396	0.947711209
107.0494571	-0.0116073	0.134572981	-0.086252829	0.931265436	0.967310672
1126.925409	0.220789859	0.05652806	3.905845304	9.38966E-05	0.001490224
2604.867515	-0.058988097	0.042757121	-1.379608727	0.167707149	0.359039321
116.7419612	0.262970565	0.118808714	2.213394597	0.026870453	0.105833468
3587.392663	0.277783748	0.122805259	2.261985764	0.023698283	0.096730801
175.2575805	-0.47505803	0.137116385	-3.464633571	0.000530954	0.005858635
568.6028541	0.158869213	0.09481425	1.675583707	0.093819762	0.246091419
13.53727967	0.374004945	0.363157931	1.029868587	0.303071699	0.517887593
32.15604242	-0.15612382	0.488290981	-0.319735212	0.749169064	0.868713392
795.0437971	0.030506634	0.060501118	0.504232569	0.614097956	0.781691371
143.6858943	-0.249139534	0.089094666	-2.796346226	0.0051684	0.03228829
21.24832498	-0.023251822	0.259102943	-0.089739706	0.928494062	0.965637301
186.8586251	-0.190038972	0.13861066	-1.371027109	0.170366505	0.362420182
172.5997496	-0.4739321	0.171573146	-2.762274345	0.005740022	0.034944985
21.77755774	-0.730492022	0.367072911	-1.990046122	0.046585855	0.154741465
483.7893279	-0.758924451	0.167266064	-4.537229072	5.69982E-06	0.00014881
235.7939555	-0.272065452	0.110514361	-2.461810838	0.013823755	0.066394328
1299.247556	-0.128598124	0.092881341	-1.384542072	0.166192535	0.35695643
14.17647994	0.270252852	0.366578143	0.737231222	0.46098175	0.663597811
62.54253022	0.053206612	0.136159078	0.390768014	0.69596872	0.836829758
1.916388418	0.28648962	0.880172118	0.325492723	0.744808189	0.866156297
6.463048226	0.987808815	0.440749776	2.241200945	0.025013062	0.10070019
4.412820977	0.684258825	0.463464663	1.47639913	0.139836776	0.318494206
507.0902933	0.114556943	0.051744198	2.213908957	0.026835042	0.105760883
127.186641	0.412538854	0.129418988	3.187622322	0.001434478	0.012705153
9.290200802	1.203560238	0.331313655	3.63269132	0.00028048	0.003574672
6.10234949	-1.043203375	0.781768166	-1.334415266	0.1820678	0.378662408
217.5711187	0.131103435	0.064157296	2.043468841	0.041006049	0.141330302
929.801693	0.085242041	0.096369344	0.884534825	0.376407572	0.589554891
265.9216077	-0.402003615	0.204108034	-1.969562921	0.048888485	0.159556541
11.1076091	-0.318552047	0.470611368	-0.676889826	0.49847586	0.694742406
141.521692	-0.266971742	0.360347901	-0.740872201	0.458770932	0.661849322
4.449593938	1.352466032	0.475785342	2.842597095	0.00447476	0.029011096
777.8541788	-0.078718319	0.068150214	-1.155070749	0.248061455	0.456791602
183.927423	0.128870589	0.139868984	0.921366442	0.356859143	0.571432496
21.52379775	0.399985959	0.260127684	1.537652406	0.124133627	0.294728391
243.1048705	0.014771234	0.071413012	0.206842328	0.836132998	0.918052187
6.985743594	1.283144678	0.44102907	2.909433336	0.003620846	0.024937214
168.6500204	-0.387594102	0.288306577	-1.344381754	0.178824971	0.374375724

11.57451344	0.020142981	0.35668335	0.056473007	0.954964998	0.97818152
40.55594588	0.348510257	0.27477774	1.268335116	0.204678302	0.407765733
182.1168341	0.036363569	0.10160983	0.357874522	0.720437218	0.851641987
99.46959052	0.112683668	0.154800703	0.727927366	0.46665805	0.668563317
2747.25896	0.036731043	0.074653107	0.492022963	0.62270311	0.787723535
49.51042872	0.134541429	0.167221643	0.804569471	0.421068165	0.629405403
134.2201662	-0.070714827	0.111546499	-0.633949323	0.526113895	0.716822193
734.4368754	-0.117212645	0.062790095	-1.866737809	0.061938211	0.187533558
1221.194025	0.34940319	0.095164454	3.671572489	0.000241063	0.003166879
246.7873467	0.185361659	0.081998245	2.260556428	0.023786738	0.09701974
528.8213341	0.119490009	0.070950957	1.684121184	0.092158251	0.243108892
5.818138258	0.509525767	0.520352272	0.979193894	0.327484185	0.542648285
136.7298023	0.124947319	0.212918656	0.58683124	0.557317053	0.741574435
35.31400101	0.199679642	0.248804374	0.802556797	0.422230948	0.630552191
44.67310002	-1.428608042	0.414125063	-3.44970196	0.000561206	0.006118666
205.3412235	0.118726726	0.096762051	1.226996787	0.219823799	0.42424757
246.3956215	-0.124462755	0.091700254	-1.357278191	0.174692834	0.368676627
83.78160177	1.156788814	0.114289684	10.12155056	4.43336E-24	6.63956E-21
2708.737269	0.025508838	0.040377069	0.631765482	0.527540132	0.717558125
398.2927871	-0.034948226	0.071821659	-0.486597313	0.626543728	0.790677612
167.8721691	-0.183059527	0.16568914	-1.104837213	0.269230133	0.480947431
5.789480216	-1.33435658	0.788423077	-1.692437244	0.090562636	0.240905679
942.530499	-0.183926742	0.064436772	-2.854375489	0.004312153	0.028268369
17.60683347	0.10732754	0.597229357	0.179709082	0.857380963	0.92936531
1.915165178	-0.510026504	1.469544182	-0.347064423	0.72854292	0.856369323
358.9831898	0.218110746	0.167648754	1.300998313	0.193259032	0.393882135
8.473307404	0.593967526	0.384702914	1.543964196	0.122597007	0.292501519
122.7124454	-0.192477027	0.149140076	-1.290578841	0.196849757	0.398342083
2453.018526	-0.066612713	0.114964211	-0.579421302	0.562304934	0.745233095
1.784400572	-1.638531711	0.770034666	-2.127867464	0.033348074	0.122290136
41.0315589	0.05845267	0.441521864	0.132389072	0.894676558	0.946865066
149.858744	-0.110053442	0.084467781	-1.30290438	0.192607409	0.393137709
423.1654199	0.387945024	0.082351285	4.710855738	2.46679E-06	7.37529E-05
538.4562375	0.063280082	0.088377881	0.716017191	0.473980734	0.674005923
92.83699453	0.100065517	0.100836688	0.992352278	0.32102571	0.536095037
99.76473875	0.040502018	0.109022891	0.371500123	0.710265064	0.845557591
895.9227649	0.149553854	0.078622036	1.902187495	0.057146647	0.177161058
953.505846	-0.05956503	0.083669143	-0.711911549	0.47651955	0.676451641
306.0074424	-0.359958407	0.073443392	-4.901168084	9.52685E-07	3.31108E-05
65.91442343	-0.400558468	0.155250224	-2.58008303	0.009877656	0.052045506
7.906741709	-0.295652727	0.359329817	-0.822789295	0.410627825	0.620670043
26.31718142	0.425319262	0.199379667	2.133212825	0.032907272	0.121115816
669.7802752	0.207926962	0.056871464	3.656085982	0.000256095	0.003330154
17.58291912	-0.058631627	0.394754225	-0.148526915	0.881926947	0.940841436
595.6031614	-1.042712673	0.139461407	-7.476711255	7.62056E-14	1.90214E-11
4.143089253	-0.968022986	0.533758088	-1.813598723	0.069739527	0.203235268
6.780134012	-0.1396691	0.361214082	-0.386665712	0.699003703	0.83858047
506.6469981	0.171548459	0.159015743	1.078814309	0.280670515	0.494308965

850.0101904	-0.068471377	0.044173118	-1.550068934	0.121124971	0.290981217
6.542487232	0.099401055	0.624677506	0.159123794	0.873571342	0.936745055
32.55347215	1.047203122	0.255996577	4.090691894	4.30088E-05	0.000790767
775.2909472	0.13320007	0.064206018	2.074572973	0.038026129	0.134199325
63.68421377	-0.022249876	0.165023149	-0.13482882	0.892747219	0.946108145
1090.570884	0.013337049	0.050733692	0.262883474	0.792640394	0.893093493
167.0917629	-0.114363557	0.094430052	-1.21109281	0.225859837	0.431435274
3.266042433	1.121376586	0.602116863	1.862390269	0.0625481	0.188824885
160.123227	-0.301293274	0.143805783	-2.09514018	0.036158533	0.129466567
1859.302531	0.048787322	0.037827094	1.289745446	0.197139055	0.398559676
5.623900879	0.635440613	0.454936677	1.396767165	0.162483599	0.351741763
66.48031604	0.11730318	0.140799068	0.833124687	0.404774412	0.615467848
490.9381116	-0.584681016	0.233133923	-2.50791909	0.012144447	0.060516522
1002.0092	-0.273252338	0.098017344	-2.787795786	0.005306798	0.032989168
1.962303	-0.540386279	0.668095182	-0.808846245	0.418603592	0.62771833
282.7506687	-0.288637269	0.069746564	-4.138372579	3.49778E-05	0.000673947
267.72635	-0.1607932	0.192193111	-0.83662312	0.402804446	0.614168857
2195.273521	-0.089761294	0.087274372	-1.028495442	0.303716834	0.518597753
39.84390191	-0.00811662	0.173199617	-0.046862808	0.96262257	0.981463149
757.1814788	0.145238939	0.049211325	2.951331599	0.00316407	0.022633475
458.0249154	0.336252513	0.132532429	2.537133856	0.011176421	0.056879939
2.797711488	-0.147906241	0.753836251	-0.196204734	0.844449923	0.922856413
257.9380711	0.033658676	0.227189837	0.148152208	0.882222648	0.940938489
7.565033856	-0.159411697	0.374774675	-0.42535344	0.670579042	0.820858905
17.42690412	0.005079177	0.248689849	0.020423741	0.983705345	0.991966796
46.02139819	-0.395193981	0.184840952	-2.138021784	0.032514975	0.120290075
12.37062182	0.023712513	0.302575105	0.078369016	0.937534519	0.970479513
6.190839467	0.591379943	0.603948338	0.979189619	0.327486297	0.542648285
55.12377742	0.189663256	0.232217231	0.816749282	0.414071722	0.623815047
10.86322871	0.009196264	0.366008656	0.02512581	0.979954613	0.989626206
7.655141834	0.122777113	0.403655952	0.304162771	0.761003886	0.875424502
9.881608294	0.290396069	0.331734067	0.875388143	0.381362749	0.594040273
707.6250638	1.02830012	0.190939532	5.385475242	7.22535E-08	3.41061E-06
11.32586645	-0.451245274	0.44415559	-1.015962163	0.309647405	0.524856539
634.9760378	-0.037164309	0.12593631	-0.295103998	0.767914435	0.879100285
116.7472662	0.230057064	0.125767879	1.829219565	0.067366723	0.198568507
83.99921702	0.226515711	0.165601631	1.367835023	0.171363722	0.363595656
360.5865329	0.05964244	0.097631304	0.610894639	0.541269334	0.728624641
467.2859775	-0.211181818	0.09515725	-2.219292986	0.026466799	0.104861481
23.52876788	0.349880125	0.273064031	1.281311653	0.200084222	0.402464892
476.8089007	-0.233464222	0.093943715	-2.48514998	0.012949686	0.063326905
330.9036923	-0.023565378	0.076515919	-0.307980073	0.758097495	0.873654994
239.8019903	-0.32524409	0.100350601	-3.241077641	0.001190787	0.011039411
43.89355112	-0.302700577	0.170923879	-1.770967164	0.076566162	0.216095761
292.8803085	0.076538532	0.073822894	1.036785843	0.299835675	0.514717479
1216.440229	-0.223783556	0.110125524	-2.032077098	0.042145851	0.144167516
40.830804	0.042982306	0.20421722	0.210473466	0.833298159	0.916833892
2.514500718	0.277507018	0.795638506	0.348785304	0.727250497	0.85562812

235.8332233	-0.122042325	0.100438768	-1.21509181	0.224331077	0.429374946
140.2563111	-0.125815285	0.084353253	-1.491528537	0.135822785	0.312398022
1744.495764	0.171564546	0.106263238	1.614523983	0.106413819	0.267602085
10.20043538	-0.305508205	0.378346065	-0.807483501	0.419387976	0.628262028
115.5807835	0.04230381	0.141582273	0.298793128	0.765097894	0.877720432
237.2393358	-0.208000147	0.084010298	-2.475888689	0.013290501	0.064605402
6.015063461	0.342558823	0.418031936	0.819456106	0.412526235	0.622273872
27.3068194	0.060956461	0.215833605	0.282423402	0.777618873	0.884312122
29.49769242	0.055712161	0.223349511	0.249439368	0.803020936	0.899097368
26.72746733	-0.620360579	0.21920658	-2.83002718	0.004654405	0.029783098
115.5304356	-0.079083511	0.146881414	-0.538417416	0.590288905	0.763627267
6.590648514	-0.086325565	0.388097996	-0.22243239	0.8239773	0.911434821
10.42601507	-0.428012729	0.337454474	-1.268356954	0.204670507	0.407765733
48.34451812	-0.062654973	0.202904884	-0.308789873	0.757481373	0.873556047
2.742066319	-0.328510608	0.658123728	-0.499162383	0.617664993	0.78385976
26.10899223	0.015414988	0.186633241	0.082595085	0.934173509	0.968872727
41.6402934	-0.503938463	0.155973942	-3.230914449	0.001233949	0.011350123
49.35574443	-0.007998446	0.189743857	-0.042153913	0.966376002	0.98333139
21.35500274	-0.029894248	0.251766022	-0.118738213	0.905482761	0.952853593
16.19956256	-0.072926647	0.250418395	-0.29121921	0.770883672	0.88038389
10.0245528	0.021167758	0.32166083	0.065807697	0.947530929	0.974992162
68.69746509	-0.100139411	0.153685909	-0.651584858	0.514669019	0.707911615
185.6597907	-0.069021191	0.149264764	-0.462407801	0.643788906	0.80219185
63.68431872	0.007211769	0.143278455	0.050333936	0.959856281	0.980448436
55.72267745	-0.061985041	0.153074614	-0.404933511	0.685526385	0.829778815
68.5918054	0.155124975	0.121964995	1.271881131	0.203415357	0.406238889
87.90122753	-0.102735286	0.190845328	-0.538317011	0.590358209	0.763627602
111.4607551	-0.096458841	0.171484141	-0.562494237	0.573779329	0.752760082
93.52030791	-0.166618764	0.149277571	-1.116167436	0.26435047	0.475731379
100.7112434	-0.042148726	0.161573166	-0.260864642	0.794196893	0.893917451
3.85350282	-0.2897459	0.49145362	-0.589569163	0.55547953	0.740249942
36.22945092	0.035886156	0.248650704	0.144323567	0.88524497	0.942747873
45.8673261	-0.153208853	0.185937766	-0.823979207	0.409951373	0.620217261
6.90295116	0.202579734	0.354147977	0.572020024	0.567308409	0.748471553
22.76507136	-0.131392021	0.238607304	-0.550662192	0.581865267	0.758210783
335.211479	-0.033028871	0.07803023	-0.423283015	0.672088785	0.821961317
108.7060317	-0.024446441	0.103440756	-0.236332781	0.813174452	0.906097599
2.146614545	0.2426209	0.685625519	0.353867955	0.723437844	0.853168805
486.4114037	0.080586879	0.131349292	0.613531123	0.53952521	0.727224539
205.2681825	0.297315951	0.083371259	3.566168429	0.000362239	0.004346336
7.205038484	-0.005413329	0.449488475	-0.01204331	0.990391061	0.99479924
685.7139068	0.120498055	0.049670272	2.425959242	0.015267985	0.0712333
49.51463891	-0.195762899	0.251765081	-0.777561759	0.436827413	0.642902204
70.56359927	-0.144303326	0.111688483	-1.292016176	0.196351543	0.397578087
2.433499359	1.502298271	0.685457852	2.191671257	0.028403253	0.109761948
1120.216608	0.209251581	0.051378295	4.072762295	4.64588E-05	0.000841058
12.61930638	-0.133378177	0.428732286	-0.311098981	0.755725376	0.872365037
3.020432379	-0.160932351	0.827631302	-0.194449329	0.845824068	0.923706045

1.865432083	-0.254769913	0.790483028	-0.3222965	0.747228084	0.867378485
880.838764	0.14643437	0.099639346	1.46964403	0.141658189	0.321045123
196.5158287	0.013920465	0.11400028	0.122109043	0.902812661	0.951311133
80.38203014	0.197671171	0.257678273	0.767123934	0.443007833	0.648528056
397.5654278	-0.23695083	0.056099242	-4.223779515	2.40239E-05	0.000498451
7.990454211	0.340336802	0.439640781	0.774124732	0.438857033	0.644424326
2.332707661	-1.934401279	1.03546759	-1.868142758	0.061742175	0.187249741
2.503839033	-1.697641901	1.236365625	-1.373090505	0.169724212	0.36172226
283.5333592	0.348111745	0.154901016	2.247317371	0.024619752	0.099505836
35.44576075	0.07677278	0.215843667	0.355686968	0.722075005	0.852415882
4.976952627	-0.967121161	0.652426597	-1.482344781	0.138248579	0.316320429
154.6376539	0.078364557	0.076435154	1.025242355	0.305248848	0.520135449
290.7550939	-0.50530716	0.113606565	-4.447869377	8.67262E-06	0.000212292
12.43300325	0.143329149	0.334998628	0.427849957	0.668760363	0.819729034
5.343339355	-0.022566296	0.52884328	-0.042671046	0.96596376	0.983090566
639.9890269	-0.051885827	0.080512599	-0.644443568	0.51928784	0.711709474
2.169913579	-0.168876647	0.787043232	-0.214570992	0.830101812	0.915395759
159.4214512	-0.023651143	0.10351704	-0.228475846	0.819276332	0.909322798
9480.721802	0.035225252	0.087634169	0.401957958	0.687714968	0.83108758
494.0485723	-0.103298817	0.083409216	-1.238458072	0.215546259	0.420053765
405.3445523	0.112237124	0.0984106	1.140498317	0.254078753	0.463389137
26.14104814	-0.061840206	0.225509136	-0.274224836	0.783911843	0.887937548
60.73709835	-0.30305986	0.224737895	-1.348503598	0.177496464	0.372768415
322.4294932	-0.0437278	0.091047471	-0.480274731	0.631032054	0.793230487
1089.25155	-0.246977412	0.047584559	-5.190284805	2.09973E-07	8.69287E-06
2.177355365	-0.425139119	0.682937715	-0.622515215	0.533603142	0.722790508
48.95107061	-0.590626846	0.214169146	-2.757758795	0.005819912	0.035304727
507.8394802	-0.075879283	0.09755861	-0.777781508	0.436697831	0.642902204
330.55423	-0.084349278	0.105994107	-0.795792151	0.426152855	0.63361391
1054.483514	-0.113295416	0.102099734	-1.10965437	0.267147993	0.47877973
9.430043109	-0.599790786	0.322042521	-1.862458364	0.062538509	0.188824885
122.8348739	-0.517006171	0.12580788	-4.109489587	3.96535E-05	0.000738972
60.88124849	-0.003178128	0.140295486	-0.022653104	0.981926984	0.990707076
474.4719213	-0.052343132	0.073101204	-0.716036524	0.473968797	0.674005923
39.81091395	-0.203459837	0.243200974	-0.836591374	0.402822296	0.614168857
232.2637245	0.296441336	0.18131813	1.634923852	0.102065012	0.26080083
88.93237207	-0.096879739	0.115199575	-0.840973053	0.400363031	0.612232486
222.3664034	0.041126987	0.075665017	0.543540316	0.586757841	0.761481698
20.98781153	0.611198462	0.225584131	2.709403628	0.006740428	0.039418466
9.592277363	0.729248558	0.318627313	2.288719542	0.022095653	0.092105817
5.512838112	1.164705533	0.436508854	2.668228888	0.007625229	0.042975937
674.2625026	-0.527498916	0.187828778	-2.808403067	0.004978787	0.03140143
5.48893388	-0.722922675	0.443630482	-1.629560421	0.103194435	0.2625116
124.1614461	0.082683615	0.11264737	0.734003955	0.462946328	0.665379323
40.09914067	-0.380550931	0.160285231	-2.374210834	0.017586505	0.07842991
44.99292239	-0.131787872	0.140110543	-0.940599255	0.346910264	0.56156035
125.5983355	-0.166788543	0.132065036	-1.262927324	0.206615299	0.410094029
8404.324584	-0.221458976	0.125251717	-1.768111305	0.077042298	0.216781036

157.1198337	-0.102807251	0.101724882	-1.010640164	0.312188685	0.526946353
189.9791934	-0.072289273	0.123015615	-0.587643066	0.556771898	0.741252646
471.6173011	0.149899676	0.064424338	2.326755402	0.019978287	0.085865459
2604.242722	-0.117242594	0.099223908	-1.181596207	0.237365959	0.445009597
17.5947195	-1.614914938	0.553705022	-2.916561839	0.003539126	0.024559206
2.153279415	0.059689489	0.934974056	0.063840797	0.949096993	0.975920679
217.6662131	-0.575028028	0.197003753	-2.918868395	0.003513045	0.024439993
64.66132325	-0.366504782	0.161577687	-2.268288332	0.023311639	0.095436366
311.7110485	-0.183711291	0.062094636	-2.958569429	0.003090706	0.022243902
1809.009463	-0.213659376	0.067829426	-3.149951118	0.001632978	0.01393812
82.57494095	-0.05198836	0.10887696	-0.477496435	0.633008659	0.794225792
3.693664247	-0.174515045	0.653449806	-0.267067254	0.78941738	0.89117124
210.1512937	-0.03442301	0.079829295	-0.431207748	0.66631732	0.818067386
12.60836407	0.189640099	0.295649577	0.641435381	0.521239852	0.713138885
96.95740788	-0.393491486	0.109970592	-3.578151917	0.000346032	0.004191571
9540.440799	-0.268118912	0.160403487	-1.671527948	0.094617443	0.247020171
164.88159	0.15095712	0.184177836	0.819626957	0.412428801	0.622273872
233.3028346	-0.100686468	0.103728168	-0.970676239	0.331709528	0.547279195
36.42644057	-0.395066872	0.184020646	-2.146861678	0.031804295	0.118432179
23.41580195	-0.254191854	0.207813576	-1.223172511	0.221264546	0.42601247
261.2545218	-0.257060418	0.082126466	-3.130055765	0.001747731	0.014615291
156.9601568	0.021695024	0.174319986	0.124455171	0.900954893	0.950575612
1022.22201	0.137468415	0.240911477	0.570617957	0.568258643	0.74897935
268.6312488	0.153347515	0.127364577	1.204004431	0.228587866	0.434644102
62721.99554	0.171372746	0.113597762	1.508592619	0.131402922	0.305494176
68.86084771	0.589966153	0.167586947	3.520358613	0.000430964	0.004968297
25.92801764	1.048282106	0.219480233	4.776202812	1.78636E-06	5.67024E-05
181.6080839	0.005394101	0.075824379	0.071139402	0.943286809	0.972629837
1318.310724	-0.182043589	0.145542575	-1.250792694	0.211010121	0.415366175
1353.159288	0.0844868	0.069207473	1.220775685	0.222170963	0.427026536
7.428301844	-2.297737261	1.00082571	-2.295841561	0.021684942	0.090946471
164.5930149	-0.444604147	0.20052392	-2.217212527	0.026608572	0.105271281
230.0071881	-0.530178271	0.320678348	-1.653302363	0.098269342	0.253664861
363.684563	-0.114692814	0.05815668	-1.972134834	0.048594216	0.158995257
14.23427794	0.298687416	0.289267174	1.032565887	0.301807099	0.516838611
496.793328	0.12015758	0.045879621	2.618974999	0.008819441	0.048157598
157.0487932	-0.004650739	0.097236348	-0.047829219	0.96185235	0.981287177
378.7000765	0.079979765	0.069110393	1.157275498	0.247159821	0.455777809
69.92138616	0.419055124	0.164047325	2.554477027	0.010634745	0.054774042
64.81306526	0.06793762	0.1354602	0.501531963	0.615996789	0.782777813
5.017659922	-1.279657377	0.536265412	-2.386238883	0.01702169	0.076658099
349.7056544	-0.203468759	0.05456261	-3.729087727	0.000192174	0.002638232
10.7118605	0.981459897	0.418250208	2.346585557	0.018946316	0.082637439
98.78270747	0.381832095	0.229714786	1.662200772	0.096472525	0.250202831
106.6451781	-0.392618042	0.29335244	-1.338383421	0.180771481	0.376918032
7.51642273	-1.00677552	0.592882804	-1.698102074	0.089488498	0.239006372
10.08709749	-0.815269259	0.429132813	-1.899806384	0.057458533	0.177793364
129.0863565	0.093950382	0.100807992	0.931973551	0.351350194	0.566504355

16.81229612	-0.234428704	0.229297639	-1.022377314	0.306602353	0.52174023
160.9327415	-0.979259662	0.14926048	-6.560743101	5.35403E-11	6.08292E-09
1268.787033	-0.140908481	0.088916691	-1.584724744	0.113028887	0.278581372
121.8468392	-0.162518127	0.170250061	-0.95458484	0.339787687	0.554608377
3.447700057	-1.502635986	0.639071757	-2.351278975	0.018709001	0.081775559
13.25460455	-0.25472146	0.302561531	-0.841883168	0.399853353	0.612021197
318.1067187	-0.062407758	0.06565941	-0.950476998	0.341869936	0.556292322
928.7577734	-0.076449141	0.084542266	-0.904271255	0.365851585	0.579264039
163.9589937	-0.460311725	0.106664586	-4.315506585	1.59237E-05	0.000353998
106.0457468	-0.157886548	0.299502589	-0.527162546	0.598080715	0.769422919
214.8944486	-0.231279782	0.203250101	-1.137907342	0.255159175	0.465090977
142.3247394	0.076816149	0.093471631	0.821812432	0.411183657	0.621111275
146.2610676	-0.077474781	0.096719522	-0.801025264	0.42311702	0.63111002
997.2481788	-0.225993668	0.04868625	-4.641837629	3.45324E-06	9.77085E-05
9.855145512	-0.478559815	0.386076842	-1.239545506	0.215143547	0.41963945
602.6673257	0.038013395	0.102699451	0.370142147	0.71127658	0.846071015
962.2889136	0.055967437	0.060156617	0.930362106	0.352183632	0.567204717
25.69218926	-0.036815943	0.30332709	-0.12137374	0.903395015	0.951456563
94.5909543	0.70548316	0.167465797	4.212699984	2.52336E-05	0.000517682
263.1334508	-0.068416198	0.107926587	-0.633914218	0.526136806	0.716822193
265.9034497	0.088178558	0.101718981	0.866884009	0.386005566	0.599289011
5.640010904	0.672923416	0.448933494	1.498937871	0.133889751	0.309528452
746.6750817	0.5918314	0.157724648	3.752307637	0.000175214	0.002458671
521.9227936	0.011593447	0.086517998	0.134000412	0.893402248	0.946123642
556.2061888	0.026898176	0.060990001	0.441026004	0.659194172	0.812416008
26.86442954	-0.346944946	0.242879101	-1.428467679	0.153157291	0.33766209
91.29747245	0.224332283	0.111752143	2.007409222	0.044706108	0.150242435
28.86202071	-0.49723723	0.314831125	-1.579377612	0.114249469	0.280540432
20.14848825	-0.177505099	0.336724416	-0.527152445	0.59808773	0.769422919
2.530332787	0.45095248	0.711901187	0.633448136	0.526441039	0.717000092
122.1713689	-0.096853419	0.11309387	-0.856398489	0.3917774	0.604967515
8.275953988	-0.728053639	0.397615341	-1.831050172	0.067093055	0.197974384
28.06152383	0.343219038	0.220780425	1.554571867	0.120048068	0.289331805
203.7975388	-0.176345747	0.098501344	-1.790287725	0.073407669	0.210535852
140.3752859	-0.269199809	0.113938308	-2.362680422	0.018143308	0.080051235
127.9156463	-0.190274662	0.12015713	-1.583548657	0.113296466	0.278948733
24.09053523	-0.187697074	0.286970819	-0.654063276	0.51307104	0.706539523
331.8057146	-0.08563344	0.087999993	-0.973107353	0.33049995	0.545820272
187.3359424	-0.268039583	0.128948077	-2.078662904	0.037648345	0.133294398
2.179507007	-1.72675869	1.17546293	-1.469003102	0.141831948	0.321247352
5.815240408	-1.245925377	0.769502123	-1.619131826	0.105418902	0.26595268
251.075812	-0.019004532	0.073769158	-0.257621643	0.796698925	0.894975662
1618.289426	-0.073901524	0.149604602	-0.493978949	0.621321046	0.786633633
18.66568648	0.387060214	0.587937441	0.658335712	0.510322435	0.704639326
2290.139905	0.055810355	0.115641077	0.482617042	0.629367677	0.79225209
7.283363023	-0.206451308	0.375743478	-0.549447482	0.582698396	0.75889593
426.572933	0.154910845	0.097211708	1.593541028	0.111038872	0.275490117
1523.772125	0.066923095	0.088191824	0.758835597	0.447950909	0.652990786

196.3228811	0.051859869	0.136674419	0.379440933	0.704360459	0.841758013
23.22117106	0.128300119	0.218119956	0.588208989	0.556392025	0.740866652
407.6962055	-0.347110551	0.099536047	-3.487284858	0.000487951	0.005468374
476.5015404	-0.133432738	0.113932094	-1.171160237	0.241534375	0.449050591
86.85939808	-0.143250573	0.162978021	-0.87895639	0.379424922	0.59230988
4.6933591	-1.225723741	0.59865199	-2.047472925	0.040611673	0.140318099
67.07087742	0.034467245	0.120211271	0.286722243	0.774325005	0.882906294
134.7268003	0.457549276	0.098679676	4.636712378	3.53994E-06	9.95171E-05
130.2389671	-0.009326558	0.1388105	-0.067189139	0.946431132	0.974651901
89.99749144	0.352475027	0.234688316	1.501885706	0.133126629	0.308209396
8.891336433	-0.538445542	0.416325417	-1.293328537	0.195897455	0.397097905
365.5052345	-0.15472859	0.080534064	-1.921281298	0.054696254	0.171435708
69.83146942	-0.025713206	0.133827229	-0.192137329	0.847634635	0.924518868
343.3045591	-0.242255559	0.076964122	-3.147642753	0.001645927	0.014012922
13.41865009	0.200905679	0.307263412	0.653854871	0.513205311	0.706547242
77.82528111	0.110337771	0.178465935	0.618256761	0.5364061	0.724858837
151.3186563	-0.234799768	0.091761965	-2.558791861	0.010503661	0.054328826
44.45627353	-0.085504136	0.180859804	-0.472764731	0.636381039	0.796591584
57.6953694	-0.05000126	0.128684012	-0.388558448	0.697602809	0.8379983
33.31427618	-0.427727546	0.259359711	-1.649167266	0.099113372	0.255075442
6.383791115	-1.89185468	1.011562733	-1.870229714	0.061451925	0.18660061
4529.479914	-0.054981615	0.138799716	-0.396121959	0.69201506	0.834043309
19.91362255	0.25472581	0.316355801	0.805187733	0.420711354	0.629081489
3.154221195	-0.812536562	0.632825611	-1.283981791	0.199148327	0.401268289
188.3927919	0.623860955	0.111295812	5.605430646	2.07738E-08	1.14457E-06
803.6514728	-0.208965796	0.100042178	-2.088776951	0.036727806	0.131068256
192.8866925	-0.086437589	0.093148927	-0.927950446	0.353433275	0.568433055
527.2934453	0.050794771	0.129178431	0.393214029	0.694161409	0.835387176
3.435354548	0.54646645	0.66368629	0.823380652	0.410291562	0.620446411
2.221317388	-0.523997621	0.644854498	-0.812582717	0.416457344	0.626035065
3015.401788	-0.161188021	0.050857101	-3.169429969	0.001527383	0.013321036
286.3073624	0.090749057	0.102995198	0.881099884	0.37826376	0.591281638
389.0900936	0.100384471	0.114334228	0.877991419	0.379948376	0.592846139
492.5655946	-0.192919274	0.110971945	-1.738450871	0.0821314	0.226297488
402.8745193	-0.092668546	0.073177973	-1.266344808	0.205389661	0.408843557
55.29496275	-0.258365373	0.220526156	-1.171586073	0.241363282	0.448985853
21.42118057	1.207779602	0.340409443	3.548020269	0.000388138	0.004581051
384.192303	0.060203955	0.055073047	1.093165504	0.27432114	0.487031626
128.4424841	-0.3238517	0.110769171	-2.923662771	0.003459393	0.024148322
98.69708721	-0.231363669	0.135769748	-1.704088519	0.088364551	0.237059479
20.62489868	0.001042824	0.226499242	0.004604095	0.996326477	0.998004635
927.5978691	-0.149597621	0.104045168	-1.437814207	0.150486777	0.333933077
108.1671431	0.231149759	0.162714075	1.420588596	0.155436396	0.34069441
352.4236063	0.077194277	0.089584173	0.861695477	0.388855115	0.602294017
27.4122618	-0.535316578	0.281135285	-1.904124475	0.056893975	0.176613806
428.6228221	0.404523356	0.111618217	3.624169651	0.000289891	0.003648334
168.0122191	0.075552611	0.073413778	1.029133948	0.303416735	0.518343743
469.3620679	0.072615566	0.110434081	0.657546709	0.510829448	0.705147867

52.83440122	-0.328289197	0.302293789	-1.085993853	0.277481712	0.490528353
12.57692552	-0.00462085	0.372264646	-0.01241281	0.990096265	0.994563772
333.5601653	-0.133572887	0.062103101	-2.150824768	0.031490033	0.117634197
69.37199419	-0.333565478	0.374757008	-0.890084695	0.37342041	0.586886402
2000.051088	-0.07326241	0.191875719	-0.381822205	0.702593247	0.840499684
696.3250592	-0.195104895	0.119889588	-1.627371461	0.103658229	0.26328487
127.6846084	0.126223774	0.23019641	0.548330767	0.583464805	0.759227189
1.775328997	0.52986131	0.782571917	0.677076826	0.498357211	0.694742406
44.50610656	-0.072185949	0.282587845	-0.255446052	0.798378606	0.895702068
872.6810739	-0.465711624	0.171204033	-2.720214103	0.006523966	0.038439133
110.0007087	0.065183275	0.142348956	0.457911859	0.647015767	0.804266033
202.6723908	0.049056883	0.096642246	0.507613234	0.611724608	0.780039684
39.34235715	-0.121579176	0.209138332	-0.581333778	0.56101552	0.744735671
3.276624544	1.119745828	0.899780272	1.244465858	0.213328157	0.417887765
2.796337807	1.376236275	0.642121328	2.143265167	0.032091812	0.119018576
2.611512922	0.323637495	0.667623432	0.48476054	0.627846217	0.791096258
8.008701637	0.323575247	0.419466866	0.771396442	0.440471984	0.646213862
2.529903485	-0.302577519	0.704011271	-0.429790731	0.667347876	0.818667728
807.8319605	0.087692688	0.103727552	0.845413653	0.397879924	0.610475354
16.56499512	1.130058245	0.716463249	1.577273148	0.114732688	0.28129174
94.44139817	-0.398868624	0.140030178	-2.848447596	0.004393309	0.028663166
373.0969182	-0.247225984	0.085811217	-2.881045068	0.00396359	0.026492884
255.5066824	0.292675568	0.081110264	3.608366608	0.000308131	0.003809753
2457.133655	-0.175947652	0.095925864	-1.834204502	0.066623643	0.197154285
114.7083118	0.128885563	0.113991953	1.130654918	0.258200363	0.468747323
3.320913372	-1.48742984	0.773498711	-1.922989422	0.054481378	0.170892275
25.79285712	0.236998421	0.235789955	1.005125181	0.314836576	0.529679101
2.700755532	-0.221285353	0.636387578	-0.347721044	0.728049691	0.856033874
12.39415853	0.207035491	0.290918381	0.711661771	0.476674246	0.676493067
2.47441743	0.171597721	0.662176261	0.259142061	0.795525633	0.89469479
883.6613739	0.32797425	0.082760387	3.962937626	7.40331E-05	0.001235686
119.3834706	-0.164876749	0.129148187	-1.276647805	0.201726616	0.404533692
5.901690761	-0.68129647	0.92365424	-0.737609856	0.460751565	0.663513594
540.1305036	-0.183604653	0.11485169	-1.598623874	0.109904202	0.273746873
751.4695641	0.175317686	0.060854035	2.880954155	0.003964733	0.026492884
1290.113696	-0.070304817	0.100669625	-0.698371699	0.48494477	0.683839419
35.31210574	0.070265678	0.237334678	0.296061574	0.767183059	0.878716103
342.1518045	-0.090512982	0.106070636	-0.853327418	0.39347776	0.606547079
3.429059157	0.851317913	0.683135336	1.246192179	0.21269385	0.417331882
12.25584659	0.174781975	0.319906633	0.546353084	0.584823253	0.760101103
173.6349296	-0.17555823	0.121270163	-1.447662199	0.147711547	0.329829955
558.8168839	-0.091223166	0.084200041	-1.083409986	0.278626485	0.491918628
1142.042753	0.051091757	0.055321594	0.923540952	0.355725369	0.57032659
166.2546714	0.059477608	0.126727603	0.469334276	0.638830719	0.798267972
634.6828206	-0.018706788	0.112383446	-0.166455014	0.867798883	0.934495951
2067.529472	-0.201155878	0.10459055	-1.923270104	0.054446137	0.170814257
1260.91615	-0.120962315	0.077255924	-1.565735132	0.117410638	0.285241535
316.181308	0.06180204	0.081144668	0.761627852	0.446282147	0.651373446

316.4084789	0.012768565	0.080066795	0.159473911	0.873295511	0.936680519
104.4950015	-0.131443255	0.116369135	-1.129537097	0.258671329	0.469363529
438.6759362	0.287500947	0.1559268	1.843819969	0.065209382	0.194400896
579.5032043	0.141904158	0.087862711	1.615066925	0.106296203	0.267537975
81.38103175	0.245081569	0.140133682	1.748912656	0.080306118	0.222908677
71.14376782	-0.004965623	0.122389385	-0.04057233	0.967636843	0.98386242
33.40066568	-0.466439115	0.287445022	-1.622707229	0.104652005	0.26483613
254.5269445	-0.190518483	0.100293394	-1.899611479	0.057484125	0.177839149
509.8503656	-0.132559842	0.060384273	-2.19527099	0.028144165	0.109119079
255.3372581	0.001478612	0.061557107	0.024020169	0.980836521	0.990108149
175.195573	-0.108291044	0.094261225	-1.148839764	0.250622059	0.45957013
238.0587288	-0.215462426	0.0780458	-2.760717769	0.005767449	0.035073075
8.813993438	-0.003949138	0.395526105	-0.009984519	0.992033638	0.995720597
863.7618716	-0.269387451	0.172162525	-1.564727584	0.117646798	0.285401367
1534.416475	-1.405014134	0.591237164	-2.37639685	0.017482649	0.078186661
3058.218294	-0.134403549	0.13105417	-1.025557212	0.305100345	0.519936183
6.356713787	0.7808839	0.431075927	1.811476472	0.070067128	0.203684164
202.3483733	0.261450195	0.078969505	3.310774119	0.000930383	0.009096217
16404.25261	-0.055342257	0.114997605	-0.481247039	0.630340933	0.79299248
3414.679624	0.311899161	0.072865832	4.280458358	1.86509E-05	0.000402301
1307.856593	0.043904031	0.046815166	0.937816401	0.348338771	0.563098118
168.1032148	0.154732953	0.105816133	1.462281298	0.143664141	0.323704153
29.15898171	0.181608097	0.211740947	0.85769002	0.391063652	0.604272476
375.1758049	-0.32341521	0.109412885	-2.955915188	0.003117428	0.022397082
930.379567	0.184583257	0.058664986	3.146395648	0.001652962	0.014058287
240.6215693	0.215604766	0.087667479	2.45934718	0.013918994	0.066715598
2916.19123	0.047071772	0.098826616	0.476306631	0.633855942	0.794736908
417.3262688	-0.193355195	0.142442992	-1.357421605	0.174647287	0.368676627
1541.630332	-0.013917503	0.067664956	-0.205682575	0.83703887	0.918557044
298.5693724	-0.18225253	0.091957468	-1.981922011	0.04748797	0.156651803
772.4345187	-0.328480972	0.156481818	-2.099163819	0.035802462	0.128498859
752.1839289	0.16967041	0.075201681	2.256205022	0.024057794	0.097834634
226.9447923	-0.125290981	0.147483207	-0.849527099	0.395588058	0.608397353
1128.595663	-0.04429833	0.136779821	-0.323865975	0.746039505	0.866955867
1421.353747	-0.010798191	0.105079009	-0.102762587	0.918151399	0.959333553
3095.160066	-0.041691533	0.081278044	-0.512949508	0.607986641	0.777456487
59.88174752	0.034487994	0.141320252	0.244041416	0.807198744	0.901735106
430.0007381	0.045710009	0.176953168	0.258316987	0.796162277	0.894773586
24.6868118	-0.093900389	0.219649989	-0.427500084	0.669015124	0.819834748
4.34235196	0.192611929	0.564042834	0.341484578	0.732738813	0.859155815
2.435477845	-0.055209667	0.917728466	-0.060159044	0.952028965	0.977124952
1598.807148	0.130019	0.120967113	1.074829324	0.282451149	0.496119014
2.432628972	0.265694489	0.690073372	0.385023535	0.700219979	0.839119929
248.1311985	0.18386214	0.134420922	1.367808949	0.171371886	0.363595656
269.4490756	0.179587551	0.073051185	2.458379712	0.013956552	0.066817856
1984.550604	0.027215843	0.083043183	0.327731213	0.743114901	0.865245523
1383.334098	0.206018936	0.064955404	3.17169819	0.001515504	0.013244783
2.076842778	0.807533167	0.798590529	1.011198027	0.31192166	0.52665752

132.8376654	0.50862328	0.107992774	4.709789952	2.47972E-06	7.40053E-05
53.20160159	0.539218626	0.159125689	3.388633414	0.000702418	0.007314565
305.1261217	0.234943828	0.107891775	2.177587944	0.029436728	0.112567469
13.28425342	0.524603639	0.259276212	2.023338874	0.043038222	0.146278453
1752.001474	0.369962013	0.087075924	4.248729115	2.14987E-05	0.000452508
773.9270218	0.191663282	0.058496276	3.276504012	0.001051008	0.009996714
8.417108927	0.170545573	0.36782052	0.463665194	0.642887638	0.801477463
1112.658257	-0.248982595	0.078016806	-3.191396918	0.001415866	0.012574113
347.8831798	0.196842921	0.067196744	2.929352088	0.003396694	0.023831832
298.243874	-0.161647652	0.100386752	-1.61024885	0.107343541	0.269036588
168.7309153	-0.096539922	0.095098738	-1.015154599	0.310032139	0.525135662
1265.471081	-0.085880688	0.134358994	-0.639188233	0.522700486	0.714069808
713.8854984	-0.071375309	0.076530838	-0.932634624	0.35100865	0.566136333
158.3160342	0.376190732	0.115586547	3.254623851	0.001135426	0.010658128
233.9920004	0.201582251	0.113991094	1.76840351	0.07699347	0.216744776
411.0263754	0.035691007	0.096510575	0.369814472	0.711520731	0.846071015
207.4657128	0.085828006	0.101571136	0.845003899	0.398108662	0.610697469
150.6240523	1.028186684	0.37565801	2.737028509	0.006199692	0.03699157
222.7797389	-0.075830568	0.100812614	-0.752193246	0.451934855	0.656598889
21.86735565	-0.1793651	0.216243299	-0.829459691	0.406844337	0.617614597
626.4518423	-0.432225701	0.236000397	-1.831461753	0.067031651	0.197899539
417.4942715	-0.125764938	0.086118256	-1.460374884	0.144187072	0.324588387
379.4717475	-0.061471219	0.06374025	-0.964401911	0.334844464	0.550070918
20.88564937	-0.169478648	0.242457993	-0.699002107	0.484550714	0.683666364
99.9066123	-1.17649796	0.445314688	-2.64194735	0.008243087	0.045753576
33.73772936	0.331458922	0.175772435	1.885727543	0.059331679	0.182056232
246.5934836	0.187201527	0.071517653	2.617556927	0.008856171	0.04827815
487.8639472	-0.594510056	0.380183961	-1.563743129	0.117877905	0.285660577
204.8051941	-0.156497594	0.203420637	-0.769329976	0.441697447	0.647608022
118.0701073	-0.044857777	0.113531509	-0.395113013	0.692759485	0.83461458
404.0722857	0.178944996	0.079360648	2.254832851	0.024143822	0.098111823
182.5514138	-0.012006434	0.108054022	-0.111115108	0.911525069	0.955549728
305.2682833	-0.324340293	0.187616838	-1.728737661	0.083856058	0.229247377
12.54773028	-1.779832165	0.603381937	-2.949760433	0.003180204	0.022706287
4.54325634	-0.725413394	0.698238122	-1.038919777	0.298842048	0.513735145
2.952892723	-1.748266213	1.514884285	-1.154059244	0.248475881	0.45711395
135.9549055	-0.026612634	0.128789785	-0.206636218	0.836293973	0.918167738
413.0190558	0.011370369	0.088584796	0.128355764	0.897867437	0.948292611
119.0185265	-0.164753948	0.133344288	-1.235553097	0.216624728	0.421392843
237.1414516	-0.130966822	0.110176195	-1.188703444	0.234556385	0.441558895
1.914716621	-0.347763711	0.68487059	-0.50778018	0.611607511	0.780039684
1332.589422	-0.118116031	0.067149591	-1.758998502	0.078577763	0.21959119
102.4810173	0.309462564	0.125436403	2.467087354	0.013621712	0.065653624
15.18038158	-0.350993787	0.417166932	-0.841374902	0.400137941	0.61222926
825.4120248	-0.029603626	0.142872488	-0.207203121	0.83585123	0.917926349
1621.161373	0.155059562	0.046219844	3.354826609	0.000794147	0.008036109
988.1261749	-0.133189784	0.597628239	-0.22286394	0.823641402	0.91138289
225.5152891	0.897211748	0.186685195	4.806014462	1.53969E-06	4.99964E-05

150.1723753	0.09500577	0.104049685	0.913080797	0.361200049	0.575339666
804.2537778	-0.000343448	0.057020821	-0.006023196	0.995194214	0.997555794
11.57919539	-0.147809316	0.318149069	-0.464591382	0.642224105	0.801090324
750.6425591	-0.160389025	0.087834289	-1.826041125	0.067844067	0.199654013
549.1163357	0.625575454	0.052841227	11.83877606	2.46017E-32	8.10577E-29
87.2860945	-0.120709391	0.107260924	-1.125380856	0.26042768	0.471512876
310.9235569	-0.07408546	0.060203926	-1.230575242	0.21848177	0.423029367
149.8078231	1.1728337	0.212734367	5.513136952	3.52494E-08	1.79783E-06
137.8685585	-0.30821207	0.103559533	-2.976182495	0.002918611	0.021350445
99.99139573	0.012096044	0.138502748	0.087334322	0.930405774	0.966851997
1476.845811	-0.065268453	0.105506285	-0.618621468	0.536165756	0.724593491
346.399529	0.602121444	0.20469114	2.941609702	0.003265112	0.023125301
438.378179	-0.225827028	0.07603704	-2.969960805	0.002978377	0.021660227
215.1921738	-0.137017973	0.095990917	-1.4274056	0.153463018	0.338123546
366.677064	-0.48934201	0.062889219	-7.781015886	7.19445E-15	1.97536E-12
1019.08667	-0.052576696	0.083282421	-0.631306047	0.527840434	0.717758424
453.4878829	-0.182280555	0.053382153	-3.414634727	0.000638676	0.006786888
12.33907309	0.111726485	0.298159922	0.374719998	0.707868718	0.844021514
265.420708	0.202111618	0.081910285	2.467475452	0.013606955	0.06563065
8.200994751	-0.98740722	0.460530874	-2.144063029	0.032027837	0.118941971
6632.898198	-0.066606936	0.096259411	-0.691952456	0.488967187	0.686897368
5.254219115	-0.300257589	0.524511965	-0.572451363	0.567016227	0.748471553
35.36987306	0.428221173	0.178937962	2.393126463	0.016705482	0.075710072
140.1961389	0.828061516	0.662514947	1.249875977	0.211344856	0.415575933
6.73833276	0.689827234	0.572239568	1.205486779	0.228015439	0.433655777
17.36420535	0.20691437	0.277673495	0.745171482	0.456168061	0.66012936
545.8728764	0.00930216	0.095371697	0.097535857	0.92230086	0.961560774
111.5362455	-0.72169907	0.276147593	-2.613454137	0.008963211	0.04869738
2.740828963	0.253732607	0.891151778	0.284724345	0.775855337	0.883652841
38.59157248	0.077938424	0.28497038	0.273496581	0.78447152	0.888266123
1.729814817	-1.05445321	0.764829951	-1.37867667	0.167994466	0.359374215
35.04565506	0.178612972	0.178660114	0.99973614	0.317438218	0.53226231
220.3963365	0.021689045	0.105166944	0.206234436	0.83660779	0.918328674
2154.375414	0.046603399	0.057441311	0.811321995	0.417180782	0.626542175
10.20143036	-0.057837899	0.304289647	-0.190075145	0.849250247	0.925628813
698.4408259	0.116589595	0.421718699	0.276462949	0.782192514	0.886843735
395.1358945	0.543915342	0.124662738	4.363094785	1.28235E-05	0.000293818
1572.873966	-0.146160147	0.109076467	-1.339978745	0.180252255	0.376311703
17630.39278	-0.031919793	0.099230622	-0.321672811	0.747700578	0.867697312
2.057198728	1.466464277	0.80196476	1.828589421	0.067461139	0.198775675
1141.212799	-0.09644676	0.056144951	-1.717817156	0.085829971	0.232674502
2021.83563	-0.105569943	0.086436608	-1.22135684	0.221950941	0.42670321
4632.670539	-0.100693283	0.083388372	-1.207521875	0.227231226	0.433043251
7.704863646	-0.382679645	0.409007559	-0.935629762	0.349463846	0.564197118
255.499099	-0.125164057	0.163466098	-0.765688165	0.443861871	0.649163748
381.9360156	-0.517153438	0.151608222	-3.41111736	0.000646972	0.006845358
13.60487638	-2.601442785	0.900883904	-2.887655972	0.003881241	0.026108439
8.651658895	-0.199465944	0.613048507	-0.325367311	0.744903093	0.866205516

352.4691981	-0.102762123	0.069526377	-1.478030745	0.139399549	0.317894265
28.04035055	-1.062631797	0.242827674	-4.376073703	1.20836E-05	0.000280769
124.2927837	-0.117969903	0.096460638	-1.222984902	0.221335399	0.426066763
851.4374426	-0.34240717	0.125166075	-2.735622819	0.006226235	0.03710962
334.5568852	-0.597944387	0.145603683	-4.106657005	4.01427E-05	0.00074656
85.13612269	-0.209610552	0.137630336	-1.522996736	0.127759522	0.29947394
2614.541494	-0.078442725	0.082644488	-0.949158573	0.342539968	0.556619
139.3444116	-0.270359923	0.137453551	-1.966918434	0.049192615	0.160253328
66.50592618	-0.101922453	0.110041314	-0.926219885	0.354331721	0.569096189
8.16133974	-0.563990588	0.543164608	-1.038341931	0.299110894	0.514082977
420.1501446	-0.13270113	0.087559271	-1.515557727	0.129631223	0.302442256
464.4370221	-0.262318912	0.08887282	-2.951621325	0.003161103	0.022622075
277.5102113	-0.148221082	0.0932885	-1.588846231	0.112095113	0.277067501
1554.030993	0.0939045	0.097761547	0.960546378	0.336780301	0.551996685
7.945523894	0.569495042	0.34988207	1.627677126	0.103593366	0.263201281
54.48600852	-0.299667981	0.267764772	-1.119146402	0.263077687	0.474432601
32.63426966	-0.490673277	0.409187984	-1.199139019	0.230473894	0.436936024
145.5023695	0.113793979	0.088257775	1.289336595	0.197281094	0.398725155
422.0257968	-0.022035237	0.059708813	-0.369044962	0.712094212	0.846212223
88.24140977	0.147469649	0.283351117	0.520448449	0.602751049	0.773169919
12.48099927	-1.809289525	0.92855845	-1.948492876	0.051356016	0.164855615
173.0850164	0.091137256	0.092789977	0.982188581	0.326006952	0.540977763
185.8975259	-0.099594932	0.087950466	-1.132397997	0.257467146	0.467957282
3064.603527	0.05330746	0.120156686	0.443649552	0.657295974	0.811359633
326.5127086	0.129077193	0.105598256	1.222342087	0.22157829	0.426335056
17.07552414	0.269809105	0.223164123	1.209016493	0.226656509	0.432268966
85.54746085	-0.13680831	0.119016381	-1.149491425	0.2503534	0.45932976
517.6507716	-0.117881855	0.070520084	-1.671606856	0.094601872	0.247020171
166.8611452	-0.132222838	0.103953962	-1.271936502	0.203395681	0.406238889
474.4264997	-0.078928565	0.088052167	-0.896384128	0.370047641	0.583496592
4.249840576	-0.170641058	0.493629228	-0.345686698	0.729578182	0.857101832
64.3148409	0.2854528	0.219603256	1.299856863	0.193650032	0.394483817
5.574444874	0.488288867	0.756408373	0.645536042	0.51857987	0.710975763
157.2345485	0.066528971	0.097566081	0.681886273	0.495310866	0.692149563
2.491451355	-1.5956623	0.69440438	-2.297886282	0.021568262	0.090757485
24168.76548	-0.169714376	0.103564887	-1.638725059	0.101270533	0.259138049
123.4369808	0.004493317	0.109138996	0.04117059	0.9671599	0.983639473
680.4166085	-0.017517398	0.05345422	-0.327708413	0.743132142	0.865245523
841.2165725	0.175830114	0.140760433	1.249144455	0.211612243	0.41593572
408.274198	-0.077176581	0.072247469	-1.068225395	0.285418852	0.499839499
33.84339647	0.38927282	0.195401682	1.992167194	0.046352716	0.154327941
172.0805281	-0.070062875	0.091959186	-0.76189099	0.446125068	0.651201876
136.5979006	0.064675689	0.124348093	0.520118062	0.60298129	0.773279575
2.407737883	0.750943412	0.733377787	1.023951673	0.305858102	0.5207427
10.26903273	-1.246127663	0.503891571	-2.473007555	0.013398131	0.06499435
319.8636223	-0.545303904	0.162094977	-3.364101183	0.000767934	0.007862403
5.564677157	0.437604664	0.648249794	0.675055616	0.499640431	0.69560352
10.68335637	0.103494725	0.449637952	0.230173465	0.817956981	0.908510201

137.0006463	0.158188758	0.081889623	1.931731421	0.053392664	0.169084461
6.849558865	0.531531247	0.410916366	1.293526594	0.195828992	0.397007977
14926.76759	-0.106298554	0.089161959	-1.192196264	0.233184298	0.440031859
26.61213689	0.476989648	0.313242457	1.52274903	0.127821506	0.299512323
25.59739354	1.035718876	0.327976396	3.157906754	0.001589064	0.013713063
341.8227778	0.246923831	0.134366637	1.837687063	0.066108531	0.196123167
8.817930448	0.687862519	0.390287833	1.762449305	0.077993414	0.218551371
299.1554794	0.183566652	0.106981018	1.715880584	0.086183901	0.233138055
37.11812836	0.147517311	0.151344637	0.974711189	0.329703541	0.545303144
2.908719127	0.380974568	0.563232235	0.676407607	0.498781889	0.694995588
698.3074985	-0.297980498	0.071500618	-4.167523381	3.07927E-05	0.000608978
6.608576992	0.532313139	0.375393158	1.418015028	0.156186377	0.341837967
386.5959818	0.134365406	0.084492797	1.590258695	0.111776505	0.276650062
1148.397592	0.070600236	0.034850682	2.025792095	0.042786094	0.145571604
4.986745571	-0.871281099	0.419817774	-2.075379256	0.037951399	0.133993003
60.06902176	-0.139686121	0.170745788	-0.818094094	0.413303461	0.6231135
664.1032232	-0.298931627	0.061554171	-4.856399185	1.1954E-06	4.01078E-05
133.8624568	-0.149359975	0.08222513	-1.816476003	0.069297386	0.202422249
24.85545303	-0.272814796	0.259321381	-1.052033562	0.292784152	0.507932405
326.4624966	-0.077008861	0.088228692	-0.872832396	0.382754439	0.595476119
338.1693059	-0.067811149	0.064254361	-1.055354806	0.291263084	0.506360072
623.4300736	0.066262734	0.070015557	0.946400146	0.343944531	0.558185618
30.3485437	0.157257096	0.202892607	0.775075537	0.438295026	0.644127981
257.5843965	-0.17376799	0.073839728	-2.353312981	0.018606965	0.081459246
265.9276717	-0.042650079	0.193333183	-0.220604027	0.825400765	0.912471628
681.2804194	0.098647995	0.075704508	1.303066323	0.19255212	0.393137709
432.8720865	-0.115205595	0.080493514	-1.43124071	0.15236124	0.336596361
69.7631876	0.168267794	0.114356746	1.471428658	0.141175228	0.320391336
74.59125641	0.565784866	0.178327231	3.172733991	0.001510108	0.013239767
61.90919014	-0.045745511	0.118712574	-0.385346802	0.699980491	0.839012442
378.5629441	-0.007624313	0.090124996	-0.084597094	0.932581709	0.968136056
4.110896055	-0.729324113	0.720354502	-1.012451662	0.311322147	0.526264641
863.5615802	0.366512326	0.059201413	6.19093887	5.98069E-10	5.18557E-08
53.62540303	0.330289972	0.136721423	2.415787993	0.0157012	0.072535495
86.0633094	-0.196158263	0.140828672	-1.392885841	0.163654315	0.353206828
4.996296312	-2.783123997	1.284158426	-2.167274645	0.030213919	0.114502897
241.5214587	0.12923077	0.10448204	1.236870658	0.216135105	0.420875853
57.07109365	-0.106303152	0.159863042	-0.664963902	0.506073582	0.701031397
19.97120546	-0.092947715	0.274824012	-0.338208128	0.735206351	0.860688745
605.4572506	0.169682321	0.068620365	2.472769127	0.013407072	0.065018578
552.3474878	0.507805821	0.097376901	5.214848845	1.83967E-07	7.79094E-06
52.70650239	0.054361646	0.166512265	0.326472324	0.744067025	0.865599899
65.42426492	-0.012518541	0.241849092	-0.051761786	0.958718505	0.979954623
5.662395004	-0.792326157	0.436485551	-1.815240287	0.069486991	0.202750388
995.5273839	0.148884475	0.561294675	0.265251893	0.790815421	0.892054639
1.754635607	0.263201226	0.729793928	0.360651433	0.718360035	0.850529195
6941.262348	0.19019608	0.589548951	0.322612872	0.746988444	0.867329872
157.4636697	-0.162238797	0.092270331	-1.758298631	0.07869671	0.219774472

1.827114544	-0.547738206	0.994188345	-0.55094008	0.581674751	0.758169938
188.4480295	0.001260665	0.100816332	0.01250457	0.990023057	0.994550877
3.64707461	0.546397559	0.582136352	0.938607521	0.34793229	0.562606649
251.7646678	0.038287595	0.077527333	0.493859316	0.621405539	0.786677541
101.2968409	-0.153939695	0.154726092	-0.994917489	0.319776396	0.534896812
6.566345729	0.350964015	0.560639005	0.62600713	0.531310265	0.720863825
244.2924174	-0.132937337	0.145722395	-0.912264291	0.361629607	0.575601075
13.14010708	0.279669111	0.30202782	0.925971358	0.354460865	0.56914116
2.149565847	1.195809594	0.880695501	1.357801411	0.174526707	0.368562104
117.9838013	0.276323752	0.220098397	1.255455543	0.209313437	0.41330811
1729.620605	0.052242137	0.087488608	0.597130743	0.550420094	0.736546229
1657.95614	0.043591445	0.075278645	0.579067871	0.562543378	0.745278458
393.7699927	0.145824176	0.095765892	1.522715161	0.127829983	0.299512323
187.333036	0.44113223	0.118971304	3.707887668	0.000208995	0.002831405
196.0160189	-0.169486594	0.118569976	-1.429422514	0.152882831	0.337385734
333.9771221	0.040537662	0.072102526	0.5622225	0.573964433	0.752765709
116.7232087	-0.130170111	0.153920581	-0.84569659	0.397722026	0.610346778
2.980338717	-0.543248239	0.621899664	-0.873530362	0.382374065	0.595109149
883.3996429	0.05933775	0.067024561	0.885313528	0.375987556	0.589177113
294.9781142	-0.073584314	0.074204108	-0.991647433	0.321369542	0.536451701
3.448909512	0.245283429	0.545838519	0.449369951	0.653164804	0.808432531
186.5081676	-0.182555951	0.08392606	-2.175199813	0.029615146	0.113092239
388.4180558	-0.186777904	0.058931358	-3.169414545	0.001527464	0.013321036
60.65076756	0.284678655	0.17989035	1.582512097	0.113532713	0.279238267
164.3392536	0.067772861	0.105030602	0.645267755	0.518753685	0.711154881
5.330719301	0.308699042	0.695944627	0.443568396	0.657354659	0.811359633
181.9540343	-0.049708497	0.078300793	-0.63484028	0.52553259	0.716334923
320.291858	-0.011774909	0.070806758	-0.166296408	0.867923693	0.934495951
188.5644641	0.128715683	0.085136723	1.511870304	0.13056686	0.304237404
1.73885937	-0.517758987	0.954067611	-0.542685845	0.587346122	0.761715809
10.8606921	-0.745798358	0.634711017	-1.175020346	0.239986576	0.447384729
163.6447841	0.478615629	0.136453391	3.507539276	0.000452272	0.00515014
23.45286613	0.264618168	0.377339921	0.701272654	0.483132876	0.682213596
83.09476495	0.370129766	0.114479027	3.233166605	0.001224261	0.011279912
160.0110889	0.56264146	0.108984568	5.162579156	2.4357E-07	9.8832E-06
3.403987187	1.231163116	1.003285507	1.227131367	0.21977322	0.42424757
300.0425542	0.252033811	0.070594146	3.570180054	0.000356736	0.004299099
815.7033272	0.137477804	0.069784368	1.970037233	0.048834104	0.159495048
145.493106	-0.547472088	0.231000364	-2.370005302	0.01778783	0.079036762
20.82689708	-0.115206773	0.596742708	-0.193059372	0.84691247	0.924220723
178.2533336	-0.270818185	0.215212356	-1.258376562	0.208255604	0.412009499
13.99298972	-3.287273494	1.164698295	-2.822424921	0.004766198	0.030292572
52.39667533	-0.035554325	0.179893382	-0.197641092	0.843325882	0.922094173
588.386537	0.144368776	0.097433294	1.481719145	0.138415041	0.316657323
146.0959795	0.067628601	0.108770211	0.62175664	0.534101901	0.723287688
24.94203923	-0.097087434	0.234804692	-0.413481661	0.679253756	0.826686346
218.2691608	0.18191907	0.072054026	2.524759262	0.011577757	0.058488798
427.8486113	0.032139754	0.090063777	0.356855502	0.721199984	0.852054542

936.956794	-0.057203845	0.089537198	-0.638883571	0.522898677	0.714222104
10.12546333	-2.223916539	0.91656825	-2.42635127	0.0152515	0.071176548
1.706874922	-0.48417659	0.878247512	-0.551298561	0.581429025	0.758146246
82.02947789	-0.255126837	0.115421951	-2.210384024	0.027078522	0.106364734
91.67069503	-0.066754166	0.111890395	-0.596603179	0.550772348	0.736778211
26.45993989	-0.101593528	0.243406785	-0.417381659	0.676399268	0.825101928
183.7503625	-0.460633831	0.102017417	-4.515246935	6.3243E-06	0.000162538
300.9458019	0.216668258	0.151675628	1.428497517	0.153148709	0.33766209
691.2628597	0.108639635	0.046594573	2.331594171	0.019722051	0.085186435
160.9135214	-0.10240957	0.086914793	-1.178275487	0.238686797	0.446360434
12.50149614	-1.028514998	0.454049613	-2.265203995	0.023500164	0.096064939
290.0137697	-0.292053365	0.069973825	-4.173751592	2.99624E-05	0.0005947
155.2269587	0.117151563	0.13753667	0.851784206	0.394333874	0.607070016
515.8991699	-0.353963486	0.09044063	-3.913766234	9.08676E-05	0.001456511
35.08782086	0.51000321	0.259711641	1.963728728	0.049561559	0.161182138
374.350623	-0.138630576	0.070716262	-1.960377607	0.049951671	0.162116594
1.875811311	0.960578338	0.802360416	1.197190588	0.231232278	0.437852936
5.842723014	0.065300418	0.538063437	0.121361932	0.903404368	0.951456563
24.10932407	-0.021082753	0.267405252	-0.078841955	0.937158332	0.970380036
8.663754064	-0.645853208	0.500508976	-1.290392858	0.196914291	0.398374803
75.95070891	-0.100340804	0.143230134	-0.70055652	0.483579821	0.68276431
275.3085928	-0.55550117	0.072026565	-7.712448413	1.23427E-14	3.33333E-12
363.8234377	0.171693152	0.087034467	1.972702977	0.048529413	0.158846323
1260.660489	0.148113461	0.159552112	0.92830774	0.35324796	0.56819048
24.5712886	0.291221534	0.210883944	1.380956413	0.167292363	0.358524052
287.5562022	-0.036977802	0.076430798	-0.483807621	0.628522406	0.791732537
857.2713219	-0.140713425	0.063576616	-2.213289022	0.026877726	0.105833468
196.8122189	0.642054132	0.169876802	3.779528013	0.000157126	0.002250863
183.1155962	0.806947698	0.11609299	6.950873587	3.63031E-12	5.98057E-10
580.3856739	-0.232626491	0.080644183	-2.884603493	0.003919069	0.026298471
237.1639675	-0.620160101	0.230031649	-2.695977283	0.007018245	0.040394218
277.6207141	-0.115677886	0.17504343	-0.660852488	0.508706918	0.703115846
1613.59802	-0.211228824	0.085465235	-2.471517497	0.013454097	0.065175576
7.280766567	-0.699474152	0.400422386	-1.746840777	0.080664961	0.223565708
63.35809929	-0.162311219	0.130215212	-1.246484309	0.212586646	0.417220921
2471.768405	-0.247434833	0.125631084	-1.969535127	0.048891673	0.159556541
333.629826	-0.447846301	0.116932829	-3.829944977	0.000128172	0.001901129
104.6197366	-0.345092805	0.1151544	-2.99678349	0.002728444	0.020366284
81.18825933	-0.175455159	0.191582983	-0.91581808	0.359762319	0.573951606
30.98405979	0.002215051	0.192970738	0.01147869	0.990841532	0.995184112
3.285386215	0.212881902	0.545796921	0.390038664	0.696507957	0.837110387
4.172153543	-0.970410874	0.544490273	-1.782237299	0.074710543	0.212922892
95.05567032	-0.410525545	0.106056507	-3.87081903	0.00010847	0.001668478
70.90117215	-0.120143927	0.160728985	-0.747493844	0.454765517	0.659025962
6.117590551	-0.515085206	0.497663885	-1.035006198	0.300666017	0.515534279
256.5199607	-0.025547074	0.078032942	-0.327388323	0.743374197	0.865441878
154.2323232	0.144007659	0.089380016	1.611184072	0.107139608	0.268729889
1.790517805	1.353673633	1.023166321	1.323024033	0.185827386	0.384015275

4859.839617	-0.320670817	0.089750558	-3.572911669	0.000353034	0.004257599
865.9649965	0.040510937	0.088366948	0.458439929	0.646636411	0.804037152
10.1692267	0.112470805	0.327488288	0.343434588	0.731271543	0.858009442
8.120104597	0.362998938	0.635773516	0.570956369	0.568029219	0.748947007
35.85584206	-0.351206792	0.194901588	-1.801969884	0.071550144	0.206828754
247.1486277	-0.138746676	0.088797667	-1.562503613	0.118169395	0.286198562
7.712709981	1.107488517	0.483930313	2.288528918	0.022106738	0.092105817
36.45068001	0.24271224	0.167294874	1.450805	0.146834167	0.328456519
119.5715194	0.53845144	0.146510831	3.675164741	0.000237696	0.003132643
9.297659076	-0.822936637	0.391791901	-2.100443205	0.035689872	0.128238793
4.597591456	0.617300597	0.484606518	1.273818189	0.202727857	0.405470119
204.1787191	-0.231142247	0.10465503	-2.208610964	0.027201712	0.106644495
1.701582552	-0.067514155	1.671539365	-0.040390407	0.967781878	0.98386242
598.2447414	0.09508958	0.085771216	1.10864209	0.267584613	0.479150969
335.1842848	0.243332755	0.059026155	4.122456501	3.74853E-05	0.000711228
169.5392072	0.070225473	0.085074154	0.825461905	0.409109402	0.619625659
1570.037503	-0.022522864	0.090190026	-0.249726767	0.802798657	0.899069005
51.02812305	-0.277624252	0.235113673	-1.180808619	0.23767876	0.445170729
599.2731602	0.478470428	0.050218319	9.527806576	1.60641E-21	1.654E-18
901.7147086	-0.085715908	0.06293652	-1.361942281	0.173216101	0.366687491
414.2247306	-0.637023197	0.105180426	-6.056480511	1.39132E-09	1.12356E-07
5.376855839	-2.359812966	0.943695551	-2.500608341	0.01239802	0.061408596
5.064228202	-2.816513013	1.030709913	-2.732595251	0.00628375	0.037321856
183.5667141	-0.08128302	0.085386304	-0.951944474	0.341125143	0.555580386
217.3105723	-0.521642015	0.141849459	-3.677433937	0.000235592	0.00311686
375.914868	-0.17289531	0.280218893	-0.617000902	0.537234131	0.725334217
3.671947814	-1.839944582	0.821557996	-2.239579667	0.025118224	0.10102481
491.5131201	-0.570999347	0.158997887	-3.591238589	0.00032911	0.004016119
12.06609672	-0.741531729	0.424428953	-1.747128049	0.080615129	0.22356189
123.8635521	0.09691835	0.443545385	0.218508304	0.827033089	0.913540506
2129.041118	0.029813587	0.051428248	0.579712281	0.56210866	0.745166619
7.394185697	0.031415309	0.389526122	0.080650069	0.935720247	0.969704969
89.55789208	-1.029447029	0.165195044	-6.231706491	4.61381E-10	4.13087E-08
805.1253916	0.165546141	0.076864325	2.153744815	0.03126019	0.11697595
2.190520798	0.83097169	0.740727618	1.121831655	0.261934024	0.473242344
1.997851712	0.155928243	1.038682921	0.15012112	0.880669058	0.940316421
684.3985309	0.005669791	0.060972159	0.092989834	0.925911638	0.964310805
695.7537971	-0.037171167	0.075960486	-0.48934872	0.624594834	0.7894411
225.2709396	-0.122627559	0.092592458	-1.324379562	0.185377019	0.38341643
34.46557673	0.103748091	0.189218237	0.548298582	0.583486901	0.759227189
10.2939402	0.452089949	0.304530808	1.484545858	0.137664167	0.315465222
10.96754232	-1.85244103	0.597624327	-3.099674742	0.001937333	0.01583116
860.3312868	0.040405342	0.085043085	0.475116144	0.634704192	0.795202438
9.674202858	-0.084939634	0.307790656	-0.275965604	0.782574485	0.887032618
127.7841217	-0.472565786	0.194532764	-2.429234934	0.015130724	0.070793397
3.056017053	0.840042423	0.811078189	1.035710779	0.300337092	0.515122671
20.31260031	0.660908164	0.311638161	2.120754922	0.033942432	0.123682288
600.812498	-0.487642236	0.195189306	-2.498304063	0.012478911	0.06167944

83.7118886	0.83884979	0.312629543	2.683207038	0.007291983	0.041667753
118.4226732	0.044597255	0.153319682	0.290877556	0.771144967	0.880499181
156.6900327	0.214805447	0.10051072	2.137139677	0.032586633	0.120446979
609.0135366	-0.027650424	0.054293895	-0.509273163	0.610560766	0.77930251
49.94253446	0.345665257	0.13959337	2.47622976	0.01327781	0.064562763
43.52692699	0.257328644	0.142056531	1.811452405	0.07007085	0.203684164
727.3143245	-0.076309791	0.098140735	-0.777554715	0.436831566	0.642902204
12.44265454	1.427584462	0.404573235	3.528618157	0.000417735	0.004863444
25.07026573	-0.123818083	0.450364199	-0.274928788	0.783370949	0.887675403
84.41712684	0.09877185	0.157843641	0.625757548	0.53147398	0.72091415
464.2981117	0.141928648	0.061895011	2.293054739	0.021844854	0.091477407
5.17653333	0.146219932	0.479997981	0.304626139	0.76065091	0.875372902
47.78338798	0.072616412	0.174611693	0.415873704	0.677502423	0.825889807
814.5301897	-0.071369391	0.125526262	-0.568561428	0.569653808	0.750099284
286.344726	0.023979739	0.082924676	0.289174953	0.772447498	0.881357441
202.4628767	-0.198040941	0.082446802	-2.402045149	0.016303696	0.074421471
38.4221798	0.399607977	0.168806119	2.367260025	0.017920337	0.079381457
191.4127392	-0.228598655	0.103156522	-2.216036862	0.026688978	0.105488056
9.600661911	-1.56124304	0.783725442	-1.992079057	0.046362384	0.154328939
396.3055471	-0.214411312	0.097094553	-2.208273334	0.027225225	0.106686098
2.802317097	-0.510852358	0.747127271	-0.683755469	0.49412959	0.69102639
40.86401664	-0.136817859	0.208819715	-0.655196081	0.512341517	0.70630244
824.4736008	-1.067189751	0.128847199	-8.282599511	1.20515E-16	5.51491E-14
148.9524301	-0.410669163	0.134656324	-3.049757708	0.00229026	0.017847565
524.5773627	0.056903926	0.079837425	0.712747517	0.476002007	0.676063201
99.4258119	-1.017741819	0.324827543	-3.133175869	0.001729258	0.014482865
12.20079953	1.044359214	0.331463717	3.150749724	0.00162852	0.013907845
1029.567742	-0.1985993	0.075438001	-2.632616147	0.008473005	0.046784039
29.40271739	-0.399745672	0.261794394	-1.52694512	0.126774659	0.29809959
45.06642837	0.461762412	0.232335392	1.987482013	0.046869007	0.155324889
372.4087634	-0.068818094	0.091023293	-0.756049272	0.449619655	0.654562937
2.610064256	-1.347753089	0.747524371	-1.802955383	0.071395222	0.206575687
276.6053101	-0.243606501	0.110127921	-2.212032133	0.026964445	0.106067875
443.6317664	0.034855753	0.069699994	0.500082584	0.617016929	0.783530169
92.23895775	-0.145022842	0.200828552	-0.722122626	0.470219089	0.670973518
3116.088297	-0.249606075	0.092305549	-2.704128596	0.006848378	0.039823572
132.0817468	-0.241866825	0.087831132	-2.753771007	0.005891297	0.035589744
27.50234156	0.095330332	0.21902357	0.435251475	0.663379903	0.815802003
4399.810862	-0.196623869	0.113464962	-1.73290385	0.08311276	0.228123893
10.07082491	0.500378052	0.320675204	1.560388973	0.118667989	0.287068495
415.1653751	-0.275717537	0.065378318	-4.21726263	2.47286E-05	0.000509861
165.2781332	0.495674894	0.136637119	3.627673791	0.000285986	0.003612989
13.2367089	0.200625943	0.660662042	0.303674088	0.761376201	0.875473689
253.9779106	0.005944614	0.450160759	0.013205536	0.989463813	0.994530438
540.1720629	0.147225419	0.065711642	2.240476957	0.025059975	0.100839773
339.6209247	0.060638812	0.066130214	0.916960769	0.359163198	0.573505333
95.8286185	-1.029660035	0.173620164	-5.930532573	3.01954E-09	2.24751E-07
19.6124429	0.010764457	0.221872614	0.048516382	0.961304709	0.980954768

10679.77299	-0.212103401	0.069510972	-3.051365782	0.002278029	0.017781276
35.02344161	-1.263681933	0.187323139	-6.746000191	1.51976E-11	2.10392E-09
113.1419786	0.011388033	0.10461809	0.10885338	0.913318784	0.956760375
15.99693461	-0.141815695	0.352034787	-0.402845686	0.68706175	0.830726992
277.3309561	0.007142584	0.062803747	0.113728632	0.909452915	0.954470747
103.9246464	-0.06522445	0.10973583	-0.594376969	0.552260009	0.737796722
2267.462036	0.161843536	0.118076385	1.37066812	0.170478437	0.362522495
3.050327009	0.645964263	0.632369928	1.02149744	0.307018821	0.522071435
12.8574429	-0.205993485	0.322770512	-0.638204163	0.523340788	0.714648221
6.421021213	-1.152321636	0.544689281	-2.115557761	0.034382437	0.124699379
24.43390179	0.182424656	0.278788241	0.654348461	0.512887331	0.706456676
442.6163107	-0.237434455	0.08227461	-2.88587762	0.003903239	0.026207498
25.15353698	0.642848888	0.550721432	1.167285038	0.243095279	0.450782488
1159.162621	0.321988866	0.106945503	3.010775178	0.002605817	0.019691849
140.2287788	0.738829405	0.135591462	5.448937505	5.06716E-08	2.46243E-06
2521.118179	-0.05556348	0.077807714	-0.714112749	0.475157463	0.675059993
3.364542761	-0.778479551	0.626338721	-1.242905037	0.213902829	0.418507745
418.4159962	-0.379946887	0.183038075	-2.075780603	0.037914247	0.133919234
278.1277102	0.189034231	0.096881568	1.951188806	0.05103459	0.164336168
143.2463934	-0.147763182	0.10612622	-1.39233435	0.163821174	0.353337265
150.3784623	-0.182177879	0.104036729	-1.751091959	0.079930071	0.22208939
1.829331147	-1.149271081	1.022389538	-1.124102935	0.26096936	0.472197561
362.8769688	0.024566815	0.091596818	0.268205982	0.78854077	0.890703684
194.9473412	0.323242388	0.144085208	2.243411323	0.024870302	0.100252501
610.1217613	0.020704452	0.098533181	0.210126703	0.833568785	0.916902912
12.89735365	-1.504251768	0.607156456	-2.477535657	0.013229319	0.064358613
7.39882387	-1.188196749	1.405419875	-0.845438983	0.397865787	0.610475354
85.35089799	0.637567291	0.166656631	3.825634109	0.000130436	0.001922005
3.251317327	-1.625866414	0.664689168	-2.446055228	0.014442887	0.068371301
16.18029513	-1.728388331	0.736511091	-2.346724104	0.018939273	0.082628597
565.625681	-0.135645102	0.069305322	-1.957210472	0.050322728	0.162775697
18.72768689	0.149178873	0.289198889	0.515834876	0.605969742	0.775901253
142.2833207	-0.102274028	0.137419494	-0.744246867	0.456727139	0.660416299
32.09170345	0.355651428	0.250009835	1.422549749	0.15486672	0.339988585
144.757749	0.789397759	0.147598492	5.348277955	8.8795E-08	4.04091E-06
472.4263868	-0.072369099	0.126479698	-0.57217957	0.567200327	0.748471553
26.39803404	-0.031142898	0.226529342	-0.137478427	0.890652643	0.945339324
182.0910051	0.095152462	0.096112257	0.990013811	0.322167368	0.537293504
188.9593043	-0.032495973	0.109192483	-0.297602652	0.76600645	0.878006201
74.12637064	0.274136528	0.168983017	1.622272662	0.10474498	0.264977062
207.4641396	0.482130886	0.12765562	3.77680895	0.00015885	0.00226768
1338.935806	-0.187635258	0.134583514	-1.394191998	0.163259634	0.352772718
57.42062799	-0.262885699	0.178778411	-1.470455512	0.141438426	0.320900239
77.37631138	-0.360851598	0.131212928	-2.750122283	0.005957303	0.035856999
1190.773386	0.247862178	0.084755212	2.924447634	0.003450681	0.024097721
553.9409248	0.106651847	0.150218744	0.709976957	0.477718426	0.6774444
153.0458769	-0.387146392	0.109806764	-3.525706248	0.000422355	0.004899915
1055.694539	-0.092985659	0.081172398	-1.145532985	0.25198844	0.461142645

4495.828992	0.024900738	0.109862308	0.22665406	0.820692752	0.909892483
11.4054203	-1.63714429	0.344992133	-4.745453976	2.08039E-06	6.43009E-05
6.640746231	0.364422526	1.016699722	0.358436732	0.720016507	0.851572398
245.4971645	-0.048403992	0.16370106	-0.295685271	0.767470447	0.878861959
53.96143602	-0.048719732	0.157902989	-0.308542176	0.757669813	0.8735899
46.21743011	0.130155511	0.138683316	0.938508795	0.347982999	0.562633422
157.0599789	0.140684013	0.142911642	0.98441255	0.32491271	0.53995884
478.5734002	0.18584481	0.059513225	3.122748081	0.00179171	0.014892349
193.5614117	-0.005394512	0.093556422	-0.057660526	0.954019037	0.977641944
314.0644221	0.48956231	0.071385657	6.857992658	6.98348E-12	1.06524E-09
3.06679053	0.411857806	0.649758671	0.633862732	0.526170409	0.716822193
3.483971582	-1.071998256	0.853490399	-1.256016773	0.209109889	0.413104247
4.115082061	0.271553382	0.520578854	0.521637366	0.601922842	0.772761603
3.224297898	-0.058228629	0.552866195	-0.105321376	0.916120802	0.958476698
18.1838928	0.01076058	0.243625908	0.044168454	0.964770128	0.982482728
141.7968881	-0.138402729	0.111242847	-1.244149474	0.213444555	0.418057973
1.979895441	-2.791172737	1.062779861	-2.626294342	0.008632014	0.047369685
82.19720114	0.11949301	0.118662874	1.006995747	0.313936818	0.528654159
451.4318506	0.000427622	0.07715815	0.005542144	0.995578031	0.9976977
365.7701959	-0.143644011	0.088135311	-1.629812267	0.10314118	0.26241665
2.149570918	-0.478484745	0.752283876	-0.636042803	0.524748524	0.715791603
2.091382277	-1.609679077	0.869890057	-1.850439678	0.064250205	0.192481882
58.02007644	-0.068365628	0.201159955	-0.339857046	0.733964185	0.859780248
631.0800945	-0.232678848	0.059452224	-3.913711394	9.08883E-05	0.001456511
155.5040182	0.01415147	0.111300011	0.127147066	0.898824	0.948877064
2.254708916	0.328932465	0.701278492	0.469046846	0.639036152	0.798267972
3585.888823	-0.042288587	0.102546742	-0.41238353	0.680058333	0.826932461
169.9071522	-0.112070123	0.084832409	-1.321076747	0.186475775	0.384866189
2744.64629	-0.13467484	0.074468312	-1.808485199	0.070531018	0.204853314
218.9900468	0.175459955	0.174933817	1.003007636	0.315857177	0.530583372
475.7822218	0.167084436	0.069585325	2.401144745	0.01634387	0.074510322
1254.462081	0.232165267	0.103097886	2.251891634	0.024329121	0.098694395
5.666634572	-0.433487948	0.417670438	-1.037870791	0.299330214	0.514175258
445.5475022	-0.032705607	0.102399072	-0.319393585	0.749428075	0.868891414
37.60331259	0.40084549	0.243273511	1.647715317	0.099411103	0.255690635
46.34374917	0.221799183	0.285548009	0.776749183	0.437306763	0.64337964
11.49188492	0.077742541	0.298178484	0.26072485	0.794304701	0.893917451
1111.687121	-0.170892024	0.061218286	-2.791519252	0.005246124	0.032724211
440.8700387	-0.190560137	0.076680315	-2.485124604	0.01295061	0.063326905
6.105577507	1.014918859	0.430863688	2.355545124	0.01849555	0.081161566
2.753150558	0.154558203	0.589601861	0.26213995	0.793213547	0.893098455
661.1914999	-0.409680995	0.059832272	-6.847157612	7.53317E-12	1.13417E-09
108.2779549	0.28585276	0.124672574	2.292827936	0.021857913	0.091508833
54.63740207	-0.713728624	0.199638099	-3.575112305	0.000350078	0.004231239
18.97327489	-0.541204653	0.243132478	-2.225966098	0.026016452	0.103500371
103.5785267	-1.178757791	0.269837268	-4.36840248	1.25159E-05	0.000287568
10.66819221	-0.981481036	0.448741718	-2.187184735	0.028729041	0.110665791
4643.456241	-0.425989246	0.089767724	-4.745461167	2.08032E-06	6.43009E-05

94.21212568	0.006415746	0.114919162	0.055828341	0.955478557	0.978293782
3798.990863	-0.324097743	0.075835518	-4.273693247	1.92261E-05	0.000410806
45.13092446	-0.021073455	0.208190506	-0.101221977	0.919374251	0.960112293
398.4186189	-0.132206934	0.084876732	-1.557634586	0.119319895	0.288180025
31.17727212	-0.196021898	0.211015754	-0.928944376	0.352917914	0.56793551
24.03566521	-0.243670602	0.233172416	-1.045023278	0.296012211	0.510695369
3856.700035	-0.658544948	0.170908986	-3.853190901	0.000116588	0.001763743
5.674795659	-1.011809172	0.567468263	-1.783023364	0.0745825	0.212683418
64.33357446	-1.55709307	0.75255885	-2.069064859	0.038540001	0.135327387
116.6016585	-2.293161328	0.771357249	-2.972891393	0.002950088	0.021519148
44.31916835	-0.102961514	0.306170059	-0.336288645	0.736653212	0.861476895
630.2201342	0.0532696	0.095463628	0.558009375	0.576837982	0.755156913
4.667653633	-0.298115641	0.438628182	-0.679654553	0.496723219	0.693461961
362.5143145	-0.29220359	0.058968246	-4.95527015	7.22299E-07	2.63255E-05
535.9481239	-0.346546601	0.109248974	-3.172081055	0.001513508	0.013241222
68.08024271	0.497653657	0.231079436	2.153604255	0.03127122	0.11697595
324.5598706	-0.294573796	0.142758116	-2.063446926	0.039070185	0.136335996
79.83963829	0.391518668	0.441094122	0.887607994	0.374751652	0.58830367
728.4975616	-0.094543906	0.087258901	-1.083487238	0.278592212	0.491910837
190.8671551	0.17514115	0.136560085	1.282520805	0.199660011	0.40190604
2634.280648	-0.013494185	0.074692873	-0.180662278	0.856632669	0.92936531
423.9780829	0.346441596	0.065494676	5.289614599	1.22574E-07	5.38477E-06
19.71260191	0.631360663	0.417240592	1.513181305	0.130233612	0.303589715
191.2431092	0.259922848	0.085617989	3.035843861	0.002398635	0.01847227
340.1089428	-0.16475294	0.074598839	-2.208518825	0.027208127	0.106644495
334.5648666	0.172040561	0.071764857	2.397281464	0.01651723	0.075116787
1438.044375	0.059694107	0.083978048	0.710829899	0.47718965	0.676999035
158.5038872	-1.285708006	0.335799919	-3.828791887	0.000128774	0.001903932
6.296841092	0.504694675	0.397494855	1.269688574	0.204195584	0.407201766
691.4065543	0.183096579	0.101580508	1.80247749	0.071470313	0.206706801
25.25460308	-0.064874131	0.187777117	-0.345484757	0.729729968	0.857101832
308.9117943	-0.065899189	0.090990586	-0.724241841	0.468917275	0.670218913
2.633676107	-1.665688802	0.970267145	-1.716732149	0.086028123	0.232886268
13.98051263	1.150208092	0.679838449	1.691884437	0.09066801	0.24095254
2.508793437	0.476042289	0.7390146	0.644158165	0.519472876	0.711844631
41.53355655	-1.258304007	0.4963509	-2.535109753	0.01124121	0.05713906
705.2174064	0.016327	0.084086763	0.194168491	0.846043954	0.923793843
152.3002863	-0.250702013	0.104667202	-2.395229909	0.016609947	0.075443137
41.01310632	0.034196634	0.161367338	0.211917942	0.832171053	0.916032128
160.9829341	-0.716709465	0.129405651	-5.53847114	3.05124E-08	1.58069E-06
12.63658838	-0.244343946	0.301800537	-0.809620648	0.418158237	0.62738969
31.25488907	-0.898549496	0.228260439	-3.936509974	8.26752E-05	0.001352855
8.804361691	-0.111446921	0.517948893	-0.215169725	0.829634994	0.915064736
223.7382268	-0.318022062	0.117789765	-2.699912513	0.006935771	0.040108248
180.810455	0.395288805	0.219350476	1.802087748	0.071531601	0.206811442
488.4160603	0.020113986	0.069288366	0.29029384	0.771591451	0.880764798
1967.259371	0.154916596	0.472647981	0.32776316	0.743090744	0.865245523
6.394562563	1.099577073	0.694578823	1.583084651	0.113402172	0.279042177

30.17519673	-0.29515382	0.954317542	-0.309282609	0.757106559	0.873294276
495.3486627	-0.372484246	0.153819031	-2.421574521	0.015453432	0.071732838
1108.008241	-0.087474632	0.06731406	-1.299500161	0.193772338	0.394586588
2.545206142	0.881544736	0.607848396	1.45027073	0.146983038	0.328621371
140.2186004	0.224404372	0.107837623	2.080946931	0.037438764	0.132672158
990.0141787	-0.252914315	0.078384406	-3.226589673	0.00125275	0.011471817
1255.016277	0.244212014	0.106466	2.293802849	0.021801826	0.091366898
548.3638929	-0.006104172	0.081854347	-0.074573583	0.940553994	0.971819273
856.2392078	0.067900867	0.074837881	0.907306115	0.364244941	0.5779081
6.063963042	1.104915732	0.520772665	2.121685346	0.03386417	0.123506384
9.020351593	-0.729952059	0.482718633	-1.512168804	0.130490925	0.30410348
1.776590721	1.859538765	1.027989706	1.808907963	0.070465303	0.204698537
37.68342552	-0.241728189	0.319342393	-0.756956152	0.449076132	0.654060667
2800.390211	0.544999022	0.086793269	6.279277507	3.4015E-10	3.14811E-08
2058.655207	0.021201581	0.152259046	0.139246775	0.889255148	0.944708151
9.811111482	1.262007452	0.372473235	3.388182916	0.000703573	0.007321962
358.595896	-0.056289077	0.073956813	-0.761107389	0.446592928	0.651711568
53.30042949	0.913738197	0.154000246	5.933355443	2.96805E-09	2.22253E-07
2511.945772	-0.007514893	0.071626244	-0.104918153	0.916440755	0.958750555
1.904447594	0.570873956	0.743982354	0.767321904	0.442890149	0.648528056
174.3342312	-0.129901627	0.111771938	-1.162202512	0.245153215	0.453424729
26.53576205	0.541787511	0.314828101	1.72089947	0.085269068	0.231764168
1834.922108	0.166873204	0.056380517	2.959767181	0.003078716	0.022196398
7.750239795	-0.415682894	0.528330393	-0.786785881	0.43140722	0.638429981
137.8199175	-0.068615719	0.225575462	-0.304180777	0.760990169	0.875424502
788.446126	0.328269573	0.174640615	1.879686306	0.060150843	0.184015782
2.883161324	-0.465151684	0.627944207	-0.740753206	0.458843092	0.661849322
5090.4962	-0.074994062	0.083892277	-0.893932842	0.371357824	0.584870822
665.561784	0.253194577	0.118285041	2.140546042	0.032310664	0.119695498
1160.735477	-0.2305813	0.061830196	-3.729266861	0.000192038	0.002638232
102.949372	0.026739462	0.101047934	0.264621561	0.791301009	0.892258236
35.78065016	-0.058479268	0.182836927	-0.319843858	0.749086699	0.868679028
295.6218141	0.212609616	0.12661358	1.679200721	0.093112932	0.244960465
310.1293428	-0.136731519	0.122881286	-1.112712306	0.265832009	0.477517884
162.9678898	-0.429040729	0.125054656	-3.430825708	0.000601747	0.006465726
3.46528239	-0.202944241	0.582647162	-0.348314132	0.727604282	0.855815575
15.24750207	-0.144358774	0.266879915	-0.540912843	0.588567658	0.76241798
2021.947393	-0.08185922	0.081832544	-1.000325988	0.317152774	0.532090262
367.9124263	-0.045215304	0.098570302	-0.458711221	0.646441555	0.80392948
181.2338167	-0.093908879	0.153477795	-0.61187274	0.540621959	0.728107108
1.73425239	-0.338546873	0.76189341	-0.444349391	0.656789997	0.810908972
82.14411088	-0.683217317	0.202879322	-3.367604498	0.000758243	0.007773051
55.289948	0.172693046	0.153462391	1.125311844	0.260456912	0.471512876
6.233372877	-0.649746947	0.408257452	-1.591512768	0.111494224	0.276203888
104.2224463	-0.085261592	0.10821371	-0.787900089	0.430755149	0.637594068
62.51395204	0.994987162	0.213537651	4.659539695	3.16917E-06	9.09564E-05
1132.682869	0.01858179	0.067556852	0.275054118	0.783274661	0.887675403
2267.290333	0.230579147	0.097690693	2.360297992	0.01826026	0.08039004

177.395252	-0.531815426	0.119409138	-4.453724695	8.43933E-06	0.000207198
31.68908322	0.000666236	0.1866613	0.003569223	0.997152178	0.998121581
414.887944	-0.053599485	0.057573082	-0.930981685	0.351863038	0.566903833
351.9810856	0.082067091	0.064660538	1.269199024	0.204370089	0.407355456
1963.420383	-0.148429416	0.141116106	-1.051824767	0.292879954	0.508045104
814.7787063	-0.023349752	0.082349563	-0.28354433	0.776759605	0.884091318
612.3811944	-0.148651864	0.064472793	-2.305652625	0.02113005	0.089438964
4.661266092	1.066533948	0.450243432	2.368794016	0.01784619	0.07918075
61.23297514	0.349165861	0.159272409	2.192255799	0.028361041	0.109624542
5.003653449	0.24866542	0.496318038	0.501020315	0.616356825	0.783174882
2.243745698	1.169575562	0.762999373	1.532865693	0.12530895	0.296332703
389.6003631	-0.389001424	0.136655411	-2.846586333	0.004419074	0.028797402
4046.190405	-0.163394852	0.060388909	-2.705709604	0.006815862	0.039709738
368.3677813	-0.256340083	0.269183372	-0.952287955	0.340950965	0.555516387
561.4382491	-0.332480559	0.083613515	-3.976397351	6.99672E-05	0.001184624
348.179711	-0.363526536	0.055746892	-6.521018909	6.98314E-11	7.72082E-09
2.578135605	0.119278953	0.75748868	0.157466317	0.874877351	0.937291376
130.2095422	0.17064682	0.116843471	1.460473722	0.144159925	0.324571627
2.785676009	-0.746568455	0.693074726	-1.077183205	0.281398427	0.495432049
145.0006387	-0.056753036	0.117563141	-0.482745152	0.6292767	0.79225209
130.8612004	-0.006173182	0.114066053	-0.054119364	0.956840065	0.978702547
4.290777997	-0.10223099	0.572583288	-0.17854344	0.858296209	0.929724191
1.944998809	0.263366306	1.134187815	0.232206961	0.816377271	0.907813535
98.85399347	-0.024829566	0.138768197	-0.178928362	0.857993952	0.929613001
36.94779395	0.101231707	0.2321628	0.436037587	0.662809459	0.815468486
1004.154801	-0.184300278	0.081141322	-2.271349212	0.023125846	0.094982596
1926.305467	0.42751818	0.197040645	2.169695394	0.030029929	0.113989183
299.5706483	-0.605720391	0.202469211	-2.991666676	0.00277459	0.020598738
2.193919721	-2.884563867	1.198000864	-2.407814512	0.016048332	0.073602513
1122.142179	-0.070148171	0.097107268	-0.722378173	0.470062003	0.670923708
506.0725685	-0.509598508	0.102547085	-4.969409977	6.71569E-07	2.46402E-05
124.1010052	-0.279285913	0.115538007	-2.417264427	0.015637652	0.072324293
221.7663824	-0.060020711	0.136796873	-0.438757916	0.660836956	0.813527725
62.46036785	-0.737632493	0.352206522	-2.094318098	0.036231653	0.129643844
183.2249225	-0.219445061	0.103014082	-2.130243324	0.03315153	0.121851474
33.48479732	0.229669724	0.267946204	0.857148639	0.391362743	0.604586444
3.048362388	0.206384824	0.854957899	0.241397646	0.809246937	0.903352275
556.8996721	-0.332685959	0.154888849	-2.147901296	0.031721597	0.118177656
3.015978852	-0.4508905	0.865821468	-0.520766135	0.602529697	0.77305874
1052.517713	0.023313813	0.052620109	0.443059005	0.657723056	0.811574386
50.09965491	-0.183698755	0.155924807	-1.178123986	0.238747181	0.446360434
908.8481136	-0.180647751	0.054748517	-3.299591657	0.000968256	0.0094051
97.58574887	-0.073785417	0.133456907	-0.552878219	0.580346803	0.757539944
278.3507587	0.008217642	0.135638639	0.060584819	0.951689864	0.977124952
6.388024457	-0.112261654	0.41584778	-0.269958528	0.78719216	0.89006202
12.09124768	-1.269169779	0.569268519	-2.229474733	0.025782335	0.102941876
8.763650608	-2.582968414	0.661708278	-3.903485115	9.48174E-05	0.001496189
144.1213295	-0.719480143	0.208089509	-3.457551262	0.000545109	0.005989193

539.2109684	-0.551543056	0.223853095	-2.463861648	0.013744916	0.066131349
292.570727	-0.066246668	0.079401823	-0.834321752	0.404099697	0.614990985
3.388307909	-1.833617375	0.830687893	-2.20734814	0.027289747	0.106786528
9.377558004	0.189863187	0.366540739	0.517986588	0.604467627	0.774579939
2415.458312	-0.235167033	0.136051611	-1.728513397	0.083896221	0.229319122
509.4410782	-0.530049459	0.181184075	-2.925474869	0.00343931	0.024068138
351.8314424	-0.046356959	0.096668704	-0.479544638	0.63155122	0.79366655
34.31538844	0.315837395	0.18320015	1.724001834	0.084707512	0.230847238
417.4579366	0.966583477	0.140867491	6.861650419	6.80695E-12	1.04802E-09
21.9410272	-0.749846847	0.19943432	-3.759868655	0.000170003	0.002397793
2.616554664	-0.566616977	0.641229207	-0.883641873	0.376889568	0.589841864
796.6374078	0.063581879	0.093939463	0.676838857	0.498508201	0.694742406
955.7740837	-0.167862132	0.069514077	-2.414793366	0.015744139	0.072708948
67.97814895	-0.316934455	0.167090633	-1.896781705	0.057856755	0.178790504
20.31466245	-1.410958646	0.58923019	-2.394579692	0.016639427	0.075472996
153.8788179	-0.153930455	0.083802911	-1.836815125	0.066237192	0.196398759
648.9375773	-0.169196333	0.073557	-2.300207095	0.02143649	0.090364569
76.05203127	-0.433109287	0.333579471	-1.298369127	0.194160523	0.395035254
34.47404529	0.220245662	0.212884784	1.034576816	0.300866586	0.51574059
5742.842476	-0.231594305	0.156871049	-1.476335544	0.139853837	0.318494206
921.3131125	-0.206819065	0.186795919	-1.107192628	0.268210654	0.480167606
343.7981195	0.041294235	0.100690849	0.410109115	0.681725907	0.828043397
3130.812497	-0.231610993	0.145298508	-1.594035586	0.110928065	0.275345405
88.80255727	-0.343118019	0.098221862	-3.493295805	0.000477098	0.005380054
730.2276916	-0.242366858	0.100642474	-2.408196535	0.016031548	0.073566497
161.5039351	-0.358777044	0.117118081	-3.063378768	0.002188529	0.01725889
3329.265615	-0.093192034	0.083540496	-1.115531245	0.264622837	0.47593243
559.5583963	-0.063116361	0.094619095	-0.667057339	0.504735509	0.700184188
70.81165529	-0.279846821	0.21442321	-1.305114409	0.191853893	0.392474982
512.0046683	-0.128950441	0.082394506	-1.565036882	0.117574262	0.285344488
142.5529517	-0.133886252	0.079832443	-1.677090755	0.093524736	0.245612388
319.8624915	-0.264076222	0.086545436	-3.05130156	0.002278516	0.017781276
87.19816269	0.179108681	0.153189596	1.169196117	0.242324625	0.449961213
20.5230036	0.800765398	0.292895324	2.733964429	0.00625768	0.037260851
40.9384979	-0.870241684	0.237202928	-3.668764519	0.000243725	0.003196762
80.84040271	-0.226867977	0.220287325	-1.029873041	0.303069608	0.517887593
31.26101665	-0.539623513	0.293708901	-1.83727327	0.066169564	0.196233554
7.158131095	-0.641553338	0.392893888	-1.632892131	0.102491685	0.261450608
3.23626542	0.527942895	0.653730379	0.807585071	0.419329484	0.628262028
456.9395126	0.022471505	0.083980929	0.267578671	0.78902365	0.891032055
439.1976872	0.081058945	0.105013798	0.77188852	0.440180457	0.645901207
6.448406066	-0.26069457	0.406762682	-0.640900903	0.521587069	0.71331773
809.829083	-0.199909684	0.066932457	-2.986737559	0.002819717	0.020849198
6.081770872	0.374042211	0.69003949	0.542059138	0.587777767	0.761885532
30.84964078	0.07300499	0.267809492	0.272600458	0.78516036	0.888692051
1304.928815	0.059424742	0.096275562	0.617235988	0.537079082	0.725232852
5.288936402	-0.85459893	0.660976415	-1.292934075	0.196033861	0.397218218
27.82778366	-0.498401254	0.559800234	-0.890319839	0.373294171	0.586798491

13.43517553	-1.275894957	0.561826504	-2.270976801	0.023148382	0.095027772
626.4825076	-0.018300042	0.092100633	-0.198696156	0.842500431	0.921757179
479.5332825	-0.280187668	0.156099618	-1.794928595	0.072665077	0.209024705
88.69658887	-0.013564564	0.107732911	-0.125909197	0.899803803	0.949728847
2.901092691	0.545121104	0.765812465	0.71182062	0.476575862	0.676469994
144.5241813	-0.362275553	0.213962164	-1.693175774	0.090422015	0.240764873
180.4915215	-0.932986735	0.349195974	-2.671814125	0.007544242	0.042753302
990.761572	0.027828825	0.058590156	0.474974413	0.634805211	0.79526852
663.8279757	-0.118072675	0.076926027	-1.534885915	0.124811854	0.295593801
267.0612547	-0.015479421	0.081449493	-0.190049326	0.849270479	0.925628813
1863.741307	0.149512487	0.042642481	3.506186432	0.000454577	0.005168183
552.2306267	0.254320048	0.069422972	3.663341414	0.000248946	0.003252294
2.25740159	0.378448251	0.687389437	0.550558723	0.581936211	0.758210783
104.3089838	0.936467822	0.155737584	6.013113847	1.81993E-09	1.41826E-07
597.5414387	0.017184673	0.138579047	0.12400629	0.901310295	0.950648941
5.611353904	0.361233681	0.525002469	0.688060918	0.49141442	0.689139271
7908.636728	0.625855446	0.126965377	4.929339503	8.25081E-07	2.94207E-05
6.477326994	0.823103043	0.645787685	1.274572219	0.202460696	0.405216559
14.4511134	-0.663771236	0.444263901	-1.494092215	0.135151519	0.311483788
84.48267771	0.041113733	0.120472526	0.341270618	0.732899865	0.859215921
2.543283752	-0.817856038	0.663000741	-1.233567307	0.217364182	0.422248697
174.2351824	-0.129339563	0.119681869	-1.080694714	0.279832935	0.4932557
7.446602143	-0.694760157	0.457784902	-1.517656336	0.129101057	0.301589735
1728.608967	-0.013651222	0.098872112	-0.138069491	0.890185498	0.945147969
6.664870864	1.229559102	1.157495045	1.062258631	0.28811829	0.502825978
591.348751	0.16745739	0.193648667	0.864748477	0.387176856	0.600236496
1.913493659	1.387146698	0.824466448	1.682478046	0.092476179	0.243713416
314.1422632	-0.417232857	0.118937089	-3.508013031	0.000451467	0.005147035
376.7740004	0.11762334	0.119448841	0.984717296	0.324762955	0.539767136
16.09369034	-1.985414034	1.037095675	-1.914398142	0.055569303	0.173161696
4.291279399	-0.59626134	0.769723574	-0.77464347	0.438550363	0.644312734
228.9373133	0.834919313	0.159160888	5.24575681	1.55642E-07	6.71217E-06
66.78606068	0.292550361	0.124336913	2.352884214	0.018628434	0.081488268
155.0977645	-0.10222949	0.087117894	-1.173461446	0.240610809	0.448041422
5.319302315	0.827825365	0.516279611	1.603443847	0.108836697	0.271827736
948.5879898	1.192957863	0.150327321	7.935735544	2.09251E-15	6.62925E-13
77.18430356	0.162270571	0.119550641	1.357337521	0.17467399	0.368676627
62.41267385	0.107721102	0.185168993	0.581744818	0.560738578	0.744504458
2.590253907	-0.824126614	0.799906698	-1.030278426	0.302879323	0.517887593
122.5909937	0.159922586	0.12912377	1.238521657	0.215522697	0.420053765
997.7232343	-0.226130892	0.068194624	-3.315963606	0.000913277	0.008987652
53.74968424	0.051759401	0.166797376	0.310313043	0.756322914	0.872831081
78.77658679	-0.533044008	0.133182595	-4.00235487	6.27151E-05	0.001081275
195.1498808	-0.040488469	0.081330132	-0.497828635	0.618604831	0.784639358
84.16554513	0.044846083	0.120537814	0.37204991	0.709855689	0.845284623
53.44361872	0.338474413	0.15409795	2.196488754	0.028056979	0.108934875
14.98489977	0.262947459	0.265819087	0.989197059	0.322566737	0.537537307
45.53981751	-0.142590189	0.283662404	-0.50267567	0.615192318	0.782406945

578.704445	0.220568901	0.077672007	2.839747672	0.004514923	0.029191069
617.6228947	-0.142556973	0.049511485	-2.879270805	0.003985959	0.026599148
187.1137359	0.070720936	0.139098828	0.508422224	0.61115727	0.779755644
146.8506479	-0.516769557	0.226016804	-2.28642096	0.022229643	0.092524291
45397.63545	-0.06254427	0.080670963	-0.7753009	0.438161877	0.644086622
173.7152843	0.058418611	0.119217127	0.490018613	0.624120728	0.789055504
1676.423349	0.007432501	0.117686752	0.06315495	0.949643118	0.976129077
1.966486889	0.457594534	1.007351646	0.454255013	0.649645292	0.80577146
11.98002827	0.716267026	0.337086095	2.124878589	0.033596745	0.122944605
1070.85927	-0.401729683	0.061591633	-6.522471668	6.91581E-11	7.69805E-09
205.8374337	-0.025809646	0.081962797	-0.314894649	0.752841644	0.871037915
337.0589286	-0.124792949	0.083815221	-1.488905578	0.136512234	0.31365447
3827.303988	0.103511453	0.089769865	1.153075732	0.248879302	0.457746747
321.1877009	0.197906514	0.060269059	3.283716663	0.001024479	0.009823784
1130.866625	0.121839179	0.07846511	1.552781612	0.120475317	0.289953536
49.9283513	-0.250607599	0.183918296	-1.362602875	0.173007702	0.366387567
1.733045327	0.452506187	0.790589228	0.572365738	0.567074222	0.748471553
526.27533	0.22437344	0.069665729	3.220714752	0.001278713	0.011644846
341.8246974	-0.010999382	0.082121663	-0.133940075	0.893449961	0.946123642
78.83281773	0.393770079	0.201551474	1.95369486	0.050737314	0.163634792
442.5569726	0.017729264	0.081616947	0.217225275	0.828032788	0.914087062
325.7144362	-0.218076331	0.192450777	-1.133153809	0.257149668	0.467787503
1.713338706	-0.087494867	1.152671195	-0.07590618	0.93949374	0.971262319
108.5724128	0.060489512	0.104769603	0.577357459	0.563698005	0.746192121
70.74476464	-0.112216415	0.154164272	-0.727901569	0.466673842	0.668563317
85.73208922	0.050186604	0.124776275	0.402212708	0.687527491	0.831033101
657.0940547	0.084437245	0.045579611	1.852522286	0.06395086	0.191758023
4.130868797	-0.132086654	0.846689534	-0.156003646	0.876030148	0.937672708
460.7024974	-0.234079315	0.058945331	-3.971125608	7.15338E-05	0.001204958
18.73935851	-0.545598431	0.449811733	-1.212948422	0.225149541	0.430441399
1309.893663	0.055711729	0.044649615	1.247753854	0.212121213	0.41657975
50.68907937	-0.104006263	0.163795705	-0.634975523	0.525444379	0.716333226
365.7704754	-0.086701492	0.069487529	-1.24772737	0.212130914	0.41657975
543.1142106	-0.357084291	0.139850452	-2.553329549	0.010669849	0.054907071
89.23961725	-0.150398382	0.11182234	-1.344976165	0.178632932	0.374375386
16.2780013	-0.702058591	0.397113773	-1.767902901	0.077077138	0.216781036
174.4997812	0.048454193	0.092076126	0.526240574	0.598721066	0.769896535
28.03005017	-0.00421257	0.182919335	-0.023029662	0.981626613	0.990525347
414.2045633	0.151277113	0.055730348	2.714447657	0.006638639	0.038975388
142.6250367	0.184416228	0.102157047	1.805222776	0.071039828	0.205823097
184.9899633	0.09033486	0.079815049	1.131802347	0.257717541	0.468200129
28.28814126	0.192516868	0.262439307	0.733567201	0.463212557	0.66558776
97.7723531	-0.194599922	0.09645845	-2.017448155	0.043648766	0.147835069
2883.674253	0.132713423	0.054173215	2.449797823	0.014293645	0.067898934
20.92603164	0.017847467	0.212067393	0.08415941	0.93292969	0.968347365
136.5769308	-0.083626064	0.098986569	-0.844822338	0.39821004	0.610697469
14575.01188	-0.576869167	0.223194273	-2.584605591	0.009749045	0.051674956
648.6830481	-0.026383585	0.196541018	-0.134239584	0.893213125	0.946108145

2114.709626	0.235063979	0.059393276	3.957754051	7.56578E-05	0.001258976
47.3576174	0.294570703	0.204665591	1.4392781	0.150071745	0.333326403
4.72861624	3.229419226	1.699639724	1.900061043	0.057425109	0.17776661
30.97506501	0.868572077	0.340196685	2.553146801	0.010675449	0.054907071
6.334968951	3.555494417	1.284912183	2.767110829	0.005655552	0.034635528
13.08968815	-0.542114631	0.586069467	-0.925000637	0.354965576	0.569609251
1.763975286	1.485161516	1.244071761	1.193790875	0.232559782	0.439306255
3.553835001	0.105716797	0.604733369	0.17481555	0.861224569	0.931215742
6.230125376	-2.579985293	0.989271327	-2.607965301	0.009108219	0.049244764
2849.102999	0.237338293	0.162291653	1.462418363	0.143626601	0.323704153
1277.724184	0.07662329	0.074790843	1.024500952	0.305598721	0.520512215
46.75219537	-0.156132903	0.190607292	-0.819133947	0.412709996	0.622446624
549.6351162	0.010830514	0.048481325	0.223395577	0.823227647	0.911263149
76.9275652	-0.206729817	0.11796934	-1.752402936	0.079704547	0.221629678
58978.26238	-0.168299635	0.131328589	-1.281515597	0.200012625	0.402370007
20.24222346	-0.557801408	0.475448328	-1.17321142	0.240711034	0.448115254
74.72578609	0.677000318	0.341108007	1.98470955	0.047176796	0.155946284
107.9001229	0.475017862	0.341342712	1.391615654	0.164038816	0.353617493
204.127795	-0.274987577	0.119104239	-2.308797569	0.020954817	0.08890806
536.9120099	0.069396608	0.103354232	0.671444277	0.501937546	0.697681331
169.8972507	0.27928607	0.216285407	1.291284854	0.196604921	0.397944399
20.0994646	-0.929240359	0.512060467	-1.814708261	0.069568755	0.202881159
7.53342688	1.47008087	0.9220585	1.594346638	0.110858417	0.275332664
52.48197954	0.264800197	0.209407407	1.264521637	0.20604286	0.409517791
2.673837202	0.287744865	0.592266308	0.485836965	0.627082764	0.790677612
80.98734951	0.439863182	0.69365218	0.634126432	0.525998314	0.716791813
97.13659492	-0.190943062	0.348923556	-0.547234657	0.584217529	0.759621118
2.266397851	-0.249946036	0.777060442	-0.321655849	0.747713429	0.867697312
155.5548	-0.158157232	0.310559009	-0.509266284	0.610565588	0.77930251
339.2005163	0.548372829	0.134095451	4.089421559	4.3245E-05	0.000794224
307.6303216	0.484400351	0.218053651	2.221473244	0.026318925	0.104401149
9.775422874	1.13370528	0.334985785	3.384338479	0.0007135	0.007392576
699.4181041	-0.084679666	0.056907327	-1.488027466	0.13674365	0.313907638
46.50004639	0.694049344	0.314714017	2.205333432	0.027430705	0.107236223
50.15269408	0.070902898	0.19844426	0.35729377	0.720871893	0.851889535
1576.093059	-0.294813848	0.152493827	-1.933283819	0.053201244	0.168642927
124.1719318	-0.10742052	0.11349932	-0.94644197	0.343923207	0.558185618
550.344845	-0.053689457	0.064665345	-0.830266314	0.40638823	0.617092791
9.040959746	0.483175329	0.380661765	1.26930355	0.204332821	0.407330456
1405.521418	0.117025834	0.071637602	1.633581124	0.102346834	0.261202439
117.0652776	-0.197236117	0.14159904	-1.392919871	0.163644023	0.353206828
74.52117996	0.017440337	0.128535135	0.135685371	0.892070013	0.94585057
686.2923964	-0.059944803	0.049707612	-1.205948152	0.227837482	0.433500443
414.344505	0.043519839	0.090543292	0.48065227	0.630763659	0.79310033
190.260842	-0.008349449	0.114562974	-0.07288087	0.941900917	0.972365984
48.44368419	0.022078775	0.441087048	0.050055368	0.960078266	0.9804928
3.478437633	0.280152983	0.80983171	0.345939754	0.729387991	0.857101832
421.1242263	0.32711319	0.072211499	4.529932142	5.90026E-06	0.000153072

35.53218845	0.07746848	0.159710099	0.485056864	0.62763601	0.790978857
30.43163424	0.189129667	0.210277269	0.899429918	0.36842371	0.582134026
277.4726363	-0.388504067	0.096678056	-4.018534131	5.85613E-05	0.001018732
58.04956126	0.199563734	0.164911314	1.210127609	0.22622993	0.431955479
404.2941923	0.118948521	0.112852609	1.05401658	0.291875326	0.507156853
9.279308447	-0.028791288	0.320180256	-0.089922123	0.928349101	0.965637301
698.9451428	0.234402507	0.068749441	3.409518721	0.000650776	0.006876771
38.49329489	-0.150486308	0.216585944	-0.694811052	0.487173721	0.685605662
2580.732152	0.085134515	0.06800014	1.251975582	0.210578762	0.414911436
16.10691257	0.063684996	0.255176577	0.249572262	0.802918152	0.899097368
100.5698022	0.256483879	0.120163443	2.134458469	0.03280527	0.120956583
379.5289032	-0.115822795	0.069983897	-1.654992069	0.097926107	0.253076725
334.1519234	-0.067809786	0.061762374	-1.097914186	0.272241963	0.484331976
531.1246703	0.211387572	0.070979689	2.978141693	0.002900019	0.021271109
2340.11906	-0.142038415	0.058257395	-2.438118201	0.014763946	0.069452096
202.4715491	0.113241357	0.117760707	0.961622603	0.336239213	0.551329232
77.06776908	0.20204016	0.137548685	1.468862899	0.141869979	0.321247352
7.096244282	0.565405466	0.457241616	1.236557317	0.216251474	0.42094764
376.1310357	0.423899093	0.094245409	4.497822199	6.86531E-06	0.000173911
12.66168259	0.565648906	0.494111972	1.144778792	0.252300803	0.461378699
105.4469069	0.441911514	0.35588895	1.241711816	0.214342909	0.419063612
445.584519	0.445780893	0.092550401	4.816628423	1.46004E-06	4.79138E-05
531.2663385	0.623917021	0.120367023	5.183454782	2.17813E-07	8.94826E-06
817.9232127	-0.157757229	0.208634194	-0.756142731	0.449563625	0.654539209
6.072729225	-0.591790332	0.547154843	-1.081577435	0.279440336	0.492716073
3.302733737	0.933432456	0.609518281	1.531426514	0.125664015	0.296716209
432.5772162	0.288476176	0.080566435	3.580599989	0.000342806	0.004158607
302.9150892	0.007156531	0.083909776	0.0852884	0.932032114	0.967778561
50.25396323	0.047128669	0.2105693	0.223815479	0.822900885	0.911237589
600.4221858	0.03976445	0.058436913	0.680468016	0.496208167	0.693050729
1517.637927	-0.075738778	0.067773805	-1.11752289	0.263770818	0.475214398
62.38689202	-0.303402921	0.137689806	-2.203524941	0.027557771	0.107590346
26.54682636	1.262830964	0.465782167	2.71120505	0.006703915	0.039246732
15.55538827	-1.083713659	0.457269816	-2.369965434	0.017789748	0.079036762
9.898589569	0.729042376	0.41076057	1.774859686	0.075921058	0.214864029
2.97878373	0.817436457	0.895388518	0.912940517	0.361273826	0.575339666
4008.078695	0.270111643	0.045129646	5.985237354	2.16075E-09	1.64037E-07
1514.580562	0.044828741	0.074173237	0.604378928	0.545591742	0.732584429
242.5790174	0.019696946	0.079357503	0.248205216	0.803975631	0.899535041
737.1859496	0.157853951	0.104112354	1.516188472	0.129471702	0.302150886
58.36263757	0.100432481	0.322611889	0.31131054	0.755564555	0.872365037
10.91361506	-0.761674561	0.578565406	-1.316488254	0.188010218	0.387125166
159.5063385	-0.084928969	0.083587143	-1.016053012	0.309604143	0.524842422
833.5215168	-0.357971512	0.07932119	-4.512936719	6.39361E-06	0.000163808
120.8574265	-0.074256768	0.119819958	-0.619736222	0.535431464	0.72395748
3.836008495	0.008757778	0.528568137	0.016568872	0.986780558	0.993287517
712.0578071	-0.136051617	0.078009397	-1.744041394	0.081151872	0.224575163
1643.833889	0.043924305	0.045999335	0.954890016	0.339633319	0.554466287

156.7480805	-0.122524285	0.082311568	-1.488542724	0.136607824	0.313786571
32.26673801	-0.308373296	0.322174425	-0.957162558	0.338485212	0.553689345
362.8648838	-0.215622057	0.115454998	-1.867585298	0.061819897	0.187313038
960.4593528	-0.055446886	0.065917172	-0.841159963	0.400258327	0.612232486
4078.911586	-0.01004724	0.083204932	-0.120752934	0.903886731	0.951599566
1690.445692	0.350537541	0.105435956	3.324648952	0.000885299	0.008759061
269.1199403	0.135728291	0.083040659	1.634479947	0.102158114	0.260922909
17137.43084	0.100137743	0.086012862	1.164218245	0.244335578	0.452267901
120.0897311	-0.139833322	0.111700509	-1.251859309	0.210621134	0.414945296
15.7527685	0.494312951	0.347435133	1.422748892	0.154808962	0.339907082
161.0619862	-0.213935305	0.133995147	-1.59658995	0.110357141	0.274418647
152.3991517	-0.140478091	0.093779828	-1.497956375	0.134144585	0.309882015
211.207361	-0.348873057	0.119777269	-2.91268167	0.003583397	0.024772506
195.4296101	-0.128851935	0.11980888	-1.075479005	0.282160327	0.495967668
88.62078066	0.141901894	0.127314812	1.114574905	0.265032629	0.476549611
488.6186076	0.157671301	0.152174959	1.036118569	0.30014683	0.51497373
3.391521617	0.787139304	0.670735669	1.173546214	0.240576836	0.448038647
8.733809864	1.166333344	0.941391357	1.238946306	0.215365383	0.419942337
3.488684668	-0.456193021	0.770079471	-0.592397328	0.553584555	0.738800394
22.95913039	0.213054478	0.380291825	0.560239438	0.57531613	0.753972704
140.9749057	-0.074555812	0.113194335	-0.658653212	0.510118484	0.704593939
20.86478194	-0.588868585	0.393815786	-1.495289438	0.13483892	0.311006321
619.3664043	0.112175954	0.073344291	1.529443565	0.126154519	0.297492663
1455.232583	0.046115157	0.070421582	0.654844091	0.51256814	0.706319325
243.6572756	0.49871535	0.129381424	3.854613233	0.000115913	0.00175955
2.074788047	-1.659886613	1.364727556	-1.216276909	0.223879455	0.429002957
3.838705467	-1.406542696	1.03031519	-1.365157681	0.172203496	0.365012917
1092.833871	-0.124854526	0.064644963	-1.931388331	0.053435047	0.169091234
94.94052941	0.335228231	0.147049487	2.2796967	0.022625683	0.093675674
6.16814146	1.560362405	0.652969416	2.389640872	0.016864855	0.076159983
55.45620823	-0.617555412	0.365069331	-1.691611319	0.090720107	0.241052103
154.0020834	0.092367012	0.092922518	0.994021828	0.320212241	0.535333514
292.7500765	-0.157807541	0.094298987	-1.673480766	0.09423269	0.246606725
392.8552956	0.003956083	0.082283626	0.048078614	0.96165359	0.981242068
194.1674207	-0.438935475	0.170359481	-2.576525075	0.009979897	0.052409569
682.0019784	0.031500867	0.12135655	0.259572861	0.795193272	0.894504197
56.57295987	-0.246805555	0.219162796	-1.126128883	0.26011097	0.471197286
137.5304487	-0.227422246	0.142777933	-1.592838903	0.111196335	0.275673202
7.864043475	-0.241196217	0.461925431	-0.522154012	0.601563103	0.772480361
768.6755117	-0.010502525	0.06110208	-0.171884908	0.863528012	0.931920109
61.06044056	-0.236488614	0.142567355	-1.658785162	0.097159091	0.251626925
16.21405829	0.126289144	0.241495534	0.522946086	0.601011773	0.772052342
297.3820252	-0.102249909	0.066808111	-1.530501414	0.125892664	0.29712833
420.0674538	-0.249254739	0.286509013	-0.869971721	0.384315859	0.597397571
141.770817	-0.149472666	0.105342695	-1.418918192	0.155922868	0.341351937
18.45782867	-0.499081096	0.383040733	-1.302945229	0.192593461	0.393137709
199.0306643	0.359861859	0.181378236	1.984041008	0.047251269	0.156026738
2.409072407	0.79900178	1.194360841	0.668978548	0.503509163	0.699200053

241.8291046	-1.621527741	0.53484844	-3.031751838	0.00243139	0.018630102
5.200528023	0.455213108	0.621808011	0.732079839	0.464119839	0.666422849
2105.925013	0.219445165	0.067787404	3.237255781	0.001206852	0.011150689
587.1963561	0.24058225	0.087551771	2.747885606	0.005998093	0.036023546
29.03363216	0.651594123	0.603614567	1.079487075	0.280370653	0.493939272
15.90263766	0.800524534	0.370322404	2.1616962	0.030641601	0.115692585
116.9109995	-0.067527349	0.101147272	-0.66761414	0.504379929	0.699836179
47.96180717	-0.153780377	0.235163106	-0.653930708	0.513156449	0.706547242
5.048973278	0.098176216	0.480569219	0.20429152	0.838125697	0.919137447
22.31797692	0.559065975	0.216144686	2.586535828	0.009694609	0.051519029
330.6559482	-0.139087231	0.072407537	-1.920894392	0.054745023	0.171458081
2148.251282	-0.177782346	0.10287831	-1.728083854	0.083973192	0.229415317
3.235586711	0.906983581	0.661410966	1.371285975	0.170285826	0.362304006
128.8892211	0.015995381	0.101544173	0.157521409	0.874833936	0.937291376
1.953451937	0.751721036	0.833905258	0.901446572	0.367350929	0.580955677
233.1943411	-0.872471183	0.257696349	-3.385655966	0.000710083	0.007366445
380.5159008	-0.230055152	0.089641336	-2.566395854	0.010276148	0.053421389
4.082310596	-1.295588322	0.663321881	-1.953181946	0.05079804	0.163734475
56.7303989	0.248641444	0.157799569	1.575678855	0.115099833	0.281621068
422.7171133	-0.0058406	0.158250308	-0.036907354	0.970558876	0.985635983
126.5255116	0.049380696	0.098874662	0.499427198	0.617478462	0.783755788
63.34315265	-0.0291441	0.213873887	-0.136267686	0.891609668	0.945641854
121.4519122	0.022436733	0.147993455	0.151606252	0.879497502	0.939674547
6.196253441	-0.444333073	0.581152745	-0.764571926	0.444526492	0.649789656
111.0270223	0.228275415	0.117701394	1.939445303	0.052447139	0.166927002
64.80136368	0.251534935	0.319869122	0.786368292	0.431651754	0.638734483
284.2375652	0.255603975	0.105623677	2.419949595	0.015522659	0.071926564
154.3626066	0.122545093	0.141897703	0.86361576	0.387799004	0.600940719
310.7614988	-0.003626772	0.061410187	-0.059058151	0.952905791	0.977165889
102.5162555	0.25109543	0.230591303	1.088919777	0.276189264	0.488820577
823.6343134	0.082130041	0.044005436	1.866361243	0.061990842	0.187589479
132.8600875	0.013533771	0.125570065	0.107778642	0.914171286	0.957227341
59.77373038	0.023442892	0.139478035	0.168075866	0.866523595	0.933868226
78.31547758	-0.241887101	0.121300321	-1.99411757	0.046139208	0.153815251
656.1043939	0.133770006	0.10994115	1.216741923	0.223702423	0.428769484
2.702075865	-0.227281096	0.631070613	-0.360151609	0.71873376	0.850727114
26.21088653	-0.179093799	0.170998895	-1.047338924	0.29494329	0.509745674
532.1580528	0.247154738	0.059626854	4.145023977	3.39778E-05	0.000658626
231.0393189	0.231753132	0.116063929	1.996771381	0.045850024	0.15318055
377.6060233	0.132223993	0.061256503	2.158529872	0.030886658	0.116303271
165.0823042	-0.11320114	0.094992312	-1.191687384	0.233383847	0.440216011
1439.641893	-0.035027073	0.100642803	-0.348033558	0.727814981	0.855941177
39.85629354	0.382773042	0.250634936	1.527213435	0.126707947	0.2980278
476.0617939	-0.076343291	0.157030828	-0.486167538	0.626848385	0.790677612
226.2223781	0.035779989	0.100560493	0.355805627	0.721986135	0.852372845
1574.104002	0.013533623	0.07854642	0.172300947	0.86320094	0.931920109
1491.473528	-0.030552138	0.054306761	-0.562584433	0.573717895	0.752760082
249.0419833	0.235519692	0.091803753	2.565469111	0.010303639	0.053519942

622.0580494	-0.086415982	0.059199588	-1.459739573	0.144361661	0.32480388
12.76778236	-0.60115932	0.377578139	-1.592145461	0.111352024	0.275976117
5.27553178	-0.112399759	0.432323635	-0.259989855	0.794871599	0.894325552
16.03453248	0.238835264	0.296984429	0.804201301	0.421280728	0.629613922
2.628867058	-0.777281169	0.619189396	-1.255320543	0.209362421	0.413355287
9.949868407	-0.037300682	0.411378225	-0.090672475	0.927752843	0.965547435
99.90571762	-0.153345729	0.188403307	-0.813922703	0.415689234	0.625357946
770.2666566	0.803714465	0.138036154	5.822492454	5.79764E-09	3.98588E-07
10.19431585	1.018513167	0.38565189	2.641016921	0.008265759	0.045863966
19.21734142	0.100589897	0.239926983	0.419252121	0.675031884	0.824166254
495.3301659	-0.141627561	0.068624888	-2.063792974	0.03903735	0.136275993
89.70289793	-0.259394771	0.130460035	-1.98830831	0.046777602	0.155178055
762.8736912	-0.24805456	0.096412309	-2.572851553	0.010086446	0.052823573
1227.068076	-0.596678333	0.146479486	-4.073460031	4.63198E-05	0.0008403
56.35193886	-0.677057922	0.301800747	-2.243393789	0.024871432	0.100252501
384.834483	0.048459241	0.078043844	0.620923299	0.534650091	0.723766845
10.01012969	-0.702927925	0.521163245	-1.348767266	0.177411732	0.372722796
15.50414513	0.607073604	0.333270816	1.821562446	0.068521403	0.200893682
285.6688882	0.128444152	0.080188797	1.601771767	0.109206088	0.272520511
241.198963	-0.017482229	0.113830755	-0.153580891	0.877940205	0.938489926
549.5046986	0.105197971	0.082373423	1.277086325	0.201571772	0.404321609
259.2589825	0.063273984	0.079121992	0.799701597	0.423883707	0.631493959
138.5466756	0.250787767	0.125092325	2.004821383	0.044982146	0.150923802
124.2579858	-0.269005528	0.21739349	-1.237412992	0.215933798	0.42053356
33.61091666	-0.358791007	0.212502864	-1.688405515	0.091333412	0.24186532
46.7367349	-0.020266954	0.268388465	-0.075513508	0.939806151	0.971351184
184.5265568	-0.078326634	0.148455524	-0.527610103	0.597769979	0.769410483
211.857616	0.09516993	0.100531572	0.94666709	0.343808445	0.558184718
4.27156384	-0.506928532	0.835668904	-0.60661409	0.544107047	0.731127202
1298.155362	0.11181902	0.066238395	1.68812997	0.091386283	0.241963616
102.3370285	0.227509557	0.117931549	1.929166194	0.053710234	0.169661869
672.0873017	0.138769914	0.056504127	2.455925277	0.014052237	0.06715885
1286.116787	0.008887988	0.061285796	0.14502525	0.884690936	0.942468039
44.79152374	0.269244637	0.507948058	0.530063327	0.596068025	0.767938112
1710.652078	-0.010753528	0.119512467	-0.089978296	0.928304462	0.965637301
941.3842802	-0.486788764	0.200744157	-2.424921213	0.015311709	0.071339235
50.73738685	0.269297107	0.232969007	1.155935334	0.247707608	0.456253929
50.17243772	1.023240967	0.282371243	3.623743536	0.00029037	0.003651563
172.3409503	-0.123105888	0.086316787	-1.426210273	0.153807655	0.33850411
17.39250121	0.378076307	0.337355982	1.120704322	0.262413742	0.473805785
303.4384234	0.602373451	0.173838332	3.46513594	0.000529964	0.005851621
678.2569433	0.172152203	0.130247913	1.321727154	0.186259023	0.384611575
489.183684	0.735110826	0.081990965	8.965754079	3.08165E-19	2.3076E-16
6.130247639	-0.861676964	0.801580464	-1.074972511	0.282387036	0.496119014
73.44191209	-0.007474806	0.125040128	-0.059779259	0.952331445	0.977124952
28.12631486	-2.92776776	1.003730487	-2.916886354	0.003535446	0.024554356
205.7961614	0.234909047	0.093482358	2.512870377	0.011975333	0.059836709
46073.14061	0.166273809	0.108316338	1.535075977	0.124765166	0.295593801

1477.189268	0.247376203	0.127314759	1.943028486	0.052012717	0.166067336
270.4821112	-0.596779814	0.107289945	-5.562308902	2.66228E-08	1.41937E-06
1.776899088	0.131701951	0.988453329	0.133240434	0.894003234	0.946388043
616.8208279	0.201503243	0.093751039	2.149344109	0.031607132	0.117911209
1.786182178	-0.29045571	0.712141287	-0.407862478	0.683374641	0.828701792
2.730961591	0.387742389	0.628130063	0.61729634	0.53703928	0.725232852
3.483460135	0.197565636	0.593027201	0.33314768	0.73902281	0.862716962
243.4310451	0.060296084	0.070038957	0.860893517	0.389296692	0.602752156
21.45036122	0.090817582	0.241978521	0.375312574	0.707428016	0.84371021
3.018718857	0.296502534	0.557054391	0.532268553	0.594540009	0.766869097
947.8584938	0.273485439	0.119213324	2.294084506	0.021785645	0.09132232
35.26872027	0.460480003	0.368258395	1.250426358	0.211143841	0.415428596
956.2992144	0.036313085	0.06573219	0.552439912	0.580646992	0.757609579
14.45546236	-0.144593802	0.262990629	-0.54980591	0.582452505	0.758823453
12.02187624	-0.69798269	0.323130967	-2.160061278	0.030767926	0.115988745
422.4243069	-0.016111807	0.157890176	-0.10204439	0.918721441	0.959711208
21.83555189	-0.926518067	0.280986553	-3.297375114	0.000975931	0.009462908
21.38318736	0.562359018	0.366853179	1.53292666	0.125293926	0.296332703
446.2304513	0.07481386	0.07405369	1.010265117	0.312368289	0.527087493
5868.525831	0.300183834	0.058019201	5.17387049	2.29294E-07	9.37317E-06
22.24095579	-0.248155444	0.233419306	-1.063131617	0.287722268	0.502378023
22.00759772	0.080887231	0.294211253	0.274929085	0.783370721	0.887675403
89.94371633	0.418467893	0.158104818	2.646775082	0.008126338	0.045258044
405.8514463	-0.427494894	0.2343087	-1.824494331	0.068077373	0.200161812
545.9473779	-0.247640528	0.310782856	-0.79682815	0.425550844	0.632924168
7.029197245	-0.068764276	0.378180005	-0.181829487	0.855716543	0.929211939
78.90020073	-0.502427167	0.221890428	-2.264303022	0.023555484	0.096195598
11.82011325	-0.033976478	0.343553918	-0.098897077	0.92121999	0.96112591
3.026922289	-1.151414648	0.885521071	-1.300267928	0.193509156	0.394294353
69.83963657	-0.25138615	0.156838498	-1.602834468	0.108971205	0.27203995
2.554749656	2.095415386	0.887948472	2.359838947	0.01828287	0.080406774
427.800386	0.112716772	0.078732457	1.431643013	0.152246013	0.336472474
56.9251922	0.318424127	0.177737432	1.791542293	0.073206314	0.210071437
3.909143628	0.235176734	0.47878465	0.491195225	0.623288376	0.788334181
350.6089915	0.230032608	0.062308587	3.691828342	0.000222648	0.002984007
221.3262328	0.898488174	0.099351501	9.043528906	1.51694E-19	1.19E-16
2980.788216	0.021362143	0.054201909	0.394121601	0.693491264	0.83500768
82.64692373	0.572426211	0.155275738	3.686514188	0.000227347	0.003035098
46.11405881	-0.211622466	0.13731437	-1.541153095	0.12327953	0.293441263
4.977020928	-0.617831891	0.856067206	-0.721709565	0.47047306	0.671219641
2.688711133	-1.426480265	0.856071873	-1.666308998	0.095651885	0.249015354
1.821286359	-3.894189098	2.107609238	-1.847680788	0.064648535	0.193183378
24.07936559	-0.787565343	0.515497244	-1.527777991	0.126567669	0.297885745
26.67570377	0.344776886	0.23078538	1.493928631	0.135194274	0.311495171
170.6272621	0.273906614	0.085378733	3.208136297	0.001335982	0.012013626
8378.519687	0.373784557	0.088326965	4.231828384	2.31799E-05	0.000481546
657.235943	-0.045146981	0.067789087	-0.665991875	0.505416294	0.700428834
130.9432466	0.411278754	0.158859906	2.588939932	0.009627189	0.051326312

382.0561194		-0.101719784	0.113020346	-0.900013032	0.368113316	0.581947768
1034.379435		-0.141363598	0.106859864	-1.322887687	0.185872731	0.384054605
12.88324233		0.702291275	0.327448115	2.144740629	0.031973591	0.118799842
491.7734176		0.222145928	0.061434833	3.615960459	0.000299236	0.003726517
8.202489734		0.321519851	0.463234183	0.694076263	0.487634384	0.685967982
604.2772318		-0.11335728	0.07115781	-1.593040593	0.111151085	0.275643982
607.6554791		-0.378005335	0.082571773	-4.577900171	4.69667E-06	0.000126633
8.510232962		0.497652332	0.384918244	1.292878006	0.196053255	0.397218218
4.005599206		-0.755974841	0.561824067	-1.345572193	0.178440525	0.374093815
460.4116189		-0.000259404	0.062482726	-0.004151605	0.996687508	0.998020302
196.5089561		-0.00281306	0.091807324	-0.030640913	0.975555913	0.987719156
145.0737668		0.052405282	0.084890413	0.617328624	0.53701799	0.725232852
177.8455931		-0.043674714	0.102612119	-0.425629199	0.67037806	0.820734852
446.5816629		0.304819931	0.06325438	4.818953744	1.44313E-06	4.75482E-05
12.56669135		-0.2318664	0.255294303	-0.908231782	0.363755776	0.577591581
823.1018129		0.010852106	0.072377596	0.149937363	0.880814033	0.940410265
35.51450799		0.549274457	0.178809613	3.071839643	0.00212744	0.016910795
24.67018862		-0.242013727	0.709885381	-0.34091944	0.733164229	0.859266193
12.42270154		-0.858126574	0.576705183	-1.487981379	0.136755804	0.313907638
25.44020667		1.524708996	0.658505883	2.315406794	0.02059068	0.087764777
33.91476202		0.203403067	0.184556906	1.102115714	0.270411369	0.482325345
10.74710089		0.990351063	0.587729051	1.685046981	0.091979508	0.242831796
56.25186994		0.056734213	0.203415074	0.278908597	0.780314971	0.885568258
257.4707965		0.467982454	0.130000963	3.59983837	0.000318415	0.00390586
733.8946945		-0.064713096	0.107746087	-0.600607386	0.548101517	0.734756644
1.890208019		-0.860423344	0.741695938	-1.160075579	0.246018036	0.454564953
12.85744862		0.111741188	0.283649094	0.393941637	0.693624129	0.835017244
93.12944922		0.121424643	0.128409936	0.945601619	0.344351818	0.558681491
1986.721035		0.354332545	0.195335048	1.813973215	0.06968185	0.203103112
245.7228553		-0.19457429	0.079458345	-2.448758397	0.014334957	0.068050177
2.628279503		-1.091021329	0.605625735	-1.80147782	0.0716276	0.206980018
28.86554282		-0.46639423	0.242075687	-1.926646312	0.054023723	0.170131231
4.625110889		-3.027217988	0.93816347	-3.226748945	0.001252053	0.01147181
2.842088103		-1.38680887	0.944156057	-1.468834374	0.141877718	0.321247352
8.943409476		-1.086502338	0.621858158	-1.747186757	0.080604948	0.22356189
7.959816529		0.177005387	0.737695617	0.239943661	0.810373933	0.903927156
11.0652596		0.085518491	0.407675314	0.20977108	0.833846345	0.916902912
4319.202478		0.026343519	0.076255228	0.345465089	0.729744751	0.857101832
1313.370957		0.109170113	0.13545324	0.805961623	0.420264977	0.628946697
142.3634584		0.079105401	0.095226404	0.83070869	0.406138216	0.616852629
464.9076495		-0.222681224	0.118888189	-1.873030666	0.061064151	0.185912182
4.398638534		-0.521710024	0.518075314	-1.007015795	0.313927184	0.528654159
203.0970805		-0.398230685	0.245020833	-1.625293162	0.104100108	0.263855836
646.6599028		0.155757354	0.0599798	2.596830185	0.009408843	0.050423317
75.23480749		0.412910882	0.39070173	1.056844262	0.29058267	0.50581772
262.4986852		0.244787641	0.12060813	2.02961144	0.042396051	0.144782866
217.1814694		-0.024537825	0.072805034	-0.337034724	0.736090724	0.861278654
28.64821975		-1.690950866	0.533053891	-3.172194958	0.001512914	0.013241222

534.5107164	0.054552251	0.066691197	0.817982777	0.413367022	0.623152299
120.1884794	-0.029365945	0.096695888	-0.30369384	0.761361151	0.875473689
65.93348102	0.049142893	0.145884191	0.336862363	0.736220659	0.861278654
6.673133003	-2.964701163	1.540638983	-1.924332174	0.05431296	0.170618383
803.7061537	0.041454434	0.057546606	0.720362804	0.471301645	0.671587519
75.05717909	-1.155871654	0.182043216	-6.349435475	2.16106E-10	2.18872E-08
143.8270975	-0.098340185	0.11576223	-0.849501476	0.395602309	0.608397353
1809.537275	-0.062371111	0.064259775	-0.970608927	0.331743059	0.547279707
144.596031	0.482449627	0.084282584	5.724191197	1.03928E-08	6.43648E-07
235.0647231	0.093632797	0.093163235	1.005040199	0.314877493	0.529693845
27.44915659	0.427651899	0.199578484	2.142775569	0.032131124	0.119110742
1229.131811	0.083379107	0.043944663	1.897365944	0.057779657	0.178585755
938.1660152	0.36799649	0.077719562	4.734927466	2.19133E-06	6.6976E-05
1315.611839	-0.013324105	0.064122354	-0.207791884	0.83539147	0.917766577
13.87618004	0.050049482	0.362269307	0.138155458	0.890117557	0.945139805
56.64002619	0.294981745	0.143402061	2.057025836	0.039683741	0.137912408
814.0531878	0.256532707	0.097211859	2.638903431	0.008317467	0.046119809
3.112351454	0.674569702	0.542060502	1.24445463	0.213332288	0.417887765
143.7998853	0.507597526	0.085894549	5.909542976	3.43058E-09	2.52301E-07
76.25150774	0.116899526	0.145406504	0.80394977	0.421425985	0.629654605
96.32533913	0.380722094	0.128130972	2.971351032	0.002964927	0.02160292
109.0613665	0.404031359	0.113950998	3.545658813	0.000391633	0.004618297
3.101192209	0.24568501	0.696100373	0.352944804	0.724129821	0.853557222
83.54137225	0.250297999	0.113000895	2.215009003	0.026759447	0.105643763
8.097929715	-0.306101988	0.323242059	-0.946974503	0.343651771	0.557985342
143.4426959	0.160174772	0.081759698	1.959092018	0.050102011	0.162387358
1118.523001	-0.163883083	0.047765704	-3.430978097	0.000601409	0.006465726
50.22803162	0.044047073	0.170688395	0.258055463	0.796364103	0.894897219
11.3282129	1.135183169	0.428307788	2.650391148	0.008039863	0.044853467
20.82318291	-1.658459279	0.795305361	-2.085311329	0.03704105	0.131881187
353.6603348	-0.202265829	0.080185308	-2.522479927	0.01165306	0.058761099
226.4033903	0.676841598	0.21372391	3.166896944	0.001540749	0.013415592
241.0443972	0.320890858	0.12081561	2.656038048	0.007906469	0.044227903
121.7822965	0.054483633	0.37891232	0.143789552	0.885666653	0.942902446
43.94507436	0.528356562	0.282068935	1.873146941	0.061048097	0.185912182
588.2144112	0.601533163	0.144613379	4.15959552	3.18812E-05	0.000628242
15.73359972	1.567092264	0.573955249	2.730338763	0.006326927	0.037519727
2.923729466	1.039527637	0.627731415	1.656007032	0.097720395	0.25264372
4.39526306	-0.781208793	0.939202816	-0.831778588	0.405533932	0.616306826
161.5891957	0.041605619	0.145957116	0.285053719	0.775602987	0.883629572
94.32754909	-0.038682564	0.115809737	-0.334018236	0.738365798	0.862255487
159.5984124	-0.112519175	0.131134433	-0.858044466	0.39086791	0.604048588
495.2270489	-0.108982819	0.062310717	-1.749022059	0.080287206	0.222893738
21.63011173	0.356775223	0.210694727	1.693327723	0.090393104	0.240738071
115.7168675	-0.216797876	0.115799309	-1.872186264	0.061180839	0.186095483
52.57083911	0.534776709	0.315016391	1.697615499	0.089580356	0.239103496
184.2319466	0.162553551	0.105091039	1.546787935	0.12191439	0.291793937
80.59529461	0.127672048	0.287855096	0.443528879	0.657383235	0.811359633

1880.81531	0.01609722	0.085261434	0.188798368	0.850250851	0.926146027
2195.982694	0.223471258	0.047406189	4.713968002	2.42939E-06	7.28993E-05
1781.036886	-0.045219434	0.109815591	-0.411776081	0.680503555	0.827343313
18702.4906	-0.030288095	0.091744315	-0.330135932	0.741297254	0.864330877
109.1326203	0.48254537	0.155591144	3.101367836	0.001926288	0.015774569
136.0462744	0.186322428	0.133807337	1.392467946	0.163780742	0.353337265
271.0742493	-0.023578099	0.10190058	-0.231383366	0.817016986	0.907955871
320.3611449	0.236072696	0.112403386	2.100227619	0.035708823	0.128238793
225.9995573	0.144370418	0.152254801	0.948215867	0.343019571	0.55723345
1409.218098	-0.013376276	0.077058061	-0.173586982	0.862190064	0.931452496
59.63279078	-0.244717326	0.169699113	-1.44206603	0.149283747	0.332337899
245.9514904	0.039729432	0.065983494	0.602111675	0.547099803	0.73377887
1221.721119	0.279437426	0.073986943	3.776847848	0.000158826	0.00226768
13.72045938	0.607871036	0.255153798	2.382371105	0.017201553	0.077277987
412.2726914	0.048792327	0.08984117	0.543095413	0.587064111	0.761579192
1114.047409	0.172156795	0.058135836	2.961285284	0.00306358	0.022126006
21.45265855	0.487123365	0.209535955	2.324772209	0.020084144	0.086207969
76.93602448	0.211322336	0.136412614	1.549140723	0.121347895	0.291158774
214.9576699	0.05595853	0.099049055	0.564957737	0.572102511	0.751595502
17.64165126	-0.392031812	0.226735545	-1.7290267	0.083804317	0.229143952
77.26253824	0.25041128	0.208459261	1.201248049	0.229654996	0.43581804
668.516679	-0.147874206	0.046367184	-3.189199641	0.001426673	0.012653687
869.0630096	-0.034279228	0.048341181	-0.709110272	0.478256049	0.67786201
215.8504997	-0.195988943	0.120376856	-1.628128112	0.103497724	0.263161214
443.1453122	0.026744145	0.133860887	0.199790589	0.841644362	0.921462698
123.4004369	-0.033001731	0.098259222	-0.33586396	0.736973456	0.8616679
13.61684722	-0.658990429	0.304609731	-2.163392573	0.030510998	0.115310435
144.9752075	0.088924311	0.096679483	0.91978472	0.35768527	0.572182625
103.988058	-0.197224637	0.114780991	-1.718269158	0.085747532	0.232545349
3518.53341	-0.104723857	0.11218616	-0.93348286	0.350570716	0.565734509
672.0508817	0.019250886	0.095185109	0.202246819	0.839723773	0.920302907
127.1402107	-0.225087114	0.213641635	-1.05357326	0.292078337	0.507295575
257.3409544	0.123605821	0.079414558	1.556462995	0.119598036	0.288555659
187.5755883	-0.249183248	0.085660171	-2.908974458	0.003626165	0.024952982
336.5318019	-0.213878637	0.1880864	-1.137129725	0.255484059	0.465528635
1559.716611	-0.015293123	0.05266276	-0.29039729	0.771512317	0.880735494
1236.976619	-0.167501613	0.092218429	-1.816357257	0.069315587	0.202429532
2.189393197	-0.483683264	1.043455159	-0.463540057	0.642977309	0.801483671
2.239645713	-0.137416176	0.684286194	-0.200816817	0.840841813	0.921196251
165.159135	0.28040289	0.135644926	2.067183034	0.03871691	0.135706889
60.3136387	-0.197099996	0.155677755	-1.266076818	0.205485581	0.408985075
13.5759572	0.308154388	0.269316222	1.144210273	0.252536444	0.461638413
80.65267626	1.17990659	0.457770891	2.577504625	0.009951655	0.052315561
328.9321496	0.146007392	0.096861609	1.507381441	0.131712913	0.305978561
1372.823793	-0.097230381	0.073205601	-1.328182261	0.184117903	0.381563184
23.70932272	1.078377132	0.235672933	4.57573604	4.74549E-06	0.00012774
1.973947062	-0.053299017	0.752089896	-0.070867881	0.943502906	0.972730889
32.54013771	-1.577151157	0.265959829	-5.930035224	3.0287E-09	2.24751E-07

137.4614165	0.175626856	0.192236096	0.913599781	0.360927181	0.575095694
143.2572139	0.577710996	0.209216181	2.761311258	0.005756978	0.035022326
191.1560928	0.324717417	0.091371441	3.553817409	0.000379683	0.004506407
345.8757964	0.045415224	0.079554092	0.570872261	0.568086236	0.748947007
481.7544456	-0.046371767	0.093286912	-0.497087594	0.619127283	0.784939422
20.86184923	0.407181425	0.247640005	1.644247364	0.100125117	0.256734287
1329.013045	0.014327207	0.068928684	0.207855513	0.835341786	0.917766577
2.546289548	-0.606441896	0.719813047	-0.842499172	0.399508603	0.611768438
228.5719352	-0.03072312	0.095004988	-0.323384284	0.74640423	0.86703309
620.4541482	0.103495018	0.070769282	1.462428539	0.143623814	0.323704153
15.06685684	0.142407874	0.30132247	0.472609539	0.636491775	0.796654422
186.3100834	0.150945918	0.08214489	1.837556996	0.066127711	0.196144743
1108.959203	0.042253379	0.070866846	0.596236206	0.551017441	0.736986387
3.331500401	-0.240955937	0.582709832	-0.413509303	0.679233508	0.826686346
209.5812687	-0.151041289	0.106525411	-1.417889758	0.156222953	0.341872599
206.4993035	-0.085598772	0.073438275	-1.16558799	0.243781066	0.451393647
431.0526285	-0.182806485	0.09054176	-2.019029516	0.043484152	0.147429084
30.42776311	-0.193649254	0.181847934	-1.06489664	0.286922706	0.501479298
761.0698777	-0.071501895	0.080515032	-0.888056462	0.374510381	0.588036982
169.1085192	-0.077420972	0.085667853	-0.903734239	0.366136339	0.579528253
883.8871072	0.018714169	0.050569972	0.37006485	0.711334172	0.846071015
25.78452035	0.027935439	0.24356172	0.114695525	0.908686461	0.954091827
1032.793321	-0.027476105	0.043104482	-0.637430357	0.52384456	0.715049508
342.5747875	-0.053738673	0.075508759	-0.711687938	0.476658039	0.676493067
727.2317535	0.443090143	0.148570836	2.982349394	0.002860453	0.021065315
519.3579474	-0.267107582	0.092961891	-2.873301952	0.004062057	0.027048635
1048.028541	-0.209405847	0.054380009	-3.85078731	0.000117739	0.001772969
20.34442575	-0.252142776	0.241509277	-1.04402936	0.296471806	0.511207508
110.5742516	-0.257461521	0.120769219	-2.131847187	0.033019411	0.121474269
107.4728867	-0.272719024	0.175598859	-1.553079698	0.120404096	0.289927645
533.8312369	-0.143741353	0.078924246	-1.821257212	0.068567767	0.200993843
106.0214012	0.027839841	0.092350019	0.301460044	0.763063714	0.876496418
246.3186922	-0.102706894	0.158765277	-0.646910306	0.517689995	0.710176534
43.34813032	-0.206500285	0.157682932	-1.30959186	0.190333943	0.390237882
143.0725176	-0.599376738	0.250627396	-2.391505267	0.016779443	0.075919952
170.8562158	-0.120450706	0.123629738	-0.974285865	0.329914621	0.545336384
1066.744616	-0.048296099	0.088738393	-0.544252573	0.586267678	0.760977281
448.8039351	-0.512202483	0.149919857	-3.416508621	0.000634297	0.006745902
261.0378775	0.21711231	0.067401598	3.221174514	0.001276664	0.011632611
403.8578543	0.220062251	0.074859752	2.93966043	0.003285721	0.023241293
1347.719851	-0.128821942	0.076904421	-1.675091517	0.093916277	0.246091419
33.16808279	0.043035273	0.210266975	0.204669674	0.837830216	0.918926885
326.9504208	-0.00654708	0.072439408	-0.090380083	0.927985183	0.965637301
34.39989667	-0.209402869	0.314156338	-0.666556244	0.505055626	0.700301859
2.159175386	0.035052725	0.710411155	0.049341463	0.960647177	0.980709029
2.912250581	-0.756207075	0.723248581	-1.045570078	0.29575957	0.510408791
1100.407358	-0.040596267	0.094587567	-0.429192428	0.667783193	0.818996435
88.00758371	0.282388902	0.116803107	2.4176489	0.015621141	0.07228727

17.75000217	0.491289385	0.255707411	1.921295058	0.05469452	0.171435708
769.1060487	0.033275218	0.05499986	0.605005502	0.545175341	0.732144662
27.08302307	-1.089410532	0.488492097	-2.230149759	0.025737502	0.102787785
317.6695517	-0.194846138	0.08535208	-2.282851673	0.022439107	0.093066931
688.5726826	-0.065508119	0.060570558	-1.081517507	0.279466978	0.492716073
8.861983587	0.322913595	0.446138248	0.723797156	0.469190276	0.670434608
391.5348589	-0.241998046	0.065081155	-3.718404264	0.000200485	0.002727328
114.0375993	0.082424518	0.09647235	0.854384884	0.392891769	0.606245849
38.28221465	-0.404859788	0.179396196	-2.256791373	0.024021114	0.097817076
216.3981739	-0.008740746	0.109474196	-0.079842983	0.936362138	0.970104387
19.37852132	0.035625998	0.276600911	0.128799278	0.897516477	0.948210116
231.6569063	0.267841741	0.089745411	2.984461703	0.002840778	0.020967281
18.126738	0.04411288	0.229121251	0.192530722	0.847326506	0.924458083
398.8240378	0.218139896	0.076143096	2.864867707	0.004171835	0.027612217
359.1943426	-0.174609967	0.071798905	-2.431930792	0.015018577	0.070348602
167.9362428	0.076559911	0.151484437	0.505397862	0.61327942	0.781093831
11.2493216	0.255121014	0.387215003	0.658861386	0.509984784	0.704468332
666.0223104	0.124834421	0.062912654	1.984249808	0.047227998	0.155981164
3.534478777	0.224370688	0.556822701	0.402948169	0.686986354	0.830726992
3.472762664	1.058153011	0.530282762	1.995450517	0.045993766	0.153505126
137.6403365	0.176475667	0.08456783	2.086794313	0.036906731	0.131545109
399.0487554	-1.636872113	0.52514249	-3.11700566	0.001826981	0.015124462
304.3360101	0.040109627	0.091477285	0.43846543	0.661048925	0.813727863
235.1477554	0.178036009	0.093315976	1.907883481	0.056406278	0.175294665
46.02963591	0.147014986	0.167979069	0.875198243	0.381466049	0.594145003
2.82443987	1.404800294	0.582129541	2.41320908	0.015812746	0.072888197
4.528749111	0.050033455	0.606828295	0.082450761	0.934288272	0.9689185
544.5075908	0.339263555	0.072499369	4.679538005	2.87522E-06	8.3983E-05
151.3705718	-0.126027329	0.114035654	-1.105157243	0.26909146	0.480856136
194.5603113	-0.016614574	0.091002354	-0.18257301	0.855133063	0.929057489
232.1185165	0.057389164	0.082090441	0.699096797	0.48449154	0.683641425
346.9462201	0.20570038	0.111815568	1.839639898	0.065821123	0.195654479
5.0759312	0.771458897	0.565234008	1.364848692	0.172300611	0.36512481
286.0065721	0.005485214	0.070195151	0.07814235	0.93771482	0.970501464
582.56869	-0.502594768	0.109542394	-4.588130214	4.47234E-06	0.000121781
222.0099323	0.043467546	0.083058677	0.523335403	0.600740868	0.771818998
37.41911266	-0.254211569	0.181823518	-1.398122595	0.162076256	0.351188949
191.4913961	-0.124970971	0.394697719	-0.316624507	0.751528538	0.870346652
6.41881283	-2.67970742	1.064890835	-2.516415141	0.011855545	0.05943647
43.73584509	0.004136628	0.242851951	0.017033537	0.986409861	0.993222667
228.0712683	0.916973345	0.231313111	3.964208258	7.36399E-05	0.00123287
41.11337223	-0.177923654	0.152540158	-1.166405336	0.243450602	0.451035225
503.0777046	0.086257372	0.092072156	0.936845355	0.348838111	0.56340775
150.8759349	0.004394503	0.105300317	0.04173305	0.966711507	0.983487023
138.9256059	-0.100710634	0.097616471	-1.031697137	0.302214019	0.517049928
345.0122838	0.014828229	0.070293541	0.21094725	0.832928433	0.916630422
13.0351079	0.98936551	0.584449706	1.692815482	0.090490595	0.240869617
30.82874116	0.107509626	0.447801769	0.240083075	0.810265855	0.903927156

3718.70569	-0.236069534	0.100095162	-2.358450984	0.018351383	0.080661869
229.2668795	0.162509198	0.084814272	1.916059586	0.055357512	0.172850578
9.150387851	-0.19831765	0.344699547	-0.575334814	0.56506488	0.747340947
433.2599432	0.308226756	0.057169955	5.391411553	6.99064E-08	3.31884E-06
2.344079769	-1.14599994	0.643531871	-1.780797488	0.07494554	0.213239062
1234.721208	-0.168409143	0.065588736	-2.567653448	0.010238946	0.053327979
662.9184706	0.04149539	0.067476608	0.614959632	0.53858138	0.726541399
509.1949909	0.040163928	0.0598713	0.670837746	0.502323899	0.698100549
789.0268269	0.085864556	0.068334697	1.256529403	0.208924092	0.412984821
22.5685933	0.524016434	0.239588122	2.187155312	0.028731188	0.110665791
666.1255065	-0.040646089	0.058787979	-0.691401356	0.489313352	0.687044432
3.4333181	0.120950649	0.533270957	0.226808993	0.820572271	0.909820137
108.7709531	-0.066863035	0.114032041	-0.586353045	0.557638291	0.741810242
38.23974998	-0.104060505	0.21410053	-0.48603572	0.62694184	0.790677612
20.95328	0.669760235	0.29639124	2.259716701	0.023838839	0.097184122
3256.429099	-0.06524777	0.062201376	-1.048976307	0.294189023	0.509065449
9086.708829	0.123605066	0.063643788	1.94213874	0.052120307	0.166175718
3.765503217	-0.37029134	0.552041042	-0.670767773	0.50236848	0.698103614
838.3988988	0.539129634	0.172338829	3.128312047	0.001758134	0.01467993
95.92195754	0.262672687	0.118493045	2.216777251	0.026638317	0.105363657
59.71203513	-0.260210753	0.141575855	-1.837959967	0.066068305	0.19603913
81.43207342	0.323321389	0.134973059	2.395451284	0.01659992	0.075439195
83.64713826	0.018591767	0.136029086	0.136674936	0.891287741	0.945526539
47.11137772	0.103863655	0.222126184	0.467588526	0.640078872	0.799140533
19.90814706	0.008835182	0.304423818	0.029022637	0.976846536	0.988158577
255.3674597	0.09625582	0.072302445	1.331294115	0.183092254	0.380360881
797.9724446	0.059120022	0.078404509	0.754038555	0.450826071	0.655739775
157.675076	-0.317661732	0.158958323	-1.998396349	0.045673707	0.152715375
118.3412944	0.112218746	0.104603924	1.072796717	0.283362334	0.497352453
39.72809531	-0.310877761	0.180083658	-1.726296347	0.084294112	0.229948866
54.34177389	0.186340234	0.187019519	0.996367836	0.319071455	0.534070902
530.4358247	-0.056567326	0.067152009	-0.842377271	0.399576811	0.611768438
22.28762767	0.942950555	0.215684685	4.371893879	1.23173E-05	0.000284196
324.3187161	0.039634064	0.082836439	0.478461714	0.632321618	0.794046377
255.0891846	0.584754392	0.148939768	3.926113209	8.63295E-05	0.001391578
17.3283209	-0.131275981	0.406259937	-0.323132972	0.74659454	0.867077573
117.5675932	-0.200454501	0.118630234	-1.6897421	0.091077305	0.241693028
309.0394481	0.057183429	0.071411151	0.800763301	0.423268688	0.631197206
19.10713948	-2.200559525	0.949145765	-2.318463196	0.020424161	0.087348813
7.790131295	-2.809286039	0.892211712	-3.148676487	0.001640117	0.0139779
2.917988701	-1.882390699	1.039191319	-1.811399561	0.070079024	0.203684164
9.416632063	-2.666093741	0.799585197	-3.334346046	0.000855002	0.008526214
6.275983063	-2.307640379	0.995732123	-2.317531318	0.020474807	0.087497267
2973.020207	-0.42209992	0.120543452	-3.501641231	0.000462402	0.005242676
6.661679991	0.344420994	0.38554706	0.89333062	0.371680145	0.5851316
64.72798945	-0.851719794	0.232970397	-3.655914252	0.000256267	0.003330154
59.14115387	-0.407580553	0.140392782	-2.903144642	0.00369436	0.025222081
1161.339927	-0.987381505	0.402471631	-2.453294663	0.014155433	0.067509546

140.6780895	0.110944263	0.185297831	0.59873482	0.549349734	0.735711529
39.94317446	-0.8797912	0.348914995	-2.52150585	0.011685373	0.058881027
32.77129044	-0.224332099	0.346494477	-0.647433404	0.517351483	0.709915873
867.0616171	-0.181281206	0.116934968	-1.55027371	0.121075835	0.290928136
72.8862479	0.005670494	0.124270355	0.045630307	0.963604913	0.982015974
1514.393747	0.040253711	0.096273775	0.418117092	0.675861509	0.824568059
144.6850339	0.157626615	0.119807864	1.315661676	0.188287623	0.387585942
2.234041503	0.963258626	0.638902372	1.507677336	0.131637129	0.305909163
211.227504	0.511887325	0.119858652	4.270758245	1.94809E-05	0.000415417
77.62472024	-0.53584224	0.291175688	-1.840271221	0.065728428	0.195417818
73.04942722	-0.189781225	0.204139959	-0.929662305	0.352545957	0.567504603
52.20469538	-0.444769316	0.177753015	-2.502175936	0.012343257	0.061229393
2146.646294	0.273852093	0.268251383	1.020878591	0.307311963	0.52240814
94.31383208	0.371225163	0.286804548	1.294348937	0.19554492	0.396578482
2913.278065	0.287682986	0.136934878	2.100874436	0.03565199	0.128182209
3.926339733	-1.035899046	0.68087059	-1.52143309	0.12815119	0.29992367
159.5171488	-0.166244639	0.144741605	-1.148561525	0.25073683	0.459623738
601.8818742	-0.330351047	0.155780551	-2.120618046	0.033953958	0.123696928
79.61225959	-0.673756957	0.306943868	-2.195049413	0.028160053	0.109129315
2279.296399	-0.040551963	0.092017323	-0.440699223	0.659430759	0.812578183
29.77204825	-0.555149304	0.246761658	-2.249738914	0.024465524	0.099085478
175.5752341	-0.359525421	0.139324014	-2.58049858	0.009865776	0.052025863
2.714360263	-1.390582558	0.94377812	-1.473421061	0.140637535	0.319655456
8.280929791	0.163549717	0.584976366	0.27958346	0.779797098	0.885224462
10.47888663	-2.463974198	0.784375036	-3.141321541	0.001681873	0.014199103
1044.954727	-0.079621987	0.106750462	-0.745870189	0.455745835	0.659864116
64.8241877	0.668265962	0.138969086	4.808738287	1.51886E-06	4.945E-05
248.28846	0.06492646	0.077319774	0.839713524	0.401069029	0.612787084
223.9114394	0.646962982	0.161231241	4.012640333	6.00434E-05	0.001041215
92.33420368	0.205471197	0.113915415	1.803717234	0.071275647	0.206288651
488.1140889	0.114322494	0.082397749	1.387446814	0.165305559	0.355700597
1404.81288	0.062473761	0.069627834	0.897252684	0.369584098	0.583080678
497.7627309	-0.029792206	0.183118856	-0.162693274	0.870759943	0.935813113
280.0425282	-1.237076221	0.46049412	-2.686410459	0.00722243	0.041342015
1450.363386	-0.130691934	0.071744349	-1.821633847	0.068510562	0.200893682
20.37988442	-0.14202697	0.262651783	-0.540742455	0.588685111	0.76241798
716.5865591	0.024402144	0.07419875	0.32887541	0.742249861	0.864890665
117.9153285	-0.419500304	0.101892721	-4.11707823	3.83706E-05	0.000724074
374.2935401	-0.043608046	0.068872657	-0.633169213	0.526623148	0.717000092
11.43239461	0.917908491	0.306780179	2.99207235	0.002770906	0.020594306
576.606081	-0.056933777	0.056517363	-1.007367893	0.313758015	0.528590495
78.26250566	-0.258166841	0.130468304	-1.978770571	0.047841843	0.157395648
126.3128728	-0.270802143	0.111575761	-2.427069648	0.015221333	0.071110984
107.5734931	0.298928967	0.191105475	1.564209332	0.117768416	0.285553549
441.5967532	-0.053262943	0.067553028	-0.788461224	0.430426971	0.637437426
58.71154641	0.015432524	0.15058879	0.10248123	0.918374711	0.959494228
585.5397024	-0.132183258	0.13733686	-0.962474732	0.33581119	0.550901568
231.813017	-0.048461909	0.072554689	-0.667936283	0.504174265	0.699609741

715.0237377	0.074118049	0.05901835	1.255847539	0.209171252	0.413162105
3.072186657	-1.106016152	1.021019086	-1.083247285	0.278698677	0.491940641
35.99908051	-0.236348984	0.163978161	-1.441344274	0.149487445	0.332560838
2447.359094	0.022415653	0.071604552	0.313047871	0.754244293	0.871653489
4.972241716	-0.766860553	0.452631534	-1.694226971	0.090222162	0.240349271
1852.676631	0.844996379	0.165432859	5.107790467	3.25948E-07	1.29296E-05
1185.452905	0.340817971	0.059091016	5.767678265	8.03711E-09	5.15188E-07
37.39432632	0.335018379	0.17488192	1.915683334	0.055405415	0.172931493
11.82592563	0.133361088	0.354616539	0.376071258	0.706863921	0.843281646
1.900016852	-3.108006633	1.768719097	-1.757207597	0.078882431	0.220159751
780.6061267	0.269026583	0.057480754	4.680289732	2.8647E-06	8.39734E-05
1053.110716	-0.119551546	0.104116637	-1.148246328	0.250866889	0.459759832
445.654027	0.213198092	0.067528839	3.157141396	0.001593241	0.013734719
48.41831357	0.133890348	0.174670217	0.766532213	0.443359692	0.648717254
1053.129594	-0.003466265	0.052365324	-0.066193905	0.94722345	0.974828397
74.097899	-0.296918591	0.142516841	-2.083393016	0.037215414	0.132159245
511.4776604	-0.122316345	0.08987542	-1.360954368	0.173528111	0.367112122
271.681975	-0.184935413	0.069048904	-2.678325113	0.007399136	0.042133898
10.00464908	-0.039223274	0.348355537	-0.112595525	0.910351233	0.955037829
684.1474684	0.024869336	0.077353852	0.321500933	0.747830805	0.86771555
5.438359115	0.61761718	0.497506368	1.241425678	0.214448538	0.419063612
858.8818598	-0.017867184	0.11323827	-0.157783971	0.87462703	0.937267006
2.675954711	-1.583558332	1.07729505	-1.469939301	0.141578194	0.320941711
1449.096967	-0.018628381	0.066509145	-0.280087509	0.779410368	0.885082046
130.5162467	-0.314595083	0.087247589	-3.605773949	0.000311224	0.003831916
464.8696977	0.472048544	0.088020868	5.362916263	8.1889E-08	3.75777E-06
62.08803412	0.131625376	0.132930486	0.990182011	0.322085163	0.537248298
530.1518675	0.428359832	0.118908319	3.602437886	0.000315247	0.003872763
3.247474295	1.091926346	0.562168113	1.942348421	0.052094935	0.166175718
15.37339419	1.18660155	0.261561617	4.536604269	5.71672E-06	0.000149015
198.1492015	-0.271332561	0.393707126	-0.689173608	0.490714023	0.688433148
234.5830098	-0.949921493	0.329372679	-2.884032448	0.003926183	0.02633548
109.7558131	0.052084516	0.10342811	0.50358182	0.614555271	0.782213052
40.99045575	0.40575151	0.200199546	2.026735414	0.042689479	0.145423175
9.884692325	0.381402405	0.33677539	1.132512695	0.25741895	0.467957282
1072.901116	0.141329414	0.078576798	1.798615069	0.072079592	0.208030693
55.50320191	0.070527694	0.150643402	0.468176458	0.639658406	0.798736646
278.7718174	0.41548552	0.16515999	2.515654797	0.011881149	0.059481626
228.3536528	-0.063240309	0.162486952	-0.389202387	0.697126438	0.837609287
156.7876114	-0.234803429	0.157096	-1.494649318	0.135005988	0.311235467
2044.007492	-0.170275352	0.120108668	-1.417677458	0.156284954	0.341962855
50.40466771	0.026683309	0.197127416	0.135360718	0.892326681	0.945865103
352.1513915	-0.018479626	0.084512232	-0.218662148	0.826913237	0.913540506
111.2858631	0.025403123	0.110952906	0.228954105	0.818904587	0.909133646
359.0511027	0.789673092	0.242017559	3.262875204	0.001102881	0.010405989
21.69943051	-0.225825878	0.3400483	-0.664099419	0.506626682	0.701476547
11.41345846	-0.579896204	0.48564262	-1.194080131	0.232446625	0.439160145
127.1754378	-0.049980691	0.242549047	-0.206064266	0.836740711	0.918413384

5.718352582	-0.258137957	0.403243209	-0.640154506	0.522072156	0.713528896
61.53469355	0.175806002	0.14549862	1.20830013	0.226931839	0.432643804
36.10732428	0.266592709	0.149894509	1.778535517	0.075315944	0.213775821
1263.167208	-0.11768395	0.058359567	-2.016532274	0.043744347	0.148006648
79.26381055	0.215777094	0.325049755	0.663827893	0.506800471	0.701658203
2948.102338	-0.039369968	0.101958948	-0.386135483	0.699396332	0.838718308
21.9674488	-1.064668938	0.39086202	-2.723899698	0.00645161	0.038149253
13.07120893	-0.317717521	0.417211465	-0.761526343	0.446342751	0.651404188
233.3233565	-0.277675894	0.098590244	-2.816464212	0.004855545	0.030741834
80.77394231	0.180974501	0.156064581	1.159612896	0.246206448	0.454811059
2.170694319	-1.529721874	1.115057008	-1.371877727	0.170101505	0.362141664
878.0651584	-0.317348326	0.082883852	-3.828831774	0.000128753	0.001903932
999.4174231	-0.284503815	0.139176551	-2.044193599	0.040934425	0.141166783
2578.893103	-0.138247791	0.122848489	-1.125351989	0.260439907	0.471512876
11.32450843	-1.126417296	0.542008386	-2.078228539	0.037688315	0.133347821
21.12026927	-0.210226261	0.246536276	-0.852719384	0.393814939	0.606781454
2073.001933	-0.105299434	0.080475761	-1.308461491	0.190716828	0.390665767
304.7638531	0.146220323	0.08584376	1.703330837	0.088506173	0.237390213
611.4461716	-0.075172081	0.113675402	-0.661287133	0.508428191	0.702907521
103.738112	-1.080985184	0.231750021	-4.664444822	3.09451E-06	8.91242E-05
351.3799365	0.039496263	0.070135404	0.563143021	0.573337499	0.752365182
2.562867277	0.223032614	0.583929744	0.38195111	0.702497628	0.840493334
380.0395395	-0.272575467	0.096731659	-2.817851659	0.004834614	0.030655386
152.5275775	-0.073231896	0.119152879	-0.614604502	0.538815941	0.726565753
153.3432184	-0.05219506	0.081262933	-0.642298504	0.520679386	0.712734217
860.4504728	0.213767635	0.109317656	1.955472178	0.050527364	0.163120846
45.20416409	0.320837451	0.14417297	2.225364793	0.026056759	0.10361068
5.807468497	-0.255768915	0.484126162	-0.52831046	0.597283873	0.769090541
11.33246127	0.203553107	0.383258444	0.531111865	0.595341263	0.767361863
5.196545033	0.445666176	0.48562949	0.917708222	0.358771642	0.573157878
95.49084697	-0.022618686	0.162209112	-0.139441525	0.889101261	0.944605583
1121.179458	-0.225368806	0.070646362	-3.190097805	0.001422247	0.01262397
18.83904591	0.232568769	0.242074455	0.960732388	0.336686742	0.551898248
106.7734459	0.036331907	0.118361015	0.306958392	0.758875042	0.874200334
26.79793945	-0.833342439	0.30592225	-2.724033439	0.006448998	0.0381475
3.508698462	0.194813006	0.552072747	0.352875608	0.724181698	0.853557222
116.5668765	-0.068259502	0.110455345	-0.617982791	0.536586683	0.724877003
66.14826458	-0.027401042	0.125903571	-0.217635144	0.8277134	0.913863048
33.82356853	-0.266208486	0.388178383	-0.685789055	0.492846139	0.690017157
13.67080729	-0.707408598	0.329965042	-2.143889526	0.03204174	0.118966784
460.1302531	-0.201271455	0.091600748	-2.197268679	0.028001263	0.108835837
3472.666935	-0.098420445	0.143446222	-0.686113889	0.492641293	0.690017157
2498.793633	0.236797261	0.091251856	2.594985691	0.009459486	0.05063725
18.0316766	1.082415773	0.739478538	1.463755494	0.143260767	0.323254058
102.7750358	-0.2528836	0.119287688	-2.119947198	0.034010498	0.123820763
2.700076975	0.65316585	0.598407878	1.091506102	0.275050248	0.487737632
212.0723733	0.173958238	0.180386714	0.964362807	0.334864063	0.550070918
2.781871711	0.091717621	0.819214963	0.111957941	0.910856755	0.95509224

103.8036188	-0.059168482	0.105068812	-0.563140298	0.573339353	0.752365182
95.31041854	0.028238661	0.117397925	0.240537989	0.809913217	0.903767699
9.196249913	-0.209380592	0.385879097	-0.542606723	0.587400609	0.761715809
1.95192054	-0.868858411	0.858280593	-1.012324429	0.311382958	0.526264641
3.736175569	-1.118106792	0.573379122	-1.950030529	0.05117248	0.164394587
1293.293568	-1.021932666	0.35837508	-2.851572903	0.004350351	0.028436719
115.4696797	-0.327556723	0.127681424	-2.565421903	0.010305042	0.053519942
294.483964	-0.207361586	0.058850827	-3.523511876	0.000425868	0.004930253
11.06068908	-1.092609089	0.46843116	-2.332485931	0.019675142	0.08507487
184.9103601	0.11765977	0.115517438	1.018545532	0.308418774	0.523533322
3.383777766	1.035177336	0.519782527	1.99155855	0.046419515	0.154380696
5.013236291	-0.08893802	0.546208855	-0.162827862	0.87065397	0.935813113
5.296527717	-1.136692011	0.459432714	-2.474120753	0.013356454	0.064830356
125.1624214	-0.157277052	0.095233855	-1.651482564	0.098640078	0.254414161
259.9344845	0.506565454	0.115247573	4.395454411	1.10541E-05	0.00025941
26.77709092	-0.014319221	0.34009186	-0.042103982	0.966415806	0.98333139
4.203883779	2.464451488	1.053375219	2.339576101	0.019305638	0.083761148
156.30255	-0.50992177	0.156831421	-3.251400571	0.001148379	0.010742988
76.99367647	-0.004497888	0.134004721	-0.033565148	0.973223915	0.986700152
401.9729598	-0.4980579	0.191881153	-2.595658269	0.009440991	0.050562707
19.47769428	-2.346931822	0.983714799	-2.385784807	0.01704272	0.076731832
64.85845716	-0.497162146	0.168927219	-2.943055295	0.003249904	0.023070625
21.70034283	-0.823761584	0.317511275	-2.594432539	0.009474721	0.050689476
2.408135507	-2.467280493	0.964752274	-2.557423868	0.010545064	0.05444042
50.01788087	0.16818228	0.136363729	1.233335879	0.217450477	0.422248697
424.4085689	-0.23815699	0.12631927	-1.88535755	0.059381581	0.182135946
446.8248233	0.017839263	0.063865402	0.279325936	0.779994704	0.885387773
26.25008985	0.046302689	0.295460848	0.15671345	0.875470687	0.937583765
621.8338842	0.231872179	0.084019125	2.759754738	0.005784477	0.03512476
81.8332429	-0.959115159	0.384836773	-2.492264839	0.012693135	0.062419914
98.55280043	-0.131807751	0.205545092	-0.64125954	0.521354072	0.713176698
31.11740404	0.736886153	0.393919667	1.870650832	0.061393494	0.186535673
194.9126613	0.031674846	0.111093667	0.285118372	0.775553455	0.883629572
1722.343625	-0.177951763	0.065864234	-2.701796605	0.006896594	0.039962886
25.76382205	0.269601598	0.221672324	1.21621677	0.223902357	0.429002957
272.3791496	-0.093664887	0.08914137	-1.050745419	0.29337553	0.508435503
351.8616607	-0.067674752	0.075200332	-0.899926245	0.368159502	0.581947768
188.1091028	-0.034786847	0.128345744	-0.271040128	0.786360169	0.889541352
240.1111883	0.035116923	0.116100736	0.302469426	0.762294237	0.875917923
4.092086918	-0.305507237	0.6082356	-0.502284373	0.6154675	0.782406945
376.5779583	-0.187698747	0.140573493	-1.335235705	0.181799214	0.378390431
447.5189511	-0.128825407	0.071739373	-1.795742036	0.072535553	0.20887095
15.60407506	-0.709667013	0.276385254	-2.567673211	0.010238362	0.053327979
1021.599052	-0.096619851	0.092011672	-1.050082545	0.293680163	0.508620235
180.6247061	-0.302146105	0.09836959	-3.071539748	0.002129578	0.016915463
329.3976731	-0.107974327	0.069192988	-1.560480776	0.11864631	0.287058203
274.8092206	0.049309731	0.130758054	0.377106645	0.706094353	0.842949182
1563.710625	0.051865271	0.117758955	0.440435897	0.65962143	0.812578183

118.5741458	-0.210352161	0.189322683	-1.111077432	0.266535021	0.478259224
344.8035815	0.002111961	0.109919268	0.019213746	0.984670592	0.992381215
1314.993755	-0.049075825	0.121164526	-0.405034596	0.685452081	0.829778815
443.9636819	-0.125220571	0.119619478	-1.046824255	0.295180641	0.509941893
517.4530197	-0.175740662	0.087182648	-2.015775685	0.043823437	0.148182944
2.028346925	0.906702862	0.742096354	1.221812851	0.221778408	0.426570635
134.6206565	-0.441464066	0.116967986	-3.774229873	0.000160503	0.002285326
759.8686327	-0.22048327	0.128261302	-1.719016306	0.085611403	0.232387914
34.93313391	-0.558748228	0.188296927	-2.967378375	0.003003511	0.021806892
64.86438984	-0.015797096	0.114215328	-0.138309769	0.889995605	0.945129092
81.46823919	0.198864013	0.116761494	1.703164344	0.088537317	0.237396446
6.157457464	0.526985827	0.405761907	1.298756287	0.194027581	0.394870964
254.7945642	-0.269479075	0.116528272	-2.312563897	0.020746628	0.08828296
11.67235795	0.003291296	0.4369214	0.007532925	0.993989653	0.996600225
498.1621299	-0.259210993	0.098860885	-2.621977274	0.008742126	0.047862341
314.0306714	-0.20094688	0.193904094	-1.036320971	0.300052426	0.514902465
406.0454468	-0.095801663	0.058229859	-1.645232607	0.099921852	0.256363898
318.8160067	0.017752321	0.098065533	0.181025085	0.856347887	0.92936531
94.09032771	-0.043196378	0.283869297	-0.152169956	0.87905289	0.939321353
14.59708053	0.05954957	0.366146549	0.162638622	0.870802976	0.935813113
905.7626292	-0.283191059	0.188179916	-1.50489523	0.132351015	0.30681677
83.88519659	0.052017685	0.140366394	0.370585038	0.710946628	0.84576363
15.5348901	-0.161692792	0.342797374	-0.471686204	0.637150786	0.797196954
1.902784657	0.85094369	0.738279967	1.15260298	0.24907338	0.458052563
168.0409363	-0.465278878	0.096162467	-4.838466502	1.30845E-06	4.36343E-05
9.494970611	0.871763839	0.689950045	1.26351733	0.206403323	0.409852144
940.8322645	-0.269425502	0.085877912	-3.137308474	0.001705066	0.014338572
2332.556473	-0.025021964	0.096986743	-0.25799365	0.796411808	0.894897219
62.18946497	-0.350356138	0.123676245	-2.832849077	0.004613516	0.029677105
2.871439012	-0.113820555	0.636171241	-0.178914964	0.858004473	0.929613001
4.03387621	-0.298436296	0.497935175	-0.599347688	0.548941054	0.735403344
9.995147353	-0.333493343	0.312409964	-1.067486254	0.285752317	0.500157637
53.99874647	-0.255346269	0.298563	-0.855250881	0.392412274	0.605583119
79.26214901	-0.271753219	0.175821086	-1.545623599	0.122195499	0.292127216
5.311990622	0.08030918	0.568159577	0.14134969	0.887593696	0.943734256
76.49633482	0.050044941	0.193669603	0.258403697	0.796095364	0.894773586
3.251478029	-0.168756694	0.823088704	-0.205028563	0.837549809	0.918811717
4.965460015	0.544435747	0.549174848	0.991370507	0.321504696	0.536568571
193.1164994	-0.188071962	0.078700254	-2.389724962	0.016860994	0.076159983
436.7698656	0.167281075	0.077457646	2.15964574	0.030800105	0.116083488
2266.373936	-0.323771123	0.1083668	-2.987733537	0.002810545	0.020800051
494.3656355	-0.065529201	0.080525874	-0.813765781	0.415779142	0.625357946
2040.078016	-0.125243902	0.151512028	-0.826626797	0.408448624	0.619157236
10.94389031	0.236781901	0.281522258	0.841077018	0.400304789	0.612232486
136.956335	0.559008251	0.112375026	4.974488305	6.54202E-07	2.41103E-05
752.1094753	0.337803369	0.116225538	2.906447013	0.003655588	0.025082115
1239.19637	0.224979252	0.232406356	0.968042592	0.333023099	0.548476551
117.0218161	-0.336727975	0.099390538	-3.387927884	0.000704228	0.007324147

1053.534162	-0.088831185	0.073684631	-1.205559192	0.227987502	0.433652708
28.24838725	-0.380249989	0.233455638	-1.628789064	0.103357681	0.262845698
593.0841867	-0.020877432	0.078135299	-0.267195913	0.789318323	0.891120481
1075.101433	-0.174585585	0.065512443	-2.664922506	0.007700607	0.043326432
12.41271835	0.420529419	0.295872947	1.421317572	0.155224457	0.340483215
100.2388918	0.082337668	0.289595411	0.284319658	0.776165423	0.883652841
635.7849845	-0.050732605	0.087414636	-0.5803674	0.561666884	0.744880072
1.862555384	-4.341245102	2.368604817	-1.832827946	0.066828162	0.19747572
186.2117202	-0.259186705	0.208353385	-1.243976455	0.213508229	0.418083271
2.811095896	0.308785455	0.641747984	0.481163109	0.630400578	0.793006957
1218.042776	-0.469453103	0.077684964	-6.043036875	1.5124E-09	1.20988E-07
454.9573644	0.309726428	0.059624185	5.194644234	2.05112E-07	8.55446E-06
17.3331315	-0.087627885	0.321652448	-0.272430338	0.785291148	0.888705528
15.65558922	0.163099203	0.25599729	0.637113006	0.524051238	0.715203387
80.13886522	-1.164691275	0.452608471	-2.573286513	0.010073777	0.052788937
235.417666	0.169767999	0.078381841	2.16590983	0.030318078	0.114765629
10.92671877	0.69959739	0.338910001	2.064257139	0.038993343	0.136269903
188.9519299	0.133813058	0.108501903	1.233278441	0.217471898	0.422248697
311.0001763	0.118880029	0.081937176	1.450868033	0.146816611	0.328456519
6.700772592	-0.346083871	0.443973424	-0.779514838	0.435676501	0.642209616
271.7566624	0.019775337	0.08399769	0.23542715	0.813877215	0.906237868
3.347117865	-0.92654333	0.52050649	-1.780080261	0.075062826	0.213356065
144.2546078	-0.118454978	0.218545679	-0.542014736	0.587808354	0.761885532
113.2208653	0.104808377	0.166662645	0.628865437	0.529437155	0.71927657
7.604316975	0.815013866	0.352935101	2.309245706	0.020929951	0.088872708
324.248722	0.113699606	0.086264396	1.318036316	0.187491491	0.386333311
969.8978475	-0.141901804	0.109027147	-1.301527263	0.193078039	0.393659358
240.7747742	-0.308110381	0.101932655	-3.022685731	0.002505423	0.019099646
344.3934299	-0.294284356	0.100280953	-2.934598723	0.003339793	0.023512713
727.4657416	-0.02286061	0.066077185	-0.345968277	0.729366556	0.857101832
4847.129955	0.092556991	0.054580821	1.695778651	0.089927807	0.239681393
49.91575692	0.069506896	0.225270398	0.308548733	0.757664825	0.8735899
22.03898164	0.416208076	0.235122356	1.770176526	0.076697739	0.216318874
106.5968268	-0.15255773	0.120111693	-1.270132215	0.204037538	0.407037346
1914.868331	-0.021397271	0.063276875	-0.338153086	0.735247827	0.860688745
3920.913688	0.035826924	0.092626275	0.386790083	0.69891162	0.838531063
681.8185509	-0.014440398	0.24532728	-0.058861771	0.953062208	0.977204631
102.0023368	-0.178230501	0.162460532	-1.097069543	0.272610993	0.484831426
175.3360061	0.055962808	0.084442264	0.662734573	0.507500563	0.702273354
6.840922791	0.689613233	0.388166762	1.776590116	0.075635703	0.214418586
561.0524521	-0.470777656	0.075692616	-6.219598136	4.9843E-10	4.43845E-08
10.92966199	-0.732176329	0.487433436	-1.502105261	0.133069926	0.30815209
107.483982	-0.731744105	0.296902269	-2.464595871	0.013716786	0.06601529
28.23609063	0.165077185	0.224722451	0.734582526	0.462593783	0.665049916
224.4846822	0.032396374	0.102650045	0.315600196	0.752305989	0.870873018
19.78166725	-0.1953538	0.263486514	-0.74141859	0.458439676	0.661672965
1305.918223	0.169073248	0.109444506	1.544830837	0.122387186	0.292415737
279.1953587	0.031369171	0.077470797	0.404916079	0.685539199	0.829778815

9.314036874	-0.647919419	0.603013686	-1.074468845	0.282612601	0.496296769
399.6066841	0.160686551	0.175865236	0.913691389	0.36087903	0.575074593
325.1326493	0.021961597	0.094490964	0.232420078	0.816211756	0.907795873
13.74637773	0.601132021	0.252925571	2.376715087	0.017467574	0.0781534
634.9524271	-0.023615855	0.105233353	-0.224414168	0.822435047	0.910976907
1528.851167	0.061338276	0.069967872	0.876663439	0.380669471	0.593610885
180.8359073	-0.132867138	0.087031779	-1.526650836	0.126847859	0.298144047
2302.426674	0.087448229	0.089657552	0.975358211	0.329382605	0.544968267
2.004829086	-1.617673174	0.982131213	-1.647104941	0.099536478	0.255829108
62.0740995	-1.555604394	0.537980977	-2.89156022	0.003833341	0.025881337
140.2409861	0.627106374	0.094109219	6.663601925	2.67197E-11	3.26059E-09
841.7168567	0.542885913	0.093942493	5.778917477	7.51828E-09	4.8955E-07
99.82676789	-0.122373258	0.126403474	-0.968116252	0.332986314	0.548476551
1184.979783	-0.10544616	0.068169602	-1.546820819	0.121906459	0.291793937
258.2288349	0.198609015	0.084184345	2.359215545	0.018313615	0.080517346
186.255767	0.015772829	0.112347741	0.140392931	0.888349543	0.944210191
17.71169099	-0.016020894	0.230311663	-0.069561801	0.944542442	0.973376216
2790.461558	-0.148679004	0.113405005	-1.311044457	0.189842744	0.389618708
11.82329459	-3.136784008	1.609607139	-1.948788579	0.051320678	0.164774285
2.455624593	-0.445374056	0.69983316	-0.636400333	0.524515524	0.715659163
5.743042988	1.100513606	0.649920301	1.693305479	0.090397336	0.240738071
37.23131276	-0.011801622	0.186792119	-0.063180513	0.949622762	0.976129077
8.267948399	-0.082103029	0.506721649	-0.162027868	0.871283908	0.935876058
16.07913353	-0.104666251	0.265661407	-0.393983652	0.693593109	0.835017244
6.385077619	-1.611479459	0.779742897	-2.066680523	0.038764268	0.13578621
21.18900684	-0.444348807	0.42222689	-1.052393435	0.292619079	0.507913467
187.7850813	0.050151352	0.092368766	0.542947084	0.587166236	0.761651699
2464.02727	-0.253580155	0.104157517	-2.434583337	0.014908946	0.069934505
212.675494	0.223660016	0.084935825	2.633282453	0.008456399	0.046717209
39.45875916	0.093883666	0.186791947	0.502610884	0.615237875	0.782406945
21.5569194	0.211181269	0.2419262	0.872916074	0.382708824	0.595461387
107.791452	0.082042412	0.148004598	0.554323402	0.579357538	0.757126454
749.2049108	0.095716553	0.052291793	1.830431645	0.067185419	0.198175935
143.5526248	-0.406020952	0.170403921	-2.38269723	0.017186323	0.077230628
51.9631534	-0.624632072	0.170359416	-3.666554434	0.000245841	0.00322194
3.585523012	-0.759917015	0.486151428	-1.563128217	0.11802244	0.285903145
2.892951312	-0.525917487	0.630557285	-0.834051877	0.404251751	0.614990985
1878.335613	0.20885005	0.05062486	4.125444468	3.70019E-05	0.00070308
831.3631187	-0.300400868	0.068142076	-4.408449001	1.04114E-05	0.000246432
17.76403427	0.366210219	0.227958017	1.606480981	0.10816827	0.270732921
164.8263386	0.300622965	0.142842762	2.104572613	0.035328528	0.12732491
455.4392694	-0.003312368	0.064731111	-0.051171193	0.959189106	0.980192379
702.6045956	-0.182602569	0.159554877	-1.144449938	0.252437088	0.461559222
322.8579524	-0.112733398	0.076225522	-1.478945565	0.139154864	0.317621792
286.6662098	-0.295445934	0.069123651	-4.274165639	1.91854E-05	0.000410737
2185.298536	-0.104085226	0.084824536	-1.22706509	0.219798128	0.42424757
104.3823526	0.013057707	0.135345818	0.096476619	0.923142042	0.962095401
10.04733245	0.139740467	0.354344613	0.394363176	0.693312926	0.834976033

23.02020472	-0.133982747	0.206424927	-0.649062828	0.516297768	0.709263628
79.71882611	0.07906575	0.136388074	0.579711606	0.562109116	0.745166619
5.261277389	0.055124687	0.43994607	0.125298738	0.900287052	0.950117161
4.477458441	0.332894059	0.555946995	0.598787406	0.549314662	0.735711529
116.0321019	0.182504302	0.371793766	0.490875099	0.62351479	0.788499474
4.295601185	-0.352789652	0.709911629	-0.496948688	0.619225236	0.785003197
121.8816267	-0.000982576	0.114220442	-0.008602457	0.993136317	0.996099098
179.1832336	-0.019402809	0.108701161	-0.178496792	0.858332841	0.929724191
193.9850674	0.210768522	0.323178337	0.652174041	0.514288904	0.707625107
2.727860177	1.689797216	0.803254367	2.103688801	0.035405602	0.127518996
2486.028398	-0.142788551	0.094263742	-1.51477703	0.129828879	0.302860515
5.585576971	0.335091069	0.495651113	0.676062375	0.499001044	0.695021779
1117.395292	-0.148816762	0.144765699	-1.02798358	0.303957552	0.518644466
19.22002584	0.190936472	0.349656214	0.546069152	0.585018403	0.7601225
176.2239126	-0.144288476	0.192940903	-0.747837672	0.454558076	0.658841258
299.651523	-0.26833026	0.135023578	-1.987284473	0.046890881	0.155332744
6.141069702	2.616797501	1.205962997	2.169882083	0.03001578	0.113961733
482.7755313	-0.087393609	0.096644805	-0.904276318	0.365848901	0.579264039
46.72779641	0.233857004	0.168063492	1.391480098	0.164079891	0.353617493
78.61442254	-0.039065377	0.132558996	-0.294701818	0.768221674	0.879172203
2.490491504	1.109176088	0.794434431	1.396183303	0.162659303	0.351983366
95.14719132	-0.177283931	0.186529993	-0.950431234	0.34189318	0.556292322
9.241782421	-0.515368106	0.322647745	-1.597308876	0.110196873	0.274187114
4461.447565	-0.175898417	0.114071474	-1.542001779	0.123073161	0.293153002
25.58530621	0.26432517	0.234632665	1.126548896	0.259933256	0.470927138
3.5246329	-0.059535184	0.519567028	-0.114586146	0.908773161	0.954122048
14.5128248	0.179373027	0.30694197	0.584387423	0.558959686	0.743154899
8.031071419	-0.185932317	0.458203662	-0.405785315	0.684900349	0.829696916
416.1460778	0.089571215	0.097485083	0.9188197	0.358189886	0.572617194
4.361084461	0.222289788	0.540296384	0.411421942	0.680763167	0.827468213
40.74956246	0.189597258	0.182105183	1.04114147	0.297809896	0.512549124
960.7574855	-0.061174504	0.072423928	-0.844672544	0.398293693	0.610712054
58.12945756	0.217356013	0.134810834	1.612303747	0.106895858	0.268363665
10.25778009	-2.189607573	0.967158124	-2.263960275	0.023576558	0.096257799
145.2599594	-0.107652665	0.301312872	-0.357278678	0.72088319	0.851889535
2.01864104	0.884872083	0.811538801	1.090363248	0.275553164	0.488166774
7.309661572	-0.731083439	0.559369954	-1.306976597	0.19122066	0.391440645
2.017019305	-1.896035083	1.232989272	-1.537754729	0.124108597	0.294728391
3111.657622	0.176365399	0.052928361	3.332153006	0.000861769	0.008578113
127.9783753	0.548008181	0.146249	3.747090116	0.000178898	0.002503715
26.21591308	0.504294379	0.196661719	2.564273226	0.010339212	0.05364667
45.19408616	-0.069302628	0.231772155	-0.29901188	0.764930981	0.877720432
15.25388656	-1.060831572	0.319092147	-3.324530486	0.000885675	0.008759061
638.7094765	-0.001635927	0.079691292	-0.020528299	0.983621937	0.991966796
1510.037759	-0.205213136	0.078113019	-2.627131039	0.008610817	0.047347995
5.992429897	0.47384052	0.578218278	0.819483813	0.412510433	0.622273872
126.457403	-1.068862449	0.296352952	-3.606721116	0.000310091	0.003823677
325.3865554	0.058933286	0.073825427	0.798278967	0.424708621	0.632265482

5894.764062	0.028972157	0.085453606	0.339039606	0.734579894	0.86021248
1.834348364	0.200813057	0.765389751	0.262367058	0.793038467	0.893098455
18.47119429	0.382093646	0.247305385	1.5450276	0.122339587	0.29234441
4.908459392	0.726742972	0.571743685	1.271099256	0.20369334	0.406646156
9.157435534	0.288972324	0.409870107	0.705033909	0.480789134	0.679756282
1242.652492	0.447517533	0.091428932	4.89470372	9.84539E-07	3.39316E-05
71.80872782	-0.368631475	0.154059795	-2.392781804	0.016721182	0.075718733
1436.691282	0.030419332	0.103925804	0.292702395	0.769749639	0.880036216
11.18931144	1.385127973	0.359725622	3.850512413	0.000117871	0.001773339
612.4374473	-0.323654403	0.183813127	-1.760779597	0.078275714	0.219044355
3.321591755	0.209820566	0.626049655	0.335150039	0.737511908	0.861788314
134.5347574	-0.053478007	0.101730515	-0.525683044	0.599108447	0.770050485
362.8055689	0.144656375	0.087284744	1.65729276	0.0974603	0.252169152
110.0014975	0.075973717	0.183122202	0.414879881	0.678229838	0.82617318
17.79374242	-0.174829068	0.300853703	-0.581109911	0.56116638	0.74475811
8.6948534	-0.492091272	0.509894232	-0.965084994	0.334502242	0.549904195
67.12164793	0.158485921	0.16471594	0.962177196	0.335960602	0.551091801
31.6368073	-0.523036547	0.289432621	-1.807109874	0.070745149	0.205366624
4.253262881	-1.177933279	0.591827522	-1.990332039	0.046554371	0.154734397
106.6682353	0.344210414	0.136300675	2.525375715	0.011557465	0.058404198
437.3848985	0.006858375	0.089097528	0.076976043	0.938642603	0.970696688
872.7752228	-0.212466117	0.078310806	-2.713113641	0.006665424	0.039049147
105.3653148	0.138721302	0.135225813	1.025849278	0.304962634	0.51975527
115.5391402	-0.008239845	0.116785622	-0.070555306	0.943751682	0.972804731
73.67557753	0.269445029	0.148791688	1.810887644	0.070158246	0.203878451
27.46519417	0.490414064	0.297758538	1.647019316	0.099554076	0.255829108
568.2698442	0.151581754	0.053300822	2.843891522	0.004456622	0.028939057
660.2346757	0.389483696	0.058915536	6.610882646	3.82035E-11	4.49546E-09
29.26103666	-0.513919243	0.277965318	-1.848861024	0.064477883	0.193023559
24.45120129	0.059302745	0.366567582	0.161778477	0.871480303	0.935937769
241.3083827	-0.155351024	0.072019267	-2.157075892	0.030999752	0.11645423
3.198362802	-0.919934226	0.606060733	-1.517891155	0.12904184	0.301579695
1897.334294	-0.062134604	0.068285264	-0.909926977	0.362861022	0.576876249
3.346082059	1.02609487	0.740306249	1.386041076	0.165734361	0.356244671
4.534766063	1.314733769	0.500858866	2.624958565	0.008665951	0.047524261
3.605322947	0.303056666	0.506086257	0.598824136	0.549290165	0.735711529
75.65484974	0.577012319	0.165646925	3.483386848	0.000495112	0.005533569
12.32626649	-0.384964413	0.273075831	-1.409734474	0.158618101	0.345782004
1.771572046	-2.206498201	1.355476127	-1.627839958	0.103558825	0.263161657
5.926688502	-1.775022847	0.998795869	-1.777162784	0.075541461	0.214268255
1.909025386	1.064777668	1.663287871	0.640164392	0.52206573	0.713528896
1294.850137	-0.085403711	0.048288175	-1.768625768	0.076956348	0.216714337
141.8427348	0.152830612	0.137835525	1.108789711	0.26752091	0.479134688
32.84534381	-0.383234296	0.220789803	-1.735742731	0.082609334	0.22727224
15.91140746	-0.296894844	0.230502631	-1.288032345	0.197734704	0.399152251
117.2192473	-0.208478517	0.148715619	-1.401860267	0.160956978	0.349393008
52.83934501	0.083164131	0.168660696	0.493085425	0.621952229	0.787067216
12.75896333	-0.165547341	0.301489754	-0.549097737	0.582938378	0.758985106

61.54005011	-0.089455225	0.206252347	-0.43371737	0.664493692	0.816747674
40.95468587	0.060194011	0.167084999	0.36025982	0.718652844	0.850692459
40.81527767	0.087274915	0.196845432	0.443367744	0.657499763	0.811359633
22.27444707	-0.378498386	0.279043687	-1.356412647	0.174967912	0.369068038
55.23860186	-0.105563154	0.151744428	-0.695664123	0.486639199	0.685332768
96.20930404	-0.049821855	0.130680813	-0.381248431	0.703018915	0.84076469
11.48709519	-0.279189214	0.314144642	-0.888728238	0.374149151	0.587693851
11.68332176	0.488575831	0.316968301	1.541402813	0.12321878	0.293381439
108.3732694	0.024727745	0.137549299	0.179773691	0.857330239	0.92936531
173.4802782	0.04348093	0.215021126	0.202217012	0.839747074	0.920302907
8.905919573	-0.401476383	0.379628408	-1.057550949	0.290260215	0.505309816
72.36226572	-0.138813034	0.242171014	-0.573202516	0.566507581	0.748319581
80.10479224	-0.015011108	0.189361572	-0.079272196	0.936816121	0.970208636
89.60203469	-0.220484221	0.209554088	-1.052159007	0.292726604	0.507915315
72.32685234	0.037373846	0.167594192	0.223002034	0.823533923	0.91138289
25.58621572	0.02884158	0.216565948	0.133176892	0.894053485	0.946388043
179.7793536	0.117571175	0.106970306	1.099101037	0.271723998	0.483723918
38.89290683	-0.207888763	0.166080084	-1.251738061	0.210665326	0.414982732
6.155797369	0.072622884	0.498199015	0.14577083	0.884102304	0.942262895
37.07278376	-0.159217651	0.160707675	-0.99072836	0.321818239	0.536983052
33.34155536	0.437938058	0.178426146	2.45445002	0.014110028	0.067376405
11.28860744	-0.119857648	0.88118221	-0.136019141	0.891806149	0.945649749
2.901212842	-1.362136757	0.906255278	-1.503038702	0.132829064	0.307680822
59.49697341	-0.342930647	0.140185752	-2.446258918	0.01443473	0.068352325
363.2124843	-0.085440926	0.058619553	-1.45754995	0.144964634	0.325798278
103.6246068	-0.234459638	0.169063286	-1.386815807	0.165497936	0.355975061
15.42438722	-0.536541039	0.478937772	-1.120272968	0.262597458	0.473929724
92.56214748	-0.107243461	0.260331647	-0.411949381	0.680376525	0.827258313
268.5854397	0.219327674	0.096270841	2.27823576	0.022712535	0.093951176
462.4232022	0.259088912	0.088111038	2.940481911	0.003277021	0.023189712
17.98489153	0.884141034	0.324750686	2.722522456	0.006478563	0.038240003
5950.198673	0.177941195	0.102535839	1.735404885	0.082669116	0.227322819
984.6145329	0.599538955	0.099712901	6.012651808	1.82513E-09	1.41826E-07
4.552717509	0.513595998	0.634152033	0.809894113	0.418001036	0.627268087
2.980710558	-0.041784387	0.550926229	-0.075843888	0.939543299	0.971262319
101.2578824	0.168735817	0.096990351	1.73971756	0.081908624	0.225948204
1211.609316	-0.199112752	0.05635492	-3.533192021	0.000410574	0.004800425
95.82890307	0.204299238	0.109452184	1.866561556	0.06196284	0.187560583
280.5983785	-0.031632498	0.086455384	-0.365882333	0.71445288	0.84797527
29.3905177	0.035773556	0.252308492	0.141784987	0.887249843	0.943568846
35.89832715	-0.198854017	0.166457276	-1.194624958	0.232233594	0.438941742
20.610534	-1.444366177	0.551902588	-2.61706723	0.008868887	0.048331474
556.1078611	0.042786853	0.094148804	0.454459864	0.649497874	0.805709937
48.53780204	0.000893649	0.18402207	0.004856208	0.996125322	0.997981838
245.0128191	0.253541988	0.079783348	3.177881035	0.001483556	0.013041677
996.4146658	-0.298222099	0.119417654	-2.497303271	0.012514188	0.061815587
23.11258905	-0.239685162	0.243189651	-0.985589482	0.324334601	0.539548668
250.699723	0.056462468	0.079541364	0.709850383	0.477796921	0.6774444

696.0696268	0.018739742	0.095592583	0.196037612	0.844580727	0.922898839
132.0402311	-0.215950415	0.102385507	-2.109189285	0.034928246	0.126197989
12.13003024	1.107603721	0.326766494	3.389587804	0.000699978	0.007294501
290.5716582	0.012071454	0.065297444	0.184868714	0.853332011	0.927600894
5.449653876	-0.38710824	0.425022274	-0.910795185	0.3624033	0.576388489
3.770143891	0.070381672	0.565834513	0.124385611	0.901009966	0.950575612
1918.099437	0.13758357	0.071753159	1.917456615	0.055179948	0.172459582
1674.751696	0.005178639	0.056383788	0.09184625	0.926820197	0.964983874
33.08652684	0.347679696	0.1884335	1.84510555	0.065022186	0.193948124
397.5400405	0.542824075	0.124589987	4.356883639	1.31927E-05	0.000301021
606.8252637	0.106405811	0.087436082	1.216955389	0.22362119	0.428713543
425.6329791	0.023464233	0.071686843	0.327315757	0.743429076	0.865441878
18.76415873	-0.279573368	0.277142855	-1.008769893	0.313085003	0.527864327
232.3022655	-0.042999805	0.1000645	-0.429720882	0.667398691	0.818669102
27.43521829	-0.478834383	0.275724935	-1.73663794	0.082451098	0.226950609
848.3121237	0.331076374	0.614598614	0.538687146	0.590102744	0.763536963
9.663714379	-1.023876743	0.351813123	-2.910285819	0.003610984	0.024900521
1419.699855	0.345909109	0.085138599	4.062894083	4.8468E-05	0.000869785
6176.156052	0.191410827	0.077631604	2.465630197	0.013677246	0.065863475
573.2484926	-0.052695561	0.129837195	-0.405858742	0.684846395	0.829696916
4.087011424	0.031421084	0.579683526	0.054203858	0.956772747	0.978702547
307.9403264	0.361814728	0.157595672	2.295841777	0.02168493	0.090946471
392.2575932	-0.095233772	0.061329428	-1.552823415	0.120465327	0.289953536
47.52644117	-0.740280461	0.309825203	-2.38934875	0.016878272	0.076178809
147.3489191	-0.057709996	0.086348431	-0.668338678	0.50391743	0.69943009
329.5512784	0.27007934	0.076046456	3.551504616	0.000383035	0.004541897
101.896437	0.005968905	0.110666722	0.053935857	0.956986268	0.978754462
487.4860167	0.089611623	0.070924772	1.263474249	0.206418795	0.409852144
94.26369327	-0.441050428	0.120041854	-3.674138751	0.000238653	0.003142742
587.2329065	0.270714049	0.069846662	3.875833723	0.00010626	0.001643691
114.5146922	-0.043060796	0.142809364	-0.301526418	0.763013109	0.876496418
437.8023126	0.212055594	0.049899552	4.249649242	2.14106E-05	0.000452202
173.3141711	-0.097005918	0.111791755	-0.86773768	0.385537953	0.5987323
177.7709918	0.540256873	0.133772888	4.038612619	5.37683E-05	0.000954503
122.249863	0.210393192	0.13361863	1.574579771	0.115353475	0.282048078
3.033978336	-0.742330183	0.723312454	-1.026292551	0.304753708	0.519506682
302.7815438	0.026486954	0.088068205	0.300755006	0.76360132	0.876869382
2.407209834	0.003055345	0.812580401	0.003760053	0.996999919	0.998110584
285.9411181	-0.169001104	0.086917481	-1.944385659	0.051848962	0.165824073
135.0310148	0.404639191	0.127006904	3.185962169	0.001442735	0.012757708
128.2767917	-0.325433939	0.105865897	-3.074020525	0.002111949	0.016840391
109.4457535	-0.123586498	0.091886117	-1.344996419	0.178626391	0.374375386
24.01923732	0.711356505	0.255717023	2.7818113	0.005405646	0.033428158
397.6426321	0.155469861	0.055768691	2.78776241	0.005307345	0.032989168
2.154021644	-1.847862419	1.320633554	-1.399224193	0.161745764	0.350743677
13.1236025	0.477830495	0.363524853	1.314436941	0.188699206	0.388013315
182.004515	0.03622801	0.167339043	0.216494667	0.828602181	0.914385171
3.507267952	-0.408274241	0.517988152	-0.788192239	0.430584268	0.637498448

54.78927343	0.91512553	0.157914243	5.795079093	6.8289E-09	4.55463E-07
13.74869875	-0.031199624	0.276592619	-0.112799914	0.910189186	0.955000105
6.204055415	0.092857503	0.435166782	0.213383712	0.831027684	0.915614595
184.4139026	0.007821328	0.079718251	0.098112141	0.921843247	0.961410841
16.91457296	-0.335435279	0.419159472	-0.800256946	0.423561943	0.631300049
56.16067588	-0.174188537	0.141143106	-1.234127135	0.217155533	0.422114485
16.90969123	-0.792296194	0.328185664	-2.414170641	0.015771074	0.072756281
389.3082598	-0.102574516	0.078187665	-1.311901502	0.189553371	0.389218774
91.14721273	0.066304218	0.113978347	0.581726439	0.56075096	0.744504458
11.58295	0.255729664	0.305135658	0.83808515	0.401982882	0.613627317
317.4018996	-0.259651208	0.071509359	-3.631010149	0.000282314	0.003588613
333.9016895	-0.280570472	0.10982203	-2.55477404	0.010625675	0.054753635
80.16720212	0.736891491	0.232316413	3.171930389	0.001514293	0.013241222
246.8499787	-0.029114957	0.085608568	-0.340093957	0.733785772	0.859681792
84.59319579	0.24285431	0.139930765	1.735531925	0.082646632	0.227322819
422.7715581	0.129525806	0.087602524	1.478562495	0.139257283	0.317745772
601.1496888	0.352412834	0.121889758	2.891242376	0.003837221	0.025896916
1514.316152	0.161076707	0.086084764	1.871140724	0.061325578	0.186398077
56.03041669	0.01073251	0.273548075	0.039234455	0.968703464	0.984297839
458.4182059	0.169164271	0.117956795	1.434120609	0.151537848	0.335677626
175.7438229	-0.054762859	0.094351543	-0.580412971	0.56163616	0.744880072
27.19798057	0.595488431	0.183791263	3.240025781	0.001195189	0.011067759
143.5961622	0.277532823	0.086330578	3.21476849	0.001305498	0.011803934
14.74339059	0.159474277	0.315306402	0.505775576	0.613014207	0.780915252
383.1832419	-0.224896404	0.132225805	-1.700851089	0.088970953	0.238171511
137.0186734	0.032270995	0.109413754	0.29494459	0.768036209	0.879106343
1918.936547	-0.0298359	0.062554597	-0.476957746	0.633392211	0.794464998
87.46578056	-0.371907306	0.153768398	-2.418619883	0.015579511	0.07213515
25.60437047	0.315703764	0.19128612	1.650426932	0.098855646	0.254699393
38.97575975	-0.40993064	0.342828169	-1.19573208	0.231801128	0.438426153
435.3356136	-0.215431822	0.088167859	-2.443428079	0.014548469	0.068707166
377.5348067	0.143904903	0.064130858	2.24392605	0.02483716	0.100199531
3.680950554	0.063030492	0.644430824	0.097808003	0.922084752	0.961540968
6.628160931	-0.527915872	0.425542733	-1.24057076	0.214764359	0.419252375
13.54319898	-0.061828057	0.352622046	-0.175338036	0.860814026	0.931184922
821.1194125	-0.07233202	0.054130491	-1.336252801	0.181466658	0.377937007
350.038761	0.114883862	0.062982467	1.824061013	0.068142849	0.200258322
142.8598881	0.897066231	0.158184847	5.670999775	1.41967E-08	8.29347E-07
1194.789967	0.111679736	0.12897942	0.865872526	0.386560071	0.599584842
582.2340763	-0.066887389	0.09875006	-0.677340238	0.498190107	0.694742406
153.2756985	-0.263651571	0.169607344	-1.554482048	0.120069476	0.289331805
797.1582585	-0.073883402	0.070316282	-1.050729642	0.293382778	0.508435503
154.2996298	0.079534392	0.086341027	0.921165687	0.35696393	0.571544735
12.71867008	-0.939104514	0.470757336	-1.994880255	0.046055942	0.153677806
261.460898	0.302215185	0.099629085	3.0334032	0.002418123	0.018550012
377.8673205	0.143115444	0.076093105	1.880793847	0.059999967	0.183641963
69.31894345	0.17675073	0.166545332	1.061277001	0.288564035	0.503240686
82.65633595	0.338047728	0.170606335	1.981448863	0.047540959	0.156667869

180.671568	0.231674019	0.122851626	1.885803437	0.059321448	0.182056232
198.1005159	0.137076547	0.093992529	1.458377059	0.144736641	0.325475608
284.4770241	0.595901667	0.190481345	3.128399095	0.001757613	0.01467993
876.8256904	-0.141384937	0.07827308	-1.806303476	0.070870949	0.205478356
221.1023282	-0.079862839	0.161793091	-0.493610938	0.621580975	0.786839171
398.0359927	0.127451562	0.073932203	1.723897801	0.084726294	0.230860234
3.925654084	-0.054746844	0.526223664	-0.104037215	0.917139816	0.95910745
3.620424681	1.2732063	1.032570587	1.233045291	0.217558867	0.422273513
9.324348456	-0.068866815	0.301787449	-0.228196419	0.819493545	0.909419782
28.70020511	0.017091954	0.196627896	0.086925375	0.93073083	0.967067782
1510.821144	0.353928765	0.055693419	6.354947654	2.08498E-10	2.14674E-08
787.2055733	-0.203746081	0.086508812	-2.355206091	0.018512435	0.081174836
13.08659268	1.481246128	0.702918746	2.107279305	0.035093376	0.126643654
4.111773943	0.100997802	0.554495008	0.182143753	0.855469913	0.929127858
4736.13242	0.072303517	0.06885335	1.050108917	0.29366804	0.508620235
210.3642138	0.038511107	0.082500315	0.466799517	0.640643322	0.79966344
1023.219755	-0.285541386	0.067796366	-4.211750601	2.53399E-05	0.000519216
109.4896503	0.01484605	0.095464063	0.15551454	0.876415692	0.93778073
416.4555493	0.050382925	0.093223952	0.540450438	0.588886432	0.76241798
3.040017325	0.440380225	0.642071238	0.685874399	0.492792315	0.690017157
15.84288572	0.65863439	0.296364428	2.222380042	0.026257633	0.104233312
228.442407	0.114306918	0.089764647	1.273406869	0.202873701	0.405698149
40.44315637	0.48195127	0.350637155	1.374501427	0.169286068	0.361245943
366.0206655	-0.031704642	0.082291891	-0.385270546	0.700036982	0.839012442
11.29768402	0.214867363	0.302856648	0.709468868	0.478033564	0.677604968
137.0674717	0.261061286	0.096976713	2.69199974	0.0071025	0.040768845
1417.19603	-0.016983804	0.063332904	-0.268167136	0.78857067	0.890703684
106.9496859	0.258226462	0.143724829	1.7966726	0.072387611	0.208663781
472.5894586	0.202166063	0.183489224	1.101787117	0.270554233	0.482423469
264.5939188	0.00582007	0.081800041	0.071149964	0.943278403	0.972629837
3.269702496	0.908162895	0.552531662	1.643639554	0.100250678	0.256927454
1891.889609	-0.196540505	0.084691308	-2.320669143	0.020304707	0.086928209
5.626934085	0.414291619	0.595324153	0.695909308	0.486485628	0.68522266
331.7146886	0.032076933	0.120905541	0.265305729	0.790773951	0.892054639
388.1201013	0.032547013	0.068157579	0.477525959	0.632987641	0.794225792
438.1555393	0.093092271	0.074646351	1.247110816	0.212356868	0.416869285
544.5383632	-0.026086875	0.072881033	-0.357937773	0.720389882	0.851641987
7.370404057	0.023972747	0.381343319	0.062863948	0.949874843	0.976186239
159.697427	0.539264641	0.139952921	3.853186035	0.000116591	0.001763743
18.17558748	-1.269613531	0.655861469	-1.935795273	0.05289278	0.167988367
53.6227584	-0.104519034	0.14592881	-0.716233033	0.473847469	0.673932765
718.8962999	-0.019015962	0.060813484	-0.312693194	0.754513767	0.871752087
576.8394411	-0.433605371	0.134558113	-3.222439444	0.00127104	0.011594196
977.8723907	0.169332773	0.075594786	2.240005993	0.025090534	0.100938084
40.45341761	0.052870296	0.368978079	0.143288448	0.886062379	0.943111133
194.2509099	-0.019438445	0.204130192	-0.095225723	0.924135539	0.962765374
228.1561446	0.138999055	0.083560243	1.663459196	0.096220552	0.249828924
8.240532018	0.399165826	0.387901491	1.029039162	0.303461273	0.518343743

47.67573558	-0.180807141	0.141572564	-1.27713404	0.201554929	0.404321609
8.09780952	0.23603376	0.329493328	0.716353687	0.473772984	0.673885006
47.14357867	0.465825674	0.143981204	3.235322821	0.001215053	0.011218172
87.16991815	0.207055006	0.120594097	1.716958059	0.085986836	0.232886268
263.6414908	-0.172451125	0.187941605	-0.91757823	0.35883972	0.573211048
1829.265906	-0.080452004	0.063077753	-1.275441825	0.202152903	0.404987157
256.7891339	-0.146490163	0.069376684	-2.111518651	0.034727756	0.125719761
12.89249796	-0.267430289	0.324687679	-0.82365395	0.410136212	0.620268425
779.7205131	-0.177020504	0.077299195	-2.290069181	0.022017307	0.091931577
50.95972183	1.135685833	0.788023191	1.441183262	0.149532916	0.332560838
103.4687674	0.436976347	0.317325549	1.377060081	0.168493673	0.35988134
6.065440136	1.759349156	1.135038757	1.550034433	0.121133252	0.290981217
9.0283566	-1.113531417	0.378982743	-2.938211401	0.003301118	0.023310169
24.40757267	0.202448942	0.352900277	0.573671814	0.566189905	0.748218036
34.21002661	0.627800882	0.181660056	3.455910424	0.000548438	0.006015286
119.9638349	-0.090026576	0.08950974	-1.005774077	0.31452426	0.52931583
1068.754787	0.11595974	0.052767208	2.197572034	0.027979618	0.108814029
174.9526729	-0.092847421	0.108832111	-0.853125239	0.393589856	0.606604855
11.1418116	0.217676481	0.374117659	0.581839633	0.560674705	0.744504458
188.9607873	0.247344431	0.080734812	3.063665174	0.002186435	0.017250635
359.3967271	-0.04790824	0.176864447	-0.270875471	0.786486811	0.889541352
1.831712119	1.109455557	0.743199798	1.492809282	0.135487117	0.311917533
161.1303446	0.110517613	0.080530972	1.372361599	0.169950898	0.362008159
1109.416394	0.759937878	0.130383751	5.828470764	5.59376E-09	3.91426E-07
8.936712588	-0.140003556	0.351008212	-0.398861198	0.689995481	0.83271258
832.5352515	0.137064587	0.056012934	2.447016746	0.014404415	0.068287288
15.33703935	0.209603095	0.247844252	0.845704886	0.397717397	0.610346778
714.1647635	0.227499147	0.056109152	4.054581772	5.02241E-05	0.000896415
442.7135418	0.129267231	0.075216452	1.718603157	0.085686656	0.232502954
361.2597321	0.144653668	0.062097862	2.329446843	0.019835406	0.08547436
21.72961988	0.678916457	0.66709844	1.017715552	0.308813154	0.523980279
3.402304594	1.677549241	0.993254314	1.688942316	0.091230485	0.241806726
55.17117983	-1.366297495	0.277657499	-4.92080171	8.61904E-07	3.06013E-05
53.05026342	0.108960809	0.142323294	0.765586617	0.44392231	0.649194509
42.74166813	-0.033676455	0.158524621	-0.212436745	0.831766321	0.915943742
1085.944272	-0.096365996	0.057148136	-1.686249153	0.09174782	0.242536042
3.07639256	-0.986152764	0.583771925	-1.689277476	0.091166268	0.241797752
197.556967	-0.117009903	0.119388088	-0.980080222	0.327046521	0.542173659
182.2907239	0.041652902	0.134116934	0.310571538	0.756126367	0.87266539
3.195602764	-1.915594481	0.737038296	-2.599043347	0.009348397	0.050164654
112.2805422	0.079181216	0.149172629	0.530802577	0.595555594	0.767505587
26.14466208	0.069823862	0.194230101	0.359490428	0.719228237	0.851006678
70.93769393	-0.101675356	0.161660403	-0.628944095	0.529385657	0.71927657
75.37939751	-0.099611232	0.14529527	-0.685577945	0.492979293	0.690017157
118.8435193	-0.152336162	0.136507444	-1.115954981	0.264441405	0.475746174
9.159073996	-0.553612813	0.388061725	-1.426610196	0.153692284	0.338357167
6.267744455	0.476890732	0.543738004	0.877059776	0.380454171	0.593354351
4.307118696	-1.615965353	0.642219423	-2.516219992	0.011862111	0.059439517

8.082816393	-0.49510065	0.422664655	-1.171379353	0.241446328	0.449038926
160.9630609	0.0536185	0.149116698	0.359574082	0.719165669	0.851006678
136.9429085	0.030889314	0.151776117	0.203518934	0.838729448	0.919615902
66.182307	0.026226487	0.142764124	0.183705024	0.854244867	0.928426219
54.77946585	-0.36108829	0.169937656	-2.124828004	0.033600967	0.122944605
258.4407117	0.134340556	0.130192674	1.031859563	0.302137911	0.517049928
85.93595841	-0.952026294	0.337741815	-2.81879901	0.004820369	0.030589661
76.18033212	-0.156784462	0.138028983	-1.135880726	0.256006486	0.466222733
75.48714628	0.512557142	0.172484434	2.9716139	0.00296239	0.021593986
933.8674302	0.035482483	0.092295848	0.384442899	0.70065021	0.83939434
812.4475896	-0.197031561	0.064891989	-3.036300208	0.002395008	0.018459493
672.3534838	0.012771576	0.062235572	0.205213442	0.837405368	0.91871444
301.0262586	-1.075332055	0.548921664	-1.958990007	0.050113957	0.162387358
304.187659	0.710818707	0.124469183	5.710800757	1.12446E-08	6.86086E-07
975.9311211	0.125244174	0.065244157	1.919622846	0.054905556	0.171830192
858.8574584	0.034827542	0.082021942	0.424612505	0.671119176	0.821214982
108.07995	-0.269795243	0.155646605	-1.733383411	0.083027544	0.228033305
13.38437852	-1.232950903	0.567669465	-2.17195213	0.029859274	0.113629401
18.40624744	0.437986965	0.217173455	2.016761046	0.043720456	0.147986602
202.2459684	-0.032507421	0.100430851	-0.323679631	0.746180594	0.866956703
301.0074539	0.329958341	0.079843605	4.132558141	3.58748E-05	0.000688813
834.9103575	0.015671251	0.152520691	0.102748364	0.918162688	0.959333553
396.223137	0.290190602	0.080016119	3.626651789	0.00028712	0.003621758
253.5344151	0.235012247	0.138882831	1.692161988	0.090615092	0.24095254
152.8996885	-0.161255809	0.083609237	-1.928684129	0.053770088	0.169727615
28.38901778	-0.144851022	0.182905072	-0.791946445	0.428391904	0.635370474
495.1837303	-0.502755188	0.094314336	-5.330633788	9.78706E-08	4.39324E-06
594.5511665	0.071255593	0.06478375	1.099899168	0.271376057	0.483320913
9.33370382	0.104723945	0.6671046	0.156982796	0.875258407	0.937516709
486.4753927	-0.333904	0.416317221	-0.802042249	0.42252852	0.630787026
166.1172057	0.092898774	0.079060609	1.175032354	0.239981772	0.447384729
492.5745449	0.111715055	0.087903159	1.270887828	0.203768557	0.406697748
560.6615487	0.230298915	0.054559394	4.221068024	2.43148E-05	0.000503595
470.1436718	0.178798659	0.073254994	2.440770902	0.014655949	0.069097227
231.2493385	-0.319514211	0.441431068	-0.723814507	0.469179622	0.670434608
3.002895245	2.164250169	1.118781818	1.934470274	0.053055334	0.168439692
1742.616607	0.150325543	0.055156934	2.725415147	0.006422068	0.03800185
48.1506842	0.254900503	0.268198338	0.950417906	0.341899949	0.556292322
19.21832258	0.745430151	0.538811656	1.383470723	0.166520578	0.357428014
10.37018266	0.129039537	0.436084787	0.295904698	0.767302863	0.878792225
51.95767357	0.494325558	0.331052471	1.493193986	0.135386416	0.311850645
125.5771255	0.869988321	0.405907464	2.143316887	0.032087662	0.119018576
115.8975295	0.210273301	0.338677845	0.620865237	0.534688295	0.723766845
241.2778266	0.382957858	0.21396642	1.789803554	0.073485498	0.21068571
4.74060959	-0.356844791	0.549367279	-0.649555962	0.515979087	0.708985891
128.7619337	0.715678648	0.430908847	1.660858562	0.096741856	0.250675642
204.9988213	0.587580551	0.476643295	1.232746913	0.217670203	0.422367364
216.3112236	0.694663699	0.511796003	1.357305833	0.174684055	0.368676627

2.539073677	3.075580081	1.991591634	1.544282487	0.122519913	0.292501519
22.66439245	-1.378176567	1.72310309	-0.799822469	0.423813663	0.631486019
4.996640946	-0.842529991	0.584451055	-1.441574935	0.149422324	0.332474776
4.50279405	0.843763392	0.659327187	1.27973396	0.200638717	0.403235601
408.709505	0.924912305	0.140302762	6.592260154	4.33181E-11	5.06115E-09
38.4143126	0.019996234	0.306450023	0.065251208	0.94797399	0.975143522
205.1093523	0.304130524	0.086142523	3.530550471	0.000414696	0.004838314
8.496049831	1.837514345	0.506292354	3.629354327	0.000284131	0.003602977
149.82184	0.04818716	0.097514608	0.49415325	0.621197953	0.786596086
849.7398672	0.035053953	0.084858745	0.413085926	0.679543661	0.826777746
17.57146466	0.604905525	0.292244396	2.069861842	0.038465284	0.135227719
373.957074	-0.063993948	0.055098873	-1.161438416	0.245463654	0.453795111
8.127948625	0.290217288	0.478241927	0.606842	0.543955772	0.730983554
40.97289485	0.15508239	0.176338783	0.879457076	0.379153497	0.592190921
172.115148	-0.002858428	0.127991542	-0.022332946	0.982182368	0.990904056
3788.81985	-0.083312941	0.121251856	-0.687106522	0.492015605	0.689578541
1252.709211	-1.013194417	0.445030703	-2.276684308	0.022805084	0.094217024
13.75908564	0.321770533	0.291203186	1.104969136	0.269172963	0.480897451
32.30810949	-0.001361541	0.251695977	-0.005409465	0.995683893	0.997743093
51.76505755	0.228611327	0.144683939	1.580073984	0.114089924	0.280232207
80.94274358	0.022651976	0.142085379	0.159425099	0.873333965	0.936680519
637.1570392	0.045378262	0.059196996	0.766563606	0.443341021	0.648717254
97.80413393	-0.100407587	0.123782984	-0.811158233	0.417274808	0.626547651
369.6333061	-0.916621538	0.402408403	-2.277838961	0.022736174	0.093991401
128.6950314	-0.543682881	0.196808098	-2.762502595	0.005736011	0.034933471
80.15566502	-0.071375002	0.159077804	-0.448679832	0.653662636	0.80858327
841.4656257	0.0425312	0.074430842	0.57141903	0.567715633	0.748773548
207.9830146	-0.148996398	0.090482844	-1.646681191	0.099623592	0.255837733
50.32861921	-0.345932612	0.176321422	-1.961943181	0.0497691	0.161651449
48.34398467	0.148010183	0.241285721	0.613422884	0.539596758	0.727261474
37.40159338	-0.26087333	0.225860667	-1.155018859	0.248082704	0.456791602
136.3456309	-0.666054966	0.307684809	-2.164731393	0.030408262	0.115080565
57.96604038	-0.011820122	0.119217283	-0.099147724	0.921020981	0.961050759
27.04978328	-0.263954638	0.218881245	-1.205926242	0.227845931	0.433500443
906.1354944	0.031891652	0.061546694	0.518170025	0.604339647	0.774536435
159.4428716	-0.084117121	0.174576468	-0.481835396	0.629922883	0.792701622
4.528278797	1.156705538	0.486470388	2.37775118	0.017418575	0.078018926
25.08062989	0.532969578	0.375604113	1.418966298	0.155908842	0.341351937
129.5041363	0.000556616	0.17463025	0.003187399	0.997456828	0.998244565
109.3162886	0.134766454	0.141566697	0.951964387	0.341115043	0.555580386
3.585963091	-0.874915109	0.603454745	-1.449843783	0.147102087	0.328814082
815.6313786	-0.013289366	0.060448029	-0.219847805	0.825989687	0.913000141
3.436638376	0.461000655	0.52637189	0.875807892	0.381134479	0.593923904
728.6305671	-0.027796382	0.064775791	-0.429116831	0.667838204	0.818996435
71.63373179	0.211229574	0.126007528	1.67632504	0.093674543	0.245849039
174.1556482	0.447199229	0.093810181	4.767064971	1.86929E-06	5.89937E-05
10.78102377	-0.822305974	0.47164541	-1.743483466	0.0812492	0.224657382
2953.381715	0.060811366	0.064770569	0.938873425	0.347795735	0.562496263

3367.990005	0.192342539	0.062305748	3.087075336	0.002021364	0.016331508
8.551267064	0.029011959	0.455395676	0.063707147	0.949203414	0.975920679
7.169189978	-0.293539386	0.491279171	-0.597500166	0.550173496	0.736515373
21.29748528	1.223837876	0.766319928	1.59703256	0.11025845	0.274256032
11.24867605	0.53329672	0.43085633	1.237759974	0.215805074	0.420332559
9.315956794	0.57097652	0.373323888	1.529440088	0.126155381	0.297492663
95.92999334	0.042517763	0.11290162	0.37659126	0.706477384	0.843064305
13.22469908	-0.192665881	0.277036252	-0.695453678	0.486771031	0.685403653
976.1274967	0.104728833	0.05369449	1.950457713	0.051121589	0.164355462
2.812798031	0.524208301	0.682380982	0.768204733	0.442365562	0.648068498
660.6015824	-0.259862433	0.292502615	-0.888410632	0.374319908	0.587904067
17.25892902	0.395592705	0.440617846	0.897813624	0.36928492	0.582831939
4.320968508	0.646942858	0.674860062	0.958632603	0.337743862	0.553111642
272.6890015	0.534174876	0.416403008	1.282831455	0.199551131	0.401856139
20.33182575	1.175294173	0.64516837	1.821685978	0.068502647	0.200893682
5.376376264	-1.004068097	0.684315737	-1.467258523	0.142305742	0.321803316
16.97688005	-0.667221486	0.47166365	-1.414612903	0.157182024	0.343532004
35.18731769	0.195149688	0.218236208	0.894213155	0.371207855	0.584690525
5.143905787	-0.546470763	0.599799536	-0.911089005	0.362248479	0.576260296
245.653	0.029380498	0.07662385	0.383438023	0.701395015	0.839737026
114.4278671	-0.077059295	0.119359256	-0.645608039	0.51853323	0.710970989
431.1278286	-0.269778418	0.095444165	-2.826557479	0.00470513	0.030043531
4.979978378	1.572687598	0.654985726	2.401102092	0.016345775	0.074510322
65.71764886	-0.003607612	0.137798041	-0.026180428	0.979113426	0.989093306
727.6456827	0.21943312	0.062600763	3.505278659	0.00045613	0.005182261
854.5942643	-0.11721498	0.204855357	-0.572184109	0.567197252	0.748471553
168.4875276	0.173063482	0.134706264	1.284747099	0.198880674	0.400876082
31.08725128	-0.029079632	0.285529069	-0.101844734	0.918879918	0.95977858
179.6219921	0.351633214	0.204211654	1.721905711	0.0850866	0.231382743
299.4146632	0.043568957	0.073292085	0.594456514	0.552206819	0.737796722
477.0798735	-0.23146399	0.099410149	-2.328373841	0.019892261	0.08564918
76.29881898	0.223193404	0.177564506	1.256970823	0.208764201	0.412817362
216.4620175	0.034560103	0.104099249	0.331991857	0.739895408	0.863255748
419.4991883	0.189995997	0.091157884	2.084251948	0.037137256	0.13196703
30.74538646	0.298666905	0.175016392	1.706508187	0.087913503	0.236380736
4.67071157	1.240803023	0.500777805	2.477751629	0.013221314	0.064354309
1086.306739	-0.003750281	0.052217292	-0.071820672	0.942744622	0.972623458
50.93115996	0.223869186	0.185802963	1.20487414	0.228251893	0.434055372
411.2180991	0.082964304	0.063541462	1.30567195	0.191664141	0.392135501
23.02386329	-0.181936191	0.273701669	-0.664724448	0.506226753	0.701102784
112.8774448	0.053215421	0.172993812	0.307614591	0.758375616	0.873792838
224.2071439	0.402125393	0.276316478	1.455307322	0.145584201	0.326573274
2.424650254	0.134488161	0.678950679	0.198082371	0.842980617	0.921896347
54.17676686	0.525739689	0.44722339	1.175563936	0.239769174	0.447181861
6.325567675	0.945446001	0.763428504	1.238421144	0.215559945	0.420053765
7.660695744	0.751582351	0.548603372	1.369992218	0.170689331	0.362830456
39.33457359	-0.046503203	0.291892665	-0.159316108	0.873419831	0.93670453
3.275119421	-0.253006952	0.553442917	-0.457150944	0.647562556	0.804581457

10.04511326	-0.558371017	0.497502142	-1.122348971	0.261714092	0.473092773
109.4236572	-0.152956997	0.108830696	-1.405458217	0.159885066	0.347551174
158.2551566	-0.049985414	0.085002477	-0.588046564	0.556501039	0.74095192
301.4810054	0.037839788	0.067561885	0.560075967	0.575427622	0.753972704
5.34987687	-0.231178485	0.464998943	-0.497159163	0.619076816	0.784935848
23.87825529	-2.802314559	0.868860266	-3.225276456	0.001258511	0.011506219
32.97588452	0.692421232	0.296932933	2.331911201	0.019705363	0.085136678
297.8010296	-0.131415695	0.088896558	-1.478299026	0.139327758	0.317818539
73.58619873	-0.112992791	0.106691364	-1.059062204	0.289571451	0.50443059
78.99344446	-0.096389627	0.121353358	-0.794288916	0.427027255	0.634341479
5.496787979	-0.30747729	0.466228555	-0.659499052	0.509575355	0.703961794
6.90701285	0.4389181	0.458683039	0.956909373	0.338613	0.553808149
162.9323818	0.443568877	0.126988077	3.492996254	0.000477633	0.005380252
8.251113426	-2.672883013	0.999621753	-2.673894407	0.007497605	0.042576888
32.94860944	0.289717358	0.293288658	0.987823259	0.323239215	0.538426979
3.422552064	0.985190948	0.879191022	1.120565297	0.262472944	0.473847416
2.687058937	3.642733078	1.985594559	1.83458051	0.066567869	0.197095447
425967.5732	0.774361771	0.143156841	5.409184523	6.33124E-08	3.04084E-06
15.63362765	-2.965805522	1.652758129	-1.794458287	0.072740051	0.20920384
4.944367663	-0.43217037	0.468913184	-0.921642608	0.356715026	0.571341394
26.49337445	-0.260807935	0.242323861	-1.07627839	0.28180277	0.495832968
1192.902523	-0.185678687	0.095412515	-1.946062177	0.051647271	0.165468134
22.98115855	0.03088138	0.513701592	0.060115407	0.952063719	0.977124952
6.36824028	-0.636846023	0.517824486	-1.229849186	0.218753585	0.423338526
10.55606576	-0.517618582	0.337391576	-1.534177554	0.124985978	0.295805174
382.3081283	0.030396064	0.096126021	0.316210569	0.751842686	0.870526878
326.7861773	-0.599646541	0.108330386	-5.535349455	3.10608E-08	1.60406E-06
1824.226091	0.593640627	0.082839941	7.166116039	7.71554E-13	1.53139E-10
455.9974219	-0.439936782	0.198193348	-2.219735354	0.026436737	0.104843239
11.76744117	-0.540773012	0.298390167	-1.812301713	0.06993959	0.203576974
4.85783517	-0.178932651	0.590930307	-0.30279823	0.762043633	0.875874332
127.3057922	0.036090196	0.122242541	0.29523434	0.767814871	0.879073054
73.0250343	-0.846334171	0.163663951	-5.171170354	2.32632E-07	9.4861E-06
592.042443	-0.732387638	0.105009111	-6.974515165	3.06927E-12	5.267E-10
310.5438046	-0.25618867	0.106797744	-2.398820984	0.016447954	0.07489119
888.1271295	-0.176936393	0.111528675	-1.586465472	0.112633759	0.278023455
2312.718565	-0.137738445	0.089443626	-1.539947016	0.123573269	0.293804883
55.76524359	-1.205798832	0.441271406	-2.732556008	0.006284499	0.037321856
101.4416627	-0.587281863	0.099992792	-5.87324197	4.27354E-09	3.03458E-07
5.309247364	-0.092292679	0.440456476	-0.209538703	0.834027725	0.916961029
473.329146	-0.043369768	0.06165676	-0.703406531	0.481802438	0.680761508
165.2009329	-0.117897092	0.090576384	-1.301631698	0.193042319	0.393635247
7.761656824	0.090146785	0.403258714	0.223545784	0.823110754	0.911237589
281.4676464	-0.16873453	0.093407697	-1.806430695	0.070851091	0.205456938
1.990564552	0.319495564	1.054229962	0.3030606	0.76184368	0.875827829
42.14527677	0.437383451	0.429379492	1.018640756	0.308373548	0.523533322
261.1333332	0.061404194	0.066298881	0.926172407	0.35435639	0.569096189
11886.70396	-0.231109571	0.067484454	-3.424634224	0.000615628	0.006572813

442.0603412	-0.236131129	0.072058225	-3.276949016	0.001049353	0.009989185
1.900742212	-0.806186102	0.872668767	-0.923816839	0.355581686	0.57021831
2.508042525	-0.948647572	0.655304609	-1.447643675	0.14771673	0.329829955
938.8902138	0.263640995	0.076645582	3.439741581	0.00058227	0.006314887
625.7109283	0.284535113	0.102050398	2.788182294	0.005300471	0.032989168
2.600749392	0.250180866	0.646464253	0.386998763	0.698757124	0.838453107
2766.761703	0.191052312	0.100890142	1.893666787	0.058269252	0.179829087
369.7459959	-0.230824277	0.065372721	-3.530895969	0.000414155	0.004835424
6.084722295	0.131990324	0.414386414	0.31851991	0.750090594	0.869353627
128.4232962	-0.165084457	0.141166173	-1.169433538	0.242229004	0.449906202
682.5758864	0.156030103	0.057499144	2.713607429	0.006655498	0.039018748
256.4495169	-0.050130208	0.086656156	-0.578495637	0.562929542	0.745493482
343.7253775	0.117188252	0.066773638	1.755007743	0.079257983	0.220668585
114.9317655	0.046069498	0.102865424	0.447861841	0.654252909	0.809049875
446.0613391	0.037632779	0.065917135	0.570910408	0.568060375	0.748947007
3.142597558	-0.274989745	0.595883439	-0.461482442	0.644452516	0.80261547
428.9778061	0.08315595	0.070278328	1.183237455	0.236715053	0.444100191
2.549058447	1.009750772	0.562966926	1.793623615	0.072873265	0.209513816
119.9519175	0.211678725	0.096073227	2.203306086	0.027573182	0.107590346
490.9272414	0.169941283	0.091589251	1.855471915	0.063528866	0.190850285
10.95675342	-1.200211426	0.356626514	-3.365457643	0.000764168	0.00782892
4.684761163	-0.297410574	0.619388555	-0.480168017	0.631107927	0.793230487
55.22827338	-0.376615514	0.238835731	-1.576880946	0.114822922	0.28135214
757.0905183	0.141906247	0.059436239	2.387537464	0.016961674	0.076471433
1238.390326	0.153192243	0.088490204	1.731177412	0.083420127	0.228624716
89.24707876	1.753768189	0.270440661	6.484853952	8.88178E-11	9.37939E-09
222.5836754	-0.098019401	0.081945592	-1.196152206	0.231637168	0.438266993
684.9956972	0.150267066	0.067163475	2.2373331	0.025264577	0.101415363
227.3551839	0.295302547	0.146921669	2.009931891	0.044438398	0.149617447
101.8898097	0.043941575	0.108011588	0.406822784	0.684138151	0.829348762
1187.608611	0.53133222	0.077614705	6.845767428	7.6067E-12	1.13417E-09
174.6956081	-0.271406631	0.11271377	-2.407927905	0.016043349	0.073600147
491.1172048	-0.046126016	0.06442743	-0.715937541	0.474029917	0.674017681
408.6632685	-0.277897942	0.125378388	-2.216474036	0.026659054	0.105420369
22.85573499	-0.511030054	0.339078097	-1.507116087	0.131780904	0.306070578
144.4126994	0.05234664	0.140545532	0.372453248	0.709555415	0.845146114
141.824883	0.124282211	0.115963604	1.071734641	0.283839237	0.497920093
383.021876	-0.137674984	0.067919381	-2.027035315	0.042658801	0.145423175
436.7555834	0.209719904	0.058103913	3.609393819	0.000306913	0.003798716
102.1339359	0.059279476	0.112717215	0.525913236	0.598948492	0.769963126
2464.4136	0.002065975	0.060416004	0.034195829	0.972720993	0.986426062
63.49694652	0.159101843	0.202721179	0.784830889	0.432552727	0.639665496
247.4743744	0.074679074	0.107770915	0.692942746	0.488345483	0.686433744
3.310974816	0.378633415	0.750748657	0.504341115	0.61402169	0.781654688
2.309143493	-1.627417769	0.714864134	-2.276541361	0.022813628	0.094217024
11.46639978	-0.383305126	0.410785497	-0.93310287	0.350766858	0.565832638
181.6371311	0.040970529	0.078658103	0.520868514	0.602458371	0.77305874
282.2577915	0.051862206	0.193094157	0.26858506	0.788249011	0.890599197

295.0660587	-0.083925389	0.077355491	-1.08493124	0.27795211	0.491096425
201.3652487	-0.190069971	0.070900003	-2.680817525	0.007344255	0.041879287
181.8849596	0.129794977	0.084717001	1.532100712	0.125497584	0.296493216
287.087204	-0.033868618	0.067858566	-0.499106	0.617704711	0.78385976
12.4211057	0.099710589	0.290711619	0.342987973	0.731607508	0.858256932
6.800772688	-0.09187824	0.365916186	-0.251090942	0.801743799	0.898498458
68.15399724	0.445543826	0.146682344	3.03747414	0.002385699	0.018417058
5.317356361	-0.348062837	0.43776363	-0.79509309	0.426559354	0.633800964
13.25562376	-0.173861971	0.311261341	-0.558572327	0.576453631	0.75482848
958.6322577	-0.016456628	0.092898926	-0.177145513	0.859394093	0.930378098
5.471412763	-0.32399482	0.638182621	-0.507683553	0.611675285	0.780039684
162.9923769	0.110017287	0.139580034	0.788202179	0.430578455	0.637498448
1.697102583	-1.741393516	1.48113751	-1.175713601	0.239709342	0.447181861
5.243417832	-3.234070297	0.997529612	-3.242079491	0.001186609	0.011025493
3.117089424	-0.249434775	0.745308949	-0.33467299	0.737871778	0.861800757
16.37335628	0.315170022	0.937839385	0.3360597	0.736825848	0.861617619
6.865323073	-1.65422808	0.799584866	-2.068858667	0.038559351	0.135327387
17.50652956	-0.864084424	0.516863728	-1.671783833	0.094566956	0.247020171
5.66700893	-2.486510445	0.987096703	-2.519014032	0.011768397	0.05910749
130.5959741	0.035907686	0.187935336	0.191064048	0.848475415	0.925190891
110.5031365	-0.024628184	0.096928549	-0.254085968	0.799429144	0.896484956
3656.96531	0.027080519	0.100832901	0.268568284	0.788261921	0.890599197
3.980574243	-1.76973762	1.31314663	-1.347707544	0.177752465	0.373078622
3.201383803	1.26077438	0.592772827	2.126909877	0.033427572	0.12251075
310.2391629	-0.001771832	0.092033207	-0.019252091	0.984640003	0.992381215
172.2531005	0.747038747	0.136600357	5.468790604	4.53117E-08	2.23158E-06
207.3811165	0.597951147	0.127249098	4.699059999	2.61362E-06	7.74401E-05
2.20852855	0.438432393	0.7004831	0.625900029	0.531380515	0.720863825
959.1166403	-0.145476007	0.074394662	-1.955462974	0.050528449	0.163120846
286.9919169	0.042686426	0.071520228	0.596844099	0.550611472	0.736682644
1407.355344	0.184065042	0.103679294	1.775330781	0.075843286	0.214717699
4.102060784	-0.005313093	0.531182677	-0.010002384	0.992019385	0.995720597
518.3911656	-0.044832291	0.100734636	-0.445053387	0.656281173	0.81076847
3.430264581	0.223292165	0.572765777	0.389848999	0.696648209	0.837156802
1366.025383	-0.039458508	0.083181004	-0.474369216	0.635236642	0.795627494
8.511786799	-0.440864058	0.579589913	-0.760648259	0.446867187	0.651970791
32.87630893	-0.020965125	1.071805647	-0.019560566	0.984393922	0.99222378
36.91804821	0.359113778	0.201722987	1.780232301	0.075037951	0.213356065
7.088964651	-0.036466163	0.472777525	-0.077131761	0.938518727	0.970690451
43.84829547	0.046675847	0.207453376	0.224994395	0.821983635	0.910734442
41.56513321	0.112588531	0.160757136	0.700364126	0.483699934	0.682875371
44.63823623	0.021000473	0.172847537	0.121497091	0.903297318	0.951456563
3.68445804	-0.041970333	0.693396045	-0.06052866	0.951734591	0.977124952
2.219810902	-0.743355149	0.778688159	-0.954624955	0.339767393	0.554608377
59.09414965	0.157338209	0.134623404	1.168728497	0.242513037	0.450158847
3.073947718	-0.17943452	0.525879227	-0.34120861	0.732946542	0.859215921
2.921112339	1.397639277	0.651801148	2.1442725	0.032011059	0.118906468
52.73671219	-0.5927409	0.187376111	-3.163374963	0.001559513	0.013521878

312.4661778	-0.91371617	0.112706452	-8.107044057	5.18661E-16	1.98707E-13
171.360822	0.097262439	0.115550372	0.841731941	0.399938015	0.612093912
35.35446351	-0.478422622	0.38972532	-1.22758927	0.219601192	0.424016647
722.1740756	-0.295064381	0.062700345	-4.70594512	2.52692E-06	7.51418E-05
406.8683529	0.078151111	0.063439023	1.231909136	0.217983028	0.422815921
8722.813153	-0.047238005	0.152385744	-0.309989663	0.756568817	0.87294769
242.8891315	-0.556519297	0.255727157	-2.176222906	0.029538597	0.112878472
412.0759947	-0.569439322	0.333536154	-1.707279151	0.087770179	0.236117088
139.8471109	0.476464318	0.147044138	3.240280934	0.00119412	0.011064078
20.99284758	0.672272788	0.274416634	2.449825207	0.014292558	0.067898934
128.8938553	-0.001778969	0.095952794	-0.01854004	0.985208035	0.992558536
791.0240553	-0.061243646	0.082571165	-0.741707416	0.458264626	0.661594202
7287.285141	-0.124231448	0.085915083	-1.445979493	0.148182954	0.330578336
89.29130754	0.545398157	0.110977112	4.914510292	8.90046E-07	3.11308E-05
416.2477324	-0.164983543	0.146320427	-1.127549631	0.259510169	0.470367534
5.551908499	-0.002701396	0.608655103	-0.004438304	0.996458758	0.998004635
46.37770123	-1.88543612	0.788758311	-2.390385107	0.016830714	0.076110125
623.833727	0.173598454	0.074191318	2.33987559	0.019290165	0.083760192
1341.89372	-0.158221029	0.049392628	-3.203332887	0.001358469	0.012182586
32.1447323	0.955289507	0.240727909	3.968337153	7.23759E-05	0.001214176
444.4909621	0.495809989	0.075567381	6.561164151	5.33893E-11	6.08292E-09
586.1338373	0.379348713	0.061217953	6.196690632	5.76627E-10	5.02611E-08
354.3182891	-0.29432868	0.089772352	-3.278611643	0.001043191	0.00995366
1492.673858	0.314298064	0.061676296	5.095929669	3.47034E-07	1.35475E-05
1321.018746	0.038467308	0.069653999	0.55226273	0.580768362	0.757707927
608.9454235	0.083941811	0.0496989	1.689007423	0.091218008	0.241806726
63.2354303	0.107035511	0.151654206	0.705786631	0.480320837	0.679394088
1222.982373	-0.148240176	0.088563358	-1.673831926	0.094163636	0.246543506
45.78280631	0.250164909	0.226981198	1.102139348	0.270401095	0.482325345
295.820939	-0.021473518	0.101194927	-0.212199553	0.831951356	0.91596382
3.237920813	0.269940202	0.582250921	0.463614898	0.642923678	0.801477463
78.19524617	0.102719991	0.179581493	0.571996534	0.567324323	0.748471553
3.209367822	1.106830254	0.656800126	1.685185813	0.091952727	0.242800005
390.4309718	0.042612229	0.059668249	0.714152503	0.475132883	0.675059993
320.9109026	-0.124824788	0.087285796	-1.430069886	0.152696962	0.337104544
178.7805189	-0.294865236	0.119459979	-2.468318162	0.01357496	0.065525583
3.481897539	0.471648994	0.606366505	0.777828244	0.436670275	0.642902204
182.8956257	0.139941066	0.088969372	1.572912816	0.115739007	0.282597362
806.0712863	0.230756646	0.067386497	3.424375164	0.000616215	0.006574821
3.642896642	-0.898578173	0.530859771	-1.692684625	0.090515513	0.24088304
299.7814779	-0.17144249	0.113594229	-1.509253529	0.131234006	0.305182102
2.10986547	-2.127880061	0.979659257	-2.172061402	0.029851032	0.113624283
34.41671773	0.753744377	0.488401085	1.543289727	0.122760495	0.292755703
19.11778616	-0.327550226	0.345079669	-0.949201751	0.342518011	0.556619
6.233846912	-0.27332318	0.381485711	-0.716470297	0.473701002	0.6738408
3.915932304	0.090666344	0.778157134	0.116514185	0.907245045	0.953303217
1805.141818	-1.458328676	0.477901444	-3.05152599	0.002276813	0.017781276
327.6531969	-0.035628275	0.058154242	-0.612651348	0.540106898	0.727710889

1423.299517	-0.025697823	0.076378595	-0.33645321	0.73652913	0.861476895
47.63780512	0.337304833	0.523079596	0.64484418	0.519028168	0.711412767
76.62748792	-0.90514011	0.270011796	-3.352224322	0.00080165	0.008102078
2.136328923	0.81901873	0.893571138	0.916568021	0.359369048	0.573611636
111.7462813	-0.871220313	0.123770389	-7.03900441	1.93618E-12	3.46703E-10
62.77764658	-0.58951847	0.303057303	-1.945237632	0.051746385	0.165689007
2426.949581	-0.069773402	0.097962298	-0.712247497	0.47631153	0.676327886
45.78739873	-0.05129293	0.152201511	-0.33700671	0.736111842	0.861278654
237.1071412	-0.223125404	0.103286936	-2.16024806	0.030753471	0.115960789
6.039430548	0.664597139	0.41054487	1.618817303	0.105486578	0.266082666
2467.404941	-0.122716281	0.097283686	-1.261427133	0.207154998	0.410670449
590.307441	0.164394611	0.114686554	1.433425325	0.151736325	0.336026915
48.90651535	0.282412739	0.958681138	0.294584641	0.768311196	0.879190026
1484.438898	-0.241355469	0.098934126	-2.439557291	0.014705271	0.069215609
150.9448452	-0.374751823	0.18174289	-2.061988914	0.039208791	0.136703837
419.3858133	0.021350028	0.060826426	0.350999221	0.725588933	0.854589253
623.8922482	-0.005927886	0.068778482	-0.086188091	0.931316898	0.967310672
663.2213234	-0.187175841	0.066027672	-2.834809047	0.004585308	0.029565883
527.0074862	0.007289728	0.092727652	0.078614391	0.937339341	0.970445469
3072.685006	-0.034933326	0.09495493	-0.367893763	0.712952451	0.846804519
231.803565	0.089138799	0.076966128	1.1581562	0.246800299	0.455397415
29.91009827	-0.075774812	0.241753826	-0.313437904	0.753947991	0.871653489
163.3658875	0.322199718	0.180856509	1.78152127	0.074827333	0.213197075
2160.285259	0.140450581	0.152160011	0.923045291	0.355983603	0.570558902
4.177577328	-4.388595698	1.089317401	-4.028757544	5.60724E-05	0.000981655
1265.655094	0.343331462	0.087133765	3.940280341	8.13864E-05	0.001339421
2.053570957	0.329145398	0.784330454	0.419651432	0.67474011	0.823993223
789.6161856	0.128307456	0.119251283	1.075941928	0.281953229	0.495844429
3.366265806	0.260288816	0.749474033	0.347295309	0.728369475	0.85622654
45.5380667	0.785526682	0.204870317	3.834263033	0.000125941	0.001877608
4.058202654	1.755642886	0.813213785	2.158894646	0.030858341	0.116249786
58.08634868	0.369811468	0.171176824	2.160406177	0.03074124	0.115960789
234.9035578	0.077939084	0.076449279	1.019487503	0.307971583	0.523034772
22.93717993	0.311512249	0.281602014	1.106214563	0.268633661	0.480491998
6.894295137	0.223059846	0.44581795	0.500338414	0.61683681	0.78342222
51.21687276	0.080377986	0.152754802	0.526189585	0.59875649	0.769896535
212.6711421	0.022848233	0.19603245	0.116553323	0.907214029	0.953303217
1736.66238	0.260793721	0.115740876	2.253255122	0.024243068	0.098369531
31584.54713	0.191646631	0.105542304	1.815827627	0.069396818	0.202559032
105.635401	-0.192103896	0.267290263	-0.718708919	0.472320286	0.672689928
245.0583231	-0.254564311	0.155586936	-1.636154795	0.101807195	0.260268734
411.554613	0.03895707	0.084495882	0.461052886	0.644760662	0.802801611
5.413725777	-1.526943281	0.689560708	-2.214371067	0.026803264	0.105707454
2.822582357	-0.43328079	0.631903254	-0.685675833	0.492917549	0.690017157
15.29435521	-0.321367696	0.268467829	-1.197043596	0.231289563	0.437911074
447.7937511	-0.089429625	0.064665962	-1.382947419	0.166680989	0.357632536
1.771000842	0.237385839	0.773746585	0.3068005	0.758995227	0.874200334
3.109952572	-0.03069495	0.594227867	-0.051655185	0.958803447	0.979970829

91.91790525	0.399558333	0.22706717	1.759648184	0.078467476	0.21943188
1977.227002	0.032465852	0.111153885	0.292080225	0.770225286	0.880181143
398.2126266	-0.229339153	0.06227704	-3.68256345	0.0002309	0.003062683
178.8648555	0.266375125	0.218982152	1.216423906	0.223823482	0.42895161
331.7778858	-0.153463499	0.08079543	-1.899408158	0.057510832	0.177888367
155.9324761	-0.100659667	0.146063569	-0.689149718	0.490729055	0.688433148
1030.681681	-0.057744893	0.138449861	-0.417081623	0.676618706	0.825308497
1.980815159	0.185834779	0.722598373	0.257175751	0.797043102	0.895203555
361.1669154	0.057676993	0.092376174	0.624370885	0.532384042	0.721851416
149.4831372	0.128178853	0.087965935	1.45714194	0.145077204	0.325924158
846.4150078	-0.135206756	0.087358359	-1.547725457	0.12168841	0.291634401
78.48612747	-0.352827063	0.116082563	-3.039449274	0.002370111	0.018339695
11.83614489	-0.486283572	0.404252455	-1.202920516	0.22900708	0.435089683
279.9807927	-0.242471218	0.078574932	-3.085859722	0.002029646	0.016366314
3826.327589	-0.067724368	0.040171259	-1.685891119	0.091816773	0.242557652
164.3492969	-0.113737751	0.086824112	-1.309978862	0.190202986	0.39011504
595.9834347	-0.007886083	0.120772578	-0.065296963	0.94793756	0.975143522
1074.569989	0.069947703	0.10170384	0.687758726	0.491604732	0.689192099
402.3549428	-0.208926674	0.097938607	-2.133241226	0.032904943	0.121115816
1723.285487	-0.140934422	0.109031925	-1.292597761	0.196150214	0.397268088
789.5692824	-0.239721296	0.074717428	-3.208371912	0.001334888	0.012013626
63.32889525	-0.854112775	0.229232306	-3.725970347	0.000194565	0.002664246
204.8793734	0.106448837	0.106238784	1.001977181	0.316354614	0.531256464
387.9271057	-0.214673278	0.077341513	-2.775653992	0.00550908	0.033940385
171.9819967	-0.207716792	0.094162069	-2.205949738	0.027387519	0.107118231
37.92230855	-0.318692246	0.249971321	-1.274915236	0.202339246	0.405117546
127.6030492	-0.107714202	0.108492208	-0.992828924	0.320793332	0.535815609
48.12869201	-0.086310969	0.172848095	-0.499345792	0.6175358	0.783766162
380.3154687	-0.109326975	0.062287599	-1.755196469	0.079225708	0.220653307
199.0480225	0.00139456	0.08452219	0.016499335	0.986836032	0.993287517
391.4128798	0.035219613	0.066699964	0.528030462	0.597478193	0.769213485
34.61916709	-0.609501463	0.162939931	-3.740651291	0.000183544	0.002553804
502.3233289	0.155357731	0.1005746	1.544701451	0.122418494	0.292448125
103.1740896	-0.286254571	0.108264617	-2.64402701	0.008192612	0.045565526
82.25223944	0.086110818	0.443901242	0.193986431	0.846186506	0.92379566
2727.7873	-0.186102542	0.115045537	-1.617642439	0.105739678	0.266633651
336.885875	-0.121012096	0.064809556	-1.867195266	0.061874324	0.187396534
56.91397081	-0.747092838	0.263013352	-2.840512971	0.004504104	0.029142828
332.4952578	-0.137506844	0.110212511	-1.247651853	0.21215858	0.41657975
44.13879748	-0.288135894	0.149460593	-1.927838559	0.05387521	0.169921742
259.5659263	0.013065917	0.070277422	0.185919131	0.852508179	0.927133598
33.61332795	-0.468100401	0.175109876	-2.673181042	0.007513569	0.042623462
2.672963669	0.243993155	0.706314824	0.345445326	0.729759607	0.857101832
686.4182709	-0.036631162	0.061869719	-0.592069314	0.553804175	0.738901334
7.391035837	-0.983794247	0.726515034	-1.354127857	0.17569559	0.370084565
534.0326854	0.323706399	0.077108482	4.198064736	2.69206E-05	0.000546843
13.65664723	0.339678382	0.247330052	1.373380951	0.169633949	0.361660369
7.843869656	0.978353453	0.499395017	1.959077324	0.050103732	0.162387358

50.21014791	0.21442678	0.1507009	1.422863299	0.154775787	0.339907082
593.6449917	-0.211323044	0.095496713	-2.212882911	0.026905719	0.10591274
1698.355275	0.241799979	0.119583895	2.02201123	0.043175192	0.146653218
140.3064481	0.216997603	0.128448184	1.689378517	0.091146915	0.24179618
706.1394303	-0.283760867	0.089343778	-3.176056267	0.00149292	0.013103017
183.0063937	-0.001859947	0.128889168	-0.014430595	0.988486451	0.993980695
10.2794568	1.109547493	0.63062314	1.759446208	0.078501749	0.219474016
528.8285739	0.224186046	0.168452981	1.330852352	0.183237598	0.38042296
378.4927387	0.063094259	0.065431722	0.964276303	0.334907418	0.550070918
114.1105676	0.048804386	0.180875751	0.269822712	0.787296649	0.890119072
27.83282044	-0.234833473	0.186296877	-1.260533596	0.207476936	0.411011911
1.969245799	-0.869362641	0.867865891	-1.001724633	0.316476606	0.531407156
1712.659657	-0.08830631	0.100517954	-0.878512801	0.379665494	0.592523458
26.19605408	-0.278046942	0.275460879	-1.009388134	0.312788527	0.527591731
1727.018605	-0.168364812	0.105713246	-1.592655771	0.111237435	0.275733599
4.08187693	-1.829801556	0.700201294	-2.613250749	0.008968547	0.04869738
305.0007098	0.163684646	0.083169261	1.968090677	0.049057605	0.160028236
22.35000656	-0.252079846	0.333510421	-0.755837989	0.449746337	0.654689507
97.80742021	-1.415546564	0.360389769	-3.927821175	8.57189E-05	0.001387164
99.13155032	-0.215731725	0.12775695	-1.688610482	0.091294101	0.24183615
606.7288139	-0.069188092	0.079413356	-0.871239999	0.383623124	0.596602223
9.661933664	0.255547865	0.306371265	0.834111729	0.404218026	0.614990985
3377.341001	0.022019608	0.065290849	0.337254116	0.735925346	0.861237063
2057.733707	-0.050513745	0.097687196	-0.517096889	0.605088525	0.775218257
12.20500936	0.290200488	0.328691353	0.882896632	0.377292124	0.590154809
224.2189801	0.116765312	0.088896778	1.313493189	0.189016815	0.388316967
360.1626435	-0.185120643	0.075025928	-2.467422232	0.013608978	0.06563065
332.974798	-0.266806219	0.082594776	-3.23030381	0.001236587	0.011368047
53.26607921	0.277833793	0.157686818	1.761934175	0.07808042	0.218609251
350.14253	0.196615658	0.106769124	1.841502971	0.065547886	0.195069681
1312.355286	-0.119707519	0.057889776	-2.06785251	0.038653894	0.135514844
245.0918418	-0.163306085	0.094790361	-1.722813199	0.084922311	0.231241348
194.1830853	-0.083618136	0.113891648	-0.734190238	0.462832802	0.665274198
714.559479	0.122234771	0.079537849	1.536812633	0.124339199	0.294940816
365.3925946	-0.220574998	0.10572618	-2.086285514	0.036952768	0.131652228
145.2128885	-0.049772155	0.132292338	-0.37622855	0.706746992	0.843214962
102.476679	0.141999438	0.159962119	0.887706657	0.374698564	0.588276389
1.741339019	-1.130197689	0.727953671	-1.552568156	0.120526338	0.290030806
13.34412105	0.511337733	0.511139733	1.000387369	0.31712308	0.532090262
130.11513	-0.378940602	0.106949619	-3.543169243	0.000395349	0.004652127
7.246924499	-0.621653472	0.428213137	-1.451738443	0.146574346	0.328079589
27.99398282	0.238439434	0.199672189	1.194154453	0.232417556	0.439160145
4.158294685	-0.061326476	0.520693091	-0.117778548	0.906243127	0.952959157
120.180035	-0.323996914	0.108907774	-2.974965894	0.002930211	0.021406785
16.1997223	0.212969293	0.288773458	0.737496079	0.460820727	0.663513594
733.1662981	-0.146235577	0.113386267	-1.289711539	0.197150832	0.398559676
27.1887337	0.226468292	0.247619695	0.914581094	0.360411588	0.57466323
53.65121804	0.435896612	0.147980268	2.945640112	0.003222871	0.022924691

14.00986721	0.740939728	0.279581402	2.650175308	0.008045001	0.044865726
247.4776649	0.051583875	0.091300713	0.564988748	0.572081417	0.751595502
735.8174839	0.519095747	0.1116169	4.650691301	3.30824E-06	9.41278E-05
7452.741702	-0.060137941	0.087224175	-0.68946414	0.490531232	0.688390112
180.1614206	0.062884113	0.108706846	0.578474265	0.562943968	0.745493482
28.05817023	0.410826644	0.212594343	1.932443913	0.053304738	0.168873511
892.3265426	-0.101662528	0.056241658	-1.807601909	0.070668481	0.205180218
520.1965277	-0.141552124	0.075101223	-1.88481783	0.059454437	0.18229153
2366.088944	-0.186887162	0.080665666	-2.316811747	0.020513989	0.087528478
499.080787	0.386930002	0.075598686	5.118210645	3.08448E-07	1.23334E-05
896.7410915	0.141473712	0.093810703	1.508076449	0.131534962	0.305714865
16.18769325	-0.959065401	0.478348097	-2.004952895	0.044968083	0.150907354
138.1825853	-0.451967146	0.18356094	-2.462218519	0.013808051	0.066338242
658.6929071	-0.025122591	0.059829257	-0.419904784	0.674555013	0.823844185
115.3552425	-0.008338011	0.125867064	-0.066244582	0.947183103	0.974828397
1487.048437	-0.139108643	0.06049646	-2.299450948	0.021479346	0.090498911
196.4196662	-0.406682804	0.169121414	-2.404679538	0.016186653	0.073989712
155.0640449	0.074983729	0.102338678	0.732701756	0.463740353	0.666055673
526.286797	-0.230183775	0.087580822	-2.628244059	0.008582691	0.047224869
469.6712737	0.108927739	0.058976633	1.846964363	0.064752305	0.193388231
40.66582237	0.28411606	0.188006026	1.511207194	0.13073567	0.304501545
270.8479485	-0.136741131	0.11193091	-1.221656563	0.221837529	0.426584738
55.52943526	0.009679821	0.161360132	0.059988926	0.952164455	0.977124952
17.08693306	-0.223986627	0.341587928	-0.65572173	0.512003185	0.706072364
1412.694378	-0.447968579	0.162503144	-2.756676381	0.005839211	0.035378876
25.79333645	0.371800796	0.285288308	1.303245821	0.192490851	0.393136763
60.75830254	-0.146887449	0.252954717	-0.580686735	0.561451603	0.74480346
1.75920442	0.158706456	0.76279385	0.208059433	0.835182561	0.917681419
34.55287785	0.589291039	0.366971787	1.605821101	0.108313223	0.270931071
52.98634393	-0.288951418	0.201738153	-1.432309229	0.152055343	0.33629222
4.657996998	-0.88317786	0.560665512	-1.575231293	0.115203066	0.281789951
88.60015661	-0.235579757	0.226086031	-1.041991654	0.297415549	0.512030907
61.0017052	0.214360462	0.140129721	1.529728746	0.126083885	0.29740921
1782.344992	0.033859525	0.105820101	0.319972521	0.748989162	0.868654689
160.1213789	-0.041611527	0.091842017	-0.453077238	0.650493125	0.80632392
1085.476548	-0.008400029	0.183832502	-0.045693926	0.963554205	0.982015974
26.38160798	-0.001997572	0.211526729	-0.009443592	0.992465216	0.995850406
128.0858103	0.399572344	0.149714486	2.668895684	0.007610108	0.042968523
44.20365526	-0.315557727	0.235200312	-1.341655224	0.179707812	0.375651122
19.28632584	0.142584232	0.244708337	0.582670102	0.560115404	0.744065931
566.8765273	0.130096342	0.083955464	1.549587565	0.121240539	0.291111594
286.0391024	-0.16635021	0.07660575	-2.171510768	0.029892584	0.113692737
516.1769215	-0.095776973	0.086891419	-1.102260433	0.270348466	0.482325345
278.7766323	0.032784282	0.092376318	0.354899211	0.722665099	0.852684776
2.026531206	-0.328636416	0.730574051	-0.449833135	0.652830763	0.808140517
91.750127	-0.327646229	0.117566721	-2.786896027	0.005321555	0.033010244
38.40294296	-0.783273456	0.266228207	-2.942112954	0.00325981	0.023117568
3.179888305	0.548243462	0.551751032	0.993642839	0.32039678	0.535424686

1083.771528	0.929156332	0.170970287	5.434607088	5.49173E-08	2.6531E-06
28.55938506	-0.805017651	0.393076444	-2.047992604	0.040560724	0.140171464
2.643993412	3.279392393	2.543980596	1.289079169	0.197370565	0.39875922
5.452054802	-1.864594108	0.585504967	-3.184591442	0.001449585	0.012790822
13.43951093	-1.155955819	2.146754197	-0.538466779	0.590254834	0.763627267
15.3142538	-2.439106556	0.88857071	-2.744977445	0.006051506	0.036278666
69.89995739	-0.220418879	0.182007472	-1.211043027	0.225878915	0.431435274
803.5942661	-0.139830805	0.06467535	-2.162041723	0.03061496	0.115650277
40.59768363	0.237359476	0.242194584	0.980036264	0.327068218	0.542173659
72.62948585	-0.111893	0.122984343	-0.909814999	0.362920083	0.576876249
171.0998535	-0.247515264	0.185870996	-1.33165082	0.182974957	0.380261063
2.147600856	1.002291256	1.243030363	0.806328861	0.420053254	0.628801209
12.84158711	0.870275913	0.338826017	2.568503799	0.010213859	0.053288494
4.909751833	0.094824217	0.446267091	0.212483104	0.831730158	0.915943742
1616.330401	0.087305434	0.103796686	0.841119668	0.400280898	0.612232486
5.688606992	0.100033533	0.40873712	0.244738069	0.80665925	0.90149274
618.9629428	-0.003850013	0.066084608	-0.058258848	0.953542447	0.977472422
2.007276586	0.148828798	0.68846859	0.216173693	0.828852359	0.914385171
423.5144352	-0.024600092	0.086954968	-0.282906111	0.77724881	0.884207847
99.40443728	-0.068799946	0.118183622	-0.582144506	0.560469349	0.744310525
497.47152	-0.085267564	0.089287434	-0.954978323	0.339588659	0.554448323
346.9410245	-0.192854089	0.075279854	-2.561828668	0.010412267	0.053991718
1307.93276	-0.055717053	0.064153843	-0.868491268	0.385125451	0.598266269
34.66079718	0.093713595	0.19964374	0.469404124	0.638780801	0.798267972
334.7890523	-0.270198968	0.12539431	-2.154794494	0.031177919	0.116759501
74.82034521	0.009872233	0.149226009	0.066156249	0.947253428	0.974828397
375.3099627	-0.089766377	0.067067715	-1.338443953	0.180751759	0.376918032
126.7605831	-0.217323698	0.108665304	-1.999936409	0.045507131	0.152282039
1120.921327	0.028800508	0.106075262	0.271510132	0.785998708	0.889337428
118.9578165	-0.096062999	0.09843198	-0.975932805	0.329097764	0.544661098
132.946653	0.18961864	0.104949739	1.806756664	0.070800228	0.205456938
12.98082348	0.114980526	0.427466092	0.26898163	0.787943819	0.890422998
335.8488858	-0.152612106	0.059060149	-2.584011532	0.009765853	0.051730759
746.700716	0.004768025	0.086483343	0.055132292	0.95603307	0.978363076
260.5348707	-0.026083678	0.099703309	-0.261612966	0.793619846	0.893466367
271.9773425	0.089222947	0.087317522	1.021821794	0.306865252	0.521971933
79.96765405	-0.247348167	0.112899988	-2.190860872	0.028461862	0.109911091
881.1638275	0.068340448	0.047124047	1.450224498	0.146995926	0.328621371
85.5247046	-0.162342616	0.120113333	-1.351578643	0.176510146	0.371559947
304.318609	0.112078309	0.127506706	0.878999323	0.379401643	0.59230988
138.234109	0.557045814	0.181139699	3.075227672	0.002103419	0.016780498
24.97937267	0.492723761	0.267857369	1.839500488	0.065841606	0.195654479
616.9483942	0.044687501	0.07544582	0.592312487	0.553641356	0.738816339
475.813392	0.066164484	0.065345017	1.012540625	0.311279633	0.526264641
215.4895416	0.20205271	0.081211917	2.487968724	0.012847503	0.063000705
81.95307342	0.64765424	0.130257526	4.972106092	6.62294E-07	2.43541E-05
7377.539848	0.178120088	0.086723444	2.053886239	0.039986704	0.138711509
330.3289367	0.123781358	0.104627322	1.183069161	0.236781739	0.444166843

5.03134725	0.511203878	0.551130197	0.92755556	0.353638159	0.568540551
329.2690318	-0.110345239	0.067007275	-1.646765072	0.099606343	0.255837733
24.54070865	0.534391282	0.50786236	1.052236441	0.292691084	0.507915315
63.71549202	0.188354503	0.170895482	1.102161981	0.270391257	0.482325345
860.8434292	-0.351150697	0.135747207	-2.58679869	0.009687217	0.051502923
4.357419528	-0.358771924	0.499115826	-0.718814964	0.472254936	0.672655008
36.50016881	-0.07558725	0.185785965	-0.406851239	0.684117251	0.829348762
67.76105074	0.410962989	0.134448622	3.056654531	0.002238221	0.017583428
16.86523533	0.578672217	0.268453149	2.155579922	0.031116482	0.116662021
1449.401349	0.04681022	0.049421598	0.947161198	0.343556644	0.5578864
188.3980962	-0.2457094	0.07951576	-3.090071722	0.002001082	0.016215356
2130.390855	0.238146847	0.074823749	3.182770859	0.00145873	0.012864625
56.23391311	0.121087376	0.141805815	0.853895704	0.39316278	0.606491244
215.3313591	0.103358912	0.11132165	0.928470899	0.353163357	0.568109866
7.614732437	-0.770539877	0.329985882	-2.33506922	0.019539805	0.084621122
6.256333571	-0.085098636	0.514209312	-0.165494155	0.86855505	0.934607931
315.4924628	-0.132192776	0.061607655	-2.14571997	0.031895327	0.118583529
29.80838135	-0.372172584	0.331413893	-1.122984257	0.261444181	0.472728727
267.2562267	-0.124854959	0.06065367	-2.058489776	0.039543142	0.137549349
5.032289985	0.107304917	0.494125739	0.217161156	0.828082755	0.914087062
2.878770984	-2.210309701	1.121462569	-1.970917053	0.048733364	0.15926075
629.9591851	0.043459034	0.054396963	0.798923897	0.424334541	0.631868578
1062.734773	0.044125999	0.041273131	1.06912167	0.285014848	0.499344317
11.25862805	0.483169265	0.312637412	1.545462079	0.122234535	0.292178137
251.6605591	-0.568386688	0.290796727	-1.954584203	0.050632167	0.163391641
1437.220301	-0.289198967	0.073904889	-3.913123636	9.11099E-05	0.001457363
5498.528075	-0.158835416	0.100670818	-1.577770188	0.114618415	0.281111176
1104.917093	0.035648633	0.062375374	0.571517746	0.567648735	0.748773548
57.90973448	0.352849109	0.124392897	2.836569593	0.004560104	0.029436972
260.9868209	-0.326769575	0.090058204	-3.628426496	0.000285154	0.003605238
40.13455889	-0.075684121	0.179113729	-0.422547852	0.672625181	0.822263819
24.91173958	-0.520585867	0.255433828	-2.038045905	0.041545342	0.142676249
942.6224667	0.303755506	0.102072689	2.975874442	0.002921544	0.021362416
1067.507892	-0.100177437	0.090043979	-1.112538979	0.26590648	0.477599581
929.8992725	-0.242893695	0.081151745	-2.993080361	0.00276177	0.020555331
39.55559362	-0.0167404	0.199391565	-0.083957415	0.93309029	0.968419923
10.49690998	0.0890192	0.333473468	0.266945375	0.78951122	0.891207713
1396.279962	-0.320091014	0.088477107	-3.61778347	0.000297137	0.003716805
39.97817574	0.599676695	0.159875401	3.750900344	0.000176201	0.002468308
464.2315347	-0.185312678	0.064542343	-2.871179893	0.004089427	0.027208895
52.73114297	0.277798515	0.16965555	1.63742663	0.101541358	0.259669718
5.175258205	-0.09465416	0.45287211	-0.209008588	0.834441534	0.917111871
51.66482053	0.381821714	0.250824699	1.522265211	0.127942641	0.299648432
500.0264921	0.158241735	0.045310397	3.492393507	0.000478712	0.005382765
501.163239	0.140020636	0.084607	1.654953336	0.097933964	0.253076725
3.110903844	-0.348219949	0.706830562	-0.49264982	0.62226004	0.787396259
11.20314599	0.559312235	0.371319445	1.506283182	0.131994494	0.306350703
319.3833083	0.028291699	0.074726418	0.378603707	0.704982169	0.842195522

143.5132763	-0.49042294	0.129811347	-3.777966662	0.000158114	0.002261086
686.274222	0.090914242	0.095430094	0.95267896	0.340752757	0.555303286
469.1758087	-0.111246987	0.072576117	-1.532831897	0.125317279	0.296332703
97.10222411	-0.35804444	0.147491864	-2.427553838	0.015201031	0.071081969
1217.344015	0.033995902	0.052407881	0.648679197	0.516545753	0.709367684
71.27112405	0.115308494	0.133443103	0.864102307	0.387531692	0.600639485
249.0364197	-0.337349696	0.085677674	-3.937428279	8.23595E-05	0.001351385
176.7340547	-0.081122246	0.116383289	-0.697026583	0.485786158	0.684588637
670.4574021	0.02313265	0.062405777	0.370681222	0.710874978	0.84576363
1.824665677	-0.356687428	0.74959347	-0.475841161	0.634187543	0.794857394
33.2971215	-0.86308014	0.276582852	-3.120512117	0.001805369	0.01498899
107.1279858	-0.173422073	0.109336476	-1.586131913	0.112709389	0.27816846
1449.028789	-0.243274391	0.094563911	-2.572592315	0.010094003	0.052823573
572.6630527	0.047140862	0.073650285	0.640063545	0.522131288	0.713528896
117.0310521	-0.076937238	0.108707391	-0.707746156	0.479102909	0.678327657
7.126701537	-0.281901402	0.406762639	-0.693036613	0.488286575	0.686409509
302.2290796	-0.0832364	0.061961349	-1.343360043	0.179155419	0.374823365
220.5163304	0.002368569	0.07497938	0.031589606	0.974799332	0.987719156
404.7844755	-0.330036261	0.071632673	-4.607342542	4.07848E-06	0.000112168
267.7877713	-0.662158206	0.143677817	-4.608632159	4.05327E-06	0.000111661
8.227398046	-1.124417413	0.475641558	-2.36400162	0.018078734	0.079911204
3.295390155	0.001774683	0.691918478	0.002564873	0.99795353	0.998559673
3.823557567	-0.123786708	0.480073177	-0.257849666	0.796522932	0.894900006
10.52725656	0.926091234	0.380521097	2.433744783	0.014943527	0.070056822
4.674449763	-0.028745874	0.503778065	-0.057060591	0.954496929	0.97793505
3.720462122	-0.058129884	0.702236034	-0.082778269	0.934027848	0.968782646
6.495433566	0.13055558	0.478329573	0.272940641	0.784898845	0.888536949
2.832096023	0.770025535	0.671385564	1.146920006	0.251414684	0.46045642
7.603419444	-0.184239228	0.503056514	-0.366239623	0.714186278	0.847730272
2.435433548	-0.508691793	0.665399532	-0.764490759	0.444574842	0.649802674
5.46576476	-0.084515576	0.407597167	-0.207350745	0.835735946	0.917860932
9.286163913	0.351807862	0.319789502	1.100123235	0.271278431	0.483274606
8.035956295	1.089406487	0.36329925	2.998647776	0.002711806	0.020260451
22.74604341	0.531682235	0.266616383	1.994184413	0.046131905	0.153815251
10.11466435	0.448079326	0.39247159	1.141686018	0.253584555	0.462865802
32.32982744	0.747582603	0.181397163	4.121247496	3.76826E-05	0.000712725
30.35077276	0.504941272	0.213282216	2.36747949	0.017909713	0.079355731
20.96997132	0.540120126	0.22402904	2.410937998	0.015911553	0.073152594
17.10890623	0.590267853	0.264142211	2.234659318	0.025439723	0.101920615
18.18406101	0.462594516	0.21986233	2.104018984	0.035376791	0.127443091
7.761466237	0.031532164	0.373541011	0.084414197	0.93272712	0.968225998
15.86460905	-0.226165227	0.243205249	-0.929935629	0.352404414	0.567443308
6.402157766	0.265381391	0.386781872	0.686126757	0.492633179	0.690017157
124.1821837	-0.140225	0.130359056	-1.075682847	0.282069122	0.495967668
29.5656576	0.553302558	0.172034736	3.216225813	0.001298886	0.011763521
46.37597271	0.414648511	0.159511081	2.599496583	0.009336061	0.050119656
55.3927455	0.391029051	0.154166442	2.536408354	0.011199605	0.056962734
41.46396857	0.190577132	0.153193676	1.244027411	0.213489475	0.418083271

55.8015371	0.431899988	0.132120768	3.268978796	0.001079364	0.010219219
95.12803637	0.259753514	0.133147151	1.950875499	0.051071858	0.164355462
72.21764605	0.252600762	0.116748956	2.163623307	0.030493271	0.11529634
80.13913981	0.399253256	0.124602828	3.204207031	0.00135435	0.012152271
78.30308223	0.699498482	0.120022447	5.828063826	5.60741E-09	3.91426E-07
90.81568401	0.522992369	0.127155101	4.113027058	3.90505E-05	0.00073438
92.01763269	0.466891064	0.12101261	3.858201765	0.000114224	0.001743959
89.82019988	0.495451159	0.120077413	4.126097861	3.6897E-05	0.000702707
86.80788366	0.584283785	0.119358008	4.895220644	9.81955E-07	3.39135E-05
122.2636572	0.554182444	0.097117517	5.706307773	1.15453E-08	6.99256E-07
95.12501472	0.579820241	0.099724456	5.814223169	6.09161E-09	4.12976E-07
102.9294193	0.506539499	0.13868372	3.652479902	0.00025972	0.003366346
89.5572081	0.50123339	0.126051295	3.97642397	6.99594E-05	0.001184624
68.51776614	0.627408107	0.133700458	4.69263991	2.69702E-06	7.96249E-05
86.85052809	0.430480204	0.124076797	3.469465811	0.000521494	0.005769711
409.4270081	-0.068840588	0.1095018	-0.628670834	0.529564576	0.719352326
42.94070496	0.322081928	0.180395529	1.785420792	0.074193089	0.21179292
29.66281629	0.417023189	0.174149628	2.394625773	0.016637336	0.075472996
1905.226931	-0.535386396	0.094667416	-5.655445347	1.55443E-08	8.98513E-07
631.16394	-0.024520445	0.075208766	-0.326031749	0.744400334	0.865910536
26.52015921	0.386707499	0.253367398	1.526271742	0.126942203	0.298280681
362.7537046	0.729532278	0.091910238	7.937442992	2.06392E-15	6.62925E-13
232.1535484	0.722948027	0.178090694	4.059437409	4.91911E-05	0.000879902
20.80013307	0.119004519	0.206701791	0.575730468	0.564797378	0.747206097
973.7665533	0.082806085	0.081857847	1.011583959	0.311737019	0.52645368
102.2422602	0.785228941	0.154869636	5.070257561	3.97278E-07	1.53633E-05
1051.402616	-0.095607869	0.066955492	-1.427931689	0.153311521	0.337880134
99.21271366	-0.258523956	0.107166188	-2.412364946	0.015849408	0.073015422
553.3233119	-0.105356934	0.051577604	-2.042687638	0.041083369	0.141532293
143.0202131	0.391990683	0.215851898	1.816016844	0.069367788	0.202546073
482.97603	-0.058285388	0.171207078	-0.34043796	0.733526736	0.859529088
10.63782731	0.015739719	0.359536694	0.043777781	0.965081538	0.982556904
256.3764534	0.305152167	0.098002711	3.113711494	0.0018475	0.015263652
2186.65534	-0.161194357	0.093724281	-1.719878289	0.085454568	0.232076912
171.6746352	0.095033418	0.087146072	1.090507189	0.275489788	0.488166774
2.109746759	-1.673783831	0.939727887	-1.781136703	0.074890121	0.213239062
4.369695963	-1.367502566	0.494564111	-2.765066317	0.005691122	0.034750019
7.821816374	-0.139563555	0.335224059	-0.416329173	0.677169148	0.825613125
762.584699	0.623756655	0.090721703	6.875495455	6.17748E-12	9.69217E-10
977.210073	-0.092152411	0.058614649	-1.572173726	0.115910267	0.282764066
6.697602101	1.108452787	0.440128372	2.518476101	0.011786388	0.059177326
423.0244193	0.013368892	0.068340306	0.195622356	0.844905761	0.92307033
501.5946228	0.195679621	0.044556744	4.391694859	1.1247E-05	0.000263187
7.623469923	-0.141054442	0.341715137	-0.412783709	0.679765088	0.826819999
11.66613322	-0.149670134	0.394262319	-0.37962069	0.704227	0.841664506
3.502071305	0.281087887	0.70977385	0.396024574	0.6920869	0.834048251
332.6202129	-0.206420089	0.15838981	-1.303240966	0.192492508	0.393136763
2.542355898	-0.77897083	0.676353387	-1.151721637	0.249435478	0.45849031

2.217155797	0.440433669	0.795900274	0.553377959	0.580004629	0.757293715
4.637689792	0.075786906	0.607277787	0.124797757	0.900683663	0.950413955
3.116430876	0.784649274	0.715798898	1.096186759	0.272997053	0.485373245
20.73785793	0.429876925	0.357430653	1.20268623	0.229097765	0.435161602
12674.19243	-0.118831686	0.11320781	-1.049677464	0.293866428	0.508791964
392.3909024	-0.130047117	0.066654325	-1.951067939	0.051048964	0.16435033
219.1771122	0.447346979	0.122226635	3.659979504	0.000252235	0.003286634
2.212782118	0.142729979	0.702969157	0.20303875	0.839104745	0.919787466
259.8051341	-0.179626679	0.073299695	-2.450578814	0.014262673	0.067790907
62.19507036	0.865629975	0.157325459	5.502160803	3.75165E-08	1.90168E-06
2.27629067	0.676951559	0.701751139	0.964660436	0.334714918	0.550047536
14.81859556	0.19561266	0.281961131	0.693757535	0.487834277	0.686007843
263.5582241	-0.161190974	0.092189022	-1.748483392	0.080380359	0.223039588
8.708261145	-0.066275295	0.334608617	-0.198068105	0.842991779	0.921896347
6.009217173	0.145713693	0.394301467	0.369548949	0.711718595	0.846071015
339.186448	0.096599616	0.074529216	1.29613085	0.194930408	0.395868287
16.76922675	-0.288794453	0.306081853	-0.943520335	0.345414819	0.559854755
622.5429176	-0.143451476	0.054391995	-2.637363733	0.008355319	0.046267402
181.0773251	-0.309709059	0.121223202	-2.554866173	0.010622863	0.054753635
813.3265606	-0.604576475	0.440333678	-1.372996221	0.169753522	0.361728045
303.3980862	-0.323501036	0.072358251	-4.470824401	7.79187E-06	0.00019449
126.8470174	-0.424479188	0.128809907	-3.295392394	0.000982843	0.00952433
135.5033436	0.043722749	0.206330671	0.211906201	0.832180213	0.916032128
84.8322895	-0.270975273	0.136167672	-1.990011797	0.046589636	0.154741465
324.3765847	0.411218779	0.10065929	4.085254112	4.40286E-05	0.000805919
277.6675923	-0.292947208	0.189871378	-1.542871865	0.122861868	0.292910391
73.97988333	0.327568926	0.114375101	2.86398807	0.004183438	0.027674804
1647.998631	-0.146791115	0.078368573	-1.873086481	0.061056444	0.185912182
72.00548133	-0.237238573	0.235385651	-1.007871856	0.313515984	0.528374662
1271.464624	-0.127177816	0.10449865	-1.217028314	0.223593443	0.42871024
24.51452383	-0.206878138	0.461175469	-0.448588773	0.653728334	0.80858327
141.2714862	-0.011877931	0.095657395	-0.124171586	0.901179419	0.950648941
317.5965575	-0.18319327	0.251457652	-0.728525332	0.466292067	0.668438524
76.57591829	0.793373749	0.137968864	5.750382549	8.90417E-09	5.57747E-07
332.7070062	0.057866101	0.066390656	0.871600086	0.383426584	0.596352865
127.2922691	-0.414376498	0.10009668	-4.139762671	3.47665E-05	0.00067066
161.9608923	-0.485532826	0.083589464	-5.808540945	6.30196E-09	4.25486E-07
2.697400007	-0.318179894	0.763134281	-0.416938279	0.676723553	0.825375274
9.128201502	0.051215615	0.357703713	0.143178873	0.886148915	0.943111133
340.8655226	-0.106250802	0.085616903	-1.241002629	0.214604778	0.419184148
230.1245108	-0.003241347	0.107797431	-0.030068867	0.97601213	0.987885465
3.27741399	-3.2132918	1.492363735	-2.153155913	0.031306427	0.117042041
165.6674819	0.276224178	0.100850995	2.738933593	0.006163882	0.03681791
156.8270901	-0.582007077	0.115167468	-5.053571853	4.33623E-07	1.66348E-05
338.0098798	0.072598172	0.113295763	0.640784523	0.52166269	0.713349603
177.3729931	-0.191186257	0.077241202	-2.475184906	0.013316721	0.064694681
170.6563357	-0.250522683	0.213325085	-1.174370481	0.240246664	0.447687352
41.76951167	-0.182357404	0.221131473	-0.824656036	0.409566898	0.619889505

98.53214653	-0.539087762	0.129944173	-4.148610516	3.34499E-05	0.000652135
35.63095042	0.02134753	0.294408145	0.072509984	0.94219606	0.972418112
533.559977	-0.075921533	0.062006523	-1.224412018	0.220796838	0.425427732
431.5732331	0.419429023	0.217431399	1.929017728	0.053728661	0.169661869
4.083804628	1.371969775	0.659552592	2.080152199	0.037511575	0.132895847
5.156178812	0.918772057	0.451611729	2.034429128	0.041908348	0.14362349
9.460744001	0.568699436	0.402014867	1.414622899	0.157179091	0.343532004
2759.708785	-0.188315969	0.110832142	-1.699109714	0.089298513	0.238700203
5.941579147	0.814688943	0.686940185	1.185967804	0.235635014	0.442880916
11.23008646	-0.349421078	0.315003512	-1.109260898	0.267317648	0.478933218
614.5514777	-0.137050413	0.067173285	-2.040251763	0.041325256	0.142127824
4.130414168	0.749120968	0.647277385	1.157341481	0.247132873	0.455777809
12.27117394	0.378444949	0.31482334	1.202086696	0.22932994	0.435369696
11.98618565	-0.606330696	0.434611384	-1.395110019	0.162982665	0.352266653
96.42555075	-0.103886458	0.658753673	-0.157701524	0.874692	0.93727566
1120.026471	0.158505774	0.420366377	0.377065775	0.706124724	0.842949182
80.35011763	0.350536151	0.404637706	0.86629631	0.38632769	0.599452311
16.275353	-0.143787604	0.317394874	-0.453024341	0.650531214	0.80632392
551.3017439	-0.092990787	0.051717441	-1.798054707	0.072168339	0.208213873
229.5150803	0.113500855	0.065747891	1.7263041	0.084292718	0.229948866
3374.654507	0.093638693	0.114819577	0.815528983	0.414769581	0.624576769
2349.805574	-0.144392281	0.104778806	-1.378067626	0.16818241	0.359542826
1455.886751	0.164960241	0.048455789	3.40434534	0.000663228	0.006973833
2381.059242	-0.273274084	0.062467743	-4.37464313	1.21631E-05	0.000281702
672.3446094	0.52382342	0.457883525	1.144010193	0.25261941	0.461738839
510.8047812	0.220785069	0.060983333	3.62041654	0.000294129	0.003687582
27.19453792	-0.51207482	0.222401778	-2.302476289	0.021308327	0.089985486
4671.961757	-0.008010807	0.134202035	-0.059692144	0.952400828	0.977124952
11.25552003	-0.407602782	0.394500129	-1.033213304	0.301504088	0.516667388
718.6646669	0.573386672	0.100231496	5.720623681	1.06134E-08	6.53438E-07
530.0788129	-0.434060815	0.197144396	-2.201740568	0.027683639	0.107892187
492.1481461	0.101074378	0.055772589	1.812259009	0.069946185	0.203576974
171.1400841	-0.337983814	0.079638425	-4.24397911	2.19591E-05	0.000460247
9.830637923	0.630928961	0.292948362	2.153720735	0.031262079	0.11697595
557.1329265	0.218358994	0.119158377	1.832510644	0.066875377	0.197579801
64.82943428	0.03141056	0.143267651	0.21924391	0.826460052	0.913336211
2342.064561	0.100727088	0.063047277	1.597643762	0.110122281	0.274082862
54.63183871	0.219733609	0.192170393	1.143431126	0.252859636	0.461985981
154.364964	0.381732438	0.098071268	3.892398298	9.92581E-05	0.001555831
10.48969082	0.132053307	0.308142076	0.428546821	0.668253053	0.819232707
4.122039486	0.29916922	0.498923159	0.599629853	0.548752947	0.735270927
20.43900362	-0.282205858	0.268802016	-1.049865111	0.293780134	0.50869602
225.0004918	-0.026565753	0.106368905	-0.249751122	0.802779821	0.899069005
37.15548065	0.433383784	0.175551055	2.468705096	0.013560292	0.065510924
697.6425543	0.53131415	0.097630131	5.442112462	5.26524E-08	2.55116E-06
517.6804236	1.008231922	0.144965233	6.954991202	3.52584E-12	5.91507E-10
57.43672921	0.10589637	0.221183971	0.478770542	0.632101875	0.793934606
412.7891131	-0.153533421	0.086847339	-1.76785406	0.077085305	0.216781036

460.573831	-0.075787342	0.058335538	-1.29916249	0.193888171	0.394773666
253.844307	-0.41310154	0.303514638	-1.361059691	0.173494827	0.367088849
336.6947245	0.084539322	0.074938161	1.128121128	0.259268768	0.470200096
86.07977742	0.154621921	0.231240349	0.668663238	0.503710323	0.699260458
634.1523871	-0.048888218	0.081373669	-0.600786702	0.547982062	0.73465629
1005.356471	-0.156130591	0.066989841	-2.330660706	0.019771258	0.085287169
1.826384966	-0.417559865	1.039293322	-0.401772874	0.687851187	0.83108758
1708.137906	0.026202542	0.063973939	0.409581507	0.682112965	0.82820821
1146.176217	-0.193884874	0.078939188	-2.456129569	0.014044251	0.06714016
120.3867295	-0.144246693	0.125670677	-1.147815046	0.251044925	0.459958458
618.3386827	-0.050356386	0.098224459	-0.512666462	0.608184654	0.777528635
3.653191316	0.383167687	0.531838427	0.720458822	0.471242544	0.67156139
1595.966945	-0.017072768	0.127004215	-0.134426779	0.893065107	0.946108145
171.3216677	0.182430332	0.138327189	1.318832061	0.187225263	0.385929552
440.2357338	-0.07269616	0.06880483	-1.05655606	0.290714243	0.50586884
16.21028197	0.950349459	0.32222485	2.949336341	0.003184572	0.022720934
110.9830366	0.022135286	0.13637505	0.162311842	0.87106029	0.935876058
11.88714359	0.717727968	0.316074182	2.27075797	0.023161634	0.095037039
845.412938	-0.266608178	0.059196782	-4.503761331	6.67612E-06	0.000169982
117.3979855	-2.226749851	0.268256766	-8.300815255	1.03399E-16	4.86686E-14
2.084640419	-2.961439254	0.998574724	-2.965666146	0.003020282	0.021890065
5.780793634	-0.112614773	0.561851352	-0.200435173	0.841140253	0.921330769
26.44813783	-1.277934718	0.30355227	-4.209932996	2.55446E-05	0.000522761
280.8900539	-0.151965522	0.072070597	-2.108564784	0.034982165	0.126297653
38.74627216	-0.587961049	0.28229544	-2.082786212	0.037270715	0.132327103
57.66767654	0.145884325	0.199562411	0.731021061	0.464766289	0.666947721
13.6699478	0.028009132	0.271506997	0.10316173	0.917834612	0.959308302
34.62871125	-3.048055931	0.555926831	-5.482836521	4.1856E-08	2.07691E-06
248.9348496	-0.353719632	0.079832216	-4.430788107	9.38893E-06	0.00022613
1359.337821	0.302641348	0.077062065	3.927241623	8.59256E-05	0.001387837
6.661123828	-0.336450371	0.369729807	-0.909989849	0.362827863	0.576876249
228.3562271	-0.413203667	0.086059692	-4.801361221	1.57591E-06	5.09049E-05
146.751513	-0.381405643	0.127562677	-2.989946989	0.002790259	0.020687093
141.2018766	-0.212255554	0.12364213	-1.716692792	0.086035318	0.232886268
207.6217558	-0.117790916	0.092616773	-1.271809758	0.203440721	0.406240296
267.3038901	-0.157362314	0.071580959	-2.198382318	0.027921873	0.108666414
6.658688932	-0.215164316	0.436866605	-0.492517198	0.622353767	0.787454375
136.2690757	-0.032375334	0.197831564	-0.163651002	0.870005892	0.935388289
213.7286424	-0.204758339	0.114217099	-1.792711782	0.073019022	0.209823018
3546.70401	0.108113769	0.119283214	0.906361973	0.364744294	0.578213769
481.3629353	-0.197109761	0.123937818	-1.590392384	0.111746386	0.27662058
63.20193511	-0.423204014	0.188174774	-2.248994408	0.024512852	0.099195461
28.16683094	0.17744743	0.255374485	0.694851836	0.487148159	0.685605662
1293.660762	0.245544847	0.050118257	4.899309367	9.61741E-07	3.33552E-05
4.796347333	0.516067119	0.553803141	0.931860225	0.351408766	0.566504355
24.16977305	-0.835609383	0.357985345	-2.334199977	0.019585253	0.084773374
49.4544126	0.039859497	0.146815638	0.271493538	0.786011469	0.889337428
4.170667628	0.26839762	0.510317876	0.52594203	0.598928485	0.769963126

35.00798798	-0.4576635	0.251733396	-1.818048405	0.069056735	0.201888314
560.5871157	-0.118676959	0.064737011	-1.833216547	0.066770374	0.197375766
293.6264008	-0.233264966	0.083200619	-2.803644612	0.005052857	0.031722851
1206.289542	-0.026697196	0.06798054	-0.392718205	0.694527623	0.835617096
233.3511139	0.142331613	0.095568442	1.489316032	0.136404168	0.313493619
90.67251312	0.085160093	0.176202322	0.483308575	0.628876653	0.791997093
14.5400246	0.529037648	0.309117175	1.711446953	0.086998636	0.234530441
1.796700534	-1.895329736	1.647852334	-1.150181783	0.250069008	0.45901246
304.412158	0.116106687	0.075642315	1.534943581	0.124797687	0.295593801
306.3701022	0.081839614	0.089220083	0.917277935	0.358997017	0.573406715
6.168201443	0.467027423	0.388521867	1.20206213	0.229339458	0.435369696
35.57619902	-0.088942564	0.30168053	-0.29482368	0.768128576	0.879126731
16.66636072	0.490101168	0.261365782	1.875154292	0.0607715	0.185360106
1838.329382	-0.205794971	0.134853667	-1.526061364	0.126994583	0.29830213
3.876351863	-0.528879405	0.839967166	-0.629642951	0.528928216	0.718979428
999.5361131	0.079291155	0.079762486	0.994090816	0.320178656	0.535331694
223.4911379	-0.125592699	0.076580107	-1.640017288	0.101001572	0.258570304
1097.945931	0.024297987	0.094964898	0.255862825	0.798056761	0.895680707
2.857528766	-0.40854876	0.53548708	-0.762947931	0.445494449	0.650630443
334.5951422	0.217515208	0.123362561	1.76321898	0.077863564	0.218389064
337.6772307	0.340098713	0.099139533	3.43050551	0.000602458	0.006465726
10.44678516	0.653303896	0.304757973	2.143681062	0.032058451	0.119000278
48.1406085	-0.250941568	0.257519942	-0.974454894	0.329830725	0.545336384
2.888386703	-0.192061309	0.688062789	-0.279133405	0.780142448	0.885448627
422.2595219	-0.069360416	0.072761129	-0.953261949	0.340457366	0.555270316
234.0558757	0.002544029	0.097329934	0.026138195	0.979147112	0.989093306
451.5023031	-0.067618575	0.103086632	-0.655939316	0.51186317	0.706056591
12.55710487	-1.32980748	0.498533604	-2.667438002	0.007643199	0.043062264
1066.588382	0.049283775	0.098828905	0.498677736	0.618006432	0.784121839
503.2510544	0.174175736	0.087647407	1.987232051	0.046896688	0.155332744
14.31536925	1.260242943	0.32270896	3.905199731	9.41476E-05	0.001491162
641.4226253	0.083929593	0.079057622	1.061625571	0.2884057	0.503091434
4.84852876	0.094452809	0.471651771	0.20025963	0.841277534	0.921330769
84.58213779	0.081477036	0.112423131	0.72473552	0.4686143	0.670018398
27494.09125	0.233143417	0.091818384	2.539180141	0.01111126	0.056653324
648.2142519	-0.285422304	0.143585761	-1.9878176	0.046831867	0.155264272
1361.873421	0.097014451	0.079914007	1.213985557	0.224753239	0.429829349
1.999123728	-4.051847639	1.982843204	-2.043453376	0.041007578	0.141330302
24.18034717	-0.832175634	0.723187822	-1.150704712	0.249853737	0.458821811
450.8287513	0.083836712	0.095256836	0.880112288	0.378798483	0.591948986
48.96090305	-0.119891749	0.283484138	-0.422922247	0.672351991	0.822173894
137.7161149	0.205843039	0.105517866	1.950788506	0.05108221	0.164355462
4.423763665	-0.660706672	0.510145678	-1.295133333	0.195274238	0.396273441
144.0600217	0.014020581	0.120411455	0.116438927	0.907304686	0.953303217
863.9979733	0.027209539	0.055839921	0.487277529	0.62606167	0.790627823
12.71217342	-0.634798394	0.416159903	-1.525371351	0.1271665	0.298509679
213.556831	-0.149429242	0.144661412	-1.03295855	0.301623297	0.516736917
8.860751991	-0.038047099	0.505974335	-0.07519571	0.940058998	0.9715198

207.7360604	0.163762372	0.087554109	1.870413325	0.061426443	0.186566966
6.704999691	-0.524709049	0.423834154	-1.238005582	0.215713991	0.420254528
1008.908282	0.219369786	0.051136198	4.289911896	1.78744E-05	0.000387451
628.2025328	0.012067162	0.053340924	0.226227096	0.821024797	0.910076874
2.190596265	0.520475594	0.762879241	0.682251614	0.495079863	0.691885448
614.7155781	0.083918446	0.260163587	0.322560305	0.74702826	0.867329872
526.4405098	0.076665942	0.051945739	1.475885103	0.139974739	0.318720643
694.2741382	0.026633065	0.078931682	0.337419203	0.735800911	0.861152614
29.14428987	0.40488933	0.184824166	2.190673112	0.028475456	0.109937817
428.8662195	0.107626156	0.05843369	1.841851099	0.065496933	0.195046363
762.1665422	0.074393202	0.063917279	1.163898145	0.244465291	0.452457163
404.7021429	0.028836865	0.059289992	0.486369862	0.626704955	0.790677612
3.689934637	-0.776106024	0.562477831	-1.379798422	0.167648719	0.359007538
284.2237518	-0.172242266	0.103522628	-1.663812723	0.096149861	0.249798581
4.847983171	-0.908379125	0.557552876	-1.629225071	0.103265381	0.26265152
373.6110475	-0.418105353	0.206068056	-2.028967327	0.042461618	0.144976725
180.5951165	-0.207472196	0.105961976	-1.957987235	0.050231511	0.162608352
367.0644317	-0.046638775	0.062237515	-0.749367566	0.453635694	0.658108374
93.33044075	-0.092249493	0.137785703	-0.66951426	0.503167487	0.69886023
9.342681828	-0.462158493	0.584705815	-0.790412001	0.429287196	0.636264261
368.4444074	-0.150430333	0.089839168	-1.67444042	0.094044074	0.246308757
144.3119305	0.111299723	0.08416779	1.3223553	0.186049866	0.384276015
200.5036465	0.036390176	0.082425154	0.441493583	0.658855706	0.812363514
301.9729896	-0.287425207	0.202517742	-1.419259389	0.155823407	0.341224885
15.68261774	-0.313187774	0.293884371	-1.065683669	0.286566663	0.501103832
3.283927833	0.134019565	0.594570165	0.225405802	0.821663599	0.910539898
1521.072773	-0.093925148	0.059701246	-1.57325271	0.115660315	0.282488958
753.9059513	0.217157639	0.107526312	2.019576742	0.04342731	0.147296994
65.75520301	-0.201905344	0.140865892	-1.433316048	0.151767538	0.336050863
580.5569001	0.104732889	0.061580606	1.70074471	0.088990936	0.238186299
56.38648272	0.087040068	0.195723921	0.444708379	0.656530513	0.81089405
172.5213069	-0.022226885	0.183923241	-0.120848703	0.903810873	0.951582251
1168.705651	-0.021510432	0.109658262	-0.19615879	0.844485882	0.922856413
51.46364326	0.046857305	0.211670337	0.221369254	0.82480493	0.911996538
180.0315584	-0.1715641	0.216347306	-0.793003171	0.427775975	0.634941316
35.03542181	-0.219125929	0.442209959	-0.495524637	0.620229836	0.785732568
35.37549431	-0.308790045	0.225981154	-1.366441581	0.171800405	0.364346019
138.4165289	-0.057295746	0.157187204	-0.364506429	0.715479874	0.848949542
30.74733437	0.124020879	0.279038381	0.444458137	0.656711389	0.81089405
5007.03368	-0.015314801	0.089045157	-0.171989153	0.863446056	0.931920109
698.0229094	-0.136967122	0.06535434	-2.095761668	0.036103338	0.129353283
37.08369253	-0.616886834	0.173750988	-3.550407632	0.000384635	0.004552068
2.457950109	0.419715525	1.375323772	0.305175795	0.760232267	0.87505484
111.1088176	-0.439948851	0.127608218	-3.447652957	0.00056548	0.00616119
169.0175069	0.279044647	0.26315981	1.060361944	0.288979968	0.503706799
547.5617363	-0.190895426	0.055659536	-3.429698493	0.000604252	0.006474886
661.0037307	0.055981524	0.068195279	0.820900287	0.411703068	0.621667859
8.15681408	0.51087345	0.339183264	1.506187083	0.132019154	0.306364777

3.585811031	0.447811156	0.555586303	0.806015472	0.420233928	0.628946697
364.9055942	-0.127297699	0.189038025	-0.673397318	0.500694555	0.696482487
23.04027846	1.329165437	0.534607335	2.486246168	0.012909863	0.063202699
246.0886575	-0.092330957	0.078001778	-1.183703239	0.236530556	0.443956293
728.3715899	0.333581226	0.101382912	3.290310167	0.00100077	0.009653987
78.47247786	0.828308691	0.35725531	2.318534304	0.020420301	0.087348813
1584.455775	0.159830951	0.03970291	4.025673496	5.68125E-05	0.000993555
58.15263564	0.137185908	0.206863899	0.663169885	0.507221756	0.70201787
18.90465747	1.778703503	0.432823517	4.109535252	3.96456E-05	0.000738972
17.30133134	-0.01692015	0.252389617	-0.067039801	0.946550018	0.974652478
125.0965928	0.120182448	0.104608925	1.148873748	0.250608044	0.45957013
19.44859342	0.760800402	0.312203367	2.436874433	0.014814823	0.069639149
1068.356904	0.100287934	0.067265084	1.490935989	0.135978301	0.312645714
1217.68694	0.064422174	0.040677325	1.583736749	0.113253638	0.278944032
10.39377324	-0.571829465	0.361300646	-1.582697044	0.113490532	0.279207634
54.15922883	-0.517200144	0.312117099	-1.65707084	0.097505154	0.252245588
5.211670915	-1.159523573	0.776683156	-1.49291711	0.135458886	0.311917533
3362.615554	-0.253299303	0.33726106	-0.751048174	0.452623664	0.657136004
158.4439069	0.220622997	0.300854155	0.733322086	0.463362007	0.665628332
84.96189125	0.000352541	0.154957576	0.002275079	0.998184751	0.998609072
16.61308998	0.634555904	0.222794528	2.848166464	0.004397192	0.028666141
121.5791275	0.104218571	0.177472914	0.587236491	0.557044888	0.74155616
122.1875075	0.099867033	0.106503851	0.937684715	0.348406461	0.563127539
52.78093603	0.125348667	0.157186259	0.797453084	0.42518794	0.632693174
222.4930386	0.341147817	0.119120174	2.863896228	0.004184651	0.027674804
104.4982247	0.383287214	0.119031311	3.220053706	0.001281666	0.011665283
370.2001687	-0.004439649	0.081583142	-0.054418709	0.956601573	0.978702547
86561.22642	-0.069509957	0.14515181	-0.478877643	0.632025677	0.793934606
58.91840795	0.082374119	0.138582576	0.594404586	0.552241542	0.737796722
82.82567158	-0.136388742	0.174466897	-0.781745673	0.434364054	0.641118471
277.0537513	-0.139434953	0.070384565	-1.981044456	0.047586288	0.156731034
53.04448552	-0.207717638	0.185103377	-1.122170982	0.261789748	0.473145837
319.9461508	-0.121819775	0.079249277	-1.53717206	0.12425118	0.294821505
1.836936219	0.200061784	0.708657616	0.28231092	0.777705113	0.884312122
91.1603564	0.141754044	0.134349677	1.055112654	0.291373806	0.506499111
312.6037684	-0.129096512	0.068255459	-1.891372694	0.058574611	0.180467204
138.7225722	-0.030104331	0.102380016	-0.294044988	0.768723527	0.879316901
24.14048391	0.425991206	0.227854975	1.869571668	0.061543323	0.186749806
277.1719333	-0.507834658	0.146180243	-3.474030734	0.000512702	0.005687716
310.5930202	-0.26729603	0.065680239	-4.069656789	4.70824E-05	0.000849547
13.12269423	0.629363268	0.26388542	2.384986898	0.01707973	0.076835463
1390.689896	0.197687534	0.045266409	4.367201549	1.25849E-05	0.000288751
981.079616	-0.172484467	0.071509154	-2.412061353	0.015862612	0.073035402
310.2857074	0.309244196	0.110920528	2.787979839	0.005303784	0.032989168
1185.462179	0.145696814	0.049184815	2.962231632	0.003054179	0.022067785
84.67759546	0.098324708	0.163035482	0.603087787	0.546450291	0.733259109
465.4362626	0.082586628	0.073169572	1.128701804	0.259023648	0.469847564
234.8160727	0.137435098	0.09260714	1.484065897	0.137791439	0.315546396

347.3896235	0.330141523	0.070873515	4.658179041	3.19019E-06	9.12416E-05
11.96559862	0.050671294	0.295599989	0.171418456	0.863894744	0.932132697
25.80654819	-0.718103144	0.224860647	-3.193547445	0.001405362	0.012514562
2237.833402	-0.056577417	0.078418163	-0.721483584	0.470612037	0.671243524
280.4845238	0.021270701	0.07529303	0.282505585	0.777555865	0.884312122
278.953443	-0.247726014	0.099820519	-2.481714351	0.013075204	0.063746942
188.0583553	0.007574056	0.073260039	0.10338591	0.917656694	0.959308302
13.14759269	0.983794142	0.50287281	1.956347854	0.050424191	0.162939403
104.1005307	-0.130956539	0.153414761	-0.853611075	0.39332052	0.606530212
277.8938143	-0.159007251	0.081526348	-1.95037868	0.051131001	0.164355462
627.3358839	-0.030508975	0.174699016	-0.174637359	0.861364592	0.931215742
117.319497	-0.01602989	0.113105649	-0.141724928	0.887297284	0.943568846
15.65064991	0.038201995	0.271723712	0.140591319	0.888192807	0.944188443
84.65975826	0.049779937	0.148735388	0.334687913	0.73786052	0.861800757
62.52078136	0.081591243	0.162293613	0.502738473	0.615148156	0.782406945
99.50409888	0.1003	0.135652517	0.739389161	0.459670722	0.662521039
200.1132027	0.253017237	0.073179433	3.457491061	0.00054523	0.005989193
358.1238777	0.086107953	0.084873993	1.014538726	0.31032576	0.525239201
299.4416316	0.4481326	0.131036014	3.419919354	0.000626397	0.006674815
562.8174339	0.250030309	0.084338825	2.964593218	0.003030834	0.021947235
383.5707732	0.455757269	0.098190448	4.641564193	3.45782E-06	9.77085E-05
16.55959882	0.514535831	0.298295883	1.724917646	0.084542315	0.230511436
5108.55837	-0.055004432	0.065334284	-0.841892318	0.39984823	0.612021197
233.4061279	0.010231348	0.11862356	0.086250559	0.93126724	0.967310672
157.4763687	-0.193940212	0.091283728	-2.124586897	0.033621098	0.122973794
1305.031496	-0.318735543	0.134248504	-2.374220456	0.017586047	0.07842991
982.6705122	-0.082327218	0.067216901	-1.224799363	0.220650825	0.425295624
89056.55683	-0.277831743	0.061326898	-4.530340709	5.88886E-06	0.000153018
17639.12088	0.234342808	0.131230154	1.78573903	0.074141523	0.211719093
176.843471	0.360873191	0.124233064	2.90480795	0.003674785	0.025161434
29.38771165	0.132814648	0.214154859	0.62018041	0.535139017	0.72384441
4.563393413	0.239432743	0.475809621	0.503211225	0.614815774	0.782242435
600.8257167	0.146208588	0.063254119	2.311447689	0.020808139	0.088462784
226.104082	0.252405709	0.083201465	3.033669045	0.002415993	0.018550012
152.7642136	0.188649055	0.146204596	1.290308651	0.196943514	0.398385002
1163.709804	-0.181183345	0.070929165	-2.554426603	0.010636285	0.054774042
35.87033147	-0.114728568	0.207525427	-0.552841016	0.58037228	0.757539944
411.8249018	-0.017053256	0.084975461	-0.200684484	0.840945293	0.921248354
240.8999897	-0.204971282	0.078512117	-2.610696149	0.009035814	0.048949686
1337.649741	-0.092727248	0.079593575	-1.165009221	0.24401526	0.451725744
114.5388643	-0.140480732	0.106848379	-1.314767083	0.188588194	0.388007045
50.08966319	-0.119215249	0.171525151	-0.695030719	0.48703605	0.685605662
287.4571314	-0.027915796	0.066426686	-0.420249719	0.674303037	0.823642636
103.0915426	-0.026892002	0.109551088	-0.245474529	0.806089028	0.901149495
5.941566735	-1.720088415	1.120147809	-1.535590573	0.124638828	0.295438856
5.834277538	-0.165957582	0.439899844	-0.377262197	0.705978763	0.842949182
124.0093806	-0.395235535	0.13494179	-2.928933536	0.003401271	0.023853787
99.35176235	0.028177656	0.121671385	0.231588193	0.816857879	0.907902872

338.2322825	0.018579057	0.104209793	0.178285135	0.858499053	0.929770219
6.333590964	0.615914084	0.613446279	1.004022855	0.315367598	0.530228099
2.007086291	-0.000237743	0.749198705	-0.00031733	0.999746807	0.999746807
498.1307648	-0.153754126	0.080405288	-1.912238989	0.055845547	0.173814386
668.2227018	0.119653978	0.057106042	2.095294542	0.036144817	0.129445592
577.8789293	0.111374313	0.126054424	0.883541483	0.37694378	0.589841864
362.9494625	0.358580563	0.092388994	3.881204322	0.00010394	0.001616917
150.044622	0.126372532	0.120298433	1.050491927	0.293492001	0.508521136
276.0387131	0.695780672	0.19253884	3.61371593	0.00030184	0.00374718
1184.60615	0.168611834	0.047794256	3.527868156	0.000418921	0.00487036
104686.787	0.17664834	0.118722771	1.487906147	0.136775646	0.313909445
180.2024586	0.527212388	0.165954593	3.17684723	0.001488854	0.013074658
36.96445231	0.63016909	0.236166846	2.668321572	0.007623126	0.042975937
3.092476042	0.019260478	1.027356058	0.018747617	0.985042442	0.9924524
263.1867445	-0.023091771	0.090339845	-0.255610032	0.798251971	0.89568199
42.72447854	0.226961212	0.569819774	0.398303504	0.690406478	0.833059131
1354.789736	-0.045839701	0.08231338	-0.556892463	0.577600905	0.755670053
309.7731108	0.085377498	0.093105773	0.91699467	0.359145433	0.573505333
1311.553042	0.304653091	0.084391856	3.609982139	0.000306218	0.00379296
5.978672465	-0.010524393	0.409635611	-0.025692085	0.979502937	0.989230713
4.43568085	0.12143102	0.540180221	0.224797236	0.822137017	0.910819449
2.237299322	0.038730133	0.820299326	0.047214635	0.962342164	0.981463149
90.83100304	-0.227024802	0.115386357	-1.967518587	0.049123455	0.160154325
94.21940161	-0.108011696	0.101980919	-1.05913633	0.289537696	0.504425128
366.0640016	0.095866495	0.077951177	1.22982742	0.218761738	0.423338526
569.0449729	-0.072523249	0.123553035	-0.586980714	0.557216659	0.741574435
52.88380548	0.021024303	0.196664932	0.106904177	0.914865	0.957710237
1465.949921	0.267767767	0.068428534	3.913101045	9.11184E-05	0.001457363
224.5168607	0.033510444	0.072452747	0.462514476	0.643712424	0.802157221
79.25193633	0.067417292	0.132453267	0.508989276	0.610759741	0.779429544
145.2882677	0.085555073	0.10633457	0.804583809	0.421059889	0.629405403
81.5627556	-0.152391867	0.428823475	-0.355372026	0.722310903	0.852432094
779.7062674	-0.302990548	0.063887989	-4.742527572	2.11068E-06	6.49932E-05
2459.267995	0.138187885	0.076844288	1.798284415	0.072131948	0.208145335
292.7179995	0.362649509	0.09894766	3.665064038	0.000247277	0.003235613
496.9702086	0.139819779	0.067481905	2.071959562	0.038269213	0.134797308
420.5894051	-0.372162765	0.214706742	-1.733353884	0.083032789	0.228033305
315.1700062	0.200536822	0.072581727	2.762910583	0.005728846	0.034915652
26.0809069	-0.17128322	0.278106082	-0.615891674	0.537966017	0.725898306
779.5246385	0.065347496	0.049450896	1.321462336	0.186347253	0.384649122
125.5182151	0.28505748	0.096285924	2.960531193	0.00307109	0.022167111
257.9435681	0.267728093	0.116892502	2.290378674	0.021999375	0.091890897
801.0000182	-0.078377038	0.093195665	-0.840994459	0.400351038	0.612232486
5.090264943	0.031893806	0.417359592	0.076418049	0.939086511	0.971033843
706.5235746	-0.239225189	0.156791544	-1.525753135	0.127071356	0.298371368
2324.59205	-0.283939031	0.098230073	-2.890550939	0.003845672	0.025932706
207.2963388	-1.160877755	0.351045803	-3.306912502	0.000943304	0.0092007
2.554245472	-0.316029236	0.653582402	-0.483533883	0.628716708	0.791856196

3.319542458	-0.9633634	0.704195787	-1.368033462	0.171301602	0.363595656
481.8708405	-0.001369741	0.091884423	-0.014907216	0.988106203	0.993893344
57.65953962	-0.513224063	0.16685913	-3.075792508	0.002099439	0.016764981
69.92320803	-0.920422425	0.181891092	-5.06029414	4.1861E-07	1.61126E-05
104.9575757	0.258224801	0.103325249	2.499145219	0.012449328	0.06160716
1.871697661	0.082618462	0.707701283	0.116741998	0.907064508	0.953303217
219.8608668	0.139403356	0.087196332	1.598729589	0.1098807	0.273729722
461.2046924	0.065814575	0.083024229	0.792715285	0.427943723	0.634941316
411.0484686	-0.226842609	0.109619863	-2.069356809	0.038512616	0.135293041
47.22981314	0.090201621	0.152912017	0.589892296	0.555262859	0.740021062
44.36279911	-0.056119315	0.163831833	-0.342542192	0.731942898	0.858466987
7.46473456	0.915760905	0.414418138	2.209751027	0.027122446	0.106485982
187.4524945	-0.26810113	0.095405564	-2.810120483	0.004952296	0.031270264
16.75529684	-0.152674813	0.244709933	-0.623901168	0.532692493	0.722031935
188.6022922	-0.315151166	0.126691208	-2.487553574	0.012862508	0.06300394
1253.231258	0.162436598	0.049932818	3.25310295	0.001141521	0.010697055
7.177118971	0.230045746	0.330412949	0.696237076	0.486280372	0.685050697
323.203455	-0.40042762	0.204041664	-1.962479688	0.049706663	0.161503179
158.58492	-0.874126196	0.10342978	-8.451397651	2.87836E-17	1.48182E-14
897.2470369	-0.027032217	0.077292876	-0.349737492	0.726535714	0.855164695
213.4848631	-0.133720293	0.098632449	-1.355743412	0.175180822	0.369375254
35.41073625	-0.550390554	0.237509204	-2.317344107	0.020484994	0.087518101
237.7288269	0.09519477	0.078309207	1.215626789	0.224127125	0.429184035
588.0218851	-0.143514463	0.092628369	-1.549357547	0.121295793	0.291158774
295.5731892	0.304981863	0.068348564	4.462154661	8.11396E-06	0.000201309
93.41884066	0.132541041	0.145195772	0.912843673	0.361324765	0.575339666
192.8895088	0.356734134	0.143526536	2.485492534	0.01293723	0.063299058
564.1348611	0.179600969	0.047998743	3.741784886	0.000182718	0.002544459
183.1739825	0.060006346	0.079460871	0.755168498	0.450147888	0.65510038
2983.359446	-0.272871506	0.07517206	-3.629959159	0.000283466	0.003600478
41529.15404	-0.061210785	0.061168671	-1.000688489	0.316977434	0.531977002
1.983549393	0.021787056	0.713799703	0.030522646	0.975650233	0.987719156
423.6703696	0.193926847	0.10119063	1.916450642	0.05530776	0.17272797
18.9287708	0.161240565	0.299788868	0.537847073	0.59068263	0.763867309
54.2870638	-0.311459265	0.386733468	-0.805358962	0.420612566	0.629066856
9199.447229	0.709078869	0.049492346	14.32704097	1.48306E-46	1.2216E-42
4.645489623	0.15998142	0.562568986	0.284376537	0.776121838	0.883652841
128.3738071	-0.432092278	0.153000804	-2.824117693	0.004741097	0.030187714
504.8209937	-0.029297149	0.405141277	-0.072313415	0.942352489	0.972429231
67.01982676	-0.193054749	0.316836165	-0.609320434	0.542312065	0.729846333
285.448791	-0.433407672	0.244102855	-1.775512505	0.075813303	0.214703661
82.63021255	0.09304447	0.186000575	0.500237537	0.61690783	0.783452019
180.9547042	-0.415558141	0.089857916	-4.624613608	3.75297E-06	0.000104437
8.088438434	0.599778273	0.557275157	1.076269533	0.28180673	0.495832968
275.89342	-0.1617578	0.130962191	-1.235148857	0.216775109	0.421573975
56.67077947	0.697712418	0.184780136	3.775905964	0.000159427	0.002273942
5.8223219	-0.137222523	0.469702684	-0.292147624	0.770173755	0.880181143
7.544903143	0.180611525	0.368646441	0.489931559	0.62418233	0.789055504

420.5005096	-0.234611744	0.1045231	-2.244592276	0.024794319	0.10011314
404.9997816	0.188753707	0.059479552	3.173421783	0.001506535	0.013215472
37.78353722	-0.118370441	0.228559373	-0.517897998	0.604529439	0.774598894
19.18135168	-0.116131315	0.254085082	-0.4570568	0.64763022	0.804604845
176.4442108	-0.144324989	0.097759458	-1.47632763	0.13985596	0.318494206
69.74954292	-0.529366444	0.21735703	-2.435469623	0.014872473	0.069842966
3082.194029	-0.054934542	0.038382214	-1.431249972	0.152358586	0.336596361
373.8549796	0.069668228	0.07799046	0.893291674	0.371700995	0.5851316
12.61315531	2.175858976	0.592407298	3.672910487	0.000239804	0.00315537
2.751645505	1.157368532	0.657548704	1.760125943	0.078386455	0.219316994
8.397041143	1.568743691	0.783413682	2.002446123	0.045236772	0.15162372
17.5880788	-0.076315901	0.321139673	-0.237640838	0.812159674	0.905367334
90.99088123	0.253663084	0.229349989	1.106008703	0.268722753	0.48051
3043.251303	0.162481861	0.106463774	1.526170407	0.126967431	0.298297413
2125.053308	0.311791257	0.074241841	4.199670341	2.67304E-05	0.00054365
2.700109786	-0.102166788	0.730578381	-0.139843706	0.88878348	0.944444948
232.8725801	0.139835774	0.139155231	1.004890534	0.314949563	0.529706901
50.56255355	-0.085386785	0.256486031	-0.332910079	0.739202161	0.862808843
5.171284014	-0.619655832	0.420702279	-1.47290819	0.140775793	0.319819079
10.71527224	0.861480679	0.901157836	0.955970913	0.33908693	0.554005032
11.62478744	-0.186942103	0.388027352	-0.481775581	0.629965378	0.792701622
4.251084114	0.834208126	0.84847862	0.98318108	0.325518325	0.540529068
5.961732296	1.596890674	0.919643473	1.736423647	0.082488954	0.226978959
32.97937118	-0.039079461	0.483729224	-0.080787884	0.935610644	0.969704969
7.014280028	0.73852299	0.604096451	1.22252496	0.221509171	0.426251849
36.02337901	0.723406792	0.53536104	1.351250347	0.176615252	0.371733698
59.01960906	-0.354594804	0.143605639	-2.469226183	0.01354056	0.065445421
13.50683608	-0.780279726	0.330059882	-2.364055033	0.018076127	0.079911204
9.601654286	-0.839182287	0.326629122	-2.569220657	0.010192753	0.053222002
7.55700642	-4.224385234	2.441416119	-1.730301198	0.083576476	0.228862843
299.1735886	0.233489452	0.10469403	2.230207888	0.025733645	0.102787785
93.87109022	0.212619188	0.155281108	1.369253416	0.170920074	0.363086692
562.1182726	0.198742193	0.06947828	2.860493868	0.004229817	0.027883958
1890.211083	0.041183391	0.081826393	0.503302047	0.614751927	0.782242435
116.2101817	-0.162163604	0.445878957	-0.363694229	0.716086353	0.849440394
218.4923854	0.264446257	0.173643548	1.522925904	0.127777244	0.29947394
59.97885007	-0.375395084	0.242317648	-1.549185905	0.121337037	0.291158774
5.666159437	-0.047906471	0.469674096	-0.101999389	0.91875716	0.959711208
2.264966904	0.6168122	0.673499951	0.915831098	0.35975549	0.573951606
2180.064703	0.007581825	0.126463369	0.059952739	0.952193275	0.977124952
18.32712652	0.559910447	0.51224126	1.09306003	0.274367444	0.487061343
490.1472024	0.061351865	0.114386468	0.536355968	0.591712556	0.764478044
101.8366714	-0.239962703	0.352444314	-0.680852815	0.495964627	0.69288681
86.27469213	-0.166163472	0.105466225	-1.575513606	0.11513794	0.281672472
614.1791779	-0.042717885	0.083808514	-0.509708171	0.610255928	0.779148737
100.0223408	0.155079314	0.111098072	1.395877632	0.162751347	0.351997597
29.85623342	-0.061961172	0.186182268	-0.33279846	0.739286421	0.862808843
1364.280543	-0.1374396	0.08678116	-1.583749276	0.113250786	0.278944032

287.478499	-2.161833526	0.283710145	-7.61986684	2.53938E-14	6.64027E-12
53.07985072	-0.178853719	0.241429069	-0.740812695	0.458807017	0.661849322
1039.307479	-0.088720894	0.06693809	-1.325417167	0.185032826	0.382895463
110.6253403	0.073649467	0.167297255	0.440231173	0.659769684	0.812578183
270.2967427	-0.255004749	0.095564651	-2.668400354	0.007621338	0.042975937
49.46122025	-0.115680827	0.171441695	-0.674753169	0.499832598	0.695753462
1511.05306	-0.170146274	0.108739443	-1.564715335	0.117649671	0.285401367
53.37238886	-0.386764863	0.15051925	-2.569537535	0.010183436	0.05319021
15.09961724	0.196279313	0.309112949	0.634976028	0.52544405	0.716333226
20.55987601	-0.617584259	0.25790847	-2.394586959	0.016639097	0.075472996
507.2383173	-0.369301346	0.221316172	-1.668659558	0.095184868	0.248139755
334.7712386	-0.142791755	0.157912165	-0.904247975	0.365863927	0.579264039
26.92354382	0.142489625	0.293666483	0.485209014	0.627528089	0.790978857
1538.352577	-0.12736965	0.175591201	-0.725376042	0.468221367	0.66992173
37.32141926	-2.367693463	0.721410266	-3.282034611	0.00103061	0.009859618
3739.589837	-0.199686556	0.140064938	-1.425671253	0.153963258	0.338591739
366.7009777	-0.431809827	0.152243958	-2.836301893	0.004563928	0.029450119
71.17613076	-0.210927636	0.132001982	-1.597912646	0.110062419	0.273975264
283.4874458	-0.023756355	0.081363331	-0.291978639	0.770302956	0.880208844
584.9028333	-0.027845453	0.097508259	-0.285570196	0.775207333	0.883490141
38.21107316	0.458530865	0.271326106	1.689962204	0.091035186	0.241655438
2563.509349	-0.061110207	0.082935023	-0.736844396	0.461216981	0.66377753
16.17828189	-0.094368584	0.320483553	-0.294456871	0.768408815	0.879190026
369.2325041	-0.076559567	0.064276361	-1.1910999	0.233614369	0.440439817
46.18712047	-0.342685241	0.212576189	-1.612058441	0.106949223	0.268415829
181.1159867	-0.084393251	0.083901282	-1.005863663	0.314481158	0.52931583
95.98623833	0.085048691	0.153697129	0.553352498	0.580022059	0.757293715
452.4785207	0.328906326	0.086689928	3.794054687	0.000148207	0.0021361
189.390151	0.280081771	0.122649728	2.283590642	0.0223956	0.092956694
162.9165296	-0.068085325	0.088720722	-0.767411755	0.442836742	0.648528056
14.26249236	-0.298135477	0.493473574	-0.604156925	0.545739316	0.732603446
171.3537396	-0.041446498	0.121869006	-0.340090556	0.733788334	0.859681792
8.957896749	-0.51343914	0.419378007	-1.224287232	0.220843892	0.425468632
436.4835407	-0.033291855	0.130509218	-0.255091983	0.798652056	0.895947836
153.6061817	-0.258424269	0.131898934	-1.959259721	0.050082378	0.162387358
300.6382221	-0.248527099	0.307025151	-0.809468208	0.418245883	0.627406908
636.377033	-0.235102805	0.085299816	-2.75619358	0.005847838	0.035379096
25.73011101	0.622536014	0.351004111	1.773586106	0.076131638	0.215164282
175.2515772	0.415994946	0.195992023	2.122509581	0.033794969	0.123390585
1251.451998	-0.056477859	0.045947658	-1.229178193	0.219005003	0.42356051
9.702338547	-0.018606026	0.312361076	-0.059565765	0.952501486	0.977124952
5049.326432	-0.489740327	0.177894853	-2.75297638	0.005905616	0.035623989
3.876362181	-0.278228941	0.616669645	-0.451179886	0.651859905	0.807424066
166.1310852	-0.069016141	0.095929885	-0.719443589	0.471867648	0.672219615
8.081124417	-0.19547669	0.333379423	-0.586348995	0.557641013	0.741810242
129.0852658	0.044319616	0.141943	0.312235306	0.754861702	0.871827159
45.38676286	0.563259108	0.177940783	3.165430093	0.001548539	0.013462072
51.51695033	0.583463873	0.1929662	3.023658409	0.002497382	0.019073657

40.45626024	0.51387183	0.210876643	2.436836167	0.014816391	0.069639149
85.55314297	0.116418682	0.210175168	0.553912642	0.579638633	0.757293715
1067.648275	0.016837042	0.067598661	0.249073599	0.803303851	0.899164189
694.7286615	-0.166751914	0.066973485	-2.489819877	0.012780785	0.062738573
7.714615419	0.684912874	0.450061746	1.521819797	0.12805424	0.299781945
20.66481624	-2.051842235	0.289469588	-7.088282579	1.35786E-12	2.5712E-10
136.6877648	-0.687607528	0.095177609	-7.224467344	5.0307E-13	1.03595E-10
691.9240767	-0.845069173	0.086117156	-9.813017647	9.89639E-23	1.2541E-19
1.880921885	-0.794849182	0.974614343	-0.815552519	0.414756115	0.624576769
100.779048	-0.328734126	0.105615027	-3.11256963	0.001854663	0.015307471
97.12272138	0.769283657	0.452383416	1.700512509	0.089034566	0.238186982
2.601829225	1.288821565	0.782562814	1.646924109	0.099573646	0.255829108
11.03498556	0.421841706	0.316337967	1.333515892	0.182362564	0.379227579
973.4230864	0.162252352	0.083287045	1.948110308	0.051401766	0.164906074
504.8868367	-0.26436922	0.214673194	-1.231496188	0.218137342	0.422825575
483.312697	-0.059169294	0.064046028	-0.923855799	0.355561399	0.57021831
313.8029278	-0.083343574	0.105771061	-0.787961973	0.430718949	0.637594068
95.33903825	-0.509121135	0.161467547	-3.153086456	0.00161554	0.013855297
264.8189983	-0.020516437	0.072534539	-0.282850592	0.77729137	0.884207847
13.15115846	-1.81995734	0.578584506	-3.145534181	0.001657838	0.014077952
889.6845058	0.085138216	0.160605981	0.530106136	0.596038345	0.767938112
407.487553	0.016216211	0.088570822	0.183087505	0.85472936	0.928813422
20.91421979	0.514862462	0.32395521	1.589301382	0.111992367	0.277021359
161.9465306	-0.373720364	0.110836681	-3.371811205	0.000746756	0.007674399
977.6407345	0.18659978	0.07233636	2.579612532	0.009891123	0.052092824
36.87669495	-0.080826699	0.237685742	-0.340056995	0.733813607	0.859681792
260.1247713	-0.227596482	0.074549477	-3.052958805	0.00226597	0.01775064
201.1970241	-0.23987317	0.191291754	-1.253965031	0.209854716	0.414034162
893.6540817	-0.449223449	0.125582821	-3.577109076	0.000347415	0.00420523
1218.563576	-0.403581658	0.064085436	-6.297556524	3.02374E-10	2.87753E-08
107.3300571	-0.000705437	0.140306282	-0.00502784	0.995988381	0.997926809
64.75592749	-0.226764672	0.146903987	-1.543625029	0.122679198	0.292646555
1.772358195	-4.300168246	2.27196904	-1.892705477	0.058397048	0.180054831
31.65612398	-0.268928689	0.365918288	-0.734941919	0.462374868	0.664876519
546.7685825	0.195252543	0.165006761	1.183300255	0.236690172	0.444100191
42.32717263	0.300012255	0.167381832	1.792382434	0.073071727	0.209828068
169.1621135	-0.560397457	0.17768879	-3.153814347	0.001611516	0.013848779
74.93877004	0.192758697	0.134382798	1.434400092	0.151458121	0.335636412
28.4691602	-0.618665577	0.346439236	-1.785783803	0.07413427	0.211719093
2.252213112	1.340160548	0.897027136	1.494002237	0.135175034	0.311494407
52.55939677	-0.877734934	0.239427971	-3.665966561	0.000246406	0.003226784
3.293196257	-0.918915519	0.688481942	-1.334698069	0.181975186	0.378613187
18.23049361	-0.338454401	0.290125655	-1.16657867	0.243380561	0.450998082
51.91348896	-1.004676133	0.40548737	-2.477700191	0.01322322	0.064354309
313.9005183	-0.259685108	0.15907344	-1.632485646	0.10257722	0.261628289
15.62256363	-0.901925066	0.557852465	-1.616780643	0.105925639	0.266918867
18.31049708	0.645195599	0.239534566	2.693538593	0.007069796	0.04063776
96.26463208	-0.415478474	0.118621419	-3.502558615	0.000460812	0.005228253

96.34258392	-0.748292033	0.386969507	-1.933723507	0.053147132	0.168596518
345.6269496	-0.119737921	0.105905289	-1.13061323	0.258217916	0.468747323
115.942779	-0.279293153	0.144797066	-1.928859204	0.053748344	0.169691493
73.83591819	0.041727598	0.247051596	0.16890236	0.865873443	0.933595071
3673.357465	0.569241877	0.187580992	3.034645836	0.002408183	0.018512555
3752.972874	-0.023733165	0.093471302	-0.253908571	0.799566193	0.896484956
25.99207577	0.399757664	0.214758753	1.86142664	0.062683951	0.189131029
542.8216763	-0.090404182	0.056854326	-1.590102068	0.1118118	0.276657794
1433.826359	0.310280103	0.08928791	3.475051705	0.000510755	0.005673753
17.30842621	0.512270091	0.277477486	1.846168129	0.064867797	0.193678961
860.4478783	-0.218444334	0.066568061	-3.281518652	0.001032497	0.009871941
38.93848436	-1.033639268	0.417061301	-2.478386906	0.013197794	0.064268538
1927.178683	-0.031414246	0.102537112	-0.306369527	0.759323307	0.87432656
31.6949517	-2.0960709	0.712392082	-2.942299548	0.003257846	0.023113591
35.08968172	0.751303915	0.212516994	3.535265118	0.000407366	0.004776478
1039.506432	-0.00168892	0.08899572	-0.018977538	0.984859024	0.992448306
9.738391879	-0.138643749	0.339112772	-0.408842603	0.682655172	0.828547438
2.399138307	-0.658695169	0.798781658	-0.824624806	0.409584634	0.619889505
252.6322175	-0.342478254	0.178287314	-1.920934512	0.054739964	0.171458081
3.093756488	-0.915107419	0.752745883	-1.21569236	0.224102136	0.429184035
17.55965506	-0.300432665	0.469927583	-0.639316943	0.522616769	0.71401465
252.412243	-0.394844295	0.445887187	-0.885525097	0.37587349	0.589110444
1.730485778	-0.270177019	0.709758554	-0.380660462	0.70345521	0.841042248
78.34208336	0.456933613	0.154610216	2.955390813	0.003122732	0.022405875
31.33935669	-0.184009054	0.439613485	-0.418570085	0.675530356	0.824469335
62.55824749	-0.236521537	0.199170066	-1.187535568	0.235016436	0.442273333
391.8381951	0.01485631	0.155518102	0.095527849	0.923895571	0.962576248
145.6931879	-0.039363791	0.081028806	-0.485799962	0.627109001	0.790677612
112.7589778	-0.077745862	0.107449826	-0.723555029	0.469338958	0.670512744
15.43784372	-0.450929401	0.489328435	-0.921527073	0.356775313	0.571353797
6.433347668	-1.65964569	0.978502077	-1.696108499	0.089865334	0.239553644
2.827101432	-2.003576105	1.281799947	-1.563095793	0.118030065	0.285903145
3.517375107	-1.601331795	1.576950043	-1.015461334	0.309885969	0.525104037
353.8236199	-0.281510394	0.244709794	-1.150384665	0.249985474	0.458961405
3026.606986	0.006963922	0.145040532	0.04801363	0.96170538	0.981242068
62.95527445	-0.526093383	0.303963831	-1.730776258	0.083491678	0.228744704
48.58571482	-0.20699357	0.139533553	-1.483468064	0.137950095	0.315856827
203.9854257	-0.103917734	0.117241974	-0.88635265	0.375427526	0.588859665
90.83877514	-0.150759271	0.094293884	-1.598823441	0.109859839	0.273719145
217.7180597	-0.178844816	0.12591108	-1.420405704	0.155489603	0.340720367
30.09712591	0.054815687	0.41285144	0.132773394	0.894372597	0.946604186
2.179745325	-0.900278311	0.992307772	-0.90725714	0.364270834	0.5779081
4.134616795	-0.620635189	0.511455894	-1.213467665	0.22495107	0.430111876
1515.502381	-0.240481033	0.044627989	-5.388569808	7.10206E-08	3.36205E-06
62.9572455	-0.344256857	0.486522797	-0.707586282	0.479202214	0.678327657
271.554339	0.029909585	0.105243438	0.2841943	0.776261484	0.883652841
8.635887574	-0.176134206	0.399996318	-0.440339567	0.659691188	0.812578183
23.05101648	0.511422989	0.248640135	2.056880269	0.039697744	0.137912408

258.4984752	-0.101242398	0.147434278	-0.686695115	0.492274876	0.689720727
121.743711	0.279927381	0.110008346	2.544601312	0.010940257	0.055902541
239.0403854	0.945537079	0.130180327	7.263287011	3.77795E-13	7.97923E-11
693.3167038	-0.408346881	0.093253532	-4.378889186	1.19286E-05	0.000277558
186.2142785	-0.324764539	0.175458485	-1.850948042	0.064177028	0.192297629
118.0563436	-0.369034552	0.111796943	-3.300935976	0.000963629	0.009371204
172.0944099	-0.085683125	0.096407677	-0.888758316	0.374132982	0.587693851
281.3647534	0.045719991	0.077662547	0.588700633	0.556062113	0.740545496
166.1504408	0.323194303	0.113437389	2.84909857	0.004384329	0.028616261
806.9114126	0.174424233	0.412127408	0.423228909	0.672128257	0.821961317
26.31654119	-0.362507229	0.299241164	-1.211421665	0.22573384	0.431358228
6.492979325	0.080829098	0.403616511	0.200262121	0.841275586	0.921330769
91.78629953	-0.144996923	0.20005476	-0.724786169	0.468583222	0.670018398
339.9288381	-0.029025916	0.064854983	-0.44755105	0.654477237	0.809205795
86.68306989	-0.141226556	0.112473883	-1.255638655	0.20924701	0.413226473
312.2857869	0.37851675	0.161892449	2.338075382	0.019383338	0.084021521
320.2369628	0.062265349	0.064688566	0.962540258	0.335778291	0.550901568
341.5463626	-0.427302758	0.403783081	-1.058248298	0.289942257	0.504916358
79.29273164	-0.084343955	0.195879695	-0.430590596	0.66676608	0.818258671
53.85502137	-0.216600133	0.563351589	-0.384484817	0.700619147	0.83939434
197.6029151	-0.148189185	0.086791187	-1.707422034	0.087743638	0.236113801
65.37767439	-0.205917242	0.189582329	-1.086162632	0.277407047	0.49044899
67.82089901	-0.451645404	0.173318302	-2.605872542	0.009164056	0.049400737
42.79883526	-0.206807121	0.332455821	-0.622058955	0.533903103	0.723137361
2.022043167	0.911572652	0.898102423	1.014998544	0.310106521	0.525137224
3.939587693	0.219405694	0.655364607	0.334784167	0.737787905	0.861800757
4164.733392	0.096457121	0.167673854	0.575266316	0.565111198	0.747342208
418.6796294	0.370746875	0.088432483	4.192428668	2.75984E-05	0.000556494
29.87866461	0.196018115	0.223391035	0.877466346	0.380233391	0.593122325
60.49428724	-0.073025425	0.189003065	-0.386371646	0.699221446	0.838658471
335.7029864	0.035126235	0.071969992	0.488067796	0.625501821	0.790284301
146.8214194	0.101930497	0.110065227	0.926091728	0.354398313	0.569096189
5.180304702	-1.855285738	0.818203035	-2.267512659	0.023358927	0.095582453
437.5616969	0.081879114	0.071316825	1.148103746	0.250925738	0.459816531
3.198272865	-1.392423917	0.806428118	-1.72665596	0.084229469	0.229924818
33.79272393	-0.840328976	0.257969713	-3.257471451	0.001124096	0.010580556
130.7227853	-0.235019545	0.102161947	-2.300460709	0.021422133	0.09032855
261.3374812	-0.228346069	0.109330908	-2.088577448	0.036745777	0.13108487
77.11382729	-0.000615306	0.119324148	-0.005156595	0.995885651	0.99788457
78.36355594	-0.292758722	0.121315337	-2.413204546	0.015812943	0.072888197
4.12844075	-0.446767951	0.657679746	-0.679309275	0.496941921	0.693546358
936.8732992	-0.534968994	0.362880717	-1.474228222	0.140420154	0.319293528
1.901750733	-0.076925754	0.783857811	-0.098137383	0.921823204	0.961410841
29668.53164	0.258710261	0.505891534	0.511394724	0.609074689	0.778180891
20.98949006	0.301816635	0.223694418	1.349236329	0.177261069	0.372537049
896.2404399	0.101282005	0.090043431	1.124812812	0.260668364	0.471750216
2919.697484	-0.004588829	0.093258749	-0.049205344	0.960755652	0.980731546
4604.653841	0.008116497	0.088890151	0.091309291	0.927246836	0.96527421

477.6863998	-0.01662906	0.062354303	-0.266686641	0.78971044	0.891207713
3.053552388	1.299215429	0.611136418	2.125900847	0.033511516	0.122709207
15.87156406	0.347178421	0.321192458	1.08090465	0.27973953	0.493143822
382.3459406	-0.307512861	0.083047608	-3.702850315	0.000213191	0.002881135
381.1685318	-0.120150014	0.11187083	-1.07400664	0.282819705	0.496600591
484.3012005	0.259021531	0.089924571	2.880431104	0.003971317	0.02651945
231.1232873	0.4350584	0.150001541	2.900359544	0.003727348	0.025394681
371.2817107	0.507753554	0.099052598	5.126100305	2.95805E-07	1.18567E-05
146.1354266	0.676320535	0.199727919	3.386209287	0.000708653	0.00735624
610.9433107	-0.065865013	0.111018726	-0.593278404	0.552994849	0.738432126
439.369295	-0.122643056	0.063229025	-1.93966387	0.052420553	0.166874627
171.3358834	-0.183939878	0.104546794	-1.759402376	0.078509189	0.219474016
98.87169505	-0.227036805	0.116655602	-1.946214341	0.051628998	0.165441765
388.636712	0.132667837	0.072783961	1.82276199	0.068339447	0.200717427
116.4377522	-0.047236537	0.12520118	-0.377285076	0.705961762	0.842949182
981.8890223	0.007837288	0.064812213	0.120923009	0.903752018	0.951582251
9.606239163	0.121351809	0.366918356	0.330732457	0.740846584	0.863925015
294.4753548	0.05692338	0.072923945	0.780585585	0.435046272	0.641743248
86.18348956	0.060143758	0.128047421	0.469699097	0.638570013	0.79822463
436.8482935	0.319768488	0.112811078	2.834548639	0.004589047	0.029565883
351.5682646	0.032790891	0.058468105	0.560833823	0.574910827	0.753826883
359.7150162	0.020980018	0.053380443	0.393028177	0.69429867	0.835430304
6.196704828	-0.21492964	0.550487204	-0.390435306	0.696214685	0.836908188
56.66621508	0.250111733	0.236717514	1.056583136	0.29070188	0.50586884
1409.42784	0.035228184	0.053613546	0.657076178	0.511131935	0.705284153
240.3042394	0.207387829	0.131838152	1.573048662	0.115707551	0.282562436
26.92957509	0.390482312	0.242336465	1.611322963	0.107109348	0.268694899
34.95137429	-0.219465881	0.194637514	-1.127562085	0.259504907	0.470367534
444.9716923	0.030665496	0.058027305	0.528466665	0.597175478	0.769064167
6.210576538	0.706031274	0.383354399	1.841719504	0.06551619	0.195068446
43.29177993	0.1190683	0.175441132	0.678679503	0.497340956	0.693868133
727.2015318	-0.170565758	0.054133023	-3.150863322	0.001627886	0.013907845
183.343423	0.113636232	0.104566249	1.086739102	0.277152128	0.490103495
777.4883711	0.002167151	0.091968232	0.023564129	0.981200285	0.990337795
347.1673504	0.067414388	0.098735909	0.682774768	0.494749176	0.691540635
72.16315987	0.655258165	0.179604612	3.648337083	0.000263943	0.00340768
1275.613863	0.43243335	0.0565775	7.643203582	2.11883E-14	5.62992E-12
3.122554668	-1.089357272	0.654263105	-1.665014065	0.09590995	0.249447772
121.4776333	-0.498696559	0.097640588	-5.107471918	3.26498E-07	1.29296E-05
124.1025416	-0.813540172	0.180514294	-4.506790856	6.58154E-06	0.00016784
146.8186145	-0.036996061	0.161083383	-0.229670249	0.818348015	0.908774216
199.1068945	0.19759583	0.130251029	1.517038537	0.129256955	0.301825524
53.44973196	-0.184880839	0.150555058	-1.227994872	0.219448893	0.423921325
68.12997352	-0.308331654	0.12877311	-2.394379177	0.016648528	0.075493489
1697.742666	-0.320240096	0.11173584	-2.866046338	0.004156334	0.027520678
410.7276042	0.048255631	0.053145036	0.907998838	0.363878836	0.577700935
47.10253949	-0.127734628	0.183524511	-0.69600855	0.486423476	0.685193701
2756.751466	0.016407164	0.093597427	0.175295035	0.860847813	0.931184922

1736.074624	-0.210980618	0.10122517	-2.084270316	0.037135586	0.13196703
47.56955975	-0.164342283	0.148927555	-1.103504872	0.269807977	0.481822939
14.82256356	0.532461262	0.29972077	1.776524404	0.075646523	0.214418586
1416.250246	-0.023103827	0.055023925	-0.419886925	0.67456806	0.823844185
29.76970707	0.488457297	0.36233717	1.348073942	0.177634602	0.372973799
537.6146149	-0.148329966	0.088469936	-1.676614366	0.093617916	0.245778732
14.06239677	0.118790403	0.308123955	0.385527972	0.699846287	0.839012442
266.7618485	-0.128683574	0.086512	-1.48746503	0.136892033	0.314024548
8.329155613	-0.77452344	0.44516966	-1.739838781	0.081887331	0.225927296
37.99062187	0.273036608	0.343982238	0.793752055	0.427339789	0.634576861
2.119066567	1.278586058	0.803444949	1.591379795	0.111524129	0.27623643
22.45520503	0.362906098	0.403662939	0.899032492	0.368635354	0.582253003
476.5051359	-0.03344024	0.061734515	-0.54167819	0.588040217	0.761885532
900.2104313	0.436699788	0.064884677	6.730399379	1.69198E-11	2.26244E-09
6.143292195	1.012509473	0.391718604	2.584787813	0.009743894	0.051664279
764.7534494	-0.042459084	0.079712261	-0.532654366	0.594272861	0.76681093
218.5620737	0.002049212	0.101157811	0.02025758	0.983837895	0.991966796
7.506448839	0.013934256	0.881916711	0.015799968	0.987393974	0.993528977
8.99806206	0.035734207	0.318146843	0.112319853	0.910569801	0.955037829
461.0552829	0.02030309	0.067473529	0.300904522	0.763487302	0.876860696
1594.832821	0.255433401	0.132283762	1.930950531	0.05348917	0.169197502
276.5677931	-0.158102399	0.112305063	-1.407794042	0.159192065	0.346574611
195.4140234	-0.049331056	0.120936434	-0.407908965	0.68334051	0.828701792
485.3896724	-0.156831105	0.069180693	-2.266977946	0.023391573	0.095692271
15.35762967	0.3843005	0.380756655	1.009307375	0.312827244	0.527591731
2885.581117	-0.545670152	0.125077572	-4.362653879	1.28494E-05	0.000294002
7.796690893	-1.230462583	0.479150182	-2.568010259	0.010228413	0.053306825
148.4192751	-0.320025455	0.192300702	-1.66419286	0.096073895	0.249719365
263.2933745	-0.118285361	0.069659988	-1.698038776	0.089500443	0.239006372
423.05003	-0.10473172	0.055009481	-1.903884893	0.056925177	0.176640679
12.75849985	-0.172867687	0.295892979	-0.584223686	0.559069828	0.74317085
249.3524695	0.140233944	0.104811733	1.337960352	0.180909362	0.377110062
29.99599623	0.049211082	0.267909718	0.183685319	0.854260327	0.928426219
26102.51393	0.28851139	0.110969115	2.599925133	0.00932441	0.050084882
89.9645471	0.393650938	0.116094422	3.390782546	0.000696934	0.007280461
3.799360593	-0.917997684	0.960401521	-0.955847803	0.339149133	0.554005032
81.15021824	-0.064619587	0.124085355	-0.520767234	0.602528931	0.77305874
270.0975459	-0.034394163	0.083973343	-0.409584304	0.682110913	0.82820821
11.55597142	-1.737103808	0.619586771	-2.803648963	0.005052789	0.031722851
354.7821505	-0.117389191	0.09383009	-1.251082578	0.210904351	0.415354248
2.314667144	-0.840400816	0.741948534	-1.13269422	0.257342686	0.467957282
68.0944737	0.123976494	0.176069004	0.70413583	0.481348189	0.68037152
1146.224185	0.32618793	0.134170867	2.431138269	0.015051469	0.070469655
12.53521133	0.902287771	0.367982993	2.451982262	0.014207166	0.067605101
14.98339563	-0.157359248	0.264606957	-0.594690519	0.552050361	0.737796722
483.7778923	0.061032085	0.094828493	0.64360492	0.519831659	0.712158565
152.1315974	0.136394099	0.085539978	1.594507051	0.110822513	0.275320729
535.4438743	-0.141731243	0.087169183	-1.625932897	0.103963931	0.263654834

4.307148019	0.37887299	0.44827273	0.845184114	0.398008051	0.61061507
124.9434602	0.228350666	0.147660185	1.546460652	0.121993356	0.291813351
271.7795589	-0.360147362	0.203848932	-1.76673656	0.077272358	0.217158788
2.098941523	0.5436973	0.708939693	0.766916151	0.443131371	0.648613612
46.44615756	-0.201158639	0.257703457	-0.78058184	0.435048476	0.641743248
52.13120355	0.053096659	0.184330879	0.288050813	0.773307843	0.882051749
642.7745888	-0.069574104	0.050277907	-1.383790781	0.166422527	0.357357235
23.65172213	-0.133003487	0.235055763	-0.565838018	0.5715039	0.751186819
12.37695185	-0.001604644	0.404041132	-0.003971486	0.996831221	0.998078933
311.8898807	-0.095976812	0.094803064	-1.012380914	0.31135596	0.526264641
19.04159257	-0.436914711	0.269513048	-1.621126378	0.104990538	0.265344941
314.7720056	-0.017846238	0.080030803	-0.222992118	0.823541641	0.91138289
674.4636498	-0.104636242	0.086092352	-1.215395325	0.22421535	0.429237897
13.09224564	-0.039370234	0.323796548	-0.121589417	0.903224195	0.951456563
2065.538384	0.060788705	0.049068629	1.238850688	0.215400798	0.419942337
118.0764391	-0.116104207	0.117374077	-0.989181002	0.322574591	0.537537307
53.81955646	-0.002090606	0.165845559	-0.01260574	0.989942341	0.994530438
1686.084414	-0.111721961	0.119396852	-0.935719485	0.349417636	0.564177804
139.205213	0.245096193	0.145030211	1.689966469	0.09103437	0.241655438
135.1214209	-0.354114261	0.148971665	-2.377057821	0.017451352	0.078102032
48.70987698	-0.542866383	0.360689678	-1.505078785	0.132303823	0.30681677
141.4539008	0.144761066	0.158406262	0.913859496	0.360790679	0.575045051
335.2089193	-0.330957665	0.099132695	-3.338531891	0.000842224	0.008424282
37.82951822	-0.247611157	0.154190799	-1.605875051	0.108301366	0.270931071
2.890556451	-0.678069911	0.571290736	-1.186908641	0.235263659	0.442536368
79.36191032	-0.106218003	0.166981894	-0.636104911	0.524708044	0.715791603
30.94466965	-0.007052905	0.226191776	-0.031181084	0.975125125	0.987719156
194.5242793	-0.020021151	0.083485661	-0.239815441	0.810473336	0.903976827
630.5562544	-0.07100262	0.102201526	-0.694731501	0.487223583	0.685617263
118.7695792	0.022648484	0.286385194	0.079083989	0.936965818	0.970302671
165.6689415	0.248119822	0.145262722	1.70807636	0.087622175	0.235864004
544.8056725	-0.171641527	0.068128566	-2.519376777	0.011756278	0.059080677
43.65700594	0.467921475	0.202600775	2.30957396	0.020911753	0.088834506
366.4139165	0.049961784	0.069092603	0.723113355	0.469610245	0.670627529
1593.798439	-0.35390532	0.097873924	-3.61593064	0.000299271	0.003726517
12.84437461	-1.625319825	0.736318469	-2.207359851	0.027288929	0.106786528
1200.970646	0.156938497	0.150191313	1.044923933	0.296058127	0.51070802
226.1540642	-0.594997787	0.226538548	-2.626474802	0.008627438	0.047369685
97.96907006	-0.143443997	0.221739804	-0.646902333	0.517695156	0.710176534
412.8182881	-0.040003357	0.062213578	-0.643000417	0.520223829	0.712399615
47.77393057	-0.389057009	0.2215856	-1.755786516	0.079124868	0.220597268
382.496069	-0.092233936	0.073106309	-1.261641252	0.207077905	0.410599683
89.39041163	-0.099580666	0.143501327	-0.693935511	0.487722652	0.685967982
743.279535	-0.05389164	0.086360211	-0.624033219	0.53260577	0.722031935
21.55862645	-0.191893294	0.292977662	-0.654975851	0.512483302	0.706319325
96.98039471	0.248897494	0.141187565	1.762885379	0.077919824	0.218450565
132.6074039	-0.064958615	0.163400399	-0.397542574	0.690967398	0.833491756
1899.557174	-0.008116737	0.096831066	-0.083823683	0.933196618	0.96844633

102.0125942	0.183422593	0.141870864	1.292884162	0.196051126	0.397218218
12.96600036	0.765202662	0.386715677	1.978721598	0.04784736	0.157395648
1.888725541	-3.113963265	1.945408052	-1.600673577	0.109449236	0.272960956
259.1917415	0.272643124	0.07729572	3.527273249	0.000419863	0.004877875
4251.87502	0.00931766	0.09352132	0.099631399	0.920636965	0.960882752
58.98617052	-0.179643946	0.138321302	-1.298743893	0.194031835	0.394870964
29.52598737	-0.248230486	0.26453842	-0.938353251	0.348062902	0.562707385
1672.282754	-0.024523617	0.183209132	-0.133855868	0.893516548	0.946123642
12.37097899	0.311333662	0.366998534	0.848323994	0.396257559	0.609075814
1233.607087	-0.167455384	0.098139549	-1.706298685	0.087952483	0.236408595
5519.357462	-0.559236039	0.226094113	-2.473465723	0.013380964	0.064930191
244.1819602	0.415440699	0.077378631	5.368933167	7.92038E-08	3.68588E-06
21.84120159	0.213098969	0.263717129	0.808058884	0.419056685	0.628199717
188.3811131	0.204935113	0.101107766	2.026897852	0.04267286	0.145423175
500.89975	-0.128302682	0.081333791	-1.577483115	0.114684404	0.281231152
211.8543132	0.011777505	0.097160772	0.121216665	0.903519425	0.951516909
49.13424532	0.039981824	0.154742573	0.258376368	0.796116453	0.894773586
108.5197753	-0.059403656	0.097393133	-0.609936795	0.541903677	0.729415899
220.0593227	-0.574177253	0.09183026	-6.252593099	4.03693E-10	3.69469E-08
19.44193703	-0.138034843	0.221458454	-0.623299046	0.533088025	0.722330327
259.2253619	0.107011361	0.093871151	1.139981346	0.254294073	0.463582742
26.02674443	-0.784815643	0.315967224	-2.483851435	0.012997002	0.063459576
63.85044566	-0.004256247	0.176451336	-0.024121366	0.980755801	0.990108149
2711.641073	-0.230800654	0.188451922	-1.224719024	0.220681104	0.425304224
20.12707273	0.344361814	0.361383097	0.952899615	0.340640935	0.555270316
374.4384006	0.113188259	0.154250961	0.733792894	0.463074972	0.665466896
3.750422983	-0.615512741	0.475018803	-1.295765002	0.195056459	0.395977832
257.8116447	0.099646878	0.073829746	1.349684687	0.177117146	0.372398541
1.936144672	-0.698730725	0.84820093	-0.82377972	0.410064733	0.620217261
576.6554888	-0.64272385	0.149336283	-4.303869344	1.67841E-05	0.000369653
14.42963399	1.073266886	0.272808875	3.934134784	8.34969E-05	0.001361909
5.556964714	0.698681059	0.52031504	1.34280389	0.179335484	0.374967985
55.79192245	-0.005096643	0.140871096	-0.036179478	0.971139249	0.985661927
229.7055913	-0.074004746	0.076754237	-0.964177984	0.3349567	0.550070918
564.5708909	-0.244803692	0.055972547	-4.373638622	1.22193E-05	0.000282328
14.41703612	0.17167358	0.268934423	0.638347364	0.523247586	0.714616913
7.708164429	0.378775509	0.367127025	1.031728758	0.302199201	0.517049928
5.643760975	0.354326961	0.471568313	0.751379919	0.452424044	0.65707782
15.02229237	0.007842311	0.278566963	0.028152338	0.977540651	0.988278901
450.7902831	0.215480008	0.101827156	2.116134996	0.034333328	0.124638
341.6518105	0.041199289	0.078258762	0.526449538	0.598575904	0.7697845
509.7488332	-0.215947491	0.066495192	-3.247565501	0.001163969	0.010857996
657.792102	-0.190994765	0.08945776	-2.135027366	0.032758775	0.120839242
4.276911819	-0.049862627	0.544093267	-0.091643529	0.926981266	0.965058732
114.7656137	0.016134765	0.218309425	0.073907781	0.941083764	0.971961872
117.944129	-0.164990906	0.132871056	-1.241736994	0.214333616	0.419063612
14.26580041	0.349753458	0.295019104	1.185528168	0.235808684	0.443055687
215.8304811	0.011126575	0.194222888	0.057287659	0.954316051	0.97782304

2166.309613	-0.139306061	0.109506356	-1.272127626	0.203327777	0.406187871
674.5750223	0.046387275	0.06032644	0.76893772	0.441930284	0.647776448
38.30099153	-0.081363271	0.230904895	-0.352367025	0.724563029	0.853884493
3.108025726	-0.337254142	0.557728854	-0.604691939	0.545383705	0.732364783
98.89504186	-0.499846514	0.12930632	-3.865600027	0.000110816	0.001701388
68.04998693	-0.543802637	0.345500105	-1.57395795	0.115497171	0.282309278
94.10173152	0.147056245	0.116744986	1.259636499	0.207800522	0.411455025
350.4179736	-0.01809104	0.065892571	-0.274553556	0.783659252	0.887762646
491.8983536	-0.191218999	0.083434193	-2.291854137	0.021914062	0.091650736
17.20185288	-0.329684865	0.274988829	-1.198902757	0.23056576	0.436936024
1042.802128	-0.270428023	0.08425985	-3.209452943	0.001329878	0.011984908
1.735576388	0.728307778	0.76678638	0.949818355	0.342204558	0.556467099
307.2472654	0.007695043	0.098660273	0.07799535	0.937831752	0.970501464
34.81966925	-0.968398703	0.39562222	-2.447786433	0.014373683	0.068180837
1.812477558	-1.639001314	1.237752315	-1.324175518	0.185444759	0.383507026
44.13135885	0.572307127	0.647618999	0.883709601	0.376852996	0.589841864
6.814345482	1.370425623	0.700053924	1.957600087	0.050276957	0.162691533
595.771525	0.107673499	0.059821469	1.799913996	0.071874219	0.207583431
73.34673078	0.344397154	0.19459973	1.769771998	0.07676513	0.216427733
185.2708518	0.057591352	0.099389335	0.579452032	0.562284204	0.745233095
67.34210486	0.162990766	0.131639735	1.238157814	0.21565755	0.420194262
210.6743108	-0.211282197	0.106087602	-1.991582373	0.046416899	0.154380696
959.5583342	-0.039480548	0.077649211	-0.508447507	0.611139544	0.779755644
187.5591306	0.07680826	0.113927397	0.674186037	0.500193046	0.696019954
1.89154829	-2.053396086	1.139440745	-1.802108705	0.071528304	0.206811442
155.9412933	0.785644129	0.193128227	4.067992232	4.742E-05	0.000854701
63.14508989	-0.040583495	0.297354392	-0.136481908	0.891440326	0.945566153
382.5901567	-0.054090054	0.052994877	-1.020665726	0.307412838	0.522484231
6.823064836	0.205083037	0.43562819	0.470775404	0.637801131	0.797573383
1421.029392	-0.25522361	0.088585127	-2.8811113	0.003962757	0.026492884
4.829415817	-0.627733812	0.525514121	-1.19451369	0.232277089	0.438973588
190.9591519	0.250734392	0.12075886	2.076322945	0.037864093	0.133770761
110.3922972	0.050324193	0.109864174	0.458058265	0.646910582	0.804256655
59.30924391	0.341293977	0.166454378	2.050375496	0.040327803	0.139571474
177.1319613	-0.195913682	0.08784517	-2.230215744	0.025733124	0.102787785
612.8904744	0.000261437	0.066585102	0.003926353	0.996867231	0.998078933
4.827631959	1.266545839	1.000914856	1.265388191	0.205732206	0.409245483
3.904521869	0.255559496	0.485028539	0.526895792	0.598265956	0.769566125
389.8677711	0.081140926	0.056780423	1.429029971	0.152995619	0.337409617
95.53106016	-0.281640202	0.10187695	-2.764513482	0.005700775	0.034783174
102.7757268	0.052099758	0.133916421	0.389046825	0.697241508	0.837686451
2.31973391	-2.074433363	1.373488453	-1.510339136	0.130956911	0.304888608
163.9822047	-0.126946196	0.103546686	-1.225980295	0.22020609	0.424786315
217.5749372	-0.015909304	0.178225168	-0.08926519	0.928871158	0.965637301
1.993771002	0.967598722	0.939352844	1.030069508	0.302977378	0.517887593
850.6313711	-0.017705132	0.063090946	-0.280628726	0.778995181	0.884794608
16.62620205	0.14482279	0.266375858	0.543678362	0.586662825	0.76141837
86.60209796	0.369202388	0.123737701	2.983750195	0.002847391	0.020997281

11.51454824	0.846165154	1.118044766	0.756825826	0.449154218	0.654116565
34.73683412	-0.10171228	0.272796392	-0.372850532	0.709259691	0.844977159
922.4399753	-0.115035915	0.072466878	-1.587427485	0.112415858	0.27765426
298.234233	0.276591308	0.073833036	3.746172753	0.000179553	0.002508868
660.861271	0.212232361	0.067309782	3.153068605	0.001615638	0.013855297
83.45985691	0.448884214	0.115781469	3.876995317	0.000105754	0.001638473
448.2208053	0.021281372	0.07605461	0.279816989	0.779617917	0.885082046
8.7669133	0.3672151	0.367120068	1.000258857	0.317185253	0.532090262
1260.948441	-0.088681528	0.063488076	-1.39682179	0.162467168	0.351741763
6.246348733	-0.533251774	0.370027593	-1.441113538	0.149552609	0.332560838
75.61426143	-0.426249689	0.148478941	-2.870775381	0.004094664	0.027221747
74.42891719	-0.255712443	0.218725674	-1.169101177	0.24236287	0.449981507
92.83898477	-0.115072244	0.116985144	-0.983648353	0.325288442	0.540256255
11.92519545	1.438817631	0.39763671	3.618422527	0.000296404	0.003710458
1074.334943	-0.104149324	0.089136971	-1.168418928	0.242637824	0.450289006
2351.364838	-0.171518448	0.086824927	-1.975451678	0.048216913	0.158263683
969.5083724	-0.26351999	0.086424486	-3.049135745	0.002295008	0.017876101
301.8423396	0.034544765	0.087621616	0.394249347	0.693396956	0.83500768
93.79547386	0.069677373	0.106095917	0.656739441	0.511348469	0.705523843
1.818958832	-0.11709828	0.750408682	-0.156046008	0.875996756	0.937672708
80.7814557	0.567223733	0.142030638	3.993671639	6.50579E-05	0.001118752
317.0933974	-0.056929131	0.078593202	-0.724351847	0.468849754	0.670180551
29.84073047	0.035801111	0.221455063	0.161663096	0.871571168	0.935940513
186.6465256	0.236704831	0.080769927	2.930605978	0.003383016	0.023745974
257.7671594	0.019930203	0.065212539	0.305619192	0.759894606	0.87474696
1443.236429	-0.104776358	0.07286432	-1.43796521	0.150443926	0.333916415
267.0974201	0.137116244	0.068636964	1.997702633	0.045748909	0.152904751
1183.08386	-0.053183546	0.05095558	-1.043723688	0.296613247	0.511344352
977.4205065	0.65617134	0.152070202	4.314923853	1.59658E-05	0.000353998
682.4613567	0.680994118	0.119834403	5.682793082	1.32513E-08	7.79647E-07
3.304230119	-0.089115836	0.610481734	-0.145976253	0.883940135	0.942221273
431.7095651	-1.087872573	0.396516126	-2.743577127	0.006077377	0.036393569
60.30470285	-0.560650882	0.243020971	-2.307006181	0.021054476	0.089210759
31.33698689	-0.244637329	0.196355371	-1.245890693	0.212804528	0.41749932
10.75794435	-1.002535874	0.339945237	-2.949109927	0.003186906	0.022727744
16.16846843	-0.163724509	0.255662857	-0.640392239	0.521917627	0.713473653
139.8159294	0.15956891	0.07851937	2.032223503	0.042131035	0.144166936
237.6560489	-0.073869239	0.077967389	-0.947437636	0.343415821	0.557767152
1670.076384	0.020026087	0.064040337	0.312710519	0.754500603	0.871752087
252.9899183	-0.268460522	0.067496558	-3.977395755	6.96742E-05	0.001182329
79.62838203	0.05941317	0.111585369	0.532445877	0.594417218	0.766830795
1335.021377	0.039219092	0.044617374	0.879009419	0.379396169	0.59230988
9.89816133	0.521817494	0.340472256	1.532628531	0.125367407	0.296351392
181.8390905	0.013755765	0.083003297	0.165725521	0.868372961	0.934546688
1.921420738	-0.07056484	0.757178611	-0.093194444	0.925749088	0.964202915
443.9072474	0.011655981	0.096319205	0.121014092	0.903679873	0.951582251
16.83047098	0.374834482	0.345515687	1.084855176	0.277985803	0.491103284
14.03449398	-0.351551455	0.392627338	-0.89538201	0.370582914	0.584208893

2.140490482	-0.240591254	0.690992539	-0.348182129	0.727703408	0.85587106
75.83090273	-0.535365575	0.209974724	-2.549666768	0.010782593	0.055285538
120.0743089	0.142009768	0.207446317	0.68456153	0.49362065	0.690666433
657.2031669	0.157367993	0.059251809	2.6559188	0.007909266	0.044228528
348.4091028	0.121375716	0.053973402	2.24880609	0.024524836	0.099219584
3051.821272	0.310288207	0.079740795	3.891210347	9.97454E-05	0.001561983
1275.401094	0.148853248	0.169927455	0.875981157	0.381040278	0.593874885
4.313064344	-1.791256611	1.636366181	-1.094655116	0.27366776	0.486358831
134.4768832	-0.068221087	0.249024895	-0.273952879	0.784120834	0.887950686
62.1849112	0.217998515	0.180107851	1.210377639	0.226134018	0.431822397
216.6258005	-0.075431289	0.104105958	-0.724562647	0.468720381	0.670053763
16.32335133	0.116433651	0.239321546	0.486515539	0.626601691	0.790677612
293.8715797	0.199282822	0.134749802	1.478909941	0.139164386	0.317621792
124.0004273	-0.073124603	0.098815239	-0.740013423	0.45929185	0.662252422
4.917358115	-2.912756038	1.542392405	-1.888466274	0.058963381	0.181297791
1128.01328	-0.062071055	0.064941563	-0.955798599	0.339173996	0.554005032
1.994294539	0.389295467	0.937741866	0.415141395	0.678038397	0.826061571
456.7331942	-0.152939691	0.082532189	-1.853091414	0.063869257	0.191584513
102.5136005	-0.249323383	0.11871616	-2.100163808	0.035714434	0.128238793
4.165598337	0.073456976	0.549473964	0.133686	0.893650876	0.946144251
139.5559804	0.095768265	0.101961964	0.939254808	0.347599935	0.562345215
1449.55624	-0.123518455	0.072166669	-1.711572068	0.086975559	0.234506606
165.3976253	0.064903373	0.109158374	0.59457988	0.552124333	0.737796722
111.3336219	0.334516193	0.143150329	2.336817492	0.019448676	0.084248616
151.7236762	0.048187031	0.115118277	0.418587147	0.675517885	0.824469335
2.657513434	-0.655261366	0.581189346	-1.127449031	0.259552679	0.47039283
4436.02108	0.136019258	0.068216177	1.993944304	0.046158142	0.153831181
356.6492561	-0.060428108	0.097547788	-0.619471842	0.535605566	0.72413048
72.54505298	-0.215080691	0.166387255	-1.292651238	0.19613171	0.397268088
201.4421238	0.098041541	0.101311066	0.967727859	0.333180301	0.548610781
162.0566786	0.002993278	0.093277695	0.032089968	0.974400304	0.987528183
78.74306684	-0.193499832	0.194980812	-0.992404482	0.321000254	0.536095037
329.1223595	0.099278794	0.094888701	1.046265712	0.295438372	0.510173138
445.9260266	-0.144234673	0.065109706	-2.215256099	0.026742491	0.105623544
2387.286341	0.164819357	0.112626418	1.463416488	0.14335345	0.323374604
68.06492716	-0.091187556	0.121949349	-0.747749425	0.454611313	0.658860453
1251.371122	0.186125712	0.054027083	3.445044598	0.000570965	0.006209332
85.28724285	0.17341168	0.172340859	1.006213392	0.314312931	0.529176416
30.37374717	0.289640791	0.227593976	1.272620638	0.203152692	0.405960388
652.2468418	0.103638138	0.070153659	1.477301953	0.139594715	0.318163162
282.3734223	-0.053595207	0.117528188	-0.456020023	0.64837558	0.805166538
265.6745099	0.115715009	0.091209709	1.268669872	0.204558832	0.407682337
82.58984429	-0.097475779	0.145769391	-0.668698542	0.503687798	0.699260458
255.4130122	0.1729008	0.083825703	2.062622719	0.039148487	0.136522477
4.731375622	0.959096997	0.467210816	2.052814198	0.040090601	0.138984125
447.1460085	0.276336668	0.067869267	4.071602392	4.66908E-05	0.00084433
134.7414174	-0.869115505	0.169867531	-5.116431037	3.11371E-07	1.24202E-05
337.1409929	-0.098281262	0.081314102	-1.208661961	0.226792741	0.432478715

350.5254052	0.149512507	0.088519194	1.689040544	0.09121166	0.241806726
390.2120197	0.037841606	0.120874855	0.313064335	0.754231784	0.871653489
407.1310864	0.002101919	0.104640231	0.020087102	0.983973889	0.992002278
144.0836889	0.140707715	0.082467237	1.70622565	0.087966075	0.236408595
5337.917661	0.071112465	0.069051348	1.029849048	0.303080872	0.517887593
917.9107315	-0.075561676	0.078996698	-0.956516886	0.338811157	0.553947499
63.14492563	0.067810657	0.149892142	0.452396342	0.650983482	0.806581068
7.485768065	0.916068629	0.414305868	2.211092574	0.027029427	0.106247383
32.61268712	-3.654144239	0.666244207	-5.484691951	4.14191E-08	2.06144E-06
116.8577199	-0.192456438	0.106092427	-1.814045011	0.069670796	0.203103112
124.4400977	0.118979666	0.106391834	1.118315774	0.263432152	0.474811956
12.84795296	-0.664534482	0.384112449	-1.730051928	0.083620998	0.228885962
107.3192662	-0.623145783	0.339441534	-1.835797098	0.066387671	0.196736818
381.8549735	-0.041740948	0.058798149	-0.709902407	0.477764658	0.6774444
82.12783844	0.130425151	0.179684968	0.725854544	0.467927945	0.669618222
202.4803262	0.029150517	0.090138929	0.323395422	0.746395796	0.86703309
290.800825	0.193372834	0.070589676	2.73939259	0.006155282	0.036779877
368.0653502	0.355861276	0.15256791	2.332477886	0.019675565	0.08507487
32.12586468	0.361553332	0.166670805	2.169266124	0.030062486	0.114043343
539.8793125	0.179034045	0.054408051	3.290580013	0.000999811	0.009653987
152.0205719	0.289077134	0.117587455	2.458401155	0.013955719	0.066817856
227.042294	0.130691338	0.076597211	1.706215352	0.087967991	0.236408595
88.76573754	0.207627491	0.122487322	1.695093722	0.090057643	0.2399498
1353.032881	0.113583448	0.056434259	2.012668349	0.044149531	0.148949289
12.42174418	0.801958284	0.278188445	2.882787901	0.003941727	0.026388992
57.88176943	-0.372211584	0.317046747	-1.173995912	0.240396663	0.447794508
65.60896884	-0.158446963	0.160773106	-0.985531512	0.324363061	0.539548668
557.2936494	0.154618724	0.060507706	2.555355918	0.010607927	0.054696396
2.247623297	0.155415334	0.645837054	0.240641711	0.809832819	0.903751921
552.6699082	0.116578145	0.137744963	0.846333274	0.39736685	0.609915354
1284.169926	-0.061260722	0.086868751	-0.70521012	0.480679484	0.679717925
5952.592789	0.29172316	0.059134547	4.933210329	8.0889E-07	2.89688E-05
158.1045047	-0.008923641	0.108789717	-0.082026514	0.93462563	0.968975621
191.4301739	-0.460693015	0.195889719	-2.351797822	0.018682927	0.081683263
524.7421044	-0.242881037	0.090857626	-2.67320475	0.007513038	0.042623462
1049.799546	0.143854641	0.04215025	3.412901247	0.000642752	0.006809454
253.5476799	-0.21022138	0.093912928	-2.238471151	0.025190347	0.101191361
330.6112002	0.139054421	0.091642449	1.517358189	0.129176274	0.301699153
145.4525381	-0.163954375	0.112645674	-1.455487541	0.145534337	0.32655035
54.95344408	-2.269099077	0.953533099	-2.37967521	0.017327903	0.077700561
468.8969201	0.038411551	0.083882864	0.457918929	0.647010687	0.804266033
823.7526949	0.898441198	0.29236134	3.073050629	0.002118825	0.016862574
51.13208938	0.109467453	0.140322268	0.78011462	0.435323412	0.641976357
181.8809775	0.487528995	0.179494278	2.716125552	0.006605086	0.038806061
57.73818364	0.163680152	0.168109525	0.973651863	0.330229426	0.545438095
144.0778512	0.068345674	0.120294418	0.568153332	0.569930857	0.750223007
575.8307503	0.249499086	0.083978548	2.970985937	0.002968454	0.021619057
15253.98762	0.13575068	0.098106622	1.38370558	0.166448624	0.357366693

620.51237	0.526926077	0.084533678	6.233327256	4.56631E-10	4.11068E-08
107.7507899	0.084975372	0.108992719	0.779642644	0.435601248	0.642156148
277.3763917	-0.058644797	0.082749052	-0.708706577	0.478506583	0.677925477
8.597794336	0.028968599	0.347904395	0.083265976	0.933640053	0.968634829
181.5750771	0.82667447	0.137710603	6.002983468	1.93724E-09	1.49132E-07
16.23069774	1.105532956	0.353828241	3.124490439	0.001781133	0.014826876
4.516524254	0.201079305	0.481034671	0.418014162	0.675936763	0.824598803
61.8198596	-0.076348044	0.148983389	-0.512460111	0.608329031	0.777532194
13.79216902	-0.194860199	0.275807477	-0.706508037	0.479872256	0.679042738
10.87277511	0.608301089	0.284841189	2.1355798	0.032713679	0.120726961
120.9574703	0.410813816	0.1292271	3.179006685	0.001477807	0.013021974
250.3323695	0.266055684	0.151332499	1.758086899	0.078732725	0.21983778
224.3350628	0.096451682	0.082406896	1.170432179	0.241827092	0.449493346
1811.636173	-0.001225238	0.073789773	-0.016604438	0.986752184	0.993287517
12.64059426	-0.178324835	0.279372	-0.638306038	0.523274482	0.714616913
164.7306168	-0.115586284	0.08400292	-1.375979359	0.168828024	0.36045527
26.22740155	0.258883805	0.237587035	1.089637759	0.275872743	0.488528712
99.66521768	-0.020280935	0.140466882	-0.144382328	0.885198571	0.942747873
1023.679729	0.122812169	0.1027158	1.195650214	0.231833087	0.438436262
182.8532501	0.291783206	0.08547658	3.413604139	0.000641097	0.006796838
298.1083103	0.087600769	0.08805051	0.994892237	0.319788679	0.534896812
2.77153818	-0.934447924	0.808988361	-1.155082037	0.248056834	0.456791602
4.530585336	-1.524254139	0.77455261	-1.967915568	0.049077753	0.160036997
726.6636928	0.108551544	0.069284084	1.566760178	0.117170759	0.28491086
23.21577747	0.420892452	0.209396762	2.010023692	0.044428681	0.14961531
30.96403542	0.232555511	0.199199502	1.167450263	0.243028583	0.450709544
563.3484525	0.015199953	0.054634762	0.278210294	0.780850933	0.885932388
819.0321466	-0.098881254	0.043483205	-2.274010252	0.02296537	0.094564673
1733.187655	-0.02094703	0.051002026	-0.410709759	0.68128537	0.827691386
81.68347244	0.033136261	0.118176142	0.280397211	0.779172776	0.884881588
620.2930487	0.020930688	0.058061038	0.360494556	0.718477327	0.850583952
195.7643521	-0.259413457	0.085937183	-3.018640455	0.002539117	0.019311823
5.770898064	-0.409617154	0.546345287	-0.749740436	0.453411049	0.658047187
13.16412267	-0.12274339	0.39158598	-0.313451952	0.753937319	0.871653489
15.27925652	1.176538248	0.68534575	1.716707587	0.086032613	0.232886268
4.154135198	-1.895846712	0.850410063	-2.229332405	0.025791796	0.1029547
2.064878976	-0.010524022	0.766897855	-0.013722847	0.989051096	0.994423421
482.1806669	0.359933131	0.117098383	3.073766868	0.002113745	0.016846561
1300.517525	-0.218935768	0.072640521	-3.013961981	0.002578601	0.019523252
16917.45551	-0.23074063	0.111544687	-2.068593631	0.038584236	0.135336351
408.3848265	-0.583589707	0.132161235	-4.415740412	1.00665E-05	0.000239646
132.0655984	0.287721439	0.120613897	2.385475021	0.017057081	0.076775505
5.597940799	0.068867364	0.482406695	0.142757893	0.886481393	0.943320904
1911.246584	0.274057203	0.058698578	4.668890021	3.02831E-06	8.76774E-05
57.01459478	0.435139446	0.129704896	3.354842113	0.000794103	0.008036109
1713.534695	0.081061659	0.097873037	0.828232789	0.407538678	0.61832678
27.81278429	0.330539577	0.208264539	1.587114053	0.112486816	0.277742279
398.5317256	-0.033327022	0.100359393	-0.332076762	0.739831297	0.863255748

217.5020494	-0.231768285	0.122930189	-1.88536508	0.059380565	0.182135946
96.55993179	-0.115727774	0.206455769	-0.560545121	0.575107672	0.753941023
157.4284599	-0.261126782	0.104612642	-2.496130263	0.012555648	0.061984343
1708.196197	-0.028239374	0.096831306	-0.291634757	0.770565898	0.880306225
116.520345	-0.106921222	0.107062223	-0.99868299	0.317948283	0.53284639
120.0879906	-0.033766449	0.124976887	-0.270181553	0.787020584	0.889929103
754.8684901	-0.138246538	0.073128518	-1.890460001	0.058696465	0.180775018
33.37068362	0.345792393	0.18004997	1.920535689	0.05479027	0.171547327
936.918009	0.105691684	0.102417133	1.031972685	0.302084914	0.517049928
6.568798008	-0.291185357	0.366190892	-0.795173675	0.426512483	0.633800964
494.7828749	0.165579547	0.058626106	2.824331329	0.004737938	0.030182826
263.9728331	0.513766662	0.186852517	2.749583839	0.0059671	0.035880292
2189.957644	-0.169613264	0.080363767	-2.110568868	0.034809386	0.125882916
140.3544884	0.048324979	0.153619913	0.314574969	0.753084387	0.871171356
3.184184957	0.033799162	0.709412501	0.047643878	0.962000062	0.981360396
6.427790886	-0.053792988	0.411426819	-0.130747403	0.895975132	0.947569772
96.31809157	0.052923213	0.136302538	0.388277533	0.697810662	0.838003561
3.849587906	-0.203705336	0.55622556	-0.366227932	0.714195001	0.847730272
1265.943616	-0.127691264	0.064108299	-1.991805507	0.046392402	0.154366477
703.2055085	0.139290188	0.081340888	1.712425214	0.086818336	0.234274408
272.2047766	0.056335027	0.093512181	0.602435174	0.546884502	0.733662483
169.7839321	-0.027136962	0.114622324	-0.236751107	0.812849884	0.906075444
18.43802545	0.258715926	0.220101688	1.175438174	0.239819459	0.447225012
554.6646512	-0.229882103	0.083101517	-2.766280474	0.005669975	0.034685169
5.982125637	1.22426759	0.512375908	2.389393358	0.016876223	0.076178809
4.611379414	1.359900163	0.989975082	1.373671104	0.169543812	0.36156178
28.40558329	-0.318208241	0.335148587	-0.949454223	0.342389644	0.556594335
255.9416488	0.065763186	0.114787242	0.57291372	0.566703114	0.748471553
1060.75272	-0.09769662	0.123010872	-0.794211269	0.427072449	0.634351414
21.47347094	-0.072725723	0.231882376	-0.313631954	0.753800588	0.871653489
699.7609977	-0.236398273	0.093449593	-2.529687561	0.011416414	0.057881436
209.1166607	-0.138937721	0.089256752	-1.556607401	0.119563726	0.288547833
503.2946485	-0.10029994	0.075952968	-1.32055327	0.186650363	0.385178263
396.6259074	0.119858424	0.079037587	1.516473725	0.129399609	0.302045521
27.68119063	-0.261105636	0.261368061	-0.998995953	0.317796652	0.532700656
2.028781708	0.329254871	0.817033865	0.402988034	0.686957027	0.830726992
34.45982357	-0.350311192	0.229463411	-1.526653816	0.126847117	0.298144047
898.4605277	0.002959141	0.100148181	0.029547627	0.976427835	0.988137991
16.51265051	0.100475764	0.27282798	0.368275145	0.712668083	0.846588838
154.870884	-0.206482175	0.138040993	-1.495803316	0.134704917	0.310889437
48.11448236	-1.379554472	0.193445754	-7.131479716	9.92956E-13	1.90209E-10
31.87866783	-0.410102896	0.345021347	-1.188630498	0.234585101	0.441562495
37.30775796	0.253086535	0.218318214	1.159255248	0.246352157	0.454978188
13.022092	0.213052238	0.29814766	0.714586316	0.474864703	0.674805193
572.3230349	-0.040973436	0.095739597	-0.427967498	0.668674784	0.819685124
344331.7078	0.087740654	0.105181959	0.834179684	0.404179737	0.614990985
80.56517082	0.153108292	0.162109371	0.944475271	0.344926833	0.559394039
768.8141903	-0.321508897	0.091954215	-3.496401959	0.000471578	0.005335695

52.40800019	-0.515801128	0.179508556	-2.873406924	0.004060707	0.027048635
210.4197623	-0.168942569	0.075663431	-2.232816642	0.025561038	0.102281405
369.5064172	-0.156715215	0.094367768	-1.660685824	0.096776562	0.250675642
525.5601179	0.172337917	0.083477344	2.064487311	0.038971536	0.136222593
140.3575191	0.096610767	0.096873909	0.997283661	0.318626842	0.53371211
1.773028247	1.489393454	1.31808333	1.129969115	0.258489239	0.469136468
38.51300536	2.015220318	0.340301959	5.921859285	3.18322E-09	2.35159E-07
32.87685739	-0.232242028	0.383253539	-0.605974909	0.544531415	0.731458784
3649.8484	0.000465504	0.140371145	0.003316239	0.997354029	0.998244565
14.44981536	0.043567784	0.558891568	0.077953912	0.937864714	0.970501464
31.97351974	0.626554776	0.712365824	0.879540758	0.379108144	0.592190921
288.4226478	-0.116214705	0.35557414	-0.326836775	0.743791344	0.865506187
4.379312635	0.588366529	0.672469901	0.874933626	0.381610021	0.594313055
391.8708596	0.081979281	0.081273283	1.008686712	0.313124906	0.52787758
15.06064527	-1.518480431	0.570548886	-2.661437906	0.007780769	0.043702826
1.918381334	0.548666968	0.734684271	0.746806472	0.455180388	0.659337177
377.0619632	0.406264326	0.098565235	4.121781121	3.75954E-05	0.000711893
461.0828461	0.583841958	0.13691302	4.264327523	2.00505E-05	0.00042676
180.9778306	-0.055772519	0.109531151	-0.509193215	0.610616799	0.779307495
741.8673679	0.158992405	0.060798227	2.615082916	0.00892058	0.048500868
561.4956502	-0.035427829	0.084715445	-0.418197993	0.675802363	0.824556964
105.6029701	-0.621245417	0.206244355	-3.012181436	0.002593775	0.019618849
19.34971296	0.652599006	0.68752521	0.949200111	0.342518845	0.556619
118.1958872	0.235743749	0.16128591	1.461651227	0.143836809	0.323976975
906.2069367	-0.174285442	0.099946753	-1.743782926	0.081196949	0.224657382
514.8614601	0.15508728	0.080249281	1.932569085	0.053289304	0.168857086
1.943561194	-0.368649257	0.718343201	-0.513193772	0.607815782	0.777358683
168.1782773	-0.108210408	0.083885787	-1.289973088	0.197060002	0.398522769
494.0575744	-0.275499845	0.071063057	-3.8768364	0.000105823	0.001638473
65.10660565	-1.629106492	0.339856382	-4.793514486	1.63885E-06	5.2424E-05
167.5267203	-0.256888599	0.167807671	-1.530851344	0.125806137	0.296966657
134.2352301	-0.5152594	0.198608209	-2.594350973	0.009476969	0.050689476
143.7338621	-0.050446406	0.150642342	-0.334875345	0.737719121	0.861800757
79.35314585	-0.133342911	0.148535786	-0.897715729	0.369337122	0.582858487
6.415405039	-1.709940815	0.46674526	-3.663541897	0.000248752	0.003252294
127.8495896	0.179676258	0.128230409	1.401198504	0.161154721	0.349671897
415.1605566	-0.230180876	0.125431647	-1.835110056	0.066489384	0.196933858
223.5684265	-0.035358086	0.149898194	-0.235880667	0.81352527	0.906196373
11.56131751	0.639197619	0.362599563	1.762819606	0.07793092	0.218450565
25.45742322	0.39542565	0.206476454	1.91511256	0.05547815	0.172997738
54.96669381	-1.382062049	0.40859069	-3.382509892	0.000718267	0.007421228
3.992642318	-0.168225184	0.612002411	-0.274876669	0.783410992	0.887675403
17.33624775	-0.331587703	0.700508068	-0.473353154	0.635961246	0.796232373
92.72181393	-0.24689655	0.10334831	-2.388975211	0.016895442	0.07623542
3.862712925	0.368637134	0.970612672	0.379798393	0.704095075	0.841624022
609.7716945	-0.221286873	0.176824489	-1.251449246	0.21077062	0.415140506
47811.39438	-0.706552363	0.138917566	-5.086126852	3.6545E-07	1.42327E-05
99.88199044	-0.100901428	0.249777427	-0.40396536	0.686238192	0.830279669

117.5048068	-0.099991717	0.22027367	-0.453943119	0.649869767	0.805867862
58.22910441	-0.200374053	0.280622666	-0.714033745	0.475206314	0.675059993
11.14168964	0.785844901	0.412143443	1.906726687	0.056555989	0.175726776
5.388991191	-2.033495425	0.7964513	-2.553194934	0.010673974	0.054907071
3.048504042	-2.374926379	1.172356008	-2.025772345	0.042788119	0.145571604
4.057400683	-1.804305327	0.800491754	-2.253996145	0.02419641	0.098252814
6.198250002	-0.211044195	1.394452153	-0.151345598	0.879703101	0.939772316
32.69504341	-0.202258158	0.167523744	-1.207340245	0.227301138	0.433097264
7112.305238	-0.056517345	0.111625074	-0.506314066	0.61263619	0.780789807
257.6428464	0.107149247	0.123831337	0.865283779	0.386883052	0.599916359
436.391097	-0.092850414	0.105040161	-0.883951552	0.376722367	0.589841864
10.76150797	-0.14716486	0.31739195	-0.463669165	0.642884792	0.801477463
205.4974854	-0.286789676	0.113142618	-2.534762599	0.011252355	0.05717807
298.5326031	-0.239228375	0.136157059	-1.757003109	0.078917279	0.220203804
388.6328758	-0.114510299	0.090743623	-1.261910141	0.206981122	0.410473939
72.69787462	-0.392297634	0.143087106	-2.741670061	0.006112771	0.036565645
8022.977162	-0.069135857	0.118308439	-0.584369617	0.558971664	0.743154899
2.004362935	1.238898383	1.321283028	0.937647996	0.348425337	0.563127539
2.344729288	0.778541788	1.082551124	0.71917323	0.472034191	0.672398726
817.7613416	0.219815574	0.071841685	3.05972184	0.002215426	0.01744646
751.0693315	-0.172399117	0.065210637	-2.643726939	0.008199878	0.045590545
51.1965756	-0.287733998	0.135888477	-2.117427508	0.034223582	0.124448658
153.5392553	0.184624325	0.088560456	2.084726442	0.037094139	0.131899168
9.312713049	-0.189833664	0.428905973	-0.442599721	0.658055287	0.811680353
616.4696796	-0.083491751	0.063122671	-1.322690387	0.185938362	0.384142033
6061.597026	-0.071899432	0.109359961	-0.65745663	0.510887349	0.705147867
152.8260421	-0.142434832	0.124889851	-1.140483638	0.254084866	0.463389137
291.3711601	-0.252948707	0.117014487	-2.161687094	0.030642303	0.115692585
605.5105459	0.316909983	0.117389099	2.699654272	0.006941157	0.040108248
1292.773343	-0.16033761	0.103714111	-1.545957526	0.122114826	0.292019109
34.60287933	0.337522186	0.383262293	0.880655864	0.378504112	0.591545085
9397.935544	-0.090910387	0.112299563	-0.809534646	0.418207683	0.627406736
14.91157476	-0.732839356	0.712718653	-1.028230918	0.303841218	0.518644466
520.1054929	0.083183023	0.07443394	1.117541587	0.263762829	0.475214398
13.8289648	0.102535094	0.368196576	0.278479216	0.780644518	0.885759197
356.3417183	-0.195147329	0.091975292	-2.121736445	0.033859876	0.123506384
87.38335439	0.055875483	0.14330356	0.38990994	0.696603143	0.837156802
620.9565319	-0.361442245	0.115224591	-3.136849884	0.001707735	0.014349094
335.9811883	-0.511995903	0.106072192	-4.826862646	1.38701E-06	4.5975E-05
253.203698	0.137812679	0.107295711	1.284419277	0.198995292	0.401058042
181.1485098	0.863740811	0.162534256	5.314207801	1.07122E-07	4.76087E-06
3.405418706	0.105583273	0.560377375	0.188414588	0.850551665	0.926289953
40.492172	-0.002088129	0.228878885	-0.009123293	0.992720766	0.995924847
463.7807891	-0.00674926	0.089185149	-0.075676949	0.939676116	0.971277722
4862.694313	0.224858891	0.101842572	2.207906636	0.027250782	0.106704405
1637.981629	-0.131778049	0.133169089	-0.989554334	0.322392	0.537439698
48.38371781	0.708348879	0.307916875	2.300454884	0.021422462	0.09032855
307.6423313	0.449312265	0.142559813	3.151745607	0.001622976	0.013898241

603.4534348	0.260665991	0.174867192	1.49065121	0.136053092	0.312774021
14.92400237	0.85690946	0.299261448	2.863414134	0.004191024	0.027686681
43.5807791	0.474941284	0.251839986	1.885885129	0.059310436	0.182056232
53.10176482	0.625357866	0.152433958	4.102483959	4.08738E-05	0.000758283
256.6010833	-0.057287068	0.109735168	-0.522048395	0.601636635	0.77251457
158.8701599	0.005984463	0.115994641	0.05159258	0.958853332	0.979970829
467.9275188	-0.097206715	0.069973475	-1.389193752	0.164773841	0.354695448
587.2954224	-0.255092592	0.070115349	-3.638184723	0.000274566	0.003515494
53.96520328	-0.260623003	0.14905015	-1.748559145	0.080367254	0.223039588
23.81220488	-0.495991897	0.221505434	-2.239186138	0.025143807	0.101062157
8.063769656	0.637835315	0.519279284	1.228308802	0.219331068	0.423903045
130.8433857	-0.112275477	0.083755304	-1.34051781	0.180077058	0.376088927
695.0574959	-0.255400165	0.116798386	-2.186675467	0.028766224	0.110748953
58.65466874	-0.291447938	0.160116574	-1.820223425	0.068724987	0.201173857
34.88184329	-0.278182278	0.23115425	-1.203448683	0.228802738	0.434802906
137.1897864	0.178680845	0.129854065	1.376012722	0.168817694	0.36045527
2564820.973	-0.052344383	0.103080376	-0.507801629	0.611592467	0.780039684
145.0616502	0.822311817	0.111224635	7.393252556	1.4328E-13	3.37199E-11
335.7228534	0.070898422	0.060282088	1.176110916	0.239550556	0.447027171
63.45251299	-0.778614104	0.391511394	-1.98873932	0.046729984	0.15512941
2.250564974	-2.654093286	1.001107589	-2.651156894	0.008021657	0.044781015
208.0561668	0.234060529	0.101764693	2.300017044	0.021447254	0.090386817
304.2370546	0.036437932	0.081612658	0.446474023	0.655254873	0.809943754
280.436012	-0.972583311	0.324952746	-2.992999208	0.002762505	0.020555331
4.806458342	0.200174444	0.444990088	0.44984023	0.652825647	0.808140517
561.1701532	0.007074017	0.071156887	0.099414367	0.920809276	0.961001712
68.78226372	-0.166615961	0.20995441	-0.79358162	0.427439036	0.634667029
497.4050878	-0.212029946	0.050520757	-4.19688774	2.70608E-05	0.000548339
235.436318	0.549670471	0.189507331	2.900523522	0.003725399	0.025391899
1057.8923	-0.18830999	0.069182475	-2.721931967	0.00649015	0.038290479
74.95939585	-0.486032037	0.143779152	-3.380406888	0.000723786	0.00746628
75.01164261	-0.013136058	0.176776789	-0.074308728	0.940764733	0.971915101
543.1629163	0.079922544	0.083096903	0.961799307	0.336150426	0.551316348
869.4192728	-0.502653851	0.074934367	-6.707921496	1.97416E-11	2.54081E-09
28.12339976	-0.313445363	0.24050508	-1.303279595	0.192479325	0.393136763
332.8804217	0.316007737	0.105294573	3.001177831	0.002689375	0.020147684
53.16311836	0.060067081	0.205451462	0.292366289	0.770006581	0.88011437
1141.976511	0.217542265	0.059768984	3.639718305	0.000272936	0.003499109
98.45057687	-0.146348725	0.157845437	-0.92716475	0.353841002	0.568700162
436.51743	-0.262322879	0.121201946	-2.164345434	0.030437848	0.11516608
93.65661173	0.138451747	0.10788108	1.28337376	0.199361163	0.401598898
1725.349451	-0.050541644	0.097897796	-0.516269484	0.605666207	0.77567875
63.17028237	-0.131068045	0.151314352	-0.866197046	0.386382113	0.599452311
11.59291241	-0.377755546	0.453128349	-0.83366125	0.4044719	0.61520359
934.8808596	-0.146546209	0.130865122	-1.119826324	0.262787779	0.47422126
111.9667459	-0.613039392	0.20070228	-3.054471483	0.002254574	0.0176866
505.1855568	-0.566408862	0.317211361	-1.7855882	0.074165959	0.211752168
112.3004444	-0.231015515	0.107104145	-2.156924133	0.031011576	0.11645423

276.4069089	0.116664623	0.106200005	1.098536887	0.27197012	0.484005158
14.33054954	0.257188055	0.327592921	0.785084287	0.432404152	0.639560603
424.481505	0.330417305	0.150676715	2.192888956	0.028315381	0.109576599
5.119020738	-0.947377605	1.022919885	-0.926150345	0.354367853	0.569096189
5.776808544	-0.698153736	0.383681394	-1.819618431	0.068817133	0.201330749
426.2593706	-0.126415735	0.079027919	-1.599633853	0.109679834	0.273412054
779.6391018	0.161910562	0.140637895	1.151258423	0.249625936	0.458607971
5.856783965	0.436236006	0.415912822	1.048864048	0.294240695	0.509065449
103.9357557	0.189231843	0.2054974	0.920847868	0.357129859	0.571675892
8.39642266	-0.66916038	0.377906386	-1.770704079	0.076609924	0.216170249
13.00804039	-0.615607097	0.355000089	-1.734104063	0.082899621	0.227880586
239.2442234	-0.298922638	0.193219107	-1.547065624	0.121847422	0.291793937
249.6817787	-0.120845383	0.074917533	-1.613045409	0.106734643	0.268081492
405.1099438	0.267095628	0.098717135	2.705666333	0.00681675	0.039709738
7.262261843	0.10004288	0.359765686	0.278077883	0.780952573	0.885986688
1193.866232	-0.142544378	0.055930432	-2.548601427	0.010815583	0.055420192
133.6201638	0.598806885	0.185412029	3.229601049	0.001239631	0.01138332
1613.599267	0.200404236	0.092486461	2.166849441	0.030246336	0.114546699
263.814711	0.031911923	0.098405033	0.324291575	0.745717297	0.866724055
402.0363801	0.151289178	0.07601375	1.990286992	0.04655933	0.154734397
1704.872712	0.044125005	0.057746734	0.764112571	0.444800161	0.650074331
295.4577143	0.044849435	0.075816451	0.591552807	0.554150084	0.73907614
1748.580905	-0.025446949	0.068590325	-0.370999101	0.710638201	0.84564098
302.0321532	0.201407476	0.065323479	3.083232548	0.002047651	0.016477006
44.272798	-0.258577957	0.352543117	-0.733464774	0.463275005	0.665596195
2.950071479	0.605119649	0.719829782	0.840642697	0.400548132	0.61245869
302.6438264	0.382257585	0.121309376	3.151096774	0.001626586	0.013905748
258.9505565	-0.38002568	0.101984321	-3.72631477	0.0001943	0.002662973
331.4419289	-0.029380757	0.071970613	-0.408232684	0.683102855	0.828677204
51.4531689	0.260356309	0.175880035	1.480306216	0.138791545	0.31721031
17.05077523	1.119463528	0.283095762	3.95436342	7.67387E-05	0.001271824
2742.231066	0.301617376	0.089703231	3.362391445	0.000772705	0.007886641
2394.801241	0.135011375	0.083669556	1.613626043	0.106608565	0.267928703
350.8041116	0.240836872	0.093754199	2.568811575	0.010204793	0.053267984
631.3371585	0.033859792	0.115694563	0.292665366	0.769777945	0.880036216
994.6430533	-0.128926349	0.070295776	-1.834055406	0.06664577	0.197184342
4.191239585	-0.667900372	0.533239724	-1.252533038	0.210375697	0.414639803
9.431255449	-1.528198425	0.769741283	-1.985340346	0.047106618	0.155767649
89.9157687	0.252507122	0.132904284	1.899917104	0.057443999	0.177781785
184.4437204	0.213934115	0.155034209	1.379915548	0.167612649	0.359007538
584.0553858	-0.065390906	0.061234041	-1.067884867	0.28557245	0.499975444
70.52851197	-0.252853554	0.126342591	-2.001332655	0.045356553	0.151896449
659.7560418	0.184894591	0.045392641	4.073228291	4.63659E-05	0.0008403
13.74063041	0.250363156	0.37537159	0.666974174	0.50478863	0.700184188
1459.852976	0.346773745	0.074236506	4.671202395	2.99442E-06	8.68486E-05
14.30991423	0.53017408	0.256172107	2.069601121	0.038489713	0.135255873
66.56308138	0.394127882	0.229746239	1.715492205	0.086255023	0.233251027
435.3925412	0.196861695	0.067981075	2.89583086	0.003781562	0.025605198

4.500288091	1.028754812	0.479259848	2.146549134	0.031829193	0.118471335
2.881464246	-0.110971401	0.724054533	-0.153263872	0.87819019	0.938643041
235.9769751	-0.117390742	0.140536153	-0.835306351	0.403545242	0.614855381
8.039717285	-0.594510392	0.397564486	-1.495381032	0.134815028	0.311006321
134.6788495	0.269776028	0.162438758	1.660786073	0.096756419	0.250675642
898.9508344	0.056129966	0.081725431	0.686811508	0.492201517	0.6896766
37.46367343	0.287522173	0.160489288	1.791534984	0.073207486	0.210071437
110.3070482	0.054733383	0.111465452	0.491034502	0.623402045	0.788417418
183.397108	-0.180871081	0.285909532	-0.632616478	0.526984124	0.717245246
2.982716729	0.80236601	0.6446388	1.244675327	0.213251119	0.417828132
10.46291336	0.92449428	0.324908069	2.845402652	0.004435531	0.028858983
4.073569896	0.485899883	0.480607577	1.0110117	0.31201083	0.526754091
160.3825647	0.935610655	0.213297244	4.386416994	1.15233E-05	0.000268888
88.40383229	-0.760076606	0.281700587	-2.69817189	0.006972143	0.040209149
6.81060581	1.132529779	0.559079004	2.025706152	0.042794906	0.145571604
138.213207	-0.135032265	0.212939553	-0.634134254	0.52599321	0.716791813
4.415753055	0.496944644	0.646455321	0.768722336	0.442058162	0.647896455
17.33851121	-0.0264129	0.430162858	-0.061402094	0.951038985	0.976640702
672.1978835	0.038658777	0.055589067	0.695438487	0.486780548	0.685403653
54.95515555	-0.25948998	0.154900773	-1.675201327	0.093894737	0.246091419
57.74947665	0.488805979	0.161061696	3.034898998	0.002406163	0.018505659
18.8101391	0.421442616	0.233911955	1.801714733	0.071590299	0.206908523
333.7429247	0.044008395	0.066237863	0.664399376	0.506434732	0.701328663
189.3270574	-0.84343195	0.27676982	-3.047413009	0.002308203	0.017944946
511.1876792	0.111904815	0.055401835	2.019875622	0.043396291	0.147222094
28.81414445	0.349286994	0.231609644	1.508084846	0.131532813	0.305714865
478.7611298	-0.169631381	0.049372879	-3.435719853	0.000590982	0.006388224
17.60719335	0.318497242	0.321670198	0.990135998	0.322107651	0.537248298
372.1864037	0.104492202	0.105923899	0.986483718	0.323895801	0.539260694
1.917066849	-2.733143606	1.865626071	-1.465000757	0.142920711	0.322751994
391.0153942	0.192237721	0.088409557	2.174399783	0.029675124	0.113216304
42.80954461	-0.083170983	0.334373317	-0.248736903	0.803564301	0.899315102
200.9109163	-0.0957343	0.070483765	-1.358246117	0.174385601	0.368358559
115.0749758	0.166604749	0.131734654	1.264699482	0.205979076	0.409517791
734.0890241	0.055126283	0.052724488	1.045553679	0.295767145	0.510408791
12.67972272	-0.035437455	0.349122728	-0.101504292	0.919150151	0.959999974
87.63542286	-0.460139807	0.14659037	-3.138949757	0.001695545	0.014270278
8.475175194	-0.285502977	0.440557102	-0.64804988	0.516952689	0.709630747
146.8412491	-0.07878051	0.228036053	-0.345473925	0.72973811	0.857101832
966.0755542	-0.127322983	0.10003323	-1.272806872	0.203086582	0.405939626
180.0527563	-0.346471332	0.103298287	-3.354085949	0.000796276	0.008052704
2.840484192	0.148005621	0.629931535	0.234955092	0.814243589	0.906341141
2.043042516	-2.153968817	0.888940105	-2.423075305	0.015389736	0.071578349
1294.572623	-0.707859112	0.128185058	-5.522165575	3.34847E-08	1.71313E-06
299.2364379	0.770569239	0.114506845	6.729460063	1.70294E-11	2.26244E-09
363.6621226	-0.008509928	0.059630165	-0.142711797	0.8865178	0.943320904
1513.367767	-0.353611832	0.052839969	-6.692127939	2.19948E-11	2.78725E-09
3.911436453	-1.217472062	0.594219727	-2.048858371	0.040475966	0.140025423

155.5519149	-0.264908046	0.161995364	-1.635281653	0.101990018	0.260655221
1354.531961	-0.117380621	0.079915374	-1.468811506	0.141883922	0.321247352
16.67578829	-0.296096731	0.282335149	-1.048742006	0.294296875	0.509109181
352.5797957	-0.380899396	0.067961709	-5.604617669	2.08715E-08	1.14612E-06
7.737667754	0.142489652	0.387842954	0.367390075	0.713328073	0.847128509
265.3577535	-0.116861743	0.275497454	-0.424184474	0.671431283	0.821413853
9243.951694	-0.101294783	0.076878496	-1.317595793	0.187638995	0.386551139
137.2370076	0.032162242	0.106258679	0.302678727	0.762134711	0.875917904
90.53497903	-0.198524257	0.167385903	-1.186027333	0.235611505	0.442880916
31.28989421	-0.077291703	0.197531351	-0.391288284	0.695584158	0.836610456
2786.56572	0.157353702	0.141811913	1.109594382	0.267173853	0.47877973
20.64142347	0.188838842	0.253535458	0.744822218	0.456379202	0.660283795
43.42902532	-0.137042843	0.185405197	-0.739153191	0.459813981	0.662623194
614.9273644	-0.354985422	0.092412858	-3.841299046	0.000122385	0.001831216
1.750450536	1.538089334	1.069483565	1.438160794	0.150388436	0.333849765
124.7409816	-0.106931684	0.140134796	-0.763063044	0.445425798	0.650630443
2.185641838	-0.145633801	0.63060292	-0.230943747	0.817358504	0.90819642
174.2335366	-0.156951307	0.0917314	-1.710987805	0.087083364	0.234605288
373.2904868	-0.361620117	0.117761041	-3.070795848	0.002134891	0.016941324
59.4211635	0.133978789	0.15623464	0.857548546	0.391141798	0.604301789
55.81893326	0.344643831	0.221099205	1.558774624	0.119049732	0.287654047
129.4192499	0.056286524	0.082169596	0.685004272	0.493341225	0.690392741
51.93569529	0.00440344	0.189140685	0.023281295	0.981425892	0.99044414
22.3224974	-0.25234307	0.286769936	-0.87994953	0.378886651	0.592030607
24.48127629	-0.010392932	0.400343492	-0.025960038	0.979289213	0.989127547
536.3106014	-0.109881506	0.07070219	-1.554145732	0.120149659	0.289462632
11.10955488	-0.11858838	0.359304294	-0.330049994	0.741362187	0.864345411
788.7496318	-0.03831481	0.085719678	-0.446978003	0.654890943	0.80965654
44.22741834	-0.085951916	0.154726805	-0.555507596	0.578547516	0.756608064
124.8716342	0.160225594	0.217472085	0.736763958	0.461265905	0.66377753
5.780254562	-0.698886645	0.548422476	-1.274358136	0.202536522	0.405319079
28.49244295	0.34229474	0.271219265	1.262059094	0.206927523	0.41041705
272.0479773	-0.276906563	0.092988188	-2.97786816	0.002902608	0.021280626
873.1213682	0.087117298	0.059500328	1.464148208	0.143153458	0.323100434
292.2238633	0.261957457	0.080268517	3.263514359	0.001100396	0.010388496
71.9387177	-0.184464169	0.139057939	-1.326527424	0.184665058	0.38237458
389.2287747	0.003348087	0.068740865	0.048705916	0.961153661	0.98086139
136.7323941	0.026787015	0.10001897	0.267819342	0.788838382	0.890883904
77.09505716	0.068242729	0.290912233	0.234581846	0.814533301	0.906602365
53.70048713	-0.452388345	0.281232858	-1.608589935	0.107706037	0.269821966
2.597304788	0.157389679	1.362181107	0.115542404	0.908015212	0.953697201
2.509998018	0.727887192	1.13360402	0.642100045	0.520808227	0.712844361
43.86669664	-0.502877477	0.324482402	-1.549783514	0.121193485	0.291041031
2633.127534	-0.338855335	0.781084515	-0.433826722	0.664414275	0.81671216
67.43174255	-0.134701496	0.738112179	-0.182494612	0.855194582	0.929057489
355.2001277	0.013016458	0.07497452	0.173611759	0.86217059	0.931452496
408.70014	0.051663302	0.158972293	0.324983058	0.745193894	0.866421357
97.33719274	-0.074340598	0.152994223	-0.485904611	0.627034799	0.790677612

69.40962349	-0.024116669	0.17915693	-0.134611982	0.892918668	0.946108145
54.98154064	0.582099078	0.181045137	3.215215217	0.001303468	0.011792051
322.5696705	-0.054404588	0.088779359	-0.612806725	0.540004144	0.727631954
210.8943235	-0.052638855	0.087687209	-0.600302549	0.54830462	0.734909309
26.01438925	1.658370502	0.642592313	2.580750605	0.009858577	0.052004548
613.3943687	0.00567253	0.390873154	0.014512457	0.988421141	0.993980695
6.056761272	0.01475249	0.407459465	0.036206032	0.971118076	0.985661927
238.3151156	0.268690742	0.060732673	4.42415471	9.68206E-06	0.000232172
475.9638734	-0.057339676	0.077014536	-0.744530571	0.456555553	0.660342071
67.0698169	0.174930486	0.207637945	0.842478413	0.399520218	0.611768438
17.32888361	-1.727818932	0.848210134	-2.037017555	0.041648283	0.142970163
35.17577608	0.209486761	0.680813735	0.30770055	0.758310201	0.873792838
148.9161326	-0.149216342	0.15923851	-0.937061909	0.348726714	0.56333829
674.5097645	-0.080098398	0.109486977	-0.731579233	0.464425427	0.666574707
22.49558194	1.49335291	0.607011743	2.460171369	0.013887069	0.066659549
17.78345085	-1.059563117	0.524819287	-2.018910403	0.043496533	0.147440716
1269.169532	-0.186047403	0.100902771	-1.843828482	0.065208141	0.194400896
189.4002491	0.11905631	0.10206086	1.166522697	0.243403177	0.450998082
59.43255341	0.095227014	0.174598088	0.545406968	0.585473649	0.76059403
62.29465881	-0.076565223	0.143387407	-0.533974529	0.593359157	0.765945679
81.58225477	0.109019446	0.118687203	0.918544231	0.358334014	0.572736445
24.2481693	-1.725435043	0.637414629	-2.706927272	0.006790913	0.03964334
22.32011957	-0.35065014	0.257604373	-1.361196384	0.173451637	0.367044607
136.1665713	-0.347220037	0.141307695	-2.457191279	0.014002811	0.066980924
14.86894696	0.073465948	0.560996112	0.130956252	0.895809915	0.947455865
38.46879745	0.213406223	0.168366155	1.267512605	0.204972059	0.408141697
13.64549229	0.440639292	0.331037852	1.331084314	0.18316127	0.380408316
111.0233767	0.052090025	0.098520974	0.52872016	0.59699959	0.76889776
3.678069013	-2.692636708	1.162095793	-2.317052281	0.020500884	0.087528478
289.062384	0.141908901	0.069603747	2.038811234	0.041468871	0.142502748
5.688800545	1.479191494	0.457715709	3.231681728	0.00123064	0.011328832
251.8626511	0.933922406	0.115820634	8.063523486	7.41263E-16	2.65469E-13
522.0363364	-1.736981309	0.236013509	-7.359669009	1.84367E-13	4.16063E-11
675.2027048	0.135552068	0.053805852	2.519281144	0.011759472	0.059080677
30.50738863	-0.42477436	0.190699448	-2.227454586	0.025916908	0.103253963
860.9343473	-0.274796293	0.086829693	-3.164773299	0.001552038	0.013482754
2355.378862	0.06391553	0.119375089	0.535417655	0.592361084	0.764957004
8.427092758	0.018999654	0.534644591	0.035536981	0.971651559	0.985894788
33.18426819	-0.571407094	0.392569592	-1.455556176	0.14551535	0.32655035
287.1681659	-0.020534011	0.13189563	-0.155683786	0.876282278	0.937737303
707.3708393	0.122530409	0.078568729	1.559531524	0.118870629	0.287389895
4.377659546	-0.579925312	0.646075673	-0.897612054	0.36939241	0.582889901
2.516309894	1.616092317	1.108873086	1.457418651	0.145000852	0.325798278
451.2091915	0.055669848	0.053506339	1.040434622	0.298138024	0.512953086
18.15124534	0.054331387	0.400980287	0.135496404	0.892219407	0.94585057
1361.823755	-0.034467072	0.063908569	-0.539318474	0.589667123	0.763213089
427.0756063	0.203778922	0.069124127	2.948014384	0.003198222	0.022778861
1.71081918	0.181640124	0.783477507	0.231838339	0.816663578	0.907813535

2.640749124	-0.09804716	0.622617296	-0.157475805	0.874869875	0.937291376
595.456595	-0.012754758	0.093665101	-0.136174072	0.891683672	0.945641854
955.3174781	0.136063971	0.111422341	1.221155199	0.222027263	0.426800132
434.6642407	-0.01221881	0.063326816	-0.192948428	0.846999358	0.924254317
636.2461777	-0.429349883	0.111252699	-3.859231169	0.000113744	0.001738241
605.0864028	-0.199476288	0.095988079	-2.078136065	0.037696829	0.133347821
12.50000277	0.169762329	0.293357045	0.578688435	0.562799421	0.745421905
1896.01555	-0.172222166	0.117165098	-1.46991015	0.14158609	0.320941711
24305.92276	-0.044185048	0.100137015	-0.441245905	0.659034984	0.812402149
388.4526707	-0.407399593	0.13671037	-2.980019689	0.002882299	0.021178852
134.8840901	-0.166251253	0.114246448	-1.455198435	0.145614334	0.326596397
111.7114164	-0.161899321	0.153348911	-1.05575788	0.291078846	0.506302775
226.1651167	0.161398535	0.084039703	1.920503396	0.054794345	0.171547327
441.0720596	0.035162408	0.091448535	0.384504883	0.700604278	0.83939434
6.531968333	-0.356579968	0.425850616	-0.837335803	0.40240384	0.613826573
1368.262241	-0.012384885	0.065482343	-0.189133206	0.849988416	0.92598262
80.48490627	-0.065304694	0.109173027	-0.598176088	0.549722445	0.736090999
579.083417	0.193069583	0.115050278	1.678132261	0.093321282	0.245234455
19.97579352	-2.67210661	0.954356401	-2.79990432	0.005111776	0.031999571
68.29574728	-0.16685031	0.137936245	-1.209619057	0.226425102	0.432121
146.6120357	-0.400689976	0.175059726	-2.288875836	0.022086568	0.092105817
135.3135491	-0.058601568	0.096474662	-0.607429623	0.543565835	0.730638345
127.9129789	-0.294911705	0.101974762	-2.892006807	0.003827897	0.025855173
391.2916943	-0.088168347	0.051631369	-1.707650758	0.087701164	0.236038062
403.6247315	-0.524484089	0.212472741	-2.468477067	0.013568935	0.065525583
1874.028371	-0.351040974	0.08577627	-4.092518523	4.26713E-05	0.000786317
3.103495638	-1.631742998	0.85703225	-1.903945852	0.056917237	0.176640679
51.46344917	-0.011000714	0.162245468	-0.067802902	0.945942534	0.974270541
646.500111	-0.343091684	0.105059437	-3.265691251	0.001091972	0.010320799
4.201612654	0.325958786	0.667218507	0.488533791	0.625171797	0.789927917
943.9323085	-0.597421851	0.075606241	-7.901753135	2.75008E-15	8.38977E-13
36.06520913	0.498666794	0.180284709	2.765996058	0.005674922	0.034690765
175.5983563	-0.47572707	0.119470944	-3.981947851	6.83528E-05	0.001165677
41.06381782	0.171432965	0.250539488	0.684255271	0.493813987	0.690760942
183.4255577	-0.030660134	0.13444542	-0.228048931	0.819608201	0.909423741
52.87486044	-0.13022282	0.171351507	-0.759974757	0.447269674	0.652410183
5.608093279	0.461016102	0.444575375	1.03698074	0.299744833	0.514642666
87.83474209	0.005612271	0.155664216	0.0360537	0.97123954	0.985661927
224.546294	0.20523991	0.074426925	2.757602983	0.005822687	0.035304727
338.8677332	-0.001109287	0.070792881	-0.015669466	0.987498086	0.993528977
1348.286647	-0.26536514	0.320520585	-0.827919182	0.407716271	0.618482307
745.1462039	-0.202970641	0.137594444	-1.475136894	0.140175743	0.3189579
12.42418931	0.586766072	0.297704473	1.970968277	0.048727504	0.15926075
20.82619984	-0.813630644	0.382179227	-2.128924304	0.033260524	0.122115861
45.76401111	-0.723088088	0.219679924	-3.291552885	0.000996359	0.009632638
34.69108677	0.528729736	0.215841509	2.449620272	0.014300694	0.067912838
145.0124801	0.494070508	0.131909656	3.745521923	0.000180019	0.002511122
7.52599222	0.548372952	0.355101862	1.544269432	0.122523075	0.292501519

20.73380654	1.104255201	0.251074904	4.398110622	1.09197E-05	0.000256988
109.0400294	-0.183993239	0.186417885	-0.986993488	0.323645831	0.538940809
136.4631463	0.328386529	0.084198795	3.900133343	9.61397E-05	0.001515604
2.13530098	-0.175226668	0.922353367	-0.189977805	0.849326523	0.925628813
414.9406673	-0.069911048	0.077151329	-0.906154812	0.364853917	0.578253801
343.8810125	-0.06252621	0.075000614	-0.83367597	0.404463603	0.61520359
491.7674312	-0.198275243	0.083031627	-2.387948424	0.016942719	0.076406887
527.6379106	0.081241936	0.078280346	1.037833121	0.299347754	0.514175258
6.249384594	-0.158386292	0.469397793	-0.33742445	0.735796956	0.861152614
59.75220451	-0.078443161	0.182037812	-0.43091685	0.666528831	0.818089403
1.96239103	-2.438624116	1.889705028	-1.290478715	0.196884497	0.39836345
101.7993456	-0.038584901	0.163972612	-0.235313081	0.813965743	0.906237868
2737.878955	-0.18835391	0.130609059	-1.442119804	0.149268579	0.332337899
78.18988454	0.215349776	0.185757214	1.15930774	0.246330768	0.454978188
40.52904351	0.142661055	0.245597841	0.580872594	0.561326324	0.74475811
321.3464673	0.224582183	0.088657258	2.533150549	0.011304239	0.05735326
981.6284582	0.04379473	0.098540414	0.444434204	0.656728689	0.81089405
944.6818575	-0.358852056	0.134321526	-2.67159008	0.007549281	0.042767142
69.38661506	-0.06406729	0.12492861	-0.512831212	0.608069394	0.777484879
2.298498865	0.255730228	0.708407006	0.360993364	0.718104408	0.850529195
38.20017064	-0.087080514	0.216750382	-0.401754838	0.687864462	0.83108758
45.28365642	-0.013812738	0.16649001	-0.082964366	0.933879874	0.968751152
32.92222051	-0.768805638	0.657174453	-1.169865376	0.242055149	0.449771264
11.8581889	-0.304639729	0.64646507	-0.471239272	0.637469877	0.797454534
24.50340404	-0.639748795	0.295599931	-2.164238648	0.030446039	0.115170618
128.7660204	0.12743551	0.101665695	1.253476014	0.210032523	0.414168188
510.8195666	-0.071339215	0.054429088	-1.310681803	0.189965289	0.389724555
6.791045129	0.481759346	0.351402013	1.370963532	0.170386324	0.362420182
1246.359295	0.047219672	0.107067673	0.441026413	0.659193876	0.812416008
1589.915764	-0.34460482	0.118279105	-2.913488557	0.003574149	0.024754305
4.182923563	-0.089169384	0.524160317	-0.170118533	0.864916919	0.932807942
335.0719552	-0.065645676	0.096098019	-0.68311164	0.494536301	0.691360405
122.6208183	-0.228506622	0.096832042	-2.359824454	0.018283585	0.080406774
21.29012891	-0.892003109	0.469362568	-1.900456427	0.057373248	0.17769635
114.3711813	0.435255023	0.19942942	2.182501572	0.029072535	0.111537249
7.010953025	0.647589797	0.410729801	1.576680813	0.114868987	0.281390587
2053.085018	-0.06338546	0.048110026	-1.317510398	0.187667598	0.386551139
431.9511262	-0.113922687	0.078823156	-1.445294655	0.148375138	0.330896444
650.7993843	-0.061797199	0.058577825	-1.054958928	0.291444109	0.50652837
2206.704464	-0.103113532	0.08910823	-1.157171802	0.247202176	0.455777809
26.93501107	0.667081264	0.373267514	1.78713989	0.073914882	0.211401694
22.25497824	0.776870658	0.238498063	3.257345771	0.001124594	0.010580556
29.7844404	0.371428472	0.188198941	1.973594914	0.048427823	0.158661514
223.7405389	-0.032924979	0.088119986	-0.373638035	0.708673632	0.844767685
9.352619674	-0.537592559	0.321462902	-1.672331565	0.094458959	0.24696348
72.96952833	-0.39926455	0.145463601	-2.744772901	0.006055278	0.036287616
308.6870139	0.108325428	0.086391531	1.253889436	0.209882195	0.414034162
49.79966269	-0.073212051	0.335903011	-0.217955923	0.827463454	0.913832067

31.95656994	0.754660068	0.239456209	3.151557729	0.001624021	0.013898241
322.0081684	0.066772742	0.117071436	0.570358957	0.568434261	0.749150881
775.3724342	-0.362103978	0.079533201	-4.552865632	5.29201E-06	0.000139712
5.534433445	-2.144736003	1.010829835	-2.121757716	0.033858089	0.123506384
145.2963573	0.162220017	0.092863728	1.746860912	0.080661468	0.223565708
28.18452578	-0.021181345	0.268480302	-0.078893477	0.937117351	0.970380036
1324.643043	-0.008529995	0.039962299	-0.213451063	0.830975155	0.915614595
100.533197	0.428848453	0.141244388	3.036215876	0.002395678	0.018459493
406.7379193	-0.112456295	0.088284254	-1.273797872	0.202735059	0.405470119
63.79930253	-0.678194683	0.219934254	-3.083624629	0.002044954	0.016473632
84.68855793	-0.02324292	0.123605119	-0.188041729	0.850843939	0.926363298
444.9827894	0.320509616	0.118552511	2.703524476	0.006860839	0.039866218
200.7094587	0.231594331	0.07851535	2.949669461	0.003181141	0.022706287
268.1967471	-0.044865826	0.083441129	-0.537694373	0.590788065	0.763924449
68.15321326	-0.289189386	0.135863085	-2.128535396	0.033292718	0.122169382
249.4082832	-0.264551386	0.12400518	-2.133389805	0.032892763	0.121115816
6.158784254	-1.711951399	0.6864817	-2.49380486	0.0126382	0.062242664
4.681862759	-0.995060725	0.722318383	-1.377592968	0.168328993	0.359716154
3.048444561	-0.439713081	0.55646791	-0.790185872	0.429419225	0.636402691
2.32264229	-1.088444907	0.755713724	-1.440287337	0.149786122	0.33291643
4.211089849	-0.13985325	0.745069749	-0.187704909	0.851107981	0.926405873
343.0959067	-0.322703899	0.15600345	-2.068568983	0.038586551	0.135336351
29.57299893	0.292183186	0.180327655	1.620290498	0.105169889	0.265568479
373.1254699	-0.238104373	0.080742837	-2.948922563	0.003188839	0.022731687
2.703310239	-1.127193407	0.672912563	-1.675096392	0.093915321	0.246091419
1011.73095	0.068730646	0.064738922	1.061658808	0.288390606	0.503091434
301.2982198	-0.044298447	0.085419891	-0.518596392	0.604042225	0.774328722
5112.97677	-0.083750962	0.097563464	-0.858425471	0.390657568	0.60400683
9.956577375	0.501764064	0.396493399	1.265504208	0.205690641	0.409244882
79.15447446	0.466903752	0.224802821	2.076947919	0.037806366	0.133624132
4.038319431	0.679041536	0.529144526	1.283281792	0.199393369	0.401614667
6.810662823	0.990083939	0.386012182	2.564903353	0.010320455	0.053583098
125.6578568	0.211288213	0.102684528	2.057644087	0.039624311	0.137744441
220.2855233	-0.042999891	0.085228896	-0.504522444	0.613894295	0.781613309
378.8469351	-0.096524533	0.124018114	-0.778309956	0.436386307	0.642738313
12.07091799	0.419437354	0.260184732	1.612075199	0.106945577	0.268415829
333.2689519	-0.008939472	0.077833506	-0.114853773	0.908561027	0.954020929
117.7451572	-0.073281351	0.107727108	-0.680249861	0.496346266	0.693126082
119.6277547	0.035350422	0.119368865	0.296144411	0.767119799	0.878716103
86.85223112	0.176724899	0.123141212	1.43514017	0.151247159	0.335304225
660.7796572	0.075724972	0.064435834	1.175199676	0.23991484	0.447352243
160.8166934	-0.451099983	0.107158799	-4.209640163	2.55778E-05	0.000522789
7.793207957	-0.358498668	0.463865957	-0.772849705	0.439611333	0.645353511
287.1441655	-0.296770243	0.2332931	-1.272091817	0.203340498	0.406187871
11.9763371	-0.180925869	0.307364916	-0.588635396	0.556105884	0.740545496
31.74168506	-0.055512657	0.180130422	-0.308180356	0.7579451	0.873603573
709.9486072	0.007874328	0.043429902	0.181311215	0.856123304	0.92936531
11.22662861	-0.024296951	0.305927713	-0.079420562	0.936698114	0.970184161

86.77756099	0.07935688	0.13057786	0.607736103	0.543362514	0.730508517
124.6191783	-0.005898329	0.312960991	-0.018846851	0.984963279	0.992448306
358.904257	0.800569506	0.126187777	6.344271405	2.23481E-10	2.24489E-08
3.499930126	1.764705054	0.570654756	3.092421528	0.001985307	0.01613515
141.7682188	-0.358720495	0.257857235	-1.391159314	0.164177122	0.353688231
26.63894611	-0.19337172	0.201887074	-0.957821204	0.338152925	0.55346724
2758.213061	-0.163463431	0.064638974	-2.528867963	0.011443107	0.057950735
88.24531576	-0.522069161	0.301440663	-1.731913522	0.083288961	0.228379218
156.1787033	-0.125137561	0.094487828	-1.324377577	0.185377678	0.38341643
585.2046366	-0.067203604	0.070533832	-0.952785387	0.34069882	0.555270316
394.1591169	0.134052846	0.055260311	2.425843153	0.015272869	0.071235915
3.087736712	-0.007687204	0.604979339	-0.012706556	0.989861908	0.994530438
79.64646645	-0.028236465	0.178257959	-0.158402268	0.874139827	0.93692775
824.9409148	-0.83353223	0.291326644	-2.861160306	0.004220936	0.027836547
40.86445294	0.026773176	0.374280823	0.071532321	0.942974102	0.972623458
886.1233026	-0.178246418	0.091537797	-1.947243925	0.051505498	0.165110249
181.8826809	-0.088653287	0.283601323	-0.312598283	0.754585883	0.871752087
387.9219646	-0.415850949	0.088404339	-4.703965361	2.55157E-06	7.57378E-05
172.1522125	-0.017850762	0.104252275	-0.17122659	0.8640456	0.932173361
230.5804833	-0.429735834	0.155475462	-2.764010665	0.005709567	0.034811033
1203.598493	0.292279863	0.063483287	4.604044246	4.14364E-06	0.000113771
4011.759536	-0.52936014	0.185996592	-2.846074398	0.004426185	0.028832335
2.046579797	0.970157013	0.78927032	1.229182182	0.219003508	0.42356051
45.66920347	0.022867696	0.170969093	0.133753392	0.893597584	0.946144251
1.781498861	0.85531957	1.06455695	0.80345121	0.421713986	0.629970639
111.5496761	0.005789677	0.102129533	0.05668955	0.954792499	0.978126461
25.22956215	0.114472034	0.226597155	0.505178602	0.613433398	0.781207622
225.3116029	-0.172574449	0.074328435	-2.321782342	0.020244659	0.086783894
4.216081722	-0.507151043	0.673308296	-0.753222625	0.451316146	0.656336704
2.432748691	-3.510923475	1.378905847	-2.54616621	0.010891332	0.055728586
73.55808239	0.028518427	0.140299862	0.203267675	0.83892582	0.919711839
1.878251506	-3.157494507	1.672174273	-1.88825684	0.058991478	0.181310749
482.0857047	-0.23156571	0.073128013	-3.166580095	0.001542429	0.013423122
116.1264094	-0.09066968	0.189814595	-0.477674964	0.632881566	0.794224511
6.681627006	0.237728383	0.529475874	0.448988131	0.653440219	0.808447666
311.3561035	0.342591267	0.092798838	3.691762466	0.000222705	0.002984007
29.58170481	-0.368009262	0.489022737	-0.752540187	0.451726272	0.656469531
7.259019845	-0.113064341	1.003366405	-0.112684997	0.910280296	0.955034874
415.7502728	-0.508452041	0.105477713	-4.820468943	1.43221E-06	4.72831E-05
47.65360639	0.005041578	0.194689869	0.025895431	0.979340744	0.989127547
494.5242326	0.417233174	0.106000539	3.936142001	8.2802E-05	0.001352855
372.245709	0.329497309	0.100915104	3.265094085	0.001094277	0.010336653
17.21886673	-0.261594985	0.370676768	-0.705722633	0.480360643	0.679394088
115.3165027	-0.054322122	0.127738513	-0.425260327	0.670646911	0.820880988
227.9163304	-0.211110671	0.121085785	-1.74348021	0.081249768	0.224657382
442.0336211	-0.326310384	0.154543063	-2.111452803	0.034733411	0.125719761
6.972806875	0.24452343	0.547870772	0.446315888	0.655369081	0.809943754
171.8483223	0.129392014	0.143457986	0.901950584	0.367083119	0.580692078

56.52669724	-0.141973165	0.195518464	-0.726136867	0.46775487	0.669428697
1223.561845	-0.111046863	0.07162394	-1.550415444	0.121041834	0.290888866
128.3325973	-0.196037714	0.128585528	-1.524570595	0.127366236	0.298780921
3.031391567	0.320783401	0.772350304	0.415334077	0.677897358	0.825950823
4.673999748	-1.311220284	0.544791161	-2.406831052	0.016091612	0.073685401
260.5959637	-0.156881633	0.118879056	-1.319674284	0.18694379	0.385590583
757.5755842	-0.078303608	0.136049417	-0.575552695	0.564917564	0.747206097
780.3947841	-0.14443393	0.059759332	-2.416926759	0.015652166	0.072329253
445.7041989	-0.640713289	0.178837231	-3.582661653	0.000340111	0.004128953
96.7147211	-0.16297412	0.112722134	-1.445804063	0.148232166	0.330622354
348.1696145	0.126831252	0.085421047	1.48477754	0.137602763	0.315387724
3.413032571	0.359199012	0.547365763	0.656232151	0.511674765	0.70585581
1.8542883	0.081694646	0.770273137	0.106059321	0.915535286	0.958290127
30.96371493	-0.046325417	0.22195692	-0.208713552	0.83467186	0.917242627
157.8815738	-0.207588204	0.094519986	-2.196236096	0.028075049	0.108979349
75.09617394	0.042437814	0.125980492	0.3368602	0.73622229	0.861278654
184.0778372	0.014833195	0.084962546	0.174585108	0.861405651	0.931215742
196.915874	-0.36884834	0.100304884	-3.677271984	0.000235742	0.00311686
112.7290892	0.157644659	0.100043614	1.575759342	0.115081276	0.281621068
109.8176735	-0.152876027	0.343797418	-0.44466892	0.656559033	0.81089405
2.385513195	-0.01984927	0.729200913	-0.027220577	0.978283803	0.988666179
6.533408533	0.745608607	0.50334877	1.481296175	0.138527667	0.316795003
296.2239224	-0.103851463	0.126598709	-0.820320081	0.412033661	0.621935878
254.3800726	-0.289312135	0.105321427	-2.746944694	0.006015328	0.036100732
278.5811146	-0.124793796	0.078897732	-1.581715883	0.113714445	0.279601757
2.000158776	4.431787689	1.903045395	2.328787165	0.019870344	0.085592894
852.3817432	-0.465194842	0.135603459	-3.430552923	0.000602352	0.006465726
11.55712148	-0.055207794	0.31430456	-0.17565063	0.860568424	0.931104966
297.6703775	-0.226213558	0.071002003	-3.186016544	0.001442463	0.012757708
337.6849704	-0.06877218	0.088293706	-0.778902413	0.436037202	0.642539879
6.50442487	0.703911387	0.42540637	1.65467994	0.097989438	0.253140663
561.7237853	-0.008373231	0.117484625	-0.07127087	0.943182178	0.972629837
700.9415518	0.601079076	0.133471077	4.503440675	6.6862E-06	0.000169982
41.45603111	0.177035925	0.195634906	0.90493015	0.365502393	0.579025524
498.7174116	-0.223374856	0.099253775	-2.25054266	0.024414519	0.098919031
206.3715768	-0.371252412	0.140854957	-2.635707117	0.008396217	0.046462639
2738.485824	-0.433217434	0.062689428	-6.910534146	4.82832E-12	7.64825E-10
142.666526	-0.221160832	0.139283413	-1.587847597	0.112320806	0.277583701
31.93853867	-0.163235157	0.187692258	-0.869695739	0.384466701	0.597519287
9.541415855	-0.546914741	0.360655183	-1.516447751	0.129406172	0.302045521
39.35264333	0.08918617	0.174624238	0.510731906	0.609538796	0.778474465
12.35158137	0.518102716	0.393012514	1.318285544	0.187408078	0.386209743
393.320119	-0.163938189	0.115993392	-1.413340758	0.157555555	0.344057558
462.7934856	0.016996048	0.155982926	0.108960949	0.913233464	0.956731834
4.886460158	-0.097895013	0.452194265	-0.216488843	0.828606721	0.914385171
164.1105598	-0.073587206	0.090442198	-0.813637959	0.415852385	0.625411009
513.1889883	-0.117935247	0.262582655	-0.44913571	0.653333761	0.808447666
311.7841329	-0.17467969	0.10742615	-1.626044401	0.10394021	0.263653388

12.93857893	-0.537766519	0.339630436	-1.5833873	0.113333216	0.278955834
20.06938119	0.206945119	0.367140761	0.563666966	0.572980803	0.752170338
3.543161984	-0.123747334	0.60399516	-0.204881334	0.837664839	0.918876719
581.0487862	0.089885804	0.051205074	1.755408147	0.07918952	0.220627117
1283.273189	0.217348241	0.063412106	3.427551192	0.000609051	0.006515269
7.753062868	-0.374327861	0.478258898	-0.782688753	0.433809908	0.640547139
55.49275967	0.806409504	0.187397679	4.303198989	1.6835E-05	0.000370279
15.51218019	0.902627647	0.274909907	3.283358011	0.001025783	0.009824855
216.0741146	0.210123342	0.072236636	2.908819591	0.003627962	0.024954922
4.290741716	-1.970330155	0.63958598	-3.080633748	0.002065606	0.016575152
39.82243614	0.054774394	0.214395745	0.255482656	0.798350338	0.895702068
18.34905625	0.408015193	0.292234222	1.396192377	0.162656572	0.351983366
257.4450695	-0.216658141	0.08865884	-2.443728582	0.014536358	0.068675641
145.510273	0.957680818	0.137719624	6.953844256	3.55464E-12	5.91507E-10
1696.213303	-0.073306858	0.068454625	-1.070882482	0.284222274	0.498334037
423.0394796	0.027228089	0.110831947	0.245670038	0.805937668	0.901149495
512.1424951	0.11016575	0.071912042	1.531951338	0.125534444	0.296537772
35.05229014	0.474215949	0.184355937	2.57228466	0.010102978	0.052853753
41.88679822	1.253345795	0.200661187	6.246079851	4.20882E-10	3.80968E-08
28.71907462	0.513862369	0.264681634	1.941435683	0.052205454	0.16638279
56.2904678	0.605611091	0.184976703	3.273985761	0.00106042	0.010080411
606.6894606	0.03051998	0.062752332	0.486356108	0.626714705	0.790677612
65.66219055	-0.153499022	0.141619712	-1.083881758	0.278417229	0.491654564
128.2096999	0.904263504	0.20282092	4.458433112	8.2561E-06	0.000203305
14.57506894	0.235128629	0.316241235	0.743510343	0.457172762	0.660712701
262.9715706	0.097787833	0.147933687	0.66102478	0.508596422	0.703081099
51.13243264	-1.532062368	0.3001605	-5.104143842	3.32295E-07	1.31022E-05
9947.88762	-0.314678178	0.143298825	-2.195957835	0.028094962	0.109030953
11.11287824	0.84503479	0.313220782	2.697888636	0.006978078	0.040209149
25.21931144	0.09064606	0.40782564	0.222266703	0.824106271	0.911434821
4.29792686	0.078893266	0.524519258	0.150410619	0.880440664	0.940144817
573.0961121	-0.131203023	0.076722663	-1.710094748	0.087248354	0.234934518
105.1791745	0.639192978	0.132697009	4.816935832	1.4578E-06	4.79138E-05
17.84993005	-0.610128204	0.477243285	-1.278442723	0.201093368	0.403803977
398.8510986	-0.148033943	0.06923691	-2.138078418	0.032510379	0.120290075
18.23442877	0.272962097	0.309493046	0.881965201	0.377795626	0.590830183
156.8866336	0.232849688	0.102429764	2.273261982	0.023010397	0.094650544
388.4618685	0.221733699	0.058116197	3.815351144	0.000135989	0.001987835
172.2927231	-0.076790501	0.112369293	-0.683376202	0.494369154	0.691185389
10.53821768	0.248329658	0.330571145	0.751213959	0.4525239	0.65711611
586.2224776	0.130002925	0.064591752	2.012686148	0.044147658	0.148949289
59.87478913	0.420862924	0.193062377	2.179932363	0.029262476	0.112187578
152.6969352	0.122426735	0.079785554	1.534447394	0.124919626	0.295727733
30.34190147	0.440782801	0.21598568	2.040796411	0.041271067	0.142089773
624.1211153	-0.164863315	0.052469153	-3.142099778	0.001677409	0.014199103
142.4301242	-0.430346629	0.119948691	-3.587755942	0.000333536	0.004061106
2652.434966	0.174370302	0.07273845	2.397223215	0.016519857	0.075116787
456.6302184	-0.050208343	0.085104117	-0.589963734	0.555214963	0.740017096

11.77424156	-0.316059761	0.348488012	-0.906945865	0.364435425	0.578113548
3.712616202	-0.171472648	0.653952729	-0.262209545	0.793159895	0.893098455
3.410879745	0.146027678	0.575017265	0.253953554	0.79953144	0.896484956
21.62660114	-0.688543347	0.358887992	-1.918546625	0.055041736	0.172184398
195.8139365	-0.313561956	0.113323667	-2.76695914	0.005658185	0.034638771
72.24630657	0.148013558	0.13912661	1.063876694	0.287384562	0.501947972
1157.234433	0.078381763	0.049186418	1.593565168	0.111033461	0.275490117
682.7200139	0.144675727	0.072458337	1.996674682	0.045860534	0.153184599
1030.046618	-0.051143627	0.115042617	-0.444562447	0.656635991	0.81089405
156.010759	-0.406528162	0.140399739	-2.895505114	0.003785489	0.025621257
533.1427395	-0.142011631	0.059771288	-2.37591718	0.017505391	0.078258838
444.7024986	-0.275762024	0.063503309	-4.342482745	1.40882E-05	0.000317494
1403.525343	0.023124264	0.071280262	0.324413281	0.745625167	0.866724055
228.6743225	0.050758237	0.116243383	0.43665485	0.662361678	0.81503931
4810.131001	-0.323818504	0.091656268	-3.532966255	0.000410925	0.004801119
577.9133615	0.20043805	0.05133473	3.904531087	9.44083E-05	0.001491162
17.07815029	0.237330449	0.258065199	0.919653055	0.357754092	0.572182625
695.5032538	-1.001416649	0.24630343	-4.065784427	4.78712E-05	0.000860011
9.052807516	0.142729087	0.329865289	0.432689017	0.66524071	0.817239035
232.8464178	-0.251919709	0.117284928	-2.147929092	0.031719389	0.118177656
133.5424044	0.180587698	0.402176868	0.44902557	0.653413211	0.808447666
1661.412101	-0.073113519	0.052274681	-1.398641135	0.161920625	0.351030447
104.9353181	-0.239307052	0.135321097	-1.7684386	0.076987608	0.216744776
73.92601566	-0.354143716	0.217899784	-1.6252596	0.104107256	0.263855836
6.171266541	0.460661598	0.402265066	1.145169285	0.252139039	0.461283628
178.8006777	0.102169166	0.094218696	1.084383141	0.278194955	0.491314719
317.6777008	0.019941496	0.067433928	0.295719034	0.76744466	0.878861959
825.8733232	-0.018339956	0.095381447	-0.192280121	0.847522789	0.924458083
15.33975006	-0.564392988	0.677456472	-0.833105907	0.404785003	0.615467848
222.1534449	0.326826992	0.146338002	2.233370595	0.025524516	0.102160075
11.22660746	0.055928127	0.288215546	0.194049654	0.846137003	0.92379566
372.9555478	-0.138006933	0.069688548	-1.980338763	0.047665476	0.156954038
6.513695332	0.156697734	0.392693158	0.399033521	0.689868505	0.83271258
316.1626453	0.227241524	0.085626503	2.653869012	0.007957471	0.044452824
213.1288176	0.244734126	0.074455023	3.287006253	0.001012586	0.009755169
1035.049585	-0.102122	0.069166613	-1.476463788	0.13981943	0.318494206
498.0152908	0.313664631	0.10378544	3.022241173	0.002509106	0.019110296
1375.321629	-0.042512766	0.077538447	-0.548279817	0.583499784	0.759227189
791.6367259	-0.052751073	0.065816159	-0.801491209	0.422847331	0.631034236
341.5644088	-0.308989389	0.081458675	-3.793204226	0.000148716	0.002141559
11.548725	-0.316694933	0.326137151	-0.97104832	0.331524217	0.547083037
3284.474862	-0.180425311	0.106269809	-1.697804036	0.089544754	0.239047201
389.5149124	-0.065677721	0.246755302	-0.266165388	0.790111838	0.891527563
329.0173217	-0.088655386	0.078904818	-1.123573791	0.26119388	0.472483582
839.3768883	0.089374863	0.135081656	0.661635827	0.508204641	0.702907521
3.826006845	0.211113592	0.571384945	0.369476993	0.711772219	0.846073709
304.9216708	0.377401435	0.163045656	2.314697887	0.020629471	0.087884643
25.84204334	0.600738665	0.194245475	3.092677781	0.001983594	0.016129183

19.46825031	0.50148439	0.228234021	2.197237674	0.028003476	0.108835837
265.6344644	-0.030771274	0.075950785	-0.405147546	0.685369059	0.829778815
5.909146734	0.483557185	0.581735093	0.831232619	0.405842231	0.61670688
435.8303726	0.061765689	0.058514541	1.055561367	0.291168659	0.506302775
26.64210288	-0.335109537	0.197998111	-1.692488557	0.09055286	0.240905679
442.4625528	0.486283712	0.129244899	3.762498292	0.000168224	0.002380867
2.331272801	1.103985029	0.776851733	1.421101328	0.155287304	0.340503534
434.1657486	0.087280497	0.139954022	0.623636932	0.53286605	0.722207759
73.83999402	-0.258617517	0.229299772	-1.127857713	0.259380015	0.470286855
8.678281392	0.466244656	0.310677267	1.500736315	0.133423776	0.308686862
880.8261663	-0.31159886	0.066780345	-4.666026537	3.0708E-06	8.87071E-05
138.3810559	0.109379213	0.100205727	1.091546521	0.275032473	0.487737632
116.4265087	-0.021019816	0.236041782	-0.089051252	0.929041179	0.965637301
564.5305377	0.027687714	0.092704609	0.298665997	0.765194904	0.877720432
1311.887559	0.0956695	0.448128369	0.213486819	0.830947269	0.915614595
271.6562621	0.07366646	0.117370757	0.627638964	0.530240478	0.719952331
425.5703355	0.013697574	0.082920494	0.16518925	0.868795027	0.934607931
509.3199474	0.091176607	0.077478949	1.176792004	0.239278535	0.446702698
6.291524911	0.057310905	0.369661088	0.155036347	0.876792663	0.93787951
882.2211747	-0.208011135	0.158254	-1.314413125	0.188707216	0.388013315
55.31485695	0.094075367	0.156788177	0.600015695	0.548495776	0.735105712
171.5280921	-1.045874485	0.21383516	-4.891031417	1.00309E-06	3.44269E-05
48.44011866	0.12834103	0.173885276	0.738078766	0.460466587	0.663319915
44.75690843	0.083948289	0.393866512	0.213138936	0.831218596	0.915631403
1208.200649	-0.182288864	0.045238295	-4.029525496	5.58896E-05	0.000980537
109.127074	-0.948918334	0.414408249	-2.289815265	0.022032028	0.09193405
80.44767534	0.212180878	0.10996996	1.92944398	0.053675768	0.169637391
140.4171122	0.777352159	0.178816341	4.347209851	1.3788E-05	0.000311156
52.76613046	-0.865862263	0.362280438	-2.390033169	0.016846851	0.076141297
228.3892076	0.058984274	0.079733254	0.739770059	0.45943953	0.662303711
16.67607664	-0.501585569	0.373039427	-1.344591302	0.178757254	0.374375724
501.729903	-0.3941796	0.164199181	-2.400618555	0.016367388	0.07457598
594.9227296	-0.165326675	0.076636553	-2.15728225	0.030983679	0.11645423
743.0176875	0.246672915	0.072873208	3.384960283	0.000711886	0.007380492
526.9659248	0.080460576	0.095888341	0.839106977	0.401409281	0.613035737
54.05740306	-0.227720187	0.158937566	-1.432765029	0.151924998	0.336128448
8.883357896	0.503580691	0.392925159	1.28161987	0.199976026	0.402370007
908.5395835	-0.228168203	0.07336879	-3.109880947	0.001871628	0.015418096
10.18596744	-0.088478773	0.503697665	-0.175658493	0.860562246	0.931104966
867.4607272	-0.012705326	0.065686101	-0.193424883	0.84662623	0.92396956
204.6973609	-0.282410849	0.071032812	-3.97578024	7.01489E-05	0.001186481
14.78076786	-1.386550339	0.662630174	-2.092495021	0.036394257	0.130027973
10.79724757	0.325231444	0.463276815	0.702024002	0.482664195	0.681763693
11325.85119	-0.147687588	0.096364249	-1.532597301	0.125375107	0.296351392
135.0541234	0.058558418	0.124120135	0.471788227	0.637077955	0.797196954
7.513349392	1.626258927	0.438390642	3.70961141	0.000207578	0.002814513
1007.421649	-0.147740122	0.088261826	-1.673884714	0.094153259	0.246543506
2.07277597	0.555688467	0.743492829	0.747402592	0.454820581	0.659047784

2.114368676	-1.261136006	1.229136513	-1.026034125	0.304875499	0.519660525
172.4311919	0.243975022	0.099822282	2.444093814	0.01452165	0.068645527
2383.540675	-0.093495704	0.113107443	-0.826609655	0.408458343	0.619157236
14.22070866	0.529796077	0.455983541	1.161875441	0.245286064	0.453568596
575.8271982	0.113441723	0.058641068	1.93450984	0.053050473	0.168439692
25.71743894	0.415993963	0.273378557	1.521677364	0.128089942	0.29982292
52.28367765	-1.567311036	0.399786875	-3.92036641	8.84144E-05	0.001422402
18.84750152	-0.069504873	0.302702342	-0.229614585	0.818391272	0.908774216
1331.509746	-0.123636998	0.19688432	-0.627967724	0.530025084	0.719778521
7.782120223	0.329532166	0.463693561	0.710667979	0.477290007	0.677000397
167.7540952	0.18173201	0.106173713	1.711647869	0.086961581	0.234506606
284.3799726	0.054652713	0.150626678	0.362835547	0.716727736	0.84963465
22.89592463	1.16840456	0.327605299	3.566500803	0.00036178	0.004345792
9.554019248	-2.673916053	1.014245564	-2.636359624	0.008380087	0.04638896
18.63714024	-0.861669174	0.313892651	-2.745107827	0.006049102	0.036278666
470.0349397	0.132723362	0.082416139	1.610405006	0.107309468	0.268992116
291.886282	-0.215705577	0.073882748	-2.919566227	0.003505189	0.024416273
5.044945653	0.54924746	0.420273603	1.306880698	0.191253232	0.391440645
264.3359985	0.133036425	0.117371571	1.133463789	0.25701954	0.467602418
486.3188344	0.025836326	0.056820615	0.454699869	0.649325175	0.805627137
2.714052901	-2.140286453	1.237020075	-1.730195408	0.083595369	0.228876533
195.2764444	0.233733787	0.069359684	3.369879648	0.00075201	0.007718764
17.92320733	-1.904681227	0.566699403	-3.361007997	0.000776586	0.007911858
40434.81785	0.826487172	0.10071824	8.205933436	2.28806E-16	9.42338E-14
258.6339505	-0.06035132	0.088620268	-0.681010352	0.49586494	0.692806295
76.23175595	-0.243311623	0.118541339	-2.052546607	0.04011657	0.139015646
30.6530112	0.029409514	0.188295682	0.156187935	0.875884886	0.937672708
107.4548319	0.167324022	0.097702912	1.712579689	0.086789893	0.234236024
5.138377149	0.744688258	0.449149642	1.657995885	0.097318297	0.251920431
156.7248974	-0.009475898	0.109110612	-0.086846712	0.930793357	0.967071756
767.9004112	-0.098979161	0.049280311	-2.008493005	0.044590929	0.149977543
327.7401545	-0.297911357	0.241048923	-1.235895819	0.216497291	0.421232594
528.9249897	0.272911338	0.070476661	3.872364741	0.000107784	0.001659478
473.3318936	0.266029065	0.053085893	5.011294938	5.4065E-07	2.03814E-05
69.22027357	-1.131913636	0.549337745	-2.060505847	0.039350207	0.13698194
28.87532834	0.500374207	0.241634304	2.070791269	0.038378307	0.135037211
188.4665697	0.071798602	0.081051551	0.885838713	0.375704445	0.589069671
12.48979689	1.321062079	0.499067795	2.647059364	0.00811951	0.045235308
15.24589034	2.500495219	0.372716165	6.708845649	1.9617E-11	2.54081E-09
242.7167877	-0.030258815	0.08756221	-0.345569341	0.729666391	0.857101832
300.5811986	0.229588906	0.082769172	2.773845649	0.005539796	0.034053206
142.5041941	0.084642982	0.100189802	0.844826318	0.398207818	0.610697469
2.190967486	-0.560841896	0.761969576	-0.736042374	0.461704911	0.66425065
3.098093573	1.347988172	0.916811829	1.470299715	0.141480598	0.320907528
112.541865	0.067804466	0.12732388	0.532535339	0.594355273	0.76681093
34.63745907	0.226746491	0.177371025	1.278373914	0.201117617	0.403803977
60.67721079	-0.489330066	0.260164005	-1.880852297	0.059992014	0.183641963
497.4917945	-0.035598786	0.072803429	-0.488971288	0.624862024	0.78965764

873.1614554	0.068453931	0.054590699	1.253948593	0.209860691	0.414034162
384.346184	-0.452155417	0.221979034	-2.036928482	0.041657209	0.142971014
2.067477379	-0.618766227	0.862445961	-0.717455069	0.47309335	0.673383433
383.8853279	0.236548174	0.168745805	1.401801801	0.16097444	0.349393008
63.58275687	0.043365852	0.149809265	0.289473766	0.772218852	0.881274949
1232.659362	-0.16067999	0.063836158	-2.517068615	0.011833578	0.059344404
936.1932593	-0.169351435	0.09347761	-1.811679137	0.070035789	0.2036663
705.8100361	0.045557847	0.051676182	0.881602421	0.377991845	0.591024835
192.8432496	0.198044239	0.112288389	1.763710751	0.07778069	0.218252272
908.7139308	0.087818852	0.066718615	1.316257123	0.188087757	0.387222907
84.42316308	-0.124191286	0.136998883	-0.90651313	0.364664318	0.578202266
2.15983114	0.673988697	0.677547079	0.994748141	0.319858774	0.534905436
1134.053464	0.318644923	0.105629742	3.016621241	0.00255609	0.01941337
51.92087297	-1.062386999	0.364717698	-2.912902239	0.003580867	0.024765408
226.4730986	-0.135369941	0.147690111	-0.916580945	0.359362273	0.573611636
541.5575705	-0.045433747	0.058249355	-0.779987138	0.435398446	0.642029538
349.4003765	0.315993028	0.08887219	3.555589513	0.000377133	0.004485835
646.7142942	-0.041385215	0.116245617	-0.356015274	0.721829126	0.852371097
18.85009201	-0.182607956	0.260404303	-0.701247844	0.483148357	0.682213596
469.8028475	0.015276499	0.057771882	0.264427925	0.791450196	0.892264031
232.9153959	0.05961439	0.098004929	0.608279513	0.543002107	0.73035734
979.7573838	-0.234230605	0.098430293	-2.379659738	0.017328631	0.077700561
86.79318717	-0.169506713	0.153909516	-1.101340042	0.27074869	0.482613495
391.0636212	0.271428003	0.098193851	2.764205697	0.005706156	0.034803112
12.800628	0.236590493	0.442569174	0.534584212	0.592937403	0.765521221
39.69290635	0.133717475	0.188002509	0.711253674	0.476927053	0.676735252
72.87072607	0.476996517	0.125641214	3.796497199	0.000146755	0.002120739
19.92762402	0.178744361	0.253136436	0.706118662	0.480114347	0.679268615
75.28737206	-0.309239965	0.140021437	-2.208518713	0.027208135	0.106644495
620.2448061	-0.02507908	0.087392899	-0.286969307	0.774135821	0.88275168
686.655789	0.350720905	0.171032454	2.050610263	0.040304916	0.139521578
92.48935037	0.284554464	0.23430821	1.214445126	0.224577792	0.429647492
124.968959	0.250783466	0.118621873	2.114141846	0.034503154	0.12508912
400.5038228	0.429139988	0.113556815	3.779077372	0.000157411	0.002252981
2.240440078	0.719887723	0.669407096	1.075410953	0.28219078	0.495967668
1.837431789	-0.165666769	0.7438982	-0.22270086	0.823768332	0.911434821
272.0312581	0.12412084	0.113895414	1.089779083	0.27581047	0.488517545
20.47761921	0.315152785	0.343307156	0.917990724	0.358623722	0.573032706
1.780751619	0.558072882	0.771647589	0.723222479	0.469543211	0.670589931
38.60151995	-0.290933479	0.186820246	-1.557290957	0.119401421	0.288250149
4.333473039	0.536518568	0.513169123	1.045500487	0.295791716	0.510408791
18.84256235	-0.577398474	0.259390283	-2.225983439	0.026015291	0.103500371
191.7010176	0.132348155	0.083392942	1.587042637	0.112502988	0.277742279
180.3536895	-0.189753759	0.12558368	-1.510974665	0.130794906	0.304596449
245.0808531	-0.006123041	0.079737215	-0.076790252	0.938790406	0.970788597
17.38559284	-0.188265496	0.318371306	-0.591339396	0.554293038	0.739147119
543.4852835	0.02233788	0.050815783	0.439585479	0.660237359	0.81303261
26.65002671	0.20638531	0.206819914	0.997898638	0.318328513	0.533375093

26.44265878	-0.107933375	0.231038824	-0.467165529	0.640381454	0.799397141
6.298430506	1.164447115	0.522861116	2.227067721	0.025942748	0.103312257
196.375814	0.050221154	0.075100393	0.668720251	0.503673947	0.699260458
1843.869448	-0.029040554	0.081095274	-0.358104154	0.720265371	0.851622172
47.24867006	-1.460433664	0.554459028	-2.633979411	0.008439061	0.046668373
48.01160122	-1.618002137	0.506804624	-3.192555988	0.001410196	0.012537274
1810.184119	-0.926048889	0.45127289	-2.052081812	0.040161712	0.139113551
12.50278198	0.085811869	0.443001898	0.193705422	0.846406546	0.923940931
458.3897538	-1.198928822	0.321793139	-3.725774971	0.000194716	0.002664246
115.6378983	0.404275683	0.108375123	3.730336554	0.000191224	0.002629572
416.1688111	-0.207643712	0.068400033	-3.035725322	0.002399579	0.01847227
2134.855748	0.150306192	0.056246043	2.672298053	0.00753337	0.042706381
462.808719	0.00053327	0.056326534	0.00946747	0.992446165	0.995850406
1267.943446	0.121619621	0.058853302	2.066487632	0.038782459	0.135792185
13836.34393	0.051628453	0.067070523	0.769763695	0.441440079	0.647288284
433.7487194	-0.24618102	0.095062606	-2.589672529	0.009606727	0.05128361
346.8396816	-0.228336025	0.137832191	-1.656623349	0.097595649	0.252440056
23.43915618	-2.631895539	0.817445451	-3.21965892	0.001283432	0.011668465
3064.473011	0.062667527	0.114080337	0.549328036	0.582780351	0.75889593
348.0083635	-0.35818565	0.077237633	-4.637449883	3.52734E-06	9.93323E-05
17.55058329	1.866481684	0.777513677	2.400577301	0.016369233	0.07457598
15.4551814	1.061337065	0.272449046	3.895543342	9.79788E-05	0.001541646
16.04984826	0.163856687	0.34586001	0.473765923	0.635666838	0.795984758
1.699643395	-1.147536236	0.741546448	-1.547490705	0.121744964	0.291692905
12.26912052	-0.425549115	0.340702644	-1.249033791	0.211652715	0.41593572
2.637394841	-1.28428696	0.770177003	-1.667521823	0.095410689	0.248662504
2.469101797	-0.004204782	0.684404187	-0.006143712	0.995098058	0.997520105
64.8688552	-0.093444145	0.139522552	-0.669742229	0.503022127	0.698776163
32.1628403	-0.278252513	0.225597564	-1.233402117	0.217425776	0.422248697
280.0094332	-0.114476637	0.129270019	-0.885562156	0.375853512	0.589110444
149.5101707	-0.354935794	0.159922682	-2.219421221	0.026458081	0.104857112
34.14107103	-0.472931328	0.269202588	-1.756785966	0.078954298	0.220269789
274.4052765	-0.288384929	0.152717934	-1.888350128	0.058978962	0.181306105
12.29162883	0.247616546	0.313080278	0.790904326	0.428999824	0.635952766
4.978937062	0.40506667	0.507855621	0.797602023	0.425101477	0.632621656
227.6833747	-0.141170199	0.170873598	-0.826167416	0.408709129	0.619247143
325.9433728	-0.223735794	0.143777866	-1.556121267	0.119679259	0.288624815
60.93684027	-0.074872169	0.169583752	-0.441505557	0.65884704	0.812363514
2.377029805	-0.520342777	0.827232118	-0.629016652	0.529338155	0.71927657
6.754555651	0.060109809	0.394961921	0.152191403	0.879035974	0.939321353
157.1676643	0.023549188	0.151623705	0.155313369	0.876574277	0.937787198
53.58590119	0.654355883	0.133254091	4.910587587	9.08039E-07	3.16929E-05
572.9774233	0.175396754	0.236024098	0.743130702	0.457402553	0.660928836
293.7165616	0.088343389	0.072756913	1.214226739	0.224661153	0.429757063
33.64270943	0.122274025	0.23722548	0.515433776	0.606249935	0.776139372
732.9320998	0.157382852	0.067503102	2.331490668	0.019727502	0.085187644
849.5972287	-0.003500082	0.065428734	-0.053494563	0.957337862	0.979025697
102.257005	0.107249362	0.120983346	0.88648037	0.375358727	0.588859665

169.3309579	-0.122495228	0.098241258	-1.246881722	0.21244087	0.416984498
311.7329829	0.070707534	0.063330989	1.116476069	0.264218408	0.475552721
974.2819934	0.057838936	0.070275539	0.823030846	0.410490453	0.620576279
1471.490259	0.386759498	0.107759003	3.589115409	0.000331802	0.00404597
633.0735638	-0.017195215	0.075230179	-0.228568043	0.819204665	0.909322798
164.050815	0.292248686	0.117962765	2.477465549	0.013231918	0.064358613
49.34476611	0.354366507	0.391120164	0.906029758	0.364920102	0.578269888
1968.89679	0.092312226	0.112128282	0.823273347	0.410352567	0.620481706
109.0162205	0.328691729	0.173640271	1.892946421	0.058364995	0.179989691
93.52710708	0.390454361	0.160806206	2.428105047	0.015177946	0.070994176
4.743328929	-0.944486671	0.695245589	-1.358493582	0.174307118	0.36828722
59.97842935	0.266852808	0.13507965	1.975521913	0.04820895	0.158263683
304.5923968	0.006378134	0.068762978	0.092755345	0.926097927	0.964382886
5.902217867	1.541164654	0.58907053	2.616265072	0.008889752	0.048397147
1039.041502	0.190217712	0.061768741	3.079514163	0.002073385	0.016621385
545.5702124	0.007824718	0.061462935	0.12730792	0.898696692	0.948864276
12.35760333	0.326187426	0.39888373	0.817750639	0.413499589	0.623295107
621.1620161	0.10094946	0.066400836	1.52030405	0.128434577	0.300458849
4.227777564	0.328635289	0.598586592	0.549018794	0.582992552	0.758986826
489.0979359	0.127299942	0.062433237	2.038977115	0.041452312	0.142475565
120.8182492	0.516976701	0.11866848	4.356478665	1.32172E-05	0.000301161
15.56558742	-0.293800758	0.614709041	-0.477950929	0.632685131	0.794224511
5.250206524	0.799736145	0.808097025	0.989653618	0.322343453	0.537439698
19534.6018	-0.179014517	0.088982328	-2.011798539	0.044241178	0.149177649
242.1301539	-0.095016451	0.090004837	-1.055681605	0.291113704	0.506302775
7.485709299	0.85202028	0.458297472	1.859098801	0.063013135	0.189672646
345.6132882	0.322982194	0.156699654	2.061154474	0.039288305	0.136847819
189.2032673	0.223344292	0.071903916	3.106149223	0.00189541	0.015581333
5.601739388	-0.555126582	0.564611071	-0.983201731	0.325508163	0.540529068
208.4448674	0.038761661	0.117831336	0.328958854	0.742186789	0.864878344
226.1805116	-0.107097479	0.083812433	-1.277823292	0.20131174	0.404095236
271.4182548	0.114055779	0.08418191	1.354872793	0.175458089	0.369817881
170.8254022	0.320320756	0.1172751	2.731362043	0.006307314	0.037416886
75.30024044	0.340098909	0.133175495	2.553764942	0.010656517	0.054861082
120.1523935	0.519025265	0.138119608	3.75779566	0.000171417	0.002411546
443.8613217	-0.041207988	0.049807056	-0.827352409	0.408037347	0.618855391
1026.298747	-0.010515487	0.043814744	-0.239998817	0.810331174	0.903927156
286.2317745	-0.174171183	0.11853551	-1.469358702	0.141735523	0.321132031
998.511617	0.173149472	0.122222919	1.416669425	0.1565796	0.342528754
638.8857545	0.24193038	0.072674181	3.328972906	0.000871669	0.00866096
67.85267369	0.490737185	0.148788477	3.29822036	0.000972997	0.009445586
620.7683812	-0.021494877	0.05137152	-0.418420113	0.675639984	0.824504834
153.7827258	0.646529453	0.164013776	3.941921644	8.08314E-05	0.001331616
62.7485382	0.255892462	0.137050254	1.867143295	0.06188158	0.187396534
48.06670772	0.2100204	0.246656281	0.851469906	0.394508373	0.607225165
824.4624399	-0.127861659	0.124226697	-1.029260716	0.303357178	0.518305968
226.022427	-0.117659863	0.099630131	-1.180966658	0.237615969	0.445170729
5.02469495	-0.07781479	0.522826951	-0.148834696	0.881684072	0.940790427

842.0656268	0.057740278	0.088319721	0.653764264	0.513263693	0.70656857
332.7605439	-0.157470427	0.092764285	-1.697532915	0.089595954	0.239106389
5432.406607	-0.135757219	0.132063577	-1.027968661	0.30396457	0.518644466
116.9777865	-0.280879311	0.140964208	-1.992557656	0.046309906	0.154216573
7.393106542	0.270169057	0.50250103	0.537648763	0.590819558	0.763924449
33.2561372	-0.139624632	0.182399036	-0.765489966	0.443979839	0.649221007
65.78116483	0.883101615	0.179904589	4.90872201	9.16718E-07	3.19281E-05
383.623284	-0.360052313	0.0835703	-4.308376444	1.64457E-05	0.000363173
310.7911793	0.047911696	0.080258716	0.596965646	0.550530317	0.736633889
271.3737034	0.070928382	0.082916535	0.855419035	0.39231921	0.605496221
5.256339244	0.345665556	0.546250139	0.632797196	0.526866088	0.717162886
446.6747304	0.347184135	0.096219496	3.608251441	0.000308268	0.003809753
273.928494	0.455757161	0.122449783	3.721992384	0.000197657	0.002695531
80.37532561	-0.671343938	0.410853393	-1.634023106	0.102253999	0.261039415
6.647788341	1.028995182	0.505961342	2.033742694	0.041977545	0.143800807
6.783239837	-1.089103895	0.537348674	-2.026810427	0.042681804	0.145423175
2.760291846	1.190572224	0.863227292	1.379210592	0.167829833	0.359180576
4855.567308	0.014143268	0.388004824	0.036451268	0.970922535	0.985661927
196.9085857	-0.21235594	0.07716061	-2.752128846	0.005920922	0.035677129
65.37260444	-0.442713642	0.137929124	-3.209718357	0.001328651	0.011984908
14.42530468	-0.809958695	0.434877887	-1.86249685	0.062533089	0.188824885
90.45343206	-0.097623036	0.124960402	-0.781231765	0.434666194	0.641466531
128.4455373	-1.022081786	0.136991572	-7.460909959	8.5927E-14	2.11278E-11
38.06441405	0.060933595	0.170843725	0.356662764	0.721344285	0.852102807
94.76516448	-0.171629199	0.129100864	-1.329419444	0.183709626	0.381066783
20.33927013	-1.00217701	0.3283658	-3.052013972	0.002273115	0.017781276
14.33332538	-1.379505154	0.691880832	-1.993847914	0.046168678	0.153831181
32510.72798	-0.414123241	0.143642893	-2.883005443	0.003939006	0.026388992
311.3464987	0.275630073	0.288380146	0.955787271	0.33917972	0.554005032
1020.938981	-0.087795297	0.105627317	-0.831179843	0.40587204	0.61670688
4.851312324	-1.397211772	0.504562683	-2.769154	0.005620206	0.034444672
329.1646304	-0.228651884	0.081975504	-2.789270847	0.005282687	0.032927347
362.9188597	-0.444979532	0.110356287	-4.032208277	5.52552E-05	0.000973555
1915.807702	0.335342018	0.148070102	2.264751715	0.02352792	0.096106857
663.3329331	0.028928718	0.086804068	0.333264548	0.738934598	0.862699071
5.557136099	0.597608651	0.509475168	1.172988771	0.240800309	0.448141017
100.8179682	0.058539451	0.103125538	0.567652319	0.570271074	0.750550905
316.4902388	-0.856639456	0.231154354	-3.705919613	0.000210625	0.0028488
135.8892424	-0.053707079	0.119453385	-0.449607007	0.652993835	0.808281646
131.0795108	-0.07819623	0.115362652	-0.677829687	0.497879686	0.694502112
3174.129913	-0.241544133	0.121130277	-1.994085533	0.046142708	0.153815251
93.33876799	-0.285544232	0.255463432	-1.117749925	0.263673814	0.475145195
1021.919967	-0.135427775	0.067862615	-1.995616791	0.04597565	0.153475758
2.370442355	-0.802288818	0.724539969	-1.107307881	0.268160838	0.4801306
3.748721398	0.078015917	0.55608885	0.140293979	0.888427721	0.944210191
12.4570447	-1.554614971	0.734259977	-2.117254132	0.034238285	0.124448658
369.4524119	-0.52478405	0.166608066	-3.149811787	0.001633757	0.01393812
124.0388077	-0.055085076	0.214427024	-0.256894282	0.797260385	0.89525023

683.8360358	-0.11371563	0.116651197	-0.974834662	0.329642281	0.545288375
2.192755638	-1.153153954	0.702403058	-1.641726842	0.100646626	0.257741726
145.8776368	0.149028491	0.119666995	1.245360018	0.212999443	0.417651121
1006.473393	0.421347813	0.078252388	5.384472243	7.26575E-08	3.41989E-06
2.707934507	-0.494882591	0.648921468	-0.762623237	0.445688122	0.650737049
2.796960111	-0.854017309	0.730820916	-1.168572615	0.242575867	0.450224745
46.97512085	-0.207698949	0.170605267	-1.217424014	0.223442931	0.428534816
310.4068628	-1.007272655	0.125069761	-8.053686549	8.03368E-16	2.8004E-13
2.608319093	1.875931394	0.825342057	2.272913852	0.023031372	0.094712636
172.0275392	0.345365238	0.099189913	3.481858461	0.000497947	0.005550187
242.5908409	0.023431193	0.078562261	0.298249988	0.765512372	0.877901205
3.089435426	-0.445604906	0.534058169	-0.834375227	0.404069572	0.614990985
648.4895926	-0.132966275	0.064967514	-2.046657879	0.040691688	0.140523894
32.32241959	0.027763258	0.288548702	0.096216886	0.92334832	0.962127781
826.5388085	-0.075382542	0.049929484	-1.509780124	0.131099538	0.305004067
205.7406302	-0.011744243	0.107091186	-0.109665818	0.91267441	0.956423806
361.6073535	-0.255009538	0.109673764	-2.325164457	0.020063168	0.086162834
6.462824083	0.588560886	0.46991181	1.252492219	0.210390562	0.414639803
322.3179912	-0.024382376	0.067872374	-0.359238592	0.719416609	0.851125005
4.157611299	0.513117437	0.500856023	1.024480916	0.30560818	0.520512215
1148.464776	-0.240440233	0.072740999	-3.305429338	0.00094831	0.009233133
230.4464987	0.139278139	0.09056302	1.537914035	0.124069635	0.294683272
2833.765532	0.126319859	0.110314331	1.145090199	0.252171795	0.461283628
151.5351168	0.468831466	0.117061764	4.004992333	6.20195E-05	0.001072098
217.6252864	-0.250292984	0.096016301	-2.606775946	0.009139915	0.049326093
181.5990369	0.011952212	0.09721359	0.122947958	0.902148309	0.951045705
2343.092654	-0.453732376	0.109562199	-4.141322283	3.45309E-05	0.000666896
3397.523467	-0.278973544	0.106491399	-2.619681466	0.008801193	0.048073892
18.37201018	0.365546123	0.272710531	1.340418067	0.180109465	0.376108928
392.5713314	-0.020376037	0.074770058	-0.272515995	0.785225294	0.888692051
16.19597838	-0.180831767	0.248846167	-0.726680941	0.467421431	0.669184033
4359.569411	-0.015488729	0.093250814	-0.166097522	0.868080205	0.934495951
363.9180201	-0.036922594	0.073791849	-0.500361413	0.616820618	0.78342222
3.207147853	1.139827836	0.634833384	1.795475577	0.07257796	0.208884779
2.856378811	-0.132409545	0.686519573	-0.192870751	0.847060193	0.924259479
448.1398544	-0.07383054	0.100209634	-0.736760899	0.461267765	0.66377753
506.1596443	-0.287954025	0.11283732	-2.551939596	0.010712509	0.055046124
599.0925532	-0.423655289	0.103961675	-4.075110249	4.59926E-05	0.000835371
7.560752265	0.010680884	0.348720892	0.030628748	0.975565616	0.987719156
522.2233149	-0.106856723	0.059901413	-1.783876503	0.074443735	0.212324461
132.1714244	-0.13233031	0.090271848	-1.465908961	0.14267309	0.322325356
491.0040263	-0.027951238	0.169329238	-0.165070355	0.868888606	0.934607931
1.974400611	1.34704009	0.712770491	1.889865121	0.058776001	0.180948038
81.7254996	0.085858362	0.119079536	0.721016935	0.470899095	0.671362327
25.81326158	-1.909506369	0.682214055	-2.798984214	0.005126364	0.032062159
118.2778747	-0.12262652	0.11047484	-1.109994994	0.267001184	0.478749089
95.47452207	0.797575236	0.260923847	3.056735692	0.002237615	0.017583428
2359.830918	-0.024448746	0.084146964	-0.290548164	0.77139691	0.880664774

1.98337599	-0.073848057	0.749993079	-0.098464985	0.921563075	0.961362088
27.10260908	-0.064219738	0.227037847	-0.282859175	0.777284791	0.884207847
54.8012353	0.254139185	0.168708809	1.506377682	0.131970247	0.306337585
112.0812304	-0.019069473	0.10299827	-0.185143625	0.853116385	0.927488902
171.1898827	0.043041149	0.085245442	0.50490851	0.613623096	0.781388799
262.2665078	0.553589367	0.075568863	7.325627806	2.37783E-13	5.22298E-11
6.745665632	0.918029993	0.383291889	2.395119799	0.016614936	0.075444997
170.5319032	0.408626445	0.109297086	3.738676495	0.000184992	0.002565279
636.0047778	0.726877021	0.12736641	5.706975829	1.15001E-08	6.99088E-07
59.19706351	-0.274917413	0.138863159	-1.979772131	0.047729139	0.157100867
106.5899903	-0.295449736	0.126210466	-2.340928966	0.019235827	0.083590347
30.73374458	-0.39976547	0.314707815	-1.270275003	0.203986689	0.40698519
195.2619926	-0.08124794	0.100875771	-0.805425714	0.420574058	0.629066856
12.14177185	0.097021945	0.279358073	0.347303172	0.728363568	0.85622654
93.98589161	-0.826626301	0.280576982	-2.946165774	0.003217399	0.022904397
7.577605386	0.171960275	0.367888144	0.467425434	0.64019553	0.79922561
551.4066071	0.0426734	0.111162528	0.383882959	0.701065197	0.839681126
445.1341139	0.002427979	0.071386417	0.034011772	0.972867763	0.986460487
759.0894504	-0.093031494	0.08281984	-1.123299614	0.261310268	0.472642221
77.95176308	0.007462533	0.146397043	0.050974619	0.959345746	0.980261259
48.55589448	-0.341622285	0.153123888	-2.231018873	0.025679879	0.102682118
6.521543608	0.70138358	0.49161731	1.426686093	0.153670396	0.338354197
9.92348385	-0.136867278	0.427788986	-0.319941099	0.749012982	0.868654689
131.9105014	0.134392804	0.101329321	1.326297285	0.184741246	0.382484263
37.35655037	0.428593141	0.350347976	1.223335571	0.221202977	0.42601247
405.2450706	0.370780882	0.102252673	3.626124116	0.000287707	0.003626386
811.846719	0.155211263	0.055082047	2.81781944	0.004835099	0.030655386
50.87030436	0.315945907	0.141771229	2.228561532	0.025843093	0.103059577
1172.075277	-0.347694105	0.091243425	-3.810620945	0.000138618	0.00201553
1062.545932	-0.201527012	0.112112318	-1.797545672	0.072249034	0.20833723
161.7922788	-0.080707891	0.10440995	-0.77299042	0.43952805	0.645288753
4814.469548	-0.387969795	0.115814764	-3.349916541	0.000808359	0.008149883
10.57670228	0.402508875	0.315887985	1.274213942	0.202587605	0.405322833
11.69142924	-0.310298662	0.69243006	-0.448129971	0.654059399	0.808871297
146.9528064	0.462602447	0.156810449	2.950074114	0.003176977	0.022706083
654.3458037	0.215891717	0.098161543	2.199351287	0.027852954	0.108441502
885.6726964	-0.579794194	0.100717958	-5.756611879	8.58189E-09	5.39816E-07
148.7440377	0.178346826	0.122774828	1.452633477	0.146325546	0.32774535
59.56259618	-0.092444768	0.167626628	-0.551492142	0.581296352	0.758096588
215.406768	0.234469511	0.095066063	2.466385004	0.013648455	0.065744047
176.1839014	-1.605220026	0.240892279	-6.663642489	2.67123E-11	3.26059E-09
316.6438481	-0.25614927	0.077268683	-3.315046409	0.000916279	0.009006433
5.530120556	-0.134406172	0.539197771	-0.249270637	0.803151443	0.899097368
236.0833361	-1.130270572	0.364953899	-3.097022874	0.001954748	0.015949736
198.0088711	0.135941927	0.101793365	1.335469435	0.181722753	0.378326883
80.76015269	0.22126429	0.121431438	1.822133499	0.068434732	0.200815147
92.47051096	-0.185706914	0.124363783	-1.493255589	0.135370296	0.311850645
10.48754591	-0.72537406	0.46365023	-1.564485496	0.117703597	0.285490143

140.2246575	-0.16674631	0.106541108	-1.565088939	0.117562057	0.285344488
470.1907163	-0.170053436	0.085043174	-1.999612998	0.045542069	0.152343891
91.87355597	0.157804649	0.253365897	0.62283303	0.533394251	0.722626389
307.5766136	0.692456594	0.123809203	5.592933127	2.23265E-08	1.2179E-06
62.42964326	0.16775831	0.170863736	0.981825132	0.326186004	0.541092361
2.157641189	1.732292255	0.697232577	2.4845257	0.012972414	0.063377092
233.7229221	0.145608891	0.107468694	1.354895883	0.175450731	0.369817881
619.1324298	0.086931204	0.119065554	0.730112121	0.465321653	0.667349167
109.3197722	-0.080263978	0.09849536	-0.814901114	0.415128916	0.624893435
444.606577	-0.088799918	0.074933575	-1.185048461	0.235998287	0.44321208
1525.180668	-0.162456352	0.130344928	-1.2463573	0.21263325	0.417262676
773.2080747	0.013117847	0.082283849	0.159421893	0.873336491	0.936680519
328.7936665	-0.288753003	0.139281393	-2.073162801	0.038157131	0.134517564
521.6272735	-0.237283017	0.102769089	-2.308894807	0.020949419	0.08890806
1891.494356	-0.184883631	0.109228265	-1.692635436	0.090524882	0.24088304
2.439759648	0.21930394	0.638612517	0.343406893	0.731292375	0.858009442
157.195205	-0.138017333	0.11635323	-1.186192544	0.23554627	0.442865699
79.37922177	-0.542880424	0.251610568	-2.15762171	0.030957255	0.11645423
9.44249177	0.362951804	0.647587796	0.560467331	0.575160717	0.753941023
24.88594417	0.036334575	0.201412972	0.180398383	0.856839824	0.92936531
11.25815931	-0.474727244	0.392561262	-1.209307413	0.226544764	0.432232145
2.498894422	-1.013378648	0.706261842	-1.434848364	0.151330313	0.33544343
16.08596551	-0.436385765	0.414443639	-1.05294357	0.292366856	0.50758263
11.12302529	-0.133213502	0.43813099	-0.304049486	0.76109019	0.875424502
129.1101048	-0.073769774	0.201265761	-0.366529177	0.713970245	0.847585632
20.84770731	-0.585991242	0.398652969	-1.469928202	0.141581201	0.320941711
7858.332658	-0.014953099	0.10518628	-0.142158262	0.886954999	0.943481414
243.8822133	-0.19649243	0.171538259	-1.145472918	0.252013308	0.461142645
140.9028388	0.083682206	0.133629924	0.626223554	0.53116832	0.720855663
44.62348182	0.572390402	0.163881012	3.492719472	0.000478129	0.005380252
525.8902051	-0.001352637	0.093756646	-0.014427107	0.988489233	0.993980695
332.0072527	-0.660362426	0.162001176	-4.076281688	4.57616E-05	0.000833012
86.54763609	-0.408538573	0.134721611	-3.032465013	0.002425652	0.018594785
156.4093626	-0.184329963	0.095732656	-1.925465892	0.054171099	0.17041301
650.81271	-0.079182151	0.085917352	-0.921608378	0.356732887	0.571341394
7.680513761	-1.008463288	0.38681795	-2.607074693	0.009131944	0.049312296
674.0191313	-0.514550778	0.241821344	-2.127813734	0.03335253	0.122290136
80.53336186	-0.339688237	0.281319462	-1.20748218	0.227246504	0.433043251
16.33718213	-0.716865422	0.415431097	-1.725594034	0.084420473	0.230255444
2510.356443	0.074095701	0.089844449	0.824710943	0.409535717	0.619889505
25.15557044	0.784031029	0.234613461	3.341799004	0.000832373	0.008346022
469.918995	0.267909738	0.155021298	1.728212454	0.083950142	0.229411954
12.97364176	-1.347379576	0.358673071	-3.756567425	0.00017226	0.002421339
257.7375585	0.041204483	0.089534752	0.460206588	0.645367943	0.803265524
121.7509751	0.466014928	0.153762459	3.030745814	0.002439505	0.018666237
66.28228534	0.190978167	0.228134447	0.837129902	0.402519554	0.613846909
226.3502706	0.017448677	0.071936255	0.242557481	0.808348223	0.902645471
1537.114624	-0.113921658	0.157185093	-0.724761208	0.468598537	0.670018398

1365.790701	-0.091460342	0.096855762	-0.94429428	0.345019288	0.559431667
28.40876793	0.025341976	0.173295785	0.146235386	0.883735572	0.942092758
31.62506828	-1.026606636	0.450642112	-2.278097424	0.022720774	0.093951312
331.9082994	-0.549475273	0.075859911	-7.243289167	4.37932E-13	9.13226E-11
6.217632653	0.546037742	0.37310198	1.463508026	0.143328419	0.323362418
22.17638513	0.049783369	0.342242982	0.145462059	0.88434607	0.942293582
83.94840111	-0.221895668	0.132222283	-1.678201762	0.093307718	0.245234455
138.0132739	-0.022937812	0.288976849	-0.079375951	0.936733596	0.970184161
21.97858975	-0.584456069	0.626382497	-0.933065773	0.35078601	0.565832638
2831.946188	-0.348718781	0.110405094	-3.158538868	0.001585622	0.01369053
3.241115813	-0.348458756	0.614284088	-0.567259942	0.570537588	0.750827482
2348.037632	0.124461252	0.093210179	1.335275323	0.181786252	0.378390431
63.06958291	-0.071939145	0.16866307	-0.426525767	0.669724777	0.820241125
252.02029	-0.048506934	0.105051509	-0.46174429	0.644264706	0.80254191
2.155839857	1.029547076	0.688546426	1.495247142	0.134849955	0.311006321
426.2076573	-0.26146635	0.192730721	-1.356640751	0.174895387	0.369009554
108.2624816	-0.33924217	0.129648035	-2.616639499	0.008880007	0.04837607
15.8666954	0.357199917	0.325589171	1.097087831	0.272602999	0.484831426
247.7752841	0.095613707	0.113370533	0.843373533	0.39901957	0.611199292
672.4962122	0.342456683	0.060149354	5.693439118	1.24506E-08	7.386E-07
26.26019109	0.493411774	0.21177282	2.329910771	0.019810868	0.085390956
209.4015536	0.083356132	0.116507078	0.715459814	0.474324964	0.674262616
296.5510659	-0.501671348	0.173054481	-2.898921463	0.003744487	0.025455973
1682.060173	-0.024966902	0.11838635	-0.210893423	0.832970437	0.916630422
18.92641832	0.048895255	0.211131571	0.231586656	0.816859073	0.907902872
17.3098232	-0.102150489	0.263393855	-0.387824116	0.698146198	0.838162109
253.3822155	0.175550609	0.149243244	1.176271733	0.239486308	0.446957905
4.091985426	0.996948646	0.888857798	1.121606456	0.262029805	0.473242344
6.386674245	0.054035078	0.465922913	0.115974287	0.90767292	0.953509862
79.65869234	-0.150641785	0.260010083	-0.579369011	0.562340209	0.745233095
126.4547041	0.022061871	0.142427335	0.154899137	0.876900834	0.937934316
5.811699304	-0.635746903	0.673669335	-0.943707648	0.345319065	0.55980971
100.3027533	0.303558732	0.118097741	2.570402534	0.010158041	0.05310808
218.8333934	0.593386124	0.106590545	5.566967743	2.5921E-08	1.3955E-06
66.87600379	-0.426575088	0.147351982	-2.894939605	0.003792315	0.025656918
3.212545168	1.227426794	0.596486799	2.057760199	0.039613158	0.137734734
2.473867247	-0.220744569	0.705113597	-0.313062421	0.754233239	0.871653489
10.12438052	-2.531383871	1.072650926	-2.359932584	0.018278256	0.080406774
9.93177135	-1.929693753	1.159723324	-1.663925966	0.096127225	0.249779165
9.328099254	-2.468114428	1.308585726	-1.886093037	0.059282418	0.182056232
246.9199222	-0.35479481	0.077108031	-4.601269216	4.19924E-06	0.000114914
60.64779117	0.024761117	0.278036396	0.089057106	0.929036527	0.965637301
26.08736906	-0.308780993	0.229237523	-1.346991489	0.177982974	0.373514842
311.2524654	0.125960415	0.159007224	0.792167878	0.428262796	0.635260337
10.07347266	-0.318351332	0.380237186	-0.837244078	0.402455386	0.613836685
121.2624887	0.059476186	0.174407617	0.341018279	0.733089821	0.859261594
25.69873161	-0.892764009	0.591273719	-1.509899696	0.13106902	0.305004067
244.8553901	-0.041790325	0.072319033	-0.577860669	0.563358191	0.745922106

125.4912896	-0.312778877	0.324453915	-0.964016344	0.335037732	0.550070918
16.98776889	-0.098032632	0.323698372	-0.302851792	0.762002811	0.875874332
418.9901599	-0.225193515	0.061646468	-3.652983231	0.000259211	0.003364076
3.725857276	0.24180282	0.52219677	0.463049244	0.64332907	0.801800809
193.175587	-0.290378471	0.107779094	-2.694200336	0.007055774	0.040585483
1282.697276	-0.000194943	0.079335769	-0.002457187	0.998039451	0.998584993
307.1038286	0.269748494	0.107234351	2.515504518	0.011886215	0.059481626
8.889643988	0.187676987	0.418626733	0.448315819	0.653925286	0.808766153
281.5960124	-0.221121524	0.08808786	-2.51023835	0.01206497	0.060156873
2692.2893	-0.218395256	0.068891239	-3.170145563	0.001523626	0.01330165
4.097062788	-0.223634165	0.509030003	-0.439333956	0.660419574	0.81313543
1601.398262	0.001388966	0.04927288	0.028189257	0.977511205	0.988278901
30.49746467	0.214713282	0.189368659	1.133837472	0.256862729	0.467472007
26.63592257	0.57973295	0.284954603	2.034474771	0.04190375	0.14362349
267.9591339	0.150032851	0.361004134	0.415598705	0.677703674	0.825950823
526.7307572	0.210148894	0.176787483	1.188709123	0.234554149	0.441558895
37.51968757	-0.90134769	0.247145611	-3.647030939	0.000265288	0.003417718
3.799220871	-1.644696464	1.050066168	-1.566278882	0.117283343	0.285057385
2.494853043	-2.236480177	1.078966831	-2.072797895	0.038191092	0.134608485
2.945848009	3.492713674	1.513048013	2.308395797	0.020977133	0.088974583
396.6774704	0.096954373	0.083096507	1.166768337	0.243303937	0.450998082
609.433308	0.106254509	0.062868213	1.690114994	0.091005957	0.241655438
20.0120534	0.678747019	0.219000334	3.099296724	0.001939806	0.015843515
245.1764215	0.830453454	0.13426239	6.185302198	6.19836E-10	5.34617E-08
1.772672452	2.732346336	1.659002864	1.646981084	0.099561934	0.255829108
5.679180961	2.953997881	0.685405296	4.309855642	1.63361E-05	0.000361236
7.224270697	1.268142009	0.8924739	1.420928959	0.155337413	0.340568079
629.4807863	0.023534137	0.078967369	0.298023568	0.765685176	0.877977141
2647.476594	0.102760874	0.065082676	1.578928215	0.114352523	0.280726901
214.521726	-0.297648382	0.072078903	-4.129479932	3.63585E-05	0.000695667
107.1654388	0.078338673	0.101618278	0.770911244	0.440759542	0.646478459
189.3844246	0.290466789	0.082460774	3.522484391	0.000427522	0.004938991
2.300944761	-1.008747006	0.840792481	-1.199757406	0.230233571	0.4366645
521.3423146	0.685128852	0.113375931	6.042983254	1.5129E-09	1.20988E-07
3.737951104	0.032487938	0.656654316	0.049474947	0.960540802	0.980661203
135.9561451	-0.401799695	0.114697204	-3.503134172	0.000459818	0.005220564
326.5001563	-0.029214134	0.068424226	-0.426955999	0.669411377	0.820160867
27.29160749	-1.051727196	0.562291304	-1.870431196	0.061423963	0.186566966
2132.706722	0.195589013	0.077320734	2.529580409	0.0114199	0.057881436
176.9574967	0.402372737	0.107046662	3.758853641	0.000170694	0.002405481
3.19054653	-3.179589429	1.072750828	-2.963958961	0.003037088	0.021973204
18.83969789	0.483041571	0.211723468	2.281473917	0.022520418	0.093310205
664.4328764	-0.35136469	0.067046977	-5.240574659	1.60077E-07	6.86748E-06
198.2682562	-0.105164851	0.098238271	-1.070507962	0.284390727	0.498515944
23.11930514	-0.772642589	0.440062738	-1.755755539	0.079130159	0.220597268
816.3481356	-0.161884959	0.055137268	-2.936035209	0.003324366	0.02341411
11.40448002	0.512879846	0.315515113	1.625531789	0.104049296	0.263790105
3.896244072	-4.26203671	2.923452645	-1.4578778	0.144874229	0.325645797

6.531303293	-2.762150017	2.347270177	-1.17674993	0.239295333	0.446702698
105.2686805	0.045449446	0.117087828	0.388165416	0.697893624	0.8380421
515.0192622	-0.123407821	0.066330197	-1.860507359	0.062813778	0.189384001
96.84973877	-0.388043607	0.266971621	-1.453501332	0.14608461	0.327339209
1242.935894	0.007455555	0.087453208	0.085251932	0.932061105	0.967778561
13.38539003	-0.527998413	0.494699968	-1.067310386	0.2858317	0.500243431
1.875862341	-2.249860425	1.505073342	-1.494851023	0.134953327	0.311157607
263.0376822	-0.097145819	0.093045772	-1.044064835	0.296455394	0.511207508
1508.055961	-0.022648713	0.079586268	-0.284580666	0.775965426	0.883652841
309.6579809	-0.031734513	0.080725296	-0.393117329	0.694232826	0.835412095
574.5809303	0.280120074	0.123532798	2.267576532	0.023355029	0.095582453
498.7205816	0.228224987	0.085821736	2.65929118	0.007830525	0.043907446
197.6883988	-0.186654625	0.095114531	-1.962419649	0.049713647	0.161503179
109.7585609	-0.248094241	0.108234906	-2.29218328	0.021895069	0.091594559
358.3326222	4.08546E-05	0.08434997	0.000484347	0.999613547	0.999734918
26.07641941	0.438799531	0.224838769	1.951618637	0.050983499	0.164235855
658.0146135	0.079075205	0.067208124	1.176572114	0.239366334	0.446784611
91.22812573	0.194010863	0.112490605	1.724685031	0.08458425	0.230587611
688.4539266	-0.035336608	0.079688179	-0.443436006	0.657450397	0.811359633
2635.112367	0.109795734	0.055218719	1.988378876	0.046769803	0.155178055
153.8345978	0.01219776	0.085794528	0.142174105	0.886942486	0.943481414
196.9396309	-0.287179786	0.094946381	-3.024652262	0.002489191	0.019019916
8.362007342	-3.474151976	1.2101091	-2.870941122	0.004092517	0.027218463
3.343985422	0.535114673	0.646158835	0.828147267	0.407587104	0.618343305
35.11598707	0.351205687	0.160201785	2.19227075	0.028359963	0.109624542
14.3751917	0.71176371	0.278378388	2.556821007	0.010563356	0.05451777
277.9729513	0.168139859	0.106316749	1.581499259	0.113763928	0.279681682
175.9278749	0.016745514	0.073800533	0.226902347	0.820499677	0.909820137
178.2871599	-0.058759025	0.079853142	-0.735838608	0.461828923	0.664302246
119.9401004	-0.174727957	0.137768171	-1.268275214	0.204699685	0.407765733
2.897247216	-1.048092057	0.739838186	-1.416650393	0.156585167	0.342528754
13.373041	-0.76472256	0.420216661	-1.819829221	0.068785016	0.201282341
206.1830963	-0.033409798	0.092574103	-0.360897885	0.718175785	0.850529195
81.60196272	-0.232442093	0.174671542	-1.330738196	0.18327517	0.380430009
103.8907671	0.422840864	0.145630895	2.903510723	0.003690043	0.025213511
143.4557803	0.249158133	0.11108654	2.242919203	0.024902025	0.100326721
2.182003967	-0.204144433	0.824890932	-0.247480516	0.804536369	0.899975022
437.7264713	0.147718918	0.170428362	0.866750791	0.38607857	0.599289396
14.96106812	-0.147673429	0.42620912	-0.346481156	0.728981144	0.856762172
568.5714792	0.239038021	0.106259567	2.249567061	0.024476441	0.099096804
199.8362026	0.353691255	0.101955434	3.469077055	0.00052225	0.005774188
53.79977893	0.126912293	0.148209423	0.856303808	0.391829755	0.604967515
462.806214	-0.115189983	0.073044175	-1.576990679	0.11479767	0.281341388
42.02083154	-0.223249324	0.207645626	-1.07514581	0.282309452	0.496081698
409.1787059	0.055364282	0.097665361	0.566877365	0.570797503	0.750883819
242.0377082	0.158321725	0.094393994	1.677243634	0.09349485	0.245573048
4373.181176	-0.058085918	0.085349864	-0.680562515	0.496148353	0.693025942
114.9337717	-0.224264592	0.133221203	-1.683400141	0.092297656	0.243320785

104.2965417	0.68994084	0.241092934	2.861721534	0.004213469	0.027798435
178.1880831	0.424152936	0.153029492	2.771707133	0.005576318	0.034226627
7.307790217	0.025352996	0.458125887	0.055340675	0.955867058	0.978293782
52.37228847	0.052901127	0.183152555	0.288836412	0.772706566	0.881532974
275.8982743	0.052933326	0.076140628	0.695204736	0.486927004	0.685551275
219.1467573	0.728750619	0.119673156	6.089507819	1.13258E-09	9.23672E-08
168.8692354	-0.018738446	0.186681978	-0.1003763	0.920045585	0.960569805
228.076875	0.392286974	0.110329364	3.555598973	0.000377119	0.004485835
224.5193849	-0.131706874	0.08003311	-1.645654818	0.099834846	0.256220479
9.805553109	0.18068113	0.317341244	0.569359116	0.569112456	0.749789631
1224.555364	0.042388269	0.060990688	0.694995753	0.487057963	0.685605662
2.208025244	0.119265123	0.636854839	0.187272069	0.851447321	0.926652783
688.7542279	-0.178192059	0.090960302	-1.959009101	0.050111721	0.162387358
38.64256253	-0.014699421	0.205848124	-0.07140906	0.943072199	0.972629837
1937.567971	0.344350714	0.408291936	0.843393374	0.399008476	0.611199292
10.74725941	0.452336349	0.295418881	1.531169394	0.125727533	0.296823643
6.55683313	1.075715775	0.45250285	2.377257457	0.017441909	0.078080983
53.15907385	-0.187789087	0.164733264	-1.139958518	0.254303584	0.463582742
513.0465329	0.107237396	0.098664681	1.086887368	0.277086589	0.490092815
6.026001057	0.472365121	0.39759496	1.188056107	0.234811305	0.441937787
634.6983062	-0.007193975	0.069331482	-0.103762021	0.917358207	0.959166505
270.2343238	0.160146522	0.097050727	1.650132117	0.098915917	0.254774988
2479.325547	-0.090268672	0.073418686	-1.229505409	0.218882371	0.423472482
751.815174	-0.064044027	0.075052667	-0.853321141	0.393481239	0.606547079
35.51444997	-0.269024438	0.187130058	-1.43763349	0.150538073	0.334001915
402.1179484	-0.29251316	0.054363931	-5.380647682	7.42183E-08	3.4834E-06
400.660895	0.457129889	0.109555165	4.172600079	3.01143E-05	0.000596996
272.6835392	0.04502104	0.070630165	0.637419429	0.523851676	0.715049508
704.7873834	-0.120317437	0.080430879	-1.495911009	0.134676847	0.310868205
1153.620389	-0.01587512	0.053762266	-0.295283688	0.767777176	0.879073054
750.7401609	-0.680756612	0.082417935	-8.259811604	1.45903E-16	6.16307E-14
83.73203493	-0.246688432	0.202428452	-1.218645055	0.222978941	0.428036626
406.6854966	0.0258019	0.059210437	0.435766076	0.663006459	0.815589039
58.9478972	-0.161016418	0.235260161	-0.68441855	0.493710906	0.690712985
338.9797886	-0.053587266	0.134884371	-0.397282986	0.691158792	0.833539527
96.10003702	-2.238405536	1.074161151	-2.083863798	0.037172558	0.132035516
155.9986978	-2.47655922	1.099989148	-2.251439685	0.024357703	0.098786017
2.067047256	0.96912602	0.680722527	1.423672615	0.154541264	0.339636178
4.083519804	-0.727107531	0.605728871	-1.200384472	0.229990057	0.436252872
165.0868702	-0.399038854	0.295576444	-1.350036047	0.17700442	0.372267435
255.7143522	-0.479677451	0.176598226	-2.716207638	0.006603449	0.038806061
53.17897505	-0.365317598	0.149514678	-2.443356085	0.014551372	0.068707166
3.781394267	0.104656593	0.597036803	0.17529337	0.860849121	0.931184922
1248.307977	0.409940081	0.095908144	4.274298979	1.9174E-05	0.000410737
6.14696888	0.55325657	0.553303799	0.999914642	0.317351818	0.532225781
118.0618122	0.020265237	0.123252156	0.164420952	0.869399771	0.935010564
11.14044902	0.420385748	0.352411939	1.192881688	0.232915712	0.439625737
477.5994771	-0.297836766	0.107910886	-2.760025218	0.00577969	0.035108633

42.09287117	0.67679963	0.197331098	3.429766709	0.0006041	0.006474886
43.80075181	1.037814075	0.182198447	5.696064327	1.22605E-08	7.34469E-07
950.5105246	0.054150938	0.066801091	0.810629541	0.417578447	0.626862341
9.449377173	-0.686225153	0.438429497	-1.565189288	0.117538533	0.285344488
29.27739606	-0.361273943	0.333584731	-1.083005032	0.278806189	0.492077691
5.159659235	0.067591319	0.420754752	0.160643033	0.87237456	0.936384156
532.4472678	-0.050711392	0.068734615	-0.737785341	0.460644903	0.663513594
1380.015135	-0.170001504	0.0609884	-2.787439965	0.00531263	0.032989168
258.7260697	-0.177642831	0.143101511	-1.241376342	0.214466755	0.419063612
150.3981387	-0.291957516	0.080449982	-3.629056319	0.000284459	0.003602977
471.9124717	0.095027933	0.051210779	1.855623668	0.063507218	0.190846024
491.5468811	0.37321071	0.091220202	4.091316399	4.28931E-05	0.000789521
1320.980246	0.058833486	0.080492114	0.730922364	0.464826575	0.666976134
580.6493034	-0.016734833	0.09176306	-0.182370045	0.855292332	0.929057489
518.1918603	-0.165728003	0.115131699	-1.439464579	0.150018939	0.333254045
3428.341039	-0.231624459	0.112236634	-2.063715297	0.039044718	0.136275993
127.2880154	0.087745432	0.097731521	0.897821209	0.369280875	0.582831939
30.38966763	0.124728054	0.183350558	0.680270927	0.49633293	0.693126082
19.60191215	0.209997487	0.345784496	0.60730741	0.543646922	0.73068772
2077.222322	-0.003929045	0.08578762	-0.045799668	0.963469924	0.982015974
445.7086045	0.062885051	0.097134898	0.647399159	0.51737364	0.709915873
7.331572867	0.361183143	0.336022964	1.074876365	0.282430085	0.496119014
526.1977447	0.140909024	0.143759116	0.980174526	0.326999976	0.542169647
278.4858213	0.088806299	0.130685803	0.679540521	0.496795442	0.693461961
3.798085269	0.021439314	0.472059633	0.045416538	0.963775299	0.982015974
898.2252193	-0.024084532	0.118623742	-0.203032983	0.839109252	0.919787466
426.0079179	-0.054981686	0.072042963	-0.763179134	0.445356571	0.650598931
11.94056403	-0.033160824	0.297512666	-0.111460211	0.911251416	0.955384448
357.183821	-0.123105255	0.094261752	-1.305993702	0.191554699	0.391960268
323.3837133	-0.125184283	0.06397147	-1.956876776	0.050361958	0.162838644
1054.801165	0.083183148	0.063186576	1.316468688	0.188016781	0.387125166
121.0980039	-1.641737663	0.453149088	-3.622952595	0.000291259	0.003657168
124.262312	-0.233790008	0.325748255	-0.71770149	0.472941365	0.673283446
158.8890074	-0.278456626	0.100345076	-2.774990424	0.005520333	0.033983146
1659.127148	0.001839053	0.068622318	0.026799641	0.978619539	0.988823497
2248.194031	0.095244266	0.062400485	1.526338546	0.126925574	0.298280681
676.3989243	0.569449714	0.130309618	4.369974539	1.24261E-05	0.000286304
283.5849155	-0.532559389	0.156135248	-3.410885079	0.000647524	0.006846795
336.453729	-0.209571699	0.064933497	-3.227482073	0.001248849	0.0114492
914.1143523	0.141779438	0.052739887	2.68827724	0.007182174	0.041140171
175.1702505	-0.153826398	0.084469005	-1.821098728	0.068591851	0.201028669
566.4928219	-0.350219276	0.112616051	-3.109852229	0.00187181	0.015418096
11.16975028	-0.13234015	0.302924158	-0.436875522	0.662201625	0.814903232
109.0009083	0.002563075	0.177670073	0.014426034	0.988490089	0.993980695
4.264137328	-0.239280353	0.582522629	-0.410765765	0.681244298	0.827691386
295.7825874	0.284732831	0.070650006	4.030188362	5.57322E-05	0.000979216
315.4710078	0.609231327	0.172730197	3.527069028	0.000420187	0.004878199
1049.846698	0.09627886	0.067340676	1.429728142	0.15279506	0.337238185

3.78305048	-1.141329303	0.489399152	-2.332103146	0.019695266	0.085130427
94.91585285	0.115665393	0.175650471	0.658497482	0.510218514	0.704622797
92.52980441	-0.153759682	0.122502022	-1.25516036	0.209420552	0.4134087
2.828707453	0.554092367	0.560009338	0.989434157	0.32245077	0.537439698
1558.042029	0.154276246	0.066619436	2.315784324	0.020570048	0.087722227
77.49424602	-0.041123281	0.163356593	-0.251739338	0.801242549	0.898120007
128.2485528	0.301154285	0.111452941	2.702075717	0.006890807	0.039943403
11.98287218	1.06098389	0.466320386	2.275225192	0.022892424	0.09440045
141.9182642	-0.380990324	0.12262825	-3.106872398	0.00189078	0.015551028
20.64276038	-0.426875025	0.31629032	-1.349630382	0.177134574	0.372398541
32.9953395	-0.392517202	0.248775284	-1.577798226	0.114611972	0.281111176
72.17836209	-0.081534561	0.133880351	-0.609010661	0.542517372	0.729950677
175.3959033	-0.599102139	0.233700442	-2.563547308	0.010360858	0.053742058
50.06185125	-0.557279793	0.380500735	-1.464595839	0.143031218	0.322957275
289.8594947	-0.825939615	0.358429275	-2.30433079	0.021204082	0.089660176
7.431415012	0.419304914	0.442257363	0.948101603	0.343077732	0.55727298
7.322972211	-1.247210856	0.619048015	-2.01472394	0.043933582	0.148433518
5123.520226	-0.114863468	0.119956414	-0.957543363	0.33829307	0.553502819
6.990638605	0.155563142	0.383035644	0.406132286	0.684645404	0.829651922
7767.835532	-0.294549224	0.123586582	-2.383343072	0.017156197	0.077116288
323.578324	0.584921676	0.083119798	7.037092163	1.96293E-12	3.47713E-10
22.72349527	0.087323929	0.242015952	0.360818897	0.718234835	0.850529195
182.4497456	0.101139751	0.139786431	0.723530534	0.469354002	0.670512744
9.67186043	0.382545399	0.395416722	0.967448712	0.333319768	0.54871497
191.6997565	-0.032810421	0.081597613	-0.402100251	0.687610248	0.831033101
250.6776918	0.002371499	0.074993413	0.031622765	0.974772889	0.987719156
22.91280988	-0.562882371	0.563545633	-0.998823056	0.317880415	0.532786851
6.221208259	0.242924455	0.514910741	0.471779739	0.637084014	0.797196954
137.8259246	0.242807656	0.11937222	2.0340382	0.041947744	0.143728606
16.13996691	0.807613683	0.256806913	3.144828439	0.001661843	0.014104685
268.2102497	-0.120498042	0.081067779	-1.486386369	0.137176953	0.314567529
552.6608627	-0.499605751	0.093479365	-5.344556561	9.06387E-08	4.11178E-06
11.35644238	-0.620995736	0.285129325	-2.177944115	0.029410198	0.112502731
103.6590532	0.10482391	0.126544952	0.828353153	0.407470529	0.61832678
196.7291103	-0.114681421	0.077862767	-1.472865978	0.140787178	0.319819079
28.43419108	0.254142561	0.309699378	0.8206105	0.411868165	0.621803166
236.9168585	0.109570593	0.094487336	1.159632573	0.246198433	0.454811059
156.2989043	0.185320984	0.136856769	1.354123627	0.17569694	0.370084565
303.5824066	0.185992935	0.085377801	2.178469508	0.0293711	0.112447014
205.3476119	-0.381216243	0.109868283	-3.469756993	0.000520929	0.005767333
3960.857725	-0.144728464	0.122863339	-1.177962964	0.238811371	0.446360434
64406.14024	0.137734451	0.084489103	1.630203729	0.103058446	0.262327695
347.653329	-0.243860527	0.052894919	-4.61028266	4.02122E-06	0.00011115
1159.115515	-0.006348852	0.106408219	-0.059665056	0.952422403	0.977124952
59.04537801	-1.370311858	0.522656435	-2.621821462	0.008746124	0.047868319
201.2375722	0.062441888	0.331261672	0.188497171	0.850486933	0.926280691
30.19305825	0.173969775	0.323889679	0.537126639	0.591180141	0.764264772
117.157214	-0.35719713	0.106584228	-3.351313202	0.000804293	0.008118823

2523.140658		-0.087708464	0.129164097	-0.679046775	0.497108225	0.693660927
2.928225058		-0.615637036	0.554721752	-1.109812322	0.267079908	0.478749089
166.4666617		-0.050739526	0.08209777	-0.618037801	0.536550421	0.724877003
123.7362649		-0.091729021	0.100328376	-0.914287906	0.360565584	0.574797506
134.4771393		-0.62846869	0.323475695	-1.942862166	0.052032814	0.16607586
467.0715975		0.035753167	0.049230436	0.726241128	0.467690963	0.669395389
4.220248419		-3.944640519	1.276275429	-3.090743916	0.001996557	0.016194627
45.24083122		-0.252301323	0.163130645	-1.546621254	0.121954601	0.291805389
122.0868638		-0.248281603	0.115104216	-2.15701572	0.03100444	0.11645423
4.911602648		-0.706238215	1.0756392	-0.656575378	0.511453985	0.70561033
348.8047016		-0.349374413	0.103470161	-3.376571641	0.000733953	0.007556959
7.293766948		0.812342439	0.985656825	0.824163561	0.409846629	0.620217261
237.9609429		0.14932319	0.126653688	1.17898809	0.238402918	0.446047664
1441.156277		-0.089948075	0.088896961	-1.01182395	0.311622237	0.526421732
42.19677274		0.664625731	0.228021543	2.914749726	0.003559739	0.024671072
307.1660735		0.449009179	0.283862788	1.58178246	0.11369924	0.279601757
72.22966492		0.398745106	0.149640354	2.664689667	0.00770594	0.043341636
7.420569946		0.781774615	0.46442947	1.683301051	0.092316827	0.243332385
95.44911012		0.509680921	0.119963375	4.248637708	2.15074E-05	0.000452508
6.633998522		-0.098029584	2.201715965	-0.044524174	0.964486583	0.982392126
193.7814102		-0.334646863	0.083146459	-4.024787932	5.70267E-05	0.000996243
19396.3444		-0.188888089	0.099761636	-1.893394057	0.058305485	0.179883228
505.2228655		-0.015244599	0.066919623	-0.227804623	0.819798132	0.909423741
10319.0958		0.175191888	0.135931728	1.288822641	0.197459753	0.398841585
130.9061927		-0.162992343	0.176071998	-0.925714164	0.354594545	0.569189373
262.4238433		-0.071065406	0.079662614	-0.892079773	0.372350176	0.585649875
492.7978412		-0.019861752	0.06824045	-0.291055406	0.771008945	0.88046592
2.477368688		0.013437228	0.669768507	0.020062496	0.983993518	0.992002278
78.48174534		-0.771090873	0.240247673	-3.209566465	0.001329353	0.011984908
2001.853931		0.035175464	0.051768881	0.679471193	0.496839354	0.693461961
49.71277336		0.007950403	0.173817607	0.04573992	0.963517546	0.982015974
177.2929124		-0.033734111	0.097105199	-0.34739758	0.728292651	0.85622654
20.50636177		-0.108871518	0.262045538	-0.415467934	0.677799384	0.825950823
25.63245206		0.490699111	0.483689768	1.014491403	0.31034833	0.525239201
274.1899268		0.213979263	0.075398084	2.837993377	0.004539812	0.029317472
6.589798674		0.012231637	0.442458923	0.027644683	0.977945543	0.988566907
88.96168792		-0.037801014	0.119798554	-0.315538151	0.752353089	0.870873018
168.5315819		0.194430343	0.136219396	1.427332293	0.153484137	0.338124855
322.2281395		0.059979157	0.094477413	0.634851813	0.525525067	0.716334923
739.1665027		-0.330978558	0.129290241	-2.559965512	0.010468255	0.054196743
101.8777331		-0.001434677	0.14164162	-0.010128919	0.99191843	0.995720597
451.2445136		-0.066127676	0.100058366	-0.66089103	0.508682199	0.703115846
251.1762747		0.183706397	0.148162307	1.239899676	0.215012504	0.419483182
79.74681295		0.053470716	0.23062518	0.231851164	0.816653617	0.907813535
223.907593		-0.192055181	0.090964992	-2.111308712	0.034745786	0.125719761
357.985778		0.066849898	0.073009693	0.915630443	0.359860759	0.573951606
85.00425181		-0.186414571	0.116774329	-1.596366022	0.110407098	0.274501438
161.9082844		0.508589206	0.104352462	4.87376338	1.09492E-06	3.7268E-05

16.32687044	0.391787636	0.2909205	1.346717183	0.178071336	0.373605093
179.6159239	-0.057803419	0.091334891	-0.63287336	0.526816346	0.717162886
160.6200146	0.274514971	0.101925948	2.693278557	0.007075313	0.04064111
503.6814437	-0.185217115	0.092048484	-2.012169088	0.044202116	0.149096162
7.215274662	-0.386249172	0.482130801	-0.801129428	0.423056722	0.63111002
4805.52494	-0.153866321	0.103082713	-1.492649121	0.135529058	0.311917533
301.1042544	-0.234707976	0.086026834	-2.728311214	0.006365952	0.037723989
44.60626123	0.419822085	0.227085527	1.848739946	0.064495373	0.193034326
3.799402173	-3.268828726	0.760080974	-4.300632217	1.70312E-05	0.000372605
12.003741	-2.88792552	1.202547783	-2.401505837	0.016327749	0.074489983
10.51755859	0.888655574	0.620595286	1.43194058	0.152160827	0.336379156
33.1341964	0.349631376	0.470065674	0.743792614	0.457001949	0.660585609
276.7948421	-0.276056114	0.107278751	-2.573259953	0.010074551	0.052788937
554.576974	-0.174064509	0.107605006	-1.617624635	0.105743517	0.266633651
126.9036233	0.053172937	0.1221843	0.43518633	0.663427184	0.815802003
48.41868896	0.192932374	0.471532944	0.409159903	0.682422318	0.8284617
12.28820723	0.984019421	0.905935723	1.086191213	0.277394404	0.49044899
12.00734621	0.205935689	0.733509923	0.280753787	0.778899251	0.884794608
104.5217941	0.64470267	0.205779905	3.13297195	0.00173046	0.014485566
2.447621285	-0.726030429	0.783003247	-0.927238082	0.353802934	0.568694462
240.919991	-0.213112686	0.097595188	-2.183639271	0.028988765	0.111319562
49.45699815	-0.044941533	0.1533275	-0.293108107	0.76943952	0.879893562
625.4883803	0.154206159	0.056177654	2.744973261	0.006051583	0.036278666
1602.495262	0.408971877	0.097652473	4.188034024	2.81381E-05	0.000564613
57.34945567	0.164713684	0.235506724	0.699401193	0.484301343	0.683548691
359.2822609	0.256129386	0.059654781	4.293526538	1.75857E-05	0.000384227
796.3065836	-0.099302938	0.0618279	-1.606118578	0.108247859	0.270849821
284.9338127	0.122876544	0.125715171	0.977420172	0.328361181	0.543715156
46.60407723	0.15830376	0.178965908	0.884547016	0.376400994	0.589554891
369.8292208	-0.151449214	0.064765066	-2.338439876	0.019364441	0.083972046
27.16662052	-0.332464452	0.254420797	-1.306750298	0.19129753	0.391482673
5.599101445	0.101212206	0.751286837	0.134718461	0.892834476	0.946108145
531.0847216	0.193662355	0.120534811	1.60669232	0.108121879	0.270699064
1180.718424	0.201389248	0.106239802	1.895610165	0.058011612	0.179168223
151.1872749	-0.15018624	0.107588093	-1.395937367	0.162733357	0.351997597
126.6744665	-0.063254581	0.148426087	-0.426168888	0.669984786	0.820436287
5.060846041	-0.114441893	0.441433899	-0.25925035	0.795442085	0.894661904
375.844674	-0.174768488	0.067699279	-2.581541368	0.00983602	0.051985434
3.206143583	-0.763601437	0.533132502	-1.432292036	0.152060261	0.33629222
690.9581466	0.088450753	0.123680604	0.715154601	0.474513518	0.67447245
113.096319	0.067057694	0.118735133	0.564767073	0.572232206	0.751690723
127.7088541	0.28368911	0.092353747	3.071766104	0.002127964	0.016910795
5318.598986	0.283082803	0.104595676	2.706448439	0.006800714	0.039672438
380.2806293	-0.167764555	0.071739259	-2.338532012	0.019359667	0.083972046
2315.978065	-0.165084346	0.159117357	-1.037500552	0.299502639	0.514334043
2.386742497	-2.132425228	1.218959371	-1.749381709	0.08022506	0.222758747
636.9149726	0.093381992	0.092131415	1.013573836	0.310786148	0.525812706
964.0856677	0.013903599	0.059966127	0.231857546	0.816648659	0.907813535

1.773996568	-2.288105489	1.214738625	-1.883619606	0.059616448	0.18265229
624.0420431	-0.581837076	0.099651865	-5.838697279	5.26106E-09	3.70387E-07
264.9304984	-0.137575086	0.078786021	-1.746186512	0.080778548	0.223729959
182.7656041	-0.011525729	0.127201269	-0.090610173	0.927802349	0.965547435
121.3649543	-0.166985367	0.110139649	-1.516124016	0.129487997	0.302150886
329.5021114	-0.098670496	0.085893325	-1.148756284	0.25065649	0.45957013
39.53160162	-0.25608008	0.315215097	-0.812397892	0.416563355	0.626095433
396.3931782	0.162966389	0.100175948	1.626801567	0.103779249	0.263517926
1.775944919	1.028929057	0.835753423	1.231139507	0.218270692	0.422984517
282.2083533	0.257106362	0.089586428	2.86992535	0.004105687	0.02727302
451.0617661	0.285591604	0.116740468	2.446380494	0.014429863	0.068348924
89.96095966	0.016479585	0.141131825	0.11676732	0.907044441	0.953303217
108.9672916	0.386133317	0.124257158	3.107533783	0.001886554	0.015524023
27.37950463	-0.066031524	0.222990162	-0.296118552	0.767139546	0.878716103
82.73540411	0.054824783	0.110498915	0.496156752	0.619783821	0.785288315
764.2188676	-0.577907169	0.129524179	-4.461770549	8.12852E-06	0.000201361
232.3849366	-0.155281019	0.133423198	-1.163823237	0.244495653	0.452462524
42.74148969	-0.973616227	0.625169155	-1.557364466	0.119383978	0.288250149
344.3959679	0.722671608	0.125539062	5.756547784	8.58515E-09	5.39816E-07
138.7904577	0.018195239	0.12564604	0.144813466	0.88485815	0.942468039
58.56654286	0.135327356	0.138959405	0.973862519	0.330124806	0.545419232
248.5018705	0.006163647	0.096010267	0.064197795	0.948812733	0.975762592
226.4181862	-0.050996832	0.105023915	-0.485573517	0.627269577	0.790819546
88.42968112	-0.491786858	0.378411381	-1.299609057	0.193734994	0.394586588
2.060368428	-0.261651948	0.94786389	-0.276043798	0.782514427	0.887032618
367.2501303	-0.649349794	0.122430494	-5.303823996	1.13402E-07	5.00852E-06
231.0876137	0.167298468	0.087568682	1.910482879	0.056071069	0.174417444
454.4748743	-0.447697558	0.115613455	-3.872365547	0.000107784	0.001659478
720.7538719	-0.010121032	0.120317633	-0.084119277	0.932961598	0.968347365
39.79891427	0.055742187	0.18630594	0.299197047	0.764789702	0.877707421
42.14342904	-0.33486572	0.160321214	-2.088717466	0.036733164	0.131068256
243.8319314	0.053246945	0.062396628	0.853362539	0.393458289	0.606547079
172.1221919	-0.018732414	0.099774069	-0.18774832	0.85107395	0.926405873
19560.6603	-0.115471883	0.108134782	-1.067851446	0.285587527	0.499975444
25.93911464	-1.088303621	0.268823516	-4.048394417	5.15702E-05	0.000919445
371.8204571	-0.198350578	0.195496896	-1.01459707	0.310297937	0.525239201
4.158499112	0.219208983	0.5409041	0.405264045	0.685283433	0.829778815
18.31069327	0.134771447	0.611350652	0.220448684	0.825521734	0.912544121
57.81970602	-0.393860224	0.236794553	-1.663299343	0.09625253	0.249828924
129.2204034	-0.38682426	0.201024911	-1.924260319	0.054321962	0.170618383
345.7479239	0.105263881	0.111772216	0.941771441	0.34630967	0.560822374
19.64747564	0.304218607	0.219497121	1.385979939	0.165753029	0.356244671
1107.68567	-0.066109304	0.146549037	-0.451107053	0.651912395	0.807428373
49.59057434	-0.280909119	0.168795588	-1.664197049	0.096073058	0.249719365
290.5707355	0.278682658	0.104278167	2.672492861	0.007528998	0.042696285
18.82469308	-0.954329866	0.460233392	-2.073578066	0.038118514	0.134467752
7.684709156	-0.468229781	0.34130445	-1.371883023	0.170099856	0.362141664
103.1121272	-0.540202125	0.189531756	-2.850193216	0.004369268	0.028529258

824.0014411	-0.131293784	0.093932078	-1.397752364	0.162187444	0.351331485
691.438975	0.314288227	0.134614378	2.334730007	0.01955753	0.084675622
30.68252333	1.646041544	0.306818117	5.364877271	8.10043E-08	3.738E-06
194.7137848	-0.545835808	0.137133219	-3.980332495	6.88189E-05	0.001172413
320.7748271	-0.120220214	0.077687878	-1.547477132	0.121748234	0.291692905
216.78066	-0.412485343	0.346759571	-1.189542778	0.234226148	0.441189294
89.26321069	0.439976491	0.222049737	1.98143216	0.04754283	0.156667869
80.92567138	-0.085895948	0.238301012	-0.360451464	0.718509547	0.850583952
1.757915248	1.509742013	0.845774845	1.785040099	0.074254813	0.211858986
2.380619457	-0.624391739	0.607378511	-1.028010915	0.303944693	0.518644466
236.5507936	-0.706262897	0.119969321	-5.887029227	3.93199E-09	2.84104E-07
235.657895	0.093850456	0.068130572	1.377508713	0.168355023	0.359725116
1037.302651	-0.039652587	0.453320485	-0.08747142	0.930296802	0.966799742
7791.933041	-0.027438175	0.07184684	-0.381898141	0.702536919	0.840493334
218.9773522	-0.398002866	0.189945088	-2.09535751	0.036139224	0.129445592
132.956898	0.180679134	0.090075523	2.005862726	0.044870896	0.150611887
32.54481491	0.172486088	0.23649344	0.729348298	0.465788635	0.667891198
91.11058605	0.115803637	0.115304659	1.00432747	0.315220797	0.530054855
3.538458097	0.747010877	0.536125562	1.393350607	0.163513794	0.353136634
28.02634151	0.235348047	0.321695363	0.731586693	0.464420873	0.666574707
9.201207628	0.555649178	0.422799796	1.314213448	0.188774384	0.388102983
1094.884602	-0.088870696	0.078939057	-1.125814014	0.260244251	0.47133507
1252.342385	-0.208886024	0.162846954	-1.282713733	0.199592387	0.401868123
30.0864016	0.985925675	0.304864046	3.233984742	0.00122076	0.011253943
88.98401829	-0.317688362	0.12999217	-2.443903825	0.014529299	0.068661983
51.17846183	-0.497328558	0.195394683	-2.545251239	0.010919914	0.055815905
136.7346119	0.383179167	0.23749423	1.613425165	0.10665217	0.267956053
33.16051841	0.038859882	0.26579688	0.146201423	0.883762382	0.942092758
351.0907758	-0.439657616	0.116795622	-3.764333017	0.000166994	0.002369563
46.99239305	-0.459014271	0.216436142	-2.12078383	0.033939998	0.123682288
127.4742338	0.326256838	0.331964966	0.982805029	0.325703406	0.540727392
155.232199	-0.652321214	0.142968989	-4.562676282	5.05057E-06	0.000134415
89.50828129	-2.141308455	0.669270754	-3.199465153	0.001376828	0.012300363
969.6796504	0.078459441	0.122510409	0.640430814	0.521892555	0.713473653
37.01280535	0.243489246	0.177628607	1.370777204	0.170444419	0.362496949
38.86862545	0.234049986	0.163852592	1.428417965	0.153171591	0.33766209
3.012096404	-1.918211427	0.995351437	-1.927169998	0.053958448	0.170028972
427.2148807	0.046040023	0.183063978	0.251496903	0.801429956	0.898268955
26.97234875	0.003787185	0.220363871	0.017186054	0.986288188	0.993222667
15.40926705	-0.106029103	0.29233398	-0.362698525	0.716830102	0.849694855
573.0593637	0.081269374	0.064476853	1.26044263	0.207509732	0.411017483
11.8180342	0.375215946	0.505147684	0.742784651	0.45761207	0.661013261
10.03602124	-0.967715506	0.580619526	-1.666694733	0.095575121	0.248894173
146.7374772	-0.480882147	0.111183775	-4.32511082	1.52455E-05	0.000341243
10.83710557	1.011194219	0.34374342	2.941712218	0.003264031	0.023125301
15.95481684	1.340630008	0.67900898	1.974392163	0.048337169	0.1585005
2472.210299	-0.162720393	0.071516846	-2.275273623	0.022889521	0.09440045
193.1438748	-0.09863897	0.079383257	-1.242566429	0.214027647	0.418702228

83.49409415	-0.62724482	0.320327522	-1.958135899	0.050214068	0.162587942
160.0404623	-0.161203874	0.156239247	-1.031775804	0.302177157	0.517049928
84.59924247	-0.080880478	0.222196353	-0.364004523	0.715854632	0.849333036
483.4930432	-0.136877367	0.085218178	-1.606199176	0.108230154	0.27084666
459.8734254	0.035390001	0.06078758	0.582191308	0.560437827	0.744310525
7.312433726	0.211582374	0.364386145	0.580654277	0.561473484	0.74480346
140.1411542	-0.306084972	0.443515277	-0.690133999	0.490109924	0.687916068
43.87343625	-0.036340374	0.169754606	-0.214075922	0.830487852	0.915614595
126.7877668	-0.170773366	0.107728766	-1.585216033	0.112917259	0.278514587
279.3179706	0.031688015	0.089309431	0.354811516	0.7227308	0.852701225
612.7591272	0.211429508	0.097985936	2.157753614	0.030946993	0.11645423
12.82092685	0.464385336	0.285832588	1.624675964	0.104231622	0.264089777
2.417027782	-0.323872156	0.664585886	-0.487329272	0.626025007	0.790627823
20.60288458	-1.808324254	0.704391809	-2.567213633	0.010251943	0.05336193
13.61444558	-0.57923238	0.305008139	-1.899071878	0.057555026	0.177991646
11.49583478	-0.362420282	0.585753606	-0.618724799	0.53609767	0.724560917
1.894494819	0.860853245	0.860338026	1.000598856	0.317020783	0.531995557
435.3628665	-0.018236187	0.092586448	-0.196963885	0.8438558	0.92241261
44.02587952	-0.226720033	0.173251642	-1.308616939	0.19066414	0.390623187
2380.599584	-0.046075493	0.112450757	-0.409739284	0.681997209	0.82820821
112.2909788	0.049413671	0.111314277	0.44391135	0.657106678	0.811239165
535.1945658	0.190977909	0.066776739	2.859946619	0.004237123	0.027909783
14.44679612	-0.28906409	0.325395627	-0.888346571	0.374354356	0.587904067
27.14024158	0.445013842	0.550186208	0.808842236	0.418605899	0.62771833
3415.564477	0.073870114	0.109889685	0.672220632	0.501443248	0.697229581
96.22211725	0.282661069	0.104177876	2.71325429	0.006662596	0.039046461
76.8038572	0.133371426	0.157225918	0.848278885	0.396282674	0.609075814
2556.234174	-0.502176067	0.150022752	-3.347332719	0.000815932	0.008206145
525.9903593	-0.435670987	0.10883289	-4.003118783	6.25129E-05	0.001079494
132.0299769	0.109458544	0.084269658	1.298908128	0.19397546	0.394853791
313.2171212	-0.130615367	0.073122604	-1.786251581	0.074058533	0.211672815
58.99954889	-0.978297148	0.277300627	-3.52792981	0.000418823	0.00487036
493.8776007	-0.081408095	0.109600808	-0.742769113	0.457621479	0.661013261
67.40305459	-0.096227639	0.217352593	-0.442725977	0.657963951	0.811680353
19.72079712	-0.232578285	0.214952639	-1.081997812	0.2792535	0.492497822
20.87468998	-1.178360386	0.330319369	-3.567336631	0.000360628	0.004336487
334.2884718	-0.359862115	0.224719933	-1.601380482	0.109292673	0.272635902
657.6543925	-0.113329199	0.067471429	-1.679662043	0.093023089	0.244836646
774.8906867	-0.258133728	0.086383502	-2.988229501	0.002805988	0.02079435
934.4163482	0.145843889	0.091538872	1.593245415	0.111105147	0.275578915
93.40989556	-0.416375615	0.208289437	-1.999024155	0.04560574	0.152519076
1089.61193	0.196541865	0.088991797	2.208539121	0.027206714	0.106644495
12.36131106	-0.705815671	0.456799079	-1.545133746	0.122313916	0.292325468
160.8273954	0.022551899	0.179225071	0.125830044	0.899866459	0.949734131
3522.09471	-0.171885179	0.137145146	-1.253308512	0.210093452	0.414202911
55.05151143	1.014646894	0.446703104	2.27141223	0.023122035	0.094982596
209.0608739	0.456377453	0.359089256	1.270930403	0.203753409	0.406697748
67.91124459	0.538200242	0.406726769	1.323247652	0.185753034	0.383999935

81.06446004	0.222357498	0.327452123	0.679053464	0.497103987	0.693660927
114.6831353	0.149874691	0.279133612	0.536928142	0.591317251	0.764264772
138.0609456	-0.169449595	0.140948805	-1.202206678	0.229283463	0.435363735
153.94451	-0.153108447	0.099848165	-1.533412731	0.125174194	0.296153909
1062.645518	-0.245870689	0.071237281	-3.451432814	0.000557619	0.006095692
282.9099725	-0.085356515	0.113362146	-0.7529543	0.451477376	0.656419939
338.184808	0.201682101	0.074725781	2.698962769	0.006955596	0.040163507
865.2450075	-0.171870397	0.063688048	-2.698628723	0.006962581	0.040189752
3800.541576	-0.01239931	0.077315149	-0.16037362	0.872586768	0.936481721
21.8720314	0.056905754	0.492541686	0.115534899	0.90802116	0.953697201
3.841111483	0.589840534	0.598353401	0.985772845	0.324244594	0.539548668
78.99776451	-0.212775052	0.131169902	-1.622133195	0.104774832	0.264977062
114.6011897	-0.785545431	0.277317935	-2.832652822	0.004616349	0.029683739
144.4387527	-0.182897636	0.087999286	-2.078399097	0.037672616	0.133347821
513.8203646	-0.158030504	0.194194407	-0.813774746	0.415774005	0.625357946
135.9861313	-0.976544799	0.213063343	-4.583354347	4.57576E-06	0.000123982
146.069852	0.127655446	0.081729274	1.561930491	0.118304365	0.286399133
135.2257821	-0.151725801	0.088351541	-1.717296604	0.085924992	0.232886268
101.939677	0.150097342	0.200916987	0.747061484	0.455026448	0.659217728
14.94476466	0.879426464	0.451077601	1.949612354	0.05122234	0.16452268
3.841648263	-1.704536888	0.582676333	-2.925358027	0.003440601	0.024068138
479.8944428	-0.109380019	0.083621703	-1.308033873	0.190861821	0.390833335
84.36797092	-0.028157104	0.161568647	-0.174273316	0.861650669	0.931286863
49.64389505	-0.159817154	0.211265598	-0.756475048	0.449364427	0.654364852
471.4962695	0.275747214	0.077922528	3.538735476	0.000402049	0.004720846
131.0090352	-0.08302437	0.099462138	-0.834733417	0.403867822	0.614990985
2.165101191	-4.577086838	1.577758252	-2.901006432	0.003719662	0.025363294
4.685005704	-0.666079434	1.007076924	-0.661398765	0.508356617	0.702907521
1.765891153	-1.229829571	0.89067541	-1.380783119	0.167345655	0.358591614
953.2667774	0.289249901	0.084207952	3.434947559	0.000592669	0.006398179
3125.8538	-0.148508905	0.238023659	-0.623924971	0.53267686	0.722031935
300.966406	0.585010973	0.101064506	5.788490874	7.10216E-09	4.69884E-07
278.2883087	0.581417269	0.103104554	5.639103679	1.70938E-08	9.74403E-07
6.934146833	-0.510198654	0.477332381	-1.068854062	0.285135434	0.499449351
57.37586275	-0.503438341	0.170225897	-2.957472099	0.003101728	0.02231348
2.333886652	-1.28358485	0.705184435	-1.820211547	0.068726795	0.201173857
596.8077882	0.127192609	0.122946412	1.034536973	0.300885201	0.51574059
563.3588596	0.074400676	0.081427428	0.913705347	0.360871694	0.575074593
2731.729985	-0.188835825	0.169026536	-1.117196327	0.263910391	0.475361883
193.4892189	0.012702069	0.127077806	0.099955056	0.920380007	0.960797176
511.0398769	-0.125044774	0.06453976	-1.937484321	0.052686168	0.167558288
570.5821355	0.013099234	0.05493797	0.238436811	0.81154232	0.904924068
17.35037619	-0.144738285	0.292374359	-0.495044387	0.620568788	0.786101516
486.2108977	-0.112260865	0.050155698	-2.238247473	0.025204921	0.101225226
22.42188693	-0.565299877	0.271884534	-2.079191003	0.037599798	0.133151134
42.93345967	0.906812427	0.154392652	5.873417001	4.26903E-09	3.03458E-07
5.140147229	0.704422509	0.406764687	1.731769083	0.083314685	0.228380373
5.775089032	-0.282009219	0.486015714	-0.580247122	0.561747981	0.744927653

435.7111951	-0.259481035	0.114094081	-2.274272532	0.022949606	0.094541586
2.906858263	0.576598703	0.642503615	0.897424839	0.369492263	0.582991624
4419.424117	0.01107477	0.133062546	0.083229809	0.933668811	0.968634829
3.915918553	0.509160661	0.455251795	1.118415495	0.26338958	0.474787169
14.7007131	-0.707905665	0.678962789	-1.042628075	0.297120581	0.511766627
5.614534018	-0.540610509	1.006694905	-0.537015243	0.591257085	0.764264772
52.52266601	-0.113796948	0.146357839	-0.777525478	0.436848809	0.642902204
194.6201131	-0.214759794	0.081779082	-2.626096911	0.008637022	0.047381387
92.29116909	-0.135507282	0.122479008	-1.106371482	0.268565764	0.480491998
4.364916033	-0.229372973	0.568870881	-0.403207442	0.686795625	0.830726992
7.634759225	0.700259183	0.34189524	2.048168856	0.040543457	0.140144732
57.82326493	-0.147588025	0.309382439	-0.477040733	0.633333117	0.794451364
610.667374	-0.062099541	0.105343657	-0.589494825	0.555529382	0.740256495
3.077734853	-0.764375522	0.593821214	-1.287214912	0.198019391	0.39958004
145.2790078	-0.873745203	0.339622505	-2.572695241	0.010091002	0.052823573
49.51112872	-0.564865412	0.341612219	-1.653528124	0.098223427	0.253625821
380.2188858	0.024662736	0.058125114	0.424304304	0.671343901	0.821390813
578.8514952	0.116751445	0.074236878	1.57268798	0.115791084	0.282613205
109.9134215	-0.134691212	0.128718887	-1.046398207	0.29537722	0.510173138
2.546710901	0.192624185	0.84833223	0.227062203	0.820375374	0.909820137
3.08615441	-1.728865132	1.292040965	-1.338088481	0.180867595	0.377070712
340.9181189	-0.678553528	0.076004244	-8.927837385	4.34418E-19	2.98191E-16
80.36269842	0.022974509	0.131984479	0.174069778	0.861810624	0.931286863
229.6606183	-0.001981964	0.097002525	-0.020432082	0.983698692	0.991966796
188.5243701	0.266656755	0.191558093	1.392041184	0.163909927	0.353482411
11.23042687	0.525835938	0.415758165	1.264763947	0.205955959	0.409517791
1641.06757	0.149175418	0.055908065	2.668227178	0.007625268	0.042975937
32.47980928	0.40729964	0.219023582	1.859615465	0.06293995	0.189592266
209.3514916	-0.109112483	0.119034552	-0.916645473	0.359328448	0.573611636
82.62809957	0.245670939	0.267299408	0.919085232	0.358050993	0.572450705
1627.9961	0.038675565	0.104794684	0.369060377	0.712082722	0.846212223
54.88933978	0.256449509	0.156749792	1.636043696	0.101830443	0.260287775
34.25282221	0.151539489	0.163788287	0.925215664	0.354853737	0.569494444
511.425973	-0.362869427	0.084582933	-4.290102194	1.78591E-05	0.000387451
741.7526352	-0.035535241	0.077230525	-0.460119116	0.645430724	0.803265524
17.87601783	0.641028312	0.277954786	2.306232325	0.021097654	0.08932479
66.03158043	-0.284991129	0.193147688	-1.475508886	0.140075781	0.318837317
1707.910061	0.13092313	0.094412973	1.386707	0.165531125	0.355999968
1307.584879	0.246741817	0.069048437	3.57345987	0.000352295	0.004251804
2.209597068	1.154623816	0.807423396	1.430010354	0.152714047	0.337104544
2951.486876	0.182943218	0.07654877	2.389891003	0.016853374	0.076149884
9.381075981	0.116703333	0.41075801	0.284116998	0.776320721	0.883652841
4.630495509	-0.558771364	0.474411669	-1.177819602	0.238868532	0.446360434
57.00104305	-0.106287579	0.146619773	-0.724919817	0.468501223	0.670018398
180.434558	0.0120942	0.080060349	0.151063541	0.879925592	0.939888103
258.0482215	-0.036504116	0.088408252	-0.412903943	0.679676992	0.826777746
303.9798233	-0.198485218	0.06968488	-2.848325449	0.004394996	0.028663166
48.6476277	-0.591088709	0.143098601	-4.130639326	3.61756E-05	0.000692973

280.1764676	-0.206109712	0.069147857	-2.980710063	0.002875809	0.021150037
391.5595134	-0.113514201	0.092802644	-1.223178516	0.221262278	0.42601247
575.8899634	-0.004788712	0.045371709	-0.105544	0.915944158	0.958430263
3.964668418	-1.408555541	0.661419195	-2.129595802	0.033204998	0.122020775
2.214982606	0.788609886	0.991802956	0.795127582	0.426539292	0.633800964
40.42808241	0.504777204	0.186490184	2.706722644	0.0067951	0.03965373
1797.955435	-0.195129623	0.108449104	-1.799273724	0.071975392	0.207802772
145.1342721	-0.046515531	0.118796076	-0.39155781	0.695384966	0.836431946
45.03427048	0.010964724	0.1877314	0.058406447	0.95342488	0.977454818
70.05354009	-0.018615803	0.111071884	-0.167601399	0.866896871	0.934032728
65.56016891	-0.038888133	0.126973574	-0.306269497	0.759399462	0.87432656
53.88008333	0.029164638	0.168571101	0.173010901	0.862642859	0.931737465
11.82155709	-0.166182469	0.271109134	-0.612972593	0.539894462	0.72754367
77.69923673	0.094956493	0.192472525	0.493350898	0.621764671	0.786950767
3.029409349	0.270503155	0.573509107	0.471663225	0.63716719	0.797196954
736.0183632	-0.010392854	0.099625264	-0.104319458	0.916915837	0.959051187
305.919662	-0.027163855	0.103636451	-0.262107148	0.793238836	0.893098455
3.981112726	-0.066684173	0.496227916	-0.134382147	0.893100399	0.946108145
10.62667628	-0.029504912	0.433312853	-0.068091477	0.945712815	0.974141328
1736.630171	-0.221096576	0.082163489	-2.690934604	0.007125216	0.040870755
3.144367387	-0.728953169	0.849162293	-0.858437987	0.39065066	0.60400683
1327.093858	-0.266501971	0.090040465	-2.959802243	0.003078366	0.022196398
3.796052644	-0.354480037	0.508013706	-0.697776522	0.485316964	0.684161532
273.9225913	-0.353453055	0.086525444	-4.084960901	4.40842E-05	0.000806042
92.3470358	-0.066563512	0.130130133	-0.511514982	0.6089905	0.778136159
55.34863948	-0.192841354	0.186301208	-1.035105224	0.300619773	0.515534279
3.748267175	-0.228874059	0.560916577	-0.408035826	0.683247373	0.828701792
14.92614319	-0.918545027	0.411273496	-2.233416538	0.025521489	0.102160075
486.6005509	-0.12067237	0.046353211	-2.603322762	0.009232499	0.049672172
409.23515	-0.321496694	0.16342664	-1.967223301	0.049157473	0.160201822
5553.883321	-0.078408392	0.117694447	-0.666202989	0.505281363	0.700428834
2.093132993	-1.676834968	1.685632656	-0.99478078	0.319842896	0.534905436
7.142565726	-2.366874175	0.853879068	-2.771907948	0.005572879	0.03421859
14.25878158	-1.526191316	0.581103156	-2.626369002	0.00863012	0.047369685
84.76808524	-0.250724765	0.130975423	-1.914288636	0.055583286	0.173161696
111.5999132	-0.207464312	0.145170902	-1.42910397	0.152974353	0.337409617
107.0279608	-0.293386111	0.142153298	-2.063871287	0.039029922	0.136275993
486.9030111	-0.036175581	0.091838367	-0.39390488	0.693651268	0.835017244
13.24687424	0.503069365	0.300260894	1.675440841	0.093847769	0.246091419
850.6915876	0.233821406	0.10362151	2.256494866	0.024039656	0.097821364
269.5289078	-0.41885515	0.119365748	-3.509006208	0.000449784	0.005134961
16.53343889	-0.192253142	0.316196407	-0.60801811	0.543175463	0.730491786
411.2904771	-0.084933689	0.14294954	-0.594151536	0.552410764	0.737831598
1.731943621	0.553704144	0.745287284	0.742940548	0.457517675	0.660985919
22.34316291	-0.695133151	0.516099384	-1.346897851	0.178013134	0.373530552
7.884321413	0.400557718	0.445202713	0.899719849	0.368269358	0.582065566
373.5859899	-0.240240317	0.111701787	-2.150729399	0.031497564	0.117635656
10.40293584	1.29843522	0.390865027	3.321952924	0.000893898	0.00882858

53.36033813	0.532607973	0.194395611	2.739814809	0.006147381	0.036745993
489.020476	-0.012040877	0.098023517	-0.122836619	0.902236476	0.95107779
3.759228106	-1.346559285	1.222033506	-1.101900462	0.270504948	0.482423469
118.4785379	-0.121583516	0.094268706	-1.289754801	0.197135806	0.398559676
73.59896396	-0.294012601	0.151860536	-1.936069822	0.05285915	0.167913929
3.814501709	-0.262518919	0.603618152	-0.434908921	0.663628539	0.815988696
1057.008047	-0.141560767	0.073569367	-1.924180845	0.054331919	0.170618383
102.7077947	0.585361153	0.218297673	2.681481409	0.007329699	0.041839729
3.301496044	1.57675471	0.576947224	2.732927111	0.006277422	0.037320193
190.7124528	0.102429379	0.089026524	1.150549011	0.24991782	0.458888338
877.9809646	0.003313748	0.059718515	0.055489453	0.955748533	0.978293782
109.1710006	-0.583487912	0.094883039	-6.149549147	7.77035E-10	6.53106E-08
29.98821368	-0.563412821	0.220417054	-2.556121729	0.010584608	0.054601691
3744.196918	-0.247669485	0.104262874	-2.375433126	0.017528368	0.078340298
83.62318896	0.396410543	0.115067898	3.445014212	0.000571029	0.006209332
534.9165671	0.042948444	0.070143571	0.612293375	0.540343672	0.727851321
27.1386554	1.825999903	0.30413106	6.003990203	1.92526E-09	1.48905E-07
3.184932534	0.750528665	0.703646367	1.066627642	0.286140013	0.500570307
1303.753586	0.528796065	0.115991843	4.558907352	5.14204E-06	0.000136409
2483.285696	0.087442753	0.091461142	0.956064529	0.339039634	0.554005032
194.8929992	-0.171057647	0.108894084	-1.570862631	0.11621456	0.283380501
1.792411042	0.408740267	0.737542253	0.55419234	0.57944722	0.757183589
24.44719904	-0.434381829	0.337966427	-1.285281006	0.198694105	0.400549032
3.835694439	-0.944475702	0.698008968	-1.353099666	0.176023792	0.370678251
458.2741127	-0.032220829	0.060321797	-0.534149026	0.593238433	0.765849851
682.9103738	-0.122226761	0.100839639	-1.212090422	0.22547777	0.430918884
202.6272767	0.172172251	0.087098331	1.976757162	0.048069086	0.158030358
153.6139586	-0.097287481	0.128155251	-0.759137689	0.447770195	0.65285976
92.5774264	-0.10015251	0.158136593	-0.633329123	0.526518739	0.717000092
733.3622752	0.12181496	0.072717676	1.675176748	0.093899558	0.246091419
59.03971852	-0.232649202	0.258717506	-0.899240278	0.368524691	0.582134026
1.81257907	-1.137898074	0.93057344	-1.222792339	0.22140814	0.426155056
282.390112	-0.344618832	0.113922198	-3.025036726	0.002486029	0.01900457
628.475932	-0.394961794	0.131862098	-2.995263983	0.002742074	0.020449493
129.6880866	0.150809932	0.193721246	0.778489378	0.436280566	0.642641632
249.3992719	0.146659939	0.07990736	1.835374613	0.066450203	0.196853199
32.25992042	-0.230645342	0.165583179	-1.392927372	0.163641754	0.353206828
865.0604306	0.241878197	0.093656162	2.582619139	0.00980535	0.051856609
235.1361464	-0.039313044	0.087093828	-0.451387259	0.651710465	0.807299661
18.90205548	0.096348166	0.261545993	0.368379437	0.712590328	0.846557515
334.4787854	-0.230683894	0.084071088	-2.74391469	0.006071131	0.036369388
476.4058933	-0.292893428	0.059341325	-4.935741313	7.98469E-07	2.87833E-05
560.8496398	-0.291715517	0.063250041	-4.612100066	3.98621E-06	0.000110368
2.020541917	0.420068913	0.700496211	0.599673355	0.548723949	0.735270927
1628.016296	-0.027687303	0.077275175	-0.358294929	0.720122613	0.851605781
296.7068955	-0.355065887	0.0639347	-5.553570869	2.79892E-08	1.47314E-06
716.4295028	0.099513303	0.051052717	1.949226389	0.051268395	0.164638506
320.1161871	0.221956567	0.26536377	0.836423779	0.40291654	0.614255698

1.900028657	0.381447546	1.176771968	0.324147377	0.74582646	0.866789778
551.3869924	-0.006973649	0.061438961	-0.11350532	0.909629945	0.954595727
1844.434848	0.072585687	0.086443111	0.839693142	0.40108046	0.612787084
1904.641327	0.147662936	0.071439044	2.066978032	0.038736224	0.135745704
695.2755935	-0.139882145	0.066076665	-2.116967373	0.034262617	0.124448658
498.9773211	-0.008477747	0.068020543	-0.124635102	0.900812437	0.950488958
57.5246557	-0.150206752	0.1959102	-0.766712257	0.443252615	0.648675808
126.5800667	-0.242568694	0.115820487	-2.094350489	0.03622877	0.129643844
2316.629699	0.0235019	0.091182123	0.257746798	0.796602326	0.894928172
1.98269725	0.499828449	0.686912815	0.727644671	0.466831128	0.668629456
102.4102112	0.014918214	0.127907264	0.116633045	0.907150851	0.953303217
362.5218912	0.35736995	0.130634224	2.735653338	0.006225657	0.03710962
4.971725719	-0.031600275	0.491512856	-0.064291858	0.948737836	0.97574648
146.1251974	0.575044411	0.229462473	2.506049913	0.012208838	0.060708845
336.900082	-0.116770364	0.164921199	-0.708037323	0.47892208	0.678280807
260.0695887	0.079491701	0.094771951	0.838768226	0.401599384	0.613212369
244.3373331	-0.083695473	0.101828443	-0.821926273	0.411118859	0.621111275
140.9928463	-0.108766851	0.17410941	-0.624704036	0.532165324	0.721614252
134.1779218	-0.040960228	0.110768602	-0.36978194	0.711544973	0.846071015
325.4223002	-0.436865236	0.114136664	-3.827562673	0.000129418	0.00191043
31.85574579	-0.19973019	0.251881412	-0.792953275	0.427805046	0.634941316
16.03344171	-0.393163792	0.310123765	-1.267764153	0.204882187	0.408030604
39.00321529	-0.305502258	0.230288375	-1.326607378	0.184638594	0.382367844
6.670011169	1.513846163	1.160387388	1.304604117	0.192027686	0.392732973
5.059927741	-0.313111834	0.448565321	-0.698029516	0.485158735	0.684040054
8.718713647	-0.049419176	0.377236774	-0.131003072	0.895772877	0.947455865
2125.528652	0.221759437	0.109332659	2.028300043	0.042529635	0.145172764
10.26023023	-0.327283749	0.325610835	-1.005137772	0.314830514	0.529679101
3077.646076	-0.295205821	0.080134078	-3.683898634	0.000229694	0.003054054
30.74336777	-0.273584733	0.250788438	-1.090898507	0.275317544	0.488011752
186.8409629	-0.046442464	0.12440844	-0.373306374	0.708920433	0.844935557
614.7521949	-0.15587502	0.079010231	-1.972846025	0.048513108	0.15882451
402.0183206	0.095184134	0.054529434	1.745555129	0.080888285	0.223920956
135.726715	0.191999889	0.120950645	1.587423445	0.112416773	0.27765426
5.501843549	-0.633012225	0.486264211	-1.301786583	0.192989352	0.393575957
759.5858019	0.126210907	0.077053647	1.637961489	0.101429728	0.259464683
9.310512103	0.409689272	0.33448101	1.224850618	0.220631509	0.425295624
555.7028047	0.351754489	0.076049646	4.625327093	3.74008E-06	0.000104254
7.366895558	1.643231526	0.792555971	2.073331836	0.038141408	0.134490915
2.443691697	3.521470711	2.091281642	1.683881616	0.09220455	0.243153154
15147.25281	-0.587512967	0.117059764	-5.018914666	5.19642E-07	1.96795E-05
114.9302483	-0.151193692	0.144505148	-1.046285849	0.295429077	0.510173138
7.060059056	-0.605027545	0.566999053	-1.067069762	0.285940335	0.5003804
45.95303846	0.325216001	0.292251682	1.112794281	0.265796792	0.477506691
33277.64905	-0.005649111	0.19129809	-0.02953041	0.976441566	0.988137991
3797.690469	0.154181497	0.067725373	2.276569181	0.022811965	0.094217024
297.9265773	-0.105486587	0.059016912	-1.787395928	0.073873519	0.211320083
248.1854663	0.181656508	0.093915861	1.934247371	0.053082721	0.168494169

511.1902638	0.33350131	0.055061763	6.056858599	1.38805E-09	1.12356E-07
866.7656101	-0.086669201	0.108864624	-0.79611905	0.425962843	0.633445737
12.78211878	0.402590518	0.3143039	1.280895714	0.200230299	0.402660393
254.0898098	-0.172920378	0.105770834	-1.634858784	0.102078654	0.26080083
2.759941422	-1.46005256	0.762077817	-1.915883821	0.055379885	0.172887669
332.6580468	-0.043172786	0.111572544	-0.386948117	0.698794618	0.838453107
43.09234918	0.525938518	0.317730042	1.655299934	0.097863672	0.252974445
56.92552609	0.696822019	0.18637827	3.738751403	0.000184936	0.002565279
5.267170879	0.787286322	0.463001807	1.700395787	0.089056504	0.238206989
126.2903609	0.081061692	0.125072978	0.648115149	0.516910476	0.709630747
39.07375096	-0.209713041	0.226264858	-0.926847602	0.354005666	0.568833517
447.6587987	-0.171875296	0.069311631	-2.479746799	0.013147571	0.064042898
243.2836152	0.066346627	0.123037236	0.539240232	0.589721103	0.763222991
5.642775761	0.241266765	0.500455388	0.482094451	0.629738852	0.7925982
267.4044269	0.0934977	0.07181276	1.301965005	0.192928349	0.393500262
2.200729318	0.671073045	0.781461449	0.858741075	0.390483383	0.60400683
3.98372016	-1.936453669	1.082033784	-1.789642521	0.073511398	0.210686634
9.864942783	2.344672603	1.243147228	1.886077972	0.059284448	0.182056232
3.857888337	2.130047653	1.198553854	1.777181431	0.075538394	0.214268255
294.9311409	0.066218186	0.102982866	0.643001979	0.520222816	0.712399615
337.6551041	0.009393843	0.073768688	0.12734187	0.898669823	0.948864276
136.2214059	0.111463934	0.112568825	0.990184759	0.32208382	0.537248298
46.84176395	-0.214317123	0.157579279	-1.360059036	0.173811243	0.367476343
81.03209612	0.329273186	0.15249069	2.159300252	0.030826881	0.11615783
226.732547	-0.290795099	0.103505115	-2.809475629	0.004962228	0.031308976
24.26346802	0.062509742	0.241653235	0.258675379	0.795885718	0.894773586
59.78260762	-0.022777626	0.14498224	-0.157106319	0.875161058	0.937516709
74.2731466	-0.057734241	0.153058653	-0.377203382	0.706022467	0.842949182
263.9683691	-0.001117967	0.086845535	-0.012873055	0.989729072	0.994530438
1102.860735	-0.016910321	0.047140319	-0.358723082	0.719802259	0.851441259
104.2516103	0.339589336	0.112881047	3.00838222	0.002626426	0.01978406
3.730389732	-2.043289195	0.750698722	-2.721849838	0.006491763	0.038290479
3.401525741	-2.816052849	1.303555647	-2.160285873	0.030750546	0.115960789
251.6062649	-0.036984135	0.104356825	-0.354400728	0.723038589	0.85299236
1437.28148	-0.16410738	0.108853833	-1.507593953	0.131658481	0.305915631
145.1800874	-0.084522854	0.08647455	-0.977430401	0.328356119	0.543715156
297.6470647	-0.086715192	0.078453682	-1.105304309	0.269027751	0.480794443
77.44259993	0.40584449	0.12992296	3.123731863	0.001785731	0.014857643
25.69771786	-0.551842225	0.249333638	-2.213268256	0.026879157	0.105833468
23.5368218	-2.876318002	1.134847628	-2.534541142	0.01125947	0.057196582
506.2204312	0.03405065	0.053889166	0.631864474	0.527475439	0.717558125
191.0966367	0.037383336	0.097429102	0.383697843	0.701202411	0.839689505
91.46856668	0.244259513	0.314881362	0.775719183	0.437914811	0.643838339
21.93306607	-0.330290607	0.418478499	-0.789265418	0.429956894	0.637008662
58.80114082	-0.118457114	0.280314347	-0.422586699	0.672596833	0.822263819
1.967530873	-0.405898605	0.955845748	-0.424648649	0.671092823	0.821214982
48.99263714	0.324002089	0.163689473	1.979370355	0.047774324	0.15721818
5.650847629	1.543306475	0.669665126	2.304594363	0.021189302	0.089620683

1102.936767	0.038679972	0.199717682	0.193673245	0.846431743	0.923940931
624.2938719	0.027292327	0.090053012	0.303069561	0.761836851	0.875827829
366.5381534	-0.168419562	0.102190931	-1.648087176	0.099334784	0.255534232
134.0205839	-0.752520618	0.197221075	-3.815619686	0.000135842	0.001987437
126.2115287	0.433653966	0.392047651	1.106125659	0.268672134	0.480491998
546.5274061	0.062876506	0.0786706	0.799237649	0.424152625	0.631666096
331.8626225	-0.229570031	0.101103055	-2.27065376	0.023167946	0.095037039
78.68188642	-0.287674438	0.121630331	-2.365153799	0.018022585	0.079749171
184.3052159	-0.056317831	0.094311156	-0.5971492	0.550407772	0.736546229
149.8951942	0.071487666	0.082348666	0.868109581	0.385334347	0.598528948
51.56570883	-0.305084584	0.345301068	-0.883532118	0.376948837	0.589841864
1371.189437	0.040085401	0.112469712	0.356410631	0.721533069	0.852264701
2.422880296	-0.709392104	0.746216223	-0.950652213	0.341780954	0.556263529
416.0363209	-0.265849037	0.098475173	-2.699655449	0.006941132	0.040108248
57.89907101	-0.292720438	0.127751569	-2.291325581	0.02194459	0.091755121
12.27741321	-2.032477761	0.800941507	-2.537610729	0.011161205	0.056820055
412.885739	0.394486702	0.101279258	3.895039419	9.81828E-05	0.001543082
544.4451691	0.030664729	0.057922803	0.529406858	0.596523242	0.768464489
12.95166943	0.441524548	0.289531215	1.524963547	0.12726819	0.298663272
53.97476537	-0.751042709	0.381884576	-1.966674633	0.049220733	0.160312843
41.13480735	-0.384761033	0.233024288	-1.651162788	0.098705339	0.254471324
1450.694959	-0.006446037	0.208644918	-0.030894771	0.97535346	0.987719156
2.723750879	-0.950711636	0.644737467	-1.47457172	0.140327723	0.319168654
421.8689035	-0.216446444	0.076847743	-2.816562152	0.004854065	0.030741834
317.1945346	-0.41970747	0.128045607	-3.277796716	0.001046207	0.009968315
181.7900458	-0.251877338	0.114259326	-2.204435704	0.027493717	0.107457057
3.296800347	-0.883023494	0.532477881	-1.658328965	0.097251086	0.251825588
941.3150327	-0.303608783	0.091665255	-3.312146804	0.000925829	0.009077896
685.0482118	0.082184873	0.085726881	0.958682647	0.337718643	0.553111642
1204.229539	-0.003258753	0.052032643	-0.062629021	0.950061919	0.976194103
300.0003449	0.240977418	0.200418576	1.202370677	0.229219945	0.4352933
4.774298724	-0.331753685	0.470142797	-0.705644513	0.480409235	0.679394088
798.1678299	0.100621337	0.071351594	1.410218504	0.158475174	0.345607626
50.52914499	0.090928136	0.147464733	0.616609368	0.537492414	0.725612556
147.5092912	-0.52742103	0.10907386	-4.835448459	1.32846E-06	4.41826E-05
872.248294	0.090139282	0.087463045	1.030598492	0.302729143	0.517785171
78.50006436	-0.01260454	0.200914395	-0.062735872	0.949976832	0.976186239
1408.010892	-0.739018511	0.202998559	-3.640511114	0.000272097	0.003491068
25.40781182	-0.268685328	0.331192719	-0.811265805	0.417213043	0.626542175
3.089473798	0.911474038	0.688000978	1.324815032	0.185232508	0.383212399
453.8435771	-0.10386425	0.082406608	-1.26038739	0.207529648	0.411017483
755.6432317	-0.539993756	0.205370789	-2.629360088	0.008554573	0.047117361
369.5550133	-1.151450027	0.267477563	-4.304847164	1.67101E-05	0.000368517
159.9622071	-0.108885702	0.10987239	-0.991019696	0.321675963	0.536800021
5818.049685	-0.087106397	0.167325348	-0.520581	0.602658688	0.773164024
4.641994465	0.763210046	0.462801679	1.649108203	0.099125469	0.255075442
25.21394971	-0.775377391	0.322606319	-2.403478618	0.016239917	0.074171441
166.0020642	0.135872083	0.111453796	1.219088876	0.222810461	0.427806473

88.86663928	-0.438828204	0.117526319	-3.733871776	0.000188559	0.002602623
220.6390279	0.092748863	0.1267548	0.731718743	0.464340254	0.666568608
17.51499948	-0.175444013	0.410501169	-0.427389802	0.669095434	0.819834748
478.4431103	0.026230151	0.195498851	0.13417036	0.893267863	0.946108145
638.9965783	-0.283610695	0.12113747	-2.341230141	0.019220315	0.083567029
187.3818183	-0.152893247	0.144488023	-1.058172465	0.289976822	0.504923176
56.63679557	0.11249258	0.164036665	0.685777052	0.492853709	0.690017157
229.2223494	0.151162751	0.124042556	1.218636221	0.222982295	0.428036626
2.261473369	-4.641262305	1.471534115	-3.1540297	0.001610327	0.013847897
9.603571946	-5.624586602	1.267181716	-4.438658269	9.05214E-06	0.000219301
19.46382395	-0.057895243	0.226358395	-0.255768038	0.798129955	0.895680707
109.6485838	-0.316939284	0.102937518	-3.078948188	0.002077328	0.016636802
359.2403931	-0.065117608	0.071740164	-0.907686911	0.364043661	0.577837465
216.2296484	-0.055507302	0.095076985	-0.583814287	0.559345265	0.743417015
146.0787913	-0.391796304	0.099719563	-3.928981351	8.53065E-05	0.00138457
488.9642716	-0.115751513	0.08778957	-1.318510997	0.187332646	0.386102591
2.026333156	-0.083061104	0.739839484	-0.112269088	0.910610051	0.955037829
7.205965851	0.195162114	0.420872884	0.463707979	0.64285698	0.801477463
16.68396969	-0.017560051	0.330105277	-0.053195307	0.957576295	0.979090857
50.89933294	0.21317429	0.188462068	1.131125706	0.258002186	0.468458946
826.0628664	-0.242855627	0.13643642	-1.779991197	0.075077401	0.213356065
93.20698817	0.289612251	0.115033829	2.517626811	0.011814843	0.059286544
2.095816455	0.221500642	0.694107333	0.319115837	0.749638675	0.869013267
260.014566	0.701749999	0.1419775	4.942684583	7.70541E-07	2.79601E-05
12.51025437	0.009316577	0.329239841	0.028297236	0.977425085	0.988278901
4.269748225	-3.1492018	1.337036986	-2.355358777	0.018504829	0.081163088
69.93057146	0.068947942	0.335055631	0.205780579	0.836962312	0.918535642
78.60546476	-1.405943139	0.484442529	-2.902187676	0.003705665	0.025288782
6.509956273	2.198577883	0.530063957	4.147759633	3.35745E-05	0.000653789
4.069229966	0.427988129	0.866414764	0.493976034	0.621323105	0.786633633
170.8787217	0.641284189	0.358441628	1.7890896	0.073600387	0.210898484
34.17964183	0.183502424	0.292312335	0.627761479	0.530160204	0.719902671
1269.782465	-0.30278723	0.078681218	-3.848278392	0.000118951	0.001786323
247.6252525	-0.174665017	0.069980481	-2.495910502	0.01256343	0.062001557
3.969419409	0.494338036	0.496202835	0.996241862	0.319132644	0.534070902
2.417062668	0.173068261	0.627001538	0.276025258	0.782528666	0.887032618
4.568644747	-0.092495556	0.459580978	-0.201260628	0.840494787	0.920938551
2.956512928	0.900862201	0.790083274	1.14021171	0.25419811	0.463493045
222.4901005	-1.990244555	0.646172937	-3.080049383	0.002069663	0.016599622
44.45859174	1.111850572	0.327444797	3.395535923	0.000684944	0.007177968
28.24500941	0.398022077	0.361041484	1.102427545	0.270275842	0.482292485
217.4377869	-0.112200966	0.079473326	-1.411806589	0.158006918	0.344951758
92.46487124	-0.488038585	0.127394716	-3.830917025	0.000127667	0.001898176
2.802518221	0.522081458	0.75887083	0.68797144	0.491470767	0.689139271
251.1061427	-0.135890961	0.080823802	-1.681323545	0.092700087	0.244186318
144.3739574	-0.278959179	0.193458304	-1.441960224	0.149313595	0.332359433
704.6935881	0.228074869	0.094606148	2.410782747	0.015918327	0.073152594
91.58568759	0.530806025	0.170275818	3.117330637	0.001824968	0.015120099

66.25338504	-1.547363864	0.227905522	-6.789497017	1.12525E-11	1.61195E-09
683.6129218	-0.008250909	0.123850031	-0.066620163	0.946884094	0.974828397
11.17657969	-0.018187913	0.372966453	-0.048765547	0.961106139	0.98086139
305.9758692	0.112222691	0.05778213	1.942169514	0.052116582	0.166175718
222.0152045	-0.197968181	0.069024721	-2.868076521	0.004129756	0.027383261
321.9750204	-0.171284214	0.204168979	-0.838933589	0.401506578	0.61312749
42.03296343	-0.368925962	0.241097864	-1.530191749	0.125969273	0.297223977
2027.355059	-0.371784988	0.103048331	-3.607870058	0.000308721	0.003812496
4093.210736	-0.073328442	0.04762225	-1.539793744	0.123610637	0.293804883
373.6461535	-0.089819846	0.078162363	-1.149144449	0.250496423	0.459489819
40.29265861	0.63503847	0.201631216	3.149504744	0.001635475	0.013945553
137.9108884	0.294778597	0.134438539	2.192664394	0.028331568	0.109587755
149.0774091	0.199697578	0.109938161	1.816453686	0.069300806	0.202422249
330.9379649	-0.225548285	0.113802876	-1.981920771	0.047488109	0.156651803
20.6491719	0.120652077	0.307252402	0.392680664	0.694555354	0.835617096
205.084102	-0.718388205	0.17965342	-3.998744943	6.36793E-05	0.001096188
91.00538101	0.130864165	0.138503805	0.94484166	0.34473972	0.559145628
12.11282311	-0.722799806	0.394229473	-1.833449439	0.066735761	0.197308853
407.2880626	0.271802441	0.127745149	2.127692858	0.033362557	0.122299682
513.3903925	-0.183041651	0.057582289	-3.178783839	0.001478943	0.013021974
119.6858759	0.063577097	0.090939709	0.699112609	0.484481659	0.683641425
1.73570291	0.248672782	0.739331636	0.336348087	0.736608392	0.861476895
672.4451491	-0.686242984	0.202596759	-3.387235743	0.000706007	0.007333391
1370.07933	-0.104583036	0.061026057	-1.713743963	0.086575759	0.233888005
1308.902115	-0.142666362	0.067002244	-2.129277389	0.033231317	0.122070771
131.6477422	0.065268367	0.16767885	0.389246272	0.697093977	0.837609287
24.81807895	-0.443371731	0.379730571	-1.167595565	0.24296994	0.450709544
1367.353311	-0.084256418	0.13414462	-0.628101357	0.529937544	0.71971897
450.2763746	-0.220519039	0.060716868	-3.631923791	0.000281316	0.003581455
38.7540797	-0.090188613	0.166889085	-0.540410492	0.588913973	0.76241798
1473.007297	-0.245475782	0.074150156	-3.310522815	0.000931219	0.009098989
278.7615894	0.044190859	0.102689946	0.430332872	0.666953518	0.818332001
27.70583972	-0.556884537	0.286476695	-1.943908691	0.051906463	0.165931889
39.11340116	0.322126718	0.185923014	1.732581194	0.083170134	0.228243344
43.31959208	0.070662509	0.180082106	0.392390511	0.694769696	0.835813931
587.2626135	-0.122347939	0.084871781	-1.441562057	0.149425959	0.332474776
18.6139418	0.268971867	0.260308254	1.033282131	0.301471887	0.516667388
73.40098943	-0.021837508	0.139923983	-0.156066942	0.875980255	0.937672708
5.373398079	0.109776272	0.414225154	0.265015948	0.790997176	0.892098821
444.0958627	0.215986653	0.084417615	2.558549562	0.010510984	0.054336482
14.97300369	-0.048101252	0.289952157	-0.165893755	0.868240563	0.934495951
339.1258883	0.134762078	0.083424521	1.615377316	0.10622901	0.267537975
1545.813438	-0.09398436	0.112223055	-0.837478187	0.402323833	0.613826573
47.51487259	-0.502938861	0.287036639	-1.752176529	0.079743458	0.221645644
4.207326336	0.681111877	0.476623444	1.429035618	0.152993996	0.337409617
466.7952381	0.045796769	0.131240884	0.348952002	0.727125344	0.85562812
67.82449618	-0.171862121	0.141409811	-1.215347929	0.224233419	0.429237897
33.09240829	0.029813844	0.20713293	0.143935798	0.885551167	0.942840427

2.171311135	-1.095922768	1.224813713	-0.894766899	0.370911708	0.584428073
1570.458364	0.156289571	0.052309877	2.987764046	0.002810264	0.020800051
210.2731654	-0.076638683	0.113206091	-0.676983744	0.498416268	0.694742406
116.6984449	0.125331683	0.155920154	0.803819643	0.421501144	0.629709789
55.58396963	0.150997128	0.209761735	0.719850682	0.471616936	0.671920558
3.677041705	0.503739157	0.544086285	0.925844248	0.354526929	0.569182771
243.5560246	-0.309640005	0.100299461	-3.087155233	0.00202082	0.016331508
487.4156556	0.144976056	0.057363913	2.527304172	0.011494191	0.058191547
12.64070407	-0.20107146	0.311262975	-0.645985794	0.518288555	0.710753822
154.5738145	-0.183924169	0.116430743	-1.57968733	0.114178489	0.280407934
58.14560376	1.541848877	0.170004563	9.069455849	1.19613E-19	9.85251E-17
124.1582782	0.131031649	0.146500545	0.894410655	0.371102214	0.584580022
32.83607087	-0.148926906	0.191432266	-0.777961362	0.436591792	0.642902204
16.19088836	-0.307217965	0.237332876	-1.294460212	0.195506504	0.396559965
14591.80515	0.048505252	0.106254402	0.456501103	0.648029678	0.804858331
57.68008082	0.22095393	0.157430173	1.403504329	0.160466502	0.348612047
193.3034199	0.018166498	0.169810425	0.10698105	0.914804013	0.957707252
114.3094118	0.106893674	0.156191444	0.684375987	0.493737775	0.690712985
2531.315265	-0.106158989	0.069316859	-1.531503165	0.125645085	0.296714038
57.34403678	-0.118114017	0.149816044	-0.788393646	0.430466486	0.637438642
27.44569126	0.118081156	0.187307033	0.630414964	0.528423123	0.71849146
417.9175229	0.908904873	0.328707481	2.76508728	0.005690757	0.034750019
17.97158267	-0.755365477	0.605252371	-1.248017379	0.212024693	0.41657975
174.5374817	-0.098233942	0.113667707	-0.864220324	0.387466869	0.600595522
98.24667296	-0.52497761	0.310269358	-1.692005982	0.090644833	0.24095254
1312.761968	0.135489182	0.190189867	0.712389069	0.476223882	0.676261722
47.82081661	0.702975144	0.2120236	3.315551395	0.000914625	0.008995544
10.89547373	0.268564908	0.319876661	0.839588943	0.401138901	0.612793235
403.5990163	0.111921802	0.072915934	1.534942988	0.124797833	0.295593801
144.7244724	-0.09990482	0.121924214	-0.819400972	0.412557681	0.622273872
338.1648916	-0.014074381	0.091611153	-0.153631746	0.877900104	0.938489926
13.98549591	-0.541259009	0.332827889	-1.626242951	0.103897982	0.263653388
6788.751606	-0.022217937	0.122809117	-0.18091439	0.856434774	0.92936531
1294.340609	-0.086724757	0.120908253	-0.717277396	0.473202952	0.673481247
4399.572453	-0.221648186	0.076937805	-2.880874825	0.003965731	0.026492884
8.212137356	-0.149350118	0.547260991	-0.27290474	0.784926443	0.888536949
238.2843678	-0.507370248	0.248906428	-2.038397528	0.041510193	0.142585264
50.30518905	0.238900354	0.15225573	1.56907299	0.116630932	0.283975463
369.2214738	-0.144933317	0.082689029	-1.752751478	0.079644675	0.221563656
9.117105745	-0.194924445	0.41025278	-0.475132539	0.634692507	0.795202438
124.9273841	-0.311882562	0.15959969	-1.954155189	0.050682867	0.163523218
341.0146688	0.06249132	0.085321325	0.732423222	0.463910289	0.666183586
728.872178	-0.027186201	0.076168263	-0.356922946	0.721149491	0.852054542
54.93181293	-0.051916593	0.135155823	-0.384123986	0.700886554	0.839555377
217.0913375	0.076041489	0.072871021	1.04350794	0.296713106	0.511392034
775.1056348	0.05548586	0.048526489	1.143413873	0.252866795	0.461985981
102.4859909	-0.050602865	0.095249943	-0.531263987	0.595235857	0.767361863
19.01637195	-0.051579684	0.300862012	-0.17143967	0.863878064	0.932132697

93.04598718	-0.284511338	0.118802164	-2.394832951	0.016627939	0.075472996
140.5431595	-0.074105347	0.102378815	-0.723834778	0.469167175	0.670434608
7.403755112	0.513632035	0.457657089	1.122307614	0.26173167	0.473092773
10.78037186	-0.027493413	0.61484092	-0.044716303	0.964333439	0.982345867
98.43013143	-0.261536489	0.131800995	-1.984328642	0.047219215	0.155981164
223.1703782	-0.388654864	0.07810915	-4.975791749	6.49815E-07	2.40024E-05
728.5945826	-0.292993418	0.109150196	-2.684314182	0.007267877	0.041558837
6.521610596	0.628327177	0.400126173	1.57032261	0.116340077	0.283560648
297.1582004	-0.326521549	0.162761867	-2.006130514	0.044842325	0.150605576
1185.403112	-0.041845136	0.085794445	-0.487737127	0.625736051	0.790458991
146.7381315	-0.494631603	0.140414216	-3.522660442	0.000427238	0.004938991
21.67658931	-1.038077148	0.302594026	-3.430593666	0.000602262	0.006465726
2665.800568	-0.198101165	0.099204556	-1.996895838	0.045836499	0.153166428
234.4795454	-0.097751147	0.146620854	-0.666693342	0.504968032	0.700298288
52.86212655	-0.429665681	0.139359174	-3.083153181	0.002048197	0.016477006
609.554549	0.034433357	0.131051983	0.262745793	0.792746519	0.893093493
132.5661488	0.076703904	0.095121402	0.806379031	0.420024334	0.628801209
22.98309093	0.143834221	0.276009027	0.521121438	0.602282177	0.77305874
430.5860372	0.013931493	0.060982451	0.228450857	0.819295756	0.909322798
517.2639732	-0.137362704	0.116812202	-1.175927703	0.239623768	0.447113144
109.5948031	-0.090600849	0.114800659	-0.789201474	0.429994261	0.637008662
181.0205713	-0.180604682	0.107119578	-1.686010019	0.09179387	0.242536042
103.4956156	0.015906363	0.097041236	0.163913437	0.869799289	0.935257064
305.98931	-0.112773585	0.100644414	-1.120515091	0.262494325	0.473847416
2969.739318	-0.062367874	0.076481136	-0.815467414	0.414804809	0.624576769
4.539469536	-1.055686005	0.500098081	-2.110957919	0.034775929	0.125801198
1270.862205	-0.148228881	0.060534073	-2.448685051	0.014337876	0.068050177
2.078957079	-0.314183296	0.655904552	-0.479007647	0.631933188	0.793934606
1597.469308	-0.334322266	0.101978546	-3.278358824	0.001044126	0.009954241
2.047162263	0.161202997	0.729362758	0.221018959	0.825077671	0.912223049
29.47082489	-0.111294314	0.181062963	-0.614671892	0.538771426	0.726565753
1764.746788	-0.144170697	0.082916364	-1.738748414	0.082079026	0.226266713
166.9868044	-0.40425015	0.236522635	-1.709139379	0.087425135	0.235372066
1162.374943	-0.256932385	0.079107353	-3.247895125	0.001162621	0.01085157
2.098847284	0.540564646	0.762717297	0.708735266	0.478488776	0.677925477
15.28489616	1.065187716	0.414914314	2.567247453	0.010250943	0.05336193
68.33141589	0.149676233	0.187722044	0.797329015	0.425259974	0.632742632
93.47489186	0.090216941	0.258698517	0.348733895	0.727289095	0.85562812
10.65993104	-0.014980037	0.343867051	-0.043563454	0.965252383	0.98260939
8.272237169	0.413388716	0.343503853	1.203447101	0.22880335	0.434802906
229.208855	-0.223059448	0.092384565	-2.414466612	0.015758267	0.072720549
7.287759179	-1.519390783	0.726093418	-2.092555509	0.036388852	0.130027973
69.00860799	-0.465436691	0.359181752	-1.295824992	0.195035786	0.395977832
4.669986852	-2.265484161	0.98953123	-2.289451906	0.022053109	0.091998714
7.700336053	0.098095501	0.591161312	0.165936943	0.868206574	0.934495951
156.7608232	0.476953245	0.278880196	1.710244226	0.087220721	0.234898504
1045.720859	0.049170088	0.107813282	0.456067073	0.648341747	0.805166538
10.46625855	-0.624758168	0.344766866	-1.81211778	0.069968	0.203576974

434.1088784	0.350050283	0.099301307	3.525132671	0.000423271	0.004907081
70.42760328	0.34393784	0.144418091	2.381542636	0.017240295	0.077430921
453.549948	0.284401523	0.079773528	3.565111512	0.000363702	0.004357392
14.463248	1.878954818	0.646837985	2.904830669	0.003674518	0.025161434
252.1252045	-0.005977528	0.066724972	-0.08958457	0.928617347	0.965637301
1679.724534	-0.235956832	0.102298742	-2.306546765	0.0210801	0.089276331
343.3124721	-0.079055977	0.064504672	-1.225585293	0.220354775	0.424860747
17.06106666	-0.39342762	0.417250248	-0.942905657	0.345729161	0.560133927
2.299349687	-0.14521715	0.596053007	-0.243631269	0.807516409	0.901900022
45.23884495	0.20262329	0.161209337	1.256895497	0.208791479	0.412821751
770.5392244	0.059641747	0.055433335	1.075918433	0.281963737	0.495844429
6.821069134	0.246924677	0.418348753	0.590236437	0.555032147	0.739858687
122.6270121	-0.135127853	0.129511214	-1.043367972	0.296777901	0.511392034
2189.132574	0.002868413	0.060608824	0.047326659	0.962252881	0.981463149
284.1044282	-0.150939379	0.076004029	-1.985939176	0.047040078	0.15561009
167.4780815	-0.218692506	0.128260514	-1.705064952	0.088182311	0.23685232
562.1181335	-0.066431804	0.055043536	-1.206895646	0.227472335	0.433254357
18.05265313	-0.134263101	0.220895525	-0.607812678	0.54331172	0.730508517
24.38629268	-0.268837136	0.207568559	-1.295172723	0.195260652	0.396273441
123.5075557	-0.007988409	0.159539596	-0.05007164	0.960065299	0.9804928
1695.068757	0.056559538	0.050521539	1.119513377	0.262921187	0.474358081
9.880489126	-0.183265086	0.379675871	-0.482688263	0.629317098	0.79225209
1112.912654	-0.468148638	0.074280563	-6.302437925	2.93E-10	2.82274E-08
369.5882276	-0.006759676	0.112243831	-0.060223141	0.951977915	0.977124952
14.89457018	-0.868956848	0.263760392	-3.294493312	0.000985993	0.009549234
50.21878653	0.194299881	0.163646316	1.187315951	0.235103019	0.442385738
104.5447014	-0.142190121	0.110604231	-1.285575783	0.198591153	0.400439493
35.42693955	-0.344630831	0.217338261	-1.585688731	0.112809937	0.278291539
23.07412765	-0.579155949	0.227175584	-2.549375857	0.010791592	0.055314466
14.35166937	-0.021359002	0.333707319	-0.064005196	0.948966089	0.975859386
548.9143015	-0.218326852	0.070038537	-3.117238884	0.001825536	0.015120099
719.5335076	-0.10051174	0.053745458	-1.870143886	0.06146384	0.18660061
218.6231157	-0.086081014	0.074388049	-1.157188747	0.247195254	0.455777809
65.72909412	-0.426930128	0.291864879	-1.462766363	0.14353132	0.323621034
29.78176216	0.165373512	0.228155231	0.72482893	0.468556986	0.670018398
48.33313752	0.079447422	0.220646243	0.36006696	0.71879706	0.850740913
2320.409801	-0.291532191	0.061512772	-4.73937661	2.14377E-06	6.57662E-05
11.91786301	-0.808322689	0.285442087	-2.831827281	0.004628284	0.029714089
6.645821321	-1.521719188	0.587653963	-2.589481708	0.009612053	0.051295419
1.806186781	-0.186239608	0.789952301	-0.235760575	0.813618463	0.906196373
35.8869004	-0.279018158	0.244399911	-1.141645906	0.253601234	0.462865802
502.0206365	0.240258864	0.065213267	3.684202247	0.00022942	0.00305288
866.8698726	0.190436511	0.065751477	2.89630773	0.003775819	0.025576829
184.8656105	-0.024414132	0.090166327	-0.270767734	0.786569678	0.889541352
108.7910001	-0.212664401	0.193844476	-1.097087753	0.272603033	0.484831426
5.149911541	0.06404975	0.49158346	0.130292727	0.896334834	0.947804865
61.41552372	0.496356464	0.152369762	3.257578523	0.001123672	0.010580556
508.2938181	-0.183997195	0.06858779	-2.682652352	0.007304087	0.041707985

48.73043487	-0.150615999	0.148833895	-1.011973783	0.311550589	0.526390425
3434.288493	0.226693928	0.078262292	2.896592004	0.0037724	0.025564179
3.376560701	0.483942123	0.518116554	0.934041037	0.350282725	0.565352955
735.2282535	0.011019536	0.091355603	0.120622444	0.903990091	0.951647566
624.6611546	0.149954171	0.042660775	3.515036233	0.000439694	0.005051268
289.2462648	-0.023385967	0.262587786	-0.089059613	0.929034534	0.965637301
28.73292812	-0.054996283	0.256268134	-0.214604455	0.83007572	0.915395759
546.8413994	0.195196089	0.066350518	2.941892457	0.003262132	0.02312408
202.9557371	-0.01109658	0.068165356	-0.162789142	0.870684457	0.935813113
78.911594	-0.041251387	0.174731911	-0.236083875	0.813367586	0.906196373
17.90636036	-1.035060298	0.637214568	-1.624351278	0.104300859	0.264183941
2.831474252	-3.045624237	1.260080649	-2.417007388	0.0156487	0.072329253
442.4049764	0.71895207	0.124557674	5.772041555	7.83168E-09	5.05957E-07
278.4984785	0.063785792	0.084071077	0.758712673	0.448024454	0.652990786
220.3696362	0.01978907	0.109684319	0.180418408	0.856824104	0.92936531
154.244751	-0.416488663	0.10581589	-3.935974676	8.28597E-05	0.001352855
49.21882976	-2.517785352	0.976205283	-2.579155631	0.009904215	0.052128449
101.5888658	0.176202576	0.102292179	1.72254201	0.084971379	0.231336722
25.39336766	-0.170382236	0.242037123	-0.703950839	0.48146339	0.680475968
185.2750912	0.017039399	0.091209589	0.186815876	0.851804998	0.926915291
194.4474656	0.067622445	0.08244173	0.820245342	0.412076258	0.621935878
43.73938156	0.374132337	0.315729551	1.184977255	0.23602644	0.44321208
4865.110506	0.282248986	0.054716899	5.158351313	2.49134E-07	1.00594E-05
52.91221446	-0.235276866	0.143492393	-1.63964696	0.101078593	0.258727275
4.23961822	-0.050048659	0.464109912	-0.107837945	0.914124243	0.957227341
76.27720611	-0.255271424	0.127426201	-2.003288356	0.045146347	0.151382234
3.457584892	-0.503843761	0.518792568	-0.971185387	0.33145597	0.54702521
2.233108354	-0.698184399	0.764988653	-0.912672882	0.36141461	0.575371501
32.4489016	0.223413565	0.19290242	1.1581688	0.246795158	0.455397415
675.3903882	-0.042128522	0.081931351	-0.514192936	0.607117106	0.776766636
73.43536818	-0.005464798	0.187866457	-0.029088736	0.976793819	0.988158577
94.14992911	-0.515130196	0.196089448	-2.627016404	0.008613718	0.047348146
455.0895943	0.164204039	0.072159953	2.275556338	0.022872577	0.094389488
5.17493393	0.433899847	0.487286464	0.890441001	0.373229135	0.586752244
197.5035834	-0.165737599	0.095234682	-1.740307164	0.081805097	0.225738218
9.620733583	-0.042819964	0.314794912	-0.136024955	0.891801553	0.945649749
130.9732221	-0.211904465	0.261053553	-0.811727951	0.416947752	0.626371962
279.9863678	-0.194346852	0.123136732	-1.578301197	0.114496431	0.280937475
353.8541292	0.300309442	0.057608928	5.212897577	1.85914E-07	7.83309E-06
2.776232503	-0.391495469	0.696078526	-0.562430033	0.573823061	0.752760082
2.515635749	0.370206561	0.682395199	0.542510501	0.587466875	0.761728322
23.15416891	-0.09549692	0.245864038	-0.388413536	0.697710029	0.838003561
15.53361904	1.050707033	0.273963578	3.835207008	0.000125459	0.001872106
333.0163021	0.099478595	0.116938802	0.850689365	0.394941932	0.607778925
7.066477384	0.538149641	0.378816377	1.42060817	0.155430702	0.34069441
366.6696809	-0.213141684	0.073158189	-2.913435774	0.003574754	0.024754305
364.4690701	0.082302364	0.072008717	1.142950019	0.253059343	0.462187196
345.4920661	-0.203407859	0.079421958	-2.561103539	0.010434025	0.054070505

499.1587239	-0.073629628	0.151531577	-0.485902867	0.627036036	0.790677612
4.61787882	-0.98232546	0.625145425	-1.571355114	0.116100186	0.28318544
10.60369349	-0.985880543	0.623546432	-1.581086015	0.113858372	0.279797768
122.0150491	0.076805798	0.099278312	0.773641256	0.439142968	0.64478086
4.332697087	0.43703907	0.541235294	0.807484425	0.419387444	0.628262028
119.1234436	-0.190431462	0.095621619	-1.991510532	0.046424789	0.154380696
2.77886011	-1.483670891	0.833034279	-1.781044223	0.074905227	0.213239062
261.4918115	-0.023957394	0.129088148	-0.185589417	0.852766753	0.927255626
132.0579147	0.60438979	0.138880211	4.351878398	1.34976E-05	0.000306281
3267.601073	-0.277660766	0.086748408	-3.2007592	0.00137066	0.012258526
9.991544292	-0.982274423	0.546961866	-1.795873689	0.072514608	0.208851218
2.776754532	-1.559386907	0.895393428	-1.741566173	0.081584385	0.225280113
3848.655595	0.05774392	0.084558231	0.682889409	0.494676727	0.691498039
509.7505723	0.301842297	0.100534894	3.002363496	0.002678921	0.020093134
320.705151	-0.475494486	0.085447912	-5.564729131	2.6256E-08	1.4068E-06
190.4737696	-0.045335986	0.102967108	-0.440295811	0.659722874	0.812578183
428.4631377	-0.144777916	0.101126829	-1.431646955	0.152244884	0.336472474
17.16365691	1.007224898	0.37204476	2.707268063	0.006783946	0.039616703
123.0837862	-0.20815328	0.101601567	-2.048721156	0.040489389	0.140042452
2512.552612	-0.141039536	0.148319837	-0.950914856	0.3416476	0.55615638
143.2820594	0.025371239	0.08544135	0.296943327	0.766509777	0.878311336
253.3032601	0.27519564	0.072472324	3.797251508	0.000146309	0.002116154
367.6440533	-0.039508228	0.058663688	-0.673469907	0.500648388	0.696477076
89.58826826	-0.118356095	0.131427627	-0.900541977	0.367831897	0.581597338
99.08130276	-0.102262977	0.10324993	-0.990441125	0.321958553	0.537162771
20.7786623	0.300832722	0.245026966	1.22775353	0.219539505	0.424016647
12.33251161	-1.734132833	0.63450443	-2.733050789	0.006275066	0.03731965
131.0447453	0.198837347	0.097348973	2.042521259	0.041099853	0.141559477
8.260739569	-0.251974587	0.4517105	-0.55782318	0.576965133	0.755177012
422.5171985	0.002629857	0.083636701	0.031443819	0.974915596	0.987719156
4.419827536	0.19759454	0.490472097	0.402866015	0.687046793	0.830726992
103.7664768	0.024597631	0.109004785	0.225656434	0.821468645	0.910446344
68.61368662	-0.061946369	0.149777911	-0.413588151	0.679175752	0.826686346
79.53595533	0.243610167	0.158395923	1.537982564	0.124052878	0.294683272
31.05373554	0.572927164	0.261007352	2.195061399	0.028159194	0.109129315
34.43270394	-1.229561108	0.570077148	-2.15683283	0.031018692	0.116454406
364.6181874	-0.03051864	0.073785868	-0.413610918	0.679159076	0.826686346
21.30607008	-0.319414905	0.225985551	-1.413430654	0.157529138	0.344045474
57.01509456	-0.132646062	0.171351222	-0.774117983	0.438861024	0.644424326
1788.653164	0.145772639	0.090445905	1.611710769	0.107024893	0.268523925
67.02653819	0.295825894	0.210116078	1.407916505	0.159155795	0.346541445
29.46666095	0.424458271	0.247887845	1.712299657	0.08684146	0.234298429
1270.318702	0.090789744	0.071876155	1.263141359	0.206538383	0.40999076
3308.618996	0.018440444	0.062496528	0.295063488	0.767945381	0.879100285
211.0044384	0.089402542	0.096752612	0.924032342	0.355469477	0.570204885
39.37292427	-0.9657316	0.488610644	-1.976484983	0.048099875	0.158036964
107.2588875	-0.03500177	0.142264495	-0.246033069	0.805656634	0.900983462
5.541663084	0.310997587	0.445955324	0.697373862	0.485568855	0.684458057

299.1646579	-0.009726064	0.079017399	-0.123087626	0.90203771	0.950989967
412.9875554	0.210331577	0.083457223	2.520232164	0.011727745	0.058981994
209.5727998	0.185890785	0.090901271	2.04497454	0.040857368	0.140989586
13.17653097	-2.201535736	0.823269759	-2.67413653	0.007492194	0.04256083
391.7526802	0.007071166	0.112605145	0.062796122	0.949928854	0.976186239
432.796023	0.294330217	0.055669749	5.287076387	1.24287E-07	5.44548E-06
2.62139351	0.010347611	0.671086434	0.015419192	0.987697752	0.993639111
342.2939835	-0.011263224	0.064003119	-0.175979305	0.860310201	0.931052885
657.5205529	-0.12367782	0.063509425	-1.947393159	0.051487618	0.165110249
539.7120604	-0.069683542	0.05845868	-1.192013606	0.23325591	0.440116581
1210.836674	0.277860316	0.068179331	4.075433301	4.59287E-05	0.000835133
93.45251667	-0.225238039	0.111164762	-2.026164012	0.04274798	0.145562263
127.4018933	-0.231682828	0.09685718	-2.392004686	0.016756628	0.075858394
9.652599216	-0.178341687	0.313772441	-0.568379066	0.569777602	0.750099284
7.855219604	0.846601652	0.428790923	1.974392662	0.048337112	0.1585005
202.8165567	0.36762903	0.07538105	4.876942286	1.07743E-06	3.67486E-05
69.16861616	0.289493905	0.164291461	1.762075175	0.078056597	0.218596475
2.270926482	0.140318201	0.899359954	0.156020068	0.876017203	0.937672708
3.479335574	2.84071382	1.002142538	2.834640496	0.004587728	0.029565883
87.99583836	-0.003626823	0.126000453	-0.028784203	0.9770367	0.988193922
2.261452918	2.133213582	1.465115342	1.456003853	0.145391555	0.326363329
301.2850798	0.344093152	0.095806968	3.591525335	0.000328748	0.004014676
3.06831211	1.068302456	0.645554392	1.654860489	0.097952801	0.253085702
194.8906189	0.545510904	0.114736704	4.75445856	1.98979E-06	6.22007E-05
22.1499155	0.760733617	0.350887092	2.168029641	0.030156431	0.114311332
76.47726771	0.458380968	0.169414157	2.705682784	0.006816412	0.039709738
366.4055602	-0.124109927	0.191599667	-0.647756486	0.517142463	0.709772968
388.5976931	-0.207035116	0.06984581	-2.964173736	0.003034969	0.021967521
752.312486	-0.373847999	0.249036308	-1.501178689	0.13330935	0.3085764
65.10650712	0.412436321	0.124376427	3.316032865	0.000913051	0.008987652
532.2936929	-0.080652268	0.08882715	-0.90796866	0.36389478	0.577700935
105.272906	0.689264225	0.20444088	3.371459872	0.000747709	0.007679403
461.0201668	0.015481465	0.087425927	0.177080936	0.859444816	0.930378098
934.7516662	-0.189839181	0.042553682	-4.461169317	8.15137E-06	0.000201361
56.36304395	-0.135905989	0.144765338	-0.938802003	0.347832411	0.562500356
28.77193627	0.272365784	0.2156976	1.262720513	0.206689639	0.41019216
581.379584	-0.078632021	0.06498199	-1.210058673	0.22625638	0.431955916
2.146550613	0.936282311	0.961036159	0.974242543	0.329936126	0.545336384
36.54055027	0.001398918	0.429281877	0.00325874	0.997399906	0.998244565
122.5556479	-0.011355556	0.157118302	-0.07227392	0.942383919	0.972429231
11.20284087	1.983454449	0.90287502	2.196820607	0.028033261	0.108903291
3.326891591	-1.017985655	0.914688144	-1.112931945	0.265737659	0.477452524
837.9889001	-0.229655229	0.056985096	-4.03009284	5.57548E-05	0.000979216
270.0485929	-0.146266141	0.106190803	-1.377389919	0.168391728	0.359756883
45.74683158	-0.097044971	0.145261942	-0.668068801	0.504089676	0.699551286
99.34801561	-0.067508815	0.114684823	-0.588646458	0.556098462	0.740545496
101.9758359	-0.062327698	0.101206327	-0.615847837	0.537994952	0.725898306
408.6314683	-0.017766622	0.058454473	-0.303939471	0.761174006	0.875424502

1760.193644	-0.026049203	0.069163261	-0.376633528	0.706445968	0.843064305
33.61076413	0.29877043	0.161733413	1.847301838	0.064703407	0.193312282
579.5914607	0.046513631	0.043777956	1.062489795	0.288013388	0.502726484
11302.7522	0.100843596	0.119448511	0.844243224	0.398533505	0.610909181
25.81269538	0.286681737	0.296683305	0.966288741	0.333899718	0.54913287
134.3495088	-0.877613198	0.324848064	-2.701611291	0.006900438	0.039971104
298.8302558	-0.331809486	0.078016448	-4.253070893	2.10859E-05	0.000445916
229.4848876	0.684679606	0.166580685	4.110198049	3.9532E-05	0.000738379
566.7040859	-0.420227252	0.140343532	-2.994275876	0.002750971	0.020497283
2286.00021	-0.819345167	0.257027991	-3.187766299	0.001433764	0.012705153
355.4762271	0.007996432	0.086179456	0.092788151	0.926071864	0.964382886
149.049649	-0.047536851	0.087899549	-0.540808818	0.588639364	0.76241798
485.3974407	-0.449016672	0.126314023	-3.554765025	0.000378317	0.004493435
1110.609001	-0.001794926	0.049977452	-0.035914714	0.971350363	0.985661927
3.848780248	-0.434584278	0.604780129	-0.71858227	0.472398341	0.672742935
371.3857382	0.58348156	0.137323952	4.248942392	2.14782E-05	0.000452508
169.9394567	0.373130724	0.403437468	0.924878709	0.355029003	0.569609251
47.96281689	-0.187080242	0.185435285	-1.008870785	0.313036608	0.527836753
197.5289522	-0.062075231	0.112541125	-0.551578192	0.581237381	0.758079694
115.4389889	0.124886314	0.114536369	1.090363833	0.275552907	0.488166774
25.38576958	0.330738191	0.199006152	1.661949583	0.096522884	0.250294032
41.33347246	0.916957344	0.194500852	4.714412994	2.42409E-06	7.2873E-05
2182.744528	0.20239303	0.077966359	2.595902029	0.009434296	0.050543283
2.35198291	0.348471971	0.761160875	0.457816452	0.647084315	0.804290554
429.8112184	0.137308735	0.079530696	1.726487274	0.084259787	0.229931378
181.7762279	-0.021913006	0.085281925	-0.256947839	0.79721904	0.89525023
28.99828347	-0.552092074	0.225032779	-2.453385135	0.014151873	0.067509546
224.4932392	-0.10679624	0.093264982	-1.145084014	0.252174357	0.461283628
6.270916501	0.551430229	0.529484052	1.041448231	0.297667568	0.512411235
38.898405	-0.118768008	0.158510943	-0.749273238	0.453692534	0.658108374
6.321512018	-0.394686984	0.404504626	-0.975729224	0.329198666	0.544751886
51.81571722	-0.940554368	0.298244147	-3.153638986	0.001612484	0.013849878
26.82431647	-1.976485687	0.224140859	-8.818051715	1.16468E-18	7.23643E-16
3.044280274	-0.395834558	0.533098737	-0.742516405	0.457774516	0.661176344
110.1705829	0.152036274	0.104626732	1.453130297	0.14618758	0.327525391
104.1854978	0.013097822	0.116465816	0.112460659	0.910458161	0.955037829
46.08514687	1.023298601	0.231397046	4.422263033	9.76724E-06	0.000233874
244.1112857	0.202053972	0.086704757	2.330367769	0.019786722	0.0853092
42.42749174	0.006561867	0.161161297	0.040716147	0.967522189	0.983825723
76.88642339	-0.434941319	0.219890136	-1.977993769	0.04792941	0.157634077
162.0918936	-0.318449149	0.161104321	-1.976664231	0.048079597	0.158033375
324.9130769	-0.104734449	0.058349102	-1.794962476	0.072659679	0.209024705
46.06102924	-0.488623249	0.240296513	-2.033417979	0.042010312	0.14385323
320.2626422	0.080354159	0.081771033	0.982672661	0.32576857	0.540781079
33.29486133	-0.164220512	0.213744922	-0.768301348	0.442308174	0.648042054
23.26209337	-0.942975645	0.242366971	-3.890693687	9.99581E-05	0.001563826
201.392334	0.037604633	0.099250709	0.378885285	0.704773052	0.842009718
46.24961263	0.081794155	0.257749612	0.317339584	0.750985944	0.870013027

166.3203188		0.209170427	0.096327226	2.171456982	0.029896646	0.113692737
403.5263116		-0.152843006	0.062978925	-2.426891294	0.015228818	0.071110984
442.3834169		-0.220505067	0.082557436	-2.67092922	0.00756416	0.042817449
16.60083192		-0.326180769	0.672412925	-0.485089974	0.627612523	0.790978857
1187.406332		0.397524312	0.089075035	4.462802764	8.08945E-06	0.000201004
707.9128465		0.290791846	0.095096434	3.057862791	0.002229216	0.017546158
183.6856519		0.469760527	0.1783672	2.633671029	0.008446728	0.046679437
14.70283053		0.031219201	0.471044695	0.066276515	0.94715768	0.974828397
208.2409319		-0.05552934	0.082602228	-0.672249908	0.501424614	0.697229581
1544.735388		-0.121544128	0.081142251	-1.497914176	0.13415555	0.309882015
314.8654004		0.083918226	0.101023692	0.830678665	0.406155182	0.616852629
22.23523112		-0.299305694	0.294814592	-1.015233651	0.309994463	0.525135662
13.71852835		-0.221581614	0.330242679	-0.670966012	0.502242181	0.69804587
4.043632463		-0.884196643	0.672981471	-1.313849907	0.188896719	0.388250202
279.2549658		0.150885152	0.069488588	2.171365925	0.029903523	0.113692737
58.88279776		0.259727423	0.145038566	1.790747316	0.073333853	0.210360769
54.52903843		0.055422678	0.148873516	0.372280305	0.709684161	0.84523835
13.21464036		0.646188349	0.372087532	1.736656815	0.082447765	0.226950609
360.7695736		0.590916683	0.110758233	5.335194203	9.54424E-08	4.29595E-06
170.9393023		0.226780825	0.101058173	2.244062182	0.024828401	0.100199531
1552.593908		0.265565718	0.052765301	5.032961297	4.82961E-07	1.83748E-05
3.44269966		-0.092157878	0.49626801	-0.18570183	0.852678593	0.927255626
153.2817043		0.492884739	0.208761904	2.360989866	0.018226229	0.080261666
104.0320997		-0.261554271	0.169858447	-1.5398367	0.123600164	0.293804883
188.1117326		0.092139205	0.084665775	1.08826979	0.276476023	0.489170443
20.18902537		-0.217808655	0.334929982	-0.65031101	0.515491347	0.708628542
4.165239492		1.647171	1.071396953	1.53740497	0.12419417	0.294766569
27.75121982		1.055598551	0.759535072	1.389795665	0.164590935	0.354394336
24.59719668		0.025222043	0.228314529	0.1104706	0.912036165	0.955963847
131.2167775		0.11194162	0.17506421	0.639431787	0.522542076	0.713971814
903.6591096		0.04895604	0.09412811	0.520100105	0.602993805	0.773279575
14.0854043		-0.174931865	0.403685711	-0.433336776	0.664770125	0.816909207
557.5622663		-0.48756988	0.175053783	-2.785257597	0.005348521	0.03312464
22.39528745		-0.288251123	0.220177652	-1.309175205	0.190475009	0.390429924
32.69203977		0.452662408	0.183122314	2.471912887	0.013439226	0.065136161
81.07559679		-0.0644559	0.11725489	-0.54970756	0.582519971	0.758851336
2533.595961		0.162931021	0.133582893	1.219699743	0.222578719	0.427651642
290.3549926		-0.350578147	0.543010962	-0.645618913	0.518526186	0.710970989
950.995547		-0.174457403	0.093039919	-1.875081208	0.060781552	0.185360106
45.13042195		-0.624847574	0.23290024	-2.682897943	0.007298726	0.041691821
27.48096412		-0.240432607	0.296082023	-0.8120473	0.416764491	0.626153965
55.3941888		-0.360999853	0.328706154	-1.098244888	0.27209757	0.484179668
1206.048539		-0.1666861	0.070921111	-2.350303012	0.018758134	0.08194683
178.8463028		-0.148581782	0.124519886	-1.193237375	0.232776421	0.43946353
188.4673102		-0.061464414	0.142717956	-0.430670504	0.666707968	0.818248311
170.595413		0.241811774	0.108052153	2.237917232	0.025226453	0.101287007
457.8475476		-0.149550748	0.080366981	-1.860848142	0.062765625	0.189273458
21.91171497		-2.327719725	0.831237731	-2.800305661	0.005105424	0.031979754

326.8797358	0.015189781	0.076020715	0.199811078	0.841628337	0.921462698
2357.014741	0.086365546	0.128575765	0.671709365	0.501768738	0.697505543
349.3936579	-0.073013832	0.068570121	-1.064805348	0.286964024	0.501479298
9.267981748	1.089620428	0.667712539	1.631870549	0.102706759	0.261918135
1014.629873	0.184268532	0.137370116	1.341401874	0.179790009	0.375771304
953.0491222	0.336036203	0.061497242	5.464248325	4.64872E-08	2.27926E-06
20.06567943	0.101757009	0.41462845	0.245417335	0.806133308	0.901149495
163.6655362	0.056515907	0.110279312	0.512479683	0.608315336	0.777532194
224.0176364	-0.484815275	0.109124297	-4.442780257	8.88038E-06	0.000216094
4.706776483	0.046738171	0.481643442	0.097038944	0.922695468	0.961690822
935.4292673	-0.812693387	0.454104086	-1.789663234	0.073508066	0.210686634
464.4547416	-0.016009801	0.066446642	-0.240942219	0.8095999	0.903582771
459.2989261	-0.030250252	0.080858625	-0.374112871	0.708320343	0.844468761
23.08154162	1.146714245	0.537143914	2.134836152	0.032774396	0.120869801
291.7813508	0.142219095	0.109335272	1.300761346	0.193340157	0.393998731
361.4010054	-0.083119822	0.093827211	-0.885881842	0.375681202	0.589069671
37.17656494	-1.104490676	0.431369578	-2.560427842	0.010454337	0.054157886
284.974717	0.177930039	0.072869482	2.44176347	0.014615719	0.068951706
289.8476681	0.067099815	0.072027095	0.93159129	0.351547786	0.566673016
1623.775334	0.081255944	0.072056339	1.127672394	0.2594583	0.470367534
1396.862226	-0.378576437	0.061244775	-6.181367112	6.35488E-10	5.45262E-08
212.7909902	0.234242237	0.134076162	1.747083405	0.080622872	0.22356189
66.93461647	-1.347513949	0.576347582	-2.338023081	0.019386051	0.084021521
106.1509839	-0.055699536	0.122855945	-0.453372733	0.650280368	0.806194971
1538.836275	0.223201771	0.096395186	2.315486695	0.020586312	0.087764777
559.4604085	0.350038743	0.073080729	4.789754378	1.66986E-06	5.32093E-05
481.2704848	0.182259543	0.071815591	2.537882657	0.011152537	0.056793477
441.3824403	0.110956462	0.09890893	1.121804293	0.26194566	0.473242344
362.4116518	0.187401161	0.059958039	3.125538544	0.001774798	0.01478161
48.76886846	-0.366000113	0.259580495	-1.409967697	0.158549221	0.34567759
124.6953811	-0.851228377	0.231461503	-3.677624001	0.000235417	0.00311686
141.2849122	-0.367881622	0.192081533	-1.915236806	0.055462311	0.172981088
190.684944	-0.389591803	0.241204684	-1.6151917	0.106269187	0.267537975
204.3425745	-0.080991219	0.083859844	-0.965792619	0.334147963	0.5493766
289.1223848	0.210939049	0.138249646	1.525783644	0.127063755	0.298371368
2186.711825	-0.209902217	0.071248751	-2.946047656	0.003218628	0.022904397
8915.698083	0.263863564	0.123507444	2.136418301	0.032645334	0.120636884
8.122750664	0.501045296	0.357034188	1.403353832	0.160511353	0.348617539
633.9279209	0.043688513	0.054295735	0.804639869	0.421027528	0.629405403
161.6239268	-0.195620616	0.16761791	-1.16706273	0.243185037	0.450898178
205.4386716	-0.404338903	0.211683516	-1.910110487	0.056118989	0.174533552
1454.088123	0.002979261	0.071129441	0.041885057	0.966590329	0.983424473
2700.577441	0.322173656	0.064576393	4.989031491	6.06828E-07	2.27202E-05
26.3170935	0.190911641	0.183140431	1.042433066	0.297210943	0.511839125
7.318113477	0.014648732	0.371669435	0.039413336	0.968560848	0.984221208
1022.91361	0.027982453	0.04289957	0.652278178	0.514221735	0.707591786
4.986830447	-0.552281615	0.477352626	-1.156967793	0.247285519	0.455782238
1.779568066	0.025938698	0.945095802	0.027445575	0.978104348	0.988606113

641.8631825	-0.167643299	0.079245557	-2.115491448	0.034388083	0.124699379
9057.292664	0.081366477	0.077558229	1.049101793	0.294131271	0.509065449
5.208493816	-0.108706563	0.460035944	-0.236300152	0.813199769	0.906097599
585.8586393	0.081241604	0.076552578	1.061252366	0.288575227	0.503240686
131.3013869	0.328204573	0.15241114	2.153415901	0.031286007	0.117004695
3.491434703	-0.095137937	0.731722374	-0.130019171	0.896551259	0.947889713
4.272712581	-0.410570053	0.660217625	-0.621870785	0.534026837	0.723245487
55.36274622	-0.346633225	0.289154975	-1.198780084	0.230613469	0.436936024
94.17696098	1.210891564	0.300075508	4.03528956	5.4535E-05	0.000963959
127.3989821	-0.160873318	0.104015417	-1.546629549	0.1219526	0.291805389
3.551136715	-0.613878058	0.678087062	-0.905308613	0.365301914	0.578763583
178.8319627	0.062742701	0.112967165	0.555406533	0.578616625	0.756621676
242.7094587	-0.701577462	0.170450414	-4.116020874	3.85469E-05	0.00072657
981.1344793	-0.24246636	0.075821916	-3.197840039	0.001384611	0.012363184
2222.431248	0.222029347	0.064620578	3.43589234	0.000590606	0.006388224
54.25558248	0.039184106	0.175759929	0.222941066	0.823581375	0.91138289
372.3152975	-0.120037881	0.080551339	-1.490203425	0.136170756	0.313000842
166.1346822	-0.131256121	0.081262814	-1.615205209	0.106266263	0.267537975
1179.75093	0.010654788	0.050712663	0.210101137	0.833588738	0.916902912
7468.105971	0.035824912	0.073519167	0.4872867	0.626055172	0.790627823
112.6142566	0.185840032	0.180775355	1.02801641	0.303942108	0.518644466
693.0055206	0.006900062	0.099560733	0.069305054	0.944746803	0.973447225
36.16135365	0.918635209	0.188529577	4.87263178	1.10121E-06	3.73893E-05
224.4491129	-0.362012423	0.453698341	-0.797914365	0.424920186	0.632424608
5.251685491	-0.37422758	0.744935775	-0.502362207	0.615412758	0.782406945
1192.19013	-0.044162425	0.090861596	-0.486040608	0.626938375	0.790677612
288.5212165	-0.035485962	0.206407765	-0.171921643	0.863499132	0.931920109
7.519134748	-2.687513018	1.155663327	-2.325515533	0.02004441	0.086104724
3.432616273	1.386010652	0.669164245	2.07125629	0.038334852	0.134913127
1.744398724	0.206616894	0.826745101	0.249916079	0.802652249	0.899069005
1910.09921	0.065027249	0.202683691	0.320831186	0.748338324	0.868055595
6.143807939	-0.770966735	0.570078439	-1.352387114	0.17625151	0.371110349
4478.916608	-0.120804161	0.125240468	-0.964577688	0.334756379	0.550047536
560.0464782	0.079560148	0.080207308	0.991931413	0.321230984	0.53627475
32.26447134	1.02011219	0.179179051	5.693255898	1.24639E-08	7.386E-07
77.12105045	0.434572867	0.149517744	2.906496955	0.003655005	0.025082115
210.9011945	-0.118649377	0.120158559	-0.987440079	0.323426944	0.538630761
3.890851191	0.30894142	0.455136546	0.678788426	0.497271928	0.693830587
1451.796662	0.110855599	0.050585539	2.191448418	0.028419359	0.109798432
383.8157574	0.100012498	0.057895144	1.727476446	0.084082132	0.229598714
2.900801002	-1.085912367	0.631284016	-1.720164521	0.08540254	0.231973858
6.095409876	-2.098904452	0.841049464	-2.495577897	0.012575214	0.062006608
86.41125859	0.624928399	0.182379767	3.426522628	0.000611363	0.006534469
7.836765392	-1.323447392	0.492706542	-2.686076353	0.007229657	0.041369004
3.217922551	0.861449682	0.546595256	1.576028465	0.115019243	0.281621068
2374.661667	-0.656663632	0.30421715	-2.158535873	0.030886192	0.116303271
433.0139512	-0.107857368	0.054651813	-1.97353688	0.048434427	0.158661514
1268.7437	0.313056426	0.050512192	6.197640902	5.73157E-10	5.02611E-08

120.7902446	0.861209338	0.108721736	7.921225019	2.35182E-15	7.31016E-13
36.33106587	-0.715322854	0.23526154	-3.040543107	0.002361519	0.018298994
4.659206536	-0.389011074	0.476289315	-0.816753729	0.41406918	0.623815047
185.83461	-0.054538919	0.276105251	-0.197529451	0.843413237	0.922112402
573.7070601	0.14818232	0.065541196	2.260903491	0.023765234	0.096956034
704.1451511	0.19799341	0.110063151	1.798907335	0.07203334	0.207933633
6.252470918	-1.981361816	0.792655228	-2.499651483	0.012431554	0.061537685
317.9211711	0.115204568	0.082715371	1.392783069	0.1636854	0.353206828
95.8275682	-0.15500106	0.156043982	-0.993316489	0.320555744	0.535581677
153.5925891	0.056829812	0.221691984	0.256345812	0.797683826	0.895532462
18.11727204	0.535853354	0.319893928	1.675096984	0.093915205	0.246091419
5.194789717	-0.053764903	0.453425821	-0.11857486	0.905612184	0.952928916
71.89694967	0.284510383	0.181664279	1.566132782	0.117317535	0.285057385
1103.047597	0.228983629	0.049674953	4.609639587	4.03368E-06	0.000111308
42.98968708	-0.511446869	0.193053772	-2.649245672	0.008067167	0.044974112
494.7001219	-0.199183314	0.11771708	-1.692051103	0.09063623	0.24095254
132.8321121	0.08426646	0.101943032	0.826603435	0.40846187	0.619157236
578.3326808	-0.031362237	0.161997993	-0.193596452	0.846491877	0.923945351
3806.912003	0.109203131	0.06132216	1.780810258	0.074943453	0.213239062
116.5841842	-0.062309544	0.275348186	-0.226293644	0.820973042	0.910076874
6.177302649	0.038085587	0.425373315	0.0895345	0.928657136	0.965637301
7.566906525	-0.226787218	0.368267953	-0.615821215	0.538012524	0.725898306
3.570168656	0.172125458	0.655629643	0.262534588	0.792909323	0.893098455
4.382356376	0.345460525	0.581106583	0.594487371	0.552186187	0.737796722
1.755423003	0.224278743	0.807001295	0.277916213	0.781076678	0.886011848
5.706314408	0.109452442	0.398739786	0.274495914	0.783703543	0.887762646
14.68414109	0.318513098	0.309584142	1.028841777	0.303554032	0.518427236
45.95747805	0.175910614	0.19357696	0.90873735	0.363488784	0.577223272
12.55729964	-0.164848648	0.344229805	-0.478891268	0.632015983	0.793934606
25.38670243	0.347869208	0.247354619	1.406358247	0.159617772	0.347134413
162.5771519	-0.190580844	0.11974482	-1.591558147	0.11148402	0.276203888
57.23409397	-0.063232236	0.195731681	-0.323055704	0.746653056	0.867077573
39.24159957	-0.366949133	0.21771808	-1.685432525	0.091905153	0.242752203
8357.739458	0.135806103	0.069093517	1.965540465	0.049351717	0.160612443
459.8765191	0.108745022	0.096035733	1.132339172	0.257491867	0.467957282
439.7292572	0.24297438	0.07857792	3.092145741	0.001987153	0.016142185
4.206380731	0.178888315	0.491743335	0.363783913	0.716019377	0.849440394
173.3344929	0.28037367	0.093390658	3.002159706	0.002680715	0.020093134
190.9229754	0.098864418	0.172237908	0.57399918	0.565968356	0.748143944
136.9622457	0.248161474	0.130311108	1.904376977	0.056861105	0.176601263
9.004084935	-0.072037183	0.381384617	-0.188883294	0.850184287	0.926134758
133.1701403	0.146663029	0.187001057	0.784289836	0.432870062	0.639905007
453.2246846	-0.054458559	0.119737422	-0.454816529	0.649241238	0.805627137
23.428892	-0.32956288	0.28082966	-1.173533024	0.240582122	0.448038647
2.175398401	1.396558326	1.178416671	1.185114197	0.235972299	0.44321208
5.369922637	0.05073828	0.462837236	0.109624455	0.912707215	0.956423806
336.7477192	0.071852816	0.11465425	0.626691251	0.53086164	0.720558182
1639.559445	-0.119533539	0.050076994	-2.386995116	0.016986717	0.076538948

185.904419	-0.784626829	0.300265377	-2.61311123	0.00897221	0.048701213
394.2703211	0.189594932	0.059651218	3.178391633	0.001480946	0.013030427
107.8033302	-0.239524932	0.137611711	-1.740585382	0.081756282	0.225701692
34.36868143	-0.268002988	0.195391118	-1.371623188	0.170180771	0.362216799
987.4132596	-0.014223239	0.080179883	-0.177391612	0.859200795	0.930236192
95.40684466	0.097183045	0.130369889	0.745440882	0.456005237	0.660067681
1447.391273	0.2369559	0.084993924	2.787915767	0.005304833	0.032989168
292.9674515	0.065640574	0.074027583	0.886704262	0.375238143	0.588730778
339.1110527	-0.208541684	0.118590359	-1.758504544	0.078661699	0.219761294
2.247895991	0.086150361	0.626200116	0.137576406	0.890575203	0.945339324
6.812595634	0.166220829	0.413391818	0.402090273	0.687617591	0.831033101
434.8094779	-0.591399158	0.288965866	-2.046605594	0.040696825	0.140523894
11832.70221	-0.095215508	0.073872339	-1.288919628	0.19742603	0.398822368
986.0660357	0.193125235	0.05308388	3.638114531	0.000274641	0.003515494
13.52070482	0.516577598	0.264712646	1.951465506	0.051001696	0.164262353
24.24655214	0.253196036	0.304756971	0.830812944	0.406079309	0.616852629
175.950938	-0.269919542	0.1338153	-2.01710523	0.043684533	0.147895396
148.6290253	-0.268690996	0.081995986	-3.276879848	0.00104961	0.009989185
534.5953661	0.882094843	0.109049733	8.08892251	6.01949E-16	2.25375E-13
1030.154435	0.222010511	0.066173311	3.354985688	0.000793691	0.008036109
205.2272773	0.135054791	0.258634251	0.522184475	0.601541894	0.772480361
1123.795023	0.181788654	0.08519163	2.133879292	0.032852663	0.12107713
198.9276046	0.006574829	0.120848844	0.054405395	0.95661218	0.978702547
176.3333728	-0.271153395	0.1126955	-2.406071178	0.016125123	0.073810856
1357.25969	-0.049816352	0.138569769	-0.359503753	0.719218271	0.851006678
1015.393812	0.202711694	0.126557255	1.601739028	0.109213331	0.272520511
8.099066344	-5.35147738	1.369833353	-3.906663077	9.35795E-05	0.001489496
2367.411761	0.191042631	0.054426824	3.510082285	0.000447968	0.005124879
410.912918	-0.149023515	0.134787152	-1.105621066	0.268890568	0.480722965
2315.226888	-0.46808325	0.100054401	-4.678287465	2.89281E-06	8.43471E-05
11.1665628	0.052013234	0.309388502	0.168116247	0.866491828	0.933868226
1063.808963	-0.027620928	0.050751436	-0.544239347	0.586276779	0.760977281
565.1018001	-0.132119079	0.06942519	-1.903042371	0.057035017	0.176848271
411.9054918	0.248181162	0.164845006	1.5055425	0.132184659	0.306705644
2.81650682	1.175127714	0.690754328	1.701223816	0.088900967	0.238171511
513.7155674	0.119916217	0.06179629	1.940508337	0.052317942	0.166612367
242.1245346	0.280162961	0.090720245	3.088207738	0.002013677	0.016293373
109.9040472	0.383390242	0.116511894	3.290567411	0.000999855	0.009653987
2759.251588	-0.033159998	0.119055419	-0.278525732	0.780608815	0.885759197
64.46836668	0.188999927	0.135049413	1.399487214	0.16166693	0.350618879
30.76860663	0.297363573	0.228366604	1.302132484	0.1928711	0.393432207
288.1245045	-0.144002077	0.081310614	-1.771012055	0.076558697	0.216095761
294.4904623	0.30866639	0.126472102	2.440588749	0.014663342	0.069097227
17.0930973	-0.053485676	0.346344383	-0.154429172	0.877271353	0.938269706
15.39085634	-0.228808106	0.269254599	-0.849783465	0.395445484	0.608310704
194.0502094	-0.022558648	0.08001165	-0.281942044	0.777987951	0.88445059
151.3874652	0.038064465	0.093938288	0.405207143	0.685325255	0.829778815
623.2925682	0.07015926	0.070662546	0.992877608	0.320769603	0.535815609

4.633712801	0.89342138	0.48864532	1.828363728	0.067494981	0.198839829
868.2181748	0.20806213	0.056246833	3.699090593	0.000216373	0.002916968
12.26012035	0.345486749	0.352948994	0.978857441	0.327650423	0.542830642
41.50586253	0.27246295	0.47966593	0.56802648	0.570016988	0.750276435
15.50681018	-0.442926456	0.248002861	-1.78597317	0.074103602	0.211719093
10.91838872	0.377008375	0.289051747	1.30429371	0.192133459	0.392803996
88.78707017	-0.232008752	0.10442276	-2.221821687	0.026295358	0.104357922
39.93542297	0.237512349	0.184486659	1.287422895	0.197946929	0.399482752
605.818297	0.113734185	0.059926459	1.897895987	0.057709785	0.178436749
317.0391749	0.142587912	0.083536692	1.706889621	0.08784257	0.236264245
49.77528306	-0.03370692	0.263352036	-0.127991873	0.898155405	0.948475137
5.165074452	2.630690016	0.656641576	4.006280006	6.16825E-05	0.001067393
123.4564408	-0.056177566	0.118984622	-0.472141403	0.636825861	0.797012022
1336.803014	0.181100971	0.051926865	3.487616103	0.000487347	0.005465323
38.28717461	0.104039394	0.235447081	0.441880159	0.658575929	0.812200918
9.254831507	0.46355597	0.407962987	1.136269674	0.255843719	0.466029348
70.77907281	-0.222810573	0.251674172	-0.885313624	0.375987504	0.589177113
74.24843742	-0.300338027	0.286747484	-1.04739551	0.294917202	0.509745674
2.668941062	0.458381786	0.786511511	0.582803658	0.560025482	0.744020951
10.76835077	0.170194806	0.301073813	0.565292625	0.571874744	0.751520783
890.6788656	0.007968092	0.106956686	0.074498309	0.940613887	0.971820205
52.45201351	-0.280165869	0.348380317	-0.804195461	0.4212841	0.629613922
5.333825547	0.433348326	0.489240162	0.885757876	0.375748013	0.589081916
565.0282069	0.119684262	0.077750361	1.539340272	0.123721247	0.294025364
12.00797979	-1.118428801	0.469827439	-2.380509755	0.017288703	0.077606021
153.189819	-0.226794578	0.093354157	-2.429399886	0.015123841	0.070781295
582.1964278	-0.0673197	0.075513962	-0.891486794	0.372668073	0.586037976
31.61168747	0.241039782	0.167507523	1.438978845	0.150156517	0.333424781
1308.080305	0.236312069	0.064167883	3.682715694	0.000230762	0.003062683
44.92187154	0.17513197	0.254616749	0.687825803	0.491562485	0.689191522
87.33069808	-0.405767004	0.186626852	-2.174215556	0.029688951	0.113242828
13.58032339	-0.367828166	0.410206424	-0.896690409	0.36988414	0.583361608
1.723613763	-0.276668753	0.780969353	-0.354263265	0.723141595	0.853002623
13.48822592	-0.583770747	0.330359117	-1.767079269	0.077214954	0.217034491
643.1672552	0.220499068	0.128291553	1.718734109	0.085662798	0.232489117
995.9494017	0.155897592	0.088591558	1.759734184	0.078452887	0.21943188
519.9665713	-0.156094233	0.167408226	-0.932416744	0.351121195	0.566262415
227.1639253	0.101148851	0.075968884	1.331451067	0.183040636	0.380301606
31.66758811	0.168509322	0.191709439	0.878982916	0.379410539	0.59230988
417.053692	0.007201622	0.063016459	0.11428161	0.90901456	0.954253862
32.67006748	-0.172763481	0.234301753	-0.737354623	0.460906723	0.663547789
411.6879379	-0.021416351	0.064240983	-0.333375199	0.738851082	0.862699071
72.65001981	0.407671563	0.158193106	2.577050126	0.00996475	0.052346712
328.0143534	-0.138483146	0.067866082	-2.04053544	0.041297025	0.142119739
9.505241534	0.406851125	0.353714072	1.150226009	0.250050796	0.45901246
502.1611837	0.219737759	0.074903554	2.933609243	0.003350457	0.02355759
6.706637448	0.098646346	0.401816142	0.245501204	0.806068376	0.901149495
272.5233764	-0.07168143	0.141226209	-0.50756464	0.611758694	0.780039684

38.19365087	0.138773258	0.160485598	0.864708481	0.387198813	0.600236496
147.3397324	0.256834381	0.118162415	2.173570859	0.029737379	0.113375047
579.7593014	0.151981465	0.095336586	1.594156777	0.110900925	0.275345405
418.8035248	-0.203817051	0.101440781	-2.009222021	0.044513593	0.149809384
774.6236281	-0.076584466	0.118224661	-0.647787575	0.517122353	0.709772968
2387.277267	0.150990317	0.077648048	1.944547497	0.051829464	0.165804784
102.7029989	0.22130803	0.111758393	1.980236338	0.047676979	0.15696054
174.8234023	0.12383147	0.152665949	0.81112698	0.417292754	0.626547651
270.0960625	0.052104931	0.060683859	0.858629169	0.39054514	0.60400683
72.83424449	0.120557233	0.118075622	1.021017127	0.307246324	0.522404371
293.8195389	-0.048568902	0.08666245	-0.560437677	0.575180938	0.753941023
68.15509361	0.710226801	0.145822608	4.870484851	1.11325E-06	3.76584E-05
190.6429019	0.018851675	0.117358407	0.160633359	0.872382179	0.936384156
52.7246396	-0.347280963	0.14055481	-2.470786751	0.013481619	0.06525536
1147.47728	-0.004501888	0.043178376	-0.104262567	0.916960983	0.959051187
27.08956629	0.508894046	0.206873341	2.459930521	0.013896392	0.06666545
38.81227747	0.745196106	0.160456179	4.644234409	3.4134E-06	9.67854E-05
63.49573812	0.512565807	0.148504544	3.451515985	0.000557447	0.006095692
2.114107316	-1.07334799	1.383677533	-0.775721195	0.437913622	0.643838339
47.88148193	0.024303741	0.191673192	0.12679781	0.899100429	0.949047253
486.6461228	0.349888172	0.104480588	3.348834259	0.000811523	0.008170485
9.641586849	-0.260328674	0.391636098	-0.664720835	0.506229065	0.701102784
123.1299622	0.569801977	0.125806269	4.52920176	5.92069E-06	0.000153262
3036.479313	0.247590831	0.156819822	1.578823573	0.11437653	0.280726901
49.10555881	0.810138649	0.210152213	3.855008888	0.000115725	0.001758728
197.2378224	-0.00811688	0.095177425	-0.085281571	0.932037542	0.967778561
391.2763183	0.001192023	0.069635212	0.0171181	0.986342399	0.993222667
10.64534685	-0.49037848	0.564636716	-0.868484933	0.385128917	0.598266269
39.81820806	-0.126965995	0.334884461	-0.37913373	0.70458856	0.84196953
164.314135	0.276654338	0.081176917	3.408041938	0.000654308	0.006905238
41.48830751	0.450870832	0.200610682	2.24749165	0.024608624	0.099485268
71.40952317	-0.057226716	0.135706368	-0.421695142	0.673247551	0.822963579
195.2707606	-0.142314031	0.089921865	-1.582641014	0.11350331	0.279207634
930.3428635	-0.065235355	0.068556537	-0.951555582	0.341322417	0.555791786
498.111791	-0.019454248	0.049311443	-0.394517929	0.693198693	0.834976033
296.5040869	0.007233703	0.088586864	0.081656606	0.934919786	0.969158606
2.380350699	-0.205736244	0.726670696	-0.283121701	0.777083547	0.884207847
8.539626028	-0.43254134	0.40102175	-1.078598206	0.280766881	0.494425826
2588.914989	0.189684501	0.066981741	2.831883704	0.004627468	0.029714089
117.3549874	-0.499924546	0.161891293	-3.088026154	0.002014907	0.016295328
4.161710216	-0.141190553	0.457945323	-0.308313124	0.75784408	0.873603573
140.0964268	-0.018223366	0.211068345	-0.086338698	0.931197177	0.967310672
130.1220303	-0.071333919	0.202097397	-0.352968027	0.72411241	0.853557222
305.1329838	-0.316824577	0.091002102	-3.481508339	0.000498598	0.00555369
139.6107467	-0.131357252	0.135036529	-0.972753467	0.330675845	0.545956491
20.93645702	-0.484203475	0.305293861	-1.586024277	0.112733802	0.278187037
867.6480868	-0.223724812	0.226361601	-0.98835143	0.322980566	0.538104959
210.8871008	-0.123774505	0.125986422	-0.982443208	0.325881549	0.540876842

174.874218	0.504727327	0.11367508	4.440087733	8.99222E-06	0.000218171
14.11524786	-0.690989587	0.401689436	-1.720208511	0.085394546	0.231973858
87.94408074	-0.03546749	0.135292242	-0.262154648	0.793202217	0.893098455
16.76658109	0.463317287	0.331912709	1.395901013	0.162744305	0.351997597
199.0396609	0.062048113	0.129812143	0.477983887	0.632661673	0.794224511
150.3690537	-0.119588946	0.111053379	-1.076860038	0.281542798	0.495580303
2392.781992	0.027619907	0.102661165	0.269039484	0.787899298	0.890422998
649.9777792	-0.002393746	0.141392269	-0.016929822	0.986492602	0.993239536
99.49486646	0.664444428	0.133287516	4.985046234	6.1947E-07	2.30886E-05
245.7979457	0.001924502	0.100835758	0.019085508	0.984772893	0.992423603
2.531693348	-0.026130792	0.696442855	-0.037520367	0.970070101	0.985321835
57.27789899	-0.514645158	0.208994537	-2.462481394	0.013797933	0.066308971
110.4054922	1.147979914	0.142574575	8.05178561	8.15947E-16	2.8004E-13
29.5874194	-0.513855406	0.276341594	-1.859493526	0.062957216	0.189592266
133.2474886	0.660415107	0.253695386	2.603181389	0.009236307	0.049676437
6918.513005	-0.098324671	0.142213566	-0.691387428	0.489322103	0.687044432
21.41520198	-0.152525799	0.330519842	-0.461472442	0.644459688	0.80261547
972.3684238	-0.434431524	0.074977029	-5.794194988	6.86497E-09	4.56022E-07
5505.740206	-0.334189554	0.168405352	-1.984435472	0.047207315	0.155981164
329.4855391	-0.41111995	0.289424437	-1.420474215	0.15546967	0.340720367
3.525667626	1.671651389	0.563231747	2.967963715	0.002997797	0.021775005
50.96612653	0.280166585	0.16417591	1.706502407	0.087914579	0.236380736
2774.366052	-0.128860897	0.136460559	-0.944308731	0.345011906	0.559431667
139.5806514	-0.177536016	0.095665467	-1.855800447	0.063482007	0.190805069
333.4167168	0.046548586	0.119877469	0.388301373	0.697793021	0.838003561
70.0120571	0.110393543	0.11889448	0.928500154	0.353148188	0.568109866
245.2780164	0.14011152	0.098393879	1.423986139	0.154450484	0.339481959
40.31713107	0.211820339	0.256043588	0.82728234	0.408077051	0.618858634
52.58576208	-0.211133686	0.228271533	-0.924923414	0.355005746	0.569609251
181.172956	-0.38121383	0.136118567	-2.800601251	0.00510075	0.031974795
174.0028561	-0.244656289	0.135616053	-1.804036354	0.071225608	0.20618005
3.443868832	0.194232141	0.509586721	0.381156205	0.703087344	0.84078549
47.27865555	-0.060982616	0.219489194	-0.277838809	0.781136098	0.886011848
87.79816032	0.140332467	0.10591218	1.324988933	0.185174822	0.383141173
221.6384027	-0.442369911	0.079760821	-5.546205595	2.91936E-08	1.52678E-06
821.4044015	-0.045204833	0.079827757	-0.566279636	0.571203702	0.751109865
291.2893708	-0.1448207	0.104056984	-1.391744166	0.163999883	0.353583833
1100.214874	-0.034238932	0.073508342	-0.465782939	0.641370879	0.800389656
211.9952945	0.121980149	0.182909499	0.666887994	0.504843681	0.700184778
134.2849905	-0.134112018	0.108743321	-1.233289702	0.217467699	0.422248697
8.942803939	0.031868988	0.341964281	0.093193908	0.925749514	0.964202915
23.67112422	-0.106299338	0.197154499	-0.539167702	0.589771144	0.763227795
5.134686576	-0.723858244	0.447008533	-1.619338762	0.105374395	0.26595268
1.703274577	-0.153632765	1.080251419	-0.142219452	0.886906667	0.943481414
513.6474347	0.508914217	0.097248889	5.233110883	1.66681E-07	7.11372E-06
1245.611246	0.076873475	0.062724281	1.225577608	0.220357669	0.424860747
823.1820046	-0.096455917	0.081300466	-1.186412844	0.235459302	0.442752716
77.93793847	0.11004473	0.129274027	0.851251662	0.39462957	0.607354964

186.7914974	-0.030396423	0.085562471	-0.355254147	0.722399203	0.852432094
157.0276438	0.097457853	0.093476596	1.042590956	0.29713778	0.511766627
2.854822969	-0.095096366	0.586787372	-0.162062734	0.871256452	0.935876058
372.1105684	-0.227069573	0.080546476	-2.819112436	0.004815665	0.030583369
11.18415438	0.121735063	0.330206838	0.368663059	0.712378887	0.846367358
267.2385331	-0.121497058	0.074873665	-1.622694154	0.104654802	0.26483613
894.4235719	-0.060194037	0.109829811	-0.54806647	0.583646262	0.759357805
124.5155295	0.118693391	0.115244243	1.029929029	0.303043323	0.517887593
95.92914941	0.05967731	0.181720439	0.328401748	0.742607922	0.86500393
321.2053195	0.355317285	0.164973688	2.153781546	0.031257308	0.11697595
8.749677168	0.261272559	0.342295657	0.763294986	0.445287492	0.650570095
560.4434756	-0.001075582	0.068395081	-0.015726008	0.987452978	0.993528977
19.49573344	0.205886647	0.290494437	0.708745575	0.478482377	0.677925477
131.2921075	-0.081424486	0.09358185	-0.87008844	0.384252074	0.597397571
374.996561	0.143414747	0.097536228	1.470374139	0.141460451	0.320906014
1758.628244	0.17468025	0.066498181	2.62684252	0.008618121	0.047356545
127.8544749	0.166310686	0.108240658	1.536489986	0.124418252	0.295043369
701.2758706	0.133283636	0.059749721	2.230698876	0.025701082	0.102741963
1348.307042	-0.014637906	0.113851246	-0.128570452	0.897697548	0.948234766
1381.267681	-0.13888018	0.071900087	-1.931571802	0.053412379	0.169084461
405.3646157	0.083019717	0.104910058	0.791341822	0.42874455	0.635631543
2103.461314	-0.051354526	0.103486997	-0.496241344	0.619724144	0.785273098
591.243292	-0.114100003	0.065188336	-1.750313154	0.080064292	0.222387313
7.110805754	0.303053938	0.356432267	0.850242714	0.395190159	0.608047322
3.960673003	0.585977595	0.464780832	1.260761105	0.207394931	0.410948291
634.82267	-0.247106208	0.070426079	-3.508731593	0.000450249	0.005136705
226.688009	0.033829501	0.130824151	0.258587585	0.795953463	0.894773586
10.86237212	0.280014666	0.337239822	0.830313173	0.406361742	0.617092791
16.47781966	0.155438057	0.369858872	0.420263157	0.674293221	0.823642636
155.6196002	-0.228359447	0.094353607	-2.420251384	0.015509781	0.071914286
2860.819942	0.024764378	0.044664178	0.554457276	0.579265937	0.757066803
72.80267194	-0.023097033	0.14037294	-0.164540497	0.869305669	0.934970399
2.601467865	-0.013513442	0.662806828	-0.020388205	0.983733693	0.991966796
48.45290356	-0.010751401	0.217263665	-0.0494855	0.960532393	0.980661203
105.9583734	-0.19615251	0.108577181	-1.806572135	0.070829017	0.205456938
163.9489217	-0.274574429	0.346350921	-0.792763676	0.427915523	0.634941316
51.97377709	0.9049716	0.216233699	4.185155256	2.84971E-05	0.000571121
5.213800129	0.413763448	0.475901881	0.86943016	0.384611892	0.59768855
230.6104395	0.14709639	0.063382772	2.320762968	0.02029964	0.086928209
376.4626909	-0.909410784	0.264626459	-3.436582982	0.000589102	0.006376389
17.75985915	-0.838440613	0.318487587	-2.632569202	0.008474176	0.046784039
1333.500349	0.26699976	0.073205132	3.64728199	0.000265029	0.003417718
17.45577878	-0.386455131	0.231427173	-1.669877937	0.094943516	0.247601629
357.021446	-0.01519621	0.07712267	-0.197039469	0.843796652	0.92240916
292.8184859	0.051088308	0.0718925	0.710620828	0.477319233	0.677000397
1201.783432	-0.112700492	0.069603889	-1.619169469	0.105410805	0.26595268
1.751289846	0.846260562	0.811116019	1.043328626	0.296796117	0.511392034
807.5133569	-0.080733168	0.072463208	-1.114126335	0.265224992	0.476791415

4.015140473	-0.607846922	0.575334267	-1.056510896	0.290734866	0.50586884
20.47205854	-0.460526584	0.378165349	-1.217791595	0.223303179	0.428452897
6.152400157	0.70027299	0.412141566	1.699107897	0.089298856	0.238700203
4418.508012	-0.277564808	0.107230933	-2.588477043	0.009640137	0.051378719
2588.481315	-0.397246846	0.11903367	-3.337264541	0.000846074	0.008452528
4.778498855	-2.425708744	0.850684568	-2.851478485	0.004351643	0.028436719
258.628154	-0.222581711	0.07391311	-3.011396912	0.002600487	0.019660588
2.774005552	0.088521532	0.93130197	0.095051374	0.924274021	0.962848756
5.334987244	-1.061173628	0.735416695	-1.442955586	0.149032983	0.331914203
70.92980696	-1.023745682	0.427025341	-2.397388597	0.016512401	0.075116787
185.6917604	-0.184036223	0.06804832	-2.704493281	0.006840865	0.039803591
30.30484338	-0.758353206	0.390164002	-1.943678047	0.051934288	0.16596808
18.22726718	0.149631852	0.259767055	0.576023208	0.564599495	0.74714532
656.5846717	0.030196404	0.096601492	0.312587344	0.754594194	0.871752087
419.3526658	0.02895988	0.052323915	0.553473113	0.579939487	0.757293715
114.4868349	0.383158398	0.106054304	3.61285099	0.000302849	0.003754048
260.0232749	-0.153692368	0.073177385	-2.100271406	0.035704973	0.128238793
121.5776978	0.180239766	0.200372312	0.899524312	0.368373452	0.582118393
214.6749587	-0.013062524	0.099704737	-0.131012068	0.895765761	0.947455865
38.07985712	-0.066974931	0.292241335	-0.22917679	0.818731511	0.909002758
10.30703849	-0.429399255	0.366570916	-1.171394775	0.241440131	0.449038926
632.8895599	-0.073632511	0.086407908	-0.852150144	0.394130764	0.607040969
2.748032201	-0.831261056	0.648270837	-1.282274334	0.199746428	0.401981756
152.4852536	-0.390063874	0.117560707	-3.317978299	0.000906715	0.008939095
282.164523	0.082986543	0.071886458	1.154411348	0.248331565	0.457077732
361.8317707	-0.025956184	0.103253139	-0.251383966	0.801517263	0.898305694
3085.289101	0.142134971	0.099809544	1.424061925	0.154428547	0.339479034
90.53129573	0.169712945	0.145358189	1.167549941	0.242988353	0.450709544
335.1716888	-0.057623043	0.064578093	-0.892300169	0.372232064	0.585520005
618.5754541	0.027903725	0.042654458	0.65418073	0.512995375	0.706494383
444.3075992	0.124702865	0.070716164	1.763428025	0.077828327	0.218348748
493.8513693	0.130118329	0.055614489	2.339647997	0.019301922	0.083761148
141.2465385	0.517114367	0.096558129	5.355472109	8.53333E-08	3.89678E-06
527.014045	-0.039929245	0.04927455	-0.810342165	0.417743548	0.626995919
658.9098947	0.037304194	0.072964847	0.511262558	0.609167219	0.778180891
37.81497465	0.199056208	0.173773279	1.145493768	0.252004676	0.461142645
682.7952402	-0.06152684	0.07208719	-0.85350588	0.393378828	0.606547079
20.19200996	0.063774718	0.227126274	0.280789698	0.778871706	0.884794608
281.4217018	-0.087011617	0.068296647	-1.274024726	0.202654653	0.405407743
185.6783784	-0.050734343	0.101156857	-0.501541311	0.615990212	0.782777813
146.967186	-0.164475398	0.099482865	-1.653303787	0.098269053	0.253664861
284.3497081	0.057499825	0.080191106	0.717034947	0.473352534	0.67363594
13.36898487	-0.338058811	0.290763694	-1.162658262	0.244968182	0.453286065
473.3593485	-0.220489255	0.130950492	-1.68376041	0.092227981	0.243176019
40.49058315	-0.075561732	0.171241307	-0.441258793	0.659025654	0.812402149
3.159993879	-0.198617535	0.566173343	-0.350806936	0.725733193	0.854652129
294.107104	-0.007578858	0.056990968	-0.132983501	0.894206429	0.946489123
541.1060954	0.106406843	0.12196006	0.872472861	0.382950467	0.595724834

16.2171784	0.041966388	0.242787574	0.172852291	0.862767533	0.931737465
5209.408264	0.029811699	0.121973744	0.244410787	0.806912688	0.901693069
212.6353445	0.08822459	0.111293814	0.792717827	0.427942242	0.634941316
245.1939347	-0.108224384	0.086946248	-1.244727485	0.213231939	0.417828132
405.7005215	0.182247117	0.080169946	2.273259817	0.023010528	0.094650544
291.8816524	0.028707302	0.06741469	0.425831556	0.670230591	0.820676287
367.9081439	0.056408641	0.061781482	0.913034772	0.361224254	0.575339666
1.838840008	-4.303468143	1.754973738	-2.452155295	0.014200336	0.067592123
17.18346942	-0.6865516	0.46146664	-1.48775998	0.136814203	0.313954198
2407.55012	-0.147568233	0.050392655	-2.92836791	0.003407466	0.02388706
809.1989907	0.009994012	0.057903456	0.172597844	0.862967547	0.931828116
254.1775393	0.290312923	0.090559865	3.20575702	0.001347077	0.012093593
684.5483909	0.153616335	0.092062555	1.668608214	0.09519505	0.248139755
94.16863794	-0.028324708	0.175126586	-0.161738483	0.871511799	0.935937769
128.7573136	0.108964584	0.103480133	1.053000039	0.292340974	0.50758263
48.36561526	-0.593294493	0.300715975	-1.972939728	0.04850243	0.158821114
70.31040129	0.271692912	0.284257464	0.95579869	0.33917395	0.554005032
872.9502138	-0.132693206	0.079590567	-1.667197639	0.095475112	0.248750551
387.7635658	-0.138123814	0.096235865	-1.435263399	0.151212053	0.335271516
155.8714534	0.110126414	0.086271084	1.276515944	0.201773194	0.404577848
4.887584951	0.075682992	0.5469741	0.138366683	0.889950626	0.945129092
161.1674329	-0.970903224	0.556970681	-1.743185515	0.081301215	0.224686498
14.29592472	0.296497176	0.306465877	0.967472066	0.333308099	0.54871497
47.6302291	-0.135399828	0.163353893	-0.828874207	0.407175592	0.617946633
327.6894225	-0.035340119	0.11352291	-0.311303848	0.755569643	0.872365037
49.38649662	-0.166815215	0.310809294	-0.536712442	0.59146626	0.764340694
410.4966366	-0.090436494	0.103026049	-0.877802209	0.380051065	0.592897872
543.7347201	0.07541502	0.044769914	1.684502222	0.09208465	0.242992556
98.24653367	0.098219729	0.126296068	0.777694278	0.436749266	0.642902204
62.8805125	-0.33806408	0.196723446	-1.71847376	0.085710237	0.232502954
1242.085876	0.162203727	0.035455639	4.574835765	4.76594E-06	0.000128082
4.925502264	0.071431176	0.444425795	0.160726891	0.872308509	0.936384156
72.17537671	0.19123088	0.163014514	1.173091128	0.240759264	0.448115254
253.5440709	0.033702956	0.104248475	0.323294472	0.74647224	0.867050954
61.16404601	0.098144555	0.120091693	0.817246824	0.413787389	0.623557761
781.5629246	-0.064947767	0.07145857	-0.908887018	0.363409767	0.577203057
7.162032088	0.493481028	0.414554925	1.190387567	0.233894098	0.440815852
141.7172946	0.601125211	0.151958079	3.955862134	7.62591E-05	0.001266424
6.659470483	0.481635787	0.469854864	1.025073534	0.305328492	0.520217352
200.5974573	0.28392375	0.0972659	2.919047166	0.003511031	0.024436301
433.8282125	0.011956498	0.077030342	0.155218025	0.876649439	0.937787198
12.23058593	1.013139121	0.28242416	3.587296219	0.000334125	0.004065266
5.064764984	-0.411750129	0.647489297	-0.635918047	0.524829839	0.715791603
71.20035842	0.298429272	0.130518565	2.286489065	0.022225663	0.092524291
5.243010949	0.030308022	0.640613497	0.047310932	0.962265415	0.981463149
303.5674561	-0.012078053	0.069139263	-0.174691673	0.861321911	0.931215742
928.9016456	-0.550515398	0.179244868	-3.071303534	0.002131264	0.016920693
146.8703288	0.109335528	0.275822684	0.396397883	0.691811527	0.834043309

777.766088	0.179548797	0.066672551	2.692994273	0.007081349	0.040661602
412.5664388	-0.18655379	0.189372098	-0.985117618	0.324566301	0.539655353
65.66444038	0.275416273	0.275117361	1.001086489	0.316784996	0.531821101
1.904510603	0.757867003	0.785587939	0.964713134	0.334688515	0.550047536
4.345572394	0.284562755	0.533721781	0.533166839	0.593918094	0.76648701
673.116072	0.116924674	0.071897711	1.626264204	0.103893463	0.263653388
371.2947865	0.271757989	0.104062505	2.611488051	0.009014914	0.048881609
3.396064919	0.058811702	0.505371177	0.116373281	0.907356711	0.953303217
341.5884462	0.035330592	0.073579002	0.480172218	0.63110494	0.793230487
2080.18688	-0.126001601	0.063197796	-1.99376575	0.04617766	0.153831181
119.3589047	-0.066540201	0.114884884	-0.579190218	0.56246083	0.745278458
2206.024407	-0.225172533	0.073875055	-3.0480185	0.002303557	0.017917283
240.0725561	-0.131409977	0.143347723	-0.916721765	0.359288458	0.573611636
19.43519064	0.257430749	0.331469507	0.776634784	0.437374273	0.64337964
723.4568691	-0.188979409	0.132533521	-1.425898954	0.153897512	0.338537536
5.535109406	1.747395042	0.879817361	1.986088386	0.047023511	0.15561009
4.390958559	0.417441601	0.500347571	0.834303241	0.404110126	0.614990985
276.766787	-0.10935451	0.184435736	-0.592913893	0.55323878	0.738578254
176.0818717	0.043053979	0.132956682	0.323819592	0.746074622	0.866955867
9.49174812	-0.584508663	0.378138686	-1.545752087	0.122164452	0.292095383
78.59109421	-0.826066315	0.363738612	-2.271043784	0.023144328	0.095027772
13.84732114	0.104378508	0.317974503	0.32826062	0.742714616	0.86500393
2.586183552	0.136205582	0.643695137	0.21159952	0.832419484	0.916234287
148.519608	0.103533847	0.087256373	1.186547682	0.235406083	0.442703175
34.84472322	-0.151326236	0.188733976	-0.80179647	0.422670701	0.630884944
58.59925803	0.133013505	0.127961567	1.039480123	0.298581497	0.513605616
144.783298	0.175420197	0.090276452	1.943144556	0.051998695	0.166067336
72.27192415	-0.121253307	0.111968155	-1.082926719	0.278840951	0.492086323
22.03773025	-2.854119942	0.591349073	-4.826455423	1.38984E-06	4.59765E-05
48.33439713	0.024169473	0.316812337	0.076289556	0.939188734	0.971078592
919.0576166	-0.064658696	0.064284325	-1.005823674	0.314500397	0.52931583
116.7752328	-0.054828	0.112746934	-0.486292603	0.626759723	0.790677612
24.44443828	-0.15731761	0.218041133	-0.721504277	0.47059931	0.671243524
775.7577	-0.133930074	0.094161051	-1.422351099	0.154924352	0.340053348
968.2146964	0.086385299	0.05678308	1.52132113	0.12817927	0.299946775
6.755158848	-0.417248643	0.404516075	-1.03147605	0.302317634	0.517173489
727.5120382	-0.817636491	0.125579803	-6.510891642	7.4706E-11	8.15038E-09
16.71935033	0.220254549	0.267013822	0.824880701	0.409439324	0.619889505
298.8815233	0.052465323	0.065455453	0.801542428	0.422817692	0.631034236
623.4386985	-0.061461886	0.084769007	-0.72505139	0.468420504	0.670018398
5.692732303	0.8209819	1.021044097	0.804061159	0.421361655	0.629615592
51.64386162	0.372987489	0.193145155	1.931125262	0.053467564	0.169161637
93.9036082	0.638930347	0.171630578	3.722706953	0.000197098	0.002690138
885.7863449	-0.110257316	0.077268381	-1.426939642	0.153597293	0.338328895
74.07251385	0.475970811	0.137901753	3.451521116	0.000557436	0.006095692
482.3193086	-0.160306596	0.088398745	-1.813448766	0.069762634	0.203260938
587.0554983	-0.242431381	0.072190709	-3.358207528	0.000784497	0.007973293
321.1796829	-0.133637583	0.075242883	-1.776082697	0.075719288	0.214551005

97.47479917	0.303637317	0.254400485	1.193540637	0.232657707	0.439370196
11.82004931	0.080991848	0.413825637	0.195714911	0.844833313	0.923052394
9.862709349	-1.997458867	0.754905652	-2.645971536	0.008145667	0.045350361
132.2822176	-0.039364484	0.108651951	-0.362298913	0.717128671	0.849987605
62.00669775	0.26593234	0.17746204	1.498530837	0.133995387	0.309729199
45.28944811	-1.423585077	0.516024389	-2.758755412	0.005802194	0.035219363
36.43810338	-0.110208057	0.157090176	-0.701559197	0.482954106	0.682114706
17.89148863	-0.053353349	0.320300842	-0.166572615	0.867706343	0.934470438
12.78187432	0.366765735	0.404222189	0.907336968	0.364228631	0.5779081
274.8218707	0.018740479	0.119578	0.156721794	0.87546411	0.937583765
821.4783336	-0.071322646	0.118038923	-0.604229895	0.545690808	0.732598025
1.720329202	-0.026506947	0.8582457	-0.030885034	0.975361226	0.987719156
742.0925893	0.115275778	0.06412029	1.797804994	0.072207916	0.208291578
54.93303448	-0.253715441	0.191644753	-1.323884097	0.185541541	0.383659011
78.15034214	-0.183301659	0.141313513	-1.29712761	0.194587283	0.395428884
433.859445	-1.117216411	0.334586863	-3.339092279	0.000840526	0.008412413
33.23426963	0.175105323	0.208100373	0.841446462	0.400097866	0.612224804
39.2864252	-0.587306457	0.213664323	-2.748734312	0.005982586	0.035956628
2.829521481	-1.210262538	0.64642978	-1.87222584	0.061175366	0.186095483
121.3862921	0.086641838	0.101482247	0.853763497	0.393236044	0.606491244
46.71764163	-0.080890668	0.227680377	-0.355281686	0.722378574	0.852432094
138.8806301	0.0968341	0.126646446	0.764601793	0.444508701	0.649789656
481.0006408	0.037240321	0.065380123	0.569596987	0.568951072	0.749725517
1067.148469	0.010274549	0.08525571	0.120514501	0.904075593	0.951676762
412.3949817	-0.277744301	0.082742473	-3.356731927	0.000788695	0.008010461
505.486647	0.001766755	0.06709347	0.026332739	0.978991942	0.989078453
249.1879415	-0.061405118	0.123208468	-0.498383908	0.618213477	0.784271046
700.4956597	0.144215356	0.128859169	1.119170308	0.26306749	0.474432601
2.360395508	0.307574592	0.662437297	0.46430748	0.642427466	0.801283319
311.4948185	-0.449474789	0.110239791	-4.077246388	4.55722E-05	0.000830483
143.1908698	0.249629797	0.231977198	1.076096271	0.281884203	0.495833718
2.25759142	0.812880063	0.992724541	0.818837481	0.412879143	0.622644728
116.5620213	-0.516285063	0.17260813	-2.991081954	0.002779909	0.020628571
220.041226	0.28371011	0.098922206	2.868012364	0.004130594	0.027383261
47.38988533	-0.143929162	0.178712156	-0.805368614	0.420606998	0.629066856
1221.151909	-0.599778769	0.237922492	-2.52089983	0.011705517	0.058899415
283.2299373	0.096210099	0.116772311	0.823911919	0.409989608	0.620217261
12.74719194	1.235669879	0.65007996	1.900796756	0.057328639	0.177591576
7.502705835	-0.695330251	0.530703101	-1.310205745	0.190126243	0.390006191
145.1993272	-0.160159655	0.13328749	-1.20161056	0.229514448	0.43565169
178.9411475	0.00927432	0.230360666	0.040259999	0.967885844	0.983871367
56.16109971	-0.029153278	0.146640257	-0.198808151	0.84241282	0.921757179
531.0165523	-0.109369268	0.06696163	-1.633312508	0.102403287	0.261306034
58.08618549	-0.756899146	0.44891959	-1.686046152	0.09178691	0.242536042
1509.174622	-0.076681748	0.05205877	-1.472984254	0.140755282	0.319819079
432.058068	0.042514789	0.075396276	0.563884467	0.572832762	0.752170338
173.5876964	-0.641762565	0.125735734	-5.104058685	3.32445E-07	1.31022E-05
498.0952755	-0.075897232	0.068983881	-1.100216899	0.271237629	0.483274606

589.2963633	-0.010916428	0.073402833	-0.148719438	0.881775022	0.940826536
766.6688257	-0.115586593	0.04143034	-2.789902119	0.005272398	0.032875656
166.8132373	0.396938624	0.101263252	3.919868429	8.85973E-05	0.001423953
18.51965132	1.0548498	0.642059048	1.642917116	0.100400083	0.257190324
12.54441768	4.243506022	0.61792943	6.867298786	6.54289E-12	1.01686E-09
2.816528909	2.670290307	0.735912675	3.628542352	0.000285026	0.003605238
112.1910704	-0.637851864	0.392092627	-1.626788721	0.103781978	0.263517926
5.641308298	1.103536276	0.412582083	2.674707216	0.007479454	0.042513555
694.7220254	0.291842845	0.094711804	3.081377751	0.002060451	0.016541844
38.86811943	-0.707591801	0.261825315	-2.70253394	0.006881316	0.039916478
2450.308435	0.24504699	0.089977755	2.723417484	0.006461035	0.038177581
337.7552253	-0.198218772	0.082012253	-2.416940925	0.015651557	0.072329253
1373.862668	-0.099134049	0.090827683	-1.091451921	0.275074077	0.487737632
14.5711994	-0.10030094	0.317164206	-0.316242938	0.751818119	0.870526878
33.6445296	0.292602509	0.226530003	1.291672206	0.196470687	0.397770442
1558.521492	-0.705304164	0.105142432	-6.708083034	1.97198E-11	2.54081E-09
392.2698311	-0.29269039	0.148806649	-1.966917414	0.049192733	0.160253328
322.68206	0.044638004	0.094081208	0.474462488	0.635170142	0.795604693
55.43311329	0.130205012	0.146899113	0.886356696	0.375425346	0.588859665
189.4323535	-0.184070312	0.097124495	-1.89519968	0.058065953	0.179302437
209.7097524	0.210459408	0.230391619	0.913485524	0.360987244	0.575135769
5.960211374	0.3055457	0.451857343	0.676199479	0.498914003	0.695021779
133.1005114	-0.118970468	0.106557547	-1.116490301	0.26421232	0.475552721
2.690735347	0.160196359	0.619983662	0.258388033	0.796107451	0.894773586
4.256261915	2.03708234	1.101556394	1.849276488	0.064417899	0.192914101
59.42697073	-0.515571963	0.195135318	-2.642125315	0.008238757	0.045744954
2258.260793	-0.09735497	0.092973442	-1.047126657	0.295041166	0.509807864
1034.820568	0.442190817	0.096413781	4.586386027	4.50984E-06	0.000122599
1288.323199	0.152756775	0.074010621	2.063984489	0.039019187	0.136275993
575.1278545	0.546799968	0.148595989	3.679776089	0.000233439	0.003093863
207.4261034	-0.136065462	0.083463663	-1.630235927	0.103051643	0.262327695
1027.971348	-0.239254245	0.089004816	-2.688104497	0.007185891	0.041147153
228.5016506	0.13756853	0.107747909	1.276762875	0.201685976	0.404501433
12.67469882	0.532355986	0.372898343	1.427616925	0.153402149	0.338034645
46.63898804	0.641373155	0.236481785	2.712146122	0.006684912	0.039149391
169.9587772	-0.180655104	0.081118649	-2.227047758	0.025944082	0.103312257
321.3188548	0.175682893	0.059175569	2.968841642	0.002989246	0.02172247
127.0799034	0.519625246	0.125415213	4.143239343	3.42434E-05	0.000662898
148.8681027	0.313635638	0.083906505	3.73791802	0.00018555	0.0025705
896.5979747	-0.109662922	0.073726586	-1.487427092	0.136902046	0.314024548
126.5425373	0.069656964	0.278293399	0.250300453	0.802355006	0.898938817
975.3055433	0.882573555	0.109214136	8.081129303	6.41697E-16	2.34918E-13
39.61422984	-0.248600298	0.176669588	-1.407148227	0.15938344	0.346807924
236.8199977	0.007821659	0.075226566	0.103974688	0.917189436	0.95910745
5.5787555	0.747315653	0.412395963	1.81213135	0.069965903	0.203576974
741.942682	0.20878359	0.051187717	4.078782997	4.52721E-05	0.000825927
275.1761656	0.055423516	0.065436223	0.846985253	0.397003342	0.609588078
98.73588284	0.198692947	0.116801991	1.70110925	0.088922474	0.238171511

40.88753537	-0.485016916	0.288995858	-1.678283279	0.093291811	0.245234455
136.5344982	-0.286747111	0.096552437	-2.969858862	0.002979366	0.021660227
620.2548719	-0.036167391	0.112699123	-0.320919892	0.748271099	0.868038735
1209.711362	-0.18810674	0.085506414	-2.199913799	0.027813011	0.108319516
660.6164031	-0.038757978	0.070343539	-0.550981345	0.581646463	0.758169938
16.60042198	-0.25775834	0.270520597	-0.952823344	0.340679584	0.555270316
364.2286767	0.258229859	0.069818903	3.698566562	0.00021682	0.002920606
258.3697178	0.144265531	0.080486922	1.792409592	0.073067379	0.209828068
715.9145805	-0.11372851	0.06506392	-1.747950486	0.080472602	0.223257939
688.56983	-0.125301535	0.158075464	-0.792669092	0.427970643	0.634941316
28.7169407	-0.008477089	0.298757336	-0.028374495	0.977363466	0.988278901
16.36063947	0.657554074	0.529992375	1.240685912	0.214721801	0.419252375
92.65286929	-0.115475285	0.113640101	-1.016149089	0.309558396	0.524818876
6.06915835	0.787251085	0.762423787	1.032563645	0.301808149	0.516838611
155.4096105	-0.227230655	0.119855499	-1.895871752	0.057977005	0.179094915
2.081259491	-0.643385402	0.848440952	-0.758314884	0.448262499	0.653164373
386.1979633	0.18891198	0.07014119	2.693310152	0.007074642	0.04064111
3.940192573	-2.109885862	1.35331163	-1.559053965	0.118983608	0.287578632
61.00054646	-3.219032563	0.819504783	-3.928021693	8.56475E-05	0.001387164
222.8889761	-0.097989159	0.083057649	-1.179772847	0.238090572	0.445647209
206.7987045	0.014623253	0.085924191	0.170187842	0.864862413	0.932807942
1616.478661	0.012308965	0.093850946	0.131154402	0.895653166	0.947455865
406.2344719	-0.170546698	0.099537833	-1.713385693	0.086641607	0.233989152
56.79690636	-0.563520509	0.217416079	-2.591898958	0.00954478	0.0510025
742.7173275	-0.432086848	0.154365699	-2.79911179	0.005124339	0.032061666
12.96620826	-1.928094469	1.144807342	-1.684208685	0.092141345	0.243103223
34.72877777	-0.126150136	0.272477479	-0.46297454	0.643382616	0.801806886
163.8877034	-0.055611127	0.107200786	-0.518756708	0.603930411	0.774252886
2206.307536	0.04438619	0.058177498	0.762944283	0.445496625	0.650630443
12.9216791	0.430217573	0.294062077	1.463016167	0.143462955	0.323577317
202.8101179	-2.317752406	0.783148968	-2.959529414	0.003081093	0.022203816
7.486071734	-0.772274835	0.687674181	-1.123024328	0.261427162	0.472728727
43.06162268	-3.10957298	0.905926686	-3.432477515	0.000598093	0.00644782
75.72523103	-0.923952311	0.257513189	-3.587980543	0.000333249	0.004060611
3.744480071	-0.883042706	0.49385887	-1.788046665	0.073768479	0.211166278
53.29260422	0.019453818	0.160051863	0.121546961	0.903257821	0.951456563
27.12929057	-0.090680225	0.250844807	-0.361499312	0.717726219	0.850267635
330.0944476	-0.012279274	0.137928636	-0.089026288	0.929061018	0.965637301
278.5931227	-0.079254275	0.104198135	-0.760611263	0.44688929	0.651970791
55.39806008	-0.33290519	0.150339599	-2.214354652	0.026804392	0.105707454
4.397828262	-0.622327303	0.501311053	-1.241399526	0.214458194	0.419063612
170.7688085	-0.403321934	0.116530727	-3.461077983	0.000538017	0.00592466
1101.165129	-0.133168998	0.088753093	-1.500443469	0.133499567	0.308799756
1303.580935	-0.168926198	0.066027847	-2.558408398	0.010515252	0.054337597
2.761445804	-1.068339302	0.627761366	-1.70182391	0.088788383	0.237953444
177.5321993	-0.210674139	0.238614139	-0.882907193	0.377286417	0.590154809
332.6018902	0.08671038	0.105557067	0.821454996	0.411387149	0.62130472
15.69439068	0.074383792	0.506420307	0.146881535	0.883225527	0.941703277

1476.424522	-0.019529479	0.103995965	-0.187790735	0.851040699	0.926405873
11.82130161	0.135038839	0.379116101	0.356193891	0.721695367	0.85233418
13.93925858	-0.056210506	0.285239988	-0.197063906	0.843777528	0.92240916
153.3217357	0.00575227	0.101730567	0.056544162	0.954908316	0.97818152
1225.182829	-0.064182502	0.092481744	-0.694001853	0.487681047	0.685967982
2.97353035	-0.008054839	0.540490526	-0.014902831	0.988109701	0.993893344
525.4919987	0.380822433	0.206803047	1.841473995	0.065552128	0.195069681
5.798831612	0.675633645	0.423537433	1.595215895	0.110663965	0.275057056
60.83542725	-0.206488073	0.32117149	-0.642921553	0.520275003	0.712410473
3.823728646	0.153636843	0.85236868	0.180246936	0.856958712	0.92936531
1210.671106	0.599194264	0.085064126	7.044030146	1.86758E-12	3.38093E-10
1406.824051	-0.092429495	0.055513721	-1.664984684	0.095915811	0.249447772
234.9661483	-0.112748862	0.074685622	-1.509646153	0.131133738	0.305040553
13.65462956	0.580320554	0.817739381	0.709664433	0.477912253	0.677549609
600.6878202	0.076415925	0.074888289	1.020398861	0.307539334	0.522484231
322.1079899	0.034735005	0.112705904	0.308191531	0.757936596	0.873603573
124.2650471	-0.298783483	0.260179852	-1.148372866	0.25081467	0.459715273
157.2808529	-0.178103513	0.12297232	-1.44832197	0.147527026	0.32954037
1.87662189	0.144237653	0.898915379	0.160457432	0.872520752	0.936471878
536.490303	-0.18172573	0.071243116	-2.550782995	0.010748123	0.055194692
37.94992724	-0.060534304	0.151356477	-0.39994525	0.689196843	0.832209103
382.8886202	-0.191040644	0.076278659	-2.504509728	0.012262123	0.060900277
36.75354062	-0.283834017	0.227917817	-1.245334924	0.213008663	0.417651121
3.012049829	-0.648997455	0.658796746	-0.985125472	0.324562443	0.539655353
7.909157441	0.427806365	0.317912952	1.345671393	0.178408516	0.374093815
93.40140001	-0.968759612	0.273480495	-3.542335305	0.000396601	0.004663529
22.21370783	-0.093658837	0.38800319	-0.241386772	0.809255364	0.903352275
20.31779617	1.052322407	0.499541331	2.106577259	0.03515424	0.126779979
678.0288593	0.092780873	0.071148417	1.304046905	0.19221759	0.392828754
883.7309712	-0.046182072	0.078082615	-0.591451399	0.55421801	0.739106897
105.934413	0.243624121	0.106080425	2.296598281	0.021641697	0.090857623
7.448770661	0.115553137	0.48493995	0.238283394	0.8116613	0.904995483
1196.294577	0.019442223	0.055306695	0.351534708	0.725187239	0.854436745
11.78952165	0.069738152	0.335938239	0.207592181	0.83554741	0.917771165
125.7307437	-0.169426278	0.098969577	-1.711902616	0.086914617	0.234457408
883.1048877	-0.01105413	0.081586866	-0.135489083	0.892225195	0.94585057
24.12273193	-1.264814827	0.521157174	-2.42693546	0.015226964	0.071110984
614.9675499	-0.145912848	0.062261633	-2.343543538	0.019101533	0.08316032
55.06425631	-0.333407952	0.247182558	-1.348832838	0.177390664	0.372722796
138.628226	-0.221308046	0.115371821	-1.918215758	0.055083659	0.172250817
199.3566829	-0.215307606	0.092374231	-2.330818928	0.01976291	0.085273487
68.81267858	-0.179397926	0.127950902	-1.402084104	0.160890133	0.349302063
218.1559783	-0.392482284	0.247258113	-1.587338346	0.112436035	0.277660207
113.445064	-0.066444584	0.298841838	-0.222340301	0.824048982	0.911434821
2138.916783	-0.001600736	0.055034376	-0.029086114	0.97679591	0.988158577
262.1360566	0.252284041	0.125892698	2.003960878	0.045074251	0.151171258
19.93497689	1.371964268	0.331859293	4.134174629	3.56233E-05	0.000684781
173.9798178	-0.050719615	0.170974252	-0.296650604	0.766733273	0.878445228

41.37521677	0.517475781	0.201601619	2.566823535	0.010263483	0.053388259
1532.279148	0.141817745	0.064882154	2.185774308	0.028832121	0.110898988
438.4481736	-0.320600205	0.110237973	-2.90825563	0.003634511	0.024979114
143.4492147	-0.195705174	0.120352883	-1.626094608	0.103929531	0.263653388
463.040372	0.217355557	0.074912735	2.901450012	0.0037144	0.025337901
2.444028227	2.057584155	0.720146664	2.857173767	0.004274318	0.028098607
2676.851754	0.524186919	0.09143631	5.732809198	9.87807E-09	6.14081E-07
16.97876089	-1.405330339	0.568617493	-2.47148629	0.013455271	0.065175576
14.24469862	-6.885387241	2.97577437	-2.313813612	0.020677948	0.088068387
2.402854551	-4.723601813	2.751356371	-1.716826604	0.086010859	0.232886268
50.83120265	-1.767398843	0.553029611	-3.19584848	0.001394203	0.012428626
446.2721921	0.140287807	0.133997858	1.046940664	0.295126946	0.509902601
210.0965124	0.336350899	0.107146451	3.139169748	0.001694273	0.014269657
74.29281161	0.323120925	0.17207236	1.877820034	0.060405789	0.184624297
14.59481047	0.192611499	0.295824792	0.651099923	0.514981986	0.708223828
8.105924232	0.270603774	0.517503624	0.522902181	0.601042326	0.772052342
6.554507333	2.19742588	1.082450886	2.030046728	0.04235179	0.144691701
159.7639025	0.864236003	0.247163991	3.496609682	0.000471211	0.005335207
262.0991038	1.910074549	0.58957047	3.2397731	0.001196248	0.011071347
436.8296872	-0.069220419	0.325460109	-0.212684802	0.831572822	0.915853099
54.72852051	0.722654019	0.336730873	2.146087804	0.031865974	0.118527897
8.194356532	-0.923062174	0.460607038	-2.004012308	0.045068742	0.151171258
78.94755045	-0.268526739	0.256330673	-1.047579426	0.294832421	0.509660997
147.5682052	0.191452759	0.11494575	1.665592314	0.095794643	0.249284405
132.7903209	-0.0220882	0.202276241	-0.109198192	0.913045295	0.956595535
147.7788202	0.209067605	0.09528533	2.194121636	0.028226666	0.109336021
1110.127013	-0.076390252	0.09272558	-0.823831487	0.410035314	0.620217261
15.7854837	-0.636472132	0.416668608	-1.527526	0.126630267	0.297930165
1120.764891	0.073781341	0.114159147	0.646302497	0.518083471	0.710531727
33.97922262	0.058124058	0.187766657	0.309554736	0.756899582	0.873190736
4.848743953	-0.174726303	0.468264478	-0.373135933	0.709047276	0.84494531
28.01059362	-0.116674866	0.172519009	-0.676301506	0.498849237	0.695021779
130.1561411	-0.334012024	0.091594425	-3.646641401	0.00026569	0.003417718
12.74108533	0.071613124	0.289485843	0.247380401	0.804613841	0.900000571
59.47775735	-0.140583678	0.154153609	-0.911971375	0.361783786	0.575736678
170.8917759	-0.101805936	0.097877799	-1.040133073	0.29827808	0.513140465
3.027203092	0.408820279	0.605311876	0.675387837	0.499429391	0.695368475
7.81884744	-1.533811076	0.753232834	-2.036304056	0.041719833	0.143156119
368.0884445	-0.261790881	0.093415109	-2.802446881	0.005071657	0.031816631
12.73217125	-0.053675048	0.28582684	-0.187788692	0.851042301	0.926405873
1835.099506	-0.11236138	0.066056051	-1.701000576	0.088942879	0.238171511
322.9356207	-0.243774529	0.139112509	-1.752355203	0.079712749	0.221629678
236.6086053	0.077822222	0.091866221	0.847125533	0.396925155	0.609578167
376.7316824	-0.348720556	0.099177076	-3.51614073	0.000437869	0.005033811
3.372242451	-0.730942766	0.748921954	-0.975993241	0.329067814	0.544661098
330.8639743	-0.258973978	0.054812107	-4.724758688	2.30389E-06	6.97688E-05
158.5975933	-0.224638653	0.093227582	-2.409572871	0.015971206	0.073371347
6.026345178	-0.289630268	0.43529338	-0.665367959	0.505815173	0.700765214

1054.815208	-0.2742537	0.116833724	-2.347384727	0.018905722	0.082504071
59.89634512	0.894284008	0.166618444	5.367256997	7.99431E-08	3.69939E-06
725.1119411	0.05003587	0.095616418	0.523297893	0.600766967	0.771818998
4.069856105	0.923863533	0.90975676	1.015506093	0.309864644	0.525104037
500.6929192	0.044260457	0.091699385	0.482669072	0.629330727	0.79225209
135.9124381	-0.024750938	0.091823178	-0.269550008	0.787506465	0.890225289
165.8961912	0.243716014	0.080619111	3.023055095	0.002502367	0.019091146
195.2520356	-0.126076537	0.105161786	-1.19888166	0.230573964	0.436936024
77.59346817	-0.010802563	0.20220253	-0.053424469	0.95739371	0.979025697
679.0882164	0.130821017	0.127132551	1.029012759	0.30347368	0.518343743
74.47902096	0.270476565	0.198168307	1.364883058	0.172289808	0.36512481
1.990331417	-0.575295287	0.798396749	-0.720563163	0.471178325	0.671530536
30.69159536	0.369060752	0.171104617	2.15693042	0.031011086	0.11645423
54.31329916	-0.068253356	0.202606882	-0.336875803	0.736210527	0.861278654
12.64017753	0.370635597	0.335301564	1.105379862	0.268995026	0.480788115
57.09668424	0.038149285	0.1296728	0.294196506	0.76860775	0.879247558
56.38291702	-0.051093987	0.171407869	-0.298084257	0.765638857	0.877977141
256.3479417	-0.137927909	0.082173666	-1.678492826	0.09325093	0.245166903
21.37982823	0.011218247	0.21330568	0.052592351	0.958056712	0.979356635
990.982738	-0.102662446	0.069942163	-1.467819146	0.142153355	0.321679732
761.2653722	0.007659636	0.05347516	0.143237262	0.886102802	0.943111133
366.463586	-0.042040499	0.105099156	-0.400007956	0.689150657	0.832209103
335.2332806	0.067759946	0.112395654	0.602869808	0.546595303	0.733393958
99.35132153	-0.093596359	0.181985034	-0.514308004	0.607036667	0.776766636
605.9489711	0.279152228	0.071862632	3.884525534	0.00010253	0.001596482
97.02369196	0.283501908	0.132475327	2.140035544	0.032351894	0.11976744
151.7330013	0.302390365	0.08731411	3.463247402	0.000533697	0.005881024
207.562614	0.098702413	0.0739635	1.334474613	0.182048361	0.378662408
1082.899413	0.079341236	0.059637966	1.330381314	0.183392668	0.380553069
126.8389196	0.214244846	0.111126321	1.92793971	0.053862626	0.169921742
1208.3809	-0.302695516	0.079325656	-3.815858949	0.00013571	0.001987277
60.84743686	0.051198091	0.137945041	0.371148473	0.710526949	0.84564098
23.46787353	-0.346378687	0.219380849	-1.578892092	0.11436081	0.280726901
1228.451125	0.278148242	0.102866713	2.703967431	0.0068517	0.039828833
3.792275232	0.753115551	0.59049934	1.275387626	0.202172076	0.404987157
404.8741652	-0.142903356	0.078286842	-1.825381537	0.067943473	0.19987514
1548.158127	-0.171007418	0.134920358	-1.267469341	0.20498752	0.408141697
4.318691721	-0.375846549	0.63766181	-0.58941361	0.555583849	0.740269195
31.93762201	0.578672152	0.290607726	1.991248338	0.046453592	0.154445303
2.448217031	-0.225396662	0.857496376	-0.262854361	0.792662834	0.893093493
11.08962374	-1.233941393	0.329941418	-3.739880248	0.000184108	0.002559489
245.6370624	0.037923343	0.0905959	0.418598892	0.675509299	0.824469335
281.1369594	0.091100574	0.064183004	1.419387814	0.155785983	0.341188286
28.05840995	0.174103584	0.19205877	0.906512023	0.364664904	0.578202266
93.87045995	0.044096237	0.118143819	0.373242018	0.708968326	0.844935557
9.329352947	0.310329996	0.352206545	0.881102298	0.378262453	0.591281638
7.501331622	-0.074855451	0.434633853	-0.172226463	0.863259495	0.931920109
11.47988369	-1.168929115	0.828729661	-1.410507153	0.158389986	0.345513324

9652.128654	0.092136597	0.078065949	1.180240537	0.237904561	0.445469394
11.82282	0.19437004	0.274682121	0.707618099	0.47918245	0.678327657
232.6173647	0.224857227	0.146695713	1.532813897	0.125321715	0.296332703
4.67906995	0.654906176	0.500442064	1.308655334	0.190651128	0.390623187
728.5010815	0.397189381	0.08970614	4.427672192	9.52555E-06	0.000228752
15.45028802	0.064617072	0.258473971	0.249994503	0.8025916	0.899069005
37.71046026	-0.884431088	0.405156415	-2.182937392	0.029040421	0.111466651
297.1509238	-0.349364438	0.157768802	-2.214407629	0.026800751	0.105707454
158.5495162	-0.115585816	0.085568399	-1.350800257	0.176759426	0.37194211
239.5696744	0.174083655	0.078429807	2.219610895	0.026445192	0.104851527
991.8688235	0.191359292	0.063959762	2.991869991	0.002772743	0.020594306
28.26244145	-0.255741493	0.446688252	-0.572527915	0.56696438	0.748471553
38.88746024	0.653567605	0.541738598	1.206426138	0.227653223	0.433427315
330.6282184	0.171182847	0.131242926	1.304320563	0.192124307	0.392803996
199.383471	0.267103966	0.129027945	2.070124934	0.038440646	0.135169949
194.6573119	0.172048896	0.081900998	2.100693529	0.035667878	0.12821135
15.43082385	-0.462580249	0.341513668	-1.35449996	0.175576926	0.369973687
86.96225947	-0.572440799	0.151089742	-3.788746949	0.000151409	0.002172746
7649.910362	0.026806904	0.084108623	0.318717668	0.749940616	0.869240956
8.405868732	0.668212333	0.304649214	2.19338276	0.028279813	0.109464672
273.0524355	0.358729403	0.09004478	3.983900041	6.77934E-05	0.001159739
3153.062568	0.037409671	0.073742906	0.507298574	0.611945339	0.780156904
2.225407644	0.166335766	0.650685937	0.255631414	0.798235459	0.89568199
403.9976155	0.036536485	0.084219326	0.433825418	0.664415222	0.81671216
133.4969274	-0.165479244	0.134364808	-1.23156686	0.218110927	0.422824124
10.97619103	0.179072001	0.392028846	0.456782717	0.64782723	0.804718256
68.05436255	0.096627918	0.198648766	0.486425969	0.626665182	0.790677612
223.5803547	0.115464026	0.148063236	0.779829142	0.43549145	0.642075928
83.47127668	-0.056207171	0.121199728	-0.463756573	0.64282216	0.801477463
429.7934966	0.233844352	0.080100406	2.919390348	0.003507168	0.024419729
12.11717259	-0.241335982	0.286895692	-0.841197652	0.400237216	0.612232486
376.2833429	0.10702975	0.116843638	0.916008363	0.359662509	0.573951606
35.9845142	0.312692542	0.187096622	1.671289083	0.094664592	0.247069785
4.063262968	-0.353488721	0.654083523	-0.540433612	0.588898033	0.76241798
42.03837043	-0.007742428	0.264896727	-0.0292281	0.97668267	0.988158577
8.547431633	-0.077252371	0.460899043	-0.167612349	0.866888255	0.934032728
185.221171	0.070485529	0.092942576	0.75837718	0.448225215	0.653164373
8.488123465	-0.122494281	0.420380097	-0.291389344	0.770753563	0.88035736
428.3719505	0.215735188	0.060555538	3.562600437	0.000367199	0.004393059
511.9744576	0.668703384	0.083782554	7.9814156	1.44664E-15	4.76641E-13
933.910136	0.004917772	0.098984933	0.049682027	0.960375779	0.980642905
300.9263099	0.246616719	0.085373381	2.888683972	0.003868577	0.026033877
2759.97516	-0.118643558	0.097111025	-1.221731093	0.221809334	0.426580314
9.075155831	0.606957008	0.374845554	1.619218907	0.105400171	0.26595268
199.9866645	-0.203261934	0.103804366	-1.958125091	0.050215336	0.162587942
4710.82592	0.108359327	0.04190553	2.585800163	0.009715324	0.051579197
1306.772128	-0.037757473	0.088106658	-0.428542792	0.668255986	0.819232707
212.7607754	-0.23278278	0.105723094	-2.201815806	0.027678322	0.107892187

147.9609862	0.147287336	0.101148417	1.456150676	0.145350971	0.326316694
268.6393889	-0.14995641	0.134818892	-1.112280395	0.266017611	0.477695021
144.2883858	-0.194565166	0.101049332	-1.925447318	0.054173421	0.17041301
360.7011032	-0.298054034	0.209959749	-1.419577021	0.155730859	0.341112907
5.46466202	-0.350737825	0.631729583	-0.555202471	0.578756179	0.756640687
96.8859058	0.054020398	0.117281868	0.46060315	0.645083351	0.803015198
1304.187777	0.266558675	0.07370046	3.616784403	0.000298286	0.003725516
5466.196293	-0.018579844	0.074744313	-0.248578699	0.803686687	0.899390971
1716.913204	0.042612555	0.046326523	0.919830637	0.35766127	0.572182625
4.825395164	0.36068423	0.460812408	0.782713799	0.433795197	0.640547139
445.4366662	0.149381775	0.054928818	2.719551984	0.006537042	0.038474894
32.30513153	0.36659592	0.322821048	1.135601046	0.256123571	0.466281325
36.72535514	-0.990179659	0.446505728	-2.217619164	0.02658081	0.105220077
151.178637	-0.037475412	0.092981982	-0.40303951	0.686919159	0.830726992
1015.784956	-0.136265223	0.130180655	-1.046739421	0.295219777	0.509956025
16.5962772	0.613251852	0.286458167	2.140807712	0.032289548	0.119644177
2028.814521	-0.245363249	0.096225741	-2.54987123	0.010776271	0.055270328
25.21954571	0.885832579	0.384319196	2.304939718	0.02116995	0.089561826
23.5605561	0.479380236	0.262259836	1.827882772	0.067567147	0.198981262
763.450041	0.019588288	0.062721414	0.31230623	0.754807806	0.871827159
124.5956976	0.05156324	0.155937491	0.330666085	0.740896723	0.863925015
1452.484044	-0.243963604	0.070700731	-3.450651778	0.000559235	0.006108533
179.2195761	0.187966424	0.099257189	1.893731089	0.058260713	0.179829087
1098.555036	-0.0328648	0.079574773	-0.413005261	0.679602759	0.826777746
44.6583775	0.068954697	0.214158478	0.321979766	0.747468023	0.867534748
6.672911836	0.513258744	0.491879317	1.043464782	0.296733083	0.511392034
1528.999073	0.498568796	0.151373876	3.293625097	0.000989043	0.009567526
611.7133316	-0.104758213	0.207602542	-0.504609489	0.613833144	0.781595858
23.13126975	1.000315908	0.227038666	4.405927532	1.05332E-05	0.000248602
800.2372849	0.138347077	0.054034989	2.56032398	0.010457462	0.054157886
14.66840678	1.226019691	0.25966795	4.721490241	2.34123E-06	7.06399E-05
37.81099968	-0.159625013	0.213080201	-0.749131134	0.453778171	0.658108374
132.8007471	-0.0268762	0.094848559	-0.283359076	0.776901596	0.884146915
1449.622367	0.162466206	0.049887343	3.256661823	0.001127307	0.010600027
14.18572976	1.443880675	0.296636781	4.867503852	1.13017E-06	3.80816E-05
166.9997477	0.233822776	0.110286464	2.120140302	0.033994215	0.123788837
5.96249403	0.11771636	0.469649476	0.250647273	0.80208683	0.898760606
238.7738586	-0.065529565	0.067418742	-0.971978457	0.331061266	0.546428544
22.72971626	-0.016911087	0.212886969	-0.079436929	0.936685097	0.970184161
8.478112465	-0.554854607	0.643172424	-0.862684074	0.388311191	0.601532943
7.043791317	0.748461136	0.459738744	1.628014056	0.103521905	0.263161558
520.0585585	-0.100551278	0.103617511	-0.970408165	0.33184308	0.547335092
181.5592672	0.002675405	0.159909144	0.016730783	0.986651389	0.993283732
1054.013594	-0.071952175	0.052100623	-1.381023312	0.167271793	0.358524052
10.46719863	-0.095544763	0.314572738	-0.30372868	0.761334606	0.875473689
62.59386107	-0.146770679	0.153513575	-0.95607622	0.339033728	0.554005032
409.1394903	0.060166664	0.058766798	1.023820682	0.30591998	0.520794228
6.704984653	0.25293001	1.012517106	0.249803197	0.802739548	0.899069005

22.74122984	1.15333316	0.461645129	2.498311124	0.012478662	0.06167944
23.79806713	-0.449833162	0.253106452	-1.777248899	0.075527297	0.214268255
10.36685199	-1.661343947	0.606915303	-2.737357153	0.006193501	0.036968021
6.598010938	0.240681324	0.443762359	0.542365344	0.587566849	0.761751497
15.67811102	0.470486047	0.273846157	1.718067007	0.085784394	0.232589221
2.938532972	-1.814352728	0.8716283	-2.081567027	0.037382036	0.132579474
10.09134453	-1.244845872	0.322796553	-3.856441029	0.00011505	0.001752916
4.288508438	0.349795078	0.895973881	0.390407673	0.696235115	0.836908188
2483.804823	-0.17599584	0.081361124	-2.163144161	0.030530093	0.115356138
1855.401724	0.105535486	0.045949014	2.296795467	0.02163044	0.090856674
1640.798002	0.049088394	0.059526387	0.824649313	0.409570716	0.619889505
542.3314121	0.141378168	0.079691031	1.774078799	0.076050117	0.215044564
554.4321884	-0.199376724	0.082220386	-2.424906195	0.015312343	0.071339235
267.0772852	0.132153032	0.090912033	1.453636303	0.146047167	0.327299826
16.43784093	-0.380733397	0.324804011	-1.172194261	0.241119072	0.448632888
2.700069069	0.525703555	0.885530393	0.593659527	0.552739858	0.738151461
41.41683091	0.160613494	0.190313347	0.843942357	0.398701617	0.611110015
803.1601867	-0.271093422	0.073610438	-3.682812234	0.000230675	0.003062683
2303.201815	-0.10865138	0.164296712	-0.661311959	0.508412273	0.702907521
278.2045907	-0.486368185	0.112524196	-4.322343144	1.54381E-05	0.00034415
152.0288589	0.127153559	0.141758376	0.896973868	0.369732861	0.583259519
591.7233686	-0.001871705	0.048689358	-0.038441774	0.969335455	0.984818519
1049.527309	0.064481816	0.054211483	1.189449399	0.234262872	0.44120802
528.6841436	0.282283298	0.093603073	3.015748204	0.00256346	0.019452071
2.479760457	0.532720089	0.637991848	0.834995135	0.403720448	0.614855381
97.59385234	0.113780553	0.128859436	0.882981924	0.377246038	0.590154809
77.47133504	-0.300175457	0.255012555	-1.1771007	0.239155316	0.446593139
1351.413686	-0.541080254	0.584453391	-0.925788544	0.354555882	0.569182771
3.078031282	-0.246541267	0.71609957	-0.344283501	0.730633091	0.857663498
1306.786278	0.182530294	0.111706373	1.634018621	0.102254941	0.261039415
32.39755561	0.635212982	0.476676005	1.332588541	0.182666868	0.37971659
57.93865619	0.102846567	0.159829196	0.643477976	0.519914001	0.712159123
2791.389756	0.078131234	0.131889918	0.592397319	0.553584561	0.738800394
26.65775298	0.066752635	0.276325853	0.241572166	0.809111692	0.903314313
163.7405943	-0.183503306	0.099563358	-1.843080735	0.065317224	0.194560608
40.27081473	-0.05885492	0.173332646	-0.339548961	0.73419622	0.859885435
175.767403	-0.23145808	0.111594016	-2.074108352	0.038069249	0.134322726
211.0006836	-0.114380297	0.083236544	-1.374159614	0.169392136	0.361378664
260.3569045	0.062367152	0.068612269	0.908979577	0.363360906	0.577203057
4964.90887	-0.078912372	0.436986077	-0.180583265	0.856694692	0.92936531
89.63737652	-0.253115246	0.155666399	-1.626010805	0.103947357	0.263653388
74.4014049	-0.093864635	0.159438646	-0.588719471	0.556049474	0.740545496
85.93507784	-0.31591773	0.200718983	-1.573930498	0.115503518	0.282309278
132.0668504	-0.422238878	0.204395377	-2.065794657	0.038847873	0.135950587
7358.01465	-0.01507021	0.098134349	-0.153567124	0.877951061	0.938489926
290.7617498	-0.194737328	0.078102963	-2.493341102	0.012654721	0.062286787
353.4338348	0.092268558	0.084173523	1.096170799	0.273004036	0.485373245
6.771107936	0.195858961	0.438601085	0.446553753	0.655197293	0.809943754

393.3092722	0.022651192	0.075334913	0.30067323	0.763663684	0.876879872
2.007244342	-0.067603325	0.758971649	-0.089072267	0.929024478	0.965637301
263.1328593	-0.109951787	0.147268068	-0.746609831	0.455299113	0.659451168
46.15797122	0.728397571	0.213369684	3.413781923	0.000640678	0.006796838
693.1763564	0.3713327	0.063045308	5.889933934	3.8635E-09	2.80385E-07
8.352306684	-1.117458713	0.795869209	-1.404073308	0.160297021	0.348335724
187.3907348	-0.075096436	0.094829719	-0.79190824	0.428414182	0.635370474
317.3466331	0.082022163	0.063740041	1.286823195	0.19815592	0.399757609
2090.298861	0.04634866	0.079276219	0.584647718	0.558784615	0.74309152
3.516930829	-0.496446426	0.689261783	-0.720258163	0.471366059	0.671621212
239.1545314	-0.198685202	0.083768797	-2.371828275	0.017700315	0.078766881
802.6390857	0.003418141	0.069524756	0.049164379	0.960788298	0.980731546
5866.952986	-0.147199896	0.112292184	-1.310865028	0.189903368	0.389684813
229.7104187	0.149335403	0.061162716	2.441608439	0.014621996	0.068961571
358.0497402	-0.051947987	0.098329986	-0.528302599	0.597289328	0.769090541
15.19677461	-2.098167251	0.770497609	-2.723132721	0.006466607	0.038196805
13.00506438	-2.299887464	0.541768943	-4.245144526	2.18453E-05	0.000458444
73.11696659	-1.201425003	0.499564469	-2.404944861	0.016174906	0.073956537
338.2172453	-0.124009991	0.079630231	-1.557323012	0.119393815	0.288250149
23.46770587	0.18733025	0.222914874	0.840366757	0.400702782	0.61246777
285.1554731	0.281143045	0.168167356	1.671805114	0.094562758	0.247020171
119.7562215	0.263282004	0.083859532	3.139559662	0.00169202	0.014257969
172.6054268	-0.060643482	0.112958606	-0.53686464	0.591361118	0.764264772
160.4952655	0.130177012	0.091693338	1.419699787	0.1556951	0.341079931
283.8793014	0.198182046	0.079506898	2.492639647	0.012679746	0.062391318
1058.994843	0.349586421	0.042244553	8.275301652	1.28131E-16	5.70494E-14
27.70223611	0.165319503	0.195110443	0.847312427	0.396821002	0.609578167
151.0338973	-0.009295079	0.111747451	-0.083179339	0.933708941	0.968634829
83.5900529	-0.330023492	0.116467179	-2.833617966	0.004602432	0.029640524
413.2640936	0.182664564	0.072207686	2.529710795	0.011415658	0.057881436
1392.002108	-0.157234258	0.050871858	-3.090790581	0.001996244	0.016194627
1334.237489	-0.516405209	0.153526051	-3.363632472	0.000769239	0.007866198
59.51542767	-0.140242942	0.137566827	-1.019453201	0.30798786	0.523034772
109.7388388	0.08846425	0.129021632	0.6856544	0.492931068	0.690017157
2206.062012	0.089215495	0.096308671	0.926349562	0.354264347	0.569096189
844.9727759	-0.166672446	0.054062085	-3.082982233	0.002049374	0.016477006
92.45944503	0.137176823	0.108051193	1.269553991	0.204243547	0.407201766
423.2465538	-0.089023652	0.075125823	-1.184994026	0.236019809	0.44321208
128.2775958	-0.322576595	0.105705506	-3.051653675	0.002275845	0.017781276
55.8119837	-0.210816837	0.143671771	-1.467350448	0.142280747	0.321803316
692.5207513	-0.236385623	0.071386428	-3.31135244	0.000928462	0.009093628
6.099729037	0.722657821	0.611789181	1.181220335	0.237515205	0.445145107
6.868997806	-0.209570818	0.565170441	-0.370809941	0.710779097	0.845747551
457.0061056	0.036165117	0.06974451	0.518537117	0.60408357	0.774328722
10.0696948	0.120875232	0.456538786	0.264764432	0.791190939	0.89219519
3011.168333	0.080550084	0.052034953	1.547999544	0.121622406	0.291561048
276.6734564	0.424064186	0.20633952	2.055176762	0.039861935	0.138424434
1981.518524	0.380384713	0.259204184	1.467509926	0.142237391	0.32178231

170.6580488	0.297256129	0.093296821	3.186133515	0.00144188	0.012757708
107.1732428	0.020242515	0.164031971	0.123405914	0.901785674	0.950906817
271.2186642	-1.52236282	0.473449674	-3.215469147	0.001302315	0.011788097
307.9133997	0.121271071	0.291975413	0.415346859	0.677888003	0.825950823
14.38945341	-0.064342322	0.375730163	-0.171246091	0.864030267	0.932173361
236.4076777	-0.347583343	0.106276057	-3.27057055	0.001073308	0.01017943
21.25459408	0.551462484	0.466753567	1.181485313	0.237409984	0.445009597
40.42366892	0.841629169	0.316269882	2.661110704	0.007788335	0.04371551
11.99014336	1.594210517	0.384033888	4.151223544	3.30703E-05	0.00064703
180.3251692	0.027876409	0.168598408	0.165342064	0.868674752	0.934607931
808.2430064	-0.086429183	0.048925212	-1.766557151	0.077302422	0.217206227
207.8977437	-0.489383045	0.183329316	-2.669420551	0.007598225	0.042926321
404.2382354	0.380208137	0.053261384	7.138532774	9.43324E-13	1.82827E-10
12.19723225	0.668798602	0.37046795	1.805280599	0.071030784	0.205823097
66.02366524	0.21686706	0.112668827	1.924818664	0.054252048	0.170530098
242.5595152	-0.037404593	0.094084178	-0.397565174	0.690950736	0.833491756
6101.328223	0.153057869	0.095822493	1.597306262	0.110197456	0.274187114
138.2305469	-0.19081777	0.113599655	-1.679738998	0.093008109	0.244836646
1025.208177	-0.108506782	0.072680867	-1.492920845	0.135457908	0.311917533
8.373066866	-0.839544685	0.374889043	-2.239448444	0.025126752	0.101034444
82.63826587	-0.208243776	0.135472695	-1.537164185	0.124253108	0.294821505
4373.381672	0.17664998	0.136846585	1.290861442	0.196751728	0.398192624
2542.520135	-0.244998816	0.086318345	-2.838316899	0.004535213	0.029299255
145.8011665	-0.407835911	0.108135541	-3.771525122	0.000162253	0.00230825
7.232030465	-0.587438072	0.379935573	-1.546151804	0.12206791	0.291949296
511.9166323	-0.24306469	0.073389025	-3.312003245	0.000926305	0.009077896
161.3334682	-0.235250546	0.14789112	-1.590700954	0.111676892	0.276531658
76.97735779	-0.037321904	0.199014884	-0.187533231	0.851242571	0.926491155
121.6169757	-0.134996395	0.100513644	-1.343065376	0.179250806	0.374886095
120.8388353	0.042797085	0.137072061	0.312223251	0.754870863	0.871827159
80.05666874	-0.006672706	0.131108414	-0.050894568	0.959409534	0.980261259
898.2096918	-0.202261813	0.074289868	-2.722602931	0.006476985	0.038240003
734.0843262	0.058026758	0.069660336	0.83299566	0.404847177	0.615490254
264.9489526	-0.219199928	0.0730894	-2.99906592	0.002708087	0.020241845
226.6968968	-0.121913481	0.102873298	-1.185083818	0.235984308	0.44321208
231.5080081	0.357253316	0.329188963	1.085253021	0.277809607	0.490949953
353.434351	0.633882015	0.11836427	5.355349322	8.53913E-08	3.89678E-06
4.963679887	1.082018835	0.622359852	1.738574287	0.082109673	0.226275468
1026.38713	0.14925331	0.095256071	1.566864028	0.117146478	0.284893869
330.3137008	0.105819286	0.075765428	1.396669815	0.162512885	0.351758937
108.7321281	0.0208258	0.107076874	0.194493908	0.845789166	0.923706045
73.79244425	-0.675940926	0.398503729	-1.69619724	0.089848533	0.239547618
2238.569501	-0.038308733	0.057989464	-0.660615405	0.508858987	0.703267027
247.7715104	0.17553628	0.105532368	1.663340674	0.096244261	0.249828924
186.6542077	0.323074553	0.122799063	2.630920341	0.008515399	0.046980137
1.81715094	1.513672718	0.824565913	1.835720703	0.066398974	0.196736818
148.0459149	-0.191106618	0.107361713	-1.780025795	0.075071739	0.213356065
33.28269399	0.20968671	0.241634042	0.867786294	0.385511335	0.5987323

1414.151433	0.004680144	0.039751335	0.117735508	0.906277231	0.952959157
71.67947434	0.115552039	0.21088145	0.547947861	0.583727704	0.759403791
378.1564896	1.014683858	0.160422052	6.325089649	2.53086E-10	2.51165E-08
5.861618542	-2.765189799	1.338723702	-2.065541826	0.038871762	0.135989257
30.59934135	-0.060470143	0.260451181	-0.232174579	0.816402422	0.907813535
4.09890228	-1.802093825	0.815216277	-2.210571448	0.027065528	0.106363908
319.8185144	-0.166842426	0.099318895	-1.679865913	0.092983407	0.244836646
5.294222049	-0.261316879	0.474579381	-0.550628388	0.581888445	0.758210783
17.14640066	0.34549641	0.310265533	1.113550728	0.265471974	0.477131279
174.7798904	0.136339487	0.075474272	1.806436597	0.070850169	0.205456938
186.4482395	-0.06952077	0.089784942	-0.774303227	0.438751496	0.644424326
11.05167606	0.39008068	0.369833974	1.054745392	0.291541786	0.506630736
1056.928978	-0.020144868	0.126719775	-0.158971775	0.873691111	0.936812508
170.5288675	-0.0898367	0.096276552	-0.933110905	0.350762709	0.565832638
254.0336471	0.11320906	0.076472352	1.480392031	0.138768655	0.317202002
1441.227854	-0.1967574	0.053398214	-3.684718738	0.000228955	0.003049159
402.8161144	-0.01358476	0.170684654	-0.079589816	0.936563495	0.970184161
96.51115073	0.252537812	0.1063633	2.37429463	0.017582514	0.07842991
384.2896846	0.242117496	0.071581271	3.382414036	0.000718518	0.007421228
976.8867208	0.426196367	0.114437498	3.724271995	0.00019588	0.002677943
32.58319435	-0.827787169	0.228606456	-3.62101396	0.000293451	0.003681878
126.5246047	-0.252659616	0.115342233	-2.190521291	0.028486452	0.109954503
862.4769397	-0.612454681	0.15996739	-3.828622086	0.000128863	0.001903932
228.2321364	-0.801419114	0.28186413	-2.843281667	0.004465159	0.028971654
3.077468444	1.135587941	0.608808855	1.865261866	0.062144708	0.188020553
7401.230385	0.045027343	0.084619415	0.532115981	0.594645669	0.766885295
4.704010842	1.689070031	0.664612515	2.541435788	0.011039822	0.056341398
843.7235336	-0.242450307	0.111690772	-2.170728175	0.029951726	0.113828295
5356.116366	-0.099432895	0.061348042	-1.620799819	0.105060577	0.2654145
11.72516281	0.282883053	0.288355041	0.981023436	0.326581184	0.541638823
162.5305375	-0.215981789	0.146839186	-1.470872968	0.141325474	0.320688135
652.2795726	0.028537113	0.096955467	0.294332174	0.768504089	0.879190026
163.9879113	-0.171679995	0.084481317	-2.032165237	0.042136931	0.144166936
110.1307672	0.035990216	0.164284279	0.219072791	0.826593345	0.913363158
52.89305888	0.163174058	0.160752008	1.015066999	0.310073891	0.525137224
27.12131155	1.059354687	0.47770556	2.217589192	0.026582855	0.105220077
103.2698004	-0.118033604	0.134529441	-0.877381211	0.380279615	0.593138267
118.5466031	-0.349597572	0.254846443	-1.371796947	0.170126658	0.362148412
2112.715556	0.036813775	0.06287125	0.585542283	0.558183145	0.742411523
14.05551049	0.332879197	0.539522393	0.616988658	0.537242207	0.725334217
7.448560893	0.958796699	0.410278232	2.336942651	0.019442166	0.084242569
46.51820861	-0.108432503	0.145505079	-0.745214553	0.456142027	0.66012936
29.63064427	-0.023035303	0.196958501	-0.116955107	0.906895628	0.953303217
817.5558915	-0.249224513	0.062656005	-3.977663622	6.95957E-05	0.001182329
52.0767061	0.704653049	0.304607124	2.313317693	0.020705179	0.08816157
209.607178	0.019416495	0.088830382	0.218579436	0.826977674	0.913540506
275.9940202	-0.299678303	0.103000528	-2.90948319	0.003620269	0.024937214
176.9887871	-0.006471145	0.115116988	-0.056213644	0.955171612	0.978241049

216.8925908	-0.579382624	0.291735082	-1.985988867	0.04703456	0.15561009
229.6956492	0.106671742	0.127396294	0.837322178	0.402411496	0.613826573
586.9945581	0.839219022	0.192097977	4.368703061	1.24987E-05	0.000287568
5.971612027	0.843132702	0.480343624	1.755269892	0.079213154	0.220653307
178.5199357	0.91685771	0.174524995	5.253446429	1.49279E-07	6.45466E-06
492.6337961	0.40832625	0.082418215	4.954320486	7.25835E-07	2.6396E-05
113.9164011	0.53377821	0.191962625	2.780636123	0.005425251	0.033525412
133.7872812	0.2051946	0.115277069	1.780012285	0.07507395	0.213356065
458.4610807	0.63260779	0.100744353	6.279337437	3.40019E-10	3.14811E-08
1.933364152	1.009805544	0.738641844	1.367111209	0.171590451	0.363947609
50.78059273	-0.183256785	0.151902275	-1.206412378	0.227658526	0.433427315
889.50427	0.432450328	0.055517235	7.789478836	6.72862E-15	1.87877E-12
831.3114778	0.093476273	0.077672247	1.203470698	0.228794224	0.434802906
22.46886545	-2.535047251	0.561961878	-4.51106623	6.45026E-06	0.000165002
103.7753868	0.374099695	0.119888446	3.120398243	0.001806067	0.01498899
462.5882683	-0.107323667	0.133640958	-0.80307466	0.421931583	0.630238544
5.468510589	-0.317434823	0.484818618	-0.654749656	0.512628949	0.706344037
14.59334838	0.486481233	0.279807457	1.738628552	0.082100121	0.226275468
50.85621168	-0.043210199	0.29658396	-0.145692975	0.884163768	0.942262895
130.8733189	-0.157780138	0.155466	-1.014885171	0.310160568	0.525137224
5.623935392	0.872274539	0.428434618	2.035957187	0.041754655	0.143245768
710.3006775	0.312921234	0.066186115	4.72789849	2.26856E-06	6.90798E-05
72.15643535	-0.265345587	0.144081995	-1.84162904	0.06552943	0.195069681
373.5333915	0.420450478	0.064827253	6.485705636	8.83175E-11	9.37939E-09
6.812472112	0.075319889	0.466396038	0.161493415	0.871704798	0.936022998
7.38098075	-0.986131302	0.604752857	-1.63063521	0.102967315	0.26222136
4.039814968	-0.488514934	0.579013682	-0.843701882	0.398836015	0.611199292
5.554968334	-0.295589001	0.455842891	-0.648444907	0.516697233	0.709516183
4.437741474	-1.204259903	0.668021141	-1.802727232	0.071431063	0.206629559
17.43615058	0.130578042	0.314864333	0.414712078	0.678352689	0.826200074
3.10552382	-1.00469108	1.362206434	-0.737546861	0.460789857	0.663513594
457.7209888	-0.015282009	0.048165956	-0.317278235	0.751032491	0.870013027
196.9221775	0.031584265	0.076158159	0.414719385	0.67834734	0.826200074
115.4420117	-0.090239113	0.088249817	-1.022541635	0.306524617	0.521661833
713.14649	-0.118764665	0.051697014	-2.297321547	0.021600434	0.090824396
163.6027298	-0.278048624	0.215859817	-1.288098117	0.197711811	0.399152251
1.884956957	0.14790408	0.821277557	0.18009025	0.857081717	0.92936531
87.06142827	-0.418180943	0.138334901	-3.022960521	0.002503149	0.019091146
386.9231696	-0.193233883	0.067939158	-2.844219582	0.004452035	0.028920675
46.19536806	-0.048965411	0.21225532	-0.230691091	0.817554795	0.908247198
17.42454838	0.141205814	0.237038376	0.595708665	0.551369869	0.737218344
3.999417817	-0.546274506	0.55713408	-0.980508149	0.326835349	0.541951237
263.2405102	0.039145953	0.083422667	0.469248399	0.638892094	0.798267972
84.51925535	0.225739152	0.211620653	1.066716074	0.286100067	0.500553579
2.857840428	0.012030529	0.734866056	0.01637105	0.986938375	0.993329839
4.572163485	0.792154562	0.502319212	1.576994354	0.114796824	0.281341388
2.502728967	-0.258701446	0.71215726	-0.363264493	0.716407314	0.849615028
67.2877989	-0.459032157	0.137217255	-3.345294717	0.000821952	0.008256606

335.5018315	-0.18842866	0.114100801	-1.651422762	0.09865228	0.254414161
47.97385405	-0.340892148	0.147548812	-2.310368634	0.020867754	0.088686106
316.6375177	-0.424413925	0.135023407	-3.143261853	0.001670763	0.014158514
210.8862605	0.029917137	0.099346346	0.301139781	0.763307907	0.876715782
25.44077857	0.41416978	0.257662957	1.607409096	0.107964656	0.270387617
308.3945486	-0.135808971	0.089116961	-1.523940776	0.127523506	0.298964315
787.0222572	0.135685151	0.083417639	1.626576253	0.103827126	0.263591937
419.3591821	-0.096381	0.053788736	-1.79184357	0.073158027	0.210002673
52.38503284	-0.149025086	0.31979909	-0.465995967	0.641218387	0.800259978
145.2187065	-0.317671784	0.096007718	-3.308815076	0.000936917	0.009149241
97.2893126	-0.046829084	0.131614563	-0.355804728	0.721986808	0.852372845
1.975483632	0.740512381	0.748334995	0.989546641	0.322395762	0.537439698
9.590561114	0.421009198	0.397054346	1.060331421	0.288993849	0.503706799
531.5210486	0.153529524	0.049082703	3.127976182	0.001760144	0.01468927
14.84452708	-0.205608039	0.29231087	-0.703388276	0.481813811	0.680761508
38.83240262	0.432671519	0.177973411	2.431102018	0.015052975	0.070469655
216.7987217	-0.128540411	0.120595299	-1.065882437	0.286476789	0.500999854
32.77168388	0.059788125	0.25134491	0.237872831	0.811979728	0.905227993
1292.893136	0.092483978	0.056093962	1.64873319	0.099202307	0.255233299
341.8544166	0.358105578	0.091604421	3.90926088	9.25789E-05	0.001474996
10.62541035	-0.214821232	0.334388932	-0.642429255	0.52059451	0.71272949
48.80830248	0.118688481	0.151322206	0.784342785	0.432839001	0.639905007
163.4429914	-0.034815715	0.114494205	-0.304082768	0.761064835	0.875424502
85.69814265	-0.271218645	0.124814897	-2.172966944	0.029782805	0.113450365
35.99961212	-0.254427145	0.219905059	-1.156986318	0.24727795	0.455782238
185.2085959	0.037898112	0.090513866	0.418699516	0.675435749	0.824469335
1424.174938	0.005834744	0.050540162	0.115447675	0.908090293	0.953709007
125.2021486	0.129013241	0.225579752	0.571918533	0.567377168	0.748476254
127.3872195	0.201539981	0.268004144	0.752003226	0.452049119	0.656706983
297.2045437	0.335097194	0.192878375	1.73734974	0.082325457	0.226794244
80.15942147	0.195830633	0.12534515	1.562331153	0.118209997	0.286233189
158.9135842	-0.028497651	0.08412125	-0.33876875	0.734783944	0.860390269
8.218269489	-0.174252243	0.348191745	-0.500449093	0.616758893	0.78342222
388.9560193	-0.05566505	0.077128562	-0.721717718	0.470468046	0.671219641
83.47969914	2.115746198	0.376176152	5.624349614	1.86208E-08	1.04696E-06
105.7493297	0.064866186	0.158524277	0.409187711	0.682401912	0.8284617
924.9076591	0.23697769	0.088745384	2.670310055	0.007578123	0.042871568
99.68708998	-0.363374937	0.135695526	-2.677869699	0.007409204	0.042176648
58.16323536	0.100640556	0.172738136	0.58261921	0.560149671	0.744065931
4.46734133	-0.185523369	0.584982696	-0.317143346	0.751134836	0.870013027
673.7186824	-0.080310189	0.048283335	-1.663310719	0.096250254	0.249828924
2.076305285	0.682796295	0.947590525	0.720560492	0.471179969	0.671530536
1724.138174	0.211033353	0.102764666	2.05355946	0.040018349	0.13879206
163.5659111	0.133671346	0.101456226	1.317527277	0.187661944	0.386551139
7538.021546	0.258100137	0.139131798	1.85507655	0.063585296	0.190941335
218.8691523	0.91083873	0.217161191	4.19429791	2.73718E-05	0.00055328
442.3591975	0.153142966	0.060404407	2.535294597	0.01123528	0.057126542
89.19824353	0.222206723	0.111762427	1.988205959	0.046788916	0.155184338

224.9292135	0.076606895	0.088909653	0.861626293	0.388893198	0.602295529
242.2733801	0.109399791	0.077640636	1.409053257	0.158819421	0.346011718
161.6278182	0.082361173	0.094949742	0.867418606	0.385712691	0.598947203
257.5466587	0.206386405	0.096970528	2.128341551	0.033308775	0.122184094
10.63834666	-0.553078186	0.303311014	-1.823468853	0.06823241	0.200438789
356.9193354	-0.079111353	0.070851524	-1.116579416	0.264174197	0.475552721
264.3918193	-0.158931154	0.082519727	-1.925977706	0.054107158	0.170302123
2317.073184	0.124438214	0.066775275	1.863537287	0.062386712	0.18860883
703.8758165	-0.029933901	0.069561175	-0.430324838	0.666959361	0.818332001
340.0252116	0.06914678	0.078248958	0.883676684	0.37687077	0.589841864
236.2928286	0.089741608	0.068326975	1.313414045	0.189043467	0.388316967
23.26820405	0.25396993	0.232544519	1.092134663	0.274773915	0.487467745
564.2932196	-0.148054205	0.052753728	-2.806516436	0.005008036	0.031513341
24.35277467	0.249920179	0.229762754	1.087731473	0.276713669	0.489538336
979.5709053	-0.286745975	0.12428728	-2.307122456	0.021047994	0.089206241
82.14475619	0.240339143	0.13721466	1.751555873	0.079850206	0.221904908
391.4180377	-0.05655496	0.075495443	-0.74911753	0.45378637	0.658108374
1349.843721	-0.013909171	0.047549118	-0.292522174	0.769887409	0.880039215
5.850391038	0.789769696	0.431886955	1.828649109	0.067452191	0.198775675
509.5522483	0.114753161	0.084214787	1.362624847	0.173000774	0.366387567
169.3262963	0.039112945	0.096638669	0.404733898	0.685673122	0.829778815
26.6467376	0.420287258	0.226340194	1.856883003	0.063327801	0.190455244
6.702797279	0.160714871	0.440852024	0.364555139	0.715443507	0.848949542
2.336585922	0.144539038	0.775165692	0.18646212	0.852082382	0.926915291
1346.637867	0.11263474	0.044972512	2.504524107	0.012261625	0.060900277
513.8989315	0.077123505	0.095094559	0.811019113	0.417354695	0.626583545
329.4120812	-0.024618182	0.057553918	-0.427741193	0.668839555	0.819765109
444.6922722	-0.019138659	0.213696157	-0.089560147	0.928636755	0.965637301
428.9536595	0.276835681	0.060895844	4.546052118	5.46615E-06	0.000143849
121.5117972	-0.108252737	0.110030119	-0.983846411	0.325191036	0.540225672
231.8590975	0.036598817	0.070907178	0.516151094	0.605748884	0.77567875
158.7383914	-0.003472839	0.091593858	-0.037915631	0.969754951	0.985183958
2338.214177	-0.017871003	0.100035065	-0.178647391	0.858214581	0.929724191
115.8723254	0.094130643	0.116911643	0.805143443	0.420736909	0.629081489
209.8460415	-0.022403397	0.078209979	-0.286451897	0.774532031	0.882959012
343.1453323	0.12659939	0.084557223	1.497203724	0.134340257	0.310221669
142.9924981	0.379054929	0.08665243	4.374429293	1.21751E-05	0.000281702
4.273704679	0.008918993	1.321789332	0.006747666	0.994616182	0.997158409
2.974677368	-0.208736116	1.472367416	-0.141769041	0.887262439	0.943568846
3.149257819	-0.290615403	0.702708368	-0.41356474	0.679192901	0.826686346
838.7570192	-0.196205057	0.080199574	-2.446460087	0.014426677	0.068348924
254.3781782	-0.098273584	0.083430263	-1.177912912	0.238831327	0.446360434
17.66481864	-0.02395449	0.241481716	-0.099197946	0.920981106	0.961050759
345.0636723	-0.023287955	0.06585931	-0.353601562	0.723637504	0.853282101
298.5462758	0.00075197	0.099978723	0.007521303	0.993998925	0.996600225
12.91407302	1.222102209	0.341709366	3.576437551	0.000348308	0.004212947
76.90892477	-0.266847047	0.127113038	-2.099289342	0.035791402	0.128487157
157.2741995	-0.139169721	0.106336605	-1.308765878	0.190613669	0.390623187

124.6455149	0.167433934	0.134790563	1.242178463	0.214170724	0.418882644
374.3347571	-0.211144205	0.080321885	-2.628725718	0.008570546	0.047189562
6.21397756	1.126503955	0.957013133	1.177103966	0.239154012	0.446593139
409.8989398	0.314536166	0.081126056	3.87712876	0.000105696	0.001638473
905.7275601	0.626500612	0.098675303	6.349112657	2.1656E-10	2.18872E-08
482.332371	-0.042332927	0.077697294	-0.544844293	0.585860613	0.760836448
130.0682334	-0.207847442	0.097552647	-2.130618169	0.033120611	0.121792177
7.134104022	-0.214519505	0.516375647	-0.415433039	0.677824924	0.825950823
127.8336955	0.139090717	0.15455717	0.899930533	0.36815722	0.581947768
1723.257128	-0.164646263	0.073645258	-2.235666872	0.025373601	0.101729057
183.0450662	-0.103855324	0.080791508	-1.285473275	0.19862695	0.400462657
289.6191543	-0.051005846	0.075901671	-0.671998995	0.501584336	0.697366896
35.91888169	0.297018306	0.189583854	1.566685663	0.117188183	0.284911177
309.9213219	-0.111524102	0.107148331	-1.040838448	0.297950533	0.512684048
9.523808382	0.419760427	0.408601354	1.027310415	0.30427432	0.518957981
12.78332352	0.249473378	0.304099998	0.82036626	0.412007344	0.621935878
405.6976144	-0.316634136	0.10417448	-3.039459711	0.002370029	0.018339695
54.52487232	-0.679067504	0.306266433	-2.217244306	0.026606401	0.105271281
214.5182907	-0.017873689	0.112503551	-0.158872217	0.87376955	0.936825644
504.3264798	-0.940644106	0.140856194	-6.678045758	2.42149E-11	3.04082E-09
66.97948355	-0.305023177	0.132522654	-2.301668183	0.021353892	0.090131697
2.410198624	0.229571878	0.732772506	0.313292155	0.754058709	0.871653489
3491.818229	0.275258271	0.049330558	5.579873449	2.40694E-08	1.30006E-06
80.48574193	-0.052719551	0.188375174	-0.279864646	0.779581352	0.885082046
56.22749957	-0.186884744	0.148526873	-1.258255426	0.208299396	0.412046619
543.9833428	-0.012264326	0.079714399	-0.153853335	0.877725379	0.938489926
1035.986832	0.117478058	0.114292138	1.027875231	0.304008522	0.518665741
47.21486136	2.764578893	0.328136043	8.425099738	3.60443E-17	1.79938E-14
297.8583421	0.177898828	0.100515335	1.769867539	0.07674921	0.21642699
15.10369637	-0.100180866	0.297835718	-0.336362832	0.736597274	0.861476895
11.72214812	0.694918948	0.36054551	1.927409796	0.05392858	0.169967366
40.75286639	-0.15871199	0.155316283	-1.021863173	0.306845665	0.521971933
206.1020306	-0.060419672	0.183111913	-0.329960354	0.741429919	0.864363207
6.516529298	-1.280648208	0.607420303	-2.108339482	0.035001635	0.126340258
446.4003713	-0.253209862	0.102641664	-2.466930608	0.013627676	0.065663159
8.58710569	0.580311002	0.316576216	1.833084652	0.066789983	0.197398311
2.860595686	-0.961700208	0.558438036	-1.722125189	0.085046843	0.231382743
129.9297382	0.567586124	0.179142368	3.168352237	0.001533057	0.013362738
158.4620355	-0.243103409	0.100449156	-2.420163774	0.015513518	0.071914286
4746.516506	-0.794833583	0.376457246	-2.111351529	0.034742108	0.125719761
375.6262688	-0.065237023	0.081414676	-0.80129316	0.42296195	0.63111002
148.0845173	0.008695174	0.114766515	0.075764033	0.939606831	0.971267048
112.6200134	0.813838379	0.199197524	4.08558481	4.39659E-05	0.000805667
125.5949247	0.295214708	0.172287139	1.71350403	0.086619853	0.233968757
27.48252715	-2.526738147	0.576303439	-4.384388456	1.16312E-05	0.000271022
434.9284216	-0.359947687	0.10803075	-3.331900274	0.000862552	0.008580722
453.5856068	-0.328871112	0.063476179	-5.18101618	2.2068E-07	9.0435E-06
166.3428584	-0.208691675	0.209989778	-0.993818256	0.320311357	0.535424686

4923.916867	-1.085389356	0.343365203	-3.1610348	0.001572097	0.013606386
7.8366035	-0.062275982	0.483987601	-0.12867268	0.897616654	0.948210116
43.30574475	-0.673981127	0.293403962	-2.297109832	0.021612505	0.090827655
486.0922166	-0.051507532	0.088151249	-0.584308585	0.559012717	0.743154899
3.199674356	-2.635948511	1.185031536	-2.224369927	0.026123565	0.103751113
67.87043641	-0.222013248	0.178248131	-1.245529181	0.212937296	0.417651121
95.40809406	-0.385945846	0.355886425	-1.084463521	0.278159333	0.491304476
236.0012827	-0.1183221	0.087987449	-1.344761118	0.17870239	0.374375724
5.972070601	-0.03335297	0.496958129	-0.067114246	0.946490752	0.974652372
361.6499461	0.030840226	0.103403079	0.298252494	0.765510459	0.877901205
43.27650604	-0.517218374	0.189625732	-2.727574828	0.006380179	0.037781115
149.3596965	-0.415040875	0.100467109	-4.13111197	3.61013E-05	0.000692354
11.44768143	-0.384268036	0.332268663	-1.156497978	0.247477526	0.455983085
205.9353903	0.049725948	0.168837185	0.294520118	0.768360493	0.879190026
27.55724803	0.314544133	0.212630191	1.479301369	0.139059787	0.317559042
3110.40877	-0.112963231	0.088373828	-1.278243036	0.201163746	0.403847374
436.9161765	0.131961085	0.079662566	1.656500557	0.097620492	0.252464677
224.4520792	0.196163458	0.16297956	1.203607726	0.228741232	0.434802906
1276.627686	-0.212866761	0.091872251	-2.316986455	0.02050447	0.087528478
257.1325379	-0.085132406	0.067767384	-1.256244549	0.20902732	0.41304019
258.1264081	-0.056513678	0.108269874	-0.521970481	0.601690884	0.772524013
4.199042675	0.781885305	0.470250019	1.662701273	0.096372247	0.25006086
186.90371	-0.106712745	0.076555551	-1.393925627	0.163340065	0.352900226
32.75298631	0.124846514	0.188261182	0.663155904	0.507230709	0.70201787
9939.918538	-0.055159289	0.153299129	-0.359814761	0.718985664	0.850903005
70.46339981	0.50502829	0.18113339	2.788156787	0.005300888	0.032989168
233.4030798	-0.171103949	0.119469119	-1.432202317	0.152085929	0.336303838
4.531058953	-1.419905175	0.731502982	-1.941079134	0.05224868	0.166456149
47.08967945	0.143062271	0.287943813	0.496840928	0.619301231	0.785039126
495.2464438	-0.222549437	0.201779056	-1.102936258	0.270054847	0.482016103
2913.209999	0.021062457	0.216506593	0.097283212	0.922501487	0.961560774
615.8287075	-0.157848449	0.064330281	-2.453719238	0.014138732	0.067474356
2709.456708	-0.266523393	0.13049415	-2.04241641	0.041110243	0.141565666
186.7023264	0.053522779	0.093258411	0.573919054	0.566022579	0.748143944
6.031719168	-0.136000449	0.504378296	-0.269639772	0.787437399	0.890217109
40.93453086	0.35192794	0.174963331	2.011438271	0.044279184	0.149233896
63.39681581	-0.050942609	0.159721397	-0.31894668	0.749766946	0.869100807
5.759667498	0.266078905	0.510711877	0.520996118	0.602369476	0.77305874
336.3971964	-0.455010765	0.190627424	-2.386911363	0.016990587	0.076538948
112.0940404	0.019101411	0.10939875	0.174603558	0.861391153	0.931215742
1210.32635	-0.30978414	0.06736539	-4.598565205	4.25411E-06	0.000116222
22.41536288	0.267202714	0.477543319	0.559536074	0.575795919	0.754267014
229.2512763	0.081339782	0.127784561	0.636538417	0.52442555	0.715595668
25.57024425	-0.087945914	0.395845015	-0.222172593	0.824179528	0.911434821
21.38722519	-0.826005494	0.436261976	-1.893370361	0.058308634	0.179883228
49.44303806	-0.032488377	0.274683104	-0.118275847	0.905849094	0.952959157
2.548917163	-0.082467524	0.638430944	-0.129172191	0.8972214	0.948096559
974.5709211	0.042346783	0.104520219	0.405153985	0.685364326	0.829778815

319.1464977	-0.435760981	0.13228844	-3.294021616	0.000987649	0.009559653
1089.430162	-0.602690613	0.412298087	-1.461783677	0.143800499	0.323939479
443.2210467	0.005087765	0.070885525	0.07177438	0.942781462	0.972623458
11.50472793	-0.382470064	0.374418692	-1.021503659	0.307015877	0.522071435
180.2689977	-0.035265059	0.099701428	-0.353706663	0.723558729	0.853250286
6.834392619	-0.579893976	0.363973306	-1.593232161	0.111108119	0.275578915
129.8104076	-0.125025479	0.097746813	-1.279074741	0.200870737	0.403455807
172.3756636	0.076509348	0.075050982	1.019431671	0.307998077	0.523034772
472.8367603	-0.181727175	0.083204772	-2.184095571	0.028955226	0.111298908
1065.59737	-0.014339437	0.052665421	-0.272274238	0.785411163	0.888780293
45.00949989	0.318596663	0.15399526	2.06887317	0.03855799	0.135327387
1580.520505	-0.239622928	0.600605876	-0.398968671	0.689916289	0.83271258
416.6009258	-0.27174768	0.112298106	-2.419877671	0.015525729	0.071926564
18.54367739	0.440031869	0.285215941	1.542802509	0.122878701	0.292910391
201.1722089	0.085413698	0.10881573	0.784938886	0.432489402	0.639629267
36.06127271	-0.606458771	0.225146445	-2.693619122	0.007068088	0.04063776
2.193561647	0.724306218	0.688424016	1.05212224	0.29274347	0.507915315
1269.299066	-0.18355846	0.047009469	-3.904712461	9.43375E-05	0.001491162
6.371439705	-0.412595557	0.45364552	-0.909510926	0.363080493	0.576964218
109.5774177	0.012455995	0.179863417	0.069252521	0.944788617	0.973447225
17635.37297	-0.243037976	0.146246151	-1.661841862	0.096544486	0.250310649
365.3131645	-0.102051406	0.062184307	-1.641111894	0.10077419	0.258028288
77.14441908	-0.345492314	0.147151788	-2.347863517	0.018881439	0.082419931
103.7018829	0.394582243	0.239683277	1.646265221	0.099709166	0.255937801
813.511966	-0.217104411	0.051913361	-4.18205269	2.88889E-05	0.000576868
61.17898227	-0.671023633	0.299957888	-2.237059463	0.025282454	0.101462399
204.7337466	-0.423715354	0.166669681	-2.542246152	0.011014258	0.056255699
679.1948059	-0.183692918	0.098327877	-1.868167232	0.061738765	0.187249741
45.8414253	0.17728479	0.230488921	0.769168377	0.441793361	0.647633371
2.081929381	-1.859093223	0.796791884	-2.333223092	0.019636439	0.08495029
209.1805483	0.134352877	0.083262026	1.613615279	0.106610901	0.267928703
1128.075581	-0.092187167	0.063383037	-1.454445417	0.145822856	0.32688607
193.4829468	0.045687243	0.08929924	0.511619618	0.608917252	0.778119086
834.2067468	-0.07091692	0.065686903	-1.079620392	0.280311257	0.493887448
151.8398715	-0.139265598	0.101638585	-1.370204025	0.170623222	0.362783552
13.19707051	-0.174529717	0.40480383	-0.431146407	0.666361919	0.818067386
214.2861857	-1.328530907	0.620176594	-2.142181629	0.032178869	0.119260898
322.9561653	-0.421420482	0.075393232	-5.589632817	2.2755E-08	1.23718E-06
16.44505314	-0.047069661	0.483440897	-0.097363837	0.922437462	0.961560774
3.24480131	1.763374033	0.581729777	3.031259704	0.002435357	0.018651821
394.9255513	-0.263069618	0.118735717	-2.215589579	0.026719623	0.105583851
266.049187	-0.305879492	0.109731622	-2.787523644	0.005311258	0.032989168
18.96303799	0.035793344	0.211534159	0.169208343	0.865632768	0.933396663
1103.029595	-0.050526204	0.087975395	-0.574321986	0.565749934	0.748001651
646.2521133	0.489105042	0.093886637	5.209527776	1.89322E-07	7.95635E-06
244.3574278	-0.034178555	0.13575232	-0.251771426	0.801217744	0.898120007
2.171781988	-1.140467868	0.816137154	-1.397397315	0.162294126	0.351508153
2196.384806	0.312818724	0.086245603	3.627068679	0.000286657	0.003618689

997.4612667	-0.179964378	0.058028252	-3.101323449	0.001926577	0.015774569
189.6218856	-0.199395923	0.113581385	-1.755533463	0.079168102	0.220604755
34.36620036	-0.619380355	0.294334001	-2.104345246	0.035348342	0.127368457
419.1800763	-0.087368614	0.115976217	-0.753332161	0.451250337	0.656298937
1172.236636	-0.139625338	0.074463517	-1.875083844	0.06078119	0.185360106
503.1310708	0.1750799	0.115609995	1.514401075	0.129924147	0.303013378
36.45430867	-0.256824021	0.186473334	-1.377269421	0.168428966	0.35978978
22.81787516	-0.247263589	0.235446493	-1.050190154	0.293630696	0.508620235
47.68725623	0.083224339	0.13934982	0.597233203	0.550351694	0.736546229
135.9717836	0.210670158	0.10042847	2.09771351	0.035930461	0.128845975
2.479662058	-0.23460742	0.668341625	-0.351029191	0.725566449	0.854589253
587.6072349	-0.013826463	0.100903008	-0.137027261	0.891009247	0.945526539
393.7878947	-0.094663578	0.05935079	-1.594984295	0.110715748	0.27514425
362.5858717	0.015058997	0.077213494	0.195030639	0.845368962	0.923392672
10.64985233	-0.305179228	0.363103466	-0.840474568	0.400642355	0.61246777
5.305378137	-1.094806039	0.474480394	-2.307378879	0.021033708	0.089168631
492.9009452	-0.191544643	0.088890291	-2.15484324	0.031174103	0.116759501
2.683803972	2.75707266	1.069832662	2.577106457	0.009963126	0.052346712
11.68162923	-0.25038149	0.34311377	-0.729733146	0.465553316	0.667611885
9.496810726	-0.70605217	0.5253797	-1.343889323	0.178984179	0.374518655
313.7130346	0.161291647	0.088684736	1.818708094	0.068955976	0.201701128
939.2791846	0.231974279	0.106225101	2.183799086	0.028977015	0.111315188
5.068944967	0.167001785	0.498045831	0.335314091	0.737388165	0.861788314
8.250357898	-1.055482604	0.674701722	-1.564369215	0.117730886	0.285514297
28.62404495	-0.003535243	0.277404921	-0.012743981	0.98983205	0.994530438
32.30831309	0.146775051	0.277892695	0.528171677	0.597380186	0.769147415
253.4076912	0.12846771	0.189001282	0.679718725	0.496682577	0.693461961
23.3947292	-0.309035608	0.197952141	-1.561163249	0.118485238	0.286752726
1357.023517	0.07237437	0.075294922	0.961211826	0.336445672	0.551557965
432.8383725	0.175874029	0.108925545	1.614626114	0.106391687	0.267587274
2112.401643	0.133668983	0.089859476	1.487533528	0.136873955	0.314024548
12.06084471	-0.49487502	0.314022067	-1.575924344	0.11504324	0.281621068
1686.880766	-0.174469542	0.160145884	-1.089441312	0.275959322	0.488528712
29.49407284	-0.690624231	0.292483406	-2.36124244	0.018213819	0.080228464
155.5306417	-0.003993663	0.132361856	-0.030172312	0.975929631	0.987885465
6.525424334	0.222147204	0.44367662	0.500696214	0.616584937	0.783310129
1442.853181	-0.332339098	0.083747593	-3.968342106	7.23744E-05	0.001214176
231.4662201	0.071282661	0.072336885	0.98542618	0.324414775	0.539566786
4.675803748	0.109765136	0.614618716	0.178590618	0.858259162	0.929724191
287.0911324	-0.31052183	0.184020314	-1.687432344	0.091520249	0.242201539
114.6912313	-0.007782766	0.140757989	-0.055291827	0.955905973	0.978293782
116.3108436	-0.048754459	0.107679112	-0.452775459	0.650710436	0.806473793
1116.634277	-0.171588995	0.097906781	-1.752575188	0.079674953	0.221604792
427.9976357	0.182407061	0.072121363	2.52916823	0.011433321	0.057918982
94.87154819	-0.284068391	0.130348538	-2.179298634	0.029309491	0.11228943
19.33249576	0.303160086	0.223563174	1.356037674	0.175087182	0.369272338
85.61971094	-0.042667209	0.113095072	-0.377268505	0.705974075	0.842949182
26.19335703	0.21881955	0.181467248	1.205834952	0.227881136	0.433500443

142.4339314	0.084689841	0.093844687	0.902446834	0.366819552	0.580392006
2.518123083	-0.77276603	0.801746751	-0.963853023	0.335119619	0.550150533
11.47562051	-0.686170751	0.390362792	-1.757777034	0.078785454	0.219947734
12.51012177	-2.751568289	0.896562654	-3.069019523	0.002147626	0.017001434
38.57339498	0.683863811	0.242820804	2.81633122	0.004857556	0.030742749
1.823175861	0.217443317	0.83738625	0.259669079	0.795119046	0.894481778
19.21935682	-3.473045757	1.326316093	-2.618565646	0.00883003	0.048193131
246.1532707	-0.260418863	0.11765646	-2.213383468	0.026871219	0.105833468
114.9101485	0.245482855	0.147738653	1.6616021	0.096592582	0.250395939
2972.293681	0.045203654	0.10377235	0.435604036	0.663124042	0.815611876
195.4919265	-0.066387581	0.131917492	-0.503250782	0.614787965	0.782242435
415.3759763	0.021922139	0.127654432	0.171730343	0.86364953	0.931990197
44.40178565	0.354644692	0.182599989	1.94219448	0.052113561	0.166175718
2.629493751	0.957737392	0.611833926	1.565355158	0.117499657	0.285344488
2.697925089	-0.116267757	0.577293719	-0.201401389	0.84038473	0.920879209
3.000747203	0.557259093	0.621438183	0.896724902	0.369865729	0.583361608
2.128803076	0.406202621	0.645322125	0.629457143	0.529049817	0.719045548
53.8981872	-0.128706949	0.193132018	-0.666419531	0.505142982	0.700364037
146.6495226	-0.157036295	0.215089009	-0.730099113	0.465329603	0.667349167
132.4302962	0.357207288	0.139297208	2.564353547	0.010336819	0.05364667
25.85890112	-0.207713957	0.228523008	-0.90894111	0.363381212	0.577203057
6.413114466	0.372973848	0.365114273	1.021526342	0.307005135	0.522071435
276.8455634	0.770404624	0.181044143	4.255341337	2.0873E-05	0.000441982
1239.382037	-0.028196064	0.106439341	-0.264902656	0.791084452	0.892136176
108.7340337	-0.007915603	0.149815276	-0.052835756	0.957862772	0.979262214
4.939846091	-1.651546686	0.59552112	-2.773279792	0.005549438	0.034099757
267.5123971	-0.005838687	0.1238898	-0.047128068	0.962411157	0.981463149
22.46076136	0.170024791	0.231167907	0.735503439	0.462032946	0.664472349
150.6523875	0.159145795	0.085867702	1.853383637	0.063827391	0.191493797
265.1930633	0.283265321	0.067120512	4.220249729	2.44032E-05	0.000503782
177.7428402	0.148555423	0.079892141	1.859449759	0.062963414	0.189592266
90.36802156	0.238521388	0.134723638	1.770449429	0.076652302	0.216227743
226.5910597	0.449014102	0.108753455	4.128734152	3.64766E-05	0.000697117
411.6413694	0.253010617	0.061443013	4.117809436	3.82491E-05	0.000722609
260.9677193	0.328524801	0.079695284	4.122261476	3.75171E-05	0.000711228
867.7666984	0.091771351	0.112770164	0.813791058	0.415764659	0.625357946
303.1772689	-0.152821832	0.126706199	-1.206111721	0.227774416	0.433500443
16.04398978	0.206549848	0.395328729	0.522476189	0.601338821	0.772372972
165.4953319	-0.09061597	0.113859329	-0.795858984	0.426114004	0.633613331
434.114609	0.195783321	0.092060528	2.126680405	0.033446647	0.122553394
193.7094606	0.118294808	0.087025683	1.359309162	0.174048642	0.367835449
158.8973014	-0.64779272	0.239922839	-2.700004396	0.006933856	0.040108248
5.417404824	0.656932103	0.487176245	1.348448553	0.177514157	0.372768415
600.9761705	0.280039287	0.080344956	3.48546195	0.000491288	0.005494555
187.3440325	-0.058641162	0.128708336	-0.455612777	0.648668454	0.805287423
48.17820081	0.370801276	0.176333492	2.102840888	0.03547968	0.127729951
95.0242432	0.905159978	0.210877273	4.292354347	1.76789E-05	0.000385627
12.745585	0.493932544	0.426300548	1.158648624	0.24659944	0.45517974

70.07745289	0.737068141	0.197266143	3.736414821	0.000186663	0.002581931
168.4902925	0.589853421	0.142943896	4.126468061	3.68377E-05	0.000702389
46.33600285	0.925702225	0.250505562	3.695336018	0.000219596	0.002950756
27.91068033	0.817665547	0.299152758	2.733270959	0.006270872	0.037308179
19.15976196	0.984157329	0.734845646	1.339270816	0.180482526	0.376649244
233.0229631	-0.254814804	0.19282148	-1.321506315	0.186332598	0.384649122
98.89679131	-0.562002389	0.243373557	-2.30921714	0.020931535	0.088872708
82.381163	-0.615243038	0.217711121	-2.825960541	0.004713907	0.030080765
383.6742833	-0.182272951	0.098040337	-1.859162838	0.06300406	0.189672646
60.55859985	-0.037780079	0.129541416	-0.291644792	0.770558225	0.880306225
211.5447834	-0.237544369	0.099321536	-2.391670306	0.016771901	0.075906674
33.32985036	-0.306716497	0.159309621	-1.925285459	0.054193656	0.170444117
5.221809611	0.465658874	0.546610653	0.851902303	0.394268319	0.607070016
1097.049336	-0.095893047	0.09137115	-1.049489332	0.293952963	0.508888305
31.53265049	-0.029767073	0.177622631	-0.167586035	0.866908958	0.934032728
75.5794155	0.078991241	0.248289438	0.318141769	0.750377401	0.869563682
1775.710373	-0.605373835	0.113831236	-5.318169744	1.04816E-07	4.67952E-06
7.465099317	-0.546589711	0.394358325	-1.386023005	0.165739879	0.356244671
24.23149128	-0.124293154	0.219251375	-0.566897943	0.570783522	0.750883819
56.05733052	-0.118796744	0.146030671	-0.813505433	0.415928332	0.625468128
2399.19173	-0.143978014	0.078210375	-1.840906839	0.065635212	0.195281648
158.7409061	-0.038415241	0.09113637	-0.421513843	0.673379905	0.823064294
120.9890605	-0.78279364	0.193676289	-4.041762915	5.30509E-05	0.000944821
104.9553515	-0.284937962	0.184625528	-1.543329163	0.122750931	0.292755703
3.77746336	-0.008485477	0.536716981	-0.015809966	0.987385998	0.993528977
8.473418488	0.672512459	0.398255049	1.688647667	0.09128697	0.24183615
13.91797564	0.073929823	0.263399693	0.280675432	0.778959354	0.884794608
8.574676689	0.011336077	0.315350488	0.035947548	0.971324182	0.985661927
51.39912659	0.353383957	0.15351852	2.301897883	0.021340932	0.090100079
30.47770878	-0.152614782	0.240327421	-0.635028584	0.525409773	0.716333226
22.69719582	0.829344054	0.235724806	3.518272296	0.000434366	0.005000526
64.58740022	0.191817974	0.14008861	1.369261742	0.170917472	0.363086692
34.64439442	0.498014135	0.180127937	2.764780092	0.005696118	0.034767637
34.04596161	0.600981457	0.18946447	3.172000841	0.001513926	0.013241222
76.69223228	0.593598527	0.113849684	5.213879436	1.84932E-07	7.8117E-06
144.186487	-0.087080581	0.108545682	-0.802248231	0.422409382	0.630666318
4.246388572	-0.274949294	0.517197456	-0.531613779	0.59499352	0.767273835
211.9205911	0.190998994	0.084742348	2.253878951	0.024203784	0.098258537
14.39533083	-0.86074119	0.369336101	-2.330509221	0.019779253	0.085299324
17.61380302	-0.853398284	0.410003451	-2.08144171	0.037393494	0.132589577
15.71175862	-1.340453008	0.546579465	-2.452439389	0.014189128	0.067580035
1460.269863	-0.002066724	0.092952382	-0.02223422	0.982261121	0.990922823
1052.530349	-0.146308636	0.069034479	-2.119355973	0.034060395	0.123947634
42.44687795	-0.126339089	0.187391501	-0.67419861	0.500185053	0.696019954
14.10977925	0.298717392	0.311384035	0.959321473	0.337396821	0.552732223
67.36756498	0.066986441	0.147672611	0.453614523	0.650106299	0.806039826
123.5349891	0.634624822	0.189343608	3.351709775	0.000803142	0.008112174
23.66126867	0.296506515	0.286480938	1.034995619	0.300670957	0.515534279

14.85783169	0.534115671	0.274771297	1.943855403	0.05191289	0.165931889
16.96191508	-0.222451401	0.269987374	-0.823932608	0.409977851	0.620217261
245.2699647	-0.270828154	0.124776893	-2.17049927	0.029969044	0.113828295
25.62671097	-0.764001009	0.246799963	-3.09562854	0.001963962	0.016001145
96.78147869	-0.07099173	0.097976627	-0.724578218	0.468710826	0.670053763
138.5031143	-0.014192372	0.091411306	-0.15525839	0.876617617	0.937787198
425.9064397	-0.196229797	0.129967156	-1.509841431	0.131083891	0.305004067
451.0070774	-0.121152768	0.078880935	-1.53589416	0.124564341	0.295345433
374.4358138	0.13298597	0.079874152	1.664943746	0.095923979	0.249447772
40.71910483	-0.173533315	0.165078247	-1.051218553	0.293158224	0.508299334
876.7097466	-0.149708093	0.051546882	-2.904309398	0.003680642	0.025180607
467.5428002	-0.188628726	0.077838288	-2.423341141	0.015378478	0.071560214
349.0133983	-0.40630069	0.141928826	-2.86270733	0.004200384	0.027734316
61.58077735	0.227946317	0.148147425	1.538645156	0.123890948	0.294343738
3519.90884	0.772555633	0.101844109	7.5856683	3.30778E-14	8.51442E-12
138.9349951	1.364260424	0.200931306	6.78968576	1.12378E-11	1.61195E-09
21.56446014	0.492320552	0.309518075	1.590603563	0.111698822	0.276544393
18.22373555	0.306498229	0.298376229	1.027220667	0.304316569	0.51897631
213.5169388	0.10595515	0.093218688	1.136629922	0.255693026	0.46580636
492.5067775	0.014561369	0.077214556	0.188583215	0.850419489	0.926268474
1119.805439	-0.032790716	0.063257289	-0.518370552	0.604199757	0.774417396
391.3263193	-0.131734401	0.124286303	-1.059926945	0.289177836	0.503958074
488.385371	-0.074375507	0.131238548	-0.56671998	0.570904444	0.750964533
54.11851882	0.24729079	0.207197994	1.193499922	0.232673643	0.439370196
359.098026	-0.082949858	0.091095107	-0.910585215	0.362513963	0.576508836
214.9790055	-0.175535475	0.078393668	-2.239153756	0.025145914	0.101062157
105.7279276	-0.068297362	0.204004931	-0.334782898	0.737788862	0.861800757
251.8090573	-0.414407644	0.151091004	-2.742768474	0.006092362	0.036456803
775.5661503	0.074937746	0.101403239	0.739007425	0.459902488	0.662623194
27.77348588	0.31647359	0.257415257	1.229428254	0.218911282	0.423478683
396.4680709	-0.339876986	0.215692016	-1.575751348	0.115083119	0.281621068
188.4303792	-0.098540448	0.101794403	-0.968034047	0.333027366	0.548476551
25.26109945	-0.330412461	0.363275296	-0.909537381	0.363066536	0.576964218
5.467221102	-0.778464737	0.566464742	-1.374250997	0.169363774	0.361364954
46.16992797	-0.752548618	0.345537554	-2.177906887	0.02941297	0.112502731
75.24094179	-0.002156358	0.285882549	-0.007542812	0.993981764	0.996600225
32.23616838	0.5570156	0.269122932	2.069744101	0.038476315	0.135237637
200.3064018	0.034970281	0.100352507	0.348474412	0.727483927	0.855796217
31.53792461	-0.90276116	0.266080629	-3.392810528	0.000691795	0.007235953
2.497689809	-1.811918487	0.970187545	-1.867596113	0.061818389	0.187313038
14.37392348	-0.424658843	0.55013721	-0.771914415	0.440165119	0.645901207
32.25602242	0.605104816	0.169564875	3.568574075	0.000358929	0.00432237
16.09096717	-0.759713423	0.440015885	-1.726559085	0.084246879	0.229931378
366.2174032	0.059979241	0.067356973	0.890468171	0.373214552	0.586752244
124.3645272	0.166664767	0.203845147	0.817604784	0.413582895	0.623363642
12.8684193	0.243061015	0.32640492	0.744661004	0.456476679	0.660285961
3059.328026	0.016414231	0.079918219	0.205387842	0.837269119	0.918686178
133.0982206	-0.205239474	0.131694036	-1.558456861	0.119124987	0.287793669

2.484345143	2.175630769	1.222757448	1.779282369	0.07519348	0.213501791
1.769577156	-2.302556315	1.537142593	-1.497945815	0.134147329	0.309882015
217.193283	0.094930235	0.11851371	0.801006351	0.423127969	0.63111002
179.571924	0.090417896	0.116672597	0.774971146	0.43835671	0.644127981
95.45520226	-0.020958499	0.117615721	-0.178194706	0.858570067	0.929770219
1.876899895	-3.563496839	1.547142166	-2.303276917	0.021263268	0.089887367
5533.940389	-0.037208727	0.104679395	-0.355454167	0.722249375	0.852432094
9.756933417	0.17908136	0.311779864	0.574383983	0.565707989	0.748001651
265.2344008	-0.308309362	0.079158108	-3.894855104	9.82574E-05	0.001543082
3.895119525	2.460344455	1.125003147	2.18696673	0.028744953	0.11069293
8.029956584	1.380779729	0.963558204	1.433000853	0.151857593	0.336094105
3614.55739	-0.039950345	0.104272498	-0.383134061	0.701620366	0.839945782
417.4437517	0.007711204	0.05615435	0.137321574	0.890776618	0.945409999
457.4060217	-0.267224282	0.060330913	-4.429309397	9.45353E-06	0.000227354
319.6276323	0.173867974	0.08373598	2.076383108	0.037858532	0.133770761
1365.021997	0.109505959	0.159758915	0.685445059	0.493063118	0.690062171
389.2625353	-0.121943453	0.110879566	-1.099782914	0.271426718	0.483344531
3737.071814	-0.04236198	0.095968991	-0.44141321	0.658913881	0.81237444
630.5232011	-0.112987356	0.117133891	-0.964600041	0.334745179	0.550047536
959.8749481	-0.069215308	0.063358003	-1.092447747	0.274636346	0.487381144
514.5766126	-0.376007428	0.214919821	-1.749524202	0.080200449	0.22272795
14.27499398	-0.399583676	0.379264416	-1.053575445	0.292077336	0.507295575
57.11569278	0.330487795	0.159920212	2.066579273	0.038773816	0.135790782
2.72754469	0.769753701	0.625334361	1.230947391	0.218342541	0.423029367
278.7863323	-0.21082443	0.063566	-3.316622578	0.000911126	0.008977208
331.5024442	-0.073953763	0.08424921	-0.877797705	0.38005351	0.592897872
191.3759175	-0.42686978	0.099726992	-4.280383584	1.86571E-05	0.000402301
318.623439	-0.149276755	0.067015424	-2.227498471	0.025913978	0.103253963
592.9684971	-0.121705739	0.099892107	-1.218371925	0.223082669	0.42812953
248.4249385	0.034021046	0.087469464	0.388947688	0.697314844	0.837713465
244.5022731	-0.236080829	0.103875265	-2.272733844	0.023042224	0.094733615
1575.628427	-0.132558882	0.090394744	-1.46644457	0.142527212	0.322128315
98.55703248	0.121617959	0.125716762	0.967396532	0.333345843	0.54871497
212.0053276	-0.246230332	0.138269738	-1.780796989	0.074945622	0.213239062
133.036202	-0.132918479	0.107609918	-1.235188001	0.216760544	0.421573975
499.4864005	-0.293044438	0.126107768	-2.323761998	0.020138254	0.086372712
369.7932805	-0.359174151	0.094367797	-3.806109325	0.00014117	0.002050824
124.7321864	0.543144071	0.192025283	2.828502909	0.004676628	0.029884703
9.120818287	0.309546531	0.413872143	0.74792792	0.454503636	0.658820316
2749.723359	0.061423978	0.10804674	0.568494506	0.569699235	0.750099284
125.3765421	0.352218981	0.298912904	1.178333143	0.23866382	0.446360434
3.30762237	-0.692613331	0.563057214	-1.230094053	0.218661887	0.423244733
2585.004991	-0.126442272	0.089700072	-1.409611704	0.158654369	0.345790832
2.389158925	-1.636796955	0.962519121	-1.700534482	0.089030436	0.238186982
2.118759617	-0.038928377	0.660087553	-0.058974567	0.952972366	0.977173332
40.01614938	0.012896704	0.180005252	0.071646266	0.942883419	0.972623458
209.95443	0.027427843	0.105455131	0.26009017	0.79479422	0.894318805
15.52121754	0.010794226	0.307151736	0.035142976	0.971965733	0.985970658

296.9639599	-0.171607879	0.064023795	-2.680376526	0.007353939	0.041919996
6501.926431	0.021340045	0.100147858	0.213085383	0.831260365	0.915631403
9.535436608	0.0087696	0.312875754	0.028029016	0.977639009	0.988317687
7.74552921	1.140111657	0.682949672	1.669393374	0.095039446	0.247812572
5.009740545	-1.075667653	0.589657005	-1.824226023	0.068117909	0.200245259
49.85124382	-0.423148305	0.286262496	-1.478182826	0.13935885	0.31784545
70.22351241	0.055212351	0.185351585	0.297879033	0.765795492	0.878006201
124.4520025	0.093152278	0.106387792	0.87559179	0.38125199	0.593923904
560.7773903	-0.289394324	0.064015334	-4.52070317	6.16346E-06	0.000158899
16.33550245	-0.056414911	0.230996714	-0.244223868	0.807057443	0.901693069
45.93430965	-0.093056985	0.171791332	-0.541686151	0.588034732	0.761885532
2.36644782	-0.717155999	0.767445801	-0.9344712	0.350060885	0.565050267
97.80460313	0.376608279	0.112735044	3.340649598	0.000835826	0.008370458
413.3167379	-0.360131012	0.081631751	-4.411653621	1.02584E-05	0.000243689
168.1932068	-0.092003306	0.131565883	-0.69929456	0.484367966	0.683584158
237.5769067	-0.143152596	0.097048382	-1.475064222	0.140195278	0.318958295
121.9111786	0.184303067	0.086561141	2.129166327	0.033240502	0.122070771
183.1718702	0.341354662	0.086160529	3.961844999	7.43728E-05	0.001240099
14.42594489	-0.043315432	0.2540866	-0.170475073	0.864636536	0.932688731
9.734137772	0.975911184	0.387122273	2.520937831	0.011704253	0.058899415
2613.413639	0.145724438	0.053904787	2.70336727	0.006864086	0.039866218
38.83393194	-0.454307799	0.168771489	-2.691851571	0.007105656	0.040772755
138.6640316	-0.05710568	0.108550298	-0.526075754	0.598835574	0.769938129
713.9428767	-0.012324404	0.086411159	-0.142625144	0.886586238	0.943332796
1119.111475	0.401778901	0.070552161	5.694778121	1.23532E-08	7.37346E-07
28.39098993	0.339690783	0.203078506	1.672706724	0.094385045	0.246887779
158.1752149	-0.07074816	0.085342279	-0.828993091	0.407108317	0.617901456
11.66464968	-0.45884093	0.611279709	-0.750623525	0.452879259	0.657391217
96.09831038	-0.078527168	0.101262612	-0.775480367	0.438055862	0.643988245
2.343168844	0.955327612	0.620136887	1.540510864	0.123435877	0.293728533
5.621698811	-0.736787206	0.710219524	-1.037407704	0.299545889	0.514354699
6.937220812	0.702968773	0.435665876	1.613550228	0.10662502	0.267928703
360.8232767	-0.008808618	0.05466754	-0.16113069	0.871990469	0.936268721
85.41884744	-0.055939401	0.125331296	-0.446332261	0.655357256	0.809943754
1220.560917	-0.151307252	0.112443185	-1.345632925	0.178420928	0.374093815
1000.065964	-0.002972312	0.088115112	-0.033732152	0.97309074	0.986625852
2738.573697	0.094710682	0.08121292	1.166202142	0.243532727	0.45113664
255.5518845	0.553289472	0.494208606	1.119546411	0.262907103	0.474358081
489.0465581	-0.046054687	0.06287797	-0.73244551	0.46389669	0.666183586
448.7143074	-0.247616537	0.125585343	-1.97169934	0.048643939	0.159094749
163.3828176	-0.111788386	0.130210128	-0.858522972	0.390603751	0.60400683
802.4028091	0.431630699	0.114747203	3.761579271	0.000168844	0.002385535
133.8429425	-0.015745087	0.089497483	-0.175927706	0.860350739	0.931052885
93.73494314	-0.165977044	0.098039653	-1.692958292	0.090463406	0.24083616
1389.008239	-0.034789684	0.050746193	-0.685562446	0.49298907	0.690017157
6.669562779	-0.110766507	0.506666576	-0.218618145	0.826947517	0.913540506
95.37576137	-0.096505116	0.139730474	-0.690651892	0.489784329	0.68751764
1.742654633	-1.622446214	0.820852912	-1.976537076	0.048093981	0.158036964

2.596352387	-2.060222786	1.229770261	-1.675290785	0.093877193	0.246091419
4.740817736	-0.22756675	0.494044655	-0.4606198	0.645071404	0.803015198
15.60662722	-0.540204463	0.296501939	-1.821925567	0.06846628	0.200839299
205.1878991	-0.195660392	0.097057402	-2.015924477	0.043807874	0.148160729
623.1140899	0.122870699	0.077939918	1.576479706	0.114915292	0.281422542
20.97475675	-2.363202224	0.882638839	-2.677428321	0.007418973	0.042217669
18.24608921	0.263388879	0.29591498	0.890082952	0.373421346	0.586886402
129.0770795	-0.271022354	0.123802107	-2.189157848	0.028585369	0.110258809
130.6931074	-0.497366567	0.113292004	-4.390129495	1.13283E-05	0.000264713
864.0589663	-0.163515143	0.060099666	-2.720732953	0.006513736	0.038392588
334.92264	0.167143261	0.086011572	1.9432648	0.051984172	0.16606307
122.7149889	0.037330149	0.100196139	0.372570733	0.709467958	0.845146114
11.08691146	-0.13470348	0.282788842	-0.476339446	0.633832568	0.794736908
472.9834628	-0.132980025	0.099775996	-1.332785741	0.182602126	0.379629913
1252.94705	0.136748446	0.057369214	2.383655579	0.017141637	0.077071869
36.83629227	0.001645584	0.177197614	0.009286717	0.992590379	0.995886705
2.030471104	0.561820825	1.047793256	0.536194351	0.591824237	0.764503449
144.1295549	-0.207790083	0.22762322	-0.91286857	0.361311669	0.575339666
259.5943065	-0.041332788	0.075073627	-0.550563359	0.581933033	0.758210783
81.76953103	0.047243828	0.135625869	0.348339355	0.727585342	0.855815575
227.4408522	-0.420294375	0.100881433	-4.166221293	3.0969E-05	0.000611731
54.78240613	0.056536078	0.132277732	0.427404348	0.669084841	0.819834748
1230.23876	0.187557197	0.069984902	2.679966565	0.007362951	0.041942345
333.5782299	-0.362280919	0.066248371	-5.468525698	4.53794E-08	2.23158E-06
903.5023914	-0.158312162	0.088315235	-1.792580437	0.073040037	0.209828068
318.1289736	-0.365813719	0.141693946	-2.581717352	0.009831006	0.05197561
10.69438235	-0.547475348	0.335745172	-1.630627611	0.102968919	0.26222136
50.42439876	0.882981638	0.333788003	2.645336651	0.008160968	0.045420198
39.56118984	-0.537885409	0.25968023	-2.071337542	0.038327263	0.134913127
67.58184968	0.110530439	0.135821654	0.813790993	0.415764696	0.625357946
22.72588296	-0.502199851	0.234696609	-2.139783155	0.032372295	0.119816039
4.858711432	-2.322356583	0.947617801	-2.450731276	0.014256634	0.067790907
222.876303	-0.138701358	0.19068818	-0.7273726	0.466997735	0.668751798
189.026188	0.083532725	0.087953154	0.949741106	0.342243818	0.556467099
5361.764815	-0.349103082	0.11044257	-3.160946746	0.001572572	0.013606386
775.7107869	-0.107580088	0.051938425	-2.071300533	0.03833072	0.134913127
55.55503934	0.067180061	0.138614255	0.484654778	0.62792125	0.791096258
25.89699434	-0.565462427	0.329344179	-1.716934633	0.085991116	0.232886268
5.23070205	-0.814550792	0.577007798	-1.411680734	0.158043989	0.344986971
2477.465439	0.23837979	0.084197103	2.831211323	0.004637207	0.029719986
712.5210606	-0.253832138	0.063880888	-3.973522411	7.08175E-05	0.001196561
131.9815593	0.70909456	0.125301654	5.659099749	1.52169E-08	8.85807E-07
105.9091837	0.264088752	0.091904572	2.873510489	0.004059376	0.027048635
225.9982684	0.143859085	0.085028578	1.691890993	0.09066676	0.24095254
408.1949245	-0.205356833	0.131333471	-1.563629069	0.117904704	0.285683497
1155.903573	0.037914481	0.114327759	0.331629708	0.740168884	0.863381874
2090.12266	-0.076144101	0.312111152	-0.243964691	0.807258166	0.901735106
314.845546	-0.062885583	0.076144791	-0.825868488	0.408878698	0.619446706

420.8694955	0.274973533	0.090339542	3.043778258	0.002336273	0.018131536
15.83474401	0.266385978	0.263662709	1.010328606	0.31233788	0.527087493
119.2348093	-0.136182284	0.24593914	-0.553723511	0.579768082	0.757293715
215.9034149	-0.112765403	0.112771719	-0.999943994	0.317337612	0.532225781
859.4520313	0.05741727	0.102438656	0.560503934	0.575135757	0.753941023
63.63129847	-0.142415707	0.257941125	-0.552124858	0.580862812	0.757771141
103.3535964	-0.08326252	0.114325665	-0.728292464	0.466434574	0.668520881
3.301582544	-1.055152657	0.803129374	-1.3138016	0.188912979	0.388250202
1.912175443	-0.872092426	1.334151422	-0.653668251	0.513325562	0.706594696
411.5614233	0.059449591	0.081383688	0.730485334	0.465093575	0.667211117
30.73539242	0.05920583	0.260992501	0.22684878	0.820541331	0.909820137
672.2868873	0.043024438	0.118962982	0.361662408	0.717604322	0.850267635
321.645029	-0.621918972	0.258120423	-2.409414045	0.015978159	0.073382826
1067.431482	0.072681119	0.115840899	0.627421921	0.530382702	0.72005197
1833.733457	0.000930402	0.072960045	0.012752213	0.989825482	0.994530438
832.9244927	-0.010003675	0.100216961	-0.099820179	0.920487087	0.960848072
142.2966416	0.101810639	0.086487196	1.177175864	0.23912532	0.446593139
1400.151093	0.102261219	0.10293582	0.993446384	0.320492467	0.535530266
20.50034127	0.12380179	0.236379704	0.523741201	0.600458555	0.771603294
154.1515165	0.001797234	0.139191055	0.012911995	0.989698005	0.994530438
624.1219374	-0.077767328	0.147510584	-0.527198293	0.598055894	0.769422919
259.0300886	-0.02619901	0.067706398	-0.386950283	0.698793015	0.838453107
100.4556393	-0.340218069	0.11098382	-3.065474499	0.002173249	0.017163042
2673.895549	0.243549067	0.069015964	3.528880164	0.000417322	0.004862068
124.6641373	0.41265176	0.145866886	2.828961187	0.004669936	0.029853523
104.6657702	0.352567935	0.099288605	3.550940552	0.000383857	0.004546127
4.240147516	-0.160273484	0.460689083	-0.347899461	0.72791569	0.855998506
385.2830317	0.843032198	0.126693601	6.654102412	2.85034E-11	3.42748E-09
639.5363692	0.044600574	0.058471388	0.76277604	0.445596973	0.650661632
205.3284239	0.175146734	0.073365999	2.387301142	0.016972582	0.076499677
385.8189234	-0.14553909	0.057595689	-2.526909432	0.011507117	0.058203331
105.5701642	0.125201143	0.116243281	1.077061332	0.281452867	0.495474944
174.4466176	0.270876514	0.207895652	1.302944589	0.19259368	0.393137709
28.90612747	0.409636674	0.223312524	1.834364985	0.066599834	0.197119235
212.3897676	0.389138554	0.156686629	2.483546655	0.013008129	0.063495088
6.150356549	0.357484139	0.413826733	0.863849797	0.387670408	0.600797959
162.3805256	-0.204556574	0.092588982	-2.209297156	0.027153979	0.106584381
1488.574827	0.016823804	0.077250413	0.217782707	0.827598419	0.913863048
46.12646112	-0.40208078	0.190212714	-2.113848078	0.034528245	0.125152543
635.1503529	-0.172088626	0.104252443	-1.650691539	0.098801576	0.254599901
4.519703552	0.831163123	0.487997095	1.70321326	0.088528166	0.237396446
96.00041013	0.060443047	0.110564848	0.546675076	0.58460198	0.759881097
858.5740341	0.108552849	0.075065994	1.446098864	0.148149474	0.330578336
409.4785916	-0.126154373	0.071644142	-1.760846998	0.078264303	0.219044355
1285.695503	-0.007204732	0.051025561	-0.141198484	0.887713144	0.943800344
1204.200337	-0.23623663	0.097704746	-2.417862394	0.01561198	0.072265173
202.063815	-0.406348933	0.111355033	-3.649129488	0.00026313	0.00340252
338.7597245	-0.082186426	0.082763068	-0.993032626	0.320694055	0.535758429

1.788035276	-1.189398504	1.149388512	-1.034809806	0.300757742	0.515629415
371.2316964	0.146834539	0.134272808	1.0935538	0.274150721	0.486781524
494.7282087	-0.230117095	0.091063891	-2.526985093	0.011504639	0.058203331
16.88447287	0.022739064	0.333608039	0.06816102	0.945657456	0.974141328
75.19177389	0.087864058	0.115894198	0.758140267	0.448367016	0.653258886
34.17262228	-0.149286485	0.172513708	-0.865360131	0.386841156	0.599907861
21.13747018	-0.247336732	0.288034247	-0.858705987	0.390502746	0.60400683
538.3720003	-0.070302403	0.112485833	-0.624988954	0.531978308	0.72142004
2506.44381	-0.159874593	0.11063976	-1.445001259	0.148457532	0.33103538
25.82216686	0.136100615	0.324568836	0.419327426	0.674976856	0.824160148
2.23125033	0.841614882	1.080592378	0.778845844	0.436070529	0.642539879
151.7639063	-0.245752172	0.109378283	-2.246809563	0.0246522	0.099588119
536.0753531	-0.311706947	0.115110969	-2.707882228	0.006771405	0.03955749
150.0347001	0.05114864	0.257585233	0.198569766	0.842599306	0.92177309
40.68996102	-0.070676265	0.159665441	-0.442652239	0.658017293	0.811680353
203.3286665	-0.082731094	0.089187733	-0.927606207	0.353611877	0.568540551
1074.339509	-0.05046106	0.092004925	-0.548460418	0.583375801	0.759227189
4.625009182	1.139759488	0.599389436	1.901534159	0.057232083	0.177359167
1968.429662	-0.166554209	0.105778812	-1.574551703	0.115359958	0.282048078
1416.545031	0.006532853	0.088289214	0.073993781	0.941015334	0.971961872
398.1445716	-0.343201874	0.222713908	-1.540998838	0.123317069	0.29348821
2.215646529	-0.094994657	1.162998534	-0.081680805	0.934900543	0.969158606
1388.323365	0.076733343	0.221897532	0.345805303	0.729489039	0.857101832
58.14070681	0.466317293	0.230011965	2.027361022	0.042625505	0.145355531
7.813823607	0.848676804	0.435105256	1.950509199	0.051115458	0.164355462
351.7082583	0.086099803	0.090246151	0.954055126	0.340055739	0.554826012
167.353692	-1.263252129	0.681160497	-1.854558705	0.06365927	0.191093806
52.98200408	-0.227802483	0.180639829	-1.261086685	0.207277617	0.41080496
77.09895349	0.361002618	0.318023676	1.135143845	0.256315053	0.466578363
128.55897	-0.213281899	0.12051811	-1.769708291	0.076775748	0.216427733
49.08833304	-0.056747629	0.198056815	-0.286521973	0.774478367	0.882958935
917.7102918	0.200620647	0.05076228	3.952159904	7.7449E-05	0.001279733
25.4865192	0.333448463	0.198535815	1.679538085	0.093047223	0.244836646
309.5240894	0.078970228	0.067395176	1.171748971	0.241297855	0.448914836
7.154073572	1.042277427	0.490846615	2.123427959	0.033718006	0.123191492
380.3854752	0.18752081	0.072533628	2.585294805	0.009729577	0.05161681
65.59304765	-0.313865095	0.128686243	-2.43899493	0.014728175	0.069303614
294.9181699	0.078223757	0.099985242	0.782353027	0.434007131	0.640723495
184.5786848	0.035744089	0.080612251	0.443407661	0.657470896	0.811359633
328.1664791	-0.014738903	0.079038311	-0.186477955	0.852069965	0.926915291
40.32161026	0.313120075	0.168189177	1.861713579	0.062643474	0.189043521
74.39460522	0.050428043	0.127852629	0.394423196	0.693268621	0.834976033
1318.645376	-0.471993566	0.471892053	-1.000215119	0.317206414	0.532090262
182.8428277	-0.005951753	0.09629938	-0.061804687	0.950718371	0.976564063
33.49016444	-0.834150517	0.370071861	-2.254023084	0.024194716	0.098252814
99.56241017	-0.112776494	0.185313746	-0.608570583	0.542809108	0.730157365
377.6555129	-0.514427608	0.078993527	-6.512275509	7.40208E-11	8.12946E-09
16.35938259	0.647528338	0.246744963	2.624281888	0.008683189	0.047587109

554.7799788	-0.195519137	0.060884826	-3.211294982	0.001321382	0.011927918
17.28236947	-0.550439707	0.398500136	-1.381278591	0.167193318	0.358468754
331.383213	0.068115455	0.081112059	0.83976977	0.401037486	0.612787084
124.9244507	-0.348521148	0.177247573	-1.966295741	0.049264458	0.160391836
192.4622409	-0.215643195	0.15552702	-1.386532035	0.165584505	0.356068286
503.8735061	0.027347464	0.06913329	0.395575908	0.692417913	0.834264076
28.36790461	0.232202931	0.190040363	1.221861122	0.22176015	0.426570635
491.3283988	-0.105837875	0.05704158	-1.855451316	0.063531805	0.190850285
17.6310859	0.502285875	0.425798021	1.179634123	0.238145765	0.445667765
2.238655987	0.206166661	0.760765426	0.270998989	0.78639181	0.889541352
8.896149193	0.968073268	0.528931869	1.830241896	0.067213775	0.198224085
17.44246631	-0.589282283	0.25733434	-2.2899481	0.022024325	0.091931577
285.5968437	0.048520195	0.09489995	0.51127735	0.609156863	0.778180891
284.6600914	0.117200075	0.113765927	1.030186091	0.302922658	0.517887593
72.73297268	0.02951495	0.138436603	0.213201925	0.831169467	0.915631403
35.20122074	0.492615999	0.266565102	1.848013848	0.064600339	0.193179523
503.9023996	0.528178067	0.169102953	3.123411252	0.001787678	0.014866331
240.3643249	-0.27645587	0.310669585	-0.889871052	0.373535129	0.587009226
83.2964145	0.1294901	0.103544365	1.25057602	0.211089204	0.415370705
7.388789	-0.050160795	0.42535807	-0.117926045	0.906126256	0.952959157
746.1083889	0.111394826	0.081234201	1.371279893	0.170287721	0.362304006
2116.247203	0.10064576	0.059062073	1.704067513	0.088368475	0.237059479
5.628704498	-2.513323523	0.910536622	-2.760266268	0.005775427	0.035108628
2750.775006	-0.139056646	0.092405719	-1.504848923	0.132362922	0.30681677
5.997903796	0.163746615	0.445004397	0.367966287	0.712898373	0.84680134
1498.552314	-0.204179558	0.077320498	-2.640691202	0.008273709	0.045892621
23.94551721	0.123611345	0.26354096	0.469040353	0.639040793	0.798267972
7.248774857	-0.090409823	0.455098547	-0.198659881	0.842528809	0.921757179
29.85760989	-0.25077441	0.174811452	-1.43454223	0.151417587	0.33559173
147.2285588	-0.214718412	0.132456177	-1.621052465	0.105006387	0.265344941
2.239357675	0.866084726	0.769571941	1.125410999	0.260414913	0.471512876
1248.835066	0.551118804	0.135530864	4.06637121	4.77509E-05	0.000859724
827.2095717	-0.107158078	0.08879133	-1.206852946	0.227488782	0.433254357
13.33590552	-0.603844412	0.297719718	-2.028231172	0.04253666	0.145172764
630.0376138	0.131753806	0.066679754	1.975919188	0.048163931	0.158184329
14.09621725	-0.472370915	0.339378892	-1.391868871	0.16396211	0.353548665
698.8013813	-0.130592354	0.052107499	-2.506210379	0.012203299	0.060708845
41.58838308	-0.577188081	0.231730234	-2.490775894	0.012746449	0.062591267
102.0516743	0.049335101	0.093593083	0.527123366	0.598107922	0.769422919
116.5942636	-0.11795752	0.223639288	-0.527445429	0.597884303	0.769420873
88.50401992	0.987874768	0.200354091	4.930644367	8.19588E-07	2.92883E-05
1210.607838	0.352603137	0.123365647	2.858195499	0.004260578	0.028038321
219.9413746	-0.325200115	0.101972157	-3.18910696	0.001427131	0.012653687
15.72278969	-0.098495417	0.265173395	-0.37143778	0.710311491	0.845557591
406.8491401	0.270840767	0.071577349	3.783889357	0.000154396	0.002213688
5.468173359	0.059761993	0.383406192	0.155871227	0.876134526	0.937723504
12.08079625	0.376076711	0.352291232	1.067516521	0.285738657	0.500157637
601.1221521	-0.163959722	0.095705019	-1.713177887	0.086679818	0.23405398

136.4498583	-0.494897115	0.223819975	-2.211139175	0.027026201	0.106247383
571.7695121	0.111296266	0.048629982	2.288634728	0.022100584	0.092105817
37.77593523	-0.08980412	0.162476918	-0.552719249	0.58045567	0.757539944
162.4524951	0.19993231	0.123962494	1.61284517	0.10677815	0.268109014
115.2240314	0.063664361	0.096806618	0.657644713	0.510766456	0.705134216
149.3849897	0.59283789	0.124785103	4.750870714	2.02543E-06	6.30722E-05
7.990274651	0.246738533	0.353260044	0.69846148	0.48488864	0.683839419
88.90065748	0.033992296	0.122620325	0.277215834	0.781614382	0.886371262
64.41036738	-0.710372921	0.534865871	-1.328132827	0.184134231	0.381563184
18.21277296	-1.060603694	0.641120149	-1.654297866	0.098067006	0.253261618
2293.561573	-0.448818147	0.075478647	-5.946292984	2.74283E-09	2.06326E-07
48.95799654	0.050898529	0.314081703	0.16205506	0.871262495	0.935876058
299.2289925	0.109590064	0.083341768	1.314947684	0.188527485	0.387934274
1022.622291	-0.199277193	0.059722218	-3.336734624	0.000847688	0.008463527
16.43029033	0.032838492	0.240066338	0.136789239	0.89119739	0.945526539
126.0369223	-0.205666198	0.109063304	-1.885750662	0.059328562	0.182056232
7047.646542	-0.302168753	0.064817788	-4.661818337	3.13428E-06	9.01119E-05
40.7666951	1.429805173	0.212290812	6.735125094	1.63789E-11	2.22997E-09
60.66765064	-0.209278271	0.133543863	-1.567112598	0.117088375	0.284836664
970.7466976	-0.006105058	0.061033715	-0.100027631	0.920322389	0.960797176
100.5271257	0.359257117	0.312527159	1.149522872	0.250340441	0.45932976
266.9345699	0.177991848	0.091630249	1.942500978	0.052076482	0.166164625
560.4535652	0.237722132	0.102125451	2.327746219	0.019925583	0.085728404
24.61175288	0.27616944	0.224429362	1.23054059	0.218494737	0.423029367
767.4082112	-0.060807625	0.073962691	-0.822139169	0.410997695	0.621058158
1319.441231	-0.024352695	0.072982598	-0.333678106	0.738622473	0.862494089
21.83048303	-0.269696914	0.335958496	-0.802768547	0.422108525	0.630445679
1512.721117	0.060683153	0.073048616	0.83072283	0.406130226	0.616852629
115.5727802	-0.117485925	0.098238538	-1.19592502	0.23172582	0.438384378
2005.304331	-0.275950698	0.11218638	-2.459752228	0.013903296	0.066673057
52.01502427	-0.198509247	0.219453771	-0.904560655	0.365698187	0.579168615
47.58538967	-0.371576204	0.470892769	-0.789088787	0.430060115	0.637008662
9.817541861	-1.233914937	0.380039752	-3.246804924	0.001167084	0.010874737
24.42063202	0.626237002	0.256660899	2.439939246	0.014689732	0.069182003
5.353285057	-1.313652008	0.691382443	-1.900036689	0.057428305	0.17776661
6.317392107	-2.104647773	1.173957483	-1.792780235	0.073008071	0.209823018
434.8436406	-0.113525	0.077229517	-1.469969049	0.141570137	0.320941711
254.0867426	0.159933701	0.148009494	1.080563804	0.279891191	0.493305603
7.614133906	0.145787848	0.597581172	0.243963256	0.807259278	0.901735106
184.3579381	-0.368143362	0.113247996	-3.250771531	0.001150923	0.010760673
44.70219992	-0.591881214	0.190038322	-3.114536093	0.001842344	0.015228689
114.3959483	-0.760385077	0.358377848	-2.12174129	0.033859469	0.123506384
698.4970897	0.300234074	0.073041677	4.110448782	3.94891E-05	0.000738379
53.22435724	-0.682081289	0.234841316	-2.90443479	0.003679168	0.025180607
178.8107386	-0.029339988	0.078056386	-0.375881971	0.707004645	0.843388452
18.904607	-0.446893146	0.287104842	-1.556550362	0.119577277	0.288547833
13.46766103	-0.301503016	0.351041239	-0.85888204	0.390405599	0.60400683
3.606447655	-0.358804117	0.59276803	-0.605302747	0.544977857	0.731939117

17.83992853	-0.553042248	0.316740946	-1.746039641	0.080804064	0.22376301
5.549763996	0.492711784	0.588028661	0.837904369	0.402084415	0.613694572
1.73007673	-0.45969324	0.862017946	-0.533275719	0.593842733	0.766449795
296.3575943	-0.381270998	0.144258309	-2.642974257	0.008218128	0.045661196
542.9648699	0.183626307	0.077911991	2.356842713	0.018431052	0.080961138
42.7896319	-1.385069878	0.37553819	-3.688226427	0.000225823	0.003017196
24.73206941	-0.504147159	0.364693642	-1.382385381	0.166853402	0.357909238
209.5845407	-0.127212089	0.156608017	-0.812296145	0.416621722	0.626095433
651.5036982	-0.102062318	0.12924287	-0.789693994	0.4297065	0.636713876
2.942127466	0.885457527	0.534104885	1.657834543	0.097350867	0.251965151
228.7142128	-0.046870481	0.102003042	-0.459500814	0.645874568	0.803575079
273.1811373	-0.154420612	0.08612816	-1.792916651	0.072986252	0.209802045
1169.637654	0.233108387	0.057935106	4.023612009	5.73123E-05	0.001000173
61.32293098	0.270923009	0.542469923	0.499424941	0.617480052	0.783755788
526.4291614	-0.226521175	0.088899089	-2.548070814	0.010832048	0.055487301
1018.475953	0.363264228	0.115262596	3.151622818	0.001623659	0.013898241
25.47985395	0.390979379	0.237811964	1.644069424	0.100161863	0.256779727
60.96937254	0.257447344	0.128155872	2.008861082	0.044551868	0.149876961
21706.29915	0.343477286	0.103738966	3.31097656	0.00092971	0.009095037
875.7363946	-0.030304925	0.052164012	-0.580954648	0.56127102	0.74475811
15.54292261	-1.402952729	0.556494195	-2.521055459	0.011700341	0.058899415
18.35569811	1.06722886	0.32334647	3.300573713	0.000964874	0.009377775
103.3333743	-0.186936885	0.171689896	-1.088805391	0.276239714	0.488857347
17.98287938	0.171515833	0.30713498	0.558437964	0.576545355	0.754888585
9.404371037	1.725018043	0.981065534	1.758310717	0.078694655	0.219774472
10.99698197	-0.057829111	0.38786348	-0.14909656	0.881477441	0.940691803
984.7802026	0.193897612	0.18441122	1.051441512	0.293055858	0.508189707
1810.682939	0.18111029	0.068131738	2.658236748	0.007855069	0.044002853
939.9677471	-0.219699514	0.073326323	-2.996188867	0.002733771	0.020396801
9.843673379	0.288796212	0.413411918	0.698567699	0.484822236	0.683839419
349.2761242	0.197937055	0.07447506	2.657762953	0.00786612	0.044047063
14.85256019	0.026924789	0.274739087	0.098001304	0.921931258	0.961441764
230.9897158	0.15864837	0.085148637	1.863193295	0.062435076	0.18860883
44.847335	0.322147035	0.194289724	1.658075522	0.097302224	0.251918409
3.39208917	0.262278082	0.737756537	0.355507636	0.722209325	0.852432094
2998.060089	0.03767676	0.061354081	0.614087267	0.539157662	0.726848063
294.7911506	0.014643765	0.084734374	0.17281965	0.862793191	0.931737465
295.6921291	0.481141601	0.103975561	4.62744895	3.70197E-06	0.000103367
3185.116595	0.339236711	0.121675624	2.788041682	0.005302772	0.032989168
242.5024346	-0.293112531	0.198655963	-1.475478139	0.140084041	0.318837317
332.1514124	-0.409318209	0.12952542	-3.160138052	0.001576944	0.013629893
978.9975833	0.138871101	0.08764505	1.584471693	0.113086418	0.278681474
287.8781299	0.284311215	0.093857106	3.029192223	0.002452086	0.018753791
69.94051489	0.191919703	0.208425006	0.920809393	0.35714995	0.571675892
26.35882694	-0.343964619	0.203993452	-1.686155198	0.091765911	0.242536042
34.78791354	0.228024145	0.304856059	0.747973142	0.454476358	0.658820316
289.8334307	0.264598779	0.137167241	1.929023123	0.053727992	0.169661869
5.649907381	-0.421921516	0.559399427	-0.754240165	0.450705024	0.655621595

392.6341317	0.01082965	0.175913976	0.06156219	0.950911487	0.976633959
17.41376936	0.813309043	0.421594923	1.929124378	0.053715423	0.169661869
253.854907	-0.148574774	0.168022083	-0.884257422	0.376557267	0.589677226
980.3156119	-0.04455832	0.1454137	-0.306424494	0.759281461	0.87432656
1.991069605	0.232896698	0.688676012	0.338180354	0.73522728	0.860688745
21.75653015	0.416973205	0.244660132	1.704295676	0.088325862	0.237022358
113.5490412	1.371975504	0.187390647	7.32147269	2.45264E-13	5.31643E-11
96.06129671	0.52300906	0.221387005	2.362419871	0.018156066	0.080059698
25.91707149	0.830955252	0.354843684	2.341750154	0.019193559	0.083491941
2491.338779	-0.015500428	0.089235193	-0.173703081	0.862098816	0.931452496
13.06288986	0.049392941	0.309377258	0.159652785	0.873154596	0.936680519
108.8440268	-0.144349059	0.093587355	-1.542399178	0.122976621	0.293101397
17.45459108	-0.018996516	0.302911713	-0.062713045	0.94999501	0.976186239
1639.805821	-0.007090592	0.126387755	-0.05610189	0.955260638	0.978241049
651.0413154	0.069051578	0.23023344	0.299919848	0.764238295	0.877295078
4.528326908	0.796192755	0.49397442	1.611809687	0.107003359	0.268510791
54.35045317	0.012459871	0.199646142	0.062409776	0.95023651	0.976312602
6.983677824	0.156709855	0.621604114	0.252105563	0.80095947	0.897924893
23.82856757	-0.473312315	0.228729192	-2.069313102	0.038516715	0.135293041
533.2016783	0.323660788	0.081775379	3.957924635	7.56038E-05	0.001258976
61.43292547	-0.567875226	0.132725999	-4.27855303	1.88112E-05	0.000405093
94.37682892	0.955702237	0.156276983	6.115438246	9.6292E-10	7.97143E-08
126.6618104	0.048708275	0.096008931	0.507330669	0.611922823	0.780156904
54.66013567	0.190553524	0.161264368	1.181621996	0.237355722	0.445009597
19.47474461	-0.403875929	0.501168121	-0.805869153	0.420318299	0.628969358
43.13783076	0.526824007	0.154752699	3.404296074	0.000663348	0.006973833
183.9104975	0.035237402	0.086623192	0.40678947	0.684162621	0.829348762
1357.049346	0.101790586	0.083495284	1.219117787	0.22279949	0.427806473
25.46816237	-0.144099511	0.194650845	-0.740297378	0.459119572	0.662074214
16.77731302	-2.125315695	0.899265847	-2.363389762	0.018108613	0.0799789
2.055623203	0.307073519	0.723143246	0.424637194	0.671101175	0.821214982
4.586808449	-0.519902692	0.63387982	-0.820191266	0.41210708	0.621935878
7.634241762	1.1400566	0.686468681	1.660755446	0.096762573	0.250675642
3.942765702	0.38073564	0.694022752	0.548592448	0.58328517	0.759227189
8.572087066	0.12135554	0.542609749	0.223651603	0.823028407	0.911237589
2.22658056	1.115799845	1.236379382	0.902473675	0.366805299	0.580392006
6.618433041	1.097981597	0.453189176	2.422788662	0.015401884	0.07161463
4.717941578	0.792465061	0.62972869	1.258422989	0.208238822	0.412009499
955.3687044	-0.258368242	0.0956108	-2.702291394	0.006886338	0.03993155
127.9070947	-0.267970668	0.12264112	-2.184998541	0.028888954	0.111091648
3.406219058	0.869196754	0.989798028	0.878155673	0.379859244	0.592763207
23.44560041	-1.427459069	0.456609697	-3.126212775	0.001770734	0.01475522
5.382709089	-1.703379183	0.875989879	-1.944519252	0.051832866	0.165804784
28.71126494	0.163026568	0.241144458	0.676053552	0.499006645	0.695021779
76.52581607	0.030869307	0.123783377	0.249381685	0.803065551	0.899097368
19.43151434	-0.727673305	0.269526837	-2.699817628	0.00693775	0.040108248
1.811962909	-2.649969597	1.094752229	-2.420611283	0.015494436	0.071882663
2.539429997	-1.636622622	0.641408658	-2.551606689	0.010722749	0.055081561

507.7995305	-0.147517275	0.114088684	-1.293005319	0.196009219	0.397218218
3.128093806	1.475994298	0.988792379	1.492724186	0.1355094	0.311917533
24.84789111	0.166218315	0.191049671	0.8700267	0.384285813	0.597397571
12.16539524	-0.241780235	0.357425272	-0.676449748	0.498755141	0.694995588
12234.806	-0.142349127	0.1058671	-1.344602117	0.178753759	0.374375724
2899.971022	0.06402998	0.108209839	0.591720498	0.554037768	0.739046008
230.3703889	0.029878705	0.117476674	0.254337343	0.799234952	0.896484956
2305.765677	0.039046269	0.087733825	0.445053759	0.656280904	0.81076847
29.39843231	0.282990904	0.176501905	1.603330591	0.108861686	0.271845468
203.9594192	-0.057901041	0.081664099	-0.709014625	0.478315401	0.677887811
402.4280839	-0.098424803	0.064336429	-1.529845613	0.126054948	0.297383534
277.0228191	0.112897353	0.065240794	1.730471772	0.08354602	0.2288492
25.91500927	0.249306975	0.240073465	1.038461187	0.299055396	0.514048267
12.22797156	-0.180588805	0.286547313	-0.630223339	0.528548471	0.718602584
561.3896613	-0.02242297	0.067364759	-0.332859048	0.739240683	0.862808843
1.899380629	-0.850855004	0.899534235	-0.94588396	0.344207776	0.558502797
45.8212799	0.002434804	0.276896787	0.008793184	0.992984144	0.996046614
247.2996215	0.045026582	0.06652474	0.676839648	0.498507699	0.694742406
13.71401001	0.744007003	0.358149083	2.077366765	0.037767721	0.133516189
144.1127769	-0.546981446	0.256982249	-2.128479487	0.033297349	0.122169382
71.9046855	-0.060904003	0.171878196	-0.354343974	0.723081116	0.85299236
730.7044681	-0.331198799	0.152680109	-2.169233442	0.030064965	0.114043343
4.26765405	0.452874788	0.996010983	0.454688548	0.649333321	0.805627137
812.7489072	0.227198138	0.075723046	3.000382964	0.002696404	0.02019116
147.9751673	-1.230387357	0.260409461	-4.724818188	2.30321E-06	6.97688E-05
137.8445874	-0.355273383	0.307764247	-1.154368602	0.248349082	0.457077732
181.5700049	-0.095460973	0.116651306	-0.818344653	0.413160416	0.623011871
483.9810622	0.093060789	0.063750185	1.459772841	0.144352515	0.32480388
7.009839623	0.473949497	0.535163336	0.88561653	0.375824201	0.589110444
5.611212607	-0.182077244	0.572812545	-0.317865322	0.750587098	0.869745506
247.0346923	-0.88877821	0.28651746	-3.102003665	0.001922156	0.015754027
132.4315797	0.141291354	0.352442056	0.400892436	0.688499319	0.831671637
13.06358724	-0.010160411	0.297702278	-0.034129437	0.972773935	0.986426062
26.14341461	-0.081196892	0.293663067	-0.276496779	0.782166533	0.886843735
155.2323516	-0.156586183	0.172197995	-0.909338015	0.36317173	0.576997886
1258.525827	-0.298467658	0.076432383	-3.904989561	9.42295E-05	0.001491162
105.9675103	-0.179031631	0.108508425	-1.649932995	0.098956641	0.254840036
93.02630342	-0.316879189	0.824751113	-0.384211896	0.700821402	0.839538378
523.4315563	-1.283985708	0.29703868	-4.322621241	1.54186E-05	0.00034415
6.027919115	2.14956814	0.497361177	4.321945985	1.54659E-05	0.000344304
283.6217049	0.180071847	0.120490343	1.494491951	0.135047084	0.311286646
750.3203659	0.039442307	0.056471355	0.698448033	0.484897046	0.683839419
109.7614037	0.821411166	0.126364794	6.500316568	8.01512E-11	8.63013E-09
13.88906689	1.179968986	0.291970479	4.04139826	5.31334E-05	0.00094527
1.9159719	0.888997178	0.76829157	1.157109115	0.247227783	0.455777809
5.556070972	-2.406536154	1.47233366	-1.63450461	0.102152939	0.260922909
3.658097663	-1.100927294	0.618589139	-1.779739127	0.075118664	0.213436508
1.726904489	-2.971401241	2.207759031	-1.345890199	0.17833793	0.374021523

75.76152078	-0.038238851	0.133406064	-0.286635025	0.774391793	0.882921337
103.0246196	-0.166618225	0.126514638	-1.316987718	0.187842742	0.386863523
235.6701038	-0.218598097	0.083898231	-2.60551497	0.009173626	0.049436154
22.77192597	-1.844887954	0.582499838	-3.1671905	0.001539195	0.013409144
1775.871002	-0.453923102	0.357007325	-1.271467195	0.203562491	0.406434187
221.6189472	0.449037113	0.101045472	4.443911299	8.8338E-06	0.000215278
4531.296696	-0.146451494	0.104125072	-1.406495966	0.159576902	0.347134413
13.67857536	0.59828983	0.297205285	2.013052462	0.044109111	0.148904403
250.7727864	-0.421481091	0.090334752	-4.665769047	3.07465E-06	8.87071E-05
9.389616356	1.639235017	0.716438354	2.288033587	0.022135565	0.092179298
253.6025913	0.053177262	0.09441118	0.563251748	0.57326347	0.752365182
747.2050801	-0.064171868	0.070609489	-0.908827825	0.363441016	0.577203057
48.1266532	-0.454486881	0.221610193	-2.050839247	0.040282604	0.139473649
3.39254551	-0.7092236	0.580650356	-1.221429717	0.221923361	0.426699983
8.594832525	-1.538955268	0.578484213	-2.660323711	0.007806558	0.043802875
55.20612508	0.183786895	0.402400831	0.456725933	0.647868049	0.804718256
2.547019383	0.281438286	0.678806787	0.414607354	0.678429364	0.826216017
327.7806717	0.015364905	0.093688658	0.163999623	0.869731441	0.935245154
319.4265202	-0.180639256	0.072033128	-2.507724713	0.012151129	0.060528614
8.040806811	0.230799716	0.382405595	0.603546912	0.546144917	0.732968746
267.8761483	0.089494296	0.080066627	1.117747799	0.263674722	0.475145195
1410.493197	-0.083155122	0.143060906	-0.581256786	0.561067401	0.74474453
1179.217252	-0.067853331	0.072690932	-0.933449736	0.350587811	0.565734509
569.4236098	0.255476572	0.117298574	2.178002371	0.02940586	0.112502731
176.9282651	-0.101313484	0.103504307	-0.978833509	0.32766225	0.542830642
706.8864008	-0.120441924	0.090049822	-1.337503197	0.181058439	0.37732533
217.1999337	0.032351485	0.06528467	0.495544905	0.620215533	0.785732568
133.3743383	-0.43806397	0.113699317	-3.852828493	0.000116761	0.001764701
7.484046037	-0.091752554	0.422946989	-0.216936298	0.82825799	0.914219238
259.4726973	-0.111871044	0.073007314	-1.532326524	0.125441879	0.296404118
9.789987234	-0.794279947	0.447615672	-1.774468582	0.075985674	0.214907011
772.5697283	0.094202429	0.056316728	1.672725535	0.09438134	0.246887779
13.1217615	0.453868568	0.289110736	1.569877945	0.11644351	0.283728794
4.417916181	0.09340217	0.437852654	0.213318726	0.831078368	0.915614595
250.0027468	-0.006088396	0.092775706	-0.065624894	0.94767647	0.975081017
21.38778123	0.319317601	0.226735687	1.408325284	0.159034772	0.346369492
132.8405549	-0.115877533	0.16373125	-0.707730099	0.479112882	0.678327657
36.77143331	0.247409894	0.296231788	0.835190227	0.403610612	0.614855381
768.7694316	0.042752917	0.073244231	0.583703541	0.559419784	0.74344875
16.80761895	0.058847278	0.254293827	0.231414496	0.816992805	0.907955871
365.9087253	-0.110461047	0.086109307	-1.282800321	0.199562042	0.401856139
2.203808144	0.55356983	0.777586408	0.711907802	0.476521871	0.676451641
2285.862559	-0.472936976	0.098230941	-4.814541831	1.47538E-06	4.8225E-05
55.95986971	0.195752964	0.18760428	1.043435491	0.296746643	0.511392034
538.3549278	-0.127233845	0.053015337	-2.399944099	0.016397576	0.074684452
75.07337105	0.245242608	0.169010986	1.451045371	0.146767227	0.328422072
16.92167774	0.528136343	0.297739858	1.773818083	0.076093246	0.215092679
360.386727	0.121291789	0.092257984	1.314702351	0.188609956	0.388007045

95.85461376	-0.371957403	0.123475636	-3.012395105	0.00259195	0.019614049
38.00697515	-0.18758892	0.161624184	-1.160648831	0.245784739	0.454286748
309.7782741	-0.255103269	0.102239685	-2.49514921	0.012590417	0.062062997
36.00076991	-0.130941117	0.213483942	-0.613353474	0.539642642	0.727263816
2.751707285	-1.727091564	1.20686289	-1.431058638	0.15241341	0.336624442
573.245272	0.256425386	0.118990291	2.155011008	0.031160973	0.116749117
43.12107112	0.523010615	0.186260291	2.807955537	0.004985711	0.031433067
43.25355165	-0.715323994	0.217810488	-3.284157717	0.001022877	0.009819854
89.09808805	-0.270656883	0.118154914	-2.290695109	0.021981054	0.091837656
631.802651	-0.239346759	0.0968055	-2.472450005	0.013419048	0.065057504
3.666497257	-0.796792895	0.552687952	-1.441668651	0.149395871	0.332474776
1147.803044	0.085752065	0.073332514	1.169359401	0.24225886	0.449906202
94.8110906	-0.06712176	0.100691462	-0.666608256	0.505022394	0.700301859
155.2326392	-0.051288135	0.083851562	-0.611653904	0.540766766	0.728242598
235.0175334	0.082453158	0.084673121	0.97378196	0.330164812	0.545419232
74.24846711	0.30527369	0.122314023	2.495819225	0.012566663	0.062001557
11.76955169	0.027568871	0.279742287	0.098550961	0.921494808	0.961351749
154.4394593	0.058596945	0.100863743	0.580951521	0.561273127	0.74475811
277.6579767	-0.060448907	0.069134351	-0.874368623	0.381917539	0.594735754
2204.015009	-0.115641351	0.072643131	-1.591910347	0.11140485	0.276065509
49.66693896	-0.643301668	0.35958528	-1.789010019	0.073613202	0.210898484
6.44424093	-0.32901104	0.39196097	-0.839397452	0.401246313	0.612843664
4.70679771	0.642854216	0.475828948	1.35101956	0.176689167	0.371841765
13.7783083	-0.323690176	0.438925774	-0.737459944	0.460842694	0.663513594
12.34243282	0.341648134	0.281102976	1.215384269	0.224219565	0.429237897
3.585712443	0.572918337	0.489951231	1.169337476	0.24226769	0.449906202
1332.60835	0.261613198	0.105713007	2.474749384	0.01333297	0.06473544
45.284029	-0.023217797	0.167827386	-0.138343318	0.889969092	0.945129092
28.34398906	0.207381253	0.250916313	0.826495698	0.408522957	0.61919286
3.658210401	-1.316695191	0.556876416	-2.364429794	0.01805785	0.079861749
288.8478898	-0.058534118	0.085782009	-0.682358909	0.495012032	0.691849343
5.469292688	0.128964924	0.576125133	0.223848807	0.822874951	0.911237589
2.933024595	0.537690318	0.623237383	0.862737589	0.38828176	0.601532943
402.153658	0.361877444	0.118349313	3.057706324	0.00223038	0.017546937
1163.250913	0.434169653	0.582125228	0.745835486	0.455766801	0.659864116
2.425812437	-0.021834935	0.748098177	-0.029187259	0.976715243	0.988158577
6.002907704	0.091052737	0.6514097	0.139777987	0.888835406	0.944444948
24.9148875	0.26490079	0.221404158	1.196458062	0.231517855	0.438141889
1227.707125	0.09185586	0.050397003	1.822645281	0.068357133	0.200733584
257.1364073	-0.170150144	0.095447713	-1.782652905	0.074642822	0.212818596
16.79084386	-0.043071365	0.285480221	-0.150873377	0.880075602	0.939892807
3.855276882	0.015369487	0.494254505	0.031096301	0.975192739	0.987719156
104.1563917	-0.042930269	0.159939795	-0.26841518	0.788379756	0.890671245
5.656034815	-0.320372819	0.526061249	-0.609002886	0.542522526	0.729950677
3.218123178	0.726332116	0.625705719	1.160820645	0.245714846	0.454208524
74.58316778	-0.068866815	0.124297286	-0.55404922	0.579545161	0.757251506
1567.87484	0.197742392	0.044931598	4.40096505	1.0777E-05	0.000253993
326.6376257	-0.07735708	0.08007266	-0.96608605	0.334001124	0.549189991

3.555524631	-0.248273299	0.55690057	-0.445812613	0.65573261	0.810332234
29.39434911	-0.291143352	0.185537718	-1.569186879	0.116604401	0.28395283
212.9539983	-0.005916745	0.111560629	-0.053036143	0.95770311	0.979159749
213.5555616	0.312193534	0.078752423	3.964240387	7.363E-05	0.00123287
24.8389319	0.46911223	0.239231781	1.960910999	0.049889406	0.162010265
153.434484	0.00181113	0.138055211	0.013118885	0.989532944	0.994530438
365.0353048	-0.82125218	0.228411512	-3.595493814	0.000323777	0.003959836
29.92341139	-0.584823514	0.561679793	-1.041204474	0.29778066	0.512549124
121.0468795	0.200161466	0.105921716	1.88971132	0.058796579	0.180948038
292.5006821	0.199841322	0.074078529	2.69769561	0.006982125	0.040218017
62.25601726	-0.145798387	0.16400955	-0.888962788	0.374023079	0.587649923
337.8467446	0.230870543	0.069256607	3.333552628	0.000857444	0.008545397
82.34816885	0.486979543	0.166502258	2.924762402	0.003447193	0.024093788
32.67339616	0.384619476	0.1661532	2.31484844	0.020621228	0.087872247
8.633244945	1.501190323	0.351515155	4.270627597	1.94924E-05	0.000415417
608.386433	-0.002058592	0.049951396	-0.041211893	0.967126973	0.983639473
1787.530918	0.13877017	0.070399951	1.971168573	0.048704597	0.15926075
11.20648512	-0.584202332	0.360138912	-1.62215832	0.104769454	0.264977062
370.5160943	0.357345339	0.086861252	4.113978664	3.88897E-05	0.000732194
85.64856174	-0.072011419	0.11715885	-0.614647712	0.538787398	0.726565753
80.45765865	-0.125977464	0.138526762	-0.909408858	0.363134348	0.57699414
6.605996788	-0.557001675	0.402123933	-1.385149277	0.166006827	0.356697074
17.29521473	-0.046885751	0.258947489	-0.181062774	0.856318304	0.92936531
62.50670639	0.159697627	0.15231602	1.048462447	0.294425595	0.509190119
2.736258964	-0.137690914	0.766585336	-0.179615899	0.857454122	0.92938346
52.84966268	0.346566235	0.207482279	1.670341377	0.094851841	0.247440892
487.2035538	0.464500069	0.299219806	1.552370729	0.120573543	0.290096969
1.786388371	1.570297972	0.993380879	1.580761221	0.113932644	0.279929363
45.59670673	-0.392843163	0.171343943	-2.29271695	0.021864306	0.091512342
22.57341429	-0.471941707	0.256641043	-1.838917503	0.065927321	0.195773161
42.95131593	-0.315867227	0.158926728	-1.98750224	0.046866768	0.155324889
31.19575264	0.015672566	0.214004773	0.073234658	0.941619388	0.972249313
5.131421994	-1.042996785	0.564224396	-1.848549606	0.064522875	0.193045791
565.802752	-0.419595199	0.083811758	-5.006400156	5.54574E-07	2.08586E-05
1.742166919	0.441295039	0.809929926	0.544855826	0.58585268	0.760836448
36.18519226	-0.198238677	0.232194504	-0.85376128	0.393237272	0.606491244
35.7465853	-0.097643318	0.158479998	-0.616123925	0.537812733	0.725807075
200.8868916	0.019967958	0.114605641	0.174231899	0.861683217	0.931286863
13.38803485	0.687156429	0.281648075	2.439769662	0.014696629	0.069194705
139.7539418	0.208255007	0.100041048	2.081695576	0.037370285	0.132575252
217.6192262	-0.201374715	0.086474002	-2.328731308	0.019873304	0.085592894
54.36206166	0.037161016	0.166246078	0.223530183	0.823122895	0.911237589
53.41953946	0.194231809	0.139459529	1.392746777	0.163696378	0.353206828
2849.847877	-0.120725481	0.067774325	-1.781286372	0.07486568	0.213239062
6.669943381	-0.466727889	0.421881505	-1.106300901	0.268596302	0.480491998
100.8855391	-0.142986912	0.103681282	-1.379100547	0.167863755	0.359187882
54.67122988	-0.062251323	0.159371008	-0.390606318	0.696088255	0.836908188
6.27871688	-0.652737103	0.740977146	-0.880913948	0.378364397	0.591382835

72.99727239	0.294859062	0.125841153	2.343105213	0.019123989	0.08323609
865.2159764	-0.072992221	0.053517606	-1.363891754	0.172601636	0.365668642
505.7147805	-0.194030702	0.06705804	-2.893474106	0.003810057	0.025755798
855.970552	0.095687257	0.066715759	1.434252702	0.151500162	0.335677626
19.44648854	0.18660607	0.233690154	0.798519179	0.424569268	0.632115148
206.3453159	-0.062337821	0.124461467	-0.500860405	0.61646937	0.783257474
59.18341514	-0.549623864	0.39400948	-1.394950861	0.163030658	0.352324159
3.395454757	-1.218582147	0.660073443	-1.846131156	0.064873164	0.193678961
1.97805035	-4.457596921	2.274102322	-1.960156708	0.049977477	0.162136462
10.64877421	-0.295675394	0.334340752	-0.884353438	0.376505449	0.589652132
6221.320129	0.091227705	0.088734907	1.028092642	0.303906251	0.518644466
704.6579149	0.020293271	0.051232857	0.396098762	0.692032172	0.834043309
2.285143944	-0.875890705	0.74004622	-1.183562163	0.236586426	0.443985278
1642.446821	0.196710007	0.220562395	0.891856507	0.37246985	0.585782177
330.0345264	0.303878293	0.082963077	3.662813675	0.00024946	0.003256422
1000.623093	0.018493643	0.073829332	0.250491816	0.802207033	0.898834161
116.9930526	0.09636713	0.107271714	0.898346136	0.369001043	0.58266301
11.57802639	0.67001629	0.43142184	1.553042122	0.120413072	0.289927645
18.67163443	0.237864968	0.265834381	0.894786322	0.370901323	0.584428073
130.4537216	0.065069748	0.122486764	0.531239013	0.595253161	0.767361863
1.754615559	-1.57888443	0.773934709	-2.040074456	0.04134291	0.142158862
34.69870374	-0.599851448	0.294683041	-2.035581845	0.041792362	0.143320323
200.3428266	-0.178883948	0.088408891	-2.023370577	0.043034955	0.146278453
232.6774929	0.573527485	0.094160448	6.090959628	1.12236E-09	9.19887E-08
42.30737805	0.41321217	0.215533349	1.917161188	0.055217457	0.17254408
249.4858964	-0.102767321	0.102387054	-1.003714011	0.315516482	0.530279479
820.3423404	0.150321199	0.040650217	3.697918706	0.000217374	0.002923287
292.4305551	0.261552272	0.110506421	2.366851352	0.017940137	0.079432005
5.696627434	1.24601933	0.469231449	2.655447184	0.007920334	0.04427539
1220.09887	-0.142648982	0.060064247	-2.374939982	0.017551804	0.078381246
261.5906914	-0.077426083	0.077180554	-1.003181227	0.315773429	0.530496785
1646.569363	-0.599466241	0.36753722	-1.631035463	0.102882837	0.262146857
813.309797	-0.054679782	0.090007977	-0.607499285	0.543519617	0.730635836
315.4952551	0.391841123	0.100526897	3.897873447	9.70411E-05	0.001528351
177.4756253	0.786899045	0.163521166	4.812215242	1.49266E-06	4.86934E-05
53.4546135	0.548642783	0.170426596	3.219232185	0.001285344	0.011679399
2.560340077	-0.249261713	0.671662764	-0.371111407	0.710554555	0.84564098
43.60236655	0.494299828	0.197625698	2.501192071	0.012377603	0.061344353
4027.476127	0.1929242	0.096075425	2.008049399	0.044638043	0.150074921
24.01662501	0.961559145	0.232946678	4.127807935	3.66238E-05	0.00069912
32.90468975	-0.040371492	0.221299628	-0.1824291	0.85524599	0.929057489
710.8429119	-0.08737256	0.05665514	-1.54218241	0.123029273	0.293122879
34.24234899	0.300974405	0.326079169	0.923010221	0.356001879	0.570558902
24.69782503	0.156890228	0.237171596	0.661505134	0.508288424	0.702907521
17.31461971	-1.160116689	0.673714053	-1.721971932	0.085074603	0.231382743
81.3676444	0.385998682	0.116454791	3.31457966	0.00091781	0.009010731
147.3647834	1.039769134	0.1600341	6.49717238	8.18437E-11	8.75515E-09
128.0489683	0.399968822	0.10465942	3.821622783	0.000132576	0.001948829

438.4084697	0.314065948	0.082736047	3.795998972	0.00014705	0.002121281
1607.206308	0.033033534	0.064097577	0.515363227	0.606299224	0.776142157
405.0342469	0.096305464	0.074297534	1.296213466	0.194901951	0.395859296
4527.90706	0.388400128	0.09781205	3.970882192	7.1607E-05	0.001204958
91.5249013	-0.048101052	0.164284241	-0.292791637	0.769681421	0.880036216
77.95678308	0.317545256	0.157701547	2.013583653	0.044053264	0.148746357
47.74797929	0.030762119	0.178259876	0.172568948	0.862990261	0.931828116
404.2779169	-0.155021267	0.084111357	-1.843047978	0.065322006	0.194560608
2.129451552	0.05892589	0.890100801	0.066201367	0.947217508	0.974828397
175.9722168	0.062202594	0.106513519	0.583987781	0.559228533	0.743321837
60.29675291	0.624675252	0.159717824	3.911117972	9.18699E-05	0.001465769
563.6652994	-0.107657835	0.080822168	-1.332033484	0.182849186	0.380047626
271.8543304	0.166610707	0.149947857	1.11112429	0.266514854	0.478259224
3.213601544	-0.581345924	0.831914181	-0.698805163	0.484673802	0.683722916
6916.860044	0.042815532	0.097761	0.437961272	0.661414358	0.814056043
12.93694295	0.626418437	0.291517674	2.148818042	0.031648827	0.118013302
85.42557664	0.130962035	0.121341535	1.07928447	0.280460934	0.49404549
112.8758337	0.068512243	0.099873928	0.685987268	0.492721137	0.690017157
72.29197221	0.279584051	0.162602984	1.719427551	0.085536549	0.232222991
191.3553901	-0.005982228	0.108127326	-0.055325771	0.955878931	0.978293782
4.52543174	-1.449410805	0.941674292	-1.539184852	0.123759175	0.294073077
110144.8963	-0.156000371	0.118269024	-1.31902984	0.187159137	0.385875174
488.5477988	0.345902477	0.113050623	3.059713145	0.002215491	0.01744646
266.5678367	0.169777539	0.08604506	1.973123602	0.048481482	0.158784082
58.92095728	-0.132257068	0.135414997	-0.976679627	0.328727784	0.544267489
37.00297743	-0.097475385	0.204320316	-0.477071428	0.633311259	0.794451364
914.2132997	-0.042927237	0.065854103	-0.651853645	0.514495591	0.707791263
324.7927187	-0.118196916	0.10105105	-1.169675291	0.242131665	0.449805712
16.90290823	0.170552033	0.254307454	0.670652905	0.502441671	0.698146431
193.1190174	0.208215653	0.098995107	2.103292372	0.03544022	0.127615778
154.8228806	0.134349336	0.129254504	1.039417053	0.298610816	0.513605616
963.4708829	0.046689535	0.05144247	0.907606786	0.364086008	0.577837465
65.18304708	-2.628804476	0.604704622	-4.347253819	1.37853E-05	0.000311156
2519.924432	-0.230330154	0.257431598	-0.894723708	0.370934802	0.584428073
481.404796	0.188161096	0.066115971	2.845925019	0.004428262	0.028834462
77.23025985	0.969154527	0.227550968	4.259065725	2.05283E-05	0.000436366
593.650164	0.671186791	0.101892165	6.587226738	4.48118E-11	5.1988E-09
1.850013677	-2.659739313	1.5253846	-1.743651609	0.081219858	0.224657382
28.67342959	0.409266092	0.339332697	1.206090942	0.227782427	0.433500443
113.7140804	0.054587743	0.128325714	0.425384289	0.670556557	0.820858905
314.5323047	-0.198666885	0.077836021	-2.552377197	0.010699062	0.054994179
129.9954609	0.020059569	0.111563471	0.179804099	0.857306365	0.92936531
82.86943446	-0.096837701	0.115357961	-0.839453994	0.401214596	0.612843664
88.33714371	0.124514572	0.110971819	1.122037768	0.261846382	0.473196281
43.41879526	0.058718644	0.175158109	0.335232231	0.73744991	0.861788314
41.20042943	0.073814265	0.159211365	0.463624343	0.642916911	0.801477463
87.48620466	-0.151877343	0.112064587	-1.355266161	0.175332772	0.369648335
38.80900258	0.577735433	0.189945654	3.041582795	0.002353378	0.018244496

34.8979801	0.099399555	0.173621951	0.57250569	0.566979432	0.748471553
26.23992322	-0.402530803	0.56985272	-0.706376908	0.479953777	0.679099762
141.2047663	0.125262453	0.102704149	1.219643559	0.222600026	0.427651642
24.75708506	-0.102465862	0.294618078	-0.347792175	0.727996267	0.856032156
78.52934611	0.398624806	0.258524056	1.54192539	0.123091725	0.293153002
32.30717188	0.372267781	0.186512204	1.995943286	0.045940096	0.153419247
564.7265768	0.004340296	0.075766841	0.057284899	0.954318249	0.97782304
191.1882365	0.464187301	0.084537517	5.490902956	3.99884E-08	1.99627E-06
2.669667165	1.299857134	0.620522917	2.094776998	0.036190821	0.129554016
188.4208684	0.032074226	0.111427778	0.287847663	0.773463351	0.882168045
14.9204962	0.43651626	0.335101272	1.302639819	0.192697757	0.393273395
43.31548335	-2.466158133	1.297782086	-1.900286774	0.057395496	0.177731843
1067.480949	-0.165808513	0.094226555	-1.759679253	0.078462205	0.21943188
7.154601001	-0.58778555	0.780665891	-0.752928438	0.451492918	0.656419939
688.8371877	0.373519756	0.154194237	2.422397639	0.015418469	0.071667959
1766.266152	-0.166183191	0.070571165	-2.354831336	0.018531115	0.081212669
44.01384148	-0.78931198	0.595265127	-1.325983908	0.184845028	0.382651041
99.94433689	0.119149648	0.155521934	0.766127614	0.443600373	0.648838902
221.138353	0.296618186	0.102784622	2.885822599	0.003903921	0.026207498
16.77181141	0.999480328	0.553999581	1.804117482	0.071212892	0.206179469
163.0410226	0.265543409	0.093729578	2.833080187	0.004610182	0.0296771
165.687298	0.112375227	0.085916111	1.307964544	0.190885336	0.390833335
648.1534282	0.023841218	0.065651202	0.363149756	0.716493018	0.849615028
36.10076899	-0.058664612	0.169907529	-0.345273765	0.729888568	0.857155993
134.3852562	0.746829606	0.150074278	4.976399787	6.47778E-07	2.39809E-05
1172.871145	0.346480556	0.072798437	4.759450466	1.94121E-06	6.10295E-05
13.66903765	0.104254758	0.369499852	0.282151015	0.777827718	0.884329456
1313.171083	0.136220021	0.072295898	1.884201241	0.05953776	0.18247908
72.18972739	-0.179100306	0.13376461	-1.338921456	0.180596244	0.376721638
1421.819702	-0.370933758	0.370149466	-1.002118851	0.316286194	0.531195714
6.64776408	0.557306185	0.406156598	1.372146083	0.170017966	0.36205739
85.55719961	0.202091057	0.161654528	1.250141643	0.21124781	0.415484291
25.11433076	0.522610533	0.202105339	2.58583239	0.009714416	0.051579197
397.4716204	-0.011133658	0.103824863	-0.107234992	0.914602556	0.957600501
46.07732056	0.178584447	0.181153164	0.98582019	0.324221356	0.539548668
1800.654282	0.001450588	0.1026656	0.014129248	0.988726866	0.994158105
193.4449261	0.098303179	0.103160959	0.952910671	0.340635333	0.555270316
8.841785851	0.50805404	0.327990762	1.548988873	0.121384395	0.291158774
44.24131206	-0.126259584	0.150235709	-0.840409945	0.400678575	0.61246777
158.587717	0.361373694	0.219921372	1.643194979	0.100342598	0.257083043
559.5829739	0.056233915	0.054113704	1.03918066	0.298720724	0.513671882
1490.359389	0.098391169	0.039488543	2.491638387	0.012715542	0.062492793
1056.646523	-0.451289964	0.081885621	-5.511223585	3.56348E-08	1.81187E-06
5.883037596	-0.061566733	0.574408407	-0.107182855	0.914643917	0.957600501
2.385329687	-3.527827485	1.196564045	-2.948298086	0.003195288	0.022767809
3.506743761	-2.823957377	1.456642389	-1.938675819	0.052540825	0.167160593
6.739519027	-1.603871876	1.200004459	-1.336554929	0.181367959	0.377779208
48.92777931	-1.252178878	0.407039537	-3.076307737	0.002095814	0.016745151

16.85694785	0.510203749	0.255816578	1.994412371	0.046107008	0.153789602
1303.602608	-0.071127729	0.065212381	-1.09070897	0.275400963	0.488107096
82.06484195	0.324276241	0.155800181	2.081359846	0.037400981	0.132589577
1.774061146	1.486786965	0.787054507	1.889052095	0.058884849	0.181152007
2.183821539	-1.893061559	1.307496694	-1.447851889	0.147658477	0.329789283
177.3310227	-0.060228073	0.084514146	-0.712638952	0.476069202	0.676100347
136.7105601	-0.167124249	0.109586197	-1.525048347	0.127247039	0.298656179
113.0230383	0.202962603	0.124683233	1.627825954	0.103561796	0.263161657
71.38951251	0.004325225	0.180080515	0.024018286	0.980838022	0.990108149
84.14095633	-0.033541078	0.142686027	-0.235069111	0.814155093	0.906303872
2808.371556	0.164995594	0.088165327	1.871434032	0.061284945	0.186343336
46.40685599	0.054218475	0.271784643	0.199490577	0.841879014	0.921597108
4.087953679	0.271053848	0.85595211	0.316669408	0.751494464	0.870346652
90.90161623	-0.682064637	0.272091018	-2.506751753	0.012184625	0.060643359
48.58011758	-0.376975232	0.175879385	-2.143373607	0.032083111	0.119018576
87.50101863	-0.139566915	0.16227992	-0.860038106	0.389768037	0.603310969
300.1569594	0.290697831	0.274794256	1.057874481	0.290112671	0.505106335
37.97355683	0.367072906	0.146403113	2.50727528	0.012166592	0.060571904
234.5236551	0.046880344	0.099011159	0.473485461	0.635866871	0.796174724
842.7719512	0.31782736	0.070724018	4.493909849	6.99272E-06	0.000176414
2057.326436	-0.131786706	0.062951273	-2.093471673	0.03630707	0.129829102
2.031150668	0.739085119	0.705767881	1.047207076	0.295004082	0.509797257
790.0583061	0.07344251	0.094234017	0.779363037	0.435765891	0.642283912
8.047998317	-0.157734159	0.35808591	-0.440492503	0.659580441	0.812578183
113.5985858	-0.251375976	0.103322041	-2.432936606	0.014976923	0.070193407
165.1766898	-0.030962999	0.09230973	-0.335425085	0.737304448	0.861788314
76.78708113	-0.304313717	0.127047785	-2.395269752	0.016608142	0.075443137
75.72384033	-0.034678675	0.108574557	-0.319399649	0.749423477	0.868891414
72.31326707	-0.128964845	0.159386048	-0.809135095	0.418437443	0.627637116
151.3152543	-0.105504293	0.089351522	-1.180777783	0.237691012	0.445170729
13.21211447	0.436958755	0.286705232	1.524069691	0.127491303	0.298931359
739.6324846	-0.060727116	0.058776937	-1.033179323	0.301519987	0.516667388
7.323463265	-4.087691831	1.372249579	-2.978825349	0.002893557	0.021242625
151.354331	0.038778428	0.100734115	0.384958249	0.700268349	0.839119929
995.6921249	-0.16653856	0.129255645	-1.288443228	0.197591719	0.399010295
53.13346839	0.601696423	0.138242392	4.352474059	1.3461E-05	0.000305871
109.7115508	0.152939538	0.099051321	1.544043392	0.122577821	0.292501519
461.0743882	-0.0640618	0.075612758	-0.847235325	0.396863967	0.609578167
1380.663052	0.047446103	0.049538374	0.957764643	0.338181452	0.55346724
1334.619803	-0.018970519	0.09865782	-0.192286017	0.847518171	0.924458083
2.862320782	-0.546887421	0.702499913	-0.778487528	0.436281656	0.642641632
202.0069476	0.733675605	0.163840819	4.477978133	7.53533E-06	0.000188945
182.5283011	0.061805863	0.128358854	0.481508375	0.630155229	0.792819401
148.0084282	-0.071202175	0.08617895	-0.82621307	0.408683235	0.619247143
320.3719503	0.124487078	0.056864409	2.189191471	0.028582926	0.110258809
322.295755	0.167936615	0.073636036	2.280630843	0.022570301	0.09349337
29.82139045	-0.252023897	0.340590702	-0.739961178	0.459323551	0.662252422
5.299099792	0.461165683	0.578507706	0.797164288	0.425355624	0.632771225

66.37725028	-0.368290597	0.213883044	-1.721925167	0.085083075	0.231382743
9.207243526	-0.272808402	0.433796627	-0.628885484	0.52942403	0.71927657
38.04059599	0.16046318	0.180844573	0.887298842	0.374918029	0.588340599
115.4333291	-0.232321646	0.120271195	-1.931648261	0.053402935	0.169084461
172.1400555	-0.048715771	0.079556783	-0.61233963	0.540313075	0.727851321
2.154924754	0.826989423	0.751751264	1.100083848	0.271295591	0.483274606
244.0226495	0.108410485	0.094858853	1.14286101	0.253096303	0.462200255
734.340388	0.079543599	0.059397333	1.339177948	0.180512749	0.376664602
11.94682995	0.580837436	0.298780344	1.944028273	0.051892042	0.165929638
28.02109514	0.440101009	0.514440384	0.855494674	0.392277352	0.605488344
1206.13838	0.031078416	0.051531277	0.603098126	0.546443413	0.733259109
1563.323191	-0.296028246	0.098508736	-3.005096375	0.002654967	0.019962541
6.788699947	0.600527304	0.381691204	1.573332833	0.115641771	0.282485547
2.227164669	-0.715250074	1.154730919	-0.619408437	0.535647324	0.72413048
9.757478018	0.395570559	0.395143445	1.00108091	0.316787693	0.531821101
976.2292169	0.098001897	0.167654224	0.584547735	0.558851859	0.743120957
612.3320649	-0.19000279	0.059363979	-3.200641083	0.001371222	0.012258526
48.10079899	0.843557825	0.410617373	2.05436467	0.03994041	0.138667716
159076.5275	0.089589178	0.095338782	0.939692921	0.347375097	0.562036668
7.557046709	1.373269767	0.369563539	3.715923308	0.000202463	0.002749691
37.93433741	-0.462709938	0.311735768	-1.484301728	0.137728892	0.315525764
78.5478746	0.257282324	0.145303824	1.770650742	0.076618798	0.216170249
40.91839888	-0.117552284	0.270630669	-0.434364238	0.664023963	0.816413968
16.15139459	0.229954529	0.338407111	0.67952038	0.496808199	0.693461961
991.8518163	-0.434360188	0.145551324	-2.984240722	0.00284283	0.020973034
16.74732498	0.358443893	0.275942265	1.298981488	0.193950282	0.394851329
56.5417157	0.190631768	0.19960881	0.955026823	0.339564133	0.554448323
269.74298	-0.2582685	0.083169053	-3.105343769	0.00190058	0.015607133
11.89466041	0.115891945	0.300161969	0.386098029	0.699424069	0.838718308
126.7027818	-0.017368123	0.25528299	-0.068034782	0.945757946	0.974141328
38.7268731	0.66280634	0.192019425	3.451767124	0.000556928	0.006095692
18.22521644	0.709605728	0.389690617	1.820946404	0.068615004	0.201034162
250.385839	0.112825352	0.134241654	0.840464561	0.400647964	0.61246777
2.84551693	0.695262796	0.623142059	1.115737232	0.264534628	0.475861921
259.0179752	-0.215253771	0.07238318	-2.973809268	0.002941278	0.021478112
88.4245682	-0.498087165	0.121680881	-4.093388881	4.25114E-05	0.000784247
232.6366807	-0.194012803	0.075176594	-2.58076075	0.009858288	0.052004548
42.23797521	-0.203149096	0.177003351	-1.147713273	0.25108695	0.459958458
45.41552044	-0.07204973	0.155330404	-0.46384821	0.6427565	0.801477463
97.1013285	0.003484787	0.112260257	0.031042034	0.975236017	0.987719156
131.2647704	0.053076301	0.099051661	0.535844634	0.592065932	0.764669523
88.78226369	-0.040682842	0.122282145	-0.332696506	0.739363387	0.862808843
240.8446137	0.31069347	0.084160912	3.691659959	0.000222795	0.002984007
64.48646604	-0.349618838	0.148049044	-2.361506897	0.018200833	0.08021735
29.99569382	-0.463039369	0.218708916	-2.117149029	0.034247202	0.124448658
41.11513612	0.100564752	0.15710796	0.640099665	0.522107807	0.713528896
477.8622092	-0.221096933	0.106731672	-2.071521319	0.038310104	0.134912496
22.77860704	0.164746927	0.2213082	0.744423057	0.456620574	0.660378136

309.8876712	-0.044808808	0.061514494	-0.72842684	0.466352338	0.668466755
115.6974122	-0.063132898	0.141855239	-0.445051581	0.656282478	0.81076847
1564.969508	-0.067416965	0.10474207	-0.643647433	0.519804084	0.712158565
1591.708953	-0.007956022	0.038102731	-0.208804518	0.834600844	0.917225771
223.3062958	-0.169649727	0.077259665	-2.195838254	0.028103523	0.10903849
113.6476216	0.077909367	0.100252983	0.777127666	0.437083453	0.643190067
2.772892271	-0.88397426	0.645369217	-1.369718662	0.170774741	0.362965176
51.37809721	0.052671413	0.267842674	0.196650563	0.844101	0.922619418
86.08691957	0.250239287	0.119088301	2.101291942	0.035615347	0.12810638
117.5068234	0.07682795	0.09912556	0.775056908	0.438306033	0.644127981
11.00270126	-0.400804954	0.423682015	-0.946004173	0.344146458	0.558458308
155.8540018	0.19161672	0.082196295	2.331208717	0.019742357	0.085219569
78.29475469	-0.099483913	0.18051852	-0.551100865	0.581564532	0.758146246
48.7839405	-0.72862408	0.349489789	-2.084822226	0.03708544	0.131896707
3.592438135	2.784686093	1.073126287	2.5949286	0.009461057	0.05063725
9.877859893	0.843369053	0.60355368	1.39733893	0.162311674	0.351508153
17.45398429	0.505536168	0.4035252	1.252799499	0.210278684	0.414518492
1.70689209	0.844032396	1.879407076	0.44909504	0.653363098	0.808447666
19.69189845	0.149600108	0.27362478	0.546734504	0.584561145	0.759881097
39.15249915	-0.382031898	0.20072354	-1.903274017	0.057004799	0.176819163
5.185138819	0.466188589	0.460182536	1.013051458	0.311035583	0.526024042
5799.056613	-0.259016704	0.187940165	-1.37818706	0.168145541	0.359542826
68.80573264	-0.255258149	0.132104535	-1.932243659	0.053329439	0.16891928
343.6638738	-0.208228529	0.081643337	-2.550465662	0.010757912	0.055225776
257.2880484	-0.122141836	0.068795256	-1.775439818	0.075825294	0.214703661
44.67249803	0.370214614	0.177563979	2.084964616	0.037072513	0.131886171
751.9438491	0.34318911	0.04907784	6.992750902	2.69548E-12	4.67424E-10
18.69846422	0.121163127	0.257746397	0.470086599	0.638293148	0.798011778
16.1696989	0.189881741	0.281532309	0.674458081	0.500020128	0.695955693
2922.367639	-0.221414031	0.102870574	-2.152355345	0.031369378	0.117210055
446.2432104	-0.051982838	0.053236032	-0.976459678	0.32883672	0.544393139
50.32572581	-0.232582481	0.189010236	-1.230528491	0.218499265	0.423029367
1.940536477	0.688022886	0.697992225	0.98571712	0.324271946	0.539548668
794.7646827	0.023827521	0.065670104	0.36283665	0.716726912	0.84963465
71.47239916	0.283583531	0.139990577	2.025732994	0.042792154	0.145571604
30.37071171	1.022073647	0.287296277	3.557559664	0.000374316	0.004458773
3.952225472	-1.128100584	0.771918241	-1.461424959	0.143898855	0.324072418
4.620169025	-0.539367178	0.553912068	-0.973741518	0.330184897	0.545419232
41.90285958	-0.627822194	0.556908185	-1.12733519	0.25960079	0.470428271
8.528216728	-0.967876382	0.660995532	-1.464270688	0.143120003	0.323069187
14.44962483	0.548442546	0.381128097	1.438997939	0.150151107	0.333424781
3.34614428	0.072076176	0.981256404	0.073452949	0.941445684	0.972130889
1.704818891	-0.884521359	1.062219436	-0.832710577	0.405007978	0.615677902
572.7265975	0.62004698	0.059180786	10.47716705	1.09989E-25	1.81196E-22
92.12553452	0.406922757	0.14668348	2.774155328	0.005534525	0.034033504
63.25602971	0.050038385	0.319353052	0.156686728	0.875491748	0.937583765
13.45450154	0.404724318	0.321385148	1.259312447	0.207917499	0.411538253
1676.067293	0.218398813	0.079920542	2.732699359	0.006281764	0.037321856

61.3041206	-0.078605065	0.157994124	-0.497518916	0.618823167	0.784795447
3.435497641	-2.111736756	0.958069837	-2.204157436	0.027513274	0.107482494
196.7883288	0.03686857	0.172793047	0.213368368	0.831039651	0.915614595
5380.308628	0.069603651	0.090948243	0.765310557	0.444086639	0.649319543
448.2177174	0.17052085	0.101820077	1.674727166	0.093987775	0.246225033
72.05038777	0.581272649	0.181506047	3.202497425	0.001362415	0.012198059
616.5220459	0.144661855	0.06255306	2.312626347	0.020743192	0.08828296
157.8351445	-0.11387874	0.120446596	-0.945470805	0.344418569	0.558734762
11528.7836	0.314991698	0.088431923	3.561968211	0.000368085	0.004400458
1046.639829	0.16910978	0.066521442	2.542184504	0.0110162	0.056255699
902.4217519	0.055190574	0.142383794	0.387618368	0.698298474	0.838283835
964.6380871	0.032748487	0.115117735	0.28447821	0.776043931	0.883652841
13.21685993	0.901240982	0.465356967	1.936665926	0.052786194	0.167779231
381.9631119	-0.208585258	0.075499752	-2.762727737	0.005732056	0.034922296
721.6587148	0.055735254	0.082331541	0.676961144	0.498430608	0.694742406
1185.774661	-0.444428341	0.423999924	-1.048180238	0.294555574	0.509342765
200.8684661	-0.144791472	0.405215362	-0.357319799	0.72085241	0.851889535
290.9317765	0.210853805	0.109632004	1.923286984	0.054444018	0.170814257
171.4575593	0.030198071	0.097949458	0.308302583	0.757852101	0.873603573
225.1896459	0.671220025	0.223020856	3.009673797	0.002615284	0.019709144
14.69811611	0.319149757	0.350297927	0.911080919	0.362252739	0.576260296
84.58771171	-0.045799703	0.119895355	-0.381997311	0.702463359	0.840493334
581.6818759	0.035095821	0.074792548	0.469242216	0.638896513	0.798267972
9.911953064	0.938058061	0.377930172	2.482093602	0.013061296	0.063716847
361.6205617	0.076167717	0.059217068	1.286245989	0.198357226	0.400024141
348.2549253	0.188441366	0.079263321	2.377409417	0.017434725	0.078070034
137.3794074	0.12718139	0.116153218	1.094945038	0.273540716	0.48625124
499.8900858	0.033261707	0.058125373	0.57224075	0.567158884	0.748471553
3.197370515	0.866645104	0.631933792	1.371417569	0.170244824	0.362304006
82.2147095	0.40890022	0.136328295	2.99937897	0.002705306	0.020232227
28.76205936	0.677633702	0.22034623	3.075313343	0.002102815	0.016780498
686.0098367	-0.051922931	0.103127371	-0.503483506	0.614624374	0.78222563
12.52326702	-1.073110952	0.600130735	-1.788128635	0.073755257	0.211165119
415.5158105	-0.18451579	0.103165605	-1.788539805	0.07368896	0.211032461
198.5709694	-0.167060347	0.131105565	-1.274242989	0.202577314	0.405322833
79.48898342	-0.132249235	0.160874584	-0.822064192	0.411040364	0.621065666
35.51509178	0.07646473	0.18164873	0.42094833	0.673792813	0.823222819
43.0233502	0.065555074	0.176234624	0.37197613	0.709910621	0.845284623
3.021427855	-0.873322921	0.549587015	-1.589053047	0.112048417	0.27702774
14.31686672	0.038816637	0.332874604	0.116610389	0.907168805	0.953303217
82.61023824	-0.285772024	0.14174906	-2.016041758	0.043795609	0.148149665
2.250627002	0.470342809	0.817137483	0.575598132	0.564886844	0.747206097
49.18421043	-0.465159446	0.143674131	-3.237600551	0.001205395	0.011143473
199.3149843	-0.275930299	0.150077603	-1.838584133	0.065976377	0.195836907
115.4080495	-0.171519073	0.125605688	-1.365535868	0.172084688	0.364854973
6.940624313	-0.343568839	0.405669294	-0.846918522	0.397040538	0.609588078
24.49781037	-0.428499989	0.283902099	-1.509323074	0.131216241	0.305182102
257.2992876	0.116514996	0.089281313	1.305032279	0.191881857	0.39248345

10.86065843	1.151951177	1.007879097	1.142945796	0.253061097	0.462187196
3.604495868	-1.25964662	1.312791415	-0.959517715	0.337298	0.552625285
1713.137536	-0.090897547	0.092760299	-0.979918652	0.327126275	0.542215339
71.69251917	0.767538422	0.187197726	4.100148222	4.12886E-05	0.000765116
6.280590693	-0.380895606	0.4201846	-0.906495873	0.364673448	0.578202266
43.15259022	-0.258193253	0.268042861	-0.963253611	0.335420267	0.55053437
29.25760321	0.773743566	0.215139085	3.596480687	0.000322552	0.003947781
95.60986741	0.174730089	0.117683513	1.484745692	0.137611203	0.315387724
6.099708762	-0.28658411	0.506510894	-0.565800487	0.571529416	0.751186819
45.44504804	0.063828111	0.133634126	0.477633313	0.632911216	0.794224511
2.59752633	-0.438231602	0.648227778	-0.676045699	0.499011631	0.695021779
2.402861104	1.646066872	0.763122834	2.157014309	0.03100455	0.11645423
6.903477874	0.833385282	0.456506043	1.825573387	0.067914548	0.199825729
3.964781363	-1.273109574	0.604514104	-2.106004751	0.03520394	0.126903655
103.6271514	0.081881459	0.127806955	0.640665122	0.52174028	0.713349603
45.6172645	-1.076833319	0.306580528	-3.51239958	0.00044408	0.005091002
35.44708724	0.512747398	0.444295501	1.1540684	0.248472128	0.45711395
2.575128859	1.002725647	0.727607678	1.378113064	0.168168383	0.359542826
77.89628706	0.199501766	0.12231925	1.63099239	0.102891925	0.262146857
6.864332688	0.574561679	0.480663818	1.195350382	0.231950163	0.438607322
4.074536233	-1.00223118	0.726459711	-1.379610136	0.167706715	0.359039321
229.1310636	-0.375121	0.075569313	-4.963932885	6.90798E-07	2.52333E-05
41.48077141	-0.261930228	0.200044758	-1.309358114	0.190413073	0.390351538
595.750242	-0.009189753	0.071395059	-0.128716926	0.897581642	0.948210116
36.94990105	0.028926374	0.173157619	0.167052275	0.867328918	0.934308284
73886.12734	0.158277127	0.137684841	1.149561032	0.250324716	0.45932976
19.90056386	1.190962994	0.805826896	1.477938998	0.139424107	0.31789814
125.9828016	-0.022553523	0.125641284	-0.179507262	0.857539415	0.929414758
561.1816364	-0.055462006	0.054965536	-1.009032388	0.312959102	0.527760082
21.68451319	0.484614486	0.469456728	1.032287872	0.301937281	0.516898552
175.164745	0.303024414	0.110062825	2.753194947	0.005901674	0.035613253
977.5175084	-0.2093053	0.097528047	-2.146103685	0.031864707	0.118527897
12.70768269	-0.079185213	0.271586794	-0.291565036	0.770619212	0.880306225
180.3144218	0.074455578	0.090525142	0.822485072	0.41080088	0.620874651
425.36417	-0.033536992	0.132034865	-0.254001031	0.799494762	0.896484956
4.034377025	0.417676802	0.474974737	0.879366353	0.37920267	0.592190921
274.0049466	-0.107870447	0.07470932	-1.443868686	0.148775917	0.331386486
4.441008976	0.588858788	0.462534583	1.273112996	0.202977948	0.405857351
702.3133623	-0.040936675	0.068249755	-0.599806925	0.548634918	0.735232379
1240.620022	-0.046600615	0.081901634	-0.568982717	0.569367869	0.749961323
236.9101866	-0.060682646	0.111324409	-0.545097401	0.58568653	0.76081059
1245.093812	0.200596864	0.065458555	3.064486568	0.00218044	0.017211578
796.1131072	0.076383512	0.090438757	0.844588256	0.398340768	0.610727391
193.3832792	0.248672085	0.10561467	2.354522207	0.018546535	0.081237869
945.0961938	-0.135628991	0.065177128	-2.080929247	0.037440383	0.132672158
3.662970248	0.919657129	0.534401242	1.720911288	0.085266923	0.231764168
44.53749264	0.149941509	0.156199458	0.959936166	0.337087343	0.552444975
764.4957391	0.051781106	0.07896537	0.655744496	0.511988534	0.706072364

57.95943177	0.056923639	0.137734399	0.413285564	0.679397406	0.826715923
56.3717192	0.292712576	0.131569187	2.224780603	0.02609597	0.103691513
170.1042997	0.106218866	0.107867736	0.984713968	0.32476459	0.539767136
1078.769344	0.273712607	0.063843952	4.287212789	1.80929E-05	0.000391672
215.2662048	-0.073150588	0.132248085	-0.553131552	0.580173332	0.757411481
725.5569043	0.42938772	0.077313574	5.55384644	2.79451E-08	1.47314E-06
96.98479807	0.057542865	0.114447011	0.502790457	0.615111604	0.782406945
320.5644796	-0.043965491	0.089776853	-0.48971967	0.624332281	0.789169799
118.1113214	-0.376873865	0.217403449	-1.733522933	0.083002765	0.228033305
702.0003236	-0.28688317	0.112889479	-2.541274634	0.011044912	0.056349919
1404.570489	-0.03542178	0.073518238	-0.481809425	0.629941334	0.792701622
9.193293612	0.71740181	0.36663263	1.95673203	0.050378982	0.162861669
2147.77501	-0.536195934	0.093396575	-5.741066377	9.40822E-09	5.87087E-07
2.22489893	-0.19656301	0.855813429	-0.229679745	0.818340636	0.908774216
48.22827134	0.280353685	0.212008564	1.322369625	0.186045098	0.384276015
137.9906046	-0.068429651	0.149103325	-0.45894115	0.646276427	0.803832228
220.5511173	-0.356901905	0.111219405	-3.208989523	0.001332023	0.011997679
9.283068185	0.258264833	0.754755505	0.342183438	0.732212848	0.858722465
4.422024508	0.676570958	0.528712385	1.279657858	0.200665492	0.403240218
52.60197952	0.470985381	0.213680276	2.204159359	0.027513139	0.107482494
54.6985459	0.744267006	0.583624915	1.275248856	0.202221174	0.405032415
523.7981544	-0.023506573	0.096553039	-0.243457619	0.807650913	0.901989095
362.2497586	-0.027621912	0.09036046	-0.305685831	0.759843863	0.87474696
8465.075089	0.026229425	0.07986874	0.328406646	0.742604218	0.86500393
20.81432431	0.117117113	0.326095693	0.359149523	0.719483236	0.851125005
2.428874182	-0.23269953	0.570168106	-0.408124424	0.68318233	0.828701792
1226.301515	0.322861746	0.115260798	2.801140986	0.005092227	0.031933514
2755.619134	-0.146020558	0.139198061	-1.04901288	0.294172191	0.509065449
41.54955394	-0.15241806	0.26322087	-0.579050056	0.562555398	0.745278458
6.3909434	0.857939447	0.450715154	1.903506993	0.056974422	0.176760194
269.9114632	-0.051630607	0.091273139	-0.565671431	0.57161716	0.751242209
6512.417548	0.023965404	0.103970449	0.230502073	0.817701653	0.908349092
1042.4434	-0.10653568	0.36239403	-0.29397747	0.76877512	0.879316901
96.25849201	0.200725306	0.112971098	1.776784593	0.075603688	0.214370939
571.3139164	-0.64757062	0.210109404	-3.082063951	0.002055707	0.016519859
8.924695689	-0.187610356	0.46204727	-0.406041478	0.684712124	0.829651922
622.6466441	0.0821732	0.0825445	0.995501822	0.319492259	0.53456383
224.3602299	0.084646029	0.140851267	0.600960362	0.547866387	0.734560988
103.7669657	0.329909607	0.224136965	1.471910739	0.141044983	0.320228094
7.657352953	0.372839678	0.441359396	0.844753009	0.398248756	0.610699991
13.99438291	0.162955724	0.324785823	0.501732872	0.615855438	0.782777813
931.4446077	-0.057196617	0.125184082	-0.456900077	0.647742868	0.804684112
310.4240574	0.6778098	0.12077287	5.612268705	1.99691E-08	1.11139E-06
405.3183795	0.074604792	0.15329622	0.486670787	0.62649165	0.790677612
52.37326682	0.150457214	0.217169337	0.69281058	0.488428432	0.686491766
2.87760024	-0.46934544	0.586020793	-0.800902367	0.42318817	0.631142667
8.336640881	0.17294334	0.31925428	0.541710327	0.588018074	0.761885532
71.96668558	0.19711049	0.140676773	1.401158742	0.161166609	0.349671897

1174.352317	-0.168797155	0.121442227	-1.389937905	0.164547734	0.354357292
349.0587854	0.191744955	0.07727869	2.481213856	0.013093579	0.063817639
1518.894706	-0.035687256	0.074215946	-0.480856986	0.630618146	0.793098965
5.072468761	-0.212483434	0.80968019	-0.262428841	0.79299084	0.893098455
235.2185372	0.026066483	0.101791254	0.25607782	0.797890748	0.895595805
11.42508859	-0.95141244	0.709825223	-1.340347467	0.180132406	0.376109159
171.7445906	-0.075635302	0.10141415	-0.745806198	0.455784495	0.659864116
17.46170176	0.021101833	0.304437979	0.069314062	0.944739633	0.973447225
411.8279886	0.031284812	0.062868	0.497626967	0.618746993	0.784759255
798.7798677	0.143832579	0.098515069	1.460005868	0.144288461	0.324772252
100.6170121	-0.264348859	0.177882004	-1.486091077	0.137255032	0.314702769
8.213837247	-0.363714951	0.433695568	-0.838641153	0.40167071	0.613264437
3.264896814	-1.091286478	0.757811121	-1.440050757	0.149853039	0.333020231
1897.376803	0.019627152	0.107137509	0.183195898	0.854644313	0.928782269
1747.311809	0.04255253	0.139885344	0.304195772	0.760978746	0.875424502
72.9624964	0.199669095	0.266554647	0.749073771	0.453812743	0.658108374
155.7297912	-0.414132789	0.365307736	-1.133654583	0.256939468	0.467560012
6.058672114	-0.808890916	0.700915085	-1.154049803	0.248479751	0.45711395
12.13296525	0.039186369	0.43114409	0.090889263	0.927580582	0.965499621
257.6553062	-0.062596669	0.181982386	-0.343971031	0.730868072	0.857755975
2694.889328	0.281198946	0.126154188	2.229009998	0.02581324	0.102990387
37.72230186	-0.061395647	0.221372775	-0.27734055	0.781518626	0.886323685
5.854402194	-0.25278815	1.079764918	-0.234114061	0.81489643	0.906822736
62.40959949	-0.06809778	0.120804567	-0.563702034	0.572956933	0.752170338
5.91851495	-3.257731941	1.356611063	-2.401375037	0.016333587	0.074495988
244.3077816	-0.040068418	0.269965562	-0.148420479	0.882010939	0.940841436
321.109172	-0.094349442	0.105641822	-0.893106918	0.371799919	0.585231403
28034.69322	0.112097599	0.113799495	0.985044778	0.324602077	0.53966037
368.8038688	-0.124098866	0.088009922	-1.410055407	0.158523323	0.345666873
144.4064806	0.047380853	0.262435116	0.180543113	0.856726211	0.92936531
196.1874217	-0.18458714	0.094983698	-1.943355999	0.05197316	0.166060092
87.64432969	0.044583242	0.168064714	0.265274257	0.790798194	0.892054639
2.248549256	-0.027146947	0.672432983	-0.04037123	0.967797167	0.98386242
30.80379605	-0.154274354	0.267330736	-0.577091721	0.563877497	0.746369748
4.977201372	-0.069660982	0.487773696	-0.142814142	0.886436968	0.943320904
6.495553551	-0.486623946	0.410343201	-1.185894989	0.235663772	0.442884438
83.70723293	-0.384148992	0.111331175	-3.450506938	0.000559535	0.006108533
534.5567671	-1.017411545	0.466087347	-2.182877421	0.029044838	0.111466651
19.05602459	-2.351432482	0.805722998	-2.918413013	0.00351818	0.024465387
1071.599437	0.514799053	0.091488983	5.626896668	1.83481E-08	1.03516E-06
22.3860056	-0.698727184	0.695631116	-1.004450732	0.315161408	0.530009088
240.7738867	-0.167118747	0.071822865	-2.326818166	0.019974945	0.085865459
4.532154701	1.388395834	0.511640504	2.713615951	0.006655327	0.039018748
2261.750226	-0.110267785	0.114034759	-0.966966433	0.333560814	0.548794652
26.40266921	0.028676897	0.231893409	0.123664133	0.901581211	0.950812936
28.16651352	0.194790446	0.194166192	1.003215049	0.315757114	0.530496785
432.4119691	-0.217480135	0.135870522	-1.600642527	0.109456117	0.272960956
6.837526044	-0.08141305	0.389175947	-0.209193427	0.834297243	0.917014464

5.259014182	0.008070613	0.636670366	0.012676282	0.989886061	0.994530438
283.0936499	-0.046822648	0.084585966	-0.553551024	0.579886152	0.757293715
583.3623084	-0.132995938	0.079087445	-1.681631487	0.092640321	0.24410695
319.9572668	-0.116082466	0.087707534	-1.323517618	0.185663301	0.383862604
8.682394624	-0.168971208	0.431011697	-0.392033925	0.695033147	0.836069811
59.14797788	0.080106114	0.144259731	0.555290889	0.578695709	0.756621676
7745.29753	-0.03084374	0.109666535	-0.281250249	0.778518466	0.884748561
1.700712747	-0.384507281	1.22763687	-0.313209297	0.754121655	0.871653489
72.62945229	0.219364173	0.171071464	1.282295531	0.199738995	0.401981756
1655.055565	-0.095651667	0.090008692	-1.062693665	0.287920895	0.502618291
432.1048513	-0.059279917	0.138224762	-0.428866116	0.66802066	0.819105647
63.72558845	-0.081246972	0.236805785	-0.343095383	0.731526704	0.858223253
4.174145469	0.020168501	0.46791814	0.043102627	0.965619725	0.982861869
3.132726306	0.217425291	0.636197064	0.341757771	0.732533192	0.859032114
123.4394139	0.264367967	0.10509168	2.515593674	0.011883209	0.059481626
9.705750321	0.750048547	0.323688043	2.317195717	0.020493073	0.087528478
7.540082462	0.760639708	0.364563269	2.086440884	0.036938705	0.131630591
2.774392841	-0.277412801	0.671306762	-0.413242972	0.679428607	0.826715923
1.996211458	0.198890239	1.068714745	0.18610227	0.852364562	0.927038608
1283.556018	0.389727076	0.070122236	5.557824443	2.73158E-08	1.45161E-06
56.71911527	0.564159746	0.167508321	3.367950576	0.000757292	0.007768134
5.328644192	-0.638331142	0.433782821	-1.471545464	0.141143662	0.320363831
439.5783525	-0.089160314	0.115057087	-0.774922405	0.438385512	0.644127981
176.4223402	-0.177097141	0.104105097	-1.701138048	0.088917068	0.238171511
154.6835222	-0.217108688	0.09159529	-2.37030406	0.017773462	0.079007019
239.4105594	-0.388943023	0.180262138	-2.157652329	0.030954873	0.11645423
4.166777233	0.015593351	0.531264649	0.029351381	0.976584348	0.988158577
134.6025818	0.11979701	0.118007604	1.015163482	0.310027905	0.525135662
1021.398656	0.310999052	0.06997485	4.444440449	8.81209E-06	0.000215067
878.9190874	-0.040080795	0.112214487	-0.357180216	0.720956895	0.851889535
85.82446106	-0.014883498	0.137201103	-0.108479436	0.913615392	0.956888548
652.159957	-0.37192297	0.0938415	-3.963310138	7.39177E-05	0.00123501
139.802272	0.045500389	0.105402155	0.431683669	0.665971339	0.817892637
222.3862849	0.288553256	0.120294199	2.398729596	0.016452059	0.07489119
104.1245985	0.04277637	0.15608965	0.274050007	0.784046192	0.887950686
182.9538648	0.109138633	0.172712102	0.631910744	0.527445203	0.717558125
49.28097125	0.265728177	0.160738252	1.65317324	0.098295611	0.253692918
173.201607	-0.16991984	0.08311008	-2.044515406	0.040902657	0.141116308
63.91264762	-0.251579348	0.146558528	-1.716579382	0.086056052	0.232904124
13.85254518	0.14484277	0.319184694	0.453789835	0.649980101	0.805944015
3.902629847	-0.191898244	0.538662737	-0.356249339	0.721653845	0.85233418
136.5265116	0.078649797	0.095555271	0.823081716	0.410461526	0.620576279
2334.329265	-0.026480678	0.080710822	-0.32809328	0.742841135	0.865090121
206.8538409	-0.073900166	0.106690181	-0.692661359	0.488522094	0.686504952
242.9126771	-0.181362305	0.070217503	-2.582864636	0.009798376	0.051836366
15522.81587	-0.177472022	0.127386532	-1.393177276	0.16356619	0.353157197
886.6257762	0.431595699	0.052699246	8.189788823	2.61685E-16	1.05146E-13
10.35721469	0.363483924	0.386673047	0.94002912	0.347202624	0.56186798

7.487037117	1.12261675	0.717715624	1.564152587	0.117781739	0.285553549
174.4589655	0.35120555	0.187466105	1.873434933	0.06100835	0.185910488
2.37982426	-0.094743817	0.594315513	-0.159416699	0.873340583	0.936680519
454.299039	0.457149627	0.079018819	5.785325983	7.23718E-09	4.76902E-07
15.86356664	1.037771376	0.269240264	3.854443465	0.000115993	0.00175955
14.75461488	0.126252878	0.466695601	0.270525109	0.786756303	0.889691332
7.803925859	-0.567301188	0.374728622	-1.513898738	0.130051524	0.303208153
732.6079756	0.340169409	0.08901956	3.821288364	0.000132756	0.001949222
1530.035138	0.164057718	0.058083617	2.824509338	0.004735307	0.030177735
264.3592143	0.161701049	0.101843289	1.587743773	0.11234429	0.277600096
79.00919609	1.952638012	0.209803799	9.306971671	1.31529E-20	1.20378E-17
3.318980882	0.374623421	0.52871156	0.708559163	0.478598085	0.677938509
671.0381695	0.108737214	0.134904798	0.806029257	0.42022598	0.628946697
14.18171879	1.226072134	0.674243856	1.818440202	0.068996879	0.201749126
3089.511573	0.07370612	0.043442269	1.696645263	0.089763747	0.239437819
71.76048807	-0.414589531	0.181450792	-2.284859312	0.022321078	0.09273626
770.4267947	-0.042732065	0.204140237	-0.20932701	0.834192968	0.916961029
56.50566948	-0.171458265	0.285586728	-0.600371967	0.548258366	0.734907105
179.836411	-0.303183558	0.091691934	-3.306545569	0.00094454	0.009207307
132.3179733	-0.384626744	0.105356691	-3.650710168	0.000261516	0.003384303
220.8021703	-0.153531278	0.080027535	-1.918480656	0.055050093	0.172184398
442.3232908	0.041924443	0.081383606	0.515146048	0.606450967	0.776215774
29.23963633	-1.047486203	0.375845697	-2.7870113	0.005319662	0.033010244
5.346462109	-2.804939143	1.01869601	-2.753460419	0.00589689	0.035597423
8.592639036	0.306399678	0.326806244	0.937557602	0.348471809	0.563147398
810.4131861	0.281657807	0.210340166	1.339058594	0.180551599	0.376697953
189.1399984	-0.078374288	0.235570141	-0.332700432	0.739360423	0.862808843
20.81650273	-0.215313369	0.377314223	-0.570647371	0.568238701	0.74897935
607.2798095	-0.092571316	0.060210248	-1.537467773	0.124178801	0.294766569
7.235072838	-1.52083593	1.0532829	-1.443900714	0.148766906	0.331386486
46.25263887	0.405785396	0.217936454	1.861943643	0.062611034	0.188980246
2106.997375	-0.577790379	0.449210411	-1.286235502	0.198360884	0.400024141
96.69002226	-0.440288286	0.114949095	-3.830289289	0.000127993	0.001901129
10.91160843	-0.749997283	0.506342904	-1.481204293	0.138552143	0.316795003
252.6688862	-0.383000901	0.129656298	-2.953970678	0.003137138	0.02248133
520.7225036	-0.023825178	0.064842498	-0.367431522	0.713297162	0.847128509
100.3083195	-0.037671324	0.119495971	-0.315251833	0.752570454	0.870932542
586.0761033	0.113643759	0.074850409	1.51827839	0.128944233	0.301437107
16.47314765	-0.088977698	0.275956788	-0.322433445	0.74712435	0.867332282
3230.852258	0.010937652	0.21504318	0.050862583	0.959435021	0.980261259
162.2402068	0.287413208	0.203276796	1.413900721	0.157391054	0.343835065
49.5418504	-0.205194103	0.240482716	-0.853259254	0.393515551	0.606547079
30.16372945	-0.632536073	0.212356019	-2.97865855	0.002895132	0.021244724
4.33028498	-0.276693754	0.476732182	-0.580396636	0.561647173	0.744880072
281.4796135	-0.131475182	0.08759026	-1.501025138	0.13334906	0.308590223
63.54919109	-0.376794617	0.14033048	-2.685051863	0.007251855	0.041481619
57.5439673	-0.619875163	0.376272074	-1.647412088	0.099473372	0.255770928
99.39937722	-0.205760029	0.115956948	-1.774451931	0.075988426	0.214907011

227.0073872	0.052305187	0.096784702	0.540428244	0.588901734	0.76241798
236.4745409	-0.184878518	0.088289198	-2.094010632	0.036259033	0.12971364
3716.626737	-0.035539452	0.094632745	-0.37555131	0.707250494	0.843620638
91.67738359	-0.029016564	0.205665071	-0.141086495	0.887801613	0.94383349
1470.686661	0.317518118	0.073366156	4.327855453	1.50568E-05	0.000337937
46.27784452	-0.164988477	0.237644599	-0.694265627	0.487515643	0.685911068
469.1096168	0.368473169	0.073431302	5.017930458	5.22311E-07	1.97352E-05
35.61390149	0.452543979	0.198826422	2.276075663	0.022841481	0.094284782
1118.531688	0.052001067	0.129974047	0.400088085	0.68909164	0.832204066
34.74010846	0.067524879	0.209432363	0.32241855	0.747135633	0.867332282
73.12523723	-0.170532688	0.125444608	-1.359426213	0.174011569	0.367804285
14.78860068	-0.240385155	0.316887205	-0.75858271	0.448102219	0.653046351
169.3207207	-0.064540589	0.087092226	-0.741060276	0.458656894	0.661797596
533.5874955	0.210937968	0.073155787	2.883407817	0.003933978	0.026372163
7293.187781	-0.164680164	0.112452353	-1.464443916	0.143072696	0.32300666
277.4120108	-0.140376926	0.104032577	-1.349355464	0.177222818	0.372536521
1512.061361	0.490730839	0.055661237	8.816384066	1.18215E-18	7.23643E-16
456.3830182	-0.306174819	0.109077643	-2.806943848	0.005001396	0.031491988
14.85994577	-0.154548545	0.405134718	-0.381474453	0.702851224	0.840686203
31.0171176	-3.457987899	0.47062638	-7.347628707	2.01754E-13	4.49148E-11
281.5994101	-0.521384595	0.145437208	-3.584946405	0.000337147	0.004098455
58.6879486	-0.531246395	0.18672013	-2.845147953	0.00443908	0.028870668
5601.006088	-0.141577269	0.090334624	-1.567253654	0.117055414	0.284798536
232.7931941	0.040602412	0.083431085	0.486658081	0.626500656	0.790677612
419.4371941	-0.844168053	0.294363404	-2.867775144	0.004133692	0.027392776
114.2225154	0.102840916	0.128370657	0.801124792	0.423059405	0.63111002
80.54635853	-0.273454126	0.123735943	-2.209981347	0.027106457	0.106448575
2206.649511	-0.021871125	0.092763797	-0.235772203	0.813609439	0.906196373
88.65150415	-0.324491117	0.230730562	-1.406363834	0.159616114	0.347134413
107.4621263	0.040758475	0.115165428	0.353912413	0.723404524	0.853168805
5.986348394	1.479409459	0.576046947	2.568209879	0.010222524	0.053292995
253.212314	-0.14179232	0.103658185	-1.367883496	0.171348547	0.363595656
5.604851065	0.06147938	0.395932049	0.155277605	0.87660247	0.937787198
187.3618086	0.09833594	0.085675177	1.147776329	0.251060912	0.459958458
6.824250266	1.131994624	0.419731747	2.696947827	0.006997823	0.040294352
18.85471013	0.379078618	0.220621176	1.718233151	0.085754097	0.232545349
8.223775543	-0.318324024	0.375694162	-0.847295637	0.396830358	0.609578167
70.6240335	0.067246692	0.169246408	0.397330098	0.691124055	0.833539527
159.5387398	0.356662009	0.204313863	1.745657409	0.0808705	0.223909348
168.6536186	0.17339609	0.145614237	1.190790775	0.233735731	0.440618198
305.5330272	-0.208944289	0.078120971	-2.674624847	0.007481291	0.042513555
3.11737083	-0.535076519	0.792102694	-0.675514075	0.499349212	0.695315605
142.760046	-0.124603182	0.142300423	-0.875634662	0.381228676	0.593923904
21.24537278	-0.197802405	0.310230972	-0.637597218	0.523735907	0.715049508
3.891074911	0.779935479	0.55392973	1.408004366	0.159129777	0.346530594
130.5099257	-0.552808469	0.300741996	-1.838148568	0.066040516	0.195991977
737.2374536	-0.100497904	0.105697343	-0.950808231	0.341701733	0.556189542
3.995131774	0.124298027	0.467818857	0.265696915	0.79047264	0.891873589

47.46606634	0.00805663	0.349536302	0.023049481	0.981610803	0.990525347
41.85940253	-0.874696518	0.253512459	-3.450309784	0.000559944	0.006108948
364.8562716	-0.318510244	0.093133432	-3.419934559	0.000626362	0.006674815
7.785513897	-1.528104472	0.561852804	-2.719759447	0.006532942	0.038464507
492.1984123	-0.36587551	0.121854972	-3.002548883	0.00267729	0.020093134
1237.513223	-0.234585897	0.156863679	-1.495476193	0.134790209	0.311006321
1.993042802	-0.39909623	1.048837553	-0.380512911	0.703564715	0.841112127
889.1916993	-0.099502574	0.105948978	-0.939155579	0.347650872	0.562372395
1130.772488	0.192046611	0.1003024	1.914676135	0.055533819	0.173110192
6.926757541	0.426411726	0.393823219	1.082749074	0.278919816	0.492172776
402.0457169	-0.19448395	0.134206686	-1.449137561	0.14729917	0.329139058
212.2064717	0.076867224	0.081484238	0.943338565	0.345507756	0.559895217
19.98973005	0.228754689	0.259506545	0.881498725	0.378047944	0.591056451
1761.095989	0.146048952	0.091577217	1.594817546	0.110753042	0.275195417
71.54289126	1.156664541	0.187238505	6.177492945	6.51275E-10	5.55912E-08
466.0050328	0.120217521	0.069408075	1.732039401	0.083266547	0.228355768
357.5017993	0.006558269	0.10899962	0.060167816	0.952021978	0.977124952
121.1953008	-0.264304853	0.105568196	-2.503640904	0.012292272	0.061013225
15.76529234	-0.478093887	0.337974302	-1.414586503	0.157189768	0.343532004
164.4569968	-0.389702285	0.117317778	-3.321766675	0.000894495	0.008829182
18.09763295	0.444837003	0.303314677	1.466585816	0.142488761	0.322085599
563.9053945	0.339610139	0.077856392	4.362007137	1.28875E-05	0.000294463
69.84213607	-0.105984998	0.145763162	-0.727104135	0.467162166	0.66892912
59.55368956	-0.069387835	0.13666329	-0.507728414	0.611643819	0.780039684
3.439309723	-1.044896911	0.668189937	-1.563772296	0.117871053	0.285660577
104.8631336	-0.162818264	0.196870667	-0.827031607	0.408219147	0.618960169
23.66157523	0.872725597	0.284079126	3.072121521	0.002125432	0.016906984
708.4715165	-0.237033795	0.4633172	-0.511601543	0.608929905	0.778119086
3.030057422	0.600084545	0.652455776	0.919732136	0.357712755	0.572182625
4.876821176	-0.1567398	0.668713142	-0.234390189	0.814682074	0.906645443
149.2185733	-0.031632371	0.107456993	-0.294372381	0.768473369	0.879190026
233.2251358	0.013703027	0.150481928	0.091060948	0.927444162	0.965418623
39.71253363	0.277406505	0.181831006	1.52562817	0.127102492	0.298401947
53.05999643	-0.659697553	0.151565401	-4.352560327	1.34557E-05	0.000305871
25.2507433	-0.264736077	0.199695035	-1.325701851	0.184938474	0.382796384
891.7784154	-0.284721952	0.087530443	-3.252833443	0.001142604	0.010700167
346.3557321	-1.923097226	0.546524861	-3.518773548	0.000433547	0.004994578
2.303822652	-0.527814544	0.889808782	-0.593177495	0.553062372	0.738462435
407.9548658	-0.300719125	0.06580915	-4.569564018	4.8874E-06	0.000130706
574.5241738	-0.384960642	0.101215239	-3.803386201	0.000142732	0.002071642
640.3266541	-0.074876028	0.066112761	-1.132550307	0.257403147	0.467957282
5.414950914	-0.148509203	0.43538856	-0.341095785	0.733031474	0.859254341
327.4219688	0.001041638	0.08133156	0.0128073	0.989781532	0.994530438
17.34303937	0.021565811	0.304690269	0.070779456	0.943573283	0.972742569
973.4453725	0.657428772	0.118878803	5.530243878	3.19786E-08	1.6463E-06
70.93263574	0.284223053	0.191475994	1.484379564	0.137708253	0.315522359
70.01435266	-0.025555773	0.17105455	-0.149401305	0.881236982	0.940617989
5.020627767	0.733954434	0.499550778	1.469228888	0.141770717	0.321167602

3.676215337	-0.34902895	0.579546366	-0.602245084	0.54701101	0.733772443
70.11281628	-0.307463648	0.153608192	-2.001609704	0.045326725	0.151863425
45.88123701	-0.400029228	0.245202096	-1.631426624	0.102800331	0.26200694
127.8055827	0.519365743	0.131634382	3.945517382	7.96279E-05	0.001313102
280.9761956	-0.072058307	0.08918625	-0.807953095	0.419117584	0.628199717
2507.610164	-0.066951066	0.093627579	-0.715078467	0.474560559	0.674481119
173.446025	0.204610178	0.116540119	1.755705929	0.079138634	0.220597268
264.178646	0.090899639	0.105113734	0.864774137	0.387162769	0.600236496
2046.810572	-0.850836944	0.396000199	-2.14857706	0.031667942	0.118057858
6.646019917	-2.344102189	0.740518427	-3.165487993	0.001548231	0.013462072
1.751688384	0.404212857	0.752664429	0.537042593	0.591238193	0.764264772
105.0820675	-0.390115588	0.207078724	-1.8838999	0.059578517	0.182570031
699.607892	-0.65536425	0.202165201	-3.24172631	0.001188081	0.011028201
120.9221319	0.116390146	0.168250911	0.691765323	0.489084717	0.686945318
10.22281788	0.301389521	0.353788157	0.851892624	0.394273691	0.607070016
2438.692344	-0.288261542	0.087923789	-3.278538674	0.001043461	0.00995366
179.2612183	-0.116186989	0.187335895	-0.620206763	0.53512167	0.72384441
42.33686373	0.610438858	0.201930931	3.023008192	0.002502755	0.019091146
867.842883	0.316129046	0.121299184	2.606192692	0.009155494	0.049386905
813.3212538	0.798425212	0.135167646	5.906925493	3.48551E-09	2.55202E-07
273.6825122	0.003409396	0.119741848	0.028472888	0.977284992	0.988263026
79.95370446	0.04197405	0.133461861	0.314502206	0.753139641	0.871171356
594.3291596	-0.176293406	0.093978895	-1.875882937	0.060671356	0.185195464
28.65673124	0.731577801	0.296105411	2.470666774	0.013486142	0.06525536
332.2519217	0.173175006	0.06133253	2.82354251	0.004749613	0.030198811
15.33434874	1.155855723	0.275782443	4.191186761	2.77499E-05	0.000558866
100.8082275	-0.064202869	0.124993918	-0.513647946	0.607498152	0.777073108
40.28897773	-0.383179497	0.152805146	-2.507634774	0.012154222	0.060528614
5.856437787	-0.375738862	0.460684013	-0.815610812	0.414722764	0.624576769
635.4304655	-0.03269055	0.138824846	-0.235480538	0.813835783	0.906237868
4.686558955	0.228031238	0.502300266	0.453973955	0.649847573	0.805867862
2.751597732	-1.212486966	1.056973816	-1.14713056	0.251327665	0.460348227
343.3759397	-0.082244643	0.06952166	-1.183007464	0.236806189	0.444166843
258.3023169	0.032354732	0.076345712	0.423792391	0.671717227	0.821580637
1113.865258	-0.774471509	0.240269777	-3.223341364	0.001267045	0.011570561
529.1327037	-0.554264572	0.308539771	-1.79641208	0.072429004	0.208746573
7.770630939	-0.818981101	0.603260499	-1.357591128	0.17459346	0.368655814
54.7831611	-0.461506121	0.147473355	-3.129420368	0.001751515	0.014639505
296.3991802	-0.304436289	0.083442594	-3.648451869	0.000263825	0.00340768
808.4429809	-0.186011881	0.088308878	-2.106378037	0.035171527	0.126814564
691.5337617	0.143206825	0.121275604	1.180837866	0.237667138	0.445170729
1101.004103	-0.010980949	0.068974716	-0.159202525	0.873509314	0.936739516
145.0026206	-0.315352382	0.113650392	-2.774758429	0.005524272	0.033983146
947.9786705	0.192855664	0.103709996	1.859566792	0.062946841	0.189592266
40.82871028	-0.541010395	0.247503269	-2.185871715	0.028824992	0.110897459
567.8860339	0.258154333	0.103769686	2.487762508	0.012854954	0.063000705
163.4358221	0.205681154	0.148221731	1.387658562	0.16524104	0.355608216
91.16845733	0.042871006	0.13464198	0.318407426	0.750175906	0.869391338

507.8637527	-0.132482481	0.154320224	-0.858490724	0.39062155	0.60400683
415.5618139	-0.419161621	0.155361302	-2.697979588	0.006976172	0.040209149
630.6061337	0.382563728	0.097942373	3.906008378	9.38333E-05	0.001490224
3.13398716	0.831106064	0.88350252	0.940694617	0.346861378	0.561536394
899.7151489	-0.150808342	0.102725264	-1.468074514	0.142083984	0.321611919
1865.110142	-0.135161157	0.067081223	-2.014888081	0.043916377	0.14841853
92.04827753	-0.504712868	0.178601728	-2.825912566	0.004714613	0.030080765
2.238423401	-0.842948152	0.899261554	-0.937378172	0.348564065	0.563185995
58.8350165	-0.667323604	0.13885936	-4.805751687	1.54171E-06	4.99964E-05
14.76409402	-0.228968818	0.277360965	-0.825526466	0.409072763	0.619625659
543.8677377	-0.18034509	0.063620219	-2.834713471	0.00458668	0.029565883
46.40078938	-0.082187822	0.149077448	-0.551309561	0.581421485	0.758146246
1778.783409	0.098543097	0.045281715	2.176222707	0.029538612	0.112878472
51.30760096	0.295852781	0.169390112	1.746576455	0.080710834	0.223617606
207.0123582	-0.001605893	0.078978955	-0.020333182	0.983777586	0.991966796
21.35668809	-0.006850972	0.236639095	-0.02895114	0.976903559	0.988158577
217.0208179	-0.406252301	0.143482073	-2.831380203	0.004634759	0.029719986
2987.972726	-0.110605658	0.084188197	-1.313790564	0.188916694	0.388250202
277.0282542	-0.541699896	0.136339118	-3.973180295	7.09193E-05	0.001197054
853.8244595	-0.063930537	0.036909937	-1.732068421	0.083261381	0.228355768
126.0995454	0.183208119	0.09911257	1.848485207	0.064532182	0.193045791
125.4859212	0.110433627	0.136900338	0.806671691	0.419855658	0.628733943
3350.503083	0.082665171	0.06639854	1.244984772	0.213137348	0.417828132
494.9596214	-0.234215907	0.126397858	-1.853005345	0.063881593	0.191586629
1.718375382	0.410577494	0.778501338	0.527394718	0.597919511	0.769420873
57.556419	0.105464258	0.181138211	0.58223087	0.560411183	0.744310525
130.466646	0.017430362	0.098958201	0.176138631	0.860185032	0.930995875
26.50017422	0.70466644	0.234265658	3.007980112	0.002629903	0.019801202
31.40042532	1.032136413	0.324691308	3.178823659	0.00147874	0.013021974
7.049438462	0.003691055	0.422132806	0.008743824	0.993023527	0.996046614
22.63588088	0.69924705	0.276690105	2.527184886	0.011498095	0.058193433
20.29678491	0.323267249	0.248207294	1.302408338	0.192776834	0.39336689
159.9106242	0.017543608	0.094837219	0.184986525	0.853239605	0.92756165
69.97429575	0.105726354	0.204829998	0.516166358	0.605738225	0.77567875
1.753092419	-0.062121774	0.750084956	-0.082819651	0.933994944	0.968782646
375.5013325	0.012616202	0.072469608	0.174089555	0.861795082	0.931286863
2.237912814	0.006569013	0.847422668	0.007751755	0.993815056	0.996600225
3.616344012	1.293393359	0.746547068	1.732500755	0.083184443	0.228244588
302.5395075	-0.186956452	0.239986478	-0.779029105	0.43596257	0.642516316
807.6126841	-0.004599345	0.072166364	-0.063732535	0.949183198	0.975920679
134.7820027	0.132248453	0.091861874	1.439644614	0.149967971	0.333185752
8.495897365	-1.401956748	0.799865961	-1.752739604	0.079646714	0.221563656
291.6116733	0.310963375	0.072690763	4.277893971	1.8867E-05	0.000405763
151.8849217	0.007158588	0.127599096	0.056102182	0.955260406	0.978241049
171.5850794	0.151306162	0.097681842	1.548969166	0.121389132	0.291158774
171.4076428	0.004034545	0.087765226	0.045969748	0.963334362	0.98199185
539.2756315	0.002880729	0.049301734	0.058430581	0.953405656	0.977454818
291.2301079	-0.045327465	0.08529546	-0.531417089	0.595129782	0.767329422

696.6303412	-0.244222718	0.054979806	-4.442044002	8.91083E-06	0.000216515
267.1508711	0.722918461	0.114398675	6.319290499	2.62767E-10	2.57668E-08
526.1142975	-0.381650834	0.075821482	-5.033544914	4.81492E-07	1.83613E-05
20.85758705	0.715573656	0.545422974	1.311960972	0.189533303	0.389218774
11.24145459	0.532228956	0.656714715	0.810441648	0.417686389	0.626967251
35.98381001	-0.580773466	0.335789827	-1.729574331	0.083706356	0.229066196
83.25355114	-0.236441992	0.190229622	-1.242929409	0.213893847	0.418507745
286.9291036	0.070380536	0.122649838	0.573833098	0.566080749	0.748143944
72.57484233	-0.0431913	0.179285431	-0.24090803	0.809626398	0.903582771
28.84826973	-0.277869747	0.286778938	-0.968933594	0.332578323	0.54821846
1487.5646	0.111469297	0.065631337	1.698415769	0.089429318	0.238932628
19.45583289	0.191546953	0.254531685	0.752546595	0.45172242	0.656469531
98.24378244	-0.002755062	0.185683494	-0.014837407	0.988161897	0.993893344
29.99989108	0.438153522	0.201858031	2.170602378	0.029961242	0.113828295
1.929266098	1.178593866	0.975610257	1.208058093	0.227024918	0.432721103
25.92878304	0.939712202	0.226235165	4.153696462	3.27147E-05	0.000640835
1252.090027	0.119009037	0.061468517	1.936097417	0.052855771	0.167913929
20.67151324	-1.208454159	0.286612771	-4.216330464	2.4831E-05	0.000510694
239.9930029	0.44816332	0.128261397	3.494140326	0.000475591	0.005370038
50.89419151	0.119970877	0.139768188	0.858356096	0.390695863	0.604009351
290.813569	0.058977711	0.080162555	0.735726434	0.461897199	0.66433512
8.941452932	-0.84704505	0.323792574	-2.616011353	0.00889636	0.048417124
23.0741456	-0.439655023	0.354101162	-1.241608531	0.214381033	0.419063612
1083.261158	0.272468647	0.069970608	3.894044285	9.85866E-05	0.001546777
177.0903589	-0.372845827	0.100583799	-3.706817894	0.00020988	0.002841051
1535.384471	-0.410068772	0.109924467	-3.730459505	0.000191131	0.002629572
246.2856666	-0.329053757	0.169353157	-1.943003386	0.052015749	0.166067336
155.2944877	-0.061764598	0.103681991	-0.595711918	0.551367695	0.737218344
131.3093926	-0.283191887	0.156047297	-1.8147824	0.069557356	0.202881159
270.964017	0.147165466	0.156990105	0.937418733	0.348543209	0.563185995
547.9121819	0.304399761	0.077327482	3.936501683	8.2678E-05	0.001352855
46.38898475	0.292855468	0.169936175	1.723326235	0.084829546	0.231062448
307.0297549	-0.454057341	0.1472757	-3.083043183	0.002048954	0.016477006
167.7652729	-0.164861536	0.096838754	-1.702433474	0.088674139	0.237724617
129.5268707	-0.255161489	0.156122535	-1.634366809	0.102181853	0.260943086
138.6420215	0.350511365	0.1693054	2.070290523	0.038425147	0.135144292
2.121442578	0.419083868	0.737351623	0.568363661	0.56978806	0.750099284
392.5823344	-0.01293144	0.068246627	-0.189481015	0.849715834	0.925869346
10615.79122	0.016073571	0.087893751	0.182875015	0.854896087	0.928872058
29.19399978	-0.033529776	0.191490147	-0.175099222	0.86100167	0.931215742
73.74435365	0.627459178	0.21151579	2.966488588	0.003012215	0.021860457
6103.32675	-0.156359561	0.099363385	-1.573613468	0.115576838	0.282368801
711.9222953	-0.586285846	0.421971833	-1.38939569	0.16471246	0.354609653
2954.070767	0.342748892	0.197619151	1.734391077	0.082848717	0.227778666
2.606822674	-0.14467982	0.628346182	-0.230254952	0.817893663	0.908501127
470.1484956	-0.257524543	0.130746673	-1.969645096	0.04887906	0.159556541
5127.723141	-0.092671722	0.112778852	-0.821711871	0.411240901	0.621140791
27.60897079	0.275058574	0.236782146	1.161652505	0.245376645	0.453685169

155.6759946	0.728400561	0.110856204	6.57067927	5.00862E-11	5.77008E-09
117.3516822	0.008630718	0.111068298	0.077706407	0.938061597	0.970644228
248.7634738	-0.245583583	0.081024685	-3.030972395	0.002437675	0.018660903
10.37729079	0.448320884	0.302275201	1.483154696	0.138033315	0.315986318
167.9532698	-0.920284096	0.305758066	-3.009844049	0.002613819	0.019709144
224.3636397	0.026700547	0.100983903	0.264403994	0.791468635	0.892264031
489.9023657	-0.155715237	0.054549383	-2.854573765	0.004309462	0.028261975
660.184435	-0.397249177	0.092890181	-4.276546492	1.89815E-05	0.000407694
10.08672671	-0.107600141	0.525565275	-0.204732211	0.837781353	0.918926885
552.0563377	-0.556925642	0.388689793	-1.432828059	0.151906981	0.336128448
4.349945552	-0.354379151	0.575042719	-0.616265783	0.537719118	0.725799652
10.71721077	-0.273021364	0.54346707	-0.502369655	0.615407521	0.782406945
3845.679018	0.022300507	0.104241574	0.213931029	0.830600842	0.915614595
340.5196902	-0.165971896	0.057082438	-2.90758247	0.003642343	0.025022501
63.61908338	0.344642356	0.214890748	1.603802672	0.108757555	0.271671261
1045.161968	0.187296979	0.069103116	2.710398466	0.006720242	0.039316932
346.3287407	0.211740305	0.089146944	2.375183003	0.017540251	0.078371071
57.34536787	-0.131304633	0.185225483	-0.708890759	0.47839227	0.677925477
145.2654244	-0.062061761	0.155718415	-0.398551202	0.690223924	0.832899863
1038.543725	-0.223521881	0.065811877	-3.396376002	0.000682845	0.007160526
234.6347206	-0.286102568	0.169451891	-1.688399971	0.091334476	0.24186532
2319.72577	0.017413234	0.066407968	0.262216032	0.793154894	0.893098455
4.919631034	1.15387051	0.464543172	2.483882186	0.01299588	0.063459576
386.9501765	0.213138373	0.087993326	2.42221068	0.015426404	0.071667959
281.4378886	-0.311614907	0.104477356	-2.982607144	0.002858046	0.021056998
240.5678156	-0.384627075	0.109216284	-3.521700808	0.000428788	0.00495014
6.584329355	0.678042767	0.565220203	1.199608159	0.230291556	0.436724197
383.2412704	-0.41656619	0.103112304	-4.03992707	5.34678E-05	0.000950193
80.45215859	-0.033412156	0.147570647	-0.226414645	0.820878939	0.910037661
338.353688	-0.184566563	0.075892035	-2.431962236	0.015017273	0.070348602
310.7289975	-0.059054661	0.063422732	-0.931127686	0.351787519	0.566893044
7.138851756	-0.403652288	0.360850355	-1.118614081	0.263304814	0.47468631
726.2991908	0.775777955	0.169450464	4.578199056	4.68996E-06	0.000126633
3.696177238	-2.688407309	1.517171111	-1.771986884	0.076396735	0.215765439
29.39219435	-1.239177585	0.455775138	-2.718835411	0.00655122	0.038544569
1.843606361	0.060603301	1.732568953	0.034978868	0.972096591	0.986031074
220.7537122	-0.025751707	0.069675731	-0.369593638	0.711685292	0.846071015
15.58361401	-2.121298315	0.675078222	-3.142300029	0.001676262	0.014197807
24.69278679	-0.441976489	0.272463156	-1.6221514	0.104770935	0.264977062
17.25805491	-0.197918439	0.410954551	-0.481606636	0.630085411	0.792792107
331.5933284	-0.187173314	0.08716559	-2.147330311	0.031766994	0.118320024
36.30151921	-0.001195997	0.157785346	-0.007579901	0.993952172	0.996600225
104.4175622	0.195979369	0.135247411	1.449043404	0.147325462	0.329139058
5.082538721	-0.245929774	0.714955843	-0.343978969	0.730862102	0.857755975
49.46238902	0.355682592	0.215447283	1.65090312	0.098758359	0.254549542
4729.292187	-0.207286384	0.111694503	-1.855833343	0.063477316	0.190805069
7.842427838	0.076825538	0.404070177	0.190129197	0.849207892	0.925628813
243.6470896	0.092932797	0.088314034	1.052299306	0.29266225	0.507915315

23.27358887	0.629247885	0.287368551	2.189689456	0.028546766	0.110161496
1677.207882	0.437664465	0.121571572	3.600055971	0.000318149	0.0039055
201.5069094	0.07650236	0.08288231	0.92302398	0.355994708	0.570558902
127.4613576	-0.544807194	0.331317872	-1.644364039	0.100101029	0.256734287
2.5151583	-0.846156366	0.724763101	-1.167493717	0.243011044	0.450709544
256.4561088	-0.185145461	0.073653901	-2.513722394	0.011946443	0.059728589
36.08005914	-0.463260093	0.335835684	-1.379424865	0.167763797	0.359113929
5.969400235	1.11024522	0.522776378	2.12374787	0.033691232	0.123156539
479.7271755	-0.120258091	0.064865224	-1.853968635	0.063743647	0.191277385
5.621310869	0.726799242	0.463139106	1.569289296	0.116580545	0.283939827
292.7226253	-0.038535423	0.074940177	-0.514215803	0.60710112	0.776766636
367.5187587	-0.140642276	0.069669479	-2.018707156	0.043517666	0.147482005
139.2865509	-0.077537376	0.099385039	-0.780171509	0.43528993	0.641976357
528.057947	-0.102906527	0.060586093	-1.698517294	0.089410171	0.238932628
990.3093678	0.264351985	0.094035343	2.811198196	0.004935737	0.031189618
13.99668023	-0.303487798	0.264178078	-1.148800082	0.250638425	0.45957013
150.2142847	0.069185479	0.105807733	0.653879227	0.513189618	0.706547242
12.98979491	-1.135155918	0.6158959	-1.843097052	0.065314842	0.194560608
65.2982872	0.05063499	0.177601684	0.285104223	0.775564295	0.883629572
127.5648614	-0.099604922	0.401509539	-0.248076104	0.804075524	0.899535041
218.9175963	0.640621405	0.11934577	5.367776376	7.97133E-08	3.69915E-06
756.5558496	0.341423502	0.067409217	5.064937954	4.08533E-07	1.57615E-05
1053.016257	0.218696132	0.06132139	3.566392279	0.000361929	0.004345792
1.817140391	0.099230434	0.885022341	0.112121953	0.910726711	0.955037829
365.6807688	0.220563985	0.077021597	2.863664136	0.004187718	0.027683974
268.2485516	-0.190653367	0.073860437	-2.581265093	0.009843896	0.051993697
5.053051687	1.15057835	0.44772315	2.569843331	0.010174452	0.053160139
85.16485695	-0.10694781	0.124412437	-0.859623145	0.389996812	0.60360837
108.1916989	-0.171297627	0.138865379	-1.233551718	0.217369994	0.422248697
163.9637883	0.210248069	0.217392569	0.967135491	0.333476305	0.548794652
9.098439383	0.990405817	0.826825165	1.197841888	0.230978577	0.437422815
1200.566219	-0.011066136	0.044547745	-0.24841068	0.803816671	0.899475332
183.219408	0.038368506	0.076382888	0.502318088	0.615443788	0.782406945
4.073233401	-0.645098704	0.558928199	-1.154170972	0.248430081	0.45711395
578.2482817	0.134582853	0.088082506	1.527918079	0.126532879	0.297871199
498.1090779	0.155076886	0.107848656	1.437912089	0.150458999	0.333916415
14.34674716	0.977614934	0.792045791	1.234290927	0.217094515	0.422095485
355.020224	0.252179846	0.082488647	3.057146118	0.002234553	0.017571374
78.30845338	0.219825531	0.107741965	2.04029628	0.041320825	0.142127824
26.83024468	1.530827544	1.000988355	1.529316037	0.126186116	0.297522552
127.6577915	0.201309012	0.124187912	1.621003273	0.105016937	0.265344941
445623.6723	0.010258942	0.099433056	0.103174361	0.917824588	0.959308302
890.3223024	0.200984459	0.085026096	2.363797339	0.018088705	0.079933831
927.3088458	-0.031258896	0.078550704	-0.397945461	0.690670388	0.833255509
814.4097855	0.136979469	0.062511109	2.191282005	0.028431391	0.109819166
2206.012244	0.088379311	0.04647491	1.901656449	0.057216083	0.177342945
92.98327846	-0.107465578	0.146308697	-0.734512575	0.462636399	0.665049916
23.97889061	-0.277835441	0.316946812	-0.876599576	0.38070417	0.593610885

46.82550813	-0.252347993	0.202739364	-1.244691649	0.213245117	0.417828132
202.0841689	0.013783553	0.087777432	0.157028442	0.875222433	0.937516709
295.7522702	0.196735478	0.155636095	1.264073595	0.206203613	0.409622371
65.15969937	0.285328834	0.132748586	2.149392645	0.031603288	0.117911209
95.12922075	0.351920604	0.129386312	2.719921433	0.006529743	0.038459415
161.275968	0.542328523	0.659109414	0.822820175	0.410610262	0.620670043
375.1165019	0.128068709	0.090171043	1.420286431	0.15552431	0.340751095
26.69004189	0.323723083	0.208143221	1.555290065	0.119877002	0.288974793
584.6391131	0.078260079	0.080428635	0.973037517	0.330534656	0.545820272
16.10543634	-0.260444634	0.348649759	-0.747009362	0.45505791	0.659217728
210.4090893	0.079432689	0.092759628	0.856328236	0.391816247	0.604967515
184975.9932	0.00613478	0.103034804	0.059540854	0.952521326	0.977124952
1505.584057	1.069592735	0.131528278	8.132036339	4.22138E-16	1.65579E-13
12.04167959	-0.022718216	0.283390595	-0.080165739	0.936105439	0.969960438
51.30797652	0.164359579	0.158169915	1.039132999	0.298742886	0.513671882
67.37068942	-0.336401103	0.141445921	-2.378301907	0.017392579	0.077963711
791.4196864	0.328277337	0.074709103	4.394074162	1.11246E-05	0.000260692
96.25583755	-0.113389545	0.096265394	-1.177884807	0.238842533	0.446360434
53.5476515	0.266082585	0.156795911	1.696999519	0.089696751	0.239336619
1.933152358	0.641643362	0.738463753	0.868889448	0.384907602	0.598035257
15.07528781	0.552084733	0.295352211	1.869241918	0.061589165	0.186854494
289.0046911	0.207483192	0.09716591	2.135349645	0.032732461	0.120769217
647.7170988	-0.006488731	0.105785112	-0.061338788	0.951089401	0.976640702
161.3957976	0.320988726	0.094893205	3.38263131	0.000717949	0.007421228
40.42139496	0.638518503	0.242801358	2.629797907	0.008543564	0.047100521
879.9965171	0.391396023	0.058996843	6.634185933	3.26298E-11	3.86722E-09
19.99225281	-0.946843511	0.50611554	-1.870805054	0.061372107	0.186505089
26.81438611	0.133472657	0.36755773	0.363133861	0.716504891	0.849615028
9.898961103	-1.145434661	0.414208413	-2.765358274	0.005686031	0.034744685
45.68166539	0.611176515	0.656880758	0.930422314	0.35215247	0.567204717
339.2444902	-0.002868108	0.065347957	-0.043889782	0.96499226	0.982543604
628.742777	0.109194612	0.075294703	1.450229667	0.146994485	0.328621371
56914.54746	0.32964481	0.10668964	3.089754633	0.002003219	0.016224696
48.42544789	1.239897685	0.474250747	2.614434861	0.00893752	0.04857694
2.451274725	0.254150718	0.625391465	0.406386611	0.684458556	0.829588747
129.1844395	0.257455814	0.092035438	2.797355246	0.005152285	0.032199826
217.0601166	-0.204265641	0.096441727	-2.118021404	0.034173255	0.124303423
127.6223717	-0.149134143	0.113869205	-1.309696879	0.1902984	0.390237882
563.8440682	-0.292933028	0.086318019	-3.393648633	0.000689681	0.00721843
54.39570202	1.011543633	0.725024747	1.395184975	0.162960067	0.352264025
539.3486193	0.162051334	0.09135691	1.773826798	0.076091804	0.215092679
45.75703642	-0.199687787	0.18084015	-1.104222637	0.269496572	0.481371195
61.53825412	0.346128509	0.12474052	2.774788088	0.005523769	0.033983146
38.54282794	-0.149642744	0.171847481	-0.870788117	0.383869855	0.596929581
41.2206535	-0.012427711	0.177351115	-0.07007405	0.944134721	0.973016916
21.99333652	-0.102035352	0.235599865	-0.433087483	0.664951216	0.817005246
37.92190636	0.091564884	0.167631286	0.546227891	0.584909296	0.760101103
229.7770021	0.178207106	0.08833087	2.017495197	0.043643862	0.147835069

334.6310198	0.198092223	0.083531899	2.37145599	0.017718157	0.078823887
52.28331234	0.232538994	0.143942973	1.615493898	0.106203781	0.267523102
2572.449689	0.145755004	0.042425558	3.435547109	0.000591359	0.006388224
24.19600623	-0.962261698	0.223733878	-4.300920838	1.7009E-05	0.000372605
46.62342412	0.354258074	0.149676467	2.366825458	0.017941392	0.079432005
64.92166043	-0.150060593	0.116520201	-1.287850438	0.197798031	0.399231164
17.37682214	-0.361865017	0.286173048	-1.264497195	0.206051627	0.409517791
737.4400053	-0.074996153	0.048202536	-1.555854928	0.119742593	0.288693031
6.148479197	-0.185508465	0.48329028	-0.383844809	0.701093474	0.839681126
281.5729335	0.419832706	0.086167122	4.872307398	1.10302E-06	3.73893E-05
115.335286	0.7470073	0.103549908	7.213983205	5.43385E-13	1.09167E-10
124.2744617	0.136103521	0.141165044	0.964144645	0.334973412	0.550070918
7.585751965	0.389903795	0.390187988	0.99927165	0.317663115	0.532585198
75.51792645	0.344331024	0.226578515	1.519698474	0.128586775	0.300729491
153.0841551	-0.016971969	0.106908084	-0.15875291	0.873863551	0.936825644
656.7481083	0.137871709	0.057326529	2.405024536	0.01617138	0.073956537
151.6501029	-0.558093493	0.171155201	-3.260745155	0.001111199	0.010472475
28.14005505	0.121145841	0.237406191	0.510289308	0.609848792	0.778749632
444.8586436	-0.2094417	0.068639431	-3.051332095	0.002278284	0.017781276
155.693069	0.157262123	0.078192434	2.01121919	0.04430231	0.149250767
398.5081446	0.271303082	0.068563952	3.956934704	7.59177E-05	0.001262026
51.40994556	0.293125609	0.131430964	2.230262953	0.025729991	0.102787785
95.44985716	-0.415453033	0.141916356	-2.927449983	0.00341754	0.023937312
1969.36522	0.007751957	0.073078551	0.106077047	0.915521222	0.958290127
168.9261851	0.326860105	0.099356757	3.289762225	0.001002721	0.009665782
10.11612569	0.237298037	0.468063236	0.506978585	0.612169844	0.78038273
64.20300891	-0.052072414	0.135111725	-0.385402631	0.699939134	0.839012442
834.2151454	0.439445143	0.07836647	5.607565891	2.05192E-08	1.13608E-06
30.22242102	0.158806884	0.192207631	0.826225695	0.408676075	0.619247143
2425.083941	0.055764633	0.100267263	0.55615992	0.578101536	0.756144875
94.06522861	-0.564917695	0.330237396	-1.710641198	0.087147369	0.234739332
21.39382517	0.324136748	0.567808927	0.570855323	0.568097718	0.748947007
20.77854267	-0.302601634	0.270908285	-1.116989224	0.263998933	0.475417405
3715.37644	-0.125439603	0.117191736	-1.070379248	0.284448636	0.498564404
49.75812733	-0.135281527	0.14663747	-0.922557704	0.356237746	0.5707853
180.8008564	0.029419511	0.07838116	0.375339061	0.70740832	0.84371021
117.1476577	0.217060268	0.179439668	1.209655984	0.226410926	0.432121
5.974105158	-0.260815854	0.574354322	-0.454102711	0.649754902	0.805846741
57.8306465	-0.06807573	0.16647223	-0.40893145	0.682589968	0.828547438
31.73007771	-0.902926174	0.280171423	-3.222763284	0.001269604	0.011587513
659.9349479	-0.028338547	0.075241065	-0.376636708	0.706443604	0.843064305
1459.774595	0.004172557	0.101626784	0.041057647	0.967249939	0.983670319
204.9073624	0.210606114	0.099957472	2.106957199	0.03512129	0.126688883
399.7869605	0.050849703	0.064221648	0.79178446	0.428486365	0.635420323
479.442089	-0.053079542	0.052447336	-1.012054108	0.311512183	0.526390425
522.9303277	-0.088942492	0.067657161	-1.314605741	0.18864244	0.388013315
50.39885904	-1.616244671	0.567991323	-2.845544654	0.004433554	0.028857516
846.9790292	-0.064841379	0.086148681	-0.752668268	0.451649283	0.656469531

301.6755677	-0.243050689	0.085549903	-2.841039921	0.004496668	0.029130206
17342.7369	-0.182957866	0.150285051	-1.217405622	0.223449925	0.428534816
515.8108752	-0.251416611	0.065116108	-3.861050938	0.0001129	0.001726946
472.7293058	-0.564047095	0.199376533	-2.829054583	0.004668574	0.029853523
20.05427646	-0.501677344	0.304186909	-1.649240416	0.099098391	0.255075442
2.199966476	0.329870408	0.814570638	0.404962311	0.685505215	0.829778815
186.3634671	-0.164230245	0.139004955	-1.181470443	0.237415889	0.445009597
217.9535848	0.161049067	0.096314557	1.672115526	0.094501544	0.246996419
350.3819123	-0.146521484	0.105229389	-1.392400781	0.163801068	0.353337265
1537.782105	-0.186536542	0.102786231	-1.814800868	0.069554517	0.202881159
418.8991994	-0.234912267	0.052992257	-4.432954572	9.29505E-06	0.000224526
106.4631108	-1.478872848	0.283272041	-5.22068059	1.78267E-07	7.56899E-06
187.6823234	0.237889685	0.179150523	1.327876029	0.184219065	0.381637624
22.07781358	0.761083144	0.294552584	2.583861713	0.009770096	0.051736599
2059.159738	-0.200649705	0.089784475	-2.234792877	0.02543095	0.101920615
175.7578972	0.126056331	0.090343846	1.395295162	0.16292685	0.352238443
965.6422872	0.071782173	0.069909239	1.026790947	0.304518913	0.519213887
39.24316262	0.296819334	0.166793257	1.779564354	0.075147284	0.213481007
385.3419473	-0.092757072	0.071118157	-1.3042671	0.192142529	0.392803996
779.9778232	-0.090127964	0.117590939	-0.766453307	0.443406625	0.648728307
286.946946	-0.116197574	0.06703417	-1.733408102	0.083023159	0.228033305
268.3597852	-0.064539062	0.077282747	-0.835103105	0.403659658	0.614855381
5.241728663	0.582034117	0.397909466	1.46273001	0.143541271	0.323621034
189.2779783	-0.378561333	0.105787798	-3.578497148	0.000345576	0.004189119
477.9878887	-0.131333384	0.089567508	-1.466306111	0.142564911	0.322169321
795.1701242	-0.111333234	0.055355631	-2.011235941	0.044300541	0.149250767
42.98272778	-0.903272502	0.505046021	-1.788495432	0.073696112	0.211032461
473.0139009	0.128274378	0.114812652	1.117249499	0.263887662	0.475361883
49.9163875	0.547850366	0.186443126	2.938431563	0.003298775	0.023303609
95.16072737	-0.132122399	0.230404189	-0.573437487	0.566348515	0.748257714
6.755343815	0.794760576	0.413948427	1.919950711	0.054864126	0.171733157
243.3563149	-2.183844051	0.784259087	-2.78459515	0.005359459	0.033179907
5.348296838	0.040769444	0.520728456	0.078293097	0.937594908	0.970479513
53.32940837	-0.033144411	0.143549513	-0.23089184	0.81739883	0.90819642
276.8813821	0.240350666	0.098709054	2.434940417	0.014894242	0.069885429
19.93898723	0.548787181	0.241846092	2.269158775	0.023258672	0.095284866
35.29974327	-0.203978816	0.174327813	-1.170087618	0.241965711	0.449700261
62.05030051	-0.624321242	0.187331767	-3.332703539	0.000860065	0.008566334
1.719623116	-0.228823203	1.085225985	-0.210853045	0.833001945	0.916630422
267.414897	0.191238244	0.104499465	1.830040408	0.067243897	0.198277422
471.0402592	-0.062029003	0.089687804	-0.69161023	0.489182136	0.686965008
1815.731567	-0.530381175	0.091429538	-5.8009828	6.59274E-09	4.41499E-07
3.249921446	-0.39650523	0.708343816	-0.559763805	0.575640555	0.754123459
6.701608706	0.050615435	0.384413673	0.131669186	0.895245959	0.947285113
7.613196584	-0.154916536	0.517821712	-0.299169641	0.764810612	0.877707421
17.35893691	-0.550162998	0.280727205	-1.959777992	0.050021745	0.162248124
72.29787099	-0.330502126	0.205890112	-1.60523555	0.108441977	0.271162377
1246.989601	-0.035165499	0.08067269	-0.435903394	0.662906823	0.815527369

4.045988158	-0.137604182	0.642236881	-0.214257677	0.830346121	0.915603882
6.63616193	0.264237918	0.381124249	0.693311745	0.488113934	0.686283942
2.165050973	0.951041799	1.058851725	0.898182225	0.369088408	0.582745106
1.759126398	0.207206983	0.705029829	0.293898179	0.76883571	0.879325151
24.46980175	-0.19511182	0.484844875	-0.402421126	0.687374125	0.830982706
336.9651913	0.251517693	0.067554934	3.723157984	0.000196746	0.002687561
46.80484318	-0.319222549	0.142476175	-2.240532847	0.025056351	0.100839773
33.88678754	1.274002916	0.307056067	4.149088889	3.33801E-05	0.000651545
2.455231693	0.378731939	0.654222196	0.578904142	0.562653855	0.745322411
164.7368603	0.151066281	0.10995116	1.373939853	0.169460356	0.361430593
2178.645612	-0.058923717	0.105480611	-0.558621311	0.576420193	0.75482848
46.82240173	0.045745474	0.162095319	0.282213419	0.77777987	0.884329456
381.8743402	0.051285861	0.116408505	0.440567986	0.659525784	0.812578183
11.22874788	-0.109943942	0.30478192	-0.360729869	0.718301394	0.850529195
7.97131819	1.112143469	0.826949566	1.344874603	0.178665733	0.374375386
497.3967359	-1.341093739	0.370580235	-3.618902502	0.000295855	0.0037064
55.33548355	0.006829196	0.396940418	0.017204586	0.986273404	0.993222667
2.489341275	-4.778489137	2.257261144	-2.11694121	0.034264838	0.124448658
2.447254493	0.407543387	0.656384028	0.620891688	0.53467089	0.723766845
108.54842	-0.023614638	0.091952942	-0.256812209	0.797323745	0.89525023
258.281996	-0.067461615	0.110803136	-0.60884211	0.542629099	0.72997483
133.4164072	0.038179014	0.103279314	0.369667577	0.711630193	0.846071015
511.0625623	0.002842657	0.071551377	0.039728894	0.968309266	0.984103224
3193.187259	-0.147464959	0.093999252	-1.568788643	0.116697196	0.284052837
1.831910314	0.39707455	0.877653177	0.452427633	0.650960944	0.806581068
128.2449871	-0.11643658	0.204522658	-0.569308951	0.569146493	0.749789631
8.544703409	0.378112745	0.427443223	0.88459174	0.376376863	0.589554891
455.4470099	0.331722521	0.24296602	1.365304174	0.172157468	0.364962311
10.46147436	0.286126005	0.707182211	0.404600116	0.685771473	0.829836877
308.6837022	-0.139843872	0.155261099	-0.900701287	0.367747164	0.581519177
6.442902289	-1.268832702	0.785806341	-1.614688805	0.106378103	0.267587274
7.949703938	0.687081927	0.920538646	0.74639118	0.455431146	0.659584412
5.893316791	1.120343388	0.663821777	1.687717134	0.091465541	0.24209565
40.41936221	0.824475669	0.248115668	3.322948837	0.000890712	0.008802398
1.978741431	-1.549168534	1.756049033	-0.882189794	0.37767418	0.59069633
231.3623883	-0.150213139	0.092137634	-1.630312521	0.103035463	0.262327695
593.5913456	-0.278023611	0.066252076	-4.196451313	2.7113E-05	0.000548721
690.5206049	0.228602382	0.084657885	2.700308212	0.006927527	0.040108248
31.55116388	0.091022854	0.217612935	0.418278692	0.675743367	0.82454605
15.04977296	0.191099404	0.400334179	0.477349709	0.633113119	0.794296362
433.6565243	0.180420085	0.058773287	3.069763408	0.002142284	0.016984602
52.16177048	0.01676379	0.208233799	0.080504654	0.935835895	0.969742139
228.5015351	0.254839304	0.088280935	2.886685602	0.00389323	0.026157047
114.4594042	-0.182990091	0.120835526	-1.514373269	0.129931195	0.303013378
29.47274735	-0.128007556	0.245992235	-0.520372345	0.602804082	0.773169919
3.756821264	0.732947687	0.815466391	0.898807964	0.368754957	0.582386076
780.3440705	-0.254066137	0.120413445	-2.109948242	0.034862814	0.126004827
149.064515	-0.039841466	0.113809162	-0.350072567	0.726284238	0.854934372

4.025006323	-0.441041535	0.551658887	-0.799482336	0.424010785	0.631569048
312.0105676	-0.226019092	0.198815259	-1.136829702	0.255609484	0.465705667
12.46488026	-1.010803903	0.490251371	-2.061807396	0.039226077	0.136735164
1528.284184	0.410867716	0.047891992	8.579048388	9.56612E-18	5.25308E-15
14.75936168	-0.475334733	0.309338245	-1.536618056	0.124386868	0.295011412
4330.303073	-0.205366154	0.147177456	-1.395364202	0.162906041	0.352238443
90.01822332	0.333342342	0.160071233	2.082462506	0.037300244	0.132403409
935.8020881	-0.608487857	0.107540466	-5.658222234	1.52949E-08	8.87212E-07
314.0308202	-0.136094168	0.095607772	-1.423463436	0.154601854	0.339724016
165.9608229	-0.198271897	0.117573711	-1.6863625	0.091726	0.242536042
152.2892809	-0.522415855	0.18450562	-2.831436009	0.00463395	0.029719986
1368.447192	-0.068036358	0.054723139	-1.243283159	0.213763508	0.418384226
21.68271156	-0.079795981	0.225402725	-0.354015156	0.723327525	0.853160854
69.49044539	0.424679664	0.146282633	2.90314479	0.003694358	0.025222081
3.947346429	-0.233432556	0.473129146	-0.493380207	0.621743965	0.786950767
32.79614135	0.220286001	0.229803566	0.95858391	0.337768401	0.553111642
724.4702889	-0.006155201	0.048456015	-0.127026553	0.898919382	0.948916949
30.33588028	0.687547627	0.214443355	3.20619693	0.001345019	0.012081703
489.5554404	-0.043092015	0.085015046	-0.506875157	0.612242418	0.780414855
506.9487777	0.211404626	0.141410196	1.494974422	0.134921118	0.311126889
9.512992099	0.79271693	0.377707069	2.098761171	0.035837959	0.128597858
4934.050287	-0.422868161	0.06561439	-6.444747225	1.15793E-10	1.21502E-08
4.855462842	-0.690931815	0.88114285	-0.784131444	0.432962987	0.63992753
63859.96375	-0.232396667	0.098687201	-2.354881525	0.018528612	0.081212669
406.055097	0.223153068	0.139521552	1.599416469	0.109728095	0.273432256
2631.998241	0.126757262	0.081672917	1.552010965	0.1206596	0.290099801
101893.4826	0.524326114	0.101557014	5.162874483	2.43186E-07	9.8832E-06
470.7876843	0.089250174	0.112170462	0.795665564	0.426226447	0.633666139
2047.413878	0.208440733	0.083454044	2.497670855	0.012501221	0.061771179
3405.734006	0.215059891	0.470185533	0.457393679	0.647388107	0.80444124
655.4411777	1.643369639	0.183679736	8.946929457	3.65508E-19	2.61799E-16
2.909149546	1.363720187	0.796151362	1.712890603	0.086732667	0.234145272
9.089603635	2.352349644	0.602257808	3.905884842	9.38812E-05	0.001490224
10.31440984	-0.029621019	0.692998357	-0.042743274	0.965906183	0.983090566
20.90505996	0.68159492	0.266306941	2.559433556	0.01048429	0.054245661
1109.45242	-0.061920741	0.065946076	-0.938960205	0.347751176	0.562479419
79.25270154	0.081468201	0.125466439	0.649322654	0.516129846	0.709092076
2.168058838	0.898479602	0.765873099	1.173144222	0.240737975	0.448115254
40.79028783	0.820323888	0.213111334	3.849273866	0.000118468	0.001780703
2131.189591	0.125960033	0.057907125	2.175207858	0.029614544	0.113092239
7.969411972	0.378884208	0.512131607	0.739818052	0.459410404	0.662303711
280.342495	0.346541753	0.121461115	2.853108601	0.004329382	0.028347474
14.00311892	-0.173426707	0.422234489	-0.410735531	0.68126647	0.827691386
2259.860639	-0.095279814	0.060409696	-1.577227177	0.114743262	0.28129174
470.3336643	-0.015617952	0.083682053	-0.186634422	0.851947276	0.926915291
1.875963624	-0.927670065	1.099942613	-0.843380422	0.399015718	0.611199292
12.70810482	-0.003218118	0.310134928	-0.010376508	0.991720893	0.995693738
15.12026871	-0.759214566	1.458794916	-0.520439547	0.602757252	0.773169919

286.8950324	0.070080138	0.105233768	0.665947245	0.505444821	0.700428834
18.67904009	0.115981572	0.278871291	0.41589642	0.677485799	0.825889807
68.05567069	-0.07140733	0.167743992	-0.425692325	0.670332055	0.820734852
84.56703044	-0.461234322	0.194501915	-2.371361345	0.017722695	0.078823887
13.34135811	-0.918708251	0.585607351	-1.568812703	0.116691588	0.284052837
379.6303895	-0.091863912	0.087559956	-1.049154386	0.294107068	0.509065449
725.730764	0.445410312	0.060462596	7.366708318	1.74893E-13	4.00164E-11
65.91179622	-0.128548454	0.16555625	-0.776463916	0.437475119	0.643391655
152.4000999	0.081186995	0.277768732	0.292282699	0.770070486	0.880126348
2.420667456	-0.368433447	0.758905101	-0.485480261	0.627335711	0.790842402
111.6303724	0.007590713	0.098283781	0.077232608	0.938438502	0.970668417
177.1819825	-0.054409467	0.286859498	-0.18967288	0.849565474	0.925766743
4.427731081	-0.174559772	0.497062726	-0.351182583	0.725451376	0.854564602
80.53144064	0.172270544	0.188029459	0.91618912	0.359567711	0.573873132
43.53049069	0.194795976	0.185367614	1.050863048	0.293321494	0.508435503
2308.75791	-0.031942591	0.058948483	-0.541873002	0.587905997	0.761885532
22.30165147	1.632661386	0.770638136	2.118583691	0.034125665	0.124157732
335.2155375	0.279243183	0.126854917	2.201279935	0.027716212	0.107993586
48.60016291	-0.065265073	0.194761126	-0.335103182	0.737547252	0.861788314
400.875735	0.16564556	0.082338296	2.011768133	0.044244385	0.149177649
298.1134772	0.002093737	0.068082899	0.030752758	0.975466716	0.987719156
213.496437	0.230147545	0.074384652	3.094019272	0.001974647	0.016072296
9.529658465	0.159553909	0.324036109	0.49239546	0.622439808	0.787502757
33.82128259	-0.105876181	0.280611276	-0.377305512	0.705946577	0.842949182
300.1346617	0.111749215	0.088177539	1.267320628	0.205040667	0.408198182
159.3410139	-0.493701593	0.078365996	-6.299946647	2.97748E-10	2.8518E-08
1.878133091	-0.762834716	0.86705306	-0.879801654	0.378966768	0.592072118
94.43911053	-0.229607175	0.217554447	-1.055400974	0.291241977	0.506360072
370.3371116	-0.200522199	0.537800624	-0.372856018	0.709255608	0.844977159
6.288807606	-1.099042963	1.045766671	-1.050944722	0.293283979	0.508424736
394.7057247	-0.116066467	0.069681805	-1.665663895	0.095780377	0.249284405
27.29976314	2.039239327	0.683494411	2.983549381	0.002849261	0.021001664
3.516464375	-0.443909282	0.818328658	-0.542458433	0.587502735	0.761728322
23.33211795	-0.434175495	0.263874328	-1.645387405	0.099889945	0.256321956
2669.421672	-0.486409377	0.123457412	-3.93989612	8.15169E-05	0.001340229
2.401850778	0.735254967	0.810167271	0.907534769	0.364124072	0.577842208
39.77210153	-0.486825811	0.192900903	-2.523709337	0.01161239	0.058608242
80.22050931	-0.133092117	0.284152661	-0.46838244	0.639511123	0.798613269
8.50613793	-0.075649391	0.394287645	-0.191863459	0.847849161	0.924691631
101.4971895	-0.53015088	0.204636801	-2.590691784	0.009578323	0.051148558
26.82296041	-0.446949216	0.229160068	-1.950380007	0.051130843	0.164355462
147.9801309	0.132028309	0.116577771	1.132534168	0.257409928	0.467957282
10.63131417	0.116490683	0.325156059	0.358260841	0.72014812	0.851605781
2.337744487	-0.448040229	0.788305203	-0.568358838	0.569791334	0.750099284
1165.713999	0.000584035	0.061303269	0.009526974	0.992398689	0.995850406
25.50796067	0.348677213	0.206716693	1.686739511	0.091653451	0.242437212
120.678516	0.100767664	0.102903036	0.979248702	0.32745711	0.542648285
7.816460708	-0.085910407	0.412486652	-0.208274392	0.835014726	0.917558204

661.2672346	-0.06883096	0.072716862	-0.946561189	0.343862428	0.558185618
573.8339026	-0.163026828	0.066279674	-2.459680587	0.013906072	0.066673057
38.03360681	0.130037727	0.203996537	0.63745066	0.523831339	0.715049508
134.2176573	0.30644602	0.212181635	1.444262694	0.148665095	0.331274013
89.82230118	-0.064267327	0.168139415	-0.382226419	0.702293427	0.840493334
250.2882714	0.882371645	0.100990615	8.737164812	2.39034E-18	1.40637E-15
437.7744298	-0.023177238	0.081925886	-0.282904944	0.777249705	0.884207847
4.175552199	-1.47103286	0.692696832	-2.123631568	0.033700964	0.123156539
130.2360347	0.024727261	0.106601696	0.231959356	0.816569582	0.907813535
263.5405924	-0.079004534	0.074391321	-1.062012779	0.288229884	0.502936087
15.50867667	-0.333284288	0.311033587	-1.071537934	0.283927624	0.498022111
2.767059212	1.840566478	0.980838775	1.876522957	0.060583505	0.18499586
2.966739432	0.271431658	0.569784236	0.476376216	0.633806376	0.794736908
706.8294853	0.12167781	0.078033782	1.559296594	0.118926198	0.287482051
3.755008579	1.041502584	0.588705256	1.769140963	0.076870353	0.216546204
3178.20055	0.017901595	0.215148322	0.083205831	0.933687877	0.968634829
2499.494802	0.338406309	0.221507983	1.527738658	0.126577438	0.297885745
18.02274782	-0.273089249	0.254052311	-1.074933139	0.282404664	0.496119014
14.30484136	0.313759511	0.325434277	0.964125581	0.334982969	0.550070918
256.7037951	-0.120836109	0.063180456	-1.912555207	0.055805019	0.173721065
193.346097	-0.135843216	0.08481782	-1.601588152	0.109246713	0.272562523
164.8623815	0.016620764	0.094977736	0.174996423	0.861082444	0.931215742
296.2625596	0.216338982	0.083268409	2.598091918	0.00937434	0.050254758
825.3305437	0.290323849	0.066768646	4.348206295	1.37255E-05	0.000310597
217.3223185	0.088814684	0.072295384	1.228497292	0.219260345	0.423903045
1299.797975	0.14472603	0.058814893	2.460703809	0.013866479	0.066580115
3.439640361	1.02468665	0.502136265	2.040654541	0.041285177	0.142108651
450.2444286	0.431274202	0.122507153	3.520400181	0.000430896	0.004968297
2.017379215	1.657359055	0.786556204	2.107108236	0.035108199	0.126669396
8.011906494	-1.084691338	0.71079903	-1.526016908	0.127005654	0.29830213
12.74376416	-2.214780396	0.763820149	-2.89960981	0.003736274	0.025427316
21.18145735	0.132544286	0.334825665	0.395860591	0.692207875	0.834133021
149.2485558	0.19230799	0.082003852	2.345109239	0.019021506	0.082899546
1299.245798	0.199576998	0.086672692	2.30265144	0.021298462	0.089985486
4516.610619	-0.093121806	0.042853217	-2.173041179	0.029777218	0.113450365
117.5442225	-0.413761034	0.116378824	-3.555294844	0.000377555	0.004487625
304.3030033	0.137104624	0.124015179	1.105547121	0.268922588	0.480722965
1175.403364	-0.120298339	0.099216294	-1.212485714	0.225326508	0.430679764
171.6178847	0.099739729	0.081110282	1.229680459	0.218816787	0.42339532
543.5975889	-0.020523599	0.077613989	-0.264431697	0.79144729	0.892264031
3.823697248	-0.16782578	0.596407072	-0.281394684	0.778407695	0.884744609
23.02137147	0.557931967	0.267155486	2.088416655	0.036760266	0.131108168
1586.332296	0.117807237	0.07372139	1.598006187	0.1100416	0.273964836
2.858764245	0.816252652	0.770370647	1.059558351	0.28934557	0.50419705
364.1115495	0.007734982	0.196299489	0.039403985	0.968568303	0.984221208
378.7335006	-0.157054756	0.076041019	-2.065395195	0.038885623	0.136008864
147.0261591	0.106012279	0.090263777	1.174471997	0.240206022	0.447687352
224.4985582	0.03984117	0.070603375	0.564295546	0.572553012	0.751992213

39.05019338	-0.298073091	0.18504086	-1.610850117	0.107212395	0.268871516
124.5105861	-0.031364312	0.10085523	-0.310983493	0.75581317	0.872365037
95.41533344	-0.088808807	0.297035764	-0.298983549	0.764952598	0.877720432
31.54704693	0.259161191	0.195144007	1.328050988	0.184161263	0.381571205
1.815175104	-0.881589047	0.897362763	-0.982422141	0.325891923	0.540876842
267.5293545	-0.165189216	0.075768324	-2.180188332	0.029243504	0.112167052
384.1335787	-0.008610395	0.106797226	-0.080623771	0.935741161	0.969704969
158.8658434	0.165396233	0.398970836	0.414557201	0.678466084	0.826216017
29148.27737	0.456827698	0.100038858	4.566502547	4.95929E-06	0.000132414
59.71379652	0.465361839	0.317255119	1.46683792	0.142420152	0.321974691
120.6864542	-0.155191058	0.125049177	-1.241040215	0.214590893	0.419184148
72.25427575	0.66340977	0.1414873	4.68882909	2.74773E-06	8.08323E-05
264.6823988	-0.106910394	0.101924736	-1.048915088	0.294217201	0.509065449
1.946891744	2.057650574	0.996081045	2.06574614	0.038852456	0.135950587
25.18780443	0.197561754	0.704151619	0.280567067	0.779042479	0.884794608
28.01664751	0.469262047	0.799464019	0.586970815	0.557223308	0.741574435
6.312262524	-2.223994123	0.937341614	-2.372661247	0.017660453	0.07865323
156.2729105	-0.150235476	0.127223244	-1.180880718	0.237650112	0.445170729
21.04624693	-0.355526543	0.226052871	-1.572758364	0.11577478	0.282613205
5.492160325	-1.599361821	0.899614173	-1.77783084	0.075431642	0.214067333
289.0700246	-0.250653284	0.096181287	-2.606050433	0.009159297	0.049391249
64.03614253	0.742360113	0.164455702	4.514043008	6.36033E-06	0.000163209
199.639858	-0.236051804	0.121033574	-1.950300206	0.051140348	0.164355462
5.074776454	0.044607927	0.450478653	0.099023399	0.921119692	0.961082133
258.3017893	-0.056428252	0.08702977	-0.648378729	0.516740024	0.709516183
340.0281209	-0.113985635	0.087098687	-1.308695221	0.190637611	0.390623187
1434.543859	0.29093207	0.095624998	3.042426923	0.002346788	0.01820197
2657.684587	-0.179144478	0.125601118	-1.426296842	0.153782676	0.33850411
299.6180568	-0.373944531	0.108156442	-3.457441119	0.000545331	0.005989193
34.19469847	-0.206402314	0.177594857	-1.162208846	0.245150642	0.453424729
269.0414115	-0.011679252	0.106871935	-0.109282683	0.912978281	0.956586161
97.52469412	0.194364155	0.156134443	1.244851239	0.213186437	0.417828132
75.00570872	0.182570463	0.158147554	1.154431154	0.248323449	0.457077732
1335.316077	0.023977424	0.035654578	0.672492164	0.501270427	0.697106957
3198.962238	0.166605045	0.087096559	1.912877464	0.055763741	0.173625378
708.2171733	1.034518615	0.106145917	9.746193187	1.91516E-22	2.25359E-19
1.927605118	-1.045189764	0.85727178	-1.219204677	0.222766517	0.427806473
738.176333	-0.247641503	0.064069862	-3.865179312	0.000111008	0.001702737
150.6433936	0.065403341	0.118147093	0.553575543	0.579869368	0.757293715
267.153389	-0.050325966	0.069428966	-0.724855466	0.468540704	0.670018398
65.11230162	-0.405820626	0.275626785	-1.472355545	0.140924891	0.320087778
4.557472653	0.958151201	0.487377639	1.965931803	0.049306489	0.160496956
107.1269729	0.126326025	0.164562675	0.767646884	0.442697	0.64838108
2.353734746	-3.522649996	1.808187749	-1.948166056	0.051395097	0.164906074
2.121452068	0.299587887	0.953474897	0.314206371	0.753364304	0.87130887
1.8321209	-0.042416946	0.710044651	-0.059738421	0.952363971	0.977124952
1008.121489	-0.21519218	0.065490853	-3.28583564	0.001016803	0.009772938
376.0735733	-0.217191665	0.066978534	-3.242705592	0.001184005	0.011007504

20.87080362	-0.875380509	0.449541139	-1.947275638	0.051501698	0.165110249
215.7197158	0.09739255	0.159948912	0.608897857	0.542592145	0.72997483
909.7434032	0.079023302	0.135865659	0.581628227	0.560817126	0.744532301
1423.788835	-0.078017985	0.059493153	-1.311377541	0.189730243	0.389476547
43.44601948	0.23712694	0.161094919	1.47197032	0.141028893	0.320228094
66.61075084	-0.225707884	0.148063497	-1.524399255	0.127409006	0.298780921
758.183578	0.15640424	0.077448009	2.019473997	0.043437977	0.147302849
90.07578372	-0.052761288	0.106636761	-0.494775793	0.620758393	0.786281237
344.9108442	-0.231172716	0.104159872	-2.21940284	0.026459331	0.104857112
216.7764779	-0.095149557	0.090038331	-1.056767216	0.29061784	0.505825493
80.90505205	-0.031623575	0.105233635	-0.300508247	0.763789507	0.876963224
72.87595708	0.246945519	0.107121243	2.305289888	0.021150343	0.089501864
405.0826984	0.164028473	0.080232459	2.044415386	0.040912529	0.141120812
2.828211796	-0.081258453	0.594184006	-0.136756379	0.891223364	0.945526539
263.6271844	-0.019181353	0.07126961	-0.26913789	0.787823574	0.890409272
137.7262343	0.034335924	0.113501644	0.302514771	0.762259675	0.875917923
933.5681493	-0.169903779	0.079552195	-2.135752247	0.032699613	0.120726961
3210.352722	-0.08602177	0.084515451	-1.017823005	0.308762077	0.523954106
10.03781791	-0.208051712	0.318836553	-0.652534063	0.514056706	0.707541998
296.1918422	0.032344618	0.09767954	0.331129913	0.740546358	0.863699872
55.02345297	-0.005600276	0.152313121	-0.036768179	0.970669846	0.985661927
9.764699737	0.693728213	0.592318878	1.171207332	0.241515449	0.449050591
464.0542031	-0.181248547	0.146967696	-1.233254331	0.217480891	0.422248697
230.99173	-0.038397201	0.109679695	-0.350084865	0.726275009	0.854934372
459.2535044	-0.088771633	0.073066781	-1.214938338	0.22438961	0.42942509
250.3315023	0.460442846	0.074777312	6.157520678	7.38926E-10	6.2426E-08
105.9053234	0.085858623	0.119139313	0.720657356	0.471120355	0.671530536
29.88583447	-0.527898688	0.418437358	-1.261595498	0.207094377	0.410599683
105.6283251	-2.2420983	0.620372532	-3.614116013	0.000301374	0.003744221
539.4445811	0.19737321	0.150380828	1.312489186	0.189355132	0.388908671
438.3687968	1.511115336	0.132959151	11.36526009	6.22787E-30	1.46568E-26
138.6513018	0.399616375	0.121593491	3.286494785	0.001014427	0.009760722
124.1845053	0.227302512	0.095800098	2.372675146	0.017659788	0.07865323
96.36769724	0.00452614	0.123991387	0.036503662	0.970880759	0.985661927
98.42938988	0.231333592	0.097189191	2.380239922	0.017301369	0.077641721
1.927929305	-0.378852399	0.819501583	-0.462296116	0.643868984	0.802230952
159.2316516	-0.11171094	0.117234546	-0.952884144	0.340648775	0.555270316
72.63898153	0.190169148	0.131970612	1.440996187	0.14958576	0.332560838
4.998037229	-0.452398999	0.660515171	-0.684918408	0.49339541	0.690409919
354.8573375	0.05041228	0.074712084	0.674754038	0.499832046	0.695753462
78.44378104	-0.256442515	0.109512931	-2.341664243	0.019197977	0.083491941
467.3198037	-0.06512852	0.064073398	-1.016467403	0.309406863	0.52466996
91.00336673	-0.09864435	0.120559234	-0.818223098	0.413229809	0.623059479
3.867894741	0.189512869	0.536605894	0.353169563	0.723961324	0.853541749
282.7125314	0.220542302	0.124880885	1.766021288	0.077392277	0.21742162
257.8609478	-0.01700776	0.10777201	-0.157812407	0.874604622	0.937267006
7.508509611	-0.440131776	0.354945483	-1.239998245	0.214976043	0.419461724
189.5486848	-0.163959753	0.086559364	-1.894188511	0.058199992	0.17968266

1819.998849	-0.213302073	0.071990546	-2.962917827	0.003047379	0.022028312
1.92604327	-0.466671968	0.781306087	-0.597297238	0.550308948	0.736546229
76.35228245	-0.362830517	0.306718334	-1.18294369	0.236831465	0.444166843
729.6578051	0.060968773	0.0580009	1.051169418	0.293180786	0.508299334
53.14195154	-0.921035588	0.29447631	-3.127706903	0.001761758	0.014695289
89.77358461	-0.217098141	0.175585027	-1.236427414	0.216299731	0.42094764
144.5605216	0.238356465	0.179058887	1.331162443	0.183135566	0.38040289
120.2662398	0.900266556	0.182472908	4.933699827	8.06864E-07	2.89688E-05
287.8282966	-0.120617219	0.125118393	-0.964024689	0.335033548	0.550070918
5.761077221	-0.372692582	0.430116669	-0.866491835	0.386220502	0.599452311
1347.914746	0.242101175	0.18989772	1.274903009	0.202343574	0.405117546
2.036614349	0.367556353	0.748006267	0.491381382	0.62315673	0.788228191
11.45238988	-0.168619859	0.349105001	-0.483006139	0.629091377	0.792206952
78.16579096	-0.29194337	0.156578964	-1.864512073	0.062249828	0.188304014
17.99311859	-0.237683287	0.276999193	-0.858064905	0.390856625	0.604048588
16.95762077	0.097379511	0.34173083	0.28495969	0.775675025	0.883650533
14.65609021	0.761774231	0.290586764	2.621503545	0.008754285	0.047897075
391.771176	0.408574159	0.129157744	3.163373305	0.001559522	0.013521878
55.94057477	-0.453927777	0.203130939	-2.234656022	0.02543994	0.101920615
500.8362661	-0.178818992	0.118300007	-1.511572123	0.130642748	0.304328143
1001.026049	-0.15778192	0.107217513	-1.471605847	0.141127346	0.320363831
28.78497231	-0.217218929	0.271573438	-0.799853367	0.423795759	0.631486019
2010.718636	-0.244436213	0.16550831	-1.476881813	0.139707321	0.318331729
40.19174759	-0.110606544	0.174046453	-0.63550013	0.525102282	0.71610389
3418.336118	0.031841754	0.077443469	0.411161252	0.680954298	0.827594328
93.81781555	0.259619604	0.141383487	1.836279537	0.066316325	0.196598008
1586.047578	0.067948361	0.110217036	0.616495993	0.537567216	0.725654074
271.861403	-0.323150385	0.069829427	-4.627710696	3.6973E-06	0.000103367
114.0079349	0.026124099	0.100149059	0.260852163	0.794206516	0.893917451
650.3612684	-0.603283835	0.254048369	-2.374681	0.017564122	0.078415001
6.044697933	-2.373161655	1.101943909	-2.153613842	0.031270468	0.11697595
124.3459423	-0.212296319	0.305762194	-0.694318404	0.487482552	0.685911068
44.5263934	-0.213383142	0.198983456	-1.072366243	0.283555564	0.497581419
43.16794812	-0.143859334	0.198077674	-0.726277379	0.467668744	0.669395389
1350.861564	-0.43556138	0.231793033	-1.87909608	0.060231375	0.184227938
88.61435566	-0.212406655	0.230832597	-0.920176171	0.357480705	0.571983016
4379.525709	0.400084881	0.110247045	3.62898509	0.000284538	0.003602977
19.39935928	-0.366118367	0.22780913	-1.607127712	0.108026355	0.270501015
247.7607443	-0.083545699	0.106721435	-0.782838974	0.433721678	0.640531683
28.04538299	0.103149313	0.228161889	0.452088266	0.651205397	0.806795345
59.93080325	-1.751690123	0.298172299	-5.874758084	4.23461E-09	3.03309E-07
129.7552954	-0.160109949	0.124201652	-1.289112875	0.197358848	0.39875922
265.5306355	-0.173059121	0.115229512	-1.501864562	0.133132091	0.308209396
35058.22768	0.130817247	0.096763553	1.351926864	0.176398713	0.37137283
12.77428726	-0.07322315	0.260585642	-0.280994567	0.778714567	0.884792834
4776.520826	-0.583254664	0.144534797	-4.035392686	5.45111E-05	0.000963959
7.092456232	-0.751081855	0.536546378	-1.39984517	0.161559688	0.35043243
30.93995967	-0.19785742	0.180824061	-1.09419852	0.273867921	0.48659406

45.06189775	-0.292696913	0.26754292	-1.094018532	0.27394685	0.486656041
8.752119562	0.639858421	0.376327491	1.700270208	0.089080112	0.238231454
306.5343771	-0.058548519	0.069022103	-0.848257529	0.396294565	0.609075814
8.530196031	-0.998647316	0.458940657	-2.175983538	0.029556491	0.112920603
318.7938941	0.127247381	0.081899031	1.553710454	0.120253498	0.289670439
589.8023673	-0.010286598	0.062310686	-0.165085611	0.868876599	0.934607931
295.4327244	-0.211316241	0.089713186	-2.355464677	0.018499556	0.081161566
140.3154835	0.219905366	0.116423149	1.88884571	0.058912506	0.181203253
124.0486238	0.061094412	0.10418919	0.586379565	0.557620473	0.741810242
12.37559346	-0.156736099	0.420309621	-0.372906285	0.709218194	0.844977159
439.7491493	0.529154052	0.128693974	4.111723615	3.92716E-05	0.000737325
11.20443017	-0.053207332	0.304030121	-0.17500678	0.861074306	0.931215742
665.7001994	0.097334951	0.11297513	0.861560869	0.388929212	0.602295529
139.9042299	-0.016965007	0.102048704	-0.166244214	0.867964766	0.934495951
9.701122928	-2.58387232	0.967673148	-2.670191194	0.007580807	0.042872025
39.37870561	0.199437511	0.230254537	0.866161048	0.386401851	0.599452311
496.8915992	-0.171511541	0.068285274	-2.511691479	0.012015408	0.059964203
1463.39585	-0.229977474	0.168943266	-1.361270441	0.173428241	0.367042245
2.389566557	0.192092554	0.597625027	0.321426556	0.747887161	0.86771555
43.42606333	-0.852461293	0.229279336	-3.718003153	0.000200804	0.002729407
56.58922728	-0.31147365	0.260241852	-1.196862255	0.231360248	0.437994568
65.87169167	-0.151783871	0.123233998	-1.231672056	0.218071612	0.422815921
268.6432006	-0.280683394	0.095120679	-2.950813598	0.003169381	0.022661626
43.91216448	-0.209463366	0.209670561	-0.999011809	0.317788971	0.532700656
26.87311856	-0.282522437	0.204847775	-1.379182352	0.167838538	0.359180576
59.80197288	-0.068928081	0.354781649	-0.194283106	0.845954213	0.923786936
81.67728208	-0.347164456	0.125312481	-2.770390101	0.005598919	0.034339759
5.738087537	-0.268109607	0.417724934	-0.641832903	0.520981683	0.712937765
121.9555216	-0.888850194	0.132049322	-6.731198484	1.68271E-11	2.26244E-09
70.05677863	-0.143240823	0.12891853	-1.111095692	0.266527162	0.478259224
1300.975766	-0.179375485	0.082134287	-2.183929406	0.028967436	0.111315188
234.0885132	0.103636865	0.117551934	0.881626197	0.377978983	0.591024835
107.4494883	0.348057484	0.160238519	2.172121198	0.029846522	0.113624283
6.718335216	1.352187839	0.389276258	3.473594424	0.000513537	0.005689309
194.5499923	-0.330780193	0.10256442	-3.22509691	0.0012593	0.011506219
2141.481393	0.477395768	0.094735111	5.039269647	4.67312E-07	1.78619E-05
84.57159493	-0.067908756	0.164591947	-0.412588571	0.679908077	0.826932461
22.75925352	0.159958921	0.413816577	0.386545464	0.699092739	0.838613574
37.37180514	0.098398426	0.184766312	0.532556097	0.5943409	0.76681093
3032.257223	-0.024326238	0.110922098	-0.219309213	0.826409185	0.913336211
64.92871223	-0.654570322	0.208913731	-3.133208709	0.001729064	0.014482865
155.5218787	-0.574849329	0.206300724	-2.786462977	0.00532867	0.03303896
122.7243344	-0.159569626	0.095921698	-1.663540463	0.096204298	0.249828924
31.3556322	-0.117583574	0.429248067	-0.273929186	0.784139042	0.887950686
49.62267617	0.23986987	0.178859358	1.34110886	0.179885111	0.375830985
34.36252058	0.795387469	0.20813173	3.821557952	0.000132611	0.001948829
120.6266789	0.195396553	0.123993362	1.57586301	0.115057377	0.281621068
12.59314644	-0.843108813	0.27943433	-3.017198394	0.002551228	0.019394982

13.20361368	-0.591327773	0.261100275	-2.264753543	0.023527808	0.096106857
1379.216989	-0.331179386	0.112527827	-2.943088789	0.003249552	0.023070625
37.24140017	-0.679187109	0.221265048	-3.069563464	0.002143719	0.016984602
89.81643536	0.606266148	0.130163903	4.657713341	3.19741E-06	9.12897E-05
6.667594786	1.094105585	0.393045339	2.783662534	0.005374893	0.033262952
344.0114887	0.050722346	0.082659277	0.613631614	0.539458788	0.727194507
11260.78222	-0.160243785	0.163903094	-0.977673944	0.328235613	0.543616516
7.709967564	0.501203842	0.432410552	1.159092534	0.246418469	0.455049642
818.6205022	-0.178796169	0.056794964	-3.148098986	0.00164336	0.013998303
29.24566217	-0.077767034	0.225610439	-0.344696079	0.730322866	0.857543756
87.28913549	-0.220910445	0.166669695	-1.325438588	0.185025725	0.382895463
10.49768248	0.273877711	0.339301614	0.80718069	0.419562389	0.628466157
82.03362941	0.121452361	0.187394843	0.648109406	0.516914191	0.709630747
139.7944608	0.020346406	0.127268794	0.159869561	0.872983827	0.936680519
18.0233327	0.091310964	0.238098442	0.383500889	0.701348412	0.839737026
238.1982935	-0.147416712	0.113115985	-1.303235005	0.192494543	0.393136763
83.75680655	-0.155633762	0.136318374	-1.14169321	0.253581564	0.462865802
337.1865246	-0.14041854	0.076161522	-1.843693997	0.065227748	0.194420468
347.7864374	0.108396818	0.072341291	1.498408669	0.134027106	0.309759054
86.5592782	-0.044890862	0.27926799	-0.160744745	0.872294447	0.936384156
2.523237717	-0.593043209	0.892860345	-0.664206012	0.506558466	0.70144105
65.59320858	-0.749298106	0.586447652	-1.277689668	0.20135887	0.404132367
1644.665609	0.345139258	0.10125826	3.408504746	0.000653199	0.006897954
7.505606295	-0.241957618	0.428545717	-0.564601648	0.572344744	0.751778609
660.7336765	-0.009555526	0.078583978	-0.12159637	0.903218688	0.951456563
863.0830248	0.030257797	0.073638113	0.410898592	0.681146895	0.827691386
343.903691	0.177413651	0.061787215	2.871365101	0.004087032	0.027203944
11.85896816	-0.146934936	0.354163145	-0.414879241	0.678230308	0.82617318
6.14368345	-1.472675344	0.55166514	-2.669509521	0.007596212	0.042926321
43.01218516	-2.821407816	0.912599062	-3.091618141	0.001990687	0.01616293
66.10016166	-0.156631802	0.126240997	-1.240736417	0.214703137	0.419252375
11.28694907	-0.452188596	0.297945103	-1.517690982	0.129092318	0.301589735
112.9125927	0.147300688	0.101414035	1.452468472	0.14637139	0.327758906
698.8484653	0.144129476	0.08026538	1.795661789	0.072548322	0.20887121
686.4797733	-0.037907397	0.09432674	-0.401873292	0.687777279	0.83108758
103.4129354	-0.148453656	0.101182409	-1.467188397	0.142324812	0.321803316
784.1570195	-0.00166117	0.083993879	-0.019777271	0.984221049	0.992142415
53.99589965	0.19922077	0.149884163	1.329164909	0.18379357	0.381192907
172.8830887	0.017944935	0.095411567	0.188079242	0.850814533	0.926363298
805.156896	-0.092428754	0.061922606	-1.492649617	0.135528928	0.311917533
996.0387975	0.002057067	0.045784925	0.044928911	0.964163972	0.982290494
292.8750664	-1.904566398	0.664263939	-2.867183188	0.004141432	0.027433033
247.3850338	0.246277387	0.080312286	3.066497022	0.00216583	0.017120863
219.9414301	0.019496683	0.075442358	0.258431515	0.796073897	0.894773586
39.93064341	0.436757994	0.175161894	2.493453245	0.012650724	0.062285724
751.043776	-0.255413008	0.076424328	-3.342038004	0.000831657	0.008343918
1391.711864	0.508626345	0.055641298	9.141166011	6.17825E-20	5.35687E-17
354.7611904	-0.021606124	0.072556252	-0.297784454	0.76586768	0.878006201

1050.773001	0.158659391	0.052228321	3.037803761	0.002383091	0.018411207
287.8409584	0.15180231	0.085479773	1.775885729	0.075751754	0.214569187
85.63113294	0.223876753	0.227348715	0.984728471	0.324757464	0.539767136
177.8996522	0.121585732	0.131212189	0.926634433	0.354116371	0.568920723
13.53680878	-0.415125964	0.371952442	-1.116072694	0.264391019	0.475731379
3.990074329	-0.573666534	0.573130965	-1.000934461	0.316858495	0.53185835
269.6107603	0.049372433	0.087781423	0.562447398	0.573811233	0.752760082
312.8163751	-0.07206673	0.077986431	-0.924093186	0.355437801	0.570204885
976.8509222	0.15026471	0.078907621	1.904311751	0.056869595	0.176601263
64.08146141	0.495182734	0.16301179	3.037711158	0.002383823	0.018411207
360.8190248	0.102139087	0.072460721	1.409578663	0.158664131	0.345790832
147.5683291	-0.053777393	0.100475261	-0.535230188	0.592490694	0.765064406
123.698082	0.256658264	0.205280822	1.250278825	0.211197711	0.415435354
409.4402963	-0.031011523	0.081584736	-0.380114285	0.703860583	0.841404777
88.40906166	-0.271394582	0.115803614	-2.343576092	0.019099866	0.08316032
162.7938887	0.184276091	0.22967249	0.802342899	0.422354634	0.630641732
5.733731926	0.551318054	0.507742107	1.085822993	0.277557312	0.490556716
516.8582309	0.413450065	0.204512877	2.021633409	0.043214238	0.146755588
229.4331701	0.074479007	0.091363794	0.815191705	0.414962585	0.624757232
570.7609243	-0.081076794	0.086223605	-0.94030856	0.34705931	0.561691234
2474.209959	-0.185551957	0.065542511	-2.831016895	0.004640027	0.029719986
45.95403874	-0.021518828	0.191772892	-0.112209956	0.910656936	0.955037829
229.4238457	0.192168287	0.0890713	2.157465845	0.030969386	0.11645423
21.62057081	-0.144723134	0.263941768	-0.548314634	0.583475881	0.759227189
486.7457063	0.131330719	0.129509316	1.014063879	0.310552272	0.52547639
379.1075198	-0.190217822	0.07271721	-2.615856999	0.008900382	0.048423019
104.9123126	0.056236153	0.098814467	0.569108495	0.569282514	0.749908854
434.700917	-0.070192913	0.072033066	-0.974454059	0.329831139	0.545336384
318.0577076	0.607114641	0.104962382	5.784116478	7.28944E-09	4.78431E-07
476.2563498	-0.023950774	0.071471872	-0.335107692	0.73754385	0.861788314
8.079170558	-3.319003096	1.168844872	-2.839558248	0.004517605	0.029196947
354.5697867	0.181108774	0.108219499	1.6735318	0.094222652	0.246606725
267.4794693	-0.226923826	0.110794081	-2.048158394	0.040544482	0.140144732
376.5865672	0.093692756	0.074219901	1.262367037	0.206816745	0.410275001
532.2919529	0.108961667	0.08671098	1.256607484	0.208895803	0.412978455
226.7620747	-0.03068765	0.077946462	-0.393701635	0.693801334	0.835136868
301.6947191	0.260370236	0.138570984	1.878966501	0.060249067	0.184247844
55.71568372	0.407068307	0.270731025	1.503589428	0.132687115	0.307395237
161.1076377	0.117247847	0.112829652	1.039158104	0.298731212	0.513671882
5.477000261	-0.300051155	0.420759189	-0.713118484	0.475772443	0.675795415
4.629564599	-1.903212889	0.584937752	-3.253701583	0.001139119	0.010680616
1572.611336	-0.174111427	0.117657218	-1.479819337	0.138921467	0.317331149
196.9427506	-0.083150682	0.090441889	-0.919382408	0.357895588	0.572313329
31.49165454	-0.011510436	0.398240497	-0.028903228	0.976941771	0.988158577
691.7121312	-0.154213549	0.051716818	-2.981883909	0.002864806	0.021087943
12.81772155	0.000152577	0.431035308	0.000353978	0.999717566	0.999746807
161.1588267	0.280963128	0.192855385	1.456859129	0.14515527	0.326034588
4.859862293	0.960061553	0.477858292	2.009092591	0.044527315	0.149824958

17.10558971	0.208154626	0.317684532	0.655224305	0.512323348	0.70630244
25.60337395	0.401163929	0.753629333	0.532309335	0.594511768	0.766869097
38.13619413	0.033255037	0.161906465	0.2053966	0.837262277	0.918686178
66.76202131	-0.185890855	0.167638552	-1.108878916	0.26748242	0.479124214
131.9910865	-0.245517547	0.10808579	-2.271506249	0.023116349	0.094982596
53.87348339	0.208223969	0.142871954	1.457416684	0.145001395	0.325798278
9.395091572	0.799177001	0.824528083	0.969253828	0.33241856	0.548064788
433.1994662	-0.064538192	0.155937708	-0.413871619	0.67896813	0.826644075
176.4089366	-0.064337208	0.121214363	-0.530772155	0.595576678	0.767505587
2.565545123	1.243018468	1.298450378	0.957309181	0.338411223	0.553623285
180.3774746	-0.310985755	0.111394763	-2.791744844	0.005242468	0.032713793
272.3720037	0.118879401	0.061881416	1.921084043	0.054721113	0.171458081
293.1179806	0.600938531	0.214799079	2.797677406	0.005147149	0.032179938
506.587558	0.188074907	0.054636847	3.442272328	0.000576849	0.006264347
34.29946554	-0.04967363	0.210021538	-0.23651684	0.813031642	0.906097599
82.48457567	0.193096547	0.127038471	1.519984812	0.128514793	0.300603819
430.4771059	0.117853922	0.059814233	1.970332426	0.048800284	0.159447816
1050.820134	0.053186437	0.064556423	0.823875217	0.410010463	0.620217261
246.5837589	0.355057313	0.123744487	2.869277835	0.004114102	0.027317905
405.026664	-0.23859627	0.060684313	-3.931761923	8.43256E-05	0.001371352
12517.43583	0.063390849	0.079176937	0.800622648	0.423350135	0.631210556
14.13333002	0.464183088	0.351223684	1.321616705	0.186295818	0.384639341
472.9604141	0.033921363	0.05629466	0.602568036	0.54679609	0.733603614
28.3965017	-0.187863579	0.180179941	-1.04264425	0.297113087	0.511766627
474.639502	-0.068449508	0.083244907	-0.822266608	0.410925177	0.621005537
2.741790376	-0.00210723	0.584057754	-0.003607913	0.997121308	0.998121581
154.9774184	0.002385762	0.072660769	0.032834257	0.97380676	0.987169562
374.4952694	0.090311292	0.084909893	1.063613313	0.287503909	0.502056191
84.37135032	-0.373227572	0.191927479	-1.944628117	0.051819753	0.165804784
41.81916457	-0.978564741	0.505430428	-1.93610176	0.052855239	0.167913929
1261.367041	0.220984979	0.228506035	0.967085964	0.333501061	0.548794652
2.842647013	1.314489042	0.589884787	2.228382677	0.025855007	0.103061398
373.7831223	-0.098555205	0.087365423	-1.128080204	0.259286049	0.470200096
615.8104425	-0.047521042	0.076654615	-0.619937139	0.535299174	0.723897438
34.05351844	0.345406065	0.223046347	1.548584276	0.121481688	0.291308491
204.3828254	0.008330163	0.093120725	0.089455517	0.928719904	0.965637301
169.8459171	-0.187420733	0.096233172	-1.947568911	0.051466567	0.165081818
98.98041488	-0.026408514	0.156067217	-0.169212439	0.865629546	0.933396663
60.38983508	-0.26221446	0.176803633	-1.48308299	0.138052363	0.315986318
106.9222785	-0.301657596	0.127371455	-2.36832968	0.017868605	0.079216202
175.9156371	0.214568161	0.132848597	1.615133059	0.106281883	0.267537975
143.6225462	-0.135499184	0.221421403	-0.611951608	0.540569775	0.728096352
248.2954846	-0.109905265	0.073858445	-1.488052789	0.136736973	0.313907638
5.470077637	0.798881477	0.44218406	1.806671811	0.070813465	0.205456938
243.1412307	0.225294398	0.110967599	2.030271903	0.042328909	0.144643526
1709.692566	-0.003960506	0.071360176	-0.055500224	0.955739951	0.978293782
794.5592961	-0.120469391	0.097695414	-1.233112037	0.217533967	0.422273513
49.08754294	-0.091246196	0.136261303	-0.669641297	0.503086482	0.698806636

46.43950352	0.109229534	0.282088593	0.387217125	0.698595473	0.838453107
23.80015407	-2.746416177	0.874302665	-3.141264789	0.001682199	0.014199103
863.7950106	-0.142099696	0.073506283	-1.93316393	0.053216007	0.168657271
640.0266241	0.167400438	0.10805331	1.549239337	0.121324196	0.291158774
698.4019115	-0.026158634	0.095678417	-0.273401616	0.784544511	0.888287716
419.8710035	0.007666426	0.280510744	0.027330242	0.978196336	0.988638432
201.4162887	-0.241137509	0.193283859	-1.247582231	0.212184089	0.41657975
189.9883729	0.056180943	0.071958606	0.780739736	0.434955585	0.641721145
1878.143102	-0.194352366	0.118659308	-1.637902405	0.101442054	0.259464683
44.90218454	0.168347227	0.283258088	0.59432452	0.552295082	0.737796722
17.29620858	-3.189365808	0.981295128	-3.250159629	0.001153402	0.010777738
146.8021232	-0.481026813	0.089066632	-5.40075225	6.6362E-08	3.15968E-06
152.3442028	-0.320240687	0.134656027	-2.378212802	0.017396783	0.077963711
1155.678564	0.033590581	0.090978572	0.369214202	0.711968071	0.846212223
3146.120696	-0.01943787	0.128836551	-0.150872322	0.880076434	0.939892807
130.0940275	-0.141337177	0.158966819	-0.889098605	0.373950088	0.58760528
216.9871732	0.20696899	0.096580299	2.142973174	0.032115252	0.119078701
179.1572782	0.28173068	0.109755273	2.566898814	0.010261255	0.053388259
749.109778	0.228152601	0.068845154	3.313996514	0.000919727	0.009024166
145.8597313	-0.059471029	0.10887596	-0.546227366	0.584909657	0.760101103
321.8468627	0.277676513	0.176137507	1.576475774	0.114916197	0.281422542
1062.050736	0.245765067	0.144162221	1.704781353	0.088235211	0.23689486
11.57683565	-1.138012696	0.525130863	-2.167103052	0.030226997	0.114526116
1947.865081	0.354052157	0.052822003	6.702740128	2.04547E-11	2.61218E-09
196.5933369	-0.047426427	0.10423649	-0.454988715	0.649117357	0.805627137
19.14089149	0.216194029	0.283404555	0.762845993	0.445555248	0.650658377
22.73898208	0.266071915	0.350416763	0.759301331	0.447672322	0.652824097
3.794902592	1.688633669	0.656386122	2.572622443	0.010093125	0.052823573
19.45503358	0.938789803	0.56183698	1.670929179	0.094735667	0.247176969
156.6547289	0.346515552	0.131565553	2.633786314	0.008443861	0.046679251
107.581324	0.218292582	0.13524139	1.614095965	0.106506613	0.267753691
7.591782782	-0.891435287	0.49018448	-1.818571014	0.068976903	0.201726523
336.7986587	0.092370972	0.068388411	1.350681647	0.176797434	0.371974576
7.669887935	0.245150135	0.338923624	0.723319703	0.469483491	0.670562773
4.595053874	0.856746077	0.561112933	1.52686924	0.12679353	0.298101414
1.91337456	0.032889804	0.723210649	0.045477488	0.963726718	0.982015974
2.533244999	0.520338744	0.670223463	0.776366053	0.437532883	0.643391655
280.8305346	0.31083851	0.08258582	3.763824214	0.000167335	0.002372349
542.3035942	-0.2621138	0.072340993	-3.623309402	0.000290858	0.00365491
171.3368724	-0.006563623	0.098605615	-0.066564392	0.946928494	0.974828397
72.34848933	-0.163405538	0.111329839	-1.467760481	0.142169296	0.321679732
174.3719214	-0.55231472	0.155638296	-3.548707059	0.000387127	0.004574992
122.0848865	-0.163054486	0.182207771	-0.894882169	0.370850079	0.584428073
504.3430313	0.159520474	0.085988709	1.855132783	0.063577267	0.190941335
490.3998998	0.046431212	0.081308776	0.571047977	0.567967122	0.748947007
76.41912272	0.003231493	0.210095731	0.015381049	0.987728183	0.993639111
8032.830537	0.278096706	0.05354989	5.193226465	2.06681E-07	8.59812E-06
63.16470695	0.134374756	0.138732122	0.968591514	0.332749039	0.548337036

259.636732	0.042477279	0.111155263	0.382143664	0.702354806	0.840493334
1197.032799	0.121334021	0.070168275	1.729186314	0.083775755	0.229143952
1558.090404	-0.355117698	0.077157449	-4.602506971	4.17436E-06	0.000114423
337.3489903	0.052274641	0.070503786	0.741444461	0.458423995	0.661672965
11.30169865	-0.114119104	0.39181	-0.291261335	0.770851456	0.88038389
366.093474	-0.26902624	0.118186892	-2.276278158	0.022829367	0.094258392
19.70631735	-2.1608384	0.734766728	-2.94084955	0.003273135	0.023172162
82.1085966	-0.398992486	0.155223307	-2.570441863	0.010156887	0.05310808
22.70665851	0.094754964	0.234043743	0.404860061	0.685580377	0.829778815
2.452106424	-1.035949223	0.85646782	-1.209560008	0.226447772	0.432121
127.6759753	-0.0949019	0.086924746	-1.091770812	0.274933851	0.487698963
1375.094416	-0.247866451	0.085556573	-2.897105876	0.003766226	0.025543356
214.0554438	-0.274419343	0.143255025	-1.915600122	0.055416014	0.172931493
79.00228001	-0.151724553	0.164403305	-0.922880186	0.356069647	0.570612001
500.311084	-0.215630226	0.087928176	-2.452345027	0.01419285	0.067580035
460.8481789	0.226724645	0.055760368	4.066053577	4.78159E-05	0.000859956
595.9040464	0.118059474	0.062755711	1.88125465	0.059937287	0.183566994
6.375852846	1.103243957	0.373480844	2.953950587	0.003137342	0.02248133
48.66775938	-0.187962914	0.177989785	-1.056032026	0.290953584	0.506142485
241.150485	-0.167090891	0.073771411	-2.264981635	0.023513807	0.096096862
119.3156917	0.053766095	0.093858847	0.572839923	0.566753085	0.748471553
181.2072463	-0.242983349	0.099046731	-2.453219281	0.0141584	0.067509546
405.8948508	-0.103090419	0.137686977	-0.748730353	0.454019745	0.658292667
17.57023649	0.401098906	0.25929938	1.546856399	0.121897877	0.291793937
977.0714684	-0.322951609	0.064838112	-4.980891653	6.3292E-07	2.34836E-05
42.53307573	-0.037893604	0.169499112	-0.223562259	0.823097933	0.911237589
7.118619157	-1.657816438	0.66193045	-2.504517565	0.012261852	0.060900277
98.06832093	0.066163409	0.140452038	0.47107475	0.637587356	0.797540934
4110.906839	0.213145723	0.13542804	1.573866999	0.115518201	0.282309278
1876.408273	-0.002744125	0.102697962	-0.026720347	0.978682785	0.988826752
110.2993156	-1.77797666	0.261720356	-6.793421374	1.09505E-11	1.59645E-09
630.4209176	-0.192246637	0.076885585	-2.50042497	0.012404441	0.061421928
7621.686888	-0.070362795	0.138623111	-0.507583438	0.611745508	0.780039684
1052.960469	-0.266944197	0.064188979	-4.158723188	3.20031E-05	0.000629289
194.1327428	0.151307379	0.108403865	1.39577476	0.162782333	0.352018398
34.22725421	0.406041213	0.208042712	1.951720435	0.050971405	0.16422901
660.205821	0.218376253	0.060111595	3.632847444	0.000280311	0.003574672
8.761709977	1.636569668	0.553031483	2.959270344	0.003083684	0.022212776
3.043871022	0.379541702	0.941311688	0.40320513	0.686797326	0.830726992
9.782979341	0.819005717	0.647163788	1.265530816	0.205681109	0.409244882
425.1433932	0.035618889	0.119192005	0.298836228	0.765065007	0.877720432
373.8517475	-0.232602415	0.124515594	-1.868058508	0.061753916	0.187250877
789.4419499	-0.114056865	0.092094157	-1.238481005	0.215537761	0.420053765
403.1340414	-0.191691937	0.072521312	-2.643249699	0.008211445	0.045639457
213.9381135	-0.314159737	0.18563845	-1.692320405	0.090584899	0.240925998
578.642446	-0.159891021	0.06761445	-2.364746323	0.018042425	0.079814958
56.06822049	-0.031061567	0.23961271	-0.129632385	0.89685728	0.947955013
10.95897166	0.374485809	0.358732088	1.043915003	0.296524716	0.51124523

27.60623283	0.393752512	0.269719879	1.459857219	0.144329319	0.32480388
745.2186751	-0.176785556	0.085945307	-2.056954141	0.039690637	0.137912408
6.241352179	0.869925375	0.511072368	1.702156933	0.088725954	0.237824823
1133.380913	0.065948037	0.046146726	1.429094611	0.152977042	0.337409617
920.1400464	-0.027784343	0.084342272	-0.329423698	0.741835458	0.86477479
698.7731854	-0.195521994	0.06543748	-2.987920603	0.002808825	0.020800051
73.4589452	-0.199968107	0.128050841	-1.561630566	0.118375044	0.286528134
1224.54591	0.137629005	0.048454084	2.840400493	0.004505693	0.029142828
151.7034291	-0.228182155	0.148706823	-1.534443084	0.124920685	0.295727733
8.835488727	-0.399684023	0.451851865	-0.884546583	0.376401228	0.589554891
55.00896571	0.138081454	0.560666863	0.246280748	0.805464912	0.900830207
1003.293727	0.007909512	0.121033227	0.065349922	0.947895395	0.975143522
20.80309621	-0.037049591	0.314320959	-0.117871842	0.906169204	0.952959157
1.761281061	0.599325341	0.947136986	0.632775776	0.526880078	0.717162886
30.3877469	-0.840061813	0.187629418	-4.477239344	7.56145E-06	0.000189312
11.26253595	-3.26476146	1.274619862	-2.561360887	0.010426299	0.054047466
1231.545993	-0.825472953	0.273797328	-3.01490507	0.002570597	0.019492114
8.425978807	-1.809705356	0.615956304	-2.93804178	0.003302925	0.023312934
95.15885396	-0.663839531	0.495815849	-1.338883241	0.180608686	0.376721638
5.606967842	-0.633246088	0.624887746	-1.013375749	0.310880718	0.525870105
179.2414524	-0.057853991	0.148190204	-0.390403615	0.696238115	0.836908188
7.317812678	0.72327353	0.375415913	1.92659263	0.054030418	0.170131231
3056.41408	0.033429967	0.064819051	0.515742935	0.606033963	0.775923177
114.8978873	-0.053785901	0.11286908	-0.476533529	0.633694327	0.794722925
519.542075	-0.221890495	0.097388208	-2.278412331	0.022702022	0.093944516
82.04869806	-0.047712651	0.116759536	-0.408640291	0.682803658	0.828558299
9.471304196	-0.410274616	0.338323805	-1.212668486	0.225256593	0.430596091
39.71594168	0.243454518	0.344150413	0.707407311	0.479313395	0.678368459
111.5687225	-0.105662694	0.217007588	-0.486907832	0.626323649	0.790677612
184.6624013	-0.227319987	0.102082619	-2.226823612	0.025959065	0.103346938
1181.35426	-0.174341101	0.101720929	-1.713915732	0.086544203	0.23387639
3.580853409	0.982531227	0.831994884	1.180934216	0.237628857	0.445170729
113.7220327	0.835504219	0.486184004	1.718493847	0.085706576	0.232502954
133.3463761	-0.003934393	0.158059154	-0.0248919	0.980141188	0.98975395
225.8501947	0.123039554	0.064992662	1.893129938	0.058340592	0.17994812
209.864432	0.060266065	0.073406036	0.820996032	0.411648529	0.621642484
530.0075806	-0.070569698	0.059472091	-1.186601924	0.235384677	0.442703175
34.40127577	0.030393746	0.294435795	0.103227076	0.917782751	0.959308302
4.97663429	2.847932787	1.322707904	2.153107862	0.031310203	0.117042041
31.86697631	0.054930474	0.379116567	0.144890724	0.88479715	0.942468039
294.7602547	-0.085032195	0.116409832	-0.73045544	0.465111841	0.667211117
3567.629656	-0.079250074	0.093008404	-0.852074331	0.394172838	0.607049017
451.6885951	-0.041531659	0.091945518	-0.451698568	0.65148615	0.807082481
114.9187497	1.041124558	0.133509679	7.798120459	6.2836E-15	1.78476E-12
108.4659436	0.759594369	0.110968957	6.845106857	7.64188E-12	1.13417E-09
8.904204486	0.938582131	0.669602126	1.401701242	0.16100448	0.349412172
398.0767954	1.181390969	0.134005038	8.816019084	1.18601E-18	7.23643E-16
19.76200747	-0.807703453	0.529819819	-1.52448705	0.127387089	0.298780921

150.3251396	0.010107712	0.091718893	0.110203167	0.912248251	0.956125314
337.6805139	0.352131936	0.113399216	3.105241368	0.001901238	0.015607133
10.06859198	0.640720884	0.369193797	1.735459505	0.082659448	0.227322819
227.9422303	0.207265513	0.076002208	2.727098558	0.006389395	0.037822097
104.7757854	0.155291234	0.119162814	1.303185354	0.192511489	0.393136763
555.3024786	-0.217899546	0.110605184	-1.970066297	0.048830773	0.159495048
69.69505881	0.075485372	0.139101662	0.542663335	0.587361623	0.761715809
166.6373189	0.168270269	0.081917034	2.054154797	0.039960712	0.138679749
14.18749883	-0.189162601	0.298723123	-0.633237223	0.526578741	0.717000092
42.37375481	-0.54276027	0.351530846	-1.543990456	0.122590645	0.292501519
125.5665619	0.298087129	0.107285969	2.778435347	0.005462138	0.03368898
2185.810273	-0.080648825	0.034646277	-2.327777491	0.019923922	0.085728404
22.95122584	0.218370669	0.219120058	0.996580008	0.318968414	0.534029791
150.6412114	0.048737224	0.090555448	0.538203109	0.590436834	0.763669342
4766.910493	0.067182918	0.107406139	0.625503518	0.53164064	0.72102148
47.19726505	-0.232635845	0.390703867	-0.595427547	0.551557716	0.737409659
3.754360508	0.283473807	0.560404566	0.50583779	0.612970527	0.780915252
1262.083717	0.721869492	0.126232427	5.718574117	1.07422E-08	6.57868E-07
3.450920479	0.18124619	0.803809211	0.225484091	0.8216027	0.910533662
122.9103565	0.197099188	0.126354252	1.559893589	0.11878503	0.287225096
15.43954649	0.125439613	0.481217807	0.260671178	0.794346095	0.893917451
87.19928663	-0.146300176	0.15494118	-0.944230426	0.34505191	0.559431667
460.4255929	0.102314887	0.072881621	1.403850326	0.160363424	0.348434061
1269.248699	-0.02243876	0.063670717	-0.352418844	0.724524172	0.853884493
1.888311415	1.171004944	0.747275392	1.567032658	0.117107058	0.284840053
242.3670686	0.00119958	0.125492311	0.00955899	0.992373146	0.995850406
22.16062524	0.471195703	0.238904259	1.972320229	0.048573062	0.158957612
110.3276761	-0.425843861	0.199673931	-2.13269634	0.032949644	0.121244682
2.201498193	-2.005582725	1.292127579	-1.552155343	0.120625058	0.290099801
42.32374981	-0.655518931	0.203686785	-3.218269313	0.001289667	0.011699322
164.9801848	-0.62402675	0.084006973	-7.428273212	1.10025E-13	2.62688E-11
3.229621672	-1.345532296	0.767283109	-1.753632108	0.079493566	0.221212331
27.74500891	-0.964806493	0.388565666	-2.482994712	0.013028302	0.063574719
3.120985229	-5.109747774	2.981453313	-1.713844638	0.086557263	0.23387639
1464.683206	-0.787059847	0.140219694	-5.613047815	1.98794E-08	1.11014E-06
331.3243847	0.010480544	0.075991227	0.137917818	0.890305368	0.945214322
50.54772402	-0.119701556	0.194689812	-0.614832153	0.538665573	0.726541399
41.97969715	-0.188355501	0.162594531	-1.158436875	0.246685798	0.455288129
22.25375876	-0.200309866	0.257806703	-0.776976951	0.437172369	0.643263452
108.431014	-0.224279907	0.127793232	-1.755021792	0.07925558	0.220668585
489.0510731	-0.324392974	0.073580599	-4.408675331	1.04005E-05	0.000246432
22.59200918	-0.274732304	0.245956979	-1.116993326	0.263997179	0.475417405
32.92800206	0.129863165	0.199930426	0.649541783	0.515988248	0.708985891
395.3331211	-0.0078229	0.06346663	-0.123260044	0.90190118	0.950958758
112.1032362	0.2038046	0.107921496	1.888452326	0.058965252	0.181297791
50.41804503	0.160566379	0.161081354	0.996803016	0.318860134	0.533994461
112.3734704	-0.447586517	0.095010508	-4.710915955	2.46606E-06	7.37529E-05
830.9419211	-0.144835751	0.080260677	-1.804566784	0.0711425	0.206084322

40.51684888	0.063221398	0.20741559	0.304805431	0.760514345	0.875276884
3.609918134	-0.0542088	0.601945026	-0.090056065	0.928242663	0.965637301
2.121656463	0.223161997	0.678001163	0.329146924	0.742044638	0.864835046
21.39687813	0.886700053	0.230602407	3.84514656	0.00012048	0.001804358
378.1784176	-0.536005179	0.116938984	-4.583631246	4.5697E-06	0.000123982
194.8243947	-0.099043993	0.088683399	-1.116826758	0.264068406	0.475490536
9.688014578	0.333057606	0.309102833	1.077497748	0.281257956	0.495237663
346.9203688	-0.200669471	0.072315319	-2.774923394	0.005521471	0.033983146
14.66506937	-0.178865157	0.286889204	-0.623464231	0.532979501	0.722242683
547.194724	0.259840054	0.068353029	3.801441705	0.000143857	0.002084339
587.4892205	0.101017279	0.055147954	1.831750257	0.066988637	0.197843458
15.53052196	1.240458114	0.720862641	1.720796784	0.085287706	0.231776587
72.98482445	0.212247564	0.125940633	1.685298536	0.091930988	0.242781516
48.74416167	-0.163001848	0.138509323	-1.176829433	0.239263592	0.446702698
12.35131631	-0.394366249	0.271831375	-1.450775319	0.146842434	0.328456519
103.754004	0.022603041	0.107654102	0.209959866	0.833698997	0.916902912
832.8208607	-0.067229787	0.061018577	-1.101792111	0.270552061	0.482423469
3.033584097	0.154698508	0.586850005	0.263608258	0.792081794	0.892771995
22.56310438	-0.185480211	0.27667747	-0.670384224	0.502612889	0.698266548
286.4332391	-0.035112377	0.085701553	-0.409705254	0.682022175	0.82820821
97.12444721	-0.062011851	0.129771215	-0.477855207	0.632753264	0.794224511
661.9494504	0.025846953	0.063784431	0.405223546	0.685313199	0.829778815
2.566235079	0.63213065	0.687393531	0.919605178	0.35777912	0.572182625
309.0763878	0.210010719	0.069776572	3.009759777	0.002614544	0.019709144
7.498450718	-0.234467285	0.413504595	-0.567024617	0.570697457	0.750883819
140.3554924	0.166013121	0.090535919	1.833671357	0.066702793	0.197307917
13.414318	-2.045063832	0.576932263	-3.544720868	0.000393029	0.004631445
355.2265894	-0.343269115	0.144529011	-2.37508797	0.017544768	0.078371071
8.121618515	-0.529655676	0.63887968	-0.829038225	0.407082778	0.617901456
171.2695623	-0.282935023	0.089061799	-3.176839295	0.001488895	0.013074658
120.5548488	-0.299557875	0.130394673	-2.297316816	0.021600703	0.090824396
879.9848136	0.01182376	0.067806261	0.174375633	0.861570263	0.931286863
16.4168777	-0.457008002	0.381188504	-1.198902899	0.230565704	0.436936024
155.9410356	-0.025292856	0.152651957	-0.165689691	0.868401159	0.934546688
207.0267399	-0.009127627	0.086498148	-0.105523956	0.915960062	0.958430263
98.09359183	-0.024688113	0.134922913	-0.182979392	0.854814189	0.928844334
528.7608409	-0.142322142	0.083821615	-1.697916961	0.089523436	0.239029024
98.54488802	-0.879725128	0.217945353	-4.036448207	5.42665E-05	0.000961276
2.414741472	-0.941853216	0.709326315	-1.327813725	0.184239652	0.381637624
43.1213421	0.013310711	0.181961915	0.073151083	0.941685892	0.972257044
4.731311071	-0.625245591	0.909622021	-0.687368574	0.491850496	0.689477967
19.00984272	-0.420852171	0.382059095	-1.101536847	0.270663077	0.482543141
257.0446702	-0.064311925	0.082009551	-0.78420042	0.432922519	0.63992513
106.7884289	0.4147973	0.159628121	2.59852273	0.009362585	0.050220357
296.2857296	-0.015722125	0.090696222	-0.17334928	0.86237689	0.931532154
294.2075499	-0.146168344	0.069598468	-2.100166109	0.035714232	0.128238793
352.5867503	0.088033619	0.103530788	0.850313424	0.395150855	0.608043638
743.904633	-0.029395945	0.063805928	-0.460708678	0.645007628	0.803015198

551.0049924	-0.150866495	0.096092771	-1.570008786	0.116413067	0.283696578
270.4466051	-0.061710466	0.070594386	-0.874155439	0.38203361	0.594860273
138.1012684	-0.152539145	0.108284048	-1.408694518	0.158925517	0.346177305
274.9057657	0.075209127	0.081404165	0.923897776	0.355539541	0.57021831
1206.916705	-0.154970292	0.103907974	-1.491418665	0.135851611	0.312398022
1147.305036	0.185721984	0.054176233	3.428108092	0.000607803	0.006506142
232.978816	0.107584827	0.140817585	0.764001358	0.444866433	0.650113512
230.9208204	-0.034285095	0.086093157	-0.398232515	0.6904588	0.833061248
291.4905484	-0.085147531	0.116318964	-0.732017624	0.464157811	0.666422849
931.0666749	-0.112725689	0.076162003	-1.480077792	0.138852488	0.317261564
2.568550064	-1.227514747	0.841480501	-1.458756021	0.144632271	0.325323871
1526.016998	-0.02909655	0.094798958	-0.306929008	0.758897409	0.874200334
1.784656654	-1.127901658	0.723784344	-1.558339398	0.119152814	0.28781869
11.2304359	-0.034858514	0.329137579	-0.105908641	0.915654838	0.958322206
347.8576273	0.112504367	0.073045654	1.540192488	0.12351344	0.293792408
92.47542597	-0.155734968	0.12911721	-1.206151904	0.227758925	0.433500443
707.1407158	-1.746733849	0.60891696	-2.868591226	0.004123043	0.027355218
176.6699648	-0.032490546	0.090082932	-0.360673717	0.718343375	0.850529195
25.47255851	0.067224232	0.213503102	0.314863021	0.752865659	0.871037915
485.9392746	-0.115630531	0.053326209	-2.168362105	0.030131147	0.114241774
93.79099886	-0.003631557	0.111623566	-0.032533966	0.974046229	0.987230071
573.4890721	0.196628562	0.065314005	3.010511468	0.002608081	0.019693319
963.4193958	-0.068216751	0.052494479	-1.299503353	0.193771243	0.394586588
7.677217916	-0.015405626	0.442142803	-0.0348431	0.972204853	0.986031074
196.1460357	-0.240052421	0.094487965	-2.54056082	0.011067484	0.056447596
2.992180475	-0.664634835	0.842858868	-0.78854819	0.430376122	0.637419423
4.345176336	-3.190947748	1.097168485	-2.90834798	0.003633438	0.024979114
163.2684241	-0.515119913	0.141942988	-3.629062073	0.000284453	0.003602977
87.56517629	-0.056102723	0.142656566	-0.39327123	0.694119165	0.835387176
5.205476566	-2.120272086	0.770291923	-2.752556559	0.005913193	0.035643594
451.86016	0.36035805	0.094343867	3.819623493	0.000133656	0.001960678
1944.126866	0.456397959	0.110058884	4.14685249	3.37077E-05	0.000654836
64.1670116	0.178179619	0.176206197	1.011199504	0.311920953	0.52665752
522.1779605	0.035772888	0.073516164	0.486598953	0.626542566	0.790677612
3.090669596	1.287995577	0.654461537	1.968023336	0.049065353	0.160028236
794.8203115	-0.210305306	0.053192418	-3.953670759	7.69613E-05	0.001274232
144.6844948	0.016871407	0.108106053	0.156063479	0.875982985	0.937672708
1151.977256	-0.200283303	0.073259447	-2.733890471	0.006259086	0.037260851
333.1388671	0.028704335	0.059047772	0.486120539	0.626881705	0.790677612
344.8561606	-0.067877795	0.067053989	-1.012285712	0.311401464	0.526264641
1389.465163	-0.099780898	0.063805114	-1.563838575	0.117855483	0.285660577
48.29752985	0.139376211	0.180471365	0.772289893	0.43994275	0.645782488
10.06097166	-0.260701142	0.39964637	-0.652329565	0.514188592	0.707591786
2066.161865	-0.1222358	0.081139935	-1.506481367	0.131943647	0.306319002
175.8501615	-0.041250933	0.110883407	-0.372020796	0.709877366	0.845284623
912.9813999	-0.056365548	0.046229919	-1.219243934	0.222751622	0.427806473
160.530442	-0.194413329	0.174780736	-1.112326987	0.265997585	0.477695021
26.76881409	-0.331767397	0.268313195	-1.236493036	0.216275353	0.42094764

14.67162428	0.25128044	0.555045459	0.452720469	0.650750038	0.806473793
261.9268548	0.045390367	0.084693357	0.535937752	0.592001572	0.764669523
1118.245223	0.246967058	0.055910096	4.417217606	9.99796E-06	0.000238359
496.0118854	-0.356038816	0.084351414	-4.220899214	2.4333E-05	0.000503595
126.3916596	-0.114678809	0.10361184	-1.106811821	0.268375296	0.480357956
310.1554315	0.385710213	0.095911502	4.021521963	5.78233E-05	0.001008023
148.0330663	-0.24998256	0.085864454	-2.911362608	0.003598561	0.02484606
34.20030362	0.089606428	0.196798224	0.455321325	0.648878086	0.805486971
27.28025967	0.030486696	0.191015741	0.159603058	0.87319377	0.936680519
565.5662303	-0.003058141	0.086609527	-0.035309527	0.971832926	0.985957361
87.91437846	0.108086207	0.109641377	0.985815847	0.324223488	0.539548668
1086.746594	-0.003020988	0.054496864	-0.055434158	0.955792583	0.978293782
191.4870808	-0.48520451	0.105907598	-4.5813947	4.61885E-06	0.000124944
1.869471674	-1.212758511	1.017854333	-1.191485335	0.23346311	0.440305813
124.7156051	0.167129337	0.116744745	1.431579105	0.152264313	0.336472474
7.009376323	-0.72675325	0.706904345	-1.028078628	0.303912843	0.518644466
193.5628838	-0.427402623	0.158032207	-2.704528599	0.006840138	0.039803591
49.23738293	-0.77608466	0.26278193	-2.953341046	0.003143545	0.02251598
6.787464453	0.044220632	0.775819295	0.056998624	0.954546291	0.97793505
147.4582088	-0.171544378	0.12521008	-1.370052457	0.170670527	0.362830456
2725.508425	-0.105728077	0.135411043	-0.780793609	0.434923894	0.641721145
214.0599281	0.305159626	0.113027909	2.699860848	0.006936848	0.040108248
172.1571391	-0.258467114	0.108913086	-2.373150221	0.017637089	0.078591671
8.944661871	-1.016414355	0.450062725	-2.258383775	0.023921743	0.097473856
674.8673755	-0.069492853	0.073369666	-0.947160558	0.34355697	0.5578864
2.024570544	-0.082867706	0.612353876	-0.135326498	0.892353735	0.945865103
458.5986993	-0.157305465	0.101031917	-1.556987832	0.119473374	0.288381593
6.92058467	-0.574466669	0.508423514	-1.129897915	0.258519243	0.469139238
125.847316	-0.124134396	0.093446178	-1.328405278	0.184044257	0.381472709
333.4192859	0.15566595	0.18456007	0.843443273	0.398980579	0.611199292
72.42501357	0.075284635	0.387801684	0.19413179	0.846072691	0.923793843
14.19611709	-0.380618977	0.37752646	-1.008191526	0.313362525	0.528170068
36.115459	0.447930537	0.16291046	2.749550494	0.005967707	0.035880292
818.0243813	0.239029736	0.056386454	4.239134013	2.24384E-05	0.000469099
824.1019981	-0.258645634	0.06026042	-4.292131285	1.76966E-05	0.000385627
1096.340138	-0.121585164	0.155679667	-0.780995784	0.434804974	0.641613861
83.50677721	-0.574841218	0.15896815	-3.616077915	0.0002991	0.003726517
784.4478882	0.056496118	0.050306628	1.123035277	0.261422512	0.472728727
346.4340635	0.195067603	0.057277791	3.40564115	0.000660089	0.006957328
3.141879114	0.370296186	0.676256708	0.547567487	0.583988919	0.759563671
122.8318945	0.02123474	0.128423827	0.165348914	0.868669361	0.934607931
643.1045703	-0.163392277	0.206039438	-0.79301457	0.427769334	0.634941316
613.4788067	0.237633229	0.132663481	1.791248264	0.073253464	0.210166766
63.73849206	-0.240614118	0.138056493	-1.742867085	0.081356835	0.224802498
134.0546011	0.116659231	0.319677265	0.364928143	0.715165046	0.84869824
50.63368149	0.402636546	0.166883163	2.412685254	0.015835488	0.072971699
636.1009622	-0.098749256	0.07759213	-1.27267103	0.203134802	0.405960388
389.6742514	-0.197500389	0.107010086	-1.845624061	0.06494681	0.193799508

428.6507644	-0.048382182	0.071453699	-0.677112345	0.498334677	0.694742406
309.0111994	-0.030609168	0.12940672	-0.236534606	0.813017857	0.906097599
54.50816135	0.022761683	0.256011903	0.08890869	0.929154477	0.965673513
862.3947612	0.154789511	0.104896513	1.475640202	0.140040507	0.318826328
3.951643981	0.05973837	0.458650096	0.130248245	0.896370026	0.947804865
8.897911236	-0.527300747	0.528992036	-0.996802808	0.318860235	0.533994461
282.4677437	0.086037259	0.081100903	1.06086684	0.288750421	0.503479513
5773.322168	-0.28859392	0.084124067	-3.430575002	0.000602303	0.006465726
80.05478531	0.459282149	0.383919342	1.196298543	0.231580077	0.438209303
2.861964949	0.829092697	0.970605474	0.854201546	0.392993327	0.606309427
87.47673711	0.07165739	0.144131611	0.497166371	0.619071734	0.784935848
16.10489622	-0.354831668	0.356649403	-0.994903299	0.319783298	0.534896812
2.783180958	0.05327893	0.580331501	0.091807751	0.926850785	0.964983874
1533.557134	-0.029968679	0.106431687	-0.281576661	0.778268138	0.884647023
957.5981012	-0.045018848	0.083927764	-0.536399943	0.591682171	0.764478044
814.7369982	0.24382009	0.049787536	4.897211404	9.72062E-07	3.36423E-05
3.32008605	0.22352619	0.614863114	0.363538136	0.71620293	0.849440394
100.1964554	0.378240608	0.125973082	3.002551039	0.002677271	0.020093134
83.44277242	-0.197620326	0.293090519	-0.674263796	0.500143617	0.696019954
87.83074709	-0.366323722	0.159972159	-2.289921719	0.022025855	0.091931577
193.1897308	0.166857478	0.152683018	1.092835868	0.27446587	0.487131087
4.6379963	-0.94981954	0.493997266	-1.922722259	0.05451494	0.170932455
643.8287828	0.239250422	0.057319436	4.173984202	2.99319E-05	0.0005947
3.474234716	0.626118765	0.647565359	0.966881191	0.333603431	0.548809958
99.48670532	-0.149540821	0.264055508	-0.566323429	0.571173937	0.751109865
65.02511083	-0.067424474	0.156452246	-0.430958815	0.666498317	0.818089403
13.65292814	0.155785801	0.313800572	0.496448428	0.619578064	0.785174138
3.065590798	0.485600663	0.804135551	0.603879113	0.545924016	0.732731974
111.0155831	0.202765646	0.211538451	0.958528556	0.337796299	0.553111642
444.1209576	0.363749494	0.080688684	4.508060812	6.54228E-06	0.000167097
90.2443008	0.220934272	0.116913906	1.889717649	0.058795732	0.180948038
922.0163532	0.046405053	0.200166709	0.231832024	0.816668483	0.907813535
23.00200699	-0.868428582	0.514758601	-1.687059878	0.091591839	0.242313145
175.5988971	-0.066114271	0.081990759	-0.806362475	0.420033877	0.628801209
30.84238579	-0.136051225	0.282042519	-0.48237842	0.629537148	0.792404873
260.2692377	-0.388031623	0.071835915	-5.401638191	6.6035E-08	3.15322E-06
9.128460735	1.435645249	0.360488783	3.982496318	6.81952E-05	0.001164194
475.8149143	0.257898359	0.087904868	2.933834776	0.003348024	0.023551034
236.7265778	-0.1279428	0.066499714	-1.923960142	0.05435958	0.170672711
282.7662745	-0.077108425	0.104791053	-0.735830235	0.461834018	0.664302246
1944.044191	0.214414053	0.057658313	3.718701462	0.00020025	0.002726373
96.06196363	-0.08278326	0.143286107	-0.577747991	0.563434273	0.74593596
1149.963866	0.156101623	0.073084772	2.135898083	0.032687722	0.120726961
3334.236827	-0.056958093	0.065006764	-0.876187187	0.380928281	0.593756505
8.751683867	0.842997544	0.320566847	2.629709064	0.008545797	0.047100521
46.85071332	0.496761022	0.197493373	2.515330085	0.011892099	0.059492994
308.304792	-0.1512727	0.138804751	-1.089823652	0.275790833	0.488517545
665.258488	-0.184847714	0.050350518	-3.671217717	0.000241398	0.003168752

1.954392943	-0.244755036	0.874104084	-0.280006741	0.779472333	0.885082046
113.0235327	0.000286775	0.099486126	0.002882565	0.997700049	0.998427324
37.32890902	0.217107274	0.15439459	1.406184465	0.159669356	0.347190237
6.618579314	1.210142648	0.456591783	2.650382008	0.008040081	0.044853467
266.9124911	-0.040014983	0.07283234	-0.549412299	0.582722535	0.75889593
175.1388493	0.249810218	0.081639725	3.059910075	0.002214035	0.01744646
32.19003294	-0.0593577	0.299637159	-0.198098594	0.842967925	0.921896347
6.19400561	0.230579595	0.428578595	0.538010059	0.590570104	0.763781747
1993.394584	0.194130155	0.04530968	4.284518303	1.83136E-05	0.000395928
647.0034813	-0.013289242	0.051663548	-0.257226663	0.797003802	0.895203555
705.9443692	-0.106463572	0.079778276	-1.334493274	0.18204225	0.378662408
51.47101146	0.139712372	0.175611823	0.795574977	0.426279116	0.633668149
49.18105739	0.253818736	0.128085923	1.981628668	0.047520816	0.156667869
360.6705847	-1.540075067	0.164213643	-9.378484259	6.69279E-21	6.48571E-18
235.5220678	0.254322993	0.107652188	2.36245076	0.018154553	0.080059698
21.69542101	0.244789643	0.216196837	1.132253585	0.257527838	0.467957282
283739.1703	-0.159336376	0.111341995	-1.431053725	0.152414818	0.336624442
555.797081	-0.040351831	0.080885188	-0.498877875	0.617865421	0.784003308
580.2231555	-0.078586182	0.080627407	-0.974683238	0.32971741	0.545303144
210.3438534	-0.042218484	0.074913967	-0.563559585	0.573053898	0.752170338
191.7214728	-0.201163053	0.100542986	-2.000766661	0.045417542	0.152044012
1420.777553	-0.223824537	0.06460897	-3.464295073	0.000531623	0.005862087
6.051565598	0.305975231	0.459367872	0.666078866	0.505360692	0.700428834
359.3389282	-0.045010763	0.07056301	-0.637880428	0.523551519	0.714876729
136.5222161	0.184555319	0.172184783	1.071844539	0.283789864	0.497886499
225.9071486	-0.135190495	0.145188148	-0.931140026	0.351781136	0.566893044
262.1420768	-0.011780676	0.074239612	-0.158684511	0.873917442	0.936825644
31.89830261	0.192146125	0.1982634	0.969145718	0.33247249	0.54809885
10.33936493	0.189066518	0.338962724	0.557779675	0.576994843	0.755177012
83.72996701	-0.523297324	0.631823299	-0.828233661	0.407538184	0.61832678
291.2621792	-0.4619636	0.087389031	-5.286288168	1.24823E-07	5.45448E-06
2180.976205	-0.120579134	0.061503102	-1.960537431	0.049933007	0.162087954
420.6173608	-0.059965733	0.071180815	-0.842442347	0.399540398	0.611768438
136.9024119	0.290245229	0.084924558	3.417683119	0.000631566	0.0067212
4035.410595	0.160864173	0.076916998	2.091399517	0.036492265	0.130321609
1097.362384	-0.128759219	0.076573785	-1.681505217	0.092664824	0.244132466
29.18697604	0.486780298	0.179115807	2.71768476	0.006574044	0.038651249
267.7874091	0.305988523	0.090451762	3.382891773	0.000717269	0.007421228
80.22411544	0.112930781	0.1385673	0.814988677	0.415078791	0.624875081
91.11312449	-0.109374022	0.118432213	-0.923515819	0.355738461	0.57032659
19.61838627	0.595596748	0.287631774	2.070691771	0.03838761	0.135041102
16.28116416	-1.034920574	0.39997633	-2.58745455	0.009668795	0.051464856
11.86837088	-0.212652433	0.58494878	-0.363540263	0.716201342	0.849440394
6.408507266	-1.243705336	0.589289266	-2.110517546	0.034813802	0.125882916
112.9583754	-0.322456042	0.102020444	-3.160700226	0.001573904	0.013610757
346.1626434	0.006288338	0.08828093	0.071230991	0.943213917	0.972629837
1173.311116	0.061783631	0.052623059	1.174079066	0.240363358	0.4477831
647.2533535	-0.028826391	0.089353175	-0.322611827	0.746989236	0.867329872

103.2386519	-0.160234814	0.132330031	-1.21087264	0.22594422	0.431509979
786.5339022	0.110381703	0.052170978	2.115768328	0.034364516	0.124675463
1152.835491	0.151843454	0.092460168	1.642258036	0.10053654	0.257499838
2846.394651	-0.356109043	0.049880082	-7.139303504	9.3805E-13	1.82827E-10
3.734424711	0.102734876	0.551823573	0.186173409	0.852308776	0.927038608
152.0956819	0.022406796	0.160187575	0.139878488	0.888755998	0.944444948
226.8371226	-0.103026291	0.118563785	-0.86895245	0.384873139	0.598035257
424.0994077	-0.06329412	0.111588761	-0.567208739	0.570572371	0.750827482
230.8956657	0.197260911	0.15226292	1.29552823	0.195138069	0.396094696
221.2927716	-0.334658943	0.122485384	-2.732235721	0.006290612	0.037344698
76.20570072	-0.273750702	0.168553991	-1.624112841	0.104351728	0.264231535
419.3905756	-0.056423688	0.08905661	-0.633571022	0.526360817	0.71698926
5.853943033	0.360411944	0.4574433	0.787883315	0.430764961	0.637594068
19.01112169	-0.734150702	0.383659309	-1.913548519	0.055677868	0.173390776
19.52399005	-0.404424516	0.306405244	-1.319900765	0.186868152	0.385482837
103.3395414	-1.751627983	0.268016063	-6.5355336	6.33831E-11	7.10322E-09
52.23491609	-0.054961763	0.193301905	-0.2843312	0.776156578	0.883652841
10.01445717	0.837467181	0.312480318	2.680063779	0.007360813	0.041942345
216.3908663	0.018755446	0.091143674	0.205778917	0.83696361	0.918535642
571.2636013	0.012633878	0.097965745	0.128962196	0.897387563	0.948210116
17.46497195	-3.107881973	1.410616469	-2.203208343	0.027580068	0.107590346
12.9442951	-0.915378118	0.930414502	-0.983839048	0.325194657	0.540225672
17.54269857	-0.57183934	0.356862303	-1.60240893	0.109065211	0.272233378
17.69472374	-2.744688891	0.99575297	-2.756395385	0.00584423	0.035379096
4.217183347	-0.240125294	0.663981966	-0.361644301	0.717617855	0.850267635
440.8508499	0.086133325	0.124249596	0.693228209	0.488166348	0.686299063
380.4105655	-0.145400935	0.090844991	-1.600538815	0.109479103	0.272976956
132.7090981	0.189175399	0.214239662	0.883008295	0.37723179	0.590154809
949.8587996	-0.081527991	0.085964607	-0.94839021	0.342930841	0.557144249
7.527103219	0.497811651	0.359786611	1.383630287	0.16647169	0.357369639
39.36090842	-0.082922971	0.169722048	-0.488581018	0.625138354	0.789927917
11.0960874	-0.181239752	0.512888762	-0.353370487	0.723810708	0.853425251
1.754443485	1.371121082	0.885212529	1.548917393	0.121401579	0.291158774
170.7148503	0.044630546	0.099084209	0.450430467	0.652400081	0.807789472
3.605471459	-0.066720363	0.563058407	-0.118496344	0.905674392	0.952933508
77.37403924	-0.330837578	0.134093889	-2.467208455	0.013617106	0.06565063
179.077529	0.340470946	0.100366445	3.392278607	0.000693139	0.007245416
33.05048178	0.484075789	0.189265458	2.557655238	0.010538051	0.054421271
155.6180255	0.308166165	0.143633175	2.14550827	0.031912231	0.118619606
323.1253189	0.063945799	0.071088116	0.899528674	0.36837113	0.582118393
127.2548973	-0.040173091	0.111387411	-0.360660959	0.718352913	0.850529195
146.7890967	0.165047283	0.102237743	1.614347876	0.106451991	0.267657219
201.9358284	-0.147969492	0.135630962	-1.090971341	0.275285493	0.488011752
137.7134246	-0.167883067	0.149670985	-1.121680779	0.261998192	0.473242344
2.352142197	0.03428519	0.690581635	0.049646832	0.960403826	0.980642905
462.2579268	-0.13895257	0.082358481	-1.687167702	0.09157111	0.242297216
9471.07657	-0.051302613	0.075248187	-0.681778718	0.495378883	0.692185897
10.52321194	-0.019191403	0.452781389	-0.042385582	0.966191322	0.983261418

79.02404537	-0.312354923	0.122663037	-2.546447008	0.010882573	0.055711471
156.559305	0.10333175	0.081020222	1.275382205	0.202173994	0.404987157
2.723382603	4.082206237	2.085260322	1.957648258	0.050271301	0.162691533
56.67201508	-0.505583226	0.235348049	-2.148236322	0.031694987	0.118131948
4.265669086	-0.759628143	0.671374111	-1.131452837	0.257864543	0.468260636
2.871648975	-0.102018195	0.607259831	-0.167997601	0.866585166	0.933873489
160.2098818	0.077558511	0.082746988	0.937297081	0.348605764	0.563198132
195.4130947	-0.512104767	0.185143673	-2.76598578	0.005675101	0.034690765
103.9790772	0.205844317	0.122881316	1.675147404	0.093905314	0.246091419
266.3713519	0.029012131	0.078683896	0.368717517	0.712338291	0.846367358
28.71523411	1.551767915	0.206832082	7.502549417	6.25884E-14	1.58628E-11
89.04559353	0.139747581	0.131594491	1.061956163	0.288255586	0.502936087
6.142847973	0.70050867	0.377634295	1.854992198	0.063597341	0.190942699
43.17247635	0.161793134	0.188532045	0.858173124	0.390796874	0.604048588
165.6473936	-1.679956983	0.489177488	-3.434248353	0.0005942	0.006410506
441.1263427	-0.268245985	0.092146199	-2.911091146	0.003601689	0.02484683
78.99499987	-0.144882868	0.132476047	-1.093653313	0.274107058	0.486764321
23.36156588	-0.376402029	0.247875338	-1.518513429	0.128885017	0.301341437
339.7337977	-0.027847689	0.104126966	-0.267439738	0.789130607	0.891087275
4.51243263	-0.164611018	0.421064855	-0.390939819	0.69584172	0.836819174
74.04787	-0.107919454	0.155933393	-0.692086869	0.488882778	0.686837359
320.7293293	-0.011564975	0.0794096	-0.145636984	0.88420797	0.942262895
143.3074691	-0.163798215	0.096564842	-1.696251059	0.089838345	0.239547618
499.1508231	-0.036308246	0.063865702	-0.568509302	0.569689191	0.750099284
41.22236392	0.227356879	0.180867983	1.257032202	0.208741975	0.412817362
13.97856611	-0.022295588	0.271372055	-0.082158748	0.934520477	0.96896052
87.28005693	0.330860164	0.122071646	2.710376856	0.00672068	0.039316932
375.3710117	-0.135033067	0.081984016	-1.64706578	0.099544526	0.255829108
412.9363432	-0.055762034	0.061840687	-0.901704631	0.367213792	0.580843016
12.60239336	0.419685762	0.415229353	1.010732406	0.312144523	0.526925798
919.113422	0.621665807	0.240123409	2.588942952	0.009627104	0.051326312
27.84987048	0.374640449	0.2898889	1.292358726	0.196232943	0.397386786
28.84256095	-0.18679296	0.254685824	-0.733425037	0.463299234	0.665596195
103.5246999	0.301380703	0.108446483	2.779073097	0.005451426	0.0336355
557.4910727	0.039693382	0.052978383	0.74923732	0.453714179	0.658108374
299.075028	-0.070272974	0.209368688	-0.335642234	0.737140672	0.861788314
50.24460372	0.145029993	0.185159171	0.783271995	0.433467405	0.640328374
430.9442471	-0.047796811	0.073894307	-0.646826708	0.517744105	0.710184544
73.31612804	0.205235966	0.243519207	0.842791699	0.399344951	0.611640826
8.392896343	-0.02398493	0.512009107	-0.046844732	0.962636977	0.981463149
2.502367558	0.088847794	0.742942905	0.119588993	0.904808739	0.952300557
3.008714349	1.182575612	0.556234084	2.126039459	0.033499974	0.122694214
7.923676867	0.872471302	0.356806381	2.445223374	0.014476245	0.068489852
98.69485356	0.297149694	0.101904283	2.91596865	0.003545861	0.024595587
271.7164905	0.154417106	0.090246574	1.711057823	0.087070439	0.234605288
1039.089701	0.141963966	0.049670596	2.858108746	0.004261743	0.028038321
1028.311945	0.08022894	0.076981158	1.042189303	0.297323921	0.51192666
1015.786173	0.204514042	0.067943746	3.010049545	0.002612051	0.019709144

4.180779723	0.398350324	0.521774473	0.763453071	0.44519324	0.650533389
590.1005044	0.003944438	0.056111487	0.070296439	0.943957717	0.972895359
115.7563477	0.05437404	0.109532415	0.49641962	0.619598385	0.785174138
104.697548	-0.132878653	0.097076253	-1.368806978	0.171059619	0.363288764
2858.371644	-0.173747722	0.066216536	-2.62393252	0.0086921	0.047620107
434.9700149	0.177522531	0.063230667	2.807538484	0.004992171	0.031461757
6.761558766	0.142750583	0.360697169	0.39576297	0.692279896	0.834158793
67.19471572	0.112300511	0.125837296	0.892426292	0.372164484	0.585510216
774.4245513	0.202327627	0.073480972	2.753469666	0.005896723	0.035597423
3.344711547	1.440459444	0.558461942	2.579333229	0.009899124	0.052118305
249.2115583	-0.281803764	0.096258239	-2.927580717	0.003416104	0.023937312
21.12582828	0.026729563	0.342861842	0.077960157	0.937859746	0.970501464
775.8788314	-0.131395171	0.097578922	-1.34655281	0.178124301	0.373668628
103.236099	-0.128357173	0.10178748	-1.261031054	0.207297659	0.41080496
1040.791828	-0.137460139	0.084467391	-1.627375215	0.103657432	0.26328487
1.990282003	0.674269264	0.704180585	0.957523224	0.33830323	0.553502819
1201.326817	0.00203408	0.04422051	0.045998558	0.963311399	0.98199185
270.1628284	-0.181801677	0.188100203	-0.966515051	0.333786519	0.549056328
21.83588122	-0.398062225	0.275611823	-1.444285735	0.148658617	0.331274013
756.6704826	-0.074674615	0.056611734	-1.31906603	0.187147039	0.385875174
634.1481559	0.766444498	0.090546792	8.464623457	2.56982E-17	1.36565E-14
30.6156939	0.168377801	0.321596571	0.523568396	0.600578768	0.771697576
186.177894	-0.115530202	0.216891764	-0.532662928	0.594266933	0.76681093
126.4325367	-0.643992818	0.208022327	-3.095787012	0.001962913	0.016000509
11.19848647	-0.503681207	0.308876807	-1.630686394	0.102956509	0.26222136
18.41994528	0.221145625	0.221200392	0.999752411	0.317430341	0.53226231
23.30195395	-0.581667452	0.226031985	-2.573385581	0.010070894	0.052788937
20.5148329	-1.024091022	0.544536582	-1.880665243	0.060017471	0.183641963
33.53864599	-1.055951022	0.232351472	-4.544628067	5.50323E-06	0.000144557
271.9360646	-0.238777398	0.091550113	-2.608160611	0.009103023	0.049240889
2.572868924	0.566207456	1.183174664	0.478549341	0.632259265	0.794046377
87.67100756	-0.148193656	0.110231736	-1.344382857	0.178824615	0.374375724
13.72337071	0.108567097	0.244135023	0.444701033	0.656535823	0.81089405
739.0384071	0.056640369	0.080057058	0.707500007	0.479255808	0.678345235
26.34439612	-0.736614609	0.415001128	-1.774970138	0.075902818	0.214849317
208.9807169	-0.072545149	0.083399901	-0.869846944	0.384384053	0.597447202
228.9305538	-2.002941677	0.480416135	-4.1691807	3.05697E-05	0.000605294
106.9912562	0.19037178	0.128486978	1.481642596	0.138435419	0.316659968
95.27284133	-0.093215289	0.134350498	-0.693821686	0.487794041	0.686007843
5.259674759	-0.401435768	0.559936308	-0.716931126	0.473416595	0.673668911
33.89012463	-0.143504742	0.219059787	-0.655093952	0.512407266	0.706319325
69.10272921	-0.603258755	0.197474783	-3.054864761	0.00225162	0.01767184
26.46550122	0.531957522	0.199171664	2.670849413	0.007565958	0.042817449
297.8386401	0.116780007	0.076234901	1.531844406	0.125560835	0.296557591
185.465971	0.084551948	0.075796622	1.115510772	0.264631605	0.47593243
110.8440545	-0.381930956	0.226500605	-1.686224879	0.091752494	0.242536042
141.9970085	-0.158907685	0.129445398	-1.227603971	0.21959567	0.424016647
829.2668162	0.103885444	0.073728729	1.409022571	0.158828494	0.346011718

800.9182803	-0.364355364	0.086450968	-4.214589757	2.50233E-05	0.000514007
2546.270859	0.128673044	0.102657069	1.253426043	0.210050699	0.414168188
20.38780323	1.06055603	0.320831686	3.305646158	0.000947577	0.009231448
422.6389999	-0.382041401	0.258181871	-1.479737515	0.13894331	0.317337042
372.0803589	-0.051537336	0.077110718	-0.668355032	0.503906993	0.69943009
41.15617945	0.325360364	0.16562932	1.9643887	0.049485032	0.161014502
674.2790049	0.145696883	0.051598488	2.823665759	0.004747787	0.030198811
326.1894122	0.002852949	0.078486576	0.03634952	0.971003665	0.985661927
115.4740816	0.034133237	0.15210445	0.224406566	0.822440962	0.910976907
21.02354277	-0.278292776	0.21740712	-1.280053644	0.20052627	0.403058782
10.69004895	-0.000322	0.316669418	-0.001016835	0.999188684	0.999431352
64.51636371	0.179202332	0.141812956	1.263652747	0.206354692	0.409823653
6.109319041	-0.355838346	0.493495527	-0.721056881	0.470874519	0.671362327
706.8553737	-0.144705805	0.064715995	-2.236012968	0.025350922	0.101662874
83.33961609	0.372889748	0.202041091	1.845613411	0.064948358	0.193799508
26.48405637	0.291246657	0.436359107	0.667447183	0.504486536	0.699925147
1921.392449	0.016785317	0.101153725	0.165938694	0.868205196	0.934495951
3.950496368	1.36970448	0.819378147	1.671639016	0.094595526	0.247020171
3181.463345	-0.219617474	0.146313061	-1.501010731	0.133352786	0.308590223
86.8462975	0.025485494	0.128188994	0.19881187	0.842409911	0.921757179
371.6845259	0.07727574	0.095949333	0.805380693	0.42060003	0.629066856
266.0627667	-1.127711298	0.456504504	-2.470318012	0.013499299	0.065273685
124.9091387	-0.501941492	0.200896447	-2.49850855	0.012471713	0.06167944
279.3967996	0.072322262	0.110029911	0.657296382	0.510990363	0.705147867
2.003525334	-3.250061609	1.341049054	-2.423521794	0.015370831	0.071551023
298.4370703	0.75983243	0.12226821	6.214472529	5.14973E-10	4.56111E-08
519.5931385	0.957639177	0.142077365	6.74026559	1.58097E-11	2.17041E-09
2.333076519	-1.032547723	0.703017411	-1.468737058	0.141904122	0.321248936
31.4444578	0.553404023	0.211215484	2.620092114	0.008790602	0.048031965
99.70539747	-0.155757609	0.173441675	-0.898040274	0.369164079	0.582808724
27.1523155	0.433072413	0.213077343	2.032465798	0.042106523	0.144122763
114.5331333	-0.084349724	0.095047296	-0.887450012	0.374836668	0.588325007
466.891675	-0.127038075	0.143138294	-0.887519839	0.37479909	0.588322078
379.9665118	0.080584327	0.073506699	1.096285492	0.272953856	0.485373245
3100.516076	-0.010809085	0.097118134	-0.111298322	0.911379786	0.955458228
322.1978598	0.025370423	0.09811038	0.258590607	0.795951132	0.894773586
96.234218	-0.089633156	0.095753933	-0.936078058	0.349233	0.563934957
566.1834305	-0.143945927	0.114565024	-1.256456129	0.208950642	0.412987748
125.7599516	-0.209307335	0.101946996	-2.053099585	0.04006292	0.138917396
21.953791	-0.258049381	0.274427756	-0.940318083	0.347054427	0.561691234
8.160800381	-1.143748474	0.578948493	-1.97556171	0.048204439	0.158263683
496.8036465	-0.144604471	0.068823338	-2.101096454	0.0356325	0.128140102
798.7141409	0.135008033	0.063423566	2.128673013	0.033281323	0.122164999
59.65535611	-1.005978766	0.189358748	-5.312555023	1.08099E-07	4.78715E-06
499.5803773	0.018165573	0.133061949	0.136519671	0.891410475	0.945566153
62.07178194	-0.229917373	0.198620543	-1.157570962	0.247039165	0.455736081
36.07102511	-0.546717831	0.359178086	-1.522135822	0.127975052	0.299681735
47.24482642	0.246790019	0.174344466	1.415531134	0.156912827	0.343154375

6.027799566	0.974420814	0.486639507	2.002346296	0.0452475	0.151628828
276.7250971	0.86273266	0.146455679	5.890742261	3.84465E-09	2.80251E-07
5.369715808	0.005473221	0.591909854	0.009246713	0.992622295	0.995886705
66.7532671	0.104266367	0.164979087	0.631997483	0.527388522	0.717558125
351.5887926	0.730073163	0.132911695	5.492918911	3.95344E-08	1.98564E-06
76.11194854	0.862427962	0.123086168	7.006700861	2.44003E-12	4.27629E-10
31.1858113	0.235693417	0.329836305	0.714576938	0.474870499	0.674805193
7.880749954	0.030304745	0.33743622	0.089808808	0.928439149	0.965637301
123.6432904	-0.332036668	0.099823071	-3.326251793	0.000880224	0.008719667
291.5617953	0.1407112	0.064828643	2.170509712	0.029968254	0.113828295
601.8490784	-0.22441547	0.046105195	-4.867466022	1.13038E-06	3.80816E-05
5.089253253	-0.248580838	0.446847891	-0.556298558	0.578006772	0.756140997
10.95078242	0.388911295	0.503947894	0.77172918	0.440274844	0.645982168
48.61686939	-0.089598348	0.164462522	-0.544794931	0.585894565	0.760836448
45.21019664	0.877346247	0.350133263	2.505749499	0.012219215	0.060742111
144.9774549	0.056817189	0.157161766	0.361520429	0.717710436	0.850267635
3.650354068	-0.389754016	0.619050598	-0.629599612	0.528956578	0.718979428
159.4743665	0.075502344	0.186820945	0.404142821	0.686107698	0.830182756
3980.492082	-0.424404453	0.102654739	-4.134289908	3.56054E-05	0.000684781
1.946863218	2.504681254	1.019108432	2.457718115	0.013982288	0.066902923
1.859888942	-0.274561858	0.993060851	-0.276480397	0.782179114	0.886843735
33.76156482	-0.121113603	0.393719684	-0.307613786	0.758376229	0.873792838
128.0290191	-0.173818996	0.109028601	-1.594251358	0.110879748	0.275344131
2.598291494	0.097364378	0.661937025	0.147090092	0.88306091	0.941588701
2.860010235	-0.222962762	0.588226505	-0.379042359	0.704656409	0.841989532
2.765766862	-0.30148816	0.650964417	-0.463140769	0.643263469	0.801779707
1813.99132	0.014827926	0.11244784	0.131864924	0.895091133	0.947182125
281.3090132	-0.571255279	0.12263087	-4.658331801	3.18782E-06	9.12416E-05
4.511398312	-0.198861787	0.479913906	-0.414369711	0.678603368	0.826322114
376.8610195	-0.104823199	0.082209001	-1.275081788	0.202280295	0.405101578
2.378798997	-0.098787447	0.763511258	-0.129385711	0.897052454	0.948048454
2518.961069	-0.115788124	0.055544711	-2.084593153	0.037106247	0.131913748
23.60767664	-0.702176514	0.246841043	-2.84465057	0.004446017	0.028899605
123.4042862	-0.349690085	0.169856998	-2.058732278	0.039519892	0.137497509
3.281591895	0.099565153	0.553162122	0.179992716	0.857158287	0.92936531
102.1924576	0.166050747	0.241610758	0.687265538	0.491915412	0.689510294
104.2613397	-0.642558052	0.332317095	-1.933569054	0.053166135	0.168596518
2831.425049	0.114827052	0.106780297	1.075358053	0.282214454	0.495967668
74.62227587	-0.0144817	0.186967304	-0.077455789	0.938260961	0.970668417
685.2933094	0.119290289	0.250618077	0.475984377	0.634085508	0.794850453
340.4522724	0.094413674	0.111253132	0.84863834	0.396082566	0.609075814
29.94952149	0.146541789	0.268349282	0.54608601	0.585006816	0.7601225
387.0215038	-0.031422587	0.085547323	-0.367312336	0.713386054	0.847136297
15.35364166	-1.68324155	0.405642046	-4.149573667	3.33095E-05	0.000650938
75.95175365	-0.027315242	0.115854037	-0.235772894	0.813608902	0.906196373
238.0271346	0.062304703	0.112881584	0.551947459	0.580984352	0.757846001
175.041752	0.049376725	0.083461017	0.591614228	0.554108944	0.73907614
160.9675321	-0.928081986	0.416810917	-2.226625909	0.025972286	0.103374592

231.4202214	-0.482319239	0.155332794	-3.105070249	0.001902338	0.015607133
517.6847812	-0.031557436	0.059834352	-0.527413346	0.597906578	0.769420873
582.1855904	-0.181905627	0.090649291	-2.006696622	0.044781976	0.150436026
1642.505307	-0.130819167	0.105407762	-1.24107718	0.214577239	0.419184148
139.8946509	-0.277183451	0.099510767	-2.785461901	0.005345152	0.033116223
1772.821288	-0.040633183	0.077998134	-0.52095071	0.602401108	0.77305874
48.31889332	0.037241998	0.175712474	0.211948516	0.8321472	0.916032128
2.91944022	-0.129891727	0.558291944	-0.232659146	0.816026095	0.907711944
1075.053043	0.296362098	0.103500828	2.863379011	0.004191489	0.027686681
19.74383937	1.163140492	0.461548226	2.520084417	0.011732669	0.058981994
2483.696787	-0.165731508	0.047021061	-3.5246229	0.000424086	0.004913076
2.734081462	0.069589807	0.68921789	0.100969241	0.919574878	0.960260937
1193.517423	-0.059151284	0.13354081	-0.442945372	0.657805248	0.811615013
1332.408825	-0.093370858	0.060850251	-1.534436683	0.124922259	0.295727733
153.8679867	-0.092366948	0.077287388	-1.195110224	0.232043968	0.438724801
140.2214694	0.267054072	0.110942047	2.407149318	0.016077595	0.073675187
9.485660563	0.300629513	0.541375935	0.555306383	0.578685114	0.756621676
1876.902255	0.24421698	0.061785164	3.952679976	7.72808E-05	0.001278236
1906.269074	0.035247792	0.095334591	0.369727208	0.711585757	0.846071015
1159.59884	0.194189639	0.093653805	2.07348371	0.038127286	0.1344699
4.651006152	1.70895553	1.318238493	1.296393285	0.194840024	0.395831121
4.848395218	1.044687036	0.981760214	1.064095918	0.28728525	0.501827718
3.118233824	-1.143365502	0.593910763	-1.925146965	0.054210975	0.170466043
30.94141193	-1.033891518	0.387473462	-2.668289883	0.007623845	0.042975937
14.13957761	-1.294581839	0.289702783	-4.468655167	7.87129E-06	0.000196175
11.24639547	0.083514667	0.50037848	0.166902995	0.867446377	0.934367069
33.20297575	0.426381135	0.177462704	2.402652083	0.016276665	0.074318675
7491.477198	0.080781063	0.08679559	0.930704695	0.35200634	0.567079254
13.48900875	-0.129597439	0.45461341	-0.285071748	0.775589174	0.883629572
18.91650466	-0.770125301	0.446666254	-1.724162714	0.084678473	0.230806282
43.25785729	-0.06384988	0.333247009	-0.19159926	0.848056122	0.92485612
1.943873107	-1.15785579	1.746894897	-0.66280793	0.507453574	0.702267321
969.0866791	0.498167289	0.267824209	1.860053247	0.062877993	0.189515886
22.25346847	-0.954003522	0.324007522	-2.94438696	0.003235951	0.023007795
2.259790997	1.710073247	0.802449097	2.131067571	0.033083576	0.12168315
112.6276404	0.115594818	0.158064113	0.73131602	0.464586147	0.666747294
939.9019225	0.247998838	0.09449302	2.624520181	0.008677115	0.047569648
499.5431414	0.082765943	0.054799641	1.510337316	0.130957375	0.304888608
282.1658171	0.160180972	0.067009978	2.390404797	0.016829812	0.076110125
349.7000631	-0.220249843	0.183371871	-1.201110299	0.229708418	0.435825382
1032.181081	0.163723187	0.056203592	2.913037791	0.003579313	0.024765056
378.0161396	0.029954185	0.071225373	0.420554974	0.674080079	0.823492415
967.9288377	0.180742756	0.060738911	2.975732559	0.002922896	0.021362819
16.58092312	-1.570561488	1.254043964	-1.25239747	0.210425068	0.414658202
5.67625406	-3.610304001	1.162811886	-3.104804864	0.001904046	0.015613366
2.071901658	-4.523635862	2.227137226	-2.031143752	0.042240414	0.144401034
4.204338597	-3.118295596	1.446257325	-2.156113952	0.031074768	0.116547057
286.7200308	0.076370242	0.08109745	0.941709529	0.346341375	0.560822374

107.9539851	0.083642837	0.121566977	0.688039129	0.491428141	0.689139271
167.6420716	-0.181086751	0.192143867	-0.942453973	0.345960266	0.560385526
10.39046035	-0.071180396	0.358859402	-0.198351765	0.842769853	0.921837233
23.32808886	-1.440783773	0.525251226	-2.743037432	0.006087375	0.036440192
14.12736032	-0.550915746	0.350731827	-1.570760633	0.11623826	0.283396343
461.7382872	-0.113984853	0.057463714	-1.98359703	0.047300781	0.15615893
198.3620186	-0.070943125	0.08917708	-0.795530927	0.426304728	0.633668149
71.32890569	-0.551998603	0.137005291	-4.029031288	5.60072E-05	0.000981555
98.47521054	-0.73220879	0.392986091	-1.863192634	0.062435169	0.18860883
1751.044713	0.487941661	0.09619975	5.07217181	3.93301E-07	1.52453E-05
1005.082145	-0.000505686	0.106882583	-0.004731227	0.996225041	0.997981838
144.1743147	0.039967191	0.083958538	0.47603486	0.634049543	0.794850453
3.974098944	0.716928707	0.50445409	1.421197133	0.155259458	0.340487794
34.54407866	-0.41834056	0.215327079	-1.942814451	0.052038581	0.16607586
140.6547913	0.054963974	0.21159467	0.259760674	0.795048387	0.89446337
47.082856	0.556674594	0.24940807	2.231983084	0.025616081	0.102451886
21.83185271	0.570661291	0.331067159	1.723702504	0.084761563	0.230918139
109.3015615	-0.411444728	0.11088612	-3.71051606	0.000206837	0.002806784
2.719233322	0.421167888	0.640676092	0.657380372	0.510936369	0.705147867
8.354908997	-0.625030782	0.470824316	-1.32752443	0.184335264	0.381787672
2318.404158	0.210207798	0.102344214	2.053929482	0.039982517	0.138711509
454.7722225	-0.128551276	0.098249567	-1.308415696	0.190732352	0.390665767
148.2291795	0.020554857	0.112896966	0.182067396	0.855529836	0.929131684
4.995645997	-0.07277041	0.409475363	-0.177716212	0.858945852	0.930082422
3135.464904	-0.007819404	0.103095134	-0.075846487	0.939541231	0.971262319
95.91566237	0.384179244	0.129230768	2.9728156	0.002950817	0.021519148
62.72898386	0.03888925	0.182705426	0.212852189	0.831442256	0.915770524
197.6348203	-0.046526478	0.100029421	-0.465127933	0.641839843	0.80079291
2241.655748	-0.506101837	0.200725679	-2.521360693	0.011690195	0.058881027
82.32772283	-0.080058411	0.136881998	-0.584871729	0.558633969	0.742951159
62.14852845	0.082180023	0.127781989	0.643126809	0.52014182	0.712399615
130.8837651	-0.056724769	0.10627622	-0.533748459	0.593515577	0.766087567
478.4294671	-0.473809667	0.250442196	-1.891892316	0.05850533	0.180354941
607.3890088	-0.140127044	0.20353051	-0.688481763	0.491149451	0.688905581
19.66224077	0.142593751	0.300057312	0.475221715	0.634628951	0.795202438
13.18515602	1.438639149	0.976090269	1.473879204	0.140514118	0.319463094
8.437587999	0.043959917	0.360049479	0.1220941	0.902824495	0.951311133
773.7572179	0.032357729	0.137243207	0.235769259	0.813611723	0.906196373
8.781645112	0.916414513	0.329302113	2.782898975	0.005387558	0.033328813
399.2537171	0.035155264	0.107919851	0.325753455	0.744610897	0.866021155
14.31655028	-0.192955129	0.300417159	-0.642290639	0.520684491	0.712734217
2882.004844	-0.04518711	0.071143818	-0.635151601	0.525329545	0.716333226
383.0602665	0.111501381	0.099193322	1.124081534	0.260978438	0.472197561
31.3015923	-0.006097463	0.361622848	-0.016861387	0.986547197	0.993239536
210.9601228	0.086622256	0.084710417	1.022569117	0.306511617	0.521661833
149.3639429	1.577947143	0.100589907	15.68693309	1.85819E-55	3.06118E-51
128.3429684	0.002761284	0.091603454	0.030143887	0.9759523	0.987885465
514.2997734	-0.051716892	0.080639404	-0.641335245	0.521304895	0.713168647

211.8356046	0.029822891	0.08006513	0.372482892	0.709533348	0.845146114
2530.845287	-0.095867978	0.087180257	-1.099652382	0.271483609	0.483348209
2141.856978	-0.09115786	0.11956176	-0.762433242	0.445801473	0.650844865
8.495876492	-0.311364708	0.372660489	-0.835518434	0.403425872	0.61474774
54.16313463	0.897935242	0.341879135	2.626469853	0.008627563	0.047369685
3.307801776	0.257210291	0.687893146	0.37391024	0.708471098	0.84458737
2.967397521	-0.756090844	0.593957904	-1.272970423	0.203028538	0.405909239
19.42277172	-0.127733017	0.362651204	-0.352220027	0.724673258	0.853953309
327.4270156	-0.242765475	0.124019577	-1.95747705	0.050291407	0.162706333
87.75493164	-0.199764091	0.154350005	-1.294227953	0.195586694	0.396614377
628.3619064	-0.215535019	0.114927233	-1.87540423	0.060737134	0.185360106
6299.538643	-0.043585312	0.105862313	-0.411716982	0.680546876	0.827343313
9.681984769	0.528436379	0.395228106	1.337041496	0.181209092	0.377543769
669.2445229	-0.370117679	0.169853681	-2.17903832	0.029328822	0.112337365
163.940014	-0.206092193	0.086982029	-2.369365201	0.017818649	0.079079856
1.796025115	-1.625238738	1.089323683	-1.491970444	0.135706894	0.312239577
718.749052	-0.110154214	0.084740849	-1.299895099	0.193636925	0.394483817
46.20082891	-0.14587247	0.231767313	-0.629391903	0.529092517	0.719045548
2.476040928	0.211677877	0.615192444	0.344084001	0.730783115	0.857755975
594.8624721	-0.117893001	0.067657367	-1.742500571	0.081420892	0.224904053
2.606531311	-1.666467941	1.028638812	-1.620071032	0.105217019	0.265606064
667.1886647	-0.037488539	0.091320112	-0.410517887	0.681426085	0.827801292
299.7166356	-0.178463462	0.099966595	-1.785230981	0.074223859	0.211815612
91.62316806	-1.515815362	0.268577419	-5.643867491	1.66272E-08	9.51099E-07
62.28408728	-1.009287153	0.298713377	-3.37878124	0.000728079	0.007505868
1987.398963	-0.321470896	0.109657687	-2.931585606	0.003372364	0.023691397
364.2579985	0.186315366	0.069753692	2.671046663	0.007561513	0.042817449
239.4254788	-0.092775827	0.078777751	-1.177690732	0.238919923	0.446360434
28.96379814	0.191669747	0.460315051	0.416388181	0.677125976	0.825613125
1231.932388	-0.225335405	0.052127559	-4.322769134	1.54083E-05	0.00034415
911.8427716	0.265756464	0.061231719	4.340176454	1.42368E-05	0.000320407
334.8222056	0.132318472	0.058679145	2.254948873	0.024136538	0.098106422
8.503944525	0.970756338	0.457293971	2.122827763	0.033768287	0.123320498
6.502732187	-0.933069867	0.651416896	-1.432369766	0.152038026	0.33629222
480.3369035	-0.11343381	0.161194187	-0.703709062	0.481613976	0.680630405
210.3060519	0.009251986	0.156032156	0.059295381	0.95271684	0.977124952
289.5115248	-0.366309453	0.088374017	-4.144990423	3.39828E-05	0.000658626
97.75097943	-0.226034611	0.111744897	-2.022773453	0.04309651	0.146416148
151.4669017	0.0262172	0.143838165	0.182268734	0.855371834	0.929082587
4.650128061	0.291522584	0.499486416	0.58364467	0.5594594	0.74344875
563.3086195	0.037632832	0.051706184	0.727820717	0.466723341	0.668563317
11.34563873	0.21564814	0.304321087	0.708620432	0.478560054	0.677938509
358.5520887	0.144756865	0.064143992	2.256748596	0.024023788	0.097817076
72.42234232	-0.060056038	0.122777767	-0.489144246	0.624739578	0.789563468
143.078166	-0.081147677	0.089510953	-0.90656701	0.364635814	0.578202266
3.725818307	0.5917746	0.511120144	1.157799406	0.246945906	0.455615058
124.2288727	0.095834731	0.175229199	0.546910739	0.584440057	0.75979398
2.296357742	0.351237853	0.742674884	0.47293622	0.636258683	0.796544232

92.75645997	0.101881687	0.137380673	0.741601312	0.458328928	0.661629054
71.8691232	0.194037109	0.204228785	0.950096773	0.342063083	0.556467099
2.68528831	0.989068781	0.73373857	1.347985266	0.177663122	0.372986143
3225.313725	-0.099962225	0.10033285	-0.996306048	0.319101466	0.534070902
223.1311764	0.142508019	0.077719799	1.833612802	0.066711491	0.197307917
817.7006975	0.233953794	0.155456742	1.504944667	0.132338303	0.30681677
444.0936072	0.261482479	0.080584597	3.244819572	0.001175251	0.010938463
82.86363153	0.159800171	0.128315707	1.245367189	0.212996808	0.417651121
242.8472509	0.041138156	0.226182085	0.181880701	0.85567635	0.929211939
3.040437297	-1.319637299	1.370590579	-0.96282385	0.33563593	0.550778594
19.23849466	0.708735988	0.272180802	2.603916154	0.00921653	0.049618666
236.8887786	0.167257935	0.090522633	1.847691887	0.064646928	0.193183378
555.1265419	-0.15932854	0.057998616	-2.747109326	0.006012309	0.036095765
78.26727978	0.086244945	0.136553382	0.631584097	0.527658681	0.717629746
505.8843161	0.228787571	0.127288495	1.797393956	0.0722731	0.208370151
204.865254	0.659020794	0.155167993	4.247143895	2.16513E-05	0.000454953
13.51064254	0.206006731	0.27889605	0.738650585	0.460119199	0.662877454
10.02693148	-0.90678542	0.501509652	-1.808111602	0.070589133	0.204985964
359.3523716	-0.36458477	0.110806929	-3.290270509	0.001000911	0.009653987
29.10217274	-0.754515251	0.247672444	-3.046423889	0.002315811	0.017995599
3.679784247	-0.948605519	0.59095969	-1.605194965	0.108450905	0.271162377
187.5022152	0.038023642	0.154861803	0.245532735	0.806043965	0.901149495
1.97726574	0.087076672	0.728830376	0.119474537	0.904899412	0.952300557
126.8283076	0.232798231	0.095604294	2.435018569	0.014891025	0.069885429
348.4932378	-0.369722927	0.150128215	-2.462714462	0.013788968	0.066304571
2.888990878	0.166301362	0.667736594	0.249052341	0.803320294	0.899164189
328.7274884	0.039115076	0.124482336	0.314221898	0.753352512	0.87130887
167.3587926	0.523304342	0.181751523	2.879229476	0.003986481	0.026599148
910.7827107	-0.222858567	0.068885271	-3.235213595	0.001215518	0.011218172
19.10589249	0.169138052	0.240784699	0.702445181	0.482401577	0.681509612
124.9763467	-0.792928429	0.251405246	-3.153985212	0.001610572	0.013847897
1176.82091	0.056147231	0.066268012	0.84727502	0.396841847	0.609578167
142.1856382	-0.434622609	0.161067912	-2.698381101	0.006967762	0.040205575
96.22306926	-0.119482838	0.117889715	-1.013513675	0.310814867	0.525812706
2101.763767	-0.31124148	0.078830172	-3.948253227	7.87235E-05	0.00129949
91.9959915	0.265158247	0.109865454	2.413481566	0.015800927	0.072873593
3.105481958	-1.473281354	0.591498441	-2.490761176	0.012746977	0.062591267
452.7501591	-0.502928381	0.098590792	-5.10116991	3.3756E-07	1.3272E-05
341.4529068	-0.024178355	0.065377235	-0.369828342	0.711510396	0.846071015
41634.02186	0.237700266	0.104181202	2.281604169	0.02251272	0.093301775
2.181706436	-0.803480729	0.781872294	-1.027636783	0.304120715	0.518803423
82.01569386	-0.221543317	0.158492584	-1.397815038	0.162168617	0.351331485
695.0771044	-0.05461461	0.050916406	-1.072632859	0.283435876	0.497424376
54.97554736	-0.043325394	0.190213668	-0.227772244	0.819823306	0.909423741
3.578563054	-0.789276333	0.713567303	-1.106099354	0.268683518	0.480491998
47.91354323	-0.176525032	0.168841374	-1.045508144	0.295788179	0.510408791
43.89687741	-0.186050092	0.184179184	-1.010158086	0.312419557	0.52712001
2926.687701	0.014402714	0.105184505	0.136928099	0.891087628	0.945526539

1.745057733	-1.697954506	0.99613326	-1.70454554	0.088279216	0.236974385
357.9101859	0.187846674	0.21382535	0.878505161	0.379669638	0.592523458
2972.728565	0.197249985	0.054802879	3.59926315	0.00031912	0.003911596
19.61432698	-1.024274361	0.562151778	-1.822060166	0.068445857	0.200815147
9.090933974	0.886305741	0.392788206	2.25644693	0.024042655	0.097821364
202.2405101	0.130405313	0.080746235	1.615001767	0.106310312	0.267537975
5.166969684	0.347111204	0.465766839	0.745246709	0.456122591	0.66012936
390.6673094	0.081807919	0.066152066	1.236664609	0.216211623	0.420925346
123.6284649	0.260386301	0.16290966	1.598347824	0.10996559	0.273816978
175.280865	-0.559184496	0.108382295	-5.159371229	2.47781E-07	1.00293E-05
586.0302358	0.036081313	0.075033696	0.480868134	0.630610222	0.793098965
192.2058074	0.266285417	0.087641169	3.038359929	0.002378697	0.018397487
3.912187789	-0.020136033	0.506018029	-0.039793114	0.968258066	0.984103224
18.93138087	-0.185471521	0.254547218	-0.728633069	0.466226144	0.668402184
372.958787	-0.002205932	0.084555773	-0.026088484	0.979186762	0.989093306
2.680054478	1.226600713	0.630758576	1.944643734	0.051817872	0.165804784
278.4274959	0.003719477	0.089934124	0.041357797	0.967010657	0.983639473
85.82443553	0.201117421	0.102189708	1.968079036	0.049058945	0.160028236
3.704149119	-0.735315942	0.537395097	-1.368296707	0.171219221	0.363512162
270.7826814	-0.186100204	0.065750274	-2.830409569	0.004648845	0.029764895
1515.372733	-0.153325517	0.106190341	-1.443874422	0.148774303	0.331386486
177.2499895	0.154270244	0.125134231	1.23283807	0.217636185	0.422351102
26.46381622	-0.833887474	0.485965931	-1.715938136	0.086173366	0.233138055
9.157249078	-1.197715086	0.464001175	-2.581275975	0.009843586	0.051993697
5827.37494	-0.030100602	0.05983896	-0.503026823	0.614945414	0.782346957
3.738031069	-1.142736926	0.588747577	-1.940962429	0.052262835	0.166469052
118.526098	0.122207709	0.115843127	1.054941388	0.291452132	0.50652837
924.3262606	0.031333684	0.075976328	0.412413766	0.680036175	0.826932461
47.16295875	0.051406862	0.134526461	0.382131976	0.702363475	0.840493334
74.80422257	0.373236885	0.129647864	2.878851026	0.003991268	0.026620305
510.2265635	-0.060362392	0.07750659	-0.778803344	0.436095568	0.642539879
60.92557311	0.553649246	0.144512135	3.831160932	0.00012754	0.001898008
608.3556434	-0.119617951	0.100114643	-1.194809747	0.232161371	0.438855586
280.9910226	-0.179456195	0.197298894	-0.909565134	0.363051893	0.576964218
6.129253913	0.374417008	0.473014726	0.791554655	0.428620396	0.635561872
297.3802664	0.035868343	0.065524621	0.547402522	0.584102223	0.759621118
224.8303493	-0.088115071	0.10062476	-0.87567981	0.381204124	0.593923904
2076.651495	0.06055291	0.098070129	0.617444988	0.536941256	0.725225076
857.4182956	0.174761339	0.079655055	2.193976753	0.028237081	0.109350651
3.41646547	0.656772961	0.644809763	1.018553066	0.308415196	0.523533322
120.7531279	-0.017671283	0.107072255	-0.165040727	0.868911926	0.934607931
744.3727483	0.996844282	0.275018297	3.624647135	0.000289356	0.003644384
1033.905654	-0.13805044	0.088215799	-1.564917406	0.117602277	0.285370439
291.2411225	-0.033871717	0.089226466	-0.379615134	0.704231125	0.841664506
19.78396805	-0.291651497	0.236999733	-1.23059842	0.218473096	0.423029367
365.4966497	0.005170196	0.117855361	0.043868991	0.965008833	0.982543604
135.1613865	-0.371291361	0.276502061	-1.342815892	0.179331596	0.374967985
3.292402785	0.941913039	0.657705325	1.432120136	0.152109443	0.336310691

75.08295899	-0.030537841	0.111235375	-0.274533535	0.783674636	0.887762646
167.2961524	-0.19604624	0.163046064	-1.20239787	0.229209414	0.4352933
210.2980037	-0.10705123	0.142801117	-0.749652608	0.453463958	0.658066001
3.61828169	-1.99987665	1.281475061	-1.560605204	0.11861693	0.287029275
52.10513589	0.153899652	0.214750564	0.716643761	0.473593935	0.673746674
1604.279022	0.009440965	0.080846822	0.116775952	0.907037601	0.953303217
745.7631143	0.319264779	0.061512425	5.190248645	2.10013E-07	8.69287E-06
268.0262254	-0.011111961	0.098914658	-0.112338868	0.910554725	0.955037829
7.375441458	-0.441418664	0.567012832	-0.778498544	0.436275165	0.642641632
235.2903901	0.041832924	0.069793775	0.599379015	0.548920168	0.735403344
84.41471273	0.450824296	0.15455483	2.916921424	0.003535048	0.024554356
18.8562242	0.123218089	0.366795895	0.335930937	0.736922947	0.8616679
200.5351484	0.1354045	0.104453224	1.296317093	0.194866262	0.39583561
357.5372116	-0.352222075	0.076229375	-4.620555729	3.82714E-06	0.000106321
306.121327	-0.245417049	0.132580668	-1.851077174	0.064158451	0.192276938
3553.997972	-0.145186245	0.057988666	-2.503700376	0.012290207	0.061013225
97.13425574	0.582144727	0.205606057	2.831359814	0.004635055	0.029719986
229.6784436	0.101890441	0.101248681	1.006338451	0.31425279	0.529129238
8.744333787	-0.238040789	0.477723101	-0.49828193	0.618285343	0.784294505
78.99803231	0.038110821	0.128050094	0.297624312	0.765989916	0.878006201
12.3994877	0.12208064	0.308102642	0.396233669	0.691932656	0.834043309
227.28941	-0.07156054	0.111083415	-0.644205442	0.519442223	0.711844631
430.4588488	-0.052963665	0.083838911	-0.631731314	0.527562463	0.717558125
218.5108618	0.154060476	0.096719727	1.592854746	0.11119278	0.275673202
140558.2233	0.130759388	0.136560231	0.957521723	0.338303987	0.553502819
2838.066176	-0.053553035	0.080294801	-0.666955195	0.504800753	0.700184188
2.380978667	0.665432492	0.657772031	1.011646072	0.311707309	0.52645368
1700.74339	-0.073837459	0.065641806	-1.124854156	0.260650841	0.471750216
2664.307766	-0.16856143	0.122003719	-1.381608944	0.167091806	0.358327313
608.6227565	0.143485142	0.161416408	0.888912993	0.374049842	0.587649923
132.0628728	0.014310022	0.115383293	0.124021615	0.901298161	0.950648941
832.0853749	-0.444647237	0.298131021	-1.491449081	0.13584363	0.312398022
3.14807122	0.00139362	0.594183829	0.002345435	0.998128615	0.998609072
974.0226238	-0.117991993	0.0535516	-2.203332717	0.027571307	0.107590346
237.1909905	0.023079628	0.089490901	0.257899155	0.796484737	0.894900006
7.495275885	0.022887457	0.407615503	0.056149623	0.955222613	0.978241049
258.992607	0.079949381	0.095507615	0.837099551	0.402536613	0.613846909
15.37466077	-1.071684194	0.777159626	-1.378975642	0.167902264	0.359223623
449.8737973	0.043931748	0.052023214	0.844464318	0.398409994	0.610776684
401.4689405	0.333169479	0.087838482	3.792978541	0.000148851	0.002141635
593.8640256	-0.15879944	0.090002174	-1.764395601	0.077665398	0.21796589
495.0667609	-0.184777328	0.087146215	-2.120313881	0.033979584	0.123762915
3.114044103	-3.258525128	1.135775468	-2.868987066	0.004117886	0.027332015
14.90172048	-3.343573089	0.85976374	-3.888944061	0.000100681	0.001570667
21.49243427	-2.280669696	0.36084758	-6.32031312	2.61034E-10	2.57501E-08
2032.214773	0.001970109	0.053371662	0.03691301	0.970554366	0.985635983
53.0389638	0.28714995	0.142076282	2.021097015	0.043269723	0.146853198
328.0953188	-0.319615962	0.229952258	-1.389923132	0.164552221	0.354357292

556.7509254	0.056652095	0.050611335	1.119355871	0.262988349	0.474375349
2.616517237	0.891234071	0.656213054	1.358147428	0.174416908	0.368377455
357.6550669	0.056748329	0.121148504	0.468419564	0.63948458	0.798613269
4.677420002	-0.274405846	0.671290096	-0.408773864	0.682705622	0.828547438
38.14222266	0.352035111	0.223850947	1.572631772	0.115804106	0.282613205
7.37077027	0.611954737	0.424703852	1.440897542	0.149613631	0.332577918
762.5618351	0.304233712	0.078985002	3.851790886	0.000117257	0.001768951
10288.02014	-0.151714662	0.051738215	-2.932352067	0.003364051	0.023643082
10.03236409	-0.266228812	0.402050196	-0.662178044	0.507857123	0.70264438
158.9659713	0.253487957	0.124529368	2.03556768	0.041793786	0.143320323
15.09685754	0.067307537	0.280877703	0.239632893	0.810614862	0.904012269
48.9194203	-0.4040299	0.156598503	-2.580036797	0.009878979	0.052045506
1032.601941	-0.091417824	0.09346635	-0.978082743	0.328033402	0.543336242
66.83344156	-0.045920141	0.115807004	-0.396522998	0.691719245	0.834041303
58.30879366	-0.235719097	0.126511759	-1.863218876	0.062431478	0.18860883
28.40612029	-0.181276628	0.396173613	-0.457568655	0.647262367	0.80444124
313.9696284	-0.210801504	0.085525583	-2.464777176	0.013709848	0.066001179
18.17151985	-0.086083994	0.360254088	-0.238953553	0.811141599	0.904538462
2.914057788	-0.630401865	0.661487376	-0.953006645	0.340586704	0.555270316
853.4944607	-0.140902094	0.073090542	-1.927774665	0.05388316	0.169921742
552.8208586	0.091355345	0.096524026	0.946451864	0.343918163	0.558185618
516.0070314	-0.349805996	0.155218712	-2.253632899	0.024219272	0.098297188
578.9653085	0.498304497	0.129863341	3.837145219	0.000124473	0.001859081
252.1728995	-0.193152462	0.088486645	-2.182843084	0.029047368	0.111466651
500.1529135	-0.232762655	0.057179982	-4.070701803	4.68717E-05	0.000846672
10.8320123	-0.042318699	0.357999203	-0.118208919	0.905902123	0.952959157
233.4575476	0.150445398	0.089622775	1.678651413	0.09322	0.245124706
352.4464704	0.104752775	0.076305254	1.372812094	0.16981077	0.361803237
289.6850029	0.014139658	0.104798816	0.134921927	0.892673603	0.946108145
116.0135491	-0.002660473	0.110869852	-0.023996361	0.980855511	0.990108149
26.56823632	0.133547531	0.308278822	0.433203715	0.66486678	0.816962432
46.48709213	-0.14244726	0.281865396	-0.505373353	0.613296631	0.781093831
12.64035127	-0.205242572	0.302754716	-0.677917012	0.497824313	0.694483676
5.230884437	0.364268897	0.6461187	0.563780149	0.572903763	0.752170338
656.8861249	0.197946462	0.136605573	1.449036501	0.147327389	0.329139058
24.7217519	0.201526371	0.204378503	0.986044855	0.324111102	0.539548668
16400.66563	-0.148124303	0.115936617	-1.277631749	0.201379301	0.404132367
225.4895354	-0.253336773	0.080839994	-3.133804951	0.001725555	0.014466563
218.4333046	-0.343476863	0.177653473	-1.933409219	0.053185806	0.168626439
676.9282537	0.237152007	0.081676497	2.903552613	0.00368955	0.025213511
191.7794106	0.341096417	0.090931516	3.751135275	0.000176036	0.002468095
13.7417666	0.14592831	0.336626068	0.433502703	0.664649603	0.816878361
140.8346939	-0.034755846	0.136775289	-0.254109105	0.799411269	0.896484956
8.747221056	-0.525608343	0.341041185	-1.5411873	0.123271208	0.293441263
260.7812458	-0.248684824	0.085968267	-2.892751392	0.003818834	0.025804544
6638.648006	-0.239779434	0.164871429	-1.45434194	0.145851528	0.326905861
84.47471566	-0.269912816	0.102291799	-2.638655481	0.008323552	0.046138021
79.83717023	-0.532727736	0.290855542	-1.831588743	0.067012715	0.197879095

429.0510689	-1.410407056	0.457681948	-3.081631389	0.002058696	0.016535816
1079.971285	-0.509509391	0.068326334	-7.456998832	8.85155E-14	2.14442E-11
1362.187725	-0.238584471	0.105490219	-2.26167385	0.023717562	0.096785511
323.5511937	-0.174224596	0.148936748	-1.169789178	0.24208582	0.449771264
1919.049857	0.167862751	0.099988888	1.678814063	0.093188287	0.245097804
896.6592622	0.103381237	0.078647948	1.314481035	0.188684376	0.388013315
393.0846978	0.309420211	0.062037446	4.987636215	6.11225E-07	2.28329E-05
42.51421528	-0.740353557	0.149925003	-4.938159371	7.88634E-07	2.84911E-05
985.4310605	-0.078866481	0.071704003	-1.099889518	0.271380262	0.483320913
25.9505095	0.043173316	0.239613656	0.180178863	0.857012152	0.92936531
22.85950697	0.307865121	0.201168759	1.530382365	0.125922111	0.297155259
18.69029368	-0.739976248	0.286789017	-2.580211254	0.009873989	0.052045506
3.954991604	-0.697723598	0.476072962	-1.465581232	0.142762407	0.322438703
6.321408157	-1.999915368	0.9158184	-2.183746655	0.028980869	0.111315188
457.9275376	-0.262832174	0.11169905	-2.353038577	0.018620702	0.081476082
151.839968	-0.298987774	0.207329991	-1.442086466	0.149277983	0.332337899
1498.70222	-0.201057978	0.111288712	-1.80663407	0.070819354	0.205456938
16.95367853	-0.458856749	0.43318925	-1.05925239	0.289484851	0.504386403
50.60627034	0.062644298	0.192328108	0.32571577	0.744639411	0.866021155
462.8685494	0.045798956	0.061417916	0.745693749	0.455852436	0.659904484
87.02925805	-0.19162636	0.102340164	-1.872445321	0.06114502	0.18608961
5.226700704	-0.60322817	0.471204107	-1.280184448	0.200480274	0.403015502
2040.894067	-0.375442037	0.147931916	-2.537938045	0.011150772	0.056793477
10.50532872	-0.326800231	0.351827529	-0.928864867	0.352959122	0.56793551
3.306821968	-2.998740629	0.994658219	-3.014845273	0.002571104	0.019492114
2.533618411	0.033809197	0.840807981	0.040210367	0.967925413	0.983871367
251.8359233	-0.177149758	0.066079183	-2.680870908	0.007343084	0.041879287
2.267762536	-0.424804359	0.619134798	-0.686125802	0.492633782	0.690017157
96.80747534	-0.0483195	0.130004758	-0.371674858	0.710134947	0.845469618
32.15171546	0.293801722	0.192184716	1.52874655	0.126327287	0.297684986
90.94150797	-0.351320343	0.123090787	-2.854156285	0.00431513	0.02827663
245.8388808	-0.08103169	0.0767637	-1.055599074	0.291151424	0.506302775
1560.711035	-0.040451322	0.111866186	-0.361604547	0.717647566	0.850267635
87.68666963	-0.447525202	0.129976371	-3.443127387	0.000575028	0.006248692
296.327394	0.326305799	0.122614445	2.661234562	0.00778547	0.043714326
10.09878385	0.191343883	0.434308147	0.440571711	0.659523086	0.812578183
12.27497933	-0.148315364	0.934755674	-0.158667519	0.87393083	0.936825644
32.21367221	0.000781154	0.164007496	0.004762917	0.996199756	0.997981838
27.38133546	0.238890734	0.195199898	1.223826122	0.221017828	0.42575394
31.90691925	-0.122615883	0.284952069	-0.430303535	0.666974856	0.818332001
10.85130771	0.095417143	0.686978273	0.138893975	0.889533932	0.944882455
3.792377503	0.468780128	0.784327036	0.597684519	0.550050457	0.736470353
20.97839918	0.14745902	0.202505002	0.728174702	0.46650665	0.668520881
176.8377153	-0.386994132	0.119972635	-3.225686702	0.001256708	0.011501675
18.02178144	0.803302465	0.329702878	2.436443597	0.014832482	0.069675026
99.91258912	-0.488767074	0.200117868	-2.442395968	0.014590134	0.068870449
1256.333977	0.084194667	0.047691037	1.76541907	0.077493361	0.217594277
36.30987701	0.359375791	0.325746839	1.103236463	0.269924491	0.481874674

338.4973307	0.024012653	0.151739797	0.158248882	0.874260686	0.936996327
7.866969666	-1.634568516	0.780664105	-2.093817949	0.036276201	0.12974688
21.14360825	-0.399169246	0.341786277	-1.167891378	0.242850582	0.450582328
24.23504455	-0.483047088	0.331636258	-1.456556925	0.145238725	0.32610914
94.2031333	-0.069431454	0.126762505	-0.547728639	0.583878244	0.759539655
15.78137163	0.437597592	0.364330192	1.201101644	0.229711775	0.435825382
133.5795832	0.24933098	0.320558744	0.777801212	0.436686213	0.642902204
119.8634354	-0.316938404	0.142473105	-2.224548998	0.02611153	0.10372832
68.27575121	-0.360051622	0.238915487	-1.507025043	0.131804238	0.306081621
48.29103611	0.02140985	0.220108413	0.097269565	0.922512325	0.961560774
744.7457064	0.057126033	0.079819583	0.715689448	0.474183128	0.674177341
132.0108236	-0.865866573	0.182602394	-4.741813913	2.11813E-06	6.51009E-05
174.569692	-1.107194984	0.184923859	-5.987301972	2.1335E-09	1.62719E-07
27.16423423	-0.160567466	0.215809599	-0.744023746	0.45686211	0.660495515
3.109496774	-2.196058572	0.965522474	-2.27447691	0.022937328	0.094514642
244.0531695	0.024494481	0.104034784	0.235445106	0.81386328	0.906237868
503.7452061	0.121587187	0.067320924	1.806083159	0.070905351	0.205513947
39.94314975	-0.086258054	0.186924966	-0.46145818	0.644469918	0.80261547
1154.19773	0.39343426	0.099957428	3.936018228	8.28447E-05	0.001352855
457.6011015	-0.08360223	0.08101791	-1.031898132	0.302119841	0.517049928
547.7313786	-0.175293802	0.067361591	-2.602281191	0.009260589	0.049790777
162.2928562	-0.060909629	0.129383742	-0.470767251	0.637806953	0.797573383
975.7473823	-0.183384242	0.079965224	-2.29329992	0.021830744	0.091460718
8.771039983	-0.392392414	0.337554194	-1.162457526	0.245049668	0.453335006
31.30901513	-0.440924261	0.264729975	-1.665562279	0.09580063	0.249284405
13.15381867	-0.144384031	0.341579593	-0.422695133	0.672517707	0.822263819
382.8597903	0.325526242	0.106050059	3.069552677	0.002143796	0.016984602
33.00144028	-0.519622419	0.203289753	-2.55606793	0.010586245	0.054601691
1.934253974	-0.27904283	0.723489217	-0.385690378	0.69972599	0.839012442
163.8932411	-0.087301861	0.115296206	-0.757196303	0.448932265	0.653908941
53.35930087	-0.7081039	0.324805143	-2.180088325	0.029250915	0.112169361
1.964992635	0.23362742	0.720018027	0.324474403	0.745578898	0.866724055
1029.23507	-0.117358433	0.048412183	-2.424150834	0.015344231	0.071467588
146.324401	-0.115225812	0.108163638	-1.065291565	0.286744009	0.50136073
20.05513194	0.009025972	0.251421019	0.035899831	0.97136223	0.985661927
300.1923433	-0.195825996	0.113359532	-1.727477095	0.084082015	0.229598714
59.14036409	-0.346535221	0.16189849	-2.14044751	0.032318618	0.119698049
10.17295543	-0.02186503	0.391278733	-0.055880957	0.955436641	0.978293782
129.6006207	0.191277772	0.146889031	1.302192347	0.192850641	0.393432207
1.703680933	-1.019180908	0.919093406	-1.108898073	0.267474155	0.479124214
706.6372726	0.195445071	0.084160504	2.322289688	0.020217342	0.086689354
68.39748939	0.339769615	0.133225964	2.550325811	0.010762229	0.055225776
41.64621959	0.409634263	0.190556645	2.149671892	0.031581177	0.117867765
37.79709003	0.533621149	0.175041357	3.048543254	0.002299538	0.017894469
240.4332217	-0.076860696	0.0762932	-1.00743835	0.31372417	0.528590495
16.826394	0.182555492	0.26486763	0.689232927	0.490676699	0.688433148
726.7208424	0.101482405	0.067106715	1.512254101	0.130469233	0.304095945
1144.779094	-0.037552136	0.057290713	-0.655466379	0.512167526	0.706239878

775.5476614	0.124562826	0.06319643	1.971042128	0.048719057	0.15926075
4043.73848	-0.033436809	0.102409589	-0.326500763	0.744045512	0.865599899
3.442571623	-0.708141616	0.625462999	-1.132187863	0.257555461	0.467957282
24.86425767	0.407303333	0.200293755	2.03352987	0.041999019	0.143844457
5679.758787	-0.107361292	0.137044821	-0.783402769	0.433390631	0.640328374
139.2744548	-0.056568197	0.104647917	-0.540557312	0.588812748	0.76241798
381.7159984	-0.402012149	0.069117986	-5.816317494	6.01582E-09	4.09855E-07
948.1728891	0.308937728	0.183030491	1.687903069	0.091429837	0.242040036
15.18684962	-0.929079117	0.915559624	-1.014766371	0.310217208	0.525179147
881.6924513	0.06196382	0.076726102	0.807597648	0.419322241	0.628262028
84.09006327	-0.172389537	0.183064327	-0.94168831	0.346352242	0.560822374
11.0012371	-0.332302908	0.528656324	-0.628580219	0.529623914	0.719352326
340.7160876	-0.082249177	0.065470107	-1.256285974	0.209012305	0.41304019
1580.457342	-1.985623443	0.369613962	-5.372154863	7.78012E-08	3.64119E-06
14.68449011	-3.672776064	1.09616785	-3.350559922	0.000806484	0.008135953
230.6795162	0.026104372	0.122364039	0.213333693	0.831066695	0.915614595
3696.895032	0.077338197	0.094103529	0.821841629	0.411167038	0.621111275
5.44519508	0.870211085	0.52198345	1.6671239	0.095489771	0.248750551
3.745059648	0.688709494	0.546657468	1.25985564	0.207721444	0.411347886
84.09091072	0.271841051	0.124273193	2.187447213	0.028709893	0.110635502
214.3436497	-0.064794892	0.08047303	-0.805175257	0.420718552	0.629081489
946.6823938	-0.380032854	0.080105868	-4.744132523	2.09402E-06	6.46008E-05
29.9432894	-0.056866946	0.212131942	-0.26807347	0.788642767	0.890724046
1105.155673	-0.203578878	0.049730455	-4.093645971	4.24643E-05	0.000784247
9.875721289	-0.341516501	0.710331247	-0.480784849	0.630669419	0.79310033
407.6618098	-0.043775424	0.104575825	-0.418599842	0.675508605	0.824469335
105.8387974	-0.060694039	0.104141127	-0.582805665	0.560024131	0.744020951
52.04387833	0.102116819	0.136429093	0.748497383	0.454160203	0.658438369
759.7117994	-0.119356529	0.068861459	-1.733284939	0.083045036	0.228033305
351.3959318	0.030618797	0.063671967	0.480883475	0.630599318	0.793098965
229.8691389	0.658889323	0.222346953	2.963338664	0.003043215	0.022007872
22.24882269	-0.725399211	0.416767052	-1.740538766	0.081764459	0.225701692
3.852896363	1.321948337	1.01890636	1.297418869	0.194487104	0.39534693
28.73188526	-0.956276599	0.408713104	-2.339725813	0.019297902	0.083761148
506.2147368	0.006555281	0.049607205	0.132143723	0.894870613	0.947009602
2.018282486	0.928713502	0.791002682	1.174096527	0.240356364	0.4477831
133.7823851	-0.380722388	0.264169157	-1.441206808	0.149526266	0.332560838
979.4879181	-0.071700985	0.132753742	-0.540105188	0.589124493	0.762630591
18.22020705	0.680646785	0.474992879	1.432962084	0.151868673	0.336094105
1535.048498	-0.032685001	0.094883212	-0.344476124	0.730488249	0.85760625
18.88280888	-0.012204725	0.275160804	-0.04435488	0.964621526	0.982392126
617.7143738	0.29878036	0.11476139	2.603491997	0.009227942	0.049663874
849.2684435	0.071782061	0.055669247	1.289438331	0.197245743	0.398702622
61.49672075	-0.508041612	0.173934246	-2.920883175	0.003490407	0.024323588
96.33597791	-0.02140687	0.107893571	-0.198407281	0.842726422	0.921837233
106.4952959	0.097765052	0.131388197	0.74409311	0.456820147	0.660492813
499.8281401	0.146709122	0.077644125	1.889507053	0.058823919	0.180998363
6.608220767	0.246181355	0.466598832	0.527608168	0.597771323	0.769410483

1022.256743	0.109417761	0.070487243	1.552305878	0.120589052	0.290096969
211.0490062	-0.030928319	0.082897035	-0.373093187	0.709079089	0.84494531
290.2307926	-0.08909762	0.063055483	-1.413003529	0.157654687	0.344228404
513.2278738	0.246165173	0.072619651	3.38978735	0.000699469	0.007294501
495.48511	-0.05572331	0.078341249	-0.711289522	0.476904843	0.676735252
154.6727774	0.268361994	0.109125101	2.459214166	0.013924153	0.066720911
116.2482598	-0.093475237	0.123909762	-0.754381539	0.450620153	0.655561717
6.692440173	0.107118666	0.598253905	0.17905218	0.85789673	0.929613001
491.2576715	-0.258415748	0.090650839	-2.850671337	0.004362704	0.028497694
62.45441948	-2.532671557	0.448696248	-5.644512451	1.6565E-08	9.50843E-07
132.3620538	0.096847849	0.101237567	0.956639438	0.338749276	0.553934685
567.6640257	-0.147215114	0.113494877	-1.297108013	0.194594025	0.395428884
19.5604712	0.30177546	0.277003502	1.089428322	0.275965047	0.488528712
252.4925121	0.011323953	0.074255304	0.152500259	0.878792386	0.939164825
34.4977287	-0.391387703	0.177961151	-2.19928732	0.027857499	0.108441502
98.94683657	-0.132022984	0.114581492	-1.152219102	0.24923105	0.458249133
3.459849617	-0.549686165	0.502194691	-1.094567855	0.273706005	0.486358831
170.3896567	0.399813263	0.124496637	3.211438246	0.001320724	0.011927918
408.5215303	-0.206156797	0.067687346	-3.045721364	0.002321228	0.018029192
162.3407026	0.121031856	0.097307624	1.243806509	0.213570784	0.418106369
47.18222944	-0.375296241	0.185162828	-2.026844398	0.042678328	0.145423175
178.3442059	0.15754343	0.096987115	1.62437484	0.104295833	0.264183941
7.999313943	0.187682762	0.368181901	0.509755535	0.610222741	0.779148737
555.7053676	0.024115968	0.085745634	0.281250097	0.778518583	0.884748561
257.0349426	0.005293487	0.14443141	0.036650522	0.97076366	0.985661927
1112.344805	0.196163221	0.103168214	1.901392034	0.057250682	0.177383438
568.2754657	-0.265313662	0.207949083	-1.275858774	0.202005447	0.404850761
133.9806351	0.094230461	0.091809615	1.026368108	0.304718105	0.519499747
630.1811402	0.283995478	0.063827337	4.449433303	8.60972E-06	0.000211066
398.1026687	0.119480535	0.073237879	1.631403535	0.1028052	0.26200694
130.6378018	-0.286770026	0.130243738	-2.20179512	0.027679784	0.107892187
76.83069963	-0.412124544	0.306592181	-1.344210876	0.178880206	0.374443775
5.248869555	0.158701533	0.554776682	0.286063814	0.774829246	0.883236715
12.95014717	-0.479864618	0.337273047	-1.422777844	0.154800566	0.339907082
164.7393883	0.241642516	0.221790369	1.089508606	0.275929662	0.488528712
45.27251752	0.576194257	0.217550595	2.648552896	0.00808372	0.04505115
820.9517895	0.276608581	0.063747486	4.339129265	1.43048E-05	0.000321498
65.65309167	-0.140889775	0.119835998	-1.175688253	0.239719475	0.447181861
2241.363218	0.121361318	0.068593692	1.769278123	0.076847472	0.216518772
111.9002331	0.32793394	0.14300149	2.293220449	0.021835316	0.091460718
15.68122214	0.174095887	0.472172666	0.368712336	0.712342153	0.846367358
398.1317239	-0.068048394	0.085650415	-0.794489951	0.426910257	0.634224869
27.05110033	-0.120368715	0.548791599	-0.219334106	0.826389796	0.913336211
4551.82081	0.065973773	0.062471737	1.056057927	0.290941751	0.506142485
156.8947954	-0.023948876	0.092599568	-0.258628377	0.795921986	0.894773586
29.66823022	0.114712119	0.205196894	0.559034384	0.576138254	0.754595452
278.4141479	-0.041928655	0.086867324	-0.482674653	0.629326764	0.79225209
1524.263183	-0.121050034	0.045476984	-2.661786763	0.00777271	0.043672451

744.1246587	-0.261040555	0.148839468	-1.75383961	0.079457994	0.221150699
1431.1989	-0.205726569	0.069263306	-2.970210081	0.002975962	0.021660227
7.775418783	0.326427498	0.392109485	0.832490694	0.405132029	0.615809655
75.76264573	0.284518178	0.195819274	1.452963094	0.146234001	0.327584843
204.3551709	0.099000229	0.079330781	1.247942193	0.212052228	0.41657975
3559.42464	0.069912347	0.100036815	0.698866186	0.484635661	0.683722916
3907.634902	0.032506499	0.075212788	0.432193776	0.665600582	0.8175592
758.1364824	-0.118024016	0.048640513	-2.426454995	0.015247141	0.071176369
841.0634702	-0.000443432	0.043687371	-0.010150125	0.991901511	0.995720597
364.2031885	-0.083375884	0.066392768	-1.255797673	0.209189336	0.413162105
132.0996516	-0.092967792	0.126020435	-0.737719974	0.460684632	0.663513594
125.7210598	-0.096470727	0.111376603	-0.866166903	0.386398641	0.599452311
241.3145409	-0.270125521	0.134611226	-2.006708727	0.044780686	0.150436026
65.93102211	0.068313561	0.177337953	0.385216811	0.700076789	0.839012442
1169.245045	0.10413337	0.078378175	1.328601611	0.18397944	0.381434344
711.8291174	-0.07997415	0.120273734	-0.664934461	0.506092413	0.701031397
3600.389944	0.014367336	0.088803699	0.161787581	0.871473133	0.935937769
125.6173308	-0.031781117	0.10565281	-0.30080711	0.763561586	0.876869382
98.80859693	0.135702611	0.107758761	1.259318593	0.20791528	0.411538253
42.31769569	0.466989562	0.21470808	2.174997612	0.029630295	0.113097656
152.9836492	-0.260918224	0.146403108	-1.78219047	0.074718176	0.212922892
8377.264915	0.235247892	0.084980037	2.768272419	0.005635433	0.034525147
171.8212009	0.489696032	0.187759353	2.608104595	0.009104513	0.049240889
4.454623212	-0.253770519	0.535283019	-0.474086624	0.635438136	0.79581936
434.946217	-0.414664157	0.132048632	-3.140238198	0.001688105	0.014232264
297.1557239	0.535991606	0.274741372	1.950895132	0.051069522	0.164355462
133.1151384	0.193982641	0.109462387	1.772139693	0.076371371	0.215730791
189.8727782	0.003436198	0.074281998	0.046258827	0.963103956	0.981878493
470.5007541	0.209962871	0.083953381	2.50094599	0.012386207	0.061368532
72.27414464	0.12588684	0.11559905	1.088995459	0.276155888	0.488814022
78.60579996	0.804048653	0.163520019	4.917126694	8.78237E-07	3.09809E-05
12.25298119	-0.157816533	0.298989744	-0.527832599	0.597615529	0.769330173
147.5651781	-0.103371485	0.100137367	-1.03229682	0.30193309	0.516898552
51.23883359	-2.548265724	1.224066829	-2.081802777	0.037360488	0.132575252
105.7061355	0.057293538	0.112174225	0.510754925	0.609522675	0.778474465
8352.958615	-0.099526042	0.086940853	-1.144755752	0.252310349	0.461378699
686.3705156	0.047477913	0.03970439	1.19578496	0.231780487	0.438426153
527.9513999	0.145139483	0.064377299	2.254513388	0.024163889	0.098169153
5.165351799	-0.389747474	0.687433105	-0.566960583	0.570740962	0.750883819
11.83093404	-0.539780572	0.930976011	-0.579800732	0.562049004	0.745166619
21.67072719	0.021753416	0.226058232	0.096229259	0.923338493	0.962127781
437.0978577	0.080149038	0.074844566	1.070873185	0.284226454	0.498334037
346.7838526	0.211878748	0.076873019	2.756217344	0.005847413	0.035379096
172.9412199	-2.738207178	1.277389099	-2.143596794	0.032065208	0.119000278
201.6134543	0.030160772	0.08157371	0.369736431	0.711578884	0.846071015
10.3542367	-0.12289991	0.303434989	-0.405028801	0.68545634	0.829778815
1108.268712	0.051922862	0.054308366	0.956074849	0.33903442	0.554005032
13.4580686	0.150070567	0.282374832	0.531458722	0.595100938	0.767329422

314.4256175	0.208108053	0.091028115	2.286195349	0.022242833	0.092531761
150.6607886	0.218740608	0.085494823	2.558524615	0.010511738	0.054336482
56.59317791	-1.519857203	0.542833225	-2.799860311	0.005112472	0.031999571
1711.469222	-0.129637117	0.539096607	-0.240471031	0.809965118	0.903767699
17.92432408	0.244694159	0.272348661	0.898459196	0.36894079	0.582623712
192.1663788	0.62708781	0.165393032	3.791500791	0.00014974	0.00215254
5.736753883	1.215434131	0.532553293	2.282276999	0.022472992	0.093184008
647.6726133	-0.755010467	0.169398005	-4.457021009	8.31065E-06	0.000204343
25.19964413	0.00160274	0.247789842	0.006468141	0.994839206	0.997321309
165.8592796	-2.795579938	1.354483045	-2.063946056	0.039022831	0.136275993
63.29858381	-0.466975952	0.142966893	-3.266322308	0.001089542	0.010303736
831.1388755	0.07629709	0.084058143	0.907670417	0.364052378	0.577837465
131.3601404	-0.022172424	0.11153003	-0.198802281	0.842417411	0.921757179
1040.33649	-0.050229081	0.117062048	-0.429080835	0.667864399	0.818996435
6.686372493	0.397461379	0.640788221	0.620269484	0.535080382	0.72384441
22.67379888	0.09540225	0.213727722	0.44637284	0.655327948	0.809943754
10.90070655	1.156569723	0.337232496	3.429591568	0.00060449	0.006474886
127.7003718	-0.607114134	0.341208662	-1.779304577	0.075189841	0.213501791
19.39527288	0.09665555	0.331559725	0.291517765	0.770655359	0.880306225
3.053505486	-0.282536574	0.662106032	-0.42672406	0.669580324	0.820241125
1087.859009	-0.038340134	0.03330158	-1.151300726	0.249608538	0.458607971
1747.17175	-0.004023556	0.08841894	-0.04550559	0.96370432	0.982015974
389.6597329	0.066414755	0.081773297	0.81218144	0.416687528	0.626095433
37.72951081	0.286648229	0.232632363	1.232194116	0.217876581	0.42271803
31.89262964	-0.214727393	0.342695921	-0.626582869	0.530932701	0.720595264
6.510347934	0.372776837	0.462181522	0.806559369	0.41992039	0.628773724
166.0877379	-0.033099736	0.156526734	-0.211463789	0.832525385	0.916289631
48.59083581	-0.093602849	0.185114315	-0.505648898	0.613103148	0.780968164
137.2524527	0.446106919	0.146129976	3.052809084	0.002267101	0.017751058
405.371698	-0.002175673	0.063101473	-0.034478957	0.972495222	0.986264854
449.2202386	-0.164226225	0.071200423	-2.306534414	0.02108079	0.089276331
263.3863512	0.015056537	0.150976399	0.09972775	0.920560469	0.960863788
38.66434383	-0.284180799	0.277419518	-1.024372045	0.305659581	0.520512215
9.87707301	0.912654013	0.343160051	2.659557866	0.007824329	0.043887638
695.7616914	0.029194306	0.058713885	0.497230008	0.619026863	0.784935848
28.22266866	-0.008865509	0.250182482	-0.035436169	0.971731944	0.985915628
2123.04768	-0.291106803	0.095476402	-3.048992192	0.002296104	0.017876193
7.717799876	0.759930115	0.441945804	1.719509741	0.085521596	0.232220663
98.45533546	-1.131116963	0.385543019	-2.933828152	0.003348095	0.023551034
874.6243208	-0.185163463	0.088809843	-2.084943029	0.037074472	0.131886171
253.2119716	0.152618757	0.119612973	1.275938164	0.201977379	0.404850761
535.3566137	-0.130897847	0.066058034	-1.981558312	0.047528697	0.156667869
98.72050073	-0.133383775	0.116643949	-1.143512176	0.252826002	0.461985981
1.814290707	1.632683658	1.217373104	1.341153055	0.179870765	0.375830985
9257.79391	0.098216735	0.097058868	1.011929524	0.311571752	0.526390425
286.2365615	0.772032275	0.135490615	5.698049813	1.21186E-08	7.28618E-07
304.1938822	0.161081551	0.22661337	0.710821036	0.477195143	0.676999035
36.21382269	-0.229062892	0.348244763	-0.657764069	0.510689746	0.705087402

213.8099652	0.023641744	0.101514233	0.232890933	0.815846099	0.907607017
3.839673862	-0.816582446	0.675751351	-1.20840668	0.226890872	0.432615766
67.83924446	-0.726745044	0.420529064	-1.728168413	0.083958035	0.229411954
4.109567733	-0.688663607	0.510637774	-1.348634281	0.177454464	0.37273809
1934.190699	-0.184004814	0.097603255	-1.885232358	0.059398474	0.182153846
74.68717306	0.043576892	0.139690671	0.311952774	0.755076415	0.872003425
332.814418	-0.234249189	0.103160433	-2.270727071	0.023163505	0.095037039
8.727351633	-0.112605646	0.322786203	-0.348855202	0.727198019	0.85562812
285.377072	0.086843571	0.075845098	1.145012315	0.252204056	0.461286735
49.7085224	-0.028865829	0.161279573	-0.178980068	0.857953353	0.929613001
8.2726022	0.512327742	0.394623771	1.298268833	0.194194972	0.395056554
3.040849423	0.70333218	0.672424918	1.045963886	0.295577707	0.510360249
262.6829138	-0.123381039	0.069799678	-1.767644799	0.077120304	0.216842445
296.6143799	-0.026121387	0.067340276	-0.387901391	0.698089009	0.838154532
2.135818742	-0.685290777	1.066351062	-0.64265025	0.520451069	0.71259233
66.03513557	0.453819515	0.185820092	2.442252132	0.014595949	0.068878161
2076.870849	-0.04395572	0.127618847	-0.344429694	0.730523161	0.85760625
11.91118039	-0.214231223	0.53188738	-0.402775534	0.687113361	0.830728424
165.1112179	-0.075194615	0.09485864	-0.792701806	0.427951578	0.634941316
3420.529086	-0.079935794	0.071812046	-1.113125143	0.265654687	0.477355509
4.441391513	0.156374443	0.498911888	0.313430983	0.753953248	0.871653489
78.43616335	0.468328494	0.198012076	2.365151177	0.018022712	0.079749171
246.1997912	-0.146615407	0.071524792	-2.049854355	0.040378646	0.139718086
1489.432153	-0.059323448	0.054401056	-1.09048339	0.275500266	0.488166774
14.3711197	0.186080234	0.264558592	0.703361145	0.481830714	0.680761508
3509.617279	-0.134157648	0.04646885	-2.88704469	0.00388879	0.026143507
11.72800131	0.092064279	0.363547336	0.253238766	0.800083711	0.897004155
3045.194146	0.042442295	0.052686378	0.805564856	0.420493796	0.629066856
40.39910426	0.224808125	0.264943379	0.848513846	0.396151865	0.609075814
166.6372719	-0.087033884	0.10907761	-0.79790788	0.42492395	0.632424608
368.5660015	-0.085393036	0.080465683	-1.061235469	0.288582904	0.503240686
1105.180774	0.042987999	0.062486971	0.687951402	0.491483386	0.689139271
47.88476616	0.492331894	0.16439134	2.994877317	0.002745552	0.020466168
2519.171268	0.306473568	0.082073359	3.734141904	0.000188356	0.002602623
1559.238102	-0.114991302	0.095707936	-1.201481367	0.22956453	0.435696552
1822.401649	0.053513139	0.088290639	0.606102071	0.544446976	0.731404997
4.403989669	0.200225444	0.505449979	0.396133056	0.692006874	0.834043309
334.6292991	0.104104037	0.077455121	1.344056209	0.178930211	0.374500864
32.15525547	-0.282562625	0.318878481	-0.886113809	0.375556203	0.589005416
4.883180505	-1.926435423	0.65306087	-2.949855844	0.003179222	0.022706287
5.86490503	0.383813037	0.397154132	0.966408268	0.333839929	0.549089355
29.87420395	0.002718297	0.252849029	0.010750671	0.991422371	0.995470966
214.9446521	-0.04536604	0.079212369	-0.572714094	0.566838293	0.748471553
1246.888577	0.20472688	0.071137113	2.877919433	0.004003074	0.026688237
595.1011284	-0.165301364	0.063984281	-2.583468347	0.009781244	0.05177899
541.3369654	-0.242942072	0.050173141	-4.842074218	1.28491E-06	4.29362E-05
6.128447487	0.837003491	0.434793946	1.925057832	0.054222124	0.170468562
4.824451766	0.08696233	0.468827495	0.185488972	0.852845529	0.927255626

2.426571472	-0.214979607	0.936678745	-0.229512635	0.818470501	0.908774216
324.8411798	-0.034433671	0.106162411	-0.324348993	0.745673832	0.866724055
1308.309453	0.131379122	0.065491394	2.006051704	0.044850732	0.150605576
112.4975334	-0.041039631	0.107424049	-0.382033922	0.702436204	0.840493334
10.98018736	0.226754081	0.305867253	0.741348015	0.458482456	0.661676739
61.1267568	-0.21200978	0.140859771	-1.505112339	0.132295197	0.30681677
18.43410147	-1.013304494	0.633485543	-1.599570039	0.109694	0.273412054
94.80322132	0.201438798	0.115815788	1.739303438	0.081981403	0.226111105
109.6187932	0.063767828	0.117051734	0.544783289	0.585902573	0.760836448
870.7947351	0.123954459	0.07637533	1.622964619	0.104596969	0.264811812
148.7547755	-0.267203223	0.119602754	-2.234089209	0.025477204	0.102020286
410.5842168	-0.673008476	0.109731824	-6.133211423	8.61226E-10	7.20195E-08
9.375079822	-1.663404385	0.612018146	-2.717900434	0.00656976	0.038639855
52.94672058	-0.078202169	0.208493311	-0.375082389	0.707599194	0.843853274
1412.192106	1.000552086	0.427579914	2.340035284	0.019281918	0.083756809
1497.048481	-0.126057042	0.085295845	-1.477880217	0.139439842	0.31789814
573.8370546	-0.131122551	0.088987906	-1.473487324	0.140619679	0.319655456
22.54437174	0.062817238	0.191316256	0.328342397	0.742652792	0.86500393
6.51245339	-1.519465734	0.722659239	-2.102603345	0.035500457	0.127760648
152.8837268	-0.110002273	0.135760266	-0.810268539	0.417785853	0.627002291
36.43645232	-0.121205349	0.193279145	-0.627099985	0.530593697	0.720253837
4958.706387	-0.39056743	0.11577955	-3.373371472	0.000742537	0.007640572
54.06514588	0.077286381	0.176445821	0.438017635	0.6613735	0.814056043
6.205968141	0.965029702	0.563409268	1.712839594	0.086742054	0.234145272
12.76589983	-1.287325987	0.421610544	-3.053353397	0.002262993	0.017735747
2.633799973	-2.916514508	1.241441374	-2.34929701	0.018808897	0.082146809
43.62214333	-0.516506483	0.288252064	-1.791857015	0.073155873	0.210002673
11.61762213	-0.889067291	0.427926517	-2.07761673	0.037744674	0.133463353
126.4300826	-0.192948793	0.121833738	-1.583705769	0.113260691	0.278944032
4.04221487	-1.850596425	0.625451287	-2.95881784	0.003088216	0.022235694
51.77616389	0.242087988	0.179428927	1.34921382	0.177268297	0.372537049
23.08446285	0.023715068	0.235540272	0.10068371	0.919801543	0.960396135
18.0098122	0.313336148	0.283070715	1.106918275	0.268329264	0.480327751
8.914043356	-0.190126209	0.342167695	-0.555652132	0.578448686	0.756538873
180.9981713	0.704381542	0.085231853	8.264299304	1.40518E-16	6.09181E-14
67.67761629	0.335902792	0.145254827	2.312506916	0.020749765	0.08828296
14.37318583	0.114924126	0.281835481	0.407770253	0.683442354	0.828722902
3.107598	-0.53912204	0.596541319	-0.90374635	0.366129916	0.579528253
7.018127966	1.066099016	0.377403855	2.824822803	0.004730677	0.030165742
66.37543965	0.606854034	0.157133955	3.862017184	0.000112455	0.001721727
7.469786783	1.007027434	0.429688658	2.343621168	0.019097558	0.08316032
22.49556167	0.899308206	0.258672325	3.476630936	0.000507756	0.00564806
131.2613653	0.131656833	0.101017619	1.30330564	0.192470436	0.393136763
6.62567648	0.093301139	0.489490891	0.190608529	0.848832308	0.92545754
134.8422467	-0.5160813	0.257723059	-2.002464593	0.045234788	0.15162372
191.0799532	0.172878535	0.078746836	2.195371171	0.028136983	0.109116917
115.288985	-0.071731555	0.153356763	-0.467743016	0.639968376	0.799063136
215.7912717	-0.519440654	0.079369652	-6.5445752	5.96647E-11	6.73231E-09

285.2832091	-0.167578825	0.072984469	-2.29608885	0.021670802	0.090941816
293.3491006	-0.207870522	0.069617598	-2.985890475	0.002827539	0.020878923
37.15569792	-0.284017305	0.161171893	-1.762201208	0.078035308	0.218596475
15.62269102	0.298976171	0.402308862	0.743150845	0.45739036	0.660928836
182.5173752	0.094069797	0.092190706	1.020382651	0.307547019	0.522484231
288.1719546	0.046123386	0.07829853	0.589070898	0.555813714	0.740455696
7.269599981	-0.241501324	0.421015632	-0.573616052	0.566227647	0.748218036
369.8299757	0.018489141	0.082186042	0.224966928	0.822005003	0.910734442
34.44448956	0.202030547	0.237020609	0.852375442	0.394005746	0.606905157
105.1732388	0.134157247	0.089213435	1.503778521	0.132638403	0.307325606
124.5976828	0.251758835	0.086812225	2.900038971	0.003731163	0.025410159
212.6191915	-0.263187043	0.078023194	-3.373189828	0.000743027	0.007640842
3.456728027	-0.069227714	0.8403611	-0.082378533	0.934345706	0.9689185
129.294451	0.009092786	0.092436444	0.098367971	0.921640107	0.961381569
9.361817738	-2.22700412	1.045953187	-2.129162326	0.033240833	0.122070771
106.0301795	0.237715189	0.116340821	2.043265526	0.04102616	0.14136477
31.08253244	0.143802177	0.443076385	0.32455392	0.745518708	0.866724055
2.548575151	0.067256273	0.653077108	0.102983664	0.917975936	0.959320958
346.1992387	-0.300388767	0.081719378	-3.67585725	0.000237052	0.003127267
577.1480475	0.608659544	0.158848574	3.831696631	0.000127263	0.001895591
5.805427971	0.38195491	0.38645005	0.988368122	0.322972394	0.538104959
62.57389639	0.204785669	0.144884752	1.413438382	0.157526867	0.344045474
73.95306402	-0.367941058	0.155346304	-2.368521482	0.017859343	0.079214443
84.24109688	0.039635331	0.102871822	0.385288514	0.70002367	0.839012442
153.1296613	-0.114547012	0.090860991	-1.260684162	0.207422663	0.410953812
6.350808318	-3.30327651	1.420741992	-2.325036163	0.020070027	0.086169825
1.779403586	1.096264466	0.90150168	1.216042621	0.223968686	0.429080142
12.12039362	0.760410552	0.376274895	2.02089101	0.043291049	0.146895312
10.13748522	-0.075337051	0.380791683	-0.197843215	0.843167734	0.922027563
112.1568063	-0.410787522	0.141382474	-2.905505261	0.003666607	0.02513678
190.5059855	-0.771296852	0.302888975	-2.546467236	0.010881943	0.055711471
515.3649198	-0.338435049	0.089786402	-3.769335218	0.000163683	0.00232458
81.40853349	0.174088363	0.1661082	1.048041956	0.294619277	0.509399451
24.72472114	0.116571825	0.196657672	0.592765205	0.553338297	0.738651252
916.0860406	0.180813013	0.098085882	1.843415263	0.065268403	0.194506454
486.8504441	-0.200395238	0.099459026	-2.014852208	0.043920137	0.14841853
639.1771789	0.263298667	0.058220213	4.522461433	6.11246E-06	0.000157832
26.96746336	0.313892445	0.243294172	1.29017659	0.196989352	0.398428801
204.8217483	-0.42328748	0.081001461	-5.225677103	1.73519E-07	7.38644E-06
2.442905033	0.31935724	0.606587677	0.526481582	0.598553645	0.7697845
190.3827617	-0.23505256	0.119006275	-1.97512744	0.048253688	0.1583213
720.9725511	-0.039638631	0.059934847	-0.661362016	0.508380179	0.702907521
7.406052976	-0.090822568	0.392876609	-0.231173263	0.817180201	0.908075995
6.716607298	-0.09898729	0.365487423	-0.270836378	0.78651688	0.889541352
34.21788249	0.231953591	0.188966143	1.227487566	0.219639393	0.424040707
5459.263722	-0.141046033	0.084598514	-1.667240075	0.095466677	0.248750551
3187.210496	-0.019517281	0.168146949	-0.116072763	0.907594875	0.953492632
2.306775188	1.064566138	0.676971836	1.572541251	0.11582508	0.282613205

376.2727218	-0.105092174	0.085911921	-1.223254851	0.221233454	0.42601247
20.79235859	-0.873284428	0.27668578	-3.156231694	0.001598219	0.013763226
11.14682529	-0.056340299	0.371491943	-0.151659545	0.879455466	0.939674547
171.4804034	-0.014548866	0.127092817	-0.114474339	0.908861787	0.954154288
387.0831239	-0.032047049	0.144302087	-0.22208306	0.824249225	0.911443263
5.163733891	-0.192786896	0.483351715	-0.398854271	0.690000585	0.83271258
290.7390817	-0.248052558	0.107497755	-2.307513842	0.021026191	0.089159711
607.1212356	-0.030923721	0.21586384	-0.143255677	0.886088259	0.943111133
54.89550065	0.6215337	0.151476857	4.103159479	4.07546E-05	0.000756924
44.45407556	0.371718313	0.192531654	1.930686749	0.053521803	0.169268223
127.3565736	0.118142544	0.095249586	1.240347063	0.214847052	0.419309363
144.949282	-0.319610407	0.087645177	-3.646639979	0.000265692	0.003417718
30.67000156	0.347869353	0.251770678	1.381691289	0.16706651	0.35831971
3.945940974	-3.25380135	1.565788568	-2.078059208	0.037703906	0.133347821
2379.755737	-0.362954296	0.093576704	-3.878682185	0.000105024	0.001630691
270.6951175	0.196197753	0.091672994	2.1401914	0.032339302	0.119747732
2645.108322	-0.008935615	0.046181016	-0.193491089	0.846574384	0.92396956
2084.772297	0.171541137	0.048545766	3.533596276	0.000409947	0.004799903
49.58883764	0.202711483	0.18999294	1.066942186	0.285997944	0.500428054
797.1436839	-0.022414773	0.067731348	-0.330936468	0.740692475	0.863809134
9.272652154	0.797854968	0.496058089	1.608390198	0.107749747	0.269890426
5.43582452	-0.434761995	0.686655471	-0.63315886	0.526629908	0.717000092
95.33518142	-0.299369065	0.199656248	-1.499422467	0.133764069	0.309281301
155.0757982	0.019818528	0.088729737	0.223358355	0.823256614	0.911263149
74.08152405	-0.264219567	0.147919751	-1.786235889	0.074061072	0.211672815
23.81529371	-0.043293462	0.232466872	-0.186234976	0.852260497	0.927038608
58.36687874	0.092087403	0.148418343	0.620458367	0.534956056	0.72384441
9.145762351	-0.167841641	0.419235762	-0.40035144	0.688897685	0.832091829
3.244384728	0.367305579	0.561235521	0.654458895	0.512816201	0.706449263
3.293503116	1.394683956	0.74673551	1.867708094	0.06180277	0.187313038
107.0102077	0.235165974	0.140546814	1.673221663	0.094283667	0.246700942
397.6664449	-0.182523664	0.1262306	-1.445954183	0.148190053	0.330578336
261.468462	0.08608529	0.187450818	0.459242007	0.646060388	0.803684878
68.53101692	-0.484320543	0.190763505	-2.538853245	0.011121646	0.056688739
76.80592345	0.168400236	0.176433783	0.95446707	0.339847271	0.554650677
251.9254478	-0.340731815	0.19786595	-1.722033609	0.08506343	0.231382743
94.0042753	-0.383942478	0.204129812	-1.880874103	0.059989047	0.183641963
3.066961008	-0.570440199	0.683444671	-0.834654542	0.403912243	0.614990985
520.8497146	-0.51133296	0.153619317	-3.328572025	0.000872924	0.008664779
6.397657	0.28924268	0.44197561	0.654431315	0.512833965	0.706449263
238.7555846	-0.334295004	0.091516087	-3.652855102	0.000259341	0.003364076
224.2439389	-0.090214634	0.101736075	-0.886751669	0.375212613	0.588730778
45.21750421	-0.380293978	0.152040837	-2.501262061	0.012375157	0.061344353
67.77689221	0.21667115	0.328260771	0.660058006	0.509216606	0.703643212
782.0980947	-0.138031087	0.068169733	-2.024814841	0.042886381	0.145852649
3.138062958	-0.635662505	0.621432484	-1.02289874	0.306355726	0.521482147
127.1119406	-0.036374105	0.13986382	-0.260068006	0.794811316	0.894318805
179.1221644	-0.264559951	0.107591739	-2.458924386	0.013935396	0.066755371

2.346977581	0.546880726	0.7237358	0.755635863	0.449867548	0.654773154
528.1122474	0.08349089	0.110675576	0.754375019	0.450624068	0.655561717
24.16936372	0.102773073	0.274291167	0.374686048	0.70789397	0.844021514
175.2039761	0.047133045	0.079787335	0.590733412	0.554699057	0.739598787
17.68662711	0.323880575	0.280039487	1.156553239	0.247454936	0.455983085
127.890579	1.116691221	0.2297489	4.860485611	1.17098E-06	3.93689E-05
1226.110221	-0.247833284	0.067964337	-3.64651955	0.000265816	0.003417718
270.6837373	-0.145764631	0.092371614	-1.578024084	0.114560077	0.281051781
166.1597308	-0.220618555	0.123455484	-1.7870292	0.073932769	0.21141615
77.68338493	-0.421634206	0.115205643	-3.659839857	0.000252373	0.003286634
37.12146145	-0.113095242	0.26128317	-0.432845493	0.665127021	0.817160306
556.5345735	-0.01712826	0.075201203	-0.227765776	0.819828335	0.909423741
707.3781533	-0.01809385	0.053247462	-0.339806798	0.734002028	0.859780248
1335.655886	-0.075374513	0.079345097	-0.949958038	0.342133575	0.556467099
8.915702662	0.278972249	0.363680457	0.767080672	0.443033553	0.648528056
22.8839284	-0.151636299	0.456907159	-0.331875516	0.739983259	0.863255748
265.2539807	-0.000907507	0.242982593	-0.003734863	0.997020018	0.998110584
531.8080495	0.366953832	0.094326541	3.890250061	0.000100141	0.001564554
27.8433155	1.333459045	0.228126223	5.845268582	5.05752E-09	3.57586E-07
17.61015952	-0.036698368	0.238646461	-0.153777131	0.877785466	0.938489926
28.2386213	1.022411878	0.652133725	1.567794824	0.116929023	0.284575082
119.3104684	-0.090199396	0.129661727	-0.695651665	0.486647002	0.685332768
126.5355608	-0.159647984	0.180215862	-0.885870882	0.375687109	0.589069671
4.261704058	0.415962274	0.501464001	0.829495781	0.406823923	0.617614597
281.0559845	0.17436722	0.098542474	1.76946257	0.076816712	0.21648971
132.0002048	0.320137497	0.20762929	1.541870596	0.123105042	0.293153002
457.6954238	0.058782329	0.090577678	0.64897147	0.516356817	0.7092856
128.6765002	0.126985493	0.11277634	1.1259941	0.260168017	0.471248808
24.94298642	0.035794247	0.205513293	0.174169985	0.861731874	0.931286863
2.298779163	-3.06527375	1.442899634	-2.124384591	0.033637997	0.123008295
15.92245317	-0.142693863	0.257241112	-0.554708625	0.579093976	0.756902107
359.7256597	0.178283592	0.228626956	0.779801275	0.435507856	0.642075928
1.798972335	0.705173535	0.754136393	0.935074267	0.349750027	0.564603816
13.81043217	-0.583638173	0.384162231	-1.51924923	0.128699774	0.300951039
7.288788746	-0.22402789	0.43711532	-0.512514387	0.608291054	0.777532194
452.5981056	0.132657791	0.090501811	1.465802613	0.142702069	0.322346618
30.34597317	0.25771749	0.231809624	1.111763548	0.266239829	0.478041955
22.77338667	-0.363397251	0.220125645	-1.650862856	0.098766582	0.254549542
493.0743383	-0.05622236	0.112295049	-0.500666419	0.616605909	0.783310129
26.78995133	-0.100284906	0.440713085	-0.22755146	0.819994959	0.909486094
92.0544121	1.321471517	0.190389121	6.940898265	3.89615E-12	6.35496E-10
775.1887938	0.48403671	0.072097178	6.713670643	1.89788E-11	2.50126E-09
243.5700023	0.268336742	0.168851405	1.589188687	0.1120178	0.27702774
114.7021154	0.196168961	0.108267368	1.811893689	0.070002625	0.203605798
266.9139598	-0.318993037	0.079084111	-4.033591992	5.49307E-05	0.000968873
23.33942543	1.20272754	0.245824923	4.892618402	9.95032E-07	3.42216E-05
39.99092582	-0.068855137	0.190516149	-0.361413649	0.717790246	0.850282341
676.8392266	-0.228396651	0.148743546	-1.535506294	0.124659512	0.295445376

32.46819635	0.169857411	0.189798209	0.894936846	0.370820849	0.584428073
68.41050561	0.416290486	0.149724251	2.780381147	0.005429513	0.033525412
111.957112	0.177559174	0.115737244	1.534157609	0.124990883	0.295805174
2.091281202	-1.261577599	0.671608178	-1.878442878	0.060320604	0.184432375
56.05485905	-0.560724645	0.178226297	-3.146138669	0.001654415	0.014063385
5.747500436	-0.217012505	0.476118163	-0.455795478	0.648537057	0.805267878
190.6070568	0.241351842	0.097788833	2.468092066	0.013583538	0.065546339
522.3439286	-0.166355592	0.091807401	-1.812006337	0.069985217	0.203591113
609.5700321	-0.10791205	0.079169623	-1.363048673	0.172867171	0.366184104
14512.56348	-0.400470637	0.324691499	-1.233388116	0.217430996	0.422248697
14.14714221	-0.270470459	0.299710051	-0.902440401	0.366822968	0.580392006
1182.894325	0.026111946	0.097917107	0.266673996	0.789720177	0.891207713
893.9093662	0.237465228	0.099928231	2.376357777	0.0174845	0.078186661
344.968389	0.151933117	0.089852135	1.690923839	0.09085135	0.241322982
483.2694468	0.059214143	0.109913207	0.538735465	0.590069399	0.763536963
221.6953498	0.113901997	0.109878209	1.036620442	0.299912782	0.514763808
2.108954637	0.52388815	0.662015206	0.791353651	0.428737649	0.635631543
19.57986485	0.625926452	0.527266296	1.187116373	0.235181721	0.442483288
4.576579157	0.672654429	0.621397642	1.082486291	0.279036504	0.492325946
4.331140235	0.097823633	0.595610187	0.164241035	0.869541399	0.935101834
450.4940327	-0.250977731	0.273350837	-0.918152413	0.358539078	0.572953028
24.51492381	-1.422027199	0.225369382	-6.30976215	2.79465E-10	2.72266E-08
496.1865997	0.116646828	0.078590882	1.484228518	0.137748307	0.315526363
126.8334663	-0.04474633	0.105895384	-0.422552225	0.67262199	0.822263819
289.4756229	-0.100253361	0.102708888	-0.976092354	0.3290187	0.544639677
53.66082799	0.488180318	0.201415566	2.42374672	0.015361315	0.071526937
255.6860974	-0.263205703	0.085269137	-3.086764007	0.002023482	0.016332601
579.7891155	-0.060817807	0.097869168	-0.621419475	0.534323661	0.723528522
1171.884107	-0.268257989	0.069634497	-3.852372042	0.000116979	0.001766374
69.28924084	-0.253634182	0.178326058	-1.422305775	0.154937503	0.340053348
282.4887544	-0.192642807	0.115846253	-1.662917892	0.096328872	0.249987688
147.7115889	0.205341677	0.166196334	1.235536741	0.216630811	0.421392843
11.79128242	1.055484342	0.383421417	2.752804866	0.00590871	0.03562961
19.35645579	-0.00258239	0.226501184	-0.011401219	0.99090334	0.995184112
2.703955725	0.729192405	0.651408029	1.119409606	0.262965435	0.474375349
173.4870152	-0.04566855	0.08670943	-0.526684924	0.598412406	0.769694408
81.90284133	-0.556794111	0.241942222	-2.301351566	0.021371767	0.09018404
60.99375885	-0.109634766	0.30693539	-0.357191674	0.720948319	0.851889535
1.722233328	-0.215088417	0.677155203	-0.317635332	0.75076157	0.86988649
6.151932648	-0.029300898	0.809663071	-0.036189001	0.971131656	0.985661927
121.8879189	0.062627976	0.12269252	0.510446566	0.609738641	0.778669331
211.4117937	-0.172368012	0.102674557	-1.678780186	0.093194892	0.245097804
77.51741958	-0.017820028	0.1225118	-0.145455607	0.884351163	0.942293582
739.8079296	-0.510216816	0.154413764	-3.304218507	0.000952416	0.009267629
339.1764309	0.092544072	0.103694261	0.892470532	0.372140781	0.585510216
6.355395791	-0.5782973	0.549987875	-1.051472818	0.293041487	0.508189707
91.09109791	0.247162813	0.159633197	1.548317126	0.121545963	0.2914202
2104.835081	0.052210981	0.091039847	0.573495927	0.566308956	0.748257714

543.8104928	-0.314994284	0.108200651	-2.911205063	0.003600376	0.02484683
381.9400297	0.143881914	0.07702693	1.867942976	0.06177002	0.187265238
33.36530294	0.222600757	0.22826731	0.9751758	0.329473064	0.545063191
372.1847659	0.027151696	0.112586188	0.241163647	0.809428285	0.903484082
3138.261775	-0.11753112	0.083909592	-1.400687547	0.161307528	0.349931554
413.2999683	0.002091905	0.062616023	0.033408464	0.97334886	0.9867661
49.78642498	0.298831075	0.146552565	2.039070927	0.041442949	0.142473111
66.78213088	0.397078096	0.135659719	2.927015479	0.003422318	0.023960593
4.919127876	1.240020362	0.869096552	1.426792408	0.15363974	0.338354197
55.08336343	0.59298096	0.575711898	1.029996015	0.303011876	0.517887593
242.2110197	0.007733428	0.075077489	0.103005944	0.917958253	0.959320958
22.93296124	-0.135197072	0.19863836	-0.680619149	0.496112507	0.693025942
1602.84915	0.034704078	0.048731812	0.712144212	0.476375479	0.676360393
611.1214706	-0.28624412	0.069382082	-4.12562024	3.69737E-05	0.00070308
1003.665566	0.195976826	0.065827749	2.97711569	0.002909742	0.021313956
57.66081427	-0.62389221	0.175791536	-3.549045788	0.00038663	0.004572391
2965.619103	-0.053333479	0.158455959	-0.336582349	0.736431764	0.861462535
245.7308467	-0.053993885	0.074877213	-0.721099021	0.470848593	0.671362327
2026.888721	-0.187755634	0.07162603	-2.621332425	0.008758681	0.047905216
13.39139625	-0.565387932	0.295343472	-1.914340369	0.055576679	0.173161696
851.1001231	-0.176135078	0.111237319	-1.583417139	0.113326419	0.278955834
463.7395814	-0.006386771	0.090381078	-0.070664916	0.943664444	0.972775674
1018.509122	-0.01357296	0.062887695	-0.215828545	0.829121397	0.914559484
608.4484437	-0.10417365	0.070328493	-1.481243892	0.138541594	0.316795003
218.1026909	0.286874542	0.069772384	4.111577187	3.92965E-05	0.000737325
51.24136444	-0.26091224	0.210536243	-1.239274702	0.215243784	0.419785261
165.5187362	0.068635373	0.092528702	0.741773865	0.458224358	0.661594047
401.5611922	-0.118935781	0.092852055	-1.280917053	0.200222802	0.402660393
28.52373076	-0.391155064	0.206887281	-1.890667525	0.05866874	0.180723415
192.7148278	-0.023176598	0.073413257	-0.315700439	0.752229893	0.870852794
461.9263601	-0.058114692	0.072708471	-0.799283648	0.424125959	0.631666096
287.7460568	-0.441490382	0.293501784	-1.504217029	0.132525494	0.307107187
14.67877875	0.510134928	0.260235092	1.960284927	0.049962497	0.16211979
302.9577004	-0.1885956	0.068329759	-2.760080004	0.005778721	0.035108633
261.228583	-0.338146404	0.13072225	-2.586754763	0.009688452	0.051502923
38.6685757	0.194536642	0.232924461	0.835191981	0.403609624	0.614855381
1867.993026	-0.205003393	0.124146837	-1.651297763	0.098677789	0.254440115
21.38290066	0.119270223	0.259181583	0.460180162	0.645386909	0.803265524
29.37761048	0.117423824	0.211106633	0.556229913	0.578053693	0.756142332
250.2842237	0.105381277	0.106718212	0.987472292	0.32341116	0.538630761
255.1360889	-0.313235333	0.137492843	-2.278193726	0.022715038	0.093951176
4.136600194	0.482458045	0.62971265	0.766155873	0.443583561	0.648838902
26.70858761	0.539902751	0.232593723	2.321226657	0.020274614	0.086867099
301.6335662	-0.254131824	0.147095816	-1.727661815	0.084048873	0.229584006
327.4550756	0.199466286	0.125139571	1.593950526	0.110947116	0.275345405
152.7791024	0.223711837	0.120053869	1.863428794	0.062401962	0.18860883
87.97191787	0.195616219	0.164295082	1.190639525	0.233795128	0.440679742
148.0622052	0.634621683	0.163323807	3.885665512	0.00010205	0.001590512

7788.177442	0.107267466	0.118810698	0.902843497	0.366608962	0.580220583
8.829942721	-0.053597719	0.314660878	-0.170334867	0.864746792	0.932746588
233.463328	-0.157365255	0.096119275	-1.637187282	0.101591344	0.259757224
43.85661605	-0.358281951	0.217827203	-1.644798933	0.100011282	0.256553389
6.040803386	-0.103722074	0.44942855	-0.230786571	0.817480615	0.908226035
1.998468082	0.021450021	1.256964699	0.017064935	0.986384813	0.993222667
4.670615844	-0.103339812	0.437148151	-0.236395399	0.813125866	0.906097599
91.50180492	-0.430308641	0.240872731	-1.786456436	0.074025385	0.211644254
8.248441359	-0.779081881	0.483882488	-1.610064219	0.107383837	0.269096644
33.62786039	-0.164799879	0.263208939	-0.626118093	0.531237485	0.720863825
540.9600661	-0.092771668	0.087005857	-1.066269225	0.286301957	0.50074726
787.8589632	-0.183059099	0.176602298	-1.036561254	0.299940378	0.514763808
78.52550527	0.096205528	0.131233634	0.73308591	0.463506033	0.665777173
3.414314052	-1.347276639	0.536471171	-2.511368201	0.012026419	0.060000975
155.1004166	-0.261377485	0.20089361	-1.301074159	0.193233072	0.393882135
10.52615645	-0.595782824	0.322423836	-1.847825	0.064627663	0.193183378
524.3607192	-0.178480085	0.183581749	-0.972210397	0.330945889	0.546292844
9.71213687	0.042682108	0.361777697	0.117978825	0.906084436	0.952959157
42.46678172	-0.057456204	0.167678875	-0.342656189	0.731857125	0.858466987
42.77467859	-0.108339586	0.171314796	-0.632400639	0.527125117	0.717377874
14.13904884	0.219188511	0.280121811	0.782475704	0.433935059	0.640674508
268.9412478	-0.127600547	0.131025533	-0.973860163	0.330125976	0.545419232
454.917254	0.57979478	0.079728114	7.272149667	3.53811E-13	7.56972E-11
322.1101447	0.073551108	0.115964786	0.634253814	0.525915193	0.716791813
18.46449044	-0.201914387	0.247278302	-0.816547126	0.414187282	0.623896487
18.90612769	0.18135925	0.226540746	0.800559073	0.423386952	0.631210556
429.6295182	-0.01897492	0.077203412	-0.245778263	0.805853885	0.901142879
844.9245436	0.186604004	0.150523235	1.239702322	0.215085518	0.419575941
52.5131087	-1.616563052	0.469735459	-3.441432875	0.000578642	0.006279676
8.63181601	-1.713494916	0.663016849	-2.584391208	0.009755107	0.05169046
146.8582491	-0.708065794	0.352995004	-2.005880497	0.044869	0.150611887
12.01213134	-1.146629206	0.539648153	-2.12477185	0.033605655	0.122944605
224.098073	-0.288530189	0.187732998	-1.536917817	0.124313436	0.294922169
1464.815402	-0.243652251	0.082172618	-2.965127045	0.00302558	0.02191882
171.0582784	0.06693419	0.121848748	0.549321939	0.582784534	0.75889593
260.5103432	-0.057588418	0.074156606	-0.7765784	0.43740755	0.64337964
443.7680065	0.483606832	0.088054505	5.492130478	3.97114E-08	1.98847E-06
162.1412168	0.648013496	0.158847758	4.079462651	4.51399E-05	0.000824429
224.2149743	-0.067625222	0.095178144	-0.710512089	0.477386637	0.677037488
6.312136026	0.210542847	0.505634829	0.416393086	0.677122387	0.825613125
69.15331331	0.62903399	0.123406802	5.097239209	3.44643E-07	1.34861E-05
2.603442697	-0.824077123	0.600523083	-1.372265523	0.169980794	0.36202503
330.0823589	-0.283417752	0.063015295	-4.497602568	6.8724E-06	0.000173911
7.774879919	0.325847686	0.419674134	0.776430234	0.437494999	0.643391655
1.909808341	-1.818227485	1.179027035	-1.542142318	0.123039013	0.293122879
5.290767677	-0.049976821	0.431184041	-0.115906007	0.907727035	0.953509862
80.09458016	-0.262888088	0.111244664	-2.363152334	0.01812022	0.079987271
15.98868507	-1.700135288	0.745106677	-2.281734068	0.022505045	0.093293437

3.249930576	-1.573991707	1.233924825	-1.275597731	0.202097757	0.404982173
65.79200857	0.213447638	0.175579714	1.215673687	0.224109252	0.429184035
1.921294077	-0.997669268	0.737059992	-1.353579463	0.175870583	0.370402963
327.0719109	-0.158678665	0.104350562	-1.520630669	0.128352546	0.300309593
1966.867015	0.328696572	0.080236224	4.096610675	4.19243E-05	0.000776024
2.90686323	1.615835132	0.934346937	1.729373821	0.083742212	0.229126259
22.72437646	0.926872466	0.493699465	1.877402208	0.060462989	0.184730579
848.4529963	0.122683073	0.055597563	2.206626798	0.027340143	0.106958328
10.38174799	1.009606488	0.357761872	2.822006945	0.004772414	0.030320384
12.52537918	-2.474630223	0.73689445	-3.358188166	0.000784552	0.007973293
294.639595	0.047716012	0.063394788	0.752680359	0.451642015	0.656469531
586.9729795	-0.191916811	0.070295388	-2.730147987	0.00633059	0.037527938
920.0806137	-0.148046447	0.109133699	-1.356560339	0.174920951	0.369016231
477.8881778	0.044448732	0.057848069	0.768370198	0.442267281	0.648039774
2411.676928	-0.176823848	0.077455849	-2.282898596	0.022436342	0.093066931
4.46356675	-0.865824252	0.555392181	-1.558942098	0.119010085	0.287600432
210.4440529	0.12582918	0.148585247	0.846848412	0.39707962	0.609588078
43.90296325	0.337200549	0.200585529	1.681081139	0.092747155	0.244271245
51.06447554	0.005335155	0.143601086	0.03715261	0.970363324	0.985558903
8.431781287	-0.686977815	0.596707456	-1.151280763	0.249616749	0.458607971
2398.270395	0.038457922	0.060715379	0.63341319	0.526463854	0.717000092
3.022785937	0.282270347	0.651395696	0.433331612	0.664773876	0.816909207
73.49937364	-0.116659337	0.130105252	-0.896653558	0.369903809	0.583361608
12203.9141	-0.110956912	0.129693496	-0.855531818	0.392256797	0.605488344
33.95276361	0.069063753	0.24774294	0.27877183	0.780419934	0.885626369
1.772412348	-0.139667085	0.986216188	-0.141619136	0.887380851	0.943568846
2849.874161	-0.097690335	1.432886104	-0.06817732	0.945644481	0.974141328
103.1185355	-0.014312519	0.174290409	-0.082118797	0.934552246	0.96896052
11.03709893	0.122036957	0.315756317	0.386490944	0.699133108	0.838613574
92.03101717	-0.160984563	0.172747931	-0.93190443	0.351385918	0.566504355
2.127010276	-0.277936288	0.801643325	-0.346708167	0.728810574	0.856622817
146.0110094	0.12779525	0.152134621	0.840014253	0.400900395	0.61271297
927.0547827	-0.135076686	0.06546823	-2.063240247	0.039089808	0.136375582
9.557864972	-0.633204425	0.487649964	-1.298481435	0.194121952	0.395005563
1227.785299	-0.205212925	0.112084874	-1.830870824	0.067119826	0.198017911
81.0910653	-0.744769894	0.139880319	-5.324336536	1.01322E-07	4.53582E-06
15.5102729	0.083306622	0.293183475	0.284145012	0.776299253	0.883652841
34.45960957	0.078448049	0.202736117	0.386946591	0.698795748	0.838453107
103.313489	-0.102681173	0.205458447	-0.49976613	0.617239762	0.783631924
52.12450837	-0.152833152	0.190872789	-0.800706864	0.423301368	0.631197206
3.576719443	-0.073642862	0.486110161	-0.151494183	0.879585899	0.939708048
1104.811018	-0.014046783	0.072999777	-0.192422268	0.847411451	0.924458083
471.5640142	-0.161091338	0.098027346	-1.643330607	0.100314549	0.257051155
3125.661946	-0.129397974	0.10552233	-1.226261531	0.220100273	0.424631911
346.644969	-0.042410027	0.068313132	-0.620818071	0.534719332	0.723766845
16.08487085	-0.656700528	0.361643207	-1.815879618	0.06938884	0.202559032
7203.773847	0.255040824	0.110465848	2.308775327	0.020956052	0.08890806
132.3827746	-0.12575702	0.118079155	-1.065023035	0.286865506	0.501479298

7.666695402	1.721337271	0.559285226	3.077744933	0.002085734	0.016696003
17.59965365	-0.19111348	0.254241548	-0.751700428	0.452231233	0.656855699
14.62655328	0.549123945	0.300924788	1.824788009	0.068033026	0.200067132
23.73642471	-0.089398294	0.20959718	-0.426524317	0.669725833	0.820241125
7.260655063	1.339713676	0.936662728	1.430305312	0.152629411	0.337030985
6.444708727	-0.461162866	0.485483149	-0.949904991	0.342160531	0.556467099
4.815445037	1.432752265	1.026358119	1.395957453	0.162727308	0.351997597
832.9365786	0.240409797	0.16874535	1.424689904	0.154246859	0.339124883
181.2649016	-0.007614482	0.114553381	-0.066471034	0.947002818	0.974828397
154.2003617	-0.028684366	0.122984174	-0.233236241	0.815577966	0.907449338
9.724479438	0.060150455	0.370370397	0.162406216	0.870985976	0.935876058
48.29386713	-0.56189009	0.328358566	-1.71120887	0.087042562	0.234572086
326.6259836	-0.501674452	0.149811305	-3.348708913	0.00081189	0.008170485
1.945872978	0.728604865	0.772157284	0.943596441	0.345375911	0.559846774
17.15114902	1.150335644	0.35450243	3.244930212	0.001174794	0.010938463
3.055261331	-0.171611948	0.618231547	-0.277585233	0.781330769	0.886171641
265.1805549	-0.361612764	0.142054859	-2.545585314	0.01090947	0.055797147
3.158246812	-0.198508932	0.541468281	-0.3666123	0.713908232	0.847585632
3.761022827	0.891561896	0.632145087	1.410375425	0.158428858	0.345552364
1.92864414	-0.723832696	0.790325142	-0.915866974	0.35973667	0.573951606
332.9932326	-0.158479439	0.144253191	-1.098619988	0.271933856	0.483992906
163.3125017	0.076299708	0.309615354	0.246433863	0.805346395	0.90075881
34.79174352	-0.03500523	0.227040245	-0.154180727	0.877467238	0.938418286
12.45065189	0.02742031	0.445888307	0.061495916	0.950964267	0.976633959
12.19038905	0.187590179	0.299631279	0.626070082	0.531268975	0.720863825
3.629807954	-0.381738515	0.62681912	-0.609009048	0.542518442	0.729950677
215.7258032	0.740767008	0.649369802	1.140747545	0.253974995	0.463342643
295.5792201	0.407107978	0.109217899	3.72748405	0.000193401	0.00265286
31.69938544	-0.306445257	0.468206174	-0.654509219	0.51278379	0.706449263
38.86995236	-0.345594301	0.158981527	-2.173801619	0.029720037	0.113335159
81.31727292	-0.265946745	0.195550979	-1.359986769	0.173834111	0.367476343
24.48693718	-0.304267984	0.272631386	-1.116041656	0.264404304	0.475731379
153.3624409	-0.077751428	0.101483261	-0.766150273	0.443586893	0.648838902
7.797765682	-0.460148548	0.585761122	-0.785556656	0.432127266	0.639208456
8.466920418	0.401492576	0.413811144	0.970231426	0.33193115	0.547370747
236.7750893	-0.100732289	0.120223665	-0.837874049	0.402101445	0.613694572
2.037439141	-0.022283115	0.826144019	-0.026972434	0.97848172	0.988805537
4.475189312	-2.120449818	0.812429767	-2.610010001	0.009053958	0.049021995
245.8758669	0.149588502	0.276024252	0.541939707	0.587860042	0.761885532
2.675583043	-0.120390886	0.752687435	-0.159948048	0.872921999	0.936680519
34.03236919	-0.128605235	0.224096384	-0.573883577	0.566046587	0.748143944
21.85405388	0.166294848	0.467327459	0.355842235	0.721958717	0.852372845
535.553958	0.158675759	0.10658442	1.488733145	0.136557653	0.313715072
55.30245593	-0.293128618	0.142210713	-2.061227404	0.03928135	0.136847819
361.5274695	-0.026292771	0.07694829	-0.341694026	0.732581168	0.859032114
9.295651861	-1.950062773	0.45070116	-4.326731199	1.51338E-05	0.000339204
259.2575186	-1.068935803	0.267973947	-3.988954205	6.63652E-05	0.00113767
28.86103127	0.103398668	0.221594821	0.466611392	0.640777935	0.79977087

9.925566701	0.797967756	0.355619426	2.243881232	0.024840044	0.100199531
101.9014297	-0.082520519	0.116933966	-0.705701879	0.480373552	0.679394088
64.46363651	0.151177662	0.155159954	0.974334283	0.329890588	0.545336384
24.40873075	0.075617545	0.239758609	0.315390323	0.752465313	0.870899202
8.818745132	-0.031766452	0.344824121	-0.092123637	0.926599809	0.964844526
60.61196639	0.057564684	0.145237348	0.39634904	0.691847554	0.834043309
312.9533599	-0.163423451	0.129096404	-1.265902422	0.205548019	0.409059926
12.64411164	0.491957664	0.285917364	1.720628848	0.085318195	0.231821202
57.7411543	0.468797967	0.188314979	2.489435361	0.012794618	0.062787769
45.17862794	-1.188508976	0.547870476	-2.169324735	0.030058039	0.114043343
17.05422227	-0.959089257	0.544279238	-1.762127215	0.078047806	0.218596475
66.4463055	-0.2654485	0.168669597	-1.573777992	0.115538784	0.282317698
62.87691158	0.530693892	0.232139085	2.28610314	0.022248225	0.092531761
66.50408536	0.573425379	0.260251593	2.203350126	0.027570081	0.107590346
100.2315076	0.349929048	0.285774146	1.224495126	0.220765504	0.425417115
153.3551222	-0.552772243	0.162868341	-3.39398215	0.000688842	0.007214228
21.49176406	-0.036552553	0.402808987	-0.090744135	0.927695901	0.965547435
3.639451244	0.973275171	0.57312048	1.698203442	0.089469371	0.239000879
45.14691828	-0.27001183	0.168254583	-1.604781421	0.108541916	0.271313213
981.3648728	-0.151891844	0.064095904	-2.369758965	0.017799685	0.079059588
26.49137472	-0.108866106	0.195252718	-0.557565127	0.577141375	0.75524879
9.4447057	0.128371016	0.331548015	0.387186803	0.698617919	0.838453107
62.91699291	-0.20809514	0.276832369	-0.751700895	0.452230952	0.656855699
3.1205457	0.022227159	0.539667071	0.041186798	0.967146978	0.983639473
17.78797523	0.475018786	0.246925096	1.923736366	0.054387637	0.17069574
9.221222109	0.340346982	0.375757582	0.905762115	0.365061778	0.578438754
290.2177169	-0.136408518	0.074675505	-1.826683571	0.06774736	0.19940504
8.710754051	1.936955916	0.408789709	4.73826976	2.15551E-06	6.60034E-05
80.09667077	-0.012114516	0.106143355	-0.114133529	0.909131944	0.954307284
259.710149	-0.668455249	0.175238526	-3.814545026	0.000136434	0.00198952
171.0166938	-0.257848343	0.099739584	-2.585215733	0.009731808	0.05161681
113.2791895	-0.001349872	0.100271266	-0.013462206	0.989259038	0.994530438
121.3869594	-0.225476353	0.162895126	-1.384181093	0.166303011	0.357147152
448.7676585	-0.028018195	0.080126725	-0.349673529	0.726583722	0.855164695
2753.009211	-0.008791206	0.112143515	-0.07839246	0.937515871	0.970479513
148.4168119	-0.209263757	0.119833853	-1.746282475	0.08076188	0.223721407
82.85905829	-0.248417691	0.125257793	-1.983251383	0.047339357	0.156254972
36.72606291	0.58376731	0.345649811	1.688898103	0.091238959	0.241806726
140.4542974	0.62159012	0.207330152	2.998069094	0.002716961	0.02028976
75.98338639	-0.106285319	0.135907751	-0.782040156	0.434190975	0.640937466
42.61589345	0.669142356	0.275914722	2.42517815	0.015300876	0.071339235
525.1042221	-0.396241897	0.094825347	-4.178649567	2.93245E-05	0.00058415
124.7169577	0.148252429	0.226368666	0.65491586	0.512521928	0.706319325
76.78110986	-0.026232007	0.130927731	-0.200354855	0.841203064	0.921330769
3.401059395	0.081648621	0.635786374	0.12842147	0.897815441	0.948292611
123.7586224	-0.689129597	0.23510047	-2.931213188	0.00337641	0.023709708
10.37472783	-0.889753302	0.340631058	-2.612073331	0.008999495	0.048817148
49.41603158	0.104444135	0.185764095	0.5622407	0.573952035	0.752765709

2.653200403	0.720867575	0.609776327	1.182183603	0.237132857	0.444681467
74.69938533	-0.058674432	0.108007159	-0.543245765	0.5869606	0.761579192
8.250243061	0.036107125	0.62299258	0.057957552	0.95378244	0.97763887
4.936130893	0.318635542	0.611718525	0.52088588	0.602446272	0.77305874
2856.911498	0.24841385	0.094786957	2.620759846	0.008773404	0.047953902
14.87814399	0.835681822	0.266047707	3.141097637	0.001683159	0.014199103
49.43516717	-0.31452064	0.181129477	-1.736440938	0.082485899	0.226978959
507.0958859	-0.442377677	0.221671984	-1.995640897	0.045973025	0.153475758
217.03208	-0.009774414	0.126520414	-0.07725563	0.938420187	0.970668417
798.0937738	-0.51215717	0.181309932	-2.824760701	0.004731594	0.030165742
173.1507521	-0.035523835	0.197054109	-0.180274519	0.856937059	0.92936531
218.5853651	-0.034407546	0.081096572	-0.424278675	0.67136259	0.821390813
702.2967303	-0.140219679	0.065722226	-2.133519937	0.032882098	0.121115816
51.42821114	0.585523193	0.18001159	3.252697193	0.001143152	0.010700167
97.76410563	0.142795956	0.100694483	1.41811101	0.156158357	0.341822054
60.07171808	-0.647092714	0.162441137	-3.983551997	6.78928E-05	0.001160235
7.303631227	-0.199386442	0.376743554	-0.529236505	0.596641396	0.7685566
544.9655723	-0.078101297	0.065969328	-1.183903186	0.236451389	0.443858271
339.8712432	-0.029470834	0.098152314	-0.300256134	0.76398179	0.877061743
6722.123671	-0.029920717	0.043193603	-0.692711782	0.488490443	0.686504952
28.60285699	-0.268181301	0.213671942	-1.255107707	0.209439663	0.4134087
2.135226274	-0.752346831	0.702286877	-1.071281346	0.284042945	0.498118317
600.957302	0.018154357	0.083878704	0.216435826	0.828648043	0.914385171
13.73870859	0.790010414	0.302307096	2.613271158	0.008968012	0.04869738
462.8558769	-0.098998779	0.101823852	-0.972255293	0.330923559	0.546292844
253.5338461	0.182135697	0.148007335	1.23058561	0.21847789	0.423029367
1144.327074	0.053777824	0.166254797	0.323466297	0.746342128	0.86703309
45.03448949	0.2904018	0.144363773	2.011597464	0.044262387	0.149207809
197.2761855	0.267103672	0.102309213	2.610748962	0.009034419	0.048949686
7.292011438	0.784844374	0.387986614	2.022864567	0.043087113	0.146414417
2.050395047	-1.028352003	0.715954304	-1.436337482	0.150906333	0.334683755
83.97068127	0.464299529	0.164805477	2.817257883	0.004843562	0.030689553
50.64617806	-0.10692873	0.151092747	-0.707702601	0.479129962	0.678327657
3.619315954	-0.628897536	0.53698585	-1.171162212	0.241533581	0.449050591
33.55606059	-0.151771341	0.185218649	-0.819417169	0.412548443	0.622273872
13.61935295	0.034992843	0.307191551	0.113912127	0.909307452	0.954378884
450.1914372	0.096295696	0.078559504	1.225767615	0.220286137	0.424860747
96.24146659	-0.00363972	0.132540263	-0.027461241	0.978091854	0.988606113
94.88505895	0.114705787	0.1355253	0.846379142	0.397341271	0.609915354
144.9798652	-0.34376078	0.150550477	-2.283358952	0.022409233	0.09298985
188.0947958	-0.188612468	0.094551837	-1.994804905	0.046064162	0.153677806
80.94158268	-0.519405384	0.165665448	-3.135266829	0.001716979	0.014409325
9.588854438	-0.33145481	0.370864252	-0.893736208	0.371463048	0.584924703
48.54032386	-0.851700143	0.357056028	-2.385340325	0.017063328	0.076782645
1441.215184	-0.351606604	0.140571177	-2.501270977	0.012374845	0.061344353
281.5616552	-0.187956353	0.118281636	-1.589057765	0.112047352	0.27702774
50.22267637	0.123540405	0.149300814	0.827459688	0.407976562	0.618820171
1169.360943	-0.437580921	0.133150946	-3.286352327	0.00101494	0.009760722

313.8827574	-0.286155266	0.295008299	-0.969990564	0.332051195	0.547513902
34.68647906	-0.765918817	0.260827066	-2.936500531	0.003319383	0.023410475
1471.308665	0.086961169	0.08300435	1.047670017	0.294790666	0.509642295
699.5033194	-0.071231353	0.071408743	-0.997515855	0.318514182	0.533631916
3.757960521	0.114527432	0.486755763	0.235287264	0.81398578	0.906237868
51.45678261	0.076074858	0.16587471	0.45862843	0.646501017	0.80392948
539.0095531	0.292917993	0.144912545	2.021343237	0.043244246	0.146827229
47.08171147	-0.092453478	0.223664706	-0.413357474	0.679344727	0.826715923
6006.172876	-0.363431074	0.103523219	-3.510623772	0.000447057	0.005118006
55.92367059	0.04227569	0.203712001	0.207526754	0.835598501	0.917771165
335.8319517	-0.148378604	0.137080833	-1.082416858	0.279067341	0.492327626
3.867418624	-0.836037533	0.616054856	-1.357082938	0.174754859	0.368760286
33.9397673	-0.739282704	0.273195024	-2.706062114	0.006808631	0.039704561
4.041857748	0.458559369	0.508697691	0.901437882	0.367355548	0.580955677
4.818602674	-0.784532829	0.500461119	-1.567619938	0.116969856	0.284632409
290.8275848	0.407921841	0.105846357	3.85390533	0.000116249	0.001761802
132.4452465	-0.421320236	0.206150769	-2.043748068	0.040978442	0.141289002
82.78658224	-0.573032908	0.17514393	-3.271782864	0.001068716	0.010147214
920.708996	0.128156565	0.080121502	1.599527731	0.109703392	0.273412054
217.6440501	-0.10874304	0.106819921	-1.018003379	0.30867635	0.523862593
423.8996805	-0.063999042	0.102007806	-0.627393575	0.530401278	0.72005197
134.0881083	0.183127837	0.184563757	0.992219925	0.321090255	0.536148476
3.665612149	-0.492152576	0.889344833	-0.553387795	0.579997894	0.757293715
217.1531155	-0.636401069	0.11214832	-5.674637573	1.38983E-08	8.14804E-07
178.7268066	0.069698152	0.078057654	0.892906058	0.371907482	0.585344785
247.3136163	-0.656552169	0.42969657	-1.527943702	0.126526516	0.297871199
24.87374725	-0.048026909	0.18214175	-0.263678748	0.792027473	0.89277185
6.947120643	-0.592063705	0.423856068	-1.396850841	0.16245843	0.351741763
20.96562294	-0.170669253	0.256261989	-0.665995196	0.505414171	0.700428834
45.83683818	-0.180958913	0.134712352	-1.343298596	0.179175306	0.374823365
73.09781119	-0.206498074	0.171131306	-1.206664513	0.227561371	0.433342507
35.58042434	-0.324256113	0.187953606	-1.725192294	0.084492824	0.23041463
1341.810154	0.084429616	0.141874159	0.595102143	0.551775196	0.737640557
164.1925522	-0.179275001	0.079845951	-2.245261015	0.024751381	0.099964269
19.20100415	-0.235495167	0.23789418	-0.989915628	0.32221536	0.537319146
17.31516333	-0.41682692	0.353410853	-1.179440066	0.238222989	0.445761644
144.0374627	-0.339737073	0.204357276	-1.66246624	0.096419327	0.250143621
145.3754657	0.159769803	0.089360775	1.787918734	0.07378912	0.211188666
106.9716511	-0.449395197	0.145157225	-3.095920285	0.001962031	0.016000509
144.0724325	-0.27426554	0.089154645	-3.076289956	0.002095939	0.016745151
75.20179873	0.017756343	0.132347167	0.134164886	0.893272192	0.946108145
6.668367406	-0.391416705	0.399987802	-0.978571604	0.327791695	0.542990487
1507.764827	-0.185055714	0.119552302	-1.547905907	0.121644952	0.291572667
13.32700383	-0.68081863	0.409772398	-1.661455563	0.096621987	0.250432757
25.5030545	-0.535218226	0.267440457	-2.001261259	0.045364242	0.151896449
53.00020539	-0.00671043	0.184395296	-0.036391547	0.970970154	0.985661927
3.086255556	-0.205065598	0.593697238	-0.345404332	0.729790421	0.857101832
598.2986092	-0.132171537	0.082066682	-1.610538325	0.107280386	0.268992116

247.1958842	0.387131128	0.072089506	5.370145349	7.86732E-08	3.67156E-06
8.208841534	0.442178285	0.348228602	1.269793126	0.20415833	0.407201766
330.2220464	-0.277153033	0.117363521	-2.361492149	0.018201557	0.08021735
78.54085876	0.029708709	0.130038263	0.228461289	0.819287647	0.909322798
4.87378408	-0.676583553	0.480775072	-1.407276694	0.159345358	0.346773473
12.35112186	-0.884877047	0.424266677	-2.085662381	0.037009216	0.131796332
126.595601	-0.038128816	2.025087971	-0.018828227	0.984978136	0.992448306
219.1594151	0.14260407	1.960970715	0.072721163	0.942028008	0.972366504
182.6543377	-0.130864141	2.26974885	-0.05765578	0.954022817	0.977641944
637.972292	-0.107014327	2.112209883	-0.050664628	0.959592764	0.980337668
202.8337332	-0.655276345	0.295323753	-2.218840642	0.026497568	0.104932917

diffexpressed	gene_symbol
not_Sig	Xkr4
not_Sig	Rp1
Sig	Sox17
not_Sig	Mrpl15
not_Sig	Lypla1
not_Sig	Tcea1
Sig	Atp6v1h
not_Sig	Rb1cc1
Sig	Pcmtd1
not_Sig	Rrs1
not_Sig	Adhfe1
not_Sig	2610203C22Rik
not_Sig	3110035E14Rik
not_Sig	Mybl1
Sig	Vcpip1
not_Sig	Sgk3
not_Sig	Mcmdc2
not_Sig	Snhg6
not_Sig	Tcf24
not_Sig	Cops5
Sig	Cspp1
not_Sig	Arfgef1
not_Sig	Prex2
Sig	A830018L16Rik
not_Sig	Sulf1
not_Sig	Slco5a1
Sig	Ncoa2
not_Sig	Tram1
not_Sig	Lactb2
not_Sig	Eya1
Sig	Msc
not_Sig	Kcnb2
not_Sig	Terf1
not_Sig	Sbspon
Sig	Rpl7
not_Sig	Rdh10
Sig	Stau2
Sig	Ube2w
not_Sig	Tceb1
not_Sig	Tmem70
not_Sig	Ly96
not_Sig	Jph1
not_Sig	Gdap1
Sig	Pi15
not_Sig	Gm16070
not_Sig	Crispld1
not_Sig	Pkhd1

not_Sig	Linc-md1
not_Sig	Mcm3
not_Sig	Paqr8
not_Sig	Efhc1
not_Sig	Tram2
not_Sig	Tmem14a
not_Sig	Gsta3
not_Sig	Kcnq5
not_Sig	Rims1
not_Sig	4933415F23Rik
not_Sig	Mir30a
not_Sig	Mir30c-2
not_Sig	Ogfrl1
not_Sig	B3gat2
not_Sig	Smap1
not_Sig	Sdhaf4
not_Sig	Fam135a
not_Sig	Col9a1
Sig	Col19a1
not_Sig	Lmbrd1
Sig	Adgrb3
not_Sig	Phf3
Sig	Ptp4a1
not_Sig	Prim2
not_Sig	1700001G17Rik
not_Sig	Rab23
not_Sig	Bag2
not_Sig	Zfp451
not_Sig	Bend6
not_Sig	Dst
not_Sig	Ccdc115
not_Sig	Imp4
not_Sig	Ptpn18
not_Sig	Arhgef4
Sig	Fam168b
not_Sig	Plekhb2
Sig	Hs6st1
Sig	Uggt1
not_Sig	Neurl3
Sig	Arid5a
not_Sig	Kansl3
not_Sig	Fer1l5
Sig	Lman2l
not_Sig	Cnnm4
not_Sig	Cnnm3
not_Sig	Ankrd23
Sig	Ankrd39
Sig	Sema4c

not_Sig	Fam178b
not_Sig	Cox5b
not_Sig	Actr1b
not_Sig	4933424G06Rik
Sig	Zap70
Sig	Tmem131
not_Sig	Cnga3
not_Sig	Inpp4a
not_Sig	Coa5
Sig	Unc50
Sig	Mgat4a
Sig	4930594C11Rik
Sig	2010300C02Rik
not_Sig	Tsga10
not_Sig	Lipt1
not_Sig	Mitd1
not_Sig	Mrpl30
not_Sig	Txndc9
not_Sig	Eif5b
not_Sig	Rev1
not_Sig	Aff3
not_Sig	Lonrf2
not_Sig	Chst10
not_Sig	Pdcl3
Sig	Npas2
not_Sig	Rpl31
not_Sig	Tbc1d8
not_Sig	Cnot11
Sig	Snord89
not_Sig	Rnf149
not_Sig	Creg2
not_Sig	Rfx8
not_Sig	Gm3646
not_Sig	Map4k4
not_Sig	Gm16894
not_Sig	Il1r2
not_Sig	Il1r1
not_Sig	Il1rl2
not_Sig	Il1rl1
not_Sig	Il18r1
not_Sig	Il18rap
not_Sig	Slc9a4
not_Sig	Slc9a2
not_Sig	Mfsd9
not_Sig	Tmem182
not_Sig	Mrps9
not_Sig	Tgfbrap1
not_Sig	AI597479

not_Sig	Fhl2
not_Sig	Nck2
not_Sig	1500015O10Rik
not_Sig	Uxs1
not_Sig	Tpp2
not_Sig	Mettl21c
not_Sig	Tex30
not_Sig	Kdelc1
not_Sig	Bivm
not_Sig	Ercc5
not_Sig	Mettl21e
not_Sig	Gulp1
not_Sig	4930521E06Rik
not_Sig	Col3a1
Sig	Col5a2
not_Sig	Wdr75
not_Sig	Slc40a1
not_Sig	Dnah7b
not_Sig	Slc39a10
not_Sig	Tmeff2
not_Sig	Sdpr
not_Sig	Nabp1
Sig	Myo1b
not_Sig	Stat4
not_Sig	Stat1
not_Sig	Gls
not_Sig	Nab1
not_Sig	Tmem194b
not_Sig	Mfsd6
not_Sig	Inpp1
not_Sig	Hibch
not_Sig	1700019D03Rik
not_Sig	Mstn
not_Sig	Pms1
not_Sig	Ormdl1
not_Sig	Osgepl1
not_Sig	Asnsd1
not_Sig	Stk17b
Sig	Hecw2
not_Sig	Ccdc150
not_Sig	Gtf3c3
not_Sig	Pgap1
not_Sig	Ankrd44
not_Sig	Sf3b1
not_Sig	Coq10b
not_Sig	Hspd1
not_Sig	Hspe1
not_Sig	Mob4

Sig	Rftn2
not_Sig	Mars2
not_Sig	Plcl1
not_Sig	9130227L01Rik
Sig	Satb2
not_Sig	9130024F11Rik
not_Sig	1700066M21Rik
not_Sig	Tyw5
not_Sig	9430016H08Rik
not_Sig	Spats2l
not_Sig	Kctd18
not_Sig	Sgol2a
not_Sig	Aox1
not_Sig	Aox3
not_Sig	Aox2
Sig	Bzw1
not_Sig	Clk1
not_Sig	Ppil3
not_Sig	Nif3l1
Sig	Orc2
Sig	Fam126b
not_Sig	Ndufb3
not_Sig	Gm20257
not_Sig	Als2cr12
not_Sig	Cflar
not_Sig	Casp8
not_Sig	Trak2
not_Sig	Stradb
not_Sig	Tmem237
not_Sig	Mpp4
not_Sig	Als2
not_Sig	Cdk15
not_Sig	Fzd7
not_Sig	Gm973
not_Sig	Sumo1
not_Sig	Nop58
not_Sig	Snord70
not_Sig	Bmpr2
not_Sig	Fam117b
not_Sig	Ica1l
Sig	Wdr12
not_Sig	Carf
not_Sig	Nbeal1
not_Sig	Cyp20a1
not_Sig	Abi2
not_Sig	Raph1
Sig	Cd28
not_Sig	Ctla4

not_Sig	Icos
not_Sig	Pard3b
not_Sig	Nrp2
Sig	4930487H11Rik
Sig	Ino80d
Sig	Ino80dos
not_Sig	Ndufs1
not_Sig	Eef1b2
not_Sig	Snora41
not_Sig	Gpr1
Sig	Zdbf2
not_Sig	Adam23
not_Sig	Fastkd2
not_Sig	4933402D24Rik
not_Sig	Gm13749
Sig	Klf7
not_Sig	Creb1
not_Sig	Mettl21a
Sig	Ccnyl1
not_Sig	Fzd5
not_Sig	Plekhm3
Sig	Idh1
not_Sig	Pikfyve
not_Sig	Map2
not_Sig	Unc80
not_Sig	Rpe
not_Sig	Kansl1l
not_Sig	Acadl
not_Sig	Myl1
not_Sig	Lancl1
not_Sig	Cps1
not_Sig	Erbb4
not_Sig	Ikzf2
not_Sig	Bard1
Sig	Atic
not_Sig	Fn1
not_Sig	Mreg
not_Sig	Pecr
not_Sig	Xrcc5
not_Sig	March4
not_Sig	Smarcal1
Sig	Rpl37a
not_Sig	Igfbp2
not_Sig	Igfbp5
not_Sig	6030407O03Rik
Sig	Tns1
not_Sig	Rufy4
not_Sig	Cxcr2

not_Sig	Arpc2
not_Sig	Aamp
Sig	Pnkd
not_Sig	Tmbim1
not_Sig	Catip
not_Sig	Slc11a1
Sig	Ctdsp1
not_Sig	Mir26b
not_Sig	Vil1
not_Sig	Usp37
not_Sig	Rqcd1
not_Sig	Plcd4
not_Sig	Zfp142
not_Sig	Bcs1l
not_Sig	Rnf25
not_Sig	Stk36
Sig	Ttll4
Sig	Cyp27a1
not_Sig	Prkag3
not_Sig	Wnt6
not_Sig	Wnt10a
not_Sig	Ihh
Sig	Nhej1
not_Sig	Slc23a3
not_Sig	Cnppd1
not_Sig	Fam134a
not_Sig	Zfand2b
not_Sig	Abcb6
not_Sig	Atg9a
not_Sig	Ankzf1
not_Sig	Glb1l
not_Sig	Stk16
not_Sig	Tuba4a
not_Sig	A630095N17Rik
not_Sig	Dnajb2
not_Sig	Ptprn
not_Sig	Dnpep
not_Sig	Des
not_Sig	Gm15179
not_Sig	Speg
not_Sig	Gmppa
not_Sig	Asic4
not_Sig	Chpf
not_Sig	Tmem198
Sig	Obsl1
Sig	Inha
not_Sig	Stk11ip
Sig	Slc4a3

Sig	Epha4
not_Sig	Pax3
not_Sig	Sgpp2
not_Sig	Farsb
not_Sig	Mogat1
Sig	Acsl3
not_Sig	Utp14b
not_Sig	Kcne4
not_Sig	Ap1s3
not_Sig	Wdfy1
not_Sig	Mrpl44
Sig	Serpine2
not_Sig	Fam124b
not_Sig	Cul3
not_Sig	Dock10
not_Sig	Irs1
not_Sig	Rhbdd1
not_Sig	Col4a4
Sig	Col4a3
not_Sig	Mff
not_Sig	Tm4sf20
not_Sig	Agfg1
not_Sig	Slc19a3
not_Sig	Sphkap
not_Sig	Pid1
not_Sig	Dner
not_Sig	Trip12
not_Sig	Fbxo36
not_Sig	Slc16a14
not_Sig	A530032D15Rik
not_Sig	Gm7609
not_Sig	C130026I21Rik
not_Sig	Sp110
not_Sig	Sp140
not_Sig	Sp100
not_Sig	A630001G21Rik
not_Sig	Cab39
Sig	Itm2c
not_Sig	4933407L21Rik
not_Sig	2810459M11Rik
not_Sig	Psmd1
not_Sig	Htr2b
not_Sig	Armc9
Sig	Ncl
not_Sig	Snora75
not_Sig	Gm24148
not_Sig	Snord82
not_Sig	C130036L24Rik

not_Sig	Ptma
not_Sig	Pde6d
not_Sig	Cops7b
not_Sig	Nppc
not_Sig	Dis3l2
Sig	Chrnd
not_Sig	Chrng
not_Sig	Eif4e2
not_Sig	Efhd1
not_Sig	Gigyf2
not_Sig	Kcnj13
Sig	Ngef
not_Sig	Neu2
not_Sig	Inpp5d
not_Sig	Atg16l1
not_Sig	Scarna6
not_Sig	Sag
not_Sig	Dgkd
not_Sig	Usp40
not_Sig	Ugt1a7c
not_Sig	Ugt1a6b
not_Sig	Ugt1a6a
not_Sig	Ugt1a5
not_Sig	Ugt1a2
not_Sig	Dnajb3
not_Sig	Ugt1a1
not_Sig	Mroh2a
not_Sig	Hjurp
not_Sig	Arl4c
not_Sig	Sh3bp4
Sig	Agap1
not_Sig	4933400F21Rik
not_Sig	Gbx2
not_Sig	Asb18
Sig	Ackr3
not_Sig	Cops8
not_Sig	Col6a3
not_Sig	Mlph
not_Sig	Rab17
Sig	Lrrfip1
not_Sig	Ramp1
not_Sig	Ube2f
not_Sig	Scly
Sig	Espnl
Sig	Klhl30
Sig	Fam132b
not_Sig	Ilkap
not_Sig	Hes6

not_Sig	Per2
not_Sig	Traf3ip1
Sig	Asb1
not_Sig	Twist2
Sig	Hdac4
not_Sig	Ndufa10
Sig	Myeov2
not_Sig	Gpc1
not_Sig	Dusp28
not_Sig	Rnpepl1
not_Sig	9430060I03Rik
not_Sig	Capn10
not_Sig	Gpr35
not_Sig	Kif1a
not_Sig	Sned1
not_Sig	Mterf4
not_Sig	Pask
not_Sig	Ppp1r7
not_Sig	Hdlbp
Sig	Sept2
not_Sig	Farp2
not_Sig	Stk25
not_Sig	Bok
not_Sig	Thap4
not_Sig	Atg4b
not_Sig	Dtymk
not_Sig	Ing5
not_Sig	D2hgdh
not_Sig	Gm9994
not_Sig	Fam174a
not_Sig	St8sia4
not_Sig	D1Ertd622e
not_Sig	Ppip5k2
not_Sig	Gin1
not_Sig	Pam
not_Sig	B230216N24Rik
not_Sig	Cdh20
not_Sig	Rnf152
not_Sig	Pign
not_Sig	2310035C23Rik
not_Sig	Tnfrsf11a
Sig	Zcchc2
not_Sig	Phlpp1
not_Sig	Bcl2
not_Sig	Kdsr
not_Sig	Vps4b
not_Sig	Serpinb10
not_Sig	Serpinb8

not_Sig	D830032E09Rik
not_Sig	Cdh19
not_Sig	Dsel
Sig	Tsn
Sig	Nifk
Sig	Clasp1
Sig	2900060B14Rik
not_Sig	Tfcp2l1
not_Sig	Gli2
not_Sig	Inhbb
not_Sig	Ralb
not_Sig	Tmem185b
not_Sig	Epb4.1l5
not_Sig	Ptpn4
not_Sig	Tmem177
Sig	Sctr
Sig	Tmem37
not_Sig	Dbi
not_Sig	3110009E18Rik
not_Sig	Steap3
not_Sig	Marco
Sig	En1
Sig	Insig2
not_Sig	Ccdc93
not_Sig	Htr5b
not_Sig	Ddx18
not_Sig	Dpp10
not_Sig	Actr3
Sig	Slc35f5
not_Sig	Nckap5
not_Sig	Mgat5
not_Sig	Tmem163
not_Sig	Acmsd
not_Sig	2900009J06Rik
not_Sig	Ccnt2
not_Sig	Map3k19
not_Sig	Rab3gap1
Sig	Zranb3
not_Sig	R3hdm1
not_Sig	Ubxn4
not_Sig	Mcm6
not_Sig	Dars
not_Sig	Cxcr4
not_Sig	Thsd7b
not_Sig	Cd55
not_Sig	C4bp
not_Sig	C4bp-ps1
not_Sig	Pfkfb2

not_Sig	Yod1
not_Sig	AA986860
not_Sig	Fcamr
not_Sig	Pigr
not_Sig	Fcmr
Sig	Mapkapk2
not_Sig	Dyrk3
not_Sig	Eif2d
not_Sig	Rassf5
not_Sig	Ikbke
not_Sig	Srgap2
not_Sig	Fam72a
not_Sig	Ctse
not_Sig	Rab7b
Sig	Pm20d1
Sig	Slc41a1
not_Sig	Rab29
not_Sig	Nucks1
not_Sig	Slc45a3
Sig	Elk4
not_Sig	Mfsd4
not_Sig	Cdk18
not_Sig	Klhdc8a
not_Sig	Nuak2
not_Sig	Tmcc2
not_Sig	Dstyk
not_Sig	Rbbp5
not_Sig	Tmem81
Sig	Cntn2
not_Sig	Nfasc
not_Sig	Lrrn2
not_Sig	Mdm4
Sig	Pik3c2b
Sig	Ppp1r15b
not_Sig	Plekha6
Sig	Gm19461
not_Sig	Etnk2
Sig	Sox13
Sig	Snrpe
not_Sig	Zc3h11a
not_Sig	Zbed6
not_Sig	Lax1
not_Sig	Atp2b4
not_Sig	Optc
not_Sig	Prelp
not_Sig	Fmod
not_Sig	Btg2
not_Sig	Chil1

not_Sig	Mybph
not_Sig	Adora1
Sig	Myog
Sig	Ppfia4
not_Sig	Tmem183a
not_Sig	Cyb5r1
not_Sig	Adipor1
not_Sig	Klhl12
not_Sig	Rabif
not_Sig	Kdm5b
not_Sig	Syt2
not_Sig	Ppp1r12b
not_Sig	Ube2t
not_Sig	Lgr6
not_Sig	Ptprv
not_Sig	Ptpn7
Sig	Arl8a
not_Sig	Gpr37l1
not_Sig	Elf3
Sig	Rnpep
not_Sig	Timm17a
not_Sig	Lmod1
not_Sig	Shisa4
not_Sig	Ipo9
Sig	Nav1
not_Sig	Csrp1
not_Sig	Phlda3
not_Sig	Tnni1
not_Sig	Lad1
not_Sig	Tnnt2
Sig	Pkp1
not_Sig	Igfn1
not_Sig	Tmem9
not_Sig	Cacna1s
not_Sig	Kif21b
not_Sig	Camsap2
not_Sig	9230116N13Rik
not_Sig	Ddx59
not_Sig	Kif14
not_Sig	Zfp281
not_Sig	Gm19705
not_Sig	Nr5a2
not_Sig	Ptprc
not_Sig	Nek7
Sig	2310009B15Rik
not_Sig	Dennd1b
not_Sig	4930596I21Rik
not_Sig	Zbtb41

Sig	Aspm
Sig	Cfh
not_Sig	Kcnt2
not_Sig	Cdc73
not_Sig	B3galt2
not_Sig	Glrx2
not_Sig	Trove2
not_Sig	Uchl5
not_Sig	Rgs2
not_Sig	Rgs1
not_Sig	Rgs18
not_Sig	Brinp3
not_Sig	Pla2g4a
Sig	Ptgs2
not_Sig	BC003331
not_Sig	Tpr
not_Sig	Prg4
Sig	Hmcn1
Sig	Ivns1abp
not_Sig	Swt1
not_Sig	Trmt1l
not_Sig	Rnf2
not_Sig	Fam129a
Sig	Edem3
not_Sig	1700025G04Rik
not_Sig	Tsen15
not_Sig	Colgalt2
not_Sig	Rgl1
not_Sig	Apobec4
not_Sig	Arpc5
not_Sig	Ncf2
not_Sig	Smg7
not_Sig	Nmnat2
Sig	Lamc2
not_Sig	Lamc1
not_Sig	E330020D12Rik
not_Sig	Dhx9
not_Sig	Npl
not_Sig	Rgs16
not_Sig	Rnasel
not_Sig	Rgsl1
not_Sig	Teddm2
not_Sig	Teddm1b
not_Sig	Teddm1a
not_Sig	Glul
not_Sig	Cacna1e
not_Sig	Ier5
not_Sig	Mr1

not_Sig	Stx6
Sig	Xpr1
Sig	Gm5532
not_Sig	Acbd6
not_Sig	Qsox1
not_Sig	Cep350
not_Sig	Tor1aip1
not_Sig	Tor1aip2
not_Sig	Tdrd5
not_Sig	Nphs2
not_Sig	Soat1
not_Sig	Abl2
not_Sig	Tor3a
Sig	Fam20b
not_Sig	Ralgps2
Sig	Angptl1
not_Sig	Rasal2
Sig	2810025M15Rik
not_Sig	BC026585
not_Sig	Sec16b
not_Sig	Brinp2
not_Sig	Astn1
not_Sig	Pappa2
not_Sig	Rfwd2
not_Sig	Tnr
not_Sig	4930523C07Rik
not_Sig	Tnn
Sig	Mrps14
not_Sig	Cacybp
not_Sig	Rabgap1l
not_Sig	Gpr52
not_Sig	Rc3h1
not_Sig	Serpinc1
not_Sig	Zbtb37
Sig	Gas5
not_Sig	Snord47
Sig	Dars2
not_Sig	Cenpl
not_Sig	Klhl20
Sig	Prdx6
not_Sig	Suco
not_Sig	Pigc
Sig	Dnm3
not_Sig	2810442N19Rik
Sig	Dnm3os
not_Sig	Mettl13
not_Sig	Vamp4
not_Sig	Myoc

Sig	Prrc2c
not_Sig	Fmo4
not_Sig	Fmo1
not_Sig	Fmo2
not_Sig	Fmo3
not_Sig	Prrx1
not_Sig	Gorab
not_Sig	Mettl11b
not_Sig	Kifap3
not_Sig	Scyl3
not_Sig	BC055324
not_Sig	Mettl18
not_Sig	Sele
not_Sig	Sell
not_Sig	Selp
Sig	F5
not_Sig	Slc19a2
not_Sig	Ccdc181
not_Sig	Blzf1
not_Sig	Nme7
not_Sig	Atp1b1
not_Sig	Dpt
Sig	Gm20743
not_Sig	Sft2d2
not_Sig	Tiprl
not_Sig	Gpr161
not_Sig	Dcaf6
not_Sig	Mpc2
not_Sig	Adcy10
Sig	Mpzl1
not_Sig	Rcsd1
Sig	Creg1
Sig	Cd247
Sig	Pou2f1
Sig	Dusp27
Sig	Ildr2
not_Sig	Tada1
not_Sig	Pogk
not_Sig	Fam78b
not_Sig	Uck2
not_Sig	Tmco1
Sig	Aldh9a1
not_Sig	Mgst3
not_Sig	Lrrc52
not_Sig	Rxrg
not_Sig	Lmx1a
not_Sig	Pbx1
not_Sig	Nuf2

not_Sig	Rgs5
not_Sig	Rgs4
not_Sig	Hsd17b7
not_Sig	3110045C21Rik
Sig	Ddr2
Sig	Uap1
not_Sig	Uhmk1
not_Sig	Gm7694
not_Sig	1700015E13Rik
not_Sig	Nos1ap
not_Sig	Olfml2b
Sig	Atf6
not_Sig	Dusp12
not_Sig	Fcrla
not_Sig	Fcgr2b
Sig	Fcgr3
not_Sig	Cfap126
not_Sig	Sdhc
not_Sig	Mpz
not_Sig	Pcp4l1
not_Sig	Nr1i3
not_Sig	Tomm40l
not_Sig	Apoa2
not_Sig	Fcer1g
not_Sig	Ndufs2
not_Sig	Adamts4
not_Sig	B4galt3
not_Sig	Ppox
not_Sig	Usp21
Sig	Ufc1
not_Sig	Dedd
Sig	Nit1
Sig	Pfdn2
not_Sig	Klhdc9
not_Sig	Pvrl4
not_Sig	Arhgap30
not_Sig	Usf1
not_Sig	Tstd1
Sig	F11r
not_Sig	Alyref2
not_Sig	Cd244
not_Sig	Ly9
not_Sig	Slamf7
not_Sig	Cd48
not_Sig	Slamf1
not_Sig	Cd84
not_Sig	Slamf6
not_Sig	Vangl2

not_Sig	Ncstn
not_Sig	Copa
not_Sig	Pex19
not_Sig	Dcaf8
not_Sig	Pea15a
not_Sig	Casq1
not_Sig	Atp1a4
not_Sig	Atp1a2
not_Sig	Igsf8
not_Sig	Kcnj10
not_Sig	Pigm
not_Sig	Slamf9
not_Sig	Igsf9
not_Sig	Tagln2
not_Sig	Slamf8
not_Sig	Dusp23
not_Sig	Crp
not_Sig	Fcer1a
not_Sig	Ackr1
Sig	Cadm3
not_Sig	Aim2
not_Sig	Pyhin1
not_Sig	Pydc3
not_Sig	AI607873
Sig	Ifi204
not_Sig	Mndal
not_Sig	Mnda
Sig	Ifi203
not_Sig	Ifi202b
not_Sig	Ifi205
Sig	Spta1
not_Sig	Fmn2
not_Sig	Grem2
Sig	Rgs7
not_Sig	Fh1
not_Sig	Kmo
not_Sig	Opn3
not_Sig	Chml
not_Sig	Exo1
not_Sig	Pld5
not_Sig	Cep170
not_Sig	Sdccag8
not_Sig	Hmga2-ps1
Sig	Akt3
not_Sig	Zbtb18
not_Sig	1700016C15Rik
not_Sig	Adss
not_Sig	Desi2

not_Sig	Cox20
Sig	Hnrnpu
not_Sig	Efcab2
not_Sig	Kif26b
Sig	Smyd3
Sig	Tfb2m
not_Sig	Cnst
not_Sig	Sccpdh
not_Sig	Ahctf1
Sig	Cdc42bpa
not_Sig	Adck3
not_Sig	Psen2
not_Sig	Gm5069
Sig	Itpkb
Sig	6330403A02Rik
not_Sig	Parp1
not_Sig	Lin9
not_Sig	Acbd3
Sig	H3f3a
Sig	Sde2
not_Sig	Pycr2
not_Sig	Lefty1
not_Sig	Tmem63a
not_Sig	Ephx1
not_Sig	Nvl
not_Sig	Cnih4
not_Sig	Wdr26
not_Sig	Cnih3
not_Sig	Lbr
not_Sig	Enah
not_Sig	Srp9
not_Sig	Degs1
Sig	Fbxo28
not_Sig	Trp53bp2
not_Sig	Capn2
Sig	Susd4
not_Sig	Tlr5
not_Sig	Disp1
not_Sig	Brox
not_Sig	Aida
not_Sig	Mia3
not_Sig	Taf1a
not_Sig	Hhipl2
not_Sig	1700056E22Rik
not_Sig	Dusp10
not_Sig	Hlx
Sig	Marc1
not_Sig	Marc2

not_Sig	C130074G19Rik
Sig	Mark1
not_Sig	Rab3gap2
not_Sig	Iars2
not_Sig	Mir194-1
not_Sig	Mir215
not_Sig	Bpnt1
not_Sig	Eprs
not_Sig	9630028B13Rik
not_Sig	Lyplal1
Sig	Tgfb2
not_Sig	Rrp15
not_Sig	Gpatch2
not_Sig	Esrrg
not_Sig	9330162B11Rik
not_Sig	Ush2a
not_Sig	Kctd3
not_Sig	Kcnk2
Sig	Cenpf
Sig	Ptpn14
not_Sig	Smyd2
not_Sig	Prox1
Sig	Rps6kc1
not_Sig	Angel2
not_Sig	Vash2
not_Sig	Mfsd7b
not_Sig	A230020J21Rik
not_Sig	Tatdn3
not_Sig	Nsl1
not_Sig	Batf3
not_Sig	Atf3
not_Sig	Nenf
not_Sig	Tmem206
not_Sig	Ppp2r5a
Sig	Dtl
not_Sig	Ints7
not_Sig	Lpgat1
Sig	Nek2
not_Sig	1700034H15Rik
not_Sig	Slc30a1
not_Sig	Rd3
not_Sig	Traf5
not_Sig	Rcor3
not_Sig	Gm10516
not_Sig	Kcnh1
not_Sig	Hhat
not_Sig	Sertad4
not_Sig	Gm15867

not_Sig	Syt14
not_Sig	Diexf
not_Sig	Irf6
not_Sig	A130010J15Rik
not_Sig	Traf3ip3
Sig	Hsd11b1
Sig	G0s2
Sig	Lamb3
not_Sig	Camk1g
Sig	Plxna2
Sig	Cd34
Sig	Gm16897
Sig	A330023F24Rik
Sig	Cd46
not_Sig	Cr1l
not_Sig	Cr2
Sig	Ppp1r14c
Sig	Plekhg1
not_Sig	Mthfd1l
Sig	Akap12
Sig	Zbtb2
not_Sig	Rmnd1
not_Sig	Gm5512
not_Sig	Armt1
not_Sig	Ccdc170
not_Sig	Esr1
not_Sig	Syne1
Sig	Myct1
not_Sig	Fbxo5
not_Sig	Mtrf1l
not_Sig	Rgs17
not_Sig	Ipcef1
Sig	Cnksr3
not_Sig	Ulbp1
not_Sig	Lrp11
not_Sig	Pcmt1
not_Sig	A630066F11Rik
not_Sig	Nup43
Sig	BC020402
not_Sig	Lats1
Sig	Katna1
not_Sig	Ginm1
not_Sig	Ppil4
not_Sig	Zc3h12d
not_Sig	Tab2
not_Sig	Ust
not_Sig	Sash1
not_Sig	Samd5

not_Sig	Stxbp5
not_Sig	Adgb
not_Sig	Rab32
not_Sig	Shprh
not_Sig	Fbxo30
not_Sig	Epm2a
not_Sig	Utrn
not_Sig	B230208H11Rik
not_Sig	Stx11
not_Sig	Sf3b5
Sig	Plagl1
not_Sig	Hymai
not_Sig	Ltv1
Sig	Phactr2
not_Sig	Fuca2
not_Sig	Pex3
not_Sig	Adat2
not_Sig	Aig1
not_Sig	Hivep2
not_Sig	Adgrg6
not_Sig	Vta1
not_Sig	1700016L04Rik
not_Sig	Nmbr
not_Sig	Gm20125
not_Sig	Cited2
not_Sig	Txlnb
not_Sig	Heca
not_Sig	Abracl
not_Sig	Reps1
Sig	Ccdc28a
not_Sig	Nhsl1
not_Sig	Hebp2
not_Sig	Arfgef3
Sig	Perp
not_Sig	Tnfaip3
not_Sig	Ifngr1
Sig	Il22ra2
not_Sig	Slc35d3
Sig	Pex7
not_Sig	Map3k5
not_Sig	Map7
not_Sig	4930405J17Rik
not_Sig	Gm6251
not_Sig	Bclaf1
not_Sig	Mtfr2
not_Sig	Pde7b
not_Sig	Ahi1
not_Sig	Myb

Sig	Hbs1l
not_Sig	4930455C13Rik
not_Sig	Aldh8a1
Sig	Gm5420
not_Sig	Sgk1
not_Sig	E030030I06Rik
not_Sig	H60b
not_Sig	C920009B18Rik
not_Sig	Gm10825
not_Sig	Slc2a12
not_Sig	Tbpl1
not_Sig	Mir7663
not_Sig	Eya4
Sig	Rps12
not_Sig	Snora33
not_Sig	Snord100
not_Sig	Slc18b1
Sig	Vnn3
Sig	Vnn1
not_Sig	Stx7
not_Sig	Moxd1
not_Sig	Ctgf
not_Sig	Enpp1
not_Sig	Enpp3
not_Sig	Med23
not_Sig	Arg1
not_Sig	Akap7
not_Sig	Epb4.1l2
not_Sig	Tmem200a
not_Sig	L3mbtl3
not_Sig	Arhgap18
not_Sig	Lama2
not_Sig	4930579H20Rik
not_Sig	Ptprk
not_Sig	Themis
Sig	4930519F09Rik
not_Sig	Soga3
not_Sig	9330159F19Rik
not_Sig	Echdc1
not_Sig	Rnf146
not_Sig	Rspo3
not_Sig	Cenpw
not_Sig	Trmt11
not_Sig	Gm20300
not_Sig	Hint3
not_Sig	Ncoa7
not_Sig	Hey2
Sig	Hddc2

not_Sig	Tpd52l1
not_Sig	Gm3258
not_Sig	Rnf217
not_Sig	Nkain2
not_Sig	Trdn
not_Sig	D830005E20Rik
not_Sig	Rsph4a
not_Sig	Zufsp
not_Sig	A830082N09Rik
not_Sig	Rwdd1
Sig	Fam26e
not_Sig	Fam26f
Sig	Dse
not_Sig	Tspyl1
not_Sig	Tspyl4
not_Sig	Nt5dc1
not_Sig	Col10a1
not_Sig	Frk
not_Sig	Hs3st5
not_Sig	Hdac2
not_Sig	Marcks
not_Sig	Lama4
not_Sig	Fam229b
not_Sig	Tube1
not_Sig	Wisp3
Sig	Fyn
not_Sig	Traf3ip2
not_Sig	E130307A14Rik
not_Sig	Rev3l
not_Sig	AA474331
Sig	G630090E17Rik
not_Sig	BC021785
not_Sig	AI317395
not_Sig	Slc16a10
not_Sig	Rpf2
not_Sig	Gtf3c6
Sig	Cdk19
Sig	Ddo
not_Sig	Mettl24
not_Sig	Cdc40
not_Sig	Wasf1
not_Sig	Fig4
not_Sig	Zbtb24
Sig	Mical1
not_Sig	Smpd2
not_Sig	Ppil6
Sig	Cd164
not_Sig	Ccdc162

not_Sig	Cep57l1
not_Sig	Sesn1
not_Sig	Armc2
not_Sig	Foxo3
not_Sig	Lace1
not_Sig	Snx3
Sig	Ostm1
not_Sig	Sec63
not_Sig	Scml4
not_Sig	Sobp
not_Sig	9030612E09Rik
not_Sig	Pdss2
not_Sig	Bend3
not_Sig	1700021F05Rik
Sig	Cd24a
not_Sig	F830002L21Rik
not_Sig	1700027J07Rik
not_Sig	Qrsl1
Sig	Rtn4ip1
not_Sig	Aim1
Sig	Atg5
not_Sig	Prdm1
not_Sig	Prep
Sig	Popdc3
not_Sig	Bves
not_Sig	Hace1
not_Sig	Grik2
Sig	Ascc3
not_Sig	Sim1
not_Sig	Lilr4b
not_Sig	Lilrb4a
not_Sig	Vgll2
not_Sig	Ros1
Sig	Dcbld1
Sig	Gopc
not_Sig	Nepn
not_Sig	Nus1
not_Sig	Zfa-ps
not_Sig	Slc35f1
not_Sig	Cep85l
not_Sig	Gm19395
not_Sig	Pln
not_Sig	Gm20597
not_Sig	Mcm9
not_Sig	Asf1a
not_Sig	Fam184a
not_Sig	Man1a
not_Sig	Gm16998

not_Sig	Tbc1d32
not_Sig	Msl3l2
not_Sig	Gja1
not_Sig	Hsf2
Sig	Serinc1
not_Sig	Pkib
Sig	Smpdl3a
not_Sig	Gcc2
not_Sig	Lims1
not_Sig	Ranbp2
not_Sig	Ccdc138
Sig	Edar
not_Sig	Sh3rf3
not_Sig	Sept10
not_Sig	Sowahc
Sig	P4ha1
not_Sig	Oit3
not_Sig	Mcu
not_Sig	Micu1
not_Sig	Dnajb12
Sig	Ddit4
not_Sig	Anapc16
not_Sig	Ascc1
Sig	Spock2
not_Sig	Chst3
not_Sig	Psap
Sig	Cdh23
not_Sig	4632428N05Rik
not_Sig	Gm17455
not_Sig	Slc29a3
Sig	Unc5b
not_Sig	Pcbd1
not_Sig	Sgpl1
not_Sig	Adamts14
not_Sig	Prf1
Sig	Pald1
not_Sig	Nodal
not_Sig	Eif4ebp2
not_Sig	Lrrc20
Sig	Ppa1
Sig	Sar1a
not_Sig	Tysnd1
not_Sig	Aifm2
not_Sig	H2afy2
Sig	Col13a1
not_Sig	2010107G23Rik
not_Sig	Tspan15
not_Sig	Hk1

not_Sig	Supv3l1
not_Sig	4930507D05Rik
not_Sig	Vps26a
not_Sig	Srgn
Sig	Kif1bp
not_Sig	Ddx21
not_Sig	Ddx50
Sig	Stox1
not_Sig	Ccar1
not_Sig	Tet1
Sig	Slc25a16
not_Sig	Dna2
not_Sig	Rufy2
Sig	Hnrnph3
not_Sig	Pbld2
not_Sig	Pbld1
not_Sig	Atoh7
not_Sig	Mypn
not_Sig	Herc4
not_Sig	Sirt1
not_Sig	Dnajc12
Sig	Ctnna3
Sig	Lrrtm3
not_Sig	Gm31763
not_Sig	Reep3
not_Sig	Jmjd1c
not_Sig	Nrbf2
not_Sig	Egr2
not_Sig	Ado
not_Sig	Zfp365
not_Sig	Rtkn2
Sig	Arid5b
not_Sig	Tmem26
Sig	Rhobtb1
Sig	Cdk1
not_Sig	Ank3
not_Sig	Ccdc6
not_Sig	Mrln
not_Sig	Slc16a9
not_Sig	Fam13c
not_Sig	Phyhipl
not_Sig	Bicc1
not_Sig	Tfam
not_Sig	Ube2d1
not_Sig	Cisd1
not_Sig	Ipmk
Sig	Zwint
Sig	Pcdh15

not_Sig	Gnaz
not_Sig	Rab36
not_Sig	Bcr
not_Sig	Specc1l
Sig	Adora2a
not_Sig	Upb1
Sig	Gucd1
Sig	Snrpd3
not_Sig	Lrrc75b
not_Sig	Ggt1
Sig	Ggt5
not_Sig	Susd2
not_Sig	Cabin1
Sig	Ddt
not_Sig	Gstt3
not_Sig	Gstt1
not_Sig	Gstt2
not_Sig	Mif
not_Sig	Derl3
not_Sig	Smarcb1
not_Sig	Mmp11
not_Sig	Chchd10
not_Sig	Gm867
not_Sig	Zfp280b
not_Sig	Prmt2
Sig	S100b
Sig	Dip2a
Sig	Pcnt
not_Sig	1700094J05Rik
not_Sig	2610028H24Rik
not_Sig	Ybey
not_Sig	Mcm3ap
not_Sig	Lss
not_Sig	4930483K19Rik
not_Sig	Ftcd
not_Sig	Col6a2
not_Sig	Col6a1
not_Sig	Pcbp3
not_Sig	Slc19a1
not_Sig	Col18a1
not_Sig	Pofut2
Sig	Adarb1
not_Sig	Gm17769
not_Sig	Fam207a
not_Sig	Itgb2
not_Sig	Pttg1ip
Sig	Sumo3
not_Sig	Ube2g2

not_Sig	Mir1930
not_Sig	Lrrc3
not_Sig	Trpm2
not_Sig	1810043G02Rik
not_Sig	Pfkl
not_Sig	Icosl
not_Sig	D10Jhu81e
not_Sig	Pwp2
not_Sig	Trappc10
not_Sig	Agpat3
not_Sig	Rrp1
not_Sig	Cstb
Sig	Pdxk
not_Sig	Ilvbl
Sig	Syde1
not_Sig	Slc1a6
not_Sig	2610008E11Rik
not_Sig	Ppap2c
not_Sig	Mier2
not_Sig	C2cd4c
Sig	Shc2
not_Sig	Odf3l2
Sig	Tpgs1
not_Sig	Cdc34
not_Sig	Gzmm
not_Sig	Bsg
not_Sig	Hcn2
not_Sig	Polrmt
not_Sig	Fgf22
not_Sig	Rnf126
not_Sig	Fstl3
not_Sig	Prss57
not_Sig	Palm
not_Sig	E130317F20Rik
not_Sig	Ptbp1
not_Sig	Lppr3
not_Sig	Prtn3
not_Sig	Elane
not_Sig	Cfd
Sig	Med16
Sig	R3hdm4
not_Sig	Kiss1r
not_Sig	Arid3a
Sig	Wdr18
not_Sig	Grin3b
Sig	Tmem259
not_Sig	Cnn2
not_Sig	Abca7

not_Sig	Hmha1
not_Sig	Polr2e
Sig	Gpx4
not_Sig	Sbno2
not_Sig	Stk11
not_Sig	Dos
not_Sig	Atp5d
Sig	Midn
Sig	Cirbp
Sig	1600002K03Rik
not_Sig	Efna2
not_Sig	Mum1
not_Sig	Ndufs7
not_Sig	Gamt
Sig	Dazap1
Sig	Rps15
Sig	Apc2
not_Sig	2310011J03Rik
not_Sig	Pcsk4
not_Sig	Reep6
not_Sig	Adamtsl5
not_Sig	Plk5
Sig	Mex3d
not_Sig	Mbd3
not_Sig	Uqcr11
not_Sig	Tcf3
Sig	Rexo1
not_Sig	Klf16
not_Sig	Abhd17a
not_Sig	Adat3
not_Sig	Scamp4
Sig	Csnk1g2
not_Sig	Btbd2
not_Sig	Mknk2
not_Sig	Mob3a
not_Sig	Izumo4
not_Sig	Ap3d1
not_Sig	Dot1l
not_Sig	Plekhj1
not_Sig	Sf3a2
Sig	Amh
not_Sig	Jsrp1
not_Sig	Lingo3
not_Sig	Lsm7
not_Sig	Sppl2b
not_Sig	Tmprss9
not_Sig	Timm13
not_Sig	Lmnb2

not_Sig	Gadd45b
not_Sig	Gng7
not_Sig	Diras1
not_Sig	Slc39a3
not_Sig	Sgta
not_Sig	Thop1
not_Sig	Creb3l3
not_Sig	Map2k2
not_Sig	Zbtb7a
not_Sig	Pias4
Sig	Eef2
not_Sig	Snord37
not_Sig	Dapk3
Sig	Atcayos
not_Sig	Nmrk2
Sig	Atcay
not_Sig	Zfr2
not_Sig	Matk
Sig	Mrpl54
not_Sig	Apba3
not_Sig	Tjp3
Sig	Pip5k1c
not_Sig	Cactin
not_Sig	Tbxa2r
Sig	Gipc3
not_Sig	Hmg20b
not_Sig	Mfsd12
not_Sig	4930404N11Rik
not_Sig	Fzr1
not_Sig	Dohh
not_Sig	Smim24
not_Sig	Nfic
not_Sig	Mir1191b
not_Sig	Celf5
not_Sig	Ncln
Sig	S1pr4
not_Sig	Gna15
not_Sig	Gna11
not_Sig	Aes
not_Sig	Tle2
not_Sig	Tle6
not_Sig	BC025920
not_Sig	Sirt6
not_Sig	Ankrd24
not_Sig	Gm10778
not_Sig	Zfp433
not_Sig	Zfp781
not_Sig	Zfp873

not_Sig	AU041133
not_Sig	Zfp938
not_Sig	1190007I07Rik
not_Sig	Glt8d2
not_Sig	Hcfc2
not_Sig	Nfyb
not_Sig	Txnrd1
Sig	Eid3
not_Sig	Chst11
not_Sig	Slc41a2
not_Sig	D10Wsu102e
not_Sig	Aldh1l2
not_Sig	A230046K03Rik
not_Sig	Appl2
not_Sig	1500009L16Rik
Sig	Nuak1
not_Sig	Ckap4
not_Sig	Tcp11l2
Sig	Polr3b
not_Sig	Ric8b
not_Sig	Fhl4
not_Sig	Tmem263
not_Sig	Mterf2
not_Sig	Cry1
not_Sig	Btbd11
not_Sig	Pwp1
not_Sig	Prdm4
not_Sig	Rtcb
not_Sig	Fbxo7
not_Sig	Syn3
not_Sig	Timp3
Sig	1810014B01Rik
Sig	Hsp90b1
not_Sig	Ttc41
not_Sig	Nt5dc3
Sig	Stab2
not_Sig	1700113H08Rik
not_Sig	Pah
Sig	Igf1
not_Sig	Parpbp
not_Sig	Nup37
Sig	Ccdc53
not_Sig	Dram1
Sig	Gnptab
Sig	Sycp3
Sig	Chpt1
not_Sig	Mybpc1
not_Sig	Spic

not_Sig	Gm4925
not_Sig	Arl1
not_Sig	Utp20
Sig	Slc5a8
not_Sig	Ano4
not_Sig	Gas2l3
Sig	Nr1h4
not_Sig	Slc17a8
not_Sig	Scyl2
not_Sig	1500026H17Rik
not_Sig	Actr6
Sig	Uhrf1bp1l
not_Sig	Anks1b
not_Sig	Apaf1
not_Sig	Ikbip
not_Sig	Slc25a3
not_Sig	Tmpo
not_Sig	Nedd1
Sig	Cdk17
not_Sig	Mir1931
Sig	Elk3
not_Sig	Gm17745
not_Sig	Lta4h
Sig	Hal
not_Sig	Amdhd1
not_Sig	Ccdc38
not_Sig	Snrpf
not_Sig	Ntn4
not_Sig	Gm15915
not_Sig	Usp44
not_Sig	Metap2
not_Sig	Vezt
not_Sig	Fgd6
not_Sig	Nr2c1
Sig	Ndufa12
not_Sig	Tmcc3
not_Sig	Mir7211
not_Sig	Cep83os
not_Sig	Cep83
not_Sig	Plxnc1
Sig	Cradd
Sig	2310039L15Rik
Sig	Socs2
Sig	5730420D15Rik
not_Sig	Mrpl42
not_Sig	Ube2n
Sig	Nudt4
not_Sig	Mir3058

not_Sig	Eea1
not_Sig	Btg1
Sig	Dcn
Sig	Lum
not_Sig	Kera
not_Sig	Atp2b1
not_Sig	Poc1b
not_Sig	Galnt4
not_Sig	Dusp6
not_Sig	Csl
Sig	Kitl
not_Sig	Tmtc3
not_Sig	Cep290
Sig	4930430F08Rik
not_Sig	Rassf9
not_Sig	Slc6a15
Sig	Tmtc2
not_Sig	Gm15663
not_Sig	Mettl25
not_Sig	Ccdc59
not_Sig	Ppfia2
not_Sig	Acss3
not_Sig	Lin7a
not_Sig	Myf5
not_Sig	Myf6
not_Sig	Ptprq
Sig	Ppp1r12a
not_Sig	Pawr
not_Sig	Syt1
not_Sig	Nav3
not_Sig	E2f7
not_Sig	Csrp2
not_Sig	Zdhhc17
Sig	Osbpl8
not_Sig	Bbs10
not_Sig	Nap1l1
not_Sig	Gm5176
not_Sig	Phlda1
not_Sig	Krr1
not_Sig	Glipr1
not_Sig	Kcnc2
not_Sig	Atxn7l3b
not_Sig	Trhde
Sig	Tph2
not_Sig	Tbc1d15
not_Sig	Rab21
Sig	Tmem19
not_Sig	Thap2

not_Sig	Zfc3h1
not_Sig	Lgr5
Sig	Tspan8
not_Sig	Ptprr
Sig	Ptprb
Sig	Cnot2
not_Sig	Rab3ip
not_Sig	4933412E12Rik
not_Sig	Best3
not_Sig	Lrrc10
Sig	Cct2
Sig	Frs2
not_Sig	Yeats4
Sig	Lyz2
not_Sig	Lyz1
not_Sig	Cpsf6
Sig	Cpm
Sig	Mdm2
not_Sig	Slc35e3
not_Sig	Nup107
not_Sig	Rap1b
not_Sig	Mdm1
not_Sig	Dyrk2
not_Sig	Cand1
not_Sig	Grip1
not_Sig	Helb
not_Sig	Irak3
not_Sig	Tmbim4
not_Sig	Llph
not_Sig	Hmga2
not_Sig	4921513I03Rik
not_Sig	Msrb3
not_Sig	Gm15910
Sig	Lemd3
not_Sig	Wif1
not_Sig	Tbc1d30
not_Sig	Gns
not_Sig	Rassf3
not_Sig	Tbk1
not_Sig	Xpot
not_Sig	BC048403
not_Sig	Srgap1
not_Sig	Gm9079
not_Sig	Tmem5
not_Sig	Avpr1a
not_Sig	Ppm1h
not_Sig	Mirlet7i
not_Sig	Mon2

not_Sig	Usp15
not_Sig	Slc16a7
not_Sig	Lrig3
not_Sig	Xrcc6bp1
Sig	Ctdsp2
not_Sig	Mir26a-2
not_Sig	Avil
not_Sig	Tsfm
not_Sig	Mettl1
not_Sig	Cyp27b1
Sig	March9
not_Sig	Cdk4
Sig	Tspan31
not_Sig	LOC100504703
Sig	Agap2
not_Sig	Os9
not_Sig	B4galnt1
not_Sig	Slc26a10
not_Sig	Arhgef25
not_Sig	Dtx3
not_Sig	F420014N23Rik
not_Sig	Pip4k2c
not_Sig	Kif5a
not_Sig	Dctn2
Sig	Mbd6
not_Sig	Ddit3
not_Sig	Mars
not_Sig	Arhgap9
not_Sig	Gli1
not_Sig	R3hdm2
not_Sig	Stac3
not_Sig	Ndufa4l2
not_Sig	Shmt2
Sig	Nxph4
not_Sig	Lrp1
not_Sig	Stat6
not_Sig	Nab2
not_Sig	1700012D01Rik
not_Sig	Tmem194
not_Sig	Zbtb39
not_Sig	Gpr182
not_Sig	Rdh1
not_Sig	Rdh9
not_Sig	Rdh16
not_Sig	Rdh7
not_Sig	Prim1
not_Sig	Naca
Sig	Ptges3

not_Sig	Atp5b
Sig	Mir677
Sig	Baz2a
not_Sig	Rbms2
not_Sig	Gls2
not_Sig	Spryd4
not_Sig	Timeless
not_Sig	Apon
Sig	Apof
not_Sig	Stat2
Sig	Pan2
Sig	Cnpy2
not_Sig	Cs
not_Sig	Coq10a
not_Sig	Ankrd52
not_Sig	Slc39a5
not_Sig	Nabp2
not_Sig	Rnf41
Sig	Smarcc2
not_Sig	Myl6
not_Sig	Myl6b
not_Sig	A430046D13Rik
not_Sig	Esyt1
not_Sig	Zc3h10
Sig	Rpl41
not_Sig	Pa2g4
not_Sig	Erbb3
not_Sig	Rps26
not_Sig	Ikzf4
Sig	Suox
not_Sig	Rab5b
not_Sig	Cdk2
not_Sig	Pmel
not_Sig	Dgka
Sig	Wibg
not_Sig	Mmp19
not_Sig	Tmem198b
not_Sig	Dnajc14
not_Sig	Ormdl2
not_Sig	Sarnp
Sig	Gdf11
Sig	Cd63
not_Sig	Rdh5
Sig	Bloc1s1
Sig	Itga7
not_Sig	Mettl7b
not_Sig	Mir684-1
Sig	Tespa1

not_Sig	Pisd-ps1
not_Sig	Sfi1
not_Sig	Eif4enif1
Sig	Drg1
Sig	Patz1
not_Sig	Gm11944
Sig	Pik3ip1
not_Sig	Limk2
not_Sig	Rnf185
not_Sig	8430429K09Rik
not_Sig	Pla2g3
Sig	Inpp5j
not_Sig	Selm
Sig	Smtn
not_Sig	Tug1
not_Sig	Morc2a
not_Sig	Osbp2
not_Sig	4921536K21Rik
Sig	Dusp18
not_Sig	Slc35e4
not_Sig	Tcn2
Sig	Pes1
not_Sig	Gal3st1
not_Sig	Sec14l4
not_Sig	Mtfp1
not_Sig	Sec14l2
not_Sig	Rnf215
not_Sig	Mir3060
not_Sig	Ccdc157
not_Sig	Sf3a1
not_Sig	Tbc1d10a
not_Sig	Gatsl3
not_Sig	Lif
not_Sig	Mtmr3
not_Sig	Ascc2
not_Sig	Uqcr10
not_Sig	Zmat5
not_Sig	Cabp7
not_Sig	Nf2
not_Sig	Nipsnap1
not_Sig	Thoc5
not_Sig	AA413626
not_Sig	Nefh
not_Sig	Ap1b1
not_Sig	Gas2l1
not_Sig	Rasl10a
Sig	Ewsr1
not_Sig	Rhbdd3

Sig	Emid1
Sig	Kremen1
not_Sig	Znrf3
not_Sig	Xbp1
not_Sig	Ccdc117
Sig	Mrps24
not_Sig	Urgcp
not_Sig	2210015D19Rik
not_Sig	Dbnl
not_Sig	Pgam2
not_Sig	Polm
not_Sig	Aebp1
not_Sig	Pold2
not_Sig	Gck
Sig	Ykt6
not_Sig	Camk2b
Sig	Nudcd3
not_Sig	Rps15a-ps6
not_Sig	Npc1l1
not_Sig	Ddx56
not_Sig	Tmed4
not_Sig	Ogdh
Sig	Zmiz2
Sig	Ppia
not_Sig	H2afv
not_Sig	Purb
not_Sig	Myo1g
not_Sig	Gm11974
Sig	Ccm2
Sig	Nacad
not_Sig	Tbrg4
Sig	Snora5c
not_Sig	Ramp3
Sig	Adcy1
Sig	Igfbp3
not_Sig	Tns3
not_Sig	Hus1
not_Sig	Upp1
not_Sig	Abca13
not_Sig	Vwc2
Sig	Zpbp
not_Sig	Ikzf1
not_Sig	Fignl1
not_Sig	Ddc
not_Sig	Grb10
Sig	Cobl
not_Sig	Vstm2a
not_Sig	Sec61g

not_Sig	Egfr
not_Sig	Fbxo48
not_Sig	Plek
not_Sig	Cnrip1
not_Sig	Ppp3r1
not_Sig	Pno1
not_Sig	Wdr92
Sig	C1d
not_Sig	Etaa1
Sig	Meis1
Sig	Spred2
Sig	Actr2
not_Sig	Rab1
not_Sig	Cep68
not_Sig	Slc1a4
not_Sig	Sertad2
not_Sig	Aftph
not_Sig	Lgalsl
not_Sig	Peli1
not_Sig	Vps54
not_Sig	Ugp2
not_Sig	Mir1933
not_Sig	4932414J04Rik
Sig	Mdh1
Sig	Wdpcp
not_Sig	Ehbp1
not_Sig	Tmem17
not_Sig	B3gnt2
Sig	Commd1
Sig	Zrsr1
not_Sig	Cct4
not_Sig	Fam161a
not_Sig	Xpo1
not_Sig	Usp34
Sig	Ahsa2
not_Sig	Gm12060
not_Sig	0610010F05Rik
Sig	Pex13
not_Sig	Pus10
Sig	Rel
not_Sig	Papolg
not_Sig	Bcl11a
not_Sig	Fancl
not_Sig	Vrk2
not_Sig	Gm12070
not_Sig	Ccdc85a
Sig	Efemp1
not_Sig	Pnpt1

not_Sig	Smek2
not_Sig	Cfap36
not_Sig	Ccdc88a
not_Sig	Prorsd1
Sig	Mtif2
not_Sig	Rps27a
not_Sig	Rtn4
Sig	Eml6
Sig	4931440F15Rik
not_Sig	Sptbn1
not_Sig	Acyp2
Sig	Psme4
not_Sig	Gpr75
not_Sig	Erlec1
not_Sig	Asb3
not_Sig	Chac2
not_Sig	Stc2
not_Sig	Bod1
not_Sig	D630024D03Rik
not_Sig	Cpeb4
not_Sig	Nsg2
not_Sig	Il9r
not_Sig	Snrnp25
not_Sig	Rhbdf1
not_Sig	Mpg
not_Sig	Nprl3
not_Sig	Hbq1b
not_Sig	Sh3pxd2b
not_Sig	Ubtd2
not_Sig	Efcab9
not_Sig	Stk10
not_Sig	Fbxw11
not_Sig	Fgf18
Sig	Npm1
not_Sig	Ranbp17
not_Sig	Kcnmb1
not_Sig	Lcp2
not_Sig	Dock2
not_Sig	Fam196b
not_Sig	Spdl1
not_Sig	Slit3
Sig	Pank3
not_Sig	Rars
Sig	Wwc1
not_Sig	Tenm2
not_Sig	Mat2b
not_Sig	Hmmr
Sig	Nudcd2

not_Sig	Ccng1
not_Sig	Gabra1
not_Sig	Gabrb2
not_Sig	Atp10b
not_Sig	Pttg1
not_Sig	Slu7
not_Sig	C1qtnf2
not_Sig	Ccnjl
not_Sig	Pwwp2a
not_Sig	Ttc1
not_Sig	Adra1b
Sig	Ublcp1
not_Sig	Rnf145
not_Sig	Ebf1
Sig	F630206G17Rik
not_Sig	Clint1
not_Sig	Lsm11
Sig	Thg1l
Sig	Adam19
not_Sig	Mir8100
not_Sig	Cyfip2
not_Sig	Fndc9
not_Sig	Itk
not_Sig	Med7
not_Sig	Havcr2
not_Sig	Timd4
Sig	Sgcd
not_Sig	Gnb2l1
not_Sig	Snord96a
not_Sig	Snord95
Sig	Trim41
Sig	Trim7
not_Sig	Irgm1
not_Sig	Gm5431
Sig	Gm12185
not_Sig	Psme2b
not_Sig	9930111J21Rik1
not_Sig	9930111J21Rik2
not_Sig	Tgtp1
Sig	Tgtp2
not_Sig	Ifi47
not_Sig	Btnl9
not_Sig	Zfp62
not_Sig	Mgat1
Sig	Flt4
not_Sig	Scgb3a1
not_Sig	Cnot6
Sig	Gfpt2

not_Sig	Mapk9
not_Sig	Rasgef1c
not_Sig	Rnf130
not_Sig	Tbc1d9b
Sig	3010026O09Rik
not_Sig	Sqstm1
not_Sig	Mgat4b
not_Sig	Ltc4s
Sig	Maml1
not_Sig	Canx
not_Sig	Hnrnph1
not_Sig	Rufy1
not_Sig	Adamts2
not_Sig	Zfp354c
not_Sig	Zfp879
not_Sig	Zfp454
not_Sig	Zfp2
not_Sig	Zfp354b
not_Sig	Zfp354a
not_Sig	Clk4
Sig	Col23a1
Sig	Phykpl
not_Sig	Hnrnpab
Sig	Nhp2
Sig	Rmnd5b
Sig	N4bp3
Sig	D930048N14Rik
Sig	0610009B22Rik
not_Sig	Sec24a
Sig	Sar1b
Sig	Jade2
not_Sig	Cdkn2aipnl
Sig	Ube2b
not_Sig	Cdkl3
not_Sig	Ppp2ca
not_Sig	Mir3061
not_Sig	Olfr1372-ps1
Sig	Skp1a
Sig	Tcf7
not_Sig	Vdac1
not_Sig	9530068E07Rik
Sig	Hspa4
not_Sig	Zcchc10
Sig	Aff4
not_Sig	Leap2
Sig	Uqcrq
not_Sig	Gdf9
not_Sig	Shroom1

not_Sig	Sowaha
not_Sig	Sept8
not_Sig	Kif3a
not_Sig	Rad50
not_Sig	Il5
not_Sig	Irf1
not_Sig	Gm12216
not_Sig	Slc22a5
not_Sig	Slc22a21
Sig	Slc22a4
not_Sig	Pdlim4
not_Sig	P4ha2
not_Sig	Acsl6
not_Sig	Fnip1
not_Sig	Rapgef6
not_Sig	Cdc42se2
not_Sig	Lyrm7
not_Sig	Hint1
Sig	Gpx3
not_Sig	Tnip1
not_Sig	Anxa6
not_Sig	Ccdc69
not_Sig	Gm2a
Sig	Slc36a2
not_Sig	Slc36a1
not_Sig	Fat2
not_Sig	Sparc
Sig	Atox1
Sig	G3bp1
not_Sig	Gm12238
Sig	Glra1
not_Sig	Gria1
not_Sig	Fam114a2
not_Sig	Mfap3
not_Sig	Galnt10
not_Sig	2010001A14Rik
not_Sig	Sap30l
not_Sig	Larp1
not_Sig	Cnot8
not_Sig	Gemin5
not_Sig	Mrpl22
not_Sig	Gm12250
not_Sig	Igtp
not_Sig	Irgm2
not_Sig	Zfp692
not_Sig	Zfp672
Sig	Sh3bp5l
not_Sig	Trim58

not_Sig	2810021J22Rik
Sig	Zfp39
not_Sig	Btnl10
not_Sig	Rnf187
not_Sig	Hist3h2ba
not_Sig	Hist3h2bb-ps
Sig	Hist3h2a
not_Sig	Trim17
Sig	Trim11
not_Sig	Obscn
not_Sig	Iba57
Sig	Gjc2
not_Sig	Guk1
Sig	2610507I01Rik
not_Sig	Mrpl55
not_Sig	2310033P09Rik
not_Sig	Arf1
not_Sig	Wnt9a
not_Sig	Snap47
not_Sig	Jmjd4
not_Sig	Zfp867
not_Sig	Zkscan17
not_Sig	4933439C10Rik
not_Sig	Nlrp3
Sig	Mprip
not_Sig	1700007J10Rik
not_Sig	Pld6
not_Sig	Flcn
not_Sig	Gm16062
not_Sig	Cops3
not_Sig	Nt5m
not_Sig	Med9os
not_Sig	Med9
Sig	Rasd1
not_Sig	Pemt
Sig	Rai1
Sig	Srebf1
Sig	Tom1l2
not_Sig	Lrrc48
Sig	Atpaf2
not_Sig	Gid4
not_Sig	Drg2
not_Sig	Myo15
not_Sig	Alkbh5
Sig	Llgl1
not_Sig	Flii
not_Sig	Mief2
not_Sig	Mir5100

not_Sig	Top3a
not_Sig	Smcr8
not_Sig	Shmt1
Sig	Dhrs7b
not_Sig	Tmem11
not_Sig	Natd1
not_Sig	Map2k3os
not_Sig	Map2k3
Sig	Kcnj12
not_Sig	Tnfrsf13b
not_Sig	Usp22
not_Sig	Aldh3a1
not_Sig	Aldh3a2
not_Sig	Slc47a1
not_Sig	Rnf112
not_Sig	Mfap4
Sig	Mapk7
not_Sig	B9d1
not_Sig	Epn2
Sig	Grap
not_Sig	Slc5a10
not_Sig	Fam83g
not_Sig	Prpsap2
Sig	Ulk2
not_Sig	Akap10
not_Sig	Specc1
not_Sig	Adora2b
Sig	Zswim7
not_Sig	Ttc19
not_Sig	Ncor1
not_Sig	Pigl
not_Sig	Cenpv
Sig	Ubb
not_Sig	Gm1821
not_Sig	Trpv2
Sig	2410006H16Rik
not_Sig	Snord49b
not_Sig	Snord49a
not_Sig	Lrrc75a
not_Sig	Mmgt2
not_Sig	Zfp287
not_Sig	Zfp286
not_Sig	Trim16
not_Sig	Fbxw10
Sig	Tvp23b
not_Sig	Pmp22
not_Sig	Hs3st3b1
not_Sig	Cox10

Sig	9630013K17Rik
not_Sig	2810001G20Rik
not_Sig	Hs3st3a1
not_Sig	Elac2
Sig	Arhgap44
not_Sig	Myocd
not_Sig	Gm12295
not_Sig	Map2k4
not_Sig	Mir744
not_Sig	Zkscan6
not_Sig	Dnah9
not_Sig	Shisa6
not_Sig	Tmem220
not_Sig	Adprm
not_Sig	Sco1
not_Sig	Myh3
not_Sig	2310065F04Rik
not_Sig	Myh2
not_Sig	Myh1
not_Sig	Myh4
not_Sig	Myh8
not_Sig	Myh13
not_Sig	Gas7
not_Sig	Glp2r
Sig	Dhrs7c
not_Sig	Usp43
not_Sig	Cfap52
not_Sig	Stx8
not_Sig	Ntn1
not_Sig	Pik3r5
Sig	Pik3r6
not_Sig	Mfsd6l
not_Sig	Myh10
not_Sig	Ndel1
Sig	Rnf222
Sig	Arhgef15
not_Sig	Slc25a35
not_Sig	Rangrf
not_Sig	Pfas
not_Sig	Ctc1
not_Sig	Aurkb
Sig	2310047M10Rik
Sig	Tmem107
Sig	Vamp2
Sig	Per1
not_Sig	Cntrob
Sig	Trappc1
not_Sig	Kcnab3

not_Sig	Chd3os
not_Sig	Chd3
not_Sig	Cyb5d1
Sig	Naa38
Sig	Tmem88
not_Sig	Kdm6b
not_Sig	Dnah2
not_Sig	Efnb3
not_Sig	Wrap53
not_Sig	Trp53
not_Sig	Atp1b2
not_Sig	Sat2
Sig	Fxr2
not_Sig	Sox15
not_Sig	Mpdu1
not_Sig	Cd68
Sig	Eif4a1
not_Sig	Senp3
not_Sig	Tnfsf13
not_Sig	Tnfsf12
not_Sig	Polr2a
Sig	Zbtb4
Sig	Chrnb1
Sig	Fgf11
Sig	Nlgn2
not_Sig	Tmem256
Sig	Plscr3
not_Sig	Tnk1
not_Sig	Kctd11
not_Sig	Acap1
not_Sig	2810408A11Rik
not_Sig	Neurl4
not_Sig	Gps2
not_Sig	Eif5a
not_Sig	Ybx2
not_Sig	Slc2a4
not_Sig	Cldn7
not_Sig	Elp5
not_Sig	Ctdnep1
Sig	Gabarap
not_Sig	Phf23
Sig	Dvl2
not_Sig	Acadvl
not_Sig	Mir324
not_Sig	Dlg4
not_Sig	Asgr2
not_Sig	Mgl2
not_Sig	Clec10a

Sig	Slc16a11
Sig	Slc16a13
Sig	Bcl6b
Sig	0610010K14Rik
not_Sig	Rnasek
not_Sig	Alox12
not_Sig	Alox15
not_Sig	Pelp1
not_Sig	Arrb2
not_Sig	Med11
not_Sig	Cxcl16
Sig	Zmynd15
not_Sig	Gltpd2
not_Sig	Psmb6
Sig	Pld2
not_Sig	Mink1
Sig	Chrne
not_Sig	4930544D05Rik
not_Sig	Gp1ba
not_Sig	Slc25a11
not_Sig	Rnf167
not_Sig	Pfn1
Sig	Eno3
not_Sig	Spag7
not_Sig	Camta2
not_Sig	Inca1
Sig	Kif1c
Sig	Zfp3
not_Sig	Rabep1
not_Sig	Nup88
not_Sig	Rpain
not_Sig	C1qbp
not_Sig	Dhx33
not_Sig	Derl2
not_Sig	Mis12
not_Sig	6330403K07Rik
not_Sig	Nlrp1b
not_Sig	Nlrp1c-ps
Sig	Wscd1
not_Sig	Fam64a
Sig	Pitpnm3
not_Sig	4933427D14Rik
not_Sig	Txndc17
not_Sig	Med31
not_Sig	4930563E22Rik
Sig	Slc13a5
not_Sig	Xaf1
not_Sig	Tekt1

not_Sig	Mir6338
not_Sig	Smtnl2
not_Sig	Ggt6
Sig	Mybbp1a
not_Sig	Spns2
Sig	Spns3
Sig	Ube2g1
not_Sig	Ankfy1
Sig	Cyb5d2
Sig	Zzef1
not_Sig	Atp2a3
not_Sig	P2rx1
not_Sig	Camkk1
not_Sig	1200014J11Rik
not_Sig	Itgae
not_Sig	Gsg2
not_Sig	P2rx5
not_Sig	Emc6
not_Sig	Tax1bp3
Sig	Ctns
not_Sig	Shpk
not_Sig	Trpv1
not_Sig	Trpv3
Sig	Aspa
not_Sig	Spata22
Sig	Rap1gap2
not_Sig	E130309D14Rik
not_Sig	Cluh
not_Sig	Pafah1b1
Sig	Mettl16
Sig	Mnt
not_Sig	Sgsm2
Sig	Tsr1
not_Sig	Snord91a
not_Sig	Srr
not_Sig	Smg6
Sig	Hic1
not_Sig	Ovca2
not_Sig	Dph1
not_Sig	Rtn4rl1
not_Sig	Rpa1
not_Sig	Smyd4
not_Sig	Serpinf1
not_Sig	Serpinf2
not_Sig	Wdr81
not_Sig	Mir22hg
not_Sig	Tlcd2
Sig	Prpf8

not_Sig	Rilp
Sig	Scarf1
not_Sig	Slc43a2
Sig	Pitpna
not_Sig	Gm12338
not_Sig	Inpp5k
not_Sig	Myo1c
Sig	Crk
not_Sig	Ywhae
not_Sig	Doc2b
not_Sig	Rph3al
not_Sig	Fam101b
not_Sig	Vps53
not_Sig	Fam57a
not_Sig	Gemin4
not_Sig	Glod4
not_Sig	Rnmtl1
not_Sig	Nxn
not_Sig	Timm22
Sig	Abr
not_Sig	Tusc5
not_Sig	Gosr1
Sig	Cpd
not_Sig	Tmigd1
not_Sig	Blmh
not_Sig	Slc6a4
not_Sig	Ccdc55
not_Sig	Efcab5
not_Sig	Ssh2
Sig	Coro6
Sig	Ankrd13b
Sig	Git1
not_Sig	Trp53i13
not_Sig	Abhd15
not_Sig	Taok1
not_Sig	Nufip2
not_Sig	Cryba1
not_Sig	Myo18a
not_Sig	Pipox
not_Sig	Sez6
Sig	Phf12
not_Sig	Dhrs13
Sig	Flot2
not_Sig	Mir144
not_Sig	Eral1
not_Sig	Fam222b
not_Sig	Traf4
not_Sig	Nek8

Sig	Tlcd1
Sig	Rpl23a
not_Sig	Snord4a
not_Sig	Snord42b
not_Sig	Rab34
not_Sig	Proca1
not_Sig	Supt6
not_Sig	Sdf2
not_Sig	2610507B11Rik
not_Sig	BC030499
Sig	Spag5
not_Sig	Aldoc
Sig	Pigs
not_Sig	Unc119
Sig	Slc46a1
not_Sig	Sarm1
not_Sig	Vtn
not_Sig	Sebox
not_Sig	Tmem199
not_Sig	Poldip2
not_Sig	Tnfaip1
not_Sig	Ift20
not_Sig	Tmem97
not_Sig	Nlk
not_Sig	Fam58b
not_Sig	Lyrm9
Sig	Nos2
not_Sig	Lgals9
Sig	Ksr1
Sig	Gm11201
not_Sig	Wsb1
not_Sig	Nf1
not_Sig	AU040972
not_Sig	Omg
Sig	Evi2a
not_Sig	Rab11fip4
not_Sig	Rab11fip4os1
not_Sig	Mir193a
not_Sig	Mir365-2
Sig	Utp6
not_Sig	Suz12
not_Sig	Crlf3
not_Sig	Atad5
not_Sig	Tefm
not_Sig	Adap2
not_Sig	Rnf135
not_Sig	Rhot1
not_Sig	Rhbdl3

not_Sig	5730455P16Rik
not_Sig	Zfp207
not_Sig	Psmd11
not_Sig	Cdk5r1
not_Sig	Myo1d
not_Sig	C030013C21Rik
not_Sig	Tmem98
not_Sig	Asic2
not_Sig	Ccl2
not_Sig	Ccl7
Sig	Ccl11
not_Sig	Ccl12
not_Sig	Ccl8
not_Sig	Tmem132e
not_Sig	Cct6b
not_Sig	Zfp830
not_Sig	Gm11423
not_Sig	Lig3
not_Sig	Rffl
not_Sig	Rad51d
not_Sig	Fndc8
not_Sig	Nle1
Sig	Unc45b
not_Sig	Slfn5os
not_Sig	Slfn5
not_Sig	Slfn9
not_Sig	Slfn8
not_Sig	Slfn10-ps
not_Sig	Slfn2
Sig	Slfn1
Sig	Slfn4
not_Sig	Slfn3
not_Sig	AI662270
Sig	Slfn14
not_Sig	AA465934
not_Sig	AI450353
Sig	Pex12
not_Sig	Ap2b1
not_Sig	Rasl10b
not_Sig	1700020L24Rik
not_Sig	Mmp28
not_Sig	Taf15
not_Sig	Heatr9
Sig	Ccl5
Sig	Ccl9
not_Sig	Ccl6
not_Sig	Wfdc17
not_Sig	Wfdc21

not_Sig	Heatr6
not_Sig	Ddx52
not_Sig	Synrg
not_Sig	Dusp14
not_Sig	Tada2a
not_Sig	Gm11437
not_Sig	Acaca
not_Sig	Aatf
not_Sig	Mrm1
not_Sig	Dhrs11
not_Sig	4930502E09Rik
not_Sig	Ggnbp2
not_Sig	Ggnbp2os
not_Sig	Pigw
not_Sig	Myo19
Sig	Znhit3
not_Sig	Car4
Sig	Usp32
not_Sig	Appbp2
not_Sig	Appbp2os
not_Sig	Ppm1d
not_Sig	Bcas3
not_Sig	Bcas3os1
not_Sig	Mir5110
not_Sig	Bcas3os2
not_Sig	2610027K06Rik
Sig	Tbx2
not_Sig	Tbx4
not_Sig	Brip1
not_Sig	Brip1os
not_Sig	Ints2
not_Sig	Med13
not_Sig	Mir467c
not_Sig	Rnft1
not_Sig	Rps6kb1
not_Sig	Tubd1
not_Sig	Vmp1
not_Sig	Mir8115
not_Sig	Ptrh2
Sig	Cltc
not_Sig	Dhx40
not_Sig	Ypel2
not_Sig	Gdpd1
Sig	Smg8
not_Sig	Prr11
Sig	Ska2
Sig	Trim37
Sig	Ppm1e

not_Sig	Rad51c
not_Sig	Tex14
Sig	Sept4
not_Sig	Mtmr4
not_Sig	Hsf5
not_Sig	Rnf43
not_Sig	1110028F11Rik
Sig	Supt4a
not_Sig	Mir142b
not_Sig	Bzrap1
Sig	Mpo
not_Sig	Mks1
not_Sig	Epx
not_Sig	Dynll2
not_Sig	Srsf1
not_Sig	Vezf1
not_Sig	2210416O15Rik
not_Sig	Cuedc1
not_Sig	Mrps23
not_Sig	Msi2
not_Sig	Mir378b
not_Sig	0610039H22Rik
not_Sig	C030037D09Rik
not_Sig	Akap1
not_Sig	4930556N13Rik
Sig	Scpep1
not_Sig	Coil
Sig	Trim25
not_Sig	Dgke
not_Sig	Nog
not_Sig	4932411E22Rik
not_Sig	Ankfn1
not_Sig	Pctp
not_Sig	Tmem100
not_Sig	Mmd
not_Sig	Hlf
not_Sig	Stxbp4
Sig	Cox11
not_Sig	Tom1l1
not_Sig	Utp18
not_Sig	Mbtd1
not_Sig	Nme2
not_Sig	Nme1
not_Sig	Spag9
not_Sig	Tob1
not_Sig	Wfikkn2
not_Sig	Luc7l3
not_Sig	Ankrd40

Sig	Abcc3
not_Sig	Cacna1g
not_Sig	Epn3
not_Sig	Rsad1
not_Sig	Acsf2
Sig	Chad
not_Sig	Lrrc59
not_Sig	Eme1
not_Sig	Mrpl27
not_Sig	Xylt2
not_Sig	Col1a1
not_Sig	Sgca
not_Sig	Hils1
not_Sig	Ppp1r9b
not_Sig	Samd14
Sig	Pdk2
not_Sig	Itga3
not_Sig	Dlx3
not_Sig	Tac4
not_Sig	Kat7
not_Sig	Fam117a
not_Sig	Slc35b1
not_Sig	Spop
not_Sig	Nxph3
not_Sig	Ngfr
not_Sig	Phb
not_Sig	Zfp652os
not_Sig	B130006D01Rik
not_Sig	Zfp652
Sig	Phospho1
Sig	Abi3
not_Sig	Gngt2
not_Sig	4833417C18Rik
Sig	B4galnt2
not_Sig	Snf8
not_Sig	Ube2z
not_Sig	Atp5g1
not_Sig	Calcoco2
not_Sig	Hoxb9
not_Sig	Hoxb8
not_Sig	Hoxb7
not_Sig	Hoxb5os
Sig	Hoxb6
not_Sig	Hoxb5
Sig	Hoxb4
not_Sig	Hoxb3
not_Sig	Hoxb2
Sig	Skap1

Sig	Snx11
not_Sig	Cbx1
not_Sig	Gm11517
not_Sig	Nfe2l1
not_Sig	Copz2
Sig	Cdk5rap3
not_Sig	Prr15l
not_Sig	Pnpo
not_Sig	D030028A08Rik
Sig	Sp2
Sig	Sp6
not_Sig	Scrn2
not_Sig	Lrrc46
Sig	Mrpl10
Sig	Osbpl7
not_Sig	Mir8103
Sig	Tbx21
Sig	Tbkbp1
not_Sig	Kpnb1
not_Sig	Npepps
not_Sig	Mrpl45
not_Sig	Gpr179
not_Sig	Socs7
Sig	Arhgap23
not_Sig	4933428G20Rik
not_Sig	Srcin1
not_Sig	E130012A19Rik
Sig	Mllt6
not_Sig	Cisd3
Sig	Pcgf2
Sig	Psmb3
Sig	Pip4k2b
not_Sig	Cwc25
not_Sig	1700001P01Rik
Sig	Rpl23
not_Sig	Snora21
not_Sig	Lasp1
not_Sig	B230217C12Rik
not_Sig	Fbxo47
Sig	Plxdc1
not_Sig	Arl5c
not_Sig	Cacnb1
not_Sig	Rpl19
not_Sig	Stac2
Sig	Fbxl20
not_Sig	Med1
Sig	Cdk12
not_Sig	Mir5119

not_Sig	Ppp1r1b
not_Sig	Stard3
Sig	Tcap
not_Sig	Pnmt
not_Sig	Pgap3
not_Sig	Erbb2
not_Sig	Mien1
not_Sig	Grb7
not_Sig	Ikzf3
not_Sig	Ormdl3
Sig	Psmd3
not_Sig	Med24
Sig	Thra
not_Sig	Nr1d1
not_Sig	Msl1
not_Sig	Gm12359
not_Sig	Casc3
not_Sig	Rapgefl1
Sig	Wipf2
Sig	Cdc6
Sig	Rara
Sig	Top2a
not_Sig	Igfbp4
not_Sig	Tns4
not_Sig	Ccr7
not_Sig	Smarce1
not_Sig	Krt222
not_Sig	Krt10
not_Sig	Krt36
not_Sig	Krt15
not_Sig	Krt19
Sig	Krt17
not_Sig	Eif1
not_Sig	Hap1
not_Sig	Jup
not_Sig	P3h4
not_Sig	Fkbp10
not_Sig	Nt5c3b
not_Sig	Klhl10
not_Sig	Klhl11
not_Sig	Acly
not_Sig	Ttc25
not_Sig	Cnp
Sig	Dnajc7
Sig	Nkiras2
Sig	Zfp385c
not_Sig	Dhx58
not_Sig	Kat2a

not_Sig	Hspb9
not_Sig	Rab5c
not_Sig	Kcnh4
not_Sig	Ghdc
not_Sig	Stat5b
not_Sig	Stat5a
not_Sig	Stat3
not_Sig	Ptrf
not_Sig	Atp6v0a1
not_Sig	Naglu
not_Sig	Hsd17b1
not_Sig	Coasy
not_Sig	Mlx
not_Sig	Psmc3ip
not_Sig	Fam134c
not_Sig	Tubg1
Sig	Tubg2
Sig	Plekhh3
not_Sig	Ccr10
not_Sig	Cntnap1
Sig	Ezh1
not_Sig	Ramp2
not_Sig	Vps25
not_Sig	Wnk4
Sig	Coa3
not_Sig	Cntd1
not_Sig	Becn1
not_Sig	Psme3
not_Sig	Aoc2
not_Sig	Aoc3
not_Sig	Aarsd1
not_Sig	Ptges3l
not_Sig	Rundc1
not_Sig	Rpl27
not_Sig	Ifi35
not_Sig	Vat1
not_Sig	Rnd2
Sig	Brca1
Sig	Nbr1
not_Sig	Tmem106a
Sig	Rdm1
not_Sig	Arl4d
not_Sig	Dhx8
not_Sig	Etv4
not_Sig	Meox1
not_Sig	Sost
not_Sig	Dusp3
Sig	Mpp3

Sig	Cd300lg
not_Sig	Mpp2
not_Sig	Tmem101
not_Sig	Lsm12
not_Sig	G6pc3
not_Sig	Hdac5
not_Sig	BC030867
not_Sig	Asb16
not_Sig	Tmub2
not_Sig	Atxn7l3
Sig	Ubtf
Sig	Slc4a1
not_Sig	Rundc3a
Sig	Slc25a39
not_Sig	Grn
not_Sig	Fam171a2
not_Sig	Itga2b
not_Sig	Gpatch8
not_Sig	Gm11627
not_Sig	Fzd2
not_Sig	2810433D01Rik
not_Sig	Gm1564
not_Sig	Ccdc43
not_Sig	Adam11
Sig	Gjc1
not_Sig	Higd1b
not_Sig	Eftud2
not_Sig	Gfap
not_Sig	Kif18b
not_Sig	Dcakd
Sig	Nmt1
not_Sig	Plcd3
not_Sig	Acbd4
Sig	Hexim1
not_Sig	Hexim2
not_Sig	Fmnl1
Sig	Map3k14
Sig	Arhgap27
Sig	Arhgap27os3
not_Sig	Plekhm1
not_Sig	Rprml
not_Sig	Gosr2
not_Sig	C130046K22Rik
not_Sig	Wnt9b
not_Sig	Nsf
not_Sig	Arf2
not_Sig	Mapt
Sig	Kansl1

not_Sig	Cdc27
Sig	Myl4
not_Sig	Gm11665
not_Sig	Itgb3
not_Sig	Mettl2
Sig	Tlk2
not_Sig	1700052K11Rik
not_Sig	Mrc2
Sig	Tanc2
not_Sig	Cyb561
not_Sig	Ace
not_Sig	Dcaf7
Sig	Taco1
not_Sig	Map3k3
not_Sig	Limd2
not_Sig	Strada
Sig	Ccdc47
not_Sig	Ddx42
not_Sig	Ftsj3
not_Sig	Psmc5
not_Sig	Smarcd2
not_Sig	Tcam1
not_Sig	Cd79b
not_Sig	Scn4a
Sig	Prr29
Sig	Icam2
not_Sig	Ern1
not_Sig	Snord104
not_Sig	Tex2
Sig	Pecam1
not_Sig	Milr1
not_Sig	Polg2
not_Sig	Ddx5
not_Sig	Mir3064
not_Sig	Cep95
Sig	Smurf2
Sig	Kpna2
not_Sig	1810010H24Rik
Sig	Bptf
not_Sig	Nol11
Sig	Pitpnc1
not_Sig	Psmd12
not_Sig	Helz
not_Sig	Cacng1
not_Sig	Cacng4
not_Sig	Prkca
not_Sig	Apoh
not_Sig	Cep112

Sig	Axin2
not_Sig	Rgs9
not_Sig	Gm11696
not_Sig	Gna13
not_Sig	Amz2
not_Sig	LOC100503496
not_Sig	Slc16a6
not_Sig	Arsg
not_Sig	Wipi1
Sig	Prkar1a
not_Sig	Fam20a
Sig	Abca8b
not_Sig	Abca8a
Sig	Abca9
not_Sig	Abca6
not_Sig	Abca5
not_Sig	Map2k6
not_Sig	Kcnj16
not_Sig	Kcnj2
not_Sig	Sox9
not_Sig	2610035D17Rik
not_Sig	Slc39a11
not_Sig	Cog1
not_Sig	Fam104a
not_Sig	D11Wsu47e
not_Sig	Cpsf4l
not_Sig	Cdc42ep4
not_Sig	Sdk2
not_Sig	4932435O22Rik
Sig	Rpl38
not_Sig	Ttyh2
not_Sig	Dnaic2
not_Sig	Kif19a
not_Sig	Btbd17
not_Sig	Gprc5c
not_Sig	Cd300a
not_Sig	Cd300lb
not_Sig	Cd300ld
not_Sig	AF251705
not_Sig	Cd300lh
not_Sig	Rab37
not_Sig	Cd300lf
not_Sig	Slc9a3r1
not_Sig	Nat9
not_Sig	Tmem104
not_Sig	Grin2c
not_Sig	Fdxr
Sig	Fads6

not_Sig	Ush1g
not_Sig	Hid1
not_Sig	Cdr2l
not_Sig	Ict1
not_Sig	Atp5h
not_Sig	Kctd2
not_Sig	Slc16a5
not_Sig	Armc7
not_Sig	Nt5c
not_Sig	Hn1
not_Sig	Sumo2
not_Sig	Nup85
not_Sig	Gga3
not_Sig	Mrps7
not_Sig	Mif4gd
Sig	Slc25a19
not_Sig	Grb2
Sig	2310067B10Rik
Sig	Caskin2
not_Sig	Tsen54
not_Sig	Llgl2
not_Sig	Recql5
not_Sig	Smim5
not_Sig	Smim6
not_Sig	Sap30bp
not_Sig	Itgb4
not_Sig	Galk1
not_Sig	H3f3b
Sig	Unk
not_Sig	Unc13d
not_Sig	Wbp2
not_Sig	Trim47
not_Sig	Trim65
not_Sig	Mrpl38
Sig	Fbf1
Sig	Acox1
not_Sig	Ten1
not_Sig	Cdk3-ps
not_Sig	Evpl
not_Sig	Srp68
not_Sig	Galr2
Sig	Exoc7
not_Sig	Rnf157
not_Sig	Qrich2
Sig	Prpsap1
not_Sig	Sphk1
not_Sig	Ube2o
not_Sig	Aanat

Sig	Rhbdf2
not_Sig	Cygb
not_Sig	Gm11744
not_Sig	1810032O08Rik
not_Sig	St6galnac2
not_Sig	Mxra7
not_Sig	Jmjd6
not_Sig	Mettl23
not_Sig	Srsf2
not_Sig	Mfsd11
not_Sig	Snhg20
not_Sig	Mir6516
Sig	Sec14l1
not_Sig	Sept9
not_Sig	2900041M22Rik
Sig	Tnrc6c
not_Sig	Tmc6
not_Sig	Tmc8
not_Sig	6030468B19Rik
not_Sig	Syngr2
not_Sig	Tk1
not_Sig	Afmid
not_Sig	Birc5
not_Sig	Tha1
not_Sig	Socs3
not_Sig	Pgs1
Sig	Dnah17
Sig	Cyth1
not_Sig	Usp36
Sig	Timp2
not_Sig	BC100451
not_Sig	Lgals3bp
not_Sig	Cant1
not_Sig	C1qtnf1
not_Sig	Gm11747
not_Sig	Engase
not_Sig	Rbfox3
not_Sig	Cbx2
not_Sig	Cbx8
not_Sig	Cbx4
not_Sig	Tbc1d16
not_Sig	Ccdc40
Sig	Gaa
not_Sig	Eif4a3
not_Sig	Card14
not_Sig	Sgsh
not_Sig	Slc26a11
not_Sig	Mir1932

not_Sig	Endov
not_Sig	Nptx1
Sig	Rptor
not_Sig	Rptoros
not_Sig	Mir5098
Sig	Chmp6
not_Sig	Baiap2
Sig	Aatk
Sig	Cep131
not_Sig	Enthd2
Sig	1810043H04Rik
not_Sig	Slc38a10
Sig	2810410L24Rik
not_Sig	Bahcc1
not_Sig	Actg1
not_Sig	Mir6935
Sig	0610009L18Rik
not_Sig	Fscn2
not_Sig	Faap100
not_Sig	Nploc4
not_Sig	Tspan10
not_Sig	Pde6g
not_Sig	Oxld1
not_Sig	Ccdc137
not_Sig	Arl16
not_Sig	Hgs
not_Sig	Mrpl12
not_Sig	Slc25a10
not_Sig	Fam195b
not_Sig	Ppp1r27
Sig	P4hb
not_Sig	Arhgdia
not_Sig	Alyref
not_Sig	Anapc11
not_Sig	Npb
not_Sig	Pcyt2
not_Sig	Sirt7
not_Sig	Mafg
not_Sig	Pycr1
not_Sig	Myadml2
not_Sig	Notum
not_Sig	Aspscr1
not_Sig	Stra13
not_Sig	Lrrc45
not_Sig	Rac3
not_Sig	Dcxr
Sig	Cbr2
Sig	Rfng

not_Sig	Gps1
not_Sig	Dus1l
not_Sig	Fasn
Sig	Ccdc57
Sig	Slc16a3
not_Sig	Csnk1d
not_Sig	Cd7
not_Sig	Uts2r
not_Sig	Ogfod3
not_Sig	Hexdc
not_Sig	BC017643
not_Sig	Narf
not_Sig	Foxk2
not_Sig	Wdr45b
Sig	Rab40b
not_Sig	Fn3krp
not_Sig	Fn3k
not_Sig	Tbcd
not_Sig	Zfp750
not_Sig	B3gntl1
not_Sig	Metrnl
not_Sig	Rab10os
not_Sig	Rab10
not_Sig	Kif3c
not_Sig	1110002L01Rik
not_Sig	Asxl2
not_Sig	Dtnb
not_Sig	Dnmt3a
not_Sig	Dnmt3aos
not_Sig	Pomc
Sig	Efr3b
not_Sig	Dnajc27
not_Sig	Adcy3
not_Sig	Cenpo
Sig	Ptrhd1
not_Sig	Ncoa1
not_Sig	Itsn2
not_Sig	Fam228a
not_Sig	Fam228b
not_Sig	Pfn4
not_Sig	Gm6682
not_Sig	Sf3b6
not_Sig	Fkbp1b
not_Sig	BC068281
not_Sig	Mfsd2b
not_Sig	Ubxn2a
not_Sig	Atad2b
not_Sig	Klhl29

not_Sig	2810032G03Rik
Sig	Apob
not_Sig	Ldah
not_Sig	Gdf7
not_Sig	Hs1bp3
not_Sig	Rhob
not_Sig	Slc7a15
not_Sig	Pum2
not_Sig	Sdc1
not_Sig	Laptm4a
not_Sig	Matn3
not_Sig	Wdr35
not_Sig	Ttc32
not_Sig	Osr1
not_Sig	Rdh14
not_Sig	Kcns3
not_Sig	9530020I12Rik
not_Sig	Gen1
not_Sig	Smc6
not_Sig	Vsnl1
not_Sig	Fam49a
not_Sig	4921511I17Rik
not_Sig	Mycn
not_Sig	Ddx1
not_Sig	Nbas
not_Sig	Fam84a
Sig	Trib2
not_Sig	Mir6387
Sig	Lpin1
not_Sig	Ntsr2
Sig	Greb1
not_Sig	E2f6
not_Sig	Rock2
not_Sig	Pqlc3
not_Sig	Kcnf1
not_Sig	Pdia6
not_Sig	Atp6v1c2
not_Sig	Nol10
Sig	Odc1
not_Sig	Hpcal1
not_Sig	5730507C01Rik
not_Sig	1700030C10Rik
not_Sig	Asap2
Sig	Itgb1bp1
not_Sig	Cpsf3
not_Sig	Iah1
Sig	Adam17
not_Sig	Ywhaq

not_Sig	Taf1b
not_Sig	Grhl1
not_Sig	Klf11
not_Sig	Cys1
not_Sig	Rrm2
not_Sig	Mboat2
Sig	Kidins220
not_Sig	Id2
not_Sig	Rnf144a
not_Sig	4930549C15Rik
not_Sig	Mir6538
not_Sig	Rsad2
not_Sig	Cmpk2
not_Sig	Sox11
not_Sig	Colec11
not_Sig	Rps7
not_Sig	Rnaseh1
not_Sig	Adi1
not_Sig	Trappc12
not_Sig	Tssc1
not_Sig	Myt1l
not_Sig	Pxdn
not_Sig	Sntg2
not_Sig	2310016D03Rik
not_Sig	Tmem18
not_Sig	Fam150b
Sig	Acp1
not_Sig	Sh3yl1
not_Sig	Fam110c
not_Sig	Lamb1
not_Sig	Dld
not_Sig	Cbll1
not_Sig	Bcap29
not_Sig	Dus4l
not_Sig	Cog5
not_Sig	Gpr22
not_Sig	Hbp1
not_Sig	Prkar2b
not_Sig	Pik3cg
Sig	Ccdc71l
not_Sig	Nampt
not_Sig	Gdap10
not_Sig	4933406C10Rik
not_Sig	Sypl
Sig	Cdhr3
not_Sig	F730043M19Rik
not_Sig	Atxn7l1
not_Sig	Atxn7l1os2

not_Sig	Twistnb
not_Sig	Twist1
not_Sig	Hdac9
not_Sig	Snx13
Sig	Ahr
Sig	Tspan13
not_Sig	Gm5434
not_Sig	Bzw2
not_Sig	Ankmy2
not_Sig	Sostdc1
not_Sig	Ispd
Sig	Meox2
not_Sig	Agmo
not_Sig	Dgkb
not_Sig	Etv1
not_Sig	Arl4a
Sig	Scin
not_Sig	Lsmem1
not_Sig	Ifrd1
not_Sig	Mir1938
not_Sig	Gm7008
Sig	Zfp277
Sig	Dock4
not_Sig	Immp2l
not_Sig	Lrrn3
Sig	Dnajb9
not_Sig	Gm2027
Sig	Pnpla8
not_Sig	Nrcam
not_Sig	Stxbp6
not_Sig	Nova1
Sig	Prkd1
not_Sig	G2e3
not_Sig	Scfd1
not_Sig	Coch
not_Sig	Strn3
not_Sig	Ap4s1
not_Sig	Hectd1
not_Sig	Heatr5a
not_Sig	Dtd2
not_Sig	Nubpl
not_Sig	Arhgap5
not_Sig	Akap6
not_Sig	Npas3
not_Sig	Egln3
not_Sig	Sptssa
not_Sig	Eapp
not_Sig	Snx6

not_Sig	Cfl2
not_Sig	Baz1a
not_Sig	2700097O09Rik
not_Sig	Srp54a
not_Sig	Srp54b
not_Sig	Ppp2r3c
not_Sig	1110008L16Rik
not_Sig	Psma6
Sig	Nfkbia
not_Sig	Insm2
not_Sig	Ralgapa1
not_Sig	Brms1l
not_Sig	Mbip
not_Sig	Pax9
not_Sig	Slc25a21
not_Sig	Prps1l3
not_Sig	Mipol1
not_Sig	Sstr1
Sig	Clec14a
not_Sig	Sec23a
not_Sig	Gemin2
not_Sig	Trappc6b
not_Sig	Pnn
not_Sig	Ctage5
not_Sig	Fbxo33
not_Sig	Lrfn5
not_Sig	Gm527
not_Sig	Klhl28
not_Sig	Fam179b
not_Sig	Prpf39
not_Sig	Fkbp3
not_Sig	Fancm
not_Sig	Mis18bp1
not_Sig	Mdga2
not_Sig	Rps29
not_Sig	Rpl36al
not_Sig	Mgat2
not_Sig	Dnaaf2
not_Sig	9330151L19Rik
not_Sig	Pole2
Sig	Klhdc1
Sig	Klhdc2
not_Sig	Nemf
not_Sig	Arf6
Sig	Vcpkmt
not_Sig	Sos2
not_Sig	L2hgdh
not_Sig	Atp5s

not_Sig	Cdkl1
not_Sig	4930512B01Rik
not_Sig	Map4k5
not_Sig	4931403G20Rik
not_Sig	Atl1
not_Sig	Gm3086
Sig	Sav1
not_Sig	Nin
Sig	Pygl
not_Sig	Trim9
Sig	Tmx1
not_Sig	Frmd6
not_Sig	Actr10
Sig	Psma3
not_Sig	3110056K07Rik
Sig	Arid4a
not_Sig	Timm9
not_Sig	2700049A03Rik
not_Sig	Dact1
not_Sig	4930404H11Rik
not_Sig	Daam1
not_Sig	Gpr135
Sig	L3hypdh
Sig	Jkamp
not_Sig	Rtn1
not_Sig	Lrrc9
Sig	Pcnxl4
not_Sig	Dhrs7
Sig	Ppm1a
not_Sig	4930447C04Rik
not_Sig	Six1
not_Sig	Six4
not_Sig	Mnat1
not_Sig	Trmt5
not_Sig	Slc38a6
not_Sig	D830013O20Rik
not_Sig	Tmem30b
not_Sig	2210039B01Rik
Sig	Prkch
not_Sig	Hif1a
not_Sig	Snapc1
Sig	Rhoj
not_Sig	Ppp2r5e
not_Sig	Wdr89
Sig	Sgpp1
not_Sig	Syne2
Sig	Esr2
not_Sig	Mthfd1

not_Sig	Akap5
not_Sig	Zbtb25
not_Sig	Zbtb1
not_Sig	Hspa2
not_Sig	Plekhg3
not_Sig	AI463170
not_Sig	Sptb
Sig	Churc1
not_Sig	Rab15
not_Sig	Fntb
not_Sig	Max
Sig	Fut8
not_Sig	Gphn
not_Sig	Mpp5
Sig	Atp6v1d
not_Sig	Eif2s1
not_Sig	Plek2
not_Sig	Tmem229b
not_Sig	Plekhh1
not_Sig	Pigh
not_Sig	Arg2
not_Sig	Vti1b
not_Sig	Rdh11
not_Sig	Rdh12
not_Sig	Zfyve26
not_Sig	Rad51b
Sig	Zfp36l1
Sig	2310015A10Rik
not_Sig	Actn1
not_Sig	Dcaf5
not_Sig	Exd2
not_Sig	2310002D06Rik
not_Sig	Galnt16
Sig	Erh
not_Sig	Slc39a9
not_Sig	Plekhd1
Sig	Susd6
not_Sig	Srsf5
Sig	Slc10a1
Sig	Smoc1
not_Sig	Slc8a3
not_Sig	Cox16
not_Sig	Gm4787
not_Sig	Adam4
not_Sig	Synj2bp
not_Sig	Adam21
not_Sig	Med6
not_Sig	Ttc9

not_Sig	Map3k9
not_Sig	Pcnx
Sig	Sipa1l1
not_Sig	Rgs6
not_Sig	Dpf3
not_Sig	Dcaf4
Sig	Zfyve1
not_Sig	Rbm25
Sig	Psen1
not_Sig	Papln
not_Sig	Numb
not_Sig	2410016O06Rik
Sig	Acot2
not_Sig	Acot1
not_Sig	Acot4
not_Sig	Acot3
not_Sig	Acot6
not_Sig	Dnal1
not_Sig	Pnma1
Sig	Elmsan1
Sig	Ptgr2
not_Sig	Zfp410
not_Sig	Fam161b
not_Sig	Coq6
not_Sig	Entpd5
not_Sig	Ccdc176
not_Sig	Rnf113a2
Sig	Aldh6a1
not_Sig	Lin52
not_Sig	Abcd4
not_Sig	Npc2
Sig	Isca2
not_Sig	Ltbp2
not_Sig	Arel1
not_Sig	Fcf1
not_Sig	Ylpm1
not_Sig	Prox2
not_Sig	Dlst
not_Sig	Rps6kl1
not_Sig	Pgf
not_Sig	Eif2b2
not_Sig	Mlh3
not_Sig	Acyp1
not_Sig	Zc2hc1c
Sig	Nek9
not_Sig	Tmed10
Sig	Fos
not_Sig	Jdp2

not_Sig	Batf
not_Sig	Mfsd7c
not_Sig	0610007P14Rik
not_Sig	Ttll5
Sig	Tgfb3
not_Sig	Ift43
not_Sig	Gpatch2l
Sig	Esrrb
Sig	Vash1
not_Sig	Angel1
Sig	Irf2bpl
not_Sig	Cipc
not_Sig	Zdhhc22
not_Sig	Tmem63c
not_Sig	Pomt2
not_Sig	Gstz1
not_Sig	Tmed8
not_Sig	Noxred1
not_Sig	Vipas39
Sig	Ahsa1
not_Sig	Ism2
not_Sig	Sptlc2
not_Sig	Alkbh1
not_Sig	Mir3068
not_Sig	Nrp
not_Sig	Slirp
not_Sig	Snw1
not_Sig	Adck1
not_Sig	3200001D21Rik
not_Sig	Nrxn3
not_Sig	Dio2
not_Sig	Cep128
not_Sig	5430427M07Rik
not_Sig	Tshr
Sig	Gtf2a1
not_Sig	Ston2
not_Sig	Sel1l
Sig	Flrt2
not_Sig	Galc
not_Sig	Gpr65
not_Sig	Spata7
not_Sig	Ptpn21
not_Sig	Zc3h14
not_Sig	Eml5
not_Sig	Ttc8
not_Sig	Foxn3
not_Sig	Tdp1
not_Sig	Kcnk13

not_Sig	Psmc1
not_Sig	Nrde2
not_Sig	Gm10433
Sig	Calm1
not_Sig	Gm10432
not_Sig	Ttc7b
not_Sig	Rps6ka5
not_Sig	9030617O03Rik
not_Sig	Gpr68
Sig	Ccdc88c
not_Sig	Mir1190
not_Sig	Smek1
not_Sig	D130020L05Rik
not_Sig	Tc2n
not_Sig	Fbln5
not_Sig	Trip11
not_Sig	Atxn3
not_Sig	Cpsf2
Sig	Rin3
not_Sig	Lgmn
not_Sig	Golga5
not_Sig	Itpk1
not_Sig	Tmem251
not_Sig	AK010878
not_Sig	Ubr7
not_Sig	Btbd7
not_Sig	Unc79
not_Sig	Prima1
not_Sig	Fam181a
not_Sig	Asb2
not_Sig	Otub2
not_Sig	Ddx24
Sig	Ifi27l2a
not_Sig	Ifi27
not_Sig	Ppp4r4
not_Sig	Serpina10
not_Sig	Serpina6
Sig	Serpina1b
Sig	Serpina1d
Sig	Serpina1a
not_Sig	Serpina1c
Sig	Serpina1e
not_Sig	Serpina3b
not_Sig	Serpina3c
not_Sig	Serpina3g
not_Sig	Serpina3f
not_Sig	Serpina3h
Sig	Serpina3m

not_Sig	Serpina3n
not_Sig	Gsc
Sig	Dicer1
not_Sig	Clmn
not_Sig	Syne3
not_Sig	Snhg10
Sig	Scarna13
Sig	Glrx5
not_Sig	D430019H16Rik
Sig	Bdkrb2
not_Sig	Atg2b
not_Sig	Gskip
not_Sig	Papola
not_Sig	Vrk1
not_Sig	Bcl11b
not_Sig	Setd3
not_Sig	Ccnk
not_Sig	Ccdc85c
not_Sig	Hhipl1
not_Sig	Cyp46a1
not_Sig	Eml1
not_Sig	Gm16596
Sig	Evl
not_Sig	Degs2
not_Sig	Yy1
not_Sig	Slc25a29
not_Sig	Mir345
not_Sig	Slc25a47
not_Sig	Wars
not_Sig	Wdr25
not_Sig	Begain
Sig	Dlk1
Sig	Meg3
Sig	Mir540
not_Sig	Mir3070a
Sig	Rtl1
Sig	Rian
not_Sig	Mir1188
Sig	Mir370
Sig	AF357359
Sig	Mirg
not_Sig	Dio3os
not_Sig	Dio3
not_Sig	Ppp2r5c
not_Sig	B930059L03Rik
Sig	Dync1h1
not_Sig	1700001K19Rik
Sig	Hsp90aa1

not_Sig	Wdr20
not_Sig	Mok
not_Sig	Zfp839
not_Sig	Cinp
not_Sig	Tecpr2
Sig	Ankrd9
not_Sig	Rcor1
not_Sig	4930595D18Rik
not_Sig	Traf3
Sig	Cdc42bpb
not_Sig	Exoc3l4
Sig	Tnfaip2
not_Sig	Gm266
not_Sig	Eif5
not_Sig	Snora28
not_Sig	2810029C07Rik
not_Sig	Mark3
not_Sig	Ckb
not_Sig	Trmt61a
not_Sig	Bag5
not_Sig	Apopt1
Sig	Klc1
Sig	Xrcc3
not_Sig	Zfyve21
not_Sig	Ppp1r13b
not_Sig	2010107E04Rik
not_Sig	Tdrd9
not_Sig	Aspg
Sig	Kif26a
not_Sig	A530016L24Rik
not_Sig	Tmem179
Sig	Inf2
not_Sig	Adssl1
not_Sig	Siva1
Sig	Akt1
Sig	Zbtb42
not_Sig	Cep170b
not_Sig	Pld4
not_Sig	BC022687
not_Sig	Cdca4
not_Sig	Gpr132
Sig	Jag2
not_Sig	Mir6941
not_Sig	Nudt14
not_Sig	Brf1
not_Sig	Btbd6
not_Sig	Pacs2
not_Sig	Tex22

not_Sig	Mta1
Sig	Crip2
not_Sig	Crip1
not_Sig	4930427A07Rik
not_Sig	Zfp386
not_Sig	Vipr2
Sig	Wdr60
not_Sig	Esyt2
not_Sig	D430020J02Rik
not_Sig	Ncapg2
not_Sig	Ptprn2
Sig	Rapgef5
not_Sig	Cdca7l
not_Sig	Dnah11
not_Sig	Sp4
not_Sig	Itgb8
Sig	Gdi2
not_Sig	Fam208b
not_Sig	Asb13
not_Sig	Calml3
Sig	Net1
not_Sig	Akr1c14
not_Sig	Akr1c18
not_Sig	Akr1c13
not_Sig	Akr1c19
not_Sig	Akr1c12
not_Sig	Akr1c6
Sig	Akr1e1
Sig	Klf6
not_Sig	Pitrm1
not_Sig	Pfkp
not_Sig	Adarb2
not_Sig	Wdr37
Sig	Idi1
not_Sig	Idi2
not_Sig	Gtpbp4
not_Sig	Larp4b
Sig	Dip2c
not_Sig	Zmynd11
not_Sig	Chrm3
not_Sig	Ryr2
not_Sig	Gm10336
not_Sig	Mtr
not_Sig	Actn2
not_Sig	Heatr1
not_Sig	Lgals8
not_Sig	Edaradd
not_Sig	Ero1lb

not_Sig	Gpr137b-ps
not_Sig	Gpr137b
not_Sig	Nid1
not_Sig	Lyst
not_Sig	Gng4
not_Sig	B3galnt2
not_Sig	Tbce
not_Sig	Ggps1
not_Sig	Arid4b
not_Sig	Hecw1
not_Sig	Mrpl32
Sig	Psma2
not_Sig	AW209491
not_Sig	Gli3
not_Sig	Inhba
not_Sig	Sugct
not_Sig	Mplkip
not_Sig	Cdk13
not_Sig	Mir466i
not_Sig	Rala
not_Sig	Yae1d1
not_Sig	Vps41
not_Sig	Amph
not_Sig	Stard3nl
not_Sig	Epdr1
Sig	Sfrp4
not_Sig	A530099J19Rik
not_Sig	Gpr141
not_Sig	Elmo1
not_Sig	Aoah
not_Sig	Trim27
not_Sig	Zscan12
not_Sig	Zkscan3
not_Sig	Pgbd1
not_Sig	Zscan26
not_Sig	Nkapl
not_Sig	Zkscan4
not_Sig	Zkscan8
not_Sig	Hist1h2bl
Sig	Hist1h2ai
not_Sig	Hist1h3h
not_Sig	Hist1h2bm
not_Sig	Hist1h4j
not_Sig	Hist1h4k
Sig	Hist1h2ak
not_Sig	Hist1h2bn
not_Sig	Hist1h1b
not_Sig	Hist1h3i

not_Sig	Hist1h2an
Sig	Hist1h2bp
Sig	Hist1h4n
not_Sig	Zfp184
not_Sig	Hist1h2ah
Sig	Hist1h2bk
Sig	Hist1h4i
not_Sig	Hist1h2ag
Sig	Hist1h2bj
not_Sig	Zfp322a
not_Sig	Abt1
not_Sig	C230035I16Rik
not_Sig	Btn1a1
not_Sig	Hist1h4h
Sig	Hist1h2af
not_Sig	Hist1h3g
not_Sig	Hist1h2bh
not_Sig	Hist1h3f
not_Sig	Hist1h4f
not_Sig	Hist1h1d
not_Sig	Hist1h3e
Sig	Hist1h2ae
Sig	Hist1h2bg
Sig	Hist1h2bf
Sig	Hist1h2ad
not_Sig	Hist1h3d
not_Sig	Hist1h4d
Sig	Hist1h2be
not_Sig	Hist1h1e
not_Sig	Hist1h2ac
Sig	Hist1h2bc
not_Sig	Hist1h4c
not_Sig	Hfe
not_Sig	Hist1h1c
Sig	Hist1h3c
Sig	Hist1h2bb
not_Sig	Hist1h2ab
not_Sig	Hist1h3b
Sig	Hist1h4b
not_Sig	Hist1h4a
not_Sig	Hist1h3a
Sig	Hist1h1a
not_Sig	Lrrc16a
not_Sig	Cmah
not_Sig	Fam65b
not_Sig	Gm11346
Sig	Gmnn
not_Sig	BC005537

not_Sig	Acot13
not_Sig	Tdp2
not_Sig	D130043K22Rik
not_Sig	Aldh5a1
not_Sig	9330162012Rik
not_Sig	Gpld1
not_Sig	Mrs2
Sig	Dcdc2a
not_Sig	2610307P16Rik
not_Sig	Sox4
not_Sig	Cdkal1
not_Sig	E2f3
not_Sig	Mboat1
not_Sig	Agtr1a
not_Sig	Uqcrfs1
not_Sig	Dusp22
not_Sig	Irf4
Sig	Exoc2
not_Sig	Hus1b
not_Sig	Foxq1
not_Sig	Foxf2
not_Sig	Foxc1
not_Sig	Gmds
Sig	Mylk4
not_Sig	Wrnip1
not_Sig	Serpinb1a
not_Sig	Serpinb1c
not_Sig	Serpinb6b
not_Sig	Serpinb9
not_Sig	Serpinb6c
not_Sig	Serpinb6a
Sig	1110046J04Rik
Sig	Nqo2
not_Sig	Ripk1
Sig	Bphl
not_Sig	Tubb2a
not_Sig	Tubb2b
Sig	Psmg4
not_Sig	Slc22a23
Sig	Pxdc1
not_Sig	Gm15908
not_Sig	Prpf4b
not_Sig	Fam217a
not_Sig	Eci3
Sig	Eci2
not_Sig	Cdyl
not_Sig	Rpp40
not_Sig	Lyrm4

not_Sig	Fars2
not_Sig	Nrn1
Sig	F13a1
not_Sig	Ly86
not_Sig	Rreb1
not_Sig	Ssr1
not_Sig	Cage1
not_Sig	Riok1
not_Sig	Dsp
not_Sig	Snrnp48
Sig	Bmp6
not_Sig	4930579J19Rik
not_Sig	Txndc5
not_Sig	Bloc1s5
Sig	Eef1e1
not_Sig	Slc35b3
not_Sig	Gcnt2
not_Sig	Pak1ip1
not_Sig	Tmem14c
not_Sig	Mak
not_Sig	Elovl2
not_Sig	Smim13
not_Sig	Nedd9
not_Sig	Tmem170b
Sig	Adtrp
not_Sig	Gm10790
Sig	Hivep1
not_Sig	Edn1
not_Sig	Phactr1
not_Sig	Tbc1d7
not_Sig	Gfod1
not_Sig	Sirt5
not_Sig	Nol7
not_Sig	Ranbp9
Sig	Mcur1
not_Sig	Cd83
not_Sig	Jarid2
not_Sig	Dtnbp1
not_Sig	Mylip
not_Sig	Gmpr
not_Sig	Atxn1
not_Sig	Rbm24
Sig	Cap2
not_Sig	Fam8a1
not_Sig	Nup153
not_Sig	Kif13a
not_Sig	Nhlrc1
Sig	Tpmt

Sig	Kdm1b
not_Sig	Dek
not_Sig	Rnf144b
not_Sig	A930002C04Rik
not_Sig	Id4
not_Sig	Zfp169
not_Sig	Mirlet7d
not_Sig	Mirlet7f-1
not_Sig	Mirlet7a-1
not_Sig	Ptpdc1
Sig	Phf2
not_Sig	Fam120a
not_Sig	Fam120aos
not_Sig	Wnk2
Sig	Ninj1
not_Sig	1110007C09Rik
not_Sig	Susd3
not_Sig	Fgd3
not_Sig	Bicd2
Sig	Ippk
not_Sig	Cenpp
not_Sig	Ecm2
Sig	Aspn
not_Sig	Omd
Sig	Ogn
not_Sig	Nol8
not_Sig	Iars
not_Sig	2310081J21Rik
not_Sig	Fbxw17
not_Sig	Nutm2
not_Sig	Gm904
not_Sig	9430083A17Rik
not_Sig	Spin1
Sig	S1pr3
Sig	Shc3
not_Sig	Cks2
not_Sig	Secisbp2
not_Sig	Sema4d
not_Sig	Gadd45g
not_Sig	Diras2
not_Sig	Syk
Sig	BB123696
not_Sig	Auh
not_Sig	Nfil3
not_Sig	Ror2
not_Sig	Sptlc1
Sig	Drd1
not_Sig	Sfxn1

not_Sig	Hrh2
not_Sig	Cplx2
not_Sig	Thoc3
not_Sig	Simc1
not_Sig	4833439L19Rik
not_Sig	Arl10
not_Sig	Nop16
not_Sig	Higd2a
Sig	Cltb
not_Sig	Faf2
not_Sig	Rnf44
not_Sig	Gprin1
not_Sig	Tspan17
not_Sig	Unc5a
Sig	Hk3
not_Sig	Uimc1
not_Sig	Zfp346
Sig	Fgfr4
not_Sig	Nsd1
not_Sig	Rab24
not_Sig	Prelid1
not_Sig	Mxd3
Sig	Lman2
not_Sig	Rgs14
not_Sig	Slc34a1
not_Sig	Grk6
not_Sig	Prr7
not_Sig	Dbn1
not_Sig	Pdlim7
not_Sig	Mir6945
not_Sig	Dok3
not_Sig	Ddx41
Sig	Fam193b
not_Sig	Tmed9
not_Sig	B4galt7
not_Sig	Caml
not_Sig	Ddx46
not_Sig	B230219D22Rik
Sig	Txndc15
not_Sig	Pcbd2
not_Sig	Catsper3
not_Sig	Pitx1
Sig	H2afy
not_Sig	Tifab
Sig	Cxcl14
not_Sig	Slc25a48
not_Sig	Fbxl21
not_Sig	Tgfbi

not_Sig	Smad5
not_Sig	Spock1
Sig	Klhl3
not_Sig	Hnrnpa0
not_Sig	Idnk
not_Sig	Ubqln1
not_Sig	Gkap1
not_Sig	Kif27
not_Sig	2210016F16Rik
not_Sig	Hnrnpk
not_Sig	Mir7-1
not_Sig	Rmi1
not_Sig	Ntrk2
Sig	Agtpbp1
not_Sig	A230056J06Rik
Sig	Naa35
not_Sig	Golm1
not_Sig	Isca1
not_Sig	Etohd2
Sig	Zcchc6
not_Sig	Gas1
not_Sig	Gm5084
not_Sig	Dapk1
not_Sig	4930486L24Rik
not_Sig	Ctla2b
not_Sig	Ctla2a
not_Sig	Zfp808
not_Sig	Gm3604
not_Sig	Zfp935
not_Sig	Zfp934
not_Sig	6720489N17Rik
not_Sig	Gm5141
Sig	Fbp2
not_Sig	Fbp1
not_Sig	2010111I01Rik
not_Sig	Gm16907
not_Sig	Fancc
not_Sig	Ptch1
not_Sig	Ercc6l2
not_Sig	Slc35d2
not_Sig	Zfp367
not_Sig	Habp4
Sig	Cdc14b
not_Sig	1810034E14Rik
not_Sig	Aaed1
not_Sig	Ctsl
not_Sig	Cdk20
not_Sig	1190003K10Rik

not_Sig	Hiatl1
not_Sig	Zfp369
not_Sig	Uqcrb
not_Sig	Gm10767
not_Sig	Mterf3
not_Sig	Ptdss1
not_Sig	4933433G19Rik
not_Sig	Zfp712
not_Sig	Zfp708
Sig	Rslcan18
not_Sig	Zfp759
not_Sig	Rsl1
not_Sig	Zfp455
not_Sig	Zfp458
not_Sig	F630042J09Rik
not_Sig	Zfp457
Sig	Zfp595
not_Sig	Zfp953
not_Sig	Zfp456
not_Sig	Zfp429
not_Sig	Zfp459
not_Sig	Zfp874a
not_Sig	Zfp874b
not_Sig	Zfp58
not_Sig	Zfp87
not_Sig	Zfp748
not_Sig	Zfp729b
not_Sig	Zfp729a
not_Sig	Zfp738
not_Sig	Zfp65
not_Sig	Zfp85os
not_Sig	Zfp85
not_Sig	Zfp493
not_Sig	4930525G20Rik
not_Sig	Zfp273
not_Sig	Mtrr
not_Sig	Fastkd3
not_Sig	1700001L19Rik
not_Sig	Adcy2
Sig	Papd7
not_Sig	Srd5a1
not_Sig	Nsun2
not_Sig	Ube2ql1
not_Sig	Med10
not_Sig	1700100L14Rik
Sig	Ice1
not_Sig	Adamts16
not_Sig	Irx1

not_Sig	Gm20554
not_Sig	Ndufs6
not_Sig	Mrpl36
not_Sig	Lpcat1
Sig	Clptm1l
not_Sig	Tert
not_Sig	Slc6a18
not_Sig	Slc6a19
not_Sig	Slc12a7
not_Sig	Nkd2
not_Sig	Trip13
not_Sig	Brd9
Sig	Tppp
not_Sig	Cep72
not_Sig	Slc9a3
not_Sig	Exoc3
not_Sig	Ahrr
not_Sig	Pdcd6
not_Sig	Sdha
not_Sig	Ccdc127
not_Sig	Lrrc14b
not_Sig	Zfp72
not_Sig	Zfp825
not_Sig	Erap1
Sig	Cast
not_Sig	Pcsk1
Sig	Mir682
not_Sig	Ell2
not_Sig	Glrx
not_Sig	Rhobtb3
not_Sig	Spata9
Sig	Rfesd
not_Sig	Arsk
not_Sig	Ttc37
not_Sig	Mctp1
not_Sig	Ankrd32
not_Sig	2210408I21Rik
not_Sig	Fam172a
Sig	Pou5f2
not_Sig	Nr2f1
Sig	A830082K12Rik
Sig	Arrdc3
not_Sig	5430425K12Rik
not_Sig	Lysmd3
not_Sig	Polr3g
not_Sig	Mblac2
not_Sig	Cetn3
not_Sig	Mef2c

not_Sig	Tmem161b
not_Sig	Ccnh
not_Sig	Rasa1
not_Sig	Cox7c
Sig	Edil3
not_Sig	Hapln1
Sig	Vcan
not_Sig	Xrcc4
Sig	Tmem167
Sig	Rps23
not_Sig	Atg10
not_Sig	A830009L08Rik
not_Sig	Ssbp2
not_Sig	4833422C13Rik
Sig	Zcchc9
not_Sig	Ckmt2
not_Sig	Rasgrf2
not_Sig	Msh3
not_Sig	Dhfr
not_Sig	Fam151b
Sig	Zfyve16
not_Sig	Serinc5
not_Sig	Thbs4
not_Sig	Mtx3
not_Sig	Cmya5
Sig	Gm4814
Sig	A630019I02Rik
not_Sig	Papd4
not_Sig	Homer1
not_Sig	Jmy
not_Sig	Bhmt
not_Sig	Arsb
not_Sig	Lhfpl2
not_Sig	Scamp1
not_Sig	Gm9776
Sig	Ap3b1
not_Sig	Tbca
not_Sig	Wdr41
not_Sig	Pde8b
not_Sig	Zbed3
not_Sig	Snora47
Sig	Aggf1
not_Sig	F2rl1
not_Sig	F2r
not_Sig	Iqgap2
not_Sig	F2rl2
not_Sig	Sv2c
not_Sig	Poc5

not_Sig	Ankdd1b
Sig	Polk
not_Sig	Col4a3bp
not_Sig	Hmgcr
not_Sig	Gcnt4
not_Sig	Fam169a
not_Sig	Nsa2
not_Sig	Gfm2
Sig	Hexb
not_Sig	Enc1
not_Sig	Arhgef28
not_Sig	Utp15
not_Sig	Ankra2
Sig	Btf3
not_Sig	Foxd1
not_Sig	Fcho2
not_Sig	Tnpo1
not_Sig	2310020H05Rik
Sig	Zfp366
not_Sig	Ptcd2
not_Sig	Mrps27
not_Sig	6430562O15Rik
Sig	Map1b
not_Sig	Mccc2
not_Sig	Bdp1
not_Sig	Serf1
not_Sig	Smn1
not_Sig	Naip2
not_Sig	Naip5
not_Sig	Naip6
not_Sig	Gtf2h2
not_Sig	Ocln
not_Sig	Marveld2
not_Sig	Rad17
not_Sig	Ak6
not_Sig	Taf9
not_Sig	Ccdc125
not_Sig	Cdk7
not_Sig	Mrps36
not_Sig	Cenph
not_Sig	Ccnb1
not_Sig	Slc30a5
not_Sig	Pik3r1
not_Sig	Cd180
not_Sig	Mast4
not_Sig	1700099I09Rik
not_Sig	Srek1
not_Sig	Erbb2ip

not_Sig	Nln
not_Sig	Sgtb
Sig	Trappc13
not_Sig	Trim23
not_Sig	Ppwd1
not_Sig	Cenpk
not_Sig	Adamts6
Sig	Cwc27
not_Sig	Srek1ip1
not_Sig	Rgs7bp
not_Sig	Rnf180
not_Sig	Ipo11
not_Sig	Dimt1
Sig	Kif2a
not_Sig	3830408C21Rik
not_Sig	Zswim6
not_Sig	Smim15
not_Sig	Ndufaf2
not_Sig	Ercc8
not_Sig	Elovl7
not_Sig	Depdc1b
not_Sig	Pde4d
not_Sig	Mir1904
not_Sig	Gapt
Sig	Plk2
not_Sig	Gpbp1
not_Sig	Mier3
not_Sig	Map3k1
not_Sig	Gm15326
not_Sig	Ankrd55
Sig	Il6st
not_Sig	Slc38a9
not_Sig	Ppap2a
not_Sig	Skiv2l2
not_Sig	Dhx29
not_Sig	Gpx8
Sig	Esm1
not_Sig	Snx18
Sig	Hspb3
not_Sig	Arl15
not_Sig	A430090L17Rik
not_Sig	Ndufs4
not_Sig	Fst
not_Sig	Mocs2
not_Sig	Itga2
Sig	Itga1
not_Sig	Pelo
not_Sig	Parp8

not_Sig	Emb
not_Sig	Hcn1
not_Sig	Mrps30
not_Sig	Fgf10
not_Sig	Nnt
not_Sig	Paip1
not_Sig	4833420G17Rik
not_Sig	3110070M22Rik
not_Sig	Gm7120
Sig	Hmgcs1
not_Sig	Nim1k
not_Sig	Zfp131
not_Sig	Gm21188
not_Sig	BC147527
not_Sig	Gm2897
not_Sig	D830030K20Rik
not_Sig	Gm3636
not_Sig	Gm3558
not_Sig	Flnb
not_Sig	Dnase1l3
Sig	Abhd6
Sig	Rpp14
Sig	Pxk
not_Sig	Pdhb
not_Sig	Kctd6
not_Sig	Acox2
not_Sig	Fam107a
not_Sig	4930452B06Rik
not_Sig	Fhit
not_Sig	Fhitos
Sig	Ptprg
not_Sig	3830406C13Rik
not_Sig	Cadps
not_Sig	Thoc7
Sig	Atxn7
not_Sig	Psmd6
not_Sig	Il3ra
not_Sig	Slc4a7
not_Sig	Nek10
not_Sig	Lrrc3b
not_Sig	Oxsm
not_Sig	Ngly1
not_Sig	Top2b
not_Sig	Rarb
not_Sig	Thrb
not_Sig	Nr1d2
not_Sig	Rpl15
not_Sig	Nkiras1

not_Sig	Ube2e1
not_Sig	Ube2e2
not_Sig	Nid2
not_Sig	2700060E02Rik
not_Sig	Gng2
not_Sig	Saysd1
not_Sig	Kcnk5
not_Sig	Nudt13
not_Sig	Ecd
not_Sig	Fam149b
not_Sig	Dnajc9
Sig	Mrps16
not_Sig	Cfap70
not_Sig	Anxa7
not_Sig	Mss51
not_Sig	Ppp3cb
not_Sig	1810062O18Rik
Sig	Usp54
not_Sig	Myoz1
Sig	Synpo2l
not_Sig	Sec24c
not_Sig	Fut11
not_Sig	6230400D17Rik
Sig	Chchd1
Sig	Zswim8
not_Sig	Ndst2
not_Sig	Camk2g
not_Sig	Plau
not_Sig	Vcl
not_Sig	Ap3m1
not_Sig	Adk
not_Sig	Kat6b
not_Sig	Dupd1
not_Sig	Dusp13
not_Sig	Samd8
not_Sig	Vdac2
not_Sig	Comtd1
not_Sig	Zfp503
not_Sig	1700112E06Rik
not_Sig	4930405A10Rik
not_Sig	Gm10248
not_Sig	Kcnma1
not_Sig	4930519K11Rik
not_Sig	Dlg5
not_Sig	Polr3a
not_Sig	Rps24
Sig	Zmiz1
not_Sig	Mir3075

Sig	4931406H21Rik
Sig	Israa
not_Sig	Ppif
not_Sig	Ppifos
Sig	Zcchc24
Sig	Anxa11
not_Sig	4933413J09Rik
Sig	Slmap
Sig	Dennd6a
not_Sig	Arf4
not_Sig	Pde12
not_Sig	Asb14
not_Sig	Appl1
Sig	Il17rd
not_Sig	Arhgef3
not_Sig	Fam208a
not_Sig	Ccdc66
Sig	Erc2
not_Sig	Wnt5a
not_Sig	Cacna2d3
not_Sig	Lrtm1
not_Sig	Selk
not_Sig	Actr8
not_Sig	Il17rb
Sig	Chdh
not_Sig	Cacna1d
Sig	Dcp1a
not_Sig	Tkt
not_Sig	Prkcd
not_Sig	Rft1
not_Sig	Sfmbt1
not_Sig	Tmem110
not_Sig	Mustn1
Sig	Itih4
not_Sig	Itih3
not_Sig	Itih1
not_Sig	Nek4
not_Sig	Spcs1
not_Sig	Glt8d1
not_Sig	Gnl3
not_Sig	Snord69
not_Sig	Pbrm1
not_Sig	Smim4
not_Sig	Nt5dc2
not_Sig	Stab1
Sig	Nisch
not_Sig	Tnnc1
Sig	Sema3g

not_Sig	Phf7
not_Sig	Bap1
not_Sig	Dnah1
not_Sig	Capn7
Sig	Sh3bp5
not_Sig	Mettl6
not_Sig	Eaf1
Sig	Colq
Sig	Hacl1
not_Sig	Btd
not_Sig	Ankrd28
Sig	Galnt15
Sig	Dph3
not_Sig	Oxnad1
Sig	Ncoa4
not_Sig	Timm23
Sig	Parg
not_Sig	Ogdhl
not_Sig	Ercc6
not_Sig	Prrxl1
not_Sig	3425401B19Rik
not_Sig	1810011H11Rik
not_Sig	Vstm4
not_Sig	Wdfy4
not_Sig	Lrrc18
not_Sig	Arhgap22
not_Sig	Mapk8
Sig	Gdf10
not_Sig	Gm5460
not_Sig	Anxa8
not_Sig	Npy4r
not_Sig	Syt15
not_Sig	Fam35a
not_Sig	Glud1
not_Sig	Gm3219
not_Sig	Sncg
Sig	Mmrn2
not_Sig	Bmpr1a
not_Sig	Ldb3
not_Sig	Opn4
not_Sig	Wapal
not_Sig	Ccser2
not_Sig	Ghitm
not_Sig	Sh2d4b
not_Sig	Tspan14
Sig	Fam213a
Sig	Mat1a
not_Sig	Ptger2

not_Sig	Txndc16
Sig	Gpr137c
not_Sig	Ero1l
Sig	Psmc6
Sig	Styx
not_Sig	Gnpnat1
not_Sig	Fermt2
not_Sig	Ddhd1
not_Sig	Bmp4
not_Sig	Cdkn3
not_Sig	Cnih1
Sig	Gmfb
not_Sig	Cgrrf1
not_Sig	Samd4
not_Sig	Gm10371
not_Sig	Gch1
not_Sig	Wdhd1
not_Sig	Socs4
not_Sig	Mapk1ip1l
not_Sig	Lgals3
not_Sig	Dlgap5
not_Sig	Fbxo34
not_Sig	Atg14
not_Sig	Ktn1
not_Sig	Peli2
not_Sig	Gm6498
not_Sig	Tmem260
not_Sig	Exoc5
not_Sig	Ap5m1
not_Sig	Naa30
not_Sig	1700011H14Rik
not_Sig	3632451O06Rik
not_Sig	Tlr11
not_Sig	Ttc5
not_Sig	Ccnb1ip1
not_Sig	Rpph1
not_Sig	Parp2
not_Sig	Tep1
not_Sig	Klhl33
not_Sig	Osgep
not_Sig	Apex1
not_Sig	Tmem55b
not_Sig	Pnp
not_Sig	Pnp2
not_Sig	Ang
Sig	Rnase4
not_Sig	Rnase6
not_Sig	Rnase2a

not_Sig	Mettl17
not_Sig	Slc39a2
not_Sig	Ndrg2
not_Sig	Tppp2
Sig	Rnase13
not_Sig	Arhgef40
not_Sig	Gm16617
not_Sig	Zfp219
not_Sig	Tmem253
not_Sig	Snord58b
Sig	Hnrnpc
not_Sig	Rpgrip1
not_Sig	Supt16
not_Sig	Chd8
not_Sig	Snord8
not_Sig	Rab2b
Sig	Tox4
not_Sig	Mettl3
not_Sig	Sall2
not_Sig	Dad1
not_Sig	Abhd4
not_Sig	Oxa1l
not_Sig	Slc7a7
not_Sig	Mrpl52
not_Sig	Mmp14
not_Sig	Lrp10
not_Sig	Prmt5
not_Sig	Haus4
Sig	Ajuba
not_Sig	4931414P19Rik
not_Sig	Psmb5
not_Sig	Cdh24
Sig	Acin1
not_Sig	4930579G18Rik
not_Sig	1700123O20Rik
not_Sig	Cebpe
not_Sig	Slc7a8
not_Sig	Homez
not_Sig	Ppp1r3e
not_Sig	Bcl2l2
Sig	Pabpn1
not_Sig	Slc22a17
not_Sig	Efs
not_Sig	Cmtm5
not_Sig	Myh6
not_Sig	Mhrt
not_Sig	Myh7
not_Sig	Ngdn

not_Sig	Gm10364
not_Sig	Zfhx2
not_Sig	Zfhx2os
not_Sig	Thtpa
not_Sig	Ap1g2
not_Sig	Jph4
not_Sig	Dhrs4
not_Sig	Lrrc16b
not_Sig	Cpne6
not_Sig	Pck2
not_Sig	Dcaf11
not_Sig	Fitm1
not_Sig	Psme1
Sig	Emc9
not_Sig	Psme2
not_Sig	Rnf31
not_Sig	Irf9
not_Sig	Rec8
not_Sig	Ipo4
not_Sig	Tm9sf1
not_Sig	Tssk4
not_Sig	Mdp1
Sig	Nedd8
not_Sig	Gmpr2
not_Sig	Tinf2
not_Sig	Tgm1
not_Sig	Rabggta
not_Sig	Dhrs1
not_Sig	Nop9
not_Sig	Ltb4r2
not_Sig	Ltb4r1
Sig	Adcy4
not_Sig	Ripk3
Sig	Nfatc4
Sig	Nynrin
not_Sig	Cbln3
Sig	Khnyn
not_Sig	Sdr39u1
not_Sig	Cma1
not_Sig	Gm5801
not_Sig	Mcpt4
Sig	Ctsg
not_Sig	Cenpj
not_Sig	Parp4
not_Sig	Mphosph8
not_Sig	Gm16973
not_Sig	Pspc1
not_Sig	Zmym5

not_Sig	Zmym2
not_Sig	Gjb2
not_Sig	Gjb6
not_Sig	Cryl1
not_Sig	Ift88
not_Sig	Il17d
Sig	N6amt2
not_Sig	Xpo4
not_Sig	Lats2
not_Sig	Sap18
not_Sig	Ska3
Sig	Mrpl57
not_Sig	Zdhhc20
Sig	Micu2
not_Sig	Fgf9
not_Sig	Rcbtb1
not_Sig	Gm6904
not_Sig	Phf11b
not_Sig	Phf11d
not_Sig	Phf11c
not_Sig	Setdb2
Sig	Cab39l
not_Sig	Cdadc1
not_Sig	Shisa2
not_Sig	Atp8a2
not_Sig	Nupl1
not_Sig	Mtmr6
Sig	Spata13
Sig	C1qtnf9
Sig	Mipep
Sig	Tnfrsf19
not_Sig	Sacs
not_Sig	Sgcg
not_Sig	Arl11
not_Sig	Ebpl
not_Sig	Kpna3
Sig	Spryd7
Sig	Trim13
not_Sig	Dleu2
not_Sig	Kcnrg
not_Sig	Mir16-1
not_Sig	Mir15a
Sig	Rnaseh2b
not_Sig	Fam124a
not_Sig	Serpine3
not_Sig	Ints6
not_Sig	Wdfy2
not_Sig	Mir6541

Sig	Ctsb
not_Sig	Fdft1
not_Sig	Neil2
not_Sig	Blk
not_Sig	Fam167a
not_Sig	Mtmr9
not_Sig	Xkr6
not_Sig	Pinx1
Sig	Sox7
not_Sig	Prss55
not_Sig	Prss51
not_Sig	Msra
not_Sig	Mir124a-1hg
not_Sig	Kif13b
Sig	Hmbox1
not_Sig	Ints9
not_Sig	Extl3
not_Sig	Fzd3
Sig	Zfp395
not_Sig	Elp3
Sig	Scara5
not_Sig	Pbk
Sig	Esco2
not_Sig	Ccdc25
Sig	Scara3
not_Sig	Clu
not_Sig	Gulo
Sig	Ephx2
not_Sig	Chrna2
not_Sig	Ptk2b
not_Sig	Trim35
not_Sig	Stmn4
Sig	Adra1a
not_Sig	Dpysl2
not_Sig	Gm5464
not_Sig	Pnma2
not_Sig	Bnip3l
not_Sig	Ppp2r2a
not_Sig	Ebf2
not_Sig	Gm6878
not_Sig	4930438E09Rik
Sig	Cdca2
not_Sig	Kctd9
not_Sig	Gnrh1
not_Sig	Dock5
not_Sig	Nefl
not_Sig	Stc1
not_Sig	Slc25a37

Sig	Entpd4
not_Sig	Loxl2
not_Sig	R3hcc1
not_Sig	Chmp7
not_Sig	4930480K23Rik
not_Sig	Tnfrsf10b
not_Sig	Rhobtb2
Sig	Pebp4
not_Sig	Egr3
not_Sig	Bin3
not_Sig	Ccar2
not_Sig	9930012K11Rik
not_Sig	Pdlim2
not_Sig	Sorbs3
not_Sig	Ppp3cc
not_Sig	Slc39a14
not_Sig	Piwil2
not_Sig	Polr3d
not_Sig	Phyhip
not_Sig	Bmp1
not_Sig	Lgi3
not_Sig	Reep4
Sig	Hr
Sig	Nudt18
not_Sig	Fam160b2
Sig	Dmtn
Sig	Npm2
Sig	Xpo7
not_Sig	Dok2
not_Sig	Gfra2
not_Sig	Fndc3a
not_Sig	Cysltr2
not_Sig	Rcbtb2
not_Sig	Rb1
not_Sig	Lpar6
Sig	Itm2b
Sig	Med4
not_Sig	Nudt15
not_Sig	Sucla2
not_Sig	Htr2a
Sig	Esd
Sig	Lrch1
not_Sig	5031414D18Rik
not_Sig	Lcp1
not_Sig	Zc3h13
not_Sig	Cog3
Sig	Slc25a30
not_Sig	Gm4285

Sig	Tpt1
Sig	Snora31
not_Sig	Gtf2f2
not_Sig	Kctd4
not_Sig	Gpalpp1
not_Sig	Nufip1
not_Sig	Tsc22d1
not_Sig	Serp2
not_Sig	Lacc1
not_Sig	Ccdc122
not_Sig	Enox1
Sig	Dnajc15
not_Sig	Epsti1
Sig	Akap11
Sig	Dgkh
not_Sig	Vwa8
not_Sig	Zfp957
not_Sig	Rgcc
not_Sig	Naa16
not_Sig	Mtrf1
not_Sig	Kbtbd7
not_Sig	Kbtbd6
not_Sig	Wbp4
not_Sig	Elf1
not_Sig	Sugt1
not_Sig	Lect1
not_Sig	Olfm4
Sig	Pcdh17
not_Sig	9630013A20Rik
not_Sig	Diap3
not_Sig	Tdrd3
not_Sig	Pcdh20
not_Sig	Gm5088
not_Sig	Pcdh9
Sig	Dach1
not_Sig	Mzt1
not_Sig	Bora
not_Sig	Dis3
not_Sig	Pibf1
not_Sig	Klf5
Sig	Klf12
not_Sig	Tbc1d4
not_Sig	Commd6
Sig	Uchl3
Sig	Lmo7
not_Sig	Kctd12
Sig	Cln5
not_Sig	Fbxl3

not_Sig	Mycbp2
Sig	Scel
not_Sig	Slain1
not_Sig	Ednrb
not_Sig	Pou4f1
not_Sig	Rnf219
not_Sig	Gm17066
Sig	Rbm26
not_Sig	Ndfip2
Sig	Spry2
not_Sig	Slitrk1
not_Sig	Slitrk6
not_Sig	Slitrk5
not_Sig	Mir18
not_Sig	Mir17hg
not_Sig	Gpc6
Sig	Dct
not_Sig	Tgds
not_Sig	Gpr180
not_Sig	Abcc4
not_Sig	Mir6391
not_Sig	Cldn10
not_Sig	Dzip1
Sig	Dnajc3
not_Sig	Uggt2
not_Sig	Hs6st3
not_Sig	Mbnl2
not_Sig	Rap2a
not_Sig	Ipo5
not_Sig	Farp1
not_Sig	Stk24
not_Sig	Dock9
not_Sig	1810041H14Rik
not_Sig	Ubac2
not_Sig	Gpr18
not_Sig	Gpr183
not_Sig	A330035P11Rik
Sig	Tm9sf2
not_Sig	Clybl
not_Sig	1700108J01Rik
not_Sig	Gm5089
Sig	Pcca
not_Sig	Ggact
Sig	Tmtc4
not_Sig	Nalcn
Sig	Itgbl1
not_Sig	Fgf14
Sig	Sepp1

not_Sig	Ccdc152
Sig	Ghr
not_Sig	Fbxo4
not_Sig	AW549877
not_Sig	A630020A06
not_Sig	BC037032
Sig	Oxct1
not_Sig	Plcxd3
not_Sig	C6
Sig	C7
not_Sig	Card6
not_Sig	Rpl37
not_Sig	Snord72
Sig	Prkaa1
Sig	Ttc33
not_Sig	Ptger4
not_Sig	Dab2
not_Sig	Fyb
not_Sig	Rictor
not_Sig	Osmr
not_Sig	Lifr
Sig	Egflam
not_Sig	Gdnf
Sig	Wdr70
not_Sig	Nup155
not_Sig	2410089E03Rik
not_Sig	Nipbl
Sig	Slc1a3
not_Sig	Ranbp3l
not_Sig	Nadk2
not_Sig	Skp2
not_Sig	Lmbrd2
not_Sig	Ugt3a2
not_Sig	Il7r
not_Sig	Spef2
not_Sig	Prlr
not_Sig	Dnajc21
not_Sig	Brix1
not_Sig	Rad1
Sig	Rai14
not_Sig	Gm10389
Sig	4930556M19Rik
not_Sig	C1qtnf3
Sig	Amacr
not_Sig	Adamts12
not_Sig	Tars
not_Sig	Npr3
not_Sig	Gm5144

not_Sig	1700047G03Rik
not_Sig	Sub1
not_Sig	Zfr
not_Sig	Mir1898
not_Sig	Mtmr12
Sig	Golph3
not_Sig	Pdzd2
not_Sig	6030458C11Rik
not_Sig	Drosha
not_Sig	Cdh6
not_Sig	Cdh9
not_Sig	Cdh10
not_Sig	Basp1
Sig	Myo10
not_Sig	Fam134b
Sig	Mir7212
not_Sig	Zfp622
Sig	Fbxl7
not_Sig	Ank
not_Sig	Mir7117
not_Sig	Otulin
not_Sig	Fam105a
Sig	Trio
not_Sig	Ctnnd2
not_Sig	Dap
not_Sig	Ankrd33b
not_Sig	Ropn1l
not_Sig	March6
not_Sig	Cmbl
Sig	Cct5
not_Sig	Fam173b
not_Sig	Snhg18
not_Sig	Sema5a
not_Sig	Sdc2
Sig	Cpq
not_Sig	1700084J12Rik
not_Sig	Tspyl5
not_Sig	Mtdh
not_Sig	Laptm4b
not_Sig	Matn2
not_Sig	Rpl30
Sig	Hrsp12
not_Sig	Pop1
not_Sig	Nipal2
not_Sig	Stk3
Sig	Osr2
not_Sig	Vps13b
not_Sig	Cox6c

Sig	Polr2k
not_Sig	Spag1
not_Sig	Rnf19a
not_Sig	Ankrd46
not_Sig	Pabpc1
not_Sig	Ywhaz
not_Sig	Zfp706
not_Sig	Grhl2
not_Sig	Ncald
not_Sig	Gm15941
not_Sig	4930447A16Rik
Sig	Rrm2b
Sig	Ubr5
not_Sig	Klf10
Sig	Azin1
Sig	Atp6v1c1
not_Sig	Baalc
not_Sig	Fzd6
not_Sig	Cthrc1
not_Sig	Slc25a32
not_Sig	Dcaf13
not_Sig	Rims2
not_Sig	Dcstamp
not_Sig	Lrp12
not_Sig	Zfpm2
not_Sig	Oxr1
not_Sig	Abra
Sig	Angpt1
not_Sig	Rspo2
not_Sig	Eif3e
Sig	Emc2
not_Sig	Nudcd1
not_Sig	Eny2
not_Sig	Pkhd1l1
not_Sig	Ebag9
not_Sig	Sybu
not_Sig	Csmd3
not_Sig	Trps1
not_Sig	Mir1907
not_Sig	Eif3h
not_Sig	Utp23
Sig	Rad21
not_Sig	Med30
not_Sig	Ext1
not_Sig	Samd12
not_Sig	Tnfrsf11b
Sig	Mal2
Sig	Nov

not_Sig	Enpp2
not_Sig	Taf2
not_Sig	Dscc1
not_Sig	Gm9920
not_Sig	Deptor
Sig	Col14a1
not_Sig	Mrpl13
not_Sig	Mtbp
Sig	Sntb1
Sig	Has2
not_Sig	Has2os
not_Sig	Zhx2
not_Sig	Derl1
not_Sig	Tbc1d31
not_Sig	Fam83a
not_Sig	9130401M01Rik
not_Sig	Zhx1
not_Sig	Atad2
not_Sig	Wdyhv1
not_Sig	Fbxo32
not_Sig	Klhl38
not_Sig	D15Ertd621e
not_Sig	Tmem65
Sig	Trmt12
not_Sig	Rnf139
Sig	Tatdn1
not_Sig	Ndufb9
not_Sig	Mtss1
not_Sig	Sqle
not_Sig	E430025E21Rik
not_Sig	Nsmce2
Sig	Trib1
not_Sig	Fam84b
Sig	9930014A18Rik
Sig	A1bg
not_Sig	Myc
not_Sig	Pvt1
not_Sig	H2afy3
not_Sig	Gsdmcl-ps
not_Sig	Fam49b
not_Sig	Asap1
not_Sig	Adcy8
Sig	Efr3a
not_Sig	Kcnq3
not_Sig	Tmem71
not_Sig	Phf20l1
not_Sig	Tg
not_Sig	Sla

not_Sig	Wisp1
not_Sig	Ndrg1
not_Sig	St3gal1
not_Sig	Zfat
not_Sig	Mir30b
not_Sig	Khdrbs3
Sig	Col22a1
not_Sig	Trappc9
not_Sig	Peg13
not_Sig	Chrac1
Sig	Ago2
Sig	Ptk2
not_Sig	Mir151
Sig	Dennd3
not_Sig	Slc45a4
not_Sig	Gpr20
Sig	Ptp4a3
not_Sig	Adgrb1
not_Sig	Arc
not_Sig	Jrk
not_Sig	4933427E11Rik
not_Sig	Them6
not_Sig	Lypd2
not_Sig	Lynx1
not_Sig	Ly6d
not_Sig	Ly6e
not_Sig	Ly6i
Sig	Ly6a
not_Sig	Ly6c1
not_Sig	Ly6c2
not_Sig	Ly6h
Sig	Gpihbp1
not_Sig	Zfp41
not_Sig	Top1mt
not_Sig	Rhpn1
Sig	Mafa
not_Sig	Zc3h3
not_Sig	Gsdmd
not_Sig	Mroh6
not_Sig	Naprt
Sig	Eef1d
not_Sig	Tigd5
Sig	Pycrl
not_Sig	Tsta3
not_Sig	Zfp623
not_Sig	Zfp707
not_Sig	Ccdc166
not_Sig	Fam83h

Sig	Scrib
Sig	Puf60
not_Sig	Nrbp2
not_Sig	Eppk1
not_Sig	Plec
not_Sig	Parp10
not_Sig	Grina
not_Sig	Spatc1
not_Sig	Smpd5
Sig	Oplah
not_Sig	Exosc4
not_Sig	Gpaa1
not_Sig	Cyc1
Sig	Sharpin
not_Sig	Maf1
not_Sig	Hgh1
not_Sig	Tssk5
Sig	Mroh1
not_Sig	Bop1
not_Sig	Scx
not_Sig	Hsf1
not_Sig	Dgat1
not_Sig	Scrt1
not_Sig	Fbxl6
not_Sig	Slc52a2
not_Sig	Adck5
not_Sig	Cpsf1
not_Sig	Slc39a4
Sig	Vps28
not_Sig	Tonsl
not_Sig	Cyhr1
not_Sig	Kifc2
not_Sig	Ppp1r16a
not_Sig	Gpt
not_Sig	Mfsd3
not_Sig	Recql4
not_Sig	Lrrc14
not_Sig	Lrrc24
Sig	C030006K11Rik
not_Sig	Arhgap39
not_Sig	Zfp251
not_Sig	Zfp7
not_Sig	Commd5
not_Sig	Rpl8
not_Sig	Zfp647
not_Sig	1110038F14Rik
not_Sig	Mb
not_Sig	Apol6

Sig	Rbfox2
not_Sig	1700109K24Rik
not_Sig	Apol7c
not_Sig	Apol10b
Sig	Gm8221
not_Sig	Apol11b
not_Sig	Apol7e
not_Sig	Apol9b
Sig	Apol8
not_Sig	Myh9
not_Sig	Txn2
not_Sig	Foxred2
not_Sig	Eif3d
not_Sig	Ift27
not_Sig	Pvalb
not_Sig	Ncf4
not_Sig	Csf2rb2
not_Sig	Csf2rb
not_Sig	Tst
not_Sig	Mpst
not_Sig	Kctd17
not_Sig	Tmprss6
not_Sig	Il2rb
not_Sig	C1qtnf6
not_Sig	Sstr3
not_Sig	Rac2
not_Sig	Cyth4
not_Sig	Elfn2
not_Sig	Mfng
Sig	Card10
not_Sig	Cdc42ep1
Sig	Lgals2
not_Sig	Gga1
not_Sig	Sh3bp1
not_Sig	Pdxp
not_Sig	Lgals1
not_Sig	Nol12
not_Sig	Triobp
not_Sig	Gm10865
not_Sig	H1f0
not_Sig	Gcat
not_Sig	Ankrd54
not_Sig	Eif3l
not_Sig	Micall1
not_Sig	Polr2f
not_Sig	Sox10
not_Sig	Gm10863
Sig	Pick1

not_Sig	Slc16a8
not_Sig	Baiap2l2
Sig	Pla2g6
not_Sig	Maff
not_Sig	Tmem184b
not_Sig	Mir1943
Sig	Csnk1e
not_Sig	Kdelr3
not_Sig	Ddx17
not_Sig	Fam227a
not_Sig	Cby1
not_Sig	Tomm22
not_Sig	Josd1
Sig	Gtpbp1
not_Sig	Sun2
not_Sig	Gm16576
not_Sig	Dnal4
not_Sig	Nptxr
Sig	Cbx6
not_Sig	D730005E14Rik
not_Sig	Apobec3
Sig	Cbx7
Sig	Pdgfb
not_Sig	Rpl3
not_Sig	Snord83b
not_Sig	Snord43
Sig	Syngr1
not_Sig	Tab1
not_Sig	Mgat3
not_Sig	Mief1
not_Sig	Atf4
not_Sig	Rps19bp1
Sig	Cacna1i
not_Sig	Grap2
not_Sig	Fam83f
not_Sig	A430088P11Rik
Sig	Tnrc6b
not_Sig	Adsl
not_Sig	Sgsm3
not_Sig	Mkl1
not_Sig	Mchr1
not_Sig	8430426J06Rik
not_Sig	Slc25a17
Sig	St13
not_Sig	Xpnpep3
not_Sig	Dnajb7
not_Sig	Rbx1
not_Sig	Ep300

not_Sig	L3mbtl2
not_Sig	Chadl
not_Sig	Rangap1
not_Sig	Zc3h7b
not_Sig	Tef
Sig	Tob2
not_Sig	Phf5a
not_Sig	Aco2
not_Sig	Polr3h
not_Sig	Csdc2
not_Sig	Pmm1
not_Sig	Desi1
Sig	Xrcc6
not_Sig	Nhp2l1
not_Sig	Ccdc134
not_Sig	Srebf2
not_Sig	Shisa8
not_Sig	Tnfrsf13c
not_Sig	Cenpm
not_Sig	Sept3
not_Sig	Naga
not_Sig	Fam109b
not_Sig	Smdt1
not_Sig	Ndufa6
Sig	Cyp2d22
not_Sig	Cyp2d10
not_Sig	Cyp2d26
not_Sig	Tcf20
not_Sig	Tbrg3
not_Sig	Gm20324
not_Sig	Nfam1
not_Sig	Serhl
not_Sig	Rrp7a
not_Sig	Poldip3
not_Sig	Rnu12
not_Sig	Cyb5r3
not_Sig	A4galt
not_Sig	Arfgap3
not_Sig	1700001L05Rik
not_Sig	Pacsin2
not_Sig	Ttll1
not_Sig	Bik
not_Sig	Mcat
not_Sig	Tspo
not_Sig	Ttll12
not_Sig	Scube1
not_Sig	Efcab6
not_Sig	Sult4a1

Sig	Pnpla3
not_Sig	Mir6392
Sig	Samm50
Sig	Parvb
not_Sig	Parvg
not_Sig	1810041L15Rik
not_Sig	Ldoc1l
not_Sig	Prr5
not_Sig	Arhgap8
not_Sig	Phf21b
not_Sig	Nup50
not_Sig	5031439G07Rik
not_Sig	Mir1249
not_Sig	Fam118a
not_Sig	Smc1b
Sig	Fbln1
not_Sig	Atxn10
not_Sig	Wnt7b
not_Sig	Lincppara
not_Sig	Ppara
not_Sig	Cdpf1
not_Sig	Pkdrej
not_Sig	Ttc38
not_Sig	Gtse1
not_Sig	Trmu
not_Sig	Celsr1
not_Sig	Gramd4
not_Sig	Cerk
not_Sig	Tbc1d22a
not_Sig	Fam19a5
not_Sig	Brd1
not_Sig	Zbed4
not_Sig	Alg12
not_Sig	Creld2
not_Sig	Pim3
not_Sig	Panx2
not_Sig	1810021B22Rik
Sig	Trabd
not_Sig	Selo
not_Sig	Tubgcp6
not_Sig	Hdac10
not_Sig	Mapk12
not_Sig	Mapk11
not_Sig	Plxnb2
not_Sig	Dennd6b
not_Sig	Ppp6r2
not_Sig	Sbf1
not_Sig	Lmf2

not_Sig	Ncaph2
not_Sig	Tymp
not_Sig	Odf3b
not_Sig	Cpt1b
not_Sig	Chkb
not_Sig	Mapk8ip2
not_Sig	Arsa
Sig	Shank3
Sig	Acr
not_Sig	Rabl2
not_Sig	Alg10b
not_Sig	Cpne8
not_Sig	Kif21a
Sig	Abcd2
not_Sig	Slc2a13
not_Sig	Lrrk2
not_Sig	Cntn1
not_Sig	Pdzrn4
not_Sig	Gxylt1
not_Sig	Yaf2
not_Sig	Zcrb1
not_Sig	Pphln1
not_Sig	Prickle1
not_Sig	Adamts20
not_Sig	Pus7l
not_Sig	Irak4
not_Sig	Twf1
not_Sig	Tmem117
not_Sig	Nell2
not_Sig	Ano6
not_Sig	E330033B04Rik
not_Sig	Arid2
not_Sig	Scaf11
not_Sig	Slc38a1
not_Sig	Slc38a2
not_Sig	Slc38a4
not_Sig	Amigo2
Sig	Pced1b
not_Sig	Rpap3
not_Sig	Endou
Sig	Rapgef3
not_Sig	Slc48a1
Sig	Hdac7
not_Sig	Vdr
not_Sig	Tmem106c
Sig	Col2a1
not_Sig	Senp1
not_Sig	Pfkm

not_Sig	Asb8
Sig	Zfp641
not_Sig	Olfr283
not_Sig	Kansl2
not_Sig	Snora34
not_Sig	Mir1291
not_Sig	Snora2b
not_Sig	Ccnt1
not_Sig	9330020H09Rik
not_Sig	4930415O20Rik
not_Sig	Adcy6
not_Sig	Cacnb3
not_Sig	Ddx23
not_Sig	Rnd1
not_Sig	Fkbp11
not_Sig	Arf3
Sig	Wnt10b
not_Sig	Ddn
not_Sig	Prkag1
Sig	Kmt2d
not_Sig	Rhebl1
not_Sig	Dhh
Sig	Lmbr1l
not_Sig	Tuba1b
not_Sig	Tuba1a
not_Sig	4930578M01Rik
not_Sig	Tuba1c
not_Sig	Prph
not_Sig	Troap
not_Sig	Spats2
not_Sig	Kcnh3
not_Sig	Mcrs1
not_Sig	1700120C14Rik
not_Sig	Fam186b
Sig	Prpf40b
Sig	Fmnl3
not_Sig	Tmbim6
Sig	Nckap5l
not_Sig	Bcdin3d
not_Sig	Faim2
not_Sig	Aqp2
not_Sig	Aqp5
not_Sig	Racgap1
not_Sig	Asic1
Sig	Smarcd1
Sig	Gpd1
not_Sig	Cox14
not_Sig	Cers5

not_Sig	Lima1
not_Sig	Larp4
not_Sig	2310068J16Rik
Sig	Dip2b
not_Sig	Mir3473a
not_Sig	Atf1
Sig	Mettl7a1
not_Sig	Mettl7a3
not_Sig	Mettl7a2
not_Sig	Slc11a2
not_Sig	Letmd1
not_Sig	Csrnp2
not_Sig	Tfcp2
Sig	Pou6f1
not_Sig	C330013E15Rik
not_Sig	Dazap2
Sig	Smagp
not_Sig	Bin2
not_Sig	Cela1
not_Sig	Galnt6
not_Sig	Slc4a8
not_Sig	Scn8a
not_Sig	Fignl2
Sig	Acvrl1
not_Sig	Acvr1b
not_Sig	Mir6962
not_Sig	A330009N23Rik
not_Sig	Grasp
not_Sig	Nr4a1
not_Sig	Atg101
not_Sig	6030408B16Rik
not_Sig	Krt80
Sig	Krt7
not_Sig	Krt75
not_Sig	Krt79
not_Sig	Krt8
not_Sig	Krt18
not_Sig	Eif4b
not_Sig	Tns2
not_Sig	Spryd3
not_Sig	Igfbp6
not_Sig	Soat2
not_Sig	Csad
not_Sig	Zfp740
not_Sig	Itgb7
not_Sig	Rarg
not_Sig	Mfsd5
Sig	Espl1

not_Sig	Pfdn5
not_Sig	Myg1
not_Sig	Aaas
not_Sig	Sp7
not_Sig	Sp1
Sig	Amhr2
not_Sig	Prr13
not_Sig	Pcbp2
Sig	Map3k12
not_Sig	Tarbp2
not_Sig	Npff
not_Sig	Atf7
Sig	Atp5g2
not_Sig	Calcoco1
not_Sig	Hotair
not_Sig	Hoxc11
Sig	Hoxc10
not_Sig	Mir196a-2
Sig	Hoxc9
not_Sig	Hoxc8
Sig	Hoxc6
Sig	Hoxc5
not_Sig	Mir615
not_Sig	Hoxc4
not_Sig	D930007P13Rik
not_Sig	Smug1
Sig	Cbx5
not_Sig	Hnrnpa1
Sig	Gm5643
not_Sig	Nfe2
not_Sig	Copz1
Sig	Zfp385a
Sig	Itga5
not_Sig	Nckap1l
not_Sig	Pde1b
Sig	Ppp1r1a
Sig	Glycam1
not_Sig	Mucl1
not_Sig	Mefv
not_Sig	Zfp263
not_Sig	Zfp174
not_Sig	Zfp597
not_Sig	Naa60
not_Sig	1700037C18Rik
not_Sig	Cluap1
not_Sig	Nlrc3
not_Sig	Slx4
not_Sig	Dnase1

not_Sig	Trap1
Sig	Crebbp
not_Sig	Adcy9
not_Sig	Srl
Sig	Tfap4
not_Sig	Glis2
not_Sig	Pam16
not_Sig	Coro7
not_Sig	Vasn
not_Sig	Dnaja3
not_Sig	Nmral1
not_Sig	Hmox2
Sig	Cdip1
not_Sig	4930562C15Rik
not_Sig	Ubald1
Sig	Mgrn1
not_Sig	Gm16861
not_Sig	Nudt16l1
not_Sig	Anks3
not_Sig	4930451G09Rik
not_Sig	Rogdi
Sig	Glyr1
not_Sig	Ubn1
not_Sig	Ppl
not_Sig	Sec14l5
not_Sig	Nagpa
not_Sig	AU021092
not_Sig	Alg1
not_Sig	Eef2kmt
not_Sig	Rbfox1
not_Sig	Mettl22
not_Sig	Abat
not_Sig	Tmem186
Sig	Pmm2
not_Sig	Carhsp1
not_Sig	Usp7
not_Sig	1810013L24Rik
not_Sig	Grin2a
not_Sig	Rpl39l
not_Sig	Emp2
not_Sig	Nubp1
not_Sig	Tvp23a
not_Sig	Ciita
not_Sig	Dexi
not_Sig	Clec16a
Sig	Socs1
not_Sig	Prm1
Sig	Rmi2

not_Sig	Litaf
Sig	Snn
Sig	Txndc11
not_Sig	Zc3h7a
not_Sig	Rsl1d1
not_Sig	2610020C07Rik
not_Sig	Gspt1
not_Sig	Tnfrsf17
not_Sig	Snx29
not_Sig	Cpped1
not_Sig	Gm9961
not_Sig	Ercc4
not_Sig	Mkl2
not_Sig	Mir365-1
not_Sig	2310015D24Rik
not_Sig	Parn
not_Sig	Bfar
not_Sig	3110001I22Rik
not_Sig	Rrn3
not_Sig	Ntan1
not_Sig	Pdxdc1
not_Sig	Mpv17l
not_Sig	Marf1
not_Sig	Nde1
Sig	Myh11
Sig	Fopnl
Sig	Abcc1
not_Sig	Snai2
not_Sig	Ube2v2
not_Sig	Mcm4
not_Sig	Prkdc
not_Sig	Mzt2
not_Sig	Cebpd
not_Sig	Spidr
not_Sig	Pkp2
not_Sig	Yars2
not_Sig	Dnm1l
Sig	Fgd4
Sig	Top3b
Sig	Ppm1f
not_Sig	Mapk1
not_Sig	Ypel1
not_Sig	Ppil2
not_Sig	Sdf2l1
not_Sig	Ccdc116
not_Sig	Ydjc
not_Sig	Ube2l3
not_Sig	Hic2

not_Sig	Tmem191c
not_Sig	Pi4ka
not_Sig	Serpind1
not_Sig	Snap29
not_Sig	Crkl
not_Sig	Aifm3
Sig	Lztr1
not_Sig	Thap7
not_Sig	Lrrc74b
not_Sig	P2rx6
not_Sig	Slc7a4
Sig	Smpd4
not_Sig	Ccdc74a
Sig	Med15
not_Sig	Klhl22
not_Sig	Scarf2
not_Sig	Car15
not_Sig	Dgcr2
not_Sig	Tssk1
not_Sig	Tssk2
not_Sig	Dgcr14
Sig	Slc25a1
not_Sig	Dgcr6
not_Sig	Prodh
not_Sig	Rtn4r
not_Sig	Mir6366
not_Sig	4933432I09Rik
Sig	Zdhhc8
not_Sig	Ranbp1
not_Sig	Trmt2a
not_Sig	Dgcr8
not_Sig	Tango2
not_Sig	Mir185
not_Sig	Arvcf
Sig	Comt
not_Sig	Txnrd2
not_Sig	Gnb1l
not_Sig	Tbx1
not_Sig	Gp1bb
not_Sig	Sept5
Sig	Cldn5
not_Sig	Cdc45
not_Sig	Ufd1l
not_Sig	2510002D24Rik
not_Sig	Mrpl40
not_Sig	Hira
not_Sig	B3gnt5
not_Sig	Klhl6

not_Sig	Klhl24
not_Sig	Yeats2
not_Sig	Map6d1
Sig	Parl
not_Sig	Cyp2ab1
not_Sig	Abcc5
not_Sig	Eif2b5
not_Sig	Dvl3
not_Sig	Ap2m1
not_Sig	Gm15760
not_Sig	Abcf3
not_Sig	Vwa5b2
not_Sig	Alg3
not_Sig	Ece2
not_Sig	Camk2n2
not_Sig	Psmd2
not_Sig	Eif4g1
not_Sig	Snord66
Sig	Fam131a
not_Sig	Clcn2
not_Sig	Polr2h
not_Sig	Thpo
not_Sig	Chrd
not_Sig	Ephb3
not_Sig	Magef1
not_Sig	Vps8
not_Sig	2510009E07Rik
not_Sig	Ehhadh
not_Sig	1300002E11Rik
not_Sig	Map3k13
not_Sig	Tmem41a
not_Sig	Senp2
Sig	Igf2bp2
not_Sig	Tra2b
Sig	Etv5
not_Sig	Dgkg
not_Sig	Tbccd1
not_Sig	Dnajb11
not_Sig	Ahsg
Sig	Fetub
not_Sig	Hrg
Sig	Kng2
not_Sig	Kng1
Sig	Eif4a2
not_Sig	Snord2
Sig	Snora81
Sig	Rfc4
not_Sig	Adipoq

not_Sig	St6gal1
not_Sig	Masp1
not_Sig	Rtp4
not_Sig	Bcl6
not_Sig	Lppos
not_Sig	Lpp
not_Sig	Morf4l1-ps1
not_Sig	A230028O05Rik
Sig	Trp63
not_Sig	P3h2
not_Sig	Cldn1
not_Sig	Il1rap
not_Sig	Ostn
not_Sig	Uts2b
not_Sig	Ccdc50
not_Sig	Fgf12
not_Sig	Mb21d2
not_Sig	Hrasls
not_Sig	Atp13a5
not_Sig	Opa1
not_Sig	4632428C04Rik
not_Sig	Hes1
not_Sig	Cpn2
not_Sig	Lrrc15
Sig	Gp5
not_Sig	Atp13a3
Sig	Tmem44
not_Sig	Lsg1
Sig	Fam43a
not_Sig	Xxylt1
not_Sig	Acap2
not_Sig	Ppp1r2
not_Sig	Apod
Sig	Bdh1
not_Sig	Dlg1
not_Sig	Pigz
not_Sig	0610012G03Rik
Sig	Ncbp2
not_Sig	Senp5
Sig	Gm15694
not_Sig	Pak2
not_Sig	Pigx
not_Sig	Cep19
not_Sig	Nrros
not_Sig	Fbxo45
not_Sig	Wdr53
not_Sig	Smco1
not_Sig	Rnf168

not_Sig	Ubxn7
not_Sig	Tm4sf19
not_Sig	Tctex1d2
not_Sig	Pcyt1a
not_Sig	Slc51a
Sig	Tfrc
Sig	Tnk2
not_Sig	Tnk2os
not_Sig	Muc4
not_Sig	Muc20
Sig	1700021K19Rik
not_Sig	Fyttd1
Sig	Lrch3
not_Sig	Iqcg
not_Sig	Rpl35a
not_Sig	Lmln
not_Sig	Osbpl11
Sig	Snx4
not_Sig	1700007L15Rik
not_Sig	Zfp148
not_Sig	Slc12a8
not_Sig	Heg1
not_Sig	Muc13
not_Sig	Itgb5
Sig	Umps
Sig	Kalrn
not_Sig	Ccdc14
Sig	Mylk
not_Sig	1700119H24Rik
Sig	E130310I04Rik
not_Sig	Hacd2
Sig	Adcy5
not_Sig	Sec22a
not_Sig	Pdia5
not_Sig	Sema5b
not_Sig	Dirc2
not_Sig	Hspbap1
not_Sig	Parp14
not_Sig	Dtx3l
not_Sig	Parp9
not_Sig	Kpna1
not_Sig	Wdr5b
not_Sig	Fam162a
not_Sig	Ccdc58
Sig	Casr
not_Sig	Cd86
not_Sig	Ildr1
not_Sig	Slc15a2

not_Sig	Eaf2
not_Sig	Iqcb1
not_Sig	4930565N06Rik
not_Sig	Golgb1
not_Sig	Hcls1
not_Sig	Fbxo40
Sig	Polq
not_Sig	Stxbp5l
Sig	Gtf2e1
not_Sig	Rabl3
not_Sig	Hgd
Sig	Ndufb4
not_Sig	Fstl1
not_Sig	Lrrc58
not_Sig	Gpr156
not_Sig	BC031361
not_Sig	Gsk3b
not_Sig	Cox17
not_Sig	Popdc2
Sig	Pla1a
not_Sig	Adprh
not_Sig	Cd80
Sig	Timmdc1
not_Sig	Poglut1
not_Sig	Tmem39a
not_Sig	Arhgap31
not_Sig	B4galt4
not_Sig	Igsf11
not_Sig	Lsamp
not_Sig	Gap43
not_Sig	Gm19522
Sig	Zbtb20
not_Sig	BC002163
not_Sig	Gm15713
Sig	Mir568
not_Sig	Tigit
not_Sig	Qtrtd1
not_Sig	2610015P09Rik
not_Sig	Zdhhc23
not_Sig	Gramd1c
not_Sig	Atp6v1a
not_Sig	Naa50
not_Sig	Gm608
not_Sig	Sidt1
not_Sig	Spice1
not_Sig	Cfap44
not_Sig	Boc
not_Sig	Mir3081

not_Sig	BC027231
not_Sig	Gtpbp8
not_Sig	Cd200r1
not_Sig	Cd200r4
not_Sig	Cd200r3
Sig	Ccdc80
not_Sig	Slc35a5
Sig	Atg3
not_Sig	Btla
not_Sig	Cd200
not_Sig	Gm609
not_Sig	Gcsam
not_Sig	Tagln3
not_Sig	Abhd10
Sig	Phldb2
not_Sig	Plcxd2
not_Sig	Cd96
Sig	Gm4737
not_Sig	Pvrl3
not_Sig	Retnla
not_Sig	Retnlg
Sig	Dzip3
not_Sig	C330027C09Rik
not_Sig	Myh15
not_Sig	Ift57
not_Sig	Cd47
not_Sig	Bbx
not_Sig	5330426P16Rik
Sig	Cblb
not_Sig	Alcam
not_Sig	Nfkbiz
not_Sig	Nxpe3
not_Sig	Cep97
Sig	Rpl24
not_Sig	Zbtb11os1
not_Sig	Zbtb11
not_Sig	Pcnp
not_Sig	Trmt10c
not_Sig	Senp7
not_Sig	Impg2
not_Sig	Abi3bp
not_Sig	Tfg
not_Sig	Tmem45a
not_Sig	Tomm70a
Sig	Nit2
not_Sig	Tbc1d23
not_Sig	Cmss1
not_Sig	Filip1l

not_Sig	Col8a1
not_Sig	Dcbld2
not_Sig	St3gal6
not_Sig	Cpox
not_Sig	Cldnd1
not_Sig	Olfr172
Sig	Mina
not_Sig	Crybg3
Sig	Arl6
not_Sig	Nsun3
not_Sig	Arl13b
not_Sig	Stx19
not_Sig	Pros1
not_Sig	Epha3
Sig	4930453N24Rik
not_Sig	Zfp654
not_Sig	Cggbp1
not_Sig	Chmp2b
not_Sig	Vgll3
not_Sig	Cadm2
not_Sig	Gbe1
not_Sig	Robo1
not_Sig	Robo2
not_Sig	Rbm11
Sig	Hspa13
not_Sig	Samsn1
Sig	Nrip1
not_Sig	Usp25
Sig	Mir99ahg
not_Sig	Mir99a
not_Sig	Mirlet7c-1
not_Sig	E330011O21Rik
not_Sig	Cxadr
not_Sig	D16Ertd472e
not_Sig	4930478L05Rik
not_Sig	Chodl
not_Sig	Ncam2
not_Sig	Mir155hg
not_Sig	Mrpl39
not_Sig	Jam2
Sig	Atp5j
not_Sig	Gabpa
not_Sig	App
not_Sig	Gm10791
Sig	Cyyr1
Sig	Adamts1
not_Sig	Adamts5
Sig	N6amt1

not_Sig	Ltn1
not_Sig	Rwdd2b
not_Sig	Usp16
Sig	Cct8
not_Sig	B130034C11Rik
not_Sig	Map3k7cl
not_Sig	Bach1
not_Sig	Cldn8
not_Sig	Tiam1
not_Sig	Gm10789
Sig	Sod1
Sig	Scaf4
not_Sig	Hunk
not_Sig	Mis18a
not_Sig	Mrap
not_Sig	Urb1
not_Sig	Eva1c
not_Sig	1110004E09Rik
not_Sig	Synj1
not_Sig	4930404I05Rik
not_Sig	Paxbp1
not_Sig	4932438H23Rik
not_Sig	Ifnar2
not_Sig	Il10rb
not_Sig	A930006K02Rik
not_Sig	Ifnar1
not_Sig	Ifngr2
Sig	Tmem50b
Sig	Dnajc28
not_Sig	Gart
not_Sig	Son
not_Sig	Donson
not_Sig	Gm10785
not_Sig	Cryzl1
not_Sig	Itsn1
not_Sig	Atp5o
not_Sig	Slc5a3
not_Sig	Mrps6
not_Sig	Smim11
not_Sig	1700048M11Rik
Sig	Rcan1
not_Sig	Clic6
not_Sig	Runx1
not_Sig	Setd4
not_Sig	Cbr1
not_Sig	Cbr3
not_Sig	Dopey2
not_Sig	Morc3

not_Sig	Chaf1b
not_Sig	Sim2
not_Sig	Hlcs
not_Sig	Ripply3
not_Sig	Pigp
not_Sig	Ttc3
not_Sig	Dscr3
not_Sig	Dyrk1a
not_Sig	Kcnj6
Sig	Kcnj15
Sig	Erg
Sig	Ets2
not_Sig	Psmg1
not_Sig	Brwd1
not_Sig	Hmgn1
not_Sig	Wrb
not_Sig	Lca5l
not_Sig	Sh3bgr
not_Sig	Itgb2l
not_Sig	Pcp4
not_Sig	Dscam
not_Sig	Bace2
not_Sig	Mx1
not_Sig	Mx2
not_Sig	Tmprss2
not_Sig	Ripk4
not_Sig	Prdm15
not_Sig	C2cd2
Sig	Zbtb21
not_Sig	B230307C23Rik
not_Sig	A630089N07Rik
not_Sig	Pisd-ps2
not_Sig	Scaf8
not_Sig	Tiam2
Sig	Tfb1m
not_Sig	Cldn20
not_Sig	1700102H20Rik
Sig	Arid1b
Sig	Tmem242
not_Sig	Ldhal6b
not_Sig	Zdhhc14
not_Sig	Snx9
not_Sig	Synj2
not_Sig	Serac1
Sig	Gtf2h5
not_Sig	Tulp4
not_Sig	Tmem181a
not_Sig	Dynlt1a

not_Sig	Dynlt1b
not_Sig	Tmem181b-ps
not_Sig	Tmem181c-ps
not_Sig	Sytl3
not_Sig	Ezr
not_Sig	Mir692-1
not_Sig	Rsph3b
not_Sig	Tagap1
not_Sig	Rnaset2b
not_Sig	Gm1604b
not_Sig	Rps6ka2
not_Sig	Gm9992
not_Sig	Fndc1
not_Sig	Tagap
not_Sig	Rsph3a
not_Sig	Fgfr1op
not_Sig	Ccr6
not_Sig	Mpc1
not_Sig	4930506C21Rik
not_Sig	Sft2d1
not_Sig	Prr18
not_Sig	Pde10a
not_Sig	1700010I14Rik
not_Sig	Qk
not_Sig	Cahm
not_Sig	1700110C19Rik
not_Sig	Pacrg
not_Sig	A230009B12Rik
not_Sig	Park2
not_Sig	D17Ertd648e
not_Sig	Agpat4
not_Sig	Map3k4
not_Sig	4732491K20Rik
not_Sig	Plg
not_Sig	Slc22a3
not_Sig	C030013G03Rik
not_Sig	Slc22a2
not_Sig	Slc22a1
Sig	Igf2r
Sig	Airn
not_Sig	Mas1
not_Sig	Mrgprh
Sig	Pnldc1
not_Sig	Mrpl18
Sig	Tcp1
not_Sig	Acat3
Sig	Acat2
not_Sig	Wtap

not_Sig	Sod2
not_Sig	Unc93a
not_Sig	2700054A10Rik
not_Sig	Smok4a
not_Sig	Tcte2
Sig	Mllt4
not_Sig	Dact2
not_Sig	Smoc2
Sig	4930474M22Rik
not_Sig	Thbs2
not_Sig	Wdr27
not_Sig	1600012H06Rik
not_Sig	Phf10
not_Sig	LOC106740
not_Sig	9030025P20Rik
not_Sig	Gm3435
not_Sig	Ermard
not_Sig	Gm5091
not_Sig	Gm10510
not_Sig	Dll1
not_Sig	Fam120b
Sig	Psmb1
not_Sig	Tbp
not_Sig	Pdcd2
not_Sig	Prdm9
not_Sig	4933401D09Rik
Sig	Chd1
Sig	Rgmb
not_Sig	Zfp960
not_Sig	Zfp97
not_Sig	Riok2
not_Sig	Lix1
not_Sig	Lnpep
not_Sig	Mir99b
Sig	Mirlet7e
not_Sig	Mir125a
Sig	Spaca6
not_Sig	Has1
not_Sig	Fpr1
not_Sig	Fpr2
not_Sig	Vmn2r96
not_Sig	Ppp2r1a
not_Sig	Zfp160
not_Sig	Zfp677
not_Sig	Zfp54
not_Sig	Zfp51
not_Sig	Zfp53
not_Sig	9330136K24Rik

not_Sig	Zfp52
not_Sig	Zfp948
not_Sig	3110052M02Rik
not_Sig	Gm10509
not_Sig	Zfp760
not_Sig	Zfp229
not_Sig	Zfp820
not_Sig	2210404O09Rik
not_Sig	Zfp942
not_Sig	Zfp943
not_Sig	Zfp947
not_Sig	Gm4944
not_Sig	Zfp944
not_Sig	Zfp758
not_Sig	Zfp946
not_Sig	Gm16386
not_Sig	Zfp945
not_Sig	Zfp40
not_Sig	6330415G19Rik
not_Sig	Zfp213
not_Sig	Zfp13
not_Sig	Mmp25
not_Sig	Ccdc64b
not_Sig	Thoc6
not_Sig	Hcfc1r1
not_Sig	Tnfrsf12a
not_Sig	Pkmyt1
not_Sig	Paqr4
not_Sig	9530082P21Rik
Sig	Flywch1
Sig	Flywch2
not_Sig	Srrm2
Sig	Tceb2
not_Sig	Prss41
not_Sig	Prss27
not_Sig	Kctd5
Sig	Pdpk1
not_Sig	Amdhd2
not_Sig	Tbc1d24
not_Sig	Ntn3
not_Sig	1600002H07Rik
Sig	Ccnf
not_Sig	Abca3
not_Sig	D330041H03Rik
Sig	Rnps1
not_Sig	Eci1
not_Sig	Dnase1l2
not_Sig	E4f1

not_Sig	Pgp
not_Sig	Mlst8
Sig	Caskin1
not_Sig	Traf7
not_Sig	Rab26os
not_Sig	Rab26
not_Sig	Pkd1
not_Sig	Tsc2
not_Sig	Nthl1
Sig	Slc9a3r2
not_Sig	Zfp598
not_Sig	Gfer
not_Sig	Noxo1
not_Sig	Tbl3
not_Sig	Snora78
not_Sig	Rps2
Sig	Snora64
Sig	Ndufb10
not_Sig	Rpl3l
not_Sig	Msrb1
not_Sig	Meiob
not_Sig	Fahd1
not_Sig	Hagh
not_Sig	Igfals
not_Sig	Nubp2
Sig	Spsb3
not_Sig	Eme2
not_Sig	Mrps34
Sig	Nme3
Sig	Mapk8ip3
not_Sig	Hn1l
Sig	Cramp1l
not_Sig	Ift140
Sig	Tmem204
not_Sig	Telo2
not_Sig	Ptx4
not_Sig	Clcn7
not_Sig	Ccdc154
not_Sig	BC003965
Sig	Unkl
Sig	Gnptg
not_Sig	Tsr3
Sig	Baiap3
Sig	Ube2i
not_Sig	Prss34
not_Sig	Tpsab1
not_Sig	Tpsb2
not_Sig	Tpsg1

Sig	Cacna1h
not_Sig	Sox8
not_Sig	2810468N07Rik
not_Sig	Lmf1
not_Sig	Chtf18
not_Sig	Rpusd1
not_Sig	Msln
not_Sig	Narfl
not_Sig	Haghl
not_Sig	Ccdc78
Sig	Fam173a
not_Sig	Metrn
Sig	Fbxl16
not_Sig	Wdr24
not_Sig	Jmjd8
not_Sig	Stub1
Sig	Rhbdl1
not_Sig	Rhot2
not_Sig	Wdr90
not_Sig	Fam195a
not_Sig	0610011F06Rik
Sig	Wfikkn1
Sig	Rab40c
not_Sig	Gm10012
not_Sig	Pigq
not_Sig	Capn15
not_Sig	Rab11fip3
not_Sig	Decr2
not_Sig	Nme4
not_Sig	Tmem8
not_Sig	Mrpl28
not_Sig	Axin1
not_Sig	Pdia2
not_Sig	Arhgdig
not_Sig	Rgs11
not_Sig	Itfg3
not_Sig	Luc7l
Sig	Neurl1b
not_Sig	Dusp1
not_Sig	Ergic1
not_Sig	Atp6v0e
not_Sig	Crebrf
not_Sig	Bnip1
not_Sig	Kifc5b
Sig	Phf1
not_Sig	Cuta
not_Sig	Syngap1
not_Sig	Zbtb9

not_Sig	Ggnbp1
not_Sig	Bak1
Sig	Itpr3
not_Sig	Uqcc2
not_Sig	Ip6k3
not_Sig	Lemd2
not_Sig	9630028I04Rik
not_Sig	Grm4
not_Sig	Hmga1
not_Sig	AI413582
Sig	Nudt3
not_Sig	Rps10
not_Sig	Pacsin1
not_Sig	Spdef
not_Sig	D17Wsu92e
not_Sig	Snrpc
Sig	Uhrf1bp1
not_Sig	Taf11
not_Sig	Anks1
not_Sig	Tcp11
not_Sig	Scube3
not_Sig	Zfp523
not_Sig	Def6
not_Sig	Ppard
not_Sig	1810013A23Rik
Sig	Fance
not_Sig	Rpl10a
not_Sig	Tead3
not_Sig	Fkbp5
not_Sig	Srpk1
not_Sig	Slc26a8
not_Sig	Mapk14
not_Sig	Mapk13
not_Sig	Brpf3
not_Sig	Kctd20
not_Sig	Stk38
not_Sig	Srsf3
not_Sig	Trp53cor1
Sig	Cdkn1a
not_Sig	Rab44
not_Sig	Ppil1
not_Sig	BC004004
Sig	Pi16
not_Sig	Mtch1
not_Sig	Fgd2
not_Sig	Pim1
not_Sig	Tbc1d22b
not_Sig	Tbc1d22bos

not_Sig	Rnf8
not_Sig	Rnf8-cmtr1
Sig	Cmtr1
not_Sig	Ccdc167
not_Sig	Mdga1
not_Sig	Zfand3
not_Sig	Btbd9
not_Sig	Gm6402
not_Sig	Glo1
not_Sig	Dnah8
not_Sig	Abcg1
not_Sig	Ubash3a
Sig	Rsph1
not_Sig	Slc37a1
Sig	Pde9a
not_Sig	Wdr4
not_Sig	Ndufv3
not_Sig	Pknox1
not_Sig	Cbs
Sig	U2af1
not_Sig	Sik1
not_Sig	Hsf2bp
not_Sig	Rrp1b
Sig	Notch3
not_Sig	Ephx3
not_Sig	Brd4
not_Sig	Gm4432
Sig	Akap8
not_Sig	Akap8l
not_Sig	Wiz
not_Sig	Rasal3
not_Sig	Pglyrp2
Sig	Cyp4f39
not_Sig	Cyp4f17
not_Sig	Cyp4f16
not_Sig	Zfp871
not_Sig	Zfp811
not_Sig	Zfp799
not_Sig	Zfp870
not_Sig	Cyp4f14
not_Sig	Cyp4f13
not_Sig	Zfp472
not_Sig	Zfp952
not_Sig	Zfp763
not_Sig	Zfp563
not_Sig	Morc2b
not_Sig	Olfr55
not_Sig	Olfr239

not_Sig	Gm4461
not_Sig	Zfp955a
not_Sig	Zfp955b
not_Sig	Zfp81
not_Sig	Zfp101
not_Sig	Adamts10
Sig	Myo1f
not_Sig	Zfp414
Sig	Pram1
not_Sig	Hnrnpm
Sig	March2
not_Sig	Rab11b
not_Sig	Angptl4
Sig	Kank3
not_Sig	Rps28
not_Sig	Ndufa7
not_Sig	Cd320
not_Sig	Kifc1
not_Sig	BC051226
not_Sig	Daxx
Sig	Zbtb22
not_Sig	Tapbp
Sig	Rgl2
not_Sig	Pfdn6
not_Sig	Wdr46
not_Sig	B3galt4
Sig	Rps18
not_Sig	Vps52
not_Sig	H2-K2
not_Sig	H2-K1
not_Sig	Ring1
not_Sig	H2-Ke6
Sig	Slc39a7
not_Sig	Rxrb
not_Sig	Col11a2
not_Sig	H2-Oa
Sig	Brd2
not_Sig	H2-DMa
not_Sig	H2-DMb2
not_Sig	H2-DMb1
not_Sig	Psmb9
not_Sig	Tap1
not_Sig	Psmb8
not_Sig	Tap2
not_Sig	H2-Ob
not_Sig	H2-Ab1
not_Sig	H2-Aa
not_Sig	H2-Eb1

not_Sig	H2-Eb2
not_Sig	H2-Ea-ps
not_Sig	Btnl2
not_Sig	BC051142
Sig	Notch4
not_Sig	Gpsm3
Sig	Pbx2
Sig	Ager
not_Sig	Rnf5
not_Sig	Agpat1
not_Sig	Egfl8
not_Sig	Ppt2
not_Sig	Prrt1
not_Sig	Fkbpl
not_Sig	Atf6b
not_Sig	Tnxb
not_Sig	C4b
not_Sig	Cyp21a1
not_Sig	C4a
not_Sig	Stk19
not_Sig	Dxo
not_Sig	Skiv2l
not_Sig	Nelfe
not_Sig	Cfb
Sig	C2
not_Sig	Zbtb12
not_Sig	Ehmt2
not_Sig	Neu1
not_Sig	1110038B12Rik
not_Sig	Hspa1b
not_Sig	Hspa1a
not_Sig	Hspa1l
not_Sig	Lsm2
not_Sig	D17H6S56E-5
not_Sig	Vars
not_Sig	Vwa7
not_Sig	Sapcd1
not_Sig	Msh5
not_Sig	Clic1
not_Sig	Ddah2
not_Sig	G6b
not_Sig	Abhd16a
not_Sig	Ly6g5b
not_Sig	Csnk2b
not_Sig	Gpank1
Sig	D17H6S53E
not_Sig	Apom
not_Sig	Bag6

Sig	Prrc2a
not_Sig	Aif1
not_Sig	Lst1
not_Sig	Ltb
not_Sig	Tnf
not_Sig	Lta
not_Sig	Nfkbil1
not_Sig	Atp6v1g2
not_Sig	Ddx39b
not_Sig	Mir8094
not_Sig	H2-D1
not_Sig	H2-Q1
not_Sig	H2-Q2
not_Sig	H2-Q4
not_Sig	H2-Q5
not_Sig	H2-Q10
not_Sig	Tcf19
not_Sig	Cchcr1
not_Sig	Cdsn
not_Sig	Vars2
not_Sig	Gtf2h4
Sig	Ddr1
not_Sig	4833427F10Rik
not_Sig	Ier3
Sig	Flot1
not_Sig	Tubb5
not_Sig	Mdc1
not_Sig	Nrm
not_Sig	Ppp1r18
not_Sig	Dhx16
not_Sig	2310061I04Rik
not_Sig	Atat1
not_Sig	Mrps18b
not_Sig	Ppp1r10
not_Sig	Mir1894
not_Sig	Gm8801
not_Sig	Abcf1
not_Sig	Prr3
not_Sig	Gnl1
not_Sig	A930015D03Rik
not_Sig	H2-T24
not_Sig	H2-T23
not_Sig	Gm6034
not_Sig	Gm11127
not_Sig	H2-Bl
not_Sig	C920025E04Rik
not_Sig	H2-T10
not_Sig	Gm7030

not_Sig	Gm8909
not_Sig	Rpp21
not_Sig	Trim39
not_Sig	Trim26
not_Sig	Trim15
Sig	Trim10
not_Sig	Rnf39
not_Sig	Ppp1r11
not_Sig	Znrd1
not_Sig	Znrd1as
not_Sig	2410137M14Rik
not_Sig	Zfp57
Sig	Gabbr1
Sig	Ubd
not_Sig	H2-M3
not_Sig	Olfr99
not_Sig	H2-M2
Sig	Rn45s
not_Sig	Rhag
not_Sig	9130008F23Rik
not_Sig	Cenpq
not_Sig	Mut
not_Sig	Ptchd4
not_Sig	Adgrf4
not_Sig	Adgrf2
not_Sig	Cd2ap
Sig	Tnfrsf21
Sig	Adgrf5
not_Sig	Pla2g7
not_Sig	1700071M16Rik
not_Sig	Tdrd6
not_Sig	Slc25a27
Sig	Cyp39a1
Sig	Rcan2
not_Sig	Enpp5
not_Sig	Enpp4
not_Sig	Clic5
Sig	Runx2
Sig	Supt3
not_Sig	4930564C03Rik
not_Sig	Cdc5l
not_Sig	B230354K17Rik
not_Sig	Spats1
not_Sig	Aars2
not_Sig	Tmem151b
not_Sig	Nfkbie
not_Sig	Slc35b2
Sig	Hsp90ab1

Sig	Slc29a1
not_Sig	Gm7325
not_Sig	Capn11
Sig	Tmem63b
Sig	Mrpl14
not_Sig	1600014C23Rik
not_Sig	Vegfa
Sig	Mrps18a
not_Sig	Rsph9
not_Sig	Mad2l1bp
Sig	Gtpbp2
not_Sig	Polh
not_Sig	Xpo5
not_Sig	Polr1c
not_Sig	Yipf3
not_Sig	Lrrc73
not_Sig	Tjap1
not_Sig	Dlk2
not_Sig	Abcc10
not_Sig	Zfp318
Sig	Crip3
not_Sig	Ttbk1
not_Sig	Dnph1
not_Sig	Cul9
not_Sig	Srf
Sig	Ptk7
not_Sig	Klc4
not_Sig	Mrpl2
not_Sig	Cul7
not_Sig	Rrp36
Sig	Klhdc3
not_Sig	Mea1
not_Sig	Ppp2r5d
not_Sig	Pex6
not_Sig	Gnmt
not_Sig	Cnpy3
not_Sig	2310039H08Rik
not_Sig	Rpl7l1
Sig	Gltscr1l
not_Sig	Tbcc
not_Sig	Ubr2
not_Sig	Trerf1
not_Sig	Mrps10
not_Sig	Guca1b
not_Sig	Guca1a
not_Sig	AI661453
not_Sig	Taf8
not_Sig	Ccnd3

not_Sig	Bysl
Sig	Med20
not_Sig	Usp49
not_Sig	Tomm6
not_Sig	Tomm6os
not_Sig	Prickle4
Sig	Frs3
not_Sig	Frs3os
not_Sig	Pgc
not_Sig	Tfeb
not_Sig	Mdfi
not_Sig	Foxp4
not_Sig	Trem1
Sig	Trem3
not_Sig	Treml4
not_Sig	Treml2
not_Sig	B430306N03Rik
not_Sig	Trem2
not_Sig	Treml1
not_Sig	Nfya
not_Sig	Oard1
not_Sig	Apobec2
not_Sig	Tspo2
not_Sig	Lrfn2
not_Sig	Mocs1
Sig	Daam2
Sig	Rftn1
not_Sig	Dazl
not_Sig	Plcl2
not_Sig	Tbc1d5
not_Sig	Satb1
not_Sig	Efhb
not_Sig	Rab5a
not_Sig	Pp2d1
not_Sig	Kat2b
not_Sig	Sgol1
not_Sig	Slc5a7
not_Sig	Pot1b
not_Sig	Adgre4
not_Sig	Zfp119a
not_Sig	Zfp959
not_Sig	Zfp119b
not_Sig	Ebi3
not_Sig	Gm16712
not_Sig	Ccdc94
not_Sig	Shd
not_Sig	Fsd1
Sig	Stap2

Sig	Mpnd
not_Sig	Sh3gl1
not_Sig	Chaf1a
Sig	Ubxn6
not_Sig	Hdgfrp2
not_Sig	Plin4
not_Sig	Plin5
not_Sig	Lrg1
Sig	Sema6b
not_Sig	Tnfaip8l1
not_Sig	Mydgf
not_Sig	Dpp9
not_Sig	Fem1a
Sig	Ticam1
not_Sig	Plin3
not_Sig	Arrdc5
not_Sig	Uhrf1
not_Sig	Kdm4b
Sig	Ptprs
Sig	Gm20219
Sig	Safb2
Sig	Safb
Sig	2410015M20Rik
Sig	Rpl36
not_Sig	Lonp1
not_Sig	Ranbp3
not_Sig	Vmac
not_Sig	Ndufa11
not_Sig	Nrtn
not_Sig	Dus3l
not_Sig	Prr22
not_Sig	Rfx2
not_Sig	1700061G19Rik
not_Sig	Mllt1
not_Sig	Clpp
not_Sig	Alkbh7
not_Sig	Pspn
not_Sig	Gtf2f1
Sig	Khsrp
not_Sig	Slc25a41
not_Sig	Slc25a23
not_Sig	Crb3
not_Sig	Dennd1c
not_Sig	Tubb4a
not_Sig	Tnfsf9
not_Sig	Tnfsf14
Sig	C3
not_Sig	Gpr108

not_Sig	Trip10
not_Sig	Vav1
Sig	Adgre1
not_Sig	Pdzph1
Sig	Nudt12
not_Sig	Efna5
not_Sig	Fbxl17
not_Sig	4930405O22Rik
not_Sig	Fer
not_Sig	1110058D11Rik
not_Sig	Pja2
not_Sig	Man2a1
not_Sig	Tmem232
not_Sig	Vapa
not_Sig	Rab31
not_Sig	Ppp4r1
not_Sig	Ralbp1
Sig	Twsg1
not_Sig	Ankrd12
not_Sig	Ndufv2
not_Sig	Wash1
not_Sig	Ddx11
not_Sig	Mtcl1
not_Sig	Rab12
Sig	Themis3
Sig	Ptprm
not_Sig	Mir5709
not_Sig	Lrrc30
not_Sig	Lama1
Sig	Arhgap28
not_Sig	Tmem200c
not_Sig	Epb4.1l3
not_Sig	Zbtb14
not_Sig	C030034I22Rik
not_Sig	Dlgap1
not_Sig	Tgif1
not_Sig	Myl12b
not_Sig	Myl12a
not_Sig	Myom1
not_Sig	Lpin2
not_Sig	Emilin2
not_Sig	Smchd1
not_Sig	Ndc80
not_Sig	Spdya
not_Sig	Trmt61b
not_Sig	Wdr43
not_Sig	Snord92
not_Sig	Snord53

not_Sig	Fam179a
not_Sig	Clip4
Sig	Ypel5
not_Sig	Lbh
not_Sig	Lclat1
not_Sig	Ehd3
not_Sig	Xdh
not_Sig	Srd5a2
not_Sig	Memo1
not_Sig	Dpy30
not_Sig	Spast
Sig	Slc30a6
not_Sig	Nlrc4
Sig	Yipf4
not_Sig	Birc6
not_Sig	Ttc27
not_Sig	Ltbp1
not_Sig	Rasgrp3
not_Sig	Fam98a
not_Sig	Crim1
Sig	Fez2
Sig	Vit
not_Sig	Strn
Sig	Heatr5b
not_Sig	Gpatch11
not_Sig	Gm6548
not_Sig	Eif2ak2
not_Sig	Cebpzos
not_Sig	Cebpz
not_Sig	Ndufaf7
not_Sig	Prkd3
Sig	Qpct
not_Sig	Cdc42ep3
Sig	Rmdn2
not_Sig	Cyp1b1
not_Sig	Atl2
not_Sig	Hnrnpll
Sig	Galm
not_Sig	Srsf7
not_Sig	Gemin6
not_Sig	Dhx57
Sig	Morn2
not_Sig	Arhgef33
not_Sig	Sos1
not_Sig	Cdkl4
Sig	Map4k3
not_Sig	C230072F16Rik
not_Sig	Tmem178

not_Sig	Thumpd2
not_Sig	Slc8a1
not_Sig	Pkdcc
not_Sig	Eml4
Sig	Cox7a2l
not_Sig	Mta3
not_Sig	Haao
not_Sig	Zfp36l2
not_Sig	Thada
not_Sig	Plekhh2
not_Sig	Dync2li1
not_Sig	Abcg8
not_Sig	Lrpprc
not_Sig	1110020A21Rik
not_Sig	Ppm1b
Sig	Slc3a1
not_Sig	Prepl
not_Sig	Camkmt
Sig	Six2
not_Sig	Srbd1
not_Sig	Prkce
not_Sig	2010106C02Rik
not_Sig	Epas1
not_Sig	Rhoq
not_Sig	Pigf
not_Sig	Cript
not_Sig	Socs5
Sig	Mcfd2
not_Sig	4833418N02Rik
not_Sig	Ttc7
not_Sig	Calm2
not_Sig	Msh2
not_Sig	Msh6
not_Sig	Fbxo11
not_Sig	Foxn2
not_Sig	Ppp1r21
not_Sig	Ston1
not_Sig	Lhcgr
not_Sig	Nrxn1
Sig	Mettl4
not_Sig	2700099C18Rik
not_Sig	Gm1976
not_Sig	Gm20939
Sig	Crem
not_Sig	Gm6225
not_Sig	Cul2
not_Sig	Bambi
Sig	Map3k8

Sig	4833419F23Rik
not_Sig	Mtpap
Sig	9430020K01Rik
not_Sig	Svil
not_Sig	Zfp438
not_Sig	Gm10125
not_Sig	Zeb1
not_Sig	Arhgap12
not_Sig	Kif5b
Sig	Epc1
not_Sig	Rab18
not_Sig	Mkx
not_Sig	Mpp7
not_Sig	Wac
not_Sig	Fzd8
not_Sig	Ccny
not_Sig	Colec12
not_Sig	Thoc1
Sig	Usp14
not_Sig	Rock1
not_Sig	Greb1l
not_Sig	Esco1
not_Sig	Snrpd1
not_Sig	Abhd3
Sig	Mib1
not_Sig	Mir133a-1
not_Sig	Mir1b
not_Sig	1010001N08Rik
not_Sig	Gata6
not_Sig	Rbbp8
not_Sig	Gm6277
not_Sig	Cables1
not_Sig	Tmem241
not_Sig	Riok3
not_Sig	3110002H16Rik
not_Sig	Npc1
not_Sig	Ankrd29
Sig	Lama3
not_Sig	Ttc39c
not_Sig	Cabyr
not_Sig	Osbpl1a
not_Sig	Impact
not_Sig	Zfp521
not_Sig	Ss18
not_Sig	Psma8
Sig	Taf4b
Sig	Kctd1
not_Sig	4933424G05Rik

Sig	Aqp4
not_Sig	Cdh2
not_Sig	Dsc3
Sig	Dsc2
not_Sig	Dsg2
Sig	Ttr
not_Sig	B4galt6
not_Sig	Trappc8
not_Sig	Rnf125
not_Sig	Rnf138
not_Sig	Garem
not_Sig	Klhl14
not_Sig	Asxl3
not_Sig	Nol4
not_Sig	Dtna
not_Sig	Gm15972
not_Sig	Mapre2
not_Sig	Mir6360
not_Sig	Zfp397
not_Sig	Zfp35
not_Sig	Zfp191
not_Sig	Ino80c
not_Sig	Galnt1
not_Sig	2700062C07Rik
not_Sig	Rprd1a
not_Sig	Slc39a6
not_Sig	Elp2
not_Sig	Mocos
not_Sig	Fhod3
not_Sig	Tpgs2
not_Sig	AW554918
not_Sig	Celf4
not_Sig	Pik3c3
not_Sig	Slc25a46
not_Sig	Sap130
not_Sig	Ammecr1l
not_Sig	Polr2d
not_Sig	Wdr33
not_Sig	Sft2d3
not_Sig	Lims2
Sig	Gpr17
not_Sig	Myo7b
not_Sig	Iws1
not_Sig	Map3k2
not_Sig	Ercc3
Sig	A830052D11Rik
not_Sig	Bin1
not_Sig	Gypc

not_Sig	Tslp
not_Sig	Wdr36
not_Sig	Camk4
not_Sig	Stard4
not_Sig	Nrep
not_Sig	Gm10549
not_Sig	Epb4.1l4aos
Sig	Epb4.1l4a
not_Sig	Gm10548
not_Sig	Apc
Sig	Srp19
Sig	Reep5
not_Sig	Pkd2l2
not_Sig	Fam13b
not_Sig	Nme5
not_Sig	4933408B17Rik
not_Sig	Brd8
not_Sig	Kif20a
not_Sig	Cdc23
not_Sig	Gfra3
not_Sig	Cdc25c
not_Sig	Fam53c
not_Sig	Kdm3b
not_Sig	Reep2
not_Sig	Egr1
Sig	Etf1
not_Sig	Hspa9
not_Sig	Ctnna1
Sig	Lrrtm2
Sig	Sil1
not_Sig	Snhg4
not_Sig	Mir1949
Sig	Snora74a
not_Sig	Matr3
Sig	Paip2
not_Sig	Slc23a1
not_Sig	Mzb1
Sig	Prob1
Sig	Spata24
Sig	Dnajc18
not_Sig	Ecscr
not_Sig	1700066B19Rik
not_Sig	Tmem173
Sig	Ube2d2a
not_Sig	Cxxc5
not_Sig	Psd2
not_Sig	Nrg2
not_Sig	Pura

Sig	Igip
not_Sig	Cystm1
not_Sig	Pfdn1
not_Sig	Hbegf
not_Sig	Ankhd1
not_Sig	Eif4ebp3
Sig	Sra1
not_Sig	Apbb3
not_Sig	Slc35a4
not_Sig	E230025N22Rik
Sig	Cd14
not_Sig	Tmco6
not_Sig	Ndufa2
not_Sig	Ik
not_Sig	Wdr55
not_Sig	Dnd1
not_Sig	Hars
not_Sig	Hars2
Sig	Zmat2
Sig	Vaultrc5
not_Sig	Pcdhac2
not_Sig	Pcdhb2
not_Sig	Pcdhb3
not_Sig	Pcdhb4
not_Sig	Pcdhb5
not_Sig	Pcdhb6
not_Sig	Pcdhb7
not_Sig	Pcdhb8
not_Sig	Pcdhb9
not_Sig	Pcdhb10
not_Sig	Pcdhb11
not_Sig	Pcdhb12
Sig	Pcdhb13
not_Sig	Pcdhb14
not_Sig	Pcdhb15
Sig	Pcdhb16
not_Sig	Pcdhb17
not_Sig	Pcdhb18
not_Sig	Pcdhb19
not_Sig	Pcdhb20
not_Sig	Pcdhb21
not_Sig	Pcdhb22
not_Sig	Slc25a2
not_Sig	Taf7
Sig	Pcdhga1
not_Sig	Pcdhga2
not_Sig	Pcdhga3
not_Sig	Pcdhgb1

Sig	Pcdhga4
not_Sig	Pcdhgb2
not_Sig	Pcdhga5
Sig	Pcdhga6
Sig	Pcdhga7
Sig	Pcdhgb4
Sig	Pcdhga8
Sig	Pcdhgb5
Sig	Pcdhga9
Sig	Pcdhgb6
Sig	Pcdhga10
Sig	Pcdhgb7
Sig	Pcdhga11
Sig	Pcdhgb8
Sig	Pcdhga12
not_Sig	Pcdhgc3
not_Sig	Pcdhgc4
not_Sig	Pcdhgc5
Sig	Diap1
not_Sig	Hdac3
not_Sig	Fchsd1
Sig	Arap3
Sig	Pcdh1
not_Sig	1700086O06Rik
not_Sig	0610009O20Rik
Sig	Pcdh12
not_Sig	Rnf14
not_Sig	Gnpda1
not_Sig	Ndfip1
not_Sig	Spry4
not_Sig	Fgf1
not_Sig	Gm5820
Sig	Arhgap26
not_Sig	Nr3c1
not_Sig	Yipf5
not_Sig	2900055J20Rik
Sig	Kctd16
not_Sig	Prelid2
Sig	Sh3rf2
not_Sig	Lars
not_Sig	Gm4013
not_Sig	Rbm27
Sig	Tcerg1
not_Sig	Gpr151
not_Sig	Ppp2r2b
not_Sig	Stk32a
not_Sig	Dpysl3
not_Sig	Jakmip2

not_Sig	Spink12
not_Sig	Spinkl
not_Sig	Spink11
not_Sig	Npy6r
not_Sig	Myot
not_Sig	Dcp2
Sig	Mcc
not_Sig	A930012L18Rik
not_Sig	Ythdc2
Sig	Kcnn2
not_Sig	A330093E20Rik
not_Sig	Trim36
not_Sig	Pggt1b
not_Sig	Ccdc112
not_Sig	Mospd4
not_Sig	Fem1c
not_Sig	Ticam2
Sig	Tmed7
not_Sig	Eif1a
not_Sig	Cdo1
Sig	Atg12
Sig	Ap3s1
not_Sig	Lvrn
not_Sig	Commd10
Sig	Sema6a
not_Sig	Eno1b
Sig	Dtwd2
not_Sig	Dmxl1
not_Sig	Tnfaip8
not_Sig	Hsd17b4
not_Sig	Prr16
not_Sig	Srfbp1
not_Sig	Lox
Sig	Sncaip
not_Sig	Snx2
Sig	Snx24
Sig	Ppic
not_Sig	Prdm6
not_Sig	Gm19466
not_Sig	Cep120
not_Sig	Csnk1g3
not_Sig	Redrum
Sig	Zfp608
Sig	Gramd3
not_Sig	Aldh7a1
not_Sig	Phax
not_Sig	Lmnb1
not_Sig	March3

Sig	C330018D20Rik
not_Sig	Megf10
not_Sig	Prrc1
not_Sig	Ctxn3
not_Sig	1700066O22Rik
not_Sig	1700011I03Rik
not_Sig	4930511M06Rik
not_Sig	Slc12a2
not_Sig	Fbn2
not_Sig	Slc27a6
not_Sig	Isoc1
not_Sig	Adamts19
not_Sig	Chsy3
not_Sig	Gm4951
not_Sig	Gm4841
not_Sig	F830016B08Rik
not_Sig	Iigp1
not_Sig	Smim3
not_Sig	Dctn4
not_Sig	Rbm22
not_Sig	Myoz3
not_Sig	Synpo
Sig	Ndst1
Sig	Rps14
not_Sig	Cd74
Sig	Tcof1
not_Sig	Arsi
not_Sig	Camk2a
not_Sig	Slc6a7
Sig	Pdgfrb
not_Sig	Csf1r
not_Sig	Hmgxb3
Sig	Slc26a2
not_Sig	Pde6a
not_Sig	Ppargc1b
not_Sig	Arhgef37
not_Sig	Csnk1a1
not_Sig	Bvht
Sig	Mir143hg
not_Sig	Mir145a
not_Sig	Mir143
not_Sig	Pcyox1l
not_Sig	Grpel2
not_Sig	1500015A07Rik
Sig	Afap1l1
Sig	Ablim3
not_Sig	Sh3tc2
not_Sig	Adrb2

not_Sig	Fbxo38
not_Sig	Apcdd1
not_Sig	Napg
not_Sig	Piezo2
not_Sig	Txnl1
not_Sig	Wdr7
not_Sig	Onecut2
not_Sig	Fech
not_Sig	Nars
not_Sig	Atp8b1
not_Sig	Nedd4l
not_Sig	Mir466p
not_Sig	Alpk2
not_Sig	Malt1
not_Sig	Zfp532
Sig	Oacyl
not_Sig	Sec11c
not_Sig	Grp
Sig	Lman1
Sig	Ccbe1
Sig	Mir694
not_Sig	Pmaip1
Sig	Gnal
not_Sig	Chmp1b
not_Sig	Mppe1
not_Sig	Impa2
not_Sig	B430212C06Rik
Sig	Cidea
Sig	Tubb6
Sig	Afg3l2
not_Sig	Slmo1
Sig	Spire1
Sig	Cep76
not_Sig	Psmg2
not_Sig	Ptpn2
not_Sig	Seh1l
not_Sig	4930549G23Rik
not_Sig	Cep192
not_Sig	Ldlrad4
not_Sig	Fam210a
not_Sig	Rnmt
not_Sig	Mc5r
not_Sig	Mc2r
Sig	Tcf4
not_Sig	Ccdc68
not_Sig	Rab27b
not_Sig	4930503L19Rik
not_Sig	Stard6

not_Sig	Poli
not_Sig	Mbd2
Sig	Mex3c
not_Sig	Smad4
not_Sig	Elac1
not_Sig	Me2
not_Sig	Mapk4
not_Sig	Ska1
not_Sig	Cxxc1
not_Sig	Mbd1
not_Sig	Cfap53
not_Sig	Myo5b
not_Sig	Scarna17
not_Sig	Acaa2
not_Sig	Lipg
not_Sig	Rpl17
not_Sig	BC031181
not_Sig	Dym
not_Sig	2010010A06Rik
not_Sig	Smad7
Sig	Ctif
not_Sig	Gm10532
not_Sig	Zbtb7c
not_Sig	2900057B20Rik
not_Sig	Smad2
not_Sig	Ier3ip1
not_Sig	Hdhd2
Sig	Katnal2
not_Sig	Pias2
not_Sig	St8sia5
Sig	Rnf165
not_Sig	8030462N17Rik
not_Sig	4930465K10Rik
not_Sig	Haus1
not_Sig	Atp5a1
not_Sig	Pstpip2
not_Sig	Epg5
not_Sig	Siglec15
not_Sig	Slc14a1
not_Sig	Setbp1
not_Sig	Pard6g
not_Sig	Adnp2
not_Sig	LOC105246506
not_Sig	Rbfa
not_Sig	Txnl4a
not_Sig	Hsbp1l1
not_Sig	Pqlc1
not_Sig	Kcng2

not_Sig	Ctdp1
not_Sig	Gm2176
Sig	Nfatc1
not_Sig	Atp9b
not_Sig	Galr1
not_Sig	Mbp
not_Sig	Zfp236
not_Sig	Zfp516
not_Sig	4930592I03Rik
not_Sig	Tshz1
not_Sig	Zadh2
not_Sig	Zfp407
not_Sig	Cndp1
not_Sig	Cndp2
not_Sig	Fam69c
not_Sig	Cyb5a
not_Sig	Timm21
not_Sig	Socs6
not_Sig	Rttn
not_Sig	Cd226
not_Sig	Tmx3
not_Sig	Ighmbp2
not_Sig	Mrpl21
not_Sig	Cpt1a
not_Sig	Mtl5
not_Sig	Gal
not_Sig	Ppp6r3
not_Sig	Lrp5
not_Sig	1810055G02Rik
not_Sig	Suv420h1
not_Sig	Chka
not_Sig	Tcirg1
not_Sig	Ndufs8
not_Sig	Aldh3b1
not_Sig	Unc93b1
not_Sig	Aldh3b2
not_Sig	Acy3
not_Sig	Nudt8
not_Sig	Doc2g
not_Sig	Ndufv1
not_Sig	Gstp1
Sig	Gstp2
not_Sig	BC021614
Sig	Cdk2ap2
not_Sig	Pitpnm1
Sig	Aip
not_Sig	Tmem134
not_Sig	Cabp4

not_Sig	Gpr152
not_Sig	Coro1b
not_Sig	Ptprcap
not_Sig	Rps6kb2
Sig	Carns1
not_Sig	Tbc1d10c
Sig	Ppp1ca
not_Sig	Rad9a
Sig	Clcf1
not_Sig	Pold4
not_Sig	Ssh3
not_Sig	Ankrd13d
not_Sig	Adrbk1
not_Sig	Kdm2a
not_Sig	Rhod
not_Sig	Syt12
not_Sig	2010003K11Rik
not_Sig	Pcx
not_Sig	Lrfn4
not_Sig	Rce1
Sig	Gm960
not_Sig	Sptbn2
not_Sig	Rbm4b
not_Sig	Rbm4
Sig	Rbm14
Sig	Ccs
not_Sig	Ctsf
not_Sig	Actn3
not_Sig	Zdhhc24
not_Sig	Bbs1
not_Sig	Dpp3
not_Sig	Peli3
not_Sig	Mrpl11
not_Sig	Npas4
not_Sig	Slc29a2
not_Sig	B4gat1
not_Sig	Brms1
not_Sig	Rin1
Sig	Cd248
Sig	Yif1a
not_Sig	Cnih2
Sig	Rab1b
not_Sig	Klc2
Sig	Pacs1
Sig	Sf3b2
not_Sig	Cst6
not_Sig	Banf1
not_Sig	Eif1ad

Sig	Sart1
not_Sig	D330050I16Rik
Sig	4930481A15Rik
not_Sig	Drap1
not_Sig	AI837181
not_Sig	Ccdc85b
not_Sig	Fibp
not_Sig	Ctsw
not_Sig	Efemp2
not_Sig	Mus81
not_Sig	Cfl1
not_Sig	Snx32
not_Sig	1700020D05Rik
not_Sig	Ovol1
not_Sig	Ap5b1
not_Sig	Rnaseh2c
Sig	Kat5
not_Sig	Rela
Sig	Sipa1
Sig	Pcnxl3
Sig	Map3k11
not_Sig	Kcnk7
not_Sig	Ehbp1l1
not_Sig	Fam89b
not_Sig	Sssca1
not_Sig	Ltbp3
not_Sig	Scyl1
Sig	Malat1
not_Sig	Neat1
Sig	Frmd8
not_Sig	Slc25a45
not_Sig	Tigd3
not_Sig	Dpf2
Sig	Cdc42ep2
not_Sig	Pola2
not_Sig	Capn1
not_Sig	Gm10814
not_Sig	Syvn1
Sig	Mrpl49
not_Sig	Fau
not_Sig	Znhit2
not_Sig	Tm7sf2
not_Sig	Vps51
not_Sig	Zfpl1
not_Sig	Cdca5
not_Sig	Naaladl1
Sig	Sac3d1
not_Sig	Snx15

not_Sig	Arl2
not_Sig	Batf2
not_Sig	Gpha2
not_Sig	Ppp2r5b
not_Sig	Atg2a
not_Sig	Ehd1
Sig	Cdc42bpg
not_Sig	Men1
Sig	Map4k2
Sig	Sf1
not_Sig	Pygm
Sig	Rasgrp2
Sig	Nrxn2
not_Sig	Slc22a12
not_Sig	Rps6ka4
not_Sig	Ccdc88b
not_Sig	Prdx5
not_Sig	Trmt112
Sig	Esrra
not_Sig	Mir6990
not_Sig	Tex40
not_Sig	Kcnk4
not_Sig	Gpr137
not_Sig	Bad
not_Sig	Plcb3
not_Sig	Ppp1r14b
not_Sig	Fkbp2
Sig	Vegfb
not_Sig	Dnajc4
not_Sig	Nudt22
not_Sig	Trpt1
not_Sig	Fermt3
Sig	Stip1
not_Sig	Macrod1
Sig	Flrt1
not_Sig	Otub1
not_Sig	Cox8a
Sig	Naa40
not_Sig	Rcor2
not_Sig	Mark2
Sig	AI846148
not_Sig	2700081O15Rik
not_Sig	Rtn3
not_Sig	Snord118
not_Sig	Atl3
Sig	Pla2g16
Sig	Lgals12
not_Sig	Hrasls5

not_Sig	Chrm1
not_Sig	Slc3a2
Sig	Snhg1
Sig	Snord22
not_Sig	Wdr74
not_Sig	1700092M07Rik
not_Sig	Stx5a
not_Sig	Nxf1
not_Sig	Tmem223
not_Sig	Tmem179b
not_Sig	Taf6l
not_Sig	Gm2518
Sig	Polr2g
not_Sig	Zbtb3
not_Sig	Ttc9c
Sig	Hnrnpul2
not_Sig	Gng3
not_Sig	Bscl2
Sig	Lrrn4cl
not_Sig	Ubxn1
not_Sig	Uqcc3
not_Sig	5730408K05Rik
not_Sig	Ints5
not_Sig	Ganab
Sig	B3gat3
not_Sig	Rom1
not_Sig	Eml3
Sig	Mta2
not_Sig	Tut1
Sig	Eef1g
not_Sig	Ahnak
not_Sig	Scgb1a1
not_Sig	Asrgl1
not_Sig	Stxbp3-ps
not_Sig	Incenp
Sig	Fth1
not_Sig	Best1
Sig	Rab3il1
not_Sig	Fads3
not_Sig	Fads2
not_Sig	Fads1
not_Sig	Fen1
Sig	Tmem258
not_Sig	Myrf
not_Sig	Dagla
Sig	Syt7
not_Sig	Lrrc10b
not_Sig	Ppp1r32

not_Sig	Sdhaf2
Sig	Cpsf7
not_Sig	Tmem216
not_Sig	Tmem138
not_Sig	Cyb561a3
not_Sig	Tkfc
not_Sig	Ddb1
not_Sig	Vps37c
Sig	Cd5
not_Sig	A430093F15Rik
not_Sig	Cd6
not_Sig	Slc15a3
not_Sig	Tmem132a
not_Sig	Tmem109
Sig	Prpf19
not_Sig	Ptgdr2
not_Sig	Ccdc86
not_Sig	AW112010
not_Sig	Ms4a8a
not_Sig	Ms4a1
not_Sig	Ms4a7
not_Sig	Ms4a4c
not_Sig	Ms4a4b
not_Sig	Ms4a6c
not_Sig	Gm8369
not_Sig	Ms4a6b
not_Sig	Ms4a4d
not_Sig	Ms4a6d
not_Sig	Ms4a2
not_Sig	Ms4a3
not_Sig	Mrpl16
not_Sig	Stx3
Sig	Patl1
not_Sig	Osbp
not_Sig	Mpeg1
not_Sig	Dtx4
not_Sig	Fam111a
not_Sig	A330040F15Rik
not_Sig	Cntf
not_Sig	Zfp91
not_Sig	Lpxn
not_Sig	Tle4
not_Sig	Psat1
not_Sig	Cep78
not_Sig	Gnaq
not_Sig	E030024N20Rik
not_Sig	Gna14
not_Sig	Vps13a

Sig	Prune2
not_Sig	Gcnt1
not_Sig	Rfk
not_Sig	Pcsk5
Sig	Ostf1
not_Sig	Nmrk1
not_Sig	2410127L17Rik
not_Sig	D030056L22Rik
not_Sig	Trpm6
not_Sig	Rorb
not_Sig	Anxa1
not_Sig	E030003E18Rik
not_Sig	C730002L08Rik
not_Sig	Aldh1a1
Sig	Aldh1a7
not_Sig	Zfand5
Sig	Gda
not_Sig	1110059E24Rik
not_Sig	Abhd17b
not_Sig	Tmem2
not_Sig	Trpm3
not_Sig	Klf9
not_Sig	Mir1192
not_Sig	Smc5
not_Sig	Mamdc2
not_Sig	Ptar1
not_Sig	Apba1
Sig	Fam189a2
not_Sig	Tjp2
not_Sig	Fxn
not_Sig	Pip5k1b
not_Sig	Fam122a
not_Sig	Tmem252
not_Sig	Pgm5
not_Sig	Cbwd1
not_Sig	Dock8
Sig	Kank1
not_Sig	2610016A17Rik
not_Sig	Dmrt2
not_Sig	Smarca2
not_Sig	4931403E22Rik
Sig	Vldlr
not_Sig	Kcnv2
not_Sig	D19Bwg1357e
not_Sig	C030016D13Rik
not_Sig	Rfx3
Sig	Glis3
Sig	Slc1a1

not_Sig	4430402I18Rik
not_Sig	Ppapdc2
not_Sig	Cdc37l1
not_Sig	Ak3
not_Sig	1700018L02Rik
Sig	Gm9895
Sig	Rcl1
Sig	Jak2
not_Sig	Insl6
Sig	Plgrkt
not_Sig	Cd274
not_Sig	Pdcd1lg2
not_Sig	A930007I19Rik
not_Sig	Ric1
not_Sig	Ermp1
not_Sig	9930021J03Rik
not_Sig	Ranbp6
Sig	Il33
not_Sig	Uhrf2
Sig	Gldc
not_Sig	Prkg1
not_Sig	Cstf2t
not_Sig	8430431K14Rik
Sig	Asah2
not_Sig	Sgms1
not_Sig	2700046G09Rik
Sig	Minpp1
not_Sig	Papss2
Sig	Atad1
Sig	Pten
not_Sig	Rnls
not_Sig	Lipo1
not_Sig	Lipf
not_Sig	Stambpl1
not_Sig	Acta2
not_Sig	Fas
Sig	Lipa
not_Sig	Ifit2
not_Sig	Ifit3
not_Sig	Ifit1bl1
Sig	Ifit3b
not_Sig	Ifit1bl2
not_Sig	Ifit1
not_Sig	Slc16a12
not_Sig	Pank1
not_Sig	Kif20b
Sig	Htr7
Sig	Rpp30

not_Sig	Ankrd1
not_Sig	Pcgf5
not_Sig	Hectd2
not_Sig	1500017E21Rik
Sig	Ppp1r3c
not_Sig	Tnks2
not_Sig	Fgfbp3
not_Sig	Btaf1
Sig	Cpeb3
not_Sig	A330032B11Rik
Sig	March5
not_Sig	4931408D14Rik
not_Sig	Ide
Sig	Kif11
Sig	Hhex
not_Sig	Exoc6
not_Sig	Cyp26c1
not_Sig	Cyp26a1
not_Sig	Myof
not_Sig	Cep55
not_Sig	Ffar4
not_Sig	Rbp4
not_Sig	Pde6c
Sig	Fra10ac1
not_Sig	Lgi1
not_Sig	Slc35g1
not_Sig	Plce1
not_Sig	Noc3l
not_Sig	Tbc1d12
not_Sig	Hells
not_Sig	Cyp2c29
not_Sig	Cyp2c37
not_Sig	Cyp2c50
not_Sig	Pdlim1
not_Sig	Sorbs1
not_Sig	Aldh18a1
not_Sig	Tctn3
not_Sig	Entpd1
not_Sig	Ccnj
not_Sig	Zfp518a
not_Sig	Blnk
not_Sig	Dntt
not_Sig	Tll2
Sig	Tm9sf3
not_Sig	Pik3ap1
not_Sig	Lcor
not_Sig	Mir8091
not_Sig	Slit1

not_Sig	Arhgap19
not_Sig	Frat1
Sig	Frat2
Sig	Rrp12
not_Sig	Pgam1
Sig	Exosc1
not_Sig	Zdhhc16
not_Sig	Mms19
Sig	Ubtd1
not_Sig	Ankrd2
not_Sig	Hoga1
not_Sig	4933411K16Rik
not_Sig	Morn4
not_Sig	Pi4k2a
not_Sig	Avpi1
not_Sig	Marveld1
not_Sig	Zfyve27
not_Sig	Sfrp5
not_Sig	Golga7b
not_Sig	Crtac1
not_Sig	R3hcc1l
not_Sig	Loxl4
Sig	Pyroxd2
not_Sig	Hps1
not_Sig	Hpse2
not_Sig	Cnnm1
not_Sig	Got1
Sig	Slc25a28
not_Sig	BC037704
not_Sig	Entpd7
not_Sig	Cox15
not_Sig	Cutc
not_Sig	Abcc2
not_Sig	Dnmbp
not_Sig	Cpn1
Sig	Cyp2c44
not_Sig	Erlin1
not_Sig	Chuk
not_Sig	Cwf19l1
not_Sig	Bloc1s2
not_Sig	Scd3
not_Sig	Scd2
not_Sig	Scd4
not_Sig	Scd1
not_Sig	Wnt8b
not_Sig	Sec31b
not_Sig	Ndufb8
not_Sig	Hif1an

not_Sig	Fam178a
not_Sig	4930414N06Rik
not_Sig	Sema4g
Sig	Mrpl43
not_Sig	Peo1
Sig	Lzts2
Sig	Pdzd7
Sig	Sfxn3
Sig	Kazald1
not_Sig	Lbx1
not_Sig	Btrc
not_Sig	Poll
not_Sig	Dpcd
not_Sig	Fbxw4
not_Sig	Npm3
not_Sig	Mgea5
not_Sig	Kcnip2
not_Sig	9130011E15Rik
not_Sig	Hps6
Sig	Ldb1
not_Sig	Pprc1
not_Sig	Nolc1
not_Sig	Elovl3
not_Sig	Pitx3
not_Sig	Gbf1
not_Sig	Nfkb2
not_Sig	Psd
not_Sig	Fbxl15
not_Sig	Cuedc2
not_Sig	2310034G01Rik
not_Sig	Tmem180
Sig	Actr1a
not_Sig	Sufu
not_Sig	Trim8
not_Sig	Arl3
Sig	Sfxn2
Sig	Wbp1l
not_Sig	Cyp17a1
Sig	2010012O05Rik
Sig	As3mt
not_Sig	Cnnm2
not_Sig	Nt5c2
not_Sig	Pcgf6
not_Sig	Taf5
Sig	Usmg5
not_Sig	Pdcd11
not_Sig	Calhm2
not_Sig	Neurl1a

not_Sig	Sh3pxd2a
not_Sig	Obfc1
not_Sig	Gm19557
not_Sig	Slk
not_Sig	Col17a1
not_Sig	Sfr1
not_Sig	Cfap43
not_Sig	Gsto1
not_Sig	Gsto2
not_Sig	Itprip
not_Sig	Sorcs3
not_Sig	Sorcs1
not_Sig	Xpnpep1
Sig	Add3
not_Sig	1700001K23Rik
not_Sig	Mxi1
not_Sig	Smndc1
not_Sig	5830416P10Rik
not_Sig	Dusp5
not_Sig	Smc3
not_Sig	Rbm20
not_Sig	Pdcd4
not_Sig	Bbip1
not_Sig	Shoc2
not_Sig	Adra2a
Sig	Gpam
not_Sig	Gucy2g
not_Sig	Acsl5
not_Sig	Zdhhc6
not_Sig	Vti1a
not_Sig	4930552P12Rik
not_Sig	Tcf7l2
not_Sig	Habp2
not_Sig	Nrap
Sig	Casp7
not_Sig	Plekhs1
not_Sig	Dclre1a
not_Sig	Nhlrc2
Sig	Adrb1
not_Sig	Ccdc186
not_Sig	Vwa2
not_Sig	Afap1l2
not_Sig	Ablim1
not_Sig	4930449E18Rik
not_Sig	B230217O12Rik
not_Sig	Fam160b1
not_Sig	Trub1
not_Sig	Atrnl1

not_Sig	Mir5623
not_Sig	Gfra1
not_Sig	Hspa12a
not_Sig	Eno4
not_Sig	Shtn1
not_Sig	Slc18a2
not_Sig	Pdzd8
not_Sig	Emx2os
not_Sig	Emx2
not_Sig	Rab11fip2
not_Sig	E330013P04Rik
not_Sig	Fam204a
not_Sig	Cacul1
not_Sig	Nanos1
not_Sig	Eif3a
not_Sig	Fam45a
not_Sig	Sfxn4
not_Sig	Prdx3
not_Sig	Grk5
not_Sig	Zfp950
not_Sig	Csf2ra
not_Sig	Fam171a1
Sig	Nmt2
not_Sig	Rpp38
not_Sig	Acbd7
not_Sig	Dclre1c
not_Sig	Suv39h2
not_Sig	Hspa14
not_Sig	Cdnf
not_Sig	Fam107b
not_Sig	Frmd4a
not_Sig	Prpf18
not_Sig	Bend7
not_Sig	Sephs1
Sig	Phyh
not_Sig	Mcm10
not_Sig	Optn
Sig	Ccdc3
not_Sig	Camk1d
not_Sig	Cdc123
not_Sig	Nudt5
not_Sig	Sec61a2
not_Sig	Dhtkd1
not_Sig	Upf2
not_Sig	Proser2
not_Sig	Echdc3
not_Sig	Usp6nl
not_Sig	Celf2

not_Sig	5031426D15Rik
not_Sig	Gata3
not_Sig	4930412O13Rik
Sig	Taf3
not_Sig	Atp5c1
not_Sig	Kin
not_Sig	Itih2
not_Sig	Itih5
not_Sig	Sfmbt2
not_Sig	Prkcq
not_Sig	Pfkfb3
Sig	Rbm17
not_Sig	Il2ra
not_Sig	Il15ra
not_Sig	Fbxo18
not_Sig	Ankrd16
not_Sig	Itga8
not_Sig	Fam188a
Sig	Pter
not_Sig	C1ql3
not_Sig	Rsu1
not_Sig	Cubn
not_Sig	Trdmt1
not_Sig	Vim
not_Sig	St8sia6
not_Sig	Hacd1
not_Sig	Stamos
not_Sig	Stam
not_Sig	Tmem236
Sig	Mrc1
Sig	Cacnb2
not_Sig	Gm13315
not_Sig	Nsun6
not_Sig	Arl5b
Sig	Plxdc2
not_Sig	Nebl
not_Sig	A930004D18Rik
not_Sig	Gm17762
not_Sig	Skida1
not_Sig	Mllt10
not_Sig	Dnajc1
Sig	Commd3
not_Sig	Bmi1
not_Sig	Spag6l
not_Sig	Pip4k2a
not_Sig	Msrb2
not_Sig	Gm3230
not_Sig	Otud1

not_Sig	Etl4
not_Sig	Arhgap21
not_Sig	Gm13375
not_Sig	Enkur
Sig	Thnsl1
not_Sig	Apbb1ip
not_Sig	Pdss1
not_Sig	Abi1
not_Sig	Acbd5
not_Sig	Mastl
Sig	Yme1l1
not_Sig	4931423N10Rik
not_Sig	Spopl
not_Sig	Hnmt
not_Sig	Il1f9
not_Sig	Psd4
not_Sig	Cacna1b
not_Sig	Ehmt1
not_Sig	Arrdc1
not_Sig	Zmynd19
not_Sig	Dph7
not_Sig	Mrpl41
not_Sig	Pnpla7
not_Sig	Nsmf
not_Sig	Entpd8
Sig	Nrarp
not_Sig	Tor4a
not_Sig	Nelfb
not_Sig	Fam166a
Sig	Tubb4b
not_Sig	Rnf208
not_Sig	Ndor1
not_Sig	Tmem203
not_Sig	Tprn
not_Sig	Ssna1
not_Sig	Anapc2
not_Sig	Grin1
not_Sig	AA543186
not_Sig	Man1b1
Sig	Dpp7
not_Sig	Uap1l1
not_Sig	Sapcd2
not_Sig	Entpd2
not_Sig	Npdc1
not_Sig	Fut7
not_Sig	Abca2
not_Sig	Clic3
Sig	BC029214

not_Sig	Ptgds
not_Sig	C8g
not_Sig	Fbxw5
Sig	Traf2
Sig	Edf1
Sig	Mamdc4
not_Sig	Phpt1
not_Sig	Gm996
not_Sig	Rabl6
not_Sig	Ccdc183
Sig	Tmem141
not_Sig	Fcna
not_Sig	Bmyc
Sig	Kcnt1
not_Sig	Camsap1
not_Sig	Ubac1
Sig	Nacc2
not_Sig	C330006A16Rik
not_Sig	Qsox2
not_Sig	4932418E24Rik
Sig	Gpsm1
not_Sig	Dnlz
not_Sig	Card9
Sig	Snapc4
not_Sig	Sdccag3
not_Sig	Pmpca
not_Sig	Inpp5e
not_Sig	Sec16a
Sig	Notch1
Sig	Egfl7
not_Sig	Mir126a
Sig	Agpat2
not_Sig	Fam69b
not_Sig	Snhg7
Sig	Snora43
not_Sig	Snora17
not_Sig	Surf6
not_Sig	Med22
not_Sig	Rpl7a
Sig	Surf1
not_Sig	Surf2
not_Sig	Surf4
not_Sig	Stkld1
not_Sig	Rexo4
not_Sig	Adamts13
not_Sig	Cacfd1
not_Sig	Slc2a6
not_Sig	Adamtsl2

not_Sig	Fam163b
not_Sig	Sardh
not_Sig	Vav2
Sig	Brd3
not_Sig	Wdr5
Sig	Rxra
not_Sig	Col5a1
not_Sig	Fcnb
not_Sig	Olfm1
not_Sig	Ppp1r26
not_Sig	Mrps2
not_Sig	Gbgt1
not_Sig	Ralgds
not_Sig	Gtf3c5
not_Sig	Gfi1b
not_Sig	Tsc1
not_Sig	Ak8
not_Sig	Gtf3c4
not_Sig	Ddx31
not_Sig	Barhl1
not_Sig	Ttf1
not_Sig	Setx
not_Sig	Ntng2
not_Sig	6530402F18Rik
not_Sig	Med27
not_Sig	Mir133c
not_Sig	Rapgef1
not_Sig	Trub2
not_Sig	Coq4
not_Sig	Slc27a4
not_Sig	Urm1
not_Sig	Cercam
not_Sig	Odf2
not_Sig	Gle1
not_Sig	Sptan1
not_Sig	Wdr34
Sig	Set
not_Sig	Pkn3
not_Sig	Zdhhc12
not_Sig	Zer1
not_Sig	Tbc1d13
not_Sig	Endog
not_Sig	D2Wsu81e
not_Sig	Ccbl1
not_Sig	1700084E18Rik
Sig	Lrrc8a
Sig	Phyhd1
not_Sig	Dolk

not_Sig	Nup188
not_Sig	Sh3glb2
not_Sig	Fam73b
not_Sig	Dolpp1
not_Sig	Crat
not_Sig	Ppp2r4
not_Sig	Ier5l
not_Sig	Cstad
Sig	1700001O22Rik
not_Sig	Ntmt1
not_Sig	Asb6
not_Sig	Prrx2
not_Sig	Ptges
not_Sig	Tor1b
not_Sig	Tor1a
not_Sig	BC005624
Sig	Usp20
not_Sig	Fnbp1
not_Sig	D330023K18Rik
Sig	Gpr107
not_Sig	Ncs1
not_Sig	Fubp3
not_Sig	Exosc2
not_Sig	Abl1
Sig	Qrfp
not_Sig	Lamc3
not_Sig	Aif1l
not_Sig	Nup214
not_Sig	Mir7674
not_Sig	Fam78a
not_Sig	Ppapdc3
Sig	Prrc2b
not_Sig	Pomt1
not_Sig	Uck1
Sig	Swi5
Sig	Golga2
not_Sig	Dnm1
not_Sig	Ciz1
not_Sig	1110008P14Rik
not_Sig	Lcn2
not_Sig	Ptges2
Sig	Slc25a25
not_Sig	Naif1
Sig	Fam102a
not_Sig	Dpm2
not_Sig	St6galnac4
Sig	St6galnac6
not_Sig	Ak1

Sig	Eng
not_Sig	Fpgs
not_Sig	Cdk9
not_Sig	Mir3960
Sig	Sh2d3c
Sig	6330409D20Rik
not_Sig	Ttc16
not_Sig	Tor2a
not_Sig	Ptrh1
not_Sig	1700019L03Rik
Sig	Stxbp1
not_Sig	Fam129b
not_Sig	Lrsam1
not_Sig	Rpl12
not_Sig	Snora65
not_Sig	Slc2a8
not_Sig	Garnl3
not_Sig	Ralgps1
not_Sig	Angptl2
Sig	Zbtb34
not_Sig	Zbtb43
not_Sig	Lmx1b
not_Sig	C130021I20Rik
not_Sig	Mvb12b
not_Sig	Nron
not_Sig	Pbx3
not_Sig	Mapkap1
not_Sig	Gapvd1
not_Sig	Hspa5
not_Sig	Rabepk
not_Sig	Fbxw2
Sig	Psmd5
not_Sig	Cutal
not_Sig	Phf19
not_Sig	Traf1
not_Sig	Hc
not_Sig	AI182371
Sig	Cntrl
Sig	Rab14
not_Sig	Gsn
Sig	Stom
not_Sig	Ggta1
not_Sig	Gm13446
Sig	Dab2ip
Sig	Ttll11
not_Sig	Ndufa8
not_Sig	Lhx6
not_Sig	Rbm18

not_Sig	Mrrf
not_Sig	Ptgs1
not_Sig	Pdcl
not_Sig	Rc3h2
not_Sig	Zbtb6
not_Sig	Zbtb26
not_Sig	Rabgap1
not_Sig	Gpr21
not_Sig	Strbp
not_Sig	Crb2
Sig	Dennd1a
Sig	Nek6
not_Sig	Psmb7
not_Sig	Nr6a1
not_Sig	Mir181a-2
not_Sig	Mir181b-2
not_Sig	Olfml2a
not_Sig	Wdr38
not_Sig	Rpl35
not_Sig	Arpc5l
not_Sig	Golga1
not_Sig	Scai
not_Sig	Gm16523
Sig	Ppp6c
not_Sig	Lrp1b
not_Sig	Kynu
not_Sig	Arhgap15
not_Sig	Gtdc1
not_Sig	Zeb2
not_Sig	Zeb2os
not_Sig	Acvr2a
not_Sig	Orc4
not_Sig	Mbd5
not_Sig	Epc2
not_Sig	Kif5c
not_Sig	Lypd6b
not_Sig	Lypd6
not_Sig	Mmadhc
not_Sig	Gm13483
not_Sig	Rnd3
Sig	Gm13490
not_Sig	Rbm43
not_Sig	Nmi
not_Sig	Tnfaip6
not_Sig	Rif1
not_Sig	Neb
not_Sig	4930573O16Rik
Sig	Arl5a

Sig	Cacnb4
not_Sig	Stam2
not_Sig	Fmnl2
not_Sig	Prpf40a
not_Sig	Arl6ip6
not_Sig	Galnt13
Sig	Kcnj3
not_Sig	Nr4a2
not_Sig	Gpd2
not_Sig	Galnt5
not_Sig	Cytip
not_Sig	Acvr1c
not_Sig	Gm13544
not_Sig	Acvr1
Sig	Upp2
not_Sig	Ccdc148
Sig	Pkp4
Sig	Tanc1
not_Sig	Wdsub1
Sig	Baz2b
not_Sig	March7
Sig	Cd302
not_Sig	Ly75
not_Sig	Pla2r1
not_Sig	Itgb6
not_Sig	Rbms1
not_Sig	Gm13582
not_Sig	Tank
Sig	Psmd14
Sig	Slc4a10
not_Sig	Dpp4
not_Sig	Fap
not_Sig	Ifih1
not_Sig	Gca
Sig	Kcnh7
not_Sig	Fign
not_Sig	Grb14
not_Sig	Cobll1
not_Sig	Slc38a11
not_Sig	Scn3a
Sig	Scn2a1
not_Sig	Csrnp3
not_Sig	Galnt3
not_Sig	Ttc21b
not_Sig	Scn1a
not_Sig	Scn7a
Sig	Xirp2
not_Sig	B3galt1

not_Sig	Stk39
not_Sig	Cers6
not_Sig	Nostrin
not_Sig	Spc25
not_Sig	Abcb11
not_Sig	Dhrs9
not_Sig	Lrp2
not_Sig	Bbs5
not_Sig	Klhl41
not_Sig	Fastkd1
not_Sig	Ppig
not_Sig	Ccdc173
not_Sig	Phospho2
not_Sig	Klhl23
not_Sig	Ssb
Sig	Mettl5
not_Sig	Ubr3
not_Sig	Myo3b
not_Sig	Gad1
Sig	Gorasp2
Sig	Tlk1
not_Sig	Mettl8
not_Sig	Dcaf17
not_Sig	Cybrd1
not_Sig	Dync1i2
not_Sig	Slc25a12
not_Sig	Hat1
not_Sig	Metap1d
Sig	Itga6
not_Sig	Pdk1
not_Sig	Rapgef4
not_Sig	Zak
not_Sig	Cdca7
not_Sig	Sp3
not_Sig	Sp3os
not_Sig	Ola1
not_Sig	Cir1
Sig	Scrn3
Sig	Gpr155
not_Sig	Wipf1
Sig	Chrna1
not_Sig	Chn1os3
not_Sig	Chn1
not_Sig	Atf2
not_Sig	Atp5g3
not_Sig	Lnp
not_Sig	Hoxd10
Sig	Hoxd9

not_Sig	Hoxd8
Sig	Hoxd3os1
not_Sig	Hoxd3
Sig	Hoxd4
not_Sig	Mtx2
not_Sig	Hnrnpa3
not_Sig	Nfe2l2
Sig	Agps
not_Sig	Ttc30b
not_Sig	Ttc30a1
not_Sig	Pde11a
not_Sig	Rbm45
not_Sig	Osbpl6
not_Sig	Prkra
not_Sig	Fkbp7
not_Sig	Plekha3
not_Sig	Ttn
Sig	Ccdc141
not_Sig	Sestd1
not_Sig	Zfp385b
Sig	Gm13944
not_Sig	Cwc22
not_Sig	Ube2e3
Sig	Itga4
not_Sig	Cerkl
not_Sig	Ssfa2
not_Sig	Pde1a
Sig	Dnajc10
Sig	Frzb
Sig	Nckap1
Sig	Dusp19
not_Sig	Nup35
not_Sig	Zc3h15
Sig	Itgav
not_Sig	Fam171b
Sig	Calcrl
not_Sig	Tfpi
Sig	Ctnnd1
not_Sig	2700094K13Rik
not_Sig	Tmx2
not_Sig	Med19
not_Sig	Zdhhc5
not_Sig	Clp1
not_Sig	Ypel4
not_Sig	Serping1
Sig	Ube2l6
not_Sig	Smtnl1
not_Sig	Timm10

not_Sig	Slc43a1
not_Sig	Rtn4rl2
not_Sig	Slc43a3
not_Sig	Prg2
not_Sig	P2rx3
not_Sig	Ssrp1
not_Sig	Tnks1bp1
not_Sig	4930443O20Rik
not_Sig	Aplnr
not_Sig	Lrrc55
not_Sig	Olfr1033
not_Sig	Ptprj
not_Sig	Nup160
Sig	Fnbp4
not_Sig	Agbl2
not_Sig	Mtch2
Sig	C1qtnf4
not_Sig	Ndufs3
not_Sig	Kbtbd4
not_Sig	Ptpmt1
not_Sig	Celf1
not_Sig	Rapsn
not_Sig	Psmc3
Sig	Slc39a13
not_Sig	Spi1
not_Sig	Mybpc3
Sig	Madd
Sig	Nr1h3
not_Sig	Acp2
not_Sig	Ddb2
Sig	A330069E16Rik
Sig	Pacsin3
not_Sig	Arfgap2
not_Sig	1110051M20Rik
not_Sig	Lrp4
not_Sig	Ckap5
not_Sig	Snord67
not_Sig	F2
not_Sig	Zfp408
not_Sig	Arhgap1
not_Sig	Atg13
not_Sig	Harbi1
Sig	Ambra1
not_Sig	Mdk
Sig	Dgkz
not_Sig	Mir6999
not_Sig	Creb3l1
Sig	Phf21a

not_Sig	Mir1955
not_Sig	Gyltl1b
not_Sig	Pex16
not_Sig	1700029I15Rik
not_Sig	Mapk8ip1
not_Sig	Cry2
not_Sig	D930015M05Rik
not_Sig	Slc35c1
not_Sig	Chst1
not_Sig	Syt13
Sig	Prdm11
not_Sig	4631405J19Rik
Sig	Trp53i11
Sig	Tspan18
not_Sig	Gm13807
not_Sig	Cd82
not_Sig	Gm10804
not_Sig	Alx4
not_Sig	Ext2
not_Sig	Accs
Sig	Gm13889
not_Sig	4921507L20Rik
not_Sig	Alkbh3
Sig	Hsd17b12
not_Sig	Ttc17
not_Sig	2810002D19Rik
Sig	Api5
not_Sig	Lrrc4c
not_Sig	B230118H07Rik
not_Sig	Rag1
not_Sig	Traf6
not_Sig	Prr5l
not_Sig	Commd9
not_Sig	Ldlrad3
not_Sig	Trim44
not_Sig	Fjx1
Sig	Pamr1
not_Sig	Slc1a2
not_Sig	Cd44
not_Sig	Pdhx
Sig	Apip
not_Sig	Ehf
not_Sig	Elf5
Sig	Cat
Sig	Abtb2
not_Sig	Nat10
Sig	Caprin1
not_Sig	Lincred1

not_Sig	Lmo2
not_Sig	Fbxo3
not_Sig	Cd59b
Sig	Cd59a
not_Sig	A930018P22Rik
not_Sig	D430041D05Rik
not_Sig	Hipk3
not_Sig	Cstf3
not_Sig	Tcp11l1
not_Sig	Depdc7
not_Sig	Qser1
not_Sig	Prrg4
not_Sig	Ccdc73
Sig	Eif3m
not_Sig	Wt1
not_Sig	Rcn1
not_Sig	Pax6
not_Sig	Elp4
Sig	Immp1l
not_Sig	Dnajc24
not_Sig	Mpped2
not_Sig	Arl14ep
not_Sig	Mettl15
not_Sig	Kif18a
not_Sig	Bdnf
not_Sig	Lin7c
not_Sig	Platr8
not_Sig	Lgr4
not_Sig	Ccdc34
not_Sig	Fibin
not_Sig	Ano3
not_Sig	Lpcat4
Sig	Nop10
not_Sig	Slc12a6
Sig	Emc4
not_Sig	Katnbl1
not_Sig	Emc7
not_Sig	Aven
not_Sig	Ryr3
not_Sig	Fmn1
not_Sig	Grem1
not_Sig	Scg5
not_Sig	Arhgap11a
not_Sig	Actc1
not_Sig	C130080G10Rik
not_Sig	Aqr
not_Sig	Zfp770
not_Sig	Dph6

not_Sig	BC052040
Sig	Meis2
not_Sig	Spred1
not_Sig	Fam98b
not_Sig	Rasgrp1
not_Sig	Thbs1
not_Sig	Gpr176
Sig	Eif2ak4
not_Sig	Srp14
not_Sig	Bmf
not_Sig	Bub1b
not_Sig	Plcb2
not_Sig	Inafm2
not_Sig	A430105I19Rik
not_Sig	Disp2
not_Sig	Knstrn
not_Sig	Ivd
not_Sig	Bahd1
not_Sig	Chst14
not_Sig	Ccdc32
not_Sig	Rpusd2
Sig	Casc5
not_Sig	Rad51
not_Sig	Rmdn3
not_Sig	Gchfr
not_Sig	Dnajc17
not_Sig	Gm14137
not_Sig	Zfyve19
not_Sig	Spint1
not_Sig	Vps18
Sig	Gm14207
Sig	Dll4
Sig	Chac1
not_Sig	Ino80
not_Sig	Exd1
Sig	Chp1
not_Sig	1700020I14Rik
not_Sig	Oip5
not_Sig	Nusap1
not_Sig	Ndufaf1
not_Sig	Rtf1
not_Sig	Itpka
not_Sig	Ltk
not_Sig	Rpap1
not_Sig	Tyro3
not_Sig	Mga
Sig	Mapkbp1
not_Sig	Jmjd7

not_Sig	Pla2g4b
not_Sig	Ehd4
not_Sig	Pla2g4e
not_Sig	Vps39
Sig	Tmem87a
not_Sig	Ganc
not_Sig	4931402G19Rik
not_Sig	Capn3
not_Sig	Zfp106
Sig	Snap23
not_Sig	Lrrc57
not_Sig	Haus2
not_Sig	Cdan1
not_Sig	Ttbk2
not_Sig	AV039307
not_Sig	Ubr1
not_Sig	Tmem62
not_Sig	Ccndbp1
Sig	Epb4.2
not_Sig	Lcmt2
not_Sig	Adal
not_Sig	Zscan29
Sig	Tubgcp4
not_Sig	Trp53bp1
not_Sig	Map1a
Sig	Ppip5k1
not_Sig	Ckmt1
not_Sig	Catsper2
Sig	Pdia3
not_Sig	Ell3
Sig	Serf2
Sig	Serinc4
Sig	Hypk
not_Sig	Mfap1b
not_Sig	Mfap1a
not_Sig	Wdr76
not_Sig	Frmd5
not_Sig	Casc4
Sig	Ctdspl2
not_Sig	Spg11
not_Sig	B2m
not_Sig	Sord
not_Sig	Duox2
not_Sig	Duoxa1
Sig	Duox1
not_Sig	Shf
Sig	Slc28a2
not_Sig	Bambi-ps1

Sig	Gm14085
not_Sig	Gatm
Sig	Spata5l1
not_Sig	AA467197
not_Sig	Slc30a4
not_Sig	Bloc1s6
not_Sig	Sqrdl
not_Sig	Sema6d
not_Sig	Slc24a5
not_Sig	Myef2
not_Sig	Slc12a1
not_Sig	Dut
not_Sig	Fbn1
not_Sig	Cep152
not_Sig	Shc4
not_Sig	Eid1
not_Sig	Secisbp2l
Sig	Cops2
not_Sig	Galk2
not_Sig	Fam227b
not_Sig	Fgf7
not_Sig	Dtwd1
not_Sig	Atp8b4
not_Sig	Slc27a2
not_Sig	Hdc
not_Sig	Gabpb1
not_Sig	Usp8
not_Sig	Usp50
not_Sig	Trpm7
Sig	Sppl2a
not_Sig	Mir3473g
not_Sig	Ap4e1
not_Sig	Blvra
not_Sig	Ncaph
not_Sig	Itpripl1
not_Sig	1810024B03Rik
not_Sig	Snrnp200
not_Sig	Ciao1
not_Sig	Tmem127
not_Sig	Stard7
not_Sig	Dusp2
Sig	Adra2b
not_Sig	Gpat2
not_Sig	Fahd2a
not_Sig	Kcnip3
Sig	Zfp661
not_Sig	Mrps5
not_Sig	Mal

Sig	Mall
not_Sig	Nphp1
Sig	1500011K16Rik
not_Sig	Bub1
not_Sig	Bcl2l11
not_Sig	Gm14005
not_Sig	Anapc1
Sig	Mertk
not_Sig	Tmem87b
Sig	Fbln7
not_Sig	Zc3h8
Sig	Zc3h6
Sig	Ttl
not_Sig	Polr1b
not_Sig	Chchd5
not_Sig	Slc20a1
not_Sig	A730036I17Rik
not_Sig	Ckap2l
not_Sig	Gm14023
not_Sig	Il1a
not_Sig	Il1b
not_Sig	Sirpa
not_Sig	4932416H05Rik
Sig	Stk35
not_Sig	Tgm3
not_Sig	Snrpb
not_Sig	Nop56
not_Sig	Idh3b
not_Sig	Ebf4
not_Sig	Cpxm1
not_Sig	1700020A23Rik
not_Sig	Tmem239
not_Sig	Pced1a
not_Sig	Vps16
not_Sig	Ptpra
not_Sig	4930473A02Rik
not_Sig	Mrps26
not_Sig	Ubox5
not_Sig	Fastkd5
not_Sig	Lzts3
not_Sig	Ddrgk1
Sig	Itpa
not_Sig	Slc4a11
not_Sig	4930402H24Rik
not_Sig	Gm14057
not_Sig	A730017L22Rik
not_Sig	Atrn
not_Sig	Gfra4

not_Sig	Adam33
not_Sig	Siglec1
Sig	Hspa12b
Sig	Mir3098
not_Sig	1700037H04Rik
not_Sig	Spef1
not_Sig	Cenpb
not_Sig	Cdc25b
not_Sig	Ap5s1
not_Sig	Mavs
not_Sig	Pank2
not_Sig	Mir103-2
not_Sig	Rnf24
Sig	Smox
not_Sig	Adra1d
not_Sig	Prnp
not_Sig	Rassf2
Sig	Slc23a2
not_Sig	Tmem230
Sig	Pcna
Sig	Cds2
Sig	Gpcpd1
not_Sig	AU019990
not_Sig	1110034G24Rik
not_Sig	Chgb
not_Sig	Trmt6
not_Sig	Mcm8
not_Sig	Crls1
not_Sig	Lrrn4
not_Sig	Fermt1
not_Sig	Bmp2
not_Sig	Hao1
Sig	Tmx4
not_Sig	Plcb1
not_Sig	4930545L23Rik
Sig	Plcb4
not_Sig	Ankef1
not_Sig	Snap25
Sig	Mkks
not_Sig	Slx4ip
Sig	Jag1
Sig	Btbd3
not_Sig	Ism1
not_Sig	Tasp1
not_Sig	Esf1
not_Sig	Ndufaf5
not_Sig	Sel1l2
not_Sig	Macrod2

not_Sig	Flrt3
not_Sig	Kif16b
not_Sig	Snrpb2
not_Sig	Pcsk2
not_Sig	Bfsp1
not_Sig	Dstn
not_Sig	Rrbp1
not_Sig	Snx5
Sig	Snord17
not_Sig	Mgme1
not_Sig	Csrp2bp
not_Sig	Zfp133-ps
not_Sig	Gm38402
not_Sig	Dzank1
not_Sig	Polr3f
not_Sig	Rbbp9
not_Sig	Sec23b
Sig	Gm561
not_Sig	Dtd1
not_Sig	Slc24a3
not_Sig	Gm14092
not_Sig	BC039771
not_Sig	Rin2
Sig	Naa20
not_Sig	Crnkl1
not_Sig	Insm1
Sig	Ralgapa2
not_Sig	A930019D19Rik
Sig	Kiz
not_Sig	Xrn2
not_Sig	Sstr4
not_Sig	Thbd
Sig	Cd93
not_Sig	Nxt1
not_Sig	Gzf1
Sig	Napb
Sig	Cst3
not_Sig	Zfp120
not_Sig	Zfp937
not_Sig	3300002I08Rik
not_Sig	Zfp442
not_Sig	Cst7
not_Sig	Apmap
not_Sig	Acss1
not_Sig	E130215H24Rik
not_Sig	Entpd6
not_Sig	Pygb
not_Sig	Abhd12

not_Sig	Gins1
not_Sig	Ninl
not_Sig	Nanp
not_Sig	Nsfl1c
Sig	Fkbp1a
not_Sig	Sdcbp2
Sig	Snph
Sig	Tmem74b
Sig	Psmf1
Sig	Angpt4
not_Sig	Fam110a
not_Sig	Slc52a3
not_Sig	Srxn1
Sig	Tcf15
not_Sig	Csnk2a1
not_Sig	Tbc1d20
not_Sig	Rbck1
not_Sig	Trib3
Sig	Sox12
not_Sig	Zcchc3
Sig	Rem1
not_Sig	H13
not_Sig	Mcts2
Sig	Id1
not_Sig	Cox4i2
not_Sig	Bcl2l1
Sig	Tpx2
not_Sig	Mylk2
Sig	Foxs1
not_Sig	Dusp15
not_Sig	Ttll9
not_Sig	Pdrg1
Sig	Ccm2l
not_Sig	Hck
not_Sig	Tm9sf4
not_Sig	Tspyl3
not_Sig	Plagl2
Sig	Pofut1
not_Sig	Kif3b
not_Sig	2500004C02Rik
not_Sig	Asxl1
not_Sig	Nol4l
not_Sig	Commd7
not_Sig	Dnmt3b
Sig	Mapre1
Sig	Cdk5rap1
not_Sig	Snta1
not_Sig	Cbfa2t2

not_Sig	Necab3
not_Sig	1700003F12Rik
not_Sig	Actl10
not_Sig	E2f1
Sig	Pxmp4
not_Sig	Zfp341
not_Sig	Chmp4b
not_Sig	Raly
not_Sig	Eif2s2
Sig	Ahcy
not_Sig	Itch
Sig	Dynlrb1
not_Sig	Map1lc3a
not_Sig	Pigu
Sig	Trp53inp2
Sig	Ncoa6
not_Sig	Ggt7
Sig	Acss2
not_Sig	Acss2os
not_Sig	Gss
not_Sig	Myh7b
not_Sig	Trpc4ap
not_Sig	Edem2
not_Sig	Procr
not_Sig	Mmp24
not_Sig	BC029722
not_Sig	Eif6
not_Sig	Uqcc1
not_Sig	Gdf5
not_Sig	Cep250
not_Sig	6430550D23Rik
not_Sig	Ergic3
not_Sig	Spag4
Sig	Cpne1
Sig	Rbm12
not_Sig	Nfs1
Sig	Romo1
not_Sig	Rbm39
not_Sig	Phf20
Sig	Scand1
not_Sig	Cnbd2
not_Sig	2900097C17Rik
not_Sig	Epb4.1l1
not_Sig	Aar2
not_Sig	Dlgap4
not_Sig	4930405A21Rik
not_Sig	Myl9
Sig	Tgif2

not_Sig	5430405H02Rik
not_Sig	1110008F13Rik
not_Sig	Sla2
not_Sig	Ndrg3
not_Sig	Dsn1
Sig	Soga1
not_Sig	Tldc2
not_Sig	Samhd1
not_Sig	Rbl1
not_Sig	Mroh8
Sig	Rpn2
not_Sig	Manbal
not_Sig	Src
not_Sig	Blcap
not_Sig	Nnat
not_Sig	Ctnnbl1
not_Sig	Tti1
not_Sig	Rprd1b
not_Sig	2010009K17Rik
not_Sig	Tgm2
not_Sig	D630003M21Rik
Sig	Lbp
not_Sig	Snhg17
not_Sig	Snhg11
Sig	Ralgapb
not_Sig	Adig
not_Sig	Actr5
Sig	Ppp1r16b
not_Sig	Fam83d
not_Sig	Dhx35
not_Sig	Gm826
not_Sig	Mafb
not_Sig	Top1
Sig	Plcg1
not_Sig	Zhx3
not_Sig	Lpin3
not_Sig	Emilin3
Sig	Chd6
not_Sig	Ptprt
not_Sig	Srsf6
Sig	Ift52
not_Sig	Mybl2
not_Sig	Tox2
not_Sig	Jph2
not_Sig	Oser1
Sig	Gdap1l1
not_Sig	Fitm2
not_Sig	2310001K24Rik

not_Sig	Hnf4a
not_Sig	Ttpal
not_Sig	Serinc3
not_Sig	0610039K10Rik
not_Sig	Pkig
not_Sig	Ada
Sig	Wisp2
not_Sig	Rims4
not_Sig	Ywhab
not_Sig	Pabpc1l
not_Sig	Tomm34
not_Sig	Stk4
Sig	Kcns1
Sig	Slpi
Sig	Matn4
not_Sig	Sdc4
Sig	Sys1
not_Sig	Trp53tg5
not_Sig	Dbndd2
not_Sig	Pigt
not_Sig	Wfdc2
Sig	Dnttip1
Sig	Ube2c
Sig	Tnnc2
not_Sig	Snx21
not_Sig	Acot8
not_Sig	Zswim3
not_Sig	Zswim1
not_Sig	Spata25
not_Sig	Neurl2
not_Sig	Ctsa
not_Sig	Pltp
Sig	Pcif1
Sig	Zfp335
not_Sig	Mmp9
not_Sig	Slc12a5
not_Sig	Ncoa5
Sig	Cd40
Sig	Cdh22
not_Sig	Slc35c2
Sig	Elmo2
not_Sig	Zfp334
not_Sig	Ocstamp
not_Sig	Slc13a3
not_Sig	Trp53rka
not_Sig	Slc2a10
not_Sig	Eya2
not_Sig	Zmynd8

not_Sig	Ncoa3
not_Sig	Sulf2
not_Sig	5031425F14Rik
not_Sig	Prex1
not_Sig	Trp53rkb
not_Sig	Arfgef2
not_Sig	Cse1l
not_Sig	Stau1
not_Sig	Ddx27
not_Sig	Znfx1
not_Sig	1500012F01Rik
not_Sig	Snord12
Sig	Kcnb1
Sig	Ptgis
not_Sig	B4galt5
not_Sig	Slc9a8
Sig	Spata2
not_Sig	Rnf114
not_Sig	Snai1
not_Sig	Ube2v1
not_Sig	Tmem189
not_Sig	Cebpb
not_Sig	A530013C23Rik
Sig	Ptpn1
not_Sig	Fam65c
not_Sig	Pard6b
Sig	Adnp
not_Sig	Dpm1
not_Sig	Mocs3
not_Sig	Nfatc2
not_Sig	Atp9a
not_Sig	Sall4
not_Sig	Zfp64
Sig	Tshz2
not_Sig	AY702102
not_Sig	A630075F10Rik
not_Sig	Zfp217
not_Sig	Bcas1
not_Sig	Bcas1os2
not_Sig	Pfdn4
not_Sig	Gm16796
not_Sig	Dok5
not_Sig	Fam210b
not_Sig	Aurka
not_Sig	Cstf1
not_Sig	Cass4
not_Sig	Rtfdc1
not_Sig	Gcnt7

not_Sig	Bmp7
not_Sig	Spo11
not_Sig	Rae1
not_Sig	Rbm38
Sig	Ctcfl
Sig	Ctcflos
not_Sig	Pck1
not_Sig	Zbp1
Sig	Pmepa1
Sig	Ppp4r1l-ps
Sig	Rab22a
Sig	Vapb
not_Sig	Stx16
not_Sig	Npepl1
not_Sig	Gnas
not_Sig	Nelfcd
not_Sig	Ctsz
Sig	Tubb1
not_Sig	Atp5e
Sig	Slmo2
not_Sig	Zfp831
Sig	Edn3
not_Sig	Gm14393
not_Sig	Gm6710
not_Sig	Gm14440
not_Sig	Gm14436
not_Sig	Gm14405
not_Sig	Gm14305
not_Sig	Gm14295
not_Sig	Gm14420
not_Sig	Gm14403
not_Sig	Gm14322
not_Sig	Gm14325
not_Sig	Gm14327
not_Sig	C330013J21Rik
not_Sig	Gm14326
not_Sig	Etohi1
not_Sig	Zfp931
not_Sig	Phactr3
not_Sig	Sycp2
not_Sig	Ppp1r3d
Sig	Fam217b
not_Sig	Cdh4
not_Sig	Taf4a
not_Sig	4921531C22Rik
not_Sig	Lsm14b
not_Sig	Psma7
not_Sig	Ss18l1

not_Sig	Mtg2
not_Sig	Osbpl2
not_Sig	Adrm1
Sig	Lama5
not_Sig	Rps21
not_Sig	Cables2
not_Sig	B230312C02Rik
not_Sig	Gm6307
not_Sig	Mir1a-1
not_Sig	Mir133a-2
not_Sig	Slco4a1
not_Sig	Mrgbp
not_Sig	Ogfr
Sig	Col9a3
Sig	Dido1
not_Sig	Gid8
not_Sig	Slc17a9
not_Sig	Ythdf1
not_Sig	Nkain4
not_Sig	Arfgap1
Sig	Col20a1
not_Sig	Chrna4
not_Sig	Kcnq2
not_Sig	Eef1a2
not_Sig	Ppdpf
not_Sig	Srms
not_Sig	Helz2
Sig	Gmeb2
not_Sig	Stmn3
not_Sig	Rtel1
not_Sig	Arfrp1
not_Sig	Zgpat
Sig	Lime1
not_Sig	Slc2a4rg-ps
Sig	Zbtb46
not_Sig	Tpd52l2
not_Sig	Dnajc5
not_Sig	Uckl1
not_Sig	Uckl1os
Sig	Znf512b
Sig	Samd10
not_Sig	Prpf6
Sig	Sox18
not_Sig	Tcea2
not_Sig	Rgs19
not_Sig	Pcmtd2
not_Sig	Polr3k
not_Sig	2700069I18Rik

not_Sig	Zfhx4
not_Sig	Pex2
not_Sig	Pkia
not_Sig	Zc2hc1a
not_Sig	Il7
not_Sig	Stmn2
Sig	Hey1
Sig	Mrps28
not_Sig	Tpd52
not_Sig	Zbtb10
not_Sig	C030034L19Rik
Sig	Zfp704
Sig	Pag1
not_Sig	Fabp5
not_Sig	Gm9833
not_Sig	Pmp2
not_Sig	Fabp9
not_Sig	Fabp4
Sig	Impa1
Sig	Zfand1
not_Sig	Chmp4c
not_Sig	Snx16
Sig	Lrrcc1
not_Sig	E2f5
not_Sig	1810022K09Rik
Sig	Car13
not_Sig	Car1
Sig	Car3
not_Sig	Car2
not_Sig	Ythdf3
Sig	Cyp7b1
Sig	Armc1
Sig	Mtfr1
not_Sig	Pde7a
not_Sig	Trim55
not_Sig	Crh
not_Sig	4632415L05Rik
Sig	Cp
not_Sig	Hps3
not_Sig	Hltf
not_Sig	Gyg
not_Sig	Cpa3
not_Sig	Tbl1xr1
not_Sig	Rprl2
not_Sig	Nlgn1
not_Sig	Ect2
Sig	Nceh1
not_Sig	Tnfsf10

not_Sig	Fndc3b
not_Sig	Mir3092
not_Sig	Pld1
Sig	Tnik
not_Sig	Mir466q
not_Sig	Slc2a2
not_Sig	Eif5a2
Sig	Rpl22l1
not_Sig	Egfem1
Sig	Mecom
not_Sig	Mynn
not_Sig	Lrrc34
not_Sig	Sec62
not_Sig	Gpr160
not_Sig	Phc3
not_Sig	Prkci
not_Sig	Skil
not_Sig	Cldn11
not_Sig	Zmat3
not_Sig	4930429B21Rik
Sig	Pik3ca
not_Sig	Zfp639
not_Sig	Mfn1
Sig	Gnb4
Sig	Actl6a
not_Sig	Mrpl47
Sig	Ndufb5
Sig	Usp13
not_Sig	Pex5l
not_Sig	Ttc14
not_Sig	Ccdc39
not_Sig	Fxr1
not_Sig	Dnajc19
not_Sig	Sox2ot
not_Sig	Sox2
not_Sig	Atp11b
not_Sig	Dcun1d1
Sig	Mccc1
not_Sig	Mccc1os
not_Sig	Acad9
not_Sig	D3Ertd254e
not_Sig	Anxa5
not_Sig	1810062G17Rik
not_Sig	Exosc9
Sig	Ccna2
not_Sig	Bbs7
Sig	Trpc3
not_Sig	4932438A13Rik

not_Sig	Cetn4
not_Sig	Bbs12
not_Sig	Fgf2
not_Sig	Nudt6
not_Sig	Spata5
Sig	Spry1
not_Sig	Gm5148
Sig	Ankrd50
Sig	Fat4
not_Sig	Intu
not_Sig	Hspa4l
not_Sig	Plk4
not_Sig	Mfsd8
not_Sig	Gm2011
Sig	3110057O12Rik
not_Sig	1700034I23Rik
not_Sig	Larp1b
not_Sig	Pgrmc2
not_Sig	Jade1
not_Sig	Sclt1
not_Sig	D3Ertd751e
not_Sig	Pcdh10
not_Sig	Pabpc4l
not_Sig	Pcdh18
not_Sig	Slc7a11
Sig	Ccrn4l
Sig	Elf2
not_Sig	4930577N17Rik
Sig	Ndufc1
not_Sig	Naa15
not_Sig	Rab33b
Sig	Setd7
not_Sig	5031434O11Rik
not_Sig	Mgst2
Sig	Maml3
not_Sig	Foxo1
Sig	Cog6
not_Sig	Lhfp
not_Sig	Nhlrc3
not_Sig	Proser1
Sig	Frem2
Sig	Ufm1
not_Sig	Trpc4
Sig	Postn
not_Sig	Supt20
not_Sig	Exosc8
not_Sig	Alg5
not_Sig	Smad9

not_Sig	Rfxap
not_Sig	Spg20
not_Sig	Dclk1
Sig	Nbea
not_Sig	Mab21l1
not_Sig	4933417G07Rik
not_Sig	Tm4sf1
not_Sig	Wwtr1
not_Sig	Commd2
not_Sig	Rnf13
not_Sig	Pfn2
not_Sig	Tsc22d2
not_Sig	Serp1
not_Sig	Eif2a
not_Sig	Selt
not_Sig	Erich6
not_Sig	Siah2
not_Sig	Med12l
not_Sig	Gpr171
not_Sig	P2ry14
not_Sig	F630111L10Rik
not_Sig	Gpr87
not_Sig	P2ry13
not_Sig	P2ry12
not_Sig	Igsf10
not_Sig	Sucnr1
not_Sig	Mbnl1
not_Sig	P2ry1
not_Sig	Rap2b
Sig	Arhgef26
not_Sig	Dhx36
Sig	Mme
Sig	E130311K13Rik
not_Sig	Slc33a1
not_Sig	Gmps
Sig	Vmn2r1
not_Sig	Kcnab1
not_Sig	A330015K06Rik
not_Sig	A730090N16Rik
not_Sig	Ssr3
Sig	4931440P22Rik
not_Sig	Tiparp
Sig	Lekr1
not_Sig	Ccnl1
Sig	Ptx3
not_Sig	Shox2
not_Sig	Rsrc1
not_Sig	Mlf1

not_Sig	Gfm1
not_Sig	Lxn
not_Sig	Rarres1
Sig	Mfsd1
not_Sig	Schip1
not_Sig	Il12a
not_Sig	Ift80
not_Sig	Smc4
not_Sig	Trim59
Sig	Kpna4
not_Sig	Gm1647
not_Sig	Ppm1l
not_Sig	B3galnt1
not_Sig	Nmd3
not_Sig	Slitrk3
not_Sig	Bche
Sig	Pdcd10
not_Sig	Serpini1
not_Sig	Golim4
Sig	Rapgef2
not_Sig	4921511C10Rik
not_Sig	Fnip2
Sig	Ppid
not_Sig	Etfdh
not_Sig	4930579G24Rik
not_Sig	Tmem144
not_Sig	Fam198b
not_Sig	Gria2
not_Sig	Glrb
not_Sig	Pdgfc
not_Sig	Ctso
not_Sig	Tdo2
not_Sig	Gucy1b3
Sig	Gucy1a3
Sig	Map9
not_Sig	Rbm46
not_Sig	Lrat
not_Sig	Fgg
not_Sig	Fga
not_Sig	Fgb
Sig	Plrg1
not_Sig	Sfrp2
not_Sig	Tlr2
not_Sig	D930015E06Rik
not_Sig	Mnd1
not_Sig	Trim2
not_Sig	Fhdc1
not_Sig	Arfip1

not_Sig	Tigd4
not_Sig	Tmem154
Sig	Fbxw7
not_Sig	Dear1
Sig	Gatb
not_Sig	Fam160a1
not_Sig	Glt28d2
not_Sig	Prss48
not_Sig	Sh3d19
Sig	Rps3a1
not_Sig	Rnu73b
not_Sig	Lrba
not_Sig	Mab21l2
not_Sig	Dclk2
not_Sig	Cd1d1
not_Sig	Kirrel
Sig	Fcrls
not_Sig	Cd5l
not_Sig	Fcrl1
not_Sig	Fcrl5
not_Sig	Etv3
not_Sig	Arhgef11
Sig	Lrrc71
Sig	Pear1
not_Sig	Ntrk1
Sig	Insrr
not_Sig	Sh2d2a
not_Sig	Prcc
not_Sig	Hdgf
Sig	Mrpl24
not_Sig	Rrnad1
Sig	Isg20l2
not_Sig	Crabp2
Sig	Nes
not_Sig	Bcan
Sig	Gpatch4
not_Sig	Apoa1bp
not_Sig	Iqgap3
not_Sig	Mef2d
Sig	1700113A16Rik
Sig	Rhbg
not_Sig	Tsacc
Sig	Cct3
not_Sig	Glmp
not_Sig	Tmem79
Sig	Smg5
not_Sig	Paqr6
not_Sig	Bglap2

not_Sig	Bglap
not_Sig	Pmf1
not_Sig	Slc25a44
not_Sig	Sema4a
not_Sig	Lmna
not_Sig	Mex3a
not_Sig	Rab25
not_Sig	Lamtor2
not_Sig	Ubqln4
not_Sig	Ssr2
not_Sig	Arhgef2
Sig	2810403A07Rik
not_Sig	Rit1
not_Sig	Syt11
not_Sig	5830417I10Rik
not_Sig	1500004A13Rik
not_Sig	Gon4l
not_Sig	Msto1
not_Sig	Dap3
not_Sig	Ash1l
not_Sig	Rusc1
Sig	Fdps
Sig	Pklr
not_Sig	Hcn3
not_Sig	Clk2
not_Sig	Scamp3
not_Sig	Fam189b
not_Sig	Gba
not_Sig	Mtx1
not_Sig	Thbs3
not_Sig	Muc1
not_Sig	Trim46
not_Sig	Krtcap2
not_Sig	Dpm3
Sig	Slc50a1
not_Sig	Efna1
not_Sig	Efna4
not_Sig	Adam15
not_Sig	Dcst1
not_Sig	Zbtb7b
Sig	Gm15417
not_Sig	Lenep
not_Sig	Flad1
not_Sig	Cks1b
not_Sig	Shc1
not_Sig	Pygo2
not_Sig	Pbxip1
not_Sig	Pmvk

Sig	Kcnn3
Sig	Adar
not_Sig	Chrnb2
not_Sig	4632404H12Rik
not_Sig	Ube2q1
Sig	She
not_Sig	Il6ra
Sig	Atp8b2
not_Sig	Hax1
not_Sig	Gm19710
not_Sig	Ubap2l
not_Sig	Mir7669
not_Sig	4933434E20Rik
not_Sig	1700094D03Rik
not_Sig	Tpm3
not_Sig	Nup210l
not_Sig	Rps27
not_Sig	Rab13
not_Sig	Jtb
not_Sig	Creb3l4
not_Sig	Slc39a1
not_Sig	Crtc2
not_Sig	Dennd4b
not_Sig	Gatad2b
not_Sig	Slc27a3
Sig	Ints3
Sig	Npr1
not_Sig	Ilf2
not_Sig	Snapin
not_Sig	Chtop
Sig	S100a1
not_Sig	S100a13
not_Sig	S100a16
not_Sig	S100a4
not_Sig	S100a6
not_Sig	S100a8
not_Sig	S100a9
not_Sig	Lor
not_Sig	Tchh
not_Sig	S100a11
Sig	S100a10
not_Sig	Them4
not_Sig	C2cd4d
Sig	Rorc
not_Sig	Lingo4
not_Sig	Tdrkh
not_Sig	Oaz3
Sig	Mrpl9

Sig	Riiad1
Sig	Celf3
not_Sig	Snx27
not_Sig	Tuft1
not_Sig	Selenbp2
not_Sig	Cgn
not_Sig	Pogz
Sig	Psmb4
not_Sig	Selenbp1
Sig	Rfx5
not_Sig	Pi4kb
Sig	Zfp687
not_Sig	Psmd4
not_Sig	Pip5k1a
not_Sig	Vps72
not_Sig	Tmod4
not_Sig	Scnm1
not_Sig	Lysmd1
not_Sig	Tnfaip8l2
not_Sig	Sema6c
not_Sig	Gabpb2
not_Sig	Gm16740
not_Sig	Mllt11
not_Sig	Cdc42se1
not_Sig	Gm128
not_Sig	Prune
not_Sig	Fam63a
not_Sig	Anxa9
not_Sig	Cers2
not_Sig	Setdb1
not_Sig	Arnt
Sig	Ctsk
not_Sig	Ctss
Sig	Golph3l
not_Sig	Ensa
not_Sig	Mcl1
Sig	Adamtsl4
Sig	Ecm1
Sig	Tars2
Sig	Rprd2
not_Sig	Prpf3
Sig	Mrps21
not_Sig	C920021L13Rik
not_Sig	Ciart
not_Sig	BC028528
Sig	Aph1a
Sig	Car14
not_Sig	Anp32e

not_Sig	Gm9054
not_Sig	Plekho1
not_Sig	Vps45
not_Sig	Mir1946b
not_Sig	Otud7b
not_Sig	Mtmr11
Sig	Sf3b4
not_Sig	Sv2a
Sig	Bola1
not_Sig	Hist2h2ab
not_Sig	Hist2h2ac
not_Sig	Hist2h2be
not_Sig	Hist2h4
not_Sig	Hist2h3b
not_Sig	Hist2h2bb
not_Sig	Fcgr1
not_Sig	Terc
not_Sig	Hfe2
not_Sig	Gm15441
not_Sig	Txnip
Sig	Polr3gl
not_Sig	Ankrd34a
not_Sig	Lix1l
not_Sig	6330549D23Rik
not_Sig	Rbm8a
not_Sig	Pex11b
not_Sig	Itga10
not_Sig	Ankrd35
not_Sig	Pias3
Sig	Nudt17
not_Sig	Polr3c
Sig	Rnf115
not_Sig	Cd160
not_Sig	Pdzk1
not_Sig	Gpr89
not_Sig	Gja5
not_Sig	Acp6
not_Sig	Bcl9
not_Sig	Chd1l
Sig	Fmo5
not_Sig	Prkab2
not_Sig	Pde4dip
Sig	Sec22b
not_Sig	Notch2
Sig	Hmgcs2
not_Sig	Phgdh
not_Sig	Zfp697
Sig	Wars2

not_Sig	Tbx15
not_Sig	Spag17
not_Sig	Wdr3
not_Sig	Gdap2
not_Sig	Fam46c
not_Sig	Man1a2
Sig	Vtcn1
not_Sig	Trim45
not_Sig	Ttf2
not_Sig	Cd101
Sig	Ptgfrn
not_Sig	Cd2
not_Sig	Igsf3
not_Sig	Atp1a1
Sig	Slc22a15
not_Sig	Casq2
Sig	Vangl1
not_Sig	Ngf
Sig	Tspan2
not_Sig	Sycp1
Sig	Sike1
not_Sig	Csde1
not_Sig	Nras
not_Sig	Ampd1
not_Sig	Dennd2c
not_Sig	Bcas2
not_Sig	Trim33
not_Sig	Atg4a
Sig	Olfml3
not_Sig	Hipk1
not_Sig	Dclre1b
not_Sig	Ap4b1
not_Sig	Bcl2l15
not_Sig	Ptpn22
Sig	Rsbn1
not_Sig	Phtf1os
not_Sig	Phtf1
not_Sig	Magi3
not_Sig	Lrig2
not_Sig	Slc16a1
not_Sig	Fam19a3
not_Sig	Ppm1j
not_Sig	Rhoc
not_Sig	Mov10
not_Sig	Capza1
not_Sig	St7l
not_Sig	Wnt2b
Sig	Cttnbp2nl

not_Sig	Kcnd3
not_Sig	Ddx20
Sig	Fam212b
not_Sig	Rap1a
not_Sig	I830077J02Rik
not_Sig	Atp5f1
Sig	Wdr77
not_Sig	Ovgp1
Sig	Chia1
not_Sig	Chil3
not_Sig	2010016I18Rik
not_Sig	Dennd2d
not_Sig	Cept1
not_Sig	Dram2
not_Sig	Lrif1
not_Sig	Cd53
not_Sig	Kcna3
not_Sig	AI504432
Sig	Kcna2
not_Sig	Kcna10
not_Sig	Lamtor5
not_Sig	Slc16a4
Sig	Rbm15
Sig	Kcnc4
not_Sig	Slc6a17
Sig	Strip1
not_Sig	Ahcyl1
not_Sig	Csf1
not_Sig	4933431E20Rik
not_Sig	Gstm5
not_Sig	Gstm7
not_Sig	Gstm6
not_Sig	Gstm2
not_Sig	Gstm1
not_Sig	Gstm4
not_Sig	Ampd2
not_Sig	Gnat2
not_Sig	Gnai3
not_Sig	Gpr61
not_Sig	Amigo1
not_Sig	Cyb561d1
Sig	Atxn7l2
Sig	Sypl2
not_Sig	Psma5
not_Sig	Sort1
not_Sig	Psrc1
not_Sig	Celsr2
not_Sig	Sars

not_Sig	5330417C22Rik
Sig	Scarna2
not_Sig	Tmem167b
not_Sig	Taf13
not_Sig	Wdr47
not_Sig	Clcc1
not_Sig	Gpsm2
not_Sig	Stxbp3
not_Sig	Fndc7
Sig	Prpf38b
not_Sig	Fam102b
not_Sig	Slc25a24
Sig	Vav3
not_Sig	Ntng1
not_Sig	Prmt6
Sig	Amy1
not_Sig	Rnpc3
not_Sig	Col11a1
Sig	S1pr1
not_Sig	A930005H10Rik
not_Sig	Dph5
not_Sig	Slc30a7
not_Sig	Extl2
not_Sig	Vcam1
not_Sig	Gpr88
Sig	Cdc14a
not_Sig	Rtca
Sig	Dbt
not_Sig	Lrrc39
not_Sig	Trmt13
not_Sig	Sass6
not_Sig	Hiat1
not_Sig	Slc35a3
not_Sig	Agl
Sig	Frrs1
not_Sig	Palmd
not_Sig	4930455H04Rik
not_Sig	Lppr4
not_Sig	Snx7
not_Sig	Dpyd
not_Sig	Ptbp2
not_Sig	Rwdd3
not_Sig	Tmem56
not_Sig	Alg14
Sig	Cnn3
not_Sig	Slc44a3
not_Sig	A730020M07Rik
Sig	F3

not_Sig	Abcd3
not_Sig	Arhgap29
Sig	Abca4
Sig	Gclm
not_Sig	Dnttip2
not_Sig	Bcar3
not_Sig	Fnbp1l
not_Sig	Pde5a
not_Sig	4930447N08Rik
not_Sig	4933405D12Rik
Sig	1810037I17Rik
not_Sig	Usp53
not_Sig	Myoz2
not_Sig	Synpo2
not_Sig	Sec24d
not_Sig	Mettl14
not_Sig	Prss12
not_Sig	Snhg8
not_Sig	Ndst3
not_Sig	Ugt8a
not_Sig	Arsj
not_Sig	Camk2d
not_Sig	Ank2
Sig	LOC100862268
not_Sig	Larp7
not_Sig	Zgrf1
not_Sig	Alpk1
not_Sig	Tifa
Sig	Ap1ar
not_Sig	5730508B09Rik
not_Sig	Pitx2
Sig	Enpep
Sig	Elovl6
not_Sig	Egf
not_Sig	6330410L21Rik
not_Sig	Gar1
not_Sig	Cfi
not_Sig	Pla2g12a
not_Sig	Casp6
not_Sig	Ccdc109b
not_Sig	Sec24b
not_Sig	Col25a1
not_Sig	Etnppl
Sig	Ostc
Sig	Rpl34
not_Sig	Lef1
not_Sig	Hadh
not_Sig	Cyp2u1

not_Sig	Sgms2
not_Sig	Papss1
not_Sig	Dkk2
not_Sig	Aimp1
not_Sig	Tbck
not_Sig	Gm29811
not_Sig	Npnt
not_Sig	Gstcd
not_Sig	Ints12
not_Sig	Ppa2
not_Sig	Tet2
not_Sig	Cxxc4
not_Sig	Tacr3
not_Sig	Cenpe
not_Sig	Bdh2
not_Sig	Slc9b2
not_Sig	Slc9b1
not_Sig	Cisd2
not_Sig	4930539J05Rik
not_Sig	Ube2d3
not_Sig	Manba
Sig	Nfkb1
not_Sig	Slc39a8
not_Sig	Bank1
not_Sig	Ppp3ca
Sig	Emcn
not_Sig	Gm4861
Sig	Ddit4l
Sig	H2afz
not_Sig	Dnajb14
not_Sig	Lamtor3
Sig	Dapp1
not_Sig	Gm5105
not_Sig	Mttp
not_Sig	Trmt10a
Sig	Adh7
not_Sig	Adh1
not_Sig	Adh5
Sig	Metap1
not_Sig	Eif4e
not_Sig	Tspan5
not_Sig	Rap1gds1
not_Sig	Unc5c
not_Sig	Bmpr1b
not_Sig	Pdlim5
not_Sig	Gbp5
not_Sig	Gbp7
not_Sig	Gbp3

not_Sig	Gbp2b
not_Sig	Gbp2
not_Sig	Ccbl2
not_Sig	Gtf2b
Sig	Pkn2
not_Sig	Lmo4
Sig	Hs2st1
Sig	Sep15
not_Sig	Sh3glb1
not_Sig	Clca3a1
not_Sig	Clca2
not_Sig	Odf2l
Sig	Col24a1
not_Sig	Znhit6
not_Sig	Cyr61
Sig	Ddah1
not_Sig	Bcl10
not_Sig	2410004B18Rik
not_Sig	Syde2
not_Sig	Mcoln2
Sig	Lpar3
not_Sig	Ssx2ip
not_Sig	Ctbs
not_Sig	Spata1
Sig	Gng5
not_Sig	Rpf1
Sig	Dnase2b
not_Sig	Uox
not_Sig	Gm16325
Sig	Prkacb
not_Sig	Ttll7
Sig	Adgrl2
Sig	Adgrl4
not_Sig	Ifi44
Sig	Ptgfr
not_Sig	Gipc2
not_Sig	Dnajb4
not_Sig	Fubp1
not_Sig	Nexn
not_Sig	Fam73a
not_Sig	Usp33
not_Sig	Zzz3
not_Sig	Ak5
not_Sig	Pigk
not_Sig	St6galnac5
Sig	St6galnac3
not_Sig	1700012D16Rik
not_Sig	Msh4

not_Sig	Rabggtb
not_Sig	Snord45b
not_Sig	Acadm
not_Sig	Slc44a5
not_Sig	Lhx8
not_Sig	AI606473
not_Sig	Tyw3
Sig	Cryz
not_Sig	Fpgt
not_Sig	Lrriq3
not_Sig	4930570G19Rik
not_Sig	Negr1
not_Sig	Zranb2
not_Sig	Mir186
not_Sig	Ptger3
not_Sig	Cth
not_Sig	Ankrd13c
not_Sig	Srsf11
not_Sig	Lrrc40
not_Sig	Lrrc7
not_Sig	Depdc1a
Sig	Wls
not_Sig	Tmem68
not_Sig	Tgs1
not_Sig	Lyn
not_Sig	6330407A03Rik
Sig	Rps20
not_Sig	Plag1
not_Sig	Chchd7
not_Sig	Penk
not_Sig	Impad1
not_Sig	Fam110b
not_Sig	Ubxn2b
Sig	Sdcbp
not_Sig	Nsmaf
not_Sig	Tox
not_Sig	Car8
not_Sig	Rab2a
Sig	Chd7
not_Sig	Clvs1
not_Sig	Asph
not_Sig	4930412C18Rik
not_Sig	Gdf6
not_Sig	2610301B20Rik
not_Sig	Plekhf2
not_Sig	Ndufaf6
Sig	Trp53inp1
Sig	Ccne2

Sig	Ints8
not_Sig	Dpy19l4
not_Sig	1110037F02Rik
not_Sig	Rad54b
not_Sig	Fsbp
Sig	Gem
not_Sig	Pdp1
not_Sig	1700123M08Rik
not_Sig	Tmem67
not_Sig	Rbm12b2
not_Sig	Rbm12b1
not_Sig	Fam92a
not_Sig	Triqk
not_Sig	Runx1t1
not_Sig	Lrrc69
not_Sig	Otud6b
not_Sig	Tmem55a
not_Sig	Gm11837
not_Sig	Necab1
Sig	Tmem64
not_Sig	Calb1
Sig	Decr1
not_Sig	Mir6400
Sig	Nbn
not_Sig	Osgin2
not_Sig	Ripk2
not_Sig	A530072M11Rik
not_Sig	Mmp16
Sig	Cpne3
not_Sig	Rmdn1
not_Sig	Wwp1
not_Sig	Slc7a13
Sig	Atp6v0d2
Sig	Ttpa
not_Sig	Ggh
not_Sig	Ccnc
not_Sig	Tstd3
not_Sig	Usp45
not_Sig	4930528A17Rik
not_Sig	Pnisr
Sig	Coq3
not_Sig	Faxc
not_Sig	Fbxl4
not_Sig	Pou3f2
not_Sig	Mms22l
not_Sig	Klhl32
not_Sig	Ndufaf4
Sig	Gpr63

Sig	Fhl5
not_Sig	Ufl1
not_Sig	Fut9
not_Sig	Manea
not_Sig	Epha7
not_Sig	4930556G01Rik
not_Sig	Map3k7
Sig	Bach2
Sig	Bach2os
not_Sig	Casp8ap2
not_Sig	Mdn1
Sig	Lyrm2
not_Sig	Ankrd6
not_Sig	Rragd
Sig	4933421O10Rik
not_Sig	Ube2j1
Sig	Gabrr2
not_Sig	Gabrr1
Sig	Pm20d2
not_Sig	Pnrc1
not_Sig	Rngtt
not_Sig	Mir8118
not_Sig	Cnr1
not_Sig	Spaca1
not_Sig	Akirin2
not_Sig	Orc3
not_Sig	Rars2
not_Sig	Slc35a1
not_Sig	Smim8
not_Sig	Zfp292
not_Sig	Mob3b
not_Sig	Ifnk
Sig	3110043O21Rik
Sig	Aco1
not_Sig	Ddx58
not_Sig	Topors
not_Sig	Toporsos
not_Sig	Ndufb6
not_Sig	Aptx
not_Sig	Gm6297
Sig	Dnaja1
Sig	Smu1
Sig	B4galt1
not_Sig	Mir5123
not_Sig	Bag1
Sig	Chmp5
not_Sig	Nfx1
not_Sig	Aqp7

not_Sig	Aqp3
not_Sig	Nol6
not_Sig	Ube2r2
not_Sig	Ubap2
not_Sig	Dcaf12
not_Sig	Ubap1
not_Sig	Kif24
not_Sig	Nudt2
not_Sig	AI464131
not_Sig	1110017D15Rik
not_Sig	Fam219aos
Sig	Fam219a
not_Sig	Dnaic1
not_Sig	Enho
not_Sig	Cntfr
not_Sig	Rpp25l
not_Sig	Dctn3
not_Sig	Sigmar1
not_Sig	Galt
Sig	Il11ra1
not_Sig	Ccl27a
not_Sig	Gm3893
not_Sig	4933409K07Rik
not_Sig	Ccl19
not_Sig	Fam205c
not_Sig	Phf24
not_Sig	Dnajb5
not_Sig	1700022I11Rik
Sig	Vcp
not_Sig	Fancg
not_Sig	Pigo
not_Sig	Stoml2
not_Sig	Fam214b
not_Sig	Unc13b
not_Sig	Atp8b5
not_Sig	Rusc2
not_Sig	Fam166b
Sig	Tesk1
not_Sig	Cd72
not_Sig	Sit1
Sig	Rmrp
not_Sig	Ccdc107
not_Sig	Arhgef39
not_Sig	Car9
not_Sig	Tpm2
not_Sig	Tln1
not_Sig	Creb3
not_Sig	Gba2

Sig	Rgp1
not_Sig	Npr2
not_Sig	Spag8
not_Sig	Hint2
not_Sig	Fam221b
not_Sig	Tmem8b
not_Sig	Hrct1
Sig	5430416O09Rik
not_Sig	A630077J23Rik
not_Sig	Reck
not_Sig	Glipr2
not_Sig	Clta
not_Sig	Gne
not_Sig	Gm12504
not_Sig	Rnf38
not_Sig	Mir5106
not_Sig	Melk
not_Sig	Pax5
not_Sig	5730488B01Rik
not_Sig	Zcchc7
not_Sig	Grhpr
not_Sig	Zbtb5
not_Sig	Polr1e
not_Sig	Fbxo10
Sig	Tomm5
not_Sig	Frmpd1
not_Sig	Trmt10b
not_Sig	Exosc3
not_Sig	Dcaf10
not_Sig	Slc25a51
Sig	Shb
Sig	Aldh1b1
not_Sig	Stra6l
not_Sig	Tdrd7
not_Sig	Tmod1
not_Sig	Tstd2
not_Sig	Ncbp1
Sig	Xpa
not_Sig	5830415F09Rik
not_Sig	Hemgn
not_Sig	Anp32b
not_Sig	Nans
not_Sig	Trim14
not_Sig	Coro2a
not_Sig	Tbc1d2
not_Sig	Gabbr2
not_Sig	Anks6
not_Sig	Galnt12

not_Sig	Col15a1
not_Sig	Tgfbr1
not_Sig	Alg2
Sig	Sec61b
not_Sig	Nr4a3
not_Sig	Stx17
not_Sig	Erp44
not_Sig	Invs
not_Sig	Tex10
not_Sig	Msantd3
not_Sig	Tmeff1
not_Sig	Murc
not_Sig	Baat
Sig	Mrpl50
not_Sig	Zfp189
not_Sig	Aldob
Sig	Tmem246
not_Sig	Rnf20
not_Sig	Grin3a
not_Sig	Smc2
not_Sig	Nipsnap3b
not_Sig	Abca1
not_Sig	AI427809
Sig	Slc44a1
Sig	Fsd1l
not_Sig	Fktn
not_Sig	Tal2
Sig	Tmem38b
not_Sig	Zfp462
not_Sig	Rad23b
not_Sig	Klf4
not_Sig	Actl7a
not_Sig	Ikbkap
not_Sig	Fam206a
Sig	Ctnnal1
not_Sig	Tmem245
not_Sig	Epb4.1l4b
Sig	Ptpn3
not_Sig	Palm2
not_Sig	Akap2
not_Sig	Txn1
Sig	Svep1
Sig	Musk
not_Sig	Lpar1
not_Sig	AI314180
not_Sig	Zkscan16
not_Sig	Ptgr1
not_Sig	Dnajc25

Sig	Gng10
not_Sig	Ugcg
not_Sig	Susd1
not_Sig	Ptbp3
not_Sig	Hsdl2
not_Sig	E130308A19Rik
not_Sig	Inip
not_Sig	Snx30
Sig	Gm2083
Sig	Mup3
not_Sig	Zfp37
Sig	Slc31a2
not_Sig	Fkbp15
not_Sig	Slc31a1
Sig	Cdc26
not_Sig	Prpf4
not_Sig	Rnf183
not_Sig	Wdr31
not_Sig	Bspry
not_Sig	Hdhd3
not_Sig	Alad
not_Sig	Pole3
not_Sig	4933430I17Rik
Sig	Rgs3
not_Sig	Zfp618
not_Sig	Ambp
not_Sig	Col27a1
Sig	Orm1
Sig	Orm3
not_Sig	Orm2
not_Sig	Akna
not_Sig	Whrn
Sig	Atp6v1g1
not_Sig	6330416G13Rik
not_Sig	Gm11213
not_Sig	1700018C11Rik
not_Sig	Tnfsf15
not_Sig	Tnfsf8
Sig	Tnc
Sig	Pappa
not_Sig	Astn2
not_Sig	Trim32
Sig	Tlr4
not_Sig	Brinp1
not_Sig	Cdk5rap2
not_Sig	Megf9
not_Sig	Tle1
Sig	C630043F03Rik

Sig	Aldoart1
not_Sig	2310002L09Rik
not_Sig	Frmd3
not_Sig	Kdm4c
Sig	Tmem261
not_Sig	Ptprd
not_Sig	Lurap1l
Sig	Mpdz
not_Sig	Nfib
not_Sig	Zdhhc21
Sig	Frem1
not_Sig	Ttc39b
not_Sig	Snapc3
not_Sig	Psip1
not_Sig	Ccdc171
Sig	Bnc2
not_Sig	Cntln
not_Sig	Sh3gl2
not_Sig	Adamtsl1
Sig	Rraga
not_Sig	Haus6
not_Sig	Scarna8
Sig	Plin2
not_Sig	Dennd4c
not_Sig	Rps6
not_Sig	Acer2
not_Sig	Slc24a2
not_Sig	Mllt3
Sig	Focad
not_Sig	Hacd4
Sig	Klhl9
not_Sig	Mtap
not_Sig	Cdkn2b
not_Sig	Tusc1
not_Sig	Caap1
not_Sig	Plaa
not_Sig	Gm12657
not_Sig	Ift74
not_Sig	Lrrc19
not_Sig	Tek
not_Sig	Eqtn
not_Sig	Mysm1
not_Sig	Jun
not_Sig	Fggy
not_Sig	9530080O11Rik
not_Sig	Hook1
not_Sig	Cyp2j6
not_Sig	Cyp2j9

not_Sig	Cyp2j5
Sig	Nfia
not_Sig	Tm2d1
not_Sig	Inadl
not_Sig	Kank4
not_Sig	Kank4os
Sig	Usp1
not_Sig	Dock7
not_Sig	Angptl3
not_Sig	Atg4c
Sig	Foxd3
not_Sig	Alg6
not_Sig	Itgb3bp
not_Sig	Efcab7
not_Sig	Pgm2
not_Sig	Ror1
not_Sig	Cachd1
not_Sig	Raver2
not_Sig	Jak1
not_Sig	E130102H24Rik
not_Sig	0610043K17Rik
Sig	Ak4
not_Sig	Dnajc6
not_Sig	Leprot
not_Sig	Lepr
not_Sig	Pde4b
Sig	Sgip1
not_Sig	Wdr78
not_Sig	Mier1
not_Sig	Slc35d1
not_Sig	Oma1
not_Sig	1700024P16Rik
not_Sig	Prkaa2
not_Sig	Ppap2b
Sig	Usp24
not_Sig	Pcsk9
not_Sig	Dhcr24
not_Sig	Pars2
not_Sig	Ttc4
not_Sig	Mroh7
not_Sig	Acot11
not_Sig	Ssbp3
not_Sig	Mrpl37
not_Sig	Cyb5rl
not_Sig	Tceanc2
not_Sig	Tmem59
not_Sig	Lrrc42
not_Sig	Hspb11

not_Sig	Yipf1
not_Sig	Ndc1
not_Sig	Glis1
not_Sig	Lrp8
not_Sig	Magoh
Sig	0610037L13Rik
Sig	Cpt2
not_Sig	Slc1a7
not_Sig	Podn
not_Sig	Scp2
Sig	Echdc2
Sig	Zyg11a
not_Sig	Zyg11b
not_Sig	Coa7
Sig	Gpx7
not_Sig	Zcchc11
not_Sig	Prpf38a
not_Sig	Orc1
not_Sig	Cc2d1b
not_Sig	Zfyve9
not_Sig	3110021N24Rik
not_Sig	Btf3l4
not_Sig	Txndc12
not_Sig	Kti12
not_Sig	Nrd1
not_Sig	Mir761
not_Sig	Osbpl9
not_Sig	Calr4
Sig	Eps15
not_Sig	Ttc39aos1
not_Sig	Ttc39a
not_Sig	Rnf11
not_Sig	Cdkn2c
Sig	Faf1
not_Sig	Dmrta2
not_Sig	Bend5
not_Sig	Spata6
not_Sig	Trabd2b
not_Sig	Foxd2
not_Sig	Foxd2os
not_Sig	Cmpk1
not_Sig	Stil
not_Sig	Tal1
not_Sig	Pdzk1ip1
not_Sig	Cyp4a32
not_Sig	Cyp4b1
not_Sig	Efcab14
not_Sig	Tex38

Sig	Atpaf1
not_Sig	Mob3c
Sig	Mknk1
Sig	Faah
not_Sig	Nsun4
not_Sig	Uqcrh
not_Sig	Lrrc41
not_Sig	Rad54l
not_Sig	2510003B16Rik
not_Sig	Lurap1
not_Sig	Pomgnt1
not_Sig	Tspan1
not_Sig	Pik3r3
not_Sig	Mast2
not_Sig	Ipp
not_Sig	Tmem69
not_Sig	Gpbp1l1
not_Sig	C530005A16Rik
not_Sig	Ccdc17
not_Sig	Nasp
not_Sig	Akr1a1
not_Sig	AV051173
Sig	Prdx1
not_Sig	Mmachc
Sig	Ccdc163
not_Sig	Tesk2
not_Sig	Toe1
not_Sig	Mutyh
not_Sig	Hpdl
not_Sig	Zswim5
Sig	Urod
not_Sig	Hectd3
not_Sig	Eif2b3
not_Sig	Ptch2
not_Sig	Btbd19
not_Sig	Plk3
Sig	Rps8
Sig	Snord55
not_Sig	Kif2c
not_Sig	Gm1661
not_Sig	Tmem53
Sig	Rnf220
Sig	Eri3
not_Sig	Dmap1
not_Sig	Slc6a9
not_Sig	Ccdc24
Sig	B4galt2
Sig	Atp6v0b

not_Sig	Dph2
Sig	Ipo13
not_Sig	Artn
not_Sig	St3gal3
Sig	Kdm4a
not_Sig	Ptprf
not_Sig	Hyi
Sig	Szt2
not_Sig	Med8
not_Sig	Elovl1
not_Sig	Cdc20
not_Sig	Mpl
Sig	Tie1
not_Sig	Ebna1bp2
not_Sig	Slc2a1
Sig	Zfp691
not_Sig	Ermap
not_Sig	Ccdc23
not_Sig	4930538K18Rik
not_Sig	AU022252
not_Sig	P3h1
not_Sig	Cldn19
Sig	Ybx1
not_Sig	Ppih
not_Sig	Ccdc30
not_Sig	Ppcs
not_Sig	Zmynd12
not_Sig	Rimkla
not_Sig	AA415398
not_Sig	Foxj3
not_Sig	Hivep3
Sig	Foxo6
not_Sig	Scmh1
not_Sig	Slfnl1
not_Sig	Ctps
not_Sig	Gm8439
not_Sig	Cited4
not_Sig	Kcnq4
Sig	Nfyc
not_Sig	Mir30c-1
not_Sig	Rims3
not_Sig	Exo5
Sig	Zfp69
not_Sig	Smap2
not_Sig	Col9a2
Sig	Zmpste24
not_Sig	Rlf
not_Sig	Ppt1

not_Sig	Cap1
not_Sig	Mfsd2a
not_Sig	Mycl
not_Sig	Trit1
not_Sig	Bmp8b
not_Sig	Ppie
not_Sig	Hpcal4
not_Sig	Nt5c1a
Sig	Heyl
Sig	Pabpc4
not_Sig	Bmp8a
not_Sig	Oxct2a
not_Sig	Macf1
Sig	D830031N03Rik
Sig	Akirin1
not_Sig	Mycbp
not_Sig	Rragc
Sig	Pou3f1
not_Sig	Utp11l
not_Sig	Fhl3
not_Sig	Sf3a3
Sig	Inpp5b
not_Sig	Mtf1
not_Sig	1110065P20Rik
not_Sig	Yrdc
not_Sig	Maneal
Sig	Cdca8
not_Sig	9930104L06Rik
not_Sig	Rspo1
not_Sig	Gnl2
not_Sig	Dnali1
not_Sig	Snip1
not_Sig	Meaf6
not_Sig	Zc3h12a
not_Sig	Grik3
not_Sig	Csf3r
not_Sig	Mrps15
not_Sig	Oscp1
not_Sig	Lsm10
not_Sig	Stk40
not_Sig	Eva1b
not_Sig	Sh3d21
not_Sig	Thrap3
not_Sig	Map7d1
not_Sig	Trappc3
not_Sig	Col8a2
not_Sig	Adprhl2
not_Sig	Tekt2

not_Sig	Ago3
not_Sig	Ago1
not_Sig	Ago4
Sig	Clspn
not_Sig	5730409E04Rik
Sig	Psmb2
not_Sig	Tfap2e
not_Sig	Ncdn
not_Sig	AU040320
not_Sig	Zmym4
Sig	Sfpq
not_Sig	Zmym1
not_Sig	Zmym6
not_Sig	Gm12942
not_Sig	Dlgap3
Sig	Smim12
not_Sig	Gja4
not_Sig	Gjb5
Sig	Csmd2
not_Sig	Zscan20
not_Sig	Tlr12
Sig	Phc2
not_Sig	A3galt2
Sig	Zfp362
not_Sig	Trim62
Sig	Azin2
not_Sig	Ak2
Sig	Rnf19b
not_Sig	Fndc5
Sig	S100pbp
not_Sig	Yars
Sig	C77080
not_Sig	Sync
Sig	Rbbp4
not_Sig	Zbtb8os
not_Sig	Zbtb8a
not_Sig	Bsdc1
not_Sig	Tssk3
not_Sig	Marcksl1
not_Sig	Hdac1
not_Sig	Lck
not_Sig	Fam167b
Sig	Eif3i
not_Sig	Tmem234
not_Sig	Dcdc2b
not_Sig	Iqcc
not_Sig	Ccdc28b
not_Sig	Txlna

not_Sig	Kpna6
not_Sig	Tmem39b
not_Sig	Khdrbs1
not_Sig	Ptp4a2
not_Sig	Adgrb2
Sig	Col16a1
Sig	Pef1
Sig	Tinagl1
Sig	Serinc2
Sig	Fabp3
not_Sig	Zcchc17
not_Sig	Snrnp40
Sig	Nkain1
not_Sig	Pum1
not_Sig	Snord85
Sig	Sdc3
not_Sig	Laptm5
not_Sig	Ptpru
not_Sig	Mecr
not_Sig	Srsf4
not_Sig	Gm12992
Sig	Tmem200b
not_Sig	Epb4.1
not_Sig	Oprd1
not_Sig	Ythdf2
not_Sig	Gmeb1
not_Sig	Rnu11
not_Sig	Taf12
not_Sig	Rab42
not_Sig	Snhg12
not_Sig	Snora16a
Sig	Snora44
Sig	Snora61
not_Sig	Snord99
not_Sig	Trnau1ap
not_Sig	Rcc1
Sig	Snhg3
not_Sig	Phactr4
not_Sig	Med18
not_Sig	Sesn2
not_Sig	Atpif1
not_Sig	Dnajc8
not_Sig	Ptafr
not_Sig	Eya3
not_Sig	Xkr8
Sig	Smpdl3b
not_Sig	Rpa2
not_Sig	Themis2

not_Sig	Ppp1r8
not_Sig	Stx12
Sig	Fam76a
not_Sig	Fgr
Sig	Ahdc1
Sig	Wasf2
Sig	Map3k6
not_Sig	Sytl1
not_Sig	Tmem222
not_Sig	Wdtc1
not_Sig	Slc9a1
not_Sig	Fam46b
not_Sig	Trnp1
not_Sig	Kdf1
not_Sig	Nudc
not_Sig	Gpatch3
not_Sig	Gpn2
not_Sig	Sfn
Sig	Zdhhc18
not_Sig	Pigv
Sig	Arid1a
not_Sig	Mir7227
not_Sig	Rps6ka1
not_Sig	Hmgn2
not_Sig	Dhdds
not_Sig	Lin28a
not_Sig	Aim1l
not_Sig	Cd52
not_Sig	Ubxn11
not_Sig	Sh3bgrl3
not_Sig	Cep85
not_Sig	Catsper4
Sig	Cnksr1
not_Sig	Zfp593
not_Sig	Grrp1
not_Sig	Pdik1l
not_Sig	Trim63
not_Sig	Slc30a2
not_Sig	Extl1
Sig	Pafah2
not_Sig	Stmn1
not_Sig	Paqr7
not_Sig	Mtfr1l
not_Sig	Sepn1
not_Sig	Man1c1
not_Sig	Ldlrap1
not_Sig	Tmem57
Sig	Rhd

not_Sig	Tmem50a
not_Sig	Rsrp1
not_Sig	Syf2
not_Sig	Runx3
not_Sig	Clic4
Sig	Srrm1
not_Sig	Ncmap
not_Sig	Rcan3
Sig	Nipal3
not_Sig	Ifnlr1
not_Sig	Myom3
not_Sig	Srsf10
not_Sig	Pnrc2
not_Sig	Cnr2
not_Sig	Fuca1
not_Sig	Hmgcl
not_Sig	Gale
not_Sig	Lypla2
not_Sig	Pithd1
not_Sig	Tceb3
Sig	Rpl11
not_Sig	Id3
not_Sig	E2f2
not_Sig	Asap3
not_Sig	Tcea3
Sig	Zfp46
not_Sig	Hnrnpr
not_Sig	Luzp1
Sig	Kdm1a
not_Sig	Ephb2
Sig	C1qb
not_Sig	C1qc
not_Sig	C1qa
not_Sig	Zbtb40
not_Sig	Wnt4
Sig	Cdc42
not_Sig	Hspg2
not_Sig	Ldlrad2
not_Sig	Usp48
not_Sig	Rap1gap
not_Sig	Alpl
not_Sig	Ece1
Sig	Eif4g3
not_Sig	1700095J12Rik
not_Sig	Mir6399
not_Sig	Hp1bp3
not_Sig	Sh2d5
not_Sig	Kif17

not_Sig	Ddost
not_Sig	Pink1
not_Sig	Cda
not_Sig	Mul1
not_Sig	Camk2n1
not_Sig	Ubxn10
not_Sig	Pla2g2f
not_Sig	Pla2g2d
Sig	Pla2g5
not_Sig	Otud3
not_Sig	Rnf186
not_Sig	Tmco4
Sig	Nbl1
Sig	Minos1
Sig	Capzb
not_Sig	Pqlc2
not_Sig	Akr7a5
not_Sig	Mrto4
not_Sig	Emc1
not_Sig	Ubr4
not_Sig	Iffo2
not_Sig	Aldh4a1
Sig	Pax7
not_Sig	Klhdc7a
not_Sig	Igsf21
not_Sig	Arhgef10l
not_Sig	Rcc2
not_Sig	Padi4
not_Sig	Padi3
not_Sig	4930515B02Rik
not_Sig	Padi2
not_Sig	Gm13031
not_Sig	Sdhb
not_Sig	Atp13a2
Sig	Mfap2
Sig	Crocc
not_Sig	Necap2
not_Sig	Spata21
not_Sig	Szrd1
not_Sig	Fbxo42
not_Sig	Rsg1
not_Sig	Arhgef19
Sig	Epha2
Sig	Fam131c
not_Sig	Clcnkb
not_Sig	Hspb7
not_Sig	Zbtb17
Sig	Spen

Sig	B330016D10Rik
Sig	Fblim1
not_Sig	Tmem82
not_Sig	Slc25a34
not_Sig	Plekhm2
not_Sig	Ddi2
not_Sig	Agmat
not_Sig	Dnajc16
not_Sig	Casp9
not_Sig	Efhd2
not_Sig	Tmem51
not_Sig	Tmem51os1
not_Sig	Kazn
Sig	Prdm2
Sig	Pdpn
not_Sig	Lrrc38
not_Sig	Pramef8
not_Sig	Dhrs3
not_Sig	Vps13d
not_Sig	Tnfrsf1b
not_Sig	Smarca5-ps
not_Sig	Gm13212
not_Sig	Gm13139
not_Sig	Gm13034
not_Sig	Gm13251
not_Sig	Gm13152
not_Sig	Gm13154
not_Sig	2610305D13Rik
not_Sig	Gm13157
not_Sig	Rps19-ps3
not_Sig	Zfp933
not_Sig	Miip
not_Sig	Fv1
not_Sig	Mfn2
not_Sig	Plod1
Sig	2510039O18Rik
not_Sig	Nppa
Sig	Clcn6
not_Sig	Mthfr
not_Sig	Agtrap
not_Sig	Draxin
not_Sig	Mad2l2
not_Sig	Fbxo6
not_Sig	Fbxo44
not_Sig	Fbxo2
not_Sig	Ptchd2
not_Sig	Ubiad1
not_Sig	Mtor

Sig	Angptl7
Sig	Exosc10
not_Sig	Srm
not_Sig	Masp2
not_Sig	Tardbp
not_Sig	Gm572
Sig	Casz1
not_Sig	Pex14
not_Sig	Dffa
not_Sig	Cort
not_Sig	Apitd1
not_Sig	Pgd
not_Sig	Kif1b
Sig	Ube4b
not_Sig	Ube4bos3
not_Sig	Rbp7
not_Sig	Nmnat1
Sig	Lzic
Sig	Ctnnbip1
Sig	Clstn1
not_Sig	Pik3cd
not_Sig	Tmem201
not_Sig	Slc25a33
not_Sig	Spsb1
not_Sig	H6pd
not_Sig	Gpr157
Sig	Slc2a5
Sig	Rere
not_Sig	Errfi1
Sig	Park7
not_Sig	Uts2
not_Sig	Per3
not_Sig	Vamp3
not_Sig	Camta1
not_Sig	Gm13090
not_Sig	Dnajc11
Sig	Thap3
Sig	Phf13
not_Sig	Klhl21
not_Sig	Zbtb48
not_Sig	Tas1r1
not_Sig	Nol9
not_Sig	Plekhg5
not_Sig	Tnfrsf25
not_Sig	Espn
not_Sig	Acot7
not_Sig	Gpr153
not_Sig	Icmt

not_Sig	Rnf207
Sig	Rpl22
not_Sig	Chd5
not_Sig	Kcnab2
not_Sig	Nphp4
not_Sig	Ajap1
not_Sig	A430005L14Rik
not_Sig	Dffb
not_Sig	Cep104
not_Sig	Lrrc47
not_Sig	Smim1
Sig	Trp73
not_Sig	Wdr8
Sig	Tprgl
not_Sig	Megf6
not_Sig	Arhgef16
not_Sig	Prdm16
not_Sig	5930403L14Rik
not_Sig	Ttc34
not_Sig	Mmel1
not_Sig	Fam213b
not_Sig	Tnfrsf14
not_Sig	Hes5
not_Sig	Pank4
not_Sig	Plch2
not_Sig	Pex10
not_Sig	Rer1
not_Sig	Morn1
Sig	Ski
not_Sig	Faap20
not_Sig	Prkcz
not_Sig	Gabrd
not_Sig	5830444B04Rik
not_Sig	Cfap74
not_Sig	Tmem52
not_Sig	Gnb1
not_Sig	Nadk
not_Sig	Slc35e2
not_Sig	Gm16023
not_Sig	Cdk11b
not_Sig	Mmp23
not_Sig	Mib2
not_Sig	B930041F14Rik
not_Sig	Ssu72
not_Sig	Tmem240
Sig	Atad3a
not_Sig	Atad3aos
not_Sig	Vwa1

not_Sig	Tmem88b
not_Sig	Mrpl20
not_Sig	Ccnl2
not_Sig	Aurkaip1
not_Sig	Mxra8
not_Sig	Dvl1
not_Sig	Tas1r3
not_Sig	Cptp
not_Sig	Cpsf3l
not_Sig	Pusl1
not_Sig	Acap3
Sig	Ube2j2
not_Sig	Fam132a
not_Sig	B3galt6
not_Sig	Sdf4
not_Sig	Tnfrsf4
Sig	Gm10560
Sig	Tnfrsf18
not_Sig	Ttll10
not_Sig	9430015G10Rik
Sig	Agrn
not_Sig	Isg15
Sig	AW011738
not_Sig	Perm1
Sig	Plekhn1
not_Sig	Klhl17
not_Sig	Noc2l
not_Sig	Samd11
not_Sig	Cdk6
Sig	Fam133b
not_Sig	1700109H08Rik
not_Sig	Rbm48
not_Sig	Pex1
not_Sig	Gatad1
not_Sig	Ankib1
not_Sig	Krit1
not_Sig	Lrrd1
not_Sig	Mterf1a
Sig	Akap9
Sig	Cyp51
not_Sig	Mterf1b
not_Sig	Fzd1
not_Sig	Cdk14
Sig	Cldn12
not_Sig	Gtpbp10
not_Sig	Steap2
not_Sig	Steap4
not_Sig	Sri

Sig	Adam22
not_Sig	Dbf4
not_Sig	Slc25a40
not_Sig	Rundc3b
not_Sig	Abcb1a
not_Sig	Abcb1b
not_Sig	Abcb4
not_Sig	Crot
Sig	Tmem243
not_Sig	Dmtf1
not_Sig	9330182L06Rik
not_Sig	Sema3d
Sig	Sema3a
not_Sig	Sema3e
Sig	Pclo
not_Sig	Cacna2d1
not_Sig	Hgf
Sig	Sema3c
not_Sig	Cd36
not_Sig	Gnai1
Sig	4921504A21Rik
not_Sig	Magi2
not_Sig	Phtf2
not_Sig	Tmem60
not_Sig	Rsbn1l
not_Sig	A630072M18Rik
not_Sig	Ptpn12
not_Sig	Gsap
not_Sig	Ccdc146
Sig	Fgl2
not_Sig	Fam185a
not_Sig	Fbxl13
not_Sig	Lrrc17
not_Sig	Armc10
Sig	Napepld
not_Sig	Pmpcb
not_Sig	Dnajc2
not_Sig	Psmc2
not_Sig	Reln
not_Sig	Orc5
not_Sig	Gm15421
not_Sig	6030443J06Rik
not_Sig	A930003O13Rik
not_Sig	Lhfpl3
Sig	5031425E22Rik
not_Sig	Kmt2e
not_Sig	Srpk2
not_Sig	AI506816

not_Sig	Pus7
not_Sig	Rint1
not_Sig	Mir3471-1
Sig	Tomm7
not_Sig	2700038G22Rik
not_Sig	Fam126a
not_Sig	Klhl7
not_Sig	Nupl2
not_Sig	Kcnh2
not_Sig	Nos3
not_Sig	Atg9b
not_Sig	Abcb8
not_Sig	Asic3
not_Sig	Cdk5
not_Sig	Slc4a2
Sig	Fastk
not_Sig	Tmub1
not_Sig	Agap3
not_Sig	Asb10
not_Sig	Abcf2
not_Sig	Chpf2
not_Sig	Smarcd3
not_Sig	Nub1
not_Sig	Wdr86
not_Sig	Crygn
Sig	Rheb
not_Sig	Prkag2
not_Sig	Prkag2os1
not_Sig	2900005J15Rik
not_Sig	Galnt11
not_Sig	Kmt2c
not_Sig	4831440E17Rik
not_Sig	Cct8l1
not_Sig	1700096K18Rik
not_Sig	Xrcc2
not_Sig	Actr3b
Sig	Dpp6
not_Sig	Speer4b
not_Sig	Paxip1
Sig	Insig1
Sig	En2
Sig	Rbm33
not_Sig	Rnf32
not_Sig	Lmbr1
not_Sig	Nom1
not_Sig	Ube3c
not_Sig	Gm5129
not_Sig	Dnajb6

not_Sig	Mir6362
not_Sig	Tyms
not_Sig	Gareml
not_Sig	Hadha
Sig	Hadhb
Sig	Adgrf3
Sig	Ept1
not_Sig	Otof
not_Sig	Gm9899
not_Sig	Kcnk3
Sig	Slc35f6
not_Sig	Cenpa
not_Sig	Dpysl5
not_Sig	Mapre3
not_Sig	Tmem214
Sig	Agbl5
not_Sig	Ost4
not_Sig	Emilin1
not_Sig	Khk
not_Sig	Cgref1
not_Sig	Abhd1
not_Sig	Preb
not_Sig	Tcf23
Sig	Slc5a6
not_Sig	Atraid
not_Sig	Cad
not_Sig	Trim54
not_Sig	Ucn
not_Sig	Mpv17
not_Sig	Gtf3c2
not_Sig	Eif2b4
not_Sig	Snx17
Sig	Zfp513
not_Sig	Ppm1g
not_Sig	Nrbp1
not_Sig	Krtcap3
not_Sig	Ift172
not_Sig	Fndc4
not_Sig	Zfp512
not_Sig	Gpn1
not_Sig	Supt7l
not_Sig	Slc4a1ap
not_Sig	AI839979
not_Sig	Mrpl33
not_Sig	Rbks
not_Sig	Mir3473e
not_Sig	Bre
not_Sig	Fosl2

not_Sig	Plb1
not_Sig	Ppp1cb
not_Sig	Yes1
not_Sig	Pisd
not_Sig	Prr14l
not_Sig	Depdc5
not_Sig	Ywhah
not_Sig	Slc5a1
not_Sig	Spon2
Sig	Ctbp1
not_Sig	Maea
Sig	Uvssa
not_Sig	Fam53a
not_Sig	Slbp
not_Sig	Tmem129
not_Sig	Tacc3
not_Sig	Fgfr3
not_Sig	Letm1
not_Sig	Whsc1
not_Sig	Nelfa
not_Sig	Gm1673
not_Sig	Nat8l
not_Sig	Poln
not_Sig	Haus3
not_Sig	Mxd4
not_Sig	Zfyve28
not_Sig	Rnf4
not_Sig	Fam193a
not_Sig	Tnip2
not_Sig	Sh3bp2
Sig	Add1
not_Sig	Mir7036b
not_Sig	Mfsd10
not_Sig	Nop14
not_Sig	Grk4
not_Sig	Htt
not_Sig	Msantd1
Sig	Rgs12
not_Sig	Hgfac
Sig	Dok7
not_Sig	Lrpap1
Sig	Adra2c
not_Sig	Cpz
not_Sig	Trmt44
not_Sig	4931431C16Rik
not_Sig	Acox3
Sig	Htra3
not_Sig	Sh3tc1

Sig	Ablim2
not_Sig	Afap1
not_Sig	Sorcs2
not_Sig	Psapl1
not_Sig	2210406O10Rik
not_Sig	Grpel1
Sig	Tada2b
not_Sig	Ccdc96
not_Sig	Tbc1d14
not_Sig	D5Ertd579e
not_Sig	Bloc1s4
Sig	Mrfap1
not_Sig	Man2b2
not_Sig	Ppp2r2c
not_Sig	Wfs1
not_Sig	Jakmip1
not_Sig	Crmp1
not_Sig	Evc
not_Sig	Evc2
not_Sig	Stk32b
not_Sig	Cytl1
not_Sig	Msx1
not_Sig	Msx1os
not_Sig	Stx18
not_Sig	Nsg1
not_Sig	Zbtb49
not_Sig	Lyar
not_Sig	Tmem128
Sig	Otop1
not_Sig	Slc2a9
not_Sig	Wdr1
not_Sig	Zfp518b
not_Sig	Hs3st1
not_Sig	Rab28
not_Sig	Bod1l
not_Sig	Gm7854
Sig	Cpeb2
not_Sig	C1qtnf7
not_Sig	Cc2d2a
not_Sig	Fbxl5
not_Sig	Bst1
not_Sig	Cd38
Sig	Prom1
not_Sig	Tapt1
Sig	Ldb2
not_Sig	Qdpr
Sig	Lap3
not_Sig	Med28

not_Sig	Fam184b
not_Sig	9630001P10Rik
Sig	Ncapg
not_Sig	Lcorl
not_Sig	Gm3414
Sig	Slit2
not_Sig	Pacrgl
not_Sig	Kcnip4
not_Sig	5730480H06Rik
not_Sig	Adgra2
not_Sig	Ppargc1a
not_Sig	Mir6417
not_Sig	Dhx15
not_Sig	9230114K14Rik
not_Sig	C130083M11Rik
Sig	Sod3
not_Sig	Ccdc149
Sig	Lgi2
not_Sig	8030423F21Rik
not_Sig	Sepsecs
not_Sig	Pi4k2b
not_Sig	Zcchc4
not_Sig	Anapc4
not_Sig	Sel1l3
Sig	Smim20
not_Sig	Rbpj
not_Sig	Tbc1d19
not_Sig	Stim2
not_Sig	4932441J04Rik
Sig	Pcdh7
not_Sig	Arap2
not_Sig	Nwd2
Sig	0610040J01Rik
Sig	Rell1
not_Sig	Pgm1
not_Sig	Tbc1d1
not_Sig	Klf3
not_Sig	Tlr1
not_Sig	Tlr6
not_Sig	Fam114a1
not_Sig	Klhl5
not_Sig	Wdr19
not_Sig	Rfc1
not_Sig	Klb
not_Sig	Rpl9
not_Sig	Lias
Sig	Ugdh
not_Sig	Smim14

not_Sig	Ube2k
Sig	Pds5a
Sig	N4bp2
not_Sig	Rhoh
Sig	Chrna9
Sig	9130230L23Rik
not_Sig	Rbm47
Sig	Nsun7
Sig	Apbb2
Sig	Uchl1
Sig	Limch1
not_Sig	Tmem33
not_Sig	Slc30a9
not_Sig	Bend4
not_Sig	Shisa3
Sig	Atp8a1
not_Sig	Yipf7
not_Sig	Guf1
not_Sig	Gnpda2
not_Sig	Commd8
not_Sig	Atp10d
not_Sig	Corin
not_Sig	Nfxl1
not_Sig	Nipal1
not_Sig	Txk
Sig	Tec
not_Sig	Slain2
Sig	Fryl
not_Sig	Ociad1
Sig	Ociad2
not_Sig	Dcun1d4
Sig	Sgcb
not_Sig	Usp46
not_Sig	Dancr
Sig	Rasl11b
not_Sig	Scfd2
Sig	Fip1l1
Sig	Lnx1
Sig	Chic2
not_Sig	Pdgfra
not_Sig	Kit
Sig	Kdr
not_Sig	Srd5a3
not_Sig	Tmem165
not_Sig	Mir1191
Sig	Clock
not_Sig	Exoc1
not_Sig	Cep135

not_Sig	C530008M17Rik
Sig	Aasdh
not_Sig	Ppat
not_Sig	Paics
not_Sig	Srp72
not_Sig	Hopx
Sig	Rest
not_Sig	Noa1
not_Sig	Polr2b
not_Sig	Igfbp7
not_Sig	Adgrl3
not_Sig	Tecrl
not_Sig	Cenpc1
not_Sig	Stap1
not_Sig	Uba6
not_Sig	Tmprss11a
Sig	Ythdc1
not_Sig	Sult1e1
Sig	Jchain
not_Sig	Utp3
not_Sig	Rufy3
not_Sig	Grsf1
not_Sig	Mob1b
not_Sig	Dck
Sig	Slc4a4
not_Sig	Gc
not_Sig	Adamts3
not_Sig	Cox18
not_Sig	Ankrd17
not_Sig	Gm9958
Sig	Alb
not_Sig	Rassf6
Sig	Ppbp
Sig	Pf4
not_Sig	Cxcl1
not_Sig	Mthfd2l
not_Sig	Btc
not_Sig	Parm1
not_Sig	Rchy1
not_Sig	Thap6
not_Sig	Gm1045
Sig	Cdkl2
not_Sig	G3bp2
not_Sig	Uso1
not_Sig	Ppef2
not_Sig	Naaa
not_Sig	Sdad1
not_Sig	Cxcl9

not_Sig	Art3
not_Sig	Cxcl10
not_Sig	Cxcl11
not_Sig	Nup54
not_Sig	Scarb2
not_Sig	Mir6415
not_Sig	Stbd1
not_Sig	Ccdc158
not_Sig	Shroom3
not_Sig	Sowahb
Sig	Sept11
not_Sig	Ccni
not_Sig	Ccng2
not_Sig	Cxcl13
not_Sig	Cnot6l
not_Sig	Mrpl1
not_Sig	Fras1
not_Sig	Anxa3
not_Sig	Gm8013
not_Sig	Bmp2k
not_Sig	Paqr3
not_Sig	Antxr2
not_Sig	Prdm8
not_Sig	Fgf5
not_Sig	Mir703
Sig	Bmp3
not_Sig	Prkg2
not_Sig	Rasgef1b
not_Sig	Hnrnpd
not_Sig	Hnrnpdl
not_Sig	Enoph1
not_Sig	Tmem150c
not_Sig	Sec31a
not_Sig	5430416N02Rik
not_Sig	Lin54
not_Sig	Cops4
not_Sig	Plac8
not_Sig	Coq2
not_Sig	Hpse
not_Sig	Helq
not_Sig	Mrps18c
not_Sig	Fam175a
not_Sig	Agpat9
not_Sig	Cds1
not_Sig	Wdfy3
not_Sig	Arhgap24
Sig	Mapk10
not_Sig	Ptpn13

not_Sig	Slc10a6
not_Sig	Aff1
Sig	Klhl8
not_Sig	Hsd17b11
Sig	Nudt9
Sig	Gm17660
Sig	Sparcl1
not_Sig	Dmp1
not_Sig	Ibsp
not_Sig	Mepe
Sig	Spp1
not_Sig	Pkd2
Sig	BC005561
not_Sig	D930016D06Rik
not_Sig	Zfp951
not_Sig	Abcg3
not_Sig	Gbp8
Sig	Gbp9
Sig	Gbp4
not_Sig	Gbp10
not_Sig	Gbp6
not_Sig	Gbp11
not_Sig	Lrrc8b
not_Sig	Lrrc8c
not_Sig	Lrrc8d
not_Sig	Zfp326
not_Sig	Zfp644
not_Sig	Cdc7
not_Sig	Tgfbr3
not_Sig	Brdt
not_Sig	Ephx4
not_Sig	Btbd8
Sig	A830010M20Rik
not_Sig	1700028K03Rik
not_Sig	Glmn
not_Sig	Rpap2
not_Sig	A930041C12Rik
not_Sig	Gfi1
Sig	Evi5
not_Sig	Ube2d2b
not_Sig	Rpl5
not_Sig	Fam69a
not_Sig	Mtf2
Sig	Tmed5
not_Sig	Ccdc18
Sig	Dr1
not_Sig	Pigg
not_Sig	Pde6b

not_Sig	Atp5k
Sig	Mfsd7a
not_Sig	Pcgf3
not_Sig	Cplx1
not_Sig	Gak
not_Sig	Tmem175
Sig	Dgkq
not_Sig	Idua
not_Sig	Slc26a1
not_Sig	Fgfrl1
not_Sig	Crlf2
not_Sig	Gm10416
not_Sig	Gm15446
not_Sig	Zfp932
not_Sig	Plcxd1
not_Sig	Gtpbp6
not_Sig	Zfp605
not_Sig	Chfr
not_Sig	Gm15787
not_Sig	Golga3
not_Sig	Ankle2
not_Sig	Pgam5
not_Sig	Pxmp2
not_Sig	Pole
Sig	Fbrsl1
not_Sig	Gm29766
Sig	Noc4l
not_Sig	Ddx51
not_Sig	Ep400
not_Sig	Pus1
Sig	Ulk1
not_Sig	Hscb
not_Sig	Chek2
Sig	Ttc28
not_Sig	Mir701
not_Sig	Pitpnb
not_Sig	Mn1
not_Sig	E130006D01Rik
not_Sig	Miat
not_Sig	Cryba4
Sig	Crybb1
not_Sig	Tpst2
not_Sig	Tfip11
not_Sig	Srrd
not_Sig	Hps4
not_Sig	Gm6583
not_Sig	Asphd2
not_Sig	Sez6l

not_Sig	Myo18b
not_Sig	1700095B10Rik
not_Sig	Adrbk2
not_Sig	Crybb3
Sig	2900026A02Rik
not_Sig	Sgsm1
not_Sig	Wscd2
not_Sig	Cmklr1
not_Sig	Ficd
not_Sig	Sart3
Sig	Iscu
not_Sig	Tmem119
not_Sig	Selplg
not_Sig	Coro1c
not_Sig	Ssh1
not_Sig	Usp30
not_Sig	Alkbh2
Sig	Ung
not_Sig	Acacb
not_Sig	Myo1h
Sig	Kctd10
not_Sig	Ube3b
not_Sig	Mir7027
not_Sig	Mmab
not_Sig	Mvk
not_Sig	Fam222a
not_Sig	Trpv4
not_Sig	Gltp
not_Sig	Tchp
Sig	Git2
not_Sig	4930515G01Rik
not_Sig	Ankrd13a
not_Sig	1500011B03Rik
not_Sig	2610524H06Rik
not_Sig	Oasl2
not_Sig	Oasl1
not_Sig	2210016L21Rik
not_Sig	Hnf1a
Sig	Sppl3
Sig	Rpl37rt
not_Sig	Acads
Sig	Unc119b
not_Sig	Mlec
not_Sig	Cabp1
not_Sig	Pop5
not_Sig	Rnf10
not_Sig	Coq5
not_Sig	Dynll1

not_Sig	Srsf9
not_Sig	Gatc
not_Sig	Triap1
not_Sig	Cox6a1
not_Sig	Msi1
not_Sig	Sirt4
Sig	Pxn
not_Sig	Rplp0
not_Sig	Gcn1l1
not_Sig	1110006O24Rik
Sig	Rab35
not_Sig	Ccdc64
not_Sig	Cit
not_Sig	Prkab1
not_Sig	Tmem233
not_Sig	Ccdc60
not_Sig	Hspb8
not_Sig	Srrm4os
not_Sig	Srrm4
not_Sig	Suds3
not_Sig	Taok3
Sig	Pebp1
not_Sig	Vsig10
not_Sig	Wsb2
not_Sig	Rfc5
not_Sig	Ksr2
Sig	Nos1
not_Sig	Fbxo21
Sig	Tesc
not_Sig	Fbxw8
Sig	Hrk
not_Sig	Rnft2
not_Sig	2410131K14Rik
Sig	Med13l
Sig	AW549542
not_Sig	Tbx3
not_Sig	Tbx5
not_Sig	Rbm19
not_Sig	Gm10390
not_Sig	Sdsl
not_Sig	Sds
not_Sig	Plbd2
not_Sig	Slc8b1
not_Sig	Tpcn1
not_Sig	Iqcd
not_Sig	Rita1
not_Sig	Ddx54
not_Sig	Rasal1

not_Sig	Dtx1
not_Sig	Oas2
Sig	Oas3
not_Sig	Oas1c
not_Sig	Oas1b
not_Sig	Oas1g
Sig	Oas1a
not_Sig	Rph3a
not_Sig	Ptpn11
not_Sig	Rpl6
not_Sig	Gm15800
not_Sig	Trafd1
not_Sig	Naa25
not_Sig	Erp29
not_Sig	Tmem116
not_Sig	Adam1b
not_Sig	Adam1a
Sig	Mapkapk5
not_Sig	Aldh2
Sig	Acad12
not_Sig	Acad10
not_Sig	Brap
not_Sig	Atxn2
Sig	Sh2b3
not_Sig	Mir7031
not_Sig	Fam109a
not_Sig	Cux2
not_Sig	Myl2
not_Sig	Ccdc63
not_Sig	Ppp1cc
not_Sig	Hvcn1
not_Sig	Tctn1
not_Sig	Pptc7
not_Sig	Rad9b
not_Sig	Vps29
not_Sig	Fam216a
not_Sig	Gpn3
not_Sig	Arpc3
not_Sig	Anapc7
not_Sig	Atp2a2
not_Sig	Ift81
not_Sig	P2rx7
not_Sig	P2rx4
not_Sig	Camkk2
not_Sig	Anapc5
not_Sig	Rnf34
not_Sig	Kdm2b
not_Sig	A930024E05Rik

not_Sig	Orai1
not_Sig	Morn3
not_Sig	Tmem120b
Sig	Rhof
Sig	Setd1b
not_Sig	Hpd
not_Sig	Psmd9
not_Sig	Bcl7a
not_Sig	Mlxip
not_Sig	B3gnt4
not_Sig	Diablo
not_Sig	Vps33a
not_Sig	Clip1
not_Sig	Zcchc8
not_Sig	Rsrc2
Sig	Kntc1
Sig	Hcar2
not_Sig	Hcar1
not_Sig	Denr
not_Sig	Ccdc62
not_Sig	Hip1r
Sig	Vps37b
not_Sig	Abcb9
not_Sig	Ogfod2
not_Sig	Arl6ip4
Sig	Pitpnm2
not_Sig	Pitpnm2os1
not_Sig	Mphosph9
Sig	2810006K23Rik
not_Sig	Cdk2ap1
Sig	Sbno1
Sig	Setd8
not_Sig	Rilpl2
not_Sig	Snrnp35
not_Sig	Rilpl1
Sig	Tmed2
not_Sig	Ddx55
not_Sig	Eif2b1
Sig	Gtf2h3
not_Sig	Tctn2
Sig	Atp6v0a2
not_Sig	Dnah10
not_Sig	Ccdc92
not_Sig	Zfp664
not_Sig	Fam101a
not_Sig	Ncor2
not_Sig	Scarb1
not_Sig	Ubc

Sig	Dhx37
not_Sig	Bri3bp
Sig	Aacs
not_Sig	Tmem132b
not_Sig	Tmem132c
Sig	Slc15a4
not_Sig	5930412G12Rik
Sig	Fzd10
Sig	Rimbp2
not_Sig	Stx2
not_Sig	Ran
Sig	Adgrd1
Sig	Sfswap
not_Sig	Mmp17
not_Sig	Zfp11
not_Sig	Mrps17
not_Sig	Gbas
not_Sig	Psph
not_Sig	Cct6a
not_Sig	Snora15
not_Sig	Sumf2
not_Sig	Phkg1
Sig	Chchd2
Sig	Zbed5
not_Sig	Nupr1l
Sig	Vkorc1l1
not_Sig	Gusb
not_Sig	Asl
not_Sig	Crcp
not_Sig	Tpst1
not_Sig	Kctd7
Sig	Rabgef1
not_Sig	Tmem248
Sig	Sbds
not_Sig	Tyw1
not_Sig	Wbscr17
Sig	Auts2
not_Sig	4930563F08Rik
not_Sig	Gatsl2
not_Sig	Wbscr16
not_Sig	Gtf2ird2
not_Sig	Ncf1
not_Sig	Gtf2i
not_Sig	Gtf2ird1
Sig	Clip2
not_Sig	Syna
not_Sig	Rfc2
not_Sig	Lat2

not_Sig	Eif4h
not_Sig	Limk1
Sig	Eln
not_Sig	Cldn13
not_Sig	Wbscr27
not_Sig	Cldn3
not_Sig	Abhd11
not_Sig	Abhd11os
not_Sig	Stx1a
not_Sig	Wbscr22
not_Sig	Dnajc30
not_Sig	Vps37d
not_Sig	Mlxipl
not_Sig	Tbl2
not_Sig	Bcl7b
Sig	Baz1b
not_Sig	Fzd9
not_Sig	Nsun5
Sig	Pom121
Sig	Hip1
Sig	Ccl24
not_Sig	Rhbdd2
Sig	Por
Sig	Tmem120a
not_Sig	Styxl1
not_Sig	Mdh2
not_Sig	Srrm3
not_Sig	Hspb1
not_Sig	Ywhag
not_Sig	Ssc4d
not_Sig	Dtx2
not_Sig	Rasa4
not_Sig	Polr2j
not_Sig	Lrwd1
not_Sig	Alkbh4
not_Sig	Orai2
not_Sig	Prkrip1
not_Sig	Sh2b2
not_Sig	Cux1
not_Sig	Myl10
not_Sig	Col26a1
not_Sig	Ift22
not_Sig	4933404O12Rik
Sig	Fis1
not_Sig	Cldn15
not_Sig	Znhit1
Sig	Plod3
not_Sig	Ap1s1

not_Sig	Serpine1
not_Sig	Trim56
Sig	Ache
not_Sig	Mir8116
Sig	Ufsp1
Sig	Srrt
Sig	Trip6
not_Sig	Slc12a9
Sig	Ephb4
not_Sig	Zan
not_Sig	Pop7
Sig	Gigyf1
not_Sig	Gnb2
Sig	Tfr2
Sig	Mospd3
not_Sig	Pcolce
not_Sig	Fbxo24
not_Sig	Gm20605
not_Sig	Irs3
not_Sig	Agfg2
not_Sig	Nyap1
Sig	Tsc22d4
not_Sig	Ppp1r35
Sig	Mepce
not_Sig	Zcwpw1
not_Sig	Pilra
not_Sig	Pilrb1
not_Sig	Pilrb2
not_Sig	Cyp3a13
not_Sig	Gjc3
not_Sig	Azgp1
not_Sig	Zkscan1
not_Sig	Zscan21
not_Sig	Zfp113
not_Sig	Cops6
not_Sig	Mcm7
not_Sig	Mir25
Sig	Ap4m1
Sig	Taf6
not_Sig	Cnpy4
not_Sig	Mblac1
not_Sig	Nxpe5
not_Sig	Lamtor4
not_Sig	BC037034
not_Sig	6330418K02Rik
not_Sig	Gal3st4
not_Sig	Gpc2
Sig	Zfp157

not_Sig	Zfp68
not_Sig	A430033K04Rik
Sig	Fam20c
not_Sig	Pdgfa
not_Sig	Prkar1b
not_Sig	Dnaaf5
not_Sig	Sun1
not_Sig	Get4
not_Sig	Adap1
Sig	Cox19
not_Sig	3110082I17Rik
not_Sig	Mir339
not_Sig	D830046C22Rik
Sig	Gpr146
not_Sig	C130050O18Rik
not_Sig	Gper1
not_Sig	Zfand2a
not_Sig	Micall2
not_Sig	Ints1
Sig	Mafk
not_Sig	Tmem184a
not_Sig	Psmg3
not_Sig	Mad1l1
not_Sig	Ftsj2
not_Sig	Nudt1
not_Sig	Snx8
not_Sig	Eif3b
not_Sig	Chst12
not_Sig	Lfng
not_Sig	Ttyh3
not_Sig	Iqce
not_Sig	Brat1
not_Sig	Amz1
not_Sig	Gna12
Sig	Card11
not_Sig	Sdk1
Sig	Foxk1
Sig	Ap5z1
not_Sig	Radil
not_Sig	Papolb
not_Sig	Wipi2
not_Sig	Slc29a4
not_Sig	Tnrc18
not_Sig	Fbxl18
not_Sig	Actb
Sig	Fscn1
not_Sig	Rnf216
not_Sig	Rbak

not_Sig	Zfp12
not_Sig	Zfp316
not_Sig	E130309D02Rik
not_Sig	Zdhhc4
not_Sig	0610040B10Rik
not_Sig	Kdelr2
not_Sig	Daglb
not_Sig	Rac1
not_Sig	Fam220a
not_Sig	Cyth3
not_Sig	Usp42
not_Sig	D130017N08Rik
Sig	Eif2ak1
not_Sig	Ankrd61
not_Sig	Aimp2
not_Sig	Pms2
not_Sig	Ccz1
not_Sig	Lmtk2
not_Sig	Bhlha15
not_Sig	Tecpr1
not_Sig	Bri3
not_Sig	Baiap2l1
not_Sig	Gm15708
not_Sig	Nptx2
not_Sig	Trrap
not_Sig	Smurf1
not_Sig	Arpc1a
not_Sig	Arpc1b
Sig	Pdap1
not_Sig	Bud31
not_Sig	Ptcd1
not_Sig	Cpsf4
not_Sig	Atp5j2
not_Sig	Zkscan14
not_Sig	Zkscan5
not_Sig	Zfp655
Sig	Zscan25
not_Sig	Cyp3a11
not_Sig	Cyp3a25
not_Sig	1700001J03Rik
not_Sig	Rnf6
not_Sig	Cdk8
not_Sig	Wasf3
not_Sig	Usp12
not_Sig	Rpl21
Sig	Rasl11a
not_Sig	Gtf3a
not_Sig	Mtif3

not_Sig	Lnx2
Sig	Polr1d
not_Sig	Flt3
Sig	Pan3
Sig	Flt1
not_Sig	Pomp
not_Sig	Slc46a3
not_Sig	Mtus2
not_Sig	Slc7a1
not_Sig	Ubl3
not_Sig	Katnal1
not_Sig	5930430L01Rik
not_Sig	Gm15408
not_Sig	Hmgb1
not_Sig	5730422E09Rik
not_Sig	Gm8615
Sig	Uspl1
not_Sig	Alox5ap
not_Sig	Medag
Sig	Hsph1
not_Sig	B3glct
not_Sig	Rxfp2
Sig	Fry
not_Sig	Brca2
not_Sig	N4bp2l1
not_Sig	N4bp2l2
not_Sig	Pds5b
Sig	Kl
not_Sig	Stard13
not_Sig	1700028E10Rik
not_Sig	Gm3704
not_Sig	Rfc3
not_Sig	Samd9l
not_Sig	Hepacam2
not_Sig	Ccdc132
not_Sig	Calcr
not_Sig	Tfpi2
Sig	Gng11
not_Sig	Bet1
not_Sig	Col1a2
not_Sig	Casd1
not_Sig	Sgce
Sig	Peg10
not_Sig	Ppp1r9a
Sig	Pon1
Sig	Pon3
Sig	Pon2
not_Sig	Asb4

Sig	Pdk4
not_Sig	Dync1i1
not_Sig	Slc25a13
not_Sig	Shfm1
not_Sig	Dlx5
not_Sig	Sdhaf3
not_Sig	Asns
not_Sig	C1galt1
not_Sig	Col28a1
not_Sig	Mios
Sig	Rpa3
Sig	Umad1
not_Sig	A430035B10Rik
not_Sig	Glcci1
not_Sig	Ica1
not_Sig	Ndufa4
not_Sig	Phf14
not_Sig	Thsd7a
not_Sig	Tmem106b
not_Sig	Tmem168
not_Sig	B630005N14Rik
not_Sig	Gpr85
not_Sig	2610001J05Rik
not_Sig	1110019D14Rik
not_Sig	Ppp1r3a
Sig	Foxp2
not_Sig	Mdfic
not_Sig	Tfec
not_Sig	Tes
not_Sig	Cav2
not_Sig	Cav1
not_Sig	Met
not_Sig	Capza2
not_Sig	St7
not_Sig	Wnt2
not_Sig	Cttnbp2
not_Sig	Lsm8
not_Sig	Kcnd2
not_Sig	Tspan12
not_Sig	Ing3
Sig	Cped1
not_Sig	Wnt16
not_Sig	Fam3c
not_Sig	Ptprz1
not_Sig	Aass
not_Sig	Cadps2
not_Sig	Iqub
not_Sig	Ndufa5

Sig	Asb15
not_Sig	Lmod2
not_Sig	Wasl
not_Sig	Tmem229a
not_Sig	Pot1a
not_Sig	Grm8
not_Sig	Zfp800
not_Sig	Gcc1
not_Sig	Arf5
not_Sig	Snd1
not_Sig	Lrrc4
not_Sig	Lep
not_Sig	Rbm28
not_Sig	Prrt4
not_Sig	Impdh1
Sig	Hilpda
not_Sig	Fam71f2
Sig	Calu
not_Sig	Opn1sw
not_Sig	Ccdc136
not_Sig	Flnc
not_Sig	Atp6v1f
not_Sig	Kcp
not_Sig	Irf5
Sig	Tnpo3
not_Sig	Tspan33
not_Sig	Smo
not_Sig	Ahcyl2
not_Sig	Strip2
not_Sig	Smkr-ps
not_Sig	Nrf1
not_Sig	Ube2h
not_Sig	Zc3hc1
not_Sig	Klhdc10
not_Sig	Tmem209
not_Sig	Cep41
not_Sig	Mest
Sig	Copg2
not_Sig	Klf14
Sig	Mir29a
not_Sig	Lincpint
Sig	2210408F21Rik
not_Sig	Mkln1os
not_Sig	Mkln1
Sig	Podxl
not_Sig	Plxna4
not_Sig	Plxna4os1
Sig	Chchd3

Sig	Exoc4
not_Sig	Slc35b4
not_Sig	Akr1b8
not_Sig	Akr1b10
not_Sig	Bpgm
not_Sig	Cald1
not_Sig	Agbl3
not_Sig	Tmem140
not_Sig	3110062M04Rik
not_Sig	Wdr91
not_Sig	2010107G12Rik
not_Sig	Cnot4
not_Sig	Nup205
not_Sig	1810058I24Rik
not_Sig	Slc13a4
not_Sig	Fam180a
not_Sig	Mtpn
not_Sig	Chrm2
not_Sig	Ptn
not_Sig	Dgki
not_Sig	Creb3l2
not_Sig	Trim24
not_Sig	Svopl
not_Sig	Atp6v0a4
not_Sig	D630045J12Rik
not_Sig	Zc3hav1l
not_Sig	Zc3hav1
not_Sig	Ttc26
not_Sig	Ubn2
not_Sig	1110001J03Rik
not_Sig	Luc7l2
not_Sig	Klrg2
not_Sig	Hipk2
not_Sig	Tbxas1
not_Sig	Parp12
not_Sig	4930599N23Rik
Sig	Kdm7a
not_Sig	Slc37a3
not_Sig	Rab19
not_Sig	Mkrn1
not_Sig	Dennd2a
not_Sig	Adck2
not_Sig	Ndufb2
not_Sig	Braf
not_Sig	Mrps33
not_Sig	Tmem178b
not_Sig	Agk
not_Sig	E330009J07Rik

not_Sig	Ssbp1
not_Sig	Tas2r137
not_Sig	Clec5a
Sig	Mgam
Sig	Ephb6
not_Sig	Trpv6
Sig	Kel
not_Sig	Gstk1
not_Sig	Casp2
not_Sig	Clcn1
not_Sig	Fam131b
not_Sig	Zyx
not_Sig	Epha1
not_Sig	2010310C07Rik
not_Sig	Tas2r143
not_Sig	Tas2r135
not_Sig	Tas2r126
not_Sig	Tcaf2
not_Sig	Tcaf1
not_Sig	Arhgef5
not_Sig	Tpk1
not_Sig	9430018G01Rik
Sig	Cntnap2
not_Sig	Cul1
not_Sig	Ezh2
Sig	Rn4.5s
not_Sig	Pdia4
not_Sig	Zfp786
not_Sig	Zfp398
Sig	Zfp282
not_Sig	Zfp212
not_Sig	Zfp783
Sig	Zfp956
Sig	Zfp777
Sig	Zfp746
not_Sig	Krba1
not_Sig	Zfp467
not_Sig	Sspo
not_Sig	Zfp862-ps
not_Sig	Atp6v0e2
not_Sig	Lrrc61
Sig	Rarres2
not_Sig	Gm5111
Sig	Repin1
not_Sig	Zfp775
not_Sig	AI854703
Sig	Gimap8
not_Sig	Gimap9

Sig	Gimap4
Sig	Gimap6
Sig	Gimap1
Sig	Gimap5
not_Sig	Gimap3
not_Sig	Tmem176b
not_Sig	Tmem176a
Sig	Gpnmb
not_Sig	Malsu1
not_Sig	Igf2bp3
not_Sig	Tra2a
not_Sig	Ccdc126
not_Sig	Fam221a
not_Sig	Mpp6
not_Sig	Dfna5
not_Sig	Osbpl3
Sig	Cycs
not_Sig	5430402O13Rik
not_Sig	4921507P07Rik
not_Sig	Nfe2l3
not_Sig	Hnrnpa2b1
not_Sig	Cbx3
Sig	Snx10
not_Sig	Skap2
not_Sig	Hoxa1
not_Sig	Hotairm1
not_Sig	Hoxa2
not_Sig	Hoxaas2
not_Sig	Hoxa3
not_Sig	Hoxa4
Sig	Hoxaas3
not_Sig	Hoxa5
Sig	Hoxa6
Sig	Mira
Sig	Hoxa7
not_Sig	Hoxa9
not_Sig	Mir196b
not_Sig	Hoxa10
not_Sig	Hoxa11
not_Sig	Hoxa11os
not_Sig	Hoxa13
not_Sig	Hibadh
not_Sig	Tax1bp1
not_Sig	Jazf1
not_Sig	9430076C15Rik
not_Sig	Creb5
Sig	Tril
not_Sig	Chn2

not_Sig	9130019P16Rik
not_Sig	Prr15
not_Sig	Wipf3
Sig	Scrn1
not_Sig	Fkbp14
not_Sig	Plekha8
not_Sig	Mturn
not_Sig	Znrf2
not_Sig	Nod1
not_Sig	Ggct
Sig	Gars
not_Sig	Crhr2
Sig	Inmt
not_Sig	Fam188b
Sig	Aqp1
Sig	Adcyap1r1
not_Sig	Pde1c
not_Sig	Lsm5
not_Sig	Avl9
not_Sig	Kbtbd2
not_Sig	Fkbp9
not_Sig	Nt5c3
not_Sig	Ppm1k
not_Sig	Herc6
not_Sig	Pyurf
not_Sig	Lancl2
not_Sig	Vopp1
Sig	Abcg2
not_Sig	Herc3
not_Sig	Nap1l5
not_Sig	Fam13a
not_Sig	Tigd2
not_Sig	Gprin3
not_Sig	A530053G22Rik
not_Sig	Snca
not_Sig	Mmrn1
not_Sig	Ccser1
not_Sig	Smarcad1
Sig	Hpgds
not_Sig	Tnip3
not_Sig	Ndnf
Sig	Prdm5
not_Sig	Mad2l1
not_Sig	Gng12
not_Sig	Gadd45a
not_Sig	E230016M11Rik
not_Sig	Serbp1
not_Sig	Il12rb2

not_Sig	Il23r
not_Sig	Tacstd2
not_Sig	Rpia
not_Sig	Eif2ak3
not_Sig	Thnsl2
not_Sig	Fabp1
not_Sig	Smyd1
not_Sig	Mir8112
Sig	Krcc1
not_Sig	Cd8b1
not_Sig	Cd8a
not_Sig	Rmnd5a
not_Sig	Rnf103
Sig	Chmp3
not_Sig	Kdm3a
not_Sig	Reep1
not_Sig	Mrpl35
not_Sig	Immt
not_Sig	Ptcd3
not_Sig	Polr1a
not_Sig	St3gal5
not_Sig	4933431G14Rik
not_Sig	Atoh8
not_Sig	Sftpb
Sig	Usp39
not_Sig	0610030E20Rik
Sig	Tmem150a
not_Sig	Rnf181
not_Sig	Vamp5
not_Sig	Vamp8
not_Sig	Ggcx
not_Sig	Mat2a
not_Sig	Particl
not_Sig	Capg
not_Sig	Elmod3
not_Sig	Retsat
Sig	Tgoln1
Sig	Tcf7l1
not_Sig	Gm15401
not_Sig	Kcmf1
not_Sig	Tmsb10
not_Sig	Dnah6
not_Sig	Suclg1
not_Sig	Lrrtm1
not_Sig	Lrrtm4
not_Sig	Gcfc2
not_Sig	Mrpl19
not_Sig	Eva1a

Sig	Pole4
not_Sig	Hk2
not_Sig	2310069B03Rik
not_Sig	Sema4f
not_Sig	M1ap
not_Sig	Dok1
not_Sig	Loxl3
not_Sig	Htra2
Sig	Aup1
not_Sig	Dqx1
not_Sig	Pcgf1
not_Sig	Lbx2
Sig	Ccdc142
Sig	Mrpl53
not_Sig	Mogs
not_Sig	Wbp1
not_Sig	Ino80b
Sig	Rtkn
not_Sig	Wdr54
not_Sig	1700003E16Rik
Sig	Dctn1
Sig	Mthfd2
not_Sig	Mob1a
not_Sig	Bola3
Sig	Tet3
not_Sig	B230319C09Rik
not_Sig	Dguok
not_Sig	Actg2
not_Sig	Stambp
not_Sig	Gm21284
not_Sig	Atp6v1b1
not_Sig	Ankrd53
not_Sig	Tex261
not_Sig	Nagk
not_Sig	Paip2b
not_Sig	Zfp638
not_Sig	Dysf
not_Sig	Cyp26b1
not_Sig	Exoc6b
not_Sig	Spr
not_Sig	Sfxn5
Sig	Rab11fip5
not_Sig	Smyd5
not_Sig	Pradc1
not_Sig	Cct7
not_Sig	Fbxo41
not_Sig	Alms1
not_Sig	Gm4477

not_Sig	Cml2
not_Sig	1700019G17Rik
not_Sig	Cml1
not_Sig	Tprkb
not_Sig	Dusp11
Sig	Add2
not_Sig	Tgfa
not_Sig	Fam136a
Sig	Snrpg
Sig	Pcyox1
not_Sig	Tia1
not_Sig	C87436
not_Sig	A430078I02Rik
not_Sig	2310040G24Rik
not_Sig	Pcbp1
not_Sig	1600020E01Rik
not_Sig	Asprv1
not_Sig	Mxd1
not_Sig	Snrnp27
not_Sig	Gmcl1
Sig	Anxa4
not_Sig	2610306M01Rik
Sig	Aak1
Sig	Nfu1
not_Sig	Gfpt1
not_Sig	Antxr1
not_Sig	Gkn3
Sig	Arhgap25
not_Sig	Prokr1
not_Sig	Aplf
not_Sig	Efcc1
not_Sig	Gp9
not_Sig	Rab43
not_Sig	Isy1
Sig	Cnbp
not_Sig	Copg1
not_Sig	Hmces
not_Sig	H1fx
not_Sig	Gm5577
Sig	Rab7
Sig	Rpn1
Sig	Gata2
Sig	Eefsec
not_Sig	Ruvbl1
not_Sig	Sec61a1
not_Sig	Kbtbd12
not_Sig	Mgll
not_Sig	Abtb1

Sig	Podxl2
not_Sig	Gm15612
not_Sig	Mcm2
not_Sig	Tpra1
not_Sig	Plxna1
not_Sig	Chchd6
not_Sig	Txnrd3
not_Sig	Chst13
not_Sig	Uroc1
not_Sig	Zxdc
not_Sig	Ccdc37
not_Sig	Klf15
not_Sig	Aldh1l1
not_Sig	Slc41a3
not_Sig	Iqsec1
not_Sig	Nup210
not_Sig	Hdac11
not_Sig	Fbln2
not_Sig	1810044D09Rik
not_Sig	Chchd4
not_Sig	Tmem43
not_Sig	Xpc
Sig	Lsm3
Sig	Slc6a6
Sig	Grip2
Sig	Ccdc174
not_Sig	4930590J08Rik
Sig	Fgd5
not_Sig	Nr2c2
not_Sig	Mrps25
not_Sig	Rbsn
not_Sig	Prickle2
not_Sig	Adamts9
not_Sig	9530026P05Rik
not_Sig	Magi1
not_Sig	4930511A08Rik
not_Sig	Slc25a26
not_Sig	Lrig1
not_Sig	Kbtbd8
not_Sig	Suclg2
not_Sig	Fam19a4
not_Sig	Eogt
not_Sig	Tmf1
not_Sig	Uba3
not_Sig	Arl6ip5
not_Sig	Lmod3
Sig	Frmd4b
not_Sig	Mitf

not_Sig	Gm765
not_Sig	Foxp1
not_Sig	Eif4e3
not_Sig	Gpr27
not_Sig	Rybp
not_Sig	Shq1
not_Sig	Gxylt2
not_Sig	Ppp4r2
not_Sig	Pdzrn3
not_Sig	Chl1
not_Sig	Il5ra
not_Sig	Trnt1
Sig	Crbn
not_Sig	Lrrn1
not_Sig	Setmar
not_Sig	Sumf1
not_Sig	Itpr1
not_Sig	0610040F04Rik
not_Sig	Bhlhe40
not_Sig	Arl8b
not_Sig	Edem1
not_Sig	Grm7
not_Sig	Lmcd1
not_Sig	5031434C07Rik
not_Sig	Ssu2
not_Sig	Cav3
not_Sig	Oxtr
not_Sig	Rad18
not_Sig	Srgap3
not_Sig	Thumpd3
not_Sig	Gt(ROSA)26Sor
Sig	Setd5
not_Sig	Lhfpl4
not_Sig	Mtmr14
not_Sig	Cpne9
not_Sig	Brpf1
not_Sig	Ogg1
not_Sig	Camk1
not_Sig	Tada3
not_Sig	Arpc4
not_Sig	Ttll3
not_Sig	Rpusd3
not_Sig	Cidec
not_Sig	Jagn1
not_Sig	Il17re
not_Sig	Il17rc
Sig	Creld1
Sig	Prrt3

Sig	Emc3
not_Sig	Fancd2
not_Sig	Brk1
not_Sig	Vhl
not_Sig	Irak2
not_Sig	Tatdn2
not_Sig	Ghrl
not_Sig	Sec13
not_Sig	Atp2b2
not_Sig	Slc6a11
not_Sig	Slc6a1
not_Sig	Hrh1
not_Sig	Atg7
not_Sig	Vgll4
not_Sig	Tamm41
not_Sig	Syn2
Sig	Timp4
not_Sig	Pparg
not_Sig	Tsen2
not_Sig	Mkrn2os
not_Sig	Mkrn2
not_Sig	Raf1
Sig	Tmem40
not_Sig	Cand2
not_Sig	Mir7660
Sig	Rpl32
not_Sig	Snora7a
not_Sig	Efcab12
not_Sig	Mbd4
not_Sig	Ift122
not_Sig	Rho
Sig	Plxnd1
not_Sig	Tmcc1
not_Sig	9530062K07Rik
not_Sig	Fam21
not_Sig	Zfand4
not_Sig	March8
not_Sig	Alox5
not_Sig	Zfp422
not_Sig	Rassf4
Sig	8430408G22Rik
Sig	Cxcl12
Sig	Zfp637
not_Sig	Zfp239
Sig	Hnrnpf
not_Sig	Fxyd4
not_Sig	Rasgef1a
not_Sig	Csgalnact2

not_Sig	Ret
not_Sig	Bms1
not_Sig	Zfp248
not_Sig	Zfp9
not_Sig	Ankrd26
Sig	Cacna1c
not_Sig	4931430N09Rik
not_Sig	Dcp1b
not_Sig	Cacna2d4
not_Sig	Lrtm2
Sig	Adipor2
not_Sig	Wnt5b
not_Sig	Fbxl14
Sig	Erc1
not_Sig	3110021A11Rik
not_Sig	Rad52
Sig	Wnk1
Sig	B4galnt3
not_Sig	Ccdc77
not_Sig	Kdm5a
not_Sig	Il17ra
not_Sig	Cecr5
not_Sig	Cecr2
not_Sig	Slc25a18
not_Sig	Atp6v1e1
not_Sig	Bcl2l13
not_Sig	Bid
not_Sig	Mical3
not_Sig	Pex26
not_Sig	Tuba8
not_Sig	Usp18
not_Sig	Slc6a13
Sig	Slc6a12
not_Sig	Iqsec3
not_Sig	A2m
not_Sig	Mug1
not_Sig	M6pr
not_Sig	Phc1
not_Sig	Rimklb
Sig	Mfap5
Sig	Apobec1
not_Sig	Slc2a3
Sig	Foxj2
Sig	C3ar1
not_Sig	Necap1
not_Sig	Clec4a1
not_Sig	Clec4a3
not_Sig	Clec4b1

not_Sig	Clec4a2
not_Sig	Clec4n
not_Sig	Clec4d
Sig	Cd163
not_Sig	Pex5
Sig	Clstn3
not_Sig	C1rl
not_Sig	C1ra
not_Sig	C1s1
not_Sig	C1s2
not_Sig	Lpcat3
not_Sig	Emg1
Sig	Phb2
not_Sig	Ptpn6
not_Sig	Grcc10
Sig	Atn1
not_Sig	Eno2
not_Sig	Spsb2
Sig	Tpi1
not_Sig	Usp5
not_Sig	Cdca3
Sig	Gnb3
not_Sig	P3h3
not_Sig	Gpr162
not_Sig	Cd4
not_Sig	Lag3
not_Sig	Ptms
Sig	Mlf2
Sig	Cops7a
not_Sig	Pianp
Sig	Zfp384
not_Sig	4930557K07Rik
not_Sig	Ing4
not_Sig	Acrbp
not_Sig	Lpar5
not_Sig	Chd4
not_Sig	Nop2
Sig	Iffo1
Sig	Gapdh
not_Sig	Ncapd2
Sig	Scarna10
not_Sig	Mrpl51
Sig	Vamp1
not_Sig	Tapbpl
not_Sig	E130112N10Rik
not_Sig	Cd27
not_Sig	Ltbr
not_Sig	Scnn1a

not_Sig	Tnfrsf1a
not_Sig	Plekhg6
not_Sig	Cd9
not_Sig	Vwf
not_Sig	Ano2
not_Sig	Ntf3
Sig	Kcna5
not_Sig	Kcna1
not_Sig	Kcna6
not_Sig	Ndufa9
not_Sig	Rad51ap1
not_Sig	D6Wsu163e
not_Sig	Fgf6
not_Sig	Tigar
not_Sig	Ccnd2
not_Sig	9330179D12Rik
not_Sig	Parp11
not_Sig	Cracr2a
not_Sig	Tspan11
Sig	Tspan9
Sig	9330102E08Rik
Sig	Tead4
not_Sig	Tulp3
not_Sig	Rhno1
not_Sig	Foxm1
Sig	Nrip2
not_Sig	Itfg2
not_Sig	Fkbp4
not_Sig	Gm10069
not_Sig	Pzp
not_Sig	Klrb1a
not_Sig	BC035044
not_Sig	Clec2i
not_Sig	Gm15987
not_Sig	BC064078
not_Sig	Klrb1f
not_Sig	Clec2e
not_Sig	Klrb1-ps1
Sig	Clec2d
not_Sig	2310001H17Rik
not_Sig	Cd69
Sig	Clec12a
not_Sig	Clec1b
not_Sig	Clec9a
not_Sig	Clec1a
Sig	Clec7a
not_Sig	Olr1
not_Sig	1700101I11Rik

not_Sig	Gabarapl1
not_Sig	Klrk1
not_Sig	Magohb
not_Sig	Styk1
not_Sig	Ybx3
not_Sig	Smim10l1
not_Sig	Etv6
not_Sig	Lrp6
not_Sig	Mansc1
not_Sig	Loh12cr1
not_Sig	Dusp16
not_Sig	Crebl2
not_Sig	Gpr19
not_Sig	2810454H06Rik
not_Sig	Cdkn1b
not_Sig	1190002F15Rik
not_Sig	Apold1
not_Sig	Ddx47
not_Sig	Gprc5a
not_Sig	Hebp1
not_Sig	8430419L09Rik
not_Sig	Emp1
not_Sig	Grin2b
Sig	Atf7ip
Sig	Plbd1
not_Sig	Hist4h4
not_Sig	H2afj
not_Sig	Wbp11
not_Sig	Smco3
not_Sig	Art4
Sig	Mgp
not_Sig	Arhgdib
not_Sig	Rerg
not_Sig	Ptpro
not_Sig	Eps8
Sig	Strap
not_Sig	Dera
not_Sig	Slc15a5
Sig	Mgst1
Sig	Rergl
not_Sig	Plekha5
not_Sig	Aebp2
Sig	Pde3a
Sig	Gm10400
not_Sig	Slco1c1
not_Sig	Slco1b2
not_Sig	Slco1a4
not_Sig	Slco1a5

not_Sig	Pyroxd1
not_Sig	Recql
Sig	Golt1b
Sig	Gys2
not_Sig	Ldhb
Sig	Kcnj8
not_Sig	Abcc9
not_Sig	5330439B14Rik
Sig	Cmas
not_Sig	St8sia1
not_Sig	C2cd5
not_Sig	Etnk1
not_Sig	Sox5
not_Sig	Sox5os3
Sig	Bcat1
not_Sig	Lrmp
not_Sig	Casc1
not_Sig	Lyrm5
not_Sig	Kras
not_Sig	Gm15706
not_Sig	Rassf8
not_Sig	Bhlhe41
not_Sig	Sspn
not_Sig	Itpr2
not_Sig	Asun
not_Sig	Fgfr1op2
not_Sig	Tm7sf3
Sig	Med21
not_Sig	Gm6654
not_Sig	Stk38l
not_Sig	Arntl2
not_Sig	Ppfibp1
not_Sig	1700034J05Rik
not_Sig	Rep15
not_Sig	Mrps35
not_Sig	Mansc4
not_Sig	Klhl42
not_Sig	Pthlh
not_Sig	Ccdc91
not_Sig	Far2
not_Sig	Ergic2
not_Sig	4732416N19Rik
Sig	Tmtc1
not_Sig	Rps4l
not_Sig	Ipo8
not_Sig	Caprin2
not_Sig	Fam60a
not_Sig	Dennd5b

Sig	Mettl20
not_Sig	Amn1
not_Sig	2810474O19Rik
not_Sig	Bicd1
not_Sig	Nlrp12
not_Sig	Myadm
Sig	Prkcg
Sig	Cacng7
not_Sig	3300002P13Rik
not_Sig	Cacng6
not_Sig	Oscar
not_Sig	Ndufa3
not_Sig	Tfpt
not_Sig	Prpf31
not_Sig	Cnot3
not_Sig	Leng1
not_Sig	Tmc4
not_Sig	Mboat7
not_Sig	Tsen34
not_Sig	Rps9
not_Sig	Pirb
not_Sig	Pira6
not_Sig	Lilra6
not_Sig	Lair1
not_Sig	Ttyh1
not_Sig	D030047H15Rik
not_Sig	Leng8
not_Sig	Leng9
not_Sig	Cdc42ep5
not_Sig	Lilra5
not_Sig	Rdh13
not_Sig	Eps8l1
not_Sig	D630041G03Rik
Sig	Ppp1r12c
not_Sig	Tnnt1
not_Sig	Tnni3
not_Sig	Dnaaf3
not_Sig	Tmem86b
not_Sig	Ppp6r1
not_Sig	Hspbp1
not_Sig	Brsk1
not_Sig	Tmem150b
not_Sig	Suv420h2
not_Sig	Cox6b2
not_Sig	Tmem190
not_Sig	Tmem238
Sig	Rpl28
not_Sig	Ube2s

not_Sig	Shisa7
not_Sig	Isoc2b
not_Sig	Isoc2a
Sig	Zfp628
not_Sig	Nat14
not_Sig	Ssc5d
Sig	Sbk2
not_Sig	Sbk3
not_Sig	Zfp579
Sig	Fiz1
not_Sig	Zfp524
Sig	Zfp865
Sig	Zfp784
not_Sig	Zfp580
Sig	Ccdc106
not_Sig	U2af2
not_Sig	Epn1
not_Sig	Rasl2-9
Sig	Zfp787
not_Sig	Zfp444
not_Sig	Zfp667
not_Sig	Zfp583
not_Sig	Zfp78
not_Sig	Zfp28
not_Sig	Gm16532
not_Sig	Zim1
not_Sig	Peg3
not_Sig	Usp29
not_Sig	Zfp954
not_Sig	Zfp773
not_Sig	Zfp418
not_Sig	Zfp772
not_Sig	Vmn2r29
Sig	Clcn4-2
not_Sig	Mir5620
not_Sig	Zik1
not_Sig	Zfp551
not_Sig	Zfp606
not_Sig	Zscan18
not_Sig	Zfp329
not_Sig	Zfp110
not_Sig	Zfp128
not_Sig	Zscan22
not_Sig	Rps5
not_Sig	Rnf225
not_Sig	Zfp324
not_Sig	Zfp446
not_Sig	Slc27a5

not_Sig	Zbtb45
not_Sig	Trim28
Sig	Chmp2a
not_Sig	Ube2m
not_Sig	Mzf1
not_Sig	6330408A02Rik
not_Sig	Lig1
not_Sig	Pla2g4c
not_Sig	Sult2a5
not_Sig	Nlrp5-ps
not_Sig	Sepw1
not_Sig	Gltscr2
not_Sig	Snord23
not_Sig	Ehd2
Sig	Gltscr1
not_Sig	Napa
not_Sig	Kptn
not_Sig	Slc8a2
not_Sig	Meis3
not_Sig	Dhx34
not_Sig	C5ar2
not_Sig	C5ar1
not_Sig	Inafm1
Sig	Ccdc9
not_Sig	Bbc3
not_Sig	Sae1
Sig	Zc3h4
not_Sig	Tmem160
Sig	Npas1
not_Sig	Arhgap35
not_Sig	Ap2s1
not_Sig	Slc1a5
not_Sig	Fkrp
Sig	Strn4
Sig	Prkd2
Sig	Dact3
not_Sig	Gng8
not_Sig	Ptgir
not_Sig	Calm3
Sig	Pnmal2
not_Sig	Ccdc8
not_Sig	Ppp5c
not_Sig	Hif3a
not_Sig	Mill2
not_Sig	Pglyrp1
Sig	Ccdc61
Sig	Nova2
Sig	Mypop

Sig	Irf2bp1
not_Sig	Sympk
not_Sig	Dmwd
Sig	Dmpk
not_Sig	Six5
not_Sig	Fbxo46
not_Sig	Qpctl
not_Sig	Snrpd2
not_Sig	Gipr
not_Sig	Eml2
Sig	Gpr4
not_Sig	Opa3
not_Sig	Vasp
not_Sig	Ppm1n
not_Sig	Rtn2
not_Sig	Fosb
not_Sig	Ercc1
not_Sig	Cd3eap
not_Sig	Ppp1r13l
not_Sig	Ercc2
not_Sig	Klc3
not_Sig	Ckm
Sig	A930016O22Rik
not_Sig	Mark4
not_Sig	Bloc1s3
not_Sig	Trappc6a
not_Sig	Ppp1r37
not_Sig	Gemin7
not_Sig	Zfp296
not_Sig	Clasrp
not_Sig	Relb
not_Sig	Clptm1
Sig	Apoc1
not_Sig	Apoe
Sig	Tomm40
Sig	Pvrl2
Sig	Bcam
not_Sig	Cblc
not_Sig	Bcl3
not_Sig	Pvr
not_Sig	Zfp180
not_Sig	Zfp112
not_Sig	Zfp235
not_Sig	Zfp111
not_Sig	Zfp109
not_Sig	Zfp108
not_Sig	Zfp93
Sig	Zfp61

not_Sig	Zfp94
not_Sig	Kcnn4
not_Sig	Smg9
not_Sig	Plaur
not_Sig	Cadm4
not_Sig	Zfp428
not_Sig	Irgq
Sig	Xrcc1
not_Sig	Zfp575
not_Sig	Ethe1
not_Sig	Phldb3
not_Sig	Cd177
not_Sig	Rps19
not_Sig	Cd79a
not_Sig	Arhgef1
not_Sig	Rabac1
not_Sig	Atp1a3
not_Sig	Grik5
Sig	Zfp574
not_Sig	Pou2f2
Sig	Dedd2
not_Sig	Zfp526
not_Sig	Gsk3a
not_Sig	9130221H12Rik
Sig	Erf
Sig	Cic
not_Sig	Pafah1b3
not_Sig	Megf8
not_Sig	4732471J01Rik
not_Sig	Lipe
not_Sig	Cxcl17
not_Sig	Ceacam1
not_Sig	Ceacam2
not_Sig	Atp5sl
not_Sig	B3gnt8
not_Sig	Bckdha
not_Sig	Exosc5
not_Sig	Tmem91
not_Sig	B9d2
not_Sig	Tgfb1
not_Sig	Ccdc97
not_Sig	Hnrnpul1
Sig	Axl
not_Sig	Cyp2s1
Sig	Cyp2b9
not_Sig	Cyp2a4
not_Sig	Cyp2a5
Sig	Cyp2f2

not_Sig	Cyp2t4
not_Sig	Egln2
not_Sig	Rab4b
not_Sig	Mir3101
not_Sig	Mia
not_Sig	Snrpa
not_Sig	BC024978
not_Sig	Itpkc
not_Sig	Adck4
not_Sig	Numbl
not_Sig	Ltbp4
not_Sig	Shkbp1
not_Sig	Sptbn4
not_Sig	Blvrb
not_Sig	Sertad3
not_Sig	Sertad1
not_Sig	Prx
not_Sig	Hipk4
not_Sig	Pld3
Sig	2310022A10Rik
not_Sig	Akt2
not_Sig	Ttc9b
not_Sig	Map3k10
not_Sig	C030039L03Rik
not_Sig	Zfp60
not_Sig	Zfp626
not_Sig	Zfp59
not_Sig	Zfp607
not_Sig	1700049G17Rik
not_Sig	Zfp780b
not_Sig	Zfp850
not_Sig	Psmc4
Sig	Fcgbp
not_Sig	Fbl
not_Sig	Dyrk1b
Sig	Eid2
not_Sig	Eid2b
not_Sig	Timm50
not_Sig	Supt5
not_Sig	Rps16
not_Sig	AF357399
Sig	Plekhg2
not_Sig	Zfp36
not_Sig	Med29
not_Sig	Paf1
not_Sig	Samd4b
not_Sig	Gmfg
not_Sig	Lrfn1

not_Sig	Pak4
not_Sig	Fbxo27
not_Sig	Fbxo17
not_Sig	Mrps12
not_Sig	Sars2
not_Sig	Gm6537
not_Sig	Nfkbib
not_Sig	Sirt2
not_Sig	Gm19897
not_Sig	Rinl
not_Sig	Hnrnpl
Sig	Ech1
not_Sig	Lgals4
not_Sig	Lgals7
not_Sig	Capn12
not_Sig	Actn4
Sig	Eif3k
not_Sig	Map4k1
not_Sig	Ryr1
Sig	Mir1963
not_Sig	Rasgrp4
not_Sig	Fam98c
not_Sig	Spred3
not_Sig	Ggn
Sig	Psmd8
not_Sig	Catsperg1
not_Sig	Kcnk6
Sig	Yif1b
not_Sig	2200002D01Rik
not_Sig	Spint2
Sig	Ppp1r14a
not_Sig	Dpf1
not_Sig	Sipa1l3
not_Sig	Catsperg2
Sig	Zfp84
Sig	Zfp30
not_Sig	Zfp790
not_Sig	Zfp940
not_Sig	Zfp420
not_Sig	Zfp27
not_Sig	Zfp383
not_Sig	Zfp74
Sig	Zfp568
not_Sig	Zfp14
not_Sig	Zfp82
not_Sig	Zfp566
not_Sig	Zfp260
not_Sig	Zfp382

not_Sig	Zfp146
not_Sig	Gm5113
not_Sig	Cox7a1
not_Sig	Capns1
not_Sig	Tbcb
not_Sig	Polr2i
not_Sig	Ovol3
not_Sig	Wdr62
not_Sig	Clip3
not_Sig	Alkbh6
not_Sig	Syne4
not_Sig	Sdhaf1
not_Sig	Lrfn3
not_Sig	Tyrobp
not_Sig	Hcst
not_Sig	Nfkbid
not_Sig	Aplp1
not_Sig	Nphs1
not_Sig	Arhgap33
not_Sig	Proser3
not_Sig	Gm38670
not_Sig	Hspb6
not_Sig	Lin37
not_Sig	Psenen
not_Sig	U2af1l4
not_Sig	Igflr1
Sig	Kmt2b
not_Sig	Zbtb32
not_Sig	Upk1a
not_Sig	Cox6b1
not_Sig	Rbm42
not_Sig	Haus5
not_Sig	Atp4a
not_Sig	Tmem147
not_Sig	Gapdhs
Sig	Sbsn
not_Sig	Dmkn
not_Sig	Krtdap
not_Sig	Ffar2
not_Sig	Cd22
not_Sig	Mag
not_Sig	Hamp2
not_Sig	Hamp
Sig	Usf2
Sig	Lsr
not_Sig	Fxyd5
not_Sig	Fxyd7
Sig	Fxyd1

not_Sig	Lgi4
not_Sig	Fxyd3
not_Sig	Hpn
not_Sig	Scn1b
not_Sig	Gramd1a
Sig	Wtip
not_Sig	Uba2
not_Sig	Pdcd2l
Sig	Gpi1
not_Sig	4931406P16Rik
not_Sig	Lsm14a
not_Sig	Kctd15
not_Sig	Chst8
Sig	Pepd
not_Sig	Cebpg
not_Sig	Cebpa
not_Sig	Slc7a10
not_Sig	Lrp3
not_Sig	Gpatch1
Sig	Rhpn2
not_Sig	Faap24
not_Sig	Cep89
not_Sig	Nudt19
not_Sig	Rgs9bp
not_Sig	Ankrd27
not_Sig	Pdcd5
not_Sig	Dpy19l3
not_Sig	Zfp507
not_Sig	E130304I02Rik
Sig	Tshz3
Sig	Zfp536
not_Sig	Uri1
not_Sig	Ccne1
not_Sig	1600014C10Rik
not_Sig	Plekhf1
not_Sig	Pop4
not_Sig	Zfp939
not_Sig	Zfp619
not_Sig	A230077H06Rik
not_Sig	Vstm2b
not_Sig	AI987944
not_Sig	Vmn2r57
Sig	AW146154
not_Sig	Zfp788
not_Sig	Zfp141
not_Sig	9830147E19Rik
not_Sig	Gm5595
not_Sig	Zfp715

not_Sig	Siglecg
not_Sig	Nkg7
not_Sig	Etfb
Sig	Vsig10l
not_Sig	Iglon5
not_Sig	Cd33
Sig	Zfp658
not_Sig	Zfp719
not_Sig	Siglece
not_Sig	Ctu1
not_Sig	Klk10
not_Sig	Klk9
not_Sig	Klk8
not_Sig	Klk1
not_Sig	2410002F23Rik
Sig	Clec11a
not_Sig	Shank1
not_Sig	1700008O03Rik
not_Sig	Syt3
not_Sig	Lrrc4b
not_Sig	Aspdh
Sig	Josd2
not_Sig	5430431A17Rik
not_Sig	Emc10
not_Sig	Fam71e1
not_Sig	Mybpc2
not_Sig	Spib
not_Sig	Pold1
not_Sig	Nr1h2
not_Sig	Napsa
Sig	Kcnc3
not_Sig	Myh14
not_Sig	Zfp473
not_Sig	Vrk3
not_Sig	Atf5
not_Sig	Nup62-il4i1
not_Sig	Nup62
not_Sig	Il4i1
not_Sig	Tbc1d17
not_Sig	Akt1s1
not_Sig	Pnkp
Sig	Ptov1
not_Sig	Med25
not_Sig	Fuz
not_Sig	Ap2a1
not_Sig	Tsks
not_Sig	Cpt1c
not_Sig	Prmt1

not_Sig	Gm15545
not_Sig	Bcl2l12
not_Sig	Irf3
Sig	Scaf1
not_Sig	Rras
Sig	Prr12
not_Sig	Prrg2
not_Sig	Nosip
not_Sig	Rcn3
not_Sig	Fcgrt
not_Sig	Rps11
not_Sig	Snord35b
Sig	Rpl13a
not_Sig	Snord35a
not_Sig	Flt3l
not_Sig	Aldh16a1
Sig	Pih1d1
not_Sig	Slc17a7
not_Sig	Ccdc155
not_Sig	Tead2
not_Sig	Cd37
not_Sig	Trpm4
not_Sig	Rpl14-ps1
not_Sig	Hrc
not_Sig	Ppfia3
not_Sig	Lin7b
Sig	Snrnp70
not_Sig	Kcna7
not_Sig	Ntf5
not_Sig	Lhb
not_Sig	Ruvbl2
not_Sig	Gys1
not_Sig	Ftl1
not_Sig	Bax
Sig	Dhdh
not_Sig	Tulp2
not_Sig	Nucb1
not_Sig	Ppp1r15a
not_Sig	Plekha4
not_Sig	Hsd17b14
not_Sig	0610005C13Rik
not_Sig	Bcat2
Sig	Rasip1
not_Sig	Mamstr
not_Sig	Fut2
not_Sig	Sec1
not_Sig	Ntn5
not_Sig	Car11

not_Sig	Dbp
not_Sig	Sphk2
not_Sig	Rpl18
not_Sig	Lmtk3
not_Sig	Cyth2
not_Sig	Kcnj14
not_Sig	Grwd1
not_Sig	Grin2d
not_Sig	Kdelr1
not_Sig	Tmem143
not_Sig	Emp3
not_Sig	Ccdc114
not_Sig	Abcc6
not_Sig	Nomo1
not_Sig	Kcnj11
not_Sig	Abcc8
not_Sig	Ush1c
not_Sig	Otog
not_Sig	Munc
not_Sig	Myod1
not_Sig	Kcnc1
not_Sig	Sergef
not_Sig	Tph1
not_Sig	Saal1
not_Sig	Saa3
not_Sig	Hps5
not_Sig	Gtf2h1
not_Sig	Ldha
not_Sig	Tsg101
not_Sig	Uevld
not_Sig	Spty2d1
not_Sig	Tmem86a
not_Sig	Mrgpra4
not_Sig	Mrgprb1
not_Sig	Mrgprx2
not_Sig	Mrgprb2
Sig	Zdhhc13
not_Sig	Csrp3
Sig	E2f8
Sig	Nav2
not_Sig	Htatip2
not_Sig	Prmt3
Sig	Nell1
not_Sig	Ano5
not_Sig	Fancf
not_Sig	Gas2
not_Sig	Svip
not_Sig	Luzp2

not_Sig	Siglech
not_Sig	Tubgcp5
not_Sig	Cyfip1
not_Sig	Nipa2
not_Sig	A230056P14Rik
not_Sig	Nipa1
not_Sig	Herc2
not_Sig	Gabrb3
not_Sig	Atp10a
not_Sig	Ube3a
not_Sig	C230091D08Rik
not_Sig	Snhg14
not_Sig	Snord64
not_Sig	A330076H08Rik
not_Sig	Ndn
not_Sig	Mkrn3
not_Sig	Peg12
not_Sig	Chrna7
not_Sig	Otud7a
Sig	Klf13
Sig	E030018B13Rik
not_Sig	Trpm1
not_Sig	Mtmr10
not_Sig	Fan1
not_Sig	Mphosph10
not_Sig	Mcee
not_Sig	Apba2
not_Sig	Ndnl2
Sig	Tjp1
not_Sig	Tarsl2
not_Sig	Tm2d3
Sig	Pcsk6
not_Sig	Snrpa1
not_Sig	Vimp
not_Sig	Chsy1
not_Sig	Lrrk1
not_Sig	Aldh1a3
not_Sig	Asb7
not_Sig	Lins
not_Sig	Adamts17
not_Sig	1700112J16Rik
not_Sig	Lysmd4
not_Sig	Mef2a
not_Sig	Lrrc28
not_Sig	Ttc23
not_Sig	Synm
Sig	Igf1r
not_Sig	Pgpep1l

not_Sig	Fam169b
not_Sig	Arrdc4
not_Sig	Gm29683
Sig	Nr2f2
Sig	B130024G19Rik
not_Sig	Mctp2
not_Sig	A730056A06Rik
Sig	Rgma
Sig	Chd2
not_Sig	1810026B05Rik
Sig	Fam174b
not_Sig	St8sia2
not_Sig	Slco3a1
not_Sig	Sv2b
not_Sig	Akap13
not_Sig	Klhl25
not_Sig	Agbl1
not_Sig	Ntrk3
Sig	Mrpl46
Sig	Mrps11
not_Sig	Det1
not_Sig	Aen
Sig	Isg20
Sig	Acan
not_Sig	Hapln3
not_Sig	Mfge8
not_Sig	Abhd2
not_Sig	Fanci
not_Sig	Polg
not_Sig	Ticrr
not_Sig	Kif7
not_Sig	Plin1
Sig	Pex11a
not_Sig	Wdr93
Sig	Anpep
not_Sig	Ap3s2
not_Sig	Arpin
not_Sig	Zfp710
not_Sig	Gm21057
not_Sig	Idh2
not_Sig	Sema4b
not_Sig	Cib1
Sig	Gdpgp1
not_Sig	Ttll13
not_Sig	Ngrn
Sig	Vps33b
not_Sig	Prc1
not_Sig	Rccd1

not_Sig	Unc45a
not_Sig	Hddc3
not_Sig	Man2a2
not_Sig	Fes
Sig	Furin
not_Sig	Blm
Sig	Crtc3
not_Sig	Gm15880
not_Sig	Iqgap1
not_Sig	Zscan2
not_Sig	Wdr73
not_Sig	Nmb
not_Sig	Sec11a
Sig	Zfp592
not_Sig	Alpk3
not_Sig	Pde8a
Sig	Rps17
Sig	Cpeb1
not_Sig	Ap3b2
Sig	BC048679
Sig	2900076A07Rik
Sig	Mir1839
not_Sig	Fsd2
not_Sig	Whamm
Sig	Homer2
not_Sig	Fam103a1
not_Sig	3110040N11Rik
not_Sig	Btbd1
not_Sig	Tm6sf1
not_Sig	Hdgfrp3
not_Sig	Sh3gl3
not_Sig	Adamtsl3
not_Sig	Saxo2
not_Sig	Eftud1
Sig	4933406J10Rik
not_Sig	Mex3b
not_Sig	Tmc3
not_Sig	Stard5
not_Sig	Il16
not_Sig	Mesdc1
Sig	Mesdc2
not_Sig	Cemip
not_Sig	Abhd17c
not_Sig	Arnt2
not_Sig	Gm2115
not_Sig	Fah
not_Sig	Zfand6
not_Sig	2610206C17Rik

not_Sig	Folh1
Sig	Nox4
Sig	Ctsc
Sig	Rab38
Sig	Tmem135
not_Sig	Fzd4
not_Sig	AI314278
not_Sig	Prss23
not_Sig	Me3
not_Sig	Ccdc81
not_Sig	l7Rn6
not_Sig	Eed
not_Sig	2310010J17Rik
not_Sig	Picalm
Sig	Sytl2
not_Sig	Crebzf
not_Sig	Tmem126a
not_Sig	Tmem126b
not_Sig	Dlg2
Sig	Ccdc90b
not_Sig	Ankrd42
Sig	Pcf11
not_Sig	4632427E13Rik
not_Sig	Rab30
not_Sig	Ddias
not_Sig	Prcp
Sig	Fam181b
not_Sig	Tenm4
not_Sig	Gm15412
not_Sig	Gm15413
not_Sig	Nars2
not_Sig	Gab2
not_Sig	Usp35
Sig	Kctd21
not_Sig	Alg8
Sig	Ndufc2
Sig	Thrsp
not_Sig	Kctd14
Sig	Ints4
Sig	Aamdc
not_Sig	Rsf1
not_Sig	Rsf1os1
not_Sig	Clns1a
not_Sig	Aqp11
Sig	Pak1
not_Sig	Myo7a
not_Sig	Capn5
not_Sig	Omp

not_Sig	Gm16938
not_Sig	Acer3
not_Sig	Tsku
Sig	Lrrc32
not_Sig	2210018M11Rik
not_Sig	Prkrir
not_Sig	Wnt11
not_Sig	Uvrag
not_Sig	Dgat2
Sig	Mogat2
not_Sig	Gm26705
not_Sig	Map6
Sig	Serpinh1
not_Sig	Gdpd5
not_Sig	Klhl35
Sig	Rps3
not_Sig	Snord15b
Sig	Snord15a
Sig	Arrb1
Sig	Gm4980
not_Sig	Slco2b1
not_Sig	Neu3
not_Sig	Spcs2
not_Sig	Xrra1
Sig	Rnf169
Sig	Chrdl2
not_Sig	Pold3
not_Sig	Lipt2
not_Sig	Kcne3
not_Sig	Pgm2l1
not_Sig	Gpx2-ps1
not_Sig	P4ha3
not_Sig	Ppme1
not_Sig	C2cd3
Sig	Ucp3
not_Sig	Ucp2
not_Sig	Dnajb13
Sig	Coa4
Sig	Mrpl48
not_Sig	Rab6a
not_Sig	Plekhb1
not_Sig	Fam168a
Sig	Relt
not_Sig	Arhgef17
not_Sig	P2ry6
not_Sig	P2ry2
not_Sig	Fchsd2
not_Sig	Atg16l2

not_Sig	Stard10
Sig	Arap1
Sig	Pde2a
not_Sig	Art2a-ps
not_Sig	Clpb
not_Sig	Inppl1
Sig	Folr2
not_Sig	Folr1
Sig	Anapc15
not_Sig	Tomt
Sig	Lamtor1
not_Sig	Lrrc51
not_Sig	Numa1
not_Sig	Il18bp
not_Sig	Rnf121
not_Sig	Xndc1
Sig	Art5
not_Sig	Art1
Sig	Chrna10
not_Sig	Nup98
not_Sig	Pgap2
not_Sig	Rhog
not_Sig	Stim1
not_Sig	Rrm1
not_Sig	Olfr543
not_Sig	Trim21
not_Sig	Trim68
Sig	Olfr558
Sig	Olfr78
not_Sig	Trim6
not_Sig	Trim34a
not_Sig	Trim34b
not_Sig	Trim12c
not_Sig	Trim30a
not_Sig	Trim30d
not_Sig	Fam160a2
not_Sig	Cnga4
not_Sig	Cckbr
not_Sig	Prkcdbp
not_Sig	Smpd1
not_Sig	Apbb1
not_Sig	Hpx
Sig	Trim3
not_Sig	Arfip2
not_Sig	Timm10b
not_Sig	Rrp8
not_Sig	Ilk
not_Sig	Taf10

Sig	Tpp1
Sig	Dchs1
Sig	Mrpl17
not_Sig	Gm4759
not_Sig	Gm1966
not_Sig	Syt9
not_Sig	Olfml1
not_Sig	Ppfibp2
not_Sig	Cyb5r2
not_Sig	Nlrp10
not_Sig	Eif3f
not_Sig	Tub
not_Sig	Ric3
not_Sig	Lmo1
not_Sig	Stk33
Sig	Trim66
not_Sig	Rpl27a
Sig	Snora3
Sig	St5
not_Sig	Akip1
not_Sig	Tmem9b
Sig	Nrip3
not_Sig	Scube2
Sig	Dennd5a
Sig	Tmem41b
Sig	Ipo7
not_Sig	Snora23
not_Sig	Zfp143
not_Sig	Wee1
not_Sig	Swap70
Sig	Sbf2
not_Sig	Adm
Sig	Ampd3
not_Sig	Rnf141
not_Sig	Lyve1
not_Sig	Mrvi1
not_Sig	Gm16336
not_Sig	Ctr9
not_Sig	Eif4g2
not_Sig	1700012D14Rik
Sig	Galnt18
not_Sig	Usp47
not_Sig	Dkk3
not_Sig	Mical2
not_Sig	Micalcl
not_Sig	Parva
not_Sig	Tead1
not_Sig	Rassf10

Sig	Arntl
not_Sig	Btbd10
Sig	Far1
not_Sig	Far1os
Sig	Spon1
not_Sig	Rras2
Sig	Copb1
Sig	Psma1
not_Sig	4933406I18Rik
not_Sig	Pde3b
not_Sig	Cyp2r1
not_Sig	Insc
not_Sig	Sox6
Sig	1110004F10Rik
not_Sig	Plekha7
Sig	Rps13
not_Sig	Pik3c2a
not_Sig	Nucb2
not_Sig	Xylt1
Sig	Rps15a
not_Sig	Arl6ip1
not_Sig	Smg1
not_Sig	Syt17
not_Sig	Itpripl2
Sig	Coq7
Sig	Tmc7
not_Sig	Tmc5
Sig	Gde1
not_Sig	Ccp110
not_Sig	9030624J02Rik
not_Sig	Knop1
not_Sig	Iqck
Sig	Gprc5b
not_Sig	Umod
Sig	Acsm5
not_Sig	Acsm1
not_Sig	Thumpd1
Sig	Acsm3
not_Sig	Eri2
not_Sig	2610020H08Rik
not_Sig	Dcun1d3
not_Sig	Lyrm1
not_Sig	Tmem159
not_Sig	Zp2
not_Sig	Crym
not_Sig	Uqcrc2
not_Sig	Pdzd9
not_Sig	BC030336

not_Sig	Vwa3a
Sig	Eef2k
not_Sig	Polr3e
not_Sig	Cdr2
not_Sig	4933427G17Rik
not_Sig	Mettl9
not_Sig	Igsf6
not_Sig	Otoa
not_Sig	Usp31
not_Sig	Scnn1b
not_Sig	Cog7
not_Sig	Gga2
not_Sig	Ears2
not_Sig	Ubfd1
Sig	Ndufab1
not_Sig	Palb2
not_Sig	Dctn5
not_Sig	Plk1
not_Sig	Chp2
not_Sig	Prkcb
Sig	4930413G21Rik
Sig	Rbbp6
Sig	Tnrc6a
not_Sig	Slc5a11
Sig	Arhgap17
not_Sig	Lcmt1
not_Sig	Zkscan2
not_Sig	Kdm8
not_Sig	Nsmce1
not_Sig	Il4ra
not_Sig	Il21r
not_Sig	Gtf3c1
not_Sig	D430042O09Rik
not_Sig	Gsg1l
not_Sig	Xpo6
not_Sig	Sbk1
not_Sig	Lat
Sig	Spns1
not_Sig	Nfatc2ip
not_Sig	Cd19
not_Sig	Rabep2
not_Sig	Atp2a1
not_Sig	Sh2b1
not_Sig	Tufm
not_Sig	Atxn2l
not_Sig	Eif3c
not_Sig	Cln3
not_Sig	Apobr

not_Sig	Nupr1
not_Sig	Ccdc101
not_Sig	Sult1a1
not_Sig	Slx1b
Sig	Bola2
not_Sig	Coro1a
not_Sig	Mapk3
not_Sig	Gdpd3
not_Sig	Ypel3
not_Sig	Tbx6
not_Sig	Ppp4c
not_Sig	Aldoa
Sig	Fam57b
not_Sig	Doc2a
not_Sig	Ino80e
not_Sig	Hirip3
Sig	Taok2
not_Sig	Tmem219
not_Sig	Kctd13
not_Sig	Asphd1
not_Sig	Sez6l2
not_Sig	Cdipt
not_Sig	Mvp
Sig	Pagr1a
Sig	Prrt2
Sig	Maz
not_Sig	Kif22
not_Sig	AI467606
Sig	Qprt
not_Sig	Spn
not_Sig	Cd2bp2
not_Sig	Tbc1d10b
Sig	Mylpf
Sig	Sept1
not_Sig	Gm4532
Sig	Zfp553
not_Sig	Zfp771
not_Sig	Dctpp1
Sig	Sephs2
not_Sig	Itgal
not_Sig	Zfp768
not_Sig	Zfp747
Sig	9130019O22Rik
not_Sig	E430018J23Rik
not_Sig	Zfp764
not_Sig	Zfp688
not_Sig	Zfp689
not_Sig	Prr14

not_Sig	Fbrs
not_Sig	1700008J07Rik
Sig	Srcap
Sig	Snora30
not_Sig	Tmem265
not_Sig	Phkg2
not_Sig	Gm166
not_Sig	Rnf40
not_Sig	1700120K04Rik
Sig	Zfp629
Sig	Bcl7c
not_Sig	Ctf1
not_Sig	Ctf2
not_Sig	Fbxl19
not_Sig	Orai3
not_Sig	Setd1a
Sig	Hsd3b7
not_Sig	Stx1b
Sig	Stx4a
not_Sig	Zfp668
Sig	Zfp646
not_Sig	Prss53
Sig	Vkorc1
not_Sig	Bckdk
Sig	Kat8
not_Sig	Prss8
not_Sig	Prss36
Sig	Fus
not_Sig	Pycard
not_Sig	Trim72
not_Sig	Itgam
not_Sig	Itgax
not_Sig	Itgad
not_Sig	Cox6a2
not_Sig	9130023H24Rik
not_Sig	Armc5
not_Sig	Tgfb1i1
not_Sig	Slc5a2
not_Sig	BC017158
Sig	Rgs10
not_Sig	Tial1
not_Sig	Bag3
not_Sig	Inpp5f
not_Sig	Mcmbp
not_Sig	Sec23ip
not_Sig	Wdr11
Sig	Fgfr2
not_Sig	Ate1

Sig	Nsmce4a
not_Sig	Tacc2
Sig	Plekha1
Sig	Htra1
not_Sig	Cuzd1
not_Sig	Fam24a
not_Sig	2310057M21Rik
not_Sig	Pstk
not_Sig	Ikzf5
not_Sig	Acadsb
Sig	Bub3
Sig	Cpxm2
not_Sig	Chst15
not_Sig	Gm10584
not_Sig	Oat
not_Sig	Lhpp
not_Sig	Fam53b
not_Sig	Mettl10
not_Sig	Fam175b
not_Sig	Zranb1
not_Sig	Ctbp2
not_Sig	Edrf1
not_Sig	Mmp21
Sig	Uros
not_Sig	Bccip
not_Sig	Dhx32
not_Sig	Adam12
not_Sig	Dock1
Sig	Fam196a
not_Sig	Ptpre
not_Sig	5830432E09Rik
Sig	Mki67
not_Sig	Gm6249
not_Sig	Mgmt
not_Sig	Ebf3
not_Sig	Gm10578
not_Sig	9430038I01Rik
Sig	Glrx3
not_Sig	Tcerg1l
not_Sig	Mapk1ip1
not_Sig	Ppp2r2d
Sig	Bnip3
not_Sig	Jakmip3
not_Sig	Dpysl4
not_Sig	Stk32c
not_Sig	Lrrc27
not_Sig	Pwwp2b
not_Sig	Inpp5a

not_Sig	Nkx6-2
not_Sig	Cfap46
not_Sig	Kndc1
not_Sig	6430531B16Rik
not_Sig	Adam8
Sig	Tubgcp2
not_Sig	Zfp511
Sig	Caly
not_Sig	Prap1
not_Sig	Fuom
not_Sig	Echs1
not_Sig	Paox
not_Sig	Mtg1
not_Sig	Sprn
not_Sig	Cd163l1
Sig	Cyp2e1
not_Sig	Syce1
not_Sig	Urah
not_Sig	Odf3
not_Sig	Bet1l
not_Sig	Ric8
not_Sig	Sirt3
not_Sig	Psmd13
not_Sig	Cox8b
not_Sig	Nlrp6
not_Sig	Athl1
not_Sig	Ifitm5
not_Sig	Ifitm2
not_Sig	Ifitm1
not_Sig	Ifitm3
not_Sig	Ifitm6
not_Sig	B4galnt4
not_Sig	Pkp3
Sig	Sigirr
not_Sig	Ano9
not_Sig	Ptdss2
Sig	Rnh1
Sig	Hras
not_Sig	Lrrc56
not_Sig	Rassf7
Sig	Phrf1
not_Sig	Irf7
Sig	Deaf1
not_Sig	Tmem80
not_Sig	Eps8l2
not_Sig	B230206H07Rik
not_Sig	Taldo1
not_Sig	Pddc1

not_Sig	Cend1
not_Sig	Slc25a22
not_Sig	Pidd1
Sig	Rplp2
not_Sig	Snora52
not_Sig	Pnpla2
not_Sig	Cracr2b
Sig	Cd151
not_Sig	Polr2l
not_Sig	Tspan4
Sig	Chid1
not_Sig	Ap2a2
not_Sig	Gm16982
Sig	Muc6
not_Sig	Muc2
not_Sig	Muc5b
not_Sig	Tollip
Sig	Brsk2
not_Sig	Mob2
not_Sig	Dusp8
not_Sig	Ifitm10
Sig	Ctsd
not_Sig	Syt8
not_Sig	Tnni2
not_Sig	Lsp1
not_Sig	Prr33
Sig	Tnnt3
not_Sig	Mrpl23
not_Sig	Nctc1
not_Sig	H19
Sig	Igf2
not_Sig	Igf2os
Sig	Th
not_Sig	Tspan32
not_Sig	R74862
not_Sig	Cd81
not_Sig	Tssc4
not_Sig	Trpm5
Sig	Kcnq1
not_Sig	Kcnq1ot1
not_Sig	4933417O13Rik
Sig	Cdkn1c
not_Sig	Slc22a18
not_Sig	Nap1l4
not_Sig	Cars
not_Sig	Tnfrsf26
not_Sig	Tnfrsf22
not_Sig	Tnfrsf23

not_Sig	Osbpl5
not_Sig	Mrgpre
not_Sig	Nadsyn1
not_Sig	Dhcr7
not_Sig	Shank2
not_Sig	Cttn
Sig	Ppfia1
not_Sig	Fadd
not_Sig	Ano1
not_Sig	Fgf4
not_Sig	Oraov1
not_Sig	Ccnd1
not_Sig	1810010D01Rik
not_Sig	Tpcn2
not_Sig	Mrgprf
not_Sig	Insr
not_Sig	A430078G23Rik
not_Sig	Arhgef18
not_Sig	Pex11g
not_Sig	Zfp358
not_Sig	Mcoln1
Sig	Pnpla6
not_Sig	C330021F23Rik
not_Sig	Camsap3
not_Sig	Xab2
Sig	Pet100
not_Sig	Pcp2
not_Sig	Stxbp2
not_Sig	Retn
not_Sig	Mcemp1
not_Sig	Trappc5
Sig	Fcer2a
not_Sig	Clec4g
not_Sig	Cd209a
Sig	Rprl3
not_Sig	Cd209e
not_Sig	Cd209d
not_Sig	Cd209b
not_Sig	Cd209c
not_Sig	Cd209f
not_Sig	Cd209g
not_Sig	Evi5l
not_Sig	Prr36
not_Sig	Lrrc8e
not_Sig	Map2k7
not_Sig	Tgfbr3l
not_Sig	Snapc2
not_Sig	Ctxn1

not_Sig	Timm44
not_Sig	Elavl1
not_Sig	Ccl25
not_Sig	Cers4
not_Sig	Zfp958
Sig	Efnb2
not_Sig	Arglu1
not_Sig	Fam155a
not_Sig	Lig4
not_Sig	Abhd13
not_Sig	Tnfsf13b
not_Sig	Myo16
not_Sig	4833411C07Rik
not_Sig	Irs2
not_Sig	9530052E02Rik
not_Sig	Col4a1
not_Sig	Col4a2
not_Sig	Rab20
not_Sig	E230013L22Rik
not_Sig	Carkd
not_Sig	Cars2
not_Sig	Ing1
not_Sig	Ankrd10
Sig	Arhgef7
not_Sig	Tubgcp3
not_Sig	Atp11a
not_Sig	Gm15350
Sig	Mcf2l
not_Sig	Gm38671
not_Sig	F7
Sig	F10
not_Sig	Proz
not_Sig	Pcid2
not_Sig	Cul4a
not_Sig	Lamp1
Sig	Grtp1
not_Sig	Adprhl1
not_Sig	Dcun1d2
not_Sig	Tmco3
not_Sig	Tfdp1
not_Sig	Grk1
not_Sig	Tmem255b
not_Sig	Gas6
not_Sig	1700029H14Rik
not_Sig	Rasa3
not_Sig	Cdc16
not_Sig	Upf3a
not_Sig	Champ1

not_Sig	Coprs
not_Sig	Fbxo25
not_Sig	Tdrp
not_Sig	Erich1
not_Sig	Dlgap2
not_Sig	Cln8
not_Sig	Arhgef10
not_Sig	Kbtbd11
Sig	Myom2
not_Sig	Csmd1
not_Sig	Mcph1
Sig	Angpt2
not_Sig	Agpat5
not_Sig	Xkr5
not_Sig	6820431F20Rik
not_Sig	2610005L07Rik
not_Sig	Atp7b
not_Sig	Alg11
not_Sig	Nek3
not_Sig	Ckap2
Sig	Vps36
Sig	Thsd1
not_Sig	Slc25a15
not_Sig	1810012K16Rik
not_Sig	Mrps31
not_Sig	Smim19
Sig	Slc20a2
not_Sig	Vdac3
Sig	Polb
not_Sig	A930013F10Rik
not_Sig	Ikbkb
not_Sig	Plat
not_Sig	Ap3m2
not_Sig	Kat6a
not_Sig	Ank1
Sig	Gm15816
not_Sig	Nkx6-3
Sig	Agpat6
not_Sig	Gins4
not_Sig	Golga7
not_Sig	Sfrp1
not_Sig	Zmat4
not_Sig	1810011O10Rik
not_Sig	Ido2
not_Sig	Adam3
not_Sig	Adam32
Sig	Adam9
Sig	Tm2d2

not_Sig	Htra4
not_Sig	Plekha2
not_Sig	Tacc1
not_Sig	Fgfr1
not_Sig	Gm16159
not_Sig	Letm2
not_Sig	Whsc1l1
not_Sig	Ppapdc1b
not_Sig	Ddhd2
not_Sig	Bag4
not_Sig	Lsm1
not_Sig	Star
not_Sig	Ash2l
not_Sig	Kcnu1
not_Sig	Hgsnat
not_Sig	Pomk
not_Sig	Fnta
not_Sig	Hook3
not_Sig	1700047A11Rik
not_Sig	Rnf170
not_Sig	Thap1
not_Sig	2310008N11Rik
not_Sig	Zfp703
not_Sig	Erlin2
not_Sig	Prosc
Sig	Adgra3
not_Sig	Brf2
not_Sig	Rab11fip1
Sig	Adrb3
not_Sig	Eif4ebp1
Sig	Dusp26
Sig	Rnf122
not_Sig	Tti2
not_Sig	Mak16
not_Sig	Fut10
not_Sig	Nrg1
not_Sig	Wrn
not_Sig	Purg
not_Sig	Tex15
not_Sig	Ppp2cb
not_Sig	Ubxn8
not_Sig	Gsr
not_Sig	Gtf2e2
not_Sig	Smim18
not_Sig	Rbpms
not_Sig	Dctn6
not_Sig	Mboat4
not_Sig	Leprotl1

Sig	Saraf
not_Sig	Mir6395
not_Sig	Dusp4
not_Sig	Tnks
Sig	Ppp1r3b
not_Sig	Eri1
not_Sig	Mfhas1
Sig	D8Ertd82e
not_Sig	Lonrf1
not_Sig	6430573F11Rik
not_Sig	Dlc1
not_Sig	G630064G18Rik
not_Sig	AI429214
not_Sig	Tusc3
not_Sig	Msr1
not_Sig	Fgf20
Sig	Mir7666
Sig	Micu3
not_Sig	Zdhhc2
not_Sig	Cnot7
not_Sig	Vps37a
not_Sig	Mtmr7
not_Sig	Slc7a2
not_Sig	Pdgfrl
not_Sig	Mtus1
not_Sig	B430010I23Rik
not_Sig	Pcm1
Sig	Asah1
not_Sig	Frg1
not_Sig	Fat1
not_Sig	Klkb1
not_Sig	Cyp4v3
not_Sig	Fam149a
not_Sig	Tlr3
not_Sig	Sorbs2
not_Sig	Sorbs2os
Sig	Pdlim3
not_Sig	1700029J07Rik
not_Sig	Ufsp2
not_Sig	Ankrd37
Sig	Lrp2bp
not_Sig	Snx25
not_Sig	Cfap97
not_Sig	Slc25a4
not_Sig	Helt
Sig	Acsl1
not_Sig	Cenpu
not_Sig	Primpol

not_Sig	Casp3
not_Sig	Gm16675
not_Sig	Irf2
not_Sig	Enpp6
not_Sig	Stox2
not_Sig	Trappc11
not_Sig	Rwdd4a
not_Sig	Ing2
not_Sig	Cdkn2aip
not_Sig	Cldn22
Sig	Wwc2
not_Sig	Dctd
not_Sig	Tenm3
not_Sig	Aga
Sig	Neil3
not_Sig	Vegfc
not_Sig	Spcs3
not_Sig	Asb5
not_Sig	Wdr17
Sig	Gpm6a
not_Sig	Hpgd
not_Sig	Cep44
Sig	Fbxo8
not_Sig	Sap30
not_Sig	2500002B13Rik
not_Sig	Hmgb2
Sig	Galnt7
not_Sig	AW046200
Sig	Mfap3l
not_Sig	2700029M09Rik
not_Sig	Clcn3
not_Sig	Nek1
not_Sig	Sh3rf1
Sig	1700001D01Rik
Sig	Cbr4
Sig	Palld
not_Sig	Ddx60
not_Sig	Spock3
not_Sig	Tll1
not_Sig	Cpe
Sig	Msmo1
Sig	Klhl2
not_Sig	Tmem192
not_Sig	March1
not_Sig	Tma16
Sig	Npy1r
not_Sig	Naf1
Sig	Nat1

not_Sig	Nat2
Sig	Psd3
Sig	Sh2d4a
Sig	Csgalnact1
Sig	1700125H03Rik
not_Sig	Ints10
not_Sig	Lpl
not_Sig	Slc18a1
Sig	Atp6v1b2
not_Sig	Lzts1
not_Sig	Zfp930
not_Sig	D130040H23Rik
not_Sig	Gm10033
not_Sig	Zfp868
not_Sig	Zfp964
not_Sig	Zfp869
not_Sig	Zfp963
not_Sig	Zfp866
not_Sig	Atp13a1
not_Sig	Gmip
not_Sig	Lpar2
not_Sig	Cilp2
Sig	Ndufa13
not_Sig	Tssk6
not_Sig	Gatad2a
not_Sig	Mau2
Sig	Sugp1
not_Sig	Tm6sf2
Sig	Hapln4
Sig	Ncan
not_Sig	Rfxank
not_Sig	Nr2c2ap
not_Sig	2310045N01Rik
not_Sig	Tmem161a
not_Sig	Slc25a42
not_Sig	Armc6
not_Sig	Sugp2
not_Sig	Homer3
not_Sig	Ddx49
not_Sig	Cope
not_Sig	Upf1
Sig	Comp
Sig	Crtc1
not_Sig	Klhl26
not_Sig	Crlf1
Sig	2810428I15Rik
Sig	Uba52
not_Sig	Kxd1

Sig	Fkbp8
not_Sig	Ell
not_Sig	Isyna1
not_Sig	Ssbp4
not_Sig	Lrrc25
not_Sig	Gdf15
not_Sig	Pgpep1
not_Sig	Lsm4
not_Sig	Jund
Sig	Pde4c
not_Sig	Rab3a
not_Sig	Mpv17l2
not_Sig	Ifi30
not_Sig	Pik3r2
not_Sig	2010320M18Rik
not_Sig	Mast3
not_Sig	Il12rb1
not_Sig	Arrdc2
not_Sig	Kcnn1
not_Sig	Ccdc124
Sig	Rpl18a
not_Sig	Snora68
not_Sig	Map1s
not_Sig	Haus8
not_Sig	Myo9b
Sig	Use1
not_Sig	Ocel1
not_Sig	Nr2f6
Sig	Ushbp1
not_Sig	Babam1
Sig	Ankle1
not_Sig	Abhd8
not_Sig	Mrpl34
not_Sig	Dda1
not_Sig	Ano8
not_Sig	Gtpbp3
not_Sig	Plvap
not_Sig	Bst2
not_Sig	Mvb12a
not_Sig	Tmem221
Sig	Nxnl1
not_Sig	Slc27a1
not_Sig	Pgls
not_Sig	Fam129c
Sig	Colgalt1
not_Sig	Unc13a
not_Sig	Jak3
not_Sig	Insl3

not_Sig	B3gnt3
not_Sig	Fcho1
not_Sig	Zfp709
not_Sig	Zfp882
not_Sig	Zfp617
not_Sig	Zfp961
not_Sig	Cyp4f18
not_Sig	Tpm4
not_Sig	Rab8a
not_Sig	Hsh2d
Sig	Fam32a
not_Sig	Ap1m1
Sig	Klf2
Sig	Eps15l1
not_Sig	Calr3
not_Sig	1700030K09Rik
not_Sig	Cherp
not_Sig	Slc35e1
Sig	Med26
Sig	Smim7
not_Sig	Tmem38a
not_Sig	Nwd1
not_Sig	Sin3b
not_Sig	F2rl3
not_Sig	Large
not_Sig	Mir28b
not_Sig	Hmgxb4
not_Sig	Tom1
not_Sig	Hmox1
not_Sig	Mcm5
not_Sig	Rasd2
not_Sig	Gm10649
not_Sig	Nr3c2
not_Sig	Arhgap10
not_Sig	0610038B21Rik
not_Sig	Prmt10
not_Sig	Tmem184c
not_Sig	Ednra
not_Sig	Rbmxl1
not_Sig	Slc10a7
not_Sig	Lsm6
not_Sig	Zfp827
not_Sig	Mmaa
not_Sig	Gm4890
not_Sig	Smad1
not_Sig	Otud4
not_Sig	Abce1
not_Sig	Anapc10

not_Sig	Hhip
Sig	Gypa
not_Sig	Smarca5
not_Sig	Gab1
not_Sig	Usp38
not_Sig	Inpp4b
not_Sig	Il15
not_Sig	Zfp330
not_Sig	Rnf150
not_Sig	Tbc1d9
Sig	Ucp1
Sig	Elmod2
not_Sig	Scoc
not_Sig	Ndufb7
not_Sig	Tecr
not_Sig	Dnajb1
not_Sig	Gipc1
not_Sig	Ptger1
Sig	Pkn1
not_Sig	Ddx39
not_Sig	Adgre5
not_Sig	Adgrl1
not_Sig	Asf1b
Sig	Prkaca
not_Sig	Samd1
not_Sig	1700067K01Rik
not_Sig	2210011C24Rik
not_Sig	Palm3
not_Sig	Il27ra
Sig	Rfx1
not_Sig	Dcaf15
not_Sig	Podnl1
not_Sig	Cc2d1a
not_Sig	4930432K21Rik
not_Sig	Mir23a
not_Sig	Mir27a
not_Sig	Mir24-2
Sig	Zswim4
Sig	D8Ertd738e
not_Sig	Mri1
not_Sig	Ccdc130
Sig	Cacna1a
not_Sig	Ier2
not_Sig	Nacc1
not_Sig	Trmt1
not_Sig	Lyl1
Sig	Nfix
not_Sig	Dand5

not_Sig	Gadd45gip1
not_Sig	Rad23a
Sig	Calr
not_Sig	Farsa
not_Sig	Syce2
not_Sig	Gcdh
Sig	Klf1
not_Sig	Dnase2a
not_Sig	Mast1
not_Sig	Gm38426
not_Sig	Rnaseh2a
Sig	Prdx2
not_Sig	Junb
not_Sig	Hook2
not_Sig	Asna1
Sig	2310036O22Rik
not_Sig	Tnpo2
Sig	A230103J11Rik
not_Sig	Fbxw9
not_Sig	Dhps
not_Sig	Wdr83
not_Sig	Wdr83os
not_Sig	Man2b1
not_Sig	Zfp791
Sig	Vps35
not_Sig	Orc6
not_Sig	Mylk3
not_Sig	4921524J17Rik
not_Sig	Gpt2
not_Sig	Dnaja2
Sig	Neto2
not_Sig	Itfg1
not_Sig	Phkb
Sig	Lonp2
not_Sig	Siah1a
not_Sig	Gm10638
Sig	N4bp1
Sig	Gm10637
not_Sig	Cbln1
not_Sig	Gm2694
not_Sig	Zfp423
not_Sig	Cnep1r1
not_Sig	Heatr3
Sig	Papd5
not_Sig	Adcy7
not_Sig	Brd7
not_Sig	Nkd1
not_Sig	Snx20

not_Sig	Nod2
not_Sig	Cyld
not_Sig	Tox3
not_Sig	Chd9
not_Sig	Rbl2
Sig	Aktip
not_Sig	Rpgrip1l
Sig	Fto
not_Sig	Irx3
not_Sig	Crnde
not_Sig	Irx5
not_Sig	Mmp2
not_Sig	Lpcat2
not_Sig	Capns2
Sig	Slc6a2
not_Sig	Ces1c
Sig	Ces1d
Sig	Ces1e
not_Sig	Ces1f
not_Sig	Ces5a
not_Sig	Gnao1
not_Sig	4930488L21Rik
not_Sig	Amfr
not_Sig	Nudt21
not_Sig	Ogfod1
not_Sig	Bbs2
not_Sig	Mt3
not_Sig	Mt2
not_Sig	Mt1
not_Sig	Nup93
not_Sig	Herpud1
not_Sig	9330175E14Rik
not_Sig	Nlrc5
not_Sig	Cpne2
not_Sig	Fam192a
not_Sig	Rspry1
not_Sig	Arl2bp
not_Sig	Pllp
not_Sig	Ccl22
not_Sig	Cx3cl1
not_Sig	Ciapin1
not_Sig	Coq9
not_Sig	Polr2c
Sig	Dok4
Sig	Ccdc102a
not_Sig	Adgrg5
Sig	Adgrg1
not_Sig	Adgrg3

not_Sig	Katnb1
Sig	Kifc3
not_Sig	Cngb1
Sig	Zfp319
not_Sig	Usb1
not_Sig	Mmp15
not_Sig	Cfap20
not_Sig	Csnk2a2
not_Sig	Gins3
not_Sig	Ndrg4
Sig	Setd6
not_Sig	Cnot1
not_Sig	4930513N10Rik
not_Sig	Slc38a7
not_Sig	Got2
not_Sig	Cdh11
not_Sig	Gm29682
Sig	Cdh5
not_Sig	Bean1
not_Sig	Tk2
not_Sig	Cklf
not_Sig	Cmtm3
not_Sig	Cmtm4
not_Sig	Dync1li2
not_Sig	Ccdc79
not_Sig	Nae1
not_Sig	Car7
not_Sig	Pdp2
not_Sig	Cdh16
Sig	Rrad
Sig	Fam96b
not_Sig	Ces2c
not_Sig	Ces2g
not_Sig	Ces3a
Sig	Cbfb
not_Sig	D230025D16Rik
not_Sig	B3gnt9
not_Sig	Tradd
not_Sig	Fbxl8
not_Sig	Hsf4
Sig	Nol3
not_Sig	4931428F04Rik
not_Sig	Exoc3l
not_Sig	E2f4
not_Sig	Elmo3
not_Sig	Lrrc29
Sig	Tmem208
not_Sig	Fhod1

not_Sig	Slc9a5
not_Sig	Plekhg4
not_Sig	Kctd19
Sig	Lrrc36
Sig	Tppp3
not_Sig	Zdhhc1
not_Sig	Hsd11b2
Sig	Atp6v0d1
not_Sig	Agrp
Sig	Fam65a
not_Sig	Ctcf
not_Sig	Rltpr
not_Sig	Acd
not_Sig	Pard6a
not_Sig	Enkd1
not_Sig	Gfod2
not_Sig	Ranbp10
not_Sig	Tsnaxip1
not_Sig	Cenpt
not_Sig	Thap11
not_Sig	Nutf2
not_Sig	Edc4
not_Sig	Nrn1l
Sig	Pskh1
not_Sig	Ctrl
not_Sig	Psmb10
Sig	Lcat
not_Sig	Slc12a4
not_Sig	Dpep2
Sig	Ddx28
not_Sig	Dus2
not_Sig	Nfatc3
not_Sig	Esrp2
not_Sig	Pla2g15
not_Sig	Slc7a6
not_Sig	Slc7a6os
not_Sig	Prmt7
Sig	Smpd3
not_Sig	4930506A18Rik
not_Sig	Zfp90
not_Sig	Cdh3
not_Sig	Cdh1
not_Sig	Tango6
not_Sig	Has3
not_Sig	Chtf8
not_Sig	Cirh1a
not_Sig	Sntb2
not_Sig	Vps4a

not_Sig	Pdf
not_Sig	Cog8
not_Sig	Nip7
not_Sig	Terf2
not_Sig	Cyb5b
Sig	Nfat5
not_Sig	Nqo1
not_Sig	Nob1
not_Sig	Wwp2
not_Sig	Psmd7
not_Sig	Lncbate1
not_Sig	Zfhx3
not_Sig	Mir3108
not_Sig	Gm1943
not_Sig	Dhx38
not_Sig	Txnl4b
Sig	Hp
not_Sig	Dhodh
not_Sig	Pkd1l3
not_Sig	Ist1
not_Sig	Zfp821
Sig	Atxn1l
not_Sig	Ap1g1
not_Sig	Snord71
not_Sig	Phlpp2
not_Sig	Marveld3
Sig	Tat
Sig	Zfp612
not_Sig	Cmtr2
Sig	Hydin
Sig	Vac14
Sig	Mtss1l
not_Sig	Il34
not_Sig	Sf3b3
not_Sig	Snord111
Sig	Cog4
not_Sig	Fuk
Sig	St3gal2
not_Sig	Ddx19a
not_Sig	Ddx19b
not_Sig	Aars
not_Sig	Exosc6
not_Sig	Clec18a
not_Sig	Pdpr
not_Sig	9430091E24Rik
not_Sig	Glg1
not_Sig	Rfwd3
not_Sig	Mlkl

not_Sig	Fa2h
not_Sig	Wdr59
Sig	Znrf1
Sig	Ldhd
not_Sig	Zfp1
Sig	Bcar1
Sig	Cfdp1
not_Sig	Tmem170
not_Sig	Tmem231
not_Sig	Gabarapl2
not_Sig	Adat1
not_Sig	Kars
Sig	Terf2ip
not_Sig	Cntnap4
not_Sig	Mon1b
not_Sig	Adamts18
Sig	Nudt7
Sig	Vat1l
not_Sig	Clec3a
not_Sig	Wwox
not_Sig	Maf
Sig	Cdyl2
not_Sig	Cmc2
not_Sig	Cenpn
not_Sig	Atmin
not_Sig	1700030J22Rik
not_Sig	Gcsh
not_Sig	Pkd1l2
not_Sig	Gan
not_Sig	Cmip
not_Sig	Plcg2
not_Sig	Sdr42e1
Sig	Mphosph6
Sig	Cdh13
Sig	Hsbp1
not_Sig	Mlycd
Sig	Osgin1
not_Sig	Mbtps1
Sig	Hsdl1
not_Sig	Dnaaf1
not_Sig	Taf1c
not_Sig	Kcng4
not_Sig	Wfdc1
not_Sig	Tldc1
not_Sig	Cotl1
not_Sig	Klhl36
not_Sig	Usp10
not_Sig	Crispld2

not_Sig	Zdhhc7
not_Sig	6430548M08Rik
not_Sig	A330074K22Rik
not_Sig	Gse1
not_Sig	Mir7687
not_Sig	Gins2
not_Sig	Emc8
Sig	Cox4i1
not_Sig	Irf8
not_Sig	Foxf1
not_Sig	Mthfsd
not_Sig	Foxc2
not_Sig	Foxl1
not_Sig	Fbxo31
not_Sig	Map1lc3b
Sig	Zcchc14
not_Sig	Jph3
Sig	Klhdc4
not_Sig	Slc7a5
not_Sig	Banp
not_Sig	Zfpm1
not_Sig	Trhr2
Sig	Zc3h18
not_Sig	Il17c
not_Sig	Cyba
not_Sig	Mvd
not_Sig	9330133O14Rik
not_Sig	Gm20735
not_Sig	Snai3
Sig	Rnf166
not_Sig	Ctu2
not_Sig	Piezo1
not_Sig	Cdt1
not_Sig	Aprt
not_Sig	Galns
Sig	Trappc2l
Sig	Pabpn1l
Sig	Cbfa2t3
not_Sig	Acsf3
not_Sig	Cdh15
Sig	Ankrd11
not_Sig	2810013P06Rik
not_Sig	Spg7
not_Sig	Rpl13
Sig	Cpne7
not_Sig	Sult5a1
not_Sig	Dpep1
Sig	Chmp1a

not_Sig	Spata33
not_Sig	Cdk10
not_Sig	Spata2l
Sig	4933417D19Rik
not_Sig	Vps9d1
Sig	Zfp276
not_Sig	Fanca
not_Sig	Spire2
Sig	Tcf25
not_Sig	Def8
not_Sig	Afg3l1
not_Sig	Dbndd1
not_Sig	Gas8
Sig	Rhou
not_Sig	Rab4a
not_Sig	Ccsap
not_Sig	Acta1
not_Sig	Nup133
not_Sig	Abcb10
not_Sig	Taf5l
not_Sig	Urb2
Sig	Galnt2
not_Sig	Pgbd5
not_Sig	Cog2
not_Sig	Agt
not_Sig	2310022B05Rik
not_Sig	Ttc13
not_Sig	Arv1
not_Sig	Fam89a
not_Sig	Trim67
Sig	2810004N23Rik
not_Sig	Gnpat
not_Sig	Exoc8
Sig	Sprtn
not_Sig	Egln1
not_Sig	Tsnax
Sig	Disc1
Sig	Sipa1l2
not_Sig	Map10
not_Sig	Ntpcr
not_Sig	Pcnxl2
not_Sig	BC021891
not_Sig	Kcnk1
not_Sig	Slc35f3
Sig	Coa6
not_Sig	Gm17296
not_Sig	Irf2bp2
not_Sig	Tomm20

not_Sig	Rbm34
not_Sig	Pard3
not_Sig	Nrp1
Sig	Itgb1
not_Sig	Ccdc7b
not_Sig	2610044O15Rik8
not_Sig	Alkbh8
not_Sig	Cwf19l2
not_Sig	Gucy1a2
Sig	Aasdhppt
not_Sig	Kbtbd3
not_Sig	Msantd4
not_Sig	Gria4
not_Sig	Casp1
not_Sig	Casp4
Sig	Casp12
not_Sig	Pdgfd
Sig	Ddi1
not_Sig	Dync2h1
not_Sig	Dcun1d5
not_Sig	Mmp13
not_Sig	Mmp12
not_Sig	Mmp3
Sig	Mmp8
not_Sig	Mmp27
not_Sig	Tmem123
not_Sig	Birc2
not_Sig	Birc3
not_Sig	Yap1
not_Sig	9230110C19Rik
not_Sig	Cep126
not_Sig	Trpc6
not_Sig	Gm32014
not_Sig	Arhgap42
not_Sig	Gm16833
not_Sig	Jrkl
Sig	Ccdc82
not_Sig	Phxr4
not_Sig	Maml2
not_Sig	Mtmr2
not_Sig	Cep57
not_Sig	Fam76b
not_Sig	Sesn3
not_Sig	Endod1
not_Sig	Kdm4d
not_Sig	Cwc15
not_Sig	Amotl1
not_Sig	Fut4

not_Sig	Ankrd49
not_Sig	Mre11a
not_Sig	Izumo1r
not_Sig	Panx1
not_Sig	Hephl1
not_Sig	Vstm5
not_Sig	Med17
Sig	4931406C07Rik
not_Sig	Taf1d
not_Sig	Cep295
Sig	Scarna9
not_Sig	Smco4
not_Sig	Ccdc67
not_Sig	Slc36a4
Sig	Fat3
Sig	Chordc1
not_Sig	Naalad2
not_Sig	Zfp558
not_Sig	Zfp317
not_Sig	Olfr856-ps1
not_Sig	Zfp560
not_Sig	Zfp26
not_Sig	Zfp426
not_Sig	Zfp266
not_Sig	Zfp846
not_Sig	Fbxl12os
Sig	Fbxl12
not_Sig	Ubl5
not_Sig	Pin1
not_Sig	Olfm2
not_Sig	Col5a3
not_Sig	A230050P20Rik
not_Sig	Angptl6
Sig	Ppan
not_Sig	Eif3g
not_Sig	Dnmt1
not_Sig	S1pr2
not_Sig	Mrpl4
not_Sig	Icam1
not_Sig	Icam4
not_Sig	Icam5
not_Sig	Zglp1
not_Sig	Fdx1l
Sig	Raver1
not_Sig	Tyk2
Sig	Cdc37
not_Sig	Pde4a
Sig	Keap1

not_Sig	S1pr5
not_Sig	Atg4d
not_Sig	Kri1
not_Sig	Cdkn2d
Sig	Slc44a2
Sig	Ilf3
not_Sig	Gm16853
not_Sig	Qtrt1
Sig	Dnm2
not_Sig	Mir199a-1
Sig	Tmed1
not_Sig	AB124611
not_Sig	Carm1
not_Sig	Yipf2
not_Sig	1810026J23Rik
not_Sig	Gm7904
not_Sig	Smarca4
not_Sig	Ldlr
not_Sig	Spc24
not_Sig	Kank2
Sig	Dock6
not_Sig	Gm6484
not_Sig	Rab3d
Sig	Tmem205
not_Sig	Ccdc159
not_Sig	Lppr2
not_Sig	Swsap1
not_Sig	Epor
Sig	Rgl3
Sig	Prkcsh
not_Sig	Elavl3
not_Sig	Zfp653
not_Sig	Gm16845
not_Sig	Ecsit
not_Sig	Cnn1
not_Sig	Elof1
Sig	Acp5
not_Sig	Pigyl
not_Sig	Zfp809
not_Sig	Zfp599
not_Sig	Zfp810
Sig	Anln
Sig	9530077C05Rik
not_Sig	Rp9
not_Sig	Bbs9
not_Sig	Bmper
not_Sig	Dpy19l1
not_Sig	Herpud2

Sig	Sept7
not_Sig	Eepd1
Sig	B3gat1
not_Sig	Glb1l2
not_Sig	Acad8
not_Sig	Thyn1
Sig	Vps26b
not_Sig	Ncapd3
not_Sig	Jam3
not_Sig	Igsf9b
not_Sig	AI414108
not_Sig	Opcml
not_Sig	Ntm
not_Sig	Snx19
not_Sig	Adamts15
not_Sig	Adamts8
not_Sig	Zbtb44
not_Sig	St14
not_Sig	Aplp2
not_Sig	Prdm10
not_Sig	Nfrkb
not_Sig	Tmem45b
not_Sig	Barx2
not_Sig	Arhgap32
not_Sig	Kcnj5
Sig	Fli1
Sig	Ets1
not_Sig	Gm27162
Sig	Kirrel3
not_Sig	St3gal4
not_Sig	4930581F22Rik
not_Sig	Dcps
not_Sig	Tirap
not_Sig	Foxred1
not_Sig	Srpr
not_Sig	Fam118b
not_Sig	Rpusd4
not_Sig	Cdon
not_Sig	Pus3
not_Sig	Hyls1
not_Sig	Chek1
not_Sig	Stt3a
not_Sig	Ei24
Sig	Fez1
not_Sig	Pknox2
not_Sig	Tmem218
not_Sig	Slc37a2
not_Sig	Ccdc15

not_Sig	Hepacam
Sig	Robo4
not_Sig	Robo3
not_Sig	Msantd2
Sig	Esam
Sig	Vsig2
not_Sig	Nrgn
not_Sig	Spa17
Sig	Siae
not_Sig	Tbrg1
Sig	Vwa5a
not_Sig	Olfr920
not_Sig	AW551984
not_Sig	Zfp202
not_Sig	Scn3b
not_Sig	Gramd1b
not_Sig	1700110K17Rik
not_Sig	Clmp
Sig	Hspa8
not_Sig	4931429I11Rik
not_Sig	Crtam
not_Sig	Ubash3b
not_Sig	3110039I08Rik
not_Sig	Mir100
not_Sig	Mirlet7a-2
not_Sig	Mir125b-1
not_Sig	Sorl1
Sig	Sc5d
not_Sig	Tecta
not_Sig	Tbcel
not_Sig	Grik4
not_Sig	Arhgef12
Sig	Tmem136
not_Sig	Oaf
not_Sig	D630033O11Rik
not_Sig	Pvrl1
not_Sig	Thy1
not_Sig	Usp2
not_Sig	Rnf26
not_Sig	Mcam
not_Sig	Cbl
not_Sig	Pdzd3
not_Sig	Nlrx1
Sig	Abcg4
not_Sig	Hinfp
not_Sig	C2cd2l
not_Sig	Dpagt1
not_Sig	H2afx

Sig	Hmbs
not_Sig	Vps11
not_Sig	Hyou1
not_Sig	Slc37a4
Sig	Trappc4
not_Sig	Rps25
not_Sig	Ccdc84
not_Sig	Upk2
Sig	Bcl9l
not_Sig	Cxcr5
Sig	Ddx6
not_Sig	Treh
not_Sig	Phldb1
not_Sig	Arcn1
not_Sig	Ift46
not_Sig	Tmem25
not_Sig	Ttc36
Sig	Kmt2a
not_Sig	Atp5l
not_Sig	Ube4a
not_Sig	Cd3g
not_Sig	Cd3d
not_Sig	Cd3e
Sig	Mpzl2
Sig	Mpzl3
not_Sig	Amica1
not_Sig	Scn2b
not_Sig	Scn4b
not_Sig	Tmprss4
not_Sig	BC049352
not_Sig	Il10ra
not_Sig	Tmprss13
not_Sig	Fxyd6
Sig	Fxyd2
not_Sig	Dscaml1
not_Sig	Cep164
Sig	Bace1
not_Sig	Rnf214
not_Sig	Pcsk7
not_Sig	Tagln
Sig	Sidt2
not_Sig	Pafah1b2
Sig	Sik3
not_Sig	Apoa1
Sig	Apoc3
not_Sig	Apoa4
not_Sig	Apoa5
not_Sig	Zpr1

not_Sig	Bud13
not_Sig	Cadm1
not_Sig	Gm10677
Sig	Nxpe2
not_Sig	Nxpe4
not_Sig	Rexo2
not_Sig	Rbm7
Sig	Gm5617
not_Sig	Nnmt
Sig	Zbtb16
not_Sig	Usp28
not_Sig	Zw10
not_Sig	Tmprss5
not_Sig	Ttc12
not_Sig	Ncam1
not_Sig	Plet1os
not_Sig	Plet1
Sig	Pts
not_Sig	Bco2
not_Sig	Il18
not_Sig	Sdhd
not_Sig	Timm8b
not_Sig	AU019823
not_Sig	Pih1d2
not_Sig	Dlat
Sig	Dixdc1
not_Sig	2310030G06Rik
not_Sig	Hspb2
not_Sig	Cryab
not_Sig	1110032A03Rik
not_Sig	Fdxacb1
not_Sig	Alg9
not_Sig	Ppp2r1b
not_Sig	Sik2
not_Sig	Layn
not_Sig	Pou2af1
not_Sig	Gm684
not_Sig	Arhgap20
Sig	Arhgap20os
not_Sig	Fdx1
not_Sig	Gm6981
not_Sig	Rdx
not_Sig	Zc3h12c
not_Sig	AI593442
not_Sig	Ddx10
Sig	Exph5
not_Sig	Kdelc2
not_Sig	Atm

not_Sig	Npat
not_Sig	Acat1
not_Sig	Cul5
not_Sig	Rab39
Sig	Sln
not_Sig	Elmod1
not_Sig	Tnfaip8l3
not_Sig	Gldn
not_Sig	Dmxl2
not_Sig	Sh2d7
not_Sig	Cib2
not_Sig	Idh3a
not_Sig	Acsbg1
not_Sig	Dnaja4
not_Sig	Wdr61
not_Sig	Crabp1
not_Sig	Ireb2
not_Sig	Hykk
not_Sig	AY074887
not_Sig	Psma4
not_Sig	Chrna5
not_Sig	Ube2q2
not_Sig	Fbxo22
Sig	Nrg4
Sig	AI118078
Sig	Etfa
Sig	Scaper
not_Sig	Rcn2
not_Sig	Pstpip1
Sig	Tspan3
Sig	Peak1
not_Sig	Hmg20a
not_Sig	Lingo1
not_Sig	Odf3l1
not_Sig	Cspg4
not_Sig	Snx33
Sig	Imp3
not_Sig	Snupn
not_Sig	Ptpn9
not_Sig	Gm10658
not_Sig	Sin3a
not_Sig	Man2c1os
not_Sig	Man2c1
not_Sig	Neil1
not_Sig	Commd4
not_Sig	Trcg1
not_Sig	1700017B05Rik
not_Sig	4930430J02Rik

not_Sig	Ppcdc
not_Sig	Scamp5
not_Sig	Rpp25
not_Sig	Cox5a
not_Sig	Fam219b
not_Sig	Mpi
Sig	Scamp2
not_Sig	Ulk3
not_Sig	Csk
not_Sig	Cyp1a2
Sig	Cyp1a1
not_Sig	Edc3
Sig	Clk3
not_Sig	Arid3b
not_Sig	Ubl7
Sig	Sema7a
not_Sig	Cyp11a1
not_Sig	Stra6
Sig	Islr
Sig	Islr2
not_Sig	1600029O15Rik
not_Sig	Pml
not_Sig	Mir6385
not_Sig	Stoml1
not_Sig	Loxl1
not_Sig	Tbc1d21
not_Sig	6030419C18Rik
Sig	Cd276
Sig	Nptn
not_Sig	Rec114
Sig	Hcn4
not_Sig	Neo1
Sig	Adpgk
not_Sig	Bbs4
Sig	Arih1
not_Sig	Gm20199
not_Sig	Tmem202
Sig	Hexa
not_Sig	Parp6
not_Sig	Pkm
not_Sig	Gramd2
not_Sig	Senp8
not_Sig	Myo9a
not_Sig	Thsd4
not_Sig	9230112J17Rik
not_Sig	Lrrc49
not_Sig	Larp6
not_Sig	Uaca

not_Sig	Gm5122
not_Sig	Tle3
Sig	Rplp1
not_Sig	Kif23
not_Sig	Paqr5
not_Sig	Glce
not_Sig	Mir5133
not_Sig	Anp32a
not_Sig	Coro2b
Sig	Itga11
not_Sig	Fem1b
Sig	Cln6
not_Sig	Gm10653
not_Sig	Calml4
not_Sig	Pias1
not_Sig	Skor1
not_Sig	Map2k5
not_Sig	2300009A05Rik
not_Sig	Iqch
Sig	Aagab
not_Sig	Smad3
not_Sig	Smad6
not_Sig	Lctl
not_Sig	Zwilch
not_Sig	Rpl4
not_Sig	Snord16a
not_Sig	Snapc5
not_Sig	Map2k1
not_Sig	Uchl4
Sig	Tipin
not_Sig	Dis3l
Sig	Megf11
not_Sig	Rab11a
not_Sig	Dennd4a
not_Sig	Slc24a1
not_Sig	Vwa9
not_Sig	Hacd3
not_Sig	Dpp8
not_Sig	Igdcc4
not_Sig	Igdcc3
not_Sig	Parp16
Sig	Cilp
not_Sig	Clpx
not_Sig	Pdcd7
not_Sig	Ubap1l
not_Sig	Kbtbd13
not_Sig	Rasl12
not_Sig	Slc51b

not_Sig	Mtfmt
not_Sig	Spg21
not_Sig	Plekho2
not_Sig	Pif1
not_Sig	Rbpms2
not_Sig	Oaz2
Sig	Zfp609
not_Sig	Trip4
not_Sig	2810417H13Rik
not_Sig	Csnk1g1
Sig	Ppib
not_Sig	Snx22
not_Sig	Snx1
Sig	Fam96a
not_Sig	Dapk2
not_Sig	Herc1
Sig	Fbxl22
not_Sig	Usp3
not_Sig	Car12
not_Sig	Aph1b
not_Sig	Aph1c
not_Sig	Rab8b
not_Sig	Rps27l
not_Sig	Lactb
not_Sig	Tpm1
not_Sig	Tln2
not_Sig	C2cd4a
not_Sig	Vps13c
not_Sig	Rora
not_Sig	9530091C08Rik
not_Sig	Ice2
not_Sig	Anxa2
not_Sig	Mir3109
not_Sig	Bnip2
not_Sig	Gtf2a2
not_Sig	Fam81a
not_Sig	Myo1e
not_Sig	Ccnb2
not_Sig	Rnf111
Sig	Sltm
not_Sig	Fam63b
not_Sig	Adam10
Sig	Lipc
Sig	Aqp9
Sig	Aldh1a2
not_Sig	Polr2m
not_Sig	Myzap
not_Sig	Cgnl1

not_Sig	Tcf12
not_Sig	Mir378a
not_Sig	Zfp280d
not_Sig	Mns1
not_Sig	Tex9
not_Sig	4930509E16Rik
Sig	Rfx7
Sig	Nedd4
not_Sig	Prtg
not_Sig	Pygo1
not_Sig	Dyx1c1
not_Sig	Ccpg1os
not_Sig	Ccpg1
not_Sig	Pigb
not_Sig	2310009A05Rik
not_Sig	Rab27a
not_Sig	Rsl24d1
not_Sig	Unc13c
not_Sig	Wdr72
not_Sig	Fam214a
not_Sig	Arpp19
not_Sig	Myo5a
Sig	Myo5c
not_Sig	Gnb5
Sig	Mapk6
not_Sig	4933433G15Rik
not_Sig	Leo1
not_Sig	Tmod3
not_Sig	Tmod2
not_Sig	Lysmd2
not_Sig	Scg3
not_Sig	Bmp5
not_Sig	Hmgcll1
not_Sig	Tinag
not_Sig	Mlip
not_Sig	Lrrc1
not_Sig	Klhl31
Sig	Gclc
not_Sig	Elovl5
Sig	Fbxo9
Sig	Ick
not_Sig	C920006O11Rik
not_Sig	Gsta4
not_Sig	Mb21d1
Sig	Mto1
not_Sig	Eef1a1
Sig	Slc17a5
not_Sig	Cd109

Sig	Col12a1
Sig	Cox7a2
not_Sig	Tmem30a
not_Sig	4930429F24Rik
not_Sig	Filip1
not_Sig	Senp6
Sig	Myo6
Sig	Irak1bp1
not_Sig	Phip
not_Sig	Hmgn3
not_Sig	Lca5
not_Sig	Sh3bgrl2
not_Sig	Elovl4
not_Sig	Ttk
not_Sig	Bckdhb
not_Sig	Fam46a
not_Sig	Ibtk
not_Sig	Tpbg
not_Sig	Ube2cbp
not_Sig	Dopey1
not_Sig	Pgm3
not_Sig	Rwdd2a
not_Sig	Me1
not_Sig	A330041J22Rik
Sig	Prss35
not_Sig	Snap91
Sig	Cyb5r4
not_Sig	Mrap2
not_Sig	Cep162
not_Sig	Tbx18
Sig	Nt5e
not_Sig	Snx14
not_Sig	Syncrip
Sig	Snhg5
Sig	Zfp949
not_Sig	Trim43a
not_Sig	9430037G07Rik
not_Sig	Mthfsl
not_Sig	Trim43c
not_Sig	9330159M07Rik
not_Sig	Trim43b
not_Sig	Bcl2a1b
not_Sig	Mthfs
Sig	Tmed3
not_Sig	Rasgrf1
not_Sig	Ctsh
not_Sig	Morf4l1
not_Sig	Adamts7

not_Sig	Tbc1d2b
not_Sig	Zic1
not_Sig	Plscr1
not_Sig	Plscr2
not_Sig	Plscr4
not_Sig	B430319G15Rik
not_Sig	Plod2
not_Sig	1190002N15Rik
not_Sig	Slc9a9
not_Sig	Chst2
not_Sig	U2surp
Sig	Paqr9
Sig	Pcolce2
not_Sig	Trpc1
not_Sig	Pls1
not_Sig	Atr
not_Sig	Xrn1
not_Sig	Gk5
Sig	Tfdp2
not_Sig	Atp1b3
Sig	Rnf7
not_Sig	Rasa2
not_Sig	Zbtb38
not_Sig	E030011O05Rik
not_Sig	Pxylp1
not_Sig	Spsb4
Sig	Slc25a36
not_Sig	Clstn2
not_Sig	4921534H16Rik
not_Sig	Nmnat3
not_Sig	Rbp1
not_Sig	4930579K19Rik
not_Sig	Copb2
not_Sig	Mrps22
not_Sig	Faim
not_Sig	Pik3cb
not_Sig	Cep70
not_Sig	Esyt3
not_Sig	Mras
not_Sig	1600029I14Rik
not_Sig	Armc8
not_Sig	Dbr1
not_Sig	Dzip1l
Sig	Il20rb
not_Sig	Nck1
not_Sig	Slc35g2
not_Sig	Stag1
not_Sig	Pccb

not_Sig	Msl2
not_Sig	Ppp2r3a
not_Sig	Gm28979
not_Sig	Ephb1
not_Sig	Ky
not_Sig	Cep63
Sig	Anapc13
not_Sig	Amotl2
not_Sig	Gm5627
not_Sig	Ryk
not_Sig	Slco2a1
not_Sig	Rab6b
not_Sig	Srprb
Sig	Trf
Sig	1300017J02Rik
not_Sig	Topbp1
not_Sig	Cdv3
not_Sig	5830418P13Rik
not_Sig	Bfsp2
not_Sig	Nphp3
not_Sig	Uba5
Sig	Acad11
not_Sig	Ackr4
Sig	Dnajc13
not_Sig	Acpp
not_Sig	Mrpl3
not_Sig	Nudt16
not_Sig	Aste1
not_Sig	Atp2c1
not_Sig	Pik3r4
Sig	Col6a6
not_Sig	Col6a5
not_Sig	Col6a4
not_Sig	Glyctk
not_Sig	Wdr82
not_Sig	Mirlet7g
not_Sig	Ppm1m
not_Sig	Twf2
not_Sig	Tlr9
not_Sig	Alas1
not_Sig	Poc1a
Sig	Dusp7
not_Sig	Rpl29
Sig	Acy1
not_Sig	Abhd14a
not_Sig	Abhd14b
not_Sig	Pcbp4
not_Sig	Gpr62

not_Sig	Parp3
not_Sig	Rrp9
not_Sig	Tex264
Sig	Rad54l2
not_Sig	Vprbp
not_Sig	Rbm15b
not_Sig	Manf
not_Sig	Dock3
Sig	Mapkapk3
Sig	Cish
not_Sig	Hemk1
not_Sig	6430571L13Rik
not_Sig	Cacna2d2
not_Sig	Tmem115
not_Sig	Cyb561d2
not_Sig	Nprl2
not_Sig	Zmynd10
Sig	Rassf1
Sig	Tusc2
not_Sig	Hyal2
not_Sig	Hyal1
not_Sig	Nat6
not_Sig	Hyal3
not_Sig	Ifrd2
not_Sig	Sema3b
not_Sig	Gnai2
not_Sig	Slc38a3
not_Sig	Sema3f
Sig	Rbm5
not_Sig	Rbm6
not_Sig	Mon1a
not_Sig	Mst1r
not_Sig	Camkv
not_Sig	Traip
not_Sig	Uba7
Sig	Fam212a
Sig	Ip6k1
not_Sig	Gmppb
not_Sig	Rnf123
not_Sig	Amigo3
not_Sig	Mst1
not_Sig	Apeh
not_Sig	Bsn
not_Sig	Dag1
not_Sig	Nicn1
not_Sig	Amt
not_Sig	Tcta
Sig	Rhoa

not_Sig	Gpx1
Sig	Usp4
not_Sig	1700102P08Rik
not_Sig	Klhdc8b
not_Sig	Ccdc71
not_Sig	Lamb2
not_Sig	Usp19
not_Sig	Qars
not_Sig	Qrich1
not_Sig	Impdh2
not_Sig	Ndufaf3
not_Sig	Dalrd3
not_Sig	Wdr6
not_Sig	P4htm
not_Sig	Arih2
not_Sig	Slc25a20
not_Sig	Prkar2a
not_Sig	Ip6k2
not_Sig	Nckipsd
not_Sig	Celsr3
not_Sig	Slc26a6
Sig	Uqcrc1
Sig	Col7a1
not_Sig	Ucn2
Sig	Pfkfb4
not_Sig	Shisa5
not_Sig	Trex1
not_Sig	Atrip
not_Sig	Tma7
not_Sig	Ccdc51
Sig	Plxnb1
not_Sig	3000002C10Rik
not_Sig	Nme6
not_Sig	Camp
not_Sig	Cdc25a
not_Sig	Map4
not_Sig	Dhx30
not_Sig	Smarcc1
not_Sig	Cspg5
not_Sig	Mir6236
not_Sig	Elp6
not_Sig	Scap
Sig	Ptpn23
not_Sig	Ngp
not_Sig	Klhl18
not_Sig	Kif9
not_Sig	Setd2
not_Sig	Nradd

not_Sig	Nbeal2
not_Sig	Ccdc12
Sig	Pth1r
not_Sig	Myl3
not_Sig	Tmie
Sig	Als2cl
not_Sig	Gm590
Sig	Lrrc2
not_Sig	Rtp3
not_Sig	Ltf
Sig	Ccrl2
not_Sig	Lrrfip2
not_Sig	Mlh1
not_Sig	Epm2aip1
not_Sig	Trank1
not_Sig	Dclk3
Sig	Stac
not_Sig	Arpp21
not_Sig	2310075C17Rik
not_Sig	2900079G21Rik
not_Sig	Pdcd6ip
not_Sig	Clasp2
not_Sig	Ubp1
not_Sig	Fbxl2
not_Sig	Susd5
not_Sig	4930520O04Rik
not_Sig	Crtap
not_Sig	Glb1
Sig	Tmppe
not_Sig	Cnot10
not_Sig	Dync1li1
not_Sig	Cmtm6
not_Sig	Cmtm7
Sig	Cmtm8
not_Sig	Gpd1l
not_Sig	Osbpl10
Sig	Stt3b
not_Sig	Stmn1-rs1
Sig	Gadl1
not_Sig	Tgfbr2
not_Sig	Rbms3
not_Sig	Azi2
not_Sig	Cmc1
not_Sig	Eomes
not_Sig	Golga4
Sig	Itga9
not_Sig	Ctdspl
not_Sig	Vill

not_Sig	Plcd1
not_Sig	Dlec1
not_Sig	Acaa1b
not_Sig	Slc22a13b-ps
not_Sig	Oxsr1
not_Sig	Myd88
not_Sig	Acaa1a
not_Sig	Xylb
not_Sig	Acvr2b
not_Sig	Exog
not_Sig	Scn5a
not_Sig	Scn11a
not_Sig	Wdr48
not_Sig	Gorasp1
not_Sig	Ttc21a
not_Sig	Csrnp1
not_Sig	Xirp1
not_Sig	Cx3cr1
not_Sig	Slc25a38
not_Sig	Rpsa
not_Sig	Snora62
not_Sig	Myrip
not_Sig	Eif1b
not_Sig	Rpl14
not_Sig	5830454E08Rik
Sig	Ctnnb1
not_Sig	Ulk4
not_Sig	Trak1
not_Sig	Vipr1
not_Sig	Sec22c
not_Sig	Deb1
not_Sig	Nktr
Sig	E530011L22Rik
Sig	Zfp651
not_Sig	Klhl40
not_Sig	Hhatl
not_Sig	Ccdc13
not_Sig	Higd1a
not_Sig	Ackr2
Sig	Cyp8b1
not_Sig	1700048O20Rik
not_Sig	Fam198a
not_Sig	Pomgnt2
Sig	Snrk
not_Sig	Ano10
Sig	Abhd5
not_Sig	A730085K08Rik
not_Sig	9530059O14Rik

not_Sig	Topaz1
not_Sig	Tcaim
not_Sig	Zfp445
not_Sig	Zkscan7
not_Sig	Zfp105
not_Sig	1110059G10Rik
not_Sig	Kif15
not_Sig	Tmem42
not_Sig	Tgm4
not_Sig	Zdhhc3
not_Sig	Exosc7
Sig	Clec3b
Sig	Cdcp1
not_Sig	Tmem158
not_Sig	Lars2
not_Sig	Limd1
not_Sig	Sacm1l
not_Sig	Slc6a20b
not_Sig	Slc6a20a
not_Sig	Lztfl1
not_Sig	Ccr9
Sig	Fyco1
not_Sig	Cxcr6
not_Sig	Xcr1
Sig	Ccr1
not_Sig	Ccr3
not_Sig	Ccr2
not_Sig	Ccr5
not_Sig	2010315B03Rik
Sig	Nlrp4g
not_Sig	4930526I15Rik
not_Sig	Ppp2r3d
not_Sig	Nudt11
not_Sig	Nudt10
Sig	Shroom4
not_Sig	Clcn5
not_Sig	Usp27x
not_Sig	Gm14379
Sig	2010204K13Rik
Sig	Ppp1r3f
not_Sig	Ppp1r3fos
Sig	Foxp3
not_Sig	Ccdc22
not_Sig	Cacna1f
not_Sig	Syp
not_Sig	Prickle3
not_Sig	Plp2
Sig	Magix

not_Sig	Gpkow
Sig	Wdr45
not_Sig	Praf2
not_Sig	Ccdc120
not_Sig	Tfe3
not_Sig	Gripap1
not_Sig	Kcnd1
not_Sig	Otud5
not_Sig	Pim2
not_Sig	Slc35a2
Sig	Pqbp1
Sig	Timm17b
not_Sig	Pcsk1n
not_Sig	Hdac6
not_Sig	Gata1
not_Sig	Suv39h1
not_Sig	Was
not_Sig	Gm6787
Sig	Wdr13
Sig	Rbm3
not_Sig	Rbm3os
not_Sig	Tbc1d25
not_Sig	Ebp
not_Sig	Porcn
not_Sig	Ftsj1
not_Sig	Slc38a5
not_Sig	Gm6592
not_Sig	B630019K06Rik
not_Sig	Lancl3
Sig	Xk
not_Sig	Cybb
Sig	Dynlt3
not_Sig	Srpx
not_Sig	Rpgr
not_Sig	Tspan7
not_Sig	Mid1ip1
Sig	Bcor
not_Sig	2900008C10Rik
Sig	Atp6ap2
not_Sig	Gm4984
not_Sig	1810030O07Rik
not_Sig	Med14
not_Sig	Gm14634
not_Sig	AA414768
not_Sig	5730405O15Rik
not_Sig	Usp9x
not_Sig	Ddx3x
not_Sig	Nyx

not_Sig	Cask
Sig	Gpr34
not_Sig	Gpr82
not_Sig	Maoa
not_Sig	Maob
not_Sig	Efhc2
not_Sig	Fundc1
not_Sig	Kdm6a
Sig	4930578C19Rik
not_Sig	Chst7
not_Sig	Slc9a7
Sig	Rp2h
not_Sig	Jade3
not_Sig	Rgn
Sig	Ndufb11
not_Sig	Rbm10
not_Sig	Uba1
Sig	Cdk16
not_Sig	Usp11
not_Sig	Araf
not_Sig	Syn1
not_Sig	Timp1
not_Sig	Cfp
not_Sig	Elk1
not_Sig	Uxt
not_Sig	A230072C01Rik
not_Sig	Zfp182
not_Sig	Zfp300
not_Sig	Gm5124
not_Sig	Agtr2
not_Sig	Klhl13
not_Sig	Wdr44
not_Sig	Dock11
not_Sig	Il13ra1
not_Sig	Lonrf3
not_Sig	Pgrmc1
not_Sig	Akap17b
not_Sig	Slc25a43
Sig	Slc25a5
not_Sig	2310010G23Rik
Sig	C330007P06Rik
not_Sig	Ube2a
not_Sig	Nkrf
not_Sig	Sept6
not_Sig	Rpl39
not_Sig	Snora69
not_Sig	Upf3b
not_Sig	Nkap

not_Sig	Akap14
not_Sig	Ndufa1
not_Sig	Rnf113a1
not_Sig	Zbtb33
not_Sig	Tmem255a
Sig	Atp1b4
Sig	Lamp2
not_Sig	Cul4b
not_Sig	Mcts1
Sig	C1galt1c1
not_Sig	Gria3
not_Sig	Thoc2
not_Sig	Xiap
not_Sig	Stag2
not_Sig	Dcaf12l1
not_Sig	Prr32
not_Sig	Smarca1
Sig	Ocrl
Sig	Apln
not_Sig	Xpnpep2
not_Sig	Sash3
not_Sig	Zdhhc9
not_Sig	Utp14a
not_Sig	9530027J09Rik
not_Sig	Bcorl1
not_Sig	Elf4
not_Sig	Aifm1
not_Sig	Zfp280c
not_Sig	Slc25a14
not_Sig	Gpr119
not_Sig	Rbmx2
not_Sig	Enox2
not_Sig	Arhgap36
not_Sig	Igsf1
not_Sig	Gm35612
not_Sig	Firre
not_Sig	Stk26
not_Sig	Frmd7
not_Sig	Rap2c
not_Sig	Mbnl3
Sig	Hs6st2
Sig	Gpc4
not_Sig	Gpc3
not_Sig	Phf6
Sig	Hprt
Sig	C430049B03Rik
not_Sig	Fam122b
not_Sig	Mospd1

not_Sig	Cxx1c
Sig	Cxx1a
not_Sig	Cxx1b
not_Sig	Xlr
Sig	Zfp449
not_Sig	Smim10l2a
not_Sig	Ddx26b
not_Sig	Mmgt1
not_Sig	Slc9a6
not_Sig	Fhl1
not_Sig	Mtap7d3
not_Sig	Htatsf1
not_Sig	Arhgef6
not_Sig	Rbmx
not_Sig	Fgf13
not_Sig	Atp11c
not_Sig	Mir505
not_Sig	Cdr1
not_Sig	Slitrk4
not_Sig	Slitrk2
not_Sig	Fmr1
Sig	Aff2
not_Sig	Ids
not_Sig	1110012L19Rik
not_Sig	BC023829
not_Sig	Mamld1
Sig	Mtm1
not_Sig	Mtmr1
Sig	Cd99l2
not_Sig	Hmgb3
not_Sig	Vma21
not_Sig	Prrg3
Sig	Gabre
not_Sig	Gabra3
not_Sig	Gabrq
not_Sig	Cetn2
not_Sig	Nsdhl
not_Sig	Zfp185
not_Sig	Pnma5
not_Sig	Xlr3b
not_Sig	F8a
not_Sig	Zfp275
not_Sig	Haus7
Sig	Bgn
not_Sig	Atp2b3
not_Sig	Dusp9
not_Sig	Pnck
not_Sig	Slc6a8

Sig	Bcap31
not_Sig	Abcd1
not_Sig	Plxnb3
not_Sig	Srpk3
not_Sig	Idh3g
not_Sig	Ssr4
not_Sig	Pdzd4
Sig	L1cam
not_Sig	Avpr2
not_Sig	Arhgap4
not_Sig	Naa10
not_Sig	Renbp
not_Sig	Hcfc1
Sig	Irak1
Sig	Mecp2
Sig	Opn1mw
not_Sig	Flna
not_Sig	Emd
Sig	Rpl10
not_Sig	Snora70
not_Sig	Dnase1l1
not_Sig	Taz
not_Sig	Atp6ap1
not_Sig	Gdi1
Sig	Fam50a
not_Sig	Plxna3
not_Sig	Lage3
not_Sig	Ubl4a
not_Sig	Slc10a3
not_Sig	Fam3a
not_Sig	Ikbkg
not_Sig	G6pdx
not_Sig	Gab3
Sig	Dkc1
not_Sig	Mpp1
not_Sig	F8
not_Sig	Fundc2
not_Sig	Cmc4
not_Sig	Mtcp1
not_Sig	Brcc3
not_Sig	Vbp1
not_Sig	Rab39b
not_Sig	4933407K13Rik
not_Sig	Pls3
not_Sig	Tbl1x
not_Sig	Prkx
not_Sig	Prrg1
Sig	Tmem47

not_Sig	Dmd
not_Sig	Tsga8
not_Sig	Tab3
not_Sig	Gyk
not_Sig	5430427O19Rik
not_Sig	Mageb18
not_Sig	Arx
not_Sig	Pola1
not_Sig	Pcyt1b
not_Sig	Pdk3
not_Sig	Zfx
not_Sig	Eif2s3x
not_Sig	Klhl15
not_Sig	Mir7673
not_Sig	Apoo
not_Sig	Gm14827
not_Sig	Maged1
not_Sig	Gspt2
not_Sig	Zxdb
not_Sig	Zxda
not_Sig	Spin4
not_Sig	Arhgef9
Sig	Amer1
not_Sig	Asb12
not_Sig	Zc4h2
not_Sig	Zc3h12b
not_Sig	Las1l
not_Sig	Msn
Sig	F630028O10Rik
not_Sig	Vsig4
not_Sig	Heph
not_Sig	Gpr165
not_Sig	Eda2r
Sig	Ar
not_Sig	Ophn1
not_Sig	Yipf6
Sig	Stard8
Sig	Efnb1
not_Sig	Pja1
not_Sig	Tmem28
Sig	Eda
not_Sig	Awat2
Sig	Igbp1
not_Sig	Dgat2l6
not_Sig	P2ry4
not_Sig	Arr3
not_Sig	Pdzd11
not_Sig	Kif4

not_Sig	Gdpd2
not_Sig	Dlg3
not_Sig	Slc7a3
not_Sig	Snx12
Sig	Foxo4
not_Sig	Gm614
not_Sig	Il2rg
not_Sig	Med12
Sig	Nlgn3
Sig	Gjb1
not_Sig	Zmym3
Sig	Nono
not_Sig	Itgb1bp2
not_Sig	Taf1
not_Sig	Ogt
not_Sig	Cxcr3
not_Sig	Nhsl2
not_Sig	Rgag4
not_Sig	Pin4
not_Sig	Ercc6l
not_Sig	Rps4x
not_Sig	Cited1
not_Sig	Hdac8
not_Sig	Phka1
not_Sig	Chic1
not_Sig	Tsix
not_Sig	Xist
not_Sig	Jpx
not_Sig	Gm9159
not_Sig	Ftx
not_Sig	Mir421
not_Sig	Slc16a2
not_Sig	Rlim
not_Sig	C77370
not_Sig	Abcb7
Sig	Uprt
not_Sig	Zdhhc15
not_Sig	5530601H04Rik
not_Sig	Pbdc1
not_Sig	Magee1
not_Sig	Fgf16
not_Sig	Atrx
not_Sig	Magt1
not_Sig	Cox7b
not_Sig	Atp7a
not_Sig	Tlr13
not_Sig	Pgk1
not_Sig	Taf9b

Sig	Fndc3c1
not_Sig	Cysltr1
not_Sig	Zcchc5
not_Sig	Lpar4
not_Sig	P2ry10
not_Sig	A630033H20Rik
not_Sig	Gpr174
not_Sig	Itm2a
not_Sig	2610002M06Rik
not_Sig	Brwd3
not_Sig	2810403D21Rik
not_Sig	Hmgn5
Sig	Sh3bgrl
not_Sig	Gm6377
Sig	Rps6ka6
not_Sig	Hdx
not_Sig	Apool
not_Sig	Satl1
not_Sig	Zfp711
not_Sig	Pof1b
not_Sig	Chm
not_Sig	Dach2
not_Sig	Klhl4
not_Sig	Pcdh11x
not_Sig	Nap1l3
not_Sig	Cldn34c1
not_Sig	Diap2
Sig	Pcdh19
not_Sig	Tnmd
not_Sig	Tspan6
not_Sig	Srpx2
not_Sig	Sytl4
not_Sig	Cstf2
not_Sig	Xkrx
not_Sig	Arl13a
not_Sig	Trmt2b
not_Sig	Tmem35
Sig	Cenpi
not_Sig	Drp2
not_Sig	Taf7l
not_Sig	Timm8a1
not_Sig	Btk
not_Sig	Rpl36a
not_Sig	Gla
not_Sig	Hnrnph2
Sig	B230119M05Rik
Sig	Armcx4
not_Sig	Armcx1

not_Sig	Armcx6
not_Sig	Armcx3
not_Sig	Armcx2
not_Sig	Zmat1
not_Sig	3632454L22Rik
not_Sig	Armcx5
not_Sig	Gprasp1
not_Sig	Gprasp2
not_Sig	Bhlhb9
not_Sig	Arxes2
not_Sig	Arxes1
not_Sig	Tceal8
not_Sig	Tceal5
not_Sig	Bex1
not_Sig	Tceal7
Sig	Wbp5
not_Sig	Ngfrap1
not_Sig	Tceal3
not_Sig	Tceal1
not_Sig	Morf4l2
not_Sig	BC065397
not_Sig	Glra4
not_Sig	Plp1
not_Sig	Tmsb15b1
not_Sig	Slc25a53
not_Sig	Zcchc18
not_Sig	Fam199x
Sig	Nrk
not_Sig	Mum1l1
Sig	Rnf128
not_Sig	Tbc1d8b
not_Sig	Morc4
not_Sig	Rbm41
not_Sig	Prps1
not_Sig	Tsc22d3
not_Sig	Mid2
not_Sig	Psmd10
not_Sig	Col4a6
Sig	Col4a5
not_Sig	Nxt2
not_Sig	Kcne1l
Sig	Acsl4
not_Sig	Tmem164
not_Sig	Ammecr1
not_Sig	Rgag1
Sig	Chrdl1
Sig	Pak3
not_Sig	Capn6

not_Sig	A730046J19Rik
not_Sig	Alg13
not_Sig	Trpc5
not_Sig	Zcchc16
Sig	Amot
Sig	Lrch2
not_Sig	Tmem29
not_Sig	Alas2
not_Sig	Apex2
Sig	Pfkfb1
not_Sig	Tro
not_Sig	Maged2
not_Sig	Gnl3l
Sig	Fgd1
not_Sig	Tsr2
Sig	Wnk3
not_Sig	A230072E10Rik
not_Sig	Fam120c
not_Sig	Phf8
not_Sig	Huwe1
not_Sig	Mir3113
not_Sig	Mir98
not_Sig	Hsd17b10
Sig	Ribc1
not_Sig	Smc1a
not_Sig	Iqsec2
not_Sig	Kdm5c
not_Sig	Kantr
Sig	Tspyl2
not_Sig	Gpr173
not_Sig	3010001F23Rik
Sig	Shroom2
not_Sig	Mageh1
not_Sig	9530051G07Rik
not_Sig	Rragb
not_Sig	Klf8
not_Sig	Ubqln2
not_Sig	Kctd12b
not_Sig	2210013O21Rik
not_Sig	Sat1
not_Sig	Acot9
Sig	Prdx4
not_Sig	Ptchd1
not_Sig	Phex
not_Sig	Sms
not_Sig	Mbtps2
not_Sig	Yy2
Sig	Smpx

not_Sig	Klhl34
Sig	Cnksr2
not_Sig	Rps6ka3
not_Sig	Eif1ax
not_Sig	Map7d2
not_Sig	A830080D01Rik
not_Sig	Sh3kbp1
not_Sig	Map3k15
Sig	Pdha1
not_Sig	Adgrg2
not_Sig	Phka2
not_Sig	Rs1
Sig	Cdkl5
not_Sig	Gja6
not_Sig	Scml2
Sig	Rai2
not_Sig	Nhs
Sig	Reps2
not_Sig	Rbbp7
not_Sig	Txlng
not_Sig	Syap1
not_Sig	Ctps2
not_Sig	S100g
Sig	Ap1s2
not_Sig	Zrsr2
not_Sig	Car5b
not_Sig	Siah1b
not_Sig	Ace2
not_Sig	Bmx
not_Sig	Pir
not_Sig	Figf
not_Sig	Piga
not_Sig	Asb11
not_Sig	Mospd2
not_Sig	Fancb
not_Sig	Gemin8
not_Sig	Gpm6b
not_Sig	Ofd1
Sig	Trappc2
Sig	Rab9
not_Sig	Tceanc
not_Sig	Egfl6
not_Sig	Tmsb4x
not_Sig	Tlr8
not_Sig	Tlr7
not_Sig	Prps2
not_Sig	Frmpd4
not_Sig	Msl3

Sig	Arhgap6
not_Sig	Amelx
not_Sig	Hccs
not_Sig	Mid1
not_Sig	4933400A11Rik
not_Sig	G530011O06Rik
not_Sig	Kdm5d
not_Sig	Eif2s3y
not_Sig	Uty
not_Sig	Ddx3y
not_Sig	Erdr1

baseMean	log2FoldChange	lfcSE	stat	pvalue	padj
8.850350593	0.907441361	0.453361152	2.001586056	0.04532927	0.188729565
25.61399584	1.115689963	0.609067112	1.831801357	0.066981021	0.239519
140.037652	0.568784723	0.182860885	3.110477797	0.001867849	0.025474217
755.2262616	-0.249782168	0.122129548	-2.045223063	0.040832872	0.177157057
872.691782	-0.406577323	0.165723261	-2.453350978	0.014153217	0.09353526
328.5078269	-0.239430792	0.076268134	-3.13932937	0.00169335	0.023835335
264.387456	-0.179205775	0.135165757	-1.325822299	0.184898565	0.430769788
1634.940347	-0.271532419	0.112010459	-2.424170215	0.015343412	0.098787387
1045.863005	-0.289017438	0.102168164	-2.82884049	0.004671698	0.04548163
238.9118613	0.251140382	0.104097456	2.412550618	0.015841338	0.100216166
466.9675715	-0.015414381	0.120311065	-0.128121058	0.898053171	0.957387882
25.78360007	-0.103636343	0.240135515	-0.43157441	0.66605076	0.837650559
13.03538926	0.060984435	0.390467341	0.156183191	0.875888626	0.948628307
11.48331436	0.465841021	0.319838661	1.456487526	0.145257895	0.37526352
545.2664202	-0.254893717	0.056279877	-4.529038267	5.92528E-06	0.000388837
129.3688075	-0.133575253	0.179768478	-0.743040461	0.457457184	0.694284175
2.910374933	0.639989864	0.570280788	1.122236409	0.261761936	0.51962191
29.86143494	-0.333481033	0.195025824	-1.709932696	0.08727832	0.279207439
4.581999578	-0.143197609	0.630663857	-0.227058531	0.820378229	0.921144748
1019.997766	-0.095977972	0.134730026	-0.712372552	0.476234107	0.70906168
104.4970949	-0.088717829	0.173582701	-0.511098335	0.609282202	0.802570863
2410.004461	-0.27962573	0.124861827	-2.239481336	0.025124614	0.131516192
605.8025468	-0.011378355	0.092696092	-0.122749025	0.902305841	0.958662553
2.96621438	-3.21161611	1.135150729	-2.829241991	0.00466584	0.04547653
290.5275212	-0.052030769	0.113031565	-0.460320701	0.645286044	0.82563765
1026.464629	-0.4373503	0.215596254	-2.028561681	0.042502955	0.181194983
774.1997745	-0.022920883	0.070582189	-0.32474033	0.745377608	0.882102347
339.8136145	-0.136879704	0.212832736	-0.643132755	0.520137962	0.743254883
272.380561	-0.175291183	0.108307473	-1.618458805	0.105563758	0.310829552
346.2104971	-0.297830584	0.152731182	-1.950031289	0.05117239	0.202418099
22.1885535	0.4736917	0.298271712	1.588121437	0.112258882	0.322584884
4.314317796	0.270366295	0.512268557	0.527782335	0.597650419	0.794878848
58.5250543	0.165100535	0.145263615	1.136558081	0.255723072	0.512836005
22.24668712	0.663429834	0.325348092	2.039138541	0.041436203	0.178687215
2636.504964	-0.17319527	0.114347732	-1.514636684	0.129864437	0.351838629
73.45831727	0.193011633	0.145559789	1.325995555	0.18484117	0.430769788
507.1962007	-0.593712726	0.18809863	-3.156390489	0.001597349	0.022984234
178.1693423	-0.473530082	0.143702941	-3.295201058	0.000983513	0.016375176
445.3826524	-0.168150109	0.133135414	-1.263000616	0.206588958	0.456725202
645.6052413	-0.005330094	0.157289575	-0.033887141	0.972967147	0.986800481
7.266447433	-0.735103112	0.679145235	-1.082394567	0.279077241	0.53751602
8565.587848	-0.180869873	0.176554645	-1.024441319	0.305626874	0.56446678
122.0571352	-0.82130353	0.271057674	-3.029995492	0.002445574	0.0301307
48.01557757	0.103185916	0.374549876	0.275493126	0.782937406	0.900791541
4.25058342	-0.453192798	0.503484861	-0.900112065	0.368060616	0.623287987
46.3951178	0.695257338	0.222162237	3.129502772	0.001751024	0.024368291
4.082211383	-0.349372101	0.708743	-0.492946104	0.62205067	0.810641951

24.27876753	0.906964784	0.53444241	1.697029964	0.089690996	0.283955102
68.21419079	0.451226221	0.249638816	1.807516265	0.070681821	0.24687097
26.79764173	0.620637377	0.257763497	2.407778385	0.01604992	0.100811755
9.106042884	-0.011339208	0.412537046	-0.02748652	0.978071692	0.988880488
56.28541934	0.249201644	0.257771058	0.966755718	0.333666166	0.591993229
16.2300543	-0.116078385	0.282898444	-0.410318217	0.68157253	0.84736731
30.38778546	-0.193342254	0.489225406	-0.395200764	0.692694729	0.853431717
784.5313957	-0.499511046	0.279867655	-1.784811633	0.074291876	0.254187125
4.515984695	-0.012840302	0.472525729	-0.027173762	0.978321143	0.988913947
49.9118149	-0.269419253	0.579742168	-0.46472254	0.642130165	0.824312819
2.738391729	0.08706004	0.566683909	0.153630691	0.877900936	0.949450031
10.859319	-0.02711902	0.387081029	-0.070060319	0.94414565	0.975492778
152.9239415	-0.275721315	0.196188439	-1.405390227	0.159905271	0.397563536
13.315543	-0.064938411	0.358114782	-0.181334071	0.856105365	0.938518178
176.4745009	0.054989726	0.124466721	0.441802639	0.658632028	0.833668367
151.6745379	0.008190415	0.205124765	0.039928943	0.968149776	0.984725103
464.3080622	0.007974865	0.131788482	0.060512609	0.951747374	0.978862788
21.14927995	2.177807273	0.653899139	3.330494171	0.00086692	0.014884084
119.4980358	-0.052426614	0.369128777	-0.142027979	0.887057905	0.95312914
722.5656081	-0.177947339	0.069318568	-2.56709486	0.010255455	0.075936734
6.154760337	2.245936407	0.525939318	4.270333726	1.95181E-05	0.000977672
881.8700541	-0.1249184	0.057723124	-2.164096321	0.030456958	0.148534492
85.02593292	-0.365964971	0.251885315	-1.452903164	0.146250642	0.3765876
30.81379165	0.398634077	0.333973276	1.193610704	0.232630285	0.487972902
102.964526	0.133485882	0.115953022	1.151206583	0.249647257	0.505482518
152.5523927	0.044554234	0.173889036	0.256222215	0.797779256	0.907614235
253.5659829	-0.025536109	0.101611881	-0.251310265	0.801574239	0.909885675
3.929167633	-0.437509162	0.722864319	-0.60524382	0.545017004	0.759994702
26581.48174	-0.281142636	0.095382562	-2.947526573	0.003203272	0.03593714
94.1706005	-0.322338192	0.172133646	-1.872604225	0.061123058	0.226311151
122.3454331	-0.087380418	0.120379232	-0.725876188	0.467914675	0.703082369
45.03806965	-0.078664292	0.413290128	-0.190336732	0.849045272	0.934802172
30.16031593	0.653076038	0.25541867	2.556884496	0.010561428	0.077296051
754.6109324	0.217229875	0.091239033	2.380887506	0.017270985	0.105400052
1103.54682	-0.105504341	0.118078203	-0.893512417	0.371582825	0.625604127
558.0520322	0.19587161	0.09592	2.042030964	0.04114846	0.178050523
1570.252262	-0.224693512	0.077831389	-2.886926646	0.003890249	0.040595255
31.58629634	1.163918503	0.519749483	2.239383666	0.025130963	0.131516192
185.9529859	0.684276386	0.186696536	3.665179865	0.000247165	0.006418101
944.3693109	-0.016260727	0.071655578	-0.226928979	0.820478968	0.921144748
60.30964447	-0.328215297	0.223913913	-1.465810199	0.142700002	0.371531678
219.993073	-0.147643177	0.143269613	-1.03052681	0.302762773	0.561492585
241.1702345	0.419150443	0.194944304	2.150103558	0.031547024	0.151309947
385.7893129	0.325099729	0.157006448	2.070613875	0.038394894	0.170445945
25415.58275	-0.040984785	0.139921556	-0.292912587	0.769588968	0.893892718
133.6807018	0.391529186	0.135431447	2.89097691	0.003840463	0.040317154
209.2052061	0.266735222	0.132230454	2.017199618	0.043674686	0.184437888
10.85596508	0.903917064	0.319509745	2.829075103	0.004668274	0.04547653

2426.436244	0.036722109	0.142728744	0.257286009	0.796957992	0.907074684
1183.192173	0.054002555	0.130558983	0.413625736	0.679148222	0.845914923
4.53361138	0.618269386	0.50242312	1.230575108	0.21848182	0.4711493
6.506929627	0.390457502	0.390961431	0.998711051	0.317934686	0.576478535
1126.228361	0.096749182	0.084748484	1.141603688	0.25361879	0.510230517
12.01117046	0.23307078	0.354839871	0.656833685	0.511287862	0.73623199
689.7690006	0.036623786	0.090183525	0.406102841	0.684667039	0.848677613
1917.661187	-0.257458529	0.134551074	-1.913463195	0.055688781	0.213974712
154.4331141	-0.129315018	0.118667674	-1.089724046	0.275834721	0.534543001
231.0974124	0.195727515	0.131743377	1.485672523	0.137365762	0.363251493
26.05170499	0.407127611	0.282047215	1.443473251	0.148887202	0.380813256
20.13312736	-0.756573092	0.310043342	-2.440217185	0.014678434	0.095684357
77.49054096	-0.024177197	0.171338961	-0.141107411	0.887785089	0.953373594
69.94626002	-0.287130717	0.175112947	-1.63968868	0.101069914	0.304491074
19.48576545	-0.124410172	0.379311981	-0.327989039	0.742919951	0.880589216
754.3072329	-0.243669755	0.095328425	-2.556108057	0.010585024	0.077396513
309.8200359	-0.178543833	0.067524331	-2.644140713	0.00818986	0.065602537
1279.317637	-0.107894048	0.094244707	-1.144828727	0.252280113	0.508385108
413.3577288	-0.021692345	0.084598812	-0.256414294	0.797630952	0.907511342
137.6914696	-0.330845041	0.275610623	-1.200407434	0.229981143	0.48527166
19.60771807	0.470226859	0.300297142	1.56587191	0.117378607	0.33099428
14.10634387	0.321931197	0.509393939	0.631988668	0.527394282	0.748701296
335.0925419	0.122152732	0.131430305	0.9294107	0.352676284	0.609517889
14.4305999	-0.886918473	0.663750095	-1.336223496	0.181476234	0.426968393
1283.838974	-0.363345956	0.152306406	-2.385624901	0.017050132	0.104499517
373.2460319	0.450050608	0.119011264	3.78157992	0.000155836	0.00460289
371.8246658	-0.02921967	0.102212994	-0.285870409	0.774977379	0.896822533
4.627579676	-0.334390838	0.464963604	-0.719176373	0.472032255	0.706224734
138.9155859	0.208517774	0.118243222	1.763464923	0.077822108	0.260784867
3.284274721	0.942917641	0.868011417	1.086296359	0.277347897	0.535639013
633.1255317	0.286224826	0.234051314	1.222914845	0.221361861	0.474540521
12.18290788	0.058511679	0.285005622	0.205300086	0.837337677	0.930780365
19.77467035	-0.944463767	0.486239268	-1.942384807	0.052090533	0.204861565
163.7708234	-0.281648757	0.305605642	-0.9216085	0.356732823	0.612881761
34.5237953	-0.364626978	0.307780267	-1.184699011	0.236136474	0.492010908
51.39245277	-0.112852677	0.333737767	-0.338147757	0.735251843	0.876856177
9.673519201	1.356290462	0.764609287	1.77383467	0.076090502	0.2579099
9.66277732	0.980837726	0.902175573	1.087191624	0.276952132	0.535402099
23.68633026	0.819130148	0.324114538	2.527286045	0.011494784	0.081245692
483.6321854	-0.163139915	0.194012583	-0.840872856	0.400419166	0.649919715
19.16045582	-0.180136352	0.303523171	-0.593484681	0.552856832	0.765246364
1445.387919	-0.155355394	0.160042077	-0.970715933	0.331689755	0.590423575
289.0256302	0.054312441	0.089423711	0.607360623	0.543611614	0.759149191
437.1029986	-0.081811379	0.095764991	-0.854293187	0.392942561	0.643530057
316.6638501	-0.294406362	0.123250811	-2.388676879	0.016909167	0.104041052
32.04461997	0.488846432	0.38715982	1.262647635	0.206715841	0.45676687
231.220224	-0.052770302	0.086044073	-0.613293855	0.539682056	0.757302797
9.408764197	1.58372461	0.794206688	1.994096289	0.046141533	0.19074428

120.3156458	-0.331719865	0.137656491	-2.409765517	0.015962776	0.100627082
836.7168343	-0.303839289	0.067181998	-4.522629531	6.10761E-06	0.000392589
315.2400246	0.221908542	0.284705578	0.779431662	0.435725479	0.679451025
27.6523941	-0.264550452	0.193598972	-1.36648686	0.171786202	0.413687851
68.59525821	0.118140251	0.141022088	0.837742885	0.402175122	0.651758072
123.6810374	-0.264887603	0.122969111	-2.154098712	0.031232432	0.150398973
263.6074908	-0.007877792	0.087768237	-0.089756752	0.928480516	0.970107815
438.4617355	-0.306056395	0.380137422	-0.805120406	0.420750202	0.665360573
76.59603201	-0.742172368	0.298706578	-2.484620104	0.012968975	0.088320666
2.675426435	-0.360732546	0.846110151	-0.426342297	0.669858441	0.840552028
4776.357499	-0.340271009	0.510304876	-0.666799448	0.504900246	0.731061249
633.8934626	-0.005862663	0.358964982	-0.016332129	0.986969425	0.992541292
195.8363312	-0.1941492	0.088942419	-2.182863949	0.029045831	0.144157627
593.6101422	0.326697945	0.112240991	2.910682999	0.003606397	0.038556736
8.394934965	0.315677279	0.377543788	0.836134214	0.403079403	0.652549272
139.3286434	-0.378817985	0.123590835	-3.065097702	0.002175989	0.027814353
67.31834164	-0.412684997	0.342872036	-1.20361229	0.228739467	0.483823304
884.9340136	0.150140654	0.181787218	0.82591425	0.408852737	0.656743786
177.9458476	-0.709857876	0.256979634	-2.7623118	0.005739364	0.051942403
366.2107059	0.571190066	0.123303213	4.632402124	3.61447E-06	0.000259459
5.636111119	0.318332089	0.652753191	0.487676036	0.625779328	0.812948148
409.137636	0.465788428	0.223790014	2.081363772	0.037400622	0.16804498
1261.543458	-0.228905493	0.112402732	-2.036476246	0.041702556	0.179293555
269.0733782	-0.089270251	0.092996905	-0.959927115	0.337091899	0.595725198
26.22549328	0.312186401	0.264053484	1.182284728	0.237092743	0.492684668
143.1703518	0.048583964	0.183006706	0.265476414	0.790642477	0.904476207
85.22909989	0.32545614	0.162038236	2.008514455	0.044588652	0.186887867
369.9653087	-0.20862973	0.126705091	-1.646577322	0.099644955	0.301938075
13.75912424	-0.367785383	0.400361564	-0.918633095	0.358287515	0.614347157
717.864887	-0.539749848	0.155928527	-3.461520847	0.000537133	0.011041136
59.31013819	0.253187369	0.15498168	1.633659988	0.102330264	0.306521747
96.64564966	-0.244472672	0.120824716	-2.02336642	0.043035384	0.18262093
308.079259	-0.24068258	0.158723316	-1.516365627	0.129426926	0.35120799
1111.144464	-0.058575963	0.114953608	-0.509561763	0.610358518	0.803167633
165.895201	-0.084711534	0.371161113	-0.228233862	0.819464438	0.920862667
234.820579	0.186901816	0.1867162	1.00099411	0.316829656	0.575733312
6.722369336	-0.534116489	0.423638896	-1.260782458	0.207387235	0.457548517
227.5386126	-0.044583546	0.086924567	-0.512899256	0.608021794	0.801631962
158.065918	-0.11877505	0.092794202	-1.279983518	0.200550932	0.449208007
211.4499094	0.430926085	0.333176621	1.293386323	0.195877478	0.444015373
1904.253091	-0.070076613	0.056098643	-1.249167714	0.211603738	0.462972839
223.5828772	-0.033865252	0.139390998	-0.242951499	0.80804297	0.913987158
1519.521947	-0.139672691	0.122633603	-1.13894306	0.254726904	0.511720904
368.7241144	-0.089886964	0.131796488	-0.682013346	0.495230512	0.722933298
410.0079994	-0.334720344	0.144413638	-2.317789022	0.02046079	0.116312806
49.12005276	0.205303001	0.238185472	0.861945943	0.388717266	0.639780065
136.0262107	0.024039757	0.138722503	0.173293853	0.862420456	0.941673797
613.2157324	-0.210390747	0.132519016	-1.587626841	0.112370745	0.322788052

12.74794822	0.652118437	0.629259148	1.036327305	0.300049472	0.558241509
14.59963514	0.063453201	0.381163811	0.166472259	0.867785313	0.944769182
10.95241142	0.618177188	0.410998534	1.504086116	0.132559195	0.355698616
38.71325842	-0.196821107	0.173656115	-1.133395777	0.257048087	0.513998503
31.6701449	-0.261929559	0.202342863	-1.294483805	0.19549836	0.443604785
166.0186681	0.010314337	0.114411551	0.090151182	0.928167077	0.969855368
48.15159588	0.410822383	0.239887009	1.712566199	0.086792376	0.278431505
66.24657075	-0.024851309	0.15621211	-0.159086955	0.873600366	0.947352611
18.95743152	1.7978627	0.504693955	3.56228301	0.000367644	0.008462615
587.312488	-0.009616373	0.15999193	-0.060105362	0.95207172	0.979038346
50.07397618	-0.984090898	0.477747673	-2.059854927	0.039412412	0.173538536
796.8908811	-0.336767385	0.079139357	-4.255371786	2.08702E-05	0.001026107
722.2962633	-0.08159556	0.10302922	-0.791965232	0.428380949	0.673125134
100.5580214	0.049529111	0.134863349	0.367254047	0.713429528	0.864200403
102.2011668	-0.221066126	0.114089789	-1.937650411	0.052665888	0.206348185
212.3551895	0.011857852	0.085671719	0.138410347	0.88991612	0.953820886
422.5993754	-0.260720977	0.105759716	-2.465220102	0.013692911	0.091816852
651.2777493	0.243762084	0.194282392	1.254679239	0.209595225	0.461244775
10.44068794	-0.183584819	0.407288748	-0.450748566	0.652170775	0.829708504
4.253220852	0.067120815	0.625956179	0.107229255	0.914607107	0.96370157
713.671592	0.014711783	0.073923383	0.19901393	0.842251848	0.93182686
75.99327936	-0.073957885	0.377036061	-0.196155999	0.844488067	0.932309255
2209.586686	-0.126534134	0.126171456	-1.002874491	0.315921422	0.575044176
843.067975	-0.219588768	0.126322216	-1.738322642	0.082153979	0.269674132
48.4693254	-0.17552264	0.182505067	-0.961741189	0.336179627	0.594703504
4.4193255	0.309119774	0.501783914	0.616041617	0.537867053	0.755431795
481.094839	-0.098696758	0.092871148	-1.062727875	0.287905376	0.547328265
3.389703045	0.767013564	0.512375163	1.496976473	0.13439938	0.358453483
1683.917525	0.069667526	0.150420181	0.463152787	0.643254855	0.824451185
21.31761894	-0.078541605	0.23231179	-0.338087037	0.735297599	0.876856177
293.1879662	-0.174413777	0.106841855	-1.632448037	0.102585137	0.306759453
165.5572502	-0.176192125	0.116726474	-1.509444414	0.13118525	0.353645491
2999.993231	-0.328079515	0.115026165	-2.852216423	0.004341553	0.043398928
348.6684595	-0.047750917	0.131380565	-0.363454951	0.716265059	0.865878001
15.22172391	-0.263371286	0.449377914	-0.586079727	0.557821939	0.768771196
180.8976319	-0.188464142	0.113697819	-1.657588021	0.097400649	0.297814105
114.3841665	-0.06258983	0.109893144	-0.569551725	0.568981779	0.775924721
1517.678256	-0.09183588	0.087774018	-1.046276355	0.295433459	0.554602454
98.55406797	-0.023198218	0.116077497	-0.199851124	0.841597017	0.93182686
317.9300093	0.325587333	0.184646465	1.763301198	0.077849703	0.260784867
762.8928357	-0.028795523	0.122805469	-0.234480786	0.814611748	0.918370519
89.19300672	-3.047097367	0.442625294	-6.884146496	5.8135E-12	5.69869E-09
3.772706408	3.066910391	1.000086304	3.066645728	0.002164753	0.027736582
353.7652999	0.260702649	0.139610175	1.867361385	0.061851138	0.227866016
439.7797682	0.447911727	0.216574401	2.068165601	0.038624454	0.171227159
13.43700207	0.316757414	0.354796929	0.892785105	0.371972264	0.626024556
990.8804107	-0.006745981	0.057965684	-0.116378873	0.907352278	0.960444971
52.06130411	0.205494186	0.13129201	1.565169021	0.117543283	0.331275626

4476.341806	0.024338037	0.160058133	0.152057486	0.879141595	0.95014173
1054.321444	-0.073884274	0.096878374	-0.762649812	0.445672269	0.685757272
3.267917961	-0.505926365	0.736615727	-0.686825364	0.492192784	0.720914422
12.08643218	-0.596115254	0.460072617	-1.295698184	0.195079487	0.443103067
91.63929267	1.469093558	0.232921894	6.307236884	2.84061E-10	1.33088E-07
61.28811119	-0.636953059	0.470557231	-1.353614432	0.17585942	0.419176162
284.4482357	-0.08876818	0.127753505	-0.694839493	0.487155895	0.717692379
6.705250893	-0.333578616	0.405744425	-0.822139739	0.410997371	0.658233714
3.375638032	0.146739526	0.564477275	0.25995648	0.794897343	0.905927111
100.3670903	0.294439437	0.151534115	1.943057086	0.052009261	0.204733671
437.4038689	-0.125430206	0.068917684	-1.820000321	0.068758956	0.242831473
116.8679401	0.00715613	0.118329207	0.060476447	0.951776174	0.978862788
260.1233383	-0.356338194	0.156853317	-2.271792536	0.023099044	0.125445085
141.8612637	0.751827549	0.149728797	5.021262196	5.1333E-07	6.00826E-05
304.3238009	0.071827363	0.084440179	0.850630161	0.394974829	0.645455477
422.3041573	-0.592549285	0.201870516	-2.935293851	0.00333232	0.036753938
381.1879396	-0.270318327	0.181612656	-1.488433311	0.136636657	0.362188015
38.16663249	-0.119161867	0.215219753	-0.553675326	0.579801064	0.782986042
6.312859465	0.484139187	0.499692585	0.968874067	0.332608026	0.591386949
145.9992136	-0.265269164	0.1383433	-1.917470264	0.055178215	0.212624484
547.6388917	-0.262765734	0.116825652	-2.249212635	0.024498971	0.129461543
2876.381197	0.144165393	0.121197378	1.189509178	0.234239362	0.489929172
57784.07069	-0.329072042	0.164400767	-2.001645421	0.04532288	0.188729565
427.0332079	-0.174026083	0.123921293	-1.404327528	0.160221341	0.397864393
17.49751033	0.857089529	0.9015496	0.95068483	0.341764391	0.59937747
31.16032048	1.02145948	0.311026158	3.284159403	0.001022871	0.016829906
406.3342443	-0.365753403	0.126175511	-2.898766975	0.003746332	0.039647418
9.384956227	0.617415418	0.709722851	0.869938762	0.384333871	0.635384466
375.0308564	-0.065645938	0.078231192	-0.839127417	0.401397812	0.650901912
3266.771862	-0.835448672	0.388460576	-2.150665278	0.031502629	0.151258267
945.9439685	0.215005811	0.155134352	1.385932952	0.165767378	0.405536664
144.9630615	0.199665137	0.17088033	1.168450095	0.242625258	0.49834135
389.0967257	-0.301011481	0.169086724	-1.780219484	0.075040048	0.25564492
7.633260656	-0.887746697	0.429992104	-2.064565113	0.038964167	0.172047859
376.6680202	-0.25027619	0.082648637	-3.028195014	0.002460193	0.030215867
1922.50323	0.047249249	0.230844577	0.204679915	0.837822213	0.930810213
3.564605225	-0.755750246	1.013688999	-0.745544488	0.455942627	0.693666133
8899.534615	-0.698118276	0.19327462	-3.612053544	0.000303782	0.007444553
11059.05323	0.234896038	0.134461005	1.746945437	0.080646803	0.266287255
6.905513996	0.505352575	0.710690225	0.711072923	0.477039048	0.709683822
7.244886646	1.908017341	1.176249652	1.622119367	0.104777792	0.309829354
784.7018654	0.053067998	0.102393107	0.518277057	0.604264978	0.798962394
857.4519266	0.17502853	0.132602449	1.319949456	0.186851894	0.433198094
287.9879672	0.246300637	0.117539172	2.095477039	0.036128607	0.16433906
594.0952536	-0.015439113	0.079298202	-0.194696889	0.845630247	0.93321593
3.047040557	-0.095853053	0.582399716	-0.164582933	0.869272266	0.945403663
64.63138024	-0.497241822	0.490371038	-1.014011398	0.310577313	0.570118748
1205.486752	0.119855199	0.10681125	1.122121487	0.261810789	0.51962191

3.732774314	0.683574561	0.569882118	1.199501685	0.23033293	0.48564897
298.4175209	-0.180403217	0.082999448	-2.173547195	0.029739158	0.146353608
273.3943904	0.099532732	0.080520719	1.236113296	0.216416453	0.469066467
1070.284628	-0.366092222	0.195991784	-1.867895752	0.061776603	0.227847604
313.7038639	-0.137922267	0.109686979	-1.257416957	0.208602692	0.459572597
171.8069159	-0.217140738	0.136660781	-1.5889031	0.112082271	0.322313594
128.0846446	0.023971719	0.088953362	0.269486377	0.787555424	0.903200814
11.74153574	-0.307606831	0.377721008	-0.814375755	0.415429723	0.661679388
248.2648073	-0.423816012	0.08683634	-4.880629608	1.05748E-06	0.000102379
659.7116032	-0.192186771	0.116130518	-1.654920466	0.097940632	0.298612622
1971.419933	-0.687330853	0.235502705	-2.918568819	0.003516422	0.037855162
14.70105998	0.76811128	0.361614711	2.12411513	0.033660518	0.157075739
4.595290247	1.577691064	0.775127638	2.035395188	0.041811125	0.179514286
32.88912365	-0.530431906	0.221870556	-2.390726897	0.016815056	0.103790035
4.207285255	-0.573248492	0.452233316	-1.267594562	0.204942775	0.454803778
273.5818488	0.236225599	0.113983198	2.07245982	0.03822258	0.17001785
237.5959622	0.213333623	0.213646551	0.998535297	0.318019857	0.576493309
101.1873943	-0.0837132	0.133864157	-0.625359332	0.531735247	0.751414479
372.8668057	0.092046783	0.109212038	0.84282635	0.399325569	0.649354145
1806.591563	-0.006301286	0.136401632	-0.04619656	0.963153584	0.98274378
110.5622359	0.380323253	0.121260603	3.136412359	0.001710285	0.023971508
40.19768259	0.19192714	0.308385275	0.622361557	0.533704153	0.752550205
206.4809255	-0.038561972	0.111315464	-0.346420614	0.729026635	0.873448484
7310.780449	-0.006623519	0.105527388	-0.062765877	0.949952939	0.977692886
639.7847632	0.016009101	0.138227485	0.115817061	0.907797529	0.960645318
7.651788245	0.314745984	0.413800233	0.760623024	0.446882264	0.686849577
399.1861324	-0.012330808	0.075979666	-0.162290894	0.871076785	0.946199847
38864.01652	0.215258589	0.140105571	1.536402783	0.124439625	0.343189051
111.1425724	-0.246001552	0.198988003	-1.236263234	0.216360732	0.469025809
12186.17297	0.105868571	0.136714623	0.774376356	0.438708262	0.681324391
272.7227688	-0.058857327	0.101900782	-0.577594461	0.563537947	0.772666244
95.66521679	0.214826194	0.183645568	1.169786982	0.242086704	0.49765417
11.29621951	0.027341602	0.38354808	0.071285984	0.94317015	0.974993168
1546.767308	0.241381301	0.249523221	0.967370089	0.333359057	0.591900483
40.68098379	0.582465054	0.21005927	2.772860503	0.005556593	0.050964685
238.55559	0.181773095	0.088328662	2.057917449	0.039598058	0.173782322
144.5129523	0.60509513	0.170851737	3.541638733	0.00039765	0.008916368
84.97023555	0.327610588	0.166046194	1.973008712	0.04849457	0.196225524
8.665904804	-0.888698838	0.7460469	-1.191210416	0.233570991	0.489137368
12.76493051	-0.152433371	0.368241901	-0.413949011	0.678911449	0.845876523
505.9336958	-0.201497238	0.097747913	-2.061396821	0.039265198	0.173036067
7.191984866	-0.956376845	0.61152817	-1.563912985	0.117838005	0.331986935
121.3455257	-0.999134278	0.242887193	-4.113573329	3.89581E-05	0.001664902
64.01589914	-0.505345146	0.276350816	-1.828636343	0.067454104	0.240348628
18.7410896	0.157082178	0.326778939	0.480698599	0.630730727	0.816068366
3.309022598	2.282746863	1.153053833	1.979740058	0.047732745	0.194553111
375.2654468	-0.399315198	0.114768894	-3.479298114	0.000502729	0.010527105
279.7799938	0.044392693	0.15839568	0.280264544	0.779274551	0.898614978

932.454383	-0.029504457	0.100854529	-0.292544696	0.769870191	0.894019225
14.40602604	-0.753798305	0.394999283	-1.908353602	0.056345529	0.215489705
3048.095744	-0.302776177	0.122800418	-2.465595647	0.013678565	0.091759886
207.0990484	0.212241448	0.385472423	0.55060086	0.581907319	0.784201399
2314.368048	-0.348320132	0.17880298	-1.948066706	0.051406982	0.203038808
161.4408334	-0.052200232	0.096094887	-0.5432155	0.586981436	0.787731397
88.91609306	0.27263792	0.233073033	1.169753172	0.242100314	0.49765417
86.79247907	0.530881488	0.210510736	2.521873699	0.011673161	0.082136321
1070.21541	-0.205229911	0.103734897	-1.97840762	0.047882741	0.194961814
4.61833906	-0.120202695	0.58312192	-0.206136472	0.83668431	0.930545789
467.4628995	-0.233218431	0.075804275	-3.076586787	0.002093853	0.027370411
2.748959192	-0.091031139	0.715928102	-0.127151231	0.898820704	0.957506447
5.752331843	0.629156885	0.462501627	1.360334425	0.173724119	0.416746572
123.7176866	-0.109404657	0.220960957	-0.495131168	0.620507534	0.809425942
3547.865657	-0.193515169	0.069573545	-2.781447587	0.005411707	0.050139147
20.35031514	-0.44089184	0.252115923	-1.748766341	0.080331417	0.265525384
8.670361681	0.336563749	0.705723008	0.476906301	0.633428845	0.817960211
4.461222068	0.071855278	0.653554669	0.109945322	0.912452739	0.962595598
12.78606856	-0.959242319	0.639479281	-1.500036588	0.133604925	0.357038617
47.91012385	0.557421203	0.530441868	1.050862002	0.293321974	0.55260803
44.63453742	0.428642829	0.470405381	0.91122008	0.362179426	0.617840126
184.5249198	0.305675167	0.292476588	1.045126959	0.295964296	0.554787142
19.10740047	0.818618696	0.592150485	1.382450434	0.16683344	0.406609306
2722.990191	-0.256107656	0.101438091	-2.524768093	0.011577466	0.08157373
492.8279798	-0.124037461	0.148042738	-0.837849006	0.402115512	0.651728809
4.517473542	0.804864555	0.54188962	1.485292438	0.137466374	0.363395013
2180.181978	-0.147605713	0.103604949	-1.424697508	0.15424466	0.38887588
52.31762602	-0.225904677	0.141338478	-1.598323963	0.109970897	0.318242753
53.88662463	0.32094869	0.176504805	1.818356675	0.069009636	0.243278743
1976.938735	-0.098807141	0.073498383	-1.344344423	0.178837037	0.423578692
2.932240469	-0.796673625	0.668483278	-1.191762983	0.233354194	0.48890291
3.659983187	-0.60438825	0.624773976	-0.967371039	0.333358583	0.591900483
20.56699969	0.31340442	0.417744217	0.750230421	0.45311594	0.691607974
545.0264302	0.296425276	0.272758531	1.086768119	0.277139301	0.535573218
222.9505855	0.008959051	0.144186039	0.062135357	0.950455041	0.978017115
147.4620127	-0.05996332	0.100561608	-0.596284416	0.55098524	0.764274809
5.345990696	3.091226311	0.623945861	4.954318165	7.25844E-07	7.69198E-05
272.2263133	0.132090822	0.109262385	1.208932264	0.22668887	0.481042922
32.95219333	0.951438554	0.45408435	2.095290347	0.03614519	0.164366819
3.042334633	-1.066888231	0.955387151	-1.116707745	0.264119305	0.521850238
609.7944976	0.026504235	0.094749455	0.279729688	0.779684899	0.898747855
62.11218907	-0.350678764	0.297972377	-1.176883464	0.239242023	0.494631148
776.1634464	-0.104050472	0.058342657	-1.783437303	0.074515145	0.254503138
28.75843646	0.185680371	0.230114116	0.806905609	0.419720867	0.664585052
5.060962979	0.532011834	0.658078145	0.808432613	0.418841584	0.663816586
115.6403535	-0.198043082	0.278126817	-0.712060362	0.476427398	0.709176698
134.5546729	0.771007166	0.392183994	1.96593226	0.049306436	0.198287729
366.6368429	0.004880777	0.065525627	0.074486544	0.940623248	0.974550053

405.8360985	-0.18507581	0.234250603	-0.790076131	0.429483309	0.674275897
4.400176105	1.124051635	0.49647055	2.264085222	0.023568874	0.126898117
994.0061164	0.128746131	0.074566159	1.726602686	0.084239043	0.274108953
400.0341996	-0.054859303	0.082105096	-0.668159532	0.504031764	0.730167397
10.34931322	0.155509673	0.540430817	0.287751305	0.773537115	0.896155718
6.274206759	0.735061664	0.664282871	1.106549177	0.268488891	0.527493394
8.435480128	0.437201901	0.425369658	1.027816376	0.304036212	0.562389898
2.907415826	0.079986104	1.119504935	0.071447746	0.943041411	0.974993168
3.30887386	0.438540168	0.708281718	0.619160649	0.535810533	0.754028927
29.10342744	0.214878211	0.204531669	1.050586507	0.293448541	0.552647324
43.19393552	0.254010549	0.207623685	1.223417981	0.221171865	0.474471281
29.40530918	-0.193200495	0.465193519	-0.415312095	0.677913448	0.845854775
88.72703914	0.230867589	0.138864988	1.662532743	0.096406004	0.295780861
702.2460306	0.246982384	0.10208985	2.419264841	0.015551913	0.099354867
38.32782636	0.089355678	0.183952981	0.485752814	0.627142433	0.813801939
3.502635108	0.364248807	0.52603597	0.692440874	0.488660505	0.718626475
293.4967103	0.182759256	0.19695574	0.927920437	0.353448842	0.610315055
234.6653979	-0.266342498	0.164661049	-1.617519747	0.105766137	0.310992892
732.8257856	-0.187386782	0.10415933	-1.79903982	0.072012382	0.249234728
1714.347298	-0.159209893	0.21300522	-0.747445971	0.454794405	0.692659256
14.10184329	-0.343753391	0.438293787	-0.784299028	0.43286467	0.677482235
1944.237533	0.11483111	0.130527816	0.879744356	0.378997815	0.631220317
208.2472469	0.949037228	0.273070004	3.475435649	0.000510024	0.010623136
316.1473319	-0.213267919	0.087198429	-2.445777066	0.014454034	0.09473342
134.6155496	0.037957379	0.10921574	0.347544949	0.728181956	0.873123696
915.470443	0.053715688	0.137228083	0.391433641	0.69547673	0.854503374
33.00218447	0.985132058	0.230030586	4.28261335	1.84711E-05	0.000935305
333.3680156	0.145553562	0.10798509	1.347904253	0.17768918	0.421963374
75.30885612	0.34148781	0.1570367	2.174573269	0.029662109	0.146203809
615.5197402	0.136961603	0.266498116	0.513930847	0.60730034	0.801442647
94.74322959	0.118012175	0.123432268	0.956088532	0.339027508	0.597335157
327.895832	0.025547008	0.121412178	0.210415528	0.833343374	0.929163639
21.05736455	-0.605123744	0.486313104	-1.244308943	0.213385881	0.465277931
941.5649442	-0.221571386	0.109477193	-2.023904529	0.042979976	0.182465501
2287.166045	0.006272958	0.128008062	0.049004401	0.960915788	0.98222115
470.3441571	-0.092944606	0.220850946	-0.420847667	0.673866322	0.843351295
5244.930841	0.195033674	0.131158769	1.487004456	0.137013634	0.362809697
249.9459177	-0.026354559	0.156264649	-0.168653365	0.866069302	0.943555914
991.5596064	0.129027771	0.071401391	1.807076418	0.070750365	0.246952274
3.840433007	0.312384145	0.522508313	0.597854881	0.549936768	0.763630657
82.33107157	0.172695603	0.121550615	1.420771117	0.15538331	0.390299781
8.868568689	0.714538175	0.648552143	1.101743603	0.270573155	0.529531989
55.95410661	1.09265915	0.387386876	2.820588969	0.004793558	0.046209073
291.5680937	-0.227884772	0.163904312	-1.390352507	0.164421861	0.403782513
142.912668	-0.278923938	0.095543396	-2.919342931	0.003507701	0.037855162
11.52177893	0.03213409	0.434923225	0.07388451	0.941102281	0.974550053
291.270797	-0.157571249	0.089587278	-1.758857409	0.078601731	0.262352803
6568.853891	-0.110538122	0.112679632	-0.980994706	0.326595352	0.585151858

1037.88364	-0.168129745	0.071145748	-2.363173466	0.018119186	0.108099837
236.221675	0.02165845	0.123689904	0.175102816	0.860998846	0.940710965
678.560363	-0.051162186	0.066443009	-0.770016078	0.441290355	0.682742007
25.93573853	0.966179646	0.279355023	3.458608462	0.000542974	0.011146594
375.5055338	-0.043064375	0.14520892	-0.296568387	0.766796049	0.892250595
263.8258768	0.024682033	0.079271557	0.311360521	0.755526563	0.887493231
116.5013436	0.126524513	0.13435289	0.941732722	0.346329497	0.604006654
145.1328163	0.229980172	0.15961635	1.440830919	0.149632456	0.382283017
177.7632078	0.000696243	0.175346139	0.003970676	0.996831867	0.998423335
4.702224765	-0.55802812	0.492367918	-1.133355971	0.257064796	0.513998503
596.5342963	0.02805982	0.19067927	0.147157162	0.883007972	0.95143563
118.4179372	-0.174595169	0.323778913	-0.539241938	0.589719926	0.789919491
268.7697668	-0.096900271	0.151126743	-0.641185466	0.521402191	0.744065636
126.3539576	0.252331124	0.177205464	1.423946635	0.154461921	0.389005157
41.8078741	-0.195768826	0.172645107	-1.13393788	0.256820606	0.513818445
835.472398	0.145777739	0.132514929	1.100085398	0.271294915	0.530481168
7.402905961	0.699677251	0.375188889	1.864866665	0.062200096	0.228700076
35.31110813	0.318962944	0.240745305	1.32489788	0.185205024	0.431116715
248.0591973	-0.137940599	0.074726421	-1.845941464	0.064900705	0.234918685
310.3215598	0.050540165	0.090140239	0.56068372	0.575013167	0.780215115
63.69183979	-0.179934687	0.378542348	-0.475335686	0.634547727	0.818575962
391.7624113	0.085960488	0.129869414	0.661899405	0.508035693	0.733544682
437.4554312	0.208323695	0.092378563	2.255108624	0.024126511	0.128620056
162.8093093	0.135156327	0.284480711	0.475098389	0.634716847	0.818618539
223.3673446	-0.331095625	0.187430213	-1.766500822	0.077311864	0.26028339
308.8572465	-0.01752093	0.098172327	-0.17847117	0.858352961	0.93958737
2.94230377	1.344070711	1.04858867	1.281790229	0.199916243	0.448439123
35.20162972	-1.035476346	0.403327312	-2.56733505	0.010248354	0.075936734
24.87819594	0.370107779	0.311055107	1.189846335	0.234106794	0.489760032
39.34081424	0.059148487	0.231368983	0.255645706	0.798224422	0.907769715
114.2019618	-0.393706939	0.182269804	-2.160022836	0.030770902	0.14918418
499.286566	-0.095949498	0.093272836	-1.028697122	0.303622023	0.562210657
166.1137968	-0.128379999	0.091356225	-1.405268217	0.159941536	0.397563536
1722.761621	0.018328627	0.081640219	0.224504879	0.822364471	0.922469201
12.60853104	-0.798425594	0.386813598	-2.064109424	0.039007343	0.172093154
648.1573444	0.131899616	0.213409702	0.618058201	0.536536974	0.75450963
50.20978967	0.420585108	0.259264207	1.622225885	0.104754991	0.309829354
92.90235145	0.260439351	0.33537824	0.776554113	0.437421883	0.680596258
180.2302276	0.178438104	0.177559083	1.004950578	0.314920648	0.573869373
81.62688519	-0.180670333	0.140284229	-1.287887701	0.197785057	0.44611812
94.04351042	-0.043170691	0.150171303	-0.2874763	0.773747646	0.896159371
541.9765869	-0.317486378	0.143509594	-2.212300717	0.026945894	0.137571418
134.1645554	0.043427098	0.144567858	0.300392486	0.763877794	0.891351784
71.96229497	-1.20152759	0.206333349	-5.823235039	5.77193E-09	1.44102E-06
353.8101709	-0.697993034	0.151463209	-4.608333862	4.05909E-06	0.000284208
724.249666	-0.186252475	0.178246186	-1.044917031	0.296061318	0.554857408
18.91093194	-0.091393619	0.236979276	-0.385660808	0.699747892	0.857008117
449.4019404	-0.144490785	0.12102901	-1.193852492	0.232535674	0.487913329

62.17764541	0.177785929	0.554822575	0.320437446	0.748636743	0.884690979
669.6763523	-0.167143652	0.122987706	-1.359027317	0.174137933	0.417483848
311.5366852	-0.056071704	0.079231653	-0.707693224	0.479135786	0.711221434
3.427635115	-0.508487911	0.61251307	-0.830166629	0.40644458	0.655022276
241.1485427	0.041211913	0.096335796	0.427794387	0.668800824	0.839560759
4.862812577	-2.020694857	1.029953444	-1.961928347	0.049770827	0.199609773
626.0714511	0.109270919	0.203547392	0.536832813	0.591383104	0.79088016
278.5774238	-0.32192588	0.116981708	-2.751933483	0.005924455	0.053140675
37.97939331	-0.457499601	0.282820218	-1.617634001	0.105741497	0.310992892
53.88894136	0.447909559	0.332469461	1.347220156	0.177909338	0.422329357
5.980987459	0.470823574	0.436698412	1.078143544	0.280969702	0.539411971
3.687321308	0.369701202	0.643650082	0.57438228	0.565709142	0.774060678
595.8317519	0.072009163	0.099323325	0.724997506	0.468453561	0.703622452
7.945122849	0.680347591	0.397862742	1.710005786	0.087264803	0.279207439
641.1861677	-0.129281285	0.052367275	-2.468741875	0.013558898	0.091230272
151.0172752	0.076836775	0.133298323	0.576427169	0.564326485	0.773206656
1180.449013	-0.215781219	0.116097213	-1.858625313	0.063080267	0.230617926
821.1618683	-0.306815031	0.091415822	-3.356257408	0.00079005	0.013922634
22.25961301	0.750741901	0.610557072	1.229601515	0.218846362	0.471611205
611.8567379	-0.110203542	0.132122788	-0.834099431	0.404224956	0.653396496
47.59493497	0.601567013	0.380194602	1.582260795	0.113590047	0.324370417
16.49112818	0.94960743	0.27632205	3.436596648	0.000589072	0.011665044
902.1993506	-0.2302946	0.154351785	-1.492011253	0.135696195	0.360905397
413.1198758	0.00991993	0.11579655	0.085666884	0.931731228	0.971304259
117.3812957	-0.005193985	0.122849707	-0.04227918	0.966276142	0.983959158
7.267805028	0.257589371	0.537470493	0.479262348	0.631752004	0.81678327
6.354424988	1.870780582	0.696216959	2.687065515	0.007208281	0.061011702
4.630918568	0.568684915	0.979314534	0.580696901	0.561444751	0.771009498
3354.211469	0.475070102	0.124522689	3.815128825	0.000136112	0.004194065
312.7233969	-0.023950409	0.099322995	-0.241136595	0.809449251	0.914983931
58.57774487	0.723510205	0.506290652	1.429041206	0.15299239	0.386958982
34.54124036	0.517791448	0.620837612	0.834020746	0.404269293	0.653396496
401.9523545	-0.095063523	0.169469966	-0.56094614	0.574834255	0.780163053
5.738380154	0.357937698	0.511303743	0.700049047	0.483896675	0.715310442
32.9677609	0.900929864	0.839477841	1.073202674	0.283180191	0.541854464
58.54960424	-0.202151378	0.232325099	-0.870122853	0.38423327	0.635352094
27.6791811	0.192275327	0.260663735	0.737637427	0.460734805	0.697145057
1086.332233	0.699319268	0.176136467	3.970326417	7.17742E-05	0.002564253
56.3708943	-0.01151844	0.155985607	-0.073842967	0.941135337	0.974550053
2143.835517	-0.120700862	0.071815717	-1.680702598	0.092820696	0.289193443
98.48588537	-0.367849842	0.203358202	-1.808876344	0.070470216	0.246268911
1040.075265	-0.008481397	0.091895119	-0.092294314	0.926464206	0.968986559
692.2662571	0.102404814	0.092168733	1.111058061	0.266543359	0.525184175
113.8141559	-0.081458327	0.203704886	-0.399884012	0.689241948	0.851119654
7.988446542	-1.241886937	0.49869116	-2.490292666	0.012763794	0.087517934
28.66562609	0.278936166	0.442999008	0.629654156	0.528920883	0.749308566
770.8742891	0.391167525	0.214758304	1.821431431	0.068541301	0.242335835
436.2280014	0.030242524	0.064790932	0.466770944	0.640663766	0.823232665

298.0078116	-0.182481768	0.073448953	-2.48447064	0.012974421	0.088320666
27.94911417	-0.0640195	0.20227492	-0.31649747	0.751624946	0.88558395
11.4918659	0.349298032	0.388064127	0.900103894	0.368064964	0.623287987
35.27616777	1.063984953	0.357482569	2.97632681	0.002917238	0.0337917
16.59526158	0.764011355	0.331254475	2.306418211	0.021087275	0.118287849
995.5733301	-0.005749466	0.084342014	-0.068168472	0.945651525	0.976005209
908.5364536	0.108320723	0.090212929	1.200722816	0.229858741	0.485078645
679.0599504	0.071264917	0.143822844	0.495504852	0.620243798	0.80924247
409.2153676	0.357989537	0.203121739	1.762438318	0.077995269	0.260963931
23.22613243	0.730091007	0.263267089	2.773195117	0.005550883	0.050942098
6.537154198	0.60046064	0.59181038	1.014616608	0.31028862	0.569788866
193.9762341	0.566144834	0.146247479	3.87114251	0.000108326	0.003536363
113.0635419	-0.041798545	0.178373919	-0.234331032	0.814727996	0.918435556
1599.95294	-0.235833425	0.058618795	-4.023170823	5.74198E-05	0.00224023
1436.076394	-0.238682425	0.069296969	-3.444341485	0.000572452	0.011505047
4.385182708	0.394841612	0.762456219	0.517854799	0.604559581	0.799020152
663.1260018	0.302742183	0.232550209	1.301835778	0.19297253	0.440314141
1099.677394	-0.234924311	0.35640218	-0.659155089	0.509796183	0.73483854
710.1256872	1.218419682	0.693137898	1.757831574	0.078776171	0.262639264
301.8836301	0.676886552	0.214018945	3.162741275	0.001562912	0.022675954
21.86386819	1.547237372	0.601680776	2.571525357	0.01012516	0.075302523
2187.448476	-0.674839475	0.505692986	-1.334484546	0.182045108	0.427527932
162.3549737	-0.687388083	0.46367631	-1.482474022	0.138214211	0.364794717
87.48005226	-0.455039338	0.352894262	-1.289449524	0.197241854	0.445691001
38.48095464	0.405157063	0.188032429	2.154719082	0.031183824	0.150211023
211.7587691	-0.04659472	0.12294327	-0.378993659	0.704692573	0.860203048
645.9359663	0.035335483	0.120709507	0.292731567	0.769727339	0.893921036
3372.657202	-0.001916283	0.088138314	-0.021741767	0.982653947	0.991070527
382.4244438	0.025266796	0.128301075	0.196933629	0.843879477	0.932271104
120.0178306	-0.142629346	0.128550468	-1.109520244	0.267205816	0.525910227
480.8324812	0.079171499	0.08642389	0.916083492	0.359623106	0.61570305
3175.124951	-0.005789796	0.131868594	-0.043905797	0.964979494	0.983605536
34.19188958	-0.211279969	0.221120948	-0.955495041	0.339327412	0.597508828
5.75177219	1.935492803	0.595581948	3.249750616	0.001155063	0.018317493
2.678143382	1.787874532	1.056901557	1.691618789	0.090718682	0.285395303
25.39738287	1.466017527	0.84564056	1.7336178	0.08298592	0.271382646
261.8544741	0.22988503	0.120169696	1.913003341	0.055747625	0.213985743
7.501980405	1.201203666	0.379226629	3.167508749	0.001537511	0.022431926
7.741570831	0.637513783	0.476020782	1.3392562	0.180487282	0.425746014
236.1594315	-0.189566313	0.121656387	-1.558210931	0.119183255	0.334192367
955.5954197	-0.035087362	0.112324138	-0.312375974	0.754754807	0.887201677
121.1330454	0.052373459	0.242058157	0.216367254	0.828701489	0.926595434
1170.374788	0.035890096	0.136717696	0.26251244	0.792926396	0.905326977
761.8601324	-0.035133148	0.066549271	-0.527926869	0.597550095	0.794878848
718.1534303	-0.079870986	0.22942423	-0.348136663	0.727737551	0.87295111
325.8421092	0.418772024	0.175531627	2.385735437	0.017045008	0.104499517
1983.69848	0.067375177	0.169419218	0.397683201	0.690863722	0.852317047
1153.919142	1.860228037	0.845078647	2.201248422	0.027718442	0.140182155

3.6420004	1.37722665	0.8604521	1.600584913	0.109468886	0.317334055
21.9801907	1.49066585	0.306443676	4.864404027	1.14802E-06	0.00010979
7.676796789	0.66889618	0.862004611	0.775977497	0.437762273	0.680661785
1511.783117	0.28014191	0.527118205	0.531459372	0.595100488	0.792928048
132.9759867	0.030909191	0.264267339	0.116961829	0.906890302	0.960313075
20226.85404	-0.067122465	0.163638052	-0.410188609	0.681667597	0.84736731
90.92194547	0.687736757	0.487130817	1.41181123	0.158005551	0.394674162
495.2083471	-0.068032154	0.132230096	-0.514498258	0.606903678	0.801235462
6.125152446	0.455984955	0.425728169	1.07107067	0.284137655	0.543001703
115.5038141	0.02907702	0.114233416	0.254540406	0.799078092	0.908235437
6.115875418	2.407050929	0.839515812	2.86718951	0.004141349	0.042232071
293.6981818	-0.359702616	0.123603218	-2.910139567	0.003612674	0.038597532
2.957061439	-0.941050508	0.600368237	-1.567455521	0.117008255	0.330301921
6.331550958	0.653409922	0.507146194	1.288405453	0.197604862	0.44611812
146.3946625	0.07501873	0.652562092	0.114960294	0.908476594	0.960992992
2229.168866	-0.168221865	0.125608844	-1.339251751	0.18048873	0.425746014
80.213662	0.054054848	0.173350105	0.31182472	0.755173736	0.887389343
853.8222588	-0.197880905	0.116455363	-1.699199583	0.089281585	0.283287958
11.93763039	0.269814227	0.308013399	0.875982108	0.38103976	0.632873833
458.1486149	-0.326648153	0.097922965	-3.335766566	0.000850646	0.014679908
11.19819568	2.08844349	0.947310378	2.204603198	0.027481951	0.139400686
600.799614	-0.929135973	0.420012254	-2.212163966	0.026955338	0.137574851
26.22534526	-0.278427252	0.297603265	-0.935565177	0.349497111	0.606632725
531.8220697	-0.226011159	0.077544768	-2.91458941	0.003561568	0.03817454
208.9580044	-0.388779083	0.297471281	-1.306946613	0.191230844	0.438494359
289.6973654	-0.264221057	0.129727805	-2.036734203	0.041676685	0.179231458
247.0006974	-0.295833141	0.096408875	-3.06852602	0.002151176	0.02767764
181.6045729	-0.03151876	0.142584701	-0.22105289	0.825051251	0.92429313
109.1288147	0.630959243	0.295658027	2.134084606	0.032835856	0.154857295
2.831930656	0.86690935	1.049246009	0.826221251	0.408678595	0.656531301
19.35293958	-0.048460822	0.70909675	-0.068341622	0.945513692	0.976005209
40.36667893	-0.162840867	0.362202275	-0.449585434	0.653009393	0.830317856
8.291114756	-0.178916265	0.539516471	-0.33162336	0.740173678	0.879355136
330.5285399	-0.236771688	0.118439224	-1.999098619	0.045597685	0.189494988
2358.151221	0.022858258	0.04960815	0.460776262	0.644959134	0.825444522
192.1779711	-0.968650452	0.617001553	-1.569931952	0.116430943	0.329345551
219.5372823	0.825422824	0.159054031	5.189575	2.10775E-07	2.89981E-05
2172.445456	-0.052742238	0.082521094	-0.639136429	0.522734183	0.744849907
132.0438562	0.024865211	0.103952627	0.239197527	0.81095242	0.915758284
221.1766507	-0.102621697	0.097308081	-1.054606116	0.291605506	0.550962625
196.6240652	-0.229883191	0.143553617	-1.601375118	0.10929386	0.317176115
249.5740072	0.343011722	0.173545027	1.976499858	0.048098192	0.19544517
797.0897447	-0.499215451	0.064834117	-7.699888178	1.36185E-14	2.13593E-11
1655.079661	0.047959205	0.16036847	0.29905632	0.764897074	0.891746525
87.81606653	0.03193899	0.153164101	0.208527913	0.83481679	0.929483538
88.71302083	0.121378391	0.135576589	0.895275448	0.370639861	0.625026387
405.1389516	-0.512648996	0.321988196	-1.592135992	0.111354152	0.320866896
280.3914213	-0.08201351	0.31581499	-0.259688465	0.795104091	0.905927111

47.69387828	-0.257553673	0.500752902	-0.514332863	0.607019289	0.801320641
1035.041025	-0.225258468	0.086531243	-2.603203893	0.009235701	0.071235615
8.545388646	0.10745758	0.441448954	0.243420172	0.807679919	0.913774208
35.78977586	-0.452440051	0.29288815	-1.544753693	0.122405852	0.339546521
2699.563709	-0.065835857	0.112891177	-0.583179827	0.55977225	0.770157539
712.9504518	-0.080127869	0.118172674	-0.67805751	0.49773523	0.72436479
69.41643331	-1.045234643	0.360081518	-2.902772268	0.003698755	0.039216336
11.26967775	0.630067554	0.397972647	1.583193115	0.113377455	0.324077445
108.3402081	-0.193165267	0.574063807	-0.336487451	0.736503313	0.877682597
5.533730758	-0.146729521	0.442864791	-0.331319003	0.740403539	0.879363902
2.723336752	1.168320535	0.637358533	1.833066436	0.066792692	0.239172735
4.632039589	0.883948803	0.546149028	1.618512087	0.105552284	0.310829552
6356.423134	-0.39327164	0.292148603	-1.346135616	0.178258784	0.422902855
7.228312215	-0.294059118	0.42792153	-0.687180002	0.491969305	0.72084769
176.3937992	0.611895849	0.199761692	3.06312909	0.002190356	0.027918062
223.2042184	-0.020124416	0.134422558	-0.149710108	0.880993333	0.950743683
167.5234276	0.258183096	0.142973427	1.805811762	0.070947748	0.247029455
1740.163202	-0.713966722	0.116027005	-6.153453014	7.58138E-10	2.73204E-07
20.37969132	0.552603525	0.515079178	1.072851609	0.283337701	0.541869101
290.6486543	0.145480701	0.124723144	1.166429072	0.24344101	0.499015555
243.8078404	0.009879386	0.144038871	0.068588335	0.945317304	0.976005209
2060.77252	-0.111887261	0.060077099	-1.862394544	0.062547498	0.229651199
2087.353011	-0.204085123	0.09729822	-2.097521653	0.035947422	0.163918072
952.4550182	-0.059524875	0.051633474	-1.152834968	0.248978129	0.504648872
8.031758508	-0.797213567	0.577480631	-1.380502693	0.167431921	0.407187604
91.97077564	-0.403739897	0.444244644	-0.908823331	0.363443389	0.618784858
922.2717436	-0.179900312	0.063304481	-2.841825861	0.004485599	0.044330263
125.8451544	0.074283535	0.134953071	0.550439751	0.58201779	0.784223971
705.8385737	-0.10538805	0.082461655	-1.278024923	0.201240639	0.450058203
60.0304836	0.965794532	0.411470494	2.347178096	0.018916211	0.110992087
99.22736574	-0.451970526	0.229011762	-1.97356905	0.048430766	0.196225299
391.3521869	-0.192838477	0.135869667	-1.419290127	0.155814449	0.39088193
44.74220761	-0.058173394	0.199162407	-0.292090232	0.770217635	0.894291781
20.23275796	-0.061162292	0.4367893	-0.140026992	0.888638664	0.953660331
72.53423744	-0.020220939	0.263211767	-0.076823841	0.938763684	0.974295153
4.435595258	1.223734609	0.712569973	1.7173536	0.085914584	0.277180856
17.23849417	0.470528947	0.494889973	0.950774864	0.341718675	0.599364314
733.7484263	-0.208716963	0.103819113	-2.010390542	0.044389871	0.186551643
78.41231335	0.369774071	0.309426917	1.19502878	0.232075786	0.487396441
6.557612023	3.434906932	0.964309382	3.562038281	0.000367987	0.008462615
289.4901286	-0.052055682	0.135462921	-0.38427993	0.700770982	0.857636942
428.8761658	-0.257543488	0.13287338	-1.938262481	0.052591207	0.206107071
299.9034999	0.194300288	0.152892353	1.270830652	0.203788902	0.453300969
10.43248525	-0.061827876	0.380877816	-0.162329948	0.871046033	0.946199847
1310.703764	-0.153227467	0.091792213	-1.669286114	0.095060691	0.292971483
9.241675088	0.639367267	0.669539165	0.954936321	0.339609901	0.597673737
133.3094476	-0.212562527	0.112608563	-1.887623117	0.059076564	0.221792559
116.0953043	0.050737965	0.187954579	0.269948012	0.787200249	0.903046278

236.1686418	-0.039274036	0.132164506	-0.297160238	0.766344177	0.892250595
10.3735523	0.43647543	0.355646241	1.227274128	0.219719576	0.472454323
219.0215209	-0.073477213	0.085888251	-0.855497834	0.392275603	0.64295648
1069.663999	-0.202356967	0.07032746	-2.877353579	0.00401026	0.041522935
323.9982263	0.115162406	0.126445229	0.910769092	0.362417051	0.618026146
159.0912535	-0.109044194	0.103688913	-1.051647577	0.292961271	0.552335575
181.2928677	0.262176207	0.191649509	1.367998322	0.171312601	0.413173434
8.129169018	0.057393989	0.485707282	0.118165799	0.905936288	0.960133527
115.0969645	0.034513644	0.31115081	0.11092256	0.911677756	0.962232976
166.5663812	0.240680951	0.158154354	1.521810466	0.128056578	0.348808506
208.1425351	0.055884636	0.107796224	0.518428508	0.604159328	0.798890052
576.0081979	-0.022081911	0.324960877	-0.067952521	0.94582343	0.976005209
1942.893611	0.06243626	0.086575566	0.721176458	0.470800954	0.705535576
3.53786465	0.044171809	0.674592921	0.065479207	0.947792462	0.976593642
385.8477745	-0.096956883	0.238356347	-0.406772818	0.684174852	0.848557456
466.2488919	0.052546833	0.222497111	0.236168608	0.813301838	0.917677859
1213.678587	-0.21081089	0.138637208	-1.520593882	0.128361783	0.349275886
53.50661014	-0.053078517	0.190968214	-0.277944248	0.781055156	0.899351668
243.7116039	-0.055420167	0.213898592	-0.25909552	0.795561541	0.906077061
567.1377893	-0.152673497	0.115287388	-1.324286201	0.185408012	0.431359424
149.9185835	0.15875187	0.135325189	1.17311397	0.240750105	0.496110133
10.57914415	0.677873872	0.483623994	1.401654758	0.161018367	0.399211518
28.40666453	-0.42722291	0.190511234	-2.242507703	0.024928578	0.130893813
5.759196266	1.403592746	0.899351524	1.560672004	0.11860116	0.333301505
4.177091351	-1.053308325	0.640403725	-1.644756713	0.100019992	0.30249008
33.00269143	0.017125536	0.400016687	0.042812053	0.965851356	0.983885343
19.72320883	0.117892124	0.656452636	0.179589689	0.8574747	0.939073885
120.3111303	0.25386734	0.172537874	1.471371673	0.14119063	0.369534051
94.04819537	-0.171493101	0.162227634	-1.057113988	0.290459568	0.549857317
290.0744574	-0.130326102	0.111123977	-1.172799113	0.240876374	0.496110133
28.11079948	-0.179052546	0.233934615	-0.765395688	0.44403596	0.685056997
2714.923052	0.629198038	0.254137146	2.475820829	0.013293027	0.089788042
609.9754964	-0.904663901	0.275251192	-3.286684776	0.001013742	0.016736354
33.33598159	-0.188289498	0.314305246	-0.599065719	0.549129061	0.763184775
619.6089876	-0.04034247	0.09830814	-0.410367543	0.681536352	0.84736731
228.8633713	-0.106949392	0.100975598	-1.05916076	0.289526573	0.549072905
28.89760251	0.253828137	0.206591114	1.228649829	0.219203124	0.472055719
3082.926544	-0.035757069	0.128569523	-0.278114663	0.78092434	0.899311917
1970.376188	0.041414054	0.182411854	0.227035978	0.820395766	0.921144748
3.357523749	-0.321868279	0.626922849	-0.513409711	0.607664754	0.801498108
87.40233729	0.884859049	0.185710398	4.7647254	1.89111E-06	0.000164779
1601.253219	-0.022970266	0.109824238	-0.209154794	0.834327401	0.929483538
585.2834436	-0.261760207	0.097776551	-2.677126608	0.007425657	0.062081025
20.42233049	0.865496729	0.394091112	2.196184339	0.028078752	0.141088009
412.8966651	0.04745766	0.077596902	0.611592196	0.540807602	0.757639765
1244.448667	0.242210217	0.134071195	1.806579087	0.070827933	0.246968718
110.7772816	-0.203128211	0.184589838	-1.100430087	0.271144776	0.530319823
106.2468407	-0.052328909	0.140061286	-0.373614369	0.708691242	0.862039895

427.0016577	-0.234794462	0.088417196	-2.655529375	0.007918404	0.064515577
40.45761382	-0.212958807	0.2308614	-0.922453071	0.356292298	0.612326146
117.9103228	0.179696592	0.164773988	1.090564073	0.275464745	0.534437044
237.3527961	-0.21686376	0.088273492	-2.45672575	0.014020968	0.092902392
189.8338153	0.072402962	0.216713728	0.33409495	0.738307911	0.878437596
1031.134256	-0.095960022	0.125518429	-0.764509426	0.444563722	0.685329016
38.85334356	0.057449603	0.442063202	0.129957896	0.896599738	0.956747655
790.2619909	-0.172062514	0.182102377	-0.944866934	0.344726814	0.60239957
7.196933304	0.347594768	0.366384633	0.948715467	0.342765345	0.600522632
612.2087858	-0.03344507	0.089176576	-0.375043215	0.707628327	0.861889372
3.568333328	0.378459059	1.41587207	0.267297496	0.789240114	0.903718901
1251.981672	0.023608558	0.165389881	0.142744875	0.886491674	0.95296336
96.08120619	-0.131866276	0.241710136	-0.545555427	0.585371571	0.786559328
246.1773037	0.312133072	0.207950073	1.501000058	0.133355547	0.356941063
794.9778545	-0.574971365	0.181777804	-3.163044942	0.001561282	0.022673288
563.4715376	-0.328054228	0.068946666	-4.758086873	1.95436E-06	0.00016553
235.1093794	-0.150264983	0.097923528	-1.534513573	0.124903357	0.343758904
18.32128484	0.811910019	0.411735372	1.971921956	0.048618516	0.196417802
21.26968129	0.431029993	0.452971931	0.951560048	0.341320151	0.59908004
84.72829248	-0.07316726	0.356638439	-0.205158086	0.837448615	0.930780365
570.8087719	-0.228534921	0.07095051	-3.221046923	0.001277232	0.019639326
73.93328634	-0.068730241	0.173411258	-0.396342441	0.691852422	0.85293695
44.09366765	-0.430569106	0.398813505	-1.079625191	0.280309119	0.53883665
2.869807081	1.636104186	1.13132405	1.446185278	0.148125242	0.379731332
60.55037994	-1.379771445	0.620165706	-2.224843187	0.026091767	0.134865403
6.180136704	-0.525192643	0.415690497	-1.263422299	0.206437454	0.456642941
2903.047376	0.151323934	0.172570644	0.876881092	0.380551227	0.632388957
335.6556954	2.076758891	0.325428237	6.381618594	1.75226E-10	8.58826E-08
179.7776301	0.750505685	0.445241616	1.685614412	0.091870091	0.287373457
6.894616378	0.467461513	0.444134879	1.052521508	0.292560348	0.552234505
305.1792526	0.149549131	0.158972567	0.940722874	0.346846894	0.604501728
15.52391588	0.540507677	0.80627789	0.670373929	0.50261945	0.728907743
40.52665507	-0.191353946	0.599595131	-0.319138592	0.74962142	0.88518749
5402.726284	-0.08328219	0.134200447	-0.620580573	0.534875625	0.753066663
7.633116209	-0.203723401	0.655387223	-0.310844329	0.755918968	0.8875741
126.2513221	0.067050674	0.160488019	0.417792393	0.676098913	0.84484991
142.3049408	0.409653256	0.246434206	1.662323031	0.096448022	0.295785247
209.1726163	-0.042307383	0.110733301	-0.382065584	0.702412719	0.858728471
457.8828237	-0.221293931	0.129243257	-1.712228056	0.086854649	0.278460408
424.2982555	-0.061110165	0.092316938	-0.661960482	0.507996548	0.733544682
183.5001125	-0.18414058	0.091931091	-2.00302832	0.045174249	0.188484415
201.2397525	0.089520398	0.145291688	0.616142599	0.537800409	0.755405841
50.98672995	-0.481557901	0.181286409	-2.656337585	0.007899449	0.064495035
5.198621119	0.14743179	0.603248418	0.244396481	0.806923767	0.91335864
82.0723485	0.231036942	0.565122964	0.408825968	0.682667382	0.847675388
199.477901	0.170965568	0.110674714	1.544757255	0.12240499	0.339546521
5.884321798	0.178823273	0.550999745	0.324543295	0.745526749	0.882212278
115.4911326	0.457956551	0.187591112	2.441248665	0.014636573	0.095548023

14.64914201	0.552287427	0.28551211	1.934374784	0.053067065	0.207557067
7.727356658	2.39969774	0.871817719	2.752522331	0.005913811	0.05312269
17.03538151	0.610375037	0.855078924	0.713823039	0.475336611	0.708263292
4.25735688	0.795323377	0.858218195	0.926714653	0.354074708	0.61085774
21.95125975	-0.856299772	0.689157105	-1.242532024	0.214040333	0.46592315
5.865530206	2.101144678	1.137835457	1.846615576	0.064802875	0.23461872
31.07231949	0.66311681	0.567434392	1.168622872	0.242555609	0.49834135
11.96632708	0.485723971	0.369854907	1.313282482	0.189087779	0.436736716
413.3124887	0.002667847	0.074213483	0.035948278	0.971323601	0.986149125
1748.132187	-0.085837406	0.05545283	-1.547935519	0.121637822	0.338467634
1114.339441	0.11484301	0.094196621	1.219183966	0.222774376	0.476215527
3702.047507	0.067468913	0.113568352	0.594081995	0.552457273	0.76490866
536.6175709	0.150996789	0.138750387	1.088262116	0.27647941	0.5350158
17256.99871	0.096401952	0.207887102	0.463722623	0.642846487	0.824387858
29.7112659	0.514008369	0.333159472	1.542829824	0.122872071	0.340481549
17383.37509	0.159509219	0.159654065	0.999092753	0.317749761	0.5764038
123.8567915	0.45568926	0.201323708	2.263465459	0.023607012	0.127059839
5.855610799	1.384764461	0.852799496	1.623786679	0.104421344	0.309651042
215.2942008	0.036492016	0.108807136	0.335382567	0.737336517	0.878218859
13.26290931	0.061262026	0.421678847	0.145281241	0.884488825	0.9521814
4.478016601	0.548640953	0.714925319	0.76741016	0.44283769	0.683810803
242.9730768	0.241562619	0.256191628	0.942898178	0.345732987	0.603475283
2.730332823	-0.917680461	0.812358605	-1.129649462	0.25862396	0.515570067
246.2371551	0.086408764	0.175320128	0.492862772	0.622109553	0.810641951
8.683650596	-1.441837434	0.629270059	-2.291285615	0.0219469	0.121159866
45.09360904	0.256008067	0.315250786	0.81207749	0.416747169	0.662355886
221.2213182	-0.298157295	0.198600127	-1.501294586	0.133279385	0.356837465
17.47251104	0.559023807	0.551545899	1.013558089	0.310793665	0.570315647
6.891105901	-7.59976E-05	1.006906157	-7.54763E-05	0.999939779	0.999939779
8.201670084	0.34485835	0.569817941	0.605207953	0.545040832	0.759994702
157.4959443	-1.148792483	0.396749117	-2.895513646	0.003785386	0.039979791
69.21153598	-0.94233479	0.295693563	-3.186862707	0.00143825	0.021397627
17.56572245	-0.283340749	0.464602769	-0.609855919	0.541957255	0.758121441
39.4750546	-0.73262033	0.351573107	-2.083834956	0.037175182	0.16733144
132.5975681	0.103712168	0.292930551	0.354050365	0.723301139	0.870759523
152.4007314	-1.119689342	0.449103106	-2.493167666	0.012660904	0.087044994
54.85174241	-1.305257535	0.530559592	-2.460152552	0.013887797	0.092490959
83.88365755	0.66440436	0.824891713	0.805444338	0.420563315	0.665360573
16.66713366	1.379321652	0.306841765	4.495221352	6.94976E-06	0.000434263
261.8001412	-0.825098459	0.288572897	-2.859237534	0.004246607	0.042776994
10.47245887	-2.655988628	0.444848076	-5.970552125	2.36452E-09	6.86762E-07
2653.374303	-0.014786388	0.142755539	-0.103578383	0.917503943	0.965132921
10.01897151	-0.014434701	0.598907546	-0.024101719	0.980771472	0.99019174
26.94848526	-0.317581597	0.307109019	-1.034100523	0.301089172	0.559711102
71.06839689	-0.087082496	0.164741348	-0.528601335	0.597082034	0.794488387
106.8438436	-0.567624227	0.214440267	-2.647003924	0.008120841	0.065428258
396.1045226	-0.403731039	0.133441708	-3.025523627	0.00248203	0.030412625
137.3569312	-0.235234536	0.098041329	-2.399340536	0.016424632	0.102164672

277.7054375	-1.0055748	0.316127881	-3.18091146	0.001468125	0.021702235
251.419815	0.353260693	0.137912697	2.561480563	0.010422707	0.076795237
1470.110128	-0.08316417	0.129710784	-0.641150778	0.521424726	0.744065636
227.1639622	-0.140562505	0.119082358	-1.180380602	0.237848874	0.493412267
764.0978515	-0.215303533	0.125950945	-1.709423729	0.087372489	0.279207439
175.2104991	-0.032527444	0.080668621	-0.403223009	0.686784175	0.849863221
2314.918297	0.127441892	0.05275333	2.415807532	0.015700358	0.099912932
119.3826583	-0.235991546	0.167073523	-1.412501165	0.157802448	0.394355257
97.35680872	0.142175598	0.226106236	0.628799986	0.529480009	0.74980955
117.8590399	0.305140918	0.152033313	2.007066164	0.044742618	0.187281352
387.0830296	-0.443660489	0.124846222	-3.553655705	0.000379916	0.008654984
1162.559512	-0.100527591	0.120756526	-0.832481647	0.405137133	0.654067923
379.3430776	0.010722619	0.14603523	0.07342488	0.94146802	0.974594762
1119.722175	-0.140277099	0.102656113	-1.366475848	0.171789656	0.413687851
848.5577029	0.082832899	0.086457137	0.958080527	0.338022154	0.596415734
9956.242537	0.628497362	0.182802729	3.438118049	0.000585772	0.011665044
155.1923738	0.061710697	0.164787934	0.374485534	0.708043117	0.861889372
5.430326199	0.746780189	0.441963326	1.689688137	0.091087634	0.285931737
692.8194092	0.887263666	0.11136223	7.967366179	1.62092E-15	3.17781E-12
148.7106929	2.313483056	0.21990599	10.52032761	6.96311E-26	1.09209E-21
966.9128934	0.120467919	0.096671808	1.246153572	0.21270802	0.464445578
53.02945954	-0.300939755	0.153517835	-1.96029181	0.049961693	0.19985689
308.7519999	0.05526334	0.068301084	0.80911366	0.418449772	0.663730402
1216.084836	-0.265268464	0.107923106	-2.457939488	0.013973672	0.092865706
198.1940337	0.156850529	0.127952048	1.225853995	0.220253624	0.473019011
130.595022	0.092678128	0.119560716	0.77515535	0.438247868	0.681014522
11.88665344	-0.044455926	0.296793626	-0.149787334	0.880932403	0.950743683
371.2902891	0.109762141	0.074645253	1.470450392	0.141439812	0.36984695
300.1521071	0.168864096	0.085073835	1.984912231	0.047154237	0.193048044
273.4065683	0.169238008	0.092856585	1.82257411	0.06836792	0.242084363
342.4020519	-0.152017983	0.113822858	-1.335566386	0.181691043	0.427200576
2791.752702	-0.081783722	0.047706902	-1.714295396	0.086474488	0.277752586
560.940048	0.306230949	0.207755265	1.473998505	0.140481994	0.368245065
913.2863327	0.183792904	0.168668985	1.08966627	0.275860179	0.534543001
390.5994692	-0.050583578	0.115615158	-0.437516833	0.661736571	0.835398185
244.1714522	-0.033873534	0.150537195	-0.225017708	0.821965499	0.922285512
380.4286606	-0.180113365	0.079841053	-2.255899175	0.024076946	0.128530573
258.5748688	0.025762082	0.115389355	0.223262203	0.823331442	0.923257803
1082.979533	-0.139022916	0.076298819	-1.822084779	0.068442123	0.242103013
6.079936527	1.068270453	0.435745237	2.451594102	0.014222499	0.093803902
32.98927452	-0.664118423	0.319244728	-2.080279999	0.037499858	0.168138301
82.14108469	0.199905983	0.189642969	1.054117557	0.291829099	0.551252269
375.9133195	-0.145854166	0.083125785	-1.754620007	0.079324327	0.263529494
317.5831551	-0.068928069	0.094054756	-0.73285044	0.463649654	0.699296945
1908.092864	-0.12696696	0.066120113	-1.920247153	0.054826688	0.211695407
79.07887212	0.07419046	0.166000385	0.446929443	0.654926004	0.831662169
7.724627464	0.145270503	0.41792204	0.347601918	0.728139165	0.873123696
378.0444856	0.296249275	0.212966825	1.391058327	0.16420774	0.403483346

137.7569028	0.225061192	0.176243511	1.276989952	0.201605795	0.450536698
20.94109869	-0.206433105	0.464207426	-0.444700136	0.656536471	0.832281264
554.6700713	0.071207392	0.130599727	0.545233851	0.585592693	0.786664905
333.8336767	0.78825116	0.213491184	3.692195362	0.000222327	0.0059344
89.57243321	0.608484088	0.273151128	2.227646258	0.025904113	0.134218736
678.5206578	-0.06965368	0.055709175	-1.250308948	0.211186711	0.462669699
1027.559007	-0.015934054	0.076202571	-0.209101265	0.834369187	0.929483538
3.857133655	0.093523902	0.549790685	0.170108197	0.864925047	0.942899493
4.683516521	0.169476803	0.435607946	0.389058108	0.697233162	0.855397756
171.5261424	-0.427571944	0.111719843	-3.827179966	0.00012962	0.004043445
3446.07126	-0.24891329	0.083074185	-2.99627724	0.002732978	0.032374647
7.502507849	-0.28190506	0.451239897	-0.624734343	0.532145429	0.751568565
35.442924	-0.500265268	0.353359855	-1.415738832	0.156851984	0.392730927
528.6650076	0.055371147	0.154442907	0.358521784	0.719952869	0.868595446
89.35072742	-0.064745301	0.14734816	-0.439403527	0.660369172	0.834476254
264.4887444	-0.138230131	0.081191605	-1.702517533	0.088658395	0.281863212
334.2250902	0.262609032	0.204779541	1.282398772	0.199702795	0.448315412
12.18720116	0.284857189	0.343842026	0.828453673	0.407413621	0.655304608
273.7153707	0.113885867	0.108596933	1.048702421	0.2943151	0.553481777
65.4816487	0.14386421	0.203518034	0.70688679	0.479636834	0.711575578
11.29903048	2.086871333	0.980987596	2.127316738	0.033393775	0.156446473
1050.093738	0.36150345	0.123853123	2.918807712	0.003513729	0.037855162
2397.832359	-0.153859618	0.142132965	-1.082504809	0.279028279	0.53751602
68.50742139	1.271079646	0.338216681	3.758181421	0.000171153	0.004889545
603.1226115	0.716207924	0.190034162	3.768837759	0.000164009	0.004773708
435.4410646	-0.093043604	0.065336974	-1.424057435	0.154429847	0.389005157
37.71896224	0.384076243	0.281321841	1.365255687	0.172172701	0.414200348
269.2379879	0.404693949	0.207033946	1.954722675	0.050615812	0.201129566
3.812330921	2.1714823	0.656415752	3.308089871	0.000939347	0.015790688
93.00117749	-0.04715439	0.156649863	-0.301017755	0.763400955	0.891126867
6.523604989	0.38039599	0.682647531	0.557236308	0.577365986	0.781716861
3.365087096	0.72154949	0.867421912	0.831832214	0.405503658	0.654312692
42.08154387	0.697337028	0.392635183	1.776043152	0.075725805	0.257074356
331.4496235	-0.109153736	0.167399234	-0.652056364	0.514364812	0.738695881
57.22192548	-0.161379131	0.208180752	-0.775187567	0.438228834	0.681014522
1258.457136	0.042286671	0.1248576	0.338679193	0.734851417	0.876677323
7.822788119	0.360570307	0.987333818	0.365195946	0.714965145	0.864906543
130.8863153	0.073597995	0.181070759	0.406459857	0.684404747	0.848557456
1408.325923	0.0682649	0.27269617	0.250333182	0.802329698	0.910590181
7.485471845	0.10200252	0.706185027	0.144441635	0.885151742	0.952291996
3.735554255	0.4626119	0.519973588	0.889683458	0.373635879	0.627431154
233.8084567	0.071575113	0.105927419	0.675699587	0.499231398	0.725734105
44.66975461	0.961908562	0.22029864	4.366384481	1.2632E-05	0.000687775
457.4229558	-0.201316958	0.120243731	-1.674240782	0.094083287	0.291474017
48.13381979	0.272191246	0.199387219	1.365138885	0.172209403	0.414200348
7.545403578	0.054944296	0.786469729	0.069861933	0.944303552	0.975591654
30.11119929	0.502611643	0.193324051	2.599840223	0.009326717	0.07188218
47.70441917	0.419433247	0.212600176	1.972873474	0.048509979	0.196225524

3.721501674	1.426902807	0.616827762	2.31329213	0.020706583	0.116904985
51.80066553	0.989573129	0.272224437	3.635137016	0.000277833	0.006983224
179.5660641	-0.003752493	0.086347395	-0.043458086	0.965336375	0.983619889
18.2662784	0.485501602	0.289841949	1.675056369	0.093923172	0.291182257
73.4714092	0.041453879	0.146415145	0.283125623	0.777080541	0.897873228
18.1496047	1.202759025	0.524612541	2.292661595	0.021867495	0.120934342
124.4120571	-1.016586205	0.397025587	-2.560505518	0.010452	0.076853809
361.6094257	-0.316755796	0.337845373	-0.937576244	0.348462224	0.605772725
16.25655674	0.28217891	0.361918914	0.779674394	0.435582555	0.679363244
605.6402825	0.220665449	0.165435908	1.333842522	0.182255472	0.427598328
623.7879668	-0.292585913	0.171693974	-1.704112884	0.08836	0.281331352
51.74439397	0.0271072	0.166336354	0.162966182	0.870545062	0.946199847
581.8789597	-0.720656039	0.155226955	-4.642595989	3.44059E-06	0.000252076
33.59178117	0.077145766	0.204469635	0.377296931	0.705952953	0.860776344
338.5769272	-0.133404433	0.088314491	-1.510561085	0.130900315	0.353093825
12.97248346	0.46182795	0.408241274	1.13126227	0.25794472	0.515109686
574.1565745	-0.560037566	0.239560218	-2.337773655	0.019398993	0.112494686
112.9840017	0.217757084	0.215146689	1.012133096	0.31147442	0.570697037
62.19909868	0.801763822	0.357256962	2.244221684	0.024818142	0.130663891
493.6189061	0.481136894	0.153945078	3.125380163	0.001775754	0.0245599
125.2495813	0.353361014	0.097163718	3.63675889	0.00027609	0.006961733
3.59117418	-0.187791698	0.511699738	-0.366995884	0.713622088	0.864250676
186.2364452	-0.332296858	0.123306415	-2.694887024	0.00704125	0.05985635
12.00339264	0.341245005	0.342054141	0.997634479	0.318456636	0.576818787
906.8232297	-0.566015694	0.1892191	-2.991324302	0.002777703	0.032731404
2653.144513	0.215864285	0.159412532	1.354123688	0.17569692	0.418979853
57.6053406	0.218202289	0.174195346	1.252629845	0.210340448	0.461824914
13.42358199	0.316704581	0.479409925	0.660613317	0.508860326	0.734084378
89.52263534	0.022668111	0.125643441	0.180416188	0.856825847	0.938857569
3.78502624	-0.909343534	0.601182065	-1.512592586	0.130383179	0.352520887
3.277910265	-1.124441063	0.700537528	-1.605111815	0.1084692	0.315276302
171.1750653	-0.208513683	0.203124901	-1.026529404	0.304642111	0.563303271
7.979171234	0.972965841	0.668075991	1.456370014	0.14529036	0.375285575
58.14945578	-0.332232574	0.182336231	-1.822087539	0.068441704	0.242103013
1112.611863	-0.035178396	0.128077917	-0.274664024	0.783574373	0.901193845
12.02553526	0.126090339	0.378063909	0.333515938	0.738744861	0.878561905
48.14731127	0.070721904	0.134640657	0.525264101	0.59939961	0.795772707
62.17057694	0.131442034	0.184507412	0.712394331	0.476220625	0.70906168
577.6258748	0.028890998	0.064732643	0.446312657	0.655371414	0.831896116
147.8388736	-0.039543698	0.110218396	-0.358775846	0.719762784	0.868512899
774.1927095	0.084375296	0.114058748	0.739752957	0.459449908	0.695896896
208.5899651	-0.083834889	0.114628378	-0.731362429	0.464557808	0.700184956
11.57515381	2.023762576	0.755156593	2.679924396	0.007363879	0.061796753
2109.377032	-0.177744336	0.111002761	-1.601260488	0.109319236	0.317176115
86.49137716	-0.164558528	0.237787437	-0.692040462	0.48891192	0.718842604
650.5335091	-0.04252212	0.210327174	-0.20217131	0.839782802	0.931173342
25.68313356	0.622896062	0.245783558	2.534327634	0.011266334	0.080163497
456.9992769	-0.220562489	0.103543985	-2.130133285	0.033160611	0.155720751

3.226294964	0.114008071	0.716773467	0.159057325	0.873623709	0.947352611
56.07574283	-0.296832429	0.447728226	-0.662974572	0.507346839	0.73318233
465.1857625	-0.048315533	0.07568869	-0.63834548	0.523248813	0.745244677
366.2581095	-0.043102474	0.115850482	-0.372052613	0.709853677	0.862515113
456.0110847	-0.089548873	0.166940298	-0.536412561	0.591673452	0.79088016
2107.968169	0.309720202	0.130188856	2.379007017	0.017359346	0.105651523
6.965750431	1.378082841	0.779876549	1.767052546	0.077219429	0.260173903
12.02306681	0.840536535	0.698412083	1.203496553	0.228784224	0.483852721
199.8332344	-0.03980643	0.106439493	-0.373981767	0.708417882	0.861905675
103.6295073	0.447239136	0.37369626	1.196798534	0.23138509	0.486759558
6.439389728	0.62838672	0.467292905	1.344738413	0.178709725	0.423524226
190.4785447	-0.171814453	0.104258872	-1.64796002	0.099360875	0.301543338
707.9523659	0.132186919	0.116503238	1.134620132	0.256534514	0.513818445
158.453243	-0.25430082	0.141513142	-1.797012045	0.072333708	0.249940927
117.1499133	-0.164303181	0.126306912	-1.300824939	0.193318383	0.440650447
49.88416861	-0.083361224	0.143446546	-0.581130922	0.56115222	0.770873642
38.60571086	-0.240413093	0.244470475	-0.983403387	0.325408944	0.584078637
2234.730182	-0.350475426	0.135612697	-2.584385042	0.009755282	0.073842588
71.85048605	0.187216466	0.222469736	0.841536782	0.400047288	0.649641838
182.032156	-0.131225751	0.071222525	-1.842475415	0.065405639	0.236146881
8.203006835	-1.134948763	0.351327679	-3.230456439	0.001235927	0.019249538
5.55294513	-0.947566028	0.597583023	-1.585664238	0.112815496	0.323444764
604.5884893	0.056155614	0.202181493	0.27774854	0.781205397	0.899392604
6687.234864	-0.114669641	0.14632787	-0.783648673	0.433246288	0.677730654
539.5798016	0.030497925	0.102875386	0.296455027	0.766882608	0.892250595
21.6468769	-0.008235943	0.477379373	-0.017252406	0.986235255	0.992541292
316.4288694	0.278126218	0.112890896	2.463672691	0.013752163	0.092056733
62.16817479	0.033826027	0.171047805	0.197757737	0.843234614	0.932221964
239.9397387	0.160787774	0.110357169	1.456976243	0.145122938	0.375211299
8.390721493	-0.335853188	0.475390023	-0.706479251	0.479890152	0.711667657
6.375145932	0.986526829	0.42054066	2.345853616	0.018983561	0.111097787
150.4649963	-1.975231528	0.494635296	-3.993308894	6.51576E-05	0.002404545
128.0557921	-0.093590305	0.205016919	-0.456500399	0.648030184	0.827595913
483.5770178	-0.353704208	0.239233665	-1.478488437	0.13927709	0.366555585
2.961872822	2.498588377	1.340152402	1.864406148	0.06226469	0.22886323
234.4407881	-0.294348291	0.131229213	-2.243008883	0.024896242	0.130840064
352.5367761	-0.176027527	0.147200789	-1.195832764	0.231761827	0.486997923
138.2208877	0.208498632	0.165054775	1.263208722	0.206514179	0.456642941
5.708900885	-0.227856347	0.483769787	-0.471001607	0.637639587	0.820943957
1052.212278	-0.02999243	0.091146109	-0.329058808	0.742111238	0.880313054
678.6059664	-0.030437743	0.173270586	-0.175665955	0.860556383	0.940613387
47.52829395	0.232766277	0.237297785	0.980903707	0.326640229	0.585151858
15.6250306	1.458080479	0.632667066	2.304656835	0.0211858	0.118658889
656.4745256	-0.15820257	0.111083856	-1.424172474	0.154396551	0.3890049
9.522577659	0.341797097	0.316681391	1.079309067	0.280449972	0.538872897
3.240772449	0.682215548	0.904062784	0.754610807	0.450482537	0.69003832
12.12834214	-0.866342195	0.488287433	-1.774246349	0.076022411	0.257802268
2448.467721	-0.203728546	0.094545485	-2.154820475	0.031175885	0.150211023

5.982979901	-0.651887275	0.450605972	-1.44669027	0.147983691	0.379505869
341.9224531	0.299105151	0.3161299	0.946146351	0.344073945	0.602015651
6.745165031	1.007140698	0.665641927	1.51303675	0.130270324	0.352451227
9.160997318	0.48215569	0.403109345	1.19609157	0.231660827	0.486916163
242.488621	0.00383738	0.192300691	0.019955103	0.984079188	0.991458113
230.1684892	-0.187864883	0.104623963	-1.795620023	0.072554969	0.250539881
3.121121363	0.250671739	0.651297984	0.384880263	0.70032613	0.857427527
577.8422221	-0.60809311	0.146464983	-4.151798589	3.29873E-05	0.001469807
1102.0952	-0.269655309	0.211371384	-1.275741796	0.202046809	0.450798249
4.918969958	-0.795122075	0.490949887	-1.619558525	0.105327145	0.310575473
89.83269953	-0.179710549	0.202010072	-0.889611826	0.373674354	0.627431154
5.622895492	-1.435423123	0.773466067	-1.855832059	0.0634775	0.231530489
13.14429054	-1.644303733	0.644726193	-2.55039077	0.010760224	0.078022814
275.0538565	-0.048888674	0.101992173	-0.479337508	0.631698543	0.81678327
14.06587756	0.611346041	0.334185067	1.82936373	0.067345137	0.240218588
304.5525137	1.059157989	0.299704408	3.534008711	0.000409308	0.009041662
81.60889535	0.613293047	0.239072902	2.565297203	0.010308746	0.076229315
53.25489059	0.10838408	0.18496877	0.585958807	0.557903197	0.768771196
265.7008963	0.023423744	0.066498882	0.352242681	0.724656271	0.871803199
8.382669429	-0.009916155	0.482393902	-0.020556137	0.983599731	0.991149398
169.1611778	0.147478045	0.114213014	1.291254291	0.196615515	0.445162804
859.238594	0.102780294	0.180269265	0.57014874	0.56857682	0.775709712
121.1983488	-0.06069293	0.122827305	-0.494132227	0.621212799	0.810102398
171.5163643	-0.055282342	0.327614699	-0.168741948	0.865999622	0.943555914
1859.343359	-0.218697282	0.229018466	-0.954932959	0.339611601	0.597673737
11.45854328	-0.327044054	0.406943978	-0.803658665	0.421594133	0.666224924
154.2491374	0.064866594	0.12644777	0.512991201	0.607957476	0.801631962
9.698682599	-0.050700531	0.637092082	-0.079581166	0.936570375	0.973575955
699.8703083	-0.294240665	0.217189422	-1.354765176	0.175492385	0.41887423
75.8090887	-0.198732044	0.152570621	-1.302557744	0.192725792	0.440233029
505.158128	-0.142547403	0.081126268	-1.757105395	0.078899846	0.262898914
19.57885606	1.02049551	0.513704915	1.986540288	0.046973364	0.192551907
9.878503305	0.424190234	0.372080522	1.140049555	0.254265656	0.511138497
56.83228082	-0.173985153	0.160743508	-1.082377479	0.279084831	0.53751602
436.1521589	-0.297540419	0.161972027	-1.836986446	0.066211896	0.238169444
309.5304337	-0.163393863	0.15388833	-1.061769033	0.288340551	0.547763227
80.13608135	0.213838985	0.345340751	0.619211559	0.535776998	0.754028927
11.0875738	0.403692046	0.406493917	0.993107224	0.320657704	0.579213006
69.34392312	0.170982595	0.183541235	0.931575922	0.351555731	0.608637468
145.9187525	-0.172238745	0.220198829	-0.78219646	0.434099125	0.678391604
3.939590849	-0.76012634	0.522557992	-1.454625806	0.145772883	0.375878512
364.8401541	0.253758878	0.140456393	1.806673748	0.070813163	0.246968718
9.326252447	0.773459718	0.326038532	2.372295426	0.01767795	0.106720924
10782.72432	-0.31279841	0.164955098	-1.896263971	0.057925148	0.219112681
151.7917919	-0.187835598	0.220386626	-0.852300349	0.394047412	0.644513465
105.6496178	0.165775916	0.114259346	1.450874014	0.146814945	0.377359161
149.0700454	-0.268795503	0.197771914	-1.359118687	0.174108982	0.417478257
460.622168	-0.297401152	0.095417266	-3.116848402	0.001827955	0.025133238

21.57544854	-0.279326804	0.323705058	-0.862905282	0.388189546	0.639466951
3.079031669	0.317257824	0.914044018	0.347092501	0.728521827	0.873219977
202.5983317	-0.72898414	0.327510057	-2.225837415	0.026025074	0.134667521
1206.039453	0.013776964	0.134026306	0.102792986	0.918127272	0.965350236
66.04168368	0.21164501	0.189253879	1.118312669	0.263433478	0.521277253
123.2637601	-0.048645006	0.098618779	-0.493263113	0.621826688	0.810633345
61.74988135	0.171565131	0.200498505	0.855692816	0.392167715	0.642846827
22.24551422	0.036120868	0.212863179	0.169690542	0.865253513	0.943060187
130.5687893	0.189797904	0.231338361	0.820434206	0.411968622	0.658847341
593.3374717	0.053840661	0.110095613	0.489035482	0.624816577	0.81216834
899.8090242	-0.129123229	0.199960233	-0.645744543	0.518444809	0.741851962
837.9242731	-0.069309461	0.179257062	-0.386648428	0.699016501	0.8567814
15.45049541	-0.256833085	0.269918542	-0.951520717	0.341340107	0.59908004
17.7573115	0.426632113	0.2808144	1.51926722	0.128695247	0.349943297
226.0947104	0.245545932	0.203622149	1.205890088	0.227859873	0.482548507
140.8085236	0.055668745	0.169534466	0.328362407	0.742637663	0.880449218
59.30722562	-0.031135567	0.142838848	-0.217976882	0.827447124	0.925836979
1417.160872	-0.172401323	0.089954821	-1.916532335	0.055297372	0.212882664
29.07425209	0.09867375	0.247098656	0.399329365	0.689650532	0.851289072
20.28876046	-0.558420082	0.266675912	-2.094002713	0.036259739	0.164702132
15.55574471	-0.035836185	0.303925751	-0.117910985	0.906138189	0.960133527
52.09667165	0.083873027	0.200898258	0.417490066	0.676319989	0.845004996
889.5352886	-0.178035658	0.138323317	-1.287097948	0.19806015	0.446254188
99.61614281	-0.160090975	0.124721688	-1.283585701	0.199286956	0.447851546
113.762836	-0.060588858	0.133671233	-0.453267745	0.650355956	0.828805376
392.2814421	-0.86805472	0.313749294	-2.766714502	0.005662432	0.051453988
323.5462057	-0.033700976	0.123601922	-0.27265738	0.785116599	0.902044615
15.78526985	0.279287989	0.295190941	0.946126558	0.344084039	0.602015651
168.4159365	0.106802673	0.095778868	1.11509642	0.264809106	0.522834476
5.762611945	-0.050296964	0.450155527	-0.111732414	0.911035577	0.962059189
204.6577686	-0.018485135	0.119222542	-0.155047316	0.876784016	0.948920022
94.41044642	0.064830877	0.116487362	0.556548591	0.577835887	0.781880601
124.1769596	0.584581394	0.250082955	2.337549926	0.019410608	0.112494686
107.2685056	0.214577821	0.114429755	1.875192518	0.060766243	0.225309163
1718.261008	-0.554715066	0.109475091	-5.067043661	4.04042E-07	5.02936E-05
8.312695903	-0.263458243	0.375940965	-0.700796846	0.483429807	0.715091304
42.14955904	0.045821826	0.150047349	0.305382443	0.760074892	0.889367077
440.8482228	-0.005610938	0.138577313	-0.040489588	0.967702808	0.984398161
4.484698992	-0.484540959	0.473329568	-1.023686225	0.305983504	0.565058904
556.5832469	0.307611475	0.197867104	1.554636767	0.120032602	0.335757327
173.194779	-0.173672408	0.160814979	-1.079951686	0.280163695	0.53868915
901.8322917	-0.226031166	0.09032092	-2.502533924	0.012330781	0.085389239
411.2150919	-0.432700209	0.130641738	-3.312113084	0.000925941	0.015632357
599.2905748	-0.136006138	0.0720114	-1.888675108	0.058935376	0.221505497
11.3942298	0.297915112	0.312311063	0.953905087	0.340131688	0.598370657
818.1986428	-0.212434637	0.145641797	-1.458610375	0.144672376	0.374855543
25.91870909	0.523272454	0.274235284	1.908114982	0.056376357	0.215502505
249.3815231	-0.08616036	0.157024165	-0.548707644	0.5832061	0.785021177

158.2167409	-0.007858679	0.109487303	-0.07177708	0.942779313	0.974993168
282.1228033	0.037184696	0.130405251	0.285147227	0.775531349	0.897140705
2535.926178	-0.174448908	0.151276648	-1.153178037	0.248837316	0.504493856
27.64482028	-0.157439504	0.388186944	-0.405576506	0.685053795	0.848888656
188.1081226	0.041883018	0.138879223	0.301578717	0.762973235	0.891042078
381.9404195	0.020238616	0.178408955	0.113439465	0.909682153	0.9616745
122.6261157	0.179648744	0.146264996	1.228241546	0.219356307	0.472320746
171.8324135	-0.172676879	0.077908687	-2.216400829	0.026664063	0.136577127
22.38503155	0.742329905	0.388969261	1.908453902	0.056332576	0.215489705
383.610101	-0.006815843	0.105134817	-0.064829552	0.948309712	0.976899148
377.45753	-0.479876345	0.115376186	-4.159232161	3.19319E-05	0.001430915
559.6801815	-0.074277403	0.155030109	-0.479115981	0.631856122	0.81678327
111.977378	0.256538453	0.196598466	1.304885322	0.1919319	0.439197537
8.954863339	0.44400627	0.315704061	1.406400249	0.159605306	0.39715209
499.1779549	-0.432399567	0.073611566	-5.874071039	4.25221E-09	1.17003E-06
25.63960685	0.520676317	0.597569047	0.871324108	0.383577211	0.634974705
45.99299588	-0.246152951	0.611982443	-0.402222243	0.687520474	0.850122158
54.61439243	-0.197569108	0.68183035	-0.289762854	0.771997667	0.895430514
606.890015	0.454994162	0.294801073	1.543393849	0.122735245	0.340282762
10.68062827	1.345954067	0.505908872	2.660467414	0.007803228	0.064091503
44.29178754	0.777319713	0.205690139	3.779081085	0.000157408	0.00461456
257.2421116	0.13177135	0.094673677	1.391847813	0.163968488	0.403299571
3.019457774	0.489882793	0.617504497	0.793326681	0.427587516	0.672443858
488.8295096	-0.308848699	0.07723032	-3.999060223	6.35945E-05	0.002380468
2.592513405	-0.565907572	0.673285583	-0.840516397	0.400618912	0.650042108
20.50561438	0.733865459	0.261489861	2.806477678	0.005008638	0.047470074
1522.443512	0.379478359	0.277992288	1.365067936	0.172231699	0.414200348
44.25388405	0.22381455	0.420856055	0.531807841	0.594859092	0.792808463
272.1827979	0.299891298	0.272357738	1.101093367	0.270856024	0.529953328
13.6578306	0.012367671	0.584667222	0.02115335	0.983123327	0.991149398
49.72446102	0.085282386	0.265257499	0.321507918	0.747825513	0.884198669
197.6433373	-0.094481712	0.108028019	-0.874603758	0.381789542	0.633716497
13.67782061	0.639021599	0.348299277	1.834691148	0.066551465	0.238799627
543.0207089	-0.362714567	0.258094257	-1.405356985	0.159915151	0.397563536
114.8568948	-0.055700386	0.128662124	-0.432919839	0.665073007	0.836844909
19.84957284	0.557392985	0.267448503	2.084113314	0.037149861	0.16733144
327.3045234	-0.334078437	0.306098989	-1.091406537	0.275094037	0.534091809
325.9080637	-0.120409344	0.1234933	-0.975027346	0.329546696	0.587876521
2198.150241	-0.363526371	0.078317482	-4.641701444	3.45552E-06	0.000252076
5.245585128	-0.05462843	0.988537953	-0.055261844	0.95592986	0.980690995
696.767238	-0.342653225	0.107579978	-3.185102201	0.001447029	0.021451042
500.294284	-0.055012435	0.100276364	-0.548608197	0.583274359	0.785040337
844.4778924	0.066923541	0.17776376	0.376474603	0.706564093	0.86122222
2854.44299	-0.258575933	0.050672687	-5.102866013	3.34548E-07	4.26589E-05
5.372760188	0.402757026	0.421448493	0.955649463	0.339249363	0.59750556
5.173179391	-0.893474668	0.57017265	-1.567024775	0.117108901	0.330526543
28.82618825	0.177317251	0.219973507	0.806084578	0.420194083	0.665067412
90.5773328	0.126121573	0.162023788	0.778413926	0.436325031	0.680183063

90.69223973	-0.278103382	0.343130742	-0.810488095	0.417659704	0.662879458
458.8464561	-0.801725822	0.09247828	-8.669341858	4.34625E-18	3.40833E-14
571.6017303	0.239817361	0.104124629	2.30317615	0.021268934	0.118841684
719.3421802	0.166429512	0.093919001	1.77205368	0.076385647	0.258307996
649.3346406	-0.148217881	0.084191219	-1.760490958	0.078324599	0.261814005
373.7290764	-0.954147529	0.478562062	-1.99378013	0.046176088	0.190786555
348.591367	-0.219837024	0.086113068	-2.552888076	0.010683382	0.077717145
154.2626007	-0.134657954	0.094973089	-1.417853783	0.156233458	0.391682474
195.6915504	0.768694138	0.21645474	3.551292695	0.000383344	0.008700962
33.17202626	1.520754545	0.419634843	3.623994931	0.000290087	0.007189519
9350.577927	-0.057116609	0.07353697	-0.776706045	0.43733222	0.680596258
215.3896899	0.653746131	0.155554009	4.20269549	2.63755E-05	0.001223887
371.0153683	0.387729266	0.360336233	1.076020756	0.281917974	0.540466121
110.8833344	-0.643083657	0.284359074	-2.261519729	0.023727092	0.127400109
141.8921763	0.977887222	0.230705113	4.238688981	2.24829E-05	0.001076665
6.608753104	0.416659678	0.558191138	0.746446244	0.455397893	0.693264981
300.7441158	-0.461689257	0.255905799	-1.804137536	0.071209749	0.247529632
3.150600712	-1.240905539	0.705599995	-1.758652986	0.078636467	0.262411564
29.91628678	0.564248553	0.266961102	2.113598382	0.034549584	0.159616584
4.049557682	1.408669127	0.763246096	1.845628998	0.064946093	0.234974515
233.6429613	0.379484733	0.102389904	3.706271021	0.000210333	0.005707381
3.041291545	0.272260759	0.571554316	0.476351506	0.633823977	0.818118933
1415.564192	0.143635505	0.070890402	2.026162924	0.042748092	0.18199269
1967.604418	-0.160410838	0.132511417	-1.210543532	0.226070397	0.480315376
341.7327234	-0.283042805	0.099624404	-2.841099102	0.004495834	0.044347584
1578.783356	-0.081114374	0.076749987	-1.056864983	0.290573212	0.549939695
277.0521993	0.303371262	0.132444473	2.290554336	0.021989203	0.121308003
287.4249292	-0.010320182	0.19655257	-0.052505961	0.958125546	0.981531095
16.2368758	0.322780788	0.342132745	0.943437286	0.345457279	0.603089043
40.61222406	1.074279398	0.314973188	3.410701096	0.000647961	0.01239914
19.13795095	0.008489518	0.255599978	0.033214082	0.973503869	0.986906773
135.1221122	0.352934848	0.137965277	2.55814256	0.010523294	0.077171427
231.1542965	0.692244196	0.336243736	2.058757151	0.039517508	0.173782322
240.1909354	-0.01091537	0.109955356	-0.099270923	0.920923165	0.966719692
4.686208215	-0.081943854	0.544419365	-0.150516053	0.880357486	0.950735166
546.335069	-0.311632535	0.092980466	-3.351591458	0.000803485	0.014096038
113.9465858	0.130117297	0.39184436	0.332063722	0.739841143	0.879332259
412.9410028	-0.136181962	0.06929967	-1.965117031	0.049400694	0.198498377
749.1169114	-0.117383664	0.069880775	-1.679770491	0.093001979	0.289643176
557.5757195	-0.016796749	0.079209434	-0.212054901	0.832064203	0.928693708
6.382868284	0.604065387	0.483691197	1.248865786	0.211714168	0.462972839
421.2300912	-0.115803789	0.092816411	-1.247665025	0.212153755	0.463622612
83.03982475	0.155544453	0.19228679	0.808919077	0.418561695	0.663815578
427.7370264	-0.433039167	0.102445683	-4.227012355	2.36815E-05	0.001122115
29.34151697	1.054745715	0.508253665	2.075234845	0.037964775	0.169592573
224.4252388	0.134143528	0.113820846	1.178549738	0.238577516	0.494147253
73.31756394	0.510624462	0.186417493	2.739144566	0.006159928	0.054575001
16.69213863	-0.052899318	0.301383186	-0.175521797	0.860669645	0.940613387

13.72388784	0.961816494	0.297429931	3.233758252	0.001221728	0.019118304
3.870281617	0.515386214	0.883119165	0.583597587	0.559491084	0.770079698
7515.624591	-0.103113065	0.134657335	-0.765744139	0.443828558	0.68493625
470.909791	-0.099270982	0.076620988	-1.295610826	0.195109597	0.443107287
227.4450538	-0.108779479	0.087974213	-1.23649278	0.216275448	0.468970568
145.1290729	0.177550192	0.180574405	0.983252264	0.325483298	0.584078637
511.0889258	0.161256008	0.209880515	0.768322909	0.442295368	0.683444389
98.95612219	0.288178002	0.240143057	1.200026378	0.230129096	0.485322676
10.67292683	0.290647926	0.297643604	0.97649646	0.328818501	0.587245431
388.8232889	-0.199647013	0.157181926	-1.270165205	0.204025789	0.453634884
1041.854653	-0.255853479	0.083749343	-3.054990868	0.002250674	0.028467393
141.0184772	0.021245259	0.104391333	0.203515541	0.8387321	0.930862799
17.17348879	1.330121295	0.288235062	4.614710246	3.93644E-06	0.000278104
678.3701615	-0.034310687	0.11163694	-0.307341702	0.758583297	0.88881073
9.966797787	0.160223818	0.36966714	0.433427267	0.664704395	0.83676248
15.0552356	1.029685147	0.643425742	1.60031699	0.10952828	0.317354801
340.8489352	0.223399755	0.141968632	1.573585317	0.11558335	0.327813609
17.85103836	-0.56881219	0.570587412	-0.996888782	0.318818497	0.577074358
522.8595535	-0.313193889	0.239191618	-1.309384887	0.190404008	0.43813035
8.417415947	0.467554533	1.053243891	0.443918581	0.65710145	0.832739103
2798.19413	0.019051749	0.138169803	0.137886486	0.890330131	0.953820886
2305.727121	-0.192840197	0.100629802	-1.91633287	0.05532274	0.21292806
278.8937488	-0.142017473	0.247021967	-0.574918395	0.565346488	0.77379303
106.8824262	-0.610152467	0.252694991	-2.414580776	0.01575333	0.100045497
118.9300873	0.19020102	0.171988298	1.105895123	0.268771916	0.527663112
205.776156	-0.269361217	0.302117346	-0.891578126	0.372619099	0.626450632
534.2555889	0.134629957	0.154048168	0.87394715	0.382147036	0.633976208
1552.953947	-0.125972263	0.167989002	-0.749883991	0.453324577	0.691759407
1482.170402	-0.037376374	0.170512631	-0.219200029	0.826494233	0.925380893
447.3825033	-0.192041478	0.08075323	-2.378127512	0.017400807	0.10571922
467.3558864	-0.236284937	0.087134281	-2.711733359	0.006693241	0.057584636
14.80009491	1.038524861	0.522613382	1.987176175	0.046902877	0.192343583
10.36377314	0.499169476	0.425603826	1.172850068	0.240855936	0.496110133
253.8520002	0.244254914	0.154944304	1.576404601	0.114932589	0.326434042
1261.368237	-0.127141442	0.066928644	-1.899656615	0.057478197	0.218064314
32.54757306	0.593965392	0.326943919	1.816719496	0.069260075	0.243608574
3.819412732	0.288148017	0.562756533	0.512029626	0.608630275	0.802097071
846.360573	0.40695207	0.118146987	3.444455758	0.00057221	0.011505047
394.806975	0.471374806	0.18269968	2.580052722	0.009878523	0.07441631
4.915100471	0.857658653	0.716050583	1.197762664	0.231009426	0.486525022
136.082269	-0.213327439	0.340934057	-0.625714665	0.531502113	0.751380545
61.49956369	0.303467517	0.258737367	1.172878586	0.240844498	0.496110133
475.960488	0.218586365	0.168163466	1.299844549	0.193654254	0.44095141
118.0856822	-0.183614769	0.113112918	-1.623287353	0.104527992	0.309733048
50.84769452	0.129301592	0.211016351	0.612756273	0.540037508	0.757466143
120.3724661	-0.225674033	0.209315501	-1.078152509	0.280965702	0.539411971
86.75246926	0.005110282	0.19743157	0.025883815	0.97935001	0.989273349
325.6145345	-0.306064535	0.157735966	-1.940359848	0.052335973	0.205414764

9.362822461	0.594029309	0.389018357	1.526995573	0.126762112	0.347308746
201.3319109	0.066706476	0.097685718	0.68286826	0.494690092	0.722813893
50.44496696	0.338314828	0.328440413	1.030064555	0.302979703	0.561618659
4598.867875	0.000420419	0.137585807	0.003055683	0.997561922	0.998771859
13.67907259	0.919954672	0.487452827	1.887269129	0.059124136	0.221798083
280.416778	-0.321564338	0.095555311	-3.365216803	0.000764835	0.01364696
65.1471423	-0.130119978	0.175343017	-0.742088168	0.458033918	0.694415566
28.59913356	0.568891705	0.28092351	2.025076882	0.04285947	0.182219554
288.2709921	0.352732248	0.118548686	2.975420984	0.002925867	0.033841667
2481.262772	0.181814534	0.142609548	1.274911369	0.202340615	0.451231367
13.14311289	-0.096736836	0.276067913	-0.35040956	0.726031353	0.872122772
5.838030321	1.257874826	0.614869167	2.045760129	0.040779977	0.177123554
101.6618041	-0.062884555	0.163822116	-0.383858763	0.701083132	0.857806901
622.7790169	0.133251405	0.073156988	1.821444647	0.068539294	0.242335835
106.247174	0.023923406	0.153400062	0.155954347	0.876069007	0.948628307
3.79170297	0.262207656	0.953233004	0.275071945	0.783260965	0.901097703
892.8345851	0.128127056	0.176811135	0.724654903	0.468663768	0.703870778
1036.725405	0.298864679	0.178835878	1.671167346	0.094688628	0.292687514
41.01139834	0.466060309	0.215881301	2.158872981	0.030860023	0.149339276
81.06664308	-0.030734614	0.142468277	-0.21572953	0.829198582	0.927019072
318.2117277	0.467663692	0.301975902	1.548678845	0.121458942	0.338118928
278.7136884	0.061784804	0.068335136	0.904144019	0.36591904	0.621852229
297.0836568	0.351733499	0.167121325	2.104659583	0.035320951	0.161791413
11.86308213	0.339743265	0.31544117	1.07704161	0.281461677	0.540059327
116.8251752	0.059312489	0.118012992	0.502592873	0.615250541	0.806889329
100.8637266	0.112740867	0.602306797	0.187181794	0.851518099	0.93609097
498.9106271	0.186216844	0.164240664	1.133804741	0.256876462	0.513818445
474.8942203	0.009086116	0.076385865	0.118950221	0.905314794	0.959974986
855.0047339	-0.125662964	0.125032875	-1.005039383	0.314877886	0.573869373
21.99074899	0.165831142	0.356328637	0.465388196	0.641653486	0.824014843
15.27536035	1.6597235	0.819746156	2.02467982	0.042900252	0.182294108
67.03265641	0.017842283	0.157804852	0.113065491	0.909978634	0.9616745
231.3098178	-0.07142225	0.211669699	-0.337423118	0.73579796	0.877119039
59.45918943	0.349564428	0.334939985	1.043662878	0.29664139	0.555344561
2069.77077	0.172342429	0.175686891	0.980963508	0.326610737	0.585151858
263.2020948	-0.015765522	0.095151563	-0.165688524	0.868402078	0.945025059
1114.499353	-0.089676444	0.050619149	-1.77159129	0.076462427	0.258511899
1395.735549	0.138203161	0.080925874	1.707774717	0.087678152	0.279614505
685.6910244	0.146704353	0.075302928	1.948189214	0.051392327	0.203032054
58.69446357	-0.469274852	0.268859521	-1.745427689	0.080910449	0.266824178
332.0262225	-0.060312774	0.152920808	-0.394405277	0.693281848	0.85351767
127.4589018	0.286493253	0.11294469	2.536580112	0.011194113	0.079949209
161.6331149	0.527457276	0.139128355	3.791155847	0.000149948	0.004505329
325.921989	-0.064309016	0.105048093	-0.612186427	0.540414421	0.757542051
20.37984448	-0.100486167	0.355214823	-0.282888439	0.777262357	0.898017149
230.5851432	0.170136491	0.115989713	1.466823972	0.142423947	0.371222252
7.727320232	-0.524039211	0.414690499	-1.263687526	0.206342204	0.456584527
96.21948484	-0.856217399	0.252078738	-3.396626805	0.000682219	0.012814286

147.0589811	-1.157837967	0.390666577	-2.963749746	0.003039153	0.03459077
160.4266315	-0.292423709	0.130945456	-2.233171865	0.025537613	0.132714354
2224.813111	-0.217657346	0.152804401	-1.424418043	0.154325494	0.388950836
58.73440474	0.012177771	0.180942748	0.067301792	0.94634145	0.976282267
2.689792891	0.080648222	0.700247328	0.115171052	0.908309543	0.960978931
4973.830968	-0.057268194	0.124637478	-0.459478122	0.645890859	0.826141921
29.93355694	0.295246415	0.199875675	1.477150306	0.139635351	0.366893988
523.4989769	0.169384108	0.11080495	1.528669145	0.126346485	0.346497337
491.630642	0.036308306	0.103742883	0.34998358	0.72635102	0.872288622
13.92067493	0.131026786	0.333269097	0.393156123	0.694204175	0.853551135
283.3144176	0.197781787	0.196955825	1.004193638	0.315285288	0.57422143
66.33536993	0.339845331	0.154052032	2.206042507	0.027381024	0.138978633
318.3312802	0.144651906	0.273012558	0.529836088	0.596225582	0.793955003
56.78826924	0.234401289	0.326799634	0.71726301	0.473211827	0.706757551
13.82837417	1.276793981	1.070749784	1.192429828	0.23309275	0.488616239
15.70183431	5.497716221	1.375871751	3.995805726	6.44746E-05	0.002395444
2507.703764	-0.860957872	0.560372983	-1.536401467	0.124439947	0.343189051
477.0749607	0.218463298	0.082206218	2.657503314	0.007872182	0.064372939
1220.926424	0.251418764	0.12238475	2.054330819	0.039943684	0.174944635
12.49803407	0.587764922	0.305763919	1.922283452	0.054570101	0.211119256
112.2932124	0.689760204	0.263859655	2.614117734	0.00894582	0.069596353
355.7292156	-0.071331185	0.093963551	-0.759136755	0.447770754	0.68770432
45.3069062	0.058652018	0.169123192	0.346800562	0.728741155	0.873219977
1016.299813	0.164717587	0.086509934	1.904030895	0.056906161	0.216525042
111.6166185	0.580392448	0.369106055	1.572427329	0.11585148	0.328233994
482.7871251	0.217322408	0.18789775	1.156599307	0.247436105	0.502785584
93.87798891	-0.881254148	0.379066531	-2.324800731	0.020082618	0.114912725
266.2264301	0.04428767	0.078278682	0.565769234	0.571550663	0.778277531
1740.918562	-0.40192899	0.088118545	-4.561230416	5.08547E-06	0.000340857
279.0166616	0.311503672	0.312997327	0.9952279	0.319625435	0.577868049
1537.159104	0.102937588	0.120109304	0.857032592	0.391426872	0.642169359
31.63130711	0.482202634	0.235565113	2.047003595	0.040657732	0.176837455
3178.424029	0.159032824	0.145414248	1.093653655	0.274106908	0.533044162
571.9949045	0.502324036	0.124820122	4.024383481	5.71248E-05	0.002234276
385.4475237	0.598378679	0.179385474	3.335714235	0.000850806	0.014679908
74.72252845	0.68013064	0.189882066	3.58185823	0.000341159	0.008012353
11.15078197	0.316073795	0.392696182	0.804881253	0.420888207	0.665511709
326.1245421	0.230775299	0.066237442	3.484061151	0.000493867	0.010369216
3461.002297	0.231217915	0.172367482	1.341424219	0.179782759	0.424747406
127.0364362	-0.258454586	0.15977956	-1.617569764	0.10575535	0.310992892
427.9987226	0.184041921	0.087801965	2.096102535	0.036073096	0.164181788
1841.937628	-0.074720339	0.086353049	-0.865288949	0.386880215	0.638114343
45.12755448	0.728024772	0.184326777	3.949641957	7.82682E-05	0.002752373
105.056237	0.277926187	0.122616705	2.26662579	0.023413095	0.126363037
28.92844616	0.294426453	0.182983874	1.609029508	0.10760989	0.314000652
48.34513057	-0.002695019	0.16585583	-0.016249166	0.987035612	0.992541292
600.0457723	0.381219413	0.156588109	2.43453615	0.01491089	0.096797353
9.405452172	0.447219331	0.430332342	1.039241738	0.298692323	0.5571706

122.386871	0.330059647	0.137337873	2.403267494	0.016249296	0.10167835
771.1925678	-0.13824988	0.104670024	-1.320816369	0.186562601	0.432655305
1465.60776	-0.058443874	0.141221199	-0.413846326	0.678986654	0.845876523
312.879012	0.505915456	0.179113239	2.824556453	0.004734611	0.045838046
373.2925521	0.209973959	0.098712632	2.127123496	0.033409824	0.156464518
61.42146878	0.662972437	0.263789804	2.51326028	0.011962104	0.083606794
724.509042	0.213244039	0.152276912	1.400370127	0.161402511	0.399906178
17.93499118	0.455244247	0.229467493	1.983916069	0.047265197	0.193300484
175.4346143	-0.265214254	0.104731155	-2.532333901	0.011330604	0.080484234
1229.670864	0.197771157	0.117952802	1.67669741	0.093601667	0.290634968
542.7712543	0.218273617	0.097499062	2.238725311	0.025173795	0.131696396
3992.523507	-0.10795189	0.119362681	-0.90440236	0.365782087	0.621686851
101.5828027	0.307283185	0.317697211	0.967220281	0.333433926	0.591900483
36.38686384	0.354449756	0.253141088	1.40020634	0.161451538	0.399906178
827.7351538	-0.090386689	0.088176143	-1.02506967	0.305330315	0.564118349
728.1741245	0.201312606	0.098548256	2.042782031	0.04107402	0.177809807
168.6894386	-0.033960574	0.12058602	-0.281629443	0.778227661	0.898338312
286.2193131	0.128137666	0.076693198	1.670782665	0.094764614	0.292749301
11.5319071	1.083306752	0.376877869	2.874423892	0.004047653	0.041710507
2916.762578	0.201172722	0.211924419	0.949266361	0.342485158	0.600305902
156.4847935	0.342225363	0.167875241	2.038569597	0.041493002	0.178833813
61.23002855	0.136312282	0.216747854	0.628897956	0.529415864	0.74980955
236.5252313	0.379047085	0.159398531	2.377983557	0.017407602	0.10571922
9.224264336	0.51863422	0.371219245	1.397110273	0.162380413	0.401193194
515.9759302	0.087635903	0.174979569	0.500835058	0.61648721	0.80702296
76.32895096	0.331523356	0.239401376	1.384801377	0.16611321	0.405813019
209.6183081	0.365210779	0.127248671	2.870055749	0.004103994	0.042064683
52.31941077	0.106467677	0.177165218	0.600951348	0.547872391	0.762398361
311.8729876	-0.017197659	0.104279725	-0.164918531	0.869008107	0.945247461
1043.131934	0.018993171	0.093418411	0.203312933	0.838890447	0.930862799
297.0837905	-0.061546604	0.109046446	-0.56440724	0.572477013	0.778738936
2.912745797	0.030784651	0.67867525	0.045359913	0.963820433	0.982935149
1716.290634	0.06649712	0.132780573	0.500804588	0.616508656	0.80702296
821.3524903	0.103514095	0.111506731	0.928321494	0.353240828	0.610139776
231.8904919	0.189754079	0.113686615	1.669097807	0.095097998	0.292986838
20332.78541	-0.178248633	0.04398076	-4.052877475	5.05915E-05	0.002030194
4.625799574	0.798089689	0.607310189	1.314138479	0.188799607	0.436423439
296.6293958	-0.113505904	0.087017175	-1.304408054	0.19209449	0.439441363
243.4502411	1.018896176	0.216246972	4.711724584	2.45629E-06	0.000190715
1512.301879	-0.114311479	0.232675061	-0.491292356	0.623219686	0.810950402
339.689726	1.360771736	0.239030745	5.692873247	1.24919E-08	2.88122E-06
58.780248	0.386380559	0.14809458	2.609012149	0.009080402	0.070368451
17.08122907	0.785048721	0.333589044	2.353340841	0.018605571	0.109702923
271.554652	-0.310151405	0.122005049	-2.54211943	0.011018252	0.079361966
692.6588608	-0.025911069	0.1213544	-0.213515694	0.830924749	0.928287183
8.503358039	0.204196813	0.476631595	0.428416444	0.668347955	0.839214838
595.3522905	0.191589572	0.097764623	1.959702461	0.050030577	0.199969311
193.7140449	0.311592554	0.120643343	2.582757946	0.009801406	0.073977505

11.91057134	1.055668411	0.433224041	2.436772459	0.014819001	0.096320436
9.540046755	1.51847006	0.391961894	3.874024699	0.000107053	0.003505245
176.0778866	0.023035324	0.12203531	0.188759496	0.850281319	0.935523059
65.35944474	0.39544175	0.284184013	1.391498931	0.164074184	0.403299571
51.52712782	0.349666221	0.25319399	1.381021017	0.167272499	0.406933747
308.5963714	0.389478244	0.174232849	2.235389288	0.025391803	0.132452965
420.5520631	0.164531766	0.16448741	1.000269661	0.317180025	0.576008142
11.43619593	0.388706079	0.306175122	1.269554746	0.204243278	0.453989735
6244.726705	-0.046501961	0.12527204	-0.371207824	0.710482746	0.862677974
7.456781769	-0.316841677	0.347152433	-0.912687475	0.361406933	0.617320505
6.686020813	0.857668551	0.444668315	1.928782693	0.053757845	0.209243639
617.6648389	0.125983349	0.113347545	1.111478409	0.266362479	0.525013602
30.55654856	1.108388517	0.544945266	2.033944667	0.041957175	0.179762383
16.78909691	0.079190819	0.572718206	0.138271873	0.890025554	0.953820886
611.0938444	0.002116023	0.058262858	0.036318552	0.971028357	0.985937912
6849.534448	0.165193655	0.140418103	1.176441294	0.239418579	0.494646201
112.618883	0.133272648	0.121521803	1.096697422	0.272773684	0.531715444
44.63723537	-0.289976889	0.162099426	-1.788882894	0.073633677	0.252928295
42.11284329	0.034511267	0.304361191	0.113389183	0.909722015	0.9616745
75.41627085	0.290339133	0.139496735	2.08133282	0.037403453	0.16804498
108.4950038	-0.063903314	0.128158341	-0.498627817	0.618041605	0.808044267
9.209152245	-0.011543559	0.375071738	-0.030776935	0.975447435	0.988030139
17.17550233	-0.519996527	0.273381847	-1.902088719	0.057159557	0.217112899
2.98756579	-0.216485577	0.697563167	-0.310345481	0.756298248	0.887592168
26.62330435	-0.124943594	0.256567721	-0.486980957	0.626271826	0.813271002
26.1948247	0.336693487	0.22279109	1.511252028	0.130724251	0.352919666
80.995974	-0.065846329	0.144744965	-0.454912745	0.649172014	0.828042767
50.01194313	0.121833704	0.160927103	0.757073865	0.44900561	0.688654515
37.94423974	0.218288425	0.246791873	0.884504106	0.376424147	0.629675376
294.1745446	-0.181622401	0.130234604	-1.394578666	0.163142932	0.402126944
465.6833214	0.07619865	0.132904653	0.573333198	0.566419111	0.774448801
1496.804862	-0.310485194	0.113383916	-2.738353068	0.006174775	0.054600664
46.42923169	0.29737875	0.153855495	1.932844514	0.053255355	0.208085945
76.08570096	-0.272975317	0.391234744	-0.697727697	0.485347504	0.716441435
45.65492643	-0.813490111	0.472200301	-1.722764915	0.084931046	0.275332477
137.471454	0.004341527	0.101113197	0.04293729	0.965751523	0.983880928
77.61964049	0.278553556	0.215304189	1.29376747	0.195745752	0.443909251
405.9744866	-0.194376804	0.090188878	-2.155219229	0.031144683	0.15019591
379.0763579	0.163334377	0.099902094	1.634944484	0.102060686	0.3060095
5.948537284	0.222723867	0.444564021	0.500993911	0.616375408	0.80702296
526.4483067	0.381003464	0.153750127	2.47806926	0.01320955	0.089455347
120.0190961	0.054834977	0.258623774	0.212026049	0.832086712	0.928693708
455.217802	0.027611174	0.113685519	0.242873274	0.80810357	0.913988445
295.6450671	-0.102878219	0.072207017	-1.424767618	0.154224386	0.38887588
695.8123234	0.027491824	0.104992566	0.261845438	0.793440606	0.905368772
5.306203509	0.164344307	0.473475235	0.347102225	0.728514522	0.873219977
214.7121001	-0.092144651	0.120059252	-0.767493134	0.442788374	0.683810803
112.6318139	-0.118516805	0.166918055	-0.710029873	0.477685612	0.709875036

229.5820804	-0.147702922	0.124577848	-1.185627495	0.235769439	0.49179517
28.76002049	0.956351112	0.268015969	3.568261681	0.000359358	0.008312925
287.4332433	0.035773564	0.075555793	0.473472161	0.635876358	0.819624255
323.6001522	-0.170992045	0.076979591	-2.221264661	0.026333041	0.13550112
447.718176	0.089618689	0.075809355	1.182158709	0.237142733	0.492684668
153.0193011	0.175573248	0.127700825	1.374879517	0.169168801	0.409957273
79.59651448	0.080514944	0.161858625	0.497439937	0.618878849	0.808403087
2450.403689	0.048532975	0.062995871	0.770415177	0.441053653	0.682669052
65.74061231	0.716996418	0.210871268	3.400161741	0.00067346	0.012725961
1576.700231	-0.173758108	0.082359924	-2.109740995	0.034880671	0.160713411
38.12433511	0.041909594	0.197266556	0.212451594	0.831754738	0.928693708
2043.042295	0.013454085	0.12955894	0.103845287	0.917292127	0.965104295
272.7858215	1.134281607	0.210181667	5.396672417	6.78882E-08	1.16123E-05
20.04548297	0.326456887	0.252878188	1.290964988	0.196715819	0.445162804
3.166727402	0.768036879	1.170612075	0.656098545	0.511760721	0.736709972
506.3756583	-0.483952605	0.230764243	-2.097173278	0.035978239	0.163940355
3.323636809	0.129439622	1.158457784	0.111734432	0.911033978	0.962059189
53.05639715	-0.158540831	0.157526162	-1.006441268	0.314203351	0.573019227
116.3497639	-0.25405815	0.103646377	-2.451201455	0.014238024	0.093866823
144.7508966	-0.426226825	0.40647015	-1.048605477	0.294359734	0.553499349
438.5435625	-0.265969822	0.122556625	-2.17017907	0.029993283	0.147004578
49.63628714	-0.657838143	0.223388315	-2.944818952	0.003231437	0.036123918
1122.038884	-0.313602078	0.122419595	-2.56169838	0.010416173	0.076795237
25086.41209	0.494324099	0.303259629	1.630035954	0.103093898	0.307458586
5.079592641	0.783476074	1.04609713	0.748951556	0.453886404	0.692078503
3.881253288	0.260084943	0.599146101	0.434092691	0.664221133	0.836652459
498.127779	-0.170174875	0.125414795	-1.356896326	0.174814154	0.417988969
474.965516	-0.087669392	0.085094925	-1.030254061	0.302890758	0.561529391
8.671393848	0.738732904	0.463867697	1.592550871	0.111260982	0.320866896
56.83556147	0.389668501	0.363966286	1.070617022	0.284341666	0.543156866
4.294297243	-0.645240879	0.687931426	-0.9379436	0.348273395	0.605634643
252.4269521	-0.161899927	0.075354226	-2.148518217	0.031672611	0.151651309
10.5037185	0.104626891	0.310581997	0.336873651	0.736212149	0.877546082
52.47878326	-0.525984702	0.213882843	-2.459218771	0.013923974	0.092628441
615.5214867	-0.571960102	0.12003443	-4.764967051	1.88885E-06	0.000164779
5.834914225	0.553275413	0.400321642	1.382077198	0.166947999	0.406609306
126.7866947	0.285162501	0.343767597	0.829521174	0.406809561	0.655071986
114.8170636	-0.243810862	0.141247025	-1.726130953	0.084323856	0.274295993
8632.551993	0.175358197	0.131971633	1.328756741	0.183928238	0.429498408
1120.538819	0.01923319	0.136570722	0.140829527	0.888004616	0.953471838
161.8643743	-0.07782878	0.089730877	-0.867357848	0.38574597	0.637114553
167.7901091	0.255767765	0.167971471	1.522685752	0.127837344	0.348467226
247.829823	0.453039744	0.189960824	2.384911446	0.017083233	0.104620627
2.69828753	0.804069222	0.573026621	1.403196978	0.160558109	0.398467573
273.8241468	0.177102457	0.187437175	0.94486303	0.344728808	0.60239957
182.264839	-1.195892455	0.398584056	-3.000351961	0.002696678	0.032212261
3.226660578	0.175828667	0.519957234	0.338159863	0.735242721	0.876856177
6.030715785	-0.395130462	0.449057969	-0.879909698	0.37890823	0.631138136

199.058038	0.084827702	0.149846817	0.56609612	0.571328441	0.778130154
4.460110683	0.504217167	0.526113416	0.958381124	0.33787061	0.596282508
5.832177135	-0.500238269	0.592840507	-0.843799071	0.398781695	0.648806234
1851.385797	0.009405616	0.093117407	0.101008134	0.919544003	0.966087273
562.19393	-0.207319325	0.078871377	-2.628574945	0.008574346	0.067815264
160.3629599	0.068307576	0.142291991	0.480052144	0.631190316	0.816460941
175.6650639	-0.072955736	0.122636236	-0.59489543	0.551913372	0.76481793
1085.599435	-0.181022361	0.190436332	-0.950566305	0.34182458	0.599416001
624.7094373	-0.126848339	0.085859626	-1.477392163	0.139570546	0.366887151
2.672665806	-0.496144518	0.59641014	-0.83188478	0.405473984	0.654312692
61.31375022	0.343706503	0.137243524	2.504354982	0.012267488	0.085209605
231.3391822	-0.087105054	0.107117381	-0.81317386	0.416118384	0.661839645
80.03014098	0.414924262	0.5079183	0.816911426	0.413979048	0.660580668
82.17354316	-0.425656131	0.240585659	-1.769249811	0.076852195	0.25938236
5.888523839	-2.659861022	0.9881797	-2.691677457	0.007109366	0.060337286
117.901602	-1.149280545	0.377153705	-3.047247132	0.002309477	0.028931186
1090.595406	-0.284883041	0.187038497	-1.523125163	0.127727394	0.348395905
424.4955608	-0.433679299	0.133239979	-3.254873667	0.001134428	0.018118504
5728.363148	0.026608922	0.196667045	0.135299342	0.892375205	0.95479462
14.20334177	1.067675153	0.3680209	2.901126412	0.003718238	0.039376673
803.3352672	-0.171292882	0.072856064	-2.351113571	0.01871732	0.110320348
364.1159233	0.18183016	0.303618505	0.598877067	0.549254865	0.763291982
4276.405902	-0.611358563	0.298535773	-2.047856966	0.040574017	0.176718377
405.6954102	-0.320047723	0.181804306	-1.760396832	0.078340546	0.261814005
27.45721872	0.717991535	0.667426772	1.075760765	0.282034264	0.540466121
1609.777269	-0.044544624	0.075974722	-0.586308475	0.557668237	0.768771196
72.55727899	0.148506378	0.132838463	1.117947123	0.263589578	0.521277253
83.14780638	0.094766903	0.168292337	0.563108844	0.57336077	0.779186406
223.9240573	0.222164985	0.201178095	1.104319958	0.269454368	0.528147648
3.639196391	-0.02552704	0.562734975	-0.045362456	0.963818406	0.982935149
495.7852727	0.08599874	0.086013888	0.999823892	0.317395741	0.576094758
215.9644791	-0.173283454	0.0798646	-2.16971543	0.03002841	0.147038898
148.5441409	0.112821827	0.108454935	1.040264573	0.298216998	0.556589137
60.78237899	-0.190357019	0.148257738	-1.28396009	0.19915592	0.447694059
29.83608136	-0.529905774	0.265177823	-1.998303513	0.045683765	0.189601527
4.783050951	-0.446068465	0.50343325	-0.886052847	0.375589051	0.628747857
55.03595562	0.216656763	0.179049864	1.210035899	0.226265118	0.480468739
3.715349871	1.107398821	0.530690263	2.086714038	0.036913992	0.166702863
62.52762012	0.29892941	0.173650996	1.721437926	0.085171387	0.275940516
88.52045701	0.125870116	0.120329251	1.046047528	0.295539089	0.554602454
11.92133469	-0.120582929	0.42236304	-0.28549593	0.775264222	0.896964006
113.1717378	-0.401406727	0.285708788	-1.404950581	0.160035974	0.397563536
34.40219414	0.11839351	0.220381869	0.537219828	0.591115777	0.79088016
12.32099803	0.365338652	0.283700616	1.287761221	0.197829095	0.44611812
715.7027608	-0.378686887	0.111328097	-3.40153921	0.000670075	0.012687447
29.65269311	0.349241858	0.360576665	0.968564779	0.332762383	0.591532966
714.516483	0.206851199	0.101251955	2.042935354	0.041058838	0.177793157
31.03791978	0.106779487	0.293590745	0.363701816	0.716080687	0.865788583

122.5758232	0.437670307	0.161212928	2.714858621	0.006630407	0.057231261
19.4888883	1.222415803	0.491379254	2.487723676	0.012856358	0.087772159
30.17396838	0.245146029	0.217144287	1.12895454	0.258917011	0.515926109
397.308137	-0.10288924	0.085197026	-1.207662335	0.227177171	0.481753212
623.6659123	-0.113679203	0.106039894	-1.072041844	0.283701238	0.542432063
32.58528573	0.498714622	0.196250065	2.541220167	0.011046633	0.079380341
739.5045911	-0.159405052	0.072364861	-2.202796346	0.027609106	0.13977444
3.755221878	-0.069607281	0.49269353	-0.141279065	0.887649488	0.953293246
67.12522504	-0.088903129	0.216285056	-0.411046102	0.68103873	0.847181914
302.9117308	-0.092600233	0.092455547	-1.001564925	0.316553768	0.575699131
3.628195735	0.939922934	0.935940836	1.004254648	0.315255888	0.57422143
19.94213154	-0.319209647	0.249547625	-1.279153217	0.200843107	0.449553772
362.5549543	0.239873564	0.107865727	2.223816308	0.026160806	0.134982713
29.83012658	-0.263855989	0.256612586	-1.028226996	0.303843062	0.562231547
6.128778847	-2.173791113	0.542242456	-4.008891389	6.10045E-05	0.002327966
459.9688689	-0.150864913	0.085591233	-1.762621093	0.077964417	0.260963931
808.3909598	-0.120303222	0.090412709	-1.330600783	0.183320405	0.428941851
292.4960598	-0.074186867	0.120018278	-0.61812974	0.53648982	0.75450963
118.7388244	-0.360969077	0.146785372	-2.459162464	0.013926158	0.092628441
610.6575476	0.076524111	0.114074803	0.670823957	0.502332684	0.728860202
752.5400107	-0.505654103	0.213394629	-2.369572776	0.01780865	0.107224073
5.323607035	-0.175461575	0.517342121	-0.339159655	0.73448946	0.876558568
2064.821325	0.328161813	0.128283644	2.558095502	0.010524718	0.077171427
374.8472162	-0.204558038	0.098674046	-2.073068323	0.038165922	0.169872686
267.708211	-0.252941595	0.134847768	-1.875756631	0.060688706	0.225206985
5.990921082	-0.118764521	0.595445044	-0.199455049	0.841906802	0.93182686
360.212976	-0.629404794	0.303739236	-2.07218798	0.038247914	0.170044074
1008.050993	-0.112354667	0.074874609	-1.500571013	0.133466554	0.356954457
439.5659952	0.019708477	0.067115526	0.293650044	0.769025331	0.893581791
115.4069957	-0.102682814	0.129518689	-0.792803064	0.427892571	0.672578929
1142.745359	-0.445139841	0.629769654	-0.706829613	0.479672369	0.711575578
29.7101306	-0.670864431	0.631174443	-1.062882755	0.287835124	0.54728446
664.6233216	0.017490091	0.065687927	0.266260362	0.790038698	0.904145475
82.14831238	0.607553565	0.21570229	2.816630104	0.004853038	0.046468286
652.5178544	-0.303621793	0.07622779	-3.983085319	6.80263E-05	0.002487004
88.24603582	-0.096086035	0.12027782	-0.798867447	0.424367276	0.669258558
127.3547951	0.250334042	0.165693774	1.510823469	0.130833434	0.353061179
485.4679539	-0.165994992	0.225196258	-0.737112566	0.461053898	0.697145057
38.96128673	0.352122201	0.275398974	1.278589371	0.201041695	0.449869874
598.9744666	-0.450538794	0.177264911	-2.541612949	0.011034229	0.079380341
534.8745833	-0.091708109	0.071597826	-1.280878406	0.200236379	0.44877213
2.886068024	0.968363893	0.869166543	1.114129278	0.26522373	0.523307206
148.0231421	0.104657854	0.12619778	0.829316129	0.406925546	0.655147881
118.5644079	-0.189776213	0.251946996	-0.75323864	0.451306524	0.690422789
475.9959434	-0.330917318	0.108878933	-3.03931449	0.002371172	0.029492038
96.22158765	0.016174438	0.140495884	0.115123931	0.908346892	0.960978931
6.560893859	-0.685630756	0.537466081	-1.275672607	0.202071276	0.450798249
23.339888	-0.237059446	0.225354864	-1.05193845	0.29282779	0.552281525

2774.25649	-0.274346987	0.136499985	-2.009868258	0.044445134	0.186583471
203.7448985	0.021041393	0.143639131	0.146487889	0.88353625	0.951745378
386.4905326	0.102674735	0.120743401	0.850354838	0.395127837	0.645455477
13.29526844	3.321564449	0.719716394	4.615101834	3.92903E-06	0.000278104
15.13510446	0.182688348	0.325713916	0.560885915	0.574875312	0.780163053
916.9951587	-0.263641129	0.175845872	-1.499273914	0.133802587	0.357207219
415.550923	0.653835923	0.190771079	3.427332525	0.000609542	0.011971582
232.3920798	0.162476536	0.12140389	1.338314087	0.180794072	0.426017761
620.4871131	-0.130043979	0.059899982	-2.171018659	0.029929762	0.146876842
40.56892018	-0.014347321	0.225402801	-0.063651921	0.949247389	0.977223239
64.26843663	-0.890436866	0.368017642	-2.419549405	0.01553975	0.099354867
5.656823769	-0.283740983	0.473082893	-0.599770119	0.54865945	0.762847578
303.8091017	0.028176831	0.13291728	0.211987716	0.832116617	0.928693708
17.95144613	0.430411897	0.485469729	0.886588537	0.375300467	0.628533105
86.24207479	0.071404134	0.316266054	0.225772363	0.821378473	0.921865923
3.460515917	1.351465696	0.746692116	1.809937011	0.070305556	0.24607729
1380.727654	-0.080911197	0.072566153	-1.114999122	0.264850798	0.522834476
2581.792723	-0.140884674	0.094518147	-1.490556884	0.136077871	0.361369003
41.19749481	-0.543920146	0.378318702	-1.437729996	0.150510679	0.38383894
124.3245344	0.26650057	0.127741998	2.086240814	0.036956815	0.166800197
26.55551361	0.185323012	0.187357253	0.989142449	0.322593451	0.581267519
2329.00642	0.275620179	0.08927584	3.08728745	0.002019922	0.026667047
110.9923893	0.3723736	0.228033901	1.632974741	0.102474309	0.306660383
183.1574429	0.130635242	0.170762185	0.76501271	0.444263976	0.685136303
73.71417162	0.131032956	0.176204707	0.743640498	0.457093996	0.694071278
24.01586182	1.551932852	0.381419781	4.068831585	4.72495E-05	0.00193995
265.9595999	0.018780424	0.155750894	0.120579877	0.904023808	0.959382191
241.2039293	-0.285804796	0.076119015	-3.754709608	0.000173543	0.004913071
3.662573042	-0.024279855	0.530310923	-0.045784188	0.963482262	0.982781984
98.57381748	0.840576592	0.306141331	2.745714174	0.006037934	0.053775673
399.7445465	0.213527414	0.169824611	1.257340808	0.208630253	0.459572597
20.92691876	-0.69112897	0.365990385	-1.888380126	0.058974937	0.221548004
40.95195161	0.203459299	0.218677508	0.930407982	0.352159887	0.609028082
164.1767561	0.156070393	0.114064393	1.36826566	0.171228936	0.413035162
248.247289	0.289791257	0.085283196	3.397987759	0.000678835	0.012765997
14.88241619	0.216265493	0.312231724	0.6926442	0.488532865	0.718626475
223.1455514	0.260425073	0.097086604	2.682399656	0.007309608	0.061558565
28.77744739	0.882489252	0.220574086	4.000874569	6.31088E-05	0.002373756
869.0061159	0.088386517	0.056022337	1.577701355	0.114634235	0.325769766
345.0262362	0.286867234	0.131026988	2.189375165	0.028569583	0.142709289
121.8663896	0.13100857	0.18584616	0.704930199	0.480853676	0.712692218
961.823813	0.020929772	0.131380661	0.159306335	0.87342753	0.947352611
44.95494901	0.93026799	0.74327948	1.251572275	0.210725762	0.462176319
30.98211831	1.138029056	0.292315933	3.893147542	9.89519E-05	0.003309148
1820.438897	-0.045700168	0.122169026	-0.37407328	0.708349797	0.861905675
1780.426105	-0.198031063	0.181711884	-1.08980799	0.275797734	0.534543001
54.78699696	0.59237362	0.201965553	2.933042846	0.003356576	0.036840121
272.2903779	0.206488377	0.10123271	2.039739702	0.041376258	0.178526888

3.040214065	1.245140756	0.61951142	2.009875389	0.044444379	0.186583471
6210.237486	-0.238572225	0.224002149	-1.06504436	0.286855856	0.546332392
424.4633094	0.017600528	0.15781472	0.111526528	0.91119883	0.962059189
84.88636358	0.851877366	0.243530859	3.498026367	0.000468715	0.009974658
5.717135971	1.36390859	0.512488875	2.661342827	0.007782967	0.064044098
74.13255401	0.51604186	0.25370672	2.034009423	0.041950645	0.179762383
261.7257079	-0.147113511	0.070437428	-2.088570179	0.036746432	0.166233354
32.52654305	0.075328018	0.269497926	0.279512424	0.779851605	0.898757629
7.137992858	0.045822784	0.417159289	0.109844813	0.912532451	0.962614942
19.80024171	0.090084787	0.345504287	0.26073421	0.794297483	0.905623853
3.205089432	-0.202148995	0.660532994	-0.306039209	0.759574793	0.889224856
19.98016567	0.39360931	0.502364692	0.783513084	0.433325873	0.677730654
7544.674346	-0.049810356	0.10761189	-0.462870372	0.643457286	0.824643248
678.31189	-0.302546431	0.092264354	-3.279125873	0.001041292	0.017065434
30176.5662	0.073465281	0.131263777	0.559676727	0.57569996	0.780736608
6.760226271	0.55781064	0.493262894	1.13085871	0.258114564	0.515243582
927.9920959	0.153453602	0.096907027	1.583513664	0.113304435	0.324077445
1097.019015	-0.215325256	0.076801726	-2.803651257	0.005052753	0.047734421
4.70065893	0.262181527	0.598802042	0.437843408	0.661499802	0.835344221
254.9355802	0.15763766	0.125757639	1.253503651	0.210022471	0.461731489
35.90095637	0.342299138	0.335577099	1.020031282	0.307713625	0.566586111
164.8533616	0.299745303	0.326302973	0.918610395	0.358299393	0.614347157
231.0904302	0.368067794	0.136522069	2.696031476	0.007017104	0.059765103
218.8298221	-0.207493878	0.116976715	-1.77380497	0.076095416	0.2579099
9266.628327	0.046976185	0.119916674	0.391740227	0.695250164	0.854463425
2771.486668	0.150158717	0.192512347	0.779995253	0.435393669	0.679203731
1553.274747	0.0716863	0.135392164	0.529471553	0.596478373	0.794021966
14.47273972	-0.05049585	0.358583841	-0.140820206	0.88801198	0.953471838
277.2244751	-0.1676474	0.108374776	-1.546922692	0.121881889	0.338875295
362.496661	0.101307286	0.063513505	1.595051092	0.110700811	0.31963025
1248.558999	0.089860942	0.067801426	1.325354748	0.185053518	0.430939775
728.1034674	-0.014062755	0.118197208	-0.118977049	0.905293539	0.959974986
108.3961242	0.481795793	0.316388746	1.522796874	0.127809532	0.348467226
316.433924	-0.026579474	0.151682653	-0.17523081	0.860898276	0.940666613
812.5765573	0.012904891	0.181609628	0.071058408	0.94335127	0.974993168
97.73612305	0.047565914	0.143350676	0.331815068	0.740028906	0.879355136
2850.078502	-0.250690483	0.142071057	-1.764542959	0.077640609	0.260752744
427.4413304	-0.071222806	0.07519037	-0.947233087	0.343520019	0.601215055
92.78635072	0.293131071	0.216699182	1.352709634	0.176148412	0.419609916
1139.778076	-0.335063641	0.11253247	-2.977484098	0.002906247	0.033739142
6.543442565	1.207597426	0.491510554	2.45691047	0.014013761	0.092902392
466.5741152	-0.296176016	0.121786389	-2.431930364	0.015018594	0.097352624
1397.639435	-0.016874141	0.089547932	-0.188436974	0.850534118	0.935533846
72.64949284	0.586415974	0.30367053	1.931092801	0.053471577	0.208618959
7.437455295	0.323077266	0.444774153	0.726384984	0.467602794	0.702883096
211.4167919	0.395559594	0.194078924	2.038137813	0.041536153	0.178921455
114.4931512	0.069360464	0.13193735	0.525707569	0.599091404	0.795609617
58.37400211	-0.960265036	0.347002988	-2.767310571	0.005652088	0.051389769

43.02817715	0.094081471	0.18124923	0.51907239	0.603710262	0.798632907
365.5972052	0.015715931	0.067179423	0.233939658	0.815031825	0.918646004
17.05243749	-0.146490775	0.290744881	-0.503846445	0.614369287	0.80620548
96.29109269	-0.174806283	0.125864274	-1.388847499	0.164879128	0.404562617
36.67482568	-0.259027196	0.344793283	-0.751253604	0.452500046	0.690975632
853.0018259	-0.248862138	0.162825097	-1.528401593	0.12641286	0.346604975
30.19452259	0.590887432	0.211525977	2.793450909	0.005214894	0.04894698
384.8133573	-0.13452345	0.125204611	-1.074428881	0.282630504	0.541176514
1432.507935	0.462224129	0.145767991	3.170957667	0.001519373	0.022246589
6.60306087	0.186560566	0.497734722	0.374819272	0.707794881	0.861889372
3.351272937	-0.738872789	0.562631784	-1.313243955	0.189100757	0.436736716
3.939788278	0.104152815	0.599078176	0.173855131	0.861979316	0.94137167
514.3366787	-0.076423508	0.165606582	-0.461476269	0.644456943	0.825182684
1512.763137	-0.041102975	0.123061677	-0.334003048	0.738377259	0.878437596
539.8247319	0.031901816	0.053912076	0.591737844	0.554026151	0.766061319
3.525981651	0.199653896	0.51113917	0.390605745	0.696088678	0.854510951
371.784681	-0.187386298	0.080455801	-2.329058884	0.019855946	0.114157135
297.2052054	0.408333464	0.100834164	4.049554719	5.13152E-05	0.002047907
31.71858502	0.130441105	0.228971099	0.56968371	0.568892241	0.775924721
553.1761023	0.100833462	0.168998334	0.596653584	0.550738688	0.764270535
318.0135846	0.183957818	0.084373188	2.180287632	0.029236147	0.144846762
289.1997992	-0.112117504	0.077498954	-1.446696999	0.147981805	0.379505869
33.81992421	0.224573237	0.204787126	1.09661794	0.272808442	0.531717112
38.14763274	0.026713686	0.421194467	0.063423639	0.949429164	0.977282077
93.00967707	0.020615281	0.290602325	0.070939834	0.94344564	0.975026451
1077.138387	0.345926974	0.113118362	3.05809744	0.002227471	0.028265098
441.8655706	-0.256484581	0.115425374	-2.222081446	0.026277802	0.135350097
564.0455313	-0.058569713	0.050504512	-1.159692687	0.246173949	0.501557834
32.71355794	0.466398083	0.271918669	1.715211699	0.086306421	0.277496905
4.29605303	1.133085048	0.504888744	2.244227193	0.024817787	0.130663891
183.216193	0.203704985	0.319740038	0.637095642	0.524062547	0.745794119
407.3649989	-0.479193229	0.234842494	-2.04048774	0.041301771	0.178500131
312.9881239	-0.525847745	0.259013327	-2.030195713	0.042336649	0.180617323
394.6594173	-0.118170604	0.061787009	-1.912547735	0.055805976	0.214104924
5.061832199	1.627929062	0.655627704	2.48300835	0.013027803	0.088530359
3.139196872	0.202199616	0.753194618	0.268456002	0.788348337	0.903504225
431.6816224	0.022100619	0.179678378	0.123000994	0.902106311	0.958662553
16.78738396	1.273658111	0.593023106	2.14773775	0.031734595	0.151884462
91.03782962	0.126984113	0.125859018	1.008939327	0.313003733	0.571976009
146.948512	0.190465417	0.104487563	1.82285252	0.068325731	0.242084363
393.9227699	0.065240216	0.075433994	0.864864928	0.38711293	0.638296803
74.76619887	0.208507165	0.202241995	1.030978582	0.302550864	0.561386357
37.55564693	0.134817615	0.222245181	0.606616594	0.544105385	0.759365444
3.151441018	1.200063834	1.042992228	1.150597101	0.249898026	0.505794379
7.888227889	0.199799471	0.405512765	0.492708217	0.622218771	0.810641951
1726.396006	0.060998419	0.071983984	0.847388755	0.39677847	0.647292857
228.7905836	-0.018225349	0.074239551	-0.245493792	0.806074114	0.913010135
2282.930409	0.034148275	0.16115238	0.211900529	0.832184638	0.928702424

73.40796069	-0.36595376	0.122238269	-2.993774076	0.002755499	0.032543107
1595.430664	-0.013186188	0.09267831	-0.142279121	0.886859538	0.953108598
21.8027898	0.267647753	0.355397915	0.753093199	0.451393911	0.690422789
214.5018439	-0.020232572	0.086677328	-0.233424041	0.815432146	0.918701084
3.659855436	-0.087073736	0.475343582	-0.183180629	0.854656293	0.937700546
5.990295438	1.507669111	0.562580829	2.679915547	0.007364074	0.061796753
886.1658032	0.047280951	0.079997332	0.591031599	0.554499249	0.766234909
132.5099548	0.253602168	0.255730872	0.991675999	0.321355602	0.579926507
3.508303318	1.317394774	0.765807768	1.72026823	0.085383696	0.27617197
1240.466196	0.199919757	0.075184418	2.659058376	0.007835938	0.06421048
273.8122317	0.258474978	0.14974608	1.726088447	0.084331502	0.274295993
50.27595876	0.762081605	0.20768247	3.669455604	0.000243068	0.006396428
1435.047735	0.416826721	0.10965551	3.801238266	0.000143975	0.004359267
240.2020592	0.052312683	0.086473341	0.604957351	0.545207335	0.76009172
812.1343469	-0.3658732	0.125418272	-2.917224046	0.00353162	0.037964309
433.0921078	-0.148525506	0.167551063	-0.886449202	0.375375516	0.628591671
500.1097717	-0.093255595	0.135972689	-0.685840628	0.492813614	0.721493215
942.1953285	0.018133894	0.122825663	0.147639295	0.882627442	0.951417787
409.3555181	-0.053706451	0.117930572	-0.455407366	0.648816197	0.827875514
236.5101635	0.226567799	0.178271306	1.270915683	0.203758646	0.453300969
13806.5358	-0.022485349	0.191589386	-0.117362186	0.906573048	0.960203382
51.86948515	0.156530148	0.232240717	0.673999587	0.500311576	0.726893893
983.3541709	-1.099239241	0.27429435	-4.007516896	6.13605E-05	0.002330212
270.0869758	0.167158522	0.102683651	1.627898124	0.103546489	0.308336615
96.83720795	-0.70433575	0.501316865	-1.404971185	0.160029847	0.397563536
564.659624	-0.097934192	0.085859294	-1.140635894	0.254021474	0.510716932
2808.219254	-0.085475213	0.166536119	-0.513253305	0.607774143	0.80150758
1550.800682	0.066287981	0.109722168	0.60414392	0.545747962	0.760687675
11.34257022	0.017339592	0.304323065	0.056977582	0.954563053	0.979993907
26.62155991	0.076579831	0.243986226	0.313869485	0.753620168	0.8866675
314.790512	0.130987661	0.098592433	1.328577233	0.183987487	0.429517084
567.0943586	-0.03526696	0.094035674	-0.375038099	0.707632132	0.861889372
13685.09089	0.019196446	0.140467126	0.136661488	0.891298372	0.954379567
817.7391316	-0.031689757	0.073104821	-0.433483813	0.664663323	0.83676248
630.6148234	-0.663943481	0.202820269	-3.27355586	0.001062034	0.017277817
385.1503978	0.08073757	0.196276438	0.411346214	0.680818687	0.847121006
1121.786345	-0.132209893	0.056355686	-2.345990299	0.018976601	0.111097787
28.24754424	-0.454814755	0.325954501	-1.395332028	0.162915738	0.401756358
12.92543231	0.226930457	0.312857291	0.725348149	0.468238474	0.70336675
251.5593269	0.219063166	0.104709898	2.092096066	0.036429923	0.165203628
51.5197704	0.676057126	0.202370693	3.34068692	0.000835714	0.014467262
591.8659238	0.123512817	0.104328393	1.183884974	0.236458599	0.492010908
29.06867919	0.208962138	0.297146529	0.703229276	0.481912877	0.713857345
6.05309151	0.423553778	0.566168225	0.748105879	0.454396296	0.69248182
101.6670631	2.046126689	0.31411058	6.514033011	7.31594E-11	4.4132E-08
323.6709716	0.48475794	0.304953929	1.589610407	0.11192265	0.322028806
568.996941	0.172904974	0.149538085	1.156260453	0.247574639	0.502785584
169.5914058	0.000718923	0.110041705	0.006533187	0.994787308	0.997203384

6.837210854	-0.324252032	0.644303223	-0.503259987	0.614781495	0.80643696
13.83193242	4.912692928	1.601488844	3.067578613	0.002158007	0.027734345
11.62913851	1.202506036	0.314210507	3.827071377	0.000129677	0.004043445
54.05583328	-0.885659377	0.356954878	-2.481152187	0.013095845	0.088915683
8.441541654	0.510473925	0.605722229	0.842752504	0.399366876	0.649354145
6.395852359	0.861217389	0.514634277	1.673455166	0.094237726	0.291686543
818.9946614	-0.10991321	0.154624351	-0.710840234	0.477183245	0.709683822
650.8868566	0.099074721	0.167329832	0.592092398	0.553788717	0.765862233
287.9049434	-0.386253498	0.142686394	-2.707010017	0.006789221	0.058186962
546.62747	-0.547588198	0.2871567	-1.906931645	0.05652944	0.215772142
5.638352895	1.129086189	0.586649261	1.924635832	0.054274933	0.210469729
81.43303352	-0.028111479	0.424617357	-0.066204263	0.947215203	0.976502838
34.44737698	-0.840467026	0.326162753	-2.576833251	0.009971004	0.074825466
1875.725504	-0.189776232	0.119728471	-1.585055173	0.112953799	0.323573952
356.9376442	-0.333898773	0.131939891	-2.530688573	0.011383887	0.080716496
90.83694847	-0.106574757	0.144327975	-0.738420649	0.46025887	0.696718475
260.6746468	-0.27136093	0.138331913	-1.961665415	0.049801451	0.199664099
59.7970639	0.343515283	0.17848808	1.924583887	0.054281437	0.210469729
121.6558048	-0.086207431	0.181941684	-0.473819022	0.635628969	0.819431592
156.6276579	0.575395674	0.101052726	5.694014364	1.24087E-08	2.88122E-06
926.1843292	-0.127733232	0.106365024	-1.200895065	0.229791909	0.485070119
1268.163513	-0.228594974	0.071909335	-3.178933222	0.001478181	0.021809781
912.1782149	0.08974491	0.112712707	0.796227084	0.425900058	0.670866377
21.50302358	0.267449368	0.273643141	0.977365508	0.328388233	0.586744252
383.3631315	0.106450622	0.090140172	1.180945405	0.237624412	0.493172063
461.6464999	-0.19599243	0.06471661	-3.028471797	0.00245794	0.03021186
1083.706343	-0.165246329	0.127016779	-1.300980314	0.193265193	0.440641269
235.2776783	0.155021428	0.107994573	1.435455723	0.151157277	0.384829693
494.8284391	-0.061935349	0.07222936	-0.857481625	0.391178766	0.641829455
6943.950713	-0.207918046	0.156221157	-1.330921175	0.183214949	0.428880565
16.4371803	0.658476014	0.422521788	1.558442743	0.119128331	0.334138972
4691.88779	-0.032934437	0.193609734	-0.17010734	0.864925721	0.942899493
49.02508033	-0.5789261	0.1716364	-3.372979742	0.000743594	0.013467127
421.4977893	-0.067877554	0.093967879	-0.722348481	0.470080253	0.705255279
32.40392215	-0.41418044	0.244243307	-1.695769862	0.089929472	0.284251076
94.74388083	-0.037680839	0.383693847	-0.098205482	0.921769129	0.966973977
303.1905694	-0.149930242	0.166791917	-0.898905923	0.368702773	0.623914531
337.9878214	-0.00335579	0.100696264	-0.033325869	0.973414725	0.986906773
1032.932144	-0.060060841	0.087106213	-0.689512716	0.490500674	0.720006182
54.74728029	0.318928819	0.210948298	1.511881449	0.130564024	0.352576817
658.0670684	-0.096240492	0.067618016	-1.423296595	0.154650194	0.389268759
2330.166584	-0.155240863	0.073070469	-2.12453629	0.033625325	0.156958213
134.6233436	-0.358441526	0.113301845	-3.163598329	0.001558317	0.022651199
318.075538	-0.321521283	0.101615388	-3.164100322	0.001555632	0.022633141
456.3182352	-0.003505653	0.155700233	-0.022515401	0.982036827	0.990754251
259.6029623	-0.082671473	0.108096223	-0.764795211	0.4443935	0.685268671
87.42097207	0.048125989	0.151628573	0.317393929	0.750944713	0.885437575
123.8328299	-0.126229193	0.116635222	-1.082256207	0.2791387	0.53751602

2.611035036	0.063378075	0.685411558	0.092467182	0.926326865	0.968954952
41.97002015	-0.006613381	0.185939021	-0.035567472	0.971627245	0.986184096
65.5955708	0.161233186	0.153913848	1.047554772	0.294843784	0.55408305
114.3507874	0.801285877	0.353486903	2.266804995	0.023402141	0.126363037
601.9630921	0.282233443	0.18604057	1.517053208	0.129253251	0.350910158
214.0530126	-0.914659884	0.292092773	-3.131401972	0.001739739	0.024275854
206.4584304	-0.031536755	0.087078474	-0.36216477	0.717228905	0.866709156
534.24499	-0.011362005	0.071386821	-0.159161103	0.873541949	0.947352611
111.2672593	-0.276453263	0.132444531	-2.087313545	0.036859801	0.166554054
140.2341635	0.108277613	0.249512326	0.433956969	0.664319689	0.836652459
119.4160021	-0.171593414	0.124082518	-1.382897583	0.166696272	0.406604094
327.7188241	-0.079986784	0.116379437	-0.687293102	0.491898045	0.720819297
828.9784366	0.416808026	0.156428445	2.664528346	0.007709637	0.063711356
6641.401981	-0.199448048	0.15404887	-1.294706333	0.195421554	0.443604785
56.86659725	0.150252432	0.125418166	1.19801171	0.230912458	0.486386113
14.57431201	0.378360045	0.350949033	1.078105392	0.280986725	0.539411971
6446.751943	0.136454752	0.142125425	0.960100924	0.337004424	0.595725198
491.8659836	-0.463212121	0.203025551	-2.281545941	0.022516161	0.123347355
5668.506957	-0.347587473	0.12068015	-2.880237343	0.003973759	0.041219865
8.51473677	0.033598268	0.34628978	0.097023561	0.922707684	0.967233479
384.6304938	-0.044990479	0.077963212	-0.57707319	0.563890015	0.772932515
143.3863056	-0.169090232	0.118660457	-1.42499225	0.154159441	0.38887588
122.8978131	-0.171383653	0.138821282	-1.234563252	0.216993092	0.469594725
95.33907847	-0.569164643	0.334882151	-1.699596832	0.089206787	0.2831079
1025.80402	0.089501314	0.154561515	0.579065974	0.562544658	0.771716083
18.70852271	1.080697621	0.322145482	3.354688118	0.000794545	0.013970454
3027.976538	-0.174875452	0.129061227	-1.354980544	0.175423756	0.418774153
65.89892809	-0.005814085	0.205310527	-0.028318493	0.977408131	0.988491024
375.0443305	0.197869003	0.100643894	1.966030876	0.049295044	0.198287729
47.32621279	0.031928242	0.212832447	0.150015858	0.880752104	0.950743683
232.4736748	-0.044964241	0.093284399	-0.482012439	0.62979711	0.815331232
4.616418527	1.704308737	1.371231415	1.242903801	0.213903285	0.46582326
190.1753276	-0.268497749	0.348069905	-0.771390301	0.440475622	0.682478371
163.0140449	-0.180409779	0.12372306	-1.458174239	0.144792523	0.374863969
7.440615551	-0.26603503	0.363805427	-0.731256354	0.464622585	0.700215299
204.3879402	0.678308665	0.316075305	2.146034991	0.031870187	0.152115647
899.4867601	-0.148664532	0.071950224	-2.066213602	0.038808315	0.171634538
16.23237476	0.459145525	0.311110425	1.475828157	0.13999003	0.367588084
845.8580848	-0.053678045	0.104495361	-0.513688306	0.607469929	0.801442647
8.844562415	0.863412922	0.409728481	2.107280706	0.035093255	0.161313777
8.4981764	1.316834938	0.448744888	2.934484542	0.003341022	0.036824025
19.87358654	-0.453399357	0.29315862	-1.546600801	0.121959536	0.338989742
194.0638147	0.530922919	0.636706787	0.833857798	0.404361121	0.653410243
50.744345	-0.031027059	0.487150677	-0.063690888	0.94921636	0.977223239
3.242290144	1.137066427	0.828079905	1.37313612	0.169710034	0.410824537
598.9673839	0.071860138	0.191456481	0.375334058	0.70741204	0.861818427
1077.382231	-0.521496961	0.228907492	-2.27819962	0.022714687	0.124128345
420.3857736	-0.062959424	0.089607101	-0.702616467	0.482294797	0.714002102

4.093796895	-0.215081241	0.643194268	-0.334395457	0.738081167	0.878437596
29.15885858	0.99506881	0.208246994	4.778310548	1.76774E-06	0.00015753
776.9902289	-0.062546403	0.1194948	-0.523423637	0.600679479	0.796908894
4.805110938	1.127134667	1.162485284	0.969590482	0.332250659	0.590885513
110.9260732	-0.04938808	0.105831698	-0.466666233	0.640738692	0.823232665
10576.09627	-0.245267101	0.145228966	-1.688830452	0.091251926	0.286181806
5.093586007	-1.281991759	0.564245452	-2.272046241	0.023083718	0.125445085
7.774441052	-1.521101565	0.475239858	-3.200702845	0.001370928	0.020754479
9.117436747	-0.200920653	0.528889143	-0.379891809	0.704025728	0.85974352
418.3001328	-0.026525181	0.084091482	-0.315432435	0.752433344	0.886266194
20.3443289	0.089866131	0.281967281	0.3187112	0.749945521	0.88518749
2.883290689	1.336161718	0.769234273	1.737002321	0.082386761	0.270091838
278.5280346	0.127214033	0.093996771	1.353387261	0.175931945	0.41928531
483.0994897	-0.183429508	0.113237213	-1.619869496	0.105260314	0.310500845
9.824647841	-0.374970346	0.357490657	-1.048895511	0.294226212	0.553447338
129.3286976	-0.269180341	0.091425955	-2.94424423	0.003237444	0.036165295
334.8600063	0.36031842	0.21230435	1.697178696	0.089662881	0.283955102
428.9471704	-0.10106577	0.221123724	-0.457055299	0.647631299	0.82742337
8.195743823	-0.410342417	0.414155381	-0.990793398	0.321786474	0.580503687
796.7837883	-0.193345315	0.073806558	-2.619622444	0.008802716	0.068961941
76.89467532	0.054412214	0.125669687	0.432978036	0.665030726	0.836844909
43.14372553	-0.581888988	0.197846715	-2.941110177	0.003270382	0.036326253
202.7238859	-0.079876258	0.365183749	-0.218728949	0.826861197	0.925638145
365.2694286	0.466060512	0.426532749	1.092672281	0.274537714	0.533628642
9.770968369	0.790681334	0.765575527	1.032793377	0.301700604	0.560316432
5.080114289	0.870000645	0.570066849	1.526137936	0.126975516	0.347432657
125.415869	-0.120651463	0.093688648	-1.287791702	0.197818482	0.44611812
2.905661486	0.809253072	0.854074693	0.947520256	0.34337374	0.601109766
39.04654216	-0.389285929	0.542888785	-0.717063863	0.473334692	0.706757551
769.7303963	-0.244758658	0.116084659	-2.108449647	0.034992114	0.160990412
2991.617915	-0.044585722	0.05283525	-0.843863181	0.398745865	0.648806234
8.321503435	0.108423908	0.405945398	0.267089881	0.789399958	0.903718901
3.354134031	-0.290882397	0.529740125	-0.549103955	0.582934111	0.784884699
368.4051787	0.131242254	0.096815311	1.355593992	0.175228384	0.418435137
240.2543791	-0.110063253	0.178226894	-0.617545705	0.536874844	0.7548494
210.5442369	0.123118637	0.180828093	0.680860121	0.495960004	0.723257713
10.77399789	-1.175512289	0.518316538	-2.267942857	0.02333269	0.126227021
14.81096371	-0.644732129	0.391334814	-1.647520499	0.099451106	0.301642072
60.68048135	-0.007662686	0.198116895	-0.038677598	0.969147435	0.985192723
29.97915513	0.222280486	0.282147657	0.787816167	0.430804243	0.675808537
6.848715011	0.099561781	0.524917201	0.1896714	0.849566633	0.935119121
32.77719495	1.325049554	0.887210846	1.493500174	0.135306309	0.360234959
32.36175677	1.474870026	0.789614491	1.867835562	0.061784995	0.227847604
28.78114567	0.583076762	0.654977051	0.890224719	0.373345234	0.627379314
48.5403163	0.095116075	0.307309517	0.3095123	0.756931857	0.887988809
335.3674651	-0.05751969	0.079954223	-0.71940778	0.471889705	0.706078814
550.7570463	0.132133546	0.067120082	1.968614206	0.04899741	0.197441962
188.989876	0.423097153	0.206918411	2.044753539	0.040879162	0.17721083

350.1645276	-0.032147115	0.107144386	-0.300035458	0.76415011	0.891536883
205.7930815	-0.274753092	0.190141896	-1.444989752	0.148460764	0.380094454
871.6417682	-0.188473247	0.093306596	-2.019934879	0.043390143	0.183579984
8.484072476	0.248902416	0.537042992	0.463468325	0.643028714	0.824387858
635.2297419	-0.178747327	0.09209058	-1.940994689	0.052258922	0.205301071
777.1111566	0.060606011	0.07219187	0.839512968	0.401181515	0.650685718
64.1582317	-0.01292806	0.154875746	-0.083473756	0.933474845	0.972410963
5184.491753	-0.052496084	0.105484819	-0.497664826	0.618720304	0.808403087
535.7327111	0.185803992	0.105701285	1.757821504	0.078777885	0.262639264
45.27779136	-1.128659525	0.518328126	-2.17750006	0.029443277	0.145474083
301.1048324	0.209802281	0.123676136	1.696384513	0.089813085	0.28416287
1720.536648	-0.182471229	0.089130584	-2.047234742	0.040635042	0.176829885
1084.686375	-0.129407219	0.083915264	-1.542117751	0.123044982	0.340780063
383.0610248	-0.039894595	0.105388453	-0.378548061	0.705023498	0.860203048
454.1521965	-0.502637206	0.394907456	-1.272797459	0.203089923	0.452130923
99.24755335	-0.30751205	0.109733229	-2.802360349	0.005073017	0.047844381
46.42602614	0.047875806	0.215236495	0.222433494	0.823976441	0.923645097
23.36710762	-0.848352486	0.258836081	-3.277566567	0.00104706	0.017088548
83.76507287	-0.101709125	0.157755721	-0.644725432	0.519105133	0.742376666
23.78247475	-0.370492693	0.242416243	-1.52833279	0.126429933	0.346604975
62.43415047	-0.337107254	0.157033724	-2.146718845	0.031815672	0.152090717
466.8492632	-0.216543163	0.076953217	-2.81395855	0.004893554	0.046742081
93.9565527	1.672429273	0.235018569	7.116158012	1.10977E-12	1.16038E-09
253.7597497	0.232653569	0.077631401	2.996900313	0.002727399	0.032370421
1198.926348	0.026447923	0.094805681	0.278969813	0.780267992	0.898986384
144.0790589	-0.437230932	0.122070705	-3.581784268	0.000341256	0.008012353
163.3404031	0.069077276	0.129249225	0.534450214	0.593030086	0.791576954
65.16885519	0.60925453	0.185853467	3.278144548	0.001044919	0.017074327
19.82171679	0.242960535	0.298139341	0.814922762	0.415116523	0.661601456
164.1199632	-0.53265272	0.147737352	-3.6054032	0.000311669	0.007578622
1018.368553	-0.20943838	0.131641906	-1.590970436	0.111616229	0.321326898
3904.343571	-0.416499444	0.172303355	-2.41724512	0.015638482	0.099704858
343.5311727	0.157106611	0.189065162	0.830965419	0.405993165	0.654824855
295.6450192	-0.233989308	0.109104525	-2.144634307	0.031982097	0.152480917
3797.883139	-0.435611725	0.159012702	-2.739477533	0.006153692	0.054558794
46.91136738	0.216323647	0.197374337	1.096006957	0.27307573	0.531877602
1402.962269	-0.104416439	0.07781392	-1.341873521	0.179637009	0.424747406
7.963632165	0.152329549	0.367914397	0.414035302	0.678848253	0.845876523
5.890987883	0.56609439	0.465074613	1.217211981	0.223523573	0.477296626
1926.858925	-0.24203313	0.131451578	-1.841234115	0.065587258	0.236600527
16.66572903	0.087389561	0.325814325	0.268218905	0.788530824	0.903520748
14700.21746	-0.021235753	0.139945453	-0.151743074	0.879389583	0.95027878
805.7386394	-0.026532728	0.068592576	-0.386816323	0.698892192	0.856695986
1517.927322	-0.238249069	0.077788816	-3.062767653	0.002193003	0.027918062
48.47205048	-0.151349677	0.161325433	-0.938163772	0.348160253	0.605516235
2054.620032	-0.272521634	0.057798483	-4.715030924	2.41674E-06	0.000188578
6.781549326	0.002117411	0.451328747	0.004691505	0.996256734	0.998186672
2678.660577	-0.219952942	0.13018571	-1.689532146	0.091117497	0.285931737

3.462773994	-1.030620931	0.565041107	-1.823975139	0.068155831	0.241954742
14.27541964	-0.200695333	0.310807736	-0.645721808	0.518459535	0.741851962
6.524829865	0.758224327	0.742971559	1.020529411	0.307477448	0.56641723
370.82663	0.026549794	0.237975145	0.111565406	0.911168003	0.962059189
124.3890917	-0.262031686	0.102266167	-2.562251967	0.010399584	0.076720167
200.7141162	0.089563999	0.09542474	0.938582579	0.347945101	0.605319822
202.6354861	0.443994887	0.280909201	1.5805637	0.113977831	0.325141561
18.09873512	0.543810235	0.299294662	1.816972716	0.069221291	0.243608574
205.979207	0.045303986	0.083132931	0.544958364	0.585782154	0.786664905
20.22228475	0.48821558	0.267137404	1.827582262	0.06761227	0.240788111
59.83598057	0.17230313	0.317075434	0.543413684	0.586845006	0.787659342
59.63125911	0.268108965	0.159771377	1.678078829	0.093331711	0.290097884
304.4402327	-0.348805691	0.17916348	-1.946857093	0.05155187	0.203355012
809.504987	-0.089220958	0.124075168	-0.71908795	0.472086731	0.706238868
1687.060495	0.045065534	0.14892455	0.30260648	0.762189775	0.890906576
426.2645915	0.166443777	0.164392305	1.012479124	0.311309024	0.570697037
356.7710166	-0.234504579	0.112139339	-2.091189233	0.036511104	0.165407324
71.26604242	-0.116548277	0.163058913	-0.714761772	0.47475626	0.707869302
738.7369591	-0.112571899	0.105734231	-1.064668445	0.287025995	0.546523698
2674.030023	-0.326233363	0.176992567	-1.843203746	0.065299268	0.235871424
391.5088038	0.156087133	0.085898038	1.81712106	0.069198579	0.243608574
2764.291125	0.038779674	0.06496243	0.596955408	0.550537153	0.764058464
40.07265301	-0.796531819	0.216985388	-3.670900737	0.000241697	0.006371057
322.7223451	-0.114330967	0.172962993	-0.661014039	0.50860331	0.733916121
90.31291544	-0.862522864	0.415916297	-2.073789536	0.038098861	0.169824595
170.1662906	-0.070337601	0.220553826	-0.318913538	0.749792078	0.88518749
2.65089681	-0.983213893	0.607714826	-1.617886961	0.105686961	0.310992892
3188.138141	0.396491545	0.352943625	1.123384915	0.261274054	0.519040185
146.3530438	-0.279549628	0.120101289	-2.327615545	0.019932527	0.114425677
1385.843533	-0.204136734	0.07382091	-2.765296912	0.005687101	0.051588482
14.3487881	-0.190902042	0.341185235	-0.559526095	0.575802728	0.780746086
3.46805123	0.205528562	0.575328206	0.357237069	0.720914337	0.869287343
596.5629748	0.018191743	0.121950881	0.149172705	0.881417357	0.950743683
217.774046	-0.028978894	0.092479923	-0.313353356	0.754012218	0.886841482
106.0653708	-0.498586981	0.261508182	-1.906582717	0.056574645	0.215820017
12.28728156	-0.095356349	0.279358873	-0.341339967	0.732847663	0.875531897
425.6971192	0.210105016	0.1555653	1.350590494	0.176826648	0.420714372
3074.180597	-0.147302264	0.089905412	-1.63841377	0.101335409	0.304822506
362.6591656	-0.087109824	0.099172822	-0.878363875	0.379746282	0.631863005
347.138902	-0.097190093	0.089339875	-1.087869136	0.276652883	0.535219417
135.4537186	-0.252464757	0.144962133	-1.741591075	0.081580024	0.268408034
15.0391166	1.354790867	0.644291342	2.10276125	0.035486645	0.162364724
158.7268403	0.230998925	0.304450724	0.758739942	0.448008138	0.687884956
290.1743093	-0.317478381	0.280950333	-1.130016034	0.258469468	0.515361701
104.1742684	0.440650723	0.160936775	2.738036253	0.006180726	0.054600664
374.3084752	-0.108941281	0.113270634	-0.961778679	0.33616079	0.594703504
416.9716422	0.255308248	0.161007265	1.585693957	0.112808751	0.323444764
39.31387657	-0.18875219	0.197094876	-0.957671727	0.338228318	0.596670665

99.32630136		-0.304090805	0.141825577	-2.144118222	0.032023415	0.152568422
1189.048998		-0.08026571	0.088885539	-0.903023266	0.366513547	0.622358722
5.662207084		0.688905125	1.305610086	0.527649972	0.597742302	0.794878848
6.125701533		0.2325089	0.549577958	0.423068096	0.672245579	0.842065303
165.6571299		-0.244522297	0.221904608	-1.101925278	0.270494158	0.529443451
15.75365203		0.057998811	0.30421033	0.190653653	0.848796952	0.934780308
214.6363174		0.14344279	0.104889632	1.367559284	0.171450069	0.413377844
101864.6296		0.106284977	0.160783001	0.661046111	0.508582743	0.733916121
148.4297429		0.025619933	0.182741699	0.140197523	0.888503929	0.953660331
5.87798073		-0.001160393	0.481411434	-0.002410397	0.998076783	0.998977228
254.0893845		-0.158232501	0.12382308	-1.277891815	0.201287575	0.45009899
192.5036855		-0.178466177	0.110809878	-1.61056198	0.107275226	0.313490711
311.9098736		-0.047997123	0.132890758	-0.361177281	0.717966924	0.867400496
51.00987726		0.49797013	0.179697659	2.77115536	0.005585777	0.051082988
2412.799807		-0.201064425	0.051360336	-3.914780185	9.04866E-05	0.003071844
199.6015212		-0.115023389	0.147280065	-0.780984099	0.434811847	0.678647416
353.4117364		0.186274346	0.12835757	1.451214338	0.146720186	0.377301097
304.7739968		-0.041987413	0.11100335	-0.378253569	0.705242234	0.860311053
123.5322677		-0.05031528	0.141190956	-0.356363338	0.721568482	0.869608119
373.6559846		0.093861365	0.078643019	1.19351173	0.232669021	0.487988891
24.67951136		0.142860897	0.208653192	0.684681102	0.493545177	0.721816725
7.560533325		-0.26364163	0.68390177	-0.385496341	0.699869717	0.857032569
1954.003093		0.162294395	0.064281017	2.524763941	0.011577603	0.08157373
20.59609825		0.747636345	0.263364578	2.838788532	0.004528516	0.044613845
6.21573783		-0.08950528	0.441952603	-0.202522352	0.839508386	0.931066348
957.4138089		0.201511949	0.12303637	1.63782424	0.101458363	0.305133839
19.54899949		-0.420281623	0.271270917	-1.549305868	0.12130821	0.337939248
553.5526932		-0.347220917	0.087229566	-3.980541622	6.87584E-05	0.002496313
156.8987006		0.007867524	0.113073927	0.069578584	0.944529083	0.975750616
2.59831144		-0.890616908	0.604421805	-1.47350228	0.140615649	0.368367437
208.3225562		-0.028347747	0.096203224	-0.294665246	0.768249615	0.892934503
77.3251721		-0.44293633	0.509136843	-0.86997501	0.384314061	0.635384466
19.48736153		0.11603393	0.323175209	0.359043415	0.719562611	0.868512899
707.8009975		0.362173572	0.106579955	3.398139675	0.000678458	0.012765997
933.391561		0.016225377	0.186284365	0.087100047	0.930591988	0.970891022
2075.827421		0.161012056	0.126351241	1.274321128	0.202549632	0.45153974
35.26008361		0.043429349	0.204423288	0.212448145	0.831757428	0.928693708
269.1811937		-0.162820789	0.095009138	-1.713738198	0.086576818	0.277910523
1283.743187		-0.175219483	0.152276522	-1.150666436	0.24986949	0.505794379
240.0240429		-0.008747611	0.075899035	-0.115253253	0.90824439	0.960978931
5.596282472		0.532037008	0.454622565	1.170282887	0.241887146	0.497476791
4221.821606		-0.108535169	0.130691442	-0.830468829	0.406273765	0.654881575
330.0643086		0.057007596	0.090879318	0.627288994	0.530469816	0.750628072
1595.524949		0.027878553	0.065076993	0.428393388	0.668364738	0.839214838
1229.857647		0.18765198	0.211135496	0.888775138	0.37412394	0.627635028
116.9348352		0.065412428	0.115237062	0.567633596	0.57028379	0.777227229
808.3257908		-0.29805385	0.07686424	-3.877665983	0.000105463	0.00348229
129.3160217		-0.062001343	0.229491066	-0.270168875	0.787030337	0.903033152

224.8543047	-0.261313989	0.116245702	-2.247945388	0.024579672	0.129844252
413.5759263	0.094982931	0.10279337	0.924018064	0.355476911	0.612138993
160.0918323	0.002210387	0.119991531	0.018421189	0.985302849	0.992070995
54.53176974	0.040439431	0.237618129	0.17018664	0.864863358	0.942899493
1519.027572	-0.017234307	0.165539053	-0.10411022	0.91708188	0.96494782
1491.37912	0.177401461	0.171907732	1.031957425	0.302092063	0.560711469
4.723003525	0.786404503	0.948002449	0.829538472	0.406799777	0.655071986
496.0886864	0.074827317	0.064702635	1.156480209	0.24748479	0.502785584
5.155174889	0.113950401	0.614312497	0.185492565	0.852842711	0.936643758
222.0201485	-0.062398689	0.120710308	-0.51692925	0.605205548	0.799666707
251.2082331	0.227619242	0.197618235	1.151812949	0.249397946	0.505173367
32.35559728	0.430435382	0.320310891	1.343805015	0.179011448	0.423878692
77.54160397	0.397570375	0.229395454	1.733122289	0.083073935	0.271557233
27.90368201	0.171029254	0.1738155	0.983970099	0.325130215	0.583983313
587.6216404	-0.037888373	0.086074773	-0.440179758	0.659806919	0.834290513
54.46451574	0.488625742	0.238683532	2.047169903	0.040641405	0.176829885
3.648155387	0.208505398	0.536256458	0.388816573	0.69741184	0.855416228
23.03808047	-0.807983263	0.26855759	-3.00860334	0.002624515	0.03154245
88.57009678	-0.003419117	0.173705303	-0.019683433	0.984295906	0.991566382
1969.727958	-0.445073851	0.112947247	-3.940546258	8.12963E-05	0.002839757
289.1011822	-0.129764649	0.083859816	-1.547399635	0.121766909	0.338615639
73.23372191	0.254932183	0.356454636	0.715188294	0.474492702	0.707610871
19.27567467	-0.430946261	0.349082451	-1.234511389	0.217012405	0.469594725
215.6075351	-0.217981641	0.133936681	-1.627497712	0.103631434	0.308358075
737.7017949	-0.274238331	0.140586058	-1.950679425	0.051095192	0.202163723
3412.022596	-0.1177481	0.0745887	-1.578631873	0.11442052	0.325525707
65.97054471	0.105298749	0.161294246	0.652836362	0.513861779	0.738414866
96.67570152	-0.046194409	0.139013453	-0.332301719	0.739661442	0.879251937
646.909294	-0.195677367	0.132772783	-1.473776197	0.140541859	0.368270499
54.4272471	-0.034468379	0.227423461	-0.151560348	0.87953371	0.950338711
44.28223887	0.38059852	0.364906366	1.043003235	0.296946795	0.555412298
70.15043958	-0.233231835	0.281375781	-0.828898047	0.407162101	0.655228755
3.300165688	-0.307993389	0.657084536	-0.468727192	0.639264648	0.821941899
4.226503513	-0.048113463	0.518459155	-0.092800874	0.926061757	0.968928443
66.93495793	-0.11538771	0.167479249	-0.688967206	0.490843905	0.720068699
28.10070171	-0.464396502	0.21848308	-2.125549043	0.033540826	0.15677578
40.37296855	0.190950103	0.234483732	0.814342647	0.415448685	0.661679388
14.91421956	-0.079903847	0.337138698	-0.23700586	0.812652244	0.917150306
644.1418186	-0.012031181	0.157564376	-0.076357241	0.939134887	0.974295153
11.84319751	1.457964537	0.36851737	3.95629801	7.61202E-05	0.002682852
70.72611346	-0.135387729	0.128039596	-1.057389531	0.290333848	0.549818409
466.0533508	0.249257507	0.225408122	1.105805349	0.268810779	0.527663112
23.37239934	0.33801711	0.418115149	0.808430671	0.418842702	0.663816586
517.668395	0.27125638	0.147719685	1.836291351	0.066314578	0.238331312
12.94387125	0.354001321	0.252286666	1.403170953	0.160565867	0.398467573
19.45006783	0.647329413	0.350403633	1.847382136	0.064691776	0.23432467
10.59366181	0.718902143	0.36305476	1.980147963	0.047686905	0.194517924
352.7674831	-0.0638669	0.099017731	-0.645004689	0.518924147	0.742203467

253.5342295	-0.595077303	0.189498522	-3.140274113	0.001687898	0.023835335
15.57324787	0.842194071	0.318861632	2.641252466	0.008260014	0.065995956
1059.398788	-0.235579147	0.113572627	-2.074259904	0.03805518	0.169824595
5.217186773	0.86858304	0.575652158	1.50886786	0.131332555	0.353920926
48.52762376	0.190700232	0.152287783	1.252235924	0.21048391	0.461904245
3.940917908	-0.050133867	0.587314252	-0.08536123	0.931974215	0.971363874
3.026529351	0.135289636	0.699141205	0.193508314	0.846560895	0.933717375
22.8650067	0.120663944	0.224424759	0.537658789	0.590812636	0.790746649
61.96985537	-0.189425578	0.149414981	-1.267781694	0.204875921	0.454803778
123.7766095	-0.035709981	0.098985411	-0.360760038	0.718278839	0.867576843
176.1549901	0.663946078	0.446750677	1.486166919	0.137234975	0.36307079
1423.998207	-0.163536853	0.25769208	-0.634621182	0.52567551	0.747140435
30247.01461	0.415471102	0.373814231	1.11143736	0.266380139	0.525013602
101733.2545	0.687129427	0.29343518	2.341673641	0.019197494	0.111869291
626447.7277	-0.249857869	0.17134849	-1.458185416	0.144789443	0.374863969
719.0764417	0.036602613	0.156702843	0.233579763	0.81531124	0.918696852
50.57327914	2.27614336	0.38290454	5.944414658	2.77446E-09	7.77048E-07
525.8943161	-0.952087342	0.336827249	-2.826633965	0.004704006	0.045730975
6.028194922	1.176118569	0.494798063	2.376966803	0.017455659	0.105949906
2007.568336	0.350432137	0.193540513	1.810639702	0.070196642	0.245766866
7.659860331	0.553656171	0.367804557	1.50529992	0.132246986	0.355061731
172.3057077	-0.051082061	0.120329531	-0.42451808	0.671188022	0.841252436
386.3572219	0.268724181	0.215185974	1.248799701	0.211738343	0.462972839
16.21352818	-0.217858596	0.27706772	-0.786300894	0.431691229	0.676575305
67.76982756	0.149754469	0.270741001	0.55312815	0.580175662	0.783198895
6.501621745	0.679778317	0.514444968	1.321381994	0.186374027	0.432345842
683.4049242	0.349250876	0.200942716	1.738061889	0.082199909	0.269712004
787.9847468	-0.069075178	0.10086695	-0.68481478	0.493460807	0.721760636
4.574326188	1.361512548	0.621500866	2.190684875	0.028474604	0.142405574
239.1645339	0.599762774	0.149796627	4.003846991	6.23207E-05	0.00236096
61.86303125	-0.098371695	0.243503868	-0.403984119	0.686224397	0.849533779
115.0531831	-0.23602582	0.232859922	-1.013595717	0.310775702	0.570315647
328.7780782	0.195336358	0.230846795	0.846173142	0.397456162	0.647859328
216.0507865	0.157349699	0.1373548	1.145571173	0.251972631	0.508197352
6.123626517	0.569499178	1.060759575	0.536878659	0.591351433	0.79088016
249.945481	0.210920244	0.157638464	1.33799987	0.180896479	0.426147734
15.62693742	-0.311898915	0.26825215	-1.162707976	0.244948004	0.500229753
515.4659668	0.042294113	0.102099128	0.414245586	0.67869426	0.845876523
1739.300485	0.299794368	0.276063046	1.085963415	0.277495179	0.535780415
48.63468195	0.45458976	0.248904023	1.826365661	0.067795201	0.241165781
149.7808402	-0.209394027	0.083524726	-2.506970526	0.012177087	0.084788555
2.950209676	-0.541413636	0.600039995	-0.902295914	0.366899695	0.622563077
3.340062775	-0.689968316	1.011053209	-0.682425327	0.494970046	0.722843347
291.078766	0.537964635	0.180979333	2.972519716	0.002953662	0.03403765
67.3989342	-0.239134299	0.139137596	-1.71868931	0.08567096	0.276758667
112.8464773	-0.21062821	0.153993571	-1.367772753	0.171383219	0.413280198
67.4435662	0.399676599	0.133707532	2.989185373	0.002797224	0.032873837
966.187783	-0.016071973	0.095829956	-0.167713454	0.866808711	0.943902508

8.762625131	0.53306441	0.426287728	1.250480311	0.211124143	0.462597243
14.53054718	0.831921456	0.342887076	2.426225762	0.015256776	0.09847213
61.88606317	0.093365425	0.17706613	0.52729127	0.597991335	0.794956442
185.7634489	0.072222183	0.152199438	0.474523323	0.63512677	0.818918798
2487.398079	-0.648731976	0.216484747	-2.996663668	0.002729517	0.032370421
28.3940215	0.057899211	0.215058701	0.26922515	0.787756428	0.903200814
1762.198484	0.293781891	0.154093571	1.906516216	0.056583264	0.215820017
3.447061809	0.268322392	0.619920988	0.432833211	0.665135944	0.836844909
93.70623702	-0.230976391	0.197106836	-1.171833489	0.241263914	0.49659462
91.61741499	-1.079902479	0.498929495	-2.164439043	0.03043067	0.148503155
938.5578729	-0.134684038	0.117137025	-1.149799025	0.250226657	0.50626353
398.5617366	-0.028154747	0.057249633	-0.491789122	0.622868427	0.810950402
5.759796069	1.061362986	0.413471074	2.566958255	0.010259496	0.075936734
39.34750803	0.257340575	0.191049663	1.346982619	0.177985831	0.422383079
1503.681153	-0.063903943	0.058493512	-1.092496249	0.274615038	0.533712796
1138.096072	0.199171406	0.108129339	1.841973768	0.065478987	0.236351047
388.3505515	-0.107051	0.156798122	-0.682731391	0.49477659	0.722813893
75.1615243	0.821018526	0.256173253	3.204934615	0.001350932	0.020534482
128.3764019	0.689959751	0.219623787	3.141552924	0.001680544	0.023767049
257.5457614	0.020882121	0.176900215	0.118044632	0.906032294	0.960133527
126.6181922	0.046748535	0.162576684	0.2875476	0.77369306	0.896159371
5.423076016	0.345874891	0.447465991	0.772963528	0.439543966	0.681475638
45.77239818	0.031179092	0.249662964	0.12488473	0.900614807	0.95813193
12.29412604	-0.095130198	0.381127841	-0.249601806	0.802895302	0.910923749
6.82985209	0.015902856	0.453925917	0.035034035	0.972052602	0.986391887
727.8343428	0.093064269	0.09016688	1.032133626	0.302009523	0.560690976
294.9967704	0.055978398	0.100705461	0.555862579	0.578304801	0.782042809
2782.239465	-0.132285147	0.073019341	-1.811645308	0.070041019	0.245589839
161.4710369	0.491264683	0.20862474	2.354776728	0.018533838	0.109444546
5753.765595	0.177918288	0.159150822	1.117922514	0.263600088	0.521277253
2.88431888	0.123327884	0.601365771	0.205079653	0.837509893	0.930780365
209.5917471	-0.192070202	0.077152985	-2.489472078	0.012793297	0.087543659
1453.842866	0.112352581	0.131842002	0.852175928	0.394116455	0.644559174
1007.327493	-0.280345016	0.093789686	-2.989081508	0.002798175	0.032873837
195.9314908	0.070883715	0.104480214	0.678441512	0.497491796	0.72427934
127.4574869	0.483195791	0.121760176	3.96842226	7.23501E-05	0.00257895
3943.509104	0.205622457	0.157937943	1.301919304	0.192943973	0.440314141
3.287094995	0.819174662	0.627037068	1.30642143	0.191409282	0.438769831
94.1231582	-0.184639704	0.116160386	-1.589523851	0.111942174	0.322028806
2.735850492	-0.680328028	0.812977383	-0.836835123	0.402685252	0.652313807
63.92546998	-0.617998422	0.221722384	-2.787262204	0.005315545	0.049535953
96.96739804	-0.73798603	0.413938457	-1.782839978	0.074612356	0.25468527
10.76196194	1.387183193	0.385102705	3.602112304	0.000315642	0.007601441
20.07990075	0.646027693	0.284693357	2.269205363	0.02325584	0.126040739
151.3998264	1.560534909	0.290597726	5.370086447	7.86989E-08	1.24678E-05
151.3479371	0.298171222	0.134125427	2.223077521	0.026210573	0.13504817
347.7770187	-0.077769452	0.094658824	-0.821576363	0.411318047	0.65849534
72.47310543	0.976369361	0.373809809	2.611941523	0.009002965	0.069922782

3.841521346	0.622351653	0.866913047	0.717893974	0.472822664	0.706665776
431.8225895	0.236345541	0.085732068	2.756792725	0.005837134	0.052682793
110.0718619	-0.037999883	0.31540684	-0.120478943	0.904103759	0.959402122
35.93713941	-0.494231815	0.275058834	-1.796822187	0.072363853	0.249990015
23.60694816	-0.008288842	0.378439486	-0.021902689	0.98252558	0.991056093
6.100093531	1.363950132	0.544455576	2.505163307	0.012239487	0.085090474
1087.73307	0.061658892	0.15012196	0.410725337	0.681273946	0.847216981
222.9725938	-0.093076473	0.08594652	-1.082958019	0.278827058	0.5373708
645.8899077	0.076851793	0.062807696	1.223604712	0.221101381	0.474384961
185.3009931	0.799151101	0.282125283	2.83261072	0.004616957	0.045088642
33.16299018	0.907961807	0.241392387	3.761352294	0.000168997	0.004845616
89.80994995	0.074607486	0.199100063	0.374723568	0.707866063	0.861889372
3127.955591	0.126048173	0.179532101	0.702092677	0.482621369	0.714303947
358.7718975	0.207935354	0.072890929	2.852691793	0.004335064	0.043389375
1638.102365	0.039347577	0.104733139	0.375693664	0.707144649	0.861693471
53464.05618	0.02466543	0.200548511	0.122989846	0.902115139	0.958662553
495.7007336	-0.103619619	0.126117153	-0.821614005	0.411296616	0.65849534
868.8222213	-0.077823765	0.069305259	-1.122912836	0.261474516	0.519306864
42.01788753	-0.005624022	0.166297874	-0.033818962	0.973021515	0.986800481
14765.35731	0.275025559	0.165597919	1.660803231	0.096752972	0.296400619
187.3339438	0.093009401	0.134390678	0.692082236	0.488885687	0.718842604
847.9964255	-0.159144181	0.092707521	-1.716626426	0.086047451	0.277180856
462.630185	0.034145961	0.073237359	0.46623693	0.641045918	0.82343687
61.8137102	-0.019419051	0.175278462	-0.110789715	0.911783101	0.962232976
641.3867817	-0.053809296	0.098771019	-0.544788302	0.585899125	0.78675016
236.9462659	-0.054007508	0.087514747	-0.617124657	0.537152507	0.754901426
172.8296268	-0.280296197	0.098349036	-2.850014687	0.004371721	0.043534014
81.86862799	0.076711833	0.171306059	0.447805718	0.654293416	0.830926149
4.049011104	1.67761275	0.575543502	2.914832229	0.003558798	0.03817454
7.595413904	0.409991274	0.774941822	0.529060715	0.596763332	0.794156453
43.27005571	0.451269023	0.20769485	2.172750182	0.029799125	0.146590087
4.707172926	2.293827822	0.786005475	2.918335681	0.003519053	0.037855162
142.7416441	0.994710795	0.251185187	3.960069492	7.4928E-05	0.002646779
63.22590661	0.222861905	0.142623144	1.562592848	0.118148392	0.332622397
174.2473267	-0.194768679	0.102664737	-1.89713318	0.057810363	0.218915758
50.13166054	-0.14167362	0.167751214	-0.84454602	0.398364359	0.648613091
12.03883354	0.034366003	0.326332541	0.105309764	0.916130016	0.964528641
39.65612964	0.389971105	0.296533362	1.315100271	0.188476206	0.435868595
25.53060748	-0.28568988	0.237816626	-1.201303228	0.229633599	0.485060385
10.26787907	-0.77855928	0.344806831	-2.257957817	0.023948289	0.128061702
3904.659974	-0.120742416	0.162267079	-0.744096814	0.456817906	0.693937939
7.563089652	-0.610698116	0.557073681	-1.096260938	0.272964599	0.531855456
1361.922317	0.126891465	0.089565957	1.416737665	0.156559641	0.392186776
841.2064713	0.289671854	0.165883683	1.746234769	0.080770166	0.266470189
15.93676547	1.674942888	0.396582109	4.223445414	2.40596E-05	0.001136597
1534.245157	-0.26777943	0.12636467	-2.119100459	0.034081978	0.158148444
472.6723466	-0.043146061	0.157832016	-0.273366978	0.784571134	0.901845062
154.6844639	-0.489830661	0.172396438	-2.841303834	0.004492948	0.044347584

1010.024443	0.058996124	0.049029378	1.203281084	0.228867567	0.483898479
403.1852708	1.11899193	0.347991514	3.215572468	0.001301846	0.019959097
16.51205113	0.220066907	0.600794885	0.36629291	0.714146519	0.864524493
20.06011996	0.431411526	0.356347526	1.210648296	0.226030226	0.480315376
198.0645007	-0.010917508	0.116442771	-0.093758574	0.92530094	0.96872171
11.02957061	1.405351491	0.704505337	1.994806026	0.04606404	0.190624908
8.925502941	1.767702581	0.811972126	2.177048356	0.02947696	0.145474083
104.0934488	0.019426957	0.171406613	0.113338433	0.909762249	0.9616745
566.2673928	-0.112563784	0.137764337	-0.817074911	0.41388562	0.660498785
41.67424617	0.208623184	0.273085126	0.763949274	0.444897472	0.685379023
113.5704114	-0.014656953	0.178716728	-0.082012208	0.934637006	0.972907108
33.76451634	-0.018031324	0.260254013	-0.06928356	0.944763912	0.975797539
14.5659983	0.02665272	0.350211595	0.07610462	0.939335864	0.974295153
68.12406345	-0.936261308	0.160645623	-5.828115898	5.60566E-09	1.44102E-06
278.2002228	0.81780699	0.176164155	4.642300766	3.44551E-06	0.000252076
7.880756564	-0.089291961	0.595951456	-0.14983093	0.880898006	0.950743683
3609.58352	0.274437079	0.114383964	2.399261841	0.016428163	0.102164672
3024.853295	-0.209780521	0.102408807	-2.048461711	0.04051478	0.176509392
175.9243035	-0.514594447	0.075780308	-6.790609061	1.11661E-11	9.21733E-09
312.9064843	0.359299305	0.095335975	3.768769416	0.000164054	0.004773708
108.6944643	0.100680718	0.108783707	0.925512842	0.354699207	0.611365136
290.9026167	-0.210704164	0.108340181	-1.944838586	0.051794408	0.204157703
3.841365285	-0.864943172	0.67725876	-1.277123637	0.201558601	0.450512341
115.4678481	-0.363336147	0.163068351	-2.228121791	0.025872395	0.13409869
600.9270727	0.091442715	0.068427002	1.33635424	0.181433516	0.426968393
209.7161597	0.600561889	0.136671954	4.394185276	1.11189E-05	0.000629562
55.64002179	-0.103286233	0.13356507	-0.773302726	0.439343243	0.681440998
74.23382848	-0.010870864	0.123909789	-0.087732084	0.930089618	0.970625163
78.79408469	-0.250845787	0.362662979	-0.691677402	0.489139941	0.719061847
906.6184984	0.00262364	0.137586491	0.019069025	0.984786041	0.991899255
111.5783506	0.065491273	0.130470262	0.501963221	0.615693393	0.80702296
543.8928084	-0.270267009	0.292797433	-0.923051159	0.355980546	0.612269826
4.245065207	0.438825328	1.010469084	0.434278826	0.664085978	0.836652459
324.073922	0.126953138	0.146120158	0.868827001	0.384941762	0.636019781
287.5060974	-0.285599789	0.120542933	-2.369278582	0.017822823	0.107224073
20.20083805	0.063844931	0.2459983	0.259534035	0.795223225	0.905927111
3685.038138	-0.121845229	0.05438447	-2.240441578	0.02506227	0.131416517
364.9007971	0.030159684	0.137000945	0.220142163	0.825760439	0.924823388
130.7551824	0.423330567	0.118908335	3.560142068	0.000370654	0.00851148
479.821973	-0.082791632	0.19548862	-0.423511262	0.671922284	0.841900389
1632.907721	0.06993817	0.146056396	0.478843597	0.632049899	0.816899103
244.9589136	0.175911832	0.14727864	1.194415105	0.232315631	0.487769526
418.010656	-0.042043278	0.093534084	-0.449496872	0.653073264	0.830317856
1329.296073	0.066251015	0.147629927	0.448764124	0.653601822	0.8307205
1176.158168	-0.424332159	0.051504764	-8.238697291	1.74096E-16	6.8263E-13
4475.372081	-0.052020173	0.125537484	-0.414379608	0.678596121	0.845876523
32.78841978	-0.313505652	0.373826607	-0.838639215	0.401671798	0.651211544
135.7650763	-0.070242857	0.166563187	-0.421718978	0.673230151	0.842875465

222.5527667	0.714288642	0.210943708	3.386157613	0.000708787	0.013120652
352.4617726	-0.033002141	0.079001958	-0.417738268	0.676138489	0.84484991
11.62719559	0.192168675	0.293089063	0.655666483	0.512038739	0.736907284
84.91674095	0.074266586	0.130250766	0.570181573	0.568554553	0.775709712
256.765855	-0.056294963	0.109574359	-0.513760366	0.607419541	0.801442647
131.1998258	0.155939407	0.178835075	0.871973279	0.383222955	0.634886324
622.276395	0.04094602	0.111678213	0.366642866	0.713885429	0.864388407
266.4023641	-0.218724399	0.150467127	-1.453635773	0.146047313	0.376249353
289.948312	0.491195061	0.183325876	2.679354778	0.007376419	0.061834184
360.8374988	-0.50943087	0.630527876	-0.807943454	0.419123135	0.664126818
353.6408632	-0.161169936	0.073674741	-2.187587409	0.02869967	0.143124204
1744.574503	-0.401491429	0.112992621	-3.553253516	0.000380498	0.008654984
294.6361551	-0.007087217	0.100275117	-0.070677727	0.943654248	0.975177784
27.06115709	-0.279626022	0.435714432	-0.641764426	0.521026151	0.743835259
116.4625581	0.078795076	0.107526897	0.732794099	0.463684021	0.699296945
8.883566935	0.271984861	0.353477903	0.769453646	0.441624052	0.682858945
1903.232875	-0.14073208	0.115919904	-1.214045865	0.224730211	0.479025363
1362.80959	0.209727643	0.165293381	1.26882057	0.204505067	0.454378449
237.312286	0.323510875	0.155953302	2.074408629	0.038041377	0.169824595
747.4592637	0.324299086	0.074156471	4.373173127	1.22454E-05	0.000673881
84.22932194	0.502212937	0.198276623	2.532890302	0.011312635	0.08042945
64.58774798	-0.325882969	0.426611089	-0.763887712	0.444934161	0.685379023
3018.041548	-0.206753731	0.067244063	-3.074676359	0.002107311	0.0274901
1209.030987	-0.235244183	0.068770824	-3.420697456	0.000624608	0.012124193
8.27500407	0.028486162	0.347045082	0.082082021	0.93458149	0.972907108
4315.669447	0.039036666	0.095534614	0.4086128	0.682823836	0.847799955
6.933688921	0.358079762	0.372016679	0.962536851	0.335780001	0.59439882
448.0684165	0.254088261	0.087953997	2.888876789	0.003866205	0.04045803
25.58744852	0.445338567	0.262849062	1.694274894	0.090213059	0.284688454
1048.337523	0.433678136	0.084685062	5.121070084	3.03807E-07	3.93794E-05
296.8519962	0.23349653	0.114912082	2.031958054	0.042157903	0.180164726
170.115207	0.056824711	0.122651899	0.4633007	0.643148844	0.824387858
83.6236329	0.230826016	0.184557007	1.250703072	0.211042829	0.462548314
21.97393496	0.799228971	0.408428172	1.956840947	0.050366171	0.200645931
35.57809448	0.108869743	0.26332707	0.413439236	0.679284833	0.845954666
1275.786675	-0.119750252	0.07159921	-1.672507995	0.094424193	0.29198522
3.017979531	0.073858111	0.54032586	0.136691794	0.891274416	0.954379567
100.3171528	-0.239458925	0.234105707	-1.02286667	0.30637089	0.565441403
26.65031648	0.051900466	0.27604568	0.188014049	0.850865638	0.935636028
1251.717503	-0.109606983	0.053598	-2.044982718	0.040856562	0.177161824
314.5422686	-0.145708959	0.11785768	-1.236312803	0.216342314	0.469025809
8084.071344	-0.171110637	0.109942044	-1.556371249	0.119619838	0.334841609
13.10533367	0.546668101	0.297054103	1.840298102	0.065724484	0.236916296
30.66646186	-0.454424814	0.383916895	-1.183654117	0.236550009	0.492049116
4.645462601	-0.113460029	0.627531308	-0.180803774	0.856521601	0.938762039
205.501555	-0.090890094	0.088298105	-1.029354982	0.303312896	0.561847107
56.49169503	0.340852232	0.355928224	0.957643168	0.338242724	0.596670665
11.98586402	-1.150844285	0.350787864	-3.280741446	0.001035346	0.017003526

7.395578053	0.192337635	0.369764896	0.520161964	0.602950693	0.798626949
166.3393457	0.554266528	0.135668214	4.085456075	4.39903E-05	0.001832455
98.78454567	-0.125178477	0.109045923	-1.147942746	0.2509922	0.507158164
1095.890819	0.111516372	0.135088166	0.825508081	0.409083196	0.656912138
988.3817049	0.088239896	0.088408542	0.998092422	0.318234544	0.576493309
90.68869715	-0.132733326	0.126437185	-1.049796586	0.293811645	0.553105021
28.07096224	0.21950277	0.284702505	0.770989951	0.440712888	0.682478371
226.4196679	-0.236352364	0.084153018	-2.808602344	0.004975706	0.047325033
115.9536412	-0.101461925	0.120083745	-0.844926386	0.398151941	0.648386984
231.8511073	-0.058283364	0.140693542	-0.414257566	0.678685487	0.845876523
56.07165824	0.692513666	0.157097122	4.408188102	1.04239E-05	0.000592349
119.9011968	0.190755984	0.290854267	0.655847296	0.511922382	0.736875059
1996.197829	0.16218698	0.112637173	1.439906352	0.149893896	0.382866618
110.8703653	0.214509351	0.149522088	1.434633194	0.15139165	0.385021347
170.8974247	0.193659923	0.265067334	0.730606521	0.465019528	0.700611554
1147.932697	-0.112393923	0.082195558	-1.367396559	0.17150104	0.413437184
17.96698529	0.121619258	0.233100445	0.521746145	0.601847091	0.797917987
27.06253868	0.124505215	0.182767176	0.681223064	0.495730357	0.723170992
45.53570595	-0.554850197	0.30672327	-1.808960229	0.070457182	0.246268911
28.81727295	0.920699883	0.523605767	1.75838377	0.078682233	0.262452603
3.999245696	0.664793913	0.588537126	1.129570053	0.258657436	0.515570067
14.5223975	-0.263627107	0.415767975	-0.634072663	0.526033402	0.747513625
567.5870631	-0.196880077	0.09833082	-2.002221447	0.04526092	0.188634008
415.3832833	0.011458485	0.078871409	0.145280588	0.884489341	0.9521814
114.548442	0.466113056	0.287195674	1.622980767	0.104593516	0.30982542
71.96057318	0.601130345	0.279267126	2.152528133	0.031355782	0.150854014
138.3714609	0.01367998	0.169887855	0.080523589	0.935820836	0.973367862
90.0420836	-0.335077874	0.345389914	-0.970143772	0.331974833	0.590796923
35.07293933	0.162873852	0.204766044	0.795414361	0.426372509	0.671340873
770.0505236	-0.157853543	0.092000826	-1.715783973	0.086201589	0.277313494
149.0666745	-0.079492278	0.18706167	-0.424952251	0.670871483	0.841015774
999.3286554	-0.172100879	0.136547316	-1.260375405	0.20753397	0.457800672
836.8812816	-0.143653142	0.091265916	-1.574006474	0.115485953	0.327655876
1159.394459	-0.141970757	0.099324849	-1.429357897	0.152901393	0.386853597
16.72121619	0.360257242	0.373395226	0.964814806	0.334637578	0.592978848
195.9011872	0.586729445	0.202002215	2.904569371	0.003677587	0.039104593
6.302393225	0.793567379	0.484996447	1.636233386	0.101790752	0.305570205
28.33591775	-0.344611966	0.495243642	-0.695843291	0.486526974	0.717237435
4.861076161	-0.516395221	0.723758364	-0.713491196	0.475541859	0.708388598
8.178334674	-0.0266585	0.574599932	-0.046394889	0.96299551	0.98274378
10.08966505	-0.500836479	0.698275514	-0.717247662	0.473221295	0.706757551
180.4194572	-1.05996615	0.228570136	-4.637378133	3.52857E-06	0.000255032
2.969916559	0.707453648	0.950842416	0.744028281	0.456859366	0.693937939
147.5397155	-3.123263531	0.991000976	-3.151625081	0.001623646	0.023255949
3.965948911	0.232232723	1.072445257	0.216545061	0.828562905	0.926506531
6.125323619	0.465986854	0.430889038	1.081454417	0.279495027	0.537667116
152.771609	0.028042734	0.113592936	0.246870406	0.80500852	0.912199526
9.286087346	0.06282648	0.384211585	0.163520525	0.870108614	0.945862776

496.9312221	0.01831834	0.096273264	0.190274427	0.849094092	0.934802172
209.6066621	0.188371329	0.159629086	1.180056427	0.237977773	0.493559008
141.9989834	0.046974587	0.155756202	0.301590474	0.762964271	0.891042078
4.849404203	0.930628311	0.531068399	1.752369965	0.079710213	0.264363496
83.53990967	0.058532458	0.127850154	0.457820784	0.647081203	0.826964966
3026.515356	-0.206295957	0.159497984	-1.293407932	0.195870008	0.444015373
7.255253257	-0.170226668	0.389258488	-0.4373101	0.661886472	0.835495165
652.5667961	-0.344328128	0.249038056	-1.382632571	0.166777557	0.406609306
11.51277057	0.598115682	0.439347643	1.361372231	0.173396087	0.416286449
41.76983738	-0.137059162	0.457499857	-0.299582961	0.764495283	0.891638188
7.517178571	0.705630158	0.55738967	1.265954855	0.205529245	0.455428184
73.80011991	0.182299095	0.634323792	0.28739123	0.773812776	0.896159371
7.884264846	2.02051659	0.864266566	2.337839585	0.019395571	0.112494686
23.21561762	2.030283747	0.853581681	2.378546533	0.017381043	0.105701543
21.78511102	0.511850376	0.330149094	1.55036129	0.121054824	0.337297995
17.57877631	-0.402300343	0.748303129	-0.537616812	0.590841621	0.790746649
3.289882894	4.977829108	2.980177111	1.670313181	0.094857417	0.292781203
12.46365291	0.461875603	0.326704073	1.413743022	0.157437369	0.393838828
13.85647236	0.02652508	0.341989163	0.077561171	0.93817713	0.974203529
135.233964	-0.26935648	0.102478888	-2.628409475	0.008578518	0.067815264
952.4753976	-0.074059869	0.071412698	-1.037068638	0.29970387	0.558061914
8.223722579	1.824052171	0.622775708	2.928907066	0.003401561	0.037074416
26.40708364	0.608555419	0.308629028	1.971802274	0.048632183	0.196417802
63.15089088	0.128174548	0.25551006	0.501641884	0.615919451	0.80702296
227.8146851	0.035141436	0.089289363	0.393567999	0.693900012	0.85351767
4.539892534	-0.194209322	0.494455371	-0.39277422	0.694486247	0.853764093
58.3188135	-0.612323201	0.362533143	-1.689013026	0.091216934	0.286129277
112.970685	-0.576283727	0.461007803	-1.250052002	0.211280552	0.462721491
16.95663767	-0.758671562	0.579611918	-1.30893023	0.190557986	0.438182526
14.16122248	2.278558066	0.869587524	2.620274558	0.0087859	0.068864595
325.2633507	-0.070264254	0.068807631	-1.021169497	0.307174141	0.566057952
230.8816925	-0.152031231	0.096215162	-1.580117193	0.114080031	0.325255626
1312.615245	-0.278150864	0.088308718	-3.149755421	0.001634072	0.023298895
55.45537556	-1.056286483	0.393175381	-2.686552955	0.00721935	0.061072433
193.6067108	-0.471886326	0.121736873	-3.876280991	0.000106065	0.003489529
727.6600941	-0.606539303	0.218999515	-2.769591994	0.005612655	0.051207287
136.3003174	0.186491987	0.099998514	1.864947592	0.062188751	0.228700076
98.14752487	0.398327039	0.180944305	2.201379247	0.027709187	0.140182155
348.1030764	0.464561786	0.150055959	3.095923603	0.001962009	0.026144562
7.671724452	0.154222616	0.336776111	0.457938109	0.646996907	0.826964966
678.2272752	0.134776795	0.056120639	2.401554895	0.016325559	0.101910875
19.67119911	0.582998963	0.241162067	2.417457152	0.015629374	0.099687313
66.38306618	-0.300466007	0.132228844	-2.272318183	0.023067299	0.125429377
72.98479506	-0.098470251	0.158006471	-0.623203912	0.533150532	0.752219683
85.24766851	-0.153743689	0.153106891	-1.004159173	0.315301897	0.57422143
62.78544282	-0.00838551	0.275204583	-0.030470094	0.975692144	0.988070826
507.8610496	-0.036603737	0.156578313	-0.233772711	0.815161436	0.918660053
220.5053756	0.101721008	0.089364134	1.138275535	0.255005446	0.512035005

4.687289343	-0.703599365	0.45181512	-1.55727273	0.119405747	0.33448111
125.1765412	0.315379677	0.136811161	2.305218921	0.021154316	0.118602989
559.7238774	-0.119898391	0.092386995	-1.297784285	0.194361471	0.442176576
48.01795407	-0.021072672	0.14676677	-0.14357931	0.885832679	0.952712045
8.666278087	-0.179416376	0.343814047	-0.521841319	0.601780818	0.797917646
15.82824809	0.360851033	0.310573053	1.161887771	0.245281055	0.500388666
79.84441369	1.244938285	0.201904384	6.16597946	7.00482E-10	2.6796E-07
15.65130031	0.274644957	0.386612633	0.710387954	0.477463591	0.709767761
5.447423518	0.230766579	0.750641435	0.307425847	0.758519257	0.888802094
139.6447785	0.049368351	0.244794311	0.201672789	0.840172537	0.931255553
132.3168688	-0.11680315	0.129993866	-0.898528164	0.368904037	0.624001322
1794.025913	-0.33570168	0.059584016	-5.634089491	1.75986E-08	3.72994E-06
359.1397565	-0.513207468	0.118714307	-4.323046476	1.53889E-05	0.000807224
621.2849777	-0.300898725	0.08832447	-3.406742502	0.000657431	0.012528737
101.6684428	-0.577728732	0.161937842	-3.567595592	0.000360272	0.008321805
728.6265541	-0.263524022	0.081074765	-3.25038281	0.001152498	0.018295316
6.589766022	-0.02039187	0.41827073	-0.048752802	0.961116296	0.98222115
166.6260734	0.145890476	0.12964825	1.125279174	0.260470752	0.517881447
3845.547265	-0.273225389	0.073032089	-3.741169045	0.000183166	0.005102628
273.6910845	-0.157313655	0.096267218	-1.634135258	0.102230453	0.306383729
229.2386598	-0.237237861	0.168498967	-1.407948461	0.159146332	0.396513275
70.1884621	-0.200831959	0.154918817	-1.296369051	0.194848369	0.442963013
179.0137892	0.055787047	0.104693374	0.532861293	0.594129601	0.792172802
7.282943026	0.967749081	0.579723393	1.669329015	0.095052193	0.292971483
32.99385623	-0.136099937	0.23834946	-0.571010051	0.56799283	0.775517738
172.1927974	-0.194285743	0.1128864	-1.721073062	0.085237567	0.276040883
37.78232042	0.551736946	0.258427707	2.134975975	0.032762973	0.154790608
6.148685726	-0.78002339	0.497322387	-1.568446162	0.116777045	0.329886739
3.130751115	-0.072731989	0.686572743	-0.105934862	0.915634033	0.96420063
124.4364467	0.256302315	0.199393914	1.285406906	0.198650129	0.447326436
373.9750599	-0.006525224	0.079944741	-0.081621685	0.934947557	0.973012718
30.62532915	0.632139643	0.25896404	2.441032523	0.014645336	0.095548023
3.259462658	0.745514522	0.555673295	1.341641804	0.179712165	0.424747406
179.3983855	-0.128532585	0.127019212	-1.011914522	0.311578925	0.570697037
4.319679087	1.886592654	1.057511	1.783993409	0.074424736	0.254495706
53.70215713	5.495484695	1.463535966	3.754936553	0.000173385	0.004913071
46.90072387	0.074070863	0.168003791	0.440888044	0.659294049	0.834044622
2479.200307	-0.017360167	0.168201544	-0.103210511	0.917795898	0.965181096
1173.736726	0.150151564	0.090681626	1.655810231	0.097760255	0.298370621
2160.766602	-0.23868672	0.138977371	-1.717450244	0.085896938	0.277180856
41.72996503	0.193038444	0.323708181	0.59633477	0.550951607	0.764274809
2546.264302	0.155660403	0.225298399	0.690907718	0.489623535	0.7193682
563.2835301	-0.026293545	0.139687546	-0.18823113	0.85069547	0.935636028
2969.829638	-0.023487334	0.157261315	-0.149352265	0.881275676	0.950743683
13.02319378	-0.124660814	0.306647721	-0.406527768	0.684354858	0.848557456
22.91860507	-0.219266508	0.25357789	-0.864690958	0.387208433	0.638304143
17.77593853	0.416164006	0.282901882	1.471054215	0.141276456	0.369534051
1032.590067	0.057905686	0.130060909	0.445219752	0.656160954	0.832064366

13.621943	0.177387834	0.277003859	0.64038037	0.521925342	0.744256234
147.5398385	-0.233913429	0.25663309	-0.911470258	0.36204765	0.617682512
74.44432112	-0.087052888	0.132855738	-0.65524372	0.51231085	0.737095988
257.8952336	0.182119393	0.163486107	1.113974736	0.265290025	0.523372171
156.8452839	0.146641464	0.141232771	1.038296303	0.299132129	0.557460589
90.74599376	0.574136286	0.284265179	2.019720768	0.04341236	0.183624447
3.145573729	2.420048692	0.750925166	3.222756144	0.001269636	0.019541678
3.683157989	2.995332983	0.801081955	3.739109294	0.000184673	0.005135493
68.14966244	-0.097546845	0.135389568	-0.720490114	0.471223285	0.705620202
233.8035488	-0.914441566	0.325562936	-2.808801197	0.004972634	0.047324512
743.6972485	-0.355044812	0.398209452	-0.891603176	0.372605667	0.626450632
355.8715238	-0.071078628	0.158030377	-0.44977826	0.652870335	0.830317856
661.3971263	-0.331248206	0.157289828	-2.105973477	0.035206656	0.161613987
271.8798468	-0.169308788	0.219507904	-0.77131067	0.44052281	0.682478371
163.2825453	-0.364199086	0.099982608	-3.642624373	0.000269873	0.006837933
144.7291169	-0.022570909	0.224906108	-0.10035703	0.920060883	0.966373094
212.3044269	-0.01657899	0.101799675	-0.162858968	0.870629478	0.946199847
2173.384162	0.004640298	0.139063354	0.033368232	0.973380942	0.986906773
104.157817	0.04208735	0.167630258	0.251072512	0.801758048	0.910028457
5004.167861	-0.171197075	0.11151544	-1.535187198	0.124737852	0.343425257
3377.079445	-0.185214656	0.188848052	-0.980760215	0.326711002	0.585151858
167.5110893	0.045737095	0.14730699	0.310488287	0.756189666	0.887592168
540.9186273	0.093316492	0.080519224	1.158934316	0.24648296	0.501861449
2747.966379	-0.128972795	0.124196718	-1.038455746	0.299057928	0.557454783
38.78466359	-0.203276921	0.438168007	-0.463924608	0.642701761	0.824387858
59.2714461	0.907485946	0.341779391	2.655180416	0.0079266	0.064527019
117.3465966	-0.066368389	0.131783517	-0.503616769	0.614530707	0.806333036
67.89287603	0.082509905	0.211004392	0.391034066	0.695772056	0.854510951
71.47796623	1.548930594	0.584950011	2.647970876	0.00809765	0.065331041
538.8636833	-0.211284202	0.109073972	-1.937072588	0.052736471	0.206469997
5.031059273	1.515797248	0.698433112	2.170282627	0.029985442	0.147004578
163.4210722	-0.173939001	0.167980446	-1.035471714	0.30044867	0.558785359
143.0327407	0.231199493	0.13250555	1.744828745	0.081014684	0.266939979
3249.324212	0.974692617	0.260063837	3.747897552	0.000178323	0.005019031
1541.856176	-0.061713272	0.097565132	-0.632534088	0.527037941	0.748534191
39.39485632	0.128064788	0.256145331	0.499969247	0.617096731	0.807352781
775.7819376	0.404355934	0.192062926	2.105330489	0.03526255	0.161646902
87.26788868	0.812652277	0.328069462	2.477073821	0.01324645	0.08955057
5986.947912	0.270757743	0.119122087	2.272943232	0.023029601	0.125328336
175.7352204	-0.108046452	0.165531412	-0.652724766	0.513933733	0.738414866
6.22073855	0.42356869	0.407234771	1.040109344	0.298289103	0.556617049
517.2459432	0.063110186	0.064863651	0.972966901	0.330569753	0.589031584
79.7025019	0.181033245	0.310440934	0.58314876	0.559793162	0.770157539
348.4338467	-0.189531275	0.08777991	-2.159164594	0.0308374	0.149321945
2462.749078	0.010891181	0.121927111	0.08932534	0.928823356	0.970144322
8.916187161	1.052193059	0.553903101	1.899597704	0.057485934	0.218064314
12.18101732	1.024814158	0.410671682	2.495458546	0.012579445	0.086761662
1121.337929	-0.08064713	0.151631859	-0.531861385	0.594822004	0.792808463

14.98212977	0.470879581	0.327622616	1.437262136	0.150643523	0.383982231
31.095391	-0.2237142	0.180407451	-1.240049666	0.214957025	0.466957774
1271.441757	-0.028846037	0.108978501	-0.264694748	0.791244624	0.904476207
203.3301359	0.285910762	0.180595225	1.583157927	0.113385473	0.324077445
64.58893209	0.578662758	0.268411998	2.155875151	0.031093415	0.150112302
15.19718088	0.408889905	0.439123333	0.931150485	0.351775727	0.608637468
7.137375786	0.787651491	0.507593794	1.551735858	0.120725439	0.336914197
124.0114771	0.165437608	0.418691226	0.395130343	0.692746697	0.853431717
171.2954472	-0.038595148	0.130122476	-0.296606316	0.766767088	0.892250595
437.5870632	0.038233833	0.061148025	0.625266852	0.531795932	0.751414479
3594.108752	0.163069617	0.139965124	1.16507321	0.24398936	0.499495198
9.731299482	-0.948278263	0.401613563	-2.361170911	0.018217333	0.108474049
3.911917154	0.080824395	0.583445129	0.138529556	0.889821913	0.953820886
4.886133542	-0.078060929	0.494368563	-0.157900269	0.874535386	0.947863958
8.098758427	0.317368433	0.372880812	0.851125675	0.394699545	0.645109177
4.572651235	-0.044073852	0.562096944	-0.078409698	0.937502159	0.973825013
16.40684709	0.110717573	0.356588406	0.310491231	0.756187427	0.887592168
12.94095445	-0.184450416	0.363108875	-0.507975509	0.611470519	0.804218333
18.96346886	0.354490577	0.318261209	1.113835324	0.26534984	0.523424335
25.62664838	0.08806352	0.273516863	0.321967425	0.747477372	0.883853672
11.47321926	-0.049872989	0.318726301	-0.156475914	0.875657904	0.948599155
157.5845689	0.059377069	0.119621383	0.496375045	0.619629829	0.809092462
220.0740347	0.093520311	0.126496452	0.739311734	0.459717726	0.696168081
26.10913576	0.16374859	0.214231267	0.7643543	0.444656134	0.685379023
8217.607352	-0.11123845	0.150839393	-0.737462859	0.460840922	0.697145057
208.6838545	-0.109178479	0.109053436	-1.00114662	0.316755929	0.575732992
283.8250565	0.106098918	0.115260629	0.920513086	0.357304698	0.613393923
6.354490291	-0.308825531	0.475535827	-0.649426424	0.516062789	0.740130678
147.0944116	0.216802539	0.179544794	1.207512259	0.227234927	0.481810544
35.54074141	0.003196957	0.194257928	0.016457281	0.986869582	0.992541292
205.0132314	0.143720054	0.104578382	1.374280717	0.16935455	0.410294394
7.283592967	1.111670826	0.429488648	2.588359045	0.009643441	0.073342536
118.9572314	0.108372105	0.235495745	0.460187106	0.645381925	0.82569297
2.654561542	0.286054414	0.624783881	0.457845381	0.647063529	0.826964966
420.0099195	-0.181973122	0.111268896	-1.635435674	0.101957749	0.305814752
114.1665165	0.175162152	0.149291819	1.173287006	0.240680731	0.496110133
16.00665712	1.29196138	0.38156899	3.385918178	0.000709405	0.013120652
182.9288884	0.956523097	0.230722084	4.145780408	3.38658E-05	0.001498918
1629.132129	-0.146381857	0.041395944	-3.53614008	0.000406019	0.009007113
1208.154514	-0.102979795	0.094302784	-1.09201225	0.274827717	0.533865621
511.6151765	-0.160111394	0.139945893	-1.144094982	0.252584249	0.508867226
[truncated: 1,583,026 more chars]
