# Supplementary material for: Age-related transcriptional drift and physiological adaptation in long-living Ames dwarf skeletal muscle
Source: NAR Mol Med. 2026 Mar 23;3(2):ugag018. doi: 10.1093/narmme/ugag018 (PMC13111926; doi:10.1093/narmme/ugag018)
Supplement: ugag018_Supplemental_Files [file ugag018_supplemental_files.zip › Suppl. Dataset 4.pdf]

Supplementary Dataset 4

Genes	log2FoldChange
Zc3h11a	-0.235833425
Zbtb7a	0.103514095
Rad21	0.03927875
Trp63	-0.554089735
Runx1	-0.450302273
Sox2	2.914051008
Ctcf	0.017853345
Gata1	1.979854346
Ar	-0.445045391

Genes	log2FoldChange
Creb1	-0.125430206
Usf1	0.170965568
Bclaf1	-0.02999243
Nfyb	0.07619865
Irf1	0.443994887
Kat2a	0.063478389
Stat3	0.070179436
Brca1	0.768535764
Ubtf	0.278773485
E2f6	-0.12803901
Max	-0.061614228
Fos	0.537492071
Yy1	-0.030362623
Zmiz1	-0.068098628
Elf1	0.204064438
Myc	-0.03928716
Cebpd	-0.481779304
Gabpa	-0.116274073
Erg	0.601025955
Chd1	0.065845053
Egr1	0.877820293
Taf7	0.165283451
Rela	0.13952947
Tcf7l2	0.005255989
Pbx3	0.263659277
Atf2	-0.129381222
Nfe2l2	-0.071135242
Spi1	0.310052125
E2f1	0.865277531
Rfx5	-0.581940162
Klf4	0.099431535
Nrf1	0.209762839
Nr2c2	0.048707544
Bhlhe40	-0.01112746
Pparg	-0.410864047
Six5	0.405569559
Usf2	0.403787479
Fli1	0.400292554
Ets1	0.724865594
Sin3a	0.096625495
Pml	0.229338475
Zbtb33	-0.122590364
Taf1	0.011431471
