# Supplementary material for: Age-related transcriptional drift and physiological adaptation in long-living Ames dwarf skeletal muscle
Source: NAR Mol Med. 2026 Mar 23;3(2):ugag018. doi: 10.1093/narmme/ugag018 (PMC13111926; doi:10.1093/narmme/ugag018)
Supplement: ugag018_Supplemental_Files [file ugag018_supplemental_files.zip › Suppl. Dataset 5.pdf]

Supplmentary Dataset 5

baseMean	log2FoldChange	lfcSE	stat	pvalue	padj
71.6992669	1.869046413	0.36770525	5.083001705	3.71516E-07	0.001362071
156.7280232	1.643698273	0.339781627	4.837513685	1.31473E-06	0.003312076
160.5669663	-0.824911028	0.115086039	-7.167776725	7.62255E-13	9.60136E-09
7997.885309	1.09464157	0.274405955	3.989131975	6.63155E-05	0.03952507
372.6384151	-0.637114022	0.110051405	-5.789240257	7.07055E-09	4.45303E-05
87.85020118	4.196823209	1.054586576	3.979590966	6.90339E-05	0.03952507
87.90953893	-0.617216674	0.153026516	-4.033396897	5.49763E-05	0.035697035
160.300307	0.905144067	0.234368322	3.862058056	0.000112436	0.048890108
2830.391764	0.930694091	0.218103682	4.267209433	1.97933E-05	0.022986064
1425.756793	1.083510809	0.229155158	4.728284619	2.26425E-06	0.004753408
1717.764837	1.074212739	0.27286142	3.93684362	8.25604E-05	0.042041001
12525.52612	1.140946081	0.275418771	4.142586493	3.43411E-05	0.028881047
1775.811991	1.037978992	0.265369902	3.91144204	9.17467E-05	0.044447736
1116.44853	1.079588798	0.260629266	4.142239329	3.43931E-05	0.028881047
2059.945165	-0.659387806	0.130467238	-5.054048953	4.32541E-07	0.001362071
373.4942826	1.174678004	0.270498235	4.342645729	1.40777E-05	0.022986064
259.5727497	0.75810975	0.187307644	4.047404238	5.17888E-05	0.035697035
581.6270217	0.767879126	0.195175777	3.934295218	8.34412E-05	0.042041001
1459.720728	1.094685719	0.281516103	3.888536772	0.00010085	0.047048576
2338.321896	1.208212992	0.283347305	4.264070884	2.00736E-05	0.022986064
3483.395334	1.029645015	0.247852642	4.154262816	3.26338E-05	0.028881047
28.44726525	1.063079886	0.363655202	2.923318242	0.003463224	0.310136233
184.0874438	-0.00301269	0.121947281	-0.024704855	0.980290383	0.999579949
762.6500042	-0.016134808	0.104892201	-0.153822759	0.877749488	0.999579949
856.8990985	-0.109975655	0.165323637	-0.665214341	0.505913409	0.999579949
332.9134171	-0.014692404	0.077030749	-0.190734269	0.848733789	0.999579949
259.320288	0.138336981	0.099196116	1.394580627	0.16314234	0.978498637
1564.290033	0.116172441	0.120744566	0.962133901	0.335982347	0.999579949
1027.336419	0.019126303	0.102255382	0.187044458	0.851625775	0.999579949
260.3128572	0.280763568	0.115856166	2.423380464	0.015376813	0.542538755
515.6542124	-0.028692198	0.121996898	-0.23518793	0.814062874	0.999579949
26.51319778	0.070899403	0.21110765	0.335844783	0.736987918	0.999579949
544.0759565	0.006894713	0.068915785	0.10004548	0.920308219	0.999579949
121.3268777	0.33372763	0.164579758	2.027756241	0.042585133	0.74969123
29.68913181	-0.069020283	0.214388092	-0.321940843	0.747497511	0.999579949
1105.694929	-0.058431455	0.12533839	-0.466189612	0.641079784	0.999579949
114.4503398	-0.074724077	0.162881051	-0.458764707	0.646403142	0.999579949
2386.674311	0.005558851	0.116208035	0.047835345	0.961847467	0.999579949
676.3567352	-0.05526185	0.084614161	-0.653104042	0.513689207	0.999579949
300.1230225	0.122000853	0.086393754	1.412148991	0.157906096	0.977304288
964.1407259	-0.018752649	0.137806182	-0.136079879	0.891758134	0.999579949
880.070669	-0.119892197	0.073489768	-1.631413455	0.102803108	0.895628172
352.9812147	0.01887637	0.151296075	0.124764437	0.900710042	0.999579949
284.960812	-0.03599299	0.117357684	-0.306694789	0.759075696	0.999579949
358.9578143	-0.144835598	0.129150899	-1.121444749	0.262098597	0.999579949
29.57322772	-0.151498704	0.198685272	-0.76250596	0.445758088	0.999579949
70.37642825	-0.100304702	0.141609414	-0.708319448	0.478746903	0.999579949

26.97657196	0.308972424	0.27824797	1.110421126	0.266817599	0.999579949
2640.44596	0.089077515	0.107054109	0.832079364	0.405364149	0.999579949
88.88381974	-0.089810901	0.139160597	-0.645375934	0.518683595	0.999579949
406.4817305	0.268605432	0.16505561	1.627363244	0.103659973	0.895628172
169.3136934	-0.096948139	0.12876966	-0.752880294	0.451521851	0.999579949
475.2119824	-0.086133037	0.157124629	-0.548182912	0.583566314	0.999579949
687.3523557	0.087506261	0.137653398	0.635699968	0.524971998	0.999579949
8981.207876	-0.051826886	0.155123538	-0.33410072	0.738303556	0.999579949
110.7736918	-0.36796664	0.217703722	-1.690217498	0.090986352	0.87821003
60.11626222	-0.280922482	0.250593133	-1.121030248	0.262274987	0.999579949
60.99287125	0.106519263	0.186312182	0.571724629	0.567508546	0.999579949
25.10570252	1.174088603	0.446427912	2.629962358	0.008539432	0.442789376
80.33393707	0.251680955	0.243901896	1.031894214	0.302121677	0.999579949
32.62186298	0.244109951	0.204514015	1.193609894	0.232630602	0.999579949
66.89406452	0.000388405	0.160751733	0.002416182	0.998072168	0.999579949
32.32031433	-0.110141404	0.491990188	-0.223869106	0.822859155	0.999579949
692.4838802	0.089733719	0.202186442	0.443816696	0.657175117	0.999579949
45.76657057	0.249314327	0.512462593	0.486502488	0.626610942	0.999579949
142.9117479	0.176894724	0.19285095	0.917261357	0.359005701	0.999579949
196.5187903	0.014337549	0.118537353	0.120953846	0.903727592	0.999579949
153.9297904	0.249729281	0.177007792	1.410837782	0.158292451	0.977304288
517.2481044	-0.034932132	0.114374143	-0.305419838	0.760046414	0.999579949
27.66761487	1.147217032	0.521459687	2.200010972	0.027806117	0.663344402
136.8440804	-0.17360461	0.222549966	-0.780070261	0.43534952	0.999579949
742.2944048	0.010356015	0.071571754	0.144694159	0.884952351	0.999579949
926.8050924	-0.00022496	0.046921128	-0.00479442	0.996174621	0.999579949
79.54465694	0.078553189	0.222148171	0.353607184	0.72363329	0.999579949
35.06801641	0.277056096	0.324814111	0.852968165	0.393676959	0.999579949
110.5333501	0.190336804	0.112959176	1.685005242	0.09198756	0.880418343
169.5366966	0.013978487	0.125996648	0.110943326	0.911661288	0.999579949
280.0709457	-0.039829697	0.08370063	-0.475858991	0.63417484	0.999579949
26436.58487	-0.009154831	0.080812265	-0.113285163	0.90980448	0.999579949
98.38293415	-0.200860622	0.186810856	-1.075208508	0.282281387	0.999579949
133.8121445	-0.084348063	0.118789828	-0.710061328	0.477666106	0.999579949
47.9562279	0.000831029	0.324924077	0.002557609	0.997959325	0.999579949
38.60975262	0.161972808	0.236230892	0.685654645	0.492930914	0.999579949
905.034197	-0.045851348	0.079125233	-0.579478203	0.56226655	0.999579949
1140.342931	0.070249839	0.098621977	0.712314238	0.476270209	0.999579949
652.4771236	0.009675458	0.080193038	0.120652095	0.903966604	0.999579949
1510.578253	0.151758709	0.065517139	2.316320767	0.020540761	0.611138086
41.12396041	0.47696593	0.460333791	1.036130607	0.300141215	0.999579949
222.7846434	0.379552684	0.140355856	2.704216933	0.006846558	0.404205857
1004.044585	0.078404368	0.061418941	1.276550314	0.201761053	0.993283621
58.75498716	0.012856297	0.157561439	0.081595451	0.934968419	0.999579949
216.2547312	0.1746209	0.080319538	2.174077492	0.029699316	0.672989344
290.3704427	0.131358473	0.106992514	1.227735168	0.2195464	0.999579949
453.0774846	0.113645061	0.150264411	0.75630058	0.449469	0.999579949
26478.62391	0.113362489	0.131168954	0.864247874	0.387451738	0.999579949

165.8481199	0.010574819	0.144864835	0.072997834	0.941807841	0.999579949
250.2914829	0.009125549	0.122786282	0.074320591	0.940755293	0.999579949
2690.033079	0.009689968	0.110762901	0.087483879	0.930286899	0.999579949
1291.086108	0.073389869	0.093477168	0.785110112	0.432389011	0.999579949
1300.425394	-0.049412114	0.077006163	-0.641664405	0.521091106	0.999579949
727.5190743	0.158930086	0.09437246	1.684072733	0.092167613	0.880418343
1999.822141	-0.116782552	0.11307261	-1.032810258	0.301692702	0.999579949
156.0424218	0.095859037	0.094809475	1.011070222	0.311982821	0.999579949
256.2223645	0.163315509	0.129398327	1.262114539	0.206907574	0.994814558
35.54028226	-0.225937452	0.265907758	-0.849683566	0.395501038	0.999579949
75.66508864	0.335295077	0.158388659	2.116913413	0.034267197	0.703403556
69.89018195	-0.008158992	0.153991734	-0.052983309	0.957745206	0.999579949
21.10954254	-0.092126204	0.299688203	-0.307406841	0.758533722	0.999579949
775.1582653	-0.062127087	0.091091941	-0.682026167	0.495222405	0.999579949
307.0114652	0.12154968	0.071145798	1.708459005	0.087551207	0.868342521
1321.385319	0.069252153	0.104400241	0.663333265	0.507117136	0.999579949
434.2824005	0.114618384	0.078989575	1.451057117	0.146763956	0.959874957
137.0807072	-0.073341159	0.267687985	-0.273980017	0.784099978	0.999579949
24.38111554	0.067334571	0.274138283	0.245622647	0.805974357	0.999579949
388.2790733	-0.032594229	0.120875877	-0.269650405	0.787429219	0.999579949
1304.096274	-0.15797104	0.150758363	-1.047842634	0.294711116	0.999579949
440.4539609	0.216164592	0.108691804	1.988784652	0.046724978	0.766764601
409.6784505	-0.035566547	0.087441138	-0.40674844	0.684192759	0.999579949
148.1766731	0.30139771	0.11297029	2.667937822	0.007631838	0.417959271
841.5351446	-0.268173172	0.173512357	-1.545556615	0.122211686	0.92236461
163.4811554	-0.023154499	0.193148932	-0.119878992	0.904579007	0.999579949
34.9263885	-0.174300019	0.254391926	-0.685163332	0.49324086	0.999579949
47.1925383	0.41722286	0.27987132	1.490766756	0.136022743	0.945791454
28.58232391	0.459052858	0.27528272	1.667568738	0.095401368	0.884235199
519.6178779	-0.102371343	0.164362554	-0.622838596	0.533390592	0.999579949
1542.115035	-0.075308879	0.125277203	-0.601137933	0.54774812	0.999579949
317.1689563	0.060509913	0.082719317	0.731508859	0.464468395	0.999579949
485.0812309	-0.116008817	0.083611056	-1.387481789	0.165294901	0.978789551
315.0220454	-0.019239883	0.113933192	-0.168869866	0.865899002	0.999579949
34.74933935	0.517034892	0.27632086	1.871139559	0.06132574	0.802971951
276.9053534	-0.288939389	0.088068347	-3.28085402	0.001034933	0.165012851
121.4730295	-0.113294322	0.109182099	-1.037663895	0.29942656	0.999579949
811.8610112	0.036238781	0.076984839	0.470726207	0.637836267	0.999579949
355.6843499	0.136566186	0.243399442	0.561078467	0.574744047	0.999579949
27.07744324	0.058564103	0.1992094	0.293982626	0.768771179	0.999579949
75.58928502	0.08772434	0.131250233	0.668374737	0.503894418	0.999579949
127.7627253	-0.091345017	0.13774079	-0.66316606	0.507224206	0.999579949
280.6011372	0.081855595	0.079011774	1.035992365	0.300205704	0.999579949
469.0463794	-0.236145942	0.258230115	-0.914478711	0.360465359	0.999579949
67.42958797	-0.195808235	0.162104836	-1.20791113	0.227081448	0.999579949
4908.574402	-0.164060687	0.224823905	-0.729729728	0.465555406	0.999579949
638.6480157	0.253380698	0.255027261	0.99354358	0.320445124	0.999579949
201.2104382	-0.008421826	0.100493489	-0.083804696	0.933211714	0.999579949

697.2650075	0.116085228	0.120080961	0.966724676	0.333681688	0.999579949
145.2450843	-0.235844788	0.121909723	-1.934585535	0.053041176	0.781231069
68.91780076	-0.246218444	0.217424126	-1.132433871	0.257452072	0.999579949
1077.587983	-0.147827173	0.14567534	-1.014771432	0.310214795	0.999579949
155.0244775	-0.125247453	0.162712698	-0.769746029	0.44145056	0.999579949
484.5738695	-0.018737348	0.123791819	-0.151361764	0.87969035	0.999579949
479.4482138	0.250584798	0.280975662	0.891838093	0.37247972	0.999579949
1301.627401	-0.056073435	0.110997662	-0.505176723	0.613434718	0.999579949
284.7874126	0.017105493	0.083438251	0.205007809	0.837566024	0.999579949
30.66891989	0.104800008	0.319520965	0.327991024	0.74291845	0.999579949
152.7999875	0.132299481	0.111794217	1.183419713	0.236642848	0.999579949
104.2039423	-0.001170258	0.147868371	-0.007914186	0.993685459	0.999579949
370.2899458	0.055262024	0.125779823	0.439355236	0.660404156	0.999579949
675.9951492	-0.143225226	0.186807821	-0.766698228	0.443260958	0.999579949
68.45442946	0.115130971	0.156812486	0.73419518	0.462829791	0.999579949
99.35705718	-0.068758218	0.107799461	-0.637834521	0.523581405	0.999579949
312.7188449	-0.021545918	0.157019999	-0.137217665	0.890858748	0.999579949
1203.711023	-0.018769604	0.115276331	-0.162822705	0.870658031	0.999579949
165.1033099	0.200683938	0.38090728	0.526857713	0.598292402	0.999579949
263.0964035	0.12973049	0.146353013	0.88642172	0.375390319	0.999579949
241.9023749	0.049966971	0.114602072	0.436004074	0.662833773	0.999579949
173.257219	-0.124744042	0.111405141	-1.119733261	0.262827447	0.999579949
276.9155713	-0.109711137	0.323917704	-0.338700652	0.734835249	0.999579949
1936.620183	0.154697008	0.07501744	2.062147259	0.039193718	0.732468953
232.9030956	0.125451292	0.137451277	0.91269644	0.361402217	0.999579949
1522.680368	0.125334462	0.099580661	1.258622521	0.208166708	0.995521169
379.1647986	0.103478442	0.123951401	0.834830757	0.403813006	0.999579949
389.8549858	0.06102247	0.159468874	0.382660693	0.701971363	0.999579949
58.71294696	-0.060700884	0.149619018	-0.405702996	0.684960841	0.999579949
146.3005425	0.096229696	0.0961687	1.000634264	0.317003658	0.999579949
625.118838	-0.000840315	0.116251014	-0.00722845	0.994232582	0.999579949
36.92665094	0.24094462	0.169528471	1.42126345	0.155240185	0.976030424
32.7774334	-0.064311775	0.185078459	-0.347483848	0.728227851	0.999579949
183.1587378	0.006285453	0.117411967	0.053533328	0.957306977	0.999579949
63.01106609	-0.125757212	0.126439345	-0.994605057	0.319928387	0.999579949
73.84791983	-0.053389626	0.147166512	-0.3627838	0.716766394	0.999579949
23.88406116	1.081546692	0.429978415	2.515351129	0.011891389	0.495973281
613.9631183	0.136046293	0.12671679	1.073624839	0.28299086	0.999579949
34.34308588	0.199749663	0.270838143	0.737524119	0.460803681	0.999579949
789.4192422	-0.059370978	0.071347951	-0.832132901	0.405333933	0.999579949
758.6162245	0.044341196	0.096627918	0.458885973	0.646316052	0.999579949
110.2086829	0.041676582	0.110919731	0.375736415	0.707112863	0.999579949
107.7408541	-0.105089773	0.109985282	-0.955489417	0.339330255	0.999579949
225.7228686	0.106240514	0.086837986	1.223433641	0.221165954	0.999579949
395.8190218	0.19467519	0.093900831	2.073199851	0.038153684	0.726381766
732.8135502	0.168661	0.122810346	1.373345198	0.169645058	0.981013142
797.909101	-0.036496366	0.06376656	-0.572343345	0.56708939	0.999579949
79.3086191	0.068236117	0.284768754	0.239619396	0.810625327	0.999579949

2241.488717	0.101889557	0.112561244	0.905192167	0.365363591	0.999579949
885.9817244	-0.098892719	0.109122321	-0.906255645	0.364800556	0.999579949
47.27304663	0.172156438	0.194899322	0.88330958	0.377069029	0.999579949
524.1637723	-0.073745955	0.083153314	-0.886867291	0.375150354	0.999579949
1979.822273	-0.124660691	0.13743534	-0.907049749	0.36438049	0.999579949
22.85116206	-0.029106694	0.21833023	-0.133314996	0.893944269	0.999579949
294.2298171	0.083738642	0.12483058	0.670818337	0.502336264	0.999579949
161.6310944	0.155334797	0.109567512	1.417708537	0.156275877	0.976030424
2952.735754	-0.030542528	0.116354859	-0.262494654	0.792940106	0.999579949
350.1931504	0.221801319	0.094503599	2.347014532	0.018924517	0.590797093
180.0950504	0.099390896	0.099555256	0.998349062	0.318110125	0.999579949
115.8861498	0.187958179	0.13187579	1.425266756	0.154080106	0.974996762
1652.458325	-0.066268335	0.085696094	-0.773294694	0.439347995	0.999579949
101.6088989	0.151303694	0.12628609	1.198102606	0.230877074	0.999579949
401.8850675	-0.10114107	0.094743692	-1.067523005	0.285735731	0.999579949
837.6788266	-0.030286707	0.09510474	-0.318456337	0.75013881	0.999579949
402.9229031	0.14187177	0.092297965	1.537106161	0.124267314	0.924973693
530.9951469	0.142213441	0.177368803	0.801795125	0.422671479	0.999579949
1113.580662	-0.070809836	0.059881665	-1.182496114	0.237008907	0.999579949
62.16154475	-0.041559763	0.165725008	-0.250775446	0.801987726	0.999579949
4836.662379	0.072936177	0.136726448	0.533445999	0.593724883	0.999579949
1088.275091	0.107044995	0.102950313	1.03977338	0.298445199	0.999579949
160.2625564	-0.22080216	0.142461124	-1.549911687	0.121162714	0.921837625
69.72626671	-0.727563536	0.391639937	-1.85773581	0.063206541	0.809095107
295.7734182	0.071790908	0.141174536	0.508525896	0.611084583	0.999579949
122.3860911	-0.02060359	0.133096446	-0.154801955	0.87697745	0.999579949
457.2024118	0.019272251	0.072663074	0.265227579	0.790834151	0.999579949
122.0482532	0.159265685	0.123258229	1.292130238	0.196312046	0.991421279
248.5807953	0.019588521	0.120021233	0.163208798	0.87035404	0.999579949
172.8961901	0.386762006	0.133071872	2.906414406	0.003655969	0.317590251
330.9391138	0.099298858	0.084519568	1.174862344	0.240049793	0.999579949
376.2617877	-0.051214342	0.161374933	-0.317362439	0.750968604	0.999579949
382.9785212	-0.027379399	0.111869997	-0.244743002	0.80665543	0.999579949
43.51803309	-0.189361971	0.241058834	-0.785542548	0.432135534	0.999579949
145.964272	-0.00049591	0.147392328	-0.00336456	0.997315474	0.999579949
563.965595	-0.087341023	0.111883831	-0.780640255	0.435014109	0.999579949
3012.609284	0.29283687	0.103378781	2.832659334	0.004616255	0.342037368
60763.89363	-0.214127331	0.144383109	-1.483049735	0.138061197	0.945791454
438.0293125	0.016422942	0.104815045	0.156684966	0.875493136	0.999579949
42.99054842	0.19263864	0.233297965	0.825719332	0.408963323	0.999579949
410.7698939	-0.147099816	0.106394697	-1.382585971	0.166791853	0.978965834
390.4513964	0.089723752	0.080745287	1.11119491	0.266484462	0.999579949
2622.243345	-0.050509371	0.218946539	-0.230692713	0.817553535	0.999579949
1129.546754	-0.034185774	0.123613174	-0.276554457	0.782122239	0.999579949
177.4790547	-0.114418408	0.137328985	-0.833170125	0.404748789	0.999579949
402.4926695	-0.139363052	0.147596585	-0.944215968	0.345059297	0.999579949
383.3318604	-0.043419432	0.063927281	-0.679200349	0.497010926	0.999579949
1981.319082	0.241944148	0.203618444	1.188223145	0.234745508	0.999579949

8594.771883	-0.385625172	0.190422157	-2.025106627	0.042856417	0.750791966
13212.56452	-0.019089972	0.135343393	-0.141048424	0.887831688	0.999579949
845.2684478	0.110183958	0.078550778	1.402709949	0.160703349	0.978356398
978.8578405	0.059745646	0.127284311	0.46938735	0.638792789	0.999579949
360.9796735	-0.142924722	0.080742416	-1.770131837	0.076705181	0.851207389
639.3454885	0.040193804	0.083339489	0.482290018	0.629599937	0.999579949
50.33699549	0.485560937	0.362570271	1.339218837	0.180499442	0.983992835
1388.868305	-0.018873091	0.053690081	-0.351519138	0.725198917	0.999579949
307.5973338	-0.001409968	0.074827825	-0.018842836	0.984966482	0.999579949
299.8419258	0.095593541	0.075534579	1.26555998	0.205670662	0.994814558
1133.741609	-0.272051542	0.153426417	-1.773172746	0.076200087	0.851207389
338.6466532	-0.084136808	0.084665392	-0.993756778	0.320341293	0.999579949
175.4033954	-0.006842529	0.115974556	-0.059000264	0.952951898	0.999579949
143.0591924	-0.020353668	0.092527409	-0.219974476	0.825891033	0.999579949
232.5133271	-0.000346791	0.092400437	-0.003753128	0.997005444	0.999579949
642.8556181	0.146545525	0.091341867	1.604363145	0.10863403	0.903743816
1929.585947	-0.408105463	0.183659903	-2.222071651	0.026278464	0.649026529
21.3865852	-0.136204503	0.315910243	-0.431149372	0.666359764	0.999579949
26.99352239	0.274455672	0.250484796	1.095697928	0.27321099	0.999579949
301.0302925	0.226906318	0.115636937	1.962230449	0.04973566	0.776295389
268.8962666	0.118385856	0.188012644	0.629669651	0.528910743	0.999579949
109.4346683	-0.046997651	0.11812943	-0.397848794	0.690741647	0.999579949
429.6736486	-0.043872286	0.083163346	-0.527543541	0.597816188	0.999579949
2041.752037	-0.087435435	0.090076963	-0.970674759	0.331710265	0.999579949
133.9318712	0.078860313	0.107075846	0.736490218	0.461432418	0.999579949
45.49587044	0.095556586	0.249845358	0.382462926	0.702118023	0.999579949
216.2578516	0.100364543	0.099541925	1.008264032	0.313327725	0.999579949
7956.315447	0.02019784	0.106901173	0.188939366	0.85014034	0.999579949
676.9030342	0.127642996	0.086104342	1.482422297	0.138227965	0.945791454
426.1812037	0.070458643	0.074022532	0.951853998	0.341171032	0.999579949
44163.39767	0.11148216	0.130738323	0.852712176	0.393818937	0.999579949
106.9356312	0.127229123	0.174025195	0.731095994	0.464720521	0.999579949
13660.59383	0.045407242	0.102773403	0.441819002	0.658620187	0.999579949
294.7818848	-0.017227592	0.100255378	-0.171837082	0.863565612	0.999579949
117.0881521	-0.111681243	0.184223944	-0.606225449	0.544365056	0.999579949
1868.559269	-0.041979944	0.188657483	-0.22251937	0.823909597	0.999579949
56.05475676	-0.109528755	0.176923958	-0.619072489	0.535868606	0.999579949
258.1709463	0.228082162	0.091767758	2.48542808	0.012939573	0.510304755
184.8821642	0.100527938	0.148479025	0.677051439	0.498373318	0.999579949
107.3139672	-0.08223142	0.118137771	-0.696063749	0.486388908	0.999579949
517.3263855	-0.00064586	0.094612345	-0.00682638	0.994553379	0.999579949
80.75202245	0.281197189	0.125699664	2.23705601	0.02528268	0.642289128
56.65153182	0.066216134	0.183708417	0.36044148	0.718517012	0.999579949
20.71571495	0.124119941	0.243345439	0.510056574	0.610011828	0.999579949
369.9454754	-0.115828504	0.113325231	-1.022089284	0.306738645	0.999579949
303.9890423	0.07867403	0.130944035	0.600821798	0.547958684	0.999579949
956.175971	0.170034124	0.077566202	2.192116135	0.028371122	0.666721367
3067.58829	-0.065828967	0.119816757	-0.549413694	0.582721578	0.999579949

227.0519138	-0.634319721	0.325993739	-1.945803385	0.051678362	0.779189484
2315.307961	-0.097390477	0.166808996	-0.583844271	0.55932509	0.999579949
181.775907	-0.132272141	0.089878514	-1.471676992	0.141108123	0.949453409
111.5372087	-0.132313831	0.182254937	-0.72598215	0.467849713	0.999579949
124.4224287	-0.288685014	0.127651196	-2.261514374	0.023727424	0.630129799
1124.574985	-0.083410845	0.107687524	-0.774563681	0.438597525	0.999579949
481.9215112	-0.060822887	0.061694114	-0.985878279	0.324192847	0.999579949
134.6669209	-0.094465088	0.164606298	-0.573885014	0.566045615	0.999579949
3677.859	-0.03328616	0.071016096	-0.468712893	0.63927487	0.999579949
56.62551626	0.302826576	0.57420294	0.527385973	0.597925582	0.999579949
48.71549903	0.439178599	0.47868912	0.917461	0.35890112	0.999579949
210.8476134	0.177600499	0.29333264	0.605457678	0.544874937	0.999579949
22.86503875	0.484271318	0.594083919	0.815156417	0.414982782	0.999579949
2650.178372	0.082770846	0.102964397	0.803878323	0.42146725	0.999579949
513.2686488	0.022989975	0.092290261	0.249105104	0.803279481	0.999579949
2296.148025	-0.029747481	0.103129164	-0.288448776	0.773003236	0.999579949
55.78464111	-0.157385053	0.167655931	-0.938738356	0.347865095	0.999579949
63.67617406	0.087247432	0.156582755	0.557196942	0.577392879	0.999579949
1994.035955	0.148534065	0.075155879	1.97634659	0.048115536	0.770705547
24.30158952	0.073387572	0.312687069	0.234699734	0.814441794	0.999579949
602.4695201	0.275298791	0.234233067	1.175319926	0.239866745	0.999579949
240.3520112	0.063705882	0.123347406	0.516475247	0.605522523	0.999579949
156.2246438	0.035231623	0.088041492	0.400170674	0.689030813	0.999579949
312.8220293	0.003378857	0.099352002	0.034008943	0.972870019	0.999579949
44.5821608	0.205361899	0.243207456	0.844389818	0.39845161	0.999579949
684.1319572	-0.032272456	0.076711456	-0.420699297	0.673974675	0.999579949
64.55368366	-0.229289632	0.168149342	-1.363607075	0.172691264	0.983601342
812.7875321	0.033800859	0.046466307	0.727427275	0.466964251	0.999579949
34.27446709	-0.048818089	0.207109507	-0.235711484	0.813656558	0.999579949
117.5279456	0.022937124	0.22780983	0.100685402	0.9198002	0.999579949
170.6029159	0.26463325	0.415576048	0.636786579	0.524263869	0.999579949
394.4385195	0.069811402	0.061592374	1.133442302	0.257028558	0.999579949
417.6297026	-0.006277084	0.164517622	-0.038154478	0.969564516	0.999579949
1150.128819	-0.025490927	0.082495933	-0.308996168	0.757324442	0.999579949
444.4025564	-0.0829816	0.081788654	-1.014585715	0.310303351	0.999579949
36.01770028	-0.144827674	0.184172168	-0.786371121	0.431650097	0.999579949
50.39069869	0.069923716	0.181507093	0.385239582	0.70005992	0.999579949
41.76919847	-0.763617074	0.49220191	-1.551430536	0.120798542	0.921837625
115.9941631	-0.269602647	0.15989444	-1.686128971	0.091770961	0.880418343
862.482555	-0.089246751	0.065502139	-1.362501325	0.173039726	0.983601342
44.66299335	-0.077396084	0.180962484	-0.427691323	0.668875868	0.999579949
337.8075273	0.042734262	0.145476569	0.293753572	0.768946215	0.999579949
241.4243047	-0.098554014	0.125885022	-0.782889117	0.433692229	0.999579949
761.5639022	-0.032387878	0.092593798	-0.349784534	0.726500407	0.999579949
1969.626263	-0.280655275	0.159697224	-1.757421124	0.078846055	0.851207389
2354.825122	-0.165139646	0.103780988	-1.591232163	0.111557337	0.913044976
277.8441874	0.240862869	0.148765872	1.619073413	0.105431468	0.896383253
332.0258599	-0.086770862	0.092412909	-0.938947411	0.347757745	0.999579949

142.4552124	0.143056077	0.109380414	1.30787654	0.190915188	0.98475336
1101.644738	-0.204086413	0.097960887	-2.083345903	0.037219705	0.717947029
54.86242037	-0.35786436	0.191701993	-1.866774332	0.061933108	0.805306145
356.5564831	0.229419295	0.103472079	2.217209683	0.026608766	0.653787748
86.15150536	0.197835824	0.144380894	1.370235479	0.170613407	0.981609963
623.191034	0.397180798	0.133286644	2.979899442	0.00288343	0.285981787
106.3695113	0.058341427	0.111086651	0.525188459	0.599452187	0.999579949
351.8104472	0.098329966	0.107209409	0.917176644	0.359050084	0.999579949
904.1710139	0.163717584	0.067503122	2.425333532	0.015294328	0.542093008
2527.982086	-0.011879165	0.110106828	-0.107887629	0.91408483	0.999579949
471.0915792	0.177074308	0.199112156	0.889319422	0.373831437	0.999579949
6291.1855	-0.064943111	0.115467592	-0.562435828	0.573819114	0.999579949
269.3072758	0.030823791	0.141587185	0.217701841	0.827661429	0.999579949
1152.464958	-0.034997626	0.051627267	-0.677890336	0.497841228	0.999579949
94.32560201	0.052830371	0.121142936	0.436099482	0.662764553	0.999579949
82.44188962	0.037644665	0.350321372	0.107457519	0.914426025	0.999579949
295.9525738	-0.013590935	0.112148532	-0.12118692	0.903542984	0.999579949
145.4641131	-0.078844264	0.096183637	-0.819726378	0.412372109	0.999579949
292.996144	0.092588161	0.087402898	1.059325981	0.289451347	0.999579949
6998.047642	-0.024910244	0.09149487	-0.27225837	0.785423364	0.999579949
1056.925506	0.042084267	0.061401234	0.68539774	0.49309297	0.999579949
261.2821233	0.004732528	0.096577435	0.049002419	0.960917368	0.999579949
762.2521769	-0.112775245	0.049709933	-2.268666207	0.023288632	0.62865781
39.32125951	-0.147809351	0.206381022	-0.71619643	0.473870067	0.999579949
419.8176561	-0.091624193	0.111757675	-0.819846986	0.412303342	0.999579949
287.6567234	0.053949912	0.075364339	0.715854634	0.474081113	0.999579949
126.0162813	0.171932944	0.147493308	1.165699966	0.243735774	0.999579949
157.7897601	0.253721212	0.148294317	1.710930113	0.087094015	0.867486202
195.5036321	-0.002539149	0.125367817	-0.020253599	0.983841071	0.999579949
638.3393335	0.106759618	0.17251231	0.618852174	0.536013748	0.999579949
130.9093501	-0.196233406	0.242744336	-0.808395404	0.418862997	0.999579949
300.829163	-0.148466713	0.156981549	-0.945759002	0.344271521	0.999579949
146.221841	0.086908727	0.16106381	0.539591898	0.589478505	0.999579949
44.96115691	-0.112016805	0.159159832	-0.703800721	0.481556885	0.999579949
964.8527766	-0.005123129	0.100386673	-0.051033951	0.959298467	0.999579949
46.38548762	-0.217388535	0.19296119	-1.126592008	0.25991502	0.999579949
254.7945284	0.05130033	0.078701355	0.651835406	0.514507358	0.999579949
341.7141256	0.039279634	0.08771	0.447835297	0.654272068	0.999579949
61.84571076	0.165968378	0.236416257	0.702017621	0.482668175	0.999579949
431.1177629	0.076410464	0.111704648	0.684040149	0.493949814	0.999579949
522.2093473	-0.035642872	0.089191695	-0.39962097	0.689435709	0.999579949
178.4927717	0.135625157	0.25573019	0.530344722	0.595872945	0.999579949
228.1856887	-0.141808515	0.141347588	-1.003260944	0.315734975	0.999579949
336.1174219	0.007551289	0.075476755	0.10004787	0.920306321	0.999579949
23.95640094	0.142094954	0.258740925	0.549178503	0.582882956	0.999579949
28.26143956	0.247324931	0.262333979	0.942786488	0.345790124	0.999579949
43.13655815	0.081757922	0.255982515	0.319388699	0.74943178	0.999579949
109.8886312	-0.054370259	0.099434831	-0.546792891	0.584521028	0.999579949

515.2881086	0.081015984	0.081103929	0.998915652	0.317835553	0.999579949
167.9789271	0.108729262	0.091304979	1.19083607	0.233717946	0.999579949
1967.287423	-0.092808678	0.066765458	-1.39007028	0.164507537	0.978498637
729.5303525	0.055864253	0.233094782	0.239663249	0.810591328	0.999579949
64.29031445	-0.04117314	0.167733647	-0.245467384	0.806094559	0.999579949
141.0802265	-0.624882171	0.289972831	-2.154967994	0.031164339	0.683621001
193.033254	0.252454464	0.129765656	1.9454644	0.05171911	0.779189484
85.13188095	-0.033849889	0.131673552	-0.257074321	0.797121401	0.999579949
99.0789155	0.076716543	0.123366036	0.621861138	0.534033181	0.999579949
588.6676226	-0.288671908	0.110615371	-2.60969074	0.009062411	0.451186274
143.8466076	0.113894954	0.103188958	1.103751372	0.269701004	0.999579949
49.64005143	-0.110121158	0.228824206	-0.481247853	0.630340354	0.999579949
275.7020365	0.213303127	0.170022262	1.254559989	0.209638536	0.996447459
798.1387489	-0.196113401	0.170829764	-1.148004875	0.250966551	0.999579949
21.07907717	-0.162650112	0.223514863	-0.727692599	0.466801782	0.999579949
478.9118232	-0.06218886	0.120092371	-0.517841887	0.604568591	0.999579949
55.12734847	0.94019499	0.454928778	2.066686116	0.03876374	0.728740425
678.2423624	0.063334919	0.098500988	0.642987649	0.520232114	0.999579949
329.2171644	0.052901771	0.073440338	0.720336706	0.47131771	0.999579949
251.0711368	0.199614479	0.095133636	2.098253445	0.035882763	0.714027297
675.9192651	0.159963494	0.192202816	0.832264049	0.405259918	0.999579949
253.4798298	0.205617906	0.103101778	1.994319691	0.046117129	0.763868825
37.14585994	-0.172877984	0.207731238	-0.832219486	0.405285067	0.999579949
63.6170014	0.204879375	0.329355458	0.622061576	0.533901379	0.999579949
640.1435219	0.140223751	0.090616429	1.547442926	0.121756477	0.922215626
649.012937	0.103998245	0.049441615	2.103455657	0.035425958	0.712820067
150.1044655	0.382993036	0.132876212	2.882329585	0.003947466	0.324982232
1235.7425	-0.084881473	0.116637674	-0.727736333	0.466775005	0.999579949
782.944637	0.083767631	0.076639657	1.093006335	0.274391018	0.999579949
26.85551266	0.457362162	0.565152921	0.809271518	0.418358986	0.999579949
637.1002125	0.044119679	0.101131962	0.436258507	0.662649183	0.999579949
73.63393609	-0.302074315	0.317021876	-0.952850066	0.340666043	0.999579949
20.86124585	0.468507267	0.23497913	1.993825012	0.046171181	0.763868825
941.4352157	-0.089666287	0.173007492	-0.518279792	0.60426307	0.999579949
436.4586524	0.125763687	0.072180568	1.742348271	0.081447522	0.85448976
119.3525568	0.235505266	0.121939417	1.931330093	0.053442244	0.781231069
4160.16444	0.087145457	0.108756753	0.801287783	0.422965062	0.999579949
339.0414297	0.018217714	0.07760455	0.23475059	0.814402319	0.999579949
74.53759943	0.21116574	0.520272298	0.405875426	0.684834135	0.999579949
43.92136433	0.040781773	0.57329085	0.07113627	0.943289302	0.999579949
453.5726266	-0.170277631	0.121072972	-1.406404981	0.159603902	0.977806784
40.82253585	0.446036739	0.869103268	0.513214891	0.60780101	0.999579949
61.29943195	-0.088427029	0.173149386	-0.510697907	0.609562607	0.999579949
30.75578369	0.188274525	0.206106305	0.913482609	0.360988776	0.999579949
1424.562032	0.105940999	0.142601845	0.742914642	0.457533361	0.999579949
57.30573635	0.201543736	0.153623769	1.311930684	0.189543523	0.983992835
2232.771825	0.030219221	0.073469597	0.411316002	0.680840837	0.999579949
93.54341543	0.030572266	0.199147902	0.153515381	0.877991862	0.999579949

1197.674582	-0.141765787	0.062536899	-2.266914256	0.023395464	0.62865781
793.695684	-0.025523203	0.071593832	-0.356500024	0.721466134	0.999579949
118.9826409	0.066805874	0.167339771	0.39922293	0.689728949	0.999579949
32.32384357	0.185864538	0.353043571	0.526463454	0.598566238	0.999579949
895.2807193	0.211958265	0.204604856	1.035939561	0.30023034	0.999579949
485.2641819	-0.010655499	0.058575317	-0.18191108	0.855652509	0.999579949
311.6332476	-0.041546954	0.067460028	-0.615875127	0.537976939	0.999579949
30.81779142	-0.074944266	0.191777696	-0.390787185	0.695954548	0.999579949
51.72911078	0.017905706	0.356445145	0.050234115	0.959935826	0.999579949
23.48977132	-0.068893944	0.266395978	-0.258614806	0.795932458	0.999579949
1057.334884	0.090705352	0.084196846	1.077301072	0.281345783	0.999579949
1109.196337	-0.191712817	0.069500824	-2.758425103	0.005808061	0.372390484
710.9344317	0.215802668	0.135466226	1.593036685	0.111151961	0.912543037
495.5122414	0.056265016	0.17355105	0.324198651	0.745787644	0.999579949
32.47143056	-0.056621279	0.224120014	-0.252638207	0.800547802	0.999579949
256.5355405	-0.026328989	0.172437109	-0.152687485	0.87864473	0.999579949
115.8144049	0.160235125	0.164024523	0.976897365	0.328619966	0.999579949
1636.827243	-0.041105388	0.063035264	-0.652101459	0.514335723	0.999579949
1483.285279	-0.072274111	0.083933009	-0.861092815	0.389186926	0.999579949
802.3847044	0.006317243	0.223526643	0.028261699	0.977453428	0.999579949
1206.169296	-0.2350451	0.414487564	-0.567073949	0.570663941	0.999579949
824.4515658	0.967220074	0.495127522	1.953476694	0.050763136	0.777437894
351.9079468	0.464673154	0.204050564	2.277245136	0.022771591	0.62865781
25.27801101	1.286329445	0.537686401	2.392341412	0.016741261	0.568390639
2324.528746	-0.59687573	0.269877012	-2.211658285	0.026990285	0.653787748
168.6096743	-0.553475466	0.320762783	-1.725497765	0.084437806	0.861032139
85.88305652	-0.16063135	0.182123592	-0.881990898	0.377781729	0.999579949
50.77962048	-0.167908862	0.182967469	-0.917697901	0.358777047	0.999579949
232.8799837	-0.049433637	0.096670253	-0.511363484	0.609096559	0.999579949
699.9496477	0.07615319	0.114436274	0.665463726	0.505753937	0.999579949
3746.509664	-0.034254549	0.091590643	-0.37399616	0.708407173	0.999579949
397.3792395	0.193993583	0.120407424	1.611143036	0.10714855	0.900529039
124.1725252	0.025984924	0.122456552	0.21219709	0.831953277	0.999579949
539.580455	0.010944782	0.076229864	0.143576044	0.885835259	0.999579949
3401.129044	0.066729378	0.104596918	0.637966964	0.523495185	0.999579949
33.98821191	0.065670984	0.225747392	0.290904729	0.771124185	0.999579949
23.11167992	-0.055989378	0.700963249	-0.079874912	0.936336744	0.999579949
298.7381317	0.10640604	0.088384766	1.203895712	0.228629889	0.999579949
239.8716467	0.026579295	0.116239996	0.228658774	0.819134139	0.999579949
1032.692934	0.011858451	0.097931927	0.121088713	0.903620769	0.999579949
138.3483218	-0.068323527	0.137543294	-0.496741974	0.619371019	0.999579949
1285.274549	0.03668474	0.118286576	0.310134431	0.75645873	0.999579949
840.7287843	-0.05120806	0.064811553	-0.790106968	0.429465301	0.999579949
887.0858521	-0.395538923	0.117074076	-3.378535507	0.00072873	0.134983569
402.0962068	0.054148178	0.159262746	0.339992746	0.733861991	0.999579949
2070.138751	0.223782882	0.173301463	1.291292514	0.196602266	0.991527744
30.30245278	0.512295769	0.261341153	1.960256792	0.049965784	0.776599223
1986.936149	-0.244540339	0.436492591	-0.560239382	0.575316168	0.999579949

160.9109742	-0.233063919	0.175166007	-1.330531664	0.183343161	0.983992835
22521.59215	-0.105600636	0.11783965	-0.896138409	0.370178845	0.999579949
153.9106647	-0.481296515	0.671314798	-0.716946082	0.473407366	0.999579949
525.6551221	0.031142863	0.101320276	0.307370487	0.758561389	0.999579949
128.9295052	-0.016139942	0.126829449	-0.127257052	0.898736951	0.999579949
287.2814906	-0.041837732	0.108962364	-0.383964982	0.701004402	0.999579949
171.7275211	-0.027189736	0.725906323	-0.03745626	0.970121215	0.999579949
2329.134639	-0.028526336	0.139294674	-0.204791294	0.837735189	0.999579949
93.21884515	-0.097214189	0.193833114	-0.501535508	0.615994295	0.999579949
892.8799901	-0.061797122	0.109480169	-0.564459503	0.572441454	0.999579949
466.3039265	-0.120196	0.10477449	-1.147187639	0.251304079	0.999579949
378.9409368	0.581422045	0.212789415	2.732382358	0.006287812	0.386347721
21.80843723	0.54638675	0.348848329	1.566258755	0.117288053	0.920479614
538.0922502	0.002100214	0.077797708	0.026995831	0.978463059	0.999579949
226.2152342	-0.356324769	0.257747393	-1.382457315	0.166831328	0.978965834
296.5268489	-0.072833321	0.139269445	-0.522966983	0.60099723	0.999579949
244.0143535	-0.010639204	0.103872231	-0.102425877	0.918418645	0.999579949
194.1205377	0.054158545	0.11214744	0.482922703	0.629150621	0.999579949
133.1582692	0.274444904	0.222945925	1.23099314	0.21832543	0.999579949
39.29563555	0.169192793	0.311707793	0.542792951	0.587272367	0.999579949
327.6882864	0.047470916	0.123535823	0.384268424	0.700779509	0.999579949
2594.778112	0.01783768	0.074639748	0.238983655	0.811118257	0.999579949
114.926784	0.709720303	0.464626871	1.527505935	0.126635253	0.933836888
311.1549347	-0.027386613	0.125176781	-0.21878349	0.826818708	0.999579949
2164.198365	0.237211892	0.076535305	3.099378661	0.00193927	0.225075621
147.3844832	-0.020061776	0.125441308	-0.159929584	0.872936544	0.999579949
216.4490927	0.245550077	0.094569169	2.596513011	0.009417534	0.458002332
200.3789668	-0.02793358	0.142525488	-0.195990068	0.84461794	0.999579949
305.9080783	0.002119178	0.168269335	0.012593962	0.989951738	0.999579949
738.9194729	-0.053558907	0.071259606	-0.751602627	0.452290063	0.999579949
1810.05569	0.060809971	0.145277568	0.418577843	0.675524686	0.999579949
94.1611961	0.087128751	0.132099128	0.659570975	0.509529186	0.999579949
103.0705064	-0.043256798	0.155355972	-0.278436659	0.780677182	0.999579949
401.0785973	-0.249382707	0.152943481	-1.630554669	0.102984321	0.895628172
281.6457403	0.177500715	0.248275895	0.71493334	0.474650234	0.999579949
47.98840845	-0.002060252	0.436590062	-0.004718963	0.996234826	0.999579949
1115.082774	-0.173744002	0.080152851	-2.167658411	0.030184686	0.677729599
30.05142584	0.272839735	0.236551386	1.153405778	0.248743871	0.999579949
3035.154557	-0.132617337	0.101736621	-1.303535888	0.192391872	0.985509566
741.8729495	0.075195604	0.094608917	0.79480462	0.426727163	0.999579949
54.79291204	-0.265601392	0.152282162	-1.744139881	0.081134701	0.85448976
88.26433143	0.755782138	0.420519557	1.797258	0.072294671	0.838669905
5216.588296	0.456139187	0.136942858	3.330872413	0.000865743	0.149382129
201.3027822	0.474266413	0.196292482	2.416121125	0.015686842	0.551931461
240.3986098	0.026714083	0.096838174	0.275863142	0.782653185	0.999579949
202.1716918	-0.032815275	0.118468001	-0.276996954	0.781782445	0.999579949
1489.648597	-0.081493816	0.146831644	-0.555015346	0.578884164	0.999579949
332.9375942	0.022342772	0.088365498	0.252844973	0.800388013	0.999579949

260.0428652	0.090807865	0.160081512	0.567260168	0.570537435	0.999579949
2192.396929	-0.021672715	0.056311578	-0.384871383	0.700332709	0.999579949
2177.105355	-0.061954083	0.099909279	-0.620103398	0.535189715	0.999579949
1009.595219	0.043853758	0.060062152	0.73013964	0.465304833	0.999579949
78.93729417	0.289096542	0.343901939	0.840636559	0.400551571	0.999579949
946.6324281	0.01013734	0.052549147	0.192911604	0.847028197	0.999579949
141.307604	0.003892874	0.110820495	0.035127745	0.971977878	0.999579949
732.673779	0.055402219	0.075304309	0.735711134	0.461906513	0.999579949
92.3565429	-0.169043658	0.423063163	-0.399570734	0.689472716	0.999579949
97.11783159	-0.168465427	0.14518151	-1.160377975	0.24589495	0.999579949
408.3565679	-0.051961549	0.115468637	-0.450005741	0.652706301	0.999579949
48.6846931	-0.053735038	0.159954804	-0.335938883	0.736916955	0.999579949
25.87927656	-0.461291795	0.346949562	-1.329564425	0.183661826	0.983992835
80.16549667	-0.04758394	0.141142691	-0.337133573	0.73601621	0.999579949
769.9683008	-0.081425585	0.081630988	-0.997483766	0.31852975	0.999579949
101.3160843	-0.123232527	0.304023468	-0.405338864	0.685228443	0.999579949
295.0959769	0.169454105	0.117809794	1.43837027	0.150329023	0.966305174
424.4734516	0.034187455	0.146386883	0.233541793	0.815340721	0.999579949
340.5457147	0.089539294	0.166730968	0.537028575	0.591247876	0.999579949
1355.160891	0.017332515	0.093098247	0.186174453	0.852307957	0.999579949
142.5945071	-0.142731095	0.104116293	-1.370881459	0.170411912	0.981568287
115.1828728	0.356921603	0.164172969	2.174058282	0.029700758	0.672989344
252.1325524	0.044166272	0.122597112	0.360255405	0.718656145	0.999579949
232.5774015	0.022401854	0.084086047	0.266415833	0.789918973	0.999579949
1065.172454	0.074674046	0.064010325	1.166593762	0.243374464	0.999579949
357.8444573	0.097411095	0.124874909	0.780069396	0.435350029	0.999579949
171.6242687	-0.054882584	0.10572671	-0.519098569	0.603692006	0.999579949
233.7971443	-0.210235204	0.143465181	-1.465409258	0.142809293	0.95210212
124.6973951	0.07307715	0.154336579	0.473492093	0.635862141	0.999579949
194.3685184	0.058885369	0.133056772	0.442558229	0.658085304	0.999579949
231.4603268	0.017306054	0.100956921	0.171420182	0.863893387	0.999579949
597.7497068	0.142636745	0.199266462	0.715809089	0.474109239	0.999579949
2216.138996	-0.048490925	0.08320377	-0.58279721	0.560029824	0.999579949
421.4381495	-0.082208309	0.204732614	-0.401539879	0.688022684	0.999579949
460.7415403	0.380543137	0.149549127	2.544602862	0.010940208	0.487387763
1196.280667	0.094023263	0.109906275	0.855485846	0.392282237	0.999579949
58.72281691	-0.066722056	0.147326137	-0.45288675	0.650630292	0.999579949
265.657672	-0.031648408	0.196155615	-0.161343369	0.871822968	0.999579949
576.2129537	0.071030141	0.121718006	0.583563131	0.559514272	0.999579949
174.3833078	-0.018312108	0.113390815	-0.161495518	0.871703141	0.999579949
27.65362283	-0.088255098	0.196424225	-0.449308621	0.65320904	0.999579949
35.85526825	0.037536506	0.303051712	0.123861719	0.901424763	0.999579949
143.4446628	0.000576194	0.132182758	0.004359068	0.996521978	0.999579949
94.52333136	0.089234571	0.174085831	0.512589512	0.608238491	0.999579949
303.484458	0.006693126	0.122319145	0.054718544	0.956362695	0.999579949
29.20622732	-0.033991567	0.208831155	-0.162770574	0.870699078	0.999579949
3250.160518	0.331772596	0.139164402	2.384033494	0.017124044	0.570162425
566.8038216	-0.518852708	0.210819384	-2.461124297	0.013850237	0.520783867

34.2460538	-0.003001314	0.255544981	-0.011744759	0.990629253	0.999579949
668.6169869	0.004834603	0.058976641	0.081974886	0.934666685	0.999579949
236.005982	0.071770232	0.104424113	0.687295592	0.491896476	0.999579949
32.7389136	0.139189414	0.185166925	0.751696956	0.452233321	0.999579949
3409.428433	-0.05536045	0.112001639	-0.494282504	0.62110668	0.999579949
2101.209638	0.130113091	0.156471834	0.831543214	0.405666826	0.999579949
125.1065842	-0.01275451	0.123555173	-0.103229268	0.917781012	0.999579949
1783.383534	-0.06248976	0.089030839	-0.701888922	0.482748438	0.999579949
563.7444695	0.103742293	0.08436407	1.22969759	0.21881037	0.999579949
44.04474816	-1.017055511	0.387418844	-2.625209191	0.008659575	0.442789376
472.4267075	-0.074503279	0.076158565	-0.978265269	0.327943143	0.999579949
1619.176602	-0.245949882	0.127535662	-1.928479282	0.053795539	0.781954831
110.0502039	0.066236258	0.146035651	0.453562247	0.650143931	0.999579949
111.3143038	0.083761275	0.124894765	0.67065481	0.502440457	0.999579949
475.7836298	-0.272552717	0.074574588	-3.654766631	0.000257416	0.075121841
40.74068215	0.030650548	0.209746589	0.146131329	0.883817715	0.999579949
128.995172	0.191512815	0.177021547	1.081861602	0.279314029	0.999579949
245.6925415	-0.044781253	0.087786434	-0.510115862	0.609970293	0.999579949
185.9382151	0.43218189	0.149573191	2.889434178	0.003859358	0.319818899
1048.931221	0.12488323	0.094022883	1.328221664	0.18410489	0.983992835
32.82243181	0.990615427	0.433937615	2.282852172	0.022439078	0.627784819
869.9111282	-0.178182405	0.162428718	-1.096988312	0.272646502	0.999579949
674.3084157	-0.04556581	0.073292305	-0.621699783	0.534139295	0.999579949
1390.03934	-0.008454239	0.142017406	-0.059529598	0.952530291	0.999579949
104.5952624	-0.105289829	0.20582479	-0.511550765	0.608965451	0.999579949
279.8891249	0.198833816	0.1986006	1.001174295	0.316742551	0.999579949
704.9003872	-0.0154066	0.076372278	-0.201730267	0.840127599	0.999579949
531.4026234	0.089668779	0.068632803	1.306500325	0.191382469	0.984804292
235.0527327	0.120347316	0.10508746	1.145211001	0.252121762	0.999579949
22.75045007	0.358704198	0.424551116	0.844902261	0.398165412	0.999579949
26.06219535	0.089640863	0.407747421	0.219844095	0.825992577	0.999579949
84.48348406	0.209738507	0.308078877	0.680794829	0.496001323	0.999579949
572.5057296	0.019174208	0.049430045	0.387905925	0.698085653	0.999579949
79.72449796	-0.025869984	0.165641189	-0.156180867	0.875890457	0.999579949
40.62315359	0.029950564	0.306417572	0.097744276	0.922135356	0.999579949
31.57301709	0.48938826	0.390168767	1.254298912	0.209733379	0.996447459
3266.578476	0.079343607	0.138920573	0.571143677	0.567902254	0.999579949
625.0360592	-0.134568796	0.333087872	-0.40400389	0.686209859	0.999579949
229.6759676	0.224653959	0.323418108	0.694623936	0.487291008	0.999579949
330.0838937	0.201414895	0.133574529	1.507883999	0.131584219	0.939056553
33.23780313	0.726538213	0.482797015	1.504852329	0.132362047	0.939285826
5698.746348	0.032447296	0.105376359	0.307918177	0.758144594	0.999579949
134.9769408	0.153624466	0.162117689	0.947610756	0.343327648	0.999579949
156.3944825	0.407575494	0.237152451	1.718622317	0.085683165	0.864463816
230.7695874	-0.054882507	0.102625778	-0.534782857	0.592800019	0.999579949
464.1025956	0.002686571	0.123705849	0.021717414	0.982673372	0.999579949
461.2787178	-0.029116768	0.084955494	-0.342729664	0.731801844	0.999579949
192.5238777	-0.054037664	0.093468689	-0.578136535	0.563171942	0.999579949

226.7424357	0.013141625	0.087215148	0.150680535	0.880227729	0.999579949
48.25369438	-0.087017254	0.198792919	-0.437728135	0.661583372	0.999579949
133.187954	0.433394363	0.746561582	0.58052058	0.561563612	0.999579949
232.3024861	-0.005688653	0.106922484	-0.053203524	0.957569748	0.999579949
158.2870227	-0.204359153	0.134004273	-1.525019679	0.127254189	0.934532219
43.91808614	-0.114353658	0.586648812	-0.194926941	0.845450143	0.999579949
454.2468144	-0.0051196	0.075884042	-0.067466094	0.946210653	0.999579949
1866.372358	-0.008765209	0.040160121	-0.218256537	0.827229237	0.999579949
1275.90424	-0.006394466	0.087884439	-0.072759938	0.941997151	0.999579949
4198.914002	-0.025739899	0.108947795	-0.236259016	0.813231688	0.999579949
645.7645735	-0.118894531	0.102284348	-1.162392225	0.24507618	0.999579949
20877.6861	-0.177813303	0.178234355	-0.997637652	0.318455096	0.999579949
46.74066435	-0.512999805	0.339168147	-1.512523537	0.13040073	0.935700262
19651.09795	0.072804018	0.104161056	0.698956217	0.484579393	0.999579949
144.2274301	0.252653304	0.172541512	1.46430445	0.143110782	0.952760787
238.1769044	0.011243758	0.09556589	0.117654512	0.90634141	0.999579949
277.0540607	0.123049434	0.238668447	0.515566409	0.606157276	0.999579949
272.5578694	0.064247751	0.226435192	0.283735714	0.776612923	0.999579949
45.42670305	0.529363079	0.291488168	1.816070553	0.06935955	0.831457432
210.5828492	0.09391567	0.120073588	0.782150941	0.434125872	0.999579949
89.84285769	0.570104312	0.273171834	2.086980575	0.036889891	0.717947029
43.2863889	0.57936389	0.22967383	2.522550736	0.011650714	0.493624274
28.3729962	0.406667782	0.254964243	1.59499927	0.110712399	0.912014923
166.3555339	-0.272384142	0.272079356	-1.001120212	0.316768694	0.999579949
99.17953379	0.182061174	0.283959403	0.641152124	0.521423852	0.999579949
30.60541851	0.37600195	0.415980395	0.903893439	0.366051908	0.999579949
76.91927528	1.288898867	0.744841606	1.7304335	0.083552853	0.859920327
24.93654299	0.18604324	0.338683833	0.549312431	0.582791058	0.999579949
260.5914621	-0.596349413	0.284104279	-2.099051144	0.035812392	0.713754582
2878.349021	0.021301259	0.119919435	0.177629751	0.859013758	0.999579949
27.1046322	-0.082808795	0.2350095	-0.352363607	0.724565591	0.999579949
73.02288918	0.086809138	0.130723386	0.664067389	0.506647181	0.999579949
100.4710844	-0.168412423	0.251491046	-0.669655742	0.503077271	0.999579949
390.2696665	-0.119149204	0.124184948	-0.959449648	0.337332274	0.999579949
142.5971691	-0.087189752	0.11889947	-0.733306483	0.463371522	0.999579949
214.8590919	-0.163812049	0.282178964	-0.580525375	0.56156038	0.999579949
307.4763508	0.019174773	0.099610753	0.192497016	0.847352905	0.999579949
1595.942281	-0.05095157	0.115417393	-0.441454865	0.65888373	0.999579949
236.2371651	0.008086344	0.106665137	0.075810564	0.939569811	0.999579949
814.8717235	-0.133584969	0.117397247	-1.137888434	0.255167072	0.999579949
191.6451377	-0.015425349	0.108734382	-0.141862665	0.887188485	0.999579949
2595.979742	0.066259233	0.04762875	1.39116044	0.16417678	0.978498637
120.0657046	0.006551625	0.152959661	0.042832369	0.965835161	0.999579949
107.2942018	0.124628458	0.175986083	0.708172237	0.478838305	0.999579949
137.9727815	0.103773466	0.13350743	0.777286072	0.436990011	0.999579949
371.2004163	-0.083441827	0.131168463	-0.636142441	0.524683585	0.999579949
1296.526926	-0.143586014	0.094236851	-1.523671601	0.127590767	0.935409261
397.6832028	0.149494719	0.136684451	1.093721471	0.274077155	0.999579949

1116.147985	0.142490145	0.059078198	2.411890512	0.015870046	0.556822021
973.1640475	-0.042282558	0.087317048	-0.484241721	0.628214331	0.999579949
11657.0707	0.405073235	0.152275303	2.660137436	0.007810877	0.42118327
177.4945732	-0.062295638	0.171431399	-0.363385226	0.716317136	0.999579949
1042.685146	-0.149323592	0.106607353	-1.400687556	0.161307526	0.978498637
258.2937234	0.154335747	0.151207719	1.02068696	0.307402775	0.999579949
1097.046542	0.024840656	0.084283065	0.294728913	0.768200974	0.999579949
50.57798286	0.094384722	0.164753741	0.572883635	0.566723486	0.999579949
341.7417313	0.035436374	0.066720012	0.531120622	0.595335195	0.999579949
1178.538849	0.083281513	0.093513038	0.890587179	0.37315068	0.999579949
222.3027627	0.094023968	0.118473656	0.793627638	0.427412238	0.999579949
143.5958345	0.082483838	0.110448688	0.746806863	0.455180152	0.999579949
440.4535616	-0.117650963	0.076552011	-1.536876189	0.124323632	0.924973693
343.0697472	0.051866347	0.102981308	0.503648171	0.614508636	0.999579949
316.641058	0.012216352	0.075170926	0.162514318	0.870900854	0.999579949
356.3848	-0.001252603	0.102344244	-0.012239117	0.990234841	0.999579949
2925.208764	0.052333193	0.056985891	0.918353519	0.358433817	0.999579949
619.8906748	0.286237273	0.201909189	1.417653518	0.156291947	0.976030424
989.4284291	0.226461031	0.106529541	2.125804999	0.0335195	0.698554126
423.5585403	-0.012609821	0.112639053	-0.111948923	0.910863905	0.999579949
278.6315122	-0.145192967	0.147464635	-0.984595166	0.324822965	0.999579949
390.2828167	0.012247013	0.06208887	0.197249741	0.843632108	0.999579949
294.3286103	-0.072559913	0.087303882	-0.831118981	0.405906418	0.999579949
1147.998662	-0.039916787	0.0807949	-0.494050831	0.621270282	0.999579949
32.45841611	-0.408663965	0.260302544	-1.56995763	0.116424969	0.918907698
105.2472222	-0.256185963	0.118763152	-2.15711657	0.030996583	0.683621001
389.5724437	0.015629626	0.073325758	0.21315328	0.831207408	0.999579949
328.1064726	0.102517982	0.071955485	1.424741736	0.15423187	0.975253331
2065.649217	-0.087427935	0.067664363	-1.292082439	0.196328597	0.991421279
86.84980869	0.077834356	0.148344403	0.524686839	0.599800908	0.999579949
492.7491255	-0.209905055	0.111870649	-1.876319281	0.060611451	0.800274462
162.7480663	-0.00193039	0.108088197	-0.017859398	0.98575102	0.999579949
27.05822111	-0.612253708	0.458612194	-1.335014018	0.181871758	0.983992835
625.9524349	-0.009298915	0.123127142	-0.075522872	0.939798701	0.999579949
553.9540428	-0.471422333	0.234029499	-2.014371414	0.043970553	0.758836007
123.4342706	-0.110740086	0.155205524	-0.713506091	0.475532646	0.999579949
722.9525352	0.016417898	0.052747857	0.311252418	0.755608737	0.999579949
1146.193719	-0.05931555	0.074516909	-0.796001212	0.426031331	0.999579949
170.6829194	-0.162710058	0.10349416	-1.572166563	0.115911928	0.918818353
3457.518364	0.001065437	0.069938296	0.015233962	0.987845527	0.999579949
37.71974856	-0.430442422	0.346316522	-1.242916219	0.213898708	0.999268361
589.523588	0.01085997	0.148090819	0.073333176	0.941540992	0.999579949
95.7568685	0.000721752	0.149922302	0.004814171	0.996158862	0.999579949
278.2649641	-0.022503199	0.076220557	-0.295237918	0.767812138	0.999579949
344.4414413	0.473577828	0.153593199	3.083325505	0.002047011	0.23021565
325.7145619	-0.112189466	0.08417429	-1.332823427	0.182589756	0.983992835
69.19637242	0.259584816	0.206795887	1.255270689	0.209380512	0.996447459
1266.843836	0.07003478	0.074287472	0.942753583	0.345806959	0.999579949

2670.428517	-0.191765953	0.133221702	-1.439449809	0.150023121	0.965606146
89.71673435	0.525374011	0.207371727	2.533489106	0.011293325	0.488767809
853.8231338	-0.101286925	0.160953035	-0.629294906	0.529156006	0.999579949
453.7466598	0.064750012	0.057590633	1.124315	0.260879417	0.999579949
48.62320978	-0.128870694	0.199540545	-0.645837135	0.518384836	0.999579949
293.6808481	0.425794959	0.170119744	2.502913239	0.012317574	0.503708792
99.32644685	0.024043341	0.138950907	0.173034791	0.862624081	0.999579949
41.75376157	1.116980081	0.405894351	2.751898565	0.005925087	0.373161982
344.7548057	0.044292784	0.152408264	0.290619308	0.771342491	0.999579949
60.2731504	-0.047522603	0.212650414	-0.223477597	0.823163818	0.999579949
1425.428401	-0.046173811	0.092797645	-0.49757524	0.618783459	0.999579949
141.7758959	0.112177691	0.172021479	0.652114442	0.514327348	0.999579949
1782.057159	-0.325508491	0.28151464	-1.156275536	0.247568472	0.999579949
252.3472416	0.125498453	0.076562669	1.639159836	0.101179976	0.895628172
64.422437	0.019694901	0.263394717	0.074773332	0.94039506	0.999579949
492.3848937	-0.149133546	0.099039939	-1.505791989	0.13212058	0.939285826
58.68955759	-0.034954272	0.148347752	-0.235623874	0.813724546	0.999579949
38.26028986	0.051985809	0.181182523	0.286925078	0.774169688	0.999579949
64.8556499	-0.238236813	0.157324535	-1.514301715	0.129949334	0.935700262
66.36934119	0.415364512	0.275974938	1.505080551	0.132303369	0.939285826
198.8027145	-0.012913211	0.087299023	-0.147919307	0.882406451	0.999579949
23.49888657	0.021850992	0.246192524	0.08875571	0.929276057	0.999579949
85.04594984	-0.099238603	0.146766629	-0.67616599	0.498935263	0.999579949
30.06287538	-0.101478586	0.638772285	-0.158865043	0.873775203	0.999579949
85.97982662	0.147720812	0.22676682	0.651421633	0.51477435	0.999579949
329.3779011	0.212536059	0.244857923	0.867997477	0.385395714	0.999579949
773.0554285	-0.212169856	0.134166275	-1.581394849	0.113787784	0.917587025
625.761991	-0.051478677	0.096419758	-0.533901749	0.593409512	0.999579949
59.28084011	-0.082289089	0.152899433	-0.538190937	0.590445236	0.999579949
491.2457893	-0.0503781	0.119942667	-0.420018177	0.674472175	0.999579949
42.57832085	-0.325053259	0.183743093	-1.769063821	0.076883225	0.851207389
354.2722064	-0.00288124	0.086554984	-0.033287976	0.973444943	0.999579949
27.33055616	-1.170817834	0.674024338	-1.737055724	0.082377335	0.856126169
535.4054088	-0.138102564	0.235950784	-0.585302415	0.558344391	0.999579949
147.7918737	-0.283465034	0.146150921	-1.939536418	0.052436055	0.780714589
72.00333544	0.605309382	0.315858009	1.916397129	0.055314566	0.785214091
664.9992344	-0.147408225	0.125946904	-1.170399753	0.241840135	0.999579949
150.626141	0.07081	0.088479987	0.800293968	0.423540497	0.999579949
176.1544889	0.083282293	0.123412236	0.674830113	0.499783706	0.999579949
864.2756141	-0.204026964	0.13854446	-1.472646131	0.140846479	0.948318761
3022.449122	0.102885667	0.148955302	0.690715034	0.48974464	0.999579949
77.9889149	-0.359886855	0.138674528	-2.59519077	0.009453843	0.458002332
94.5985994	0.142580083	0.118266044	1.205587657	0.227976521	0.999579949
172.0399662	0.040357891	0.086920066	0.464310397	0.642425376	0.999579949
59.02354344	-0.129236357	0.172270052	-0.750196308	0.453136482	0.999579949
1212.318672	-0.012270862	0.114605115	-0.107070807	0.914732806	0.999579949
52.66534141	0.07863693	0.136639001	0.575508675	0.564947325	0.999579949
76.68136951	-0.213051799	0.161530367	-1.318958183	0.187183093	0.983992835

646.1274997	-0.027997767	0.054178723	-0.516766828	0.605318939	0.999579949
154.8170809	0.100234728	0.091016147	1.101285113	0.270772588	0.999579949
895.0502205	-0.063557263	0.125963463	-0.504569033	0.613861564	0.999579949
227.3974821	-0.071957841	0.122545741	-0.587191696	0.557074969	0.999579949
21.16657294	-0.106245153	0.800841093	-0.13266696	0.894456774	0.999579949
2241.22856	-0.086131948	0.094738547	-0.909154203	0.363268734	0.999579949
94.00180679	-0.148762407	0.180120707	-0.825903973	0.408858567	0.999579949
773.1159248	-0.261291716	0.123303628	-2.119091867	0.034082704	0.703403556
36.51343164	-0.159230809	0.212514773	-0.749269366	0.453694868	0.999579949
457.8053675	0.034529894	0.099188207	0.348124998	0.727746311	0.999579949
51.41901542	0.207581421	0.369589382	0.561654179	0.574351659	0.999579949
528.0878156	-0.143370185	0.062313159	-2.300801115	0.021402875	0.622105864
382.2558067	0.102798074	0.10441733	0.984492464	0.324873435	0.999579949
505.6581859	-0.117360563	0.123016848	-0.954020242	0.340073396	0.999579949
2706.219449	-0.154059827	0.125359265	-1.228946479	0.219091873	0.999579949
217.1693353	-0.012381143	0.104898256	-0.118030017	0.906043874	0.999579949
148.5746266	-0.337472737	0.252887581	-1.334477306	0.18204748	0.983992835
204.1528426	-0.11211411	0.082435855	-1.360016341	0.173824753	0.983601342
863.8099847	-0.170853919	0.089055021	-1.918520902	0.055044994	0.785214091
153.7113215	0.093738758	0.143867259	0.65156422	0.514682336	0.999579949
121.3410163	-0.003321719	0.136671852	-0.02430434	0.980609851	0.999579949
55.19256738	-0.103158849	0.165325755	-0.62397325	0.532645152	0.999579949
40.92754546	-0.152900351	0.240863224	-0.634801563	0.525557843	0.999579949
2154.352428	0.004945389	0.084714901	0.058376851	0.953448453	0.999579949
82.16713419	0.074228907	0.181243854	0.409552681	0.682134114	0.999579949
190.6385337	-0.002865364	0.082203175	-0.034857092	0.972193695	0.999579949
717.6589991	-0.166024497	0.131746994	-1.260176734	0.207605614	0.995145345
6938.856832	0.047086277	0.130049247	0.362064971	0.717303479	0.999579949
597.6222457	0.001886363	0.08393541	0.022473986	0.982069863	0.999579949
22.56582367	0.138394703	0.397469933	0.348189111	0.727698165	0.999579949
360.4233973	0.162619168	0.103203218	1.575717999	0.115090808	0.918818353
59.76849217	0.454250603	0.173049822	2.624970076	0.008665658	0.442789376
269.5298043	0.084994204	0.10809714	0.786276155	0.431705719	0.999579949
70.50480123	-0.147305785	0.22368274	-0.65854784	0.510186167	0.999579949
159.3791586	-0.431216464	0.193556475	-2.227858629	0.025889944	0.648237123
479.2939071	-0.079691313	0.171716471	-0.464086599	0.642585703	0.999579949
230.3809003	0.016064714	0.127955889	0.125548843	0.90008906	0.999579949
369.2475759	-0.041988228	0.134192054	-0.312896532	0.754359272	0.999579949
143.3757366	0.390220331	0.172894982	2.256978931	0.024009391	0.634610194
1141.328212	0.004930483	0.098985336	0.049810235	0.960273611	0.999579949
744.8523355	-0.028031881	0.135230926	-0.207288981	0.835784179	0.999579949
56.20894389	0.002802945	0.263847668	0.010623344	0.991523957	0.999579949
20.82354898	0.677508626	0.616170708	1.099546955	0.271529565	0.999579949
680.2563832	0.007471695	0.108414948	0.068917568	0.945055235	0.999579949
2489.692155	0.010448131	0.133545818	0.078236305	0.937640084	0.999579949
427.9216326	-0.093901024	0.196116704	-0.478801768	0.632079659	0.999579949
256.9375266	0.113181217	0.157673198	0.717821536	0.472867333	0.999579949
233.9514404	0.028693432	0.112202435	0.255729141	0.798159992	0.999579949

528.9863398	-0.144623195	0.164409491	-0.879652353	0.379047669	0.999579949
1037.61341	0.165674608	0.123893679	1.337232131	0.181146877	0.983992835
98.29776482	-0.176775698	0.154329769	-1.145441344	0.25202638	0.999579949
295.349689	0.01959703	0.076776601	0.255247434	0.798531997	0.999579949
360.1854428	0.752892154	0.264947866	2.841661512	0.004487911	0.33947676
90.12536669	0.595930599	0.209630169	2.842771165	0.004472317	0.33947676
69.90924166	-0.400703837	0.140650585	-2.848931172	0.004386637	0.337942157
291.9068577	0.010996761	0.080501993	0.13660234	0.891345127	0.999579949
184.1690398	0.167755615	0.097270978	1.724621453	0.084595715	0.861032139
1080.633007	-0.278185844	0.181006717	-1.536881327	0.124322373	0.924973693
136.3715253	-0.129278061	0.115463439	-1.119644988	0.262865076	0.999579949
203.2136047	-0.265463159	0.106551731	-2.491401659	0.012724019	0.510304755
1808.674646	0.124587354	0.12576562	0.990631253	0.321865671	0.999579949
181.888523	-0.133546038	0.115717877	-1.154065743	0.248473217	0.999579949
701.3665881	-0.044355244	0.173232269	-0.256044928	0.797916146	0.999579949
77.87540563	-0.007404264	0.133725984	-0.055368929	0.955844549	0.999579949
529.2116296	-0.008417248	0.07373703	-0.114152256	0.909117098	0.999579949
22.82043771	0.747475107	0.362891533	2.059775548	0.039420003	0.734990441
58.19455204	0.029700487	0.168227731	0.176549293	0.859862426	0.999579949
417.6909703	0.083919624	0.194595197	0.431252287	0.666284939	0.999579949
320.5723952	0.004156361	0.142736306	0.029119157	0.976769557	0.999579949
88.44550884	0.193640893	0.295427195	0.655460621	0.512171232	0.999579949
71.69479199	0.354098561	0.163951431	2.159777197	0.030789922	0.683621001
140.0948808	0.225484156	0.17342274	1.300199477	0.19353261	0.986374671
439.6538278	-0.023271449	0.12824132	-0.181466074	0.85600176	0.999579949
10683.00961	-0.032813273	0.192079554	-0.17083168	0.864356118	0.999579949
162.6862231	-0.119937017	0.196222066	-0.611231036	0.54104664	0.999579949
119.9214112	0.052437412	0.109138486	0.480466736	0.630895551	0.999579949
148.4289701	-0.002921986	0.178645633	-0.016356324	0.986950123	0.999579949
477.3573193	-0.140631096	0.097851553	-1.437188188	0.150664528	0.96651114
171.5268888	-0.070708378	0.182762209	-0.3868873	0.698839643	0.999579949
1283.744669	0.106961529	0.127252347	0.840546615	0.400601977	0.999579949
75.13335827	0.093251493	0.176531259	0.528243515	0.597330331	0.999579949
130.4276997	0.040652782	0.108190614	0.375751462	0.707101675	0.999579949
72.14943214	-0.021283953	0.177638449	-0.119816141	0.904628796	0.999579949
24.72447762	-0.01317547	0.188617201	-0.069852963	0.944310692	0.999579949
155.6507238	-0.053356226	0.198793804	-0.268399843	0.78839156	0.999579949
656.070528	0.034657395	0.110991603	0.312252407	0.754848707	0.999579949
966.9725996	-0.070511138	0.108687659	-0.64875018	0.516499864	0.999579949
990.4229643	-0.271680556	0.137956139	-1.969325592	0.048915714	0.773861498
271.4533457	-0.028401037	0.164400538	-0.172755134	0.862843905	0.999579949
153.7769776	0.068611112	0.125882601	0.545040472	0.585725682	0.999579949
64.42172056	0.002960563	0.122089552	0.024249108	0.980653907	0.999579949
1497.993021	-0.068154204	0.095943496	-0.710357722	0.477482334	0.999579949
33.03673884	0.013045641	0.221892159	0.058792707	0.953117218	0.999579949
51.11663614	0.459093093	0.233271785	1.968060962	0.049061024	0.774401825
963.6289831	-0.140554407	0.099357862	-1.414627937	0.157177613	0.977304288
101.8404287	0.048279201	0.126985861	0.380193519	0.70380177	0.999579949

129.2126752	-0.153107811	0.141932894	-1.07873381	0.280706409	0.999579949
309.60388	-0.04345685	0.173388384	-0.250632992	0.802097872	0.999579949
327.5095593	0.212136222	0.112336717	1.88839614	0.058972789	0.79629879
187.0907388	0.070465195	0.101727703	0.692684427	0.488507614	0.999579949
219.9729788	0.033337784	0.094430966	0.353038685	0.724059439	0.999579949
107.0428457	-0.026947844	0.107674441	-0.250271499	0.802377395	0.999579949
166.000706	-0.047177382	0.2185648	-0.215850778	0.829104066	0.999579949
120.0238174	0.157686047	0.104213228	1.513109701	0.130251796	0.935700262
1551.93649	-0.04468942	0.089526782	-0.499173754	0.617656983	0.999579949
45.22167257	0.146825715	0.143796374	1.021066879	0.307222754	0.999579949
464.8297651	0.113014462	0.101361031	1.11496954	0.264863475	0.999579949
610.8367915	0.312807671	0.120413099	2.597787723	0.009382648	0.458002332
180.1449856	-0.016993943	0.115283611	-0.147409881	0.882808507	0.999579949
921.4427206	-0.024561889	0.085679511	-0.28667168	0.774363724	0.999579949
375.3624189	0.072500642	0.131850608	0.549869606	0.582408812	0.999579949
637.7769842	-0.048967469	0.066618244	-0.73504593	0.462311522	0.999579949
26.05281598	-0.302230716	0.652802257	-0.462974374	0.643382736	0.999579949
913.7996334	-0.256730719	0.129569916	-1.981406847	0.047545667	0.770705547
35.02213959	-0.120867051	0.223535631	-0.540705973	0.588710261	0.999579949
264.2468122	0.018316687	0.110754319	0.165381245	0.868643915	0.999579949
183.2144118	-0.15699231	0.094841541	-1.655311669	0.097861293	0.890209389
307.4715029	0.062293379	0.116243483	0.535887067	0.592036603	0.999579949
2333.274768	0.352028113	0.141961699	2.479740056	0.013147819	0.514046841
25.5714613	0.381097675	0.249614283	1.526746267	0.126824118	0.933836888
211.8572769	-0.024771072	0.117819067	-0.210246713	0.833475123	0.999579949
392.4709664	0.221496155	0.130792867	1.693488028	0.090362612	0.876222021
151.231228	-0.158812517	0.169193339	-0.93864521	0.347912933	0.999579949
171.4794782	0.105314025	0.0896191	1.175129237	0.239943015	0.999579949
32.43397153	-0.128726013	0.266088633	-0.483771185	0.628548267	0.999579949
417.4958126	0.023457792	0.080871719	0.290061751	0.771768997	0.999579949
354.7108005	-0.066953952	0.11638319	-0.575288849	0.565095962	0.999579949
607.3807467	-0.039471779	0.140081253	-0.281777738	0.778113942	0.999579949
120.7146699	0.315785521	0.195518061	1.61512199	0.10628428	0.898494488
462.2854566	0.024110111	0.089415429	0.269641508	0.787436064	0.999579949
29.25511282	0.400870234	0.433248002	0.925267358	0.354826853	0.999579949
35.92781497	0.834108162	0.522000668	1.597906309	0.11006383	0.909091147
39.73214741	1.230108194	0.552955018	2.22460807	0.02610756	0.648237123
614.7053704	0.750391945	0.198695419	3.776594085	0.000158988	0.057217337
59.15973966	0.118007229	0.146670826	0.804571923	0.42106675	0.999579949
300.8751973	-0.054122924	0.090145549	-0.60039486	0.548243112	0.999579949
473.1829714	0.042708823	0.077656975	0.549967646	0.582341565	0.999579949
30.64623554	-0.232446687	0.26100852	-0.890571261	0.373159223	0.999579949
1630.593256	0.463116096	0.159548037	2.90267498	0.003699904	0.319205419
40.45399904	0.886682456	0.322912154	2.74589372	0.006034631	0.374444391
304.0838184	0.245328024	0.187052849	1.311543903	0.189674071	0.983992835
54.01396519	0.106969224	0.261173893	0.409570889	0.682120755	0.999579949
215.5223133	-0.066741518	0.095830647	-0.696452748	0.486145339	0.999579949
538.7118562	-0.093059117	0.183771204	-0.506385738	0.612585884	0.999579949

129.1632369	-0.118901101	0.134600162	-0.883365213	0.37703898	0.999579949
24.83019329	0.098689191	0.215358532	0.458255312	0.646769026	0.999579949
329.0030428	-0.095781179	0.162261045	-0.59029066	0.5549958	0.999579949
329.6966848	0.118331834	0.11546705	1.024810403	0.305452657	0.999579949
2112.13708	-0.000518508	0.083580525	-0.006203689	0.995050204	0.999579949
680.5670073	-0.025228189	0.105837932	-0.238366228	0.811597058	0.999579949
516.0717249	0.129864283	0.106513707	1.219226024	0.222758417	0.999579949
860.6212522	0.296463506	0.116390825	2.547138109	0.010861044	0.486853058
2827.536181	0.02728615	0.073843517	0.369513146	0.711745276	0.999579949
31.4613536	0.177765164	0.236463612	0.751765412	0.452192145	0.999579949
105.9802975	-0.062179251	0.134347284	-0.462824772	0.643489974	0.999579949
96.59980304	-0.195369549	0.303431076	-0.643867965	0.519661057	0.999579949
365.7293362	0.018456223	0.083735512	0.220410937	0.825551128	0.999579949
673.6052613	0.027976877	0.10384582	0.269407826	0.787615864	0.999579949
794.2307185	0.150036035	0.084951836	1.766130571	0.077373946	0.851207389
691.4552228	-0.060912279	0.076131463	-0.800093377	0.423656698	0.999579949
248.4976518	0.360809584	0.284443085	1.268477256	0.204627568	0.994814558
345.292974	0.068804725	0.079752793	0.862724953	0.38828871	0.999579949
157.3083566	0.081578886	0.11155	0.731321261	0.464582947	0.999579949
244.3628268	0.308235797	0.203009649	1.518330773	0.128931034	0.935700262
56.85457779	-0.136700805	0.495232955	-0.276033336	0.782522462	0.999579949
10111.9148	-0.013135779	0.072877183	-0.180245431	0.856959894	0.999579949
323.1178257	-0.294612153	0.108242474	-2.72177956	0.006493144	0.39001336
438.52249	0.152442724	0.301069187	0.506337847	0.612619498	0.999579949
102.4807688	-0.213448086	0.25151655	-0.848644295	0.396079252	0.999579949
218.7755589	-0.154681579	0.187203459	-0.826275214	0.40864799	0.999579949
280.5263741	-0.030794458	0.199399259	-0.154436169	0.877265837	0.999579949
35.75080653	0.264298209	0.249584124	1.058954411	0.289620542	0.999579949
310.7678668	-0.187016987	0.10649647	-1.756086257	0.079073681	0.851207389
1671.225971	-0.067637583	0.081606709	-0.828823799	0.407204119	0.999579949
2055.629652	-0.020682037	0.110358101	-0.18740842	0.851340421	0.999579949
345.0853035	-0.052077687	0.078476681	-0.663607146	0.506941782	0.999579949
1648.409125	0.064508587	0.074146106	0.870019893	0.384289533	0.999579949
327.675869	0.076362093	0.118937604	0.6420349	0.520850523	0.999579949
335.2778338	-0.17892245	0.180584573	-0.990795872	0.321785265	0.999579949
59.65516386	0.033098498	0.259737358	0.127430642	0.898599565	0.999579949
187.5893046	-0.334635165	0.134502529	-2.487947021	0.012848287	0.510304755
276.7568136	0.385187729	0.314362594	1.225297588	0.220463117	0.999579949
244.7789844	0.212536364	0.098268143	2.162820592	0.030554981	0.683621001
518.4172068	0.091256577	0.088218311	1.034440309	0.300930368	0.999579949
117.2334114	0.331691757	0.369580289	0.897482272	0.369461629	0.999579949
425.3087158	0.050869381	0.064147711	0.793003833	0.427775589	0.999579949
781.5183854	0.029222879	0.050495715	0.57871998	0.562778133	0.999579949
594.3548289	0.074280607	0.091528315	0.811558767	0.417044859	0.999579949
454.6080277	-0.070663316	0.100345548	-0.704199809	0.48130835	0.999579949
107.8452812	-0.314063109	0.190270342	-1.65061515	0.098817183	0.894181927
403.580672	-0.025022429	0.091710808	-0.272840566	0.784975775	0.999579949
31.98447474	1.094580645	0.479806545	2.281295777	0.02253095	0.627784819

246.0589019	0.136099489	0.084292066	1.614618024	0.10639344	0.898814062
86.30298559	0.276562898	0.143253777	1.930580149	0.053534995	0.781231069
8303.521865	-0.119972684	0.111384785	-1.077101181	0.281435065	0.999579949
471.7310659	0.171234754	0.081786292	2.093685271	0.036288026	0.716432561
232.2404492	0.094843693	0.084835967	1.117965602	0.263581685	0.999579949
168.2009669	0.020931155	0.178260114	0.117419175	0.90652789	0.999579949
597.6954109	-0.023439547	0.179990175	-0.130226816	0.896386979	0.999579949
126.4539562	-0.154342899	0.215455831	-0.716355173	0.473772067	0.999579949
399.7979034	-0.018323952	0.111992712	-0.16361736	0.870032377	0.999579949
1045.944966	-0.009333412	0.083291413	-0.112057315	0.910777962	0.999579949
154.8722393	0.025416221	0.100673812	0.252461092	0.800684684	0.999579949
26.1099561	0.08870371	0.227337755	0.390184684	0.696399986	0.999579949
764.8162579	-0.10857444	0.097488956	-1.113710154	0.265403552	0.999579949
406.6679161	-0.025790049	0.090862257	-0.283836758	0.776535484	0.999579949
444.9657172	0.442237398	0.203280291	2.175505535	0.029592254	0.672989344
3135.263566	-0.038517922	0.101627386	-0.379011241	0.704679516	0.999579949
2485.064182	-0.141189983	0.09003603	-1.568149805	0.116846176	0.920479614
295.2906856	-0.040039251	0.222099424	-0.180276249	0.8569357	0.999579949
93.67872696	-0.032311626	0.206320465	-0.156608926	0.875553068	0.999579949
137.7272697	0.027643403	0.161201579	0.171483452	0.863843641	0.999579949
210.1150862	-0.069757574	0.13823221	-0.504640519	0.613811345	0.999579949
580.8833206	0.167827149	0.146555848	1.145141261	0.252150646	0.999579949
1709.166596	-0.131894884	0.166588692	-0.791739717	0.428512459	0.999579949
1569.164085	0.070242765	0.146924565	0.478087273	0.63258809	0.999579949
452.680913	0.043157171	0.088848384	0.485739517	0.627151862	0.999579949
479.416333	-0.054395146	0.056451219	-0.963577891	0.335257596	0.999579949
295.2764868	0.068442606	0.14806947	0.462233075	0.643914187	0.999579949
1350.763781	-0.057006099	0.063168927	-0.902438933	0.366823747	0.999579949
48.71751826	-0.342149723	0.275336756	-1.24265909	0.213993485	0.999268361
1036.089333	0.066590441	0.104801465	0.63539609	0.525170117	0.999579949
497.0187936	0.041509808	0.160630951	0.258417247	0.796084907	0.999579949
144.7735108	-0.127689569	0.15181326	-0.84109629	0.400293993	0.999579949
73.27934841	0.04201206	0.161148186	0.260704517	0.794320382	0.999579949
545.0611288	0.089960731	0.155932301	0.576921718	0.563992338	0.999579949
118.6017761	0.065191462	0.100159751	0.650874839	0.515127285	0.999579949
62.42259126	-0.211928733	0.16758918	-1.264572885	0.206024478	0.994814558
130.960851	-0.208775976	0.217004787	-0.96208005	0.336009395	0.999579949
99.64861247	-0.127885526	0.167291949	-0.764445191	0.444601987	0.999579949
335.7108536	-0.136681289	0.118004549	-1.158271362	0.246753315	0.999579949
227.4852836	-0.022944853	0.111661045	-0.205486641	0.837191935	0.999579949
62.30593538	-0.036788942	0.204673401	-0.179744616	0.857353065	0.999579949
5009.339904	0.024378029	0.108134824	0.225441061	0.821636172	0.999579949
281.8427017	-0.074127946	0.088433824	-0.838230705	0.401901145	0.999579949
71.95551631	-0.154157188	0.168674795	-0.913931383	0.360752902	0.999579949
34.68476496	0.255334808	0.226398664	1.127810579	0.259399925	0.999579949
368.2311406	-0.110091529	0.100515894	-1.095264881	0.273400609	0.999579949
2732.726447	0.173374933	0.086137575	2.012767749	0.044139068	0.758836007
107.9035175	0.043151524	0.139295901	0.309783156	0.756725862	0.999579949

693.4212869	0.094260695	0.05773066	1.632766627	0.102518089	0.895628172
123.3797137	-0.138358398	0.156687844	-0.883019345	0.37722582	0.999579949
1132.538415	-0.278902569	0.179972377	-1.549696537	0.12121437	0.921837625
1349.595231	-0.201981727	0.138859491	-1.454576316	0.145786592	0.957420545
57.76724177	-0.289248579	0.199117641	-1.452651699	0.146320484	0.959294798
93.55308248	-0.170508613	0.119304463	-1.429188892	0.15294995	0.972669728
469.4250196	-0.397152558	0.288143027	-1.378317435	0.168105302	0.980087289
308.9042	0.034156207	0.082154081	0.415757887	0.677587178	0.999579949
360.8361458	0.041221235	0.147535566	0.279398633	0.77993892	0.999579949
130.480777	0.00061161	0.118521669	0.00516032	0.995882679	0.999579949
110.7398231	0.130900115	0.625265725	0.209351176	0.834174104	0.999579949
561.8698948	0.107507814	0.132491857	0.811429599	0.417119007	0.999579949
532.4754576	-0.056770887	0.058101163	-0.977104139	0.328517599	0.999579949
863.4092509	0.114589336	0.090254161	1.26962939	0.204216675	0.994814558
28.38058123	-0.286183793	0.338448034	-0.845576762	0.397788894	0.999579949
20.50432257	0.765562625	0.73801882	1.037321278	0.299586153	0.999579949
72.02158434	0.071948797	0.129647949	0.554955147	0.57892534	0.999579949
256.4279872	-0.096379676	0.154208465	-0.624996017	0.531973672	0.999579949
83.03767158	-0.350361543	0.436683974	-0.80232288	0.422366211	0.999579949
2321.704453	0.109540665	0.149518189	0.732624342	0.46378758	0.999579949
279.9868355	0.076984091	0.099199417	0.776053864	0.437717183	0.999579949
1205.337099	-0.045481668	0.048774597	-0.932486809	0.351085	0.999579949
1591.882036	0.025156467	0.072250836	0.348182364	0.727703231	0.999579949
762.3902533	0.112160258	0.048054156	2.334038668	0.019593697	0.601494972
57.78381131	-0.189458408	0.178738869	-1.059973183	0.2891568	0.999579949
333.0063157	0.210694779	0.09868764	2.134966228	0.032763769	0.695900535
142.9121403	0.215301337	0.111479744	1.931304556	0.0534454	0.781231069
214.3941302	-0.065239855	0.122033083	-0.53460794	0.592920992	0.999579949
358.3417739	-0.070772777	0.119926492	-0.590134637	0.555100389	0.999579949
22.73210749	-0.150020318	0.329361118	-0.455488852	0.648757586	0.999579949
273.8999928	-0.055990749	0.110622533	-0.506142354	0.61275672	0.999579949
70.93675892	0.165559639	0.253296236	0.653620605	0.513356266	0.999579949
89.62884911	0.333723066	0.489641148	0.681566628	0.495513023	0.999579949
162.0442898	-0.066484629	0.115922694	-0.573525564	0.566288895	0.999579949
2234.092769	0.033526573	0.13013387	0.257631418	0.79669138	0.999579949
63.82007552	0.033034477	0.186546711	0.17708421	0.859442244	0.999579949
5543.659038	-0.098232967	0.107819409	-0.911087975	0.362249022	0.999579949
38.64616145	-0.187056987	0.225528945	-0.829414543	0.406869875	0.999579949
616.7429301	-0.036849178	0.091971858	-0.400657104	0.688672597	0.999579949
554.1669271	-0.039520985	0.105111856	-0.375989798	0.706924481	0.999579949
348.1052974	-0.133104343	0.179559323	-0.741283388	0.458521632	0.999579949
80.4901922	0.04658274	0.123778097	0.376340736	0.706663599	0.999579949
370.8719056	-0.031220745	0.235155801	-0.132766213	0.894378276	0.999579949
67.23806917	0.002138239	0.30547354	0.006999751	0.994415053	0.999579949
20.86688853	2.149242204	1.201211481	1.789228822	0.073577972	0.842343849
2229.862255	-0.359037184	0.531174386	-0.675930906	0.499084514	0.999579949
580.4717606	-0.085914026	0.08319675	-1.032660841	0.301762645	0.999579949
1485.915122	-0.055121471	0.09362264	-0.588762192	0.556020812	0.999579949

129.6915769	0.508176578	0.252447884	2.012995993	0.044115051	0.758836007
378.2037674	0.019335207	0.075183106	0.257174893	0.797043764	0.999579949
48.93820278	0.113818776	0.1700147	0.669464319	0.503199335	0.999579949
1192.858478	-0.030556011	0.057296345	-0.533297734	0.593827496	0.999579949
153.1957584	-0.053862233	0.387594076	-0.138965573	0.889477353	0.999579949
542.2835917	0.148495971	0.178181162	0.833398822	0.404619839	0.999579949
125.1198161	-0.960792724	0.432601627	-2.220964195	0.026353387	0.649603244
297.2181706	-0.010581673	0.086243579	-0.122695201	0.902348464	0.999579949
1695.796485	-0.083674677	0.080010401	-1.045797497	0.295654537	0.999579949
312.4011254	0.247634518	0.253337579	0.977488296	0.328327469	0.999579949
1687.145865	0.106158119	0.099290921	1.069162394	0.284996501	0.999579949
40.68417274	0.000300533	0.229967874	0.001306847	0.998957287	0.999840121
3643.698009	0.031811864	0.115046319	0.276513532	0.782153668	0.999579949
703.6489979	0.134672749	0.112415826	1.197987454	0.230921901	0.999579949
525.1983121	-0.086115076	0.155675089	-0.553171842	0.580145746	0.999579949
93.56588233	0.232417771	0.222395719	1.045064051	0.295993367	0.999579949
388.0230596	-0.006806318	0.074553124	-0.091294879	0.927258287	0.999579949
4033.593064	0.052072309	0.136457861	0.38159992	0.702758143	0.999579949
144.6074624	-0.35135059	0.137376055	-2.557582474	0.010540256	0.483019182
500.650729	-0.001226149	0.077733485	-0.01577375	0.987414891	0.999579949
2017.414336	-0.067200737	0.06594204	-1.019087924	0.308161227	0.999579949
67.83816928	-0.272226969	0.192712689	-1.412605313	0.157771806	0.977304288
119.4350513	0.181758725	0.127636151	1.424037974	0.15443548	0.975561336
33.16924728	0.173250168	0.183452289	0.944388151	0.344971334	0.999579949
58.69456138	-0.271544585	0.20632449	-1.316104477	0.188138978	0.983992835
701.7733141	0.180576368	0.11189233	1.613840454	0.106562037	0.899087397
152.951266	-0.075735302	0.118479152	-0.639228936	0.52267401	0.999579949
824.3545209	-0.062383973	0.078962312	-0.790047442	0.429500062	0.999579949
1553.73617	0.043535884	0.103233348	0.421723061	0.67322717	0.999579949
402.0444906	0.008291263	0.178677171	0.046403595	0.962988571	0.999579949
449.9441833	-0.064829404	0.094183353	-0.688331872	0.491243815	0.999579949
82.0666293	0.027024131	0.259516699	0.104132532	0.917064174	0.999579949
855.4566523	-0.002299685	0.139550467	-0.016479235	0.986852068	0.999579949
20.60400257	0.312875982	0.234162108	1.3361512	0.181499858	0.983992835
180.5762549	-0.082968041	0.097886387	-0.847595293	0.396663397	0.999579949
1473.101764	-0.058674246	0.091799712	-0.639155011	0.522722096	0.999579949
635.1744176	0.028176698	0.078925502	0.357003726	0.721089016	0.999579949
4033.661523	0.133248987	0.107067847	1.244528496	0.213305118	0.999268361
119.5298138	0.089994633	0.34199301	0.263147581	0.792436832	0.999579949
41.03436768	0.274169455	0.256453509	1.069080536	0.285033381	0.999579949
898.1793312	-0.057113786	0.085051758	-0.671517996	0.501890599	0.999579949
839.247029	0.057154109	0.073760313	0.774862609	0.438420848	0.999579949
177.9288909	0.090835595	0.120910348	0.751264022	0.452493777	0.999579949
320.8765645	0.068211942	0.085226734	0.800358518	0.423503108	0.999579949
3299.713716	0.112123968	0.177046485	0.633302423	0.526536171	0.999579949
202.8490274	-0.157263251	0.130440631	-1.205630863	0.227959853	0.999579949
62.49154655	0.365888779	0.21174351	1.727981079	0.083991617	0.860148832
281.8952716	0.125830196	0.14739017	0.853721761	0.393259174	0.999579949

557.1000212	0.140723377	0.159477338	0.882403595	0.377558592	0.999579949
89.97986474	0.130689304	0.185795287	0.703404841	0.48180349	0.999579949
259.7748627	-0.008717734	0.125823893	-0.069285204	0.944762603	0.999579949
59.24899532	-0.003853977	0.137162466	-0.028097899	0.977584069	0.999579949
351.2644636	-0.08730721	0.078803229	-1.107914123	0.267898906	0.999579949
1173.479502	-0.048864061	0.078951336	-0.618913672	0.535973231	0.999579949
333.1124927	-0.116980269	0.088786924	-1.317539388	0.187657887	0.983992835
1936.567858	-0.011256067	0.115747901	-0.097246401	0.92253072	0.999579949
927.1352796	0.024564376	0.07573651	0.324339952	0.745680676	0.999579949
262.1451938	0.106420461	0.115452448	0.921768767	0.356649202	0.999579949
20660.11866	0.040734373	0.049892154	0.816448464	0.414243688	0.999579949
314.1426133	-0.007272917	0.093717195	-0.077604936	0.938142316	0.999579949
343.7396557	0.117219329	0.188790271	0.620897085	0.534667339	0.999579949
1595.989085	-0.001664079	0.150433624	-0.011061882	0.991174075	0.999579949
525.7869375	0.061890916	0.209993982	0.294727093	0.768202364	0.999579949
70.13539765	0.139376704	0.149947793	0.929501535	0.35262923	0.999579949
23.18968485	0.066465345	0.313092358	0.212286704	0.831883367	0.999579949
265.1332545	0.017870542	0.116691147	0.153143941	0.878284764	0.999579949
753.0218882	0.004880195	0.086963094	0.056117998	0.955247806	0.999579949
724.4247419	-0.109649117	0.070704169	-1.550815434	0.120945921	0.921837625
231.4168054	0.061925668	0.089674389	0.690561355	0.48984124	0.999579949
195.6216166	-0.013952862	0.123062026	-0.113380726	0.909728719	0.999579949
75.74418994	0.217419641	0.279968029	0.776587388	0.437402245	0.999579949
55.04363164	0.427095636	0.232167373	1.839602313	0.065826645	0.82135988
350.5627782	0.28150767	0.162144468	1.736153409	0.082536713	0.85707538
469.0676992	0.119621608	0.151923484	0.78738063	0.431059082	0.999579949
7179.667917	-0.173967632	0.091527218	-1.900720187	0.057338673	0.792796844
697.0973876	0.045779644	0.084595971	0.541156315	0.588399845	0.999579949
47.2342307	-0.082184609	0.583820434	-0.140770354	0.888051364	0.999579949
693.212911	-0.086410847	0.064725156	-1.335042703	0.18186237	0.983992835
8126.692303	-0.061438719	0.102124661	-0.601605118	0.547437021	0.999579949
127.8187609	0.046345458	0.10472842	0.442529911	0.658105791	0.999579949
44.12010692	0.006375386	0.163904346	0.038896993	0.968972514	0.999579949
43.82033868	0.220533734	0.221027185	0.997767462	0.318392132	0.999579949
91.24695177	-0.007061911	0.129597033	-0.054491301	0.956543739	0.999579949
117.0331804	-0.026107816	0.098517348	-0.265007292	0.791003844	0.999579949
29.92425255	-0.190882306	0.227258311	-0.839935427	0.400944593	0.999579949
30.93441556	0.086860286	0.206664994	0.420295106	0.674269885	0.999579949
91.38664582	-0.132629262	0.121187978	-1.0944094	0.273775463	0.999579949
57.27982232	0.011450723	0.17782341	0.064393789	0.948656676	0.999579949
42.81931767	0.1097945	0.208119998	0.527553821	0.597809051	0.999579949
307.6961239	-0.042449514	0.1048469	-0.404871426	0.685572023	0.999579949
543.5663054	-0.098811194	0.11999405	-0.823467444	0.410242224	0.999579949
1435.56125	0.065186067	0.088185563	0.739192058	0.459790383	0.999579949
59.08413023	-0.140884906	0.148025503	-0.951761041	0.341218185	0.999579949
66.28318689	0.403940757	0.34411341	1.173859388	0.240451352	0.999579949
36.3735167	-0.010222081	0.310638366	-0.032906691	0.973748997	0.999579949
154.6346802	-0.060433966	0.087309296	-0.692182497	0.488822729	0.999579949

86.22894364	0.247077183	0.23540018	1.049604905	0.293899801	0.999579949
397.9853513	0.123415569	0.074675064	1.652701206	0.098391689	0.89325335
421.297555	0.123055004	0.08170397	1.506108014	0.132039448	0.939285826
645.0168604	0.040382669	0.126301457	0.319732405	0.749171192	0.999579949
129.0592269	0.108639993	0.20677717	0.525396458	0.599307616	0.999579949
492.6630608	0.071445774	0.113720674	0.628256687	0.5298358	0.999579949
297.8349272	0.145293501	0.078454946	1.851935509	0.064035084	0.814813368
784.4913495	-0.046772123	0.100261632	-0.466500712	0.640857138	0.999579949
232.3461	-0.054018978	0.09131363	-0.591576283	0.55413436	0.999579949
112.7736072	0.155605167	0.1785276	0.871602862	0.383425069	0.999579949
238.8982722	0.006230964	0.094639382	0.065839021	0.947505989	0.999579949
41.29962656	0.000972252	0.205486266	0.004731467	0.99622485	0.999579949
308.7997298	0.102917948	0.0724998	1.419561817	0.155735288	0.976030424
331.1210917	0.025125943	0.070907748	0.354346935	0.723078898	0.999579949
506.7342911	-0.000995113	0.06821947	-0.014586935	0.988361723	0.999579949
170.6031458	0.132685315	0.129442816	1.025049665	0.305339754	0.999579949
85.76525873	0.142969493	0.151208918	0.945509668	0.344398738	0.999579949
2732.204371	0.004471415	0.076029815	0.058811338	0.953102377	0.999579949
86.85089161	0.094606734	0.157361248	0.601207315	0.547701913	0.999579949
1577.41658	0.090444907	0.053926865	1.677177162	0.093507843	0.880418343
39.3058555	0.222790013	0.233223869	0.95526249	0.339444972	0.999579949
2307.652589	-0.066536653	0.11617741	-0.572715929	0.56683705	0.999579949
399.0251136	0.092161382	0.161372379	0.571110014	0.567925071	0.999579949
24.6644342	-0.00780641	0.276896833	-0.028192486	0.97750863	0.999579949
444.9167535	0.119048347	0.17552658	0.678235435	0.497622428	0.999579949
53.99291204	0.050862386	0.172856355	0.294246549	0.768569513	0.999579949
112.4549117	0.111480471	0.106806481	1.043761297	0.296595842	0.999579949
147.3532605	-0.233539966	0.36808253	-0.634477181	0.525769454	0.999579949
466.0344393	-0.177259018	0.106969252	-1.657102531	0.097498748	0.890209389
43.74592442	-0.097152382	0.240530611	-0.403908596	0.686279935	0.999579949
1116.686233	-0.045990814	0.10485495	-0.438613671	0.66094149	0.999579949
26818.71141	0.590758573	0.175789805	3.36059633	0.000777744	0.137990252
510.7838382	0.024397663	0.125086609	0.195046165	0.845356807	0.999579949
498.3739352	0.046498014	0.070281852	0.661593473	0.508231792	0.999579949
66.51053124	0.177667191	0.293432063	0.605479813	0.544860234	0.999579949
253.1656579	0.102194976	0.091931367	1.111644257	0.266291136	0.999579949
47.92028647	-0.024996199	0.217873634	-0.114727965	0.908660748	0.999579949
503.3341921	0.231101886	0.115522473	2.000492887	0.045447067	0.761238375
131.9310919	0.461286424	0.270694278	1.704086351	0.088364956	0.870840008
118.1800207	-0.072984572	0.118854814	-0.614064926	0.539172425	0.999579949
9850.038809	0.060916853	0.11735358	0.519088149	0.603699272	0.999579949
1180.76589	0.143491031	0.111816787	1.283269131	0.199397804	0.992481822
163.3446077	0.16228922	0.102594403	1.581852565	0.113683232	0.917331191
204.6280378	-0.057409093	0.164746831	-0.34846857	0.727488314	0.999579949
332.0119022	-0.153137062	0.133179358	-1.149855841	0.250203251	0.999579949
282.8182504	0.371011513	0.180403684	2.056562839	0.039728296	0.73515901
108.1036751	0.355458198	0.30980223	1.147371334	0.251228184	0.999579949
218.3915703	0.080885929	0.106835889	0.757104474	0.448987274	0.999579949

2006.017398	0.048506292	0.084933008	0.571112371	0.567923473	0.999579949
571.7165334	0.011006138	0.091894297	0.119769542	0.90466571	0.999579949
170.5828065	0.167383361	0.11876241	1.4093968	0.15871787	0.977304288
187.6217215	0.004216303	0.121025896	0.034838024	0.9722089	0.999579949
1202.150854	-0.203040544	0.159629305	-1.271950315	0.203390773	0.994409048
694.5380938	-0.15853424	0.087920152	-1.803161571	0.071362844	0.834620594
74.15756451	0.034276429	0.131138473	0.261375844	0.793802683	0.999579949
235.4741503	0.138302452	0.099614402	1.388378078	0.165021948	0.978789551
85.6216023	0.49852891	0.501014228	0.995039426	0.319717089	0.999579949
65.68186256	0.508561169	0.261837159	1.942280354	0.05210317	0.780444402
62.98274134	0.825730245	0.401036293	2.058991317	0.03949507	0.734990441
1127.367333	-0.12126001	0.15819511	-0.766521862	0.443365849	0.999579949
417.7618161	-0.143928901	0.125351998	-1.148197904	0.250886875	0.999579949
6549.307235	-0.087504296	0.169581883	-0.516000266	0.605854224	0.999579949
824.0820279	0.020144686	0.080417612	0.250500923	0.802199991	0.999579949
445.1802358	-0.13289545	0.290125236	-0.458062357	0.646907642	0.999579949
3780.166874	-0.049946525	0.111986558	-0.446004643	0.655593892	0.999579949
376.3148258	0.151471964	0.188671704	0.802833498	0.422070978	0.999579949
30.79953308	0.635880758	0.576656077	1.102703646	0.270155883	0.999579949
1728.940253	0.018879949	0.070141031	0.26917125	0.787797903	0.999579949
79.41756239	0.141610226	0.14257215	0.99325307	0.320586642	0.999579949
96.60623185	-0.079943697	0.144726567	-0.552377483	0.580689755	0.999579949
261.0236658	0.036718031	0.130526111	0.28130794	0.778474221	0.999579949
565.5077287	-0.023184418	0.072967547	-0.317736018	0.750685187	0.999579949
225.6164154	-0.025674812	0.084481195	-0.303911568	0.761195264	0.999579949
163.0616424	0.117301476	0.141239193	0.830516468	0.406246841	0.999579949
61.72247341	0.031684829	0.144526563	0.219231875	0.826469427	0.999579949
31.30922577	-0.432771679	0.200729454	-2.155994905	0.031084063	0.683621001
59.8806472	0.243519481	0.204938698	1.188255239	0.234732867	0.999579949
72.83585937	0.097237601	0.156569282	0.621051583	0.534565684	0.999579949
101.5870019	0.003175494	0.114772212	0.027667796	0.977927109	0.999579949
106.4132685	0.013225619	0.233472859	0.056647353	0.954826113	0.999579949
41.10812132	-0.112718102	0.253788676	-0.444141574	0.65694023	0.999579949
658.5302329	0.110242724	0.112932128	0.976185665	0.328972466	0.999579949
29.8439534	0.661146924	0.275650374	2.398498195	0.016462458	0.563098469
840.3686843	0.00117398	0.082785207	0.014181032	0.988685552	0.999579949
32.11092532	0.28320595	0.320541754	0.883522808	0.376953865	0.999579949
147.9348073	0.131173652	0.155933595	0.841214827	0.400227595	0.999579949
22.70321083	0.954918753	0.488634283	1.954260653	0.0506704	0.777437894
36.12242514	-0.019704438	0.229260911	-0.085947657	0.931508027	0.999579949
420.1906026	0.005347569	0.097727761	0.05471904	0.9563623	0.999579949
620.3905289	0.172969302	0.093401415	1.851891666	0.064041381	0.814813368
36.055278	0.477204154	0.198009925	2.410001179	0.015952469	0.558159164
767.7822154	-0.00428889	0.066984347	-0.064028247	0.948947735	0.999579949
68.80444898	0.097233275	0.209446544	0.4642391	0.64247645	0.999579949
313.571498	0.082518761	0.105955931	0.778802659	0.436095971	0.999579949
21.02563919	-0.242931372	0.219277121	-1.107873777	0.267916332	0.999579949
421.3627727	0.06559634	0.086158907	0.761341364	0.4464532	0.999579949

36.08282424	-0.528629038	0.212564662	-2.486909308	0.012885824	0.510304755
462.1996271	0.102645593	0.100847264	1.017832202	0.308757706	0.999579949
842.6883523	0.027877743	0.083216475	0.335002694	0.737623054	0.999579949
291.5141277	0.2088885	0.102681803	2.034328319	0.041918504	0.74969123
108.8691857	0.152750416	0.132802596	1.150206553	0.250058808	0.999579949
685.7410183	0.008913507	0.090170773	0.098851399	0.921256258	0.999579949
712.138049	-0.1165385	0.186998976	-0.623203945	0.53315051	0.999579949
2712.986675	-0.200771338	0.104660592	-1.918308828	0.055071864	0.785214091
405.3356977	-0.162323605	0.105268468	-1.541996466	0.123074452	0.92236461
267.0505987	0.01889771	0.117766139	0.160468109	0.872512342	0.999579949
301.3849743	0.090731835	0.215268817	0.42148155	0.673403481	0.999579949
1043.526885	0.058501973	0.05766639	1.014489939	0.310349028	0.999579949
503.2261814	-0.098523615	0.073959152	-1.332135543	0.182815653	0.983992835
113.2017478	0.228637233	0.136187651	1.678839683	0.093183292	0.880418343
823.0357693	0.864609446	0.460453384	1.877735021	0.060417423	0.800274462
29.47853292	-0.437007905	0.58351069	-0.748928705	0.453900178	0.999579949
735.6955825	-0.006313467	0.075278086	-0.083868596	0.933160909	0.999579949
111.0753037	-0.041512217	0.150914359	-0.275071355	0.783261419	0.999579949
627.3045588	0.064877441	0.095421972	0.679900445	0.4965675	0.999579949
91.23877658	0.058421229	0.112817686	0.517837505	0.604571648	0.999579949
145.7049936	0.118166466	0.153762426	0.768500271	0.44219003	0.999579949
477.8816516	0.148469445	0.148683458	0.998560609	0.31800759	0.999579949
43.14803977	0.295083412	0.246411542	1.197522689	0.231102889	0.999579949
653.8683092	-0.437682903	0.11046661	-3.962128487	7.42845E-05	0.040682091
556.6286973	0.058263139	0.067268906	0.866122887	0.386422775	0.999579949
165.738497	0.044516991	0.124293258	0.358160949	0.72022287	0.999579949
115.7732872	0.141755165	0.178882635	0.79244788	0.428099571	0.999579949
490.2075669	-0.158662457	0.104931607	-1.512055917	0.130519638	0.935700262
107.3305218	-0.023837295	0.139614294	-0.170736781	0.86443074	0.999579949
23.79137925	-0.011981438	0.243935812	-0.049117176	0.960825916	0.999579949
2859.780446	-0.103237258	0.147718854	-0.698876654	0.484629118	0.999579949
237.47688	-0.141348244	0.141754304	-0.997135469	0.318698759	0.999579949
438.1571815	0.011295932	0.074254375	0.152124796	0.879088507	0.999579949
922.606278	-0.023373066	0.080349674	-0.29089186	0.771134027	0.999579949
512.2695643	0.258463446	0.130757409	1.976663867	0.048079638	0.770705547
264.2832901	0.058676061	0.090838287	0.645939755	0.518318372	0.999579949
649.7015435	0.006128368	0.065184221	0.09401613	0.925096344	0.999579949
44.65804344	-0.031820922	0.182233906	-0.174615817	0.86138152	0.999579949
56.44965864	-0.368255727	0.26528874	-1.388131766	0.165096925	0.978789551
341.4563149	-0.036419988	0.131621202	-0.276703051	0.782008129	0.999579949
21.53991789	0.102481557	0.260528527	0.393360213	0.694053451	0.999579949
99.0815267	-0.066656434	0.288262992	-0.231234796	0.817132399	0.999579949
1509.809482	-0.068662756	0.06918404	-0.992465247	0.320970624	0.999579949
2694.84869	0.0026406	0.091702273	0.028795362	0.9770278	0.999579949
41.31355109	-0.309751064	0.357001161	-0.867647216	0.38558749	0.999579949
147.3516205	0.035001072	0.105917474	0.330456069	0.741055381	0.999579949
30.15815135	0.096742321	0.176584411	0.547853121	0.58379276	0.999579949
2870.613757	-0.07123538	0.066527056	-1.070773063	0.284271482	0.999579949

128.6712471	0.2037206	0.184370631	1.104951469	0.269180619	0.999579949
205.8304942	0.061785542	0.142957985	0.432193707	0.665600633	0.999579949
86.97908768	-0.068514137	0.168394566	-0.406866677	0.684105912	0.999579949
40.4504599	-0.096412146	0.275016878	-0.350568107	0.725912387	0.999579949
282.565429	0.115638812	0.13543699	0.85382001	0.393204726	0.999579949
236.6229659	0.011021271	0.071807146	0.153484316	0.878016358	0.999579949
126.0457017	0.301245204	0.278523597	1.081578749	0.279439751	0.999579949
461.5280772	0.060269277	0.128168641	0.470234188	0.638187711	0.999579949
53.4802932	-0.147853641	0.775399227	-0.190680666	0.848775787	0.999579949
48.89756192	-0.026695822	0.206017235	-0.12958053	0.896898308	0.999579949
193.446103	-0.039857744	0.102813844	-0.387669036	0.698260973	0.999579949
295.9713583	0.032856684	0.073051734	0.449772811	0.652874264	0.999579949
250.3984025	0.186119082	0.09553874	1.948100665	0.051402919	0.779189484
45.95025866	-0.319714146	0.249149237	-1.283223457	0.1994138	0.992481822
974.9491816	0.026729433	0.047626629	0.561228738	0.574641614	0.999579949
410.7821808	0.039035278	0.126364757	0.308909532	0.757390346	0.999579949
136.5792496	0.078062385	0.124923007	0.624883976	0.53204721	0.999579949
1046.292583	0.050140202	0.111740685	0.4487193	0.65363416	0.999579949
33.13397616	-1.439096599	0.547591879	-2.628045909	0.008587692	0.442789376
41.77958719	0.364700379	0.246332711	1.480519487	0.138734664	0.945791454
2008.472554	-0.058263832	0.091857978	-0.634281668	0.525897018	0.999579949
1852.31088	-0.047948591	0.133998149	-0.357830253	0.720470349	0.999579949
72.72837492	-0.021786129	0.210557293	-0.103468889	0.91759084	0.999579949
309.975822	0.098068606	0.085641249	1.145109481	0.252163809	0.999579949
6368.145884	-0.050803259	0.128155232	-0.3964197	0.691795435	0.999579949
474.4231831	-0.035151586	0.155610107	-0.225895264	0.821282881	0.999579949
112.620392	0.184389331	0.159399342	1.15677599	0.247363894	0.999579949
86.79689986	0.305421514	0.220945223	1.382340431	0.166867197	0.978965834
284.6194473	-0.127061726	0.075723713	-1.677964816	0.093353968	0.880418343
33.40562454	0.275757586	0.229813545	1.199918769	0.23017089	0.999579949
23.06823642	-0.072711794	0.279037664	-0.260580573	0.794415973	0.999579949
20.55217759	0.61171597	0.47734573	1.28149459	0.200019999	0.992610246
8383.05291	-0.083246906	0.10052784	-0.82809803	0.407614985	0.999579949
653.2254899	0.061747421	0.103499509	0.596596268	0.550776963	0.999579949
33850.88235	0.011811276	0.103099782	0.114561603	0.908792616	0.999579949
1078.243093	-0.01301757	0.094170438	-0.138234142	0.890055372	0.999579949
1152.510483	-0.095951701	0.070738767	-1.356423156	0.17496457	0.983992835
283.4322891	0.121742711	0.122968272	0.990033518	0.322157736	0.999579949
44.92339049	0.016003705	0.243377067	0.065756832	0.947571425	0.999579949
188.3951182	0.172043586	0.318203743	0.540671157	0.588734262	0.999579949
279.9069986	0.055451849	0.130142139	0.426086814	0.670044588	0.999579949
220.7521826	0.033639784	0.115251664	0.291881111	0.770377526	0.999579949
10284.00141	0.016621534	0.091910354	0.180845061	0.856489193	0.999579949
3070.96363	0.124104761	0.142279564	0.872259919	0.383066598	0.999579949
1713.400892	0.059041037	0.072123936	0.818605309	0.413011637	0.999579949
313.4440866	-0.246488174	0.108511082	-2.271548384	0.023113801	0.62865781
405.7412972	0.04249827	0.057161123	0.743482064	0.457189877	0.999579949
1479.016738	-0.126743544	0.070203184	-1.805381694	0.071014974	0.834620594

801.744639	-0.022868501	0.114450578	-0.199811149	0.841628282	0.999579949
118.9733834	0.482510663	0.224693706	2.147415125	0.031760248	0.689986698
334.2964676	0.089131996	0.134776079	0.661333946	0.508398177	0.999579949
942.3324249	-0.142521334	0.189157617	-0.753452787	0.451177872	0.999579949
105.6834608	0.099682499	0.126766956	0.786344506	0.431665685	0.999579949
2816.160788	0.04301683	0.091310403	0.471105465	0.637565423	0.999579949
443.714542	0.093850227	0.069578556	1.348838376	0.177388885	0.983992835
101.0310639	0.325967091	0.159641531	2.041868982	0.041164529	0.745838643
1097.274901	0.023909139	0.094571275	0.252816082	0.800410339	0.999579949
482.288641	-0.132084787	0.095568717	-1.382092296	0.166943364	0.978965834
1565.714631	-0.072784446	0.076041944	-0.957161831	0.338485579	0.999579949
85.17818653	0.355759239	0.29034462	1.225299917	0.22046224	0.999579949
270.3721612	-0.070375163	0.269296382	-0.261329776	0.793838206	0.999579949
133.8582024	-0.117579702	0.125663318	-0.935672429	0.34944187	0.999579949
52.03610507	-0.483150763	0.304895782	-1.584642332	0.113047621	0.916629381
48.87458784	-0.012342415	0.163983074	-0.075266395	0.940002759	0.999579949
407.2252959	-0.022908644	0.070343452	-0.325668466	0.744675205	0.999579949
99.74631797	-0.000432518	0.131676833	-0.00328469	0.997379201	0.999579949
36.41074965	0.007619718	0.28534797	0.026703249	0.978696421	0.999579949
838.0949129	0.060974244	0.128933744	0.472911449	0.636276357	0.999579949
37.25871665	0.212675105	0.194299171	1.094575462	0.27370267	0.999579949
398.1522891	0.031047846	0.115251002	0.269393284	0.787627054	0.999579949
1811.106553	0.019871468	0.139196961	0.142757919	0.886481372	0.999579949
526.3296018	0.127404584	0.137556928	0.926195326	0.354344481	0.999579949
1613.167997	0.042521783	0.13579846	0.313124193	0.754186309	0.999579949
603.2609283	-0.018548374	0.057748832	-0.321190454	0.748066066	0.999579949
376.8489413	0.036152787	0.079831843	0.45286174	0.650648303	0.999579949
362.0972927	0.079480921	0.09190109	0.86485286	0.387119554	0.999579949
35.57972231	0.060757539	0.250656852	0.24239329	0.808475433	0.999579949
587.4244381	0.202071215	0.144834228	1.395189649	0.162958658	0.978498637
360.7540573	0.083723318	0.076029553	1.101194402	0.270812058	0.999579949
305.1653139	-0.00112553	0.076504621	-0.014711922	0.988262008	0.999579949
41.39906039	-0.080828572	0.192844907	-0.419137708	0.675115494	0.999579949
34.0599478	0.711970284	0.412026702	1.727971222	0.083993384	0.860148832
99.18657748	0.086546513	0.176854383	0.489365947	0.62458264	0.999579949
1342.401074	-0.038646378	0.099112278	-0.389925233	0.696591835	0.999579949
445.1716525	-0.023684088	0.098586349	-0.240236993	0.810146537	0.999579949
604.6531088	0.012711588	0.064910466	0.195832646	0.844741157	0.999579949
37.03875642	0.339000872	0.256999311	1.319073079	0.187144682	0.983992835
173.0616742	0.721844107	0.235061253	3.070876619	0.002134313	0.233772259
382.5670644	-0.068789162	0.111185631	-0.618687517	0.536122235	0.999579949
276.3823705	0.055952512	0.157651608	0.354912407	0.722655213	0.999579949
414.506638	3.06628E-07	0.059442528	5.15839E-06	0.999995884	0.999995884
453.404135	0.156925381	0.152935126	1.026091161	0.304848616	0.999579949
20.84637152	0.749517128	0.588662661	1.273254069	0.2029279	0.994409048
104.5824641	-0.017771515	0.112228162	-0.15835165	0.874179711	0.999579949
167.0078627	0.086859862	0.095976564	0.905011161	0.365459474	0.999579949
443.8945699	-0.008841649	0.067176745	-0.131617703	0.895286683	0.999579949

83.65471682	0.141120539	0.167185312	0.844096515	0.398615475	0.999579949
47.49501813	-0.265725857	0.20237537	-1.313034574	0.189171298	0.983992835
1914.512711	0.033043849	0.059604101	0.554388846	0.579312758	0.999579949
248.0333999	0.015820575	0.070657597	0.223904792	0.822831387	0.999579949
2434.584941	0.122673145	0.154698567	0.79298178	0.427788438	0.999579949
71.50863367	-0.031157098	0.1120566	-0.27804786	0.78097562	0.999579949
1707.724955	0.061973885	0.068432744	0.905617421	0.365138385	0.999579949
26.65859884	-0.063107044	0.328131507	-0.192322415	0.847489661	0.999579949
228.2535212	0.071515801	0.077700221	0.920406662	0.357360288	0.999579949
986.1739057	0.00594436	0.050176262	0.11846957	0.905695605	0.999579949
158.9745863	-0.015039644	0.242955196	-0.061902954	0.950640115	0.999579949
1424.44255	0.065108371	0.080160968	0.812220376	0.41666519	0.999579949
317.3096103	0.094696287	0.13775261	0.68743733	0.491807181	0.999579949
71.42193381	-0.068136804	0.143457772	-0.474960702	0.634814984	0.999579949
1783.545222	0.030737402	0.076802482	0.400213657	0.688999156	0.999579949
291.4907966	-0.225980542	0.079015516	-2.85995148	0.004237058	0.335660291
759.2766061	0.075406759	0.100382677	0.751192949	0.452536543	0.999579949
447.4345293	0.025606739	0.135793345	0.188571384	0.850428762	0.999579949
512.3072176	0.109380024	0.12893641	0.848325348	0.396256805	0.999579949
1038.987462	0.00623238	0.079717965	0.078180372	0.937684575	0.999579949
448.5829207	-0.045737999	0.107054302	-0.427241112	0.66920372	0.999579949
267.7111125	0.133007891	0.172985237	0.768897354	0.441954249	0.999579949
15695.36075	-0.118829444	0.141278679	-0.841099622	0.400292127	0.999579949
63.3773303	-0.165475876	0.165428532	-1.000286192	0.317172028	0.999579949
700.8419011	-0.057557596	0.120587505	-0.477309788	0.633141542	0.999579949
308.9891553	0.046838679	0.098236524	0.476794953	0.63350814	0.999579949
68.09547598	0.546858366	0.475581729	1.149872529	0.250196377	0.999579949
577.4516621	0.110352773	0.084895547	1.299865268	0.193647151	0.986374671
2963.576926	0.028637759	0.128269569	0.223262304	0.823331364	0.999579949
1742.343979	0.000234761	0.088307937	0.002658433	0.99787888	0.999579949
28.88154814	0.133257001	0.217569995	0.612478759	0.540221047	0.999579949
351.0086617	0.087112329	0.072445892	1.202446766	0.229190479	0.999579949
621.7840598	-0.03008176	0.085135713	-0.353338908	0.72383438	0.999579949
15090.68268	0.007619171	0.096626745	0.078851578	0.937150679	0.999579949
960.5922864	-0.217664484	0.070339697	-3.094475706	0.001971611	0.225767362
566.7583234	-0.158149184	0.144965133	-1.090946357	0.275296487	0.999579949
408.4852349	0.188302802	0.189766516	0.992286765	0.321057657	0.999579949
1203.733536	-0.068610511	0.057008535	-1.203512968	0.228777876	0.999579949
52.34716832	-1.259696525	0.438223019	-2.87455581	0.004045963	0.327922523
301.0885251	-0.034944763	0.095559111	-0.365687404	0.714598347	0.999579949
66.31264492	0.147172917	0.19407196	0.758341992	0.448246275	0.999579949
684.1271398	-0.024880593	0.083480598	-0.298040432	0.765672305	0.999579949
29.75631334	0.400543962	0.227721105	1.758923317	0.078590534	0.851207389
170.2020764	0.156255238	0.232289178	0.672675495	0.501153761	0.999579949
418.4109483	-0.031484045	0.218205873	-0.144285965	0.885274661	0.999579949
626.2170831	0.165556492	0.13298367	1.244938513	0.213154353	0.999268361
176.1112905	0.163757103	0.107518662	1.523057478	0.127744326	0.935409261
68.14827556	-0.904384234	0.460475932	-1.96402064	0.049527698	0.775350081

924.2893222	-0.185321325	0.100905625	-1.836580716	0.066271816	0.822423447
728.7441824	0.043519459	0.146884098	0.296284346	0.76701294	0.999579949
272.5505842	0.008934587	0.118014994	0.075707215	0.939652036	0.999579949
486.5339015	0.004109069	0.185591749	0.022140364	0.982335989	0.999579949
80.00542934	0.22527798	0.43298674	0.520288405	0.602862576	0.999579949
31.20657586	-0.390183734	0.211596457	-1.843999373	0.065183232	0.817776877
1975.473702	-0.07406056	0.13180578	-0.561891598	0.574189879	0.999579949
347.1370998	-0.001215698	0.144084286	-0.008437412	0.993267999	0.999579949
98.77635711	-0.086890029	0.125405367	-0.692873289	0.488389074	0.999579949
263.9464865	-0.051869625	0.123187005	-0.421064098	0.673708277	0.999579949
70.88608724	0.116308621	0.141669708	0.820984401	0.411655154	0.999579949
143.7309596	-0.286043095	0.159188526	-1.796882609	0.072354258	0.838669905
208.6182266	-0.043371697	0.117623569	-0.368733046	0.712326715	0.999579949
912.8684153	0.187525484	0.099641459	1.882002585	0.059835665	0.798635337
1291.386217	-0.019193404	0.080890655	-0.237275919	0.812442742	0.999579949
1032.257174	0.003235955	0.091783076	0.03525655	0.97187517	0.999579949
27.53530018	-0.169670667	0.243290919	-0.697398275	0.485553581	0.999579949
427.0115441	0.064035683	0.081307867	0.787570568	0.430947936	0.999579949
457.4592052	0.09859722	0.082540634	1.194529465	0.232270922	0.999579949
1147.005178	-0.063021782	0.138591355	-0.454730975	0.649302794	0.999579949
261.8068871	0.118575995	0.102596086	1.155755542	0.247781162	0.999579949
516.619635	0.08880283	0.084195453	1.054722395	0.291552306	0.999579949
6924.300892	0.064401451	0.175597232	0.366756643	0.713800552	0.999579949
4611.574329	0.303633044	0.149760052	2.027463533	0.04261503	0.74969123
42.82234622	0.005684319	0.158382801	0.03588975	0.971370269	0.999579949
477.2571615	-0.154907624	0.102040583	-1.518098189	0.128989648	0.935700262
31.59781686	-0.106917482	0.21698851	-0.492733381	0.622200988	0.999579949
97.9463601	0.136884913	0.304689853	0.449259834	0.653244229	0.999579949
305.0319006	0.100425088	0.12612708	0.796221461	0.425903326	0.999579949
343.6285807	0.230079154	0.093013907	2.473599488	0.013375956	0.516515866
1068.560684	0.115067186	0.099696985	1.154169163	0.248430822	0.999579949
66.8095487	-0.002547804	0.181283375	-0.014054259	0.988786693	0.999579949
659.9425569	0.167236222	0.100862615	1.658059548	0.097305448	0.890093987
2478.869211	-0.066777614	0.076683036	-0.870826416	0.383848939	0.999579949
128.6700317	0.021230745	0.110292854	0.192494291	0.847355039	0.999579949
330.8463661	-0.176948463	0.102116043	-1.732817474	0.083128116	0.859673028
438.6575279	0.412160004	0.169514647	2.431412328	0.015040088	0.541405219
268.7919092	0.091130332	0.120088045	0.758862646	0.447934726	0.999579949
96.37771029	0.033328955	0.155978089	0.213677159	0.830798824	0.999579949
131.0530267	-0.018003503	0.113260834	-0.158956127	0.87370344	0.999579949
44.16237065	0.119560632	0.167089258	0.71554948	0.474269578	0.999579949
67.22963597	0.369338545	0.169881954	2.174089346	0.029698426	0.672989344
136.83655	0.489324203	0.307940905	1.589019828	0.112055916	0.913563961
628.6935974	0.450087156	0.142893009	3.14981929	0.001633715	0.214762992
180.0273076	-0.277992857	0.186852503	-1.487766301	0.136812536	0.945791454
224.8324592	0.001760563	0.104718683	0.016812306	0.986586352	0.999579949
583.7594197	-0.001711413	0.068900695	-0.02483884	0.980183511	0.999579949
109.7807235	0.018974233	0.125825367	0.150798151	0.880134945	0.999579949

157.6821029	0.034892561	0.195036463	0.178902756	0.858014058	0.999579949
121.8327499	0.029167197	0.120336006	0.242381299	0.808484724	0.999579949
342.0884114	0.071323555	0.111193272	0.64143768	0.521238359	0.999579949
919.8099372	0.384115206	0.150506873	2.552143949	0.010706227	0.483266726
6862.549492	-0.030171271	0.125018015	-0.241335388	0.809295186	0.999579949
72.36300431	-0.25283232	0.126800745	-1.993934019	0.046159266	0.763868825
20.60873449	-0.325643993	0.298268591	-1.091781042	0.274929354	0.999579949
8027.675847	-0.221977769	0.146056821	-1.519804193	0.128560195	0.935700262
482.8097515	-0.170418068	0.17786575	-0.958127511	0.337998464	0.999579949
5509.387817	-0.01604994	0.122020255	-0.13153505	0.895352062	0.999579949
421.1151714	-0.038299298	0.085072737	-0.450194732	0.652570034	0.999579949
149.229436	-0.018763776	0.099153904	-0.189238905	0.849905576	0.999579949
124.1474061	0.072427704	0.119433003	0.60642956	0.544229544	0.999579949
85.8653476	-0.057864008	0.193150115	-0.2995805	0.76449716	0.999579949
1130.717598	0.079303611	0.142185757	0.557746516	0.577017489	0.999579949
24.55436322	0.398517705	0.285704773	1.394858409	0.163058541	0.978498637
3279.140497	-0.137082309	0.106885738	-1.282512631	0.199662877	0.992493287
72.81678428	-0.022593946	0.162964411	-0.138643439	0.889731916	0.999579949
448.3651197	-0.05536181	0.099075511	-0.558783994	0.576309147	0.999579949
53.29177399	-0.054489587	0.184943977	-0.294627529	0.76827843	0.999579949
252.0837277	-0.007227626	0.092440015	-0.078187198	0.937679146	0.999579949
196.9494371	-0.112314339	0.208762674	-0.538000097	0.590576982	0.999579949
175.3645364	-0.132692026	0.113395834	-1.170166682	0.241933898	0.999579949
272.4734433	0.038971573	0.306583658	0.127115623	0.898848886	0.999579949
961.3543013	-0.073893845	0.068284664	-1.08214409	0.279188507	0.999579949
833.4517036	0.270612423	0.102909029	2.629627587	0.008547845	0.442789376
55.73693783	0.46147305	0.762053374	0.605565261	0.544803476	0.999579949
194.3443264	-0.432949777	0.637912535	-0.678697711	0.497329417	0.999579949
48.93248245	0.362621265	0.50348266	0.720225927	0.471385903	0.999579949
713.2708718	-0.162096035	0.197296672	-0.821585247	0.411312989	0.999579949
974.9111558	-0.010608955	0.176521348	-0.060100125	0.95207589	0.999579949
452.8768041	-0.004679483	0.081582521	-0.057358895	0.954259306	0.999579949
42.33462669	0.033648572	0.179836497	0.18710647	0.851577155	0.999579949
829.9184044	0.017253179	0.104354537	0.165332331	0.868682412	0.999579949
111.0656879	0.217511945	0.108556101	2.003682355	0.045104098	0.760273172
10607.84318	0.00624546	0.148786165	0.041976081	0.966517766	0.999579949
452.7944754	0.010622085	0.091833591	0.115666666	0.907916726	0.999579949
23.78419087	-0.10961189	0.246300981	-0.445032293	0.656296417	0.999579949
309.2953079	0.097236824	0.087684625	1.108938134	0.267456872	0.999579949
491.6000733	0.033138172	0.116580467	0.284251495	0.776217655	0.999579949
126.867089	0.041475571	0.106710738	0.388672894	0.697518136	0.999579949
393.4867862	0.145560825	0.207471879	0.701593036	0.482932996	0.999579949
491.8157243	-0.221344062	0.160541402	-1.378735071	0.167976452	0.980087289
832.2068262	-0.055075375	0.059148386	-0.931139109	0.35178161	0.999579949
90.03366116	-0.112988219	0.114398613	-0.987671231	0.323313688	0.999579949
39.23523405	-0.08597773	0.181598065	-0.473450698	0.635891667	0.999579949
238.4842867	-0.266124447	0.181583712	-1.465574439	0.142764259	0.95210212
360.0737638	0.870987238	0.338228782	2.575142285	0.010019886	0.472698434

127.3647997	0.118908446	0.105523065	1.126847912	0.259806789	0.999579949
30.95618579	-0.91720323	0.466709061	-1.965256958	0.049384505	0.775350081
746.5903399	0.105223338	0.098434425	1.068968882	0.285083692	0.999579949
3211.962035	0.019057523	0.055811946	0.341459571	0.732757636	0.999579949
412.4997784	0.074644002	0.084695694	0.881319914	0.378144691	0.999579949
263.3685969	-0.105156769	0.109097939	-0.963874936	0.335108631	0.999579949
224.7428116	0.207332648	0.209903488	0.987752272	0.323273987	0.999579949
68.02599485	-0.069746113	0.184667368	-0.3776851	0.705664538	0.999579949
34.13694882	0.086263858	0.332717154	0.259270846	0.795426272	0.999579949
42.1683515	0.649848019	0.929172252	0.699383798	0.48431221	0.999579949
42.83158685	0.648291611	0.902597819	0.718250806	0.472602657	0.999579949
31.42114999	0.592933478	0.639914733	0.926582007	0.354143601	0.999579949
59.57929753	-0.226256015	0.233481525	-0.96905318	0.332518656	0.999579949
350.4329177	0.0924776	0.103874164	0.890284898	0.373312928	0.999579949
635.4170584	-0.01481057	0.074935459	-0.19764435	0.843323333	0.999579949
239.7804606	-0.024662838	0.137049579	-0.179955587	0.857187435	0.999579949
377.428841	0.019614909	0.095059913	0.206342597	0.836523307	0.999579949
187.7914253	0.246950652	0.140703944	1.755108243	0.079240795	0.851207389
956.943899	-0.187283041	0.0861411	-2.174142667	0.029694423	0.672989344
678.8440497	-0.106372903	0.082697515	-1.286288991	0.198342223	0.992481822
879.7614535	-0.026225673	0.058574667	-0.44773063	0.654347613	0.999579949
64.07907136	0.269884995	0.158860971	1.698875398	0.089342663	0.870840008
5536.592441	0.028394367	0.103966037	0.273111945	0.784767165	0.999579949
640.1656286	-0.065786308	0.096673939	-0.680496825	0.496189932	0.999579949
33.76813585	-0.229285412	0.409847192	-0.559441218	0.575860638	0.999579949
356.0057813	-0.010916875	0.099686138	-0.109512469	0.912796032	0.999579949
1747.977297	0.036037764	0.054217522	0.664688516	0.506249741	0.999579949
1097.172438	0.11036013	0.064889876	1.700729553	0.088993783	0.870840008
407.1513924	0.052804377	0.07913199	0.667294948	0.504583753	0.999579949
435.6159467	-0.152088463	0.184278097	-0.825320344	0.409189745	0.999579949
104.0648608	-0.180234098	0.142762745	-1.262472909	0.206778668	0.994814558
50.34855	0.080202359	0.209710832	0.382442615	0.702133086	0.999579949
21.25120218	-0.386309596	0.229478385	-1.683424763	0.092292893	0.880418343
90.93066571	-0.057383222	0.124235917	-0.461889147	0.644160817	0.999579949
27.15402601	-0.43547034	0.226283384	-1.924446827	0.0542986	0.783576392
62.03336126	-0.0537835	0.217122018	-0.247710944	0.804358063	0.999579949
462.747308	0.068905867	0.091323393	0.75452592	0.450533487	0.999579949
146.3554015	0.204245761	0.20101193	1.016087753	0.3095876	0.999579949
305.4885756	-0.039247994	0.068507006	-0.572904824	0.566709138	0.999579949
1309.125949	0.044766124	0.079843444	0.560673757	0.575019961	0.999579949
140.6291647	-0.129799218	0.104371087	-1.243631943	0.213635054	0.999268361
174.8798735	0.14703919	0.106121541	1.385573453	0.165877189	0.978789551
99.2324263	-0.367796561	0.206470163	-1.781354536	0.07485455	0.849430556
27.90728121	-0.45031293	0.255742836	-1.760803693	0.078271635	0.851207389
158.9789594	-0.208499003	0.140758529	-1.481253072	0.138539149	0.945791454
1063.550636	-0.07069674	0.113392463	-0.623469479	0.532976053	0.999579949
3625.082451	0.038922411	0.170633439	0.22810541	0.819564294	0.999579949
392.1202167	0.041968071	0.158716176	0.264422139	0.791454654	0.999579949

311.9688873	-0.130314368	0.09770482	-1.333755776	0.182283909	0.983992835
3522.171626	0.019604127	0.156715424	0.125093792	0.900449298	0.999579949
51.76360314	0.21071215	0.170177239	1.238192315	0.215644761	0.999579949
1448.037534	0.074387887	0.074307116	1.001086987	0.316784756	0.999579949
1862.169696	0.119886602	0.136309252	0.879519183	0.379119837	0.999579949
31.64520718	-1.177677864	0.520533089	-2.262445726	0.023669878	0.630129799
15849.76673	0.032205824	0.104370426	0.308572314	0.757646884	0.999579949
871.8842359	0.013596057	0.058394112	0.23283267	0.815891343	0.999579949
1467.788365	0.122188937	0.077425481	1.578148901	0.114531406	0.918818353
52.42035839	-0.1001774	0.162178574	-0.617698119	0.536774352	0.999579949
2062.802306	-0.025513692	0.057733191	-0.441924153	0.658544092	0.999579949
2668.47853	0.053988121	0.120047943	0.449721334	0.652911387	0.999579949
404.7291063	0.043223201	0.181017625	0.238778966	0.811276981	0.999579949
129.0607261	-0.121395863	0.105417304	-1.151574346	0.249496028	0.999579949
222.1303747	0.064287722	0.106168558	0.605525055	0.544830182	0.999579949
250.6324043	0.065676595	0.286721708	0.22906042	0.818821956	0.999579949
22.92254223	0.112068992	0.243390202	0.46044989	0.645193331	0.999579949
231.9110999	-0.02787426	0.08489246	-0.328347884	0.742648643	0.999579949
27.23410864	-0.17125962	0.206172467	-0.830661934	0.406164636	0.999579949
63.35477926	0.29637556	0.280352735	1.057152379	0.29044205	0.999579949
69.63211496	0.060419436	0.151080993	0.399914209	0.689219706	0.999579949
285.9623538	0.084414212	0.079007235	1.068436477	0.28532367	0.999579949
875.4549387	-0.046326807	0.125091396	-0.370343668	0.711126441	0.999579949
1753.976731	0.211035342	0.143578112	1.469829482	0.141607942	0.949966262
490.4928913	0.024047057	0.188463771	0.127595117	0.898469395	0.999579949
367.433856	-0.060071704	0.0758635	-0.791839347	0.428454356	0.999579949
77.36871663	-0.065053989	0.135115975	-0.481467782	0.630184072	0.999579949
791.4082614	-0.041764147	0.096504334	-0.432769658	0.665182119	0.999579949
2367.903564	0.290561151	0.091300251	3.182479205	0.0014602	0.203876378
451.3378248	0.011587423	0.072719517	0.159344064	0.873397807	0.999579949
3138.494932	-0.057140432	0.071323962	-0.801139337	0.423050986	0.999579949
40.52619557	-0.616882144	0.211948554	-2.910527732	0.003608189	0.315616349
348.8148775	-0.073448225	0.152813194	-0.480640596	0.630771958	0.999579949
84.91414177	-0.496002778	0.323493982	-1.533267405	0.125209982	0.927500246
174.6214026	0.123186079	0.166567412	0.739556904	0.4595689	0.999579949
3726.947749	0.194298611	0.275759304	0.704594943	0.481062347	0.999579949
149.3832189	-0.097127492	0.10754289	-0.903151212	0.366445647	0.999579949
1406.044374	0.017406971	0.057865698	0.300816754	0.763554232	0.999579949
653.5468055	0.02703395	0.116188599	0.232673001	0.816015336	0.999579949
254.3631989	-0.200765608	0.081487111	-2.463771345	0.013748379	0.520783867
112.981527	-0.429068763	0.296433425	-1.447437189	0.147774516	0.962301096
500.1917037	0.008647378	0.129693963	0.066675251	0.946840238	0.999579949
3283.572048	-0.069371483	0.070082078	-0.989860536	0.322242291	0.999579949
374.6147563	0.095326961	0.093991847	1.014204573	0.310485147	0.999579949
369.1546815	-0.002304563	0.074944414	-0.030750292	0.975468683	0.999579949
140.6691922	-0.101917623	0.140258196	-0.726642904	0.467444739	0.999579949
180.0128753	0.133927575	0.414333475	0.323236192	0.746516374	0.999579949
301.7333325	-0.175037183	0.491070334	-0.356440149	0.721510967	0.999579949

126.8739612	0.120065424	0.143466453	0.836888494	0.402655249	0.999579949
390.774444	0.036207696	0.089627541	0.403979576	0.686227738	0.999579949
502.4858943	-0.022788805	0.118061491	-0.193024881	0.846939482	0.999579949
39.61846521	0.027223666	0.210672784	0.129222512	0.897181584	0.999579949
92.07639484	0.177266511	0.143561901	1.234774059	0.216914604	0.999579949
1278.259252	-0.018622324	0.088178188	-0.211189693	0.832739253	0.999579949
153.6985262	0.167280986	0.20148902	0.830223831	0.406412244	0.999579949
245.2278631	0.021881995	0.104283219	0.20983237	0.833798508	0.999579949
113003.7973	0.076872841	0.123276111	0.623582622	0.532901726	0.999579949
150.0028342	0.288827322	0.154147433	1.873708288	0.060970643	0.802493437
261.8245739	0.024020776	0.112943229	0.21268009	0.831576497	0.999579949
197.1423707	0.018276943	0.107424923	0.170136895	0.864902479	0.999579949
337.0406657	0.003211601	0.123410171	0.026023798	0.979238357	0.999579949
60.74643625	0.230097379	0.167561644	1.373210321	0.169686972	0.981013142
2527.971824	-0.070923156	0.052929727	-1.339949403	0.180261795	0.983992835
218.0637964	-0.105017473	0.113062807	-0.928841905	0.352971024	0.999579949
405.7462289	0.054190009	0.119946898	0.45178333	0.65142508	0.999579949
323.8785897	0.056793199	0.094736666	0.599484885	0.548849586	0.999579949
138.5595743	-0.115623576	0.117438068	-0.984549373	0.324845468	0.999579949
422.8606289	0.008698008	0.07348758	0.11836024	0.905782228	0.999579949
27.24418125	0.086101507	0.199028227	0.432609525	0.665298469	0.999579949
2357.06761	-0.111837123	0.062987721	-1.775538502	0.075809014	0.851207389
27.98068255	0.048242329	0.216227859	0.223108757	0.823450863	0.999579949
1087.648181	0.098001091	0.120170589	0.815516441	0.414776758	0.999579949
530.1672694	0.030511261	0.108701681	0.280688036	0.778949686	0.999579949
180.5308564	-0.126876248	0.095785971	-1.324580695	0.185310263	0.983992835
234.6625807	-0.095039027	0.093009413	-1.021821601	0.306865344	0.999579949
75.46729587	-0.138277268	0.468603304	-0.295083852	0.767929825	0.999579949
22.54601924	-0.021539786	0.224256711	-0.096049683	0.923481114	0.999579949
898.1257349	-0.076081812	0.083694883	-0.909037796	0.363330175	0.999579949
1017.792075	0.036376277	0.172799595	0.210511353	0.833268592	0.999579949
2443.091898	-0.042501351	0.090952299	-0.467292761	0.640290435	0.999579949
42.53592378	-0.200260834	0.182164257	-1.099342082	0.271618883	0.999579949
293.0203031	-0.138056304	0.075801089	-1.821297123	0.068561704	0.831064901
1395.807636	-0.147063722	0.123180894	-1.19388419	0.232523273	0.999579949
267.09468	-0.04751555	0.082883681	-0.573279927	0.566455175	0.999579949
4493.332734	-0.019551855	0.111775762	-0.174920343	0.861142225	0.999579949
383.6145349	-0.107000477	0.077007883	-1.389474332	0.164688561	0.978498637
1759.509708	0.01583655	0.047320493	0.334665786	0.737877214	0.999579949
1404.397183	0.070888381	0.161051343	0.440160137	0.659821129	0.999579949
132.620991	-0.023533215	0.10774955	-0.218406617	0.82711231	0.999579949
812.7385792	-0.05797146	0.060754192	-0.954196872	0.339983998	0.999579949
149.7822275	-0.2028764	0.172538242	-1.175834397	0.239661058	0.999579949
232.9841279	-0.099249731	0.102671901	-0.966668872	0.333709593	0.999579949
454.9155595	0.094714593	0.095613867	0.99059473	0.321883512	0.999579949
180.2832657	-0.070787423	0.106008483	-0.667752442	0.504291629	0.999579949
59.25437229	0.074795293	0.188513859	0.396762836	0.691542359	0.999579949
1664.769679	-0.011370593	0.098293321	-0.115680217	0.907905986	0.999579949

1752.333854	-0.023083785	0.104923625	-0.220005595	0.825866798	0.999579949
539.2829191	0.102485146	0.05942775	1.724533514	0.084611574	0.861032139
233.1531449	0.055052217	0.089098739	0.617878745	0.536655271	0.999579949
286.4234697	0.116044765	0.157394797	0.737284634	0.460949275	0.999579949
38.72418925	0.141304262	0.235607473	0.599744399	0.548676594	0.999579949
100.3734613	-0.114936879	0.192949065	-0.595685081	0.551385627	0.999579949
31.64315182	0.047859573	0.164535771	0.290876402	0.77114585	0.999579949
667.1942835	-0.12969656	0.073999274	-1.752673402	0.079658084	0.852823173
75.77549626	-0.231861811	0.254640626	-0.910545245	0.362535032	0.999579949
98.03810019	-0.030326386	0.153918542	-0.197028806	0.843804996	0.999579949
1814.315143	0.028652452	0.099567239	0.287769872	0.773522901	0.999579949
306.0348033	-0.028302653	0.079539569	-0.355831105	0.721967054	0.999579949
83.67988114	0.162926299	0.308674854	0.527824983	0.597620816	0.999579949
214.3285069	0.068298479	0.137862041	0.495411782	0.62030948	0.999579949
743.5717474	-0.038711127	0.111412393	-0.347457998	0.728247268	0.999579949
3624.73247	-0.024276012	0.068379524	-0.355018731	0.722575559	0.999579949
73.93683945	0.04297891	0.154390742	0.278377509	0.780722583	0.999579949
101.2933195	0.099390699	0.137683766	0.721876674	0.470370304	0.999579949
703.8826982	-0.168232624	0.087008747	-1.933513936	0.053172918	0.781231069
54.50582998	0.237830676	0.214389679	1.109338273	0.26728428	0.999579949
51.47703623	0.193184648	0.362361321	0.533127123	0.593945585	0.999579949
64.84816545	0.2539816	0.210970252	1.203873991	0.228638286	0.999579949
70.48407515	0.019231265	0.128737853	0.149383146	0.88125131	0.999579949
25.36859197	0.023540385	0.233345008	0.100882317	0.91964388	0.999579949
46.08526718	0.062562424	0.195667433	0.319738566	0.749166522	0.999579949
698.8578952	0.022972698	0.114044511	0.201436242	0.840357479	0.999579949
72.55232223	0.059525888	0.134773319	0.441674128	0.658725034	0.999579949
573.4220912	-0.091565281	0.186196022	-0.49176819	0.622883226	0.999579949
28.57107151	-0.008525879	0.400186931	-0.021304741	0.983002562	0.999579949
614.9978017	0.033798139	0.092600046	0.364990522	0.715118481	0.999579949
23.02834498	0.379735413	0.305203572	1.244203698	0.213424603	0.999268361
377.1906182	0.014523561	0.081374575	0.178477869	0.8583477	0.999579949
211.2774833	0.147965979	0.16819404	0.879733779	0.379003546	0.999579949
21.34869537	0.109889494	0.339219661	0.323947892	0.745977485	0.999579949
1089.24205	-0.053510529	0.114115132	-0.46891703	0.639128943	0.999579949
55.54510667	0.046906704	0.151204449	0.310220399	0.756393359	0.999579949
25.98612352	0.008006056	0.207363264	0.038608845	0.96920225	0.999579949
63.86121648	-0.036219044	0.171245404	-0.211503743	0.832494212	0.999579949
127.4771629	0.155844016	0.104187024	1.49581023	0.134703115	0.945791454
180.218072	0.942146829	0.266305306	3.537844755	0.000403407	0.097717629
1551.486327	-0.140878179	0.221247451	-0.636744866	0.524291044	0.999579949
29959.1967	0.791209916	0.251478166	3.146237021	0.001653859	0.214762992
114646.7161	0.592968798	0.223338147	2.655026951	0.007930207	0.425059108
698974.7409	-0.291773592	0.145079279	-2.011132075	0.044311508	0.758967899
770.9445766	0.107849547	0.144109978	0.748383617	0.454228801	0.999579949
71.66765683	0.850331585	0.383910727	2.214920096	0.026765549	0.653787748
399.2978818	-0.042352137	0.171554661	-0.24687255	0.805006861	0.999579949
2540.134728	-0.077820244	0.122592264	-0.634789186	0.525565917	0.999579949

185.0975041	0.010937657	0.123406336	0.088631243	0.929374978	0.999579949
440.0093011	0.150516549	0.121635931	1.237434926	0.215925659	0.999579949
34.74233253	-0.078261026	0.621656803	-0.125891047	0.89981817	0.999579949
82.28933584	-0.149503752	0.228830945	-0.653337124	0.513538964	0.999579949
745.0556769	0.373217113	0.130609712	2.85749894	0.004269941	0.336151074
844.8108173	0.002040804	0.084051181	0.02428049	0.980628876	0.999579949
335.5798933	-0.161153853	0.11896795	-1.354598888	0.175545388	0.983992835
70.01918119	-0.174494147	0.21065535	-0.828339501	0.407478258	0.999579949
124.3224914	-0.191688448	0.191469886	-1.001141498	0.316758405	0.999579949
387.5467351	-0.017429079	0.142383155	-0.122409697	0.902574559	0.999579949
240.2131177	0.129970261	0.083896659	1.549170885	0.121340646	0.921837625
295.8472031	-0.011266674	0.151549136	-0.074343371	0.940737168	0.999579949
564.9096946	0.049816797	0.066881072	0.744856431	0.456358516	0.999579949
2010.25611	0.140051282	0.126655471	1.105765755	0.268827921	0.999579949
56.86902352	0.238862397	0.198661683	1.202357665	0.229224984	0.999579949
150.3203297	0.042118248	0.108556965	0.387982917	0.698028675	0.999579949
392.4602412	-0.104447266	0.195412631	-0.534495983	0.592998428	0.999579949
71.25176858	-0.128940744	0.115258041	-1.118713646	0.263262322	0.999579949
111.4043861	0.095253925	0.146455442	0.650395257	0.51543694	0.999579949
93.01791709	-0.267245034	0.138704572	-1.926721158	0.05401439	0.782044005
1124.810071	-0.178677841	0.091479737	-1.953195833	0.050796395	0.777437894
20.59950069	-0.010210478	0.264154224	-0.038653472	0.96916667	0.999579949
70.87791981	-0.014904765	0.150400111	-0.099100758	0.92105827	0.999579949
217.7878684	-0.119890177	0.13500928	-0.888014342	0.374533037	0.999579949
2282.697003	-0.196083429	0.158211634	-1.239374281	0.215206922	0.999579949
34.18977244	-0.20548826	0.201214458	-1.021240037	0.307140728	0.999579949
2088.832742	0.060022277	0.122586758	0.489630997	0.624395038	0.999579949
96.68281707	-0.060453791	0.139921125	-0.43205621	0.66570056	0.999579949
59.43418107	0.249957118	0.298670357	0.836899651	0.402648976	0.999579949
945.2953733	0.114507826	0.075784713	1.510962068	0.130798115	0.936571507
441.2401963	-0.054712352	0.05266091	-1.038955678	0.29882535	0.999579949
46.67015051	-0.001184652	0.179226478	-0.006609805	0.994726177	0.999579949
1658.421037	-0.077597976	0.059559928	-1.302855454	0.192624115	0.985859356
1368.621709	-0.068080596	0.066369751	-1.025777472	0.304996487	0.999579949
452.7396315	-0.26831436	0.169511126	-1.58287168	0.113450715	0.916629381
113.9459428	-0.207422905	0.227963395	-0.909895662	0.362877538	0.999579949
182.6165289	-0.142293001	0.134682505	-1.056506944	0.29073667	0.999579949
266.7527557	0.19703291	0.172318203	1.143424822	0.252862251	0.999579949
141.8221503	-0.019017915	0.104275751	-0.182380997	0.855283738	0.999579949
50.56991964	0.005992366	0.174537881	0.034332752	0.972611808	0.999579949
30.62559687	-1.220350964	0.521422479	-2.340426457	0.019261732	0.594658752
830.9941947	-0.01818305	0.051348514	-0.354110549	0.723256037	0.999579949
331.6453923	-0.009204785	0.098048069	-0.093880327	0.925204222	0.999579949
3010.540252	-0.090439943	0.071111879	-1.271797964	0.203444912	0.994409048
208.2142729	-0.008160671	0.164632695	-0.049568959	0.960465883	0.999579949
6705.444839	0.001204824	0.105376684	0.011433497	0.990877588	0.999579949
218.678777	-0.047183729	0.076306518	-0.618344672	0.53634816	0.999579949
1689.174227	-0.051449488	0.101630371	-0.506241266	0.612687289	0.999579949

968.9088825	0.087794183	0.068418786	1.283188264	0.199426127	0.992481822
220.4781988	0.000545022	0.095462793	0.00570926	0.995444694	0.999579949
157.8012606	0.094047207	0.126533606	0.743258733	0.457325051	0.999579949
4302.51557	0.226786485	0.128997902	1.758063362	0.078736729	0.851207389
105.7473989	-0.246371466	0.125817273	-1.958168869	0.050210201	0.776599223
62.24000421	-0.329186366	0.236674731	-1.390880914	0.164261541	0.978498637
76.91422296	0.100644522	0.225600625	0.446118101	0.655511939	0.999579949
29.96732517	-0.283035806	0.258297533	-1.095774329	0.273177545	0.999579949
246.3664933	0.025850577	0.181911807	0.142105001	0.886997068	0.999579949
186.8583512	-0.055127946	0.1155099	-0.477257326	0.633178894	0.999579949
387.6219708	-0.117359327	0.094797282	-1.238003079	0.215714919	0.999579949
84.37472365	0.74657431	0.341977466	2.183109663	0.029027735	0.672989344
503.7620859	0.05331453	0.081496078	0.654197495	0.512984575	0.999579949
115.9605288	0.086321078	0.261756827	0.329775843	0.741569342	0.999579949
34.61080819	-0.170728641	0.21452432	-0.795847488	0.426120686	0.999579949
28.71145309	-0.295804466	0.382890851	-0.772555585	0.439785439	0.999579949
1223.749495	-0.007550088	0.141381485	-0.053402239	0.957411421	0.999579949
257.7408478	-0.24118059	0.086598594	-2.785040485	0.005352104	0.360508567
758.7123169	-0.11957845	0.073382882	-1.629514211	0.103204209	0.895628172
272.3679572	-0.148193123	0.184899795	-0.801478027	0.42285496	0.999579949
44.26447694	0.209398368	0.244431909	0.856673618	0.391625287	0.999579949
96.68584047	0.142884079	0.190247895	0.751041577	0.452627634	0.999579949
3540.690622	0.037365833	0.143988028	0.259506526	0.795244448	0.999579949
420.6972883	0.00835716	0.075982031	0.109988642	0.912418383	0.999579949
1886.331227	-0.09522143	0.093766259	-1.015519137	0.309858429	0.999579949
64478.61104	-0.235684163	0.133212767	-1.769231041	0.076855326	0.851207389
509.0119498	0.087781648	0.097360555	0.901614086	0.367261906	0.999579949
954.7679115	-0.077031808	0.061026252	-1.262273296	0.206850462	0.994814558
46.96905122	-0.053450608	0.168916778	-0.316431611	0.751674927	0.999579949
17663.17624	0.01595084	0.133205294	0.119746293	0.904684128	0.999579949
218.8070349	-0.086740168	0.118043298	-0.734816542	0.462451231	0.999579949
881.7775948	-0.004713384	0.090837124	-0.051888298	0.958617699	0.999579949
509.2175967	0.02700212	0.070490187	0.383062117	0.701673707	0.999579949
62.32534799	0.211869041	0.163443929	1.296279667	0.194879151	0.988443849
682.9169442	0.037349028	0.099642772	0.374829275	0.707787441	0.999579949
263.093321	-0.08633593	0.08023504	-1.076037728	0.281910384	0.999579949
174.7423557	-0.054251812	0.085171637	-0.636970408	0.524144119	0.999579949
85.05762652	0.248755109	0.174089962	1.4288883	0.153036341	0.972669728
60.85771228	-0.288200553	0.184778577	-1.559707612	0.118828993	0.921521033
219.4583734	-0.143350116	0.169072756	-0.847860524	0.396515651	0.999579949
72.5172875	0.074638673	0.142368436	0.524264193	0.600094798	0.999579949
182.7702442	-0.078821862	0.09031075	-0.872784937	0.382780311	0.999579949
52.63461285	-0.012189248	0.145405322	-0.083829451	0.933192032	0.999579949
45.73832136	0.231939846	0.252618656	0.91814219	0.358544429	0.999579949
22.02623837	0.410927441	0.279275691	1.471404259	0.141181822	0.949453409
4302.890943	-0.129944985	0.141301015	-0.919632352	0.357764915	0.999579949
1569.810652	-0.014880448	0.071982781	-0.206722324	0.836226722	0.999579949
1074.19918	-0.157579088	0.162752857	-0.968210889	0.332939058	0.999579949

24.78501627	0.234129398	0.351165915	0.666720169	0.504950893	0.999579949
1513.294759	0.030578201	0.127159639	0.240470965	0.809965169	0.999579949
499.5364294	0.066582987	0.134646419	0.494502472	0.620951361	0.999579949
147.6607456	-0.116531917	0.171470369	-0.679603814	0.496755354	0.999579949
1154.633954	-0.057839064	0.044596061	-1.296954546	0.194646827	0.988443849
547.6132011	0.31164797	0.344330227	0.905084555	0.365420594	0.999579949
22.10237128	0.391387745	0.281649483	1.389627071	0.164642151	0.978498637
214.2522412	0.034384703	0.112402624	0.305906582	0.759675774	0.999579949
112.4560938	0.070918432	0.119030933	0.595798339	0.551309953	0.999579949
586.6913361	0.056578645	0.13206294	0.428421819	0.668344042	0.999579949
52.81516458	-0.21831187	0.240225781	-0.908777855	0.363467398	0.999579949
130.8820674	-0.155155319	0.11767592	-1.318496754	0.187337411	0.983992835
38.05381601	-0.092124045	0.218109127	-0.42237593	0.672750644	0.999579949
51.9785523	-0.036579887	0.210000086	-0.174189867	0.861716249	0.999579949
444.030927	-0.356365717	0.172295645	-2.068338504	0.038608204	0.72691918
4356.366851	-0.010510013	0.084395188	-0.124533317	0.900893022	0.999579949
3050.603597	0.029246273	0.101981351	0.286780603	0.774280316	0.999579949
158.1724578	0.021974587	0.080427201	0.273223328	0.784681549	0.999579949
387.7900882	-0.011403877	0.081598306	-0.139756299	0.888852543	0.999579949
126.5609958	-0.076542798	0.115562272	-0.662351103	0.507746233	0.999579949
283.5228465	0.132054527	0.111787111	1.181303693	0.237482101	0.999579949
114.3608564	-0.080194474	0.147187594	-0.544845331	0.585859898	0.999579949
671.2378254	0.039418842	0.066180222	0.595628733	0.551423277	0.999579949
281.3666006	-0.049211354	0.135987976	-0.361880184	0.717441568	0.999579949
59.19970172	-0.031953413	0.132457004	-0.241236113	0.809372123	0.999579949
80.84363798	0.010150232	0.123550606	0.082154445	0.934523898	0.999579949
91.44016401	-0.399608049	0.360195735	-1.109419157	0.267249402	0.999579949
970.4750766	0.077528418	0.094534152	0.82011016	0.412153311	0.999579949
124.1449663	0.030234411	0.114065661	0.265061463	0.790962114	0.999579949
523.2314772	0.098956451	0.159607305	0.61999951	0.53525811	0.999579949
353.2666958	0.144336356	0.116072625	1.243500405	0.213683492	0.999268361
280.0772849	0.048239933	0.133254579	0.362013323	0.717342074	0.999579949
23.91573283	-0.160625144	0.225870924	-0.711136878	0.476999419	0.999579949
3921.722543	-0.032819109	0.038647471	-0.849191615	0.395774678	0.999579949
401.0543014	0.027076119	0.104620171	0.258804	0.795786471	0.999579949
163.4142283	0.022222785	0.096640563	0.22995297	0.818128315	0.999579949
459.383124	0.329394967	0.171518313	1.920465291	0.054799153	0.785214091
2030.058322	-0.275172577	0.125696936	-2.189174901	0.02858413	0.666751303
290.2977132	-0.045336725	0.131570355	-0.344581614	0.73040893	0.999579949
445.3567784	0.042791548	0.075112946	0.569696045	0.568883873	0.999579949
1555.600232	-0.115725143	0.136035222	-0.85069985	0.394936106	0.999579949
1123.227469	-0.0522883	0.058502067	-0.893785504	0.371436666	0.999579949
4922.21732	-0.055624235	0.120279186	-0.462459356	0.643751942	0.999579949
26.18434753	0.671280621	0.313209704	2.143230599	0.032094586	0.689986698
150.709234	-0.095408972	0.1323926	-0.720651848	0.471123745	0.999579949
293.7301373	0.099696898	0.20906092	0.476879648	0.633447825	0.999579949
388.5966985	-0.048741645	0.076284095	-0.638948981	0.522856122	0.999579949
97.08747549	-0.026414583	0.114019812	-0.23166661	0.816796967	0.999579949

277.820143	-0.009957512	0.108583712	-0.091703552	0.926933575	0.999579949
145.4935837	0.130021671	0.142812789	0.910434367	0.362593481	0.999579949
666.2295178	0.117758723	0.124595738	0.945126416	0.344594341	0.999579949
274.0720822	-0.040453122	0.112963095	-0.358109189	0.720261603	0.999579949
359.0384309	0.106190853	0.140641435	0.755046711	0.450220956	0.999579949
371.8081549	-0.352540768	0.576408535	-0.611616148	0.540791752	0.999579949
375.4188319	-0.057281056	0.057245554	-1.000620158	0.317010481	0.999579949
1693.41341	-0.072910416	0.057550985	-1.266883902	0.205196806	0.994814558
318.1086037	0.048031196	0.097295183	0.493664688	0.621543008	0.999579949
25.59395303	0.125988935	0.390022985	0.323029512	0.746672891	0.999579949
133.2214765	-0.03832952	0.119964784	-0.319506432	0.749342515	0.999579949
1996.953756	-0.013414873	0.07183684	-0.186740859	0.851863818	0.999579949
1463.149474	0.280619533	0.136632801	2.053822594	0.039992865	0.73515901
297.3526263	-0.073466996	0.112250206	-0.654493199	0.512794107	0.999579949
913.5147463	-0.00189889	0.070463334	-0.026948622	0.978500713	0.999579949
103.4708591	0.13212159	0.175637543	0.752240029	0.451906725	0.999579949
66.35430141	-0.149627935	0.391912006	-0.381789617	0.702617421	0.999579949
3085.899688	-0.007449155	0.070438815	-0.10575355	0.915777892	0.999579949
1221.188464	-0.002995676	0.082386015	-0.036361459	0.970994145	0.999579949
4705.583012	0.06026313	0.095374512	0.631857808	0.527479795	0.999579949
539.1881051	-0.014505518	0.071426682	-0.20308262	0.839070456	0.999579949
30.31520876	0.199308249	0.252980956	0.787838943	0.430790919	0.999579949
1363.993273	-0.086882223	0.074449404	-1.16699689	0.243211625	0.999579949
339.0265936	0.116396562	0.096169852	1.210322774	0.226155062	0.999579949
193.6022568	-0.037923425	0.103834844	-0.365228308	0.714940989	0.999579949
101.1576316	-0.062861887	0.160716019	-0.391136412	0.695696407	0.999579949
29.54414703	0.122636327	0.301603381	0.406614562	0.6842911	0.999579949
39.6391382	0.07773113	0.251491873	0.309080087	0.757260606	0.999579949
1351.289237	-0.018070241	0.057006474	-0.316985778	0.751254394	0.999579949
106.7000349	-0.161721687	0.207018807	-0.781193211	0.434688866	0.999579949
31.3268982	-0.12701316	0.270947382	-0.468774264	0.639230998	0.999579949
1333.810451	-0.026285725	0.044980643	-0.584378598	0.558965623	0.999579949
330.3253565	-0.023770598	0.09149372	-0.259805782	0.795013591	0.999579949
8700.259679	-0.115740321	0.088486875	-1.307994228	0.190875267	0.98475336
23.34740565	0.442130055	0.412079772	1.072923462	0.283305458	0.999579949
220.5769066	-0.032301803	0.097138261	-0.33253429	0.739485852	0.999579949
71.57353308	-0.09442309	0.331977311	-0.284426336	0.776083679	0.999579949
228.7235948	-0.147210806	0.155803327	-0.94485021	0.344735354	0.999579949
109.0822722	-0.130674233	0.105320729	-1.240726626	0.214706755	0.999579949
1254.968212	-0.010629909	0.101682997	-0.104539686	0.916741076	0.999579949
1163.320398	-0.109369069	0.084062716	-1.301041349	0.193244302	0.986374671
98.01492795	-0.092253675	0.120638038	-0.764714644	0.444441484	0.999579949
33.31495089	-0.028644492	0.285558282	-0.100310492	0.920097828	0.999579949
233.6261626	-0.06997023	0.081652755	-0.856924293	0.391486726	0.999579949
126.1765218	-0.072689811	0.109535198	-0.663620574	0.506933186	0.999579949
251.7868503	-0.027591086	0.121861439	-0.226413591	0.820879758	0.999579949
94.65705801	-0.576039864	0.154011492	-3.740239478	0.000183845	0.059377225
137.9568	0.046898908	0.252658653	0.185621618	0.852741499	0.999579949

2502.294492	-0.216351619	0.113294629	-1.909637032	0.056179964	0.785214091
139.7509409	-0.190366989	0.124360523	-1.530767034	0.12582698	0.930115397
199.4258272	0.010069332	0.177424989	0.056752615	0.954742261	0.999579949
1295.939425	-0.190314617	0.067965788	-2.80015316	0.005107837	0.357404857
29.64780448	0.119019118	0.19303287	0.616574357	0.537515513	0.999579949
39.55699491	0.067215156	0.190503268	0.352829411	0.724216333	0.999579949
44.80744881	-0.208832146	0.440949618	-0.473596388	0.635787752	0.999579949
574.7957196	0.029799033	0.090650722	0.328723618	0.742364601	0.999579949
444.0774527	0.088736736	0.093967786	0.944331456	0.345000296	0.999579949
131.207545	0.322349629	0.305131023	1.056430204	0.290771713	0.999579949
80.09770766	0.557158685	0.272574908	2.04405713	0.040947904	0.745346523
150.0951865	0.052388447	0.142320783	0.368101172	0.712797797	0.999579949
88.89648634	-0.054042787	0.270366066	-0.199887463	0.841568596	0.999579949
41.75052189	-0.096004919	0.20213218	-0.47496108	0.634814715	0.999579949
789.6778912	0.039081217	0.096199916	0.406250006	0.684558915	0.999579949
173.8355897	-0.242508949	0.161759273	-1.499196586	0.133822641	0.943801781
1038.782357	-0.017577965	0.140522405	-0.125090123	0.900452202	0.999579949
860.5035323	0.039987736	0.102754561	0.389157766	0.697159444	0.999579949
1160.916991	0.125474853	0.102787335	1.220722897	0.222190955	0.999579949
259.7865501	-0.015944572	0.198260882	-0.080422178	0.935901489	0.999579949
28.82001933	-0.146625841	0.250005342	-0.586490833	0.55754572	0.999579949
129.9948991	-0.046270623	0.143285958	-0.322925034	0.746752017	0.999579949
26.80132665	0.713952592	0.519483935	1.374349704	0.169333143	0.981013142
162.5627326	0.125568341	0.115534765	1.08684465	0.277105471	0.999579949
559.2958603	-0.051252274	0.081893643	-0.625839461	0.531420246	0.999579949
230.0983982	0.19119549	0.14903989	1.282847767	0.199545415	0.992493287
157.7486834	0.007638625	0.134174695	0.056930443	0.954600604	0.999579949
91.84810229	0.062134318	0.124231869	0.500147982	0.616970883	0.999579949
3043.116787	0.041941287	0.136378268	0.30753644	0.758435091	0.999579949
622.5367522	0.04122958	0.157218422	0.262243948	0.793133373	0.999579949
44.52988177	-0.06770949	0.489846201	-0.138226018	0.890061793	0.999579949
71.30649976	0.602526478	0.542120541	1.111425287	0.266385333	0.999579949
23.84742201	2.497153034	0.832204668	3.000647713	0.002694061	0.27147509
24.67495235	0.409748594	0.330987276	1.237958747	0.215731358	0.999579949
26.87327127	1.254329724	0.845129066	1.484187179	0.13775927	0.945791454
128.2452699	0.15251011	0.103468897	1.473970585	0.140489511	0.948318761
991.5863332	0.078627847	0.043297355	1.815996548	0.069370902	0.831457432
33.5850738	0.132830528	0.274227536	0.484380708	0.628115708	0.999579949
82.19793369	-0.343031905	0.24895507	-1.377886799	0.168238241	0.980087289
245.8975032	0.089271392	0.096122194	0.928728205	0.35302996	0.999579949
49.99979064	0.022869528	0.303844643	0.075267174	0.940002139	0.999579949
88.3768739	0.359586681	0.377161313	0.953402878	0.340385984	0.999579949
348.5417208	-0.00037168	0.05677023	-0.006547093	0.994776213	0.999579949
243.0895167	-0.040523098	0.100906415	-0.401590899	0.687985129	0.999579949
1345.488742	-0.090926448	0.081295307	-1.118471055	0.263365862	0.999579949
41.1546005	-0.105854621	0.256268071	-0.413062077	0.679561133	0.999579949
180.8762702	-0.037658144	0.12070014	-0.311997521	0.755042408	0.999579949
700.9545818	-0.278556878	0.172847314	-1.611577704	0.107053866	0.900529039

153.516889	0.1127707	0.092370419	1.220852966	0.222141696	0.999579949
116.8692529	0.150490222	0.162848335	0.924112745	0.355427619	0.999579949
416.5726043	0.188527902	0.123258102	1.529537601	0.126131225	0.930818754
751.5505908	0.107268289	0.06870768	1.561227065	0.118470185	0.921521033
24.27280892	0.208623524	0.232695256	0.896552545	0.36995773	0.999579949
66.60308588	-0.045933105	0.136520599	-0.336455491	0.73652741	0.999579949
80.87015253	-0.122240225	0.158445256	-0.771498166	0.440411709	0.999579949
86.56871336	0.060574038	0.141036608	0.429491598	0.667565507	0.999579949
77.66922381	-0.332623094	0.199264259	-1.669256173	0.095066622	0.883081984
515.3394044	0.198113322	0.150383547	1.317386942	0.187708956	0.983992835
243.8889325	0.07237933	0.082681421	0.875400165	0.38135621	0.999579949
152.8428929	-0.008079255	0.09958064	-0.081132788	0.935336351	0.999579949
585.6730835	0.018012614	0.088239218	0.204133881	0.838248878	0.999579949
53.34972526	-0.049799057	0.138784021	-0.358824142	0.719726651	0.999579949
120.3950914	0.069830781	0.170880353	0.408653069	0.682794279	0.999579949
145.8796921	0.194745677	0.218197854	0.892518753	0.372114946	0.999579949
139.5863693	-0.007372084	0.14508177	-0.050813304	0.959474289	0.999579949
1811.751325	-0.11104881	0.060419462	-1.837964227	0.066067677	0.821508842
346.2532599	-0.180798387	0.123878476	-1.459481845	0.144432534	0.955875831
635.1131975	-0.107893696	0.094961195	-1.136187218	0.255878219	0.999579949
97.70097674	-0.228032526	0.140029027	-1.628466114	0.103426089	0.895628172
725.6042365	0.006260185	0.104242271	0.060054193	0.952112473	0.999579949
184.8956295	0.114081924	0.117141061	0.973885011	0.330113637	0.999579949
3726.401981	0.07546321	0.047540368	1.587350149	0.112433363	0.914735409
278.6512711	0.053265732	0.077025587	0.691532963	0.489230673	0.999579949
228.0449326	0.035278694	0.121167903	0.291155437	0.770932443	0.999579949
72.15893162	-0.025220397	0.153158431	-0.164668681	0.869204769	0.999579949
207.1625488	-0.099918122	0.093251329	-1.071492735	0.283947936	0.999579949
33.26744254	0.128215194	0.198718436	0.645210359	0.518790874	0.999579949
173.3488994	0.053715039	0.116845268	0.459710859	0.645723774	0.999579949
48.16445607	0.089456768	0.204331577	0.437801976	0.661529838	0.999579949
170.0014893	-0.357327094	0.131960431	-2.707835159	0.006772365	0.404205857
410.1324452	-0.001196087	0.080627068	-0.014834803	0.988163974	0.999579949
35.2069061	0.465754109	0.217015356	2.146180421	0.031858587	0.689986698
179.2908255	0.141069467	0.121761851	1.158568682	0.246632041	0.999579949
61.29149801	4.067027652	1.418138458	2.867863592	0.004132537	0.331550518
51.11740848	0.085943941	0.159833311	0.537709821	0.590777398	0.999579949
2693.81331	0.014058529	0.114933206	0.122319126	0.902646285	0.999579949
1268.585242	0.19954688	0.10165755	1.962932226	0.04965405	0.775983138
2305.3869	-0.160977159	0.133260899	-1.207984939	0.227053056	0.999579949
45.76226716	0.20891672	0.247502954	0.844097883	0.398614711	0.999579949
2807.328491	0.144606646	0.155415812	0.930450026	0.352138127	0.999579949
603.2315096	0.047407301	0.131817886	0.359642403	0.71911457	0.999579949
3295.454644	-0.052843353	0.127118097	-0.415702834	0.677627467	0.999579949
23.62109439	-0.01441703	0.232339677	-0.062051519	0.950521805	0.999579949
22.32429493	0.00431124	0.215910862	0.019967683	0.984069152	0.999579949
1142.534617	0.036568862	0.11270315	0.324470629	0.745581755	0.999579949
144.1762164	0.093245376	0.151705688	0.614646538	0.538788173	0.999579949

84.22518532	-0.146625743	0.111455453	-1.315554675	0.188323555	0.983992835
304.0840932	-0.030919593	0.130680975	-0.236603631	0.812964303	0.999579949
185.5333542	-0.066984399	0.121103523	-0.553116846	0.580183402	0.999579949
108.3252531	0.292981498	0.252855797	1.15869006	0.246582544	0.999579949
75.93775162	-0.132073435	0.133646848	-0.988227084	0.323041447	0.999579949
204.0150859	-0.372633719	0.308834886	-1.206579103	0.227594278	0.999579949
821.1378383	-0.371674823	0.40500887	-0.917695513	0.358778298	0.999579949
383.792724	-0.01553167	0.143706471	-0.108079126	0.913932926	0.999579949
644.6512876	-0.004164882	0.163162581	-0.025525962	0.97963544	0.999579949
282.1377323	-0.011421486	0.149936156	-0.076175663	0.939279345	0.999579949
156.6413748	-0.005265478	0.140868082	-0.037378784	0.970182989	0.999579949
152.4103391	0.101953643	0.193015917	0.528213653	0.597351054	0.999579949
235.1513376	-0.04546206	0.100647297	-0.451696782	0.651487437	0.999579949
2325.885733	0.080978357	0.115681747	0.700009799	0.483921185	0.999579949
107.1186741	0.23527703	0.168749052	1.394242082	0.163244514	0.978498637
5191.047208	-0.012236996	0.099918943	-0.122469228	0.902527415	0.999579949
3522.424206	-0.04169783	0.124568298	-0.334738699	0.737822206	0.999579949
187.3380776	0.000729996	0.130179322	0.00560762	0.99552579	0.999579949
609.2807277	0.019362801	0.105116975	0.184202414	0.853854666	0.999579949
2945.684721	-0.061116947	0.100529005	-0.607953369	0.543218402	0.999579949
33.4674235	0.522840465	0.392469959	1.332179579	0.182801185	0.983992835
79.33950954	0.19751826	0.234827817	0.841119519	0.400280981	0.999579949
124.3680216	0.030623739	0.120314427	0.254530896	0.799085438	0.999579949
78.70302928	-0.074125303	0.196830024	-0.376595508	0.706474226	0.999579949
83.44122353	1.236703886	0.457627155	2.702426795	0.006883534	0.404205857
566.6412469	-0.094383132	0.109779974	-0.859748171	0.389927875	0.999579949
169.3908101	-0.015474764	0.155888305	-0.099268282	0.920925261	0.999579949
171.6152696	-0.043513052	0.103301446	-0.421224038	0.673591493	0.999579949
4405.326851	0.260227392	0.244186522	1.065691052	0.286563324	0.999579949
1712.649897	-0.093205049	0.092076131	-1.012260706	0.311413417	0.999579949
37.8462618	0.57335353	0.22592795	2.537771572	0.011156077	0.487909795
1025.904994	-0.156132812	0.180730844	-0.863896876	0.387644543	0.999579949
104.5491484	0.490395883	0.292480845	1.676676923	0.093605676	0.880418343
7539.024398	-0.133425034	0.092003709	-1.450213644	0.146998951	0.959874957
213.9310337	-0.373768145	0.129325833	-2.89012748	0.003850856	0.319818899
577.0707534	0.016255736	0.060237595	0.269860301	0.78726773	0.999579949
92.77567289	0.034572985	0.266765199	0.129600807	0.896882265	0.999579949
368.5450965	-0.086152501	0.093568496	-0.920742606	0.357184826	0.999579949
2619.333084	0.106116259	0.112667819	0.941850658	0.346269105	0.999579949
1215.159988	-0.04171888	0.119778902	-0.348299071	0.727615591	0.999579949
20.76144921	-0.221038505	0.262951937	-0.840604211	0.400569699	0.999579949
32.43411113	-0.045318537	0.196533086	-0.23058986	0.817633446	0.999579949
1407.967733	-0.051658652	0.0927213	-0.557138989	0.577432471	0.999579949
253.7708142	-0.092180473	0.148340055	-0.621413227	0.534327771	0.999579949
86.81435559	-0.052931536	0.25011871	-0.211625657	0.832399091	0.999579949
158.9479762	-0.273240576	0.41718389	-0.65496435	0.512490707	0.999579949
186.1687966	-0.019522471	0.097136576	-0.200979612	0.840714516	0.999579949
482.5995347	0.024614805	0.060071735	0.409756849	0.681984323	0.999579949

4080.989258	0.064036254	0.141463057	0.452671218	0.650785508	0.999579949
23.58985511	-0.030281874	0.259981403	-0.116477076	0.907274453	0.999579949
30.07863047	-0.095265519	0.26601345	-0.358122941	0.720251312	0.999579949
184.3949317	-0.126304971	0.113074073	-1.117010892	0.263989668	0.999579949
244.5675233	0.061038953	0.121162388	0.503778061	0.614417346	0.999579949
28.6087573	0.201359075	0.200103892	1.006272653	0.314284432	0.999579949
8196.02222	0.169462878	0.122469879	1.383710674	0.166447064	0.978789551
219.8271943	0.007471668	0.115361597	0.064767377	0.948359216	0.999579949
339.3078484	-0.141245529	0.100758705	-1.401819608	0.160969122	0.978498637
163.1656399	0.190975295	0.145302285	1.314330985	0.188734845	0.983992835
38.41845459	0.034930397	0.193601317	0.180424375	0.85681942	0.999579949
242.892835	-0.080312203	0.096590182	-0.831473767	0.405706042	0.999579949
135.8771982	-0.003557217	0.175462819	-0.02027334	0.983825323	0.999579949
437.8734663	-0.036229762	0.098432063	-0.368068701	0.712822008	0.999579949
139.0524761	-0.136289075	0.1249672	-1.090598775	0.275449469	0.999579949
28.22283531	-0.351089049	0.321511153	-1.091996485	0.274834646	0.999579949
273.7758416	-0.094875961	0.145338132	-0.652794689	0.513888648	0.999579949
1731.849905	-0.054697269	0.039067375	-1.400075349	0.161490756	0.978498637
1236.374893	0.100775227	0.08168989	1.233631562	0.217340227	0.999579949
529.2947415	0.008871026	0.118006999	0.075173728	0.940076487	0.999579949
1693.49193	-0.063066483	0.098863053	-0.637917616	0.52352731	0.999579949
445.9799897	-0.034309239	0.205189079	-0.167207921	0.867206452	0.999579949
116.8432456	-0.051792143	0.1731653	-0.299090772	0.764870787	0.999579949
2279.073338	-0.061373603	0.056496141	-1.086332661	0.277331842	0.999579949
155.5786323	-0.001150758	0.133532778	-0.008617793	0.993124081	0.999579949
83.51076492	0.028224665	0.14778831	0.190980363	0.848540978	0.999579949
647.9480884	0.046710281	0.075972427	0.614832023	0.538665659	0.999579949
1756.354699	0.004461245	0.082712059	0.053937054	0.956985315	0.999579949
184.8213657	-0.017369439	0.089260321	-0.194593061	0.845711536	0.999579949
1217.638311	-0.011844485	0.068452373	-0.173032504	0.862625878	0.999579949
38.88264614	0.198439358	0.254594744	0.779432267	0.435725123	0.999579949
541.823353	-0.147095573	0.240047983	-0.612775709	0.540024655	0.999579949
75.78437747	-0.375224804	0.20168297	-1.860468456	0.062819277	0.807133028
2122.462391	-0.215224609	0.09498265	-2.265936038	0.0234553	0.62865781
1475.632485	-0.0566931	0.072303946	-0.784094133	0.432984878	0.999579949
827.1554255	-0.0513156	0.057763557	-0.888373276	0.374339996	0.999579949
877.4882232	0.062665246	0.074683007	0.839083063	0.4014227	0.999579949
668.9210336	0.027778942	0.122064107	0.227576661	0.819975366	0.999579949
235.6585868	0.081995931	0.090331752	0.907719926	0.364026213	0.999579949
20358.6062	0.56066644	0.201586268	2.781272982	0.005414619	0.362779445
43.27679975	0.027667373	0.175770045	0.157406645	0.874924377	0.999579949
144.2356685	0.188141775	0.107623768	1.748143348	0.080439208	0.85448976
142.5172979	0.073325515	0.154385701	0.474950171	0.63482249	0.999579949
22.5012764	-0.665667118	0.860467815	-0.77361071	0.439161036	0.999579949
527.1332496	-0.186939831	0.147261969	-1.269437266	0.204285152	0.994814558
1639.153925	-0.00222608	0.077942527	-0.028560535	0.977215088	0.999579949
735.2683273	-0.051953638	0.071058819	-0.731135678	0.464696284	0.999579949
1203.53374	-0.101537933	0.087267661	-1.163523032	0.244617359	0.999579949

906.6115621	0.00721207	0.07629786	0.094525193	0.924691971	0.999579949
461.146366	0.060407279	0.076389055	0.790784479	0.429069769	0.999579949
348.0343396	-0.015628336	0.075237315	-0.207720538	0.83544718	0.999579949
352.4519875	-0.110669373	0.10627757	-1.041323892	0.297725252	0.999579949
41.40506826	0.957754283	0.90002373	1.064143368	0.287263757	0.999579949
1470.520697	-0.026120615	0.215280011	-0.12133321	0.903427116	0.999579949
332.4816417	-0.01307958	0.067299356	-0.194349261	0.845902416	0.999579949
99.58183146	0.242470907	0.257369621	0.942111603	0.346135504	0.999579949
25.86035716	0.541484008	0.215628414	2.511190418	0.012032477	0.500201597
4286.142767	-0.004123485	0.098358497	-0.041923013	0.966560071	0.999579949
1364.601644	-0.045074802	0.079099968	-0.569846021	0.568782139	0.999579949
132.9856819	0.218006238	0.110689949	1.969521537	0.048893232	0.773861498
83.23661821	0.235782305	0.195842027	1.203941306	0.228612265	0.999579949
93.28199985	-0.268635345	0.137471925	-1.954110597	0.050688139	0.777437894
142.244797	0.018785924	0.087816907	0.213921496	0.830608277	0.999579949
534.5470541	-0.200307912	0.184901077	-1.083324746	0.278664305	0.999579949
189.3194896	-0.137801166	0.175918484	-0.783323974	0.433436888	0.999579949
1028.83263	-0.023492823	0.076439077	-0.307340492	0.758584218	0.999579949
117.7944696	0.110769782	0.100965378	1.097106594	0.272594798	0.999579949
22.52460435	0.228532513	0.403412101	0.566498904	0.571054678	0.999579949
223.1181282	-0.027468601	0.086873235	-0.316191759	0.751856963	0.999579949
565.174156	-0.079482345	0.100279359	-0.792609228	0.428005531	0.999579949
76.93289767	0.014918119	0.133869453	0.111437812	0.911269177	0.999579949
3731.046498	-0.105491359	0.10141084	-1.040237501	0.298229573	0.999579949
335.7881996	-0.046043996	0.073280908	-0.628321862	0.529793113	0.999579949
1024.088099	0.04007797	0.105397037	0.380257083	0.70375459	0.999579949
5063.631339	-0.199087675	0.170820187	-1.16548096	0.243824363	0.999579949
1070.014945	-0.092536311	0.069420843	-1.332975915	0.182539708	0.983992835
136.1921297	0.000184693	0.111933471	0.001650027	0.99868347	0.999794706
150.487717	-0.11989723	0.081074674	-1.478849359	0.13918058	0.947119714
125.170836	0.124240463	0.11892293	1.044714115	0.296155119	0.999579949
621.5317072	0.030132147	0.069920338	0.430949678	0.666504961	0.999579949
295.7521667	-0.20883088	0.106175435	-1.966847407	0.049200805	0.77466668
34.07781063	-0.027787468	0.212321552	-0.130874458	0.895874621	0.999579949
851.0457666	-0.011029243	0.13125539	-0.084028874	0.933033475	0.999579949
74.31069661	-0.27736228	0.173384879	-1.599691281	0.109667087	0.908196337
766.3246905	0.010128249	0.078824875	0.128490519	0.897760801	0.999579949
164.9799088	0.018640129	0.139412901	0.133704479	0.893636263	0.999579949
302.6773938	-0.032762454	0.063905593	-0.512669597	0.60818246	0.999579949
23.08488938	0.131910852	0.305577798	0.431676821	0.665976316	0.999579949
295.9840155	0.12289742	0.106259318	1.156580168	0.247443929	0.999579949
609.5872877	0.025179487	0.081849474	0.307631633	0.758362647	0.999579949
764.4787405	0.106988759	0.081524555	1.312350111	0.189402031	0.983992835
1269.911646	-0.699940153	0.476073216	-1.470236361	0.14149775	0.949966262
264.2045719	0.100132843	0.119985433	0.834541667	0.403975818	0.999579949
193.1270907	0.192613988	0.164996708	1.167380798	0.243056622	0.999579949
137.1449407	0.03559011	0.107793082	0.330170631	0.741271037	0.999579949
769.4959412	-0.134913401	0.132938586	-1.014855096	0.310174906	0.999579949

57.76034617	-0.083047537	0.252555962	-0.328828257	0.742285504	0.999579949
463.0588022	-0.099314942	0.161377027	-0.61542181	0.538276192	0.999579949
24.703842	-0.351886946	0.254245441	-1.384044271	0.166344899	0.978789551
1589.294965	0.029079101	0.066696969	0.435988343	0.662845187	0.999579949
61.05102429	-0.066536674	0.210084655	-0.31671363	0.751460905	0.999579949
230.2588445	0.053605055	0.109433036	0.489843437	0.624244691	0.999579949
64.79791301	0.066518193	0.198567178	0.334990875	0.73763197	0.999579949
369.1803467	-0.041829387	0.066385474	-0.630098496	0.528630143	0.999579949
36.5415846	-0.117856973	0.203124241	-0.580221113	0.561765518	0.999579949
43.3963622	-0.124037926	0.172069793	-0.720858228	0.470996745	0.999579949
2491.060038	-0.094162278	0.096469672	-0.97608166	0.329023999	0.999579949
286.208131	-0.095085939	0.171704443	-0.553776815	0.579731597	0.999579949
524.4790378	-0.244550724	0.148358096	-1.648381391	0.099274432	0.895628172
74.9728656	0.248936627	0.231497732	1.075330737	0.28222668	0.999579949
128.9838481	0.142004377	0.14839354	0.95694447	0.338595284	0.999579949
688.33045	-0.084761185	0.109411081	-0.774703846	0.438514678	0.999579949
430.9351909	-0.103728494	0.106913036	-0.970213719	0.331939974	0.999579949
1430.281682	0.010544567	0.078053143	0.135094717	0.892536987	0.999579949
1158.550821	-0.098822614	0.157871696	-0.625967901	0.531335995	0.999579949
212.4060307	-0.029982323	0.091808465	-0.326574715	0.74398957	0.999579949
352.1228005	-0.01540129	0.125710074	-0.122514366	0.902491669	0.999579949
1036.034445	0.025694864	0.088294277	0.291013919	0.771040674	0.999579949
156.8166607	2.19468066	0.900821604	2.436309976	0.014837963	0.539131676
502.1793816	0.022413037	0.120594989	0.185853798	0.852559415	0.999579949
562.9560304	0.043258786	0.209285388	0.206697591	0.836246039	0.999579949
49.57112654	-0.015582161	0.143527447	-0.108565722	0.91354695	0.999579949
49.56146027	0.424667653	0.448144878	0.947612422	0.3433268	0.999579949
1067.517606	-0.065761217	0.090721195	-0.724871598	0.468530807	0.999579949
91.11331161	0.257282219	0.202586786	1.269985195	0.204089904	0.994814558
442.8356661	-0.126851276	0.111003829	-1.142764869	0.253136228	0.999579949
37.62629984	-0.398797866	0.32836596	-1.214492105	0.224559863	0.999579949
108.297771	-0.338139095	0.172141429	-1.964309792	0.049494177	0.775350081
20.88154396	-0.349106075	0.272257183	-1.282265801	0.199749421	0.99252217
479.018024	-0.000269549	0.082422936	-0.003270315	0.997390671	0.999579949
100.8038575	0.107260102	0.126684317	0.846672298	0.397177804	0.999579949
562.5932794	-0.004317034	0.05832307	-0.074019314	0.940995017	0.999579949
191.4777918	-0.061797348	0.14049863	-0.439843063	0.660050776	0.999579949
352.625549	-0.212635377	0.098067712	-2.168250615	0.030139624	0.677729599
591.9252105	-0.136936563	0.10630743	-1.28811846	0.19770473	0.992338959
44.20742436	-0.109240349	0.182647043	-0.598095363	0.549776304	0.999579949
261.2414268	0.235427012	0.458209641	0.513797596	0.607393509	0.999579949
176.9696804	0.15322929	0.180206639	0.850297694	0.395159598	0.999579949
191.0791183	0.074931113	0.209343879	0.357933147	0.720393344	0.999579949
29.37979511	0.015766799	0.331964895	0.04749538	0.962118412	0.999579949
570.6998912	0.018650362	0.108112697	0.172508528	0.863037758	0.999579949
839.4386612	-0.053296881	0.055057371	-0.96802444	0.333032164	0.999579949
77.89347537	0.127331181	0.133701704	0.952352715	0.340918131	0.999579949
324.614313	-0.012653347	0.095396584	-0.132639412	0.894478561	0.999579949

236.3974257	-0.00486253	0.074746919	-0.065053247	0.948131605	0.999579949
868.7935166	-0.059159038	0.120060601	-0.492743146	0.622194087	0.999579949
1076.374769	0.042279632	0.044372327	0.95283785	0.340672234	0.999579949
618.1596665	-0.077904929	0.086624018	-0.899345593	0.368468609	0.999579949
168.4016981	0.188788859	0.336918504	0.560339834	0.575247662	0.999579949
98.99728203	0.403908727	0.400790314	1.00778066	0.313559773	0.999579949
97.34659788	0.214351529	0.100016459	2.143162553	0.032100048	0.689986698
870.3040999	-0.0056519	0.079125903	-0.0714292	0.943056171	0.999579949
78.945769	-0.039374191	0.165710358	-0.237608511	0.812184748	0.999579949
379.9285863	0.085731234	0.203851198	0.420557912	0.674077933	0.999579949
425.2391617	-0.422670079	0.119382076	-3.540481899	0.000399397	0.097717629
64.06319841	0.23775716	0.214770577	1.10702855	0.268281585	0.999579949
823.9334824	-0.107545049	0.179995182	-0.597488488	0.550181291	0.999579949
963.1645136	0.025765774	0.061597016	0.418295819	0.675730846	0.999579949
506.7349418	-0.006652351	0.138030391	-0.048194825	0.961560974	0.999579949
568.3881678	-0.002083514	0.088183602	-0.023626998	0.981150137	0.999579949
191.1385079	-0.105057327	0.424300335	-0.247601329	0.804442882	0.999579949
200.9772072	-0.033659803	0.102047505	-0.329844449	0.7415175	0.999579949
1172.40191	0.084081667	0.107676095	0.780875892	0.434875492	0.999579949
724.739433	0.078741337	0.053826313	1.462878148	0.143500724	0.953645321
416.5060259	0.097586245	0.074810528	1.304445343	0.192081784	0.984804292
1028.739356	0.018185625	0.103736425	0.175306072	0.860839141	0.999579949
300.1311059	0.03597211	0.145670998	0.246940781	0.804954055	0.999579949
12158.792	-0.118144583	0.103597861	-1.140415272	0.254113333	0.999579949
73.56669744	0.016428724	0.169614041	0.096859456	0.922838007	0.999579949
85.39877332	-0.226529004	0.216031438	-1.048592771	0.294365584	0.999579949
386.4797861	0.173530805	0.11245134	1.543163518	0.122791107	0.92236461
1328.561742	0.121607287	0.091111202	1.334712786	0.181970367	0.983992835
1118.053345	-0.129128491	0.126195537	-1.023241348	0.306193747	0.999579949
57.85959352	0.004463823	0.17366933	0.025703001	0.97949423	0.999579949
4585.627078	0.07305727	0.07703225	0.948398498	0.342926624	0.999579949
52.32574405	0.121675283	0.232021822	0.524413102	0.599991246	0.999579949
110.4432802	0.112402728	0.112872863	0.995834823	0.319330408	0.999579949
568.6879918	0.030697119	0.060287771	0.509176552	0.610628477	0.999579949
120.7005451	0.230410297	0.23814033	0.967540008	0.333274151	0.999579949
1449.232794	-0.133881908	0.09628256	-1.390510477	0.16437392	0.978498637
228.5940565	0.031218137	0.084333772	0.370173617	0.711253133	0.999579949
1647.022917	0.058391165	0.094099041	0.620528804	0.534909696	0.999579949
881.7057219	-0.03840491	0.093567198	-0.410452713	0.681473885	0.999579949
740.5852448	0.052874653	0.05226715	1.011623038	0.311718326	0.999579949
2227.127829	-0.103728625	0.132262897	-0.784260951	0.432887008	0.999579949
1391.653588	-0.14953966	0.109833012	-1.361518335	0.173349943	0.983601342
41.96938382	0.014011869	0.183437944	0.076384789	0.939112971	0.999579949
281.6144758	-0.111511487	0.109639012	-1.017078543	0.309116067	0.999579949
20.81676897	0.18816257	0.243481195	0.772801242	0.439640018	0.999579949
909.3730453	0.03190373	0.077971315	0.409172649	0.682412965	0.999579949
650.3629109	-0.061293524	0.095332057	-0.642947674	0.520258054	0.999579949
102.664261	0.322749395	0.226257808	1.42646743	0.153733462	0.973567967

62.00793207	0.013549672	0.15096445	0.089754057	0.928482658	0.999579949
597.8209295	0.072849221	0.119580098	0.609208572	0.542386199	0.999579949
2754.22482	0.067010785	0.126124062	0.53130849	0.595205023	0.999579949
54.49965391	-0.063366124	0.153701419	-0.412267658	0.680143251	0.999579949
974.5373951	0.066444211	0.084477956	0.78652721	0.431558685	0.999579949
1079.654674	0.087288881	0.146702785	0.595004936	0.551840172	0.999579949
659.4286311	0.07898412	0.069133693	1.142483743	0.253253	0.999579949
230.0675769	0.167833782	0.134969445	1.243494651	0.213685611	0.999268361
518.3673747	-0.025061126	0.146752178	-0.170771749	0.864403244	0.999579949
496.4239341	0.194515836	0.186878206	1.040869556	0.297936093	0.999579949
1568.279406	0.091989005	0.150005696	0.613236749	0.539719809	0.999579949
36.58052427	0.519223168	0.225811656	2.299363888	0.021484285	0.622105864
61.47418389	0.089710458	0.270662314	0.331447909	0.740306182	0.999579949
369.9524258	0.005806656	0.059899086	0.096940653	0.922773525	0.999579949
525.5431176	-0.068976058	0.088503752	-0.779357446	0.435769184	0.999579949
63.55408401	0.118992072	0.159481788	0.746116991	0.455596747	0.999579949
323.9090927	0.052196196	0.194803543	0.267942744	0.788743391	0.999579949
22.0368175	0.399455729	0.457507498	0.873112969	0.382601506	0.999579949
899.1808854	0.152516218	0.140314168	1.086962351	0.277053449	0.999579949
406.1971037	-0.366192507	0.118026858	-3.102620134	0.001918157	0.225075621
28.87550618	0.285922237	0.236625192	1.208333882	0.226918861	0.999579949
575.7803992	-0.233715639	0.124242332	-1.881127263	0.059954609	0.799141014
47.30852886	0.576876753	0.306813416	1.880220104	0.060078087	0.799940361
75.90971952	0.402502409	0.37149423	1.083468805	0.27860039	0.999579949
105.401906	0.186355614	0.125396775	1.486127646	0.137245361	0.945791454
191.1419651	-0.079882996	0.116649706	-0.684810945	0.493463228	0.999579949
71.12671153	0.2077586	0.146970942	1.413603247	0.157478427	0.977304288
60.60338858	0.082507201	0.193085492	0.427309169	0.669154156	0.999579949
60.88969547	-0.171176081	0.189769555	-0.902020777	0.367045831	0.999579949
76.42852414	-0.001211305	0.192401104	-0.00629573	0.994976768	0.999579949
313.3405934	0.072342671	0.104713239	0.690864611	0.489650628	0.999579949
2624.068834	0.231460639	0.202142783	1.145035385	0.252194499	0.999579949
263.9087151	-0.083720731	0.116545303	-0.718353542	0.472539324	0.999579949
32.504282	0.084592383	0.343406764	0.246332898	0.805424546	0.999579949
57.24358435	-0.025760737	0.265317242	-0.097094093	0.922651672	0.999579949
360.8917231	-0.022129716	0.10558159	-0.209598244	0.83398125	0.999579949
319.4611701	-0.154263171	0.097448676	-1.583019675	0.11341698	0.916629381
300.2698849	0.085528951	0.09390098	0.910841943	0.362378659	0.999579949
310.3674047	0.060325871	0.072269662	0.83473299	0.403868063	0.999579949
634.4302154	0.00830382	0.102505192	0.081008778	0.935434972	0.999579949
347.285554	-0.187275029	0.0799032	-2.343773817	0.019089744	0.590797093
381.601215	-0.140262731	0.080154916	-1.749895557	0.080136337	0.85448976
743.8178942	0.059744408	0.050149858	1.191317587	0.233528932	0.999579949
2490.792581	-0.067253046	0.09828713	-0.684250781	0.493816821	0.999579949
638.188646	-0.069813295	0.098558587	-0.7083431	0.478732218	0.999579949
114.8797938	-0.00789708	0.116803753	-0.06760981	0.946096246	0.999579949
218.6540371	-0.041206523	0.115497926	-0.356772841	0.72126187	0.999579949
185.3092766	-0.037649107	0.104450885	-0.360447942	0.71851218	0.999579949

23.50293741	1.19616387	0.352211915	3.396148227	0.000683413	0.128481718
169.7110986	0.031165098	0.096726588	0.322197838	0.747302822	0.999579949
467.3390801	0.144395543	0.192310688	0.750845131	0.452745865	0.999579949
71.1085363	0.006686711	0.183292764	0.036481043	0.970898794	0.999579949
816.5578232	0.216376514	0.122239783	1.770098969	0.076710655	0.851207389
245.3359257	0.016423837	0.096613981	0.169994417	0.865014527	0.999579949
31.816612	0.239596453	0.609596449	0.393041091	0.694289132	0.999579949
868.7926162	-0.12088632	0.085889422	-1.407464591	0.15928967	0.977762491
218.7598235	0.261419229	0.166479343	1.570280276	0.116349921	0.918907698
92.16224141	-0.036264007	0.151788002	-0.238912207	0.81117366	0.999579949
509.5849692	0.067376496	0.081003807	0.831769496	0.405539065	0.999579949
225.3262834	0.006964908	0.067796343	0.10273279	0.918175048	0.999579949
2183.007658	0.075269731	0.114096822	0.659700509	0.50944604	0.999579949
74.44088386	0.015556266	0.157876636	0.098534314	0.921508026	0.999579949
1203.514459	0.024905486	0.062177718	0.400553241	0.688749078	0.999579949
545.8761731	-0.040073773	0.08265961	-0.484804766	0.627814842	0.999579949
297.5513724	-0.214442713	0.118129099	-1.815325061	0.06947397	0.831457432
204.242352	-0.103764351	0.103520554	-1.002355051	0.316172143	0.999579949
28.64846419	1.259137471	0.552130052	2.280508851	0.022577526	0.627784819
1074.639611	0.139677052	0.131440564	1.062663213	0.287934709	0.999579949
488.573798	0.130987767	0.074394445	1.760719722	0.078285853	0.851207389
387.1443779	-0.005086424	0.124187109	-0.040957745	0.967329582	0.999579949
46.41911438	0.280612271	0.169392445	1.656580787	0.097604259	0.890209389
78.20867576	-0.178029862	0.137431027	-1.295412444	0.195177988	0.988599841
90.74689133	0.019090878	0.214030097	0.089197167	0.928925217	0.999579949
289.9235862	0.021024953	0.06366335	0.330252065	0.74120951	0.999579949
255.8116163	0.06090593	0.122546975	0.497000686	0.619188568	0.999579949
1311.832771	0.126781952	0.094627881	1.339794893	0.180312036	0.983992835
241.0031367	-0.100941074	0.089476541	-1.128128929	0.259265473	0.999579949
66.23862926	0.226861322	0.222492161	1.019637371	0.307900474	0.999579949
161.3517773	0.199533106	0.325022342	0.613905816	0.539277567	0.999579949
1345.545396	-0.033290134	0.08338058	-0.399255241	0.689705144	0.999579949
354.5223426	-0.037674605	0.237574353	-0.158580271	0.873999573	0.999579949
1393.985528	0.013047179	0.055131292	0.236656501	0.812923283	0.999579949
139.9203902	0.431301811	0.335897321	1.284028731	0.199131903	0.992481822
41.59610831	-0.150522542	0.454869926	-0.330913374	0.74070992	0.999579949
1803.778261	0.011877364	0.090727243	0.130912869	0.895844235	0.999579949
121.242538	0.401956601	0.21332128	1.884278026	0.059527378	0.798635337
66.34346216	0.108562948	0.20242025	0.536324544	0.591734271	0.999579949
80.28155234	0.302827247	0.149346714	2.027679339	0.042592986	0.74969123
78.02936661	0.333072878	0.234357387	1.421217748	0.155253467	0.976030424
329.2905942	0.033792032	0.08643067	0.390972691	0.695817422	0.999579949
50.96697141	-0.129669512	0.237674652	-0.545575688	0.58535764	0.999579949
844.3973622	-0.060085744	0.05968629	-1.006692562	0.314082538	0.999579949
555.1974481	-0.023398533	0.05981034	-0.391212171	0.695640413	0.999579949
1717.874591	0.034995973	0.075971906	0.460643614	0.645054315	0.999579949
44.69503449	-0.025482792	0.226882175	-0.112317294	0.91057183	0.999579949
245.693358	-0.12315671	0.236161073	-0.521494537	0.60202231	0.999579949

308.2761494	-0.08060316	0.101383772	-0.795030195	0.426595938	0.999579949
196.3956536	-0.291827805	0.194903074	-1.497297086	0.134315973	0.945791454
135.3821071	0.055396315	0.115805733	0.478355547	0.632397167	0.999579949
33.79303699	0.008502908	0.273919685	0.031041609	0.975236357	0.999579949
33.14181885	-0.047260911	0.192763301	-0.245175877	0.806320254	0.999579949
113.4548171	-0.103604272	0.345311442	-0.300031391	0.764153212	0.999579949
1179.744614	-0.101829917	0.159442565	-0.638662065	0.523042796	0.999579949
3031.794491	0.020731984	0.095261011	0.217633465	0.827714708	0.999579949
362.1709613	0.050757752	0.076839642	0.660567262	0.50888987	0.999579949
106.9564231	-0.202506838	0.178918839	-1.131836303	0.257703262	0.999579949
74.88922817	-0.19242099	0.119286983	-1.613092935	0.106724319	0.899238988
135.8665868	0.158363876	0.115380002	1.372541798	0.169894836	0.981013142
1157.637356	-0.051053386	0.055305489	-0.923116077	0.355946717	0.999579949
296.6928937	-0.065388175	0.092970701	-0.703320228	0.481856207	0.999579949
87.86624067	0.015555843	0.254251569	0.061182879	0.951213565	0.999579949
263.6676466	0.047476448	0.191151883	0.248370284	0.803847923	0.999579949
182.1950107	0.061398866	0.133471397	0.460015161	0.645505338	0.999579949
100.0901591	-0.063211653	0.127926541	-0.494124619	0.621218172	0.999579949
131.3891174	0.2307695	0.176336485	1.308688332	0.190639946	0.98475336
984.1001505	0.046290707	0.087318542	0.530136054	0.596017603	0.999579949
66.17729398	-0.018334176	0.135501227	-0.135306348	0.892369667	0.999579949
511.5638604	-0.343985967	0.098735143	-3.48392637	0.000494115	0.111276918
1426.605119	-0.022561006	0.138444836	-0.16296026	0.870549725	0.999579949
242.6062257	0.008087248	0.154039637	0.052501084	0.958129431	0.999579949
37.80242509	0.282699256	0.343519995	0.822948474	0.410537295	0.999579949
328.7679353	-0.033287191	0.081047011	-0.410714597	0.681281823	0.999579949
1710.320955	-0.014087639	0.088796911	-0.158650102	0.873944553	0.999579949
104.1382383	0.307244978	0.192444738	1.596536135	0.110369145	0.909823133
81.07790828	0.059537037	0.122647112	0.48543366	0.62736876	0.999579949
48.47445324	-0.068363227	0.160188178	-0.426768245	0.669548138	0.999579949
440.2536224	0.072067792	0.069352675	1.039149424	0.298735248	0.999579949
1013.499289	0.001799322	0.119545229	0.015051387	0.987991184	0.999579949
239.6159306	-0.569578358	0.313244606	-1.81831817	0.069015517	0.831457432
252.8614836	0.133208752	0.134958726	0.987033262	0.323626333	0.999579949
580.5105025	-0.006390403	0.167294227	-0.038198586	0.969529348	0.999579949
2253.268552	-0.042473995	0.042384618	-1.00210871	0.316291091	0.999579949
1099.891644	-0.063198827	0.106422127	-0.593850433	0.552612154	0.999579949
289.248005	0.035311562	0.098996934	0.356693494	0.721321276	0.999579949
284.3925845	0.090528362	0.138646215	0.65294507	0.513791692	0.999579949
354.8688154	-0.01297679	0.066156639	-0.196152504	0.844490803	0.999579949
108.0720451	0.221004218	0.25493658	0.866898811	0.385997455	0.999579949
403.8653618	0.037176907	0.085323993	0.435714576	0.663043829	0.999579949
36.94639183	0.050310122	0.178167778	0.282374978	0.777655999	0.999579949
1066.44118	0.018677434	0.119016004	0.156932121	0.875298344	0.999579949
34.27710562	0.012822912	0.219688179	0.058368692	0.953454952	0.999579949
311.7254995	0.090096213	0.073049269	1.233362275	0.217440633	0.999579949
123.6860683	-0.155731189	0.112031021	-1.39007203	0.164507006	0.978498637
122.3914525	0.073749636	0.137063988	0.538067199	0.590530657	0.999579949

109.2429082	0.055584232	0.175314795	0.317053857	0.751202737	0.999579949
238.3304241	0.144857766	0.21304026	0.679954888	0.496533025	0.999579949
227.6365119	-0.116742756	0.087910238	-1.327976787	0.184185776	0.983992835
492.6766126	-0.010141621	0.075415067	-0.134477378	0.893025099	0.999579949
382.4991867	0.078112581	0.112157633	0.69645355	0.486144837	0.999579949
3876.67817	-1.088302292	0.403636987	-2.696240255	0.007012707	0.404205857
161.7778377	-0.063942284	0.133062179	-0.480544396	0.630840343	0.999579949
4100.90665	-0.17528075	0.141990422	-1.234454741	0.217033502	0.999579949
1132.358469	0.093566635	0.05730337	1.632829535	0.102504854	0.895628172
26.81591172	0.112120499	0.212843901	0.52677337	0.598350978	0.999579949
90.57714341	-0.014461501	0.128665946	-0.112395712	0.910509655	0.999579949
92.72023767	-0.057008434	0.109471121	-0.520762314	0.602532359	0.999579949
76.59608763	0.227211005	0.254533591	0.892656267	0.372041277	0.999579949
2152.910495	-0.001749976	0.114688265	-0.015258549	0.987825912	0.999579949
853.7853191	-0.112530999	0.057198652	-1.967371533	0.049140394	0.77466668
263.4771927	0.006385383	0.065727285	0.097149648	0.922607554	0.999579949
163.7260799	0.146346745	0.122744795	1.19228473	0.23314962	0.999579949
121.0836402	0.355030599	0.16645164	2.132935427	0.032930024	0.695900535
213.1631384	0.382986358	0.192809664	1.9863442	0.046995118	0.767769787
362.4470586	0.03672716	0.110188414	0.333312355	0.738898514	0.999579949
26.75023367	0.366303552	0.265366289	1.380369574	0.167472883	0.979790264
71.54621772	0.064532987	0.142553759	0.452692282	0.650770338	0.999579949
86.0684962	0.124701364	0.180866984	0.689464499	0.490531007	0.999579949
91.82672772	0.017155699	0.12760745	0.134441199	0.893053705	0.999579949
4970.020992	0.006023889	0.116713677	0.051612535	0.958837431	0.999579949
750.1242942	-0.036479451	0.117609156	-0.310175268	0.756427677	0.999579949
34.17152283	-0.179523128	0.168180548	-1.067442876	0.285771896	0.999579949
813.6246293	-0.02253834	0.06456063	-0.349103465	0.727011635	0.999579949
51.40664082	0.203206686	0.292942293	0.693674798	0.487886174	0.999579949
1929.556796	-0.087803234	0.082273366	-1.067213342	0.285875509	0.999579949
21.21749969	-0.158939627	0.280504549	-0.566620495	0.570972047	0.999579949
296.625294	-0.135847097	0.263887203	-0.51479229	0.606698174	0.999579949
1153.905809	0.107317649	0.10648414	1.007827542	0.313537262	0.999579949
102.8174752	-0.258324561	0.231419234	-1.116262274	0.264309885	0.999579949
45.68587448	0.0383753	0.249109283	0.154050062	0.877570263	0.999579949
48.36265108	0.182816314	0.156869495	1.165403849	0.24385556	0.999579949
962.7004795	-0.120335399	0.075517187	-1.593483606	0.111051743	0.912513443
622.7778241	-0.011852121	0.093703438	-0.126485442	0.899347672	0.999579949
129.885471	0.011111067	0.08729917	0.127275748	0.898722155	0.999579949
84.85575697	0.228984879	0.124237691	1.843119241	0.065311603	0.818572086
35.8802516	0.275189715	0.321462299	0.856055951	0.391966832	0.999579949
2151.628251	-0.141164562	0.151244189	-0.93335528	0.350636562	0.999579949
536.8704601	0.060186592	0.085510163	0.70385308	0.481524274	0.999579949
198.806365	-0.10450159	0.109514394	-0.954226985	0.339968758	0.999579949
36.03759667	1.397021445	1.03165064	1.35416137	0.1756849	0.983992835
168.797415	-0.055047459	0.107231416	-0.513351973	0.607705134	0.999579949
116.3058126	0.164939792	0.104255872	1.582067168	0.113634238	0.917331191
816.4517504	0.165233978	0.149583598	1.104626315	0.269321544	0.999579949

48.20912542	0.188911742	0.280454704	0.67359092	0.500571428	0.999579949
2030.256709	-0.008731362	0.065623267	-0.133052847	0.894151586	0.999579949
91.3115851	0.187863617	0.234325894	0.80171941	0.422715286	0.999579949
2877.915326	-0.096199975	0.09627538	-0.999216779	0.317689689	0.999579949
240.1936255	0.050058901	0.115527361	0.433307753	0.664791206	0.999579949
103.523214	0.018921121	0.191971439	0.098562169	0.921485908	0.999579949
59.31122958	-0.126934963	0.236523069	-0.536670539	0.59149521	0.999579949
416.8439539	0.018905651	0.139069922	0.135943491	0.891865954	0.999579949
48.02599107	-0.703501121	0.453469602	-1.551374378	0.120811992	0.921837625
41.74815173	0.106828402	0.26659897	0.400708231	0.68863495	0.999579949
29.59458206	0.289010366	0.277342143	1.042071583	0.297378492	0.999579949
685.7163278	-0.006993331	0.089065414	-0.078519044	0.937415182	0.999579949
137.0533518	-0.351395301	0.228352316	-1.538829592	0.123845904	0.924045374
21.69918455	0.044786606	0.409964522	0.109245077	0.913008108	0.999579949
1455.092377	0.005429215	0.112556793	0.048235341	0.961528685	0.999579949
803.2100778	-0.012416013	0.066413761	-0.186949397	0.851700309	0.999579949
473.0266233	0.065129009	0.07463401	0.872645183	0.382856505	0.999579949
5731.885487	-0.088569666	0.09268618	-0.955586538	0.339281166	0.999579949
31.92081846	0.04138119	0.257585898	0.16065006	0.872369025	0.999579949
1457.460883	0.102362972	0.10848309	0.943584596	0.345381967	0.999579949
3451.338972	-0.026981692	0.09242037	-0.291945294	0.770328452	0.999579949
97.01086803	0.088262001	0.218622196	0.403719306	0.686419141	0.999579949
36.36077307	0.212126584	0.284888253	0.744595755	0.456516135	0.999579949
525.719514	0.051191296	0.092707516	0.552180642	0.580824596	0.999579949
182.6210768	-0.014315003	0.094351426	-0.151720053	0.87940774	0.999579949
510.7869201	0.310492753	0.169250797	1.8345128	0.06657791	0.822978752
84.60941644	0.104630322	0.225082806	0.464852573	0.642037036	0.999579949
286.4738577	-0.065308809	0.093390613	-0.699308069	0.484359525	0.999579949
309.6378015	-0.116265124	0.088188212	-1.318374888	0.187378183	0.983992835
231.6225699	-0.12159295	0.117872877	-1.03156004	0.302278268	0.999579949
37.29351637	-0.09389474	0.28218142	-0.332746004	0.73932602	0.999579949
327.4348816	0.09813839	0.099490759	0.986407088	0.323933388	0.999579949
227.6111396	-0.164077841	0.1710597	-0.959184668	0.337465723	0.999579949
90.41553392	0.107960294	0.121222953	0.890592839	0.373147642	0.999579949
23.04950885	0.319791504	0.284360795	1.124597728	0.260759537	0.999579949
195.2389747	0.089294266	0.163751244	0.545304352	0.585544211	0.999579949
45.8947597	0.575028016	0.18518958	3.105077597	0.001902291	0.225075621
28.60391379	0.780434183	0.582234137	1.340412959	0.180111125	0.983992835
1368.18346	0.023973109	0.057222972	0.41894205	0.675258485	0.999579949
45.41826479	0.100806024	0.348420629	0.289322776	0.772334385	0.999579949
200.0195312	-0.29376594	0.175053341	-1.678150999	0.093317625	0.880418343
77.46082165	0.629659213	0.447947963	1.405652587	0.159827312	0.978261482
63.53984631	0.563268614	0.276683862	2.035784123	0.041772037	0.74969123
1085.054624	-0.053021213	0.058299122	-0.909468475	0.363102891	0.999579949
64.92366622	-0.060135896	0.138180363	-0.435198562	0.663418306	0.999579949
656.8861593	-0.089192235	0.135020547	-0.660582684	0.508879977	0.999579949
427.789076	-0.068128978	0.063606965	-1.071093058	0.284127589	0.999579949
72.30026932	0.084890121	0.125346732	0.677242391	0.498252175	0.999579949

640.5548403	-0.442405548	0.161758289	-2.73497916	0.006238423	0.385192012
27.16741583	0.052597316	0.246826479	0.213094301	0.83125341	0.999579949
136.7485557	-0.109403625	0.101690978	-1.075843964	0.281997046	0.999579949
207.7658214	0.10919688	0.109627621	0.996070875	0.31921571	0.999579949
1060.509107	-0.164057052	0.097573984	-1.68136059	0.092692896	0.880418343
2599.548913	0.024683709	0.125644663	0.196456486	0.844252888	0.999579949
201.1918173	-0.079453179	0.119908936	-0.662612662	0.507578659	0.999579949
321.2353293	-0.007446105	0.150445298	-0.049493768	0.960525803	0.999579949
36.88239718	0.124287662	0.178911264	0.694688861	0.48725031	0.999579949
241.2773798	0.011355689	0.066997595	0.169493974	0.865408112	0.999579949
30.19582862	-0.085106733	0.272520812	-0.312294436	0.754816768	0.999579949
1056.322763	0.02122115	0.101289136	0.209510625	0.834049642	0.999579949
437.8916395	-0.089183263	0.511814704	-0.174249123	0.861669682	0.999579949
83.81415345	0.715182221	0.538084234	1.329126884	0.183806112	0.983992835
208.3617251	0.363233021	0.110257019	3.294420849	0.000986247	0.161334665
1531.150645	0.159644355	0.129332576	1.234370801	0.217064764	0.999579949
180.4255544	0.226859852	0.141370143	1.604722511	0.108554886	0.903743816
1774.327602	-0.091646878	0.117532046	-0.779760762	0.435531706	0.999579949
25.16314756	0.236804012	0.235559354	1.005283842	0.314760193	0.999579949
272.9919638	0.011918581	0.100046344	0.119130602	0.905171886	0.999579949
148.5089737	-0.092705176	0.100611449	-0.921417766	0.356832357	0.999579949
46.02713243	0.219761757	0.226043079	0.972211836	0.330945173	0.999579949
322.7166383	-0.034741219	0.113798532	-0.305287053	0.760147536	0.999579949
1072.272827	-0.00043616	0.111115684	-0.003925281	0.996868087	0.999579949
120.0028452	-0.431718817	0.115080518	-3.751450053	0.000175815	0.058895284
345.4785187	-0.099519569	0.118896782	-0.837024918	0.402578562	0.999579949
1156.137262	0.063661712	0.115478176	0.551287821	0.581436386	0.999579949
101.7691715	-0.265105309	0.116996893	-2.265917512	0.023456434	0.62865781
55.33531771	0.03467727	0.191432618	0.181146086	0.856252911	0.999579949
260.7928633	-0.087025042	0.113520218	-0.766603896	0.443317058	0.999579949
73.92612383	-0.295400715	0.172814818	-1.709348304	0.087386451	0.867715687
24.70008998	0.095078981	0.250394213	0.379717165	0.704155377	0.999579949
65.52141476	0.535814067	0.19670405	2.723960519	0.006450422	0.39001336
375.9430326	-0.056518482	0.155003269	-0.364627679	0.71538935	0.999579949
64.38495777	0.096762515	0.150337575	0.643634933	0.519812192	0.999579949
324.7399541	0.00190658	0.185072215	0.010301815	0.991780486	0.999579949
570.0344123	0.179395417	0.096176566	1.865271592	0.062143345	0.805306145
100.3149906	-0.006407456	0.110940005	-0.057756045	0.953942951	0.999579949
855.7954628	-0.136604343	0.104340973	-1.309210936	0.190462909	0.98475336
66.72744236	0.020039993	0.159638355	0.125533695	0.900101051	0.999579949
42.25991315	0.166042497	0.156994789	1.057630626	0.290223874	0.999579949
326.3292429	0.132875675	0.146938335	0.904295502	0.365838731	0.999579949
1716.947289	-0.008618136	0.117315232	-0.073461354	0.941438996	0.999579949
139.1522808	0.023522115	0.124486311	0.188953427	0.850129319	0.999579949
156.7645471	-0.15534059	0.196602665	-0.790124536	0.429455042	0.999579949
192.6629804	-0.144827895	0.194192043	-0.745797268	0.455789891	0.999579949
117.6337051	0.290728143	0.188770993	1.54011026	0.123533479	0.922910858
223.812916	-0.012139312	0.113542048	-0.106914682	0.914856666	0.999579949

22.51643228	0.520627166	0.291235192	1.787651977	0.073832174	0.843146024
2583.863095	0.084452183	0.115220476	0.732961588	0.463581858	0.999579949
500.8344408	0.071686485	0.132578712	0.540708863	0.588708269	0.999579949
5237.950235	0.072904674	0.075111288	0.970622069	0.331736512	0.999579949
958.7947387	0.047172776	0.080138424	0.588641173	0.556102008	0.999579949
104.1260423	-0.051240258	0.137757596	-0.371959586	0.70992294	0.999579949
94.75203416	0.088134788	0.147507359	0.59749418	0.550177491	0.999579949
1188.133556	0.002650601	0.127038029	0.020864624	0.983353647	0.999579949
2449.502781	0.121321661	0.122423648	0.990998583	0.321686272	0.999579949
433.9313127	0.321268055	0.266677816	1.204704838	0.228317268	0.999579949
207.1533577	0.08537851	0.086480091	0.987262026	0.323514201	0.999579949
151.3552665	0.00446247	0.108541693	0.041112962	0.967205841	0.999579949
312.7250293	0.051700528	0.088714679	0.582773101	0.560046055	0.999579949
3079.959413	0.102173793	0.146419946	0.697813352	0.485293928	0.999579949
173.4525899	0.481562801	0.387947496	1.241309213	0.214491543	0.999579949
74.67497256	0.034754529	0.181082135	0.191926882	0.84779948	0.999579949
146.2570112	-0.11198481	0.124633927	-0.898509845	0.368913799	0.999579949
124.5831678	0.132547346	0.155429813	0.852779422	0.393781637	0.999579949
901.8420967	0.098436498	0.095516382	1.03057189	0.302741624	0.999579949
389.7546705	-0.007919302	0.110601792	-0.071601936	0.9429187	0.999579949
1453.702735	0.082397504	0.087051064	0.946542188	0.343872115	0.999579949
208.2216955	-0.038695184	0.121483841	-0.318521244	0.750089583	0.999579949
88.33656494	0.175907475	0.144069473	1.220990622	0.222089571	0.999579949
428.4436634	-0.055995054	0.097798817	-0.572553497	0.566947054	0.999579949
36.17630975	-0.074405714	0.19151317	-0.388514871	0.697635052	0.999579949
185.8044996	-0.061937432	0.121383887	-0.510260743	0.609868802	0.999579949
1482.085363	-0.036105315	0.13110671	-0.275388762	0.783017577	0.999579949
50.92037031	0.183044898	0.173540456	1.054767872	0.291531502	0.999579949
321.7022577	-0.127404465	0.086036571	-1.480817559	0.138655196	0.945791454
417.6746328	0.086109972	0.114719086	0.750615918	0.452883838	0.999579949
901.7797139	-0.003426091	0.090296384	-0.037942732	0.969733342	0.999579949
79.63792715	0.09940906	0.193549852	0.513609587	0.607524976	0.999579949
23.31927523	-0.131828289	0.216626017	-0.60855243	0.542821144	0.999579949
184.0735245	0.136309275	0.10886854	1.252053855	0.210550241	0.996581389
561.1929849	-0.068521824	0.112482071	-0.609179965	0.542405158	0.999579949
359.1084024	0.039852265	0.119036471	0.334790373	0.737783223	0.999579949
584.7189495	0.074122634	0.156482579	0.473679783	0.635728272	0.999579949
93.42240603	0.067676252	0.154116427	0.439124199	0.660571546	0.999579949
1608.995666	-0.109716537	0.087145051	-1.259010533	0.208026527	0.995521169
486.2741848	-0.118503687	0.107941724	-1.097848752	0.272270539	0.999579949
196.4289619	0.083642118	0.123964853	0.674724453	0.499850846	0.999579949
40.43777721	0.144787003	0.248151103	0.583463065	0.559581614	0.999579949
35.56596784	0.078517121	0.214423809	0.366177253	0.714232814	0.999579949
222.4795325	-0.064679366	0.149788746	-0.431803905	0.665883941	0.999579949
1378.334749	0.041979233	0.137434861	0.305448215	0.760024805	0.999579949
482.8287288	0.030609311	0.078776393	0.388559433	0.697602081	0.999579949
482.5273576	0.042431509	0.134998389	0.314311222	0.753284676	0.999579949
72.91895768	0.028200114	0.162754089	0.173268238	0.862440589	0.999579949

1403.129799	-0.054922398	0.098954117	-0.555028937	0.578874868	0.999579949
675.1512255	0.09253313	0.149731456	0.617993925	0.536579343	0.999579949
102.2204764	-0.111708093	0.15824995	-0.705896544	0.480252477	0.999579949
679.552103	0.126727472	0.066729869	1.899111646	0.057549798	0.793865133
41.25231355	0.082841932	0.157159186	0.527121157	0.598109456	0.999579949
49.67856043	-0.007158339	0.172625954	-0.041467337	0.966923332	0.999579949
577.2933615	-0.124374019	0.10123439	-1.228574782	0.219231275	0.999579949
1139.303901	-0.005076701	0.121016481	-0.041950495	0.966538163	0.999579949
195.248017	0.489269667	0.371005757	1.318765703	0.187247454	0.983992835
222.5069151	0.021107764	0.115847408	0.18220316	0.855423293	0.999579949
591.3952823	0.079968355	0.081314438	0.983445962	0.325387999	0.999579949
522.9697236	0.065621569	0.092635118	0.708387601	0.47870459	0.999579949
839.8287287	0.072934169	0.115810207	0.629773232	0.528842961	0.999579949
43.26165549	0.002808074	0.183635861	0.015291533	0.987799597	0.999579949
388.7982926	-0.011637707	0.092380276	-0.125976098	0.899750845	0.999579949
138.7314528	0.092422148	0.146755151	0.62977107	0.528844376	0.999579949
184.1841475	0.020412251	0.10153515	0.201036306	0.840670186	0.999579949
143.6946665	0.031612959	0.128087599	0.246807335	0.805057334	0.999579949
401.3494575	-0.036880424	0.114314444	-0.322622603	0.746981074	0.999579949
30.88833138	-0.258330397	0.265795766	-0.971913141	0.331093762	0.999579949
98.08781234	0.006531469	0.107380575	0.060825427	0.95149824	0.999579949
73.45557842	0.008567243	0.158497909	0.054052716	0.956893164	0.999579949
127.66135	0.073004822	0.111417726	0.655235251	0.512316301	0.999579949
188.9058549	0.07860083	0.127408398	0.616920322	0.537287282	0.999579949
2589.203032	0.045971101	0.107221579	0.428748597	0.668106191	0.999579949
1144.806379	-0.005339589	0.119600111	-0.044645354	0.964389991	0.999579949
556.4841989	-0.047308692	0.113474749	-0.416909425	0.67674466	0.999579949
238.0014579	0.020715058	0.122543871	0.169041975	0.865763625	0.999579949
77.00026197	0.086883701	0.163948358	0.529945538	0.596149692	0.999579949
56.18983838	-0.050703712	0.197676479	-0.256498456	0.797565972	0.999579949
261.5762692	-0.08421812	0.144754408	-0.581800037	0.560701379	0.999579949
514.0655778	-0.018844585	0.07077175	-0.266272702	0.790029195	0.999579949
71.67375424	0.068461506	0.155413114	0.44051306	0.659565555	0.999579949
241.4205495	-0.081489891	0.101394964	-0.803687758	0.421577326	0.999579949
771.0149125	0.019474087	0.075945143	0.256423076	0.797624171	0.999579949
333.2945028	0.06713212	0.07370598	0.91080968	0.362395661	0.999579949
1685.538985	-0.142041565	0.086458267	-1.642891638	0.100405356	0.895628172
44.25593252	-0.159141137	0.37425924	-0.42521632	0.670678989	0.999579949
700.2941238	0.049600445	0.071129423	0.697326694	0.485598366	0.999579949
41.79791793	0.059734425	0.223575286	0.267178122	0.78933202	0.999579949
93.95126732	-0.047182601	0.128969996	-0.36584169	0.714483209	0.999579949
325.7737016	-0.028978562	0.074674907	-0.388062917	0.697969474	0.999579949
98.52411332	0.217786591	0.149778135	1.454061305	0.14592931	0.957855963
312.8871542	0.075849744	0.15648747	0.484701704	0.627887958	0.999579949
11777.89662	0.051400163	0.092074499	0.558245368	0.576676845	0.999579949
270.0981081	0.07060324	0.150056722	0.470510344	0.637990446	0.999579949
51.6426889	0.456163618	0.229618151	1.986618289	0.046964713	0.767769787
210.1540602	-0.02696046	0.074219998	-0.363250624	0.716417674	0.999579949

341.6155072	0.059050161	0.097956524	0.60282009	0.546628381	0.999579949
434.7601506	0.034071413	0.089431348	0.380978414	0.703219266	0.999579949
634.012148	-0.094223751	0.097302834	-0.968355671	0.33286677	0.999579949
791.2358752	0.23978011	0.139793441	1.715245789	0.086300173	0.866133472
625.7948294	0.179536186	0.116727652	1.538077586	0.124029646	0.924424507
360.6002261	0.05487884	0.093796764	0.585082444	0.558492283	0.999579949
81.61217067	-0.00025368	0.318178749	-0.000797289	0.999363855	0.999840121
26.40662054	0.305425492	0.262779997	1.162285925	0.245119342	0.999579949
487.3255575	0.036744893	0.102114646	0.3598396	0.718967088	0.999579949
46.39763969	0.311585855	0.22118444	1.408715074	0.158919436	0.977416608
575.6067189	0.042952249	0.0572506	0.750249747	0.453104302	0.999579949
674.1854402	0.070736067	0.113219671	0.624768352	0.532123105	0.999579949
446.9188441	0.130876393	0.129340042	1.011878382	0.311596207	0.999579949
343.768019	0.255110813	0.279633309	0.912304811	0.361608282	0.999579949
1139.890484	-0.020336527	0.084300789	-0.24123768	0.809370909	0.999579949
514.259039	0.039357275	0.129951884	0.302860363	0.76199628	0.999579949
132.2535192	-0.147490025	0.199510345	-0.739260037	0.459749111	0.999579949
155.6623393	0.04766917	0.115137467	0.414019616	0.678859741	0.999579949
259.0919306	-0.029927712	0.079610488	-0.375926752	0.706971351	0.999579949
1211.146158	0.05839481	0.060339053	0.96777803	0.333155239	0.999579949
1439.294903	0.154901081	0.095160524	1.627787175	0.103570021	0.895628172
45.11420298	0.414463349	0.173398082	2.390241837	0.016837282	0.570113982
150.7909605	-0.081326266	0.411756632	-0.197510519	0.843428051	0.999579949
1850.19017	-0.004088167	0.14519767	-0.028155872	0.977537833	0.999579949
83.10070259	-0.118788711	0.167256224	-0.710219973	0.477567737	0.999579949
42.03311271	-0.258279311	0.184587028	-1.399227852	0.161744667	0.978498637
58.37243693	0.033393208	0.151536749	0.220363763	0.825587864	0.999579949
648.3925219	0.058296956	0.115588956	0.504347112	0.614017476	0.999579949
74.52762545	0.034611702	0.142052494	0.243654308	0.807498564	0.999579949
62.57151144	0.526274553	0.236738763	2.223018092	0.02621458	0.648720728
70.12492877	0.883586877	0.397283326	2.224072392	0.026143574	0.648237123
2559.046177	0.006333812	0.103039995	0.061469449	0.950985345	0.999579949
668.2033645	-0.218868764	0.121963916	-1.794537033	0.072727493	0.838669905
259.3677957	0.004422034	0.107514326	0.041129722	0.96719248	0.999579949
276.0503121	-0.075200515	0.102453056	-0.73399972	0.462948909	0.999579949
691.6261977	-0.003475821	0.092079982	-0.037747842	0.96988873	0.999579949
820.7804158	0.103982871	0.083984826	1.238114974	0.215673433	0.999579949
386.1500729	0.027864963	0.105348579	0.264502503	0.791392736	0.999579949
84.1408001	0.182200376	0.137503802	1.325057011	0.185152243	0.983992835
309.2829066	0.087483254	0.090357599	0.968189229	0.332949873	0.999579949
78.13190646	0.237577675	0.177106624	1.34143868	0.179778066	0.983992835
270.1442121	0.187796344	0.126586442	1.483542319	0.137930381	0.945791454
44.3256549	0.232201463	0.20188803	1.15014973	0.250082207	0.999579949
149.6401021	0.091360158	0.120118238	0.760585228	0.446904846	0.999579949
631.3204613	-0.000496259	0.095403893	-0.005201662	0.995849693	0.999579949
877.7850671	0.048820733	0.101735655	0.479878298	0.631313934	0.999579949
243.7276078	0.013144186	0.084607473	0.155354905	0.876541533	0.999579949
27.35955312	0.06661791	0.216393088	0.307855999	0.758191908	0.999579949

242.2951606	0.152565995	0.114774334	1.329269269	0.183759149	0.983992835
324.7819024	-0.036645871	0.077243947	-0.474417373	0.635202307	0.999579949
36.11358773	-0.161382781	0.1889123	-0.854273547	0.392953441	0.999579949
99.77653902	0.046811708	0.116572219	0.401568306	0.688001759	0.999579949
2183.585184	0.075896436	0.112560935	0.674269771	0.500139819	0.999579949
155.9776454	0.217298141	0.134879657	1.611052	0.107168389	0.900529039
102.911773	-0.094528065	0.119994435	-0.787770409	0.430831013	0.999579949
517.4874962	0.032635505	0.164367049	0.1985526	0.842612735	0.999579949
216.8456464	0.009463596	0.116626897	0.081144202	0.935327274	0.999579949
44.82999982	-0.420890545	0.608999581	-0.691117954	0.489491418	0.999579949
705.5367792	0.012881928	0.073362214	0.175593507	0.860613303	0.999579949
95.77324961	-0.004378636	0.10862322	-0.040310317	0.967845729	0.999579949
761.7999893	-0.056675676	0.06186923	-0.916055936	0.359637558	0.999579949
49.39369082	-0.128453618	0.168784691	-0.761050173	0.4466271	0.999579949
3709.667682	0.004021548	0.093252606	0.043125316	0.965601639	0.999579949
21.72903599	-0.236186116	0.238735353	-0.989321912	0.322505667	0.999579949
21.53470113	0.423208491	0.303406937	1.394854367	0.16305976	0.978498637
391.8142242	-0.081475584	0.111113562	-0.73326408	0.463397379	0.999579949
423.6183643	0.009620186	0.109710267	0.0876872	0.930125293	0.999579949
112.7261303	0.150300617	0.191307141	0.785650845	0.432072068	0.999579949
47.50061074	0.115899746	0.165064552	0.702148002	0.482586869	0.999579949
3053.185405	0.044126446	0.082511955	0.534788509	0.59279611	0.999579949
1174.296999	-0.05120826	0.049477016	-1.034990885	0.300673168	0.999579949
84.6500808	0.842830885	0.291345051	2.892895839	0.003817079	0.319818899
91.20273814	0.354892068	0.203329374	1.74540481	0.080914429	0.85448976
41.89374258	-0.39015581	0.193910571	-2.01203992	0.044215729	0.758836007
198.4518651	0.041212187	0.087776763	0.469511358	0.638704169	0.999579949
575.1696126	0.167071216	0.120955858	1.381257749	0.167199724	0.979101686
56.18213841	0.080742558	0.16329732	0.494451214	0.620987553	0.999579949
471.3143116	-0.023867198	0.065086049	-0.36670221	0.713841158	0.999579949
95.80943229	0.886519855	0.240744494	3.682409676	0.00023104	0.071755818
215.5343669	-0.051549639	0.168461538	-0.306002422	0.759602802	0.999579949
143.1287023	0.013970667	0.133102087	0.104962042	0.916405929	0.999579949
800.3551447	0.057735258	0.070734001	0.816230632	0.41436824	0.999579949
755.4130516	0.107529431	0.105974494	1.014672745	0.31026185	0.999579949
181.3627009	0.028594389	0.093459084	0.305956228	0.759637974	0.999579949
572.9799863	-0.226647497	0.217378654	-1.042639163	0.297115444	0.999579949
168.3947961	0.00592964	0.168461019	0.035198883	0.971921153	0.999579949
196.9901948	0.07778564	0.095252401	0.816626559	0.414141872	0.999579949
734.1545618	0.088133619	0.081138763	1.08620857	0.277386726	0.999579949
169.578742	0.04834083	0.161826404	0.298720288	0.765153476	0.999579949
66.70951225	0.091102028	0.143708774	0.633935044	0.526123214	0.999579949
141.8673513	-0.190875552	0.262826834	-0.72624073	0.467691207	0.999579949
615.2447301	0.210605419	0.090923775	2.316285459	0.020542688	0.611138086
314.266133	0.04271275	0.113154453	0.377472991	0.705822132	0.999579949
23.55307555	-0.07744813	0.240494509	-0.322037002	0.747424663	0.999579949
52.52864514	0.56840086	0.248867444	2.283950249	0.022374455	0.627784819
56.90529036	0.18828535	0.300341067	0.626905109	0.530721438	0.999579949

109.974386	-0.594714033	0.492518805	-1.207495077	0.22724154	0.999579949
461.6451803	0.018084372	0.085602853	0.211258983	0.832685187	0.999579949
2208.747862	-0.099449538	0.122922252	-0.809044229	0.418489706	0.999579949
1295.9489	-0.005752613	0.046764766	-0.123011697	0.902097835	0.999579949
208.3234295	0.28230891	0.144963023	1.947454626	0.051480254	0.779189484
108.1715141	0.141901287	0.136554923	1.039151744	0.298734169	0.999579949
58.46416355	0.128334193	0.132580866	0.967969182	0.333059761	0.999579949
1191.570399	-0.12101648	0.084211133	-1.437060345	0.150700848	0.96651114
620.7690943	-3.09328E-05	0.071664688	-0.000431633	0.999655607	0.99989122
268.4849969	0.028630784	0.087138186	0.328567591	0.742482548	0.999579949
73.24704322	-0.08374052	0.146527337	-0.571500999	0.567660084	0.999579949
2118.412409	-0.037530722	0.097841742	-0.383585998	0.701285319	0.999579949
65.89724983	0.049753182	0.151479087	0.328449184	0.74257206	0.999579949
1035.451982	-0.024179063	0.092444273	-0.261552852	0.793666197	0.999579949
286.1318444	-0.0096407	0.112995103	-0.085319623	0.932007291	0.999579949
6497.399924	-0.174899066	0.124287426	-1.407214486	0.159363798	0.977762491
1787.394718	-0.05609777	0.105422974	-0.532120924	0.594642246	0.999579949
214.0921835	0.249542379	0.150714024	1.655734306	0.097775637	0.890209389
196.5282315	0.28114227	0.185412864	1.516304011	0.129442498	0.935700262
33.49979351	-0.023817712	0.416107161	-0.05723937	0.954354517	0.999579949
1340.453403	-0.300918546	0.118410157	-2.541323765	0.01104336	0.487909795
383.091732	0.029055203	0.109711939	0.264831729	0.791139093	0.999579949
23.17300308	0.017870659	0.264452025	0.067576186	0.946123013	0.999579949
354.6028453	0.003821867	0.105657804	0.036172119	0.971145118	0.999579949
1207.264651	0.036218123	0.071691303	0.505195494	0.613421535	0.999579949
215.5750647	0.045892657	0.107081313	0.428577645	0.668230617	0.999579949
390.289171	0.112221646	0.066790022	1.680215733	0.092915349	0.880418343
245.1930467	0.255458196	0.288427975	0.885691466	0.375783808	0.999579949
451.3191247	0.166526892	0.119266383	1.396260106	0.162636182	0.978498637
456.7923345	-0.067101159	0.081769115	-0.820617408	0.411864229	0.999579949
156.8233342	-0.24816365	0.128168494	-1.936229736	0.05283957	0.781231069
59.97402264	0.089054509	0.126415711	0.7044576	0.481147846	0.999579949
31.24212592	-0.080418663	0.210529432	-0.381982994	0.702473979	0.999579949
338.7488193	0.175195501	0.086381744	2.028154253	0.042544507	0.74969123
470.5637891	-0.077901106	0.068802446	-1.132243272	0.257532173	0.999579949
89.43169134	-0.0961336	0.274338895	-0.350419143	0.726024162	0.999579949
8184.444525	-0.058926183	0.119264935	-0.494078019	0.621251081	0.999579949
197.8035247	0.142018931	0.217122682	0.654095324	0.513050394	0.999579949
1231.648687	0.066056607	0.07036286	0.938799348	0.347833774	0.999579949
319.4880981	-0.096856649	0.476776556	-0.203148934	0.839018626	0.999579949
371.3350044	-0.149902557	0.204551644	-0.73283477	0.463659212	0.999579949
31.25884156	0.319789483	0.540896544	0.591221161	0.554372246	0.999579949
98.47409556	-0.345058186	0.327338193	-1.054133593	0.291821758	0.999579949
22.81380109	0.615865767	0.771817867	0.797941837	0.424904243	0.999579949
236.8318035	-0.113635219	0.5263707	-0.215884393	0.829077862	0.999579949
1308.933842	-0.038362639	0.058715875	-0.653360594	0.513523837	0.999579949
208.4789064	-0.203673628	0.195020594	-1.044369846	0.296314309	0.999579949
338.9975186	0.114104878	0.148305504	0.769390711	0.441661401	0.999579949

111.3155291	-0.058195431	0.194467021	-0.29925604	0.764744693	0.999579949
649.0076398	0.185629573	0.141148633	1.315135467	0.188464379	0.983992835
33.36084815	-0.158304702	0.520052187	-0.304401569	0.760821972	0.999579949
985.6776486	-0.04353296	0.045326206	-0.960436874	0.336835387	0.999579949
106.6641848	-0.117369261	0.123580956	-0.949735826	0.342246501	0.999579949
901.8374532	0.002562552	0.096300793	0.026609871	0.9787709	0.999579949
127.5450547	0.304836118	0.173897219	1.752967188	0.079607639	0.852823173
1791.445773	-0.010040389	0.095289499	-0.105367213	0.916084431	0.999579949
1533.785066	-0.195412752	0.169730636	-1.151311022	0.249604304	0.999579949
496.0269183	-0.086633664	0.143298741	-0.604566818	0.54546686	0.999579949
63.87157532	0.4221835	0.198157811	2.130541809	0.033126908	0.695900535
1486.065384	-0.070916476	0.106374045	-0.666670858	0.504982397	0.999579949
90.04613878	-0.223441168	0.304784329	-0.733112389	0.463489884	0.999579949
503.3982452	0.006185299	0.061507922	0.100561012	0.919898948	0.999579949
184.0841296	0.091449187	0.102196143	0.894839913	0.370872671	0.999579949
23.38873557	0.413963267	0.213697981	1.937141683	0.052728026	0.781231069
600.492791	0.01713373	0.142579689	0.120169501	0.904348877	0.999579949
52.43140007	0.168717606	0.155764708	1.083156823	0.278738821	0.999579949
126.7850036	0.301103744	0.357586427	0.842044667	0.399762952	0.999579949
563.898906	0.020281717	0.181343187	0.111841629	0.910948979	0.999579949
30.22393348	-0.062663179	0.263776442	-0.237561696	0.812221062	0.999579949
274.1664976	0.08619737	0.271085553	0.317971096	0.750506862	0.999579949
1360.680857	0.042458917	0.079582582	0.533520227	0.593673513	0.999579949
6850.087343	0.003056689	0.053049722	0.057619331	0.954051851	0.999579949
32.03882571	-0.243329116	0.234669418	-1.036901691	0.299781676	0.999579949
388.8956531	0.183297811	0.139706284	1.312022663	0.189512488	0.983992835
196.9141565	-0.003606808	0.077489824	-0.046545567	0.962875416	0.999579949
240.3800912	0.247081408	0.103639262	2.384052172	0.017123175	0.570162425
182.1675165	-0.035751997	0.100158126	-0.356955536	0.721125093	0.999579949
613.1449662	-0.06051246	0.121549291	-0.497842971	0.618594726	0.999579949
1829.059255	-0.066108249	0.155187009	-0.425990871	0.670114498	0.999579949
168.5945227	0.17400708	0.134833558	1.290532439	0.196865856	0.992244407
447.9070554	0.039518751	0.100799395	0.392053455	0.695018718	0.999579949
1188.140145	-0.069732103	0.080733916	-0.86372749	0.387737609	0.999579949
23.59950569	-0.09138215	0.350882837	-0.260434939	0.794528295	0.999579949
500.9905909	-0.021996781	0.192663558	-0.114171984	0.90910146	0.999579949
2569.343612	0.036273504	0.093649339	0.387333257	0.698509508	0.999579949
31.83132585	0.099024885	0.207272948	0.477751131	0.632827347	0.999579949
947.7894806	0.000175923	0.049907896	0.003524955	0.997187499	0.999579949
159.9067884	-0.07797869	0.198527986	-0.392784371	0.694478749	0.999579949
52.14995507	-0.024617637	0.210391106	-0.117008924	0.906852982	0.999579949
232.1231522	0.047429866	0.106586773	0.444988296	0.656328213	0.999579949
223.7311639	-0.128482929	0.098494528	-1.304467689	0.192074169	0.984804292
1045.029256	-0.03137595	0.083793835	-0.374442226	0.708075332	0.999579949
133.507874	-0.022079275	0.108542215	-0.203416475	0.838809524	0.999579949
199.6908927	0.126275675	0.160102737	0.78871653	0.430277704	0.999579949
281.0838128	-0.044977034	0.109096963	-0.412266597	0.680144029	0.999579949
1282.554502	0.280326758	0.16879865	1.660716829	0.096770332	0.887131806

303.9706301	-0.362396873	0.134443735	-2.695528158	0.007027714	0.404205857
141.8906244	-0.494019373	0.328824661	-1.502379327	0.132999172	0.941167035
35.06677119	-0.563059657	0.410500149	-1.371643003	0.1701746	0.981464863
492.8396455	-0.047497381	0.143319564	-0.331408913	0.740335633	0.999579949
11042.31921	0.095529694	0.131423763	0.726882966	0.467297653	0.999579949
148.543744	0.129909465	0.170633306	0.761337095	0.446455749	0.999579949
723.9613183	0.038575932	0.106835504	0.361077833	0.718041264	0.999579949
34.62515691	0.213624003	0.28220617	0.756978499	0.449062744	0.999579949
427.4808433	-0.128889725	0.095749633	-1.346111955	0.178266414	0.983992835
99.35602017	0.151216139	0.347100592	0.435655087	0.663086996	0.999579949
74.26597165	0.374035297	0.267664282	1.397404591	0.162291939	0.978498637
951.7580279	-0.131978116	0.107235365	-1.230733127	0.218422694	0.999579949
75.87974254	0.156374782	0.176689509	0.885025843	0.376142693	0.999579949
442.5100606	0.007380218	0.093274296	0.07912381	0.936934145	0.999579949
251.1919418	0.072668379	0.080692592	0.900558246	0.367823243	0.999579949
1091.507716	-0.054835055	0.068870881	-0.796200866	0.425915294	0.999579949
750.7945326	-0.131206108	0.053848613	-2.436573602	0.014827152	0.539131676
351.4601892	-0.153435486	0.123876866	-1.238612912	0.215488884	0.999579949
282.6717983	0.120912033	0.139340622	0.867744317	0.385534319	0.999579949
393.3303274	-0.037740158	0.117222082	-0.32195434	0.747487286	0.999579949
176.5008971	-0.10676527	0.221507425	-0.481994092	0.629810143	0.999579949
212.6578728	-0.01744736	0.113621899	-0.153556311	0.877959587	0.999579949
1052.754505	0.040759729	0.05560323	0.733046065	0.463530334	0.999579949
60.18258168	0.08789132	0.226573247	0.387915701	0.698078418	0.999579949
152.3584344	0.098995267	0.133265815	0.74284067	0.45757815	0.999579949
353.4513794	0.041457861	0.141630202	0.292719073	0.76973689	0.999579949
30.94245733	0.198729906	0.287666848	0.690833538	0.489670157	0.999579949
1468.895301	0.044350122	0.062636083	0.708060281	0.478907824	0.999579949
502.2711578	0.033601712	0.10429244	0.322187416	0.747310717	0.999579949
237.7551241	0.033278763	0.12544035	0.265295519	0.790781816	0.999579949
593.399675	0.274643384	0.164276028	1.671840916	0.094555696	0.880418343
33.06605362	-0.34531349	0.208223736	-1.658377169	0.097241362	0.890093987
37.23918589	0.017686999	0.187658816	0.094250827	0.92490991	0.999579949
1001.254957	0.091668646	0.071677696	1.278900568	0.200932073	0.993208272
541.2503697	0.011821794	0.057769381	0.204637711	0.83785519	0.999579949
145.0111192	-0.053990515	0.220563197	-0.244784785	0.806623075	0.999579949
639.2679212	0.017271845	0.081009349	0.213208043	0.831164695	0.999579949
41.04847346	-0.245209386	0.162502918	-1.508953742	0.131310604	0.938166973
464.5501739	0.066364194	0.085196082	0.778958288	0.436004287	0.999579949
1627.483238	0.064109488	0.070912509	0.904064588	0.365961155	0.999579949
901.812042	-0.076898145	0.085969023	-0.894486669	0.371061559	0.999579949
1428.746787	-0.095108307	0.091745509	-1.036653547	0.299897348	0.999579949
21.70334377	0.52943535	0.322461399	1.641856517	0.100619743	0.895628172
30.12209318	-0.309287615	0.376886881	-0.820637784	0.411852619	0.999579949
192.9469953	0.040548036	0.11356322	0.357052538	0.721052474	0.999579949
576.2969825	0.084588226	0.055820074	1.515372866	0.129678005	0.935700262
15978.67848	0.50563765	0.178137569	2.838467224	0.004533077	0.33947676
342.6082581	0.000984535	0.075421035	0.013053858	0.989584824	0.999579949

408.0978354	-0.008922021	0.066646603	-0.133870607	0.893504893	0.999579949
52.33604875	-0.00373249	0.258317817	-0.014449217	0.988471594	0.999579949
86.52369283	0.10694013	0.200835988	0.532474938	0.594397096	0.999579949
63.01404098	0.103293165	0.223365248	0.462440627	0.64376537	0.999579949
2831.118208	-0.16617696	0.168280873	-0.987497613	0.323398752	0.999579949
630.9509747	0.261571752	0.29880082	0.875405066	0.381353544	0.999579949
1156.749823	-0.27184816	0.160692643	-1.691727485	0.090697945	0.877608448
179.8617	-0.129410575	0.088796065	-1.457390876	0.145008514	0.955875831
121.0885936	-0.122306326	0.140600923	-0.86988281	0.38436445	0.999579949
425.5287947	0.042490009	0.093251109	0.455651517	0.648640591	0.999579949
161.2787562	-0.030170946	0.197621861	-0.152670084	0.878658454	0.999579949
581.512965	0.007788601	0.069504353	0.112059188	0.910776477	0.999579949
165.1465793	-0.042338099	0.112400932	-0.376670357	0.706418595	0.999579949
93.44081786	-0.052436982	0.126255538	-0.415324211	0.67790458	0.999579949
96.36124895	0.134496678	0.113258639	1.18751805	0.235023341	0.999579949
922.2536315	0.087149064	0.076113837	1.14498319	0.252216121	0.999579949
416.3048386	-0.082509182	0.088474559	-0.932575223	0.351039331	0.999579949
145.6861541	-0.037091908	0.117903669	-0.314595027	0.753069155	0.999579949
638.3888311	-0.090503673	0.08764171	-1.032655266	0.301765255	0.999579949
81.01613817	-0.103479474	0.117693113	-0.87923134	0.379275855	0.999579949
1776.781995	0.093341042	0.173426483	0.538216774	0.590427401	0.999579949
82.58986346	0.153146581	0.237496975	0.644835922	0.51903352	0.999579949
188.6618699	0.223682006	0.288775104	0.774588956	0.438582585	0.999579949
272.1155498	0.07624434	0.105488624	0.722773098	0.469819298	0.999579949
122.2076419	0.065699636	0.148830914	0.441438099	0.658895866	0.999579949
259.7729594	0.01119508	0.078644841	0.142349839	0.886803681	0.999579949
20.77708043	0.272654253	0.243931049	1.117751327	0.263673215	0.999579949
409.4398118	0.063125968	0.124508066	0.507003041	0.612152685	0.999579949
338.6725152	-0.070534923	0.087388196	-0.80714474	0.419583098	0.999579949
21.6963593	1.210914302	0.773281043	1.565943344	0.117361882	0.920479614
30.64029829	0.966695706	0.79365749	1.218026313	0.223213974	0.999579949
44.0141167	1.60566224	1.031925964	1.55598589	0.119711448	0.921521033
24.80848872	1.122724403	1.136926842	0.987508046	0.323393641	0.999579949
35.48079086	1.6433654	0.700913682	2.344604537	0.01904727	0.590797093
35.92379509	0.140327479	0.52956438	0.264986627	0.791019764	0.999579949
21.91120663	0.707727923	0.732703162	0.965913565	0.334087434	0.999579949
72.07920695	0.367362059	0.245860908	1.494186536	0.135126871	0.945791454
173.2871359	0.017679313	0.135718201	0.130264861	0.89635688	0.999579949
133.6072958	0.085469932	0.126478315	0.675767482	0.499188284	0.999579949
683.7404644	0.62374559	0.263021347	2.37146375	0.017717785	0.573039104
82.15520087	0.318619308	0.369711853	0.861804416	0.388795154	0.999579949
88.08611016	0.597736969	0.491781193	1.215453088	0.224193331	0.999579949
125.1011484	0.436671379	0.357671911	1.220871322	0.222134744	0.999579949
178.4185615	0.171182361	0.213893418	0.800316168	0.423527638	0.999579949
26.52364118	0.429182749	0.335332756	1.279871235	0.200590426	0.993204353
513.9931231	0.859048421	0.410122172	2.094615898	0.036205151	0.716432561
174.3205454	0.619560446	0.351726196	1.76148508	0.078156336	0.851207389
528.7955949	0.146057969	0.114376398	1.276993951	0.201604383	0.993283621

293.1773383	0.160972877	0.192833424	0.83477684	0.403843368	0.999579949
471.3242121	0.365555293	0.218592347	1.672315145	0.094462195	0.880418343
192.2953934	-0.166925602	0.222785391	-0.749266373	0.453696671	0.999579949
2148.503391	0.60913607	0.241510724	2.522190573	0.011662651	0.493624274
28.04601409	1.285682885	0.776004101	1.656799085	0.097560102	0.890209389
22.61076018	0.792844674	0.636521876	1.245589043	0.212915307	0.999268361
126.4252041	1.269116553	0.648742955	1.956270264	0.050433325	0.776599223
36.31150053	1.136164974	0.749989043	1.514908764	0.129795511	0.935700262
65.83883722	0.080077846	0.303244155	0.264070534	0.791725568	0.999579949
118.3931652	0.017130506	0.441166086	0.038830061	0.969025878	0.999579949
168.2636991	0.061786692	0.583267214	0.10593205	0.915636265	0.999579949
24.87609169	0.629803031	0.38931793	1.617708775	0.105725374	0.896383253
1248.868211	0.101356492	0.058556359	1.730922027	0.083465672	0.859920327
534.1235621	-0.060641047	0.128467453	-0.472034323	0.63690229	0.999579949
113.1873692	0.073934607	0.148310673	0.498511704	0.618123422	0.999579949
231.105852	-0.061260033	0.128956223	-0.475045189	0.634754765	0.999579949
397.1644858	-0.10744665	0.09731932	-1.104062894	0.269565856	0.999579949
465.7376451	0.047890007	0.102604128	0.466745423	0.640682028	0.999579949
22.76692657	-0.009797643	0.25097607	-0.039038157	0.968859967	0.999579949
100.6725176	-0.386215155	0.242187055	-1.594697768	0.110779837	0.912014923
455.8389552	-0.075228002	0.119062712	-0.631835113	0.527494627	0.999579949
280.6304836	-0.126085893	0.108572346	-1.161307619	0.245516822	0.999579949
38.72367818	-0.189579336	0.207603294	-0.913180771	0.361147476	0.999579949
74.37217874	-0.280805647	0.178526655	-1.572906002	0.115740585	0.918818353
4928.251462	0.125243206	0.157688376	0.79424501	0.42705281	0.999579949
463.3687668	0.016910115	0.112867055	0.1498233	0.880904026	0.999579949
60.1169489	-0.011918514	0.378759888	-0.031467203	0.974896948	0.999579949
394.067306	0.094775097	0.071229131	1.33056652	0.183331685	0.983992835
29.69476044	0.709761291	0.502218088	1.413253143	0.157581306	0.977304288
90.99260896	0.273955737	0.230685108	1.187574436	0.235001114	0.999579949
38.13588499	0.215454706	0.245780906	0.876612872	0.380696945	0.999579949
18335.2661	0.106976827	0.169091988	0.632654619	0.526959211	0.999579949
161.3857402	-0.025597543	0.09449183	-0.270896885	0.786470341	0.999579949
58.68002265	0.017184893	0.344133791	0.049936662	0.960172862	0.999579949
65.89563549	-0.130436117	0.28054626	-0.464936217	0.641977133	0.999579949
72.0731825	-0.140578361	0.184926437	-0.760185313	0.447143823	0.999579949
1761.497826	-0.063467714	0.142395055	-0.445715714	0.655802612	0.999579949
312.6112083	0.114124021	0.124187558	0.918965015	0.358113871	0.999579949
137.9645932	0.083807839	0.205990922	0.406852099	0.684116619	0.999579949
216.2441242	0.04257103	0.115069282	0.369959985	0.711412306	0.999579949
104.2151763	-0.082011023	0.22805209	-0.35961531	0.719134833	0.999579949
24.27103419	-0.254523998	0.241564716	-1.053647248	0.292044449	0.999579949
55.86326628	-0.126506383	0.146361103	-0.864344288	0.387398788	0.999579949
385.9669281	0.078920091	0.142750205	0.552854486	0.580363055	0.999579949
156.9326815	0.010778801	0.20542368	0.052471075	0.958153342	0.999579949
630.5182426	-0.026568721	0.086077298	-0.30866119	0.757579269	0.999579949
649.827156	0.158811502	0.094069017	1.688244506	0.091364303	0.879835445
183.7943738	-0.002149338	0.123980946	-0.017336034	0.986168539	0.999579949

53.7220863	-0.047927016	0.170623142	-0.280893996	0.778791706	0.999579949
70.90673668	0.091662137	0.122564386	0.74786926	0.454539021	0.999579949
346.054349	0.095663301	0.095277803	1.004046045	0.31535642	0.999579949
491.3693249	0.14770443	0.20562209	0.718329582	0.472554094	0.999579949
1316.746443	-0.153691839	0.07556	-2.034037041	0.041947861	0.74969123
984.0686731	0.021292862	0.060522752	0.351815821	0.724976392	0.999579949
150.1930387	0.044582951	0.10092342	0.441750296	0.65866991	0.999579949
95.9915013	0.120725072	0.120556565	1.001397745	0.316634554	0.999579949
269.0702814	-0.045166816	0.130044746	-0.347317498	0.728352807	0.999579949
827.9669224	0.017106171	0.087046386	0.196517878	0.844204841	0.999579949
140.1129626	-0.012822355	0.085230863	-0.15044263	0.88041541	0.999579949
97.5725507	0.051526812	0.145916062	0.353126392	0.723993688	0.999579949
58.53168274	-0.204470012	0.189458605	-1.079233176	0.280483794	0.999579949
177.6609976	0.438462066	0.178686668	2.453804027	0.014135399	0.528336764
317.7993588	0.091983794	0.084378116	1.090138044	0.27565234	0.999579949
359.1578572	0.153769778	0.115544688	1.330825159	0.183246547	0.983992835
1084.167435	-0.023084999	0.127847343	-0.180566904	0.856707535	0.999579949
280.5392511	0.139693645	0.293537265	0.475897482	0.634147416	0.999579949
977.4137558	-0.000469149	0.075879738	-0.006182793	0.995066877	0.999579949
724.3786072	-0.078048508	0.106511591	-0.732770088	0.463698668	0.999579949
75.05552412	-0.20520595	0.190580578	-1.076741143	0.281595926	0.999579949
105.5758048	0.087488856	0.126230319	0.69308908	0.48825365	0.999579949
232.1571819	0.057027376	0.160986798	0.354236352	0.723161763	0.999579949
182.4676027	0.277456838	0.169175248	1.640055752	0.100993575	0.895628172
518.1147061	0.104278338	0.119190873	0.87488526	0.38163634	0.999579949
627.0674731	0.076400167	0.0791134	0.965704511	0.334192062	0.999579949
627.97704	6.50036E-06	0.070603932	9.20679E-05	0.99992654	0.999995884
23.79303088	-0.575814508	0.591266018	-0.973867076	0.330122543	0.999579949
782.4152102	-0.032510525	0.078676177	-0.413219435	0.67944585	0.999579949
464.0799329	0.039731958	0.084114802	0.472353941	0.636674175	0.999579949
375.4402809	-0.032065066	0.132258278	-0.24244279	0.808437082	0.999579949
1920.405012	-0.00389147	0.125328851	-0.031050071	0.975229608	0.999579949
2081.692618	-0.013041788	0.083478427	-0.156229437	0.875852174	0.999579949
2651.70542	0.011826921	0.113917578	0.10381998	0.91731221	0.999579949
3150.002623	0.060540459	0.148129784	0.408698762	0.682760742	0.999579949
528.0741486	0.051778342	0.158551095	0.326571964	0.743991651	0.999579949
1122.474271	-0.041443428	0.057457006	-0.721294595	0.470728282	0.999579949
2291.542094	-0.007350237	0.091954297	-0.079933586	0.936290078	0.999579949
137.1063071	-0.051374196	0.164992985	-0.311372004	0.755517835	0.999579949
39.61520764	-0.034611733	0.179042053	-0.193316221	0.846711323	0.999579949
247.3142034	-0.034099116	0.101737662	-0.335167093	0.737499044	0.999579949
401.9161103	0.166356228	0.164119395	1.013629302	0.31075967	0.999579949
565.002689	0.120234803	0.11035611	1.089516499	0.275926183	0.999579949
89.19809409	-0.040449655	0.173792627	-0.232746668	0.815958129	0.999579949
112.5355532	-0.000499063	0.133346162	-0.003742615	0.997013832	0.999579949
1235.505697	-0.107701547	0.064558946	-1.668266806	0.095262774	0.883604099
2710.845596	0.014805659	0.047746291	0.310090251	0.756492326	0.999579949
133.3588633	-0.025223229	0.11907103	-0.21183347	0.832236955	0.999579949

4043.64628	-0.079963604	0.093399409	-0.856146782	0.391916595	0.999579949
159.1445522	0.085261765	0.142190178	0.599631887	0.548751591	0.999579949
632.7415759	-0.007906365	0.128006838	-0.061765176	0.950749836	0.999579949
33.03186727	0.091342959	0.616766067	0.148099845	0.882263972	0.999579949
829.9745352	-0.067850733	0.056232521	-1.206610201	0.227582296	0.999579949
145.1747489	0.1358022	0.101015633	1.344368159	0.178829365	0.983992835
22.18963519	0.020237598	0.315002072	0.064245921	0.948774413	0.999579949
192.9816047	0.128974928	0.141713407	0.910110982	0.362763983	0.999579949
182.8551486	0.480122822	0.204629002	2.346308766	0.018960393	0.590797093
20.94459526	0.532150125	0.403029756	1.320374282	0.186710086	0.983992835
418.8491524	0.271651534	0.185717938	1.46271026	0.143546677	0.953645321
230.0110355	0.052274079	0.109651536	0.476729109	0.633555032	0.999579949
1309.218917	-0.00715266	0.080147011	-0.089244248	0.928887801	0.999579949
65.6673071	-0.050520139	0.145422436	-0.347402646	0.728288845	0.999579949
560.8299241	0.015294157	0.055509045	0.2755255	0.782912538	0.999579949
143.4986386	-0.188177534	0.146318869	-1.28607838	0.198415709	0.992481822
246.0581396	0.071144642	0.081945286	0.868196882	0.385286561	0.999579949
991.7255703	-0.06017614	0.105395029	-0.570958043	0.568028084	0.999579949
308.0879018	0.624760143	0.18387353	3.39777098	0.000679373	0.128481718
135.2160492	-0.006797538	0.452069543	-0.015036487	0.988003071	0.999579949
803.2826208	0.011604578	0.118874218	0.097620644	0.922233531	0.999579949
236.2520082	0.415936946	0.149010589	2.791324756	0.005249277	0.357404857
25.04445581	-0.085048142	0.288562923	-0.29472997	0.768200167	0.999579949
255.3639029	-0.035096825	0.075388306	-0.465547336	0.641539547	0.999579949
140.3685961	-0.274517357	0.345868214	-0.793705074	0.427367146	0.999579949
45.12583835	-0.15955852	0.251560522	-0.63427488	0.525901447	0.999579949
201.3671253	0.094116102	0.11139764	0.844866213	0.398185541	0.999579949
104.4586259	-0.133744606	0.146227111	-0.914636176	0.36038266	0.999579949
2782.441411	-0.164509884	0.108660102	-1.513986105	0.130029364	0.935700262
58.2438445	0.166106594	0.2156626	0.770215116	0.441172299	0.999579949
142.3559829	0.070210084	0.121225208	0.579170666	0.562474022	0.999579949
717.4601756	-0.114962234	0.101797077	-1.129327452	0.258759725	0.999579949
1379.188751	-0.142581791	0.123556811	-1.15397759	0.248509357	0.999579949
275.8870816	-0.000869867	0.094119899	-0.009242113	0.992625966	0.999579949
583.0322314	0.013909955	0.086992413	0.159898487	0.872961041	0.999579949
143.1870044	-0.155333451	0.200964594	-0.77293939	0.439558252	0.999579949
35.3912572	-0.381830026	0.278613751	-1.370463678	0.170542206	0.981609963
32.72502773	0.662739316	0.459258536	1.443063687	0.149002531	0.962301096
229.203233	0.04500562	0.105776841	0.425477069	0.670488934	0.999579949
257.7664068	0.088326018	0.083681922	1.055497003	0.29119808	0.999579949
114.8760136	-0.221028226	0.137075872	-1.612451726	0.106863677	0.899769299
2871.313254	-0.016570321	0.047344511	-0.349994557	0.726342783	0.999579949
427.4571471	0.077843745	0.120801799	0.644392264	0.5193211	0.999579949
178.0265392	0.205833834	0.206067152	0.998867755	0.317858758	0.999579949
904.7589031	-0.081644813	0.060438707	-1.35086961	0.176737205	0.983992835
411.7975692	0.031720251	0.247101693	0.128369219	0.89785679	0.999579949
111.8773042	-0.223985566	0.149806214	-1.495168732	0.134870412	0.945791454
2379.54117	-0.185594341	0.119242639	-1.55644275	0.119602846	0.921521033

45.39849213	0.090581629	0.456819474	0.198287582	0.842820067	0.999579949
152.5665147	0.009267145	0.108478442	0.08542845	0.931920776	0.999579949
509.7673524	0.018692958	0.110940812	0.168494876	0.866193975	0.999579949
600.7136527	0.094847281	0.12351364	0.76790937	0.44254103	0.999579949
95.62319537	-0.188352343	0.113349986	-1.661688285	0.096575291	0.88663438
107.348727	0.006577197	0.11605732	0.056671967	0.954806505	0.999579949
1003.071655	0.016750681	0.068555612	0.244337124	0.806969733	0.999579949
977.2705392	-0.030534843	0.079614882	-0.383531848	0.701325461	0.999579949
290.8911689	0.069207136	0.121847867	0.567979869	0.570048638	0.999579949
62.50198859	0.113453691	0.137712798	0.823842751	0.410028913	0.999579949
35.75374106	0.320607853	0.202356545	1.584371056	0.113109304	0.916629381
776.7836804	0.069736134	0.078331132	0.890273535	0.373319028	0.999579949
25.46154078	0.882602996	0.637651919	1.384145441	0.166313925	0.978789551
411.9934869	0.006570596	0.20628064	0.031852705	0.974589515	0.999579949
555.5608185	-0.058896451	0.164793225	-0.357396072	0.720795317	0.999579949
858.8048648	-0.06945284	0.050183906	-1.383966409	0.16636874	0.978789551
46.8004232	0.010882961	0.211445801	0.05146927	0.958951589	0.999579949
903.1307552	0.149111287	0.126596621	1.177845716	0.23885812	0.999579949
66.92815189	0.026179983	0.195833564	0.133684863	0.893651775	0.999579949
1139.150105	-0.051871101	0.06220236	-0.833908892	0.404332326	0.999579949
169.8017982	-0.00806947	0.099540271	-0.08106739	0.935388359	0.999579949
145.4980961	-0.116571633	0.139428606	-0.836066836	0.403117304	0.999579949
2014.262977	0.02463324	0.036513477	0.674634171	0.499908218	0.999579949
73.89870222	0.047428682	0.130728961	0.362801646	0.716753062	0.999579949
373.4546351	-0.319717737	0.239499353	-1.334941966	0.181895341	0.983992835
956.703587	-0.008159393	0.089773237	-0.09088893	0.927580846	0.999579949
268.8850715	0.000435665	0.081073281	0.005373714	0.995712417	0.999579949
264.0935956	-0.03010479	0.104456079	-0.288205249	0.773189631	0.999579949
2202.326544	-0.060460302	0.083359865	-0.725292701	0.468272482	0.999579949
44.21620186	0.136079445	0.175981882	0.773258268	0.439369548	0.999579949
858.4728449	0.063250771	0.103277737	0.612433746	0.54025082	0.999579949
533.5642936	-0.035514347	0.177086463	-0.200548059	0.841051976	0.999579949
250.625861	-0.282075497	0.222683959	-1.266707753	0.205259807	0.994814558
51.03013726	0.520392657	0.237640243	2.189833885	0.028536286	0.666751303
63.13530571	0.032287906	0.195137539	0.165462298	0.868580123	0.999579949
975.6749548	-0.084085948	0.160412009	-0.524187359	0.600148232	0.999579949
691.2942005	0.115640766	0.085988673	1.344837198	0.178677815	0.983992835
126.7515548	-0.145145971	0.106987666	-1.356660795	0.174889015	0.983992835
97.64568692	0.07416053	0.152007178	0.487875186	0.625638252	0.999579949
375.8438337	-0.094540461	0.105876282	-0.892933331	0.371892876	0.999579949
455.8060556	-0.013568983	0.095980683	-0.141372023	0.887576055	0.999579949
25.35346965	-0.265653539	0.268785517	-0.98834767	0.322982407	0.999579949
466.4950531	0.015582571	0.130804413	0.119128784	0.905173327	0.999579949
343.8483622	-0.029613097	0.094132194	-0.314590536	0.753072565	0.999579949
244.3063895	0.223159893	0.111155274	2.007641066	0.044681447	0.759209569
43.63619735	0.128368151	0.195352978	0.657108748	0.511110994	0.999579949
311.3082088	-0.072851463	0.119378777	-0.610254727	0.541693082	0.999579949
1157.936819	-0.069423062	0.100446937	-0.691141653	0.489476526	0.999579949

41.85879103	-0.154296211	0.202666911	-0.761329071	0.446460541	0.999579949
255.091806	0.190672548	0.143354783	1.330074548	0.18349371	0.983992835
137.7022636	-0.072650606	0.097025701	-0.748776925	0.45399167	0.999579949
2002.423044	0.070493604	0.126663663	0.55654165	0.57784063	0.999579949
120.0421864	-0.1035772	0.125052845	-0.828267442	0.407519057	0.999579949
242.429517	0.113994865	0.085085181	1.339773426	0.180319018	0.983992835
31.06343078	0.115958102	0.215176503	0.538897606	0.589957509	0.999579949
32.30322177	0.035890633	0.262197503	0.136883964	0.891122515	0.999579949
21.6277832	-0.10078976	0.250757635	-0.401940944	0.687727489	0.999579949
117.4789341	-0.034868195	0.19492557	-0.178879533	0.858032294	0.999579949
48.64592409	-0.091187519	0.208593109	-0.437154993	0.661998947	0.999579949
27.75614683	-0.024099621	0.276785966	-0.087069519	0.930616253	0.999579949
38.35958821	0.155736235	0.181908513	0.856123951	0.391929222	0.999579949
52.4319504	0.035102967	0.189652434	0.185091044	0.853157626	0.999579949
75.72434992	0.207016959	0.180378636	1.147680033	0.251100677	0.999579949
198.4919773	0.100003506	0.162790461	0.614308144	0.539011722	0.999579949
65.89249771	-0.060183742	0.138674912	-0.433991567	0.664294565	0.999579949
57.48092805	-0.011271157	0.158496659	-0.071112902	0.9433079	0.999579949
78.25429921	0.062200976	0.114944741	0.541138081	0.588412412	0.999579949
90.39023312	0.024906761	0.178453736	0.13956985	0.888999863	0.999579949
112.6774594	-0.027709023	0.213623437	-0.12970966	0.896796139	0.999579949
95.92412185	0.075306055	0.176869054	0.425772926	0.670273317	0.999579949
101.6441496	-0.098962114	0.164224351	-0.602603168	0.546772713	0.999579949
35.17447186	-0.308023748	0.206537585	-1.491368981	0.135864647	0.945791454
42.60673144	-0.222690313	0.23561269	-0.945154155	0.344580181	0.999579949
358.4362683	0.061957089	0.118404419	0.523266692	0.600788677	0.999579949
106.6265609	-0.214099728	0.116083817	-1.844354656	0.065131471	0.817776877
540.3010748	0.060012375	0.121198747	0.495156728	0.620489493	0.999579949
238.8482259	0.011360193	0.101595865	0.111817478	0.910968129	0.999579949
750.2326089	-0.01982279	0.071580688	-0.276929297	0.781834396	0.999579949
48.08512872	-0.066453751	0.171457543	-0.387581377	0.698325853	0.999579949
72.2674373	0.0509769	0.129409548	0.393919156	0.693640727	0.999579949
1237.593939	-0.076026439	0.049020313	-1.550917029	0.120921569	0.921837625
998.732377	0.057562338	0.113750046	0.506042327	0.612826936	0.999579949
213.693163	0.057743325	0.139373195	0.414307245	0.678649109	0.999579949
106.8504592	0.457479944	0.356127821	1.284594792	0.198933919	0.992481822
387.7217271	0.016900843	0.077854424	0.217082629	0.828143951	0.999579949
329.6390612	-0.046706539	0.162115756	-0.288106104	0.773265521	0.999579949
40.00909863	0.096213296	0.21266088	0.452425926	0.650962173	0.999579949
174.4033831	0.11110733	0.099305153	1.118847575	0.263205172	0.999579949
257.0195095	0.02793864	0.112798934	0.24768532	0.804377891	0.999579949
660.8849855	-0.017992259	0.076867063	-0.234069809	0.814930784	0.999579949
164.4339168	-0.03226792	0.089034522	-0.36242032	0.717037957	0.999579949
10216.33423	0.021501697	0.100857646	0.213188568	0.831179884	0.999579949
515.9995123	0.068887918	0.110859834	0.621396546	0.534338744	0.999579949
438.3701314	-0.045472887	0.149521325	-0.304123085	0.76103412	0.999579949
25.86507698	-0.136448292	0.271638874	-0.502315041	0.615445931	0.999579949
54.43292953	-0.16697784	0.255565854	-0.653365218	0.513520857	0.999579949

332.5923188	-0.024182094	0.106433972	-0.227202781	0.820266065	0.999579949
1069.116416	0.040644083	0.068009946	0.597619687	0.550093725	0.999579949
41.15988529	-0.024389956	0.215745066	-0.11304989	0.909991002	0.999579949
525.1926644	0.016636224	0.096703572	0.172033194	0.863411433	0.999579949
342.9972948	0.032142884	0.144983131	0.221700852	0.824546767	0.999579949
1071.834115	0.002088307	0.139078397	0.015015326	0.988019953	0.999579949
106.9335084	-0.010757024	0.132974699	-0.080895272	0.93552524	0.999579949
61.43725435	-0.142378812	0.166245204	-0.856438616	0.391755213	0.999579949
467.0242063	-0.159935103	0.065574465	-2.438984501	0.0147286	0.539131676
49.00690407	0.61679276	0.377188504	1.63523743	0.101999285	0.895628172
274.07986	0.033159037	0.159543435	0.20783705	0.835356202	0.999579949
96.7704615	0.166078253	0.176081095	0.943191844	0.345582784	0.999579949
243.0957937	0.05465769	0.093105844	0.587048977	0.557170813	0.999579949
25.06084582	-0.178553519	0.226302177	-0.789004867	0.430109162	0.999579949
677.3584163	0.401045202	0.175829329	2.280877738	0.022555683	0.627784819
128.6733987	-0.134085995	0.148926015	-0.900353069	0.367932387	0.999579949
36.58345167	0.007203802	0.167032001	0.043128273	0.965599281	0.999579949
49.85072095	0.244087655	0.157054925	1.554154738	0.120147511	0.921837625
133.5778992	0.176975657	0.103929473	1.702843775	0.088597308	0.870840008
8158.323922	-0.014894333	0.150070493	-0.099248913	0.92094064	0.999579949
151.3018913	-0.189241564	0.105426627	-1.795007295	0.072652538	0.838669905
196.6386899	0.006468348	0.125719559	0.051450612	0.958966456	0.999579949
522.4098383	-0.017310142	0.064993545	-0.266336329	0.789980197	0.999579949
2626.643403	-0.015403123	0.134202109	-0.114775569	0.908623014	0.999579949
195.2323955	0.159100795	0.240130482	0.662559764	0.507612546	0.999579949
62.59361651	0.104695202	0.172141407	0.60819302	0.543059464	0.999579949
308.2539181	-0.008434004	0.07834765	-0.107648457	0.914274557	0.999579949
1778.571995	0.014602184	0.062015299	0.235460999	0.813850946	0.999579949
85.93278666	0.012665419	0.135637416	0.093377029	0.925604039	0.999579949
223.168985	0.042821452	0.080988931	0.52873215	0.596991271	0.999579949
88.92548524	0.018231194	0.117605775	0.155019549	0.876805906	0.999579949
10263.58025	0.31581382	0.156257399	2.021112747	0.043268095	0.754854468
169.6720807	-0.233021932	0.150700602	-1.546257476	0.122042397	0.92236461
240.1878482	0.024105937	0.106126613	0.227143189	0.820312402	0.999579949
35.14341353	0.189077939	0.248836335	0.759848592	0.447345093	0.999579949
21.94140838	-0.092933434	0.227236146	-0.408972937	0.682559522	0.999579949
250.4291295	-0.014665208	0.082881782	-0.176941279	0.859554514	0.999579949
178.1198502	0.192328047	0.225193786	0.854055748	0.393074101	0.999579949
1157.004784	0.064151461	0.171640521	0.373754753	0.708586786	0.999579949
310.2377436	0.097913715	0.11608073	0.843496725	0.398950697	0.999579949
67110.48981	-0.133249335	0.136608073	-0.975413329	0.329355275	0.999579949
84.1225784	-0.135804343	0.18166656	-0.747547279	0.454733275	0.999579949
34.31750698	-0.211568327	0.189922061	-1.113974464	0.265290142	0.999579949
194.3030522	0.037186532	0.079750701	0.466284698	0.641011731	0.999579949
1281.835871	-0.053541792	0.163807656	-0.326857692	0.743775522	0.999579949
1532.572339	0.111977144	0.08895503	1.258806214	0.208100335	0.995521169
158.166771	0.212695296	0.226002276	0.941120149	0.346643291	0.999579949
177.1218531	-0.354696338	0.32674736	-1.085536965	0.277683901	0.999579949

376.91015	0.05564094	0.088550982	0.628349211	0.5297752	0.999579949
543.228168	-0.015299859	0.057969249	-0.26393061	0.791833388	0.999579949
166.8571704	0.024986263	0.090906308	0.274857303	0.783425871	0.999579949
400.9142867	-0.080265712	0.076989739	-1.042550768	0.297156401	0.999579949
84.86593928	-0.002832425	0.165916024	-0.017071436	0.986379626	0.999579949
74.93971191	0.190526613	0.15240709	1.250116466	0.211257006	0.996937328
359.3715711	0.122250297	0.086227628	1.417762492	0.156260118	0.976030424
124.1907179	0.142186756	0.19786016	0.718622467	0.472373567	0.999579949
110.3116655	0.344140712	0.378531526	0.909146765	0.36327266	0.999579949
145.6997024	0.083456082	0.12086061	0.690515147	0.489870288	0.999579949
121.0816927	0.158516978	0.109596137	1.446373771	0.148072394	0.962301096
1279.636904	0.006782808	0.078280748	0.086647202	0.930951946	0.999579949
134.7537558	0.2929886	0.180460177	1.62356374	0.104468949	0.895628172
327.697515	-0.010587186	0.083860241	-0.126247977	0.899535635	0.999579949
957.9551841	0.005148023	0.106445951	0.04836279	0.961427114	0.999579949
154.056945	0.175797831	0.187127288	0.939455877	0.347496736	0.999579949
119.7909883	0.335763426	0.315850114	1.063046713	0.287760768	0.999579949
188.3320253	-0.316134743	0.188878377	-1.673747666	0.094180202	0.880418343
160.8147296	0.11917583	0.096980212	1.228867499	0.219121489	0.999579949
154.1519376	0.064977664	0.088777954	0.731912155	0.464222187	0.999579949
984.3673481	0.037357451	0.061922437	0.603294257	0.546312953	0.999579949
658.0433182	0.054390441	0.108529354	0.5011588	0.616259367	0.999579949
1021.181416	-0.04482582	0.068062012	-0.658602629	0.510150974	0.999579949
25.57543413	-0.124897855	0.289624402	-0.431240786	0.666293301	0.999579949
113.7150737	-0.269474966	0.125558004	-2.146218944	0.031855515	0.689986698
274.7495195	0.031359387	0.11285561	0.27787176	0.781110803	0.999579949
304.5622375	0.135915912	0.127370198	1.067093519	0.285929608	0.999579949
960.2941658	0.034229727	0.126773997	0.270005895	0.787155718	0.999579949
572.0906667	0.091505581	0.116052266	0.788485947	0.430412516	0.999579949
593.4497505	0.003126298	0.066333192	0.047130218	0.962409444	0.999579949
22.94204684	-0.224580339	0.290796012	-0.772295113	0.439939659	0.999579949
105.43662	0.041471579	0.113503613	0.365376735	0.714830205	0.999579949
33.50904728	0.749406499	0.518389781	1.445642885	0.148277392	0.962301096
22.78572223	0.390203146	0.578752142	0.674214604	0.500174886	0.999579949
125.32256	0.004851116	0.138877637	0.034930867	0.972134867	0.999579949
33.05558358	0.004016993	0.222669888	0.018040125	0.985606844	0.999579949
211.5725711	0.120513826	0.10000972	1.205021124	0.228195147	0.999579949
132.7526075	-0.050417646	0.148879249	-0.338647904	0.734874991	0.999579949
128.6960113	0.038555925	0.135133364	0.285317581	0.775400844	0.999579949
23.62574114	0.024892007	0.407105263	0.061143909	0.9512446	0.999579949
342.2886735	0.010175432	0.078569713	0.129508327	0.896955437	0.999579949
181.6182374	0.029321391	0.138878855	0.211129269	0.832786402	0.999579949
261.0085776	-0.022478326	0.096552267	-0.232809922	0.815909008	0.999579949
1894.650776	0.350835999	0.222477769	1.576948566	0.11480736	0.918818353
2149.890146	-0.428382646	0.134791796	-3.178106217	0.001482404	0.203876378
474.1226164	-0.004424023	0.094632255	-0.046749631	0.962712774	0.999579949
1656.088775	0.014801489	0.102628834	0.144223495	0.885323988	0.999579949
206.8556911	-0.06576911	0.114820861	-0.572797567	0.566781767	0.999579949

22.85821338	-0.368002523	0.214073362	-1.719048649	0.085605514	0.864463816
384.943589	0.037046612	0.122404534	0.30265719	0.762151126	0.999579949
482.7515913	0.012311505	0.142235917	0.086556935	0.931023699	0.999579949
89.32302462	0.083575949	0.178510514	0.468185022	0.639652282	0.999579949
75.12009706	0.135479767	0.123217278	1.099519231	0.27154165	0.999579949
157.9416043	-0.140403399	0.092149261	-1.523651914	0.127595687	0.935409261
140.7678477	0.060445288	0.13574535	0.44528441	0.656114233	0.999579949
106.084941	-0.00683611	0.295483315	-0.02313535	0.981542308	0.999579949
369.3585873	0.022600932	0.07858993	0.287580514	0.773667863	0.999579949
72.00380061	-0.045210497	0.149287987	-0.302840823	0.762011171	0.999579949
331.7883537	0.001307694	0.106226587	0.012310424	0.990177951	0.999579949
89.63751756	0.156553068	0.269473545	0.580958949	0.561268121	0.999579949
146.4261289	-0.026130116	0.100130355	-0.260960981	0.794122598	0.999579949
45.82135798	0.011352132	0.212506727	0.053420107	0.957397185	0.999579949
58.88592117	-0.061455881	0.12812847	-0.479642669	0.631481501	0.999579949
26.98076809	-0.322233014	0.308956819	-1.042971037	0.296961707	0.999579949
4948.569802	0.147704011	0.119913168	1.231758054	0.218039476	0.999579949
27.27086336	0.422803757	0.352746456	1.198605257	0.230681473	0.999579949
217.4925944	-0.311128892	0.107628568	-2.890764946	0.003843054	0.319818899
788.2100307	0.001648273	0.113326288	0.014544487	0.988395587	0.999579949
215.1218928	0.236069702	0.145362056	1.624011849	0.10437328	0.895628172
555.0083426	-0.056527793	0.160850676	-0.351430246	0.725265595	0.999579949
2924.657126	-0.082486402	0.060964579	-1.35302177	0.176048676	0.983992835
326.523194	0.123362168	0.095036497	1.298050455	0.194269998	0.987898624
431.5323406	0.034782062	0.116003081	0.299837399	0.764301187	0.999579949
496.5336083	0.062609562	0.102202049	0.612605737	0.540137064	0.999579949
406.1200413	-0.051532002	0.097616657	-0.527901725	0.597567547	0.999579949
49.12641809	-0.186828114	0.194029988	-0.962882675	0.335606405	0.999579949
30.33873639	-0.081246347	0.320724335	-0.253321429	0.800019838	0.999579949
415.3708955	0.015106103	0.10365617	0.145732795	0.884132331	0.999579949
133.5442443	0.265450588	0.135602738	1.95756069	0.050281584	0.776599223
96.43580574	0.031455833	0.133334934	0.235915914	0.813497919	0.999579949
21.00542845	-0.052902167	0.284037028	-0.186250951	0.85224797	0.999579949
984.413375	0.159499437	0.126177898	1.264083803	0.206199949	0.994814558
116.4864183	-0.172567044	0.13495883	-1.278664339	0.201015283	0.993208272
368.3335526	-0.117120495	0.112551512	-1.040594592	0.298063742	0.999579949
29.73817792	0.622412595	0.292947794	2.124653633	0.033615525	0.698554126
491.128633	-0.146578282	0.153828334	-0.952869208	0.340656343	0.999579949
188.5564151	0.086913717	0.083132455	1.045484785	0.295798969	0.999579949
504.6121931	-0.02637027	0.103516067	-0.254745672	0.798919539	0.999579949
64.67332559	0.704955626	0.408251039	1.72676995	0.084208987	0.861032139
340.0440739	0.027999385	0.10349882	0.270528546	0.786753659	0.999579949
54.555181	-0.540236846	0.346826027	-1.557659473	0.119313992	0.921521033
2436.827512	0.450167706	0.170351362	2.642583549	0.008227616	0.43544141
697.3227264	0.044119493	0.136551191	0.323098558	0.746620603	0.999579949
149.2064529	0.163394559	0.321840229	0.507688426	0.611671867	0.999579949
39.16448665	-0.506935322	0.284537524	-1.781611488	0.07481261	0.849430556
738.5581835	-0.142408607	0.126001342	-1.130215003	0.25838564	0.999579949

117.0141689	-0.04891699	0.182389658	-0.268200457	0.788545023	0.999579949
220.0172057	0.037216216	0.09024584	0.412387053	0.680055751	0.999579949
42.92045844	0.222623767	0.249921605	0.890774397	0.373050213	0.999579949
878.1593913	-0.001269871	0.101625661	-0.01249557	0.990030237	0.999579949
88.55909882	0.089225903	0.186705261	0.477897101	0.632723445	0.999579949
365.6427612	0.034879424	0.106749874	0.326739716	0.743864758	0.999579949
283.4979238	-0.133264815	0.130819708	-1.018690669	0.308349844	0.999579949
2477.690089	0.045961417	0.110937613	0.414299675	0.678654652	0.999579949
121.940212	-0.113175905	0.112947553	-1.002021753	0.316333087	0.999579949
36.15229377	0.533338637	0.307522284	1.734308908	0.082863288	0.859050183
1025.191195	-0.040662686	0.0979077	-0.41531653	0.677910202	0.999579949
116.1012324	-0.071909803	0.15418826	-0.466376641	0.640945929	0.999579949
523.7731087	-0.061816885	0.135281857	-0.456948821	0.647707832	0.999579949
821.2587123	-0.079667556	0.068029559	-1.171072661	0.241569572	0.999579949
1356.583592	0.054376284	0.146665632	0.370750003	0.710823743	0.999579949
38.70740566	0.039744692	0.225530957	0.176227212	0.860115443	0.999579949
373.3921166	0.173680535	0.127463656	1.362588686	0.173012176	0.983601342
172.496025	-0.003991818	0.146489354	-0.027249887	0.978260426	0.999579949
560.035848	-0.067998851	0.09295288	-0.731541093	0.464448714	0.999579949
1233.44551	0.010113766	0.056191656	0.179986972	0.857162796	0.999579949
175.8627125	-0.067761219	0.117085279	-0.57873389	0.562768745	0.999579949
627.39689	-0.18088689	0.150323658	-1.203316178	0.228853991	0.999579949
2096.856689	0.08730375	0.129555269	0.673872634	0.500392292	0.999579949
1304.741673	0.061171314	0.099021557	0.617757545	0.536735173	0.999579949
352.701821	0.083985373	0.126921156	0.661712955	0.5081552	0.999579949
317.9510188	-0.164510709	0.141608157	-1.161731866	0.245344398	0.999579949
100.5618016	-0.127188106	0.129899137	-0.979129729	0.327515884	0.999579949
463.0419768	-0.288944075	0.127695644	-2.262755931	0.023650739	0.630129799
623.5847996	-0.090041282	0.107448556	-0.837994341	0.402033882	0.999579949
91.69023474	-0.030608816	0.220182843	-0.139015444	0.889437945	0.999579949
69.62758375	-0.232770566	0.13452426	-1.730324074	0.083572391	0.859920327
36.06621985	0.543789262	0.264553162	2.055500902	0.039830649	0.73515901
258.5719008	0.073355302	0.10409504	0.704695458	0.480999778	0.999579949
501.382211	-0.075840882	0.08119709	-0.934034481	0.350286107	0.999579949
258.8075253	-0.127358013	0.074142459	-1.717747362	0.085842706	0.864463816
182.7765727	0.080184796	0.128251511	0.625215211	0.53182982	0.999579949
232.7814338	-0.008917515	0.121904853	-0.073151435	0.941685612	0.999579949
835.6345732	0.026520349	0.151874567	0.174620077	0.861378172	0.999579949
974.1422217	0.253106731	0.646648675	0.391413052	0.695491947	0.999579949
3087.939572	0.006042444	0.137261478	0.04402141	0.964887338	0.999579949
262.0797682	0.324358183	0.127612821	2.541736653	0.011030325	0.487909795
16442.92613	-0.098108693	0.111027074	-0.883646567	0.376887033	0.999579949
3933.759258	-0.047535047	0.096493468	-0.492624505	0.62227793	0.999579949
1384.307045	-0.037169856	0.049994704	-0.743475874	0.457193623	0.999579949
188.1007402	0.008841574	0.09598004	0.092118884	0.926603585	0.999579949
32.70964452	-0.047412096	0.176619623	-0.268441837	0.78835924	0.999579949
351.8910997	-0.00641364	0.14641981	-0.043803087	0.965061367	0.999579949
1048.601558	0.009385194	0.052500094	0.17876528	0.858122009	0.999579949

270.5536505	-0.019657027	0.103816106	-0.189344676	0.849822682	0.999579949
3101.651095	-0.028709774	0.122981859	-0.233447225	0.815414146	0.999579949
390.547814	-0.157974985	0.160890812	-0.981876984	0.326160456	0.999579949
1596.998412	-0.042246057	0.097630417	-0.432714097	0.665222488	0.999579949
299.8305343	0.036264377	0.140586755	0.257950169	0.796445365	0.999579949
775.4232301	0.191374972	0.14065933	1.360556543	0.173653873	0.983601342
807.5813108	-0.12630367	0.078087721	-1.617458777	0.105779287	0.896383253
215.2643662	-0.191183226	0.241282638	-0.792362134	0.428149552	0.999579949
1150.318408	-0.061633159	0.143587391	-0.429237959	0.667750061	0.999579949
1465.800237	-0.061467254	0.128538158	-0.47820239	0.632506162	0.999579949
3159.571004	-0.059346649	0.098061661	-0.605197266	0.545047932	0.999579949
65.40365772	0.090800523	0.13754197	0.660165931	0.509147352	0.999579949
449.6590738	-0.077400138	0.183013353	-0.422920713	0.67235311	0.999579949
26.17900454	0.111575352	0.217050178	0.514053259	0.607214755	0.999579949
1817.227959	0.080688886	0.12832698	0.628775695	0.529495914	0.999579949
274.0691422	-0.046770975	0.131948835	-0.354462965	0.722991954	0.999579949
298.0169918	-0.047480667	0.079662755	-0.596020898	0.551161266	0.999579949
2134.357732	0.023825919	0.092735108	0.256924479	0.797237073	0.999579949
1464.072928	-0.201386969	0.101916037	-1.976008627	0.0481538	0.770705547
156.3917764	-0.139198182	0.151473625	-0.91895987	0.358116563	0.999579949
62.61120141	-0.181958614	0.189641092	-0.959489378	0.337312268	0.999579949
359.397004	0.083401926	0.128185082	0.650636757	0.515280997	0.999579949
2084.291716	-0.005952873	0.079660668	-0.074727886	0.94043122	0.999579949
883.7415645	0.036759726	0.068783393	0.534427344	0.593045905	0.999579949
1122.071907	0.121147098	0.078173684	1.549717134	0.121209424	0.921837625
384.6073141	-0.048413131	0.071316134	-0.678852434	0.497231367	0.999579949
297.0122131	0.006721407	0.109374968	0.061452881	0.950998539	0.999579949
168.4323285	-0.053576865	0.095776762	-0.559393151	0.575893435	0.999579949
1273.504183	-0.055743989	0.143017075	-0.389771562	0.696705474	0.999579949
718.2266454	-0.073538408	0.102034221	-0.720722981	0.47107997	0.999579949
182.3491077	-0.108150731	0.122337272	-0.884037464	0.376675989	0.999579949
260.1997952	-0.03822456	0.137744766	-0.277502814	0.781394046	0.999579949
446.9283449	0.051698936	0.10726973	0.481952704	0.629839545	0.999579949
229.2680737	0.033614782	0.101281428	0.331894827	0.739968677	0.999579949
235.5284364	0.268268572	0.275081363	0.975233544	0.329444426	0.999579949
210.3704791	-0.272571098	0.132140884	-2.062731006	0.039138192	0.732468953
23.93835115	0.29827046	0.252440771	1.181546307	0.237385769	0.999579949
570.0584637	0.031969849	0.253564807	0.12608157	0.899667356	0.999579949
418.4460283	-0.028707241	0.136842572	-0.209782968	0.833837067	0.999579949
401.0898496	0.051470206	0.086370316	0.595924713	0.551225523	0.999579949
59.18702278	-0.254068734	0.424193321	-0.598945624	0.549209146	0.999579949
37.8445572	-0.126329351	0.225351635	-0.560587684	0.575078649	0.999579949
270.7594282	-0.057710809	0.07737224	-0.745885202	0.455736765	0.999579949
402.8066515	-0.053308673	0.300949613	-0.177134879	0.859402446	0.999579949
216.8425222	0.16694039	0.317588663	0.525649712	0.59913161	0.999579949
120.9906433	-0.041191279	0.105839918	-0.389184722	0.697139505	0.999579949
438.359688	-0.100165148	0.070628347	-1.418200371	0.156132274	0.976030424
193.8266917	0.031843909	0.102563115	0.310481103	0.756195128	0.999579949

319.8504695	0.298837717	0.12644689	2.363345734	0.018110765	0.578992883
131.8491649	-0.229588251	0.148175912	-1.549430322	0.121278309	0.921837625
446.0552167	0.047265491	0.118927123	0.397432395	0.691048631	0.999579949
121.1032572	0.057575179	0.118599642	0.485458289	0.627351294	0.999579949
249.8693525	0.126259956	0.098673554	1.279572397	0.200695563	0.993204353
1330.43289	-0.042639629	0.069851219	-0.610435003	0.541573687	0.999579949
127.8642695	0.166443038	0.093837324	1.773740248	0.076106126	0.851207389
865.4903135	0.009616718	0.156135102	0.061592284	0.950887522	0.999579949
1833.260793	0.045116934	0.057512284	0.784474738	0.432761599	0.999579949
1782.577207	1.367250127	0.379587038	3.601941029	0.00031585	0.08288431
290.3341845	-0.197992226	0.173379735	-1.141957137	0.253471837	0.999579949
159.9316088	-0.081350542	0.117016058	-0.695208356	0.486924737	0.999579949
849.0468887	-0.006922292	0.078338188	-0.088364207	0.929587208	0.999579949
744.8931451	-0.019847056	0.0871	-0.227865163	0.819751066	0.999579949
642.743103	-0.282209962	0.091054758	-3.099343393	0.001939501	0.225075621
91.78666221	0.121072282	0.106665902	1.135060785	0.256349851	0.999579949
313.6782879	-0.054851891	0.080261083	-0.683418281	0.494342572	0.999579949
210.5468346	-0.132278951	0.146258005	-0.904421952	0.365771702	0.999579949
122.1393934	-0.190248098	0.113501192	-1.676177089	0.09370351	0.880418343
101.8220822	-0.108673275	0.137026783	-0.79308054	0.4277309	0.999579949
1547.167383	0.038842385	0.109237581	0.35557712	0.722157281	0.999579949
450.536752	0.057056626	0.198285562	0.287749777	0.773538284	0.999579949
414.9236076	-0.090278555	0.112249212	-0.804268938	0.421241673	0.999579949
214.9922982	-0.026893026	0.111959681	-0.240202772	0.810173065	0.999579949
343.1523017	0.186229697	0.094509087	1.970495145	0.048781651	0.773861498
1033.254622	-0.071004903	0.084716978	-0.838142542	0.401950652	0.999579949
457.830076	0.051052528	0.069406461	0.735558721	0.461999291	0.999579949
297.8289519	-0.020177069	0.068525245	-0.294447231	0.76841618	0.999579949
7045.635539	0.080805548	0.128939193	0.626695008	0.530859177	0.999579949
42.04095619	-0.033177832	0.198493942	-0.167147834	0.86725373	0.999579949
120.3880117	0.0389031	0.727427365	0.053480391	0.957349154	0.999579949
569.0070001	-0.053746221	0.108452625	-0.495573264	0.62019552	0.999579949
87.64113517	-0.029675317	0.292518799	-0.101447556	0.919195187	0.999579949
39.50085205	-0.164328616	0.372397179	-0.44127245	0.659015768	0.999579949
43.38442157	0.27946616	0.172942249	1.61595077	0.106104957	0.897580956
234.6610353	-0.01090942	0.131732218	-0.082815126	0.933998542	0.999579949
2338.186279	0.029119066	0.073177705	0.397922638	0.690687213	0.999579949
621.6482805	-0.691004339	0.509679098	-1.35576354	0.175174415	0.983992835
465.5199756	-0.184721588	0.132497334	-1.394153241	0.163271335	0.978498637
1547.664348	-0.05774349	0.111293036	-0.518841896	0.603870999	0.999579949
18142.7927	-0.044613342	0.110249364	-0.4046585	0.685728551	0.999579949
1179.75467	0.036496289	0.065956811	0.553336172	0.580033236	0.999579949
2023.1911	-0.051967778	0.107579278	-0.483064948	0.629049621	0.999579949
4687.993457	-0.022976475	0.097954476	-0.23456279	0.814548093	0.999579949
254.1875175	-0.034152725	0.190353506	-0.17941737	0.857609994	0.999579949
354.4311429	0.188026368	0.138247402	1.360071615	0.173807263	0.983601342
374.6506765	0.11286684	0.103442508	1.091106963	0.275225819	0.999579949
24.03921978	0.576548761	0.312181449	1.846838636	0.06477053	0.817776877

129.3114113	0.092257402	0.139383676	0.661895314	0.508038315	0.999579949
817.103028	0.082112386	0.149801971	0.548139559	0.58359608	0.999579949
274.358673	-0.075739034	0.169293377	-0.447383328	0.654598312	0.999579949
85.15603449	0.065230979	0.164067985	0.397585056	0.690936078	0.999579949
2600.726118	-0.097205689	0.094966266	-1.023581242	0.306033109	0.999579949
137.4521787	0.102711187	0.180853264	0.567925539	0.57008553	0.999579949
71.11381093	0.124612532	0.143890548	0.866023057	0.386477518	0.999579949
408.5144765	-0.116202099	0.096717988	-1.201452815	0.229575599	0.999579949
457.1581285	0.068945989	0.093116089	0.740430464	0.45903884	0.999579949
264.7228095	-0.158203026	0.095841914	-1.650666379	0.098806717	0.894181927
1740.551929	0.07278349	0.122368106	0.594791341	0.551982956	0.999579949
65.52121045	0.641993579	0.367717021	1.745890296	0.080830016	0.85448976
31.16332413	0.272275285	0.319149723	0.853127124	0.393588811	0.999579949
160.8769085	0.026612707	0.105185711	0.253006866	0.800262907	0.999579949
443.2648144	0.007366066	0.060695749	0.121360489	0.903405511	0.999579949
123.7658833	0.613487219	0.296246718	2.070865874	0.038371332	0.72680496
187.5651991	-0.012720039	0.145112634	-0.087656316	0.930149841	0.999579949
194.4760082	0.069114832	0.101168154	0.68316787	0.494500773	0.999579949
3350.074337	0.04435955	0.132124428	0.335740713	0.737066401	0.999579949
355.5301081	-0.041635562	0.083831258	-0.496659153	0.619429432	0.999579949
20.66938985	0.115807423	0.204952792	0.565044378	0.57204358	0.999579949
87.41156031	0.071393612	0.150789114	0.473466621	0.63588031	0.999579949
528.6128716	0.015250232	0.078235158	0.194928113	0.845449225	0.999579949
171.5105493	0.052935863	0.099934982	0.529703035	0.596317843	0.999579949
479.3023249	-0.053480859	0.106376772	-0.502749409	0.615140467	0.999579949
82.16479095	0.26007642	0.198427249	1.31068904	0.189962843	0.984512516
173.8777653	0.064315744	0.116027254	0.554315836	0.579362715	0.999579949
25409.82354	0.155388518	0.188810057	0.822988566	0.410514496	0.999579949
134.1610625	0.082961689	0.119602416	0.693645598	0.48790449	0.999579949
698.1246042	-0.066122098	0.058396782	-1.132290095	0.257512493	0.999579949
914.5012841	-0.090705246	0.18472003	-0.491041747	0.623396921	0.999579949
422.189894	0.010270228	0.125163301	0.082054624	0.934603276	0.999579949
39.39598776	-0.088200591	0.155043444	-0.568876623	0.569439871	0.999579949
180.2427649	0.05831574	0.100810698	0.578467774	0.562948349	0.999579949
161.8747125	0.263609113	0.229092755	1.150665431	0.249869903	0.999579949
278.3222654	0.033737192	0.135561741	0.248869572	0.803461673	0.999579949
145.8743479	-0.132874232	0.093286582	-1.424365958	0.154340563	0.975450945
15845.60497	0.11899568	0.10532087	1.1298395	0.258543861	0.999579949
32.46520179	-0.033395197	0.25568584	-0.130610271	0.896083617	0.999579949
32.15371715	-0.372363561	0.284126988	-1.310553296	0.190008727	0.984512516
377.3225721	-0.111228735	0.134057647	-0.829708246	0.406703758	0.999579949
322.33125	-0.1271228	0.078334615	-1.622817715	0.104628378	0.895628172
37.71923565	-0.31512216	0.159404595	-1.976869981	0.048056329	0.770705547
689.1064647	0.114681969	0.072245617	1.587389992	0.112424345	0.914735409
457.6153464	0.192782102	0.118108669	1.632243466	0.102628208	0.895628172
1246.917148	0.00991611	0.034346155	0.288710913	0.772802611	0.999579949
62.41233012	0.119908734	0.160248271	0.748268501	0.45429822	0.999579949
628.4985385	-0.010030093	0.080801823	-0.124132017	0.901210748	0.999579949

139.0864553	0.087019802	0.091372266	0.952365583	0.340911608	0.999579949
24.84414574	0.16570466	0.215246744	0.769835848	0.441397272	0.999579949
331.5282512	-0.03451755	0.077627794	-0.444654532	0.656569432	0.999579949
352.6540547	0.034109797	0.083695905	0.407544395	0.683608194	0.999579949
675.3562391	-0.001957469	0.070926484	-0.027598561	0.977982329	0.999579949
38.43153575	0.379964668	0.317240861	1.197716671	0.231027337	0.999579949
269.3585235	0.144806108	0.214548952	0.674932722	0.49971851	0.999579949
272.9823184	-0.034240936	0.213943228	-0.160046834	0.872844181	0.999579949
724.3476178	-0.076448086	0.084813232	-0.901369795	0.367391735	0.999579949
444.9912737	0.029853743	0.08809605	0.338877198	0.734702242	0.999579949
81.24197093	0.124411422	0.153793328	0.808952014	0.418542748	0.999579949
90.85130252	-0.102318511	0.203686189	-0.502334066	0.61543255	0.999579949
65.49912197	0.023967429	0.136842839	0.175145656	0.860965184	0.999579949
406.5489517	0.051972469	0.132135243	0.393327837	0.694077361	0.999579949
1011.580411	-0.040481337	0.074678891	-0.542072013	0.587768898	0.999579949
65.20705153	0.093691939	0.141593689	0.661695723	0.508166246	0.999579949
87.39637415	0.107269432	0.13651119	0.785792227	0.431989221	0.999579949
268.5834003	0.022142531	0.124407337	0.177984126	0.858735442	0.999579949
57.73808657	-0.018521006	0.159632461	-0.116022807	0.907634466	0.999579949
682.5634214	0.015176152	0.074875408	0.202685402	0.839380934	0.999579949
708.2093413	0.094548739	0.095160436	0.993571933	0.320431314	0.999579949
56.40327513	-0.005670626	0.15300315	-0.037062155	0.970435447	0.999579949
63.57291207	-0.208790206	0.148689633	-1.404201496	0.160258857	0.978261482
166.1547643	0.149129369	0.117456309	1.269658233	0.204206397	0.994814558
188.4277153	-0.17551159	0.086391762	-2.031577848	0.042196411	0.74969123
259.6805196	-0.11999016	0.115879286	-1.03547549	0.300446907	0.999579949
99.08796731	-0.03178556	0.20778623	-0.152972409	0.878420033	0.999579949
267.659688	0.224504211	0.113883175	1.971355399	0.048683239	0.773861498
146.3228255	0.209322982	0.225342694	0.928909556	0.35293596	0.999579949
1849.559539	-0.019677073	0.110724975	-0.177711241	0.858949756	0.999579949
1749.929658	-0.047442409	0.108130426	-0.438751706	0.660841457	0.999579949
417.083589	-0.134055975	0.102712306	-1.305159817	0.191838434	0.984804292
229.2797339	0.011655195	0.13966615	0.083450393	0.93349342	0.999579949
200.4734047	0.090649009	0.114670316	0.790518518	0.429225012	0.999579949
373.8316284	0.132616953	0.07962769	1.665462758	0.095820468	0.88463985
131.0948238	0.305827905	0.242696764	1.260123542	0.207624799	0.995145345
957.0863369	0.008672914	0.092544566	0.093716076	0.925334699	0.999579949
314.1243763	0.096959784	0.088231505	1.098924744	0.271800893	0.999579949
185.9689074	0.012364306	0.097732553	0.126511649	0.899326928	0.999579949
398.4100481	0.100376122	0.102004275	0.984038387	0.325096639	0.999579949
67.81403298	-0.081171102	0.202528628	-0.400788289	0.688576002	0.999579949
186.5575264	0.135188709	0.13566079	0.996520135	0.318997489	0.999579949
193.7199599	0.062192265	0.101936968	0.610105111	0.541792181	0.999579949
347.1425251	0.073608597	0.092172095	0.798599584	0.424522629	0.999579949
205.9324408	-0.024040977	0.088326354	-0.272183508	0.785480922	0.999579949
200.1395794	-0.011305433	0.170378826	-0.066354683	0.947095449	0.999579949
27.26996432	0.043045419	0.40136034	0.107248809	0.914591595	0.999579949
104.6129458	0.148359309	0.143505703	1.033821693	0.301219528	0.999579949

192.5719634	-0.135005289	0.116737849	-1.156482586	0.247483819	0.999579949
354.6907534	0.089091034	0.105241027	0.846542804	0.397250006	0.999579949
899.3126001	-0.015248889	0.112894631	-0.13507187	0.892555051	0.999579949
125.625824	0.02095208	0.183335298	0.114282848	0.909013579	0.999579949
20.83943143	-0.061378063	0.570871893	-0.107516351	0.914379354	0.999579949
158.3785329	-0.224195767	0.205938106	-1.08865606	0.276305585	0.999579949
60.10878072	0.297308497	0.188419748	1.577905181	0.114587395	0.918818353
679.8322075	0.112558918	0.078822564	1.428003762	0.153290776	0.973156976
163.105846	0.074393164	0.110308961	0.674407255	0.500052432	0.999579949
25.31229011	-0.00743462	0.241524402	-0.030782068	0.975443342	0.999579949
253.1173333	0.094530047	0.082195821	1.150059035	0.250119557	0.999579949
455.4127981	-0.014585532	0.102916191	-0.141722428	0.887299259	0.999579949
937.7250694	-0.102320144	0.084018396	-1.217830243	0.223288489	0.999579949
79.46066963	0.019389845	0.140865393	0.137648041	0.890518585	0.999579949
94.04093799	-0.011478057	0.117667864	-0.097546236	0.922292618	0.999579949
30.56436237	0.340840833	0.237056178	1.437806162	0.15048906	0.96651114
162.9308145	-0.020240955	0.163000977	-0.124176893	0.901175217	0.999579949
376.8257835	0.261303558	0.11744108	2.224975768	0.026082865	0.648237123
763.889654	0.021023805	0.054273102	0.387370607	0.69848186	0.999579949
164.9193063	0.016825907	0.103757743	0.16216531	0.871175677	0.999579949
276.5968616	0.009977756	0.094306407	0.105801469	0.915739871	0.999579949
168.5504397	-0.029073471	0.171176571	-0.169844918	0.8651321	0.999579949
510.9123207	0.179083535	0.101531149	1.763828515	0.077760855	0.851207389
40.62098074	-0.230754991	0.216549879	-1.065597415	0.286605668	0.999579949
384.0575544	0.044362371	0.091016392	0.487410784	0.625967253	0.999579949
28.14801908	0.304955006	0.269639633	1.130972486	0.258066672	0.999579949
78.42091517	0.050780768	0.156838391	0.323777664	0.746106368	0.999579949
242.6584681	0.079838694	0.081174356	0.983545768	0.325338902	0.999579949
417.5716462	0.063121601	0.092195251	0.684651329	0.493563969	0.999579949
1396.924329	-0.008525794	0.116622704	-0.073105783	0.94172194	0.999579949
28.9707558	0.057440763	0.193771994	0.296434802	0.766898051	0.999579949
302.2860222	0.018464475	0.087323227	0.211449754	0.832536336	0.999579949
846.4895257	-0.056107904	0.091517104	-0.613086529	0.539819127	0.999579949
244.9510017	-0.089388028	0.227249195	-0.393348051	0.694062433	0.999579949
229.4755107	-0.217426484	0.121277309	-1.792804331	0.073004217	0.838669905
583.1962251	0.091510086	0.106918094	0.855889613	0.392058841	0.999579949
232.9309007	0.441033174	0.205416674	2.147017402	0.031791896	0.689986698
282.8362037	0.007065103	0.126066646	0.056042601	0.955307869	0.999579949
1594.563955	0.022832004	0.117116752	0.194950798	0.845431466	0.999579949
60.50075084	-0.151742834	0.151651786	-1.000600373	0.317020049	0.999579949
2342.892829	-0.056412996	0.139568456	-0.404195886	0.686068679	0.999579949
292.384422	-0.066267123	0.137308669	-0.482614272	0.629369643	0.999579949
93.19717336	-0.150063566	0.150398764	-0.997771276	0.318390282	0.999579949
85.35357953	0.183591728	0.16630229	1.10396392	0.269608789	0.999579949
31.33289941	-0.167424076	0.205622733	-0.814229409	0.415513541	0.999579949
90.98497384	0.160280021	0.119488775	1.341381401	0.179796653	0.983992835
76.49983111	0.173662441	0.195537594	0.888128149	0.374471822	0.999579949
275.1056101	0.067342697	0.090818511	0.741508494	0.458385184	0.999579949

166.2810305	-0.079721645	0.09067459	-0.879206017	0.379289583	0.999579949
4497.960827	-0.043702169	0.082350486	-0.530685017	0.59563707	0.999579949
956.3175912	0.083776019	0.092873368	0.902045667	0.367032609	0.999579949
32.23299298	-0.081798904	0.257606182	-0.317534707	0.750837909	0.999579949
244.0526561	-0.050189943	0.083140356	-0.603677265	0.546058232	0.999579949
39.96022182	-0.112763768	0.168326723	-0.66991008	0.502915113	0.999579949
135.807838	-0.302980501	0.1200258	-2.524294782	0.011593067	0.493624274
203.6260286	0.079329093	0.127775149	0.620849149	0.534698881	0.999579949
636.7018187	-0.071418605	0.076016941	-0.93950906	0.347469443	0.999579949
364.4939269	-0.146850867	0.069803033	-2.103789189	0.03539684	0.712820067
183.575361	-0.000389681	0.085539341	-0.004555577	0.996365188	0.999579949
1561.92494	-0.156838009	0.085457241	-1.835280508	0.066464138	0.822978752
46.99466987	-0.07145591	0.214664231	-0.332872924	0.739230209	0.999579949
695.9731033	-0.174537715	0.096154956	-1.81517129	0.06949759	0.831457432
939.7584405	0.042944209	0.106112275	0.40470539	0.68569408	0.999579949
352.255007	0.078901066	0.121812359	0.647726277	0.517162006	0.999579949
183.1793319	-0.084353552	0.092170682	-0.915188543	0.360092658	0.999579949
202.1071406	0.200445728	0.160374201	1.249862679	0.211349715	0.996937328
398.6593687	0.187023714	0.257931247	0.725091342	0.468395996	0.999579949
428.3530409	0.070072296	0.146949841	0.476844995	0.633472503	0.999579949
140.6205226	0.113126282	0.586849934	0.192768672	0.84714014	0.999579949
2198.47909	-0.099765295	0.110821572	-0.900233529	0.367995986	0.999579949
60.54009648	-0.080619809	0.215743134	-0.373684238	0.708639254	0.999579949
862.5326857	-0.122657842	0.078378106	-1.564950324	0.117594557	0.920584864
761.349895	0.140483873	0.077471763	1.813355825	0.069776958	0.833880992
714.7244965	-0.046657559	0.089493888	-0.521349112	0.602123594	0.999579949
220.6326869	-0.098678161	0.112780917	-0.874954415	0.381598709	0.999579949
36.04661121	-0.135629431	0.202491569	-0.669802857	0.502983472	0.999579949
923.6645561	0.002338873	0.119378871	0.01959202	0.98436883	0.999579949
141.056353	0.593932803	0.278648652	2.131475601	0.03304998	0.695900535
26.50418487	0.065660913	0.347897139	0.188736571	0.850299287	0.999579949
555.2140795	0.141280432	0.21012457	0.672365123	0.50135128	0.999579949
92.89767118	-0.65043517	0.31767049	-2.04751524	0.040607522	0.741293258
128.6751399	0.028690095	0.193453214	0.148305085	0.882102003	0.999579949
176.6234123	-0.033165025	0.120128686	-0.276079146	0.782487278	0.999579949
637.4404625	-0.002936279	0.107749601	-0.027250953	0.978259576	0.999579949
56.4538909	-0.140569214	0.157679889	-0.891484735	0.372669177	0.999579949
46.44272852	-0.198004955	0.151796713	-1.304408709	0.192094267	0.984804292
733.6780808	-0.061369577	0.097933244	-0.626647038	0.530890628	0.999579949
28.55453221	0.338919807	0.466304109	0.726821405	0.467335369	0.999579949
96.43840491	0.142206811	0.177357901	0.801807029	0.422664592	0.999579949
506.1438456	-0.040590596	0.059245968	-0.685119972	0.493268218	0.999579949
52.50201082	0.061524518	0.257741696	0.238706114	0.811333476	0.999579949
936.7026553	0.298461347	0.103637852	2.87984883	0.003978659	0.325423296
312.4654356	0.050594939	0.079865124	0.633504795	0.52640405	0.999579949
200.2497316	0.024101312	0.115115292	0.209366732	0.834161962	0.999579949
46.90012033	0.012113012	0.168554926	0.071863887	0.94271023	0.999579949
202.5780903	0.216210206	0.105095433	2.057274996	0.039659781	0.73515901

377.1238663	-0.077187068	0.137824726	-0.560037886	0.575453596	0.999579949
43.97772158	0.209434445	0.329298635	0.63600156	0.524775406	0.999579949
541.9966981	-0.112607262	0.227108093	-0.495831131	0.620013559	0.999579949
138.7909387	0.082866865	0.151521608	0.546898003	0.584448808	0.999579949
553.5397628	-0.063505765	0.095886892	-0.662298714	0.507779801	0.999579949
72.5774556	0.138065774	0.428287852	0.322366776	0.747174851	0.999579949
1090.363817	0.206632822	0.148827582	1.388404078	0.165014035	0.978789551
26.50383389	-0.001513443	0.41323732	-0.003662407	0.997077829	0.999579949
50.35135275	-0.256390272	0.301942243	-0.849136806	0.395805172	0.999579949
378.3266776	-0.036203756	0.129562444	-0.279430941	0.779914129	0.999579949
257.4219792	-0.10387405	0.090079023	-1.153143608	0.248851445	0.999579949
482.4766868	0.05220545	0.104490386	0.499619649	0.61734292	0.999579949
83.25159475	-0.30208034	0.201380936	-1.500044375	0.133602908	0.943308426
2970.338161	-0.036317185	0.089572437	-0.405450452	0.685146432	0.999579949
139.1958088	0.221478083	0.104344267	2.122570695	0.033789843	0.698878259
31.76119865	0.135282802	0.233096241	0.580373159	0.561663001	0.999579949
4478.362843	0.093735353	0.137092035	0.683740331	0.494139151	0.999579949
396.9772233	0.002825021	0.059133624	0.047773505	0.961896752	0.999579949
211.4175973	0.108933539	0.161882764	0.672916228	0.501000587	0.999579949
266.59847	-0.023819075	0.616054395	-0.038663915	0.969158344	0.999579949
608.5657618	0.037661961	0.072450673	0.519829002	0.603182763	0.999579949
370.1070691	0.026118908	0.068518149	0.381196926	0.70305713	0.999579949
70.07015979	0.150996646	0.14689547	1.02791901	0.303987926	0.999579949
21.56744004	0.13623043	0.230155274	0.591906621	0.55391312	0.999579949
10064.28603	-0.11260493	0.103456996	-1.088422572	0.276408601	0.999579949
24.97850075	0.356824248	0.262988242	1.356806845	0.174842592	0.983992835
120.093493	-0.010394731	0.101005263	-0.102912766	0.918032206	0.999579949
292.2125841	-0.022842943	0.076572716	-0.298316998	0.765461232	0.999579949
114.9618782	0.164482746	0.113891371	1.444207274	0.148680679	0.962301096
2626.950101	0.105539026	0.104837126	1.006695148	0.314081295	0.999579949
27.95307937	0.063390944	0.267341163	0.237116287	0.812566577	0.999579949
415.6865359	-0.094714403	0.120133919	-0.788406833	0.430458775	0.999579949
28.79268695	-0.341062084	0.619545861	-0.550503369	0.581974167	0.999579949
1318.605553	-0.093950604	0.100643972	-0.933494596	0.35056466	0.999579949
178.0296703	-0.105023582	0.16979106	-0.618546004	0.536215483	0.999579949
2608.532011	-0.005787714	0.093703021	-0.061766564	0.950748731	0.999579949
420.6357337	0.253099433	0.207864631	1.217616637	0.223369689	0.999579949
299.9543423	-0.133734137	0.109623053	-1.219945385	0.222485579	0.999579949
145.8263817	0.027258318	0.145044343	0.187930929	0.850930797	0.999579949
159.9777017	0.204659192	0.115508266	1.771814245	0.076425397	0.851207389
388.4201404	0.004625272	0.099793999	0.046348195	0.963032727	0.999579949
224.942428	-0.044625008	0.239034794	-0.186688335	0.851905003	0.999579949
660.2509247	0.043996548	0.127539398	0.344964371	0.730121156	0.999579949
97.20950976	-0.385027028	0.1365733	-2.819196936	0.004814397	0.346526541
558.7396576	-0.059835851	0.098634402	-0.606642809	0.544087984	0.999579949
150.1755336	0.09616159	0.113905629	0.84422158	0.398545598	0.999579949
37.30956143	-0.03437016	0.235578176	-0.145897047	0.884002662	0.999579949
183.5704823	-0.160084563	0.148065516	-1.081173845	0.27961979	0.999579949

472.8046475	-0.088053875	0.14530865	-0.605978203	0.544529227	0.999579949
33.06313401	0.515753068	0.299114054	1.72426892	0.084659307	0.861032139
201.9841843	0.053155772	0.092500481	0.574654007	0.565525319	0.999579949
213.5321204	0.20797903	0.134104672	1.550870872	0.120932632	0.921837625
92.59740178	0.211485028	0.199701383	1.059006329	0.289596897	0.999579949
260.1348468	0.048106948	0.135238937	0.355718174	0.722051633	0.999579949
1390.049995	0.140154061	0.130211843	1.076354176	0.281768888	0.999579949
54.47397843	-0.044771593	0.154418114	-0.289937442	0.771864097	0.999579949
71.51348119	-0.006008483	0.165876356	-0.03622266	0.971104817	0.999579949
1410.694741	0.087967632	0.088463737	0.994391994	0.320032064	0.999579949
627.4201073	0.09690072	0.204228431	0.474472232	0.635163195	0.999579949
147.7966522	0.156343701	0.122176774	1.279651565	0.200667706	0.993204353
1120.389218	0.101945083	0.103236701	0.987488769	0.323403086	0.999579949
4646.300923	-0.090843127	0.104366797	-0.870421712	0.384069985	0.999579949
244.1278026	-0.131517655	0.132502801	-0.992565097	0.320921941	0.999579949
57.60391348	0.081277684	0.220447696	0.368693735	0.712356019	0.999579949
52.5068007	0.09405576	0.146125701	0.643663361	0.519793753	0.999579949
168.4844078	-0.098059579	0.17006934	-0.576585874	0.564219244	0.999579949
526.7164004	-0.069078033	0.064970798	-1.063216638	0.287683719	0.999579949
192.1204181	-0.188112562	0.1021328	-1.841842791	0.065498149	0.819279724
355.558869	-0.265088474	0.110257455	-2.404268026	0.016204887	0.560760331
22.21310169	0.391592088	0.265921254	1.472586646	0.140862528	0.948318761
146.8777708	0.082894806	0.129516481	0.640032882	0.522151222	0.999579949
89.75264751	-0.022309137	0.13981926	-0.159556965	0.873230082	0.999579949
455.9626099	-0.139314876	0.121320876	-1.148317429	0.250837547	0.999579949
370.4114983	0.030477049	0.087812286	0.347070446	0.728538396	0.999579949
60.96487095	0.067106006	0.212350954	0.31601462	0.751991411	0.999579949
609.7464125	-0.012407582	0.07921995	-0.156621933	0.875542816	0.999579949
159.5421572	-0.115370807	0.123575516	-0.933605705	0.350507322	0.999579949
3676.968248	-0.044835459	0.116153934	-0.386000352	0.699496408	0.999579949
173.408062	0.010549475	0.111797344	0.094362484	0.924821216	0.999579949
2901.582221	0.134762123	0.087435687	1.541271385	0.12325075	0.92236461
243.028166	-0.019007272	0.155774626	-0.122017768	0.902884947	0.999579949
546.7224769	0.084426969	0.108845518	0.775658664	0.437950552	0.999579949
1358.228611	-0.15653516	0.086809957	-1.803193599	0.071357815	0.834620594
458.3457802	-0.048638314	0.084828208	-0.57337429	0.566391294	0.999579949
44.97108871	-0.000599137	0.269187572	-0.002225723	0.998224131	0.999644252
51.99018135	-0.035911334	0.334812324	-0.1072581	0.914584224	0.999579949
1141.296745	0.087117684	0.062566635	1.392398429	0.16380178	0.978498637
416.5657574	-0.129673579	0.121524318	-1.06705869	0.285945335	0.999579949
605.5244114	0.026043685	0.072633173	0.35856461	0.719920825	0.999579949
117.7521464	-0.216242938	0.130908623	-1.651861687	0.09856275	0.893805902
44.8487319	0.07767862	0.199451237	0.389461709	0.696934631	0.999579949
80.11950823	0.481027146	0.274665427	1.751320327	0.079890748	0.854007036
4104.602759	-0.057808729	0.125722706	-0.459811367	0.645651623	0.999579949
99.9003244	0.010104092	0.128040053	0.078913529	0.937101403	0.999579949
3463.856412	-0.084641023	0.122431046	-0.691336277	0.48935424	0.999579949
47.05718055	-0.004982734	0.234793945	-0.02122173	0.983068781	0.999579949

403.9931533	0.015356578	0.104764606	0.146581737	0.88346217	0.999579949
31.87964374	0.092516043	0.229772618	0.402641725	0.68721181	0.999579949
27.98879308	0.493548719	0.262176325	1.882506821	0.059767235	0.798635337
3501.628017	0.281956482	0.165702026	1.70158742	0.088832737	0.870840008
31.82488154	-0.245178603	0.745214332	-0.329004143	0.742152556	0.999579949
59.55001909	0.892496669	0.821089867	1.086965879	0.27705189	0.999579949
46.39379113	0.096161949	0.352391957	0.272883497	0.784942773	0.999579949
707.5220385	0.116098708	0.135353917	0.85774177	0.39103507	0.999579949
342.6568234	-0.015155348	0.065822086	-0.230247158	0.817899719	0.999579949
521.2468391	0.121164205	0.141761384	0.854705291	0.39271432	0.999579949
81.86681355	-0.074378296	0.214522921	-0.346714912	0.728805506	0.999579949
297.4326332	-0.100473224	0.153417081	-0.654902467	0.512530552	0.999579949
88.39676851	-0.232248911	0.624499559	-0.371896037	0.709970256	0.999579949
733.1625437	-0.043657159	0.090989602	-0.479803821	0.631366896	0.999579949
218.478362	0.059805479	0.187459388	0.319031657	0.749702507	0.999579949
2815.578215	0.045795332	0.069473232	0.659179525	0.509780493	0.999579949
481.610411	-0.129162513	0.100033461	-1.29119308	0.196636734	0.991527744
33.19475434	0.696581066	0.341707962	2.038527467	0.041497211	0.746712667
204.3331549	-0.234480399	0.082980573	-2.825726439	0.004717353	0.3435258
344.7907719	0.05939602	0.080256594	0.740076512	0.45925357	0.999579949
362.5513607	-0.097857421	0.072112905	-1.357002882	0.174780295	0.983992835
1565.437046	0.025995996	0.072266458	0.35972423	0.719053371	0.999579949
91.0194203	-0.190243448	0.347865178	-0.54688845	0.584455371	0.999579949
820.2930245	0.151745689	0.136152414	1.114528085	0.265052703	0.999579949
29.89205116	0.397234173	0.318418024	1.247524144	0.212205373	0.999268361
312.0146606	-0.07675848	0.107243903	-0.715737474	0.474153467	0.999579949
24.99465829	-0.072080272	0.359304618	-0.200610481	0.841003162	0.999579949
759.7889418	0.035094986	0.122981863	0.285367167	0.775362859	0.999579949
150.8474483	0.075150824	0.09817315	0.76549264	0.443978247	0.999579949
47.2468735	0.235091619	0.171067138	1.374265228	0.169359357	0.981013142
138.6816983	0.210671769	0.178645935	1.179269874	0.23829073	0.999579949
26.20516159	0.339970174	0.255081049	1.332792753	0.182599824	0.983992835
217.544204	0.083280376	0.130193848	0.639664447	0.522390774	0.999579949
184.7423805	-0.500127722	0.228014709	-2.193401135	0.028278491	0.666721367
513.5089141	-0.040050297	0.065063939	-0.615552918	0.538189633	0.999579949
1846.082797	-0.545297785	0.474016985	-1.150376045	0.249989022	0.999579949
23.95295769	-0.579694875	0.852359376	-0.680106176	0.496437234	0.999579949
455.8960733	-0.002567144	0.119936437	-0.021404205	0.982923219	0.999579949
1131.415528	-0.011457045	0.070166892	-0.163282781	0.870295791	0.999579949
166.1409709	0.122242221	0.168220719	0.726677557	0.467423505	0.999579949
963.6240893	0.024481881	0.085365311	0.286789572	0.774273448	0.999579949
1336.544991	-0.219214617	0.115139107	-1.903911044	0.056921771	0.789632843
572.0076492	-0.034152906	0.084872352	-0.402403194	0.68738732	0.999579949
912.2726661	-0.047096483	0.074421968	-0.632830391	0.526844408	0.999579949
35.36155484	-0.062694519	0.374584546	-0.16737081	0.867078291	0.999579949
3311.004903	-0.176380852	0.093429441	-1.887850879	0.059045973	0.79629879
2559.246809	0.417563905	0.168943116	2.471624265	0.01345008	0.516515866
392.6447734	0.150794863	0.119482688	1.262064537	0.206925565	0.994814558

69.19220811	-0.207068269	0.161209732	-1.284465067	0.198979279	0.992481822
2724.095725	0.08004609	0.094246402	0.849327808	0.395698912	0.999579949
190.5777408	0.213336526	0.118166166	1.805394335	0.071012997	0.834620594
32.28631361	-0.067681297	0.38527824	-0.17566862	0.86055429	0.999579949
1984.1212	-0.100121162	0.072467153	-1.381607489	0.167092253	0.978999491
133.7841856	-0.173459308	0.277151888	-0.625863707	0.531404341	0.999579949
988.2244632	0.175420242	0.130390065	1.345349762	0.178512311	0.983992835
5116.300441	-0.069868688	0.101927837	-0.685472095	0.493046064	0.999579949
729.1402534	-0.142837305	0.167656379	-0.851964628	0.394233725	0.999579949
1117.251403	-0.02977077	0.075413958	-0.39476472	0.693016534	0.999579949
109.0484393	-0.034705612	0.12628027	-0.274830041	0.783446817	0.999579949
35.03074328	-0.158238797	0.216232983	-0.731797689	0.46429206	0.999579949
312.2795148	-0.212986566	0.160233537	-1.329225896	0.183773454	0.983992835
295.4569823	-0.158575901	0.106419063	-1.490108039	0.136195831	0.945791454
140.8018337	-0.105201784	0.108620613	-0.968525044	0.332782218	0.999579949
2123.878995	0.063258642	0.107086223	0.590726246	0.554703859	0.999579949
362.4874862	-0.16182394	0.117866493	-1.372942688	0.169770164	0.981013142
168.4945785	-0.284661537	0.124419515	-2.287917112	0.022142349	0.627784819
67.93875783	0.038239528	0.165401961	0.231191501	0.817166032	0.999579949
58.71875387	-0.13309074	0.151762757	-0.876965748	0.380505242	0.999579949
105.5302324	-0.017070686	0.100991858	-0.169030321	0.865772792	0.999579949
75.09617292	-0.499099693	0.214249831	-2.329521987	0.01983143	0.601919728
1219.781491	0.039006831	0.086511402	0.450886593	0.652071287	0.999579949
2671.190168	0.090745481	0.11590473	0.782931645	0.433667253	0.999579949
151.8896198	-0.031184856	0.155998058	-0.199905413	0.841554557	0.999579949
35.97984262	0.217520992	0.20768041	1.047383294	0.294922833	0.999579949
442.7230023	0.089501401	0.077140517	1.160238541	0.245951699	0.999579949
376.4674623	-0.048726739	0.087354233	-0.557806271	0.57697668	0.999579949
2047.18479	0.113092901	0.199994276	0.565480686	0.571746858	0.999579949
886.9206043	0.108312363	0.129535413	0.836160245	0.40306476	0.999579949
607.1855277	-0.030719697	0.070129781	-0.438040678	0.661356796	0.999579949
74.92299853	0.08887578	0.153576716	0.578706087	0.562787508	0.999579949
361.7164966	0.038236593	0.1286935	0.29711363	0.766379759	0.999579949
4031.635941	-0.002829879	0.055174131	-0.051289965	0.959094465	0.999579949
344.283832	-0.086266892	0.26629108	-0.323957121	0.745970498	0.999579949
552.7071013	0.144744331	0.126800789	1.141509703	0.253657876	0.999579949
342.7983801	0.165141076	0.078586811	2.101384106	0.035607262	0.713050989
136.2630304	-0.208036829	0.123029547	-1.690950143	0.090846325	0.877608448
146.830671	-0.075937641	0.11002747	-0.690169841	0.490087387	0.999579949
139.8896016	0.045580837	0.120124501	0.379446632	0.704356228	0.999579949
99.36732189	-0.115112492	0.144583242	-0.796167589	0.425934633	0.999579949
44.13811448	0.246698211	0.238430124	1.034677194	0.30081969	0.999579949
985.7087567	-0.02458311	0.099971449	-0.24590131	0.80575863	0.999579949
2528.545291	0.221055344	0.148009914	1.493517146	0.13530187	0.945791454
268.7951547	0.190934375	0.149461092	1.277485483	0.201430902	0.993208272
1154.930178	-0.007531328	0.109378325	-0.068855764	0.94510443	0.999579949
451.5243489	0.063231037	0.135398687	0.466998889	0.640500673	0.999579949
116.4044589	-0.054285559	0.11631932	-0.466694261	0.640718637	0.999579949

234.8517227	0.063771835	0.138781691	0.459511878	0.645866625	0.999579949
77.22532641	1.095889598	0.492340426	2.225877747	0.026022371	0.648237123
175.5541532	-0.075952376	0.10566768	-0.718785307	0.472273212	0.999579949
33.27334259	-0.412759518	0.216275018	-1.908493736	0.056327432	0.785214091
514.328706	-0.028134493	0.086904545	-0.32374018	0.746134748	0.999579949
1121.241306	-0.004045453	0.08576823	-0.047167266	0.962379916	0.999579949
50.82626001	0.079035587	0.142739131	0.553706519	0.579779713	0.999579949
943.8069905	0.138665078	0.075030177	1.848124096	0.064584393	0.817660046
102.2361048	0.039942805	0.12936501	0.308760502	0.757503717	0.999579949
292.1567949	-0.019573047	0.116186335	-0.168462554	0.8662194	0.999579949
124.57408	0.216518925	0.189885475	1.140260595	0.25417775	0.999579949
438.6632361	-0.153253904	0.242982774	-0.630719211	0.528224135	0.999579949
295.5802172	-0.056059781	0.078524843	-0.713911407	0.475281964	0.999579949
2101.786949	-0.33432737	0.163821957	-2.040797075	0.041271001	0.745838643
443.64536	0.020083291	0.194241678	0.103393315	0.917650817	0.999579949
364.6228314	-0.015685775	0.105121814	-0.149215224	0.881383807	0.999579949
43.94245529	0.277377998	0.211065802	1.314177829	0.188786368	0.983992835
518.5883721	-0.361829302	0.187551342	-1.929228005	0.053702563	0.781954831
772.5843974	-0.330265385	0.076304369	-4.328263091	1.5029E-05	0.022986064
942.8372916	-0.022444104	0.070773436	-0.317126108	0.751147915	0.999579949
63.38545466	0.018855441	0.187684882	0.100463293	0.919976523	0.999579949
149.885896	-0.093236872	0.116866856	-0.797804226	0.424984108	0.999579949
639.3682124	-0.030855056	0.080572361	-0.382948394	0.701758028	0.999579949
70.78541289	0.117653836	0.308740862	0.381076336	0.703146606	0.999579949
39.76034158	0.043728076	0.211042881	0.207199955	0.835853702	0.999579949
6498.787401	0.409887769	0.190390139	2.152883399	0.031327844	0.685079023
1034.447156	0.365674691	0.22490143	1.62593315	0.103963877	0.895628172
361.3852236	-0.053618892	0.091925416	-0.58328691	0.559700173	0.999579949
3279.249731	0.208007597	0.137995243	1.507353381	0.131720102	0.939285826
86.92688301	0.12909508	0.128748653	1.002690724	0.316010107	0.999579949
701.5275077	-0.024174384	0.103146856	-0.234368594	0.814698838	0.999579949
150.7523416	0.021638593	0.127636142	0.169533428	0.865377081	0.999579949
3429.117702	0.020227765	0.119919881	0.168677327	0.866050454	0.999579949
565.3685472	-0.062451797	0.08240347	-0.757878235	0.448523881	0.999579949
60.27643533	-0.31136908	0.255072552	-1.220707903	0.222196634	0.999579949
518.1717166	0.002728207	0.071276207	0.03827655	0.969467187	0.999579949
158.8479995	0.261408625	0.103004178	2.537844882	0.011153741	0.487909795
334.6679391	0.235074241	0.098417486	2.388541409	0.016915402	0.570162425
94.16519748	-0.117520305	0.134906491	-0.871124172	0.383686357	0.999579949
24.21799687	-0.386109844	0.209356562	-1.844269132	0.065143928	0.817776877
33.40130022	0.230471488	0.235418174	0.978987665	0.327586074	0.999579949
90.36084792	0.399384439	0.218819337	1.825178915	0.067974035	0.829912169
28.00127356	0.135606488	0.305654299	0.443659678	0.657288652	0.999579949
503.7193525	0.096539929	0.089699783	1.076255992	0.281812784	0.999579949
502.1913178	0.147857195	0.22191781	0.666270074	0.50523849	0.999579949
770.2566099	-0.102153889	0.065879066	-1.550627452	0.120990989	0.921837625
38.27569343	0.382817119	0.34079083	1.123319895	0.261301657	0.999579949
1401.9173	-0.01316763	0.09092554	-0.144817731	0.884854783	0.999579949

28.28897454	0.423959636	0.725308784	0.584522958	0.558868524	0.999579949
674.3581903	0.071222609	0.141059052	0.504913421	0.613619647	0.999579949
454.4986504	-0.01630289	0.190641541	-0.085515939	0.931851224	0.999579949
90.99905133	-0.069389872	0.120792146	-0.574456816	0.565658715	0.999579949
149.4551758	0.296639165	0.187994241	1.577916239	0.114584854	0.918818353
186.2067955	0.88206709	0.373720949	2.360229183	0.018263648	0.580931591
1046.335803	-0.035426661	0.069386745	-0.510568137	0.609653492	0.999579949
684.0626366	0.047411892	0.076202072	0.622186386	0.533819317	0.999579949
284.722426	0.029045555	0.103970647	0.279363034	0.779966237	0.999579949
2018.86606	-0.074221399	0.071311747	-1.040801857	0.297967518	0.999579949
603.2091224	-0.159984768	0.079952367	-2.001001024	0.04539228	0.761238375
145.0877654	0.012869644	0.260566594	0.049390998	0.960607702	0.999579949
613.4295468	-0.100914643	0.081000227	-1.245856307	0.212817153	0.999268361
10499.89603	0.09851162	0.103907058	0.948074389	0.343091585	0.999579949
88.83090038	-0.05256417	0.119332261	-0.440485831	0.659585272	0.999579949
176.8735704	0.007208654	0.134437979	0.053620665	0.957237392	0.999579949
1826.704997	0.012736883	0.126549831	0.100647172	0.919830549	0.999579949
663.3055455	0.013992325	0.252288543	0.055461595	0.955770726	0.999579949
313.1195681	0.268583386	0.189059082	1.420632023	0.155423764	0.976030424
407.3748541	-0.047125922	0.155380184	-0.303294283	0.761665603	0.999579949
283.5583996	-0.2716523	0.156009788	-1.741251649	0.081639477	0.85448976
70.75112522	-0.271024194	0.12500408	-2.168122781	0.030149346	0.677729599
157.5411185	-0.029139493	0.125881119	-0.23148422	0.816938643	0.999579949
1291.320179	-0.232805208	0.231213508	-1.006884115	0.313990467	0.999579949
87.21706553	0.047864522	0.122312648	0.391329291	0.695553851	0.999579949
72.09367343	0.164918522	0.312839774	0.527166094	0.598078252	0.999579949
134.5784781	-0.047887542	0.153695465	-0.311574199	0.755364145	0.999579949
1031.377403	0.165714305	0.136864249	1.210793222	0.225974664	0.999579949
59.55840861	0.068063276	0.167653901	0.405974901	0.684761042	0.999579949
75.40635568	0.285378164	0.133772485	2.133309882	0.032899314	0.695900535
206.9816762	0.047251029	0.093606985	0.504781015	0.613712651	0.999579949
88.28233056	-0.064942388	0.123180833	-0.527211798	0.598046517	0.999579949
60.78420079	-0.129780759	0.134953792	-0.961668117	0.336216343	0.999579949
50.37078715	0.279199535	0.177491981	1.573026187	0.115712755	0.918818353
670.4170962	0.05429539	0.104329596	0.520421743	0.602769658	0.999579949
627.5662241	0.034918618	0.059559158	0.586284621	0.557684264	0.999579949
205.3578235	0.041836202	0.15890808	0.263272966	0.792340195	0.999579949
133.8000976	0.136742395	0.211328205	0.647061736	0.517591989	0.999579949
48981.28729	0.120900372	0.113782143	1.062560162	0.287981461	0.999579949
192.9397897	0.104134545	0.146182661	0.712359073	0.476242453	0.999579949
1687.983168	-0.149895068	0.102746029	-1.45888916	0.144595617	0.955875831
1016.580981	0.115806268	0.099749056	1.160976071	0.245651631	0.999579949
212.2133045	-0.052114488	0.101864227	-0.511607362	0.608925832	0.999579949
344.5066377	0.03009902	0.078598174	0.382948075	0.701758265	0.999579949
4214.26268	0.016777541	0.121608333	0.137963743	0.890269073	0.999579949
350.1774975	-0.102759388	0.060218911	-1.706430532	0.08792795	0.870023926
1269.965333	0.052896862	0.151343841	0.349514467	0.726703112	0.999579949
50.88560987	0.175614528	0.187864196	0.934795092	0.349893909	0.999579949

614.824116	0.071376658	0.073540449	0.970576864	0.331759032	0.999579949
357.6458022	-0.020496289	0.083051757	-0.246789345	0.805071257	0.999579949
104.952847	0.272823719	0.213280306	1.279179142	0.200833979	0.993208272
476.7834647	0.031229141	0.054614856	0.57180671	0.567452931	0.999579949
308.0979656	-0.086026774	0.171027187	-0.503000579	0.614963865	0.999579949
120.1944406	0.088929392	0.130901147	0.679362969	0.496907907	0.999579949
70.29140652	-0.064526186	0.17177228	-0.375649587	0.707177421	0.999579949
98.88264212	0.186349146	0.137856105	1.351765635	0.176450301	0.983992835
733.0482559	0.073450804	0.060363404	1.216810172	0.223676449	0.999579949
443.5348722	-0.026534341	0.09028194	-0.293905299	0.768830269	0.999579949
1410.392096	-0.0025317	0.101507386	-0.02494104	0.980101993	0.999579949
54.97148536	0.175996274	0.167579567	1.050225136	0.293614615	0.999579949
375.4323356	0.002300343	0.121616195	0.018914775	0.984909093	0.999579949
495.0015355	-0.047928505	0.120761947	-0.396884167	0.69145288	0.999579949
92.44293974	0.08701003	0.124196212	0.700585211	0.483561911	0.999579949
190.429166	0.054125958	0.092154784	0.587337476	0.556977077	0.999579949
31.31340188	0.152490805	0.19949319	0.76439103	0.444634253	0.999579949
471.6568569	0.059074462	0.092396756	0.639356449	0.522591074	0.999579949
160.5274143	0.012253921	0.119181704	0.102817132	0.918108108	0.999579949
216.4495002	0.196460172	0.125060595	1.570919864	0.116201264	0.918907698
31.08510116	-0.025666409	0.228240375	-0.112453413	0.910463906	0.999579949
108.7452468	0.319490208	0.15317702	2.085758082	0.037000542	0.717947029
3240.844255	0.049177745	0.095274806	0.516167354	0.605737529	0.999579949
21.87872866	-0.037090945	0.253296108	-0.146433141	0.883579467	0.999579949
144.9140403	0.091520633	0.100214739	0.913245235	0.361113578	0.999579949
17028.91838	0.811770706	0.212433285	3.821297141	0.000132752	0.050670878
700.734414	0.088837026	0.177483795	0.500535987	0.616697723	0.999579949
2396.153622	-0.026806063	0.093842053	-0.28565086	0.775145545	0.999579949
50.83184576	-0.254058048	0.215455264	-1.179168442	0.23833111	0.999579949
43.23163491	0.066656667	0.459268425	0.14513662	0.884603006	0.999579949
3428.936189	0.144717847	0.249014341	0.581162701	0.561130804	0.999579949
1401.843144	0.030753163	0.099531627	0.308978805	0.75733765	0.999579949
48.75224375	0.147131018	0.268272171	0.548439361	0.583390256	0.999579949
575.4182478	-0.033132429	0.056758933	-0.583739467	0.55939561	0.999579949
79.89599521	0.148337321	0.131901753	1.12460462	0.260756615	0.999579949
56335.54988	-0.120960661	0.190538431	-0.634836031	0.525535361	0.999579949
113.1226011	0.411821557	0.454091902	0.906912357	0.364453146	0.999579949
150.836398	0.35256088	0.513312631	0.686834608	0.492186958	0.999579949
195.8620748	0.009775076	0.179363309	0.054498748	0.956537806	0.999579949
587.1023152	0.027372361	0.126901792	0.215697198	0.829223785	0.999579949
178.1178881	-0.293370227	0.190610733	-1.539106542	0.123778288	0.924045374
62.89896604	0.122440345	0.243869286	0.502073658	0.615615709	0.999579949
49.41478237	-1.019575411	0.511514418	-1.993248625	0.046234229	0.763868825
105.7944354	0.27802878	0.462107996	0.601653255	0.547404972	0.999579949
134.8298109	-0.43861155	0.302588589	-1.449531032	0.147189341	0.960339903
404.8744082	-0.148590769	0.135656441	-1.095346207	0.273364991	0.999579949
345.9766852	-0.276654999	0.20575904	-1.344558176	0.178767958	0.983992835
722.9835076	0.019249854	0.051077695	0.376873983	0.706267257	0.999579949

64.21680156	0.172640617	0.424594545	0.406601118	0.684300976	0.999579949
50.30518271	-0.254676878	0.190261085	-1.338565256	0.180712243	0.983992835
1346.321333	-0.321851767	0.178460886	-1.80348632	0.071311872	0.834620594
120.8810037	-0.122560129	0.102490106	-1.195824013	0.231765243	0.999579949
574.9629789	0.021796533	0.075770053	0.287666853	0.773601765	0.999579949
1563.095717	0.031290392	0.110227884	0.283870023	0.77650999	0.999579949
119.0238506	0.087718972	0.12698169	0.69080016	0.489691135	0.999579949
83.70089753	0.170922204	0.169557972	1.008045815	0.313432469	0.999579949
701.0149183	-0.031559128	0.06526496	-0.483553937	0.628702472	0.999579949
450.8272505	0.049792861	0.122883451	0.405203958	0.685327596	0.999579949
203.4786544	0.044524742	0.108852464	0.409037519	0.682512128	0.999579949
48.54044582	-0.155514269	0.293183717	-0.530432832	0.595811867	0.999579949
512.6074361	0.093091648	0.12419514	0.749559513	0.453520043	0.999579949
42.73905232	0.265058505	0.185217043	1.431069731	0.152410231	0.972669728
35.86954385	0.098750951	0.265496725	0.371947906	0.709931636	0.999579949
259.544019	0.058530416	0.078146576	0.748982479	0.453867766	0.999579949
82.107645	0.57994608	0.168571165	3.440363492	0.000580933	0.121957286
414.4358456	-0.203668734	0.101519211	-2.00620879	0.044833976	0.759209569
808.5293704	0.03250774	0.081984464	0.396510974	0.691728114	0.999579949
40.48177342	0.147649417	0.186087125	0.79344241	0.42752011	0.999579949
2851.643509	0.04584501	0.078993388	0.580365155	0.561668398	0.999579949
105.5877086	-0.269325214	0.129602727	-2.078082918	0.037701723	0.723919053
417.4142672	0.21881694	0.110409797	1.981861619	0.047494731	0.770705547
349.3265558	0.048237663	0.081669244	0.590646615	0.554757224	0.999579949
609.4768414	0.040344699	0.076686151	0.526101502	0.598817685	0.999579949
2406.462734	0.067629392	0.06477153	1.044122202	0.296428855	0.999579949
225.553569	0.035972465	0.135576364	0.265329916	0.79075532	0.999579949
88.43183035	0.040327546	0.119203783	0.338307607	0.735131392	0.999579949
452.1477664	-0.019865074	0.082585598	-0.240539209	0.809912271	0.999579949
143.3466059	0.311715393	0.431387563	0.722587806	0.469933162	0.999579949
529.7593959	-0.070917245	0.120614724	-0.587965074	0.556555736	0.999579949
640.075578	-0.187389426	0.106573256	-1.758315674	0.078693812	0.851207389
874.8556226	0.193954484	0.220409928	0.879971629	0.378874679	0.999579949
488.8178993	-0.088154488	0.136994784	-0.643487918	0.519907552	0.999579949
325.7557696	0.033197474	0.093706664	0.354270149	0.723136436	0.999579949
52.46743493	-0.086053439	0.194853986	-0.441630375	0.6587567	0.999579949
640.5612583	-0.018481747	0.065801874	-0.280869614	0.778810407	0.999579949
1584.807078	0.038499955	0.063035409	0.610767119	0.541353764	0.999579949
57.78735447	-0.051725624	0.155952191	-0.331676163	0.740133802	0.999579949
37.52111313	-0.150115416	0.498654555	-0.3010409	0.763383307	0.999579949
4550.838071	-0.051265206	0.064137443	-0.799302306	0.424115142	0.999579949
1658.774547	0.055303878	0.110928203	0.498555616	0.61809248	0.999579949
258.9263565	-0.006699594	0.099938767	-0.067036988	0.946552257	0.999579949
717.7489888	-0.420950867	0.098141573	-4.289220697	1.79301E-05	0.022986064
73.05079867	0.366930069	0.267868723	1.369813039	0.170745271	0.981609963
163.8400622	0.008841698	0.096505257	0.091618825	0.927000894	0.999579949
783.2698139	0.040707855	0.078073589	0.521403659	0.602085603	0.999579949
116.2744823	-0.197896334	0.103971559	-1.903369878	0.056992299	0.789741468

740.8080306	0.087824777	0.12592365	0.697444661	0.48552456	0.999579949
1733.215683	-0.049451022	0.085982134	-0.57513136	0.56520246	0.999579949
158.2293138	0.021133348	0.09137475	0.231282141	0.81709562	0.999579949
33.99299145	0.334619837	0.254561698	1.314494048	0.18868	0.983992835
385.3907514	0.239250541	0.12388953	1.931160301	0.053463232	0.781231069
968.1310844	-0.077639817	0.066299485	-1.171047061	0.241579861	0.999579949
4378.147241	0.054017046	0.122699725	0.440237708	0.659764952	0.999579949
2089.199222	0.120329468	0.124851154	0.963783386	0.335154538	0.999579949
323.4346226	0.22318606	0.144830855	1.541011819	0.123313909	0.92236461
18526.44912	-0.032876042	0.091115003	-0.360819193	0.718234614	0.999579949
124.413228	0.088224475	0.105515127	0.836131059	0.403081177	0.999579949
160.5180046	0.061080923	0.115788992	0.527519253	0.59783305	0.999579949
153.4430291	0.022320278	0.11109784	0.200906495	0.840771689	0.999579949
209.7368327	0.181366639	0.08923751	2.032403619	0.042112812	0.74969123
199.3067922	0.023711038	0.093309933	0.25411054	0.799410161	0.999579949
93.04507323	-0.155352212	0.143799086	-1.080342134	0.279989853	0.999579949
517.0170685	-0.148301148	0.187713696	-0.790039041	0.429504968	0.999579949
24.10488884	-0.237384731	0.342966141	-0.692152091	0.488841822	0.999579949
144.7607918	0.002666999	0.151468742	0.017607589	0.985951902	0.999579949
28.68315669	1.205808212	0.539178262	2.236381355	0.025326801	0.642289128
706.6243067	0.11072272	0.161908957	0.6838579	0.494064901	0.999579949
1528.899584	-0.064480657	0.113777844	-0.566724196	0.570901579	0.999579949
300.9756023	-0.008659908	0.084764216	-0.102164664	0.918625975	0.999579949
1093.896749	-0.024137472	0.107038117	-0.22550352	0.821587588	0.999579949
114.9302057	0.096649368	0.182232879	0.530361858	0.595861066	0.999579949
50.63297282	0.26731434	0.477041972	0.560358116	0.575235195	0.999579949
175.5939452	0.101620921	0.10679862	0.951519046	0.341340955	0.999579949
279.0353885	-0.129146156	0.094936279	-1.360345659	0.173720566	0.983601342
427.1509135	0.08469327	0.106482374	0.79537361	0.426396206	0.999579949
194.2593633	0.297113014	0.232495729	1.277928908	0.201274494	0.993208272
701.0308808	-0.111468842	0.136076003	-0.819166049	0.412691682	0.999579949
53.30215524	-0.073909309	0.226393906	-0.32646333	0.744073829	0.999579949
128.8755894	-0.09614973	0.170976707	-0.562355723	0.57387368	0.999579949
848.4640696	0.131866261	0.054282533	2.429257695	0.015129774	0.541405219
58.41933338	-0.012021633	0.145160207	-0.082816311	0.933997599	0.999579949
301.5953479	-0.019941854	0.12871293	-0.154932792	0.876874302	0.999579949
360.0819186	-0.362825432	0.277963177	-1.305300351	0.191790595	0.984804292
140.078158	-0.023296004	0.12362061	-0.188447576	0.850525807	0.999579949
21.05566759	0.684449044	0.428598671	1.596946257	0.110277687	0.909823133
258.1826566	0.236077717	0.103427361	2.282546074	0.022457121	0.627784819
113.7766762	-0.291814493	0.568653243	-0.513167728	0.607833998	0.999579949
2387.371682	-0.008907601	0.101079576	-0.088124638	0.929777615	0.999579949
718.5970321	0.187518159	0.120907627	1.550920842	0.120920655	0.921837625
35.1442193	-0.167986984	0.628626993	-0.267228398	0.789293313	0.999579949
21.92634477	0.1004844	0.339123111	0.296306552	0.766995983	0.999579949
116.303856	-0.108966801	0.113413082	-0.96079569	0.336654906	0.999579949
51.91147477	0.228732096	0.260564512	0.877832881	0.380034418	0.999579949
27.58586021	-0.085077403	0.188741755	-0.450760898	0.652161886	0.999579949

346.3803228	0.123982802	0.106172462	1.167749145	0.242907967	0.999579949
2119.482728	-0.016115981	0.12684999	-0.127047554	0.898902761	0.999579949
135.868362	-0.022413282	0.121965983	-0.183766665	0.854196508	0.999579949
184.4956544	0.166895291	0.178427514	0.935367464	0.349598959	0.999579949
377.3265028	0.061220951	0.094976001	0.644593902	0.519190388	0.999579949
60.60588816	-0.170329818	0.196058401	-0.86877082	0.384972497	0.999579949
531.4595044	0.468384497	0.129421482	3.61906301	0.000295672	0.079280286
136.5429358	0.009401022	0.134141173	0.070083047	0.94412756	0.999579949
77.70861997	0.42330935	0.192564158	2.198276946	0.027929376	0.663770609
140.5648088	0.224501506	0.130729522	1.717297693	0.085924793	0.864463816
126.3462267	-0.015911326	0.138479813	-0.11489997	0.908524409	0.999579949
81.08570583	0.254287002	0.411041842	0.618640187	0.536153421	0.999579949
310.6716597	-0.143549312	0.122480202	-1.172020536	0.241188811	0.999579949
173.8339268	0.070456598	0.230586747	0.305553544	0.759944596	0.999579949
339.0936657	0.098482301	0.058422612	1.685688096	0.091855891	0.880418343
123.1494479	0.122208414	0.219075892	0.557835975	0.576956395	0.999579949
906.5751575	0.038085383	0.04645926	0.819758705	0.412353676	0.999579949
129.0560196	-0.27142146	0.125051976	-2.170469189	0.02997132	0.676556901
63.67612184	0.008703766	0.151759374	0.057352414	0.954264469	0.999579949
80.21941536	0.187028059	0.196342183	0.952561778	0.340812151	0.999579949
761.5280471	0.135087097	0.136057229	0.992869675	0.32077347	0.999579949
27.7061893	0.205794711	0.173438358	1.186558229	0.235401921	0.999579949
616.2647397	0.023144287	0.098243979	0.235579694	0.813758832	0.999579949
267.3284386	0.023397434	0.097450302	0.240096066	0.810255784	0.999579949
425.1773466	0.051368146	0.068898812	0.745559242	0.455933712	0.999579949
166.0912264	-0.023644186	0.109832304	-0.215275338	0.829552656	0.999579949
1541.354682	0.070554656	0.145000663	0.486581609	0.626554859	0.999579949
45.31048503	-0.144351953	0.313944255	-0.459801224	0.645658904	0.999579949
498.5670733	0.055545258	0.15848722	0.350471529	0.725984853	0.999579949
258.6790065	0.175238905	0.101320027	1.729558407	0.083709203	0.860033538
1656.010901	-0.029957001	0.075451208	-0.397038054	0.691339399	0.999579949
1577.855324	0.031605867	0.05902386	0.535476114	0.59232067	0.999579949
281.0240395	-0.037508063	0.109448068	-0.342701922	0.731822717	0.999579949
667.4671527	0.130611826	0.099701	1.310035266	0.190183906	0.984610142
95.42310063	-0.117104466	0.185690482	-0.63064334	0.528273754	0.999579949
978.8437243	-0.16474626	0.126570033	-1.301621377	0.193045848	0.986064814
22.47577749	0.186775624	0.220196313	0.848223214	0.396313672	0.999579949
496.7200166	-0.007815631	0.095697898	-0.081669825	0.934909275	0.999579949
85.38154023	0.000802376	0.178067025	0.004506032	0.996404719	0.999579949
721.5080518	-0.066934675	0.107550116	-0.622358002	0.53370649	0.999579949
939.7803544	-0.283134426	0.145240643	-1.949415951	0.051245771	0.779189484
42.43653347	-0.218010138	0.216067238	-1.00899211	0.312978418	0.999579949
427.0606556	0.094293415	0.150798452	0.625294317	0.531777909	0.999579949
317.374446	0.025909596	0.094823388	0.273240563	0.784668301	0.999579949
249.8291533	-0.056049892	0.073600953	-0.761537582	0.44633604	0.999579949
634.4268872	0.145823365	0.170486812	0.85533516	0.392365628	0.999579949
276.1325025	-0.042108154	0.078852257	-0.534013306	0.593332328	0.999579949
161.8933049	0.043850147	0.094848278	0.462318857	0.643852679	0.999579949

125.4149755	0.140464896	0.182287451	0.770568108	0.440962971	0.999579949
31.34527446	0.033284434	0.190826862	0.174422163	0.861533698	0.999579949
53.30984178	0.261015565	0.267864334	0.97443195	0.329842112	0.999579949
182.9377264	-0.096480978	0.173956532	-0.554626935	0.579149862	0.999579949
226.0463199	-0.074938188	0.150925498	-0.496524372	0.619524496	0.999579949
1373.639607	-0.110638343	0.060651497	-1.824165085	0.068127118	0.829912169
124.5082289	0.188156436	0.103618829	1.815851789	0.06939311	0.831457432
740.2772585	-0.013438897	0.086781662	-0.154858718	0.876932699	0.999579949
1373.41794	0.019675057	0.055185442	0.356526209	0.721446528	0.999579949
53.72923123	0.12020511	0.647972908	0.185509469	0.852829454	0.999579949
1795.177948	-0.009155395	0.126522114	-0.072362012	0.942313815	0.999579949
676.6946269	-0.657973942	0.209536234	-3.140143974	0.001688648	0.214855947
59.41576659	0.04444929	0.249416674	0.178212984	0.858555713	0.999579949
67.83678329	-0.129829088	0.330606599	-0.392699627	0.694541346	0.999579949
190.6341517	0.227871821	0.125424421	1.816805841	0.069246848	0.831457432
393.1730878	0.044843555	0.135394502	0.331206617	0.740488422	0.999579949
718.8000981	-0.16307774	0.116589099	-1.398739178	0.161891211	0.978498637
610.5694714	-0.165904227	0.107195248	-1.547682672	0.121698716	0.922215626
75.17258918	-0.091661521	0.134974401	-0.679103007	0.497072598	0.999579949
237.9968506	0.042700641	0.14020224	0.304564612	0.760697776	0.999579949
50628.47453	-0.048754116	0.154555822	-0.315446646	0.752422555	0.999579949
1719.292877	0.039672782	0.12983674	0.305558985	0.759940453	0.999579949
238.9647409	0.131955797	0.123648734	1.067182758	0.285889317	0.999579949
706.9882477	0.033006538	0.111818198	0.295180376	0.767856092	0.999579949
255.4113186	-0.090098647	0.086972518	-1.035943871	0.300228329	0.999579949
24.55592784	0.124596589	0.248611027	0.501170807	0.616250918	0.999579949
1040.440576	-0.153717279	0.127681442	-1.20391246	0.228623415	0.999579949
33.30949302	-0.864228656	0.35447543	-2.438049533	0.01476675	0.539131676
1021.790171	-0.007749151	0.09254616	-0.083732821	0.933268862	0.999579949
433.4454849	-0.064479965	0.154094435	-0.418444471	0.675622178	0.999579949
26.92591618	-0.017993823	0.400650002	-0.044911576	0.96417779	0.999579949
494.7816684	0.055085291	0.067415866	0.817096828	0.413873096	0.999579949
6611.83944	-0.101601317	0.057342299	-1.771838928	0.076421299	0.851207389
21.79864435	0.032348773	0.26843769	0.120507568	0.904081085	0.999579949
25.91979622	0.226826116	0.307527254	0.737580535	0.460769387	0.999579949
108.0127454	-0.011057405	0.158010807	-0.069978788	0.944210543	0.999579949
350.9575186	-0.117925385	0.311785498	-0.378226011	0.705262704	0.999579949
465.5953926	-0.387264526	0.319071686	-1.213722629	0.22485366	0.999579949
79.74188532	0.407250444	0.396089221	1.028178557	0.303865843	0.999579949
57.99360128	-0.482203081	0.171306265	-2.814859588	0.004879855	0.347269229
491.4247262	0.124784873	0.103840158	1.2017015	0.2294792	0.999579949
65.85468876	-0.056801798	0.169006702	-0.336091985	0.736801502	0.999579949
403.1194878	0.010335953	0.085782713	0.120489928	0.904095058	0.999579949
306.0624162	0.020497317	0.134938768	0.151900873	0.87926512	0.999579949
3220.2419	0.04197113	0.081583675	0.514455001	0.606933914	0.999579949
101.0546785	-0.082955831	0.171508008	-0.483684884	0.628609522	0.999579949
47.61113194	0.140167017	0.155802685	0.89964443	0.368309505	0.999579949
25.28163751	-0.680446946	0.276368172	-2.46210315	0.013812494	0.520783867

194.6697509	-0.028971398	0.100913481	-0.287091451	0.774042298	0.999579949
9089.585907	-0.288076474	0.104307586	-2.761797922	0.005748404	0.372390484
708.162573	0.09842919	0.160997861	0.611369553	0.540954954	0.999579949
148.2583958	-0.174937662	0.174490942	-1.002560132	0.31607314	0.999579949
395.2700073	0.050000048	0.121073211	0.412973669	0.679625906	0.999579949
1064.420795	0.064761623	0.101803779	0.636141636	0.52468411	0.999579949
548.9765069	-0.057288665	0.086201604	-0.664589308	0.50631321	0.999579949
609.8784789	-0.020263017	0.079854511	-0.253749182	0.799689335	0.999579949
569.1981974	0.058781672	0.080025882	0.734533266	0.462623793	0.999579949
470.1304477	-0.100696324	0.064505182	-1.561057889	0.118510093	0.921521033
203.3625965	-0.052554634	0.087061398	-0.603650244	0.546076201	0.999579949
151.3209536	-0.097576718	0.096147353	-1.014866393	0.31016952	0.999579949
181.306802	-0.056062404	0.0996433	-0.562630941	0.573686218	0.999579949
503.2708663	-0.115450459	0.076327757	-1.512561916	0.130390974	0.935700262
863.1788621	-0.032940106	0.115371854	-0.285512498	0.77525153	0.999579949
43.60770672	-0.015648164	0.179817057	-0.087022692	0.930653475	0.999579949
27.87660023	-1.318629293	0.746078084	-1.767414593	0.077158821	0.851207389
45.05331599	0.427449756	0.205122858	2.083871883	0.037171823	0.717947029
60.42350701	0.017401679	0.190928365	0.091142449	0.927379403	0.999579949
304.5359533	-0.104158813	0.126822108	-0.821298546	0.411476236	0.999579949
796.3368867	0.135931946	0.124321893	1.093387034	0.274223904	0.999579949
107.7906097	0.144801622	0.108059618	1.340016041	0.18024013	0.983992835
2415.223199	0.070299537	0.191948429	0.366241791	0.71418466	0.999579949
234.3580817	-0.111495845	0.094487273	-1.180009137	0.237996581	0.999579949
30.15697276	0.434859754	0.292028647	1.489099644	0.136461132	0.945791454
4711.872271	0.064191926	0.092680756	0.692613325	0.488552246	0.999579949
1437.389925	-0.004862201	0.119085187	-0.040829604	0.967431739	0.999579949
153.0173521	-0.013325433	0.125360635	-0.106296787	0.915346881	0.999579949
485.6977326	0.191543112	0.144614943	1.324504289	0.18533562	0.983992835
168.4867344	-0.278117831	0.197064169	-1.411305931	0.158154426	0.977304288
739.3179178	0.074164815	0.087391305	0.848652106	0.396074904	0.999579949
91.27078107	0.020808699	0.503193976	0.041353236	0.967014293	0.999579949
292.1192436	-0.076365949	0.130059269	-0.587162679	0.557094455	0.999579949
231.5373593	0.043558354	0.073924897	0.589224415	0.555710741	0.999579949
588.8769159	0.058807831	0.103562139	0.567850675	0.570136367	0.999579949
123.9118967	-0.029382114	0.109142014	-0.269209932	0.787768138	0.999579949
75.66490087	0.218388604	0.182746221	1.195037592	0.232072343	0.999579949
868.728739	0.02732511	0.095885773	0.284975645	0.775662801	0.999579949
53.04843648	0.211302291	0.236413276	0.893783523	0.371437727	0.999579949
142.1802248	-0.066696803	0.144185803	-0.462575379	0.64366876	0.999579949
1768.995936	-0.164915976	0.105171691	-1.568064308	0.116866125	0.920479614
182.8452914	0.06225444	0.083666776	0.744075992	0.456830503	0.999579949
251.8963177	-0.054580197	0.085263537	-0.640135266	0.522084664	0.999579949
33.80760676	0.06550612	0.185000049	0.354087043	0.723273652	0.999579949
1284.670899	-0.119166802	0.089119221	-1.337161618	0.181169887	0.983992835
1056.12923	-0.163768208	0.166844364	-0.981562724	0.326315319	0.999579949
1390.327162	0.01003937	0.094675255	0.10604006	0.915550568	0.999579949
61.69741979	-0.221334092	0.17790975	-1.244080731	0.213469852	0.999268361

886.8736864	-0.164080551	0.123507182	-1.328510195	0.184009618	0.983992835
173.7793837	-0.078358605	0.118930605	-0.658859886	0.509985747	0.999579949
79.93784506	-0.140744226	0.190954292	-0.73705715	0.461087596	0.999579949
110.7699308	-0.129571955	0.147485269	-0.878541678	0.37964983	0.999579949
121.1639941	-0.242196267	0.128691297	-1.881994147	0.05983681	0.798635337
91.09617742	-0.179120254	0.12245626	-1.462728443	0.1435417	0.953645321
162.4228475	0.035200147	0.093614537	0.376011553	0.706908307	0.999579949
1168.188832	0.133074233	0.07004303	1.899892567	0.05744722	0.793426732
61.21743455	0.342646421	0.173125127	1.979183655	0.047795332	0.770705547
371.32508	0.190067691	0.08591231	2.212345242	0.026942819	0.653787748
275.0596753	-0.201186067	0.238011829	-0.845277595	0.397955867	0.999579949
309.6865294	0.250819609	0.143447399	1.748512767	0.080375277	0.85448976
152.4716297	0.410056265	0.673691246	0.608670912	0.542742592	0.999579949
52.71216293	-0.093327408	0.411897723	-0.226579081	0.82075106	0.999579949
782.7171376	0.123912721	0.170588711	0.726382894	0.467604075	0.999579949
184.6914918	0.180872103	0.192078638	0.941656525	0.34636852	0.999579949
94.78114021	-0.111174038	0.15141861	-0.734216474	0.462816814	0.999579949
168.8036827	0.110869535	0.114235833	0.97053203	0.331781367	0.999579949
496.9407847	-0.039757152	0.091205022	-0.435909684	0.662902259	0.999579949
26.56962572	0.090701133	0.206117322	0.440046143	0.659903688	0.999579949
117.3632274	0.09968306	0.122325734	0.814898525	0.415130398	0.999579949
68.21798286	0.108969737	0.362976311	0.300211704	0.764015678	0.999579949
209.5492196	0.061097138	0.125026003	0.48867545	0.625071487	0.999579949
82.29445124	-0.240481802	0.213785812	-1.124872599	0.260643025	0.999579949
1980.352701	-0.027435367	0.101316942	-0.270787559	0.786554429	0.999579949
2377.216712	-0.149329059	0.068548776	-2.178435106	0.029373659	0.672989344
1864.686018	0.017125327	0.101679395	0.168424755	0.866249135	0.999579949
19395.09604	-0.023992713	0.104823091	-0.228887674	0.818956221	0.999579949
133.248247	-0.021078397	0.214380292	-0.098322458	0.921676246	0.999579949
151.2006873	-0.031178803	0.129969168	-0.239893845	0.810412553	0.999579949
289.7098965	0.046837583	0.110174416	0.425122135	0.670747642	0.999579949
365.9604949	0.003952663	0.181517864	0.021775614	0.982626947	0.999579949
254.9657042	0.054051725	0.203080456	0.266159164	0.790116631	0.999579949
1463.568118	-0.039326924	0.091455271	-0.430012653	0.667186437	0.999579949
60.6973777	0.160200263	0.193074674	0.829732141	0.406690245	0.999579949
273.0517013	0.098068516	0.103428928	0.948172988	0.343041396	0.999579949
1367.997371	-0.105329717	0.080909574	-1.301820178	0.192977864	0.986064814
419.8269435	-0.160489607	0.087013643	-1.844418882	0.065122118	0.817776877
1226.441175	-0.051994402	0.068231793	-0.762026022	0.446044473	0.999579949
21.55687692	-0.632867613	0.226589608	-2.793012522	0.005221967	0.357404857
93.00547277	0.166072642	0.145586692	1.140713064	0.253989348	0.999579949
229.0616935	-0.0355478	0.106648021	-0.333318892	0.73889358	0.999579949
90.60909909	0.073085419	0.223075873	0.327625835	0.743194586	0.999579949
684.4617954	0.060700843	0.051024698	1.189636483	0.2341893	0.999579949
928.5506735	0.062875838	0.063431886	0.991233943	0.32157136	0.999579949
225.129082	0.162797569	0.137724141	1.182055437	0.237183705	0.999579949
444.8272208	-0.18405204	0.154629679	-1.190276289	0.233937817	0.999579949
128.9129761	0.011025608	0.110261188	0.099995363	0.920348007	0.999579949

156.1951335	-0.051831838	0.09096509	-0.569799222	0.568813884	0.999579949
103.6197396	0.033100974	0.134599737	0.245921533	0.805742975	0.999579949
3418.349565	-0.139471966	0.146279896	-0.953459564	0.340357275	0.999579949
702.6769612	-0.053276667	0.111421862	-0.478152722	0.63254151	0.999579949
126.0905075	0.047981129	0.163226391	0.293954483	0.768792685	0.999579949
284.4798138	0.006622869	0.086390734	0.076661802	0.938892592	0.999579949
183.3151364	0.048919235	0.090602883	0.539930221	0.589245156	0.999579949
338.1608048	0.081639816	0.226036749	0.361179396	0.717965344	0.999579949
1675.591629	0.062443797	0.074124432	0.842418558	0.399553709	0.999579949
1272.94092	0.091277276	0.091202169	1.000823524	0.316912135	0.999579949
176.1790912	-0.230997438	0.122138932	-1.891267879	0.058588594	0.79580383
56.32967379	-0.111470017	0.191514318	-0.582045342	0.56053614	0.999579949
103.3642251	-0.416412635	0.638425025	-0.652249862	0.514239998	0.999579949
369.9782921	0.029958312	0.13264846	0.225847416	0.821320097	0.999579949
1355.071754	-0.101701656	0.101627934	-1.000725414	0.316959577	0.999579949
30.0232163	-0.357037683	0.280842762	-1.271308117	0.203619056	0.994814558
156.2318978	0.058048628	0.250366421	0.231854687	0.81665088	0.999579949
161.8944173	-0.337688338	0.259136004	-1.303131685	0.192529808	0.985815229
217.2185955	-0.081503616	0.115007709	-0.708679587	0.478523335	0.999579949
373.0194092	0.023997825	0.135741068	0.176791187	0.859672411	0.999579949
521.4363036	0.108398478	0.105036768	1.032005081	0.302069737	0.999579949
22.87320242	-0.271126604	0.282661729	-0.959191062	0.337462502	0.999579949
1388.231101	-0.050995639	0.086341285	-0.590628676	0.554769247	0.999579949
248.297231	0.109829396	0.118845006	0.924139766	0.355413551	0.999579949
670.6509939	-0.039962765	0.089440804	-0.446806867	0.655014513	0.999579949
209.4033208	0.026146353	0.081240324	0.321839593	0.747574218	0.999579949
1095.197609	-0.251056293	0.084486821	-2.971543842	0.002963066	0.290143966
194.8133717	-0.226573225	0.165377227	-1.370038844	0.170674777	0.981609963
210.8780498	0.005750478	0.074020792	0.077687328	0.938076774	0.999579949
431.3626992	0.024231781	0.106073838	0.228442575	0.819302194	0.999579949
31.16248943	0.115008415	0.184676852	0.6227549	0.5334456	0.999579949
793.258926	0.027793967	0.107141305	0.259414115	0.79531574	0.999579949
177.2856429	0.059159195	0.123556387	0.478803213	0.632078631	0.999579949
930.3311998	-0.034271041	0.059689689	-0.574153452	0.565863965	0.999579949
24.59025626	-0.362188182	0.222919678	-1.624747468	0.104216378	0.895628172
1075.001549	-0.016820088	0.045004252	-0.37374442	0.708594474	0.999579949
354.0433613	-0.00891885	0.091437834	-0.097540038	0.92229754	0.999579949
846.3934139	-0.131332226	0.16454009	-0.79817767	0.424767394	0.999579949
506.1320989	0.040133417	0.115526552	0.347395611	0.72829413	0.999579949
1051.263859	0.060445063	0.068877172	0.877577596	0.380172992	0.999579949
107.890609	0.025804721	0.126602987	0.203823952	0.838491074	0.999579949
121.6299995	0.468783147	0.294514022	1.59171758	0.111448176	0.913044976
527.9123094	-0.042451224	0.081093974	-0.52348186	0.600638971	0.999579949
111.2946979	-0.054937698	0.102830613	-0.534254311	0.593165598	0.999579949
310.8096332	0.564515557	0.182213866	3.098093289	0.001947701	0.225075621
48.07217799	0.366533743	0.244223732	1.500811328	0.133404368	0.942434895
109.7151011	-0.277758323	0.262382086	-1.058602465	0.289780864	0.999579949
183.5911637	0.164585903	0.137708014	1.195180283	0.2320166	0.999579949

1114.039244	0.013406104	0.130798289	0.102494489	0.918364187	0.999579949
397.6153361	0.048511995	0.135315483	0.358510307	0.719961456	0.999579949
296.5403575	0.001709629	0.077617852	0.022026237	0.982427026	0.999579949
442.2682549	-0.11422305	0.081837684	-1.39572681	0.162796778	0.978498637
1402.920774	0.086738228	0.092146957	0.941303229	0.346549489	0.999579949
35.19846258	-0.010334709	0.183779732	-0.056234213	0.955155227	0.999579949
346.7588745	0.017766673	0.069103951	0.257100685	0.797101049	0.999579949
35.26829327	0.188193425	0.251350153	0.748730096	0.4540199	0.999579949
1112.932729	-0.088521925	0.098438446	-0.899261713	0.368513276	0.999579949
100.6483062	-0.033104527	0.110208081	-0.300382033	0.763885767	0.999579949
21.12026989	-0.10052482	0.268951182	-0.373766047	0.708578382	0.999579949
791.1333134	-0.108120479	0.074231926	-1.456522621	0.145248201	0.955875831
20.98754279	0.391261372	0.241555736	1.619756078	0.105284685	0.896383253
309.9712771	-0.034549089	0.080295651	-0.43027348	0.666996716	0.999579949
724.5040912	0.050772005	0.070691593	0.718218427	0.472622618	0.999579949
387.0902334	0.064763279	0.068308423	0.948100926	0.343078077	0.999579949
116.847524	-0.176678892	0.106509876	-1.658802897	0.097155516	0.89001519
40.45675211	0.383411379	0.219790567	1.744439643	0.081082457	0.85448976
234.3179953	0.072493976	0.129972228	0.557765123	0.577004782	0.999579949
21.74481713	0.163665677	0.29665137	0.551710505	0.581146712	0.999579949
266.2823782	0.000305413	0.098459944	0.003101898	0.997525048	0.999579949
22.87245699	0.425956668	0.241252266	1.76560691	0.07746182	0.851207389
445.2694111	-0.048422897	0.100371555	-0.48243645	0.629495933	0.999579949
363.4362449	0.0460472	0.076170475	0.604528192	0.545492532	0.999579949
194.5911298	0.19483427	0.18919638	1.029799147	0.303104301	0.999579949
726.8981648	-0.027621485	0.05808095	-0.475568763	0.634381634	0.999579949
148.782974	-0.105063776	0.090888626	-1.15596176	0.247696799	0.999579949
172.0565507	-0.55673989	0.29898094	-1.862125028	0.062585469	0.807133028
338.26995	0.102674023	0.119377971	0.860075126	0.389747632	0.999579949
304.387879	0.388500185	0.127067823	3.057423789	0.002232484	0.23630562
45.21293585	-0.409116962	0.182913325	-2.236671178	0.025307839	0.642289128
600.6181457	-0.206036975	0.064460834	-3.196312582	0.001391962	0.203873911
156.0760161	0.077604262	0.113985009	0.680828668	0.495979908	0.999579949
212.4875101	0.117865793	0.128916073	0.914283147	0.360568084	0.999579949
252.3481192	0.038588292	0.100319414	0.384654279	0.700493574	0.999579949
428.1926181	0.240071186	0.110013066	2.182206111	0.029094324	0.672989344
304.0364269	0.001159186	0.080713842	0.01436167	0.988541439	0.999579949
502.3747766	-0.036362804	0.105143962	-0.345838254	0.729464275	0.999579949
242.1919306	0.045039713	0.100946067	0.446176009	0.655470112	0.999579949
38.65175636	0.18717018	0.190143673	0.984361862	0.324937623	0.999579949
249.2716475	0.654122188	0.338022905	1.935141605	0.052972921	0.781231069
54.73509851	0.469548421	0.3555037	1.320797562	0.186568873	0.983992835
274.2432754	-0.437379237	0.231884966	-1.886190571	0.059269279	0.797602387
46.49697991	0.398373509	0.290099433	1.37323091	0.169680573	0.981013142
542.2357643	-0.031796956	0.090582381	-0.35102804	0.725567313	0.999579949
175.9846921	0.246563321	0.120800202	2.041083691	0.041242509	0.745838643
138.3296735	-0.08103063	0.113459715	-0.714179736	0.475116045	0.999579949
368.7321424	0.01209294	0.078187629	0.154665638	0.87708492	0.999579949

41.7720212	0.57735748	0.599693867	0.962753684	0.335671149	0.999579949
3667.167454	0.045824674	0.108639585	0.421804577	0.673167664	0.999579949
252.4319352	-0.025030331	0.090641392	-0.276146811	0.782435308	0.999579949
491.8061295	-0.096852694	0.080966904	-1.196201032	0.231618118	0.999579949
1212.951558	-0.038465288	0.076377921	-0.503617896	0.614529914	0.999579949
712.7266459	-0.000547226	0.080357619	-0.006809877	0.994566546	0.999579949
545.7301674	-0.00480702	0.07039509	-0.068286299	0.94555773	0.999579949
847.5324456	-0.044345337	0.061618646	-0.719673992	0.471725743	0.999579949
26.62348445	-0.147331741	0.239211972	-0.615904546	0.537957521	0.999579949
716.525721	0.083157248	0.087363398	0.951854554	0.34117075	0.999579949
106.1759522	-0.170610348	0.129410183	-1.318368798	0.187380221	0.983992835
39.17251765	0.022408682	0.180590714	0.124085461	0.90124761	0.999579949
27.77929472	0.043973702	0.274560363	0.160160417	0.872754709	0.999579949
3375.300519	0.008995778	0.097099722	0.092644732	0.926185805	0.999579949
9894.769878	-0.034983411	0.07077272	-0.494306436	0.62108978	0.999579949
1034.987046	-0.042532171	0.159367006	-0.26688191	0.789560086	0.999579949
112.3102839	0.044926668	0.112224932	0.40032698	0.688915698	0.999579949
57.25614449	-0.0269287	0.162495559	-0.165719607	0.868377616	0.999579949
100.177901	0.102564093	0.166500161	0.61599996	0.537894547	0.999579949
94.05010195	0.145840136	0.1418977	1.027783652	0.304051608	0.999579949
51.42682169	0.015833872	0.210324763	0.075282965	0.939989575	0.999579949
268.2633152	-0.109206794	0.076525227	-1.427069191	0.153559952	0.973447989
811.5432032	-0.178319949	0.111231029	-1.603149325	0.10890169	0.904832251
150.9740989	0.047686392	0.160032373	0.297979658	0.765718689	0.999579949
137.0425158	0.146350291	0.104827325	1.396108229	0.162681906	0.978498637
36.68757316	-0.069892487	0.223647243	-0.312512179	0.754651309	0.999579949
60.84998856	-0.016586527	0.148285978	-0.111854994	0.910938382	0.999579949
543.6769528	-0.027639758	0.06649971	-0.415637277	0.677675445	0.999579949
27.71587511	-0.327936864	0.198124937	-1.655202366	0.097883455	0.890209389
366.0300627	0.14782286	0.114980799	1.285630829	0.198571932	0.992481822
318.7476003	-0.045252951	0.142675355	-0.317174265	0.751111376	0.999579949
25.79792995	1.012194182	0.533100837	1.898691787	0.057605012	0.793865133
120.789208	0.130820386	0.130120011	1.005382535	0.314712687	0.999579949
331.200379	-0.004837285	0.078660412	-0.061495802	0.950964358	0.999579949
2760.266655	0.078325806	0.12836999	0.610156675	0.541758027	0.999579949
57.27876518	0.412832359	0.252085006	1.63767122	0.101490297	0.895628172
59.37236267	0.277111836	0.15968232	1.735394609	0.082670934	0.857762017
722.4818036	-0.402585391	0.509912434	-0.789518678	0.429808917	0.999579949
152.5924849	-0.030226699	0.225413435	-0.134094488	0.893327858	0.999579949
35.55208455	0.47115011	0.255442821	1.844444514	0.065118385	0.817776877
34.45831212	0.237100111	0.371184422	0.638766331	0.522974954	0.999579949
796.3482239	-0.227611691	0.163929192	-1.388475648	0.164992255	0.978789551
77.26583973	-0.01466594	0.173476383	-0.084541423	0.93262597	0.999579949
1538.395956	-0.156308952	0.11854388	-1.318574622	0.187311363	0.983992835
155.9275627	-0.10118471	0.131825776	-0.767563922	0.442746303	0.999579949
232.8929273	-0.357458099	0.15539711	-2.300287941	0.021431912	0.622105864
84.70238784	0.617451794	0.228795995	2.698700186	0.006961086	0.404205857
80.41559966	0.290912533	0.226194023	1.286119455	0.198401375	0.992481822

51.49587656	0.285969945	0.20822523	1.373368372	0.169637857	0.981013142
2464.344128	-0.021708358	0.368823264	-0.05885843	0.953064869	0.999579949
103.8712598	-0.243486695	0.349588231	-0.696495686	0.486118458	0.999579949
3111.643691	-0.253905446	0.139184262	-1.824239621	0.068115854	0.829912169
152.7677515	-0.121660826	0.136031962	-0.894354706	0.371132139	0.999579949
528.0901488	-0.196308141	0.159981663	-1.227066508	0.219797595	0.999579949
74.40385751	0.368943205	0.298342001	1.236645208	0.216218829	0.999579949
2349.958334	-0.031218436	0.112756109	-0.276866915	0.781882298	0.999579949
29.84424034	0.420512923	0.228288638	1.842023004	0.065471785	0.819279724
170.9181813	0.138879629	0.122266502	1.135876352	0.256008317	0.999579949
1060.697818	-0.03475788	0.085070557	-0.408577082	0.682850052	0.999579949
75.0654882	-0.347212194	0.159501086	-2.176864129	0.029490707	0.672989344
269.3470441	-0.003234732	0.105845739	-0.030560811	0.975619795	0.999579949
265.1720723	-0.250717633	0.164375163	-1.525276858	0.127190057	0.934532219
103.0849974	-0.025862745	0.12400266	-0.208566053	0.834787013	0.999579949
554.7910494	0.101963518	0.077993593	1.307331989	0.191099984	0.984804292
1534.670778	0.030221999	0.099302778	0.304341932	0.760867402	0.999579949
574.1112871	0.269919232	0.232645991	1.160214415	0.245961519	0.999579949
269.0988141	1.039369021	0.464294424	2.238598972	0.025182021	0.642289128
1429.905926	-0.068996159	0.085884677	-0.803358195	0.421767731	0.999579949
775.1469105	0.038662067	0.138112629	0.279931435	0.779530109	0.999579949
109.6412286	0.102655413	0.105493171	0.973100076	0.330503566	0.999579949
383.9687111	-0.041015658	0.078303024	-0.523806827	0.600412904	0.999579949
603.5930146	0.03255759	0.117497593	0.277091548	0.781709811	0.999579949
71.32540412	-0.155353678	0.147341565	-1.054377814	0.291709976	0.999579949
124.536346	0.080570276	0.112384059	0.716919074	0.473424032	0.999579949
131.7839555	0.148091563	0.320103893	0.462635931	0.643625349	0.999579949
435.3369983	-0.150290742	0.083342739	-1.803285361	0.07134341	0.834620594
64.336417	0.07874585	0.137050663	0.574574745	0.565578936	0.999579949
590.7637355	-7.80558E-05	0.168672035	-0.000462767	0.999630766	0.99989122
242.9764195	0.036894425	0.087798951	0.420214876	0.674328488	0.999579949
773.6018617	-0.003961314	0.075868327	-0.052213015	0.958358963	0.999579949
36.45210548	0.104414543	0.198353003	0.526407674	0.598604985	0.999579949
2648.210627	0.044221135	0.086042476	0.513945407	0.60729016	0.999579949
2587.229762	0.080488944	0.156112563	0.515582749	0.606145861	0.999579949
1315.527531	-0.18767172	0.09556505	-1.963811241	0.049551986	0.775350081
43.934925	-0.026624989	0.196989034	-0.135159754	0.892485566	0.999579949
830.2792338	-0.252394271	0.073949735	-3.41305173	0.000642397	0.128142583
1055.644943	-0.032440715	0.134346642	-0.241470233	0.809190685	0.999579949
497.0609519	-0.048964091	0.06315566	-0.775292194	0.438167021	0.999579949
56.81395798	0.137161481	0.208479581	0.657913262	0.510593868	0.999579949
1142.347583	0.076252991	0.094131488	0.810068896	0.41790058	0.999579949
74.97224917	0.180624941	0.136976022	1.318661017	0.187282465	0.983992835
519.0328774	0.008630362	0.09738555	0.088620564	0.929383465	0.999579949
270.1213101	0.023611472	0.080409147	0.293641621	0.769031768	0.999579949
721.2162097	-0.033522461	0.082172528	-0.407952171	0.68330879	0.999579949
855.2423525	-0.157492554	0.137813578	-1.142794168	0.253124061	0.999579949
1622.848651	0.178262597	0.105959796	1.682360708	0.092498916	0.880418343

126.4436286	0.10320335	0.116317273	0.887257306	0.374940386	0.999579949
573.1829867	0.010994444	0.111949913	0.098208601	0.921766653	0.999579949
73.3336177	0.156259343	0.1548546	1.009071368	0.312940409	0.999579949
645.8604195	0.01526213	0.152726213	0.099931308	0.92039886	0.999579949
20.6677002	-0.276346605	0.243796932	-1.133511415	0.25699955	0.999579949
172.8858722	-0.281615215	0.427637437	-0.658537328	0.510192919	0.999579949
160.5015669	-0.153925295	0.190732461	-0.807022014	0.4196538	0.999579949
113.3389117	-0.129233176	0.096359985	-1.341149818	0.179871815	0.983992835
48.96679265	-0.031186419	0.198888321	-0.156803672	0.875399579	0.999579949
1217.649779	0.063626574	0.12089734	0.526285968	0.598689531	0.999579949
64.20168146	0.182068807	0.160416029	1.134978889	0.256384164	0.999579949
336.6773276	0.006469167	0.240059422	0.026948191	0.978501057	0.999579949
242.9159606	0.090847117	0.184617197	0.492083718	0.622660162	0.999579949
157.1088575	0.096984474	0.240530944	0.403209965	0.686793769	0.999579949
2024.037436	-0.01288719	0.120023469	-0.107372248	0.91449367	0.999579949
53.84938493	0.006822451	0.184742218	0.036929569	0.970541163	0.999579949
370.5509079	0.004181016	0.118686371	0.035227429	0.971898391	0.999579949
119.2659142	0.006652181	0.113426185	0.058647669	0.953232742	0.999579949
416.3941687	-0.449099661	0.289520762	-1.551182922	0.120857854	0.921837625
21.64819027	0.076869454	0.437753833	0.175599729	0.860608415	0.999579949
128.1054644	-0.088009632	0.2825838	-0.311446135	0.755461486	0.999579949
63.67467164	-0.232220428	0.150401896	-1.543999334	0.122588494	0.92236461
42.01833545	0.01684438	0.196768449	0.085605086	0.931780355	0.999579949
1273.051588	-0.016381441	0.066348927	-0.246898353	0.804986891	0.999579949
102.4375613	0.36543998	0.469210716	0.778839799	0.43607409	0.999579949
3018.672191	-0.052160814	0.126577069	-0.412087393	0.680275368	0.999579949
237.0509948	0.161460931	0.120282935	1.342342792	0.179484875	0.983992835
90.91290641	0.005229511	0.136352099	0.03835299	0.969406242	0.999579949
804.5722262	-0.08011773	0.130454478	-0.614143192	0.539120709	0.999579949
938.8956131	-0.042571225	0.142919164	-0.297869252	0.765802956	0.999579949
2575.987803	-0.02079082	0.098854838	-0.210316662	0.833420532	0.999579949
20.77518926	-0.00988826	0.212999732	-0.046423815	0.962972456	0.999579949
2139.713808	0.037774585	0.068212197	0.553780512	0.579729067	0.999579949
355.4797262	0.136016008	0.180790473	0.752340575	0.451846273	0.999579949
617.3814971	-0.057564777	0.122229337	-0.470957127	0.637671351	0.999579949
71.58208189	0.054303272	0.251860716	0.215608346	0.82929305	0.999579949
386.7298748	0.07421889	0.092218514	0.804815505	0.420926153	0.999579949
382.0094189	0.13550582	0.122276953	1.108187736	0.267780746	0.999579949
152.9596243	-0.074486936	0.127429603	-0.584534004	0.558861095	0.999579949
162.5546566	0.070046331	0.083735116	0.836522774	0.40286087	0.999579949
935.2735178	-0.126723229	0.100325483	-1.263121053	0.206545679	0.994814558
51.18717956	-0.082759164	0.191890233	-0.431283881	0.666261969	0.999579949
96.46712174	-0.131734408	0.151994981	-0.866702357	0.386105114	0.999579949
1092.033975	-0.002943864	0.096263638	-0.030581265	0.975603484	0.999579949
119.9555376	0.12831183	0.129167151	0.993378179	0.320525692	0.999579949
22.07047955	0.20189954	0.392712826	0.514114963	0.607171616	0.999579949
124.8721558	0.091981027	0.113534906	0.810156362	0.417850314	0.999579949
72.98793214	0.142442893	0.126969998	1.121862614	0.261920859	0.999579949

42.64558794	0.694209088	0.279662717	2.482308322	0.013053427	0.512214868
471.2592778	0.121219815	0.085245464	1.422008972	0.155023646	0.976030424
3581.021839	0.029230315	0.14931857	0.195758072	0.844799529	0.999579949
2706.162826	-0.161801702	0.094794748	-1.706863561	0.087847415	0.869910409
23.56174579	-0.250413558	0.698484574	-0.35850979	0.719961843	0.999579949
107.8361592	0.241973062	0.148013596	1.634802941	0.102090364	0.895628172
238.024002	0.012187346	0.15528246	0.078485011	0.937442253	0.999579949
106.022926	-0.017316201	0.124959689	-0.138574293	0.889786558	0.999579949
103.851121	0.048178743	0.116069803	0.415084214	0.678080255	0.999579949
872.5757127	-0.096215573	0.37469599	-0.256783033	0.797346269	0.999579949
111.3107583	0.093596024	0.163660633	0.57189088	0.567395903	0.999579949
288.57125	0.000548656	0.068259862	0.008037756	0.993586868	0.999579949
210.0965039	0.098074873	0.113035076	0.867649905	0.385586018	0.999579949
130.7012532	0.124505745	0.108418683	1.148379052	0.250812118	0.999579949
307.0200852	-0.148906273	0.072663525	-2.049257481	0.040436944	0.741027736
26.07957355	-0.21796196	0.356058891	-0.612151432	0.540437572	0.999579949
135.982032	-0.01591381	0.125537615	-0.126765273	0.899126182	0.999579949
83.56472503	0.080273322	0.129951265	0.617718668	0.536760804	0.999579949
316.2790304	-0.329539284	0.210573251	-1.564962701	0.117591655	0.920584864
60.35719963	0.182203366	0.18871254	0.965507463	0.3342907	0.999579949
23.26015777	0.906445977	0.508509547	1.782554493	0.074658853	0.848739093
61.19702497	0.261337268	0.136610152	1.913014986	0.055746134	0.785214091
441.9995363	0.204691975	0.219537012	0.932380255	0.351140045	0.999579949
459.2076401	-0.094935725	0.072370262	-1.311805725	0.189585693	0.983992835
26.43923745	-0.161130592	0.250064357	-0.644356493	0.51934429	0.999579949
730.7069985	0.080275622	0.083721383	0.958842529	0.337638081	0.999579949
57.03324939	-0.074882554	0.344932074	-0.217093625	0.828135382	0.999579949
108.8750371	0.262885006	0.208123238	1.263121832	0.206545399	0.994814558
44.32040449	0.220658382	0.421068066	0.524044447	0.600247626	0.999579949
200.9633511	-0.097465621	0.108122559	-0.901436492	0.367356286	0.999579949
1682.642602	-0.044161666	0.081467287	-0.542078512	0.587764421	0.999579949
29.42690195	-0.018386097	0.195740347	-0.093931051	0.925163928	0.999579949
289.7302609	0.109175535	0.080517608	1.355921246	0.175124227	0.983992835
361.0650355	-0.022600776	0.094448546	-0.239291943	0.810879212	0.999579949
197.9461463	0.011634469	0.125151651	0.092962973	0.925932977	0.999579949
256.7793903	0.000266688	0.133784301	0.001993414	0.998409486	0.999644252
384.7534453	0.099011866	0.154084367	0.642582165	0.520495259	0.999579949
444.8155936	-0.053403721	0.083709206	-0.63796712	0.523495083	0.999579949
1070.822391	0.071125752	0.097226947	0.73154361	0.464447177	0.999579949
161.9134753	-0.162034486	0.143453053	-1.12952971	0.258674444	0.999579949
333.3727335	-0.025774947	0.08179625	-0.315111596	0.752676924	0.999579949
293.4249386	-0.018606074	0.16582857	-0.112200653	0.910664311	0.999579949
1688.448176	0.010109625	0.123374936	0.081942293	0.934692603	0.999579949
126.5664674	0.238126063	0.197104076	1.208123487	0.226999767	0.999579949
358.7937714	-0.049201434	0.110294666	-0.446090786	0.655531669	0.999579949
1269.508382	-0.220481365	0.154290236	-1.429004002	0.153003083	0.972669728
510.0553125	0.353987628	0.170738914	2.073268599	0.038147289	0.726381766
485.0542872	-0.177376581	0.098459357	-1.801520806	0.071620831	0.836066794

125.7508247	0.115045047	0.113449152	1.014067046	0.310550761	0.999579949
757.9667097	0.063180769	0.172691596	0.365858965	0.714470318	0.999579949
30.47297453	0.058259401	0.176572174	0.32994667	0.741440259	0.999579949
68.0543435	-0.031353781	0.143477911	-0.218526885	0.827018614	0.999579949
92.68924416	0.017171461	0.1279847	0.134168073	0.893269671	0.999579949
241.144378	-0.028272125	0.131461673	-0.215059834	0.829720669	0.999579949
461.7315049	-0.114264662	0.10585482	-1.07944695	0.280388531	0.999579949
307.9349845	-0.009359408	0.183828195	-0.050913887	0.95939414	0.999579949
422.9235526	0.065859348	0.075503238	0.872271836	0.383060098	0.999579949
334.1637205	-0.0450631	0.121441884	-0.371067197	0.710587482	0.999579949
89.49970926	-0.275615197	0.278584065	-0.989343009	0.322495348	0.999579949
977.131166	0.336869109	0.14118672	2.38598296	0.01703354	0.570162425
90.43670284	0.008262776	0.166752102	0.049551254	0.960479993	0.999579949
151.6883643	0.064506607	0.103450108	0.623552827	0.532921299	0.999579949
926.5294506	0.077850732	0.106229091	0.732856989	0.463645658	0.999579949
2446.441287	0.002373517	0.085476466	0.027768082	0.977847123	0.999579949
54.67435375	-0.162471235	0.141141161	-1.151125824	0.249680475	0.999579949
51.63805129	-7.00307E-05	0.208849604	-0.000335316	0.999732456	0.99989122
81.02223278	0.190070361	0.180040785	1.055707247	0.291101985	0.999579949
87.38415591	0.156721729	0.236347129	0.66309978	0.507266651	0.999579949
192.1991367	0.029494993	0.102966529	0.286452243	0.774531766	0.999579949
478.6158976	-0.060937958	0.079681146	-0.764772609	0.444406961	0.999579949
2160.168631	0.042883185	0.132247429	0.324264793	0.745737572	0.999579949
519.0144533	0.043668864	0.088681201	0.49242527	0.622418739	0.999579949
2047.663418	-0.020464463	0.150408675	-0.13605906	0.891774592	0.999579949
165.4024792	-0.128295293	0.124846019	-1.027628225	0.304124742	0.999579949
939.069663	0.15784381	0.096974029	1.627691571	0.103590301	0.895628172
1233.60831	-0.419245423	0.236246595	-1.774609376	0.075962408	0.851207389
109.3320113	0.005326636	0.119239384	0.04467178	0.964368928	0.999579949
1088.938288	0.021662325	0.081084154	0.267158551	0.789347088	0.999579949
25.51526225	-0.03777978	0.236004093	-0.160081036	0.87281724	0.999579949
610.1401838	-0.062745453	0.088852973	-0.706171677	0.480081382	0.999579949
1099.596482	0.083197311	0.088424738	0.940882753	0.346764947	0.999579949
118.7087349	0.232704693	0.270199649	0.861232404	0.389110056	0.999579949
689.2627957	0.120068258	0.094321191	1.272972242	0.203027892	0.994409048
195.4411015	0.245717573	0.235858949	1.041798811	0.297504965	0.999579949
1084.626876	0.014598878	0.088592273	0.164787261	0.869111431	0.999579949
520.4081155	-0.062055714	0.07286514	-0.851651613	0.394407484	0.999579949
22.00545959	0.646557889	0.352426094	1.834591425	0.06656625	0.822978752
42.76040531	-0.637501597	0.467165557	-1.364616009	0.172373771	0.983601342
263.437814	0.010275778	0.086864015	0.118297291	0.905832104	0.999579949
205.7982693	-0.057581153	0.12696409	-0.453523145	0.650172081	0.999579949
368.2326345	0.205172996	0.133680576	1.534800353	0.124832876	0.927119638
296.5421562	0.076374061	0.100314145	0.761348871	0.446448718	0.999579949
152.6988451	0.135364144	0.291408938	0.464516103	0.642278025	0.999579949
117.1849205	-0.15548225	0.213187212	-0.729322588	0.465804358	0.999579949
354.9154756	-0.001850753	0.095698412	-0.019339437	0.984570323	0.999579949
930.7966823	-0.136051606	0.123534998	-1.101320343	0.27075726	0.999579949

221.4795326	-0.066416736	0.128753543	-0.515843952	0.605963402	0.999579949
338.0572726	0.09157448	0.114770554	0.797891767	0.424933302	0.999579949
769.8549751	0.027919611	0.074470796	0.37490684	0.707729752	0.999579949
5321.208329	0.017884737	0.040989889	0.436320686	0.662604076	0.999579949
58.65124751	0.255430006	0.297155101	0.859584793	0.39001796	0.999579949
27.52626977	0.086812963	0.212531706	0.408470643	0.682928179	0.999579949
108.1021659	0.031435578	0.102959984	0.305318405	0.760123659	0.999579949
1998.545203	-0.015414752	0.077550993	-0.198769236	0.842443262	0.999579949
4156.058089	-0.027163128	0.099434059	-0.273177301	0.784716927	0.999579949
625.7620296	-0.421673996	0.272001002	-1.550266333	0.121077605	0.921837625
92.1760135	-0.27426578	0.187223493	-1.464911138	0.142945164	0.952161439
197.6974637	0.116123357	0.214269013	0.541951237	0.587852099	0.999579949
544.7300905	0.24808286	0.110540543	2.244270329	0.024815013	0.640512108
110.3072985	0.676928002	0.368109442	1.838931371	0.065925281	0.82135988
34.24761488	0.226053806	0.221042288	1.022672213	0.306462854	0.999579949
234.699722	-0.065020327	0.11073785	-0.587155402	0.557099342	0.999579949
1367.743832	-0.197461886	0.087638298	-2.253146057	0.024249941	0.635241199
298.1526242	0.011226927	0.106740051	0.105180077	0.916232919	0.999579949
409.8663608	-0.25582503	0.163651286	-1.563232624	0.117997889	0.921521033
352.8331197	0.051436705	0.082629968	0.622494551	0.533616726	0.999579949
634.7192807	-0.142043147	0.142446559	-0.997167978	0.318682981	0.999579949
1642.078828	-0.01668103	0.079470772	-0.20990144	0.833744598	0.999579949
178.5424537	-0.055240473	0.091443181	-0.604096144	0.545779723	0.999579949
2515.5337	0.008343845	0.079889522	0.104442297	0.916818358	0.999579949
34.83945718	0.139814724	0.580822271	0.240718601	0.809773222	0.999579949
175.5801838	-0.063653399	0.122411519	-0.519995172	0.60306694	0.999579949
982.9759977	-0.214359796	0.086602174	-2.475224185	0.013315256	0.516058371
98.06405828	-0.090996037	0.143147947	-0.635678251	0.524986155	0.999579949
1228.732792	0.049392349	0.054879978	0.900006724	0.368116673	0.999579949
303.3501274	0.119086914	0.104946172	1.134742811	0.256483094	0.999579949
196.9141492	-0.016032707	0.144028272	-0.111316387	0.911365461	0.999579949
2725.63564	-0.075736387	0.138861828	-0.545408252	0.585472766	0.999579949
39.63949499	0.046460276	0.214036894	0.217066672	0.828156387	0.999579949
195.5756193	-0.085588241	0.087334653	-0.980003219	0.32708453	0.999579949
2339.557899	-0.044587187	0.110541697	-0.40335175	0.686689477	0.999579949
235.718513	-0.076977582	0.087981913	-0.874925073	0.381614675	0.999579949
44.97398971	0.10295571	0.169543829	0.6072513	0.543684152	0.999579949
23.43119785	-0.126708035	0.237403004	-0.533725491	0.59353147	0.999579949
120.5110589	0.088012781	0.157339366	0.559381819	0.575901167	0.999579949
826.1178229	0.025959058	0.052669334	0.492868549	0.622105472	0.999579949
156.5634196	0.488200647	0.167476672	2.915036705	0.003556467	0.315473661
45.46744847	0.154578037	0.152615137	1.012861768	0.311126192	0.999579949
2032.379498	-0.136872074	0.060053027	-2.279186941	0.022655955	0.628577996
808.0594677	0.072207777	0.16518885	0.437122587	0.662022448	0.999579949
180.6568935	-0.176507068	0.095042359	-1.85714106	0.063291088	0.809353757
474.9317292	-0.039235297	0.068002562	-0.576967921	0.563961126	0.999579949
806.4819091	0.396894156	0.15754642	2.519220401	0.011761501	0.494354131
328.6754321	-0.00100281	0.1021373	-0.009818257	0.99216629	0.999579949

279.8075498	0.072194449	0.126675151	0.569917998	0.568733317	0.999579949
2172.14003	-0.073313846	0.116387417	-0.629912132	0.528752075	0.999579949
111.2871011	0.013670597	0.112501355	0.121514955	0.90328317	0.999579949
24.48446623	0.112494207	0.203845027	0.551861422	0.581043301	0.999579949
87.58039831	0.037080359	0.152937484	0.242454354	0.808428122	0.999579949
118.2854808	-0.285629065	0.386813228	-0.738415968	0.460261714	0.999579949
129.658597	0.018756434	0.128319833	0.146169412	0.883787652	0.999579949
190.6960172	0.027748978	0.11975844	0.231707915	0.816764884	0.999579949
197.0272539	-0.338791417	0.270362395	-1.253101111	0.210168912	0.996581389
2557.317042	0.066838145	0.108208722	0.617677982	0.536787629	0.999579949
1071.381086	-0.132135077	0.136138222	-0.970594999	0.331749997	0.999579949
24.46756988	0.357482585	0.440066079	0.812338422	0.41659747	0.999579949
172.0816745	-0.079613299	0.195428558	-0.407378017	0.68373037	0.999579949
310.97083	0.216906942	0.123466139	1.756813201	0.078949654	0.851207389
490.3383054	-0.028993254	0.120152537	-0.241303723	0.809319725	0.999579949
54.98412089	0.082633985	0.162813492	0.507537699	0.611777592	0.999579949
82.59575175	0.042727782	0.165519317	0.258143777	0.796295947	0.999579949
94.2133489	-0.00388779	0.152271526	-0.025531954	0.979630661	0.999579949
4691.084261	0.162313527	0.107907855	1.504186396	0.13253338	0.939443134
33.95201793	0.354312768	0.260306894	1.361134784	0.173471099	0.983601342
471.2092519	0.103554172	0.125649134	0.824153485	0.409852353	0.999579949
51.94087339	0.376644755	0.263080774	1.431669633	0.152238391	0.972669728
982.3353419	-0.036913561	0.067176894	-0.549497875	0.582663823	0.999579949
63.80291996	-0.098056152	0.153781403	-0.637633358	0.523712376	0.999579949
132.4078419	-0.33414463	0.304085646	-1.098850389	0.271833329	0.999579949
3423.839611	-0.056369807	0.072292959	-0.779741319	0.435543153	0.999579949
146.3219282	-0.29471942	0.127316039	-2.314864827	0.020620331	0.611138086
31.16296739	-0.131480066	0.229922906	-0.57184414	0.567427571	0.999579949
46.36332905	-0.027920691	0.246827791	-0.113118102	0.909936924	0.999579949
701.4185863	0.106170416	0.126868437	0.836854445	0.40267439	0.999579949
1531.748306	0.090880554	0.100951848	0.900236654	0.367994324	0.999579949
81.88175742	-0.138023966	0.210136326	-0.656830587	0.511289854	0.999579949
358.8033211	0.063755734	0.076248541	0.836156774	0.403066712	0.999579949
6174.499303	-0.054731883	0.12290318	-0.445325201	0.656084758	0.999579949
22.06931713	-0.043696492	0.307304246	-0.142192932	0.886927614	0.999579949
1338.840263	-0.38316034	0.104437041	-3.668816522	0.000243676	0.073079555
68.26064431	0.070657683	0.232732975	0.303599794	0.761432808	0.999579949
1549.048661	0.026028703	0.167634551	0.155270513	0.876608061	0.999579949
613.1502494	0.18332744	0.190891036	0.960377417	0.336865299	0.999579949
143.4103478	0.071224	0.115196406	0.618283175	0.536388691	0.999579949
412.7947895	0.062821741	0.092888365	0.676314428	0.498841034	0.999579949
120.5838455	0.016849055	0.209187579	0.080545197	0.935803651	0.999579949
74.46293001	-0.011155157	0.160179491	-0.069641604	0.944478922	0.999579949
29.93855781	0.231073444	0.320011164	0.722079323	0.47024571	0.999579949
119.2643321	-0.159763083	0.134668245	-1.186345623	0.235485836	0.999579949
513.3197029	0.277521489	0.088127343	3.149096306	0.001637762	0.214762992
875.1813862	0.063783991	0.076224443	0.836791835	0.402709588	0.999579949
111.7835423	-0.110205202	0.143612096	-0.767381059	0.442854986	0.999579949

127.3263529	0.114742037	0.162524067	0.706000279	0.480187964	0.999579949
82.32802988	-0.118030912	0.138217102	-0.853953025	0.393131018	0.999579949
30.69877588	-0.319136837	0.331655799	-0.962253149	0.335922457	0.999579949
641.8160533	0.048229301	0.061325089	0.78645301	0.431602138	0.999579949
755.6625013	-0.139945986	0.116691334	-1.199283448	0.230417749	0.999579949
28.50333047	0.291221271	0.286056622	1.018054639	0.308651991	0.999579949
27.63840137	0.111718597	0.376788307	0.296502294	0.766846515	0.999579949
239.1455197	-0.035226883	0.096924117	-0.363448066	0.716270201	0.999579949
2002.427854	0.057977625	0.066836468	0.867454942	0.385692789	0.999579949
93.50012241	-0.060941716	0.155386807	-0.392193634	0.694915147	0.999579949
1346.645682	0.037367542	0.066054848	0.565704767	0.571594494	0.999579949
155.1138568	-0.060135457	0.121843635	-0.493546148	0.621626741	0.999579949
27.66136619	-0.22140989	0.274786312	-0.805752979	0.420385295	0.999579949
115.7938919	0.013132192	0.176052472	0.074592487	0.940538952	0.999579949
57.64435087	0.031559844	0.174624525	0.180729732	0.85657972	0.999579949
69.33611153	0.262654937	0.208139403	1.261918378	0.206978158	0.994814558
45.60940264	0.09661199	0.207283563	0.466086115	0.641153862	0.999579949
45.4892481	0.08192783	0.184521863	0.444000667	0.657042101	0.999579949
57.06851327	0.049576979	0.150732513	0.328907005	0.74222598	0.999579949
100.1500905	-0.000813878	0.162567851	-0.005006388	0.996005497	0.999579949
106.3955697	-0.250669899	0.154051136	-1.627186308	0.103697535	0.895628172
182.9026133	-0.053243373	0.20774302	-0.2562944	0.797723521	0.999579949
71.50062049	-0.05194354	0.247284395	-0.210055877	0.833624062	0.999579949
79.66616292	-0.172575931	0.169380559	-1.01886505	0.308267038	0.999579949
90.03197387	0.077368772	0.183579883	0.421444718	0.673430371	0.999579949
80.20907673	0.091102453	0.192605726	0.472999713	0.636213384	0.999579949
26.65623464	-0.05022854	0.22145474	-0.226811764	0.820570116	0.999579949
192.7850102	-0.081826152	0.150216853	-0.544720182	0.585945982	0.999579949
37.39490769	-0.070145255	0.215528657	-0.325456747	0.744835413	0.999579949
36.00879719	-0.07795709	0.197351869	-0.395015719	0.692831288	0.999579949
42.2852822	0.078586007	0.19992178	0.39308377	0.694257611	0.999579949
56.17432888	0.022384153	0.142406266	0.157185171	0.875098915	0.999579949
374.0548237	0.002851681	0.116254648	0.02452961	0.980430166	0.999579949
102.5912993	0.053505928	0.218429161	0.244957805	0.806489103	0.999579949
88.00121034	-0.190247527	0.292617986	-0.650156642	0.515591045	0.999579949
306.6050321	0.001240008	0.091253694	0.013588574	0.98915822	0.999579949
561.8231271	0.15144487	0.146512735	1.033663526	0.30129349	0.999579949
24.11662161	-0.060221811	0.292928405	-0.205585427	0.837114762	0.999579949
6622.81776	-0.022667609	0.085385958	-0.265472333	0.79064562	0.999579949
1161.891776	-0.2293899	0.123344241	-1.859753633	0.06292039	0.807133028
113.697339	-0.000360598	0.097855812	-0.003684991	0.997059809	0.999579949
1189.68314	-0.00697986	0.055700708	-0.125310085	0.900278068	0.999579949
110.9405543	0.065368458	0.135371757	0.482881067	0.629180186	0.999579949
308.5768097	0.15579834	0.100079739	1.55674207	0.119531736	0.921521033
30.12036614	-0.084055378	0.238577165	-0.352319462	0.724598694	0.999579949
41.35248395	0.436343002	0.199000703	2.192670652	0.028331117	0.666721367
597.7227509	0.013307319	0.115564619	0.115150463	0.908325862	0.999579949
49.99084412	-0.082588235	0.189894562	-0.434916272	0.663623203	0.999579949

283.0719905	0.006150795	0.073278481	0.083937261	0.933106314	0.999579949
1009.862589	0.185033324	0.114063844	1.622190854	0.104762489	0.895628172
23.85935323	0.203965769	0.217886915	0.936108389	0.349217384	0.999579949
277.7185426	0.081751211	0.08351336	0.978899792	0.327629495	0.999579949
763.4731809	0.08505492	0.158152501	0.537803195	0.590712926	0.999579949
123.9961562	-0.121801063	0.105433106	-1.155244955	0.24799013	0.999579949
303.551102	-0.035278525	0.116651898	-0.302425643	0.762327609	0.999579949
2154.454582	0.041177702	0.09348173	0.440489305	0.659582756	0.999579949
1738.626498	-0.058924553	0.071327058	-0.826117808	0.408737267	0.999579949
36.64949007	-0.175028789	0.165050813	-1.060453961	0.288938124	0.999579949
476.2083966	-0.133756255	0.137994586	-0.969286249	0.332402388	0.999579949
658.2033396	-0.03501606	0.07612512	-0.459980352	0.645530323	0.999579949
461.8998116	0.043183484	0.119885108	0.360207239	0.718692161	0.999579949
255.1892045	0.144958363	0.096226779	1.506424347	0.131958275	0.939285826
28.44481259	0.442545725	0.289809885	1.527020809	0.126755837	0.933836888
1633.92056	1.184933494	0.564227106	2.100100264	0.035720022	0.713754582
1681.14686	0.001475714	0.117559861	0.012552876	0.989984517	0.999579949
6580.85137	-0.166392866	0.095619714	-1.740152312	0.081832277	0.85448976
665.1419777	0.306941671	0.149414717	2.054293422	0.039947301	0.73515901
430.3578017	0.435890492	0.196750625	2.215446544	0.02672943	0.653787748
429.3211048	0.195322081	0.07911777	2.46875111	0.013558549	0.518640006
34.34144468	-0.271980529	0.229795618	-1.183575786	0.23658103	0.999579949
146.7704823	-0.101725306	0.110270377	-0.922508008	0.356263655	0.999579949
373.9720836	-0.051676248	0.080860957	-0.639075401	0.522773881	0.999579949
115.511421	0.199872497	0.12617505	1.584088906	0.113173489	0.916629381
538.3237162	0.036399058	0.140837053	0.258448022	0.796061159	0.999579949
88.44285032	0.145883977	0.160374424	0.909646146	0.363009154	0.999579949
702.6625727	0.105409993	0.068713596	1.534048555	0.125017708	0.927398735
115.6461886	-0.068817222	0.167713557	-0.410325933	0.681566871	0.999579949
495.2994477	-0.006648957	0.068174813	-0.097528063	0.922307049	0.999579949
176.1203972	-0.000128406	0.131319918	-0.000977814	0.999219817	0.999840121
212.1019163	-0.149730003	0.097148798	-1.541243999	0.123257413	0.92236461
142.1813532	0.072687727	0.11855433	0.613117441	0.539798689	0.999579949
325.0448174	0.022450214	0.093195049	0.240894919	0.80963656	0.999579949
319.8644731	0.3183057	0.163044163	1.952266767	0.050906541	0.778178142
158.4105823	-0.067970306	0.115888022	-0.586517098	0.557528075	0.999579949
118.9571667	-0.012868211	0.093370783	-0.137818385	0.890383954	0.999579949
115.8917495	0.111442945	0.13769439	0.809349928	0.418313895	0.999579949
30.53445435	-0.073644917	0.237969913	-0.309471545	0.756962855	0.999579949
455.7489042	0.080769939	0.066711829	1.210728893	0.225999325	0.999579949
192.8290601	-0.026784912	0.19621502	-0.13650796	0.891419733	0.999579949
73.23550097	-0.088600002	0.204628954	-0.432978816	0.66503016	0.999579949
190.5139703	-0.084508545	0.085021478	-0.993967012	0.320238928	0.999579949
21.90474505	0.876629988	0.54846572	1.598331411	0.10996924	0.909091147
54.58288212	-0.045153385	0.144991531	-0.311420844	0.75548071	0.999579949
406.4746328	0.05968854	0.093746395	0.636702239	0.524318815	0.999579949
103.863479	0.120051937	0.115408983	1.040230436	0.298232855	0.999579949
309.7681832	0.044130829	0.107502742	0.410508869	0.681432699	0.999579949

302.4542097	-0.157906058	0.13561902	-1.164335636	0.24428802	0.999579949
260.924417	0.023651667	0.097488014	0.242611023	0.808306741	0.999579949
90.80198191	-0.174898448	0.128559449	-1.360448024	0.17368819	0.983601342
463.8320107	-0.022410054	0.132665654	-0.168921291	0.865858552	0.999579949
676.3742353	-0.15777854	0.158131581	-0.997767421	0.318392152	0.999579949
1650.393622	-0.070272452	0.112866476	-0.622615807	0.533537021	0.999579949
70.3485615	0.47503286	0.313148103	1.516959084	0.129277015	0.935700262
524.9216871	0.064625373	0.126537223	0.51072223	0.609545573	0.999579949
178.9066487	-0.060190335	0.138108053	-0.435820598	0.662966898	0.999579949
33.89150733	-0.075473273	0.164967631	-0.45750353	0.647309165	0.999579949
164.9738938	-0.021654323	0.086185593	-0.251252239	0.801619098	0.999579949
419.8093747	0.326728843	0.225685447	1.447717819	0.147695984	0.962301096
153.7842125	0.152922733	0.164043325	0.93220942	0.351228308	0.999579949
2014.133436	0.008190277	0.09131369	0.089693851	0.928530502	0.999579949
82.16507879	0.062360754	0.130580558	0.477565386	0.632959572	0.999579949
28.88997738	-0.086352672	0.22420555	-0.385149572	0.700126602	0.999579949
43.32461892	0.556319723	0.377484213	1.473756263	0.140547228	0.948318761
454.5335403	0.180703676	0.14543115	1.24253762	0.214038269	0.999268361
407.1069408	-0.080312666	0.069886982	-1.149179203	0.250482094	0.999579949
872.0497788	0.079560636	0.099524497	0.799407568	0.424054124	0.999579949
398.0805607	0.095185947	0.083735165	1.136749976	0.255642821	0.999579949
178.3172478	-0.291952082	0.161298998	-1.810005552	0.070294926	0.834620594
1373.713305	0.129722748	0.10175013	1.27491481	0.202339397	0.994409048
555.9891813	-0.228394007	0.131764487	-1.733350258	0.083033433	0.859571728
144.4067131	-0.045356204	0.208721713	-0.217304674	0.827970915	0.999579949
839.341843	0.061224454	0.093996183	0.65135043	0.514820302	0.999579949
163.385098	-0.091456198	0.096097454	-0.951702618	0.341247821	0.999579949
313.8290191	0.081269548	0.125292272	0.648639751	0.516571256	0.999579949
431.6075939	0.082875189	0.140506408	0.589832097	0.555303221	0.999579949
73.43988666	-0.140394002	0.178812514	-0.7851464	0.432367737	0.999579949
99.19022167	0.05207469	0.159902378	0.325665514	0.744677439	0.999579949
220.1082433	0.1752272	0.137104974	1.27805137	0.201231314	0.993208272
222.0451384	0.045411528	0.090318978	0.502790541	0.615111545	0.999579949
348.2621526	-0.108111401	0.227286195	-0.475661977	0.634315214	0.999579949
846.2830589	-0.117161098	0.123289131	-0.950295429	0.341962161	0.999579949
282.4490141	0.572265524	0.207777929	2.754217089	0.005883273	0.372390484
462.056657	0.143073204	0.138966469	1.029551988	0.303220363	0.999579949
27.79388079	-0.241185991	0.239227172	-1.008188112	0.313364164	0.999579949
1826.196513	0.052809905	0.118297885	0.446414621	0.655297773	0.999579949
752.0714667	-0.075481287	0.088208766	-0.855711857	0.39215718	0.999579949
5084.378432	-0.026385592	0.09618817	-0.274312235	0.783844683	0.999579949
221.8283872	-0.063372056	0.097251409	-0.651631233	0.514639094	0.999579949
982.5436723	0.022822909	0.075723022	0.301399865	0.763109597	0.999579949
118.5148747	0.040701934	0.103310739	0.393975834	0.693598881	0.999579949
476.9928812	0.182247801	0.112630761	1.618099708	0.105641112	0.896383253
242.2773141	-0.112562821	0.075316873	-1.494523293	0.135038898	0.945791454
59.6126795	0.488744332	0.471625819	1.03629681	0.300063694	0.999579949
367.5349314	-0.108847234	0.085994714	-1.265743317	0.205604995	0.994814558

170.5014703	0.216156649	0.147125823	1.469195853	0.141779675	0.949966262
1509.907213	0.041425444	0.093629668	0.442439293	0.65817135	0.999579949
125.1369997	0.048965256	0.122218019	0.4006386	0.688686223	0.999579949
641.9972679	0.489830597	0.316766915	1.54634393	0.122021528	0.92236461
275.0038509	-0.041042091	0.100732819	-0.40743515	0.683688415	0.999579949
1878.275336	0.0226386	0.079553685	0.284570103	0.775973519	0.999579949
352.5376589	-0.007160853	0.180578331	-0.039655108	0.968368092	0.999579949
423.4500988	0.065882102	0.093272817	0.706337646	0.479978187	0.999579949
468.2897752	-0.051974398	0.131224032	-0.396073779	0.692050601	0.999579949
609.9342703	0.189846667	0.094356883	2.01200655	0.044219247	0.758836007
195.7325875	-0.051351344	0.151249756	-0.339513568	0.734222878	0.999579949
58.97695408	0.254039884	0.159826529	1.589472565	0.111953743	0.913563961
750.7707874	-0.019614372	0.092557446	-0.211915654	0.832172838	0.999579949
504.7028764	-0.081015159	0.149646801	-0.541375814	0.588248575	0.999579949
1090.166547	-0.00975884	0.091782102	-0.106326177	0.915323563	0.999579949
45.65300861	0.146376685	0.461907254	0.316896268	0.751322314	0.999579949
214.1931836	0.141706335	0.268774199	0.527231913	0.59803255	0.999579949
240.2433182	-0.153533735	0.085676411	-1.792018755	0.073129962	0.838669905
50.34262314	0.188074021	0.204183717	0.921101954	0.3569972	0.999579949
58.74075368	0.074422275	0.127705384	0.582765369	0.560051261	0.999579949
93.88333369	-0.120286076	0.131144678	-0.917201354	0.359037137	0.999579949
261.0973893	-0.013403404	0.210942818	-0.063540464	0.949336139	0.999579949
1866.984544	-0.020462221	0.072895062	-0.28070792	0.778934434	0.999579949
259.5804168	0.034802855	0.097230149	0.357943036	0.720385944	0.999579949
794.4488581	0.073783625	0.133377559	0.553193698	0.580130782	0.999579949
31.36685346	0.994083361	0.574328844	1.730860937	0.08347657	0.859920327
126.181163	0.012932004	0.411596672	0.031419117	0.974935296	0.999579949
30.02795055	0.243158086	0.445427517	0.545898214	0.585135906	0.999579949
41.07753086	-0.183625979	0.260348252	-0.70530905	0.48061793	0.999579949
126.9735458	0.101844608	0.096821021	1.051885296	0.292852179	0.999579949
1164.15976	-0.030389058	0.098720624	-0.307828869	0.758212553	0.999579949
179.5746941	0.016122618	0.094385549	0.170816594	0.864367981	0.999579949
228.2386049	0.134657265	0.095028136	1.417025209	0.156475557	0.976692824
415.2741095	0.294609802	0.17546876	1.678987194	0.09315454	0.880418343
177.4052936	0.027354515	0.094055694	0.29083316	0.771178923	0.999579949
1386.131152	-0.183270887	0.137143914	-1.336339916	0.181438195	0.983992835
898.7527942	-0.069837397	0.09987356	-0.699258107	0.484390743	0.999579949
814.142395	0.000725369	0.055493011	0.01307136	0.98957086	0.999579949
489.3287629	0.002369726	0.085957569	0.027568557	0.97800626	0.999579949
382.3181729	-0.143081744	0.069273195	-2.065470559	0.038878498	0.728740425
25.64796354	-0.354579787	0.824808674	-0.429893378	0.667273203	0.999579949
44.27064721	0.793346874	0.335465426	2.364913977	0.018034259	0.578992883
53.83401285	-0.204072192	0.142437393	-1.432715019	0.151939296	0.971979364
39.75184519	-0.322442684	0.23567903	-1.368143293	0.171267228	0.983264357
1112.173683	0.007918209	0.062694455	0.1262984	0.899495723	0.999579949
212.7671096	0.163197945	0.167318343	0.975373906	0.329374822	0.999579949
190.2707647	-0.089150683	0.144377634	-0.617482647	0.536916424	0.999579949
123.1121184	0.038220601	0.123830167	0.308653391	0.757585203	0.999579949

29.02568399	0.072121909	0.215406944	0.334817012	0.737763127	0.999579949
72.39213795	-0.002479095	0.14180613	-0.017482287	0.986051864	0.999579949
73.70128487	-0.129198635	0.200966442	-0.642886614	0.520297676	0.999579949
125.7604622	0.148848551	0.149086357	0.998404912	0.318083053	0.999579949
168.6511504	-0.088067807	0.144582742	-0.609117009	0.542446884	0.999579949
146.2065238	-0.012381916	0.166748466	-0.074255054	0.94080744	0.999579949
66.18901742	-0.191454033	0.164689374	-1.162516005	0.245025927	0.999579949
53.96138122	0.176907292	0.171338351	1.032502594	0.301836733	0.999579949
265.5143545	-0.214482199	0.175719258	-1.220595858	0.222239075	0.999579949
73.54608126	0.474616586	0.423606868	1.12041759	0.262535853	0.999579949
83.98976345	0.2886387	0.137498185	2.099218255	0.035797666	0.713754582
111.5332248	0.478929209	0.248506249	1.927232053	0.053950717	0.782044005
1068.307104	0.18741246	0.115041866	1.62908049	0.103295981	0.895628172
818.7603288	0.068993946	0.086869975	0.794220852	0.427066871	0.999579949
689.3690283	-0.102134128	0.08721867	-1.171012214	0.241593868	0.999579949
160.7594172	-0.809206933	0.502645972	-1.609894395	0.107420913	0.900848082
398.001395	-0.010223695	0.116086228	-0.088069838	0.929821169	0.999579949
1071.438377	-0.00668801	0.066141596	-0.101116553	0.919457938	0.999579949
946.6599235	0.082841556	0.094406994	0.877493843	0.380218461	0.999579949
108.4924267	0.128636231	0.151406131	0.849610448	0.395541701	0.999579949
218.7997761	0.098208657	0.089708396	1.094754351	0.27362427	0.999579949
349.7500204	-0.03877591	0.098226329	-0.394760859	0.693019384	0.999579949
875.619873	-0.036047645	0.162473434	-0.221867934	0.824416693	0.999579949
489.3774455	0.172545453	0.109654129	1.573542686	0.115593212	0.918818353
267.3460509	-0.239294295	0.136309587	-1.755520648	0.079170292	0.851207389
156.0536096	0.078177999	0.105310205	0.7423592	0.457869732	0.999579949
31.04582061	0.222738768	0.199819434	1.114700223	0.264978905	0.999579949
450.5463381	0.117316317	0.116844638	1.004036798	0.315360877	0.999579949
635.288394	-0.041293689	0.070024555	-0.589702975	0.5553898	0.999579949
526.3373817	0.396268332	0.346105969	1.144933537	0.25223669	0.999579949
187.5570772	0.102190481	0.088515329	1.154494729	0.248297398	0.999579949
572.8468897	0.163196431	0.188949993	0.863701705	0.387751777	0.999579949
626.6542149	-0.056681469	0.068340603	-0.829396672	0.406879984	0.999579949
549.7189391	0.114892916	0.070211378	1.636385999	0.101758828	0.895628172
221.3006645	0.056475977	0.207959187	0.271572405	0.78595082	0.999579949
1955.90258	0.026835575	0.065916677	0.407113587	0.683924565	0.999579949
52.62616723	-0.142126581	0.353348761	-0.402227478	0.687516622	0.999579949
22.48661873	-0.425098135	0.613074416	-0.693387498	0.488066406	0.999579949
70.59743595	0.280860079	0.405556474	0.692530134	0.488604468	0.999579949
182.0741571	0.171966298	0.530440961	0.324194983	0.74579042	0.999579949
142.4463436	0.234067859	0.37812244	0.619026628	0.535898817	0.999579949
323.4330077	0.318309753	0.236471459	1.346081066	0.178276374	0.983992835
139.4706805	-0.621004644	0.625901648	-0.992176081	0.321111638	0.999579949
234.5874224	-0.314230227	0.592052156	-0.530747542	0.595593736	0.999579949
239.7340393	-0.519588368	0.638476702	-0.813793779	0.4157631	0.999579949
36.55454296	2.171946856	0.958770558	2.265345799	0.023491468	0.62865781
552.7678634	-0.071107784	0.122366332	-0.581105791	0.561169156	0.999579949
49.51377464	0.531054864	0.481601412	1.102685436	0.270163794	0.999579949

242.5536068	0.05894395	0.099886348	0.59011017	0.555116791	0.999579949
161.3954208	0.014995371	0.099356219	0.150925338	0.880034612	0.999579949
896.7109666	-0.041165369	0.077559331	-0.530759716	0.595585299	0.999579949
23.02930054	0.105389641	0.304064303	0.346603134	0.728889491	0.999579949
379.2179676	-0.059256219	0.079717642	-0.743326287	0.45728416	0.999579949
45.3172634	-0.03580592	0.163144481	-0.219473685	0.826281077	0.999579949
183.4252786	0.046313812	0.173293306	0.267256785	0.789271458	0.999579949
3884.089674	-0.00405123	0.130488894	-0.031046551	0.975232415	0.999579949
802.2761805	-0.269477306	0.319863475	-0.842476017	0.399521559	0.999579949
33.35812355	-0.073611418	0.244020147	-0.301661232	0.762910325	0.999579949
63.41255997	0.199352579	0.165879398	1.201792272	0.229444021	0.999579949
88.5150549	0.102349855	0.153307758	0.667610406	0.504382313	0.999579949
674.2892898	-0.043563834	0.081548655	-0.534206651	0.593198568	0.999579949
104.9290741	0.161032123	0.112796404	1.427635256	0.15339687	0.973156976
250.0681381	-0.236263503	0.387674075	-0.609438491	0.542233832	0.999579949
107.0733128	-0.089123772	0.193378163	-0.46087816	0.644886022	0.999579949
92.56787348	0.308242848	0.191879935	1.606436066	0.108178132	0.903743816
883.4470234	-0.059177774	0.096534722	-0.613020608	0.539862713	0.999579949
217.086047	0.112797016	0.107069092	1.053497458	0.292113059	0.999579949
50.09708897	0.180463997	0.1807252	0.998554695	0.318010456	0.999579949
52.68593818	-0.055725668	0.233713111	-0.238436206	0.811542789	0.999579949
48.18384122	0.746362289	0.237845374	3.13801474	0.001700963	0.214855947
65.66709063	0.220061538	0.136065137	1.617324929	0.105808161	0.896383253
65.75784876	2.016611136	0.557239383	3.618931465	0.000295822	0.079280286
1008.254158	0.117436532	0.104696183	1.121688759	0.261994798	0.999579949
180.1309541	0.253391077	0.145872054	1.7370776	0.082373475	0.856126169
34.51331713	0.288320702	0.480881157	0.599567477	0.548794528	0.999579949
154.8810397	0.34962519	0.257434623	1.358112541	0.174427976	0.983992835
123.9647767	0.079426874	0.152644434	0.520339143	0.602827218	0.999579949
835.9069345	-0.07781942	0.064031883	-1.215323002	0.224242922	0.999579949
769.0905767	0.019836247	0.078678604	0.252117431	0.800950297	0.999579949
84.07042332	0.08496944	0.126931058	0.669414102	0.503231358	0.999579949
206.2949627	-0.101252401	0.093884694	-1.078476132	0.280821328	0.999579949
3187.065719	-0.00112007	0.064711499	-0.017308663	0.986190375	0.999579949
3729.389543	-0.050565685	0.057076383	-0.88593009	0.3756552	0.999579949
27.27236507	-0.545620369	0.790997128	-0.689788053	0.490327483	0.999579949
111.1074408	0.209976483	0.1668724	1.258305643	0.208281241	0.995521169
1084.590364	0.041958442	0.066493777	0.631013071	0.528031978	0.999579949
630.9838227	-0.018471147	0.333534708	-0.055379986	0.955835741	0.999579949
380.9994437	0.307090932	0.458895288	0.669196088	0.503370402	0.999579949
31.58944161	0.189940704	0.670626366	0.283228804	0.77700145	0.999579949
21.18780551	1.090892904	0.535104104	2.03865546	0.041484426	0.746712667
43.8320785	0.315041405	0.276375236	1.13990461	0.254326044	0.999579949
273.466281	0.11759917	0.093675274	1.255391782	0.209336571	0.996447459
118.9902479	0.017231154	0.149999803	0.114874514	0.908544586	0.999579949
409.8261849	-0.023474747	0.096873575	-0.242323533	0.808529481	0.999579949
83.93463577	0.500725863	0.179741423	2.785812277	0.005339378	0.360508567
813.2596878	-0.055936408	0.05389242	-1.037927207	0.299303946	0.999579949

948.0631058	0.25187733	0.265365267	0.949172183	0.342533047	0.999579949
193.8731184	0.09608935	0.177952413	0.539972169	0.589216226	0.999579949
33.75428242	0.156129182	0.407085851	0.383528884	0.701327658	0.999579949
216.72722	0.057761831	0.298020704	0.193818181	0.846318251	0.999579949
321.360938	0.011075286	0.101207699	0.109431256	0.912860444	0.999579949
467.3714635	0.01508351	0.081964056	0.184025907	0.853993131	0.999579949
82.11441414	-0.145349754	0.193252814	-0.752122317	0.451977504	0.999579949
232.6496763	0.00391571	0.116793602	0.033526754	0.973254531	0.999579949
533.7823301	0.329464788	0.127879938	2.57636023	0.009984656	0.472698434
38.11097154	0.136166452	0.164926129	0.825620857	0.4090192	0.999579949
1158.417795	0.026891803	0.077404287	0.347420076	0.728275753	0.999579949
62.37986429	0.217023041	0.148975635	1.456768689	0.145180242	0.955875831
459.2143038	0.072451404	0.12255919	0.591154395	0.554416976	0.999579949
24.08091274	0.196748983	0.319193477	0.616394124	0.537634431	0.999579949
143.166109	0.455139565	0.342400424	1.329261103	0.183761842	0.983992835
291.3429605	0.219576099	0.329155517	0.667089227	0.504715141	0.999579949
56.41362783	-0.563120896	0.434444179	-1.296186998	0.194911068	0.988443849
42.36000229	0.120443958	0.29339046	0.410524452	0.68142127	0.999579949
109.3258198	-0.003050452	0.138925154	-0.021957522	0.98248184	0.999579949
167.1991911	0.066893964	0.10449587	0.640158925	0.522069283	0.999579949
312.9787552	-0.084412002	0.087350476	-0.966359949	0.333864098	0.999579949
38.71624621	-0.322350803	0.318496916	-1.012100232	0.311490132	0.999579949
299.0681723	-0.012852638	0.106377899	-0.120820565	0.903833161	0.999579949
74.48515824	0.008879832	0.122945003	0.072226047	0.942422016	0.999579949
81.11060751	-0.001414823	0.138403351	-0.010222463	0.991843797	0.999579949
205.6798322	0.100636098	0.165169784	0.609288793	0.542333034	0.999579949
40.20532828	0.154737179	0.38606863	0.400802259	0.688565716	0.999579949
638380.5519	0.319080822	0.206669181	1.543920677	0.12260755	0.92236461
29.88200943	0.41783189	0.273746372	1.526346767	0.126923527	0.933836888
1184.120982	0.011166307	0.117561908	0.094982355	0.924328842	0.999579949
23.22871879	-0.170194516	0.40448952	-0.420763722	0.673927625	0.999579949
419.1583606	0.075037709	0.108284148	0.692970401	0.488328127	0.999579949
262.3473262	-0.12522078	0.168984592	-0.74101892	0.458681968	0.999579949
2157.850425	-0.216812243	0.122712947	-1.766824517	0.077257621	0.851207389
422.7405171	0.092280667	0.279136105	0.330593804	0.740951327	0.999579949
139.3522197	0.052155012	0.136903674	0.380961376	0.703231909	0.999579949
62.32686755	0.324149459	0.183845454	1.763162765	0.077873042	0.851207389
538.1389802	0.365959228	0.16253176	2.251616719	0.024346504	0.63537469
287.3415256	-0.116007747	0.129161552	-0.898160056	0.369100225	0.999579949
879.1825678	-0.007613687	0.126108772	-0.060373966	0.951857793	0.999579949
2302.824036	-0.031836372	0.094668246	-0.33629409	0.736649106	0.999579949
39.76240814	0.321902566	0.358445168	0.898052463	0.369157581	0.999579949
90.45688942	0.14937971	0.124087789	1.203822807	0.228658073	0.999579949
499.7064656	0.034392261	0.071943918	0.478042654	0.632619847	0.999579949
168.7378706	0.009828521	0.119098301	0.082524444	0.934229681	0.999579949
286.2638012	0.053358234	0.138239664	0.38598353	0.699508866	0.999579949
48.1477024	-0.16756831	0.526723516	-0.318133336	0.750383797	0.999579949
279.6393812	-0.028250549	0.101242225	-0.279039198	0.780214743	0.999579949

11744.89421	0.046162122	0.078525336	0.587862781	0.5566244	0.999579949
446.2620386	0.117932171	0.101164259	1.165749372	0.243715792	0.999579949
1088.639938	0.011848389	0.095497523	0.12407012	0.901259756	0.999579949
773.0642731	0.175001063	0.090868232	1.925877273	0.0541197	0.782654121
3371.096081	0.217933818	0.119461351	1.824303982	0.068106129	0.829912169
374.4123707	0.108976718	0.118018121	0.923389707	0.355804154	0.999579949
144.3768389	0.317170904	0.165788792	1.913102211	0.055734969	0.785214091
778.6904945	0.070596757	0.076061987	0.928147692	0.353330963	0.999579949
261.5821464	-0.056083638	0.086399522	-0.649119769	0.516260965	0.999579949
372.5784089	-0.031293898	0.075184956	-0.416225535	0.677244976	0.999579949
126.7538262	0.105908679	0.096578002	1.096612855	0.272810666	0.999579949
459.5671599	-0.105929158	0.053359062	-1.985214043	0.047120663	0.768027574
466.4444346	-0.004763347	0.08542722	-0.05575912	0.955533702	0.999579949
136.323489	0.01511324	0.10914804	0.138465518	0.889872519	0.999579949
522.7459391	-0.145497642	0.108321777	-1.34319845	0.179207723	0.983992835
49.13498901	-0.099661662	0.291291135	-0.342137643	0.73224731	0.999579949
834.0041316	-0.025026767	0.075488998	-0.331528667	0.740245191	0.999579949
1355.173084	-0.050666042	0.096769907	-0.523572294	0.600576056	0.999579949
145.9377781	0.012309072	0.2185709	0.056316151	0.955089952	0.999579949
234.4276441	0.080916856	0.100093835	0.808409988	0.418854604	0.999579949
780.744299	0.06888875	0.125715179	0.547974802	0.583709205	0.999579949
263.1381749	-0.010857873	0.128781618	-0.084312287	0.932808144	0.999579949
113.19014	0.089307664	0.136524776	0.65414987	0.513015255	0.999579949
1510.266831	0.049370229	0.09277773	0.532134476	0.594632861	0.999579949
165.7187831	-0.031151335	0.125922858	-0.247384272	0.804610845	0.999579949
518.5070308	0.043066609	0.059923885	0.718688529	0.472332853	0.999579949
435.5176651	0.302333271	0.160655516	1.881872953	0.059853268	0.798635337
29.2519406	0.950689314	0.275650665	3.44889179	0.000562892	0.12017272
154.9060134	0.00865486	0.14296519	0.060538233	0.951726966	0.999579949
151.9095622	-0.097913587	0.140591072	-0.696442424	0.486151802	0.999579949
382.6514568	-0.011369395	0.056963907	-0.199589446	0.841801682	0.999579949
469.1553714	-0.15961516	0.105337395	-1.515275362	0.129702685	0.935700262
119.9889567	0.224677595	0.158791325	1.414923606	0.157090896	0.977304288
2691.279558	0.090596693	0.057846357	1.566160719	0.117310996	0.920479614
72.99550775	0.096172646	0.245882172	0.391133059	0.695698886	0.999579949
276.1254466	0.075042682	0.164209408	0.456993802	0.647675501	0.999579949
198.0973836	0.054697434	0.101509096	0.538842691	0.589995404	0.999579949
352.0382923	0.40534961	0.323002973	1.254940801	0.209500251	0.996447459
303.686206	0.003437274	0.075560008	0.045490655	0.963716223	0.999579949
212.1270077	0.175747842	0.088936055	1.976114655	0.048141793	0.770705547
202.4690763	0.036856794	0.10144201	0.363328704	0.716359353	0.999579949
307.0888815	0.059116517	0.138221464	0.427694193	0.668873778	0.999579949
80.85075534	-0.069614968	0.175335048	-0.397039658	0.691338216	0.999579949
1022.510071	0.041057645	0.099385289	0.413115919	0.679521687	0.999579949
167.3189791	-0.181004748	0.179901398	-1.006133085	0.314351555	0.999579949
148.7357167	0.189656325	0.226945297	0.835691803	0.403328308	0.999579949
116.7841888	0.026140982	0.118706198	0.220215813	0.825703082	0.999579949
3914.276959	0.009521592	0.107964904	0.088191544	0.929724438	0.999579949

341.7377224	0.130731164	0.076302224	1.713333596	0.086651185	0.867486202
214.3554935	-0.177911841	0.123145462	-1.444729162	0.148533975	0.962301096
242.2349016	-0.260947913	0.15641874	-1.668265021	0.095263129	0.883604099
977.7710644	0.042708069	0.093030234	0.459077301	0.646178656	0.999579949
303.0106202	-0.043182346	0.091441126	-0.472242061	0.636754021	0.999579949
1521.048716	-0.117132879	0.110218652	-1.062731913	0.287903544	0.999579949
547.6832578	0.03733281	0.108276228	0.344792299	0.730250523	0.999579949
1406.67449	-0.037650006	0.086928518	-0.433114546	0.664931556	0.999579949
27.33746947	-0.781370813	1.139652617	-0.685621918	0.492951556	0.999579949
46.04359489	0.168032601	0.286816748	0.585853518	0.557973956	0.999579949
46.56544684	-0.042872507	0.210114129	-0.204043903	0.838319191	0.999579949
45.40788391	-0.020946537	0.174672426	-0.119918969	0.904547338	0.999579949
47.9724136	0.022228842	0.193339443	0.114973135	0.908466416	0.999579949
67.49928468	0.093327435	0.137795362	0.677290105	0.498221908	0.999579949
43.42244393	-0.023850479	0.205779547	-0.115903059	0.907729371	0.999579949
234.1807909	0.071609885	0.120113636	0.59618448	0.551051993	0.999579949
190.4259263	0.065509256	0.124418088	0.526525177	0.598523364	0.999579949
31.40420181	0.059810771	0.33682335	0.177573113	0.859058242	0.999579949
691.3774045	0.02449073	0.108957572	0.224773093	0.822155799	0.999579949
449.8655614	0.049834147	0.055103612	0.904371683	0.365798348	0.999579949
9348.976358	0.0873744	0.171508863	0.509445394	0.610440064	0.999579949
248.9078794	0.476903388	0.178088143	2.677906456	0.007408391	0.411084085
386.4197579	0.268234394	0.294617666	0.910449118	0.362585704	0.999579949
172.5188667	0.014899945	0.165840396	0.08984509	0.928410317	0.999579949
24.62324506	-0.287593759	0.278208411	-1.033734953	0.301260088	0.999579949
135.4166887	-0.006409029	0.101192467	-0.063335038	0.949499715	0.999579949
803.9616597	-0.049286703	0.086628179	-0.568945384	0.569393205	0.999579949
7553.44781	0.070288141	0.115736984	0.60730925	0.543645701	0.999579949
116.3511554	0.107087789	0.120611071	0.887876943	0.374606949	0.999579949
399.872862	-0.102415899	0.132841327	-0.770964136	0.44072819	0.999579949
25.10094834	0.50849547	0.633502273	0.802673473	0.422163489	0.999579949
701.5659984	0.008220059	0.070846903	0.116025668	0.907632199	0.999579949
1359.656017	0.043065079	0.076748178	0.561121842	0.574714479	0.999579949
42.19738642	-0.173648199	0.190318716	-0.912407369	0.361554312	0.999579949
559.8426174	0.054627661	0.098045227	0.557167978	0.577412666	0.999579949
698.3190134	-0.003281418	0.074490053	-0.04405176	0.964863145	0.999579949
323.3903078	-0.116183749	0.100304132	-1.158314684	0.246735641	0.999579949
1741.487519	-0.0101788	0.100814817	-0.100965315	0.919577994	0.999579949
1426.790007	0.024613153	0.11106269	0.221614957	0.824613638	0.999579949
669.9301605	0.024926457	0.070254442	0.354802579	0.722737496	0.999579949
66.933795	-0.105431761	0.150495913	-0.700562288	0.48357622	0.999579949
1233.265399	0.015761486	0.109758283	0.143601792	0.885814926	0.999579949
54.18289792	0.085118778	0.225882209	0.376828164	0.706301309	0.999579949
320.0616175	0.081284183	0.0772333	1.052449954	0.29259316	0.999579949
98.22046388	0.376268036	0.247543601	1.520007118	0.128509187	0.935700262
401.8249989	-0.11874704	0.052405755	-2.265916032	0.023456525	0.62865781
339.3138215	0.120100451	0.113050276	1.06236318	0.288070842	0.999579949
189.8918625	0.302749767	0.153765516	1.968905473	0.048963947	0.773861498

201.5977148	-0.003620342	0.103957591	-0.034825178	0.972219144	0.999579949
936.0264472	0.05726292	0.096627904	0.592612666	0.553440401	0.999579949
283.0954584	-0.149261357	0.108873804	-1.37095749	0.170388208	0.981568287
39.79873348	-0.414634771	0.612473587	-0.676983922	0.498416155	0.999579949
1132.201584	0.292951343	0.530486688	0.552231282	0.580789905	0.999579949
329.56052	-0.096915206	0.05670302	-1.709171871	0.087419118	0.867715687
1546.76393	0.101975881	0.099662247	1.023214747	0.306206322	0.999579949
39.42217164	-1.293407467	0.480581164	-2.691340326	0.007116556	0.407455156
60.77334962	0.218468829	0.332214135	0.65761449	0.510785882	0.999579949
87.01171138	0.118219448	0.123792205	0.954982978	0.339586305	0.999579949
50.75096956	-0.096246677	0.265248778	-0.362854364	0.716713679	0.999579949
2519.835727	0.018825471	0.125211298	0.150349623	0.880488785	0.999579949
51.47475146	0.213556744	0.153445002	1.391747793	0.163998784	0.978498637
224.6938726	-0.088223281	0.105444018	-0.836683607	0.402770436	0.999579949
2501.87923	0.00557438	0.106567373	0.052308501	0.958282879	0.999579949
673.0323173	0.059026807	0.166645181	0.354206507	0.723184128	0.999579949
55.05386457	-0.091240839	0.855462297	-0.106656763	0.915061285	0.999579949
1457.640508	0.037218413	0.134524756	0.276665902	0.782036656	0.999579949
147.8750927	0.181298161	0.227221381	0.79789217	0.424933068	0.999579949
442.8217681	-0.022696629	0.091101763	-0.249134905	0.80325643	0.999579949
657.1792709	-0.003216491	0.067278695	-0.047808463	0.961868892	0.999579949
635.9107432	-0.092655098	0.074744931	-1.239617146	0.215117036	0.999579949
563.5204544	0.026504863	0.090374273	0.293278849	0.769309019	0.999579949
3225.82978	0.016039897	0.107992659	0.148527659	0.881926359	0.999579949
255.3903035	0.028170392	0.072621381	0.387907682	0.698084353	0.999579949
31.47723174	0.133388985	0.336064774	0.39691451	0.691430504	0.999579949
196.7549118	0.06430151	0.194687282	0.330280998	0.741187649	0.999579949
2400.251763	0.007594096	0.132240679	0.057426321	0.954205596	0.999579949
1440.151865	-0.114447589	0.078347132	-1.460775733	0.144076998	0.955657641
923.6226309	0.161434206	0.096393223	1.674746435	0.093983993	0.880418343
53.02146425	-0.423111074	0.192617925	-2.196633947	0.0280466	0.665301263
68.21629976	-0.040445524	0.168916294	-0.239441226	0.810763466	0.999579949
265.3121993	0.106830463	0.076770993	1.391547223	0.16405955	0.978498637
29.81844417	0.276699474	0.24690917	1.120652887	0.262435644	0.999579949
53.3100725	-0.105770371	0.180218229	-0.586901621	0.557269781	0.999579949
237.3807171	0.140625134	0.236797996	0.593861168	0.552604973	0.999579949
2113.817596	0.153322564	0.091712056	1.671781992	0.094567319	0.880418343
35593.44377	0.00060811	0.117486568	0.005175999	0.995870169	0.999579949
121.7181058	0.426050754	0.391972112	1.086941496	0.277062666	0.999579949
244.6816443	0.107366518	0.224578018	0.478081155	0.632592444	0.999579949
453.1662846	0.089435057	0.132340796	0.675793555	0.499171727	0.999579949
480.4921168	0.12471122	0.076433401	1.631632472	0.102756933	0.895628172
110.3437927	-0.007644029	0.182966119	-0.041778385	0.966675366	0.999579949
2124.943925	0.014811265	0.11749846	0.126054969	0.899688413	0.999579949
390.7837681	0.019544173	0.061951199	0.315476923	0.75239957	0.999579949
198.661563	-0.106538557	0.29937703	-0.355867507	0.721939791	0.999579949
332.6202756	0.004755767	0.090710406	0.052428023	0.958187645	0.999579949
163.0133623	0.072364926	0.239839148	0.301722745	0.762863428	0.999579949

1133.102592	0.169623258	0.201900449	0.84013314	0.40083374	0.999579949
374.9195992	-0.111555097	0.114816548	-0.971594239	0.33125245	0.999579949
160.0299361	-0.108701611	0.102875087	-1.056636883	0.290677341	0.999579949
844.4526675	-0.029703754	0.135040354	-0.219962059	0.825900703	0.999579949
73.79820189	0.042416457	0.138879582	0.305418957	0.760047085	0.999579949
286.525302	0.150841878	0.126805827	1.189550045	0.23422329	0.999579949
3894.864515	-0.040168561	0.050775014	-0.791108823	0.428880491	0.999579949
171.5336183	0.073250884	0.096370939	0.760093084	0.447198946	0.999579949
610.8977201	-0.079897811	0.130897464	-0.610384716	0.54160699	0.999579949
1083.96836	-0.213728918	0.12103055	-1.765908832	0.077411145	0.851207389
406.6698611	0.082582493	0.11197703	0.737494941	0.460821418	0.999579949
1917.737551	0.27813313	0.122146585	2.277043856	0.022783607	0.62865781
782.8912874	0.060636617	0.101944496	0.594800297	0.551976969	0.999579949
52.3074071	0.277843079	0.25404331	1.093683904	0.274093636	0.999579949
220.6083143	-0.036316093	0.145539763	-0.249526949	0.802953198	0.999579949
381.6632848	0.020121086	0.095164113	0.211435647	0.832547343	0.999579949
172.3590977	0.058837061	0.105392696	0.558265076	0.576663389	0.999579949
40.70871585	0.40222683	0.187816024	2.141600178	0.032225669	0.690330837
130.968362	0.046858272	0.140867773	0.332640112	0.739405961	0.999579949
46.35627987	-0.195621124	0.214391908	-0.912446398	0.361533775	0.999579949
400.6280174	0.090937643	0.087669936	1.037272836	0.299608722	0.999579949
215.3796674	0.057961203	0.111421193	0.520199092	0.602924818	0.999579949
399.694388	-0.129297368	0.117764638	-1.097930328	0.272234914	0.999579949
27.39211697	-0.194862405	0.183845889	-1.059922557	0.289179833	0.999579949
540.7815571	-0.103553304	0.082593245	-1.253774496	0.209923982	0.996550653
100.6328445	0.047933189	0.156578955	0.306127914	0.759507256	0.999579949
89.67854365	0.021010397	0.475361952	0.044198736	0.96474599	0.999579949
2756.557918	0.061844423	0.118190351	0.523261187	0.600792507	0.999579949
352.1090274	0.092260906	0.066384804	1.389789531	0.164592798	0.978498637
41.7479517	-0.200424269	0.260835006	-0.768394825	0.442252654	0.999579949
373.6461559	0.304842493	0.118281783	2.577256498	0.009958802	0.472698434
42.72533158	0.022546265	0.165716534	0.136053199	0.891779225	0.999579949
284.0117723	0.079936032	0.083429611	0.958125432	0.337999513	0.999579949
32.30950467	0.212936994	0.17828746	1.194346443	0.232342477	0.999579949
719.6303099	0.006975898	0.067909803	0.102722988	0.918182829	0.999579949
624.6543395	-0.017209515	0.090217373	-0.190756106	0.84871668	0.999579949
55.35852576	-0.068012845	0.14697131	-0.462762732	0.643534447	0.999579949
596.838146	0.069061247	0.138910635	0.497163135	0.619074015	0.999579949
1886.863576	-0.090061016	0.151857246	-0.593063677	0.553138538	0.999579949
158.3630214	-0.007409427	0.19558683	-0.037883054	0.969780924	0.999579949
711.1742751	0.14981067	0.092264371	1.623710948	0.104437513	0.895628172
193.6173228	0.010630797	0.150340603	0.070711416	0.943627435	0.999579949
588.3773737	-0.063492574	0.194647003	-0.326193431	0.744278011	0.999579949
401.7018746	-0.045380179	0.083826283	-0.541359799	0.588259611	0.999579949
117.7496385	-0.113220025	0.136820574	-0.82750731	0.407949581	0.999579949
27.99549063	0.087291498	0.223639972	0.390321542	0.696298796	0.999579949
1851.397734	0.149443738	0.113309985	1.318892925	0.187204912	0.983992835
28.2741908	0.314270268	0.185727937	1.692100135	0.090626883	0.877608448

1821.668718	0.162147978	0.100971205	1.605883359	0.108299541	0.903743816
341.9633561	0.004534543	0.088189598	0.051418114	0.958992351	0.999579949
22.69475096	0.181603253	0.270174134	0.672171131	0.501474757	0.999579949
70.50549247	0.577926249	0.516163409	1.119657533	0.262859728	0.999579949
110.7421394	0.386662629	0.127851933	3.024300213	0.00249209	0.255470986
614.5219113	-0.056343535	0.087602732	-0.643170976	0.520113164	0.999579949
3691.287375	0.073857753	0.094411103	0.782299439	0.434038617	0.999579949
2094.226696	-0.059083423	0.108111716	-0.546503424	0.584719935	0.999579949
246.8838821	-0.012573876	0.112714708	-0.111554882	0.911176347	0.999579949
365.5657497	0.070527256	0.094054704	0.749853567	0.453342903	0.999579949
317.9974258	-0.011585384	0.088781042	-0.130493893	0.896175686	0.999579949
58.84970573	-0.150816702	0.162541554	-0.927865509	0.353477338	0.999579949
411.1959225	0.1188305	0.136695345	0.869309043	0.384678118	0.999579949
1297.857769	-0.068511259	0.075982246	-0.901674562	0.36722977	0.999579949
263.3727148	0.200467655	0.10384633	1.930426003	0.053554076	0.781231069
209.1295064	0.149560746	0.134090424	1.115372306	0.264690912	0.999579949
769.3711159	-0.070806336	0.081864771	-0.864918275	0.387083647	0.999579949
368.4061936	0.088266765	0.103178049	0.855480079	0.392285428	0.999579949
154.8257798	0.082850697	0.157187923	0.527080551	0.598137652	0.999579949
112.788161	-0.007371691	0.161864622	-0.045542323	0.963675041	0.999579949
122.6900064	0.077129652	0.127683438	0.60406935	0.545797536	0.999579949
29.07210255	-0.269918121	0.194501217	-1.387745154	0.165214661	0.978789551
120.8119614	0.186967153	0.130801793	1.429392895	0.152891339	0.972669728
731.3964396	-0.018673942	0.12604767	-0.148149839	0.882224518	0.999579949
32.16301576	0.11100533	0.263175361	0.421792257	0.673176658	0.999579949
65.96710877	0.05651955	0.184487917	0.306359087	0.759331256	0.999579949
265.9846402	-0.003022623	0.09805607	-0.030825454	0.975408741	0.999579949
873.8637413	-0.140560472	0.093604017	-1.50165	0.133187523	0.941431
7928.040318	0.078745309	0.087582082	0.899102967	0.368597818	0.999579949
185.5968364	-0.148547256	0.125300922	-1.18552405	0.235810312	0.999579949
35.23147378	0.099187349	0.233806787	0.424227843	0.671399657	0.999579949
890.2951873	-0.067013639	0.063713589	-1.051795067	0.292893583	0.999579949
496.7259861	-0.151889832	0.074985404	-2.025591975	0.042806615	0.750791966
2426.250157	0.103135854	0.092523728	1.114696258	0.264980605	0.999579949
599.7917905	0.010388213	0.074630523	0.139195234	0.889295875	0.999579949
1009.520051	0.04102919	0.108580619	0.377868448	0.705528322	0.999579949
121.8906663	-0.024987602	0.185600602	-0.134631037	0.892903601	0.999579949
700.2954524	0.043072495	0.058302952	0.738770405	0.460046426	0.999579949
119.3492846	-0.051091388	0.147845699	-0.345572363	0.729664119	0.999579949
1471.368286	-0.050879814	0.079725248	-0.638189472	0.52335035	0.999579949
191.9620601	0.214870829	0.142768534	1.505029315	0.13231654	0.939285826
168.9751872	0.00361409	0.096474577	0.037461575	0.970116977	0.999579949
532.8715431	0.110226598	0.097701212	1.128200932	0.25923507	0.999579949
533.4895324	0.10693478	0.11656611	0.917374525	0.358946417	0.999579949
48.42822703	0.098324167	0.162324597	0.60572562	0.544696968	0.999579949
264.9573069	-0.084522873	0.118768862	-0.711658529	0.476676254	0.999579949
60.62248448	0.096086178	0.184726273	0.52015437	0.602955986	0.999579949
1337.902701	0.160978471	0.125635047	1.281318195	0.200081924	0.992610246

29.26455334	-0.163319825	0.297128294	-0.549660967	0.582551934	0.999579949
58.36452256	-0.135326259	0.211941782	-0.63850675	0.523143862	0.999579949
36.82341794	-0.546607396	0.376900487	-1.450269805	0.146983296	0.959874957
50.45786684	0.041628758	0.282802031	0.147201057	0.882973327	0.999579949
83.42830449	-0.076678675	0.163795514	-0.468136602	0.639686906	0.999579949
67.54007441	-0.071421329	0.146281317	-0.488246418	0.62537531	0.999579949
1890.661557	-0.024076729	0.092360446	-0.260682252	0.794337554	0.999579949
173.1363808	0.112889347	0.096152106	1.174070462	0.240366804	0.999579949
1150.929555	0.017747005	0.155239719	0.114319999	0.90898413	0.999579949
27.50603734	-0.035314503	0.26927706	-0.131145604	0.895660126	0.999579949
150.9694414	-0.045002964	0.121238327	-0.371194203	0.71049289	0.999579949
41.76788969	0.010685854	0.226268625	0.047226406	0.962332782	0.999579949
22.16118137	0.074309994	0.241236334	0.308038148	0.758053305	0.999579949
637.6608964	0.051806934	0.057497347	0.901031738	0.367571445	0.999579949
286.8334585	0.023428789	0.083648483	0.280086239	0.779411342	0.999579949
519.6431943	-0.049430313	0.07960294	-0.620960895	0.534625353	0.999579949
286.5077924	-0.098872289	0.118393069	-0.835118894	0.403650769	0.999579949
92.52320398	0.201170975	0.123854891	1.624247325	0.104323034	0.895628172
32.90018056	0.302199854	0.236076483	1.280093002	0.200512429	0.993204353
1416.099124	-0.175090637	0.139932219	-1.251253203	0.210842113	0.996581389
27.22779163	0.603257771	0.410874567	1.468228554	0.14204215	0.950166185
77.30662873	0.360249525	0.320614378	1.123622489	0.261173211	0.999579949
803.0177667	-0.018835432	0.07253964	-0.259657092	0.795128293	0.999579949
43.43148039	-0.191732834	0.238115706	-0.805208682	0.420699268	0.999579949
72.60375957	-0.054492189	0.149656416	-0.364115288	0.715771921	0.999579949
182.7974637	0.277131621	0.158265304	1.751057332	0.079936035	0.854007036
1746.914854	-0.021974734	0.119470908	-0.183933762	0.854065419	0.999579949
637.9802941	-0.067791605	0.083361317	-0.813226167	0.4160884	0.999579949
454.9635661	0.069776854	0.098899885	0.70553018	0.480480357	0.999579949
103.1597453	0.020388453	0.137590421	0.148182212	0.88219897	0.999579949
506.3291437	-0.026347655	0.102067939	-0.258138401	0.796300096	0.999579949
339.8210927	-0.02730939	0.089417504	-0.305414367	0.760050581	0.999579949
1320.71752	-0.078621173	0.073839967	-1.064750925	0.286988658	0.999579949
34.28199119	-0.269222969	0.199918281	-1.346665089	0.178088121	0.983992835
307.9845728	-0.120117503	0.141086892	-0.851372523	0.39456245	0.999579949
76.54771548	-0.072119845	0.171792156	-0.419808716	0.674625197	0.999579949
393.5030731	0.070299631	0.067697632	1.038435604	0.299067301	0.999579949
127.6238262	0.098591131	0.114541693	0.860744485	0.389378786	0.999579949
1165.960782	-0.076309101	0.134184777	-0.568686727	0.569568757	0.999579949
117.3427528	-0.100098676	0.109379733	-0.915148295	0.360113784	0.999579949
145.0908691	-0.089019312	0.106069766	-0.839252466	0.401327651	0.999579949
340.3352297	0.035560622	0.069036076	0.515102016	0.606481734	0.999579949
782.1515219	-0.035137661	0.105327088	-0.333605168	0.738677518	0.999579949
267.4274196	-0.051485903	0.138949885	-0.370535769	0.710983331	0.999579949
298.7525309	0.033697191	0.080626927	0.417939665	0.675991231	0.999579949
81.08280438	0.144842802	0.124783002	1.160757469	0.245740544	0.999579949
961.5993498	0.020792366	0.04755426	0.437234566	0.661941244	0.999579949
93.52676325	0.248225177	0.160545302	1.546137903	0.122071267	0.92236461

312.4512416	-0.203641549	0.121082873	-1.68183612	0.092600623	0.880418343
167.8791905	-0.105439781	0.130315376	-0.809112363	0.418450518	0.999579949
27.0225523	-0.437962266	0.269962092	-1.622310237	0.104736938	0.895628172
672.8174508	0.041345595	0.064966796	0.636411176	0.524508458	0.999579949
508.3682943	-0.032762547	0.062824047	-0.521496921	0.60202065	0.999579949
243.3050556	-0.003469951	0.09224459	-0.037616849	0.969993174	0.999579949
101.2858064	-0.150602979	0.146887561	-1.025294302	0.305224344	0.999579949
8251.654333	-0.008722642	0.140695352	-0.061996659	0.950565492	0.999579949
359.3332486	-0.034911897	0.143506162	-0.243278036	0.807790018	0.999579949
344.6661897	0.076131278	0.082099413	0.927305995	0.353767682	0.999579949
28.97826589	-0.152815625	0.566222082	-0.269886376	0.787247669	0.999579949
73.40296477	0.073898051	0.200384161	0.368781897	0.712290299	0.999579949
786.5834835	-0.048992883	0.19501296	-0.251228855	0.801637177	0.999579949
42.83762646	0.345242443	0.296523632	1.164299926	0.244302486	0.999579949
76.06906645	-0.208779043	0.164119498	-1.272116022	0.203331899	0.994409048
21.00481604	-0.06630134	0.226165662	-0.293153874	0.769404539	0.999579949
1508.84442	-0.09505601	0.062725178	-1.515436286	0.129661954	0.935700262
188.7076008	0.08648584	0.108341299	0.798272127	0.424712589	0.999579949
2335.209745	-0.127061299	0.120687295	-1.05281421	0.292426151	0.999579949
64.03124959	0.104330577	0.130884606	0.797118777	0.425382052	0.999579949
237.3085159	0.040086673	0.126994751	0.31565614	0.752263521	0.999579949
330.7143845	0.123018437	0.104753368	1.174362595	0.240249821	0.999579949
37.91651211	0.849621835	0.436942521	1.944470483	0.051838741	0.779189484
276.9248472	0.079903823	0.065901656	1.212470633	0.225332278	0.999579949
682.6515016	0.02282278	0.081183342	0.281126395	0.778613457	0.999579949
1130.550821	-0.021741593	0.052858759	-0.411314858	0.680841676	0.999579949
241.8541814	0.329661997	0.250582322	1.315583615	0.188313836	0.983992835
1406.107893	0.077837156	0.082374365	0.944919639	0.344699904	0.999579949
5622.327458	0.067558479	0.098456863	0.686173381	0.492603781	0.999579949
1167.017822	-0.031783986	0.064850068	-0.490114919	0.624052582	0.999579949
63.97368647	-0.207449321	0.137186503	-1.512170052	0.130490608	0.935700262
242.0832041	-0.032939944	0.094696046	-0.347849196	0.727953441	0.999579949
43.69412134	0.171824514	0.200606324	0.856525907	0.391706949	0.999579949
20.69882954	-0.125626608	0.289953244	-0.433265056	0.664822221	0.999579949
1107.178271	0.014918817	0.152415649	0.097882446	0.922025639	0.999579949
1131.969678	0.107881022	0.09766936	1.104553384	0.26935316	0.999579949
921.1908266	0.063381719	0.105436801	0.601134697	0.547750275	0.999579949
41.08279688	-0.044913871	0.171905727	-0.261270361	0.793884022	0.999579949
1419.861652	0.216717797	0.138376342	1.566147753	0.117314031	0.920479614
49.22334398	-0.104038116	0.147597493	-0.704877257	0.480886625	0.999579949
460.2450568	0.000209228	0.083221562	0.002514108	0.997994034	0.999579949
60.43991432	-0.019257127	0.184299856	-0.104488019	0.916782076	0.999579949
60.47373616	-0.043903218	0.30633833	-0.143316109	0.886040534	0.999579949
543.2709388	-0.080068094	0.056026195	-1.42911889	0.152970065	0.972669728
486.3730306	-0.412362826	0.113053887	-3.647489158	0.000264815	0.075121841
331.5682294	-0.083748786	0.11629271	-0.720155084	0.471429515	0.999579949
123.8259793	-0.059663486	0.168206362	-0.3547041	0.722811279	0.999579949
728.1056747	-0.082444604	0.10840135	-0.760549603	0.446926131	0.999579949

448.8323715	-0.18475251	0.09926936	-1.861123216	0.062726778	0.807133028
94.9983033	0.156510607	0.23027959	0.679654705	0.496723122	0.999579949
1280.444772	-0.047421407	0.064077373	-0.74006478	0.459260688	0.999579949
76.5570518	-0.053994053	0.137774038	-0.391902955	0.695129919	0.999579949
239.0039646	0.073666609	0.095263962	0.773289362	0.43935115	0.999579949
190.6254402	0.129439326	0.108742141	1.1903327	0.233915653	0.999579949
699.8929422	-0.061719197	0.059489075	-1.037487933	0.299508517	0.999579949
25.43399787	0.08329689	0.232050721	0.358959842	0.719625131	0.999579949
106.7668239	0.029773871	0.128134719	0.232363809	0.816255456	0.999579949
1532.515794	0.244774434	0.206550242	1.185060021	0.235993717	0.999579949
616.4249145	0.00633802	0.071252866	0.088951088	0.929120782	0.999579949
125.922895	0.118752522	0.108445349	1.095044857	0.273496985	0.999579949
311.6751484	0.01163369	0.087177088	0.133448941	0.893838342	0.999579949
237.878272	0.046715749	0.100976048	0.462641879	0.643621085	0.999579949
398.1895242	0.138711221	0.093972863	1.476077427	0.139923108	0.948318761
216.5300675	-0.042307328	0.200238106	-0.211285101	0.832664808	0.999579949
23.87190736	-0.579177938	0.2199104	-2.633699622	0.008446017	0.441435814
39.72502449	-0.223281216	0.155632973	-1.43466524	0.151382514	0.969895293
37.89977742	0.033460269	0.183733389	0.182113167	0.855493916	0.999579949
24.8635415	-0.169519652	0.281759304	-0.601647043	0.547409108	0.999579949
22.95668531	0.12548056	0.240160968	0.522485234	0.601332524	0.999579949
20.43573654	-0.245724395	0.258665401	-0.949970094	0.342127449	0.999579949
135.7156948	0.211786537	0.143782269	1.472966995	0.140759935	0.948318761
35.64402837	-0.133852228	0.203885433	-0.65650707	0.51149792	0.999579949
63.0581942	0.292660668	0.170133211	1.720185408	0.085398745	0.864463816
71.62480889	0.199104282	0.188778495	1.054697896	0.291563514	0.999579949
50.17504462	0.190767354	0.135363041	1.409301631	0.158745997	0.977304288
71.38029925	0.123917723	0.176062065	0.703829772	0.48153879	0.999579949
110.6912953	0.012539327	0.122093692	0.102702494	0.918199094	0.999579949
89.8620167	0.211051728	0.124444995	1.695943883	0.089896508	0.874391055
91.99378852	-0.149332022	0.153741463	-0.971319117	0.331389392	0.999579949
104.8192035	0.060191835	0.117864154	0.51068822	0.609569391	0.999579949
108.3834383	-0.130161144	0.132437972	-0.982808347	0.325701773	0.999579949
118.6213856	0.11027068	0.120776485	0.913014484	0.361234924	0.999579949
106.4443944	-0.122726012	0.143823528	-0.853309704	0.39348758	0.999579949
107.418859	-0.072536901	0.110895793	-0.654099665	0.513047597	0.999579949
153.3541709	-0.022410698	0.140489056	-0.15951917	0.873259856	0.999579949
120.3686892	-0.01512964	0.138102168	-0.109553964	0.912763122	0.999579949
127.3144019	-0.010747481	0.13667132	-0.078637427	0.937321017	0.999579949
107.3585214	-0.072619031	0.127187782	-0.570959175	0.568027317	0.999579949
85.43913173	-0.10614642	0.155395265	-0.683073711	0.494560266	0.999579949
104.4102911	-0.028289728	0.17685112	-0.159963522	0.87290981	0.999579949
407.7416585	-0.099369335	0.087648691	-1.13372298	0.256910767	0.999579949
51.33029632	0.048271576	0.178571397	0.270320873	0.786913409	0.999579949
36.27983434	0.066844913	0.160051795	0.417645506	0.67620632	0.999579949
1807.535767	0.26115787	0.101531527	2.572184994	0.010105888	0.473799407
655.4721471	-0.030849559	0.094871162	-0.325173196	0.745049994	0.999579949
30.44827782	-0.07797963	0.307855243	-0.253299665	0.800036655	0.999579949

467.8514647	-0.061952752	0.125406014	-0.494017391	0.621293897	0.999579949
268.8468789	-0.399930424	0.142665247	-2.803278538	0.005058596	0.355966903
23.21944986	0.048298082	0.216334477	0.223256518	0.823335867	0.999579949
1064.079206	0.011258408	0.093492295	0.120420706	0.90414989	0.999579949
117.8450047	-0.466858398	0.165774182	-2.816231052	0.004859071	0.347269229
1084.203569	0.021448485	0.075114755	0.285542898	0.775228244	0.999579949
104.9994373	0.277795865	0.123664956	2.24635882	0.024681034	0.639857539
565.8829342	0.004235555	0.074785673	0.056635921	0.95483522	0.999579949
176.2966031	0.078151378	0.137628672	0.567842272	0.570142073	0.999579949
550.495262	0.262335546	0.143253083	1.831273303	0.06705976	0.825081178
298.0444791	0.007516107	0.151244155	0.049695188	0.960365291	0.999579949
2127.562629	-0.07334758	0.097702484	-0.750723802	0.452818895	0.999579949
193.7036041	0.088897573	0.087740556	1.013186799	0.310970945	0.999579949
986.0387406	0.02538645	0.126118421	0.201290578	0.840471369	0.999579949
1024.03411	0.063760198	0.072021837	0.885289807	0.376000346	0.999579949
441.6927903	-0.048405449	0.089177017	-0.542801838	0.587266247	0.999579949
555.0351257	-0.052492789	0.044906479	-1.168935751	0.242429518	0.999579949
334.6873721	0.079110123	0.139935212	0.565333929	0.571846655	0.999579949
27.85290391	0.261489941	0.346926825	0.753732264	0.451010005	0.999579949
13439.14337	0.128617377	0.137139148	0.937860404	0.348316154	0.999579949
398.227632	0.02066266	0.079808203	0.258903966	0.795709338	0.999579949
247.2491207	-0.235486922	0.130300156	-1.807265078	0.070720958	0.834620594
265.7198314	0.085372322	0.089517392	0.953695362	0.340237869	0.999579949
82.77194943	-0.085551297	0.199019156	-0.429864633	0.667294114	0.999579949
272.0349797	0.088922483	0.097281569	0.914073285	0.360678339	0.999579949
382.9563691	0.092242507	0.071533641	1.289498272	0.197224917	0.992338959
632.5658895	0.029447027	0.071221453	0.413457259	0.679271631	0.999579949
179.8266211	0.149301166	0.117188887	1.274021532	0.202655785	0.994409048
552.2908728	-0.688092161	0.439857364	-1.564352941	0.117734706	0.921109536
282.4478363	-0.02345928	0.083763455	-0.280065813	0.779427013	0.999579949
110.8562839	-0.0837651	0.203053395	-0.412527455	0.679952861	0.999579949
165.5869851	0.349127532	0.182712323	1.910804522	0.056029706	0.785214091
80.47824886	-0.033917943	0.136332486	-0.248788413	0.803524455	0.999579949
359.4430677	-0.258327791	0.104963765	-2.461114002	0.013850635	0.520783867
246.9859888	-0.188868181	0.121607372	-1.553098117	0.120399696	0.921837625
90.10604792	0.06812746	0.17305837	0.393667524	0.693826521	0.999579949
1637.254782	-0.028405445	0.089422337	-0.317654915	0.750746713	0.999579949
73.21243404	0.153754693	0.350594728	0.43855392	0.660984792	0.999579949
1273.995392	-0.024715685	0.115595737	-0.21381139	0.830694143	0.999579949
22.27750187	-0.231381396	0.476505539	-0.485579656	0.627265223	0.999579949
156.5831887	0.139404438	0.128568643	1.084280235	0.278240566	0.999579949
352.0111366	0.309686095	0.317741945	0.974646566	0.329735607	0.999579949
95.76371797	-0.219337918	0.113366532	-1.934767815	0.053018794	0.781231069
341.6966218	-0.131003254	0.07692813	-1.702930448	0.088581085	0.870840008
110.3969832	-0.15658564	0.131172792	-1.193735666	0.232581384	0.999579949
149.1106886	0.125926477	0.118259195	1.064834553	0.286950806	0.999579949
363.5489025	0.131888179	0.07753091	1.701104487	0.088923368	0.870840008
249.7937976	0.080494244	0.102189679	0.787694455	0.43087545	0.999579949

194.801696	0.053383465	0.132531269	0.40279902	0.687096082	0.999579949
146.4101127	0.269261073	0.164081474	1.641020565	0.100793146	0.895628172
389.119378	0.171823063	0.162380355	1.058151789	0.289986247	0.999579949
176.8496377	0.034701753	0.091648513	0.37863956	0.704955541	0.999579949
209.807429	0.641748773	0.250124869	2.565713583	0.010296381	0.47773736
47.30892857	0.388108126	0.327325634	1.185694264	0.23574306	0.999579949
91.28423027	0.196425922	0.152503589	1.288008522	0.197742997	0.992338959
44.08985819	0.39989703	0.216700794	1.845387928	0.064981128	0.817776877
562.751609	0.068124568	0.067899455	1.003315382	0.315708717	0.999579949
595.5845496	0.35805521	0.240709724	1.487497904	0.136883356	0.945791454
2674.365121	-0.056214905	0.121606005	-0.462270802	0.643887136	0.999579949
594.7472063	-0.112781411	0.085152907	-1.324457559	0.185351129	0.983992835
35.10255957	0.318541398	0.343799095	0.926533558	0.354168766	0.999579949
967.0147918	-0.865297124	0.423463629	-2.043380031	0.041014833	0.745487492
212.3965927	0.361907723	0.791913627	0.457004035	0.647668145	0.999579949
23.35153037	0.906646164	0.434995935	2.084263532	0.037136203	0.717947029
559.4653566	-0.019139241	0.056621342	-0.338021673	0.735346855	0.999579949
256.9852053	0.053985831	0.06266301	0.861526299	0.388948243	0.999579949
3666.415521	-0.01310729	0.130987528	-0.100065174	0.920292584	0.999579949
2579.985874	0.246993061	0.108611428	2.274098278	0.022960078	0.62865781
1612.088105	-0.028123005	0.068832471	-0.408571773	0.682853949	0.999579949
2363.037159	0.102863463	0.127600121	0.806139227	0.420162576	0.999579949
777.830256	-0.224002468	0.562045409	-0.398548701	0.690225767	0.999579949
567.4012036	-0.073115203	0.059907938	-1.220459356	0.222290788	0.999579949
24.3062373	0.072472208	0.245430881	0.295285613	0.767775706	0.999579949
4669.884037	-0.156965972	0.135065371	-1.162148161	0.245175287	0.999579949
854.0928153	-0.182420434	0.120775362	-1.510410992	0.130938586	0.936571507
482.8351221	0.04054702	0.231799509	0.174922803	0.861140292	0.999579949
541.1406385	0.009327012	0.053833822	0.173255623	0.862450504	0.999579949
158.2471404	-0.002526249	0.091635786	-0.027568371	0.978006408	0.999579949
644.5594562	0.04727197	0.123465117	0.382877129	0.70181087	0.999579949
70.25453972	0.043843423	0.144195339	0.304055759	0.761085412	0.999579949
2540.344849	-0.025252063	0.072453337	-0.348528646	0.727443204	0.999579949
57.15795115	-0.254588714	0.212310689	-1.199132813	0.230476307	0.999579949
166.8028428	-0.294121067	0.110336041	-2.665684421	0.007683178	0.418949383
240.7214824	0.054301625	0.094937471	0.571972526	0.567340588	0.999579949
44.41092638	-0.06120342	0.174633827	-0.350467153	0.725988137	0.999579949
789.1444358	-0.307173448	0.075854654	-4.049500355	5.13271E-05	0.035697035
656.3854995	-0.331585687	0.123287474	-2.689532645	0.007155215	0.407814869
70.30102846	0.313548919	0.359811534	0.871425426	0.383521908	0.999579949
449.9182539	0.232150821	0.110321333	2.10431486	0.035350991	0.712820067
480.2601366	0.040442822	0.060796469	0.665216622	0.505911951	0.999579949
227.681276	-0.027521159	0.332001031	-0.082894801	0.933935188	0.999579949
364.2034697	-0.018921147	0.092219559	-0.205174988	0.83743541	0.999579949
99.51187591	0.106040794	0.206919453	0.512473779	0.608319467	0.999579949
677.7722049	0.077357667	0.09627425	0.803513584	0.421677949	0.999579949
989.2447935	-0.048245753	0.06513842	-0.740665071	0.458896543	0.999579949
1884.4426	0.096458603	0.081971345	1.176735638	0.239301039	0.999579949

1159.552117	0.071376269	0.095789731	0.745134871	0.456190191	0.999579949
134.5921056	0.292267253	0.126218018	2.315574734	0.0205815	0.611138086
641.1416746	-0.006548661	0.063556827	-0.103036313	0.917934151	0.999579949
1790.544063	0.184091268	0.116747191	1.576836814	0.114833078	0.918818353
194.4304256	0.02912386	0.182127699	0.159909011	0.87295275	0.999579949
436.2728002	-0.112906814	0.105814292	-1.067028014	0.285959186	0.999579949
24.68273152	0.230031915	0.305376889	0.753272179	0.451286373	0.999579949
122.9758932	0.125280912	0.152816359	0.819813484	0.412322443	0.999579949
797.2047375	-0.051240218	0.071627144	-0.715374292	0.474377794	0.999579949
55.24341431	0.610842601	0.277122062	2.204236637	0.027507706	0.660154442
24.62479565	1.041622045	0.299414941	3.478857945	0.000503555	0.111276918
278.1725085	-0.047566316	0.072064321	-0.660053625	0.509219417	0.999579949
42.49863498	0.708038828	0.3893758	1.818394536	0.069003853	0.831457432
66.17914155	0.101767822	0.184705742	0.550972703	0.581652387	0.999579949
234.1268084	0.04319512	0.089217234	0.484156679	0.628274679	0.999579949
1593.576694	0.011486974	0.101065203	0.113659037	0.909508086	0.999579949
227.3509465	0.270464159	0.172066279	1.571860334	0.115982945	0.918818353
144.4685293	0.203088149	0.139507413	1.45575167	0.14546128	0.956456209
140.2290482	0.029487271	0.10476891	0.28145059	0.778364821	0.999579949
218.6541911	0.105224014	0.094594923	1.112364296	0.265981549	0.999579949
277.2805574	0.111728753	0.070890812	1.57606818	0.115010091	0.918818353
151.2731484	0.181180996	0.278784479	0.64989628	0.515759221	0.999579949
217.9059717	0.099800358	0.11103939	0.898783377	0.368768056	0.999579949
3885.810322	-0.001814099	0.113059281	-0.016045554	0.98719805	0.999579949
494.5086156	0.116147049	0.130295346	0.891413646	0.372707299	0.999579949
55.12476514	-0.106698527	0.21871109	-0.48785147	0.625655051	0.999579949
26.72429692	-0.515188041	0.36932216	-1.394955671	0.163029208	0.978498637
1410.148374	-0.151347465	0.05996211	-2.524051671	0.011601088	0.493624274
27.33402618	1.005270398	0.526287944	1.910114815	0.056118432	0.785214091
56.68052972	0.194960043	0.199629343	0.976610153	0.328762191	0.999579949
30.4402064	-0.051376252	0.297206736	-0.172863685	0.862758577	0.999579949
574.5430253	0.034636556	0.075564351	0.458371645	0.646685459	0.999579949
296.8057569	0.117953397	0.09481477	1.244040319	0.213484724	0.999268361
1285.548085	0.046683264	0.087703288	0.532286358	0.594527679	0.999579949
261.8121223	0.020873967	0.110568324	0.188787951	0.850259016	0.999579949
98.68218632	0.012518023	0.271642841	0.046082655	0.963244371	0.999579949
350.1240607	0.144633251	0.087339173	1.655995203	0.09772279	0.890209389
343.8823449	0.095579726	0.077904978	1.226875727	0.219869303	0.999579949
39.9675362	0.256788844	0.232153802	1.106115176	0.268676671	0.999579949
23.0901779	0.347060532	0.225064333	1.54205034	0.123061361	0.92236461
1958.459451	0.226936469	0.108903277	2.083835089	0.03717517	0.717947029
1048.889276	-0.099198232	0.093026746	-1.066340988	0.286269527	0.999579949
230.3935703	0.055605594	0.118025793	0.471130868	0.637547283	0.999579949
1161.927406	-0.023446966	0.088134114	-0.266037352	0.790210442	0.999579949
415.1800664	0.24336178	0.136038608	1.788917011	0.073628182	0.842343849
372.4699679	-0.207952511	0.124833673	-1.665836678	0.095745949	0.88463985
46.6329852	0.024139338	0.236447194	0.102091879	0.918683747	0.999579949
427.2174345	-0.048048169	0.075636513	-0.635250983	0.525264737	0.999579949

236.9597186	-0.144566063	0.113150588	-1.277643053	0.201375313	0.993208272
474.3743084	0.04499815	0.087621264	0.513552846	0.607564654	0.999579949
1085.290132	-0.164286989	0.115658745	-1.420445897	0.155477909	0.976030424
536.3980651	-0.153043164	0.139527448	-1.096867792	0.27269919	0.999579949
676.2490001	-0.095088373	0.107042881	-0.888320379	0.374368441	0.999579949
96.40374445	0.115497832	0.122340644	0.944067547	0.345135132	0.999579949
31803.7892	0.037357293	0.116459977	0.320773654	0.748381926	0.999579949
622.0365683	0.018426673	0.130098471	0.141636351	0.887367252	0.999579949
1445.076995	-0.08683235	0.078125595	-1.111445622	0.266376584	0.999579949
488.9835639	-0.008595006	0.127084434	-0.067632246	0.946078385	0.999579949
46.61572404	-0.135702735	0.20414746	-0.664728991	0.506223848	0.999579949
163.3908005	0.156919569	0.112694432	1.392434091	0.163790987	0.978498637
155.2787441	0.031895703	0.118175364	0.269901459	0.787236065	0.999579949
940.5022718	0.055531941	0.079813322	0.695772838	0.486571102	0.999579949
241.0362481	0.328187712	0.157243306	2.087133114	0.036876103	0.717947029
236.2814356	0.063928422	0.121165578	0.527612075	0.59776861	0.999579949
1132.663394	-0.036874946	0.059311216	-0.621719604	0.534126258	0.999579949
650.7139274	-0.071952077	0.062343164	-1.154129383	0.248447129	0.999579949
671.2390172	0.012426775	0.300051841	0.041415426	0.966964715	0.999579949
564.3623104	-0.023304618	0.059864414	-0.389290013	0.697061623	0.999579949
748.8774252	0.033228537	0.084626866	0.392647611	0.69457977	0.999579949
35.70914756	0.03089469	0.172616902	0.178978362	0.857954692	0.999579949
463.9795913	-0.039831564	0.050369536	-0.790786808	0.429068409	0.999579949
834.7397103	0.024190182	0.089023794	0.271727153	0.785831821	0.999579949
435.7714268	0.026045039	0.059442043	0.438158545	0.661271357	0.999579949
286.0986504	0.040598492	0.117220169	0.346343915	0.729084269	0.999579949
325.8309329	-0.10410763	0.218226855	-0.477061495	0.633318332	0.999579949
181.2822897	0.062067233	0.103710865	0.598464138	0.549530281	0.999579949
392.7230232	0.07949665	0.105488429	0.753605405	0.451086198	0.999579949
104.2807321	0.277358186	0.142916152	1.94070567	0.052293988	0.780444402
367.7796679	-0.011845538	0.103476445	-0.114475693	0.908860714	0.999579949
161.9692888	0.064012891	0.105780607	0.6051477	0.545080863	0.999579949
214.8913026	-0.003886862	0.092547532	-0.041998552	0.966499853	0.999579949
300.2367263	0.126496216	0.166465279	0.759895497	0.447317053	0.999579949
1556.205426	0.001119799	0.058283187	0.01921307	0.984671131	0.999579949
839.7067285	-0.055599598	0.133863209	-0.415346372	0.677888359	0.999579949
61.49721525	-0.149424062	0.199459024	-0.749146662	0.453768813	0.999579949
627.1705154	-0.039049001	0.071973571	-0.542546382	0.587442164	0.999579949
69.41714339	0.363473926	0.205285491	1.770577764	0.076630942	0.851207389
193.5821697	0.200393653	0.293740354	0.68221356	0.495103922	0.999579949
1225.621626	-0.000884168	0.121207964	-0.00729464	0.994179771	0.999579949
59.85254387	0.252838084	0.300928664	0.840192756	0.400800319	0.999579949
187.6797759	0.141574568	0.340977506	0.415202076	0.677993979	0.999579949
28.99912588	-0.512348832	0.567450116	-0.90289669	0.366580728	0.999579949
30.41820225	-0.266173294	0.232268857	-1.145970658	0.251807293	0.999579949
155.035147	0.207388448	0.147597856	1.405091199	0.159994161	0.978261482
40.1269689	0.464239332	0.173122665	2.681562987	0.007327912	0.410232786
5446.495878	0.097194026	0.138146047	0.70355995	0.48170686	0.999579949

739.6617284	0.14478017	0.181145115	0.799249649	0.424145668	0.999579949
34.66621717	0.28434745	0.290108472	0.980141833	0.327016112	0.999579949
109.7068197	0.277685483	0.205179679	1.353377124	0.175935182	0.983992835
193.4445886	-0.031007342	0.383007206	-0.080957593	0.935475677	0.999579949
531.8951958	-0.045981052	0.064395219	-0.714044508	0.475199658	0.999579949
694.0072533	-0.074408517	0.082585369	-0.900989095	0.367594118	0.999579949
359.8717619	-0.062649008	0.209266812	-0.299373833	0.764654825	0.999579949
26.85657758	-0.857476958	0.582000292	-1.473327367	0.140662785	0.948318761
252.6442325	-0.001156547	0.077609782	-0.014902079	0.988110301	0.999579949
834.6099398	-0.084644698	0.150690379	-0.561712688	0.574311788	0.999579949
109.5767067	0.097837464	0.45157173	0.216659852	0.828473438	0.999579949
1742.930341	-0.041713134	0.065911969	-0.632861292	0.526824227	0.999579949
65.49623161	0.065203332	0.2322928	0.28069459	0.778944658	0.999579949
27.61788574	-0.315557409	0.450890192	-0.699854232	0.484018343	0.999579949
137.3852884	-0.004227087	0.117665664	-0.035924554	0.971342517	0.999579949
25.08797476	-0.029174033	0.285167649	-0.102304849	0.918514707	0.999579949
1172.590635	0.012241952	0.105978985	0.115513018	0.908038503	0.999579949
1329.999037	0.034246067	0.056993799	0.600873571	0.547924198	0.999579949
44.83220398	-0.130856163	0.334861514	-0.390776957	0.695962109	0.999579949
3355.54497	0.097788967	0.292079996	0.334802	0.737774451	0.999579949
190.8261876	0.158759955	0.277986581	0.571106543	0.567927423	0.999579949
88.07439932	-0.05587859	0.165599846	-0.337431414	0.735791707	0.999579949
136.0597393	0.063661509	0.126319616	0.503971684	0.614281276	0.999579949
134.9863524	0.044221117	0.105097151	0.420764187	0.673927285	0.999579949
58.03206553	0.006584192	0.181313245	0.036313904	0.971032064	0.999579949
265.6206449	0.047791539	0.113724938	0.420237988	0.674311606	0.999579949
129.8467775	0.110858785	0.128111929	0.865327575	0.38685902	0.999579949
393.1198941	0.01480753	0.072280671	0.204861541	0.837680304	0.999579949
82910.56784	-0.221643538	0.163435918	-1.356149497	0.175051607	0.983992835
64.97824269	0.052819658	0.203930002	0.259008768	0.795628475	0.999579949
86.57456226	0.105381804	0.146738162	0.718162216	0.472657272	0.999579949
282.0109346	0.037928668	0.106270746	0.356906014	0.721162167	0.999579949
51.42305164	-0.042652868	0.197781082	-0.215656965	0.829255149	0.999579949
326.370188	0.015518985	0.107838996	0.143908842	0.885572454	0.999579949
104.7775194	0.094787077	0.12143685	0.780546245	0.435069419	0.999579949
315.5426053	-0.012937258	0.106961391	-0.120952598	0.903728581	0.999579949
145.6210505	0.021507504	0.101850699	0.211166973	0.832756982	0.999579949
26.30931241	-0.293093843	0.299035597	-0.980130278	0.327021815	0.999579949
221.1949588	-0.269695573	0.161378979	-1.671193942	0.094683376	0.880418343
298.1679874	-0.001091412	0.080528051	-0.013553194	0.989186446	0.999579949
1568.157124	-0.004597102	0.084283897	-0.054543059	0.956502504	0.999579949
953.6182861	-0.066173879	0.110299274	-0.599948451	0.548540591	0.999579949
351.9772813	-0.084629898	0.130763658	-0.64719739	0.517504201	0.999579949
1324.922771	0.023770339	0.066517417	0.357355121	0.72082597	0.999579949
86.63193289	-0.215821145	0.137109098	-1.574083328	0.115468186	0.918818353
515.3450684	0.051183528	0.10481869	0.488305357	0.625333568	0.999579949
256.4313845	-0.048987887	0.087100707	-0.562428127	0.57382436	0.999579949
423.4741067	0.099514532	0.118935839	0.836707702	0.402756889	0.999579949

2084.099741	-0.326091666	0.139042215	-2.345270946	0.019013257	0.590797093
285.2410817	-0.133271253	0.083671323	-1.592794849	0.111206221	0.912543037
287.1429277	0.169100528	0.210869616	0.801919837	0.42259933	0.999579949
193.9889991	-0.08818271	0.111865358	-0.788293279	0.430525178	0.999579949
98.74947837	-0.17247693	0.126632231	-1.362030249	0.173188339	0.983601342
270.5839117	-0.078078573	0.093562407	-0.834507954	0.403994807	0.999579949
681.0438207	0.109797313	0.277172855	0.396132996	0.692006918	0.999579949
123.9463401	0.005423609	0.089549291	0.060565622	0.951705153	0.999579949
86.89758037	-0.146058332	0.198613815	-0.735388585	0.462102872	0.999579949
74.85695437	0.291039396	0.278031438	1.046785924	0.295198324	0.999579949
118.2847832	0.241880376	0.125927156	1.920795993	0.054757432	0.785214091
226.9710785	-0.032861346	0.0852494	-0.38547305	0.69988697	0.999579949
386.3912575	-0.021471134	0.102087919	-0.210320031	0.833417903	0.999579949
368.0691519	0.030692282	0.256931636	0.119456999	0.904913306	0.999579949
631.0623324	-0.064182983	0.126024287	-0.509290587	0.610548555	0.999579949
464.0071337	-0.025133046	0.11169374	-0.225017501	0.82196566	0.999579949
22.94595739	0.283005416	0.242703562	1.166053823	0.243592685	0.999579949
5439.137167	0.075972225	0.10830818	0.701444943	0.483025383	0.999579949
240.9904168	-0.071848454	0.123822522	-0.580253518	0.561743668	0.999579949
161.6318205	0.09817696	0.130754154	0.750851557	0.452741997	0.999579949
1213.829786	-0.032997573	0.088733361	-0.371873354	0.709987145	0.999579949
984.509183	-0.072505451	0.073432399	-0.987376852	0.323457928	0.999579949
87958.42244	0.095182324	0.059398979	1.602423562	0.109061978	0.905566693
21096.38688	0.129793013	0.096017288	1.351767126	0.176449824	0.983992835
211.1421968	0.026249185	0.16503829	0.159049061	0.873630221	0.999579949
31.48420945	-0.064362433	0.330747145	-0.194597092	0.84570838	0.999579949
667.2518753	-0.009799908	0.054967579	-0.178285237	0.858498972	0.999579949
270.6513694	0.106782587	0.108082354	0.987974287	0.323165241	0.999579949
174.0554073	0.047770779	0.16266397	0.293677691	0.769004203	0.999579949
1187.020762	0.081807982	0.104613558	0.782001716	0.434213565	0.999579949
38.97066264	0.175217799	0.175120467	1.000555798	0.317041609	0.999579949
433.4501211	0.006753044	0.09095763	0.074243845	0.940816359	0.999579949
230.9343718	-0.07651314	0.109302842	-0.700010524	0.483920732	0.999579949
1436.759322	0.139451683	0.155412592	0.897299771	0.369558978	0.999579949
122.858143	0.168561775	0.113684306	1.48271807	0.138149332	0.945791454
50.54346899	-0.001092367	0.178234131	-0.006128833	0.99510993	0.999579949
309.5399505	0.077721852	0.075016455	1.036064041	0.300172266	0.999579949
104.5441534	-0.079999617	0.132778154	-0.602505868	0.546837459	0.999579949
122.8039492	0.228290304	0.197077309	1.158379446	0.246709223	0.999579949
105.2638664	-0.002999248	0.123767957	-0.024232832	0.98066689	0.999579949
373.7933034	0.100999924	0.130852755	0.771859364	0.440197727	0.999579949
489.2069454	-0.056688276	0.083482436	-0.679044345	0.497109765	0.999579949
737.5045319	0.00272553	0.052203298	0.052209911	0.958361436	0.999579949
636.0417747	0.010367555	0.12898922	0.08037536	0.935938723	0.999579949
417.1873239	-0.097220643	0.103229153	-0.94179445	0.346297887	0.999579949
158.7187825	-0.10396387	0.140167052	-0.741714036	0.458260614	0.999579949
360.7120427	0.003105843	0.270245573	0.011492671	0.990830377	0.999579949
1302.383355	-0.043445942	0.066593849	-0.652401726	0.514142051	0.999579949

110222.6099	-0.187646415	0.137882271	-1.360917642	0.173539718	0.983601342
229.7119419	0.071851855	0.273403941	0.262804751	0.792701073	0.999579949
48.04092368	0.030706503	0.176863757	0.173616705	0.862166703	0.999579949
283.7001539	0.076167165	0.100544276	0.757548497	0.448721322	0.999579949
49.56525809	0.067277342	0.576113126	0.116778006	0.907035973	0.999579949
1449.486864	0.080762666	0.104291678	0.774392239	0.438698872	0.999579949
354.9556333	0.138431754	0.153754106	0.900345083	0.367936636	0.999579949
1579.260631	0.085998792	0.12692572	0.677552129	0.498055707	0.999579949
92.09492934	0.119882843	0.123524408	0.97051947	0.331787625	0.999579949
96.11300636	0.005080355	0.117380034	0.043281256	0.965477333	0.999579949
408.878331	0.067934374	0.081469754	0.833860059	0.404359846	0.999579949
537.1697373	-0.266923823	0.109649121	-2.434345299	0.014918756	0.53999036
60.77165751	0.234531377	0.195330826	1.200687989	0.229872255	0.999579949
1726.765406	0.057444681	0.101901787	0.563725941	0.57294066	0.999579949
231.1177051	-0.114724665	0.103942436	-1.103732702	0.269709106	0.999579949
88.94656904	0.099160315	0.14814627	0.669340611	0.503278226	0.999579949
158.3827127	-0.003356191	0.113984959	-0.029444154	0.976510358	0.999579949
84.44219775	0.105108038	0.483728227	0.217287378	0.827984393	0.999579949
750.5558642	0.048978765	0.061431249	0.797293975	0.425280319	0.999579949
2736.607516	0.0139173	0.14402749	0.096629473	0.92302065	0.999579949
332.7663324	-0.140532376	0.170306195	-0.825174773	0.409272373	0.999579949
546.4214912	-0.033188174	0.078541583	-0.422555451	0.672619635	0.999579949
372.3963018	-0.111891776	0.183784218	-0.608821463	0.542642786	0.999579949
350.9328351	-0.051915947	0.07837671	-0.662389976	0.507721326	0.999579949
23.69310402	-0.232663156	0.339593629	-0.685122264	0.493266772	0.999579949
835.3815131	-0.02028084	0.065495676	-0.309651586	0.756825923	0.999579949
146.4510209	0.020641169	0.111093303	0.185800296	0.852601372	0.999579949
288.8296097	-0.085226927	0.113248284	-0.752567047	0.451710126	0.999579949
807.8310608	-0.053020984	0.103511382	-0.512223708	0.608494453	0.999579949
655.6807453	-0.1249091	0.164933824	-0.757328586	0.448853029	0.999579949
2240.57563	0.031381879	0.075821133	0.413893569	0.678952054	0.999579949
126.3134186	-0.201590383	0.436024652	-0.462337122	0.643839583	0.999579949
509.9546848	0.010856864	0.122291754	0.08877838	0.92925804	0.999579949
58.31071454	0.42783996	0.174841693	2.447013365	0.01440455	0.533646201
56.97877978	0.294303125	0.240935348	1.221502478	0.221895828	0.999579949
120.0959299	-0.012508027	0.098446612	-0.127053913	0.898897728	0.999579949
251.8540666	0.100222991	0.11441422	0.875966212	0.381048402	0.999579949
496.3755133	-0.006220185	0.129340762	-0.048091452	0.961643359	0.999579949
396.2009103	-0.034353204	0.169641265	-0.202504997	0.839521952	0.999579949
49.58845885	-0.066019155	0.182795894	-0.361163226	0.717977431	0.999579949
47.33953907	0.088312772	0.153669853	0.574691589	0.565499897	0.999579949
185.7700129	0.084986488	0.108170119	0.785674348	0.432058295	0.999579949
183.7605926	0.096490485	0.103310216	0.93398783	0.350310171	0.999579949
1434.343525	0.070138766	0.084777023	0.827332259	0.408048764	0.999579949
269.341835	-0.26292892	0.167478365	-1.569927678	0.116431938	0.918907698
135.5696742	0.359346897	0.128592981	2.794451876	0.005198778	0.357404857
952.6085754	0.039728268	0.163167512	0.243481486	0.807632425	0.999579949
221.0476927	0.074154865	0.140480875	0.527864489	0.597593394	0.999579949

36.18199004	0.47676412	0.326477933	1.460325714	0.144200578	0.955875831
264.5275245	0.062537128	0.074384901	0.840723412	0.400502901	0.999579949
589.2290416	0.002040953	0.113657391	0.017957068	0.985673103	0.999579949
357.6679481	0.096703629	0.104721415	0.923436991	0.355779522	0.999579949
103.7584532	0.004585233	0.140654727	0.032599212	0.973994199	0.999579949
229.0473893	0.012579332	0.179071284	0.070247625	0.943996569	0.999579949
654.8931882	0.105787117	0.111570117	0.948167122	0.343044382	0.999579949
202.3306794	0.067631712	0.088866989	0.761044258	0.446630633	0.999579949
2861.635763	0.00596718	0.097590787	0.061144912	0.951243802	0.999579949
43182.83539	0.015576497	0.094766684	0.164366802	0.869442397	0.999579949
471.2494084	-0.042254677	0.103867259	-0.40681421	0.684144449	0.999579949
21.97025173	0.134226221	0.284639168	0.471566237	0.637236431	0.999579949
52.69929338	0.108635327	0.296956814	0.365828706	0.714492898	0.999579949
11873.50366	-0.046622677	0.059870213	-0.778729104	0.436139308	0.999579949
132.0509104	0.380558705	0.289722579	1.313527949	0.189005109	0.983992835
461.9255389	-0.415511696	0.415869914	-0.99913863	0.31772754	0.999579949
61.52975994	-0.182462334	0.224198472	-0.813842896	0.415734957	0.999579949
236.0850141	-0.23568356	0.151211378	-1.558636421	0.119082458	0.921521033
111.5959851	0.582584676	0.278380468	2.092764197	0.036370209	0.716931389
152.8235481	-0.194734161	0.134625751	-1.446485228	0.148041152	0.962301096
249.7687729	-0.298533675	0.141214282	-2.114047324	0.034511226	0.703403556
72.69844629	-0.063756441	0.184987263	-0.34465314	0.730355151	0.999579949
423.4245312	0.098830121	0.109638095	0.901421361	0.367364328	0.999579949
449.9347804	-0.031058287	0.068408219	-0.454013966	0.649818775	0.999579949
38.8618971	0.052253431	0.2191579	0.238428232	0.811548973	0.999579949
21.03188832	0.215707926	0.242195101	0.890637033	0.373123925	0.999579949
178.6914038	0.044447597	0.151485649	0.293411272	0.769207811	0.999579949
61.70780269	0.078098296	0.172281805	0.453317146	0.650320388	0.999579949
3207.945941	0.010991081	0.042811035	0.256734757	0.797383538	0.999579949
399.4156906	-0.043573636	0.102635436	-0.424547675	0.671166444	0.999579949
22.67943874	0.602912934	0.536478464	1.123834366	0.261083299	0.999579949
101.7551431	-0.070993375	0.187104902	-0.379430866	0.704367933	0.999579949
3365.512554	-0.027113807	0.165007084	-0.164319045	0.86947999	0.999579949
2491.334017	0.002857222	0.099183745	0.028807361	0.97701823	0.999579949
251.3396636	-0.088516352	0.111552309	-0.793496367	0.427488685	0.999579949
58.23142498	0.325964767	0.309578906	1.052929514	0.292373298	0.999579949
38.22292756	0.312014434	0.59994897	0.520068288	0.60301598	0.999579949
34.3363827	-1.143939747	0.567210884	-2.016780316	0.043718444	0.758836007
53.92664533	-0.046101648	0.19630655	-0.234845185	0.814328896	0.999579949
345.8580107	0.02691309	0.112353549	0.239539297	0.810687429	0.999579949
110.8261578	0.1087385	0.173056021	0.628342775	0.529779416	0.999579949
619.6569817	-0.07687325	0.063462016	-1.211326943	0.225770126	0.999579949
2035.308006	0.012063081	0.085715193	0.140734455	0.888079725	0.999579949
150.4877858	0.677334558	0.691971745	0.978847132	0.327655518	0.999579949
235.0887296	-0.196203785	0.140790069	-1.393591082	0.163441123	0.978498637
71.06256739	0.671839667	0.482730427	1.391749161	0.16399837	0.978498637
2244.510986	-0.08425929	0.146287954	-0.575982426	0.56462706	0.999579949
528.7364827	-0.005894921	0.095127657	-0.061968532	0.950587891	0.999579949

89.03622137	-0.307559644	0.353223527	-0.870722421	0.383905733	0.999579949
93.23950685	0.225504359	0.112374325	2.006724921	0.044778961	0.759209569
619.0693079	-0.090829333	0.092059614	-0.98663604	0.323821096	0.999579949
112.6548091	0.039687419	0.126653309	0.313354771	0.754011142	0.999579949
32.81255354	0.194017539	0.198962518	0.975146178	0.329487755	0.999579949
1389.689963	0.034865239	0.100931547	0.345434504	0.729767741	0.999579949
179.5417148	1.160207922	0.487279328	2.380991466	0.017266112	0.570162425
58.86329401	0.332745253	0.373105442	0.891826319	0.372486032	0.999579949
1056.829001	-0.023539903	0.114545931	-0.205506233	0.837176628	0.999579949
111.2789951	-0.219730795	0.16013675	-1.372144709	0.170018394	0.981013142
269.1547302	0.103433511	0.153053429	0.675800022	0.499167621	0.999579949
47.79394453	-0.134549434	0.192597863	-0.698602944	0.484800204	0.999579949
1511.885476	0.016353588	0.127805	0.127957344	0.89818273	0.999579949
57.82893938	0.481806069	0.183594421	2.624295805	0.008682834	0.442789376
525.1730925	0.318172449	0.391398811	0.812911128	0.416269014	0.999579949
367.796489	0.245864746	0.226188858	1.086988758	0.277041778	0.999579949
33.81803838	0.337209145	0.385618111	0.874463972	0.381865632	0.999579949
1732.035486	0.297623372	0.224763097	1.324164754	0.185448334	0.983992835
3944.796989	0.195072821	0.146953714	1.327443972	0.184361862	0.983992835
388.182641	0.440936244	0.237334547	1.857867929	0.063187772	0.809095107
69.28102644	-0.026490352	0.154525532	-0.17143026	0.863885463	0.999579949
294.1727494	-0.040455991	0.096573654	-0.418913329	0.675279475	0.999579949
593.018976	-0.088691265	0.088613919	-1.000872839	0.316888289	0.999579949
45.00097065	-0.091460864	0.26553853	-0.344435376	0.730518888	0.999579949
2792.623732	0.144813663	0.114285612	1.267120686	0.20511214	0.994814558
388.7693583	0.064838189	0.063763271	1.016857945	0.309221012	0.999579949
44.64651113	0.113112816	0.261336829	0.432823865	0.665142735	0.999579949
182.9158648	-0.045080584	0.083589167	-0.539311321	0.589672058	0.999579949
105.6637032	0.040061427	0.148705567	0.269400992	0.787621123	0.999579949
526.7747884	-0.033774177	0.13742262	-0.245768685	0.805861299	0.999579949
206.4687479	-0.171020013	0.106771151	-1.601743647	0.109212309	0.905620964
163.3404625	-0.081129244	0.137266051	-0.591036478	0.55449598	0.999579949
176.0790893	-0.038305818	0.11600159	-0.330218048	0.741235211	0.999579949
493.5932876	0.219482999	0.095581336	2.29629558	0.021658987	0.625726138
150.1351066	0.03394635	0.143975056	0.23577938	0.813603869	0.999579949
330.6337328	0.353707264	0.536593928	0.659171201	0.509785838	0.999579949
621.9393907	0.014874912	0.078011145	0.190676752	0.848778854	0.999579949
26.77194108	-0.683765517	0.429108254	-1.59345692	0.111057725	0.912513443
180.6412075	-0.49957533	0.261144226	-1.913024607	0.055744902	0.785214091
1330.262002	0.073010323	0.051905772	1.406593526	0.159547954	0.977806784
4544.148235	0.066725052	0.148036373	0.450734169	0.652181153	0.999579949
171.3013431	0.010933044	0.095274836	0.114752695	0.908641145	0.999579949
136.0178592	-0.049866426	0.145039052	-0.343813791	0.730986328	0.999579949
54.82562403	-0.088503969	0.190724184	-0.464041672	0.64261789	0.999579949
61.47279232	-0.182086493	0.206766058	-0.880640152	0.378512619	0.999579949
43.35545784	-0.447760334	0.298152131	-1.501784784	0.133152699	0.941431
88.70627726	-0.14666058	0.216364497	-0.677840318	0.497872944	0.999579949
1154.107405	0.045127876	0.065912565	0.684662725	0.493556776	0.999579949

675.4151797	-0.068530359	0.098020857	-0.699140587	0.484464176	0.999579949
110.301196	0.00659094	0.109449897	0.060218787	0.951981383	0.999579949
536.4063437	0.075003001	0.072475349	1.034876029	0.300726811	0.999579949
96.77453406	0.058260366	0.113343145	0.514017549	0.607239721	0.999579949
133.6879275	0.092753564	0.566873325	0.163623089	0.870027867	0.999579949
1021.033868	-0.188218792	0.07863492	-2.393577697	0.016684947	0.568009711
461.8154616	-0.14308304	0.192705771	-0.742494836	0.457787579	0.999579949
496.5387569	-0.022939241	0.072948327	-0.314458767	0.753172629	0.999579949
325.2087807	0.01818572	0.1048259	0.173484986	0.862270228	0.999579949
97.87418799	0.435386808	0.185692337	2.344667611	0.019044049	0.590797093
293.4063968	0.161470665	0.086477785	1.867192414	0.061874723	0.805306145
1096.401253	0.338717971	0.173057139	1.957260895	0.050316803	0.776599223
443.6469108	0.063388299	0.072384297	0.875718927	0.381182854	0.999579949
26.62133506	0.049239131	0.258862183	0.190213691	0.849141683	0.999579949
151.2391779	0.034822893	0.108609771	0.320623939	0.748495394	0.999579949
1084.010162	-0.046273616	0.075683349	-0.611410786	0.540927664	0.999579949
41.08942048	0.178109509	0.216592112	0.82232685	0.4108909	0.999579949
277.6372921	0.254799224	0.135762321	1.876803683	0.060545005	0.800274462
195.5557137	0.017152622	0.17602792	0.097442621	0.922374899	0.999579949
844.0010094	0.153893842	0.131590428	1.169491156	0.242205802	0.999579949
1128.295744	0.052495813	0.06536896	0.803069415	0.421934615	0.999579949
119.4909273	0.132504326	0.166138342	0.797554157	0.425129263	0.999579949
59.97025429	-0.145531371	0.189216509	-0.769126181	0.441818407	0.999579949
27.91280029	-0.224612825	0.429217305	-0.523307942	0.600759975	0.999579949
587.3110485	-0.141637301	0.195578866	-0.724195329	0.468945826	0.999579949
50.17231957	0.052932503	0.212414952	0.249193865	0.803210824	0.999579949
146.3155431	0.044641777	0.174015469	0.256539132	0.797534568	0.999579949
84.45697389	0.030800414	0.176331584	0.174673269	0.861336373	0.999579949
26.0934837	0.321433304	0.307981308	1.043677963	0.296634409	0.999579949
48.85944168	0.585536121	0.278564273	2.101978534	0.035555156	0.713050989
23.65033814	0.878870477	0.32295807	2.721314492	0.006502287	0.39001336
36.15672232	-0.026916012	0.31597114	-0.085185034	0.932114289	0.999579949
378.6076364	0.59008649	0.168352528	3.505064624	0.000456496	0.106482026
22.91657467	-0.125072511	0.224805806	-0.556358009	0.577966139	0.999579949
87.74783198	0.006715243	0.132820699	0.050558707	0.959677168	0.999579949
375.8304746	0.188615981	0.107410242	1.756033483	0.079082692	0.851207389
112.0596834	0.028108127	0.154976367	0.18137041	0.856076843	0.999579949
98.87319563	0.579851305	0.270738634	2.141738313	0.032214546	0.690330837
4419.567348	-0.141547011	0.245354632	-0.576907838	0.564001716	0.999579949
3975.645855	0.030206957	0.106022682	0.284910323	0.775712847	0.999579949
31.47610689	-0.001395637	0.24898593	-0.005605284	0.995527654	0.999579949
561.9687621	0.037814517	0.095227942	0.397094767	0.691297579	0.999579949
1639.400717	-0.068959161	0.122894853	-0.561123261	0.574713512	0.999579949
826.5309934	-0.052338613	0.056799148	-0.921468277	0.356805996	0.999579949
25.27555122	-0.182169864	0.471950801	-0.385993335	0.699501605	0.999579949
2019.628165	0.006440571	0.121837361	0.052862041	0.957841829	0.999579949
42.72100882	-0.233882875	0.309518849	-0.755633704	0.449868842	0.999579949
1098.996288	5.89804E-05	0.100514764	0.000586784	0.999531814	0.99989122

231.890532	-0.037392822	0.191216601	-0.195552175	0.844960696	0.999579949
186.369743	-0.693507357	0.405024462	-1.712260425	0.086848686	0.867486202
100.5205478	0.123458688	0.17231522	0.716470017	0.473701175	0.999579949
47.95492988	1.050525088	0.322401615	3.258436188	0.001120281	0.174210561
54.73572009	-0.281267888	0.222788298	-1.262489507	0.2067727	0.994814558
387.0790953	-0.217731721	0.14235607	-1.529486737	0.126143824	0.930818754
155.9851871	0.07211483	0.097423217	0.740222223	0.459165165	0.999579949
115.4175157	-0.030783034	0.133937052	-0.229832101	0.81822224	0.999579949
406.3322942	0.491642492	0.193992204	2.534341491	0.011265888	0.488767809
3430.773048	0.18945256	0.137496335	1.377873522	0.168242341	0.980087289
59.62059863	0.273029674	0.34755933	0.78556278	0.432123677	0.999579949
50.76851836	0.181945692	0.164858272	1.103649149	0.269745362	0.999579949
221.3425742	0.188069437	0.161659135	1.163370304	0.244679293	0.999579949
97.83332455	0.208456635	0.104576562	1.993339906	0.046224239	0.763868825
221.2956954	0.073711115	0.145639651	0.506119826	0.612772533	0.999579949
34.19320338	0.163164137	0.534744785	0.305125252	0.760270759	0.999579949
1448.501655	-0.038639018	0.052659246	-0.733755628	0.463097688	0.999579949
65.25483402	0.30166831	0.578153159	0.52177923	0.601824053	0.999579949
281.0184579	-0.094853922	0.095799434	-0.990130293	0.322110438	0.999579949
25.48753454	-0.383176561	0.261875587	-1.463200773	0.143412449	0.953645321
301.7553279	0.370463815	0.152446695	2.430120337	0.01509381	0.541405219
148.7309977	0.138691318	0.146335274	0.94776409	0.343249565	0.999579949
319.0923529	-0.120876964	0.155756147	-0.776065451	0.437710341	0.999579949
636.7159707	0.028414855	0.118931681	0.238917457	0.811169589	0.999579949
179.6821212	0.094483697	0.186185393	0.50747105	0.611824344	0.999579949
108.0154771	-0.011028699	0.129994472	-0.084839753	0.93238879	0.999579949
174.8980598	-0.023780353	0.093670609	-0.253872088	0.799594379	0.999579949
308.2108857	0.06006285	0.085410771	0.703223364	0.481916561	0.999579949
188.0351734	-0.099071271	0.098732467	-1.003431538	0.315652693	0.999579949
1190.831263	0.708743845	0.226460539	3.129657153	0.001750104	0.218260535
28.34814285	0.37643122	0.196965741	1.911150731	0.055985213	0.785214091
82.74166634	-0.336714726	0.188306957	-1.788116235	0.073757257	0.843054815
376.8374947	0.155124805	0.070419515	2.202866719	0.027604144	0.661030033
87.30055812	-0.014743558	0.139578287	-0.105629307	0.915876471	0.999579949
370.6452276	-0.016666654	0.174732919	-0.095383595	0.924010146	0.999579949
362.9432174	0.135482244	0.103820776	1.304962735	0.191905537	0.984804292
315.3892253	0.067240163	0.355056006	0.189379032	0.849795757	0.999579949
81.82909465	0.021891535	0.267153066	0.081943789	0.934691413	0.999579949
51.65845538	-0.054776103	0.471332493	-0.11621542	0.907481816	0.999579949
200.58312	0.051060286	0.07815671	0.653306497	0.513558705	0.999579949
60.77435879	-0.130687368	0.20191954	-0.647224969	0.517486354	0.999579949
61.10379575	0.050130786	0.168201581	0.29803992	0.765672695	0.999579949
44.3714345	0.176658213	0.386528282	0.457038258	0.647643547	0.999579949
4792.222609	0.147343876	0.156668516	0.940481729	0.346970517	0.999579949
484.5961514	-0.093272658	0.092764187	-1.005481333	0.314665134	0.999579949
37.3490778	0.25846254	0.199691614	1.294308436	0.195558904	0.989265933
64.9400834	0.145968688	0.232420643	0.628036676	0.529979914	0.999579949
373.1805794	0.102007219	0.08213633	1.241925705	0.214263975	0.999554723

160.7936321	-0.00129093	0.143928103	-0.008969272	0.992843652	0.999579949
463.1254334	-0.074703025	0.064952006	-1.150126516	0.250091767	0.999579949
26.27393446	0.068524439	0.31165148	0.219875222	0.825968334	0.999579949
125.0429962	-0.032047407	0.121381104	-0.26402303	0.791762173	0.999579949
260.6280208	0.072452381	0.100749447	0.719134282	0.472058186	0.999579949
87.44997802	0.20365012	0.153751927	1.324537025	0.185324755	0.983992835
77.3232693	0.109601394	0.162976046	0.672500023	0.501265425	0.999579949
746.7323491	-0.237305795	0.20274335	-1.170473877	0.241810321	0.999579949
22433.42152	-1.674436056	0.489868594	-3.418133101	0.000630523	0.128097794
20.50066021	-0.613427478	0.260899665	-2.351200713	0.018712937	0.590797093
957.2317132	-0.071476021	0.115211267	-0.620390892	0.535000467	0.999579949
3071.241728	-0.008984718	0.111227635	-0.080777747	0.935618706	0.999579949
4859.078583	-0.012521583	0.134526515	-0.093078924	0.925840861	0.999579949
514.9505377	0.075832124	0.077644498	0.976658051	0.328738469	0.999579949
368.5773549	0.056803194	0.103844496	0.547002455	0.584377045	0.999579949
399.2134696	0.090308342	0.114713977	0.787247939	0.431136739	0.999579949
579.807292	0.111485093	0.093205472	1.19612176	0.231649047	0.999579949
239.2128839	-0.505708578	0.163151257	-3.099630283	0.001937623	0.225075621
430.1445463	-0.20171594	0.127392974	-1.583414958	0.113326916	0.916629381
177.7369569	-0.201977523	0.242537992	-0.832766534	0.404976412	0.999579949
631.6774179	0.000968411	0.177853888	0.005444982	0.995655555	0.999579949
467.9758708	0.140113928	0.08579696	1.633087325	0.102450632	0.895628172
179.5360623	0.150358662	0.129532471	1.160779691	0.245731504	0.999579949
100.7674634	0.122668087	0.13994753	0.876529134	0.380742445	0.999579949
448.4261109	0.119886272	0.137201219	0.873798889	0.382227786	0.999579949
120.9468523	0.006127768	0.114068533	0.05372006	0.9571582	0.999579949
1069.907819	0.079195138	0.086978186	0.910517244	0.362549792	0.999579949
320.0805497	0.0087276	0.07901634	0.110453107	0.912050038	0.999579949
96.65905732	0.100101358	0.123389732	0.811261656	0.417215425	0.999579949
523.0379385	0.060775855	0.1306998	0.465003423	0.641929006	0.999579949
384.270463	0.071364115	0.08619956	0.827894183	0.407730429	0.999579949
392.8318748	0.077956576	0.062695559	1.243414642	0.213715078	0.999268361
73.30487384	0.332018181	0.23936983	1.387051079	0.165426188	0.978789551
1490.025135	-0.036024465	0.055558539	-0.648405546	0.516722684	0.999579949
260.153307	-0.124701845	0.147814949	-0.843634866	0.398873475	0.999579949
32.23106314	0.024753143	0.280436859	0.08826637	0.929664968	0.999579949
34.55368546	0.033071223	0.209824134	0.157614009	0.874760964	0.999579949
473.6962227	-0.015055467	0.076260917	-0.197420483	0.843498502	0.999579949
49.63179351	0.132871623	0.151226677	0.878625554	0.379604336	0.999579949
707.4353233	-0.062894814	0.078813276	-0.798023097	0.424857087	0.999579949
200.4417366	-0.005667031	0.090228979	-0.062807222	0.949920015	0.999579949
795.0727691	-0.100229759	0.097232852	-1.030821958	0.302624319	0.999579949
378.0923961	0.018707405	0.156274732	0.119708445	0.90471411	0.999579949
96.16402838	0.083894726	0.205561409	0.408124882	0.683181994	0.999579949
1547.424282	-0.003992872	0.062166079	-0.064229118	0.948787792	0.999579949
109.907585	0.078882544	0.112284418	0.70252441	0.482352185	0.999579949
111.416312	0.422699129	0.177685874	2.378912406	0.017363802	0.570162425
153.8161409	0.021894175	0.165030639	0.132667332	0.89445648	0.999579949

235.0999157	0.127084773	0.141592193	0.897540818	0.369430403	0.999579949
50.50365631	-0.12004325	0.172797453	-0.694704972	0.487240212	0.999579949
75.40281994	0.439719631	0.150205124	2.927460922	0.00341742	0.310136233
1636.719193	0.071418411	0.15381296	0.464319857	0.642418599	0.999579949
453.948489	0.079471405	0.068024904	1.168269261	0.242698171	0.999579949
51.50603189	0.229677447	0.271889537	0.84474544	0.398252983	0.999579949
2846.342084	-0.085312884	0.121293827	-0.703357179	0.481833185	0.999579949
1709.122838	0.014170116	0.093597134	0.15139476	0.879664323	0.999579949
50.69049646	0.172829329	0.147022039	1.175533477	0.239781352	0.999579949
1548.390777	0.118036752	0.060484614	1.951517	0.050995576	0.77827144
34.36764988	-0.154701968	0.175077412	-0.883620375	0.376901176	0.999579949
526.3934925	-0.070897236	0.097164555	-0.729661513	0.465597112	0.999579949
276.7515021	0.078307105	0.107636499	0.727514417	0.466910887	0.999579949
59.35016439	0.784150011	0.437630137	1.791809895	0.073163423	0.838669905
25.48487842	-0.156270857	0.388344852	-0.402402288	0.687387986	0.999579949
498.6102163	0.009990733	0.089736288	0.111334371	0.911351201	0.999579949
1036.432291	-0.164468973	0.065740565	-2.501788232	0.012356781	0.503708792
822.9974329	0.09146776	0.111206422	0.822504298	0.410789941	0.999579949
245.8722671	0.163875623	0.089866222	1.823550811	0.068220009	0.830240803
504.182956	0.080326946	0.071861876	1.117796389	0.263653964	0.999579949
1833.700252	-0.001605699	0.13142091	-0.012217989	0.990251698	0.999579949
285.7929805	0.105713306	0.126112364	0.838246957	0.40189202	0.999579949
203.7386957	0.005825354	0.138153736	0.042165734	0.966366579	0.999579949
485.9955931	0.001948872	0.077136351	0.025265281	0.979843367	0.999579949
2586.111779	0.118087372	0.153722439	0.768185654	0.442376895	0.999579949
148.1248704	0.179542961	0.255475607	0.702779271	0.482193318	0.999579949
261.8233381	-0.055991676	0.101447586	-0.55192714	0.580998273	0.999579949
437.9924625	0.040901105	0.070735211	0.578228356	0.563109957	0.999579949
276.6298166	0.008075721	0.084327271	0.095766426	0.923706083	0.999579949
32.56665303	-0.008632771	0.287266791	-0.030051408	0.976026054	0.999579949
30828.56441	0.046547589	0.110170117	0.422506489	0.672655366	0.999579949
105.7217334	-0.051530307	0.14755614	-0.349225098	0.726920326	0.999579949
85.17689604	0.059076043	0.135256708	0.43676978	0.662278317	0.999579949
290.2325546	0.086262404	0.067814872	1.272027826	0.203363233	0.994409048
353.781001	-0.055481448	0.081595433	-0.679957765	0.496531204	0.999579949
75.69735292	0.018481023	0.164383167	0.11242649	0.910485252	0.999579949
1337.148554	-0.023073392	0.130664608	-0.176584865	0.859834483	0.999579949
543.9097638	0.117040807	0.074411179	1.572892785	0.115743646	0.918818353
171.7642403	0.042450413	0.10828785	0.392014553	0.695047461	0.999579949
528.6789039	-0.051685549	0.108327201	-0.477124382	0.633273553	0.999579949
140.8621231	-0.039692941	0.197334943	-0.201145019	0.840585181	0.999579949
235.0832555	-0.200277982	0.194114035	-1.031754256	0.302187253	0.999579949
45.92336743	0.035105407	0.246347014	0.142503886	0.88668201	0.999579949
55.19455077	-0.027953936	0.192784494	-0.145000956	0.884710117	0.999579949
679.3012991	0.07538366	0.086416552	0.872328949	0.383028949	0.999579949
25.91696582	0.220896531	0.217382107	1.016167032	0.309549853	0.999579949
324.1589285	0.04114449	0.102017762	0.403307122	0.686722303	0.999579949
341.3119822	0.082952155	0.140616929	0.58991585	0.555247067	0.999579949

693.6193461	0.02391793	0.080496857	0.297128743	0.766368221	0.999579949
2323.315235	0.115611597	0.060907108	1.898162641	0.05767466	0.79395631
125.3219103	0.138715867	0.099259681	1.39750466	0.162261866	0.978498637
56.55585944	-0.009759498	0.187401895	-0.052077907	0.958466617	0.999579949
1723.665326	0.017119143	0.130552247	0.131128671	0.895673521	0.999579949
143.0419155	-0.319258092	0.179476895	-1.778825577	0.075268363	0.851207389
126.760627	0.034029172	0.202282232	0.168226203	0.866405328	0.999579949
41.99109048	0.02601172	0.454112565	0.057280336	0.954321884	0.999579949
161.5627335	0.078727052	0.126099911	0.624322821	0.5324156	0.999579949
284.1562206	-0.296559251	0.111507209	-2.659552262	0.007824459	0.42118327
37.72975971	0.091341221	0.190731142	0.478900406	0.632009482	0.999579949
81.19719388	0.033509121	0.203538115	0.164633152	0.869232736	0.999579949
29.33960942	-0.299179519	0.27059949	-1.10561745	0.268892134	0.999579949
213.1159055	0.128138991	0.114712036	1.117049227	0.263973278	0.999579949
639.5729072	-0.0514543	0.138206104	-0.37230121	0.709668598	0.999579949
119.8795411	-0.148934372	0.337139707	-0.441758621	0.658663885	0.999579949
191.9234816	0.037498659	0.199924365	0.187564225	0.851218273	0.999579949
557.7179377	0.078516212	0.077938195	1.007416358	0.313734734	0.999579949
48.14198866	-0.336476123	0.179916522	-1.870179125	0.061458947	0.80388048
384.1053399	-0.075681285	0.077010614	-0.982738378	0.325736217	0.999579949
1530.484765	0.09693722	0.108258403	0.895424438	0.370560242	0.999579949
1377.123728	0.080386497	0.135667213	0.592527074	0.553497697	0.999579949
190.7900696	0.011228271	0.191554525	0.058616577	0.953257507	0.999579949
100.4851265	0.06640541	0.255188851	0.260220654	0.794693573	0.999579949
435.7328805	0.028796119	0.075790606	0.379943111	0.703987645	0.999579949
45.14346447	0.138004729	0.222691725	0.619711977	0.535447429	0.999579949
385.4105977	-0.042426312	0.104969856	-0.404176144	0.686083195	0.999579949
96.54242785	0.156532807	0.150001092	1.043544451	0.296696205	0.999579949
760.3983268	-0.043214493	0.079565059	-0.543134056	0.587037506	0.999579949
21.58472514	0.100974982	0.285654572	0.353486316	0.723723886	0.999579949
104.4226821	-0.192173442	0.158640708	-1.21137534	0.225751586	0.999579949
144.6456845	0.164048649	0.195096228	0.840860177	0.40042627	0.999579949
1911.150002	-0.136594763	0.114081995	-1.197338483	0.231174651	0.999579949
108.0351703	-0.177379048	0.18024027	-0.984125509	0.325053806	0.999579949
287.8970412	-0.122338101	0.080315612	-1.523216948	0.127704437	0.935409261
4524.250417	0.010267001	0.105419717	0.097391659	0.922415369	0.999579949
64.0324193	0.253005117	0.132834093	1.90467003	0.056822977	0.789131444
31.63455665	0.289513736	0.259004875	1.117792614	0.263655577	0.999579949
1567.054654	-0.342702323	0.149025541	-2.299621398	0.021469679	0.622105864
1258.148279	0.066607648	0.097185365	0.685367058	0.493112326	0.999579949
4179.483269	-0.371799686	0.242977666	-1.530180498	0.125972057	0.930641662
288.9925344	-0.046842816	0.066812382	-0.701109815	0.483234486	0.999579949
23.1833118	-0.189392682	0.294857096	-0.642320247	0.520665271	0.999579949
213.3364649	-0.002881622	0.118620654	-0.024292751	0.980619095	0.999579949
494.7246104	-0.057282225	0.062283615	-0.919699737	0.35772969	0.999579949
231.660707	0.078434546	0.1142067	0.686777097	0.492223204	0.999579949
54.29336422	0.099036613	0.196478798	0.504057508	0.614220966	0.999579949
108.5536548	-0.082411004	0.114523036	-0.719601984	0.47177009	0.999579949

183.0486254	-0.069610071	0.14760221	-0.471605883	0.637208126	0.999579949
267.4312423	-0.167781016	0.140619663	-1.193154729	0.232808781	0.999579949
67.28103305	0.004574954	0.14423356	0.031719066	0.97469609	0.999579949
2538.907553	-0.111103128	0.222110707	-0.500215092	0.616923633	0.999579949
25.90448352	0.274691584	0.339659653	0.808725973	0.418672785	0.999579949
383.4786147	-0.210631264	0.145614069	-1.446503527	0.148036024	0.962301096
284.8444283	0.025767956	0.078400414	0.32867117	0.742404248	0.999579949
471.0708189	-0.027108499	0.169986194	-0.159474712	0.87329488	0.999579949
20.89056432	0.038747603	0.299396776	0.129418907	0.897026188	0.999579949
58.96182741	0.001716869	0.143079655	0.011999394	0.990426099	0.999579949
236.1252349	-0.012017216	0.102829155	-0.116865847	0.906966363	0.999579949
536.7165806	-0.052350931	0.086324661	-0.606442355	0.54422105	0.999579949
517.7781144	0.029930053	0.076374962	0.391883046	0.695144631	0.999579949
363.2925748	-0.015240564	0.075058663	-0.203048705	0.839096964	0.999579949
512.0924016	0.069418809	0.105423307	0.658476871	0.510231754	0.999579949
645.341791	-0.02124742	0.118978529	-0.17858197	0.858265953	0.999579949
131.4703129	0.219329394	0.321578583	0.682039805	0.495213781	0.999579949
119.1858923	0.037807007	0.143413027	0.26362324	0.792070249	0.999579949
2204.435717	0.032886349	0.134786477	0.24398849	0.807239734	0.999579949
717.0585487	-0.031350376	0.082920765	-0.378076302	0.705373912	0.999579949
42.84475318	0.247505162	0.197034428	1.256151855	0.209060918	0.996447459
91.44364092	0.128155305	0.148073007	0.865487289	0.38677139	0.999579949
72.86127694	0.584233271	0.47994183	1.217300171	0.223490029	0.999579949
109.0795979	0.101558051	0.111459682	0.911164013	0.362208962	0.999579949
367.4706795	0.003456742	0.120956517	0.028578384	0.977200852	0.999579949
493.8711395	0.041477815	0.09052189	0.45820757	0.646803322	0.999579949
1005.817154	0.01873682	0.09481461	0.197615326	0.843346043	0.999579949
327.0512394	0.008222418	0.123890955	0.066368186	0.947084699	0.999579949
21.96211466	-0.34885008	0.422327364	-0.82601818	0.408793779	0.999579949
36.78106484	-1.627830881	0.613671409	-2.652609943	0.007987211	0.426300473
645.1336654	-0.035925477	0.051693287	-0.69497374	0.487071759	0.999579949
86.84028026	0.027606866	0.259204099	0.106506288	0.915180666	0.999579949
197.050062	-0.041972907	0.140858971	-0.297978233	0.765719777	0.999579949
71.11111705	-0.151392923	0.132950334	-1.138717883	0.254820841	0.999579949
224.8692767	0.230644024	0.11855336	1.945487028	0.05171639	0.779189484
1020.730763	0.056788717	0.112241144	0.505952764	0.61288981	0.999579949
204.9288229	0.030221604	0.084773889	0.356496614	0.721468687	0.999579949
196.8843363	-0.178386627	0.123810752	-1.440800765	0.149640977	0.963638931
72.86440635	0.300717195	0.373842645	0.804395108	0.421168826	0.999579949
410.6980412	0.100430155	0.095353508	1.053240277	0.292230883	0.999579949
1299.281501	-0.152975127	0.080179297	-1.907913052	0.056402455	0.785214091
214.1897704	-0.061263366	0.117152708	-0.522935978	0.601018806	0.999579949
127.7612402	0.194220965	0.153305371	1.266889499	0.205194804	0.994814558
68.15147435	-0.064322602	0.160397822	-0.401019172	0.688406009	0.999579949
185.8553903	0.162667438	0.136502698	1.191679283	0.233387024	0.999579949
648.2655497	0.005312806	0.100782304	0.052715668	0.957958455	0.999579949
418.505274	-0.034855411	0.066249635	-0.526122317	0.598803224	0.999579949
94.66500993	0.127185736	0.113810957	1.1175175	0.263773121	0.999579949

116.596687	0.149703812	0.175320908	0.853884532	0.393168971	0.999579949
162.6182881	-0.052354564	0.129096285	-0.405546632	0.685075748	0.999579949
216.3456352	-0.161160634	0.16974979	-0.94940108	0.342416661	0.999579949
879.4812431	-0.040954224	0.069461505	-0.589595974	0.555461551	0.999579949
20.59828619	0.334222717	0.253463811	1.31862105	0.187295833	0.983992835
99.9867409	-0.073667839	0.124044246	-0.593883568	0.55258999	0.999579949
31.21225562	-0.431251175	0.195267424	-2.208515711	0.027208344	0.656544634
926.3184945	-0.033648776	0.096969915	-0.347002219	0.728589652	0.999579949
315.5861375	-0.279218816	0.102338365	-2.72838848	0.006364461	0.387278966
737.0929508	-0.049282624	0.112483372	-0.438132524	0.661290219	0.999579949
100.6566161	-0.018083541	0.106447554	-0.169882165	0.865102807	0.999579949
497.8920196	0.117610627	0.175969554	0.668357817	0.503905215	0.999579949
1302.816178	0.023156255	0.077163367	0.300093887	0.764105542	0.999579949
66.22893618	-0.07578726	0.177544419	-0.426863659	0.669478637	0.999579949
82.98598389	0.417406322	0.262290222	1.591391091	0.111521588	0.913044976
95.8264278	0.051155464	0.14956014	0.342039423	0.732321224	0.999579949
1103.542141	0.021793971	0.08216793	0.265236947	0.790826934	0.999579949
2338.842763	0.001830414	0.098304563	0.018619827	0.985144386	0.999579949
910.3975763	-0.066222551	0.082485518	-0.802838519	0.422068075	0.999579949
334.4467832	0.107775811	0.11408828	0.944670313	0.344827217	0.999579949
106.2906924	0.137927154	0.1105798	1.247308768	0.212284304	0.999268361
100.2085689	-0.035435297	0.163829431	-0.216293842	0.828758708	0.999579949
333.7265533	0.044956229	0.110265668	0.407708304	0.68348784	0.999579949
43.62245537	0.845561642	0.289268909	2.923098946	0.003465664	0.310136233
221.1478586	0.100816768	0.083011166	1.214496466	0.224558199	0.999579949
268.9359269	-0.048740596	0.089712577	-0.543297251	0.586925157	0.999579949
1470.146741	-0.000601657	0.105424895	-0.005706971	0.995446521	0.999579949
282.0532037	-0.137933222	0.062744474	-2.198332593	0.027925413	0.663770609
1235.754536	0.022915538	0.060691059	0.377576841	0.705744972	0.999579949
1190.662783	-0.175484613	0.157992608	-1.110714071	0.266691443	0.999579949
844.4548506	-0.150875053	0.095312898	-1.582944776	0.113434052	0.916629381
258.4995979	-0.371231988	0.269466179	-1.377657075	0.16830919	0.980087289
58.69925272	0.361387262	0.197353445	1.83116774	0.06707551	0.825081178
33.5882616	0.287715648	0.176850697	1.626884447	0.103761642	0.895628172
155.966186	-0.004416466	0.0906903	-0.048698325	0.961159711	0.999579949
248.9618097	0.056067988	0.105835876	0.529763543	0.596275885	0.999579949
1743.975075	-0.054090497	0.058247822	-0.928626946	0.353082453	0.999579949
241.3121415	-0.011518101	0.069689923	-0.165276427	0.868726412	0.999579949
82.14842587	-0.130135437	0.113782591	-1.143720101	0.252739734	0.999579949
1401.976245	-0.058439713	0.051408618	-1.136768799	0.25563495	0.999579949
183.6854274	-0.145166674	0.089756062	-1.617346737	0.105803456	0.896383253
468.2851976	-0.022494972	0.112311912	-0.200290169	0.841253651	0.999579949
21.92879885	0.280371364	0.355525445	0.788611245	0.430339256	0.999579949
68.76220694	0.161615783	0.208148943	0.776442972	0.437487481	0.999579949
135.64444	0.068494205	0.176928133	0.387130096	0.698659898	0.999579949
734.9947769	0.0091281	0.068643676	0.132978013	0.894210769	0.999579949
387.4252648	0.031071984	0.072870685	0.426398956	0.669817162	0.999579949
3387.407776	-0.156724519	0.084842547	-1.847239677	0.064712411	0.817776877

1383.356419	-0.069883373	0.196704777	-0.35527034	0.722387073	0.999579949
127.8050876	-0.246374889	0.28721202	-0.857815382	0.390994414	0.999579949
76.72487872	0.209959157	0.15663806	1.340409591	0.180112219	0.983992835
216.9448431	-0.082204974	0.112068054	-0.733527267	0.463236903	0.999579949
304.5810117	-0.256069235	0.143967016	-1.778665986	0.075294539	0.851207389
129.5912934	0.06862764	0.093790113	0.731715078	0.464342491	0.999579949
1208.254624	0.095563704	0.081375414	1.174355976	0.240252471	0.999579949
450.0964199	-0.050372114	0.073870746	-0.6818953	0.495305157	0.999579949
99.4190363	0.013729727	0.110786451	0.123929656	0.901370972	0.999579949
151.5206386	0.005682976	0.108798899	0.052233768	0.958342427	0.999579949
1501.341065	0.06676696	0.064989802	1.027345178	0.304257956	0.999579949
182.9979053	0.069769749	0.161031098	0.43326879	0.664819509	0.999579949
141.8935927	0.228731553	0.175870189	1.300570345	0.193405563	0.986374671
161.1465715	-0.054777296	0.087636188	-0.625053387	0.531936019	0.999579949
4758.60431	-0.092039486	0.070039432	-1.314109547	0.188809342	0.983992835
383.333408	0.095407302	0.100839547	0.946129809	0.344082382	0.999579949
78.66651109	0.271419193	0.155977452	1.740118136	0.081838276	0.85448976
218.3818325	-0.004951219	0.131608109	-0.037620928	0.969989922	0.999579949
180.925993	0.135664539	0.114881375	1.180909778	0.237638566	0.999579949
83.18551377	0.210547079	0.241946534	0.870221515	0.384179361	0.999579949
366.7648481	0.06628613	0.121139762	0.54718722	0.584250115	0.999579949
453.3873691	0.033617654	0.073475862	0.457533304	0.64728777	0.999579949
2517.686597	-0.170233448	0.118963857	-1.430967794	0.152439445	0.972669728
73.11290389	0.112333551	0.131538668	0.853996413	0.393106977	0.999579949
1443.831059	0.072102899	0.083545218	0.863040403	0.388115253	0.999579949
102.4168187	0.201982844	0.159968741	1.262639453	0.206718783	0.994814558
34.36231511	-0.0890301	0.227120755	-0.391994558	0.695062235	0.999579949
706.5480974	-0.035562146	0.066917578	-0.531432054	0.595119414	0.999579949
278.2974126	-0.139568039	0.127643949	-1.093416809	0.274210837	0.999579949
302.1849305	0.095777979	0.158921934	0.602673127	0.546726162	0.999579949
88.91336525	0.14825126	0.149145453	0.994004562	0.320220646	0.999579949
275.5321066	-0.122859631	0.13426623	-0.915044918	0.36016805	0.999579949
540.5512768	0.12374123	0.119221106	1.037913795	0.29931019	0.999579949
116.6202396	0.395704589	0.204948291	1.930753305	0.053513567	0.781231069
343.2556912	-0.019931706	0.098484263	-0.202384676	0.839616007	0.999579949
406.0105648	0.123616075	0.105723392	1.169240529	0.242306736	0.999579949
391.138895	-0.190717854	0.115671523	-1.648788302	0.099191012	0.895628172
422.4173259	-0.053744811	0.09155022	-0.587052777	0.557168262	0.999579949
154.9879918	-0.098117556	0.095363515	-1.028879395	0.303536352	0.999579949
5694.401588	-0.043689857	0.090127029	-0.484758646	0.627847561	0.999579949
966.1051493	0.066004244	0.111439304	0.592288728	0.553657263	0.999579949
69.34975211	0.053266553	0.139130298	0.382853728	0.701828222	0.999579949
113.7563334	-0.047084294	0.138017304	-0.34114776	0.732992348	0.999579949
134.2499541	-0.030258953	0.141315875	-0.214122815	0.830451285	0.999579949
78.11783023	-0.396982518	0.201022467	-1.974816668	0.048288957	0.771887944
392.9488871	-0.038742315	0.08497464	-0.455927973	0.648441774	0.999579949
86.1653115	-0.131250059	0.188112428	-0.697721361	0.485351466	0.999579949
213.7114782	-0.033538453	0.086428316	-0.388049368	0.6979795	0.999579949

339.0306529	0.089466074	0.097919677	0.913667984	0.360891332	0.999579949
434.9326577	-0.008319632	0.181143725	-0.045928348	0.963367359	0.999579949
36.65087616	-0.149000219	0.235276205	-0.633299142	0.526538313	0.999579949
598.5690706	-0.038045361	0.060723824	-0.626531054	0.530966675	0.999579949
160.0322249	-0.314226333	0.146512871	-2.144701215	0.031976744	0.689986698
253.4865757	0.027571836	0.079907283	0.345047848	0.7300584	0.999579949
103.0022084	0.077535658	0.115461257	0.671529656	0.501883174	0.999579949
1493.854756	0.012871839	0.068731861	0.187276163	0.851444112	0.999579949
54.7443021	0.095917244	0.255033773	0.376096243	0.706845347	0.999579949
63.27368759	-0.115956664	0.145471339	-0.797110035	0.425387129	0.999579949
607.0411599	-0.05912445	0.081099072	-0.729039777	0.465977331	0.999579949
562.7130447	-0.232634224	0.119599969	-1.945102704	0.051762619	0.779189484
1237.993138	-0.212381816	0.129839499	-1.63572578	0.101896992	0.895628172
6737.480157	-0.080430011	0.068670183	-1.171250862	0.241497956	0.999579949
160.053231	-0.10539395	0.108397908	-0.972287675	0.330907453	0.999579949
157.5396506	-0.222187907	0.131401526	-1.690908122	0.090854352	0.877608448
528.2689874	0.111996074	0.10964052	1.021484338	0.307025026	0.999579949
1161.245247	-0.009265985	0.056070133	-0.165257048	0.868741665	0.999579949
243.769368	-0.045885931	0.098400517	-0.466317985	0.640987907	0.999579949
361.6826943	-0.025178911	0.096568988	-0.260734962	0.794296903	0.999579949
131.5971805	-0.283780953	0.119574359	-2.37325924	0.017631884	0.573039104
47.83580355	1.927351273	1.009990876	1.908285825	0.056354284	0.785214091
499.4033114	-0.017554765	0.112809979	-0.155613584	0.876337617	0.999579949
1189.363392	0.135971576	0.247710636	0.548912951	0.58306519	0.999579949
55.8631512	-0.01452204	0.139907836	-0.103797191	0.917330296	0.999579949
218.9854465	-0.063186459	0.213018611	-0.296624125	0.76675349	0.999579949
61.61600831	-0.101458971	0.158181957	-0.641406728	0.521258463	0.999579949
160.8493812	0.098473218	0.161368457	0.610238334	0.541703939	0.999579949
647.5123791	-0.064191491	0.088343262	-0.726614449	0.467462174	0.999579949
16434.96899	-0.077389578	0.119001892	-0.650322246	0.51548409	0.999579949
742.6009546	-0.120253154	0.112799137	-1.066082219	0.286386477	0.999579949
112.3332733	-0.131108281	0.127442376	-1.028765194	0.303590026	0.999579949
290.1608001	0.030733109	0.094625054	0.324788284	0.745341312	0.999579949
248.732939	0.042904306	0.192063254	0.223386333	0.82323484	0.999579949
22.90834802	-0.064146573	0.247320962	-0.259365695	0.795353096	0.999579949
67.18492409	0.163400803	0.170223836	0.959917289	0.337096844	0.999579949
136.6236635	-0.187922946	0.133800503	-1.40450104	0.160169702	0.978261482
281.4942979	-0.07067464	0.155216802	-0.455328539	0.648872897	0.999579949
245.0113142	-0.010828765	0.084967759	-0.127445572	0.898587749	0.999579949
1932.113904	0.028152518	0.105918978	0.265792953	0.790398671	0.999579949
167.3147155	0.002391979	0.090980297	0.026291175	0.979025094	0.999579949
28.98001719	-0.093584058	0.241260742	-0.387895923	0.698093055	0.999579949
110.2582711	0.149981345	0.163370361	0.918045011	0.358595302	0.999579949
1059.91404	-0.183271139	0.092070686	-1.990548215	0.046530578	0.765142511
207.4390694	-0.066818834	0.109671714	-0.609262235	0.542350635	0.999579949
320.6592381	-0.044017341	0.084088362	-0.523465319	0.600650479	0.999579949
779.3895057	-0.067924534	0.073677294	-0.921919505	0.356570564	0.999579949
26.59142338	-0.111773668	0.253422997	-0.441055743	0.659172642	0.999579949

38.54268347	0.250621203	0.228216204	1.098174444	0.272128323	0.999579949
588.0891221	-0.059692988	0.088274135	-0.676222863	0.498899159	0.999579949
874.6106603	0.131361639	0.063487381	2.069098405	0.038536853	0.72691918
1860.964949	0.066512116	0.04144826	1.604702266	0.108559343	0.903743816
84.53975983	-0.064325464	0.122382674	-0.525609238	0.599159737	0.999579949
656.8035617	-0.023291727	0.063636265	-0.366013416	0.714355065	0.999579949
202.4372831	0.2088056	0.091413314	2.284192421	0.022360225	0.627784819
582.1925871	0.049432718	0.136695635	0.361626164	0.71763141	0.999579949
1321.687983	0.110372278	0.075682779	1.458353928	0.144743013	0.955875831
15091.99465	-0.260256554	0.135777735	-1.916783734	0.055265412	0.785214091
381.7304817	0.279730614	0.212546035	1.316094249	0.188142411	0.983992835
159.6222942	0.130301561	0.155188684	0.839633132	0.401114117	0.999579949
2107.817325	-0.141248791	0.072768751	-1.941063836	0.052250535	0.780444402
72.13077907	0.092310315	0.16997523	0.543080984	0.587074045	0.999579949
1917.393046	0.083100383	0.143121147	0.580629657	0.56149008	0.999579949
32.25351496	-0.038227564	0.228981244	-0.166946267	0.867412328	0.999579949
420.962074	0.02594635	0.126460359	0.205173779	0.837436355	0.999579949
208.1830488	-0.046481583	0.130532944	-0.356090821	0.721772551	0.999579949
105.6591077	0.236835002	0.250496518	0.945462251	0.344422934	0.999579949
151.0262423	0.004328405	0.103039333	0.04200731	0.966492871	0.999579949
1722.599822	-0.109786743	0.094771409	-1.158437386	0.24668559	0.999579949
121.8860224	0.074738755	0.114116373	0.654934549	0.512509895	0.999579949
129.6398288	0.092470311	0.133671863	0.691770946	0.489081185	0.999579949
776.3261542	0.058138097	0.083379206	0.697273331	0.485631755	0.999579949
37.34693686	-0.144350747	0.194953506	-0.740436781	0.459035008	0.999579949
986.5237046	-0.11646352	0.129075158	-0.902292288	0.366901621	0.999579949
533.009578	-0.113604202	0.07881057	-1.441484324	0.149447903	0.963629286
311.3410084	-0.156323637	0.169517915	-0.922165882	0.356442057	0.999579949
2167.60142	-0.015296834	0.126796639	-0.12064069	0.903975638	0.999579949
155.3412152	0.075765223	0.170215085	0.445114619	0.656236924	0.999579949
99.00219685	-0.135873724	0.138667905	-0.979849834	0.327160249	0.999579949
1266.431108	-0.027227287	0.07462742	-0.36484294	0.71522865	0.999579949
804.7210083	0.086598176	0.130324172	0.664482842	0.506381327	0.999579949
285.6165838	-0.081953502	0.08659256	-0.946426599	0.343931044	0.999579949
175.3624471	-0.036686648	0.109377283	-0.335413785	0.737312971	0.999579949
544.7978354	0.025952153	0.106086431	0.244632159	0.806741261	0.999579949
26.78326349	0.114579745	0.564029561	0.203144929	0.839021755	0.999579949
276.3677896	-0.01398369	0.115932207	-0.120619545	0.903992387	0.999579949
991.6195535	-0.265285827	0.152172605	-1.74332185	0.081277411	0.85448976
655.1395954	-0.109115445	0.097284249	-1.121614719	0.262026291	0.999579949
216.0231498	0.071918782	0.141353296	0.508787443	0.610901222	0.999579949
503.2754204	-0.060327849	0.093505154	-0.645182077	0.5188092	0.999579949
420.4725861	-0.112666755	0.08704803	-1.294305626	0.195559874	0.989265933
24.25869583	-0.262398154	0.286629454	-0.915461237	0.359949543	0.999579949
32.13074072	-0.004570159	0.280657229	-0.016283774	0.987008002	0.999579949
981.3799873	0.087749705	0.134764147	0.651135388	0.514959094	0.999579949
155.5954189	0.066074033	0.124975975	0.528693878	0.597017825	0.999579949
29.635614	0.105974196	0.330662605	0.320490418	0.748596593	0.999579949

34.27217651	0.490392011	0.365117121	1.34310878	0.179236753	0.983992835
42.21099115	-0.01296788	0.217436124	-0.059639952	0.952442398	0.999579949
623.3657333	0.125838759	0.08294488	1.517137153	0.12923206	0.935700262
365384.4631	-0.07504724	0.129133272	-0.581161141	0.561131855	0.999579949
84.80770979	-0.172211816	0.151373346	-1.137662746	0.255261335	0.999579949
720.8251132	-0.007641841	0.095096123	-0.08035912	0.935951639	0.999579949
45.4356381	-0.008497109	0.208979363	-0.040660037	0.967566921	0.999579949
203.7629518	-0.067625488	0.087053434	-0.776827345	0.437260641	0.999579949
350.8050462	-0.147737733	0.0981052	-1.505911344	0.132089934	0.939285826
607.9015896	0.08832374	0.080702505	1.094436166	0.273763729	0.999579949
154.4903795	0.036918292	0.108861531	0.339130743	0.73451124	0.999579949
73.66240513	0.315829512	0.293497651	1.076088722	0.281887579	0.999579949
33.63423715	0.138989605	0.285233746	0.487283174	0.62605767	0.999579949
3518.774658	-0.278630084	0.183074699	-1.521947523	0.12802223	0.935700262
24.65019529	0.04729419	0.727481993	0.065010805	0.948165397	0.999579949
233.9347362	-0.736240029	0.533970361	-1.378803176	0.167955447	0.980087289
415.350239	-0.083615514	0.101583113	-0.823124153	0.410437395	0.999579949
474.5925446	0.126793598	0.111461066	1.137559533	0.255304452	0.999579949
553.6467497	-0.159888905	0.110082338	-1.452448306	0.146376993	0.959294798
187.5960557	-0.011450991	0.131571932	-0.087032172	0.930645939	0.999579949
828.269446	0.00676938	0.062201931	0.108829094	0.913338047	0.999579949
599.1424182	0.058832192	0.081819495	0.719048588	0.472110983	0.999579949
85.66075552	-0.062485324	0.202884664	-0.30798446	0.758094157	0.999579949
23.73032354	-0.117778906	0.731984639	-0.160903521	0.872169389	0.999579949
133.9244466	-0.026174876	0.159324527	-0.164286542	0.869505576	0.999579949
929.9354501	0.092544112	0.161865785	0.571733625	0.567502451	0.999579949
562.3607623	-0.054678919	0.098303903	-0.556223272	0.578058232	0.999579949
166.55655	-0.066129387	0.138677268	-0.476858163	0.633463125	0.999579949
481.2374969	0.046030537	0.103324049	0.445496837	0.655960745	0.999579949
29.81744506	-0.373399812	0.403054443	-0.926425247	0.354225029	0.999579949
147.1245117	-0.277115321	0.174604871	-1.587099602	0.112490088	0.914735409
124.3623731	0.189485816	0.188172883	1.006977272	0.313945696	0.999579949
150.5115209	0.044045587	0.15332904	0.287261874	0.773911813	0.999579949
87.6327178	0.268459164	0.213181172	1.259300534	0.2079218	0.995521169
129.8489598	-0.294647903	0.13975696	-2.108287863	0.035006097	0.71004216
446.3256564	0.272491965	0.174681523	1.55993582	0.118775049	0.921521033
242.1432496	0.104841232	0.184774274	0.567401674	0.570441313	0.999579949
33.00516901	0.188617271	0.201234905	0.93729898	0.348604788	0.999579949
32.48573549	0.019926877	0.351817767	0.05663977	0.954832154	0.999579949
22.81808032	0.873821585	0.537073628	1.627005198	0.103735994	0.895628172
96.29610053	0.209964329	0.1138123	1.844829852	0.065062293	0.817776877
567.9949494	-0.134891322	0.211844698	-0.636746273	0.524290127	0.999579949
41116.64066	0.192126418	0.156156963	1.230341662	0.218569189	0.999579949
117.6032233	0.385312296	0.150845515	2.554350365	0.010638615	0.483266726
121.8157887	0.058239957	0.164880661	0.353224915	0.723919831	0.999579949
66.02280688	0.401679721	0.365311137	1.099555091	0.271526018	0.999579949
34.32672539	0.179743908	0.184644228	0.973460746	0.33032436	0.999579949
7702.869124	0.125214579	0.12692681	0.986510093	0.323882865	0.999579949

279.6802435	-0.034434928	0.137465725	-0.250498279	0.802202036	0.999579949
451.7021248	0.034242003	0.123002796	0.278383938	0.780717648	0.999579949
196.5965234	0.004789586	0.11378002	0.042095143	0.966422852	0.999579949
299.5752024	0.088807168	0.143457629	0.619048072	0.535884691	0.999579949
402.4113623	0.055239935	0.103098412	0.535798115	0.592098085	0.999579949
71.49417984	0.19680826	0.166534819	1.181784453	0.237291238	0.999579949
8242.734921	-0.011303076	0.126053483	-0.08966889	0.928550338	0.999579949
899.2736993	-0.100784731	0.08163995	-1.234502603	0.217015678	0.999579949
721.2581195	-0.101181426	0.080953045	-1.249877958	0.211344132	0.996937328
52.23751752	0.189427808	0.146234558	1.295369646	0.195192744	0.988599841
172.0072059	0.003435308	0.112027499	0.030664866	0.975536811	0.999579949
647.9737975	0.063136779	0.068942784	0.915785174	0.359779581	0.999579949
6259.855552	0.00638517	0.13575691	0.047033846	0.962486252	0.999579949
166.4622759	0.227103013	0.133662825	1.699073873	0.089305265	0.870840008
287.0323988	0.060946006	0.126892982	0.480294534	0.631017975	0.999579949
695.4714416	-0.056429866	0.127218794	-0.443565488	0.657356762	0.999579949
1371.582339	0.169570266	0.125382528	1.352423407	0.176239906	0.983992835
37.8870019	-0.210483129	0.486146408	-0.432962428	0.665042066	0.999579949
9769.921491	0.044852241	0.133058154	0.337087504	0.736050937	0.999579949
565.241372	0.005324566	0.094164155	0.05654557	0.954907194	0.999579949
339.9617933	-0.09765634	0.1330479	-0.733993841	0.462952492	0.999579949
100.8848291	0.184350126	0.139399099	1.322462825	0.186014081	0.983992835
567.1783256	-0.039011156	0.144308844	-0.270331014	0.786905607	0.999579949
287.0756137	-0.061534388	0.14543336	-0.423110546	0.672214608	0.999579949
284.3923154	0.046355065	0.171641203	0.270069565	0.787106736	0.999579949
250.5296096	0.028274574	0.159237003	0.177562837	0.859066313	0.999579949
40.16515996	-0.144487565	0.181479475	-0.796164772	0.42593627	0.999579949
498.9222809	0.055450294	0.074202405	0.747284322	0.454891954	0.999579949
5633.58962	0.048753605	0.124927936	0.390253825	0.696348864	0.999579949
1669.298443	0.02898667	0.152495453	0.190082191	0.849244725	0.999579949
54.50015848	-0.492682306	0.348655205	-1.413093218	0.157628317	0.977304288
319.8752331	-0.510100937	0.239797878	-2.127212061	0.033402467	0.698554126
625.4495388	-0.324340547	0.231172714	-1.403022619	0.160610094	0.978261482
51.50388986	-0.121061555	0.284182199	-0.425999784	0.670108003	0.999579949
65.94057993	-0.108303155	0.197985816	-0.547024816	0.584361683	0.999579949
270.8035395	0.044839455	0.111882537	0.400772596	0.688587557	0.999579949
165.1928942	-0.055625597	0.143181922	-0.388495952	0.697649049	0.999579949
503.5218527	0.142836615	0.096822131	1.475247595	0.14014599	0.948318761
596.7180234	0.152229574	0.080889114	1.881953772	0.059842293	0.798635337
56.47254954	0.199247138	0.169731718	1.173894549	0.240437266	0.999579949
24.18854185	0.363545282	0.240671004	1.510548742	0.130903462	0.936571507
139.9218731	0.174673715	0.091489965	1.909211731	0.056234784	0.785214091
622.8668333	-0.21916464	0.148591382	-1.474948532	0.140226381	0.948318761
57.32208139	0.099961647	0.192588165	0.519043562	0.603730364	0.999579949
33.02340189	-0.016223253	0.227162291	-0.071417018	0.943065866	0.999579949
153.8283897	-0.001784295	0.132909817	-0.013424853	0.989288839	0.999579949
2662483.651	0.001939903	0.114649433	0.016920299	0.986500199	0.999579949
180.5544459	-0.243585616	0.154831422	-1.573231147	0.115665306	0.918818353

357.0317975	-0.063974187	0.064513814	-0.991635484	0.321375373	0.999579949
66.16295177	0.749541048	0.292213962	2.565041871	0.010316335	0.47773736
237.3281097	-0.002721342	0.096148235	-0.028303608	0.977420003	0.999579949
319.6363145	-0.054507607	0.101396935	-0.53756662	0.59087628	0.999579949
235.9146838	0.444831724	0.57679784	0.771209067	0.440583021	0.999579949
576.1677279	-0.092441587	0.066295641	-1.394384095	0.163201649	0.978498637
63.66684294	-0.196898788	0.249281284	-0.789865911	0.429606081	0.999579949
490.5198833	0.015805484	0.064480274	0.245121232	0.806362564	0.999579949
279.9316433	-0.161044438	0.170878823	-0.942448192	0.345963224	0.999579949
1019.40015	-0.074200692	0.082201874	-0.902664244	0.366704119	0.999579949
62.38852755	-0.178149914	0.164691854	-1.081716615	0.279378468	0.999579949
84.87351732	0.201488801	0.151709209	1.32812505	0.184136799	0.983992835
602.7711411	0.053126943	0.106162511	0.500430353	0.616772085	0.999579949
765.2000196	0.019195755	0.066697218	0.287804434	0.773496443	0.999579949
28.64790291	0.209472993	0.266722423	0.785359516	0.43224281	0.999579949
385.1634169	-0.042682733	0.105145682	-0.405938997	0.684787424	0.999579949
62.99370297	0.277760191	0.241688873	1.14924691	0.250454183	0.999579949
1269.95093	-0.060863937	0.061341338	-0.992217308	0.321091531	0.999579949
99.94062639	0.03349094	0.222172371	0.150743046	0.880178415	0.999579949
401.3424166	-0.132289006	0.130150102	-1.016434134	0.309422698	0.999579949
96.86362561	-0.213748042	0.119205022	-1.793112734	0.0729549	0.838669905
1670.880002	-0.209111207	0.110764373	-1.887892299	0.059040411	0.79629879
64.12462876	0.02598998	0.198133493	0.131174086	0.895637595	0.999579949
937.480571	-0.000121117	0.147634405	-0.000820388	0.999345425	0.999840121
140.6211732	1.01005981	0.426067613	2.370656157	0.017756541	0.573039104
115.9293165	0.14854436	0.134512524	1.104316202	0.269455997	0.999579949
342.7678932	0.338365808	0.159897778	2.116138281	0.034333049	0.703403556
535.0636272	0.191623122	0.226794824	0.844918409	0.398156395	0.999579949
463.6795776	0.216418883	0.115898458	1.8673146	0.061857668	0.805306145
850.6655332	-0.064042697	0.130114967	-0.492200844	0.622577368	0.999579949
92.54206472	-0.780657579	0.262787985	-2.970674553	0.002971465	0.290143966
264.8387105	0.422597282	0.299036465	1.413196488	0.157597959	0.977304288
266.2250632	0.150428633	0.095300359	1.578468695	0.114457974	0.918818353
467.1863751	0.001678747	0.125444901	0.013382345	0.989322752	0.999579949
1189.124355	-0.027388952	0.085597176	-0.319974947	0.748987322	0.999579949
167.1451451	-0.062334355	0.253190253	-0.246195715	0.805530733	0.999579949
1818.218875	-0.009588697	0.109908809	-0.087242295	0.930478921	0.999579949
281.0468099	-0.013044569	0.092751599	-0.140639824	0.888154486	0.999579949
442.5239704	-0.030311692	0.105034953	-0.288586712	0.772897666	0.999579949
1767.636649	-0.102482355	0.061519799	-1.665843464	0.095744597	0.88463985
320.3881976	0.025197875	0.147402536	0.170946006	0.864266222	0.999579949
1839.512751	0.010549198	0.113563068	0.092892855	0.925988682	0.999579949
335.3182149	-0.053674828	0.079573401	-0.674532281	0.49997297	0.999579949
41.47692668	-0.045645377	0.445808108	-0.102387947	0.918448751	0.999579949
349.6900493	-0.100024742	0.140553455	-0.711649111	0.476682088	0.999579949
225.7019619	-0.147443477	0.131001924	-1.1255062	0.260374592	0.999579949
340.7167845	-0.052045587	0.087692199	-0.593503045	0.552844546	0.999579949
61.03421305	0.089354914	0.200552065	0.445544722	0.655926148	0.999579949

21.55367696	-0.476159593	0.268967193	-1.770325917	0.076672863	0.851207389
3319.870019	0.103922198	0.143477909	0.724308002	0.468876665	0.999579949
2673.99736	0.026020142	0.100140517	0.259836302	0.794990048	0.999579949
404.2051002	0.013969271	0.106506337	0.13115906	0.895649481	0.999579949
681.8728759	0.030216846	0.124433738	0.242834833	0.808133351	0.999579949
1062.544758	0.157614173	0.071972988	2.189907304	0.02853096	0.666751303
100.3877483	-0.071852372	0.142743208	-0.503368066	0.614705519	0.999579949
197.561467	-0.16151517	0.17616844	-0.916822388	0.359235719	0.999579949
609.9854516	0.023433768	0.076502291	0.306314592	0.75936513	0.999579949
73.0067502	0.188293492	0.113145128	1.66417675	0.096077113	0.88463985
737.677476	-0.015876201	0.047937823	-0.331183184	0.740506121	0.999579949
1717.883274	-0.016303046	0.073238518	-0.222602068	0.823845226	0.999579949
75.32779921	-0.170825012	0.241433398	-0.707545075	0.479227812	0.999579949
471.060384	-0.12525683	0.070242844	-1.783197029	0.074554236	0.848695362
239.5988317	0.007011699	0.122938427	0.057034237	0.954517922	0.999579949
141.6941155	-0.274318339	0.15202834	-1.804389494	0.071170269	0.834620594
983.2160794	0.038639262	0.107176448	0.360520086	0.718458239	0.999579949
41.12479215	-0.181450006	0.171024889	-1.060956724	0.288709569	0.999579949
127.9884102	0.228441949	0.138394847	1.650653573	0.098809333	0.894181927
167.3743352	-0.239079766	0.335942869	-0.711667929	0.476670432	0.999579949
200.0802213	-0.322586437	0.204765018	-1.575398183	0.115164563	0.918818353
61.96833395	-0.321118829	0.218033592	-1.472795207	0.140806265	0.948318761
163.965828	0.452113883	0.320477264	1.410751817	0.158317806	0.977304288
703.4321402	-0.067562874	0.067237869	-1.00483366	0.314976953	0.999579949
54.92119719	0.11077711	0.15521323	0.713709198	0.475407018	0.999579949
68.60956282	-0.120058567	0.200909659	-0.597574891	0.550123623	0.999579949
22.81849024	0.01284297	0.234420825	0.054785962	0.956308985	0.999579949
355.6682731	-0.030408342	0.088894474	-0.342072356	0.73229644	0.999579949
131.6629206	-0.242109151	0.229191494	-1.056361853	0.290802927	0.999579949
544.3690026	-0.091926118	0.076197601	-1.206417486	0.227656557	0.999579949
30.38162001	-0.354526832	0.273784471	-1.294912128	0.195350544	0.989001389
483.8554859	0.047896071	0.090253261	0.530685212	0.595636935	0.999579949
413.0742533	0.03074625	0.165284931	0.18601968	0.852429329	0.999579949
423.1280244	-0.124463068	0.083594491	-1.488890793	0.136516128	0.945791454
42.83239185	-0.0424994	0.25909578	-0.164029689	0.869707772	0.999579949
206.447823	-0.004872716	0.100574144	-0.048448988	0.961358418	0.999579949
138.5042221	0.204011477	0.169423408	1.204151658	0.228530967	0.999579949
775.4358321	-0.054344489	0.077504079	-0.701182306	0.483189251	0.999579949
80.37749599	0.084175335	0.139907568	0.601649619	0.547407392	0.999579949
151.8462663	0.025479797	0.296231527	0.086013119	0.931455989	0.999579949
993.2674653	0.04839052	0.120005847	0.403234687	0.686775585	0.999579949
167.7616384	-0.001345044	0.09960363	-0.013503962	0.989225724	0.999579949
1091.093195	0.138195254	0.14379818	0.961036179	0.336533977	0.999579949
405.4638166	0.045500985	0.165215205	0.275404342	0.783005609	0.999579949
406.9186155	0.153374438	0.064441406	2.380060369	0.017309802	0.570162425
1389.660218	-0.025612174	0.076784955	-0.333557187	0.73871373	0.999579949
178.2221143	0.482973087	0.293441424	1.645892667	0.099785859	0.895628172
1396.483686	0.045500435	0.097462351	0.466851396	0.640606201	0.999579949

318.57964	-0.052108748	0.094958738	-0.54875148	0.583176012	0.999579949
281.7020153	0.126647024	0.435553572	0.290772554	0.771225278	0.999579949
9315.49276	-0.034224203	0.091300068	-0.374854074	0.707768996	0.999579949
151.1467592	0.086784954	0.112014184	0.774767541	0.438477033	0.999579949
92.69582583	0.10263772	0.136102932	0.754118359	0.450778154	0.999579949
35.09509474	0.226604879	0.226975658	0.998366438	0.318101702	0.999579949
2913.182661	-0.190252345	0.173395046	-1.097219033	0.272545655	0.999579949
20.45375905	-0.383465053	0.252430314	-1.519092724	0.128739158	0.935700262
44.18499721	0.009925438	0.212582283	0.046689863	0.96276041	0.999579949
610.5559488	0.185731651	0.144523678	1.285129562	0.198747013	0.992481822
136.6234325	0.218170915	0.151691459	1.438254446	0.150361872	0.966305174
172.7066001	-0.025656057	0.111776003	-0.229530992	0.818456236	0.999579949
349.6763309	0.028976283	0.16142894	0.179498691	0.857546145	0.999579949
68.32035306	0.112613063	0.174643863	0.644815461	0.519046782	0.999579949
69.86738531	0.160325253	0.192237223	0.833996926	0.404282716	0.999579949
135.645309	-0.084938134	0.0971194	-0.87457433	0.38180556	0.999579949
53.10584477	-0.111083058	0.16311551	-0.681008559	0.495866075	0.999579949
25.08428432	0.450101858	0.336978643	1.335698471	0.18164785	0.983992835
31.76025587	0.565815731	0.328957331	1.720027728	0.085427401	0.864463816
565.2318371	0.09948212	0.084465689	1.177781423	0.238883756	0.999579949
823.7045999	0.001921844	0.119849742	0.016035444	0.987206115	0.999579949
45.94802187	0.066850743	0.21398771	0.312404589	0.754733063	0.999579949
144.0382984	0.100443113	0.232307369	0.432371618	0.665471343	0.999579949
32.51692478	-0.041773091	0.35067391	-0.119122324	0.905178445	0.999579949
250.5460261	-0.109786454	0.14744721	-0.744581429	0.456524798	0.999579949
956.1537911	0.018079918	0.081696493	0.221305925	0.824854237	0.999579949
351.031029	0.109623702	0.150417959	0.728793972	0.466127699	0.999579949
73.9545368	0.110610322	0.150815771	0.733413495	0.463306271	0.999579949
404.0410644	-0.065058898	0.083432379	-0.779779975	0.435520395	0.999579949
136.6837587	-0.199340998	0.114282688	-1.744279916	0.081110292	0.85448976
95.38199121	0.365697923	0.182140486	2.007779443	0.044666734	0.759209569
48.05622736	0.030010931	0.332737694	0.090193963	0.928133081	0.999579949
37.58010543	-0.019779378	0.320246243	-0.061763029	0.950751546	0.999579949
1697.446976	-1.384160584	0.467559442	-2.960394893	0.003072449	0.295424219
59.64866412	-0.402680248	0.887921836	-0.453508667	0.650182504	0.999579949
389.0107362	0.090584628	0.0935032	0.968786394	0.332651777	0.999579949
469.0508324	0.177901046	0.144827114	1.228368365	0.219308718	0.999579949
100.447636	-0.007821365	0.172384769	-0.045371555	0.963811153	0.999579949
76.30481568	0.1410714	0.150879002	0.934996906	0.349789894	0.999579949
56.69795729	-0.642503386	0.251867309	-2.550959822	0.010742671	0.483266726
346.6876047	0.108620208	0.086514685	1.255511797	0.209293028	0.996447459
229.9120598	0.118749164	0.106037318	1.119880871	0.262764531	0.999579949
725.236005	0.304104359	0.420188178	0.723733735	0.469229218	0.999579949
277.4522677	0.039345452	0.065530735	0.600412189	0.548231566	0.999579949
502.2127789	0.051911354	0.143534578	0.361664451	0.717602795	0.999579949
71.01370252	-0.149282644	0.169989903	-0.878185359	0.379843136	0.999579949
39.37246679	-0.024014106	0.867591827	-0.027679037	0.977918143	0.999579949
156.5892796	0.146834951	0.206551088	0.710889264	0.477152859	0.999579949

713.7248957	0.08113672	0.109934778	0.738044153	0.460487619	0.999579949
28.5578384	-0.66205169	0.666728614	-0.992985267	0.320717135	0.999579949
20.63462216	1.293082139	0.617424257	2.094317036	0.036231748	0.716432561
1299.268529	0.099059236	0.096990733	1.021326808	0.307099629	0.999579949
206.7668636	-0.012265837	0.13835688	-0.088653611	0.9293572	0.999579949
63.94544356	-0.035076852	0.164152812	-0.213684138	0.830793381	0.999579949
66.19400794	0.095626756	0.135130615	0.707661665	0.479155389	0.999579949
93.30741689	0.099431874	0.107201063	0.92752694	0.353653011	0.999579949
25.0628176	0.560892668	0.327673884	1.711740531	0.086944496	0.867486202
133.8415851	0.169759449	0.140455052	1.20863897	0.226801578	0.999579949
45.50220423	0.134684633	0.201678923	0.667817095	0.504250353	0.999579949
117.0715589	-0.055250286	0.116863357	-0.472776815	0.636372417	0.999579949
332.4919987	0.101264037	0.087323213	1.15964625	0.246192862	0.999579949
326.1236339	-0.215152814	0.107018832	-2.01042013	0.044386742	0.758967899
353.2860832	0.826852142	0.309591177	2.67078717	0.007567361	0.416237905
745.5999815	-0.011008289	0.059728417	-0.184305714	0.853773632	0.999579949
28.86862866	0.100604091	0.248472743	0.404889848	0.685558481	0.999579949
791.250829	-0.114762404	0.110255742	-1.040874624	0.29793374	0.999579949
2498.339268	-0.053195044	0.147183273	-0.361420446	0.717785166	0.999579949
29.62723871	0.183828688	0.324822975	0.565934992	0.571437973	0.999579949
311.5770185	0.090790446	0.156458326	0.580285166	0.561722329	0.999579949
784.8394615	0.015426622	0.083779628	0.184133334	0.853908856	0.999579949
485.0186638	-0.014549454	0.075805161	-0.191932238	0.847795285	0.999579949
1445.780839	0.047088357	0.065725487	0.716439837	0.473719804	0.999579949
473.1437852	-0.061893816	0.085525974	-0.723684433	0.469259492	0.999579949
623.0540532	-0.013737102	0.090404651	-0.151951277	0.879225365	0.999579949
1040.699056	-0.047877092	0.116622817	-0.410529376	0.681417659	0.999579949
475.6259246	0.106319156	0.071591658	1.485077435	0.137523313	0.945791454
567.9043259	-0.028971477	0.110019507	-0.263330367	0.792295956	0.999579949
562.0168158	-0.173531923	0.140213242	-1.237628639	0.21585379	0.999579949
1865.695647	-0.028991501	0.146934714	-0.197308724	0.843585953	0.999579949
26049.97994	0.084330073	0.115044255	0.733022896	0.463544465	0.999579949
373.5526874	0.158875078	0.142530688	1.114672774	0.264990672	0.999579949
137.6706392	0.053863469	0.12596385	0.427610534	0.668934696	0.999579949
121.7279541	0.22958327	0.155286843	1.478446373	0.139288341	0.947341221
253.1507577	0.024479743	0.103160411	0.237297844	0.812425734	0.999579949
463.9376398	-0.047934807	0.067097427	-0.714406043	0.474976136	0.999579949
1425.893167	-0.030735326	0.085430987	-0.359767897	0.719020712	0.999579949
85.65909457	0.080025834	0.113902987	0.702578885	0.482318225	0.999579949
678.1214462	0.105939458	0.207201063	0.511288196	0.609149269	0.999579949
70.84819177	0.117209739	0.142611796	0.821879691	0.411145372	0.999579949
139.6339288	0.1220444	0.186411434	0.654704471	0.512658046	0.999579949
143.1925109	0.079407702	0.136452271	0.581944887	0.560603804	0.999579949
123.6734465	0.075897955	0.106125357	0.715172671	0.474502354	0.999579949
397.9017028	-0.029665972	0.057937998	-0.512029631	0.608630272	0.999579949
421.5999881	0.495491072	0.212644354	2.330139792	0.019798764	0.601919728
1883.775352	0.220093356	0.114141023	1.928258136	0.053823026	0.781954831
58.47957787	0.1897327	0.163887175	1.157703156	0.246985196	0.999579949

620.479281	0.090501675	0.113752739	0.795599968	0.426264585	0.999579949
824.0449513	0.102346412	0.106797245	0.958324458	0.337899174	0.999579949
40.36533634	-0.327062399	0.187370118	-1.745541933	0.08089058	0.85448976
158.9508085	0.058614478	0.136510685	0.429376483	0.667649265	0.999579949
44.13928602	-0.125552764	0.261779643	-0.479612405	0.631503024	0.999579949
186.8152318	-0.087149904	0.149024163	-0.584803844	0.558679619	0.999579949
61.76374558	0.418765801	0.248370359	1.686053849	0.091785428	0.880418343
98.12821138	0.144483345	0.158814995	0.909758837	0.362949707	0.999579949
254.322456	0.000833948	0.093482442	0.008920904	0.992882243	0.999579949
349.0549639	-0.065300148	0.079669596	-0.81963699	0.41242308	0.999579949
1281.669308	-0.027273553	0.384187458	-0.070990222	0.943405538	0.999579949
764.0189428	0.119032665	0.15080561	0.789311916	0.429929724	0.999579949
37.52052336	0.073790315	0.27017761	0.273117803	0.784762662	0.999579949
45.42504162	0.133179847	0.223811409	0.595053879	0.551807457	0.999579949
175.6097283	-0.054883555	0.164679621	-0.33327472	0.73892692	0.999579949
27.61770231	-0.20702314	0.233175475	-0.887842689	0.374625377	0.999579949
111.5732421	0.100788175	0.221047625	0.455956833	0.64842102	0.999579949
173.4786283	0.213800956	0.214095636	0.998623606	0.31797706	0.999579949
410.3607207	-0.130482198	0.081158012	-1.607754971	0.107888854	0.903569152
353.2945624	-0.026814979	0.083792983	-0.320014614	0.748957252	0.999579949
475.2046414	-0.054208066	0.084853092	-0.638846087	0.522923063	0.999579949
564.6498193	-0.046549239	0.05828265	-0.798680891	0.42447547	0.999579949
61.83483348	0.039740778	0.215245178	0.18463028	0.853519034	0.999579949
117.7331244	0.29707029	0.224374566	1.323992713	0.185505465	0.983992835
2426.153857	-0.333165458	0.163307358	-2.040112959	0.041339076	0.745998574
94.21655155	0.194575814	0.24389449	0.797786838	0.4249942	0.999579949
46.89422764	0.12308918	0.278954233	0.441252238	0.659030399	0.999579949
383.3740131	0.127161992	0.139769731	0.909796356	0.362929917	0.999579949
1035.2054	-0.051116564	0.105321567	-0.485338047	0.627436571	0.999579949
893.5589228	0.059783217	0.1454298	0.411079552	0.681014203	0.999579949
69.73210293	-0.068985059	0.155881814	-0.442547192	0.658093288	0.999579949
42.63040405	0.242824824	0.219680148	1.105356247	0.269005254	0.999579949
49.29878155	0.084045402	0.171345851	0.490501526	0.62377905	0.999579949
31.250052	0.532111305	0.727686186	0.731237332	0.464634201	0.999579949
33.34496497	1.213305997	0.53331518	2.275026182	0.022904359	0.62865781
149.7782845	0.157919611	0.112022573	1.409712398	0.158624622	0.977304288
523.9421449	-0.012857679	0.068358617	-0.188091566	0.850804872	0.999579949
1350.635622	0.024099375	0.096341189	0.250146119	0.802474352	0.999579949
1467.479539	-0.025594011	0.131562952	-0.194538134	0.84575454	0.999579949
346.2075809	-0.000968877	0.090043647	-0.010760082	0.991414862	0.999579949
121.6991256	0.05425472	0.113515999	0.477947785	0.632687369	0.999579949
121.9401504	-0.397237737	0.192272703	-2.066012129	0.038827334	0.728740425
2152.726614	0.039220381	0.053230294	0.736805653	0.461240545	0.999579949
444.0004454	0.031187283	0.10356509	0.301137023	0.76331001	0.999579949
678.9994499	0.025998737	0.061311952	0.424040277	0.67153644	0.999579949
2431.720325	0.216794619	0.118493511	1.829590635	0.067311175	0.826962196
34.89159254	0.016547485	0.462800775	0.035755093	0.971477641	0.999579949
26.10084376	-0.40775689	0.229499195	-1.776724707	0.075613545	0.851207389

34.8694486	-0.078724903	0.181750781	-0.433147535	0.664907591	0.999579949
237.6658589	0.041107646	0.124814263	0.32935055	0.74189074	0.999579949
68.55418362	0.078093684	0.155213252	0.503137989	0.614867259	0.999579949
334.3825348	-0.035718983	0.088782289	-0.402321043	0.68744777	0.999579949
50.12281563	-0.072425646	0.325583686	-0.222448633	0.823964656	0.999579949
39.40649652	-0.209360277	0.282796578	-0.740321111	0.459105174	0.999579949
335.3444832	-0.116438991	0.135914271	-0.856709085	0.391605681	0.999579949
771.7668717	0.202187922	0.221638147	0.912243332	0.361640638	0.999579949
168.5916819	0.123426311	0.120001018	1.028543864	0.303694068	0.999579949
29.45923748	0.003435051	0.340123917	0.010099409	0.991941975	0.999579949
1399.41396	0.004286417	0.036278418	0.11815337	0.905946137	0.999579949
123.9096933	0.051362691	0.250693698	0.20488226	0.837664116	0.999579949
413.9853541	0.001248675	0.063297128	0.019727206	0.984260988	0.999579949
56.57541971	0.266853015	0.211082551	1.264211625	0.206154079	0.994814558
89.93958935	0.020697148	0.148183734	0.1396722	0.888918992	0.999579949
521.6618801	-0.009175721	0.127092178	-0.072197371	0.942444837	0.999579949
228.7187413	-0.005347823	0.082090751	-0.065145256	0.948058348	0.999579949
290.8214313	0.107515931	0.092573181	1.161415539	0.245472953	0.999579949
65.76524089	0.039293727	0.151464664	0.259425047	0.795307306	0.999579949
270.2531201	0.333204367	0.200371556	1.66293247	0.096325954	0.88499031
361.0456091	0.315570751	0.251996703	1.252281271	0.210467391	0.996581389
35.59998621	0.073154818	0.176519724	0.414428575	0.678560266	0.999579949
398.0221282	0.257744049	0.104435172	2.467981286	0.013587742	0.518640006
1073.468634	-0.058314044	0.096339643	-0.605296448	0.544982041	0.999579949
334.2060616	0.179932116	0.083917121	2.144164543	0.032019705	0.689986698
5388.618744	0.076338763	0.115990818	0.658144884	0.510445037	0.999579949
93.98582115	-0.085620952	0.181474769	-0.471806367	0.637065006	0.999579949
141.5969571	-0.01933035	0.104744064	-0.184548408	0.853583255	0.999579949
229.6457647	0.013770221	0.109826612	0.125381463	0.900221563	0.999579949
382.1094258	-0.031692717	0.138509029	-0.228813363	0.81901398	0.999579949
369.4605407	0.137805461	0.172641686	0.798216607	0.424744802	0.999579949
119.4861984	-0.050165706	0.098141509	-0.511156859	0.609241224	0.999579949
125.7780355	-0.057182836	0.126278104	-0.452832552	0.650669321	0.999579949
104.9620643	0.22086486	0.152762348	1.445806921	0.148231364	0.962301096
723.5206452	0.02765168	0.104987576	0.263380496	0.792257322	0.999579949
143.9292962	0.010308958	0.11490269	0.089719034	0.92851049	0.999579949
249.566494	-0.260005999	0.215251464	-1.207917447	0.227079018	0.999579949
31.70278676	-0.105603963	0.189479623	-0.55733678	0.577297351	0.999579949
744.9810058	-0.02653405	0.050770311	-0.522629253	0.60123228	0.999579949
93.91979769	-0.010960015	0.15361702	-0.071346356	0.943122102	0.999579949
139.9842963	0.183538259	0.524226514	0.350112507	0.726254265	0.999579949
447.424088	-0.225963536	0.133423184	-1.693585241	0.090344125	0.876222021
127.7923483	-0.06328727	0.237539317	-0.26642861	0.789909134	0.999579949
29.69748047	0.312726923	0.242173705	1.291333108	0.196588195	0.991527744
2822.614618	0.074861449	0.096598673	0.774973885	0.438355091	0.999579949
83.89495708	0.269719538	0.304108771	0.886917983	0.375123059	0.999579949
169.900053	0.184969004	0.103740801	1.782991861	0.074587628	0.848695362
637.6144441	0.147419899	0.071793183	2.053396883	0.040034101	0.73515901

429.0025724	-0.046415971	0.067665754	-0.685959556	0.492738612	0.999579949
81.46415303	-0.04423595	0.225843291	-0.195870111	0.844711832	0.999579949
561.5689999	-0.335363692	0.146310868	-2.292131106	0.021898079	0.627784819
43.18057732	-0.009734426	0.271892042	-0.035802542	0.971439806	0.999579949
862.1088582	-0.054515056	0.096470198	-0.565097381	0.57200753	0.999579949
200.3959897	0.209989418	0.390510456	0.537730591	0.590763057	0.999579949
360.7288367	0.080501557	0.115757007	0.695435713	0.486782286	0.999579949
184.1378389	0.045107131	0.129776194	0.347576311	0.728158399	0.999579949
239.1601928	0.393633554	0.230089223	1.710786579	0.087120518	0.867486202
1419.014724	0.037813128	0.098867577	0.382462373	0.702118433	0.999579949
3458.034776	-0.009955962	0.177711589	-0.056023146	0.955323368	0.999579949
53.05562125	0.238835547	0.142246263	1.679028619	0.093146466	0.880418343
117.9584526	-0.011728586	0.122325652	-0.095880022	0.923615861	0.999579949
26.64519319	-0.078464572	0.225623036	-0.347768443	0.728014091	0.999579949
225.3422573	0.011519559	0.128938803	0.089341292	0.928810679	0.999579949
77.83849471	-0.00325605	0.145363699	-0.022399332	0.982129413	0.999579949
491.5148791	0.134855464	0.087280846	1.545075115	0.122328095	0.92236461
111.7819513	-0.166549328	0.145509703	-1.144592592	0.252377963	0.999579949
355.511977	-0.105040543	0.130631299	-0.804099349	0.421339601	0.999579949
20.49111458	-0.955017741	0.581741145	-1.641654107	0.100661708	0.895628172
367.8096008	0.037475836	0.138704739	0.270184252	0.787018507	0.999579949
45.56946233	-0.304462198	0.263888199	-1.153754502	0.248600832	0.999579949
576.6919966	-0.104098738	0.120044393	-0.867168675	0.385849597	0.999579949
419.1565993	-0.138571152	0.08893772	-1.558069536	0.119216765	0.921521033
124.8583673	0.120145558	0.129430806	0.928260906	0.353272248	0.999579949
235.2631716	0.141900391	0.154294108	0.91967472	0.357742767	0.999579949
417.8552153	0.022610054	0.179860619	0.125708751	0.899962474	0.999579949
191.5541778	0.02414523	0.211090773	0.11438316	0.908934063	0.999579949
55.65660809	-0.036845851	0.161710365	-0.227850894	0.819762159	0.999579949
1274.828548	0.069603635	0.092153547	0.755300657	0.450068605	0.999579949
132.2538816	0.127960501	0.139763603	0.915549526	0.359903215	0.999579949
268.0731582	0.096342206	0.146924085	0.655727798	0.51199928	0.999579949
756.0569451	-0.083167327	0.151704226	-0.54822024	0.583540686	0.999579949
777.5327907	-0.026057092	0.073531507	-0.354366348	0.723064351	0.999579949
389.1125474	0.155188488	0.263783632	0.588317351	0.556319301	0.999579949
101.4524849	0.140951405	0.13857574	1.017143442	0.309085197	0.999579949
394.2465163	0.065726458	0.0992309	0.662358785	0.507741311	0.999579949
32.33220494	-0.009158072	0.219426579	-0.041736386	0.966708848	0.999579949
162.8493159	0.134836075	0.107763113	1.251226616	0.21085181	0.996581389
80.44087708	-0.022468848	0.130049493	-0.172771515	0.862831028	0.999579949
187.3222836	-0.105161875	0.102474954	-1.026220274	0.304787767	0.999579949
186.114416	0.053759918	0.166425581	0.323026767	0.746674971	0.999579949
129.6668453	0.086339494	0.154377077	0.559276645	0.575972932	0.999579949
93.6261654	-0.499011296	0.330135928	-1.511532836	0.130652749	0.936121746
287.0927088	-0.135023067	0.09587754	-1.408286718	0.159046187	0.977718776
243.5140995	0.013377112	0.123005793	0.108751887	0.913399286	0.999579949
285.4942075	0.035919677	0.094033455	0.381988273	0.702470063	0.999579949
757.3197159	0.007343083	0.130258567	0.056373128	0.955044563	0.999579949

288.5502479	-0.018205529	0.103123116	-0.176541687	0.859868401	0.999579949
349.6989261	0.01566144	0.109850162	0.142570936	0.886629052	0.999579949
574.5326039	-0.079931379	0.121566081	-0.657513829	0.510850582	0.999579949
828.0762826	-0.226064466	0.159722484	-1.415357811	0.156963613	0.977304288
48.31993068	0.132885643	0.215007533	0.618051102	0.536541654	0.999579949
532.3090573	0.244471439	0.093469264	2.615527598	0.008908972	0.447081326
195.380884	0.069033878	0.131489508	0.525014346	0.599573218	0.999579949
2472.232886	0.013320046	0.068084704	0.195639326	0.844892477	0.999579949
138.767333	-0.017202831	0.145714861	-0.11805818	0.906021559	0.999579949
33.03165465	0.100329172	0.251733201	0.398553595	0.690222161	0.999579949
42.04827002	-0.073017673	0.188818076	-0.386709125	0.69897156	0.999579949
368.9793168	-0.177695962	0.10033607	-1.771007791	0.076559406	0.851207389
583.6263638	0.456387119	0.112298452	4.064055308	4.82274E-05	0.035697035
167.6470724	-0.021195446	0.09621681	-0.220288388	0.825646563	0.999579949
466.4074869	-0.330532235	0.292272682	-1.130903623	0.258095658	0.999579949
303.6485402	-0.05060964	0.105769805	-0.478488543	0.632302527	0.999579949
25.95816313	0.399858668	0.416356613	0.960375447	0.336866291	0.999579949
622.5859465	-0.053787278	0.082131333	-0.654893523	0.512536311	0.999579949
1410.801952	-0.097389562	0.099930101	-0.97457684	0.329770207	0.999579949
73.00147987	-0.081117476	0.184917141	-0.438669316	0.660901163	0.999579949
21.50195944	-0.022717443	0.253956825	-0.089453955	0.928721145	0.999579949
243.5787179	-0.00695315	0.096484721	-0.072064778	0.942550357	0.999579949
43.62248163	0.051464075	0.20422864	0.25199245	0.8010469	0.999579949
22.48334998	0.066103593	0.260567351	0.253691005	0.799734284	0.999579949
269.411657	0.181562929	0.095436334	1.9024508	0.057112245	0.790533889
203.5422497	0.002583263	0.191385834	0.01349767	0.989230745	0.999579949
1767.864445	0.032334567	0.086804248	0.372499817	0.709520748	0.999579949
417.0253845	-0.236849482	0.118167485	-2.004354087	0.045032144	0.760273172
558.6008261	-0.010222271	0.088147684	-0.115967553	0.907678258	0.999579949
36.16585253	-0.5610223	0.264124547	-2.124082393	0.033663255	0.698554126
56.7421464	-0.274779147	0.232006648	-1.184358936	0.236271008	0.999579949
38.68391697	0.25993296	0.261776361	0.992958109	0.32073037	0.999579949
66.45523883	-0.244600648	0.141553745	-1.727970159	0.083993574	0.860148832
620.995413	-0.118434284	0.087059914	-1.360376771	0.173710726	0.983601342
66.26392269	0.034542293	0.136202241	0.253610311	0.79979663	0.999579949
208.9718131	0.454885327	0.182737966	2.48927651	0.012800337	0.510304755
346.0762578	0.5016458	0.241730135	2.075230712	0.037965158	0.726381766
29.57774744	0.201663269	0.364214616	0.553693511	0.579788617	0.999579949
9630.645655	0.078016184	0.114928508	0.678823601	0.497249638	0.999579949
29.95431411	0.226793511	0.43894415	0.516679652	0.605379802	0.999579949
593.8292502	0.071581018	0.132156655	0.541637635	0.58806816	0.999579949
133.9507563	-0.034919239	0.12373406	-0.282212022	0.777780941	0.999579949
34.89098541	1.894213299	0.702449068	2.696584543	0.007005461	0.404205857
390.6044922	-0.061890904	0.090241279	-0.685838062	0.492815231	0.999579949
21.28754371	0.039791985	0.358793493	0.110904979	0.911691697	0.999579949
185.9177519	0.112458863	0.173164579	0.649433412	0.516058273	0.999579949
437.918667	-0.025506585	0.06628809	-0.384783823	0.700397585	0.999579949
177.4152789	0.020477304	0.147491576	0.138837108	0.88957887	0.999579949

658.2927362	0.05432298	0.088050567	0.616952071	0.537266339	0.999579949
67.21157753	-0.205188885	0.219163656	-0.936235908	0.349151739	0.999579949
171.9406996	0.045981467	0.130557284	0.352193808	0.724692921	0.999579949
38.04551839	0.079757956	0.217286336	0.367063837	0.713571401	0.999579949
635.6345012	0.068956444	0.066424133	1.038123357	0.299212629	0.999579949
139.8103924	0.229007603	0.13943072	1.642447258	0.100497348	0.895628172
2984.323924	0.010973969	0.105703637	0.103818272	0.917313566	0.999579949
456.8715054	-0.112604472	0.07559444	-1.489586707	0.136332939	0.945791454
27.88958756	1.170112055	0.530872411	2.204130467	0.02751517	0.660154442
183.7248235	-0.017458095	0.118719628	-0.147053148	0.88309007	0.999579949
78.29474022	-0.042923783	0.141519077	-0.303307397	0.761655609	0.999579949
1261.789427	0.008731473	0.092434107	0.094461591	0.924742492	0.999579949
770.1157115	0.051077303	0.089548111	0.570389501	0.568413549	0.999579949
1070.566902	0.001948958	0.136120878	0.014317844	0.988576404	0.999579949
143.6137384	0.029521983	0.143866335	0.205204249	0.83741255	0.999579949
540.3426277	0.028081088	0.079112308	0.354952208	0.722625395	0.999579949
404.0062648	-0.148041236	0.105222803	-1.406931113	0.159447817	0.977801703
1496.902298	0.001966762	0.088514075	0.022219769	0.982272648	0.999579949
244.4737317	-0.020790866	0.129537238	-0.160501074	0.872486376	0.999579949
4539.129239	0.015180815	0.138601402	0.109528584	0.912783251	0.999579949
625.1142382	-0.131950058	0.1045046	-1.262624396	0.206724196	0.994814558
556.9877266	0.350569743	0.126565841	2.76986065	0.005608028	0.37178274
248.6610486	0.273506279	0.126637883	2.159750883	0.03079196	0.683621001
216.4067482	0.909827847	0.24269791	3.748807912	0.000177677	0.058895284
1746.646377	0.055323711	0.075438696	0.733359851	0.46333898	0.999579949
110.0254752	0.233563466	0.171469496	1.362128379	0.173157373	0.983601342
68.83348743	0.022143198	0.211664006	0.10461485	0.916681431	0.999579949
190.0116958	-0.083710426	0.094094885	-0.889638438	0.37366006	0.999579949
338.329454	0.001452615	0.117131052	0.012401624	0.990105189	0.999579949
882.413805	0.047846611	0.11192654	0.427482266	0.669028099	0.999579949
265.1481084	0.053134283	0.183942138	0.288864114	0.772685366	0.999579949
372.3515327	-0.012613255	0.076766452	-0.16430686	0.869489582	0.999579949
348.0001021	-0.093798299	0.083908732	-1.117860999	0.263626365	0.999579949
251.6337722	0.085136846	0.098655022	0.862975287	0.388151055	0.999579949
1054.936266	-0.002326171	0.113613367	-0.020474449	0.983664894	0.999579949
595.9287934	0.064015806	0.18229375	0.351168411	0.725462007	0.999579949
1490.592893	0.113552661	0.066720178	1.701923826	0.088769649	0.870840008
801.7861148	-0.07368533	0.064588365	-1.140845257	0.253934323	0.999579949
346.8901523	0.206427281	0.162282581	1.272023649	0.203364717	0.994409048
3348.114662	0.080693297	0.135697813	0.594654371	0.552074528	0.999579949
358.7430395	-0.349434221	0.222225472	-1.572431003	0.115850629	0.918818353
347.6839366	0.074621421	0.130641018	0.571194425	0.567867857	0.999579949
891.9898572	-0.075327873	0.0930253	-0.809756841	0.418079942	0.999579949
345.4740422	-0.153335997	0.183704411	-0.834688703	0.403893004	0.999579949
29.94697989	-0.27011481	0.242392054	-1.114371553	0.265119822	0.999579949
24.19151165	0.001448229	0.248859945	0.005819454	0.995356774	0.999579949
280.104584	0.028302238	0.124960196	0.226490028	0.820820315	0.999579949
456.6498033	-0.089383641	0.076350129	-1.17070714	0.241716514	0.999579949

28.56007316	0.407762973	0.305298393	1.335621093	0.181673152	0.983992835
521.040541	-0.135498574	0.134685617	-1.00603596	0.314398272	0.999579949
502.3820319	0.173488615	0.22181006	0.782149443	0.434126753	0.999579949
87.25997359	0.562002292	0.344341853	1.632105676	0.102657226	0.895628172
822.8019424	-0.019128128	0.064164648	-0.29811007	0.765619156	0.999579949
140.7205425	-0.235582628	0.119039645	-1.979026635	0.047813007	0.770705547
119.3638838	-0.049029788	0.23911597	-0.205046061	0.837536138	0.999579949
568.9607098	-0.1707649	0.10049443	-1.699247416	0.089272576	0.870840008
1220.301315	-0.505646148	0.516534667	-0.978920062	0.327619479	0.999579949
297.7583643	0.034059645	0.091176618	0.373556788	0.708734088	0.999579949
451.9495336	-0.007941525	0.092224802	-0.086110516	0.931378564	0.999579949
552.6977522	-0.012091411	0.077166402	-0.156692691	0.875487047	0.999579949
864.0892419	-0.000992759	0.18523391	-0.005359488	0.995723767	0.999579949
59.85069106	-0.058630705	0.164355038	-0.356732021	0.721292431	0.999579949
137.9974419	0.418611955	0.210534073	1.988333523	0.046774816	0.766764601
50.47344495	-0.129851034	0.212115532	-0.612171266	0.54042445	0.999579949
36.9922545	-0.926893645	0.382271852	-2.42469761	0.015321142	0.542093008
1156.051934	-0.100340669	0.059381532	-1.689762214	0.091073455	0.878377675
66.92437068	-0.500395694	0.412054921	-1.214390775	0.224598537	0.999579949
93.21852847	0.068324285	0.133343225	0.512394122	0.608375204	0.999579949
173.4402747	-0.228004623	0.196624456	-1.15959443	0.24621397	0.999579949
36.83301675	-0.207805862	0.326772948	-0.635933493	0.524819771	0.999579949
256.6622166	0.105339393	0.084822452	1.241881016	0.214280465	0.999554723
538.9878831	0.436707216	0.113266378	3.855576762	0.000115457	0.048890108
609.9731719	0.085048386	0.067167415	1.266214951	0.205436136	0.994814558
796.3356481	-0.199682275	0.090198764	-2.213802784	0.026842348	0.653787748
536.6707186	-0.191436949	0.099359356	-1.92671286	0.054015424	0.782044005
49.56243835	-0.142923444	0.169010764	-0.845646988	0.397749705	0.999579949
924.1489158	0.127496601	0.089051449	1.431718432	0.152224419	0.972669728
932.8116281	0.065733119	0.079021971	0.831833449	0.405502961	0.999579949
197.7013811	0.044156891	0.082724728	0.533781035	0.593493037	0.999579949
11187.64256	-0.043852777	0.154814324	-0.283260463	0.776977183	0.999579949
143.8902335	-0.045062104	0.123063033	-0.366170923	0.714237538	0.999579949
1013.662937	0.006960586	0.087658911	0.079405349	0.936710214	0.999579949
198.6509479	0.025891943	0.182593001	0.141801398	0.88723688	0.999579949
2547.933881	0.126192208	0.166968219	0.755785793	0.449777636	0.999579949
643.3722317	0.037858433	0.117140967	0.323186958	0.746553657	0.999579949
31.32689372	0.067834026	0.26458868	0.256375388	0.79766099	0.999579949
33.59016052	0.49861544	0.415080891	1.201248843	0.229654688	0.999579949
1239.139109	-0.250299095	0.190814874	-1.311737863	0.189608597	0.983992835
186.4713986	-0.022044383	0.114543806	-0.19245373	0.847386809	0.999579949
323.421285	0.159220415	0.223983483	0.71085784	0.477172334	0.999579949
30.91630527	-0.253528844	0.412400255	-0.614764033	0.538710565	0.999579949
535.6427886	0.08893493	0.089487412	0.993826145	0.320307515	0.999579949
293.7081949	0.081079864	0.091634117	0.884821796	0.376252752	0.999579949
303.9565857	0.107917573	0.172154839	0.62686343	0.530748761	0.999579949
495.8005375	-0.133945372	0.069310172	-1.932549975	0.05329166	0.781231069
222.7032442	0.010606491	0.074768835	0.141857111	0.887192872	0.999579949

53847.86281	-0.042498596	0.165111465	-0.257393366	0.796875124	0.999579949
270.0121182	0.020307382	0.093892163	0.216284099	0.828766303	0.999579949
77.62066228	0.157442937	0.135748455	1.159813839	0.246124609	0.999579949
33.01134485	0.049110045	0.188050691	0.261153226	0.793974347	0.999579949
116.2633514	-0.107277732	0.105425336	-1.017570687	0.308882023	0.999579949
162.6173314	-0.053639548	0.149852607	-0.357948715	0.720381694	0.999579949
758.7067703	-0.090475597	0.064974669	-1.392474904	0.163778636	0.978498637
282.7468775	-0.285548005	0.215342221	-1.326019598	0.184833206	0.983992835
625.9200378	0.06452603	0.084436453	0.764196364	0.444750233	0.999579949
542.1476831	-0.016220119	0.060980443	-0.265988869	0.790247781	0.999579949
67.21367003	0.949849238	0.575998162	1.649049078	0.099137581	0.895628172
30.57849189	-0.459874371	0.224508116	-2.048364125	0.040524334	0.741027736
206.1471325	0.043281317	0.089670949	0.48266821	0.629331338	0.999579949
28.03600859	0.075076665	0.286677801	0.261885171	0.793409972	0.999579949
244.054056	-0.105620868	0.079505658	-1.328469836	0.184022942	0.983992835
327.8333644	-0.126005394	0.081756442	-1.541228943	0.123261076	0.92236461
159.0899017	0.075333194	0.114380176	0.658621071	0.510139129	0.999579949
123.7023613	0.037431809	0.194985088	0.191972673	0.847763611	0.999579949
36.83547815	-0.146935625	0.194765005	-0.754425185	0.450593954	0.999579949
59.22551139	0.29719129	0.274634949	1.082132085	0.279193841	0.999579949
521.1476667	0.019744889	0.09800078	0.20147686	0.840325722	0.999579949
943.5172608	-0.002467966	0.106769773	-0.023114843	0.981558666	0.999579949
328.2289663	-0.128745569	0.246371669	-0.522566453	0.601275991	0.999579949
445.2922722	0.046495274	0.237132056	0.19607334	0.844552763	0.999579949
74.6854469	0.224890391	0.173134164	1.29893711	0.193965513	0.987144791
1240.192778	0.022997253	0.07228996	0.318125128	0.750390024	0.999579949
935.8760372	0.012683471	0.102271108	0.124018128	0.901300922	0.999579949
756.5225452	-0.005586678	0.051878323	-0.107688107	0.914243104	0.999579949
212.9081909	-0.045890463	0.103281901	-0.444322407	0.656809503	0.999579949
985.3837243	-0.006694397	0.094397664	-0.070916973	0.943463835	0.999579949
91.11262743	0.173292897	0.182736466	0.948321374	0.342965873	0.999579949
1168.053398	-0.401793544	0.104521994	-3.844105246	0.000120993	0.049162218
50.19416626	0.886654334	0.352116147	2.518073485	0.01179987	0.494354131
229.3055883	0.014298619	0.14016026	0.102016214	0.918743806	0.999579949
565.5075046	0.006172344	0.059801203	0.103214382	0.917792826	0.999579949
379.0061559	-0.23384475	0.068061743	-3.435773762	0.000590864	0.122008606
684.3873346	0.043065933	0.089334455	0.482075288	0.629752464	0.999579949
493.9521818	-0.036055962	0.073495211	-0.490589265	0.623716981	0.999579949
245.9884031	-0.057294256	0.114547928	-0.500177147	0.616950348	0.999579949
1134.776939	0.470782945	0.144688351	3.253772267	0.001138835	0.174936221
96.61627194	0.284438233	0.164317618	1.731026995	0.08344695	0.859920327
465.8621534	0.087064272	0.140525508	0.619562053	0.535546157	0.999579949
46.21116975	0.165812052	0.198223218	0.836491575	0.402878414	0.999579949
94.89378337	0.163727507	0.166995034	0.980433387	0.326872236	0.999579949
23.82533122	0.125665253	0.237765283	0.528526498	0.59713396	0.999579949
83.51695648	0.420514077	0.166756948	2.521718469	0.011678313	0.493624274
661.7333339	0.047897943	0.086617595	0.552981678	0.580275956	0.999579949
804.4471922	-0.032281801	0.1429779	-0.225781751	0.821371171	0.999579949

109.4528096	0.039487835	0.158246059	0.249534395	0.802947439	0.999579949
139.6667524	-0.07986363	0.119504723	-0.668288488	0.50394946	0.999579949
469.2227716	-0.099626743	0.124605956	-0.799534361	0.423980631	0.999579949
289.607742	-0.089331314	0.181964317	-0.490927647	0.623477622	0.999579949
24.12589439	0.001156375	0.402292768	0.002874462	0.997706515	0.999579949
39.70261329	0.209718323	0.235962555	0.888777981	0.374122412	0.999579949
206.6076673	-0.060382587	0.099960316	-0.604065589	0.545800036	0.999579949
180.0531368	0.03416253	0.123907858	0.275709148	0.782771469	0.999579949
255.2291794	-0.036916861	0.085640514	-0.431067715	0.666419135	0.999579949
573.9375437	-0.026193361	0.065989906	-0.396929815	0.691419217	0.999579949
31.07900469	0.09359131	0.222394413	0.420834805	0.673875714	0.999579949
26.62471498	0.011672732	0.25069235	0.04656198	0.962862335	0.999579949
213.1635221	0.027825852	0.078756218	0.353316257	0.723851359	0.999579949
1973.12215	0.064330093	0.097702701	0.658426963	0.510263814	0.999579949
26.01937405	-0.061447055	0.543208892	-0.113118647	0.909936492	0.999579949
25.24476465	0.03919299	0.619081049	0.063308334	0.949520979	0.999579949
1213.356681	-0.251856875	0.57279809	-0.439695732	0.660157494	0.999579949
301.6402803	0.073890526	0.260393474	0.28376489	0.776590562	0.999579949
135.5031943	-0.087592406	0.111988355	-0.782156376	0.434122678	0.999579949
400.6768277	-0.055085031	0.073245003	-0.752065381	0.452011741	0.999579949
2338.933899	-0.043427193	0.100543013	-0.431926515	0.665794823	0.999579949
489.4736629	-0.0010704	0.068468531	-0.015633458	0.987526813	0.999579949
1380.757669	-0.03356189	0.074896515	-0.448110169	0.654073689	0.999579949
14922.43808	0.007420639	0.077351833	0.095933594	0.923573313	0.999579949
444.7629003	0.164421392	0.108629479	1.513598267	0.130127761	0.935700262
349.2280816	0.101044024	0.144797625	0.697829295	0.485283956	0.999579949
3530.64156	0.181810453	0.242126391	0.750890691	0.452718443	0.999579949
322.2623228	0.000577625	0.107631991	0.00536667	0.995718037	0.999579949
62.24127662	-0.18980886	0.210731762	-0.900713107	0.367740878	0.999579949
28.80281169	-0.219587218	0.274566981	-0.799758284	0.423850857	0.999579949
284.7400103	0.000852198	0.163207294	0.005221566	0.995833812	0.999579949
135.1879204	-0.078477209	0.19203068	-0.408670161	0.682781734	0.999579949
26.97834986	-0.370599925	0.272402697	-1.360485519	0.173676332	0.983601342
269.7829988	0.0861768	0.177766159	0.484776185	0.627835118	0.999579949
234.0254299	0.057962085	0.155918549	0.371745927	0.710082027	0.999579949
336.1937924	0.154342412	0.120397011	1.281945547	0.199861749	0.992610246
65.53168571	0.089941973	0.153186498	0.587140343	0.557109455	0.999579949
167.988925	0.009400447	0.132928402	0.070718124	0.943622096	0.999579949
65.63861032	-0.148810319	0.128228447	-1.160509414	0.245841463	0.999579949
552.1683618	-0.475565106	0.217047951	-2.191060103	0.028447443	0.666751303
318.3887119	-0.001213473	0.08710751	-0.01393075	0.988885229	0.999579949
37.21367447	0.044334707	0.214536667	0.206653286	0.836280642	0.999579949
803.8007753	-0.050131353	0.090580572	-0.553444867	0.579958823	0.999579949
921.0999773	0.071072458	0.086704876	0.819705431	0.412384053	0.999579949
111.0762267	-0.041207615	0.125630465	-0.328006546	0.742906713	0.999579949
165.6520896	-0.102364194	0.110539131	-0.926044857	0.354422669	0.999579949
340.0678858	0.018257291	0.082170187	0.222188746	0.824166955	0.999579949
1052.413973	0.003692323	0.1000957	0.036887929	0.970574365	0.999579949

1699.794245	-0.10771278	0.089132022	-1.208463336	0.22686909	0.999579949
676.1498913	0.048140348	0.130947487	0.367630941	0.71314844	0.999579949
178.7427432	-0.188439751	0.117770256	-1.600062326	0.109584758	0.908111583
44.5625609	-0.930842507	0.312341819	-2.980204538	0.00288056	0.285981787
2112.440421	-0.049005929	0.098621823	-0.496907553	0.619254245	0.999579949
129.0506416	0.006570804	0.179215674	0.036664225	0.970752734	0.999579949
113.238571	0.017074647	0.129710236	0.131636852	0.895271535	0.999579949
66.4049524	-0.10660414	0.153571421	-0.694166525	0.487577783	0.999579949
330.7762384	0.07165474	0.106970583	0.66985463	0.502950464	0.999579949
1177.881232	0.020650615	0.080244937	0.257344768	0.796912636	0.999579949
594.1950815	0.07460677	0.081431549	0.916189988	0.359567256	0.999579949
689.4634775	0.035178258	0.066137339	0.531897089	0.594797274	0.999579949
538.6053637	-0.010270243	0.079997062	-0.12838275	0.897846082	0.999579949
160.9999234	0.186792869	0.142056607	1.31491856	0.188537274	0.983992835
19060.0014	-0.04588937	0.114126143	-0.40209341	0.687615283	0.999579949
237.4435393	-0.126921259	0.148857833	-0.852634059	0.393862269	0.999579949
414.536745	0.064228385	0.256627027	0.250279116	0.802371506	0.999579949
216.7976504	0.030270596	0.094494222	0.320343355	0.748708061	0.999579949
233.4134197	0.137203348	0.105643082	1.298744277	0.194031703	0.987144791
232.7141173	0.033022684	0.081590367	0.404737534	0.685670449	0.999579949
309.6901877	0.105194417	0.127291617	0.826404908	0.40857444	0.999579949
204.2792142	0.051392619	0.134136932	0.383135492	0.701619305	0.999579949
88.27304127	-0.008440989	0.151907369	-0.055566684	0.955687005	0.999579949
146.0025154	-0.065259082	0.107978084	-0.604373407	0.545595412	0.999579949
482.2636074	0.109726076	0.061167654	1.793857843	0.072835862	0.838669905
1089.296735	0.019333743	0.0495138	0.390471797	0.696187706	0.999579949
291.2842335	0.067299665	0.101411316	0.663630718	0.506926692	0.999579949
1122.0971	0.007412154	0.091000968	0.081451373	0.935082995	0.999579949
694.5819632	-0.157743845	0.069065173	-2.28398537	0.022372391	0.627784819
75.99579698	-0.277902518	0.123770222	-2.245310001	0.024748238	0.640100228
655.2731454	0.019003423	0.060769922	0.312711004	0.754500235	0.999579949
189.157974	-0.138170332	0.192441549	-0.71798597	0.472765938	0.999579949
74.61164212	0.086355978	0.144158258	0.599035944	0.549148916	0.999579949
63.39938506	0.430967852	0.373471448	1.15395127	0.248520148	0.999579949
877.4894376	0.145442161	0.138496042	1.050153915	0.293647354	0.999579949
230.2323874	0.004572014	0.125083403	0.036551721	0.970842439	0.999579949
913.3328728	0.018659476	0.091054351	0.204926795	0.83762932	0.999579949
331.4574122	-0.016318129	0.107199987	-0.152221369	0.87901234	0.999579949
5323.908174	-0.079343612	0.158127335	-0.501770374	0.615829055	0.999579949
115.0815079	0.0852286	0.150788153	0.565220796	0.571923593	0.999579949
36.32171676	0.236057879	0.159894464	1.476335531	0.13985384	0.948318761
83.13284443	-0.24581179	0.177529952	-1.384621502	0.166168233	0.978789551
355.3774327	-0.000364377	0.113466624	-0.003211316	0.997437745	0.999579949
339.4692333	0.040345434	0.102404293	0.393981856	0.693594435	0.999579949
301.0039128	0.056011316	0.085729304	0.653350871	0.513530103	0.999579949
505.2706933	-0.124944976	0.091055792	-1.372180436	0.170007274	0.981013142
327.6657233	-0.05580133	0.189261747	-0.294836809	0.768118546	0.999579949
55.05794973	-0.537679736	0.299612274	-1.794585146	0.072719822	0.838669905

4399.216289	-0.501560754	0.383355921	-1.308342265	0.190757246	0.98475336
186.4713044	-0.094759362	0.087447501	-1.083614297	0.278535849	0.999579949
60.83613117	0.088428244	0.136209798	0.649206186	0.516205114	0.999579949
91.27482262	-0.044692526	0.126278205	-0.353921139	0.723397985	0.999579949
101.8875241	0.356073595	0.13923202	2.557411687	0.010545433	0.483019182
39.62567519	-0.067646035	0.163647712	-0.413363771	0.679340114	0.999579949
91.04249602	-0.107178565	0.201672532	-0.5314485	0.59510802	0.999579949
30476.36718	0.097054092	0.185068901	0.524421397	0.599985477	0.999579949
464.9414512	0.664320995	0.327526993	2.028293879	0.042530263	0.74969123
1032.784586	-0.037750665	0.11147049	-0.33866062	0.73486541	0.999579949
327.0556096	0.052970739	0.090590249	0.584728927	0.558730001	0.999579949
359.6237947	0.280921963	0.122512454	2.293007403	0.021847579	0.627784819
2308.82779	0.062996698	0.172889381	0.364375753	0.715577439	0.999579949
696.7682339	-0.04877196	0.124339895	-0.39224707	0.694875668	0.999579949
113.0505849	0.097946982	0.114689861	0.854016044	0.3930961	0.999579949
266.6635048	0.321233323	0.226026539	1.421219492	0.15525296	0.976030424
141.9166986	0.035181282	0.185067889	0.190099329	0.849231296	0.999579949
134.0209402	0.002190915	0.1517171	0.014440789	0.988478318	0.999579949
3149.641668	0.06878049	0.123906518	0.555099854	0.578826363	0.999579949
82.68041398	-0.214360639	0.246465146	-0.869740175	0.384442412	0.999579949
1039.828069	0.029376213	0.067731899	0.433713112	0.664496784	0.999579949
362.4879535	0.337535368	0.13909324	2.426684197	0.015237513	0.542093008
122.6159635	-0.126343035	0.276051173	-0.457679763	0.647182529	0.999579949
675.3392354	-0.079737274	0.140402618	-0.567918709	0.570090167	0.999579949
166.7233764	0.099448795	0.110340226	0.901292288	0.367432933	0.999579949
1183.462516	-0.084000087	0.082785959	-1.014665872	0.310265128	0.999579949
45.08362028	-0.056655897	0.210963269	-0.268558109	0.788269752	0.999579949
229.8576468	0.144751394	0.157244525	0.920549659	0.357285595	0.999579949
198.733625	-0.083156254	0.096769522	-0.859322769	0.390162466	0.999579949
252.0808192	-0.078312773	0.087004208	-0.90010328	0.368065291	0.999579949
671.3957537	0.074732098	0.066084328	1.130859626	0.258114179	0.999579949
35.42762767	0.092685568	0.35286397	0.262666567	0.792807588	0.999579949
874.2793738	0.076081093	0.056499082	1.346589892	0.178112351	0.983992835
202.7769532	-0.186234007	0.116261706	-1.601851666	0.109188415	0.905620964
371.0626055	0.177587876	0.141893218	1.251559995	0.210730239	0.996581389
326.0034946	-0.107271808	0.091143045	-1.176960987	0.239211078	0.999579949
1109.685571	-0.009701095	0.07735749	-0.125406018	0.900202124	0.999579949
260.856681	0.051426901	0.088346352	0.582105541	0.560495593	0.999579949
3213.396634	0.07855479	0.110994493	0.707735922	0.479109265	0.999579949
176.2136846	-0.161657163	0.121985293	-1.325218466	0.185098702	0.983992835
221.5402746	0.142589584	0.09145852	1.559062887	0.118981497	0.921521033
202.2257819	0.116596527	0.120432344	0.968149617	0.332969653	0.999579949
2172.706419	0.109740258	0.140991814	0.778344893	0.436365716	0.999579949
3340.903215	0.082978816	0.11045992	0.751211985	0.452525089	0.999579949
414.9069361	0.026441229	0.094832698	0.278819749	0.780383158	0.999579949
4586.398312	0.001981475	0.134212239	0.014763741	0.988220667	0.999579949
371.3580445	-0.072900654	0.096304618	-0.756979833	0.449061945	0.999579949
484.7601477	0.141617816	0.113709629	1.245433809	0.212972332	0.999268361

473.33006	-0.051486919	0.122184426	-0.421386916	0.673472571	0.999579949
553.815288	0.068823313	0.106168658	0.648245107	0.516826432	0.999579949
538.2615945	0.031027328	0.091730689	0.338243707	0.735179542	0.999579949
127.7317105	-0.108441705	0.154215162	-0.703184461	0.481940802	0.999579949
486.9166796	-0.157203306	0.157484292	-0.998215786	0.318174733	0.999579949
93.94317303	0.116550143	0.113060319	1.030866925	0.302603229	0.999579949
118.6840343	-0.034422236	0.175398006	-0.196252152	0.84441281	0.999579949
131.346219	0.057944391	0.16209332	0.357475503	0.720735862	0.999579949
2417.281619	-0.066561629	0.085003897	-0.783042084	0.433602399	0.999579949
28.1320225	0.041956515	0.212755037	0.197205742	0.843666538	0.999579949
56.36892023	-0.320697771	0.160999696	-1.99191538	0.046380343	0.763868825
112.921962	-0.127983871	0.118505181	-1.079985446	0.280148661	0.999579949
186.2145836	0.045730421	0.082887793	0.551714788	0.581143776	0.999579949
333.9545198	0.025356369	0.133625708	0.189756669	0.849499812	0.999579949
197.1371516	-0.129688396	0.102499778	-1.265255387	0.205779793	0.994814558
793.8162158	-0.160473519	0.126389913	-1.269670297	0.204202097	0.994814558
63.10224118	0.303022068	0.148449475	2.041247159	0.041226266	0.745838643
103.0428642	0.077898017	0.173260486	0.449600593	0.652998461	0.999579949
34.79976009	0.616773593	0.410532863	1.502373252	0.13300074	0.941167035
197.5849904	-0.04413975	0.12431265	-0.355070459	0.722536807	0.999579949
81.87666456	0.360855279	0.214585244	1.681640693	0.092638535	0.880418343
577.3754476	-0.07310828	0.109321049	-0.668748434	0.503655966	0.999579949
459.1158512	-0.070238672	0.0632684	-1.110169881	0.266925829	0.999579949
746.2603357	-0.11777205	0.115019882	-1.023927761	0.305869397	0.999579949
85.73101996	0.116097248	0.14816108	0.783588024	0.433281885	0.999579949
46.01066188	0.064994489	0.185222046	0.350900397	0.725663074	0.999579949
136.8282067	-0.18939298	0.102137165	-1.854300338	0.063696204	0.812062129
45.34769294	0.00654767	0.38067705	0.017200065	0.986277011	0.999579949
510.608664	0.15396467	0.138109857	1.114798562	0.264936753	0.999579949
920.810729	0.045612833	0.061871208	0.737222285	0.460987183	0.999579949
63.83569666	0.160145155	0.13654061	1.172875635	0.240845682	0.999579949
1061.871516	-0.077539592	0.139467824	-0.555967603	0.578233002	0.999579949
1021.717735	-0.065841541	0.116783201	-0.563792912	0.572895076	0.999579949
169.0933954	0.035087817	0.105070403	0.333945769	0.738420481	0.999579949
4401.790971	-0.003918472	0.11135624	-0.035188613	0.971929342	0.999579949
179.4489862	-0.008826006	0.142871758	-0.061775721	0.950741439	0.999579949
767.4312863	0.094552966	0.154107408	0.61355237	0.539511166	0.999579949
770.9389874	0.074390504	0.100932438	0.737032668	0.461102484	0.999579949
171.590968	0.094648588	0.122305791	0.773868412	0.439008611	0.999579949
62.65383432	0.082236154	0.126112124	0.652087614	0.514344654	0.999579949
236.3686858	-0.124100889	0.106761177	-1.162415899	0.245066568	0.999579949
113.5281841	0.548227352	0.277391119	1.976369521	0.048112941	0.770705547
306.4471638	0.004775979	0.098292282	0.04858956	0.96124639	0.999579949
226.7387872	0.927991754	0.475606596	1.951175111	0.051036219	0.77827144
225.5835328	0.088884361	0.124606831	0.713318524	0.475648678	0.999579949
94.62257285	0.098676547	0.132223775	0.746284446	0.455495605	0.999579949
93.93069771	0.074666739	0.143519453	0.520255184	0.602885727	0.999579949
142.4081081	0.049985432	0.149248931	0.334913165	0.737690591	0.999579949

465.2617788	-0.023112119	0.091697913	-0.252046287	0.801005286	0.999579949
87.32917794	-0.488636223	0.328930815	-1.485528874	0.13740378	0.945791454
378.4788553	-0.174433168	0.083547502	-2.087832233	0.036812971	0.717947029
67.5195815	-0.099278799	0.178728342	-0.555473171	0.578571056	0.999579949
251.4191442	-0.098849074	0.13381979	-0.738673061	0.460105548	0.999579949
639.9197414	-0.155083049	0.094225234	-1.645875966	0.099789298	0.895628172
104.7282832	-0.220499249	0.101412083	-2.174289726	0.029683383	0.672989344
462.8996569	0.041819149	0.101477812	0.412101404	0.6802651	0.999579949
1567.821224	0.085338087	0.157533863	0.541712657	0.588016469	0.999579949
783.3154468	-0.141184126	0.08366119	-1.687570137	0.091493776	0.880409168
324.1432199	0.110935926	0.177977007	0.623316054	0.53307685	0.999579949
501.6311286	-0.027551077	0.114316523	-0.241006957	0.809549724	0.999579949
1863.371052	-0.012106366	0.115074424	-0.105204661	0.916213412	0.999579949
160.7076986	0.031996585	0.130499439	0.24518561	0.806312718	0.999579949
69.62635259	0.073374636	0.335892842	0.21844656	0.827081191	0.999579949
28.75413877	0.210120305	0.233249668	0.900838601	0.36767414	0.999579949
131.4641194	-0.026087352	0.223970897	-0.116476528	0.907274887	0.999579949
7868.725649	-0.143887048	0.118755304	-1.211626287	0.225655467	0.999579949
265.8376672	0.278662374	0.152696725	1.824940088	0.068010071	0.829912169
148.8597634	-0.071019101	0.167899591	-0.422985551	0.672305803	0.999579949
53.286104	-0.163392412	0.160578646	-1.017522665	0.308904856	0.999579949
571.5379693	0.077883889	0.097525455	0.798600625	0.424522025	0.999579949
257.723653	-0.156613705	0.17633039	-0.888183286	0.374442168	0.999579949
82.76867818	0.142402354	0.145143357	0.981115207	0.326535932	0.999579949
153.8056597	-0.023337708	0.100248138	-0.232799417	0.815917165	0.999579949
692.2509852	0.092825279	0.093048325	0.997602903	0.318471953	0.999579949
553.6978525	-0.172445282	0.237859217	-0.724988854	0.468458868	0.999579949
72.60793338	-0.104605392	0.278080776	-0.376169088	0.706791194	0.999579949
2697.756685	-0.025016396	0.133073543	-0.187989257	0.850885073	0.999579949
34.5166336	0.037302985	0.250507707	0.14890953	0.881625021	0.999579949
536.1978	-0.03544235	0.185084946	-0.191492342	0.848139879	0.999579949
287.1569478	0.122232304	0.110976642	1.101423705	0.270712293	0.999579949
156.8620386	0.128601106	0.180519498	0.712394547	0.476220492	0.999579949
69.53733735	-0.193858111	0.160982934	-1.20421529	0.228506378	0.999579949
238.5451609	-0.017333045	0.106641719	-0.162535309	0.870884325	0.999579949
1586.291358	0.047726663	0.159208806	0.299774013	0.764349539	0.999579949
1393.418442	-0.009793105	0.115162612	-0.085037189	0.932231825	0.999579949
30.83729673	0.083472444	0.198642888	0.420213604	0.674329418	0.999579949
24.58877623	0.333567153	0.527604544	0.632229493	0.527236928	0.999579949
288.8237929	0.053242849	0.083795394	0.635391119	0.525173359	0.999579949
22.19267891	-0.211128744	0.35284743	-0.59835704	0.549601724	0.999579949
87.62834329	0.210293379	0.175879203	1.195669386	0.231825602	0.999579949
143.5507467	-0.012434573	0.308160689	-0.040350938	0.967813344	0.999579949
32.43239736	1.331207747	0.755147919	1.762843694	0.077926856	0.851207389
2617.594394	-0.017101755	0.121581964	-0.140660293	0.888138315	0.999579949
2548.364449	-0.045972215	0.13102864	-0.350856235	0.725696206	0.999579949
64.66388368	0.002373293	0.177773031	0.01335013	0.989348454	0.999579949
260.7718978	-0.020766689	0.101083254	-0.205441437	0.837227249	0.999579949

373.3410762	-0.278825567	0.264691148	-1.053399667	0.292157857	0.999579949
106.9857057	0.153503186	0.126953035	1.209133646	0.226611505	0.999579949
20.56707929	0.249057221	0.271355111	0.917827641	0.358709109	0.999579949
271.8765174	0.030868416	0.144251309	0.213990542	0.830554433	0.999579949
758.7906791	-0.137786406	0.083870957	-1.642838127	0.100416429	0.895628172
29.15681893	-0.26213171	0.207220765	-1.264987657	0.205875753	0.994814558
230.2542608	0.039463504	0.194094438	0.203321146	0.838884028	0.999579949
276.9728236	0.181687585	0.156453701	1.161286593	0.24552537	0.999579949
1844.454411	0.127639024	0.100857217	1.265541804	0.205677173	0.994814558
22.34975098	0.29397327	0.302766579	0.970956804	0.331569789	0.999579949
267.1277755	-0.18643863	0.134053102	-1.390781915	0.164291569	0.978498637
77.80785423	-0.060239344	0.21578962	-0.279157747	0.780123768	0.999579949
130.1120832	-0.10455722	0.191430751	-0.546188216	0.584936565	0.999579949
110.7583373	-0.168097218	0.145918254	-1.151995812	0.249322794	0.999579949
264.4918334	-0.148045662	0.111851904	-1.323586429	0.185640435	0.983992835
51.94357226	-0.473622013	0.17965613	-2.63626971	0.008382308	0.441435814
242.3338356	0.156304272	0.083375209	1.874709202	0.06083274	0.801515896
56.57331625	-0.387382641	0.252358293	-1.535050169	0.124771505	0.927119638
26.56856071	0.251795003	0.355405089	0.708473261	0.478651411	0.999579949
385.4947091	0.320430301	0.183961587	1.741832664	0.081537729	0.85448976
124.8430608	-0.116322231	0.149061648	-0.780363242	0.435177097	0.999579949
260.6108847	0.043507013	0.080547793	0.540139106	0.589101103	0.999579949
144.6659892	0.528303975	0.337001863	1.567658913	0.116960755	0.920479614
23.85516265	0.843918462	0.575593281	1.466171495	0.142601572	0.951955082
406.0893326	-0.023422289	0.082326845	-0.284503665	0.776024426	0.999579949
196.4842818	0.190562426	0.118349463	1.610167213	0.107361357	0.900848082
1320.141934	-0.077959859	0.121068745	-0.643930511	0.519620495	0.999579949
349.706331	-0.048271133	0.146591071	-0.329291083	0.741935683	0.999579949
265.1722422	-0.091500054	0.085203203	-1.073903921	0.282865746	0.999579949
2766.343169	0.14242405	0.109498811	1.300690379	0.193364457	0.986374671
1689.601658	-0.0072371	0.064392402	-0.112390592	0.910513715	0.999579949
36.2238604	0.156953804	0.189260139	0.829301956	0.406933564	0.999579949
32.23487813	-0.088740345	0.281423223	-0.315327014	0.752513376	0.999579949
242.90742	-0.66343605	0.524306433	-1.265359357	0.205742538	0.994814558
550.8939141	-0.24120389	0.170850295	-1.411785033	0.158013267	0.977304288
25.52832693	-0.238586148	0.229342912	-1.040303125	0.298199093	0.999579949
430.2188986	-0.026021302	0.109601724	-0.237416904	0.812333377	0.999579949
648.875266	-0.085864369	0.056599213	-1.517059404	0.129251687	0.935700262
27.05453166	0.099503611	0.209930306	0.473984024	0.6355113	0.999579949
324.0914376	-0.065677817	0.134599557	-0.487949726	0.625585451	0.999579949
666.9652964	-0.019920366	0.088693796	-0.224597065	0.822292748	0.999579949
2885.563424	-0.012616251	0.109511307	-0.115205012	0.908282626	0.999579949
214.3520821	0.1502274	0.119115458	1.261191473	0.20723987	0.994814558
128.2790825	0.264801567	0.10807291	2.450212244	0.014277203	0.532058131
219.2641557	0.002215905	0.099230369	0.022330918	0.982183986	0.999579949
685.386036	0.022252323	0.132066	0.168493957	0.866194698	0.999579949
129.3211811	0.125085149	0.129019673	0.96950447	0.332293551	0.999579949
346.5909786	0.04075717	0.114021835	0.357450571	0.720754523	0.999579949

2382.863874	-0.030707841	0.080222569	-0.382783071	0.701880615	0.999579949
201.015152	-0.17964653	0.11421823	-1.572835887	0.115756824	0.918818353
21.3114271	-0.268662345	0.215056309	-1.249265117	0.211568122	0.997347329
614.256949	-0.017436705	0.064934061	-0.268529409	0.78829184	0.999579949
201.265518	-0.005873642	0.092202343	-0.063703824	0.94920606	0.999579949
797.4681267	-0.062774922	0.057082658	-1.099719666	0.271454283	0.999579949
125.8854806	0.297481024	0.166537311	1.786272529	0.074055143	0.844926247
502.0354243	-0.104294704	0.078173861	-1.334137805	0.182158698	0.983992835
99.14512765	0.318477191	0.428201445	0.743755525	0.457024391	0.999579949
1381.350788	0.135023918	0.109436319	1.233812684	0.217272712	0.999579949
277.85624	0.088105883	0.131007301	0.672526509	0.50124857	0.999579949
1562.072525	-0.03711521	0.092549771	-0.401029734	0.688398232	0.999579949
316.1527257	-0.061002262	0.081362846	-0.749755755	0.453401821	0.999579949
686.5636858	0.088944119	0.148754858	0.59792413	0.549890558	0.999579949
543.9215798	-0.127512761	0.096137343	-1.326360368	0.18472036	0.983992835
198.3129762	0.039494903	0.097292588	0.405939486	0.684787065	0.999579949
107.3090281	0.043781099	0.154379353	0.283594265	0.776721333	0.999579949
369.5595042	-0.076719418	0.074962675	-1.023434903	0.306102264	0.999579949
31.28065627	-0.025437487	0.27109817	-0.093831276	0.925243187	0.999579949
690.6351768	-0.09569896	0.058539985	-1.634762272	0.102098893	0.895628172
98.33486004	-0.137242956	0.111034157	-1.236042672	0.216442702	0.999579949
738.9286639	0.076975655	0.128195893	0.600453368	0.54820413	0.999579949
2791.175772	-0.103853585	0.061133728	-1.69879358	0.089358084	0.870840008
169.2070639	0.100389339	0.095422967	1.052045875	0.292778503	0.999579949
181.2503691	-0.093706088	0.100962914	-0.928123852	0.353343328	0.999579949
37.6624014	-0.307688772	0.155889936	-1.973756482	0.048409439	0.772833077
299.7926181	-0.113286059	0.108420834	-1.044873525	0.296081427	0.999579949
182.7766567	-0.060199737	0.103499897	-0.581640548	0.560808825	0.999579949
191.9914032	0.108856135	0.093293983	1.166807671	0.243288048	0.999579949
116.0702317	-0.07417575	0.153945866	-0.481830084	0.629926657	0.999579949
206.2542788	-0.119147997	0.099917386	-1.192465116	0.233078921	0.999579949
80.02217164	0.027949854	0.2268323	0.123218139	0.901934362	0.999579949
117.5251521	-0.192111558	0.136877453	-1.403529605	0.16045897	0.978261482
153.7138737	-0.213940414	0.12474749	-1.714987725	0.086347477	0.866133472
484.6343119	-0.008113668	0.18382642	-0.044137661	0.964794673	0.999579949
587.1559578	-0.308493036	0.119661205	-2.578053896	0.00993585	0.472698434
230.1669179	-0.097730941	0.138604421	-0.705106956	0.480743678	0.999579949
59.13086027	-0.000121554	0.140858018	-0.000862958	0.99931146	0.999840121
470.6999255	0.005151452	0.095706941	0.05382527	0.957074376	0.999579949
42.09198521	0.120577004	0.274076625	0.439939029	0.659981267	0.999579949
437.7822904	-0.008636881	0.123392519	-0.069995176	0.944197499	0.999579949
273.5751799	0.042764555	0.14646598	0.29197603	0.770304951	0.999579949
4604.086675	0.04663265	0.10985947	0.42447547	0.671219091	0.999579949
113.9976702	0.058443686	0.161094783	0.36279068	0.716761255	0.999579949
134.1718041	-0.05633722	0.167347301	-0.336648511	0.736381882	0.999579949
226.4461663	0.145734332	0.257397424	0.566184111	0.57126863	0.999579949
56.98613472	0.035532951	0.172078547	0.206492623	0.836406126	0.999579949
300.4500662	0.038531232	0.095623446	0.402947539	0.686986818	0.999579949

266.1909727	-0.248993037	0.117274588	-2.123162749	0.033740216	0.698878259
207.9630766	0.430198761	0.288462285	1.491351846	0.135869144	0.945791454
271.5551549	-0.011585013	0.152979193	-0.07572934	0.939634433	0.999579949
228.0598174	0.016903577	0.107495329	0.157249414	0.875048286	0.999579949
1261.533726	-0.116985111	0.103926666	-1.125650566	0.260313456	0.999579949
692.8056275	0.038092988	0.112554238	0.338441172	0.735030752	0.999579949
42.29719977	0.115020007	0.211592627	0.543591752	0.586722437	0.999579949
53.68046843	0.054888964	0.167946505	0.326824092	0.743800936	0.999579949
576.1766891	0.062557705	0.162100652	0.385918901	0.699556732	0.999579949
658.1492845	-0.050659727	0.066844911	-0.757869607	0.448529047	0.999579949
297.785518	-0.030651119	0.106498087	-0.287809104	0.773492868	0.999579949
2548.234317	0.010263515	0.102194885	0.100430813	0.920002308	0.999579949
755.1738113	-0.084677593	0.079451517	-1.065776913	0.2865245	0.999579949
34.05840153	-0.005251589	0.207395726	-0.025321589	0.979798454	0.999579949
400.6356051	0.141660195	0.066960818	2.115568472	0.034381526	0.703403556
460.7941653	-0.183940748	0.109619541	-1.677992311	0.0933486	0.880418343
300.0610021	0.07430196	0.076386095	0.972715786	0.330694577	0.999579949
698.0581575	-0.066640384	0.141575631	-0.47070519	0.637851278	0.999579949
1248.685994	0.0813051	0.076706679	1.059948117	0.289168204	0.999579949
613.7299703	0.018018509	0.088242825	0.204192337	0.8382032	0.999579949
75.95005566	-0.177369745	0.218786222	-0.810698881	0.417538616	0.999579949
416.1132306	-0.127819768	0.093375675	-1.368876512	0.171037879	0.982395406
56.3871662	-0.104068574	0.296954062	-0.350453445	0.725998423	0.999579949
352.9858677	0.01315485	0.161951503	0.081227095	0.935261352	0.999579949
82.20632406	3.625384474	1.142334151	3.173663739	0.00150528	0.203876378
121.2646254	3.258354613	1.114897663	2.922559372	0.003471674	0.310136233
145.280433	-0.09880713	0.407992556	-0.242178757	0.808641654	0.999579949
219.9077381	-0.067480478	0.232476152	-0.29026839	0.771610919	0.999579949
53.45180332	0.214384747	0.213926684	1.002141216	0.316275394	0.999579949
1575.786129	0.127859372	0.106697383	1.198336528	0.230786032	0.999579949
126.9501038	0.019678309	0.16799922	0.117133336	0.906754393	0.999579949
484.4066051	0.182075389	0.104561044	1.741331018	0.081625572	0.85448976
53.79493148	-0.065848751	0.202736478	-0.324799718	0.745332658	0.999579949
58.83899539	-0.165134812	0.156828111	-1.05296692	0.292356154	0.999579949
1015.075133	-0.025836071	0.074714795	-0.345795915	0.729496095	0.999579949
27.11542967	0.007516342	0.285675152	0.026310801	0.97900944	0.999579949
548.6490806	-0.019100633	0.087464619	-0.218381252	0.827132072	0.999579949
1401.336326	0.058611301	0.113501191	0.516393711	0.605579457	0.999579949
266.4482225	0.109182849	0.126278206	0.864621482	0.387246578	0.999579949
140.4425331	-0.028076934	0.117890552	-0.238161016	0.811756212	0.999579949
509.6557001	-0.030695291	0.058734174	-0.522613819	0.601243022	0.999579949
547.7846491	-0.202704862	0.073770435	-2.747779119	0.006000041	0.374141199
1466.250271	0.079852088	0.116005332	0.68834843	0.491233391	0.999579949
597.9794318	-0.063238219	0.079121838	-0.79925114	0.424144804	0.999579949
512.4452666	-0.026824136	0.116352812	-0.230541364	0.817671125	0.999579949
3275.935593	-0.05024879	0.126366599	-0.397642972	0.69089338	0.999579949
144.7303725	0.100030561	0.132506939	0.7549081	0.450304125	0.999579949
36.52866219	0.185457253	0.170132073	1.090078135	0.275678727	0.999579949

25.32237431	0.392573608	0.433806039	0.90495192	0.365490859	0.999579949
2190.427148	-0.002999322	0.144995298	-0.020685651	0.983496416	0.999579949
504.3373047	0.135537316	0.131138527	1.033543074	0.301349824	0.999579949
560.4467545	-0.121008697	0.156949459	-0.771004229	0.440704425	0.999579949
321.7647259	0.172434063	0.180982732	0.952765279	0.34070901	0.999579949
977.1198209	0.103984083	0.103534552	1.004341851	0.315213868	0.999579949
462.4161627	0.128226874	0.08284389	1.547813293	0.121667255	0.922215626
365.9093811	0.043218054	0.160664811	0.268995147	0.787933417	0.999579949
330.959637	0.038506942	0.064609209	0.595997731	0.551176743	0.999579949
1168.59565	0.048797275	0.066981297	0.7285209	0.466294779	0.999579949
68.61406222	0.28867821	0.201215099	1.434674692	0.151379819	0.969895293
124.0632386	0.083169897	0.403318462	0.20621396	0.836623784	0.999579949
153.50418	0.039617856	0.14510911	0.273021146	0.784836961	0.999579949
1785.653704	0.047850054	0.102005712	0.469091909	0.639003942	0.999579949
2661.364285	0.223777356	0.092970546	2.406970442	0.016085472	0.559034656
951.0344539	0.291586615	0.116680682	2.499013639	0.012453952	0.504405069
259.4832378	0.167138879	0.153024859	1.092233511	0.274730476	0.999579949
341.835358	0.099127456	0.085167471	1.163912174	0.244459606	0.999579949
983.6561854	-0.08900738	0.068471012	-1.299927919	0.193625675	0.986374671
179.7545901	0.081128295	0.097044077	0.835994297	0.403158111	0.999579949
524.8445051	-0.010490694	0.136930589	-0.076613226	0.938931237	0.999579949
105.9502148	-0.267445475	0.17195554	-1.55531758	0.119870452	0.921521033
321.0809271	-0.205837206	0.07627109	-2.698757881	0.006959879	0.404205857
396.0885388	-0.049571681	0.142854905	-0.347007205	0.728585906	0.999579949
1189.792454	0.101685073	0.107819255	0.943106797	0.345626279	0.999579949
108.7185794	0.121304556	0.214347399	0.56592502	0.571444752	0.999579949
88.83341555	-0.104674885	0.116333055	-0.899786265	0.368234005	0.999579949
1762.145392	0.044286908	0.099439189	0.445366751	0.656054736	0.999579949
89.72500477	0.287883674	0.153812548	1.871652715	0.061254665	0.802875925
142.0549817	-0.134711629	0.12317655	-1.093646716	0.274109952	0.999579949
137.267334	0.132513182	0.166228337	0.797175648	0.425349027	0.999579949
20.85079677	0.359834126	0.353468289	1.018009643	0.308673374	0.999579949
47.64445596	1.143035322	0.480421941	2.37923214	0.017348747	0.570162425
77.14268379	0.12487486	0.127584196	0.978764329	0.327696439	0.999579949
158.4735915	0.210341055	0.218125347	0.964312756	0.334889148	0.999579949
43.57172454	0.081684285	0.345628877	0.236335243	0.813172541	0.999579949
237.6950334	0.208153268	0.30265055	0.687767683	0.49159909	0.999579949
4995.825381	-0.117829209	0.137148844	-0.859133807	0.390266698	0.999579949
7043.522261	-0.134221886	0.178005481	-0.754032322	0.450829814	0.999579949
401.5053403	-0.062923626	0.120248997	-0.523277763	0.600780973	0.999579949
22.52116313	-0.330283319	0.227254243	-1.453364807	0.146122492	0.958624434
197.1863012	-0.037856872	0.142802512	-0.265099484	0.790932825	0.999579949
207.7836955	0.111449723	0.091476415	1.218343794	0.223093354	0.999579949
261.4824423	-0.04780945	0.087146776	-0.548608361	0.583274247	0.999579949
30.98823951	1.154744305	0.392283172	2.943649864	0.003243667	0.304904733
154.7219843	-0.055019412	0.128643706	-0.42768833	0.668878047	0.999579949
20.42941334	-0.215325736	0.258320823	-0.833559345	0.404529343	0.999579949
263.5042359	-0.087727225	0.079506594	-1.103395587	0.269855412	0.999579949

483.0099147	-0.006894736	0.132270878	-0.052125882	0.95842839	0.999579949
112.228587	-0.03951663	0.105985274	-0.372850192	0.709259944	0.999579949
194.3158484	-0.067653623	0.096958779	-0.697756549	0.485329457	0.999579949
33.46756545	0.083266407	0.276120678	0.301558027	0.762989009	0.999579949
250.0679299	-0.112855331	0.089666809	-1.258607647	0.208172084	0.995521169
166.0617969	-0.154502427	0.146751727	-1.05281505	0.292425766	0.999579949
342.1246588	-0.005127661	0.09816607	-0.052234553	0.958341801	0.999579949
198.1194689	0.145725295	0.093360603	1.560886394	0.118550558	0.921521033
3786.180072	-0.144512433	0.166316239	-0.868901518	0.384900999	0.999579949
72499.09138	0.047492421	0.110091209	0.431391583	0.666183669	0.999579949
337.0441518	-0.003429309	0.065581833	-0.052290537	0.958297193	0.999579949
1243.533997	0.051834113	0.174351067	0.297297365	0.766239493	0.999579949
37.67361398	0.226453382	0.428030911	0.529058479	0.596764884	0.999579949
193.1499411	-0.358040993	0.352913395	-1.014529338	0.310330237	0.999579949
37.6221175	0.290975054	0.339113806	0.858045436	0.390867375	0.999579949
112.7907655	0.112838698	0.099604055	1.132872536	0.257267785	0.999579949
2701.627017	0.123771559	0.132280241	0.935676852	0.349439592	0.999579949
175.3031367	0.045924174	0.098843471	0.464615146	0.642207084	0.999579949
121.9922884	-0.080521187	0.093291882	-0.863110323	0.388076813	0.999579949
175.7975297	1.106907269	0.584464658	1.893882297	0.058240635	0.79580383
491.9880958	-0.040407181	0.059325189	-0.681113401	0.495799738	0.999579949
47.17829322	0.237108671	0.157377503	1.506623669	0.131907147	0.939285826
114.9807412	-0.047435609	0.153302998	-0.309423883	0.756999105	0.999579949
329.4460643	0.052349484	0.09943436	0.526472777	0.598559762	0.999579949
257.1528704	-0.074766785	0.175808877	-0.425273094	0.670637605	0.999579949
1475.478331	0.002597113	0.079168412	0.03280492	0.973830155	0.999579949
56.33335115	0.114971227	0.323385317	0.355523955	0.722197102	0.999579949
488.9245905	0.687568866	0.240977022	2.85325489	0.00432739	0.336488715
85.31782013	-0.030978225	0.152985959	-0.20249064	0.839533175	0.999579949
117.2161082	-0.027216889	0.14488724	-0.187848761	0.85099521	0.999579949
185.7508973	0.080254296	0.09225841	0.869885961	0.384362729	0.999579949
19069.47746	-0.012885126	0.122483322	-0.105199029	0.916217881	0.999579949
535.8151203	0.023481786	0.075774696	0.309889547	0.756644952	0.999579949
10869.32436	-0.185194958	0.182461688	-1.01497997	0.310115376	0.999579949
133.233345	0.044174541	0.162879222	0.271210411	0.786229206	0.999579949
280.5449938	0.092044682	0.107425135	0.856826306	0.391540885	0.999579949
512.8427349	-0.027908942	0.05826324	-0.47901459	0.631928249	0.999579949
74.99369391	0.550940111	0.232400505	2.37064937	0.017756867	0.573039104
2250.54368	0.140972852	0.154127707	0.914649642	0.360375589	0.999579949
58.90635676	0.333305255	0.244897924	1.360996653	0.173514747	0.983601342
198.977334	0.207905609	0.081433542	2.553070943	0.010677775	0.483266726
20.95946568	0.062292541	0.245870139	0.253355456	0.799993546	0.999579949
26.22078472	-0.587422522	0.566965414	-1.036081755	0.300164003	0.999579949
320.2122707	0.085139693	0.069158209	1.231085857	0.218290754	0.999579949
92.72850694	0.00953803	0.158723036	0.060092285	0.952082134	0.999579949
195.8476714	0.097173479	0.159662911	0.608616482	0.542778677	0.999579949
339.5080286	-0.065154363	0.098284371	-0.662916818	0.50738383	0.999579949
830.9864516	0.487346971	0.168337218	2.895063704	0.003790816	0.319818899

106.0558691	-0.061183893	0.157283922	-0.389002843	0.697274044	0.999579949
458.5199347	-0.053787152	0.122703127	-0.438351929	0.661131188	0.999579949
280.3734152	-0.020054602	0.11464422	-0.174929027	0.861135402	0.999579949
84.13314984	-0.030540691	0.255905873	-0.119343453	0.905003259	0.999579949
227.8714468	0.096527564	0.1710073	0.564464583	0.572437997	0.999579949
380.0047354	-0.061233385	0.075082277	-0.815550464	0.414757291	0.999579949
88.52121542	0.133819081	0.127259627	1.051543872	0.293008871	0.999579949
199.9248568	-0.005791351	0.128139762	-0.045195584	0.963951414	0.999579949
198.1614604	0.155717531	0.110913757	1.403951465	0.160333303	0.978261482
186.6407724	0.000853045	0.098709697	0.00864196	0.993104799	0.999579949
507.6925746	0.05193633	0.088627486	0.586007032	0.55787079	0.999579949
4888.56855	0.047804719	0.117327869	0.407445553	0.683680776	0.999579949
301.3241849	0.081253962	0.094340873	0.861280573	0.389083533	0.999579949
51.14005679	-0.146128315	0.210873184	-0.692967747	0.488329792	0.999579949
33.31681065	-0.48045708	0.501599015	-0.957850924	0.338137936	0.999579949
258.7845696	-0.063709123	0.100447068	-0.63425568	0.525913975	0.999579949
540.0868755	-0.0591943	0.124934098	-0.473804196	0.635639542	0.999579949
138.0031956	0.023075718	0.158360486	0.145716391	0.884145282	0.999579949
49.79988778	-0.272919473	0.611685661	-0.446176019	0.655470104	0.999579949
120.1688192	-0.351916957	0.236202725	-1.489893724	0.136252182	0.945791454
238.4796322	0.027927898	0.104961325	0.266077983	0.790179151	0.999579949
54.10147065	0.131503963	0.131602485	0.999251366	0.317672938	0.999579949
708.8014036	0.051450696	0.083909996	0.613165277	0.539767062	0.999579949
1839.391651	-0.146018757	0.135685488	-1.076156038	0.281857477	0.999579949
63.84368717	-0.002275901	0.210957068	-0.010788456	0.991392224	0.999579949
402.788712	-0.076164713	0.067185933	-1.133640767	0.256945265	0.999579949
806.9368362	-0.011751283	0.071947106	-0.163332256	0.870256839	0.999579949
308.9975539	-0.038714688	0.168113322	-0.230289231	0.817867028	0.999579949
49.88495199	-0.103250696	0.166193615	-0.621267528	0.534423615	0.999579949
366.6650579	-0.040310827	0.110581744	-0.364534199	0.715459141	0.999579949
26.80091629	0.137917502	0.214075409	0.644247291	0.519415089	0.999579949
564.4024356	-0.17357	0.136962722	-1.267279133	0.205055499	0.994814558
1232.572732	-0.240582353	0.111617947	-2.155409233	0.031129824	0.683621001
150.1799188	-0.031140396	0.137502132	-0.226472095	0.82083426	0.999579949
129.6324689	-0.00845426	0.244454701	-0.034584157	0.972411335	0.999579949
390.2173914	0.136526347	0.116567989	1.17121646	0.241511781	0.999579949
700.3409291	-0.219151842	0.131079809	-1.671896251	0.094544782	0.880418343
121.4785306	-0.004403426	0.121260454	-0.036313788	0.971032156	0.999579949
139.6598411	-0.182877454	0.103285245	-1.770605798	0.076626277	0.851207389
5698.21191	-0.239726181	0.107724744	-2.225358555	0.026057177	0.648237123
397.3234738	0.124950311	0.105663012	1.182535957	0.236993107	0.999579949
2094.82489	-0.293362409	0.141638019	-2.071212311	0.03833896	0.72680496
664.654224	-0.130356223	0.122553105	-1.063671324	0.287477619	0.999579949
1049.224085	0.067394139	0.078246062	0.861310297	0.389067166	0.999579949
593.1748184	0.31882737	0.189780106	1.6799831	0.092960604	0.880418343
268.9988019	0.027248734	0.06817931	0.399662805	0.689404891	0.999579949
196.8181459	0.05196167	0.191481116	0.271367071	0.786108725	0.999579949
121.3725232	0.013920205	0.102728571	0.135504712	0.892212839	0.999579949

352.1117051	0.122108338	0.107867743	1.132019036	0.257626432	0.999579949
50.52871512	0.758467582	0.401102679	1.890956161	0.058630196	0.79580383
446.5537156	0.02116489	0.100330958	0.210950745	0.832925706	0.999579949
327.5008397	0.039650592	0.115059884	0.344608307	0.73038886	0.999579949
519.3445	-0.024937827	0.142337132	-0.175202541	0.860920488	0.999579949
103.1459533	0.210575008	0.216876552	0.970944096	0.331576118	0.999579949
134.2593071	0.090140963	0.166796242	0.540425623	0.588903541	0.999579949
30.26475267	0.194217952	0.20087698	0.96685022	0.333618915	0.999579949
94.65056554	0.16675074	0.113729366	1.466206539	0.142592028	0.951955082
661.3142311	0.061074757	0.213107543	0.286591249	0.774425316	0.999579949
242.6478582	0.119527102	0.108330719	1.103353716	0.269873588	0.999579949
105.2609578	2.560237464	0.794209555	3.223629641	0.00126577	0.187572206
432.5772807	-0.13949783	0.171591415	-0.81296509	0.416238074	0.999579949
138.3866138	-0.201244212	0.149502967	-1.346088414	0.178274005	0.983992835
68.73973249	0.136288999	0.133047616	1.024362583	0.305664048	0.999579949
260.7281915	-0.014492712	0.134835214	-0.107484624	0.914404523	0.999579949
233.5961279	-0.031534689	0.109912358	-0.286907585	0.774183082	0.999579949
83.0698273	0.198873	0.38939519	0.510722795	0.609545176	0.999579949
338.4803538	0.310118975	0.136375378	2.274010016	0.022965384	0.62865781
249.2482124	-0.118942846	0.106638553	-1.115383158	0.264686264	0.999579949
446.1628263	0.258170823	0.113412329	2.27639116	0.022822608	0.62865781
748.9068952	-0.041612094	0.137924473	-0.301702035	0.762879217	0.999579949
43.3048079	0.01828956	0.162675372	0.1124298	0.910482628	0.999579949
43.53135405	0.310056704	0.2116109	1.465220858	0.142860671	0.95210212
265.7050929	0.040280021	0.08366221	0.481460165	0.630189485	0.999579949
179.5729163	-0.005747652	0.087799646	-0.065463276	0.947805145	0.999579949
19543.32442	-0.044251626	0.136903636	-0.32323193	0.746519601	0.999579949
22.62768598	0.680280213	0.506162814	1.343994847	0.178950053	0.983992835
351.3950296	-0.121784833	0.176303942	-0.690766365	0.489712376	0.999579949
50.86739978	-0.094825342	0.194413646	-0.487750444	0.625726616	0.999579949
119.6549667	0.028157522	0.16589769	0.169728233	0.865223869	0.999579949
375.7706104	-0.026947894	0.111930627	-0.240755324	0.809744757	0.999579949
23.68280504	0.095613825	0.223192045	0.428392619	0.668365298	0.999579949
1081.710701	-0.165706952	0.175091451	-0.946402299	0.343943433	0.999579949
46.6374345	-0.030883041	0.159467158	-0.19366396	0.846439013	0.999579949
336.0333648	0.004097443	0.108494032	0.037766528	0.969873832	0.999579949
81.16953046	-0.247911135	0.18358625	-1.350379641	0.176894238	0.983992835
863.4559123	0.104000624	0.103427049	1.005545697	0.314634157	0.999579949
745.0080159	-0.250898792	0.13568009	-1.849193875	0.064429823	0.817660046
57.07988703	0.401686798	0.395155655	1.016528026	0.309378008	0.999579949
197.7596145	0.459530098	0.238078885	1.930158981	0.053587142	0.781231069
324.5613882	-0.010156884	0.087004773	-0.116739389	0.907066576	0.999579949
172.7612448	-0.399134033	0.298294752	-1.33805248	0.18087933	0.983992835
104.6380206	-0.083552898	0.168638569	-0.495455449	0.620278662	0.999579949
98.85141753	0.476355811	0.251772549	1.89200853	0.058489844	0.79580383
219.0686069	0.376451424	0.123293626	3.053291861	0.002263457	0.237587514
252.6657969	-0.054818381	0.072834179	-0.75264638	0.451662439	0.999579949
1366.83633	0.612550469	0.251461386	2.435962353	0.01485223	0.539131676

8287.44034	0.045565115	0.096958586	0.469944096	0.638394959	0.999579949
193.6506288	-0.08472345	0.195584	-0.433181904	0.664882624	0.999579949
146.9728353	-0.059427244	0.090248211	-0.658486676	0.510225456	0.999579949
34.60522374	-0.158842185	0.264747842	-0.599975372	0.548522649	0.999579949
102.1038182	0.045389179	0.156265517	0.2904619	0.771462895	0.999579949
30.1694432	-0.18724543	0.350558492	-0.53413463	0.593248392	0.999579949
1062.974892	-0.161174877	0.074251702	-2.170655658	0.029957211	0.676556901
1246.146479	0.042896868	0.160357975	0.267506923	0.789078885	0.999579949
36.49738729	-0.454993932	0.234587536	-1.939548619	0.05243457	0.780714589
88.13488659	0.141393819	0.158708467	0.890902807	0.372981314	0.999579949
57.75575822	0.692861211	0.342480368	2.023068399	0.043066096	0.753417424
182.6119565	0.300776975	0.213305738	1.410074471	0.158517695	0.977304288
39.23431841	0.289411776	0.332899856	0.86936588	0.38464704	0.999579949
315.720367	0.004286992	0.122935226	0.034871955	0.972181844	0.999579949
48.64669026	0.432883536	0.304638268	1.420975569	0.155323862	0.976030424
162.3752274	0.211735543	0.171940508	1.231446537	0.218155901	0.999579949
127.1776149	-0.011217521	0.127240045	-0.088160308	0.929749264	0.999579949
34.46145697	-0.021829175	0.218787091	-0.099773597	0.92052407	0.999579949
976.4712638	-0.226603268	0.17195656	-1.31779368	0.187572724	0.983992835
37.00773832	-0.439379364	0.232106895	-1.893004355	0.05835729	0.79580383
50.82631825	0.364210781	0.168770727	2.158021047	0.030926195	0.683621001
492.6537009	0.195926144	0.15355904	1.275901078	0.20199049	0.99346826
28.93520198	0.041879732	0.205658782	0.203636976	0.838637196	0.999579949
606.8852322	-0.074107488	0.070430357	-1.052209462	0.292703459	0.999579949
130.5412374	0.033268872	0.113379344	0.29342974	0.769193696	0.999579949
2563.389477	0.108846266	0.087057521	1.25027988	0.211197326	0.996937328
194.5717198	-0.054505917	0.100793667	-0.540767272	0.588668003	0.999579949
92.0081784	0.73709208	0.438282301	1.681774686	0.092612539	0.880418343
152.6980473	-0.109879301	0.112377966	-0.977765526	0.328190305	0.999579949
126.6391998	0.929857407	0.297585768	3.124670285	0.001780045	0.219818056
501.0494794	0.077367734	0.127985908	0.604501971	0.545509959	0.999579949
483.3387786	-0.056704538	0.063719517	-0.88990846	0.373515041	0.999579949
127.7463223	-0.10385395	0.408132789	-0.254461178	0.799139293	0.999579949
48.33683005	0.199473946	0.211610341	0.942647438	0.345861267	0.999579949
129.9320859	0.080983365	0.101847892	0.795140315	0.426531886	0.999579949
308.2334797	0.087994279	0.107846709	0.815919926	0.414545935	0.999579949
698.0648178	0.010939237	0.137669333	0.079460232	0.936666562	0.999579949
451.4214558	-0.045220199	0.110748218	-0.408315361	0.683042163	0.999579949
43.35116515	0.039822875	0.190298054	0.209265803	0.834240746	0.999579949
2489.355352	0.015461668	0.107648035	0.143631681	0.885791323	0.999579949
115.3950981	-0.119936454	0.09979761	-1.201796848	0.229442247	0.999579949
600.8825402	-0.005439136	0.094586724	-0.057504222	0.954143543	0.999579949
26.79457451	-0.656322468	0.590065107	-1.112288222	0.266014246	0.999579949
3662.271001	-0.03128687	0.155066337	-0.201764424	0.840100895	0.999579949
104.6945713	-0.17730021	0.137232974	-1.291965074	0.19636924	0.991421279
84.25345208	-0.036254563	0.193415387	-0.187444045	0.851312492	0.999579949
2216.740964	-0.025940366	0.13530107	-0.191723292	0.84795896	0.999579949
502.7353052	0.180690204	0.140393304	1.287028651	0.198084302	0.992481822

139.2116161	-0.11932706	0.091676202	-1.301614338	0.193048256	0.986064814
320.2044041	0.023276029	0.087128606	0.267145664	0.78935701	0.999579949
49.00096195	0.440419205	0.357546524	1.231781531	0.218030704	0.999579949
480.0269768	-0.167645147	0.138786087	-1.20793914	0.227070673	0.999579949
66.46216419	-0.121686099	0.186764369	-0.651548791	0.514692292	0.999579949
306.1928619	-0.03167556	0.146281159	-0.21653889	0.828567714	0.999579949
673.8483711	0.022436252	0.094794292	0.236683578	0.812902276	0.999579949
760.3499984	0.051349381	0.106344496	0.482858851	0.629195961	0.999579949
1009.850995	-0.080804873	0.104107179	-0.776170039	0.437648594	0.999579949
91.74283643	0.238279736	0.256178736	0.930130814	0.352303358	0.999579949
1187.104039	-0.106167288	0.115659568	-0.917929137	0.358655966	0.999579949
180.6338316	0.140890415	0.160224069	0.879333645	0.379220398	0.999579949
3774.045777	0.209858765	0.15743941	1.33294939	0.182548413	0.983992835
89.92597956	0.390283662	0.603284948	0.64693088	0.517676679	0.999579949
285.5550719	0.305012289	0.532007622	0.573323155	0.56642591	0.999579949
97.76270013	0.384699416	0.472865353	0.813549593	0.415903025	0.999579949
104.062136	0.332794165	0.399894039	0.832205866	0.405292753	0.999579949
128.1041228	0.018076429	0.381967778	0.047324488	0.962254611	0.999579949
149.0762364	0.228080856	0.110012592	2.073224995	0.038151345	0.726381766
149.870964	-0.080388129	0.100231768	-0.802022464	0.422539964	0.999579949
1029.151187	0.001707141	0.06618385	0.025793922	0.97942171	0.999579949
285.8040269	-0.04204142	0.104042285	-0.404080129	0.686153797	0.999579949
376.5306069	-0.057110129	0.086494316	-0.660276093	0.509076669	0.999579949
844.7083111	-0.052943812	0.073040058	-0.724859934	0.468537963	0.999579949
3863.520134	-0.101636244	0.100127377	-1.015069479	0.310072709	0.999579949
34.57181829	0.975009227	0.574004997	1.69860756	0.089393151	0.870840008
80.28759897	0.102148658	0.13245916	0.771170964	0.440605602	0.999579949
95.0516013	0.187870997	0.293263517	0.640621783	0.521768444	0.999579949
145.5915934	0.043144909	0.139313799	0.309695872	0.756792242	0.999579949
557.698038	0.2287603	0.193834108	1.180185995	0.237926248	0.999579949
102.8256148	0.160879852	0.189753266	0.847837062	0.39652872	0.999579949
158.588261	-0.041433302	0.092078454	-0.449978259	0.652726117	0.999579949
132.4075639	-0.063020073	0.110176948	-0.57198964	0.567328994	0.999579949
107.0344835	-0.159053374	0.312458745	-0.509037998	0.61072559	0.999579949
491.9323738	0.015559285	0.10086288	0.154261755	0.877403351	0.999579949
91.90459041	0.113334303	0.163905323	0.691462002	0.489275252	0.999579949
50.79805382	0.07169707	0.197839248	0.362400638	0.717052663	0.999579949
544.9496839	-0.006436974	0.112040323	-0.057452295	0.954184906	0.999579949
133.3397935	-0.025438973	0.104843114	-0.242638468	0.808285479	0.999579949
1089.252264	-0.053758847	0.067317221	-0.798589807	0.4245283	0.999579949
2970.579172	-0.157954277	0.203663928	-0.775563342	0.438006852	0.999579949
352.0695545	-0.242453413	0.122581626	-1.977893592	0.047940712	0.770705547
332.2782163	-0.180022655	0.136663923	-1.317265391	0.187749682	0.983992835
56.41982231	0.311574085	0.156362017	1.992645598	0.046300268	0.763868825
698.5895589	0.163188939	0.098118857	1.663176116	0.096277187	0.88499031
640.1444626	0.134719794	0.101911133	1.32193402	0.186190123	0.983992835
2685.813392	-0.013172712	0.167882923	-0.078463683	0.937459218	0.999579949
217.6097531	0.15629604	0.127132964	1.229390351	0.218925486	0.999579949

531.7775559	0.07911288	0.070629494	1.120111106	0.262666418	0.999579949
587.3903847	-0.091240671	0.0579864	-1.57348395	0.115606802	0.918818353
486.8639322	-0.052453891	0.075597943	-0.693853413	0.487774142	0.999579949
54.72216483	-0.229248805	0.160233785	-1.430714535	0.152512046	0.972669728
460.3341359	0.254494429	0.129513843	1.9649979	0.049414481	0.775350081
4993.943616	0.177097471	0.125121693	1.415401813	0.156950718	0.977304288
53.26537217	-0.014711625	0.137005093	-0.10738013	0.914487417	0.999579949
195.4374046	0.066855311	0.102968343	0.649280248	0.51615725	0.999579949
93.86928438	0.010567954	0.129960887	0.081316417	0.935190319	0.999579949
47.99215564	-0.590711635	0.34081027	-1.733256556	0.083050079	0.859571728
647.2847806	0.069000073	0.116772769	0.590891814	0.554592911	0.999579949
94.08416503	-0.438492396	0.409949521	-1.06962534	0.284787984	0.999579949
51.18983292	0.496538686	0.217171237	2.286392488	0.022231307	0.627784819
391.6355111	-0.100459652	0.057419128	-1.749585124	0.080189929	0.85448976
641.0086724	0.021937274	0.081234282	0.270049464	0.7871222	0.999579949
115.1549531	0.105277307	0.140270775	0.75052916	0.452936068	0.999579949
297.1617821	0.196368095	0.102042656	1.924372646	0.054307891	0.783576392
83.86913611	-0.04883721	0.133481133	-0.365873508	0.714459466	0.999579949
242.340389	-0.00044679	0.098309696	-0.004544724	0.996373848	0.999579949
218.2408775	0.018010202	0.305525757	0.058948227	0.952993345	0.999579949
1874.177094	0.078199327	0.140213071	0.557717815	0.577037091	0.999579949
40.19117488	0.089929414	0.199505863	0.450760756	0.652161988	0.999579949
233.1844619	0.245865905	0.173243023	1.419196578	0.155841713	0.976030424
103.858291	0.262709837	0.238785322	1.100192571	0.271248227	0.999579949
1753.193736	0.01473594	0.136214004	0.108182269	0.91385111	0.999579949
56.18884348	-0.343764002	0.171463041	-2.004886879	0.044975142	0.760273172
39.27286432	0.101416233	0.253829352	0.39954494	0.689491717	0.999579949
513.4014305	0.235109644	0.121357937	1.937324008	0.052705749	0.781231069
776.0509986	0.00149293	0.068020451	0.021948254	0.982489233	0.999579949
22.64984498	-0.019748358	0.365946958	-0.053965082	0.956962984	0.999579949
70.25466875	0.298344772	0.18397624	1.621648385	0.104878656	0.895628172
1881.424951	-0.009398492	0.118851317	-0.079077729	0.936970798	0.999579949
1472.421127	-0.053213522	0.070433179	-0.75551782	0.449938344	0.999579949
3244.654022	-0.064432957	0.128472911	-0.501529519	0.615998509	0.999579949
60.62890318	0.103510059	0.149069369	0.694375107	0.487447	0.999579949
204.0286468	0.182302959	0.115593954	1.577097701	0.114773046	0.918818353
279.5959749	0.095538244	0.142677359	0.669610405	0.503106179	0.999579949
303.6893635	0.054254122	0.099488767	0.545329121	0.585527179	0.999579949
40.83621141	-0.019784021	0.228518914	-0.086574983	0.931009352	0.999579949
282.9829296	0.076539425	0.07290942	1.04978787	0.293815653	0.999579949
407.095514	0.061369135	0.112474089	0.545629094	0.585320921	0.999579949
604.5448612	-0.013237138	0.05135368	-0.257764168	0.79658892	0.999579949
44.26255604	-0.348454882	0.185499441	-1.878468641	0.060317083	0.800274462
1939.496377	0.249279686	0.131906656	1.889818861	0.05878219	0.79629879
154.6969861	0.054532562	0.184598119	0.295412341	0.767678907	0.999579949
48.23024109	0.023909177	0.149674923	0.159740699	0.873085339	0.999579949
79.84612832	0.229833327	0.135658968	1.694199293	0.090227419	0.876222021
72.23066689	0.160333481	0.153345362	1.045571112	0.295759092	0.999579949

57.95716583	0.011962241	0.158941449	0.075261933	0.940006309	0.999579949
82.63456637	-0.073192105	0.141644498	-0.516731013	0.605343943	0.999579949
758.4009754	-0.06702586	0.108913422	-0.615404957	0.538287319	0.999579949
306.8120041	-0.131899882	0.112527506	-1.172156805	0.241134107	0.999579949
1790.042912	0.152374128	0.126317955	1.206274504	0.227711665	0.999579949
1306.495354	0.071778974	0.112907211	0.635734186	0.52494969	0.999579949
254.3426298	-0.006631384	0.094256083	-0.070354968	0.943911133	0.999579949
99.17255599	0.108250732	0.116419621	0.929832368	0.352457885	0.999579949
54.11608758	-0.02494176	0.195865191	-0.127341462	0.898670146	0.999579949
499.2824163	0.045216491	0.089734422	0.503892376	0.614337008	0.999579949
410.1045945	0.177569273	0.165735776	1.071399775	0.283989714	0.999579949
5559.862129	-0.077447473	0.134948308	-0.573904738	0.566032267	0.999579949
74.43780864	-0.288249018	0.135078577	-2.133935854	0.032848032	0.695900535
113.605673	0.094070202	0.132772228	0.708508119	0.478629772	0.999579949
106.576811	0.134413069	0.157239287	0.854831327	0.392644533	0.999579949
507.5146902	-0.005169075	0.103816853	-0.04979033	0.960289473	0.999579949
972.5714546	0.002528929	0.106773719	0.023684941	0.981103918	0.999579949
250.8470054	0.07429603	0.151958967	0.488921656	0.624897164	0.999579949
395.6221758	-0.19393405	0.125667017	-1.543237477	0.122773167	0.92236461
353.9681812	-0.07394863	0.113534397	-0.651332392	0.514831943	0.999579949
62.00349943	-0.226607727	0.199031347	-1.138552949	0.254889662	0.999579949
498.0505282	-0.100037211	0.101218461	-0.988329702	0.322991203	0.999579949
115.0705426	-0.110597544	0.128445675	-0.861045296	0.389213096	0.999579949
77.49426973	0.269146145	0.16009892	1.681124053	0.092738821	0.880418343
1043.67923	-0.054508264	0.07658746	-0.711712642	0.476642738	0.999579949
129.4504419	-0.015240994	0.196494482	-0.077564491	0.93817449	0.999579949
205.8715429	-0.032582171	0.10258431	-0.317613594	0.750778061	0.999579949
917.8883411	-0.02786979	0.062448585	-0.446283762	0.655392284	0.999579949
96.14201944	0.126326118	0.10368795	1.218329777	0.223098679	0.999579949
32.09558621	0.59864128	0.229013963	2.613994678	0.008949043	0.447310104
3612.718636	-0.004340842	0.139436996	-0.031131208	0.975164901	0.999579949
100.8282629	0.006891973	0.113802085	0.060561044	0.951708799	0.999579949
582.105828	0.039724497	0.080687247	0.492326834	0.622488313	0.999579949
49.74841673	0.288539816	0.395870012	0.728875154	0.466078035	0.999579949
1760.400576	0.216725839	0.133621263	1.62194125	0.104815928	0.895628172
2720.937772	0.017241761	0.113755429	0.151568682	0.879527136	0.999579949
192.9146778	-0.007030007	0.0991422	-0.070908323	0.943470719	0.999579949
21.54067086	-0.007209917	0.359268762	-0.020068309	0.98398888	0.999579949
500.5120128	0.116025685	0.089084769	1.302418889	0.192773229	0.985859356
694.0678517	0.011046807	0.107235526	0.103014438	0.917951512	0.999579949
220.6876746	-0.090952148	0.132883442	-0.684450574	0.49369069	0.999579949
160.8642112	0.062016423	0.097841968	0.633842761	0.526183444	0.999579949
95.79313139	0.026733778	0.1950794	0.137040497	0.890998785	0.999579949
788.8897063	-0.069803376	0.069376288	-1.006156102	0.314340485	0.999579949
55.20375586	-0.0927263	0.268573164	-0.345255269	0.729902472	0.999579949
259.8721299	-0.042634762	0.102690296	-0.415178101	0.678011529	0.999579949
552.6193176	-0.107266071	0.103726352	-1.03412556	0.301077469	0.999579949
144.4546411	0.019807673	0.213120822	0.092941049	0.925950394	0.999579949

277.9190165	-0.001378476	0.076402921	-0.018042186	0.9856052	0.999579949
30.7009172	-0.064444792	0.22684778	-0.284088266	0.776342739	0.999579949
1013.091141	0.061636383	0.138237843	0.445871994	0.655689713	0.999579949
246.9640564	0.009062465	0.085534317	0.105951213	0.915621061	0.999579949
325.0729009	0.001779211	0.120333309	0.014785687	0.988203159	0.999579949
470.3680722	0.101350093	0.074386572	1.362478327	0.173046979	0.983601342
582.9134992	0.252611037	0.143175136	1.764349896	0.077673088	0.851207389
1715.486056	0.018754267	0.1154508	0.162443806	0.870956377	0.999579949
275.6376034	-0.003772293	0.070196056	-0.053739382	0.957142806	0.999579949
798.3260661	0.047604031	0.062218449	0.765111185	0.44420534	0.999579949
305.3832803	-0.561491113	0.213131648	-2.634480232	0.008426621	0.441435814
556.8674409	-0.131787426	0.058885455	-2.23803017	0.025219088	0.642289128
2012.561115	0.020994401	0.128019843	0.163993336	0.86973639	0.999579949
1988.30664	-0.186906383	0.097812584	-1.91086234	0.056022274	0.785214091
736.375154	0.148391036	0.104300752	1.422722581	0.154816592	0.976030424
512.3156336	-0.075877845	0.0690818	-1.098376781	0.272039997	0.999579949
62.1909147	0.245274728	0.291212275	0.842254084	0.399645746	0.999579949
119.339772	-0.07588451	0.160395866	-0.473107643	0.636136384	0.999579949
2414.791392	-0.064181059	0.119401203	-0.537524388	0.590905444	0.999579949
112.7349463	0.088081589	0.190519365	0.46232355	0.643849314	0.999579949
420.6256663	-0.071928526	0.135818198	-0.52959417	0.596393337	0.999579949
171.9902208	-0.226398125	0.192544317	-1.175823458	0.23966543	0.999579949
326.8070368	-0.129093292	0.143814649	-0.897636592	0.369379324	0.999579949
293.9292128	0.10200399	0.146788922	0.694902509	0.4871164	0.999579949
245.7944439	-0.048980145	0.117386841	-0.417254141	0.676492528	0.999579949
130.3909073	-0.286956008	0.195310828	-1.469227338	0.141771138	0.949966262
141.7735315	0.037998519	0.111248445	0.341564495	0.73267866	0.999579949
306.1967747	0.128855504	0.170195281	0.757103858	0.448987643	0.999579949
32.09453659	0.057112959	0.26339511	0.216833788	0.828337879	0.999579949
40.04706586	0.210809784	0.259578738	0.812122694	0.416721233	0.999579949
2371.141255	-0.058752584	0.109850471	-0.534841439	0.592759505	0.999579949
2962.383818	0.039843	0.092217505	0.43205463	0.665701709	0.999579949
31.86105102	0.257563254	0.324902744	0.792739547	0.427929584	0.999579949
197.3583242	0.057887922	0.123405292	0.469087843	0.639006849	0.999579949
637.875876	0.101165687	0.122803615	0.823800561	0.410052889	0.999579949
448.1876346	0.059670488	0.07960035	0.749625958	0.453480013	0.999579949
154.6453304	0.036946936	0.090407291	0.40867209	0.682780319	0.999579949
885.3096109	0.154295454	0.070532649	2.187574911	0.028700581	0.668230165
641.1803109	-0.083836979	0.088019019	-0.952487089	0.34085001	0.999579949
12855.28021	0.013406226	0.154292269	0.086888512	0.930760131	0.999579949
119.0870529	0.097229531	0.166351512	0.584482397	0.558895805	0.999579949
51.7739677	-0.128242998	0.250422662	-0.512106201	0.608576685	0.999579949
41187.45239	0.42977638	0.18134019	2.370000714	0.017788051	0.573039104
4422.29063	0.127943908	0.117175587	1.091899014	0.274877491	0.999579949
308.4725135	0.039613319	0.108329563	0.365674128	0.714608254	0.999579949
279.2620115	-0.001084287	0.094855345	-0.011430949	0.990879621	0.999579949
612.1063682	0.039625857	0.06080382	0.651700126	0.514594641	0.999579949
956.7344351	0.202885194	0.09586091	2.116453878	0.034306224	0.703403556

272.588733	0.212403586	0.164893767	1.288123805	0.19770287	0.992338959
380.417971	0.25076726	0.116033547	2.161161717	0.03068285	0.683621001
46.72169564	-0.437556312	0.306730859	-1.426515457	0.153719609	0.973567967
65.64061546	-0.371515789	0.201918918	-1.839925614	0.065779159	0.82135988
133.9802972	-0.062953739	0.124613025	-0.50519389	0.613422661	0.999579949
50.48185204	0.724212735	0.430485391	1.682316636	0.092507457	0.880418343
480.7263463	0.220132314	0.117275962	1.877045473	0.060511862	0.800274462
254.7512449	-0.077858953	0.115990854	-0.671250799	0.502060772	0.999579949
294.7024916	0.040828989	0.142873319	0.285770563	0.775053856	0.999579949
324.0142459	0.045970652	0.103621835	0.443638663	0.657303848	0.999579949
368.6526397	0.083361568	0.139344324	0.598241575	0.549678755	0.999579949
145.8602778	-0.055635823	0.096445505	-0.57686279	0.564032149	0.999579949
45.12567404	-0.001621308	0.16006384	-0.010129134	0.991918259	0.999579949
99.77673081	0.149403418	0.203059684	0.735761109	0.461876093	0.999579949
217.785202	0.014966509	0.131922954	0.113448862	0.909674703	0.999579949
25.71504659	-0.013408264	0.250197703	-0.053590676	0.957261285	0.999579949
60.28496013	-0.095655249	0.126071753	-0.758736564	0.44801016	0.999579949
78.91815435	0.087477383	0.133843438	0.653579918	0.513382486	0.999579949
285.1195431	0.061603668	0.083848595	0.73470126	0.462521453	0.999579949
1107.285647	-0.132941823	0.06919506	-1.92126178	0.054698713	0.785214091
125.8120144	0.064646993	0.125170817	0.516470168	0.605526069	0.999579949
256.4600925	-0.063858045	0.10290168	-0.620573396	0.534880348	0.999579949
1428.252171	-0.00983711	0.111072375	-0.088564862	0.929427735	0.999579949
150.8980101	0.041869998	0.093745467	0.446634905	0.655138689	0.999579949
307.7004326	0.030965519	0.085652586	0.361524628	0.717707298	0.999579949
88.96409132	-0.155728852	0.159731015	-0.974944361	0.32958786	0.999579949
28.45451419	0.698622111	0.320073794	2.182690753	0.029058591	0.672989344
579.4509462	0.198595722	0.123297652	1.610701572	0.107244783	0.90057019
203.222607	-0.013229716	0.10005991	-0.13221795	0.894811904	0.999579949
87.53285024	-0.566019707	0.32205106	-1.757546481	0.078824706	0.851207389
23.73487204	0.405767725	0.661411467	0.613487588	0.539553987	0.999579949
58.88538291	-0.002399612	0.279293318	-0.008591727	0.993144878	0.999579949
54.96422408	-0.155083235	0.184002853	-0.842830602	0.39932319	0.999579949
978.7533518	-0.605733179	0.183808623	-3.295455736	0.000982622	0.161334665
650.412239	-0.070576012	0.087789012	-0.803927622	0.421438777	0.999579949
382.8689147	0.133007349	0.130360691	1.020302577	0.307584981	0.999579949
109.2086262	0.105738321	0.190833464	0.554086891	0.579519381	0.999579949
236.3811335	1.059468771	0.544786487	1.94474128	0.051806125	0.779189484
586.7089184	-0.02576191	0.071783194	-0.358884982	0.719681135	0.999579949
312.9444218	-0.093549049	0.092882264	-1.007178816	0.313848851	0.999579949
73.94584471	-0.016520924	0.130326021	-0.126766122	0.89912551	0.999579949
195.4622318	0.062522214	0.10478798	0.596654446	0.550738112	0.999579949
156.5852831	-0.110292767	0.103772102	-1.062836402	0.287856148	0.999579949
41.97734544	-0.457654346	0.39114464	-1.17003865	0.241985415	0.999579949
1434.299526	-0.072082669	0.12761205	-0.564857855	0.572170452	0.999579949
418.2625177	0.127178882	0.11395279	1.116066421	0.264393704	0.999579949
62.46403394	0.37426101	0.123764112	3.023986538	0.002494675	0.255470986
470.0421636	-0.164108683	0.131982956	-1.243408157	0.213717466	0.999268361

591.1971154	0.047182081	0.065448741	0.720901274	0.470970259	0.999579949
44.41172736	0.151905539	0.379978284	0.399774263	0.689322789	0.999579949
36.43134334	-0.077174954	0.235502166	-0.327703797	0.743135632	0.999579949
1437.946227	-0.183793714	0.217885587	-0.843533144	0.398930337	0.999579949
412.1737712	-0.001471637	0.069522853	-0.021167666	0.983111907	0.999579949
279.0629295	-0.08661778	0.161637324	-0.535877342	0.592043325	0.999579949
167.5471477	-0.143654769	0.104489882	-1.374819908	0.169187285	0.981013142
928.404413	0.115899963	0.1230019	0.942261562	0.346058742	0.999579949
722.7374803	-0.091870282	0.101757262	-0.902837593	0.366612096	0.999579949
1257.944473	-0.032416337	0.071396133	-0.454034914	0.649803698	0.999579949
372.16798	0.222984572	0.191202909	1.16621956	0.243525686	0.999579949
895.1343415	0.06632118	0.099317145	0.667771719	0.504279322	0.999579949
53.10024797	-0.074133452	0.173182107	-0.428066461	0.668602734	0.999579949
133.07141	0.098532262	0.107436492	0.917120995	0.359079241	0.999579949
922.2847049	-0.092479458	0.084847733	-1.089946123	0.275736878	0.999579949
78.79734062	-0.125610013	0.20212353	-0.621451708	0.534302459	0.999579949
1483.527755	0.73202252	0.24866554	2.943803632	0.003242056	0.304904733
22.36737139	-0.194133978	0.291300142	-0.666439693	0.505130099	0.999579949
476.0077083	0.086291903	0.095865382	0.900136222	0.368047762	0.999579949
630.4621545	-0.092547586	0.212257609	-0.436015399	0.662825557	0.999579949
242.6053898	0.000721178	0.187800434	0.003840131	0.996936026	0.999579949
166.1160494	0.058514014	0.109086961	0.536397877	0.591683598	0.999579949
6052.152503	0.04265757	0.147366764	0.289465341	0.772225299	0.999579949
22.4504156	0.42449352	0.353512828	1.200786751	0.229833932	0.999579949
176.7782385	-0.126336285	0.094404655	-1.338242109	0.180817526	0.983992835
76.5223962	-0.099705491	0.154410744	-0.645716017	0.518463285	0.999579949
253.0314257	0.146441686	0.18889264	0.775264119	0.438183607	0.999579949
25.93290608	1.012311934	0.590008027	1.715759595	0.086206052	0.866133472
547.9700074	0.203481435	0.272028185	0.748016003	0.454450505	0.999579949
648.1821301	0.171517528	0.093923487	1.826140983	0.067829028	0.829912169
175.0886002	-0.207858238	0.099678615	-2.085284163	0.037043514	0.717947029
62.31523118	0.039784675	0.204003934	0.195019153	0.845377953	0.999579949
264.1452085	0.107359032	0.13036651	0.823516959	0.410214077	0.999579949
20.8174597	0.092050021	0.219705595	0.418969853	0.675238165	0.999579949
107.201122	0.126039252	0.165524094	0.761455619	0.446384978	0.999579949
382.6466584	0.09484461	0.103041192	0.920453351	0.3573359	0.999579949
225.0000271	0.003106449	0.080228606	0.038719963	0.969113658	0.999579949
143.1487287	0.181976282	0.179315318	1.014839582	0.310182302	0.999579949
507.0226566	0.058117987	0.098354476	0.590903331	0.554585194	0.999579949
52.90366571	-0.252868263	0.224065502	-1.128546169	0.25908933	0.999579949
931.665992	0.413679704	0.177768947	2.327063935	0.019961863	0.604422168
106.6700683	-0.028830802	0.133973902	-0.21519715	0.829613613	0.999579949
312.0466382	-0.257427594	0.142367527	-1.808190394	0.070576873	0.834620594
83.14646834	0.285154492	0.340325187	0.837888299	0.402093441	0.999579949
39.62975987	-0.421294572	0.603276374	-0.698344225	0.484961948	0.999579949
241.9582887	0.265026333	0.555283926	0.477280757	0.633162212	0.999579949
36.72460842	-0.108403452	0.329017307	-0.329476444	0.741795596	0.999579949
1171.850693	-0.072628703	0.143038211	-0.50775735	0.611623523	0.999579949

243.6999699	-0.026191524	0.085957272	-0.304703997	0.760591605	0.999579949
205.5916026	1.74576326	0.651709204	2.678745749	0.007389848	0.411084085
61.37855485	-0.112338549	0.257811931	-0.435738365	0.663026567	0.999579949
36.19920472	0.181598227	0.248829887	0.729808742	0.4655071	0.999579949
219.9515554	-0.012537908	0.108467875	-0.115590981	0.907976711	0.999579949
94.31572374	0.410954123	0.203964497	2.014831649	0.043922292	0.758836007
266.8971992	0.163994393	0.108385515	1.51306559	0.130262999	0.935700262
139.07536	0.038062563	0.245621546	0.154964268	0.876849487	0.999579949
856.2921444	0.176200367	0.147468991	1.19482995	0.232153476	0.999579949
117.9223488	0.069030353	0.154774221	0.446006785	0.655592345	0.999579949
36.93519144	0.078624879	0.227736342	0.345245199	0.729910041	0.999579949
722.6520019	0.005021802	0.126368052	0.039739491	0.968300818	0.999579949
335.3740618	-0.000647674	0.061112396	-0.01059808	0.991544114	0.999579949
225.9611735	0.086767399	0.121795268	0.712403698	0.476214826	0.999579949
323.4583492	0.037827086	0.18551021	0.203908379	0.838425097	0.999579949
38.13136949	-0.070460514	0.225602375	-0.312321684	0.754796062	0.999579949
1896.921707	0.044246126	0.092011687	0.480875063	0.630605297	0.999579949
4127.458487	-0.062127281	0.08298632	-0.748644849	0.454071293	0.999579949
367.8219193	-0.120185185	0.10830579	-1.109683836	0.267135291	0.999579949
46.96698475	-0.282018459	0.189264964	-1.490072185	0.136205257	0.945791454
171.0553362	0.186608461	0.17252597	1.081625338	0.279419041	0.999579949
165.6914405	-0.05455347	0.105260527	-0.518270919	0.60426926	0.999579949
331.9555117	0.089463066	0.128764927	0.694778218	0.487194301	0.999579949
22.26647897	-0.062051179	0.32296378	-0.192130459	0.847640016	0.999579949
162.3928969	-0.018602215	0.134066373	-0.138753773	0.889644724	0.999579949
99.10127495	-0.023206355	0.14486352	-0.160194609	0.872727776	0.999579949
470.3834802	-0.00739988	0.159298935	-0.046452791	0.962949361	0.999579949
505.8487278	-0.018714757	0.087496008	-0.213892697	0.830630735	0.999579949
134.9848007	0.111477364	0.118163805	0.943413803	0.345469286	0.999579949
514.2738742	-0.170741713	0.161738503	-1.055665222	0.291121191	0.999579949
1360.209354	-0.07713605	0.078039063	-0.988428703	0.322942736	0.999579949
1374.177893	0.126061226	0.090447206	1.393754786	0.163391666	0.978498637
169.2384268	0.463525659	0.256066133	1.81017948	0.070267959	0.834620594
28.37550212	0.625831476	0.3625993	1.725958863	0.084354814	0.861032139
1348.223544	-0.117445793	0.152922757	-0.768007296	0.44248285	0.999579949
445.2738236	0.03391031	0.070770742	0.479157193	0.631826805	0.999579949
38.92621005	-0.045381966	0.276506535	-0.164126197	0.869631799	0.999579949
1508.285683	0.159442137	0.097072011	1.642514007	0.100483526	0.895628172
278.1338372	-0.218587431	0.149864383	-1.458568247	0.144683978	0.955875831
23.17766101	-0.06055395	0.400763308	-0.151096542	0.87989956	0.999579949
49.84790571	0.249634939	0.241551984	1.033462591	0.301387468	0.999579949
50.0172372	0.186237139	0.201688658	0.923389252	0.355804391	0.999579949
604.2976437	0.041559829	0.099946975	0.415818782	0.677542614	0.999579949
22.15678184	0.051779386	0.249103123	0.207863256	0.83533574	0.999579949
73.96918721	-0.120954439	0.182806301	-0.661653555	0.508193277	0.999579949
470.71906	-0.208109969	0.093955458	-2.214985422	0.026761065	0.653787748
378.2852184	0.027885212	0.094424588	0.295317271	0.767751524	0.999579949
1801.245136	0.354651039	0.12081385	2.935516415	0.00332993	0.308410274

44.9669412	0.222907871	0.290808721	0.766510269	0.443372744	0.999579949
474.9792356	-0.161645224	0.176609163	-0.915270878	0.360049443	0.999579949
65.10738167	-0.09201511	0.176682312	-0.520794126	0.602510196	0.999579949
30.80900664	-0.382602565	0.209877067	-1.822984146	0.068305792	0.830482391
1641.181728	-0.189919745	0.090627569	-2.095606753	0.03611709	0.716426553
223.6837323	0.085644374	0.147656119	0.580025903	0.56189715	0.999579949
131.6090899	0.072823655	0.120586756	0.603910887	0.54590289	0.999579949
61.81322132	0.024526489	0.193952629	0.126456079	0.899370914	0.999579949
250.7449078	0.249737574	0.130748613	1.910059066	0.056125609	0.785214091
517.8087895	-0.130615256	0.068910942	-1.895421134	0.058036631	0.79580383
157.2784077	0.072757325	0.12297099	0.591662512	0.554076605	0.999579949
90.46031749	-0.039465072	0.246716143	-0.159961449	0.872911443	0.999579949
140.1960671	0.056299813	0.156170345	0.360502587	0.718471322	0.999579949
33.76662972	0.030940276	0.21564303	0.143479138	0.885911787	0.999579949
15278.37113	-0.075436429	0.163921309	-0.460199038	0.645373361	0.999579949
68.22712746	0.134628806	0.161882133	0.831647097	0.40560817	0.999579949
200.1720785	-0.080281756	0.14232937	-0.564056147	0.572715922	0.999579949
116.6942842	-0.215844679	0.228478983	-0.944702558	0.344810751	0.999579949
2508.155878	-0.079189772	0.038257609	-2.069909053	0.038460862	0.72691918
58.55013762	0.009626593	0.154724007	0.062217836	0.950389359	0.999579949
29.32813293	-0.100623756	0.207914851	-0.483966179	0.628409872	0.999579949
594.3437209	0.087726806	0.241516705	0.363232869	0.716430936	0.999579949
172.0053318	-0.082616186	0.138001581	-0.598661157	0.549398864	0.999579949
112.4777625	0.717767333	0.339476341	2.114336836	0.034486508	0.703403556
1360.913782	-0.1945041	0.159933122	-1.216158963	0.223924373	0.999579949
56.32308009	-0.335239638	0.177195584	-1.891918698	0.058501814	0.79580383
444.779222	0.014914713	0.084969601	0.175529992	0.860663206	0.999579949
146.7401962	-0.034727681	0.122894075	-0.282582225	0.777497107	0.999579949
350.1477157	-0.047063009	0.110324508	-0.426587077	0.669680112	0.999579949
6952.975176	-0.068619804	0.144769357	-0.473993982	0.635504199	0.999579949
1288.840271	-0.084587749	0.110495029	-0.76553443	0.443953372	0.999579949
4354.499725	0.040253131	0.077510149	0.519327227	0.60353257	0.999579949
200.0126372	-0.108755058	0.175872429	-0.618374688	0.536328379	0.999579949
59.00329174	0.075716146	0.160612674	0.471420745	0.637340305	0.999579949
369.3127136	-0.018862696	0.094288706	-0.200052544	0.841439488	0.999579949
131.0933428	0.293238996	0.179569894	1.633007569	0.102467405	0.895628172
359.5209361	-0.056822713	0.086998526	-0.653145702	0.513662351	0.999579949
796.1714034	0.119315688	0.127245633	0.937680026	0.348408872	0.999579949
56.50048717	-0.035080401	0.15483106	-0.226572118	0.820756475	0.999579949
229.3254725	-0.07821188	0.089334332	-0.875496324	0.38130391	0.999579949
829.9025563	-0.019474975	0.064868512	-0.300222315	0.764007585	0.999579949
107.6056943	0.039579909	0.106592705	0.371319118	0.71039986	0.999579949
20.47559017	0.070275421	0.26754648	0.262666214	0.79280786	0.999579949
88.08520039	0.001426003	0.129377394	0.011022044	0.99120586	0.999579949
142.2129652	-0.042524474	0.112880043	-0.376722699	0.706379692	0.999579949
99.04379162	0.121901113	0.131224235	0.928952737	0.35291358	0.999579949
215.2752747	0.139090471	0.108315627	1.284121928	0.199099297	0.992481822
712.1085559	0.079141957	0.107163179	0.738518191	0.460199617	0.999579949

288.2265857	0.095439895	0.114122561	0.836292969	0.402990108	0.999579949
1245.94378	0.025219633	0.085217981	0.295942625	0.767273898	0.999579949
130.2482317	0.037278384	0.141846862	0.262807254	0.792699144	0.999579949
2703.666766	0.084322063	0.123020468	0.685431169	0.493071881	0.999579949
231.9239334	-0.100881385	0.129830472	-0.777023943	0.437144645	0.999579949
45.81367761	-0.120487569	0.164510055	-0.732402462	0.463922956	0.999579949
649.0087814	-0.012712591	0.117176607	-0.10849086	0.91360633	0.999579949
143.0861658	-0.014866887	0.088678174	-0.167649897	0.866858715	0.999579949
28.29408603	0.328026322	0.35582318	0.921880138	0.356591101	0.999579949
457.3804746	0.000457083	0.081666339	0.005596955	0.9955343	0.999579949
529.026346	0.041788318	0.09973398	0.418997801	0.675217739	0.999579949
118.4387676	0.134735104	0.16924282	0.796105289	0.42597084	0.999579949
181.5255923	0.02639895	0.104499746	0.252622149	0.800560213	0.999579949
108.1964914	-0.031053286	0.100262439	-0.309720033	0.756773868	0.999579949
323.2972701	0.107077994	0.173986825	0.615437367	0.53826592	0.999579949
3093.270719	0.020115415	0.085974083	0.233970689	0.815007734	0.999579949
1260.906886	-0.031473261	0.086928265	-0.362060148	0.717307084	0.999579949
1492.834324	-0.001890496	0.094623753	-0.019979088	0.984060055	0.999579949
31.75468748	0.210297818	0.223677172	0.940184535	0.347122913	0.999579949
1741.900664	-0.051198128	0.102734285	-0.498354837	0.618233963	0.999579949
158.061621	0.126207954	0.216696761	0.582417351	0.560285597	0.999579949
1158.859619	0.096503205	0.121794216	0.79234637	0.428158741	0.999579949
23.56012522	0.228480738	0.285888558	0.799195113	0.424177285	0.999579949
76.54084643	0.033832335	0.225118282	0.150286929	0.880538245	0.999579949
90.57678043	-0.352882052	0.239018014	-1.476382667	0.139841193	0.948318761
225.2395783	0.023889904	0.078937296	0.302644061	0.762161133	0.999579949
67.80877153	0.294206936	0.267203664	1.101058766	0.270871082	0.999579949
177.5407524	-0.248091142	0.25158478	-0.986113477	0.324077431	0.999579949
1075.164775	-0.131708188	0.133811592	-0.984280849	0.324977443	0.999579949
519.4753476	0.02533797	0.153762484	0.164786425	0.869112089	0.999579949
79.91507723	-0.10988444	0.171698754	-0.63998391	0.52218306	0.999579949
521.6783343	-0.026954677	0.083995515	-0.320906146	0.748281516	0.999579949
270.8533239	0.047301229	0.0995465	0.475167174	0.634667822	0.999579949
1706.492762	0.128414643	0.141955649	0.90461101	0.3656715	0.999579949
359.6717085	0.062529337	0.057447017	1.088469689	0.27638781	0.999579949
22.51529743	0.968098878	0.540073082	1.792533103	0.073047611	0.838669905
51.5725839	-0.005176049	0.170649765	-0.030331417	0.975802741	0.999579949
839.1559983	0.021258734	0.091900576	0.231323185	0.817063736	0.999579949
121.4551749	-0.048610962	0.164323529	-0.295824722	0.767363942	0.999579949
2434.818026	0.138440291	0.06900725	2.006170244	0.044838087	0.759209569
292.6853884	0.069551347	0.12341397	0.563561381	0.573052676	0.999579949
176.6380722	0.204478127	0.155249203	1.317096153	0.187806396	0.983992835
582.0682916	0.002064048	0.064822533	0.031841513	0.974598441	0.999579949
22.49252492	-0.097313255	0.219194423	-0.443958628	0.657072496	0.999579949
141.5882344	0.251954059	0.155437627	1.620933511	0.105031899	0.896329133
1805.388083	-0.032996618	0.088005479	-0.37493822	0.707706414	0.999579949
975.1046763	-0.035207304	0.091877644	-0.383197728	0.701573162	0.999579949
370.7139224	-0.150341479	0.150211316	-1.00086653	0.31689134	0.999579949

59.53839829	0.131235246	0.194823002	0.673612687	0.500557585	0.999579949
103.2764355	-0.042142828	0.119463913	-0.352766175	0.724263744	0.999579949
30.26794862	-0.232819718	0.234425404	-0.993150546	0.320636594	0.999579949
564.1509502	0.14263405	0.110850569	1.286723665	0.198190622	0.992481822
725.7377843	-0.036634224	0.075467885	-0.485427998	0.627372776	0.999579949
224.15364	-0.013257008	0.087338503	-0.151788815	0.879353504	0.999579949
58.03108102	-0.053688618	0.290118899	-0.185057293	0.853184098	0.999579949
34.37540168	0.093207448	0.224202862	0.415728183	0.677608916	0.999579949
56.48214079	0.207954726	0.194979891	1.066544477	0.286177584	0.999579949
2180.066115	-0.031372822	0.062248985	-0.503989288	0.614268904	0.999579949
36.82493232	0.218371551	0.213646427	1.022116562	0.306725735	0.999579949
588.8004366	0.062900628	0.104469879	0.60209343	0.547111948	0.999579949
981.0492188	0.01020861	0.102799637	0.099305898	0.920895396	0.999579949
196.0102406	0.036794825	0.091785328	0.400879159	0.688509095	0.999579949
123.5179499	0.41853029	0.302634672	1.38295552	0.166678505	0.978965834
74.0870377	-0.086298655	0.153866557	-0.560866876	0.574888292	0.999579949
503.5693664	0.003884892	0.069916413	0.055564801	0.955688506	0.999579949
53.37382878	0.239399505	0.148956986	1.60717205	0.108016631	0.903743816
3859.463321	-0.041225476	0.102293118	-0.403013193	0.686938519	0.999579949
786.8958955	0.023005502	0.095415609	0.241108373	0.809471123	0.999579949
706.0426317	0.050317107	0.075153344	0.669525851	0.503160096	0.999579949
280.6075867	-0.225525018	0.185206412	-1.21769552	0.2233397	0.999579949
29.75136346	0.017107455	0.300462858	0.056937005	0.954595376	0.999579949
637.8611176	0.103509516	0.073884552	1.400962887	0.161225171	0.978498637
216.239569	0.02287601	0.072554113	0.315295845	0.752537039	0.999579949
84.68428335	0.111538863	0.272032663	0.410020113	0.681791193	0.999579949
546.5889339	-0.18610645	0.127850735	-1.455654124	0.145488258	0.956456209
300.0765159	-0.015452686	0.096953635	-0.159382219	0.873367747	0.999579949
243.7121233	0.105913787	0.133965671	0.790603935	0.42917515	0.999579949
142.2026283	0.055978717	0.12370501	0.452517784	0.650896013	0.999579949
26.53060894	1.288230611	1.008020216	1.277980928	0.201256151	0.993208272
106.3855282	-0.216247373	0.115523888	-1.871884472	0.061222588	0.802875925
25.67894913	0.03908339	0.251056996	0.155675369	0.876288913	0.999579949
192.3615603	-0.082851524	0.103177484	-0.803000041	0.421974711	0.999579949
209.8170497	-0.011451963	0.086652862	-0.132159081	0.894858466	0.999579949
52.6138164	0.020326718	0.370672437	0.054837415	0.956267992	0.999579949
5498.455333	-0.075859	0.083517631	-0.908299235	0.363720147	0.999579949
54.54826411	0.16882537	0.158046706	1.068199238	0.285430648	0.999579949
75.47920867	0.057660745	0.166129185	0.347083775	0.728528382	0.999579949
34.5132106	-0.175144026	0.196593763	-0.890893094	0.372986526	0.999579949
710.8601231	0.024935176	0.089575316	0.278371064	0.780727529	0.999579949
81.47434308	0.150413773	0.240501568	0.625417016	0.531697397	0.999579949
83.8096365	0.06445008	0.251914221	0.255841372	0.798073327	0.999579949
498.0596799	-0.062523151	0.079697382	-0.784506966	0.432742696	0.999579949
201.1217491	0.055837545	0.095630084	0.583890991	0.559293655	0.999579949
119.7751601	-0.208962991	0.234810178	-0.889923055	0.373507204	0.999579949
289.43356	0.132604316	0.126937721	1.044640753	0.296189037	0.999579949
395.3022211	-0.134462033	0.089932386	-1.49514584	0.134876385	0.945791454

23.66155852	0.043429103	0.25882399	0.167793964	0.866745371	0.999579949
395.5054525	0.235606558	0.123088828	1.914118144	0.055605062	0.785214091
369.5083207	0.09071615	0.088781844	1.021787189	0.306881634	0.999579949
400.1002163	0.028739099	0.081230005	0.353799056	0.723489481	0.999579949
342.326055	0.027622061	0.092453411	0.298767356	0.76511756	0.999579949
546.9699191	0.178584161	0.236903599	0.753826289	0.450953536	0.999579949
134.3389458	0.031116637	0.109203053	0.284942921	0.775687872	0.999579949
115.3051203	-0.068596307	0.090535029	-0.757676978	0.448644385	0.999579949
283.5964951	0.089968005	0.113120917	0.795325985	0.426423902	0.999579949
154.9047748	-0.256147651	0.171521371	-1.49338622	0.135336118	0.945791454
3217.726885	0.086282092	0.082268664	1.048784409	0.294277355	0.999579949
4148.15053	0.00062293	0.125697789	0.004955778	0.996045877	0.999579949
563.1675043	-0.16282223	0.090071134	-1.80770711	0.070652097	0.834620594
273.5796971	-0.098538335	0.093523794	-1.053617811	0.292057931	0.999579949
206.073136	0.098393055	0.156878983	0.627190801	0.530534173	0.999579949
422.8149008	-0.04784561	0.10882875	-0.43964127	0.660196945	0.999579949
24.09425495	-0.046467774	0.256416903	-0.181219621	0.856195194	0.999579949
127.5463537	0.148175123	0.095883628	1.545364174	0.122258201	0.92236461
2678.819723	0.16449797	0.138916347	1.184151281	0.236353183	0.999579949
157.6054549	0.096865306	0.09838154	0.984588225	0.324826376	0.999579949
284.4472893	-0.076866781	0.090608541	-0.848339247	0.396249067	0.999579949
378.1247715	-0.038035857	0.064066763	-0.59369094	0.552718844	0.999579949
95.43618839	0.128009888	0.217241987	0.589250218	0.555693434	0.999579949
108.5113003	0.192609447	0.109404699	1.760522628	0.078319234	0.851207389
25.42599999	0.127040426	0.227813814	0.557650232	0.577083248	0.999579949
146.1904061	-0.056668948	0.111727758	-0.507205635	0.612010542	0.999579949
452.448599	0.029189391	0.108063615	0.270113039	0.787073291	0.999579949
115.1754601	0.124163602	0.100255769	1.238468404	0.21554243	0.999579949
75.61904531	0.179994489	0.13650241	1.31861766	0.187296967	0.983992835
92.05602836	0.03334936	0.226039899	0.147537494	0.882707788	0.999579949
35.68376657	-0.256126545	0.251628713	-1.017874875	0.308737423	0.999579949
32.56459162	1.009813757	0.606261568	1.665640394	0.095785061	0.88463985
391.7838802	0.073558194	0.09239415	0.796134759	0.425953713	0.999579949
53.15865009	-0.210577986	0.151956445	-1.385778578	0.165814526	0.978789551
1956.044366	-0.04522265	0.10265931	-0.440511926	0.659566377	0.999579949
76.51098073	-0.04749861	0.208476476	-0.227836784	0.819773129	0.999579949
35.47508421	-0.012819899	0.228875462	-0.056012554	0.955331807	0.999579949
1383.353703	-0.002168754	0.064389543	-0.033681767	0.973130918	0.999579949
3390.153116	-0.11056731	0.085215721	-1.297498964	0.194459561	0.988064798
230.5295604	0.008395808	0.077831302	0.107871864	0.914097336	0.999579949
46.3120169	1.199288723	0.472799757	2.536567977	0.011194501	0.487909795
108.9082441	-0.087404439	0.140773021	-0.620889136	0.53467257	0.999579949
301.9770814	-0.122944663	0.091088874	-1.349722063	0.177105152	0.983992835
447.1963562	-0.125296933	0.091532031	-1.368886191	0.171034853	0.982395406
243.4121505	0.084117827	0.121318124	0.693365709	0.488080076	0.999579949
415.4504342	-0.003828402	0.137925303	-0.027757064	0.977855911	0.999579949
496.3513391	-0.041240791	0.064787406	-0.63655568	0.524414301	0.999579949
361.7288376	0.005826283	0.074080189	0.078648329	0.937312346	0.999579949

675.1191059	0.037549136	0.057625713	0.651603845	0.514656767	0.999579949
558.7690848	0.007473315	0.069808947	0.107053836	0.914746271	0.999579949
1412.949664	0.023758763	0.095350466	0.249173016	0.803226951	0.999579949
92.89984487	0.064169494	0.114290344	0.561460326	0.574483769	0.999579949
133.5527142	0.212486917	0.106884842	1.987998613	0.046811843	0.766764601
247.7916446	0.070345855	0.124332937	0.565786162	0.571539155	0.999579949
78.37625548	-0.081473887	0.180432273	-0.451548302	0.65159442	0.999579949
95.4662642	0.086825715	0.120490493	0.720602212	0.471154292	0.999579949
350.1390926	-0.03816188	0.121230135	-0.314788729	0.75292207	0.999579949
231.6098878	-0.152600391	0.121303169	-1.258008285	0.20838876	0.995521169
25.94645987	-0.399330476	0.257293084	-1.552045123	0.120651427	0.921837625
92.95423942	-0.014662013	0.168824305	-0.086847763	0.930792522	0.999579949
358.3272532	-0.093344593	0.167450881	-0.557444623	0.577223685	0.999579949
393.8497158	0.103104713	0.084543462	1.219546863	0.2226367	0.999579949
767.9921872	0.284277555	0.156583234	1.815504434	0.069446425	0.831457432
78.94555722	0.027412292	0.141808796	0.193304598	0.846720425	0.999579949
567.7929251	0.102747787	0.135368757	0.759021425	0.44783974	0.999579949
141.425249	0.10033336	0.159365627	0.629579675	0.528969625	0.999579949
477.5718668	-0.079187962	0.146937098	-0.538924223	0.589939142	0.999579949
939.6606542	0.051851432	0.057525234	0.901368479	0.367392435	0.999579949
60.39473978	0.161496393	0.162218289	0.995549844	0.319468915	0.999579949
31.02394276	-0.181794007	0.228498422	-0.795602899	0.426262881	0.999579949
626.7931432	0.135836837	0.090728527	1.49717891	0.134346711	0.945791454
44.39033557	0.405633642	0.50076848	0.810022311	0.417927353	0.999579949
126.8334751	-0.02542968	0.176636123	-0.143966475	0.885526943	0.999579949
807.0871652	-0.03323179	0.066052625	-0.503110826	0.614886356	0.999579949
276.0425366	0.056651567	0.121826958	0.465016675	0.641919515	0.999579949
48.48765721	0.106735666	0.140966181	0.757172147	0.448946735	0.999579949
106.407769	0.087533374	0.108429856	0.80728111	0.419504544	0.999579949
106.7834305	0.033979863	0.10626565	0.319763377	0.749147712	0.999579949
434.1680878	0.045321652	0.094812285	0.478014551	0.632639849	0.999579949
1827.922802	-0.025807968	0.089303853	-0.288990535	0.772588621	0.999579949
37.57376019	-0.091756834	0.158068909	-0.580486287	0.561586731	0.999579949
640.1959133	0.078269689	0.076302591	1.025780221	0.304995191	0.999579949
11663.1377	-0.172511553	0.172263708	-1.001438754	0.316614736	0.999579949
29.29496481	-0.062742062	0.299539325	-0.209461852	0.834087713	0.999579949
133.07432	0.738709989	0.331375224	2.229225168	0.025798927	0.648237123
287.9304945	0.089928654	0.078969792	1.138772829	0.254797917	0.999579949
309.3975623	0.101893415	0.172950381	0.589148256	0.555761825	0.999579949
479.5580744	-0.200033376	0.119575544	-1.672861929	0.09435448	0.880418343
2109.888663	0.501011358	0.201294875	2.488942438	0.012812371	0.510304755
378.5663544	0.007352448	0.126553443	0.05809757	0.95367091	0.999579949
168.0880067	0.229828195	0.113796473	2.019642513	0.043420482	0.756465272
438.5767095	0.028454205	0.17765871	0.160162175	0.872753324	0.999579949
1152.306718	-0.0506129	0.064923505	-0.779577431	0.435639644	0.999579949
477.9630124	0.048705112	0.197691674	0.246369059	0.805396556	0.999579949
218.3618458	0.209446037	0.620204566	0.337704765	0.735585684	0.999579949
55.24253905	0.441130997	0.225169207	1.959108895	0.050100035	0.776599223

202.1747159	-0.033571787	0.100739929	-0.333252045	0.738944035	0.999579949
128.7106152	0.043111758	0.12795102	0.336939543	0.736162476	0.999579949
30.84493936	0.09994278	0.194605043	0.513567268	0.607554569	0.999579949
54.76851402	-0.110778849	0.213689991	-0.518409163	0.604172823	0.999579949
2502.206204	0.040280735	0.10891694	0.369829845	0.711509276	0.999579949
479.580625	0.018231163	0.067482898	0.270159754	0.787037353	0.999579949
190.6245348	0.006984199	0.113277628	0.061655588	0.950837108	0.999579949
25.00405714	0.009425529	0.302136059	0.031196307	0.975112985	0.999579949
229.7900436	0.013172741	0.124045473	0.106192838	0.915429353	0.999579949
44.7429525	0.359495465	0.173771992	2.068776802	0.038567036	0.72691918
40.26480688	0.169842509	0.284594959	0.596786779	0.550649746	0.999579949
122.5833486	-0.003604952	0.111334942	-0.032379336	0.974169541	0.999579949
115.4261897	0.149892186	0.168418815	0.889996677	0.37346767	0.999579949
63.98569769	-0.056596579	0.240029206	-0.235790387	0.813595328	0.999579949
269.1247618	-0.069120052	0.1277934	-0.540873413	0.588594837	0.999579949
44.44709187	-0.000579572	0.157551976	-0.003678607	0.997064903	0.999579949
66.7653037	-0.089579854	0.200419703	-0.446961312	0.654902994	0.999579949
166.3918701	0.256045641	0.195760555	1.307953184	0.190889189	0.98475336
340.8667182	0.083097303	0.063630434	1.305936463	0.191574165	0.984804292
41.71165948	0.095935093	0.310206645	0.309261889	0.757122319	0.999579949
344.5482187	-0.024230612	0.103405056	-0.234327146	0.814731013	0.999579949
34.7997641	0.142556392	0.234817485	0.607094452	0.543788232	0.999579949
218.9159396	0.038075614	0.125425409	0.303571772	0.76145416	0.999579949
59.38032448	0.457530482	0.378246168	1.209610356	0.226428442	0.999579949
183.309789	-0.090350124	0.100220671	-0.901511869	0.367316226	0.999579949
411.9955601	0.050525808	0.071388202	0.707761323	0.479093488	0.999579949
464.2402242	0.194528654	0.096397211	2.017990476	0.043592253	0.758408869
1345.643537	-0.174553195	0.092129156	-1.894657491	0.058137793	0.79580383
819.9704149	-0.008337083	0.1285855	-0.064836881	0.948303877	0.999579949
266.0165792	0.445083833	0.239116084	1.861371373	0.06269175	0.807133028
21.38461779	0.843754115	0.538518585	1.566805935	0.11716006	0.920479614
221.5883851	0.077864862	0.078262568	0.994918314	0.319775995	0.999579949
1575.159289	0.019239199	0.088237882	0.218037862	0.827399611	0.999579949
339.0473125	-0.016238402	0.125543909	-0.129344405	0.897085136	0.999579949
28.21305646	0.741399331	0.32073939	2.311531897	0.020803493	0.612244866
309.0661825	-0.012439482	0.08225202	-0.151236186	0.879789406	0.999579949
69.51001604	0.052424925	0.172233554	0.304382765	0.760836297	0.999579949
63.53895237	0.230618014	0.151442151	1.522812587	0.1278056	0.935409261
446.6973479	-0.073749722	0.10050653	-0.733780401	0.463082588	0.999579949
190.7199899	-0.067104541	0.109480329	-0.612936968	0.539918018	0.999579949
1732.556521	-0.094633331	0.097997348	-0.96567237	0.334208149	0.999579949
201.0648706	0.174568079	0.305914193	0.570643935	0.56824103	0.999579949
133.1502963	0.751413027	0.286934613	2.618760491	0.008824988	0.444638218
204.0076543	-0.005306916	0.103368263	-0.051339898	0.959054676	0.999579949
23.73803983	0.53889119	0.353048935	1.526392341	0.126912184	0.933836888
26.51283544	0.069241784	0.258951745	0.267392613	0.789166886	0.999579949
157.6851158	0.255432086	0.237221865	1.07676451	0.281585484	0.999579949
991.7956455	0.05705274	0.084505354	0.675137578	0.499588361	0.999579949

552.0392183	0.320013769	0.163188215	1.961010294	0.049877822	0.776589675
41.90723267	0.151902036	0.237917226	0.638465901	0.523170444	0.999579949
86.98425355	0.119192234	0.148950258	0.800215023	0.423586227	0.999579949
2942.024968	0.111290409	0.159901088	0.695995322	0.48643176	0.999579949
271.9575988	0.024472301	0.503773959	0.04857794	0.961255651	0.999579949
988.1519166	0.125451369	0.113748377	1.102884919	0.270077144	0.999579949
42.07936898	0.335465556	0.24424037	1.373505765	0.16959517	0.981013142
25.13942203	-0.173380236	0.319974961	-0.541855635	0.587917962	0.999579949
52.14018932	0.054919028	0.304221351	0.180523253	0.856741801	0.999579949
1205.657076	0.007555705	0.114289049	0.066110492	0.947289858	0.999579949
180.0266079	0.018694597	0.113560829	0.164621874	0.869241613	0.999579949
201.9054341	0.105303762	0.166392906	0.632862088	0.526823707	0.999579949
195.2676889	0.01369626	0.176778841	0.077476807	0.938244241	0.999579949
494.148904	0.204480992	0.078570313	2.602522288	0.00925408	0.457115261
342.5303337	-0.026498771	0.092199627	-0.287406487	0.773801095	0.999579949
2714.796171	0.159562499	0.129538583	1.231775858	0.218032824	0.999579949
362.8334637	0.020543212	0.08068675	0.254604534	0.799028557	0.999579949
1158.229632	0.046006828	0.108655026	0.42342108	0.671988068	0.999579949
1125.808317	0.003434446	0.067734528	0.050704503	0.959560988	0.999579949
24.4743761	0.279189586	0.408018282	0.684257539	0.493812554	0.999579949
176.0689947	-0.007698178	0.095096994	-0.080950807	0.935481074	0.999579949
208.3599754	0.160469306	0.10390506	1.544383942	0.122495348	0.92236461
508.6629603	0.120617235	0.07299038	1.652508653	0.098430903	0.89325335
495.5936497	0.092548049	0.084548306	1.094617426	0.273684278	0.999579949
35.32885276	0.152274067	0.7080233	0.215069288	0.829713298	0.999579949
321.1326614	-0.022784956	0.109676591	-0.207746755	0.835426709	0.999579949
392.3103304	0.15454229	0.154129353	1.002679155	0.31601569	0.999579949
26.01234855	0.16065122	0.342786353	0.468662823	0.639310664	0.999579949
323.4084385	0.031843493	0.067913199	0.468885191	0.639151702	0.999579949
314.9248873	0.004885247	0.105918909	0.046122519	0.963212597	0.999579949
1712.688425	-0.089131591	0.08091887	-1.101493273	0.270682031	0.999579949
1346.316669	0.13583391	0.121240048	1.120371625	0.262555431	0.999579949
257.9032033	0.165284077	0.174071241	0.949519724	0.342356345	0.999579949
51.20189632	0.657082472	0.606025275	1.084249287	0.278254284	0.999579949
101.3547403	-0.263773746	0.128113837	-2.058901307	0.039503694	0.734990441
1737.887041	-0.024197007	0.151484527	-0.159732529	0.873091775	0.999579949
685.2736299	0.092349671	0.104241092	0.885923861	0.375658557	0.999579949
548.1420275	0.042912169	0.06495213	0.660673769	0.508821549	0.999579949
469.6195036	-0.090575649	0.116527674	-0.77728874	0.436988437	0.999579949
407.0949294	-0.002760072	0.069498102	-0.039714345	0.968320865	0.999579949
42.02078665	-0.20010535	0.254152771	-0.787342782	0.431081232	0.999579949
108.1364179	0.393450612	0.251969672	1.561499882	0.118405852	0.921521033
138.8402833	0.188013421	0.265983886	0.706860193	0.479653364	0.999579949
188.7766216	0.227654563	0.242983657	0.936913068	0.348803277	0.999579949
201.8915699	-0.117284021	0.089052469	-1.317021547	0.187831402	0.983992835
312.0974385	-0.149754466	0.176207215	-0.849877041	0.395393451	0.999579949
2139.39609	-0.005225793	0.08298824	-0.062970285	0.949790167	0.999579949
9560.055865	-0.218152428	0.113582895	-1.920645081	0.054776468	0.785214091

691.7100033	0.055998283	0.064867319	0.863274202	0.387986725	0.999579949
185.964155	0.417331448	0.182409134	2.287886782	0.022144115	0.627784819
212.977634	0.36294268	0.259985489	1.396011295	0.162711094	0.978498637
1534.573544	-0.006147531	0.085445133	-0.071947115	0.942643995	0.999579949
3070.991176	-0.09495879	0.072477045	-1.310191255	0.190131143	0.984610142
33.24000785	0.268895063	0.232837389	1.154862046	0.248146924	0.999579949
1156.995132	0.164379579	0.059545977	2.760548859	0.005770433	0.372390484
645.3930708	0.037816475	0.070067264	0.539716732	0.589392399	0.999579949
10322.09804	0.134249656	0.148933148	0.901408836	0.367370985	0.999579949
651.6499561	0.062145671	0.106635561	0.582785609	0.560037634	0.999579949
161.6111978	0.126229084	0.177646155	0.710564685	0.477354034	0.999579949
50.49197173	-0.04699402	0.344634165	-0.136359145	0.89153737	0.999579949
134.1202286	-0.104015426	0.372431672	-0.279287273	0.780024373	0.999579949
129.943527	0.058016235	0.097801969	0.593201097	0.553046579	0.999579949
189.882809	-0.04423965	0.163563013	-0.270474657	0.786795111	0.999579949
218.9900389	0.316556921	0.154745664	2.045659398	0.040789893	0.743544853
1008.957642	0.166479577	0.124013454	1.342431582	0.1794561	0.983992835
2474.773991	-0.064396241	0.09804845	-0.656779794	0.511322518	0.999579949
62.84580458	0.208687901	0.217329936	0.960235415	0.336936747	0.999579949
375.9317444	-0.009240852	0.12696707	-0.072781488	0.941980002	0.999579949
178.4320375	0.172165851	0.138502268	1.243054385	0.213847793	0.999268361
1246.632337	-0.01124692	0.07383569	-0.152323626	0.878931691	0.999579949
7979.249309	-0.004219271	0.057957385	-0.072799544	0.941965634	0.999579949
121.7920664	-0.107695831	0.243706	-0.441908822	0.658555186	0.999579949
769.0627185	0.131331509	0.142958359	0.91866967	0.358268378	0.999579949
44.16980392	-0.427514319	0.184221748	-2.320650654	0.020305706	0.608885423
174.1398405	-0.556055042	0.505766908	-1.099429467	0.271580784	0.999579949
1232.605522	-0.020971062	0.115948623	-0.180865126	0.856473442	0.999579949
332.6889625	0.282237498	0.272545841	1.035559734	0.300407585	0.999579949
2274.116925	0.267226457	0.183393865	1.457117755	0.145083878	0.955875831
4789.053625	0.153454955	0.148024085	1.036689103	0.299880772	0.999579949
608.443425	0.004100254	0.090395154	0.045359228	0.963820979	0.999579949
45.07390594	-0.046406366	0.162436921	-0.285688533	0.775116688	0.999579949
91.42541008	-0.06678862	0.136330314	-0.489902931	0.624202589	0.999579949
216.4282538	0.049850621	0.116238053	0.428866625	0.66802029	0.999579949
1581.750842	-0.022213639	0.106411165	-0.208752894	0.834641147	0.999579949
408.1430657	-0.079777906	0.092058565	-0.866599496	0.38616149	0.999579949
118.9489585	0.209473653	0.187133594	1.119380274	0.262977943	0.999579949
455.5852402	0.090178568	0.079214241	1.138413579	0.254947827	0.999579949
1444.023699	-0.081686263	0.070138294	-1.164645699	0.244162437	0.999579949
162.5594298	-0.051566296	0.16336157	-0.315657445	0.752262531	0.999579949
30.54578419	0.181790562	0.285171091	0.63747893	0.52381293	0.999579949
638.6962943	0.004138422	0.08271847	0.050030207	0.960098317	0.999579949
791.4614477	-0.016572021	0.138236225	-0.1198819	0.904576703	0.999579949
355.7233447	0.042760009	0.078677607	0.543483857	0.586796702	0.999579949
98.3453602	0.080351311	0.173496615	0.463128983	0.643271916	0.999579949
182.0117393	0.274636147	0.23288494	1.179278259	0.238287393	0.999579949
22.28009924	-0.000656705	0.264657212	-0.00248134	0.998020179	0.999579949

79.09943737	-0.153066283	0.141874146	-1.078887785	0.280637755	0.999579949
1215.638317	-0.103843921	0.055943187	-1.85623892	0.063419511	0.809353757
38.96912858	0.142323761	0.150499244	0.945677577	0.344313062	0.999579949
479.7459403	-0.045649951	0.120539373	-0.378714026	0.704900237	0.999579949
145.7693501	0.004593809	0.117366279	0.039140792	0.968778138	0.999579949
583.0977302	-0.103264233	0.145391348	-0.710250198	0.477548997	0.999579949
4130.943503	-0.031651686	0.060172208	-0.526018347	0.59887546	0.999579949
131.2687838	0.242350405	0.333242392	0.727249625	0.467073051	0.999579949
50.57352367	-0.049959397	0.21195492	-0.235707655	0.813659529	0.999579949
25.74629729	-0.507968355	0.207477444	-2.448306407	0.014352954	0.533303265
159.7908395	-0.020110005	0.10935339	-0.183899234	0.854092506	0.999579949
62.89197657	0.177284606	0.181518864	0.976673177	0.328730979	0.999579949
39.72457561	0.254448902	0.232188335	1.095872896	0.273134402	0.999579949
8974.456807	-0.087973108	0.093800598	-0.937873635	0.348309354	0.999579949
474.5956395	-0.178747614	0.086267569	-2.072014047	0.038264131	0.72680496
530.6256308	0.147464215	0.122406945	1.204704642	0.228317343	0.999579949
194.3017404	-0.089816111	0.107079125	-0.838782635	0.401591297	0.999579949
200.1631319	-0.121714844	0.16397346	-0.742283811	0.457915398	0.999579949
168.1967226	0.207064182	0.128102242	1.616397791	0.106008336	0.897366261
146.9088173	-0.022599502	0.146924169	-0.153817458	0.877753668	0.999579949
481.6120685	0.064612919	0.099734847	0.647846977	0.517083928	0.999579949
26.72584488	0.512778676	0.306826078	1.671235639	0.094675143	0.880418343
377.9151307	0.097272353	0.111832869	0.869801104	0.384409108	0.999579949
1690.504921	0.046936095	0.053914404	0.870566898	0.383990677	0.999579949
234.5708811	1.173331752	0.349146385	3.360572533	0.000777811	0.137990252
453.6902285	0.065616095	0.061573477	1.065655186	0.286579543	0.999579949
117.7804868	0.336428857	0.152563977	2.205165752	0.027442465	0.660154442
35.90828036	0.262433285	0.202508204	1.295914339	0.195004999	0.988443849
1087.73805	0.13201657	0.081278098	1.624257612	0.10432084	0.895628172
105.7917587	0.031534488	0.132187138	0.238559428	0.811447229	0.999579949
1630.665125	-0.045416542	0.102213956	-0.444328188	0.656805324	0.999579949
313.0221722	-0.03631932	0.081383177	-0.446275521	0.655398236	0.999579949
342.7019712	0.080496203	0.121193259	0.664197036	0.50656421	0.999579949
375.6838081	0.074598104	0.286894481	0.260019305	0.794848882	0.999579949
11899.00148	-0.046761801	0.10306659	-0.453704743	0.650041353	0.999579949
1115.693969	0.009846831	0.060930607	0.161607297	0.87161511	0.999579949
29.50623989	0.17259038	0.245620592	0.702670645	0.482261026	0.999579949
167.0946394	-0.031692491	0.146171413	-0.216817302	0.828350727	0.999579949
146.1925571	0.066689332	0.093769592	0.711204247	0.476957677	0.999579949
680.4795615	-0.222717134	0.152768271	-1.457875601	0.144874835	0.955875831
1158.597193	-0.036641119	0.064494763	-0.568125491	0.56994976	0.999579949
228.1290882	0.019035888	0.366391767	0.051955009	0.958564542	0.999579949
1265.148091	0.00312396	0.091005223	0.034327255	0.972616191	0.999579949
226.423423	0.190025596	0.149889213	1.267773665	0.204878789	0.994814558
196.2857959	0.401288245	0.117700984	3.409387333	0.00065109	0.128142583
1344.056527	-0.140577322	0.119801254	-1.173421119	0.240626972	0.999579949
1192.365672	0.104062546	0.112285184	0.926770053	0.354045937	0.999579949
2596.083255	-0.079257402	0.084787636	-0.934775466	0.349904026	0.999579949

444.9857817	0.216264353	0.116107321	1.86262461	0.062515099	0.807133028
1990.459977	-0.092272555	0.118780706	-0.776831171	0.437258383	0.999579949
1164.922378	0.124735545	0.087253695	1.429573223	0.152839545	0.972669728
566.2561965	-0.023238037	0.088667031	-0.262082049	0.793258186	0.999579949
518.7462615	0.254200539	0.127584964	1.992402008	0.046326967	0.763868825
576.6373035	0.052648113	0.075981081	0.692910811	0.488365525	0.999579949
292.0874022	0.10408933	0.128662902	0.8090081	0.418510487	0.999579949
127.5377536	-0.075031958	0.117768787	-0.637112434	0.52405161	0.999579949
3139.928135	0.237058201	0.158664618	1.494083577	0.135153776	0.945791454
74.85924747	0.094305143	0.139040869	0.678254846	0.497610123	0.999579949
36.06731227	-0.002469579	0.189873265	-0.013006461	0.989622638	0.999579949
283.4773162	-0.060216472	0.093722803	-0.642495424	0.520551559	0.999579949
331.8697549	-0.110111862	0.1127399	-0.976689375	0.328722957	0.999579949
20.48157474	0.419010199	0.353344314	1.185841068	0.23568507	0.999579949
215.7376348	0.161747284	0.110271167	1.466813932	0.142426679	0.951955082
165.1953926	0.053970087	0.13338745	0.404611434	0.685763152	0.999579949
674.0149971	-0.006989273	0.072027309	-0.09703643	0.922697464	0.999579949
1041.434289	0.158700252	0.119117546	1.332299542	0.182761778	0.983992835
55.57912526	0.407586089	0.723829923	0.563096491	0.573369181	0.999579949
92.23475556	0.175157683	0.131894609	1.328012454	0.184173993	0.983992835
44.07541875	-0.082842307	0.17160596	-0.48274726	0.629275203	0.999579949
657.3662077	-0.043050869	0.065896564	-0.653309764	0.513556599	0.999579949
370.0710797	0.147525809	0.131828147	1.119076705	0.263107417	0.999579949
51.90092076	0.027555629	0.251238666	0.109679093	0.912663882	0.999579949
132.4589626	0.095024263	0.162579859	0.58447746	0.558899126	0.999579949
1471.015004	-0.061159224	0.096451556	-0.634092662	0.526020351	0.999579949
42.94130796	0.088687664	0.264858954	0.334848653	0.737739257	0.999579949
66.28725756	-0.11206397	0.176584972	-0.634617822	0.525677702	0.999579949
70.38522897	0.017127538	0.195537997	0.087591867	0.930201067	0.999579949
897.4821311	-0.15041987	0.159170352	-0.945024426	0.344646406	0.999579949
52.96049665	0.178998935	0.36430361	0.491345487	0.623182113	0.999579949
640.7080339	0.083840977	0.068248879	1.2284594	0.219274561	0.999579949
162.3669079	0.228803004	0.098629911	2.319813552	0.020350966	0.608885423
598.228644	-0.014651273	0.100436521	-0.145875949	0.884019318	0.999579949
35.91167448	-0.037662213	0.187694807	-0.200656662	0.840967049	0.999579949
1461.94741	-0.065180778	0.066077662	-0.986426822	0.323923708	0.999579949
48.6797871	-0.058149967	0.260237387	-0.223449704	0.823185524	0.999579949
94.76897155	0.481173977	0.141504188	3.400422149	0.000672819	0.128481718
739.2019423	0.030000302	0.143417483	0.209181625	0.834306456	0.999579949
1072.472083	-0.095914049	0.096359283	-0.99537944	0.319551754	0.999579949
500.7790809	-0.103696701	0.197275869	-0.525643112	0.599136197	0.999579949
253.8448942	0.069194314	0.084246078	0.821335737	0.411455057	0.999579949
37.39219773	0.138310998	0.164982245	0.838338686	0.401840514	0.999579949
445.5905738	0.022770283	0.068491939	0.332452012	0.73954797	0.999579949
31.73417295	-0.04571667	0.307276484	-0.148780243	0.881727041	0.999579949
437.304212	0.037372519	0.071594165	0.522005091	0.601666786	0.999579949
86.08943429	-0.066064895	0.178632353	-0.369837234	0.711503771	0.999579949
337.6458531	0.053097718	0.080287132	0.661347796	0.508389296	0.999579949

582.3793805	0.054067168	0.078477785	0.688948704	0.490855548	0.999579949
267.4032241	-0.154274328	0.147891809	-1.043156677	0.296875735	0.999579949
41.91228788	-0.048265322	0.157772121	-0.305917939	0.759667127	0.999579949
163.1766577	-0.116433091	0.124001115	-0.938968093	0.347747126	0.999579949
654.3307422	0.043635194	0.101485785	0.429963606	0.667222115	0.999579949
410.8280534	-0.003469563	0.094379576	-0.036761794	0.970674937	0.999579949
774.1981257	-0.085577948	0.09453176	-0.905282501	0.365315744	0.999579949
2577.572525	-0.087432855	0.091946379	-0.950911343	0.341649383	0.999579949
109.690065	-0.180078037	0.109939623	-1.637972113	0.101427511	0.895628172
186.6758442	-0.098762132	0.117235417	-0.842425736	0.399549692	0.999579949
282.1502913	-0.085870895	0.065090825	-1.319247301	0.187086449	0.983992835
82.43370164	0.085449026	0.116120377	0.735865904	0.461812309	0.999579949
314.2802309	0.092270526	0.106069195	0.869908795	0.384350249	0.999579949
95.48001972	0.178512043	0.196433811	0.908764346	0.36347453	0.999579949
205.2493226	0.04693901	0.123662696	0.379572912	0.704262471	0.999579949
53.56502907	0.252306417	0.169973169	1.484389673	0.137705573	0.945791454
1224.872551	0.03145374	0.06701882	0.469326971	0.63883594	0.999579949
32.32343891	-0.113947114	0.208774303	-0.545790893	0.585209684	0.999579949
48.59699748	-0.20311185	0.175902831	-1.1546821	0.248220632	0.999579949
76.83076888	-0.098298317	0.145479632	-0.675684395	0.499241046	0.999579949
52.08714385	0.102872562	0.185645194	0.554135333	0.579486231	0.999579949
581.4636385	0.028524519	0.101979949	0.279707132	0.779702206	0.999579949
152.0089935	-0.066251935	0.10161569	-0.651985289	0.514410663	0.999579949
3711.740526	0.17760167	0.128714572	1.37981013	0.167645113	0.980087289
77.05280081	0.42222929	0.157106418	2.68753686	0.007198116	0.408412008
206.1152679	-0.022304462	0.114983174	-0.193980225	0.846191366	0.999579949
429.8869745	0.107970227	0.095961311	1.12514331	0.260528311	0.999579949
53.12542495	0.742924781	0.439866602	1.688977469	0.091223748	0.879154037
190.5452607	0.001232458	0.103278191	0.011933383	0.990478764	0.999579949
52.47493379	0.081104932	0.174410307	0.465023732	0.641914462	0.999579949
78.62348963	0.155408367	0.141359473	1.099384173	0.271600531	0.999579949
194.1059372	-0.037413654	0.098327619	-0.380499949	0.703574334	0.999579949
962.1545308	-0.000855292	0.092996675	-0.009197021	0.992661943	0.999579949
530.8220875	0.037961944	0.054638668	0.694781641	0.487192155	0.999579949
317.772983	0.031397322	0.092250939	0.340346911	0.733595293	0.999579949
2936.200387	0.018999523	0.080035044	0.237390045	0.812354212	0.999579949
103.0341153	0.018263698	0.099463864	0.183621441	0.854310442	0.999579949
147.1932639	0.014779428	0.20492516	0.072121101	0.942505534	0.999579949
121.8626391	-0.27876685	0.181832657	-1.533095624	0.125252296	0.927500246
305.439205	0.167079793	0.09806828	1.703708811	0.088435501	0.870840008
144.5310267	0.058330831	0.145141326	0.401889887	0.687765066	0.999579949
20.80383605	0.345699309	0.258162528	1.339076247	0.180545852	0.983992835
753.7372662	-0.353299169	0.225014485	-1.570117452	0.116387789	0.918907698
211.922918	-0.006833757	0.188268245	-0.036297979	0.971044761	0.999579949
233.1535065	0.18968655	0.12574399	1.508513837	0.131423068	0.938438189
88.53212382	-0.112795827	0.130132481	-0.866776886	0.386064269	0.999579949
22.82418825	0.286201506	0.380593621	0.751987135	0.452058796	0.999579949
204.501933	-0.144053795	0.130389689	-1.10479438	0.269248696	0.999579949

151.6400135	-0.014581202	0.108177457	-0.134789656	0.892778185	0.999579949
2513.258679	-0.045976475	0.096657972	-0.475661488	0.634315562	0.999579949
659.2409349	-0.120271551	0.132391105	-0.908456438	0.363637119	0.999579949
129.8347205	0.000447567	0.13906717	0.00321835	0.997432132	0.999579949
264.8535385	0.060457598	0.118005945	0.512326714	0.608422372	0.999579949
56.8938976	0.377219749	0.196729765	1.917451323	0.055180619	0.785214091
149.8730736	-0.207721075	0.210479641	-0.986893903	0.323694653	0.999579949
26.9415119	0.115885204	0.269057259	0.430708333	0.666680459	0.999579949
152.0885775	-0.381470284	0.284300358	-1.341786156	0.179665342	0.983992835
6823.134063	-0.100746343	0.149596573	-0.673453547	0.500658792	0.999579949
856.2839386	-0.063434103	0.135844058	-0.466962658	0.640526595	0.999579949
5026.287197	-0.068837733	0.177049274	-0.38880551	0.697420025	0.999579949
263.7952357	-0.383972268	0.329094552	-1.166753646	0.243309871	0.999579949
61.06682763	0.096512711	0.160819516	0.600130587	0.54841921	0.999579949
2803.711568	0.002857495	0.152272218	0.018765698	0.985028018	0.999579949
138.0310241	-0.016981833	0.118493939	-0.14331394	0.886042247	0.999579949
348.1148629	-0.081039477	0.121754521	-0.665597273	0.50566855	0.999579949
81.19352153	0.146141955	0.134906743	1.083281318	0.278683575	0.999579949
286.1476671	0.145707071	0.149395922	0.97530822	0.329407394	0.999579949
50.28212961	0.281643621	0.309094024	0.911190768	0.362194867	0.999579949
47.85360563	-0.234904726	0.233405876	-1.006421648	0.314212785	0.999579949
178.8934096	0.21097807	0.112979865	1.867395303	0.061846405	0.805306145
177.3775775	0.142412667	0.135071063	1.054353635	0.291721041	0.999579949
47.30236771	-0.073604013	0.272933011	-0.269677944	0.78740803	0.999579949
104.37319	0.20337007	0.106468224	1.910148051	0.056114154	0.785214091
208.5851454	0.137900633	0.106739567	1.29193547	0.196379493	0.991421279
863.6152278	0.029035277	0.111791017	0.259728173	0.795073459	0.999579949
306.4079859	0.123878388	0.107094292	1.1567226	0.247385713	0.999579949
1169.484662	0.048626649	0.099930028	0.486606976	0.626536879	0.999579949
255.1940086	0.251133871	0.23477812	1.069664718	0.284770253	0.999579949
138.4584812	0.061728711	0.115918812	0.532516768	0.594368131	0.999579949
24.52183232	0.03996111	0.210585585	0.18976185	0.849495752	0.999579949
667.7437578	0.12554955	0.123551679	1.016170327	0.309548284	0.999579949
1375.858155	0.05115728	0.098997174	0.516754957	0.605327227	0.999579949
857.083305	0.050364953	0.079509598	0.633444938	0.526443127	0.999579949
85.59607064	0.038230687	0.171072208	0.223476904	0.823164356	0.999579949
197.923268	0.02879791	0.092407132	0.311641642	0.755312883	0.999579949
168.6152753	-0.050751811	0.102591106	-0.494699908	0.620811967	0.999579949
368.0661997	0.045902102	0.113241116	0.405348371	0.685221456	0.999579949
281.4009144	0.117281191	0.158020241	0.742190938	0.457971658	0.999579949
966.4706257	0.120012969	0.115871234	1.035744283	0.300321457	0.999579949
135.5570518	-0.041010761	0.138267405	-0.296604691	0.766768329	0.999579949
96.48688092	-0.20788031	0.189500361	-1.09699163	0.272645051	0.999579949
346.133143	-0.287192577	0.128641622	-2.232501206	0.025581856	0.647046292
594.7143433	0.00904282	0.086921498	0.104034333	0.917142102	0.999579949
24.22031612	0.22666592	0.244888589	0.92558792	0.354660174	0.999579949
140.1154096	0.107633484	0.108098961	0.995693973	0.319398859	0.999579949
403.9518514	-0.078034668	0.08946595	-0.872227574	0.38308424	0.999579949

1999.340933	0.040511633	0.102320002	0.395930726	0.692156133	0.999579949
140.2120466	-0.070824044	0.114486811	-0.618621856	0.5361655	0.999579949
789.2085452	0.046782113	0.101541157	0.460720699	0.644999002	0.999579949
1418.81403	0.000360617	0.13054155	0.00276247	0.997795871	0.999579949
1363.428318	-0.057251384	0.094421707	-0.606337096	0.54429093	0.999579949
439.0198308	-0.010510165	0.108979783	-0.09644142	0.923169997	0.999579949
2070.114901	-0.158929654	0.110827155	-1.434031709	0.151563214	0.970559353
623.7449743	0.101882551	0.062641374	1.626441822	0.1038557	0.895628172
625.7190867	0.051270184	0.101835683	0.503459916	0.614640956	0.999579949
237.5138069	-0.059261241	0.135051661	-0.438804235	0.660803391	0.999579949
151.9045889	-0.004533252	0.092517288	-0.048998969	0.960920117	0.999579949
2975.031986	-0.072273631	0.042527693	-1.699448642	0.089234684	0.870840008
79.91033735	0.110354457	0.160974405	0.685540396	0.493002979	0.999579949
46.92673342	-0.243392326	0.167956554	-1.449138601	0.14729888	0.960339903
103.036839	-0.048850379	0.134224036	-0.363946581	0.7158979	0.999579949
157.3942489	0.011541371	0.286360854	0.040303593	0.967851089	0.999579949
65.05884777	-0.301886325	0.176286191	-1.712478571	0.08680851	0.867486202
261.9967106	0.074585373	0.104659209	0.712649879	0.476062438	0.999579949
254.2249825	-0.249090243	0.198616102	-1.254129149	0.209795067	0.996447459
1520.834633	-0.036063534	0.084816323	-0.425195671	0.670694039	0.999579949
383.3230643	0.051873646	0.13504622	0.384117719	0.7008912	0.999579949
318.0370482	0.017388073	0.113992983	0.15253635	0.878763923	0.999579949
1212.563601	-0.02064962	0.080086287	-0.257842147	0.796528735	0.999579949
855.4776941	0.083761794	0.095169608	0.88013175	0.378787941	0.999579949
25.11059618	0.866402868	0.478218293	1.811730919	0.070027784	0.834620594
4500.141526	0.179017212	0.102760717	1.742078272	0.081494749	0.85448976
2558.687098	0.223000024	0.120123088	1.856429336	0.063392387	0.809353757
257.9643786	0.05572514	0.072013119	0.773819275	0.439037672	0.999579949
39.69604035	-0.701328491	0.334433746	-2.097062569	0.035988037	0.714992608
180.884386	-0.041189306	0.101022357	-0.407724662	0.683475829	0.999579949
39.83386951	1.261141663	0.495677438	2.544278932	0.01095036	0.487387763
692.726608	-0.035908472	0.116657896	-0.307810042	0.75822688	0.999579949
439.1753791	-0.052767045	0.069603733	-0.758106548	0.4483872	0.999579949
135.1520291	-0.019003791	0.103418046	-0.183757013	0.854204081	0.999579949
265.059896	0.04925443	0.115793691	0.425363676	0.670571582	0.999579949
155.4032239	0.366118753	0.253557333	1.443928867	0.148758985	0.962301096
223.2915732	-0.044767155	0.116065203	-0.385706944	0.69971372	0.999579949
36.09146989	-0.269470663	0.262454997	-1.026730933	0.30454718	0.999579949
655.9889543	0.013763188	0.066905738	0.205710123	0.837017351	0.999579949
135.6302515	-0.061949127	0.132238695	-0.468464449	0.639452489	0.999579949
306.804553	-0.015804217	0.087833291	-0.179934241	0.857204193	0.999579949
391.1337167	0.10311188	0.113412899	0.909172422	0.363259119	0.999579949
3457.634846	0.029815283	0.104752513	0.284625949	0.775930729	0.999579949
104.1338251	0.054936201	0.170641108	0.321940018	0.747498136	0.999579949
350.2901552	0.018947929	0.122159469	0.155108148	0.876736059	0.999579949
662.4845267	0.016463893	0.072676099	0.226537927	0.820783064	0.999579949
509.4176544	0.104348894	0.098456915	1.059843226	0.289215928	0.999579949
549.8997946	0.024002729	0.110162963	0.217883839	0.827519619	0.999579949

170.0337985	-0.087141249	0.101308022	-0.860161386	0.389700087	0.999579949
576.599723	0.134108627	0.092054765	1.456835263	0.145161859	0.955875831
703.1025599	-0.011408213	0.07263941	-0.157052668	0.87520334	0.999579949
42.33221973	0.020136223	0.193047084	0.104307317	0.916925472	0.999579949
714.6854428	0.032239084	0.082441205	0.391055469	0.695756235	0.999579949
21.90471243	0.0120306	0.228304061	0.052695513	0.957974514	0.999579949
296.717565	0.08643944	0.068322381	1.265170202	0.205810322	0.994814558
189.615804	-0.055252434	0.100670064	-0.548846713	0.58311065	0.999579949
150.045992	0.072462056	0.108728837	0.666447451	0.505125142	0.999579949
313.262511	0.055114562	0.109996856	0.501055793	0.616331857	0.999579949
509.5173902	0.265999981	0.208237061	1.277390199	0.201464523	0.993208272
40.91231902	-0.027807104	0.159839219	-0.173969219	0.861889653	0.999579949
307.9278412	-0.013893732	0.088317029	-0.157316565	0.874995366	0.999579949
588.1336682	-0.018038224	0.137656825	-0.131037627	0.895745542	0.999579949
5776.716828	0.107578737	0.134938978	0.797239895	0.42531172	0.999579949
233.2825935	0.013008146	0.090032079	0.144483461	0.885118716	0.999579949
249.5906752	0.01178895	0.071227301	0.165511673	0.868541263	0.999579949
443.9839864	-0.069779695	0.134592424	-0.518451879	0.604143026	0.999579949
313.155405	0.019452063	0.077907684	0.249680932	0.802834105	0.999579949
411.6970357	0.115850365	0.094101621	1.231119756	0.218278077	0.999579949
2448.708513	0.040109817	0.098228393	0.40833221	0.683029795	0.999579949
871.0332358	0.038511924	0.081283968	0.473794836	0.635646217	0.999579949
302.1870834	0.067953085	0.086097496	0.789257393	0.429961584	0.999579949
746.9338838	-0.062526786	0.133557324	-0.468164414	0.639667019	0.999579949
113.7196015	0.402239119	0.270470597	1.487182426	0.136966636	0.945791454
130.4997646	-0.235314753	0.108241309	-2.173982883	0.029706421	0.672989344
61.8814861	1.045810286	0.440659282	2.373285504	0.01763063	0.573039104
95.10809784	0.439317247	0.27029507	1.625324673	0.104093397	0.895628172
886.862834	0.022305129	0.091134377	0.244749892	0.806650094	0.999579949
410.1515148	0.150714436	0.16752659	0.899644861	0.368309275	0.999579949
171.9370129	0.022753784	0.110423462	0.206059326	0.83674457	0.999579949
99.98615452	-0.431241126	0.471885332	-0.913868469	0.360785964	0.999579949
48.34941192	0.042524064	0.169415142	0.251005094	0.801810171	0.999579949
336.6918228	-0.040455005	0.111874913	-0.361609265	0.71764404	0.999579949
46.97308026	-0.131672086	0.293241907	-0.449022063	0.653415741	0.999579949
422.126107	0.017757762	0.102396708	0.173421217	0.862320349	0.999579949
593.378701	0.02194429	0.071863588	0.305360347	0.760091719	0.999579949
113.5310048	0.150475661	0.104385406	1.441539266	0.149432393	0.963629286
62.43677629	0.192055694	0.300483776	0.639154953	0.522722133	0.999579949
1411.92063	0.051825338	0.068192282	0.759988323	0.447261565	0.999579949
84.33245495	0.135542361	0.232477913	0.58303328	0.559870897	0.999579949
287.905528	0.154798369	0.115094625	1.344966103	0.178636181	0.983992835
65.91329384	-0.042463391	0.12414878	-0.342036315	0.732323564	0.999579949
805.8511195	-0.00233883	0.099609636	-0.023479953	0.981267429	0.999579949
177.8944901	-0.032306132	0.205249535	-0.157399294	0.87493017	0.999579949
238.1445036	0.054845926	0.108674762	0.504679513	0.613783953	0.999579949
445.6939071	-0.09514601	0.067448904	-1.410638334	0.158351283	0.977304288
81.25735222	-0.055854614	0.138187724	-0.404193751	0.686070249	0.999579949

318.8824409	-0.000807785	0.083332266	-0.009693542	0.992265794	0.999579949
763.2397711	-0.127343002	0.1498906	-0.84957297	0.395562545	0.999579949
146.1856883	-0.287985281	0.261123117	-1.102871642	0.270082911	0.999579949
866.1609601	-0.02780504	0.105339619	-0.263956153	0.791813706	0.999579949
392.5385353	-0.108534518	0.162655176	-0.667267532	0.504601261	0.999579949
86.03910947	0.365158241	0.180312847	2.025137129	0.042853285	0.750791966
742.7273151	0.007274958	0.085743076	0.084846006	0.932383818	0.999579949
400.9458586	-0.206581549	0.090377662	-2.28575895	0.022268364	0.627784819
360.0703375	-0.041469119	0.07635174	-0.543132598	0.58703851	0.999579949
2133.247574	0.040579857	0.073050888	0.555501208	0.578551884	0.999579949
126.5295633	0.086815774	0.128319929	0.676557219	0.49868693	0.999579949
2300.029938	0.189496761	0.188264355	1.006546146	0.314152925	0.999579949
237.3349845	-0.051777373	0.153525277	-0.337256338	0.735923671	0.999579949
22.42115202	-0.003064352	0.275118635	-0.011138295	0.99111311	0.999579949
771.9492179	0.212765812	0.122864873	1.731705788	0.08332596	0.859920327
327.3970827	0.407495525	0.13942431	2.92270068	0.003470099	0.310136233
211.6912365	0.309673938	0.123661792	2.504200633	0.012272842	0.503708792
63.12663391	0.169277357	0.283169749	0.597794638	0.549976969	0.999579949
152.6727569	-0.173371831	0.086775924	-1.997925503	0.045724737	0.76284608
34.89715269	0.032751486	0.22385047	0.146309659	0.883676941	0.999579949
67.76251046	0.115740257	0.131697004	0.878837431	0.379489428	0.999579949
164.1784037	0.02417002	0.08159686	0.296212622	0.76706771	0.999579949
73.74280291	0.013352749	0.109689214	0.121732564	0.903110822	0.999579949
53.56538632	0.119025252	0.354723594	0.335543658	0.737215018	0.999579949
978.6869982	0.088500392	0.097457416	0.908092946	0.363829117	0.999579949
117.5417005	-0.080424901	0.114351528	-0.703312871	0.481860791	0.999579949
23.40765001	-0.100491538	0.251889821	-0.398950373	0.689929771	0.999579949
790.4740528	0.028490768	0.117695895	0.242071039	0.808725119	0.999579949
1061.860197	0.021473611	0.060840946	0.352946697	0.724128402	0.999579949
569.7241585	0.063705529	0.1384665	0.460079006	0.645459513	0.999579949
312.89907	-0.093099449	0.074018123	-1.257792626	0.208466763	0.995521169
642.6240528	-0.015720842	0.08860532	-0.177425482	0.859174193	0.999579949
62.74048051	0.061043282	0.204955751	0.297836397	0.765828033	0.999579949
125.9090309	0.128096244	0.201993511	0.634160192	0.525976284	0.999579949
901.0590212	0.000479593	0.079156489	0.006058793	0.995165812	0.999579949
86.706527	-0.154808297	0.134045236	-1.154895924	0.248133049	0.999579949
482.9587984	0.004332711	0.122649579	0.035325932	0.971819846	0.999579949
570.0242562	0.007129989	0.10623755	0.067113643	0.946491233	0.999579949
318.2948202	-0.055422954	0.101252397	-0.547374243	0.584121647	0.999579949
115.560884	0.039379485	0.248632258	0.158384456	0.874153861	0.999579949
134.4289676	-0.06089211	0.096119335	-0.633505311	0.526403714	0.999579949
69.42585415	-0.064607482	0.201318328	-0.320922006	0.748269497	0.999579949
47.71497534	1.426231179	0.598433076	2.383275987	0.017159324	0.570162425
34.85670018	-0.152084844	0.170890098	-0.889957031	0.373488959	0.999579949
287.4093435	-0.047507109	0.113255977	-0.419466684	0.674875098	0.999579949
903.0200577	0.178375603	0.142214751	1.254269351	0.20974412	0.996447459
840.9838977	0.086990083	0.067682136	1.285273905	0.198696586	0.992481822
55.40475093	0.120605971	0.187926765	0.641771124	0.521021801	0.999579949

73.83267951	-0.145027738	0.184847371	-0.784581015	0.432699265	0.999579949
263.2937487	-0.285404741	0.323401266	-0.882509658	0.377501259	0.999579949
33.76354085	-0.267407952	0.198378027	-1.347971628	0.177667508	0.983992835
30.76318654	-0.232742952	0.253968656	-0.916423924	0.359444592	0.999579949
131.1754427	-0.031796568	0.108824426	-0.292182268	0.770147268	0.999579949
50.70517363	0.18517959	0.24559223	0.754012413	0.450841768	0.999579949
158.2164762	0.104836832	0.110231011	0.951064779	0.341571493	0.999579949
518.6792608	0.01417738	0.087195294	0.162593402	0.870838582	0.999579949
1168.232994	0.08722581	0.111478595	0.782444468	0.433953409	0.999579949
399.2668655	0.032474658	0.127854346	0.253997299	0.799497645	0.999579949
530.0304972	-0.026113506	0.080629361	-0.323870935	0.74603575	0.999579949
246.0421266	-0.142540254	0.143655661	-0.992235553	0.321082633	0.999579949
727.9391534	-0.198981632	0.100614768	-1.977658307	0.047967267	0.770705547
275.8842809	-0.016539978	0.149054249	-0.110966163	0.911643179	0.999579949
152.6355567	-0.2119415	0.253703086	-0.835391888	0.403497096	0.999579949
105.9351474	0.120469202	0.184616264	0.652538402	0.514053908	0.999579949
261.4065323	0.064735964	0.09669949	0.669455075	0.503205229	0.999579949
48.75641026	0.110897964	0.196623279	0.564012385	0.572745703	0.999579949
1298.374778	0.613795885	0.234203827	2.620776499	0.008772976	0.444337558
323.3186735	0.133744663	0.199974105	0.668809909	0.503616745	0.999579949
137.5871288	-0.146237011	0.149292007	-0.979536775	0.327314827	0.999579949
170.9245006	-0.311599785	0.24910416	-1.250881495	0.210977716	0.996802442
56.1187643	-0.093071308	0.16685415	-0.557800376	0.576980706	0.999579949
545.8950519	0.031815231	0.06897748	0.46124085	0.644625816	0.999579949
36.29286691	-0.737584428	0.519313718	-1.420306076	0.155518593	0.976030424
1546.278656	-0.009537787	0.055504414	-0.171838348	0.863564617	0.999579949
480.3876529	0.09706698	0.098349592	0.986958641	0.323662914	0.999579949
154.6478829	0.221724846	0.136050584	1.629723596	0.103159928	0.895628172
501.2112072	-0.061517632	0.067817459	-0.90710612	0.364350682	0.999579949
616.1746193	-0.021142177	0.091893395	-0.230072874	0.818035143	0.999579949
786.9367006	0.036679214	0.076622912	0.478697724	0.632153685	0.999579949
204.182745	0.05635707	0.127662333	0.441454174	0.65888423	0.999579949
22.49460867	-0.34482192	0.35456218	-0.972528767	0.330787561	0.999579949
82.77609005	-0.339705658	0.419981378	-0.808858859	0.418596336	0.999579949
769.4771946	-0.149040006	0.073454633	-2.029007556	0.042457521	0.74969123
44.90766685	0.912605418	0.365986063	2.493552377	0.012647192	0.508958578
2865.130763	0.055337553	0.101781225	0.543691168	0.586654011	0.999579949
329.0364552	-0.02422808	0.095422389	-0.253903519	0.799570096	0.999579949
1389.852338	-0.026640843	0.11034693	-0.241428041	0.809223382	0.999579949
40.12828154	0.07006647	0.240492784	0.291345414	0.770787158	0.999579949
1307.836582	0.122007136	0.110351824	1.105619572	0.268891215	0.999579949
393.7250189	0.150307845	0.139837585	1.074874435	0.282430949	0.999579949
345.66238	-0.012612497	0.088389298	-0.142692586	0.886532973	0.999579949
59.65356259	-0.087529575	0.148637202	-0.588880666	0.555941328	0.999579949
185.9391114	-0.027471028	0.082690397	-0.332215459	0.739726572	0.999579949
224.7426079	-0.163368997	0.296144771	-0.55165248	0.581186473	0.999579949
135.2966252	0.026048825	0.117872009	0.220992458	0.825098306	0.999579949
59.76452217	0.4066046	0.222277432	1.829266235	0.067359734	0.826962196

2307.366964	0.000631217	0.096677208	0.006529114	0.994790557	0.999579949
1230.610451	-0.065208131	0.145944737	-0.446800154	0.65501936	0.999579949
1466.932906	0.063691931	0.107294452	0.593618117	0.552767561	0.999579949
714.9048628	-0.027913721	0.266340208	-0.104804757	0.916530736	0.999579949
204.692865	-0.071813893	0.120758692	-0.594689225	0.552051226	0.999579949
984.4334992	-0.036553487	0.122587987	-0.298181636	0.765564537	0.999579949
250.2137938	-0.02360157	0.083596285	-0.282327975	0.777692037	0.999579949
65.09735146	0.228001062	0.250185646	0.911327511	0.362122835	0.999579949
173.8905286	0.0862072	0.089715481	0.960895487	0.33660472	0.999579949
361.4677617	0.008546825	0.076032446	0.112410229	0.910498145	0.999579949
148.2593047	-0.186947965	0.147533126	-1.267159246	0.205098355	0.994814558
174.2972806	-0.007190513	0.080042817	-0.089833331	0.928419661	0.999579949
870.742143	-0.134688957	0.074562678	-1.806385736	0.070858108	0.834620594
127.9162526	-0.180456359	0.23420282	-0.770513179	0.44099554	0.999579949
1207.282535	-0.311210567	0.112935074	-2.755659119	0.005857401	0.372390484
41.89514308	0.28937936	0.213079413	1.358082211	0.174437599	0.983992835
252.3965861	0.020544799	0.102942816	0.199574868	0.841813085	0.999579949
731.4660993	-0.437686128	0.120109684	-3.644053603	0.000268377	0.075121841
293.0160508	-0.025583526	0.075377475	-0.339405448	0.734304315	0.999579949
115.8363143	0.128712532	0.163159761	0.788874236	0.430185515	0.999579949
38.21030383	0.188583486	0.24726867	0.762666318	0.445662423	0.999579949
133.3983942	0.083339708	0.110803855	0.752137261	0.451968518	0.999579949
642.5262944	-0.024752495	0.160421936	-0.154296198	0.877376195	0.999579949
1183.308007	-0.029725888	0.104051583	-0.285684149	0.775120046	0.999579949
688.1069793	-0.005185961	0.090696438	-0.057179323	0.954402349	0.999579949
407.1329629	-0.086881603	0.057080679	-1.522084243	0.127987974	0.935700262
283.8937896	-0.033022907	0.102375017	-0.322568023	0.747022414	0.999579949
736.2156517	0.032495373	0.071494739	0.454514192	0.64945878	0.999579949
709.5692063	0.057602084	0.154021778	0.373986618	0.708414272	0.999579949
23.50712873	-0.807295054	0.262059688	-3.080577019	0.002065999	0.230294913
28.38851043	0.748815464	0.348703404	2.147428031	0.031759221	0.689986698
97.79581685	0.127731805	0.170081432	0.751003821	0.452650356	0.999579949
150.0280134	-0.018725128	0.118984316	-0.157374759	0.874949505	0.999579949
438.830265	0.024994697	0.070053439	0.356794717	0.721245491	0.999579949
240.6861468	0.156148607	0.121160489	1.28877498	0.197476327	0.992338959
213.9472469	-0.065184543	0.08258282	-0.789323287	0.429923079	0.999579949
1763.068128	0.07668454	0.113976333	0.672811085	0.501067484	0.999579949
398.0653017	-0.049873892	0.118079422	-0.422375814	0.672750729	0.999579949
51.27777977	0.189664563	0.240449969	0.788790131	0.430234679	0.999579949
627.6970865	-0.188449106	0.161896294	-1.164011243	0.244419456	0.999579949
32.66409748	-0.15966985	0.307230653	-0.519706771	0.603267967	0.999579949
173.7428071	0.04985106	0.098713615	0.505006933	0.613553967	0.999579949
2336.077768	-0.040174058	0.052648465	-0.763062297	0.445426244	0.999579949
209.7848171	1.289770966	0.863793914	1.493146623	0.135398811	0.945791454
74.73666425	0.779077996	0.400194379	1.946748973	0.051564837	0.779189484
53.90500711	-0.144692903	0.166929073	-0.866792706	0.386055599	0.999579949
27.21900905	-0.056853008	0.256021071	-0.222063786	0.824264229	0.999579949
313.2724767	-0.312226636	0.155984924	-2.001646232	0.045322793	0.7611812

294.0851598	0.071374689	0.10143486	0.703650492	0.481650459	0.999579949
54.8142505	0.152649905	0.157931885	0.966555327	0.333766376	0.999579949
150.5242281	-0.100321601	0.127431956	-0.787256224	0.43113189	0.999579949
1103.680671	-0.018691266	0.086107852	-0.217068079	0.82815529	0.999579949
1306.81025	0.018709295	0.076584774	0.244295234	0.807002174	0.999579949
169.9214603	-0.062884604	0.268926879	-0.233835326	0.815112824	0.999579949
373.6446901	0.085216554	0.098099183	0.868677512	0.385023545	0.999579949
1529.831718	-0.037911141	0.133760384	-0.28342578	0.776850469	0.999579949
155.5560777	-0.104297697	0.110379221	-0.944903364	0.344708214	0.999579949
1242.996064	-0.055506084	0.110804523	-0.500936988	0.61641547	0.999579949
579.5011145	-0.243902136	0.206210036	-1.182784992	0.23689437	0.999579949
71.34394631	0.487675976	0.306632823	1.590423263	0.11173943	0.913348385
1486.515974	-0.106644175	0.114406515	-0.932151234	0.351258374	0.999579949
1473.973818	0.06886529	0.06298304	1.093394194	0.274220761	0.999579949
251.3440916	0.144999097	0.088734078	1.634085801	0.102240836	0.895628172
644.7505781	-0.028925597	0.076217764	-0.37951253	0.704307301	0.999579949
342.5610862	-0.018608887	0.127603734	-0.145833408	0.884052902	0.999579949
136.0075219	0.385303892	0.233122547	1.652795479	0.098372495	0.89325335
167.8490055	0.211607564	0.182612773	1.158777457	0.246546909	0.999579949
526.7334854	-0.021528634	0.130186593	-0.165367523	0.868654715	0.999579949
38.56177027	-0.077110604	0.173342888	-0.444844347	0.656432243	0.999579949
369.1674027	-0.061941765	0.087333112	-0.709258654	0.478163981	0.999579949
33.08792317	-0.196621745	0.302696142	-0.649568056	0.515971273	0.999579949
68.91680431	0.097367333	0.293582272	0.33165263	0.740151574	0.999579949
24.01385391	0.192988766	0.307519123	0.627566715	0.530287819	0.999579949
26.51428014	-0.255936577	0.552603682	-0.463146709	0.643259212	0.999579949
753.8372417	0.051944851	0.083831098	0.619637007	0.535496797	0.999579949
952.031388	0.098353903	0.081020123	1.213944132	0.224769059	0.999579949
124.6532608	0.06042078	0.157832635	0.382815508	0.701856562	0.999579949
1236.991604	-0.085817651	0.057918595	-1.481694289	0.138421657	0.945791454
132.5371449	0.168004192	0.143918125	1.167359512	0.243065215	0.999579949
924.4646616	-0.020562561	0.101775615	-0.202038193	0.839886866	0.999579949
20.68353408	0.849402981	0.518239985	1.639014754	0.101210187	0.895628172
629.8580006	0.056228757	0.13576107	0.414174379	0.678746404	0.999579949
55.12274418	0.200992875	0.35738658	0.562396258	0.573846068	0.999579949
141.6400546	0.133734101	0.139455154	0.958975678	0.337570998	0.999579949
198.7043922	0.041199894	0.132532843	0.310865544	0.75590284	0.999579949
71.68275284	0.152358957	0.147990704	1.029517073	0.303236761	0.999579949
203.3332346	0.060166524	0.268612515	0.223990026	0.822765064	0.999579949
134.3030579	0.37265349	0.294020771	1.267439334	0.204998243	0.994814558
2203.819388	-0.071198404	0.064090552	-1.110903274	0.266609986	0.999579949
297.7601161	-0.036011903	0.104968718	-0.343072718	0.731543755	0.999579949
29.6901775	-0.067638411	0.244716636	-0.276394821	0.782244834	0.999579949
195.0716034	0.219085503	0.166869424	1.312915803	0.189211321	0.983992835
51.48325857	-0.011260458	0.248038398	-0.045398044	0.96379004	0.999579949
1693.246228	-0.008191112	0.078409491	-0.104465821	0.91679969	0.999579949
412.2688277	-0.004145517	0.13917148	-0.029787117	0.976236833	0.999579949
138.4185363	-0.045878513	0.157472456	-0.291343097	0.77078893	0.999579949

534.2020615	0.038713415	0.097008612	0.399071943	0.689840195	0.999579949
3035.949846	-0.28691838	0.102557155	-2.797643717	0.005147686	0.357404857
46.70438997	1.516559674	0.605859206	2.503155288	0.012309152	0.503708792
480.7167518	-0.081790653	0.133677578	-0.611850199	0.540636874	0.999579949
233.0096476	-0.191299294	0.113974505	-1.678439352	0.093261361	0.880418343
87.39900069	-0.01024803	0.170071086	-0.060257331	0.951950685	0.999579949
225.0038722	0.091763314	0.343102476	0.267451625	0.789121456	0.999579949
509.5635377	0.410302669	0.754904459	0.543516022	0.586774563	0.999579949
491.0009338	0.238047267	0.30137833	0.789861923	0.429608411	0.999579949
70.38148843	-0.067690852	0.365121876	-0.185392486	0.852921202	0.999579949
75.39000213	-0.00462313	0.256489812	-0.018024613	0.985619219	0.999579949
168.9606915	0.047898813	0.167235406	0.286415504	0.774559902	0.999579949
127.1876761	-0.253971128	0.202350075	-1.255107654	0.209439682	0.996447459
167.8937503	0.004097864	0.090674486	0.045193128	0.963953372	0.999579949
1150.755866	0.021158245	0.08647704	0.244668937	0.806712782	0.999579949
1185.861119	-0.068765122	0.110383439	-0.622965934	0.533306908	0.999579949
34.05993463	-0.199497888	0.194979724	-1.023172485	0.3062263	0.999579949
32.6952011	0.368234664	0.179333058	2.053356299	0.040038034	0.73515901
128.0098994	0.144597435	0.134112731	1.07817829	0.280954198	0.999579949
62.55963129	0.122632262	0.210925635	0.581400463	0.560970585	0.999579949
171.5724259	-0.042890147	0.14017744	-0.305970396	0.759627186	0.999579949
372.6433324	0.14864937	0.116459973	1.276398802	0.201814579	0.993283621
1890.741215	0.042762591	0.061601674	0.694179042	0.487569934	0.999579949
306.2988438	-0.064604531	0.128015562	-0.504661542	0.613796577	0.999579949
260.099211	0.035182827	0.097790935	0.359775952	0.719014688	0.999579949
352.4506795	0.014004187	0.119008696	0.117673646	0.906326249	0.999579949
327.0131043	0.06633508	0.074503184	0.89036571	0.373269549	0.999579949
158.4357166	0.068643391	0.10666433	0.643545891	0.519869947	0.999579949
1007.870872	-0.003957202	0.132511416	-0.029863106	0.97617623	0.999579949
78.70037892	-0.141867562	0.167151955	-0.848734086	0.396029275	0.999579949
735.226917	-0.176718011	0.07580289	-2.331283293	0.019738427	0.601919728
536.6120535	0.003156676	0.123843506	0.025489233	0.979664737	0.999579949
138.6756055	-0.07634289	0.110079385	-0.693525765	0.487979663	0.999579949
193.5891168	0.057705905	0.075574864	0.763559499	0.445129793	0.999579949
209.4277477	0.158183827	0.111349097	1.420611678	0.155429682	0.976030424
84.47155064	0.094174749	0.156576736	0.601460672	0.547533198	0.999579949
744.4885744	-0.021532848	0.118272988	-0.182060571	0.855535192	0.999579949
90.37138144	0.146743402	0.220961564	0.664112793	0.506618123	0.999579949
39.45855386	0.206041044	0.214405383	0.960988205	0.336558098	0.999579949
56.16552362	0.006585074	0.181439246	0.036293546	0.971048296	0.999579949
61.99286188	0.024830566	0.177948057	0.139538283	0.889024806	0.999579949
59.60177713	0.042254501	0.180101686	0.234614688	0.814507808	0.999579949
259.4154634	0.019296264	0.089165047	0.216410631	0.82866768	0.999579949
23.58728345	0.115869707	0.207497809	0.558414123	0.576561631	0.999579949
1029.14689	0.048652111	0.064411663	0.755330775	0.450050538	0.999579949
805.206817	-0.00888814	0.08301177	-0.107070841	0.91473278	0.999579949
393.069126	0.076984344	0.117505023	0.655157894	0.512366101	0.999579949
385.1269286	0.164402018	0.116499512	1.411182038	0.158190945	0.977304288

119.2960674	0.450515229	0.238784522	1.886701973	0.059200422	0.797527826
727.3155188	0.095800542	0.083082233	1.153080972	0.248877151	0.999579949
110.9198571	-0.041814614	0.129023483	-0.324085301	0.745873455	0.999579949
177.1299104	-0.011591631	0.091473717	-0.126720888	0.899161313	0.999579949
227.7510453	0.013101001	0.07539953	0.17375441	0.862058475	0.999579949
1158.293076	-0.039547192	0.116005857	-0.340906859	0.733173701	0.999579949
147.1544795	0.066678311	0.114396043	0.582872527	0.559979116	0.999579949
1185.5521	0.100745223	0.080217513	1.255900599	0.209152011	0.996447459
66.55873109	0.062895023	0.136243645	0.461636383	0.644342099	0.999579949
20.96123214	-0.079243011	0.231859694	-0.341771392	0.73252294	0.999579949
1337.103609	-0.186584823	0.088770167	-2.101886577	0.035563213	0.713050989
411.9350728	0.033510969	0.07797581	0.429761093	0.667369438	0.999579949
1796.54384	0.415816884	0.148729235	2.7957979	0.005177176	0.357404857
40.20384932	-0.039843582	0.338556936	-0.117686503	0.906316061	0.999579949
272.6230113	0.101823921	0.110374502	0.922531198	0.356251565	0.999579949
304.2355683	-0.019497907	0.074832134	-0.260555272	0.794435486	0.999579949
30.81603048	-0.042088106	0.185903538	-0.226397551	0.820892232	0.999579949
100.7360598	0.034182746	0.139645649	0.244782034	0.806625205	0.999579949
10594.57901	0.018785648	0.103582129	0.181359933	0.856085066	0.999579949
250.5935794	-0.147962136	0.12667005	-1.168090925	0.242770091	0.999579949
847.7904237	-0.095972135	0.085210526	-1.126294351	0.260040948	0.999579949
26.44392735	-0.145949368	0.456811942	-0.319495519	0.749350789	0.999579949
274.219369	-0.014170529	0.205032916	-0.069113436	0.944899327	0.999579949
156.7686242	-0.083028031	0.101453122	-0.818388124	0.413135601	0.999579949
282.5656405	0.1416945	0.124132382	1.141478934	0.253670674	0.999579949
1076.203957	-0.115196133	0.096764135	-1.190483771	0.233856305	0.999579949
30.60788832	0.350472469	0.396222513	0.884534466	0.376407765	0.999579949
45.52068107	-0.284694816	0.624215802	-0.456083962	0.648329603	0.999579949
370.4598357	0.006891327	0.165958764	0.041524334	0.966877894	0.999579949
219.1411446	-0.144026654	0.138680587	-1.038549493	0.299014306	0.999579949
202.9884057	-0.21031868	0.091340681	-2.302574023	0.021302822	0.622105864
85.74928773	0.404784155	0.170017867	2.380833044	0.017273539	0.570162425
8430.213015	0.093288997	0.102427589	0.910779967	0.36241132	0.999579949
319.5351835	-0.03701944	0.130437073	-0.283810719	0.776555439	0.999579949
3364.046226	-0.010580694	0.090658954	-0.116708755	0.907090853	0.999579949
414.7738388	-0.125528975	0.11790662	-1.064647379	0.287035531	0.999579949
129.5618316	-0.078485486	0.131454307	-0.597055263	0.550470485	0.999579949
74.77280154	0.035562172	0.188723111	0.188435702	0.850535115	0.999579949
232.8895325	-0.152725045	0.164642454	-0.927616429	0.353606573	0.999579949
87.15488104	0.042321626	0.120976238	0.349834205	0.726463127	0.999579949
481.315434	-0.054158521	0.122345843	-0.442667442	0.658006295	0.999579949
435.8849657	0.162870045	0.201280953	0.809167698	0.418418692	0.999579949
39.38710015	-0.1894043	0.235698124	-0.803588488	0.421634674	0.999579949
50.47733356	0.380385367	0.316181847	1.203058842	0.228953551	0.999579949
211.9746302	0.150071019	0.115750925	1.296499527	0.194803443	0.988443849
476.0134267	-0.059971205	0.058628953	-1.022894011	0.306357961	0.999579949
641.277996	-0.097504196	0.134500253	-0.724936897	0.468490744	0.999579949
1019.356478	0.086057931	0.145146737	0.592902965	0.553246094	0.999579949

325.5537155	-0.175102733	0.099467628	-1.760399213	0.078340143	0.851207389
2636.063016	-0.17577167	0.082135025	-2.140033063	0.032352095	0.691862456
199.3996431	0.038705356	0.173261383	0.223392859	0.823229762	0.999579949
5177.292342	0.006141079	0.063882799	0.096130398	0.923417009	0.999579949
1400.41142	0.075404299	0.110422152	0.682872932	0.494687139	0.999579949
208.8707464	0.043136734	0.127222825	0.339064424	0.734561198	0.999579949
166.3074772	0.042113936	0.134242188	0.313716106	0.753736667	0.999579949
277.1062282	0.079645202	0.133973415	0.594485123	0.55218769	0.999579949
143.0314656	-0.000806622	0.111768927	-0.007216872	0.994241819	0.999579949
354.9572608	0.109577416	0.186176089	0.588568688	0.556150644	0.999579949
106.9763067	0.077472166	0.110504866	0.701074705	0.483256395	0.999579949
1528.354185	0.042728613	0.059823281	0.714247237	0.475074313	0.999579949
5631.344633	-0.054621706	0.049452294	-1.104533296	0.269361868	0.999579949
1899.219098	0.08646331	0.082448701	1.048692213	0.294319799	0.999579949
487.8346775	-0.041045438	0.057925706	-0.708587617	0.478580423	0.999579949
36.84091769	-0.120220686	0.332389687	-0.361685968	0.717586714	0.999579949
28.81456199	0.30623435	0.481557639	0.635924603	0.524825566	0.999579949
163.668921	0.12783044	0.155137229	0.823983006	0.409949214	0.999579949
968.7039243	-0.16322271	0.142346671	-1.146656324	0.251523688	0.999579949
26.05531126	0.485238918	0.29954783	1.61990463	0.105252765	0.896383253
1874.254557	-0.13661176	0.103245051	-1.323179749	0.185775609	0.983992835
31.28208451	-0.301943983	0.519069111	-0.581702855	0.560766848	0.999579949
28.92164889	-0.007297417	0.310128443	-0.023530304	0.981227266	0.999579949
809.0403675	-0.015378833	0.086702268	-0.17737521	0.859213678	0.999579949
137.9453402	0.096173781	0.274936545	0.349803557	0.72648613	0.999579949
1389.196854	-0.034887424	0.097012674	-0.359617176	0.719133437	0.999579949
205.1636667	0.046584727	0.119674492	0.389261961	0.697082372	0.999579949
1176.09751	0.066607828	0.100450879	0.663088555	0.50727384	0.999579949
53.99940802	0.322127556	0.288918079	1.114944265	0.264874306	0.999579949
1926.787595	0.051161563	0.157023298	0.325821476	0.744559429	0.999579949
602.2245051	-0.099663748	0.158132738	-0.630253726	0.528528593	0.999579949
33.2914054	0.08812087	0.24221532	0.363812122	0.71599831	0.999579949
864.3961235	-0.075121455	0.076432207	-0.982850781	0.325680885	0.999579949
22.06281774	0.058802266	0.299182369	0.196543219	0.844185008	0.999579949
36.02531256	-0.091492078	0.184398993	-0.496163653	0.619778953	0.999579949
135.6705737	-0.080646575	0.113822815	-0.708527332	0.478617845	0.999579949
1593.208934	-0.043847858	0.062748585	-0.698786415	0.484685519	0.999579949
21.59254334	-0.076358779	0.248942386	-0.306732735	0.759046811	0.999579949
202.8521618	0.17658365	0.150194297	1.175701431	0.239714207	0.999579949
250.2849657	0.037069964	0.088190881	0.420337834	0.674238675	0.999579949
24.59096996	0.096985514	0.210392393	0.460974435	0.644816947	0.999579949
560.1661816	0.15214495	0.094276842	1.613810427	0.106568552	0.899087397
184.2386713	-0.109507084	0.200349148	-0.546581232	0.584666466	0.999579949
1096.696699	0.029703564	0.065606291	0.452754806	0.65072531	0.999579949
63.72451196	0.022845101	0.172144938	0.132708527	0.894423899	0.999579949
437.1738743	-0.021882645	0.077294155	-0.283108667	0.777093538	0.999579949
33.85374034	0.074546292	0.524721612	0.142068271	0.88702608	0.999579949
27.20586125	0.67426197	0.45650343	1.477014027	0.139671877	0.948318761

20.95877738	0.273339819	0.351074273	0.778581172	0.436226474	0.999579949
2468.227003	0.00351226	0.100979038	0.034782072	0.972253516	0.999579949
2065.218739	0.044911436	0.099742068	0.450275762	0.652511614	0.999579949
1883.58812	0.186680253	0.143115991	1.304398283	0.19209782	0.984804292
624.2530798	0.109220356	0.06333944	1.724365671	0.084641851	0.861032139
554.8593628	0.042630338	0.0825873	0.516185159	0.605725094	0.999579949
294.4300405	0.001987529	0.091551399	0.021709431	0.982679741	0.999579949
46.62194214	0.037342895	0.202385303	0.184513867	0.85361035	0.999579949
777.6731759	0.028144257	0.112211428	0.250814529	0.801957508	0.999579949
2422.946513	0.097036216	0.106299467	0.912857038	0.361317735	0.999579949
255.2916752	0.115193128	0.132835939	0.867183451	0.385841502	0.999579949
178.1923276	0.159217703	0.156564252	1.016947995	0.309178169	0.999579949
631.6394459	0.030333772	0.05512995	0.550223088	0.58216637	0.999579949
1109.426119	-0.065043569	0.071641259	-0.907906554	0.363927594	0.999579949
615.7097026	0.013389862	0.112348566	0.119181424	0.905131623	0.999579949
112.762556	0.146902295	0.126023673	1.165672225	0.243746994	0.999579949
69.80638875	-0.154119336	0.254387961	-0.60584367	0.544618568	0.999579949
1451.212356	-0.034368706	0.133907676	-0.256659712	0.797441475	0.999579949
44.10002966	0.185201699	0.554865787	0.33377747	0.738547486	0.999579949
66.34201566	0.153871624	0.1614522	0.953047557	0.340565976	0.999579949
3159.596049	0.118355997	0.128686949	0.919720277	0.357718954	0.999579949
30.17249596	0.136419563	0.228402529	0.597276938	0.550322499	0.999579949
165.0734897	0.045737068	0.084526105	0.541099919	0.588438714	0.999579949
43.40118816	0.137656518	0.177625578	0.774981393	0.438350655	0.999579949
167.8147207	-0.060291276	0.143117713	-0.421270541	0.673557539	0.999579949
217.7761218	0.062507412	0.147045103	0.42509006	0.670771024	0.999579949
286.5831201	0.050712143	0.071383425	0.710419029	0.477444326	0.999579949
99.61927325	0.370770251	0.17195296	2.15623069	0.031065656	0.683621001
77.13617293	0.054301189	0.173516701	0.312945027	0.754322428	0.999579949
83.86065537	0.122413042	0.229231495	0.534014935	0.593331201	0.999579949
129.43613	0.242093057	0.294857098	0.82105216	0.411616558	0.999579949
7520.933981	-0.081996204	0.121356769	-0.675662387	0.499255022	0.999579949
291.1894656	0.03789003	0.100416637	0.377328213	0.705929708	0.999579949
389.8750379	0.024837225	0.082321148	0.301711356	0.76287211	0.999579949
415.3415466	-0.033015885	0.11415747	-0.289213534	0.772417975	0.999579949
274.0818464	0.084758092	0.113941933	0.743870935	0.456954561	0.999579949
60.13866673	-0.02325634	0.268358075	-0.086661598	0.930940502	0.999579949
796.3028384	-0.10459879	0.097575525	-1.071977731	0.283730035	0.999579949
203.7225166	0.151808831	0.097000126	1.56503746	0.117574126	0.920584864
363.2143133	0.148559957	0.079866478	1.86010403	0.062870809	0.807133028
2194.503259	-0.065857636	0.075091689	-0.877029618	0.380470551	0.999579949
253.5377184	0.204732642	0.089444462	2.288935925	0.022083076	0.627784819
838.5283032	-0.040757898	0.087629701	-0.465115112	0.641849024	0.999579949
6150.321182	0.124948916	0.100226683	1.246663188	0.212521022	0.999268361
263.1097112	0.097122296	0.083072965	1.169120374	0.242355136	0.999579949
391.1063346	0.141480712	0.108487238	1.304123092	0.192191617	0.984884298
40.09288197	-0.479781578	0.48475899	-0.989732192	0.322305036	0.999579949
344.7691497	0.009401307	0.10056456	0.09348529	0.925518035	0.999579949

24.7813432	-0.185207083	0.21512683	-0.860920431	0.389281868	0.999579949
326.3855538	-0.030885171	0.130415378	-0.236821546	0.812795235	0.999579949
136.8530969	-0.005492828	0.178676204	-0.030741801	0.975475454	0.999579949
175.433624	-0.047826633	0.132670483	-0.360491887	0.718479323	0.999579949
182.2269362	0.083279327	0.150312388	0.554041674	0.579550326	0.999579949
327.5317146	0.057855695	0.113386736	0.510250998	0.609875628	0.999579949
1205.394845	-0.117688297	0.051825244	-2.270868181	0.023154959	0.62865781
30.41238731	-0.069184614	0.181817332	-0.380517154	0.703561565	0.999579949
164.9312214	0.116819269	0.128609102	0.908328156	0.363704871	0.999579949
80.11308596	0.076436242	0.125131724	0.610846231	0.541301384	0.999579949
452.9578696	-0.075378777	0.114066111	-0.660834105	0.508718708	0.999579949
1378.299072	-0.026511525	0.048094459	-0.551238663	0.581470079	0.999579949
1167.859044	0.018844477	0.17081422	0.110321477	0.912154425	0.999579949
59.13985552	-0.007171701	0.18265725	-0.039263163	0.968680576	0.999579949
121.2986742	0.034746007	0.118379048	0.293514835	0.769128662	0.999579949
2389.409918	-0.017392615	0.110467808	-0.157445098	0.874894074	0.999579949
822.9755565	-0.069558702	0.07643229	-0.910069583	0.362785815	0.999579949
111.3309709	0.229149791	0.103514316	2.213701449	0.026849323	0.653787748
423.2411165	-0.076332198	0.093766155	-0.81406983	0.415604948	0.999579949
126.0221672	0.120609061	0.107376574	1.123234387	0.261337962	0.999579949
55.11261388	0.04733904	0.141490162	0.334574778	0.737945873	0.999579949
668.3164563	-0.017105211	0.071377657	-0.239643775	0.810606426	0.999579949
484.7755336	-0.018246903	0.053872947	-0.338702519	0.734833843	0.999579949
3221.233367	-0.044020061	0.055710089	-0.790163173	0.42943248	0.999579949
314.0125539	-0.184825044	0.183440236	-1.007549097	0.313670976	0.999579949
2231.915047	-0.17533534	0.370820612	-0.47283062	0.636334027	0.999579949
196.7151085	-0.019344092	0.073714427	-0.262419348	0.792998157	0.999579949
115.8902289	0.074380872	0.154467424	0.481531118	0.630139069	0.999579949
144.2319727	-0.068093333	0.238409013	-0.285615599	0.775172555	0.999579949
321.0957078	-0.161967781	0.303948907	-0.532878314	0.594117818	0.999579949
224.6295126	0.070648681	0.11963914	0.590514783	0.554845578	0.999579949
28.32589115	0.156786753	0.293506534	0.53418488	0.593213629	0.999579949
55.15387247	0.001907737	0.284716542	0.006700477	0.994653833	0.999579949
196.5118977	0.074655176	0.208092399	0.358759746	0.719774829	0.999579949
859.4553767	0.106259148	0.087153385	1.219219976	0.222760712	0.999579949
184.5885591	0.028610632	0.176027642	0.162534884	0.87088466	0.999579949
487.0318726	0.030959613	0.066449155	0.465914326	0.641276826	0.999579949
71.93311519	-0.119779465	0.155292282	-0.771316273	0.440519489	0.999579949
257.1636163	0.042974839	0.10146628	0.423538137	0.67190268	0.999579949
6813.148632	0.010289156	0.117923851	0.087252542	0.930470776	0.999579949
135.3038372	-0.026089055	0.128931801	-0.202347709	0.839644905	0.999579949
1075.627774	0.086233216	0.108540013	0.794483196	0.426914188	0.999579949
77.54516425	-0.129356523	0.137808951	-0.938665611	0.347902455	0.999579949
4608.148947	-0.185489801	0.1617746	-1.146594095	0.251549418	0.999579949
2504.777779	0.05119492	0.122056222	0.419437198	0.674896643	0.999579949
132.697563	-0.005749208	0.163062253	-0.035257747	0.971874215	0.999579949
494.6689605	-0.011831091	0.076612993	-0.154426686	0.877273314	0.999579949
153.8674989	-0.037022129	0.200897256	-0.184283894	0.853790749	0.999579949

76.27695205	-0.130466927	0.150844423	-0.864910508	0.38708791	0.999579949
119.4072112	-0.077617352	0.125858308	-0.616704234	0.537429828	0.999579949
121.7916376	-0.187225965	0.153216054	-1.221973542	0.221717633	0.999579949
81.46900621	-0.107557268	0.138260141	-0.777934022	0.43660791	0.999579949
867.7974491	-0.053028345	0.104880761	-0.505606026	0.61313325	0.999579949
780.1860877	-0.045474387	0.076418962	-0.595066799	0.55179882	0.999579949
266.0251827	0.079288903	0.082261073	0.963869057	0.335111579	0.999579949
228.9302693	-0.003069141	0.110255678	-0.027836579	0.977792491	0.999579949
264.9626201	-0.107703802	0.223229878	-0.482479328	0.62946548	0.999579949
1143.063086	0.005170641	0.106137494	0.048716445	0.96114527	0.999579949
370.8110193	0.059616225	0.124612917	0.478411279	0.632357507	0.999579949
119.4751871	0.107348075	0.102599618	1.046281429	0.295431117	0.999579949
74.149864	0.566374179	0.538416485	1.051925775	0.292833605	0.999579949
2336.552982	0.001401248	0.072697812	0.019274965	0.984621756	0.999579949
265.1287161	-0.137016598	0.104065236	-1.316641406	0.187958853	0.983992835
210.5687678	-0.114078205	0.139051859	-0.820400432	0.411987869	0.999579949
148.8709409	0.074396731	0.145605087	0.510948708	0.609386973	0.999579949
38.02757364	0.046940785	0.307817843	0.152495336	0.878796269	0.999579949
1536.725844	0.076146699	0.06913591	1.101405903	0.270720038	0.999579949
76.31313476	-0.074283827	0.250126571	-0.296984952	0.766477998	0.999579949
502.977879	-0.18421808	0.207243401	-0.888897208	0.374058326	0.999579949
33.70986734	0.207010334	0.353090536	0.586281174	0.55768658	0.999579949
318.8803284	0.004138995	0.130021636	0.031833126	0.974605129	0.999579949
189.5477371	-0.071359819	0.086475098	-0.825206569	0.409254325	0.999579949
200.1754703	0.106960023	0.108775273	0.983311917	0.325453947	0.999579949
1099.898902	-0.024082664	0.133315106	-0.180644675	0.856646487	0.999579949
169.823643	-0.066869876	0.13245128	-0.504863949	0.613654396	0.999579949
267.5591783	-0.120581121	0.068805648	-1.752488694	0.079689812	0.852823173
1479.605357	0.120406672	0.051006372	2.360620219	0.018244404	0.580931591
436.460493	0.081887393	0.196409726	0.416921274	0.676735992	0.999579949
113.4357507	0.051382124	0.131865982	0.389654128	0.696792321	0.999579949
427.8627559	-0.076273242	0.07189532	-1.060892998	0.288738532	0.999579949
1127.815581	-0.143593611	0.123112075	-1.166364964	0.243466918	0.999579949
24.55733617	-0.040969088	0.271209266	-0.151060799	0.879927755	0.999579949
124.5014725	0.069154772	0.138875237	0.497963304	0.618509907	0.999579949
752.8902002	0.125280966	0.159390093	0.786002215	0.431866189	0.999579949
169.0494572	-0.113463868	0.267903237	-0.423525559	0.671911855	0.999579949
7897.248011	-0.016667778	0.107905	-0.154467154	0.877241407	0.999579949
875.0062358	0.190902418	0.109308468	1.74645589	0.080731765	0.85448976
5453.84355	-0.006099922	0.082935292	-0.073550375	0.941368159	0.999579949
150.6391405	-0.150131972	0.125200644	-1.19913099	0.230477016	0.999579949
705.7758015	0.037932591	0.096838952	0.391707988	0.695273987	0.999579949
158.7114078	-0.076357823	0.114307219	-0.668005257	0.504130237	0.999579949
123.2962951	0.151271459	0.198395586	0.762473913	0.445777207	0.999579949
59.15105702	0.017513594	0.187115282	0.093597879	0.925428594	0.999579949
41.60974437	0.216622723	0.616356497	0.351456866	0.725245627	0.999579949
101.1647522	-0.092072847	0.116825552	-0.788122504	0.430625053	0.999579949
112.5288244	0.07189792	0.298694422	0.240707273	0.809782002	0.999579949

2218.464298	-0.056428255	0.071546478	-0.788693672	0.430291067	0.999579949
21.56240427	0.672362621	0.381220766	1.763709328	0.07778093	0.851207389
46.67551894	-0.035195887	0.147900818	-0.237969521	0.811904734	0.999579949
32.0203116	0.135434401	0.191225566	0.708244217	0.478793611	0.999579949
816.5205908	0.092053782	0.139453355	0.66010446	0.509186797	0.999579949
59.75285572	-0.400073239	0.263371525	-1.519045151	0.128751131	0.935700262
227.5809372	0.052511377	0.147115773	0.356939134	0.721137372	0.999579949
252.5367545	-0.093504024	0.136378797	-0.685619952	0.492952796	0.999579949
177.9531874	-0.124076623	0.089694621	-1.38332289	0.166565883	0.978965834
218.1664705	0.457468251	0.273173387	1.674644281	0.094004046	0.880418343
261.1917105	0.118439513	0.168550284	0.702695425	0.48224558	0.999579949
761.1842124	-0.132422022	0.177110058	-0.747682113	0.454651923	0.999579949
236.9030813	-0.11946559	0.186919575	-0.639128299	0.522739472	0.999579949
583.3212926	-0.04316865	0.108009156	-0.399675842	0.689395288	0.999579949
135.8979751	-0.125752083	0.141734609	-0.887236251	0.374951719	0.999579949
162.531133	0.200954883	0.121938835	1.6479974	0.099353205	0.895628172
570.5804585	-0.091755348	0.094730007	-0.968598558	0.332745523	0.999579949
50.75494294	0.011728326	0.183348473	0.063967405	0.948996181	0.999579949
1080.961689	0.001025045	0.077050999	0.013303457	0.98938569	0.999579949
897.4536452	-0.023021926	0.083635413	-0.275265287	0.783112432	0.999579949
120.0547064	-0.082825514	0.126632344	-0.654062871	0.513071301	0.999579949
460.7416183	-0.058060517	0.114944474	-0.505117953	0.613475993	0.999579949
46.26635582	-0.405084048	0.469414009	-0.862956879	0.388161176	0.999579949
144.513103	0.290062319	0.210337929	1.379030019	0.167885499	0.980087289
823.6648347	-0.031594083	0.110488832	-0.285948201	0.774917795	0.999579949
76.64237617	0.291271223	0.18808064	1.548650741	0.121465701	0.922215626
454.1741776	0.014378464	0.078072767	0.184167464	0.853882083	0.999579949
20.90258147	0.229612389	0.295784929	0.776281571	0.437582752	0.999579949
486.5583633	0.029205867	0.067555028	0.432327059	0.665503723	0.999579949
210.2410021	-0.010282125	0.083973654	-0.122444651	0.902546878	0.999579949
124.5702321	0.156830751	0.108606763	1.444023796	0.148732282	0.962301096
697.5968594	-0.104747572	0.102444004	-1.02248611	0.306550883	0.999579949
157.4375777	0.036939041	0.178715869	0.206691441	0.836250842	0.999579949
83.16837749	0.17302336	0.140485475	1.231610315	0.218094686	0.999579949
374.5696976	-0.048818672	0.08164862	-0.597911786	0.549898795	0.999579949
44.47629569	-0.177043024	0.175950059	-1.006211796	0.314313699	0.999579949
21.90523493	0.3289925	0.220533203	1.49180484	0.135750315	0.945791454
296.6514235	0.144475729	0.154349664	0.93602879	0.349258365	0.999579949
108.2635327	0.34053735	0.278553697	1.222519586	0.221511202	0.999579949
62.9870876	0.155819507	0.170091132	0.916094244	0.359617467	0.999579949
343.6893384	0.098501498	0.101549416	0.96998587	0.332053535	0.999579949
47.78840807	0.201838379	0.192176755	1.050274672	0.293591846	0.999579949
277.1887901	-0.089147101	0.106932414	-0.833677064	0.404462986	0.999579949
222.3125669	-0.049308106	0.11656695	-0.423002456	0.672293469	0.999579949
29.93616996	-0.051714707	0.252013631	-0.205205992	0.837411188	0.999579949
328.3800118	0.147650156	0.105776154	1.395873735	0.162752521	0.978498637
857.4309151	-0.045176182	0.105699491	-0.427402075	0.669086496	0.999579949
427.7880455	-0.008012359	0.071949117	-0.111361469	0.911329713	0.999579949

53.7183356	0.085081206	0.428143339	0.198721312	0.842480752	0.999579949
137.6449068	0.01367187	0.095908177	0.142551661	0.886644277	0.999579949
99.09435297	-0.070569573	0.157367788	-0.44843722	0.653837685	0.999579949
563.0338137	-0.143511235	0.050297723	-2.853235235	0.004327657	0.336488715
43.94509592	-0.216769967	0.172319823	-1.257951423	0.208409325	0.995521169
219.7540838	0.00937925	0.103803049	0.090356208	0.928004155	0.999579949
34.56147839	-0.046329094	0.205930751	-0.224974142	0.82199939	0.999579949
1404.554722	-0.010543656	0.077149625	-0.136665028	0.891295574	0.999579949
431.1736186	0.169789479	0.128990942	1.316289939	0.188076747	0.983992835
55.77316365	0.102658428	0.156001738	0.658059514	0.510499889	0.999579949
173.4430426	0.051184454	0.116055375	0.441034752	0.659187839	0.999579949
86.26403046	0.131578989	0.164418399	0.800269255	0.423554812	0.999579949
38.01358613	0.251728553	0.239594065	1.050646028	0.293421193	0.999579949
210.2999881	0.16321755	0.09523906	1.713766902	0.086571544	0.867486202
1532.666847	0.043997361	0.07996037	0.550239592	0.582155051	0.999579949
129.4370774	-0.18616419	0.274099713	-0.679184185	0.497021167	0.999579949
145.3191264	0.034331163	0.338526642	0.101413475	0.91922224	0.999579949
344.5123828	-0.041681801	0.238675252	-0.174638135	0.861363982	0.999579949
79.90190711	-0.35541606	0.187815622	-1.892366872	0.058442117	0.79580383
164.8899358	-0.032615667	0.103084111	-0.316398586	0.75169999	0.999579949
393.3387395	-0.068096369	0.099285679	-0.685862955	0.492799532	0.999579949
106.6578966	-1.017318941	0.396271228	-2.567228882	0.010251492	0.47773736
116.4726419	0.049603316	0.165152708	0.300348186	0.763911582	0.999579949
1028.498049	-0.080554884	0.068778488	-1.171222074	0.241509525	0.999579949
99.43339718	0.229454812	0.127131767	1.804858199	0.071096874	0.834620594
61.11376658	-0.119950066	0.192095995	-0.624427729	0.532346719	0.999579949
702.9782323	0.042348412	0.059436578	0.71249747	0.476156777	0.999579949
1933.304633	-0.031872654	0.112144404	-0.284210833	0.776248814	0.999579949
175.3776972	-0.106041873	0.094324849	-1.124219904	0.260919748	0.999579949
8667.546431	-0.002671701	0.230229601	-0.011604507	0.990741151	0.999579949
268.1152404	-0.366381049	0.238910413	-1.533549938	0.125140412	0.927500246
485.77511	-0.038210955	0.059648323	-0.640604014	0.521779991	0.999579949
103.4765873	0.061202553	0.112422709	0.544396712	0.586168508	0.999579949
237.9809395	-0.078083364	0.091107562	-0.857045913	0.391419511	0.999579949
272.2707249	0.056469536	0.130836661	0.431603311	0.666029752	0.999579949
172.8126515	-0.040802573	0.106979648	-0.381405006	0.702902747	0.999579949
288.0923615	-0.047999669	0.075090068	-0.639227933	0.522674663	0.999579949
362.315848	-0.02474254	0.07144693	-0.346306557	0.729112342	0.999579949
272.6688568	0.091289606	0.105941368	0.861699331	0.388852994	0.999579949
2565.621535	0.010980556	0.054754958	0.200539932	0.841058331	0.999579949
765.7185995	0.106175041	0.082431979	1.288032178	0.197734762	0.992338959
375.7730621	0.06579147	0.091417731	0.719679529	0.471722333	0.999579949
247.0061727	-0.114149395	0.072075273	-1.583752514	0.113250049	0.916629381
25.73344381	-0.129613988	0.214275126	-0.604895164	0.545248656	0.999579949
579.3322118	0.071053349	0.096137568	0.739079949	0.459858452	0.999579949
30.42123214	0.264064437	0.275184558	0.959590314	0.337261446	0.999579949
857.7812755	-0.253634767	0.148243653	-1.710931713	0.08709372	0.867486202
94.24926714	0.023535656	0.137053343	0.171726247	0.86365275	0.999579949

391.3584206	-0.10900144	0.102841344	-1.059899018	0.289190543	0.999579949
1405.61197	-0.032352074	0.040679953	-0.795282978	0.426448913	0.999579949
534.3516518	-0.138917172	0.100270899	-1.38541863	0.165924498	0.978789551
179.2385206	-0.042174209	0.096209958	-0.438355969	0.661128259	0.999579949
33.85339717	0.15155594	0.165792779	0.914128715	0.360649216	0.999579949
1476.031806	-0.001228988	0.051413716	-0.02390389	0.980929271	0.999579949
550.5597773	-0.033832647	0.100840545	-0.335506388	0.737243127	0.999579949
355.786934	0.09373549	0.066602287	1.407391454	0.159311344	0.977762491
498.8182049	0.189151408	0.269473724	0.701928951	0.482723473	0.999579949
499.6116337	0.015240954	0.07090219	0.214957449	0.829800494	0.999579949
121.2670062	-0.059024026	0.10376303	-0.568834833	0.569468234	0.999579949
250.1707729	0.022078151	0.085699247	0.257623631	0.79669739	0.999579949
177.2713834	0.172115825	0.111317436	1.546171301	0.122063203	0.92236461
2530.460699	0.085055248	0.125187478	0.679422968	0.496869901	0.999579949
122.9847799	-0.099172852	0.114694962	-0.864666156	0.38722205	0.999579949
227.7879115	0.081412113	0.102207922	0.796534279	0.42572156	0.999579949
383.0268193	0.028774348	0.085434126	0.336801566	0.736266492	0.999579949
168.8210881	-0.046872151	0.095834974	-0.489092336	0.624776327	0.999579949
849.8305154	0.07658188	0.111199222	0.688690785	0.491017876	0.999579949
270.4255942	0.110397212	0.081774518	1.350019726	0.177009656	0.983992835
348.2889241	-0.108967847	0.0700681	-1.555170572	0.119905451	0.921521033
310.9113085	-0.040800989	0.106653958	-0.382554846	0.702049855	0.999579949
72.15824207	-0.065234671	0.136265894	-0.478730733	0.632130199	0.999579949
157.3016541	-0.035094923	0.118786334	-0.295445794	0.767653355	0.999579949
132.8090438	-0.13306787	0.193652547	-0.687147531	0.491989765	0.999579949
371.7212753	0.03210826	0.087207632	0.368181762	0.712737709	0.999579949
487.8694054	0.049514673	0.133418259	0.371123666	0.710545425	0.999579949
1154.477282	-0.018379263	0.113689023	-0.161662599	0.871571558	0.999579949
523.979881	0.118747775	0.11155862	1.064442841	0.287128135	0.999579949
137.508889	0.199123312	0.102802612	1.936947979	0.052751703	0.781231069
164.6667411	0.413013555	0.218608541	1.889283706	0.058853824	0.79629879
1800.927873	0.132601774	0.132888593	0.997841653	0.318356149	0.999579949
187.1733884	0.01712689	0.087754741	0.195167685	0.845261676	0.999579949
302.6675355	0.016424394	0.146582466	0.11204883	0.91078469	0.999579949
37.7396654	-0.336814743	0.204550239	-1.646611337	0.099637959	0.895628172
316.1577527	0.013492311	0.107043276	0.126045382	0.899696001	0.999579949
377.765664	-0.039636828	0.093867401	-0.422264039	0.672832303	0.999579949
47.51692157	0.21650111	0.353011603	0.613297435	0.539679689	0.999579949
211.0445778	-0.188571485	0.088722864	-2.125398985	0.033553335	0.698554126
365.0810383	0.00298812	0.117359578	0.025461239	0.979687065	0.999579949
62.72208203	-0.019899821	0.206515581	-0.0963599	0.923234739	0.999579949
3913.906589	-0.093909171	0.080580156	-1.165413116	0.243851811	0.999579949
94.64485299	0.360064479	0.243422789	1.479173253	0.139094016	0.947119714
57.19046895	0.106222329	0.189221497	0.561365018	0.574548726	0.999579949
590.7984703	0.088646555	0.078399599	1.130701639	0.258180691	0.999579949
1114.784611	-0.064699206	0.121348526	-0.533168453	0.593916977	0.999579949
90.44563971	0.101570245	0.358065879	0.283663568	0.776668217	0.999579949
325.1515776	-0.077020396	0.095702773	-0.804787507	0.420942312	0.999579949

42.88958518	0.18010684	0.212568591	0.84728811	0.396834552	0.999579949
218.3124069	0.073681222	0.308913068	0.238517659	0.811479621	0.999579949
440.7743061	0.064468092	0.116190335	0.554849009	0.578997942	0.999579949
165.2933094	0.016863735	0.176813141	0.095376029	0.924016155	0.999579949
153.998054	0.005440201	0.111046859	0.048990141	0.960927153	0.999579949
3869.707475	0.15096538	0.207822311	0.726415654	0.467583998	0.999579949
378.7809666	-0.075150541	0.086495862	-0.868833947	0.384937963	0.999579949
150.2794745	-0.118728171	0.129858672	-0.914287579	0.360565756	0.999579949
133.1543967	-0.40614216	0.199647355	-2.034297724	0.041921586	0.74969123
132.6303048	-0.283401174	0.144485583	-1.961449493	0.049826611	0.776589675
389.6327327	-0.094015118	0.132764528	-0.708134317	0.47886185	0.999579949
432.3466979	0.04428543	0.087134134	0.508244331	0.611282005	0.999579949
177.7882675	0.233434964	0.196983663	1.185047335	0.235998732	0.999579949
4736.785342	0.882476421	0.277892189	3.175607151	0.001495233	0.203876378
45.64381994	0.658606728	0.185727187	3.546097584	0.000390981	0.097717629
550.0992493	0.239676743	0.188359635	1.272442173	0.203216058	0.994409048
63.89236167	-0.112216156	0.214239448	-0.523788486	0.600425662	0.999579949
88.55969341	0.051374685	0.429134773	0.119716901	0.904707412	0.999579949
234.4624769	-0.057350325	0.098814501	-0.580383694	0.561655899	0.999579949
396.7910273	0.068350012	0.099825551	0.684694564	0.49353668	0.999579949
41.52042817	0.263844648	0.188779998	1.397630316	0.16222411	0.978498637
131.1006754	-0.090623409	0.095291073	-0.951016776	0.34159586	0.999579949
212.7764423	-0.108793372	0.179320058	-0.606699403	0.544050419	0.999579949
31.73839652	-0.011921739	0.259025321	-0.046025379	0.963290022	0.999579949
3211.451432	0.047294511	0.113296099	0.417441658	0.67635539	0.999579949
486.000622	0.015353666	0.082699573	0.185655926	0.852714593	0.999579949
243.8698679	-0.114565145	0.148863231	-0.769600015	0.441537196	0.999579949
1269.734219	0.043743479	0.124518027	0.351302376	0.725361513	0.999579949
273.1841453	0.093728823	0.068046194	1.377429309	0.168379557	0.980087289
257.1749653	-0.113587095	0.129337533	-0.87822222	0.379823136	0.999579949
188.7166047	-0.020393503	0.11320192	-0.180151562	0.857033584	0.999579949
32.3874893	-0.330559755	0.190096716	-1.738903034	0.082051821	0.855566831
10270.1962	-0.008821421	0.164006776	-0.053786929	0.957104923	0.999579949
89.62913943	0.060183659	0.200192898	0.300628342	0.763697917	0.999579949
227.1206475	-0.064236958	0.11783187	-0.545157755	0.585645023	0.999579949
52.33296033	0.036345034	0.346720502	0.104825165	0.916514541	0.999579949
446.4883408	-0.235877815	0.236852826	-0.995883476	0.319306765	0.999579949
2798.109094	-0.313228794	0.23811058	-1.315476171	0.188349921	0.983992835
627.2133296	0.058842587	0.087343013	0.673695408	0.500504982	0.999579949
2506.251571	-0.107571032	0.140733973	-0.764357248	0.444654378	0.999579949
200.0422993	-0.028228122	0.077195052	-0.36567269	0.714609327	0.999579949
50.08315617	0.083211382	0.186728891	0.445626714	0.655866911	0.999579949
70.03200874	0.181068511	0.187181127	0.967343842	0.333372174	0.999579949
292.3376469	-0.07047944	0.169980308	-0.41463297	0.678410609	0.999579949
119.722664	0.014013436	0.117550573	0.119211974	0.905107421	0.999579949
1161.707301	0.047483365	0.104830536	0.452953566	0.650582178	0.999579949
31.16668031	0.482174202	0.420161497	1.147592546	0.251136808	0.999579949
255.5180969	0.077902378	0.086592667	0.899641739	0.368310937	0.999579949

28.80941198	0.309529152	0.317882049	0.973723281	0.330193954	0.999579949
48.26411548	-0.167542006	0.179711931	-0.932280928	0.351191361	0.999579949
1093.004531	0.124520366	0.110851106	1.123311893	0.261305054	0.999579949
306.9439871	0.188497777	0.163091017	1.155782708	0.247770048	0.999579949
484.9889929	0.086535008	0.08871542	0.975422409	0.329350772	0.999579949
189.5969687	0.018837972	0.120850967	0.155877709	0.876129416	0.999579949
137.8094368	0.151975168	0.126556851	1.200845048	0.229811314	0.999579949
186.5771588	-0.000927194	0.075640046	-0.012257977	0.990219794	0.999579949
514.0112703	0.262979198	0.139067985	1.891011787	0.058622771	0.79580383
1112.393888	-0.018134366	0.064695927	-0.280301507	0.779246195	0.999579949
52.91074534	0.02678432	0.175915565	0.152256683	0.878984488	0.999579949
1345.488491	-0.40481059	0.678960838	-0.596220823	0.551027717	0.999579949
412.9874536	0.09705318	0.115641713	0.839257539	0.401324805	0.999579949
21.47168712	-0.123097388	0.252856021	-0.486827987	0.626380235	0.999579949
213.5990417	-0.059297384	0.113561087	-0.522162873	0.601556933	0.999579949
27.78519747	-0.256186934	0.239227111	-1.070894236	0.284216988	0.999579949
1262.920692	0.01603333	0.049163965	0.326119555	0.744333902	0.999579949
126.4211876	0.217539446	0.186366442	1.167267262	0.243102455	0.999579949
18618.4689	0.240705719	0.139539283	1.725003265	0.084526884	0.861032139
362.4631108	-0.08235434	0.094966763	-0.867191182	0.385837267	0.999579949
76.63254704	0.154880167	0.172874917	0.895908844	0.37030145	0.999579949
132.5273773	0.175484123	0.277315524	0.632795888	0.526866943	0.999579949
805.8852829	0.034914921	0.051441629	0.678728915	0.497309642	0.999579949
57.54137229	0.400581697	0.300739955	1.331986956	0.182864475	0.983992835
194.3069203	0.156544867	0.115849393	1.351279128	0.176606036	0.983992835
680.7783153	0.039452032	0.124648801	0.316505503	0.751618849	0.999579949
50.65318806	-0.031984099	0.244490368	-0.130819463	0.895918126	0.999579949
238.7274804	0.107775822	0.116426794	0.925696034	0.354603969	0.999579949
1180.306978	0.062707042	0.072378691	0.866374361	0.3862849	0.999579949
208.1855597	0.000795412	0.132192327	0.006017083	0.995199092	0.999579949
857.6247944	-0.010663831	0.088656396	-0.120282709	0.904259201	0.999579949
154.2532746	0.021906414	0.104884161	0.208862942	0.834555234	0.999579949
137.6153787	0.180797666	0.36244804	0.498823683	0.617903602	0.999579949
301.5420295	0.095211048	0.063237099	1.505620118	0.132164721	0.939285826
415.8635345	0.249724076	0.109061016	2.289764801	0.022034955	0.627784819
252.6315098	0.002211096	0.136193602	0.016234946	0.987046956	0.999579949
1145.397159	-0.002214886	0.097195836	-0.022787866	0.981819487	0.999579949
763.9756195	-0.126714117	0.107332662	-1.180573696	0.237772118	0.999579949
268.0541036	0.142416614	0.134182087	1.061368306	0.288522555	0.999579949
2542.366917	-0.03393069	0.106243012	-0.319368672	0.749446964	0.999579949
970.7760646	-0.055959532	0.0813829	-0.68760799	0.491699675	0.999579949
174.5573767	-0.181939996	0.153520683	-1.185117166	0.235971125	0.999579949
30.0386294	0.164369037	0.256349681	0.641190723	0.521398777	0.999579949
445.0221271	0.097011017	0.124644297	0.778302889	0.436390473	0.999579949
1197.25096	0.042282331	0.090582492	0.466782602	0.640655425	0.999579949
544.1488782	-0.101035395	0.127204831	-0.79427326	0.427036367	0.999579949
35.52453275	0.054757984	0.2036868	0.268834229	0.788057252	0.999579949
25.36074536	0.36020035	0.219755281	1.639097586	0.101192938	0.895628172

50.60180723	-0.063399824	0.143449603	-0.441965835	0.658513929	0.999579949
153.969197	0.006308342	0.157486495	0.040056403	0.968048159	0.999579949
613.2991772	-0.024388429	0.118456111	-0.205885781	0.836880133	0.999579949
415.5879794	0.103960168	0.071335487	1.457341544	0.145022124	0.955875831
400.881876	0.108985352	0.066635804	1.635537438	0.101936433	0.895628172
496.8829418	0.067499705	0.139348715	0.48439417	0.628106155	0.999579949
337.5190167	-0.099910566	0.075369869	-1.325603554	0.184971049	0.983992835
1045.412373	-0.076706945	0.077572763	-0.988838637	0.322742097	0.999579949
25.93378099	-0.443167764	0.273156796	-1.622393333	0.104719156	0.895628172
35.7165749	0.039335841	0.234186065	0.167968325	0.866608198	0.999579949
301.4632647	0.2157978	0.25537213	0.845032697	0.398092583	0.999579949
23.38083391	0.14509845	0.22209005	0.653331612	0.513542517	0.999579949
1462.991639	-0.015733151	0.069884664	-0.225130239	0.821877958	0.999579949
519.5797728	0.186577845	0.189026211	0.987047478	0.323619364	0.999579949
2386.023238	0.06036266	0.092284398	0.654093883	0.513051322	0.999579949
1605.831453	-0.124507032	0.129781403	-0.959359572	0.337377634	0.999579949
28.28010495	0.474920501	0.466660622	1.01769997	0.308820562	0.999579949
175.5633944	0.194353661	0.13028279	1.491783072	0.135756023	0.945791454
1415.440403	0.135026915	0.086529011	1.560481431	0.118646155	0.921521033
240.7985222	-0.102278304	0.068373141	-1.495884255	0.13468382	0.945791454
340.2777303	0.602194846	0.303356746	1.985104512	0.047132845	0.768027574
122.4968475	0.048529116	0.134969236	0.359556871	0.719178541	0.999579949
120.7273528	0.017696828	0.095308104	0.185680199	0.852695557	0.999579949
1139.834441	0.074155167	0.141683918	0.523384501	0.600706708	0.999579949
464.7158872	-0.098149794	0.104082059	-0.943003968	0.345678874	0.999579949
90.23808215	-0.013454252	0.163088357	-0.082496704	0.934251739	0.999579949
90.95133073	0.074156636	0.114120723	0.649808675	0.515815815	0.999579949
28.74510329	-0.07839297	0.27674372	-0.283269193	0.776970491	0.999579949
152.9217791	-0.047718864	0.08541747	-0.558654621	0.576397455	0.999579949
48.8847053	-0.044781489	0.247246626	-0.181120727	0.856272816	0.999579949
243.7394678	0.085662456	0.124215506	0.689627725	0.490428328	0.999579949
131.869231	0.029621465	0.203255238	0.145735308	0.884130347	0.999579949
3077.175685	-0.107001766	0.110248263	-0.970552858	0.331770991	0.999579949
192.2120008	-0.149602678	0.193962161	-0.771298266	0.44053016	0.999579949
472.1620123	0.186248357	0.227254526	0.819558406	0.412467894	0.999579949
56.89564745	0.211773856	0.170385792	1.242907948	0.213901757	0.999268361
49.84074829	-0.231296066	0.195917803	-1.180577072	0.237770776	0.999579949
138.6503083	-0.160496191	0.197365341	-0.813193388	0.41610719	0.999579949
150.117779	-0.114510479	0.09591114	-1.193922607	0.232508243	0.999579949
26.6090099	0.155200225	0.206260534	0.752447513	0.451781983	0.999579949
348.661147	-0.168716852	0.202481169	-0.833247127	0.404705369	0.999579949
1288.71343	-0.019564522	0.100895944	-0.193907915	0.846247986	0.999579949
125.9835909	0.280993403	0.23497967	1.195820064	0.231766784	0.999579949
274.7124182	-0.063641717	0.141691259	-0.449157676	0.653317916	0.999579949
21.44555085	-0.528972121	0.211608686	-2.499765635	0.012427549	0.504405069
164.6060426	-0.072220567	0.103296393	-0.699158653	0.484452887	0.999579949
302.6511843	-0.056017399	0.080421582	-0.696546843	0.486086432	0.999579949
208.9555709	0.150665884	0.106038658	1.420858077	0.155358023	0.976030424

107.5346814	0.113988287	0.120156017	0.948668986	0.342788992	0.999579949
273.7333792	-0.037793168	0.138075717	-0.273713355	0.784304914	0.999579949
473.8579611	-0.004016978	0.094252615	-0.042619272	0.966005033	0.999579949
295.8485731	-0.103339922	0.070353671	-1.468863254	0.141869883	0.950022886
974.4907988	0.080338787	0.119260604	0.67364062	0.500539822	0.999579949
289.46964	-0.13706069	0.109350259	-1.253409831	0.210056596	0.996562289
171.8304814	0.048249638	0.090486959	0.533222005	0.59387991	0.999579949
498.7781373	0.042336243	0.110687669	0.382483825	0.702102524	0.999579949
222.4155954	0.117036833	0.110190319	1.062133538	0.288175066	0.999579949
122.885841	-0.187041998	0.160401771	-1.16608437	0.243580335	0.999579949
676.3816147	-0.090245586	0.110214678	-0.818816403	0.412891171	0.999579949
202.5831916	0.109682668	0.130318388	0.841651505	0.399983051	0.999579949
57.31230804	-0.012492191	0.218701888	-0.057119722	0.954449826	0.999579949
121.9902047	-0.204397796	0.287488652	-0.710976919	0.477098539	0.999579949
93.13422673	0.021875561	0.269688236	0.081114258	0.935351086	0.999579949
195.0722383	-0.272555788	0.197789005	-1.378012837	0.168199325	0.980087289
56.56425205	-0.384328471	0.306571484	-1.253634112	0.209975026	0.996550653
37.04911875	-0.016682833	0.325816845	-0.051203102	0.95916368	0.999579949
250.9293303	0.309434575	0.185119922	1.671535788	0.094615896	0.880418343
94.30695929	0.317232101	0.251474604	1.261487627	0.207133215	0.994814558
72.63466993	0.153899337	0.222594239	0.691389579	0.489320751	0.999579949
394.518299	0.100488417	0.158351444	0.634591103	0.525695132	0.999579949
60.15552941	-0.143629046	0.189358809	-0.758502057	0.448150482	0.999579949
232.0585005	0.337766074	0.140867742	2.397753155	0.016495978	0.563098469
31.21441932	-0.033052246	0.171155535	-0.193112339	0.846870989	0.999579949
1118.03182	-0.008870678	0.114171906	-0.077695804	0.938070032	0.999579949
33.4423616	0.05835431	0.295614548	0.197399995	0.843514533	0.999579949
102.7034614	0.58173744	0.19125429	3.041696158	0.002352492	0.244892502
1491.301741	0.004881511	0.140351404	0.034780633	0.972254664	0.999579949
21.65938117	-0.416314542	0.251250644	-1.656969053	0.097525732	0.890209389
56.12735442	-0.005201513	0.140958144	-0.036901114	0.970563851	0.999579949
2566.397984	0.177646926	0.104539705	1.699324931	0.089257978	0.870840008
173.7617457	0.133738712	0.150875724	0.886416369	0.375393202	0.999579949
98.45225639	0.148821997	0.218400223	0.681418706	0.49560659	0.999579949
112.4412892	0.338516843	0.274890727	1.231459664	0.218150994	0.999579949
57.46482468	-0.19265962	0.173637867	-1.109548413	0.267193672	0.999579949
32.16220281	0.12600117	0.254239355	0.495600572	0.620176249	0.999579949
28.1673825	-0.284221006	0.250611493	-1.134110025	0.256748399	0.999579949
67.44232821	-0.225004131	0.162932557	-1.380964828	0.167289775	0.979173796
40.05209792	-0.187579722	0.190527836	-0.984526595	0.324856662	0.999579949
42.79054235	-0.050767819	0.18601205	-0.272927582	0.784908884	0.999579949
94.47118761	-0.099953517	0.153863137	-0.649626148	0.515933738	0.999579949
142.6805384	-0.109250492	0.106739803	-1.023521583	0.306061301	0.999579949
227.3548633	-0.16030309	0.107564976	-1.490290762	0.136147801	0.945791454
1583.357746	0.074638622	0.118630569	0.629168544	0.52923872	0.999579949
1062.254763	0.016928779	0.079892792	0.211893697	0.832189968	0.999579949
42.73089525	0.035290828	0.201685671	0.174979354	0.861095856	0.999579949
77.20138408	0.168042131	0.175829566	0.955710318	0.339218608	0.999579949

144.5819722	-0.290063243	0.204340529	-1.419509113	0.155750642	0.976030424
27.78918185	0.000348843	0.263547625	0.001323642	0.998943886	0.999840121
251.9234495	0.19105382	0.140132852	1.363376381	0.172763921	0.983601342
21.66295284	0.190599366	0.265073308	0.719043977	0.472113824	0.999579949
95.99396271	-0.115555114	0.111288382	-1.038339419	0.299112063	0.999579949
142.3696009	-0.045676691	0.107354889	-0.425473782	0.67049133	0.999579949
416.3620275	-0.02136339	0.131746293	-0.16215553	0.871183378	0.999579949
471.4075135	0.083461723	0.089550666	0.932005611	0.351333625	0.999579949
413.0627103	-0.003357959	0.095345832	-0.035218734	0.971905324	0.999579949
41.24519531	0.040261782	0.176981986	0.227490848	0.820042085	0.999579949
899.1745945	0.06205022	0.078933041	0.786112112	0.431801809	0.999579949
451.938426	-0.068684994	0.105013846	-0.654056552	0.513075372	0.999579949
358.4783408	0.333299958	0.204126304	1.632812389	0.102508461	0.895628172
69.14395883	-0.022535947	0.156576349	-0.143929447	0.885556182	0.999579949
4639.7121	-0.03260373	0.10900716	-0.299097145	0.764865925	0.999579949
192.8398041	-0.272227145	0.26110016	-1.042615776	0.29712628	0.999579949
25.9696928	-0.080841957	0.247714963	-0.326350723	0.744159016	0.999579949
20.95486869	-0.045557176	0.272981103	-0.166887654	0.867458448	0.999579949
225.8182619	-0.095487037	0.113069001	-0.844502346	0.398388753	0.999579949
537.8095083	0.07392556	0.092745888	0.797076414	0.425406653	0.999579949
1135.750676	-0.085662866	0.090480144	-0.946758729	0.343761737	0.999579949
418.7015039	0.163435583	0.11715202	1.395072689	0.162993921	0.978498637
574.555548	0.358677204	0.125941682	2.847962634	0.004400009	0.337942157
67.01181689	0.232954588	0.301502489	0.772645655	0.439732117	0.999579949
366.8161067	-0.022871093	0.103470939	-0.221038813	0.825062212	0.999579949
221.4424595	0.100536031	0.088337984	1.138083827	0.255085481	0.999579949
129.885092	0.478339666	0.246438525	1.941010101	0.052257052	0.780444402
247.2083578	0.225275988	0.144165172	1.562624217	0.118141009	0.921521033
882.1769339	0.131162011	0.089276114	1.469172492	0.141786009	0.949966262
29.15041129	-0.331009951	0.230300533	-1.437295636	0.150634008	0.96651114
378.367398	0.070261631	0.176265999	0.398611366	0.690179585	0.999579949
203.1014497	0.143369962	0.104154242	1.376515825	0.168661991	0.981013142
21.32073094	-0.298321747	0.379570192	-0.785946191	0.431899012	0.999579949
58.26615426	1.151293386	0.525116925	2.192451492	0.028346922	0.666721367
87.0740708	0.264007206	0.322741599	0.818014184	0.413349088	0.999579949
39.44722765	-0.052547705	0.184661621	-0.284562135	0.775979624	0.999579949
214.5726217	0.006374992	0.10046908	0.063452277	0.94940636	0.999579949
26.08736598	0.334576338	0.246404382	1.357834368	0.174516247	0.983992835
40.86316579	-0.017692118	0.176891882	-0.100016563	0.920331176	0.999579949
383.3636945	-0.077309836	0.097185003	-0.795491422	0.426327699	0.999579949
144.6196788	0.121998772	0.175305294	0.695921778	0.486477818	0.999579949
3242.76077	-0.007718313	0.105758913	-0.072980257	0.941821828	0.999579949
130.1668279	-0.021843734	0.16780857	-0.130170552	0.896431492	0.999579949
239.0293952	0.032596498	0.122205852	0.266734344	0.789673709	0.999579949
202.756986	0.117623047	0.138008393	0.852289087	0.394053661	0.999579949
99.85507313	-0.011450833	0.135941855	-0.084233316	0.93287093	0.999579949
5709.642013	-0.031587505	0.119474913	-0.264386089	0.79148243	0.999579949
246.6701104	-0.049281847	0.078897259	-0.624633203	0.532211823	0.999579949

3785.895578	0.014191766	0.120448314	0.117824527	0.906206695	0.999579949
457.1494678	0.08882002	0.086817545	1.023065326	0.30627696	0.999579949
438.2090654	0.000204036	0.053997602	0.003778604	0.996985117	0.999579949
360.734347	0.021591525	0.080155555	0.269370284	0.787644751	0.999579949
1383.205914	-0.239546515	0.192999591	-1.241176286	0.214540633	0.999579949
401.7192891	0.052210157	0.135926894	0.384104684	0.70090086	0.999579949
3870.528083	-0.015356167	0.113163922	-0.135698436	0.892059684	0.999579949
657.7032781	0.074276659	0.112232834	0.661808637	0.50809387	0.999579949
1015.40859	0.070523455	0.080716928	0.873713314	0.382274399	0.999579949
625.1320989	0.7159787	0.286001715	2.503407016	0.0123004	0.503708792
77.00990156	0.375039817	0.14772976	2.538688327	0.01112689	0.487909795
283.3443719	0.093768617	0.084096082	1.115017672	0.264842849	0.999579949
350.4201242	0.07624426	0.088832299	0.858294353	0.390729947	0.999579949
174.8753421	0.039721876	0.099676272	0.398508844	0.69025514	0.999579949
308.6049614	-0.095275911	0.083523107	-1.140713201	0.253989291	0.999579949
625.9062265	0.118112481	0.159443524	0.740779422	0.458827194	0.999579949
281.7566659	0.167037894	0.159063202	1.050135366	0.293655881	0.999579949
229.3811436	-0.092659531	0.090235554	-1.026862773	0.304485086	0.999579949
1540.565978	-0.090052861	0.090242001	-0.997904081	0.318325873	0.999579949
109.9596805	0.024112424	0.124674941	0.193402329	0.846643892	0.999579949
204.9387053	0.010459773	0.167289118	0.062525124	0.950144655	0.999579949
145.3442106	0.224100659	0.099349362	2.255682929	0.024090495	0.634610194
483.3114544	0.0509986	0.150475334	0.338916676	0.734672501	0.999579949
342.5307217	0.000757846	0.080831156	0.009375661	0.992519414	0.999579949
140.675618	-0.317838628	0.176981283	-1.795888367	0.072512273	0.838669905
2932.766871	-0.035113927	0.116341954	-0.30181655	0.762791914	0.999579949
132.8351385	-0.336504543	0.34790663	-0.96722659	0.333430773	0.999579949
2624.366623	0.012950485	0.106085234	0.122076223	0.902838652	0.999579949
42.43134606	0.0186324	0.1996713	0.093315365	0.925653026	0.999579949
219.6416429	-0.050110618	0.085273262	-0.587647479	0.556768935	0.999579949
308.2490837	0.120536266	0.104076185	1.158154152	0.246801134	0.999579949
7503.759596	0.224435298	0.124095454	1.808569859	0.070517855	0.834620594
51.51275069	0.388543682	0.364293924	1.066566465	0.28616765	0.999579949
77.78758024	0.114640118	0.194194794	0.590335692	0.554965615	0.999579949
145.2767604	0.211879177	0.144158221	1.469768254	0.14162453	0.949966262
556.0613383	0.120956787	0.057162758	2.116006831	0.034344227	0.703403556
46.14719261	-0.039678221	0.173203285	-0.229084689	0.818803094	0.999579949
118.1132245	0.040968886	0.130582954	0.313738391	0.75371974	0.999579949
410.4800128	0.190492604	0.144535621	1.317963019	0.187516028	0.983992835
180.2314791	0.150618235	0.163897558	0.91897791	0.358107127	0.999579949
254.842506	0.182141208	0.111431999	1.634550315	0.10214335	0.895628172
138.621838	0.052088637	0.092657469	0.562163396	0.574004698	0.999579949
205.1307979	-0.160065097	0.123142503	-1.299836308	0.193657079	0.986374671
2925.137598	0.023117931	0.104850853	0.220483958	0.825494265	0.999579949
40.64223622	0.477324271	0.277186336	1.722033912	0.085063376	0.864079257
149.3194272	0.12672789	0.19612833	0.646147806	0.518183637	0.999579949
707.2874725	-0.180127576	0.135192267	-1.332380762	0.1827351	0.983992835
1286.227131	-0.132146163	0.081069637	-1.630032757	0.103094574	0.895628172

34.45681438	0.093293145	0.178687938	0.522100967	0.601600033	0.999579949
165.1379147	0.027827944	0.086763201	0.320734404	0.748411673	0.999579949
94.50773428	-0.149450233	0.103030641	-1.450541614	0.146907543	0.959874957
375.883076	-0.041236951	0.063770691	-0.646644254	0.51786221	0.999579949
89.71567483	0.055554453	0.12677377	0.438217255	0.661228802	0.999579949
1304.385321	0.180230619	0.124909399	1.442890777	0.149051242	0.962301096
1060.936937	0.012129412	0.088324666	0.137327569	0.89077188	0.999579949
2933.753897	-0.055540547	0.103072102	-0.538851407	0.589989389	0.999579949
301.4989372	-0.189322846	0.470942191	-0.402008675	0.687677642	0.999579949
499.5894884	-0.056519744	0.05820293	-0.971080741	0.331508073	0.999579949
449.8647737	0.104543612	0.201891415	0.517820988	0.604583173	0.999579949
154.1173731	-0.195496631	0.119908284	-1.630384694	0.103020217	0.895628172
998.596222	0.069294841	0.111702411	0.620352245	0.535025906	0.999579949
143.129222	0.053621734	0.102131736	0.525025188	0.599565681	0.999579949
93.00648612	-0.018232093	0.107738934	-0.169224735	0.865619875	0.999579949
1499.865441	0.095633131	0.113691702	0.841161925	0.400257227	0.999579949
108.6382222	0.331800847	0.177832582	1.865804584	0.062068711	0.805306145
199.1292448	-0.049969344	0.11234288	-0.444793157	0.65646924	0.999579949
682.7624892	-0.015311628	0.109648343	-0.139643041	0.888942032	0.999579949
20.46820615	-0.054826795	0.326695969	-0.167822073	0.866723257	0.999579949
126.3880838	0.072134564	0.106981074	0.674274073	0.500137084	0.999579949
116.1809785	0.072506726	0.129179787	0.561285382	0.574603005	0.999579949
815.467052	-0.163758464	0.072738094	-2.2513439	0.024363764	0.63537469
356.7782252	-0.144576787	0.091685079	-1.57688457	0.114822088	0.918818353
130.288967	-0.011623319	0.103948335	-0.111818229	0.910967534	0.999579949
460.5469233	-0.109441977	0.174918988	-0.625672365	0.531529863	0.999579949
1366.423795	-0.039745908	0.069742781	-0.569892787	0.568750417	0.999579949
40.98450123	0.158847968	0.189834623	0.836770266	0.402721714	0.999579949
214.1567272	1.029474849	0.383499431	2.684423404	0.007265503	0.409474612
267.3652236	-0.040767544	0.103709238	-0.393094629	0.694249591	0.999579949
86.24360805	-0.037843721	0.154093489	-0.245589359	0.806000127	0.999579949
207.7563606	0.034012744	0.121450042	0.280055434	0.779434976	0.999579949
58.44662157	-0.056857073	0.156963887	-0.362230279	0.717179954	0.999579949
1360.40746	-0.051809626	0.092240362	-0.56168064	0.574333627	0.999579949
317.112836	0.074189285	0.09294305	0.798223051	0.424741063	0.999579949
925.9486844	0.072962215	0.115366925	0.632436154	0.527101916	0.999579949
306.9101026	0.127949986	0.137236481	0.932332168	0.351164888	0.999579949
71.2408709	0.094119517	0.448954309	0.209641638	0.833947379	0.999579949
37.54646561	0.269781596	0.263247181	1.024822357	0.305447015	0.999579949
74.41601859	0.043761727	0.169127382	0.2587501	0.795828061	0.999579949
21.4688504	0.226059751	0.225126151	1.004147009	0.31530776	0.999579949
258.0197166	0.387443258	0.276345002	1.402027374	0.160907073	0.978498637
209.113624	0.044580024	0.10890293	0.409355598	0.68227872	0.999579949
5325.539561	0.185428757	0.14810921	1.251973172	0.21057964	0.996581389
771.1845254	-0.058741058	0.059851049	-0.981454114	0.326368852	0.999579949
60.72638911	0.035253524	0.166726318	0.211445467	0.832539681	0.999579949
22.43490968	0.077204833	0.26530993	0.290998656	0.771052347	0.999579949
2877.090942	0.042886764	0.107100307	0.400435491	0.688835788	0.999579949

698.8176247	0.048254927	0.073202181	0.659200675	0.509766914	0.999579949
172.3756339	-0.009677468	0.142769286	-0.067783964	0.945957609	0.999579949
118.2451196	-0.081682838	0.099194836	-0.823458564	0.410247271	0.999579949
247.4206611	-0.042241989	0.101752364	-0.415145039	0.67803573	0.999579949
402.5253081	0.019569608	0.172348397	0.113546793	0.909597068	0.999579949
1245.063624	0.015349641	0.146792453	0.104566963	0.91671943	0.999579949
1854.278293	-0.464980453	0.27318645	-1.702062653	0.088743624	0.870840008
332.3686945	0.063552313	0.120255628	0.528476826	0.597168427	0.999579949
481.3174577	-0.040422814	0.171586337	-0.235582941	0.813756312	0.999579949
124.5696789	0.119680768	0.208011527	0.575356424	0.565050269	0.999579949
225.2678855	0.078133869	0.101812136	0.767431781	0.442824839	0.999579949
833.0673117	-0.321441211	0.087356749	-3.679637967	0.000233565	0.071755818
56.13090869	-0.388591091	0.233501296	-1.664192441	0.096073978	0.88463985
99.64552149	-0.20080697	0.135051951	-1.486886853	0.137044697	0.945791454
455.042189	0.067195742	0.05558578	1.2088657	0.226714446	0.999579949
34.2039456	0.097642386	0.275473372	0.354453081	0.72299936	0.999579949
799.4470619	0.283873519	0.132801716	2.13757418	0.032551319	0.693767202
241.1863007	-0.31475218	0.172747861	-1.822032283	0.068450087	0.830633239
1066.927281	-0.243728181	0.156684586	-1.555533871	0.119818974	0.921521033
1866.408486	-0.111093928	0.073270608	-1.516214078	0.12946523	0.935700262
889.5525206	0.041398669	0.117628101	0.351945397	0.724879213	0.999579949
158.8321878	0.052879089	0.147211537	0.359204788	0.719441895	0.999579949
1538.961594	0.010638923	0.116427899	0.091377783	0.927192415	0.999579949
22.07909875	-0.111911756	0.238064399	-0.470090264	0.63829053	0.999579949
169.1157252	0.093622488	0.142184879	0.658456009	0.510245156	0.999579949
585.7759183	-0.27410397	0.176254975	-1.55515593	0.119908937	0.921521033
275.5389963	0.049768578	0.067565511	0.736597383	0.461367227	0.999579949
95.24245359	0.055709087	0.108452602	0.513672205	0.607481188	0.999579949
2892.093427	-0.172367655	0.076097559	-2.26508783	0.02350729	0.62865781
141.9416744	-0.188698292	0.103864449	-1.816774603	0.069251633	0.831457432
119.3977693	-0.139857982	0.101717802	-1.37496072	0.169143623	0.981013142
483.3447534	-0.237324354	0.115399887	-2.056538887	0.039730602	0.73515901
687.3149366	-0.001251062	0.056812757	-0.02202079	0.982431371	0.999579949
229.7005542	-0.017370825	0.090891458	-0.191116148	0.848434597	0.999579949
375.1354722	-0.097884226	0.069038819	-1.41781431	0.156244985	0.976030424
116.8919831	0.001426796	0.134009717	0.010646959	0.991505116	0.999579949
210.274365	0.128864561	0.228754556	0.563331123	0.573209429	0.999579949
32.87135442	-0.186145376	0.206527891	-0.901308655	0.367424233	0.999579949
250.0462225	-0.048602006	0.143206407	-0.339384302	0.734320243	0.999579949
167.1217844	0.124451152	0.128440986	0.968936444	0.332576901	0.999579949
1617.956591	0.060999169	0.094537981	0.645234531	0.518775212	0.999579949
41.37507965	-0.016481614	0.188384313	-0.087489312	0.930282581	0.999579949
615.8628287	-0.069520537	0.095429516	-0.728501409	0.466306706	0.999579949
99.85966884	-0.101812166	0.140581738	-0.724220427	0.46893042	0.999579949
897.7036831	-0.142519665	0.093072466	-1.531276344	0.12570111	0.929730579
417.3431061	0.017246557	0.086559018	0.199246213	0.842070151	0.999579949
1343.465821	-0.028053023	0.117327123	-0.239100917	0.81102733	0.999579949
1275.058144	0.2403189	0.138124106	1.739876605	0.081880688	0.85448976

190.8855796	0.120213761	0.151109432	0.795541083	0.426298823	0.999579949
344.7831672	-0.033663501	0.085235004	-0.394949249	0.692880343	0.999579949
409.00782	-0.017904986	0.169996473	-0.10532563	0.916117426	0.999579949
464.4905255	-0.103254928	0.084710799	-1.218911047	0.222877956	0.999579949
23.64528574	0.703106501	0.297390007	2.36425732	0.018066259	0.578992883
81.85783449	0.03247383	0.13814659	0.235067907	0.814156027	0.999579949
40.37839266	0.442641586	0.171224788	2.585148979	0.009733693	0.467960289
23.78375177	0.448324755	0.308935452	1.451192317	0.146726316	0.959874957
564.5876175	0.045113417	0.139108745	0.324303239	0.745708468	0.999579949
2491.535759	-0.013032153	0.144172378	-0.090392854	0.927975035	0.999579949
25.24550017	-0.381247128	0.29049828	-1.31239031	0.189388475	0.983992835
153.0009888	0.116177579	0.119871859	0.969181425	0.332454677	0.999579949
524.3342775	0.101569194	0.120077028	0.845866983	0.397626953	0.999579949
207.9752595	0.653768836	0.199245846	3.281216886	0.001033602	0.165012851
42.1060979	-0.019912167	0.202580038	-0.098292838	0.921699765	0.999579949
214.6308984	0.075263981	0.091964858	0.818399362	0.413129186	0.999579949
1086.179727	-0.075421755	0.083596626	-0.902210508	0.366945054	0.999579949
1929.054169	-0.04821478	0.11288471	-0.427115236	0.669295396	0.999579949
1494.84096	-0.0123021	0.111565851	-0.110267615	0.91219714	0.999579949
375.770398	0.041253926	0.264246022	0.156119384	0.87593892	0.999579949
1658.618153	0.265525091	0.156967643	1.691591255	0.090723935	0.877608448
87.39547489	0.537723278	0.23253225	2.312467533	0.020751933	0.61215771
373.3067195	-0.08188056	0.11369053	-0.720205627	0.4713984	0.999579949
92.44217343	-0.367347417	0.619592719	-0.592885303	0.553257915	0.999579949
52.2753026	0.073325147	0.240751235	0.304568102	0.760695117	0.999579949
99.07114569	0.230401586	0.287825401	0.800490802	0.423426491	0.999579949
127.5693677	0.04022824	0.11927269	0.337279555	0.73590617	0.999579949
48.68991534	-0.135034248	0.197654632	-0.683182816	0.49449133	0.999579949
1025.054866	-0.032594923	0.063397109	-0.51413895	0.607154847	0.999579949
34.08709976	0.329136867	0.186064257	1.768941933	0.076903565	0.851207389
343.9867083	0.067063857	0.074984166	0.894373583	0.371122042	0.999579949
421.3443122	-0.041936283	0.095969925	-0.436973176	0.662130801	0.999579949
62.28787085	0.022346824	0.130902139	0.170713972	0.864448676	0.999579949
314.4816448	-0.050611049	0.090764124	-0.557610725	0.577110231	0.999579949
198.8125048	0.018844709	0.083154481	0.226622896	0.820716987	0.999579949
343.7686941	0.005200289	0.109677713	0.047414275	0.962183052	0.999579949
48.92081003	0.141299404	0.198912436	0.710359831	0.477481026	0.999579949
79.43550252	-0.034039776	0.125253376	-0.27176733	0.785800927	0.999579949
1078.587591	-0.239200106	0.585800004	-0.408330666	0.683030928	0.999579949
189.623287	-0.05805769	0.115554974	-0.50242485	0.615368703	0.999579949
22.45405381	-0.369838568	0.315776122	-1.171204984	0.241516392	0.999579949
96.1592201	-0.138907459	0.19544395	-0.710727852	0.477252897	0.999579949
326.2629533	-0.024329394	0.100827029	-0.241298337	0.8093239	0.999579949
532.4700167	-0.079145899	0.085924572	-0.921109031	0.356993506	0.999579949
360.6038969	0.02490445	0.072030255	0.345749857	0.729530712	0.999579949
116.0072825	-0.003979868	0.156889277	-0.025367369	0.979761939	0.999579949
189.7902809	0.027485045	0.196016514	0.140218007	0.888487745	0.999579949
532.0618949	-0.035110336	0.055852119	-0.62863033	0.529591099	0.999579949

33.8695133	0.123069373	0.177004048	0.695291291	0.486872771	0.999579949
501.6201386	0.010306292	0.06086245	0.169337454	0.865531217	0.999579949
296.2267781	-0.089118042	0.07888889	-1.129665312	0.258617279	0.999579949
308.1192708	-0.043646926	0.177409481	-0.246023636	0.805663936	0.999579949
82.50135059	0.175228883	0.210145419	0.833845839	0.404367861	0.999579949
53.53745368	0.567858197	0.403323514	1.407947163	0.159146716	0.977762491
624.6378849	-0.016873122	0.233891903	-0.072140686	0.942489948	0.999579949
207.9967844	-0.297891091	0.358420488	-0.831121829	0.405904809	0.999579949
92.70231359	0.01377848	0.111152876	0.123959723	0.901347165	0.999579949
779.9766814	-0.148172476	0.104653838	-1.415834131	0.156824074	0.977304288
2256.313055	-0.076769834	0.070268504	-1.092521248	0.274604056	0.999579949
2746.894891	-0.021977075	0.115844162	-0.189712405	0.849534499	0.999579949
1414.260091	-0.11387629	0.078288361	-1.45457497	0.145786964	0.957420545
27.72273904	0.128025711	0.252291676	0.507451191	0.611838275	0.999579949
33.1378664	0.399366566	0.21197142	1.884058544	0.059557057	0.798635337
145.9016383	0.031235227	0.119518098	0.261343076	0.793827951	0.999579949
1413.272148	-0.318520721	0.095798345	-3.324908403	0.000884476	0.150552137
858.2823831	0.053901026	0.083349527	0.646686646	0.517834767	0.999579949
705.0388183	0.034428145	0.059103647	0.58250457	0.560226864	0.999579949
714.5223119	0.041098387	0.084158698	0.488343896	0.625306274	0.999579949
38.9920332	0.297212227	0.354083566	0.839384416	0.401253626	0.999579949
109.8003844	0.012730955	0.113465274	0.112201335	0.910663771	0.999579949
129.3004089	0.259772768	0.309222145	0.84008462	0.400860943	0.999579949
133.9711707	0.209123224	0.251800866	0.830510344	0.406250302	0.999579949
1473.938522	0.078004297	0.1831216	0.42596994	0.67012975	0.999579949
214.658841	0.100279008	0.125954053	0.796155473	0.425941675	0.999579949
470.7201255	0.012852296	0.118671328	0.108301613	0.913756443	0.999579949
598.95514	-0.00500062	0.099781562	-0.05011567	0.960030212	0.999579949
106.1664822	-0.375968991	0.286769278	-1.311050448	0.18984072	0.98445192
622.9694931	-0.029099677	0.067813156	-0.429115508	0.667839167	0.999579949
41.05168141	0.131621813	0.18653615	0.705610217	0.480430569	0.999579949
175.6423174	-0.140090545	0.161197713	-0.869060378	0.384814107	0.999579949
124.5909216	0.007583669	0.09972008	0.076049566	0.939379664	0.999579949
173.6065374	-0.268243038	0.167226667	-1.604068553	0.108698943	0.903743816
95.46562565	-0.002385491	0.121677294	-0.019605059	0.984358428	0.999579949
43.88302668	-0.500941009	0.275984962	-1.81510255	0.069508151	0.831457432
2023.796981	-0.035635869	0.064513309	-0.552380111	0.580687954	0.999579949
58.05585195	0.280115166	0.266742247	1.050134234	0.293656401	0.999579949
319.8601007	-0.061744333	0.113734255	-0.542882465	0.587210729	0.999579949
1005.093019	-0.006294562	0.073868503	-0.085213076	0.932091995	0.999579949
128.3745272	0.09813292	0.092774517	1.057757278	0.290166115	0.999579949
6536.176008	-0.059341534	0.06399143	-0.92733565	0.35375229	0.999579949
55.89507508	-0.356827029	0.235911641	-1.512545235	0.130395214	0.935700262
63.59403796	0.183896324	0.144984937	1.268382277	0.204661468	0.994814558
1020.600525	-0.011357536	0.06362212	-0.178515526	0.858318129	0.999579949
116.6617647	-0.056137989	0.372724748	-0.150615137	0.88027932	0.999579949
317.2864624	0.158008163	0.118393756	1.334598783	0.182007697	0.983992835
640.4812374	-0.005251549	0.123986592	-0.042355779	0.96621508	0.999579949

32.76296932	0.372183159	0.214166576	1.737820939	0.08224237	0.856126169
801.5074587	0.02086835	0.065142967	0.320346933	0.748705349	0.999579949
1422.745685	0.077303624	0.073122994	1.057172571	0.290432836	0.999579949
24.41612948	0.44577361	0.520397802	0.856601639	0.391665079	0.999579949
1629.865735	-0.002870219	0.084101519	-0.034128027	0.97277506	0.999579949
116.2360384	-0.02104265	0.108377189	-0.194161247	0.846049626	0.999579949
1827.559508	-0.141482357	0.122603447	-1.153983513	0.248506928	0.999579949
50.6753628	0.016048809	0.257744527	0.062266344	0.95035073	0.999579949
37.38267354	-0.508182644	0.407573179	-1.246850063	0.21245248	0.999268361
28.41821899	-0.321707681	0.235707552	-1.364859455	0.172297228	0.983601342
450.976364	0.069598149	0.092017852	0.75635486	0.449436464	0.999579949
259.9344296	-0.262831227	0.126014243	-2.085726357	0.037003418	0.717947029
170.7086855	0.012883669	0.161225067	0.079911082	0.936307976	0.999579949
39.01929448	0.130270392	0.310581016	0.419440935	0.674893913	0.999579949
134.63655	1.003396852	0.413762137	2.425057204	0.015305975	0.542093008
790.8541756	-0.091055676	0.069236023	-1.31514885	0.188459882	0.983992835
39.81804419	-0.239193774	0.302482618	-0.790768659	0.429079002	0.999579949
182.2655147	-0.067198403	0.088187805	-0.761992011	0.446064772	0.999579949
24.19701417	1.137397312	0.645157356	1.762976586	0.077904439	0.851207389
285.0166447	0.137845563	0.13105654	1.051802241	0.292890291	0.999579949
608.8670859	-0.006720312	0.107231751	-0.062670918	0.950028556	0.999579949
26.45257152	0.172141074	0.260248965	0.661447681	0.508325256	0.999579949
22.46894146	0.143282548	0.469912944	0.304912963	0.760432442	0.999579949
213.8297811	0.037712956	0.160244212	0.235346762	0.813939604	0.999579949
653.1196378	-0.045584727	0.135259399	-0.337017075	0.736104029	0.999579949
230.0644173	-0.083072807	0.174003248	-0.477421014	0.633062354	0.999579949
292.5140733	0.187572948	0.126213923	1.486151002	0.137239184	0.945791454
1376.048657	0.085185213	0.087233387	0.976520757	0.328806467	0.999579949
49.97086138	-1.269921275	0.51622326	-2.460023355	0.013892797	0.520814515
513.7173324	0.003864665	0.111326145	0.034714799	0.97230716	0.999579949
1110.967152	-0.26207933	0.089002254	-2.944637	0.003233338	0.304904733
26.79965918	-0.371825937	0.240768436	-1.544330077	0.12250839	0.92236461
67.31606826	-0.112572032	0.1447554	-0.777670689	0.436763176	0.999579949
24045.61403	-0.193104496	0.123713472	-1.560901109	0.118547086	0.921521033
931.0514456	0.047384704	0.085010381	0.557399029	0.577254829	0.999579949
23.1738101	-0.435445267	0.297756617	-1.462420119	0.143626119	0.953645321
106.5202561	0.129718567	0.18638819	0.695959156	0.486454409	0.999579949
20.64923226	0.104211395	0.249547333	0.417601717	0.676238341	0.999579949
1061.608024	-0.131683125	0.16561914	-0.795096053	0.42655763	0.999579949
2016.071038	-0.025125474	0.11119856	-0.225951437	0.821239191	0.999579949
900.1868617	-0.058920226	0.106342278	-0.554062102	0.579536345	0.999579949
386.0029714	-0.067954693	0.069332254	-0.98013102	0.327021449	0.999579949
257.8442088	0.002872972	0.077186708	0.037221078	0.970308732	0.999579949
52.47467723	0.009271884	0.180532852	0.051358428	0.95903991	0.999579949
3234.720075	0.023093902	0.069928798	0.330248807	0.741211971	0.999579949
319.7045806	0.056512235	0.094496885	0.598032779	0.549818062	0.999579949
351.5118759	-0.10835844	0.09684876	-1.118841789	0.26320764	0.999579949
3494.053482	-0.220148114	0.119763341	-1.838192828	0.066033996	0.821508842

249.2491851	0.227597823	0.121973254	1.865965004	0.062046262	0.805306145
285.8705929	-0.158216622	0.164908976	-0.959417889	0.337348266	0.999579949
1120.625229	0.091055308	0.138200555	0.658863549	0.509983395	0.999579949
327.5827866	-0.068657339	0.077517779	-0.885697961	0.375780307	0.999579949
79.98715905	0.05620025	0.253983605	0.221275111	0.824878228	0.999579949
25.89961731	0.137407679	0.211341551	0.650168784	0.515583203	0.999579949
36.31810675	-0.253168855	0.338614055	-0.747661981	0.454664069	0.999579949
323.2753597	-0.101081319	0.100709637	-1.003690626	0.315527757	0.999579949
413.2781762	-0.018186658	0.181729161	-0.100075618	0.920284292	0.999579949
284.0338697	0.311200719	0.145540875	2.138235872	0.032497604	0.693767202
1141.238586	0.309461897	0.159428494	1.941070185	0.052249765	0.780444402
24.54467637	-0.248612835	0.30648234	-0.8111816	0.417261391	0.999579949
173.5498918	0.010286341	0.185717999	0.055386885	0.955830244	0.999579949
113.0298765	-0.172378594	0.221840284	-0.777039188	0.43713565	0.999579949
32.04536153	-0.256032844	0.285652589	-0.8963085	0.370088021	0.999579949
2642.813011	0.025840767	0.107220804	0.241005161	0.809551116	0.999579949
112.3830914	0.082949803	0.110471637	0.750869681	0.452731088	0.999579949
21.40025304	0.386605305	0.300375449	1.287073581	0.198068643	0.992481822
1708.397384	-0.035505147	0.13663523	-0.259853527	0.79497676	0.999579949
642.8631034	-0.27454627	0.263735289	-1.04099179	0.297879359	0.999579949
58.18860882	0.058678357	0.240328518	0.244158943	0.807107723	0.999579949
21.56616103	0.072116061	0.231479478	0.31154408	0.755387038	0.999579949
587.8341042	-0.18841912	0.100493587	-1.874936752	0.060801425	0.801515896
48.57359386	-0.216810073	0.218624489	-0.991700766	0.321343517	0.999579949
116.2353757	-0.402904553	0.178671128	-2.255006484	0.024132922	0.634610194
137.4666149	0.020363105	0.094210386	0.216145008	0.828874718	0.999579949
69.86943114	0.35408059	0.166648987	2.124708931	0.033610908	0.698554126
51.99822525	-0.072145801	0.155144738	-0.465022544	0.641915312	0.999579949
186.6509453	-0.156170388	0.088650653	-1.761638331	0.078130423	0.851207389
1513.412267	0.055088764	0.124650219	0.441946788	0.658527712	0.999579949
27.14328793	0.140013147	0.188385133	0.743228219	0.457343521	0.999579949
945.2365966	0.077814178	0.083218833	0.935054912	0.349760001	0.999579949
124.1469859	0.032196339	0.13319484	0.241723619	0.808994327	0.999579949
31.87217132	0.006902092	0.288257167	0.023944216	0.980897105	0.999579949
83.11052801	0.028794732	0.138886965	0.207324941	0.835756097	0.999579949
560.0924648	0.260736643	0.094648004	2.754803405	0.005872741	0.372390484
26.57023798	-0.140547722	0.205399469	-0.684265265	0.493807677	0.999579949
13007.51151	0.159186592	0.147138183	1.081884997	0.279303632	0.999579949
3055.543131	-0.073191895	0.136913929	-0.534583267	0.592938057	0.999579949
240.5428889	-0.048629485	0.17246996	-0.28195916	0.777974826	0.999579949
2453.640153	-0.020107058	0.072997281	-0.275449407	0.78297099	0.999579949
31.34214636	-0.217671027	0.190964946	-1.13984808	0.254349599	0.999579949
211.6424238	-0.009638992	0.121788344	-0.079145439	0.936916941	0.999579949
432.8167262	0.139522171	0.055417132	2.517672188	0.011813321	0.494354131
297.8447064	-0.066046528	0.094931244	-0.695730145	0.486597843	0.999579949
27.70098366	-0.18878796	0.231554507	-0.815306783	0.414896727	0.999579949
604.6363607	0.075462156	0.112186623	0.672648433	0.501170981	0.999579949
69.93899135	0.912469464	0.264362446	3.451585039	0.000557304	0.12017272

273.7072689	0.082341225	0.091204084	0.902823882	0.366619374	0.999579949
122.6821183	-0.017724567	0.246888179	-0.071791882	0.942767534	0.999579949
77.22687841	0.141236069	0.226414319	0.623794774	0.532762372	0.999579949
682.017796	-0.00750685	0.155259049	-0.048350482	0.961436922	0.999579949
958.1336682	0.09335055	0.071450512	1.306506379	0.191380411	0.984804292
119.832436	0.648444818	0.55668632	1.164829806	0.244087891	0.999579949
162.3859791	0.633826762	0.380691375	1.664935963	0.095925532	0.88463985
200.8960731	0.228825979	0.160278349	1.427678663	0.15338437	0.973156976
531.1927294	0.012473761	0.065955157	0.189124882	0.84999494	0.999579949
205.2034713	0.318837726	0.235352279	1.354725466	0.175505041	0.983992835
148.0302975	0.030661978	0.448607799	0.068349185	0.945507671	0.999579949
25.97987775	-0.063621338	0.36075076	-0.176358155	0.860012577	0.999579949
155.1640946	0.005653794	0.160942924	0.035129189	0.971976726	0.999579949
1193.15823	-0.001571674	0.109431869	-0.014362125	0.988541076	0.999579949
105.3216949	0.02562365	0.111929975	0.228925723	0.818926648	0.999579949
100.3879543	0.374263055	0.843828517	0.443529755	0.657382602	0.999579949
311.6916351	-0.094046425	0.356667695	-0.263680806	0.792025887	0.999579949
322.7879085	0.038118697	0.081645012	0.466883352	0.640583337	0.999579949
789.6227322	-0.053034378	0.05109103	-1.03803697	0.299252844	0.999579949
143.2684331	-0.09449	0.153324793	-0.616273457	0.537714054	0.999579949
79.04770557	-0.009158336	0.13610552	-0.067288501	0.946352031	0.999579949
103.0587438	0.036325912	0.115364374	0.314879805	0.752852915	0.999579949
226.0733899	-0.054795883	0.090738428	-0.603888395	0.545917845	0.999579949
2017.323155	0.630359482	0.179242992	3.516787316	0.000436804	0.103810916
265.6524656	-0.055554197	0.125990725	-0.44093878	0.659257318	0.999579949
4472.928535	-0.047115288	0.111434406	-0.422807366	0.672435813	0.999579949
249.0351511	0.2676838	0.124239762	2.154574315	0.031195161	0.683621001
278.7370034	0.072096328	0.131171468	0.549634223	0.582570281	0.999579949
790.4875826	0.069289109	0.083423767	0.830567976	0.406217732	0.999579949
39.09393685	-0.243570053	0.211344712	-1.152477631	0.249124857	0.999579949
60.27438946	-0.071496221	0.539736593	-0.132465025	0.894616485	0.999579949
358.2357685	0.073186907	0.128239045	0.570706895	0.568198344	0.999579949
328.9363816	0.111915665	0.121515714	0.920997471	0.357051747	0.999579949
278.1550109	-0.150648296	0.101561562	-1.483319995	0.137989412	0.945791454
1377.191274	-0.149123014	0.103485182	-1.441008382	0.149582315	0.963638931
1239.30949	0.049883585	0.103049152	0.48407565	0.628332182	0.999579949
647.0146737	-0.030946665	0.136873908	-0.226096163	0.821126629	0.999579949
180.4852464	0.009742504	0.1052672	0.092550236	0.92626088	0.999579949
708.109986	-0.036450269	0.094441038	-0.385957941	0.699527818	0.999579949
241.8204521	0.120690191	0.086672697	1.392482231	0.163776419	0.978498637
121.0516761	0.01650617	0.158850976	0.103909779	0.917240947	0.999579949
255.5852449	-0.097959508	0.081742391	-1.198392984	0.230764063	0.999579949
814.5489258	-0.101003192	0.088257197	-1.14441876	0.252450012	0.999579949
278.6596134	0.145938112	0.086558296	1.686009532	0.091793963	0.880418343
26.97059877	0.132459434	0.208218089	0.636157186	0.524673976	0.999579949
133.0759996	-0.025481463	0.175851731	-0.144903112	0.88478737	0.999579949
39.80825514	-0.173065166	0.277689913	-0.623231734	0.53313225	0.999579949
826.0580444	0.000732245	0.109979326	0.006658022	0.994687706	0.999579949

381.0502969	0.062736767	0.099770496	0.628810819	0.529472916	0.999579949
2070.120334	0.064755968	0.123962722	0.522382596	0.601403971	0.999579949
60.55250027	-0.136309006	0.201079311	-0.677886773	0.497843487	0.999579949
536.4691102	-0.039385939	0.06171313	-0.638210035	0.523336966	0.999579949
79.71865164	-0.244526544	0.179463603	-1.362541154	0.173027166	0.983601342
412.4262574	0.102764445	0.07640052	1.345075206	0.178600949	0.983992835
92.41539976	0.127739867	0.164628661	0.775927264	0.437791934	0.999579949
38.54076411	0.094407382	0.159228707	0.592904282	0.553245212	0.999579949
332.4201232	0.300095422	0.161331393	1.860117958	0.062868839	0.807133028
43.2270627	0.461047089	0.208560971	2.210610579	0.027062816	0.654286419
655.5725548	-0.014943049	0.168698468	-0.088578452	0.929416933	0.999579949
50.90119046	-0.162180831	0.22310949	-0.726911397	0.467280235	0.999579949
32.25247072	-0.201930186	0.209226178	-0.965128684	0.334480362	0.999579949
83.97507589	-0.05522871	0.201149337	-0.274565707	0.783649916	0.999579949
614.7005852	0.006418482	0.120988732	0.053050245	0.957691874	0.999579949
1250.469662	0.000995011	0.096396007	0.010322117	0.991764288	0.999579949
101.4891719	0.087158334	0.128130356	0.680231732	0.496357743	0.999579949
169.4441289	0.146303222	0.106077791	1.379206903	0.16783097	0.980087289
267.1473747	0.14087954	0.109651006	1.28479934	0.198862413	0.992481822
87.39719615	0.008765008	0.131186887	0.066813138	0.946730464	0.999579949
168.8820281	0.05644578	0.134751446	0.418888123	0.675297897	0.999579949
298.3652058	0.107934735	0.115045767	0.938189537	0.348147014	0.999579949
2398.198584	0.196889353	0.141527945	1.391169444	0.164174051	0.978498637
63.36110526	1.076206391	0.493294365	2.181671769	0.029133766	0.672989344
1566.210802	0.056840366	0.12266509	0.463378501	0.643093086	0.999579949
47.07382517	-0.025114847	0.147544676	-0.170218591	0.864838231	0.999579949
28.46788312	-0.338941636	0.266442413	-1.272100912	0.203337267	0.994409048
305.1533522	0.046000833	0.108415168	0.424302554	0.671345177	0.999579949
504.7715541	0.152663315	0.143018564	1.067437053	0.285774524	0.999579949
31.74808441	0.309681643	0.310807944	0.996376214	0.319067386	0.999579949
1380.906107	0.091145427	0.076994394	1.183793026	0.236495003	0.999579949
260.6197027	0.054601989	0.146505929	0.372694738	0.709375653	0.999579949
111.958986	0.1222488	0.205775959	0.594086893	0.552453997	0.999579949
74.48686621	-0.095368751	0.140306641	-0.679716584	0.496683933	0.999579949
1797.103666	0.043595682	0.054543811	0.799278253	0.424129086	0.999579949
353.7969693	0.142984086	0.133613383	1.070132966	0.284559462	0.999579949
30.48643012	0.286642408	0.192119113	1.492003598	0.135698202	0.945791454
236.6365455	0.148104448	0.166932732	0.887210354	0.374965659	0.999579949
234.729144	-0.181596757	0.077824489	-2.333414066	0.019626424	0.601494972
29.96222714	-0.053730308	0.219313771	-0.24499286	0.80646196	0.999579949
169.5096293	0.121694289	0.136238275	0.893245961	0.37172547	0.999579949
341.6164307	0.532660701	0.266035965	2.002213127	0.045261814	0.761171983
31.19029355	0.53354076	0.43011638	1.240456736	0.214806507	0.999579949
142.738274	0.118830459	0.145002687	0.819505218	0.412498226	0.999579949
331.7718452	0.006168722	0.080829389	0.076317809	0.939166257	0.999579949
62.49649382	-0.002459175	0.15109514	-0.016275675	0.987014464	0.999579949
382.9758833	-0.022750165	0.0949349	-0.239639636	0.810609635	0.999579949
103.9195501	0.044822349	0.143383571	0.312604496	0.754581162	0.999579949

39.38371048	0.040027567	0.169421154	0.236260738	0.813230351	0.999579949
654.6355359	0.063095364	0.072681975	0.868101947	0.385338525	0.999579949
1957.014901	-0.03378221	0.06685904	-0.505275133	0.613365606	0.999579949
424.4425921	-0.110785872	0.131516799	-0.842370507	0.399580596	0.999579949
95.57860002	0.220358034	0.16188162	1.361229485	0.173441179	0.983601342
76.87227171	-0.165992398	0.158219361	-1.049128227	0.294119106	0.999579949
64.04943614	-0.260120355	0.159490385	-1.630946931	0.102901518	0.895628172
57.84586051	-0.243989969	0.214202478	-1.13906231	0.254677166	0.999579949
565.9471946	-0.15986054	0.273137419	-0.585275135	0.558362731	0.999579949
41.82721511	-0.005210484	0.190510859	-0.027350064	0.978180527	0.999579949
20.59691928	0.121049812	0.232181413	0.521358753	0.602116879	0.999579949
39.41621885	-0.110237516	0.176964171	-0.622936923	0.533325973	0.999579949
29.85475759	-0.322917811	0.203364658	-1.587875756	0.112314437	0.914735409
522.320278	0.057595122	0.099688586	0.57775041	0.56343264	0.999579949
38.78612466	0.213826729	0.162424363	1.316469557	0.18801649	0.983992835
35.69157658	-0.084753936	0.213488905	-0.396994572	0.691371464	0.999579949
213.4573499	-0.018600647	0.121649161	-0.152904028	0.87847396	0.999579949
158.5750909	0.001890926	0.099676992	0.018970533	0.984864612	0.999579949
221.9241638	0.100046991	0.093558905	1.069347606	0.284913067	0.999579949
56.2339552	-0.108395296	0.156202477	-0.693940952	0.48771924	0.999579949
58.32428225	-0.076393895	0.156648808	-0.487676197	0.625779215	0.999579949
2896.791683	0.011404212	0.073854686	0.1544142	0.877283158	0.999579949
102.573826	0.039753184	0.113506437	0.350228458	0.726167251	0.999579949
59.10909628	0.126789497	0.148581319	0.85333404	0.393474088	0.999579949
86.80462546	0.074300919	0.120377104	0.617234642	0.537079969	0.999579949
921.047	0.099267716	0.11012676	0.901395054	0.36737831	0.999579949
510.6358665	0.066579272	0.124626612	0.534229975	0.593182433	0.999579949
926.7704611	-0.029765177	0.077027075	-0.386424857	0.699182044	0.999579949
22.10782543	0.028716885	0.244704124	0.117353498	0.906579933	0.999579949
228.509689	0.185406593	0.148982386	1.244486671	0.213320502	0.999268361
53.5410803	0.175115593	0.317354934	0.55179729	0.581087244	0.999579949
6464.68291	-0.141921112	0.148816073	-0.953667898	0.340251775	0.999579949
772.9852902	0.08633871	0.103886035	0.831090628	0.405922434	0.999579949
1649.748099	-0.357868088	0.210050409	-1.703724782	0.088432516	0.870840008
357.5110317	-0.222234975	0.087021203	-2.553802611	0.010655364	0.483266726
1088.693778	0.063156654	0.0914688	0.690472095	0.489897352	0.999579949
127.7547505	0.000427396	0.126217285	0.003386193	0.997298214	0.999579949
21.89246377	0.066687246	0.247784074	0.269134513	0.787826172	0.999579949
140.7540801	0.005215811	0.129917656	0.040147054	0.967975888	0.999579949
39.07731334	0.7825281	0.443459422	1.764599108	0.077631165	0.851207389
233.5211325	0.435286212	0.161412103	2.696738382	0.007002226	0.404205857
309.2742761	0.145914762	0.118194787	1.234527903	0.217006256	0.999579949
48.55853196	-0.109480054	0.188949493	-0.579414383	0.562309602	0.999579949
265.898262	0.136555398	0.09372497	1.456979896	0.14512193	0.955875831
939.7656224	0.087735168	0.049584305	1.769414071	0.076824799	0.851207389
351.4280085	0.119931504	0.204672694	0.585967289	0.557897497	0.999579949
1193.669627	-0.078092959	0.075815125	-1.030044583	0.302989077	0.999579949
283.042078	0.150521488	0.141808541	1.061441619	0.288489251	0.999579949

1278.072966	-0.237839355	0.367595742	-0.647013359	0.517623298	0.999579949
837.6671495	-0.020949699	0.10685654	-0.19605444	0.844567556	0.999579949
374.9337452	-0.01742292	0.114585003	-0.152052362	0.879145637	0.999579949
235.0894709	-0.019235903	0.251590306	-0.076457252	0.939055322	0.999579949
63.16705064	-0.191768565	0.22733722	-0.843542315	0.39892521	0.999579949
58.87209783	0.256828223	0.229986576	1.116709624	0.264118501	0.999579949
4481.000959	-0.038417006	0.130000391	-0.295514543	0.767600844	0.999579949
30.41800525	-0.280388465	0.233541781	-1.200592305	0.229909387	0.999579949
35.11196379	0.104860644	0.196195819	0.534469312	0.593016876	0.999579949
767.4124166	0.141797307	0.071873539	1.972872214	0.048510123	0.773460139
52.62839507	0.716395676	0.404019264	1.773172072	0.076200198	0.851207389
25.29815966	-0.205845932	0.231370218	-0.889682057	0.373636632	0.999579949
101.2351075	0.117709761	0.165209506	0.712487823	0.476162749	0.999579949
193.2763909	-0.245403587	0.142824729	-1.718214966	0.085757413	0.864463816
143.6946726	-0.210969927	0.120305797	-1.753613976	0.079496675	0.852823173
528.6825743	0.084279751	0.174014835	0.484325092	0.628155171	0.999579949
1791.380483	0.116658977	0.09484361	1.230014097	0.218691826	0.999579949
458.8722944	0.098486441	0.073517139	1.3396392	0.180362673	0.983992835
5445.796041	0.011237063	0.097047303	0.115789544	0.907819337	0.999579949
93.12276975	-0.050427392	0.150811764	-0.334373068	0.738098059	0.999579949
88.94005542	-0.051413525	0.1637454	-0.313984546	0.753532776	0.999579949
49.21277139	-0.093881975	0.200010857	-0.469384397	0.638794899	0.999579949
400.8547416	-0.025906104	0.098925472	-0.261874959	0.793417846	0.999579949
191.8908537	0.037222436	0.12543937	0.296736468	0.766667713	0.999579949
78.32847676	0.036053574	0.149001224	0.24196831	0.808804719	0.999579949
569.9585345	-0.021150714	0.093101377	-0.227179393	0.820284251	0.999579949
318.6790847	0.139368901	0.262019439	0.531902906	0.594793245	0.999579949
6965.835874	-0.187760854	0.119127133	-1.576138442	0.114993901	0.918818353
94.29671557	-0.012474222	0.137005771	-0.09104888	0.927453752	0.999579949
127.8474474	0.116072144	0.100305668	1.157184299	0.247197071	0.999579949
85.09579679	0.059065001	0.154285106	0.382830216	0.701845656	0.999579949
199.2038719	-0.044723388	0.108504216	-0.41218111	0.680206682	0.999579949
101649.7304	-0.240636099	0.138507594	-1.737349505	0.082325498	0.856126169
530.1842683	-0.259445144	0.127636574	-2.032686525	0.042084203	0.74969123
285.0626343	-0.128990701	0.097305113	-1.325631275	0.184961862	0.983992835
60.30431762	0.024261448	0.201300909	0.12052329	0.904068632	0.999579949
38.15419672	0.055708155	0.194754993	0.286042244	0.774845767	0.999579949
975.7980267	0.069510484	0.098815529	0.703436848	0.48178355	0.999579949
322.2466654	-0.064128632	0.10904223	-0.588108226	0.556459652	0.999579949
227.0488241	0.101959004	0.114343861	0.89168761	0.372560396	0.999579949
154.1708986	-0.314260623	0.140890985	-2.230523294	0.025712723	0.648237123
1012.208927	-0.06799668	0.065143839	-1.04379295	0.296581194	0.999579949
2343.980471	-0.131441255	0.232894137	-0.564381985	0.572494197	0.999579949
547.0652232	0.029858069	0.085325879	0.349929818	0.726391369	0.999579949
100.3022853	-0.214586643	0.159701222	-1.343675651	0.179053294	0.983992835
780.0204846	0.032662807	0.140327795	0.232760778	0.815947171	0.999579949
36.2784356	0.161962923	0.441211953	0.367086436	0.713554545	0.999579949
117.0907124	-0.145239549	0.136372592	-1.06502008	0.286866843	0.999579949

304.6562498	-0.047633894	0.070207521	-0.678472811	0.497471957	0.999579949
139.7621658	0.010868818	0.135580663	0.080164959	0.936106059	0.999579949
81.99325257	-0.102748124	0.136722649	-0.751507703	0.452347166	0.999579949
92.99333768	-0.121251181	0.115230273	-1.0522511	0.29268436	0.999579949
46.65803573	-0.030154653	0.189843839	-0.158839248	0.873795526	0.999579949
41.27683543	-0.23378702	0.235221753	-0.993900512	0.320271305	0.999579949
88.66313986	0.034713822	0.164136901	0.211493097	0.832502518	0.999579949
47.28839252	-0.131350431	0.183071378	-0.71748207	0.473076696	0.999579949
36.46700132	-0.105918879	0.182297065	-0.581023502	0.561224614	0.999579949
24.30721559	0.081635472	0.608639185	0.134127861	0.893301469	0.999579949
155.082703	0.002565564	0.10706544	0.023962578	0.980882459	0.999579949
33.19259361	0.735509086	0.472452378	1.55678989	0.119520378	0.921521033
89.84386948	-0.152955956	0.284417735	-0.53778628	0.590724605	0.999579949
41.40012133	0.200581361	0.230065021	0.871846404	0.383292175	0.999579949
599.3852788	0.002989413	0.091046507	0.032833907	0.973807039	0.999579949
219.8607503	-0.173409874	0.076697838	-2.260948653	0.023762437	0.630129799
209.5161548	0.110187363	0.125488598	0.878066732	0.379907506	0.999579949
28.16223929	2.795731888	1.323705785	2.112049308	0.03468222	0.705746763
1072.632142	0.028546868	0.080766945	0.353447422	0.72375304	0.999579949
828.7060828	0.026188956	0.228704416	0.114510059	0.908833473	0.999579949
1741.694302	-0.031007343	0.107269452	-0.289060325	0.772535214	0.999579949
21.45058138	0.015297936	0.498676302	0.030677086	0.975527065	0.999579949
114.0620185	0.117254752	0.144958969	0.808882357	0.418582818	0.999579949
254.2634473	-0.040724725	0.094323141	-0.431757514	0.665917661	0.999579949
194.0162161	0.097793708	0.156627746	0.62437027	0.532384445	0.999579949
180.1413209	-0.023619822	0.110814015	-0.213148328	0.83121127	0.999579949
661.8669149	-0.129896602	0.088070052	-1.474923639	0.140233074	0.948318761
38.65312116	0.100497405	0.169717883	0.592143872	0.553754251	0.999579949
176.0573021	-0.029216954	0.156029274	-0.187253029	0.851462249	0.999579949
1370.019331	-0.036090927	0.093148868	-0.387454276	0.698419929	0.999579949
1442.568139	-0.020775506	0.112273209	-0.185044202	0.853194366	0.999579949
69.95227147	-0.077187473	0.147554537	-0.523111485	0.600896674	0.999579949
1129.489812	-0.465783233	0.415165817	-1.121920962	0.261896047	0.999579949
102.017207	0.168250828	0.25763373	0.653062113	0.513716236	0.999579949
30.15933463	-0.112701793	0.222083551	-0.507474743	0.611821754	0.999579949
435.5301454	0.109928738	0.106601789	1.031209129	0.302442761	0.999579949
57.22688925	0.280016389	0.148035447	1.891549591	0.058551018	0.79580383
1946.515607	0.063115483	0.115405409	0.546902295	0.584445859	0.999579949
213.4986147	0.030653113	0.134584191	0.227761621	0.819831564	0.999579949
50.40117649	0.332252585	0.185171021	1.794301196	0.072765107	0.838669905
211.0508342	0.316812282	0.352316814	0.899225554	0.368532531	0.999579949
616.0421714	0.060393378	0.089146939	0.677458795	0.498114905	0.999579949
1646.936952	0.033283639	0.060129304	0.553534413	0.579897523	0.999579949
989.2543233	0.135109028	0.101876823	1.326199859	0.184773506	0.983992835
27.90264069	-0.261918397	0.503314296	-0.520387358	0.60279362	0.999579949
21.99445109	0.127410888	0.268965385	0.473707382	0.635708588	0.999579949
1324.783396	-0.042755554	0.113430987	-0.376930108	0.706225546	0.999579949
94.36183739	-0.033200442	0.186804248	-0.177728516	0.858936189	0.999579949

178.307776	-0.076696738	0.125478635	-0.611233441	0.541045048	0.999579949
136.7752514	0.003168974	0.116484341	0.027205152	0.978296107	0.999579949
128.954237	0.018076123	0.115648287	0.156302559	0.875794539	0.999579949
77.36549764	0.087145567	0.162895978	0.534976791	0.592665907	0.999579949
89.34451713	0.053868376	0.14013911	0.384392169	0.700687803	0.999579949
3071.94379	-0.061695939	0.153576045	-0.401728925	0.687883534	0.999579949
52.15688027	0.1505389	0.397565107	0.378652193	0.704946159	0.999579949
72.30140773	-0.037973605	0.260816724	-0.145594977	0.884241134	0.999579949
48.1082859	0.214648087	0.191018566	1.123702744	0.261139152	0.999579949
94.56838329	0.184683622	0.165482411	1.116031736	0.26440855	0.999579949
366.6364663	0.137610609	0.326448851	0.421538041	0.673362239	0.999579949
46.19226889	0.039993961	0.184643581	0.216600877	0.828519402	0.999579949
247.6239751	-0.047251041	0.121474125	-0.388980298	0.697290721	0.999579949
971.7791171	-0.05425218	0.091445195	-0.593275353	0.552996891	0.999579949
2022.058865	-0.075191405	0.069911637	-1.075520586	0.282141721	0.999579949
836.656442	-0.069295412	0.116304101	-0.595812283	0.551300637	0.999579949
111.5753579	0.049551434	0.108792324	0.455468104	0.648772509	0.999579949
179.0698233	0.094133231	0.098214839	0.958442039	0.337839905	0.999579949
73.31957999	0.019421619	0.164995574	0.117709938	0.906297492	0.999579949
82.18509263	0.104222167	0.115223843	0.904519103	0.365720209	0.999579949
75.47258426	0.084829772	0.179369004	0.472934397	0.636259983	0.999579949
145.39239	-0.166274894	0.11268726	-1.475542965	0.140066626	0.948318761
763.6239923	-0.013096276	0.077469141	-0.169051514	0.865756122	0.999579949
167.0126291	0.09791656	0.123872338	0.790463489	0.429257137	0.999579949
1003.364669	0.034405754	0.135093284	0.254681457	0.798969139	0.999579949
72.72676751	0.185587944	0.136257824	1.362035139	0.173186796	0.983601342
126.3733578	0.096185368	0.125237913	0.768021163	0.442474612	0.999579949
460.2922711	-0.103102491	0.094579194	-1.090118096	0.275661126	0.999579949
1472.972281	-0.0229803	0.050104634	-0.458646194	0.646488259	0.999579949
1358.290516	-0.087844306	0.088593296	-0.991545747	0.321419166	0.999579949
254.0874598	-0.13567754	0.206838941	-0.655957431	0.511851514	0.999579949
215.8213917	0.258251437	0.143037838	1.805476376	0.071000169	0.834620594
151.3632225	-0.024354034	0.097681883	-0.24931987	0.803113363	0.999579949
353.1815196	-0.003332062	0.063594746	-0.052395234	0.958213771	0.999579949
352.2765582	-0.058166192	0.102745737	-0.566117815	0.571313694	0.999579949
38.54604642	0.777201057	0.547898155	1.418513734	0.156040832	0.976030424
61.86710398	0.04965947	0.184985243	0.268450982	0.788352201	0.999579949
39.53007724	-0.238863788	0.197751193	-1.207900617	0.227085492	0.999579949
114.6125504	0.064389467	0.142497721	0.451863136	0.651367583	0.999579949
189.5707328	0.150316389	0.123287569	1.219233945	0.222755411	0.999579949
277.8833867	0.101997112	0.129322594	0.788702958	0.430285639	0.999579949
801.3257097	0.010601472	0.096811882	0.109505897	0.912801245	0.999579949
42.23971453	0.581265859	0.59747747	0.972866574	0.33061962	0.999579949
1325.309635	0.081944114	0.060277699	1.359443303	0.174006157	0.983992835
1537.953965	0.100883476	0.075397913	1.338014174	0.180891816	0.983992835
1014.417747	-0.14620499	0.176151609	-0.8299952	0.406541497	0.999579949
605.4743188	0.005113493	0.074628961	0.06851888	0.945372591	0.999579949
65.60588155	0.022196397	0.591603642	0.037519033	0.970071164	0.999579949

172130.4971	-0.019841001	0.119683881	-0.165778388	0.868331355	0.999579949
37.70773234	0.330701934	0.390881747	0.846040871	0.397529944	0.999579949
95.9388817	0.145220168	0.189352611	0.766929842	0.44312323	0.999579949
38.40790572	-0.247317169	0.226403041	-1.092375651	0.274668021	0.999579949
900.5339536	0.027058647	0.106296072	0.254559239	0.799063545	0.999579949
66.15989664	0.121734242	0.246071602	0.494710649	0.620804383	0.999579949
264.8591947	0.058417018	0.086460411	0.675650487	0.499262579	0.999579949
135.7241448	0.065382446	0.183455278	0.356394468	0.721545172	0.999579949
47.53381475	-0.151072376	0.208415134	-0.724862794	0.468536208	0.999579949
23.34905007	-0.079083315	0.293099968	-0.269816869	0.787301145	0.999579949
262.6311791	-0.125460471	0.148287989	-0.846059563	0.397519518	0.999579949
246.8126523	-0.091999777	0.06643594	-1.384789272	0.166116912	0.978789551
82.42056149	0.151386511	0.162875974	0.929458826	0.352651354	0.999579949
230.5313819	0.00083986	0.09193018	0.009135846	0.992710751	0.999579949
41.28971278	-0.011335399	0.169264806	-0.066968431	0.946606835	0.999579949
48.42441503	0.073683422	0.175321751	0.420275418	0.674284265	0.999579949
98.41333997	-0.117820809	0.127836569	-0.921651837	0.356710211	0.999579949
147.5443488	0.116772372	0.135360634	0.862676019	0.388315621	0.999579949
90.56500333	-0.069042375	0.118935848	-0.580500973	0.561576831	0.999579949
275.0262098	-0.07207243	0.097529189	-0.738983178	0.459917212	0.999579949
62.97220527	0.13100068	0.145115194	0.902735795	0.366666134	0.999579949
28.51128986	0.177856562	0.24088001	0.738361652	0.460294711	0.999579949
41.8919108	-0.194650065	0.177163846	-1.09870083	0.271898581	0.999579949
455.752095	-0.072587309	0.105933266	-0.685217323	0.493206794	0.999579949
24.15116408	-0.116526228	0.222700244	-0.523242481	0.600805523	0.999579949
324.1594634	0.01519567	0.073130641	0.207788001	0.835394502	0.999579949
122.1987943	0.039578722	0.13908712	0.284560657	0.775980757	0.999579949
1657.905308	0.074079772	0.140744707	0.526341441	0.598650995	0.999579949
1628.809061	-0.085275462	0.050921822	-1.674634948	0.094005878	0.880418343
226.5463582	0.048972592	0.150212556	0.326021961	0.74440774	0.999579949
123.1356011	-0.025330796	0.137556903	-0.184147767	0.853897535	0.999579949
56.36705189	0.071131182	0.35258792	0.20174027	0.840119779	0.999579949
95.26147911	-0.123280809	0.146706771	-0.840321193	0.400728322	0.999579949
128.1698772	-0.012429609	0.095551299	-0.130083098	0.896500682	0.999579949
180.0540143	0.058840434	0.11388222	0.516677972	0.605380976	0.999579949
79.93966695	-0.020644364	0.144821024	-0.142550879	0.886644894	0.999579949
55.42296725	0.908801603	0.554421989	1.639187516	0.101174213	0.895628172
20.94305732	-0.067183611	0.321581149	-0.20891651	0.834513415	0.999579949
38.99419519	0.237795658	0.167659756	1.41832282	0.156096537	0.976030424
6150.119333	0.268390981	0.199017746	1.348578141	0.177472506	0.983992835
70.51358055	0.203785797	0.141002911	1.445259505	0.148385008	0.962301096
341.7738503	0.035711992	0.127701146	0.27965287	0.779743841	0.999579949
263.6717107	0.026320746	0.07678003	0.342807189	0.731743518	0.999579949
53.7411447	0.048317469	0.176617541	0.273571178	0.784414186	0.999579949
916.3519007	0.090144	0.077537935	1.162579331	0.245000221	0.999579949
3003.062683	0.145152744	0.130995612	1.108073334	0.267830146	0.999579949
464.0544027	0.001289309	0.104314961	0.012359769	0.990138582	0.999579949
52.99048649	0.254692832	0.229619623	1.109194539	0.267346268	0.999579949

793.1423472	-0.199527369	0.099256384	-2.010222017	0.044407696	0.758967899
81.92286983	-0.064595632	0.140653209	-0.459254593	0.646051351	0.999579949
36.32960392	-0.539146136	0.25536811	-2.111250838	0.034750757	0.706000868
30.72377119	-0.373573336	0.556370256	-0.671447353	0.501935587	0.999579949
728.5784887	-0.017919468	0.07279976	-0.246147358	0.805568164	0.999579949
113.7318948	0.066357736	0.115840827	0.572835478	0.566756095	0.999579949
69.18185541	0.066846591	0.375781228	0.17788699	0.858811729	0.999579949
1862.705563	-0.067266362	0.109506557	-0.614267887	0.53903832	0.999579949
64.37860853	0.054461148	0.198120542	0.274888952	0.783401555	0.999579949
226.1765069	0.190048946	0.215650944	0.881280384	0.37816608	0.999579949
5745.085467	-0.039081257	0.145068128	-0.269399332	0.7876224	0.999579949
520.3189945	0.104113524	0.11843717	0.879061228	0.379368079	0.999579949
88.79138209	-0.081197707	0.192018925	-0.422863044	0.672395187	0.999579949
677.3996379	-0.033075484	0.068601108	-0.482142117	0.629704992	0.999579949
164.9169305	0.08032196	0.155489176	0.516575895	0.605452246	0.999579949
12815.07136	-0.156120129	0.090776517	-1.71982947	0.085463444	0.864463816
1135.824781	-0.09044689	0.071913457	-1.257718562	0.208493557	0.995521169
970.9229693	-0.001851354	0.087588871	-0.021136866	0.983136476	0.999579949
966.5773133	-0.194935256	0.109781984	-1.775657979	0.075789307	0.851207389
387.0231603	0.091503585	0.095109917	0.962082482	0.336008173	0.999579949
782.8950555	0.01716499	0.094222355	0.18217534	0.855445125	0.999579949
871.7087179	-0.637330336	0.439261229	-1.450914156	0.146803766	0.959874957
177.5873718	-0.391651485	0.579015505	-0.676409322	0.4987808	0.999579949
310.671375	-0.172928177	0.122625533	-1.410213467	0.15847666	0.977304288
183.8911765	0.00513316	0.109755078	0.046769224	0.962697158	0.999579949
287.5127637	-0.049046415	0.21101529	-0.232430621	0.816203568	0.999579949
87.69578235	0.012281114	0.139370348	0.088118557	0.929782448	0.999579949
622.4225811	-0.003681455	0.122894485	-0.029956226	0.976101964	0.999579949
402.0942047	0.06743368	0.08826098	0.764025956	0.444851775	0.999579949
398.8708064	0.041972067	0.143246439	0.293006007	0.76951756	0.999579949
144.7651999	-0.124651573	0.091150437	-1.36753675	0.171457126	0.98345809
523.8998253	-0.060031783	0.080070602	-0.749735631	0.453413943	0.999579949
96.76525715	-0.086605814	0.133784911	-0.647351133	0.517404715	0.999579949
36.45342832	-0.10636263	0.197065051	-0.539733606	0.58938076	0.999579949
683.7053396	-0.123040496	0.114982717	-1.070078175	0.284584122	0.999579949
400.4476776	-0.073753086	0.108405113	-0.680346933	0.496284814	0.999579949
214.7100085	0.218580886	0.148367739	1.473237295	0.140687062	0.948318761
79.3169091	-0.029492103	0.145496741	-0.202699408	0.839369986	0.999579949
37.71641565	-0.077685672	0.189231434	-0.410532595	0.681415298	0.999579949
43.27425421	-0.220674783	0.218775986	-1.008679185	0.313128518	0.999579949
81.72943706	0.091885206	0.178635977	0.514371222	0.606992475	0.999579949
45.91099253	0.122155281	0.174091514	0.701672807	0.482883235	0.999579949
189.3895709	-0.019085768	0.147189869	-0.129667672	0.896829361	0.999579949
119.7963559	0.129082369	0.114254336	1.129780921	0.258568549	0.999579949
21.93239375	-0.023821939	0.364243719	-0.065401098	0.94785465	0.999579949
278.3753246	-0.05783804	0.096348217	-0.60030213	0.5483049	0.999579949
1884.455703	0.200899718	0.128235785	1.566643171	0.117198121	0.920479614
87.36034188	-0.281370561	0.172472645	-1.631392388	0.10280755	0.895628172

59.81192469	0.92046209	0.511628952	1.79908132	0.072005818	0.838248876
36.84316657	-0.163299278	0.255366736	-0.63946965	0.522517451	0.999579949
104.1604451	-0.081194585	0.14804352	-0.548450784	0.583382414	0.999579949
50.94147157	0.103097566	0.166172685	0.620424262	0.534978503	0.999579949
110.1680541	-0.025511018	0.141074811	-0.180833262	0.856498454	0.999579949
45.37909255	0.967923435	0.342770378	2.823824631	0.004745434	0.3435258
41.03553597	-0.198311063	0.465283798	-0.426215277	0.669950986	0.999579949
73.9558941	-0.588528942	0.181858189	-3.236197094	0.001211337	0.181642887
229.5277848	0.235295124	0.104881897	2.243429328	0.024869142	0.640596561
45.53242333	0.355887932	0.208171426	1.70959069	0.087341588	0.867715687
624.6188817	-0.014408605	0.123826605	-0.116361143	0.907366329	0.999579949
43.71747837	0.239336839	0.214544589	1.115557563	0.264611566	0.999579949
77655.48643	-0.174720185	0.170668452	-1.023740372	0.305957921	0.999579949
28.74552423	-0.038850266	1.024785652	-0.037910627	0.96975894	0.999579949
151.7373715	0.394309212	0.229574967	1.717561878	0.085876559	0.864463816
583.796747	0.008710223	0.059623788	0.146086369	0.883853207	0.999579949
31.5018291	0.459901924	0.535502722	0.858822757	0.39043831	0.999579949
176.0526256	-0.475294186	0.167464055	-2.838186294	0.004537069	0.33947676
1044.622129	0.237555886	0.130458605	1.820929222	0.068617616	0.831064901
187.1652359	-0.117125326	0.09794847	-1.195785146	0.231780414	0.999579949
467.9958972	0.145681531	0.140428369	1.037408125	0.299545693	0.999579949
277.9477734	0.003879399	0.085895498	0.045164169	0.963976454	0.999579949
765.3919898	0.125113259	0.137003731	0.913210595	0.361131793	0.999579949
1294.806878	0.009287522	0.111379215	0.083386495	0.933544226	0.999579949
260.4439295	0.183218082	0.144050289	1.271903608	0.20340737	0.994409048
1412.895743	0.009993662	0.093952437	0.106369378	0.915289288	0.999579949
823.1387635	-0.143319333	0.076819452	-1.865664619	0.062088303	0.805306145
226.7349875	0.056030676	0.156011807	0.359143818	0.719487504	0.999579949
948.271399	-0.013775892	0.057130815	-0.241128932	0.80945519	0.999579949
50.57671955	0.08872666	0.162052401	0.547518332	0.584022679	0.999579949
803.9438262	-0.071097247	0.105763249	-0.672230169	0.501437178	0.999579949
60.9308943	-0.083634716	0.14438869	-0.579233152	0.562431864	0.999579949
64.2640662	-0.058232962	0.129523118	-0.449595125	0.653002404	0.999579949
192.3145048	0.097076342	0.107072284	0.906643046	0.36459559	0.999579949
1241.257835	-0.015968385	0.058524849	-0.272847954	0.784970095	0.999579949
209.7715748	-0.156909841	0.144830777	-1.083401227	0.278630371	0.999579949
817.1426078	-0.217719584	0.070541712	-3.086394951	0.002025995	0.229904825
100.2320667	-0.114049877	0.127549386	-0.894162491	0.371234958	0.999579949
356.5488035	0.181458519	0.10115477	1.793870112	0.072833903	0.838669905
121.8549999	0.329344922	0.222038216	1.483280342	0.137999943	0.945791454
653.7633273	-0.062547226	0.135174115	-0.462716	0.643567948	0.999579949
1372.519891	-0.194606347	0.127629408	-1.524776692	0.127314805	0.934532219
1890.111447	0.058189262	0.097751852	0.595275292	0.551659469	0.999579949
54.99952896	-0.039966757	0.267140698	-0.14960939	0.881072799	0.999579949
153.5582847	0.229070615	0.155499824	1.473124596	0.140717442	0.948318761
199.0425965	-0.081042287	0.098957285	-0.818962312	0.412807916	0.999579949
69.07173846	0.227459803	0.238455137	0.953889297	0.340139682	0.999579949
57.88113987	-0.730607635	0.782863489	-0.933250363	0.350690717	0.999579949

570.213623	0.102964665	0.115202165	0.893773691	0.371442988	0.999579949
354.8653304	-0.186850069	0.115835066	-1.613069994	0.106729302	0.899238988
8929.51792	-0.031504545	0.117738862	-0.26757983	0.789022758	0.999579949
22.10134623	-0.112382806	0.284885663	-0.394483893	0.693223816	0.999579949
1496.169288	0.108028864	0.089018477	1.213555509	0.224917505	0.999579949
2871.678767	0.107235157	0.14669658	0.73099971	0.46477933	0.999579949
37.7696774	-0.295714746	0.208878185	-1.415728243	0.156855086	0.977304288
281.5666113	0.020718081	0.077789473	0.266335278	0.789981006	0.999579949
7108.374796	0.068887441	0.120180778	0.573198496	0.566510302	0.999579949
1091.662726	0.081230204	0.279404095	0.290726606	0.771260422	0.999579949
110.6746435	0.058250883	0.134808988	0.432099404	0.665669168	0.999579949
465.1144259	-0.03211374	0.221194237	-0.145183437	0.884566042	0.999579949
667.3394281	-0.034287498	0.095879173	-0.357611534	0.720634045	0.999579949
264.0864089	0.221202377	0.190498284	1.161177792	0.245569604	0.999579949
119.2241432	-0.076493741	0.247425258	-0.309158983	0.757200594	0.999579949
1016.911989	0.148417859	0.13129854	1.130384695	0.258314161	0.999579949
396.3171199	-0.057163179	0.10854946	-0.526609518	0.598464781	0.999579949
416.0492246	-0.166138951	0.155467357	-1.068642023	0.285231005	0.999579949
57.10904675	-0.078225666	0.234729767	-0.333258396	0.738939242	0.999579949
75.13295774	-0.241910288	0.159702858	-1.514752401	0.129835119	0.935700262
1158.632133	-0.025921623	0.224288971	-0.115572436	0.907991409	0.999579949
373.115674	-0.152142545	0.105383654	-1.443701557	0.148822943	0.962301096
1602.160274	0.03131567	0.095780717	0.326951715	0.743704406	0.999579949
256.602704	0.072168136	0.088310352	0.817210379	0.413808212	0.999579949
188.5004374	0.177485171	0.138301537	1.283320306	0.199379881	0.992481822
437.3269703	-0.025093417	0.06350777	-0.395123569	0.692751695	0.999579949
872.6287197	-0.046548528	0.122925083	-0.378673963	0.704929991	0.999579949
102.2008542	0.173813404	0.239860351	0.724644163	0.468670358	0.999579949
2007.8092	-0.016690079	0.10244125	-0.162923419	0.870578732	0.999579949
1789.64332	-0.136200765	0.158357032	-0.860086623	0.389741295	0.999579949
81.18015177	-0.041072932	0.271153402	-0.15147489	0.879601117	0.999579949
141.7182657	0.010225377	0.336106765	0.030423003	0.9757297	0.999579949
283.266164	0.169958288	0.123556242	1.375554035	0.168959746	0.981013142
3117.02103	-0.007416872	0.143407674	-0.051718796	0.95875276	0.999579949
41.10105397	0.142258906	0.195181251	0.728855389	0.466090126	0.999579949
67.9031703	0.159075579	0.130127372	1.222460556	0.221533511	0.999579949
244.9828937	-0.112585729	0.218645048	-0.514924669	0.606605662	0.999579949
322.8158034	-0.051711046	0.08648743	-0.597902446	0.549905027	0.999579949
28901.38772	-0.187046723	0.127395884	-1.46823208	0.142041193	0.950166185
372.458225	-0.019219717	0.094249121	-0.203924626	0.838412401	0.999579949
146.8294639	-0.161212156	0.231698875	-0.695783076	0.486564689	0.999579949
200.8496148	0.110660773	0.125289919	0.883237645	0.377107886	0.999579949
101.743903	0.223467283	0.14935541	1.496211508	0.134598547	0.945791454
30.5922287	-0.024769404	0.236736905	-0.104628402	0.916670677	0.999579949
79.29960314	0.10957551	0.15068037	0.727204941	0.46710042	0.999579949
543.76626	0.93378869	0.330572127	2.824765348	0.004731525	0.3435258
1260.490917	-0.162874904	0.102162859	-1.594267281	0.110876183	0.912211885
243.9847415	0.045976053	0.121836932	0.37735728	0.70590811	0.999579949

2252.285865	-0.059673907	0.155079621	-0.384795285	0.700389092	0.999579949
29.49306668	0.135805809	0.258343809	0.525678588	0.599111543	0.999579949
34.20355196	0.187414855	0.218141575	0.859143218	0.390261506	0.999579949
440.7920075	0.11707908	0.143330568	0.816846546	0.414016128	0.999579949
291.942074	-0.022988079	0.094257261	-0.243886563	0.807318676	0.999579949
577.9039571	-0.044654931	0.092995804	-0.480182214	0.631097832	0.999579949
322.0565219	-0.028958448	0.108134265	-0.267800853	0.788852614	0.999579949
68.20085756	0.204026249	0.167015413	1.221601322	0.221858428	0.999579949
8164.439302	0.023776207	0.11911663	0.199604434	0.84178996	0.999579949
80.94352718	-0.0426031	0.212466665	-0.200516633	0.841076551	0.999579949
1711.16488	0.032150144	0.10612648	0.302941775	0.761934235	0.999579949
433.6710209	-0.092761562	0.159923229	-0.580038079	0.561888939	0.999579949
72.812662	0.286522511	0.284813808	1.005999367	0.314415875	0.999579949
143.8009447	0.037732976	0.147135358	0.256450771	0.797602789	0.999579949
1523.355678	-0.023789382	0.097035764	-0.245160969	0.806331797	0.999579949
71.72337032	0.002613899	0.169487069	0.01542241	0.987695185	0.999579949
427.0669908	-0.159530459	0.107113398	-1.48936045	0.136392477	0.945791454
173.4558091	-0.040375762	0.116229773	-0.347378822	0.728306742	0.999579949
155.3317231	0.076420152	0.117009585	0.653110182	0.513685249	0.999579949
226.4708766	0.090323902	0.117097677	0.771355196	0.440496424	0.999579949
154.6469761	0.106044233	0.121705186	0.87132058	0.383579137	0.999579949
1120.101763	-0.194391728	0.086931931	-2.236137233	0.025342783	0.642289128
923.3352236	0.020663831	0.146142833	0.14139476	0.887558093	0.999579949
86.11003119	-0.142438402	0.150090753	-0.949015178	0.342612893	0.999579949
585.8846856	-0.071952395	0.092022106	-0.78190338	0.434271359	0.999579949
162.0700119	0.213710678	0.137373641	1.55568912	0.119782035	0.921521033
272.7961751	0.14619363	0.156350477	0.935037954	0.34976874	0.999579949
117.7801633	0.177077787	0.174915571	1.012361481	0.311365248	0.999579949
204.0456867	0.053525539	0.228837217	0.233902247	0.815060869	0.999579949
57.82349082	0.061735942	0.165486205	0.373057932	0.709105328	0.999579949
169.9374933	-0.03335719	0.098519657	-0.33858411	0.734923054	0.999579949
66.08174991	0.207766322	0.137058863	1.515891177	0.129546872	0.935700262
144.7767983	-0.080193747	0.11623491	-0.689928244	0.490239313	0.999579949
2428.154826	-0.019886316	0.094249476	-0.210996567	0.83288995	0.999579949
207.2203441	-0.096486579	0.100575496	-0.959344799	0.337385074	0.999579949
228.6640021	-0.148272885	0.099815315	-1.485472287	0.137418759	0.945791454
15417.01723	0.00404732	0.151126793	0.026780956	0.978634443	0.999579949
1118.942289	0.114745664	0.110435196	1.039031648	0.298790018	0.999579949
200.1888326	-0.088356436	0.143381279	-0.616234119	0.537740013	0.999579949
542.1677915	-0.075136372	0.082919987	-0.906131021	0.364866508	0.999579949
21.44292768	-0.197521899	0.273730771	-0.72159187	0.470545439	0.999579949
20.58155835	0.642069612	0.611115892	1.050651145	0.293418842	0.999579949
778.562925	-0.323135068	0.121430927	-2.661060699	0.007789491	0.42118327
1643.765045	-0.114426159	0.071275399	-1.605408879	0.108403852	0.903743816
315.4758402	0.18076671	0.083851014	2.15580829	0.031098638	0.683621001
153.2081326	0.385867028	0.303667075	1.270691029	0.203838588	0.994814558
746.0196411	0.039007602	0.145888001	0.267380469	0.789176236	0.999579949
21.58228403	0.062455296	0.501209431	0.12460918	0.900832961	0.999579949

3430.45742	0.068572481	0.052021935	1.318145524	0.187454938	0.983992835
71.11617708	0.265500252	0.229879791	1.154952557	0.248109855	0.999579949
887.994136	0.277758071	0.21108194	1.315877954	0.188215009	0.983992835
47.70261956	-0.482699947	0.334512905	-1.442993499	0.149022302	0.962301096
178.4353407	0.131643333	0.149111937	0.882849058	0.37731783	0.999579949
119.247839	-0.034670928	0.126807703	-0.273413422	0.784535437	0.999579949
219.1650087	-0.025834728	0.110978016	-0.232791407	0.815923386	0.999579949
465.6823299	-0.062177426	0.104988688	-0.592229764	0.553696741	0.999579949
23.41583317	0.443724594	0.406128928	1.092570767	0.274582304	0.999579949
887.5916816	-0.168999298	0.190974507	-0.884931189	0.376193745	0.999579949
190.4362359	-0.051664308	0.243589973	-0.212095379	0.832032625	0.999579949
24.99158435	0.591527972	0.422764188	1.399191295	0.161755626	0.978498637
651.6421914	0.14176138	0.068953652	2.055893723	0.039792761	0.73515901
50.85329771	-0.259456543	0.220246293	-1.178029101	0.238785005	0.999579949
1451.633381	-0.677696234	0.516539568	-1.311992877	0.189522538	0.983992835
87.66130309	0.040714912	0.148792097	0.273636254	0.78436417	0.999579949
227.1785297	-0.05290338	0.12062463	-0.438578592	0.660966912	0.999579949
543.1914821	-0.019643648	0.10869291	-0.180726119	0.856582556	0.999579949
105.7931895	0.027250651	0.138134701	0.197275926	0.843611617	0.999579949
655.9172103	0.055532374	0.09309224	0.59653065	0.550820785	0.999579949
4213.717986	0.545751737	0.141625004	3.853498486	0.000116442	0.048890108
189.3210714	0.024940703	0.212385937	0.117431049	0.906518481	0.999579949
49.02075596	0.054166424	0.288493056	0.187756423	0.851067597	0.999579949
27.58416459	0.324999951	0.194260941	1.673007186	0.094325881	0.880418343
292.9745754	0.090550261	0.106297715	0.851855196	0.394294467	0.999579949
59.48820743	0.098369931	0.164665535	0.597392347	0.550245462	0.999579949
48.13140617	0.042113713	0.263206602	0.160002493	0.87287911	0.999579949
101.6181755	0.131471963	0.117063496	1.123082493	0.261402461	0.999579949
247.7421795	0.044857194	0.123432494	0.363414789	0.716295056	0.999579949
226.2659918	-0.10607208	0.091922178	-1.153933498	0.248527434	0.999579949
3753.473611	-0.097200431	0.095399036	-1.01888273	0.308258643	0.999579949
98.49530935	0.090699171	0.279812149	0.324143078	0.745829714	0.999579949
1618.35137	-0.189223999	0.059488261	-3.180862815	0.001468371	0.203876378
56.28527207	0.559587835	0.334680013	1.672008524	0.094522641	0.880418343
528.4497963	-0.170388455	0.072415991	-2.352912021	0.018627041	0.590797093
39.69950701	-0.287362334	0.176338996	-1.629601738	0.103185697	0.895628172
1244.371768	0.097456135	0.199850457	0.487645297	0.625801105	0.999579949
39.41626381	0.126286264	0.189436094	0.666643093	0.505000136	0.999579949
76.57238659	0.153813629	0.137483941	1.118775243	0.263236036	0.999579949
177.8783323	0.056602824	0.115321661	0.490825605	0.623549798	0.999579949
616.865198	0.054767622	0.077464819	0.706999937	0.479566517	0.999579949
7671.636262	0.152173569	0.108367691	1.40423375	0.160249255	0.978261482
292.0417598	0.127806415	0.132068458	0.967728528	0.333179967	0.999579949
1808.139369	-0.094509919	0.082289783	-1.148501261	0.250761693	0.999579949
399.2887793	-0.239694489	0.103611426	-2.313398226	0.020700755	0.612081465
245.5847159	0.011473369	0.193389061	0.059327913	0.952690929	0.999579949
54.98037225	0.211883251	0.217142947	0.975777727	0.329174625	0.999579949
5556.501145	-0.037551642	0.120635184	-0.311282666	0.755585743	0.999579949

255.0505208	0.058123922	0.084954821	0.684174498	0.493864984	0.999579949
420.8230099	0.725491227	0.227463277	3.189487273	0.001425254	0.203876378
121.5551771	-0.084422005	0.133367914	-0.633000868	0.526733076	0.999579949
80.97822455	0.118128589	0.133913548	0.882125751	0.377708808	0.999579949
2396.171604	0.097187291	0.104783284	0.927507586	0.353663055	0.999579949
93.40016814	0.328567002	0.297486298	1.104477769	0.269385942	0.999579949
117.0828978	0.056430133	0.141738768	0.398127725	0.690536039	0.999579949
262.8438716	0.085193685	0.139705418	0.609809457	0.541988036	0.999579949
208.1246033	0.049414015	0.086539806	0.57099753	0.568001317	0.999579949
74.95028435	-0.033524573	0.146836777	-0.228311826	0.819403831	0.999579949
188.2243231	-0.010429126	0.289413501	-0.036035383	0.971254146	0.999579949
202.3180775	0.209115112	0.198748307	1.052160471	0.292725932	0.999579949
306.2094335	0.064131881	0.096227694	0.666459706	0.50511731	0.999579949
137.7522144	-0.12267698	0.102087922	-1.201679664	0.229487663	0.999579949
24.36192986	0.44751432	0.352538974	1.26940382	0.204297074	0.994814558
101.5155658	-0.286844334	0.283074861	-1.013316172	0.310909165	0.999579949
750.966422	-0.005079616	0.127389038	-0.039874829	0.968192919	0.999579949
47.50029314	-0.172394606	0.485763286	-0.354894269	0.722668802	0.999579949
32.65114909	0.124005956	0.233051228	0.532097415	0.594658528	0.999579949
341.6830957	-0.006686219	0.118484578	-0.056431132	0.954998357	0.999579949
414.0607711	-0.280930311	0.113061791	-2.484750217	0.012964236	0.510304755
1162.298757	-0.09794186	0.183368959	-0.534124535	0.593255376	0.999579949
788.3721521	-0.439521605	0.195080947	-2.253021687	0.024257782	0.635241199
1284.460987	0.020416073	0.121922166	0.167451691	0.867014656	0.999579949
401.7068998	0.035719108	0.158657198	0.225133867	0.821875135	0.999579949
233.5705861	0.037739684	0.082389351	0.458065064	0.646905697	0.999579949
22.67961235	-0.034239625	0.282055211	-0.121393344	0.903379488	0.999579949
2023.216842	0.097416813	0.112960104	0.862400172	0.388467346	0.999579949
99.82186583	-0.142725435	0.253865949	-0.562207873	0.573974398	0.999579949
544.6496481	0.172070816	0.113149996	1.520731961	0.128327115	0.935700262
359.9849621	-0.152298848	0.141708527	-1.074733125	0.282494229	0.999579949
114.7499044	-0.057279624	0.118169325	-0.48472498	0.627871444	0.999579949
151.6828591	0.011933204	0.158907757	0.075095161	0.940138998	0.999579949
21.06786106	-0.14110565	0.28035269	-0.503314772	0.614742982	0.999579949
670.0537513	0.020959909	0.089709785	0.233641276	0.815263481	0.999579949
76.9129705	0.205574606	0.171282925	1.200204898	0.230059774	0.999579949
60.40584929	-0.039986187	0.178271931	-0.224298836	0.822524782	0.999579949
104.2504489	0.012260732	0.231011184	0.053074193	0.957672793	0.999579949
29.45319507	-0.292192461	0.267828249	-1.090969541	0.275286286	0.999579949
545.6381584	-0.766446649	0.505226594	-1.517035442	0.129257736	0.935700262
163.8206946	0.124031113	0.096468266	1.285719307	0.198541041	0.992481822
246.7088584	-0.0107533	0.14007762	-0.076766725	0.938809122	0.999579949
43.25433784	-0.186465167	0.164528735	-1.133328883	0.257076167	0.999579949
44.0946008	0.027739756	0.177680385	0.156121656	0.875937128	0.999579949
24.72042992	0.092357267	0.250234862	0.369082335	0.712066355	0.999579949
891.0573103	0.132282936	0.150013182	0.881808744	0.377880242	0.999579949
152.2882697	-0.003484078	0.339386668	-0.010265807	0.991809215	0.999579949
385.9903965	-0.004817485	0.082626432	-0.0583044	0.953506163	0.999579949

502.7848026	-0.140830027	0.161050367	-0.874447102	0.381874816	0.999579949
650.9406807	-0.039247633	0.083469528	-0.47020313	0.638209898	0.999579949
328.6385486	-0.153860008	0.069159136	-2.224724257	0.026099755	0.648237123
1159.21184	-0.248978391	0.139429726	-1.785690888	0.074149322	0.845235162
89.70145953	0.246775371	0.274489592	0.899033618	0.368634754	0.999579949
80.79907563	0.289373229	0.206168709	1.40357492	0.160445468	0.978261482
72.60358622	0.262356822	0.246962322	1.062335421	0.288083439	0.999579949
46.47777036	0.317001634	0.241206358	1.314234154	0.188767418	0.983992835
153.3933982	-0.101901921	0.167044657	-0.610028019	0.541843247	0.999579949
289.0590308	-0.006028327	0.145213676	-0.041513491	0.966886537	0.999579949
2539.50921	-0.056813263	0.106192491	-0.53500264	0.592648031	0.999579949
197.9696641	0.008677846	0.129697705	0.066908248	0.946654747	0.999579949
279.3222931	-0.081130916	0.129218469	-0.627858513	0.530096631	0.999579949
1439.447475	-0.208173192	0.422765505	-0.492408179	0.622430819	0.999579949
89.31778057	-0.205469112	0.216695138	-0.948194382	0.343030507	0.999579949
549.5092116	-0.125109429	0.177035485	-0.706691251	0.479758368	0.999579949
128.8671484	-0.096489384	0.152093938	-0.634406508	0.525815563	0.999579949
2439.80588	0.142142659	0.106402464	1.335896314	0.181583166	0.983992835
203.6992976	0.303425551	0.243980444	1.243647014	0.213629505	0.999268361
56.22792021	0.109746609	0.233917352	0.469168311	0.638949334	0.999579949
1017.731054	0.005912913	0.166507548	0.035511382	0.97167197	0.999579949
1045.66655	-0.126591125	0.201644291	-0.627794241	0.530138739	0.999579949
270.8884531	-0.194409513	0.130238578	-1.49271833	0.135510933	0.945791454
90.59437466	0.168140903	0.13169098	1.276783744	0.201678606	0.993283621
607.2550937	0.08271047	0.119119539	0.694348474	0.487463698	0.999579949
35.42715146	-0.201016221	0.296200529	-0.678649094	0.497360228	0.999579949
375.9657129	0.029295059	0.08386734	0.349302349	0.726862335	0.999579949
20.55016128	-0.217173295	0.276525595	-0.78536417	0.432240082	0.999579949
115.9049649	0.325120168	0.190619031	1.705601831	0.088082238	0.870840008
41.07763543	0.277914185	0.169090246	1.643584958	0.100261963	0.895628172
640.2866367	-0.107790193	0.142493332	-0.756457806	0.449374761	0.999579949
347.9125132	-0.046940004	0.079705246	-0.588919883	0.555915019	0.999579949
270.0964921	-0.047672982	0.128421774	-0.371221958	0.71047222	0.999579949
996.6845002	0.37328884	0.258985973	1.441347714	0.149486474	0.963629286
541.3350365	0.475954882	0.531991688	0.894666012	0.370965652	0.999579949
52.76484966	0.231105275	0.147868425	1.562911592	0.118073391	0.921521033
281.6830699	0.010323846	0.100078412	0.103157568	0.917837916	0.999579949
789.9077423	-0.036530361	0.11282537	-0.3237779	0.746106189	0.999579949
813.1867572	0.162089792	0.177421228	0.913587364	0.360933709	0.999579949
1132.632722	-0.070090738	0.070031203	-1.000850129	0.31689927	0.999579949
137.0025094	0.02044472	0.175192042	0.116698907	0.907098657	0.999579949
1037.766409	-0.086782218	0.089120975	-0.973757511	0.330176954	0.999579949
33.57596549	-0.13437147	0.271755164	-0.494457834	0.620982878	0.999579949
657.6613727	0.012940663	0.125512522	0.103102569	0.917881566	0.999579949
177.6493092	-0.115814011	0.203457364	-0.569229879	0.569200146	0.999579949
101.4881211	0.117423961	0.149039024	0.787873923	0.430770456	0.999579949
536.2738767	0.131588538	0.155001456	0.848950336	0.395908928	0.999579949
413.6478133	0.269689541	0.209347822	1.28823667	0.197663591	0.992338959

722.5841859	-0.124043823	0.136797744	-0.906768049	0.364529469	0.999579949
914.2570493	0.040918586	0.11090585	0.36894885	0.712165851	0.999579949
1960.418515	0.119344712	0.090710414	1.315667158	0.188285782	0.983992835
77.26815941	-0.099849918	0.190705072	-0.523582917	0.600568666	0.999579949
49.72706936	0.113550789	0.200635359	0.56595602	0.571423678	0.999579949
535.061282	-0.020708956	0.07999946	-0.258863695	0.795740411	0.999579949
46.81457651	-0.030824718	0.149876842	-0.205666983	0.837051051	0.999579949
1984.507002	0.060843054	0.074993919	0.811306503	0.417189676	0.999579949
59.90409525	0.038677562	0.227585618	0.169947302	0.865051581	0.999579949
213.4541219	-0.064378105	0.094923403	-0.678211093	0.49763786	0.999579949
25.74586292	0.344102654	0.243666073	1.412189433	0.157894191	0.977304288
239.5874043	0.507232125	0.17652913	2.873362171	0.004061282	0.327922523
3127.494862	0.083556794	0.118053926	0.707784963	0.479078805	0.999579949
236.3098077	-0.028505179	0.183985986	-0.15493125	0.876875517	0.999579949
853.8804273	-0.094916602	0.076926167	-1.233866257	0.217252745	0.999579949
137.4349075	-0.072621352	0.092658977	-0.783748691	0.433187586	0.999579949
137.3421446	0.007322712	0.245230597	0.029860514	0.976178297	0.999579949
3723.422122	0.062195469	0.100732088	0.61743453	0.536948152	0.999579949
534.6085385	0.296197259	0.155490253	1.904924931	0.05678983	0.789131444
60.84931548	-0.067991578	0.214343274	-0.317208825	0.751085154	0.999579949
139.5074394	0.011873063	0.097846113	0.121344249	0.903418373	0.999579949
30.7388375	-0.388604037	0.194990196	-1.992941412	0.046267863	0.763868825
45.27664509	0.027259842	0.219845418	0.123995498	0.901318839	0.999579949
28.18884182	-0.140364203	0.357715718	-0.39239037	0.6947698	0.999579949
23.60076348	0.009269806	0.296031524	0.031313576	0.975019464	0.999579949
175.3536524	0.09907141	0.15173175	0.6529379	0.513796314	0.999579949
81.21262386	0.186462896	0.281524207	0.662333439	0.507757551	0.999579949
409.0399178	0.074533599	0.088183428	0.845210945	0.397993073	0.999579949
258.6158251	-0.453477679	0.273029241	-1.660912497	0.096731022	0.887131806
845.6639821	-0.027029698	0.086578072	-0.312200271	0.754888326	0.999579949
147.7510735	-0.028003564	0.093951513	-0.298063998	0.765654319	0.999579949
328.8305322	-0.10568555	0.073966133	-1.42883703	0.15305108	0.972669728
164.1207264	0.072688814	0.089508653	0.812087004	0.41674171	0.999579949
191.4103278	0.005215966	0.117159696	0.044520135	0.964489802	0.999579949
180.779574	-0.015079961	0.087313209	-0.172711113	0.862878508	0.999579949
554.707965	-0.069283879	0.096327361	-0.719254402	0.471984185	0.999579949
306.0495284	0.036631821	0.085963235	0.426133581	0.670010512	0.999579949
663.666123	-0.047442493	0.078616837	-0.603464791	0.546199532	0.999579949
337.3229522	-0.123301159	0.148504182	-0.830287456	0.406376279	0.999579949
511.1158679	0.159157519	0.1262816	1.260338153	0.207547402	0.995145345
31.29910166	0.373213709	0.644675599	0.57891707	0.562645132	0.999579949
32.03766074	0.129447801	0.360903412	0.358677132	0.719836638	0.999579949
71.73361398	-0.362848087	0.230349296	-1.575208143	0.115208408	0.918818353
345.4536044	0.289500073	0.144513551	2.003272851	0.04514801	0.760273172
83.91605659	0.269472177	0.177729553	1.516192287	0.129470738	0.935700262
30.46771268	0.274836234	0.261393659	1.051426552	0.293062726	0.999579949
1674.419746	0.069666856	0.101174154	0.68858353	0.491085388	0.999579949
21.60686486	-0.067417131	0.271396148	-0.248408578	0.803818298	0.999579949

103.7323593	-0.011679691	0.147977737	-0.078928706	0.937089331	0.999579949
35.39406582	-0.074626862	0.198332501	-0.376271469	0.706715088	0.999579949
35.17574367	-0.066972348	0.238409139	-0.280913508	0.77877674	0.999579949
1386.430204	0.021842929	0.063896346	0.341849415	0.73246422	0.999579949
267.7312464	-0.260333895	0.152686833	-1.70501863	0.08819095	0.870840008
54.27434255	-0.094518618	0.146925611	-0.643309341	0.520023397	0.999579949
312.6231024	-0.023966721	0.077602254	-0.308840528	0.757442838	0.999579949
27.7000281	0.752349444	0.289729928	2.596726713	0.009411677	0.458002332
1234.927436	-0.041364486	0.103241009	-0.400659456	0.688670865	0.999579949
166.7153399	0.057476755	0.095974326	0.598876359	0.549255337	0.999579949
1465.432795	0.140814933	0.094666423	1.487485521	0.136886625	0.945791454
253.6610747	0.254007852	0.221868576	1.144857267	0.252268288	0.999579949
157.4505467	-0.055772425	0.097492647	-0.572068015	0.567275897	0.999579949
149.1962714	0.478974106	0.143364061	3.34096358	0.000834882	0.146057922
304.411116	0.040705622	0.178148955	0.228492063	0.819263726	0.999579949
609.0477502	-0.147684785	0.103627548	-1.425149849	0.15411389	0.974996762
46.43546748	-0.428269431	0.161926576	-2.644837196	0.008173023	0.434377203
312.9213542	0.367369592	0.148611954	2.472005671	0.013435739	0.516515866
167.8265766	0.012016137	0.11137462	0.107889368	0.914083451	0.999579949
131.9734979	0.162125168	0.213567405	0.759128801	0.447775512	0.999579949
182.7060536	0.296157301	0.226752318	1.306082795	0.191524403	0.984804292
401.3966344	-0.087523645	0.071360681	-1.226496775	0.220011788	0.999579949
11077.12293	-0.052783225	0.096552229	-0.546680544	0.584598223	0.999579949
32.02603098	0.105156656	0.222052286	0.473567093	0.635808646	0.999579949
92.74951897	-0.041202751	0.171386428	-0.24040848	0.810013605	0.999579949
6303.070533	0.09273083	0.116530134	0.795766955	0.426167502	0.999579949
818.6421914	0.782022391	0.427017378	1.831359636	0.067046882	0.825081178
3050.142744	-0.419399172	0.232120419	-1.806817227	0.070790782	0.834620594
429.8376662	-0.148809503	0.135940438	-1.094666945	0.273662575	0.999579949
5430.800805	0.098828956	0.124144693	0.796078776	0.425986249	0.999579949
29.78016407	-0.191647749	0.259690659	-0.737984759	0.460523711	0.999579949
120.1811521	-0.100948704	0.111943874	-0.901779616	0.36717395	0.999579949
256.8920026	0.193203831	0.156820017	1.232010015	0.217945343	0.999579949
132.6105047	0.228580365	0.182905324	1.249719584	0.211402001	0.996937328
226.5523209	-0.165733472	0.110298876	-1.502585321	0.132946011	0.941167035
490.5836903	0.010526839	0.070667965	0.148961962	0.881583648	0.999579949
626.2486039	0.109024594	0.097538997	1.117753904	0.263672114	0.999579949
427.3439537	-0.301719057	0.406532567	-0.742176842	0.457980197	0.999579949
3907.368769	-0.1391315	0.106619148	-1.304939148	0.19191357	0.984804292
333.29863	-0.052724769	0.069478842	-0.758860795	0.447935833	0.999579949
72.37422472	-0.105198091	0.216410661	-0.486104015	0.62689342	0.999579949
1157.050887	-0.048498713	0.054105205	-0.896377944	0.370050943	0.999579949
391.776038	-0.006432904	0.083489809	-0.077050174	0.93858363	0.999579949
57.48534136	0.006847712	0.200364523	0.034176271	0.972736588	0.999579949
143.772745	-0.111100198	0.164930814	-0.673616989	0.500554849	0.999579949
1012.251775	-0.000633834	0.080842445	-0.007840358	0.993744363	0.999579949
244.2863769	0.258352851	0.260867895	0.990358936	0.321998709	0.999579949
2486.902306	0.024585772	0.072438923	0.339400024	0.734308401	0.999579949

445.6979421	0.043183496	0.113670723	0.379899896	0.704019725	0.999579949
273.270617	0.081171726	0.136447664	0.594892749	0.551915164	0.999579949
220.6400394	-0.008079697	0.145244086	-0.055628405	0.955637836	0.999579949
337.2542219	-0.081768289	0.09725309	-0.840778314	0.400472138	0.999579949
91.15029069	0.252088094	0.183584338	1.373145969	0.169706973	0.981013142
329.1316527	-0.045537425	0.077057233	-0.590955881	0.554549982	0.999579949
310.0972084	-0.106358444	0.069919878	-1.521147445	0.128222841	0.935700262
927.2480151	-0.131383867	0.155503726	-0.844892085	0.398171094	0.999579949
20.54999164	0.302984085	0.44077494	0.687389543	0.491837285	0.999579949
231.6614785	-0.00709484	0.067123258	-0.105698686	0.915821423	0.999579949
21.67459786	0.007492837	0.233445454	0.032096734	0.974394908	0.999579949
334.3837497	0.057607137	0.095481446	0.603333313	0.546286976	0.999579949
37.58956447	-0.086176273	0.160726153	-0.536168334	0.591842216	0.999579949
130.5914495	0.271919671	0.131114494	2.07391008	0.038087663	0.726381766
56.95463548	-0.075368636	0.196488351	-0.383578139	0.701291145	0.999579949
4708.915839	0.043031824	0.117774492	0.36537474	0.714831694	0.999579949
258.2707121	-0.083602945	0.091048996	-0.918219298	0.358504067	0.999579949
28.63387009	-0.11886933	0.264172767	-0.449968146	0.652733409	0.999579949
1978.795804	-0.088643147	0.118298507	-0.749317547	0.453665834	0.999579949
215.6771936	-0.037859968	0.090390089	-0.418850872	0.675325123	0.999579949
134.249513	0.539639255	0.553673938	0.974651718	0.32973305	0.999579949
263.657441	0.106669284	0.081184245	1.313916077	0.188874448	0.983992835
35.15189562	0.284562017	0.432986087	0.657208224	0.511047038	0.999579949
502.1158307	0.090951114	0.05793295	1.569937554	0.11642964	0.918907698
309.4604557	0.037983857	0.090900616	0.417861378	0.676048472	0.999579949
364.4263068	-0.035897708	0.084722427	-0.423709629	0.671777592	0.999579949
144.9612098	0.023906164	0.109405818	0.218509076	0.827032487	0.999579949
540.1921761	0.003902641	0.078410859	0.049771691	0.960304326	0.999579949
1158.391978	0.039488025	0.112873899	0.349841947	0.726457317	0.999579949
164.3870863	0.040446848	0.132063594	0.306267965	0.759400628	0.999579949
62.36309129	-0.362787573	0.180840674	-2.006117122	0.044843754	0.759209569
143.6560423	0.276770554	0.630248702	0.439144981	0.660556488	0.999579949
298.993748	0.167628352	0.2264615	0.740206845	0.459174495	0.999579949
875.1521044	-0.06366716	0.060754202	-1.047946608	0.294663207	0.999579949
1156.750578	-0.100063509	0.087402884	-1.144853634	0.252269793	0.999579949
408.0467164	-0.057010442	0.074061646	-0.769770117	0.441436269	0.999579949
276.8234472	0.115323253	0.129081869	0.893411708	0.371636735	0.999579949
91.72090948	0.161128403	0.139937992	1.151427146	0.249556551	0.999579949
112.1992134	0.11864288	0.189278753	0.626815627	0.530780099	0.999579949
189.264375	0.057902795	0.249682247	0.231905934	0.816611076	0.999579949
1278.585094	0.031350086	0.079073119	0.39646958	0.691758645	0.999579949
192.0281175	-0.062226657	0.087513101	-0.711055325	0.477049953	0.999579949
663.0006967	0.103942711	0.10283218	1.01079945	0.312112426	0.999579949
531.100417	-0.128712802	0.100360303	-1.282507107	0.199664813	0.992493287
404.9594159	-0.025077068	0.110580258	-0.22677708	0.820597087	0.999579949
86.1703943	-0.07164352	0.145667844	-0.491827967	0.622840964	0.999579949
29.27664996	-1.347888073	1.21329196	-1.110934645	0.266596482	0.999579949
149.0222842	0.10705301	0.127020864	0.842798628	0.399341075	0.999579949

457578.6805	-0.094611806	0.125985559	-0.750973424	0.45266865	0.999579949
986.7652952	-0.058268061	0.072140355	-0.8077041	0.419260943	0.999579949
984.4112334	0.042008763	0.102408018	0.410209707	0.681652121	0.999579949
886.9544649	-0.042004486	0.11002754	-0.381763382	0.702636882	0.999579949
2370.544627	-0.040829693	0.058327311	-0.700009853	0.483921151	0.999579949
94.65020279	0.019092673	0.210354749	0.090764164	0.927679986	0.999579949
23.59482968	0.130809349	0.398932777	0.327898224	0.742988617	0.999579949
42.51994043	-0.144667056	0.206199138	-0.701589043	0.482935487	0.999579949
211.6504518	-0.058041288	0.081949993	-0.708252506	0.478788465	0.999579949
325.2157347	-0.066799344	0.164425756	-0.406258397	0.68455275	0.999579949
70.35240189	-0.203963944	0.137665799	-1.481587627	0.138450053	0.945791454
113.1396138	0.013827431	0.206694583	0.06689789	0.946662993	0.999579949
97.42615266	-1.037203924	0.4904168	-2.114943707	0.034434745	0.703403556
405.7920201	-0.050374887	0.106512512	-0.472948073	0.636250227	0.999579949
32.95444649	0.127908994	0.179408829	0.712947046	0.475878526	0.999579949
633.1339984	-0.008017274	0.100456491	-0.079808423	0.936389625	0.999579949
242.8820869	0.181902591	0.133535865	1.362200272	0.17313469	0.983601342
188407.1289	-0.114230629	0.125257281	-0.911967979	0.361785574	0.999579949
1971.797234	-0.269573799	0.118627737	-2.272434808	0.023060261	0.62865781
56.81517532	-0.004036045	0.159559757	-0.025294882	0.979819756	0.999579949
65.1241387	0.125120786	0.167906147	0.74518288	0.456161171	0.999579949
919.4511147	-0.034011027	0.080480126	-0.422601561	0.672585988	0.999579949
95.70023967	-0.075198791	0.104380843	-0.720427125	0.471262054	0.999579949
61.54085178	-0.016658228	0.164801689	-0.101080448	0.919486599	0.999579949
326.2001286	-0.008331508	0.108847832	-0.076542713	0.938987333	0.999579949
703.8794841	0.083872571	0.126726645	0.661838486	0.508074738	0.999579949
190.2028702	0.02135284	0.120226443	0.177605189	0.859033049	0.999579949
47.86200456	-0.235333816	0.219466069	-1.072301597	0.28358459	0.999579949
1062.906176	0.025721426	0.075122923	0.342391185	0.732056521	0.999579949
32.27815125	0.272810687	0.499749798	0.545894541	0.585138431	0.999579949
45.19657895	0.469052397	0.799897937	0.586390307	0.557613257	0.999579949
366.3632189	0.071553565	0.071192546	1.005071029	0.314862649	0.999579949
691.0826683	0.009084665	0.091909354	0.098843751	0.92126233	0.999579949
62340.75426	-0.215146714	0.113733645	-1.891671658	0.058534742	0.79580383
58.25483391	-0.679524053	0.514102435	-1.321767816	0.186245478	0.983992835
144.4187624	-0.099241429	0.100196509	-0.990467932	0.321945456	0.999579949
223.1138539	0.117761895	0.123930503	0.95022526	0.341997806	0.999579949
126.9546652	-0.020471825	0.135035775	-0.151602971	0.87950009	0.999579949
551.1194566	0.080390175	0.074377063	1.08084633	0.279765476	0.999579949
39.52262113	-0.86636361	0.539724682	-1.605195461	0.108450796	0.903743816
605.1820014	0.010497535	0.114038565	0.092052499	0.926656329	0.999579949
46.64751726	0.110587383	0.166036266	0.666043545	0.505383268	0.999579949
66.37855912	-0.291531847	0.13939551	-2.091400554	0.036492173	0.717947029
40.97147189	0.149945392	0.194953401	0.769134528	0.441813453	0.999579949
42.285675	-0.054467361	0.148451792	-0.366902685	0.713691609	0.999579949
22.07768922	0.005463989	0.231936605	0.023558112	0.981205084	0.999579949
44.81036024	0.261137734	0.195273024	1.337295492	0.181126202	0.983992835
259.9454659	0.030441824	0.117430959	0.259231678	0.795456491	0.999579949

372.1627173	-0.038277277	0.103393934	-0.37020815	0.711227404	0.999579949
57.09742054	-0.145948783	0.153254241	-0.952331125	0.340929078	0.999579949
2807.621946	-0.047483998	0.037793373	-1.256410682	0.208967111	0.996447459
54.42893401	-0.051024583	0.14050935	-0.363140122	0.716500215	0.999579949
63.90975587	-0.032500793	0.141561584	-0.22958766	0.818412197	0.999579949
786.8805536	0.105637366	0.063035173	1.675847949	0.093767979	0.880418343
338.9270029	-0.021604399	0.077429614	-0.279019845	0.780229596	0.999579949
151.0127666	-0.035370344	0.109826856	-0.322055513	0.74741064	0.999579949
134.8330795	-0.079023463	0.180237324	-0.438441168	0.661066509	0.999579949
92.23041843	0.118248586	0.204608291	0.577926656	0.563313638	0.999579949
159.4203947	-0.021322936	0.11772735	-0.181121348	0.856272329	0.999579949
716.7062655	-0.041860601	0.063674461	-0.657415869	0.51091355	0.999579949
132.3801074	0.076289039	0.158257575	0.482056161	0.629766051	0.999579949
31.61404967	0.041954816	0.253800846	0.165306052	0.868703096	0.999579949
438.7085816	0.01475133	0.077166089	0.191163372	0.848397599	0.999579949
172.8569538	-0.009908013	0.114307236	-0.086678793	0.930926834	0.999579949
445.7281603	-0.091721816	0.089164062	-1.02868593	0.303627284	0.999579949
58.89230126	-0.052439144	0.133227937	-0.393604712	0.693872902	0.999579949
86.50619505	-0.011908255	0.153169447	-0.077745631	0.938030395	0.999579949
2075.660178	-0.017356741	0.096850327	-0.179212001	0.857771241	0.999579949
194.0906682	-0.085522119	0.091660749	-0.933028799	0.350805099	0.999579949
64.84259789	-0.092009759	0.154597545	-0.595156666	0.551738754	0.999579949
1004.698557	-0.025295287	0.066587903	-0.379878113	0.704035895	0.999579949
31.37184848	-0.17667134	0.238046345	-0.742172034	0.45798311	0.999579949
2877.434281	0.266316019	0.133236208	1.998826174	0.045627165	0.762524133
92.72088693	0.401517901	0.41079534	0.97741591	0.32836329	0.999579949
20.42917747	-0.667018842	0.780999464	-0.854058002	0.393072853	0.999579949
28.55583618	0.919166082	0.323998552	2.836945027	0.004554745	0.33947676
3977.081016	0.160277871	0.114212546	1.403329822	0.160518509	0.978261482
49.97118276	-0.01001312	0.150776465	-0.066410366	0.947051118	0.999579949
189.2611273	-0.051413214	0.111220326	-0.46226455	0.643891618	0.999579949
130.0037068	-0.059130398	0.192976513	-0.30641241	0.759290661	0.999579949
60.11882088	0.0588593	0.204635423	0.287630064	0.773629929	0.999579949
28.09451718	0.526651691	0.420406372	1.252720527	0.210307433	0.996581389
693.8064447	0.013777095	0.097544861	0.141238556	0.887681488	0.999579949
1614.729345	0.123366861	0.093625812	1.317658651	0.187617942	0.983992835
234.7006318	0.025496283	0.099089231	0.257306292	0.796942336	0.999579949
432.805597	0.027651241	0.072336581	0.382258054	0.702269965	0.999579949
492.2127272	-0.032669588	0.065571046	-0.498231921	0.618320586	0.999579949
545.8542888	0.047064093	0.066263463	0.710257067	0.477544738	0.999579949
24.38358969	-0.182794624	0.451849299	-0.404547764	0.685809962	0.999579949
856.9746732	-0.06208433	0.109986042	-0.56447463	0.572431161	0.999579949
311.8487311	0.181635414	0.116756014	1.555683583	0.119783352	0.921521033
18703.24207	0.238017115	0.137581987	1.73000202	0.083629914	0.859920327
484.4874291	-0.085087761	0.083231156	-1.022306602	0.30663581	0.999579949
374.7323429	-0.217617292	0.184817637	-1.177470371	0.239007817	0.999579949
20.57848382	0.423318315	0.39027071	1.084678671	0.278063998	0.999579949
208.7069166	0.312462417	0.143470245	2.177890037	0.029414224	0.672989344

255.9988011	0.135733274	0.143294076	0.947235771	0.343518651	0.999579949
347.9884508	-0.034374378	0.108153986	-0.317828116	0.750615322	0.999579949
1598.937163	0.142004149	0.120343745	1.179987789	0.238005072	0.999579949
415.6255748	0.061695689	0.067212304	0.917922541	0.35865942	0.999579949
79.9543919	0.764129994	0.310208811	2.463276242	0.01376738	0.520783867
213.1417004	-0.010112056	0.185899414	-0.054395309	0.956620216	0.999579949
33.37292759	0.383015027	0.336341135	1.138769502	0.254799305	0.999579949
2126.903867	0.137736464	0.104371781	1.31967149	0.186944723	0.983992835
194.7554015	0.011868687	0.127810802	0.092861374	0.926013692	0.999579949
1011.141517	-0.099060041	0.087797838	-1.128274258	0.259204111	0.999579949
46.05870898	0.007561716	0.149448545	0.050597456	0.95964629	0.999579949
396.6644177	0.009884828	0.093800845	0.105381016	0.916073479	0.999579949
818.5826889	0.067427893	0.122781072	0.549171723	0.582887608	0.999579949
282.0001393	-0.08344447	0.074127093	-1.12569462	0.260294802	0.999579949
280.1932293	0.026614163	0.081908842	0.324924182	0.745238455	0.999579949
180.0152475	0.107655013	0.163344996	0.65906526	0.509853863	0.999579949
498.6946536	0.089147856	0.139649079	0.638370525	0.523232513	0.999579949
784.2274947	-0.092242337	0.098311603	-0.938265004	0.348108239	0.999579949
31.57884031	0.01055705	0.572146065	0.01845167	0.985278533	0.999579949
513.5769127	-0.043454224	0.109361009	-0.397346589	0.691111896	0.999579949
58.69077389	-0.174292474	0.162124691	-1.075052004	0.282351446	0.999579949
105.6870982	0.277973692	0.366247985	0.758976714	0.447866487	0.999579949
140.6476626	1.023945228	0.811001532	1.262568797	0.206744188	0.994814558
53.90529365	-0.100732193	0.169064682	-0.595820439	0.551295188	0.999579949
297.8909382	-0.182047099	0.100986872	-1.802680831	0.071438354	0.834728675
24.85283331	-0.043667976	0.249825637	-0.174793815	0.861241649	0.999579949
33.5522527	-0.107003387	0.180183178	-0.593858916	0.55260648	0.999579949
56.14780885	0.225148951	0.221645893	1.015804751	0.309722373	0.999579949
301.9348611	-0.001026668	0.093285576	-0.01100565	0.991218939	0.999579949
489.8632613	0.007410362	0.08065067	0.091882209	0.926791627	0.999579949
1713.378962	0.240930074	0.116225134	2.072960167	0.038175987	0.726381766
69.63401164	0.08806138	0.198869793	0.442809229	0.657903727	0.999579949
1336.231934	0.072515067	0.102694019	0.706127464	0.480108874	0.999579949
26.59251198	0.276977824	0.63928843	0.43325956	0.664826214	0.999579949
382.1908914	-0.03808529	0.071338436	-0.533867739	0.593433044	0.999579949
44.88823091	0.073877791	0.186168033	0.396833923	0.691489933	0.999579949
49.39988194	-0.078689705	0.484429402	-0.162437922	0.87096101	0.999579949
195.8951756	0.17034875	0.156545789	1.088172038	0.276519166	0.999579949
2437.663786	0.216317719	0.136397103	1.58594072	0.112752758	0.915689061
50.46829226	0.023811407	0.17744253	0.134192219	0.893250578	0.999579949
416.7042416	0.035349596	0.127618107	0.276995149	0.781783831	0.999579949
290.8034923	-0.065192652	0.462388558	-0.140991059	0.887877006	0.999579949
41.14813497	-1.292055461	0.541195878	-2.387408171	0.016967641	0.570162425
109.2492346	-0.115928985	0.10978753	-1.05593946	0.290995875	0.999579949
274.1914851	0.086141586	0.124842065	0.690004496	0.49019136	0.999579949
151.901561	0.164485487	0.131410726	1.251689969	0.210682857	0.996581389
563.0541961	0.106206257	0.107027438	0.992327378	0.321037852	0.999579949
3320.551912	0.102242656	0.148304195	0.689411758	0.490564186	0.999579949

125.7297875	-0.083699301	0.227760063	-0.367488924	0.713254352	0.999579949
473.9441939	-0.37741799	0.276776455	-1.363620288	0.172687103	0.983601342
313.1604012	0.03476007	0.215150652	0.161561539	0.871651147	0.999579949
50.02347112	-0.241933961	0.239808946	-1.008861282	0.313041166	0.999579949
253.2246402	0.252361863	0.12817448	1.968893212	0.048965355	0.773861498
566.9507091	0.002726772	0.097513618	0.027962991	0.977691669	0.999579949
739.7101691	-0.185618073	0.115654549	-1.604935347	0.108508034	0.903743816
34.13546279	-0.00437139	0.206097173	-0.021210336	0.983077869	0.999579949
505.4533352	0.115683347	0.097875569	1.181943038	0.237228304	0.999579949
62.24121024	0.35507532	0.365856384	0.970531978	0.331781393	0.999579949
248.7078547	-0.157095999	0.110049994	-1.427496665	0.153436786	0.973156976
117.6036265	0.100160513	0.12011496	0.833872092	0.404353065	0.999579949
30.66924895	0.109354828	0.293154426	0.373028062	0.709127558	0.999579949
721.9099596	-0.112641976	0.092520223	-1.217484917	0.223419772	0.999579949
147.5378515	-0.153142283	0.132415502	-1.156528355	0.247465108	0.999579949
319.1874129	0.140278978	0.192368235	0.729221113	0.465866418	0.999579949
1877.792689	0.053485212	0.094957729	0.563252855	0.573262716	0.999579949
4011.759746	-0.171044504	0.140152858	-1.220413957	0.222307989	0.999579949
106.5547105	0.013552572	0.138945651	0.097538656	0.922298637	0.999579949
754.9057092	-0.108177376	0.113833761	-0.950310129	0.341954694	0.999579949
309.1083513	-0.070616725	0.171341943	-0.412139162	0.680237426	0.999579949
174.3782963	0.1757704	0.108499589	1.620009818	0.105230168	0.896383253
129.3455481	-0.056519784	0.162822418	-0.347125322	0.728497171	0.999579949
1407.916416	-0.011316101	0.053762621	-0.210482696	0.833290956	0.999579949
22.70765683	0.089121372	0.264976	0.336337526	0.736616355	0.999579949
77.6453119	-0.211001763	0.184081269	-1.146242441	0.251694851	0.999579949
45.01134492	0.481444648	0.152881689	3.149132179	0.001637561	0.214762992
791.6280612	0.099895136	0.064821683	1.54107593	0.123298307	0.92236461
39.98662336	0.052017156	0.185822325	0.279929531	0.77953157	0.999579949
519.2042778	0.044791946	0.092679908	0.483297258	0.628884687	0.999579949
583.2072289	0.040783815	0.15782876	0.258405472	0.796093994	0.999579949
4558.071189	0.065279398	0.081601668	0.799976272	0.423724545	0.999579949
62782.28841	0.033427601	0.156441869	0.21367426	0.830801085	0.999579949
465.3014846	0.022410716	0.207586894	0.107958244	0.914028815	0.999579949
2755.493157	-0.155851054	0.09821914	-1.586768664	0.112565048	0.914754419
121607.7134	-0.128434044	0.11463141	-1.120408837	0.262539581	0.999579949
477.417028	-0.220268208	0.111192805	-1.980957389	0.047596052	0.770705547
2329.595333	0.010838964	0.108270539	0.100110004	0.920256993	0.999579949
4698.370837	0.516393596	0.18638423	2.77058631	0.005595547	0.37178274
1016.422016	-0.093891564	0.227319743	-0.413037436	0.679579186	0.999579949
26.90832573	-0.013405274	0.260622542	-0.051435591	0.958978425	0.999579949
1112.483261	-0.087919874	0.065094804	-1.350643516	0.176809654	0.983992835
92.31363381	0.20540074	0.15654001	1.312129339	0.189476498	0.983992835
52.84576883	-0.119585089	0.179559938	-0.665989811	0.505417613	0.999579949
2376.89192	0.031377181	0.083939581	0.373806733	0.70854811	0.999579949
324.4958721	-0.063791604	0.181171725	-0.352105739	0.724758965	0.999579949
2344.711213	0.041857249	0.088090259	0.475163204	0.634670652	0.999579949
531.4552607	0.195613235	0.094009013	2.080792355	0.037452916	0.720239593

310.0406377	-0.004587978	0.085313453	-0.053777897	0.95711212	0.999579949
21.69564663	0.116805502	0.221664745	0.526946685	0.598230613	0.999579949
71.71069331	0.055594638	0.144627212	0.384399569	0.70068232	0.999579949
78.17301933	0.125020126	0.159148278	0.785557515	0.432126762	0.999579949
392.0416769	0.034093553	0.080131304	0.42547109	0.670493292	0.999579949
896.9416336	0.035661032	0.072657989	0.490806757	0.62356313	0.999579949
66.18669169	0.004927275	0.120897689	0.040755739	0.967490625	0.999579949
167.2769765	0.045705474	0.153467938	0.297817736	0.765842277	0.999579949
114.8873244	-0.104807122	0.128534846	-0.815398509	0.414844237	0.999579949
186.4649029	0.056430032	0.190367961	0.2964261	0.766904695	0.999579949
89.75368867	0.004581318	0.24279696	0.018868925	0.984945669	0.999579949
43.62701559	-0.377822586	0.187509034	-2.014956705	0.043909186	0.758836007
2555.535697	0.159872638	0.071441822	2.237801813	0.025233982	0.642289128
388.0608133	0.003886652	0.15783894	0.024624162	0.980354747	0.999579949
48.20399568	-0.106899397	0.163131043	-0.655297699	0.512276102	0.999579949
438.2543367	-0.073220653	0.104930591	-0.69780082	0.485301766	0.999579949
314.5193143	-0.013248613	0.07014314	-0.188879669	0.850187128	0.999579949
233.7944459	-0.10070602	0.093478298	-1.077319786	0.281337426	0.999579949
34.43971288	0.017813208	0.241402248	0.073790562	0.941177037	0.999579949
332.1373571	0.03051793	0.089112886	0.342463712	0.732001948	0.999579949
145.7619159	0.104869239	0.127332426	0.823586277	0.410174676	0.999579949
101.2717289	0.286588238	0.350046219	0.818715421	0.412948796	0.999579949
296.2300603	-0.669996543	0.572649796	-1.169993508	0.242003581	0.999579949
400.1971026	-0.004063747	0.088963202	-0.045678968	0.963566127	0.999579949
34.92063762	-0.975047216	0.658339658	-1.481070151	0.138587881	0.945791454
2752.122875	0.424757172	0.273915023	1.55068958	0.120976093	0.921837625
32.41907976	-0.245986132	0.283807074	-0.86673714	0.386086051	0.999579949
74.57091833	-0.237554202	0.45419555	-0.523021862	0.60095904	0.999579949
83.42812506	-0.149980948	0.234653955	-0.639157981	0.522720164	0.999579949
24.4822727	0.061659646	0.22036415	0.279807974	0.779624833	0.999579949
162.1379811	-0.029950651	0.115887867	-0.258445102	0.796063412	0.999579949
1226.517148	-0.014713263	0.053605969	-0.274470613	0.783722984	0.999579949
32.15473818	0.161808448	0.172972486	0.935457725	0.34955246	0.999579949
134.1701528	0.033425455	0.10795602	0.309621035	0.756849158	0.999579949
704.1173842	0.0870821	0.087149118	0.999230992	0.317682806	0.999579949
559.0618852	-0.060737797	0.05734305	-1.059200679	0.289508396	0.999579949
39.4487172	-0.189543383	0.191918999	-0.987621778	0.323337916	0.999579949
147.1810394	-0.18964433	0.19970588	-0.949618155	0.34230631	0.999579949
95.39823426	0.065578049	0.152048228	0.431297689	0.66625193	0.999579949
305.948476	-0.354163481	0.109388592	-3.237663778	0.001205127	0.181642887
441.5279516	-0.117505799	0.091707051	-1.281316953	0.200082361	0.992610246
131.0582872	-0.171100841	0.128627138	-1.330207945	0.183449767	0.983992835
275.5174497	0.037657579	0.073933172	0.509346178	0.610509595	0.999579949
828.1790872	0.173845044	0.137228104	1.266832658	0.205215132	0.994814558
3109.858546	-0.250436217	0.188321742	-1.329831667	0.18357374	0.983992835
2658.134831	-0.31794562	0.185955362	-1.709795388	0.087303716	0.867715687
20.69844224	0.502201493	0.259925046	1.932101199	0.053347016	0.781231069
257.7343759	-0.022275213	0.08517786	-0.261514124	0.793696059	0.999579949

199.3235428	0.065008066	0.112265071	0.579058695	0.562549569	0.999579949
183.6331924	0.146321782	0.102393174	1.429018911	0.152998798	0.972669728
347.2331718	0.087079588	0.077040973	1.130302283	0.258348873	0.999579949
924.6951179	-0.109653502	0.078077316	-1.404422027	0.160193216	0.978261482
241.2420716	0.061195371	0.078382055	0.780731912	0.434960187	0.999579949
1406.970281	-0.074576358	0.055171367	-1.351722136	0.176464221	0.983992835
535.7552575	-0.055408513	0.122016957	-0.454105027	0.649753235	0.999579949
23.13994508	-0.043315272	0.255292109	-0.16966945	0.865270101	0.999579949
160.624457	-0.138861581	0.077115828	-1.800688438	0.071752001	0.836066794
1481.201637	0.023591929	0.086214874	0.273641052	0.784360482	0.999579949
4584.351977	-0.022512552	0.075305465	-0.298949774	0.764978368	0.999579949
110.774607	0.094717779	0.120843497	0.78380535	0.433154334	0.999579949
358.4351308	0.17022381	0.147273919	1.155831332	0.247750155	0.999579949
1154.367591	-0.090248648	0.117702802	-0.766750211	0.443230044	0.999579949
180.48251	-0.11496001	0.089844145	-1.279549261	0.200703704	0.993204353
575.6135675	0.026110732	0.088825294	0.293956044	0.768791492	0.999579949
29.17504684	0.015600139	0.232098568	0.06721342	0.946411801	0.999579949
1802.107908	0.092534526	0.055593797	1.664475723	0.096017399	0.88463985
390.5936734	0.039714249	0.242460557	0.163796741	0.869891158	0.999579949
382.3588363	0.027786044	0.081397629	0.341361829	0.732831207	0.999579949
166.1875868	0.090895991	0.119278074	0.762051128	0.44602949	0.999579949
237.238165	-0.035695583	0.089358324	-0.399465668	0.689550116	0.999579949
34.33708073	-0.25302792	0.218137633	-1.159946207	0.246070708	0.999579949
131.6715075	0.034539895	0.110817106	0.311683783	0.755280854	0.999579949
88.0871663	-0.314352662	0.337189839	-0.932272048	0.35119595	0.999579949
35.69614831	-0.041344433	0.183922337	-0.224792888	0.822140399	0.999579949
267.9068409	0.022255468	0.095077434	0.234077292	0.814924975	0.999579949
374.7274205	-0.232033754	0.118505254	-1.958003935	0.050229551	0.776599223
163.4455268	-0.244003758	0.50893281	-0.479441987	0.63162423	0.999579949
36172.09101	0.04247794	0.138732333	0.306186305	0.759462799	0.999579949
77.02369498	0.148121843	0.431277356	0.343449154	0.731260586	0.999579949
125.5750641	0.125025135	0.169583209	0.73724949	0.460970642	0.999579949
90.89782237	-0.071052182	0.172209327	-0.412591949	0.679905601	0.999579949
272.0479865	0.024522877	0.106429019	0.230415329	0.81776905	0.999579949
24.60161704	-1.215265943	0.817838891	-1.485947851	0.137292915	0.945791454
154.6185138	-0.04446585	0.140242102	-0.317064911	0.75119435	0.999579949
287.2232297	0.075832476	0.096058974	0.789436663	0.429856835	0.999579949
80.01395076	-0.18784189	0.140084356	-1.340919824	0.179946485	0.983992835
194.4703271	-0.003696221	0.118249508	-0.031257817	0.975063931	0.999579949
267.9342212	-0.003708383	0.117166951	-0.031650417	0.974750836	0.999579949
367.75633	0.164579267	0.117932104	1.39554254	0.162852296	0.978498637
1692.524895	0.038979913	0.102448108	0.38048446	0.70358583	0.999579949
2684.367766	0.053463204	0.140811132	0.379680236	0.704182793	0.999579949
294.9256301	0.177972913	0.128231612	1.387902019	0.165166883	0.978789551
33.39517097	-0.029096837	0.20093389	-0.144808009	0.884862458	0.999579949
283.2893559	0.008608188	0.153181883	0.056195864	0.955185776	0.999579949
103.1503369	-0.20565655	0.164267285	-1.251962917	0.210583377	0.996581389
79.30083381	-0.163437148	0.195400664	-0.836420634	0.402918308	0.999579949

1392.93391	-0.056627454	0.050851799	-1.113578188	0.265460188	0.999579949
3530.047115	-0.036797995	0.121962343	-0.30171604	0.76286854	0.999579949
976.877067	-0.08461362	0.150901673	-0.560720225	0.574988278	0.999579949
711.9070285	-0.007135697	0.073777767	-0.096718795	0.922949713	0.999579949
170.680928	0.127631987	0.138462551	0.921779834	0.356643428	0.999579949
276.7719643	-0.011022716	0.085382975	-0.129097352	0.897280617	0.999579949
65.16882313	0.257779036	0.239052205	1.07833783	0.280883021	0.999579949
126.8988848	0.209782063	0.178941196	1.172351966	0.241055776	0.999579949
1009.596721	0.064529055	0.09600072	0.672172614	0.501473813	0.999579949
380.5854402	0.086805832	0.115184534	0.753624023	0.451075015	0.999579949
240.0528107	0.06081866	0.185583814	0.327715326	0.743126915	0.999579949
1007.057597	0.057629319	0.15799693	0.364749611	0.715298322	0.999579949
1448.24442	-0.030897002	0.084174038	-0.367060941	0.713573561	0.999579949
48.40185014	-0.077708914	0.183115194	-0.424371746	0.671294723	0.999579949
69.04007696	0.17674626	0.166359015	1.062438726	0.288036561	0.999579949
842.1532004	-0.004073116	0.124420003	-0.032736823	0.973884459	0.999579949
91.9504316	-0.017769378	0.136724375	-0.129964958	0.896594151	0.999579949
334.2673979	0.000656503	0.080926504	0.008112341	0.99352736	0.999579949
218.3112553	-0.037752483	0.117318721	-0.321794189	0.747608617	0.999579949
78.6323727	-0.234643937	0.129165747	-1.816611158	0.069276674	0.831457432
81.23715335	-0.103019838	0.124027522	-0.83062079	0.406187886	0.999579949
456.6353084	0.022504589	0.082343691	0.273300706	0.784622073	0.999579949
276.6583346	-0.010393172	0.079108427	-0.131378822	0.895475641	0.999579949
142.2117864	-0.102732491	0.148372052	-0.692397858	0.48868751	0.999579949
941.218407	0.037191568	0.13280026	0.280056442	0.779434202	0.999579949
3245.947285	-0.040889433	0.108503441	-0.376849181	0.706285689	0.999579949
293.4755798	-0.236237667	0.131415683	-1.797636799	0.072234583	0.838669905
54.9713621	-0.153537991	0.174718355	-0.878774245	0.379523693	0.999579949
511.6705267	0.295190899	0.15152785	1.948096661	0.051403398	0.779189484
234.7376738	-0.073327594	0.095132456	-0.770794714	0.440828621	0.999579949
492.5431062	0.124621414	0.089958805	1.385316468	0.16595572	0.978789551
291.4633474	-0.144871179	0.10036948	-1.443378791	0.148913795	0.962301096
115.3615541	-0.006050551	0.133394903	-0.04535819	0.963821806	0.999579949
44.29354362	1.301115449	0.425125184	3.060546625	0.002209334	0.23630562
39.20374308	0.027654143	0.571244555	0.048410339	0.961389219	0.999579949
625.1743713	0.07687428	0.110833739	0.693599992	0.487933099	0.999579949
733.6801073	0.184408675	0.174235761	1.058385914	0.289879538	0.999579949
169.0885253	0.023342016	0.126015729	0.185230973	0.853047877	0.999579949
146.320264	0.093209232	0.084710928	1.100321219	0.27119219	0.999579949
102.012103	-0.001822988	0.11433361	-0.015944465	0.987278696	0.999579949
109.1853734	-0.112527474	0.114476424	-0.982975098	0.325619695	0.999579949
179.1839055	0.294697236	0.185291024	1.590456078	0.111732038	0.913348385
80.18691503	-0.047887625	0.12929378	-0.370378414	0.711100555	0.999579949
388.5654616	0.046848651	0.074764647	0.626615028	0.530911615	0.999579949
79.02654891	0.129287238	0.11150497	1.159475109	0.246262577	0.999579949
489.7218418	0.044632455	0.08999293	0.495955128	0.61992607	0.999579949
95.79909077	0.102884375	0.133480358	0.770782876	0.440835639	0.999579949
304.6050724	-0.161580769	0.120849081	-1.337045905	0.181207653	0.983992835

282.9483603	0.111499083	0.104965911	1.062240897	0.288126338	0.999579949
190.7569643	0.029087942	0.088663689	0.328070518	0.742858345	0.999579949
1802.254081	0.031721448	0.103120411	0.307615611	0.75837484	0.999579949
63.06999543	-0.322676236	0.217962042	-1.480423994	0.138760131	0.945791454
793.349656	0.018340545	0.057298414	0.320088181	0.748901485	0.999579949
39.81468838	0.113848704	0.222615197	0.511414789	0.609060642	0.999579949
92.8826118	0.193579947	0.225253717	0.859386248	0.390127454	0.999579949
165.2938086	0.007787772	0.212561148	0.036637797	0.970773806	0.999579949
154.9730521	-0.187435518	0.171304286	-1.094167122	0.273881688	0.999579949
334.658418	0.369865641	0.204272336	1.810649681	0.070195097	0.834620594
1448.763802	-0.188148815	0.179757895	-1.046679006	0.29524765	0.999579949
70.90333282	-0.10975987	0.176730083	-0.621059351	0.534560573	0.999579949
454.9119658	-0.114381428	0.177099434	-0.64585993	0.518370072	0.999579949
46.89201385	-0.167403578	0.266968149	-0.627054498	0.530623512	0.999579949
497.9526272	0.003736087	0.111201937	0.03359732	0.973198259	0.999579949
1006.491736	0.017252806	0.118698567	0.145349742	0.884434743	0.999579949
28.16798731	0.039348194	0.211347521	0.186177692	0.852305417	0.999579949
2354.866836	0.50729326	0.172696876	2.93747792	0.003308937	0.308410274
45.6270664	0.312664929	0.177542345	1.761072433	0.078226144	0.851207389
3666.471044	0.01086342	0.104417605	0.104038207	0.917139028	0.999579949
102.2123094	-0.181506387	0.147698789	-1.22889557	0.219110963	0.999579949
1678.79014	-0.063810311	0.125540447	-0.508284881	0.611253571	0.999579949
259.350213	0.05297191	0.07539887	0.702555758	0.482332642	0.999579949
128.0712628	0.142660095	0.134215477	1.062918362	0.287818975	0.999579949
568.6630299	0.120854372	0.254322174	0.475201865	0.634643098	0.999579949
107.6211098	-0.375883021	0.324185043	-1.159470581	0.246264422	0.999579949
41.4956378	-0.131949691	0.201143592	-0.655997488	0.511825741	0.999579949
40.02645001	-0.230269686	0.231617336	-0.994181565	0.320134481	0.999579949
1220.68845	0.01696889	0.214838176	0.078984518	0.937044937	0.999579949
84.48340399	-0.080985954	0.243082758	-0.333162067	0.73901195	0.999579949
5073.497388	-0.109672196	0.150285519	-0.729758907	0.465537567	0.999579949
20.88582384	0.411812587	0.238860589	1.724070883	0.084695048	0.861032139
248.6707894	-0.071330711	0.12977534	-0.549647656	0.582561065	0.999579949
30.9937086	0.007652958	0.229169099	0.033394372	0.973360098	0.999579949
31.00527809	0.18341005	0.296989553	0.61756398	0.536862794	0.999579949
131.1566442	0.041313844	0.135075681	0.305857011	0.759713519	0.999579949
271.3103688	0.07669046	0.11946232	0.641963592	0.520896823	0.999579949
39877.96004	0.083786802	0.141647048	0.591518163	0.554173289	0.999579949
4286.462513	0.165946484	0.128700049	1.28940498	0.197257331	0.992338959
30.97430474	0.082121313	0.226879021	0.361960805	0.71738132	0.999579949
47.23368064	0.31755771	0.361297512	0.878936887	0.379435497	0.999579949
312.0819963	-0.05025062	0.072845455	-0.689825062	0.490304206	0.999579949
344.5860089	-0.06749848	0.083424649	-0.80909516	0.418460412	0.999579949
625.2882522	0.01279512	0.068316377	0.187292136	0.851431589	0.999579949
297.1786077	0.07141195	0.100137637	0.713137955	0.475760395	0.999579949
165.7558695	0.091676469	0.153740845	0.596305225	0.550971341	0.999579949
140.3256601	0.114908074	0.103226208	1.113167643	0.265636437	0.999579949
528.3631533	-0.111740904	0.099008859	-1.128595012	0.259068716	0.999579949

740.3305307	0.047909559	0.109749861	0.436534121	0.662449249	0.999579949
143.4995694	-0.078116582	0.150761558	-0.518146556	0.604356019	0.999579949
46.11753497	0.094711251	0.225568456	0.419878081	0.674574521	0.999579949
514.9038794	0.118266538	0.067952369	1.740432895	0.081783034	0.85448976
1438.638885	0.029619588	0.115589063	0.256249054	0.797758533	0.999579949
36.54897348	0.299988313	0.25509692	1.175977793	0.23960375	0.999579949
51.52381807	-0.089446435	0.357326763	-0.250321119	0.802339026	0.999579949
65.17398746	-0.051859387	0.135463851	-0.382828236	0.701847124	0.999579949
257.1348596	-0.008129179	0.114682047	-0.070884491	0.943489686	0.999579949
49.49985916	0.391279267	0.212751075	1.839141199	0.065894421	0.82135988
26.86291719	0.133653173	0.202158103	0.661131914	0.50852772	0.999579949
74.60144435	0.503682287	0.340797552	1.4779516	0.139420733	0.947729929
77.00673765	0.065853739	0.203457012	0.323673968	0.746184881	0.999579949
92.6989171	0.073552856	0.200600973	0.36666251	0.713870775	0.999579949
75.01955811	0.208767221	0.148777398	1.403218663	0.160551644	0.978261482
1303.018357	0.030503884	0.093668879	0.325656553	0.74468422	0.999579949
254.7849202	-0.022233291	0.12540502	-0.177291874	0.859279134	0.999579949
127.4355473	0.02141631	0.148960315	0.143771915	0.885680581	0.999579949
188.6432925	0.093081101	0.140057498	0.664592058	0.506311451	0.999579949
2429.889591	-0.243041904	0.093578379	-2.597201473	0.009398678	0.458002332
88.76270435	0.062067054	0.209232059	0.296642178	0.766739706	0.999579949
25.64227858	0.015063334	0.373431153	0.040337648	0.967823939	0.999579949
44.06737432	0.210040634	0.190098216	1.104905865	0.269200381	0.999579949
3000.571575	-0.1699238	0.151508784	-1.121544219	0.26205628	0.999579949
52.58628833	-0.020649007	0.224408079	-0.092015436	0.926685776	0.999579949
129.9371788	-0.039043261	0.280189157	-0.139346081	0.889176677	0.999579949
116.7619678	-0.156384515	0.105466819	-1.482784028	0.138131801	0.945791454
35.45588933	0.32224822	0.538128216	0.598831673	0.549285139	0.999579949
53.42385648	-0.193028697	0.144066851	-1.339855044	0.180292476	0.983992835
47.31541128	0.06598604	0.224443238	0.293998786	0.768758831	0.999579949
138.6844065	0.041903278	0.143193086	0.292634783	0.769801324	0.999579949
1173.485876	-0.28932958	0.14905062	-1.941149787	0.052240112	0.780444402
34.06114973	0.353417935	0.235834104	1.498587052	0.133980794	0.944388404
107.5941473	-0.172015161	0.120895916	-1.422836818	0.154783466	0.976030424
372.4096844	0.024787685	0.074167432	0.334212528	0.738219191	0.999579949
11291.90447	0.013612647	0.177888903	0.076523306	0.939002773	0.999579949
862.8069775	0.170666834	0.072950731	2.3394808	0.019310564	0.594708717
35.21806156	0.410272189	0.235776122	1.740092193	0.081842831	0.85448976
85.66290401	0.017327472	0.187863437	0.092234401	0.926511807	0.999579949
86.95773631	-0.11733767	0.177183536	-0.662237997	0.507818706	0.999579949
151.9560649	0.058518198	0.125700625	0.465536255	0.64154748	0.999579949
238.1230102	-0.012815806	0.113891248	-0.112526698	0.910405803	0.999579949
86.13878386	0.072046893	0.146709687	0.491084769	0.623366494	0.999579949
348.5805179	0.082488972	0.089473604	0.921936401	0.35656175	0.999579949
374.0114511	-0.044509036	0.087802698	-0.50692105	0.612210215	0.999579949
109.7732743	0.533355144	0.453069075	1.177204918	0.239113726	0.999579949
76.23155372	0.968080269	0.549667188	1.761211675	0.078202583	0.851207389
2007.336919	0.089269654	0.130563315	0.683726925	0.494147618	0.999579949

685.9349601	-0.037853926	0.097440127	-0.388483954	0.697657926	0.999579949
938.6990979	0.04762168	0.040347169	1.180297937	0.237881739	0.999579949
382.2453963	-0.03106096	0.061724574	-0.503218694	0.614810523	0.999579949
69.59135134	0.138751623	0.116895939	1.186967005	0.235240636	0.999579949
122.3096758	-0.071950045	0.126747236	-0.567665597	0.570262056	0.999579949
764.4089915	-0.041887502	0.098932945	-0.423392853	0.672008658	0.999579949
776.0018332	0.21965085	0.105068718	2.090544686	0.036568899	0.717947029
108.2521728	0.107377601	0.118889403	0.903172177	0.366434522	0.999579949
864.5720461	0.117262606	0.093946931	1.248179209	0.211965436	0.998846476
61.43186966	0.025878521	0.144738872	0.178794548	0.858099027	0.999579949
178.9150701	-0.079145314	0.097262832	-0.813726193	0.415801825	0.999579949
807.6343906	-0.0607326	0.09615048	-0.631641151	0.527621391	0.999579949
1071.16486	0.052301369	0.050772682	1.03010844	0.302959104	0.999579949
219.9003988	1.248042359	0.483291484	2.582380199	0.009812142	0.469938188
257.8806049	-0.277130799	0.082124374	-3.374525566	0.00073943	0.134983569
235.299117	0.0018745	0.098813913	0.018970004	0.984865034	0.999579949
48.65657286	-0.025813495	0.157564276	-0.163828347	0.869866275	0.999579949
744.0104807	0.077953774	0.159243981	0.489524143	0.624470666	0.999579949
1656.006568	-0.123204627	0.083307397	-1.478915802	0.139162819	0.947119714
377.4429656	0.025925355	0.100809568	0.257171574	0.797046327	0.999579949
1193.877789	0.055731185	0.100794105	0.552921073	0.580317457	0.999579949
319.3002827	-0.003321636	0.100439801	-0.033070909	0.973618041	0.999579949
94.86962271	-0.051781934	0.246965775	-0.20967251	0.833923282	0.999579949
201.2909387	0.084838085	0.140917062	0.602042671	0.547145734	0.999579949
298.3283725	0.082152217	0.090154281	0.911240326	0.362168761	0.999579949
341.919876	0.161224185	0.146234864	1.102501693	0.270243623	0.999579949
1184.819756	0.241989605	0.12922043	1.872688445	0.06111142	0.802668869
82.27313495	0.091913488	0.169515094	0.542214181	0.587670967	0.999579949
391.9415912	-0.028297672	0.121513905	-0.232876002	0.815857694	0.999579949
150.4175042	-0.050250247	0.116282531	-0.432139261	0.665640201	0.999579949
143.274083	0.026788765	0.262763291	0.10195018	0.918796219	0.999579949
424.917998	-0.021453277	0.07469416	-0.287214916	0.773947766	0.999579949
85.32102311	0.019942911	0.134898183	0.147836767	0.882471592	0.999579949
191.1927881	0.129178729	0.320627642	0.402893302	0.687026719	0.999579949
763.0138179	0.532366656	0.23783343	2.238401284	0.025194898	0.642289128
249.3491983	-0.005824684	0.114683283	-0.050789299	0.959493418	0.999579949
592.1092715	0.027118247	0.16215357	0.167238054	0.867182744	0.999579949
2504.588492	0.06624889	0.108481469	0.610693146	0.541402744	0.999579949
49.61196904	0.067295748	0.21962568	0.30641111	0.75929165	0.999579949
267.4781456	0.105192798	0.132490113	0.79396715	0.427214555	0.999579949
23.76572899	0.302342839	0.39850356	0.758695454	0.448034757	0.999579949
558.5547456	0.110622658	0.183372407	0.603267743	0.546330588	0.999579949
398.1731074	0.172657248	0.145049514	1.190333174	0.233915467	0.999579949
110.7612849	-0.052994192	0.116556021	-0.454667138	0.649348726	0.999579949
450.6253013	0.016392378	0.143584666	0.114165238	0.909106808	0.999579949
386.1360746	-0.150802625	0.10262039	-1.469519116	0.141692041	0.949966262
512.8646061	0.076533702	0.106034747	0.721779452	0.470430085	0.999579949
404.7082331	0.045977594	0.129324645	0.355520741	0.722199509	0.999579949

262.0646316	0.015088477	0.118710543	0.127103091	0.898858805	0.999579949
402.217129	-0.06956038	0.063267751	-1.099460299	0.271567342	0.999579949
579.7997509	-0.02140243	0.147808934	-0.144797948	0.884870403	0.999579949
244.8496455	0.086107102	0.111574364	0.7717463	0.440264703	0.999579949
373.6473175	0.207937044	0.188460023	1.103348289	0.269875944	0.999579949
68.68374762	0.093137784	0.298204004	0.312329086	0.754790437	0.999579949
174.6798517	-0.040594478	0.137376968	-0.295496971	0.767614266	0.999579949
1737.717822	0.293454334	0.106037519	2.767457567	0.00564954	0.372390484
211.70381	0.139865952	0.130173191	1.074460501	0.282616339	0.999579949
42.68239871	0.684951326	0.398681031	1.718043429	0.085788694	0.864463816
708.561269	0.067925343	0.060222967	1.127897649	0.259363147	0.999579949
206.8521749	0.282811728	0.279333058	1.012453485	0.311321276	0.999579949
29.74674085	-0.0873868	0.961761306	-0.090861214	0.92760287	0.999579949
41.46372535	0.049483812	0.169126237	0.292585069	0.769839328	0.999579949
64.6996248	-0.062502848	0.168021545	-0.371993054	0.709898021	0.999579949
133.8336594	0.120662251	0.118467434	1.018526759	0.308427691	0.999579949
60.06090555	-0.027549486	0.176433031	-0.156146983	0.875917165	0.999579949
459.9370675	0.081485209	0.213442642	0.381766307	0.702634713	0.999579949
182.8089986	0.025647304	0.170575047	0.150357887	0.880482265	0.999579949
172.7477487	0.058285606	0.142118179	0.410120691	0.681717415	0.999579949
297.3611617	-0.012371461	0.104520594	-0.118363863	0.905779358	0.999579949
423.0970144	0.33995546	0.17190339	1.977596029	0.047974298	0.770705547
563.944946	-0.031922856	0.069867582	-0.456905118	0.647739246	0.999579949
34.88016958	-0.089228833	0.218126239	-0.409069691	0.682488518	0.999579949
91.60429238	-0.041479808	0.135826529	-0.305388117	0.760070571	0.999579949
474.0869834	0.012710696	0.074192422	0.171320684	0.863971617	0.999579949
1155.56838	0.057660001	0.065305823	0.882922819	0.377277974	0.999579949
300.0218306	0.071095021	0.113816022	0.624648619	0.532201702	0.999579949
397.2818876	0.02853166	0.075269458	0.379060253	0.704643121	0.999579949
13520.97592	0.00051732	0.117848335	0.004389711	0.996497528	0.999579949
503.7647738	-0.003090787	0.066995529	-0.046134232	0.963203262	0.999579949
29.32385905	0.13754951	0.269799963	0.509820344	0.610177332	0.999579949
498.1714639	0.041915743	0.11403061	0.367583257	0.713184001	0.999579949
164.2750852	0.018888474	0.100394012	0.188143432	0.850764215	0.999579949
431.8502762	0.150723107	0.10685778	1.410501944	0.158391523	0.977304288
92.68626923	0.480810689	0.34704776	1.385430895	0.165920749	0.978789551
33.90138407	0.377212033	0.522771619	0.721561805	0.470563929	0.999579949
1414.804369	-0.041301006	0.232389722	-0.177723034	0.858940495	0.999579949
397.9086171	0.115600876	0.132776579	0.870642078	0.383949614	0.999579949
666.7912982	0.11098316	0.09868508	1.124619451	0.260750328	0.999579949
44.34425186	0.22899791	0.22842708	1.002498961	0.316102668	0.999579949
225.3746506	0.094764182	0.089493986	1.058888822	0.289650416	0.999579949
156.3351034	-0.21651078	0.164638846	-1.315064976	0.188488066	0.983992835
102.2561814	-0.030150364	0.1464904	-0.20581802	0.836933065	0.999579949
65.05492858	0.352100357	0.180064491	1.955412506	0.050534401	0.777205517
100.9926605	0.02439898	0.115296017	0.211620317	0.832403258	0.999579949
211.1069686	0.157160222	0.177118957	0.887314517	0.374909592	0.999579949
166.3563555	0.37726238	0.136935104	2.755045042	0.005868406	0.372390484

260.4746031	0.092303276	0.104777335	0.880946967	0.378346525	0.999579949
270.5647103	-0.065299029	0.109661137	-0.595461904	0.551534757	0.999579949
1799.724581	-0.009380174	0.074967425	-0.125123327	0.900425916	0.999579949
809.3518595	0.015836611	0.101387159	0.156199377	0.875875867	0.999579949
51.14589315	0.057330924	0.170887706	0.335488872	0.737256338	0.999579949
57.80279728	0.359556288	0.161630262	2.22456045	0.02611076	0.648237123
935.5879555	0.205776498	0.098591001	2.087173238	0.036872478	0.717947029
788.6513258	0.267802825	0.112897011	2.372098445	0.017687377	0.573039104
737.2964686	0.019614907	0.079022624	0.248218878	0.803965061	0.999579949
494.3004659	0.288659585	0.179887903	1.604663679	0.108567839	0.903743816
195.2292605	0.001332016	0.20742615	0.006421642	0.994876306	0.999579949
206.4956328	0.024940513	0.082842783	0.301058364	0.76336999	0.999579949
1816.090274	-0.056527227	0.116632461	-0.484661188	0.627916702	0.999579949
49.70461946	-0.006742107	0.348022374	-0.019372626	0.984543848	0.999579949
132.1691913	0.071497669	0.095220267	0.75086608	0.452733256	0.999579949
145.4185976	0.031711106	0.144010332	0.220200213	0.825715231	0.999579949
1303.10897	0.149654285	0.154315897	0.969791762	0.3321503	0.999579949
3174.581525	-0.116016201	0.157195802	-0.738036256	0.460492418	0.999579949
144.8877637	0.29873317	0.184715017	1.617265207	0.105821046	0.896383253
259.4733927	0.15681634	0.132394167	1.184465625	0.236228796	0.999579949
203.8028804	-0.046501792	0.141727142	-0.328107883	0.742830094	0.999579949
847.9967113	-0.017539755	0.107842807	-0.162641861	0.870800425	0.999579949
160.1691742	0.179358561	0.160166046	1.119828861	0.262786698	0.999579949
383.2464313	0.083636702	0.259229237	0.322636069	0.746970874	0.999579949
1136.783246	-0.207485648	0.108173107	-1.91808902	0.055099725	0.785214091
2286.841635	-0.029117582	0.090522771	-0.321660309	0.74771005	0.999579949
202.557625	-0.013263927	0.149885752	-0.088493583	0.929484384	0.999579949
22.42899317	0.076052952	0.269016693	0.282707183	0.77740131	0.999579949
23.37759742	-0.375305882	0.379839842	-0.988063494	0.323121553	0.999579949
190.4199155	0.086307766	0.152306424	0.566671867	0.570937138	0.999579949
126.2642095	0.106044947	0.190912049	0.555464924	0.578576695	0.999579949
362.7169393	-0.039342915	0.089979236	-0.437244378	0.661934129	0.999579949
321.1757598	-0.072378512	0.075490391	-0.958777813	0.337670689	0.999579949
514.1254529	-0.045815641	0.09003077	-0.508888691	0.610830247	0.999579949
182.2440991	0.016864669	0.139936357	0.120516708	0.904073845	0.999579949
73.07078082	0.029620677	0.120417794	0.245982562	0.805695731	0.999579949
143.1264383	-0.127720843	0.149743009	-0.852933594	0.393696131	0.999579949
143.9598673	0.454216702	0.205259241	2.212892828	0.026905035	0.653787748
532.4042291	-0.157432003	0.100484617	-1.566727406	0.117178422	0.920479614
534.0890156	0.034068996	0.123840187	0.275104524	0.783235936	0.999579949
95.91446527	0.471064752	0.37851289	1.244514426	0.213310293	0.999268361
9003.972256	-0.096534263	0.092282967	-1.046068041	0.295529619	0.999579949
66.97032262	-0.120843821	0.158863489	-0.760677122	0.446849943	0.999579949
286.3213807	0.076708701	0.12745474	0.60185052	0.547273643	0.999579949
1341.969453	0.048717938	0.102786216	0.473973452	0.635518839	0.999579949
1431.388649	-0.022056442	0.071699731	-0.307622376	0.758369692	0.999579949
363.2666331	0.003484268	0.076828903	0.04535101	0.963827529	0.999579949
383.6210142	0.244104651	0.149268036	1.63534442	0.101976867	0.895628172

82.91782278	0.286497443	0.203141722	1.410332848	0.158441423	0.977304288
27.10202666	0.27363291	0.302860867	0.903493781	0.366263888	0.999579949
133.5549206	0.075905331	0.141123711	0.537863769	0.590671103	0.999579949
1415.437583	0.178827199	0.12364617	1.446281742	0.148098195	0.962301096
227.25092	0.291011908	0.142077349	2.048263917	0.040534147	0.741027736
78.19093672	-0.045512154	0.167093055	-0.272376098	0.785332849	0.999579949
498.5189742	0.050017027	0.09998926	0.500223997	0.616917363	0.999579949
533.1470014	0.042007025	0.086160555	0.487543574	0.625873171	0.999579949
650.3395825	-0.021700077	0.055013672	-0.394448801	0.69324972	0.999579949
51.88268187	0.216866839	0.168326443	1.288370595	0.19761699	0.992338959
246.1476306	0.063413752	0.086275699	0.735012904	0.462331636	0.999579949
138.1520809	0.186120763	0.154784106	1.202453972	0.229187689	0.999579949
171.5360364	-0.069291153	0.152505104	-0.454353012	0.649574767	0.999579949
430.3002179	0.115692034	0.232206448	0.498229204	0.618322501	0.999579949
947.8326936	0.089013462	0.075407974	1.180425064	0.237831198	0.999579949
52.81422178	0.48555261	0.215861646	2.24936954	0.024488995	0.637321032
106.1753944	-0.011905276	0.153699474	-0.077458146	0.938259086	0.999579949
4133.458577	-0.373106922	0.161526788	-2.309876438	0.020894996	0.613233439
1992.787141	0.016038334	0.105934306	0.15139887	0.879661081	0.999579949
52.91054166	0.012379241	0.265097481	0.046696941	0.962754768	0.999579949
606.5870464	-0.076265684	0.10724403	-0.711141532	0.476996536	0.999579949
7425.887986	-0.167909388	0.157175144	-1.068294794	0.285387556	0.999579949
1014.677742	0.010745458	0.060291426	0.178225318	0.858546027	0.999579949
214.1518607	-0.02963416	0.123887499	-0.239202177	0.810948815	0.999579949
40.91273034	-0.039647033	0.233995502	-0.169435023	0.865454477	0.999579949
746.295584	-0.012183037	0.05929431	-0.205467213	0.837207112	0.999579949
448.5478834	-0.042925243	0.114729693	-0.374142402	0.708298374	0.999579949
365.5788809	0.013114178	0.130453536	0.100527582	0.919925487	0.999579949
815.1940477	0.046963154	0.142208763	0.330240929	0.741217923	0.999579949
390.8254335	-0.054258125	0.061212752	-0.886385987	0.375409568	0.999579949
210.7861849	0.132957912	0.316292356	0.420363975	0.674219581	0.999579949
576.6227054	-0.002351212	0.065891252	-0.035683225	0.971534947	0.999579949
54.8152571	-0.193703378	0.247693329	-0.782029047	0.434197503	0.999579949
32.33947558	-0.028323033	0.342987735	-0.082577394	0.934187577	0.999579949
772.834706	0.119348374	0.072694151	1.641787845	0.100633979	0.895628172
1214.415105	-0.026013949	0.062512143	-0.416142339	0.67730585	0.999579949
1015.187229	0.146737368	0.101257352	1.449152738	0.147294933	0.960339903
683.4038977	-0.026295531	0.111748509	-0.235309902	0.81396821	0.999579949
70.68032362	-0.057943673	0.165482806	-0.350149202	0.726226728	0.999579949
1348.602861	-0.018253808	0.085042801	-0.214642605	0.830045974	0.999579949
145.669698	-0.033505344	0.222277098	-0.150736825	0.880183323	0.999579949
52.04408722	-0.497702514	0.569922123	-0.873281619	0.382509597	0.999579949
1096.687932	0.088544636	0.117325762	0.754690488	0.450434715	0.999579949
21.15448606	-0.029752942	0.406702268	-0.073156567	0.941681528	0.999579949
26.1900187	0.334587488	0.318199811	1.051501217	0.293028451	0.999579949
818.7374391	-0.424240371	0.378621987	-1.120485301	0.262507013	0.999579949
54.94932998	-1.266227545	0.542867138	-2.332481482	0.019675376	0.601531641
197.7989268	0.171043202	0.116979968	1.462158045	0.143697906	0.953645321

3238.359411	-0.026798549	0.089384011	-0.299813672	0.764319286	0.999579949
119.3020017	0.016489154	0.13368466	0.123343648	0.901834979	0.999579949
515.6428377	0.046405131	0.107287207	0.432531826	0.665354927	0.999579949
85.75902207	0.024149885	0.128363842	0.188136193	0.85076989	0.999579949
57.55966898	0.616300177	0.465356267	1.324362044	0.185382834	0.983992835
132.6698329	0.425608581	0.270002917	1.576311047	0.114954137	0.918818353
183.9788633	0.079053433	0.112180654	0.704697562	0.480998469	0.999579949
1202.981583	0.070175374	0.090453733	0.775815123	0.437858154	0.999579949
183.0817273	0.464323179	0.642465063	0.72272129	0.469851133	0.999579949
139.8765773	-0.005115998	0.205181436	-0.024934019	0.980107592	0.999579949
243.9097107	-0.060685849	0.082800198	-0.732919131	0.463607754	0.999579949
233.1574619	0.087987647	0.097576995	0.901725324	0.367202797	0.999579949
537.8377417	-0.053825268	0.077033912	-0.698721733	0.48472595	0.999579949
32.65954108	-0.363709505	0.293624258	-1.23869025	0.215460231	0.999579949
34.19932827	0.026150239	0.24912016	0.104970384	0.916399309	0.999579949
321.1057922	0.172682945	0.090192333	1.914607801	0.055542539	0.785214091
3692.184418	0.019603133	0.112723153	0.173905116	0.861940032	0.999579949
476.6319176	0.031260437	0.140420769	0.222619752	0.823831462	0.999579949
152.1100721	-0.21972552	0.121822141	-1.803658338	0.071284885	0.834620594
128.1980799	-0.380180315	0.156446186	-2.430102799	0.015094541	0.541405219
569.3861473	-0.071043949	0.142324742	-0.499167947	0.617661073	0.999579949
26.52950117	1.351858077	0.634527513	2.130495605	0.033130718	0.695900535
164.0965097	0.086651306	0.091294078	0.949144872	0.342546935	0.999579949
394.4820395	-0.043186996	0.120211637	-0.359258035	0.719402065	0.999579949
253.9605003	-0.049873124	0.09377039	-0.531864311	0.594819977	0.999579949
118.831828	0.064458251	0.120364258	0.535526509	0.592285832	0.999579949
501.460057	-0.239442839	0.109150032	-2.193703792	0.028256711	0.666721367
79.68081848	0.176399682	0.149501237	1.179921222	0.238031549	0.999579949
185.1490369	0.007467444	0.0872599	0.085577043	0.931802648	0.999579949
46.65303597	0.637889481	0.370713171	1.720708975	0.085303647	0.864463816
144.944118	-0.037612933	0.134403109	-0.27985166	0.779591315	0.999579949
2215.240157	-0.039245452	0.047601929	-0.824450888	0.409683411	0.999579949
27.18358592	0.095922502	0.229619376	0.417745677	0.676133072	0.999579949
158.3346325	-0.061471747	0.087113158	-0.705653986	0.480403343	0.999579949
5239.071973	0.046742044	0.119888626	0.389878888	0.696626106	0.999579949
48.7730352	0.207810575	0.253067981	0.821165028	0.411552274	0.999579949
1476.683067	-0.36144687	0.127696586	-2.830513178	0.00464734	0.342326845
131.2748578	-0.151423569	0.163438753	-0.926485101	0.354193936	0.999579949
88.39171705	0.053597921	0.174599813	0.306975821	0.758861776	0.999579949
497.6276457	-0.03580109	0.066546461	-0.537986392	0.590586443	0.999579949
1344.436576	0.024730886	0.062162684	0.397841341	0.690747142	0.999579949
260.6515337	0.040543196	0.114417871	0.354343209	0.723081689	0.999579949
24.85432749	-0.239432339	0.243054509	-0.985097293	0.324576283	0.999579949
110.0709096	0.290822873	0.164620949	1.766621283	0.077291674	0.851207389
37.95307865	0.225852796	0.214146613	1.054664342	0.291578865	0.999579949
138.9081069	0.031924241	0.120173547	0.265651148	0.79050789	0.999579949
20.883466	0.171843438	0.529633385	0.324457337	0.745591817	0.999579949
1131.726402	-0.010523958	0.186020601	-0.056574156	0.954884422	0.999579949

350.1029087	-0.01792409	0.076960256	-0.232900604	0.815838589	0.999579949
56.78343277	0.291206359	0.194178877	1.499680928	0.133697073	0.943444446
41.0337261	0.008084759	0.21658684	0.037328023	0.970223462	0.999579949
21.76654862	0.01428434	0.2873635	0.049708261	0.960354873	0.999579949
108.588278	0.063587972	0.148285202	0.4288221	0.668052694	0.999579949
465.3134625	0.033852888	0.12240321	0.27656863	0.782111355	0.999579949
24.18345152	0.284329783	0.205802316	1.381567465	0.16710455	0.978999491
38.25922847	0.173358562	0.191738729	0.904139522	0.365921424	0.999579949
420.5441136	0.037372337	0.115114148	0.324654592	0.745442505	0.999579949
127.8144003	0.03958498	0.142282328	0.278214313	0.780847848	0.999579949
60.32162724	0.211436507	0.203964093	1.036635931	0.299905561	0.999579949
103.0768916	0.071148895	0.106217269	0.669843002	0.502957878	0.999579949
826.0843524	-0.030454081	0.079423396	-0.383439677	0.701393789	0.999579949
43.79048633	-0.012323745	0.200914016	-0.061338404	0.951089706	0.999579949
29.19964448	-0.046876411	0.220387639	-0.212699821	0.831561106	0.999579949
345.8536212	0.157104126	0.128885623	1.218942204	0.22286613	0.999579949
204.0088028	0.073217134	0.091728634	0.79819278	0.424758627	0.999579949
355.9201543	0.121583141	0.083278428	1.459959606	0.144301176	0.955875831
611.085529	-0.091750647	0.082305141	-1.114762046	0.264952405	0.999579949
649.7885653	0.032583772	0.064443089	0.505620882	0.61312282	0.999579949
82.75811549	0.013331131	0.145730784	0.091477796	0.927112948	0.999579949
48.53232519	-0.013037946	0.141153103	-0.092367405	0.926406136	0.999579949
111.4927686	0.023343788	0.167472977	0.139388386	0.88914325	0.999579949
935.0264151	0.233878535	0.132748025	1.761823079	0.078099194	0.851207389
26.22806787	0.486326833	0.362318402	1.342263683	0.179510514	0.983992835
302.1227107	0.023660705	0.113037283	0.209317705	0.834200231	0.999579949
99.68451184	-0.028452209	0.159454787	-0.178434333	0.858381888	0.999579949
721.1120939	0.058414348	0.051235958	1.140104533	0.254242753	0.999579949
334.4239805	-0.137544398	0.060176527	-2.285681876	0.022272876	0.627784819
162.4901828	0.116456036	0.138186683	0.842744277	0.399371478	0.999579949
352.5321589	0.178799154	0.150669358	1.186698847	0.23534643	0.999579949
169.0757925	0.088871884	0.116709507	0.761479387	0.446370786	0.999579949
121.5122496	0.174319702	0.128452484	1.357075369	0.174757264	0.983992835
893.626751	-0.127262712	0.061010633	-2.085910387	0.036986741	0.717947029
158.7853478	-0.074798272	0.144248424	-0.518537879	0.604083039	0.999579949
211.7459048	-0.084121383	0.125688603	-0.669284095	0.50331427	0.999579949
105.9747032	0.081554042	0.154515053	0.527806449	0.59763368	0.999579949
537.5893543	0.030500809	0.122173268	0.249652068	0.802856429	0.999579949
84.34129655	0.401408235	0.228546384	1.756353475	0.079028072	0.851207389
44.88583794	-0.025564369	0.161605011	-0.158190445	0.874306733	0.999579949
265.9120016	-0.006343965	0.101626736	-0.062424169	0.950225048	0.999579949
114.4360729	-0.378206899	0.115554176	-3.272983384	0.001064187	0.167556296
318.0267319	0.075000939	0.103804294	0.722522506	0.469973294	0.999579949
295.9838858	0.005865328	0.087690775	0.066886489	0.946672069	0.999579949
380.815646	-0.019258664	0.127132775	-0.151484653	0.879593417	0.999579949
753.0898862	-0.102193901	0.072302232	-1.413426637	0.157530318	0.977304288
558.4370064	0.030887331	0.133662888	0.231083821	0.817249684	0.999579949
286.7914578	0.066917383	0.084827616	0.788863176	0.43019198	0.999579949

142.0549067	0.062798382	0.128146744	0.490050543	0.624098134	0.999579949
299.9171678	0.014700958	0.086723995	0.169514305	0.865392122	0.999579949
1126.258661	-0.207213963	0.11874167	-1.745082099	0.080970579	0.85448976
1235.888925	-0.125570442	0.09186875	-1.3668461	0.171673549	0.983601342
257.7724107	0.021000399	0.139925791	0.150082402	0.880699604	0.999579949
247.1560435	0.060923625	0.076614095	0.795201261	0.426496438	0.999579949
319.5854152	0.197466416	0.145645472	1.355801956	0.175162189	0.983992835
976.1171597	0.08711844	0.101317206	0.859858294	0.389867161	0.999579949
1584.697641	-0.020389014	0.086469521	-0.235794225	0.81359235	0.999579949
378.0552239	-0.023080725	0.095798436	-0.240930082	0.809609307	0.999579949
93.38375031	0.028258402	0.103741422	0.272392663	0.785320114	0.999579949
407.8490964	0.461323934	0.624923182	0.738209027	0.460387438	0.999579949
193.4677186	0.126765864	0.121664643	1.041928542	0.29744481	0.999579949
35.2499276	0.62990797	0.212651968	2.962154442	0.003054945	0.295424219
532.3255313	0.209868642	0.067316366	3.117646645	0.001823012	0.222938473
103.5970196	0.135286331	0.138661435	0.975659383	0.329233286	0.999579949
627.9445596	-0.087624946	0.07433716	-1.178750242	0.238497643	0.999579949
992.9366221	-0.005236687	0.057324801	-0.091351165	0.927213564	0.999579949
207.8605179	0.25858943	0.151727647	1.704300012	0.088325053	0.870840008
157.97149	0.282981091	0.18979823	1.49095748	0.135972659	0.945791454
91.73654081	0.016492516	0.141458694	0.116588916	0.907185822	0.999579949
511.6580144	-0.145620488	0.082681994	-1.761211623	0.078202591	0.851207389
2262.669904	-0.146665955	0.149188586	-0.983090989	0.325562659	0.999579949
68.60362796	-0.121489652	0.18250053	-0.665694791	0.505606204	0.999579949
570.96503	0.072049044	0.107045326	0.67307044	0.500902479	0.999579949
779.8199248	0.000591281	0.075995721	0.007780454	0.993792159	0.999579949
155.0314359	0.036318415	0.111119685	0.326840516	0.743788513	0.999579949
1145.499647	0.031199861	0.093071046	0.335226288	0.737454394	0.999579949
358.0016427	0.020239082	0.059593876	0.3396168	0.734145125	0.999579949
341.4522502	-0.131803948	0.085996809	-1.532660927	0.125359421	0.927748102
1436.523543	0.036162606	0.077801345	0.464806948	0.642069712	0.999579949
54.95311514	0.090003952	0.240637557	0.37402288	0.708387294	0.999579949
2123.922085	0.043413741	0.096964666	0.447727432	0.654349921	0.999579949
176.8878244	-0.112500181	0.150300932	-0.748499559	0.454158891	0.999579949
940.1883806	-0.01426264	0.045455026	-0.313774773	0.753692106	0.999579949
173.5615062	0.272172105	0.214844681	1.266831942	0.205215388	0.994814558
26.19398899	0.19657717	0.301814077	0.651318757	0.514840743	0.999579949
270.0861721	-0.117259254	0.072435944	-1.618799268	0.10549046	0.896383253
1330.884435	0.10157273	0.089742328	1.13182634	0.257707451	0.999579949
468.1389186	0.050756796	0.138140209	0.367429563	0.713298623	0.999579949
136.8780538	0.172746777	0.126693445	1.363502084	0.172724328	0.983601342
355.5336588	-0.130584999	0.069846631	-1.869596234	0.061539909	0.804104449
143.3048918	0.019416292	0.094767579	0.204883279	0.837663319	0.999579949
35.84373927	-0.111190297	0.230612156	-0.4821528	0.629697404	0.999579949
27.3969737	-0.155629418	0.212603255	-0.732018039	0.464157558	0.999579949
603.2347313	0.025513465	0.128255475	0.198926905	0.842319923	0.999579949
97.70179429	0.024737334	0.140429395	0.176154953	0.86017221	0.999579949
1113.269751	-0.09071777	0.075118191	-1.207667123	0.227175329	0.999579949

179.4052575	0.169527891	0.11562922	1.46613365	0.14261188	0.951955082
143.4114874	0.092421414	0.126221674	0.732215091	0.464037295	0.999579949
193.2278244	0.2749219	0.135189844	2.033598761	0.041992067	0.74969123
38.40227807	0.019179997	0.356876137	0.053744129	0.957139024	0.999579949
140.9930495	-0.102346659	0.128055778	-0.799234992	0.424154166	0.999579949
2567.213642	-0.234073414	0.16744057	-1.39794922	0.162128317	0.978498637
243.2267405	-0.083892229	0.121082234	-0.692853331	0.4884016	0.999579949
175.4540138	0.152562653	0.159447962	0.956817832	0.33865921	0.999579949
700.5074601	0.017194646	0.107072027	0.160589527	0.872416704	0.999579949
455.8038082	-0.021658817	0.118334979	-0.183029707	0.854774709	0.999579949
127.6830335	0.017494794	0.105161303	0.166361516	0.867872458	0.999579949
382.971643	0.09204494	0.237707651	0.387219089	0.69859402	0.999579949
83.68524487	0.187946662	0.484626296	0.387817713	0.698150937	0.999579949
41.79911816	-0.130074071	0.163754629	-0.794323019	0.427007407	0.999579949
912.5996263	-0.077434268	0.109770274	-0.705421099	0.480548217	0.999579949
781.0327618	-0.047464708	0.083303338	-0.569781581	0.56882585	0.999579949
1209.820893	0.237180148	0.148549492	1.596640583	0.110345847	0.909823133
76.96497839	0.234592273	0.163707194	1.432999167	0.151858075	0.971953413
821.1642402	-0.082052	0.044398331	-1.848087465	0.064589691	0.817660046
384.9635804	-0.048243052	0.061995307	-0.778172638	0.436467245	0.999579949
131.1598622	0.017722703	0.11041668	0.160507477	0.872481332	0.999579949
602.0692228	-0.187406884	0.218712184	-0.856865312	0.391519325	0.999579949
662.0403838	-0.17636154	0.118734126	-1.485348368	0.137451565	0.945791454
61.87775895	0.001772742	0.148310176	0.011952938	0.990463162	0.999579949
168.6876127	0.373373406	0.510868266	0.730860439	0.464864402	0.999579949
58.75250745	-0.102971229	0.194039092	-0.530672596	0.595645679	0.999579949
627.4737128	-0.099413002	0.057848473	-1.718506928	0.085704192	0.864463816
402.8791135	0.140505928	0.134632018	1.043629372	0.296656898	0.999579949
448.5796746	0.013618669	0.068930692	0.197570462	0.843381147	0.999579949
373.1938649	0.389307626	0.173314538	2.2462491	0.024688057	0.639857539
68.63936663	0.43835482	0.215908919	2.030276572	0.042328434	0.74969123
948.5726524	-0.033379474	0.100630833	-0.331702253	0.740114099	0.999579949
296.5052548	-0.103595791	0.104812781	-0.98838891	0.322962217	0.999579949
5738.573227	0.123902381	0.130679558	0.948138962	0.343058716	0.999579949
102.8061757	0.145858279	0.574124233	0.254053514	0.799454216	0.999579949
98.70779937	0.092089848	0.118317974	0.778325087	0.43637739	0.999579949
1705.783253	0.172045404	0.113769986	1.512221369	0.130477557	0.935700262
1018.206357	0.060118414	0.078768421	0.763229897	0.445326302	0.999579949
916.8614852	-0.053849424	0.062092001	-0.867252199	0.385803841	0.999579949
119.1502494	0.016090136	0.106414611	0.15120232	0.87981612	0.999579949
92.29559022	0.333838536	0.25438013	1.312360895	0.189398395	0.983992835
95.20845892	0.45001665	0.194882888	2.309164518	0.020934454	0.613233439
209.4667367	-0.080782137	0.149861756	-0.539044377	0.589856234	0.999579949
746.4304644	0.037397741	0.065492045	0.571027228	0.567981186	0.999579949
120.0286122	0.508808554	0.363546945	1.399567679	0.161642818	0.978498637
66.83581333	0.010611459	0.165545184	0.064100078	0.94889054	0.999579949
179.2372255	0.884158743	0.293322694	3.014286857	0.002575841	0.261655622
513.1201405	-0.090020364	0.108880896	-0.826778315	0.408362723	0.999579949

109.0372427	0.168718891	0.108097063	1.560809212	0.118568773	0.921521033
908.8749157	-0.257040354	0.195783712	-1.312879154	0.189223672	0.983992835
21.02637252	0.564697126	0.498425083	1.132962896	0.257229835	0.999579949
187.7415887	0.092583995	0.136671454	0.677420136	0.498139426	0.999579949
33.72028625	0.285809414	0.30547985	0.935608074	0.349475016	0.999579949
250.586893	0.131928223	0.112551477	1.172158964	0.24113324	0.999579949
521.9505342	-0.138730113	0.107534582	-1.290097662	0.197016752	0.992252301
243.9709267	0.05356412	0.083289796	0.643105422	0.520155697	0.999579949
296.9045106	0.051674295	0.133203832	0.387933994	0.698064881	0.999579949
2244.758047	0.047202074	0.058252944	0.810295076	0.417770605	0.999579949
101.4200414	0.077537191	0.143910645	0.538787045	0.590033804	0.999579949
1284.421013	0.004566709	0.098484353	0.046369891	0.963015434	0.999579949
3419.537431	-0.028648509	0.075175929	-0.381086204	0.703139284	0.999579949
57.39010393	-0.039092924	0.172999643	-0.225971126	0.821223877	0.999579949
291.5301321	-0.166244952	0.149115727	-1.114872024	0.264905267	0.999579949
673.2874906	0.064398528	0.092294732	0.697748682	0.485334377	0.999579949
125.6524015	0.122159387	0.125485138	0.973496856	0.330306422	0.999579949
42.09587848	-0.029052055	0.165117088	-0.175948201	0.860334637	0.999579949
279.174959	0.002141229	0.07656645	0.027965631	0.977689563	0.999579949
215.1945296	0.198265817	0.121923324	1.626151664	0.103917396	0.895628172
34.7652969	0.152291035	0.286660799	0.531258671	0.595239541	0.999579949
2152.807043	-0.129899157	0.054148266	-2.398953233	0.016442014	0.563098469
680.990474	-0.005129988	0.075470465	-0.067973446	0.945806772	0.999579949
734.6684336	0.060857894	0.08795837	0.691894298	0.489003712	0.999579949
57.20002054	0.002637055	0.154357933	0.017084028	0.986369581	0.999579949
53.99310345	-0.13856565	0.137081725	-1.010825111	0.312100143	0.999579949
231.9381442	0.466946594	0.181567309	2.571754773	0.010118454	0.473799407
258.0174966	-0.145491794	0.115310043	-1.26174434	0.207040796	0.994814558
25.5332823	0.129971065	0.270466469	0.480544098	0.630840554	0.999579949
286830.5017	0.035984435	0.115993794	0.310227242	0.756388155	0.999579949
597.2881056	0.083410499	0.064225642	1.298710245	0.194043387	0.987144791
648.8101151	0.2366287	0.169607609	1.395153799	0.162969466	0.978498637
217.9750266	-0.006511681	0.077678384	-0.083828742	0.933192596	0.999579949
189.4327061	0.014057507	0.087176526	0.161253346	0.871893866	0.999579949
1377.810068	-0.015037814	0.069106922	-0.217602145	0.827739113	0.999579949
383.5235888	0.065427119	0.081382853	0.803942316	0.42143029	0.999579949
145.5823573	-0.14498793	0.123975558	-1.169488026	0.242207063	0.999579949
222.6704185	-0.058451083	0.168876957	-0.346116392	0.729255245	0.999579949
273.5845249	-0.019546995	0.092431314	-0.2114759	0.832515936	0.999579949
37.16305485	0.104022119	0.177424594	0.586289178	0.557681202	0.999579949
62.42994407	-0.475130015	0.71262658	-0.666730695	0.504944168	0.999579949
256.3528368	-0.028806353	0.102700273	-0.28048955	0.779101942	0.999579949
2251.533769	0.053013762	0.070609872	0.750798163	0.452774135	0.999579949
450.6145972	0.099313706	0.087987319	1.128727497	0.259012806	0.999579949
151.9549787	-0.124777853	0.101906904	-1.224429823	0.220790125	0.999579949
4477.57856	-0.016486963	0.118208923	-0.139473085	0.889076323	0.999579949
1135.653077	0.066954937	0.102382977	0.653965519	0.513134021	0.999579949
34.19979528	-0.135772038	0.203921529	-0.665805315	0.505535547	0.999579949

316.4874525	0.035991716	0.144663105	0.248796791	0.803517973	0.999579949
90.04917818	0.047524019	0.146054485	0.325385551	0.74488929	0.999579949
90.77619274	-0.069258328	0.128260217	-0.539982936	0.589208801	0.999579949
25.39727016	-0.003810081	0.216972777	-0.017560181	0.985989722	0.999579949
105.3829349	-0.023079209	0.140085567	-0.164750801	0.86914013	0.999579949
375.9312681	0.062790266	0.135934813	0.461914534	0.644142611	0.999579949
1347.923588	0.177659222	0.096883283	1.833744849	0.066691878	0.823579307
692.3586982	0.056009031	0.083650767	0.669557883	0.50313967	0.999579949
110.9304774	0.195220225	0.130958947	1.490697885	0.136040832	0.945791454
821.0079784	-0.149891199	0.084768044	-1.768251244	0.077018911	0.851207389
1240.125705	-0.098282608	0.107864599	-0.911166495	0.362207655	0.999579949
2644.421307	0.005265019	0.056069236	0.093902091	0.925186933	0.999579949
166.0765298	0.062247587	0.171964947	0.361978344	0.717368214	0.999579949
229.2228609	-0.030559304	0.121672666	-0.25115998	0.801690425	0.999579949
449.7145924	0.068371455	0.127044431	0.538169632	0.590459943	0.999579949
255.4277948	-0.062393165	0.150941862	-0.413358918	0.679343669	0.999579949
214.0666898	0.089674713	0.155460618	0.576832346	0.564052716	0.999579949
80.24125806	0.24813569	0.159041455	1.560195048	0.118713796	0.921521033
451.9330989	0.101569524	0.103366259	0.982617787	0.325795587	0.999579949
53.81559543	0.214288147	0.221118984	0.969107867	0.332491373	0.999579949
50.48581438	-0.180610337	0.205532968	-0.878741443	0.379541483	0.999579949
234.7626453	0.056133998	0.074737064	0.751086475	0.452600615	0.999579949
634.5235422	0.124960315	0.162959132	0.76681996	0.443188567	0.999579949
451.9561478	-0.174189009	0.173207036	-1.005669357	0.314574648	0.999579949
402.1105782	0.144806165	0.10209746	1.418313099	0.156099374	0.976030424
140.2792905	-0.172721065	0.265161305	-0.65138111	0.514800502	0.999579949
983.3247495	0.023785129	0.091300374	0.260515134	0.794466443	0.999579949
44.42029034	0.255002366	0.199305016	1.279457844	0.200735875	0.993204353
181.9627535	-0.022825235	0.114583196	-0.199202288	0.84210451	0.999579949
75.12159246	0.101538548	0.146478716	0.693196599	0.488186183	0.999579949
221.4349562	0.131301633	0.143236036	0.91668016	0.359310266	0.999579949
38.97562644	-0.115475543	0.213000745	-0.542136805	0.587724266	0.999579949
184.8959201	0.049779192	0.164800937	0.30205649	0.762609	0.999579949
340.8900515	-0.066380994	0.093064927	-0.713276167	0.475674883	0.999579949
132.9551993	0.024950636	0.143073304	0.17439058	0.861558517	0.999579949
166.9943268	0.06030376	0.148594726	0.405827056	0.684869678	0.999579949
199.9883566	-0.029644906	0.131546926	-0.225356128	0.821702239	0.999579949
149.3029781	0.248325947	0.22361114	1.110525831	0.266772504	0.999579949
449.8810784	-0.099566173	0.097843863	-1.017602632	0.308866836	0.999579949
9840.691594	0.002849517	0.108730299	0.026207203	0.979092071	0.999579949
72.28448439	-0.069722037	0.132406885	-0.52657411	0.598489375	0.999579949
169.1558544	-0.038144778	0.087717126	-0.434861245	0.663663147	0.999579949
57.79142443	0.414741429	0.287430968	1.442925343	0.149041503	0.962301096
181.621072	0.132617613	0.081555365	1.626105309	0.103927255	0.895628172
165.093823	-0.085598263	0.136065806	-0.629094597	0.529287127	0.999579949
118.2510247	0.019992866	0.125930842	0.158760677	0.873857431	0.999579949
289.3269187	0.057039432	0.13564913	0.420492429	0.674125759	0.999579949
45.32835062	0.001221874	0.183585751	0.006655606	0.994689634	0.999579949

93.99421165	-0.14352187	0.140057513	-1.024735249	0.305488126	0.999579949
47.8935576	0.011964897	0.213075706	0.056153266	0.955219711	0.999579949
130.1253805	1.089546165	0.445500233	2.445669124	0.014458362	0.534068996
420.4690141	-0.02174468	0.098488689	-0.220783523	0.825260993	0.999579949
81.34834317	0.07252619	0.149269088	0.485875478	0.627055456	0.999579949
353.2109778	-0.023574935	0.114911384	-0.205157527	0.837449052	0.999579949
74.04573413	-0.068946974	0.137725501	-0.500611533	0.616644544	0.999579949
345.274173	0.060178612	0.077956694	0.771949265	0.440144477	0.999579949
138.8767135	-0.081550428	0.101727222	-0.801657873	0.422750891	0.999579949
516.6831703	-0.028851549	0.073982209	-0.38997956	0.696551661	0.999579949
44.58834689	-0.159013753	0.175531708	-0.905897596	0.364990057	0.999579949
105.3638546	0.065472905	0.131461114	0.498040093	0.618455784	0.999579949
373.4486865	-0.036483933	0.08700297	-0.419341236	0.674966764	0.999579949
418.8083415	-0.065668938	0.087156469	-0.753460288	0.451173367	0.999579949
942.9701412	-0.733119949	0.191659329	-3.825120085	0.000130708	0.050670878
32.45346737	-0.056778094	0.31574934	-0.179820151	0.857293763	0.999579949
29.99678594	0.12445031	0.297224217	0.418708513	0.675429173	0.999579949
124.4809114	0.104325252	0.126583411	0.824162123	0.409847446	0.999579949
620.8264332	0.1140163	0.139447345	0.817629761	0.413568629	0.999579949
365.5673715	0.462995186	0.284663407	1.626465416	0.103850685	0.895628172
54.15742083	-0.058844871	0.197425281	-0.298061479	0.765656241	0.999579949
449.4245051	0.005879638	0.094449083	0.062251936	0.950362204	0.999579949
89.37067901	0.226874785	0.32042409	0.708045344	0.478917099	0.999579949
111.3406755	-0.079841972	0.140524903	-0.56816956	0.569919839	0.999579949
300.2902569	-0.005710447	0.13803146	-0.041370619	0.967000436	0.999579949
1178.382265	0.065638302	0.089447876	0.733816216	0.463060756	0.999579949
1061.817483	-0.150772335	0.10985877	-1.372419647	0.169932837	0.981013142
1159.661768	0.023571774	0.091081049	0.258799987	0.795789568	0.999579949
604.8887691	-0.097971789	0.070085963	-1.397880331	0.162149006	0.978498637
128.7073605	0.108388961	0.131726887	0.822830958	0.410604129	0.999579949
124.5383221	0.457022792	0.210191308	2.174318229	0.029681244	0.672989344
2793.249218	-0.047626316	0.077375634	-0.615520855	0.538210801	0.999579949
487.8950329	0.00905972	0.063305884	0.143110229	0.886203127	0.999579949
75.78878017	0.072487598	0.142438687	0.50890386	0.610819614	0.999579949
854.3125959	-0.067167803	0.121640538	-0.552182721	0.580823171	0.999579949
244.8004676	0.0761118	0.12106816	0.628669007	0.529565773	0.999579949
24.89002674	0.301373053	0.421527269	0.714955058	0.474636814	0.999579949
740.2645532	-0.165320605	0.087759569	-1.883790085	0.059593375	0.798635337
106.1747617	0.049569592	0.111262118	0.445520836	0.655943405	0.999579949
1066.050836	0.047206691	0.128498122	0.367372618	0.713341093	0.999579949
1247.745203	-0.049052124	0.070433782	-0.696428942	0.486160243	0.999579949
262.5902938	-0.049957632	0.218862712	-0.228260134	0.819444015	0.999579949
24.80997305	0.619886959	0.310150921	1.998662317	0.045644903	0.762524133
757.8040735	-0.079308409	0.060374066	-1.313617156	0.188975072	0.983992835
830.7129329	-0.048505428	0.096655497	-0.50183828	0.615781283	0.999579949
31.26065667	-0.247030675	0.227906227	-1.083913671	0.278403078	0.999579949
188.3108165	-0.00021221	0.249500564	-0.00085054	0.999321367	0.999840121
97.95311228	-0.179167254	0.178566014	-1.003367048	0.315683797	0.999579949

20.73728871	0.158093426	0.25593032	0.617720581	0.536759543	0.999579949
22.52879339	-0.029254308	0.266262129	-0.109870331	0.912512212	0.999579949
273.44094	0.116901766	0.106509909	1.097567047	0.272393589	0.999579949
90.72697556	0.104153219	0.128523052	0.810385509	0.417718644	0.999579949
778.4907629	-0.071138422	0.09535845	-0.746010678	0.455660964	0.999579949
213.2144595	-0.027486237	0.105978215	-0.259357429	0.795359473	0.999579949
145.3969415	1.010161616	0.680742208	1.483912124	0.137832235	0.945791454
115.5558863	-0.139215338	0.171028349	-0.813989833	0.415650775	0.999579949
95.72735603	-0.027678311	0.158716137	-0.174388763	0.861559945	0.999579949
30.55468921	-0.292161013	0.253948673	-1.150472689	0.249949236	0.999579949
81.43901895	0.877140249	0.334740589	2.620358204	0.008783745	0.444337558
30.58362412	-0.198533415	0.21617894	-0.918375377	0.358422377	0.999579949
316.731399	-0.096865386	0.098519451	-0.983210769	0.325503716	0.999579949
207.8783994	0.085575534	0.105155782	0.813797705	0.41576085	0.999579949
105.3171615	0.098508708	0.23698782	0.415669919	0.677651556	0.999579949
149.1069417	0.158368657	0.209343206	0.756502492	0.449347979	0.999579949
891.4354476	-0.05754792	0.087096447	-0.66073786	0.508780439	0.999579949
753.0580058	0.050112893	0.094160306	0.532208267	0.594581758	0.999579949
2732.475876	-0.083244555	0.10071129	-0.826566272	0.408482941	0.999579949
25.60524146	-0.4195107	0.318876274	-1.315590823	0.188311415	0.983992835
329.8525505	-0.510413539	0.346174028	-1.474442036	0.140362613	0.948318761
398.8199328	0.082569747	0.115218448	0.716636521	0.473598404	0.999579949
44.18672429	-0.27219974	0.162568021	-1.674374442	0.094057032	0.880418343
737.9292435	-0.04982276	0.047306505	-1.05319047	0.292253705	0.999579949
364.378453	0.156095622	0.119346312	1.307921626	0.190899894	0.98475336
128.0228121	0.091128034	0.118020189	0.772139367	0.440031889	0.999579949
66.90386453	-0.260412901	0.16701455	-1.559222839	0.118943647	0.921521033
708.7945457	-0.008906564	0.064198183	-0.138735449	0.889659205	0.999579949
90.29452506	-0.274396912	0.146502562	-1.87298371	0.061070635	0.802668869
2055.36063	0.01755269	0.119104476	0.147372212	0.882838237	0.999579949
2872.02722	-0.235726223	0.109895913	-2.144995356	0.031953218	0.689986698
96.21874474	0.11915554	0.157888539	0.754681376	0.450440184	0.999579949
398.1829163	-0.043094213	0.085795958	-0.502287225	0.615465494	0.999579949
155.5980534	-0.391041182	0.595671761	-0.656470908	0.51152118	0.999579949
116.8154141	0.179112804	0.244826099	0.731591956	0.464417659	0.999579949
305.3689678	0.028758388	0.090324191	0.318390761	0.750188546	0.999579949
376.2701352	-0.153028514	0.168488812	-0.908241398	0.363750696	0.999579949
652.1239284	-0.3210478	0.208240414	-1.54171707	0.123142362	0.92236461
35.82403802	-0.258721323	0.203266914	-1.272815718	0.203083442	0.994409048
104.8791316	0.169250639	0.184633734	0.916683183	0.359308681	0.999579949
33.47494283	0.063026903	0.276879502	0.227632968	0.819931588	0.999579949
121.436175	0.093469596	0.107614328	0.868560884	0.385087357	0.999579949
435.3836756	-0.241887059	0.137459736	-1.759693896	0.078459721	0.851207389
405.1505692	-0.056568456	0.08849144	-0.639253417	0.522658087	0.999579949
3201.127307	-0.0572659	0.116890098	-0.489912324	0.624195942	0.999579949
329.1801159	-0.127152714	0.113835647	-1.116985036	0.264000724	0.999579949
99.37637336	0.025520532	0.099731072	0.255893493	0.798033079	0.999579949
548.7804988	-0.107859809	0.083217096	-1.29612561	0.194932213	0.988443849

125.8502555	0.061496677	0.125455476	0.490187268	0.624001389	0.999579949
23.61540489	0.342706255	0.242697254	1.412073061	0.15792845	0.977304288
493.23567	-0.022374741	0.068931561	-0.32459357	0.745488695	0.999579949
855.6938044	-0.09762224	0.086543256	-1.128016724	0.259312856	0.999579949
46.32627685	0.252171556	0.192535168	1.309742831	0.190282849	0.984717651
526.3462512	-0.029171198	0.132346859	-0.220414735	0.825548171	0.999579949
61.70006466	0.041099118	0.231900661	0.177227255	0.859329888	0.999579949
34.88345321	0.348804196	0.376268845	0.927007911	0.353922427	0.999579949
54.99319766	0.061380594	0.152273339	0.403094819	0.686878472	0.999579949
351.3134354	-0.214764203	0.094597847	-2.270286377	0.023190213	0.62865781
74.47574005	0.048727528	0.14354227	0.339464662	0.734259714	0.999579949
440.4902288	-0.151982168	0.131222016	-1.158206316	0.246779851	0.999579949
98.1471844	-0.167657481	0.128723429	-1.30246282	0.19275822	0.985859356
39.11142211	0.278399579	0.33166771	0.839393075	0.401248769	0.999579949
113.0751762	-0.079221814	0.098190878	-0.806814396	0.419773424	0.999579949
331.9749858	0.068618233	0.064582923	1.062482624	0.288016642	0.999579949
592.6811766	0.030315868	0.071613354	0.423327025	0.672056679	0.999579949
51.61591387	0.090143663	0.154095564	0.58498545	0.5585575	0.999579949
61.26571131	-0.03893187	0.331317197	-0.117506337	0.906458822	0.999579949
168.2290213	0.218579377	0.16202861	1.349017166	0.177331452	0.983992835
153.8853271	-0.353426219	0.200265427	-1.76478898	0.077599237	0.851207389
3522.111366	-0.056459555	0.086392372	-0.653524776	0.513418022	0.999579949
42.19518178	0.574899799	0.437868058	1.312952131	0.189199079	0.983992835
125.6372152	-0.018834755	0.131486412	-0.143244875	0.88609679	0.999579949
2039.893694	0.161102968	0.088114106	1.828344818	0.067497817	0.827850544
226.6674995	-0.158352828	0.128323616	-1.234011581	0.217198589	0.999579949
412.0589012	0.193608574	0.103183985	1.876343253	0.060608161	0.800274462
2524.934831	-0.034445204	0.056762225	-0.606833228	0.543961595	0.999579949
114.154947	-0.008943181	0.168920736	-0.052943061	0.957777275	0.999579949
105.8874949	-0.213220727	0.167070719	-1.27623038	0.201874092	0.993283621
76.8271606	-0.342110001	0.401466715	-0.852150347	0.394130651	0.999579949
3386.535791	0.23391673	0.120828901	1.935933607	0.052875833	0.781231069
81.50105592	0.121228706	0.292197159	0.41488667	0.678224869	0.999579949
682.3938211	-0.304284802	0.216983938	-1.402337909	0.160814365	0.978498637
377.5705803	0.053127812	0.196273722	0.270682248	0.786635431	0.999579949
35.27692913	0.134722313	0.293988896	0.458256466	0.646768196	0.999579949
412.3427962	0.052420802	0.089593623	0.585095238	0.558483681	0.999579949
86.80202019	0.244735959	0.114680096	2.134075287	0.032836618	0.695900535
254.831621	-0.02228231	0.093650112	-0.237931485	0.811934235	0.999579949
189.8799321	0.019774345	0.081074379	0.243903752	0.807305363	0.999579949
133.2732676	0.357965656	0.308440951	1.160564625	0.245818998	0.999579949
220.2712427	0.222843894	0.126851113	1.756735821	0.078962849	0.851207389
537.0222393	-0.026099244	0.05960897	-0.43784088	0.661501635	0.999579949
576.116447	0.004564646	0.082449056	0.055363229	0.955849089	0.999579949
1517.960528	-0.266101611	0.145264814	-1.831838038	0.066975554	0.825081178
140.8936871	0.150552635	0.114692082	1.312668081	0.189294818	0.983992835
1788.444812	-0.094855575	0.095225015	-0.996120354	0.319191671	0.999579949
52.53943371	0.063788978	0.178359742	0.35764224	0.720611063	0.999579949

1181.961408	-0.172753714	0.103658822	-1.666560651	0.095601799	0.88463985
26.99359598	-0.185208707	0.536583442	-0.345162919	0.729971894	0.999579949
2462.097568	-0.014588002	0.039258737	-0.37158612	0.710201025	0.999579949
1233.191832	-0.004114167	0.105016822	-0.039176267	0.968749855	0.999579949
1337.324101	-0.055217596	0.078879366	-0.700025858	0.483911156	0.999579949
154.0002699	-0.070785165	0.088484945	-0.799968463	0.423729069	0.999579949
167.9231847	0.087970423	0.148791538	0.591232699	0.554364516	0.999579949
2161.065713	0.015182241	0.083871177	0.18101858	0.856352992	0.999579949
2120.770515	0.109781046	0.111843175	0.981562314	0.326315521	0.999579949
1379.896213	0.150060931	0.110463413	1.358467271	0.174315461	0.983992835
21.90922165	0.106835836	0.282883199	0.377667661	0.705677494	0.999579949
45.01589567	0.291837486	0.210768738	1.384633645	0.166164518	0.978789551
8078.194102	-0.021335543	0.12296192	-0.173513415	0.862247884	0.999579949
51.15611863	0.375175593	0.442224476	0.848382695	0.396224877	0.999579949
1423.540041	0.457219895	0.216934861	2.107636792	0.035062419	0.71004216
122.9312751	0.0027778	0.187102464	0.014846408	0.988154715	0.999579949
971.094871	-0.323353105	0.076885884	-4.205623794	2.60363E-05	0.027329439
537.7961594	-0.034815515	0.063138004	-0.551419318	0.581346261	0.999579949
314.4716374	-0.003380762	0.079690656	-0.042423571	0.966161038	0.999579949
340.1851708	-0.003859083	0.196446773	-0.019644421	0.984327028	0.999579949
1151.767497	0.002200159	0.060617117	0.03629601	0.971046331	0.999579949
405.7484998	0.025303074	0.065523117	0.386170178	0.699370639	0.999579949
1079.947892	-0.02376016	0.055503505	-0.428083956	0.668589997	0.999579949
324.9551814	0.120784804	0.092539369	1.305226145	0.191815854	0.984804292
122.6104864	0.108506805	0.118483683	0.915795337	0.35977425	0.999579949
166.059189	0.010808979	0.127965953	0.084467619	0.932684647	0.999579949
473.9859174	0.029127838	0.089528677	0.325346456	0.744918875	0.999579949
199.4359554	-0.066604088	0.083810477	-0.79469883	0.426788713	0.999579949
67.09512005	0.251245592	0.163082012	1.540608852	0.123412012	0.922550567
61.45918898	-0.802826991	0.364734099	-2.201129515	0.027726856	0.662708696
2341.427973	0.223771564	0.128310216	1.743988676	0.081161064	0.85448976
1051.675373	-0.030651157	0.102178948	-0.299975259	0.764196027	0.999579949
158.7565569	0.085270857	0.102548228	0.831519557	0.405680185	0.999579949
32.22987162	0.106566307	0.232313195	0.45871827	0.646436492	0.999579949
167.5575391	0.291612955	0.227928368	1.279406144	0.200754071	0.993204353
59.79996965	0.031985166	0.368285072	0.086848935	0.93079159	0.999579949
25.80006364	-0.197422191	0.325128908	-0.607212052	0.543710195	0.999579949
98.66800872	0.015329421	0.113372644	0.135212696	0.892443709	0.999579949
2601.979386	-0.029390026	0.105428585	-0.27876715	0.780423526	0.999579949
449.8678702	-0.064830329	0.111033401	-0.583881323	0.55930016	0.999579949
156.0866953	-0.046413633	0.100144867	-0.463464927	0.643031149	0.999579949
3257.382563	-0.041475972	0.13725195	-0.30218858	0.76250831	0.999579949
110.1361664	-0.109685651	0.150748551	-0.727606667	0.466854398	0.999579949
71.38514125	0.144428505	0.198836306	0.726368881	0.467612663	0.999579949
208.4010479	0.036121802	0.147881801	0.244261306	0.807028449	0.999579949
2421.52515	0.560051516	0.13910097	4.026222934	5.668E-05	0.035697035
82.37149583	-0.081885157	0.169046727	-0.484393627	0.62810654	0.999579949
69.82968395	0.068828829	0.154631277	0.445115828	0.656236051	0.999579949

131.7931303	-0.079634206	0.12183405	-0.653628492	0.513351183	0.999579949
392.8157035	-0.22007411	0.218837576	-1.005650466	0.314583738	0.999579949
537.0486937	-0.395095458	0.200482915	-1.970718844	0.048756044	0.773861498
22.82730874	0.098322772	0.29561065	0.33260903	0.739429426	0.999579949
737.3699529	-0.353547738	0.139623247	-2.532155249	0.011336379	0.488767809
438.8372534	0.070954465	0.174075506	0.407607402	0.683561929	0.999579949
2992.672326	-0.006173952	0.087239377	-0.070770244	0.943580614	0.999579949
386.1402474	-0.259764668	0.111852404	-2.32238789	0.020212059	0.608885423
45.28804339	0.778402711	0.335218603	2.322074924	0.020228902	0.608885423
240.422777	0.129370935	0.089902903	1.439007326	0.150148447	0.965919223
221.7249791	-0.197299445	0.204082898	-0.966761285	0.333663383	0.999579949
131.8142273	-0.075781342	0.107468125	-0.705151802	0.480715772	0.999579949
551.5026189	0.088301713	0.086173275	1.024699511	0.305504994	0.999579949
229.323402	0.047559051	0.095270279	0.499201341	0.61763755	0.999579949
2676.271569	0.096731816	0.095916836	1.008496736	0.313216054	0.999579949
2218.783281	0.034523579	0.148171275	0.232997788	0.815763123	0.999579949
75.62406885	0.054057971	0.331844937	0.162901298	0.870596149	0.999579949
21.34119551	0.2269448	0.371886597	0.6102527	0.541694425	0.999579949
339.2570907	0.191152747	0.153941164	1.241726006	0.214337671	0.999554723
79.79532879	-0.236823534	0.173145966	-1.367768124	0.171384668	0.98345809
589.6140952	-0.122577127	0.129455865	-0.946864223	0.343707971	0.999579949
6362.535119	-0.087669759	0.123612977	-0.70922779	0.478183131	0.999579949
685.7123128	0.28919284	0.143075183	2.021264866	0.043252354	0.754854468
169.4524928	0.13977645	0.108645283	1.286539519	0.198254836	0.992481822
732.3147703	0.005021979	0.093757254	0.053563636	0.957282829	0.999579949
44.75819244	-0.091685082	0.217835559	-0.420891258	0.673834489	0.999579949
586.6380513	-0.081379267	0.093761699	-0.867937199	0.385428713	0.999579949
679.9138142	-0.072181225	0.110318324	-0.654299509	0.512918862	0.999579949
287.6070009	-0.095613236	0.105004047	-0.910567155	0.362523483	0.999579949
47.93736651	-0.118621055	0.325880751	-0.364001415	0.715856953	0.999579949
45.0310867	0.092250113	0.324973302	0.283869821	0.776510145	0.999579949
1983.710286	0.16898485	0.112335048	1.504293207	0.132505887	0.939443134
415.9594795	0.033142692	0.078894938	0.420086419	0.674422324	0.999579949
247.0783928	0.014771522	0.105982277	0.139377285	0.889152021	0.999579949
32.84710438	0.035897608	0.483995547	0.074169294	0.940875678	0.999579949
1192.545665	-0.02194125	0.084937406	-0.258322578	0.796157963	0.999579949
969.8976388	-0.245523225	0.074003297	-3.317733628	0.00090751	0.152413255
353.1880364	-0.141228314	0.058777527	-2.402760399	0.016271845	0.561534683
477.5584229	-0.058352065	0.138837177	-0.420291355	0.674272625	0.999579949
212.4023983	-0.146006139	0.158649155	-0.92030833	0.357411657	0.999579949
278.7352474	0.109412777	0.114785356	0.95319456	0.340491502	0.999579949
98.70156374	0.083867591	0.128351329	0.653422073	0.513484213	0.999579949
157.9587828	-0.058126496	0.153041325	-0.379809155	0.704087086	0.999579949
597.3482719	-0.023735963	0.062382234	-0.380492357	0.703579969	0.999579949
404.4248343	0.053791307	0.061348308	0.876818099	0.380585447	0.999579949
76.43991048	0.033649275	0.136737884	0.246085972	0.805615682	0.999579949
148.797839	0.026145864	0.11605066	0.225296988	0.821748243	0.999579949
138.3752345	0.069874514	0.270300414	0.258506869	0.796015749	0.999579949

96.78122658	-0.124371897	0.12689781	-0.980094906	0.327039274	0.999579949
72.29378572	-0.340981596	0.1992216	-1.711569406	0.08697605	0.867486202
3380.334904	0.076761076	0.128391029	0.597869469	0.549927033	0.999579949
246.6781742	-0.007323882	0.083267136	-0.087956453	0.929911287	0.999579949
991.1637536	0.166803825	0.155785481	1.070727664	0.2842919	0.999579949
478.785817	-0.198824441	0.079275521	-2.508018107	0.012141045	0.503054596
93.15064879	0.043533471	0.142464014	0.305575212	0.759928096	0.999579949
261.791653	0.023122555	0.312525436	0.073986155	0.941021402	0.999579949
28.84512882	0.36682869	0.51381935	0.713925409	0.475273305	0.999579949
256.7809953	-0.084681669	0.101227551	-0.836547639	0.402846888	0.999579949
570.1906459	0.081457997	0.078963641	1.031588671	0.30226485	0.999579949
86.33887745	0.063364076	0.191278753	0.331265629	0.740443851	0.999579949
555.6464725	-0.116144205	0.084561314	-1.373491003	0.169599756	0.981013142
261.976583	-0.0366415	0.194767849	-0.188129099	0.850775451	0.999579949
336.9777223	0.046556558	0.11400026	0.408389925	0.682987429	0.999579949
26.80748182	0.435934212	0.240975634	1.809038552	0.070445014	0.834620594
211.9452859	0.162959681	0.252540701	0.645280863	0.518745192	0.999579949
142.4023008	-0.050214682	0.143752189	-0.349314209	0.726853432	0.999579949
299.4134151	-0.207818732	0.175641522	-1.1831982	0.236730606	0.999579949
369.766221	0.133130674	0.167201083	0.79623093	0.425897823	0.999579949
212.4644221	0.043259381	0.160648952	0.269278948	0.787715031	0.999579949
860.5743965	-0.09265213	0.089047979	-1.040474257	0.298119618	0.999579949
21.62360049	0.0946557	0.364834112	0.259448602	0.795289134	0.999579949
112.7436556	0.413084691	0.191737836	2.154424498	0.031206897	0.683621001
1258.386137	-0.025504025	0.085589341	-0.297981327	0.765717415	0.999579949
139.3419623	0.250104269	0.216052846	1.157606918	0.247024485	0.999579949
100.8162526	0.09104254	0.104281672	0.873044497	0.382638824	0.999579949
2040.226397	0.079562155	0.07215475	1.102659974	0.270174855	0.999579949
106.9073746	0.021656952	0.102005523	0.212311565	0.831863973	0.999579949
413.5470466	0.125543888	0.105677511	1.187990588	0.234837118	0.999579949
340.3510161	-0.149147173	0.091198614	-1.635410533	0.101963016	0.895628172
43759.91644	-0.254663223	0.143450639	-1.775267255	0.07585377	0.851207389
84.33780116	0.1391491	0.150290049	0.925870345	0.354513364	0.999579949
732.3988331	0.047575495	0.059257832	0.802855816	0.422058076	0.999579949
63.65576356	0.315850377	0.156841151	2.013823378	0.04402808	0.758836007
52.68169528	0.297755764	0.160844313	1.851204797	0.064140096	0.815245861
44.92107359	0.093120349	0.173707249	0.536076357	0.59190578	0.999579949
3168.101078	0.053838668	0.129745918	0.414954623	0.678175122	0.999579949
401.5534125	-0.005484332	0.234690091	-0.023368402	0.981356409	0.999579949
3397.83846	0.036367219	0.087746214	0.41445913	0.678537893	0.999579949
211.4781234	-0.15583471	0.081233518	-1.918354826	0.055066035	0.785214091
432.7216811	0.059599063	0.076297816	0.781137213	0.434721797	0.999579949
138.9876096	-0.073049789	0.146768397	-0.497721516	0.618680341	0.999579949
152.5038557	0.061974402	0.153700287	0.403215915	0.686789393	0.999579949
615.9743596	-0.05432864	0.060947525	-0.891400278	0.372714469	0.999579949
219.4308891	-0.022398386	0.079430949	-0.281985631	0.777954528	0.999579949
406.4352632	0.086717859	0.09385137	0.923991398	0.355490795	0.999579949
292.1279277	-0.020849364	0.079083506	-0.263637331	0.79205939	0.999579949

95.73004728	-0.03589281	0.14165209	-0.253387082	0.799969109	0.999579949
279.6243249	0.127701822	0.095686546	1.334584921	0.182012236	0.983992835
1433.569621	-0.166893009	0.087049146	-1.917227413	0.055209047	0.785214091
191.1371908	-0.096924693	0.130360391	-0.743513361	0.457170935	0.999579949
5956.660757	-0.066593613	0.065322901	-1.019452783	0.307988059	0.999579949
129.2873806	-0.023811658	0.121063664	-0.196687079	0.844072422	0.999579949
980.7300506	-0.022183769	0.12651504	-0.175344918	0.860808619	0.999579949
48.38632648	-0.155656271	0.142917927	-1.089130484	0.276096349	0.999579949
90.38508516	0.040058236	0.137535878	0.29125663	0.770855054	0.999579949
521.7810036	-0.033728019	0.089857543	-0.375349889	0.707400268	0.999579949
77.44375127	0.035298858	0.174189059	0.202646814	0.839411097	0.999579949
612.4969669	-0.021477999	0.096508824	-0.222549589	0.823886075	0.999579949
332.0992396	0.473554491	0.374410342	1.264800777	0.205942753	0.994814558
314.9739288	-0.039682087	0.073012022	-0.543500721	0.586785095	0.999579949
231.2639501	0.016782329	0.104815408	0.160113186	0.872791914	0.999579949
2173.984598	-0.089236895	0.105338261	-0.847146077	0.396913705	0.999579949
975.0678851	0.039296178	0.108135933	0.363396113	0.716309005	0.999579949
129.7969523	0.070379555	0.091265846	0.771148878	0.440618692	0.999579949
853.4375499	-0.669119604	0.209819004	-3.189032409	0.001427499	0.203876378
1116.805753	0.19495447	0.119204355	1.635464318	0.101951749	0.895628172
318.9151699	0.128798253	0.141140882	0.912551002	0.361478734	0.999579949
398.6316847	0.079869666	0.182047083	0.438730822	0.660856591	0.999579949
112.079432	-0.318562075	0.173517151	-1.835911163	0.066370796	0.822841095
80.85509674	0.095045036	0.120591005	0.788160246	0.430602979	0.999579949
158.7742165	-0.096305534	0.159332417	-0.604431514	0.545556789	0.999579949
233.4903876	0.249264355	0.251219177	0.992218659	0.321090872	0.999579949
54.57622945	-0.165939923	0.256635994	-0.646596449	0.517893157	0.999579949
1703.785092	0.003255604	0.117924825	0.027607456	0.977975235	0.999579949
839.0049544	-0.124139348	0.068031389	-1.824736353	0.068040825	0.829912169
288.2917514	0.054984542	0.096847853	0.567741463	0.570210533	0.999579949
250.7981213	-0.009427345	0.091459629	-0.10307657	0.917902201	0.999579949
102.6661783	0.021432605	0.151714128	0.141269672	0.887656908	0.999579949
25.45702065	0.594439372	0.392962269	1.512713611	0.130352421	0.935700262
223.2383306	0.035977567	0.127257449	0.282714824	0.777395452	0.999579949
341.3771451	0.072075598	0.109658228	0.657274869	0.511004193	0.999579949
341.143563	0.379879826	0.145906043	2.60359213	0.009225247	0.457115261
3712.682175	0.113391095	0.062729005	1.807634195	0.070663453	0.834620594
115.0907428	-0.204019509	0.179000326	-1.139771714	0.254381421	0.999579949
248.2798921	-0.02965766	0.113199076	-0.261995601	0.793324833	0.999579949
84.53205666	0.017070065	0.143068179	0.119314199	0.905026434	0.999579949
250.8587894	0.198524389	0.213096265	0.931618343	0.3515338	0.999579949
423.6774373	-0.161112314	0.155064256	-1.039003562	0.29880308	0.999579949
234.6657364	-0.120792787	0.117413124	-1.028784369	0.303581014	0.999579949
144778.0695	-0.20884233	0.168628225	-1.238477901	0.215538911	0.999579949
2843.714508	-0.10229421	0.089792242	-1.13923216	0.254606335	0.999579949
1728.450643	-0.036657311	0.065165537	-0.562526031	0.573757673	0.999579949
2565.878039	-0.096281812	0.119191473	-0.807791105	0.419210847	0.999579949
642.1943325	-0.150649468	0.143478972	-1.049975934	0.293729178	0.999579949

143.8925296	0.07224313	0.121596285	0.594122838	0.552429957	0.999579949
682.7135616	-0.263602137	0.297598924	-0.885763072	0.375745213	0.999579949
983.8598542	-0.010996651	0.060488535	-0.181797281	0.855741818	0.999579949
247.47421	-0.064655281	0.10923194	-0.591908198	0.553912064	0.999579949
273.7452733	-0.06915804	0.102466186	-0.674935239	0.49971691	0.999579949
475.4424413	-0.041326903	0.073894346	-0.559270166	0.575977353	0.999579949
463.418627	-0.059550707	0.088139186	-0.675643946	0.499266733	0.999579949
600.7958789	0.039631172	0.095647827	0.414344724	0.678621665	0.999579949
504.1913227	0.089132011	0.171027535	0.521155909	0.602258166	0.999579949
2118.410992	-0.044029291	0.085788673	-0.513229652	0.607790687	0.999579949
66.92100363	0.249103712	0.154901989	1.608137591	0.107805048	0.903467985
283.3412069	-0.252116708	0.194289043	-1.297637295	0.194412	0.988064798
596.1135747	-0.01691312	0.067919531	-0.249017031	0.803347607	0.999579949
391.680733	0.042944798	0.127953596	0.335627906	0.737151477	0.999579949
42.79148813	-0.156873053	0.21783539	-0.720144938	0.471435762	0.999579949
875.0815286	-0.054787023	0.081645235	-0.671037605	0.502196574	0.999579949
10140.75482	-0.045819872	0.082819782	-0.553247914	0.580093662	0.999579949
181.1320718	-0.03208303	0.151623781	-0.211596295	0.832422	0.999579949
44.99731908	-0.005417486	0.18798331	-0.028818972	0.97700897	0.999579949
1105.883244	0.12795137	0.084784183	1.509141981	0.131262503	0.938166973
69.27369345	-0.008665246	0.146617285	-0.05910112	0.952871567	0.999579949
57.19439602	0.032541129	0.151112986	0.215343038	0.829499877	0.999579949
43.15765087	1.074700236	0.534867356	2.009283653	0.044507061	0.759209569
307.9853419	0.001584106	0.085445535	0.018539368	0.985208572	0.999579949
917.4051434	0.185522573	0.107327247	1.728569192	0.083886227	0.860148832
640.2745014	0.16485679	0.105614924	1.560923252	0.11854186	0.921521033
485.4527866	0.040315777	0.155445159	0.259356915	0.79535987	0.999579949
666.4911118	-0.218138999	0.157646883	-1.383719068	0.166444493	0.978789551
254.4129418	0.060085901	0.111147644	0.540595362	0.588786515	0.999579949
461.6907564	-0.155624262	0.076676989	-2.029608412	0.042396359	0.74969123
242.0496093	-0.213379631	0.088863627	-2.401203269	0.016341256	0.562389248
393.0996724	0.059238634	0.129732534	0.456621265	0.647943292	0.999579949
291.9669964	-0.158911982	0.132550694	-1.198877027	0.230575766	0.999579949
126.0674254	0.085104596	0.15292227	0.556521924	0.577854111	0.999579949
23.4746699	-0.726972794	0.403692864	-1.800806652	0.07173336	0.836066794
51.41329475	0.28159221	0.257058793	1.095438935	0.273324385	0.999579949
748.6643001	0.024734304	0.145771009	0.169679168	0.865262458	0.999579949
30.25644046	0.27674363	0.261795672	1.057097802	0.290466954	0.999579949
15172.22044	-0.241729006	0.142468003	-1.696724884	0.089748686	0.873627856
237.5961987	0.24851869	0.177822614	1.397565164	0.162243685	0.978498637
202.9530194	0.001921615	0.186734695	0.010290614	0.991789423	0.999579949
803.2487975	0.101047109	0.11329475	0.891895777	0.372448798	0.999579949
219.0324238	-0.096018845	0.094816224	-1.012683702	0.311211265	0.999579949
156.2651034	0.166348725	0.159977492	1.039825807	0.298420837	0.999579949
248.3657131	-0.048957097	0.105228041	-0.465247632	0.641754132	0.999579949
6708.851987	0.118899081	0.184429449	0.644685985	0.5191307	0.999579949
87.59189136	0.208766671	0.107210416	1.947261087	0.051503441	0.779189484
80.45694908	0.430708666	0.273679848	1.573768287	0.115541028	0.918818353

534.1543477	1.727291485	0.56359313	3.064784491	0.002178269	0.23630562
941.4482957	-0.001857554	0.115474604	-0.016086257	0.987165577	0.999579949
1299.62121	-0.048326544	0.108910641	-0.443726563	0.657240288	0.999579949
332.9050581	0.097482477	0.165893188	0.58762194	0.556786081	0.999579949
2152.060573	0.007290019	0.101931238	0.071518986	0.942984715	0.999579949
968.6903602	-0.042011618	0.069477555	-0.604678996	0.545392307	0.999579949
451.546995	-0.058290343	0.067544929	-0.862986211	0.388145048	0.999579949
40.87576964	0.51160239	0.206389383	2.478821253	0.013181735	0.514046841
1027.990543	0.043339947	0.082993145	0.522211168	0.601523311	0.999579949
27.25547414	-0.041848028	0.274914329	-0.152222068	0.879011789	0.999579949
25.89678059	-0.074495187	0.213618159	-0.348730591	0.727291576	0.999579949
460.4108181	0.128458558	0.109537086	1.17274033	0.240899953	0.999579949
152.6493737	0.176652045	0.225737308	0.782555823	0.433887992	0.999579949
1500.043129	0.050488979	0.110797742	0.455685991	0.648615797	0.999579949
54.7400668	-0.01668661	0.195658256	-0.085284467	0.93203524	0.999579949
496.3349285	-0.005444254	0.073616626	-0.073954132	0.941046883	0.999579949
89.96938397	0.149202548	0.105890037	1.409032918	0.158825435	0.97731567
1815.714369	-0.09781169	0.162016147	-0.603715692	0.54603268	0.999579949
250.888372	-0.001289004	0.077916236	-0.016543454	0.986800835	0.999579949
94.88855721	-0.153532926	0.145334439	-1.056411183	0.290780399	0.999579949
34.90700192	-0.179368944	0.199177605	-0.900547749	0.367828827	0.999579949
84.33412895	0.013109732	0.177981228	0.073657948	0.941282561	0.999579949
253.7392905	0.006666255	0.079426547	0.083929808	0.93311224	0.999579949
1600.800991	-0.046468192	0.105369784	-0.441001115	0.65921219	0.999579949
80.85607983	0.104863745	0.132930024	0.788864259	0.430191347	0.999579949
350.4528643	0.019547414	0.13869565	0.14093747	0.887919341	0.999579949
35.24680118	0.072921643	0.200878804	0.363013128	0.716595078	0.999579949
28.36996098	-0.32291167	0.261534122	-1.234682751	0.216948598	0.999579949
32.73705486	0.025920289	0.31565066	0.082117011	0.934553666	0.999579949
21.33954172	-0.224993029	0.220774261	-1.01910897	0.308151236	0.999579949
166.4529075	0.095370922	0.14162555	0.67340195	0.500691608	0.999579949
31.0208314	0.652024533	0.347491823	1.876373745	0.060603977	0.800274462
92.25731059	0.153445701	0.297084604	0.51650506	0.605501706	0.999579949
1361.841461	-0.01246515	0.078877551	-0.158031659	0.874431852	0.999579949
38.18823116	-0.33896494	0.194538352	-1.742406756	0.081437295	0.85448976
366.8860224	0.058169522	0.168128881	0.345981736	0.72935644	0.999579949
21.18980557	0.346086637	0.351315257	0.985117014	0.324566597	0.999579949
22.45560395	0.178076671	0.229060754	0.777421135	0.436910347	0.999579949
97.306756	0.000261466	0.134926165	0.001937848	0.998453822	0.999644252
145.5239928	-0.147392425	0.244514425	-0.60279644	0.546644116	0.999579949
112.1799512	-0.001965969	0.168548127	-0.011664138	0.990693575	0.999579949
61.66349248	-0.051761853	0.334444993	-0.154769407	0.87700311	0.999579949
56.74750719	0.295255755	0.306731177	0.962588017	0.335754313	0.999579949
847.4207476	0.149465709	0.079597222	1.877775448	0.06041189	0.800274462
112.4098959	0.361106454	0.238002312	1.517239265	0.129206286	0.935700262
124.7051687	0.179202223	0.166910794	1.073640708	0.282983745	0.999579949
30.48037239	0.306406682	0.197960148	1.547820028	0.121665633	0.922215626
265.7258927	0.064079753	0.116798246	0.548636262	0.583255095	0.999579949

555.2601642	0.002266925	0.058375143	0.038833738	0.969022946	0.999579949
39.73927382	-0.053413995	0.214445276	-0.249079842	0.803299022	0.999579949
1408.426587	0.048193759	0.108531826	0.444051862	0.657005089	0.999579949
467.5739977	-0.005918401	0.102200635	-0.057909635	0.953820609	0.999579949
562.5847225	0.090171628	0.131409867	0.686186131	0.492595742	0.999579949
164.0969	-0.058287506	0.191112724	-0.30499019	0.760373624	0.999579949
960.7469815	-0.016795407	0.124532685	-0.134867458	0.892716669	0.999579949
38.1060604	0.809151577	0.35871867	2.255671771	0.024091195	0.634610194
435.6577809	-0.096873785	0.106648104	-0.908349809	0.363693435	0.999579949
27.15113784	-0.166829643	0.229375894	-0.727319859	0.467030035	0.999579949
171.4555296	0.060453386	0.135353197	0.446634339	0.655139098	0.999579949
44.66901195	0.14079252	0.163082004	0.863323458	0.38795965	0.999579949
1057.482856	0.039901752	0.063426271	0.629104498	0.529280646	0.999579949
153.7424161	0.103494659	0.15191313	0.68127527	0.495697328	0.999579949
22.57827667	0.173245034	0.23770521	0.728823039	0.466109917	0.999579949
294.2086676	-0.007100401	0.106190466	-0.06686477	0.94668936	0.999579949
63.09094112	0.377689486	0.160697719	2.350310182	0.018757772	0.590797093
147.1614876	0.021125847	0.142537405	0.148212653	0.882174947	0.999579949
794.6585275	-0.013260735	0.075645556	-0.175300911	0.860843196	0.999579949
81.56766803	0.014804795	0.119032196	0.124376389	0.901017267	0.999579949
49.05224601	-0.058000028	0.159391402	-0.363884297	0.715944411	0.999579949
42.79163602	-0.291056251	0.162442932	-1.791744626	0.073173883	0.838669905
241.8278961	-0.064803658	0.082179986	-0.788557657	0.430370587	0.999579949
805.3060227	0.034498326	0.066006324	0.522651833	0.601216563	0.999579949
1192.926526	-7.83947E-05	0.080561535	-0.000973104	0.999223576	0.999840121
860.2617624	0.015951599	0.071568815	0.222884774	0.823625187	0.999579949
4145.902158	-0.054128365	0.122171621	-0.443051872	0.657728216	0.999579949
26.7690314	-0.338271351	0.221727635	-1.525616558	0.127105386	0.934532219
5327.523102	-0.245030504	0.168761006	-1.451937924	0.146518867	0.959725243
142.8785331	-0.025664222	0.100863461	-0.254445189	0.799151643	0.999579949
358.0557411	0.08533525	0.152114715	0.560992737	0.574802488	0.999579949
1070.05595	-0.103666381	0.15324607	-0.676470077	0.498742238	0.999579949
934.7916627	-0.056245977	0.112294879	-0.500877486	0.616457348	0.999579949
88.08671932	0.171979569	0.174724456	0.984290196	0.324972849	0.999579949
348.6738226	-0.009339517	0.084329403	-0.110750419	0.911814263	0.999579949
690.9341077	0.074321089	0.245399443	0.30285761	0.761998378	0.999579949
258.4358429	0.152045885	0.20667982	0.735659074	0.461938202	0.999579949
4115.295276	0.072344931	0.125448493	0.576690321	0.564148672	0.999579949
96.47930708	-0.009076006	0.124124842	-0.073119977	0.941710645	0.999579949
216.3148897	-0.068718448	0.08556717	-0.803093608	0.421920632	0.999579949
885.1951009	0.050573045	0.09841223	0.513889833	0.607329016	0.999579949
30.99033824	0.050843921	0.217026365	0.234275317	0.814771247	0.999579949
1101.464155	0.042279971	0.054547184	0.77510822	0.438275715	0.999579949
444.1190325	0.124039529	0.11949729	1.038011229	0.299264827	0.999579949
119.5852882	0.234818428	0.133206382	1.762816651	0.077931418	0.851207389
57.37261518	0.052458759	0.142327794	0.368577055	0.712443001	0.999579949
805.0445777	0.124909152	0.065284829	1.913295227	0.055710268	0.785214091
375.7893197	0.002848683	0.062001341	0.045945498	0.96335369	0.999579949

268.8963206	-0.306156231	0.127187116	-2.407132429	0.016078338	0.559034656
22.18221903	0.190911557	0.268467879	0.711115077	0.477012928	0.999579949
539.2317884	0.024396567	0.075752101	0.322057962	0.747408784	0.999579949
124.811133	0.055185583	0.301888418	0.182801261	0.854953959	0.999579949
1127.659598	0.302204196	0.110759272	2.728477619	0.006362741	0.387278966
23.70290086	0.008520242	0.560995401	0.015187721	0.987882418	0.999579949
1647.997119	0.076847586	0.098817822	0.777669301	0.436763995	0.999579949
22.0182649	0.341607267	0.367547759	0.929422798	0.352670017	0.999579949
769.828725	0.188730764	0.12296639	1.534815849	0.124829069	0.927119638
911.6789969	-0.022302602	0.070718531	-0.315371402	0.752479677	0.999579949
62.93752744	0.445684208	0.14567834	3.059371813	0.002218017	0.23630562
98.30266612	-0.076710241	0.12318716	-0.622712963	0.533473163	0.999579949
114.2609114	-0.049753691	0.168475844	-0.295316466	0.767752139	0.999579949
566.2490007	0.050500486	0.096711267	0.522177898	0.601546473	0.999579949
1075.876669	-0.121660268	0.100946002	-1.20520146	0.228125539	0.999579949
226.4374329	0.068348974	0.129806216	0.526546236	0.598508736	0.999579949
297.5112766	-0.00267692	0.084469815	-0.031690853	0.97471859	0.999579949
574.3819363	-0.070311683	0.075119423	-0.935998715	0.349273849	0.999579949
532.4328557	0.099068421	0.077798662	1.273394928	0.202877936	0.994409048
185.840188	0.126378656	0.138324754	0.913637311	0.360907455	0.999579949
122.9966155	0.117982082	0.151555814	0.778472825	0.436290321	0.999579949
505.0564576	0.183758078	0.134048256	1.37083527	0.170426313	0.981568287
44.02128141	1.819258006	0.510117185	3.566353102	0.000361984	0.093051939
153.0467479	0.133352885	0.114489633	1.164759475	0.244116367	0.999579949
553.7646141	-0.085106071	0.090057742	-0.945016715	0.344650343	0.999579949
21.23374476	-0.240866883	0.279244214	-0.86256714	0.388375504	0.999579949
261.7314715	-0.064213237	0.084720958	-0.757938041	0.448488075	0.999579949
32.08586094	0.058944083	0.211322924	0.27892896	0.780299343	0.999579949
97.95172875	-0.067439697	0.102644398	-0.657022673	0.511166338	0.999579949
201.418896	-0.056905321	0.131312335	-0.433358535	0.664754319	0.999579949
395.6420969	-0.04091434	0.118299411	-0.345854127	0.729452345	0.999579949
167.2207685	-0.192622786	0.113743362	-1.693485963	0.090363005	0.876222021
41.51333779	-0.091654289	0.173084722	-0.529534252	0.59643489	0.999579949
195.0524366	-0.060664675	0.112893009	-0.537364322	0.591015983	0.999579949
623.9572991	0.146254298	0.129767039	1.127052754	0.259720177	0.999579949
292.5062208	0.196623523	0.120079656	1.637442425	0.10153806	0.895628172
1253.161556	-0.001913724	0.155738714	-0.012288045	0.990195805	0.999579949
605.5231321	0.286963657	0.163118426	1.759235084	0.078537587	0.851207389
154.7091476	0.160812424	0.151525227	1.061291424	0.288557482	0.999579949
734.0712569	0.015613549	0.073516382	0.212381897	0.831809108	0.999579949
445.4499749	0.056489671	0.077195508	0.73177407	0.464306478	0.999579949
124.0311231	-0.010253291	0.114817841	-0.089300505	0.928843093	0.999579949
66.26916632	-0.138227746	0.313837576	-0.440443581	0.659615867	0.999579949
212.8841502	0.337990813	0.359052246	0.941341592	0.346529835	0.999579949
54.89600778	-0.101060526	0.254172689	-0.397605762	0.690920812	0.999579949
990.2461709	0.116136579	0.090421623	1.284389456	0.19900572	0.992481822
65.91456477	0.007072371	0.150575343	0.046968987	0.962537945	0.999579949
2465.234532	-0.004642062	0.075852222	-0.061198764	0.951200914	0.999579949

140.3909491	0.178463201	0.134445162	1.327405156	0.184374695	0.983992835
427.6820383	0.121089949	0.127126044	0.95251882	0.340833926	0.999579949
29.6139908	0.240267257	0.621727307	0.386451189	0.699162546	0.999579949
4825.354492	-0.056930059	0.097652014	-0.582989092	0.559900644	0.999579949
163.4896497	-0.024975241	0.107353574	-0.232644707	0.816037308	0.999579949
31.53658045	-0.120335922	0.178960408	-0.672416445	0.501318616	0.999579949
299.301813	0.082817869	0.107222122	0.772395351	0.439880306	0.999579949
1535.762605	-0.011873002	0.049715453	-0.238819142	0.811245826	0.999579949
683.2173906	-0.132872037	0.175529807	-0.756977057	0.449063608	0.999579949
1438.943444	0.064991698	0.060944674	1.06640488	0.286240657	0.999579949
85.67904138	-0.069642122	0.157533167	-0.442079106	0.658431963	0.999579949
217.2089827	-0.073551876	0.082268377	-0.894047976	0.371296223	0.999579949
3835.071437	-0.013499906	0.10702518	-0.126137661	0.899622956	0.999579949
4179.209018	0.001475848	0.102382609	0.014415023	0.988498874	0.999579949
776.2429227	0.027548953	0.074511995	0.369725074	0.711587347	0.999579949
882.3377436	-0.023786776	0.059667239	-0.39865722	0.690145793	0.999579949
382.6978766	0.069671028	0.072794243	0.957095306	0.338519153	0.999579949
143.2408668	0.160629091	0.119075035	1.348973705	0.177345412	0.983992835
134.0320572	0.129059748	0.102360557	1.260834752	0.20736839	0.995052284
238.4299988	0.093505991	0.147605484	0.633485886	0.526416395	0.999579949
73.56299255	0.069120259	0.160144476	0.431611882	0.666023521	0.999579949
1274.457283	-0.017003214	0.097039203	-0.175220048	0.860906732	0.999579949
805.3546364	0.262139415	0.111824596	2.344201763	0.019067853	0.590797093
3790.832847	-0.025456321	0.115923493	-0.219595881	0.8261859	0.999579949
133.3672076	0.050170678	0.123652784	0.405738363	0.684934851	0.999579949
106.839926	-0.082705919	0.114736158	-0.720835702	0.471010607	0.999579949
47.93472428	-0.212138715	0.247807902	-0.856061141	0.391963961	0.999579949
135.9698872	-0.246295634	0.154016881	-1.599147009	0.109787942	0.908599811
9704.171428	0.03929147	0.123704826	0.317622778	0.750771094	0.999579949
242.0841937	0.358842299	0.170539683	2.104157187	0.035364738	0.712820067
385.8484171	-0.057229029	0.075977994	-0.753231641	0.451310729	0.999579949
345.2207103	-0.219018822	0.344016334	-0.636652393	0.52435129	0.999579949
144.5928021	-0.103216054	0.089857732	-1.148660797	0.250695877	0.999579949
215.7815138	0.187539919	0.107167135	1.749976038	0.080122449	0.85448976
541.7783874	0.041410087	0.114751263	0.36086825	0.71819794	0.999579949
80.95300702	0.030661191	0.166408743	0.184252281	0.853815547	0.999579949
103.7511377	-0.042094303	0.208100248	-0.202278964	0.839698644	0.999579949
154.2178428	0.054304987	0.126326067	0.429879505	0.667283295	0.999579949
35.60531194	3.470438767	1.191451793	2.912781522	0.003582251	0.315538728
122.7157005	0.226494776	0.171312889	1.322111708	0.186130956	0.983992835
8720.508122	0.065144006	0.08601888	0.757322191	0.44885686	0.999579949
728.436701	-0.041404212	0.058300289	-0.710188803	0.477587063	0.999579949
569.7057427	-0.070852449	0.071243327	-0.994513485	0.319972943	0.999579949
24.00971435	0.097577836	0.283308421	0.344422645	0.730528461	0.999579949
465.9704048	-0.047237742	0.087586434	-0.539327152	0.589661137	0.999579949
389.3298839	-0.031183525	0.080103743	-0.389289241	0.697062194	0.999579949
121.8942164	3.64259843	1.357319012	2.68367156	0.00728186	0.409474612
220.2567561	0.063333249	0.111922185	0.565868595	0.571483112	0.999579949

1200.717662	0.02221663	0.068353113	0.325027337	0.745160382	0.999579949
391.4501097	0.268566024	0.231112418	1.162057956	0.245211924	0.999579949
179.7102507	0.136389836	0.116202127	1.173729256	0.240503488	0.999579949
31.66311543	0.093116708	0.331280673	0.281081016	0.778648261	0.999579949
2676.511191	1.070231886	0.284240771	3.765230026	0.000166396	0.058220051
23.3979275	0.417736444	0.262879811	1.589077696	0.112042853	0.913563961
237.3889422	-0.10557511	0.172676637	-0.611403554	0.54093245	0.999579949
621.4159772	0.524990897	0.167344237	3.137191384	0.001705747	0.214855947
28.08391855	0.128128563	0.23253605	0.551005161	0.581630137	0.999579949
101.0502615	3.469389479	1.441734633	2.406399486	0.016110637	0.559034656
56.90752995	0.063748267	0.172609434	0.369320874	0.711888568	0.999579949
920.7633566	0.055885776	0.089949761	0.621299882	0.534402331	0.999579949
144.0587366	0.12411787	0.118309699	1.049092942	0.294135344	0.999579949
1096.896281	0.042004151	0.106120204	0.395816718	0.692240243	0.999579949
24.11378279	-0.115952337	0.206565213	-0.561335255	0.574569012	0.999579949
104.9016036	-0.060549676	0.305625755	-0.19811706	0.842953477	0.999579949
23.82159667	0.287471367	0.255065771	1.127048001	0.259722187	0.999579949
1131.834046	-0.003012676	0.038612971	-0.078022376	0.937810254	0.999579949
1859.006151	0.022148356	0.085696643	0.258450684	0.796059105	0.999579949
432.8958388	0.084709818	0.113214757	0.748222405	0.454326019	0.999579949
45.43819832	0.110886007	0.241115272	0.459887945	0.645596653	0.999579949
32.1521519	0.12007848	0.295962391	0.405722092	0.684946808	0.999579949
167.0233349	-0.102402868	0.132516028	-0.772758361	0.439665401	0.999579949
48.92411155	-0.010495508	0.246501021	-0.04257795	0.966037973	0.999579949
157.943561	-0.159542279	0.161439268	-0.988249514	0.323030464	0.999579949
427.3410789	-0.009327013	0.070398282	-0.132489214	0.894597354	0.999579949
460.8511362	0.078081946	0.068240428	1.144218292	0.252533119	0.999579949
290.2411234	0.114005703	0.192508775	0.592210421	0.553709692	0.999579949
48.5986155	0.743202867	0.441016331	1.685204868	0.091949052	0.880418343
751.911103	0.032133102	0.074159253	0.433298612	0.664797846	0.999579949
27.78633846	-0.208777107	0.265077414	-0.787608057	0.430926	0.999579949
1989.749811	-0.041079288	0.056698902	-0.724516475	0.468748717	0.999579949
63.16320311	-0.0990266	0.338476791	-0.292565408	0.769854358	0.999579949
897.8931176	0.108081204	0.101550292	1.064312099	0.287187339	0.999579949
262.5576469	-0.20552288	0.125002683	-1.644147752	0.100145686	0.895628172
571.1827419	0.15238607	0.061526278	2.476764013	0.013257953	0.515423382
100.7429934	0.005859994	0.144252034	0.040623305	0.967596205	0.999579949
10265.85731	0.042203042	0.120305765	0.350798167	0.725739773	0.999579949
372.4444433	-0.070718538	0.145253866	-0.486861661	0.62635637	0.999579949
323.6952206	-0.133775683	0.210593881	-0.635230625	0.525278012	0.999579949
40.37617269	0.402700697	0.25339818	1.589201222	0.112014971	0.913563961
236.951863	0.112388	0.096960605	1.159109924	0.246411381	0.999579949
49.77123016	-0.242593317	0.317248714	-0.764678646	0.444462925	0.999579949
1955.795717	0.060780798	0.108965465	0.557798727	0.576981832	0.999579949
84.13910856	0.160703907	0.222830598	0.721193177	0.470790669	0.999579949
329.3753915	0.055778095	0.10952193	0.509286998	0.61055107	0.999579949
312.0554963	0.008952851	0.152254588	0.058801846	0.953109938	0.999579949
51.20478963	-0.02537615	0.165443717	-0.153382376	0.878096743	0.999579949

271.5612644	0.070377526	0.082041052	0.857833063	0.39098465	0.999579949
317.6117432	0.057510837	0.120207414	0.478430032	0.632344163	0.999579949
86.55071154	0.203727663	0.198165253	1.02806955	0.303917113	0.999579949
2315.905286	0.192402475	0.105572655	1.82246506	0.068384451	0.830633239
183.8970325	0.2306691	0.149922794	1.538585923	0.123905417	0.924045374
3512.584446	-0.000843338	0.075778767	-0.011128947	0.991120568	0.999579949
98.85075381	0.08124901	0.247480024	0.328305326	0.742680818	0.999579949
245.9873939	-0.008663709	0.088309533	-0.098106153	0.921848001	0.999579949
1532.345169	-0.018453827	0.057389759	-0.321552606	0.747791653	0.999579949
3488.550561	-0.039767346	0.05261796	-0.75577514	0.449784024	0.999579949
3201.631002	-0.058450493	0.070405926	-0.8301928	0.406429785	0.999579949
42.31463848	-0.231369732	0.249128106	-0.928717904	0.3530353	0.999579949
164.5618109	-0.091337733	0.126619695	-0.721354867	0.470691207	0.999579949
368.9715508	-0.080787284	0.109432429	-0.738238976	0.460369242	0.999579949
1179.315319	-0.013462672	0.069720359	-0.193095279	0.84688435	0.999579949
63.6145108	0.177603153	0.157264622	1.1293268	0.258759999	0.999579949
2863.053895	-0.082088025	0.12000188	-0.684056157	0.493939706	0.999579949
1564.141053	-0.034886777	0.119276782	-0.292485901	0.769915138	0.999579949
1899.001598	-0.09592287	0.122550412	-0.782721722	0.433790544	0.999579949
351.8742811	-0.127122701	0.117339893	-1.083371542	0.278643542	0.999579949
29.9562181	-0.083574835	0.339178646	-0.246403588	0.805369829	0.999579949
33.1549718	0.13108337	0.222936177	0.587986083	0.556541634	0.999579949
219.9088994	-0.056840475	0.098974255	-0.574295552	0.565767819	0.999579949
1433.83995	0.045322906	0.075863387	0.597427931	0.55022171	0.999579949
572.6951747	-0.105323622	0.062527856	-1.684427213	0.092099135	0.880418343
536.4483572	0.067800232	0.080491515	0.842327692	0.399604554	0.999579949
339.1165456	-0.005605191	0.139441093	-0.040197556	0.967935626	0.999579949
1468.505555	0.04493327	0.071087064	0.63208786	0.527329468	0.999579949
115.4807729	-0.044305985	0.134140394	-0.330295624	0.741176599	0.999579949
63.21313274	0.147493395	0.167521424	0.880444968	0.378618305	0.999579949
102.3622438	-0.155485804	0.136194591	-1.141644488	0.253601824	0.999579949
114.5401939	-0.100106551	0.134721379	-0.743063586	0.457443184	0.999579949
933.4485503	-0.086048308	0.067542974	-1.273978669	0.202670976	0.994409048
142.9002118	-0.00287633	0.153094141	-0.018787982	0.985010241	0.999579949
332.2405218	-0.018698637	0.124356067	-0.150363685	0.88047769	0.999579949
62.31440429	0.377558532	0.192866254	1.957618421	0.050274804	0.776599223
2101.562113	0.153459906	0.196887111	0.779430939	0.435725905	0.999579949
1489.381415	-0.049302931	0.071347586	-0.691024509	0.489550139	0.999579949
566.3568527	-0.066484172	0.098436837	-0.675399307	0.499422106	0.999579949
24.03794311	-0.046775129	0.217855469	-0.214707161	0.829995639	0.999579949
157.4809003	0.029521234	0.129138642	0.228601084	0.819178982	0.999579949
35.82676363	-0.099705325	0.209701139	-0.475463919	0.634456345	0.999579949
4692.107634	0.09609561	0.139556496	0.688578555	0.49108852	0.999579949
57.20025307	-0.080245523	0.187164636	-0.428742975	0.668110282	0.999579949
42.25164356	0.306846854	0.552987497	0.554889316	0.57897037	0.999579949
126.6057233	0.03561049	0.155408778	0.229140789	0.818759492	0.999579949
61.70452747	0.102395255	0.201703986	0.507651125	0.61169803	0.999579949
25.64162552	0.146861528	0.210851893	0.696515101	0.486106303	0.999579949

234.4199271	-0.035090839	0.110803278	-0.31669495	0.751475081	0.999579949
72.65301726	-0.290853018	0.184389808	-1.577381211	0.114707836	0.918818353
94.02935613	0.258394104	0.187854387	1.375502097	0.168975836	0.981013142
30.51617705	-0.039876061	0.28685394	-0.139011724	0.889440885	0.999579949
140.2198509	-0.108041894	0.096717361	-1.117088931	0.263956303	0.999579949
103.6354396	-0.383401129	0.263022544	-1.457674022	0.144930416	0.955875831
221.9388331	0.101396757	0.102848657	0.985883147	0.324190458	0.999579949
121.8071192	0.07334111	0.196998845	0.372292082	0.709675393	0.999579949
208.9098285	0.290546534	0.105676744	2.74938953	0.005970638	0.374141199
287.1743678	0.030583059	0.062115095	0.492361136	0.622464068	0.999579949
282.6529571	-0.051268731	0.086192956	-0.594813469	0.551968163	0.999579949
34.00286507	-0.105393504	0.174585777	-0.60367749	0.546058083	0.999579949
201.6425358	0.043013677	0.109596165	0.392474291	0.694707803	0.999579949
320.4761194	0.095954327	0.070549549	1.360098371	0.173798797	0.983601342
435.2136185	0.28790785	0.152088121	1.893033125	0.058353464	0.79580383
39.51042833	0.046304048	0.231967344	0.199614511	0.841782078	0.999579949
121.5720662	0.114878797	0.098388523	1.167603633	0.242966684	0.999579949
138.8774218	-0.081063976	0.084791422	-0.956039817	0.339052118	0.999579949
209.9745791	0.077828999	0.148762358	0.5231767	0.600851294	0.999579949
137.006485	-0.010541831	0.103981569	-0.10138173	0.919247439	0.999579949
128.0844615	0.172034151	0.170825763	1.00707381	0.313899306	0.999579949
32.32376028	-0.166939637	0.565399608	-0.295259555	0.767795611	0.999579949
353.6320278	0.211814518	0.098215883	2.156621837	0.031035142	0.683621001
715.3469139	-0.085673285	0.126277731	-0.67845125	0.497485623	0.999579949
70.88222839	0.005420074	0.149341519	0.036293148	0.971048613	0.999579949
70.86679329	0.126273834	0.165248901	0.764143264	0.444781873	0.999579949
89.59650023	-0.026302383	0.109694044	-0.239779503	0.810501198	0.999579949
161.9942827	0.103144625	0.084603204	1.219157427	0.222784447	0.999579949
125.6358351	0.56441391	0.162166817	3.480452531	0.000500568	0.111276918
162.093601	0.243477309	0.401822019	0.605933218	0.5445591	0.999579949
466.0427622	-0.095046887	0.136862466	-0.694470074	0.487387462	0.999579949
87.60708382	-0.102176271	0.147299955	-0.69366125	0.487894673	0.999579949
29.01011519	0.205333704	0.220270183	0.932190191	0.351238244	0.999579949
1053.132888	0.064669376	0.104527251	0.618684366	0.536124312	0.999579949
575.7772097	0.494186052	0.130049376	3.799987883	0.000144703	0.053608266
727.1497643	-0.039503257	0.080738939	-0.489271442	0.624649537	0.999579949
31.41847195	-0.020519275	0.234530763	-0.087490762	0.930281428	0.999579949
205.5301483	0.301356651	0.112766651	2.672391607	0.00753127	0.416069642
191.9455003	0.10972204	0.131571142	0.833936974	0.4043165	0.999579949
748.2999851	-0.016135719	0.090594356	-0.178109545	0.858636946	0.999579949
36.70313497	-0.189865155	0.195794084	-0.969718553	0.3321868	0.999579949
5285.030239	-0.110309812	0.108978951	-1.01221209	0.311436657	0.999579949
3305.099002	-0.035051644	0.171225293	-0.204710669	0.837798184	0.999579949
367.3374027	-0.119408645	0.080553621	-1.482349808	0.138247242	0.945791454
160.7003138	-0.350125011	0.189425634	-1.848350738	0.06455162	0.817660046
395.2296257	-0.065573927	0.149766852	-0.437840057	0.661502231	0.999579949
277.5972951	-0.035430107	0.169008831	-0.209634647	0.833952836	0.999579949
650.9060805	0.071893793	0.239383265	0.300329235	0.763926036	0.999579949

66.86342734	-0.180340416	0.184544806	-0.977217513	0.32846148	0.999579949
47.0854017	-0.380011722	0.227407689	-1.671059247	0.094709976	0.880418343
135.0427631	-0.130220786	0.136139442	-0.956525044	0.338807038	0.999579949
135.9514011	0.010416755	0.149987193	0.069450963	0.944630664	0.999579949
33.88316984	-0.190266127	0.243483017	-0.7814349	0.434546752	0.999579949
2226.727944	0.033908625	0.118752022	0.285541452	0.775229351	0.999579949
313.1675569	0.08142908	0.101937786	0.798811543	0.424399697	0.999579949
2782.543431	-0.004312285	0.054745847	-0.07876917	0.937216227	0.999579949
2306.779393	-0.03555058	0.077413661	-0.459228765	0.646069896	0.999579949
58.50247012	0.132110632	0.265052185	0.498432533	0.618179212	0.999579949
851.8785883	0.048902946	0.069744798	0.701169795	0.483197058	0.999579949
93.62951256	0.118006705	0.27797469	0.424523199	0.67118429	0.999579949
153.263039	-0.205075607	0.126004144	-1.627530653	0.103624444	0.895628172
66.37368769	-0.234194241	0.186525281	-1.255562997	0.209274454	0.996447459
27.7118301	0.323043898	0.249639938	1.294039329	0.195651836	0.989333814
67.54059855	0.182338106	0.163870171	1.112698578	0.265837906	0.999579949
111.9005169	-0.277237066	0.130141243	-2.130278299	0.033148644	0.695900535
407.3965837	0.095013797	0.137697186	0.690019887	0.490181681	0.999579949
274.8239398	-0.096166207	0.266661777	-0.360629888	0.718376143	0.999579949
63.23503984	0.156272636	0.239785368	0.651718814	0.514582583	0.999579949
99.15102075	0.377697964	0.189625992	1.991804814	0.046392478	0.763868825
230.3604256	-0.052158728	0.162625758	-0.320728578	0.748416088	0.999579949
91.14213523	0.161213436	0.229303395	0.703057344	0.482020013	0.999579949
451.1197802	-0.010036845	0.178810776	-0.056131096	0.955237372	0.999579949
217.6947272	-0.056882844	0.088880602	-0.639991667	0.522178017	0.999579949
226.3924703	-0.050313347	0.115009255	-0.437472161	0.661768961	0.999579949
44.83180436	0.23495017	0.153125193	1.534366528	0.124939507	0.927364781
74.51900827	-0.076644949	0.356497735	-0.21499421	0.829771833	0.999579949
770.7334027	-0.057802196	0.088829073	-0.650712585	0.515232038	0.999579949
139.7022929	0.150526243	0.140387392	1.072220523	0.283620994	0.999579949
178.0586373	0.091910459	0.126552912	0.726261117	0.467678711	0.999579949
570.9638956	-0.018364297	0.109335128	-0.167963375	0.866612092	0.999579949
28.99573913	0.261518378	0.234813189	1.113729509	0.265395246	0.999579949
185.9913789	-0.030041529	0.08403621	-0.357483144	0.720730143	0.999579949
20.58622571	-0.002149841	0.236860595	-0.009076398	0.992758181	0.999579949
220.2249165	0.461218622	0.167092828	2.760253847	0.005775646	0.372390484
1187.370799	0.005682671	0.084109884	0.06756247	0.946133932	0.999579949
284.5110228	0.132116265	0.110247161	1.198364336	0.23077521	0.999579949
167.5939865	0.086840366	0.135182399	0.642394028	0.520617376	0.999579949
71.0910006	0.053888734	0.135271238	0.398375402	0.690353487	0.999579949
38.02548623	0.04568242	0.333475362	0.136988891	0.891039576	0.999579949
593.6137816	0.044077513	0.084771868	0.519954489	0.603095296	0.999579949
740.4542791	-0.007528985	0.052610727	-0.143107421	0.886205344	0.999579949
1414.256018	0.080448495	0.09096759	0.884364359	0.376499556	0.999579949
260.1711599	-0.221885398	0.289381713	-0.766756808	0.443226121	0.999579949
590.7422542	-0.207956487	0.127438077	-1.631823801	0.10271661	0.895628172
41.67588481	-0.053128247	0.216826647	-0.245026372	0.806436012	0.999579949
27.45723273	0.251718424	0.701174139	0.358995591	0.719598388	0.999579949

121.0662979	-0.014128564	0.118313071	-0.119416767	0.904945178	0.999579949
139.7141383	0.281392264	0.151057339	1.862817563	0.062487938	0.807133028
302.7094647	-0.103237189	0.10960987	-0.941860331	0.346264152	0.999579949
166.014512	0.201254879	0.322792566	0.623480525	0.532968796	0.999579949
511.8226041	0.102067239	0.129757985	0.786596978	0.431517829	0.999579949
142.2857433	0.015061627	0.119148716	0.126410316	0.899407137	0.999579949
24.30753298	-0.269754372	0.206137607	-1.308613095	0.190665443	0.98475336
383.7572157	-0.150561512	0.189749764	-0.79347404	0.427501688	0.999579949
513.381183	0.06896846	0.088726603	0.777314329	0.436973343	0.999579949
41.78277549	0.520747822	0.213488765	2.439228232	0.014718669	0.539131676
22.58361482	0.201011935	0.26737994	0.751783905	0.452181022	0.999579949
500.5147278	-0.062197599	0.113807847	-0.546514153	0.584712562	0.999579949
36.84774008	0.790323137	0.594795248	1.328731424	0.183936594	0.983992835
138.9986259	0.004277673	0.234804089	0.018218051	0.985464902	0.999579949
927.2617973	-0.088157952	0.082397087	-1.069915878	0.284657175	0.999579949
280.9851337	-0.004853416	0.152752137	-0.031773146	0.974652962	0.999579949
128.7079155	-0.00338434	0.1133074	-0.029868659	0.976171801	0.999579949
263.7605719	0.12672505	0.104105424	1.217276153	0.223499164	0.999579949
32.81685374	-0.132096747	0.205445399	-0.642977392	0.52023877	0.999579949
42.23411201	0.108941343	0.205017406	0.531376067	0.595158203	0.999579949
663.2240429	0.018061909	0.151306625	0.119372892	0.904979937	0.999579949
34.45274425	-0.126776112	0.249194979	-0.508742642	0.610932629	0.999579949
78.23481885	-0.161757713	0.132274966	-1.222889847	0.221371304	0.999579949
124.4754242	-0.037691479	0.10743792	-0.350821	0.725722641	0.999579949
46.72469625	-0.07383972	0.195564753	-0.37757172	0.705748776	0.999579949
224.3074468	0.088201073	0.132960651	0.663362221	0.507098595	0.999579949
518.7595369	-0.012421616	0.136368321	-0.091088722	0.927422094	0.999579949
604.7114482	-0.077204883	0.103963957	-0.742612007	0.457716617	0.999579949
16935.18255	0.643808834	0.209639313	3.071031029	0.00213321	0.233772259
1276.745466	0.032120558	0.120566668	0.26641325	0.789920962	0.999579949
1015.993258	-0.02072065	0.086289599	-0.240129169	0.810230122	0.999579949
390.5062898	0.049522935	0.080997497	0.611413151	0.540926099	0.999579949
520.0935534	-0.0119447	0.140276228	-0.085151279	0.932141123	0.999579949
240.777257	-0.028856651	0.106819724	-0.270143471	0.78704988	0.999579949
411.875868	-0.160519359	0.230834802	-0.6953863	0.486813244	0.999579949
532.9992941	-0.074624008	0.080505833	-0.926939142	0.353958133	0.999579949
128.1666692	-0.090475159	0.134983529	-0.670268135	0.502686876	0.999579949
269.7903084	-0.277608291	0.107292833	-2.587388956	0.009670636	0.466710082
57.54377315	-0.440239872	0.206792811	-2.128893505	0.033263072	0.697140863
260.1482335	0.15142195	0.110829739	1.366257384	0.171858192	0.983601342
596.9464952	-0.017929893	0.106548519	-0.168279142	0.866363682	0.999579949
1145.561363	0.053964141	0.120108631	0.449294445	0.653219265	0.999579949
72.56872129	0.252124641	0.224426011	1.12341987	0.261259215	0.999579949
288.7999031	0.092309214	0.136422752	0.676640904	0.49863382	0.999579949
173.4524483	0.102121845	0.213202585	0.47898971	0.631945949	0.999579949
20.84363225	0.090821129	0.258987733	0.35067734	0.725830427	0.999579949
182.9702948	0.027353096	0.125914019	0.217236304	0.828024194	0.999579949
68.51372655	-0.037173108	0.146488319	-0.253761582	0.799679756	0.999579949

55.42664375	-0.325832527	0.312429936	-1.042897907	0.296995579	0.999579949
133.019246	0.018992441	0.129938216	0.146165171	0.883791	0.999579949
213.3078843	0.046387702	0.112049642	0.413992413	0.678879663	0.999579949
81.06996836	0.015911146	0.127279687	0.125009315	0.900516176	0.999579949
721.3245673	0.309507562	0.207266277	1.493284709	0.135362677	0.945791454
370.6509496	0.000453132	0.133813586	0.003386294	0.997298134	0.999579949
108.6634653	0.101072851	0.204290802	0.494749887	0.620776682	0.999579949
2274.694094	0.011472433	0.1152446	0.099548548	0.920702744	0.999579949
522.099837	0.053309036	0.101975478	0.522763284	0.601138993	0.999579949
417.5179337	-0.034700519	0.078018705	-0.444771785	0.656484687	0.999579949
43.6229025	0.372301361	0.207491463	1.79429725	0.072765737	0.838669905
400.725579	0.017692658	0.117926936	0.150030672	0.880740416	0.999579949
3154.036731	-0.025660042	0.101156268	-0.253667342	0.799752566	0.999579949
442.2163669	0.029853495	0.128582048	0.232174677	0.816402345	0.999579949
57.80658842	0.01536757	0.164775914	0.093263447	0.92569427	0.999579949
74.45353233	-0.208652683	0.219468515	-0.95071807	0.341747513	0.999579949
49.98238053	-1.212973396	0.522827053	-2.320027988	0.020339363	0.608885423
253.5407791	-0.036883805	0.099798287	-0.369583549	0.71169281	0.999579949
25.1626291	0.27681432	0.270528274	1.02323619	0.306196185	0.999579949
1704.313662	-0.015222039	0.07947448	-0.191533673	0.848107502	0.999579949
576.0728883	-0.022237287	0.081156596	-0.274004673	0.784081031	0.999579949
1135.804231	0.002887191	0.064228678	0.044951738	0.964145778	0.999579949
59.16826931	0.552037356	0.218098721	2.531135233	0.0113694	0.488767809
3266.32552	0.16906537	0.26499842	0.637986334	0.523482576	0.999579949
249.1603603	-0.077291935	0.085109446	-0.908147545	0.363800273	0.999579949
2024.153429	0.030395349	0.099468751	0.305576862	0.75992684	0.999579949
822.4831302	-0.080016217	0.148204708	-0.539903343	0.589263692	0.999579949
498.9175531	0.055534676	0.085480687	0.649675131	0.515902091	0.999579949
1055.456923	-0.044185473	0.072601459	-0.608603102	0.542787548	0.999579949
638.8247937	0.091387861	0.082967369	1.10149161	0.270682754	0.999579949
255.4876945	0.015001841	0.0858869	0.174669718	0.861339164	0.999579949
50.1552402	0.08705313	0.183887219	0.473405006	0.635924259	0.999579949
173.7408365	-0.089075856	0.126621869	-0.703479236	0.481757142	0.999579949
395.1681184	-0.089806459	0.121944298	-0.736454762	0.461453989	0.999579949
26.61243542	0.098877782	0.274531183	0.360169586	0.718720318	0.999579949
199.6979002	-0.036925353	0.080728465	-0.457401893	0.647382204	0.999579949
484.466419	0.030725593	0.069391173	0.442788198	0.657918941	0.999579949
245.4601114	-0.140770575	0.358908027	-0.392219078	0.694896349	0.999579949
309.3012607	0.095418213	0.08996721	1.060588778	0.288876825	0.999579949
239.2063849	-0.045573054	0.191695991	-0.237736082	0.812085797	0.999579949
41.49211778	-0.131676973	0.389223518	-0.338306825	0.735131981	0.999579949
1804.131211	-0.048382561	0.148078298	-0.326736339	0.743867313	0.999579949
23.94124707	0.004590997	0.21883444	0.020979315	0.983262156	0.999579949
33.28500478	0.08057116	0.226351736	0.355955564	0.721873843	0.999579949
259.5229238	-0.169700758	0.122593208	-1.384259054	0.166279147	0.978789551
259.401564	0.206881927	0.158257741	1.307246808	0.191128903	0.984804292
31.96838747	-0.072731868	0.170398266	-0.426834553	0.669499837	0.999579949
290.4434403	0.002436284	0.115455686	0.021101463	0.983164718	0.999579949

334.4616728	-0.30205241	0.118060271	-2.558459391	0.01051371	0.483019182
168.7793827	-0.089439012	0.111065709	-0.805280166	0.420658025	0.999579949
98.26389259	-0.024425493	0.184834747	-0.132147733	0.894867441	0.999579949
189.9676274	-0.007787897	0.149383414	-0.052133615	0.958422229	0.999579949
8913.49307	0.122477384	0.139898049	0.875475997	0.381314965	0.999579949
239.5465112	0.078451543	0.112457136	0.697612846	0.485419346	0.999579949
46.05020477	0.363882528	0.189826656	1.916920081	0.055248086	0.785214091
95.02144273	0.407653847	0.31593013	1.290329122	0.19693641	0.992244407
36.88959965	0.298531725	0.331645426	0.900153303	0.368038673	0.999579949
565.3262493	0.058756227	0.083951336	0.699884355	0.483999528	0.999579949
781.7873076	0.007100654	0.196162866	0.036197748	0.971124681	0.999579949
86.06082283	-0.002613716	0.117034229	-0.022332919	0.98218239	0.999579949
143.7725986	-0.109762227	0.232672299	-0.471746003	0.637108097	0.999579949
509.7806841	-0.055168586	0.134651239	-0.409714652	0.682015281	0.999579949
41.8068094	-0.138535101	0.210340705	-0.658622407	0.510138271	0.999579949
42.40625115	-0.07003385	0.191573364	-0.36557196	0.714684501	0.999579949
266.9336619	-0.056580872	0.140631909	-0.402333103	0.687438896	0.999579949
541.236344	-0.192900453	0.110573419	-1.744546329	0.08106387	0.85448976
337.7287967	-0.103911386	0.137032827	-0.758295571	0.448274058	0.999579949
21.09240713	-0.023469742	0.234808696	-0.099952611	0.920381948	0.999579949
464.144668	0.078467525	0.089784817	0.87395094	0.382144972	0.999579949
978.8454516	0.085798576	0.20947147	0.409595523	0.682102682	0.999579949
40.8282312	1.008865614	0.631338196	1.597979689	0.110047497	0.909091147
107.2233176	-0.279307827	0.389966585	-0.716235281	0.473846081	0.999579949
225.5373037	0.152502936	0.179871354	0.847844486	0.396524584	0.999579949
1353.79688	-0.136990586	0.125171478	-1.094423332	0.273769356	0.999579949
181.2670197	-0.051372881	0.122085169	-0.420795428	0.67390447	0.999579949
268.6105556	-0.015109518	0.08102702	-0.18647506	0.852072235	0.999579949
512.7152957	-0.193720324	0.081445453	-2.378528425	0.017381897	0.570162425
194.5098732	-0.21230258	0.135054182	-1.571980796	0.115955005	0.918818353
223.1000359	-0.098172605	0.104404173	-0.940313039	0.347057013	0.999579949
82.89277826	-0.218581465	0.11753459	-1.859720318	0.062925106	0.807133028
309.6190502	-0.048463396	0.082853503	-0.584928749	0.558595627	0.999579949
78.94309723	0.082805811	0.134705156	0.614718938	0.538740351	0.999579949
67.94912243	-0.25040561	0.136339607	-1.83663145	0.066264321	0.822423447
328.2289123	0.007027591	0.08709414	0.080689597	0.935688811	0.999579949
2365.324614	0.061193954	0.138135145	0.443000614	0.657765291	0.999579949
30.66446825	-0.182020582	0.516805618	-0.352203181	0.724685892	0.999579949
927.2518406	-0.023701923	0.065541453	-0.361632546	0.71762664	0.999579949
315.2750062	-0.006738986	0.071047064	-0.094852425	0.924432046	0.999579949
587.3309837	0.054441198	0.077002151	0.707008797	0.479561011	0.999579949
955.0300623	0.096851899	0.131400937	0.737071596	0.461078811	0.999579949
516.1188397	0.027568562	0.080346013	0.343122967	0.731505953	0.999579949
2391.2469	-0.001204442	0.094698229	-0.012718743	0.989852185	0.999579949
259.230903	0.308076292	0.258353331	1.192461081	0.233080502	0.999579949
52.30974628	0.070079371	0.243889403	0.28734078	0.773851401	0.999579949
54.71603065	0.039677614	0.156366256	0.253747929	0.799690303	0.999579949
2742.065956	0.183938506	0.152207937	1.208468557	0.226867083	0.999579949

74.38094873	-0.017132817	0.136686821	-0.125343591	0.900251543	0.999579949
11574.23854	-0.20627683	0.156796468	-1.315570638	0.188318194	0.983992835
35.17270487	-0.124915391	0.254091395	-0.491615985	0.622990841	0.999579949
2681.979384	-0.245629055	1.487312725	-0.165149568	0.868826259	0.999579949
106.790824	-0.042793557	0.196944024	-0.217287916	0.827983973	0.999579949
89.48526509	-0.067160029	0.161042147	-0.417033864	0.676653639	0.999579949
167.2266957	0.102186314	0.111146686	0.919382462	0.357895559	0.999579949
952.3906135	0.056059422	0.065939562	0.850163707	0.395234076	0.999579949
1201.248724	-0.010257542	0.134057596	-0.076515933	0.939008639	0.999579949
68.34680439	0.181342642	0.186968473	0.969910265	0.332091223	0.999579949
36.15031728	-0.064306401	0.198667115	-0.323689209	0.746173342	0.999579949
103.1780647	-0.055452343	0.200211859	-0.276968325	0.781804428	0.999579949
52.52593414	0.033349508	0.180880766	0.184372879	0.853720945	0.999579949
1178.36156	0.044351514	0.07665932	0.578553454	0.56289052	0.999579949
479.5617338	0.048201159	0.097974358	0.491977291	0.622735397	0.999579949
3125.335427	-0.027215819	0.139236963	-0.195464036	0.84502969	0.999579949
357.9503238	-0.021013721	0.071082728	-0.29562345	0.767517664	0.999579949
22.216104	1.268054268	0.540332857	2.346802071	0.018935311	0.590797093
7899.087973	-0.140384459	0.129268146	-1.085994217	0.277481551	0.999579949
128.3177301	-0.12346417	0.131235128	-0.940785989	0.346814543	0.999579949
24.00502327	-0.027279736	0.25758082	-0.10590748	0.915655759	0.999579949
987.5856089	0.099131246	0.201546596	0.491852742	0.622823448	0.999579949
186.2473615	-0.077112069	0.125206929	-0.615877014	0.537975694	0.999579949
160.3839646	-0.011891578	0.116868863	-0.101751462	0.918953954	0.999579949
45.45222951	0.273437629	0.320442156	0.853313536	0.393485456	0.999579949
284.850294	-0.004234101	0.200732415	-0.021093261	0.983171261	0.999579949
24.29328116	-0.047698188	0.263318829	-0.181142336	0.856255855	0.999579949
251.3975312	0.063839984	0.163252414	0.391050783	0.695759699	0.999579949
329.5671914	-0.028618843	0.165961312	-0.172442858	0.863089381	0.999579949
195.0470673	0.262965896	0.281518166	0.934099207	0.350252721	0.999579949
31.03140892	-0.50333351	0.263828379	-1.907806552	0.056416224	0.785214091
307.7302059	0.212840729	0.225300836	0.944695694	0.344814256	0.999579949
346.3189266	-0.084727236	0.116889661	-0.724847986	0.468545293	0.999579949
39.79044275	0.730691087	0.428011104	1.707177874	0.087788996	0.869910409
38.204748	0.165375971	0.15195405	1.088328813	0.276449975	0.999579949
75.26068695	-0.08770011	0.214006717	-0.409800736	0.681952126	0.999579949
24.08570193	0.156428709	0.259997181	0.601655405	0.54740354	0.999579949
157.3392696	0.010328919	0.135731227	0.076098325	0.939340873	0.999579949
244.8075363	0.034229386	0.091742681	0.373102084	0.709072467	0.999579949
282.0168656	0.088272303	0.300690183	0.29356563	0.769089842	0.999579949
33.96027184	-0.04916644	0.269531957	-0.182414141	0.855257729	0.999579949
31.06415732	0.636126479	0.668902924	0.950999699	0.341604529	0.999579949
604.0462066	0.036968907	0.131130072	0.281925469	0.77800066	0.999579949
54.4884814	0.102611666	0.150266842	0.682862995	0.494693419	0.999579949
398.9409263	0.145098142	0.079796766	1.818346152	0.069011243	0.831457432
208.3512891	0.439114845	0.250215567	1.75494615	0.079268519	0.851207389
32.17942222	0.070467079	0.211939117	0.332487367	0.739521278	0.999579949
107.4897608	0.063597259	0.098704543	0.64431948	0.519368286	0.999579949

73.72734054	0.103774824	0.152380473	0.681024425	0.495856036	0.999579949
27.94321847	0.145551805	0.231376474	0.629069166	0.529303775	0.999579949
63.35261384	-0.105532197	0.144315519	-0.731260213	0.464620228	0.999579949
318.386176	0.049975526	0.121527469	0.411228232	0.680905189	0.999579949
74.95710264	0.152691655	0.179658553	0.849899167	0.395381149	0.999579949
29.77485022	0.073462266	0.672984857	0.109158869	0.913076483	0.999579949
65.8073978	0.103438231	0.165043668	0.626732503	0.530834594	0.999579949
93.91248085	0.461737512	0.221807409	2.081704635	0.037369457	0.719733456
93.27704516	0.279662105	0.317072411	0.882013368	0.377769578	0.999579949
139.6591685	0.430337955	0.277752271	1.54935891	0.121295466	0.921837625
141.2632763	0.204885444	0.162439898	1.261300003	0.20720078	0.994814558
25.66415918	0.425539591	0.311877961	1.364442647	0.172428293	0.983601342
43.64851546	0.00219249	0.18347357	0.011949898	0.990465588	0.999579949
974.1151687	-0.028174963	0.090343957	-0.311863279	0.755144431	0.999579949
28.72266679	0.173903718	0.212936944	0.816691154	0.414104948	0.999579949
66.55851541	0.211694532	0.297689164	0.711126093	0.477006102	0.999579949
22.38346494	0.055023022	0.224328042	0.245279287	0.806240188	0.999579949
286.4238165	-0.066252086	0.118141525	-0.560785771	0.574943588	0.999579949
80.62479065	-0.135487585	0.18112351	-0.748039745	0.454436184	0.999579949
218.7261087	0.086558491	0.207104254	0.417946467	0.675986258	0.999579949
168.0935929	0.051151067	0.085927155	0.595284073	0.5516536	0.999579949
118.5091132	-0.040317347	0.139382679	-0.289256504	0.772385095	0.999579949
123.9979719	0.13111968	0.160751525	0.815666792	0.414690737	0.999579949
460.4751809	-0.061272529	0.104140386	-0.588364717	0.556287515	0.999579949
3033.928312	0.126402927	0.12501813	1.011076768	0.311979688	0.999579949
150.281091	0.087161037	0.128458687	0.678514151	0.497445754	0.999579949
84.43598704	0.135945732	0.132971107	1.02237046	0.306605596	0.999579949
45.77847211	-0.015301457	0.207682751	-0.073677073	0.941267342	0.999579949
159.8803005	-0.361078087	0.179404135	-2.012651982	0.044151254	0.758836007
77.43430177	0.027371195	0.143523492	0.190708815	0.848753732	0.999579949
68.99165761	0.569467632	0.291048383	1.95660813	0.050393558	0.776599223
479.9624945	-0.002224114	0.09732257	-0.022853018	0.981767517	0.999579949
155.9503275	0.337543013	0.264895838	1.274248081	0.20257551	0.994409048
83.17446811	0.1031614	0.17225325	0.598893778	0.549243721	0.999579949
100.2740298	0.013462644	0.285378933	0.047174626	0.96237405	0.999579949
62.42142936	0.383570642	0.241018106	1.591459861	0.111506122	0.913044976
83.21798051	0.209235573	0.125822229	1.662945995	0.096323246	0.88499031
3252.128777	-0.023463578	0.110180464	-0.212955879	0.831361376	0.999579949
49.3206046	0.155248033	0.179763165	0.863625386	0.387793714	0.999579949
620.8899183	0.795187839	0.414858187	1.916770269	0.055267124	0.785214091
236.2900614	0.091314077	0.118771652	0.768820464	0.441999899	0.999579949
781.9864171	0.321454312	0.197856164	1.624686869	0.104229297	0.895628172
194.9023861	0.205869524	0.227557483	0.904692393	0.365628372	0.999579949
266.0398091	0.407396504	0.144852268	2.812496543	0.004915855	0.347865798
706.8879024	-0.000205963	0.09993198	-0.002061033	0.998355535	0.999644252
64.25186326	-0.029464129	0.169353776	-0.173979757	0.861881371	0.999579949
110.5183565	0.043913091	0.113387911	0.387281948	0.698547488	0.999579949
55.79782897	0.308715023	0.180020538	1.714887789	0.086365802	0.866133472

564.453852	0.019200292	0.058620747	0.327534081	0.74326397	0.999579949
349.6069451	-0.045143933	0.108569096	-0.415808316	0.677550273	0.999579949
6901.263131	-0.054235888	0.052587816	-1.031339419	0.302381679	0.999579949
30.07633465	0.238199762	0.254663745	0.93535011	0.349607899	0.999579949
629.930126	-0.040020205	0.111711236	-0.358246907	0.720158547	0.999579949
483.2139646	0.074360381	0.103412782	0.719063734	0.472101651	0.999579949
274.8367419	-0.103653234	0.114835104	-0.902626722	0.366724039	0.999579949
1260.893677	0.066705536	0.161159131	0.413910992	0.678939294	0.999579949
50.30825195	-0.121274123	0.167940601	-0.722125099	0.470217568	0.999579949
217.7023991	-0.127084342	0.106294054	-1.195592204	0.231855735	0.999579949
107.5087628	0.12478213	0.111792073	1.11619837	0.264337232	0.999579949
52.11204617	0.017180051	0.154389558	0.111277288	0.911396465	0.999579949
36.8996022	0.25576901	0.193057259	1.324834982	0.18522589	0.983992835
478.1699969	-0.084913306	0.093373432	-0.909394716	0.36314181	0.999579949
100.384851	-0.017848226	0.134191023	-0.133006108	0.89418855	0.999579949
104.1093456	-0.008800977	0.164577434	-0.053476209	0.957352486	0.999579949
145.2097386	0.213385749	0.239827925	0.88974522	0.373602707	0.999579949
190.6953469	0.076419396	0.089103878	0.857643881	0.391089137	0.999579949
70.73189821	0.042369966	0.135224802	0.313329846	0.754030077	0.999579949
44.56100215	0.548519901	0.314177662	1.745890835	0.080829923	0.85448976
1358.166775	0.041167418	0.198359381	0.207539554	0.835588505	0.999579949
277.3773232	-0.015640988	0.113498356	-0.137808054	0.890392119	0.999579949
56.4270152	0.018015236	0.150840172	0.119432611	0.904932627	0.999579949
1127.884182	0.198579072	0.157940801	1.257300651	0.208644789	0.995865766
376.8850593	0.603457027	0.208714364	2.891305679	0.003836448	0.319818899
32.62644279	0.468538633	0.285199558	1.642844876	0.100415033	0.895628172
1525.744327	-0.145491691	0.077052747	-1.888209004	0.058997897	0.79629879
701.1972573	-0.084005467	0.111438919	-0.753825213	0.450954182	0.999579949
58.33686343	0.131245651	0.206832643	0.634549991	0.525721953	0.999579949
643.8316351	0.0754803	0.126298221	0.597635494	0.550083176	0.999579949
50.57711264	0.163809018	0.186725161	0.877273408	0.380338153	0.999579949
5757.35171	0.103492967	0.124719772	0.829804006	0.406649606	0.999579949
60.65719272	0.05549532	0.327066055	0.169676182	0.865264806	0.999579949
327.0663052	-0.079153554	0.134669978	-0.587759465	0.556693755	0.999579949
29.4021747	0.255880114	0.242417827	1.055533402	0.291181441	0.999579949
336.6743067	-0.127653307	0.117349161	-1.087807578	0.276680063	0.999579949
127.7813189	0.180002636	0.212801465	0.845871223	0.397624588	0.999579949
73.08778436	0.115732122	0.139582525	0.829130448	0.407030596	0.999579949
1033.866304	0.045609952	0.084665085	0.538710291	0.590086772	0.999579949
220.6403498	-0.021008018	0.100878678	-0.208250329	0.835033513	0.999579949
420.644211	-0.119914759	0.105875394	-1.132602722	0.257381125	0.999579949
152.3342818	0.042648937	0.199389765	0.213897322	0.830627129	0.999579949
208.365648	0.394295819	0.137721449	2.862994981	0.004196572	0.334557103
195.6799154	0.033925074	0.097209678	0.348988646	0.727097833	0.999579949
174.4279342	-0.469083983	0.408269612	-1.148956398	0.25057396	0.999579949
25.76377437	0.029229064	0.229259438	0.127493393	0.898549902	0.999579949
25.66939628	0.574886142	0.342757382	1.677239276	0.093495702	0.880418343
47.8608914	0.158878399	0.144632069	1.098500495	0.271986001	0.999579949

75.74744071	0.158336698	0.181365362	0.873026119	0.382648841	0.999579949
41.43210249	0.589566861	0.236346307	2.494504222	0.012613323	0.508958578
1516.105575	0.107706986	0.149823991	0.718890115	0.472208627	0.999579949
168.4034465	0.104004033	0.100409987	1.035793709	0.300298393	0.999579949
136.9319119	0.06056856	0.187854917	0.322422009	0.747133013	0.999579949
156.814911	-0.095087165	0.097753273	-0.972726151	0.330689424	0.999579949
97.69211383	0.054899405	0.135809508	0.404238303	0.68603749	0.999579949
143.2702668	0.118244074	0.112570374	1.050401361	0.29353362	0.999579949
78.58507574	-0.078801317	0.124644976	-0.632206122	0.527252198	0.999579949
1564.198177	0.136562952	0.203652451	0.67056866	0.502495353	0.999579949
22.80554295	0.145550932	0.364230212	0.399612463	0.689441975	0.999579949
56.02470281	0.027757596	0.251269094	0.110469599	0.912036958	0.999579949
604.4701533	0.00412207	0.111642712	0.036921978	0.970547216	0.999579949
298.9942559	0.022263518	0.102419402	0.217375978	0.827915351	0.999579949
324.5541277	0.078766176	0.123662899	0.636942662	0.524162192	0.999579949
84.01140966	0.003546548	0.149018532	0.023799378	0.981012636	0.999579949
140.2564484	0.170861549	1.869010154	0.091418203	0.927160299	0.999579949
243.8271902	0.010325306	2.312858072	0.004464306	0.996438011	0.999579949
180.3753605	-0.060829417	2.263253201	-0.026876982	0.978557852	0.999579949
613.8283607	-0.165097521	2.246004006	-0.073507225	0.941402495	0.999579949
201.8080601	0.512914267	0.28332238	1.810355633	0.070240655	0.834620594

diffexpressed	gene_symbol	delabel
UP	Adamts8	Adamts8
UP	Ankrd1	Ankrd1
DOWN	Arntl	Arntl
UP	Atp2a2	Atp2a2
DOWN	Auts2	Auts2
UP	Col2a1	Col2a1
DOWN	Fbxl16	Fbxl16
UP	Hspa1l	Hspa1l
UP	Hspb7	Hspb7
UP	Lmod2	Lmod2
UP	Myh6	Myh6
UP	Myh7	Myh7
UP	Myl2	Myl2
UP	Myom3	Myom3
DOWN	Nr1d1	Nr1d1
UP	Otud1	Otud1
UP	Slc25a34	Slc25a34
UP	Smtnl1	Smtnl1
UP	Tnni1	Tnni1
UP	Tnnt1	Tnnt1
UP	Tpm3	Tpm3
NO	Rp1	
NO	Sox17	
NO	Mrpl15	
NO	Lypla1	
NO	Tcea1	
NO	Atp6v1h	
NO	Rb1cc1	
NO	Pcmtd1	
NO	Rrs1	
NO	Adhfe1	
NO	2610203C22Rik	
NO	Vcpip1	
NO	Sgk3	
NO	Snhg6	
NO	Cops5	
NO	Cspp1	
NO	Arfgef1	
NO	Prex2	
NO	Sulf1	
NO	Slco5a1	
NO	Ncoa2	
NO	Tram1	
NO	Lactb2	
NO	Eya1	
NO	Msc	
NO	Terf1	

NO	Sbspon
NO	Rpl7
NO	Rdh10
NO	Stau2
NO	Ube2w
NO	Tceb1
NO	Tmem70
NO	Jph1
NO	Gdap1
NO	Pi15
NO	Crispld1
NO	Linc-md1
NO	Mcm3
NO	Paqr8
NO	Tram2
NO	Gsta3
NO	Kcnq5
NO	4933415F23Rik
NO	Ogfrl1
NO	Smap1
NO	Sdhaf4
NO	Fam135a
NO	Col9a1
NO	Col19a1
NO	Lmbrd1
NO	Phf3
NO	Ptp4a1
NO	Prim2
NO	Rab23
NO	Bag2
NO	Zfp451
NO	Dst
NO	Ccdc115
NO	Imp4
NO	Ptpn18
NO	Arhgef4
NO	Fam168b
NO	Plekhb2
NO	Hs6st1
NO	Uggt1
NO	Neurl3
NO	Arid5a
NO	Kansl3
NO	Fer1l5
NO	Lman2l
NO	Cnnm4
NO	Cnnm3
NO	Ankrd23

NO	Ankrd39
NO	Sema4c
NO	Cox5b
NO	Actr1b
NO	Tmem131
NO	Inpp4a
NO	Coa5
NO	Unc50
NO	Mgat4a
NO	4930594C11Rik
NO	Tsga10
NO	Lipt1
NO	Mitd1
NO	Mrpl30
NO	Txndc9
NO	Eif5b
NO	Rev1
NO	Aff3
NO	Lonrf2
NO	Pdcl3
NO	Rpl31
NO	Tbc1d8
NO	Cnot11
NO	Rnf149
NO	Map4k4
NO	Il1r1
NO	Il1rl2
NO	Il1rl1
NO	Slc9a4
NO	Slc9a2
NO	Tmem182
NO	Mrps9
NO	Tgfbrap1
NO	AI597479
NO	Fhl2
NO	Nck2
NO	Uxs1
NO	Tpp2
NO	Mettl21c
NO	Tex30
NO	Kdelc1
NO	Bivm
NO	Ercc5
NO	Mettl21e
NO	Gulp1
NO	Col3a1
NO	Col5a2
NO	Wdr75

NO	Slc40a1
NO	Slc39a10
NO	Tmeff2
NO	Sdpr
NO	Nabp1
NO	Myo1b
NO	Stat1
NO	Gls
NO	Nab1
NO	Tmem194b
NO	Mfsd6
NO	Inpp1
NO	Hibch
NO	Mstn
NO	Pms1
NO	Ormdl1
NO	Osgepl1
NO	Asnsd1
NO	Stk17b
NO	Hecw2
NO	Gtf3c3
NO	Pgap1
NO	Ankrd44
NO	Sf3b1
NO	Coq10b
NO	Hspd1
NO	Hspe1
NO	Mob4
NO	Rftn2
NO	Mars2
NO	Plcl1
NO	1700066M21Rik
NO	Tyw5
NO	9430016H08Rik
NO	Spats2l
NO	Kctd18
NO	Sgol2a
NO	Aox1
NO	Aox3
NO	Bzw1
NO	Clk1
NO	Ppil3
NO	Nif3l1
NO	Orc2
NO	Fam126b
NO	Ndufb3
NO	Cflar
NO	Casp8

NO	Trak2
NO	Stradb
NO	Tmem237
NO	Als2
NO	Fzd7
NO	Gm973
NO	Sumo1
NO	Nop58
NO	Bmpr2
NO	Fam117b
NO	Wdr12
NO	Carf
NO	Nbeal1
NO	Cyp20a1
NO	Abi2
NO	Raph1
NO	Pard3b
NO	Nrp2
NO	Ino80d
NO	Ino80dos
NO	Ndufs1
NO	Eef1b2
NO	Zdbf2
NO	Adam23
NO	Fastkd2
NO	Klf7
NO	Creb1
NO	Mettl21a
NO	Ccnyl1
NO	Fzd5
NO	Plekhm3
NO	Idh1
NO	Pikfyve
NO	Map2
NO	Rpe
NO	Kansl1l
NO	Acadl
NO	Myl1
NO	Lancl1
NO	Erbb4
NO	Ikzf2
NO	Atic
NO	Fn1
NO	Mreg
NO	Pecr
NO	Xrcc5
NO	Smarcal1
NO	Rpl37a

NO	Igfbp5
NO	Tns1
NO	Arpc2
NO	Aamp
NO	Pnkd
NO	Tmbim1
NO	Slc11a1
NO	Ctdsp1
NO	Usp37
NO	Rqcd1
NO	Plcd4
NO	Zfp142
NO	Bcs1l
NO	Rnf25
NO	Ttll4
NO	Cyp27a1
NO	Prkag3
NO	Wnt6
NO	Nhej1
NO	Cnppd1
NO	Fam134a
NO	Zfand2b
NO	Abcb6
NO	Atg9a
NO	Ankzf1
NO	Glb1l
NO	Stk16
NO	Tuba4a
NO	Dnajb2
NO	Dnpep
NO	Des
NO	Gm15179
NO	Speg
NO	Gmppa
NO	Chpf
NO	Obsl1
NO	Inha
NO	Stk11ip
NO	Slc4a3
NO	Epha4
NO	Farsb
NO	Acsl3
NO	Utp14b
NO	Kcne4
NO	Wdfy1
NO	Mrpl44
NO	Serpine2
NO	Cul3

NO	Dock10
NO	Irs1
NO	Rhbdd1
NO	Col4a4
NO	Col4a3
NO	Mff
NO	Agfg1
NO	Pid1
NO	Trip12
NO	Sp110
NO	Sp140
NO	Sp100
NO	A630001G21Rik
NO	Cab39
NO	Itm2c
NO	Psmd1
NO	Htr2b
NO	Armc9
NO	Ncl
NO	C130036L24Rik
NO	Ptma
NO	Pde6d
NO	Cops7b
NO	Dis3l2
NO	Chrnd
NO	Eif4e2
NO	Efhd1
NO	Gigyf2
NO	Kcnj13
NO	Neu2
NO	Inpp5d
NO	Atg16l1
NO	Scarna6
NO	Dgkd
NO	Usp40
NO	Mroh2a
NO	Hjurp
NO	Arl4c
NO	Sh3bp4
NO	Agap1
NO	4933400F21Rik
NO	Asb18
NO	Ackr3
NO	Cops8
NO	Col6a3
NO	Lrrfip1
NO	Ramp1
NO	Ube2f

NO	Scly
NO	Klhl30
NO	Fam132b
NO	Ilkap
NO	Hes6
NO	Per2
NO	Traf3ip1
NO	Asb1
NO	Hdac4
NO	Ndufa10
NO	Myeov2
NO	Gpc1
NO	Dusp28
NO	Rnpepl1
NO	Capn10
NO	Kif1a
NO	Sned1
NO	Mterf4
NO	Ppp1r7
NO	Hdlbp
NO	Sept2
NO	Farp2
NO	Stk25
NO	Bok
NO	Thap4
NO	Atg4b
NO	Dtymk
NO	Ing5
NO	D2hgdh
NO	Fam174a
NO	St8sia4
NO	D1Ertd622e
NO	Ppip5k2
NO	Gin1
NO	Pam
NO	Rnf152
NO	Pign
NO	2310035C23Rik
NO	Tnfrsf11a
NO	Zcchc2
NO	Phlpp1
NO	Bcl2
NO	Kdsr
NO	Vps4b
NO	Serpinb8
NO	D830032E09Rik
NO	Cdh19
NO	Dsel

NO	Tsn
NO	Nifk
NO	Clasp1
NO	Tfcp2l1
NO	Gli2
NO	Inhbb
NO	Ralb
NO	Tmem185b
NO	Epb4.1l5
NO	Ptpn4
NO	Tmem177
NO	Sctr
NO	Tmem37
NO	Dbi
NO	3110009E18Rik
NO	Steap3
NO	En1
NO	Insig2
NO	Ccdc93
NO	Ddx18
NO	Actr3
NO	Slc35f5
NO	Nckap5
NO	Mgat5
NO	Ccnt2
NO	Rab3gap1
NO	Zranb3
NO	R3hdm1
NO	Ubxn4
NO	Mcm6
NO	Dars
NO	Cxcr4
NO	Thsd7b
NO	Cd55
NO	Pfkfb2
NO	Yod1
NO	Mapkapk2
NO	Eif2d
NO	Rassf5
NO	Ikbke
NO	Srgap2
NO	Ctse
NO	Rab7b
NO	Pm20d1
NO	Slc41a1
NO	Rab29
NO	Nucks1
NO	Slc45a3

NO	Elk4
NO	Mfsd4
NO	Cdk18
NO	Nuak2
NO	Tmcc2
NO	Dstyk
NO	Rbbp5
NO	Tmem81
NO	Nfasc
NO	Lrrn2
NO	Mdm4
NO	Pik3c2b
NO	Ppp1r15b
NO	Plekha6
NO	Gm19461
NO	Sox13
NO	Snrpe
NO	Zc3h11a
NO	Zbed6
NO	Atp2b4
NO	Prelp
NO	Fmod
NO	Btg2
NO	Chil1
NO	Mybph
NO	Adora1
NO	Myog
NO	Ppfia4
NO	Tmem183a
NO	Cyb5r1
NO	Adipor1
NO	Klhl12
NO	Rabif
NO	Kdm5b
NO	Ppp1r12b
NO	Ube2t
NO	Ptpn7
NO	Arl8a
NO	Rnpep
NO	Timm17a
NO	Lmod1
NO	Shisa4
NO	Ipo9
NO	Nav1
NO	Csrp1
NO	Phlda3
NO	Tnnt2
NO	Igfn1

NO	Tmem9
NO	Cacna1s
NO	Kif21b
NO	Camsap2
NO	Ddx59
NO	Zfp281
NO	Ptprc
NO	Nek7
NO	2310009B15Rik
NO	Dennd1b
NO	Zbtb41
NO	Cfh
NO	Kcnt2
NO	Cdc73
NO	B3galt2
NO	Glrx2
NO	Trove2
NO	Uchl5
NO	Rgs2
NO	Pla2g4a
NO	BC003331
NO	Tpr
NO	Prg4
NO	Hmcn1
NO	Ivns1abp
NO	Swt1
NO	Trmt1l
NO	Rnf2
NO	Fam129a
NO	Edem3
NO	1700025G04Rik
NO	Tsen15
NO	Colgalt2
NO	Rgl1
NO	Arpc5
NO	Ncf2
NO	Smg7
NO	Lamc2
NO	Lamc1
NO	Dhx9
NO	Npl
NO	Rnasel
NO	Glul
NO	Ier5
NO	Mr1
NO	Stx6
NO	Xpr1
NO	Acbd6

NO	Qsox1
NO	Cep350
NO	Tor1aip1
NO	Tor1aip2
NO	Soat1
NO	Abl2
NO	Tor3a
NO	Fam20b
NO	Ralgps2
NO	Angptl1
NO	Rasal2
NO	2810025M15Rik
NO	BC026585
NO	Sec16b
NO	Rfwd2
NO	4930523C07Rik
NO	Mrps14
NO	Cacybp
NO	Rabgap1l
NO	Rc3h1
NO	Zbtb37
NO	Gas5
NO	Dars2
NO	Klhl20
NO	Prdx6
NO	Suco
NO	Pigc
NO	Dnm3
NO	Dnm3os
NO	Mettl13
NO	Vamp4
NO	Myoc
NO	Prrc2c
NO	Fmo1
NO	Fmo2
NO	Prrx1
NO	Gorab
NO	Mettl11b
NO	Kifap3
NO	Scyl3
NO	Mettl18
NO	Selp
NO	Slc19a2
NO	Ccdc181
NO	Blzf1
NO	Nme7
NO	Atp1b1
NO	Dpt

NO	Gm20743
NO	Sft2d2
NO	Tiprl
NO	Gpr161
NO	Dcaf6
NO	Mpc2
NO	Mpzl1
NO	Rcsd1
NO	Creg1
NO	Cd247
NO	Pou2f1
NO	Dusp27
NO	Ildr2
NO	Tada1
NO	Pogk
NO	Fam78b
NO	Uck2
NO	Tmco1
NO	Aldh9a1
NO	Mgst3
NO	Lrrc52
NO	Rxrg
NO	Pbx1
NO	Rgs5
NO	Rgs4
NO	Hsd17b7
NO	Ddr2
NO	Uap1
NO	Uhmk1
NO	Gm7694
NO	Nos1ap
NO	Olfml2b
NO	Atf6
NO	Dusp12
NO	Fcgr2b
NO	Fcgr3
NO	Sdhc
NO	Mpz
NO	Pcp4l1
NO	Tomm40l
NO	Fcer1g
NO	Ndufs2
NO	B4galt3
NO	Ppox
NO	Usp21
NO	Ufc1
NO	Dedd
NO	Nit1

NO	Pfdn2
NO	Klhdc9
NO	Arhgap30
NO	Usf1
NO	F11r
NO	Cd84
NO	Ncstn
NO	Copa
NO	Pex19
NO	Dcaf8
NO	Pea15a
NO	Casq1
NO	Atp1a4
NO	Atp1a2
NO	Igsf8
NO	Pigm
NO	Tagln2
NO	Dusp23
NO	Ackr1
NO	Cadm3
NO	AI607873
NO	Ifi204
NO	Mnda
NO	Ifi203
NO	Ifi202b
NO	Ifi205
NO	Spta1
NO	Fmn2
NO	Grem2
NO	Fh1
NO	Opn3
NO	Chml
NO	Pld5
NO	Cep170
NO	Sdccag8
NO	Hmga2-ps1
NO	Akt3
NO	Zbtb18
NO	Adss
NO	Desi2
NO	Cox20
NO	Hnrnpu
NO	Efcab2
NO	Kif26b
NO	Smyd3
NO	Tfb2m
NO	Cnst
NO	Sccpdh

NO	Ahctf1
NO	Cdc42bpa
NO	Adck3
NO	Psen2
NO	Itpkb
NO	6330403A02Rik
NO	Parp1
NO	Lin9
NO	Acbd3
NO	H3f3a
NO	Sde2
NO	Pycr2
NO	Tmem63a
NO	Ephx1
NO	Nvl
NO	Cnih4
NO	Wdr26
NO	Lbr
NO	Enah
NO	Srp9
NO	Degs1
NO	Fbxo28
NO	Trp53bp2
NO	Capn2
NO	Tlr5
NO	Disp1
NO	Brox
NO	Aida
NO	Mia3
NO	Taf1a
NO	Dusp10
NO	Hlx
NO	Marc1
NO	Marc2
NO	C130074G19Rik
NO	Mark1
NO	Rab3gap2
NO	Iars2
NO	Bpnt1
NO	Eprs
NO	Lyplal1
NO	Tgfb2
NO	Rrp15
NO	Gpatch2
NO	Esrrg
NO	Kctd3
NO	Kcnk2
NO	Ptpn14

NO	Smyd2
NO	Prox1
NO	Rps6kc1
NO	Angel2
NO	Vash2
NO	Mfsd7b
NO	Tatdn3
NO	Atf3
NO	Nenf
NO	Tmem206
NO	Ppp2r5a
NO	Ints7
NO	Lpgat1
NO	Slc30a1
NO	Traf5
NO	Rcor3
NO	Gm10516
NO	Hhat
NO	Sertad4
NO	Syt14
NO	Diexf
NO	Irf6
NO	A130010J15Rik
NO	Traf3ip3
NO	Hsd11b1
NO	G0s2
NO	Plxna2
NO	Cd34
NO	Gm16897
NO	A330023F24Rik
NO	Cd46
NO	Cr1l
NO	Cr2
NO	Ppp1r14c
NO	Plekhg1
NO	Mthfd1l
NO	Akap12
NO	Zbtb2
NO	Armt1
NO	Esr1
NO	Syne1
NO	Myct1
NO	Mtrf1l
NO	Cnksr3
NO	Lrp11
NO	Pcmt1
NO	Nup43
NO	BC020402

NO	Lats1
NO	Katna1
NO	Ginm1
NO	Ppil4
NO	Zc3h12d
NO	Tab2
NO	Ust
NO	Sash1
NO	Samd5
NO	Stxbp5
NO	Rab32
NO	Shprh
NO	Fbxo30
NO	Epm2a
NO	Utrn
NO	Sf3b5
NO	Plagl1
NO	Ltv1
NO	Phactr2
NO	Fuca2
NO	Pex3
NO	Adat2
NO	Aig1
NO	Hivep2
NO	Adgrg6
NO	Vta1
NO	Cited2
NO	Txlnb
NO	Heca
NO	Abracl
NO	Reps1
NO	Ccdc28a
NO	Nhsl1
NO	Perp
NO	Tnfaip3
NO	Ifngr1
NO	Pex7
NO	Map3k5
NO	Map7
NO	Bclaf1
NO	Pde7b
NO	Ahi1
NO	Myb
NO	Hbs1l
NO	Sgk1
NO	H60b
NO	Slc2a12
NO	Tbpl1

NO	Eya4
NO	Rps12
NO	Slc18b1
NO	Stx7
NO	Ctgf
NO	Enpp1
NO	Enpp3
NO	Med23
NO	Akap7
NO	Epb4.1l2
NO	L3mbtl3
NO	Arhgap18
NO	Lama2
NO	Ptprk
NO	9330159F19Rik
NO	Echdc1
NO	Rnf146
NO	Rspo3
NO	Trmt11
NO	Gm20300
NO	Hint3
NO	Ncoa7
NO	Hddc2
NO	Tpd52l1
NO	Rnf217
NO	Trdn
NO	D830005E20Rik
NO	Zufsp
NO	A830082N09Rik
NO	Rwdd1
NO	Dse
NO	Tspyl1
NO	Tspyl4
NO	Nt5dc1
NO	Col10a1
NO	Frk
NO	Hs3st5
NO	Hdac2
NO	Marcks
NO	Lama4
NO	Fyn
NO	Traf3ip2
NO	E130307A14Rik
NO	Rev3l
NO	AA474331
NO	AI317395
NO	Slc16a10
NO	Rpf2

NO	Gtf3c6
NO	Cdk19
NO	Ddo
NO	Cdc40
NO	Fig4
NO	Zbtb24
NO	Mical1
NO	Smpd2
NO	Cd164
NO	Cep57l1
NO	Sesn1
NO	Foxo3
NO	Lace1
NO	Snx3
NO	Ostm1
NO	Sec63
NO	Scml4
NO	Sobp
NO	9030612E09Rik
NO	Pdss2
NO	Bend3
NO	1700021F05Rik
NO	Cd24a
NO	1700027J07Rik
NO	Qrsl1
NO	Rtn4ip1
NO	Aim1
NO	Atg5
NO	Prdm1
NO	Prep
NO	Popdc3
NO	Bves
NO	Hace1
NO	Ascc3
NO	Sim1
NO	Lilr4b
NO	Lilrb4a
NO	Vgll2
NO	Dcbld1
NO	Gopc
NO	Nus1
NO	Slc35f1
NO	Cep85l
NO	Gm19395
NO	Pln
NO	Mcm9
NO	Asf1a
NO	Man1a

NO	Tbc1d32
NO	Msl3l2
NO	Gja1
NO	Hsf2
NO	Serinc1
NO	Smpdl3a
NO	Gcc2
NO	Lims1
NO	Ranbp2
NO	Sh3rf3
NO	Sept10
NO	Sowahc
NO	P4ha1
NO	Mcu
NO	Micu1
NO	Dnajb12
NO	Ddit4
NO	Anapc16
NO	Ascc1
NO	Spock2
NO	Chst3
NO	Psap
NO	Cdh23
NO	4632428N05Rik
NO	Slc29a3
NO	Unc5b
NO	Sgpl1
NO	Adamts14
NO	Pald1
NO	Eif4ebp2
NO	Lrrc20
NO	Ppa1
NO	Sar1a
NO	Tysnd1
NO	Aifm2
NO	Col13a1
NO	Tspan15
NO	Hk1
NO	Supv3l1
NO	Vps26a
NO	Srgn
NO	Kif1bp
NO	Ddx21
NO	Ddx50
NO	Ccar1
NO	Tet1
NO	Slc25a16
NO	Dna2

NO	Rufy2
NO	Hnrnph3
NO	Mypn
NO	Herc4
NO	Sirt1
NO	Dnajc12
NO	Ctnna3
NO	Lrrtm3
NO	Reep3
NO	Jmjd1c
NO	Nrbf2
NO	Egr2
NO	Ado
NO	Arid5b
NO	Rhobtb1
NO	Ank3
NO	Ccdc6
NO	Mrln
NO	Slc16a9
NO	Fam13c
NO	Bicc1
NO	Tfam
NO	Ube2d1
NO	Cisd1
NO	Ipmk
NO	Zwint
NO	Bcr
NO	Specc1l
NO	Adora2a
NO	Gucd1
NO	Snrpd3
NO	Ggt5
NO	Susd2
NO	Cabin1
NO	Ddt
NO	Gstt3
NO	Gstt1
NO	Gstt2
NO	Mif
NO	Smarcb1
NO	Mmp11
NO	Chchd10
NO	Zfp280b
NO	Prmt2
NO	S100b
NO	Dip2a
NO	Pcnt
NO	Ybey

NO	Mcm3ap
NO	Lss
NO	Col6a2
NO	Col6a1
NO	Pcbp3
NO	Slc19a1
NO	Col18a1
NO	Pofut2
NO	Adarb1
NO	Fam207a
NO	Itgb2
NO	Pttg1ip
NO	Sumo3
NO	Ube2g2
NO	Lrrc3
NO	Trpm2
NO	1810043G02Rik
NO	Pfkl
NO	Icosl
NO	D10Jhu81e
NO	Pwp2
NO	Trappc10
NO	Agpat3
NO	Rrp1
NO	Cstb
NO	Pdxk
NO	Ilvbl
NO	Syde1
NO	2610008E11Rik
NO	Ppap2c
NO	Mier2
NO	Shc2
NO	Odf3l2
NO	Tpgs1
NO	Cdc34
NO	Gzmm
NO	Bsg
NO	Hcn2
NO	Polrmt
NO	Rnf126
NO	Palm
NO	E130317F20Rik
NO	Ptbp1
NO	Lppr3
NO	Elane
NO	Cfd
NO	Med16
NO	R3hdm4

NO	Arid3a
NO	Wdr18
NO	Grin3b
NO	Tmem259
NO	Cnn2
NO	Abca7
NO	Hmha1
NO	Polr2e
NO	Gpx4
NO	Sbno2
NO	Stk11
NO	Dos
NO	Atp5d
NO	Midn
NO	Cirbp
NO	1600002K03Rik
NO	Mum1
NO	Ndufs7
NO	Gamt
NO	Dazap1
NO	Rps15
NO	Apc2
NO	2310011J03Rik
NO	Pcsk4
NO	Reep6
NO	Adamtsl5
NO	Mex3d
NO	Mbd3
NO	Uqcr11
NO	Tcf3
NO	Rexo1
NO	Klf16
NO	Abhd17a
NO	Adat3
NO	Scamp4
NO	Csnk1g2
NO	Btbd2
NO	Mknk2
NO	Mob3a
NO	Izumo4
NO	Ap3d1
NO	Dot1l
NO	Plekhj1
NO	Sf3a2
NO	Jsrp1
NO	Lingo3
NO	Lsm7
NO	Sppl2b

NO	Timm13
NO	Lmnb2
NO	Gadd45b
NO	Gng7
NO	Slc39a3
NO	Sgta
NO	Thop1
NO	Map2k2
NO	Zbtb7a
NO	Pias4
NO	Eef2
NO	Dapk3
NO	Atcayos
NO	Nmrk2
NO	Atcay
NO	Zfr2
NO	Matk
NO	Mrpl54
NO	Apba3
NO	Pip5k1c
NO	Cactin
NO	Hmg20b
NO	Mfsd12
NO	4930404N11Rik
NO	Fzr1
NO	Dohh
NO	Nfic
NO	Ncln
NO	S1pr4
NO	Gna11
NO	Aes
NO	Tle2
NO	Tle6
NO	BC025920
NO	Sirt6
NO	Ankrd24
NO	Zfp873
NO	AU041133
NO	Zfp938
NO	1190007I07Rik
NO	Glt8d2
NO	Hcfc2
NO	Nfyb
NO	Txnrd1
NO	Eid3
NO	Chst11
NO	Slc41a2
NO	D10Wsu102e

NO	Aldh1l2
NO	A230046K03Rik
NO	Appl2
NO	Nuak1
NO	Ckap4
NO	Tcp11l2
NO	Polr3b
NO	Ric8b
NO	Tmem263
NO	Mterf2
NO	Cry1
NO	Btbd11
NO	Pwp1
NO	Prdm4
NO	Rtcb
NO	Fbxo7
NO	Syn3
NO	Timp3
NO	1810014B01Rik
NO	Hsp90b1
NO	Ttc41
NO	Nt5dc3
NO	Stab2
NO	1700113H08Rik
NO	Igf1
NO	Nup37
NO	Ccdc53
NO	Dram1
NO	Gnptab
NO	Sycp3
NO	Chpt1
NO	Mybpc1
NO	Arl1
NO	Utp20
NO	Gas2l3
NO	Scyl2
NO	Actr6
NO	Uhrf1bp1l
NO	Apaf1
NO	Ikbip
NO	Slc25a3
NO	Tmpo
NO	Nedd1
NO	Cdk17
NO	Elk3
NO	Lta4h
NO	Hal
NO	Ntn4

NO	Metap2
NO	Vezt
NO	Fgd6
NO	Nr2c1
NO	Ndufa12
NO	Tmcc3
NO	Cep83os
NO	Cep83
NO	Plxnc1
NO	Cradd
NO	Socs2
NO	Mrpl42
NO	Ube2n
NO	Nudt4
NO	Eea1
NO	Btg1
NO	Dcn
NO	Lum
NO	Kera
NO	Atp2b1
NO	Poc1b
NO	Galnt4
NO	Dusp6
NO	Kitl
NO	Tmtc3
NO	Cep290
NO	4930430F08Rik
NO	Rassf9
NO	Tmtc2
NO	Mettl25
NO	Ccdc59
NO	Acss3
NO	Lin7a
NO	Myf6
NO	Ptprq
NO	Ppp1r12a
NO	Pawr
NO	Nav3
NO	E2f7
NO	Csrp2
NO	Zdhhc17
NO	Osbpl8
NO	Bbs10
NO	Nap1l1
NO	Phlda1
NO	Krr1
NO	Kcnc2
NO	Atxn7l3b

NO	Trhde
NO	Tbc1d15
NO	Rab21
NO	Tmem19
NO	Thap2
NO	Zfc3h1
NO	Tspan8
NO	Ptprb
NO	Cnot2
NO	Rab3ip
NO	Best3
NO	Cct2
NO	Frs2
NO	Yeats4
NO	Lyz2
NO	Lyz1
NO	Cpsf6
NO	Cpm
NO	Mdm2
NO	Slc35e3
NO	Nup107
NO	Rap1b
NO	Mdm1
NO	Dyrk2
NO	Cand1
NO	Helb
NO	Irak3
NO	Tmbim4
NO	Llph
NO	4921513I03Rik
NO	Msrb3
NO	Gm15910
NO	Lemd3
NO	Gns
NO	Rassf3
NO	Tbk1
NO	Xpot
NO	BC048403
NO	Srgap1
NO	Tmem5
NO	Avpr1a
NO	Ppm1h
NO	Mon2
NO	Usp15
NO	Slc16a7
NO	Lrig3
NO	Xrcc6bp1
NO	Ctdsp2

NO	Avil
NO	Tsfm
NO	Mettl1
NO	March9
NO	Cdk4
NO	Tspan31
NO	Agap2
NO	Os9
NO	B4galnt1
NO	Slc26a10
NO	Arhgef25
NO	Dtx3
NO	Pip4k2c
NO	Kif5a
NO	Dctn2
NO	Mbd6
NO	Ddit3
NO	Mars
NO	Arhgap9
NO	Gli1
NO	R3hdm2
NO	Stac3
NO	Ndufa4l2
NO	Shmt2
NO	Lrp1
NO	Stat6
NO	Nab2
NO	Tmem194
NO	Zbtb39
NO	Gpr182
NO	Rdh9
NO	Prim1
NO	Naca
NO	Ptges3
NO	Atp5b
NO	Baz2a
NO	Rbms2
NO	Spryd4
NO	Timeless
NO	Stat2
NO	Pan2
NO	Cnpy2
NO	Cs
NO	Coq10a
NO	Ankrd52
NO	Nabp2
NO	Rnf41
NO	Smarcc2

NO	Myl6
NO	Myl6b
NO	A430046D13Rik
NO	Esyt1
NO	Zc3h10
NO	Rpl41
NO	Pa2g4
NO	Erbb3
NO	Rps26
NO	Suox
NO	Rab5b
NO	Cdk2
NO	Dgka
NO	Wibg
NO	Mmp19
NO	Tmem198b
NO	Dnajc14
NO	Sarnp
NO	Gdf11
NO	Cd63
NO	Rdh5
NO	Bloc1s1
NO	Itga7
NO	Pisd-ps1
NO	Sfi1
NO	Eif4enif1
NO	Drg1
NO	Patz1
NO	Gm11944
NO	Pik3ip1
NO	Limk2
NO	Rnf185
NO	8430429K09Rik
NO	Inpp5j
NO	Selm
NO	Smtn
NO	Tug1
NO	Morc2a
NO	Osbp2
NO	Dusp18
NO	Slc35e4
NO	Tcn2
NO	Pes1
NO	Mtfp1
NO	Sec14l2
NO	Rnf215
NO	Ccdc157
NO	Sf3a1

NO	Tbc1d10a
NO	Gatsl3
NO	Mtmr3
NO	Ascc2
NO	Uqcr10
NO	Zmat5
NO	Nf2
NO	Nipsnap1
NO	Thoc5
NO	Ap1b1
NO	Gas2l1
NO	Ewsr1
NO	Rhbdd3
NO	Emid1
NO	Kremen1
NO	Znrf3
NO	Xbp1
NO	Ccdc117
NO	Mrps24
NO	Urgcp
NO	2210015D19Rik
NO	Dbnl
NO	Pgam2
NO	Polm
NO	Aebp1
NO	Pold2
NO	Gck
NO	Ykt6
NO	Camk2b
NO	Nudcd3
NO	Npc1l1
NO	Ddx56
NO	Tmed4
NO	Ogdh
NO	Zmiz2
NO	Ppia
NO	H2afv
NO	Purb
NO	Myo1g
NO	Ccm2
NO	Nacad
NO	Tbrg4
NO	Snora5c
NO	Adcy1
NO	Igfbp3
NO	Tns3
NO	Hus1
NO	Ikzf1

NO	Grb10
NO	Cobl
NO	Sec61g
NO	Egfr
NO	Plek
NO	Cnrip1
NO	Ppp3r1
NO	Pno1
NO	Wdr92
NO	C1d
NO	Etaa1
NO	Meis1
NO	Spred2
NO	Actr2
NO	Rab1
NO	Cep68
NO	Slc1a4
NO	Sertad2
NO	Aftph
NO	Lgalsl
NO	Peli1
NO	Vps54
NO	Ugp2
NO	Mdh1
NO	Wdpcp
NO	Ehbp1
NO	Tmem17
NO	B3gnt2
NO	Commd1
NO	Zrsr1
NO	Cct4
NO	Fam161a
NO	Xpo1
NO	Usp34
NO	Ahsa2
NO	0610010F05Rik
NO	Pex13
NO	Pus10
NO	Rel
NO	Papolg
NO	Fancl
NO	Vrk2
NO	Gm12070
NO	Ccdc85a
NO	Efemp1
NO	Pnpt1
NO	Smek2
NO	Cfap36

NO	Ccdc88a
NO	Prorsd1
NO	Mtif2
NO	Rps27a
NO	Rtn4
NO	Eml6
NO	4931440F15Rik
NO	Sptbn1
NO	Acyp2
NO	Psme4
NO	Erlec1
NO	Asb3
NO	Chac2
NO	Stc2
NO	Bod1
NO	D630024D03Rik
NO	Cpeb4
NO	Snrnp25
NO	Rhbdf1
NO	Mpg
NO	Nprl3
NO	Sh3pxd2b
NO	Ubtd2
NO	Stk10
NO	Fbxw11
NO	Npm1
NO	Lcp2
NO	Dock2
NO	Fam196b
NO	Slit3
NO	Pank3
NO	Rars
NO	Tenm2
NO	Mat2b
NO	Nudcd2
NO	Ccng1
NO	Slu7
NO	C1qtnf2
NO	Pwwp2a
NO	Ttc1
NO	Ublcp1
NO	Rnf145
NO	Ebf1
NO	Clint1
NO	Lsm11
NO	Thg1l
NO	Adam19
NO	Cyfip2

NO	Med7
NO	Timd4
NO	Sgcd
NO	Gnb2l1
NO	Trim41
NO	Trim7
NO	Irgm1
NO	Psme2b
NO	9930111J21Rik1
NO	Tgtp1
NO	Tgtp2
NO	Ifi47
NO	Btnl9
NO	Zfp62
NO	Mgat1
NO	Flt4
NO	Cnot6
NO	Gfpt2
NO	Mapk9
NO	Rnf130
NO	Tbc1d9b
NO	3010026O09Rik
NO	Sqstm1
NO	Mgat4b
NO	Ltc4s
NO	Maml1
NO	Canx
NO	Hnrnph1
NO	Rufy1
NO	Adamts2
NO	Zfp354c
NO	Zfp879
NO	Zfp454
NO	Zfp2
NO	Zfp354b
NO	Zfp354a
NO	Clk4
NO	Col23a1
NO	Phykpl
NO	Hnrnpab
NO	Nhp2
NO	Rmnd5b
NO	N4bp3
NO	D930048N14Rik
NO	0610009B22Rik
NO	Sec24a
NO	Sar1b
NO	Jade2

NO	Cdkn2aipnl
NO	Ube2b
NO	Cdkl3
NO	Ppp2ca
NO	Skp1a
NO	Tcf7
NO	Vdac1
NO	9530068E07Rik
NO	Hspa4
NO	Zcchc10
NO	Aff4
NO	Uqcrq
NO	Sept8
NO	Kif3a
NO	Rad50
NO	Irf1
NO	Gm12216
NO	Slc22a5
NO	Slc22a21
NO	Slc22a4
NO	Pdlim4
NO	P4ha2
NO	Acsl6
NO	Fnip1
NO	Rapgef6
NO	Cdc42se2
NO	Lyrm7
NO	Hint1
NO	Gpx3
NO	Tnip1
NO	Anxa6
NO	Ccdc69
NO	Gm2a
NO	Slc36a2
NO	Slc36a1
NO	Sparc
NO	Atox1
NO	G3bp1
NO	Fam114a2
NO	Mfap3
NO	Galnt10
NO	Sap30l
NO	Larp1
NO	Cnot8
NO	Gemin5
NO	Mrpl22
NO	Igtp
NO	Irgm2

NO	Zfp692
NO	Zfp672
NO	Sh3bp5l
NO	2810021J22Rik
NO	Zfp39
NO	Rnf187
NO	Hist3h2a
NO	Trim11
NO	Obscn
NO	Iba57
NO	Guk1
NO	2610507I01Rik
NO	Mrpl55
NO	2310033P09Rik
NO	Arf1
NO	Wnt9a
NO	Snap47
NO	Jmjd4
NO	Zfp867
NO	Zkscan17
NO	4933439C10Rik
NO	Mprip
NO	1700007J10Rik
NO	Flcn
NO	Cops3
NO	Nt5m
NO	Med9
NO	Rasd1
NO	Pemt
NO	Rai1
NO	Srebf1
NO	Tom1l2
NO	Lrrc48
NO	Atpaf2
NO	Gid4
NO	Drg2
NO	Alkbh5
NO	Llgl1
NO	Flii
NO	Mief2
NO	Top3a
NO	Smcr8
NO	Shmt1
NO	Dhrs7b
NO	Tmem11
NO	Natd1
NO	Map2k3os
NO	Map2k3

NO	Kcnj12
NO	Usp22
NO	Aldh3a2
NO	Slc47a1
NO	Mfap4
NO	Mapk7
NO	B9d1
NO	Epn2
NO	Grap
NO	Prpsap2
NO	Ulk2
NO	Akap10
NO	Specc1
NO	Zswim7
NO	Ttc19
NO	Ncor1
NO	Pigl
NO	Cenpv
NO	Ubb
NO	Gm1821
NO	Trpv2
NO	2410006H16Rik
NO	Lrrc75a
NO	Mmgt2
NO	Zfp287
NO	Trim16
NO	Tvp23b
NO	Pmp22
NO	Hs3st3b1
NO	Cox10
NO	2810001G20Rik
NO	Elac2
NO	Arhgap44
NO	Myocd
NO	Map2k4
NO	Zkscan6
NO	Tmem220
NO	Adprm
NO	Sco1
NO	Myh3
NO	2310065F04Rik
NO	Myh2
NO	Myh1
NO	Myh4
NO	Myh8
NO	Myh13
NO	Gas7
NO	Dhrs7c

NO	Stx8
NO	Ntn1
NO	Pik3r5
NO	Pik3r6
NO	Myh10
NO	Ndel1
NO	Arhgef15
NO	Slc25a35
NO	Rangrf
NO	Pfas
NO	Ctc1
NO	2310047M10Rik
NO	Vamp2
NO	Per1
NO	Cntrob
NO	Trappc1
NO	Chd3
NO	Cyb5d1
NO	Naa38
NO	Tmem88
NO	Kdm6b
NO	Efnb3
NO	Wrap53
NO	Trp53
NO	Atp1b2
NO	Sat2
NO	Fxr2
NO	Mpdu1
NO	Cd68
NO	Eif4a1
NO	Senp3
NO	Tnfsf12
NO	Polr2a
NO	Zbtb4
NO	Chrnb1
NO	Fgf11
NO	Nlgn2
NO	Tmem256
NO	Plscr3
NO	Kctd11
NO	Acap1
NO	Neurl4
NO	Gps2
NO	Eif5a
NO	Ybx2
NO	Slc2a4
NO	Elp5
NO	Ctdnep1

NO	Gabarap
NO	Phf23
NO	Dvl2
NO	Acadvl
NO	Dlg4
NO	Mgl2
NO	Clec10a
NO	Slc16a13
NO	Bcl6b
NO	0610010K14Rik
NO	Rnasek
NO	Alox12
NO	Pelp1
NO	Arrb2
NO	Med11
NO	Cxcl16
NO	Psmb6
NO	Pld2
NO	Mink1
NO	Chrne
NO	4930544D05Rik
NO	Gp1ba
NO	Slc25a11
NO	Rnf167
NO	Pfn1
NO	Eno3
NO	Spag7
NO	Camta2
NO	Inca1
NO	Kif1c
NO	Zfp3
NO	Rabep1
NO	Nup88
NO	Rpain
NO	C1qbp
NO	Dhx33
NO	Derl2
NO	Mis12
NO	Wscd1
NO	Pitpnm3
NO	4933427D14Rik
NO	Txndc17
NO	Med31
NO	Xaf1
NO	Tekt1
NO	Smtnl2
NO	Mybbp1a
NO	Spns2

NO	Spns3
NO	Ube2g1
NO	Ankfy1
NO	Cyb5d2
NO	Zzef1
NO	Atp2a3
NO	Camkk1
NO	1200014J11Rik
NO	P2rx5
NO	Emc6
NO	Tax1bp3
NO	Ctns
NO	Shpk
NO	Aspa
NO	Rap1gap2
NO	Cluh
NO	Pafah1b1
NO	Mettl16
NO	Mnt
NO	Sgsm2
NO	Tsr1
NO	Srr
NO	Smg6
NO	Hic1
NO	Ovca2
NO	Dph1
NO	Rtn4rl1
NO	Rpa1
NO	Smyd4
NO	Serpinf1
NO	Wdr81
NO	Mir22hg
NO	Tlcd2
NO	Prpf8
NO	Rilp
NO	Scarf1
NO	Slc43a2
NO	Pitpna
NO	Gm12338
NO	Inpp5k
NO	Myo1c
NO	Crk
NO	Ywhae
NO	Doc2b
NO	Rph3al
NO	Fam101b
NO	Vps53
NO	Gemin4

NO	Glod4
NO	Rnmtl1
NO	Nxn
NO	Timm22
NO	Abr
NO	Tusc5
NO	Gosr1
NO	Cpd
NO	Blmh
NO	Slc6a4
NO	Ccdc55
NO	Ssh2
NO	Coro6
NO	Ankrd13b
NO	Git1
NO	Trp53i13
NO	Abhd15
NO	Taok1
NO	Nufip2
NO	Myo18a
NO	Phf12
NO	Dhrs13
NO	Flot2
NO	Eral1
NO	Fam222b
NO	Traf4
NO	Nek8
NO	Tlcd1
NO	Rpl23a
NO	Rab34
NO	Proca1
NO	Supt6
NO	Sdf2
NO	2610507B11Rik
NO	Spag5
NO	Pigs
NO	Unc119
NO	Vtn
NO	Tmem199
NO	Poldip2
NO	Tnfaip1
NO	Ift20
NO	Tmem97
NO	Nlk
NO	Fam58b
NO	Lyrm9
NO	Nos2
NO	Lgals9

NO	Ksr1
NO	Gm11201
NO	Wsb1
NO	Nf1
NO	Omg
NO	Evi2a
NO	Rab11fip4
NO	Utp6
NO	Suz12
NO	Crlf3
NO	Atad5
NO	Tefm
NO	Adap2
NO	Rnf135
NO	Rhot1
NO	Rhbdl3
NO	5730455P16Rik
NO	Zfp207
NO	Psmd11
NO	Myo1d
NO	Tmem98
NO	Ccl11
NO	Ccl8
NO	Zfp830
NO	Lig3
NO	Rffl
NO	Rad51d
NO	Nle1
NO	Unc45b
NO	Slfn5
NO	Slfn8
NO	Slfn2
NO	Slfn4
NO	Slfn3
NO	AI662270
NO	Pex12
NO	Ap2b1
NO	1700020L24Rik
NO	Mmp28
NO	Taf15
NO	Ccl9
NO	Ccl6
NO	Heatr6
NO	Ddx52
NO	Synrg
NO	Dusp14
NO	Tada2a
NO	Acaca

NO	Aatf
NO	Mrm1
NO	Dhrs11
NO	Ggnbp2
NO	Ggnbp2os
NO	Pigw
NO	Myo19
NO	Znhit3
NO	Car4
NO	Usp32
NO	Appbp2
NO	Ppm1d
NO	Bcas3
NO	Bcas3os1
NO	Tbx2
NO	Brip1os
NO	Ints2
NO	Med13
NO	Rnft1
NO	Rps6kb1
NO	Tubd1
NO	Vmp1
NO	Ptrh2
NO	Cltc
NO	Dhx40
NO	Ypel2
NO	Gdpd1
NO	Smg8
NO	Ska2
NO	Trim37
NO	Ppm1e
NO	Sept4
NO	Mtmr4
NO	Rnf43
NO	Supt4a
NO	Mpo
NO	Mks1
NO	Dynll2
NO	Srsf1
NO	Vezf1
NO	2210416O15Rik
NO	Cuedc1
NO	Mrps23
NO	Msi2
NO	0610039H22Rik
NO	C030037D09Rik
NO	Akap1
NO	Scpep1

NO	Coil
NO	Trim25
NO	Dgke
NO	Nog
NO	Pctp
NO	Tmem100
NO	Mmd
NO	Hlf
NO	Stxbp4
NO	Cox11
NO	Tom1l1
NO	Utp18
NO	Mbtd1
NO	Nme2
NO	Nme1
NO	Spag9
NO	Tob1
NO	Wfikkn2
NO	Luc7l3
NO	Ankrd40
NO	Abcc3
NO	Cacna1g
NO	Rsad1
NO	Acsf2
NO	Chad
NO	Lrrc59
NO	Mrpl27
NO	Xylt2
NO	Col1a1
NO	Sgca
NO	Hils1
NO	Ppp1r9b
NO	Samd14
NO	Pdk2
NO	Itga3
NO	Kat7
NO	Fam117a
NO	Slc35b1
NO	Spop
NO	Phb
NO	Zfp652os
NO	B130006D01Rik
NO	Zfp652
NO	Phospho1
NO	Abi3
NO	B4galnt2
NO	Snf8
NO	Ube2z

NO	Atp5g1
NO	Hoxb4
NO	Hoxb3
NO	Snx11
NO	Cbx1
NO	Gm11517
NO	Nfe2l1
NO	Copz2
NO	Cdk5rap3
NO	Pnpo
NO	D030028A08Rik
NO	Sp2
NO	Scrn2
NO	Mrpl10
NO	Osbpl7
NO	Tbx21
NO	Tbkbp1
NO	Kpnb1
NO	Npepps
NO	Mrpl45
NO	Socs7
NO	Arhgap23
NO	Srcin1
NO	Mllt6
NO	Cisd3
NO	Pcgf2
NO	Psmb3
NO	Pip4k2b
NO	Cwc25
NO	Rpl23
NO	Snora21
NO	Lasp1
NO	Plxdc1
NO	Cacnb1
NO	Rpl19
NO	Fbxl20
NO	Med1
NO	Cdk12
NO	Stard3
NO	Tcap
NO	Pgap3
NO	Erbb2
NO	Mien1
NO	Ikzf3
NO	Ormdl3
NO	Psmd3
NO	Med24
NO	Thra

NO	Msl1
NO	Casc3
NO	Wipf2
NO	Rara
NO	Top2a
NO	Igfbp4
NO	Smarce1
NO	Krt222
NO	Krt10
NO	Eif1
NO	Jup
NO	P3h4
NO	Fkbp10
NO	Nt5c3b
NO	Klhl11
NO	Acly
NO	Cnp
NO	Dnajc7
NO	Nkiras2
NO	Dhx58
NO	Kat2a
NO	Rab5c
NO	Ghdc
NO	Stat5b
NO	Stat5a
NO	Stat3
NO	Ptrf
NO	Atp6v0a1
NO	Naglu
NO	Coasy
NO	Mlx
NO	Fam134c
NO	Tubg1
NO	Tubg2
NO	Plekhh3
NO	Cntnap1
NO	Ezh1
NO	Ramp2
NO	Vps25
NO	Wnk4
NO	Coa3
NO	Becn1
NO	Psme3
NO	Aoc3
NO	Aarsd1
NO	Ptges3l
NO	Rundc1
NO	Rpl27

NO	Ifi35
NO	Vat1
NO	Rnd2
NO	Nbr1
NO	Tmem106a
NO	Rdm1
NO	Arl4d
NO	Dhx8
NO	Etv4
NO	Meox1
NO	Dusp3
NO	Mpp3
NO	Cd300lg
NO	Mpp2
NO	Tmem101
NO	Lsm12
NO	G6pc3
NO	Hdac5
NO	Asb16
NO	Tmub2
NO	Atxn7l3
NO	Ubtf
NO	Slc4a1
NO	Slc25a39
NO	Grn
NO	Fam171a2
NO	Itga2b
NO	Gpatch8
NO	Fzd2
NO	Ccdc43
NO	Adam11
NO	Gjc1
NO	Higd1b
NO	Eftud2
NO	Dcakd
NO	Nmt1
NO	Plcd3
NO	Acbd4
NO	Hexim1
NO	Hexim2
NO	Fmnl1
NO	Map3k14
NO	Arhgap27
NO	Arhgap27os3
NO	Plekhm1
NO	Gosr2
NO	C130046K22Rik
NO	Nsf

NO	Arf2
NO	Mapt
NO	Kansl1
NO	Cdc27
NO	Myl4
NO	Itgb3
NO	Mettl2
NO	Tlk2
NO	1700052K11Rik
NO	Mrc2
NO	Tanc2
NO	Cyb561
NO	Ace
NO	Dcaf7
NO	Taco1
NO	Map3k3
NO	Limd2
NO	Strada
NO	Ccdc47
NO	Ddx42
NO	Ftsj3
NO	Psmc5
NO	Smarcd2
NO	Scn4a
NO	Prr29
NO	Icam2
NO	Ern1
NO	Tex2
NO	Pecam1
NO	Polg2
NO	Ddx5
NO	Mir3064
NO	Cep95
NO	Smurf2
NO	Kpna2
NO	Bptf
NO	Nol11
NO	Pitpnc1
NO	Psmd12
NO	Helz
NO	Cacng1
NO	Prkca
NO	Cep112
NO	Axin2
NO	Gm11696
NO	Gna13
NO	Amz2
NO	Slc16a6

NO	Arsg
NO	Wipi1
NO	Prkar1a
NO	Fam20a
NO	Abca8b
NO	Abca8a
NO	Abca9
NO	Abca6
NO	Abca5
NO	Map2k6
NO	Kcnj2
NO	Sox9
NO	Slc39a11
NO	Cog1
NO	Fam104a
NO	D11Wsu47e
NO	Cdc42ep4
NO	Sdk2
NO	Rpl38
NO	Ttyh2
NO	Kif19a
NO	Gprc5c
NO	Cd300a
NO	Slc9a3r1
NO	Nat9
NO	Tmem104
NO	Fdxr
NO	Fads6
NO	Hid1
NO	Cdr2l
NO	Ict1
NO	Atp5h
NO	Kctd2
NO	Armc7
NO	Nt5c
NO	Hn1
NO	Sumo2
NO	Nup85
NO	Gga3
NO	Mrps7
NO	Mif4gd
NO	Slc25a19
NO	Grb2
NO	2310067B10Rik
NO	Caskin2
NO	Tsen54
NO	Llgl2
NO	Recql5

NO	Smim5
NO	Sap30bp
NO	Itgb4
NO	Galk1
NO	H3f3b
NO	Unk
NO	Unc13d
NO	Wbp2
NO	Trim47
NO	Trim65
NO	Mrpl38
NO	Fbf1
NO	Acox1
NO	Ten1
NO	Srp68
NO	Exoc7
NO	Rnf157
NO	Prpsap1
NO	Sphk1
NO	Ube2o
NO	Rhbdf2
NO	Cygb
NO	1810032O08Rik
NO	St6galnac2
NO	Mxra7
NO	Jmjd6
NO	Mettl23
NO	Srsf2
NO	Mfsd11
NO	Snhg20
NO	Mir6516
NO	Sec14l1
NO	Sept9
NO	Tnrc6c
NO	Tmc6
NO	Tmc8
NO	Syngr2
NO	Tk1
NO	Afmid
NO	Tha1
NO	Socs3
NO	Pgs1
NO	Dnah17
NO	Cyth1
NO	Usp36
NO	Timp2
NO	BC100451
NO	Lgals3bp

NO	Cant1
NO	C1qtnf1
NO	Engase
NO	Cbx2
NO	Cbx8
NO	Cbx4
NO	Tbc1d16
NO	Gaa
NO	Eif4a3
NO	Sgsh
NO	Slc26a11
NO	Endov
NO	Rptor
NO	Chmp6
NO	Baiap2
NO	Aatk
NO	Cep131
NO	Enthd2
NO	1810043H04Rik
NO	Slc38a10
NO	2810410L24Rik
NO	Bahcc1
NO	Actg1
NO	0610009L18Rik
NO	Fscn2
NO	Faap100
NO	Nploc4
NO	Oxld1
NO	Ccdc137
NO	Arl16
NO	Hgs
NO	Mrpl12
NO	Slc25a10
NO	Fam195b
NO	Ppp1r27
NO	P4hb
NO	Arhgdia
NO	Alyref
NO	Anapc11
NO	Pcyt2
NO	Sirt7
NO	Mafg
NO	Pycr1
NO	Myadml2
NO	Notum
NO	Aspscr1
NO	Stra13
NO	Lrrc45

NO	Dcxr
NO	Cbr2
NO	Rfng
NO	Gps1
NO	Dus1l
NO	Fasn
NO	Ccdc57
NO	Slc16a3
NO	Csnk1d
NO	Uts2r
NO	Ogfod3
NO	Hexdc
NO	BC017643
NO	Narf
NO	Foxk2
NO	Wdr45b
NO	Rab40b
NO	Fn3krp
NO	Fn3k
NO	Tbcd
NO	Zfp750
NO	B3gntl1
NO	Metrnl
NO	Rab10os
NO	Rab10
NO	Kif3c
NO	1110002L01Rik
NO	Asxl2
NO	Dtnb
NO	Dnmt3a
NO	Dnmt3aos
NO	Efr3b
NO	Dnajc27
NO	Adcy3
NO	Cenpo
NO	Ptrhd1
NO	Ncoa1
NO	Itsn2
NO	Sf3b6
NO	BC068281
NO	Mfsd2b
NO	Ubxn2a
NO	Atad2b
NO	Klhl29
NO	Apob
NO	Ldah
NO	Hs1bp3
NO	Rhob

NO	Slc7a15
NO	Pum2
NO	Sdc1
NO	Laptm4a
NO	Wdr35
NO	Ttc32
NO	Osr1
NO	Rdh14
NO	Kcns3
NO	9530020I12Rik
NO	Gen1
NO	Smc6
NO	Fam49a
NO	Mycn
NO	Ddx1
NO	Nbas
NO	Trib2
NO	Lpin1
NO	Ntsr2
NO	E2f6
NO	Rock2
NO	Pqlc3
NO	Kcnf1
NO	Pdia6
NO	Nol10
NO	Odc1
NO	Hpcal1
NO	Asap2
NO	Itgb1bp1
NO	Cpsf3
NO	Iah1
NO	Adam17
NO	Ywhaq
NO	Taf1b
NO	Grhl1
NO	Klf11
NO	Cys1
NO	Rrm2
NO	Kidins220
NO	Id2
NO	Rnf144a
NO	Rsad2
NO	Cmpk2
NO	Rps7
NO	Rnaseh1
NO	Adi1
NO	Trappc12
NO	Tssc1

NO	Pxdn
NO	2310016D03Rik
NO	Tmem18
NO	Acp1
NO	Lamb1
NO	Dld
NO	Cbll1
NO	Bcap29
NO	Dus4l
NO	Cog5
NO	Gpr22
NO	Hbp1
NO	Prkar2b
NO	Pik3cg
NO	Ccdc71l
NO	Nampt
NO	Gdap10
NO	Sypl
NO	Cdhr3
NO	Atxn7l1
NO	Twistnb
NO	Twist1
NO	Hdac9
NO	Snx13
NO	Ahr
NO	Tspan13
NO	Bzw2
NO	Ankmy2
NO	Ispd
NO	Meox2
NO	Agmo
NO	Dgkb
NO	Etv1
NO	Arl4a
NO	Scin
NO	Lsmem1
NO	Ifrd1
NO	Zfp277
NO	Dock4
NO	Immp2l
NO	Lrrn3
NO	Dnajb9
NO	Pnpla8
NO	Stxbp6
NO	Nova1
NO	Prkd1
NO	G2e3
NO	Scfd1

NO	Coch
NO	Strn3
NO	Ap4s1
NO	Hectd1
NO	Heatr5a
NO	Dtd2
NO	Nubpl
NO	Arhgap5
NO	Akap6
NO	Egln3
NO	Sptssa
NO	Eapp
NO	Snx6
NO	Cfl2
NO	Baz1a
NO	2700097O09Rik
NO	Ppp2r3c
NO	1110008L16Rik
NO	Psma6
NO	Nfkbia
NO	Ralgapa1
NO	Brms1l
NO	Mbip
NO	Prps1l3
NO	Mipol1
NO	Clec14a
NO	Sec23a
NO	Gemin2
NO	Trappc6b
NO	Pnn
NO	Ctage5
NO	Fbxo33
NO	Gm527
NO	Klhl28
NO	Fam179b
NO	Prpf39
NO	Fkbp3
NO	Fancm
NO	Rps29
NO	Rpl36al
NO	Mgat2
NO	Dnaaf2
NO	9330151L19Rik
NO	Klhdc1
NO	Klhdc2
NO	Nemf
NO	Arf6
NO	Vcpkmt

NO	Sos2
NO	L2hgdh
NO	Atp5s
NO	Map4k5
NO	4931403G20Rik
NO	Atl1
NO	Sav1
NO	Nin
NO	Pygl
NO	Tmx1
NO	Frmd6
NO	Actr10
NO	Psma3
NO	3110056K07Rik
NO	Arid4a
NO	Timm9
NO	2700049A03Rik
NO	Dact1
NO	Daam1
NO	L3hypdh
NO	Jkamp
NO	Rtn1
NO	Pcnxl4
NO	Dhrs7
NO	Ppm1a
NO	Six1
NO	Six4
NO	Mnat1
NO	Trmt5
NO	Slc38a6
NO	Prkch
NO	Hif1a
NO	Snapc1
NO	Rhoj
NO	Ppp2r5e
NO	Sgpp1
NO	Syne2
NO	Esr2
NO	Mthfd1
NO	Akap5
NO	Zbtb25
NO	Zbtb1
NO	Hspa2
NO	Plekhg3
NO	Sptb
NO	Churc1
NO	Rab15
NO	Fntb

NO	Max
NO	Fut8
NO	Gphn
NO	Mpp5
NO	Atp6v1d
NO	Eif2s1
NO	Tmem229b
NO	Plekhh1
NO	Vti1b
NO	Rdh11
NO	Zfyve26
NO	Zfp36l1
NO	2310015A10Rik
NO	Actn1
NO	Dcaf5
NO	Exd2
NO	Galnt16
NO	Erh
NO	Slc39a9
NO	Susd6
NO	Srsf5
NO	Slc10a1
NO	Smoc1
NO	Slc8a3
NO	Cox16
NO	Gm4787
NO	Adam4
NO	Synj2bp
NO	Med6
NO	Ttc9
NO	Map3k9
NO	Pcnx
NO	Sipa1l1
NO	Dpf3
NO	Dcaf4
NO	Zfyve1
NO	Rbm25
NO	Psen1
NO	Papln
NO	Numb
NO	2410016O06Rik
NO	Acot2
NO	Acot6
NO	Dnal1
NO	Elmsan1
NO	Ptgr2
NO	Zfp410
NO	Fam161b

NO	Coq6
NO	Entpd5
NO	Ccdc176
NO	Rnf113a2
NO	Aldh6a1
NO	Lin52
NO	Abcd4
NO	Npc2
NO	Isca2
NO	Ltbp2
NO	Arel1
NO	Fcf1
NO	Ylpm1
NO	Prox2
NO	Dlst
NO	Rps6kl1
NO	Pgf
NO	Eif2b2
NO	Mlh3
NO	Acyp1
NO	Zc2hc1c
NO	Nek9
NO	Tmed10
NO	Fos
NO	Jdp2
NO	0610007P14Rik
NO	Ttll5
NO	Tgfb3
NO	Ift43
NO	Gpatch2l
NO	Esrrb
NO	Vash1
NO	Angel1
NO	Irf2bpl
NO	Cipc
NO	Pomt2
NO	Gstz1
NO	Tmed8
NO	Vipas39
NO	Ahsa1
NO	Sptlc2
NO	Alkbh1
NO	Slirp
NO	Snw1
NO	Adck1
NO	3200001D21Rik
NO	Dio2
NO	Cep128

NO	Tshr
NO	Gtf2a1
NO	Ston2
NO	Sel1l
NO	Flrt2
NO	Galc
NO	Spata7
NO	Ptpn21
NO	Zc3h14
NO	Eml5
NO	Ttc8
NO	Foxn3
NO	Tdp1
NO	Psmc1
NO	Nrde2
NO	Calm1
NO	Ttc7b
NO	Rps6ka5
NO	9030617O03Rik
NO	Gpr68
NO	Ccdc88c
NO	Smek1
NO	D130020L05Rik
NO	Fbln5
NO	Trip11
NO	Atxn3
NO	Cpsf2
NO	Rin3
NO	Lgmn
NO	Golga5
NO	Itpk1
NO	Tmem251
NO	AK010878
NO	Ubr7
NO	Btbd7
NO	Prima1
NO	Asb2
NO	Otub2
NO	Ddx24
NO	Ifi27l2a
NO	Ifi27
NO	Serpina1b
NO	Serpina3c
NO	Serpina3g
NO	Serpina3n
NO	Dicer1
NO	Clmn
NO	Syne3

NO	Scarna13
NO	Glrx5
NO	D430019H16Rik
NO	Atg2b
NO	Gskip
NO	Papola
NO	Vrk1
NO	Setd3
NO	Ccnk
NO	Ccdc85c
NO	Hhipl1
NO	Eml1
NO	Evl
NO	Yy1
NO	Slc25a29
NO	Slc25a47
NO	Wars
NO	Wdr25
NO	Dlk1
NO	Meg3
NO	Rtl1
NO	Rian
NO	Ppp2r5c
NO	Dync1h1
NO	1700001K19Rik
NO	Hsp90aa1
NO	Wdr20
NO	Zfp839
NO	Cinp
NO	Tecpr2
NO	Ankrd9
NO	Rcor1
NO	Traf3
NO	Cdc42bpb
NO	Exoc3l4
NO	Tnfaip2
NO	Eif5
NO	Snora28
NO	Mark3
NO	Ckb
NO	Trmt61a
NO	Bag5
NO	Apopt1
NO	Klc1
NO	Xrcc3
NO	Zfyve21
NO	Ppp1r13b
NO	2010107E04Rik

NO	Kif26a
NO	A530016L24Rik
NO	Tmem179
NO	Inf2
NO	Adssl1
NO	Siva1
NO	Akt1
NO	Zbtb42
NO	Cep170b
NO	Pld4
NO	Cdca4
NO	Jag2
NO	Nudt14
NO	Brf1
NO	Btbd6
NO	Pacs2
NO	Mta1
NO	Crip2
NO	Crip1
NO	Zfp386
NO	Vipr2
NO	Wdr60
NO	Esyt2
NO	Ncapg2
NO	Rapgef5
NO	Sp4
NO	Itgb8
NO	Gdi2
NO	Fam208b
NO	Asb13
NO	Net1
NO	Akr1c14
NO	Akr1e1
NO	Klf6
NO	Pitrm1
NO	Pfkp
NO	Wdr37
NO	Idi1
NO	Gtpbp4
NO	Larp4b
NO	Dip2c
NO	Zmynd11
NO	Chrm3
NO	Ryr2
NO	Gm10336
NO	Mtr
NO	Actn2
NO	Heatr1

NO	Lgals8
NO	Ero1lb
NO	Gpr137b-ps
NO	Gpr137b
NO	Nid1
NO	Lyst
NO	B3galnt2
NO	Tbce
NO	Ggps1
NO	Arid4b
NO	Mrpl32
NO	Psma2
NO	AW209491
NO	Gli3
NO	Mplkip
NO	Cdk13
NO	Rala
NO	Yae1d1
NO	Vps41
NO	Stard3nl
NO	Epdr1
NO	Sfrp4
NO	Elmo1
NO	Trim27
NO	Zscan12
NO	Zkscan3
NO	Pgbd1
NO	Zscan26
NO	Zkscan8
NO	Hist1h4j
NO	Hist1h4k
NO	Hist1h2ak
NO	Hist1h2bn
NO	Hist1h1b
NO	Hist1h3i
NO	Hist1h4n
NO	Hist1h4i
NO	Zfp322a
NO	Abt1
NO	Hist1h4h
NO	Hist1h3f
NO	Hist1h4f
NO	Hist1h1d
NO	Hist1h3e
NO	Hist1h3d
NO	Hist1h4d
NO	Hist1h2be
NO	Hist1h1e

NO	Hist1h2bc
NO	Hist1h4c
NO	Hfe
NO	Hist1h1c
NO	Hist1h3c
NO	Hist1h2bb
NO	Hist1h4a
NO	Hist1h1a
NO	Lrrc16a
NO	Cmah
NO	Fam65b
NO	Gmnn
NO	BC005537
NO	Acot13
NO	Tdp2
NO	Aldh5a1
NO	Gpld1
NO	Mrs2
NO	2610307P16Rik
NO	Sox4
NO	Cdkal1
NO	E2f3
NO	Mboat1
NO	Agtr1a
NO	Uqcrfs1
NO	Dusp22
NO	Irf4
NO	Exoc2
NO	Foxq1
NO	Foxc1
NO	Gmds
NO	Mylk4
NO	Wrnip1
NO	Serpinb1a
NO	Serpinb1c
NO	Serpinb9
NO	Serpinb6a
NO	Nqo2
NO	Ripk1
NO	Bphl
NO	Tubb2a
NO	Tubb2b
NO	Psmg4
NO	Slc22a23
NO	Pxdc1
NO	Prpf4b
NO	Eci2
NO	Cdyl

NO	Rpp40
NO	Lyrm4
NO	Fars2
NO	F13a1
NO	Rreb1
NO	Ssr1
NO	Riok1
NO	Snrnp48
NO	Bmp6
NO	Txndc5
NO	Bloc1s5
NO	Eef1e1
NO	Slc35b3
NO	Gcnt2
NO	Pak1ip1
NO	Tmem14c
NO	Smim13
NO	Nedd9
NO	Tmem170b
NO	Hivep1
NO	Phactr1
NO	Tbc1d7
NO	Gfod1
NO	Sirt5
NO	Nol7
NO	Ranbp9
NO	Mcur1
NO	Cd83
NO	Jarid2
NO	Dtnbp1
NO	Mylip
NO	Gmpr
NO	Atxn1
NO	Rbm24
NO	Cap2
NO	Fam8a1
NO	Nup153
NO	Kif13a
NO	Nhlrc1
NO	Tpmt
NO	Kdm1b
NO	Dek
NO	Rnf144b
NO	Zfp169
NO	Ptpdc1
NO	Phf2
NO	Fam120a
NO	Fam120aos

NO	Wnk2
NO	Ninj1
NO	1110007C09Rik
NO	Fgd3
NO	Bicd2
NO	Ippk
NO	Cenpp
NO	Ecm2
NO	Aspn
NO	Omd
NO	Ogn
NO	Nol8
NO	Iars
NO	Fbxw17
NO	Spin1
NO	S1pr3
NO	Secisbp2
NO	Sema4d
NO	Gadd45g
NO	Syk
NO	Auh
NO	Nfil3
NO	Ror2
NO	Sptlc1
NO	Sfxn1
NO	Cplx2
NO	Thoc3
NO	Simc1
NO	4833439L19Rik
NO	Arl10
NO	Nop16
NO	Higd2a
NO	Cltb
NO	Faf2
NO	Rnf44
NO	Tspan17
NO	Unc5a
NO	Hk3
NO	Uimc1
NO	Zfp346
NO	Fgfr4
NO	Nsd1
NO	Rab24
NO	Prelid1
NO	Lman2
NO	Grk6
NO	Dbn1
NO	Pdlim7

NO	Dok3
NO	Ddx41
NO	Fam193b
NO	Tmed9
NO	B4galt7
NO	Caml
NO	Ddx46
NO	B230219D22Rik
NO	Txndc15
NO	Pcbd2
NO	Pitx1
NO	H2afy
NO	Tifab
NO	Cxcl14
NO	Tgfbi
NO	Smad5
NO	Klhl3
NO	Hnrnpa0
NO	Idnk
NO	Ubqln1
NO	Gkap1
NO	2210016F16Rik
NO	Hnrnpk
NO	Rmi1
NO	Ntrk2
NO	Agtpbp1
NO	Naa35
NO	Golm1
NO	Isca1
NO	Etohd2
NO	Zcchc6
NO	Gas1
NO	Dapk1
NO	Ctla2a
NO	Zfp935
NO	Fbp2
NO	2010111I01Rik
NO	Gm16907
NO	Fancc
NO	Ptch1
NO	Ercc6l2
NO	Slc35d2
NO	Zfp367
NO	Habp4
NO	Cdc14b
NO	1810034E14Rik
NO	Aaed1
NO	Ctsl

NO	Cdk20
NO	Hiatl1
NO	Zfp369
NO	Uqcrb
NO	Mterf3
NO	Ptdss1
NO	Zfp712
NO	Zfp708
NO	Rslcan18
NO	Zfp759
NO	Zfp458
NO	Zfp457
NO	Zfp595
NO	Zfp953
NO	Zfp874a
NO	Zfp874b
NO	Zfp58
NO	Zfp87
NO	Zfp748
NO	Zfp729b
NO	Zfp729a
NO	Zfp738
NO	Zfp65
NO	Zfp85
NO	Zfp493
NO	Mtrr
NO	Fastkd3
NO	Adcy2
NO	Papd7
NO	Nsun2
NO	Ube2ql1
NO	Med10
NO	Ice1
NO	Ndufs6
NO	Mrpl36
NO	Lpcat1
NO	Clptm1l
NO	Slc12a7
NO	Nkd2
NO	Brd9
NO	Tppp
NO	Exoc3
NO	Pdcd6
NO	Sdha
NO	Ccdc127
NO	Lrrc14b
NO	Zfp72
NO	Zfp825

NO	Erap1
NO	Cast
NO	Mir682
NO	Ell2
NO	Glrx
NO	Rhobtb3
NO	Rfesd
NO	Arsk
NO	Ttc37
NO	Mctp1
NO	Ankrd32
NO	2210408I21Rik
NO	Fam172a
NO	Pou5f2
NO	Arrdc3
NO	Lysmd3
NO	Polr3g
NO	Mblac2
NO	Cetn3
NO	Mef2c
NO	Tmem161b
NO	Ccnh
NO	Rasa1
NO	Cox7c
NO	Vcan
NO	Xrcc4
NO	Tmem167
NO	Rps23
NO	Atg10
NO	Ssbp2
NO	Zcchc9
NO	Ckmt2
NO	Rasgrf2
NO	Msh3
NO	Dhfr
NO	Fam151b
NO	Zfyve16
NO	Serinc5
NO	Thbs4
NO	Mtx3
NO	Cmya5
NO	Gm4814
NO	A630019I02Rik
NO	Papd4
NO	Homer1
NO	Jmy
NO	Arsb
NO	Lhfpl2

NO	Scamp1
NO	Ap3b1
NO	Tbca
NO	Wdr41
NO	Pde8b
NO	Zbed3
NO	Aggf1
NO	F2r
NO	Iqgap2
NO	Poc5
NO	Polk
NO	Col4a3bp
NO	Hmgcr
NO	Nsa2
NO	Gfm2
NO	Hexb
NO	Enc1
NO	Arhgef28
NO	Utp15
NO	Ankra2
NO	Btf3
NO	Fcho2
NO	Tnpo1
NO	2310020H05Rik
NO	Zfp366
NO	Ptcd2
NO	Mrps27
NO	Map1b
NO	Mccc2
NO	Bdp1
NO	Serf1
NO	Smn1
NO	Naip2
NO	Naip5
NO	Gtf2h2
NO	Marveld2
NO	Rad17
NO	Ak6
NO	Taf9
NO	Ccdc125
NO	Cdk7
NO	Mrps36
NO	Slc30a5
NO	Pik3r1
NO	Mast4
NO	Srek1
NO	Erbb2ip
NO	Nln

NO	Sgtb
NO	Trappc13
NO	Trim23
NO	Ppwd1
NO	Adamts6
NO	Cwc27
NO	Srek1ip1
NO	Rgs7bp
NO	Ipo11
NO	Dimt1
NO	Kif2a
NO	Zswim6
NO	Smim15
NO	Ndufaf2
NO	Ercc8
NO	Elovl7
NO	Pde4d
NO	Mir1904
NO	Plk2
NO	Gpbp1
NO	Mier3
NO	Map3k1
NO	Il6st
NO	Slc38a9
NO	Ppap2a
NO	Skiv2l2
NO	Dhx29
NO	Gpx8
NO	Esm1
NO	Snx18
NO	Hspb3
NO	Arl15
NO	A430090L17Rik
NO	Ndufs4
NO	Fst
NO	Mocs2
NO	Itga2
NO	Itga1
NO	Pelo
NO	Parp8
NO	Emb
NO	Mrps30
NO	Fgf10
NO	Nnt
NO	Paip1
NO	4833420G17Rik
NO	Gm7120
NO	Hmgcs1

NO	Nim1k
NO	Zfp131
NO	Gm21188
NO	Flnb
NO	Abhd6
NO	Rpp14
NO	Pxk
NO	Pdhb
NO	Kctd6
NO	Fam107a
NO	Ptprg
NO	3830406C13Rik
NO	Thoc7
NO	Atxn7
NO	Psmd6
NO	Il3ra
NO	Slc4a7
NO	Oxsm
NO	Ngly1
NO	Top2b
NO	Rarb
NO	Thrb
NO	Nr1d2
NO	Rpl15
NO	Nkiras1
NO	Ube2e1
NO	Ube2e2
NO	Nid2
NO	2700060E02Rik
NO	Gng2
NO	Saysd1
NO	Kcnk5
NO	Nudt13
NO	Ecd
NO	Fam149b
NO	Dnajc9
NO	Mrps16
NO	Anxa7
NO	Mss51
NO	Ppp3cb
NO	Usp54
NO	Myoz1
NO	Synpo2l
NO	Sec24c
NO	Fut11
NO	6230400D17Rik
NO	Chchd1
NO	Zswim8

NO	Ndst2
NO	Camk2g
NO	Plau
NO	Vcl
NO	Ap3m1
NO	Adk
NO	Kat6b
NO	Dupd1
NO	Dusp13
NO	Samd8
NO	Vdac2
NO	Comtd1
NO	Zfp503
NO	1700112E06Rik
NO	Kcnma1
NO	Dlg5
NO	Polr3a
NO	Rps24
NO	Zmiz1
NO	4931406H21Rik
NO	Israa
NO	Ppif
NO	Zcchc24
NO	Anxa11
NO	Slmap
NO	Dennd6a
NO	Arf4
NO	Pde12
NO	Asb14
NO	Appl1
NO	Il17rd
NO	Arhgef3
NO	Fam208a
NO	Ccdc66
NO	Erc2
NO	Wnt5a
NO	Cacna2d3
NO	Lrtm1
NO	Selk
NO	Actr8
NO	Chdh
NO	Cacna1d
NO	Dcp1a
NO	Tkt
NO	Prkcd
NO	Rft1
NO	Sfmbt1
NO	Tmem110

NO	Mustn1
NO	Nek4
NO	Spcs1
NO	Glt8d1
NO	Gnl3
NO	Pbrm1
NO	Smim4
NO	Stab1
NO	Nisch
NO	Tnnc1
NO	Sema3g
NO	Phf7
NO	Bap1
NO	Capn7
NO	Sh3bp5
NO	Mettl6
NO	Eaf1
NO	Colq
NO	Hacl1
NO	Btd
NO	Ankrd28
NO	Galnt15
NO	Dph3
NO	Oxnad1
NO	Ncoa4
NO	Timm23
NO	Parg
NO	Ercc6
NO	3425401B19Rik
NO	Vstm4
NO	Wdfy4
NO	Mapk8
NO	Gdf10
NO	Anxa8
NO	Syt15
NO	Fam35a
NO	Glud1
NO	Sncg
NO	Mmrn2
NO	Bmpr1a
NO	Ldb3
NO	Wapal
NO	Ccser2
NO	Ghitm
NO	Tspan14
NO	Fam213a
NO	Txndc16
NO	Gpr137c

NO	Ero1l
NO	Psmc6
NO	Styx
NO	Gnpnat1
NO	Fermt2
NO	Ddhd1
NO	Bmp4
NO	Cnih1
NO	Gmfb
NO	Cgrrf1
NO	Samd4
NO	Gch1
NO	Wdhd1
NO	Socs4
NO	Mapk1ip1l
NO	Lgals3
NO	Fbxo34
NO	Atg14
NO	Ktn1
NO	Peli2
NO	Gm6498
NO	Tmem260
NO	Exoc5
NO	Ap5m1
NO	Naa30
NO	3632451O06Rik
NO	Ttc5
NO	Rpph1
NO	Parp2
NO	Tep1
NO	Klhl33
NO	Osgep
NO	Apex1
NO	Tmem55b
NO	Pnp
NO	Rnase4
NO	Mettl17
NO	Ndrg2
NO	Tppp2
NO	Rnase13
NO	Arhgef40
NO	Zfp219
NO	Tmem253
NO	Hnrnpc
NO	Supt16
NO	Chd8
NO	Rab2b
NO	Tox4

NO	Mettl3
NO	Sall2
NO	Dad1
NO	Abhd4
NO	Oxa1l
NO	Slc7a7
NO	Mrpl52
NO	Mmp14
NO	Lrp10
NO	Prmt5
NO	Haus4
NO	Ajuba
NO	4931414P19Rik
NO	Psmb5
NO	Acin1
NO	4930579G18Rik
NO	1700123O20Rik
NO	Slc7a8
NO	Homez
NO	Bcl2l2
NO	Pabpn1
NO	Slc22a17
NO	Efs
NO	Ngdn
NO	Zfhx2
NO	Thtpa
NO	Ap1g2
NO	Dhrs4
NO	Pck2
NO	Dcaf11
NO	Fitm1
NO	Psme1
NO	Emc9
NO	Psme2
NO	Rnf31
NO	Irf9
NO	Ipo4
NO	Tm9sf1
NO	Mdp1
NO	Nedd8
NO	Gmpr2
NO	Tinf2
NO	Rabggta
NO	Dhrs1
NO	Nop9
NO	Adcy4
NO	Ripk3
NO	Nfatc4

NO	Nynrin
NO	Khnyn
NO	Sdr39u1
NO	Cma1
NO	Gm5801
NO	Mcpt4
NO	Cenpj
NO	Parp4
NO	Mphosph8
NO	Gm16973
NO	Pspc1
NO	Zmym5
NO	Zmym2
NO	Cryl1
NO	Ift88
NO	Il17d
NO	N6amt2
NO	Xpo4
NO	Lats2
NO	Sap18
NO	Mrpl57
NO	Zdhhc20
NO	Micu2
NO	Fgf9
NO	Rcbtb1
NO	Phf11d
NO	Setdb2
NO	Cab39l
NO	Cdadc1
NO	Shisa2
NO	Atp8a2
NO	Nupl1
NO	Mtmr6
NO	Spata13
NO	C1qtnf9
NO	Mipep
NO	Tnfrsf19
NO	Sacs
NO	Sgcg
NO	Ebpl
NO	Kpna3
NO	Spryd7
NO	Trim13
NO	Dleu2
NO	Kcnrg
NO	Rnaseh2b
NO	Fam124a
NO	Ints6

NO	Wdfy2
NO	Ctsb
NO	Fdft1
NO	Fam167a
NO	Mtmr9
NO	Pinx1
NO	Sox7
NO	Msra
NO	Kif13b
NO	Hmbox1
NO	Ints9
NO	Extl3
NO	Fzd3
NO	Zfp395
NO	Elp3
NO	Scara5
NO	Ccdc25
NO	Scara3
NO	Clu
NO	Ephx2
NO	Ptk2b
NO	Trim35
NO	Adra1a
NO	Dpysl2
NO	Bnip3l
NO	Ppp2r2a
NO	Ebf2
NO	Gm6878
NO	Kctd9
NO	Dock5
NO	Stc1
NO	Slc25a37
NO	Entpd4
NO	Loxl2
NO	R3hcc1
NO	Chmp7
NO	4930480K23Rik
NO	Tnfrsf10b
NO	Rhobtb2
NO	Egr3
NO	Bin3
NO	Ccar2
NO	Pdlim2
NO	Sorbs3
NO	Ppp3cc
NO	Slc39a14
NO	Piwil2
NO	Polr3d

NO	Bmp1
NO	Reep4
NO	Hr
NO	Nudt18
NO	Fam160b2
NO	Dmtn
NO	Xpo7
NO	Dok2
NO	Gfra2
NO	Fndc3a
NO	Rcbtb2
NO	Rb1
NO	Lpar6
NO	Itm2b
NO	Med4
NO	Nudt15
NO	Sucla2
NO	Esd
NO	Lrch1
NO	Lcp1
NO	Zc3h13
NO	Cog3
NO	Slc25a30
NO	Gm4285
NO	Tpt1
NO	Snora31
NO	Gtf2f2
NO	Gpalpp1
NO	Nufip1
NO	Tsc22d1
NO	Enox1
NO	Dnajc15
NO	Epsti1
NO	Akap11
NO	Dgkh
NO	Vwa8
NO	Rgcc
NO	Naa16
NO	Mtrf1
NO	Kbtbd7
NO	Wbp4
NO	Elf1
NO	Sugt1
NO	Pcdh17
NO	Tdrd3
NO	Gm5088
NO	Pcdh9
NO	Dach1

NO	Mzt1
NO	Bora
NO	Dis3
NO	Pibf1
NO	Klf5
NO	Klf12
NO	Tbc1d4
NO	Commd6
NO	Uchl3
NO	Lmo7
NO	Kctd12
NO	Cln5
NO	Fbxl3
NO	Mycbp2
NO	Ednrb
NO	Pou4f1
NO	Rnf219
NO	Gm17066
NO	Rbm26
NO	Ndfip2
NO	Spry2
NO	Mir17hg
NO	Gpc6
NO	Tgds
NO	Gpr180
NO	Abcc4
NO	Dzip1
NO	Dnajc3
NO	Uggt2
NO	Mbnl2
NO	Rap2a
NO	Ipo5
NO	Farp1
NO	Stk24
NO	Dock9
NO	Ubac2
NO	Gpr18
NO	Gpr183
NO	Tm9sf2
NO	Clybl
NO	Pcca
NO	Ggact
NO	Tmtc4
NO	Itgbl1
NO	Sepp1
NO	Ccdc152
NO	Ghr
NO	Fbxo4

NO	AW549877
NO	A630020A06
NO	BC037032
NO	Oxct1
NO	C6
NO	C7
NO	Card6
NO	Rpl37
NO	Prkaa1
NO	Ttc33
NO	Ptger4
NO	Dab2
NO	Fyb
NO	Rictor
NO	Osmr
NO	Lifr
NO	Egflam
NO	Gdnf
NO	Wdr70
NO	Nup155
NO	2410089E03Rik
NO	Nipbl
NO	Slc1a3
NO	Nadk2
NO	Skp2
NO	Lmbrd2
NO	Prlr
NO	Dnajc21
NO	Brix1
NO	Rad1
NO	Rai14
NO	4930556M19Rik
NO	Amacr
NO	Adamts12
NO	Tars
NO	Npr3
NO	1700047G03Rik
NO	Sub1
NO	Zfr
NO	Mtmr12
NO	Golph3
NO	Pdzd2
NO	6030458C11Rik
NO	Drosha
NO	Basp1
NO	Myo10
NO	Fam134b
NO	Zfp622

NO	Fbxl7
NO	Ank
NO	Otulin
NO	Fam105a
NO	Trio
NO	Dap
NO	Ankrd33b
NO	March6
NO	Cmbl
NO	Cct5
NO	Fam173b
NO	Snhg18
NO	Sema5a
NO	Sdc2
NO	Cpq
NO	Mtdh
NO	Laptm4b
NO	Matn2
NO	Hrsp12
NO	Pop1
NO	Stk3
NO	Osr2
NO	Vps13b
NO	Cox6c
NO	Polr2k
NO	Spag1
NO	Rnf19a
NO	Ankrd46
NO	Pabpc1
NO	Ywhaz
NO	Zfp706
NO	Ncald
NO	Rrm2b
NO	Ubr5
NO	Klf10
NO	Azin1
NO	Atp6v1c1
NO	Fzd6
NO	Slc25a32
NO	Dcaf13
NO	Lrp12
NO	Zfpm2
NO	Oxr1
NO	Abra
NO	Angpt1
NO	Eif3e
NO	Emc2
NO	Nudcd1

NO	Eny2
NO	Pkhd1l1
NO	Ebag9
NO	Sybu
NO	Trps1
NO	Eif3h
NO	Utp23
NO	Rad21
NO	Med30
NO	Ext1
NO	Nov
NO	Enpp2
NO	Taf2
NO	Deptor
NO	Col14a1
NO	Mrpl13
NO	Mtbp
NO	Sntb1
NO	Zhx2
NO	Derl1
NO	Tbc1d31
NO	9130401M01Rik
NO	Zhx1
NO	Atad2
NO	Wdyhv1
NO	Fbxo32
NO	Klhl38
NO	D15Ertd621e
NO	Tmem65
NO	Trmt12
NO	Rnf139
NO	Tatdn1
NO	Ndufb9
NO	Mtss1
NO	Sqle
NO	E430025E21Rik
NO	Nsmce2
NO	Trib1
NO	Fam84b
NO	9930014A18Rik
NO	A1bg
NO	Myc
NO	Pvt1
NO	Fam49b
NO	Asap1
NO	Efr3a
NO	Tmem71
NO	Phf20l1

NO	Sla
NO	Ndrg1
NO	St3gal1
NO	Zfat
NO	Khdrbs3
NO	Col22a1
NO	Trappc9
NO	Peg13
NO	Chrac1
NO	Ago2
NO	Ptk2
NO	Dennd3
NO	Slc45a4
NO	Ptp4a3
NO	Jrk
NO	Them6
NO	Lynx1
NO	Ly6e
NO	Ly6a
NO	Ly6c1
NO	Gpihbp1
NO	Zfp41
NO	Top1mt
NO	Mafa
NO	Zc3h3
NO	Gsdmd
NO	Naprt
NO	Eef1d
NO	Tigd5
NO	Pycrl
NO	Tsta3
NO	Zfp623
NO	Zfp707
NO	Fam83h
NO	Scrib
NO	Puf60
NO	Nrbp2
NO	Eppk1
NO	Plec
NO	Parp10
NO	Grina
NO	Oplah
NO	Exosc4
NO	Gpaa1
NO	Cyc1
NO	Sharpin
NO	Maf1
NO	Hgh1

NO	Mroh1
NO	Bop1
NO	Scx
NO	Hsf1
NO	Dgat1
NO	Fbxl6
NO	Slc52a2
NO	Adck5
NO	Cpsf1
NO	Vps28
NO	Cyhr1
NO	Kifc2
NO	Ppp1r16a
NO	Gpt
NO	Mfsd3
NO	Lrrc14
NO	Lrrc24
NO	C030006K11Rik
NO	Arhgap39
NO	Zfp251
NO	Zfp7
NO	Commd5
NO	Rpl8
NO	Zfp647
NO	1110038F14Rik
NO	Mb
NO	Apol6
NO	Rbfox2
NO	1700109K24Rik
NO	Apol10b
NO	Myh9
NO	Txn2
NO	Foxred2
NO	Eif3d
NO	Ift27
NO	Pvalb
NO	Csf2rb2
NO	Csf2rb
NO	Tst
NO	Mpst
NO	Kctd17
NO	C1qtnf6
NO	Rac2
NO	Cyth4
NO	Mfng
NO	Card10
NO	Cdc42ep1
NO	Gga1

NO	Sh3bp1
NO	Pdxp
NO	Lgals1
NO	Nol12
NO	Triobp
NO	H1f0
NO	Gcat
NO	Ankrd54
NO	Eif3l
NO	Micall1
NO	Polr2f
NO	Sox10
NO	Pick1
NO	Slc16a8
NO	Baiap2l2
NO	Pla2g6
NO	Maff
NO	Tmem184b
NO	Csnk1e
NO	Kdelr3
NO	Ddx17
NO	Cby1
NO	Tomm22
NO	Josd1
NO	Gtpbp1
NO	Sun2
NO	Gm16576
NO	Dnal4
NO	Cbx6
NO	Apobec3
NO	Cbx7
NO	Pdgfb
NO	Rpl3
NO	Syngr1
NO	Tab1
NO	Mgat3
NO	Mief1
NO	Atf4
NO	Rps19bp1
NO	Cacna1i
NO	Tnrc6b
NO	Adsl
NO	Sgsm3
NO	Mkl1
NO	Mchr1
NO	Slc25a17
NO	St13
NO	Xpnpep3

NO	Rbx1
NO	Ep300
NO	L3mbtl2
NO	Chadl
NO	Rangap1
NO	Zc3h7b
NO	Tef
NO	Tob2
NO	Phf5a
NO	Aco2
NO	Polr3h
NO	Pmm1
NO	Desi1
NO	Xrcc6
NO	Nhp2l1
NO	Ccdc134
NO	Srebf2
NO	Sept3
NO	Naga
NO	Fam109b
NO	Smdt1
NO	Ndufa6
NO	Cyp2d22
NO	Tcf20
NO	Tbrg3
NO	Nfam1
NO	Serhl
NO	Rrp7a
NO	Poldip3
NO	Rnu12
NO	Cyb5r3
NO	A4galt
NO	Arfgap3
NO	Pacsin2
NO	Ttll1
NO	Mcat
NO	Tspo
NO	Ttll12
NO	Scube1
NO	Efcab6
NO	Pnpla3
NO	Samm50
NO	Parvb
NO	Parvg
NO	1810041L15Rik
NO	Ldoc1l
NO	Prr5
NO	Phf21b

NO	Nup50
NO	5031439G07Rik
NO	Fam118a
NO	Fbln1
NO	Atxn10
NO	Lincppara
NO	Ppara
NO	Cdpf1
NO	Pkdrej
NO	Ttc38
NO	Trmu
NO	Celsr1
NO	Gramd4
NO	Cerk
NO	Tbc1d22a
NO	Fam19a5
NO	Brd1
NO	Zbed4
NO	Alg12
NO	Creld2
NO	Pim3
NO	1810021B22Rik
NO	Trabd
NO	Selo
NO	Tubgcp6
NO	Hdac10
NO	Mapk12
NO	Mapk11
NO	Plxnb2
NO	Dennd6b
NO	Ppp6r2
NO	Sbf1
NO	Lmf2
NO	Ncaph2
NO	Arsa
NO	Shank3
NO	Rabl2
NO	Alg10b
NO	Cpne8
NO	Kif21a
NO	Abcd2
NO	Slc2a13
NO	Lrrk2
NO	Gxylt1
NO	Yaf2
NO	Zcrb1
NO	Pphln1
NO	Prickle1

NO	Adamts20
NO	Pus7l
NO	Irak4
NO	Twf1
NO	Tmem117
NO	Ano6
NO	E330033B04Rik
NO	Arid2
NO	Scaf11
NO	Slc38a1
NO	Slc38a2
NO	Slc38a4
NO	Amigo2
NO	Pced1b
NO	Rpap3
NO	Rapgef3
NO	Slc48a1
NO	Hdac7
NO	Tmem106c
NO	Senp1
NO	Pfkm
NO	Asb8
NO	Zfp641
NO	Kansl2
NO	Ccnt1
NO	9330020H09Rik
NO	Adcy6
NO	Cacnb3
NO	Ddx23
NO	Arf3
NO	Ddn
NO	Prkag1
NO	Kmt2d
NO	Rhebl1
NO	Dhh
NO	Lmbr1l
NO	Tuba1b
NO	Tuba1a
NO	Tuba1c
NO	Spats2
NO	Mcrs1
NO	1700120C14Rik
NO	Prpf40b
NO	Fmnl3
NO	Tmbim6
NO	Nckap5l
NO	Bcdin3d
NO	Asic1

NO	Smarcd1
NO	Gpd1
NO	Cox14
NO	Cers5
NO	Lima1
NO	Larp4
NO	Dip2b
NO	Atf1
NO	Mettl7a1
NO	Slc11a2
NO	Letmd1
NO	Csrnp2
NO	Tfcp2
NO	Pou6f1
NO	Dazap2
NO	Smagp
NO	Galnt6
NO	Slc4a8
NO	Fignl2
NO	Acvrl1
NO	Acvr1b
NO	Grasp
NO	Nr4a1
NO	Atg101
NO	Krt80
NO	Eif4b
NO	Tns2
NO	Spryd3
NO	Igfbp6
NO	Csad
NO	Zfp740
NO	Itgb7
NO	Rarg
NO	Mfsd5
NO	Pfdn5
NO	Myg1
NO	Aaas
NO	Sp1
NO	Amhr2
NO	Prr13
NO	Pcbp2
NO	Map3k12
NO	Tarbp2
NO	Npff
NO	Atf7
NO	Atp5g2
NO	Calcoco1
NO	Hoxc11

NO	Hoxc10
NO	Hoxc9
NO	Hoxc8
NO	Hoxc6
NO	Hoxc5
NO	Hoxc4
NO	Smug1
NO	Cbx5
NO	Hnrnpa1
NO	Copz1
NO	Zfp385a
NO	Itga5
NO	Nckap1l
NO	Pde1b
NO	Ppp1r1a
NO	Zfp263
NO	Zfp174
NO	Zfp597
NO	Naa60
NO	1700037C18Rik
NO	Cluap1
NO	Nlrc3
NO	Slx4
NO	Dnase1
NO	Trap1
NO	Crebbp
NO	Adcy9
NO	Srl
NO	Tfap4
NO	Glis2
NO	Pam16
NO	Coro7
NO	Vasn
NO	Dnaja3
NO	Nmral1
NO	Hmox2
NO	Cdip1
NO	Ubald1
NO	Mgrn1
NO	Gm16861
NO	Nudt16l1
NO	Anks3
NO	Rogdi
NO	Glyr1
NO	Ubn1
NO	Ppl
NO	Sec14l5
NO	Nagpa

NO	Alg1
NO	Eef2kmt
NO	Rbfox1
NO	Mettl22
NO	Abat
NO	Tmem186
NO	Pmm2
NO	Carhsp1
NO	Usp7
NO	1810013L24Rik
NO	Emp2
NO	Nubp1
NO	Ciita
NO	Dexi
NO	Clec16a
NO	Socs1
NO	Litaf
NO	Snn
NO	Txndc11
NO	Zc3h7a
NO	Rsl1d1
NO	2610020C07Rik
NO	Gspt1
NO	Snx29
NO	Cpped1
NO	Ercc4
NO	Mkl2
NO	2310015D24Rik
NO	Parn
NO	Bfar
NO	3110001I22Rik
NO	Rrn3
NO	Ntan1
NO	Pdxdc1
NO	Mpv17l
NO	Marf1
NO	Nde1
NO	Myh11
NO	Fopnl
NO	Abcc1
NO	Ube2v2
NO	Mcm4
NO	Prkdc
NO	Mzt2
NO	Cebpd
NO	Spidr
NO	Pkp2
NO	Yars2

NO	Dnm1l
NO	Fgd4
NO	Top3b
NO	Ppm1f
NO	Mapk1
NO	Ypel1
NO	Ppil2
NO	Sdf2l1
NO	Ube2l3
NO	Hic2
NO	Tmem191c
NO	Pi4ka
NO	Serpind1
NO	Snap29
NO	Crkl
NO	Lztr1
NO	Thap7
NO	Lrrc74b
NO	P2rx6
NO	Slc7a4
NO	Smpd4
NO	Ccdc74a
NO	Med15
NO	Klhl22
NO	Scarf2
NO	Dgcr2
NO	Dgcr14
NO	Slc25a1
NO	Dgcr6
NO	Prodh
NO	Rtn4r
NO	Zdhhc8
NO	Ranbp1
NO	Trmt2a
NO	Dgcr8
NO	Tango2
NO	Arvcf
NO	Comt
NO	Txnrd2
NO	Gnb1l
NO	Tbx1
NO	Sept5
NO	Cldn5
NO	Cdc45
NO	Ufd1l
NO	2510002D24Rik
NO	Mrpl40
NO	Hira

NO	Klhl6
NO	Klhl24
NO	Yeats2
NO	Parl
NO	Abcc5
NO	Eif2b5
NO	Dvl3
NO	Ap2m1
NO	Gm15760
NO	Abcf3
NO	Alg3
NO	Ece2
NO	Camk2n2
NO	Psmd2
NO	Eif4g1
NO	Fam131a
NO	Clcn2
NO	Polr2h
NO	Thpo
NO	Chrd
NO	Ephb3
NO	Vps8
NO	2510009E07Rik
NO	Ehhadh
NO	1300002E11Rik
NO	Map3k13
NO	Tmem41a
NO	Senp2
NO	Igf2bp2
NO	Tra2b
NO	Etv5
NO	Dgkg
NO	Tbccd1
NO	Dnajb11
NO	Eif4a2
NO	Snora81
NO	Rfc4
NO	Adipoq
NO	St6gal1
NO	Masp1
NO	Rtp4
NO	Bcl6
NO	Lppos
NO	Lpp
NO	Morf4l1-ps1
NO	Trp63
NO	P3h2
NO	Cldn1

NO	Il1rap
NO	Ostn
NO	Uts2b
NO	Ccdc50
NO	Mb21d2
NO	Hrasls
NO	Atp13a5
NO	Opa1
NO	4632428C04Rik
NO	Hes1
NO	Atp13a3
NO	Tmem44
NO	Lsg1
NO	Fam43a
NO	Xxylt1
NO	Acap2
NO	Ppp1r2
NO	Apod
NO	Bdh1
NO	Dlg1
NO	0610012G03Rik
NO	Ncbp2
NO	Senp5
NO	Pak2
NO	Pigx
NO	Cep19
NO	Nrros
NO	Fbxo45
NO	Wdr53
NO	Smco1
NO	Rnf168
NO	Ubxn7
NO	Tctex1d2
NO	Pcyt1a
NO	Tfrc
NO	Tnk2
NO	Tnk2os
NO	1700021K19Rik
NO	Fyttd1
NO	Lrch3
NO	Iqcg
NO	Rpl35a
NO	Lmln
NO	Osbpl11
NO	Snx4
NO	Zfp148
NO	Heg1
NO	Itgb5

NO	Umps
NO	Kalrn
NO	Ccdc14
NO	Mylk
NO	E130310I04Rik
NO	Hacd2
NO	Adcy5
NO	Sec22a
NO	Pdia5
NO	Dirc2
NO	Hspbap1
NO	Parp14
NO	Dtx3l
NO	Parp9
NO	Kpna1
NO	Wdr5b
NO	Fam162a
NO	Ccdc58
NO	Casr
NO	Cd86
NO	Slc15a2
NO	Iqcb1
NO	4930565N06Rik
NO	Golgb1
NO	Hcls1
NO	Fbxo40
NO	Gtf2e1
NO	Rabl3
NO	Ndufb4
NO	Fstl1
NO	Lrrc58
NO	BC031361
NO	Gsk3b
NO	Cox17
NO	Popdc2
NO	Pla1a
NO	Adprh
NO	Timmdc1
NO	Poglut1
NO	Tmem39a
NO	Arhgap31
NO	B4galt4
NO	Gm19522
NO	Zbtb20
NO	Gm15713
NO	Mir568
NO	Qtrtd1
NO	2610015P09Rik

NO	Zdhhc23
NO	Atp6v1a
NO	Naa50
NO	Gm608
NO	Sidt1
NO	Spice1
NO	Boc
NO	BC027231
NO	Gtpbp8
NO	Ccdc80
NO	Slc35a5
NO	Atg3
NO	Cd200
NO	Abhd10
NO	Phldb2
NO	Plcxd2
NO	Gm4737
NO	Pvrl3
NO	Retnla
NO	Dzip3
NO	C330027C09Rik
NO	Ift57
NO	Cd47
NO	Bbx
NO	5330426P16Rik
NO	Cblb
NO	Alcam
NO	Nfkbiz
NO	Nxpe3
NO	Cep97
NO	Rpl24
NO	Zbtb11os1
NO	Zbtb11
NO	Pcnp
NO	Trmt10c
NO	Senp7
NO	Abi3bp
NO	Tfg
NO	Tomm70a
NO	Nit2
NO	Tbc1d23
NO	Cmss1
NO	Filip1l
NO	Col8a1
NO	Dcbld2
NO	St3gal6
NO	Cpox
NO	Cldnd1

NO	Mina
NO	Crybg3
NO	Arl6
NO	Nsun3
NO	Arl13b
NO	Pros1
NO	4930453N24Rik
NO	Zfp654
NO	Cggbp1
NO	Chmp2b
NO	Vgll3
NO	Gbe1
NO	Robo1
NO	Hspa13
NO	Nrip1
NO	Usp25
NO	Mir99ahg
NO	Cxadr
NO	D16Ertd472e
NO	Chodl
NO	Mrpl39
NO	Jam2
NO	Atp5j
NO	Gabpa
NO	App
NO	Cyyr1
NO	Adamts1
NO	Adamts5
NO	N6amt1
NO	Ltn1
NO	Rwdd2b
NO	Usp16
NO	Cct8
NO	Map3k7cl
NO	Bach1
NO	Tiam1
NO	Sod1
NO	Scaf4
NO	Mis18a
NO	Mrap
NO	Urb1
NO	1110004E09Rik
NO	Synj1
NO	Paxbp1
NO	Ifnar2
NO	Il10rb
NO	Ifnar1
NO	Ifngr2

NO	Tmem50b
NO	Dnajc28
NO	Gart
NO	Son
NO	Donson
NO	Gm10785
NO	Cryzl1
NO	Itsn1
NO	Atp5o
NO	Slc5a3
NO	Mrps6
NO	Smim11
NO	Rcan1
NO	Runx1
NO	Setd4
NO	Cbr1
NO	Dopey2
NO	Morc3
NO	Sim2
NO	Hlcs
NO	Pigp
NO	Ttc3
NO	Dscr3
NO	Dyrk1a
NO	Kcnj15
NO	Erg
NO	Ets2
NO	Psmg1
NO	Brwd1
NO	Hmgn1
NO	Wrb
NO	Sh3bgr
NO	Bace2
NO	Prdm15
NO	C2cd2
NO	Zbtb21
NO	B230307C23Rik
NO	A630089N07Rik
NO	Pisd-ps2
NO	Scaf8
NO	Tiam2
NO	Tfb1m
NO	Arid1b
NO	Tmem242
NO	Zdhhc14
NO	Snx9
NO	Synj2
NO	Serac1

NO	Gtf2h5
NO	Tulp4
NO	Tmem181a
NO	Dynlt1b
NO	Tmem181b-ps
NO	Ezr
NO	Rsph3b
NO	Tagap1
NO	Rnaset2b
NO	Rps6ka2
NO	Fndc1
NO	Tagap
NO	Rsph3a
NO	Fgfr1op
NO	Mpc1
NO	4930506C21Rik
NO	Sft2d1
NO	Pde10a
NO	Qk
NO	Cahm
NO	Park2
NO	Agpat4
NO	Map3k4
NO	4732491K20Rik
NO	Slc22a3
NO	Igf2r
NO	Airn
NO	Mas1
NO	Mrgprh
NO	Mrpl18
NO	Tcp1
NO	Acat2
NO	Wtap
NO	Sod2
NO	2700054A10Rik
NO	Mllt4
NO	Dact2
NO	Smoc2
NO	Thbs2
NO	1600012H06Rik
NO	Phf10
NO	LOC106740
NO	Ermard
NO	Gm5091
NO	Dll1
NO	Fam120b
NO	Psmb1
NO	Tbp

NO	Pdcd2
NO	Prdm9
NO	4933401D09Rik
NO	Chd1
NO	Rgmb
NO	Zfp960
NO	Zfp97
NO	Riok2
NO	Lnpep
NO	Spaca6
NO	Ppp2r1a
NO	Zfp160
NO	Zfp677
NO	Zfp51
NO	Zfp53
NO	Zfp52
NO	Zfp948
NO	3110052M02Rik
NO	Zfp760
NO	Zfp229
NO	Zfp942
NO	Zfp943
NO	Gm4944
NO	Zfp944
NO	Zfp758
NO	Zfp946
NO	Gm16386
NO	Zfp945
NO	Zfp40
NO	Zfp213
NO	Zfp13
NO	Thoc6
NO	Hcfc1r1
NO	Tnfrsf12a
NO	Paqr4
NO	9530082P21Rik
NO	Flywch1
NO	Flywch2
NO	Srrm2
NO	Tceb2
NO	Kctd5
NO	Pdpk1
NO	Amdhd2
NO	Tbc1d24
NO	Ntn3
NO	1600002H07Rik
NO	Abca3
NO	D330041H03Rik

NO	Rnps1
NO	Eci1
NO	Dnase1l2
NO	E4f1
NO	Pgp
NO	Mlst8
NO	Traf7
NO	Pkd1
NO	Tsc2
NO	Nthl1
NO	Slc9a3r2
NO	Zfp598
NO	Gfer
NO	Tbl3
NO	Snora78
NO	Rps2
NO	Ndufb10
NO	Rpl3l
NO	Msrb1
NO	Fahd1
NO	Hagh
NO	Igfals
NO	Nubp2
NO	Spsb3
NO	Eme2
NO	Mrps34
NO	Nme3
NO	Mapk8ip3
NO	Hn1l
NO	Cramp1l
NO	Ift140
NO	Tmem204
NO	Telo2
NO	Clcn7
NO	BC003965
NO	Unkl
NO	Gnptg
NO	Tsr3
NO	Baiap3
NO	Ube2i
NO	Tpsb2
NO	Cacna1h
NO	Lmf1
NO	Chtf18
NO	Rpusd1
NO	Narfl
NO	Haghl
NO	Fam173a

NO	Metrn
NO	Wdr24
NO	Jmjd8
NO	Stub1
NO	Rhbdl1
NO	Rhot2
NO	Wdr90
NO	Fam195a
NO	0610011F06Rik
NO	Wfikkn1
NO	Rab40c
NO	Pigq
NO	Capn15
NO	Rab11fip3
NO	Decr2
NO	Nme4
NO	Tmem8
NO	Mrpl28
NO	Axin1
NO	Itfg3
NO	Luc7l
NO	Neurl1b
NO	Dusp1
NO	Ergic1
NO	Atp6v0e
NO	Crebrf
NO	Bnip1
NO	Phf1
NO	Cuta
NO	Syngap1
NO	Zbtb9
NO	Ggnbp1
NO	Bak1
NO	Itpr3
NO	Uqcc2
NO	Ip6k3
NO	Lemd2
NO	AI413582
NO	Nudt3
NO	Rps10
NO	D17Wsu92e
NO	Snrpc
NO	Uhrf1bp1
NO	Taf11
NO	Anks1
NO	Zfp523
NO	Def6
NO	Ppard

NO	Fance
NO	Rpl10a
NO	Tead3
NO	Fkbp5
NO	Srpk1
NO	Mapk14
NO	Brpf3
NO	Kctd20
NO	Stk38
NO	Srsf3
NO	Cdkn1a
NO	Ppil1
NO	BC004004
NO	Pi16
NO	Mtch1
NO	Fgd2
NO	Pim1
NO	Tbc1d22b
NO	Rnf8
NO	Cmtr1
NO	Ccdc167
NO	Mdga1
NO	Zfand3
NO	Btbd9
NO	Glo1
NO	Dnah8
NO	Abcg1
NO	Slc37a1
NO	Pde9a
NO	Wdr4
NO	Ndufv3
NO	Pknox1
NO	U2af1
NO	Sik1
NO	Rrp1b
NO	Notch3
NO	Brd4
NO	Akap8
NO	Akap8l
NO	Wiz
NO	Rasal3
NO	Cyp4f39
NO	Cyp4f17
NO	Cyp4f16
NO	Zfp871
NO	Zfp799
NO	Zfp870
NO	Cyp4f13

NO	Zfp472
NO	Zfp952
NO	Zfp763
NO	Zfp563
NO	Zfp955a
NO	Zfp955b
NO	Zfp81
NO	Zfp101
NO	Adamts10
NO	Myo1f
NO	Zfp414
NO	Pram1
NO	Hnrnpm
NO	March2
NO	Rab11b
NO	Angptl4
NO	Kank3
NO	Rps28
NO	Ndufa7
NO	Cd320
NO	Daxx
NO	Zbtb22
NO	Tapbp
NO	Rgl2
NO	Pfdn6
NO	Wdr46
NO	B3galt4
NO	Rps18
NO	Vps52
NO	H2-K1
NO	Ring1
NO	H2-Ke6
NO	Slc39a7
NO	Rxrb
NO	Col11a2
NO	Brd2
NO	H2-DMa
NO	H2-DMb2
NO	Psmb9
NO	Tap1
NO	Psmb8
NO	Tap2
NO	H2-Ab1
NO	H2-Aa
NO	H2-Eb1
NO	H2-Ea-ps
NO	Notch4
NO	Gpsm3

NO	Pbx2
NO	Rnf5
NO	Agpat1
NO	Egfl8
NO	Ppt2
NO	Fkbpl
NO	Atf6b
NO	Tnxb
NO	C4b
NO	C4a
NO	Stk19
NO	Dxo
NO	Skiv2l
NO	Nelfe
NO	Cfb
NO	C2
NO	Zbtb12
NO	Ehmt2
NO	Neu1
NO	1110038B12Rik
NO	Hspa1b
NO	Hspa1a
NO	Lsm2
NO	D17H6S56E-5
NO	Vars
NO	Vwa7
NO	Msh5
NO	Clic1
NO	Ddah2
NO	Abhd16a
NO	Csnk2b
NO	Gpank1
NO	D17H6S53E
NO	Bag6
NO	Prrc2a
NO	Ltb
NO	Nfkbil1
NO	Ddx39b
NO	H2-D1
NO	H2-Q4
NO	H2-Q5
NO	Tcf19
NO	Cchcr1
NO	Vars2
NO	Gtf2h4
NO	Ddr1
NO	Ier3
NO	Flot1

NO	Tubb5
NO	Mdc1
NO	Nrm
NO	Ppp1r18
NO	Dhx16
NO	2310061I04Rik
NO	Atat1
NO	Mrps18b
NO	Ppp1r10
NO	Mir1894
NO	Abcf1
NO	Prr3
NO	Gnl1
NO	A930015D03Rik
NO	H2-T24
NO	H2-T23
NO	Gm11127
NO	H2-T10
NO	Rpp21
NO	Trim39
NO	Trim26
NO	Rnf39
NO	Ppp1r11
NO	Znrd1
NO	Znrd1as
NO	Gabbr1
NO	H2-M3
NO	Rn45s
NO	Cenpq
NO	Mut
NO	Ptchd4
NO	Cd2ap
NO	Tnfrsf21
NO	Adgrf5
NO	Pla2g7
NO	Slc25a27
NO	Cyp39a1
NO	Rcan2
NO	Enpp5
NO	Enpp4
NO	Clic5
NO	Runx2
NO	Supt3
NO	Cdc5l
NO	B230354K17Rik
NO	Aars2
NO	Nfkbie
NO	Slc35b2

NO	Hsp90ab1
NO	Slc29a1
NO	Tmem63b
NO	Mrpl14
NO	Vegfa
NO	Mrps18a
NO	Mad2l1bp
NO	Gtpbp2
NO	Polh
NO	Xpo5
NO	Polr1c
NO	Yipf3
NO	Tjap1
NO	Abcc10
NO	Zfp318
NO	Dnph1
NO	Cul9
NO	Srf
NO	Ptk7
NO	Klc4
NO	Mrpl2
NO	Cul7
NO	Rrp36
NO	Klhdc3
NO	Mea1
NO	Ppp2r5d
NO	Pex6
NO	Gnmt
NO	Cnpy3
NO	2310039H08Rik
NO	Rpl7l1
NO	Gltscr1l
NO	Tbcc
NO	Ubr2
NO	Trerf1
NO	Mrps10
NO	Taf8
NO	Ccnd3
NO	Bysl
NO	Med20
NO	Usp49
NO	Tomm6
NO	Frs3
NO	Tfeb
NO	Foxp4
NO	Nfya
NO	Oard1
NO	Apobec2

NO	Mocs1
NO	Daam2
NO	Rftn1
NO	Plcl2
NO	Tbc1d5
NO	Satb1
NO	Rab5a
NO	Kat2b
NO	Slc5a7
NO	Pot1b
NO	Zfp119a
NO	Zfp959
NO	Zfp119b
NO	Ccdc94
NO	Stap2
NO	Mpnd
NO	Sh3gl1
NO	Chaf1a
NO	Ubxn6
NO	Hdgfrp2
NO	Plin4
NO	Plin5
NO	Lrg1
NO	Sema6b
NO	Tnfaip8l1
NO	Mydgf
NO	Dpp9
NO	Fem1a
NO	Ticam1
NO	Plin3
NO	Uhrf1
NO	Kdm4b
NO	Ptprs
NO	Gm20219
NO	Safb2
NO	Safb
NO	2410015M20Rik
NO	Rpl36
NO	Lonp1
NO	Ranbp3
NO	Vmac
NO	Ndufa11
NO	Nrtn
NO	Dus3l
NO	Rfx2
NO	Mllt1
NO	Clpp
NO	Alkbh7

NO	Gtf2f1
NO	Khsrp
NO	Slc25a23
NO	Dennd1c
NO	C3
NO	Gpr108
NO	Trip10
NO	Vav1
NO	Adgre1
NO	Nudt12
NO	Efna5
NO	Fbxl17
NO	4930405O22Rik
NO	Fer
NO	Pja2
NO	Man2a1
NO	Tmem232
NO	Vapa
NO	Rab31
NO	Ppp4r1
NO	Ralbp1
NO	Twsg1
NO	Ankrd12
NO	Ndufv2
NO	Wash1
NO	Ddx11
NO	Mtcl1
NO	Rab12
NO	Ptprm
NO	Lrrc30
NO	Arhgap28
NO	Epb4.1l3
NO	Zbtb14
NO	C030034I22Rik
NO	Tgif1
NO	Myl12b
NO	Myl12a
NO	Myom1
NO	Lpin2
NO	Emilin2
NO	Smchd1
NO	Wdr43
NO	Fam179a
NO	Clip4
NO	Ypel5
NO	Lbh
NO	Lclat1
NO	Ehd3

NO	Xdh
NO	Memo1
NO	Dpy30
NO	Spast
NO	Slc30a6
NO	Yipf4
NO	Birc6
NO	Ttc27
NO	Ltbp1
NO	Rasgrp3
NO	Fam98a
NO	Crim1
NO	Fez2
NO	Vit
NO	Strn
NO	Heatr5b
NO	Gpatch11
NO	Gm6548
NO	Eif2ak2
NO	Cebpzos
NO	Cebpz
NO	Ndufaf7
NO	Prkd3
NO	Qpct
NO	Cdc42ep3
NO	Rmdn2
NO	Cyp1b1
NO	Atl2
NO	Hnrnpll
NO	Galm
NO	Srsf7
NO	Gemin6
NO	Dhx57
NO	Morn2
NO	Sos1
NO	Map4k3
NO	Thumpd2
NO	Slc8a1
NO	Pkdcc
NO	Eml4
NO	Cox7a2l
NO	Mta3
NO	Zfp36l2
NO	Thada
NO	Plekhh2
NO	Dync2li1
NO	Lrpprc
NO	1110020A21Rik

NO	Ppm1b
NO	Prepl
NO	Camkmt
NO	Six2
NO	Srbd1
NO	Prkce
NO	Epas1
NO	Rhoq
NO	Cript
NO	Socs5
NO	Mcfd2
NO	4833418N02Rik
NO	Ttc7
NO	Calm2
NO	Msh2
NO	Msh6
NO	Fbxo11
NO	Foxn2
NO	Ppp1r21
NO	Ston1
NO	Mettl4
NO	Gm1976
NO	Crem
NO	Cul2
NO	Bambi
NO	Map3k8
NO	Mtpap
NO	9430020K01Rik
NO	Svil
NO	Zfp438
NO	Gm10125
NO	Zeb1
NO	Arhgap12
NO	Kif5b
NO	Epc1
NO	Rab18
NO	Mpp7
NO	Wac
NO	Fzd8
NO	Ccny
NO	Colec12
NO	Thoc1
NO	Usp14
NO	Rock1
NO	Greb1l
NO	Esco1
NO	Snrpd1
NO	Mib1

NO	Mir133a-1
NO	Mir1b
NO	Gata6
NO	Rbbp8
NO	Cables1
NO	Tmem241
NO	Riok3
NO	3110002H16Rik
NO	Npc1
NO	Ankrd29
NO	Lama3
NO	Ttc39c
NO	Cabyr
NO	Osbpl1a
NO	Impact
NO	Zfp521
NO	Ss18
NO	Taf4b
NO	Kctd1
NO	Aqp4
NO	Cdh2
NO	B4galt6
NO	Trappc8
NO	Rnf125
NO	Rnf138
NO	Garem
NO	Dtna
NO	Mapre2
NO	Zfp397
NO	Zfp35
NO	Zfp191
NO	Ino80c
NO	Galnt1
NO	2700062C07Rik
NO	Rprd1a
NO	Slc39a6
NO	Elp2
NO	Mocos
NO	Fhod3
NO	Tpgs2
NO	AW554918
NO	Pik3c3
NO	Slc25a46
NO	Sap130
NO	Ammecr1l
NO	Polr2d
NO	Wdr33
NO	Sft2d3

NO	Lims2
NO	Gpr17
NO	Myo7b
NO	Iws1
NO	Map3k2
NO	Ercc3
NO	A830052D11Rik
NO	Bin1
NO	Gypc
NO	Wdr36
NO	Camk4
NO	Stard4
NO	Nrep
NO	Epb4.1l4aos
NO	Epb4.1l4a
NO	Gm10548
NO	Apc
NO	Srp19
NO	Reep5
NO	Pkd2l2
NO	Fam13b
NO	Brd8
NO	Kif20a
NO	Cdc23
NO	Fam53c
NO	Kdm3b
NO	Egr1
NO	Etf1
NO	Hspa9
NO	Ctnna1
NO	Lrrtm2
NO	Sil1
NO	Snhg4
NO	Mir1949
NO	Snora74a
NO	Matr3
NO	Paip2
NO	Slc23a1
NO	Prob1
NO	Spata24
NO	Dnajc18
NO	Ecscr
NO	Tmem173
NO	Ube2d2a
NO	Cxxc5
NO	Pura
NO	Igip
NO	Cystm1

NO	Pfdn1
NO	Hbegf
NO	Ankhd1
NO	Eif4ebp3
NO	Sra1
NO	Apbb3
NO	Slc35a4
NO	Cd14
NO	Tmco6
NO	Ndufa2
NO	Ik
NO	Wdr55
NO	Hars
NO	Hars2
NO	Zmat2
NO	Vaultrc5
NO	Pcdhb14
NO	Pcdhb16
NO	Pcdhb17
NO	Pcdhb18
NO	Pcdhb19
NO	Pcdhb20
NO	Taf7
NO	Pcdhga1
NO	Pcdhga2
NO	Pcdhga3
NO	Pcdhgb1
NO	Pcdhga4
NO	Pcdhgb2
NO	Pcdhga5
NO	Pcdhga6
NO	Pcdhga7
NO	Pcdhgb4
NO	Pcdhga8
NO	Pcdhgb5
NO	Pcdhga9
NO	Pcdhgb6
NO	Pcdhga10
NO	Pcdhgb7
NO	Pcdhga11
NO	Pcdhgb8
NO	Pcdhga12
NO	Pcdhgc3
NO	Pcdhgc4
NO	Pcdhgc5
NO	Diap1
NO	Hdac3
NO	Fchsd1

NO	Arap3
NO	Pcdh1
NO	1700086O06Rik
NO	0610009O20Rik
NO	Pcdh12
NO	Rnf14
NO	Gnpda1
NO	Ndfip1
NO	Spry4
NO	Fgf1
NO	Arhgap26
NO	Nr3c1
NO	Yipf5
NO	Sh3rf2
NO	Lars
NO	Rbm27
NO	Tcerg1
NO	Dpysl3
NO	Npy6r
NO	Myot
NO	Dcp2
NO	Mcc
NO	Ythdc2
NO	Kcnn2
NO	Pggt1b
NO	Fem1c
NO	Tmed7
NO	Eif1a
NO	Cdo1
NO	Atg12
NO	Ap3s1
NO	Lvrn
NO	Commd10
NO	Sema6a
NO	Eno1b
NO	Dtwd2
NO	Dmxl1
NO	Tnfaip8
NO	Hsd17b4
NO	Prr16
NO	Srfbp1
NO	Lox
NO	Sncaip
NO	Snx2
NO	Snx24
NO	Ppic
NO	Cep120
NO	Csnk1g3

NO	Zfp608
NO	Gramd3
NO	Aldh7a1
NO	Phax
NO	Lmnb1
NO	March3
NO	C330018D20Rik
NO	Megf10
NO	Prrc1
NO	Ctxn3
NO	Slc12a2
NO	Isoc1
NO	Gm4841
NO	F830016B08Rik
NO	Iigp1
NO	Smim3
NO	Dctn4
NO	Rbm22
NO	Myoz3
NO	Synpo
NO	Ndst1
NO	Rps14
NO	Cd74
NO	Tcof1
NO	Arsi
NO	Camk2a
NO	Pdgfrb
NO	Csf1r
NO	Hmgxb3
NO	Slc26a2
NO	Ppargc1b
NO	Arhgef37
NO	Csnk1a1
NO	Bvht
NO	Mir143hg
NO	Grpel2
NO	1500015A07Rik
NO	Afap1l1
NO	Ablim3
NO	Sh3tc2
NO	Adrb2
NO	Fbxo38
NO	Apcdd1
NO	Napg
NO	Piezo2
NO	Txnl1
NO	Wdr7
NO	Fech

NO	Nars
NO	Atp8b1
NO	Nedd4l
NO	Alpk2
NO	Malt1
NO	Zfp532
NO	Oacyl
NO	Sec11c
NO	Lman1
NO	Ccbe1
NO	Gnal
NO	Chmp1b
NO	Mppe1
NO	Impa2
NO	Tubb6
NO	Afg3l2
NO	Spire1
NO	Cep76
NO	Psmg2
NO	Ptpn2
NO	Seh1l
NO	Cep192
NO	Ldlrad4
NO	Fam210a
NO	Rnmt
NO	Mc5r
NO	Mc2r
NO	Tcf4
NO	Rab27b
NO	4930503L19Rik
NO	Poli
NO	Mbd2
NO	Mex3c
NO	Smad4
NO	Elac1
NO	Me2
NO	Cxxc1
NO	Mbd1
NO	Myo5b
NO	Scarna17
NO	Acaa2
NO	Rpl17
NO	BC031181
NO	Dym
NO	Smad7
NO	Ctif
NO	Zbtb7c
NO	Smad2

NO	Ier3ip1
NO	Hdhd2
NO	Pias2
NO	St8sia5
NO	8030462N17Rik
NO	Haus1
NO	Atp5a1
NO	Pstpip2
NO	Epg5
NO	Setbp1
NO	Pard6g
NO	Adnp2
NO	Rbfa
NO	Txnl4a
NO	Pqlc1
NO	Ctdp1
NO	Nfatc1
NO	Atp9b
NO	Mbp
NO	Zfp236
NO	Zfp516
NO	4930592I03Rik
NO	Tshz1
NO	Zadh2
NO	Zfp407
NO	Cndp2
NO	Cyb5a
NO	Timm21
NO	Socs6
NO	Rttn
NO	Tmx3
NO	Ighmbp2
NO	Mrpl21
NO	Cpt1a
NO	Ppp6r3
NO	Lrp5
NO	1810055G02Rik
NO	Suv420h1
NO	Chka
NO	Tcirg1
NO	Ndufs8
NO	Aldh3b1
NO	Unc93b1
NO	Aldh3b2
NO	Acy3
NO	Nudt8
NO	Doc2g
NO	Ndufv1

NO	Gstp1
NO	Gstp2
NO	Cdk2ap2
NO	Pitpnm1
NO	Aip
NO	Tmem134
NO	Coro1b
NO	Ptprcap
NO	Rps6kb2
NO	Carns1
NO	Tbc1d10c
NO	Ppp1ca
NO	Rad9a
NO	Clcf1
NO	Ssh3
NO	Ankrd13d
NO	Adrbk1
NO	Kdm2a
NO	Syt12
NO	Pcx
NO	Lrfn4
NO	Rce1
NO	Sptbn2
NO	Rbm4b
NO	Rbm4
NO	Rbm14
NO	Ccs
NO	Ctsf
NO	Actn3
NO	Zdhhc24
NO	Bbs1
NO	Dpp3
NO	Peli3
NO	Mrpl11
NO	Slc29a2
NO	B4gat1
NO	Brms1
NO	Rin1
NO	Cd248
NO	Yif1a
NO	Rab1b
NO	Klc2
NO	Pacs1
NO	Sf3b2
NO	Cst6
NO	Banf1
NO	Eif1ad
NO	Sart1

NO	Drap1
NO	AI837181
NO	Ccdc85b
NO	Fibp
NO	Efemp2
NO	Mus81
NO	Cfl1
NO	Snx32
NO	Ovol1
NO	Ap5b1
NO	Rnaseh2c
NO	Kat5
NO	Rela
NO	Sipa1
NO	Pcnxl3
NO	Map3k11
NO	Kcnk7
NO	Ehbp1l1
NO	Fam89b
NO	Sssca1
NO	Ltbp3
NO	Scyl1
NO	Malat1
NO	Neat1
NO	Frmd8
NO	Slc25a45
NO	Dpf2
NO	Cdc42ep2
NO	Pola2
NO	Capn1
NO	Gm10814
NO	Syvn1
NO	Mrpl49
NO	Fau
NO	Znhit2
NO	Tm7sf2
NO	Vps51
NO	Zfpl1
NO	Sac3d1
NO	Snx15
NO	Arl2
NO	Ppp2r5b
NO	Atg2a
NO	Ehd1
NO	Cdc42bpg
NO	Men1
NO	Map4k2
NO	Sf1

NO	Pygm
NO	Rasgrp2
NO	Nrxn2
NO	Rps6ka4
NO	Ccdc88b
NO	Prdx5
NO	Trmt112
NO	Esrra
NO	Gpr137
NO	Bad
NO	Plcb3
NO	Ppp1r14b
NO	Fkbp2
NO	Vegfb
NO	Dnajc4
NO	Nudt22
NO	Trpt1
NO	Fermt3
NO	Stip1
NO	Macrod1
NO	Flrt1
NO	Otub1
NO	Cox8a
NO	Naa40
NO	Rcor2
NO	Mark2
NO	AI846148
NO	2700081O15Rik
NO	Rtn3
NO	Atl3
NO	Pla2g16
NO	Lgals12
NO	Slc3a2
NO	Snhg1
NO	Snord22
NO	Wdr74
NO	Stx5a
NO	Nxf1
NO	Tmem223
NO	Tmem179b
NO	Taf6l
NO	Polr2g
NO	Ttc9c
NO	Hnrnpul2
NO	Bscl2
NO	Lrrn4cl
NO	Ubxn1
NO	Uqcc3

NO	5730408K05Rik
NO	Ints5
NO	Ganab
NO	B3gat3
NO	Rom1
NO	Eml3
NO	Mta2
NO	Tut1
NO	Eef1g
NO	Ahnak
NO	Asrgl1
NO	Stxbp3-ps
NO	Incenp
NO	Fth1
NO	Rab3il1
NO	Fads3
NO	Fads2
NO	Fads1
NO	Fen1
NO	Tmem258
NO	Dagla
NO	Syt7
NO	Sdhaf2
NO	Cpsf7
NO	Tmem216
NO	Tmem138
NO	Cyb561a3
NO	Tkfc
NO	Ddb1
NO	Vps37c
NO	Slc15a3
NO	Tmem132a
NO	Tmem109
NO	Prpf19
NO	Ccdc86
NO	AW112010
NO	Ms4a6c
NO	Ms4a6b
NO	Ms4a4d
NO	Mrpl16
NO	Stx3
NO	Patl1
NO	Osbp
NO	Mpeg1
NO	Dtx4
NO	Fam111a
NO	Zfp91
NO	Tle4

NO	Psat1
NO	Cep78
NO	Gnaq
NO	E030024N20Rik
NO	Gna14
NO	Vps13a
NO	Prune2
NO	Gcnt1
NO	Rfk
NO	Pcsk5
NO	Ostf1
NO	Nmrk1
NO	2410127L17Rik
NO	D030056L22Rik
NO	Anxa1
NO	E030003E18Rik
NO	C730002L08Rik
NO	Aldh1a1
NO	Zfand5
NO	Gda
NO	1110059E24Rik
NO	Abhd17b
NO	Tmem2
NO	Trpm3
NO	Klf9
NO	Smc5
NO	Mamdc2
NO	Ptar1
NO	Apba1
NO	Fam189a2
NO	Tjp2
NO	Fxn
NO	Fam122a
NO	Pgm5
NO	Cbwd1
NO	Dock8
NO	Kank1
NO	2610016A17Rik
NO	Dmrt2
NO	Smarca2
NO	Vldlr
NO	D19Bwg1357e
NO	Rfx3
NO	Glis3
NO	Slc1a1
NO	4430402I18Rik
NO	Ppapdc2
NO	Cdc37l1

NO	Ak3
NO	Rcl1
NO	Jak2
NO	Plgrkt
NO	Cd274
NO	Ric1
NO	Ermp1
NO	9930021J03Rik
NO	Ranbp6
NO	Il33
NO	Uhrf2
NO	Prkg1
NO	Cstf2t
NO	8430431K14Rik
NO	Asah2
NO	Sgms1
NO	2700046G09Rik
NO	Minpp1
NO	Papss2
NO	Atad1
NO	Pten
NO	Rnls
NO	Lipo1
NO	Stambpl1
NO	Acta2
NO	Fas
NO	Lipa
NO	Ifit2
NO	Ifit3
NO	Ifit3b
NO	Ifit1
NO	Slc16a12
NO	Pank1
NO	Htr7
NO	Rpp30
NO	Pcgf5
NO	Hectd2
NO	1500017E21Rik
NO	Ppp1r3c
NO	Tnks2
NO	Fgfbp3
NO	Btaf1
NO	Cpeb3
NO	March5
NO	4931408D14Rik
NO	Ide
NO	Hhex
NO	Exoc6

NO	Myof
NO	Rbp4
NO	Fra10ac1
NO	Lgi1
NO	Slc35g1
NO	Plce1
NO	Noc3l
NO	Tbc1d12
NO	Pdlim1
NO	Sorbs1
NO	Aldh18a1
NO	Tctn3
NO	Entpd1
NO	Ccnj
NO	Zfp518a
NO	Blnk
NO	Tm9sf3
NO	Pik3ap1
NO	Lcor
NO	Slit1
NO	Arhgap19
NO	Frat1
NO	Frat2
NO	Rrp12
NO	Pgam1
NO	Exosc1
NO	Zdhhc16
NO	Mms19
NO	Ubtd1
NO	Ankrd2
NO	Hoga1
NO	Morn4
NO	Pi4k2a
NO	Avpi1
NO	Marveld1
NO	Zfyve27
NO	Sfrp5
NO	Golga7b
NO	Crtac1
NO	R3hcc1l
NO	Loxl4
NO	Pyroxd2
NO	Hps1
NO	Got1
NO	Slc25a28
NO	BC037704
NO	Entpd7
NO	Cox15

NO	Cutc
NO	Dnmbp
NO	Cyp2c44
NO	Erlin1
NO	Chuk
NO	Cwf19l1
NO	Bloc1s2
NO	Scd2
NO	Scd1
NO	Wnt8b
NO	Sec31b
NO	Ndufb8
NO	Hif1an
NO	Fam178a
NO	Mrpl43
NO	Peo1
NO	Lzts2
NO	Pdzd7
NO	Sfxn3
NO	Kazald1
NO	Lbx1
NO	Btrc
NO	Poll
NO	Dpcd
NO	Fbxw4
NO	Npm3
NO	Mgea5
NO	9130011E15Rik
NO	Hps6
NO	Ldb1
NO	Pprc1
NO	Nolc1
NO	Pitx3
NO	Gbf1
NO	Nfkb2
NO	Psd
NO	Fbxl15
NO	Cuedc2
NO	Tmem180
NO	Actr1a
NO	Sufu
NO	Trim8
NO	Arl3
NO	Sfxn2
NO	Wbp1l
NO	2010012O05Rik
NO	As3mt
NO	Cnnm2

NO	Nt5c2
NO	Pcgf6
NO	Taf5
NO	Usmg5
NO	Pdcd11
NO	Calhm2
NO	Neurl1a
NO	Sh3pxd2a
NO	Obfc1
NO	Slk
NO	Col17a1
NO	Sfr1
NO	Gsto1
NO	Itprip
NO	Sorcs1
NO	Xpnpep1
NO	Add3
NO	Mxi1
NO	Smndc1
NO	Smc3
NO	Rbm20
NO	Pdcd4
NO	Bbip1
NO	Shoc2
NO	Gpam
NO	Acsl5
NO	Zdhhc6
NO	Vti1a
NO	Tcf7l2
NO	Habp2
NO	Nrap
NO	Casp7
NO	Dclre1a
NO	Nhlrc2
NO	Ccdc186
NO	Afap1l2
NO	Ablim1
NO	Fam160b1
NO	Trub1
NO	Atrnl1
NO	Gfra1
NO	Hspa12a
NO	Shtn1
NO	Slc18a2
NO	Pdzd8
NO	Emx2os
NO	Rab11fip2
NO	Fam204a

NO	Cacul1
NO	Eif3a
NO	Fam45a
NO	Sfxn4
NO	Prdx3
NO	Grk5
NO	Zfp950
NO	Csf2ra
NO	Fam171a1
NO	Nmt2
NO	Rpp38
NO	Dclre1c
NO	Suv39h2
NO	Hspa14
NO	Cdnf
NO	Fam107b
NO	Frmd4a
NO	Prpf18
NO	Bend7
NO	Sephs1
NO	Phyh
NO	Optn
NO	Ccdc3
NO	Camk1d
NO	Cdc123
NO	Nudt5
NO	Sec61a2
NO	Dhtkd1
NO	Upf2
NO	Proser2
NO	Echdc3
NO	Usp6nl
NO	Celf2
NO	5031426D15Rik
NO	Taf3
NO	Atp5c1
NO	Kin
NO	Itih2
NO	Itih5
NO	Prkcq
NO	Pfkfb3
NO	Rbm17
NO	Il2ra
NO	Il15ra
NO	Fbxo18
NO	Ankrd16
NO	Itga8
NO	Fam188a

NO	Pter
NO	Rsu1
NO	Trdmt1
NO	Vim
NO	St8sia6
NO	Hacd1
NO	Stam
NO	Mrc1
NO	Cacnb2
NO	Nsun6
NO	Arl5b
NO	Plxdc2
NO	Mllt10
NO	Dnajc1
NO	Commd3
NO	Bmi1
NO	Pip4k2a
NO	Msrb2
NO	Etl4
NO	Arhgap21
NO	Gm13375
NO	Thnsl1
NO	Apbb1ip
NO	Pdss1
NO	Abi1
NO	Acbd5
NO	Yme1l1
NO	Spopl
NO	Hnmt
NO	Psd4
NO	Ehmt1
NO	Arrdc1
NO	Zmynd19
NO	Dph7
NO	Mrpl41
NO	Pnpla7
NO	Nsmf
NO	Nrarp
NO	Tor4a
NO	Nelfb
NO	Tubb4b
NO	Ndor1
NO	Tmem203
NO	Tprn
NO	Ssna1
NO	Anapc2
NO	Man1b1
NO	Dpp7

NO	Uap1l1
NO	Entpd2
NO	Npdc1
NO	Abca2
NO	Clic3
NO	BC029214
NO	C8g
NO	Fbxw5
NO	Traf2
NO	Edf1
NO	Mamdc4
NO	Phpt1
NO	Rabl6
NO	Tmem141
NO	Fcna
NO	Bmyc
NO	Camsap1
NO	Ubac1
NO	Nacc2
NO	C330006A16Rik
NO	Qsox2
NO	Gpsm1
NO	Dnlz
NO	Card9
NO	Snapc4
NO	Sdccag3
NO	Pmpca
NO	Inpp5e
NO	Sec16a
NO	Notch1
NO	Egfl7
NO	Agpat2
NO	Fam69b
NO	Snhg7
NO	Surf6
NO	Med22
NO	Rpl7a
NO	Surf1
NO	Surf2
NO	Surf4
NO	Rexo4
NO	Cacfd1
NO	Slc2a6
NO	Sardh
NO	Vav2
NO	Brd3
NO	Wdr5
NO	Rxra

NO	Col5a1
NO	Olfm1
NO	Ppp1r26
NO	Mrps2
NO	Ralgds
NO	Gtf3c5
NO	Tsc1
NO	Gtf3c4
NO	Ddx31
NO	Ttf1
NO	Setx
NO	Ntng2
NO	6530402F18Rik
NO	Med27
NO	Rapgef1
NO	Trub2
NO	Coq4
NO	Slc27a4
NO	Urm1
NO	Cercam
NO	Odf2
NO	Gle1
NO	Sptan1
NO	Wdr34
NO	Set
NO	Pkn3
NO	Zdhhc12
NO	Zer1
NO	Tbc1d13
NO	Endog
NO	D2Wsu81e
NO	Ccbl1
NO	Lrrc8a
NO	Phyhd1
NO	Dolk
NO	Nup188
NO	Sh3glb2
NO	Fam73b
NO	Dolpp1
NO	Crat
NO	Ppp2r4
NO	Ier5l
NO	Ntmt1
NO	Asb6
NO	Ptges
NO	Tor1b
NO	Tor1a
NO	BC005624

NO	Usp20
NO	Fnbp1
NO	D330023K18Rik
NO	Gpr107
NO	Ncs1
NO	Fubp3
NO	Exosc2
NO	Abl1
NO	Lamc3
NO	Aif1l
NO	Nup214
NO	Fam78a
NO	Ppapdc3
NO	Prrc2b
NO	Pomt1
NO	Uck1
NO	Swi5
NO	Golga2
NO	Dnm1
NO	Ciz1
NO	1110008P14Rik
NO	Lcn2
NO	Ptges2
NO	Slc25a25
NO	Naif1
NO	Fam102a
NO	Dpm2
NO	St6galnac4
NO	St6galnac6
NO	Ak1
NO	Eng
NO	Fpgs
NO	Cdk9
NO	Sh2d3c
NO	6330409D20Rik
NO	Tor2a
NO	Stxbp1
NO	Fam129b
NO	Lrsam1
NO	Rpl12
NO	Slc2a8
NO	Garnl3
NO	Ralgps1
NO	Angptl2
NO	Zbtb34
NO	Zbtb43
NO	Mvb12b
NO	Nron

NO	Pbx3
NO	Mapkap1
NO	Gapvd1
NO	Hspa5
NO	Rabepk
NO	Fbxw2
NO	Psmd5
NO	Cntrl
NO	Rab14
NO	Gsn
NO	Stom
NO	Ggta1
NO	Dab2ip
NO	Ttll11
NO	Ndufa8
NO	Lhx6
NO	Rbm18
NO	Mrrf
NO	Ptgs1
NO	Pdcl
NO	Rc3h2
NO	Zbtb6
NO	Zbtb26
NO	Rabgap1
NO	Gpr21
NO	Strbp
NO	Dennd1a
NO	Nek6
NO	Psmb7
NO	Nr6a1
NO	Olfml2a
NO	Rpl35
NO	Arpc5l
NO	Golga1
NO	Scai
NO	Ppp6c
NO	Arhgap15
NO	Gtdc1
NO	Zeb2
NO	Acvr2a
NO	Orc4
NO	Mbd5
NO	Epc2
NO	Kif5c
NO	Lypd6
NO	Mmadhc
NO	Rnd3
NO	Gm13490

NO	Rbm43
NO	Nmi
NO	Rif1
NO	Neb
NO	4930573O16Rik
NO	Arl5a
NO	Cacnb4
NO	Stam2
NO	Fmnl2
NO	Prpf40a
NO	Arl6ip6
NO	Kcnj3
NO	Nr4a2
NO	Gpd2
NO	Cytip
NO	Acvr1c
NO	Acvr1
NO	Pkp4
NO	Tanc1
NO	Wdsub1
NO	Baz2b
NO	March7
NO	Cd302
NO	Ly75
NO	Pla2r1
NO	Itgb6
NO	Rbms1
NO	Tank
NO	Psmd14
NO	Slc4a10
NO	Dpp4
NO	Fap
NO	Ifih1
NO	Gca
NO	Fign
NO	Grb14
NO	Cobll1
NO	Scn3a
NO	Scn2a1
NO	Galnt3
NO	Ttc21b
NO	Scn7a
NO	Xirp2
NO	B3galt1
NO	Stk39
NO	Cers6
NO	Bbs5
NO	Klhl41

NO	Fastkd1
NO	Ppig
NO	Phospho2
NO	Klhl23
NO	Ssb
NO	Mettl5
NO	Ubr3
NO	Gorasp2
NO	Tlk1
NO	Mettl8
NO	Dcaf17
NO	Dync1i2
NO	Slc25a12
NO	Hat1
NO	Metap1d
NO	Itga6
NO	Pdk1
NO	Rapgef4
NO	Zak
NO	Sp3
NO	Ola1
NO	Cir1
NO	Scrn3
NO	Gpr155
NO	Wipf1
NO	Chrna1
NO	Chn1
NO	Atf2
NO	Atp5g3
NO	Lnp
NO	Hoxd10
NO	Hoxd9
NO	Hoxd8
NO	Hoxd3
NO	Hoxd4
NO	Mtx2
NO	Hnrnpa3
NO	Nfe2l2
NO	Agps
NO	Ttc30b
NO	Ttc30a1
NO	Rbm45
NO	Osbpl6
NO	Prkra
NO	Fkbp7
NO	Plekha3
NO	Ttn
NO	Ccdc141

NO	Sestd1
NO	Zfp385b
NO	Cwc22
NO	Ube2e3
NO	Itga4
NO	Ssfa2
NO	Pde1a
NO	Dnajc10
NO	Frzb
NO	Nckap1
NO	Dusp19
NO	Nup35
NO	Zc3h15
NO	Itgav
NO	Fam171b
NO	Calcrl
NO	Tfpi
NO	Ctnnd1
NO	2700094K13Rik
NO	Tmx2
NO	Med19
NO	Zdhhc5
NO	Clp1
NO	Serping1
NO	Ube2l6
NO	Timm10
NO	Slc43a1
NO	Slc43a3
NO	Ssrp1
NO	Tnks1bp1
NO	Aplnr
NO	Ptprj
NO	Nup160
NO	Fnbp4
NO	Mtch2
NO	C1qtnf4
NO	Ndufs3
NO	Kbtbd4
NO	Ptpmt1
NO	Celf1
NO	Rapsn
NO	Psmc3
NO	Slc39a13
NO	Spi1
NO	Madd
NO	Nr1h3
NO	Acp2
NO	Ddb2

NO	A330069E16Rik
NO	Pacsin3
NO	Arfgap2
NO	1110051M20Rik
NO	Lrp4
NO	Ckap5
NO	Zfp408
NO	Arhgap1
NO	Atg13
NO	Harbi1
NO	Ambra1
NO	Dgkz
NO	Creb3l1
NO	Phf21a
NO	Pex16
NO	Mapk8ip1
NO	Cry2
NO	D930015M05Rik
NO	Slc35c1
NO	Chst1
NO	Trp53i11
NO	Tspan18
NO	Cd82
NO	Ext2
NO	Accs
NO	Gm13889
NO	4921507L20Rik
NO	Alkbh3
NO	Hsd17b12
NO	Ttc17
NO	2810002D19Rik
NO	Api5
NO	B230118H07Rik
NO	Traf6
NO	Prr5l
NO	Commd9
NO	Ldlrad3
NO	Trim44
NO	Pamr1
NO	Cd44
NO	Pdhx
NO	Apip
NO	Cat
NO	Abtb2
NO	Nat10
NO	Caprin1
NO	Lmo2
NO	Fbxo3

NO	Cd59a
NO	D430041D05Rik
NO	Hipk3
NO	Cstf3
NO	Tcp11l1
NO	Depdc7
NO	Qser1
NO	Prrg4
NO	Ccdc73
NO	Eif3m
NO	Rcn1
NO	Elp4
NO	Immp1l
NO	Dnajc24
NO	Mpped2
NO	Arl14ep
NO	Mettl15
NO	Kif18a
NO	Bdnf
NO	Lin7c
NO	Lgr4
NO	Ccdc34
NO	Fibin
NO	Lpcat4
NO	Nop10
NO	Slc12a6
NO	Emc4
NO	Katnbl1
NO	Emc7
NO	Aven
NO	Ryr3
NO	Fmn1
NO	Arhgap11a
NO	Actc1
NO	C130080G10Rik
NO	Aqr
NO	Zfp770
NO	Dph6
NO	BC052040
NO	Meis2
NO	Spred1
NO	Fam98b
NO	Thbs1
NO	Eif2ak4
NO	Srp14
NO	Bmf
NO	Plcb2
NO	Inafm2

NO	A430105I19Rik
NO	Disp2
NO	Knstrn
NO	Ivd
NO	Bahd1
NO	Chst14
NO	Ccdc32
NO	Rpusd2
NO	Rad51
NO	Rmdn3
NO	Dnajc17
NO	Zfyve19
NO	Vps18
NO	Dll4
NO	Chac1
NO	Ino80
NO	Exd1
NO	Chp1
NO	1700020I14Rik
NO	Nusap1
NO	Ndufaf1
NO	Rtf1
NO	Rpap1
NO	Mga
NO	Mapkbp1
NO	Ehd4
NO	Pla2g4e
NO	Vps39
NO	Tmem87a
NO	Ganc
NO	Capn3
NO	Zfp106
NO	Snap23
NO	Lrrc57
NO	Haus2
NO	Cdan1
NO	Ttbk2
NO	Ubr1
NO	Tmem62
NO	Ccndbp1
NO	Lcmt2
NO	Adal
NO	Zscan29
NO	Tubgcp4
NO	Trp53bp1
NO	Map1a
NO	Ppip5k1
NO	Catsper2

NO	Pdia3
NO	Serf2
NO	Serinc4
NO	Hypk
NO	Mfap1b
NO	Mfap1a
NO	Wdr76
NO	Casc4
NO	Ctdspl2
NO	Spg11
NO	B2m
NO	Sord
NO	Duox1
NO	Shf
NO	Slc28a2
NO	Gm14085
NO	Gatm
NO	Spata5l1
NO	Slc30a4
NO	Bloc1s6
NO	Sqrdl
NO	Sema6d
NO	Myef2
NO	Dut
NO	Fbn1
NO	Cep152
NO	Shc4
NO	Eid1
NO	Secisbp2l
NO	Cops2
NO	Galk2
NO	Fgf7
NO	Dtwd1
NO	Atp8b4
NO	Hdc
NO	Gabpb1
NO	Usp8
NO	Trpm7
NO	Sppl2a
NO	Ap4e1
NO	Blvra
NO	Itpripl1
NO	Snrnp200
NO	Ciao1
NO	Tmem127
NO	Stard7
NO	Dusp2
NO	Adra2b

NO	Gpat2
NO	Fahd2a
NO	Zfp661
NO	Mrps5
NO	Mal
NO	Mall
NO	Nphp1
NO	1500011K16Rik
NO	Bcl2l11
NO	Gm14005
NO	Anapc1
NO	Mertk
NO	Tmem87b
NO	Fbln7
NO	Zc3h8
NO	Zc3h6
NO	Ttl
NO	Polr1b
NO	Chchd5
NO	Slc20a1
NO	Sirpa
NO	4932416H05Rik
NO	Stk35
NO	Snrpb
NO	Nop56
NO	Idh3b
NO	Cpxm1
NO	Pced1a
NO	Vps16
NO	Ptpra
NO	Mrps26
NO	Ubox5
NO	Fastkd5
NO	Lzts3
NO	Ddrgk1
NO	Itpa
NO	4930402H24Rik
NO	A730017L22Rik
NO	Atrn
NO	Adam33
NO	Siglec1
NO	Hspa12b
NO	1700037H04Rik
NO	Spef1
NO	Cenpb
NO	Cdc25b
NO	Ap5s1
NO	Mavs

NO	Pank2
NO	Rnf24
NO	Smox
NO	Adra1d
NO	Prnp
NO	Rassf2
NO	Slc23a2
NO	Tmem230
NO	Pcna
NO	Cds2
NO	Gpcpd1
NO	1110034G24Rik
NO	Trmt6
NO	Mcm8
NO	Crls1
NO	Bmp2
NO	Tmx4
NO	Plcb1
NO	Plcb4
NO	Ankef1
NO	Mkks
NO	Slx4ip
NO	Jag1
NO	Btbd3
NO	Tasp1
NO	Esf1
NO	Ndufaf5
NO	Macrod2
NO	Flrt3
NO	Kif16b
NO	Snrpb2
NO	Dstn
NO	Rrbp1
NO	Snx5
NO	Snord17
NO	Mgme1
NO	Csrp2bp
NO	Dzank1
NO	Polr3f
NO	Rbbp9
NO	Sec23b
NO	Gm561
NO	Dtd1
NO	Slc24a3
NO	Rin2
NO	Naa20
NO	Crnkl1
NO	Ralgapa2

NO	Kiz
NO	Xrn2
NO	Thbd
NO	Cd93
NO	Nxt1
NO	Gzf1
NO	Napb
NO	Cst3
NO	Zfp120
NO	Zfp937
NO	Zfp442
NO	Apmap
NO	Acss1
NO	Entpd6
NO	Pygb
NO	Abhd12
NO	Ninl
NO	Nsfl1c
NO	Fkbp1a
NO	Snph
NO	Tmem74b
NO	Psmf1
NO	Fam110a
NO	Slc52a3
NO	Srxn1
NO	Tcf15
NO	Csnk2a1
NO	Tbc1d20
NO	Rbck1
NO	Trib3
NO	Sox12
NO	Zcchc3
NO	Rem1
NO	H13
NO	Mcts2
NO	Id1
NO	Bcl2l1
NO	Tpx2
NO	Mylk2
NO	Dusp15
NO	Pdrg1
NO	Ccm2l
NO	Hck
NO	Tm9sf4
NO	Tspyl3
NO	Plagl2
NO	Pofut1
NO	Kif3b

NO	Asxl1
NO	Nol4l
NO	Commd7
NO	Dnmt3b
NO	Mapre1
NO	Cdk5rap1
NO	Snta1
NO	Cbfa2t2
NO	E2f1
NO	Pxmp4
NO	Zfp341
NO	Chmp4b
NO	Raly
NO	Eif2s2
NO	Ahcy
NO	Itch
NO	Dynlrb1
NO	Map1lc3a
NO	Pigu
NO	Trp53inp2
NO	Ncoa6
NO	Acss2
NO	Gss
NO	Myh7b
NO	Trpc4ap
NO	Edem2
NO	Procr
NO	BC029722
NO	Eif6
NO	Uqcc1
NO	Cep250
NO	Ergic3
NO	Cpne1
NO	Rbm12
NO	Nfs1
NO	Romo1
NO	Rbm39
NO	Phf20
NO	Scand1
NO	2900097C17Rik
NO	Epb4.1l1
NO	Aar2
NO	Dlgap4
NO	Myl9
NO	Tgif2
NO	5430405H02Rik
NO	1110008F13Rik
NO	Ndrg3

NO	Dsn1
NO	Soga1
NO	Samhd1
NO	Rbl1
NO	Rpn2
NO	Manbal
NO	Src
NO	Blcap
NO	Nnat
NO	Ctnnbl1
NO	Tti1
NO	Rprd1b
NO	Tgm2
NO	D630003M21Rik
NO	Lbp
NO	Snhg17
NO	Snhg11
NO	Ralgapb
NO	Adig
NO	Actr5
NO	Ppp1r16b
NO	Fam83d
NO	Dhx35
NO	Mafb
NO	Top1
NO	Plcg1
NO	Zhx3
NO	Lpin3
NO	Chd6
NO	Srsf6
NO	Ift52
NO	Jph2
NO	Oser1
NO	Fitm2
NO	Ttpal
NO	Serinc3
NO	Pkig
NO	Ada
NO	Wisp2
NO	Ywhab
NO	Tomm34
NO	Stk4
NO	Kcns1
NO	Sdc4
NO	Sys1
NO	Dbndd2
NO	Pigt
NO	Dnttip1

NO	Tnnc2
NO	Snx21
NO	Acot8
NO	Zswim3
NO	Zswim1
NO	Neurl2
NO	Ctsa
NO	Pltp
NO	Pcif1
NO	Zfp335
NO	Mmp9
NO	Slc12a5
NO	Ncoa5
NO	Cdh22
NO	Slc35c2
NO	Elmo2
NO	Zfp334
NO	Trp53rka
NO	Slc2a10
NO	Eya2
NO	Zmynd8
NO	Ncoa3
NO	Sulf2
NO	Prex1
NO	Trp53rkb
NO	Arfgef2
NO	Cse1l
NO	Stau1
NO	Ddx27
NO	Znfx1
NO	1500012F01Rik
NO	Kcnb1
NO	Ptgis
NO	B4galt5
NO	Slc9a8
NO	Spata2
NO	Rnf114
NO	Ube2v1
NO	Tmem189
NO	Cebpb
NO	A530013C23Rik
NO	Ptpn1
NO	Pard6b
NO	Adnp
NO	Dpm1
NO	Mocs3
NO	Nfatc2
NO	Atp9a

NO	Sall4
NO	Zfp64
NO	Tshz2
NO	Zfp217
NO	Bcas1
NO	Pfdn4
NO	Fam210b
NO	Aurka
NO	Cstf1
NO	Rtfdc1
NO	Gcnt7
NO	Bmp7
NO	Rae1
NO	Rbm38
NO	Ctcfl
NO	Ctcflos
NO	Pck1
NO	Pmepa1
NO	Ppp4r1l-ps
NO	Rab22a
NO	Vapb
NO	Stx16
NO	Npepl1
NO	Gnas
NO	Nelfcd
NO	Ctsz
NO	Atp5e
NO	Slmo2
NO	Gm14305
NO	Gm14295
NO	Gm14420
NO	Gm14403
NO	Gm14322
NO	Gm14325
NO	Gm14326
NO	Etohi1
NO	Zfp931
NO	Ppp1r3d
NO	Fam217b
NO	Cdh4
NO	Taf4a
NO	4921531C22Rik
NO	Lsm14b
NO	Psma7
NO	Ss18l1
NO	Mtg2
NO	Osbpl2
NO	Adrm1

NO	Lama5
NO	Rps21
NO	Cables2
NO	B230312C02Rik
NO	Gm6307
NO	Mir1a-1
NO	Mir133a-2
NO	Mrgbp
NO	Ogfr
NO	Dido1
NO	Gid8
NO	Ythdf1
NO	Arfgap1
NO	Col20a1
NO	Eef1a2
NO	Ppdpf
NO	Helz2
NO	Gmeb2
NO	Rtel1
NO	Arfrp1
NO	Zgpat
NO	Lime1
NO	Slc2a4rg-ps
NO	Zbtb46
NO	Tpd52l2
NO	Dnajc5
NO	Uckl1
NO	Uckl1os
NO	Znf512b
NO	Samd10
NO	Prpf6
NO	Sox18
NO	Tcea2
NO	Rgs19
NO	Pcmtd2
NO	Polr3k
NO	Zfhx4
NO	Pex2
NO	Pkia
NO	Zc2hc1a
NO	Stmn2
NO	Hey1
NO	Mrps28
NO	Tpd52
NO	Zbtb10
NO	Zfp704
NO	Pag1
NO	Fabp5

NO	Fabp4
NO	Impa1
NO	Zfand1
NO	Snx16
NO	Lrrcc1
NO	E2f5
NO	1810022K09Rik
NO	Car3
NO	Car2
NO	Ythdf3
NO	Armc1
NO	Mtfr1
NO	Pde7a
NO	Trim55
NO	4632415L05Rik
NO	Cp
NO	Hps3
NO	Hltf
NO	Gyg
NO	Cpa3
NO	Tbl1xr1
NO	Nceh1
NO	Tnfsf10
NO	Fndc3b
NO	Pld1
NO	Tnik
NO	Eif5a2
NO	Rpl22l1
NO	Mecom
NO	Mynn
NO	Sec62
NO	Gpr160
NO	Phc3
NO	Prkci
NO	Skil
NO	Zmat3
NO	Pik3ca
NO	Zfp639
NO	Mfn1
NO	Gnb4
NO	Actl6a
NO	Mrpl47
NO	Ndufb5
NO	Usp13
NO	Ttc14
NO	Fxr1
NO	Dnajc19
NO	Atp11b

NO	Dcun1d1
NO	Mccc1
NO	Acad9
NO	D3Ertd254e
NO	Anxa5
NO	Exosc9
NO	Bbs7
NO	Trpc3
NO	4932438A13Rik
NO	Bbs12
NO	Fgf2
NO	Nudt6
NO	Spata5
NO	Spry1
NO	Ankrd50
NO	Fat4
NO	Intu
NO	Hspa4l
NO	Plk4
NO	Mfsd8
NO	3110057O12Rik
NO	Larp1b
NO	Pgrmc2
NO	Jade1
NO	Sclt1
NO	D3Ertd751e
NO	Pcdh18
NO	Slc7a11
NO	Ccrn4l
NO	Elf2
NO	4930577N17Rik
NO	Ndufc1
NO	Naa15
NO	Rab33b
NO	Setd7
NO	Maml3
NO	Foxo1
NO	Cog6
NO	Lhfp
NO	Nhlrc3
NO	Proser1
NO	Frem2
NO	Ufm1
NO	Postn
NO	Supt20
NO	Exosc8
NO	Alg5
NO	Rfxap

NO	Spg20
NO	Dclk1
NO	Nbea
NO	Mab21l1
NO	Tm4sf1
NO	Wwtr1
NO	Commd2
NO	Rnf13
NO	Pfn2
NO	Tsc22d2
NO	Serp1
NO	Eif2a
NO	Selt
NO	Siah2
NO	Med12l
NO	P2ry14
NO	Igsf10
NO	Mbnl1
NO	P2ry1
NO	Rap2b
NO	Arhgef26
NO	Dhx36
NO	Mme
NO	E130311K13Rik
NO	Slc33a1
NO	Gmps
NO	Kcnab1
NO	A330015K06Rik
NO	Ssr3
NO	4931440P22Rik
NO	Tiparp
NO	Ccnl1
NO	Ptx3
NO	Shox2
NO	Rsrc1
NO	Mlf1
NO	Gfm1
NO	Lxn
NO	Rarres1
NO	Mfsd1
NO	Il12a
NO	Ift80
NO	Smc4
NO	Trim59
NO	Kpna4
NO	Ppm1l
NO	B3galnt1
NO	Nmd3

NO	Bche
NO	Pdcd10
NO	Serpini1
NO	Golim4
NO	Rapgef2
NO	4921511C10Rik
NO	Fnip2
NO	Ppid
NO	Etfdh
NO	4930579G24Rik
NO	Fam198b
NO	Pdgfc
NO	Ctso
NO	Gucy1b3
NO	Gucy1a3
NO	Map9
NO	Plrg1
NO	Sfrp2
NO	Tlr2
NO	D930015E06Rik
NO	Trim2
NO	Arfip1
NO	Tigd4
NO	Tmem154
NO	Fbxw7
NO	Gatb
NO	Fam160a1
NO	Glt28d2
NO	Sh3d19
NO	Rps3a1
NO	Lrba
NO	Mab21l2
NO	Dclk2
NO	Cd1d1
NO	Kirrel
NO	Fcrls
NO	Etv3
NO	Arhgef11
NO	Lrrc71
NO	Pear1
NO	Prcc
NO	Hdgf
NO	Mrpl24
NO	Rrnad1
NO	Isg20l2
NO	Nes
NO	Gpatch4
NO	Apoa1bp

NO	Mef2d
NO	1700113A16Rik
NO	Tsacc
NO	Cct3
NO	Glmp
NO	Smg5
NO	Pmf1
NO	Slc25a44
NO	Sema4a
NO	Lmna
NO	Lamtor2
NO	Ubqln4
NO	Ssr2
NO	Arhgef2
NO	2810403A07Rik
NO	Rit1
NO	Syt11
NO	5830417I10Rik
NO	1500004A13Rik
NO	Gon4l
NO	Msto1
NO	Dap3
NO	Ash1l
NO	Rusc1
NO	Fdps
NO	Clk2
NO	Fam189b
NO	Gba
NO	Mtx1
NO	Thbs3
NO	Krtcap2
NO	Dpm3
NO	Slc50a1
NO	Efna1
NO	Adam15
NO	Zbtb7b
NO	Gm15417
NO	Lenep
NO	Flad1
NO	Cks1b
NO	Shc1
NO	Pygo2
NO	Pbxip1
NO	Pmvk
NO	Kcnn3
NO	Adar
NO	4632404H12Rik
NO	Ube2q1

NO	She
NO	Il6ra
NO	Atp8b2
NO	Hax1
NO	Ubap2l
NO	4933434E20Rik
NO	1700094D03Rik
NO	Rab13
NO	Jtb
NO	Slc39a1
NO	Crtc2
NO	Dennd4b
NO	Gatad2b
NO	Slc27a3
NO	Ints3
NO	Npr1
NO	Ilf2
NO	Snapin
NO	Chtop
NO	S100a1
NO	S100a13
NO	S100a16
NO	S100a4
NO	S100a6
NO	S100a8
NO	S100a9
NO	S100a11
NO	S100a10
NO	Them4
NO	Rorc
NO	Tdrkh
NO	Mrpl9
NO	Riiad1
NO	Celf3
NO	Snx27
NO	Selenbp2
NO	Pogz
NO	Psmb4
NO	Selenbp1
NO	Rfx5
NO	Pi4kb
NO	Zfp687
NO	Psmd4
NO	Pip5k1a
NO	Vps72
NO	Tmod4
NO	Scnm1
NO	Lysmd1

NO	Tnfaip8l2
NO	Sema6c
NO	Gabpb2
NO	Mllt11
NO	Cdc42se1
NO	Prune
NO	Fam63a
NO	Cers2
NO	Setdb1
NO	Arnt
NO	Ctsk
NO	Ctss
NO	Golph3l
NO	Ensa
NO	Mcl1
NO	Adamtsl4
NO	Ecm1
NO	Tars2
NO	Rprd2
NO	Prpf3
NO	Mrps21
NO	Ciart
NO	Aph1a
NO	Car14
NO	Anp32e
NO	Plekho1
NO	Vps45
NO	Otud7b
NO	Mtmr11
NO	Sf3b4
NO	Bola1
NO	Hist2h2ab
NO	Hist2h2ac
NO	Hist2h2be
NO	Hist2h4
NO	Hist2h3b
NO	Hist2h2bb
NO	Hfe2
NO	Txnip
NO	Polr3gl
NO	Ankrd34a
NO	Lix1l
NO	Rbm8a
NO	Pex11b
NO	Itga10
NO	Pias3
NO	Nudt17
NO	Polr3c

NO	Rnf115
NO	Pdzk1
NO	Gpr89
NO	Gja5
NO	Acp6
NO	Bcl9
NO	Chd1l
NO	Fmo5
NO	Prkab2
NO	Pde4dip
NO	Sec22b
NO	Notch2
NO	Hmgcs2
NO	Phgdh
NO	Zfp697
NO	Wars2
NO	Tbx15
NO	Wdr3
NO	Gdap2
NO	Fam46c
NO	Man1a2
NO	Trim45
NO	Ttf2
NO	Ptgfrn
NO	Igsf3
NO	Atp1a1
NO	Slc22a15
NO	Casq2
NO	Vangl1
NO	Tspan2
NO	Sike1
NO	Csde1
NO	Nras
NO	Ampd1
NO	Dennd2c
NO	Bcas2
NO	Trim33
NO	Olfml3
NO	Hipk1
NO	Dclre1b
NO	Ap4b1
NO	Bcl2l15
NO	Ptpn22
NO	Rsbn1
NO	Phtf1
NO	Magi3
NO	Lrig2
NO	Slc16a1

NO	Fam19a3
NO	Ppm1j
NO	Rhoc
NO	Mov10
NO	Capza1
NO	St7l
NO	Wnt2b
NO	Cttnbp2nl
NO	Ddx20
NO	Fam212b
NO	Rap1a
NO	Atp5f1
NO	Wdr77
NO	Ovgp1
NO	Dennd2d
NO	Cept1
NO	Dram2
NO	Lrif1
NO	Cd53
NO	Kcna2
NO	Lamtor5
NO	Slc16a4
NO	Rbm15
NO	Kcnc4
NO	Slc6a17
NO	Strip1
NO	Ahcyl1
NO	Csf1
NO	4933431E20Rik
NO	Gstm5
NO	Gstm7
NO	Gstm2
NO	Gstm1
NO	Gstm4
NO	Ampd2
NO	Gnai3
NO	Amigo1
NO	Cyb561d1
NO	Atxn7l2
NO	Sypl2
NO	Psma5
NO	Sort1
NO	Celsr2
NO	Sars
NO	Scarna2
NO	Tmem167b
NO	Taf13
NO	Wdr47

NO	Clcc1
NO	Gpsm2
NO	Stxbp3
NO	Prpf38b
NO	Fam102b
NO	Slc25a24
NO	Vav3
NO	Ntng1
NO	Prmt6
NO	Amy1
NO	Rnpc3
NO	Col11a1
NO	S1pr1
NO	A930005H10Rik
NO	Dph5
NO	Slc30a7
NO	Extl2
NO	Vcam1
NO	Cdc14a
NO	Rtca
NO	Dbt
NO	Lrrc39
NO	Trmt13
NO	Sass6
NO	Hiat1
NO	Slc35a3
NO	Agl
NO	Frrs1
NO	Palmd
NO	Snx7
NO	Dpyd
NO	Ptbp2
NO	Rwdd3
NO	Tmem56
NO	Alg14
NO	Cnn3
NO	F3
NO	Abcd3
NO	Arhgap29
NO	Abca4
NO	Gclm
NO	Dnttip2
NO	Bcar3
NO	Fnbp1l
NO	Pde5a
NO	1810037I17Rik
NO	Usp53
NO	Myoz2

NO	Synpo2
NO	Sec24d
NO	Mettl14
NO	Prss12
NO	Snhg8
NO	Ugt8a
NO	Camk2d
NO	Ank2
NO	LOC100862268
NO	Larp7
NO	Zgrf1
NO	Alpk1
NO	Tifa
NO	Ap1ar
NO	5730508B09Rik
NO	Pitx2
NO	Enpep
NO	Elovl6
NO	Egf
NO	6330410L21Rik
NO	Gar1
NO	Pla2g12a
NO	Casp6
NO	Sec24b
NO	Ostc
NO	Hadh
NO	Cyp2u1
NO	Sgms2
NO	Papss1
NO	Dkk2
NO	Aimp1
NO	Tbck
NO	Npnt
NO	Gstcd
NO	Ints12
NO	Ppa2
NO	Tet2
NO	Cisd2
NO	4930539J05Rik
NO	Ube2d3
NO	Manba
NO	Nfkb1
NO	Bank1
NO	Ppp3ca
NO	Emcn
NO	Gm4861
NO	Ddit4l
NO	H2afz

NO	Dnajb14
NO	Lamtor3
NO	Dapp1
NO	Gm5105
NO	Mttp
NO	Adh1
NO	Adh5
NO	Metap1
NO	Eif4e
NO	Tspan5
NO	Rap1gds1
NO	Bmpr1b
NO	Pdlim5
NO	Gbp5
NO	Gbp7
NO	Gbp3
NO	Gbp2b
NO	Gbp2
NO	Ccbl2
NO	Gtf2b
NO	Pkn2
NO	Lmo4
NO	Hs2st1
NO	Sep15
NO	Sh3glb1
NO	Clca3a1
NO	Odf2l
NO	Col24a1
NO	Znhit6
NO	Cyr61
NO	Ddah1
NO	Bcl10
NO	2410004B18Rik
NO	Syde2
NO	Ssx2ip
NO	Ctbs
NO	Spata1
NO	Gng5
NO	Rpf1
NO	Prkacb
NO	Ttll7
NO	Adgrl2
NO	Adgrl4
NO	Ptgfr
NO	Dnajb4
NO	Fubp1
NO	Nexn
NO	Fam73a

NO	Usp33
NO	Zzz3
NO	Pigk
NO	St6galnac3
NO	Rabggtb
NO	Acadm
NO	Tyw3
NO	Cryz
NO	Fpgt
NO	Negr1
NO	Zranb2
NO	Ptger3
NO	Cth
NO	Ankrd13c
NO	Srsf11
NO	Lrrc40
NO	Wls
NO	Tmem68
NO	Tgs1
NO	Lyn
NO	Rps20
NO	Plag1
NO	Chchd7
NO	Penk
NO	Impad1
NO	Fam110b
NO	Ubxn2b
NO	Sdcbp
NO	Nsmaf
NO	Tox
NO	Car8
NO	Rab2a
NO	Chd7
NO	Asph
NO	2610301B20Rik
NO	Plekhf2
NO	Ndufaf6
NO	Trp53inp1
NO	Ccne2
NO	Ints8
NO	Dpy19l4
NO	1110037F02Rik
NO	Gem
NO	Pdp1
NO	1700123M08Rik
NO	Tmem67
NO	Rbm12b2
NO	Rbm12b1

NO	Fam92a
NO	Runx1t1
NO	Otud6b
NO	Tmem55a
NO	Tmem64
NO	Decr1
NO	Nbn
NO	Osgin2
NO	Ripk2
NO	Cpne3
NO	Rmdn1
NO	Wwp1
NO	Ggh
NO	Ccnc
NO	Tstd3
NO	Usp45
NO	Pnisr
NO	Coq3
NO	Fbxl4
NO	Ndufaf4
NO	Fhl5
NO	Ufl1
NO	Manea
NO	Epha7
NO	Map3k7
NO	Bach2
NO	Casp8ap2
NO	Mdn1
NO	Lyrm2
NO	Ankrd6
NO	Rragd
NO	4933421O10Rik
NO	Ube2j1
NO	Gabrr2
NO	Pm20d2
NO	Pnrc1
NO	Rngtt
NO	Cnr1
NO	Akirin2
NO	Orc3
NO	Rars2
NO	Slc35a1
NO	Smim8
NO	Zfp292
NO	Mob3b
NO	3110043O21Rik
NO	Aco1
NO	Ddx58

NO	Topors
NO	Toporsos
NO	Ndufb6
NO	Aptx
NO	Dnaja1
NO	Smu1
NO	B4galt1
NO	Bag1
NO	Chmp5
NO	Nfx1
NO	Aqp7
NO	Nol6
NO	Ube2r2
NO	Ubap2
NO	Dcaf12
NO	Ubap1
NO	Kif24
NO	Nudt2
NO	AI464131
NO	Fam219aos
NO	Fam219a
NO	Enho
NO	Cntfr
NO	Rpp25l
NO	Dctn3
NO	Sigmar1
NO	Galt
NO	Il11ra1
NO	Ccl27a
NO	4933409K07Rik
NO	Dnajb5
NO	Vcp
NO	Fancg
NO	Pigo
NO	Stoml2
NO	Fam214b
NO	Unc13b
NO	Rusc2
NO	Tesk1
NO	Rmrp
NO	Ccdc107
NO	Car9
NO	Tpm2
NO	Tln1
NO	Creb3
NO	Gba2
NO	Rgp1
NO	Npr2

NO	Hint2
NO	Tmem8b
NO	Hrct1
NO	5430416O09Rik
NO	Reck
NO	Glipr2
NO	Clta
NO	Gne
NO	Rnf38
NO	Zcchc7
NO	Grhpr
NO	Zbtb5
NO	Polr1e
NO	Fbxo10
NO	Tomm5
NO	Frmpd1
NO	Trmt10b
NO	Exosc3
NO	Dcaf10
NO	Slc25a51
NO	Shb
NO	Tdrd7
NO	Tmod1
NO	Tstd2
NO	Ncbp1
NO	Xpa
NO	5830415F09Rik
NO	Anp32b
NO	Nans
NO	Trim14
NO	Coro2a
NO	Tbc1d2
NO	Anks6
NO	Col15a1
NO	Tgfbr1
NO	Alg2
NO	Sec61b
NO	Nr4a3
NO	Stx17
NO	Erp44
NO	Invs
NO	Tex10
NO	Msantd3
NO	Tmeff1
NO	Murc
NO	Mrpl50
NO	Zfp189
NO	Tmem246

NO	Rnf20
NO	Smc2
NO	Nipsnap3b
NO	Abca1
NO	Slc44a1
NO	Fsd1l
NO	Fktn
NO	Tmem38b
NO	Zfp462
NO	Rad23b
NO	Klf4
NO	Ikbkap
NO	Fam206a
NO	Ctnnal1
NO	Tmem245
NO	Epb4.1l4b
NO	Ptpn3
NO	Palm2
NO	Txn1
NO	Svep1
NO	Musk
NO	Lpar1
NO	AI314180
NO	Ptgr1
NO	Dnajc25
NO	Gng10
NO	Ugcg
NO	Susd1
NO	Ptbp3
NO	Hsdl2
NO	E130308A19Rik
NO	Inip
NO	Snx30
NO	Zfp37
NO	Slc31a2
NO	Fkbp15
NO	Slc31a1
NO	Cdc26
NO	Prpf4
NO	Hdhd3
NO	Alad
NO	Pole3
NO	Rgs3
NO	Col27a1
NO	Orm1
NO	Akna
NO	Whrn
NO	Atp6v1g1

NO	6330416G13Rik
NO	Tnc
NO	Pappa
NO	Astn2
NO	Trim32
NO	Tlr4
NO	Cdk5rap2
NO	Megf9
NO	Tle1
NO	C630043F03Rik
NO	Aldoart1
NO	2310002L09Rik
NO	Kdm4c
NO	Tmem261
NO	Ptprd
NO	Lurap1l
NO	Mpdz
NO	Nfib
NO	Zdhhc21
NO	Frem1
NO	Ttc39b
NO	Snapc3
NO	Psip1
NO	Ccdc171
NO	Bnc2
NO	Cntln
NO	Adamtsl1
NO	Rraga
NO	Haus6
NO	Plin2
NO	Dennd4c
NO	Rps6
NO	Acer2
NO	Slc24a2
NO	Mllt3
NO	Focad
NO	Hacd4
NO	Klhl9
NO	Mtap
NO	Cdkn2b
NO	Tusc1
NO	Caap1
NO	Plaa
NO	Gm12657
NO	Ift74
NO	Tek
NO	Mysm1
NO	Jun

NO	Fggy
NO	Hook1
NO	Cyp2j6
NO	Cyp2j9
NO	Nfia
NO	Tm2d1
NO	Inadl
NO	Kank4
NO	Usp1
NO	Dock7
NO	Atg4c
NO	Foxd3
NO	Alg6
NO	Itgb3bp
NO	Pgm2
NO	Ror1
NO	Cachd1
NO	Raver2
NO	Jak1
NO	E130102H24Rik
NO	0610043K17Rik
NO	Ak4
NO	Leprot
NO	Lepr
NO	Pde4b
NO	Sgip1
NO	Mier1
NO	Slc35d1
NO	Oma1
NO	Prkaa2
NO	Ppap2b
NO	Usp24
NO	Dhcr24
NO	Pars2
NO	Ttc4
NO	Acot11
NO	Ssbp3
NO	Mrpl37
NO	Cyb5rl
NO	Tceanc2
NO	Tmem59
NO	Lrrc42
NO	Hspb11
NO	Yipf1
NO	Ndc1
NO	Magoh
NO	0610037L13Rik
NO	Cpt2

NO	Podn
NO	Scp2
NO	Echdc2
NO	Zyg11b
NO	Coa7
NO	Gpx7
NO	Zcchc11
NO	Prpf38a
NO	Orc1
NO	Cc2d1b
NO	Zfyve9
NO	3110021N24Rik
NO	Btf3l4
NO	Txndc12
NO	Kti12
NO	Nrd1
NO	Osbpl9
NO	Eps15
NO	Ttc39a
NO	Rnf11
NO	Cdkn2c
NO	Faf1
NO	Bend5
NO	Spata6
NO	Trabd2b
NO	Cmpk1
NO	Tal1
NO	Cyp4b1
NO	Efcab14
NO	Atpaf1
NO	Mob3c
NO	Mknk1
NO	Nsun4
NO	Uqcrh
NO	Lrrc41
NO	Rad54l
NO	Lurap1
NO	Pomgnt1
NO	Pik3r3
NO	Mast2
NO	Ipp
NO	Tmem69
NO	Gpbp1l1
NO	Ccdc17
NO	Nasp
NO	Akr1a1
NO	Prdx1
NO	Mmachc

NO	Tesk2
NO	Toe1
NO	Mutyh
NO	Urod
NO	Hectd3
NO	Eif2b3
NO	Ptch2
NO	Btbd19
NO	Plk3
NO	Rps8
NO	Tmem53
NO	Rnf220
NO	Eri3
NO	Dmap1
NO	Slc6a9
NO	B4galt2
NO	Atp6v0b
NO	Dph2
NO	Ipo13
NO	St3gal3
NO	Kdm4a
NO	Ptprf
NO	Hyi
NO	Szt2
NO	Med8
NO	Elovl1
NO	Tie1
NO	Ebna1bp2
NO	Slc2a1
NO	Zfp691
NO	Ermap
NO	Ccdc23
NO	4930538K18Rik
NO	AU022252
NO	P3h1
NO	Cldn19
NO	Ybx1
NO	Ppih
NO	Ppcs
NO	AA415398
NO	Foxj3
NO	Hivep3
NO	Foxo6
NO	Scmh1
NO	Ctps
NO	Cited4
NO	Kcnq4
NO	Nfyc

NO	Exo5
NO	Smap2
NO	Zmpste24
NO	Rlf
NO	Ppt1
NO	Cap1
NO	Trit1
NO	Ppie
NO	Nt5c1a
NO	Heyl
NO	Pabpc4
NO	Macf1
NO	D830031N03Rik
NO	Akirin1
NO	Mycbp
NO	Rragc
NO	Pou3f1
NO	Utp11l
NO	Fhl3
NO	Sf3a3
NO	Inpp5b
NO	Mtf1
NO	1110065P20Rik
NO	Yrdc
NO	Maneal
NO	9930104L06Rik
NO	Gnl2
NO	Snip1
NO	Meaf6
NO	Zc3h12a
NO	Grik3
NO	Csf3r
NO	Mrps15
NO	Lsm10
NO	Stk40
NO	Eva1b
NO	Sh3d21
NO	Thrap3
NO	Map7d1
NO	Trappc3
NO	Col8a2
NO	Adprhl2
NO	Ago3
NO	Ago1
NO	Ago4
NO	5730409E04Rik
NO	Psmb2
NO	Ncdn

NO	AU040320
NO	Zmym4
NO	Sfpq
NO	Zmym1
NO	Zmym6
NO	Smim12
NO	Gja4
NO	Zscan20
NO	Phc2
NO	Zfp362
NO	Trim62
NO	Azin2
NO	Ak2
NO	Rnf19b
NO	Fndc5
NO	S100pbp
NO	Yars
NO	C77080
NO	Sync
NO	Rbbp4
NO	Zbtb8os
NO	Zbtb8a
NO	Bsdc1
NO	Marcksl1
NO	Hdac1
NO	Eif3i
NO	Tmem234
NO	Dcdc2b
NO	Iqcc
NO	Ccdc28b
NO	Txlna
NO	Kpna6
NO	Tmem39b
NO	Khdrbs1
NO	Ptp4a2
NO	Adgrb2
NO	Col16a1
NO	Pef1
NO	Tinagl1
NO	Serinc2
NO	Fabp3
NO	Zcchc17
NO	Snrnp40
NO	Nkain1
NO	Pum1
NO	Sdc3
NO	Laptm5
NO	Ptpru

NO	Mecr
NO	Srsf4
NO	Gm12992
NO	Tmem200b
NO	Epb4.1
NO	Ythdf2
NO	Gmeb1
NO	Rnu11
NO	Taf12
NO	Snhg12
NO	Snora44
NO	Trnau1ap
NO	Rcc1
NO	Snhg3
NO	Phactr4
NO	Med18
NO	Sesn2
NO	Atpif1
NO	Dnajc8
NO	Ptafr
NO	Eya3
NO	Xkr8
NO	Rpa2
NO	Themis2
NO	Ppp1r8
NO	Stx12
NO	Fam76a
NO	Ahdc1
NO	Wasf2
NO	Map3k6
NO	Sytl1
NO	Tmem222
NO	Wdtc1
NO	Slc9a1
NO	Fam46b
NO	Nudc
NO	Gpatch3
NO	Gpn2
NO	Zdhhc18
NO	Pigv
NO	Arid1a
NO	Rps6ka1
NO	Hmgn2
NO	Dhdds
NO	Lin28a
NO	Ubxn11
NO	Sh3bgrl3
NO	Cep85

NO	Cnksr1
NO	Grrp1
NO	Pdik1l
NO	Trim63
NO	Slc30a2
NO	Extl1
NO	Pafah2
NO	Stmn1
NO	Paqr7
NO	Mtfr1l
NO	Sepn1
NO	Man1c1
NO	Ldlrap1
NO	Tmem57
NO	Tmem50a
NO	Rsrp1
NO	Syf2
NO	Clic4
NO	Srrm1
NO	Ncmap
NO	Rcan3
NO	Nipal3
NO	Srsf10
NO	Pnrc2
NO	Cnr2
NO	Fuca1
NO	Hmgcl
NO	Gale
NO	Lypla2
NO	Pithd1
NO	Tceb3
NO	Rpl11
NO	Id3
NO	E2f2
NO	Asap3
NO	Tcea3
NO	Zfp46
NO	Hnrnpr
NO	Luzp1
NO	Kdm1a
NO	Ephb2
NO	C1qb
NO	C1qc
NO	C1qa
NO	Zbtb40
NO	Wnt4
NO	Cdc42
NO	Hspg2

NO	Usp48
NO	Rap1gap
NO	Alpl
NO	Ece1
NO	Eif4g3
NO	1700095J12Rik
NO	Hp1bp3
NO	Ddost
NO	Pink1
NO	Mul1
NO	Camk2n1
NO	Pla2g2d
NO	Pla2g5
NO	Otud3
NO	Tmco4
NO	Nbl1
NO	Minos1
NO	Capzb
NO	Pqlc2
NO	Akr7a5
NO	Mrto4
NO	Emc1
NO	Ubr4
NO	Iffo2
NO	Aldh4a1
NO	Pax7
NO	Klhdc7a
NO	Arhgef10l
NO	Rcc2
NO	Padi2
NO	Sdhb
NO	Atp13a2
NO	Mfap2
NO	Crocc
NO	Necap2
NO	Szrd1
NO	Fbxo42
NO	Epha2
NO	Zbtb17
NO	Spen
NO	B330016D10Rik
NO	Fblim1
NO	Plekhm2
NO	Ddi2
NO	Dnajc16
NO	Casp9
NO	Efhd2
NO	Tmem51

NO	Kazn
NO	Prdm2
NO	Pdpn
NO	Lrrc38
NO	Pramef8
NO	Dhrs3
NO	Vps13d
NO	Tnfrsf1b
NO	2610305D13Rik
NO	Rps19-ps3
NO	Zfp933
NO	Miip
NO	Fv1
NO	Mfn2
NO	Plod1
NO	2510039O18Rik
NO	Clcn6
NO	Mthfr
NO	Agtrap
NO	Mad2l2
NO	Fbxo6
NO	Fbxo44
NO	Ubiad1
NO	Mtor
NO	Angptl7
NO	Exosc10
NO	Srm
NO	Masp2
NO	Tardbp
NO	Gm572
NO	Casz1
NO	Pex14
NO	Dffa
NO	Pgd
NO	Kif1b
NO	Ube4b
NO	Rbp7
NO	Nmnat1
NO	Lzic
NO	Ctnnbip1
NO	Clstn1
NO	Pik3cd
NO	Tmem201
NO	Slc25a33
NO	Spsb1
NO	H6pd
NO	Gpr157
NO	Rere

NO	Errfi1
NO	Park7
NO	Per3
NO	Vamp3
NO	Camta1
NO	Dnajc11
NO	Thap3
NO	Phf13
NO	Klhl21
NO	Zbtb48
NO	Tas1r1
NO	Nol9
NO	Plekhg5
NO	Tnfrsf25
NO	Acot7
NO	Gpr153
NO	Icmt
NO	Rpl22
NO	Kcnab2
NO	A430005L14Rik
NO	Dffb
NO	Cep104
NO	Lrrc47
NO	Smim1
NO	Wdr8
NO	Tprgl
NO	Megf6
NO	Prdm16
NO	5930403L14Rik
NO	Fam213b
NO	Tnfrsf14
NO	Pank4
NO	Pex10
NO	Rer1
NO	Morn1
NO	Ski
NO	Faap20
NO	Prkcz
NO	Tmem52
NO	Gnb1
NO	Nadk
NO	Slc35e2
NO	Gm16023
NO	Cdk11b
NO	Mmp23
NO	Mib2
NO	B930041F14Rik
NO	Ssu72

NO	Atad3a
NO	Vwa1
NO	Tmem88b
NO	Mrpl20
NO	Ccnl2
NO	Aurkaip1
NO	Mxra8
NO	Dvl1
NO	Tas1r3
NO	Cptp
NO	Cpsf3l
NO	Pusl1
NO	Acap3
NO	Ube2j2
NO	Fam132a
NO	B3galt6
NO	Sdf4
NO	Tnfrsf4
NO	Gm10560
NO	Tnfrsf18
NO	9430015G10Rik
NO	Agrn
NO	AW011738
NO	Perm1
NO	Plekhn1
NO	Klhl17
NO	Noc2l
NO	Cdk6
NO	Fam133b
NO	1700109H08Rik
NO	Rbm48
NO	Pex1
NO	Gatad1
NO	Ankib1
NO	Krit1
NO	Akap9
NO	Cyp51
NO	Fzd1
NO	Cdk14
NO	Cldn12
NO	Gtpbp10
NO	Steap2
NO	Steap4
NO	Sri
NO	Adam22
NO	Slc25a40
NO	Rundc3b
NO	Abcb1a

NO	Abcb1b
NO	Abcb4
NO	Crot
NO	Tmem243
NO	Dmtf1
NO	Sema3d
NO	Sema3a
NO	Sema3e
NO	Pclo
NO	Cacna2d1
NO	Sema3c
NO	Cd36
NO	Gnai1
NO	Magi2
NO	Phtf2
NO	Tmem60
NO	Rsbn1l
NO	A630072M18Rik
NO	Ptpn12
NO	Gsap
NO	Ccdc146
NO	Fgl2
NO	Fam185a
NO	Lrrc17
NO	Armc10
NO	Napepld
NO	Pmpcb
NO	Dnajc2
NO	Psmc2
NO	Reln
NO	Orc5
NO	6030443J06Rik
NO	5031425E22Rik
NO	Kmt2e
NO	Srpk2
NO	AI506816
NO	Pus7
NO	Rint1
NO	Tomm7
NO	Fam126a
NO	Klhl7
NO	Nupl2
NO	Kcnh2
NO	Nos3
NO	Abcb8
NO	Asic3
NO	Cdk5
NO	Slc4a2

NO	Fastk
NO	Tmub1
NO	Agap3
NO	Asb10
NO	Abcf2
NO	Chpf2
NO	Smarcd3
NO	Nub1
NO	Rheb
NO	Prkag2
NO	Galnt11
NO	Kmt2c
NO	4831440E17Rik
NO	1700096K18Rik
NO	Xrcc2
NO	Actr3b
NO	Dpp6
NO	Paxip1
NO	Insig1
NO	Rbm33
NO	Lmbr1
NO	Nom1
NO	Ube3c
NO	Dnajb6
NO	Tyms
NO	Hadha
NO	Hadhb
NO	Ept1
NO	Kcnk3
NO	Slc35f6
NO	Cenpa
NO	Mapre3
NO	Tmem214
NO	Agbl5
NO	Ost4
NO	Emilin1
NO	Khk
NO	Cgref1
NO	Preb
NO	Slc5a6
NO	Atraid
NO	Cad
NO	Trim54
NO	Ucn
NO	Mpv17
NO	Gtf3c2
NO	Eif2b4
NO	Snx17

NO	Zfp513
NO	Ppm1g
NO	Nrbp1
NO	Krtcap3
NO	Ift172
NO	Fndc4
NO	Zfp512
NO	Gpn1
NO	Supt7l
NO	Slc4a1ap
NO	Mrpl33
NO	Rbks
NO	Bre
NO	Fosl2
NO	Ppp1cb
NO	Yes1
NO	Pisd
NO	Prr14l
NO	Depdc5
NO	Ywhah
NO	Ctbp1
NO	Maea
NO	Uvssa
NO	Fam53a
NO	Slbp
NO	Tmem129
NO	Tacc3
NO	Fgfr3
NO	Letm1
NO	Whsc1
NO	Nelfa
NO	Nat8l
NO	Haus3
NO	Mxd4
NO	Zfyve28
NO	Rnf4
NO	Fam193a
NO	Tnip2
NO	Sh3bp2
NO	Add1
NO	Mfsd10
NO	Nop14
NO	Grk4
NO	Htt
NO	Rgs12
NO	Dok7
NO	Lrpap1
NO	Trmt44

NO	Acox3
NO	Htra3
NO	Sh3tc1
NO	Ablim2
NO	Afap1
NO	Sorcs2
NO	Grpel1
NO	Tada2b
NO	Tbc1d14
NO	D5Ertd579e
NO	Bloc1s4
NO	Mrfap1
NO	Man2b2
NO	Ppp2r2c
NO	Wfs1
NO	Evc
NO	Evc2
NO	Cytl1
NO	Stx18
NO	Nsg1
NO	Zbtb49
NO	Lyar
NO	Tmem128
NO	Slc2a9
NO	Wdr1
NO	Zfp518b
NO	Hs3st1
NO	Rab28
NO	Bod1l
NO	Cpeb2
NO	Cc2d2a
NO	Fbxl5
NO	Cd38
NO	Prom1
NO	Tapt1
NO	Ldb2
NO	Qdpr
NO	Lap3
NO	Med28
NO	Fam184b
NO	Lcorl
NO	Gm3414
NO	Slit2
NO	Pacrgl
NO	Adgra2
NO	Ppargc1a
NO	Dhx15
NO	9230114K14Rik

NO	C130083M11Rik
NO	Sod3
NO	Ccdc149
NO	Lgi2
NO	Sepsecs
NO	Pi4k2b
NO	Zcchc4
NO	Anapc4
NO	Sel1l3
NO	Smim20
NO	Rbpj
NO	Tbc1d19
NO	Stim2
NO	Pcdh7
NO	Arap2
NO	0610040J01Rik
NO	Rell1
NO	Pgm1
NO	Tbc1d1
NO	Klf3
NO	Fam114a1
NO	Klhl5
NO	Wdr19
NO	Rfc1
NO	Klb
NO	Rpl9
NO	Lias
NO	Ugdh
NO	Smim14
NO	Ube2k
NO	Pds5a
NO	N4bp2
NO	Chrna9
NO	Rbm47
NO	Apbb2
NO	Uchl1
NO	Limch1
NO	Tmem33
NO	Slc30a9
NO	Shisa3
NO	Atp8a1
NO	Yipf7
NO	Guf1
NO	Gnpda2
NO	Commd8
NO	Atp10d
NO	Nfxl1
NO	Tec

NO	Slain2
NO	Fryl
NO	Ociad1
NO	Ociad2
NO	Dcun1d4
NO	Sgcb
NO	Usp46
NO	Rasl11b
NO	Scfd2
NO	Fip1l1
NO	Lnx1
NO	Chic2
NO	Pdgfra
NO	Kit
NO	Kdr
NO	Srd5a3
NO	Tmem165
NO	Clock
NO	Exoc1
NO	Cep135
NO	C530008M17Rik
NO	Aasdh
NO	Ppat
NO	Paics
NO	Srp72
NO	Rest
NO	Noa1
NO	Polr2b
NO	Igfbp7
NO	Adgrl3
NO	Tecrl
NO	Cenpc1
NO	Uba6
NO	Ythdc1
NO	Utp3
NO	Rufy3
NO	Grsf1
NO	Mob1b
NO	Dck
NO	Slc4a4
NO	Adamts3
NO	Cox18
NO	Ankrd17
NO	Alb
NO	Pf4
NO	Mthfd2l
NO	Btc
NO	Parm1

NO	Rchy1
NO	Thap6
NO	Cdkl2
NO	G3bp2
NO	Uso1
NO	Naaa
NO	Sdad1
NO	Art3
NO	Nup54
NO	Scarb2
NO	Stbd1
NO	Shroom3
NO	Sept11
NO	Ccni
NO	Ccng2
NO	Cnot6l
NO	Mrpl1
NO	Fras1
NO	Anxa3
NO	Bmp2k
NO	Paqr3
NO	Antxr2
NO	Prdm8
NO	Bmp3
NO	Prkg2
NO	Rasgef1b
NO	Hnrnpd
NO	Hnrnpdl
NO	Enoph1
NO	Sec31a
NO	Lin54
NO	Cops4
NO	Plac8
NO	Coq2
NO	Hpse
NO	Helq
NO	Mrps18c
NO	Fam175a
NO	Agpat9
NO	Cds1
NO	Wdfy3
NO	Arhgap24
NO	Mapk10
NO	Ptpn13
NO	Slc10a6
NO	Aff1
NO	Klhl8
NO	Hsd17b11

NO	Nudt9
NO	Sparcl1
NO	Spp1
NO	Pkd2
NO	BC005561
NO	D930016D06Rik
NO	Gbp9
NO	Gbp4
NO	Gbp10
NO	Gbp6
NO	Lrrc8b
NO	Lrrc8c
NO	Lrrc8d
NO	Zfp326
NO	Zfp644
NO	Tgfbr3
NO	Brdt
NO	Btbd8
NO	A830010M20Rik
NO	Glmn
NO	Rpap2
NO	Evi5
NO	Rpl5
NO	Fam69a
NO	Mtf2
NO	Tmed5
NO	Dr1
NO	Pigg
NO	Atp5k
NO	Mfsd7a
NO	Pcgf3
NO	Gak
NO	Tmem175
NO	Dgkq
NO	Idua
NO	Slc26a1
NO	Fgfrl1
NO	Crlf2
NO	Gm15446
NO	Zfp932
NO	Gtpbp6
NO	Zfp605
NO	Chfr
NO	Gm15787
NO	Golga3
NO	Ankle2
NO	Pgam5
NO	Pxmp2

NO	Pole
NO	Fbrsl1
NO	Gm29766
NO	Noc4l
NO	Ddx51
NO	Ep400
NO	Pus1
NO	Ulk1
NO	Hscb
NO	Chek2
NO	Ttc28
NO	Pitpnb
NO	Mn1
NO	Miat
NO	Tpst2
NO	Tfip11
NO	Srrd
NO	Hps4
NO	Myo18b
NO	Adrbk2
NO	2900026A02Rik
NO	Wscd2
NO	Cmklr1
NO	Ficd
NO	Sart3
NO	Iscu
NO	Tmem119
NO	Selplg
NO	Coro1c
NO	Ssh1
NO	Usp30
NO	Ung
NO	Acacb
NO	Kctd10
NO	Ube3b
NO	Mmab
NO	Mvk
NO	Trpv4
NO	Gltp
NO	Tchp
NO	Git2
NO	Ankrd13a
NO	1500011B03Rik
NO	Oasl2
NO	2210016L21Rik
NO	Sppl3
NO	Rpl37rt
NO	Acads

NO	Unc119b
NO	Mlec
NO	Pop5
NO	Rnf10
NO	Coq5
NO	Dynll1
NO	Srsf9
NO	Gatc
NO	Triap1
NO	Cox6a1
NO	Sirt4
NO	Pxn
NO	Rplp0
NO	Gcn1l1
NO	Rab35
NO	Ccdc64
NO	Cit
NO	Prkab1
NO	Tmem233
NO	Ccdc60
NO	Hspb8
NO	Srrm4os
NO	Srrm4
NO	Suds3
NO	Taok3
NO	Pebp1
NO	Vsig10
NO	Wsb2
NO	Rfc5
NO	Nos1
NO	Fbxo21
NO	Tesc
NO	Fbxw8
NO	Hrk
NO	Rnft2
NO	2410131K14Rik
NO	Med13l
NO	AW549542
NO	Tbx3
NO	Rbm19
NO	Gm10390
NO	Plbd2
NO	Slc8b1
NO	Tpcn1
NO	Rita1
NO	Ddx54
NO	Dtx1
NO	Oas2

NO	Oas1b
NO	Ptpn11
NO	Rpl6
NO	Gm15800
NO	Trafd1
NO	Naa25
NO	Erp29
NO	Adam1a
NO	Mapkapk5
NO	Aldh2
NO	Acad12
NO	Acad10
NO	Brap
NO	Atxn2
NO	Sh2b3
NO	Fam109a
NO	Cux2
NO	Ppp1cc
NO	Hvcn1
NO	Tctn1
NO	Pptc7
NO	Rad9b
NO	Vps29
NO	Fam216a
NO	Gpn3
NO	Arpc3
NO	Anapc7
NO	Ift81
NO	P2rx7
NO	P2rx4
NO	Camkk2
NO	Anapc5
NO	Rnf34
NO	Kdm2b
NO	Orai1
NO	Tmem120b
NO	Rhof
NO	Setd1b
NO	Psmd9
NO	Bcl7a
NO	Mlxip
NO	Diablo
NO	Vps33a
NO	Clip1
NO	Zcchc8
NO	Rsrc2
NO	Hcar1
NO	Denr

NO	Ccdc62
NO	Hip1r
NO	Vps37b
NO	Abcb9
NO	Ogfod2
NO	Arl6ip4
NO	Pitpnm2
NO	Pitpnm2os1
NO	Mphosph9
NO	2810006K23Rik
NO	Cdk2ap1
NO	Sbno1
NO	Setd8
NO	Rilpl2
NO	Snrnp35
NO	Rilpl1
NO	Tmed2
NO	Ddx55
NO	Eif2b1
NO	Gtf2h3
NO	Tctn2
NO	Atp6v0a2
NO	Zfp664
NO	Ncor2
NO	Scarb1
NO	Ubc
NO	Dhx37
NO	Bri3bp
NO	Aacs
NO	Tmem132b
NO	Slc15a4
NO	5930412G12Rik
NO	Fzd10
NO	Stx2
NO	Ran
NO	Adgrd1
NO	Sfswap
NO	Zfp11
NO	Mrps17
NO	Gbas
NO	Psph
NO	Cct6a
NO	Sumf2
NO	Phkg1
NO	Chchd2
NO	Zbed5
NO	Vkorc1l1
NO	Gusb

NO	Asl
NO	Crcp
NO	Tpst1
NO	Kctd7
NO	Rabgef1
NO	Tmem248
NO	Sbds
NO	Tyw1
NO	Wbscr17
NO	Gatsl2
NO	Wbscr16
NO	Gtf2ird2
NO	Ncf1
NO	Gtf2i
NO	Gtf2ird1
NO	Clip2
NO	Rfc2
NO	Lat2
NO	Eif4h
NO	Limk1
NO	Eln
NO	Wbscr27
NO	Abhd11
NO	Wbscr22
NO	Dnajc30
NO	Mlxipl
NO	Tbl2
NO	Bcl7b
NO	Baz1b
NO	Fzd9
NO	Nsun5
NO	Pom121
NO	Hip1
NO	Ccl24
NO	Rhbdd2
NO	Por
NO	Tmem120a
NO	Mdh2
NO	Hspb1
NO	Ywhag
NO	Dtx2
NO	Rasa4
NO	Polr2j
NO	Lrwd1
NO	Alkbh4
NO	Orai2
NO	Prkrip1
NO	Sh2b2

NO	Cux1
NO	Myl10
NO	Ift22
NO	4933404O12Rik
NO	Fis1
NO	Cldn15
NO	Znhit1
NO	Plod3
NO	Ap1s1
NO	Serpine1
NO	Trim56
NO	Ache
NO	Ufsp1
NO	Srrt
NO	Trip6
NO	Slc12a9
NO	Ephb4
NO	Pop7
NO	Gigyf1
NO	Gnb2
NO	Mospd3
NO	Pcolce
NO	Irs3
NO	Agfg2
NO	Tsc22d4
NO	Ppp1r35
NO	Mepce
NO	Gjc3
NO	Zkscan1
NO	Zscan21
NO	Zfp113
NO	Cops6
NO	Mcm7
NO	Ap4m1
NO	Taf6
NO	Cnpy4
NO	Mblac1
NO	Lamtor4
NO	BC037034
NO	Zfp157
NO	Zfp68
NO	A430033K04Rik
NO	Fam20c
NO	Pdgfa
NO	Prkar1b
NO	Dnaaf5
NO	Sun1
NO	Get4

NO	Adap1
NO	Cox19
NO	3110082I17Rik
NO	Gpr146
NO	Gper1
NO	Zfand2a
NO	Micall2
NO	Ints1
NO	Mafk
NO	Psmg3
NO	Mad1l1
NO	Ftsj2
NO	Nudt1
NO	Snx8
NO	Eif3b
NO	Chst12
NO	Lfng
NO	Ttyh3
NO	Iqce
NO	Brat1
NO	Gna12
NO	Card11
NO	Sdk1
NO	Foxk1
NO	Ap5z1
NO	Radil
NO	Wipi2
NO	Tnrc18
NO	Fbxl18
NO	Actb
NO	Fscn1
NO	Rnf216
NO	Rbak
NO	Zfp12
NO	Zfp316
NO	E130309D02Rik
NO	Zdhhc4
NO	Kdelr2
NO	Daglb
NO	Rac1
NO	Fam220a
NO	Cyth3
NO	Usp42
NO	D130017N08Rik
NO	Eif2ak1
NO	Ankrd61
NO	Aimp2
NO	Pms2

NO	Ccz1
NO	Lmtk2
NO	Tecpr1
NO	Bri3
NO	Baiap2l1
NO	Trrap
NO	Smurf1
NO	Arpc1a
NO	Arpc1b
NO	Pdap1
NO	Bud31
NO	Ptcd1
NO	Cpsf4
NO	Atp5j2
NO	Zkscan14
NO	Zkscan5
NO	Zfp655
NO	Zscan25
NO	Rnf6
NO	Cdk8
NO	Usp12
NO	Rpl21
NO	Gtf3a
NO	Mtif3
NO	Lnx2
NO	Polr1d
NO	Pan3
NO	Flt1
NO	Pomp
NO	Slc46a3
NO	Slc7a1
NO	Ubl3
NO	Katnal1
NO	5930430L01Rik
NO	Gm15408
NO	Hmgb1
NO	Uspl1
NO	Alox5ap
NO	Medag
NO	Hsph1
NO	B3glct
NO	Fry
NO	Brca2
NO	N4bp2l1
NO	N4bp2l2
NO	Pds5b
NO	Kl
NO	Stard13

NO	Rfc3
NO	Samd9l
NO	Ccdc132
NO	Gng11
NO	Bet1
NO	Col1a2
NO	Casd1
NO	Sgce
NO	Peg10
NO	Ppp1r9a
NO	Pon3
NO	Pon2
NO	Asb4
NO	Pdk4
NO	Slc25a13
NO	Shfm1
NO	Sdhaf3
NO	Asns
NO	C1galt1
NO	Mios
NO	Rpa3
NO	Umad1
NO	Glcci1
NO	Ica1
NO	Ndufa4
NO	Phf14
NO	Thsd7a
NO	Tmem106b
NO	Tmem168
NO	B630005N14Rik
NO	2610001J05Rik
NO	1110019D14Rik
NO	Ppp1r3a
NO	Foxp2
NO	Mdfic
NO	Tes
NO	Cav2
NO	Cav1
NO	Met
NO	Capza2
NO	St7
NO	Cttnbp2
NO	Lsm8
NO	Tspan12
NO	Ing3
NO	Cped1
NO	Wnt16
NO	Fam3c

NO	Ptprz1
NO	Cadps2
NO	Ndufa5
NO	Asb15
NO	Wasl
NO	Pot1a
NO	Zfp800
NO	Gcc1
NO	Arf5
NO	Snd1
NO	Lrrc4
NO	Lep
NO	Rbm28
NO	Prrt4
NO	Impdh1
NO	Hilpda
NO	Calu
NO	Ccdc136
NO	Flnc
NO	Atp6v1f
NO	Kcp
NO	Irf5
NO	Tnpo3
NO	Tspan33
NO	Smo
NO	Ahcyl2
NO	Strip2
NO	Nrf1
NO	Ube2h
NO	Zc3hc1
NO	Klhdc10
NO	Tmem209
NO	Mest
NO	Copg2
NO	Lincpint
NO	2210408F21Rik
NO	Mkln1
NO	Podxl
NO	Plxna4
NO	Chchd3
NO	Exoc4
NO	Slc35b4
NO	Akr1b8
NO	Akr1b10
NO	Bpgm
NO	Cald1
NO	Agbl3
NO	Tmem140

NO	3110062M04Rik
NO	Wdr91
NO	Cnot4
NO	Nup205
NO	1810058I24Rik
NO	Mtpn
NO	Creb3l2
NO	Trim24
NO	D630045J12Rik
NO	Zc3hav1l
NO	Zc3hav1
NO	Ttc26
NO	Ubn2
NO	1110001J03Rik
NO	Luc7l2
NO	Hipk2
NO	Tbxas1
NO	Parp12
NO	Kdm7a
NO	Slc37a3
NO	Mkrn1
NO	Dennd2a
NO	Adck2
NO	Ndufb2
NO	Braf
NO	Mrps33
NO	Agk
NO	E330009J07Rik
NO	Ssbp1
NO	Ephb6
NO	Gstk1
NO	Casp2
NO	Clcn1
NO	Fam131b
NO	Zyx
NO	Epha1
NO	Tcaf2
NO	Tcaf1
NO	Arhgef5
NO	Tpk1
NO	Cntnap2
NO	Cul1
NO	Ezh2
NO	Pdia4
NO	Zfp786
NO	Zfp398
NO	Zfp282
NO	Zfp212

NO	Zfp783
NO	Zfp956
NO	Zfp777
NO	Zfp746
NO	Krba1
NO	Zfp467
NO	Zfp862-ps
NO	Atp6v0e2
NO	Lrrc61
NO	Rarres2
NO	Repin1
NO	Zfp775
NO	AI854703
NO	Gimap8
NO	Gimap4
NO	Gimap6
NO	Gimap1
NO	Gimap5
NO	Tmem176b
NO	Tmem176a
NO	Gpnmb
NO	Malsu1
NO	Igf2bp3
NO	Tra2a
NO	Ccdc126
NO	Mpp6
NO	Dfna5
NO	Osbpl3
NO	Cycs
NO	4921507P07Rik
NO	Nfe2l3
NO	Hnrnpa2b1
NO	Cbx3
NO	Snx10
NO	Skap2
NO	Hoxa3
NO	Hoxa4
NO	Hoxaas3
NO	Hoxa5
NO	Hoxa6
NO	Mira
NO	Hoxa7
NO	Hoxa9
NO	Hoxa10
NO	Hibadh
NO	Tax1bp1
NO	Jazf1
NO	Creb5

NO	Tril
NO	Chn2
NO	Wipf3
NO	Scrn1
NO	Fkbp14
NO	Plekha8
NO	Mturn
NO	Znrf2
NO	Nod1
NO	Ggct
NO	Gars
NO	Crhr2
NO	Inmt
NO	Fam188b
NO	Aqp1
NO	Adcyap1r1
NO	Pde1c
NO	Lsm5
NO	Avl9
NO	Kbtbd2
NO	Fkbp9
NO	Nt5c3
NO	Ppm1k
NO	Herc6
NO	Pyurf
NO	Lancl2
NO	Vopp1
NO	Abcg2
NO	Herc3
NO	Nap1l5
NO	Fam13a
NO	Tigd2
NO	Gprin3
NO	Snca
NO	Mmrn1
NO	Ccser1
NO	Smarcad1
NO	Hpgds
NO	Prdm5
NO	Gng12
NO	Gadd45a
NO	Serbp1
NO	Il12rb2
NO	Rpia
NO	Eif2ak3
NO	Thnsl2
NO	Smyd1
NO	Krcc1

NO	Rmnd5a
NO	Rnf103
NO	Chmp3
NO	Kdm3a
NO	Reep1
NO	Mrpl35
NO	Immt
NO	Ptcd3
NO	Polr1a
NO	St3gal5
NO	Atoh8
NO	Usp39
NO	0610030E20Rik
NO	Tmem150a
NO	Rnf181
NO	Vamp5
NO	Vamp8
NO	Ggcx
NO	Mat2a
NO	Particl
NO	Capg
NO	Elmod3
NO	Retsat
NO	Tgoln1
NO	Tcf7l1
NO	Kcmf1
NO	Tmsb10
NO	Suclg1
NO	Gcfc2
NO	Mrpl19
NO	Pole4
NO	Hk2
NO	Dok1
NO	Loxl3
NO	Htra2
NO	Aup1
NO	Pcgf1
NO	Ccdc142
NO	Mrpl53
NO	Mogs
NO	Wbp1
NO	Ino80b
NO	Rtkn
NO	Dctn1
NO	Mthfd2
NO	Mob1a
NO	Bola3
NO	Tet3

NO	B230319C09Rik
NO	Dguok
NO	Stambp
NO	Tex261
NO	Nagk
NO	Paip2b
NO	Zfp638
NO	Dysf
NO	Cyp26b1
NO	Exoc6b
NO	Spr
NO	Sfxn5
NO	Rab11fip5
NO	Smyd5
NO	Pradc1
NO	Cct7
NO	Alms1
NO	Tprkb
NO	Dusp11
NO	Tgfa
NO	Fam136a
NO	Snrpg
NO	Pcyox1
NO	Tia1
NO	C87436
NO	2310040G24Rik
NO	Pcbp1
NO	1600020E01Rik
NO	Mxd1
NO	Snrnp27
NO	Gmcl1
NO	Anxa4
NO	2610306M01Rik
NO	Aak1
NO	Nfu1
NO	Gfpt1
NO	Antxr1
NO	Arhgap25
NO	Prokr1
NO	Aplf
NO	Efcc1
NO	Rab43
NO	Isy1
NO	Cnbp
NO	Copg1
NO	Hmces
NO	H1fx
NO	Rab7

NO	Rpn1
NO	Gata2
NO	Eefsec
NO	Ruvbl1
NO	Sec61a1
NO	Kbtbd12
NO	Mgll
NO	Abtb1
NO	Podxl2
NO	Mcm2
NO	Tpra1
NO	Plxna1
NO	Chchd6
NO	Txnrd3
NO	Zxdc
NO	Ccdc37
NO	Klf15
NO	Aldh1l1
NO	Slc41a3
NO	Iqsec1
NO	Nup210
NO	Hdac11
NO	Fbln2
NO	1810044D09Rik
NO	Chchd4
NO	Tmem43
NO	Xpc
NO	Lsm3
NO	Slc6a6
NO	Grip2
NO	Ccdc174
NO	Fgd5
NO	Nr2c2
NO	Mrps25
NO	Rbsn
NO	Prickle2
NO	Adamts9
NO	9530026P05Rik
NO	Magi1
NO	Slc25a26
NO	Lrig1
NO	Kbtbd8
NO	Suclg2
NO	Eogt
NO	Tmf1
NO	Uba3
NO	Arl6ip5
NO	Lmod3

NO	Frmd4b
NO	Mitf
NO	Foxp1
NO	Eif4e3
NO	Gpr27
NO	Rybp
NO	Shq1
NO	Gxylt2
NO	Ppp4r2
NO	Pdzrn3
NO	Chl1
NO	Trnt1
NO	Crbn
NO	Lrrn1
NO	Setmar
NO	Sumf1
NO	Itpr1
NO	Bhlhe40
NO	Arl8b
NO	Edem1
NO	Lmcd1
NO	5031434C07Rik
NO	Cav3
NO	Oxtr
NO	Rad18
NO	Srgap3
NO	Thumpd3
NO	Gt(ROSA)26Sor
NO	Setd5
NO	Lhfpl4
NO	Mtmr14
NO	Brpf1
NO	Ogg1
NO	Camk1
NO	Tada3
NO	Arpc4
NO	Ttll3
NO	Rpusd3
NO	Cidec
NO	Jagn1
NO	Il17re
NO	Il17rc
NO	Creld1
NO	Emc3
NO	Brk1
NO	Vhl
NO	Irak2
NO	Tatdn2

NO	Ghrl
NO	Sec13
NO	Atg7
NO	Vgll4
NO	Tamm41
NO	Syn2
NO	Timp4
NO	Pparg
NO	Tsen2
NO	Mkrn2
NO	Raf1
NO	Cand2
NO	Rpl32
NO	Snora7a
NO	Mbd4
NO	Ift122
NO	Plxnd1
NO	Tmcc1
NO	Fam21
NO	March8
NO	Alox5
NO	Zfp422
NO	Rassf4
NO	8430408G22Rik
NO	Cxcl12
NO	Zfp637
NO	Hnrnpf
NO	Csgalnact2
NO	Ret
NO	Bms1
NO	Zfp248
NO	Zfp9
NO	Ankrd26
NO	Cacna1c
NO	Dcp1b
NO	Cacna2d4
NO	Adipor2
NO	Wnt5b
NO	Fbxl14
NO	Erc1
NO	Rad52
NO	Wnk1
NO	B4galnt3
NO	Ccdc77
NO	Kdm5a
NO	Il17ra
NO	Cecr5
NO	Cecr2

NO	Slc25a18
NO	Atp6v1e1
NO	Bcl2l13
NO	Bid
NO	Mical3
NO	Pex26
NO	Tuba8
NO	Usp18
NO	Slc6a13
NO	Iqsec3
NO	M6pr
NO	Phc1
NO	Mfap5
NO	Apobec1
NO	Slc2a3
NO	Foxj2
NO	C3ar1
NO	Necap1
NO	Clec4a2
NO	Cd163
NO	Pex5
NO	Clstn3
NO	C1rl
NO	C1ra
NO	C1s1
NO	Lpcat3
NO	Emg1
NO	Phb2
NO	Ptpn6
NO	Grcc10
NO	Atn1
NO	Eno2
NO	Spsb2
NO	Tpi1
NO	Usp5
NO	Gnb3
NO	P3h3
NO	Gpr162
NO	Ptms
NO	Mlf2
NO	Cops7a
NO	Zfp384
NO	Ing4
NO	Acrbp
NO	Chd4
NO	Nop2
NO	Iffo1
NO	Gapdh

NO	Ncapd2
NO	Scarna10
NO	Mrpl51
NO	Vamp1
NO	Tapbpl
NO	E130112N10Rik
NO	Cd27
NO	Ltbr
NO	Tnfrsf1a
NO	Cd9
NO	Vwf
NO	Ntf3
NO	Kcna5
NO	Kcna1
NO	Kcna6
NO	Ndufa9
NO	D6Wsu163e
NO	Fgf6
NO	Tigar
NO	Ccnd2
NO	Parp11
NO	Tspan11
NO	Tspan9
NO	9330102E08Rik
NO	Tead4
NO	Tulp3
NO	Rhno1
NO	Nrip2
NO	Itfg2
NO	Fkbp4
NO	Gm10069
NO	Clec2d
NO	2310001H17Rik
NO	Clec9a
NO	Clec1a
NO	Gabarapl1
NO	Magohb
NO	Ybx3
NO	Smim10l1
NO	Etv6
NO	Lrp6
NO	Mansc1
NO	Loh12cr1
NO	Dusp16
NO	Crebl2
NO	Gpr19
NO	Cdkn1b
NO	Apold1

NO	Ddx47
NO	Hebp1
NO	8430419L09Rik
NO	Emp1
NO	Atf7ip
NO	Plbd1
NO	Hist4h4
NO	H2afj
NO	Wbp11
NO	Mgp
NO	Arhgdib
NO	Ptpro
NO	Eps8
NO	Strap
NO	Dera
NO	Slc15a5
NO	Mgst1
NO	Plekha5
NO	Aebp2
NO	Pde3a
NO	Pyroxd1
NO	Recql
NO	Golt1b
NO	Ldhb
NO	Kcnj8
NO	Abcc9
NO	Cmas
NO	C2cd5
NO	Etnk1
NO	Sox5
NO	Lrmp
NO	Lyrm5
NO	Kras
NO	Rassf8
NO	Bhlhe41
NO	Sspn
NO	Itpr2
NO	Asun
NO	Fgfr1op2
NO	Tm7sf3
NO	Med21
NO	Stk38l
NO	Ppfibp1
NO	Mrps35
NO	Mansc4
NO	Klhl42
NO	Pthlh
NO	Ccdc91

NO	Ergic2
NO	Tmtc1
NO	Rps4l
NO	Ipo8
NO	Caprin2
NO	Dennd5b
NO	Mettl20
NO	Amn1
NO	2810474O19Rik
NO	Bicd1
NO	Myadm
NO	Prkcg
NO	Cacng7
NO	3300002P13Rik
NO	Cacng6
NO	Ndufa3
NO	Tfpt
NO	Prpf31
NO	Cnot3
NO	Leng1
NO	Mboat7
NO	Tsen34
NO	Rps9
NO	Pirb
NO	Leng8
NO	Leng9
NO	Cdc42ep5
NO	Rdh13
NO	Ppp1r12c
NO	Tmem86b
NO	Ppp6r1
NO	Hspbp1
NO	Suv420h2
NO	Tmem238
NO	Rpl28
NO	Ube2s
NO	Isoc2b
NO	Isoc2a
NO	Zfp628
NO	Nat14
NO	Ssc5d
NO	Sbk2
NO	Sbk3
NO	Zfp579
NO	Fiz1
NO	Zfp524
NO	Zfp865
NO	Zfp784

NO	Zfp580
NO	U2af2
NO	Epn1
NO	Zfp787
NO	Zfp444
NO	Zfp667
NO	Zfp28
NO	Zim1
NO	Peg3
NO	Zfp954
NO	Zfp773
NO	Zfp418
NO	Zfp772
NO	Clcn4-2
NO	Zik1
NO	Zfp551
NO	Zfp606
NO	Zfp329
NO	Zfp110
NO	Zfp128
NO	Zscan22
NO	Rps5
NO	Zfp324
NO	Zfp446
NO	Zbtb45
NO	Trim28
NO	Chmp2a
NO	Ube2m
NO	Mzf1
NO	6330408A02Rik
NO	Lig1
NO	Sepw1
NO	Gltscr2
NO	Ehd2
NO	Gltscr1
NO	Napa
NO	Kptn
NO	Meis3
NO	Dhx34
NO	C5ar1
NO	Inafm1
NO	Ccdc9
NO	Bbc3
NO	Sae1
NO	Zc3h4
NO	Tmem160
NO	Arhgap35
NO	Ap2s1

NO	Slc1a5
NO	Fkrp
NO	Strn4
NO	Prkd2
NO	Dact3
NO	Ptgir
NO	Calm3
NO	Pnmal2
NO	Ccdc8
NO	Ppp5c
NO	Hif3a
NO	Mill2
NO	Ccdc61
NO	Nova2
NO	Mypop
NO	Irf2bp1
NO	Sympk
NO	Dmwd
NO	Dmpk
NO	Six5
NO	Fbxo46
NO	Qpctl
NO	Snrpd2
NO	Eml2
NO	Gpr4
NO	Opa3
NO	Vasp
NO	Rtn2
NO	Ercc1
NO	Cd3eap
NO	Ppp1r13l
NO	Ercc2
NO	Ckm
NO	A930016O22Rik
NO	Mark4
NO	Bloc1s3
NO	Trappc6a
NO	Ppp1r37
NO	Gemin7
NO	Clasrp
NO	Relb
NO	Clptm1
NO	Apoe
NO	Tomm40
NO	Pvrl2
NO	Bcam
NO	Bcl3
NO	Pvr

NO	Zfp180
NO	Zfp112
NO	Zfp235
NO	Zfp111
NO	Zfp109
NO	Zfp108
NO	Zfp93
NO	Zfp61
NO	Zfp94
NO	Kcnn4
NO	Smg9
NO	Plaur
NO	Cadm4
NO	Zfp428
NO	Irgq
NO	Xrcc1
NO	Ethe1
NO	Cd177
NO	Rps19
NO	Arhgef1
NO	Rabac1
NO	Atp1a3
NO	Grik5
NO	Zfp574
NO	Dedd2
NO	Zfp526
NO	Gsk3a
NO	9130221H12Rik
NO	Erf
NO	Cic
NO	Megf8
NO	4732471J01Rik
NO	Lipe
NO	Ceacam1
NO	Ceacam2
NO	Atp5sl
NO	B3gnt8
NO	Bckdha
NO	Exosc5
NO	B9d2
NO	Tgfb1
NO	Ccdc97
NO	Hnrnpul1
NO	Axl
NO	Cyp2f2
NO	Cyp2t4
NO	Egln2
NO	Rab4b

NO	Snrpa
NO	BC024978
NO	Itpkc
NO	Adck4
NO	Numbl
NO	Ltbp4
NO	Shkbp1
NO	Blvrb
NO	Sertad3
NO	Sertad1
NO	Prx
NO	Hipk4
NO	Pld3
NO	2310022A10Rik
NO	Akt2
NO	Map3k10
NO	Zfp60
NO	Zfp626
NO	Zfp59
NO	Zfp607
NO	1700049G17Rik
NO	Zfp780b
NO	Psmc4
NO	Fbl
NO	Dyrk1b
NO	Eid2
NO	Eid2b
NO	Timm50
NO	Supt5
NO	Rps16
NO	Plekhg2
NO	Zfp36
NO	Med29
NO	Paf1
NO	Samd4b
NO	Gmfg
NO	Pak4
NO	Fbxo17
NO	Mrps12
NO	Sars2
NO	Nfkbib
NO	Sirt2
NO	Rinl
NO	Hnrnpl
NO	Ech1
NO	Actn4
NO	Eif3k
NO	Map4k1

NO	Ryr1
NO	Rasgrp4
NO	Fam98c
NO	Spred3
NO	Psmd8
NO	Kcnk6
NO	Yif1b
NO	Spint2
NO	Ppp1r14a
NO	Dpf1
NO	Sipa1l3
NO	Zfp84
NO	Zfp30
NO	Zfp790
NO	Zfp940
NO	Zfp420
NO	Zfp27
NO	Zfp383
NO	Zfp74
NO	Zfp568
NO	Zfp14
NO	Zfp82
NO	Zfp566
NO	Zfp260
NO	Zfp382
NO	Zfp146
NO	Gm5113
NO	Cox7a1
NO	Capns1
NO	Tbcb
NO	Polr2i
NO	Wdr62
NO	Clip3
NO	Alkbh6
NO	Sdhaf1
NO	Lrfn3
NO	Tyrobp
NO	Arhgap33
NO	Proser3
NO	Hspb6
NO	Lin37
NO	Psenen
NO	U2af1l4
NO	Igflr1
NO	Kmt2b
NO	Cox6b1
NO	Rbm42
NO	Haus5

NO	Tmem147
NO	Gapdhs
NO	Sbsn
NO	Ffar2
NO	Usf2
NO	Lsr
NO	Fxyd5
NO	Fxyd1
NO	Lgi4
NO	Hpn
NO	Scn1b
NO	Gramd1a
NO	Wtip
NO	Uba2
NO	Pdcd2l
NO	Gpi1
NO	4931406P16Rik
NO	Lsm14a
NO	Kctd15
NO	Pepd
NO	Cebpg
NO	Cebpa
NO	Slc7a10
NO	Lrp3
NO	Gpatch1
NO	Rhpn2
NO	Cep89
NO	Nudt19
NO	Ankrd27
NO	Pdcd5
NO	Dpy19l3
NO	Zfp507
NO	Tshz3
NO	Zfp536
NO	Uri1
NO	1600014C10Rik
NO	Plekhf1
NO	Pop4
NO	Zfp939
NO	Zfp619
NO	AI987944
NO	AW146154
NO	Zfp788
NO	Zfp141
NO	Gm5595
NO	Zfp715
NO	Etfb
NO	Vsig10l

NO	Cd33
NO	Zfp658
NO	Zfp719
NO	Ctu1
NO	2410002F23Rik
NO	Clec11a
NO	Shank1
NO	Syt3
NO	Josd2
NO	5430431A17Rik
NO	Emc10
NO	Fam71e1
NO	Mybpc2
NO	Spib
NO	Pold1
NO	Nr1h2
NO	Napsa
NO	Kcnc3
NO	Myh14
NO	Vrk3
NO	Atf5
NO	Nup62
NO	Tbc1d17
NO	Akt1s1
NO	Pnkp
NO	Ptov1
NO	Med25
NO	Fuz
NO	Ap2a1
NO	Cpt1c
NO	Prmt1
NO	Gm15545
NO	Bcl2l12
NO	Irf3
NO	Scaf1
NO	Rras
NO	Prr12
NO	Prrg2
NO	Nosip
NO	Rcn3
NO	Fcgrt
NO	Rps11
NO	Rpl13a
NO	Flt3l
NO	Aldh16a1
NO	Pih1d1
NO	Tead2
NO	Cd37

NO	Trpm4
NO	Rpl14-ps1
NO	Hrc
NO	Ppfia3
NO	Snrnp70
NO	Kcna7
NO	Ntf5
NO	Ruvbl2
NO	Gys1
NO	Ftl1
NO	Bax
NO	Dhdh
NO	Nucb1
NO	Ppp1r15a
NO	Plekha4
NO	Bcat2
NO	Rasip1
NO	Mamstr
NO	Fut2
NO	Car11
NO	Dbp
NO	Sphk2
NO	Rpl18
NO	Cyth2
NO	Grwd1
NO	Kdelr1
NO	Tmem143
NO	Emp3
NO	Nomo1
NO	Kcnj11
NO	Abcc8
NO	Ush1c
NO	Myod1
NO	Kcnc1
NO	Sergef
NO	Saal1
NO	Hps5
NO	Gtf2h1
NO	Ldha
NO	Tsg101
NO	Uevld
NO	Spty2d1
NO	Tmem86a
NO	Mrgprb1
NO	Zdhhc13
NO	Csrp3
NO	Nav2
NO	Prmt3

NO	Ano5
NO	Fancf
NO	Gas2
NO	Svip
NO	Tubgcp5
NO	Cyfip1
NO	Nipa2
NO	Nipa1
NO	Herc2
NO	Atp10a
NO	Ube3a
NO	C230091D08Rik
NO	Snhg14
NO	Ndn
NO	Klf13
NO	E030018B13Rik
NO	Mtmr10
NO	Fan1
NO	Mphosph10
NO	Mcee
NO	Ndnl2
NO	Tjp1
NO	Tarsl2
NO	Tm2d3
NO	Pcsk6
NO	Snrpa1
NO	Vimp
NO	Chsy1
NO	Lrrk1
NO	Aldh1a3
NO	Asb7
NO	Lins
NO	Lysmd4
NO	Mef2a
NO	Lrrc28
NO	Ttc23
NO	Synm
NO	Igf1r
NO	Arrdc4
NO	Nr2f2
NO	B130024G19Rik
NO	Mctp2
NO	Rgma
NO	Chd2
NO	1810026B05Rik
NO	Fam174b
NO	Slco3a1
NO	Sv2b

NO	Akap13
NO	Klhl25
NO	Agbl1
NO	Ntrk3
NO	Mrpl46
NO	Mrps11
NO	Det1
NO	Aen
NO	Isg20
NO	Mfge8
NO	Abhd2
NO	Fanci
NO	Polg
NO	Kif7
NO	Plin1
NO	Pex11a
NO	Anpep
NO	Ap3s2
NO	Arpin
NO	Zfp710
NO	Idh2
NO	Sema4b
NO	Cib1
NO	Gdpgp1
NO	Ngrn
NO	Vps33b
NO	Prc1
NO	Rccd1
NO	Unc45a
NO	Hddc3
NO	Man2a2
NO	Fes
NO	Furin
NO	Blm
NO	Crtc3
NO	Gm15880
NO	Iqgap1
NO	Zscan2
NO	Wdr73
NO	Sec11a
NO	Zfp592
NO	Alpk3
NO	Pde8a
NO	Rps17
NO	Cpeb1
NO	2900076A07Rik
NO	Mir1839
NO	Fsd2

NO	Whamm
NO	Homer2
NO	Fam103a1
NO	3110040N11Rik
NO	Btbd1
NO	Tm6sf1
NO	Hdgfrp3
NO	Adamtsl3
NO	Eftud1
NO	Stard5
NO	Il16
NO	Mesdc1
NO	Mesdc2
NO	Abhd17c
NO	Arnt2
NO	Fah
NO	Zfand6
NO	Folh1
NO	Nox4
NO	Ctsc
NO	Tmem135
NO	Fzd4
NO	Prss23
NO	Me3
NO	l7Rn6
NO	Eed
NO	2310010J17Rik
NO	Picalm
NO	Sytl2
NO	Crebzf
NO	Tmem126a
NO	Tmem126b
NO	Ccdc90b
NO	Ankrd42
NO	Pcf11
NO	4632427E13Rik
NO	Rab30
NO	Prcp
NO	Fam181b
NO	Tenm4
NO	Nars2
NO	Gab2
NO	Usp35
NO	Kctd21
NO	Alg8
NO	Ndufc2
NO	Thrsp
NO	Ints4

NO	Aamdc
NO	Rsf1
NO	Clns1a
NO	Pak1
NO	Myo7a
NO	Capn5
NO	Acer3
NO	Tsku
NO	Lrrc32
NO	2210018M11Rik
NO	Prkrir
NO	Wnt11
NO	Uvrag
NO	Dgat2
NO	Map6
NO	Serpinh1
NO	Gdpd5
NO	Rps3
NO	Snord15b
NO	Snord15a
NO	Arrb1
NO	Gm4980
NO	Slco2b1
NO	Neu3
NO	Spcs2
NO	Xrra1
NO	Rnf169
NO	Chrdl2
NO	Pold3
NO	Lipt2
NO	Pgm2l1
NO	Ppme1
NO	C2cd3
NO	Ucp3
NO	Ucp2
NO	Coa4
NO	Mrpl48
NO	Rab6a
NO	Plekhb1
NO	Fam168a
NO	Relt
NO	Arhgef17
NO	P2ry6
NO	P2ry2
NO	Fchsd2
NO	Atg16l2
NO	Stard10
NO	Arap1

NO	Pde2a
NO	Clpb
NO	Inppl1
NO	Folr2
NO	Anapc15
NO	Lamtor1
NO	Lrrc51
NO	Numa1
NO	Il18bp
NO	Rnf121
NO	Xndc1
NO	Art5
NO	Art1
NO	Chrna10
NO	Nup98
NO	Pgap2
NO	Rhog
NO	Stim1
NO	Rrm1
NO	Trim21
NO	Trim68
NO	Olfr558
NO	Olfr78
NO	Trim34a
NO	Trim34b
NO	Trim12c
NO	Trim30a
NO	Fam160a2
NO	Prkcdbp
NO	Smpd1
NO	Apbb1
NO	Trim3
NO	Arfip2
NO	Timm10b
NO	Rrp8
NO	Ilk
NO	Taf10
NO	Tpp1
NO	Dchs1
NO	Mrpl17
NO	Gm4759
NO	Syt9
NO	Olfml1
NO	Ppfibp2
NO	Cyb5r2
NO	Nlrp10
NO	Eif3f
NO	Tub

NO	Ric3
NO	Lmo1
NO	Trim66
NO	Rpl27a
NO	St5
NO	Akip1
NO	Tmem9b
NO	Scube2
NO	Dennd5a
NO	Tmem41b
NO	Ipo7
NO	Snora23
NO	Zfp143
NO	Wee1
NO	Swap70
NO	Sbf2
NO	Adm
NO	Ampd3
NO	Rnf141
NO	Lyve1
NO	Mrvi1
NO	Ctr9
NO	Eif4g2
NO	1700012D14Rik
NO	Galnt18
NO	Usp47
NO	Dkk3
NO	Mical2
NO	Parva
NO	Tead1
NO	Rassf10
NO	Btbd10
NO	Far1
NO	Spon1
NO	Rras2
NO	Copb1
NO	Psma1
NO	Pde3b
NO	Sox6
NO	1110004F10Rik
NO	Plekha7
NO	Rps13
NO	Pik3c2a
NO	Nucb2
NO	Xylt1
NO	Rps15a
NO	Arl6ip1
NO	Smg1

NO	Itpripl2
NO	Coq7
NO	Tmc7
NO	Gde1
NO	Ccp110
NO	9030624J02Rik
NO	Knop1
NO	Gprc5b
NO	Acsm5
NO	Thumpd1
NO	Eri2
NO	Dcun1d3
NO	Lyrm1
NO	Tmem159
NO	Crym
NO	Uqcrc2
NO	BC030336
NO	Vwa3a
NO	Eef2k
NO	Polr3e
NO	Cdr2
NO	Mettl9
NO	Igsf6
NO	Usp31
NO	Cog7
NO	Gga2
NO	Ears2
NO	Ubfd1
NO	Ndufab1
NO	Dctn5
NO	Chp2
NO	Prkcb
NO	4930413G21Rik
NO	Rbbp6
NO	Tnrc6a
NO	Arhgap17
NO	Lcmt1
NO	Kdm8
NO	Nsmce1
NO	Il4ra
NO	Gtf3c1
NO	D430042O09Rik
NO	Xpo6
NO	Sbk1
NO	Spns1
NO	Nfatc2ip
NO	Cd19
NO	Rabep2

NO	Atp2a1
NO	Sh2b1
NO	Tufm
NO	Atxn2l
NO	Eif3c
NO	Cln3
NO	Apobr
NO	Nupr1
NO	Ccdc101
NO	Sult1a1
NO	Slx1b
NO	Bola2
NO	Coro1a
NO	Mapk3
NO	Gdpd3
NO	Ypel3
NO	Ppp4c
NO	Aldoa
NO	Fam57b
NO	Ino80e
NO	Hirip3
NO	Taok2
NO	Tmem219
NO	Kctd13
NO	Cdipt
NO	Mvp
NO	Pagr1a
NO	Prrt2
NO	Maz
NO	AI467606
NO	Spn
NO	Cd2bp2
NO	Tbc1d10b
NO	Mylpf
NO	Sept1
NO	Zfp553
NO	Zfp771
NO	Dctpp1
NO	Sephs2
NO	Itgal
NO	Zfp768
NO	Zfp747
NO	9130019O22Rik
NO	E430018J23Rik
NO	Zfp764
NO	Zfp688
NO	Zfp689
NO	Prr14

NO	Fbrs
NO	1700008J07Rik
NO	Srcap
NO	Tmem265
NO	Phkg2
NO	Rnf40
NO	Zfp629
NO	Bcl7c
NO	Ctf1
NO	Fbxl19
NO	Orai3
NO	Setd1a
NO	Hsd3b7
NO	Stx1b
NO	Stx4a
NO	Zfp668
NO	Zfp646
NO	Prss53
NO	Vkorc1
NO	Bckdk
NO	Kat8
NO	Prss36
NO	Fus
NO	Pycard
NO	Trim72
NO	Itgam
NO	Itgax
NO	Itgad
NO	Cox6a2
NO	9130023H24Rik
NO	Armc5
NO	Tgfb1i1
NO	BC017158
NO	Rgs10
NO	Tial1
NO	Bag3
NO	Inpp5f
NO	Mcmbp
NO	Sec23ip
NO	Wdr11
NO	Fgfr2
NO	Ate1
NO	Nsmce4a
NO	Tacc2
NO	Plekha1
NO	Htra1
NO	Cuzd1
NO	2310057M21Rik

NO	Pstk
NO	Ikzf5
NO	Acadsb
NO	Bub3
NO	Cpxm2
NO	Chst15
NO	Gm10584
NO	Oat
NO	Lhpp
NO	Fam53b
NO	Mettl10
NO	Fam175b
NO	Zranb1
NO	Ctbp2
NO	Edrf1
NO	Uros
NO	Bccip
NO	Dhx32
NO	Adam12
NO	Dock1
NO	Fam196a
NO	Ptpre
NO	Mki67
NO	Mgmt
NO	Ebf3
NO	Gm10578
NO	9430038I01Rik
NO	Glrx3
NO	Mapk1ip1
NO	Ppp2r2d
NO	Bnip3
NO	Pwwp2b
NO	Inpp5a
NO	Adam8
NO	Tubgcp2
NO	Zfp511
NO	Caly
NO	Fuom
NO	Echs1
NO	Paox
NO	Mtg1
NO	Cyp2e1
NO	Syce1
NO	Bet1l
NO	Ric8
NO	Sirt3
NO	Psmd13
NO	Cox8b

NO	Athl1
NO	Ifitm2
NO	Ifitm3
NO	Sigirr
NO	Ptdss2
NO	Rnh1
NO	Hras
NO	Lrrc56
NO	Phrf1
NO	Irf7
NO	Deaf1
NO	Tmem80
NO	Eps8l2
NO	Taldo1
NO	Pddc1
NO	Slc25a22
NO	Rplp2
NO	Pnpla2
NO	Cracr2b
NO	Cd151
NO	Polr2l
NO	Tspan4
NO	Chid1
NO	Ap2a2
NO	Gm16982
NO	Muc6
NO	Muc5b
NO	Tollip
NO	Brsk2
NO	Mob2
NO	Dusp8
NO	Ctsd
NO	Tnni2
NO	Lsp1
NO	Prr33
NO	Tnnt3
NO	Mrpl23
NO	Nctc1
NO	H19
NO	Igf2
NO	R74862
NO	Cd81
NO	Tssc4
NO	Kcnq1
NO	Kcnq1ot1
NO	Cdkn1c
NO	Nap1l4
NO	Cars

NO	Osbpl5
NO	Mrgpre
NO	Nadsyn1
NO	Dhcr7
NO	Cttn
NO	Ppfia1
NO	Fadd
NO	Ano1
NO	Oraov1
NO	Ccnd1
NO	Tpcn2
NO	Mrgprf
NO	Insr
NO	Arhgef18
NO	Pex11g
NO	Zfp358
NO	Mcoln1
NO	Pnpla6
NO	Camsap3
NO	Xab2
NO	Pet100
NO	Stxbp2
NO	Retn
NO	Trappc5
NO	Fcer2a
NO	Rprl3
NO	Cd209d
NO	Cd209b
NO	Cd209f
NO	Cd209g
NO	Evi5l
NO	Map2k7
NO	Tgfbr3l
NO	Snapc2
NO	Timm44
NO	Elavl1
NO	Ccl25
NO	Cers4
NO	Zfp958
NO	Efnb2
NO	Arglu1
NO	Lig4
NO	Abhd13
NO	Irs2
NO	Col4a1
NO	Col4a2
NO	E230013L22Rik
NO	Carkd

NO	Cars2
NO	Ing1
NO	Ankrd10
NO	Arhgef7
NO	Tubgcp3
NO	Atp11a
NO	Mcf2l
NO	Proz
NO	Pcid2
NO	Cul4a
NO	Lamp1
NO	Grtp1
NO	Adprhl1
NO	Dcun1d2
NO	Tmco3
NO	Tfdp1
NO	Tmem255b
NO	Gas6
NO	Rasa3
NO	Cdc16
NO	Upf3a
NO	Champ1
NO	Coprs
NO	Fbxo25
NO	Tdrp
NO	Erich1
NO	Cln8
NO	Arhgef10
NO	Kbtbd11
NO	Myom2
NO	Csmd1
NO	Mcph1
NO	Angpt2
NO	Agpat5
NO	2610005L07Rik
NO	Alg11
NO	Vps36
NO	Thsd1
NO	Slc25a15
NO	Mrps31
NO	Smim19
NO	Slc20a2
NO	Vdac3
NO	Polb
NO	A930013F10Rik
NO	Ikbkb
NO	Plat
NO	Ap3m2

NO	Kat6a
NO	Ank1
NO	Gm15816
NO	Agpat6
NO	Gins4
NO	Golga7
NO	Sfrp1
NO	1810011O10Rik
NO	Adam9
NO	Tm2d2
NO	Plekha2
NO	Tacc1
NO	Fgfr1
NO	Gm16159
NO	Letm2
NO	Whsc1l1
NO	Ppapdc1b
NO	Ddhd2
NO	Bag4
NO	Lsm1
NO	Star
NO	Ash2l
NO	Hgsnat
NO	Pomk
NO	Fnta
NO	Hook3
NO	Rnf170
NO	Thap1
NO	Zfp703
NO	Erlin2
NO	Prosc
NO	Adgra3
NO	Brf2
NO	Rab11fip1
NO	Adrb3
NO	Eif4ebp1
NO	Dusp26
NO	Rnf122
NO	Tti2
NO	Mak16
NO	Fut10
NO	Wrn
NO	Purg
NO	Ppp2cb
NO	Ubxn8
NO	Gsr
NO	Gtf2e2
NO	Rbpms

NO	Dctn6
NO	Leprotl1
NO	Saraf
NO	Dusp4
NO	Tnks
NO	Ppp1r3b
NO	Eri1
NO	Mfhas1
NO	D8Ertd82e
NO	Lonrf1
NO	Dlc1
NO	Tusc3
NO	Micu3
NO	Zdhhc2
NO	Cnot7
NO	Vps37a
NO	Mtmr7
NO	Slc7a2
NO	Pdgfrl
NO	Mtus1
NO	B430010I23Rik
NO	Pcm1
NO	Asah1
NO	Frg1
NO	Fat1
NO	Cyp4v3
NO	Fam149a
NO	Tlr3
NO	Sorbs2
NO	Sorbs2os
NO	Pdlim3
NO	1700029J07Rik
NO	Ufsp2
NO	Ankrd37
NO	Lrp2bp
NO	Snx25
NO	Cfap97
NO	Slc25a4
NO	Acsl1
NO	Primpol
NO	Casp3
NO	Irf2
NO	Stox2
NO	Trappc11
NO	Rwdd4a
NO	Ing2
NO	Cdkn2aip
NO	Wwc2

NO	Tenm3
NO	Aga
NO	Vegfc
NO	Spcs3
NO	Asb5
NO	Gpm6a
NO	Hpgd
NO	Cep44
NO	Fbxo8
NO	Sap30
NO	2500002B13Rik
NO	Hmgb2
NO	Galnt7
NO	Mfap3l
NO	2700029M09Rik
NO	Clcn3
NO	Nek1
NO	Sh3rf1
NO	Cbr4
NO	Palld
NO	Ddx60
NO	Spock3
NO	Tll1
NO	Cpe
NO	Msmo1
NO	Klhl2
NO	Tmem192
NO	March1
NO	Tma16
NO	Npy1r
NO	Naf1
NO	Psd3
NO	Sh2d4a
NO	Csgalnact1
NO	Ints10
NO	Lpl
NO	Atp6v1b2
NO	Lzts1
NO	Zfp930
NO	Gm10033
NO	Zfp868
NO	Zfp869
NO	Zfp963
NO	Zfp866
NO	Atp13a1
NO	Gmip
NO	Cilp2
NO	Ndufa13

NO	Gatad2a
NO	Mau2
NO	Sugp1
NO	Rfxank
NO	2310045N01Rik
NO	Tmem161a
NO	Slc25a42
NO	Armc6
NO	Sugp2
NO	Homer3
NO	Ddx49
NO	Cope
NO	Upf1
NO	Comp
NO	Crtc1
NO	Klhl26
NO	Crlf1
NO	2810428I15Rik
NO	Uba52
NO	Kxd1
NO	Fkbp8
NO	Ell
NO	Isyna1
NO	Ssbp4
NO	Pgpep1
NO	Lsm4
NO	Jund
NO	Pde4c
NO	Rab3a
NO	Mpv17l2
NO	Ifi30
NO	Pik3r2
NO	2010320M18Rik
NO	Mast3
NO	Arrdc2
NO	Kcnn1
NO	Ccdc124
NO	Rpl18a
NO	Snora68
NO	Map1s
NO	Haus8
NO	Myo9b
NO	Use1
NO	Ocel1
NO	Nr2f6
NO	Ushbp1
NO	Babam1
NO	Abhd8

NO	Mrpl34
NO	Dda1
NO	Ano8
NO	Gtpbp3
NO	Plvap
NO	Bst2
NO	Mvb12a
NO	Slc27a1
NO	Pgls
NO	Fam129c
NO	Colgalt1
NO	Jak3
NO	Fcho1
NO	Zfp709
NO	Zfp882
NO	Zfp617
NO	Zfp961
NO	Tpm4
NO	Rab8a
NO	Fam32a
NO	Ap1m1
NO	Klf2
NO	Eps15l1
NO	Calr3
NO	1700030K09Rik
NO	Cherp
NO	Slc35e1
NO	Med26
NO	Smim7
NO	Tmem38a
NO	Sin3b
NO	F2rl3
NO	Large
NO	Hmgxb4
NO	Tom1
NO	Hmox1
NO	Mcm5
NO	Rasd2
NO	Nr3c2
NO	Arhgap10
NO	0610038B21Rik
NO	Prmt10
NO	Tmem184c
NO	Ednra
NO	Rbmxl1
NO	Slc10a7
NO	Lsm6
NO	Zfp827

NO	Mmaa
NO	Smad1
NO	Otud4
NO	Abce1
NO	Anapc10
NO	Hhip
NO	Smarca5
NO	Gab1
NO	Usp38
NO	Inpp4b
NO	Il15
NO	Zfp330
NO	Rnf150
NO	Tbc1d9
NO	Elmod2
NO	Scoc
NO	Ndufb7
NO	Tecr
NO	Dnajb1
NO	Gipc1
NO	Ptger1
NO	Pkn1
NO	Ddx39
NO	Adgre5
NO	Adgrl1
NO	Prkaca
NO	Samd1
NO	1700067K01Rik
NO	2210011C24Rik
NO	Rfx1
NO	Dcaf15
NO	Cc2d1a
NO	Zswim4
NO	D8Ertd738e
NO	Mri1
NO	Ccdc130
NO	Cacna1a
NO	Ier2
NO	Nacc1
NO	Trmt1
NO	Lyl1
NO	Nfix
NO	Dand5
NO	Gadd45gip1
NO	Rad23a
NO	Calr
NO	Farsa
NO	Gcdh

NO	Dnase2a
NO	Mast1
NO	Rnaseh2a
NO	Prdx2
NO	Junb
NO	Hook2
NO	Asna1
NO	2310036O22Rik
NO	Tnpo2
NO	Fbxw9
NO	Dhps
NO	Wdr83
NO	Wdr83os
NO	Man2b1
NO	Vps35
NO	Orc6
NO	4921524J17Rik
NO	Gpt2
NO	Dnaja2
NO	Neto2
NO	Itfg1
NO	Phkb
NO	Lonp2
NO	Siah1a
NO	Gm10638
NO	N4bp1
NO	Zfp423
NO	Cnep1r1
NO	Heatr3
NO	Papd5
NO	Adcy7
NO	Brd7
NO	Nkd1
NO	Nod2
NO	Cyld
NO	Chd9
NO	Rbl2
NO	Aktip
NO	Rpgrip1l
NO	Fto
NO	Irx3
NO	Irx5
NO	Mmp2
NO	Lpcat2
NO	Slc6a2
NO	Ces1d
NO	Ces1f
NO	Gnao1

NO	Amfr
NO	Nudt21
NO	Ogfod1
NO	Bbs2
NO	Mt2
NO	Mt1
NO	Nup93
NO	Herpud1
NO	Nlrc5
NO	Cpne2
NO	Fam192a
NO	Rspry1
NO	Arl2bp
NO	Pllp
NO	Cx3cl1
NO	Ciapin1
NO	Coq9
NO	Polr2c
NO	Dok4
NO	Ccdc102a
NO	Adgrg1
NO	Adgrg3
NO	Katnb1
NO	Kifc3
NO	Zfp319
NO	Usb1
NO	Mmp15
NO	Cfap20
NO	Csnk2a2
NO	Ndrg4
NO	Setd6
NO	Cnot1
NO	4930513N10Rik
NO	Slc38a7
NO	Got2
NO	Cdh11
NO	Cdh5
NO	Tk2
NO	Cmtm3
NO	Cmtm4
NO	Dync1li2
NO	Nae1
NO	Car7
NO	Pdp2
NO	Rrad
NO	Fam96b
NO	Ces2g
NO	Cbfb

NO	D230025D16Rik
NO	B3gnt9
NO	Tradd
NO	Fbxl8
NO	Hsf4
NO	Nol3
NO	4931428F04Rik
NO	Exoc3l
NO	E2f4
NO	Elmo3
NO	Lrrc29
NO	Tmem208
NO	Fhod1
NO	Slc9a5
NO	Lrrc36
NO	Tppp3
NO	Zdhhc1
NO	Atp6v0d1
NO	Fam65a
NO	Ctcf
NO	Acd
NO	Pard6a
NO	Gfod2
NO	Ranbp10
NO	Cenpt
NO	Thap11
NO	Nutf2
NO	Edc4
NO	Pskh1
NO	Psmb10
NO	Slc12a4
NO	Ddx28
NO	Dus2
NO	Nfatc3
NO	Pla2g15
NO	Slc7a6
NO	Slc7a6os
NO	Prmt7
NO	Smpd3
NO	Zfp90
NO	Tango6
NO	Has3
NO	Chtf8
NO	Cirh1a
NO	Sntb2
NO	Vps4a
NO	Pdf
NO	Cog8

NO	Nip7
NO	Terf2
NO	Cyb5b
NO	Nfat5
NO	Nqo1
NO	Nob1
NO	Wwp2
NO	Psmd7
NO	Zfhx3
NO	Dhx38
NO	Txnl4b
NO	Hp
NO	Dhodh
NO	Pkd1l3
NO	Ist1
NO	Zfp821
NO	Atxn1l
NO	Ap1g1
NO	Phlpp2
NO	Zfp612
NO	Cmtr2
NO	Vac14
NO	Mtss1l
NO	Il34
NO	Sf3b3
NO	Cog4
NO	Fuk
NO	St3gal2
NO	Ddx19a
NO	Ddx19b
NO	Aars
NO	Exosc6
NO	Pdpr
NO	9430091E24Rik
NO	Glg1
NO	Rfwd3
NO	Mlkl
NO	Wdr59
NO	Znrf1
NO	Ldhd
NO	Zfp1
NO	Bcar1
NO	Cfdp1
NO	Tmem170
NO	Tmem231
NO	Gabarapl2
NO	Adat1
NO	Kars

NO	Terf2ip
NO	Mon1b
NO	Nudt7
NO	Vat1l
NO	Wwox
NO	Maf
NO	Cdyl2
NO	Cmc2
NO	Atmin
NO	Gcsh
NO	Gan
NO	Cmip
NO	Plcg2
NO	Mphosph6
NO	Cdh13
NO	Hsbp1
NO	Mlycd
NO	Osgin1
NO	Mbtps1
NO	Hsdl1
NO	Taf1c
NO	Kcng4
NO	Wfdc1
NO	Tldc1
NO	Cotl1
NO	Klhl36
NO	Usp10
NO	Crispld2
NO	Zdhhc7
NO	6430548M08Rik
NO	A330074K22Rik
NO	Gse1
NO	Emc8
NO	Cox4i1
NO	Irf8
NO	Mthfsd
NO	Fbxo31
NO	Map1lc3b
NO	Zcchc14
NO	Klhdc4
NO	Slc7a5
NO	Banp
NO	Zfpm1
NO	Zc3h18
NO	Cyba
NO	Mvd
NO	Snai3
NO	Rnf166

NO	Ctu2
NO	Piezo1
NO	Cdt1
NO	Aprt
NO	Galns
NO	Trappc2l
NO	Cbfa2t3
NO	Acsf3
NO	Cdh15
NO	Ankrd11
NO	2810013P06Rik
NO	Spg7
NO	Rpl13
NO	Sult5a1
NO	Dpep1
NO	Chmp1a
NO	Cdk10
NO	Spata2l
NO	Vps9d1
NO	Zfp276
NO	Fanca
NO	Tcf25
NO	Def8
NO	Afg3l1
NO	Dbndd1
NO	Gas8
NO	Rhou
NO	Rab4a
NO	Ccsap
NO	Acta1
NO	Nup133
NO	Abcb10
NO	Taf5l
NO	Urb2
NO	Galnt2
NO	Cog2
NO	Agt
NO	2310022B05Rik
NO	Ttc13
NO	Arv1
NO	Trim67
NO	2810004N23Rik
NO	Gnpat
NO	Exoc8
NO	Sprtn
NO	Egln1
NO	Tsnax
NO	Disc1

NO	Sipa1l2
NO	Map10
NO	Ntpcr
NO	Pcnxl2
NO	Coa6
NO	Gm17296
NO	Irf2bp2
NO	Tomm20
NO	Rbm34
NO	Pard3
NO	Nrp1
NO	Itgb1
NO	2610044O15Rik8
NO	Alkbh8
NO	Cwf19l2
NO	Gucy1a2
NO	Aasdhppt
NO	Kbtbd3
NO	Msantd4
NO	Casp12
NO	Pdgfd
NO	Dync2h1
NO	Dcun1d5
NO	Tmem123
NO	Birc2
NO	Birc3
NO	Yap1
NO	Cep126
NO	Arhgap42
NO	Jrkl
NO	Ccdc82
NO	Phxr4
NO	Maml2
NO	Mtmr2
NO	Cep57
NO	Fam76b
NO	Sesn3
NO	Endod1
NO	Cwc15
NO	Amotl1
NO	Ankrd49
NO	Mre11a
NO	Panx1
NO	Med17
NO	4931406C07Rik
NO	Taf1d
NO	Cep295
NO	Scarna9

NO	Smco4
NO	Slc36a4
NO	Fat3
NO	Chordc1
NO	Naalad2
NO	Zfp317
NO	Zfp560
NO	Zfp26
NO	Zfp426
NO	Zfp266
NO	Zfp846
NO	Fbxl12
NO	Ubl5
NO	Pin1
NO	Col5a3
NO	A230050P20Rik
NO	Angptl6
NO	Ppan
NO	Eif3g
NO	Dnmt1
NO	S1pr2
NO	Mrpl4
NO	Icam1
NO	Raver1
NO	Tyk2
NO	Cdc37
NO	Pde4a
NO	Keap1
NO	Atg4d
NO	Kri1
NO	Cdkn2d
NO	Slc44a2
NO	Ilf3
NO	Qtrt1
NO	Dnm2
NO	Tmed1
NO	AB124611
NO	Carm1
NO	Yipf2
NO	1810026J23Rik
NO	Smarca4
NO	Ldlr
NO	Spc24
NO	Kank2
NO	Dock6
NO	Gm6484
NO	Rab3d
NO	Tmem205

NO	Swsap1
NO	Rgl3
NO	Prkcsh
NO	Zfp653
NO	Ecsit
NO	Elof1
NO	Acp5
NO	Pigyl
NO	Zfp809
NO	Zfp810
NO	Anln
NO	9530077C05Rik
NO	Rp9
NO	Bbs9
NO	Bmper
NO	Dpy19l1
NO	Herpud2
NO	Sept7
NO	Eepd1
NO	B3gat1
NO	Glb1l2
NO	Acad8
NO	Thyn1
NO	Vps26b
NO	Ncapd3
NO	Jam3
NO	Opcml
NO	Snx19
NO	Adamts15
NO	Zbtb44
NO	Aplp2
NO	Prdm10
NO	Nfrkb
NO	Tmem45b
NO	Barx2
NO	Arhgap32
NO	Fli1
NO	Ets1
NO	Kirrel3
NO	St3gal4
NO	4930581F22Rik
NO	Dcps
NO	Tirap
NO	Foxred1
NO	Srpr
NO	Fam118b
NO	Rpusd4
NO	Cdon

NO	Pus3
NO	Hyls1
NO	Stt3a
NO	Ei24
NO	Fez1
NO	Pknox2
NO	Tmem218
NO	Slc37a2
NO	Ccdc15
NO	Robo4
NO	Msantd2
NO	Esam
NO	Vsig2
NO	Nrgn
NO	Siae
NO	Tbrg1
NO	Vwa5a
NO	Zfp202
NO	Scn3b
NO	Gramd1b
NO	Clmp
NO	Hspa8
NO	Ubash3b
NO	3110039I08Rik
NO	Sorl1
NO	Sc5d
NO	Tbcel
NO	Arhgef12
NO	Oaf
NO	Pvrl1
NO	Thy1
NO	Usp2
NO	Rnf26
NO	Mcam
NO	Cbl
NO	Pdzd3
NO	Nlrx1
NO	Hinfp
NO	C2cd2l
NO	Dpagt1
NO	H2afx
NO	Hmbs
NO	Vps11
NO	Hyou1
NO	Slc37a4
NO	Trappc4
NO	Rps25
NO	Ccdc84

NO	Bcl9l
NO	Cxcr5
NO	Ddx6
NO	Phldb1
NO	Arcn1
NO	Ift46
NO	Tmem25
NO	Kmt2a
NO	Atp5l
NO	Ube4a
NO	Mpzl2
NO	Scn2b
NO	Scn4b
NO	Il10ra
NO	Fxyd6
NO	Cep164
NO	Bace1
NO	Rnf214
NO	Pcsk7
NO	Tagln
NO	Sidt2
NO	Pafah1b2
NO	Sik3
NO	Zpr1
NO	Bud13
NO	Cadm1
NO	Rexo2
NO	Rbm7
NO	Gm5617
NO	Nnmt
NO	Zbtb16
NO	Usp28
NO	Zw10
NO	Ttc12
NO	Ncam1
NO	Plet1os
NO	Plet1
NO	Pts
NO	Sdhd
NO	Timm8b
NO	AU019823
NO	Dlat
NO	Dixdc1
NO	2310030G06Rik
NO	Hspb2
NO	Cryab
NO	1110032A03Rik
NO	Fdxacb1

NO	Alg9
NO	Ppp2r1b
NO	Sik2
NO	Layn
NO	Arhgap20
NO	Fdx1
NO	Rdx
NO	Zc3h12c
NO	AI593442
NO	Ddx10
NO	Exph5
NO	Kdelc2
NO	Atm
NO	Npat
NO	Acat1
NO	Cul5
NO	Sln
NO	Gldn
NO	Dmxl2
NO	Sh2d7
NO	Cib2
NO	Idh3a
NO	Dnaja4
NO	Wdr61
NO	Ireb2
NO	Hykk
NO	Psma4
NO	Ube2q2
NO	Fbxo22
NO	Nrg4
NO	AI118078
NO	Etfa
NO	Scaper
NO	Rcn2
NO	Pstpip1
NO	Tspan3
NO	Peak1
NO	Hmg20a
NO	Cspg4
NO	Snx33
NO	Imp3
NO	Snupn
NO	Ptpn9
NO	Sin3a
NO	Man2c1
NO	Neil1
NO	Commd4
NO	1700017B05Rik

NO	Ppcdc
NO	Scamp5
NO	Cox5a
NO	Fam219b
NO	Mpi
NO	Scamp2
NO	Ulk3
NO	Csk
NO	Cyp1a1
NO	Edc3
NO	Clk3
NO	Arid3b
NO	Ubl7
NO	Sema7a
NO	Islr
NO	Islr2
NO	Pml
NO	Stoml1
NO	Loxl1
NO	6030419C18Rik
NO	Cd276
NO	Nptn
NO	Rec114
NO	Hcn4
NO	Neo1
NO	Adpgk
NO	Bbs4
NO	Arih1
NO	Gm20199
NO	Hexa
NO	Parp6
NO	Pkm
NO	Senp8
NO	Myo9a
NO	Thsd4
NO	Lrrc49
NO	Larp6
NO	Uaca
NO	Tle3
NO	Rplp1
NO	Glce
NO	Anp32a
NO	Coro2b
NO	Itga11
NO	Fem1b
NO	Cln6
NO	Pias1
NO	Map2k5

NO	2300009A05Rik
NO	Aagab
NO	Smad3
NO	Smad6
NO	Rpl4
NO	Snapc5
NO	Map2k1
NO	Uchl4
NO	Tipin
NO	Dis3l
NO	Megf11
NO	Rab11a
NO	Dennd4a
NO	Vwa9
NO	Hacd3
NO	Dpp8
NO	Igdcc4
NO	Parp16
NO	Cilp
NO	Clpx
NO	Pdcd7
NO	Kbtbd13
NO	Rasl12
NO	Mtfmt
NO	Spg21
NO	Plekho2
NO	Rbpms2
NO	Oaz2
NO	Zfp609
NO	Trip4
NO	Csnk1g1
NO	Ppib
NO	Snx22
NO	Snx1
NO	Fam96a
NO	Dapk2
NO	Herc1
NO	Fbxl22
NO	Usp3
NO	Aph1b
NO	Rab8b
NO	Rps27l
NO	Lactb
NO	Tpm1
NO	Tln2
NO	Vps13c
NO	Rora
NO	9530091C08Rik

NO	Ice2
NO	Anxa2
NO	Bnip2
NO	Gtf2a2
NO	Myo1e
NO	Rnf111
NO	Sltm
NO	Fam63b
NO	Adam10
NO	Polr2m
NO	Myzap
NO	Cgnl1
NO	Tcf12
NO	Zfp280d
NO	Tex9
NO	Rfx7
NO	Nedd4
NO	Pygo1
NO	Ccpg1os
NO	Ccpg1
NO	Pigb
NO	2310009A05Rik
NO	Rab27a
NO	Rsl24d1
NO	Fam214a
NO	Arpp19
NO	Myo5a
NO	Myo5c
NO	Gnb5
NO	Mapk6
NO	Leo1
NO	Tmod3
NO	Tmod2
NO	Lysmd2
NO	Scg3
NO	Bmp5
NO	Mlip
NO	Lrrc1
NO	Klhl31
NO	Gclc
NO	Elovl5
NO	Fbxo9
NO	Ick
NO	Gsta4
NO	Mto1
NO	Eef1a1
NO	Slc17a5
NO	Cd109

NO	Col12a1
NO	Cox7a2
NO	Tmem30a
NO	4930429F24Rik
NO	Filip1
NO	Senp6
NO	Myo6
NO	Irak1bp1
NO	Phip
NO	Hmgn3
NO	Lca5
NO	Bckdhb
NO	Fam46a
NO	Ibtk
NO	Ube2cbp
NO	Dopey1
NO	Pgm3
NO	Me1
NO	Cyb5r4
NO	Cep162
NO	Tbx18
NO	Nt5e
NO	Snx14
NO	Syncrip
NO	Snhg5
NO	Zfp949
NO	Mthfsl
NO	Trim43c
NO	9330159M07Rik
NO	Mthfs
NO	Tmed3
NO	Rasgrf1
NO	Ctsh
NO	Morf4l1
NO	Adamts7
NO	Tbc1d2b
NO	Plscr1
NO	Plscr2
NO	Plscr4
NO	Plod2
NO	1190002N15Rik
NO	Slc9a9
NO	Chst2
NO	U2surp
NO	Paqr9
NO	Pcolce2
NO	Trpc1
NO	Atr

NO	Xrn1
NO	Gk5
NO	Tfdp2
NO	Atp1b3
NO	Rnf7
NO	Rasa2
NO	Zbtb38
NO	Pxylp1
NO	Slc25a36
NO	Clstn2
NO	Nmnat3
NO	Rbp1
NO	Copb2
NO	Mrps22
NO	Faim
NO	Pik3cb
NO	Cep70
NO	Mras
NO	Armc8
NO	Dbr1
NO	Dzip1l
NO	Il20rb
NO	Nck1
NO	Stag1
NO	Pccb
NO	Msl2
NO	Ppp2r3a
NO	Ephb1
NO	Ky
NO	Cep63
NO	Anapc13
NO	Amotl2
NO	Ryk
NO	Slco2a1
NO	Srprb
NO	Trf
NO	Topbp1
NO	Cdv3
NO	Nphp3
NO	Uba5
NO	Acad11
NO	Ackr4
NO	Dnajc13
NO	Mrpl3
NO	Nudt16
NO	Aste1
NO	Atp2c1
NO	Pik3r4

NO	Col6a6
NO	Glyctk
NO	Wdr82
NO	Ppm1m
NO	Twf2
NO	Tlr9
NO	Alas1
NO	Poc1a
NO	Dusp7
NO	Rpl29
NO	Acy1
NO	Abhd14a
NO	Abhd14b
NO	Pcbp4
NO	Parp3
NO	Rrp9
NO	Tex264
NO	Rad54l2
NO	Vprbp
NO	Rbm15b
NO	Manf
NO	Mapkapk3
NO	Cish
NO	Hemk1
NO	6430571L13Rik
NO	Cacna2d2
NO	Tmem115
NO	Cyb561d2
NO	Nprl2
NO	Rassf1
NO	Tusc2
NO	Hyal2
NO	Hyal1
NO	Nat6
NO	Ifrd2
NO	Sema3b
NO	Gnai2
NO	Slc38a3
NO	Sema3f
NO	Rbm5
NO	Rbm6
NO	Mon1a
NO	Mst1r
NO	Uba7
NO	Fam212a
NO	Ip6k1
NO	Gmppb
NO	Rnf123

NO	Amigo3
NO	Apeh
NO	Bsn
NO	Dag1
NO	Nicn1
NO	Amt
NO	Tcta
NO	Rhoa
NO	Gpx1
NO	Usp4
NO	Klhdc8b
NO	Ccdc71
NO	Lamb2
NO	Usp19
NO	Qars
NO	Qrich1
NO	Impdh2
NO	Ndufaf3
NO	Dalrd3
NO	Wdr6
NO	P4htm
NO	Arih2
NO	Slc25a20
NO	Prkar2a
NO	Ip6k2
NO	Nckipsd
NO	Celsr3
NO	Slc26a6
NO	Uqcrc1
NO	Col7a1
NO	Pfkfb4
NO	Shisa5
NO	Trex1
NO	Atrip
NO	Tma7
NO	Ccdc51
NO	Plxnb1
NO	Nme6
NO	Camp
NO	Cdc25a
NO	Map4
NO	Dhx30
NO	Smarcc1
NO	Elp6
NO	Scap
NO	Ptpn23
NO	Ngp
NO	Klhl18

NO	Setd2
NO	Nbeal2
NO	Ccdc12
NO	Pth1r
NO	Myl3
NO	Tmie
NO	Als2cl
NO	Lrrc2
NO	Rtp3
NO	Ltf
NO	Ccrl2
NO	Lrrfip2
NO	Mlh1
NO	Epm2aip1
NO	Dclk3
NO	Arpp21
NO	2310075C17Rik
NO	Pdcd6ip
NO	Clasp2
NO	Ubp1
NO	Fbxl2
NO	Susd5
NO	Crtap
NO	Glb1
NO	Tmppe
NO	Cnot10
NO	Dync1li1
NO	Cmtm6
NO	Cmtm7
NO	Gpd1l
NO	Osbpl10
NO	Stt3b
NO	Gadl1
NO	Tgfbr2
NO	Rbms3
NO	Azi2
NO	Cmc1
NO	Golga4
NO	Itga9
NO	Ctdspl
NO	Vill
NO	Plcd1
NO	Acaa1b
NO	Oxsr1
NO	Myd88
NO	Acaa1a
NO	Acvr2b
NO	Exog

NO	Wdr48
NO	Gorasp1
NO	Csrnp1
NO	Xirp1
NO	Slc25a38
NO	Rpsa
NO	Myrip
NO	Eif1b
NO	Rpl14
NO	Ctnnb1
NO	Trak1
NO	Vipr1
NO	Sec22c
NO	Deb1
NO	Nktr
NO	E530011L22Rik
NO	Zfp651
NO	Klhl40
NO	Hhatl
NO	Higd1a
NO	Ackr2
NO	Fam198a
NO	Pomgnt2
NO	Snrk
NO	Ano10
NO	Abhd5
NO	Tcaim
NO	Zfp445
NO	Zkscan7
NO	1110059G10Rik
NO	Tmem42
NO	Tgm4
NO	Zdhhc3
NO	Exosc7
NO	Clec3b
NO	Tmem158
NO	Lars2
NO	Limd1
NO	Sacm1l
NO	Slc6a20b
NO	Lztfl1
NO	Ccr9
NO	Fyco1
NO	Cxcr6
NO	Ccr2
NO	2010315B03Rik
NO	4930526I15Rik
NO	Ppp2r3d

NO	Shroom4
NO	Clcn5
NO	Ppp1r3f
NO	Foxp3
NO	Ccdc22
NO	Syp
NO	Prickle3
NO	Plp2
NO	Magix
NO	Gpkow
NO	Wdr45
NO	Praf2
NO	Tfe3
NO	Gripap1
NO	Otud5
NO	Pim2
NO	Slc35a2
NO	Pqbp1
NO	Timm17b
NO	Hdac6
NO	Suv39h1
NO	Was
NO	Wdr13
NO	Rbm3
NO	Tbc1d25
NO	Ebp
NO	Porcn
NO	Ftsj1
NO	Xk
NO	Cybb
NO	Dynlt3
NO	Srpx
NO	Rpgr
NO	Tspan7
NO	Mid1ip1
NO	Bcor
NO	2900008C10Rik
NO	Atp6ap2
NO	1810030O07Rik
NO	Med14
NO	5730405O15Rik
NO	Usp9x
NO	Ddx3x
NO	Cask
NO	Maoa
NO	Maob
NO	Fundc1
NO	Kdm6a

NO	4930578C19Rik
NO	Chst7
NO	Slc9a7
NO	Rp2h
NO	Jade3
NO	Ndufb11
NO	Rbm10
NO	Uba1
NO	Cdk16
NO	Usp11
NO	Araf
NO	Cfp
NO	Elk1
NO	Uxt
NO	A230072C01Rik
NO	Zfp182
NO	Klhl13
NO	Wdr44
NO	Dock11
NO	Il13ra1
NO	Lonrf3
NO	Pgrmc1
NO	Akap17b
NO	Slc25a5
NO	C330007P06Rik
NO	Ube2a
NO	Nkrf
NO	Sept6
NO	Rpl39
NO	Upf3b
NO	Nkap
NO	Ndufa1
NO	Rnf113a1
NO	Zbtb33
NO	Tmem255a
NO	Atp1b4
NO	Lamp2
NO	Cul4b
NO	Mcts1
NO	C1galt1c1
NO	Gria3
NO	Thoc2
NO	Xiap
NO	Stag2
NO	Smarca1
NO	Ocrl
NO	Apln
NO	Sash3

NO	Zdhhc9
NO	Utp14a
NO	Bcorl1
NO	Elf4
NO	Aifm1
NO	Zfp280c
NO	Slc25a14
NO	Enox2
NO	Firre
NO	Stk26
NO	Frmd7
NO	Rap2c
NO	Mbnl3
NO	Hs6st2
NO	Gpc4
NO	Gpc3
NO	Phf6
NO	Hprt
NO	C430049B03Rik
NO	Fam122b
NO	Mospd1
NO	Cxx1c
NO	Cxx1a
NO	Cxx1b
NO	Zfp449
NO	Ddx26b
NO	Mmgt1
NO	Slc9a6
NO	Fhl1
NO	Htatsf1
NO	Arhgef6
NO	Rbmx
NO	Fgf13
NO	Atp11c
NO	Fmr1
NO	Ids
NO	1110012L19Rik
NO	BC023829
NO	Mamld1
NO	Mtm1
NO	Mtmr1
NO	Cd99l2
NO	Hmgb3
NO	Vma21
NO	Prrg3
NO	Gabra3
NO	Cetn2
NO	Nsdhl

NO	Zfp185
NO	F8a
NO	Zfp275
NO	Haus7
NO	Bgn
NO	Atp2b3
NO	Pnck
NO	Slc6a8
NO	Bcap31
NO	Abcd1
NO	Plxnb3
NO	Srpk3
NO	Idh3g
NO	Ssr4
NO	Pdzd4
NO	L1cam
NO	Arhgap4
NO	Naa10
NO	Renbp
NO	Hcfc1
NO	Irak1
NO	Mecp2
NO	Opn1mw
NO	Flna
NO	Emd
NO	Rpl10
NO	Dnase1l1
NO	Taz
NO	Atp6ap1
NO	Gdi1
NO	Fam50a
NO	Plxna3
NO	Lage3
NO	Ubl4a
NO	Slc10a3
NO	Fam3a
NO	Ikbkg
NO	G6pdx
NO	Dkc1
NO	Mpp1
NO	F8
NO	Fundc2
NO	Cmc4
NO	Mtcp1
NO	Brcc3
NO	Vbp1
NO	4933407K13Rik
NO	Pls3

NO	Tbl1x
NO	Prkx
NO	Prrg1
NO	Tmem47
NO	Dmd
NO	Tab3
NO	Gyk
NO	Pola1
NO	Pdk3
NO	Zfx
NO	Eif2s3x
NO	Klhl15
NO	Apoo
NO	Maged1
NO	Zxdb
NO	Zxda
NO	Arhgef9
NO	Amer1
NO	Asb12
NO	Zc3h12b
NO	Las1l
NO	Msn
NO	F630028O10Rik
NO	Heph
NO	Eda2r
NO	Ar
NO	Ophn1
NO	Yipf6
NO	Stard8
NO	Efnb1
NO	Pja1
NO	Eda
NO	Igbp1
NO	Pdzd11
NO	Dlg3
NO	Snx12
NO	Foxo4
NO	Il2rg
NO	Med12
NO	Zmym3
NO	Nono
NO	Itgb1bp2
NO	Taf1
NO	Ogt
NO	Nhsl2
NO	Rgag4
NO	Pin4
NO	Rps4x

NO	Hdac8
NO	Phka1
NO	Chic1
NO	Xist
NO	Jpx
NO	Ftx
NO	Slc16a2
NO	Rlim
NO	Abcb7
NO	Uprt
NO	5530601H04Rik
NO	Pbdc1
NO	Magee1
NO	Atrx
NO	Magt1
NO	Cox7b
NO	Atp7a
NO	Tlr13
NO	Pgk1
NO	Taf9b
NO	Lpar4
NO	Itm2a
NO	2610002M06Rik
NO	Brwd3
NO	Hmgn5
NO	Sh3bgrl
NO	Rps6ka6
NO	Apool
NO	Chm
NO	Dach2
NO	Klhl4
NO	Diap2
NO	Pcdh19
NO	Tnmd
NO	Tspan6
NO	Srpx2
NO	Sytl4
NO	Cstf2
NO	Trmt2b
NO	Drp2
NO	Timm8a1
NO	Btk
NO	Rpl36a
NO	Gla
NO	Hnrnph2
NO	Armcx4
NO	Armcx1
NO	Armcx3

NO	Armcx2
NO	Zmat1
NO	Armcx5
NO	Gprasp1
NO	Bhlhb9
NO	Arxes2
NO	Tceal8
NO	Tceal5
NO	Bex1
NO	Tceal7
NO	Wbp5
NO	Ngfrap1
NO	Tceal1
NO	Morf4l2
NO	BC065397
NO	Plp1
NO	Slc25a53
NO	Fam199x
NO	Mum1l1
NO	Rnf128
NO	Tbc1d8b
NO	Morc4
NO	Rbm41
NO	Prps1
NO	Tsc22d3
NO	Mid2
NO	Psmd10
NO	Col4a6
NO	Col4a5
NO	Nxt2
NO	Kcne1l
NO	Acsl4
NO	Tmem164
NO	Ammecr1
NO	Chrdl1
NO	Capn6
NO	Alg13
NO	Amot
NO	Tmem29
NO	Alas2
NO	Apex2
NO	Pfkfb1
NO	Tro
NO	Maged2
NO	Gnl3l
NO	Fgd1
NO	Tsr2
NO	Wnk3

NO	Fam120c
NO	Phf8
NO	Huwe1
NO	Mir3113
NO	Hsd17b10
NO	Smc1a
NO	Iqsec2
NO	Kdm5c
NO	Kantr
NO	Tspyl2
NO	Shroom2
NO	Mageh1
NO	Rragb
NO	Ubqln2
NO	Kctd12b
NO	2210013O21Rik
NO	Sat1
NO	Acot9
NO	Prdx4
NO	Phex
NO	Sms
NO	Mbtps2
NO	Yy2
NO	Smpx
NO	Klhl34
NO	Cnksr2
NO	Rps6ka3
NO	Eif1ax
NO	A830080D01Rik
NO	Sh3kbp1
NO	Map3k15
NO	Pdha1
NO	Adgrg2
NO	Phka2
NO	Cdkl5
NO	Rai2
NO	Nhs
NO	Reps2
NO	Rbbp7
NO	Txlng
NO	Syap1
NO	Ctps2
NO	Ap1s2
NO	Zrsr2
NO	Car5b
NO	Siah1b
NO	Bmx
NO	Pir

NO	Figf
NO	Piga
NO	Asb11
NO	Mospd2
NO	Gpm6b
NO	Ofd1
NO	Trappc2
NO	Rab9
NO	Tceanc
NO	Tmsb4x
NO	Tlr7
NO	Prps2
NO	Msl3
NO	Arhgap6
NO	Hccs
NO	Mid1
NO	Kdm5d
NO	Eif2s3y
NO	Uty
NO	Ddx3y
NO	Erdr1

baseMean	log2FoldChange	lfcSE	stat	pvalue	padj
761.5660907	-0.907405806	0.207167182	-4.380065402	1.18644E-05	0.005665045
149.1321178	-1.319037848	0.262162323	-5.031378393	4.86966E-07	0.000450504
13.00354569	-22.5447518	2.975761164	-7.576129453	3.56017E-14	5.26976E-10
355.1270421	-1.036518953	0.167204972	-6.199091681	5.67899E-10	2.10151E-06
397.4331283	-1.128261444	0.222348471	-5.074293685	3.88938E-07	0.000411219
240.8095511	-1.56299614	0.317457261	-4.923485235	8.50163E-07	0.000740242
166.2065565	-1.528508449	0.276448889	-5.529081546	3.21912E-08	4.76494E-05
263.0378706	-0.659670023	0.118174399	-5.582173722	2.37531E-08	4.50773E-05
26.96003345	-1.417581755	0.265034305	-5.348672719	8.86016E-08	0.000114607
29.39462961	-1.131197167	0.203553956	-5.557234989	2.74082E-08	4.50773E-05
23.35937108	-3.296175858	0.828628513	-3.977869223	6.95356E-05	0.01583486
48.76933724	-1.83826469	0.483362843	-3.803074062	0.000142912	0.023504196
31.72492516	-0.927625768	0.249260261	-3.721514876	0.000198031	0.030203532
63.8076732	-0.847578123	0.232038352	-3.652750146	0.000259447	0.034597561
26.47582127	-0.892352677	0.231466946	-3.855205641	0.000115632	0.019959647
42.19029134	-4.70108896	1.146522221	-4.100303397	4.12609E-05	0.012620566
125.8193239	-0.68120868	0.156873101	-4.342418663	1.40923E-05	0.00613511
77.55160961	-2.342711469	0.645706237	-3.628138205	0.000285472	0.035213026
27.39335229	-1.206073855	0.312388082	-3.860819041	0.000113008	0.019934809
6.828729556	-1.64844836	0.451029544	-3.654856723	0.000257326	0.034597561
5.999121071	-1.802155533	0.505581613	-3.564519531	0.000364523	0.040876318
39.26017455	-0.923342011	0.263249362	-3.507480526	0.000452371	0.046179327
137.3195298	-0.727101278	0.18459615	-3.938875638	8.18643E-05	0.017310801
457.8224345	-0.632286977	0.154025066	-4.10509142	4.04155E-05	0.012620566
13.07162321	-1.636538348	0.448615017	-3.647979415	0.000264311	0.03464515
23.46803358	-4.750606394	1.11774065	-4.250186653	2.13592E-05	0.008319988
10.39169317	-1.592649549	0.394423617	-4.037916296	5.39281E-05	0.013808293
710.0525809	-0.707279211	0.177450402	-3.985785338	6.72573E-05	0.015610676
35.95214346	-0.987732179	0.266689603	-3.703677114	0.000212497	0.031142338
729.8519291	-1.171815706	0.331302894	-3.536992063	0.000404712	0.044167969
194.3606432	-0.827684885	0.211117235	-3.920498884	8.83658E-05	0.018166545
52.08383445	-0.754486061	0.212436881	-3.551577565	0.000382929	0.042299378
20.98158164	-1.020518453	0.288743821	-3.534338672	0.000408797	0.044167969
226.8316987	-0.889131405	0.21717755	-4.094029998	4.23939E-05	0.012620566
91.08350178	-0.66242778	0.171181784	-3.869732897	0.000108955	0.019934809
27.27293191	-0.869346125	0.243200022	-3.574613679	0.000350745	0.039936411
32.94967867	-1.28230901	0.327597449	-3.914282653	9.06734E-05	0.018385578
48.81766559	-0.892756914	0.230455546	-3.87387907	0.000107117	0.019934809
66.48387575	-1.093350351	0.263522423	-4.148984129	3.33954E-05	0.010984859
10.18600005	-1.123423648	0.298614621	-3.762118698	0.00016848	0.027106975
24.20566883	-1.342923149	0.317846615	-4.225066705	2.3887E-05	0.008839385
36.93345776	-2.168170421	0.522504253	-4.149574689	3.33094E-05	0.010984859
44.04112203	-2.079416923	0.590511523	-3.521382468	0.000429303	0.044626692
20.09708605	-2.745815548	0.686247525	-4.001202845	6.30213E-05	0.015156055
25.81242644	-0.786379744	0.188002636	-4.182812337	2.87925E-05	0.010147302
47.99345715	-0.850441898	0.218263176	-3.896405761	9.76308E-05	0.019268409
72.68526601	-0.616410911	0.165743952	-3.719055218	0.000199969	0.030203532

63.60050417	-0.959097758	0.225249958	-4.257926462	2.06332E-05	0.008254387
22.59432176	-1.14081718	0.294958138	-3.86772573	0.000109855	0.019934809
10.92297798	-1.48846627	0.405940696	-3.666708674	0.000245692	0.034435888
372.8333922	-0.651472115	0.156287894	-4.16841062	3.06731E-05	0.01055868
123.5343766	-0.756212907	0.214695838	-3.52225229	0.000427897	0.044626692
162.6943636	-0.714192382	0.190857843	-3.74201223	0.000182553	0.028506312
11.27841474	-2.618447835	0.706111547	-3.708263726	0.000208685	0.030889591
9.306299801	-1.176817924	0.387333997	-3.038251049	0.002379556	0.115994263
16.27352026	-0.182683483	0.687137586	-0.265861577	0.790345817	0.953061804
96.29361859	0.302527342	0.222707453	1.358406908	0.174334604	0.618034305
768.9958026	0.028807794	0.100696703	0.286084776	0.774813192	0.949820378
881.4274218	0.197045876	0.08753609	2.251024414	0.024383991	0.309236976
326.4162318	0.094703358	0.074204968	1.276240134	0.201870646	0.645481631
265.2533884	0.00488823	0.13066707	0.037409812	0.97015825	0.992623378
1643.056467	0.087635439	0.114113282	0.767968792	0.442505726	0.824977821
1094.675077	-0.016319229	0.080650823	-0.202344237	0.839647619	0.966440785
210.5754685	-0.050383698	0.088578656	-0.568801784	0.569490664	0.8841274
457.8647439	-0.081621315	0.104886029	-0.778190533	0.436456697	0.822078703
24.85367711	0.071537023	0.229418198	0.311819303	0.755177853	0.944498739
12.13374504	-0.036286086	0.33725617	-0.107592059	0.914319296	0.980657455
10.9646551	-0.405925753	0.412949744	-0.982990687	0.325612022	0.756297513
553.5035868	0.048648224	0.049456025	0.983666288	0.325279621	0.756297513
122.6792201	0.139372458	0.144426778	0.965004272	0.334542672	0.761596529
30.39781169	0.080900823	0.187775392	0.430838262	0.666585976	0.916235862
981.427075	0.046075092	0.113736335	0.405104422	0.685400756	0.922971433
92.47029562	0.313100278	0.113603475	2.756080115	0.005849867	0.167161634
2484.234252	0.020987137	0.113155843	0.185471083	0.852859559	0.969966486
578.5111698	-0.018156729	0.110768608	-0.163915836	0.8697974	0.97270634
6.676668342	-0.655698731	0.873075623	-0.751021691	0.452639602	0.830105164
265.3966781	0.156929623	0.093019441	1.68706264	0.091591308	0.499235543
1046.981051	0.197270265	0.204278831	0.965691179	0.334198735	0.761545389
731.4373839	0.033746692	0.037979837	0.888542308	0.374249109	0.786475871
328.886439	0.072069535	0.180565042	0.399133378	0.689794929	0.92405254
268.5806077	0.053274883	0.097166805	0.548282752	0.583497769	0.88906731
337.8143665	0.199453993	0.1271196	1.569026278	0.116641816	0.538641109
16.51085923	0.210159585	0.344254124	0.610478046	0.541545182	0.870731238
55.3883983	-0.135776793	0.132398763	-1.025514057	0.305120696	0.741701149
18.16782808	-0.315751564	0.33810233	-0.933893487	0.350358839	0.770421231
2571.574302	0.081825085	0.074260868	1.101860067	0.270522511	0.713265802
70.16652381	-0.200296803	0.112042852	-1.787680337	0.073827596	0.467806538
575.1381499	0.012200601	0.175036291	0.069703265	0.944429843	0.984876985
196.0425113	0.001269505	0.113554477	0.011179699	0.991080077	0.998213079
429.9663054	0.10299584	0.077487112	1.329199622	0.18378212	0.629653329
599.2044654	0.062496689	0.12595845	0.496169087	0.619775119	0.901976704
6.944251574	0.702427788	0.662720394	1.059915756	0.289182927	0.730209089
7954.296274	0.242122913	0.143261321	1.69007874	0.091012892	0.498583575
145.0616503	0.043977388	0.284653725	0.154494335	0.877219978	0.974191592
42.36409954	0.096746526	0.336355652	0.287631634	0.773628727	0.949369326

40.72771275	-0.493192035	0.434295259	-1.135614596	0.256117898	0.699328006
6.083577409	-0.875228439	0.85867518	-1.019277672	0.308071161	0.743405059
12.15423433	0.916452423	0.554459095	1.652876527	0.098355995	0.509935356
64.62991412	-0.432947423	0.327364269	-1.322524978	0.185993397	0.631625529
21.30733195	-0.199158381	0.223137037	-0.89253843	0.372104404	0.78594312
8.489996979	0.033428277	0.347171485	0.096287507	0.923292233	0.982590996
48.33907895	0.025419104	0.283162705	0.089768545	0.928471145	0.984049111
16.53625581	-0.070699501	0.238433042	-0.296517213	0.766835123	0.947349028
27.80382495	0.331959387	0.343000792	0.96780939	0.333139574	0.761183657
747.5608432	0.47894822	0.250137813	1.914737378	0.055526004	0.424533014
4.708720601	-0.305713586	0.538009992	-0.568230312	0.569878591	0.884207852
37.82576563	1.086844114	0.553748981	1.962701786	0.049680835	0.404942943
9.317717107	0.275591124	0.359951957	0.765633074	0.443894659	0.825650759
168.2083536	-0.147916633	0.281173001	-0.526069829	0.59883969	0.897895573
12.60896703	0.088090793	0.349253313	0.252226078	0.800866322	0.955697618
167.0427425	-0.049977537	0.100322365	-0.498169441	0.61836462	0.901976704
147.9532038	-0.099651052	0.181681595	-0.548492831	0.58335355	0.88906731
412.0923129	0.179092086	0.110489391	1.620898482	0.105039413	0.519411311
13.11767125	-1.369961446	0.530049214	-2.584592922	0.009749403	0.213793574
90.82309097	0.784919622	0.37369338	2.100437589	0.035690365	0.357328889
729.2639978	-0.017223018	0.083627237	-0.205949862	0.836830076	0.966316856
831.1733721	0.137938189	0.067405645	2.046389269	0.040718087	0.374528048
103.0200195	-0.342645764	0.207386543	-1.652208279	0.098492099	0.510283533
29.42537158	-0.369009432	0.301362606	-1.224469875	0.220775024	0.664764193
86.93360455	0.195059939	0.1544539	1.2629007	0.206624868	0.651884304
146.1257276	-0.078540216	0.159183356	-0.493394647	0.621733764	0.902299404
240.8434528	0.018113381	0.08379988	0.216150435	0.828870487	0.964918675
5.263757224	-0.525227476	0.608491583	-0.863163092	0.388047803	0.792781092
26997.56122	0.066242023	0.09804529	0.67562677	0.499277641	0.855692311
106.6920879	-0.208427399	0.155422687	-1.341035878	0.179908804	0.625077039
124.2920685	-0.100087029	0.123116901	-0.812943053	0.416250709	0.810170019
41.5095809	0.174406588	0.402799501	0.432986109	0.665024862	0.916049829
22.9764671	-0.067990254	0.191823471	-0.354441788	0.723007822	0.931432762
660.105354	0.002810371	0.063922682	0.043965168	0.964932169	0.990562866
1077.229129	0.014622329	0.104883742	0.139414636	0.889122507	0.976961229
487.5935246	0.029305569	0.086918062	0.337163166	0.735993902	0.938503341
1520.947504	0.153695503	0.085457446	1.798503349	0.072097278	0.463154629
23.11986234	-0.587136544	0.38493762	-1.525277119	0.127189992	0.556444786
127.6998106	0.169655576	0.187460338	0.905021177	0.365454168	0.780279438
913.0675164	-0.04604681	0.071302448	-0.645795643	0.51841171	0.862002027
61.8846893	0.090169378	0.217376249	0.414807867	0.67828256	0.921517986
220.52682	-0.019770513	0.160533852	-0.123154794	0.901984523	0.979381499
205.181905	-0.137716744	0.219635005	-0.627025479	0.530642534	0.866123834
331.0139264	-0.069905119	0.078422973	-0.891385731	0.37272227	0.786266676
24279.49135	0.015445804	0.136826412	0.112886126	0.910120835	0.980530316
106.1018206	0.080077041	0.119127843	0.672194163	0.501460097	0.85676495
172.1533172	0.131566266	0.141485166	0.92989442	0.352425753	0.772144167
10.71633598	-0.975919814	0.313340507	-3.114566395	0.001842155	0.102581855

2240.794712	0.035705484	0.126513369	0.282226964	0.777769484	0.951371283
1085.774856	0.035671876	0.109882142	0.324637608	0.745455361	0.940455922
4.466969549	0.584886487	0.472029905	1.239087779	0.215312992	0.660219973
991.6711907	0.113131198	0.055069281	2.054343109	0.039942495	0.372633565
13.44850908	-0.667055409	0.337674142	-1.975441188	0.048218103	0.399398071
637.1361443	0.035076725	0.088581471	0.395982642	0.692117834	0.924581226
1950.771966	0.039051712	0.104249482	0.374598614	0.707959004	0.928420247
160.1855593	-0.135470556	0.11394538	-1.188907845	0.234475933	0.677212245
187.8131776	0.245562197	0.106035954	2.315839	0.020567061	0.286111125
20.52735365	0.107872502	0.234979572	0.459071831	0.646182584	0.909300678
22.48391173	0.242505352	0.430166569	0.563747556	0.572925948	0.885236101
74.29093679	-0.026328722	0.192885309	-0.136499365	0.891426526	0.977283948
72.22363431	0.023295642	0.163331631	0.142627869	0.886584086	0.976961229
19.58534295	-0.037400443	0.329311728	-0.11357155	0.909577441	0.980530316
742.2327998	0.121757428	0.076418667	1.593294324	0.111094179	0.531325251
297.1713286	0.126867875	0.070798609	1.791954341	0.07314028	0.465821429
1182.059833	0.178781783	0.074534837	2.398633854	0.016456361	0.263145637
379.2640504	0.107537679	0.107327399	1.001959236	0.316363281	0.749887496
148.3218391	-0.056010035	0.255785315	-0.218972832	0.826671211	0.964482326
15.17083799	0.068058585	0.247714577	0.274745982	0.783511401	0.95173434
12.18276106	-0.04655941	0.369971038	-0.125846095	0.899853753	0.978996707
300.6544154	0.024994585	0.091965646	0.271781765	0.785789827	0.952591608
12.80144568	1.173002613	0.654944488	1.790995472	0.073294022	0.465821429
1272.564627	0.217128763	0.07741328	2.804799944	0.005034782	0.155909709
315.8354429	-0.152749094	0.089846952	-1.700103236	0.089111509	0.493693668
359.0920689	-0.030471557	0.090826406	-0.33549227	0.737253775	0.938737429
6.451740245	-0.732781914	0.384949372	-1.903579972	0.056964909	0.428680279
125.7559596	-0.071637892	0.112075507	-0.639193111	0.522697313	0.863362704
595.5153291	-0.27003063	0.228448686	-1.182018751	0.237198261	0.679753163
11.73124608	-0.134526096	0.418653049	-0.321330744	0.74795976	0.941487306
17.50318908	1.274092254	0.497516446	2.560904798	0.010439996	0.221076998
155.5886372	0.270000558	0.277327848	0.973578962	0.330265636	0.759333947
32.50572799	0.39059617	0.329084274	1.186918369	0.235259821	0.678901985
48.35062934	0.140965729	0.279066312	0.505133451	0.613465108	0.901444946
6.597753531	-0.569675815	0.462774552	-1.231000736	0.218322589	0.662774346
8.37251301	-0.759223958	0.786616637	-0.965176583	0.334456374	0.761596529
20.34727057	-0.601852488	0.361559422	-1.664601866	0.095992213	0.50540715
489.2667159	-0.034498124	0.132221218	-0.260912165	0.794160243	0.953454328
19.06007951	0.033977174	0.2762692	0.122985748	0.902118384	0.979381499
1484.149233	-0.081734962	0.129515833	-0.631080845	0.527987665	0.865762435
272.4106525	-0.048412491	0.097386962	-0.497114703	0.619108167	0.901976704
414.6579608	0.072763385	0.094250903	0.772017901	0.440103825	0.823715107
316.1701522	0.126244402	0.108031908	1.168584396	0.242571118	0.685720081
29.96741228	-0.458344701	0.400293959	-1.145020279	0.252200757	0.694913553
236.7138349	-0.16837824	0.093291842	-1.804854918	0.071097388	0.462488289
7.237429007	-1.173094301	0.82407397	-1.423530342	0.154582473	0.592506562
127.640731	-0.016819011	0.133040372	-0.126420354	0.899399191	0.978996707
839.8101564	0.119724178	0.081894743	1.461927509	0.143761076	0.578069441

309.803825	-0.322293678	0.339614752	-0.948997875	0.342621693	0.766114448
26.6286496	0.241730225	0.191700958	1.260975571	0.207317649	0.652224408
62.34470918	0.016076324	0.1494344	0.107581147	0.914327952	0.980657455
123.005577	0.107060561	0.093299286	1.147496034	0.251176671	0.694735013
237.8150777	0.151316008	0.083808732	1.805492145	0.070997704	0.462488289
427.4989308	0.216588372	0.38029053	0.569533962	0.56899383	0.8841274
77.54942038	0.498566896	0.293350451	1.699560694	0.089213589	0.493844258
3963.101704	-0.723535167	0.256673562	-2.818892453	0.004818966	0.154050722
676.1765251	-0.322430022	0.329090026	-0.979762367	0.327203432	0.757588801
186.3471761	0.159486855	0.098585092	1.617758337	0.105714688	0.521075197
500.4496096	-0.008974305	0.114040985	-0.078693681	0.937276272	0.984334593
8.910407077	-0.561188308	0.378645645	-1.482093655	0.138315377	0.569973331
153.1321786	-0.067148854	0.104423026	-0.643046435	0.52019397	0.863188882
66.74410543	0.255169241	0.332978957	0.766322421	0.443484481	0.825536387
862.5810743	-0.229084758	0.181145114	-1.264647733	0.205997634	0.651161956
189.6838572	0.297135106	0.244589185	1.214833375	0.224429649	0.667854242
278.0480888	0.007309696	0.123676076	0.059103553	0.952869629	0.986647305
4.92865116	-0.063456898	0.621875467	-0.102041166	0.918724	0.981661203
322.0110999	0.036083638	0.12197702	0.295823245	0.76736507	0.94737077
1253.155348	0.080980248	0.096903088	0.835682844	0.403333349	0.802494375
262.1217591	0.006032963	0.086707424	0.069578392	0.944529236	0.984876985
21.31801972	0.145340478	0.279412363	0.520164809	0.602948711	0.899375001
137.4418802	-0.079827556	0.189356736	-0.421572305	0.673337225	0.92071479
75.50989912	-0.126188527	0.126097709	-1.000720218	0.31696209	0.75042752
375.4083154	0.004218868	0.098913789	0.042651967	0.96597897	0.990895439
14.37519801	0.03275182	0.33472371	0.097847327	0.922053526	0.982590996
735.7107658	0.272954436	0.200085526	1.364188812	0.172508148	0.616428289
55.90254701	-0.241721695	0.134267139	-1.800304199	0.071812618	0.463154629
100.0096762	-0.027912295	0.128875048	-0.216584164	0.828532428	0.964814045
311.9471154	0.032722626	0.127128141	0.257398764	0.796870957	0.9546972
1066.01989	0.018606577	0.080744042	0.230439008	0.817750652	0.961348992
148.164217	0.264299386	0.217579024	1.214728245	0.224469756	0.667854242
172.5407803	0.599097387	0.182928914	3.27502839	0.001056514	0.078080172
6.791604824	0.333978543	0.489903375	0.681723295	0.495413934	0.853825991
214.0321212	0.049998594	0.093865715	0.53266088	0.594268351	0.894609104
149.3725465	0.115304394	0.108707034	1.060689357	0.288831099	0.729902555
168.5792524	0.033351867	0.226523269	0.147233733	0.882947535	0.975884268
1799.227625	0.074681199	0.049414326	1.511326891	0.130705186	0.563723241
218.4599888	-0.058481897	0.118526552	-0.49340756	0.621724642	0.902299404
1499.252117	0.017555186	0.127241674	0.137967263	0.89026629	0.976961229
346.6215966	0.107515099	0.124735547	0.861944343	0.388718146	0.792862891
415.1932337	0.119545311	0.102612642	1.165015425	0.244012749	0.686277164
41.24136452	0.139012152	0.237036682	0.586458397	0.557567511	0.879365746
127.5541676	-0.004392103	0.158763578	-0.027664423	0.977929799	0.994457054
585.4186981	0.182367114	0.112516977	1.620796418	0.105061307	0.519411311
10.86346734	-0.400228809	0.682522161	-0.586396796	0.557608897	0.879365746
21.93287936	-1.137554513	0.539948836	-2.106782044	0.035136477	0.355098128
10.18966178	-0.541957016	0.45032128	-1.203489686	0.22878688	0.671658747

37.93571314	0.111856843	0.163752907	0.683083097	0.494554336	0.853198001
30.76357289	0.169388619	0.178817276	0.947272116	0.343500136	0.766809956
152.038505	0.074478814	0.093502008	0.796547746	0.425713736	0.816273895
43.09729285	-0.263515873	0.245389899	-1.073866017	0.282882736	0.724058492
64.371775	-0.037610106	0.140955716	-0.26682214	0.789606107	0.953026618
13.20595908	-1.159032501	0.472324365	-2.453890985	0.014131982	0.25138514
580.3790404	-0.11117428	0.160213731	-0.693912308	0.487737204	0.849513564
50.24619728	0.7389788	0.529816948	1.394781354	0.163081783	0.601684318
821.1129461	0.074651923	0.067845762	1.100318156	0.271193524	0.713807699
680.0612705	0.097957629	0.101284612	0.967152135	0.333467986	0.761455418
95.00886006	-0.057625681	0.132323473	-0.435490995	0.663206074	0.915744058
102.5229523	0.038783377	0.113261301	0.342423905	0.732031901	0.936065863
208.0815398	-0.105535058	0.073662479	-1.432684041	0.151948152	0.589764517
413.1289526	0.158717229	0.12007883	1.321775276	0.186242993	0.631908584
570.8168316	-0.03139334	0.19038988	-0.164889753	0.869030758	0.972216255
11.46397438	-0.212104156	0.418183976	-0.507202973	0.612012409	0.901124807
5.694041282	-0.889287868	0.543959221	-1.634842897	0.102081986	0.513951548
663.7162429	0.034789679	0.059518195	0.58452174	0.558869343	0.880046199
73.79803918	0.004489137	0.330458752	0.013584562	0.989161421	0.99764435
2123.684795	0.081865526	0.094619306	0.865209541	0.38692379	0.792352176
828.4940566	0.103569835	0.121466631	0.852660803	0.393847434	0.796193624
47.95285327	0.017774688	0.158569548	0.112093955	0.910748911	0.980530316
4.504298397	-0.461357334	0.54880548	-0.840657302	0.400539947	0.801079895
472.6994044	-0.013910357	0.071779635	-0.193792527	0.846338338	0.968496334
1573.694232	-0.032622643	0.1291431	-0.252608484	0.800570774	0.955697618
22.55308335	-0.222443098	0.261457328	-0.850781654	0.394890655	0.797136213
277.0271706	0.168788387	0.103562721	1.629817997	0.103139969	0.515595346
158.7696424	0.144850194	0.084149483	1.72134384	0.085188449	0.488696192
2974.427122	0.181635593	0.10244413	1.773020998	0.076225227	0.471783446
298.9443006	0.359375026	0.129075421	2.784225091	0.005365578	0.159160905
11.73425496	1.006913948	0.360835704	2.790505307	0.005262584	0.158649215
182.8810651	-0.013463189	0.107136824	-0.125663508	0.899998289	0.978996707
110.9009684	-0.019981794	0.120376495	-0.165994147	0.868161556	0.972204994
1433.834882	0.095772859	0.087090348	1.099695453	0.271464837	0.714058914
92.89984098	0.051974305	0.139421577	0.372785232	0.709308295	0.92902743
260.2657469	0.07543829	0.252113939	0.299223003	0.764769899	0.946826103
681.7177018	0.199725115	0.132278568	1.509882646	0.131073372	0.563912594
125.717044	0.585699879	0.358974071	1.631593829	0.102765079	0.515110188
306.6990227	-0.021058568	0.125227321	-0.168162732	0.866455259	0.971979594
367.4360921	-0.118504425	0.189730835	-0.624592335	0.532238652	0.866797125
10.60486033	0.192152381	0.365621605	0.525549853	0.599201007	0.897965712
894.8110165	0.14926792	0.04771062	3.128609949	0.001756353	0.100967155
40.58440086	0.324109412	0.187033089	1.732898786	0.08311366	0.485269233
4283.911707	-0.054304794	0.152297507	-0.356570474	0.721413385	0.931135539
1008.941882	0.042409703	0.097775385	0.433746214	0.664472744	0.916049829
12.88609318	0.183041285	0.495376825	0.369499087	0.711755754	0.929376205
38.32081382	0.568452286	0.296596526	1.916584437	0.055290747	0.423973339
61.94616072	0.404996652	0.418528034	0.967669115	0.333209647	0.761183657

271.6271605	0.05556218	0.10575388	0.525391409	0.599311124	0.897965712
6.18816006	0.407977243	0.355137447	1.148786888	0.250643867	0.694543766
85.88703487	-0.014643461	0.124287756	-0.117819019	0.906211059	0.979768942
411.283926	0.145364805	0.084968915	1.710799828	0.087118071	0.491740726
106.8899209	0.090030566	0.103058485	0.873587129	0.382343138	0.789880775
274.0109354	0.024019307	0.166736214	0.144055732	0.885456461	0.976804762
110.9078733	-0.278153953	0.139398972	-1.995380223	0.046001426	0.391329374
279.4048649	0.012911559	0.076005431	0.169876792	0.865107033	0.971917805
528.4552337	-0.259232864	0.203827992	-1.271821702	0.203436476	0.647584242
376.8089267	0.136937622	0.165932131	0.825262843	0.409222382	0.806739148
45.61021473	-0.498200087	0.280176909	-1.778162546	0.075377162	0.470687737
6.329819903	-0.617694603	0.596894805	-1.034846672	0.300740523	0.739093677
154.3868665	-0.048708319	0.135094361	-0.360550345	0.718435615	0.930765281
537.5351434	0.149464464	0.096204043	1.553619362	0.120275238	0.545941145
2641.985332	-0.059943484	0.142704981	-0.4200518	0.674447613	0.921149714
60902.88958	0.00123705	0.164644217	0.007513473	0.994005172	0.998877139
424.8082482	0.022152241	0.090655189	0.244357117	0.806954251	0.957805921
19.53031912	-1.206555585	0.866885694	-1.391827773	0.163974558	0.602869201
24.50565739	-0.578362653	0.339555047	-1.703295705	0.088512744	0.493295644
420.8045625	0.083503254	0.123769224	0.674668963	0.499886107	0.856061604
11.16553264	-1.095467143	0.758424368	-1.444398663	0.148626867	0.58556691
372.0525436	-0.070014964	0.067656718	-1.03485604	0.300736147	0.739093677
3701.530166	0.201236059	0.395248979	0.509137455	0.61065588	0.900471044
839.8986489	-0.03901344	0.145740531	-0.267691079	0.788937117	0.953026618
129.4045471	-0.04338032	0.147310978	-0.294481243	0.768390194	0.947586561
385.1652594	0.161199224	0.122636241	1.314450138	0.188694767	0.634590162
8.983474993	0.156707208	0.412865509	0.379559941	0.704272102	0.9269128
372.5994288	0.115246992	0.079209514	1.454964017	0.145679223	0.579974144
1788.183095	0.001439501	0.122047841	0.011794564	0.990589517	0.99821004
10137.21036	0.082145474	0.185348558	0.443194569	0.657625007	0.91380052
9394.603178	0.069659239	0.115832129	0.601380974	0.547586268	0.874460238
6.848257139	-0.555477545	0.591266786	-0.939470233	0.347489368	0.768780165
6.359926134	-1.73897388	0.913302479	-1.904050323	0.056903631	0.428680279
743.6427175	-0.05424463	0.079375663	-0.683391196	0.494359682	0.853055725
767.6699057	-0.017570105	0.058228765	-0.301742713	0.762848205	0.946826103
246.3832954	0.032472013	0.103151215	0.314800099	0.752913437	0.943990537
561.4593206	0.018907367	0.08832632	0.214062661	0.830498193	0.965541183
63.5696843	0.365486846	0.420253919	0.869680993	0.384474762	0.790387368
1069.741497	0.06591979	0.107165944	0.615118826	0.53847625	0.869675597
287.0880131	0.132412703	0.082724795	1.600641054	0.109456444	0.527744066
250.8334274	-0.012035159	0.072719954	-0.165500085	0.868550383	0.972204994
1219.626498	-0.186088379	0.203454774	-0.914642479	0.36037935	0.777564105
292.1747422	0.192454113	0.104652751	1.838978048	0.065918415	0.450826177
178.2234599	-0.05618219	0.140792628	-0.399042125	0.689862165	0.92405254
122.8097062	-0.062185171	0.102217502	-0.608361287	0.542947881	0.871203645
11.81183954	0.117240643	0.383786373	0.305484121	0.759997462	0.946795592
258.772538	0.123281677	0.071107351	1.733740254	0.082964181	0.485197868
681.1697082	-0.064445636	0.154414699	-0.41735428	0.676419291	0.921393988

2201.557622	0.132499657	0.257276736	0.515008312	0.606547212	0.900307811
14.44811152	-0.855206386	0.319829449	-2.673945094	0.007496472	0.189027811
37.14110099	-0.016639349	0.206707246	-0.080497171	0.935841846	0.984334593
233.9212446	0.051738018	0.081981303	0.631095333	0.527978193	0.865762435
219.4781486	-0.142686741	0.169882437	-0.839914614	0.400956263	0.801262941
100.4194235	-0.05368764	0.122794925	-0.437213834	0.661956278	0.915138866
357.6920849	-0.130138549	0.094821388	-1.372459856	0.169920328	0.612975504
1701.478916	0.022554224	0.12621282	0.178699946	0.858173312	0.969966486
93.26201377	-0.061285439	0.134112461	-0.456970504	0.647692246	0.910632659
39.6262949	-0.284458142	0.271855941	-1.046356169	0.295396621	0.735238067
199.2546381	-0.005981155	0.093175541	-0.064192332	0.948817083	0.985917197
6810.763972	0.053754186	0.096117047	0.559257566	0.575985951	0.886202779
602.1572338	0.00197373	0.133496348	0.014784902	0.988203785	0.997338733
5.916386746	0.162286879	0.615677118	0.263590889	0.792095179	0.953153173
386.3819491	-0.051206352	0.06784272	-0.754780349	0.450380787	0.828449907
33767.04684	0.025453767	0.100093731	0.254299318	0.799264326	0.955167977
115.1478532	-0.02912959	0.202907946	-0.143560616	0.885847442	0.976926973
11427.94588	-0.078095328	0.131392715	-0.594365733	0.552267523	0.876603606
250.55165	0.149379719	0.084733052	1.762945094	0.07790975	0.473730652
103.1512759	-0.539740332	0.168508358	-3.203047839	0.001359814	0.089856988
9.121339244	0.484334872	0.331106176	1.462778129	0.143528099	0.578069441
1192.663377	0.365673231	0.205685031	1.777831029	0.075431611	0.470687737
28.66201395	0.189997502	0.245382987	0.77428963	0.438759535	0.822562228
217.0316841	-0.07256847	0.082015967	-0.884809057	0.376259624	0.786851292
108.450947	-0.007380451	0.154934356	-0.047635985	0.962006352	0.989412036
68.86597298	0.09916824	0.215930164	0.459260708	0.64604696	0.909300678
8.247827692	0.866504114	0.827663628	1.046927864	0.29513285	0.735234129
11.55152958	0.339929478	0.343807091	0.988721544	0.322799399	0.755427147
510.3847696	0.007000047	0.09005084	0.077734394	0.938039335	0.984334593
13.89846821	-1.036878033	0.532483266	-1.947249987	0.051504771	0.410734915
154.9896305	-0.036749632	0.259464249	-0.141636593	0.887367061	0.976961229
66.19657129	0.219666794	0.342743849	0.640906597	0.52158337	0.863328113
15.84108249	0.174072816	0.327806566	0.53102297	0.595402862	0.895281711
373.0883899	0.237382078	0.0896264	2.648573156	0.008083235	0.194233844
256.4733238	0.044876554	0.137645881	0.326029035	0.744402388	0.940317814
930.4717601	-0.120196604	0.084881592	-1.416050301	0.156760778	0.595862142
13.88061265	0.663673745	0.442176227	1.500925884	0.133374733	0.566220634
3056.101717	0.124051406	0.111614322	1.111429102	0.266383692	0.708792271
181.6771505	-0.000242103	0.244796973	-0.000988996	0.999210895	0.999761804
2426.874945	0.034256458	0.178237169	0.192195929	0.847588735	0.968938143
154.8636421	0.009407574	0.099183515	0.094850182	0.924433827	0.982590996
74.25122664	0.07968055	0.313496792	0.254167033	0.799366518	0.955212982
66.32688324	0.033031843	0.271624725	0.121608381	0.903209176	0.979381499
1035.046601	0.137594405	0.086115362	1.597791631	0.110089358	0.528668387
4.619610584	-0.110765368	0.509718607	-0.217306896	0.827969183	0.964626741
463.7194874	0.088288962	0.071499374	1.234821477	0.216896952	0.661222104
6.703507084	-1.073624507	0.450484274	-2.383267453	0.017159722	0.266768098
120.7924364	0.018730531	0.229146567	0.081740393	0.934853157	0.984334593

3499.283295	0.069164135	0.057640509	1.199922356	0.230169497	0.673001336
19.92119238	0.297897005	0.26119322	1.140523499	0.254068268	0.696717102
5.429189367	1.04726056	0.46926947	2.231682705	0.025635941	0.314125165
11.3498513	1.250992716	0.572976294	2.183323691	0.029011981	0.328042225
34.0584114	0.236727976	0.277616661	0.852715303	0.393817202	0.796193624
37.52256659	-0.096062579	0.315160318	-0.304805439	0.760514339	0.946826103
146.2262974	0.201656606	0.192682446	1.046574872	0.295295696	0.735234129
12.33679296	0.217016482	0.486257188	0.446299791	0.655380707	0.912518505
2652.264202	0.16741623	0.097325171	1.720174013	0.085400815	0.488858859
518.9735574	-0.179598101	0.161926072	-1.109136404	0.267371342	0.709634322
2035.263071	0.189666636	0.077719028	2.440414402	0.014670422	0.256113483
46.24451609	0.444647828	0.139075177	3.197176072	0.001387802	0.090494464
50.31214016	-0.248476876	0.180001595	-1.380414852	0.16745895	0.609023925
1957.419694	-0.033528066	0.056416092	-0.594299688	0.552311688	0.876603606
17.94652953	-0.129608112	0.369330625	-0.350927064	0.725643067	0.932699607
486.1657099	-0.134249991	0.254784693	-0.526915449	0.598252305	0.897417322
198.8491916	0.168673728	0.113321806	1.488448991	0.136632525	0.569036171
138.9640213	0.075075424	0.131974682	0.568862325	0.569449575	0.8841274
244.4432934	0.016343994	0.099039804	0.165024504	0.868924695	0.972204994
22.17340254	-0.117281889	0.447485019	-0.262091207	0.793251126	0.953153173
568.9314201	0.015654754	0.084660157	0.184912889	0.853297362	0.969966486
73.32980592	-0.297599389	0.292338767	-1.017994954	0.308680354	0.744049104
735.6516448	0.099330541	0.073772364	1.346446503	0.178158562	0.622545571
24.86311617	0.045518418	0.24659725	0.184586073	0.85355371	0.969966486
9.397578391	-1.880030733	0.82238802	-2.28606289	0.02225058	0.298957038
108.9631378	0.213893235	0.249949228	0.855746731	0.392137886	0.795344613
123.0828856	0.244302102	0.343606695	0.710993428	0.477088308	0.843597766
345.6717328	0.00301291	0.058985661	0.051078678	0.959262826	0.988444717
378.2668322	0.230503103	0.231571909	0.995384563	0.319549264	0.752460739
981.9389824	-0.240784409	0.074991116	-3.21083913	0.00132348	0.088643218
376.5898504	0.070553671	0.078885066	0.894385651	0.371115587	0.785086883
10.50140679	-0.258305644	0.459676372	-0.561929348	0.574164157	0.885935354
7.655845178	-1.280620954	0.693540845	-1.846496805	0.064820103	0.446824324
7.462992681	-0.240381831	0.408723408	-0.588128369	0.556446133	0.878642775
26.34106305	-0.122601153	0.238571498	-0.513896898	0.607324076	0.900307811
40.9901449	-0.234147363	0.200876957	-1.165625796	0.243765773	0.686012467
28.01714073	0.17926225	0.411768588	0.435347075	0.66331052	0.915802846
81.15614395	-0.116111432	0.157282338	-0.73823567	0.46037125	0.834673974
625.7333231	-0.081684387	0.09987714	-0.817848681	0.413443597	0.808272153
34.03106473	0.122184391	0.185175291	0.659830963	0.509362311	0.859636085
270.8210998	-0.114234807	0.17622222	-0.648242923	0.516827844	0.862002027
263.894701	-0.23627511	0.149992851	-1.575242479	0.115200485	0.536394333
736.7517863	0.006300158	0.082231054	0.076615308	0.93892958	0.984334593
1772.385544	-0.098245611	0.201537264	-0.487481119	0.62591742	0.903948193
13.96957708	0.208847618	0.295778035	0.706095768	0.480128584	0.845350695
1789.003451	-0.035010219	0.119260281	-0.293561434	0.769093049	0.947650255
169.1199024	-0.597334168	0.29553491	-2.021196642	0.043259413	0.380979267
305.0815868	0.150441521	0.084266699	1.785302174	0.074212316	0.468839397

131.9345551	-0.115699199	0.100005888	-1.15692387	0.247303466	0.690937316
843.4588545	0.024557338	0.11865247	0.206968616	0.836034368	0.966316856
21.57966092	-0.040432979	0.268778757	-0.150432198	0.88042364	0.975430592
289.3022033	0.101344166	0.060132259	1.68535438	0.09192022	0.499719658
65.56270789	-0.132376348	0.127802046	-1.035792092	0.300299147	0.739093677
508.3559198	0.267879931	0.265939831	1.007295258	0.313792908	0.747661118
84.30211272	0.065579855	0.121764963	0.538577384	0.590178496	0.892788725
322.5666014	-0.131868109	0.091353557	-1.443491775	0.148881988	0.586104038
20.68591552	0.460588432	0.423155673	1.088460962	0.276391661	0.718547195
899.072127	0.194423516	0.11033165	1.762173547	0.07803998	0.474004015
2184.738659	-0.031336726	0.11152941	-0.280972758	0.778731294	0.951560132
461.1415995	-0.015270648	0.109037842	-0.140049066	0.888621223	0.976961229
4786.576843	-0.093728966	0.120989488	-0.774686857	0.438524719	0.822520267
233.1563192	0.06806422	0.1034562	0.657903737	0.510599989	0.860200661
915.7408668	-0.061718102	0.083229182	-0.741544023	0.45836365	0.833531988
78.42132246	-0.184476326	0.119812433	-1.539709371	0.123631212	0.551533814
6.656037898	-0.080775731	0.595294159	-0.135690448	0.892065999	0.977738002
50.24766662	-0.982960392	0.386215024	-2.545111739	0.010924277	0.226714336
272.9648739	0.264110563	0.142580527	1.852360691	0.063974046	0.444821962
142.9955261	0.121423315	0.092987438	1.30580342	0.191619417	0.637569428
12.46494426	-0.353383435	0.53188101	-0.664403182	0.506432297	0.859011504
277.9069377	0.119875452	0.080180616	1.495067735	0.134896766	0.567901571
6321.14425	0.062948071	0.087920159	0.715968572	0.474010755	0.841713532
1030.118802	0.024687716	0.062615452	0.394275137	0.693377917	0.924581226
220.4514152	0.017747458	0.103850304	0.170894615	0.864306631	0.971852533
656.3264451	-0.014476094	0.076012411	-0.190443823	0.84896136	0.969175232
21.88828858	-0.705397429	0.264625201	-2.665647217	0.007684028	0.189660453
356.2033849	0.035252883	0.120816919	0.29178763	0.770449004	0.948321877
244.8616736	0.049131952	0.079502345	0.617993741	0.536579464	0.869429425
111.8247375	-0.13667054	0.119859848	-1.140252905	0.254180952	0.696717102
133.3475457	-0.146825842	0.120878446	-1.214656929	0.224496966	0.667854242
173.8470219	-0.094422644	0.14011286	-0.673904191	0.500372227	0.856182624
559.0324752	0.001714724	0.09831423	0.017441254	0.986084598	0.996587752
109.8266707	0.237582868	0.291285871	0.815634714	0.414709089	0.809297816
266.2226735	-0.034594689	0.13663409	-0.253192221	0.800119677	0.955456191
115.6742494	-0.161822157	0.128165563	-1.262602476	0.206732078	0.651884304
41.24531786	0.075014253	0.171673148	0.436959733	0.662140551	0.915224518
837.3922797	-0.298729272	0.157443043	-1.897379939	0.057777811	0.431279458
6.135324358	-0.347638824	0.541159624	-0.642396086	0.520616041	0.863188882
31.89249645	-0.199275606	0.312018665	-0.638665657	0.523040459	0.863484457
242.639744	0.059370383	0.068128802	0.87144322	0.383512196	0.790043276
288.27268	0.004892001	0.064228736	0.076165306	0.939287584	0.984334593
63.91873797	0.011693629	0.382815445	0.030546388	0.975631299	0.993405995
335.732857	0.209549737	0.101869483	2.057041335	0.03968225	0.371757382
397.6329259	-0.09748672	0.068732175	-1.418356397	0.156086739	0.595002809
145.8537611	0.023616673	0.219158793	0.107760556	0.914185633	0.980657455
229.249333	0.082494663	0.176462644	0.467490802	0.640148771	0.907798398
280.9807764	0.144204276	0.094551751	1.525135969	0.127225187	0.556444786

38.73674477	0.450436282	0.420986073	1.069955306	0.284639427	0.725564801
18.26231677	0.341447296	0.443228224	0.770364516	0.441083696	0.82417628
36.99026938	-0.039333492	0.257013243	-0.153040722	0.878366162	0.974491263
117.6475936	0.125441304	0.199295609	0.629423322	0.529071953	0.866063379
493.0519817	-0.032473372	0.078742486	-0.412399635	0.68004653	0.922112199
172.2949458	-0.141739021	0.090971636	-1.55805729	0.119219668	0.54348307
1571.419253	0.089281366	0.07874814	1.133758412	0.2568959	0.700073143
15.58566174	-0.077030088	0.357359524	-0.215553477	0.829335823	0.96513412
562.1420603	0.119742483	0.338504247	0.353739971	0.723533764	0.931597925
49.35762032	-0.511899326	0.261271028	-1.959265556	0.050081695	0.4059744
104.3914325	-0.68470744	0.406384196	-1.684877137	0.092012279	0.499719658
156.9376575	0.063684881	0.174034067	0.365933415	0.714414762	0.930012765
84.63459197	-0.076009795	0.135374191	-0.561479217	0.574470895	0.886131532
94.09269241	-0.121297276	0.137081209	-0.884857062	0.376233729	0.786851292
564.6295177	0.022437276	0.128357669	0.174802765	0.861234616	0.970240869
125.557622	-0.015739732	0.126300017	-0.124621772	0.900822991	0.979217606
87.73526237	0.234898095	0.184118978	1.275795126	0.202027951	0.645481631
371.1914579	0.337186355	0.204838811	1.646105801	0.099741978	0.511758204
782.7509935	-0.194864085	0.21623777	-0.90115656	0.367505084	0.781703698
19.67912837	-0.16078696	0.248959323	-0.64583627	0.518385396	0.862002027
467.8846477	-0.128657319	0.089173515	-1.442775006	0.149083862	0.586588013
57.81771674	-0.138388435	0.447930036	-0.308951006	0.757358796	0.945625212
674.2173916	-0.017412691	0.094747012	-0.183780902	0.854185339	0.969966486
286.1411094	0.14272013	0.066662431	2.140937963	0.032279041	0.340364617
220.3048089	0.051992566	0.080590953	0.645141475	0.518835509	0.862414733
5.71301713	0.857608275	0.996297265	0.860795573	0.389350644	0.793111346
570.4189277	-0.000558999	0.113057326	-0.004944389	0.996054964	0.998981303
279.1308893	0.140093659	0.133276467	1.051150762	0.293189353	0.733939814
34.4476181	0.599276033	0.274414101	2.183838337	0.028974129	0.328011759
49.80841989	-0.362283973	0.274101391	-1.321715191	0.186263008	0.631908584
5.350829692	-0.292878486	0.411467196	-0.711790608	0.476594449	0.843597766
540.4064819	0.050236599	0.085887696	0.584910312	0.558608024	0.880046199
5.550566198	0.126609351	0.373871063	0.338644426	0.734877611	0.937620779
595.1084873	0.187614127	0.063121354	2.972276649	0.002956002	0.128901255
142.9164741	-0.092545541	0.111744031	-0.828192253	0.407561631	0.805288281
1134.694099	0.167304049	0.106790374	1.566658523	0.117194531	0.539200101
794.4150017	0.23530806	0.092858459	2.534050877	0.011275236	0.23081568
32.43574686	-1.62783717	0.655150388	-2.484677106	0.012966899	0.243883149
556.3445001	0.232608373	0.124823564	1.863497286	0.062392334	0.44188166
40.16193641	-0.298097263	0.358855909	-0.830687903	0.406149962	0.804437981
11.142646	-0.196902942	0.333324685	-0.590724152	0.554705262	0.877791979
845.75191	0.259403783	0.111748281	2.321322348	0.020269453	0.283580759
386.051691	0.028500665	0.123612712	0.230564188	0.817653392	0.961335246
101.2278796	0.298241988	0.099227864	3.00562742	0.002650335	0.121833113
7.552539293	-0.461552765	0.455564805	-1.013144035	0.310991367	0.745771331
6.684177137	-1.471953437	0.99161003	-1.484407571	0.137700827	0.569036171
2808.163959	-0.15459141	0.085202792	-1.814393725	0.069617131	0.460514862
297.3855662	0.0098229	0.095342735	0.103027253	0.917941341	0.981376661

43.44846509	-0.105391703	0.299732479	-0.351619229	0.725123842	0.932356073
28.49628607	-0.157985217	0.470504821	-0.335778104	0.737038203	0.938705858
373.3011413	0.154100889	0.174238968	0.884422647	0.376468102	0.786851292
4.576138502	0.120730634	0.485737319	0.248551283	0.803707896	0.957233889
52.24155726	-1.002570793	0.886411465	-1.131044478	0.258036371	0.701275747
60.64823586	-0.066446736	0.223696384	-0.297039831	0.7664361	0.947349028
35.691523	-0.945509495	0.382898269	-2.469349099	0.013535909	0.249823606
860.7312322	-0.256688518	0.158151365	-1.623055974	0.10457744	0.518619353
53.17544464	0.022474889	0.179257529	0.125377657	0.900224576	0.978996707
2039.980236	0.10472056	0.054945417	1.905901621	0.056662971	0.428139506
98.04328134	0.209135611	0.220091839	0.950219744	0.342000609	0.765506277
926.1266628	0.1906765	0.086331466	2.208655877	0.027198586	0.322849611
657.0828777	-0.11324979	0.100549204	-1.126312147	0.260033418	0.702759659
104.2491391	0.181898708	0.167383907	1.08671563	0.277162504	0.719218718
7.620090326	1.251487587	0.576273233	2.171691335	0.029878953	0.331039114
23.65734671	0.111362915	0.40336487	0.276084814	0.782482924	0.95173434
720.7104209	-0.355125417	0.096116291	-3.694747412	0.000220105	0.03194119
407.0114037	0.008722357	0.06773638	0.128769164	0.897540306	0.97893802
291.0683534	0.087504347	0.074101193	1.180876356	0.237651846	0.679753163
25.41955458	0.214527539	0.209412876	1.024423821	0.305635135	0.742035396
9.335437974	0.107887415	0.437575915	0.246557023	0.805251068	0.957233889
29.47476427	-0.796042589	0.312892528	-2.544140612	0.010954698	0.226714336
12.46808008	-0.211808042	0.325329412	-0.651057156	0.515009592	0.861826223
896.3731292	0.155445227	0.066798649	2.32707142	0.019961464	0.28166787
780.930115	0.170608305	0.096198358	1.773505372	0.076145003	0.471783446
642.9866359	-0.068164354	0.087896727	-0.775505024	0.438041298	0.82251807
387.0101313	-0.354653892	0.162664955	-2.180272277	0.029237285	0.329102882
17.641071	-0.230449389	0.249195519	-0.924773406	0.355083787	0.773833221
5.926710858	-0.478207636	0.497269088	-0.961667734	0.336216535	0.763059975
146.7297033	0.029904795	0.1518305	0.196961711	0.843857501	0.968131661
118.457072	-0.246755208	0.13446621	-1.835072231	0.066494988	0.452949291
1627.511844	0.017908936	0.064855669	0.276135243	0.782444192	0.95173434
1403.540611	0.138448741	0.068387247	2.024481859	0.042920597	0.37951653
630.3752981	-0.311754199	0.201994044	-1.543383125	0.122737846	0.548899724
1250.196105	-0.289119756	0.324407044	-0.891225272	0.372808329	0.786266676
619.8125008	-1.04816513	0.659625663	-1.589030246	0.112053564	0.532835074
229.7330602	-0.120776279	0.165872807	-0.728125853	0.466536549	0.837672118
11.69756853	-0.283347813	0.574712118	-0.493025646	0.621994467	0.902358335
2332.989467	0.266684158	0.479348291	0.556347364	0.577973414	0.887079361
195.8445262	-0.087849035	0.436414873	-0.20129707	0.840466294	0.966484002
72.46748969	0.958301824	0.370686436	2.585208765	0.009732005	0.213793574
30.49415357	0.092620117	0.187637153	0.493612886	0.621579599	0.902299404
205.8641049	-0.028730579	0.095607465	-0.3005056	0.763791525	0.946826103
597.7083813	0.028439087	0.090618601	0.313832775	0.75364805	0.944021193
3154.840607	0.03643552	0.076600958	0.47565358	0.634321197	0.906920638
349.8129846	0.071352522	0.095545703	0.746789436	0.455190674	0.831550545
119.2178193	0.015046149	0.093357772	0.161166541	0.871962233	0.972881041
449.3512567	-0.044516705	0.068478296	-0.650084886	0.515637392	0.861826223

2939.044796	0.072140692	0.119208705	0.605162952	0.54507073	0.872850885
33.08008173	0.136323833	0.235430309	0.579041134	0.562561418	0.881820831
6.07482436	-2.088021151	0.643011642	-3.247252482	0.00116525	0.082322747
15.92575614	-0.566043468	0.715175738	-0.791474652	0.428667062	0.818292445
212.7001552	0.208222841	0.12511098	1.664305088	0.096051476	0.505422662
6.483140538	-1.009018291	0.376878912	-2.677301012	0.007421792	0.188434598
7.497440318	-0.66912699	0.516976491	-1.294308351	0.195558933	0.640835592
245.2961619	-0.076145363	0.101816104	-0.747871505	0.454537667	0.831343944
926.3944375	-0.035305086	0.089899713	-0.392716337	0.694529003	0.924581226
124.4020511	-0.274652372	0.238879772	-1.149751483	0.250246243	0.694208707
1158.52217	-0.160844079	0.119455885	-1.346472627	0.178150142	0.622545571
738.4352276	-0.03360064	0.052417685	-0.641017244	0.52151148	0.863328113
672.9141102	0.109073215	0.235757429	0.462650172	0.64361514	0.908494289
302.503985	-0.362782427	0.177507573	-2.043757466	0.040977513	0.374892505
1903.301267	-0.105040594	0.115738355	-0.907569439	0.364105747	0.779728871
634.0608198	-0.750809659	0.965790353	-0.777404389	0.436920224	0.822078703
12.49521261	-0.387947824	0.363422472	-1.067484411	0.28575315	0.726643643
10.26137086	-1.380338171	0.546413538	-2.526178572	0.011531085	0.23260487
1127.953601	0.42651188	0.545783118	0.781467704	0.434527464	0.821579957
129.2338573	-0.098286292	0.269079463	-0.365268651	0.714910877	0.930012765
19876.96113	-0.039401583	0.14838325	-0.265539294	0.790594044	0.953153173
79.94681469	-0.505129169	0.267129785	-1.890950384	0.058630968	0.433061669
460.688853	0.12383213	0.108101543	1.145516764	0.251995155	0.694735013
4.747815647	0.113192964	0.476239806	0.237680603	0.812128829	0.959924214
103.3812495	0.111130041	0.121286092	0.91626368	0.359528613	0.777290907
10.87814133	-1.591244509	0.961284935	-1.655330745	0.097857426	0.509133783
282.3557593	0.312818745	0.10235433	3.056233617	0.002241365	0.11246336
6.187405906	-0.726995551	0.64995084	-1.11853929	0.263336736	0.705730729
130.9258776	0.087991884	0.417817068	0.210599066	0.833200141	0.966227926
2105.673378	0.173688658	0.093351125	1.860595239	0.062801358	0.442595858
67.46107329	0.286463444	0.120220979	2.382807442	0.017181179	0.266768098
802.5290051	0.219280156	0.118217054	1.854894439	0.063611302	0.443511303
9.287834383	0.35140748	0.353760868	0.993347518	0.320540628	0.752639971
461.8309215	0.128553264	0.089221124	1.440838876	0.149630208	0.587712444
622.4902405	0.574881915	0.393012036	1.462759055	0.14353332	0.578069441
26.46835393	0.081191362	0.266289902	0.304898388	0.760443543	0.946826103
514.418788	0.153469308	0.071038274	2.160374937	0.030743656	0.33384793
246.4714844	-0.259949824	0.268258378	-0.969027792	0.332531323	0.760742564
280.5258183	0.186757765	0.102887702	1.815161199	0.069499141	0.460346985
251.9435515	0.062308377	0.086922249	0.71682886	0.473479702	0.841511024
167.7578644	0.105309486	0.15209424	0.692396281	0.488688501	0.849736271
94.49480636	-0.413346638	0.296423651	-1.394445538	0.163183105	0.601694699
18.60111327	0.008715653	0.497105434	0.017532807	0.986011561	0.996581982
41.16342091	-0.050329364	0.264060312	-0.190597989	0.848840566	0.969123636
9.494408361	-0.382551487	0.55033027	-0.695130737	0.486973374	0.849484378
337.2007886	0.009980254	0.097010697	0.102877874	0.918059899	0.981376661
2179.984784	0.043941513	0.04846311	0.906700232	0.364565341	0.779728871
218.0141037	0.300686847	0.651307557	0.461666449	0.644320534	0.908524911

168.3255244	-0.319439759	0.152808444	-2.090458807	0.036576605	0.360198137
2182.914308	-0.118589776	0.084119849	-1.409771614	0.158607131	0.598751021
118.7787384	0.129152534	0.101795997	1.268738831	0.204534228	0.648992858
208.1392362	0.121302383	0.100833292	1.202999338	0.228976577	0.671940348
198.4400104	0.03148842	0.116592517	0.270072391	0.787104562	0.95288876
213.7318949	-0.071494226	0.119440541	-0.598575874	0.549455749	0.875273783
825.7621218	0.203650746	0.059551224	3.419757504	0.00062677	0.057623892
1526.139025	0.028277465	0.135402008	0.208840811	0.834572511	0.966316856
81.81780337	-0.000447116	0.158348377	-0.00282362	0.99774708	0.999382914
82.81746437	-0.101911178	0.136260386	-0.747914936	0.454511468	0.831343944
396.6262232	0.394434539	0.31621418	1.247365123	0.212263649	0.657306805
278.5105299	-0.056391539	0.262292735	-0.214994665	0.829771478	0.965360168
49.42694811	-0.009494269	0.403684974	-0.023519004	0.981236279	0.994880431
1030.38775	0.072474184	0.073521398	0.98575634	0.324252695	0.756297513
10.44578688	-0.674935994	0.339947096	-1.985414793	0.047098342	0.395875469
44.79383776	-0.357878315	0.300740224	-1.189991514	0.234049727	0.676905835
2739.875142	-0.133269464	0.11405897	-1.168425973	0.242634983	0.685720081
670.9761653	0.098637953	0.091575026	1.077127224	0.281423432	0.722987449
69.39384489	0.816973161	0.364021175	2.244301206	0.024813028	0.312049653
10.28836288	-0.535492418	0.396079263	-1.351982971	0.176380763	0.62005562
93.88708801	0.481241949	0.505952429	0.951160469	0.341522922	0.765195212
4.481672222	0.631799277	0.528701374	1.195002147	0.232086191	0.674917024
5305.267322	0.858070194	0.291526461	2.943369843	0.003246603	0.133489497
8.473039862	-0.304904049	0.561425641	-0.543088927	0.587068576	0.890984353
140.8443185	-0.184066943	0.130736515	-1.407922978	0.159153878	0.599744325
210.1182183	0.027653081	0.137321379	0.201374916	0.840405428	0.966484002
149.4212181	-0.088260408	0.139425561	-0.633028893	0.526714775	0.865020759
2030.085037	0.023686631	0.104258419	0.227191545	0.820274802	0.962543699
23.2901466	-0.994155051	0.601290092	-1.653370085	0.098255567	0.509712212
265.5345696	-0.04516833	0.106747013	-0.423134371	0.672197227	0.920198876
235.0331441	-0.058677026	0.142118089	-0.41287514	0.679698095	0.922072151
1939.21954	0.129826376	0.069325864	1.87269755	0.061110162	0.437941099
2042.619526	0.10256007	0.086254552	1.189039511	0.23442412	0.677194734
909.5653175	0.028238011	0.064007846	0.441164836	0.659093668	0.914197671
6.810898589	1.356098427	0.565569234	2.39775848	0.016495738	0.263145637
90.08287888	0.295125691	0.352880723	0.836332708	0.402967758	0.802357916
874.3922886	0.170768161	0.071650224	2.383358368	0.017155484	0.266768098
119.6456373	-0.078941317	0.116689878	-0.676505263	0.498719905	0.855634722
705.9927814	-0.05757204	0.082177278	-0.700583431	0.483563022	0.847822935
47.42327827	-0.564553864	0.259632478	-2.174434677	0.029672506	0.330732257
116.8163646	-0.207607635	0.219996919	-0.943684283	0.345331008	0.767851822
365.4972184	0.235768951	0.10692988	2.204893065	0.027461599	0.323635822
37.34794111	0.468982509	0.210216695	2.230947969	0.025684576	0.314205151
23.71279022	-0.497138257	0.423269077	-1.174520614	0.24018656	0.682387996
73.96292143	-0.182157907	0.329072513	-0.553549445	0.579887233	0.887483205
14.09928158	-0.059643128	0.423156049	-0.140948305	0.887910782	0.976961229
757.969511	-0.04992586	0.105573268	-0.47290248	0.636282756	0.907353194
68.7406738	-0.157096257	0.223053789	-0.704297641	0.481247435	0.845711093

4.552821203	-2.87234698	1.045233205	-2.748044135	0.005995194	0.168912649
297.9030573	-0.185781327	0.122684867	-1.514297015	0.129950525	0.562106276
422.7267554	0.130091623	0.092108881	1.412367868	0.157841672	0.597576311
270.7288277	-0.057821224	0.113696618	-0.508557113	0.611062697	0.900509492
9.749830617	0.154232768	0.358962522	0.429662594	0.667441097	0.916775933
1247.294352	0.138047619	0.092188185	1.497454569	0.134275018	0.567632616
8.636560571	-0.614676137	0.74193841	-0.828473266	0.407402529	0.805288281
127.324871	0.191699063	0.104657473	1.831680598	0.066999021	0.454159121
114.9488612	-0.169240266	0.160361571	-1.055366727	0.291257634	0.732859559
240.373916	-0.166113456	0.09390771	-1.768901157	0.076910371	0.472050486
10.31721321	-0.49782268	0.465961816	-1.068376556	0.285350688	0.726606035
207.3074431	0.081502606	0.076865875	1.060322364	0.288997968	0.729902555
1083.219113	0.001224772	0.076875945	0.015931801	0.9872888	0.996928555
291.8400423	0.030920609	0.073301669	0.421826806	0.673151438	0.920554864
150.7308932	0.094812599	0.081768709	1.15952177	0.246243568	0.689705283
146.6649679	0.185143295	0.183641433	1.008178234	0.313368905	0.747296731
8.233078852	-0.223567703	0.354510736	-0.630637327	0.528277686	0.865762435
91.97577523	0.494787851	0.300730961	1.645284041	0.099911249	0.511833836
144.402566	-0.002749565	0.123240711	-0.022310526	0.982200253	0.995244259
195.5843604	-0.051478837	0.092285344	-0.557822455	0.576965628	0.88676146
538.0872324	0.062706097	0.326815322	0.191870128	0.847843937	0.968938143
1731.808008	0.113021269	0.049238349	2.295391124	0.02171072	0.295327604
355.8599687	0.179809755	0.198320617	0.906661937	0.364585598	0.779728871
382.9222227	0.380329863	0.186381594	2.040597757	0.041290825	0.374892505
1206.965006	0.060537776	0.138687439	0.436505111	0.662470292	0.915556436
52.92339095	-0.074751884	0.197103782	-0.37925139	0.704501193	0.9269128
227.4889674	0.091124884	0.173655845	0.524744124	0.59976108	0.898108141
540.4143208	0.12780165	0.098912823	1.292063512	0.196335151	0.641418933
131.7008289	0.062432948	0.123288063	0.506398969	0.612576598	0.901417518
7.793017935	0.067551297	0.450082968	0.150086322	0.880696511	0.975430592
28.8080796	0.192492309	0.189934745	1.013465486	0.310837874	0.745771331
9.16246621	-1.256759326	0.796843705	-1.577171681	0.114756027	0.535291597
32.35642279	-0.116627258	0.346385105	-0.33669825	0.736344383	0.938503341
39.2617672	-1.752197707	0.741046703	-2.364490254	0.018054902	0.27123243
100.4344023	0.098091181	0.211923358	0.462861583	0.643463586	0.908494289
95.8842028	-0.056423899	0.118986975	-0.474202315	0.635355643	0.907169387
289.5187222	-0.032916299	0.092538229	-0.35570487	0.722061598	0.931135539
30.38208348	-0.175472655	0.195473717	-0.897679019	0.369356698	0.783381264
2109.4115	-0.12216535	0.282352598	-0.432669473	0.66525491	0.916049829
768.8612806	-0.060301326	0.25742947	-0.234244067	0.814795506	0.960909552
36.8635405	-0.261601222	0.2335	-1.120347846	0.26256556	0.705380597
616.1584719	-0.099735183	0.098789621	-1.009571479	0.31270064	0.746754295
225.3528021	-0.022937022	0.076929748	-0.298155432	0.765584536	0.94709722
25.41934489	-0.019671553	0.250438675	-0.078548385	0.937391844	0.984334593
2792.456356	0.168319335	0.114380606	1.471572329	0.141136402	0.575193014
1934.25232	-0.142325612	0.137394512	-1.035890082	0.300253425	0.739093677
4.768770436	-0.651314454	0.54648288	-1.191829565	0.233328081	0.676392617
66.63042557	-0.355164084	0.192194484	-1.847941087	0.064610866	0.446418062

1482.497751	0.088946015	0.100411711	0.885813155	0.37571822	0.786839429
608.4120758	-0.019176805	0.085443852	-0.224437507	0.822416889	0.963169453
14.63192415	-0.176677047	0.33860168	-0.521784319	0.601820509	0.898995577
371.7892102	0.096624661	0.072131405	1.339564386	0.180387009	0.625606491
1060.937389	0.049477229	0.106444327	0.464817902	0.642061866	0.908064184
126.6290038	-0.338224475	0.167737013	-2.016397385	0.043758439	0.383085971
97.66780887	0.160202206	0.117264718	1.36615862	0.171889182	0.616109027
421.0962065	0.105587974	0.076063659	1.388152703	0.165090551	0.605350304
43.70221578	-0.145389096	0.233272027	-0.623259881	0.533113757	0.867356078
111.6761434	-0.175153633	0.112222706	-1.560768217	0.118578449	0.542229905
237.368138	0.049266455	0.073710281	0.668379694	0.503891254	0.857575309
188.9636269	-0.207977088	0.230899244	-0.900726585	0.36773371	0.781731205
1005.416271	0.008267719	0.121087245	0.068279021	0.945563524	0.985511286
32.28624199	0.33196251	0.466133535	0.712161827	0.476364572	0.843597766
777.8764577	0.053090778	0.151951211	0.349393585	0.726793848	0.933287286
5.77075068	0.031757993	0.413933457	0.076722461	0.938844336	0.984334593
580.5416315	0.022922047	0.076598635	0.299248762	0.764750246	0.946826103
5.117471601	-1.271048893	1.029017	-1.235206894	0.216753514	0.661222104
1152.364264	0.056138828	0.153651139	0.365365519	0.714838577	0.930012765
93.0461302	0.056147655	0.222612299	0.252221712	0.800869697	0.955697618
187.5000789	0.313981588	0.228771312	1.372469235	0.16991741	0.612975504
860.37498	0.134153867	0.175103898	0.766138667	0.443593798	0.825536387
594.0208972	-0.009323659	0.062135342	-0.150054038	0.880721981	0.975430592
227.5409969	0.083515989	0.080084507	1.042848265	0.297018573	0.736647043
17.82108059	-0.854381389	0.366358707	-2.332089757	0.01969597	0.281261453
26.23904299	-1.01602026	0.342060147	-2.97029709	0.002975119	0.128901255
93.63735687	-0.347555191	0.308995229	-1.124791447	0.26067742	0.70310069
573.8788624	0.043894974	0.069186385	0.634445261	0.52579028	0.864850467
67.7188092	0.157409979	0.1362962	1.154910991	0.248126878	0.69187626
43.35857521	0.318096341	0.354242956	0.89796095	0.369206369	0.783246787
59.40555561	1.20011352	0.551777423	2.174995694	0.029630439	0.330732257
7.484741011	-0.218967156	0.381133552	-0.574515561	0.565618974	0.883265494
2601.598835	0.003842967	0.14327804	0.026821742	0.978601912	0.99459552
222.4713213	-1.325390509	0.460787387	-2.876360216	0.004022903	0.144354659
10.20942262	-1.496107349	0.705122547	-2.121769267	0.033857119	0.348624664
281.9990831	-0.080815306	0.138950361	-0.581612776	0.560827535	0.880936184
20.37050999	-1.261868184	0.886885055	-1.422809164	0.154791484	0.592842063
33.00262974	0.711982078	0.487927864	1.459195365	0.144511344	0.578069441
5275.103037	-0.006231169	0.124572278	-0.050020512	0.960106043	0.989015817
7.453727505	0.123326379	0.643891935	0.191532729	0.848108241	0.968987638
108.9876686	0.23225663	0.096628953	2.403592524	0.016234858	0.263145637
122.8403361	-0.156704312	0.106142654	-1.476355698	0.139848429	0.57262419
191.6591168	0.137069141	0.107309174	1.277329196	0.20148605	0.645261037
468.1379214	-0.014592416	0.080794964	-0.180610469	0.856673338	0.969966486
391.345323	0.134537903	0.085905705	1.566111384	0.117322543	0.539200101
190.4498004	-0.084738075	0.078570033	-1.07850374	0.280809013	0.722498699
166.5039279	0.310513108	0.146109661	2.125205856	0.03356944	0.347119785
50.15488937	0.360352579	0.16075397	2.24164031	0.024984629	0.312413884

7.020038134	-0.925882897	0.683704223	-1.35421556	0.175667616	0.619005807
69.98390442	0.064204469	0.414494495	0.154898243	0.876901539	0.973973449
180.8027127	-0.043920386	0.092926011	-0.472638231	0.636471302	0.907353194
5.222028118	0.024522884	0.680371472	0.036043375	0.971247773	0.992799208
100.7430661	-0.244621304	0.191188204	-1.279479063	0.200728408	0.645045462
12.36697169	-0.314939985	0.295491953	-1.065815777	0.286506928	0.727013824
12.24712734	0.132201989	0.704439319	0.187669803	0.851135503	0.969309003
22.03236629	0.640926115	0.529113048	1.211321696	0.225772137	0.668749399
42.9835002	-0.106545435	0.605008087	-0.176105804	0.860210821	0.969966486
11.41192258	-0.470328837	0.451071217	-1.042693082	0.297090463	0.736647043
382.8649623	0.064319694	0.072519289	0.886932217	0.375115395	0.786801485
1672.515338	0.053200913	0.050551739	1.052405185	0.292613691	0.733939814
1046.445871	-0.093792789	0.066190437	-1.417014195	0.156478777	0.595721049
3391.171531	0.027281516	0.090148655	0.302628103	0.762173295	0.946826103
513.648047	-0.181155551	0.146268157	-1.238516673	0.215524544	0.660219973
16429.0148	-0.11067886	0.158182749	-0.699689825	0.484121032	0.847993853
28.78546506	-0.584774844	0.256975527	-2.275605194	0.02286965	0.302517035
16728.07779	-0.201982118	0.157826513	-1.279773052	0.200624964	0.645045462
108.1047473	-0.245135643	0.182231146	-1.345190701	0.178563658	0.623105158
4.848025725	-1.077516757	0.726527404	-1.483105456	0.138046395	0.569816714
195.4381625	0.088413639	0.084137683	1.050820936	0.293340838	0.733949599
16.26907813	-0.707748429	0.417001702	-1.697231512	0.0896529	0.494630299
5.181056816	-0.915473416	0.720183312	-1.271167217	0.203669167	0.647823067
221.7041431	-0.139039006	0.192858549	-0.72093774	0.470947822	0.840276866
219.1673374	0.091647282	0.234114628	0.391463289	0.695454819	0.924581226
8.832245836	1.119286332	0.613506181	1.824409219	0.068090229	0.456050486
37.47843167	0.112138362	0.209433343	0.535437007	0.592347706	0.893683696
237.6254454	-0.078683223	0.195554001	-0.402360588	0.687418671	0.923064029
12.14729656	0.304654948	0.51763855	0.588547641	0.556164766	0.878449141
5.44118761	0.589441834	0.721625784	0.816824796	0.41402856	0.808821113
5.176306713	1.029965179	0.487409166	2.113142822	0.034588545	0.352363583
68.07881005	0.789527254	0.27058048	2.917901741	0.003523954	0.135713888
13.59010419	1.035796473	0.475538627	2.178154233	0.029394556	0.330351122
35.99199471	0.888678214	0.378946648	2.345127523	0.019020573	0.276929913
108.2809435	0.336664066	0.178021391	1.89114389	0.058605139	0.433061669
145.4828864	1.077873718	0.409484552	2.632269552	0.008481654	0.199913127
52.38265297	1.254235138	0.475623219	2.637035133	0.008363417	0.198674451
143.9228646	-1.015664643	0.891487319	-1.139292305	0.254581257	0.696832138
10.1383748	-0.381252105	0.305074521	-1.249701558	0.211408588	0.656444287
289.3732118	0.275007904	0.237125245	1.159758015	0.246147342	0.689659845
16.60002963	0.141961461	0.412587465	0.34407604	0.730789101	0.935657839
2646.429065	-0.132105351	0.138324449	-0.95503978	0.33955758	0.764202418
8.327774427	0.443186323	0.533854852	0.830162584	0.406446867	0.804663634
30.88039145	-0.214617421	0.294608366	-0.728483796	0.466317484	0.837628265
68.39114562	0.049486035	0.16787811	0.2947736	0.768166835	0.947580311
4.751137331	-2.723559058	0.961556575	-2.832448061	0.004619307	0.150941896
119.5251145	0.026816499	0.143432494	0.186962509	0.851690028	0.969398357
397.9362126	0.212789702	0.114658096	1.855862856	0.063473109	0.443016545

133.6925225	0.147735239	0.117053138	1.262121128	0.206905204	0.651884304
286.0164486	0.66210886	0.372097248	1.779397356	0.07517464	0.470687737
201.7029321	0.109371356	0.135496075	0.807192058	0.419555841	0.811799419
1438.519228	-0.014522866	0.10917	-0.133029824	0.894169794	0.978305957
225.4661335	0.006866716	0.08066224	0.085129246	0.932158639	0.984334593
752.45683	0.092589473	0.109760598	0.843558381	0.398916229	0.799994313
166.7290079	0.003840869	0.104869455	0.036625241	0.970783818	0.992796176
1997.09571	0.141243795	0.049167511	2.872705818	0.004069729	0.144354659
119.3992709	0.053114897	0.152968149	0.347228473	0.728419682	0.934240371
83.17554697	0.148194129	0.217424165	0.681589966	0.495498261	0.853825991
92.06072235	0.247970086	0.13432609	1.846030704	0.064887747	0.446824324
423.9445036	-0.015667444	0.096397682	-0.162529255	0.870889092	0.972881041
1168.621833	-0.074084667	0.098210962	-0.754342136	0.450643807	0.828630047
348.5382873	0.071901581	0.092219534	0.779678424	0.435580182	0.822078703
1041.674817	0.194507369	0.106652901	1.823741934	0.068191096	0.456313112
763.3485597	0.062715524	0.068603688	0.914171316	0.360626834	0.777605482
8366.821413	-0.332632191	0.192794129	-1.725323236	0.084469237	0.487641826
149.3454132	-0.108636793	0.142231686	-0.763801625	0.444985468	0.826302547
475.1333311	-0.090141583	0.097299236	-0.9264367	0.354219079	0.773422825
43.54616185	0.227047933	0.259905595	0.873578472	0.382347854	0.789880775
908.8990943	-0.100728666	0.07254617	-1.388476688	0.164991939	0.605350304
55.7628459	0.015446435	0.145039271	0.106498298	0.915187006	0.981139861
282.9926978	0.036744647	0.073542991	0.499634929	0.617332158	0.901976704
1223.799272	0.07777183	0.084274501	0.922839403	0.356090903	0.774305138
166.0517418	0.195166444	0.092875657	2.101373502	0.035608192	0.356853392
131.0307958	-0.242525646	0.113154178	-2.143320291	0.032087389	0.340059659
12.47634926	-0.271158874	0.259449652	-1.045130996	0.29596243	0.735715302
352.4417237	-0.113397488	0.095780185	-1.183934735	0.236438899	0.679171082
287.8251269	-0.207514315	0.094939318	-2.185757373	0.028833361	0.328011759
248.6765113	-0.055433163	0.098785301	-0.561147882	0.574696729	0.88616072
325.3473205	0.138982915	0.091984484	1.510938688	0.130804073	0.563821166
2610.432023	0.118250456	0.052901446	2.235297227	0.025397842	0.313432167
490.3550202	-0.08593426	0.111424009	-0.771236476	0.440566777	0.823778083
778.2180903	0.116452749	0.189906423	0.613211219	0.539736687	0.869675597
365.9727707	0.077325141	0.074473439	1.038291541	0.299134346	0.738631505
259.5333923	-0.285746023	0.140909498	-2.027869142	0.042573605	0.377880689
384.2528032	-0.013909589	0.072639055	-0.191489122	0.848142402	0.968987638
236.8750904	0.070402364	0.11665506	0.603508876	0.546170213	0.873065405
1109.584458	-0.090448291	0.073076315	-1.237723756	0.215818507	0.660219973
33.52112843	0.426933516	0.305773718	1.396240067	0.162642215	0.601684318
79.60592719	-0.260438079	0.18333276	-1.420575787	0.155440122	0.593486898
358.3852128	0.120813475	0.075513725	1.599887643	0.109623511	0.528197178
292.24396	0.150988545	0.095239559	1.585355357	0.112885618	0.533844383
1822.144611	0.099620268	0.07946631	1.253616377	0.209981476	0.655248746
74.16923826	-0.048759718	0.126211709	-0.386332758	0.699250243	0.925900484
7.801572966	-0.331043225	0.427343311	-0.774654046	0.438544112	0.822520267
358.4200341	-0.302044687	0.205951487	-1.466581723	0.142489875	0.577212679
119.7375439	0.003942902	0.175201095	0.022505009	0.982045116	0.99517046

555.7489203	-0.22866566	0.091057643	-2.511218745	0.012031512	0.236404198
308.0578852	-0.728119111	0.228951644	-3.18023098	0.001471577	0.09327498
59.67447614	0.366173064	0.305844135	1.197253835	0.231207632	0.673555476
653.9664442	0.017843669	0.054602853	0.326790053	0.743826683	0.940317814
991.4739961	-0.039323533	0.051783845	-0.759378392	0.447626235	0.827180558
183.843487	0.04222633	0.098280206	0.429652442	0.667448483	0.916775933
3424.675137	0.099374709	0.082500508	1.204534507	0.228383053	0.671005548
6.935774085	0.376745706	0.425976937	0.884427473	0.376465498	0.786851292
40.08878606	-0.049230912	0.289431024	-0.170095491	0.864935039	0.971917805
449.4745139	0.261381165	0.125550735	2.081876815	0.037353723	0.361278136
83.06183287	0.099528588	0.111462254	0.892935361	0.371891789	0.785830444
254.1774356	0.086910191	0.071953623	1.207864002	0.227099578	0.669850929
301.7870858	-0.136204203	0.266067157	-0.511916632	0.608709357	0.900307811
11.41671919	-0.301262194	0.352033575	-0.855776879	0.392121207	0.795344613
265.257627	-0.176712218	0.10515019	-1.680569641	0.092846537	0.500506422
69.15260311	-0.422298593	0.216901218	-1.946962755	0.0515392	0.410734915
32.91400073	-2.400357092	1.055910543	-2.273258002	0.023010637	0.303197947
873.7509081	-0.010097647	0.108679634	-0.092912046	0.925973436	0.98316181
2238.31976	0.196339596	0.146552674	1.339720324	0.180336288	0.625591734
43.26523799	-0.357863566	0.381528837	-0.937972524	0.34825853	0.768780165
433.3576861	-0.007474091	0.118297826	-0.063180294	0.949622937	0.986108475
420.1606907	0.033396371	0.060435827	0.552592268	0.580542637	0.887818175
31.1386797	-0.026004452	0.318720341	-0.081590188	0.934972604	0.984334593
236.7566193	-0.215304043	0.217147447	-0.991510817	0.321436213	0.753905693
89.65537796	-0.015391588	0.129817444	-0.118563323	0.905621324	0.979768942
8.204036589	-0.980089431	0.630702919	-1.553963683	0.12019308	0.545735572
35.61967573	-0.428800156	0.321332327	-1.3344445	0.182058224	0.627539984
328.1619085	-0.027558592	0.094393165	-0.291955373	0.770320745	0.948321877
54.87382772	0.145901858	0.152201211	0.958611679	0.337754407	0.7637398
1314.152309	-0.308217433	0.164973794	-1.868281167	0.06172289	0.439326285
15.20944626	-1.846964357	0.827134654	-2.232966962	0.025551123	0.313865332
132.2467687	-0.236808165	0.116549628	-2.031822573	0.042171621	0.376265422
1482.287354	-0.353524831	0.248349041	-1.423499887	0.154591295	0.592506562
12.74900134	-1.397533072	0.645779154	-2.164103724	0.03045639	0.332880971
209.8611118	0.081703913	0.110930455	0.736532747	0.461406546	0.835033586
36.40628447	-0.598002657	0.175521214	-3.407010719	0.000656785	0.059321946
456.142563	0.038303078	0.116343739	0.329223371	0.741986859	0.939964277
44.54038613	-0.234942342	0.212493081	-1.105647022	0.268879329	0.711435249
9.217307892	-0.663455625	0.89468476	-0.741552393	0.458358577	0.833531988
25.55470643	-0.223177611	0.19661876	-1.135077912	0.256342676	0.699425674
42.99041695	-0.252741456	0.282839998	-0.893584563	0.371544209	0.785656768
41.28230836	-0.602231937	0.251785143	-2.391848577	0.016763757	0.264538519
169.5612246	0.009926829	0.085910563	0.11554841	0.908010452	0.980014717
21.35017897	-0.955972016	0.406021206	-2.354487899	0.018548248	0.273710839
69.07873823	-0.02551387	0.116968109	-0.218126722	0.827330378	0.964553924
11.63708374	-0.274896335	0.438407514	-0.627033813	0.530637071	0.866123834
156.2959199	0.025565104	0.447111367	0.05717838	0.9544031	0.987292586
384.3765589	-0.034548619	0.30174617	-0.114495633	0.908844908	0.98010679

24.15058521	-1.294729946	0.428760071	-3.019707369	0.00253019	0.119399986
506.6574888	0.131682936	0.139241517	0.945716044	0.344293437	0.767389167
670.6071468	-0.088586383	0.160338765	-0.552495109	0.580609184	0.887828217
43.97423908	0.28650838	0.165793763	1.728101071	0.083970106	0.486282844
650.0254233	0.157348627	0.152975372	1.028587969	0.303673334	0.741377649
29.63731626	0.129762075	0.215358427	0.602540037	0.546814721	0.873510846
333.6352267	0.020697051	0.061818758	0.334802117	0.737774363	0.938868945
10.76359616	-0.094460863	0.441537064	-0.21393643	0.830596631	0.965541183
650.2100423	-0.012093423	0.145890271	-0.082893965	0.933935853	0.984334593
88.78211306	0.341647468	0.227101851	1.504379934	0.132483568	0.564647789
48.52977081	-0.320337599	0.316034514	-1.013615872	0.310766081	0.745771331
418.7659757	-0.200058466	0.127142331	-1.573500065	0.115603073	0.537086218
99.57161604	0.135862502	0.096559877	1.40702854	0.159418926	0.600573799
185.4979598	0.173343115	0.099621974	1.740008829	0.081857468	0.483200119
11.14521911	-0.270316312	0.288361006	-0.937423253	0.348540884	0.768780165
959.9532299	0.19738972	0.18979186	1.040032589	0.29832476	0.738522837
2305.012711	0.023269014	0.132313033	0.175863355	0.860401295	0.969966486
53.23459132	-0.158114847	0.145915331	-1.083606817	0.278539167	0.720309746
16.39978331	-0.887179516	0.53358339	-1.662682035	0.0963761	0.505983706
83.14014796	0.022910184	0.132791103	0.172528001	0.86302245	0.971275942
6.830976551	-1.070054286	0.799792276	-1.337915255	0.180924064	0.626148701
140.8405545	0.688977625	0.208687144	3.301485719	0.000961742	0.075321215
6.442477655	-0.507166678	0.562714703	-0.901285634	0.36743647	0.781703698
63.20330605	-0.07937581	0.161404798	-0.491780981	0.622874183	0.902441895
1010.386477	0.158053449	0.096048144	1.645564849	0.099853381	0.511826735
11.56559055	-0.212404089	0.326471618	-0.650605067	0.515301459	0.861826223
44.9560281	-0.027385863	0.133416737	-0.205265572	0.837364641	0.966384462
53.1018704	0.131474471	0.162567675	0.80873686	0.418666521	0.811455684
533.8173093	0.040357491	0.069215851	0.583067178	0.559848078	0.880280849
150.6840722	-0.164056227	0.108515918	-1.511817153	0.130580385	0.563357211
738.7729757	-0.104446773	0.074852606	-1.395365897	0.16290553	0.601684318
194.1130425	0.135703823	0.099454521	1.364481199	0.172416168	0.616428289
6.81548979	-1.051764169	0.535498853	-1.964082954	0.049520473	0.40452651
2094.264258	0.035409899	0.109139688	0.324445668	0.74560065	0.940455922
84.68703639	0.070734494	0.247223167	0.286115961	0.774789307	0.949820378
601.5559346	0.110873312	0.224168724	0.494597596	0.6208842	0.902250925
22.79486001	-0.479434919	0.255072734	-1.879600818	0.060162502	0.435320275
443.4529522	0.153501408	0.069562715	2.206662121	0.027337673	0.323603282
5.283380959	-1.300536416	0.576804649	-2.254725962	0.024150535	0.308436782
53.29881348	0.289656566	0.388769225	0.745060431	0.456235189	0.83202452
456.7392848	-0.058614928	0.08155459	-0.718720155	0.472313363	0.840757256
349.1491056	0.022758061	0.118703565	0.191721801	0.847960128	0.968938143
450.9218817	-0.039546964	0.159606461	-0.247777963	0.804306207	0.957233889
1925.404565	-0.214675275	0.122909174	-1.746617178	0.080703765	0.480706878
4.525370665	-0.497699746	0.53204891	-0.935439837	0.349561675	0.769742921
11.91165462	-0.924466152	0.574290681	-1.609753009	0.107451787	0.524524053
202.594353	-0.162177027	0.074251597	-2.184155394	0.028950831	0.328011759
83.78975039	-0.027091051	0.360581421	-0.075131578	0.940110023	0.984334593

5.332068502	-0.294443386	0.493536909	-0.596598513	0.550775464	0.875773813
194.3568286	-0.054991567	0.106244782	-0.517593108	0.604742191	0.900089886
642.5888837	-0.013920662	0.104782116	-0.132853416	0.894309309	0.978386282
153.560345	0.184045162	0.101655676	1.810476003	0.070222002	0.461430171
120.1935443	-0.064895354	0.101847833	-0.637179533	0.524007908	0.863484457
48.60532027	-0.016016778	0.1530548	-0.104647344	0.916655646	0.981290994
40.82504729	-0.074275834	0.178178097	-0.416862876	0.676778709	0.921393988
2249.337597	0.157785311	0.15126266	1.043121361	0.296892089	0.736647043
63.23971131	0.032744184	0.227666355	0.143825308	0.885638418	0.976914812
180.0924631	-0.005114513	0.080945522	-0.063184634	0.949619481	0.986108475
10.14860273	0.155210466	0.302121113	0.513735913	0.607436639	0.900307811
5.554671537	0.737387791	0.618887679	1.191472727	0.233468057	0.676472759
593.466534	-0.158969602	0.201915588	-0.787307231	0.431102038	0.818841807
6663.424016	-0.034252298	0.132511547	-0.258485383	0.796032328	0.95451485
502.5258797	0.012006011	0.085669149	0.140143927	0.888546274	0.976961229
21.21989326	-0.064637562	0.409175778	-0.157970157	0.874480314	0.973456991
281.2973076	-0.113292222	0.118132614	-0.959025775	0.337545761	0.7637398
62.75231027	-0.223101698	0.132607021	-1.68242749	0.092485975	0.499809199
221.9428417	-0.086375036	0.146212476	-0.590750106	0.55468787	0.877791979
10.8486681	-0.516480499	0.399507559	-1.292792808	0.196082728	0.641398981
6.421318509	-1.028291761	0.551762956	-1.863647694	0.062371195	0.44188166
203.6136336	0.331143165	0.637211336	0.51967557	0.603289717	0.899375001
129.3793268	-0.091463766	0.179285915	-0.510155891	0.609942252	0.900307811
456.7130337	0.361119589	0.200647217	1.799773721	0.071896375	0.463154629
257.4218337	-0.147715916	0.109670538	-1.346906089	0.17801048	0.622328585
342.6505658	0.104542719	0.134280721	0.778538557	0.436251585	0.822078703
118.2576751	0.069603487	0.209160892	0.332774863	0.739304234	0.939500078
5.485248585	0.203672095	0.456448163	0.446210789	0.65544499	0.912518505
952.1284279	0.163706561	0.06724919	2.434327611	0.014919485	0.256113483
642.8815285	0.028108222	0.167775602	0.167534623	0.866949407	0.972138679
46.2262477	-0.26809219	0.162784166	-1.646918105	0.09957488	0.511744973
18.02333683	-1.80563298	0.614182364	-2.93989715	0.003283212	0.133812668
666.3061347	-0.047240138	0.076293953	-0.61918588	0.535793913	0.869002283
8.208617019	-0.066995278	0.304536275	-0.219991126	0.825878066	0.964118059
17.01718007	-0.372761224	0.423500687	-0.880190365	0.378756192	0.787072097
2293.085731	0.237379762	0.130476824	1.819325111	0.068861845	0.458541286
6.759941345	0.153005629	0.417336937	0.366623741	0.713899697	0.929846524
316.772509	-0.245626488	0.278833215	-0.880908282	0.378367464	0.787072097
5.63264404	-0.664406315	0.412661833	-1.610050319	0.107386872	0.524456614
7.495692189	-0.10841542	0.513232769	-0.211240253	0.832699802	0.966227926
219.414662	0.127218234	0.176177194	0.72210387	0.470230619	0.839923915
210.4617931	0.288259348	0.09689732	2.97489494	0.002930889	0.128732997
599.4766416	0.294317242	0.16599613	1.773036768	0.076222614	0.471783446
1062.223655	0.211955678	0.190672376	1.111622363	0.266300553	0.708743266
6.126491909	-0.132073811	0.503885257	-0.262110886	0.793235955	0.953153173
99.77142559	-0.27310951	0.195383119	-1.397815284	0.162168543	0.601569453
8.799010663	-0.282564165	0.624509123	-0.452458026	0.650939053	0.910782566
16.70296165	0.367013034	0.600213046	0.61147127	0.540887632	0.870290562

245.1910053	0.231057625	0.100803789	2.29215219	0.021896863	0.296249025
15.80681323	-0.975464523	0.406897912	-2.397320051	0.016515491	0.263145637
221.8765714	-0.454030637	0.311780478	-1.456251016	0.145323241	0.579024123
78.06693918	-0.623778968	0.298340284	-2.090830507	0.03654326	0.360198137
55.10795482	-0.346089997	0.183982288	-1.881104976	0.05995764	0.435320275
242.9783072	0.093004159	0.074514227	1.248139613	0.211979934	0.656840481
10.19493112	-0.578076701	0.487942691	-1.184722533	0.23612717	0.678901985
151.3468114	0.012739838	0.116594073	0.109266599	0.912991038	0.980530316
874.8081435	-0.297078593	0.17443325	-1.703107598	0.088547934	0.493295644
111.4255216	0.157317966	0.108673928	1.447614612	0.147724862	0.58409168
175.2351744	-0.163917683	0.33100216	-0.495216355	0.620447407	0.901976704
1842.616153	0.078168572	0.22016736	0.355041601	0.722558426	0.931322694
9.514232128	0.750928088	0.386524542	1.942769493	0.052044015	0.412837898
155.8016021	-0.235493631	0.169697508	-1.387725922	0.16522052	0.605350304
16.66640096	-1.364206023	0.435936898	-3.129365811	0.001751841	0.100967155
719.1195611	0.042383618	0.201264586	0.210586566	0.833209896	0.966227926
79.77389469	-0.107811393	0.190209882	-0.566802271	0.570848527	0.884692692
489.8061534	0.066469647	0.07229159	0.919465826	0.357851972	0.776106212
13.16848942	-0.203771097	0.542465204	-0.375639019	0.707185278	0.92811888
8.348757776	-0.07443405	0.358448488	-0.207656198	0.835497421	0.966316856
55.50416238	0.086597902	0.143364909	0.604038343	0.54581815	0.872896521
448.4540131	0.040322356	0.113363936	0.355689447	0.722073149	0.931135539
306.2423708	0.02790507	0.135102956	0.206546702	0.836363888	0.966316856
72.54188361	-0.080947092	0.274144089	-0.295272066	0.767786054	0.947386869
9.322133143	-0.050118289	0.394945289	-0.12689932	0.899020085	0.978996707
69.4698813	-0.347964972	0.158223095	-2.199204693	0.027863371	0.324735857
142.127588	0.083271727	0.234693251	0.354810915	0.72273125	0.931403637
317.5761896	-0.023966796	0.118715	-0.201885151	0.840006511	0.966440785
7.405244143	-0.398664837	0.344223327	-1.158157525	0.246799758	0.690311795
11200.54561	0.02846311	0.135454609	0.210130246	0.833566019	0.966316856
137.4306569	0.323509799	0.185891113	1.740318799	0.081803055	0.483179896
94.16683405	-0.008241717	0.115703501	-0.071231355	0.943213627	0.98464854
144.3270146	0.18642148	0.165169477	1.128667858	0.259037973	0.702208457
477.3543485	0.015477871	0.086681048	0.178561192	0.858282269	0.969966486
22.47239526	-0.008933346	0.289948682	-0.030810092	0.975420992	0.993405995
228.1343504	0.138244397	0.319344413	0.432900628	0.665086963	0.916049829
1076.830282	0.159275291	0.073211651	2.175545685	0.029589248	0.330732257
60.13718271	-0.105627272	0.188599864	-0.560060168	0.575438398	0.886202779
113.5578061	0.133389283	0.095737565	1.393280504	0.163534984	0.602187485
52.74953495	0.154502952	0.170842571	0.904358624	0.365805271	0.780433787
21.20900327	-0.016591444	0.258141846	-0.064272588	0.94875318	0.985917197
129.028057	-0.310059528	0.17588346	-1.762869164	0.077922559	0.473730652
557.542516	-0.037262866	0.058879249	-0.632869252	0.526819029	0.865095991
842.9409807	0.160312479	0.203450982	0.787966111	0.430716529	0.818841807
877.5804966	-0.215310344	0.181788044	-1.184403216	0.236253488	0.678901985
15.83306651	0.007326787	0.244833963	0.029925535	0.976126441	0.993487297
16.34517722	-0.34223691	0.255851399	-1.337639393	0.181014016	0.626190428
182.1821778	0.214902546	0.155633956	1.38082043	0.16733418	0.609023925

122.1158084	0.206130326	0.175018567	1.177762619	0.238891255	0.681087839
52.19120928	0.261835471	0.160051961	1.635940414	0.101852059	0.51366752
1300.814449	0.267285345	0.091427929	2.923454017	0.003461714	0.135044319
25.0561665	0.18546881	0.206987089	0.89604048	0.370231144	0.784153324
23.52776396	-0.090140185	0.268633499	-0.335550797	0.737209633	0.938737429
14.13432172	0.118498551	0.257851266	0.459561642	0.645830897	0.909300678
50.70717717	-0.17811219	0.187106453	-0.951929702	0.341132635	0.765180569
897.6019352	-0.012125544	0.154601256	-0.078431084	0.937485148	0.984334593
97.2224329	0.058457523	0.112634079	0.519003872	0.603758041	0.899439062
107.8658195	0.046527214	0.107154925	0.434205092	0.664139515	0.916049829
499.4455755	-0.114483139	0.381264269	-0.300272406	0.76396938	0.946826103
315.5168766	-0.054897713	0.109290519	-0.50230993	0.615449525	0.901900555
15.14338304	-0.360238753	0.365403261	-0.985866278	0.324198737	0.756297513
158.0607743	-0.082239944	0.098111308	-0.83823104	0.401900957	0.801317531
6.709564013	-0.47914725	0.400511346	-1.196338766	0.231564386	0.674196705
196.8838502	-0.034477576	0.103385903	-0.333484306	0.738768734	0.939226436
93.21835437	-0.156679979	0.105214065	-1.489154315	0.136446738	0.569036171
100.2143017	-0.175246822	0.18251274	-0.960189529	0.336959836	0.7637398
100.2261031	-0.188073555	0.113956829	-1.650393022	0.098862577	0.51077273
1865.942218	0.107080718	0.104420702	1.025474024	0.305139576	0.741701149
8.470730828	0.030351816	0.345848012	0.087760562	0.930066984	0.984334593
37.47936149	0.109348001	0.220406728	0.49611916	0.619810342	0.901976704
413.7872015	0.03050856	0.211479052	0.144262797	0.885292955	0.976804762
5.165809012	-0.001289638	0.452162142	-0.002852159	0.997724309	0.999382914
452.7274541	0.120143913	0.193403312	0.621209182	0.534461999	0.867840168
163.8906707	0.174290982	0.151937525	1.14712269	0.251330917	0.694735013
910.9999332	0.028419485	0.083218524	0.341504317	0.732723956	0.936434121
457.6526134	-0.063907692	0.10382013	-0.615561669	0.538183856	0.869675597
565.6380917	0.145730416	0.059189517	2.462098395	0.013812677	0.250625508
11.84174783	-0.457154953	0.379835474	-1.203560448	0.228759515	0.671658747
822.8331275	0.029367922	0.144928989	0.20263663	0.839419058	0.966440785
21.08064086	-0.171500394	0.278863945	-0.614996656	0.538556929	0.869675597
233.0570195	0.120920284	0.16053622	0.753227428	0.45131326	0.828948899
162.0241121	-0.216928084	0.103770378	-2.09046251	0.036576273	0.360198137
264.0133275	-0.008674969	0.112597564	-0.077044018	0.938588528	0.984334593
2380.299107	0.20211128	0.103217936	1.958102328	0.050218007	0.40663399
21.28432969	0.893402169	0.410805172	2.174758814	0.029648195	0.330732257
175.0119745	-0.003703091	0.141390869	-0.026190452	0.979105431	0.994602886
379.5647944	-0.155512139	0.170873238	-0.910102371	0.362768525	0.779676999
107.9354444	0.048890057	0.175827642	0.278056718	0.78096882	0.95173434
172.0467558	0.000121843	0.077317975	0.001575872	0.998742637	0.99966493
19.30690038	-0.511416643	0.388075794	-1.317826699	0.187561668	0.633746703
368.8255203	-0.034268454	0.091036908	-0.376423751	0.706601891	0.928011279
402.2899322	0.095566875	0.106596073	0.896532791	0.369968275	0.783892128
514.0826879	0.162195979	0.125176232	1.295741026	0.195064722	0.640317357
96.6438266	0.005515823	0.152852441	0.036085934	0.971213838	0.992799208
8.470457224	-0.462059121	0.331618396	-1.393345865	0.163515227	0.602187485
521.6505267	0.124493938	0.073417265	1.695703839	0.089941981	0.495390485

19.58295829	0.04740674	0.595201417	0.079648231	0.936517034	0.984334593
37.99546919	0.712097669	0.500000762	1.424193169	0.154390562	0.592506562
43.7411195	0.775308028	0.532094385	1.457087406	0.145092254	0.578290974
476.4042857	0.052572223	0.326030055	0.161249622	0.8718968	0.972881041
4.86138908	0.460910406	0.598644148	0.769923847	0.441345067	0.82422323
27.33113193	0.369605802	0.250896515	1.47314044	0.140713171	0.574417086
230.1041572	0.017107339	0.072196024	0.236956805	0.812690301	0.96005245
464.5514118	0.290230354	0.087753664	3.307330326	0.000941898	0.074555974
16.3615034	-0.334218627	0.255683567	-1.307157246	0.191159312	0.637278237
1112.966384	0.368284987	0.261821261	1.406627505	0.159537873	0.600573799
34.71836071	0.327047605	0.376322728	0.869061528	0.384813478	0.790453664
205.2466538	0.364230938	0.278758292	1.306619207	0.19134207	0.637278237
12.92212562	-0.005569376	0.572160368	-0.009733942	0.992233561	0.998439236
45.64016518	0.008094805	0.185926555	0.043537647	0.965272955	0.99063789
193.7513975	-0.006561885	0.089259042	-0.073515076	0.941396248	0.984334593
12.36775589	-0.529240194	0.404060293	-1.309805005	0.19026181	0.636441877
521.6573115	0.31400763	0.231782527	1.354751089	0.175496875	0.619005807
111.2926774	0.000181538	0.109761631	0.001653933	0.998680353	0.99966493
18.18263732	-0.45475447	0.238802116	-1.904315074	0.056869162	0.428680279
334.5292209	0.094328139	0.304559566	0.309719835	0.756774018	0.945306795
300.8201032	0.189504884	0.129141921	1.467415712	0.142263003	0.576962874
2240.307878	0.129294859	0.066985835	1.930182097	0.053584279	0.417128004
728.8118897	0.033949501	0.087611585	0.387500135	0.698385985	0.925802378
485.5497371	-0.022175986	0.104135326	-0.212953539	0.831363202	0.965997183
783.2573841	-0.008460001	0.184262258	-0.045912825	0.963379732	0.989954388
2796.915439	0.151626623	0.075489185	2.008587358	0.044580913	0.387423459
5.567350399	-0.640649759	0.420130501	-1.524882761	0.127288342	0.556444786
7.725896787	-0.454755971	0.54973871	-0.827222029	0.408111228	0.805770628
26.16698439	-0.037184422	0.248582971	-0.149585556	0.881091605	0.975487608
86.02822178	-0.134262187	0.128068725	-1.048360462	0.294472563	0.734915339
111.5103417	-0.448434204	0.363344725	-1.234183882	0.217134392	0.66132166
545.427817	0.03474737	0.123721258	0.280852056	0.778823875	0.951560132
500.7170885	-0.030152338	0.081570769	-0.369646359	0.711646004	0.929376205
653.6601225	-0.055989336	0.082225471	-0.680924483	0.495919276	0.853954993
637.3521615	0.038254591	0.086396983	0.442776932	0.65792709	0.91380052
336.2723337	0.15180753	0.087707917	1.73083041	0.083482016	0.485778721
146.5419754	0.109651797	0.090535145	1.211151721	0.225837262	0.668749399
115.8063613	0.531258692	0.189356183	2.805605198	0.005022218	0.155846704
21.80987536	-0.730433419	0.332218909	-2.198650946	0.027902751	0.324735857
9230.876666	-0.063945188	0.055172952	-1.158995236	0.246458127	0.689929208
157.3628096	0.014922211	0.171082748	0.087222182	0.930494908	0.984334593
320.2064969	-0.143780798	0.285388949	-0.503806466	0.614397384	0.901900555
114.4485015	0.350110054	0.224849965	1.557082982	0.119450785	0.54413566
103.0357289	-0.3492681	0.204635377	-1.706782592	0.087862469	0.492827855
9.185704807	-1.284506979	0.712635695	-1.802473535	0.071470935	0.463154629
301.5874411	0.271705465	0.224760657	1.208865773	0.226714417	0.669424857
28.12754673	-0.534385162	0.251878498	-2.121598974	0.033871429	0.348624664
202.605606	-0.135386372	0.109662409	-1.234574117	0.216989047	0.661222104

1300.094255	-0.061161829	0.11419175	-0.535606373	0.592230623	0.893683696
1985.277888	-0.027460512	0.135470276	-0.202705074	0.839365557	0.966440785
364.3317504	-0.07489576	0.088373074	-0.847495243	0.396719137	0.798330977
1536.687679	-0.003157059	0.051392697	-0.061430105	0.951016678	0.98625342
258.3690498	-0.260733713	0.104089088	-2.504909197	0.012248283	0.237458144
321.8522439	-0.439395079	0.205113352	-2.142206126	0.032176899	0.34013779
14.76890204	-0.18872395	0.288166971	-0.6549118	0.512524542	0.860200661
25.84016656	-0.121053173	0.31931775	-0.379099417	0.704614039	0.9269128
18.03278244	0.005171441	0.314044153	0.016467243	0.986861635	0.996928555
127.4130242	-0.339047217	0.187616947	-1.807124684	0.070742841	0.462488289
182.280738	-0.232213059	0.193812924	-1.1981299	0.23086645	0.673080052
217.6013411	0.134952192	0.108886989	1.239378499	0.21520536	0.660219973
533.8213142	0.204947593	0.08395228	2.441239167	0.014636958	0.256113483
103.7725506	-0.017358624	0.227778723	-0.076208277	0.939253398	0.984334593
413.3258765	-0.02986945	0.066096174	-0.451908902	0.651334611	0.910908439
710.312478	0.115425199	0.073405731	1.572427631	0.11585141	0.537228251
522.396933	0.037477076	0.059794377	0.626765883	0.53081271	0.866123834
399.3010915	0.112788088	0.074148306	1.521114829	0.128231024	0.55891508
89.00591115	-0.476671712	0.19031263	-2.504677235	0.012256318	0.237458144
464.8838445	-0.00100109	0.079612506	-0.012574534	0.989967238	0.997999732
21.51315354	-0.431785275	0.399678548	-1.080331375	0.279994643	0.721663546
201.0639528	0.016941156	0.107861759	0.157063598	0.875194726	0.973587929
50.47588142	0.376066708	0.17620672	2.134235908	0.032823475	0.342874434
17.9097117	-0.302562728	0.364020694	-0.831169033	0.405878146	0.804391391
15.19188685	-1.31545464	0.474853709	-2.770231371	0.005601649	0.163219689
7044.638772	0.134093167	0.11755464	1.140687997	0.253999783	0.696717102
440.1726385	0.138456813	0.069781671	1.984142996	0.047239901	0.396172814
208.2605175	0.210274139	0.113179007	1.85788995	0.063184644	0.443016545
127.9388878	0.013521466	0.112512917	0.120177006	0.904342932	0.979589029
424.6872187	0.217336237	0.186411614	1.165894292	0.243657187	0.686012467
80.60396637	0.126355773	0.221419937	0.570661227	0.568229306	0.8841274
9.419064203	-0.147852291	0.281036226	-0.526096914	0.598820873	0.897895573
379.5916167	0.104621175	0.128307962	0.815391137	0.414848456	0.80946307
1031.467228	0.118664755	0.058384941	2.032454821	0.042107633	0.376265422
132.4233033	-0.006344319	0.103164143	-0.061497331	0.95096314	0.98625342
13.62175133	-0.932506662	0.318645587	-2.926469718	0.003428329	0.134809333
644.6405864	0.020975642	0.090933935	0.230669023	0.817571941	0.961335246
10.25499498	-0.362022072	0.360717747	-1.003615915	0.315563781	0.749486634
12.05578231	-0.66440995	0.55855756	-1.189510263	0.234238935	0.677056183
292.8072422	0.047387979	0.139554664	0.339565717	0.7341836	0.937386243
19.6911064	0.075048135	0.538482437	0.139369699	0.889158015	0.976961229
604.6097255	-0.268186924	0.249163836	-1.076347706	0.28177178	0.723215865
14.40677435	-1.699332744	0.937227832	-1.813147972	0.069809002	0.460758859
2484.461687	0.17186378	0.133739427	1.285064423	0.198769772	0.64421391
2306.497523	0.027483757	0.111167118	0.247229195	0.804730852	0.957233889
260.1073089	0.18322899	0.186242142	0.983821321	0.325203374	0.756297513
114.4796725	0.192959795	0.244217481	0.790114588	0.429460851	0.818650305
96.3639899	0.256502418	0.17285231	1.483939774	0.137824899	0.569378775

208.4738559	0.059669759	0.291503142	0.204696796	0.837809024	0.966431512
490.6782011	-0.052006453	0.129931538	-0.400260427	0.688964712	0.923813534
1510.474044	0.045582887	0.141873438	0.321292606	0.747988659	0.941487306
1454.451314	-0.06585969	0.126321655	-0.521365005	0.602112525	0.899159563
432.8719322	0.123992166	0.071417387	1.736162184	0.082535162	0.484988275
476.2964923	0.010053217	0.089253115	0.112637159	0.910318224	0.980530316
11.83383247	-0.637899708	0.395948682	-1.611066629	0.107165201	0.524305548
8.578097573	-0.14507585	0.391000099	-0.371037885	0.710609315	0.929359766
244.0618244	-0.277517306	0.141011181	-1.968051775	0.049062081	0.402791699
1241.354943	0.00970281	0.049100631	0.197610704	0.843349659	0.968131661
24.91689909	-0.043797993	0.2913995	-0.150302224	0.880526178	0.975430592
696.7876466	-0.032995322	0.113998985	-0.289435223	0.772248344	0.948706053
298.7680424	0.139231182	0.15235896	0.913836518	0.360802755	0.777606636
5.836960472	-1.289248738	0.726033864	-1.775741878	0.075775472	0.471272491
119.7725897	0.449630001	0.330734819	1.359487949	0.173992019	0.618034305
54.83331327	-0.147799871	0.256931317	-0.575250511	0.565121886	0.883265494
423.2186416	-0.040767984	0.120403573	-0.338594467	0.734915251	0.937620779
127.7439503	-0.195995796	0.150589392	-1.301524586	0.193078955	0.639147095
53.01443726	-0.383696634	0.24996079	-1.535027287	0.124777125	0.553310668
132.7380667	-0.212496451	0.169309782	-1.255074859	0.209451586	0.654704857
84.70701628	-0.08846123	0.216229771	-0.409107542	0.682460742	0.922776218
311.4313469	0.274523539	0.1241286	2.211605849	0.026993911	0.321192821
7.440216234	-0.093255041	0.350400499	-0.266138436	0.790132594	0.953026618
185.7376454	0.020378745	0.07574564	0.269041825	0.787897497	0.953026618
47.03415625	-0.319358291	0.315794691	-1.011284544	0.311880261	0.746220174
4624.751271	-0.169145205	0.125023658	-1.352905582	0.176085796	0.619691382
10.16655607	-0.321257614	0.370573933	-0.866919081	0.385986348	0.791597811
273.5620723	0.216653222	0.144144918	1.50302366	0.132832943	0.565075221
64.9419729	-0.015772583	0.144229019	-0.109357903	0.912918622	0.980530316
243.6771138	-0.051077083	0.109436993	-0.466725933	0.640695973	0.907798398
2054.600929	0.207425872	0.137506571	1.508479711	0.131431795	0.564647789
10.22069159	0.761233861	0.313672267	2.42684464	0.015230777	0.257947316
93.07735761	0.158827627	0.139168645	1.141260139	0.253761684	0.696490986
567.9430338	-0.028994393	0.069395463	-0.417813962	0.676083142	0.921393988
116.2161431	-0.396870811	0.167170029	-2.374054803	0.017593939	0.269492791
923.7432638	-0.364683722	0.150104989	-2.429524318	0.015118651	0.25781828
958.4610573	-0.236808659	0.162238075	-1.459636761	0.144389931	0.578069441
37.31308938	-0.363877513	0.215474657	-1.688725336	0.091272078	0.498962299
86.35008006	-0.275754821	0.133724003	-2.06211911	0.039196397	0.370488554
335.8863703	-0.728587266	0.310751587	-2.344597086	0.019047651	0.276929913
255.1077328	0.03561517	0.070862635	0.502594485	0.615249407	0.901900555
241.7479562	0.065481317	0.152284918	0.429992134	0.667201363	0.916690146
9.784499458	0.012440115	0.387497565	0.032103724	0.974389334	0.993301399
112.7709413	-0.114144609	0.094152467	-1.212337943	0.225383045	0.668524251
101.4470686	-0.279257167	0.428006656	-0.652459869	0.514104553	0.860931734
452.13069	-0.066269871	0.151384434	-0.437758817	0.661561127	0.915002321
432.3726784	0.110056198	0.07530726	1.4614288	0.143897802	0.578069441
776.731682	0.252146983	0.118879345	2.121032751	0.033919046	0.348624664

21.39276113	-0.243109806	0.372953798	-0.651849658	0.514498163	0.861298554
10.91825362	-1.081668479	0.727086219	-1.487675672	0.136836446	0.569036171
67.79628319	-0.190787389	0.166453163	-1.146192633	0.251715456	0.694735013
265.9474586	-0.451328825	0.269005264	-1.677769491	0.093392107	0.500506422
50.89346089	-0.078494683	0.15877185	-0.494386648	0.621033142	0.902299404
1851.601195	-0.012972591	0.128917657	-0.100626952	0.919846601	0.981939232
253.5275183	-0.043383437	0.077178815	-0.56211588	0.57403707	0.885935354
1083.040518	0.011606063	0.042288791	0.274447735	0.783740564	0.951880071
1285.802965	-0.063740412	0.068659162	-0.928359882	0.353220921	0.772599211
619.989814	-0.014383329	0.075784051	-0.189793612	0.849470862	0.969251033
67.78284681	-0.139317822	0.270667447	-0.514719533	0.606749022	0.900307811
335.1353038	-0.118906207	0.157264594	-0.756090128	0.44959516	0.828449907
107.8824762	0.008220579	0.117142228	0.070176052	0.944053535	0.984843572
135.0986966	-0.210549139	0.156122473	-1.348615189	0.1774606	0.621574017
309.3644892	0.046459935	0.090941206	0.510878811	0.609435919	0.900307811
24.62071512	-0.528644975	0.407969102	-1.295796599	0.19504557	0.640317357
211.9459647	-0.093303314	0.09440246	-0.988356813	0.322977931	0.755505673
8.383597239	0.01227322	0.464920793	0.026398518	0.978939476	0.99459552
116.7772312	0.007358543	0.309198622	0.023798759	0.98101313	0.994880431
154.8380647	0.712045598	0.461787042	1.541934988	0.123089393	0.549779478
184.3305015	-0.271893233	0.094208289	-2.886086105	0.003900654	0.143269191
2205.385086	0.078681297	0.121131238	0.64955414	0.515980264	0.862002027
51.70859756	0.192866677	0.165499298	1.165362506	0.243872288	0.686012467
4823.58269	-0.011647032	0.100205641	-0.116231305	0.907469227	0.979768942
29.76928108	-0.400756808	0.25325558	-1.582420446	0.11355362	0.533883941
480.1531352	-0.083645005	0.092365664	-0.905585494	0.36515529	0.779833877
486.2446437	-0.163046501	0.113501334	-1.436516167	0.150855519	0.589528489
15.32965665	-0.512108323	0.266819774	-1.919304238	0.054945842	0.422717439
278.8454499	-0.299740925	0.168598806	-1.777835392	0.075430894	0.470687737
60.93962096	-0.283921099	0.178832872	-1.587633725	0.112369187	0.533148601
302.614269	-0.153448442	0.21449548	-0.715392426	0.474366591	0.841713532
50.58368563	-0.052308624	0.254706529	-0.20536821	0.837284456	0.966384462
23.37063487	-2.342891075	0.995220164	-2.354143495	0.018565443	0.273710839
11.63655731	-2.636036195	1.265167665	-2.083546922	0.037201399	0.361102242
3364.16392	-0.260235705	0.511778582	-0.508492764	0.611107813	0.900509492
421.7499574	-0.044288666	0.054127974	-0.81822139	0.413230784	0.808272153
1039.217962	0.045504931	0.097663284	0.465936933	0.641260644	0.907798398
9.179840564	0.099634492	0.332882254	0.299308511	0.764704661	0.946826103
80.56359101	0.043907397	0.151636852	0.28955624	0.772155749	0.948706053
361.8637964	-0.140781779	0.084165673	-1.672674539	0.094391384	0.502328657
41.39525565	0.022280651	0.183747518	0.12125688	0.903487573	0.979381499
900.9845575	0.018293725	0.08946285	0.20448404	0.837975263	0.966437851
105.6644473	-0.573417611	0.283096589	-2.025519322	0.042814066	0.379065918
417.0610603	0.043948437	0.097129437	0.452472884	0.650928352	0.910782566
127.7350703	-0.283537639	0.442543949	-0.640699393	0.521718009	0.863328113
271.6406021	-0.248953808	0.072142624	-3.450856038	0.000558812	0.054062279
1899.518797	-0.034818751	0.059913219	-0.581153061	0.5611373	0.881268362
241.8556827	-0.072433478	0.252770915	-0.286557805	0.774450927	0.949748607

1465.264822	-0.120870269	0.084398012	-1.432145914	0.152102067	0.589839569
28.63251933	-0.35994333	0.21155951	-1.701380997	0.088871467	0.493693668
2842.144698	0.002377871	0.120175771	0.019786612	0.984213598	0.995852736
448.1520904	0.002066252	0.079523691	0.025982842	0.979271024	0.994602886
295.5592871	-0.054954341	0.160531788	-0.342326849	0.732104932	0.936065863
51.89997343	0.11679636	0.166725077	0.700532654	0.48359472	0.847822935
9.898808254	-0.15061718	0.363299055	-0.414581811	0.678448066	0.921573669
311.1049414	-0.248585313	0.071319082	-3.485537207	0.00049115	0.048791953
3157.928307	-0.130102627	0.137199041	-0.948276507	0.342988708	0.766327374
144.5640312	-0.273885567	0.166222621	-1.647703337	0.099413563	0.51147708
373.4915915	0.056991441	0.088993192	0.640402256	0.521911117	0.863358108
1603.760248	0.336084795	0.082503706	4.07357211	4.62975E-05	0.013047858
37.34892915	-0.410126683	0.163559314	-2.507510413	0.0121585	0.236908623
93.87369714	-0.106006664	0.130739141	-0.810825765	0.417465736	0.811361647
26.42085524	-0.170234411	0.193597901	-0.879319511	0.37922806	0.787394269
490.9185078	0.013947329	0.15599383	0.089409495	0.928756478	0.984080734
9.866403801	-0.674498839	0.38860727	-1.735682504	0.082619989	0.485011749
101.3353262	0.028412009	0.122256239	0.23239721	0.816229516	0.961335246
778.9947324	-0.051146952	0.085175606	-0.600488267	0.548180878	0.874640417
1479.670466	-0.123969417	0.132489058	-0.935695512	0.349429982	0.769567415
264.6231172	-0.208484129	0.122616861	-1.700289238	0.089076534	0.493693668
322.5870145	0.057698841	0.067683667	0.852478055	0.393948814	0.796289819
48.69204301	-0.181776063	0.200326965	-0.907396881	0.364196958	0.779728871
678.6187681	-0.183303243	0.102487342	-1.788545193	0.073688091	0.467122552
14.95229729	-0.095494553	0.287636684	-0.331997127	0.739891429	0.939690097
181.0142991	0.009375046	0.097020834	0.096629206	0.923020862	0.982590996
1092.770312	-0.020271543	0.087253732	-0.232328662	0.816282752	0.961335246
512.2789154	-0.20970555	0.08112166	-2.585074689	0.00973579	0.213793574
3823.447759	0.074386531	0.080605647	0.922845153	0.356087907	0.774305138
89.99205075	-0.118765688	0.183839866	-0.646027929	0.518261268	0.862002027
31.66998442	-0.111728658	0.22530279	-0.495904457	0.619961821	0.901976704
803.2271819	0.020168552	0.067564531	0.298507986	0.765315482	0.946857219
649.8663484	-0.037397481	0.10352743	-0.361232577	0.717925591	0.930765281
153.0913722	0.16787244	0.095156605	1.764170131	0.07770334	0.473513725
261.1476505	-0.024355756	0.079681812	-0.305662676	0.759861494	0.946795592
6.304530657	0.260813979	0.412824943	0.631778636	0.527531536	0.86540195
2605.011847	-0.038229136	0.141627297	-0.269927735	0.787215849	0.95288876
160.9780735	-0.553318916	0.177867601	-3.110847122	0.001865515	0.103380352
54.65144792	0.008523514	0.17265078	0.049368522	0.960625613	0.989126211
212.5774844	-0.226357617	0.098449938	-2.299215439	0.021492709	0.292942058
7.080406907	0.083912311	0.461967504	0.181641155	0.855864349	0.969966486
477.3132351	-0.019900125	0.101882491	-0.195324285	0.845139087	0.968131661
68.69980784	-0.173830493	0.204205355	-0.851253353	0.394628631	0.796902183
200.0395852	-0.388067385	0.127420727	-3.045559342	0.002322479	0.114974383
52.76225692	-0.282264103	0.172344758	-1.63778758	0.101466013	0.513342798
298.2609706	-0.010675057	0.10134831	-0.105330394	0.916113646	0.981280425
994.5919875	-0.039395616	0.081191569	-0.48521807	0.627521665	0.904512355
294.0283544	-0.068196814	0.097829038	-0.697101959	0.485738988	0.848867592

5.254782772	-1.472871943	0.968464707	-1.52083182	0.128302047	0.559060024
1647.039288	-0.103888299	0.101322104	-1.025327105	0.30520887	0.741701149
744.7339986	0.021307499	0.095973614	0.22201414	0.824302876	0.963628181
215.5784309	-0.141805796	0.084229385	-1.683566793	0.092265421	0.499801846
20355.07134	0.011900246	0.065392125	0.181982855	0.855596181	0.969966486
294.5614152	-0.022349247	0.084854263	-0.263383904	0.792254695	0.953153173
165.5655356	-0.238500528	0.222575965	-1.071546643	0.28392371	0.724842834
1453.156449	0.069470765	0.228627002	0.303860717	0.761234007	0.946826103
207.5444477	-0.387433087	0.198442894	-1.952365637	0.05089481	0.409258179
54.23125484	-0.342211119	0.142124987	-2.407818118	0.016048174	0.262799497
13.3142434	-0.299487848	0.259734623	-1.153053239	0.248888533	0.692441932
279.7803703	0.052732965	0.09040001	0.583329196	0.559671712	0.880280849
693.2887168	-0.130388391	0.105702034	-1.23354666	0.21737188	0.661500527
8.082975049	-0.174875596	0.303323928	-0.57653083	0.564256438	0.883075047
518.5217209	0.0448472	0.111229885	0.403193797	0.686805662	0.923064029
169.338476	-0.087166671	0.105860297	-0.823412305	0.410273568	0.807613405
9.557391515	-0.626686009	0.385204846	-1.626890254	0.103760408	0.517004034
5.724715309	-0.534268005	0.441633495	-1.209754266	0.2263732	0.669184942
167.1253211	-0.018503801	0.11649335	-0.158839977	0.873794951	0.973206386
67.93109901	-0.59886437	0.261075059	-2.293839836	0.0217997	0.295493739
50.45996462	-0.40337994	0.287764054	-1.401773205	0.160982982	0.600875279
277.8625022	-0.250112985	0.117536376	-2.12796237	0.033340204	0.346560178
386.2670432	-0.076776636	0.085160797	-0.901549059	0.367296462	0.781703698
9.137570798	0.025665385	0.354278627	0.072444068	0.942248515	0.98446865
5990.230993	0.009430986	0.111997687	0.084206974	0.932891874	0.984334593
7.933002891	-0.070147138	0.340945116	-0.205743195	0.836991516	0.966316856
5.153421352	-0.313975796	0.551969712	-0.568827943	0.56947291	0.8841274
572.3761137	-0.065157073	0.098740926	-0.65987909	0.509331423	0.859636085
18.61598191	-0.02173939	0.274550398	-0.079181783	0.936888034	0.984334593
17.50766066	-0.30827817	0.528726533	-0.583057878	0.559854338	0.880280849
580.6947103	-0.015603396	0.053364217	-0.292394364	0.769985117	0.948279493
6307.25271	-0.087122965	0.119852354	-0.7269191	0.467275516	0.838019653
106.672809	-0.123555248	0.12914122	-0.956745242	0.338695857	0.764007173
41.65145215	0.344745735	0.161429317	2.135583181	0.032713404	0.342207632
38.88254874	0.045718586	0.296104621	0.154400109	0.877294268	0.974191592
67.45614534	-0.14771904	0.130266051	-1.133979565	0.25680312	0.700073143
103.523353	0.042979083	0.148859856	0.288721786	0.77279429	0.948892665
8.139271803	0.201678793	0.347558056	0.58027368	0.561730073	0.881435293
20.03694317	-0.167133706	0.256095258	-0.652623198	0.513999226	0.860852744
27.75345218	-0.143430644	0.248668934	-0.576793576	0.56407891	0.883035769
23.12013094	-0.14992367	0.262533065	-0.571065859	0.567955	0.8841274
74.52519615	0.129700218	0.158102722	0.820354112	0.412014267	0.808272153
48.0516073	-0.168441974	0.162111661	-1.039049092	0.298781905	0.738522837
35.80285989	-0.218945661	0.219074578	-0.999411536	0.317595374	0.751021269
288.1472809	0.081438514	0.10067944	0.808889223	0.418578868	0.811455684
444.1440853	-0.100398692	0.131746109	-0.762061912	0.446023054	0.826302547
1481.031897	0.171771713	0.112888211	1.521608958	0.128107092	0.558663295
36.90710352	0.181265119	0.200737531	0.902995662	0.366528197	0.781525551

82.1013375	-0.101756605	0.361476447	-0.281502726	0.778324838	0.951560132
49.11629707	0.350711192	0.457103106	0.76724745	0.442934406	0.825028589
136.4969302	-0.118680828	0.087800996	-1.351702526	0.176470497	0.62005562
70.07293486	-0.160035499	0.179881064	-0.889673964	0.373640978	0.786373874
386.9321642	0.173506335	0.077537308	2.237714187	0.0252397	0.313430481
330.1988996	0.070978675	0.087785353	0.808548039	0.418775163	0.811455684
5.603377873	-0.09667766	0.494918338	-0.195340631	0.845126291	0.968131661
448.8152	-0.102869358	0.126603107	-0.812534228	0.416485155	0.810413206
120.2597505	-0.205150817	0.259585978	-0.790300072	0.429352545	0.818650305
416.296199	0.072310982	0.101485337	0.712526397	0.476138871	0.843341818
290.6453185	0.001514725	0.082042803	0.018462618	0.985269799	0.996308482
677.0789323	-0.107814618	0.078475485	-1.373863673	0.169484009	0.612177234
216.4512637	-0.094731668	0.108537428	-0.872801845	0.382771094	0.789880775
109.5025139	0.033758722	0.156687303	0.215452825	0.829414288	0.96513412
208.1810491	0.274225013	0.139188924	1.970164035	0.048819574	0.40203769
21.14703696	-0.326497576	0.32974226	-0.99015994	0.32209595	0.754735515
262.4547551	0.057902937	0.076700085	0.754926631	0.450293006	0.828449907
324.0659383	-0.00274584	0.082777095	-0.033171494	0.97353783	0.993248159
415.5821211	-0.032787851	0.070812315	-0.463024705	0.643346659	0.908494289
144.145703	-0.149123394	0.090557981	-1.646717303	0.099616166	0.511744973
69.94564866	0.134621226	0.173248022	0.777043363	0.437133187	0.822181608
2239.180933	0.054024295	0.079710243	0.677758504	0.497924825	0.855318935
45.59472879	0.071329248	0.298786231	0.238730035	0.811314925	0.959320734
1550.69774	0.059434004	0.083210818	0.714258136	0.475067574	0.842250597
38.06951603	-0.236099214	0.159403064	-1.481146022	0.138567666	0.570638112
1821.601894	0.163800103	0.137446796	1.191734603	0.233365325	0.676392617
186.9720246	-0.392713032	0.23616282	-1.662891017	0.096334253	0.505983706
17.09572771	-0.090231947	0.233732218	-0.386048392	0.69946083	0.925900484
585.1115268	-0.129375082	0.236193293	-0.54775087	0.583862977	0.889312594
5.229018777	-1.258776688	1.094460637	-1.150134272	0.250088573	0.694208707
53.31601148	0.016162145	0.14908417	0.108409533	0.913670839	0.980550322
128.4805715	-0.198034949	0.084931797	-2.331693858	0.019716802	0.281261453
194.4789786	-0.561171944	0.375766075	-1.493407686	0.135330502	0.568673186
477.0544255	-0.138272336	0.095705787	-1.444764626	0.14852401	0.585550863
65.70505139	-0.347990877	0.157802829	-2.205225847	0.02743825	0.323618312
1274.193666	-0.219004344	0.111771797	-1.959388229	0.050067338	0.4059744
21644.63453	-0.254463433	0.330180764	-0.770679157	0.440897129	0.824114068
491.316901	0.04193883	0.070398233	0.595736969	0.551350957	0.875936124
456.4745549	0.042760731	0.094423291	0.45286211	0.650648036	0.910782566
6.155681533	-0.055909669	0.462039414	-0.121006277	0.903686063	0.979381499
55.26215627	-0.458804216	0.316902445	-1.447777456	0.1476793	0.58409168
243.8327296	0.102719564	0.07067436	1.453420502	0.146107038	0.581364616
8.481063002	0.285170954	0.466275286	0.611593543	0.540806711	0.870290562
57.05695117	0.089617024	0.186339783	0.480933392	0.63056384	0.905295366
656.1595283	0.177953736	0.092104473	1.932085714	0.053348927	0.416932852
5.124202391	-0.37180727	0.498211682	-0.746283725	0.45549604	0.831550545
107.414949	0.023321835	0.268948526	0.086714866	0.93089816	0.984334593
113.7593373	0.105896059	0.13079044	0.809662076	0.41813442	0.811361647

7596.313377	0.031338705	0.111556112	0.280923248	0.778769269	0.951560132
968.4895443	0.256808571	0.106747961	2.405746849	0.016139444	0.262858884
153.388782	0.076661047	0.073139134	1.048153599	0.294567845	0.735006489
146.1661803	-0.018747016	0.094191167	-0.199031568	0.842238051	0.967531499
195.9915201	0.028574558	0.171381852	0.166730361	0.867582215	0.972138679
240.3118409	0.041369615	0.095924522	0.431272572	0.666270191	0.916129249
188.2121633	0.814661444	0.356662194	2.28412615	0.022364118	0.300121194
5.711830794	-2.589145758	0.95604095	-2.708195458	0.006765017	0.180424826
6.529052803	0.067374123	0.428262704	0.157319613	0.874992964	0.973587929
198.1434101	-0.221343745	0.167426072	-1.322038693	0.186155267	0.631843673
5.472659512	0.508518111	0.451357583	1.126641337	0.259894154	0.702516818
1726.268081	0.033883443	0.11022856	0.307392596	0.758544563	0.946310714
534.8600217	0.185441867	0.066590193	2.784822474	0.005355704	0.159160905
147.9531718	0.0147575	0.120389301	0.122581488	0.902438514	0.979381499
170.0089958	-0.003326635	0.093267734	-0.035667592	0.971547412	0.992799208
957.7737612	0.40922745	0.156974018	2.606975691	0.009134585	0.20735117
629.4938917	-0.057947854	0.075401363	-0.76852528	0.442175178	0.824946967
54.71281262	-0.178851962	0.130204442	-1.373624123	0.169558404	0.612296535
221.2181939	0.049200507	0.105472021	0.466479227	0.640872514	0.907798398
63.95557667	0.047695601	0.340059139	0.140256785	0.888457108	0.976961229
89.89226334	-0.025994162	0.188680049	-0.137768472	0.890423402	0.976961229
8.428805993	0.451850924	0.753334059	0.599801534	0.548638511	0.874685073
166.6987168	-0.221906208	0.287445573	-0.771993827	0.440118084	0.823715107
1079.068888	0.148681498	0.136992106	1.085328944	0.277775991	0.719824968
432.383785	0.197567828	0.107840645	1.832034924	0.066946218	0.454159121
5045.607495	0.189501041	0.139245132	1.360916808	0.173539981	0.617336891
9.781972639	-0.373936868	0.369180621	-1.012883254	0.311115928	0.745771331
774.7633768	0.112068824	0.07304575	1.534227851	0.124973608	0.553850105
308.4403319	0.136509152	0.239942724	0.56892391	0.569407779	0.8841274
4414.532544	0.312704474	0.291285077	1.073534137	0.283031531	0.724063726
464.5106951	-0.232852142	0.187370528	-1.242736223	0.213965051	0.659367121
31.17690787	-1.120199623	0.619239386	-1.808992853	0.070452114	0.461430171
1492.220312	0.103666713	0.071428534	1.451334731	0.146686675	0.582320297
70.45488651	-0.205223659	0.122194926	-1.679477745	0.093058973	0.500506422
80.71708476	-0.152858249	0.15492454	-0.986662597	0.323808072	0.756297513
200.3149655	-0.063604993	0.220055795	-0.2890403	0.772550538	0.948706053
430.0226759	0.16764159	0.082870461	2.02293541	0.043079807	0.380532797
213.1632812	0.041678064	0.085676347	0.486459402	0.626641483	0.904257122
136.2884468	-0.02059476	0.099296616	-0.207406463	0.835692435	0.966316856
65.0142583	-0.166337888	0.127697074	-1.302597488	0.192712216	0.638943964
33.66765945	-0.036617838	0.26409928	-0.13865179	0.889725316	0.976961229
58.15584178	-0.472769153	0.164957714	-2.866002094	0.004156915	0.144861011
52.9214834	-0.029688693	0.164002113	-0.181026284	0.856346945	0.969966486
76.40221947	0.139738829	0.120188061	1.162668138	0.244964174	0.687646444
13.17324618	-0.275807085	0.354691168	-0.777597837	0.436806137	0.822078703
112.3414019	0.25058649	0.24857894	1.008076106	0.313417928	0.747296731
29.12891125	0.200150666	0.207267046	0.965665646	0.334211515	0.761545389
10.8353862	-0.186727624	0.357636534	-0.522115628	0.601589826	0.898837114

699.5229901	0.271363457	0.117681806	2.305908328	0.021115755	0.290209293
26.28701534	-0.19129974	0.370037489	-0.516973945	0.605174347	0.90025894
610.9225556	0.082362837	0.07621838	1.080616478	0.27986775	0.721663546
29.97363277	-0.141329574	0.272616471	-0.51841906	0.604165919	0.89973814
121.5904097	-0.552919926	0.142958482	-3.86769585	0.000109869	0.019934809
14.14800505	-0.578717652	0.471149334	-1.228310454	0.219330448	0.663637842
27.72280958	-0.12537244	0.202543218	-0.618991052	0.535922254	0.869002283
368.6087468	0.158754061	0.077680353	2.043683564	0.040984818	0.374892505
605.9325162	0.036066805	0.076301858	0.472685803	0.636437357	0.907353194
28.35554289	-0.293580188	0.173189386	-1.695139606	0.090048941	0.495390485
730.0066692	0.033252238	0.09761771	0.340637351	0.733376607	0.936944635
66.70696887	-0.023250832	0.303554792	-0.076595175	0.938945598	0.984334593
292.125771	0.029703833	0.063200735	0.469991896	0.638360808	0.907684986
22.80411133	-0.241539124	0.274028902	-0.881436674	0.378081515	0.787072097
324.2808953	-0.078119443	0.106242393	-0.735294458	0.462160183	0.835473731
33.42983752	-0.218020558	0.227550467	-0.958119582	0.338002462	0.764007173
16.10878674	-1.250295186	0.862166604	-1.450178167	0.147008842	0.582502337
439.2563987	0.120642043	0.064739608	1.863496641	0.062392425	0.44188166
784.4618442	0.042527699	0.092675625	0.458887644	0.646314852	0.909300678
284.6647689	-0.015647044	0.109105323	-0.143412288	0.885964579	0.976961229
110.6261734	0.412813554	0.140671191	2.934599121	0.003339789	0.133812668
556.3489364	0.035234641	0.101387872	0.347523237	0.728198264	0.934037323
876.8536301	-0.134011066	0.193790172	-0.691526639	0.489234646	0.849736271
5.572580071	-0.11832342	0.43257785	-0.273530927	0.784445122	0.951928436
1644.428464	0.155969025	0.129200337	1.207187446	0.227359964	0.669923811
346.598749	0.28093095	0.061792866	4.546333051	5.45886E-06	0.003412027
271.9691238	0.037684741	0.13481185	0.279535822	0.779833651	0.95173434
5.770024469	0.067683399	0.482935164	0.140150074	0.888541417	0.976961229
372.5096483	0.327613161	0.300734228	1.089377696	0.275987363	0.718081375
985.9179479	0.014655068	0.07392601	0.198239667	0.842857554	0.967880334
399.6432377	0.09835259	0.062205885	1.581081757	0.113859345	0.533883941
108.7278969	0.139315269	0.101344135	1.374675207	0.169232161	0.611731367
1046.925643	0.553787056	0.511889429	1.08184898	0.279319639	0.721299597
29.77047464	0.472670133	0.492787097	0.959177172	0.337469499	0.7637398
595.4467062	0.143002321	0.060224727	2.374478531	0.017573758	0.269492791
55.95164624	0.291134961	0.187474615	1.552930039	0.12043985	0.546018577
670.9111287	0.047546886	0.070287127	0.676466482	0.49874452	0.855634722
82.09968514	0.133565701	0.110114964	1.212965941	0.225142842	0.668374464
115.4611907	-0.134075728	0.11173633	-1.199929583	0.23016669	0.673001336
450.1122843	0.229670211	0.189736581	1.210468796	0.226099057	0.668749399
31.27825704	0.120024655	0.254666889	0.47130059	0.637426094	0.907684986
639.8954163	0.066311895	0.167288794	0.396391737	0.691816061	0.924581226
511.8382716	0.064228764	0.070886697	0.906076407	0.364895412	0.779728871
135.8180077	-0.00624995	0.096615001	-0.064689234	0.948421435	0.985917197
114.2443253	0.135032672	0.208428547	0.647860734	0.517075029	0.862002027
482.0973371	0.118465916	0.069755566	1.698300556	0.08945105	0.494293655
85.82638816	0.146010491	0.118189112	1.235397139	0.216682737	0.661222104
6.493568208	0.482741236	0.532456042	0.906631155	0.364601881	0.779728871

20.05509348	0.548495978	0.235296615	2.33108316	0.019748975	0.281261453
2866.669956	0.008624968	0.127064878	0.067878457	0.945882388	0.98568891
176.5609844	0.233111825	0.156929612	1.485454667	0.137423424	0.569036171
328.4628672	0.212732676	0.116239225	1.830128131	0.067230781	0.45461399
9.325256279	-2.844932439	0.921372732	-3.087710695	0.002017047	0.10701195
13.03075799	0.134136699	0.346119715	0.387544231	0.698353346	0.925802378
915.5149205	0.099288047	0.168540457	0.589105127	0.555790754	0.878240444
333.4465266	-0.239417511	0.199173575	-1.202054599	0.229342375	0.672274238
203.3710947	0.062023695	0.101858081	0.608922676	0.542575693	0.871069897
600.9658458	0.055558344	0.05778481	0.961469696	0.336316056	0.763168826
38.34779365	0.023881113	0.223573049	0.106815704	0.914935189	0.981012001
73.64001769	0.212428249	0.367953787	0.577323177	0.56372116	0.882610599
6.188407723	-0.080508132	0.450994024	-0.178512635	0.858320399	0.969966486
281.7956345	0.024308244	0.077220815	0.314788747	0.752922056	0.943990537
13.73888219	0.153030718	0.517785051	0.295548737	0.767574727	0.94737077
77.06578065	0.102085098	0.264858464	0.385432645	0.6999169	0.926034765
1306.538597	0.080707275	0.051232714	1.57530744	0.115185497	0.536394333
2510.832273	0.060374687	0.092968404	0.649410818	0.516072874	0.862002027
54.65384317	-0.432850516	0.324933245	-1.33212136	0.182820312	0.628481684
109.2775965	-0.064281011	0.105550789	-0.609005495	0.542520796	0.871069897
24.40232581	-0.071633521	0.197232599	-0.36319311	0.716460634	0.93026757
2030.769304	-0.052069029	0.078780128	-0.660941164	0.508650046	0.859636085
86.90249938	0.146067806	0.249520073	0.585395014	0.55828214	0.879865017
165.3762883	0.001100735	0.128465694	0.008568315	0.993163558	0.998685608
67.9539792	-0.069688892	0.161505975	-0.4314942	0.666109069	0.916091953
14.60824127	-0.593234917	0.430938734	-1.376610803	0.168632609	0.610890818
258.5956009	-0.086991042	0.133035814	-0.653891909	0.513181447	0.860406963
249.7622117	0.032300224	0.092244333	0.350159438	0.726219046	0.933034834
64.29551483	0.13001385	0.227612825	0.57120617	0.567859896	0.8841274
370.3937159	-0.152566511	0.170827814	-0.893101115	0.371803026	0.785830444
36.27434179	-0.959369982	0.556905311	-1.722680613	0.084946298	0.488446247
34.35895486	0.121710778	0.241446343	0.504090376	0.61419787	0.901900555
143.6260102	0.059474078	0.113486458	0.524063214	0.600234574	0.898108141
222.5437507	-0.145518799	0.096164174	-1.51323297	0.130220492	0.562578903
12.48345874	0.155413052	0.302677034	0.513461658	0.607628425	0.900307811
202.8786978	-0.138891446	0.090925796	-1.527525209	0.126630464	0.556378986
24.84636808	-0.657474474	0.238990078	-2.751053436	0.005940395	0.168522396
794.4634553	0.009510698	0.056027999	0.169749024	0.865207518	0.971917805
291.4767141	0.036384281	0.083400621	0.436259118	0.66264874	0.915556436
117.0295144	-0.16894841	0.216122333	-0.781725828	0.43437572	0.821579957
909.0971668	-0.016430786	0.105738541	-0.155390698	0.876513317	0.973964714
32.41614053	-0.276958689	0.513016288	-0.539863344	0.589291279	0.891958928
1711.388627	0.067920066	0.098245535	0.691329793	0.489358313	0.849736271
1720.275109	0.136974673	0.157229877	0.871174585	0.383658835	0.790043276
43.28635781	-0.129264029	0.172596598	-0.748937296	0.453895	0.831004392
250.462571	-0.123988729	0.093998792	-1.319045983	0.18715374	0.633489517
6489.41785	-0.057295169	0.227689654	-0.25163712	0.801321564	0.956046055
403.0390338	-0.022175534	0.113584351	-0.195234061	0.845209715	0.968131661

69.17222564	-0.499735751	0.223425985	-2.236694856	0.02530629	0.313430481
58.36379022	-0.017871788	0.189700099	-0.094210745	0.924941749	0.982590996
246.36223	0.164647455	0.076021582	2.165798843	0.030326562	0.332880971
26.08366437	0.455802205	0.256818524	1.774802685	0.075930473	0.471783446
5.486279965	0.615269504	0.492300036	1.249785618	0.211377871	0.656444287
18.68673471	-0.115252808	0.422939291	-0.272504377	0.785234226	0.952464208
17.53913228	-0.138902067	0.361524188	-0.384212376	0.700821047	0.926034765
6766.950251	0.213545201	0.1148165	1.859882516	0.062902149	0.442595858
691.4617648	0.068496135	0.053688332	1.275810454	0.202022531	0.645481631
28274.44415	-0.043124502	0.115559506	-0.373180049	0.709014444	0.928992812
830.824726	0.00615624	0.05602595	0.109881942	0.912503004	0.980530316
1093.242308	0.056857264	0.053775794	1.057302172	0.290373702	0.731967225
9.789426732	-1.932175068	0.712376175	-2.712296024	0.006681889	0.179562132
240.6772706	-0.155985945	0.111687056	-1.396634047	0.162523646	0.601684318
33.41832712	-0.279090928	0.380272579	-0.73392336	0.462995449	0.835927584
7.055671337	-2.261536144	1.262954572	-1.790671015	0.073346104	0.465952372
127.9674248	0.274557182	0.221455384	1.239785535	0.21505473	0.660219973
190.2591409	0.013801501	0.108690742	0.126979546	0.898956587	0.978996707
225.2020288	-0.03504092	0.095196302	-0.368091185	0.712805244	0.929680431
8701.025609	-0.022618548	0.104282767	-0.216896321	0.828289145	0.964626741
2631.728657	-0.157240339	0.181933206	-0.864275097	0.387436787	0.792728629
1373.489094	0.128134057	0.143130716	0.895224032	0.370667341	0.784587155
12.426573	0.349774393	0.34754133	1.006425315	0.314211021	0.748102226
282.1199676	-0.048220979	0.086549876	-0.557146715	0.577427193	0.886969751
325.2070761	0.055602715	0.058958714	0.94307883	0.345640583	0.767958555
1152.950705	-0.015793949	0.061573722	-0.256504696	0.797561155	0.954840537
701.4768602	-0.032399506	0.110992082	-0.291908267	0.770356763	0.948321877
83.48927666	0.073494864	0.304916936	0.241032409	0.809529998	0.958681749
300.6513066	0.012061337	0.125220601	0.09632071	0.923265863	0.982590996
849.6334161	-0.284608409	0.172592185	-1.649022575	0.09914301	0.51147708
85.26758172	0.199639164	0.123538071	1.616013282	0.106091442	0.521361916
2896.653284	0.034579356	0.13333747	0.259337123	0.795375139	0.954063436
406.1189383	0.065678548	0.057294489	1.146332732	0.251657504	0.694735013
95.59232799	-0.495700996	0.280693313	-1.765987905	0.077397878	0.472757172
1133.951704	0.175984777	0.103183175	1.705556915	0.088090607	0.492827855
4.692049632	-0.586781735	0.463833571	-1.265069568	0.205846391	0.650916103
493.0077888	-0.03715529	0.099797045	-0.372308519	0.709663157	0.92902743
1318.219837	0.02814819	0.067989942	0.414005212	0.67887029	0.921716417
61.44508526	-0.29094593	0.190650609	-1.526068711	0.126992753	0.556444786
5.778980991	0.271028822	0.424550857	0.638389529	0.523220145	0.863484457
172.2662113	0.025894729	0.11275929	0.229646082	0.818366795	0.961844156
98.75267162	0.218181195	0.113300701	1.925682661	0.05414401	0.42005114
71.57019908	0.060869668	0.342104234	0.177927259	0.858780103	0.969966486
44.17181588	-0.341429077	0.177778087	-1.920535224	0.054790328	0.422619301
348.4567909	-0.029588686	0.064892604	-0.455963927	0.648415918	0.910701841
17.40355249	-0.053991159	0.257067036	-0.210027547	0.833646174	0.966316856
99.44599184	-0.076948663	0.145579534	-0.528567863	0.597105259	0.896930388
46.82314858	-0.565776242	0.28965135	-1.953300899	0.050783951	0.409258179

880.8799883	-0.015061369	0.127662044	-0.117978444	0.906084737	0.979768942
24.65764563	-0.190065948	0.207248876	-0.917090365	0.35909529	0.777095745
400.6600443	-0.142544928	0.105156813	-1.355546294	0.17524357	0.619005807
1180.005701	-0.097633287	0.099157359	-0.984629763	0.324805965	0.756297513
7.19625934	-0.521958789	0.471134194	-1.107877109	0.267914893	0.710235608
481.6424825	0.11038351	0.169205069	0.652365271	0.514165563	0.8609365
1379.189381	0.156345393	0.117204411	1.333954848	0.182218654	0.627539984
498.4807542	0.040054333	0.056347391	0.71084627	0.477179504	0.843597766
370.1290056	0.022995593	0.071034389	0.323724793	0.746146399	0.940513992
245.2384088	-0.044432705	0.094981182	-0.467805352	0.639923793	0.907798398
28.77494784	-0.021057849	0.199683471	-0.105456144	0.916013868	0.981266182
543.3150444	-0.204481006	0.127005003	-1.610023232	0.107392785	0.524456614
297.4614835	-0.154049544	0.070919546	-2.172173302	0.029842594	0.330883948
266.2122822	0.182529564	0.062211665	2.934008679	0.003346149	0.133812668
28.70261343	0.061479094	0.225859799	0.272200253	0.785468048	0.952523189
42.18620255	-0.441673475	0.320815001	-1.376723262	0.168597824	0.610890818
88.32520641	-0.03455852	0.261983048	-0.131911282	0.895054465	0.978783502
944.4215101	-0.142553219	0.099189698	-1.437177662	0.150667519	0.589528489
426.9716686	0.191636727	0.10685923	1.793356812	0.072915889	0.465015503
524.7264352	0.107746188	0.060834552	1.771134736	0.076538299	0.471783446
29.75249406	-0.364756402	0.244493705	-1.491884633	0.135729392	0.568673186
184.5291555	-0.365485823	0.371590477	-0.983571555	0.325326217	0.756297513
402.642354	0.337338556	0.235844982	1.430340185	0.152619407	0.589990195
314.5174742	0.324270024	0.246520436	1.315388002	0.188379536	0.634590162
405.2582542	-0.116252314	0.064998254	-1.78854518	0.073688093	0.467122552
4.672573556	-1.23266312	0.929796578	-1.325734197	0.184927756	0.630643427
428.1821015	-0.152765149	0.165172795	-0.924880812	0.355027908	0.773833221
19.10095215	-1.615004387	0.466576811	-3.46139017	0.000537393	0.052678783
83.30322299	-0.008618588	0.128360591	-0.067143565	0.946467412	0.985728152
127.5792272	0.056680967	0.098923848	0.572975757	0.566661108	0.883661792
368.3342024	-0.022194431	0.068372546	-0.324610282	0.745476044	0.940455922
69.13078762	-0.149324464	0.156539107	-0.953911565	0.340128409	0.76465745
35.53689098	-0.121512066	0.205891156	-0.590176232	0.555072505	0.877811471
8.898520989	-0.617745621	0.45600229	-1.354698505	0.175513634	0.619005807
1608.818673	-0.016268951	0.061112261	-0.266214189	0.790074256	0.953026618
216.7473856	0.018395692	0.08476149	0.217028894	0.828185827	0.964626741
2134.230335	0.001834104	0.096644866	0.018977775	0.984858835	0.996051197
76.93373605	0.050634495	0.138941252	0.364430973	0.715536211	0.930241908
1506.945057	0.020228762	0.076961818	0.262841529	0.792672725	0.953153173
25.60988833	-0.779133363	0.400264072	-1.946548336	0.051588908	0.410734915
203.9231949	-0.000592979	0.082202685	-0.007213617	0.994244416	0.998877139
826.7268129	-0.007810277	0.074812076	-0.10439862	0.916853018	0.981290994
127.9309148	-0.299288863	0.196876125	-1.520188709	0.128463554	0.559434401
1030.313944	0.178642632	0.073865578	2.418482821	0.015585381	0.259816378
240.8518063	-0.062721442	0.108537468	-0.57787825	0.563346321	0.882294272
35.1828339	0.021770327	0.224956302	0.096775803	0.92290444	0.982590996
1154.487308	0.022248672	0.109688063	0.202835852	0.839263335	0.966440785
232.0955163	-0.118193759	0.079352216	-1.489482771	0.136360287	0.569036171

812.5993193	0.190220158	0.1322168	1.438698853	0.150235865	0.588460245
426.9874532	0.026512092	0.148380125	0.178676839	0.858191457	0.969966486
509.4437279	-0.119032177	0.084630646	-1.406490238	0.159578601	0.600573799
897.3738792	-0.035788214	0.119068091	-0.30056931	0.763742937	0.946826103
380.3419316	0.103449325	0.121017763	0.854827607	0.392646592	0.795831146
211.3405505	-0.057285416	0.098982267	-0.578744224	0.562761771	0.881830918
13490.56229	-0.066757846	0.165585881	-0.403161462	0.686829449	0.923064029
48.63435768	-0.119560335	0.227654462	-0.525183357	0.599455733	0.898091474
1042.546187	0.635340087	0.293758667	2.162796057	0.030556869	0.333310814
251.0978905	-0.124299394	0.087395455	-1.42226383	0.154949675	0.592842063
90.66140852	0.74593452	0.316293809	2.358359535	0.018355905	0.272290126
550.5839761	0.006612892	0.059749164	0.110677565	0.911872037	0.980530316
2488.895792	0.289746278	0.135031306	2.145771129	0.031891243	0.339363178
1368.774728	0.138383605	0.085621747	1.616220293	0.106046693	0.521361916
9.758766981	0.273258666	0.297966263	0.917079213	0.359101133	0.777095745
20.40168276	0.54431765	0.28157185	1.933139448	0.053219022	0.416932852
278.1561285	0.062100007	0.103961382	0.597337257	0.550282235	0.875690724
533.3376365	0.050189585	0.069898118	0.718039148	0.472733149	0.840932108
12922.727	-0.010483821	0.134821678	-0.077760649	0.938018449	0.984334593
766.2275591	0.06540975	0.083948069	0.779169205	0.435880048	0.822078703
724.9912605	0.028100017	0.178765321	0.15718942	0.875095566	0.973587929
354.1403745	0.002851324	0.087970688	0.032412201	0.974143332	0.993248159
1073.712072	0.099436583	0.062648824	1.587205881	0.112466023	0.533148601
37.08674077	-0.474268367	0.390022445	-1.216002754	0.223983873	0.667854242
11.15219619	0.030429525	0.294017569	0.103495601	0.917569641	0.981376661
219.4024069	0.018123615	0.087797678	0.20642477	0.836459124	0.966316856
36.60600181	0.098456894	0.156647426	0.628525453	0.529659778	0.866063379
557.8854409	-0.112821999	0.097168749	-1.161093451	0.245603898	0.688707558
29.10586294	-0.385890495	0.283160618	-1.362797195	0.172946436	0.616559041
55.57811058	-0.975618709	0.345359432	-2.824937203	0.004728988	0.15195001
274.1909101	1.313518697	0.423654785	3.100445798	0.001932296	0.104224472
504.5940609	0.014085266	0.146370243	0.096230393	0.923337593	0.982590996
158.4756306	0.035568915	0.087151722	0.408126362	0.683180907	0.922776218
6.60130298	0.299530218	0.613187158	0.488480905	0.625209248	0.903303786
21.5043552	-2.526977602	1.413947221	-1.787179581	0.073908468	0.46791837
7.591583016	-0.350526542	0.375350733	-0.933864013	0.350374044	0.770421231
76.64470112	-0.384490653	0.438576191	-0.876679267	0.380660871	0.78844599
11.21913746	-1.219746136	0.647868571	-1.882706138	0.059740203	0.435320275
745.1555291	0.232364099	0.211669632	1.097767767	0.27230591	0.714971121
570.7380173	0.12369609	0.101081488	1.223726449	0.221055439	0.664764193
288.8131006	0.195201527	0.1345159	1.451140922	0.146740624	0.582320297
518.2062981	0.540932893	0.279311308	1.936666642	0.052786106	0.415276565
120.2342575	0.292456593	0.47610775	0.614265558	0.539039858	0.869675597
33.81778811	0.69152956	0.316070446	2.187896935	0.028677111	0.327277249
1825.914521	0.103990647	0.094711806	1.09796921	0.272217934	0.714934326
351.2747223	0.20295788	0.097316396	2.085546623	0.037019711	0.361102242
91.18504067	-0.080012398	0.148134647	-0.54013291	0.589105376	0.891958928
275.9718148	-0.07005415	0.11689667	-0.599282687	0.548984391	0.874898106

55.49386892	-0.281201487	0.192987236	-1.457098889	0.145089085	0.578290974
110.0957692	0.229402436	0.141291342	1.623612833	0.104458465	0.518555962
125.3611609	-0.134478713	0.098402407	-1.366620163	0.171744394	0.616109027
924.2461102	-0.024678759	0.088892912	-0.277623477	0.781301408	0.95173434
1223.658537	0.166032267	0.051421084	3.228875298	0.001242781	0.084772537
840.5510168	-0.014063591	0.10052966	-0.139894938	0.888743	0.976961229
22.83042618	-0.51467502	0.292848453	-1.757479045	0.078836191	0.475716793
335.3868056	0.122822453	0.083709962	1.467238187	0.142311272	0.576962874
447.9938669	0.111320221	0.051100282	2.178465864	0.029371371	0.330351122
1107.95013	-0.062523344	0.111309136	-0.561709004	0.574314299	0.886074656
213.2911926	-0.037872101	0.0948131	-0.399439535	0.689569368	0.92405254
457.8675186	0.126935299	0.075995451	1.670301267	0.094859773	0.502934579
6610.604258	0.191212611	0.159634927	1.197811877	0.230990263	0.673187215
11.09381766	0.210052831	0.307800983	0.682430669	0.494966668	0.853605572
4880.851002	-0.228421313	0.199401486	-1.145534656	0.251987748	0.694735013
52.16749433	0.199439708	0.153310247	1.300889613	0.193296242	0.639147095
391.6359404	0.117424592	0.071244921	1.648181924	0.099315345	0.51147708
34.95898942	-0.024966946	0.204543222	-0.122061958	0.90284995	0.979381499
83.48203793	0.257827593	0.303231768	0.850265771	0.395177342	0.797298333
307.7047947	-0.056315012	0.138025299	-0.408005002	0.683270002	0.922776218
320.8386142	-0.008814758	0.096773406	-0.091086574	0.927423801	0.983502443
999.0843706	-0.005913566	0.060431534	-0.09785563	0.922046933	0.982590996
49.82088758	-0.22992313	0.164427437	-1.398325814	0.16201525	0.601439633
634.9334992	0.041939344	0.051236798	0.818539527	0.413049181	0.808272153
2229.536925	0.122668091	0.061018678	2.010336736	0.044395561	0.386697566
140.2987774	0.069568481	0.10905683	0.637910351	0.523532039	0.863484457
329.7565234	0.03781984	0.080673646	0.468800434	0.639212289	0.907684986
439.1907276	-0.04511914	0.126179189	-0.357579884	0.720657735	0.931135539
242.1274689	0.11714751	0.076229815	1.536767604	0.124350229	0.552139359
70.62595731	0.452534663	0.145199346	3.116643944	0.001829224	0.102581855
118.0065559	0.115407813	0.103944408	1.110284	0.266876666	0.709341177
6.472147855	-2.088018115	0.978941369	-2.132934802	0.032930075	0.34350315
38.08575581	0.149169966	0.160729011	0.928083641	0.353364185	0.772599211
60.48282012	-0.065995246	0.141205959	-0.467368702	0.640236111	0.907798398
75.79539019	0.125227795	0.248011074	0.504928239	0.613609239	0.901444946
461.3169327	0.331172256	0.17701389	1.870882874	0.061361317	0.438354353
243.6071234	0.249395345	0.254922996	0.978316386	0.327917868	0.758221793
200.1216009	-0.039095587	0.077345853	-0.50546456	0.613232584	0.901444946
489.7818045	0.105731517	0.068952504	1.533396328	0.125178233	0.55425911
106.6221831	0.22907291	0.131656591	1.739927397	0.081871767	0.483200119
131.5552069	-0.079285671	0.223711539	-0.354410288	0.723031425	0.931432762
115.764071	0.083655097	0.138389545	0.60449001	0.545517909	0.872896521
326.3150074	-0.068668279	0.089027367	-0.771316515	0.440519345	0.823778083
678.5422109	-0.028190247	0.151147123	-0.186508661	0.852045888	0.969560739
6841.814708	-0.050701779	0.12269781	-0.41322481	0.679441913	0.922072151
46.24005427	0.272639451	0.163395197	1.668589133	0.095198834	0.503423693
11.31105979	0.17154742	0.280817671	0.610885416	0.54127544	0.870670743
6079.716744	-0.125746974	0.132263835	-0.950728327	0.341742305	0.765195212

543.6682384	-0.020758562	0.167438275	-0.123977399	0.90133317	0.979381499
6063.310702	-0.025585292	0.134246051	-0.190585065	0.848850692	0.969123636
9.451113826	-0.439080981	0.303836579	-1.445122185	0.148423568	0.585389198
366.9001944	0.011247155	0.063733448	0.176471786	0.859923312	0.969966486
134.1481082	0.202396457	0.123623059	1.637206349	0.101587361	0.513342798
116.6530626	0.160968803	0.163528019	0.984349986	0.32494346	0.756297513
88.67653582	0.639883091	0.398188012	1.60698733	0.108057148	0.525102396
962.1605529	-0.064288054	0.083914771	-0.766111298	0.443610081	0.825536387
12.45213797	-0.285219487	0.363903461	-0.783777889	0.43317045	0.820529587
2966.608597	0.071452938	0.1244314	0.574235586	0.565808392	0.883265494
60.04285672	0.123863089	0.208260173	0.594751683	0.552009469	0.876603606
347.0624136	-0.134293213	0.107761544	-1.246207211	0.212688332	0.657796217
50.54454711	-0.341991261	0.175469788	-1.949003674	0.051294986	0.410734915
219.1574629	0.061647497	0.067974148	0.906925623	0.36444613	0.779728871
190.878374	0.089176105	0.360228269	0.247554433	0.804479171	0.957233889
149.5376003	0.260744991	0.099682466	2.615755815	0.00890302	0.204313961
7.810072795	-0.046763792	0.384011119	-0.121777183	0.903075485	0.979381499
157.9857608	-0.162982461	0.223229222	-0.730112571	0.465321378	0.837170481
856.0858181	0.12993693	0.05298604	2.452286086	0.014195175	0.251563345
13.62185649	-0.145844066	0.35079265	-0.415755764	0.677588731	0.921393988
791.9000886	0.085455493	0.088945462	0.960762812	0.336671441	0.763390114
7.439205568	-0.521010835	0.409833727	-1.271273693	0.203631298	0.647823067
5.231270749	-0.295825608	0.434950137	-0.680136831	0.496417826	0.854371062
30.94579253	0.386970433	0.496739936	0.779020177	0.435967829	0.822078703
143.9145251	0.125281564	0.378942505	0.330608369	0.740940324	0.939831839
36.37053376	1.03812287	0.408244652	2.542893986	0.010993858	0.226961068
557.2434097	-0.004968794	0.19196416	-0.025883966	0.979349889	0.994602886
1250.466198	-0.110592644	0.231963374	-0.476767701	0.633527548	0.906724287
395.9829261	0.078374169	0.071026784	1.103445269	0.269833847	0.712556401
12.30579226	-2.241399529	0.680610117	-3.293220999	0.000990466	0.075960346
758.5259943	-0.028801338	0.104793747	-0.274838325	0.783440453	0.95173434
7.74111167	-2.106573887	1.004846779	-2.096413037	0.036045567	0.358452687
108.8913547	-0.07418129	0.101970619	-0.727477094	0.466933743	0.837677571
10737.60839	0.033873157	0.135200734	0.25053974	0.802169976	0.956478169
6.340646322	0.32304962	0.511482818	0.631594275	0.527652028	0.865503693
7.891439921	1.200468016	0.417217067	2.877322411	0.004010656	0.144354659
11.92564165	-0.601812285	0.397951905	-1.512273914	0.130464194	0.563176146
400.9422452	-0.017243346	0.083900806	-0.205520625	0.837165386	0.966384462
21.96412938	-0.438310413	0.260864964	-1.680219549	0.092914607	0.500506422
253.0855072	-0.0049033	0.078670534	-0.062327022	0.95030241	0.98625342
474.0255955	0.070506843	0.10872367	0.648495794	0.51666433	0.862002027
13.45704198	-0.661708252	0.434707644	-1.522191434	0.127961121	0.558465847
133.1269453	0.016294313	0.090821721	0.179409869	0.857615883	0.969966486
284.9970835	-0.0727984	0.176865098	-0.411604103	0.680629624	0.922420774
431.4887225	-0.075321911	0.211282113	-0.356499232	0.721466727	0.931135539
9.301610078	-0.15421583	0.362454945	-0.425475861	0.670489815	0.918943541
772.2663002	0.12285698	0.062486156	1.966147187	0.049281611	0.403465933
72.98323483	-0.046186851	0.114417007	-0.403671201	0.686454519	0.923064029

52.29673265	-0.151748428	0.220941222	-0.686827142	0.492191664	0.851383417
179.6087006	0.282140627	0.344684671	0.818547069	0.413044877	0.808272153
321.2037313	-0.273008945	0.553568906	-0.493179697	0.621885622	0.902358335
7.63441589	-0.290047858	0.734076839	-0.395119206	0.692754915	0.924581226
119.2096899	0.102150219	0.095980034	1.06428613	0.287199099	0.728056357
36.08447716	0.471184767	0.385141053	1.223408317	0.221175514	0.664764193
791.0919584	-0.00450263	0.088916203	-0.05063903	0.959613161	0.988599249
2734.866418	0.149828478	0.053226773	2.814908195	0.004879117	0.154120605
7.127813296	0.164347005	0.405122174	0.405672698	0.684983105	0.922907843
321.2165152	0.107371275	0.074851148	1.434463978	0.151439902	0.589528489
259.3030485	-0.257934347	0.169112129	-1.525226777	0.127202544	0.556444786
183.7955269	0.108487319	0.111965918	0.968931629	0.332579303	0.760742564
11.6102748	0.637416997	0.485472007	1.312984039	0.189188327	0.63500354
19.16801147	-0.290426133	0.387032989	-0.75039116	0.453019154	0.830105164
59.40037813	-0.086096192	0.172241666	-0.499856937	0.617175816	0.901932427
23.50159966	0.361386593	0.221353074	1.63262514	0.10254786	0.514997052
6.076925709	0.089965207	0.564679406	0.159320858	0.873416089	0.97300406
12.87278893	1.114649135	0.770918241	1.445872047	0.148213093	0.585106808
11.83012455	1.104738572	0.613712248	1.800092104	0.071846096	0.463154629
15.42198367	1.293556679	0.518578841	2.494426257	0.012616094	0.240861519
46.84946646	-0.155260915	0.346408255	-0.448202122	0.654007331	0.911976263
305.5178458	0.165160497	0.102120079	1.617316587	0.105809961	0.521197683
503.704023	-0.036956177	0.052925015	-0.698274287	0.485005676	0.848765348
137.6060955	0.324731914	0.18833592	1.724216575	0.084668753	0.487842306
323.4736691	0.110652434	0.093587516	1.182341818	0.237070099	0.679753163
216.2729776	-0.038647431	0.197554276	-0.195629431	0.844900223	0.968131661
866.8315003	0.038876377	0.086669769	0.448557527	0.653750879	0.911945899
7.122115366	0.121230803	0.449298796	0.269822231	0.787297019	0.95288876
610.3749564	0.130484293	0.070146935	1.860156719	0.062863356	0.442595858
754.6328075	-0.128901659	0.067479521	-1.910233767	0.056103121	0.426020528
67.59086751	-0.28433971	0.157909876	-1.800645515	0.07175877	0.463154629
4809.87932	0.112549343	0.106994654	1.051915575	0.292838285	0.733939814
505.9985962	-0.182718095	0.103286224	-1.769046144	0.076886174	0.472050486
58.98780176	-0.010212007	0.523846164	-0.019494286	0.984446795	0.995884463
255.8825546	0.0957399	0.104239425	0.918461513	0.358377299	0.77666298
1665.065592	0.116231228	0.094658989	1.227894244	0.219486671	0.663637842
1009.886872	0.183367214	0.080898296	2.266638776	0.023412301	0.304792334
343.119242	0.201552439	0.09294163	2.168591615	0.030113702	0.332282647
411.6915716	-0.576129626	0.204902889	-2.81172037	0.004927732	0.154506093
94.40583198	0.285579707	0.128139628	2.228660333	0.025836513	0.314500059
44.18636421	-0.098795502	0.176303668	-0.560371223	0.575226256	0.886202779
27.83677052	0.068565018	0.277984298	0.246650687	0.805178574	0.957233889
82.8856582	-0.031396952	0.142108942	-0.220935796	0.825142425	0.963909899
25.88114338	-0.03317646	0.207078574	-0.16021194	0.872714124	0.972881041
62.12610088	0.151707864	0.149307668	1.016075498	0.309593436	0.745259723
460.3299381	0.088318468	0.091168804	0.968735626	0.332677112	0.760742564
47.13853185	-0.301379609	0.208631615	-1.444553881	0.148583235	0.585550863
221.304957	-0.006651875	0.0894101	-0.074397359	0.94069421	0.984334593

1087.759599	0.101134849	0.069287793	1.459634445	0.144390567	0.578069441
161.4744649	-0.075951771	0.115815967	-0.655797064	0.511954706	0.860200661
157.9436372	-0.129498409	0.095295856	-1.358909126	0.174175387	0.618034305
52.30596027	-0.175471675	0.187227396	-0.937211532	0.348649759	0.768780165
17.37956975	-0.012277528	0.267580548	-0.045883483	0.963403119	0.989954388
183.8078033	-0.008854293	0.108278019	-0.08177369	0.934826679	0.984334593
924.4577626	0.343215251	0.118211226	2.903406577	0.003691271	0.138675611
4142.13323	0.058763741	0.125961404	0.466521798	0.640842049	0.907798398
274.9484938	0.346376137	0.158057794	2.191452426	0.028419069	0.326184223
284.9550259	0.174632504	0.106890014	1.633758825	0.102309501	0.514571946
4021.967898	0.081473626	0.126396781	0.64458624	0.519195354	0.862777252
42.27292567	-0.07477637	0.164121376	-0.455616277	0.648665936	0.910701841
1322.048133	0.116944491	0.095527615	1.224195651	0.220878429	0.664764193
5.917583571	0.535481524	0.44000811	1.216981034	0.223611432	0.667854242
4.711135341	-0.228507984	0.493297128	-0.463225855	0.643202486	0.908494289
1918.7066	0.085666072	0.082600669	1.037111125	0.299684071	0.738923503
14.35078251	0.217204986	0.290992558	0.746427977	0.455408924	0.831550545
14352.7256	-0.066045624	0.127475564	-0.518104193	0.604385575	0.89980354
734.0420068	0.137132765	0.067331375	2.036684451	0.041681674	0.375287186
1585.241039	-0.055410847	0.066478803	-0.833511499	0.404556315	0.8031996
45.32878376	0.151870106	0.142328423	1.067039892	0.285953823	0.726889933
2083.561973	0.060793268	0.063125632	0.963052033	0.335521412	0.762456424
6.124961143	0.17446019	0.400117755	0.436022116	0.662820684	0.915638988
2818.869568	-0.091589735	0.126151382	-0.726030373	0.467820151	0.838388803
17.82685494	-0.505031229	0.433589154	-1.164769054	0.244112488	0.686342349
9.445395874	-1.609397438	0.742413402	-2.167791466	0.030174556	0.332282647
331.6923082	0.140434274	0.195458786	0.718485351	0.472458077	0.84082619
119.955626	0.190661882	0.102747577	1.855633857	0.063505764	0.443016545
190.2822965	-0.082057914	0.082574221	-0.993747358	0.32034588	0.752619929
175.9170648	-0.215100678	0.153012974	-1.405767572	0.159793154	0.600578247
15.0339464	-0.192578492	0.291356344	-0.660972365	0.508630036	0.859636085
199.9072945	-0.123403398	0.093869182	-1.314631657	0.188633725	0.634590162
16.87956296	-0.152875275	0.276759397	-0.552376095	0.580690705	0.887861153
68.60725036	-0.632975486	0.217533624	-2.909782282	0.003616806	0.137979288
55.21548388	-0.189541923	0.170783545	-1.109837155	0.267069206	0.709341177
298.489429	0.23733723	0.18536478	1.280379312	0.200411766	0.645045462
737.3677275	0.202558733	0.134299023	1.508266616	0.131486304	0.564647789
1576.000155	-0.007668649	0.126649942	-0.060549958	0.951717628	0.98625342
370.2824553	0.080290795	0.094706705	0.847783638	0.396558477	0.79829438
357.4395571	0.06205705	0.118524935	0.523578018	0.600572074	0.898289446
75.32094799	-0.199722885	0.175188534	-1.140045414	0.254267382	0.696717102
684.3994011	0.177471051	0.076769064	2.311752167	0.020791345	0.287889135
2624.389515	0.212404279	0.191897336	1.106864134	0.268352674	0.71058252
360.5451997	-0.079998168	0.105483282	-0.758396656	0.448213559	0.82753699
2758.008801	-0.185123022	0.073108196	-2.532178769	0.011335618	0.231434235
50.69739072	-0.114722979	0.209884922	-0.546599429	0.584653962	0.889519618
350.7999786	-0.270175476	0.186166431	-1.451257751	0.146708101	0.582320297
138.6431203	-0.593283694	0.43319066	-1.369567143	0.170822062	0.614754125

168.3343528	-0.052791139	0.205788036	-0.256531622	0.797540366	0.954840537
9.723085461	-2.211202122	0.787267849	-2.808703701	0.00497414	0.155144013
3476.584106	-0.740137089	0.33789606	-2.190428291	0.02849319	0.326184223
158.5153928	-0.108895965	0.120854325	-0.901051452	0.367560964	0.781703698
1398.895774	0.013352599	0.080560211	0.165746826	0.868356194	0.972204994
13.97645256	0.034912484	0.318837479	0.109499311	0.912806468	0.980530316
561.2236368	-0.002509306	0.096134699	-0.026101984	0.979175994	0.994602886
215.6124605	-0.095559804	0.076529678	-1.248663355	0.211788229	0.656521334
117.9473644	-0.00241076	0.214632023	-0.01123206	0.991038301	0.998213079
12.55660752	-0.126725602	0.257446134	-0.492241229	0.622548821	0.902441895
384.7768669	-0.084047679	0.100613091	-0.835355303	0.403517688	0.802589199
3017.142677	0.040097339	0.092201792	0.434886764	0.663644622	0.916049829
357.0979143	-0.029891128	0.112258377	-0.266270806	0.790030655	0.953026618
338.3572491	0.007362028	0.074777212	0.098452822	0.921572732	0.982590996
139.2886335	-0.004883781	0.10802857	-0.045208236	0.963941329	0.990149113
6.052016308	1.124137427	0.519261192	2.164878572	0.030396986	0.332880971
122.3370803	0.388006469	0.214460757	1.809218968	0.070416992	0.461430171
261.8931721	0.484815976	0.127781709	3.794095268	0.000148183	0.024103312
85.54051737	-0.053268304	0.159739364	-0.33347011	0.738779448	0.939226436
355.5074355	0.096214664	0.100352286	0.958769029	0.337675115	0.7637398
352.0018682	0.067144582	0.13347804	0.503038421	0.614937259	0.901900555
6.28839951	-1.486252197	1.395005375	-1.065409656	0.286690589	0.727140867
39.31944895	0.030307859	0.173749342	0.174434379	0.861524098	0.970324122
104.279242	-0.008310283	0.127662247	-0.065095851	0.948097684	0.985917197
5.379314183	-2.200363164	2.499620567	-0.880278868	0.378708257	0.787072097
1174.258441	-0.041310331	0.043679715	-0.945755496	0.34427331	0.767389167
8.303398102	-1.57250365	1.021817368	-1.538928286	0.123821805	0.55188031
7.399828031	-0.725684057	0.404541435	-1.793843583	0.072838138	0.465015503
198.0310192	-0.404151891	0.253249503	-1.595864498	0.110519049	0.529761324
15.27452807	-0.135956699	0.242504333	-0.560636166	0.575045591	0.886202779
188.5619472	0.077660807	0.06772512	1.146706075	0.251503119	0.694735013
94112.69071	-0.035902415	0.136853886	-0.262341214	0.79305839	0.953153173
140.3290859	-0.019930545	0.170913722	-0.116611732	0.90716774	0.979768942
4.739659938	0.463809645	0.444111137	1.044354907	0.296321218	0.73593065
260.4143695	-0.080012009	0.115349457	-0.693648772	0.487902499	0.849539207
200.621729	-0.098086886	0.082106539	-1.194629403	0.232231856	0.674945206
292.9942928	0.064634826	0.118645865	0.544770997	0.585911028	0.890690669
44.71251467	-0.280463949	0.191385927	-1.465436639	0.142801827	0.57754946
2385.780726	0.069235518	0.045577208	1.519082057	0.128741843	0.559880723
202.205409	-0.086806087	0.130725355	-0.664034053	0.506668517	0.859011504
314.5921997	-0.019215612	0.09891001	-0.194273687	0.845961588	0.968214909
274.3826413	0.184945649	0.101248467	1.82665135	0.067752207	0.455435138
118.1715706	0.011938315	0.157641012	0.075731024	0.939633093	0.984334593
338.9598182	0.020966908	0.074008602	0.283303669	0.776944065	0.950754469
21.57255617	0.104492259	0.216338208	0.483004181	0.629092768	0.904638489
7.500059065	0.135166989	0.622737457	0.217052929	0.828167097	0.964626741
1747.579761	-0.004991196	0.053068635	-0.094051711	0.925068079	0.982590996
15.30618098	-0.162789527	0.282757889	-0.575720547	0.564804085	0.883265494

5.435780941	0.323432953	0.425666764	0.759826653	0.447358209	0.827089297
845.4907084	-0.011445099	0.096810574	-0.118221583	0.905892089	0.979768942
18.10833821	0.481172309	0.277367831	1.734780515	0.082779689	0.485197868
564.5742979	0.108342014	0.091824445	1.179882042	0.238047133	0.679832851
145.6311835	0.038702262	0.112189765	0.344971419	0.730115858	0.935281257
200.6475674	-0.028207931	0.087903127	-0.320897919	0.748287751	0.941542093
113.3066127	-0.753533729	0.438135575	-1.719864287	0.085457114	0.488858859
22.70887553	-0.641010029	0.395846928	-1.619338142	0.105374528	0.520612072
603.4051887	-0.079498229	0.092748504	-0.857137588	0.39136885	0.794437976
1098.02949	-0.584219263	0.215156335	-2.715324481	0.006621086	0.178841823
1843.780811	0.01875854	0.116322072	0.161263807	0.871885628	0.972881041
30.63589244	0.162199777	0.206061751	0.787141607	0.431198975	0.818841807
284.9036945	-0.161552232	0.080038124	-2.018441004	0.043545353	0.38184734
1333.860483	-0.09596294	0.133824264	-0.71708177	0.473323643	0.841511024
231.0290851	-0.048834084	0.079612894	-0.613394157	0.539615749	0.869675597
4114.168723	0.02375809	0.098814069	0.240432264	0.809995168	0.958722103
303.1418441	0.026103026	0.07820893	0.33376017	0.738560541	0.939226436
1471.679052	0.045847711	0.067480456	0.679422071	0.496870469	0.854509261
1090.32547	-0.00332434	0.166179262	-0.020004541	0.98403975	0.995803025
113.9657374	-0.132695199	0.10475928	-1.266667726	0.205274125	0.65021776
787.3827771	0.209134769	0.067309806	3.107047561	0.00188966	0.103380352
129.2777574	-0.093117134	0.257071515	-0.362222686	0.717185628	0.930525309
257.2035017	-0.286481036	0.093326317	-3.069670428	0.002142951	0.110908954
382.1260379	-0.029101078	0.09140509	-0.318374811	0.750200643	0.942174606
154.6718509	-0.056266163	0.105224189	-0.53472651	0.592838988	0.893969305
47.49295202	0.202872448	0.193346532	1.049268614	0.294054507	0.734367271
1382.578165	0.135277978	0.15416288	0.877500327	0.380214941	0.788305094
1249.633372	0.175536916	0.171292207	1.024780511	0.305466764	0.742035396
471.6602716	-0.088282679	0.059612691	-1.480937645	0.13862319	0.570638112
230.1525444	-0.190771079	0.154136451	-1.237676602	0.215835998	0.660219973
220.0847021	-0.016261116	0.204792101	-0.079403045	0.936712047	0.984334593
27.11640704	-0.100716623	0.384749564	-0.261771896	0.793497308	0.953153173
62.67038516	0.022099935	0.181059951	0.122058658	0.902852563	0.979381499
26.45515744	-0.177682465	0.190719901	-0.931640925	0.351522126	0.770952892
566.1804561	-0.014072804	0.076956037	-0.182868101	0.854901512	0.969966486
42.19844613	0.057967018	0.226536019	0.255884332	0.798040153	0.954840537
26.38531417	0.155645812	0.275127853	0.565721757	0.571582942	0.88481183
86.87854506	-0.099435	0.118474544	-0.839294218	0.401304227	0.801291311
2137.404771	0.017110296	0.101593053	0.16841994	0.866252922	0.971979594
283.8498651	0.024632455	0.061187895	0.402570713	0.687264058	0.923064029
87.04866197	-0.804685692	0.362478302	-2.219955476	0.02642179	0.317447514
17.76600931	0.474960654	0.265385308	1.789702142	0.073501808	0.466541063
218.4155139	0.007709368	0.098560569	0.078219597	0.937653374	0.984334593
706.1092162	0.238851355	0.124662368	1.915986032	0.055366874	0.423973339
3283.938063	0.069213067	0.045477504	1.521918748	0.128029441	0.558529851
61.91244332	-0.082018651	0.126585126	-0.647932765	0.517028438	0.862002027
93.03460443	-0.00547859	0.176684184	-0.031007814	0.975263308	0.993340811
554.5666745	0.521675875	0.147820899	3.529107709	0.000416963	0.044477829

47.96921764	0.230812261	0.215973378	1.068707004	0.285201714	0.726354285
36.52729312	0.004023189	0.251088609	0.016022984	0.987216055	0.996928555
70.36920899	0.047242073	0.228645599	0.206617022	0.836308965	0.966316856
4.672975521	-0.288097991	0.529672394	-0.543917325	0.586498366	0.890984353
59.04365255	0.343509265	0.195329483	1.75861452	0.078643005	0.475132147
29.96492469	0.102880599	0.234699448	0.438350409	0.661132289	0.914896282
33.46993028	0.204910174	0.217277254	0.943081575	0.345639179	0.767958555
13.10964555	0.311287287	0.347400889	0.896046317	0.370228027	0.784153324
619.7862844	-0.035814685	0.158403461	-0.226097871	0.821125301	0.962824423
6.493549403	-0.267484498	0.445715303	-0.600124107	0.548423527	0.874664912
76.83149535	-0.235534618	0.127352647	-1.849467789	0.064390295	0.445584453
471.6881139	-0.423876748	0.212357013	-1.996057214	0.045927695	0.391329374
19.29215074	0.076487541	0.485525091	0.157535712	0.874822665	0.973587929
445.8599891	-0.012599599	0.150135216	-0.083921677	0.933118705	0.984334593
9.284466645	0.44905957	0.313097066	1.43425033	0.151500839	0.589528489
13.77915588	0.129622133	0.340596587	0.380573788	0.703519534	0.9269128
6.967057737	0.249118165	0.476135311	0.523208759	0.600828987	0.898289446
345.4495803	-0.033003358	0.099302906	-0.332350379	0.739624703	0.939690097
286.5968307	0.024087082	0.194683217	0.123724491	0.90153342	0.979381499
10.24832997	0.07934973	0.399354796	0.198694822	0.842501475	0.967631986
1018.410231	0.186848219	0.112089869	1.666950104	0.095524327	0.504442057
42.00750637	0.072131381	0.160912926	0.448263435	0.653963086	0.911976263
22.1282348	-0.154944236	0.253520402	-0.611170678	0.541086593	0.870466662
63.69754116	-0.054471647	0.12285911	-0.443366772	0.657500466	0.91380052
116.235701	0.04877726	0.105425853	0.462668871	0.643601735	0.908494289
149.4051324	-0.398538499	0.456980733	-0.872112258	0.38314714	0.789880775
1361.668718	0.134885616	0.229824362	0.586907388	0.557265908	0.879365746
31846.26474	-0.670934567	0.522421705	-1.284277742	0.199044792	0.64421391
72825.82509	0.041435288	0.300106856	0.138068449	0.890186321	0.976961229
631717.8718	0.058524435	0.16143826	0.36251899	0.716964235	0.930431756
720.2277896	-0.195468118	0.161097643	-1.213351818	0.224995339	0.668193468
21.51369743	-0.716827747	0.38188986	-1.877053627	0.060510744	0.436434491
534.6675621	0.665997072	0.329770485	2.019577563	0.043427224	0.381263212
1674.243401	-0.003944584	0.212952329	-0.018523321	0.985221373	0.996308482
7.244591952	-0.536598335	0.395761635	-1.355862436	0.175142941	0.619005807
163.326674	0.026468627	0.083370181	0.317483136	0.750877033	0.94246433
358.835191	-0.217579848	0.218237956	-0.996984447	0.318772059	0.752066309
28.75185721	0.266836262	0.578039438	0.461622936	0.644351744	0.908524911
56.51173034	0.217945122	0.208242191	1.046594453	0.295286661	0.735234129
5.123019692	-0.164284014	0.540162326	-0.304138231	0.761022581	0.946826103
567.0716336	0.011588379	0.25814109	0.04489165	0.964193673	0.990149113
729.7119058	0.133882518	0.105227284	1.272317523	0.203260325	0.647391537
172.9056859	0.112661035	0.143008314	0.787793603	0.430817444	0.818841807
66.21567967	-0.232670543	0.195457051	-1.190392173	0.233892288	0.676714943
124.5936993	-0.163865855	0.236733426	-0.692195682	0.48881445	0.849736271
309.8476674	-0.184171742	0.274476219	-0.670993441	0.502224708	0.857134801
197.0341084	-0.049174761	0.132932872	-0.369921755	0.711440792	0.929376205
9.93200162	-1.68052059	0.963549024	-1.744094538	0.081142606	0.481584947

211.8873189	0.097906404	0.091692565	1.067768193	0.285625089	0.726643643
18.78524611	-0.34413304	0.277143423	-1.241714618	0.214341875	0.659648752
448.533783	0.20700315	0.096840564	2.137566535	0.03255194	0.341836409
1287.414392	0.428601312	0.273075302	1.569535248	0.116523273	0.538641109
43.40095225	-0.27464541	0.266335352	-1.031201481	0.302446346	0.740307837
162.6074789	-0.198513968	0.081876669	-2.424548644	0.01532743	0.258245094
254.1309488	-0.328190327	0.133005805	-2.467488746	0.01360645	0.250121471
63.33260098	0.260065767	0.163634325	1.589310593	0.111990288	0.532835074
119.5465738	-0.113364178	0.139096371	-0.815004575	0.415069692	0.809574591
55.65146772	-0.031650952	0.133659629	-0.236802632	0.81280991	0.96005245
964.9127642	-0.133837231	0.102060452	-1.311352524	0.189738691	0.635553768
7.494371497	-0.181955871	0.487292007	-0.373402125	0.708849178	0.928992812
11.42862855	-0.417652156	0.395861092	-1.05504725	0.291403715	0.732961888
59.08434518	-0.093467164	0.149082374	-0.626949793	0.530692147	0.866123834
176.2407116	-0.070506765	0.111285328	-0.633567484	0.526363127	0.864850467
2772.319031	0.108791762	0.207994207	0.523051884	0.600938148	0.898312105
30.2768652	-0.394061649	0.192916224	-2.042656866	0.041086418	0.374892505
1442.633605	0.113736885	0.130387875	0.872296489	0.383046653	0.789880775
97.62191338	-0.044775114	0.217492036	-0.205870131	0.836892358	0.966316856
96.94558406	0.627763345	0.493532291	1.271980286	0.203380124	0.647544115
919.7533111	0.032924258	0.10686807	0.308083212	0.758019015	0.945887766
378.6127224	0.018839079	0.057923344	0.325241558	0.744998259	0.940455922
32.386328	0.154130815	0.209252049	0.736579715	0.461377974	0.835033586
1484.094809	-0.055385243	0.045183886	-1.225774223	0.22028365	0.664595064
951.8500382	0.156758247	0.109272493	1.434562745	0.151411737	0.589528489
318.812462	0.569423668	0.112925178	5.042486329	4.59522E-07	0.000450504
50.63144692	0.035373692	0.242239145	0.146027977	0.883899303	0.97623321
96.15746984	-0.096966134	0.210857554	-0.459865594	0.645612698	0.909300678
222.2948721	0.256060345	0.08236456	3.108865584	0.001878071	0.103380352
110.7665568	0.193588844	0.154527824	1.252776617	0.210287014	0.655397227
5.117326711	-0.290893058	0.418066655	-0.695805452	0.486550675	0.849283383
43.17338981	-0.00060838	0.246505263	-0.002468021	0.998030806	0.999394422
16.7618372	-0.817177734	0.544345269	-1.501212156	0.133300697	0.566220634
6.964869865	-0.189197857	0.42092058	-0.449485879	0.653081192	0.911800397
669.1889879	-0.009333914	0.091911086	-0.101553736	0.919110903	0.981861979
274.0800894	-0.003261887	0.090946005	-0.035866189	0.971389056	0.992799208
2704.900144	0.054723684	0.048906545	1.118943981	0.263164038	0.70568009
134.7446774	-0.163928344	0.224705165	-0.729526373	0.465679741	0.837531001
5149.582932	-0.019596368	0.140589909	-0.139386734	0.889144555	0.976961229
213.3291558	-0.021958099	0.072213463	-0.304072095	0.761072966	0.946826103
1358.489203	-0.075061757	0.112492009	-0.667263014	0.504604147	0.857734335
1030.569984	0.040076005	0.084513996	0.474193711	0.635361778	0.907169387
181.6927024	-0.006283682	0.074135856	-0.084759018	0.932452975	0.984334593
103.3911309	-0.072984429	0.111367885	-0.655345382	0.512245408	0.860200661
3683.182254	-0.167971566	0.154267032	-1.088836443	0.276226018	0.718449748
96.36620946	-0.052338353	0.105654087	-0.49537462	0.620335706	0.901976704
77.89501179	-0.073019466	0.173103537	-0.421825383	0.673152477	0.920554864
102.2759342	0.408433099	0.4229205	0.965744386	0.334172103	0.761545389

7.244590799	-0.643774857	0.45039241	-1.429364357	0.152899537	0.59076454
16.0755701	-0.200488752	0.247623235	-0.809652424	0.418139969	0.811361647
88.79379266	-0.537746467	0.320772106	-1.676412807	0.093657362	0.500655931
132.3226199	-0.086792292	0.09566945	-0.907210111	0.364295698	0.779728871
344.2880422	-0.058974672	0.078882036	-0.747631205	0.454682637	0.831501359
54.47966469	-0.432179076	0.354322856	-1.219732425	0.222566326	0.666984161
384.2142722	-0.068052982	0.068986103	-0.986473779	0.323900676	0.756297513
108.2752571	-0.071367706	0.260515094	-0.273948448	0.784124239	0.95189998
40.72698572	-0.055853042	0.23960577	-0.233103911	0.815680718	0.961129278
22.30150479	0.045794141	0.260380456	0.175873955	0.860392968	0.969966486
1021.03438	-0.037260679	0.074870691	-0.497667087	0.61871871	0.901976704
213.6753175	0.058311723	0.078047772	0.7471286	0.454985938	0.831550545
591.9726873	0.017146954	0.084628789	0.20261372	0.839436966	0.966440785
141.105383	-0.272391671	0.323969429	-0.840794366	0.400463144	0.801079895
21.25809629	0.060962512	0.363790251	0.167575993	0.866916859	0.972138679
87.06574336	-0.12849896	0.172652867	-0.744261953	0.456718014	0.832142746
2880.619756	-0.047409695	0.165355564	-0.286713636	0.774331595	0.949748607
329.875656	-0.119862418	0.062630341	-1.913807536	0.055644752	0.425001869
1591.842656	-0.10998563	0.073221688	-1.502090881	0.13307364	0.56585924
48981.28147	0.07272438	0.191914252	0.378942051	0.704730897	0.9269128
461.7010339	0.148423506	0.107555338	1.379973399	0.167594835	0.609207556
871.6881377	-0.090648343	0.081397937	-1.113644232	0.265431843	0.708627777
38.80845334	0.057889288	0.15479144	0.37398249	0.708417343	0.928564109
12612.49338	0.010076171	0.120934355	0.083319341	0.933597621	0.984334593
156.8284833	0.25639216	0.1173723	2.184434993	0.028930299	0.328011759
853.0600922	-0.023214906	0.086551305	-0.268221326	0.788528961	0.953026618
427.9742106	0.025331008	0.063355651	0.399822388	0.68928734	0.924002102
57.80191392	0.069679927	0.156816031	0.444341863	0.656795439	0.913625833
590.780023	0.1331169	0.075188745	1.770436516	0.076654451	0.471783446
225.720785	0.040255706	0.078627751	0.511978346	0.608666164	0.900307811
175.4397948	0.075381482	0.079352476	0.949957518	0.342133839	0.765688703
77.82504036	-0.07623714	0.124013133	-0.614750535	0.538719481	0.869675597
6.723700775	-0.228916972	0.597479576	-0.383137736	0.701617641	0.926243544
34.47101953	-0.016822325	0.170631306	-0.098588736	0.921464814	0.982590996
95.4564019	-0.165601982	0.280399027	-0.590593995	0.554792489	0.877791979
56.02056869	-0.0203312	0.143265243	-0.141912998	0.887148728	0.976961229
179.8575515	-0.062235047	0.110325182	-0.564105546	0.572682304	0.885217571
52.91639484	-0.176218919	0.159590581	-1.104193733	0.269509108	0.711988901
10.2740214	0.305858518	0.301760996	1.013578702	0.310783825	0.745771331
30.92880108	0.163584971	0.243960508	0.670538736	0.502514422	0.857172233
24.41373722	0.243161007	0.378665277	0.642152904	0.520773908	0.863188882
12.06751385	0.02683957	0.36529196	0.073474298	0.941428696	0.984334593
4229.194714	-0.258468241	0.180183374	-1.434473313	0.151437239	0.589528489
6.933600027	0.706634986	0.569868908	1.239995683	0.214976991	0.660219973
1228.842163	0.009012993	0.073026063	0.123421598	0.901773255	0.979381499
722.702697	-0.023610296	0.26397919	-0.089439991	0.928732243	0.984080734
9.262732875	-0.588791766	0.344120867	-1.711002798	0.087080596	0.491740726
1598.137139	-0.020904579	0.115774093	-0.180563531	0.856710184	0.969966486

448.0566784	0.041986063	0.113985823	0.368344605	0.712616297	0.929589333
177.996755	-0.116361838	0.166243586	-0.699947835	0.483959882	0.847905053
942.4056517	-0.017263579	0.042901925	-0.402396383	0.687392332	0.923064029
288.6578746	-0.480100388	0.232430452	-2.065565779	0.038869498	0.368339511
14.8971609	-0.044338373	0.423343058	-0.104733907	0.916586956	0.981290994
16.46546512	-0.001284869	0.363874629	-0.003531077	0.997182614	0.999248121
184.4408166	0.065655803	0.089842049	0.730791464	0.464906538	0.837170481
8.941270848	-1.03180758	0.540516206	-1.90892996	0.056271128	0.426485013
5.512800082	-0.816183177	0.658853321	-1.238793447	0.215422001	0.660219973
103.7914617	-0.167239005	0.195744882	-0.854372303	0.392898737	0.795848318
553.7489971	0.016905893	0.088400379	0.191242317	0.848335751	0.969058943
43.84701698	-0.481507425	0.227019732	-2.120993717	0.033922331	0.348624664
96.99289691	0.331786142	0.167296867	1.983217908	0.047343094	0.396588842
38.93960888	-0.496305288	0.26733959	-1.85646012	0.063388002	0.443016545
13.34704027	0.076384697	0.37487846	0.203758566	0.838542173	0.966440785
87.11040332	-0.064834872	0.181090801	-0.358024107	0.720325273	0.931135539
215.1950277	-0.328834007	0.183438188	-1.792614778	0.073034542	0.465571614
10.21839668	-0.688663147	0.693453396	-0.993092182	0.320665034	0.75269328
3248.895558	-0.137732944	0.098094896	-1.404078589	0.160295449	0.600672338
3002.325567	0.066062704	0.086656583	0.762350674	0.445850737	0.826302547
182.2583078	0.212911159	0.07811245	2.725700673	0.006416516	0.174970531
258.2328217	0.012884801	0.093408625	0.137940168	0.890287705	0.976961229
104.9574362	-0.154799122	0.10102634	-1.532264976	0.12545706	0.554664099
293.6138361	0.012789534	0.097325794	0.1314095	0.895451374	0.978798764
124.3175504	-0.026765815	0.136628929	-0.195901519	0.844687248	0.968131661
563.3817271	-0.060745019	0.049353551	-1.230813548	0.218392607	0.662774346
176.1480581	-0.299132131	0.131746002	-2.270521516	0.023175959	0.303584558
56.02391438	-0.062649058	0.14733078	-0.425227223	0.670671041	0.918962624
69.67304478	0.037344109	0.133141049	0.280485313	0.779105192	0.951703357
96.71323955	-0.469523533	0.323850682	-1.449814868	0.147110152	0.582537313
814.1407872	0.153540105	0.116816626	1.314368598	0.188722193	0.634590162
104.7591547	-0.061496158	0.135882333	-0.452569192	0.650858987	0.910782566
556.8582844	0.031444077	0.297993005	0.105519514	0.915963586	0.981266182
305.9510036	-0.116764293	0.126101992	-0.925951218	0.354471332	0.773422825
299.632995	-0.012250707	0.150121201	-0.081605439	0.934960476	0.984334593
24.1881896	-0.651943678	0.229277948	-2.843464378	0.0044626	0.149960163
3613.717219	0.018553784	0.045365219	0.408986988	0.68254921	0.922776218
360.7145651	-0.150054491	0.121873343	-1.23123308	0.218235702	0.662774346
99.51026286	0.176976522	0.12110942	1.461294443	0.143934654	0.578069441
446.6485748	0.133615431	0.188311498	0.709544728	0.477986505	0.844290722
1517.019654	-0.016397214	0.122730575	-0.133603336	0.893716246	0.978111861
218.3867349	-0.005121747	0.131890827	-0.03883323	0.969023351	0.992218786
376.1963934	0.189171683	0.082944821	2.280693127	0.022566612	0.300162997
1255.914248	-0.059366177	0.14990569	-0.396023508	0.692087686	0.924581226
1207.022889	0.166255566	0.056574061	2.938724275	0.003295661	0.133812668
4181.37615	0.091241215	0.085096669	1.072206659	0.28362722	0.724631363
25.28110239	1.102890232	0.348464531	3.164999973	0.00155083	0.094686033
128.1027559	0.082167856	0.131031272	0.627085847	0.530602964	0.866123834

183.7553926	-0.368942611	0.169049988	-2.182446839	0.029076571	0.328042225
328.1957793	0.075250167	0.06374602	1.180468477	0.237813941	0.679803171
10.5749046	-0.051882431	0.307442715	-0.168754792	0.865989519	0.971929232
80.09474764	-0.053552395	0.157850154	-0.33926096	0.73441315	0.937386243
252.6533144	-0.058003593	0.066466657	-0.872672042	0.382841861	0.789880775
122.8613389	-0.134017017	0.191370671	-0.700300711	0.483739528	0.847905053
562.0320719	0.095710834	0.080806394	1.184446292	0.236236445	0.678901985
266.4575453	0.049643453	0.129916406	0.382118431	0.702373521	0.926349622
219.1865407	0.124457004	0.163941736	0.759153872	0.447760516	0.827180558
522.6422516	-0.682629024	0.556014345	-1.227718367	0.219552709	0.663637842
349.8180019	0.028687941	0.076714548	0.373956977	0.708436325	0.928564109
1950.561688	-0.105403777	0.118535893	-0.889214016	0.373888071	0.786373874
278.8169474	0.008297251	0.087518131	0.094806083	0.924468855	0.982590996
27.50040839	0.085642599	0.353529535	0.242250196	0.808586303	0.95852687
104.0470299	0.088153648	0.096932198	0.90943618	0.363119931	0.779728871
8.268454187	-0.222590441	0.349916448	-0.636124544	0.524695249	0.864004793
1818.388525	0.112582966	0.117669257	0.95677469	0.33868099	0.764007173
1331.382341	-0.293093038	0.155722524	-1.882149288	0.059815749	0.435320275
202.5036297	-0.042815836	0.144917697	-0.295449327	0.767650657	0.94737077
657.222039	-0.127593058	0.049852264	-2.559423526	0.010484592	0.221704189
70.79665928	-0.212026378	0.141803633	-1.49521118	0.134859337	0.567901571
82.37146692	-0.499228665	0.380390986	-1.312409292	0.189382073	0.635477898
2964.821681	0.093691319	0.064802418	1.445799728	0.148233383	0.585106808
1176.254405	0.150332863	0.065525625	2.294260634	0.021775532	0.29543669
7.027991496	0.314744308	0.384570212	0.818431324	0.413110942	0.808272153
4005.420908	0.018077044	0.069101774	0.261600296	0.793629615	0.953153173
6.251746874	-0.265178163	0.444015586	-0.597227149	0.550355735	0.875690724
393.2526078	-0.038129069	0.079014186	-0.482559785	0.629408339	0.904638489
21.12156346	-0.055154428	0.272306315	-0.202545532	0.839490266	0.966440785
894.4972986	-0.163457441	0.066667664	-2.451824945	0.014213379	0.251563345
262.4321252	-0.045520081	0.091747692	-0.496144151	0.619792711	0.901976704
156.2148863	0.033488833	0.125672383	0.266477267	0.789871665	0.953026618
77.56131946	-0.152249164	0.198993245	-0.765097145	0.4442137	0.826036581
19.77056395	-0.652849062	0.369273177	-1.767929823	0.077072636	0.472393028
44.52098946	-0.780757509	0.283910357	-2.750014189	0.005959268	0.16865983
1131.8258	0.323052943	0.072374866	4.463606764	8.05914E-06	0.004406037
121.8619822	-0.446988047	0.271245951	-1.647906802	0.099371797	0.51147708
27.05802568	-0.198674767	0.262226227	-0.75764644	0.448662671	0.827801949
1215.557022	0.032588146	0.057757303	0.56422555	0.572600642	0.885183781
315.2460827	-0.029786673	0.110724283	-0.269016625	0.78791689	0.953026618
8149.691061	-0.014458961	0.092894436	-0.155649378	0.876309401	0.973881805
12.52659589	-0.589591091	0.370320031	-1.59211234	0.111359465	0.531325251
44.14375869	-0.670565645	0.324273283	-2.067902845	0.03864916	0.367408361
9.59191852	-1.626543323	0.687691377	-2.365222798	0.018019227	0.27123243
217.6287753	-0.221086121	0.097991419	-2.256178371	0.024059463	0.308336073
57.35408683	-0.508718682	0.310730731	-1.63716888	0.101595188	0.513342798
17.67051565	-0.329647016	0.249615412	-1.320619645	0.186628219	0.63228906
8.082136851	-0.558574809	0.328053357	-1.702694994	0.088625162	0.493540124

156.2878586	-0.532941901	0.299490929	-1.77949263	0.075159032	0.470687737
92.5356693	0.136795232	0.102852094	1.330018938	0.183512032	0.629366333
985.8581909	0.034248252	0.108057706	0.316944098	0.75128602	0.942897716
910.3643269	-0.012706232	0.084262349	-0.150793709	0.880138449	0.975430592
97.52707767	-0.225134495	0.132421018	-1.700141705	0.089104274	0.493693668
27.92614692	-0.327224944	0.245884189	-1.330809217	0.183251794	0.628765197
233.7444639	-0.013886624	0.093553059	-0.148435814	0.881998838	0.975487608
112.5958227	0.020407963	0.120026677	0.170028558	0.864987677	0.971917805
211.3751681	0.159140398	0.128212745	1.241221363	0.214523985	0.659783094
42.08723861	-0.10789716	0.209954883	-0.513906411	0.607317425	0.900307811
110.7201697	-0.116337495	0.237333221	-0.490186306	0.62400207	0.902875034
1819.675409	-0.057925819	0.107629688	-0.538195546	0.590442055	0.892788725
94.28737791	0.080444075	0.146310692	0.549816787	0.582445044	0.888664491
148.618255	0.045983603	0.235534079	0.195231208	0.845211948	0.968131661
1106.711111	0.056276021	0.076291475	0.737644947	0.460730235	0.834673974
15.73555802	0.199889969	0.258564595	0.773075558	0.439477666	0.823332288
22.88720392	0.215964261	0.201959818	1.069342721	0.284915267	0.725996864
49.0287729	0.13361714	0.284481145	0.469687155	0.638578546	0.907684986
27.95871252	-0.964006222	0.520725275	-1.851276035	0.064129852	0.444821962
13.73616553	0.26370426	0.34521559	0.763882826	0.444937073	0.826302547
592.6220979	-0.094304071	0.076033651	-1.240293856	0.214866724	0.660219973
382.9239097	0.075545374	0.063291146	1.193616778	0.232627908	0.675563167
105.4205828	-0.382047049	0.201828897	-1.892925415	0.058367789	0.432845697
58.87452756	-0.204768017	0.210569599	-0.972448149	0.330827648	0.759701198
132.536254	-0.059067267	0.145753874	-0.405253499	0.685291184	0.922907843
91.27615701	0.119008854	0.353723694	0.336445809	0.73653471	0.938503341
31.5092745	0.014313619	0.174667419	0.081947846	0.934688187	0.984334593
750.5629547	0.065668634	0.081063303	0.810090774	0.417888006	0.811361647
138.6990921	0.130908942	0.230804078	0.567186432	0.570587525	0.884612839
977.7441612	0.071435707	0.1166242	0.612529018	0.540187805	0.869829343
809.2034452	0.079419475	0.0531357	1.494653775	0.135004824	0.568194883
1068.729234	0.220205708	0.073790438	2.984203829	0.002843173	0.126760985
13.3562509	0.149776276	0.320826726	0.466844761	0.640610949	0.907798398
166.4090181	-0.308248856	0.17923752	-1.719778634	0.085472688	0.488858859
32.09244989	-0.187762506	0.478864684	-0.392099298	0.694984846	0.924581226
9.117131451	-0.435301398	0.591820208	-0.735529798	0.462016899	0.835391063
9.991309257	0.340473003	0.78478673	0.433841438	0.664403588	0.916049829
224.5973951	0.07287355	0.202736838	0.359448984	0.719259236	0.931124297
6.559236364	-0.753196605	0.362734975	-2.076437774	0.037853481	0.363363957
142.0679517	0.016574191	0.090918383	0.182297465	0.855349288	0.969966486
8.733767161	-0.066039258	0.364281934	-0.181286118	0.856143001	0.969966486
466.2870416	0.005267387	0.086040425	0.06121991	0.951184074	0.98625342
195.3154049	-0.133029658	0.116294055	-1.143907638	0.252661943	0.69506877
143.7990621	-0.228329931	0.167242783	-1.36526029	0.172171255	0.616109027
76.75841261	0.004317455	0.132717008	0.03253129	0.974048364	0.993248159
3188.173712	-0.106455906	0.12989351	-0.819562936	0.41246531	0.808272153
6.697048536	0.211878908	0.400981822	0.528400282	0.597221542	0.896990756
628.232138	0.287604329	0.211331294	1.3609169	0.173539952	0.617336891

12.29345499	-0.785662279	0.516879405	-1.520010802	0.128508261	0.559464493
39.59639963	0.151075111	0.371439947	0.406728227	0.684207606	0.922907843
5.846430448	-0.131399792	0.439063425	-0.299272916	0.764731818	0.946826103
35.06119531	0.033888808	0.291831582	0.116124538	0.907553841	0.979768942
20.3034921	-1.874356659	0.816794082	-2.294772576	0.021746161	0.295327604
15.9918037	0.213930042	0.298430054	0.716851532	0.473465711	0.841511024
19.28858202	-0.047439946	0.55828401	-0.084974574	0.932281604	0.984334593
9.946807389	-2.529000182	2.29804794	-1.100499314	0.271114628	0.713807699
9.659430134	0.098754193	0.314142626	0.314361009	0.753246866	0.944021193
12.4074815	0.102883834	0.30412866	0.338290491	0.735144289	0.937623649
133.0226593	0.136096744	0.104145801	1.306790514	0.191283868	0.637278237
906.7954843	0.05809046	0.073751288	0.787653505	0.430899409	0.818841807
23.79349846	-0.450201374	0.34540388	-1.303405668	0.192436303	0.638906868
65.32094623	-0.353636717	0.258829947	-1.366289804	0.17184802	0.616109027
220.6407833	-0.084860523	0.090213732	-0.940660816	0.346878705	0.768780165
69.95177021	-0.052992786	0.330049174	-0.160560274	0.872439745	0.972881041
115.6326388	0.341522508	0.382591523	0.892655711	0.372041575	0.785922562
14.90939573	1.071888967	0.647980876	1.65419846	0.098087196	0.509712212
8.529947537	-1.45214091	0.595503339	-2.438510103	0.014747946	0.256113483
320.0869023	-0.044939829	0.085757642	-0.524032939	0.60025563	0.898108141
218.3047477	0.150188947	0.09364708	1.603776077	0.10876342	0.526116385
1281.421734	0.179930517	0.070243951	2.561509051	0.010421853	0.221076998
65.61555263	0.23286041	0.413363177	0.563331286	0.573209318	0.885477387
202.4519431	0.139611671	0.150076021	0.930273001	0.352229753	0.771828987
960.103975	-0.388002294	0.372069017	-1.042823446	0.29703007	0.736647043
125.0428857	-0.109282934	0.087459651	-1.249524013	0.211473476	0.656508052
87.82737888	-0.242991323	0.130921748	-1.856004265	0.06345295	0.443016545
283.7480239	-0.068557014	0.140573707	-0.487694435	0.625766294	0.903932145
6.82920212	0.027984954	0.41511267	0.067415321	0.946251072	0.985728152
602.8743505	0.037746881	0.051778927	0.729000828	0.466001156	0.837543146
15.04322675	-0.013418869	0.253306063	-0.05297492	0.95775189	0.987864832
62.52443571	0.286847466	0.131916049	2.174469815	0.02966987	0.330732257
73.42922868	-0.073197218	0.114863071	-0.637256321	0.523957897	0.863484457
94.61605935	-0.307599212	0.128677555	-2.390465159	0.016827046	0.264972265
55.9214474	0.194715105	0.280210421	0.694888879	0.487124942	0.849484378
492.4392897	-0.028155593	0.073021764	-0.3855781	0.699809155	0.926034765
190.4642011	0.162995579	0.102699218	1.587116071	0.112486359	0.533148601
5.492184798	0.105180971	0.430236796	0.244472282	0.806865066	0.957805921
110.458671	-0.122160624	0.125085119	-0.97661996	0.328757334	0.758664748
535.8760091	0.086367024	0.079934456	1.080473026	0.279931592	0.721663546
42.18227089	0.269482622	0.151384431	1.780121116	0.075056142	0.470687737
7.40805602	0.468104587	0.319986123	1.462890273	0.143497406	0.578069441
12.04069623	0.247859955	0.35726853	0.693763748	0.48783038	0.849513564
52.75711895	-0.427448589	0.167916675	-2.545599407	0.01090903	0.226714336
15.65802612	-0.424494435	0.380113029	-1.116758446	0.264097621	0.70690289
6.296115586	-0.638483066	0.617373357	-1.034192775	0.301046051	0.739230864
131.8157259	-0.034787759	0.198240954	-0.175482202	0.860700754	0.970088938
137.3916358	-0.138927913	0.090031168	-1.543109086	0.122804311	0.548917042

1806.190185	0.142616768	0.065249955	2.185699092	0.028837627	0.328011759
373.3663585	0.197031352	0.087233916	2.25865536	0.023904831	0.307486383
611.2939256	0.177654961	0.077071316	2.305072352	0.021162522	0.290582231
108.0868243	0.197922348	0.146157405	1.354172566	0.175681329	0.619005807
706.1683846	0.185913888	0.046484567	3.999475558	6.3483E-05	0.015156055
4.631354808	1.041107027	0.532749854	1.954213632	0.050675958	0.409222874
144.8397629	0.092850248	0.117645246	0.789239269	0.429972174	0.818650305
3764.146333	0.168487593	0.068119356	2.473417273	0.013382778	0.248838234
271.7231733	0.013428336	0.092953317	0.144463229	0.885134691	0.976723103
226.4247219	0.107031738	0.173777195	0.615913601	0.537951544	0.869675597
67.47476434	0.137531301	0.158720429	0.866500309	0.386215857	0.791768942
160.3858384	0.099354372	0.112978825	0.879407026	0.379180624	0.787394269
8.279490486	-1.285228052	0.54555375	-2.355822964	0.018481723	0.27356647
36.43152015	-0.264318426	0.198161363	-1.333854499	0.182251546	0.627539984
177.9610652	-0.045243585	0.09122647	-0.495947997	0.619931101	0.901976704
28.85469127	0.02995929	0.254175346	0.117868591	0.90617178	0.979768942
6.123125662	0.641655457	0.437839331	1.465504378	0.142783359	0.57754946
116.5470958	-0.22694939	0.199002928	-1.140432415	0.254106195	0.696717102
375.1127363	-0.150148176	0.064446516	-2.329810589	0.019816164	0.281261453
28.24115895	-0.55216716	0.302020419	-1.828244468	0.06751287	0.45536467
189.0093095	-0.180505116	0.112711623	-1.601477394	0.109271223	0.527269235
6.337004936	-2.618776947	1.068877986	-2.450024214	0.014284661	0.252016149
41.84872668	0.108241223	0.172494049	0.627506999	0.53032695	0.866123834
2468.698991	-0.124886557	0.143401744	-0.870885897	0.383816458	0.790043276
1044.156162	0.025903425	0.063440261	0.40831209	0.683044564	0.922776218
2153.143245	0.082009039	0.113619708	0.721785341	0.470426463	0.839938793
33.88125632	0.2468768	0.291951669	0.845608454	0.397771208	0.799213984
2310.79113	-0.035733662	0.220060777	-0.162380879	0.871005927	0.972881041
526.6311374	0.05840172	0.097612808	0.598299765	0.549639934	0.875472969
2652.034664	0.199437943	0.128906554	1.547151303	0.121826765	0.547875652
11.76738373	0.223231383	0.294108619	0.759010001	0.447846574	0.827180558
20.4874087	0.406326564	0.267057338	1.5214956	0.128135515	0.558663295
16.79186111	-0.403293281	0.302280087	-1.334170851	0.18214787	0.627539984
1014.089894	-0.158929903	0.116054711	-1.369439474	0.170861942	0.614754125
12.21606106	-0.010001659	0.254220665	-0.039342433	0.968617376	0.99207545
150.3497713	0.013544352	0.235190395	0.057588883	0.954076105	0.987171945
68.20593933	0.182229499	0.137056283	1.32959609	0.183651387	0.629406769
221.227325	0.103899201	0.144571191	0.718671542	0.472343322	0.840757256
140.3502678	-0.001252426	0.131184928	-0.009547028	0.992382689	0.998453546
68.0276892	0.026440416	0.230260226	0.114828412	0.908581128	0.980020248
68.02823851	-0.064675064	0.13560911	-0.476922711	0.633417159	0.906724287
241.0997315	0.58111375	0.371542747	1.564056236	0.117804362	0.539880626
1000.354269	-0.615104598	0.371867825	-1.654094701	0.098108273	0.509712212
354.2538433	-0.074067171	0.171083761	-0.432929287	0.665066143	0.916049829
671.7071768	0.112829311	0.153298814	0.736009028	0.461725204	0.835201817
236.4282988	0.440623643	0.184052588	2.394009496	0.016665317	0.263828906
173.8144786	0.015285279	0.103849392	0.14718699	0.882984429	0.975884268
144.274804	-0.120730223	0.211955561	-0.569601582	0.568947955	0.8841274

202.8256814	-0.015714616	0.084199895	-0.186634628	0.851947115	0.969560739
1981.318789	0.107047637	0.104182364	1.027502472	0.304183922	0.741640846
97.41814434	0.014841185	0.142873965	0.103876062	0.917267704	0.981376661
4830.208061	0.112561025	0.107089348	1.051094504	0.293215188	0.733939814
3139.28017	0.243414337	0.186814722	1.302971921	0.192584348	0.638906868
154.3997926	0.032716539	0.148135514	0.220855475	0.825204967	0.963909899
481.08997	0.088653412	0.083864398	1.057104257	0.290464009	0.732070195
2645.626433	0.079236651	0.108756835	0.728567088	0.466266517	0.837628265
52.14839787	-0.719099871	0.454575635	-1.581914682	0.113669049	0.533883941
42.33871022	-0.214972111	0.370611602	-0.580046901	0.56188299	0.881435293
114.2734807	-0.028931641	0.143043669	-0.202257402	0.8397155	0.966440785
74.17395108	-0.443034139	0.257157276	-1.722813939	0.084922177	0.488446247
90.71786751	-2.089912608	0.772496317	-2.705401387	0.00682219	0.181059331
540.0575334	0.041080366	0.110306909	0.372418792	0.709581065	0.92902743
161.6390761	0.039837371	0.124546681	0.319858955	0.749075254	0.941640077
131.2711183	-0.139432512	0.14176061	-0.98357726	0.325323411	0.756297513
4291.593429	-1.689346963	0.492992356	-3.426720398	0.000610918	0.057176062
1496.419843	-0.011012864	0.09611435	-0.114580852	0.908777358	0.98010679
31.7954552	0.318357988	0.282490886	1.126967288	0.259756312	0.702516818
725.6879974	-0.372139973	0.178261507	-2.087607019	0.036833298	0.360625126
66.97965526	-0.290779277	0.255042563	-1.140120589	0.254236065	0.696717102
5097.26439	0.023660974	0.115561901	0.204747183	0.837769655	0.966431512
182.0387967	-0.145146466	0.186983369	-0.776253349	0.437599412	0.82251807
5.016609445	0.032574223	0.452602709	0.071970896	0.942625069	0.98446865
467.4770288	0.071232251	0.058542457	1.216762247	0.223694688	0.667854242
85.89100614	-0.502707507	0.287847308	-1.746438106	0.080734853	0.480706878
354.0515717	-0.028536699	0.074286457	-0.38414403	0.700871699	0.926034765
2280.300994	0.053967958	0.094342082	0.57204544	0.56729119	0.8841274
5.164113461	0.17907541	0.647411494	0.276602149	0.782085614	0.95173434
9.431696465	-0.563110023	0.50312368	-1.119227827	0.263042957	0.705491334
1148.313359	-0.14284802	0.130647497	-1.093385051	0.274224774	0.716722874
13.10591315	-0.304396475	0.307833875	-0.988833588	0.322744568	0.755418262
30.35088882	0.128089946	0.208899713	0.613164778	0.539767392	0.869675597
1220.086654	-0.011457668	0.108491224	-0.105609169	0.915892449	0.981266182
169.0467894	0.076950641	0.178977776	0.429945228	0.667235484	0.916690146
46.47559671	0.189618524	0.188340836	1.006783916	0.314038625	0.747932379
12.4764851	0.006307573	0.435254613	0.014491686	0.988437712	0.997370523
4.894671645	0.040975971	0.460832584	0.08891726	0.929147666	0.984152592
94.87962655	0.482933306	0.344871109	1.400329844	0.161414568	0.601071304
158.6359902	0.099168807	0.112931531	0.878132147	0.379872009	0.787992438
402.8267465	0.048468581	0.072357218	0.669851355	0.502952552	0.857172233
3225.208515	-0.01231256	0.110565727	-0.111359647	0.911331158	0.980530316
8.556899337	1.319442136	0.464866602	2.838324223	0.004535109	0.149960163
6.911244419	-0.079584777	0.379038514	-0.209964882	0.833695082	0.966316856
11.43484589	0.848443558	0.352218889	2.408853091	0.016002738	0.262608127
10.96489763	0.560325807	0.295882433	1.893744762	0.058258897	0.432392203
14.41723255	0.302856087	0.30759118	0.984605886	0.324817697	0.756297513
20.50701255	0.424075086	0.281877027	1.504468423	0.132460798	0.564647789

9.264098876	0.53964112	0.385941432	1.398246147	0.162039163	0.601439633
143.8470271	0.045038395	0.08116272	0.554914804	0.578952936	0.887274009
205.2885836	-0.050493421	0.095137616	-0.53074087	0.59559836	0.895484706
22.67474265	0.059973694	0.19813344	0.302693448	0.762123491	0.946826103
7551.335351	0.203464899	0.127334558	1.597876515	0.110070462	0.528668387
199.8030824	0.078695642	0.071959353	1.093612415	0.274125002	0.716722874
260.6340476	-0.020963288	0.09347387	-0.224268968	0.822548021	0.963169453
6.30821161	0.210250278	0.521195135	0.403400308	0.68665376	0.923064029
122.2126876	0.147971165	0.198979293	0.743651075	0.457087595	0.832305398
33.61157753	0.047070245	0.222265672	0.211774696	0.83228281	0.966227926
173.9083567	0.175725783	0.105466571	1.666175182	0.095678528	0.504685054
115.4644857	-0.177761171	0.194779017	-0.912629983	0.361437179	0.778294025
434.219395	-0.073491114	0.084260042	-0.872194139	0.383102476	0.789880775
92.9267447	0.259195514	0.134293984	1.930060498	0.053599342	0.417128004
11.37054258	-0.692871796	0.369687658	-1.874208623	0.060901676	0.437392823
125.0905463	-0.166453205	0.342911853	-0.485411058	0.62738479	0.904512355
1621.170962	0.002681945	0.043732534	0.061326071	0.951099528	0.98625342
1178.685907	0.013617754	0.104385556	0.130456309	0.896205419	0.978807264
503.3037045	0.044429244	0.116370572	0.381791062	0.702616349	0.926349622
21.14187639	1.238185493	0.452847246	2.734223305	0.006252762	0.173972533
1556.375233	0.12529073	0.13569749	0.923309121	0.355846136	0.774251728
276.5742018	-0.031584622	0.178536982	-0.176908006	0.85958065	0.969966486
6.398057628	0.558060726	0.385303713	1.448365813	0.14751477	0.583827174
83.04894704	-0.603984771	0.287211545	-2.102926505	0.035472195	0.35645582
12.47226737	0.276731654	0.401397333	0.689420759	0.490558523	0.850315507
1672.931244	-0.01401623	0.076006901	-0.184407339	0.853693913	0.969966486
143.1881371	0.08901372	0.13859978	0.642235647	0.520720191	0.863188882
54.08830377	0.086718217	0.221550616	0.391414921	0.695490565	0.924581226
627.9826244	0.050297384	0.053807792	0.934760236	0.349911876	0.770219235
1382.462543	0.069873802	0.085229345	0.819832677	0.4123115	0.808272153
146.9875065	0.238147223	0.101204994	2.353117302	0.01861676	0.274194313
972.7139977	0.283880903	0.086758962	3.272064305	0.001067653	0.078234644
33.76304699	-0.54194061	0.24151101	-2.243958192	0.024835091	0.312061989
399.8217943	-0.311022788	0.255159406	-1.218935225	0.222868779	0.666984161
5.9713124	-0.138276332	0.37651446	-0.367253708	0.713429781	0.929705922
42.82983563	-0.371114732	0.21363295	-1.737160548	0.082358836	0.484721867
5.681578209	-0.753106448	0.571532529	-1.31769656	0.187605247	0.633746703
1897.741544	0.097661552	0.138059433	0.70738775	0.479325548	0.845076955
1363.412701	0.195229781	0.09109133	2.143231209	0.032094537	0.340059659
6.341915517	-1.106884552	0.555808228	-1.991486447	0.046427434	0.393251403
607.6016446	0.026246591	0.087293022	0.300672269	0.763664417	0.946826103
786.8484215	0.134056285	0.073144201	1.832767094	0.066837215	0.454159121
536.1222784	0.227904915	0.080550621	2.829337782	0.004664444	0.151645037
179.8265743	-0.15085141	0.089896362	-1.678059121	0.093335558	0.500506422
16991.42058	-0.147221249	0.184531694	-0.797810103	0.424980698	0.815987051
10.56167775	0.449800751	0.526505326	0.854313773	0.392931158	0.795848318
32.56930567	-0.10650625	0.178916251	-0.595285502	0.551652645	0.87625782
99.35674493	-0.251632087	0.171723565	-1.465332304	0.142830277	0.57754946

118.1115624	0.049100895	0.12190954	0.402764994	0.687121115	0.923064029
7.903540876	-1.941878185	0.615365941	-3.155647814	0.001601421	0.096358673
8.728559081	-1.238122934	0.676483213	-1.830234527	0.067214877	0.45461399
505.0810125	-0.298153615	0.193605938	-1.540002434	0.12355976	0.551381239
1173.369256	0.090368693	0.095165107	0.949599028	0.342316032	0.765749118
601.113029	0.049989006	0.086788358	0.575987461	0.564623657	0.883265494
849.5838831	-0.184495433	0.075674833	-2.438002519	0.014768671	0.256113483
1799.397031	-0.502251998	0.263838517	-1.9036341	0.056957854	0.428680279
697.9854726	-0.030193101	0.050638352	-0.596249679	0.551008442	0.875900621
13.04363538	0.028911062	0.256420317	0.112748717	0.910229777	0.980530316
367.257604	0.120349741	0.058864085	2.044536011	0.040900624	0.374892505
5.327183448	-0.205407658	0.473706614	-0.43361788	0.664565948	0.916049829
262.2110164	0.009011702	0.077208349	0.116719269	0.90708252	0.979768942
16.98274188	-1.128243289	0.802620935	-1.405698804	0.159813582	0.600578247
223.0700226	-0.123964191	0.112012217	-1.106702415	0.268422611	0.710640581
72.3496685	-2.206043175	1.010809434	-2.182452104	0.029076182	0.328042225
1244.311555	-0.067889196	0.261547926	-0.259566944	0.795197837	0.95400538
5.193323301	-0.393322301	0.667205763	-0.58950675	0.555521385	0.87817235
273.5971187	0.029967379	0.075005196	0.399537374	0.689497291	0.92405254
89.30786799	0.09210803	0.21022661	0.438136875	0.661287065	0.914896282
20.26949316	-0.040707753	0.198175541	-0.205412599	0.837249777	0.966384462
11.56898123	-0.602923733	0.432826565	-1.392991517	0.163622356	0.602187485
5.149329137	-2.138791491	0.838324719	-2.551268551	0.010733159	0.224713175
3928.293196	0.148253838	0.109920382	1.348738386	0.177421011	0.621574017
5.612258698	-0.633026841	0.508806215	-1.244141331	0.213447552	0.659145589
1096.331468	0.059967715	0.081731013	0.733720442	0.463119137	0.835927584
101.0198018	-0.398615043	0.164130223	-2.428651082	0.015155109	0.257824025
70.91278467	-0.039810918	0.362291922	-0.109886299	0.912499548	0.980530316
78.17907243	0.076169015	0.136873702	0.556491231	0.577875087	0.887079361
11.21550004	-0.323928686	0.314478438	-1.030050543	0.30298628	0.740555303
104.3024937	-0.078567054	0.127995093	-0.613828642	0.539328568	0.869675597
696.9469035	-0.670771755	0.503375281	-1.33254806	0.18268016	0.628481684
5.072085411	-0.461084777	0.628318915	-0.733838765	0.463047011	0.835927584
128.4411422	-0.3796134	0.154722637	-2.453509122	0.014146995	0.25138514
758.4725316	0.047142599	0.095057703	0.49593665	0.619939107	0.901976704
75.1556078	0.225669755	0.159318194	1.41647196	0.156637368	0.595721049
6.838061655	0.333880453	0.448661295	0.744170395	0.456773396	0.832142746
17.09483546	-0.149736918	0.293336272	-0.510461652	0.609728074	0.900307811
188.8314915	-0.093537196	0.096852525	-0.965769313	0.334159627	0.761545389
510.2856433	0.004379507	0.145714435	0.030055413	0.97602286	0.993473413
74.57528721	-0.053506179	0.108073188	-0.495092078	0.620535126	0.901976704
3369.711735	-0.153780181	0.102857767	-1.495076017	0.134894604	0.567901571
299.8408572	-0.363034541	0.158642942	-2.288374989	0.022115693	0.298061592
844.7334742	0.02757845	0.158320776	0.174193497	0.861713396	0.970324122
3412.17649	-0.254483655	0.168178935	-1.513171995	0.130235976	0.562578903
984.9811003	0.063860421	0.096400331	0.662450223	0.507682726	0.859144938
117.0374799	-0.202218394	0.161465024	-1.252397507	0.210425054	0.65559075
137.7815765	-0.250456527	0.164445113	-1.523040253	0.127748635	0.558127301

112.9227891	0.09584575	0.092429018	1.036966015	0.299751696	0.738923503
14.66104626	-0.33786205	0.313887425	-1.076379692	0.281757481	0.723215865
560.1316844	-0.053651429	0.074141801	-0.723632668	0.46929128	0.839503873
286.5328356	-0.105409002	0.069251081	-1.522127884	0.12797704	0.558465847
20.69697769	0.129534785	0.275421766	0.470314263	0.638130509	0.907684986
616.9693381	0.026908873	0.122357639	0.219919841	0.825933583	0.964118059
12.55153165	0.205910989	0.280195825	0.73488243	0.4624111	0.835625577
53.28502763	-0.13718377	0.223879925	-0.612756012	0.54003768	0.869829343
592.6977483	0.016101315	0.064400582	0.250018163	0.802573302	0.956727875
147.7589785	0.068279083	0.137949289	0.494957846	0.620629877	0.901976704
266.5039352	-0.013750655	0.076920804	-0.178763794	0.858123176	0.969966486
13.85282348	-0.284102573	0.330856043	-0.858689388	0.390511907	0.794005116
298.6601309	-0.049057578	0.103852762	-0.472376247	0.636658255	0.907489155
14.67651188	0.327287704	0.258425191	1.266469817	0.205344929	0.650249702
530.7740464	0.107215019	0.05872216	1.825801691	0.067880138	0.455754696
702.9836171	0.034834574	0.050402495	0.691127964	0.489485128	0.849736271
13.39980088	-0.156163253	0.320992769	-0.486500843	0.626612108	0.904257122
1314.649359	-0.583931617	0.419521856	-1.391897963	0.163953299	0.602869201
214.4832647	0.112302083	0.129776806	0.865347872	0.386847883	0.792352176
191.920632	0.053601102	0.215691967	0.248507642	0.803741658	0.957233889
129.9865005	-0.069741563	0.105991114	-0.657994435	0.510541707	0.860200661
758.9094511	0.199014407	0.069290341	2.872181095	0.004076493	0.144354659
50.86744813	-0.062040288	0.252061313	-0.246131734	0.805580258	0.957233889
432.4528117	0.254822729	0.277643244	0.917806339	0.358720263	0.777078492
19.12735009	-0.362977033	0.274829565	-1.320735024	0.186589732	0.63228906
15.36621876	-1.827733476	0.833435401	-2.193011568	0.028306546	0.325561673
1559.079913	0.054806135	0.047462749	1.154718946	0.248205538	0.69187626
86.45451569	0.519196917	0.375877768	1.381291902	0.167189227	0.609023925
250.2393857	-0.164713655	0.103878971	-1.585630405	0.112823175	0.533719601
62.68173015	0.070962269	0.176400564	0.40227915	0.687478598	0.923064029
317.766603	0.01074871	0.068632859	0.156611723	0.875550863	0.973587929
23.85839061	-0.38851061	0.339624404	-1.14394197	0.252647704	0.69506877
26.38903007	-0.331397784	0.262546463	-1.262244328	0.206860882	0.651884304
2443.916072	0.189747926	0.138913285	1.365945137	0.171956184	0.616109027
209.9188822	0.326097707	0.160485149	2.031949431	0.042158776	0.376265422
314.2155385	0.119999104	0.162211469	0.73976954	0.459439844	0.834180976
51.44889523	-0.28653675	0.280386577	-1.021934621	0.306811845	0.743405059
98.67477361	-0.099003949	0.139963341	-0.707356285	0.479345096	0.845076955
660.845489	0.010210041	0.081953769	0.124582936	0.900853738	0.979217606
408.57023	-0.101631985	0.089710067	-1.132893878	0.257258821	0.70050498
1153.086653	-0.127385162	0.067566411	-1.885332677	0.059384937	0.434941039
848.9757979	-0.170630073	0.144052926	-1.184495714	0.236216892	0.678901985
182.4779887	0.152893174	0.093214575	1.640228188	0.10095773	0.512774612
275.5896663	-0.023879301	0.103397188	-0.230947297	0.817355746	0.961335246
741.6806412	0.00591857	0.060340365	0.098086414	0.921863676	0.982590996
339.0227487	-1.342181058	1.085371572	-1.23660974	0.216232002	0.660475876
11.27466746	-0.366019418	0.286338239	-1.278276415	0.201151981	0.645045462
490.7465964	-0.187852502	0.134028737	-1.40158376	0.161039579	0.600875279

605.0230752	0.133462505	0.311940064	0.42784663	0.668762785	0.917626349
32.52381947	-0.224796872	0.270827424	-0.830037332	0.406517677	0.804663634
53.95190267	-1.234427237	0.522209936	-2.363852451	0.018086014	0.27123243
875.76016	0.176994175	0.079812752	2.217617762	0.026580906	0.318325702
74.23970637	-0.641416666	0.188624535	-3.400494355	0.000672641	0.059977924
484.804161	0.023889729	0.17249511	0.13849511	0.889849134	0.976961229
17.31286454	-0.460922769	0.330771355	-1.393478492	0.163475145	0.602187485
63.56421228	0.089731668	0.197957752	0.453286964	0.650342118	0.910782566
14.04013587	0.172264018	0.36440525	0.472726498	0.636408319	0.907353194
398.9200705	-0.067130472	0.056619921	-1.185633442	0.23576709	0.678901985
9.520743232	-2.058414903	1.047558634	-1.964963905	0.049418415	0.404138887
90.20141865	-0.271498685	0.129334314	-2.099200717	0.035799211	0.357462935
580.3627203	-0.020783934	0.053801593	-0.386307038	0.699269289	0.925900484
127.2746699	-0.402725879	0.177522024	-2.268596707	0.023292862	0.304263956
304.47478	-0.462612039	0.176505556	-2.620948874	0.008768541	0.203117285
453.9197972	-0.04126644	0.075490227	-0.546646126	0.584621874	0.889519618
44.6446147	-0.243685507	0.180324555	-1.35137173	0.176576385	0.620182124
130.1381713	-0.105735643	0.186758153	-0.566163464	0.571282664	0.884717095
118.9990575	0.198810135	0.140993675	1.410064216	0.158520722	0.598577482
92.75471471	0.338071607	0.23746168	1.423689108	0.154536488	0.592506562
14.36918287	0.476870364	0.350566479	1.360285116	0.173739717	0.617663245
520.2324192	-0.112658152	0.119028038	-0.94648415	0.343901703	0.767121103
17.73524777	-0.177826411	0.342343249	-0.519438931	0.603454689	0.899398548
748.6766844	0.000215209	0.076742973	0.00280428	0.997762512	0.999382914
58.34534145	0.077142802	0.192768889	0.400182844	0.68902185	0.923813534
301.1949661	0.097263108	0.064601054	1.505596295	0.13217084	0.564647789
230.6074484	0.11585389	0.082924299	1.397104234	0.162382229	0.601647497
805.3871596	0.158180473	0.091895445	1.721309183	0.085194734	0.488696192
835.0254881	0.108993522	0.052639851	2.070551489	0.03840073	0.36671458
626.9469513	0.031801209	0.08387643	0.379143567	0.704581256	0.9269128
69.01334047	0.31225696	0.250065946	1.248698454	0.211775387	0.656521334
6.301095052	0.051231059	0.382429682	0.133962036	0.893432594	0.978111861
123.1462387	-1.191924816	0.511497519	-2.330265096	0.019792144	0.281261453
85.04593048	-0.058567302	0.114510502	-0.511457908	0.609030455	0.900307811
669.8020349	0.154547687	0.065532749	2.358327538	0.018357488	0.272290126
68.56361445	-0.330617149	0.145770377	-2.268068144	0.023325054	0.304263956
376.3839059	-0.546836279	0.210062239	-2.603210751	0.009235516	0.208708566
338.2452671	0.050287886	0.11836014	0.424871804	0.67093013	0.919117083
70.11509449	0.163539529	0.253125636	0.646080464	0.518227246	0.862002027
926.3273333	0.475952585	0.321746398	1.479278674	0.13906585	0.571316324
797.9166914	0.086472385	0.065739534	1.315378729	0.188382651	0.634590162
519.2563458	-0.237439696	0.150545642	-1.577194087	0.114750873	0.535291597
485.5399045	-0.028176092	0.073594738	-0.382854707	0.701827496	0.926243544
92.66579026	0.012477011	0.19075249	0.065409426	0.94784802	0.985917197
160.308949	0.092669641	0.093770983	0.988254977	0.323027789	0.755505673
1207.618506	0.063655064	0.095370704	0.667448822	0.50448549	0.857729637
613.4812253	0.075650826	0.057521307	1.315179182	0.18844969	0.634590162
350.2341546	-0.065143889	0.076097051	-0.856063246	0.391962797	0.795207417

909.296729	0.131924061	0.082644509	1.59628343	0.110425528	0.529628512
243.6694436	0.040539187	0.183223118	0.22125585	0.824893225	0.963851398
9.174838911	-1.657069883	0.680566102	-2.434840462	0.014898357	0.256113483
10625.51418	0.000725576	0.139476696	0.005202132	0.995849318	0.998981303
34.92124249	-0.190871437	0.176631682	-1.080618347	0.279866918	0.721663546
45.56140874	-0.073742769	0.192444799	-0.383189203	0.701579483	0.926243544
292.1350873	0.126155729	0.103831105	1.21500902	0.224362651	0.667854242
15.29329712	-0.234175955	0.31934094	-0.733310159	0.46336928	0.836122746
1284.439669	-0.026918514	0.126917885	-0.212093936	0.83203375	0.966227926
704.5977481	0.026858632	0.144755912	0.185544286	0.852802148	0.969966486
10.69555405	-0.060063815	0.341126496	-0.176074904	0.860235096	0.969966486
43.60106867	-0.128021621	0.193870197	-0.660347095	0.509031115	0.859636085
3857.922215	0.179094121	0.065446248	2.736507064	0.006209526	0.173421522
57.7252394	-0.011818976	0.125300979	-0.094324692	0.924851236	0.982590996
88.42045311	0.061272785	0.119514333	0.51268148	0.608174146	0.900307811
609.9347188	0.077395695	0.064341471	1.202889749	0.229018988	0.671940348
136.9407503	-0.091478139	0.119122764	-0.767931639	0.4425278	0.824977821
14.66708217	0.097464778	0.257810925	0.37804751	0.7053953	0.926977731
1151.070419	0.126069634	0.043278032	2.913016781	0.003579554	0.137265682
209.3595711	0.053830838	0.088383042	0.609062961	0.542482707	0.871069897
1153.711273	-0.028756847	0.155795146	-0.184581151	0.853557571	0.969966486
805.7242085	0.032185513	0.078292121	0.411095174	0.681002748	0.922615232
644.1668602	0.083247837	0.056239051	1.480249677	0.138806628	0.570638112
2531.81473	0.195789992	0.164632008	1.189258359	0.234338016	0.677194734
1225.137249	0.024724552	0.108441632	0.227998706	0.819647247	0.96243206
11.16154229	-1.105056049	0.657607222	-1.680419577	0.09287571	0.500506422
35.23739988	-0.020949869	0.192626086	-0.10875925	0.913393446	0.980550322
201.7406313	-0.020232806	0.111765802	-0.181028596	0.856345131	0.969966486
6.312932623	-0.638593917	0.455501764	-1.401957069	0.160928067	0.600875279
11.25529245	-0.392181647	0.345273213	-1.135858886	0.256015628	0.69930118
755.389642	0.130398734	0.088092624	1.480245765	0.138807671	0.570638112
601.4579351	0.026323182	0.085256163	0.308754005	0.757508659	0.945625212
81.20771409	0.075530706	0.183930523	0.410648026	0.681330643	0.922658753
50.43486531	0.046471419	0.207123204	0.224366069	0.822472471	0.963169453
507.6016672	0.160622063	0.105949003	1.516031848	0.129511299	0.561354686
1915.579643	0.041670085	0.088118243	0.472888287	0.636292882	0.907353194
45.4705278	-0.19949808	0.236087844	-0.845016316	0.398101729	0.799250874
1048.802783	-0.140374984	0.147598014	-0.951062827	0.341572483	0.765195212
1006.464679	0.272817534	0.131181693	2.079692131	0.03755378	0.362130981
736.6868079	0.208720395	0.210865529	0.989827008	0.322258681	0.75479971
216.0830741	0.089185533	0.18207821	0.489819913	0.624261339	0.902875034
473.2068967	-0.082833332	0.159510407	-0.519297349	0.603553402	0.899405764
422.9512082	0.013841504	0.252774415	0.054758326	0.956331002	0.98762377
1435.347339	-0.072275389	0.185304249	-0.39003633	0.696509682	0.925326625
30.89442431	-0.69427581	0.351831971	-1.973316433	0.048459522	0.400922142
4.58500348	-0.107222987	0.540176805	-0.198496097	0.842656938	0.967725037
49.58468521	-0.112783743	0.149761905	-0.753086998	0.451397637	0.828948899
310.1462545	0.013748423	0.071989351	0.190978564	0.848542388	0.969123636

439.5668186	0.009475396	0.074679562	0.126880711	0.899034813	0.978996707
57.56635215	0.066737232	0.138181413	0.482968228	0.629118296	0.904638489
6.471184815	-0.15533083	0.370720667	-0.418996952	0.67521836	0.921149714
196.8882521	-0.178342841	0.17430278	-1.023178409	0.3062235	0.742703628
15.77687451	-1.482785211	0.541814634	-2.736702034	0.006205848	0.173421522
685.5346153	0.377721345	0.088461241	4.269907831	1.95554E-05	0.008040519
358.7941832	-0.226801575	0.158506438	-1.430866644	0.152468438	0.589868747
17.35101561	-0.914744924	0.333001332	-2.746970769	0.00601485	0.168940801
629.1320673	-0.083883872	0.1789298	-0.468808838	0.639206282	0.907684986
43.16324945	0.0849057	0.430400563	0.197271351	0.843615198	0.968131661
19.00461041	-0.480813953	0.492691808	-0.975891917	0.329118028	0.758888028
16.94674606	-0.465608866	0.486179995	-0.957688244	0.338219986	0.764007173
9.245132722	0.15921921	0.681848035	0.233511284	0.815364408	0.960909552
6.312287263	0.202931977	0.484430998	0.418907911	0.675283436	0.921149714
7.985379095	-0.322970204	0.507372686	-0.63655418	0.524415279	0.863946059
6.103122529	-0.680633004	0.977523387	-0.69628309	0.486251561	0.849161822
57.41082902	-0.521401398	0.368912394	-1.413347465	0.157553584	0.597210795
87.15331748	-0.154039607	0.150853263	-1.021122142	0.307196574	0.743405059
180.4231849	0.126807315	0.120762262	1.050057462	0.293691695	0.734036718
4.779213073	-0.651016899	0.516549035	-1.260319649	0.207554074	0.652275034
55.43704849	-0.274023725	0.146180939	-1.874551681	0.060854426	0.437265637
65.85771385	-0.325810366	0.24983437	-1.304105459	0.192197628	0.638587944
7.586182128	-0.876539484	0.546604265	-1.603608937	0.10880028	0.52612275
40.27578356	-0.416111972	0.251686282	-1.653296189	0.098270598	0.509712212
58.86267597	0.226798336	0.327727126	0.692034067	0.488915935	0.849736271
276.2193282	0.003604762	0.101865668	0.035387407	0.971770826	0.992799208
2313.453091	-0.130922856	0.128744767	-1.016917881	0.309192496	0.744779061
203.2033791	-0.078282121	0.095873905	-0.81651124	0.414207797	0.808821113
5.41340129	-0.610269363	0.686097269	-0.889479364	0.373745509	0.786373874
20.11468933	-0.396245524	0.277882158	-1.42594806	0.153883335	0.59219701
51.66995605	0.160390177	0.272364027	0.588881649	0.555940669	0.878240444
346.2184731	0.124696465	0.156133605	0.798652316	0.424492044	0.815487505
322.9679532	0.092020139	0.067479749	1.363670448	0.172671309	0.616428289
245.90304	0.042501139	0.092390261	0.460017521	0.645503645	0.909300678
251.4826391	0.110962719	0.081229561	1.366038634	0.171926838	0.616109027
580.2999422	0.025909697	0.077038639	0.33632081	0.736628959	0.938503341
315.595436	-0.240394449	0.06767561	-3.552157863	0.000382086	0.042299378
371.7744098	-0.236618174	0.129505378	-1.827091489	0.067686015	0.455435138
614.4165886	0.032019537	0.072357581	0.442518068	0.658114359	0.91380052
1775.495465	-0.00140986	0.143357991	-0.009834542	0.992153297	0.998426345
417.4365873	0.049537499	0.089140699	0.555722579	0.578400519	0.887251177
93.67181724	-0.146307603	0.125408689	-1.166646458	0.243353174	0.686012467
170.0784399	-0.230951684	0.087869104	-2.628360538	0.008579753	0.200844982
155.5570165	-0.060513714	0.089801027	-0.673864386	0.500397536	0.856182624
14.68497385	-0.187546328	0.329692232	-0.568852735	0.569456084	0.8841274
8.702048038	-0.381347074	0.337584322	-1.129635022	0.258630047	0.701528671
142.9182441	0.010615683	0.079558233	0.133432869	0.893851052	0.978111861
56.17898309	-0.301734927	0.254967321	-1.183425884	0.236640404	0.679408314

633.7979045	0.016740194	0.160721003	0.104156851	0.917044876	0.981290994
179.5794766	-0.006996026	0.096825965	-0.072253614	0.942400078	0.98446865
19.68814344	-1.235848067	0.468737016	-2.636548905	0.008375413	0.198674451
745.5881946	0.010617753	0.102890345	0.103194839	0.917808336	0.981376661
143.8871307	-0.10810035	0.161497817	-0.669361061	0.503265184	0.857172233
85.74098141	-0.143596825	0.165056478	-0.869986003	0.384308054	0.790375007
446.1336298	-0.028882763	0.120708668	-0.239276631	0.810891085	0.959197877
164.8153571	0.094205065	0.13328421	0.706798387	0.479691777	0.845119155
2399.042553	-0.072638035	0.10575023	-0.686882996	0.492156463	0.851383417
68.34947656	0.018990384	0.13327967	0.142485225	0.886696749	0.976961229
10.45702165	-0.547821344	0.496517158	-1.103328122	0.269884699	0.712556401
1054.78148	0.016978647	0.080587978	0.210684612	0.833133383	0.966227926
12.19830621	0.411741467	0.405816779	1.014599415	0.310296818	0.745771331
755.0976251	0.092894068	0.105013018	0.884595734	0.376374708	0.786851292
238.5873407	-0.169556078	0.169629053	-0.999569802	0.317518743	0.751021269
144.3783818	0.095813546	0.111298318	0.860871458	0.389308843	0.793111346
14.30392047	-0.78781519	0.526890071	-1.495217378	0.13485772	0.567901571
838.0757072	-0.056368656	0.074929818	-0.752286047	0.451879057	0.829548482
8.841894071	0.246051767	0.323381517	0.76087146	0.446733847	0.826805895
328.0694714	0.13434734	0.08814229	1.524209769	0.127456318	0.557014593
349.9369706	0.253190675	0.200464874	1.263017656	0.206582834	0.651884304
39.88774822	-0.159366294	0.169687039	-0.939177768	0.347639481	0.768780165
54.93078854	-0.13137	0.15872776	-0.827643505	0.407872422	0.805729026
84.79624139	-0.068682997	0.145919148	-0.470692149	0.637860592	0.907684986
236.3404265	0.123073401	0.084696377	1.453112933	0.146192401	0.581547948
247.2105784	0.030227346	0.13077991	0.23113142	0.817212707	0.961335246
1048.84855	0.029676159	0.047093209	0.630157935	0.528591258	0.86594688
246.7313845	0.083288956	0.098015984	0.849748715	0.395464808	0.797502736
40.68898304	-0.005597409	0.175525413	-0.031889452	0.97456021	0.993301399
97.76611092	0.167051318	0.189751705	0.880367941	0.378660018	0.787072097
928.9649668	0.131993704	0.089342947	1.477382471	0.139573142	0.572130062
219.9996204	-0.160519954	0.207144384	-0.774918202	0.438387996	0.82251807
6.948639692	0.604824558	0.411880049	1.4684483	0.14198249	0.576571115
945.4900812	0.255691161	0.08013968	3.190568779	0.00141993	0.091780828
78.34513395	-0.601556479	0.245140482	-2.453925498	0.014130625	0.25138514
20.45978204	-0.526002688	0.341725765	-1.539253814	0.123742344	0.551842421
1548.054338	0.159128375	0.093954128	1.693681573	0.090325808	0.49573697
89.23925673	-0.216741355	0.130107597	-1.665862416	0.095740821	0.504685054
47.52046586	0.108735741	0.21486329	0.506069423	0.612807915	0.901444946
12.46242621	-1.97016691	0.981993514	-2.0062932	0.044824975	0.38775713
69.81723829	0.017046586	0.130170806	0.130955526	0.895810489	0.978798764
53.65160217	0.048827184	0.312865211	0.1560646	0.875982102	0.97359116
272.0719935	-0.09326138	0.070617348	-1.320658218	0.186615351	0.63228906
32.60382779	0.004881168	0.178761459	0.027305485	0.978216082	0.9945432
603.6846755	-0.04690902	0.095557716	-0.490897249	0.623499123	0.902854913
448.493759	0.074323285	0.090601981	0.820327375	0.412029505	0.808272153
2029.673038	0.206381264	0.250549813	0.823713504	0.410102365	0.807613405
32.77068018	0.00358635	0.284588368	0.012601887	0.989945415	0.997999732

289.147644	-0.302407994	0.302703975	-0.999022209	0.317783933	0.751051855
241.0278035	-0.061922032	0.122253079	-0.506506935	0.612500822	0.901395623
241.01249	0.044564622	0.245301977	0.181672496	0.855839752	0.969966486
132.5793669	-0.210440812	0.150575151	-1.397579953	0.162239242	0.601569453
20.41325462	-0.20365151	0.311466263	-0.653847735	0.513209909	0.860406963
25.98707141	-0.065226555	0.218953996	-0.297900728	0.765778932	0.94709722
82.19974003	-0.328504025	0.260016305	-1.263397789	0.206446258	0.651884304
936.3099139	0.086438027	0.156678292	0.551691149	0.581159975	0.887898616
3219.455164	0.097160389	0.115329804	0.842456902	0.399532254	0.80037241
314.9832811	-0.096218681	0.066865049	-1.438998137	0.150151051	0.588453924
13.58949467	0.433636854	0.420071817	1.032292185	0.301935261	0.739990763
91.177899	-0.016543688	0.180041274	-0.09188831	0.92678678	0.983320043
63.74522071	0.05741855	0.156140138	0.367737281	0.713069139	0.929705922
100.6701835	-0.127211621	0.09766288	-1.302558563	0.192725512	0.638943964
907.1235181	-0.07522117	0.062704567	-1.199612306	0.230289945	0.673001336
15.5501718	0.203021014	0.292345	0.694456939	0.487395696	0.849513564
10.99614175	0.45387254	0.328274314	1.382601442	0.166787107	0.608824353
228.527922	0.047783476	0.093736322	0.509764787	0.610216258	0.900307811
88.13567933	-0.122032836	0.272098719	-0.448487362	0.653801505	0.911945899
120.2075863	-0.250048594	0.090646946	-2.758488901	0.005806928	0.166644523
77.81831724	0.162900149	0.163917951	0.993790786	0.320324733	0.752619929
122.022141	-0.131226172	0.11597927	-1.131462308	0.257860559	0.701069052
866.3260152	-0.417418415	0.12116708	-3.444982038	0.000571097	0.054892096
40.97928594	0.018880078	0.142684504	0.132320454	0.89473083	0.978702759
381.2377128	-0.211675938	0.127341428	-1.662270801	0.09645849	0.506056944
1127.852445	-0.12096465	0.08944492	-1.352392619	0.176249749	0.619973572
11.4483998	-0.013017171	0.389256026	-0.033441156	0.97332279	0.993248159
159.9970128	-0.03421485	0.138743768	-0.246604591	0.805214251	0.957233889
20.82361072	0.374455559	0.375284258	0.99779181	0.318380322	0.751465423
282.5614033	0.029057598	0.171192666	0.169736231	0.865217579	0.971917805
1392.265255	0.205257429	0.093778298	2.188751906	0.028614877	0.327071359
5.3509714	0.163663811	0.367404237	0.445459782	0.655987518	0.912934239
85.15808073	-0.13509762	0.14988706	-0.901329439	0.367413186	0.781703698
65.59459545	-0.036920683	0.134850838	-0.273789048	0.784246741	0.95189998
39.13781133	0.182329438	0.18344062	0.993942551	0.320250837	0.752619929
384.1532066	-0.004719197	0.096347772	-0.048980862	0.960934547	0.989165233
901.1556857	-0.0475013	0.105233248	-0.45139061	0.65170805	0.911172434
338.6350093	-1.208004542	0.407724594	-2.962795379	0.003048592	0.129722664
185.3461664	-0.120036565	0.098771867	-1.215291048	0.224255105	0.667854242
580.1812426	0.043347249	0.19114075	0.226781832	0.820593392	0.962685375
2187.257901	0.06146603	0.095602768	0.642931496	0.520268551	0.863188882
902.8714394	0.027421079	0.111411467	0.246124388	0.805585945	0.957233889
262.1525991	-0.189454442	0.098964498	-1.914367742	0.055573184	0.42467438
259.9838443	-0.167267501	0.093722095	-1.784717907	0.074307085	0.469237829
302.632752	-0.212846798	0.130026074	-1.63695474	0.101639927	0.513342798
80.03679399	-0.253008426	0.172841684	-1.463816024	0.143244223	0.578069441
382.8934894	-0.003896883	0.102696896	-0.037945477	0.969731154	0.992511035
30.55485406	-0.081218015	0.201473887	-0.403119316	0.686860452	0.923064029

943.7999619	-0.047133237	0.119793487	-0.393454084	0.693984131	0.924581226
20.45194323	0.151629333	0.297426672	0.509804088	0.610188722	0.900307811
268.1614102	-0.06299771	0.075741731	-0.831743725	0.405553614	0.804137887
121.686726	-0.182124487	0.117916299	-1.54452343	0.12246158	0.548630842
83.62894407	-0.128393032	0.143072195	-0.897400309	0.369505348	0.783514907
12.14478276	-0.32277608	0.267320757	-1.207448624	0.227259419	0.669850929
86.07724938	-0.268826073	0.129024065	-2.083534365	0.037202543	0.361102242
372.3761686	0.175496236	0.432274067	0.405983725	0.684754558	0.922907843
166.7061086	0.029206841	0.119724876	0.243949645	0.80726982	0.957929122
451.5552808	-0.095166655	0.082754073	-1.149993616	0.250146501	0.694208707
321.2067051	-0.014065451	0.08483361	-0.16580046	0.868313984	0.972204994
5715.729196	-1.036130299	0.627020889	-1.652465361	0.098439721	0.510190738
113.2692999	0.313780395	0.165316952	1.898053352	0.057689054	0.431275905
3168.140387	0.281433822	0.183070423	1.537298144	0.124220316	0.552139359
913.1113929	0.035950804	0.08056621	0.446226826	0.655433407	0.912518505
19.51183008	0.228894456	0.310822606	0.736415085	0.461478127	0.835061032
88.51582925	-0.102788474	0.12334138	-0.833365682	0.404638523	0.8031996
81.63193907	-0.250800227	0.140893283	-1.780072278	0.075064133	0.470687737
70.42495871	-0.050213767	0.190245364	-0.263942131	0.791824511	0.953153173
1848.310439	-0.001622146	0.158425749	-0.010239156	0.991830478	0.998372984
761.7226773	0.128390842	0.075012978	1.711581709	0.086973781	0.491556286
266.0559542	0.129077063	0.1290782	0.999991195	0.317314769	0.751000532
241.4823564	0.337334697	0.189759681	1.777694268	0.075454082	0.470687737
110.1629892	-0.036867331	0.135728932	-0.271624705	0.785910601	0.952591608
149.8201367	0.005261453	0.126152986	0.041706927	0.966732332	0.990966732
316.6659318	-0.017685612	0.108519224	-0.162972161	0.870540354	0.972881041
22.46883278	-0.205099722	0.305599766	-0.671138348	0.502132399	0.857076081
64.56515279	-0.182012602	0.129831921	-1.401909487	0.160942277	0.600875279
87.60646634	0.004661885	0.265884897	0.017533472	0.98601103	0.996581982
72.79824941	0.080176523	0.135311657	0.592532267	0.55349422	0.877205564
4619.273302	0.209843979	0.109716281	1.912605642	0.055798557	0.425517897
719.3569558	0.034320809	0.14668226	0.23398064	0.815000009	0.960909552
31.61541986	0.043143406	0.177360141	0.243253112	0.807809325	0.958108464
715.2886609	0.113269676	0.056375003	2.00921807	0.044514012	0.387130675
37.11953586	-0.312853088	0.329006878	-0.950901362	0.34165445	0.765195212
1534.428702	-0.124737974	0.110569336	-1.128142562	0.259259717	0.702208457
15.71308884	-0.075020934	0.293252853	-0.255823371	0.798087226	0.954840537
147.9109882	-0.002804482	0.281219586	-0.009972571	0.992043171	0.998383398
937.6976997	-0.003733253	0.139136922	-0.026831505	0.978594125	0.99459552
89.78224805	-0.155159695	0.260019133	-0.596724148	0.550691567	0.875749053
42.09086849	-0.052079569	0.18349824	-0.28381509	0.776552089	0.950667772
51.26484967	-0.089216496	0.158307463	-0.563564692	0.573050422	0.885324323
869.6992689	0.058974443	0.084025176	0.701866345	0.482762519	0.846865467
531.6935705	0.104559543	0.074459141	1.404253947	0.160243243	0.600672338
6.480397038	0.400942713	0.446031032	0.898912147	0.368699457	0.782820132
16.58688539	-0.159561813	0.325682813	-0.489930101	0.624183362	0.902875034
7.322392351	-0.288489822	0.450647341	-0.640167589	0.522063651	0.863362704
126.2786802	0.128593351	0.131736345	0.976141786	0.328994207	0.758767879

6.715895174	0.097731839	0.716468418	0.136407742	0.891498954	0.977283948
79.49179545	0.047661774	0.144254314	0.330401032	0.741096962	0.939831839
29.0912157	-0.40660011	0.526683461	-0.772000907	0.44011389	0.823715107
1957.851007	0.161280666	0.115781191	1.392978124	0.163626406	0.602187485
469.4501299	0.113343975	0.055770556	2.03232643	0.042120621	0.376265422
161.1102665	-0.087028862	0.126913528	-0.685733533	0.492881157	0.851795317
6.487015529	-0.076082129	0.444367943	-0.17121426	0.864055295	0.971717556
44.57585239	-1.793267	0.738293003	-2.428936739	0.015143174	0.257824025
170.6718365	-0.098026093	0.112379979	-0.872273636	0.383059117	0.789880775
114.4496274	-0.106936672	0.137329514	-0.778686742	0.436164268	0.822078703
653.7796503	0.213853544	0.214342545	0.9977186	0.318415831	0.751465423
33.78440197	-0.077560986	0.380746466	-0.203707697	0.838581927	0.966440785
2055.43785	0.109448223	0.061865324	1.769136835	0.076871042	0.472050486
75.5566806	-0.844892896	0.278995805	-3.028335485	0.002459049	0.118178076
2822.564255	0.092427764	0.053021119	1.743225463	0.081294239	0.481991357
212.2037356	0.091993307	0.095466753	0.963616169	0.335238397	0.762359618
94.45686967	0.165258738	0.140877262	1.173068921	0.240768169	0.682548146
65.74668861	0.543473736	0.365846566	1.485523676	0.137405156	0.569036171
395.1368978	-0.100984421	0.089889838	-1.123424219	0.261257368	0.703498557
39.86702627	-0.572199552	0.404747109	-1.413721159	0.15744379	0.597100432
20.48402405	-0.458515355	0.3199181	-1.433227299	0.15179289	0.589757816
24.10819366	0.084346275	0.28119455	0.299957006	0.764209951	0.946826103
562.0441417	-0.046944235	0.067503512	-0.695433955	0.486783388	0.849484378
105.1513474	0.184696805	0.200196705	0.922576646	0.356227871	0.774305138
5.07267457	0.28075099	0.456487562	0.615024403	0.538538605	0.869675597
12.04077794	-1.139166424	0.558344608	-2.040256872	0.041324748	0.374892505
1362.49376	-0.013041167	0.082939453	-0.157237191	0.875057919	0.973587929
773.3879454	0.036814609	0.082677767	0.44527822	0.656118705	0.913027083
11.65499645	0.366278677	0.36584333	1.001189983	0.316734968	0.750009759
310.6200172	-0.11335262	0.137189404	-0.826249091	0.408662806	0.806247273
4688.350392	0.232897376	0.090291093	2.579405882	0.009897042	0.215114309
22.86414796	0.212190195	0.300378913	0.706408426	0.479934182	0.845119155
35.84649098	-0.468216392	0.630726621	-0.742344428	0.457878681	0.83323337
1430.689642	-0.018609649	0.141654836	-0.131373196	0.895480092	0.978798764
3468.623474	0.201457904	0.091340199	2.205577671	0.027413584	0.323603282
91.19390499	0.225538936	0.173939084	1.296654725	0.194750014	0.640229004
43.65846219	0.252716756	0.594465326	0.425116057	0.670752073	0.918962624
527.6389289	-0.054126455	0.102498963	-0.528068313	0.597451923	0.89699598
7.614331473	-0.29080865	0.619553263	-0.469384422	0.638794882	0.907684986
160.9475881	0.032592796	0.083746933	0.389181971	0.697141539	0.92544754
519.9177041	-0.306141959	0.206750312	-1.480732756	0.138677802	0.570638112
62.49367169	-0.139938246	0.250911534	-0.55771946	0.577035967	0.88676146
9.893344085	-1.156772964	0.399198684	-2.897737416	0.003758651	0.140493831
191.5780539	-0.291888409	0.155030434	-1.882781341	0.059730007	0.435320275
313.0431418	-0.032214226	0.079068234	-0.407423111	0.683697256	0.922776218
210.6003626	-0.054305226	0.08916775	-0.609023167	0.542509083	0.871069897
42.54921628	-0.633100685	0.312693915	-2.024665826	0.04290169	0.37951653
312.1107289	-0.08360765	0.079388978	-1.053139255	0.292277174	0.733939814

207.4872889	-0.066787552	0.180606942	-0.369795045	0.711535208	0.929376205
87.05557705	0.109054002	0.133638819	0.816035358	0.414479913	0.809062418
21.32784705	-0.414681349	0.382305315	-1.08468633	0.278060604	0.720058269
152.3965249	-0.214047887	0.221232295	-0.9675255	0.3332814	0.761183657
39.62725078	-0.241439455	0.256186243	-0.942437235	0.345968831	0.76823142
34.652713	-1.598493019	0.707745226	-2.258571248	0.023910068	0.307486383
18.67185235	-0.670899826	0.328594926	-2.041723022	0.041179014	0.374892505
1291.877887	0.004480826	0.05463812	0.082009147	0.934639439	0.984334593
36.51312769	-0.035649606	0.373124547	-0.09554345	0.92388318	0.982590996
142.9102723	-0.012314189	0.328998711	-0.037429292	0.970142717	0.992623378
4.756072301	0.48564831	0.423820824	1.14588119	0.251844315	0.694735013
93.55605242	-0.226795609	0.540953688	-0.419251433	0.675032387	0.921149714
49.58023011	-0.244459174	0.278785924	-0.876870577	0.380556939	0.78844599
8.190442414	0.592573652	0.453636176	1.306275124	0.191459014	0.637278237
7.158275122	-0.154008062	0.389578289	-0.395319932	0.692606792	0.924581226
968.1450379	0.107021066	0.086195471	1.24160893	0.214380886	0.659648752
56.14993638	0.019810628	0.133733531	0.148135087	0.88223616	0.975560757
607.525129	-0.025293295	0.155306202	-0.162860815	0.870628024	0.972881041
369.9944945	-0.038983092	0.080344998	-0.485196252	0.62753714	0.904512355
58.1240815	0.042089678	0.140086902	0.300454054	0.763830838	0.946826103
466.4454897	-0.245248833	0.219038625	-1.119660208	0.262858588	0.705491334
6.503620278	0.042647659	0.497478254	0.085727684	0.931682894	0.984334593
14.85423264	-0.281105235	0.352838505	-0.796696593	0.425627263	0.816273895
118.8841027	-0.084099657	0.11001193	-0.764459431	0.444593504	0.826302547
9.70591922	0.468860573	0.399622404	1.173258978	0.240691967	0.682548146
197.3866012	-0.041068557	0.079417882	-0.51711977	0.605072554	0.90025894
14.53613954	-0.276392134	0.3336717	-0.828335558	0.407480491	0.805288281
6.456300381	-0.976230818	0.630612297	-1.548068159	0.121605887	0.547270993
858.93485	-0.035026476	0.196538676	-0.178216709	0.858552788	0.969966486
2521.194133	-0.027054673	0.130179942	-0.207825202	0.835365454	0.966316856
181.3353838	0.133971168	0.115051716	1.164443025	0.24424452	0.686537673
287.3067723	0.229818301	0.110890116	2.072486784	0.038220067	0.365460878
38.10268181	-0.144617383	0.15300554	-0.94517743	0.3445683	0.767770582
222.9312265	0.015762027	0.083203629	0.189439181	0.849748619	0.969251033
23.81596142	0.606633837	0.249956191	2.426960638	0.015225908	0.257947316
1026.037452	-0.067740569	0.085809813	-0.789426833	0.429862578	0.818650305
571.2414557	-0.311181927	0.455619105	-0.682987003	0.494615055	0.853198001
74.17636888	0.071583716	0.408914745	0.175057801	0.861034216	0.970088938
220.1459978	0.239910727	0.112876421	2.125428193	0.0335509	0.347119785
1603.382609	0.254824887	0.091802767	2.775786557	0.005506835	0.162022566
152.6601323	0.417653609	0.114957515	3.633112722	0.000280023	0.035213026
11.14265576	0.501766586	0.364433401	1.376840277	0.168561635	0.610890818
1957.874771	0.150127382	0.13428346	1.11798863	0.263571849	0.70613403
7.325517896	0.719803572	0.481926215	1.493597047	0.135280972	0.568673186
8.788346019	0.554593653	0.492255965	1.126636734	0.259896101	0.702516818
236.2095178	0.003438795	0.080859624	0.042527956	0.966077826	0.990895439
12.56330052	0.440808793	0.296884378	1.484782718	0.137601391	0.569036171
138.6337237	0.10872682	0.107198384	1.014258021	0.310459649	0.745771331

47.18431964	0.153309476	0.283474826	0.540822187	0.588630148	0.891761618
279.1538693	-0.208667553	0.117811868	-1.771192984	0.076528616	0.471783446
1138.381402	0.183249188	0.088923459	2.06075191	0.039326714	0.370654063
92.1679972	0.177085897	0.219183927	0.80793286	0.419129234	0.811610533
197.8230112	-0.040523882	0.092641372	-0.437427478	0.66180136	0.915084888
6.454030439	0.160369346	0.382290465	0.419496065	0.67485363	0.921149714
933.3894686	-0.008012113	0.095923637	-0.083525952	0.933433343	0.984334593
104.1166179	-0.332057295	0.132025007	-2.515109098	0.011899555	0.235782022
49.72990939	0.054572022	0.154087939	0.354161539	0.723217825	0.931442639
179.2920518	-0.167090395	0.096637713	-1.729039217	0.083802076	0.486282844
81.13708044	0.020810735	0.22778896	0.091359717	0.927206769	0.983502443
18.54134005	-0.029138131	0.283032707	-0.102949695	0.918002896	0.981376661
61.17832338	0.35374796	0.231028745	1.531185913	0.125723451	0.554908265
427.8132448	0.216052404	0.111479771	1.93804132	0.052618182	0.41516755
70.00132141	-0.111277089	0.201176424	-0.553131858	0.580173123	0.887483205
269.329331	-0.072641831	0.154305188	-0.470767261	0.637806946	0.907684986
504.8509964	0.27535591	0.096470345	2.854306258	0.004313093	0.14642753
8.801760864	0.036720316	0.398512571	0.092143431	0.926584082	0.983240215
106.7147921	-0.084979237	0.105171266	-0.808008119	0.419085909	0.811610533
642.4618514	0.053802442	0.102840317	0.523164884	0.600859516	0.898289446
75.3693873	-0.010491508	0.144445293	-0.072633089	0.942098095	0.98446865
41.51212543	-0.00320668	0.160575277	-0.019969946	0.984067348	0.995803025
374.5217059	0.236449126	0.11384928	2.076860974	0.037814393	0.363296636
1940.129852	0.026782428	0.103018122	0.259977837	0.794880869	0.953773263
19.31663825	0.266135974	0.470034207	0.566205543	0.571254062	0.884717095
117.0393924	-0.153044881	0.130855575	-1.169570963	0.242173668	0.685171875
201.2739383	0.484733104	0.311055151	1.558350996	0.119150066	0.54348307
112.776346	0.100475267	0.201318477	0.499086166	0.617718683	0.901976704
104.1499883	0.057604146	0.162606101	0.354255745	0.72314723	0.931432762
218.1633318	-0.041096628	0.080963624	-0.507593739	0.611738282	0.900810789
18.6292899	-0.007637584	0.242884477	-0.031445337	0.974914385	0.993301399
2683.291599	0.175806742	0.10520072	1.671155313	0.094691004	0.50255154
454.4015434	0.043473576	0.094116994	0.461909952	0.644145897	0.908494289
4728.058493	0.01646252	0.076073868	0.216401778	0.828674581	0.964814045
892.508783	0.030808558	0.08067005	0.381908248	0.702529422	0.926349622
80.88979346	-0.076413358	0.134925149	-0.566338881	0.571163435	0.884717095
94.25367636	-0.01275312	0.130826939	-0.097480839	0.92234455	0.982590996
1140.225448	0.054648634	0.109051352	0.501127521	0.616281379	0.901900555
2314.517981	0.044716071	0.148571166	0.300974085	0.763434256	0.946826103
533.2287106	0.154219275	0.348549405	0.442460302	0.658156151	0.91380052
178.0254147	0.036262816	0.097750388	0.370973633	0.710657171	0.929359766
139.7964593	0.178942028	0.092028351	1.944422851	0.05184448	0.411696352
308.4722297	0.159467941	0.100009192	1.594532833	0.110816743	0.530615767
2821.336313	0.128252449	0.119317814	1.074880984	0.282428017	0.723520163
108.8296241	0.000595069	0.251426261	0.002366774	0.998111589	0.999394422
61.66413949	0.072418402	0.182447613	0.396927098	0.691421221	0.924455975
9.337991358	-0.199897585	0.313432666	-0.637768829	0.523624173	0.863484457
19.11416857	-1.090578036	0.536676634	-2.032095246	0.042144015	0.376265422

153.7122013	-0.05253177	0.12619973	-0.416258972	0.677220511	0.921393988
119.0202649	0.090963607	0.160368728	0.567215369	0.570567868	0.884612839
856.5639825	0.030019759	0.081418659	0.368708593	0.712344943	0.929589333
324.4824362	0.534352183	0.172296761	3.101347812	0.001926419	0.104224472
1452.799341	0.170462828	0.063614257	2.679632457	0.007370304	0.187448858
199.5442946	0.215919352	0.097949213	2.204401099	0.027496148	0.323773476
72.84106975	0.107836283	0.131237199	0.821689916	0.4112534	0.808165706
4.789571007	-1.603075527	0.99698697	-1.607920238	0.107852649	0.5247978
472.591003	0.106234778	0.113020406	0.939961038	0.347237546	0.768780165
40.42830961	0.003974343	0.174527911	0.022771962	0.981832174	0.99517046
108.0850792	0.075684546	0.151338931	0.500099643	0.617004917	0.901900555
1557.243848	0.033290415	0.134386782	0.247720904	0.804350357	0.957233889
50.80641457	-0.123754507	0.132818741	-0.931754857	0.351463229	0.770952892
267.9950978	0.193180546	0.089501378	2.158408627	0.030896076	0.334089034
300.3068038	0.083992987	0.076714719	1.094874468	0.273571636	0.716229876
801.0696041	0.12960455	0.082387667	1.573106204	0.115694229	0.53716935
71.13064792	0.160100247	0.201803229	0.793348292	0.427574928	0.817479701
4.890789035	-0.521909437	0.636628662	-0.819801979	0.412329002	0.808272153
22.23688148	-0.022626896	0.20523937	-0.110246373	0.912213986	0.980530316
173.8224121	0.177326447	0.104688835	1.693842964	0.090295128	0.49573697
519.3809224	0.144261448	0.109949438	1.312070802	0.189496247	0.635553768
311.4079134	0.146745101	0.083963003	1.747735257	0.080509881	0.480333436
604.6579529	-0.017266405	0.142609133	-0.12107503	0.903631607	0.979381499
77.53448495	-0.154186854	0.126174383	-1.222013934	0.221702358	0.665526839
7.482105982	-0.810487575	0.90085107	-0.899690971	0.368284729	0.782648499
1611.651982	0.159102887	0.119717055	1.328990982	0.183850945	0.629653329
466.9501058	0.238408375	0.091916508	2.593749266	0.009493569	0.211314005
187.5391249	-0.055146154	0.171062731	-0.322373865	0.747169481	0.941343425
31.90655005	-0.009025551	0.188094464	-0.047984142	0.961728881	0.989264135
33.24654221	0.043844494	0.249271065	0.17589083	0.86037971	0.969966486
9.274425825	-0.181849318	0.446295061	-0.407464329	0.683666988	0.922776218
274.662438	0.147644131	0.128304489	1.150732387	0.249842348	0.693839857
1386.831512	-0.03948209	0.127796792	-0.3089443	0.757363897	0.945625212
449.7003646	0.102034063	0.068161234	1.496951519	0.134405873	0.567732615
397.9277869	-0.079889838	0.128873728	-0.619907868	0.535318446	0.868931202
48.11627614	0.24705338	0.161672739	1.528107848	0.126485763	0.556378986
1307.294809	0.095263944	0.101322731	0.940203084	0.3471134	0.768780165
633.2221018	0.091985271	0.149826296	0.613946106	0.539250942	0.869675597
99.23946403	0.211480736	0.16432849	1.286938958	0.198115565	0.643940842
10.30933807	-0.80382509	0.540813455	-1.486325982	0.137192918	0.569036171
14.26992568	0.197074568	0.284310911	0.693165687	0.488205579	0.849736271
576.6481128	0.001665767	0.071302838	0.023361868	0.981361622	0.994939365
25.35027692	-0.086091073	0.232309822	-0.370587315	0.710944932	0.929376205
38.70766361	-0.051941077	0.176766611	-0.293839863	0.768880273	0.947650255
9.260963095	-0.155716471	0.331888311	-0.469183355	0.638938582	0.907684986
674.6979331	0.106608727	0.103438994	1.030643505	0.302708027	0.74043163
888.1047095	-0.042726973	0.106798702	-0.400070149	0.68910485	0.923841137
297.3765007	-1.195666021	0.456631686	-2.618447332	0.008833093	0.20396848

243.4139645	0.03800911	0.082819239	0.45894058	0.646276837	0.909300678
486.8637856	0.028098459	0.074654722	0.376378859	0.70663526	0.928011279
483.2828259	0.066008159	0.080529615	0.819675577	0.412401077	0.808272153
911.4115833	0.028210864	0.065184336	0.432785939	0.66517029	0.916049829
34.9399739	-0.034645914	0.21601308	-0.160388038	0.872575412	0.972881041
278.6230704	0.015277344	0.081091143	0.188397194	0.850565299	0.969251033
102.1806753	0.192380636	0.166920605	1.152527793	0.249104256	0.692699829
152.4331869	0.132689649	0.106706004	1.243506876	0.213681109	0.659274529
116.6069199	0.02665555	0.146624965	0.181794077	0.855744333	0.969966486
343.3688929	0.093840233	0.089814398	1.044823936	0.29610435	0.735885928
5.563711379	-0.445604686	0.526457407	-0.846421155	0.397317841	0.79867172
44.49989076	-0.384330709	0.332412342	-1.15618664	0.247604823	0.691387775
100.5551548	-0.191723852	0.118090018	-1.623539873	0.104474047	0.518555962
57.95744561	0.164405387	0.195234145	0.842093409	0.39973567	0.80037241
5.171909843	0.362646858	0.552173469	0.656762554	0.511333605	0.860200661
141.5271064	0.099502567	0.108969947	0.913119354	0.361179773	0.778029658
185.7206718	-0.339800701	0.16861273	-2.015273111	0.043876041	0.383634512
2488.3793	-0.051024964	0.093849861	-0.543687159	0.58665677	0.890984353
1126.294271	-0.001114594	0.098021971	-0.01137086	0.990927562	0.998210818
572.6311273	0.065780289	0.121709884	0.540467931	0.588874371	0.891897927
215.0259439	-0.036988346	0.123416915	-0.299702404	0.764404165	0.946826103
79.82482162	-0.020522871	0.108534161	-0.18909135	0.850021221	0.969251033
64.49351038	0.081273743	0.234802207	0.346137051	0.72923972	0.93467002
5.81134681	0.067003006	0.528366954	0.1268115	0.899089593	0.978996707
12.41191326	-1.056638516	0.656236319	-1.610149401	0.107365245	0.524456614
141.1261378	0.076168295	0.164231414	0.463786393	0.642800793	0.908494289
503.5078208	0.025280692	0.129939993	0.194556669	0.845740028	0.968131661
56.02641368	-0.166879022	0.152069817	-1.097384249	0.272473456	0.714971121
188.8127069	0.340644823	0.239990473	1.419409774	0.155779585	0.594137957
707.7611602	-0.038740285	0.063117993	-0.613775611	0.539363616	0.869675597
18.46422307	-0.422069635	0.241626845	-1.746782879	0.080675008	0.480706878
374.6195826	0.15742865	0.128752941	1.222718863	0.2214359	0.66525151
1250.47896	-0.078643297	0.135471549	-0.58051523	0.561567219	0.881303315
8.451382072	0.514654742	0.456754819	1.126763684	0.259842409	0.702516818
605.3652796	-0.023385207	0.116911502	-0.200024861	0.841461138	0.967269438
31.71327938	-0.261677336	0.194683813	-1.3441145	0.178911364	0.623528077
71.40304733	0.033434889	0.121531734	0.275112414	0.783229874	0.95173434
307.2403115	0.006226351	0.060649155	0.102661786	0.918231404	0.981460609
86.73523191	-0.251757366	0.210526201	-1.195848141	0.231755826	0.67448874
223.1229836	-0.017917268	0.171634448	-0.104392028	0.916858249	0.981290994
7.422092826	-0.143150157	0.401958652	-0.356131547	0.721742053	0.931135539
9392.321296	-0.014366929	0.104214902	-0.13785868	0.890352107	0.976961229
279.7844618	-0.104092586	0.129066959	-0.80650065	0.419954233	0.812193479
53.44483791	-0.311930414	0.446454242	-0.698683951	0.484749566	0.848641404
192.2293517	0.078235011	0.101047469	0.774240185	0.438788769	0.822562228
288.5134376	0.260087657	0.112729865	2.307176152	0.021045002	0.290044804
432.3940129	-0.046746743	0.084751885	-0.551571721	0.581241816	0.887898616
619.2761595	-0.057348269	0.080387156	-0.713400894	0.47559772	0.842988558

663.8743523	0.087110937	0.131481192	0.662535347	0.507628189	0.859144938
624.8414096	0.039270971	0.086204467	0.455556097	0.64870922	0.910701841
367.9384312	0.067252359	0.095204481	0.706399097	0.479939982	0.845119155
59.60102569	0.153953928	0.269000505	0.572318361	0.567106313	0.883983535
13.01231473	-0.202768511	0.257781096	-0.786591857	0.431520827	0.818841807
5.069941321	-0.335440062	0.583098543	-0.575271652	0.565107591	0.883265494
441.0506364	0.098265829	0.107377389	0.915144517	0.360115767	0.777407022
44.20862093	0.058162674	0.199715347	0.291227864	0.770877053	0.948571355
6.05591269	-0.13409662	0.410260798	-0.326857015	0.743776034	0.940317814
497.7755506	0.052039011	0.056818061	0.915888538	0.359725359	0.777370801
8.40154024	-0.606557239	0.459598917	-1.319753412	0.186917361	0.6329789
481.0354872	0.257547129	0.170513462	1.510421085	0.130936012	0.56389725
241.0322329	0.044584814	0.154965564	0.287707881	0.773570357	0.949369326
231.0576366	-0.387343969	0.176399343	-2.195835671	0.028103707	0.325247129
1083.558678	0.01485657	0.095141882	0.156151735	0.87591342	0.973587929
513.0271497	-0.091510431	0.140657893	-0.650588666	0.515312048	0.861826223
137.6911197	0.479735861	0.349109133	1.374171616	0.16938841	0.611981266
157.8027793	-0.026143422	0.111485003	-0.234501688	0.814595523	0.960909552
242.0329163	0.090948012	0.102177457	0.890098598	0.373412946	0.786373874
4.621743898	-0.831873837	0.883383598	-0.941690381	0.346351181	0.768681576
1237.666629	0.0317453	0.085128093	0.372912154	0.709213826	0.92902743
1129.070222	-0.043464197	0.114485932	-0.379646615	0.704207753	0.9269128
46.65748587	-0.752466517	0.278931381	-2.697676092	0.006982534	0.182836032
249.8457924	-0.366526373	0.400379871	-0.915446555	0.359957248	0.777407022
1441.514499	0.161435664	0.215844229	0.747926706	0.454504368	0.831343944
84.9733267	-0.137841903	0.132658608	-1.039072433	0.29877105	0.738522837
39.00586656	0.372955075	0.198839039	1.87566323	0.060701538	0.436582661
56.10736713	0.283791713	0.153059243	1.854129864	0.063720583	0.4439753
664.8777368	0.060137988	0.094990149	0.633097105	0.526670233	0.865020759
4.869372301	-0.059952996	0.433490892	-0.13830278	0.890001128	0.976961229
67.69341836	-0.095433374	0.122111611	-0.781525799	0.434493309	0.821579957
59.62873617	-0.125714078	0.445941826	-0.281906901	0.778014898	0.951435601
29.83146305	-0.067744035	0.421803077	-0.160605833	0.87240386	0.972881041
2277.119725	-0.02502168	0.089046294	-0.280996313	0.778713228	0.951560132
470.6921314	-0.205648906	0.153208819	-1.342278515	0.179505707	0.624452051
10.23624541	-0.406092916	0.313352491	-1.295961983	0.194988583	0.640317357
202.7048048	0.152093648	0.158689596	0.958434907	0.3378435	0.763824574
251.6595911	-0.0339202	0.08314127	-0.407982704	0.683286373	0.922776218
504.3081459	0.144528318	0.077622956	1.861927506	0.062613309	0.442595858
643.0557177	0.165036084	0.058663548	2.813264605	0.004904128	0.154448721
384.2629395	0.089214088	0.073663736	1.211099154	0.225857406	0.668749399
67.17522247	-0.541251199	0.185032145	-2.925173883	0.003442638	0.134809333
274.1080293	0.093851688	0.083474662	1.124313473	0.260880065	0.703138216
55.21083458	-0.260087059	0.150196886	-1.731640829	0.083337532	0.485656938
290.6524173	-0.135805783	0.163687157	-0.829666698	0.406727255	0.804970829
15.62074619	-0.010020837	0.41512306	-0.024139436	0.980741387	0.994807512
11.51463626	0.010061401	0.530937201	0.018950265	0.984880781	0.996051197
44.30027835	0.080263981	0.14759612	0.543808207	0.586573461	0.890984353

143.3150974	-0.048516904	0.103803318	-0.467392609	0.640219009	0.907798398
7.478296104	-0.094784933	0.321971698	-0.29438902	0.768460656	0.947586561
435.7833362	-0.078480124	0.082469464	-0.951626463	0.341286456	0.765180569
898.6628114	-0.088549751	0.111938101	-0.79105997	0.428908997	0.818343771
215.0176474	0.155149354	0.069205538	2.241863282	0.02497021	0.312413884
25.87828371	0.10271407	0.195898207	0.524323688	0.600053424	0.898108141
214.3871043	-0.080617312	0.096023246	-0.839560367	0.401154929	0.801291311
307.5380767	-0.012173899	0.128716284	-0.09457932	0.924648977	0.982590996
31.08492989	0.20720143	0.164810022	1.257213781	0.208676234	0.653304912
88.71988232	-0.006102772	0.123722491	-0.049326297	0.960659263	0.989126211
2199.468619	-0.002844325	0.075165564	-0.037840803	0.969814611	0.992511035
119.3918086	-0.044133894	0.11514814	-0.383279258	0.701512717	0.926243544
97.93740901	0.116682202	0.109010259	1.070378166	0.284449123	0.725308513
521.6514019	0.018272457	0.222001828	0.082307687	0.934402042	0.984334593
225.9366499	-0.166292096	0.092513197	-1.797495939	0.072256922	0.463408563
80.54418777	-0.994576115	0.540165202	-1.841244329	0.065585762	0.449670505
686.3894775	0.113896057	0.065624062	1.735583777	0.082637456	0.485011749
97.69235527	0.205112294	0.117184561	1.75033547	0.080060444	0.479003512
639.6714788	0.04070543	0.051735762	0.786794818	0.431401987	0.818841807
39.52758543	-0.087836199	0.156777414	-0.560260541	0.575301738	0.886202779
3258.621223	-0.05890645	0.121812382	-0.483583436	0.628681532	0.904638489
16.89845193	0.399413514	0.292006785	1.367822715	0.171367576	0.615974468
18.13588427	-0.28291337	0.300914384	-0.940178949	0.347125778	0.768780165
317.4727972	0.012023991	0.082714524	0.145367342	0.884420848	0.976398641
389.7373029	0.168832316	0.099063751	1.704279453	0.088328892	0.492827855
96.99647955	0.161238669	0.184650185	0.873211521	0.382547796	0.789880775
44.07454406	-0.118169139	0.170525721	-0.692969587	0.488328638	0.849736271
3217.329334	0.094911881	0.084331868	1.125456883	0.260395478	0.703096291
1080.629063	0.205860835	0.070940791	2.901868332	0.003709444	0.13900555
92.07435722	-0.885325855	0.400555225	-2.210246677	0.027088047	0.321795565
75.05973673	0.062875294	0.184762185	0.340303913	0.733627671	0.937022761
13.41617096	-0.609137533	0.391266809	-1.556834156	0.119509866	0.54413566
40.88040744	-0.15354059	0.204290696	-0.751578966	0.452304296	0.829925399
160.0930376	0.192867395	0.108389071	1.77939891	0.075174385	0.470687737
571.9011984	-0.058517667	0.122989278	-0.475794865	0.634220528	0.906920638
58.5022042	-0.209566912	0.168633437	-1.242736411	0.213964982	0.659367121
375.9366655	0.01325865	0.072656214	0.182484733	0.855202335	0.969966486
98.25471929	-0.261890949	0.342341245	-0.764999699	0.444271724	0.826040706
116.8395901	-0.445519423	0.148634913	-2.997407641	0.002722864	0.12394732
126.6081784	-0.117803382	0.115754343	-1.017701613	0.30881978	0.744244609
700.6829386	-0.191779266	0.105738426	-1.813714017	0.069721766	0.460518331
744.7547403	0.134389144	0.103666054	1.296365963	0.194849433	0.640317357
159.6756074	0.126552015	0.088877054	1.423899756	0.154475492	0.592506562
641.7732645	-0.345358947	0.207407873	-1.665119761	0.095888865	0.505105686
131.652912	-0.083692718	0.126306417	-0.662616517	0.507576189	0.859144938
15.60669523	0.646666541	0.298700219	2.164934942	0.030392668	0.332880971
164.6674988	0.020053623	0.095614911	0.209733221	0.833875896	0.966316856
718.3042072	-0.033610817	0.107155607	-0.313663631	0.753776526	0.944022349

153.6309641	0.087375876	0.225364926	0.387708403	0.698231837	0.925802378
56.26904361	0.109120765	0.174851736	0.624075957	0.532577703	0.867049621
6.755594659	0.223545847	0.402748646	0.555050524	0.578860103	0.887274009
160.6025805	0.118280208	0.109115559	1.0839903	0.2783691	0.720249062
548.2053453	0.090260899	0.069441647	1.299809297	0.193666338	0.639258351
284.5320567	-0.147629804	0.106177273	-1.390408696	0.164404807	0.603851107
22.74854322	-0.152483689	0.229549099	-0.664274828	0.506514429	0.859011504
42.05638706	-0.642846239	0.323635038	-1.986330788	0.046996606	0.395490636
47.1587164	-0.43661032	0.349917036	-1.247753825	0.212121223	0.657003211
120.6018666	-0.313569776	0.493560865	-0.635321393	0.525218824	0.864408528
512.3755431	0.104203122	0.058003722	1.796490254	0.072416581	0.463687475
2252.994395	0.048248776	0.146593758	0.329132539	0.742055511	0.939964277
1159.807971	0.019834378	0.060120095	0.329912621	0.741465986	0.939959274
226.305272	-0.014493974	0.238273902	-0.060829046	0.951495358	0.98625342
111.5576435	0.128464663	0.189271042	0.678733852	0.497306513	0.854836211
5.377628142	0.333860451	0.587215515	0.568548415	0.569662641	0.8841274
51.85916612	-0.037757249	0.130264396	-0.289850871	0.771930328	0.948706053
1012.179512	0.090584325	0.090786049	0.997778029	0.318387006	0.751465423
590.1167266	0.238584056	0.058803336	4.057321805	4.96387E-05	0.013359118
213.2700447	0.004040893	0.093834898	0.043063857	0.965650631	0.99075072
60.2536913	0.007928026	0.120898443	0.065575914	0.947715466	0.985917197
1755.775674	-0.001480099	0.107770632	-0.013733787	0.989042368	0.99764435
51.44631349	-0.135277091	0.141491564	-0.956078841	0.339032404	0.764007173
16.20150139	0.350234757	0.58084593	0.602973593	0.546526257	0.873332792
923.2443636	0.008463435	0.101982636	0.082988984	0.933860299	0.984334593
231.9564431	-0.128969642	0.078409468	-1.64482232	0.100006458	0.512035832
7873.539057	0.15448779	0.127845958	1.208390098	0.226897247	0.669697517
5.0344048	0.355962998	0.436615456	0.815278051	0.41491317	0.809482633
1456.197792	-0.029689818	0.106872162	-0.277806844	0.781160637	0.95173434
195.7392761	0.072613777	0.127733978	0.568476594	0.569711394	0.8841274
183.3774445	0.021141348	0.124615089	0.169653194	0.865282886	0.971917805
21.0305385	-0.140177386	0.223729809	-0.626547647	0.530955795	0.866123834
926.1964126	-0.120965763	0.125716933	-0.962207397	0.335945434	0.762938488
335.7753842	0.208495059	0.080963643	2.575168937	0.010019114	0.215870336
18.17321215	-0.040267489	0.227135214	-0.177284218	0.859285147	0.969966486
8.470781072	0.370301145	0.465907758	0.794794974	0.426732775	0.816487562
303.3692516	0.080347216	0.247825066	0.324209401	0.745779505	0.940455922
1070.927432	0.178618677	0.094658913	1.886971552	0.059164152	0.434680797
196.4514031	0.14778304	0.12050974	1.226316149	0.220079727	0.664549187
336.9875702	0.067744606	0.077348631	0.875834578	0.381119969	0.788667382
133.8811394	0.435457175	0.225957754	1.9271619	0.053959456	0.419371005
494.1101678	0.430319591	0.35119832	1.225289435	0.220466188	0.664595064
410.108253	0.075899663	0.070270597	1.080105578	0.280095168	0.721665567
145.8852956	-0.172641024	0.15586331	-1.107643766	0.268015694	0.710235608
52.08978945	-0.032320557	0.139917956	-0.230996495	0.817317525	0.961335246
34.48963013	0.358762107	0.17971961	1.996232396	0.045908633	0.391329374
309.4092209	-0.02596614	0.064010345	-0.405655358	0.684995848	0.922907843
453.6547595	0.069868262	0.053457788	1.306980048	0.191219488	0.637278237

13.73810142	-1.642154811	0.763829146	-2.149898074	0.031563278	0.338966233
98.30170712	1.063473644	0.500314071	2.125612103	0.033535571	0.347119785
7399.685652	0.118376537	0.132791797	0.891444649	0.372690673	0.786266676
211.4648176	0.347455034	0.166185353	2.090768098	0.036548857	0.360198137
1022.097422	0.080974829	0.055222012	1.466350576	0.142552804	0.577309603
664.8298584	-0.391954894	0.441166206	-0.888451764	0.374297791	0.786475871
431.8230078	-0.286100476	0.217525034	-1.315253106	0.188424852	0.634590162
9.715205736	-0.199008524	0.357614906	-0.556488336	0.577877066	0.887079361
6.207324801	-1.589373047	0.720397899	-2.206243312	0.027366968	0.323603282
24.12477255	-2.957524835	0.986950429	-2.996629564	0.002729822	0.12394732
118.5480678	-0.138390163	0.317691712	-0.435611498	0.663118627	0.915744058
7.617247227	0.478472778	0.601493406	0.795474686	0.42633743	0.816487562
11.81836408	1.122595949	0.613983852	1.828380251	0.067492503	0.45536467
5.491950195	0.462074185	0.908939287	0.508366391	0.611196418	0.900550406
242.8947071	-0.73555844	0.360293017	-2.041556191	0.041195575	0.374892505
1303.526834	0.092667097	0.064532463	1.435976456	0.151009041	0.589528489
178.5166612	-0.475623468	0.297454016	-1.598981498	0.109824714	0.528327039
257.1050039	-0.332858193	0.179030518	-1.859225996	0.062995111	0.442595858
8.402594758	-0.064361855	0.364106392	-0.176766617	0.859691712	0.969966486
162.3589713	-0.375224244	0.178546547	-2.101548589	0.035592837	0.356853392
570.9851464	-0.265068148	0.086007407	-3.081922333	0.002056685	0.108338257
34.76677341	-1.290958121	0.476796894	-2.707564032	0.006777899	0.180443287
867.5898103	0.098524678	0.068721173	1.433687365	0.151661499	0.589675207
112.599677	-0.069472728	0.1228668	-0.565431247	0.571780476	0.884932518
881.0099692	0.241924999	0.051166886	4.728155611	2.26569E-06	0.001765088
92.37520237	-0.074094965	0.130962947	-0.565770446	0.571549839	0.88481183
6.359838855	-1.082788697	0.696787464	-1.553972699	0.120190929	0.545735572
1587.609234	0.071078213	0.093097738	0.763479485	0.445177493	0.826302547
1501.571182	0.036380865	0.144241365	0.252222137	0.800869368	0.955697618
354.8526505	-0.25378633	0.152924924	-1.659548514	0.097005313	0.507375494
47.80348551	0.650367582	0.212474079	3.060926708	0.002206531	0.111533785
5.848668669	-0.897834195	0.479736852	-1.871513915	0.061273883	0.437941099
1254.650702	-0.07684147	0.129421587	-0.59372993	0.552692761	0.876718018
12.10415624	0.060749193	0.276125583	0.220005667	0.825866741	0.964118059
42.06280981	-0.106044789	0.253135415	-0.418925137	0.675270845	0.921149714
10.40510549	-0.601975618	0.389109492	-1.547059711	0.121848848	0.547875652
414.5777041	0.202404707	0.075057259	2.696670649	0.00700365	0.182836032
147.9452259	0.015595217	0.178795499	0.087223768	0.930493647	0.984334593
19.05414996	-0.372810562	0.407646131	-0.914544584	0.360430762	0.777564105
458.3223759	0.170021291	0.109283476	1.555782236	0.119759884	0.54443667
49.85995522	-0.003256235	0.158969986	-0.020483334	0.983657807	0.995742202
8.28579183	-0.589562486	0.359253405	-1.641076962	0.10078144	0.512774612
14.55529784	0.250091934	0.536676903	0.466000926	0.641214838	0.907798398
167.2255735	-0.014420995	0.202165424	-0.071332648	0.943133012	0.98464204
8.253384505	0.600538878	0.333941044	1.798338023	0.072123458	0.463154629
83.44402247	0.089030353	0.239697155	0.371428495	0.710318405	0.929220772
6.118329461	-0.044585787	0.583100766	-0.076463263	0.93905054	0.984334593
1212.91457	0.09856364	0.079625486	1.237840357	0.215775261	0.660219973

8.646424471	0.042044727	0.328457587	0.128006564	0.898143779	0.97893802
6712.671001	0.058641569	0.035284703	1.661954446	0.096521909	0.506088677
23.52981558	0.434219917	0.235206224	1.846124262	0.064874165	0.446824324
435.5666963	0.04379865	0.084359277	0.519191861	0.603626954	0.899424822
153.7040617	0.181172747	0.115511708	1.568436227	0.116779362	0.538641109
10.99155315	0.100804077	0.266618185	0.378084027	0.705368174	0.926977731
186.9087117	-0.00863936	0.100728077	-0.085769139	0.93164994	0.984334593
149.1409814	-0.0730915	0.075519455	-0.967849933	0.333119322	0.761183657
645.6663863	0.573411007	0.22248295	2.577325615	0.009956811	0.215114309
1562.549565	0.526773318	0.221413595	2.379137187	0.017353216	0.26838663
156.849836	-0.038596757	0.09340004	-0.413241331	0.67942981	0.922072151
377.8091041	-0.079007075	0.119850936	-0.659211159	0.509760182	0.859882646
803.0672605	-0.041708276	0.061796153	-0.674933207	0.499718202	0.855916319
21.5491947	-0.288196555	0.289160693	-0.996665736	0.318926786	0.752071737
543.6404717	-0.154562408	0.175528001	-0.88055699	0.378557646	0.787072097
2287.931612	0.111786017	0.056701609	1.97147875	0.048669141	0.401561108
34.5037042	0.07528529	0.196458558	0.383212068	0.701562531	0.926243544
864.1882166	0.013467132	0.067425024	0.199734924	0.841687898	0.967343794
120.4717308	-0.340318873	0.291283962	-1.168340581	0.242669412	0.685720081
45.03848235	0.038974779	0.164566092	0.236833593	0.812785889	0.96005245
188.3372077	0.182961228	0.075820562	2.413081926	0.015818264	0.261113561
207.3408842	0.115775583	0.076836711	1.506774327	0.131868512	0.564647789
830.5862713	0.119849218	0.05395235	2.221390146	0.026324548	0.317309412
98.12945541	0.104153281	0.106142654	0.981257552	0.32646575	0.757054149
143.2692495	0.040624247	0.12654214	0.321033349	0.748185119	0.941542093
204.0777138	-0.070116134	0.087243532	-0.803682895	0.421580135	0.813907546
1204.059164	0.020307752	0.115879911	0.175248252	0.860884571	0.970088938
9.692999311	-1.962531899	0.681814546	-2.878395468	0.003997037	0.144354659
160.4768311	-0.102356868	0.132843234	-0.770508705	0.440998193	0.82417628
128.1991706	-0.732567869	0.412424709	-1.776246312	0.075692328	0.471272491
28.88092873	-1.112050489	0.469301242	-2.369587782	0.017807927	0.270629301
303.5200819	0.035321853	0.148870659	0.237265375	0.812450922	0.96005245
8404.310928	-0.05026429	0.154734422	-0.324842328	0.745300407	0.940455922
101.4618484	-0.098451353	0.116059895	-0.84828056	0.396281742	0.79798834
552.7984263	0.179193475	0.167323171	1.070942379	0.284195339	0.725036093
15.69589327	-0.125441625	0.338164419	-0.370948622	0.7106758	0.929359766
325.8334893	-0.099943611	0.085674304	-1.166552942	0.243390957	0.686012467
126.9431242	0.752230909	0.486629493	1.545798024	0.122153354	0.548564727
9.092631446	0.001636464	0.529414085	0.003091084	0.997533676	0.99935658
51.12659173	-0.177874862	0.245103948	-0.725711941	0.46801538	0.838388803
618.1858702	0.020713765	0.127602225	0.162330751	0.8710454	0.972881041
50.37087938	-0.109556066	0.162508638	-0.67415534	0.500212559	0.856182624
333.0211981	-0.038370306	0.058586145	-0.654938221	0.512507531	0.860200661
191.5977443	-0.054275729	0.091765467	-0.591461369	0.554211332	0.877583363
932.5278218	-0.021510803	0.077695116	-0.27686172	0.781886287	0.95173434
614.5749468	-0.056108462	0.093553009	-0.599750484	0.548672538	0.874685073
221.4192436	-0.099858757	0.090152667	-1.107662812	0.268007465	0.710235608
281.1213222	0.140022777	0.200508535	0.698338236	0.484965692	0.848765348

5.138671641	-2.024657574	0.834911461	-2.424996743	0.015308524	0.258245094
402.5283906	0.019735106	0.106556491	0.185207918	0.853065959	0.969966486
137.4461461	-0.036676351	0.28063168	-0.130692125	0.896018862	0.978807264
238.1779388	0.259657165	0.10962516	2.368590974	0.017855988	0.271081375
999.8973223	0.205420143	0.03846771	5.340066853	9.29123E-08	0.000114607
14.66503224	-0.326801062	0.297061195	-1.100113606	0.271282626	0.713807699
64.01672513	-0.605152583	0.35184127	-1.719959067	0.085439882	0.488858859
93.0168608	0.450605186	0.201032581	2.241453524	0.024996713	0.312413884
11.95985691	-1.007654675	0.65362417	-1.541642309	0.123160538	0.549931309
323.9944741	0.152940767	0.101208075	1.511151815	0.130749776	0.56375129
1454.721037	0.003771348	0.089135556	0.042310255	0.96625137	0.990895439
441.3423066	0.026176355	0.067062139	0.390329848	0.696292655	0.925326625
221.9333545	0.169090631	0.115826018	1.459867423	0.144326514	0.578069441
747.3460401	0.205202743	0.121917205	1.68313195	0.092349551	0.499801846
25.47098868	-0.365490484	0.424313636	-0.861368697	0.389035011	0.792962851
10.01938514	-0.368341738	0.55515062	-0.663498741	0.507011185	0.859028158
53.47484967	-0.158335554	0.227225045	-0.69682263	0.485913802	0.848972627
700.7720009	0.390021204	0.147842346	2.63808858	0.00833748	0.198674451
500.2206329	0.087856848	0.060934539	1.441823474	0.149352179	0.587330222
139.0403853	-0.336892943	0.278500818	-1.2096659	0.22640712	0.669184942
590.3314915	0.108510195	0.079690557	1.361644329	0.173310158	0.616964156
51.5565224	-0.20473155	0.164520556	-1.244413192	0.21334753	0.659008794
429.2924421	0.094651632	0.071998255	1.314637855	0.188631641	0.634590162
1446.834765	-0.034666868	0.090523478	-0.382959964	0.70174945	0.926243544
696.6955057	-0.032397634	0.080453234	-0.402689017	0.687177015	0.923064029
1276.794455	0.064338915	0.06091942	1.056131441	0.290908168	0.732815299
11.71801639	-0.298858001	0.39530743	-0.756014125	0.449640727	0.828449907
31.26686369	-0.866925116	0.456891807	-1.897440712	0.057769797	0.431279458
188.0966745	0.150347917	0.120165474	1.251174003	0.210871001	0.656013567
531.8729983	-0.01663656	0.092478874	-0.17989579	0.85723438	0.969966486
10972.71187	-0.118111726	0.283539585	-0.416561679	0.676999044	0.921393988
336.093334	0.142486142	0.089913364	1.584704825	0.113033415	0.533883941
378.3426913	0.016677585	0.057334438	0.290882511	0.771141177	0.948706053
51.05496514	0.174983339	0.13229171	1.322708278	0.18593241	0.631625529
65.99400563	0.148171264	0.266827951	0.555306383	0.578685114	0.887274009
45.54280703	-0.129004972	0.260509764	-0.495202062	0.620457495	0.901976704
2697.198022	0.006140482	0.269568491	0.022778931	0.981826614	0.99517046
496.1342177	-0.044333522	0.140426741	-0.315705695	0.752225904	0.943629947
1264.11103	0.136210817	0.158160698	0.861217856	0.389118068	0.793022943
181.5801856	-0.023298689	0.07517563	-0.30992343	0.756619185	0.945306795
124.5983251	0.092986474	0.106819469	0.870501178	0.384026575	0.790043276
377.149782	0.055153498	0.071224694	0.774359219	0.438718393	0.822562228
159.4513077	-0.012298628	0.111715013	-0.110089306	0.912338549	0.980530316
598.3545606	0.053691266	0.066027891	0.813160391	0.416126106	0.810170019
143.6483944	0.127644928	0.136486427	0.935220672	0.349674587	0.769877025
79.34164499	-0.116171624	0.225255286	-0.515733175	0.606040781	0.900307811
13.39212574	-0.488150446	0.422526275	-1.155313823	0.247961937	0.691857602
15.47604212	-0.303417208	0.38344358	-0.791295573	0.428771531	0.818292445

75.7866936	-0.060137352	0.114139015	-0.52687814	0.598278215	0.897417322
726.2280218	0.032484438	0.043303766	0.750152722	0.453162729	0.830265469
368.4444141	0.222318693	0.086172626	2.579922472	0.00988225	0.215114309
143.1183298	0.147133129	0.109326545	1.345813398	0.178362704	0.623105158
604.9750292	0.057951644	0.07261123	0.798108567	0.42480749	0.815987051
8.477448469	-0.89022886	0.370910179	-2.400119785	0.016389708	0.263145637
75.3867521	-0.196122157	0.192940889	-1.016488307	0.309396913	0.744907792
1352.172389	-0.085182001	0.184394275	-0.461955777	0.644113034	0.908494289
143.1938852	-0.172047337	0.353331852	-0.486928465	0.626309026	0.904098518
6.593043608	0.532795435	0.539873697	0.986889041	0.323697037	0.756297513
4.525527108	-0.81158572	0.744549671	-1.090035697	0.27569742	0.717918564
126.3126732	-0.017428014	0.223391186	-0.078015672	0.937815586	0.984334593
17.66212017	0.729966518	0.451651657	1.616215741	0.106047677	0.521361916
230.7418108	-0.03102297	0.078155547	-0.396938808	0.691412585	0.924455975
104.1267399	0.071457764	0.12548692	0.569443922	0.569054917	0.8841274
240.9144817	0.020706837	0.077795088	0.26617152	0.790107116	0.953026618
17.53684593	0.297597627	0.344249754	0.864481742	0.387323306	0.792728629
380.9665652	0.164089152	0.10401891	1.577493474	0.114682022	0.535291597
5.375541675	-0.432551726	0.486878412	-0.888418372	0.374315747	0.786475871
13.30368422	-0.369243121	0.31907882	-1.157216016	0.247184116	0.690864668
334.9614554	0.059506149	0.078456719	0.758458295	0.448176671	0.82753699
26.7689518	-1.744244311	0.963783295	-1.809788902	0.07032853	0.461430171
13.99750058	-2.379240304	1.04902612	-2.268046771	0.023326356	0.304263956
15.21201224	-1.87167314	1.184836007	-1.579689619	0.114177964	0.534829818
21.32565327	-2.031570515	1.116673649	-1.819305503	0.068864834	0.458541286
14.25982526	-1.914096355	0.687362344	-2.784697726	0.005357764	0.159160905
40.17297345	-1.704254663	0.986178439	-1.728140259	0.083963081	0.486282844
53.64614607	-2.078038286	1.065633943	-1.950048888	0.051170292	0.410527191
36.86247616	-1.937789	1.021949607	-1.896168839	0.057937722	0.431712666
68.9207783	-2.287343419	0.918771956	-2.489565995	0.012789917	0.242092523
39.80186052	-0.976867278	0.544357839	-1.794531477	0.072728379	0.465015503
4.831355655	-1.72527403	1.227604352	-1.405399082	0.15990264	0.600578247
30.13414754	-0.796839344	0.418721372	-1.903030028	0.057036627	0.428680279
5.431350051	0.205242686	0.606189455	0.33857845	0.734927319	0.937620779
4.744541778	-3.017971148	1.249329073	-2.415673512	0.015706137	0.259890973
16.51024958	-1.820963196	1.053851351	-1.727912759	0.084003866	0.486282844
105.6591236	-0.201634253	0.146679541	-1.374658333	0.169237395	0.611731367
16.71599264	-2.168805767	1.007276906	-2.153137588	0.031307867	0.337277327
28.27892471	-1.631338162	0.694724994	-2.348178311	0.018865487	0.275935717
159.1800375	0.018895944	0.097804679	0.193200817	0.846801699	0.968775098
119.426698	0.090144297	0.094300052	0.955930517	0.33910734	0.764007173
445.0764707	-0.050092073	0.164970074	-0.303643392	0.761399589	0.946826103
29.95098193	-1.540850286	0.824702303	-1.868371508	0.061710306	0.439326285
15.37695116	-2.257668673	0.737533731	-3.061105651	0.002205213	0.111533785
27.10574876	-1.39232261	0.731507457	-1.90336079	0.056993484	0.428680279
85.60547855	-0.826378257	0.499791261	-1.653446793	0.098239966	0.509712212
109.5395032	-1.532985715	0.699082281	-2.192854485	0.028317865	0.325561673
144.9146559	-1.172510253	0.514102186	-2.280694937	0.022566505	0.300162997

145.1271007	-0.675307585	0.362997863	-1.860362426	0.062834267	0.442595858
15.92919293	-1.540368367	0.813206398	-1.894191156	0.058199642	0.432392203
25.78993828	-1.544573141	0.756032053	-2.042999546	0.041052483	0.374892505
14.7739857	-1.940648304	0.893154439	-2.17280262	0.029795176	0.330878164
4.637783688	-1.644376743	1.017174066	-1.616612926	0.10596186	0.521361916
29.70319416	-0.711059151	0.486252122	-1.462326062	0.14365188	0.578069441
336.7727364	-0.358588892	0.303655322	-1.180907646	0.237639414	0.679753163
154.9620325	-0.274618511	0.161426077	-1.701202909	0.088904892	0.493693668
467.3485442	-0.37420139	0.227405669	-1.645523579	0.099861884	0.511826735
9.36725216	-0.658692674	0.617723247	-1.066323272	0.286277533	0.727013824
310.3128996	-0.101455517	0.106608273	-0.95166645	0.341266169	0.765180569
293.6274798	0.011057067	0.148015453	0.074702111	0.940451728	0.984334593
189.0962303	0.094780298	0.191096219	0.495982067	0.619907064	0.901976704
1463.686309	-0.426392895	0.143491306	-2.971559086	0.002962919	0.128901255
53.35075183	-1.782003672	1.036526694	-1.719206733	0.085576735	0.489075998
34.54693797	-1.102075626	0.735448283	-1.498508666	0.134001143	0.567033996
15.76497398	-1.957685932	1.239085847	-1.579943744	0.11411975	0.534726351
9.233633921	-1.484769648	1.302789221	-1.139685242	0.254417458	0.69674139
18.39475099	-1.967993934	0.936078553	-2.10238118	0.035519898	0.356693037
78.3758289	-0.780206949	0.447574557	-1.743188785	0.081300644	0.481991357
18.61912067	-1.971086011	0.999096034	-1.972869418	0.048510442	0.400922142
65.30927514	-2.172410212	1.057344199	-2.054591318	0.039918495	0.372633565
64.397284	-0.026084841	0.254446131	-0.102516165	0.918346983	0.981460609
122.6288509	0.452937484	0.421081836	1.075651917	0.282082959	0.723387381
143.6645687	-0.092641708	0.283701605	-0.326546295	0.744011069	0.940317814
33.65519316	-0.44595578	0.229003919	-1.947371824	0.051490173	0.410734915
1052.25812	0.020769147	0.056450066	0.367920688	0.712932374	0.929705922
452.0690344	0.081558506	0.124788782	0.653572416	0.513387321	0.860509469
98.13810761	-0.058478775	0.110822994	-0.527677276	0.597723348	0.8971305
186.4108445	-0.10632591	0.10674985	-0.996028666	0.319236217	0.752202243
5.526068313	-0.083781664	0.556691841	-0.150499176	0.8803708	0.975430592
364.4647109	0.178547377	0.12174313	1.466590986	0.142487354	0.577212679
444.1614672	0.061667283	0.105995814	0.581789795	0.560708278	0.880936184
17.5079203	-0.496480348	0.30219045	-1.642938579	0.100395642	0.512774612
74.12332697	-0.522241146	0.303353314	-1.721560708	0.085149126	0.488696192
448.0579745	0.066134298	0.104611516	0.632189463	0.527263082	0.865153325
276.1533815	-0.061053185	0.078605203	-0.776706669	0.437331851	0.822330546
35.50595398	-0.214224835	0.281621895	-0.760682456	0.446846756	0.826805895
48.85478381	-0.491633629	0.296543954	-1.657877772	0.097342139	0.507880983
4176.395092	-0.040202937	0.150706957	-0.266762319	0.789652169	0.953026618
454.4273634	-0.040356532	0.10033627	-0.4022128	0.687527423	0.923064029
63.50719432	0.330035408	0.254283119	1.297905302	0.194319878	0.640085942
410.7620003	-0.056492015	0.057636501	-0.980143027	0.327015523	0.757457651
5.947179455	0.175919677	0.385046465	0.456879087	0.647757957	0.910638548
13.15124685	0.069346297	0.512633713	0.135274555	0.892394803	0.977809451
55.94771846	-0.559613041	0.261943881	-2.13638524	0.032648026	0.341836409
35.92477964	0.355341914	0.250220161	1.420117037	0.155573611	0.593569201
23216.91392	0.15944133	0.238050924	0.669778244	0.502999164	0.857172233

140.7271891	-0.193089571	0.07813636	-2.471187186	0.013466531	0.249224048
80.31626284	0.050200551	0.275838971	0.181992237	0.855588818	0.969966486
43.69183376	0.465412998	0.34901697	1.333496759	0.182368838	0.627772918
14.29769825	-0.439132531	0.276564735	-1.587811011	0.112329081	0.533148601
60.33929399	0.090758661	0.183658578	0.494170554	0.621185734	0.902299404
5.751166654	-0.703957514	0.471600859	-1.492697693	0.135516338	0.568673186
2309.213592	0.095279246	0.283193143	0.336446163	0.736534444	0.938503341
14.77466614	-0.005032183	0.332270995	-0.015144814	0.987916648	0.997281745
338.536595	-0.135806086	0.113407489	-1.197505445	0.231109606	0.67340244
126.6659472	0.155436317	0.239819326	0.648139245	0.516894893	0.862002027
209.64001	-0.169259776	0.109315303	-1.548363055	0.121534911	0.547270993
80.66653127	-0.011601631	0.236420161	-0.04907209	0.960861846	0.989165233
16.28186319	-0.314146981	0.284142433	-1.105596856	0.268901051	0.711435249
40.4323539	0.220174685	0.212586224	1.035695921	0.300344026	0.739093677
339.3790393	-0.170443137	0.116669712	-1.460903042	0.144042052	0.578069441
93.013717	0.064287495	0.205926425	0.312186722	0.754898623	0.944498739
518.5853093	0.142614186	0.05790475	2.462909983	0.013781451	0.250625508
6.555591142	-0.267695003	0.729306236	-0.367054318	0.713578501	0.929705922
622.6578544	-0.116992528	0.095783598	-1.221425492	0.22192496	0.665909844
158.4563209	-0.010183624	0.104331952	-0.097607917	0.922243638	0.982590996
56.96284503	0.191181246	0.170929315	1.118481319	0.263361481	0.705730729
65.03212842	-0.047464224	0.157917427	-0.300563563	0.76374732	0.946826103
297.2663542	-0.052861624	0.087465405	-0.604371797	0.545596482	0.872896521
21.6126313	-0.731285445	0.438351325	-1.66826334	0.095263462	0.503423693
1010.647716	1.223060015	0.585939449	2.087348815	0.036856615	0.360625126
27.16987138	0.760671394	0.610134659	1.246727066	0.212497591	0.65748105
1105.884417	-0.025602241	0.095024678	-0.269427282	0.787600894	0.953026618
825.9476023	0.115859666	0.056810599	2.039402294	0.041409894	0.374892505
6.236583701	-0.173762881	0.388354906	-0.447433206	0.654562304	0.912480225
111.1368828	0.030572649	0.115386	0.264959782	0.791040444	0.953153173
4.792014865	-1.271573597	0.958658469	-1.326409392	0.184704129	0.630643427
87.43461794	0.197812795	0.111609315	1.772368156	0.076333464	0.471783446
161.1057937	0.159100669	0.147570274	1.078134946	0.280973538	0.722699559
652.3143077	-0.09054105	0.117688884	-0.769325418	0.441700152	0.824601978
129.2382787	-0.02751069	0.108672745	-0.253151699	0.80015099	0.955456191
114.1034427	0.122428267	0.126755239	0.965863562	0.334112458	0.761545389
47.28071386	0.014522183	0.191024207	0.076022738	0.939401009	0.984334593
172.9154245	-0.175359859	0.208715985	-0.840184132	0.400805154	0.801262941
270.1373831	0.062183412	0.08783504	0.707956777	0.478972099	0.844762442
315.7086639	-0.113656294	0.108013794	-1.052238692	0.292690052	0.733939814
1060.797565	0.086741957	0.120355014	0.720717431	0.471083386	0.840276866
187.7588869	-0.765432855	0.32218545	-2.375752395	0.01751321	0.269470416
994.2126501	0.132170253	0.105255679	1.255706617	0.209222359	0.654527686
6.319527741	-0.734379027	0.725547813	-1.012171788	0.311455923	0.745938435
572.1821254	0.073969598	0.057330354	1.290234441	0.196969271	0.642286481
19.59013868	-0.014984238	0.247427499	-0.060560117	0.951709537	0.98625342
45.75557032	-0.312203781	0.229391915	-1.36100604	0.173511781	0.617336891
101.3509901	-0.166917932	0.140243185	-1.190203512	0.233966413	0.676797116

135.4531111	-0.327455657	0.177988895	-1.839753302	0.065804464	0.450526216
147.9148846	0.057524205	0.177324813	0.324400203	0.745635066	0.940455922
422.1949993	0.23526763	0.090471767	2.600453577	0.009310061	0.208799287
580.6639591	0.096278828	0.092601618	1.039709989	0.298474659	0.738522837
591.0616686	-0.184663247	0.074221414	-2.488004978	0.012846193	0.242721162
22.75657651	-0.023685632	0.372489349	-0.063587406	0.94929876	0.986108475
685.1839918	-0.149574939	0.084378741	-1.772661419	0.076284828	0.471783446
390.0111568	0.007148559	0.060470313	0.118216002	0.905896511	0.979768942
325.0532838	-0.07583123	0.199756897	-0.379617579	0.70422931	0.9269128
1436.994527	-0.111558967	0.171833129	-0.649228515	0.516190683	0.862002027
2026.519893	0.108989889	0.157769814	0.690815857	0.489681269	0.849736271
2339.464522	0.211750987	0.133646046	1.584416397	0.113098993	0.533883941
3616.743912	0.070485144	0.116695504	0.604009078	0.545837607	0.872896521
463.3125006	-0.016787037	0.084147087	-0.199496357	0.841874493	0.967351827
1044.374979	0.041460004	0.063277581	0.655208423	0.512333572	0.860200661
2215.448481	0.072721872	0.099612916	0.730044603	0.465362921	0.837170481
130.1974967	-0.003906982	0.111020085	-0.035191665	0.971926908	0.992799208
47.57360383	-0.209016301	0.188475348	-1.108984829	0.267436727	0.709680608
249.1345518	-0.128094293	0.075898445	-1.687706413	0.0914676	0.499042911
311.6417215	0.051768266	0.086971299	0.595233902	0.551687132	0.87625782
484.9097932	0.317489734	0.125253682	2.534773662	0.011252	0.230681587
13.44457224	-0.01784207	0.360502713	-0.049492194	0.960527058	0.989126211
13.23906168	-0.301357801	0.53829803	-0.559834486	0.575592339	0.886202779
76.05552149	0.05610867	0.124415166	0.450979341	0.652004439	0.911246717
7.626222737	-0.034180303	0.396987476	-0.086099197	0.931387562	0.984334593
6.712281199	-0.149686116	0.42247092	-0.354311051	0.723105787	0.931432762
95.49765918	-0.091622971	0.144912992	-0.63226195	0.527215722	0.865153325
961.0972383	0.032932171	0.057714008	0.570609677	0.568264257	0.8841274
2451.986107	0.032117604	0.056489169	0.568562163	0.569653308	0.8841274
100.5992956	0.096125836	0.150853582	0.637212819	0.523986229	0.863484457
2979.762286	0.019257606	0.147779366	0.130313229	0.896318614	0.978829031
189.5206221	0.043628458	0.169138675	0.257944895	0.796449435	0.954648719
496.3597044	0.037299118	0.079435975	0.469549447	0.638676949	0.907684986
12.48115437	0.366405299	0.578369656	0.633514042	0.526398014	0.864850467
27.04772274	-0.434915173	0.362081482	-1.201152763	0.229691949	0.672581648
726.4600478	0.036352375	0.088862915	0.409083762	0.682478192	0.922776218
144.2380786	0.027444947	0.092139489	0.297863025	0.76580771	0.94709722
20.76786961	0.133091722	0.220339747	0.604029564	0.545823987	0.872896521
170.1542303	-0.202594837	0.188314402	-1.075832939	0.282001978	0.723387381
190.9968203	0.111175105	0.233068447	0.477006246	0.633357674	0.906724287
21.49541107	-0.489919333	0.383327411	-1.278070179	0.201224683	0.645045462
640.1558032	0.38328566	0.271337954	1.412576655	0.157780237	0.597576311
240.2490248	0.062460909	0.122546409	0.509691877	0.610267345	0.900307811
1238.231848	1.56986E-05	0.10920932	0.000143748	0.999885306	0.999885306
11.72583047	0.000135069	0.390636561	0.000345767	0.999724118	0.999799058
57.44136458	0.057965634	0.145346489	0.398810005	0.690033204	0.92405254
482.0417236	0.223576768	0.05633882	3.968431824	7.23471E-05	0.016150506
98.06538447	-0.058443373	0.222193145	-0.263029596	0.792527768	0.953153173

6.058867311	-0.677060427	0.927104794	-0.730295465	0.4652096	0.837170481
197.5643927	0.118900975	0.075573707	1.573311398	0.115646731	0.537120464
782.5838258	-0.14959703	0.118849311	-1.258711803	0.208134447	0.652874199
384.3466226	0.834939362	0.365609893	2.283689197	0.022389803	0.300162997
9.083948908	-0.695167885	0.454389774	-1.529893332	0.126043134	0.555429245
139.1381571	-0.047256992	0.399802636	-0.118200801	0.905908555	0.979768942
5.013190194	1.76378787	0.646675544	2.727469572	0.006382214	0.174970531
727.8912718	-0.002632147	0.085926941	-0.030632387	0.975562713	0.993405995
15.66675525	-0.292127883	0.282668561	-1.033464361	0.30138664	0.739331412
225.9961311	0.061500745	0.079666403	0.771978439	0.440127198	0.823715107
158.4212168	-0.295885808	0.357673883	-0.827250246	0.408095238	0.805770628
10.03680196	-0.15747709	0.342370185	-0.459961461	0.645543883	0.909300678
27.04261452	-0.617872254	0.291378592	-2.120513557	0.033962759	0.348624664
160.5667012	-0.061498736	0.097776672	-0.628971458	0.529367742	0.866063379
94.28886513	0.212234849	0.129384205	1.640345888	0.100933269	0.512774612
2465.379422	-0.011325926	0.104601344	-0.108277061	0.913775918	0.980550322
42.31853352	-0.08547669	0.162539574	-0.525882332	0.598969965	0.897911345
130.3073864	0.114788506	0.135638014	0.846285657	0.397393407	0.79867172
670.5466261	-0.114365024	0.083550068	-1.368820252	0.171055469	0.615232513
1036.682221	0.158213782	0.092833792	1.704269307	0.088330786	0.492827855
224.8933882	0.177066284	0.087667947	2.019737992	0.043410572	0.381263212
453.4252141	-0.057754014	0.075670843	-0.763226779	0.445328161	0.826302547
112.8704804	-0.162160697	0.16534989	-0.980712458	0.326734559	0.757203811
30.27959965	0.040820346	0.288760704	0.141363924	0.887582453	0.976961229
50.13627726	-0.551756528	0.478105762	-1.154047015	0.248480894	0.69187626
183.1977401	0.056462989	0.08123746	0.695036358	0.487032517	0.849484378
230.6056412	-0.053674378	0.087377116	-0.614284157	0.53902757	0.869675597
50.30821933	0.215621285	0.227843181	0.9463583	0.343965867	0.767121103
2510.671456	0.069386105	0.036855026	1.882676852	0.059744174	0.435320275
355.0952225	-0.102200717	0.083888663	-1.218289974	0.223113799	0.667582464
164.5859433	0.019477015	0.254944776	0.076396996	0.939103259	0.984334593
6.272131898	-0.598571821	0.849088634	-0.704957995	0.480836377	0.84566728
881.9950186	-0.108176888	0.0644216	-1.679202135	0.093112656	0.500506422
13.26132898	-0.524406157	0.535362068	-0.979535511	0.327315451	0.757729639
291.4298659	-0.117979951	0.140435061	-0.840103247	0.4008505	0.801262941
4.666041804	-1.02559761	0.51645071	-1.985857682	0.047049129	0.395693866
114.3632948	-0.14440431	0.234143591	-0.616733986	0.537410201	0.869675597
2193.503639	-0.127175134	0.15010917	-0.847217619	0.396873835	0.79837903
35.65784151	-0.098097259	0.414440872	-0.236697839	0.812891212	0.96005245
141.2451775	-0.029527014	0.106610218	-0.27696233	0.781809031	0.95173434
338.8154059	0.035389901	0.078112905	0.453060876	0.650504907	0.910782566
479.0676285	-0.010553944	0.099308633	-0.106274181	0.915364816	0.981173687
86.80225369	0.170843546	0.108435455	1.575532148	0.115133664	0.536394333
95.43967117	0.158947991	0.146348472	1.086092591	0.27743803	0.719218718
797.9326644	0.078611347	0.08173357	0.961799999	0.336150078	0.76302614
900.9040058	0.038194948	0.066946001	0.570533683	0.568315783	0.8841274
310.7806375	-0.029484612	0.061938813	-0.476028039	0.634054402	0.906920638
64.68958356	-0.176841292	0.161246536	-1.096713742	0.272766547	0.715316121

9.798864814	0.569836542	0.33119827	1.720529946	0.085336155	0.488858859
21.40180372	0.110352958	0.238540705	0.462616886	0.643639003	0.908494289
577.5316312	0.053071707	0.087705528	0.60511245	0.545104282	0.872850885
15.55375231	-0.187895375	0.511276207	-0.367502678	0.713244095	0.929705922
873.3205976	0.469174789	0.195837441	2.395735901	0.016587037	0.263395465
5.618647685	0.342647979	0.660197632	0.519008191	0.603755029	0.899439062
6.397143878	-0.120282211	0.373865868	-0.321725574	0.747660602	0.941482887
556.8690285	0.639135893	0.343276927	1.861866738	0.062621877	0.442595858
828.4414798	0.116554717	0.05734632	2.032470733	0.042106024	0.376265422
19.00825346	-0.058167409	0.276893674	-0.210071281	0.83361204	0.966316856
645.9960245	-0.132413163	0.059388745	-2.229600273	0.025773992	0.314428791
55.00645771	-0.185531004	0.19388833	-0.95689619	0.338619655	0.764007173
1031.649099	0.116301196	0.068626973	1.694686364	0.090134934	0.49542417
162.1477367	0.065771786	0.091337435	0.720096698	0.47146546	0.840318577
150.0353735	-0.218310954	0.1311578	-1.664490823	0.096014383	0.50540715
1702.852284	0.056386868	0.045479322	1.239835288	0.215036323	0.660219973
58.78591825	0.035233789	0.135455875	0.260112671	0.794776865	0.953773263
460.6276787	-0.488499148	0.298387977	-1.637127453	0.101603842	0.513342798
1041.797851	0.114234297	0.119163705	0.958633309	0.337743506	0.7637398
276.5167094	-0.072576306	0.064673565	-1.122194298	0.261779837	0.704520935
240.5467954	-0.212136404	0.244362853	-0.868120509	0.385328365	0.791181919
2026.297819	-0.056553249	0.117134862	-0.482804591	0.629234491	0.904638489
40.29235925	0.244421571	0.177215741	1.379231723	0.16782332	0.609749824
668.8731688	0.047860806	0.055943088	0.855526712	0.392259623	0.795482523
357.6288086	-0.103607924	0.181743049	-0.570079156	0.568624012	0.8841274
290.88681	0.581905654	0.410797831	1.416525625	0.156621667	0.595721049
7.183405685	-0.0513335	0.498529288	-0.102969878	0.917986878	0.981376661
16.84079874	0.141710973	0.299230754	0.473584251	0.635796408	0.907353194
42.2620421	-0.409070049	0.190913206	-2.142701689	0.03213706	0.34013779
10.47643871	-0.210419532	0.304686403	-0.690610183	0.489810546	0.849762741
5.613830335	0.554255682	0.539449736	1.027446386	0.304210319	0.741640846
50.90847723	0.204279415	0.210534733	0.970288425	0.331902745	0.760616881
8.543037289	-0.40758252	0.317233374	-1.284803409	0.198860991	0.64421391
7.461562614	0.269348312	0.375183987	0.717909936	0.472812821	0.840972769
7.804193967	-0.161655605	0.405366911	-0.39878836	0.690049155	0.92405254
475.2582729	0.308535155	0.264597428	1.166055004	0.243592207	0.686012467
12.4498546	0.088530399	0.547214245	0.161783799	0.871476111	0.972881041
562.3980864	-0.126835732	0.177266018	-0.715510697	0.474293533	0.841713532
88.30202186	-0.319982077	0.183284191	-1.745824752	0.080841409	0.480761161
67.51430441	-0.142034647	0.16123025	-0.880942923	0.378348714	0.787072097
477.996708	0.120680726	0.082125046	1.469475297	0.141703917	0.576553431
19.75760275	0.083344428	0.298483409	0.279226333	0.780071136	0.95173434
477.9081347	0.046626881	0.104290343	0.447087233	0.654812077	0.912480225
299.1798153	-0.048357049	0.083252053	-0.580851131	0.561340791	0.881273052
246.1975941	0.028104509	0.09554247	0.294157237	0.768637756	0.947586561
42.86648683	-0.106071356	0.170575996	-0.621842219	0.534045622	0.86753109
268.8339816	0.090438176	0.069907502	1.293683413	0.195774797	0.640835592
1171.520247	-0.062801558	0.174566319	-0.359757589	0.719028421	0.931011662

35.2663083	-0.131343709	0.172920836	-0.75955976	0.44751778	0.827089297
216.7250435	-0.108345794	0.121557918	-0.891310053	0.372762857	0.786266676
135.3792331	0.033201433	0.083773293	0.396324793	0.691865439	0.924581226
1590.002845	0.168399764	0.164150978	1.025883405	0.304946546	0.741672489
9.120636777	-0.370471474	0.338496065	-1.094463162	0.273751895	0.716547401
115.5658146	0.285126311	0.1420255	2.007571255	0.044688872	0.387512993
227.0789471	-0.060356397	0.08876717	-0.679940536	0.496542113	0.854371062
4.895424576	0.05370887	0.547964508	0.098015235	0.921920196	0.982590996
27.78874118	0.325914601	0.193970896	1.680224237	0.092913695	0.500506422
29.73879376	0.321176474	0.169391784	1.896056976	0.057952511	0.431712666
28.09531396	-0.124442845	0.226938062	-0.548355986	0.583447492	0.88906731
114.8728406	0.161230177	0.105380162	1.529986046	0.126020183	0.555429245
6.53951184	0.126702594	0.401020456	0.31595045	0.752040118	0.943524142
11.41116579	0.113870083	0.37540398	0.303326788	0.761640833	0.946826103
50.22264734	0.273897715	0.183655414	1.491367496	0.135865037	0.568673186
25.30199007	0.204991576	0.209667835	0.977696824	0.328224293	0.758342203
43.48070396	-0.078714209	0.150799064	-0.521980757	0.601683729	0.89888197
48.33418471	0.183000227	0.159204069	1.149469533	0.250362423	0.694351765
20.18342637	0.092098326	0.232916823	0.395412942	0.692538159	0.924581226
16.44303164	0.231423277	0.23105098	1.001611318	0.316531352	0.749887496
8.78857845	-0.11153688	0.31875236	-0.349917032	0.726400964	0.933106576
65.70052665	0.035719766	0.134872657	0.264840681	0.791132197	0.953153173
177.1031233	0.056264578	0.143049722	0.393321829	0.694081798	0.924581226
61.41940021	0.167993816	0.145879934	1.15158961	0.249489753	0.693250858
55.34022448	0.158038181	0.174095785	0.907765691	0.364002029	0.779728871
63.71281045	0.178944301	0.148279839	1.206801288	0.22750868	0.670086571
85.29132871	0.057578757	0.161815184	0.355830373	0.721967602	0.931135539
109.2407841	0.102066042	0.119555516	0.853712525	0.393264293	0.796041628
93.65123297	0.080656646	0.113407191	0.711212804	0.476952375	0.843597766
99.68303081	0.175973196	0.140225703	1.25492825	0.209504808	0.654704857
35.80613492	0.254488911	0.258123808	0.98591801	0.324173348	0.756297513
50.10036025	0.351906185	0.160337952	2.194777842	0.028179538	0.325561673
5.608295401	-0.136005845	0.400897572	-0.339253353	0.73441888	0.937386243
22.00412764	0.043878577	0.191483223	0.229151027	0.818751534	0.962089609
315.4480087	0.059643362	0.097002165	0.614866299	0.53864302	0.869675597
103.9741227	0.108777926	0.119369975	0.911267059	0.362154679	0.778957081
448.1424293	0.106504732	0.16466651	0.646790487	0.51776755	0.862002027
179.1865581	0.174277976	0.076384372	2.281592047	0.022513437	0.300162997
6.356291358	-0.179551767	0.494219473	-0.363303708	0.716378024	0.930241908
608.6182912	0.045790933	0.052891978	0.865744386	0.386630354	0.792206879
50.52949566	0.123254603	0.329233249	0.374368636	0.708130074	0.928491572
64.12422112	-0.180288119	0.157831797	-1.142280088	0.253337615	0.695646467
976.0023048	0.082000198	0.088835527	0.923056364	0.355977833	0.774305138
12.73580325	0.128662503	0.576526128	0.223168555	0.823404324	0.963417933
4.640763369	1.09703432	0.907467464	1.208896587	0.226702578	0.669424857
5.975900789	-0.504966832	1.024048238	-0.493108443	0.621935965	0.902358335
791.1096445	0.104034729	0.127741581	0.81441554	0.415406939	0.809711016
185.7267851	0.115344545	0.090227258	1.278378029	0.201116167	0.645045462

61.50273576	-0.33277203	0.278306473	-1.195703521	0.231812277	0.674520409
390.1224844	-0.024818646	0.103131544	-0.240650387	0.809826094	0.958681749
5.013280672	-0.859917755	0.488366594	-1.7608038	0.078271616	0.474223305
241.1974768	0.156880111	0.197379585	0.794814269	0.426721549	0.816487562
30.66087511	-0.097010423	0.308376775	-0.314584078	0.75307747	0.944021193
136.796769	-0.014783829	0.085762302	-0.172381435	0.863137666	0.971275942
287.2363849	-0.243944662	0.117902871	-2.069030707	0.038543205	0.36712775
9.772069954	-0.284123451	0.307140928	-0.925058906	0.354935267	0.773813861
4.542579282	-0.315500333	0.509956838	-0.618680464	0.536126882	0.869194974
610.0036193	0.072243387	0.085301378	0.846919335	0.397040085	0.798395236
146.6510148	-0.004501079	0.112705453	-0.039936658	0.968143626	0.991727471
8471.976322	-0.025714154	0.140896308	-0.182504102	0.855187135	0.969966486
483.7445879	0.103753039	0.083818554	1.23782903	0.215779462	0.660219973
369.2828074	0.109306463	0.147766791	0.739722791	0.459468216	0.834180976
29.18449494	0.497679366	0.172070173	2.892304683	0.003824269	0.140813003
62.54541162	0.032623985	0.152179441	0.214378397	0.830251987	0.965541183
305.2420427	0.064221116	0.087436667	0.734487233	0.462651839	0.835754517
1111.72699	0.080779304	0.044196169	1.827744503	0.067587906	0.45536467
50.81004438	-0.19128782	0.320454412	-0.596926778	0.550556268	0.875741796
546.4180266	0.378428456	0.146550453	2.582240099	0.009816127	0.214620838
324.5557609	0.128603971	0.107753427	1.193502379	0.232672681	0.675563167
1060.029719	0.163975552	0.107202203	1.529591251	0.126117936	0.555429245
10.62278927	0.067678563	0.320327922	0.211279	0.832669569	0.966227926
123.4174902	-0.204325253	0.107784184	-1.895688642	0.058001228	0.431858239
59.8784319	0.200283399	0.128037524	1.56425549	0.11775758	0.539880626
454.4343564	0.085737597	0.077005646	1.11339365	0.265539401	0.708627777
38.96422923	-0.02398425	0.308530691	-0.077736999	0.938037263	0.984334593
210.1734522	0.278560796	0.172868407	1.611403727	0.107091755	0.524257131
85.42554086	0.057292691	0.112247895	0.510412167	0.609762735	0.900307811
214.0922532	0.180126178	0.083101423	2.167546239	0.030193228	0.332282647
14.89902488	-0.03698676	0.256592904	-0.144145685	0.88538543	0.976804762
7.30568039	0.218256218	0.388881003	0.561241656	0.574632809	0.88616072
975.2797041	0.752186362	0.267577359	2.811098685	0.004937264	0.154506093
7.07341672	0.353835776	0.33744616	1.048569573	0.294376266	0.734810234
117.8059885	0.18740288	0.12090772	1.549966209	0.121149627	0.546889761
43.74744807	0.140834289	0.169503613	0.830863054	0.406050997	0.804391743
43.82017748	0.063109832	0.147050226	0.429171949	0.667798095	0.91697539
122.243406	0.020724849	0.132494832	0.156420054	0.875701931	0.973587929
9183.227815	0.291211931	0.16740818	1.739532267	0.081941182	0.483334978
147.1725596	-0.013728817	0.105605439	-0.130001046	0.896565599	0.978829031
192.5167972	0.220427737	0.110774096	1.989885235	0.046603579	0.393961267
450.8441442	0.2746437	0.067528043	4.06710589	4.76006E-05	0.013047858
2473.728521	0.000561463	0.103416114	0.005429161	0.995668178	0.998981303
17.31082229	-1.222855683	0.635163259	-1.925261996	0.05419659	0.420229397
246.0216038	0.092538192	0.308269544	0.30018597	0.764035306	0.946826103
64.35526478	-0.095250181	0.145157225	-0.65618629	0.511704269	0.860200661
312.2429012	0.080375371	0.060071319	1.337999109	0.180896727	0.626148701
1769.220308	-0.008907873	0.114818955	-0.077581906	0.938160636	0.984334593

76.31881535	-0.027867491	0.147877662	-0.188449632	0.850524196	0.969251033
188.7717072	-0.081450357	0.085018956	-0.958025843	0.338049727	0.764007173
10.31697429	-0.122058599	0.318090448	-0.383722931	0.701183815	0.926243544
98.8316328	-0.046579171	0.10244178	-0.454689198	0.649332853	0.910782566
8795.745832	-0.236403243	0.224668282	-1.052232388	0.292692943	0.733939814
137.9629364	-0.095968031	0.187029903	-0.513115976	0.607870197	0.900307811
216.8711861	-0.045612965	0.124598568	-0.366079372	0.71430585	0.930012765
36.705109	-0.08544421	0.174901185	-0.488528479	0.625175559	0.903303786
24.16214799	0.106675766	0.20174534	0.528764459	0.596968855	0.896816502
260.7821241	0.015127393	0.082661916	0.183003173	0.854795529	0.969966486
127.2790354	-0.352928813	0.259811178	-1.358405041	0.174335196	0.618034305
735.1384867	-0.614334925	0.370435801	-1.65841132	0.097234473	0.507880983
233.6513774	0.019846305	0.113974198	0.174129802	0.861763452	0.970324122
56566.7072	0.138103944	0.120413432	1.146914776	0.251416845	0.694735013
17.71370463	0.379181134	0.33419179	1.134621332	0.256534011	0.69968978
176.656032	0.17162581	0.137970658	1.243929778	0.213525409	0.659145589
1351.129909	0.154510153	0.167145225	0.924406627	0.355274646	0.773939082
1260.287428	0.142243547	0.080028251	1.777416666	0.075499711	0.470694355
7.743104864	-1.454680446	0.979613869	-1.48495289	0.137556304	0.569036171
149.3544125	-0.472668247	0.241811402	-1.954697932	0.050618734	0.4089839
193.0060958	-0.849283054	0.356262032	-2.383871917	0.017131564	0.266768098
367.7531542	0.178082606	0.056480486	3.152993527	0.001616054	0.096583507
13.6771729	0.400143759	0.337536472	1.185483027	0.235826522	0.678901985
453.4337264	0.114625647	0.078513099	1.459955704	0.144302248	0.578069441
144.8741892	0.027618464	0.10223352	0.270150771	0.787044264	0.95288876
352.0451264	0.131343179	0.067294056	1.951779808	0.050964353	0.409318691
58.19262448	0.192520165	0.165959337	1.160044191	0.246030815	0.689463862
53.91774869	-0.230895085	0.172518336	-1.338379963	0.180772607	0.626148701
5.432031946	-0.646863251	0.503570806	-1.284552725	0.198948628	0.64421391
331.8126658	-0.092565597	0.069474096	-1.332375705	0.182736761	0.628481684
7.307459296	0.249624674	0.438410737	0.569385403	0.56909462	0.8841274
87.25606174	0.294451209	0.364391384	0.808063038	0.419054294	0.811610533
109.1976868	-0.041754471	0.363050259	-0.115010166	0.908437064	0.980020248
7.451122215	-0.70454698	0.608965992	-1.1569562	0.247290256	0.690937316
9.115181953	-0.866761874	0.494547417	-1.752636541	0.079664415	0.478180319
117.6552277	0.093077878	0.087695589	1.061374687	0.288519656	0.729654527
16.35590148	-0.030261191	0.253192575	-0.119518476	0.904864603	0.979768942
195.3509906	0.003100573	0.186009991	0.016668853	0.986700796	0.996928555
1263.30072	0.110697747	0.091120981	1.214843665	0.224425723	0.667854242
106.7223406	-0.295239894	0.178315385	-1.655717452	0.097779051	0.509133783
15.32001738	0.391083415	0.235602529	1.659928762	0.096928784	0.507281065
311.0997765	0.143079813	0.085306074	1.677252342	0.093493148	0.500506422
884.3991871	0.048906557	0.124867218	0.391668509	0.695303161	0.924581226
167.5277911	-0.116706784	0.171298656	-0.681305896	0.495677954	0.853896962
104.5354805	0.063087419	0.333554672	0.189136666	0.849985704	0.969251033
187.0311206	-0.387610097	0.171897825	-2.254886573	0.024140449	0.308436782
133.3995245	0.154899032	0.088398376	1.75228369	0.079725039	0.478268331
141.6105669	0.091948592	0.09545755	0.963240639	0.335426775	0.762456424

992.0804071	0.028028764	0.066331961	0.42255293	0.672621475	0.920302871
581.1616661	0.187353047	0.106100742	1.765803356	0.077428845	0.472757172
897.098275	0.11107369	0.042144263	2.635558946	0.008399883	0.19893612
22.24830862	-0.206492683	0.283430091	-0.728548905	0.466277643	0.837628265
68.876381	0.155309487	0.210530822	0.737704274	0.460694175	0.834673974
249.5310445	0.051378158	0.101633972	0.505521503	0.6131926	0.901444946
234.8137931	-0.001311301	0.105605843	-0.012416934	0.990092975	0.99804932
563.433274	0.099074766	0.226132442	0.438127165	0.661294103	0.914896282
453.9668042	-0.130434728	0.117985253	-1.105517211	0.268935541	0.711435249
535.9128832	0.182707103	0.077397888	2.360621309	0.01824435	0.272230718
27.69141457	0.038395944	0.262157302	0.146461471	0.883557104	0.976013852
80.77983721	0.129497128	0.120007405	1.079076147	0.280553784	0.722218627
33.12157495	0.186833357	0.388873939	0.480447103	0.630909508	0.905353615
22.96011375	0.410644823	0.464609065	0.883850217	0.376777074	0.787072097
115.4787699	0.019732023	0.10984431	0.179636275	0.857438125	0.969966486
24.06534482	0.159747068	0.242401126	0.659019497	0.509883249	0.859992234
207.2888679	0.135342447	0.089946818	1.504694103	0.13240274	0.564647789
146.8527768	0.142325096	0.115904535	1.227951062	0.21946534	0.663637842
131.7946156	0.148379519	0.131893044	1.124998824	0.260589533	0.70310069
21.42664017	-0.258605833	0.256101086	-1.009780304	0.312600558	0.746754295
316.7911686	0.044064185	0.085754774	0.513839438	0.607364253	0.900307811
195.345895	0.131448673	0.135602837	0.969365218	0.332363	0.760742564
5.662067202	-0.834018669	1.029430746	-0.810174626	0.417839819	0.811361647
228.5105489	-0.032155254	0.083155923	-0.386686277	0.698988477	0.925900484
1917.039126	0.614447979	0.239898618	2.561281866	0.010428671	0.221076998
14.7303321	-0.051624707	0.516725458	-0.09990742	0.920417826	0.982262997
2009.157515	-0.058077242	0.161187603	-0.360308367	0.718616543	0.930765281
7.152014747	0.05535309	0.392534303	0.141014656	0.887858364	0.976961229
398.9721145	0.221608902	0.0729584	3.037469325	0.002385737	0.115994263
1488.082726	0.251023496	0.094373095	2.659905315	0.007816262	0.190918007
164.2647854	-0.211689667	0.185949334	-1.138426593	0.254942395	0.697376909
18.49177069	-0.31894074	0.255004911	-1.250723912	0.211035223	0.656114597
409.5472832	-0.050954796	0.073341002	-0.694765474	0.487202289	0.849513564
446.5997649	-0.054257614	0.105802997	-0.512817364	0.608079083	0.900307811
88.97294586	0.184324862	0.124728496	1.477808742	0.139458978	0.572130062
5.298437343	-0.366486248	0.508604382	-0.720572338	0.471172678	0.840276866
63.07462145	0.113320448	0.179090972	0.632753551	0.526894594	0.865114435
110.3174057	0.192704004	0.115308979	1.671196859	0.0946828	0.50255154
119.3245645	0.010780319	0.127481697	0.084563664	0.932608287	0.984334593
71.70500954	-0.016451168	0.311618012	-0.052792737	0.957897048	0.987864832
10.32735134	0.17088856	0.477549856	0.357844439	0.720459732	0.931135539
358.8711394	0.061898592	0.081782861	0.756865081	0.449130697	0.828415275
67.79306256	0.161871159	0.138785492	1.166340636	0.24347675	0.686012467
344.882927	0.037312558	0.066009619	0.56525941	0.571897333	0.884938196
11.24800311	-0.001532836	0.306677204	-0.004998206	0.996012025	0.998981303
70.02108714	0.04931562	0.245510281	0.200869877	0.840800322	0.966642825
151.1172206	0.026134745	0.116616465	0.224108538	0.822672849	0.963169453
45.54387902	0.264051958	0.145453564	1.815369459	0.069467151	0.460346985

57.15087873	0.17877794	0.144751109	1.235071301	0.216803969	0.661222104
29.30771523	-0.533378106	0.275139925	-1.938570373	0.052553674	0.415101112
5.596465198	-2.217225465	1.229288651	-1.803665447	0.071283769	0.462578849
4629.790794	0.260122419	0.167765682	1.550510308	0.121019081	0.546802333
16.21814434	-0.042940101	0.385947359	-0.111258959	0.911410999	0.980530316
136.3377351	0.009285515	0.265245604	0.035007236	0.972073971	0.992799208
822.6650088	0.127637614	0.099207855	1.286567624	0.198245034	0.644078796
196.9292605	0.232397736	0.080315235	2.89356978	0.003808896	0.140808558
450.8339475	-0.145043274	0.168117283	-0.862750525	0.388274647	0.792781092
3039.385474	0.125715334	0.06981058	1.800806326	0.071733411	0.463154629
257.0298598	0.041918193	0.115308407	0.363531108	0.716208179	0.930241908
357.1191295	0.117455075	0.109540525	1.072252256	0.283606745	0.724631363
494.2548677	0.085232816	0.09696202	0.879033	0.379383383	0.787606289
387.6380462	0.06310298	0.083467439	0.756019116	0.449637734	0.828449907
52.88268156	-0.136151123	0.269994285	-0.504274092	0.614068781	0.901875538
9.539895981	-0.742125132	0.470278892	-1.578053247	0.114553378	0.535291597
337.3034202	-0.062908267	0.068188853	-0.922559405	0.356236859	0.774305138
136.9662079	0.146785543	0.156719958	0.936610402	0.348958999	0.769102309
97.64298346	-0.002539258	0.15617458	-0.016259098	0.987027688	0.996928555
19.9078054	0.170074566	0.215660383	0.788622201	0.430332851	0.818841807
914.8921816	0.077546257	0.119008567	0.651602312	0.514657756	0.861468291
102.6655804	0.326164989	0.342102332	0.953413521	0.340380593	0.76465745
311.8844383	-0.01862531	0.101821184	-0.182921757	0.854859411	0.969966486
24.46378929	-0.63274285	0.322899706	-1.959564651	0.050046696	0.4059744
346.000549	0.081291029	0.137281123	0.59215009	0.553750087	0.877205564
148.7880833	-0.009493587	0.09608324	-0.098805855	0.92129242	0.982590996
406.3697518	-0.078903504	0.128527582	-0.613903281	0.539279242	0.869675597
67.94283123	0.605157518	0.496431814	1.219014375	0.222838736	0.666984161
9.028716032	-0.835915447	0.393360957	-2.125059522	0.033581647	0.347119785
306.9483788	-0.102293529	0.059057258	-1.732107657	0.083254396	0.485552235
67.83921697	-0.120269858	0.398403352	-0.301879633	0.762743823	0.946826103
1810.82912	-0.097806795	0.218541304	-0.447543752	0.654482505	0.912480225
690.2315983	0.050842379	0.105949007	0.479875937	0.631315613	0.905585202
123.1656582	0.277942264	0.222072287	1.251584643	0.210721253	0.655685514
49.13360711	0.45195757	0.314857569	1.435434983	0.151163183	0.589528489
801.9967841	-0.443071151	0.198744669	-2.229348611	0.025790719	0.314428791
90.39363375	-0.240200107	0.160268759	-1.498733188	0.133942863	0.567033996
197.8048378	0.241386996	0.099538343	2.425065449	0.015305627	0.258245094
37.18729088	-0.037454502	0.257708115	-0.145336914	0.884444871	0.976398641
8.51048009	0.72045527	0.464865702	1.549813778	0.121186219	0.546889761
680.3161138	-0.149875506	0.115807201	-1.29418123	0.195602829	0.640835592
7.523333647	-0.776072099	0.55723703	-1.392714512	0.163706138	0.602331161
101.7912428	0.112491738	0.153071797	0.734895258	0.462403286	0.835625577
365.2306358	-0.030265002	0.115180567	-0.26276136	0.79273452	0.953153173
223.6119674	0.186177921	0.084744913	2.196921493	0.028026054	0.325247129
2345.526857	-0.040918393	0.12981021	-0.315217062	0.752596852	0.94390261
106.1923096	0.162717807	0.104514364	1.556894191	0.119495609	0.54413566
4.510493181	-0.018237292	0.544655072	-0.033484113	0.973288535	0.993248159

36.95679592	1.284817017	0.40863157	3.144194211	0.001665449	0.098215026
11.180711	0.208590529	0.295115289	0.706810309	0.479684367	0.845119155
746.6083838	0.124983507	0.081829841	1.527358536	0.126671881	0.556378986
109.9434002	-0.131600745	0.114988169	-1.14447204	0.252427927	0.69506877
5.083217429	-0.949378643	1.033761256	-0.91837321	0.358423511	0.77666298
525.8593202	0.008929127	0.099427302	0.089805589	0.928441707	0.984049111
674.5834615	0.132718427	0.084665829	1.567555974	0.116984794	0.53905189
1229.625713	0.055336092	0.087900567	0.629530551	0.529001774	0.866063379
34.99779238	0.276530621	0.357904788	0.77263739	0.43973701	0.823505469
318.7017052	-0.033390396	0.110137195	-0.303170933	0.761759599	0.946826103
8.499537449	-0.605848826	0.329720476	-1.837461943	0.06614173	0.45158205
171.6200932	0.067323164	0.100053656	0.672870604	0.501029615	0.856727618
530.5494391	0.023434418	0.080340108	0.291690141	0.770523548	0.948321877
1030.894873	0.016914264	0.057021443	0.296629891	0.766749088	0.947349028
148.7080625	0.001391106	0.121426559	0.011456355	0.990859351	0.99821004
621.7333429	0.181112016	0.137598308	1.316237232	0.188094431	0.634208147
2064.550613	0.064420993	0.135511593	0.475391013	0.634508299	0.906920638
1238.883698	0.092592634	0.076425103	1.211547382	0.225685687	0.668749399
291.6083538	0.096713647	0.124983248	0.773812878	0.439041456	0.8229317
289.2620603	0.019802678	0.077063829	0.256964628	0.797206079	0.954840537
93.93019408	-0.186403048	0.123403951	-1.510511177	0.13091304	0.56389725
331.3129399	-0.260460956	0.217408958	-1.198023112	0.230908019	0.673080052
562.9558331	0.311425987	0.091065268	3.41981079	0.000626647	0.057623892
82.33487618	0.492327781	0.254814661	1.932101472	0.053346983	0.416932852
69.11685951	0.161012618	0.131001431	1.229090532	0.219037865	0.663637842
32.17532852	-0.312420286	0.325441998	-0.95998761	0.337061451	0.7637398
237.0645774	-0.126486359	0.145266879	-0.870717122	0.383908627	0.790043276
482.5286124	-0.021652751	0.073399873	-0.294997123	0.767996078	0.947560052
230.1067555	-0.025905519	0.068328127	-0.379134039	0.70458833	0.9269128
161.2846478	-0.090429118	0.102552364	-0.881784826	0.377893179	0.787072097
241.3007943	0.087948165	0.08012417	1.09764837	0.272358063	0.714971121
8.774541884	0.211510649	0.336328566	0.628881014	0.529426956	0.866063379
876.0443299	0.045602651	0.170992071	0.266694538	0.789704359	0.953026618
1745.05466	-0.481634656	0.553242239	-0.870567397	0.383990405	0.790043276
3122.104682	0.185571797	0.135577757	1.368748093	0.171078031	0.615232513
6.610380942	1.085461611	0.440885823	2.462001622	0.013816404	0.250625508
203.2977003	0.511910791	0.139646445	3.665763141	0.000246602	0.034435888
15449.71389	0.035363192	0.132798301	0.266292504	0.790013946	0.953026618
2949.268557	0.165248311	0.148515955	1.112663699	0.265852892	0.708743266
1179.728325	0.00767777	0.048797709	0.157338747	0.874977885	0.973587929
147.9963796	0.045806201	0.130402472	0.351267888	0.725387383	0.932532921
25.96591985	0.121059755	0.239264756	0.50596568	0.612880743	0.901444946
389.1212425	0.061799861	0.117144439	0.527552662	0.597809856	0.897163483
812.4729412	0.059998828	0.061876615	0.969652729	0.332219621	0.760742564
213.0610668	0.124994515	0.130929216	0.954672448	0.339743367	0.764202418
2586.329855	-0.035209112	0.126054756	-0.279316016	0.780002316	0.95173434
458.4431898	0.326076648	0.292856991	1.113433033	0.265522494	0.708627777
1390.338072	-0.048870103	0.069527486	-0.702888975	0.482124942	0.846449222

299.525312	0.0891805	0.100180329	0.890199709	0.373358661	0.786373874
749.8876766	-0.134615883	0.177520036	-0.758313747	0.44826318	0.82753699
667.3975501	0.09411767	0.084842854	1.109317595	0.267293197	0.709554144
237.2568663	0.251929711	0.197292409	1.276935653	0.201624966	0.645339044
1117.572099	0.184373941	0.129395614	1.424885551	0.154190287	0.59219701
1461.821789	0.317293751	0.125977253	2.518659075	0.011780266	0.235319155
2874.403844	0.008392684	0.088809372	0.094502237	0.924710206	0.982590996
53.88870194	-0.013544097	0.143204201	-0.094578906	0.924649305	0.982590996
403.7389742	0.124813935	0.286545366	0.435581762	0.663140206	0.915744058
25.38784943	0.235593241	0.195302844	1.206297036	0.22770298	0.670429007
5.269698971	0.848999692	0.441902301	1.921238451	0.054701653	0.422375515
1406.114157	0.025813239	0.159485209	0.1618535	0.871421221	0.972881041
207.5598587	-0.064990463	0.115468046	-0.56284371	0.573541313	0.88589779
231.3616186	0.010010861	0.077750117	0.128756866	0.897550038	0.97893802
1990.775298	0.284142856	0.116571593	2.437496545	0.014789356	0.256113483
1202.116815	0.069944981	0.096484666	0.724933648	0.468492737	0.838629761
104.2087753	0.126223914	0.130534337	0.966978628	0.333554718	0.761455418
45.53483419	0.347990676	0.149598496	2.326164261	0.020009789	0.282080861
257.0067708	0.020109986	0.115407275	0.174252322	0.861667168	0.970324122
10.64164359	0.229273963	0.278373951	0.823618597	0.410156306	0.807613405
1474.020235	0.153871566	0.110880745	1.387721243	0.165221945	0.605350304
676.9980772	0.073652938	0.078903281	0.933458496	0.35058329	0.770560164
7.524822808	0.156909923	0.377728741	0.415403716	0.677846387	0.921393988
1121.655075	0.049006095	0.080986082	0.6051175	0.545100927	0.872850885
317.1122949	0.191166955	0.077971052	2.451768324	0.014215616	0.251563345
296.9255996	0.085257933	0.143498514	0.594138091	0.552419756	0.876603606
159.9066727	0.023765273	0.090852356	0.261581253	0.793644298	0.953153173
1144.738361	-0.113360001	0.150951576	-0.750969311	0.452671125	0.830105164
691.4696978	0.097446601	0.086367791	1.128274783	0.25920389	0.702208457
124.020216	-0.049820464	0.103122266	-0.483120334	0.629010297	0.904638489
228.014972	0.366772847	0.206322791	1.777665209	0.075458857	0.470687737
391.8317825	0.152105018	0.094525331	1.609145573	0.107584514	0.524527661
195.3378872	0.170306775	0.104528532	1.629285056	0.103252688	0.515984568
112.1031928	0.563000176	0.402346452	1.39929201	0.161725435	0.601319304
180.4114508	-0.453023889	0.126939475	-3.568818041	0.000358595	0.040518538
19.73058752	-0.212342828	0.296468217	-0.716241459	0.473842267	0.841691256
621.4036957	-0.162868234	0.258506046	-0.630036462	0.528670728	0.86594688
408.7808731	0.074493013	0.078903172	0.944106693	0.34511513	0.767851822
362.3156915	0.066185235	0.06723669	0.984361891	0.324937609	0.756297513
19.25778349	-0.144913837	0.242990906	-0.596375561	0.550924363	0.875900621
104.4870024	-0.613227807	0.502739477	-1.219772536	0.222551115	0.666984161
31.71112153	0.39608921	0.346448449	1.143284699	0.252920406	0.695085007
228.1653304	0.218228838	0.080095746	2.724599603	0.006437951	0.175173802
434.7041744	-0.659382653	0.377362169	-1.747346999	0.080577165	0.480347644
206.7345059	0.130363094	0.297328898	0.438447438	0.661061965	0.914896282
104.1336308	-0.142075451	0.127753764	-1.11210383	0.26609351	0.708743266
377.0689643	0.236143403	0.07025531	3.36121789	0.000775996	0.065635944
165.740109	-0.026963931	0.10947671	-0.246298327	0.805451305	0.957233889

351.5361701	0.343878281	0.267592019	1.28508422	0.198762855	0.64421391
15.07907506	-0.597468287	0.770600763	-0.77532792	0.438145915	0.82251807
5.893598447	0.266098612	0.666295497	0.399370269	0.689620398	0.92405254
127.1126861	0.057264227	0.124639123	0.459440229	0.645918065	0.909300678
408.8046333	0.235181855	0.064805119	3.629062928	0.000284452	0.035213026
119.1730997	0.100680571	0.145079652	0.693967553	0.487702557	0.849513564
242.1407869	0.188754282	0.102261867	1.845793428	0.064922205	0.446824324
1309.00907	0.094537061	0.047656084	1.983735392	0.047285346	0.396329383
86.9025564	0.112240925	0.161191619	0.696319857	0.48622854	0.849161822
13.40830787	-0.505887774	0.527497362	-0.959033751	0.337541743	0.7637398
886.6769345	0.409883411	0.303548577	1.350305824	0.176917905	0.620701311
1446.731397	0.090931573	0.061324335	1.48279754	0.13812821	0.569849584
731.1296921	-0.867923822	0.964769823	-0.899617506	0.368323838	0.782648499
145.5702499	0.035409476	0.232210204	0.152488888	0.878801354	0.974821466
132.5267244	0.011789866	0.123102189	0.095773002	0.92370086	0.982590996
741.101271	0.030139718	0.092954244	0.324242518	0.745754435	0.940455922
10.74339292	-0.089439747	0.345325436	-0.259001329	0.795634215	0.954296868
731.5049534	0.033348495	0.084651867	0.393948722	0.693618898	0.924581226
374.2313992	-0.158303955	0.136985638	-1.155624468	0.247834795	0.691768931
84.92722397	0.054560966	0.108583865	0.502477656	0.615331566	0.901900555
285.9371597	-0.057743187	0.065921914	-0.875933104	0.381066402	0.78866679
88.36368487	0.144550068	0.278590468	0.518862217	0.603856827	0.899495699
126.4246915	-0.296501144	0.132656046	-2.235112176	0.025409985	0.313432167
83.01602864	-0.275571749	0.167907776	-1.641208976	0.100754043	0.512774612
1379.446046	0.001786771	0.129363445	0.013812021	0.988979952	0.99764435
311.2548823	0.561971151	0.292273615	1.92275704	0.05451057	0.421780163
439.4599008	0.050424566	0.078868817	0.639347312	0.522597017	0.863362704
197.9869029	-0.104085068	0.137167383	-0.758817916	0.447961487	0.827289573
384.8443226	-0.049820762	0.07936167	-0.627768574	0.530155556	0.866123834
963.8475179	0.050442004	0.081945508	0.615555445	0.538187965	0.869675597
436.0870562	-0.019215051	0.071211618	-0.269830281	0.787290826	0.95288876
10.35973443	-0.114893836	0.436461285	-0.263239467	0.792366013	0.953153173
231.5015931	0.079518341	0.081438413	0.976423017	0.32885488	0.758682969
7.738677732	-0.87724997	0.377329252	-2.324892556	0.020077706	0.282442098
6038.070476	-0.074908405	0.138771222	-0.539797832	0.589336463	0.891958928
6.398981438	0.476083067	0.54292091	0.876892117	0.380545238	0.78844599
26.22238017	-0.162427751	0.31248075	-0.519800822	0.603202407	0.899375001
83.25881531	-0.319588493	0.401193628	-0.79659414	0.425686782	0.816273895
4.758341127	0.024201861	0.543685928	0.044514415	0.964494362	0.990319475
12.80300648	-0.45340491	0.30413701	-1.490791633	0.136016209	0.568673186
506.1984158	0.054901822	0.086521613	0.634544605	0.525725467	0.864850467
113.3180716	-0.343187837	0.293894945	-1.167722829	0.242918585	0.685806007
40.02769372	0.398512522	0.484741427	0.822113605	0.411012243	0.808156644
28.50306567	-0.179488282	0.223211045	-0.804119178	0.42132815	0.813626324
197.2722213	-0.028864675	0.131545162	-0.219427874	0.826316759	0.964296804
1925.287907	-0.013286139	0.058967732	-0.225312023	0.821736547	0.963169453
10.6272745	0.315794698	0.290023887	1.088857548	0.276216709	0.718449748
516.330332	-0.538939836	0.404664185	-1.331819954	0.182919359	0.628528754

286.7977029	-0.060135103	0.118613264	-0.506984637	0.612165598	0.901171077
1570.217315	0.113313799	0.115498269	0.981086561	0.326550057	0.757054149
17020.73327	0.127240183	0.150531024	0.845275476	0.39795705	0.799250874
1124.766494	0.124053326	0.046282094	2.680374108	0.007353992	0.187448858
1952.920963	0.058638054	0.077372857	0.757863369	0.448532782	0.827725001
4328.788865	-0.031540656	0.104025332	-0.303201685	0.761736165	0.946826103
10.10379685	0.675131389	0.374821135	1.8012095	0.071669866	0.463154629
231.0025825	-0.153447524	0.167333846	-0.917014271	0.359135162	0.777095745
366.6593921	-0.344166752	0.160358995	-2.146226675	0.031854898	0.339220292
14.374951	-1.560757564	0.956679842	-1.631431431	0.102799317	0.515110188
7.708651355	-0.29788487	0.641901541	-0.464066296	0.642600249	0.908494289
325.7792814	-0.068169429	0.093579441	-0.728465873	0.466328452	0.837628265
30.41399363	-0.379420286	0.279189339	-1.359007075	0.174144347	0.618034305
114.1081727	-0.100218621	0.088186216	-1.136443156	0.255771143	0.698767895
879.0097436	0.030942981	0.075910044	0.407626971	0.68354756	0.922776218
355.8789768	-0.091946218	0.150790498	-0.609761351	0.542019907	0.870853624
90.92533621	0.23710713	0.101461619	2.336914513	0.01944363	0.280640059
2369.158827	-0.099331828	0.087323763	-1.137511995	0.255324313	0.697933607
131.3838695	-0.178006054	0.177242491	-1.004308015	0.315230172	0.749348681
64.56901728	0.067704834	0.111297007	0.608325737	0.542971454	0.871203645
7.044263648	-0.74330885	0.549836324	-1.351872943	0.176415964	0.62005562
410.9908526	0.071102728	0.088682231	0.801769729	0.422686172	0.814538241
477.1237875	0.086735609	0.126134182	0.687645553	0.491676015	0.851287798
272.8527146	0.075658352	0.113241797	0.668113312	0.504061265	0.857575309
1408.301417	0.073570121	0.096462598	0.762680271	0.445654099	0.826302547
4.970076054	-0.49134432	0.425372657	-1.155091454	0.248052978	0.691857602
50.09900002	-0.274001658	0.291729132	-0.939233104	0.347611076	0.768780165
28.37485688	-0.663634557	0.440408464	-1.506861496	0.131846163	0.564647789
134.3617595	0.134880601	0.087338731	1.544338914	0.12250625	0.548664905
402.0117963	0.097552173	0.065806574	1.482407712	0.138231843	0.569957848
86.58029275	0.331397465	0.377971854	0.876778155	0.380607147	0.78844599
10.49008706	-2.437862523	1.353604765	-1.801015027	0.071700511	0.463154629
151.0489863	-0.03861513	0.101703892	-0.379681933	0.704181533	0.9269128
166.9861558	-0.15331938	0.077843188	-1.969592776	0.04888506	0.402221602
2921.041194	0.173704589	0.137017809	1.267751916	0.204886558	0.649546121
289.7405786	0.047753412	0.151574678	0.315048746	0.752724643	0.943915745
16.67353981	0.451028123	0.253694066	1.777842622	0.075429706	0.470687737
82.06991449	0.006113751	0.119212854	0.051284324	0.959098959	0.988344667
521.6215423	0.160035204	0.058685241	2.727009389	0.006391122	0.174970531
168.5336991	0.151858259	0.103981361	1.460437312	0.144169925	0.578069441
461.864445	0.10765805	0.094210608	1.142738089	0.253147351	0.695504133
69.4043862	0.693349188	0.391598394	1.770561876	0.076633586	0.471783446
133.92832	-0.152351556	0.093869357	-1.62301694	0.104585784	0.518619353
25924.18629	0.286654622	0.192556724	1.488676255	0.136572641	0.569036171
114.5613666	0.059672114	0.145302328	0.410675551	0.681310457	0.922658753
625.9653715	0.001671797	0.052896979	0.031604765	0.974787243	0.993301399
689.1899768	-0.132703477	0.227921624	-0.582232938	0.56040979	0.880686454
386.0923603	0.026277898	0.0887779	0.295995937	0.767233184	0.94737077

29.09903754	0.246553497	0.234252858	1.052510091	0.292565583	0.733939814
157.0232336	-0.07536161	0.096677493	-0.779515563	0.435676074	0.822078703
133.0779967	0.236598497	0.179994714	1.314474693	0.188686509	0.634590162
12.12772148	-0.276482006	0.469035991	-0.589468636	0.555546946	0.87817235
444.8343153	0.647468935	0.397226547	1.629973979	0.103106997	0.515595346
4.950133852	0.314475369	0.527816424	0.595804441	0.551305877	0.875936124
9.556523692	0.029917807	0.404964413	0.07387762	0.941107764	0.984334593
117.1346772	-0.034204046	0.103962931	-0.329002323	0.742153932	0.939964277
6.895928462	0.767607498	0.445625098	1.722540992	0.084971564	0.488446247
13905.10425	-0.045941319	0.116208571	-0.395335036	0.692595646	0.924581226
31.68457817	1.092933016	0.418179747	2.613548421	0.008960739	0.204686499
24.70454352	1.156679993	0.424689043	2.723592739	0.006457608	0.175386271
256.7349187	-0.32868183	0.152409951	-2.156564098	0.031039644	0.334875231
6.245185531	0.049971466	0.453791319	0.110119925	0.912314267	0.980530316
237.193665	-0.225992625	0.116358083	-1.942216813	0.052110859	0.413146721
29.41018626	-0.260928843	0.193567743	-1.347997546	0.177659172	0.62197518
706.850957	0.016994666	0.066215161	0.25665823	0.797442619	0.954840537
4.97206827	0.01504601	0.437202647	0.03441427	0.972546804	0.992939564
342.4706277	0.04747329	0.096810605	0.490372822	0.623870105	0.902875034
1007.184615	-0.045475235	0.037761771	-1.204266489	0.228486594	0.671176537
5.267412685	-0.346185263	0.469382513	-0.737533362	0.460798063	0.834673974
56.87451756	-0.04704461	0.178073313	-0.264186751	0.791636019	0.953153173
671.9730548	0.014594134	0.067729512	0.215476727	0.829395655	0.96513412
129.0290186	0.020294904	0.09159087	0.221582179	0.824639157	0.963782772
20.81308207	-0.609679547	0.312556419	-1.950622384	0.051101982	0.410201488
312.0118605	0.056336906	0.088228516	0.638533983	0.52312614	0.863484457
310.5149172	-0.058274408	0.111389077	-0.523160879	0.600862303	0.898289446
550.3560287	-0.029241083	0.082841091	-0.352978003	0.724104932	0.931855434
28.67543965	0.221295041	0.342679683	0.645778117	0.518423062	0.862002027
243.7532233	-0.074714597	0.071051224	-1.0515596	0.293001651	0.733939814
314.6362276	0.632532268	0.470588122	1.344131394	0.178905902	0.623528077
578.7731033	-0.109190959	0.077747516	-1.404430193	0.160190785	0.600672338
410.6422765	0.003019406	0.089881537	0.03359317	0.973201569	0.993248159
56.88561277	-0.13917433	0.120708201	-1.152981564	0.248917952	0.692441932
50.45350991	-0.253342372	0.222588786	-1.138163231	0.255052329	0.697447732
52.50569207	-0.246973969	0.144643035	-1.707472248	0.087734312	0.492827855
343.8014786	-0.020014992	0.106635385	-0.187695597	0.851115282	0.969309003
742.2972465	0.208144059	0.092958592	2.239105107	0.025149078	0.313430481
44.80831033	0.100823256	0.14531649	0.693818414	0.487796093	0.849513564
80.34820066	-0.126891255	0.123469683	-1.027711837	0.304085398	0.741640846
4.519307051	-2.849569795	1.570803231	-1.814084501	0.069664717	0.460514862
50.34389939	-0.222779526	0.133082616	-1.673994196	0.09413174	0.501922917
17.59364861	-0.211652913	0.232267751	-0.911245375	0.362166101	0.778957081
504.7656503	-0.084106239	0.089191811	-0.942981624	0.345690302	0.767958555
442.748541	0.172624614	0.093755219	1.841226723	0.065588341	0.449670505
40.01270326	-0.532238881	0.185719536	-2.865820657	0.004159298	0.144861011
54.85106047	-0.289310752	0.292294814	-0.989790917	0.322276325	0.75479971
4.786555206	-1.153615524	0.462746739	-2.492973861	0.012667817	0.241324362

686.5038916	-0.759054783	0.913580222	-0.830857285	0.406054257	0.804391743
4823.536086	-0.671929751	0.993522698	-0.676310417	0.49884358	0.855692311
151.0022694	-0.02731796	0.086849985	-0.314541912	0.753109489	0.944021193
187.8527015	0.250311729	0.135173587	1.851779884	0.064057437	0.444821962
227.6661951	0.009223755	0.084114847	0.109656685	0.912681654	0.980530316
94.43078891	-0.096355298	0.149829723	-0.643098689	0.520160065	0.863188882
218.6739632	-0.194617229	0.179457471	-1.084475489	0.278154029	0.720174207
10.05948633	-0.244964181	0.301187668	-0.813327392	0.416030376	0.810170019
88.20056101	-0.311643719	0.243581711	-1.27942167	0.200748607	0.645045462
1572.794027	0.042203661	0.113886169	0.370577579	0.710952184	0.929376205
1532.322724	0.079610749	0.138571039	0.574512176	0.565621263	0.883265494
318.8520284	-0.195021313	0.072558051	-2.687797023	0.00719251	0.186457572
150.3739188	0.104993296	0.145671426	0.720754227	0.471060742	0.840276866
177.1471032	-0.202252554	0.114530625	-1.765925517	0.077408346	0.472757172
295.0935596	-0.050917347	0.104148317	-0.488892651	0.624917699	0.90314702
105.3909604	-0.178082931	0.169009726	-1.053684511	0.292027383	0.733761555
787.7608353	-0.003673284	0.088293346	-0.04160318	0.966815038	0.990966732
268.2966334	-0.079809823	0.084765725	-0.941534122	0.346431211	0.768681576
181.193882	0.0016854	0.091887109	0.018342069	0.985365967	0.996337662
385.3820683	0.058886634	0.067930034	0.866871852	0.386012228	0.791597811
50.50131964	0.022581028	0.189689031	0.119042347	0.905241806	0.979768942
144.0524308	-0.040962042	0.123354912	-0.332066563	0.739838998	0.939690097
4.878514324	0.269316111	0.712228302	0.378131717	0.705332748	0.926977731
161.5073331	-0.136675468	0.07696233	-1.775874859	0.075753546	0.471272491
284.0772935	-0.093925461	0.101314625	-0.927067151	0.35389167	0.773295616
168.3670153	0.066285119	0.093340759	0.710141207	0.477616575	0.843939423
9.887325518	-0.755427815	0.62389517	-1.210824912	0.225962515	0.668749399
132.4400035	0.173584216	0.191131097	0.908194528	0.363775455	0.779728871
17.70391231	-0.323723687	0.459014264	-0.705258446	0.480649415	0.845612509
63.66381644	-0.126323773	0.209121266	-0.604069475	0.545797453	0.872896521
117.1189461	-0.025023203	0.14969823	-0.167157639	0.867246014	0.972138679
225.992933	-0.320815725	0.085627716	-3.746634157	0.000179223	0.028506312
711.5850601	0.012516301	0.107706495	0.116207487	0.907488103	0.979768942
168.9531385	0.186181281	0.379854277	0.490138699	0.624035755	0.902875034
12.85395814	-1.740799599	1.114636234	-1.561764768	0.118343415	0.54159575
189.8160327	0.174477517	0.332366397	0.524955345	0.599614234	0.898108141
13.46426521	-2.630018337	1.585500506	-1.658793754	0.097157359	0.507880983
51.71092904	0.191649268	0.182202548	1.051847359	0.292869587	0.733939814
544.8442102	0.181959395	0.10320892	1.763020038	0.07789711	0.473730652
134.9958808	0.104268315	0.094943562	1.098213633	0.272111214	0.714780869
22.31958266	-0.142314639	0.2130316	-0.668044735	0.504105037	0.857575309
186.4122065	-0.011150274	0.080495219	-0.138520948	0.889828715	0.976961229
390.8799843	0.031857275	0.073149375	0.435509874	0.663192374	0.915744058
907.4632778	0.112084025	0.088343341	1.268732007	0.204536663	0.648992858
8.493543257	-3.079776323	1.04405775	-2.949814148	0.003179651	0.132817478
77.35002297	-0.161736249	0.116087165	-1.393231113	0.163549914	0.602187485
91.59150853	0.193992755	0.101491843	1.911412289	0.055951619	0.42580764
20.85627266	-0.599242746	0.331336924	-1.808560114	0.07051937	0.461462295

179.8207367	-0.236702085	0.10930393	-2.165540478	0.030346319	0.332880971
248.1988312	-0.068710835	0.204512199	-0.33597426	0.736890277	0.938598217
637.9405736	0.142205912	0.085127521	1.67050456	0.094819578	0.502934579
151.3281689	-0.00789368	0.080499601	-0.098058622	0.921885744	0.982590996
13.16449933	-0.433207877	0.39648142	-1.092630965	0.274555862	0.716878153
291.4311377	0.004506836	0.068233997	0.066049721	0.94733824	0.985833846
136.8618896	0.013730541	0.205778375	0.0667249	0.946800711	0.985759593
526.9497415	-0.006159089	0.099778658	-0.061727519	0.950779825	0.98625342
27.66249449	0.131297043	0.426169769	0.308086243	0.758016709	0.945887766
355.0014872	-0.03146378	0.070944345	-0.443499471	0.657404502	0.91380052
6.600556863	0.533233558	0.572701786	0.931084154	0.351810035	0.771251797
25.25709999	0.33949805	0.33037863	1.027602936	0.304136643	0.741640846
8.88307273	-0.251538898	0.379421684	-0.662953406	0.507360396	0.859144938
72.2919551	0.022602806	0.139984049	0.161467012	0.871725591	0.972881041
289.5478932	-0.093925422	0.061342124	-1.531173279	0.125726573	0.554908265
330.0156412	0.158469889	0.107037818	1.480503735	0.138738865	0.570638112
1248.291607	0.364288005	0.174759558	2.084509761	0.037113823	0.361102242
18.62030243	-0.277673265	0.282630027	-0.98246201	0.32587229	0.756399818
279.3322306	0.146604876	0.063638373	2.303718167	0.021238469	0.291354795
846.8942476	0.089504566	0.062470717	1.43274434	0.151930913	0.589764517
127.5765424	-0.298769744	0.1814455	-1.646608726	0.099638496	0.511744973
126.2443656	0.115528175	0.132300006	0.873228796	0.382538382	0.789880775
570.1854155	-0.008094207	0.12166543	-0.066528406	0.946957143	0.985783785
231.6216721	-0.38407805	0.311145966	-1.234398294	0.217054524	0.661222104
249.5067551	-0.159989412	0.226730114	-0.705638122	0.48041321	0.845611089
1539.879634	-0.075594918	0.126417736	-0.597977155	0.549855177	0.875627361
7.974404445	-0.161117506	0.535391067	-0.300934243	0.763464637	0.946826103
55.4535182	-0.290725724	0.160274882	-1.813919441	0.069690129	0.460514862
2490.840207	0.047969394	0.116314272	0.412411933	0.680037518	0.922112199
373.9814452	0.172254609	0.126451546	1.362218291	0.173129005	0.616656352
96.64191274	-0.306243272	0.179689807	-1.704288499	0.088327203	0.492827855
79.13687554	0.021267529	0.196086135	0.108460135	0.913630702	0.980550322
27.4596928	-0.090957727	0.200459959	-0.453745115	0.650012291	0.910782566
4.848532651	-0.152674705	0.504013892	-0.302917653	0.761952618	0.946826103
97.45582377	-0.050395158	0.101387757	-0.497053681	0.619151197	0.901976704
90.53288268	0.749452819	0.299889146	2.499099516	0.012450934	0.239038556
7.22808652	0.274057096	0.389453002	0.703697478	0.481621191	0.845969003
246.9776761	0.134527434	0.087822612	1.531808623	0.125569668	0.554830513
136.634054	0.045687386	0.111878472	0.408366195	0.683004848	0.922776218
4837.255697	-0.053767328	0.156572222	-0.343402729	0.731295507	0.93601881
747.5687156	-0.118470235	0.096635949	-1.225943725	0.220219853	0.664595064
8.509865994	-0.128833645	0.336531715	-0.382827648	0.70184756	0.926243544
4.636222727	-1.263946922	0.509967339	-2.478486023	0.013194128	0.24658174
32.44880175	-0.416400653	0.201565834	-2.065829536	0.038844578	0.368339171
233.8664084	-0.034406705	0.094536678	-0.363950859	0.715894705	0.930241908
33.08152333	0.239513721	0.164151397	1.459102541	0.144536887	0.578069441
86.74203333	-0.067840504	0.170863436	-0.397045181	0.691334143	0.924455975
8.148484409	-1.014289951	0.430017591	-2.358717348	0.018338217	0.272290126

191.3767828	-0.145648634	0.12292006	-1.184905328	0.23605488	0.678901985
498.7036254	-0.175059586	0.100485058	-1.742145447	0.081482997	0.48207474
282.1959607	0.025217633	0.056920493	0.443032579	0.65774217	0.91380052
148.773053	-0.049153711	0.088362029	-0.556276399	0.578021918	0.887079361
1358.268268	-0.181828531	0.094869917	-1.916608932	0.055287632	0.423973339
447.194187	-0.063933023	0.070298577	-0.909449743	0.363112775	0.779728871
829.7293459	-0.059281872	0.082955013	-0.714626757	0.474839707	0.842149214
459.0773201	-0.013226041	0.176244512	-0.075043705	0.940179938	0.984334593
5.042612592	-2.138247355	1.201792393	-1.779215252	0.075204479	0.470687737
4.869433955	-2.263358581	1.285254268	-1.761020086	0.078235003	0.474223305
175.1824651	0.055361893	0.140346599	0.394465511	0.693237386	0.924581226
234.0693579	-0.003023347	0.330234916	-0.009155141	0.992695356	0.998618424
307.499838	-0.530383138	0.258109418	-2.05487712	0.039890875	0.372633565
491.8627667	-0.259767424	0.177351712	-1.464702092	0.143002214	0.57754946
10.33842271	-0.982454191	0.446798133	-2.198877118	0.027886661	0.324735857
88.02687171	-0.714465686	0.35029585	-2.03960648	0.041389536	0.374892505
1878.940833	-0.066967919	0.083664175	-0.800437221	0.423457523	0.814538241
6.76383673	0.008101477	0.378845894	0.021384624	0.982938839	0.995529925
105.8549121	-0.101008167	0.274189992	-0.368387506	0.712584312	0.929589333
687.65233	-0.025931017	0.107604904	-0.240983597	0.80956783	0.958681749
642.3788076	0.083779194	0.097090981	0.862893683	0.388195924	0.792781092
662.97701	0.08754161	0.081439668	1.074925914	0.282407899	0.723520163
206.1250575	-0.12877313	0.152720235	-0.843196257	0.399118692	0.800075146
29.72067039	-0.057403883	0.174452754	-0.329051172	0.74211701	0.939964277
8.5637678	0.24608551	0.336146681	0.732077762	0.464121106	0.836383228
11.73329651	-0.975451444	0.645221014	-1.511809788	0.130582259	0.563357211
811.6808443	0.135341977	0.109169654	1.239739907	0.215071611	0.660219973
9.363272092	0.080930404	0.332365825	0.243497971	0.807619657	0.958108464
121.3140368	-0.337748435	0.200691516	-1.682923329	0.092389935	0.499809199
16.8311029	0.394047261	0.55727242	0.70709988	0.479504411	0.845088483
624.0327499	-0.076354627	0.265746406	-0.287321392	0.773866245	0.949500013
63.3461881	0.396506921	0.311660892	1.272238294	0.203288465	0.647391537
116.6322538	0.253724919	0.33945425	0.747449529	0.454792258	0.831501359
135.8726581	0.074035332	0.09010283	0.82167599	0.411261327	0.808165706
548.0956578	-0.063090021	0.079890311	-0.789708041	0.429698294	0.818650305
40.64151206	0.06298478	0.167508899	0.376008561	0.706910531	0.928109779
34.33473784	-0.161748264	0.186001177	-0.869608818	0.384514217	0.790387368
670.1049442	-0.04698305	0.129506473	-0.36278534	0.716765244	0.930336648
5.72197427	-0.1616763	0.442227485	-0.365595322	0.714667066	0.930012765
15.73189394	-1.694808892	0.495867409	-3.41786708	0.000631139	0.057667424
75.21162919	0.013130699	0.247587303	0.053034622	0.957704322	0.987864832
387.3605878	-0.118895117	0.078979606	-1.505390087	0.132223816	0.564647789
43.05295535	0.011329405	0.254461215	0.044523109	0.964487432	0.990319475
812.1036248	0.179444264	0.170459105	1.052711522	0.292473226	0.733939814
261.3023283	0.027052374	0.104982312	0.257685064	0.796649974	0.954664258
211.5317329	0.187799003	0.109299821	1.718200469	0.085760056	0.489179326
31.94542055	0.163412101	0.184004935	0.888085423	0.374494803	0.786726096
184.3295753	-0.063907485	0.102658934	-0.622522392	0.533598425	0.867356078

9.07652233	-1.339553747	0.679285511	-1.972004001	0.04860915	0.401289813
353.2916387	-0.295566218	0.160213077	-1.844832047	0.065061974	0.447304849
36.66761302	-0.192457252	0.219174341	-0.878101201	0.379888801	0.787992438
1171.154658	0.378326281	0.24933194	1.517359872	0.12917585	0.560557293
165.3706681	0.181813894	0.226791834	0.801677429	0.422739575	0.814538241
491.0176614	0.132212922	0.081934588	1.61363991	0.106605555	0.523092509
92.5492713	-0.934064236	0.416635373	-2.241922546	0.024966379	0.312413884
6.704955844	-0.168189588	0.45773886	-0.367435677	0.713294064	0.929705922
988.1176478	-0.046442741	0.077944257	-0.595845574	0.551278395	0.875936124
29.14318463	-0.167669344	0.36934293	-0.453966573	0.649852886	0.910782566
31.94187627	-0.254041098	0.18703348	-1.358265362	0.174379497	0.618034305
361.464579	0.101697879	0.181569878	0.560103251	0.575409013	0.886202779
255.3396533	-0.20788828	0.111064522	-1.871779361	0.061237135	0.437941099
447.8512432	0.306675115	0.093088403	3.294450267	0.000986144	0.075960346
84.08987481	-0.158140004	0.236079364	-0.669859499	0.50294736	0.857172233
3100.796796	0.009421467	0.145746635	0.064642778	0.948458424	0.985917197
137.2441043	0.137781967	0.09841725	1.399977823	0.161519959	0.601161287
26.77248415	0.277831286	0.231726372	1.198962739	0.230542435	0.673080052
4237.292592	-0.035460204	0.147311813	-0.240715277	0.809775797	0.958681749
7.079763936	-0.168125739	0.341422165	-0.492427721	0.622417007	0.902441895
394.6281061	-0.152576478	0.08713177	-1.75110041	0.079928616	0.478696768
131.5237486	0.130253597	0.172852329	0.75355419	0.45111696	0.828948899
12.55236443	0.276248704	0.665681206	0.414986485	0.678151797	0.921509492
225.0975252	-0.084764083	0.446954073	-0.189648305	0.849584732	0.969251033
470.0368261	0.017592423	0.073034579	0.240877995	0.809649677	0.958681749
313.0075745	0.090509288	0.070125717	1.290671837	0.196817494	0.642224847
121.0441638	0.087731848	0.192549468	0.455632775	0.648654071	0.910701841
17.35950583	-0.120465517	0.287734338	-0.41866924	0.675457879	0.921149714
11197.30926	0.189275709	0.072758002	2.601441803	0.009283281	0.208799287
38.4671682	-0.537640733	0.199281592	-2.697894613	0.006977953	0.182836032
105.5824213	0.069094134	0.102926628	0.671295029	0.502032601	0.857004561
15.50129406	0.028032761	0.357814901	0.078344308	0.937554173	0.984334593
260.6131915	0.083738332	0.085686997	0.977258334	0.328441275	0.758556366
96.32243519	-0.038394759	0.108013726	-0.355461855	0.722243617	0.931160179
1985.970845	0.046284846	0.106109371	0.43619942	0.662692049	0.915556436
12.34810484	-0.087968641	0.416523639	-0.211197234	0.832733369	0.966227926
6.939930197	-0.509814445	0.537579635	-0.948351485	0.342950549	0.766327374
19.86959449	-0.169685513	0.306258735	-0.554059342	0.579538234	0.887483205
448.4961741	0.071546179	0.087496805	0.817700473	0.413528241	0.808272153
19.84150822	0.257531537	0.456100866	0.564637247	0.572320525	0.885183781
975.0878872	0.102321661	0.10747414	0.952058432	0.341067349	0.765180569
101.5217022	0.159413358	0.168915419	0.943746636	0.345299136	0.767851822
2420.758591	0.090645042	0.107646605	0.842061322	0.39975363	0.80037241
454.0596194	0.141703397	0.180499512	0.785062494	0.432416928	0.819823391
257.0172573	0.226205137	0.084738219	2.669458225	0.007597372	0.189660453
137.0105653	0.004302951	0.133945696	0.032124596	0.974372689	0.993301399
148.3917512	0.054217443	0.111479961	0.486342503	0.626724349	0.904257122
333.9239128	0.048109928	0.075178061	0.639946389	0.522207454	0.863362704

162.2547337	0.065166175	0.199824207	0.326117523	0.74433544	0.940317814
591.5236508	0.190479112	0.099293525	1.918343744	0.055067439	0.423212999
12.40621504	-1.207012017	0.62726276	-1.924252633	0.054322925	0.420709255
5.609638617	-2.372403935	1.661984444	-1.427452552	0.153449493	0.591551404
60.36271153	-0.019682824	0.371501001	-0.052981888	0.957746339	0.987864832
15.71631199	-1.372069055	0.862967356	-1.589943172	0.111847615	0.532679666
557.7238622	0.09183562	0.062170476	1.477158066	0.139633271	0.572218074
17.7564016	0.211689698	0.325307905	0.650736408	0.515216656	0.861826223
132.4985217	-0.031841018	0.154755397	-0.205750616	0.836985719	0.966316856
26.01972442	-0.01096764	0.368216651	-0.029785835	0.976237856	0.993487297
99.97433841	0.102723512	0.186197583	0.551690898	0.581160147	0.887898616
456.6097405	0.092563174	0.127193554	0.727734787	0.466775951	0.837677571
24.1922606	-0.059797447	0.234112485	-0.255421863	0.798397286	0.95501603
175.4402629	0.249792184	0.076773727	3.25361543	0.001139464	0.081479948
177.7613054	0.057239025	0.100958955	0.566953422	0.570745828	0.884626151
61.50914083	0.003979947	0.193492345	0.020569015	0.983589458	0.995742202
172.3025423	0.244960941	0.213384029	1.14798161	0.250976156	0.694642681
1349.713011	0.104969187	0.123302205	0.851316381	0.394593628	0.796902183
56.29981714	-0.045208017	0.165289856	-0.273507514	0.784463117	0.951928436
78.05008562	-0.029885148	0.139446195	-0.214313115	0.830302891	0.965541183
1047.615001	0.149586446	0.102442699	1.460196251	0.144236145	0.578069441
513.5243139	0.14610529	0.197539228	0.739626712	0.459526529	0.834180976
161.8430235	0.047204734	0.108033047	0.436947164	0.662149666	0.915224518
998.0120213	0.010031051	0.075010932	0.133727856	0.893617777	0.978111861
4001.313102	-0.048864128	0.113270034	-0.431395011	0.666181177	0.916091953
14.46289384	-0.305944917	0.31802768	-0.962007196	0.336045989	0.763023889
226.7356118	-0.019852199	0.235556906	-0.084277719	0.932835627	0.984334593
55.99452068	0.312541945	0.168979102	1.849589337	0.064372761	0.445584453
40.8361384	0.019589826	0.150644175	0.130040382	0.896534478	0.978829031
142.6746828	0.121777762	0.100913523	1.206753651	0.227527031	0.670086571
427.8840425	0.122890627	0.053106344	2.314047974	0.020665091	0.286677294
180.5399724	0.059258006	0.079915293	0.741510209	0.458384144	0.833531988
236.4297569	-0.027081309	0.087944507	-0.307936332	0.758130779	0.945887766
4.576678891	0.662492687	0.488830749	1.355259847	0.175334783	0.619005807
17.67001167	0.205966176	0.233845981	0.880777061	0.378438498	0.787072097
132.9321941	-0.064969287	0.202897653	-0.320207186	0.748811276	0.941640077
69.40759043	-0.108681834	0.116423197	-0.933506694	0.350558416	0.770560164
392.6976497	-0.136820517	0.078896724	-1.734172356	0.082887506	0.485197868
352.8120271	0.018873046	0.103487746	0.182369865	0.855292474	0.969966486
53.26112695	-0.086107665	0.199862771	-0.430833939	0.66658912	0.916235862
644.5965145	0.094451958	0.081277558	1.162091485	0.245198306	0.688173175
142.5675848	0.018649085	0.105531895	0.17671515	0.85973214	0.969966486
3636.525328	0.251096633	0.117967167	2.128529825	0.03329318	0.34631458
160.3109688	-0.019471378	0.12640749	-0.154036584	0.87758089	0.97425769
2675.77794	0.058485245	0.088564881	0.660366092	0.509018926	0.859636085
177.6425091	-0.168359765	0.205189232	-0.820509746	0.411925575	0.808272153
397.5919515	-0.086842735	0.051833395	-1.675420527	0.093851752	0.50133296
1043.867504	-0.025095299	0.103720845	-0.241950393	0.808818602	0.958618312

5.879279072	-0.022434696	0.423742528	-0.052944169	0.957776392	0.987864832
393.6066778	-0.131679068	0.116982959	-1.125626067	0.26032383	0.703096291
29.92012734	0.043360964	0.22423599	0.19337201	0.846667635	0.968723377
43.89957654	0.303068648	0.18058008	1.678306088	0.09328736	0.500506422
10.11071815	-0.05031265	0.343331317	-0.14654256	0.883493095	0.976013852
1138.557593	0.16001058	0.050382795	3.175897239	0.001493739	0.09329248
423.1140291	-0.038251346	0.104919865	-0.364576774	0.715427355	0.930241908
5.316635644	0.916190002	0.439974063	2.082372755	0.037308435	0.36125228
654.3648267	-0.149220353	0.080787906	-1.847063026	0.064738006	0.446529343
92.34932656	0.096126171	0.125522042	0.765811084	0.443788718	0.825650759
49.94693923	-0.721130085	0.227059288	-3.175955024	0.001493441	0.09329248
22.25654304	0.178832776	0.307507787	0.581555277	0.560866275	0.880936184
131.7466952	0.0064343	0.362880159	0.017731198	0.985853292	0.996565278
14.19928678	0.25800819	0.426558731	0.60485971	0.545272216	0.872896521
4880.015166	0.011254556	0.142988001	0.078709792	0.937263457	0.984334593
90.92210046	0.151540524	0.203147535	0.745962898	0.455689828	0.831550545
3792.031423	-0.048242424	0.071848235	-0.671448974	0.501934555	0.857004561
43.25877037	0.106249496	0.226514008	0.469063687	0.639024114	0.907684986
376.726999	-0.029970403	0.091085889	-0.329034528	0.74212959	0.939964277
28.84985216	-0.128394032	0.24279529	-0.528815992	0.596933103	0.896816502
21.71098064	-0.30835594	0.21887632	-1.408813616	0.158890288	0.59901927
4074.88109	-0.165979667	0.207230568	-0.800942006	0.42316522	0.814538241
8.989476071	0.641004709	0.561935382	1.140708931	0.253991069	0.696717102
79.63935366	-0.296525922	0.820702236	-0.36130756	0.717869543	0.930765281
284.5603217	1.14837443	0.858299649	1.337964465	0.180908021	0.626148701
44.68681984	0.173967802	0.268088943	0.648918229	0.516391231	0.862002027
629.2488391	0.294025417	0.106397676	2.763457139	0.00571926	0.16566828
367.8740506	0.025058307	0.061476851	0.407605577	0.683563269	0.922776218
570.4685529	0.116134819	0.114908012	1.010676419	0.312171327	0.746608496
47.407021	-0.262828751	0.326043069	-0.806116662	0.420175585	0.812361417
458.2663546	0.852766715	0.433618493	1.966629026	0.049225995	0.403233631
71.82703379	0.346012652	0.455031129	0.760415343	0.447006355	0.826805895
735.8687927	0.191421473	0.089378401	2.141697219	0.032217855	0.340148848
191.6422689	0.424240275	0.258440733	1.641537966	0.100685793	0.512774612
2547.694454	0.149422836	0.087068652	1.716149641	0.086134657	0.49014457
337.5769537	-0.019172701	0.122125774	-0.156991438	0.875251596	0.973587929
18.39755246	0.672417451	0.501285853	1.341385253	0.179795403	0.624872401
172.4310234	0.227388358	0.091298837	2.490594236	0.012752967	0.241702202
323.0068367	-0.04234364	0.080519575	-0.525880077	0.598971532	0.897911345
286.9254278	-0.00818542	0.084628359	-0.096721953	0.922947206	0.982590996
1345.319646	0.126667618	0.074161637	1.707993839	0.087637486	0.492827855
225.0796809	0.25684718	0.436775988	0.588052428	0.556497103	0.878642775
4.503417272	-0.123086872	0.443567571	-0.277492946	0.781401622	0.95173434
590.8892242	0.002735756	0.105669177	0.025889825	0.979345216	0.994602886
24.36466467	0.061788249	0.192306371	0.321301102	0.747982221	0.941487306
300.8933668	0.120359057	0.10252935	1.173898576	0.240435653	0.682548146
8.937900459	0.280060168	0.406454073	0.689032751	0.490802658	0.850315507
44.35504562	-0.597210506	0.543681169	-1.098457221	0.272004888	0.714755255

646.007863	0.029851475	0.099646753	0.299572985	0.764502893	0.946826103
150.3729078	-0.016208103	0.083052959	-0.195153824	0.845272527	0.968131661
41.7898702	0.346295214	0.144310269	2.399657458	0.01641042	0.263145637
194.5558857	0.16956621	0.10672319	1.588841277	0.112096232	0.532835074
13.35765042	0.206060727	0.276171007	0.74613454	0.455586146	0.831550545
41.18919276	0.266965706	0.213501676	1.25041504	0.211147973	0.65618566
7.963609446	-0.162221231	0.489367157	-0.331491864	0.740272985	0.939732486
204.6818135	-0.301676884	0.142742527	-2.113433814	0.034563654	0.352363583
151.9870287	0.173112375	0.296513499	0.583826288	0.55933719	0.880215722
461.852566	0.116637564	0.072846139	1.601149579	0.109343793	0.527372704
1366.536871	-0.718950802	0.42582545	-1.688369734	0.091340277	0.498962299
19.88172049	-1.684279949	0.87678036	-1.920982751	0.054733882	0.422404026
512.187104	0.009576031	0.179879499	0.053235812	0.957544022	0.987864832
1076.598575	0.092121857	0.056882992	1.6194974	0.105340285	0.520612072
129.1591937	0.237170051	0.159348408	1.488374149	0.13665225	0.569036171
1009.301036	0.076100327	0.073569328	1.034402906	0.300947847	0.739230864
1019.648218	-0.081394978	0.130635347	-0.623070094	0.533238461	0.867356078
524.4077388	0.127508353	0.082502778	1.545503757	0.122224461	0.548564727
748.3902284	-0.053652747	0.102344429	-0.52423711	0.600113633	0.898108141
11.70772661	0.305411909	0.388295958	0.786544136	0.431548773	0.818841807
36.23053826	-0.108373199	0.386949971	-0.280070312	0.779423561	0.95173434
2222.199344	0.186376315	0.085937601	2.168740041	0.030102426	0.332282647
2090.681204	0.311310812	0.127873933	2.434513462	0.014911825	0.256113483
8.224096704	1.114617843	0.469774819	2.372664092	0.017660317	0.269492791
325.7736107	-0.068818772	0.080654908	-0.853249648	0.393520876	0.796185896
34.32009127	0.070213461	0.174933138	0.401373129	0.68814543	0.92352421
2409.820198	0.133866547	0.113353519	1.180965071	0.2376166	0.679753163
164.7327343	-0.027618964	0.144380216	-0.191293274	0.848295831	0.969058943
24.49628572	0.542744083	0.396910942	1.36742031	0.1714936	0.616109027
1622.690113	0.079547466	0.072068714	1.103772512	0.269691832	0.712344485
6.684591747	-0.636546228	0.519543778	-1.225202293	0.220499011	0.664595064
121.5625056	-0.179458797	0.247315785	-0.725626135	0.468067995	0.838388803
645.8947712	0.034732227	0.231206633	0.150221584	0.880589797	0.975430592
4885.98703	0.069919355	0.099386245	0.703511389	0.481737112	0.845969003
520.4884363	-0.187774339	0.121615376	-1.544001628	0.122587938	0.548680826
1111.556532	-0.084010739	0.064470928	-1.303079428	0.192547646	0.638906868
93.69591658	0.029313095	0.104199908	0.281315942	0.778468084	0.951560132
32.23148401	-0.133448675	0.231643529	-0.576094981	0.564550983	0.883265494
241.7382139	-0.101436908	0.270686127	-0.374739956	0.707853873	0.928420247
290.2796337	-0.06922759	0.153929125	-0.44973679	0.652900241	0.911644397
172.0347725	0.010498591	0.149615381	0.070170533	0.944057928	0.984843572
13.83243162	-0.200367193	0.296619205	-0.675503103	0.499356181	0.855692311
1997.714192	0.144361905	0.07510924	1.922025915	0.054602497	0.422126133
354.4747252	0.112373151	0.112612663	0.997873131	0.318340882	0.751465423
85.10996865	-0.245180455	0.194939579	-1.257725371	0.208491093	0.652885502
47.27332396	-0.024402152	0.168674859	-0.144669765	0.884971612	0.976688771
7.649406383	0.214386128	0.506819622	0.423002817	0.672293206	0.920198876
101.8307364	0.097573647	0.148865147	0.655449908	0.512178127	0.860200661

38.5235541	0.017724659	0.287112525	0.061734188	0.950774514	0.98625342
1066.320771	0.103528807	0.071034766	1.457438566	0.144995358	0.578290974
2067.753803	0.229184252	0.137131297	1.67127605	0.094667165	0.50255154
187.8049334	-0.064077583	0.140049134	-0.457536445	0.647285513	0.910320206
30.69236913	0.162793441	0.212474115	0.766180111	0.443569141	0.825536387
384.2442725	-0.003074088	0.065847837	-0.046684717	0.962764511	0.989916664
316.1540003	0.03437609	0.058821797	0.584410746	0.558943999	0.880046199
1775.356394	-0.178353323	0.229012593	-0.778792643	0.436101872	0.822078703
764.8931484	0.055046913	0.12109502	0.45457619	0.649414167	0.910782566
612.9376635	0.121099402	0.052396718	2.311202067	0.020821696	0.288001684
46.22741251	-0.178150549	0.163718855	-1.088149248	0.276529226	0.718591762
4.567694896	0.295529504	0.508903136	0.580718576	0.56143014	0.881273052
412.3242777	0.064706433	0.132601672	0.487975993	0.625566845	0.903732232
3914.416159	0.009482636	0.074234928	0.127738205	0.898356154	0.978978708
354.0639074	-0.10006401	0.258859361	-0.386557431	0.699083878	0.925900484
547.6996337	-0.123978587	0.087420455	-1.41818739	0.156136062	0.595037589
346.8904804	-0.093792206	0.069054058	-1.35824322	0.174386521	0.618034305
114.0552179	0.055569519	0.138430963	0.401424058	0.68810794	0.92352421
140.0964644	0.104352513	0.085741544	1.217058943	0.22358179	0.667854242
120.5110893	0.014906417	0.115084782	0.12952553	0.896941825	0.97893802
96.27166818	0.152656374	0.118929981	1.283581922	0.199288278	0.644243885
37.13354244	0.330864978	0.227077309	1.457058737	0.145100167	0.578290974
1024.098721	0.137843528	0.078841805	1.748355798	0.080402437	0.479885835
1913.607567	0.634298721	0.253392885	2.503222302	0.012306822	0.237814066
309.2292264	-0.192687736	0.212066751	-0.908618327	0.363551629	0.779728871
1132.770519	0.210191613	0.075164843	2.796408594	0.005167403	0.157953926
535.0277578	-0.0421758	0.095818282	-0.44016444	0.659818012	0.914735059
125.8396572	0.049811178	0.122763595	0.405748772	0.684927203	0.922907843
219.4198255	0.166153824	0.098305792	1.690173301	0.090994805	0.498583575
63.50213768	-0.35239339	0.447031724	-0.788296158	0.430523494	0.818841807
186.1980887	0.099963685	0.113173541	0.883277875	0.377086155	0.787072097
30.5915015	0.234614429	0.295738792	0.793316382	0.427593515	0.817479701
565.312391	-0.004763414	0.157350549	-0.030272626	0.975849628	0.993433714
997.8030836	0.126119471	0.05219975	2.416093404	0.015688036	0.259890973
50.07826068	0.075568849	0.171040457	0.441818561	0.658620506	0.914102272
856.4836111	-0.084242706	0.054855877	-1.535709769	0.124609578	0.55306476
91.83604574	0.020217893	0.114821689	0.176080786	0.860230475	0.969966486
252.9405849	-0.009849526	0.175613521	-0.056086374	0.955272998	0.987483032
6.483952732	0.243461552	0.372708718	0.653222049	0.513613137	0.860641318
12.00547673	-0.854102209	0.490195844	-1.742369339	0.081443837	0.48207474
9.93490915	-1.09360757	0.489130565	-2.235819323	0.025363609	0.313430481
147.9296466	-0.313028314	0.229310988	-1.365082053	0.172227262	0.616109027
518.3817389	-0.375820996	0.24963143	-1.505503517	0.132194673	0.564647789
279.2854103	0.065768689	0.065636183	1.002018797	0.316334515	0.749887496
6.792481997	-0.452328641	0.394761317	-1.145828177	0.251866254	0.694735013
2456.615775	0.085763564	0.175889587	0.487598872	0.625833994	0.903941724
619.3490419	0.324882634	0.303468083	1.07056607	0.284364586	0.725217885
339.914619	0.115827787	0.109200103	1.060693027	0.28882943	0.729902555

28.48053424	0.036115574	0.196056273	0.184210247	0.853848521	0.969966486
239.1207862	-0.228592635	0.258824612	-0.883195123	0.377130856	0.787072097
20.51358989	-0.665971081	0.40837365	-1.630788571	0.10293494	0.515192869
722.7674677	0.04682526	0.105728694	0.44288128	0.657851608	0.91380052
923.1696674	0.00023149	0.072164785	0.003207792	0.997440557	0.999330927
71.43717083	0.097312886	0.155672763	0.625111833	0.531897662	0.866797125
21.26840933	-0.776183089	0.620144551	-1.251616398	0.210709677	0.655685514
143.016525	-0.101471365	0.077264837	-1.313292941	0.189084256	0.634942187
661.4967891	0.148592046	0.07078274	2.099269488	0.035793152	0.357462935
70.33094478	-0.387934494	0.373538814	-1.038538644	0.299019354	0.738631505
28.75385907	-0.054498727	0.224663414	-0.242579446	0.808331205	0.958420258
5335.429382	-0.17651284	0.224976697	-0.784582768	0.432698236	0.820076734
797.5284366	-0.376285231	0.176138611	-2.136301797	0.032654823	0.341836409
319.0400008	0.084767577	0.160495824	0.528160641	0.597387844	0.896990756
3026.569697	-0.05734904	0.174203988	-0.329206243	0.741999805	0.939964277
81.39534162	-0.315015365	0.141321593	-2.229067463	0.025809416	0.314428791
718.3211475	-0.014737763	0.103705181	-0.142112117	0.886991447	0.976961229
167.5873725	0.038360585	0.156445529	0.245200905	0.806300875	0.95754698
3103.136316	-0.031132581	0.125722077	-0.247630182	0.804420556	0.957233889
518.8557031	-0.020106638	0.16171292	-0.124335384	0.901049733	0.979318463
58.43486945	-0.64662864	0.27125906	-2.383804763	0.01713469	0.266768098
507.009291	0.105544968	0.089000416	1.185892975	0.235664568	0.678901985
137.1181522	0.022970882	0.086741748	0.264819214	0.791148734	0.953153173
314.3466973	-0.032499381	0.119811304	-0.271254712	0.786195136	0.952606765
71.63019795	-0.130777181	0.168547623	-0.775906408	0.437804249	0.82251807
14.44615764	0.132743129	0.350624177	0.378590918	0.704991667	0.926977731
48.59868953	0.0129766	0.219501593	0.059118476	0.952857742	0.986647305
85.58015957	0.198157836	0.291184156	0.680524102	0.496172666	0.854102145
41.85715163	0.564597257	0.238738363	2.364920532	0.01803394	0.27123243
8.222506466	0.118544157	0.348342992	0.340308717	0.733624053	0.937022761
435.5660135	0.137834311	0.057296299	2.405640724	0.016144133	0.262858884
404.6064692	0.102322991	0.15208261	0.672811912	0.501066959	0.856727618
5.526763204	-0.435797419	0.515484886	-0.845412603	0.397880511	0.799216626
809.6746839	0.066844867	0.065904195	1.014273332	0.310452346	0.745771331
4.678291001	-0.056829048	0.856392832	-0.066358622	0.947092312	0.985785839
29.25652274	0.150850095	0.306848874	0.491610393	0.622994795	0.902441895
1262.427028	0.219348604	0.076185972	2.879120616	0.003987858	0.144354659
26.27826685	-0.398945551	0.492650673	-0.809793983	0.418058591	0.811361647
13.73301797	-0.823049679	0.600087928	-1.371548468	0.170204045	0.61328147
497.3788877	-0.461762155	0.10163094	-4.543519485	5.53227E-06	0.003412027
465.4931167	-0.093617102	0.20541835	-0.455738752	0.648577852	0.910701841
79.79814148	-0.035133637	0.148583691	-0.236456889	0.813078156	0.960091267
127.7871719	-0.470692782	0.273750676	-1.719421442	0.085537661	0.489041504
183.769968	-0.514975598	0.434345299	-1.185636405	0.235765919	0.678901985
881.6471612	-0.04288838	0.104628023	-0.40991294	0.681869812	0.922658753
664.4119942	0.148306033	0.085499776	1.734578042	0.082815571	0.485197868
261.0191456	0.175881876	0.091247556	1.927524251	0.053914329	0.419358851
1641.533114	0.051851617	0.077205036	0.671609267	0.501832477	0.856959429

486.6605181	0.161837931	0.060485815	2.675634475	0.007458794	0.188726623
65.6413479	0.018882414	0.145955265	0.129371241	0.897063903	0.97893802
488.7073771	-0.318332819	0.166743892	-1.909112325	0.056247603	0.426485013
4.601981251	0.057020694	0.624440391	0.091314872	0.927242402	0.983502443
6358.926204	0.31051598	0.134103247	2.315499351	0.02058562	0.286111125
16.47734142	0.059280754	0.407268061	0.145557091	0.884271043	0.976398641
72.90007155	-0.134891286	0.141510825	-0.953222386	0.340477407	0.76465745
155.5758986	-0.197997342	0.138343234	-1.431203649	0.152371858	0.589866872
7.50320514	-0.345631195	0.482401637	-0.716480147	0.473694922	0.841530513
1408.601975	-0.363479136	0.179444579	-2.025578812	0.042807965	0.379065918
492.4064858	-0.093812493	0.175260194	-0.535275532	0.592459343	0.893761027
392.328869	0.475768002	0.218882522	2.173622624	0.029733488	0.330878164
401.7480375	0.520328113	0.175320874	2.967861741	0.002998792	0.128901255
14.09673704	-2.323085909	0.967091254	-2.402137233	0.016299592	0.263145637
174.9054546	0.412785877	0.231093793	1.786226586	0.074062578	0.468493282
52.26292534	-0.159117504	0.161400886	-0.985852732	0.324205385	0.756297513
146.1270279	-4.72144E-05	0.094679201	-0.000498677	0.999602113	0.999799058
507.9354025	-0.105685119	0.185117808	-0.570907357	0.568062444	0.8841274
66.30137205	6.94081E-05	0.123233044	0.000563226	0.99955061	0.999799058
59.50539216	0.210400927	0.27461676	0.766162006	0.443579912	0.825536387
93.13721091	-0.389344982	0.127180138	-3.061366255	0.002203294	0.111533785
968.0204848	-0.039134764	0.070751748	-0.553127874	0.580175851	0.887483205
47.95590552	0.000757342	0.156811914	0.004829623	0.996146533	0.998981303
80.45895159	-0.162124987	0.139890163	-1.158944868	0.246478658	0.689929208
178.8116454	-0.023072132	0.087278967	-0.264349273	0.791510796	0.953153173
82.09463369	0.218733585	0.115941031	1.886593415	0.059215033	0.434680797
41.73210516	-0.106562454	0.164972625	-0.645940222	0.51831807	0.862002027
11.19653183	-0.289941134	0.297745955	-0.973786982	0.330162318	0.759333947
37.15169269	-0.513076883	0.300906568	-1.705103634	0.088175098	0.492827855
479.5705547	-0.04965764	0.088309325	-0.562314792	0.573901562	0.885935354
570.0224217	-0.117248124	0.050702112	-2.312489934	0.020750699	0.287595367
154.8137917	-0.224094236	0.143279565	-1.564034875	0.117809379	0.539880626
140.0416084	-0.370749671	0.318513609	-1.163999467	0.244424228	0.686810508
40783.77379	-0.109953149	0.116204042	-0.946207615	0.344042703	0.767165886
142.7002943	-0.26165767	0.130706524	-2.001871538	0.04529855	0.389378124
1517.530854	-0.0169772	0.139032826	-0.122109291	0.902812464	0.979381499
10.42075735	0.544140972	0.310342515	1.753356197	0.079540885	0.477933724
1015.916696	-0.273237885	0.136583024	-2.000525956	0.0454435	0.390179471
188.823545	-0.001026687	0.088457144	-0.011606603	0.990739479	0.99821004
332.5225083	0.105446291	0.101663019	1.037213846	0.299636207	0.738923503
3278.671841	-0.078133624	0.150279859	-0.519920795	0.603118781	0.899375001
274.9561427	0.020386104	0.061107914	0.333608245	0.738675196	0.939226436
994.3165755	0.016683654	0.074959728	0.222568229	0.823871566	0.963494265
45.81595523	-0.23555699	0.162425195	-1.450249083	0.146989072	0.582502337
470.8074668	0.168140988	0.08361235	2.010958751	0.044329814	0.386436929
310.8099131	-0.019455421	0.105852577	-0.183797333	0.854172448	0.969966486
59.96465041	-0.117732859	0.190768068	-0.617151811	0.537134597	0.869675597
405.2945486	0.025599937	0.095930214	0.266860002	0.789576954	0.953026618

288.1358783	-0.316032587	0.225465194	-1.40169124	0.161007468	0.600875279
96.19396279	-0.029209324	0.114022875	-0.256170739	0.797819002	0.954840537
64.28750435	-0.123767783	0.126223101	-0.980547786	0.326815793	0.757203811
74.72072366	-0.0980496	0.120847719	-0.811348371	0.417165639	0.811097568
558.5506523	-0.120974305	0.065370231	-1.850602381	0.064226777	0.445077131
436.4266052	-0.124928791	0.065516456	-1.906830717	0.056542512	0.427447531
14.71580253	-1.184015403	0.480258948	-2.465368753	0.013687231	0.250121471
1134.629571	-0.094776286	0.090999172	-1.041507128	0.297640247	0.737350783
50.55413701	0.149910399	0.153255251	0.978174631	0.327987961	0.758221793
321.4011034	-0.198844806	0.099631667	-1.995799252	0.045955778	0.391329374
81.42556256	-0.146987498	0.142804489	-1.029291858	0.303342548	0.740856504
14.35945213	-0.822562175	0.436628845	-1.883893346	0.059579404	0.435320275
154.19796	-0.035109691	0.090493416	-0.387980608	0.698030384	0.925802378
25.35341908	-0.016202271	0.224742045	-0.072092747	0.942528098	0.98446865
361.1189442	0.030478887	0.125973957	0.241945933	0.808822058	0.958618312
119.8547828	-0.044584445	0.116724567	-0.38196282	0.702488943	0.926349622
164.2939086	0.008853428	0.07923517	0.111736085	0.911032667	0.980530316
22.84066205	-0.159326635	0.25425435	-0.62664271	0.530893465	0.866123834
93.87075195	-0.050930554	0.101481291	-0.501871361	0.615758012	0.901900555
2590.468679	0.087932523	0.065079289	1.351159871	0.176644226	0.620182124
19.51330503	0.053196923	0.243888032	0.218120269	0.827335405	0.964553924
119.34258	-0.23101724	0.102621409	-2.251160277	0.024375388	0.309236976
14868.18061	-0.206553854	0.28871008	-0.715436931	0.474339099	0.841713532
549.1349085	-0.254553908	0.212920091	-1.195537292	0.231877175	0.67457664
1794.186839	0.034605199	0.110591223	0.312910899	0.754348357	0.94414502
36.38742488	-0.201877884	0.215465926	-0.936936467	0.348791239	0.768961562
20.98910172	0.14720232	0.37271073	0.394950582	0.692879359	0.924581226
17.31554765	0.526743752	0.772373087	0.681980977	0.49525098	0.853825991
6.221850418	-1.812625128	1.21610522	-1.490516691	0.136088431	0.568673186
2184.512393	-0.26908895	0.165868891	-1.622299084	0.104739325	0.518635678
1143.466428	0.022517589	0.107775402	0.208930689	0.834502346	0.966316856
51.99445959	0.370501768	0.345586101	1.072096841	0.283676537	0.724631363
503.5255132	0.023812825	0.061758697	0.385578479	0.699808874	0.926034765
77.41605035	0.069830273	0.122105269	0.571885833	0.567399322	0.8841274
54911.17644	-0.107905604	0.157422907	-0.685450461	0.493059711	0.852004418
16.87864986	-0.932753984	0.498644182	-1.870580299	0.061403277	0.438421236
66.39638924	0.605550509	0.421452371	1.436818371	0.150769609	0.589528489
96.55747479	0.418641278	0.482480108	0.867686089	0.385566204	0.791450693
174.6495075	-0.48940508	0.121173815	-4.038868302	5.37097E-05	0.013808293
462.8441043	-0.094928976	0.119401756	-0.795038351	0.426591194	0.816487562
127.5360018	-0.287356454	0.232063855	-1.238264586	0.215617971	0.660219973
15.929844	-1.612198414	0.603312235	-2.672245515	0.00753455	0.189027811
46.59540214	0.171755996	0.37164209	0.462154316	0.643970661	0.908494289
52.65148872	-0.568930664	0.519271741	-1.095631861	0.273239913	0.716221441
106.2639042	0.313468698	0.463906584	0.67571513	0.499221528	0.855692311
124.8492673	-0.583591733	0.334477392	-1.744786782	0.081021991	0.481062093
241.8901007	-0.116248033	0.161650009	-0.719134094	0.472058302	0.84064088
210.8279827	-0.318067456	0.244877514	-1.298883881	0.193983782	0.639689082

5.95686504	0.176547103	0.421002364	0.419349434	0.674960773	0.921149714
629.2237171	-0.125463047	0.064444105	-1.946850649	0.051552643	0.410734915
32.32244409	-0.029248175	0.392532383	-0.074511496	0.940603394	0.984334593
42.79475953	-0.132147536	0.209502198	-0.63076921	0.528191438	0.865762435
1486.803724	-0.191511684	0.152153529	-1.258673956	0.208148122	0.652874199
127.1917273	0.228110241	0.104985741	2.172773554	0.029797365	0.330878164
470.8004278	-0.255484529	0.109684081	-2.329276302	0.019844433	0.281261453
7.018779543	0.064038446	0.437531356	0.146363101	0.883634755	0.976013852
1221.014253	-0.022958687	0.073147503	-0.313868368	0.753621016	0.944021193
112.1059527	-0.045488106	0.158299658	-0.28735442	0.773840958	0.949500013
68.33898776	0.023562826	0.142104997	0.165812793	0.868304278	0.972204994
656.7215999	0.074000638	0.057599361	1.284747558	0.198880513	0.64421391
389.7764457	0.12335726	0.126981479	0.971458677	0.331319921	0.759815544
177.2709911	0.05385489	0.132886403	0.405270131	0.685278959	0.922907843
30.13577698	-1.427052643	0.472630035	-3.019386278	0.002532874	0.119399986
349.6544797	0.07557982	0.110977443	0.681037682	0.495847647	0.853930992
28.37677799	-0.285617824	0.229393945	-1.245097482	0.21309592	0.65850643
25.50388761	-0.024312076	0.313225438	-0.077618459	0.938131559	0.984334593
276.9468163	-0.10261182	0.128145097	-0.800747145	0.423278043	0.814538241
50.15596902	0.031129666	0.242482592	0.128378972	0.897849071	0.97893802
345.3310972	-0.069363572	0.121302841	-0.571821495	0.567442913	0.8841274
7.458952411	-0.382995166	0.358745997	-1.067594255	0.285703576	0.726643643
612.0317543	0.123316053	0.095100955	1.296685747	0.194739336	0.640229004
38.27556817	0.077392343	0.304068562	0.254522673	0.79909179	0.955167977
2326.556059	0.049183379	0.065011454	0.756534058	0.44932906	0.828449907
15.773828	0.285936823	0.243496952	1.174293234	0.240277592	0.682515625
84.4297546	0.022470437	0.141037951	0.159321915	0.873415256	0.97300406
364.5504172	0.027060264	0.065907975	0.410576484	0.68138311	0.922658753
301.1132488	-0.103959151	0.057963049	-1.793541818	0.07288633	0.465015503
457.9731605	0.056754929	0.076114163	0.745655298	0.455875669	0.831633185
2180.96302	-0.07911402	0.069714442	-1.134829708	0.256446676	0.699580483
175.0426256	-0.038186632	0.112146781	-0.34050582	0.733475641	0.936990285
66.94149857	0.051347218	0.135444984	0.37910018	0.704613473	0.9269128
4.977467986	-0.157697241	0.496382484	-0.317693	0.750717822	0.942359611
283.9466337	-0.103688669	0.123130161	-0.842106182	0.399728521	0.80037241
11.67099712	0.568474223	0.519180411	1.09494544	0.27354054	0.716229876
84.01452685	0.076762796	0.361225913	0.212506339	0.831712033	0.966174974
347.6417387	0.022291866	0.082366812	0.270641361	0.786666881	0.952886718
345.1806375	-0.301580363	0.134883584	-2.235856676	0.025361161	0.313430481
774.736363	-0.048068879	0.265477213	-0.181065932	0.856315825	0.969966486
4.9607069	-0.99687179	0.511991378	-1.947048003	0.05152898	0.410734915
368.7204558	0.109065264	0.118260022	0.922249653	0.356398369	0.774323527
258.9538318	-0.187852134	0.097549128	-1.925718236	0.054139566	0.42005114
33.3425055	-1.059184896	0.32741944	-3.234948098	0.001216648	0.084153413
551.769261	0.061769298	0.087678151	0.704500466	0.48112116	0.84566728
1410.669188	-0.023617557	0.079792023	-0.29598895	0.76723852	0.94737077
58.54929695	-0.224831316	0.14305009	-1.571696426	0.116020971	0.537362993
28.08102913	1.562497525	0.483484336	3.231743844	0.001230373	0.084706887

15.50267966	-0.739720586	0.466027403	-1.58728989	0.112447004	0.533148601
7.689440617	0.281750953	0.531442202	0.530162925	0.595998974	0.895953399
3447.903686	0.110774458	0.068221873	1.623738156	0.104431704	0.518555962
1345.883502	-0.031049415	0.090524731	-0.342993728	0.731603179	0.936065863
216.3163009	-0.037981265	0.082622786	-0.459694803	0.645735301	0.909300678
592.7903001	-0.211895676	0.145108175	-1.460260088	0.144218607	0.578069441
51.27611077	-0.007420775	0.360026955	-0.020611719	0.983555392	0.995742202
13.05158196	0.094198848	0.710398405	0.132600028	0.89450971	0.9785332
145.0513439	-0.107762886	0.09855652	-1.093412044	0.274212928	0.716722874
856.2943491	0.007808425	0.080732385	0.096719859	0.922948868	0.982590996
112.2191375	-0.013492786	0.160964229	-0.083824746	0.933195772	0.984334593
5.120916285	0.925262628	0.409921085	2.257172566	0.023997294	0.308072806
692.6991703	0.051033852	0.092726757	0.550368137	0.582066898	0.888496878
1541.136654	0.120457862	0.051199053	2.352736163	0.018635852	0.27420266
144.9903262	-0.089725587	0.089448554	-1.003097122	0.315814003	0.749627786
25.65117079	-0.802131091	0.341763791	-2.347033571	0.01892355	0.275966879
346.7119709	-0.075904812	0.1450613	-0.523260252	0.600793158	0.898289446
857.2695838	-0.122116173	0.066716431	-1.830376293	0.06719369	0.45461399
3713.783484	-0.017185591	0.104541541	-0.164390068	0.869424082	0.972532991
1912.323276	0.863814039	0.304552395	2.836339668	0.004563388	0.149960163
259.5762173	0.289254698	0.130076228	2.22373221	0.026166467	0.316367615
14849.22754	-0.048611626	0.122419112	-0.397091802	0.691299765	0.924455975
111.6801265	-0.080938763	0.108744125	-0.744304697	0.45669216	0.832142746
9.698473437	-0.645796371	0.4524492	-1.427334541	0.153483489	0.591551404
150.4154993	-0.147179611	0.147671084	-0.996671838	0.318923824	0.752071737
139.2430899	-0.137194251	0.098807186	-1.388504779	0.164983391	0.605350304
213.0029535	-0.034056935	0.150718511	-0.22596385	0.821229536	0.962824423
193.8584476	0.108856744	0.13912386	0.782444829	0.433953197	0.821238123
89.78215662	0.422704435	0.159407998	2.651714096	0.008008432	0.193378164
387.5264016	-0.258164153	0.135020305	-1.912039482	0.05587113	0.425680221
15.65058719	-0.748372299	0.348179118	-2.149388805	0.031603592	0.338966233
128.3364387	-0.092707434	0.201666667	-0.459706284	0.645727059	0.909300678
17.31926641	-0.984255431	0.460506248	-2.137333503	0.032570876	0.341836409
562.61936	0.099218962	0.113208942	0.876423371	0.380799918	0.78844599
1359.645804	0.112890955	0.103403707	1.091749591	0.274943181	0.716878153
180.1447192	-0.07131679	0.163628793	-0.435844991	0.662949199	0.91573106
959.9035041	-0.242304742	0.078778589	-3.075769004	0.002099604	0.109046821
84.56675314	0.257981402	0.148076307	1.74221931	0.081470076	0.48207474
64.9986184	0.155347419	0.536436682	0.289591344	0.77212889	0.948706053
131.2810632	-0.102393881	0.102619517	-0.997801236	0.318375751	0.751465423
269.6051118	-0.1364582	0.080339376	-1.698522023	0.08940928	0.494293655
353.2234347	-0.044274393	0.108712146	-0.407262619	0.683815115	0.922851142
214.5716779	0.133865117	0.299405982	0.447102348	0.654801164	0.912480225
571.6636848	-0.224355328	0.145105251	-1.546155822	0.12206694	0.548564727
61.41719374	0.243225367	0.365474096	0.665506448	0.505726621	0.858853309
135.3281021	-0.014969023	0.211797363	-0.070676153	0.943655501	0.98479534
9.378766362	0.475678724	0.641112897	0.741957815	0.458112895	0.833454655
690.7491389	-0.056149514	0.072363805	-0.775933688	0.43778814	0.82251807

61.50527603	0.076990012	0.140062605	0.549682846	0.582536924	0.888664491
14.94278933	0.126466178	0.299016071	0.422941073	0.672338255	0.920198876
260.0845049	-0.226322688	0.084932501	-2.664735949	0.007704879	0.189660453
377.5827569	-0.297027814	0.273789465	-1.08487671	0.277976264	0.719965821
130.5849402	-0.116707224	0.113332776	-1.029774681	0.303115789	0.740731811
138.5474417	-0.431528095	0.209043626	-2.06429683	0.038989581	0.369004977
244.1226234	-1.095777989	0.581656233	-1.8838928	0.059579477	0.435320275
6.316742541	1.134807559	0.673703048	1.684432871	0.092098043	0.499719658
1816.533751	0.064809366	0.127450225	0.508507268	0.611097643	0.900509492
494.9863261	0.021937565	0.107281024	0.204486913	0.837973018	0.966437851
16.81059966	-0.679241375	0.600899724	-1.130373917	0.2583187	0.701275747
12.48517203	0.460827008	0.482240735	0.95559536	0.339276707	0.764030704
106.4447905	-0.08452478	0.104366185	-0.809886651	0.418005325	0.811361647
47.02822732	0.037561949	0.263339963	0.14263672	0.886577096	0.976961229
5.312534959	0.424768309	0.497252961	0.854229823	0.392977662	0.795848318
16.6969613	0.022901695	0.264986467	0.086425903	0.931127857	0.984334593
320.5380041	0.043396399	0.076773811	0.565250026	0.571903714	0.884938196
2000.115273	-0.117670586	0.122746341	-0.958648419	0.337735892	0.7637398
115.8181962	-0.039059743	0.089777093	-0.435074716	0.663508195	0.915990329
259.7313294	-0.177383016	0.28894704	-0.613894558	0.539285007	0.869675597
357.2701601	-0.146064761	0.089963032	-1.623608701	0.104459347	0.518555962
48.25716057	0.054237569	0.193879107	0.279749428	0.779669754	0.95173434
419.1638255	0.226465605	0.270816111	0.836233869	0.403023348	0.802360672
115.9259124	0.0634036	0.104766432	0.605190024	0.545052744	0.872850885
59.5485973	0.06023651	0.288457425	0.208822878	0.834586511	0.966316856
100.5900255	-0.26506726	0.134474751	-1.971130333	0.04870897	0.401665835
4.828125301	-1.026081713	0.684818618	-1.498326252	0.134048507	0.567072305
88.91508756	-0.141590812	0.134665333	-1.051427334	0.293062367	0.733939814
41.5326549	-0.905234854	0.305682085	-2.961360504	0.003062832	0.129902693
222.5356253	-0.19543666	0.100953282	-1.93591191	0.052878491	0.415302038
151.4315969	0.308643282	0.273948537	1.126646941	0.259891784	0.702516818
278.9744704	-0.055544991	0.100195858	-0.554364148	0.579329657	0.887467795
96.64958284	0.329428389	0.200396654	1.643881683	0.100200645	0.512774612
736.0815349	0.022249462	0.058085801	0.383044772	0.701686568	0.926243544
124.47397	0.094767122	0.143392521	0.660893062	0.508680895	0.859636085
55.44383581	0.055529173	0.128175705	0.433226975	0.664849884	0.916049829
74.05923979	-0.158125047	0.122850094	-1.287138194	0.198046125	0.643940842
534.3300106	-0.199429042	0.12244925	-1.628666908	0.103383552	0.516137077
23.59354115	-0.203193004	0.219499642	-0.925709955	0.354596733	0.773466084
452.8149396	0.059839667	0.086523835	0.69159748	0.489190145	0.849736271
195.2529702	0.019566643	0.130410937	0.15003836	0.880734351	0.975430592
325.1883396	-0.027694097	0.072010373	-0.384584834	0.700545032	0.926034765
145.5988296	-0.21919777	0.093931074	-2.3336023	0.019616556	0.281261453
1326.22055	-0.008083219	0.107809921	-0.074976583	0.940233344	0.984334593
28.25787995	-0.347043315	0.230249	-1.50725221	0.131746022	0.564647789
435.4176927	-0.074134601	0.296882705	-0.24971007	0.80281157	0.956857788
199.2141878	-0.060216803	0.116877146	-0.515214526	0.60640312	0.900307811
1416.534561	-0.026784155	0.101981629	-0.26263706	0.792830333	0.953153173

1368.413379	-0.014470789	0.062444624	-0.231737949	0.816741554	0.961335246
211.8215325	0.047039985	0.111685043	0.421184284	0.67362052	0.920866983
565.0139758	-0.096689117	0.058235678	-1.660307216	0.096852665	0.507281065
10.31026357	-1.121384486	0.373003567	-3.006363974	0.002643923	0.121833113
4.663141688	-0.211226014	0.448256232	-0.471217127	0.63748569	0.907684986
13.69688538	0.065412613	0.30558672	0.214055811	0.830503534	0.965541183
7.057936864	-0.898724519	0.448544603	-2.003645821	0.045108014	0.388856211
91.55824878	-0.150527728	0.203701817	-0.738961146	0.459930591	0.834673974
528.0581789	0.088195599	0.154932205	0.569252847	0.569184561	0.8841274
6.387518914	0.160176054	0.447639546	0.357823734	0.720475228	0.931135539
18.89545864	0.301068311	0.232369213	1.295646298	0.195097371	0.640317357
483.0802151	0.05298	0.063914314	0.828922297	0.407148377	0.805288281
93.17458902	0.106832392	0.268725218	0.397552534	0.690960055	0.924455975
774.5481377	0.070946508	0.1119926	0.63349282	0.526411868	0.864850467
1212.067107	-0.324939171	0.154815209	-2.098884041	0.035827124	0.357462935
55.25230967	-0.430383626	0.367415342	-1.17138175	0.241445365	0.683864196
334.5073746	-0.095260896	0.065234321	-1.460288011	0.144210936	0.578069441
11.62130304	0.045518502	0.405218842	0.112330665	0.910561229	0.980530316
9.712432418	-0.454613374	0.425589938	-1.068195776	0.28543221	0.726643643
262.5300218	0.144486859	0.074645042	1.935652463	0.05291028	0.415302038
235.6650813	0.176401414	0.122040078	1.445438392	0.148334786	0.585194964
513.0103603	0.170263453	0.089760983	1.896853704	0.057847249	0.431580132
238.520215	0.07792922	0.080622338	0.966595885	0.333746093	0.761463483
117.6788319	0.060567836	0.161066204	0.376043111	0.706884846	0.928109779
170.2205143	0.805185814	0.359867588	2.237450221	0.025256929	0.313430481
34.41741147	-0.012819288	0.21393087	-0.05992257	0.952217304	0.98648845
43.779459	0.034824405	0.338225979	0.102961945	0.917993174	0.981376661
177.580401	0.067434884	0.203682019	0.331079218	0.740584649	0.939732486
204.4902746	0.246215024	0.137646418	1.788749963	0.073655093	0.467122552
1181.004136	0.103392613	0.076492783	1.351664944	0.176482524	0.62005562
88.21699993	0.089610021	0.176364607	0.508095262	0.611386538	0.900561602
609.4141774	0.120224347	0.063592851	1.890532442	0.058686786	0.433257759
1196.923708	0.060374151	0.047960647	1.258826855	0.208092878	0.652874199
32.83904141	-0.393302272	0.408493926	-0.962810575	0.335642593	0.76257585
1873.594219	0.4763829	0.121138191	3.932557492	8.40469E-05	0.017521997
1002.325346	-0.002042503	0.241770375	-0.008448112	0.993259462	0.998685608
45.43929092	0.213274933	0.218057186	0.978068817	0.328040289	0.758221793
30.38551442	-0.008870043	0.217784769	-0.040728482	0.967512355	0.991458658
162.0485249	-0.028370633	0.083014867	-0.34175364	0.732536301	0.936275134
12.96052052	-0.174639164	0.437469835	-0.399202757	0.689743812	0.92405254
220.5119286	0.010723953	0.194048336	0.055264339	0.955927872	0.987483032
545.3719183	-0.194259471	0.120626912	-1.610415675	0.107307141	0.524456614
352.1087732	0.135281289	0.149561166	0.904521489	0.365718945	0.78036534
6.88976349	-0.16279921	0.889788486	-0.182963943	0.85482631	0.969966486
65.74186724	-0.067929018	0.118372826	-0.573856519	0.566064899	0.883491972
177.1135726	0.073017676	0.101119443	0.722093336	0.470237095	0.839923915
39805.06011	0.012140733	0.17618624	0.068908518	0.945062438	0.985197142
1270.348138	0.085596717	0.137834159	0.621012363	0.534591489	0.867893827

296.6598695	-0.003313923	0.131115252	-0.025274882	0.979835708	0.994755018
545.2195584	0.116401287	0.108181376	1.075982682	0.281935002	0.723387381
228.1601752	0.135976582	0.078290211	1.736827371	0.082417646	0.484875199
18.14479198	-0.109421883	0.231393521	-0.472882223	0.636297208	0.907353194
758.8943646	-0.092792169	0.139640488	-0.664507624	0.506365471	0.859011504
21.4177159	-0.764916766	0.252951843	-3.023962023	0.002494878	0.119126379
873.4732092	0.037677831	0.075043732	0.502078327	0.615612425	0.901900555
13.01942398	-0.142358109	0.290043804	-0.490815896	0.623556666	0.902854913
11.01450257	-0.681099536	0.340725252	-1.998969937	0.045611607	0.390707756
372.2982474	-0.121922802	0.186233845	-0.654675858	0.512676472	0.860200661
22.17954667	-0.541663481	0.398552129	-1.359078127	0.174121833	0.618034305
405.3240815	0.061886045	0.078864359	0.78471499	0.432620692	0.820034765
5022.252621	0.127389944	0.067777498	1.879531509	0.060171955	0.435320275
19.27059724	-0.405402471	0.223599541	-1.813073811	0.069820438	0.460758859
15.27624859	-0.811685557	0.384967914	-2.108449895	0.034992092	0.354276985
70.47662324	-0.000766391	0.169746024	-0.004514925	0.996397623	0.998981303
353.9878191	-0.588329637	0.253059905	-2.324863104	0.020079281	0.282442098
440.77256	-0.670152383	0.320129093	-2.093381696	0.036315095	0.359796544
5.582784657	-0.505213595	0.423414007	-1.193190558	0.232794752	0.67578504
68.25440105	-0.708732733	0.278616018	-2.543761614	0.01096659	0.226714336
9.221153095	-0.556375629	0.374591635	-1.485285778	0.137468138	0.569036171
64.70863406	-0.22718178	0.19339925	-1.174677667	0.240123697	0.682340366
381.7565768	0.050024054	0.108394065	0.46150178	0.644438645	0.908560894
49.05416203	0.176544425	0.255879291	0.689951985	0.490224383	0.850131243
296.5396338	0.028866238	0.10526657	0.274220371	0.783915274	0.95189998
138.1544305	-0.056282662	0.109112453	-0.515822538	0.60597836	0.900307811
2645.041314	-0.060001211	0.081228962	-0.738667704	0.460108801	0.834673974
57.16542239	-0.196491392	0.151090847	-1.300485084	0.193434765	0.639147095
45.95803356	0.045478471	0.13265956	0.342820909	0.731733195	0.936065863
20.34757925	-1.127951305	0.617035487	-1.828016912	0.067547014	0.45536467
18.24227985	-0.479042364	0.242253562	-1.97744198	0.047991693	0.399398071
136.0276188	-0.118224393	0.112802477	-1.04806557	0.294608398	0.735006489
6626.145787	-0.015336383	0.187026609	-0.082001075	0.934645858	0.984334593
621.2957825	0.0570671	0.112202637	0.508607477	0.611027387	0.900509492
109.2522049	0.163820441	0.237308335	0.690327379	0.489988334	0.84997156
371.9030101	0.07797093	0.232911048	0.334766986	0.737800866	0.938868945
1026.711728	0.102735909	0.08634629	1.189812657	0.234120034	0.676914641
9.017257499	0.044707359	0.40590426	0.110142621	0.912296267	0.980530316
431.8229719	0.114797477	0.119838006	0.957938807	0.338093617	0.764007173
6.481252017	-0.062689464	0.389353812	-0.16100899	0.87208632	0.972881041
590.4422591	0.087422825	0.06963786	1.255392175	0.209336429	0.654541152
637.2647452	0.047691979	0.094674762	0.503745433	0.614440277	0.901900555
7.106754618	0.310766068	0.390246684	0.796332372	0.425838874	0.816273895
422.0934671	0.017012064	0.068601208	0.247984905	0.804146086	0.957233889
180.5363516	0.013363614	0.111961738	0.119358762	0.904991131	0.979768942
125.6898426	-0.097938139	0.106905817	-0.916116093	0.359606008	0.777290907
166.4820626	0.03110614	0.113959927	0.272956831	0.7848864	0.952285942
364.1264625	-0.002562437	0.07171916	-0.035728768	0.971498632	0.992799208

11.50250316	-0.250444892	0.268719081	-0.931995194	0.351339009	0.770952892
754.8888294	0.022896084	0.102280877	0.223854985	0.822870144	0.963260435
32.90580463	0.550909108	0.295819951	1.862312217	0.062559094	0.442595858
19.11190055	-0.839157879	0.638682802	-1.313888329	0.188883787	0.634700979
11.88957685	-0.701160744	0.642203288	-1.091804973	0.274918833	0.716878153
14.94979328	0.553846634	0.634488638	0.872902368	0.382716296	0.789880775
26.43317111	-0.201902865	0.283903871	-0.711166297	0.476981191	0.843597766
4.966367899	-0.90273189	0.665248732	-1.356984007	0.174786292	0.61853318
47.29221302	-0.198582624	0.241747809	-0.821445393	0.411392617	0.808165706
213.0984745	0.214947092	0.136280255	1.577243104	0.114739598	0.535291597
776.4770555	0.343610293	0.174209056	1.972402008	0.048563733	0.401138601
10.69248156	-0.162885616	0.30586155	-0.532546886	0.594347277	0.894609104
75.58529115	-0.232034586	0.148320205	-1.564416565	0.117719773	0.539880626
1778.477822	0.30388723	0.321084232	0.94644084	0.343923784	0.767121103
236.870973	-0.02139766	0.118459075	-0.180633358	0.856655371	0.969966486
30.09412537	-0.062310569	0.279411241	-0.223006666	0.823530319	0.96344756
4.517568646	-2.202700248	0.91703745	-2.401974149	0.016306861	0.263145637
7.178184445	-1.767402903	0.630540943	-2.802994669	0.005063051	0.156457777
7.023474102	0.081324746	0.886023954	0.09178617	0.926867932	0.98333566
11.72396023	0.469723465	0.497276742	0.944591665	0.344867384	0.767851822
4116.605547	0.14694457	0.076658965	1.916860866	0.05525561	0.423973339
1133.287036	-0.052512457	0.144502232	-0.363402394	0.716304314	0.930241908
116.3474988	-0.251152358	0.100698084	-2.494112575	0.012627249	0.240861519
430.7260579	-0.18223948	0.213448783	-0.853785518	0.39322384	0.796041628
188.1799804	-0.347855704	0.274315893	-1.268084398	0.204767813	0.64944786
550.7022311	-0.041502707	0.077436503	-0.535957918	0.591987635	0.893683696
54.74465887	-0.235423552	0.219142245	-1.074295613	0.282690211	0.723887399
203.8146366	-0.23236868	0.146938658	-1.58139922	0.113786786	0.533883941
209.1086236	0.132819216	0.086348855	1.53816998	0.124007059	0.55188031
28.78586561	-1.185193834	0.578812078	-2.047631484	0.040596122	0.373696391
519.0537066	0.217749225	0.07851996	2.77317036	0.005551305	0.162116465
110.4676039	-0.017224537	0.103590307	-0.166275568	0.867940093	0.972204994
58.55227131	-0.028256649	0.144236919	-0.195904411	0.844684985	0.968131661
5.867162622	-3.562803902	2.49237634	-1.429480711	0.152866115	0.59076454
744.0917166	0.077528341	0.094208864	0.822941039	0.410541523	0.8079824
97.57830898	0.089529873	0.182879795	0.489555845	0.624448228	0.902875034
139.506831	0.060883142	0.149100649	0.408335863	0.683027114	0.922776218
1650.526488	-0.063558138	0.060339615	-1.05334013	0.292185133	0.733939814
119.0216901	0.207712	0.114821731	1.808995539	0.070451696	0.461430171
208.5553394	0.014322572	0.116688113	0.122742343	0.902311133	0.979381499
22.95141813	0.203986833	0.210464356	0.969222708	0.332434083	0.760742564
1132.926466	0.111495009	0.043743616	2.548829289	0.010808519	0.225971333
839.6039829	0.317292852	0.109604348	2.894892916	0.003792879	0.140808558
1165.777385	-0.099658965	0.064522337	-1.544565332	0.122451437	0.548630842
11.55839833	-0.252041949	0.438169151	-0.575216097	0.565145158	0.883265494
51.8103277	0.291067325	0.209732024	1.387805831	0.165196179	0.605350304
708.3837381	0.129361043	0.122451807	1.056424119	0.290774492	0.732603239
109.7515844	0.033844351	0.092913333	0.364257206	0.715665953	0.930241908

69.0936344	0.105311372	0.154452875	0.681834973	0.495343307	0.853825991
82.22284072	0.202441535	0.124606274	1.624649612	0.10423724	0.518542662
91.84079757	0.176308026	0.121592779	1.449987637	0.147061967	0.582502337
71.86977194	0.08292784	0.163940209	0.505841981	0.612967585	0.901444946
6.227435035	-0.934765454	0.428677068	-2.180581894	0.029214356	0.329102882
122.9993708	-0.02237107	0.086633217	-0.258227392	0.796231419	0.954549078
1089.089725	0.024718621	0.062913623	0.392897749	0.694395004	0.924581226
47.25436011	0.120872358	0.182106505	0.66374542	0.506853264	0.859011504
5.866267828	-0.248885781	0.613513257	-0.405673028	0.684982863	0.922907843
18.69552416	-1.774506267	0.890567093	-1.992557643	0.046309907	0.392824783
345.4648469	-0.005868205	0.077421014	-0.075796024	0.939581379	0.984334593
164.4547765	0.090038047	0.299666609	0.300460726	0.76382575	0.946826103
197.4521205	0.021346253	0.159297074	0.134002793	0.893400366	0.978111861
123.8921539	0.340175424	0.461354883	0.737340032	0.460915594	0.834757447
31.86565594	-0.105154641	0.363696196	-0.289127689	0.772483665	0.948706053
434.3412576	0.050784847	0.233211039	0.21776348	0.827613401	0.964626741
5.372400928	-1.048402851	0.533730335	-1.964293168	0.049496104	0.40452651
146.9898987	0.02267095	0.120734485	0.187775265	0.851052827	0.969309003
87.12535638	0.002669677	0.113103816	0.023603775	0.981168661	0.994880431
166.3952518	0.270955589	0.13926686	1.945585537	0.051704546	0.410906597
473.2558563	0.031387189	0.102410823	0.30648313	0.759236823	0.946465822
18.59850552	0.204998725	0.228116338	0.898658672	0.368834497	0.78283456
106.5992838	-0.193745616	0.104337092	-1.856919838	0.06332256	0.443016545
39.85687499	0.048736601	0.238801811	0.20408807	0.838284677	0.966440785
168.2976182	0.162196934	0.10914759	1.486033122	0.13727036	0.569036171
68.23458816	-0.086588835	0.315189753	-0.2747197	0.783531595	0.95173434
1721.086488	0.024812481	0.102785768	0.241399962	0.809245142	0.958681749
1914.617937	0.096615203	0.043786354	2.206513979	0.027348033	0.323603282
1649.644882	-0.001941193	0.127084852	-0.015274783	0.987812961	0.997281745
16917.0955	-0.056844463	0.14001302	-0.405994119	0.684746921	0.922907843
89.34894664	0.187722767	0.12705467	1.477496007	0.139542728	0.572130062
106.9694579	-0.266904696	0.142596014	-1.871754256	0.06124061	0.437941099
258.3698193	0.096705473	0.10039887	0.96321277	0.335440758	0.762456424
262.1828913	-0.080514732	0.073708488	-1.092340033	0.274683671	0.716878153
192.3574504	-0.060225474	0.160727517	-0.37470543	0.707879553	0.928420247
1328.850299	0.081213884	0.104348116	0.778297565	0.436393611	0.822078703
54.0699794	-0.274160398	0.179057535	-1.531130192	0.125737219	0.554908265
226.6749108	0.059904195	0.070126505	0.854230427	0.392977327	0.795848318
1031.750057	0.070306506	0.100426288	0.700080693	0.483876912	0.847905053
10.43066119	0.165268939	0.298739369	0.553221156	0.580111982	0.887483205
372.0285463	0.019785618	0.108019247	0.183167524	0.854666575	0.969966486
933.9756436	-0.068318802	0.062673552	-1.090073887	0.275680598	0.717918564
16.77477182	0.123111524	0.227234601	0.54178159	0.587968976	0.891256285
67.29415009	0.084610654	0.132943481	0.636440789	0.524489163	0.863946059
185.8059294	-0.111103329	0.093053982	-1.193966406	0.232491109	0.675362916
16.14597237	-0.358997109	0.253714261	-1.414966219	0.157078401	0.59632585
59.36781704	-0.248406605	0.18741948	-1.325404405	0.185037057	0.630643427
643.5990821	0.007257687	0.044174785	0.164294797	0.869499078	0.972532991

798.5934977	-0.016022977	0.051719171	-0.309807306	0.756707495	0.945306795
213.6690206	0.035162829	0.16068539	0.21883028	0.826782258	0.96453586
386.2295283	-0.108411972	0.176488479	-0.614272232	0.539035449	0.869675597
114.5263552	0.003468354	0.118087762	0.02937099	0.97656871	0.993619057
13.20127369	-0.413923353	0.295771184	-1.399471537	0.161671628	0.601319304
126.5773231	-0.035403802	0.099346063	-0.356368449	0.721564654	0.931135539
100.2898711	-0.033842416	0.114062878	-0.296699648	0.766695826	0.947349028
3502.636239	0.143797484	0.113148653	1.270872256	0.203774098	0.647823067
614.1161532	0.02493297	0.120181371	0.20746119	0.835649699	0.966316856
101.3345522	-0.694480493	0.281091655	-2.470654967	0.013486587	0.249224048
236.778012	0.151489509	0.130661926	1.159400555	0.246292951	0.689705283
174.2967109	-0.193566991	0.094283017	-2.053041974	0.040068507	0.372780665
298.8937435	-0.30428928	0.231532989	-1.314237257	0.188766374	0.634594566
1453.820809	0.04428595	0.048181721	0.919144216	0.358020145	0.776357191
1222.123299	0.065604808	0.100036507	0.655808668	0.511947239	0.860200661
132.397928	-0.086631729	0.137572168	-0.629718424	0.528878826	0.866063379
56.42395851	-0.135686998	0.16139344	-0.840721892	0.400503754	0.801079895
9.534369247	-0.471764455	0.318857435	-1.479546667	0.138994269	0.571180778
45.50205816	0.009324538	0.198023828	0.047087958	0.962443125	0.989751022
283.6358545	-0.01219969	0.110909668	-0.109996628	0.912412049	0.980530316
1322.375319	0.060551067	0.097990757	0.617926306	0.536623918	0.869429425
15.0094082	0.145008073	0.392850492	0.369117709	0.71203999	0.929527063
34.72032737	-0.825394667	0.262619987	-3.14292403	0.001672692	0.098250769
109.3168398	-0.23928856	0.223204878	-1.072057935	0.283694011	0.724631363
99.39222385	-0.168829024	0.2235399	-0.7552523	0.450097614	0.828449907
166.0168768	0.190435197	0.09296814	2.048392036	0.040521601	0.373696391
324.2385815	0.115873258	0.088433694	1.31028404	0.190099765	0.636043561
459.7557815	0.078598229	0.064325562	1.221881729	0.221752356	0.665526839
17.17339892	0.125669574	0.253869453	0.495016525	0.620588456	0.901976704
1257.991226	0.117614339	0.068228232	1.723836816	0.084737306	0.488047319
216.676759	0.065875837	0.141548091	0.465395445	0.641648295	0.908064184
561.8984823	0.086242359	0.100583038	0.857424474	0.391210339	0.794437976
14.08230017	0.19942858	0.263031071	0.758194002	0.448334852	0.82756609
164.8365041	0.066117296	0.092551544	0.714383502	0.474990071	0.842250597
1042.302632	0.123839305	0.08652401	1.431270978	0.152352568	0.589866872
201.8602078	0.008945246	0.159712475	0.056008439	0.955335084	0.987483032
194.3384832	-0.011419287	0.094358024	-0.121020836	0.903674532	0.979381499
416.4375237	-0.011557109	0.095708671	-0.120752996	0.903886681	0.979381499
29.71637917	-0.001356631	0.213168912	-0.006364112	0.994922208	0.998981303
746.8508331	0.137181865	0.083528017	1.642345537	0.100518415	0.512774612
160.2625198	0.037973598	0.081023601	0.468673291	0.639303181	0.907684986
797.8431486	-0.013249488	0.046468894	-0.285125964	0.775547639	0.950224001
20.61493526	-0.357790383	0.268146022	-1.334311729	0.182101715	0.627539984
957.7105507	0.01866875	0.044609141	0.418496058	0.675584468	0.921149714
319.812826	0.009294921	0.069170083	0.134377765	0.893103863	0.978111861
546.8956218	-0.081851959	0.172798909	-0.473683308	0.635725758	0.907353194
512.2286071	-0.030431734	0.111908709	-0.271933563	0.785673104	0.952591608
1021.40787	-0.011365292	0.063767159	-0.178231112	0.858541477	0.969966486

18.23047031	-0.318790864	0.377470067	-0.844546075	0.398364328	0.799587373
111.2608709	0.034706439	0.09090237	0.381799061	0.702610415	0.926349622
97.85939403	-0.291142081	0.20070365	-1.450606803	0.14688938	0.582502337
499.9552384	-0.064810995	0.06702316	-0.96699401	0.333547028	0.761455418
95.24882503	-0.020331257	0.094440522	-0.215281079	0.82954818	0.965176243
339.8493958	0.93913997	0.31755673	2.95739275	0.003102527	0.130836463
45.46484771	0.177780906	0.246098691	0.722396798	0.470050555	0.839894775
144.8642222	-0.251135887	0.236412001	-1.062280624	0.288108308	0.729194145
164.3818728	0.036537036	0.124868195	0.29260482	0.769824229	0.948234854
1019.17833	0.084089884	0.088653457	0.94852346	0.342863036	0.766311221
511.6743833	0.168410494	0.16246246	1.036611746	0.299916837	0.738923503
224.2356019	0.05313898	0.077637579	0.684449209	0.493691552	0.852282384
326.1484288	-0.123777352	0.067651546	-1.8296308	0.067305165	0.454909156
1346.343884	0.131931722	0.073348839	1.798688611	0.072067951	0.463154629
27.19371315	-0.28134611	0.226437307	-1.242490089	0.214055795	0.659367121
296.7819552	-0.029251567	0.067381332	-0.434119748	0.664201486	0.916049829
34.6801077	0.072869456	0.343699368	0.212015099	0.832095255	0.966227926
1080.844342	0.166981938	0.091142869	1.832089982	0.066938016	0.454159121
72.24070427	-0.005456843	0.148066955	-0.036853886	0.970601508	0.992796176
11.84615672	-0.415008759	0.30370705	-1.3664772	0.171789232	0.616109027
713.0432765	0.072787614	0.06232322	1.167905218	0.242844999	0.685806007
26.60550442	-0.788827667	0.492584008	-1.601407382	0.109286719	0.527269235
323.6461301	0.122413562	0.104620973	1.170067131	0.241973955	0.684968154
655.8763719	0.06170037	0.06881626	0.896595816	0.369934631	0.783892128
5.517495744	-0.841792579	0.465058735	-1.810077986	0.070283694	0.461430171
380.5179179	-0.045010683	0.068123275	-0.660724009	0.508789324	0.859636085
105.7452922	0.107751446	0.105950928	1.016993885	0.309156338	0.744779061
32.52225914	-0.670200495	0.198568866	-3.375153956	0.000737744	0.063860162
196.1642426	-0.025089831	0.140172971	-0.178991936	0.857944033	0.969966486
15.52728467	-0.407307076	0.283061845	-1.438933165	0.15016946	0.588453924
192.0944659	0.009235581	0.076808352	0.120241888	0.904291537	0.979589029
17.11506269	0.152645056	0.239570419	0.637161535	0.52401963	0.863484457
337.8106207	0.007413199	0.089837277	0.082518077	0.934234744	0.984334593
332.0106256	-0.130740818	0.076584464	-1.70714544	0.087795023	0.492827855
139.4513215	-0.212403129	0.225966838	-0.93997478	0.347230497	0.768780165
9.473037919	0.065119995	0.475156002	0.137049716	0.890991498	0.977283948
583.0139521	0.005532945	0.0708669	0.078075165	0.937768263	0.984334593
116.6547529	-0.043576044	0.097823472	-0.445455918	0.65599031	0.912934239
362.789506	-1.648953856	0.538919886	-3.059738372	0.002215304	0.111533785
274.1878443	0.006691586	0.094130241	0.071088589	0.94332725	0.98464854
207.390069	0.0841197	0.189386056	0.444170502	0.656919318	0.913625833
38.84878991	-0.04947787	0.235022841	-0.210523664	0.833258984	0.966227926
441.8743791	0.025809233	0.104120287	0.247879003	0.804228026	0.957233889
145.420588	0.019861622	0.122818408	0.161715353	0.871530014	0.972881041
174.21611	-0.089488149	0.107680826	-0.831049986	0.405945392	0.804391391
207.8441622	0.003974982	0.081165521	0.048973773	0.960940197	0.989165233
304.5495887	0.103410968	0.122945494	0.841112305	0.400285022	0.801079895
241.9596997	-0.236097544	0.072966064	-3.235717155	0.001213376	0.084153413

571.6691165	-0.263707751	0.136516115	-1.931696866	0.053396932	0.417087803
199.8767347	0.007362859	0.105526408	0.069772664	0.944374605	0.984876985
33.27616743	-0.32541464	0.169826471	-1.916159698	0.055344771	0.423973339
197.5484627	0.219804069	0.513771189	0.427824825	0.668778662	0.917626349
6.735645596	-1.619789469	0.913862692	-1.772464816	0.076317431	0.471783446
36.77452535	-0.283068573	0.296353268	-0.955172775	0.339490331	0.764202418
132.4493553	-0.260128815	0.313910369	-0.828672261	0.407289886	0.805288281
37.27914986	-0.1912865	0.157103074	-1.21758598	0.223381345	0.667842994
459.9267931	0.087551862	0.065349263	1.339752863	0.180325705	0.625591734
139.2125285	0.04156026	0.130061572	0.31954296	0.74931482	0.941713091
133.3007846	0.055132935	0.087714886	0.628547071	0.529645621	0.866063379
320.0461513	0.054335556	0.077672055	0.699550903	0.484207813	0.847993853
5.558970506	-1.380642027	0.577099448	-2.392381471	0.016739434	0.264538519
24.91148071	-0.263863892	0.422229242	-0.624930406	0.532016735	0.866797125
3650.837993	-0.016209221	0.088216388	-0.183743876	0.854214387	0.969966486
205.0259873	0.095215855	0.105982692	0.898409473	0.368967288	0.782987934
7.026850969	-0.771023907	0.412226655	-1.870388284	0.061429918	0.438421236
362.7804173	0.082693713	0.06976593	1.185302229	0.235897972	0.678901985
1208.602626	0.039425056	0.115136451	0.342420284	0.732034626	0.936065863
625.2113701	0.134795101	0.078094322	1.726055084	0.084337503	0.487641826
451.5161941	-0.040087575	0.062544459	-0.640945271	0.521558242	0.863328113
723.0013004	0.095551418	0.072001886	1.327068262	0.184486103	0.630643427
19.05893176	0.302642299	0.231191746	1.309053219	0.190516324	0.636683496
618.4669328	0.012888828	0.079473469	0.162177739	0.87116589	0.972881041
98.890362	-0.070019563	0.163699915	-0.427731212	0.668846823	0.917626349
36.96750427	0.052148576	0.188899243	0.276065565	0.782497709	0.95173434
13.87543668	-0.234701915	0.314685001	-0.745831275	0.455769345	0.831550545
3077.363387	0.035568821	0.057183464	0.622012353	0.533933745	0.867452088
8120.511567	0.064742957	0.09889098	0.654690215	0.512667227	0.860200661
615.5302659	-0.038548895	0.214000035	-0.180134992	0.857046592	0.969966486
69.57695338	-0.41326346	0.14571878	-2.836034302	0.004567754	0.149960163
55.21397132	-0.203362214	0.157380641	-1.292167915	0.196299001	0.641418933
68.85054736	0.136225591	0.157188301	0.866639504	0.386139561	0.791749243
69.77128356	-0.253038896	0.126233862	-2.00452472	0.045013882	0.388284077
39.30592033	-0.186821789	0.337486001	-0.553569002	0.579873845	0.887483205
15.96578947	-0.404295124	0.315035795	-1.283330754	0.199376223	0.644285509
224.0251271	-0.015533752	0.090422714	-0.171790379	0.863602329	0.971503396
737.216128	0.09577883	0.117803548	0.813038587	0.416195935	0.810170019
144.6062841	-0.301849074	0.167267008	-1.804594206	0.071138205	0.462488289
107.7950453	0.097327782	0.096995056	1.003430347	0.315653268	0.749486634
39.27345988	-0.090438232	0.162615589	-0.556147368	0.578110116	0.887122738
46.47721918	0.025936656	0.206259421	0.125747742	0.899931609	0.978996707
520.9091287	0.152417846	0.092320027	1.650972725	0.098744144	0.510766594
15.66288407	0.270396225	0.354195513	0.763409515	0.445219207	0.826302547
309.9131159	0.162655235	0.060241258	2.700063723	0.00693262	0.182267559
182.4645134	-0.062671308	0.211298228	-0.296601202	0.766770993	0.947349028
12.87393166	-0.830589279	0.455607751	-1.823035882	0.068297957	0.456821669
109.2830345	-0.143175726	0.09421849	-1.519613884	0.128608046	0.559569754

282.66176	0.063566154	0.094225448	0.674617685	0.499918694	0.856061604
17.33140515	-2.287455745	0.858237801	-2.665293631	0.007692113	0.189660453
5.206652074	0.183491998	0.54287558	0.338000096	0.735363116	0.937782789
11.29704063	-0.955132577	0.919389124	-1.038877393	0.298861762	0.738522837
7.378746571	-0.93645596	0.981406239	-0.954198092	0.33998338	0.764459061
3211.190419	0.093149378	0.098424455	0.946404819	0.343942149	0.767121103
5.192029127	-0.110811186	0.517801971	-0.214003021	0.830544702	0.965541183
77.46586391	0.041793988	0.324107835	0.128950873	0.897396522	0.97893802
60.04125879	-0.062766391	0.143182842	-0.438365307	0.661121492	0.914896282
1212.97006	-0.470130285	0.422447845	-1.112871779	0.265763502	0.708743266
121.1541814	-0.065998186	0.247567347	-0.266586795	0.789787324	0.953026618
36.1669303	-0.917003061	0.388561599	-2.359994049	0.018275228	0.272290126
31.38031396	-0.082637394	0.343328049	-0.240695145	0.809791402	0.958681749
907.4831876	0.212356297	0.201280039	1.055029092	0.29141202	0.732961888
63.57031597	-0.108509984	0.171391923	-0.633110256	0.526661646	0.865020759
1461.314037	0.195176137	0.098511081	1.981260738	0.047562041	0.397298719
139.3708333	0.306883616	0.101759153	3.015783892	0.002563159	0.119684149
69.28519295	-0.623922191	0.345614891	-1.805252631	0.071035158	0.462488289
58.36649274	-0.638650736	0.283017471	-2.256577069	0.024034515	0.308283262
54.3545287	-0.055659789	0.209033833	-0.266271675	0.790029986	0.953026618
1792.289577	0.027361903	0.367054188	0.074544588	0.940577064	0.984334593
81.79206028	0.241340013	0.355056838	0.679722194	0.496680381	0.854371062
2611.129079	0.238489369	0.12634743	1.887568033	0.059083965	0.434680797
146.7212321	-0.145537501	0.118279942	-1.23044954	0.218528812	0.662774346
603.1929041	-0.042077716	0.173553266	-0.242448427	0.808432714	0.95846384
96.72249979	0.214576889	0.224343018	0.956467872	0.338835909	0.764007173
2147.593039	0.051367827	0.107780569	0.476596362	0.633649574	0.906724287
26.91138903	-0.58721021	0.354156021	-1.658055134	0.097306339	0.507880983
159.2941761	-0.382112799	0.176246971	-2.168053138	0.030154644	0.332282647
5.089443806	0.834196902	0.597871743	1.395277353	0.162932219	0.601684318
9.715896734	-2.33816496	0.804876896	-2.90499699	0.003672566	0.138642962
1002.910798	0.064910712	0.146104054	0.44427728	0.656842126	0.913625833
43.71154484	-0.139777129	0.152404866	-0.91714348	0.35906746	0.777095745
215.8025626	-0.070867719	0.074192562	-0.955186294	0.339483496	0.764202418
167.7184601	0.13673275	0.239725714	0.570371646	0.568425657	0.8841274
79.53902936	0.044220761	0.138849116	0.31848068	0.750120347	0.942153702
456.110615	0.17461163	0.131524238	1.327600392	0.184310155	0.63049663
1335.463604	0.175665214	0.069021784	2.545069176	0.010925609	0.226714336
554.0682446	0.497849354	0.427147393	1.165521227	0.243808073	0.686012467
319.1114526	-0.36228289	0.468590712	-0.773132888	0.439443739	0.823332288
1394.996402	0.024967309	0.083907267	0.297558357	0.766040261	0.947120611
20.18717812	0.064039967	0.311083724	0.205860873	0.83689959	0.966316856
634.5857098	-0.061059498	0.10278924	-0.594026166	0.552494612	0.876623995
123.3453053	-0.008623584	0.124848119	-0.069072596	0.944931834	0.985130371
375.3261978	0.221273604	0.056941463	3.885983789	0.000101916	0.019849542
7.199290188	-0.002791881	0.335503666	-0.008321461	0.993360511	0.998685608
558.9031448	0.111323969	0.081953272	1.35838346	0.17434204	0.618034305
78.4442095	0.018003569	0.178098894	0.101087483	0.919481015	0.981892218

121.6523994	-0.105191074	0.103687009	-1.014505822	0.310341453	0.745771331
103.9017196	0.433346464	0.375745549	1.153297665	0.248788228	0.692441932
413.6757986	0.026743658	0.077325496	0.34585822	0.729449269	0.93467002
54.01932176	0.012871099	0.151583953	0.084910694	0.93233239	0.984334593
552.9394088	-0.029167353	0.161281646	-0.180847316	0.856487422	0.969966486
218.4749442	0.046662326	0.070312515	0.663641825	0.506919582	0.859011504
668.6452295	0.1384958	0.066727559	2.075541232	0.037936402	0.363923926
37.97362192	0.176441017	0.162623998	1.084962975	0.277938054	0.719965821
2278.549668	0.079150608	0.087389361	0.905723619	0.365082158	0.779790202
4.67521188	-0.590284851	0.478364432	-1.233964759	0.217216036	0.661397028
1168.582526	-0.102251193	0.1785422	-0.572700419	0.566847553	0.883766325
875.4345937	-0.259726509	0.092836208	-2.797685454	0.005147021	0.157735413
27.0414546	-0.301050806	0.230694192	-1.304977831	0.191900397	0.637743528
8.840787609	-0.473270611	0.412243703	-1.148035997	0.250953704	0.694642681
666.2278368	0.084861498	0.062987664	1.347271719	0.177892737	0.622328585
1040.377401	0.108275451	0.084026605	1.28858534	0.197542283	0.64297162
400.0874629	0.165926353	0.0773459	2.145250776	0.031932802	0.33936609
43.45071477	0.107444874	0.206044151	0.521465294	0.602042677	0.899145969
1006.879028	0.123939668	0.055742227	2.223443056	0.026185939	0.316367615
72.56685801	-0.069585667	0.132168233	-0.526493131	0.598545623	0.897636506
498.9283548	0.070772003	0.10361472	0.683030393	0.494587637	0.853198001
255.5223271	-0.109122769	0.079758643	-1.368162313	0.171261275	0.615741899
9.029601327	-0.039008489	0.309214145	-0.126153638	0.89961031	0.978996707
654.5653488	0.153387665	0.060950977	2.516574355	0.011850189	0.235730372
4.808577582	0.518689226	0.497384531	1.042833448	0.297025437	0.736647043
816.3531273	0.095357111	0.149497678	0.637850111	0.523571256	0.863484457
1236.419655	-0.221357044	0.069039776	-3.206224814	0.001344889	0.089546488
124.5721747	-0.167145913	0.084671074	-1.97406158	0.048374741	0.400471432
389.8328142	0.259214973	0.116042789	2.233787863	0.025497035	0.313568464
55.87172388	0.09402175	0.134555199	0.698759696	0.48470222	0.848641404
398.447608	-0.097183474	0.120532739	-0.806282793	0.42007981	0.812282344
9.882700767	0.410562007	0.317819111	1.29181032	0.196422841	0.641538148
164.3187762	-0.597508383	0.404292879	-1.477909739	0.139431939	0.572130062
244.905057	-0.444887716	0.389626538	-1.141831145	0.253524214	0.695969104
102.0123353	0.09642388	0.106755261	0.903223679	0.366407193	0.78149269
34.92684814	0.218791544	0.29666304	0.737508602	0.460813114	0.834673974
7.560790495	-0.134870767	0.402891241	-0.334757259	0.737808205	0.938868945
946.1993866	0.046818184	0.095509993	0.490191476	0.623998412	0.902875034
50.73481959	0.058657722	0.139746836	0.419742754	0.674673388	0.921149714
234.1165682	0.195543006	0.220937108	0.885061853	0.376123272	0.786851292
217.7685342	0.059937278	0.145323188	0.412441253	0.680016031	0.922112199
145.2188431	-0.189163074	0.155913303	-1.213258075	0.225031166	0.668193468
2090.923527	0.159450298	0.135884531	1.173424943	0.240625439	0.682548146
44.11268029	-0.107673844	0.155192373	-0.693808865	0.487802082	0.849513564
327.5598246	0.036026335	0.080618558	0.446873968	0.654966061	0.912480225
100.9856974	0.022227963	0.132584372	0.167651455	0.866857489	0.972138679
276.5866606	0.383221669	0.374114834	1.024342352	0.3056736	0.742035396
25.62691304	0.465059798	0.470996176	0.987396123	0.323448484	0.756002222

9.103999852	-1.117470415	0.716593292	-1.559420703	0.118896839	0.542885034
109.9467644	-0.214998448	0.359792337	-0.597562611	0.550131818	0.875690724
6.086259129	0.180697695	0.367459535	0.491748554	0.622897109	0.902441895
54.49976965	0.06897919	0.142220034	0.485017392	0.627664009	0.904512355
31.5319159	0.159812188	0.15502112	1.030905902	0.302584949	0.740307837
1198.742716	-0.00478455	0.073421148	-0.065165831	0.948041966	0.985917197
72.13193659	0.198979749	0.328989664	0.60482067	0.545298158	0.872896521
2826.61515	0.101653958	0.159504678	0.6373102	0.523922808	0.863484457
19.99807799	-1.066143048	0.424584735	-2.511025385	0.012038104	0.236404198
9.885167771	-0.979211759	0.453002711	-2.161602428	0.030648834	0.333576502
230.8661598	-0.025562811	0.09806825	-0.260663479	0.794352032	0.953454328
70.61572009	0.065850674	0.165945595	0.396820863	0.691499564	0.924455975
900.7045228	0.035721688	0.09846194	0.362796916	0.716756596	0.930336648
1089.181265	0.230716621	0.287365605	0.802867904	0.422051089	0.814164202
2645.205679	0.194436398	0.147541277	1.317844079	0.187555849	0.633746703
11.14297345	-0.82221988	0.527719441	-1.558062518	0.119218429	0.54348307
18.25147886	-0.334669651	0.265964264	-1.258325633	0.208274015	0.652874199
2047.528662	0.12145859	0.07140418	1.701001116	0.088942778	0.493693668
274.745294	0.114819056	0.128536573	0.893279262	0.371707641	0.785777849
596.0124593	0.114567584	0.15930357	0.719177755	0.472031403	0.84064088
140.2496021	0.233748665	0.336620052	0.694399111	0.487431951	0.849513564
316.6334615	0.007323166	0.077290672	0.094748385	0.924514685	0.982590996
373.2945491	-0.044776863	0.125514697	-0.356745973	0.721281986	0.931135539
149.695319	0.127687855	0.083646037	1.526526067	0.126878904	0.556444786
139.7893333	-0.067056521	0.093290696	-0.71879109	0.472269648	0.840757256
720.7577619	-0.02602053	0.140923084	-0.184643492	0.853508671	0.969966486
37.60895704	0.081800549	0.225147352	0.363320059	0.716365811	0.930241908
4.597025107	-0.732676016	0.60977795	-1.201545607	0.229539627	0.672401653
9.056009016	-0.198148035	0.389962858	-0.508120277	0.611368996	0.900561602
83.34205303	-0.161058917	0.14731221	-1.093316822	0.27425472	0.716722874
1135.469113	0.082454813	0.063070416	1.30734532	0.191095459	0.637278237
15.58470065	-0.019972261	0.221340231	-0.090233305	0.928101819	0.984009965
106.4826903	0.287657987	0.133929349	2.147833835	0.031726958	0.339077569
33.1155242	0.143161811	0.225258686	0.635544021	0.525073666	0.864339457
115.1803116	0.156771424	0.11768801	1.332093422	0.182829491	0.628481684
57.07728899	-0.183781965	0.134610715	-1.365284808	0.172163552	0.616109027
36.4793482	0.21890042	0.454337662	0.481801177	0.629947193	0.904849913
15.09819004	-0.056540642	0.403307549	-0.140192372	0.888507999	0.976961229
462.6631522	0.078700629	0.119748847	0.657214087	0.511043269	0.860200661
3678.873186	0.314851097	0.15230422	2.067251307	0.03871048	0.367538502
2311.669886	0.272024009	0.1166858	2.331252032	0.019740074	0.281261453
8.502178396	-0.704759777	0.600475371	-1.173669748	0.240527332	0.682548146
96.32865126	-0.169437729	0.101861818	-1.663407664	0.09623086	0.505827127
172.107007	-0.174736414	0.208865758	-0.836596749	0.402819274	0.802278107
103.6353737	0.198177657	0.108146	1.83250104	0.066876807	0.454159121
90.46895683	0.142423192	0.130370464	1.092449833	0.27463543	0.716878153
10.51369816	0.372299331	0.473332404	0.786549428	0.431545674	0.818841807
1433.230401	-0.300707992	0.349525387	-0.860332334	0.389605874	0.79322193

115.6940928	-0.053805056	0.133235298	-0.403834844	0.686334172	0.923064029
289.630209	0.020424942	0.063602659	0.321133451	0.748109262	0.941542093
11.81731036	-0.472962053	0.424678093	-1.113695434	0.265409869	0.708627777
158.8122973	-0.049983082	0.149443345	-0.334461748	0.738031151	0.93892025
6.715900553	0.058729534	0.537782212	0.109206911	0.913038379	0.980530316
126.9273461	0.143182803	0.122033959	1.173302942	0.240674343	0.682548146
203.4396509	0.107266686	0.194260912	0.552178434	0.580826108	0.887898616
30.93531418	0.580087715	0.38863367	1.492633709	0.135533095	0.568673186
149.4649688	-0.348041204	0.168891141	-2.060742808	0.039327583	0.370654063
68.20248324	-0.099627949	0.15126843	-0.65861693	0.510141788	0.860200661
574.1375946	0.741103014	0.544055693	1.36218226	0.173140373	0.616656352
16.40377278	-3.265585545	1.091372287	-2.992182946	0.002769902	0.12485574
66.87263641	-0.110328309	0.165706095	-0.665807187	0.505534351	0.858625297
21.67815898	-0.492016501	0.327366459	-1.502953306	0.132851086	0.565075221
45.74315782	0.175270644	0.140960674	1.24340101	0.213720098	0.659274529
412.992381	-0.048407631	0.161367523	-0.299983729	0.764189566	0.946826103
411.7064029	0.045932641	0.054744359	0.839038801	0.401447536	0.801291311
19.5714943	-0.61040922	0.330173086	-1.848755235	0.064493164	0.445879408
580.1364292	0.292845194	0.10185143	2.875219274	0.00403747	0.144354659
73.87370076	-1.006566935	0.381025217	-2.641733117	0.008248302	0.19712288
92.7095022	-0.051381129	0.241574916	-0.212692317	0.83156696	0.966158096
23.1947702	0.236216886	0.249174831	0.947996576	0.343131197	0.76653003
188.9342666	0.201886538	0.092760617	2.176425121	0.029523487	0.330564792
1724.555342	0.091878547	0.079611759	1.154082616	0.2484663	0.69187626
20.53749779	-0.122855603	0.21579242	-0.56932307	0.569136913	0.8841274
273.31093	0.179850158	0.104175613	1.726413244	0.084273095	0.487460082
355.5502026	0.22264416	0.070028712	3.179326809	0.001476176	0.09327498
174.7983415	0.019490321	0.102879334	0.189448355	0.849741429	0.969251033
227.5434915	0.142743158	0.085999776	1.659808475	0.096952988	0.507281065
344.9803024	-0.180633949	0.198995929	-0.907726855	0.364022552	0.779728871
438.0594097	0.074045383	0.06650652	1.113355242	0.26555589	0.708627777
15.48036574	-0.461251846	0.340681666	-1.353908623	0.175765532	0.619005807
982.9564516	0.057084851	0.08520968	0.669933872	0.502899946	0.857172233
176.1517834	-0.086182173	0.110437063	-0.780373637	0.435170981	0.822078703
314.5408992	0.021502608	0.078090485	0.27535503	0.78304349	0.95173434
234.2428468	-0.147461012	0.173692992	-0.848975024	0.39589519	0.797935812
1321.566123	-0.174290344	0.15256012	-1.142437119	0.25327237	0.695646467
104.3498005	-0.337062537	0.207766633	-1.622313129	0.104736319	0.518635678
318.5999009	0.028412152	0.108001541	0.263071727	0.792495296	0.953153173
1271.527646	0.116115403	0.14133482	0.821562609	0.411325877	0.808165706
417.2746414	-0.036125559	0.137511437	-0.262709488	0.792774503	0.953153173
500.1630792	-0.002695827	0.090417511	-0.029815316	0.976214344	0.993487297
133.8568557	-0.154763975	0.135872151	-1.139041178	0.25468598	0.696832138
783.3227505	0.138150142	0.14058861	0.982655294	0.325777121	0.756297513
37.42983497	-0.058559865	0.202020105	-0.289871471	0.771914568	0.948706053
61.04879492	0.085950571	0.126737839	0.678176081	0.497660056	0.855161847
71.15217941	0.074062747	0.120131347	0.616514747	0.537554842	0.869675597
5.568650612	0.473248315	0.400971373	1.18025462	0.237898961	0.679803171

304.3979598	0.47641026	0.346194155	1.376136058	0.168779514	0.611123866
9.704318759	-0.286625418	0.46487908	-0.616559079	0.537525592	0.869675597
524.8263652	0.161937548	0.092026228	1.759689086	0.078460537	0.474223305
324.1728314	0.156376228	0.151169086	1.034445817	0.300927795	0.739230864
396.3176375	0.089338802	0.074991424	1.191320257	0.233527885	0.676472759
308.3493019	0.179555314	0.063473079	2.828842028	0.004671675	0.151645037
118.235619	0.772985085	0.460823691	1.677398754	0.093464533	0.500506422
11.73224031	-0.322638445	0.355999231	-0.906289723	0.364782523	0.779728871
951.1462834	0.13247499	0.214756858	0.61686035	0.537326842	0.869675597
66.01106598	-0.405907554	0.164703575	-2.464473248	0.013721481	0.25043817
14.00916483	-0.16443717	0.282374016	-0.582338178	0.560338915	0.880668572
186.0930541	0.123248042	0.091924801	1.340748539	0.180002109	0.625150451
6.386325757	0.046035748	0.607010435	0.075840127	0.939546291	0.984334593
1093.29079	0.41146323	0.240278162	1.712445386	0.086814621	0.491219427
2215.460378	0.087990711	0.090556015	0.971671631	0.331213934	0.759815544
65.0839867	0.044912916	0.154963156	0.28982964	0.771946571	0.948706053
7.785933149	-0.856843931	0.356794993	-2.401502119	0.016327914	0.263145637
39.78623001	-1.065577652	0.318025358	-3.350605938	0.00080635	0.067053858
75.40998783	-0.150914585	0.151366469	-0.997014635	0.318757406	0.752066309
4.761673186	0.055356424	0.524559764	0.105529298	0.915955823	0.981266182
63.67364109	-0.220578215	0.231883134	-0.95124734	0.341478832	0.765195212
191.9980635	0.057648971	0.099534566	0.579185439	0.562464055	0.881820831
366.741787	-0.061671559	0.093777484	-0.657637165	0.510771307	0.860200661
2212.321485	-0.113971287	0.151586578	-0.751856062	0.452137624	0.829825308
476.8760992	0.090175199	0.072578052	1.242458242	0.214067538	0.659367121
1815.531794	-0.204037008	0.17026348	-1.198360379	0.23077675	0.673080052
11.67642059	0.623164344	0.279327809	2.230942724	0.025684923	0.314205151
111.4893527	0.283379658	0.163381877	1.734462005	0.082836142	0.485197868
661.6654707	0.241276404	0.153835806	1.56840212	0.116787317	0.538641109
1049.52915	0.016391745	0.26874798	0.060992997	0.951364786	0.98625342
114.9180347	-0.111251055	0.11926985	-0.932767629	0.350939958	0.770952892
1009.807807	0.05326603	0.072450505	0.735205769	0.462214186	0.835473731
27.15882829	-0.20031661	0.184807467	-1.083920538	0.278400033	0.720249062
573.8758876	0.145605556	0.072086318	2.019877836	0.043396061	0.381263212
1051.04681	0.030655419	0.073983106	0.414357008	0.678612669	0.921628107
10.87219161	0.330000387	0.303210154	1.088355327	0.276438274	0.718547195
88.83080437	-0.003653018	0.305334816	-0.011963975	0.990454357	0.998209668
641.7736697	0.225538488	0.05638331	4.000093115	6.33176E-05	0.015156055
163.136595	-0.396400222	0.21973969	-1.803953677	0.071238569	0.462488289
1210.147325	-0.194973612	0.082053519	-2.376176107	0.017493112	0.269441247
395.7711298	0.188818917	0.063631671	2.967373255	0.003003561	0.128901255
14.96059492	-0.284935157	0.351134855	-0.811469305	0.417096213	0.811069121
14.23968787	0.113469884	0.288782372	0.392925245	0.694374695	0.924581226
79.35057191	-0.812944901	0.514150252	-1.581142668	0.113845421	0.533883941
211.7414459	0.126368678	0.099796622	1.266262076	0.20541927	0.650249702
8.76323696	0.360460733	0.385079876	0.93606744	0.349238466	0.769374576
167.5790831	0.066499404	0.132838088	0.500604944	0.616649182	0.901900555
273.2415557	0.012251593	0.132598523	0.092396153	0.926383296	0.983240215

6.546239182	-0.092402105	0.406138419	-0.227513825	0.82002422	0.962543699
248.9434332	0.033143048	0.071299517	0.464842531	0.642044228	0.908064184
4.473858193	0.225506367	0.48819601	0.461917678	0.644140356	0.908494289
126.9016831	-0.238308354	0.299017171	-0.796972137	0.425467214	0.816273895
103.3301312	0.09282168	0.254903773	0.364144003	0.715750479	0.930241908
5.384546754	0.242120774	0.462238439	0.523800604	0.600417233	0.898259135
296.3806027	0.110768947	0.154757312	0.715759053	0.47414014	0.841713532
922.9724516	-0.016316188	0.122290764	-0.133421261	0.893860232	0.978111861
232.6393661	-0.125382777	0.07859353	-1.595332048	0.110638003	0.53015983
333.1241494	-0.110184811	0.139504124	-0.789831925	0.429625932	0.818650305
663.581952	-0.022977845	0.065715704	-0.34965531	0.726597397	0.933204828
4525.85024	0.154825346	0.049946459	3.09982629	0.001936342	0.104224472
43.20640289	-0.093584021	0.186382061	-0.502108523	0.615591185	0.901900555
18.7335862	0.248994341	0.228069362	1.091748315	0.274943742	0.716878153
110.3024162	0.195329823	0.108541913	1.799579694	0.07192703	0.463154629
1787.987976	0.044689267	0.075391353	0.592763826	0.55333922	0.877205564
3549.985519	0.012809714	0.112871609	0.113489248	0.909642687	0.980530316
573.1967964	-0.264341196	0.268821799	-0.983332444	0.325443847	0.756297513
97.29755059	-0.036169793	0.154813282	-0.233634948	0.815268394	0.960909552
162.8212087	0.090576177	0.102315555	0.88526302	0.37601479	0.786851292
4.93656297	0.097951139	0.439942627	0.222645257	0.82381161	0.963494265
579.9473684	-0.075663305	0.134074377	-0.564338291	0.572523927	0.885183781
9.188569518	-1.099457558	0.412355022	-2.666288754	0.007669379	0.189660453
141.3890494	0.383050781	0.440332346	0.869912884	0.384348014	0.790375007
23.83012157	-0.062692927	0.233642626	-0.268328293	0.78844663	0.953026618
199.1489304	-0.052798592	0.112611056	-0.468857982	0.639171152	0.907684986
20.8445757	0.187464855	0.266956106	0.702231005	0.482535113	0.846691049
1093.752904	-0.074064362	0.12477499	-0.593583396	0.552790789	0.876718018
262.7642651	0.11321355	0.079383056	1.426167693	0.153819943	0.59219701
8.542457907	-0.656273779	0.767924638	-0.85460701	0.392768746	0.795848318
371.7835428	0.213788817	0.241318047	0.885921376	0.375659896	0.786839429
292.0503105	-0.023046167	0.120779508	-0.190811897	0.848672968	0.969123636
11.17845596	0.30651395	0.306371112	1.000466224	0.317084935	0.750598307
602.2875655	0.086757087	0.095181289	0.91149308	0.36203563	0.778909683
1360.227573	-0.012216721	0.064380977	-0.189756687	0.849499798	0.969251033
176.9664539	0.057809495	0.127274003	0.454212912	0.649675591	0.910782566
2150.291699	0.150934148	0.094180932	1.60259773	0.109023495	0.526514119
61.22030029	-1.156556934	0.606250671	-1.907720665	0.056427329	0.427168741
98.9301761	-0.040554279	0.124930987	-0.324613455	0.745473643	0.940455922
653.6506314	0.122539263	0.108398007	1.130456783	0.258283799	0.701275747
90.16607635	-0.141836248	0.135026824	-1.050430156	0.293520387	0.734026791
1139.97834	0.047271302	0.073755811	0.640916301	0.521577065	0.863328113
230.3282152	0.133684072	0.101661042	1.314998048	0.188510558	0.634590162
170.1382487	0.020472415	0.129201827	0.158452986	0.874099865	0.973366431
17.49069463	0.186634877	0.241448032	0.77298156	0.439533294	0.823332298
2844.090094	0.169134223	0.195082358	0.866988818	0.385948136	0.791597811
10.22096555	-4.177233455	1.424404583	-2.932617253	0.00336118	0.133812668
7.341582699	1.778420865	0.730723811	2.43377982	0.014942081	0.256113483

26.95993745	-0.77472582	0.237735603	-3.258770706	0.001118961	0.080402212
7.675803383	-0.082963079	0.5996046	-0.138362979	0.889953554	0.976961229
14.21890559	-0.198476368	0.29639103	-0.669643639	0.503084988	0.857172233
5.412657787	-2.128452712	1.010638115	-2.106048327	0.035200155	0.355411111
28.63692268	0.648517523	0.584330609	1.109846913	0.267065	0.709341177
179.5539545	0.182842991	0.132489251	1.380059057	0.167568462	0.609207556
2592.985569	0.164309291	0.162872589	1.00882102	0.313060478	0.747045171
193.7796962	0.221944617	0.079313036	2.798337168	0.005136646	0.157735413
36.1404294	0.099465325	0.163949479	0.606682779	0.544061453	0.87184125
19.31369386	0.124607836	0.219768038	0.566997083	0.570716164	0.884626151
99.16619084	0.099275726	0.165367924	0.60033242	0.548284717	0.874640417
708.3018763	0.194273164	0.061358782	3.166183519	0.001544533	0.094686033
180.8560779	0.506494834	0.195213077	2.594574311	0.009470814	0.211314005
56.57825329	-0.033649533	0.172788226	-0.194744365	0.845593079	0.968131661
1582.601014	-0.012594271	0.05522323	-0.228061102	0.819598739	0.96243206
808.0314676	-0.106744771	0.122725692	-0.869783406	0.384418781	0.790387368
13.78177334	-0.117214205	0.257312237	-0.455532959	0.648725862	0.910701841
141.5503034	0.136283411	0.18924854	0.720129261	0.471445413	0.840318577
435.9381587	0.127395891	0.082901588	1.536712304	0.124363777	0.552139359
651.3934444	-0.135364482	0.218599641	-0.619234694	0.53576176	0.869002283
319.6546217	0.121153774	0.07420271	1.632740553	0.102523575	0.514997052
276.9362437	-0.122278922	0.077261671	-1.582659549	0.113499083	0.533883941
2151.090831	0.112887863	0.081326103	1.388088924	0.165109969	0.605350304
97.10365199	0.067979901	0.14073476	0.483035614	0.629070449	0.904638489
9.729511873	0.262229321	0.338428507	0.77484407	0.438431805	0.82251807
21.26100945	-0.080407464	0.267527785	-0.300557432	0.763751996	0.946826103
73.61337615	0.111328394	0.120572574	0.923330996	0.35583474	0.774251728
5.443196829	0.358355912	0.419183752	0.854889796	0.39261216	0.795831146
80.10276294	-0.70684785	0.331554645	-2.131919613	0.033013456	0.343888226
5.851808671	0.642960256	0.634194082	1.013822541	0.310667437	0.745771331
109.3587882	-0.044951755	0.135961694	-0.330620731	0.740930985	0.939831839
185.1365876	0.324615825	0.125881957	2.578731964	0.00991637	0.215114309
189.4025627	0.384447463	0.317134842	1.212252369	0.225415791	0.668524251
2308.541532	-0.090606037	0.157263971	-0.57613983	0.564520671	0.883265494
4.961970793	0.245974868	0.550588576	0.44674895	0.655056334	0.912487801
1113.551717	0.104028375	0.225479692	0.46136472	0.644536958	0.908612958
15.51044521	-0.187301643	0.263358898	-0.711203016	0.47695844	0.843597766
168.7420459	0.003075239	0.173523594	0.017722312	0.985860381	0.996565278
295.1846353	-0.032962724	0.131711588	-0.250264419	0.80238287	0.956577903
472.9843926	0.114588284	0.121307055	0.944613519	0.344856223	0.767851822
41.93554725	0.197079236	0.163560178	1.204934102	0.228228742	0.671005548
73.44237907	0.012591274	0.227523999	0.055340421	0.95586726	0.987483032
95.68293032	0.098874118	0.213119455	0.463937552	0.642692487	0.908494289
9.66603733	-0.087587257	0.322707001	-0.271414183	0.786072495	0.952606765
4352.820518	0.021167884	0.119980684	0.176427431	0.859958156	0.969966486
21.15052369	-0.039839576	0.23825159	-0.167216412	0.867199771	0.972138679
12.52049711	0.036846954	0.286375333	0.128666649	0.897621427	0.97893802
7.856985112	0.040445815	0.455190176	0.088854763	0.929197335	0.984152592

381.4506455	0.099607599	0.077826007	1.279875492	0.200588928	0.645045462
5.445286434	0.938999543	0.468836296	2.002830309	0.045195505	0.389170373
32.32328004	-0.271896885	0.241701115	-1.124930202	0.260618612	0.70310069
855.9389263	-0.132304405	0.082075546	-1.611983247	0.106965585	0.524099502
51.9978069	0.142104727	0.179771207	0.790475455	0.429250151	0.818650305
10.78762061	-1.317525595	0.996247563	-1.322488148	0.186005653	0.631625529
110.3716533	-0.727117244	0.340321975	-2.136556845	0.032634053	0.341836409
5.383946085	-1.853708591	0.634304492	-2.922427027	0.00347315	0.135044319
2669.109202	0.003398407	0.080233117	0.042356661	0.966214377	0.990895439
103.0134625	0.227953661	0.137835854	1.653805267	0.098167086	0.509712212
21.28053303	0.198785517	0.203714735	0.975803331	0.329161934	0.758888028
50.21894259	0.466363229	0.221119061	2.109104598	0.034935554	0.353946661
15.86010306	-0.574873016	0.431952538	-1.330870791	0.18323153	0.628765197
592.6498808	0.048214172	0.137327872	0.351088027	0.72552231	0.932625378
1490.96333	0.028435218	0.064460494	0.441126286	0.659121574	0.914197671
8.562235828	-0.176726939	0.653201977	-0.270554814	0.786733453	0.952886718
128.8661296	-0.622817007	0.340038695	-1.831606275	0.067010101	0.454159121
307.6187256	0.150415106	0.058028701	2.592081214	0.009539725	0.211704658
5367.435297	0.022341834	0.124315818	0.179718353	0.857373684	0.969966486
15.1275038	0.125513914	0.292219796	0.429518862	0.66754567	0.916775933
4.559270615	0.805943175	0.507578794	1.587818848	0.112327308	0.533148601
7.749833201	0.119843712	0.392669077	0.305202826	0.76021168	0.946795592
933.8140134	-0.080079095	0.102987873	-0.77755849	0.43682934	0.822078703
69.09043924	-0.211577785	0.261530112	-0.808999712	0.418515312	0.811455684
1241.469048	-0.132831104	0.116169423	-1.143425698	0.252861888	0.695085007
8.505566061	0.9929199	0.349929939	2.837481993	0.004547091	0.149960163
746.8843369	0.485989144	0.355982398	1.365205546	0.172188456	0.616109027
128.6197021	0.0747738	0.102117481	0.732233101	0.464026304	0.836383228
331.2570096	0.1406565	0.070505431	1.994973956	0.04604572	0.391422191
98.45962899	0.028330032	0.157583054	0.179778414	0.857326531	0.969966486
17.48531345	0.04594839	0.256085813	0.179425755	0.85760341	0.969966486
8.728371627	-0.205083496	0.425060519	-0.482480698	0.629464507	0.904638489
61.8353359	0.181575343	0.135187705	1.343135039	0.179228252	0.624367282
39.30606027	0.401034387	0.26987007	1.486027653	0.137271807	0.569036171
7.079060527	0.692711465	0.525344054	1.318586287	0.187307461	0.633720007
83.94936514	-0.071124322	0.207739351	-0.342372892	0.732070286	0.936065863
412.1220928	0.098594571	0.115786569	0.851519926	0.394480599	0.796838286
908.3321217	0.167390131	0.083715635	1.999508592	0.045553353	0.390450954
92.35371903	0.019347347	0.104731553	0.184732744	0.853438662	0.969966486
101.801969	-0.097525432	0.120792399	-0.807380537	0.419447276	0.811695462
61.59063599	0.051546566	0.184358496	0.279599622	0.779784697	0.95173434
21.43077862	0.089090997	0.25524443	0.349041886	0.727057864	0.933383392
514.5415845	0.131116173	0.054248256	2.41696567	0.015650493	0.259890973
535.5524061	0.078264179	0.087215801	0.897362389	0.369525575	0.783514907
31.55148433	-0.014448226	0.191530839	-0.075435505	0.93986821	0.984334593
20.51738638	-0.196040738	0.395558939	-0.495604369	0.62017357	0.901976704
231.8735124	0.001124842	0.085685911	0.013127497	0.989526074	0.997749656
4.453799871	0.342638001	0.441521447	0.776039314	0.437725773	0.82251807

1866.214264	0.15052845	0.073958977	2.035296525	0.041821046	0.375856173
54.90259959	-0.028471615	0.179830528	-0.158324706	0.874200941	0.973366431
12.18326072	-0.11941723	0.287258108	-0.415714046	0.677619262	0.921393988
5.635282418	-1.56202933	1.127260785	-1.38568586	0.165842848	0.606616156
1197.764368	-0.048127991	0.053076798	-0.906761399	0.364532987	0.779728871
122.2507289	-0.007505816	0.123361056	-0.060844294	0.951483214	0.98625342
35.69567529	0.143566003	0.160413914	0.894972258	0.370801918	0.784591562
18.49290177	0.414852047	0.241914333	1.714871712	0.08636875	0.490195644
118.3701131	0.081205262	0.113816321	0.713476429	0.475550994	0.842988558
49.22557466	0.158365058	0.15976169	0.991258032	0.3215596	0.753956155
11.72074658	-0.168352317	0.310327553	-0.542498773	0.587474952	0.891054846
40.88231638	0.295459261	0.161127389	1.833699796	0.066698569	0.454127055
35.13151799	-0.101005123	0.20191757	-0.500229491	0.616913495	0.901900555
22.74810302	-0.063755117	0.213529672	-0.29857732	0.765262573	0.946857219
50.88133564	-0.103374299	0.199329552	-0.518610002	0.604032733	0.899667188
91.76015101	0.085646934	0.102868735	0.832584685	0.405078999	0.803642856
11.03095426	-0.169071508	0.352977739	-0.478986319	0.631948362	0.906005577
9.672142551	0.154318838	0.380780138	0.405270187	0.685278918	0.922907843
106.2413166	0.211962975	0.096208463	2.203163512	0.027583226	0.323780263
176.3064636	0.326613534	0.163630726	1.996040371	0.045929529	0.391329374
11.18275005	0.491478171	0.373333313	1.316459457	0.188019877	0.634170926
73.06329724	0.132074057	0.206700699	0.638962797	0.522847134	0.863362704
75.5068599	0.065377418	0.161531662	0.404734385	0.685672764	0.923064029
90.26766206	0.065867087	0.163740487	0.402265122	0.68748892	0.923064029
69.3959042	0.159795614	0.133203465	1.199635564	0.230280908	0.673001336
23.9651283	0.07584221	0.199897237	0.379405993	0.704386401	0.9269128
154.5535983	-0.058249403	0.105331576	-0.553009885	0.580256641	0.887483205
35.15805676	-0.24326166	0.194043766	-1.253643265	0.209971698	0.655248746
5.14303403	-0.273872378	0.445642949	-0.614555619	0.538848231	0.869675597
33.6095759	-0.16387887	0.187348404	-0.874727871	0.381721992	0.789361403
26.69710662	0.085616288	0.195024671	0.439002345	0.660659837	0.914896282
6.181992508	-2.771761944	0.994840655	-2.786136583	0.005334039	0.159160905
63.55483159	0.138603187	0.127851889	1.084091817	0.278324091	0.720249062
344.8781867	0.025496599	0.073975649	0.344662053	0.730348449	0.935399183
108.7791351	0.165452227	0.174367516	0.948870701	0.342686378	0.766114448
12.43607311	-1.007705417	0.450706826	-2.235833493	0.02536268	0.313430481
77.4461217	-0.376528218	0.229650864	-1.639568042	0.101095012	0.512787322
219.3981201	-0.089598399	0.104105173	-0.8606527	0.389429351	0.793111346
379.6438761	-0.034394479	0.086561892	-0.397339727	0.691116955	0.924455975
11.87097161	0.05286137	0.370883229	0.142528338	0.886662698	0.976961229
5481.192785	0.202168351	0.097207099	2.079769416	0.037546688	0.362130981
823.0380026	0.383984202	0.121018416	3.172940232	0.001509036	0.093851886
4.449023833	0.667638846	0.572543316	1.166093163	0.243576781	0.686012467
88.45990846	0.042230705	0.115203074	0.366576196	0.713935167	0.929846524
1225.19018	0.102028917	0.057697268	1.768349203	0.077002543	0.472354597
82.1758226	0.024266595	0.115070213	0.210885114	0.832976921	0.966227926
257.7451395	-0.014904709	0.088226684	-0.168936519	0.865846574	0.971917805
24.31554189	-0.249306605	0.243419281	-1.02418594	0.305747458	0.742035396

32.95210401	-0.207202909	0.206897164	-1.001477763	0.316595886	0.749920355
18.78466696	-1.434709317	0.647831142	-2.214634684	0.026785149	0.319793729
490.728491	-0.054387145	0.091014625	-0.597564901	0.55013029	0.875690724
43.28142346	-0.037664369	0.219167517	-0.171851965	0.863553911	0.971503396
216.2674378	0.171079231	0.094831006	1.804043203	0.071224535	0.462488289
945.4002503	-0.175373062	0.136605352	-1.283793485	0.199214224	0.644243885
19.77327406	-0.43824348	0.255181815	-1.717377389	0.08591024	0.489470122
221.3971004	-0.033087029	0.073756979	-0.448595235	0.653723672	0.911945899
587.3884653	-0.213631272	0.137533346	-1.553305273	0.120350221	0.546018577
124.7123111	-0.090705125	0.133413126	-0.679881564	0.496579456	0.854371062
6.791209977	-0.078446544	0.384432904	-0.20405783	0.838308308	0.966440785
259.9472002	-0.05577073	0.088624695	-0.629291081	0.52915851	0.866063379
4.711953793	-0.676331575	0.461682897	-1.464926639	0.142940934	0.57754946
1635.24883	-0.056938626	0.062645127	-0.908907504	0.363398953	0.779728871
1492.104247	-0.063048783	0.101020318	-0.624119823	0.532548896	0.867049621
26.15270684	-0.043419664	0.262948261	-0.165126263	0.868844602	0.972204994
319.3909833	0.222303245	0.1834501	1.211791352	0.22559226	0.668749399
534.9060328	0.007371247	0.089015468	0.082808605	0.934003727	0.984334593
409.7690168	0.174579301	0.060980187	2.862885635	0.004198021	0.145184815
18.35199155	-0.048286207	0.277308053	-0.174124791	0.86176739	0.970324122
211.7080977	-0.04057009	0.098948714	-0.410011295	0.681797662	0.922658753
23.5785192	-0.706843792	0.264498403	-2.672393425	0.007531229	0.189027811
536.3092547	-0.818035935	0.436179523	-1.875456991	0.060729881	0.436582661
9.514508122	-0.70365373	0.384873113	-1.828274584	0.067508352	0.45536467
1226.373589	0.202033434	0.155347484	1.300525951	0.193420768	0.639147095
5477.541809	0.112861035	0.150981214	0.747517073	0.454751501	0.831501359
489.2774281	-0.258289505	0.128803837	-2.00529357	0.044931672	0.387840011
5.603372576	1.005246422	0.512079493	1.963067134	0.049638374	0.404942943
254.3457747	0.100256967	0.148186866	0.676557709	0.49868662	0.855634722
361.7060494	-0.065941954	0.074516508	-0.884930813	0.376193948	0.786851292
42.77342574	-0.786136905	0.33821873	-2.324344682	0.020107028	0.282442098
134.7241986	-0.071439853	0.089564689	-0.797634134	0.425082837	0.815987051
271.0635817	-0.019787829	0.068574666	-0.288558888	0.77291896	0.948967025
84.36569218	-0.288281203	0.120773248	-2.38696241	0.016988228	0.265843192
438.6206698	0.052843872	0.080294416	0.658126368	0.510456934	0.860200661
91.35538257	-0.25677468	0.124181495	-2.067737068	0.038664754	0.367408361
480.7385631	-0.030882188	0.089219541	-0.346137045	0.729239725	0.93467002
99.25626168	-0.204179257	0.161532482	-1.264013616	0.20622514	0.651556995
368.5725501	-0.003631979	0.077245735	-0.047018509	0.962498476	0.989751022
158.8135105	-0.087064343	0.106300092	-0.819042964	0.412761901	0.808272153
138.6914078	0.142878989	0.145635023	0.981075746	0.32655539	0.757054149
99.81710313	-0.106371505	0.12677937	-0.839028499	0.401453317	0.801291311
275.6168241	0.012206921	0.093096645	0.131120958	0.895679622	0.978798764
278.3592099	0.027440372	0.107257297	0.255836879	0.798076796	0.954840537
105.7804154	-0.015027839	0.124276566	-0.120922545	0.903752385	0.979381499
135.8348634	0.110111532	0.107319575	1.02601535	0.304884349	0.741672489
105.6290057	0.027780293	0.088763023	0.312971463	0.754302343	0.94414502
13.73565249	-0.597826467	0.315014713	-1.897773161	0.05772597	0.431279458

363.6666203	0.152524941	0.067676682	2.25372961	0.024213183	0.308436782
10.21096612	0.066800439	0.587210092	0.113759011	0.909428832	0.980530316
184.9304546	0.32072813	0.326057359	0.983655547	0.325284903	0.756297513
33.6444445	-0.074589708	0.194003758	-0.384475586	0.700625988	0.926034765
9.13816231	-1.177273444	0.425506561	-2.766757438	0.005661686	0.164644953
5.400916259	-0.020146642	0.548910729	-0.036702947	0.970721859	0.992796176
160.4220442	-0.126083028	0.097792752	-1.289288068	0.197297958	0.642424591
13.86172804	-0.706060335	0.41387719	-1.705965811	0.088014445	0.492827855
51.36576316	-0.18533591	0.144990976	-1.278258246	0.201158385	0.645045462
14.23357218	-1.203052716	0.494213168	-2.434278958	0.01492149	0.256113483
347.9629495	-0.164133782	0.107424527	-1.527898588	0.126537719	0.556378986
76.83538746	-0.143866943	0.166876829	-0.862114554	0.388624483	0.792781092
9.335472595	-0.153324357	0.318444748	-0.481478679	0.630176329	0.905091212
291.0988477	-0.244819484	0.119945776	-2.041084665	0.041242412	0.374892505
299.85909	-0.333719496	0.133954726	-2.49128571	0.012728172	0.241702202
50.65319648	-0.223210289	0.338549108	-0.659314363	0.509693921	0.859882646
220.3335187	-0.090854754	0.110571402	-0.821684019	0.411256757	0.808165706
75.03793078	0.16544748	0.17515487	0.944578245	0.344874238	0.767851822
356.1477466	-0.100509864	0.088828177	-1.131508802	0.257841001	0.701069052
472.5894303	-0.055103158	0.177408517	-0.310600409	0.756104416	0.944952618
1279.96091	-0.055191823	0.152211106	-0.362600499	0.716903338	0.930431756
43.52272167	-0.502042689	0.295004099	-1.701815976	0.088789871	0.493693668
400.1968646	0.048244735	0.141392179	0.34121219	0.732943847	0.936634277
166.3333777	0.060420154	0.131975833	0.457812257	0.647087329	0.910195219
20.45135414	0.04250009	0.240612421	0.176632985	0.859796683	0.969966486
116.5682874	-0.059948798	0.098796744	-0.606789204	0.543990814	0.871822436
13.45137147	0.157332984	0.300097708	0.524272528	0.600089002	0.898108141
348.0696632	-0.238577941	0.133826952	-1.782734633	0.074629511	0.470671504
123.6461016	0.005674827	0.117336628	0.048363642	0.961426435	0.989220207
1786.146173	0.028078751	0.105357144	0.266510179	0.789846321	0.953026618
89.01959023	-0.029754769	0.180347252	-0.164985988	0.868955011	0.972204994
22.38281126	0.198563106	0.264574501	0.750499783	0.452953754	0.830105164
54.5949446	0.746862382	0.508967491	1.467406849	0.142265412	0.576962874
402.0705657	-0.175830235	0.134532872	-1.306968567	0.191223387	0.637278237
325.0435102	-0.020481712	0.060834733	-0.336677938	0.736359697	0.938503341
6.958489383	-0.063843861	0.429198722	-0.148751284	0.881749893	0.975487608
12.58825449	-0.001270126	0.365085435	-0.003478984	0.997224178	0.999248121
751.0682539	-0.060770173	0.06854932	-0.886517521	0.375338717	0.786839429
317.1117085	0.089270703	0.080585637	1.10777436	0.267959276	0.710235608
93.85546603	0.086305101	0.136190831	0.633707135	0.526271968	0.864850467
1230.089148	0.429947419	0.258603201	1.662575782	0.096397382	0.505983706
544.9930922	0.008659307	0.101598787	0.085230416	0.93207821	0.984334593
150.3619156	-0.050991261	0.14670348	-0.347580444	0.728155295	0.934037323
768.8139687	0.081940373	0.080831995	1.01371212	0.310720139	0.745771331
134.3413188	-0.051742468	0.091614948	-0.56478194	0.572222093	0.885183781
13.57427991	-0.333592991	0.466543185	-0.715031323	0.474589688	0.841806659
211.3398448	-0.037102399	0.088958717	-0.417074354	0.676624023	0.921393988
334.9251627	0.060176679	0.075887397	0.792973281	0.42779339	0.817479701

60.0794975	0.016862796	0.206462272	0.08167495	0.934905199	0.984334593
63.71311856	-0.117737337	0.166106274	-0.708807282	0.478444078	0.844359716
150.6418231	-0.009088082	0.163155312	-0.055702031	0.955579182	0.987483032
175.2691842	0.05032074	0.111078179	0.453020931	0.650533669	0.910782566
195.9771804	-0.161216583	0.152161751	-1.059507937	0.289368517	0.730553093
871.815542	0.106097861	0.103956178	1.02060179	0.307443141	0.743405059
349.8302781	1.248628543	0.374677248	3.332544342	0.000860558	0.07116186
332.2914812	-0.128022281	0.116885699	-1.09527754	0.273395064	0.716229876
6.910701448	-0.728298974	0.344652632	-2.113139161	0.034588859	0.352363583
27.60874846	0.12416447	0.333663165	0.372125193	0.70979964	0.929034775
1255.118488	0.10444419	0.087949512	1.187547134	0.235011877	0.678363065
790.474896	0.074137004	0.11932817	0.621286694	0.534411007	0.867840168
4171.167481	-0.029512299	0.119471726	-0.247023289	0.8048902	0.957233889
191.3157534	0.028076424	0.086576136	0.324297501	0.745712812	0.940455922
950.2873098	-0.227682674	0.087531203	-2.60116012	0.009290908	0.208799287
101.1209726	0.060272197	0.110019914	0.547829887	0.583808715	0.889312594
367.8563606	-0.045378734	0.093298845	-0.486380449	0.62669745	0.904257122
12.41300936	0.303981871	0.323805993	0.938777778	0.347844851	0.768780165
188.6435886	-0.168953615	0.094605338	-1.785878244	0.074118974	0.468649747
29.60657904	-0.119184482	0.265555006	-0.448812787	0.653566713	0.911945899
357.3363168	0.158227867	0.157415609	1.005159955	0.314819834	0.748708739
9.745088449	0.107457357	0.326907845	0.328708406	0.7423761	0.940084783
114.2402899	0.000823724	0.112157353	0.007344363	0.994140099	0.998877139
1416.587229	0.232959999	0.110238459	2.113237074	0.034580482	0.352363583
95.00436999	0.180419612	0.143774127	1.254882335	0.209521477	0.654704857
252.4376826	0.132487907	0.063172365	2.097244689	0.03597192	0.35807422
1887.615436	0.066346916	0.085207959	0.778646936	0.436187723	0.822078703
284.404404	-0.152033756	0.129305741	-1.175769578	0.239686966	0.681681953
354.1990368	0.029516187	0.072195153	0.408838905	0.682657887	0.922776218
405.942298	0.136331389	0.086386962	1.578147743	0.114531672	0.535291597
495.6663048	-0.034560006	0.071843086	-0.481048456	0.63048206	0.905295366
6.530712201	-0.067302662	0.368746511	-0.18251742	0.855176685	0.969966486
15.09064623	-1.788836054	0.684103278	-2.614862566	0.008926337	0.20453194
50.41067429	-0.039884728	0.18358189	-0.21725851	0.828006889	0.964626741
670.198459	0.042568299	0.063654894	0.668735689	0.503664097	0.857416442
590.813984	-0.07314498	0.252878706	-0.289249267	0.772390633	0.948706053
828.9216013	-0.04256731	0.082832738	-0.513894757	0.607325573	0.900307811
43.18528787	0.452152369	0.507760647	0.890483285	0.37320644	0.786363234
164.4250727	-0.250546562	0.254758645	-0.983466377	0.325377956	0.756297513
194.4122003	-0.050291495	0.108100651	-0.465228422	0.641767887	0.908064184
6.912918588	0.217353198	0.328206261	0.662245738	0.507813746	0.859144938
43.24347513	-0.195952072	0.186283428	-1.051902868	0.292844116	0.733939814
6.030331685	-0.303626568	0.369126221	-0.822554864	0.410761175	0.808156644
40.44333614	0.282708221	0.219478617	1.28809004	0.197714622	0.643215458
75.57585664	0.049290431	0.126553126	0.389484102	0.696918069	0.92544754
215.2444165	-0.53499932	0.261239323	-2.047927985	0.040567056	0.373696391
1718.078371	0.004534049	0.098614736	0.045977401	0.963328262	0.989954388
250.0718235	0.042258059	0.067632458	0.624819218	0.532089716	0.866797125

12.01459695	-0.223330248	0.282771068	-0.789791718	0.429649416	0.818650305
765.7757174	0.040705024	0.115215231	0.353295515	0.723866908	0.931711128
22.27085425	-0.916846725	0.496313232	-1.847314689	0.064701545	0.446485908
71.46298014	-0.351910455	0.306081903	-1.149726432	0.250256564	0.694208707
9.991395199	-0.350241106	0.408346122	-0.857706459	0.391054573	0.794437976
17.89280254	-0.463045206	0.291824398	-1.586725475	0.112574834	0.533397149
25.89802125	0.16666091	0.193231625	0.862492932	0.388416321	0.792781092
118.929052	0.142018506	0.092935669	1.528137778	0.126478333	0.556378986
946.7333542	0.034623461	0.091042327	0.380300708	0.70372221	0.9269128
166.4889692	0.022075937	0.152793595	0.144482084	0.885119803	0.976723103
7.96714554	-0.368229377	0.44537574	-0.826783645	0.408359702	0.806126449
170.518866	0.212946003	0.095400104	2.232135962	0.025605978	0.314017971
432.5774153	0.672416815	0.297589023	2.259548443	0.02384929	0.307360833
146.2501199	0.091559773	0.087289897	1.048916041	0.294216762	0.734648534
699.8708033	-0.212613016	0.106786756	-1.991005475	0.046480285	0.393368315
8.411279659	-0.003096693	0.372945899	-0.008303331	0.993374977	0.998685608
754.3463221	0.113712778	0.053552988	2.12336943	0.033722907	0.34809377
14.78223406	0.366722929	0.239744766	1.529638941	0.126106125	0.555429245
628.7729083	0.129050545	0.054046474	2.387769925	0.01695095	0.265843192
392.6813089	0.050647782	0.069903011	0.724543642	0.468732045	0.838954848
317.149606	0.03820357	0.087141207	0.438409919	0.661089157	0.914896282
12.0857067	-0.795598658	0.439172849	-1.811584346	0.070050445	0.461281109
70.96094995	-0.055271135	0.306555111	-0.180297547	0.856918981	0.969966486
45.35079459	-0.11859542	0.198937974	-0.596142697	0.551079903	0.875900621
38.21437131	-0.115275504	0.287245996	-0.401312832	0.688189817	0.92352421
1051.987857	0.07419486	0.066496537	1.115770289	0.264520475	0.707695868
175.4244223	-0.18818779	0.105403134	-1.785409819	0.074194867	0.468839397
186.5024823	0.348632746	0.126391406	2.758358005	0.005809254	0.166644523
103.7912479	0.121651756	0.137319045	0.885905928	0.375668221	0.786839429
25.5661046	0.223364557	0.188472743	1.185129231	0.235966355	0.678901985
69.47605598	0.103037999	0.133519477	0.77170763	0.440287611	0.823778083
67.25294382	-0.156891634	0.174914968	-0.896959457	0.369740552	0.78374619
114.2959035	-0.001820817	0.150867533	-0.012068982	0.99037058	0.998194285
10.22900784	0.070654856	0.374836171	0.1884953	0.8504884	0.969251033
5.404563515	0.335847234	0.402358515	0.834696474	0.403888627	0.802866253
4.520245297	-0.883046769	0.624036422	-1.415056459	0.157051943	0.59632585
8.15601853	-0.187698378	0.427791855	-0.438760989	0.66083473	0.914896282
143.6321331	-0.00683162	0.152101062	-0.044915002	0.964175059	0.990149113
130.7287906	0.152592853	0.146874372	1.038934505	0.298835198	0.738522837
57.57245408	-0.126587423	0.145044586	-0.872748346	0.38280026	0.789880775
55.06533473	-0.055167918	0.152782904	-0.36108698	0.718034426	0.930765281
205.7889559	-0.273699564	0.127777831	-2.141995699	0.032193828	0.34013779
84.16071596	-0.676579057	0.330186789	-2.049079732	0.040454319	0.373696391
67.68962031	-0.247961789	0.181573202	-1.365629877	0.172055164	0.616109027
58.46335356	0.09178941	0.191365807	0.479654186	0.631473309	0.905723636
839.1875621	-0.008966917	0.083361562	-0.107566568	0.914339518	0.980657455
736.0315229	-0.219847171	0.059202335	-3.713488167	0.000204422	0.03056419
619.0116845	0.036750804	0.068426305	0.537085914	0.591208271	0.893201436

293.0743537	-0.805372132	0.551613311	-1.46003027	0.144281755	0.578069441
225.9121659	0.198539024	0.173168011	1.146510969	0.251583791	0.694735013
926.8464705	0.22847683	0.119645677	1.909612086	0.056183178	0.426254945
802.7676516	0.101842299	0.074866006	1.360327669	0.173726256	0.617663245
98.29112375	-0.272690281	0.154225909	-1.768122378	0.077040447	0.472391342
11.6442873	-1.456360127	0.68337253	-2.131136477	0.0330779	0.344317217
14.18971445	-0.017673428	0.249329814	-0.070883732	0.943490291	0.984737222
181.2724024	-0.087859858	0.097721698	-0.899082393	0.368608775	0.782820132
243.8198128	0.012242539	0.093994755	0.130247047	0.896370974	0.978829031
699.8952353	-0.241294393	0.152437275	-1.582909384	0.113442119	0.533883941
317.9042763	-0.069910196	0.096179149	-0.726874766	0.467302677	0.838019653
224.5173641	0.156226868	0.152880069	1.021891667	0.306832177	0.743405059
144.4303293	-0.055654172	0.078970495	-0.704746399	0.480968071	0.84566728
22.52957443	-0.616418266	0.241283127	-2.554750824	0.010626384	0.223743575
506.3428536	-0.140754607	0.152378405	-0.923717552	0.355633391	0.774130215
541.5392675	0.068406627	0.067011051	1.020826057	0.307336857	0.743405059
7.315654723	-0.322667981	0.562817644	-0.573308219	0.566436021	0.883595206
386.9310313	-0.826169252	0.369359242	-2.236763447	0.025301805	0.313430481
150.4105292	0.074960969	0.121778812	0.615550175	0.538191444	0.869675597
434.360599	0.013931964	0.095149377	0.146422015	0.883588249	0.976013852
499.3168511	0.160264481	0.099861638	1.604865325	0.108523446	0.52564018
415.4870605	0.089422535	0.130232018	0.686640169	0.492309509	0.851383417
186.7943683	-0.750629302	0.425235847	-1.765207018	0.07752898	0.472757172
1472.075866	-0.072390689	0.052581854	-1.37672379	0.168597661	0.610890818
40.96942757	0.02947092	0.177001921	0.166500567	0.867763037	0.972194102
9.704502209	-1.132972505	0.394324455	-2.873198681	0.004063385	0.144354659
7.961625457	-0.474102758	0.498528121	-0.951005044	0.341601816	0.765195212
41.54216986	0.149912793	0.299551596	0.500457333	0.616753092	0.901900555
80.25113759	-0.02722801	0.353850227	-0.076947838	0.938665041	0.984334593
89.33430855	-0.284191062	0.323596028	-0.878227905	0.379820051	0.787992438
185.9631993	-0.079534462	0.214545519	-0.370711363	0.710852526	0.929376205
94.84127804	0.174628249	0.310738385	0.561978364	0.574130761	0.885935354
147.8808644	-0.035303926	0.300689063	-0.117410075	0.9065351	0.979768942
152.1294852	0.023769156	0.315056078	0.075444206	0.939861288	0.984334593
15.98700522	-3.808296531	1.995468826	-1.908472075	0.056330229	0.426714458
4.581338177	-0.891107511	0.582114561	-1.530811238	0.125816051	0.555090669
292.1408289	0.348873252	0.220412667	1.582818518	0.113462835	0.533883941
31.33158839	-0.357667543	0.342527341	-1.044201442	0.2963922	0.735983449
168.4024824	-0.000653775	0.106665646	-0.006129199	0.995109637	0.998981303
140.581578	0.135553045	0.109567545	1.237164212	0.216026125	0.660326119
746.5996705	-0.073684885	0.127009197	-0.580153929	0.561810819	0.881435293
11.88052078	-0.16204899	0.301796758	-0.536947417	0.591303937	0.893201436
337.7657455	-0.09229387	0.093829541	-0.983633402	0.325295796	0.756297513
5.76175109	-0.503505129	0.513363123	-0.980797231	0.326692744	0.757203811
36.0563421	0.058732452	0.182162198	0.322418438	0.747135718	0.941343425
150.005971	-0.140717995	0.134423433	-1.046826376	0.295179663	0.735234129
3645.709483	0.06841374	0.220332602	0.310502119	0.756179149	0.944952618
2274.751031	0.980040501	0.633131893	1.547924709	0.121640425	0.547270993

11.24387983	-0.029252239	0.309439456	-0.094532996	0.924685772	0.982590996
38.18235359	0.654145507	0.428277785	1.527386032	0.126665048	0.556378986
45.0251725	0.114955395	0.152933198	0.751670641	0.45224915	0.829925399
70.08741905	-0.12023748	0.139890172	-0.85951342	0.390057319	0.793516828
555.2257822	-0.091098013	0.056946068	-1.59972439	0.109659739	0.528197178
86.99944574	-0.177147788	0.136828939	-1.294666095	0.195435441	0.640835592
397.3070505	-0.322011704	0.353281721	-0.911487023	0.36203882	0.778909683
152.6172328	0.243082181	0.287068325	0.846774652	0.397120739	0.798448952
72.58581351	-0.09142665	0.156419138	-0.584497854	0.558885409	0.880046199
744.0352936	-0.050945096	0.071827738	-0.709267711	0.478158362	0.844298425
200.6962382	0.00634206	0.094945617	0.066796764	0.9467435	0.985759593
54.40757851	0.136751417	0.262242903	0.521468514	0.602040434	0.899145969
42.959285	0.071818709	0.371995145	0.193063566	0.846909186	0.968775098
33.92280666	-0.273643294	0.310122881	-0.882370541	0.377576461	0.787072097
131.2628546	-0.470483156	0.330825969	-1.422146991	0.154983584	0.592842063
54.62156773	0.067467216	0.120477886	0.559996677	0.575481704	0.886202779
23.19247817	-0.470921137	0.21495665	-2.190772594	0.028468253	0.326184223
796.6721389	-0.07462612	0.077087596	-0.968069105	0.333009858	0.761183657
148.9633921	-0.016388391	0.188981212	-0.086719685	0.930894329	0.984334593
17.27155203	-0.274557746	0.423018997	-0.649043537	0.516310236	0.862002027
121.6024089	0.068242563	0.27325673	0.249737903	0.802790044	0.956857788
88.62489145	-0.238678996	0.212068688	-1.125479663	0.260385831	0.703096291
727.471442	-0.078993247	0.07089249	-1.114268185	0.265164152	0.708476493
658.5952153	-0.052903863	0.081802541	-0.646726407	0.517809029	0.862002027
62.21486152	0.088465075	0.188968262	0.468147793	0.639678903	0.907798398
140.1027212	0.120841617	0.124347329	0.971807096	0.331146525	0.759815544
10.59739217	-0.555554096	0.452319898	-1.228232713	0.219359622	0.663637842
2582.791895	-0.062147802	0.07845708	-0.792124843	0.428287886	0.81800223
2882.672319	0.013969091	0.070191426	0.199014201	0.842251636	0.967531499
6.778496409	-0.494900759	0.479681257	-1.031728366	0.302199385	0.740098494
6.159290883	-0.594215097	0.585576956	-1.014751504	0.310224296	0.745771331
7.998620245	-1.449946354	0.623959938	-2.32378117	0.020137225	0.282531954
5.845795233	-1.444709503	0.543512354	-2.658098739	0.007858287	0.19131309
7.785689486	0.365045836	0.357533765	1.021010801	0.307249322	0.743405059
81.86961003	-0.14133893	0.112287678	-1.258721641	0.208130892	0.652874199
11.92617188	-0.14735462	0.268627744	-0.548545799	0.583317191	0.88906731
866.5245706	0.028284557	0.055377812	0.510756136	0.609521827	0.900307811
630.2864719	-0.122951664	0.286697738	-0.42885467	0.66802899	0.917099343
12.2908521	-0.319025167	0.428578568	-0.744379655	0.456646824	0.832142746
4.443916986	0.89743197	0.559180357	1.604906108	0.108514469	0.52564018
188.18856	-0.22934061	0.317201535	-0.723012296	0.46967233	0.839786136
11.63739917	0.084134224	0.574033665	0.146566707	0.883474034	0.976013852
6.024992738	-0.264479418	0.70089262	-0.377346558	0.705916077	0.927395914
14.35300245	-0.971570229	0.53636394	-1.811401097	0.070078786	0.461281109
25.91965186	-0.484591139	0.282949059	-1.712644464	0.086777968	0.491199802
216.1830418	-0.074333015	0.101068857	-0.735469035	0.462053891	0.835391063
107.9789139	0.034107214	0.138876527	0.245593799	0.80599669	0.957416179
411.8073817	-0.125399172	0.111411922	-1.125545366	0.260358005	0.703096291

65.5878052	0.243594405	0.150164607	1.622182551	0.104764267	0.518635678
611.7654699	-0.007106219	0.065061295	-0.109223452	0.913025259	0.980530316
776.9029336	-0.130342989	0.255490846	-0.510166965	0.609934494	0.900307811
146.5825579	0.032179365	0.156844886	0.205166812	0.837441798	0.966384462
25.17383451	-0.423696787	0.317294298	-1.335343213	0.181764041	0.62744201
141.3232644	-0.066290095	0.21866554	-0.303157486	0.761769847	0.946826103
261.5130529	-0.084206635	0.094653678	-0.889628765	0.373665255	0.786373874
452.6012666	-0.111468288	0.11016892	-1.011794328	0.311636402	0.746032519
57.84730833	-0.339002967	0.212388064	-1.596148865	0.110455561	0.529628512
202.0535637	0.102704844	0.104609567	0.981792082	0.32620229	0.756809764
381.2285539	0.183271631	0.129838552	1.411534777	0.158086989	0.59773962
24.94987538	-0.010464876	0.192486828	-0.054366712	0.956643	0.987725711
991.9024739	-0.002734379	0.068366563	-0.039995849	0.968096436	0.991727471
39.3051572	-0.277252999	0.204659406	-1.3547044	0.175511755	0.619005807
349.1484519	-0.120793394	0.081930982	-1.474331078	0.140392471	0.574057835
19.85436977	-0.377878934	0.269628934	-1.401477686	0.161071276	0.600875279
99.19467898	-0.05937061	0.202242978	-0.293560798	0.769093535	0.947650255
182.6627934	0.099957395	0.244868525	0.408208424	0.683120664	0.922776218
44.55983415	0.264368049	0.384750256	0.687115978	0.492009646	0.851383417
5.502320021	0.060690912	0.554588329	0.109434168	0.912858134	0.980530316
35.9718244	-0.053514011	0.274539688	-0.194922677	0.845453481	0.968131661
8.607464648	-0.789298099	0.429659798	-1.837030373	0.066205412	0.4517497
108.1992542	0.082350412	0.101764978	0.809221536	0.41838773	0.811455684
148.4196124	0.032589973	0.081366109	0.400534987	0.688762519	0.923813534
282.4007297	0.10329658	0.0866783	1.191723657	0.233369619	0.676392617
5.644182572	0.196358613	0.436412184	0.449938432	0.652754835	0.911644397
24.30146138	-1.86728489	0.91829336	-2.033429589	0.042009141	0.376265422
25.44515988	0.276403192	0.309225961	0.893855067	0.371399441	0.785462856
278.6179553	-0.055808932	0.103544021	-0.538987484	0.58989549	0.892531233
68.96439936	-0.012547184	0.149339398	-0.084017912	0.93304219	0.984334593
72.31202412	-0.059904802	0.121865704	-0.491564075	0.623027545	0.902441895
5.486117231	-0.100338274	0.498047141	-0.201463407	0.84033624	0.966484002
6.760621013	0.636726324	0.523779497	1.215638121	0.224122806	0.667854242
127.4428055	0.030789944	0.158791563	0.193901639	0.8462529	0.968473437
7.152276418	-3.32518378	1.016922598	-3.269849433	0.001076047	0.07846135
27.29133958	0.017929026	0.270952984	0.066170249	0.947242282	0.985833846
357850.7545	0.580641516	0.279508454	2.077366562	0.03776774	0.363296636
15.78295824	-2.022126737	1.753697767	-1.153064556	0.248883888	0.692441932
25.92200493	-0.044235018	0.203892431	-0.216952723	0.828245189	0.964626741
1154.598535	-0.009432596	0.109259547	-0.086332007	0.931202495	0.984334593
19.69552445	-0.159992881	0.624818027	-0.256063165	0.797902064	0.954840537
7.164158057	0.008418785	0.412089118	0.020429525	0.983700731	0.995742202
11.003463	-0.046313924	0.373742998	-0.123919175	0.90137927	0.979381499
355.0736843	0.075904712	0.115458657	0.657418973	0.510911556	0.860200661
326.3711294	-0.288420545	0.085655737	-3.367206404	0.000759338	0.064596126
1405.097227	0.162031616	0.123306257	1.314058341	0.188826572	0.634652797
458.2705062	-0.134002916	0.212358522	-0.631022078	0.528026089	0.865762435
14.46881902	0.306024426	0.314805804	0.972105413	0.33099811	0.759815544

122.139712	0.174861338	0.118529544	1.475255306	0.140143918	0.573199853
85.08545689	-0.013615067	0.171431819	-0.079419717	0.936698786	0.984334593
676.8093864	0.007301796	0.122385793	0.059662121	0.952424741	0.98648845
295.6515913	-0.125272233	0.119923583	-1.044600491	0.296207652	0.73593065
912.0390216	0.165525387	0.155035165	1.067663502	0.285672328	0.726643643
2215.436888	0.005247601	0.118265039	0.044371533	0.964608253	0.990354707
50.35400153	-1.26530228	0.463608023	-2.729250181	0.006347853	0.174970531
104.2067259	-0.211710527	0.135110946	-1.566938381	0.117129095	0.539200101
5.466322091	0.233668653	0.395939235	0.590162914	0.555081433	0.877811471
447.6325477	0.060613101	0.059012184	1.027128578	0.304359924	0.741672489
158.6788898	0.025940032	0.093970415	0.276044669	0.782513758	0.95173434
7.051661049	0.096820469	0.420368035	0.2303231	0.817840712	0.961378511
257.5055986	-0.152880203	0.14760288	-1.035753521	0.300317146	0.739093677
29.98744555	-0.289496074	0.452898931	-0.639206794	0.522688413	0.863362704
223.0211893	-0.125640439	0.08702664	-1.443700905	0.148823127	0.586028179
11895.0641	0.043356825	0.079834343	0.543084883	0.587071361	0.890984353
421.9919211	-0.103206209	0.079548754	-1.29739567	0.194495082	0.640229004
812.5824381	0.122841423	0.101302826	1.212615952	0.225276687	0.668379539
544.3780791	0.15816823	0.125886016	1.256440031	0.208956475	0.653905655
2404.299462	0.055503042	0.108219115	0.512876516	0.608037702	0.900307811
367.3329718	0.022864874	0.069401915	0.32945596	0.741811076	0.939964277
5.577585978	0.14746238	0.387464518	0.380582926	0.703512753	0.9269128
133.1050021	0.196062321	0.13745339	1.42639131	0.153755421	0.59219701
569.8326903	-0.095305841	0.085855891	-1.110067572	0.26696991	0.709341177
237.5268125	-0.002194621	0.102595919	-0.021390918	0.982933819	0.995529925
302.3582278	0.005673578	0.089766264	0.063203898	0.94960414	0.986108475
111.3775385	0.213443266	0.117033723	1.823775751	0.068185981	0.456313112
400.3956517	-0.012112789	0.104089709	-0.116368743	0.907360307	0.979768942
391.947548	0.092229369	0.078043901	1.181762672	0.237299883	0.679753163
103.2589177	0.052708974	0.119896123	0.439622005	0.660210901	0.914896282
434.277153	0.084237978	0.069248179	1.216464884	0.22380788	0.667854242
10.81576311	-0.897893523	0.377092302	-2.381097459	0.017261145	0.267336898
53.10095413	-0.203855303	0.230829239	-0.883143327	0.377158837	0.787072097
646.2015409	-0.04657239	0.061172493	-0.761328951	0.446460613	0.826590213
1031.709937	-0.106592754	0.124878413	-0.853572299	0.393342013	0.796041628
41.18967689	-0.629437286	0.471202135	-1.335811616	0.181610855	0.62739604
196.2216395	-0.19411941	0.099352277	-1.953849637	0.050719002	0.409258179
589.7161945	-0.010362466	0.101740712	-0.10185171	0.91887438	0.981751016
167.4389878	-0.334431613	0.178693459	-1.871538081	0.061270537	0.437941099
94.3920124	0.077235329	0.11172841	0.691277433	0.489391211	0.849736271
918.3857901	0.102163449	0.171141662	0.596952537	0.55053907	0.875741796
166.657127	-0.131603388	0.094384206	-1.394336973	0.163215871	0.601694699
439.5480322	-0.104215156	0.067869146	-1.535530685	0.124653526	0.553093971
388.7512035	-0.146043232	0.153277985	-0.952799791	0.34069152	0.76465745
24.44649237	-0.046570739	0.413599412	-0.112598658	0.910348749	0.980530316
120.9738741	-0.209635754	0.144452448	-1.451244039	0.146711918	0.582320297
124.4326169	0.011450591	0.120710251	0.094860139	0.924425918	0.982590996
372.0602199	0.044935887	0.069922024	0.642657122	0.520446609	0.863188882

396.9110315	0.203781715	0.062354334	3.268124289	0.001082628	0.078554222
86.31030958	-0.167161219	0.110964137	-1.506443649	0.131953323	0.564647789
2318.369444	0.089124755	0.101180471	0.880849376	0.378399351	0.787072097
56.40276713	0.069117391	0.263872145	0.261935153	0.793371437	0.953153173
228.3943334	0.113648652	0.098639074	1.152166665	0.249252593	0.692982135
11.06644194	-0.18261482	0.421838797	-0.432901908	0.665086034	0.916049829
165.6120874	0.029995195	0.084251666	0.356019015	0.721826325	0.931135539
218.6146332	-0.461170463	0.209073477	-2.205781759	0.027399284	0.323603282
275.7273439	-0.006843872	0.089180531	-0.076741775	0.938828971	0.984334593
190.5491823	-0.069981828	0.070663926	-0.990347298	0.322004396	0.754640447
160.3959073	0.034285495	0.090584778	0.378490694	0.705066105	0.926977731
257.6652727	-0.079271763	0.088370675	-0.897036978	0.369699186	0.78374619
11.588231	0.099358339	0.300063017	0.331124909	0.740550137	0.939732486
6.461087215	0.079007765	0.436698184	0.180920754	0.856429778	0.969966486
52.77945642	-0.000677326	0.143572017	-0.004717671	0.996235857	0.998981303
4.54867933	-0.639798393	0.418853772	-1.527498223	0.126637169	0.556378986
14.42352073	0.335579858	0.343088689	0.978114024	0.328017932	0.758221793
900.2853203	0.066749715	0.158520636	0.421079026	0.673697376	0.920866983
4.81281869	-0.51250179	0.767634745	-0.667637562	0.504364975	0.857703709
144.2516759	0.021998594	0.133277201	0.165058943	0.868897588	0.972204994
4.841948322	-3.155036193	1.069388134	-2.95031906	0.003174459	0.132817478
8.153240065	-2.174684471	0.897460512	-2.423153379	0.015386429	0.258245094
5.989579258	-1.975548235	0.848871298	-2.327264734	0.01995118	0.28166787
21.79269967	0.139573432	0.711171955	0.196258347	0.844407962	0.968131661
4.999860304	-2.847816558	1.319429145	-2.158370208	0.03089906	0.334089034
117.5112937	-0.011568602	0.191144359	-0.060522854	0.951739214	0.98625342
105.2047756	0.097804577	0.137491512	0.711349928	0.47686742	0.843597766
3158.10536	-0.135556056	0.155237324	-0.873218193	0.38254416	0.789880775
278.6749162	-0.053463955	0.128700346	-0.41541423	0.677838691	0.921393988
105.5655237	-0.324474888	0.1593951	-2.03566413	0.041784093	0.375856173
157.3405822	0.122840319	0.17819214	0.689370019	0.490590445	0.850315507
929.1537347	0.032055687	0.103110122	0.310887875	0.755885863	0.944905206
272.4157423	0.147382207	0.070798157	2.081723762	0.037367709	0.361278136
1208.187968	0.013888673	0.119092483	0.116620904	0.907160472	0.979768942
497.1007263	0.093550731	0.078356482	1.193911831	0.232512459	0.675362916
1288.659145	0.054930155	0.089075256	0.616671316	0.537451544	0.869675597
7.626867153	-0.50950969	0.610534946	-0.834529937	0.403982425	0.802866253
19.66132626	-1.731406533	1.362695415	-1.270574858	0.203879936	0.64788124
34.40904043	0.387725476	0.21537348	1.800247069	0.071821635	0.463154629
6.492907385	-0.079800723	0.529786724	-0.150628016	0.88026916	0.975430592
40.98105188	0.098259956	0.189182854	0.519391442	0.603487798	0.899398548
40.09446369	0.24480966	0.145183843	1.686204573	0.091756403	0.499697676
42.43685505	0.118531258	0.149092622	0.795017598	0.426603265	0.816487562
52.40811624	0.079534748	0.145033678	0.548388132	0.583425424	0.88906731
54.62956919	-0.164427854	0.209298863	-0.785612742	0.432094397	0.81951323
351.3539829	-0.167798894	0.098401476	-1.705247733	0.088148231	0.492827855
152.3027168	0.010216884	0.138478545	0.073779546	0.941185803	0.984334593
29.44919296	-0.819336137	0.435495351	-1.881388942	0.05991903	0.435320275

704.7334704	-0.085947957	0.058323809	-1.473634149	0.140580122	0.574349147
355.2285719	-0.050706198	0.079263437	-0.639717371	0.52235636	0.863362704
8009.328945	-0.030324924	0.249362126	-0.121609984	0.903207906	0.979381499
251.5473734	-0.141743511	0.268342254	-0.528219125	0.597347257	0.896990756
455.525411	0.036972404	0.514394257	0.071875617	0.942700895	0.98446865
112.8110333	0.152768335	0.200441721	0.762158367	0.445965491	0.826302547
14.480648	-0.118687707	0.294238125	-0.403372972	0.686673867	0.923064029
122.250041	0.1005523	0.10373557	0.969313613	0.332388739	0.760742564
740.5602619	0.015124481	0.109214586	0.138484071	0.889857857	0.976961229
6842.144404	-0.039969502	0.129803953	-0.307922068	0.758141633	0.945887766
77.01624611	0.410093097	0.169211639	2.423551356	0.01536958	0.258245094
385.0260351	-0.124271246	0.201027633	-0.618179922	0.536456744	0.86935733
8.674845161	1.280007002	0.734736737	1.742130124	0.081485678	0.48207474
41.01092657	-2.101383379	0.791310496	-2.655573748	0.007917362	0.192434798
524.3670275	-0.058844884	0.069106979	-0.851504215	0.394489323	0.796838286
1175.498146	-0.286868748	0.106596432	-2.691166495	0.007120265	0.185553103
23.50304507	0.426922607	0.22344145	1.910668796	0.056047157	0.426020528
356.7934627	0.16736685	0.090424584	1.850899867	0.06418396	0.444988746
488.9979343	0.141604563	0.107411328	1.318339186	0.187390129	0.633746703
335.4872045	-0.18030851	0.106969282	-1.68561018	0.091870907	0.499719658
1379.033759	0.342732344	0.076684937	4.469356786	7.84552E-06	0.004406037
1157.092497	-0.080225366	0.111530745	-0.719311665	0.47194891	0.84064088
533.0916441	-0.034611532	0.053781069	-0.64356348	0.519858538	0.863188882
52.27283601	-0.187959485	0.17223851	-1.091274446	0.275152139	0.717295167
1123.846145	-0.126943792	0.121544312	-1.044423961	0.296289282	0.73593065
37.60338042	-0.03940662	0.242707413	-0.162362657	0.871020276	0.972881041
264.788181	-0.076359377	0.110487843	-0.691111118	0.489495714	0.849736271
66.11608513	-0.124492638	0.188505589	-0.660418818	0.5089851	0.859636085
343.0564073	-0.069058802	0.082191869	-0.84021452	0.400788119	0.801262941
303.4827259	-0.01231802	0.100858757	-0.122131391	0.902794962	0.979381499
163.3047645	-0.292891453	0.193987209	-1.50984931	0.131081879	0.563912594
158.3159611	-0.006768281	0.09992493	-0.067733661	0.945997654	0.98568891
666.2915804	-0.048382197	0.081672485	-0.592392855	0.55358755	0.877205564
4.542313926	0.17853353	0.457869992	0.389921885	0.696594311	0.925326625
265.2649064	-0.264871789	0.137814243	-1.921947859	0.054612319	0.422126133
22.02103862	-0.209541757	0.435292875	-0.481381087	0.630245676	0.905102988
18.40501007	-0.181084693	0.339534018	-0.53333299	0.593803095	0.894602891
5.602081327	-0.375921272	0.459857214	-0.817473904	0.413657657	0.808311635
2253.730231	-0.206879692	0.413558186	-0.500243252	0.616903806	0.901900555
309.4736917	0.058451823	0.082243994	0.710712355	0.477262502	0.843615901
1381.570538	0.149136144	0.106904752	1.395037558	0.163004514	0.601684318
39.41537314	0.05922263	0.531963753	0.111328318	0.911356001	0.980530316
114.8244587	0.595692652	0.426569723	1.396471948	0.162572422	0.601684318
118.0845317	-0.332121632	0.122796742	-2.704645299	0.006837736	0.181059331
59.58173192	-0.451279332	0.330172252	-1.366799691	0.1716881	0.616109027
2378.066169	0.133053722	0.131578313	1.011213158	0.31191442	0.746220174
44.60860564	0.153078974	0.177161577	0.864064188	0.387552631	0.792728629
238.3285076	0.065919165	0.111646769	0.590426089	0.554905024	0.877791979

4.813009887	0.349366325	0.417018891	0.837770981	0.40215934	0.801659902
2436.02973	0.104184661	0.100001842	1.041827419	0.2974917	0.737229556
553.5471909	0.237602603	0.12397918	1.916471809	0.055305068	0.423973339
53.0549595	0.701016349	0.903786438	0.775643802	0.43795933	0.82251807
1495.192083	0.052173203	0.096982381	0.537965784	0.590600671	0.892788725
166.8582258	0.190544546	0.332261872	0.573477014	0.566321758	0.883595206
381.4760446	0.003635626	0.065179695	0.055778499	0.955518263	0.987483032
569.213618	-0.013632773	0.062016213	-0.219825946	0.826006712	0.964118059
687.9282806	0.180326276	0.085373225	2.112211122	0.034668345	0.352445634
496.9583582	0.100059878	0.094783024	1.05567299	0.291117641	0.732859559
2948.683839	0.107876672	0.105732451	1.020279681	0.307595837	0.743405059
197.8507213	-0.102855101	0.081533543	-1.261506583	0.20712639	0.651884304
23.47354976	-0.558427213	0.269786039	-2.069889221	0.03846272	0.366996019
123.3737377	-0.217505976	0.264893231	-0.821108095	0.411584699	0.808208639
2025.405058	0.212728242	0.143954412	1.477747291	0.139475431	0.572130062
7.721710854	0.129925115	0.951707294	0.136517935	0.891411847	0.977283948
1040.586236	0.063207239	0.100621036	0.62817122	0.529891782	0.866123834
30.87278757	0.016463274	0.244231162	0.067408573	0.946256444	0.985728152
45.75846354	-0.059689642	0.198136368	-0.301255358	0.76321978	0.946826103
221.9749212	0.179855166	0.115284378	1.560100068	0.118736235	0.542464536
19.07707905	0.06976183	0.274179253	0.254438762	0.799156608	0.955167977
6.0702888	0.12208762	0.373397357	0.326964337	0.743694859	0.940317814
47.47729997	0.1110042	0.283513332	0.391530795	0.695404931	0.924581226
194.3389687	0.01978833	0.214431984	0.092282548	0.926473554	0.983240215
1550.065882	0.200744436	0.153353753	1.309028519	0.19052469	0.636683496
28500.3199	0.160262427	0.116435398	1.376406393	0.16869585	0.610970386
93.87757386	-0.283428884	0.291394725	-0.972663057	0.330720792	0.759672433
260.7950796	0.187226451	0.253654474	0.738116102	0.4604439	0.834673974
391.0533022	0.151720658	0.090395814	1.678403577	0.09326834	0.500506422
4.531723062	-2.057559851	0.932898552	-2.205555841	0.027415114	0.323603282
12.96110034	-0.596514552	0.315362322	-1.891521307	0.05855479	0.433061669
445.2842414	0.154135839	0.062174123	2.479099523	0.013171455	0.24658174
69.33965691	-0.12435288	0.345693165	-0.359720391	0.719056242	0.931011662
1778.949258	-0.008249883	0.166867067	-0.049439848	0.960568773	0.989126211
391.6420538	-0.00605205	0.095265954	-0.063527944	0.949346108	0.986108475
142.2214581	-0.129338523	0.317957504	-0.406779274	0.68417011	0.922907843
329.5895325	0.099167882	0.095616397	1.037143056	0.299669192	0.738923503
148.6036042	0.032859086	0.196106527	0.167557327	0.866931544	0.972138679
1146.830853	0.474767757	0.24024363	1.976192908	0.048132933	0.399398071
335.507259	0.111902369	0.115087032	0.972328224	0.330887287	0.759701198
139.9825334	0.198862803	0.090888541	2.187985421	0.028670664	0.327277249
822.1699197	0.050173741	0.092298648	0.543602121	0.5867153	0.890984353
80.94413447	0.011920882	0.117821991	0.101177058	0.919409909	0.981892218
16.05658593	0.6000565	0.478293898	1.25457695	0.209632375	0.654774936
273.0262749	-0.046002121	0.072166507	-0.637444201	0.523835545	0.863484457
3631.60689	0.044700617	0.036358385	1.229444542	0.218905179	0.663574535
156.6284808	0.018795987	0.113270283	0.165939263	0.868204748	0.972204994
595.9057937	0.241607475	0.14226901	1.69824388	0.089461742	0.494293655

927.9916231	-0.088558527	0.126363242	-0.700825066	0.483412193	0.847804181
408.7386573	0.109623846	0.10429779	1.051065852	0.293228346	0.733939814
1619.094562	-0.0534772	0.149580064	-0.357515556	0.720705883	0.931135539
776.9037632	-0.012318397	0.097563322	-0.126260532	0.899525697	0.978996707
64.37149867	-0.47735068	0.269866966	-1.768837023	0.076921076	0.472050486
175.243883	-0.085765602	0.129500591	-0.662279618	0.507792037	0.859144938
374.9971548	-0.04283761	0.07746408	-0.552999658	0.580263644	0.887483205
166.9332242	-0.031141996	0.09712709	-0.320631408	0.748489733	0.941636803
36.20340336	-0.206036543	0.314849209	-0.654397524	0.512855729	0.860307567
113.7031574	-0.190649343	0.132757923	-1.436067534	0.150983125	0.589528489
43.70551466	-0.10479908	0.173873952	-0.602730188	0.546688196	0.873402987
366.6317997	0.047158162	0.064315991	0.733226079	0.463420552	0.836122746
178.7159588	-0.040026795	0.092617533	-0.432172982	0.665615694	0.916080289
357.3298671	0.028732479	0.123377321	0.232882985	0.815852271	0.961254901
41.8255983	0.231379214	0.288312993	0.802527877	0.422247669	0.81434658
422.6305475	-0.073656055	0.108735989	-0.677384331	0.498162138	0.855428766
100.3151208	-0.073456534	0.11586524	-0.633982499	0.526092244	0.864850467
65.97904961	-0.294733883	0.400084814	-0.736678505	0.461317882	0.835033586
2917.851445	0.265690515	0.168720433	1.574738227	0.11531688	0.536514073
322.6654159	0.019093238	0.064959535	0.293925106	0.768815133	0.947650255
55.96816952	-0.483736768	0.273943581	-1.765826259	0.077425002	0.472757172
329.9100353	0.105730332	0.096501767	1.095631045	0.27324027	0.716221441
41.48511348	-0.18887379	0.173371876	-1.089414239	0.275971255	0.718081375
245.3878933	0.105479822	0.070318893	1.500021072	0.133608944	0.566832787
30.95963527	-0.399390385	0.184570216	-2.163894013	0.030472485	0.332880971
657.2529257	0.103444291	0.073826325	1.401184341	0.161158956	0.600875279
7.423998413	-0.623326238	0.623692571	-0.999412638	0.31759484	0.751021269
432.8819712	0.003785025	0.069004164	0.054852124	0.956256274	0.987615501
10.8607838	0.020566845	0.318139002	0.064647354	0.948454781	0.985917197
4.896206657	0.129857934	0.578544829	0.22445613	0.822402399	0.963169453
44.99185627	0.146516337	0.216367482	0.677164309	0.49830171	0.855506503
639.7414628	0.263356869	0.159243726	1.653797458	0.098168673	0.509712212
1315.039194	-0.232062893	0.167899978	-1.382149634	0.166925761	0.608828473
115.6948868	-0.089231323	0.134671388	-0.662585603	0.507595993	0.859144938
718.5174554	0.042825392	0.083032362	0.515767473	0.606016823	0.900307811
158.4794314	-0.165395247	0.118328437	-1.397764156	0.162183902	0.601569453
5.395415991	-0.120956574	0.652397508	-0.185403183	0.852912812	0.969966486
440.622195	-0.0340949	0.262100223	-0.130083443	0.896500409	0.978829031
351.3432983	0.111667496	0.060105353	1.857862732	0.06318851	0.443016545
86.21100045	-0.554246588	0.22658886	-2.446045181	0.01444329	0.254511403
26.47642872	-0.139598936	0.174012098	-0.802236959	0.422415901	0.814538241
1587.489417	-0.042376709	0.127842665	-0.331475484	0.740285357	0.939732486
22.24005228	-0.505434255	0.287678608	-1.756940698	0.078927918	0.475882296
1624.312257	-0.077270447	0.139189813	-0.555144413	0.578795887	0.887274009
4.459493858	-0.867071411	0.602835	-1.438322942	0.150342445	0.588721925
263.0591995	0.01295369	0.103592221	0.125045005	0.900487921	0.979139221
21.94055832	-0.012974555	0.307956586	-0.042131118	0.966394174	0.990895439
108.4040346	-0.580270381	0.563241196	-1.030234267	0.302900048	0.740555303

96.66836459	-0.02257587	0.133088814	-0.169630112	0.86530104	0.971917805
577.4286973	0.054491751	0.100815865	0.540507701	0.588846951	0.891897927
9.556861665	0.434799193	0.369044066	1.1781769	0.23872609	0.681087839
2870.477741	-0.18884336	0.094830736	-1.991372925	0.046439904	0.393251403
1992.719466	0.118214379	0.096316173	1.227357516	0.219688247	0.663637842
10.73811407	0.153221764	0.339756526	0.45097519	0.65200743	0.911246717
212.2841211	0.22445385	0.103539791	2.167802807	0.030173693	0.332282647
348.2676252	-0.006654093	0.087367267	-0.076162311	0.939289967	0.984334593
327.1985165	-0.044166588	0.089760404	-0.492049788	0.622684147	0.902441895
42.824964	-0.094883898	0.168308048	-0.563751404	0.572923329	0.885236101
298.4003481	0.000613218	0.109982866	0.005575575	0.995551358	0.998981303
1325.221365	0.168857557	0.070169576	2.406421232	0.016109678	0.262858884
229.8378993	-0.085415925	0.102906106	-0.830037479	0.406517594	0.804663634
181.0810672	-0.017052368	0.123822272	-0.137716481	0.890464494	0.976961229
653.7254343	0.130565172	0.076479266	1.707196979	0.087785446	0.492827855
345.0653361	-0.118400114	0.144626374	-0.818661981	0.412979294	0.808272153
135.0062084	-0.007859479	0.239581251	-0.032805068	0.973830037	0.993248159
91.75261585	0.091056077	0.184887537	0.492494402	0.622369878	0.902441895
7.453604817	-0.988160425	0.58745886	-1.682092982	0.092550811	0.499977045
136.4388422	0.05263535	0.096779311	0.543869861	0.58653103	0.890984353
7.156099462	-0.331560771	0.419321502	-0.790707772	0.42911454	0.818630419
25.08495289	0.185263052	0.209722663	0.883371636	0.377035511	0.787072097
137.9122739	0.340323966	0.120480017	2.824733712	0.004731992	0.15195001
13.28278775	-0.061642133	0.266340376	-0.231441189	0.816972069	0.961335246
725.5899819	0.08989094	0.111033299	0.809585422	0.41817849	0.811361647
19.99871307	-0.449058196	0.321843635	-1.395268224	0.16293497	0.601684318
48.28116533	0.370834915	0.306715147	1.209053152	0.226642426	0.669424857
9.44968252	-0.027377788	0.335468271	-0.081610662	0.934956322	0.984334593
236.6646242	0.179934076	0.078866449	2.281503454	0.022518673	0.300162997
572.1430175	0.102468372	0.112192671	0.913325003	0.361071636	0.777983744
6893.490361	-0.021299107	0.111058396	-0.19178295	0.847912227	0.968938143
162.7284628	0.037075576	0.123910893	0.299211595	0.764778603	0.946826103
23.80804688	0.19813543	0.223509792	0.88647315	0.375362617	0.786839429
864.273661	0.069049735	0.063222844	1.09216434	0.274760873	0.716878153
493.7228552	-0.024387352	0.07535306	-0.323641159	0.746209723	0.940513992
2342.272958	0.05331681	0.066720227	0.799110134	0.424226554	0.815162241
429.0132513	0.231845728	0.069814161	3.32089829	0.000897282	0.072975674
842.6228052	0.221999036	0.090571151	2.451100963	0.014242	0.251563345
14.21284413	-1.103209846	0.653164831	-1.689022119	0.091215191	0.498962299
126.6327319	-0.440470965	0.174717045	-2.5210532	0.011700416	0.234397729
621.4628134	0.070348261	0.0663149	1.060821353	0.288771096	0.729902555
92.92043232	-0.383713164	0.141951408	-2.703130376	0.00686898	0.181431117
1434.730417	0.025243678	0.069542005	0.362998999	0.716605632	0.930292629
213.4052303	0.114910738	0.27343062	0.420255559	0.674298771	0.921149714
152.8137181	0.272397135	0.105284367	2.587251479	0.009674496	0.213098041
537.1813902	0.096658998	0.065261693	1.481098535	0.138580318	0.570638112
425.353135	0.081113283	0.078339193	1.035411271	0.300476884	0.739093677
36.59620017	0.232221333	0.192326711	1.207431522	0.227266002	0.669850929

258.466834	-0.009464044	0.104217828	-0.090810221	0.927643388	0.983664835
54.14514283	0.175214618	0.145365098	1.205341726	0.228071408	0.670888907
16.48218982	-0.059876383	0.389038974	-0.153908443	0.877681927	0.97425769
1704.437898	0.367967675	0.247542733	1.486481424	0.137151827	0.569036171
61.76368595	0.171866022	0.258032301	0.666063983	0.505370205	0.858594831
24.2196106	-0.136172612	0.354316899	-0.384324351	0.700738062	0.926034765
52.2950179	-0.064851448	0.242466879	-0.26746518	0.78911102	0.953026618
5.073624961	-0.209777353	0.573384438	-0.365858121	0.714470948	0.930012765
86.78805408	-0.028501538	0.243606319	-0.116998351	0.90686136	0.979768942
55.57354491	0.212471646	0.152059691	1.397291055	0.162326065	0.601589987
1742.325413	0.222387241	0.095979858	2.317019906	0.020502647	0.285762888
143.39787	-0.104942541	0.109246655	-0.960601865	0.336752391	0.763456714
1030.077527	0.101361424	0.243889573	0.415603763	0.677699973	0.921393988
22.60966578	-0.211643629	0.237613448	-0.8907056	0.37308713	0.786266676
96.23601416	-0.135756928	0.163016275	-0.832781441	0.404968003	0.803530347
41.94136028	-0.191419043	0.221892484	-0.862665736	0.388321276	0.792781092
16.50972258	-0.059332467	0.268025054	-0.221369109	0.824805043	0.963824445
495.9592007	0.01165601	0.099511345	0.117132471	0.906755079	0.979768942
289.7587483	0.133346557	0.135920955	0.981059598	0.326563352	0.757054149
505.2836955	0.104495152	0.096828945	1.07917268	0.280510756	0.722218627
250.8843685	-0.012430717	0.108045995	-0.115050233	0.908405306	0.980020248
93.0792704	0.004831612	0.105821591	0.045658097	0.963582763	0.990001531
38.08159656	-0.470046019	0.294395209	-1.596649688	0.110343816	0.529498712
710.5696065	0.120169511	0.223382327	0.537954427	0.590608512	0.892788725
23.0381522	-1.344453094	0.4817542	-2.790744939	0.005258689	0.158649215
4.817345393	-2.080944233	0.635591915	-3.27402565	0.00106027	0.078080172
8.943766121	-4.076123399	1.455675673	-2.800159043	0.005107743	0.157182574
15.35943898	-1.78478853	0.74959937	-2.380989902	0.017266185	0.267336898
69.98149161	0.036402818	0.337849795	0.107748529	0.914195173	0.980657455
781.0626713	0.047022346	0.057784478	0.813753928	0.415785933	0.81011206
32.65068023	-0.126957841	0.270543078	-0.469270334	0.638876417	0.907684986
67.8500865	-0.043818772	0.143490805	-0.305376864	0.760079141	0.946795592
177.5832969	0.137235659	0.192506734	0.712887573	0.47591533	0.843248589
7.14129154	-0.463608164	0.369907922	-1.253306937	0.210094025	0.655249
1514.03379	0.15982445	0.130486391	1.224836158	0.220636959	0.664764193
5.285795267	0.130883599	0.411845977	0.317797444	0.750638589	0.942359611
634.3262402	0.315414156	0.154014797	2.047947096	0.040565183	0.373696391
405.8071796	0.112625683	0.063195938	1.782166474	0.074722088	0.470687737
97.70923283	0.116907568	0.135263276	0.864296445	0.387425063	0.792728629
483.426379	0.098498429	0.10375252	0.949359391	0.342437857	0.765905886
345.7062174	0.068917946	0.068365873	1.00807527	0.313418329	0.747296731
1243.811541	0.063161956	0.066736646	0.946435875	0.343926315	0.767121103
30.38185803	-0.017003334	0.188038634	-0.090424684	0.927949742	0.983919204
341.9611857	0.069247789	0.1277575	0.542025235	0.587801122	0.891256285
66.47338084	-0.095396505	0.23784608	-0.401085041	0.688357514	0.923592089
349.4348203	-0.035108793	0.065209495	-0.538400007	0.590300922	0.892788725
118.7806936	-0.144009995	0.123492676	-1.166141994	0.24355704	0.686012467
1047.995512	0.098248083	0.153204194	0.641288466	0.521335281	0.863328113

114.089874	0.049084606	0.125135332	0.392252171	0.694871899	0.924581226
117.4985745	0.09040253	0.10146794	0.890946735	0.372957746	0.786266676
11.86806233	0.086876052	0.451224682	0.192533909	0.847324009	0.96887524
326.5698295	0.035244711	0.0894145	0.39417221	0.693453901	0.924581226
717.3041237	0.150613284	0.087985987	1.711787166	0.086935898	0.491556286
229.2260756	-0.140307291	0.094658156	-1.48225253	0.138273114	0.569957848
247.1950155	0.074542063	0.093538743	0.796911112	0.425502657	0.816273895
78.63276059	-0.039693036	0.108365652	-0.366287982	0.714150197	0.930012765
807.9983235	0.083712473	0.046389205	1.804567952	0.071142317	0.462488289
82.13107407	-0.031148941	0.129915613	-0.239762873	0.81051409	0.959016558
269.1693995	0.02608247	0.151245184	0.172451577	0.863082527	0.971275942
122.6925683	0.493215866	0.237820183	2.073902474	0.038088369	0.364672731
20.37063664	0.218375268	0.291473738	0.749210785	0.45373017	0.831004392
604.2353405	0.23810628	0.077593816	3.068624421	0.002150468	0.110910179
456.0211815	0.198685351	0.076016995	2.613696451	0.008956857	0.204686499
188.9617131	0.09156722	0.078824159	1.161664416	0.245371805	0.688399064
64.38442087	0.291309086	0.146951078	1.982354191	0.047439613	0.397042795
6552.47991	0.103411351	0.123321126	0.838553416	0.401719961	0.801291311
282.6350721	-0.064796208	0.213336735	-0.303727382	0.761335595	0.946826103
5.506748182	0.890772958	0.629999226	1.413927069	0.157383317	0.597024055
317.3268516	0.048297467	0.056200028	0.85938511	0.390128082	0.79355172
12.87336047	-1.321840383	0.473631382	-2.790863178	0.005256769	0.158649215
47.19808689	-0.454389618	0.233457003	-1.946352482	0.051612413	0.410734915
35.54902688	0.089239929	0.187962449	0.474775305	0.634947136	0.90701218
47.14012345	-0.339948384	0.14814189	-2.294748525	0.02174754	0.295327604
13.95916578	0.337790514	0.304278605	1.110135609	0.266940595	0.709341177
1333.563406	0.069200426	0.051694063	1.338653267	0.180683577	0.626148701
188.8154509	0.034269538	0.081124106	0.422433481	0.672708644	0.920302871
1731.424083	-0.087843313	0.101667716	-0.864023672	0.387574887	0.792728629
50.16926739	0.054765339	0.179778249	0.30462717	0.760650124	0.946826103
193.2830306	0.051094403	0.13157384	0.388332535	0.697769963	0.925648951
9.165405757	0.180050049	0.316655061	0.568599942	0.569627664	0.8841274
5.388394979	-0.306876001	0.617647052	-0.496846865	0.619297044	0.901976704
298.0793578	-0.034226628	0.059577268	-0.574491392	0.565635324	0.883265494
26.68197898	-0.42975252	0.349144789	-1.23087193	0.218370768	0.662774346
251.8232301	-0.037927191	0.065400819	-0.579919206	0.561969103	0.881435293
571.3654439	0.027609842	0.069803299	0.395537777	0.692446048	0.924581226
935.4934834	-0.065534737	0.038543965	-1.700259367	0.08908215	0.493693668
7.962174463	-0.256115812	0.368494249	-0.695033403	0.487034369	0.849484378
200.5632197	-1.122085115	0.462355329	-2.426889114	0.01522891	0.257947316
1529.172638	0.16297251	0.064106508	2.54221474	0.011015247	0.227085924
5253.252031	-0.02258825	0.123318474	-0.183170041	0.8546646	0.969966486
1028.764459	0.090492159	0.071264805	1.269801532	0.204155335	0.648477955
50.18173636	0.228280183	0.14591403	1.564484123	0.117703919	0.539880626
255.7147218	-0.102674262	0.091646953	-1.120323793	0.262575807	0.705380597
35.49664286	-0.181134477	0.213012371	-0.850347221	0.395132071	0.797298333
26.2724908	-0.090936991	0.322916662	-0.281611331	0.778241551	0.951560132
988.8373101	0.646826064	0.211239646	3.062048603	0.002198278	0.111533785

1031.403061	0.062697666	0.073627158	0.851556249	0.394460431	0.796838286
940.7749753	0.063895684	0.067700375	0.94380103	0.345271334	0.767851822
34.70304358	-0.11999142	0.242430803	-0.494951214	0.620634559	0.901976704
8.953251282	-0.085658684	0.319761114	-0.267883368	0.788789097	0.953026618
1329.137913	-0.187507401	0.100878872	-1.858738092	0.063064271	0.442826065
29.60177302	0.063953616	0.173374586	0.368875377	0.712220617	0.929589333
453.5204128	0.014154998	0.072156358	0.196171182	0.844476183	0.968131661
43.22648156	-0.021664692	0.200075859	-0.10828239	0.913771692	0.980550322
49.99706235	0.509844464	0.376547746	1.353996854	0.175737381	0.619005807
449.7687156	0.119033004	0.05280966	2.254000584	0.024196131	0.308436782
404.8259818	-0.218902181	0.106767293	-2.050273775	0.040337722	0.373696391
9.342841897	0.275280584	0.487816843	0.564311355	0.572542255	0.885183781
282.7470935	-0.057554145	0.070076581	-0.821303548	0.411473387	0.808165706
143.9170898	-0.181490237	0.12960254	-1.40036018	0.161405488	0.601071304
621.8037079	0.069453431	0.099573506	0.69750915	0.485484215	0.848867592
444.5737588	-0.000232593	0.065782623	-0.003535778	0.997178863	0.999248121
104.6337864	0.145083249	0.216384879	0.670487003	0.502547389	0.857172233
1133.008837	0.089079401	0.051508565	1.729409477	0.083735835	0.486282844
63.47645531	0.051530853	0.130453734	0.395012479	0.692833679	0.924581226
262.3485857	0.089630338	0.08928121	1.003910438	0.315421785	0.749417858
165.7324174	-0.00129042	0.121403486	-0.010629187	0.991519296	0.998263407
612.0831168	0.025645613	0.07040593	0.364253596	0.715668648	0.930241908
46.41242436	0.437201589	0.482305715	0.906482291	0.364680634	0.779728871
96.00400571	-0.205378558	0.106406743	-1.930127283	0.053591069	0.417128004
1360.734339	-0.154524337	0.115090754	-1.342630329	0.179391705	0.624452051
539.3024497	0.137331545	0.070026502	1.961136742	0.049863074	0.405560777
107.7114025	-0.048935149	0.107193187	-0.456513618	0.648020681	0.910701841
6.869383509	-0.084201397	0.444158198	-0.189575239	0.849641992	0.969251033
277.4997078	-0.072591923	0.066813765	-1.086481548	0.277266	0.719218718
198.5818149	-0.026820252	0.083849129	-0.319863215	0.749072024	0.941640077
400.2239123	-0.085307615	0.085463498	-0.998176019	0.318194013	0.751465423
302.7429081	0.025967201	0.369477362	0.070280898	0.943970086	0.984843572
13.27529979	0.633416344	0.605213345	1.046600095	0.295284058	0.735234129
7.873637199	0.467300114	0.429975859	1.086805466	0.277122791	0.719218718
4.806194426	0.229888508	0.570874041	0.402695677	0.687172115	0.923064029
5.355619032	-0.226508576	0.692958712	-0.326871676	0.743764945	0.940317814
9.291469069	0.585020697	0.611073463	0.957365574	0.338382768	0.764007173
8.070652642	0.199651095	0.388869641	0.513413941	0.607661796	0.900307811
6.244586682	0.735134332	0.408661817	1.798881866	0.07203737	0.463154629
16.5942015	-0.046218703	0.295942475	-0.156174619	0.875895382	0.973587929
6.938050896	-0.354101011	0.452415203	-0.782690344	0.433808974	0.821182113
24.90636701	0.303999827	0.286694269	1.060362415	0.288979754	0.729902555
25.07230948	0.230579621	0.286158466	0.805775989	0.420372025	0.812422864
15.75474036	0.003710265	0.248733727	0.014916614	0.988098705	0.997338733
12.95171382	0.127517575	0.307118346	0.415206635	0.677990642	0.92145969
14.09055591	0.023895304	0.254493684	0.093893505	0.925193754	0.98261591
6.496572134	-0.273450681	0.475409314	-0.575189995	0.565162808	0.883265494
15.26558344	-0.091458033	0.292632343	-0.312535627	0.754633491	0.944376474

5.977332914	0.325505975	0.372425311	0.874016792	0.382109109	0.789880775
114.7668045	-0.100062397	0.119876166	-0.834714692	0.403878367	0.802866253
25.1598478	0.339985363	0.18037762	1.884853362	0.059449638	0.435199577
40.60477287	0.302095429	0.147943995	2.04195803	0.041155695	0.374892505
50.39926263	0.382410888	0.169624757	2.254452095	0.024167741	0.308436782
37.07705764	0.125221048	0.181104101	0.691431324	0.489294525	0.849736271
48.809194	0.326332337	0.156049839	2.091205852	0.036509615	0.360198137
89.99117018	0.351513173	0.168741483	2.083146171	0.037237902	0.361202768
64.18161715	0.197701772	0.138201824	1.430529394	0.152565136	0.589934467
66.93707326	0.153786084	0.13149242	1.169543342	0.242184789	0.685171875
64.84127137	0.478832771	0.159386773	3.004219004	0.002662636	0.122019616
76.20589037	0.325353228	0.129759669	2.507352489	0.012163934	0.236908623
76.21485197	0.225996731	0.14259961	1.584834151	0.113004021	0.533883941
72.41221014	0.173849907	0.115490787	1.505314071	0.132243349	0.564647789
68.85860578	0.219515174	0.140133236	1.566474737	0.117237518	0.539200101
98.76665788	0.242299678	0.115640274	2.095288001	0.036145398	0.359076636
80.68194853	0.398103041	0.119822794	3.322431624	0.000892365	0.072975674
82.20766628	0.17333565	0.126104158	1.374543498	0.169273016	0.611731367
72.55815499	0.200969054	0.152357223	1.319064826	0.187147441	0.633489517
54.15500294	0.289621211	0.171580723	1.687958914	0.091419116	0.498962299
69.1771857	0.076148817	0.134472469	0.566278125	0.571204728	0.884717095
374.5847449	-0.068779673	0.143621449	-0.478895552	0.632012935	0.906005577
35.55627661	0.070224159	0.1868325	0.375866931	0.707015826	0.928109779
23.88714927	0.082605442	0.216792208	0.381035108	0.703177198	0.926841396
2044.63876	-0.019680429	0.126962791	-0.155009424	0.876813888	0.973973449
582.2312411	0.008468798	0.076209181	0.111125691	0.911516677	0.980530316
21.60871403	0.047428662	0.259777897	0.182573894	0.85513237	0.969966486
253.9666271	0.058704405	0.095724196	0.613266103	0.539700403	0.869675597
158.0244545	-0.029708524	0.186156442	-0.159589019	0.873204829	0.97300406
16.37231942	-0.303183454	0.231364069	-1.310417193	0.19005474	0.636043561
852.5119366	-0.034999502	0.098058778	-0.356923705	0.721148923	0.931135539
64.97342185	-0.158772095	0.15543785	-1.021450664	0.307040972	0.743405059
1011.801385	0.059667354	0.098551837	0.605441321	0.544885803	0.872850885
109.8539035	0.277920685	0.144442928	1.924086486	0.054343744	0.420709255
531.3441884	0.040402063	0.079976617	0.505173447	0.613437018	0.901444946
108.77922	-0.110143594	0.285031658	-0.386425827	0.699181325	0.925900484
451.4919473	0.012273634	0.230702657	0.053201093	0.957571685	0.987864832
10.42082642	0.255278933	0.389923126	0.654690414	0.512667098	0.860200661
258.9689487	0.547594423	0.226493613	2.417703599	0.015618794	0.259816378
2172.815715	0.087821356	0.124293588	0.70656385	0.479837561	0.845119155
160.1357526	0.151238741	0.094975769	1.592392905	0.111296449	0.531325251
6.124592832	0.208990744	0.476344983	0.438738208	0.660851239	0.914896282
7.271196819	-0.041631378	0.356454128	-0.116793086	0.907024023	0.979768942
605.7506019	0.276286661	0.136388949	2.025726149	0.042792855	0.379065918
906.6636402	-0.04252826	0.062483674	-0.680629941	0.496105676	0.854102145
4.942619404	0.679700948	0.476952266	1.425092186	0.154130555	0.59219701
406.7508503	0.163577119	0.064245721	2.546116935	0.010892869	0.226714336
445.4022356	0.126575213	0.063410144	1.996135081	0.045919221	0.391329374

7.133015656	-0.033547484	0.333669743	-0.100540983	0.919914848	0.98194127
9.661823976	-0.482681464	0.371916461	-1.297822265	0.194348417	0.640085942
307.2970954	-0.174713988	0.234123409	-0.746247411	0.455517973	0.831550545
4.641071805	0.318469002	0.653463466	0.487355481	0.626006436	0.903948193
23.14001537	0.91605443	0.550537874	1.663926268	0.096127165	0.505641185
12764.19588	0.162471181	0.133132115	1.22037557	0.222322534	0.666697356
371.858933	-0.017166453	0.057689451	-0.29756659	0.766033977	0.947120611
177.8669233	0.13307685	0.14493781	0.918165178	0.358532396	0.776785206
261.6446467	0.114029823	0.08410975	1.355726574	0.175186181	0.619005807
40.41808763	0.031465367	0.270931835	0.116137577	0.907543508	0.979768942
11.31642747	-0.364251056	0.338166996	-1.077133664	0.281420556	0.722987449
247.9560139	-0.070178781	0.08467952	-0.828757434	0.407241679	0.805288281
7.739805069	-0.162188375	0.33163535	-0.489056355	0.624801799	0.90314702
5.424419909	0.104638633	0.408139823	0.256379375	0.797657912	0.954840537
326.3876658	0.247052405	0.079473413	3.108617027	0.001879652	0.103380352
22.33935445	0.692920237	0.576590565	1.20175438	0.229458705	0.672297655
633.9960514	0.164290302	0.052403551	3.135098657	0.001717963	0.100115323
190.1190219	0.105031677	0.178981374	0.58683021	0.557317745	0.879365746
682.8570319	-0.924634098	0.493509101	-1.873590774	0.060986851	0.437579914
302.8445858	-0.04764781	0.094360101	-0.504957175	0.613588915	0.901444946
134.9843845	0.046700941	0.20185596	0.231357749	0.817036886	0.961335246
181.1183063	0.980536233	0.340066444	2.883366622	0.003934492	0.144154343
84.04788337	-0.039968593	0.171027579	-0.233696769	0.815220396	0.960909552
250.9152036	-0.037386187	0.169957721	-0.219973457	0.825891826	0.964118059
245.2525031	-0.399241962	0.202947065	-1.967222153	0.049157605	0.402896385
69.88227301	0.415136861	0.130538109	3.180196694	0.001471751	0.09327498
1614.978844	0.060397545	0.063268165	0.954627737	0.339765985	0.764202418
68.71577736	-0.113045114	0.226989927	-0.498018196	0.618471217	0.901976704
1160.159009	-0.126660571	0.12079481	-1.048559713	0.294380806	0.734810234
18.89784965	-0.788759305	0.461864939	-1.707770474	0.08767894	0.492827855
133.2500287	0.083356021	0.105956248	0.786702276	0.431456171	0.818841807
311.1980272	0.023294751	0.36788637	0.063320507	0.949511286	0.986108475
55.60258221	0.24317455	0.163095438	1.490995415	0.135962699	0.568673186
334.8023935	0.314964585	0.211348688	1.490260424	0.136155774	0.568673186
126.0606698	-0.162954976	0.114602085	-1.421919819	0.155049529	0.592881201
163.5416695	-0.162892648	0.180087668	-0.904518616	0.365720468	0.78036534
7.4905168	-0.246638893	0.375663039	-0.656542879	0.511474888	0.860200661
339.0158199	0.138008352	0.089030671	1.550121447	0.121112369	0.546889761
220.074033	0.125779977	0.105537053	1.191808699	0.233336264	0.676392617
133.6191016	-0.076146637	0.139693387	-0.545098368	0.585685865	0.890439828
178.8415568	0.107703417	0.142432262	0.756172903	0.449545537	0.828449907
307.344557	0.063189119	0.124677014	0.506822526	0.612279349	0.901248899
175.7744341	0.035424277	0.083487967	0.424303987	0.671344132	0.919433368
142.4229721	-0.549096799	0.222454304	-2.468357719	0.01357346	0.250121471
40.11240142	-0.028376511	0.227396066	-0.124788926	0.900690654	0.979217606
107.1250351	0.02651869	0.132795247	0.199696074	0.841718284	0.967343794
24.76374847	-0.874687415	0.394815442	-2.215433649	0.026730314	0.319793729
501.138357	0.008494428	0.069905842	0.121512424	0.903285175	0.979381499

469.3056226	0.835064758	0.25228257	3.310037461	0.000932835	0.0742356
8.415268827	0.523624518	0.319099003	1.640946895	0.100808439	0.512774612
2759.747743	0.080482318	0.106668638	0.754507787	0.450544372	0.828630047
12.31756412	0.156265164	0.446922496	0.349647121	0.726603543	0.933204828
560.7124517	-0.139039908	0.104559364	-1.329770017	0.183594057	0.629406769
9.79385076	0.009332803	0.35038877	0.026635566	0.978750406	0.99459552
17.35073292	0.694922146	0.477260248	1.456065428	0.145374534	0.579072618
104.1349465	0.356790636	0.315513946	1.130823663	0.258129318	0.701275747
1237.177102	0.642014935	0.238936275	2.686971394	0.007210313	0.186457572
85.94587689	0.7365807	0.465130254	1.583600924	0.113284564	0.533883941
15.98516545	0.033484491	0.410858204	0.081498899	0.935045201	0.984334593
530.5213458	0.060586026	0.044793686	1.352557264	0.176197113	0.619935742
201.3802473	-0.006416306	0.0767147	-0.083638552	0.933343814	0.984334593
3158.762885	0.163172191	0.180924397	0.901880533	0.367120334	0.781703698
2329.873112	0.096690667	0.136844396	0.706573819	0.479831364	0.845119155
1210.756854	-0.098947508	0.049934326	-1.981552873	0.047529306	0.397249458
2373.561996	-0.007370054	0.119506629	-0.061670671	0.950825097	0.98625342
477.6571577	-0.15833159	0.308554189	-0.513140303	0.607853181	0.900307811
427.0162333	-0.024397594	0.068433327	-0.356516261	0.721453977	0.931135539
30.86378613	0.11929203	0.314049547	0.379850986	0.704056032	0.9269128
4213.53256	-0.042913873	0.152100967	-0.282140699	0.777835627	0.951373571
11.5404001	-0.108909903	0.410580562	-0.265258303	0.790810484	0.953153173
529.8175709	0.010127977	0.148944667	0.067998252	0.945787026	0.985674826
701.5008692	0.605129268	0.436182458	1.387330593	0.165340979	0.605636517
439.740266	0.047710874	0.079155094	0.602751789	0.546673823	0.873402987
166.2857283	-0.154183168	0.087351039	-1.76509827	0.077547251	0.472757172
8.261619235	0.446500835	0.303056501	1.473325381	0.14066332	0.574417086
446.1171927	-0.153310047	0.127920269	-1.198481274	0.230729709	0.673080052
71.03756521	0.512605594	0.133183328	3.848872093	0.000118663	0.019959647
2157.046007	0.12648786	0.050375406	2.510905052	0.012042208	0.236404198
46.38925909	0.016375423	0.188833754	0.086718729	0.930895089	0.984334593
127.2060443	0.111979195	0.117753035	0.950966528	0.341621368	0.765195212
9.141640064	0.010450794	0.335576673	0.031142789	0.975155665	0.993340811
17.56911224	-0.519683248	0.327358171	-1.587506571	0.11239796	0.533148601
220.5482047	0.177289538	0.117020826	1.515025528	0.12976594	0.561938079
31.06193122	0.204847376	0.184270621	1.111665955	0.266281803	0.708743266
544.1444873	0.125428141	0.100357474	1.249813647	0.21136763	0.656444287
326.5216166	0.117219797	0.166375125	0.704551216	0.481089567	0.84566728
40.78460044	-0.686179883	0.282009262	-2.433182085	0.014966772	0.256113483
377.2139592	-0.149033508	0.104561969	-1.425312751	0.154066816	0.59219701
423.0595632	-0.056885999	0.051456549	-1.105515235	0.268936397	0.711435249
223.1670639	-0.541445361	0.329721118	-1.642131277	0.100562801	0.512774612
311.5056487	0.124807829	0.07479212	1.668729669	0.095170967	0.503423693
68.16727495	-0.267360352	0.297450529	-0.898839726	0.368738037	0.782820132
620.4011541	0.148763461	0.084483149	1.760865492	0.078261172	0.474223305
984.6911827	0.051596171	0.071819736	0.718412159	0.472503192	0.84082619
1586.925941	0.075525798	0.059186372	1.276067355	0.20193171	0.645481631
1116.817773	0.002265741	0.07700272	0.029424173	0.976526294	0.993619057

120.0541601	0.107664335	0.197818589	0.544257926	0.586263995	0.890984353
566.4623321	-0.040024579	0.118946631	-0.33649191	0.736499951	0.938503341
1506.668847	0.080456229	0.125063502	0.643323016	0.520014525	0.863188882
141.6356651	-0.106948936	0.101877217	-1.049782668	0.293818046	0.734036718
406.6996418	-0.031446724	0.102341843	-0.307271423	0.758636785	0.946346005
10.82047517	0.188953739	0.460585311	0.410246994	0.681624771	0.922658753
106.2500421	0.144369448	0.107930187	1.33761881	0.18102073	0.626190428
6.201615389	-0.942668711	0.406194119	-2.320734516	0.020301177	0.28375639
833.2339081	-0.030906971	0.059633699	-0.518280287	0.604262724	0.89973814
220.5775092	0.58014646	0.266993755	2.172884008	0.029789048	0.330878164
6.06331461	0.247244283	0.482321973	0.512612522	0.608222392	0.900307811
38.24807464	0.292065163	0.375432559	0.777943085	0.436602567	0.822078703
288.4769334	0.18220523	0.080427862	2.265449135	0.023485132	0.305392039
33.83232854	-0.771110334	0.287237443	-2.684574564	0.007262218	0.186457572
46.11369833	-0.234519088	0.234030109	-1.002089385	0.316300424	0.749887496
10.24131807	-0.583315308	0.339993095	-1.715668102	0.086222806	0.49014457
48.92398826	-0.429280097	0.744465873	-0.576628309	0.564190572	0.883065332
272.3786348	0.177015456	0.164869076	1.073672887	0.282969317	0.724063726
1159.02244	0.120866808	0.139162259	0.868531515	0.385103427	0.790829763
6.971018092	0.087007719	0.561785828	0.154877027	0.876918265	0.973973449
237.0305037	-0.012588745	0.09826162	-0.128114571	0.898058305	0.97893802
147.9627577	-0.058897409	0.126230184	-0.466587366	0.640795128	0.907798398
138.9671337	0.019572697	0.126334871	0.154927114	0.876878778	0.973973449
207.6427251	0.137544433	0.080536065	1.707861349	0.087662072	0.492827855
260.4496479	0.029284421	0.090214244	0.324609725	0.745476466	0.940455922
5.388434124	-0.574236351	0.416814919	-1.377677057	0.168303018	0.61083652
120.2855286	-0.137431073	0.22438464	-0.612479862	0.540220318	0.869829343
194.4894474	-0.209240948	0.118543058	-1.765105036	0.077546114	0.472757172
3176.979836	0.05556566	0.141810599	0.391830096	0.695183756	0.924581226
483.2625301	0.08307084	0.094813985	0.876145431	0.380950978	0.78862793
62.47595224	-0.176065308	0.268439814	-0.655883735	0.511898934	0.860200661
22.72686612	-0.199094002	0.263964933	-0.754244132	0.450702642	0.828630047
1113.241243	0.083847493	0.080973802	1.03548915	0.300440531	0.739093677
25.72325704	-0.316738788	0.42401299	-0.747002557	0.455062018	0.831550545
45.89431442	0.065584313	0.219473689	0.298825402	0.765073268	0.946857219
31.25184736	-0.588846186	0.220726428	-2.667764761	0.00763577	0.189660453
530.6952613	-0.015249103	0.074087204	-0.205826414	0.836926508	0.966316856
294.9066371	0.050404655	0.075286866	0.669501307	0.503175747	0.857172233
1119.118901	0.019798147	0.061877333	0.319957987	0.749000179	0.941640077
207.7818828	0.068120049	0.076655193	0.888655373	0.374188322	0.786475871
80.94006118	0.007376857	0.242062001	0.030475071	0.975688175	0.993405995
11.04978644	0.035030355	0.418168833	0.083770841	0.933238632	0.984334593
271.1327859	0.05117837	0.098305768	0.520603933	0.602642709	0.899375001
276.3495436	0.045916111	0.096633485	0.475157358	0.634674818	0.906954229
4.991100169	0.184126253	0.44407134	0.414632147	0.678411211	0.921573669
28.50605117	-0.506190131	0.315559917	-1.604101482	0.108691685	0.525941264
14.19121601	0.319336605	0.348320723	0.916788994	0.359253221	0.777095745
1663.099716	-0.233282098	0.164655808	-1.416786334	0.156545407	0.595721049

987.1174423	0.29025608	0.110574112	2.624991279	0.008665119	0.20191478
208.6636598	-0.063486302	0.083128298	-0.763714689	0.445037285	0.826302547
1039.423407	0.127914822	0.10243593	1.248730038	0.211763831	0.656521334
288.4521562	0.063696861	0.124748719	0.510601326	0.609630247	0.900307811
264.3008423	-0.081092991	0.133415672	-0.607822078	0.543305485	0.871203645
9.394340703	0.566278737	0.358534	1.579428277	0.114237856	0.534941076
398.4093878	0.025161272	0.085535706	0.294161037	0.768634852	0.947586561
229.5537684	0.202432604	0.085665444	2.36306023	0.018124724	0.271266087
434.0327057	0.083450811	0.133311184	0.625985074	0.531324731	0.866248339
17.4717411	0.161739436	0.599094342	0.269973232	0.787180847	0.95288876
1023.051623	0.18737966	0.090997866	2.059165442	0.039478392	0.371577411
411.4511729	-0.137405171	0.142419256	-0.964793492	0.334648256	0.761719744
7.917506145	0.029801498	0.441946215	0.067432408	0.94623747	0.985728152
587.0374193	0.092546329	0.088046257	1.051110318	0.293207926	0.733939814
75.97506572	0.047912218	0.130519563	0.367088402	0.713553078	0.929705922
23400.52204	0.040286814	0.147086494	0.273898801	0.784162393	0.95189998
643.6461775	-0.030723947	0.135887031	-0.226099182	0.82112428	0.962824423
1234.73519	0.078668788	0.080844957	0.973082195	0.330512452	0.759429574
22.92505674	-0.700400561	0.717218356	-0.976551361	0.328791309	0.758664748
392.8983407	-0.047887673	0.088623057	-0.540352298	0.588954098	0.891927415
43.75261148	-0.1996879	0.313296201	-0.637377342	0.523879084	0.863484457
119.8774087	0.062102446	0.150751599	0.411952154	0.680374493	0.922243887
128.3920102	-0.056590798	0.109956036	-0.514667502	0.606785386	0.900307811
787.1763362	0.022910265	0.058573216	0.391138933	0.695694544	0.924718987
11.67388039	-0.638391009	0.454394884	-1.404925608	0.160043401	0.6006497
189.9711358	-0.231276983	0.160151244	-1.444116057	0.148706332	0.585724089
7.329997741	-0.32262341	0.490947856	-0.657143944	0.511088365	0.860200661
191.5979896	0.190592705	0.092956948	2.050333078	0.040331939	0.373696391
5.237232666	-1.055596513	0.603567656	-1.748928231	0.080303426	0.479488225
888.5117645	0.125696768	0.106928601	1.175520553	0.23978652	0.681681953
562.4604731	-0.046081648	0.070063411	-0.657713454	0.510722275	0.860200661
480.5740856	0.080311992	0.065677367	1.222825998	0.221395424	0.66525151
638.6405239	0.049196744	0.076035485	0.647023474	0.517616751	0.862002027
27.85617012	0.512337772	0.213493696	2.399779392	0.016404955	0.263145637
368.9785508	-0.067970453	0.086133182	-0.789132032	0.430034842	0.818650305
684.4234022	0.02896073	0.102967095	0.281261991	0.778509461	0.951560132
374.4628565	0.071774751	0.075994935	0.944467562	0.344930771	0.767851822
4.733608549	0.326628955	0.470679579	0.693951831	0.487712417	0.849513564
292.2917427	0.167348518	0.16980256	0.985547675	0.324355125	0.756297513
7.562100108	0.636224933	0.519956061	1.223612881	0.221098298	0.664764193
361.2182669	-0.23310397	0.231609614	-1.006452046	0.314198168	0.748102226
170.475749	-0.093398566	0.132758605	-0.703521754	0.481730655	0.845969003
371.1790546	0.247010744	0.086350933	2.860545172	0.004229133	0.145919876
90.70305918	0.075936145	0.136140663	0.557777105	0.576996598	0.88676146
8.303912598	-0.578821367	0.445979395	-1.297865717	0.194333482	0.640085942
362.2976524	0.06947739	0.062301054	1.115188023	0.264769857	0.70780629
131.9512032	0.123554795	0.104418743	1.183262612	0.236705086	0.679408314
188.5078323	0.121571538	0.08163894	1.489136656	0.136451387	0.569036171

287.236728	-0.159751845	0.309989242	-0.515346416	0.606310969	0.900307811
12.36277032	-0.821417369	0.374614181	-2.192702282	0.028328836	0.325561673
1471.702597	0.074470422	0.054137952	1.37556778	0.168955488	0.61146189
631.195032	-0.023725668	0.11430193	-0.20757014	0.835564621	0.966316856
62.7938532	-0.048106274	0.153095655	-0.314223642	0.753351188	0.944021193
533.4250214	0.120930363	0.057250311	2.112309284	0.03465993	0.352445634
49.24847424	-0.036601898	0.235684246	-0.15530057	0.876584367	0.973964714
148.925555	-0.200157734	0.296223555	-0.675698236	0.499232256	0.855692311
1062.158156	-0.032757767	0.143307748	-0.228583365	0.819192755	0.962187758
42.770425	-0.26270166	0.326375468	-0.804906269	0.420873771	0.813074073
192.8262172	0.275195823	0.353303957	0.778920861	0.436026334	0.822078703
28.17179891	-0.648453537	0.494626196	-1.310997158	0.189858724	0.635668136
31.01142663	-0.462493682	0.271256079	-1.705007621	0.088193003	0.492827855
125.5045893	-0.06823339	0.165478585	-0.412339702	0.680090453	0.922112199
27.53752636	0.075593727	0.296606849	0.254861703	0.798829916	0.955167977
4406.8017	-0.122685492	0.137745535	-0.890667651	0.373107494	0.786266676
653.5661771	-0.059837497	0.119400483	-0.501149537	0.616265886	0.901900555
45.77598412	0.300296849	0.1591885	1.886423005	0.059237975	0.434680797
112.4566456	-0.124027584	0.127812659	-0.970385753	0.331854247	0.760616881
128.1134177	-0.260235516	0.228047164	-1.141147783	0.25380843	0.696490986
536.2080677	0.018264213	0.105356685	0.173355997	0.862371611	0.970782918
577.5215979	-0.06488501	0.059295055	-1.094273544	0.273835025	0.716624931
6.277934203	0.069912765	0.382691779	0.182686875	0.855043715	0.969966486
338.8432915	-0.079462664	0.244908798	-0.324458185	0.745591175	0.940455922
10.81277282	-0.285496143	0.331946037	-0.860067937	0.389751594	0.79322193
235.1022274	0.050597416	0.091868685	0.550758028	0.58179956	0.888272005
543.4917903	-0.236102055	0.146558307	-1.610976959	0.107184745	0.524305548
48.56079982	-0.158461639	0.167066539	-0.948494176	0.342877937	0.766311221
1370.050084	0.011264394	0.048834245	0.23066588	0.817574383	0.961335246
49.20233527	-0.090100512	0.188760169	-0.47732799	0.633128583	0.906724287
6.872974888	-0.393487197	0.402158619	-0.978437807	0.327857837	0.758221793
15.17638211	-0.140945386	0.251831468	-0.559681388	0.575696779	0.886202779
108.0882478	-0.043296374	0.129397694	-0.334599271	0.737927395	0.938868945
13.68415767	0.055903534	0.340294897	0.16427967	0.869510985	0.972532991
931.9718525	-0.027694469	0.057435903	-0.482180441	0.62967777	0.904638489
1094.027925	0.021827453	0.049781544	0.438464762	0.661049409	0.914896282
10.84031786	-0.131944718	0.333236381	-0.395949318	0.692142417	0.924581226
47.49207842	-0.651959944	0.340984854	-1.911990917	0.055877359	0.425680221
3273.362423	-0.053218918	0.383570221	-0.138746219	0.889650694	0.976961229
110.7405818	-0.600149941	0.29586985	-2.028425478	0.042516842	0.377880689
71.31735575	-0.249992909	0.17149375	-1.457737721	0.144912851	0.578290974
12.71594331	0.212738459	0.256148619	0.830527446	0.406240637	0.804437981
106.2072659	-0.013425964	0.18354671	-0.073147396	0.941688826	0.98438404
103.5571145	-0.115466939	0.112367206	-1.027585742	0.304144734	0.741640846
48.66453809	0.155045233	0.149403162	1.037764064	0.299379911	0.738776886
186.6413502	0.113870654	0.144588796	0.787548258	0.43096099	0.818841807
86.37185779	0.120469541	0.129272083	0.931906861	0.351384661	0.770952892
342.2632859	0.026628025	0.091070292	0.29238981	0.769988598	0.948279493

78280.87571	-0.097185902	0.164399445	-0.591157118	0.554415152	0.877695516
55.22667423	0.138016077	0.150438725	0.917423866	0.358920572	0.777095745
81.9648357	0.096421057	0.165756593	0.581702697	0.560766955	0.880936184
244.5212781	-0.247742762	0.084081627	-2.946455372	0.003214388	0.132903271
50.16576543	-0.098192607	0.152932437	-0.642065273	0.520830803	0.863188882
300.0087781	-0.040017109	0.079906036	-0.50080208	0.616510421	0.901900555
83.12552254	0.141380044	0.169479642	0.834200748	0.40416787	0.803019169
283.6361863	-0.150162407	0.065828642	-2.2811105	0.022541909	0.300162997
127.8118807	-0.019571511	0.096648269	-0.202502447	0.839523946	0.966440785
18.71458836	-0.016757302	0.293972987	-0.057002865	0.954542913	0.987292586
270.8913788	-0.288116531	0.275088735	-1.047358519	0.294934255	0.735234129
302.3200445	-0.068269624	0.083129692	-0.821242352	0.411508237	0.808165706
9.685645012	0.029913262	0.312770244	0.095639731	0.923806709	0.982590996
1179.709874	-0.005722	0.093405806	-0.061259575	0.951152485	0.98625342
934.7515845	-0.042592045	0.159749001	-0.266618536	0.789762882	0.953026618
237.0806016	-0.196413162	0.139427698	-1.40870978	0.158921002	0.59901927
1032.670086	0.012198107	0.061090598	0.199672405	0.841736797	0.967343794
81.94080096	0.257641234	0.183945984	1.400635274	0.161323167	0.601052447
422.0452526	0.066697042	0.134262368	0.496766462	0.619353748	0.901976704
213.0960519	0.126801023	0.092473075	1.37122101	0.170306071	0.613499746
288.545203	0.078768843	0.074492536	1.057405843	0.290326406	0.731967225
12.69196206	0.382506255	0.265776483	1.439202788	0.150093076	0.588453924
40.08163574	0.801953293	0.309547954	2.590723931	0.009577429	0.211763732
2181.343163	0.131088656	0.10749187	1.219521594	0.222646285	0.666984161
260.5143323	0.076708578	0.075263185	1.019204507	0.308105887	0.743405059
253.6651995	-0.261585508	0.095973888	-2.725590408	0.00641866	0.174970531
172.4470122	0.014612047	0.086865443	0.168214725	0.866414357	0.971979594
7.966734282	-0.064505603	0.386253385	-0.167003334	0.867367426	0.972138679
103.8853995	0.110052844	0.309428469	0.355664893	0.722091539	0.931135539
264.7176568	-0.02546681	0.099210806	-0.256693915	0.797415069	0.954840537
562.3978886	-0.086493524	0.258199663	-0.334986975	0.737634912	0.938868945
103.4045854	-0.115262927	0.15162328	-0.760192813	0.44713934	0.826805895
14.86777969	0.157547559	0.275673846	0.57149984	0.567660869	0.8841274
79.95740935	0.152314195	0.135448699	1.124515751	0.260794292	0.703138216
51.26639226	-0.252675798	0.205586063	-1.229051202	0.219052609	0.663637842
91.58111169	0.12825439	0.130265052	0.984564843	0.324837866	0.756297513
179.8433513	0.203436256	0.083584665	2.433894485	0.014937349	0.256113483
308.8915883	-0.079740305	0.10262524	-0.777004815	0.43715593	0.822181608
213.5758265	-0.249275438	0.167842466	-1.485175016	0.137497468	0.569036171
463.7024696	-0.033246804	0.085383859	-0.389380434	0.696994743	0.92544754
289.812672	-0.054872465	0.098959852	-0.554492195	0.579242046	0.887467795
15.97626045	0.679119691	0.277040947	2.451333276	0.01423281	0.251563345
4693.019968	-0.035878907	0.11324661	-0.316821024	0.751379411	0.942934976
203.7447758	-0.129550292	0.127899968	-1.012903236	0.311106383	0.745771331
146.1732087	-0.139960696	0.092876523	-1.50695452	0.131822315	0.564647789
1140.737309	-0.463345865	0.134681356	-3.440311835	0.000581044	0.055487857
912.7454902	-0.028703559	0.0725088	-0.395863114	0.692206014	0.924581226
93095.75567	0.123985412	0.082737643	1.498536918	0.133993808	0.567033996

16899.02347	0.360707448	0.15045274	2.397480083	0.016508278	0.263145637
145.0342651	0.065167675	0.129838042	0.50191511	0.615727236	0.901900555
25.6347222	-0.008856886	0.393882463	-0.022486115	0.982060188	0.99517046
543.6331777	0.126020956	0.085289372	1.477569284	0.139523101	0.572130062
196.2723167	0.12179693	0.081238678	1.499248046	0.133809295	0.567033996
119.8407194	-0.257708599	0.151035199	-1.706281719	0.08795564	0.492827855
1105.535759	-0.060993942	0.090710713	-0.672400645	0.501328672	0.85676495
33.64691142	-0.051868095	0.215896584	-0.240245095	0.810140256	0.958722103
362.7688361	-0.122263846	0.065395077	-1.869618506	0.061536813	0.43854979
220.4334516	-0.190703536	0.083203071	-2.292025195	0.021904189	0.296249025
1305.11235	0.096394653	0.137185264	0.702660405	0.482267409	0.846598931
106.0355318	-0.086228006	0.125995372	-0.684374393	0.493738781	0.852282384
49.22816135	0.101936231	0.164692199	0.618949966	0.53594932	0.869002283
256.4140968	-0.098268294	0.075197119	-1.306809307	0.191277484	0.637278237
92.5584832	-0.08987711	0.100671844	-0.892773059	0.371978716	0.785901935
117.5607215	-0.268190827	0.150908962	-1.777169651	0.075540331	0.470694355
91.45121535	0.061824988	0.115992102	0.533010325	0.594026433	0.894609104
325.3435247	0.16904752	0.136675939	1.236849157	0.216143089	0.660340557
5.569797758	0.515448517	0.597687393	0.862404867	0.388464763	0.792781092
448.4972803	-0.191356293	0.097255175	-1.967569253	0.04911762	0.402791699
584.299267	-0.001689696	0.06332296	-0.026683774	0.978711955	0.99459552
527.7563232	0.112023106	0.175089247	0.639805747	0.522298896	0.863362704
309.5653343	0.184024612	0.097178893	1.89366854	0.05826902	0.432392203
126.6263041	-0.108440031	0.130391138	-0.83165185	0.405605487	0.804137887
198.9744669	0.100162773	0.183348991	0.546295739	0.584862665	0.889736605
1044.706089	0.071389799	0.076104119	0.938054337	0.348216487	0.768780165
88921.46074	-0.025680201	0.154336797	-0.166390658	0.867849525	0.972204994
128.8579939	-0.132727955	0.201316684	-0.65929933	0.509703573	0.859882646
26.40896731	-0.031831059	0.263195789	-0.120940608	0.903738078	0.979381499
226.4840913	-0.202158702	0.075245961	-2.686638576	0.0072175	0.186457572
27.37758196	-0.942589045	0.502245067	-1.876751225	0.060552198	0.436434491
1192.327818	-0.154153236	0.086673387	-1.778553284	0.075313029	0.470687737
283.7780188	0.09911608	0.100263257	0.988558348	0.322879274	0.755494627
1074.155007	0.009127087	0.171627626	0.053179589	0.957588818	0.987864832
4.46910454	-0.650139889	0.497948072	-1.305637926	0.191675717	0.637569428
4.483150986	0.459044885	0.556085358	0.825493566	0.409091434	0.806739148
87.04695922	-0.074358645	0.112434669	-0.661349789	0.508388018	0.859636085
87.992094	-0.057676759	0.108295244	-0.532588105	0.594318738	0.894609104
316.284657	-0.064646511	0.080013283	-0.807947245	0.419120952	0.811610533
537.9528528	0.028982137	0.15412715	0.188040443	0.850844948	0.969307082
46.6767077	-0.082703105	0.206527197	-0.400446556	0.68882764	0.923813534
1178.589338	-0.086338583	0.127445996	-0.677452293	0.498119029	0.855428766
192.4662781	-0.143824934	0.075261678	-1.910998242	0.056004807	0.425993397
67.13194842	-0.146259501	0.140947015	-1.037691369	0.299413764	0.738776886
120.8773169	-0.177289034	0.137202639	-1.292169271	0.196298531	0.641418933
73.98189409	-0.182033063	0.487751352	-0.373208731	0.708993099	0.928992812
776.8228642	-0.033100394	0.120868763	-0.273853999	0.784196824	0.95189998
2135.506262	-0.001473049	0.122066107	-0.012067631	0.990371657	0.998194285

233.8606225	0.003344238	0.131143648	0.02550057	0.979655694	0.994668345
433.5270151	0.01590237	0.076053309	0.209095043	0.834374044	0.966316856
398.1947067	-0.252871567	0.211664159	-1.194682974	0.232210917	0.674945206
264.7188527	-0.034021252	0.100564975	-0.338301201	0.735136219	0.937623649
19.01049894	-1.006421754	0.347868574	-2.893109144	0.003814487	0.140808558
679.9090249	-0.062535738	0.063930248	-0.978186999	0.327981845	0.758221793
108.6882687	0.127498473	0.100319869	1.270919451	0.203757306	0.647823067
204.5072267	-0.133711341	0.143634475	-0.930913981	0.351898062	0.771330536
738.8526887	-0.047069063	0.084574992	-0.556536414	0.577844209	0.887079361
4.977227234	0.247713142	0.425151754	0.582646408	0.560131357	0.880622873
669.6564409	-0.124538712	0.18594001	-0.669778992	0.502998687	0.857172233
2097.236647	-0.321419904	0.125803784	-2.554930339	0.010620905	0.223743575
216.8752386	-0.602412391	0.404706991	-1.488514911	0.136615153	0.569036171
445.0810696	0.029821255	0.138798494	0.214852871	0.82988203	0.965412906
59.06525627	-0.150566356	0.183706079	-0.819604649	0.412441523	0.808272153
73.89363333	-0.39694051	0.303814011	-1.3065247	0.191374185	0.637278237
92.11572716	0.173071287	0.121204855	1.42792372	0.153313815	0.591551404
199.4667428	0.110811017	0.090564257	1.223562372	0.221117362	0.664764193
430.9316279	0.131268355	0.062034041	2.116069702	0.03433888	0.351267519
386.2526197	-0.133432656	0.105587418	-1.263717388	0.206331482	0.651753862
47.43772816	0.31557125	0.18205159	1.73341661	0.083021647	0.485235749
40.59196067	-0.066488291	0.176933213	-0.375781854	0.707079079	0.928109779
5.795142416	0.465877671	0.456394121	1.020779299	0.307359014	0.743405059
183.9180209	-0.046127519	0.081735865	-0.564348577	0.572516928	0.885183781
14.72825631	-0.248997941	0.246579844	-1.009806548	0.312587982	0.746754295
205.2422286	0.203119517	0.105101697	1.932599775	0.05328552	0.416932852
1125.756308	0.119907975	0.078013888	1.537008062	0.124291335	0.552139359
5.497626617	-0.278757912	0.374836384	-0.743678906	0.457070754	0.832305398
293.6157687	-0.417525097	0.227000919	-1.839310163	0.065869579	0.450763528
199.8420996	0.173389803	0.264807173	0.654777592	0.51261096	0.860200661
849.0174777	0.07665442	0.088041512	0.870662237	0.383938603	0.790043276
208.2253891	0.055914493	0.122810136	0.455292171	0.648899058	0.910772223
37.88146753	-0.064322787	0.243332589	-0.264341031	0.791517146	0.953153173
206.9416904	-0.038046588	0.100294162	-0.379349974	0.704427994	0.9269128
536.2434912	-0.14693257	0.099580578	-1.475514343	0.140074315	0.573073524
262.8056662	0.233514017	0.066903433	3.490314437	0.000482453	0.048580016
79.76105448	-0.043890148	0.142298348	-0.308437508	0.757749445	0.945717309
151.7776045	-0.057783202	0.18498267	-0.312370895	0.754758667	0.944453275
489.1659589	0.025804462	0.071638656	0.360203042	0.7186953	0.930765281
156.3549038	-0.141486642	0.09271425	-1.526050655	0.12699725	0.556444786
2967.415068	-0.011720103	0.068450971	-0.171218949	0.864051608	0.971717556
38844.46381	0.009358236	0.063070592	0.148377173	0.882045114	0.975487608
376.8617518	0.127457147	0.092260176	1.381496906	0.167126228	0.609023925
17.58988453	0.213697335	0.239647019	0.891717062	0.372544606	0.786266676
46.0007669	-0.569358792	0.424102961	-1.342501335	0.179433498	0.624452051
6964.543459	0.245418045	0.110911422	2.212739142	0.026915635	0.320777159
4.479376633	0.3075371	0.473379436	0.649662991	0.515909935	0.862002027
145.8622104	0.212034309	0.377929914	0.561041351	0.574769348	0.88616072

381.0097668	-0.649799789	0.388071864	-1.67443159	0.094045809	0.501896522
48.16161739	-1.073353147	0.329359706	-3.258908504	0.001118417	0.080402212
269.2321374	-0.325689694	0.280548915	-1.160901634	0.245681904	0.688746884
70.14581377	-0.115653318	0.232321685	-0.497815424	0.618614143	0.901976704
179.9652639	-0.1344037	0.091716541	-1.465424859	0.142805039	0.57754946
5.81103585	-0.005613261	0.583537795	-0.009619362	0.992324978	0.998453546
253.4632261	-0.137561289	0.169013628	-0.813906493	0.41569852	0.81005391
41.61932682	0.164997488	0.186433374	0.885021195	0.3761452	0.786851292
4.862277878	-0.458403042	0.637380391	-0.719198533	0.472018603	0.84064088
6.756730654	0.118731106	0.37353967	0.317854073	0.750595632	0.942359611
404.9341197	-0.071238325	0.088982254	-0.800590242	0.423368901	0.814538241
357.5807028	0.094310228	0.074860752	1.25980871	0.207738377	0.65243867
33.7680265	-0.187487038	0.281504995	-0.666016736	0.505400403	0.858594831
17.38459427	-0.155216296	0.23709079	-0.654670288	0.512680059	0.860200661
158.6020444	-0.199339706	0.082525737	-2.415485313	0.015714256	0.259890973
59.47900269	-0.772835021	0.27415863	-2.818933768	0.004818346	0.154050722
2858.484638	-0.010140732	0.039830106	-0.25459967	0.799032314	0.955167977
335.6989249	0.024900678	0.089159565	0.279282188	0.780028274	0.95173434
13.09019238	-0.799933504	0.421143689	-1.899431299	0.057507792	0.430349004
61.16213197	-0.70086377	0.232381772	-3.016001494	0.00256132	0.119684149
2645.460365	0.026586999	0.164946263	0.161185825	0.871947045	0.972881041
1760.701222	0.050190594	0.08981548	0.558818965	0.576285278	0.886344003
206.8866227	0.06704285	0.157683692	0.425173011	0.670710557	0.918962624
47.01261973	-0.016770202	0.188861103	-0.088796485	0.929243651	0.984152592
6.37659251	0.282408687	0.46800784	0.603427257	0.546224494	0.873065405
4.989365269	-1.19882336	0.836396133	-1.433320064	0.151766391	0.589757816
20.10711005	1.332749883	0.496606559	2.683713814	0.00728094	0.186457572
28.82704623	-0.179617368	0.327700265	-0.548114808	0.583613073	0.889114936
5.897422547	0.50930877	0.58602955	0.869083768	0.384801314	0.790453664
27.5436401	0.279204987	0.233666328	1.194887553	0.232130967	0.674917024
71.39766107	0.4255415	0.198617655	2.142515984	0.032151984	0.34013779
30.94686208	1.565547031	0.642012593	2.438498946	0.014748402	0.256113483
11.64939364	0.039561685	0.36507758	0.108365145	0.913706049	0.980550322
7.16564437	-3.385120333	2.516953676	-1.344927547	0.178648633	0.623228157
278.8397059	0.292753706	0.1283282	2.281288971	0.022531353	0.300162997
95.5152012	0.487821822	0.115294693	4.231086534	2.32565E-05	0.008826743
510.9750974	0.18826881	0.068228265	2.759396148	0.005790829	0.166644523
1647.118933	-0.093614716	0.086086189	-1.087453375	0.276836493	0.718959505
111.402533	-0.023790861	0.459830468	-0.051738331	0.958737195	0.988123044
184.2731921	0.039891445	0.347301111	0.114861266	0.908555087	0.980020248
60.43495004	-0.081318285	0.295961537	-0.27475964	0.783500908	0.95173434
4.599136185	-0.389755368	0.612563977	-0.636268835	0.524601214	0.863946059
2121.057298	0.185748035	0.112031427	1.657999371	0.097317593	0.507880983
16.65995377	0.528495458	0.498423559	1.060334024	0.288992665	0.729902555
474.0201994	0.222536142	0.149571052	1.487828955	0.136796008	0.569036171
80.99338895	-0.715606538	0.36561287	-1.957279399	0.050314628	0.407193618
79.1272031	-0.142603727	0.131199163	-1.086925586	0.277069698	0.719218718
548.2640839	-0.10994828	0.107812898	-1.019806372	0.307820301	0.743405059

89.71205052	0.115553299	0.12085652	0.956119697	0.339011765	0.764007173
29.01696644	0.114042138	0.215564722	0.529038966	0.59677842	0.896731622
1367.089147	0.133676337	0.097546769	1.370382002	0.170567687	0.614254682
492.5136666	0.372846074	0.648467402	0.574965022	0.565314953	0.883265494
57.15187247	0.272956179	0.414567089	0.658412561	0.510273066	0.860200661
1008.152583	0.08679914	0.08172004	1.062152445	0.288166484	0.729194145
104.1621216	0.147294882	0.285229526	0.516408256	0.6055693	0.900307811
268.7938915	-0.004137898	0.113143906	-0.036571991	0.970826276	0.992796176
46.88971133	-0.013627292	0.14329381	-0.095100354	0.924235117	0.982590996
1464.796975	0.009275453	0.126210837	0.073491729	0.941414826	0.984334593
56.54401119	0.072472487	0.146418722	0.494967351	0.620623167	0.901976704
12.30987856	-0.139168243	0.348547601	-0.399280451	0.68968657	0.92405254
23.30977908	0.044611083	0.267476963	0.166784767	0.867539405	0.972138679
509.6219723	-0.072715264	0.257576788	-0.282305189	0.777709507	0.951371283
342.1917202	0.177849746	0.12741568	1.395823075	0.16276778	0.601684318
31.65191483	0.781284432	0.257262217	3.036918673	0.002390099	0.115994263
1556.928138	0.167365213	0.170211676	0.983276927	0.325471163	0.756297513
35.38682945	-2.077779273	0.759719996	-2.734927717	0.006239398	0.173927613
3665.78756	0.012639493	0.125993691	0.100318459	0.920091503	0.982059016
373.4874044	-0.085700329	0.144663866	-0.592410057	0.553576033	0.877205564
68.70752787	-0.052901815	0.115036045	-0.459871642	0.645608356	0.909300678
267.2469978	0.067000669	0.070210988	0.954276119	0.339943893	0.764459061
561.7194078	0.118683994	0.106958124	1.109630479	0.267158292	0.70945049
30.63978734	0.106486353	0.276925373	0.384530865	0.700585024	0.926034765
2520.552021	0.150278278	0.105370106	1.426194616	0.153812173	0.59219701
17.41783508	0.364802809	0.337265762	1.081647918	0.279409004	0.721404513
341.2726503	-0.041766563	0.065344437	-0.639175508	0.522708763	0.863362704
41.5343834	-0.410220004	0.194036423	-2.11413918	0.034503382	0.352363583
182.240158	0.195055999	0.088589261	2.201801847	0.027679309	0.32400917
92.69059323	0.231708226	0.223550295	1.036492598	0.299972391	0.738923503
356.5096349	-0.075024061	0.176649451	-0.424705881	0.671051096	0.919117083
139.2736798	-0.350805517	0.120654373	-2.907524255	0.003643021	0.138266655
152.7137997	0.00700205	0.091473811	0.076547048	0.938983885	0.984334593
11.40008048	-0.783972146	0.570081491	-1.375193124	0.16907158	0.611582972
158.4330692	-0.001283736	0.118226262	-0.0108583	0.9913365	0.998215162
9.186597206	-0.152871627	0.34670581	-0.440926061	0.659266526	0.914227386
367.6484877	-0.2778015	0.212456689	-1.30756768	0.191019985	0.637278237
149.8168881	-0.048210061	0.131320535	-0.36711746	0.713531404	0.929705922
267.5551429	-0.33174924	0.308327995	-1.075962109	0.281944203	0.723387381
629.6968106	0.006786357	0.097858777	0.069348476	0.94471224	0.984970809
18.13032204	-0.056146704	0.376558471	-0.149104876	0.881470878	0.975487608
124.0062165	-0.307275751	0.19909566	-1.543357355	0.122744095	0.548899724
1185.12447	0.05112605	0.081503534	0.627286296	0.530471584	0.866123834
8.322679242	-0.173926399	0.344122485	-0.505420037	0.613263849	0.901444946
5080.292093	-0.173266541	0.239806701	-0.722525852	0.469971237	0.839894775
151.9683197	-0.073045177	0.107026315	-0.68249736	0.494924511	0.853605572
7.700277764	-0.020681272	0.380874802	-0.054299397	0.95669663	0.987725711
118.8138461	0.070708291	0.102924091	0.686994569	0.492086151	0.851383417

38.00694319	0.320421716	0.339695126	0.943262626	0.345546587	0.767958555
42.95566875	0.373629712	0.239493116	1.560085389	0.118739704	0.542464536
33.46405594	0.27012668	0.265242127	1.018415449	0.308480563	0.743911583
76.37184063	0.047521112	0.296293	0.160385537	0.872577382	0.972881041
1047.225732	0.218560232	0.092668363	2.358520478	0.018347947	0.272290126
658.4623918	-0.052960735	0.09636851	-0.549564743	0.582617947	0.888696501
6.058663062	0.305333958	0.502319433	0.607848191	0.543288164	0.871203645
26.22764346	-0.486446887	0.243268253	-1.999631603	0.045540059	0.390450954
139.9704969	-0.284447978	0.095412958	-2.981230053	0.00287093	0.127232068
776.3885239	-0.129661891	0.105517342	-1.228820672	0.219139049	0.663637842
99.39537998	-0.092437756	0.127360333	-0.725797067	0.467963185	0.838388803
69.53561726	0.171095124	0.442852939	0.386347495	0.69923933	0.925900484
10.61346808	0.608289665	0.295456887	2.05881024	0.03951242	0.371577411
849.9408592	0.038745098	0.098618985	0.392876663	0.694410578	0.924581226
450.0773291	-0.34236635	0.212178197	-1.613579314	0.106618707	0.523092509
461.1555453	0.073197363	0.05830016	1.255525925	0.209287903	0.654527686
300.9864276	0.066408403	0.115873034	0.573113525	0.56656783	0.883661792
95.77594658	-0.187510501	0.13684073	-1.370282816	0.170598635	0.614254682
255.7300738	0.140490241	0.111500257	1.259999262	0.207669629	0.652433255
14.55105377	-0.844229705	0.597250298	-1.413527474	0.157500689	0.597163217
722.0450633	-0.263653333	0.183863915	-1.433959094	0.151583937	0.589528489
373.505675	0.030030977	0.092689355	0.323995963	0.74594109	0.940495743
17.63197828	0.303719427	0.289896947	1.04768067	0.294785756	0.73520114
154.6795162	-0.225132215	0.106825336	-2.107479588	0.035076029	0.354884063
916.2593191	0.260773505	0.101559826	2.567683657	0.010238054	0.218571568
32.05181684	-0.226417586	0.257172124	-0.88041263	0.378635817	0.787072097
254.8449918	-0.017879313	0.07755485	-0.230537647	0.817674013	0.961335246
196.6296219	-0.037053158	0.24540312	-0.150988944	0.879984437	0.975430592
970.0432748	0.084850503	0.128585537	0.65987595	0.509333439	0.859636085
1328.892003	0.133251608	0.072542282	1.836881954	0.066227324	0.4517497
98.9089032	0.035986842	0.165704072	0.217175364	0.828071684	0.964626741
67.85813124	0.176656034	0.138959559	1.271276591	0.203630267	0.647823067
27.5059383	-0.451403862	0.424978011	-1.062181689	0.288153211	0.729194145
429.5096143	-0.239404221	0.223956429	-1.068976775	0.285080135	0.726291937
42.42911227	0.543832667	0.211315052	2.57356332	0.010065723	0.216530925
187.9378774	0.055637402	0.306344011	0.181617399	0.855882994	0.969966486
66.97133003	0.116541724	0.258781449	0.450348062	0.652459489	0.911448221
28.48433529	-0.311676726	0.372225236	-0.837333679	0.402405034	0.801884667
65.08152647	0.143247319	0.23982891	0.59728962	0.550314034	0.875690724
18.99378853	0.077026757	0.342324446	0.225010973	0.821970738	0.963169453
47.55124705	-0.996977906	0.449075183	-2.220069032	0.026414082	0.317447514
286.7224013	-0.25748942	0.182332629	-1.412196058	0.157892241	0.597576311
13.6699101	-1.089493955	0.602781893	-1.807443069	0.070693224	0.462395537
13.9688614	0.248093674	0.291931759	0.849834478	0.395417118	0.797502736
99.63425046	-0.025764217	0.127437643	-0.202171164	0.839782915	0.966440785
191.4001503	1.304118714	0.456625414	2.855992402	0.004290254	0.145986988
329.1270563	0.007686936	0.117261081	0.06555403	0.947732889	0.985917197
125.4189657	0.217479087	0.11685732	1.861065168	0.062734974	0.442595858

74.6371027	0.329572117	0.255413255	1.290348525	0.196929676	0.642286481
3510.021926	0.046129419	0.104093058	0.443155577	0.657653208	0.91380052
21.53537589	0.132856567	0.270213153	0.491673206	0.622950383	0.902441895
537.8204624	0.147669834	0.049709379	2.97066343	0.002971572	0.128901255
1202.937525	0.083365916	0.10518228	0.792585173	0.42801955	0.817595223
13.01203733	0.035740276	0.353336957	0.101150689	0.919430841	0.981892218
846.3588011	0.005345835	0.065142289	0.082063974	0.934595841	0.984334593
37.76149428	-0.773712092	0.414794537	-1.865289975	0.06214077	0.440943275
1933.266842	0.231685122	0.102325068	2.264206868	0.023561394	0.305392039
29.41936856	-1.965470593	0.906217232	-2.168873559	0.030092285	0.332282647
21.18871852	-0.399057554	0.311563464	-1.280822692	0.200255952	0.645045462
944.307444	-0.015027022	0.111496443	-0.134775794	0.892789144	0.977951966
8.191730554	-0.41655634	0.404255349	-1.030428764	0.302808776	0.740488272
265.7837433	0.08099371	0.30147798	0.268655474	0.788194819	0.953026618
13.63607238	-0.896436411	0.657533509	-1.363331903	0.172777932	0.616450149
186.704612	-1.227310725	0.432770467	-2.835939186	0.004569115	0.149960163
67.34163708	0.315824305	0.186720034	1.691432342	0.09075426	0.497535019
28.61836801	-0.178538244	0.607877033	-0.29370783	0.76898117	0.947650255
61.98331408	-0.001406601	0.215739624	-0.006519901	0.994797909	0.998981303
338.485387	-0.151040097	0.192726171	-0.783703098	0.433214344	0.820529587
141.8941281	0.145853669	0.087390018	1.668996906	0.095117993	0.503423693
106.0679409	-0.004987789	0.10110756	-0.049331517	0.960655103	0.989126211
12.23253288	-0.991962476	0.527733867	-1.879664236	0.060153853	0.435320275
5.796752339	-1.618688915	0.913708106	-1.771560201	0.076467591	0.471783446
341.7298932	-0.108896852	0.301248423	-0.361485218	0.717736753	0.930765281
2659.059175	-0.105440275	0.140795632	-0.748888822	0.453924218	0.831004392
60.55362849	-0.364401667	0.336976187	-1.081386998	0.279525003	0.721452326
46.29361385	-0.083250485	0.149818871	-0.555674224	0.578433581	0.887251177
193.8950616	0.007841867	0.18877862	0.041540015	0.966865393	0.990966732
88.75820174	0.046571866	0.102926462	0.45247709	0.650925322	0.910782566
211.8484273	0.01628382	0.101061451	0.161127904	0.871992663	0.972881041
23.98354529	-0.380210782	0.342354581	-1.110576002	0.266750897	0.709341177
1506.738346	0.016964849	0.038378129	0.442044724	0.658456843	0.914041253
65.16046315	0.02844121	0.539192422	0.052747793	0.957932859	0.987864832
275.7757734	0.320018637	0.090740147	3.526759075	0.000420679	0.044477829
7.881164682	-0.150399266	0.360755393	-0.41690095	0.676750859	0.921393988
17.11746471	-0.011557368	0.251198897	-0.046008831	0.963303211	0.989954388
235.2728906	-0.10582914	0.188689809	-0.560863038	0.574890909	0.886202779
96.90765609	-0.093051905	0.205979755	-0.451752671	0.651447169	0.910978693
164.6533519	0.268014205	0.313314405	0.855416159	0.392320801	0.795497602
724.1309317	0.012246833	0.103198504	0.118672583	0.905534759	0.979768942
180.6485011	-0.132580859	0.19942271	-0.664823276	0.506163533	0.859011504
119.8950562	-0.044349989	0.103077125	-0.430260246	0.667006342	0.916630571
165.1204322	0.058271747	0.098685987	0.590476409	0.554871297	0.877791979
256.9732634	0.040663832	0.088834985	0.457745702	0.64713515	0.910195219
145.0925846	0.20883063	0.10535014	1.98225299	0.047450933	0.397042795
646.0580589	-0.206057081	0.526291397	-0.3915266	0.695408031	0.924581226
26.49899212	-0.060851746	0.307777876	-0.197713192	0.843269468	0.968131661

6.914092028	0.544685772	0.46412309	1.173580422	0.240563127	0.682548146
84.38729517	-0.126511406	0.22822472	-0.554328237	0.579354229	0.887467795
315.537245	0.012170831	0.062120694	0.195922321	0.844670966	0.968131661
81.2603716	-0.063204465	0.13438416	-0.470326752	0.638121587	0.907684986
236.463192	-0.141986482	0.194512938	-0.729959065	0.465415207	0.837170481
271.3160787	-0.148038884	0.075216419	-1.968172467	0.049048197	0.402791699
262.4834188	-1.096682274	0.357801929	-3.065054112	0.002176306	0.111533785
69.6688675	-0.202529512	0.222881675	-0.908686245	0.363515767	0.779728871
49.56063929	-0.191286512	0.619199663	-0.308925413	0.757378265	0.945625212
196.277792	0.095601391	0.088925931	1.075067645	0.282344444	0.723520163
62.66704482	-0.079651174	0.304702524	-0.261406348	0.793779162	0.953153173
70.8309224	-0.04469065	0.193334043	-0.231157689	0.817192299	0.961335246
36.75100913	-0.421090048	0.319984985	-1.315968148	0.188184734	0.634368123
3719.68227	0.035534831	0.233409774	0.15224226	0.878995864	0.974940935
335.3126256	0.023295202	0.091513484	0.254554864	0.799066924	0.955167977
26.65075353	0.108140533	0.219434426	0.492814801	0.622143452	0.902441895
54.891386	-0.113016312	0.20812131	-0.543030947	0.587108495	0.890984353
305.008237	0.021094481	0.085995046	0.245298788	0.80622509	0.957533802
132.2701427	0.070007127	0.115982464	0.603600961	0.546108974	0.873065405
6.325013884	-0.570658635	0.760407242	-0.750464493	0.452975001	0.830105164
395.7666587	0.053676855	0.080820309	0.664150578	0.506593941	0.859011504
5.83533339	0.690760986	0.589167743	1.172435175	0.241022384	0.68279681
38.80748041	-0.049606231	0.189224104	-0.262155982	0.793201188	0.953153173
134.7463351	0.124073506	0.104832073	1.183545293	0.236593107	0.679408314
251.2908635	-0.076202267	0.102454717	-0.743765334	0.457018456	0.832305398
78.71226458	0.300893339	0.100605188	2.990833224	0.002782174	0.12485574
75.3469473	-0.135428511	0.113043188	-1.198024516	0.230907473	0.673080052
18788.00903	-0.926240061	0.508640967	-1.821009556	0.068605404	0.45784364
16.49611965	-0.124116423	0.243663768	-0.509375786	0.610488846	0.900314426
836.1304027	0.163721588	0.134587102	1.216473085	0.223804758	0.667854242
2637.113142	-0.034658011	0.119411318	-0.290240585	0.77163219	0.948706053
4213.261182	0.01554345	0.130686181	0.118937212	0.9053251	0.979768942
467.7565989	0.177248593	0.068731973	2.578837546	0.009913339	0.215114309
12.24224361	-0.018669032	0.33488536	-0.055747532	0.955542934	0.987483032
366.814626	-0.14528644	0.098332116	-1.477507505	0.139539648	0.572130062
350.9952285	-0.091657027	0.144479624	-0.634394143	0.525823631	0.864850467
410.0404363	0.052793879	0.127592089	0.413770791	0.679041977	0.921716417
177.1292385	-0.037717958	0.201670219	-0.187027902	0.851638756	0.969398357
294.9726593	0.14191933	0.118968481	1.192915377	0.232902516	0.675965303
90.37949786	-0.397854523	0.250016021	-1.591316114	0.111538452	0.53158829
522.6045589	-0.262423769	0.145386319	-1.805010064	0.071073107	0.462488289
404.5775415	-0.088725028	0.086980561	-1.020055833	0.307701982	0.743405059
163.8113587	-0.040997669	0.147452941	-0.278039006	0.780982416	0.95173434
92.27958927	-0.143725111	0.148766901	-0.966109464	0.333989409	0.761545389
338.7949406	0.008715725	0.087967802	0.099078584	0.921075876	0.982590996
108.9266994	0.008903201	0.133085732	0.066898241	0.946662713	0.985759593
916.933973	0.07162223	0.060199339	1.1897511	0.234144235	0.676914641
7.211361091	-0.495632209	0.455413964	-1.0883114	0.276457659	0.718547195

269.3842253	0.073351842	0.108342018	0.677039649	0.498380798	0.855506503
73.7707213	-0.127887142	0.124618072	-1.02623271	0.304781907	0.741672489
340.826139	-0.123768318	0.122879605	-1.007232392	0.31382311	0.747661118
339.5108079	0.190345413	0.109196718	1.743142248	0.08130877	0.481991357
329.7943089	0.035885088	0.082804597	0.433370717	0.664745471	0.916049829
7.056048987	0.420162923	0.556047391	0.755624305	0.449874479	0.828449907
48.33942811	0.059816819	0.29551615	0.202414721	0.839592521	0.966440785
1321.96694	0.112781987	0.065796269	1.714109137	0.086508684	0.490801664
219.8041861	0.206342446	0.16951922	1.217221537	0.223519938	0.667854242
19.65499649	-0.243891375	0.254390217	-0.958729381	0.337695093	0.7637398
31.03764497	-0.307772626	0.182181553	-1.689373162	0.091147941	0.498962299
401.3577528	-0.007169761	0.055801936	-0.12848588	0.897764472	0.97893802
6.585446809	1.005764091	0.408855297	2.459951231	0.01389559	0.251082651
38.25945916	0.037566347	0.182560949	0.205774274	0.836967238	0.966316856
689.2524488	-0.058054668	0.049150968	-1.18115004	0.237543124	0.679753163
157.624787	-0.058725123	0.10583704	-0.554863614	0.578987951	0.887274009
657.3995169	-0.226141206	0.091449333	-2.472857911	0.013403742	0.248838234
302.618345	-0.061512585	0.107244811	-0.573571668	0.566257688	0.883595206
51.725074	0.041916889	0.150787966	0.27798564	0.781023382	0.95173434
1178.464251	0.464488184	0.159602978	2.910272657	0.003611136	0.137979288
129.784869	-0.000744576	0.098462678	-0.007562017	0.993966441	0.998877139
143.6282051	-0.014842063	0.202511839	-0.073289854	0.941575466	0.98438404
134.9056703	-0.011160083	0.16632653	-0.067097434	0.946504137	0.985728152
167.0164902	-0.044827278	0.176069736	-0.254599563	0.799032397	0.955167977
52.38391171	0.049597827	0.167857633	0.295475553	0.767630625	0.94737077
70.68372627	0.084196556	0.135589217	0.620967935	0.534620721	0.867893827
1775.247822	0.08738666	0.138136096	0.632612785	0.526986536	0.865114435
371.3911998	0.026577127	0.067729344	0.392401961	0.694761237	0.924581226
44.11443358	-0.089085307	0.177861233	-0.500869727	0.616462809	0.901900555
2411.201806	-0.107692286	0.157962855	-0.681757027	0.495392601	0.853825991
1668.599611	-0.05523253	0.117302853	-0.470854101	0.637744927	0.907684986
45.38061611	-0.031635733	0.14194676	-0.222870416	0.823636362	0.96344756
9.882940173	-0.333515105	0.325251489	-1.02540685	0.305171257	0.741701149
1328.723834	0.057854405	0.070716964	0.818112117	0.413293171	0.808272153
21.62398482	-0.144264507	0.399658468	-0.360969473	0.718122267	0.930765281
521.6985043	0.032848686	0.090467619	0.363098825	0.716531062	0.930277413
12.71791987	0.10923452	0.314108606	0.347760355	0.728020166	0.934037323
251.6358764	-0.024230883	0.094911691	-0.25529924	0.798491987	0.95501603
7.596034431	-0.787236635	0.39604777	-1.987731518	0.046841391	0.395069101
29.50348532	-0.207597958	0.427855681	-0.485205567	0.627530533	0.904512355
12.43704366	-1.34196969	0.467808395	-2.868631055	0.004122524	0.144755559
426.2894711	-0.089477094	0.050847837	-1.759703053	0.078458168	0.474223305
716.7967919	0.073322199	0.10832751	0.676856679	0.498496893	0.855606564
687.3905126	-0.087324873	0.081074564	-1.07709334	0.281438568	0.722987449
218.4249797	0.250431224	0.098582363	2.540324818	0.011074956	0.227999302
7.929542426	-0.153749798	0.391833603	-0.392385433	0.694773448	0.924581226
420.4181191	0.016097144	0.058663881	0.274396171	0.783780185	0.951880071
1484.375951	0.307276458	0.141442951	2.17244095	0.029822419	0.330883948

253.7322515	-0.137409047	0.100141272	-1.372151999	0.170016125	0.612975504
186.0383651	0.078035082	0.09839085	0.793113203	0.427711871	0.817479701
469.5307996	0.017106267	0.065818327	0.259901281	0.794939923	0.953773263
10.1406647	-0.55293326	0.41603941	-1.329040585	0.183834581	0.629653329
2842.841623	-0.288707095	0.17489995	-1.650698556	0.098800143	0.51077273
7.151721023	-1.20519626	0.576751708	-2.089627551	0.03665127	0.360329398
143.4866381	-0.149148286	0.237215158	-0.628746863	0.529514792	0.866063379
254.2243109	0.04717814	0.072035803	0.654926273	0.512515224	0.860200661
402.9962891	0.021816754	0.071235626	0.306261846	0.759405287	0.946465822
12.01344235	-0.098246056	0.275713267	-0.356334163	0.721590328	0.931135539
227.9293762	0.138204363	0.155770444	0.887230976	0.374954559	0.786801485
22.00533765	-0.660726901	0.334879455	-1.973029077	0.04849225	0.400922142
22095.4452	0.083627364	0.136388099	0.613157341	0.539772308	0.869675597
66.63890798	-0.191259598	0.136696188	-1.39915824	0.161765536	0.601319304
73.17729427	-0.106253468	0.13479873	-0.788237901	0.430557564	0.818841807
254.2504964	0.05424037	0.087059292	0.623027921	0.533266174	0.867356078
12.20559687	-0.967056552	0.620581089	-1.558308122	0.119160225	0.54348307
354.1809258	0.136020607	0.079466338	1.711675798	0.086956431	0.491556286
51.38745519	-0.445337306	0.187499078	-2.375143976	0.017542106	0.269492791
950.7086275	0.065759552	0.150245611	0.437680352	0.661618014	0.915002321
8.658810525	0.240928203	0.386718619	0.623006474	0.533280268	0.867356078
15.72224508	0.274280548	0.297458327	0.922080584	0.356486544	0.774392988
464.1217213	0.20182768	0.121808211	1.656930014	0.097533625	0.508377369
135.112312	0.067705425	0.088942545	0.761226526	0.446521777	0.826590213
489.3249089	-0.139982591	0.118619711	-1.180095534	0.237962221	0.679827763
100.1526846	-0.158108551	0.140660024	-1.124047523	0.260992866	0.703170077
243.4735599	-0.424942315	0.241969575	-1.756180771	0.079057547	0.476247807
51.23264125	0.31515156	0.329511808	0.956419624	0.338860274	0.764007173
51.04781999	0.215788077	0.175621899	1.22870826	0.219181208	0.663637842
591.9495223	-0.047215995	0.050240431	-0.939800752	0.347319773	0.768780165
23.55139799	0.101219503	0.431811177	0.234406861	0.814669132	0.960909552
13.24707184	0.405811764	0.530382076	0.765130992	0.444193546	0.826036581
306.8064944	0.120136208	0.089183562	1.347066727	0.177958743	0.622328585
22.02613547	0.234856115	0.361856392	0.649031272	0.516318163	0.862002027
293.4752211	0.044457801	0.094852385	0.468705145	0.639280409	0.907684986
669.4074199	0.138749458	0.094977404	1.460868076	0.144051649	0.578069441
11.64184821	-0.150917656	0.33395562	-0.451909318	0.651334311	0.910908439
1958.48092	0.16550688	0.04060555	4.075966917	4.58235E-05	0.013047858
119.317162	0.171015725	0.133632829	1.279743353	0.200635412	0.645045462
48.23602567	-0.032477348	0.160462547	-0.20239831	0.839605349	0.966440785
1620.051359	0.038242597	0.126153201	0.303144088	0.761780057	0.946826103
108.4604066	-0.215884404	0.207900932	-1.038400366	0.299083699	0.738631505
149.0898309	0.192229691	0.10696807	1.797075434	0.072323645	0.463635598
48.42971681	-0.271898473	0.458663136	-0.592806467	0.55331068	0.877205564
121.846037	-0.027214504	0.179629467	-0.15150356	0.879578503	0.975321146
351.0665176	0.083912112	0.106894462	0.784999618	0.432453792	0.819823391
40.20588906	0.179569466	0.16605114	1.08141062	0.2795145	0.721452326
85.00780274	0.32666784	0.290717988	1.123658848	0.261157781	0.703358346

29.96819512	0.158146377	0.216938657	0.728991225	0.46600703	0.837543146
170.7137217	-0.138067846	0.099978164	-1.380980009	0.167285107	0.609023925
589.848041	0.003879366	0.145264705	0.026705495	0.97869463	0.99459552
87.56454174	-0.679189533	0.282421559	-2.404878493	0.016177844	0.262858884
134.7369263	-0.085460874	0.181630267	-0.470521107	0.637982758	0.907684986
522.3020604	-0.022683358	0.080184493	-0.28288958	0.777261483	0.950985656
32.47561349	-0.081723962	0.21365843	-0.382498189	0.702091872	0.926349622
320.4989733	-0.075187973	0.065738883	-1.143736691	0.252732852	0.695085007
1528.352822	-0.196403576	0.13061561	-1.503676139	0.132664776	0.56493211
11.41865425	-1.808785895	0.73515324	-2.460420219	0.013877442	0.251082651
1040.4579	0.009232591	0.128405748	0.071901694	0.942680142	0.98446865
91.2700906	-0.096340051	0.274134026	-0.351434121	0.725262688	0.932453601
383.0858724	0.007198643	0.060445772	0.119092575	0.905202013	0.979768942
47.55334461	-0.108995687	0.226999569	-0.480158122	0.631114962	0.905472877
368.1190655	0.067759638	0.093897398	0.721634887	0.470518984	0.839938793
80.37993052	-0.145795161	0.142101396	-1.025993872	0.304894473	0.741672489
686.4668796	-0.021273725	0.067979908	-0.312941367	0.754325209	0.94414502
17.98639003	-0.484002085	0.345563198	-1.400618146	0.161328292	0.601052447
78.6508035	-0.075363143	0.183886691	-0.409834682	0.681927223	0.922658753
111.2444931	-0.327647287	0.199689931	-1.640780207	0.100843048	0.512774612
1832.08382	0.147284985	0.10779491	1.36634452	0.171830853	0.616109027
92.50554592	0.166291384	0.164789857	1.009111771	0.312921033	0.746832817
7.979616513	-0.193604667	0.367425995	-0.526921529	0.598248083	0.897417322
221.6627707	0.098254023	0.099797023	0.984538622	0.324850751	0.756297513
4041.512999	0.123612648	0.117100109	1.055615135	0.291144083	0.732859559
54.85001812	-0.124208559	0.126542334	-0.981557356	0.326317965	0.756959492
23.92253618	-0.622012911	0.315860561	-1.969264252	0.048922754	0.402308114
1369.486805	-0.361324053	0.250109324	-1.444664465	0.148552156	0.585550863
7.452327247	-1.016928247	0.405800512	-2.505980688	0.012211229	0.237458144
1203.533183	0.029552516	0.11247544	0.262746391	0.792746058	0.953153173
5060.137145	-0.544111852	0.407703176	-1.334578399	0.182014372	0.627539984
202.905631	0.173802117	0.094370413	1.841701351	0.065518846	0.449610554
17.76246766	-0.123858201	0.260958871	-0.474627286	0.635052654	0.90701218
182.4913403	0.359868964	0.096212339	3.740361887	0.000183755	0.028506312
485.2075809	0.044302016	0.103136895	0.429545766	0.667526096	0.916775933
190.9393574	-0.017613365	0.087323553	-0.201702341	0.840149432	0.966440785
39.60901428	-0.317183959	0.233360736	-1.359200196	0.174083159	0.618034305
102.0810937	0.01392779	0.106772047	0.130444163	0.896215028	0.978807264
223.7719236	-0.216517382	0.08402289	-2.57688568	0.009969492	0.215114309
17.90150324	-0.093500754	0.256188987	-0.364967888	0.715135377	0.930178722
235.6067651	0.089728252	0.112811943	0.795379017	0.426393062	0.816487562
22.86049046	-0.957522699	0.372961191	-2.567352103	0.010247849	0.218571568
55.89629408	-0.121485447	0.182442069	-0.665885054	0.505484575	0.858625297
2420.977887	-0.302337448	0.199457926	-1.515795611	0.129571042	0.561449229
18.24106346	0.341090194	0.372544451	0.915569117	0.359892936	0.777407022
324.9746317	-0.026541932	0.170873659	-0.155330741	0.876560582	0.973964714
242.6490873	0.185013315	0.084608701	2.186693722	0.02876489	0.328011759
1062.206867	1.207577289	0.584030717	2.067660576	0.038671952	0.367408361

9.189164515	0.207339226	0.341411051	0.607300864	0.543651265	0.871466973
50.689646	-0.008683821	0.145387116	-0.05972896	0.952371506	0.98648845
213.5485677	-0.00560795	0.077383241	-0.072469821	0.942228021	0.98446865
548.806197	-0.059201612	0.109087894	-0.542696442	0.587338824	0.891054846
10.56620673	-0.478675742	0.28912937	-1.655576331	0.097807647	0.509133783
5.326465135	-0.361973354	0.398298271	-0.908799714	0.363455858	0.779728871
12.47629292	-0.290317027	0.31544841	-0.920331243	0.357399687	0.775579852
405.1432534	0.173200496	0.101833946	1.700812973	0.088978112	0.493693668
313.7153374	0.058691423	0.077572413	0.756601742	0.449288497	0.828449907
500.5996808	0.002758135	0.078695373	0.035048247	0.972041269	0.992799208
670.3756862	0.131824648	0.085326063	1.544951718	0.122357942	0.548630842
113.2288002	0.220582725	0.306502009	0.719677911	0.471723329	0.840549985
106.5087358	-0.186462112	0.176329738	-1.05746265	0.290300492	0.731967225
10.4284737	-0.3095262	0.353122751	-0.876539955	0.380736565	0.78844599
206.8867859	0.152590401	0.262378513	0.58156592	0.560859104	0.880936184
2169.753755	0.129179315	0.130575342	0.989308648	0.322512154	0.754993659
619.7862207	0.06590305	0.065041944	1.013239241	0.310945901	0.745771331
31.34167696	-0.442357459	0.23571267	-1.87668087	0.060561846	0.436434491
103.9529887	-0.04758181	0.125983939	-0.377681558	0.70566717	0.927151202
86.25571114	0.414478278	0.456208764	0.908527654	0.36359951	0.779728871
86.36495361	0.167125839	0.129496886	1.290578056	0.196850029	0.642224847
336.5420589	0.121470234	0.121075908	1.003256852	0.315736949	0.749565087
482.0844568	0.016184628	0.081733151	0.198017917	0.843031045	0.968004463
16.55862747	-0.145469615	0.272810635	-0.5332256	0.593877422	0.894609104
1063.559673	0.062259872	0.083179744	0.748497996	0.454159834	0.831063649
299.4445102	0.191104818	0.083819692	2.279951322	0.022610576	0.300162997
39.69489471	-0.163587116	0.363581915	-0.449931937	0.652759518	0.911644397
24.67415642	-0.956427063	0.525382869	-1.820438235	0.068692294	0.458216917
539.8637983	0.08413093	0.063492355	1.325056059	0.185152559	0.630643427
59.15403593	-0.012976932	0.226423669	-0.057312611	0.954296175	0.987292586
163.8181373	-0.024862259	0.114870491	-0.2164373	0.828646894	0.964814045
56.67491312	-0.075457967	0.140912238	-0.535496189	0.592306792	0.893683696
193.9044974	-0.180109742	0.105468688	-1.707708177	0.087690504	0.492827855
845.1969151	-0.145029148	0.081136376	-1.787473828	0.073860939	0.467817549
158.1429433	-0.166405969	0.136488901	-1.219190481	0.222771904	0.666984161
91.3952622	-0.415331333	0.268265679	-1.54820898	0.12157199	0.547270993
56.86522975	-0.097311132	0.395412594	-0.246100235	0.805604641	0.957233889
338.9056223	-0.141076613	0.058236726	-2.422468143	0.015415477	0.258245094
6.515285817	0.369164545	0.387441718	0.952826007	0.340678235	0.76465745
1512.385587	0.193674777	0.12946164	1.496001258	0.134653327	0.567901571
157.5043664	-0.042278216	0.136954589	-0.308702446	0.757547883	0.945625212
98.38761124	-0.015164981	0.1014695	-0.149453594	0.881195725	0.975487608
47.35378268	-0.038462362	0.191429053	-0.200922279	0.840759347	0.966642825
167.1412637	-0.098685288	0.105232533	-0.937783078	0.348355899	0.768780165
530.4880479	-0.156578394	0.058422726	-2.680093948	0.00736015	0.187448858
346.1984682	0.00064373	0.058150626	0.011070036	0.991167569	0.998213079
102.9180901	0.18679363	0.136840183	1.365049545	0.172237478	0.616109027
97.84472666	0.151232643	0.241483458	0.626265024	0.531141123	0.866139794

162.8836344	0.107153845	0.241233499	0.444191394	0.656904213	0.913625833
189.7147403	-0.156899376	0.206529048	-0.759696409	0.447436076	0.827089297
730.5118633	-0.200781216	0.064631182	-3.106568848	0.001892722	0.103380352
11.37517481	-0.824842779	0.321215301	-2.567881349	0.010232217	0.218571568
64.75431105	-0.172272682	0.130843913	-1.316627404	0.187963548	0.634170926
28.07465516	-0.488869561	0.285414743	-1.7128392	0.086742126	0.491184756
841.0629306	-0.117261937	0.071997859	-1.628686433	0.103379416	0.516137077
243.1203104	-0.038784177	0.104839471	-0.369938697	0.711428167	0.929376205
576.1090396	0.086556654	0.102176674	0.847127334	0.396924151	0.79837903
67.99053062	0.155775836	0.127299048	1.223699928	0.221065447	0.664764193
389.5524284	-0.119703337	0.100151636	-1.195220993	0.232000699	0.674803368
4.600543805	-1.583537707	0.56205133	-2.817425427	0.004841036	0.154050722
1206.690819	0.05035815	0.134576367	0.374197577	0.708257326	0.92855044
7.544449415	0.342413057	0.336322426	1.0181095	0.308625921	0.744049104
82.6693179	0.123766649	0.159678478	0.775099129	0.438281086	0.82251807
76.56664395	0.08478606	0.271836618	0.311900804	0.755115912	0.944498739
84.13340781	-0.150229721	0.136309213	-1.102124484	0.270407556	0.713216794
5.095999694	-0.391270752	0.503005906	-0.777865126	0.436648529	0.822078703
1034.418818	0.052982917	0.092964779	0.56992463	0.568728819	0.8841274
2203.598604	-0.091362957	0.126388398	-0.722874552	0.469756959	0.839786136
948.4317963	-0.050536182	0.109019958	-0.46354982	0.642970313	0.908494289
256.9593341	-0.179930075	0.114486346	-1.571629119	0.116036589	0.537362993
84.99042848	0.053573715	0.146320777	0.366138806	0.714261502	0.930012765
56.99063975	-0.146271227	0.14722078	-0.993550137	0.32044193	0.752619929
305.9959841	0.108607553	0.087477161	1.241553243	0.214401442	0.659648752
27.24949202	0.022938182	0.229147755	0.100102147	0.920263231	0.982171487
154.7345035	-0.023289499	0.095512255	-0.243837813	0.807356434	0.957955105
241.9973673	0.100849907	0.074459069	1.354434167	0.175597903	0.619005807
1362.357066	-0.004695216	0.088738227	-0.052910862	0.95780293	0.987864832
226.2715193	-0.073431651	0.090617472	-0.810347603	0.417740423	0.811361647
1117.158462	0.04596992	0.053458637	0.859915685	0.389835522	0.793258985
684.712246	-0.034336013	0.160737797	-0.213615052	0.830847261	0.96562509
455.7010455	-0.144469333	0.153993085	-0.938154674	0.348164928	0.768780165
388.4717685	-1.147112483	0.435042648	-2.636781677	0.008369668	0.198674451
27.1755567	-0.435043468	0.255156318	-1.705007626	0.088193002	0.492827855
12.73928911	-0.157698502	0.321070879	-0.491164141	0.623310359	0.902674878
15.10602858	-0.153847455	0.295668792	-0.520337145	0.602828611	0.899375001
116.8040542	-0.09487944	0.089023188	-1.065783448	0.286521545	0.727013824
204.0257468	-0.273536295	0.088520161	-3.090101645	0.00200088	0.106536068
1649.54014	0.23673479	0.084503333	2.801484655	0.005086806	0.156864381
236.490085	-0.187023736	0.072197644	-2.590440965	0.009585306	0.211763732
75.96611008	0.178260641	0.133893667	1.331359759	0.183070664	0.628580831
1251.416627	0.111262533	0.051498193	2.160513327	0.030732953	0.33384793
7.247991945	-0.084951512	0.386005943	-0.220078248	0.825810215	0.964118059
158.9737457	-0.112932945	0.088997472	-1.26894553	0.204460492	0.648992858
398.097545	-0.039440421	0.097494307	-0.404540764	0.685815108	0.923064029
11.19844212	-0.538268181	0.396313502	-1.358187844	0.174404087	0.618034305
16.04791398	0.313433285	0.42478662	0.737860541	0.460599199	0.834673974

87.70516947	-0.975696304	0.403575642	-2.417629318	0.015621982	0.259816378
92.6317997	-0.373966575	0.217619957	-1.718438786	0.085716611	0.489179326
571.6862911	0.027092006	0.057740457	0.46920318	0.638924413	0.907684986
298.5028776	-0.056681199	0.06418548	-0.883084457	0.377190641	0.787072097
2511.457687	0.028519663	0.111378121	0.256061628	0.797903251	0.954840537
1012.165706	-0.26548174	0.256900741	-1.033402003	0.301415809	0.739331412
108.7593509	-0.460102321	0.245760682	-1.872155945	0.061185032	0.437941099
51.69392683	-0.028413293	0.190913792	-0.14882787	0.881689458	0.975487608
197.4777468	-0.081092992	0.097424113	-0.832370849	0.405199651	0.803717932
18.1032473	0.569815416	0.381989324	1.491705081	0.135776477	0.568673186
260.6397719	0.115018541	0.223784713	0.51396961	0.607273238	0.900307811
113.0824828	-0.0673015	0.120643173	-0.557855857	0.576942817	0.88676146
5.345740301	-1.50183197	1.448884861	-1.036543352	0.299948726	0.738923503
1065.65522	0.039980223	0.073859889	0.541298176	0.588302077	0.891487239
430.6144449	-0.058052643	0.074470543	-0.779538337	0.435662663	0.822078703
100.1344198	-0.042111299	0.108664375	-0.38753546	0.698359839	0.925802378
125.8973924	0.055967099	0.104800703	0.534033618	0.593318275	0.894509789
1379.302987	-0.000120988	0.07895989	-0.001532278	0.99877742	0.99966493
157.0375594	0.180602894	0.14334295	1.259935655	0.207692575	0.652433255
102.4506058	0.371656702	0.137401328	2.704898911	0.006832518	0.181059331
139.2151758	0.065072489	0.131149168	0.496171572	0.619773366	0.901976704
3875.826246	0.013349175	0.072606409	0.183856698	0.854125876	0.969966486
331.8652503	0.000264747	0.10578256	0.00250275	0.998003097	0.999394422
71.49713015	0.017153408	0.145115075	0.118205554	0.90590479	0.979768942
175.4911784	-0.030027229	0.12496414	-0.240286764	0.810107955	0.958722103
137.4775171	-0.207499798	0.116764561	-1.777078555	0.075555316	0.470694355
67.99446311	-0.383990307	0.206336251	-1.860992948	0.062745173	0.442595858
294.1318658	0.033790956	0.103832878	0.325436	0.744851113	0.940455922
415.7738616	-0.078675418	0.071941266	-1.093606236	0.274127713	0.716722874
2014.572688	-0.058129855	0.118123397	-0.492111272	0.622640684	0.902441895
63.12317498	-0.034098202	0.12826118	-0.265849748	0.790354928	0.953061804
1108.348679	0.106184985	0.063065223	1.683732803	0.092233319	0.499801846
75.9971224	0.110636054	0.224258343	0.493341979	0.621770972	0.902299404
24.97887213	0.001085545	0.218561763	0.004966766	0.99603711	0.998981303
562.3405825	-0.056133851	0.089315295	-0.628490909	0.5296824	0.866063379
249.8966763	-0.148986929	0.111835196	-1.332200722	0.182794239	0.628481684
230.3138893	-0.031479433	0.142359867	-0.221125756	0.824994516	0.963893664
77.79970241	0.001268079	0.118531538	0.010698237	0.991464204	0.998263407
203.7149299	-0.20515455	0.15246118	-1.345618276	0.178425655	0.623105158
411.1700749	0.298357985	0.133901049	2.228197513	0.025867347	0.314616654
152.8376069	-0.121015916	0.23547551	-0.513921453	0.607306908	0.900307811
307.072348	-0.097918119	0.105656387	-0.92676005	0.354051131	0.773422825
305.256536	0.015293805	0.086644952	0.176511204	0.859892348	0.969966486
329.2408732	-0.19580794	0.135254879	-1.447695947	0.147702104	0.58409168
373.6313162	0.017258154	0.146430053	0.117859368	0.906179088	0.979768942
123.738883	-0.037347808	0.095117822	-0.392647845	0.694579596	0.924581226
4711.697122	-0.024432162	0.09048382	-0.270016918	0.787147238	0.95288876
787.8679782	-0.264255365	0.068637375	-3.850021414	0.000118107	0.019959647

49.53815557	-0.423033819	0.209125953	-2.022866187	0.043086946	0.380532797
53.14583456	-0.112073434	0.907550779	-0.123489987	0.901719103	0.979381499
106.5681147	-0.201547652	0.137258434	-1.468380823	0.142000808	0.576571115
106.3626969	-0.080848386	0.129444461	-0.624579724	0.532246931	0.866797125
12.53423597	-0.455382887	0.394550788	-1.154180655	0.248426112	0.69187626
95.28640322	-0.732247959	0.356590269	-2.053471513	0.04002687	0.372780665
366.1498823	0.103740215	0.063057011	1.645181307	0.099932427	0.511833836
72.9082022	0.038972431	0.183842213	0.211988477	0.832116024	0.966227926
191.5006145	0.13539374	0.096058421	1.409493702	0.158689235	0.598908223
253.9693521	0.0770191	0.09079911	0.848236285	0.396306394	0.79798834
287.0331393	-0.083106073	0.145838918	-0.569848389	0.568780533	0.8841274
26.86236727	0.130598608	0.190610679	0.685158927	0.493243639	0.852046514
480.513676	0.111750345	0.080128342	1.394641924	0.163123846	0.601684318
124.594806	-0.008981281	0.096789254	-0.092792133	0.926068701	0.983192434
200.1573449	0.033421119	0.074067568	0.451224739	0.651827581	0.911246717
76.07038248	0.040454274	0.119026362	0.339876588	0.733949468	0.937191168
1126.547493	-0.154458392	0.058180451	-2.654815977	0.007935168	0.192551414
9.052290761	0.260519438	0.323716504	0.80477651	0.420948659	0.813112626
49.47716144	-0.604002008	0.379435304	-1.591844517	0.111419645	0.531325251
75.58087198	0.463951871	0.319480617	1.45220663	0.14644416	0.582080143
483.6652358	0.018138221	0.056268308	0.322352349	0.747185779	0.941343425
475.7180337	-0.049289345	0.169728953	-0.290400336	0.771509987	0.948706053
1210.307706	0.032771541	0.1736565	0.18871474	0.850316398	0.969251033
4899.055429	0.008362416	0.068014892	0.122949773	0.902146871	0.979381499
150.2551282	0.106371507	0.135913032	0.782643913	0.433836247	0.821182113
174.418968	-0.467384017	0.206110327	-2.267639979	0.023351159	0.304263956
523.3921992	0.022761256	0.090356189	0.25190589	0.801113807	0.955911855
922.910605	0.039959709	0.072215985	0.553336067	0.580033308	0.887483205
251.8825659	0.03423017	0.231203915	0.14805186	0.882301841	0.975560757
293.8720113	0.060307366	0.093896094	0.64227768	0.520692904	0.863188882
144.2843283	0.075823799	0.12071475	0.628123728	0.52992289	0.866123834
65.92890114	-0.769757357	1.051111016	-0.732327362	0.463968783	0.836383228
409.7595643	-0.085538891	0.131460469	-0.650681471	0.515252127	0.861826223
1069.141101	1.618833209	0.600297462	2.696718395	0.007002646	0.182836032
42.22089758	-0.17937157	0.156626877	-1.145215769	0.252119788	0.694819791
125.349935	-0.308364956	0.24578915	-1.25459141	0.209627123	0.654774936
53.25117487	0.172428073	0.170142823	1.01343136	0.310854167	0.745771331
130.2115917	0.036947173	0.121290762	0.304616542	0.76065822	0.946826103
472.4382625	-0.0460665	0.096554583	-0.477103188	0.633288645	0.906724287
13179.81884	-0.019017581	0.139126214	-0.13669301	0.891273455	0.977283948
492.7866592	0.16866247	0.136961078	1.231462786	0.218149827	0.662774346
92.10459772	-0.117792579	0.096600263	-1.219381546	0.222699411	0.666984161
247.0228218	-0.140344119	0.076808756	-1.827189049	0.06767135	0.455435138
6.196983828	-0.694014598	0.385816206	-1.798821791	0.072046875	0.463154629
124.368641	0.116573989	0.175860216	0.66287869	0.507408251	0.859144938
10.95030439	0.434246911	0.464879549	0.934106289	0.350249068	0.770421231
60.84805595	0.145636621	0.153747127	0.947247753	0.343512547	0.766809956
13.84693129	0.09642759	0.288846844	0.333836399	0.738503015	0.939226436

10.19133848	0.648521629	0.371451262	1.745913114	0.080826051	0.480761161
89.09539785	-0.200549379	0.193584358	-1.035979257	0.30021182	0.739093677
196.2377557	-0.176026808	0.206282732	-0.853327888	0.393477499	0.796185896
198.4355755	0.011233846	0.087542059	0.128325131	0.897891678	0.97893802
1770.494306	0.187913218	0.132096139	1.422548907	0.154866964	0.592842063
11.47132485	-0.233220635	0.45118442	-0.516907555	0.605220694	0.90025894
149.7418936	-0.137883428	0.098612295	-1.398237694	0.162041701	0.601439633
23.98641715	0.223207031	0.398523693	0.560084721	0.575421652	0.886202779
86.4687782	-0.173523051	0.116650723	-1.487543724	0.136871264	0.569036171
922.2271826	0.085678633	0.128022006	0.66924926	0.503336488	0.857172233
156.4030143	0.115193575	0.10784854	1.068105093	0.285473109	0.726643643
267.3997014	0.039037007	0.08709709	0.448201044	0.654008109	0.911976263
5.56028501	-0.230934299	0.648318098	-0.356205232	0.721686874	0.931135539
610.57152	-0.128025754	0.106018751	-1.207576519	0.227210196	0.669850929
16.63443304	-0.314338096	0.258481481	-1.21609523	0.223948647	0.667854242
28.08742905	0.213202949	0.231695972	0.920184097	0.357476563	0.775632965
499.5182561	-0.060170253	0.10390752	-0.57907506	0.562538527	0.881820831
780.7489329	0.028891965	0.036494368	0.791682831	0.428545636	0.818177802
1617.815301	0.043620688	0.067368208	0.647496633	0.517310573	0.862002027
72.4277761	-0.041958656	0.109083223	-0.384648112	0.700498143	0.926034765
586.783295	0.118326576	0.057713996	2.050223248	0.04034265	0.373696391
199.1489813	0.064644109	0.074886871	0.863223526	0.388014581	0.792781092
5.420384211	-0.322017966	0.52562151	-0.612642292	0.540112888	0.869829343
12.95169366	0.075022741	0.3582903	0.209390935	0.834143069	0.966316856
8.288693883	-0.117284833	0.481735814	-0.243462972	0.807646767	0.958108464
7.911066199	0.577836311	0.932847365	0.61943286	0.535631239	0.869002283
393.1460624	0.054478305	0.075411202	0.722416608	0.470038379	0.839894775
1313.179744	0.078945506	0.05963901	1.3237226	0.18559519	0.631625529
15916.78399	-0.13758612	0.178701042	-0.769923433	0.441345313	0.82422323
437.4889251	-0.061520064	0.150583349	-0.408544928	0.682873653	0.922776218
107.894635	-0.027566038	0.176225543	-0.156424759	0.875698223	0.973587929
5.207358348	0.185686804	0.611560211	0.303628001	0.761411317	0.946826103
1554.842723	-0.044988761	0.120966193	-0.371911852	0.709958481	0.929036752
51.90851549	0.444089559	0.165395652	2.685013498	0.007252688	0.186457572
1508.186357	-0.021668568	0.123697881	-0.175173319	0.860943448	0.970088938
24.72011665	0.271669939	0.223022016	1.218130587	0.223174352	0.667628691
388.7796839	0.15469296	0.078302513	1.975581028	0.048202249	0.399398071
212.4532355	-0.021867775	0.097538196	-0.224197044	0.822603983	0.963169453
94.46670719	0.077342531	0.278217477	0.277993073	0.781017677	0.95173434
150.1004266	-0.141088523	0.106990788	-1.31869785	0.187270146	0.633720007
1655.616416	0.141825087	0.095528269	1.484639986	0.137639217	0.569036171
119.1195728	0.198314666	0.101866377	1.946811803	0.051557301	0.410734915
123.6644965	0.30667988	0.111157654	2.758963235	0.005798506	0.166644523
735.1821634	0.050857978	0.067916489	0.748831077	0.453959026	0.831004392
29.12919791	0.223167317	0.197646846	1.129121569	0.258846554	0.70186028
861.9045909	0.128710548	0.108894946	1.181969898	0.237217645	0.679753163
5.493878991	-0.58452452	0.403906011	-1.447179551	0.147846642	0.584359412
444.1665331	0.124233915	0.073116946	1.699112466	0.089297995	0.493984514

210.7311205	0.16598903	0.272970649	0.608083801	0.543131895	0.871203645
2067.774913	-0.067350376	0.084633556	-0.795788091	0.426155215	0.816457351
130.5596858	0.095650641	0.150569159	0.635260507	0.525258526	0.864408528
5.810635967	-0.019656926	0.47031194	-0.041795508	0.966661716	0.990966732
80.91653307	-0.214770014	0.144684984	-1.4843974	0.137703524	0.569036171
1325.745886	0.257832842	0.138605981	1.860185545	0.062859279	0.442595858
668.760015	0.253691875	0.118169972	2.146838742	0.031806122	0.339220292
254.0210231	0.11807441	0.080175865	1.472692693	0.140833918	0.574459705
147.7859407	-0.175624548	0.128896997	-1.362518535	0.173034299	0.616575275
15.39914945	0.067643424	0.289204408	0.233894857	0.815066606	0.960909552
556.0538836	0.050776856	0.08104172	0.626552046	0.530952911	0.866123834
31.1057488	0.190699145	0.409212023	0.466015498	0.641204407	0.907798398
228.3395372	-0.000405882	0.102786277	-0.0039488	0.996849322	0.999212004
1044.20091	0.117782157	0.20100687	0.585960853	0.557901822	0.879607943
18.54956638	-0.275576016	0.28300818	-0.973738695	0.330186299	0.759333947
723.0915381	0.127161149	0.101277737	1.255568615	0.209272416	0.654527686
213.2527021	0.17407352	0.075961999	2.291586866	0.021929494	0.296249025
490.0577294	0.088372164	0.094102532	0.939105054	0.34767681	0.768780165
374.2630107	0.205988357	0.070109217	2.938106634	0.003302234	0.133812668
33.11212412	-0.17175158	0.305541861	-0.562121273	0.574033395	0.885935354
816.7569158	-0.006933918	0.110974362	-0.062482162	0.950178867	0.98625342
13.9441661	-0.092542074	0.248884276	-0.371827723	0.710021121	0.929036752
152.4283934	0.013353237	0.181289916	0.073656811	0.941283465	0.984334593
50.69761858	-0.738706283	0.308012465	-2.398299966	0.01647137	0.263145637
32.21175563	-0.107020187	0.349486483	-0.306221248	0.759436196	0.946465822
32.57707514	0.124019216	0.216530687	0.572755844	0.56681002	0.883766325
15.30215176	0.807072344	0.389292278	2.073178403	0.03815568	0.36508104
552.30267	0.114483213	0.088340809	1.295926687	0.195000744	0.640317357
325927.3196	0.187294944	0.12330251	1.518987274	0.128765699	0.559880723
78.70728137	0.349742247	0.28290779	1.236241132	0.216368946	0.660621521
800.5047116	0.076150454	0.078497577	0.970099421	0.331996938	0.760714965
52.6524894	-0.195872554	0.169573397	-1.155090114	0.248053526	0.691857602
199.7805856	-0.05489766	0.100691273	-0.545207725	0.585610659	0.890416947
354.5165253	-0.009604697	0.101074432	-0.095025977	0.924294193	0.982590996
470.3749834	0.116253047	0.061453672	1.891718487	0.058528499	0.433061669
129.4241846	0.129997828	0.150868856	0.861661123	0.388874025	0.792962851
14.10996796	0.087474835	0.517628896	0.168991405	0.865803402	0.971917805
25.56391373	-0.77647048	0.41114829	-1.88854119	0.058953334	0.434575322
3322.462517	-0.006959144	0.164757009	-0.042238836	0.966308304	0.990895439
11.52745029	-0.365650513	0.601684714	-0.607711156	0.543379062	0.871219333
17.90251136	-0.840321167	0.514066376	-1.634654991	0.102121393	0.51397513
244.3301084	-0.351947919	0.422175256	-0.833653594	0.404476216	0.8031996
4.722891574	0.955418467	0.513802715	1.859504513	0.06295566	0.442595858
352.0013557	0.042259672	0.087635339	0.482221817	0.62964838	0.904638489
13.59189503	-1.55501825	0.628031092	-2.476021123	0.013285572	0.247362306
317.3780456	0.196688962	0.131025865	1.501146067	0.133317786	0.566220634
352.9500169	0.131124802	0.166160703	0.789144485	0.430027564	0.818650305
171.2219744	0.039563889	0.130166887	0.303947415	0.761167953	0.946826103

647.5955481	0.034795671	0.070015967	0.496967653	0.619211862	0.901976704
548.6646368	0.153843226	0.088495052	1.738438726	0.082133538	0.483592928
127.4168101	0.232638409	0.334201791	0.696101623	0.48636519	0.849260062
11.19201306	-0.687448792	0.61179039	-1.123667195	0.261154238	0.703358346
105.3329896	0.162758807	0.212104824	0.767350806	0.442872969	0.825028589
909.5510335	0.104024613	0.204450361	0.508801316	0.610891497	0.900509492
459.8498965	0.091953514	0.124219071	0.740252787	0.459146623	0.834175979
164.3684856	0.087519147	0.081892731	1.068704707	0.28520275	0.726354285
481.2172874	-0.079154395	0.072857285	-1.086430758	0.27728846	0.719218718
92.80841582	0.090967012	0.372574726	0.244157765	0.807108636	0.957814642
195.2489718	0.422353111	0.318643022	1.325474222	0.185013914	0.630643427
136.931613	-0.161535464	0.241778701	-0.668112879	0.504061541	0.857575309
133.1904928	-0.017558642	0.168334333	-0.104308144	0.916924815	0.981290994
90.1163047	0.466313666	0.127992147	3.64329905	0.000269166	0.03464515
6.539779831	-1.129144119	0.522738212	-2.160056589	0.030768289	0.33384793
113.3065125	0.103206592	0.116457014	0.886220494	0.375498722	0.786839429
356.9772293	-0.420610633	0.158042447	-2.661377639	0.007782162	0.190812837
197.9673467	-0.122857942	0.144680951	-0.8491646	0.395789708	0.797831848
8.669464567	0.155007973	0.362631408	0.427453247	0.669049232	0.917818974
24.66880081	0.543959789	0.207940223	2.61594309	0.008898139	0.204313961
69.41501947	-0.126259571	0.361833383	-0.348943952	0.727131387	0.933396826
12.32606828	-1.35344526	0.76002523	-1.780789908	0.074946779	0.470687737
83.71568613	-0.294851378	0.124591707	-2.366540963	0.017955187	0.27123243
666.8920945	0.292422191	0.309464642	0.944929245	0.344695	0.767851822
53629.00815	-0.026565412	0.182642674	-0.145450191	0.884355439	0.976398641
98.10465841	0.11425274	0.341429206	0.334630836	0.737903581	0.938868945
102.4859435	-0.246750602	0.246952861	-0.999180978	0.317707029	0.751021269
55.50374486	-0.083011579	0.390540887	-0.212555412	0.831673753	0.966174974
9.494069935	0.612584697	0.478047885	1.281429573	0.200042822	0.645045462
5.89904257	-1.001821821	0.825082894	-1.214207479	0.224668505	0.667913881
5.671860779	-0.328248585	1.131724459	-0.290042848	0.771783458	0.948706053
34.22078817	0.185488098	0.154605862	1.199748161	0.230237163	0.673001336
6894.225265	0.114632593	0.136067716	0.842467235	0.399526472	0.80037241
241.9066211	0.191779371	0.16110043	1.190433638	0.233875998	0.676714943
413.2225224	0.012118385	0.067352167	0.179925691	0.857210906	0.969966486
10.2399708	-0.008568342	0.29363145	-0.029180601	0.976720553	0.99363695
211.3780595	0.072555778	0.129664601	0.559565042	0.575776155	0.886202779
377.9820599	0.063740152	0.067034619	0.950854253	0.341678368	0.765195212
70.78358637	-0.185114447	0.133215278	-1.389588712	0.164653805	0.604615635
7967.344797	0.169441847	0.112384291	1.507700457	0.131631209	0.564647789
752.1493435	0.241434179	0.084332202	2.862894287	0.004197906	0.145184815
744.5323816	0.068962005	0.084472821	0.81638099	0.414282266	0.808821113
53.69773781	0.121619784	0.144897498	0.839350474	0.401272667	0.801291311
141.0068427	0.190558748	0.098342103	1.937712759	0.052658276	0.41526255
6.693919496	-1.136245383	0.508214389	-2.235759965	0.025367499	0.313430481
595.0923886	0.076548704	0.052789103	1.450085328	0.147034726	0.582502337
5621.026846	-0.025408702	0.161214276	-0.157608262	0.874765492	0.973587929
147.9159997	0.031384564	0.106484043	0.294734901	0.7681964	0.947580311

276.9990119	-0.127183335	0.080871664	-1.572656345	0.115798413	0.537228251
440.2332121	-0.343304613	0.146035	-2.350837908	0.018731192	0.274872481
1200.793417	-0.106556755	0.107449145	-0.99169477	0.321346443	0.753905693
21.8688561	-0.817699304	0.320406534	-2.552068132	0.010708558	0.224713175
9704.958947	0.25497657	0.134336297	1.89804673	0.057689926	0.431275905
12.07153681	-1.272622797	0.738030842	-1.724349071	0.084644846	0.487842306
497.8555236	0.207293551	0.100182346	2.069162471	0.038530843	0.36712775
13.37189669	0.225240236	0.366794444	0.614077558	0.539164077	0.869675597
351.097991	0.038633739	0.107870938	0.358147796	0.720232712	0.931135539
82.50551575	0.14021307	0.12504836	1.121270764	0.262172626	0.7049341
671.8224722	0.149516473	0.135575721	1.102826315	0.270102598	0.712807244
377.3191731	0.131645303	0.12221629	1.077150213	0.281413163	0.722987449
236.9284248	0.196825443	0.177208216	1.110701562	0.26669683	0.709341177
123.3690376	0.150093521	0.331155791	0.453241419	0.65037491	0.910782566
32.09992668	-0.43794934	0.315482533	-1.388188865	0.165079542	0.605350304
452.7994686	0.176041771	0.073739931	2.387332994	0.016971112	0.265843192
4003.66008	-0.064342641	0.186024479	-0.345882654	0.729430905	0.93467002
1602.136058	0.069525046	0.16610493	0.418561004	0.675536994	0.921149714
34.7441593	0.083732026	0.330565605	0.25329927	0.80003696	0.955456191
236.189937	-0.014510748	0.201567113	-0.071989661	0.942610136	0.98446865
516.3844135	0.085629161	0.258490773	0.331265833	0.740443697	0.939732486
11.94513135	0.548344152	0.343788756	1.595003161	0.110711529	0.530340469
36.80870171	0.271869043	0.264031647	1.029683549	0.30315858	0.740731811
45.85352289	0.511395108	0.225830274	2.264510861	0.023542713	0.305392039
230.2830006	-0.107538473	0.106200478	-1.01259876	0.311251852	0.745938435
154.5819102	0.176338238	0.092452516	1.907338442	0.056476775	0.427168741
459.9294571	0.115314499	0.093526937	1.232954939	0.217592577	0.662036037
584.7771927	0.007346799	0.045929174	0.159959305	0.872913131	0.97300406
49.18983284	-0.28918884	0.162826792	-1.776051942	0.075724356	0.471272491
26.0487821	0.084146054	0.214778966	0.391779772	0.695220943	0.924581226
5.861409469	0.045844718	0.649699209	0.070562989	0.943745568	0.98479534
122.4293073	-0.047771807	0.09552096	-0.500118585	0.616991581	0.901900555
665.2758626	-0.107009051	0.172218264	-0.62135716	0.534364653	0.867840168
55.77316347	-0.166275695	0.202625909	-0.820604312	0.411871691	0.808272153
35.99265239	0.056604207	0.302399874	0.187183302	0.851516916	0.969398357
120.7872133	0.072730306	0.173068206	0.420240709	0.674309619	0.921149714
2495529.606	0.129195309	0.124994606	1.033607073	0.301319891	0.739331412
106.1127722	0.289868886	0.210886171	1.37452771	0.169277914	0.611731367
315.8311072	0.151892723	0.10419187	1.457817412	0.144890878	0.578290974
65.50655628	-0.340953751	0.533552894	-0.639025211	0.522806531	0.863362704
183.9338116	0.138730185	0.084052775	1.650512844	0.098838088	0.51077273
265.2700271	-0.088651249	0.081908182	-1.082324704	0.279108273	0.720879541
386.7899867	0.358462719	0.222239825	1.612954468	0.1067544	0.523584703
516.7476112	0.034916714	0.07091683	0.492361463	0.622463837	0.902441895
66.16280934	-0.019143742	0.247918809	-0.077217788	0.938450291	0.984334593
486.2376995	-0.009327825	0.065863372	-0.141623857	0.887377122	0.976961229
151.7351949	-0.431115972	0.336820413	-1.279957971	0.200559918	0.645045462
1038.981221	0.026775566	0.074769226	0.358109441	0.720261414	0.931135539

75.35205374	-0.158628359	0.17332463	-0.915209559	0.360081627	0.777407022
72.74962471	0.166146636	0.178228788	0.932209878	0.351228071	0.770952892
524.9897738	0.238083075	0.075860389	3.138437309	0.001698513	0.099373059
952.5581486	0.066493687	0.096409254	0.689702335	0.490381397	0.850155241
28.40072341	-0.034974782	0.277602448	-0.125988739	0.899740839	0.978996707
290.5432654	0.194204118	0.120186398	1.615857708	0.106125081	0.521361916
48.53077491	0.054350974	0.27854449	0.195124928	0.845295148	0.968131661
972.2726033	0.023245799	0.098557212	0.235860966	0.813540558	0.960442442
86.87377374	-0.266924413	0.161954003	-1.648149524	0.099321992	0.51147708
419.0717709	-0.104124729	0.121417916	-0.857573016	0.39112828	0.794437976
85.60342227	0.144196354	0.11456945	1.258593403	0.208177231	0.652874199
1566.331224	-0.0662638	0.08958177	-0.739701833	0.459480935	0.834180976
60.94522973	0.037966944	0.13137163	0.289004131	0.772578216	0.948706053
10.05601984	-0.561709019	0.49394321	-1.137193525	0.255457393	0.698168452
1052.783284	0.429880662	0.315206771	1.363805291	0.172628855	0.616428289
111.9866804	-0.304486865	0.326229653	-0.933351284	0.350638625	0.770560164
461.5971497	-0.562505939	0.40573947	-1.386372243	0.165633268	0.606257079
111.0328976	0.015036597	0.119646642	0.125675045	0.899989156	0.978996707
227.5921175	-0.196152908	0.203767667	-0.962630188	0.335733142	0.762664514
11.32449024	-0.158888793	0.383904842	-0.41387546	0.678965316	0.921716417
406.7566152	0.452979745	0.188131805	2.407778658	0.016049908	0.262799497
6.212704703	-0.145950672	0.349672067	-0.417392997	0.676390976	0.921393988
389.2121897	-0.128637336	0.079239232	-1.623404635	0.104502934	0.518555962
629.0643144	-0.197413522	0.131699247	-1.498972286	0.133880822	0.567033996
5.619938671	0.55035316	0.45854784	1.200208814	0.230058253	0.673001336
72.9433046	-0.637760917	0.335111135	-1.903132573	0.057023249	0.428680279
8.607671403	-0.232848738	0.32926489	-0.707177549	0.479456148	0.845088483
13.3577664	-0.250514985	0.500453826	-0.500575622	0.616669822	0.901900555
255.0323431	0.155320998	0.310270964	0.500597917	0.616654129	0.901900555
236.6384919	-0.003651356	0.084372346	-0.043276689	0.965480973	0.99075072
346.1780114	0.08689223	0.073857584	1.176483505	0.23940172	0.681681953
4.836795732	-1.00141708	0.494410515	-2.025476906	0.042818417	0.379065918
1139.217365	-0.009914353	0.072166365	-0.137381905	0.890728933	0.977069043
95.07290543	-0.057895708	0.262240442	-0.220773378	0.825268893	0.963909899
1416.845136	0.095516624	0.119456991	0.799590069	0.423948343	0.815063803
237.3941556	-0.003507118	0.097588451	-0.035937841	0.971331923	0.992799208
349.9084584	0.019269122	0.105145635	0.183261263	0.854593027	0.969966486
1604.003216	0.129701265	0.061223247	2.118497006	0.034132998	0.349886869
271.2531544	0.061866962	0.089521279	0.691086662	0.489511081	0.849736271
1649.324365	0.068176602	0.061962341	1.100290932	0.271205382	0.713807699
262.858612	0.070137382	0.104243419	0.672823121	0.501059826	0.856727618
36.67119378	-0.623930096	0.467138334	-1.33564311	0.181665952	0.62739604
242.6850468	0.029977799	0.108654573	0.27590002	0.782624858	0.95173434
264.878541	-0.025395389	0.135409595	-0.187544971	0.851233367	0.969309003
313.3460078	0.062349091	0.064051649	0.97341899	0.330345104	0.759368073
43.63931288	0.049789598	0.148077026	0.336241205	0.736688982	0.938503341
11.55189482	0.441898839	0.363645704	1.215190595	0.224293407	0.667854242
2445.925922	0.241671588	0.140914339	1.715024812	0.086340678	0.490195644

2206.804544	0.162036635	0.099009312	1.636579748	0.10171831	0.513342798
326.7844257	0.29880144	0.128067812	2.333150197	0.019640264	0.281261453
511.6422849	-0.328389501	0.15761225	-2.083527774	0.037203143	0.361102242
973.5336783	0.074583667	0.066605696	1.119779112	0.262807903	0.705491334
9.98867728	-0.841424819	0.754490874	-1.115221997	0.264755302	0.70780629
71.67148401	-0.134977597	0.116422721	-1.159375041	0.246303346	0.689705283
151.063515	-0.099151662	0.20437359	-0.485149092	0.627570591	0.904512355
556.9326347	0.057839425	0.073447831	0.787489895	0.430995142	0.818841807
72.10760404	0.072317161	0.129526916	0.558317634	0.576627506	0.886593989
579.3074409	0.073638255	0.045114338	1.632258345	0.102625074	0.514997052
10.89978395	-0.167127175	0.429301655	-0.389300096	0.697054165	0.92544754
1134.539484	-0.097576961	0.104695442	-0.932007733	0.351332529	0.770952892
10.95727116	0.075532538	0.266272504	0.283666307	0.776666117	0.950670616
56.30947519	0.183428441	0.372382901	0.492580193	0.622309246	0.902441895
369.0164221	-0.009180304	0.074983081	-0.122431668	0.902557159	0.979381499
202.1744531	-0.312879897	0.144659376	-2.162873268	0.030550928	0.333310814
7.068543037	-0.717416947	0.45717429	-1.56924167	0.116591638	0.538641109
832.5920175	0.099388194	0.080766171	1.230567119	0.21848481	0.662774346
28.7930666	-0.173515988	0.171441034	-1.012103023	0.311488797	0.745938435
96.46372835	-0.068792854	0.114528055	-0.600663774	0.548063952	0.874640417
157.8492182	-0.366999985	0.326333983	-1.124614673	0.260752353	0.703138216
6.510857484	-0.113645067	0.425310306	-0.267205064	0.789311278	0.953026618
89.44241006	-0.364718324	0.434782792	-0.838851791	0.401552484	0.801291311
79.90170626	-0.803609644	0.314971358	-2.551373715	0.01072992	0.224713175
4.750217242	0.485724337	0.455453693	1.066462617	0.286214569	0.727013824
120.4621839	-0.28408281	0.19967411	-1.422732324	0.154813767	0.592842063
14.80597341	-0.224876782	0.445551459	-0.504715622	0.613758587	0.901543575
589.0127398	-0.078380179	0.067389844	-1.163085925	0.244794642	0.687431283
51.85248615	-0.175589176	0.136559752	-1.285804736	0.198511217	0.64421391
46.22711243	0.147864416	0.215054217	0.687568084	0.491724812	0.851287798
15.70285802	0.211226491	0.260804357	0.809903998	0.417995354	0.811361647
299.1667808	-0.006406262	0.058466198	-0.109572064	0.912748767	0.980530316
199.1950858	-0.330921396	0.31904513	-1.03722441	0.299631285	0.738923503
469.4721545	0.134706674	0.069103949	1.949333939	0.051255558	0.410734915
26.58178694	0.355294041	0.227157003	1.564090195	0.117796388	0.539880626
490.0257408	0.155944649	0.054596984	2.856286906	0.004286276	0.145986988
13.47970983	-0.209352285	0.374455644	-0.559084335	0.576104165	0.886202779
331.5856391	0.042332531	0.139764122	0.302885533	0.761977097	0.946826103
355.84947	0.184803009	0.083905193	2.202521715	0.027628477	0.323871531
32.51474193	-0.711462331	0.410502445	-1.733150045	0.083069003	0.485235749
198.8274435	0.130901301	0.080068799	1.634860297	0.102078337	0.513951548
106.6111	0.200401953	0.140743535	1.423880347	0.154481112	0.592506562
660.9139849	0.010085349	0.071994394	0.140085193	0.888592679	0.976961229
11.72280407	-0.041429784	0.380892027	-0.108770414	0.913384591	0.980550322
108.1539453	0.404659661	0.384414019	1.052666242	0.292493985	0.733939814
5.297872444	-1.66549661	0.54721554	-3.043584272	0.00233778	0.115346043
122.5535363	-0.364645731	0.243626323	-1.49674192	0.134460424	0.567732615
911.6224305	-0.027029732	0.115210851	-0.234610987	0.814510681	0.960909552

176.6031614	-0.117214428	0.123306362	-0.950595132	0.341809941	0.765195212
1352.923422	-0.240719908	0.158407484	-1.519624589	0.128605354	0.559569754
192.9330793	-0.13086414	0.14273498	-0.916832998	0.359230158	0.777095745
340.400914	0.062848709	0.067528874	0.930693868	0.352011942	0.771465912
1507.400044	-0.081152962	0.05694185	-1.425190129	0.154102249	0.59219701
150.8412032	-0.090475704	0.292364773	-0.309461716	0.75697033	0.945462393
1251.573147	-0.079095791	0.104335702	-0.75808941	0.448397459	0.827578453
16.20182179	-0.115290342	0.316535703	-0.364225397	0.715689703	0.930241908
330.7730729	-0.292564432	0.083152542	-3.518406372	0.000434147	0.044626692
6.01267779	-0.299190845	0.443933755	-0.673953809	0.500340681	0.856182624
233.186854	-0.232077133	0.270993942	-0.856392329	0.391780806	0.794947155
9094.617474	0.11443325	0.092443833	1.237867863	0.21576506	0.660219973
124.5472225	0.018144877	0.106992635	0.16958996	0.865332619	0.971917805
91.22659977	0.097071761	0.169835522	0.571563357	0.567617826	0.8841274
28.37655678	-0.061079454	0.217185628	-0.281231564	0.778532796	0.951560132
2564.916028	0.180338148	0.13353009	1.350543153	0.176841821	0.62058147
15.70741141	-0.33734015	0.266430166	-1.266148481	0.205459928	0.650249702
41.99547186	0.031237126	0.149637876	0.208751468	0.83464226	0.966316856
614.3912639	-0.073688623	0.095700897	-0.769988843	0.441306511	0.82422323
110.9256318	-0.198072942	0.245052666	-0.808287235	0.418925249	0.811610533
167.7802438	0.00300386	0.095497462	0.031454871	0.974906782	0.993301399
379.7808857	-0.027779193	0.101004803	-0.275028432	0.783294394	0.95173434
53.02733126	0.083650962	0.149499666	0.559539461	0.575793608	0.886202779
47.05317469	0.14495253	0.357353593	0.405627739	0.685016144	0.922907843
113.8681796	-0.048409129	0.085702098	-0.564853492	0.572173419	0.885183781
53.48657356	0.347431406	0.175425984	1.980501391	0.047647217	0.397561505
21.68106356	-0.068116556	0.297173807	-0.229214533	0.818702177	0.962089609
20.98605541	-0.186862187	0.401521526	-0.465385227	0.641655612	0.908064184
536.3094525	0.15197785	0.058167915	2.61274367	0.008981864	0.204852927
12.40614822	0.432459416	0.343768056	1.257997679	0.208392596	0.652885502
717.4920422	-0.044533323	0.099621192	-0.447026601	0.654855854	0.912480225
39.5871839	-0.167015615	0.175186577	-0.953358515	0.340408453	0.76465745
135.2438886	0.599009616	0.242925409	2.465817051	0.013670114	0.250121471
5.967038915	-0.275089409	0.546311813	-0.503539193	0.614585232	0.901900555
20.76019766	-0.325945972	0.358336867	-0.909607698	0.363029437	0.779728871
282.3676936	0.103742519	0.084459682	1.228308197	0.219331295	0.663637842
787.2776502	0.049156945	0.066013537	0.74464946	0.45648366	0.83202452
263.0975408	0.231326204	0.128299743	1.803013773	0.071386052	0.463039587
71.89656473	0.066683671	0.141801355	0.47026117	0.638168436	0.907684986
363.2490152	0.067671684	0.065131568	1.03899976	0.298804848	0.738522837
124.5703532	0.025923236	0.124894	0.207561902	0.835571054	0.966316856
53.66694421	-0.836463482	0.34888593	-2.397527127	0.016506159	0.263145637
62.50719433	0.259713813	0.408140223	0.636334766	0.52455825	0.863946059
41.70242066	-0.368988149	0.368485619	-1.001363769	0.316650974	0.749930834
1110.617475	0.447685413	0.70354388	0.636329056	0.52456197	0.863946059
67.03077806	0.11371457	0.966670252	0.117635326	0.906356613	0.979768942
340.8826325	0.152411522	0.069233113	2.201425226	0.027705935	0.32400917
403.475766	0.268003455	0.166907677	1.605698796	0.108340106	0.525558244

94.46768039	0.09538765	0.146706939	0.650191807	0.515568333	0.861826223
67.5044276	0.166029252	0.196374118	0.845474208	0.397846127	0.799216626
46.03521253	0.348911432	0.275904957	1.264607334	0.206012123	0.651161956
327.1806609	0.234617458	0.205403535	1.14222697	0.253359688	0.695646467
199.2243845	0.058498385	0.129516243	0.451668333	0.651507935	0.910978693
10.4354265	-0.284951073	0.435029639	-0.655015309	0.512457898	0.860200661
437.1446697	-0.828161197	0.518256237	-1.597976324	0.110048246	0.528668387
4.962511011	-0.29928582	0.454823723	-0.658025968	0.510521445	0.860200661
203.8882583	0.08544567	0.10971889	0.778769001	0.436115802	0.822078703
465.7215533	0.139765706	0.082925662	1.685433703	0.091904926	0.499719658
61.50840577	0.168857144	0.348676521	0.484280226	0.628187007	0.904512355
14.33974599	-2.463922173	1.173989483	-2.098760005	0.035838062	0.357462935
19.87631915	-1.588968917	0.582477603	-2.727948524	0.006372955	0.174970531
143.7195846	0.014318204	0.159668369	0.089674644	0.928545766	0.984057738
613.51317	-0.090519073	0.107961034	-0.838442071	0.401782469	0.801291311
14.28706206	0.726284327	0.75938479	0.956411475	0.338864389	0.764007173
15.83146984	-1.157970449	0.521383837	-2.220955784	0.026353956	0.31740542
1183.896198	-0.118778862	0.134652943	-0.882111148	0.377716704	0.787072097
162.9629358	-0.056559841	0.114169552	-0.495402145	0.620316281	0.901976704
44.91940034	-0.513573478	0.175204144	-2.931286136	0.003375617	0.133956791
56.51115128	-0.096307976	0.139184243	-0.691945967	0.488971262	0.849736271
72.98535475	0.06821894	0.140889829	0.4842006	0.628243511	0.904512355
23.18195903	-1.448327446	0.74679682	-1.939386199	0.05245433	0.414846075
20.83780067	-0.259092579	0.26804113	-0.96661501	0.333736528	0.761463483
127.8020745	-0.257607369	0.160520897	-1.604821384	0.108533118	0.52564018
9.674407771	-1.121899103	0.615924696	-1.821487449	0.068532793	0.45784364
30.44677734	-0.175995819	0.192321446	-0.915112809	0.360132411	0.777407022
11.24399599	0.12880183	0.291811544	0.441387026	0.658932833	0.914192876
98.02103568	-0.039850398	0.103156188	-0.386311268	0.699266156	0.925900484
257.0035877	0.067762312	0.076320637	0.887863564	0.374614146	0.786753489
147.5917137	-0.191950332	0.170215184	-1.127692177	0.259449943	0.702208457
658.126349	-0.331770308	0.206634492	-1.605590161	0.108363989	0.525558244
615.2321276	0.132540156	0.070516684	1.879557404	0.060168423	0.435320275
33.04102469	0.119084921	0.202985526	0.586667057	0.557427337	0.879365746
843.0103536	-0.059278628	0.115353742	-0.513885608	0.60733197	0.900307811
2114.921652	0.017539703	0.158702246	0.110519564	0.911997335	0.980530316
7.060550669	-0.215150714	0.58538557	-0.367536757	0.713218679	0.929705922
29.29508062	-0.706134591	0.433979997	-1.627113222	0.103713053	0.517004034
273.5607896	0.104515476	0.122571644	0.852688866	0.393831867	0.796193624
630.1854976	0.056162075	0.090736412	0.618958519	0.535943686	0.869002283
383.5987099	-0.151949282	0.078047539	-1.946881132	0.051548987	0.410734915
13.16177927	-0.747546498	0.511333851	-1.461953859	0.143753854	0.578069441
1324.63157	0.144280895	0.068872697	2.094892478	0.036180552	0.359128126
363.8728246	0.018171363	0.066010794	0.275278663	0.783102156	0.95173434
605.5098894	0.281300596	0.162559094	1.730451307	0.083549674	0.485778721
904.1990837	0.235541364	0.144214425	1.633271874	0.102411829	0.514767228
398.3732791	0.00356598	0.057849316	0.061642559	0.950847484	0.98625342
661.5283143	-0.024204492	0.125324755	-0.193134165	0.846853897	0.968775098

581.2005206	-0.045358758	0.12465826	-0.363864843	0.715958939	0.930241908
12.35105819	0.3601901	0.304886103	1.181392318	0.237446908	0.679753163
1869.12566	0.054917476	0.128367203	0.42781548	0.668785466	0.917626349
22529.10088	0.000119306	0.130478258	0.000914372	0.999270437	0.999761804
404.063635	-0.002509561	0.090213068	-0.027818151	0.977807189	0.994440017
127.6641845	-0.046294897	0.105328353	-0.439529296	0.660278059	0.914896282
105.6472805	-0.06099634	0.123150579	-0.495298849	0.620389183	0.901976704
192.4758683	-0.033551743	0.087866917	-0.381847275	0.70257465	0.926349622
425.3433558	0.187656494	0.084409044	2.223179951	0.026203668	0.316367615
6.543412283	-0.095139979	0.418783922	-0.227181546	0.820282577	0.962543699
1320.167315	0.144182598	0.06517736	2.212157687	0.026955771	0.320997046
72.1046025	-0.116113897	0.114913622	-1.010445019	0.312282128	0.746752838
551.8108962	0.307983987	0.111003014	2.774555173	0.005527726	0.162022566
22.37354806	-1.257794704	1.111650967	-1.131465487	0.257859222	0.701069052
63.33621082	-0.12673285	0.130093028	-0.974170963	0.32997166	0.759333947
147.5175225	-0.092186207	0.115050551	-0.801266978	0.422977104	0.814538241
129.6944175	0.070822688	0.107870927	0.656550281	0.511470127	0.860200661
123.979656	-0.1069671	0.109815511	-0.974061856	0.330025827	0.759333947
373.5151459	0.050604811	0.07175811	0.70521382	0.480677182	0.845612509
402.3165317	-0.234786558	0.229663941	-1.022304838	0.306636644	0.743340256
1912.598737	-0.007172	0.121728524	-0.058917989	0.95301743	0.986667305
49.00922804	0.114006351	0.189837641	0.600546603	0.548142012	0.874640417
666.4112474	0.024266529	0.178946565	0.135607683	0.892131432	0.977738002
992.7831296	-0.131438931	0.103731049	-1.267112714	0.20511499	0.650131067
31.60964034	0.387579437	0.213228143	1.817674865	0.069113842	0.459166554
171.0585047	-0.259649572	0.171431094	-1.514600218	0.129873677	0.561938079
35.91077776	0.079431728	0.159143904	0.499118888	0.617695632	0.901976704
177.1543743	0.113532701	0.131999838	0.860097272	0.389735425	0.79322193
46.69812005	-0.235316879	0.184365225	-1.276362603	0.201827369	0.645481631
72.91223756	-0.282143589	0.285502259	-0.988235927	0.323037117	0.755505673
203.3876807	0.183432099	0.100996148	1.81622867	0.069335302	0.46001844
308.735296	-0.01683467	0.060468725	-0.278402933	0.780703069	0.95173434
1293.701158	-0.111151396	0.312525593	-0.355655342	0.722098693	0.931135539
687.0716342	-0.169081664	0.12891443	-1.311580591	0.189661685	0.635553768
14.23525188	1.062106698	0.323752966	3.280608398	0.001035835	0.077436478
30.33233814	0.56428297	0.384244866	1.468550446	0.141954764	0.576571115
63.54684137	0.515683794	0.315403641	1.634996323	0.102049819	0.513951548
25.50112332	-0.067422294	0.250503563	-0.269147047	0.787816528	0.953026618
114.3569121	0.108970908	0.153190513	0.711342409	0.476872078	0.843597766
4.830551793	-0.49342057	0.443829151	-1.111735381	0.266251942	0.708743266
14.02426094	0.382600136	0.286448309	1.335669032	0.181657476	0.62739604
98.94475001	-0.209718521	0.375369899	-0.558698292	0.576367646	0.886378586
110.5898608	0.015447905	0.102262819	0.151060817	0.879927741	0.975430592
416.3193864	0.194403658	0.085554876	2.272268598	0.023070292	0.303197947
331.7296497	0.09646219	0.075064178	1.285062895	0.198770306	0.64421391
477.2835219	-0.011034023	0.087690773	-0.125828786	0.899867455	0.978996707
479.5746178	0.06815066	0.105320309	0.647079946	0.517580204	0.862002027
5.151698003	-0.486985218	0.540527814	-0.900943866	0.367618167	0.781703698

49.91066581	-0.364059819	0.188920132	-1.92705677	0.053972555	0.419371005
84.27528259	-0.339162035	0.18054013	-1.87859638	0.060299626	0.435605203
2517.970448	-0.166177717	0.333187734	-0.498751005	0.617954807	0.901976704
63.28491978	-0.132010638	0.195089777	-0.6766661	0.49861783	0.855634722
281.3803523	0.103231253	0.103620924	0.996239452	0.319133815	0.752200376
840.998623	-0.144783416	0.096687105	-1.497442873	0.13427806	0.567632616
1028.058072	0.164356543	0.100220825	1.639944023	0.101016806	0.512774612
65.06303882	-0.005006418	0.158994249	-0.031488043	0.974880328	0.993301399
37.12510317	0.085971804	0.330027847	0.260498637	0.794479166	0.953529605
44.82001162	0.238781684	0.156102575	1.529646031	0.126104369	0.555429245
25.30626881	-1.571904299	0.562228644	-2.795845277	0.005176418	0.157953926
10.78984214	-0.283983987	0.69986677	-0.405768639	0.684912603	0.922907843
27.60959656	0.02059721	0.345287602	0.059652331	0.952432539	0.98648845
116.2988795	0.078626832	0.107138151	0.733882671	0.463020249	0.835927584
495.8308545	0.100382318	0.076910499	1.305183537	0.191830359	0.637743528
5.021375313	-0.177004058	0.434710026	-0.407177307	0.683877768	0.922851556
1135.930552	0.041405041	0.090180539	0.459134991	0.64613723	0.909300678
1653.038539	0.051660478	0.084724995	0.609743062	0.542032024	0.870853624
4.660229528	0.491246185	0.478486717	1.026666296	0.304577625	0.741672489
321.3613116	0.079242497	0.092658575	0.855209536	0.392435158	0.795620491
119.7315578	-0.033417003	0.095833835	-0.348697333	0.727316546	0.933553548
18.37142908	-1.134447975	0.52840021	-2.146948379	0.031797391	0.339220292
92.44663462	0.116112049	0.208525345	0.556824632	0.577647253	0.887079361
5.733889776	0.406519473	0.416693884	0.97558301	0.329271148	0.758888028
1942.343987	0.041130217	0.060088026	0.684499396	0.493659871	0.852282384
405.1524602	-0.032425102	0.075702988	-0.428319982	0.668418173	0.917463445
601.0571033	-0.032173719	0.07055602	-0.456002458	0.648388211	0.910701841
2012.86334	-0.104878458	0.12071689	-0.868796891	0.384958234	0.79064129
17.80639292	-0.214990149	0.343377928	-0.626103577	0.531247006	0.866217029
15.35891065	0.112976708	0.269195174	0.41968326	0.674716856	0.921149714
22.47585835	-0.156742846	0.199247751	-0.786673098	0.431473256	0.818841807
201.1237682	-0.069387499	0.147078389	-0.471772222	0.63708938	0.907622426
8.416051847	-0.624644051	0.321345091	-1.943841898	0.05191452	0.412031484
78.68406303	0.097114184	0.139926371	0.694037753	0.487658533	0.849513564
273.4624379	0.028625888	0.100609834	0.284523755	0.776009032	0.95044075
25.20019152	0.368089592	0.201372682	1.827902316	0.067564213	0.45536467
286.1290926	-0.009239384	0.110898224	-0.083314088	0.933601799	0.984334593
754.2758919	-0.160037968	0.092807612	-1.724405624	0.084634643	0.487842306
5.554006802	-1.524326497	1.186834671	-1.284362964	0.199014985	0.64421391
126.4960851	0.017029964	0.206605731	0.082427356	0.934306883	0.984334593
25.93941988	-0.018645864	0.202205341	-0.092212522	0.926529189	0.983240215
1207.068052	-0.012783364	0.046280357	-0.27621576	0.782382354	0.95173434
102.6408637	0.698579605	0.295137467	2.366963474	0.017934703	0.27123243
386.4626029	0.005047306	0.094633766	0.053335151	0.957464873	0.987864832
72.33700117	0.009372335	0.289230258	0.032404405	0.974149549	0.993248159
81.37962449	0.12482796	0.11821872	1.055906883	0.291010759	0.732859559
353.292233	-0.062723997	0.117121282	-0.535547396	0.592271393	0.893683696
178.8595466	0.170740131	0.091909793	1.857692469	0.063212699	0.443016545

257.9846617	0.11139051	0.110342687	1.009496073	0.312736784	0.746754295
66.89121212	-0.089269033	0.121276503	-0.736078551	0.461682895	0.835201817
241.9500815	-0.073290013	0.11700014	-0.626409621	0.531046301	0.866139794
7.635771057	-0.396051887	0.728428299	-0.543707442	0.586642811	0.890984353
4.695075701	-0.489136399	0.932986883	-0.52426932	0.600091232	0.898108141
349.740251	0.009427799	0.291410543	0.032352292	0.974191108	0.993248159
24.07573795	0.026842531	0.199931223	0.134258826	0.89319791	0.978111861
336.6216768	-0.270989656	0.129645416	-2.090237083	0.036596508	0.360198137
924.3719197	0.072566508	0.104169802	0.696617507	0.486042196	0.849012775
274.9106629	-0.039397312	0.100188826	-0.393230602	0.69414917	0.924581226
4723.833016	-0.047801603	0.125899518	-0.379680589	0.704182531	0.9269128
7.19257898	-0.171215476	0.439083838	-0.389938006	0.696582389	0.925326625
66.55178664	0.246687671	0.410847969	0.600435415	0.548216091	0.874640417
110.8017449	0.128803374	0.095067869	1.354857068	0.1754631	0.619005807
195.0508486	-0.135988215	0.081832188	-1.661793715	0.096554143	0.506088677
342.3003346	-0.130963439	0.139672651	-0.937645546	0.348426597	0.768780165
11.12710266	0.41887402	0.347328034	1.205989669	0.227821474	0.670429007
296.1914073	-0.079912343	0.080261607	-0.995648425	0.319420997	0.752398091
109.7120622	-0.011190257	0.107879635	-0.103729095	0.917384337	0.981376661
109.7733886	0.061506021	0.131845344	0.46650127	0.640856738	0.907798398
71.22988389	-0.144349646	0.135697999	-1.063756628	0.287438964	0.72841492
598.3214558	0.053526876	0.091736512	0.583484971	0.559566871	0.880280849
162.6964778	-0.125962001	0.10633401	-1.184588086	0.23618035	0.678901985
7.302802084	-0.305630668	0.376037188	-0.812767137	0.41635158	0.810259807
256.5388829	-0.366565283	0.243552727	-1.505075666	0.132304624	0.564647789
12.97037107	0.261768509	0.36569689	0.715807315	0.474110335	0.841713532
30.62862785	0.119905154	0.213251418	0.562271312	0.573931181	0.885935354
649.1319379	0.012980736	0.066710949	0.194581798	0.845720354	0.968131661
9.672979976	-0.265649865	0.394109226	-0.674051373	0.500278653	0.856182624
83.77056294	0.226057479	0.31241372	0.72358371	0.469321345	0.839503873
115.8218033	0.036713588	0.263063013	0.139561955	0.889006102	0.976961229
255.7187241	0.195254969	0.125860195	1.551363949	0.12081449	0.54632081
124.5994612	-0.488335655	0.284667005	-1.715462795	0.086260411	0.49014457
24.64295898	-0.142468081	0.206226327	-0.690833624	0.489670103	0.849736271
2420.484866	-0.285194266	0.064073269	-4.451064705	8.54456E-06	0.004406037
75.89691001	-0.74472735	0.391329952	-1.903067595	0.057031726	0.428680279
143.0451466	-0.120532431	0.100940123	-1.194098313	0.232439513	0.675362916
524.1176415	-0.122030109	0.108139007	-1.128455985	0.259127395	0.702208457
350.3606512	0.061395677	0.059403022	1.03354469	0.301349068	0.739331412
71.73496539	-0.072276121	0.203014899	-0.356013878	0.721830172	0.931135539
991.0348167	0.076133703	0.34995477	0.217552979	0.827777424	0.964626741
31.97455298	-0.449583253	0.394668211	-1.139142299	0.254643807	0.696832138
895.0870221	0.117997658	0.145085229	0.813298906	0.416046704	0.810170019
184.4931869	0.203559734	0.342020513	0.595168203	0.551731042	0.87625782
397.9913287	-0.051915209	0.109290443	-0.475020575	0.634772308	0.906954229
172.4403673	0.242971953	0.104094004	2.334158966	0.0195874	0.281261453
228.919888	-0.160920227	0.153195631	-1.050423079	0.293523639	0.734026791
995.7759419	0.023882609	0.071867213	0.332315776	0.739650828	0.939690097

4267.490381	-0.040015899	0.16098882	-0.248563219	0.803698663	0.957233889
44.26583784	0.185671827	0.170914374	1.086344129	0.27732677	0.719218718
102.2329956	0.001159753	0.115578987	0.010034293	0.991993927	0.998383398
23.98137525	0.20452968	0.245079879	0.834542926	0.403975108	0.802866253
217.0157567	-0.016675973	0.071203373	-0.234202015	0.81482815	0.960909552
62.49866042	-0.227964331	0.193268934	-1.179518752	0.238191674	0.679855961
467.2595877	-0.045588147	0.091804618	-0.496577929	0.619486721	0.901976704
97.10837664	-0.362792482	0.220156026	-1.647888036	0.099375649	0.51147708
6.274935057	0.332482253	0.488071889	0.68121574	0.49573499	0.853896962
264.1397111	0.153653088	0.119367618	1.287225893	0.198015565	0.643940842
25.04324738	-0.64388208	0.489701571	-1.314845852	0.188561714	0.634590162
427.3211529	-0.121885064	0.111871921	-1.089505415	0.275931068	0.718081375
44.94792649	0.101313776	0.157911984	0.641583835	0.521143432	0.863287843
356.8390452	-0.238128427	0.11661167	-2.042063437	0.041145239	0.374892505
317.1559384	0.1485432	0.09468778	1.568768425	0.116701908	0.538641109
17.76698014	0.061370627	0.521742912	0.117626183	0.906363858	0.979768942
110.9340829	0.092222951	0.128217063	0.719272065	0.471973304	0.84064088
215.8603582	-0.093160233	0.079900093	-1.165959005	0.24363102	0.686012467
427.0183267	-0.145147608	0.16817292	-0.863085495	0.388090462	0.792781092
6.062066642	0.12532057	0.541261951	0.231534048	0.816899938	0.961335246
154.4205638	0.092689494	0.167245429	0.554212418	0.57943348	0.887483205
54.63627107	0.042952764	0.219937383	0.195295421	0.845161681	0.968131661
1130.59093	-0.07199017	0.104615117	-0.688143093	0.491362676	0.850960752
130.0890734	0.09602272	0.125810515	0.763232866	0.445324531	0.826302547
5.052971212	-0.662858378	0.512707677	-1.292858303	0.196060071	0.641398981
244.8133853	-0.077728733	0.182198958	-0.426614587	0.669660071	0.918061348
675.7929066	-0.149731057	0.166516498	-0.899196532	0.368547987	0.782820132
799.2173167	0.184007212	0.111773134	1.646256174	0.099711028	0.511758204
529.4692945	0.18190013	0.364181493	0.499476589	0.617443675	0.901976704
94.08562278	0.033250048	0.180826632	0.183878047	0.854109127	0.969966486
316.6745715	0.121673612	0.096448225	1.261543296	0.207113171	0.651884304
29.96364817	0.116758916	0.224490313	0.520106699	0.602989209	0.899375001
153.3679749	-0.031936629	0.082455855	-0.387317903	0.698520873	0.925898269
69.32494163	0.078947887	0.132264396	0.596894473	0.550577838	0.875741796
167.7657747	-0.00236255	0.122415957	-0.019299365	0.984602291	0.995973697
201.7644733	-0.008572666	0.118088544	-0.07259524	0.942128215	0.98446865
102.791506	0.15100823	0.101058985	1.494258323	0.135108114	0.568467964
86.01103721	-0.664842847	0.411422465	-1.615961458	0.106102646	0.521361916
5.472159568	0.538550272	0.5559171	0.96876004	0.332664928	0.760742564
308.2643298	0.257459819	0.362826249	0.709595351	0.477955103	0.844290722
265.861323	0.112869264	0.088254111	1.278912251	0.200927958	0.645045462
270.3064677	0.052129107	0.066568406	0.783090819	0.433573782	0.821000272
881.7010642	-0.065735163	0.16947274	-0.387880453	0.698104504	0.925802378
10.65484057	0.003613965	0.307628433	0.011747824	0.990626808	0.99821004
292.6832056	0.001115831	0.084628421	0.013185058	0.989480151	0.997749656
317.6835747	0.011170804	0.097221625	0.114900409	0.908524061	0.980020248
5.070064962	0.363305854	0.440261878	0.82520398	0.409255794	0.806739148
531.6879772	0.094793875	0.132924957	0.713138278	0.475760195	0.843074633

520.2638526	0.06474541	0.213930695	0.302646658	0.762159153	0.946826103
37.40837489	0.145870433	0.172618394	0.845045707	0.39808532	0.799250874
511.3872221	0.119391938	0.117000515	1.020439421	0.307520106	0.743405059
228.4851152	0.199849963	0.175291258	1.140102277	0.254243693	0.696717102
3000.291533	0.122068571	0.144460676	0.844995153	0.398113545	0.799250874
144.3213494	0.072141181	0.108909047	0.662398422	0.507715915	0.859144938
30.21971348	-0.070920249	0.189242875	-0.37475783	0.707840579	0.928420247
9.352173891	-0.345154096	0.354090955	-0.97476112	0.329678767	0.759107774
37.17632032	0.190153729	0.165360545	1.149934099	0.250171015	0.694208707
8.775346491	-0.133523403	0.353210469	-0.378027873	0.705409888	0.926977731
348.4647237	-0.252846151	0.133662784	-1.891672031	0.058534692	0.433061669
395.3279326	-0.176205491	0.192444846	-0.915615536	0.35986858	0.777407022
148.3291911	-0.099984373	0.109673052	-0.911658531	0.3619485	0.778909683
413.4257405	-0.522580196	0.283243831	-1.844983503	0.065039938	0.447304849
295.5825165	-0.063478384	0.118838113	-0.534158469	0.5932319	0.894470672
14.46151264	0.079391342	0.288615024	0.275076955	0.783257116	0.95173434
14.93311765	-0.419093521	0.397119088	-1.055334619	0.291272314	0.732859559
531.8098619	0.101942805	0.052521121	1.940986849	0.052259873	0.413884769
1114.251238	0.079047422	0.066870125	1.182103708	0.237164553	0.679753163
6.702886937	-0.558090742	0.510881609	-1.092407189	0.274654165	0.716878153
39.28474018	0.204001606	0.25084519	0.813256997	0.416070727	0.810170019
9.525009095	-0.05335554	0.337180461	-0.158240307	0.874267443	0.973366431
178.6164115	-0.070556722	0.073098836	-0.965223604	0.334432827	0.761596529
6.207191844	-0.023102979	0.513369556	-0.045002627	0.964105215	0.990149113
37.15530856	0.118093678	0.228259617	0.517365616	0.604900957	0.90021313
18.77292072	0.680055129	0.473869861	1.435109479	0.151255903	0.589528489
282.076626	0.308359033	0.141210249	2.183687343	0.02898523	0.328011759
84.37520642	-0.189065146	0.143606713	-1.31654811	0.187990142	0.634170926
1701.514685	0.194151307	0.082146334	2.363481084	0.018104151	0.27123243
370.5960973	-0.095962393	0.13888545	-0.690946337	0.489599265	0.849736271
444.0638947	-0.041194823	0.066200517	-0.622273423	0.533762094	0.867356078
26.57442593	-0.047037757	0.239641113	-0.196284171	0.84438775	0.968131661
19.5858287	-0.428305755	0.20870695	-2.052187309	0.040151462	0.373317806
20.24918477	-0.228570321	0.315644686	-0.724138031	0.468980999	0.839199316
41.23895247	0.034828099	0.194607725	0.178965655	0.85796467	0.969966486
553.3608165	0.023621848	0.077849308	0.303430413	0.761561871	0.946826103
60.47197912	-0.112024455	0.160479669	-0.698060109	0.485139604	0.848765348
89.26344656	0.254722149	0.249704714	1.020093471	0.307684133	0.743405059
10.8802054	-0.334291649	0.34093516	-0.980513858	0.326832533	0.757203811
254.792913	0.260044538	0.210586244	1.234860041	0.216882597	0.661222104
56.99740611	-0.627475432	0.291381932	-2.153446605	0.031283596	0.337261319
10869.84873	0.213751457	0.186867763	1.143864805	0.252679709	0.69506877
6.130200337	-0.471362793	0.441427752	-1.067814136	0.28560436	0.726643643
16.29226875	-1.13948383	0.386363074	-2.949256559	0.003185394	0.132817478
538.5806883	-0.040810218	0.103618252	-0.393851636	0.693690579	0.924581226
83.58654214	0.294436276	0.143945146	2.045475551	0.040807997	0.374689316
17.92526921	-0.312730167	0.461408861	-0.677772348	0.497916046	0.855318935
374.8675868	-0.059088288	0.061492333	-0.960904962	0.336599955	0.763390114

17.28657295	0.359428344	0.252150813	1.42544987	0.154027201	0.59219701
128.9294173	-0.06097633	0.105132074	-0.579997402	0.56191637	0.881435293
332.5048825	0.039745023	0.083579143	0.475537575	0.634403857	0.906920638
167.1918103	0.086882977	0.161725546	0.537224818	0.59111233	0.893201436
8.564558165	-0.079030196	0.345965746	-0.228433587	0.819309181	0.962187758
500.8392009	-0.057695385	0.091014971	-0.633910934	0.526138949	0.864850467
40.79699378	-0.43093999	0.212488874	-2.028059081	0.042554218	0.377880689
128.4045325	-0.116089436	0.082625277	-1.40501116	0.16001796	0.6006497
24.39886275	0.085185797	0.230506884	0.369558582	0.711711417	0.929376205
604.737867	0.011312584	0.062800769	0.180134479	0.857046995	0.969966486
142.6328306	-0.122611107	0.109930411	-1.115352037	0.264699594	0.70780629
2359.110193	0.103728059	0.135123467	0.767653918	0.44269282	0.824977821
440.4346229	0.106614584	0.090322946	1.180370987	0.237852696	0.679803171
11.05592763	-0.253186856	0.401872353	-0.630018099	0.528682743	0.86594688
[truncated: 878,995 more chars]
