# Supplementary material for: Age-related transcriptional drift and physiological adaptation in long-living Ames dwarf skeletal muscle
Source: NAR Mol Med. 2026 Mar 23;3(2):ugag018. doi: 10.1093/narmme/ugag018 (PMC13111926; doi:10.1093/narmme/ugag018)
Supplement: ugag018_Supplemental_Files [file ugag018_supplemental_files.zip › Supplementary Table primers.pdf]

Name	Sequence	Scale	Purification
6330403A02Rik_F	TCTGGCTGAATATCGCTGC	25nM	STD
6330403A02Rik_R	CCCTGATCCTTGTACCCTTG	25nM	STD
Akirin1_F	ACTTCAGAAACTCAGCCTTCC	25nM	STD
Akirin1_R	TTGTCGAAGGGTAAAGGTGG	25nM	STD
Atp1b4_F	CATAGCCTTAACTTCAACTTCAACG	25nM	STD
Atp1b4_R	CTTGGATGAAGTACCGACCC	25nM	STD
Bgn_F	CTGAGTTTTCTGCCTACCCTG	25nM	STD
Bgn_R	AGAAGTCATTGATGCCCACC	25nM	STD
Ccbe1_F	ATCAAGAACTCGCTCTATGGC	25nM	STD
Ccbe1_R	GAGTATCGAATGCCCAGTCTG	25nM	STD
Ccl11_F	GCTCCATCCCAACTTCCTG	25nM	STD
Ccl11_R	AGATCTCTTTGCCCAACCTG	25nM	STD
Ctsk_F	TGACCACTGCCTTCCAATAC	25nM	STD
Ctsk_R	CTCTGTACCCTCTGCATTTAGC	25nM	STD
Efcab6_F	GTGACGAAGGGAGATCTGAAG	25nM	STD
Efcab6_R	CTGGAGAGGAATTCAAGGTACG	25nM	STD
Exoc7_F	CGACCAGCTCACTAAGAACATG	25nM	STD
Exoc7_R	AGCCTCTGTAGGTTCTCTGTC	25nM	STD
Exph5_F	CCACCCCTTACATATAGGCTAC	25nM	STD
Exph5_R	GAGAACAGTGAAGCAAAGGATG	25nM	STD
Fn1_F	AGTGTCTGTGTCTGGGAAATG	25nM	STD
Fn1_R	CAGCATGATCAAAACACTTCTCA	25nM	STD
Fth1_F	TCAACCGCCAGATCAACC	25nM	STD
Fth1_R	TCAGTTTCTCGGCATGCTC	25nM	STD
Hspg2_F	CATTCAGGTGGTCGTCCTCTCA	25nM	STD
Hspg2_R	AGGTCAAGCGTCTGTCCTTCAG	25nM	STD
Itpkb_F	CTGACTTCGATTCGCCCTG	25nM	STD
Itpkb_R	CTTCTGGTACATGTCCTTCCG	25nM	STD
Jak2_F	TTTCAGAGCTGTCATCCGTG	25nM	STD
Jak2_R	CTCTTCAAACTGTGTAGGGTCC	25nM	STD
Kl_F	GAACAACCTCTCGTCTCTTCTG	25nM	STD
Kl_R	TGGCGGAACTTCATGTTAGG	25nM	STD
Lrp2bp_F	GATCCTCGACTCTTCATGCC	25nM	STD
Lrp2bp_R	CTCTGCTTCCTCATCTGATCG	25nM	STD
Mgp_F	GAGACACCATGAAGAGCCTG	25nM	STD
Mgp_R	GTGTTGGCATTTCTCCTGTTG	25nM	STD
Myl4_F	CATGTCCTTGCTACCCTGG	25nM	STD
Myl4_R	CCCTGGAGAAACGTGCTTTAC	25nM	STD
P4ha1_F	GAAGAGGACAAGTTAGAGCAAATC	25nM	STD
P4ha1_R	CTCAGTGTTCAGACGTTTCATTAAC	25nM	STD
Rhou_F	CCCACCGAGTACATCCCTAC	25nM	STD
Rhou_R	TGTCTGTGTTGGTGTAGCAG	25nM	STD
Rps6kc1_F	AGGTGTACAAGGAGAGTCGAG	25nM	STD

Rps6kc1_R	CTTGAACTTAGTGATCCTGGAGG	25nM	STD
Sh3bp5_F	TGGCAAAGAAAATCGGCAAAG	25nM	STD
Sh3bp5_R	GATTGTTTCCTTGGCTGCAC	25nM	STD
Sparc_F	AATGGGAGAATTTGAGGACGG	25nM	STD
Sparc_R	GGTGTTGCTCTCGTCCAG	25nM	STD
Spp1_F	GTGATTTGCTTTTGCCTGTTTG	25nM	STD
Spp1_R	GAGATTCTGCTTCTGAGATGGG	25nM	STD
Thbs4_F	TGTCAGAATGGAGCGTGTG	25nM	STD
Thbs4_R	TCCTTTCAGTCTTGCATCCC	25nM	STD
Vim_F	TGACCTTGAACGGAAAGTGG	25nM	STD
Vim_R	ACATCGATCTGGACATGCTG	25nM	STD
Wls_F	ATTGGGTCAGTGGCTCATAAG	25nM	STD
Wls_R	CCATACCTTAGTGAAACCTCCG	25nM	STD
