# Supplementary material for: Age-related transcriptional drift and physiological adaptation in long-living Ames dwarf skeletal muscle
Source: NAR Mol Med. 2026 Mar 23;3(2):ugag018. doi: 10.1093/narmme/ugag018 (PMC13111926; doi:10.1093/narmme/ugag018)
Supplement: ugag018_Supplemental_Files [file Suppl. Dataset 2.pdf › Suppl. Dataset 2.pdf]

Supplementary Dataset 2

Term
Cholesterol Homeostasis (GO:0042632)
Extracellular Matrix Organization (GO:0030198)
Sterol Homeostasis (GO:0055092)
Negative Regulation Of Lipid Metabolic Process (GO:0045833)
Regulation Of Lipid Catabolic Process (GO:0050994)
fatty-acyl-CoA Biosynthetic Process (GO:0046949)
High-Density Lipoprotein Particle Remodeling (GO:0034375)
Regulation Of Nitric Oxide Biosynthetic Process (GO:0045428)
Regulation Of Chromosome Segregation (GO:0051983)
NAD Biosynthetic Process (GO:0009435)
Long-Chain fatty-acyl-CoA Biosynthetic Process (GO:0035338)
Sterol Metabolic Process (GO:0016125)
Negative Regulation Of Lipid Catabolic Process (GO:0050995)
Triglyceride Metabolic Process (GO:0006641)
NAD Biosynthesis Via Nicotinamide Riboside Salvage Pathway (GO:0034356)
Response To Fatty Acid (GO:0070542)
Negative Regulation Of Leukocyte Cell-Cell Adhesion (GO:1903038)
Nitrogen Compound Transport (GO:0071705)
Positive Regulation Of Lipid Storage (GO:0010884)
Monocarboxylic Acid Biosynthetic Process (GO:0072330)
Proton Transmembrane Transport (GO:1902600)
Very-Low-Density Lipoprotein Particle Assembly (GO:0034379)
Sister Chromatid Segregation (GO:0000819)
Intracellular Iron Ion Homeostasis (GO:0006879)
Regulation Of Tumor Necrosis Factor Production (GO:0032680)
Cholesterol Transport (GO:0030301)
Positive Regulation Of Metabolic Process (GO:0009893)
Extracellular Structure Organization (GO:0043062)
External Encapsulating Structure Organization (GO:0045229)
Long-Chain Fatty Acid Transport (GO:0015909)
Long-Chain fatty-acyl-CoA Metabolic Process (GO:0035336)
Negative Regulation Of Lymphocyte Activation (GO:0051250)
Triglyceride Catabolic Process (GO:0019433)
Low-Density Lipoprotein Particle Remodeling (GO:0034374)
Positive Regulation Of Vascular Endothelial Growth Factor Production (GO:0010575)
Positive Regulation Of Cell Cycle Process (GO:0090068)
Regulation Of Fatty Acid Biosynthetic Process (GO:0042304)
Regulation Of Vascular Endothelial Growth Factor Production (GO:0010574)
Positive Regulation Of Tumor Necrosis Factor Production (GO:0032760)
Positive Regulation Of Nitric Oxide Biosynthetic Process (GO:0045429)
Regulation Of Exit From Mitosis (GO:0007096)
Blood Coagulation, Fibrin Clot Formation (GO:0072378)
Negative Regulation Of Fatty Acid Biosynthetic Process (GO:0045717)
Protein Activation Cascade (GO:0072376)
Fatty Acid Metabolic Process (GO:0006631)
Positive Regulation Of Nitric Oxide Metabolic Process (GO:1904407)
Positive Regulation Of Tumor Necrosis Factor Superfamily Cytokine Production (GO:1903557)

Ammonium Homeostasis (GO:0097272)
Regulation Of Morphogenesis Of A Branching Structure (GO:0060688)
Regulation Of Very-Low-Density Lipoprotein Particle Remodeling (GO:0010901)
Negative Regulation Of Lipoprotein Lipase Activity (GO:0051005)
Negative Regulation Of Monocyte Chemotaxis (GO:0090027)
Positive Regulation Of Mitotic Sister Chromatid Separation (GO:1901970)
Mitotic Nuclear Division (GO:0140014)
Skeletal System Development (GO:0001501)
Cellular Response To Fatty Acid (GO:0071398)
Reverse Cholesterol Transport (GO:0043691)
Positive Regulation Of Homotypic Cell-Cell Adhesion (GO:0034112)
Regulation Of Mitotic Cell Cycle Phase Transition (GO:1901990)
Endodermal Cell Differentiation (GO:0035987)
Cellular Response To Lipid (GO:0071396)
Nicotinamide Nucleotide Metabolic Process (GO:0046496)
Negative Regulation Of T Cell Activation (GO:0050868)
Glucan Catabolic Process (GO:0009251)
Positive Regulation Of Phospholipid Biosynthetic Process (GO:0071073)
Positive Regulation Of Unsaturated Fatty Acid Biosynthetic Process (GO:2001280)
Mitotic Sister Chromatid Segregation (GO:0000070)
Positive Regulation Of Chromosome Segregation (GO:0051984)
Positive Regulation Of Neuroblast Proliferation (GO:0002052)
SMAD Protein Signal Transduction (GO:0060395)
Steroid Metabolic Process (GO:0008202)
Lipid Biosynthetic Process (GO:0008610)
Sprouting Angiogenesis (GO:0002040)
NADP Metabolic Process (GO:0006739)
Regulation Of Neuroinflammatory Response (GO:0150077)
Regulation Of Nuclear Division (GO:0051783)
Embryonic Digestive Tract Development (GO:0048566)
Negative Regulation Of Fatty Acid Metabolic Process (GO:0045922)
DNA Ligation (GO:0006266)
SREBP Signaling Pathway (GO:0032933)
Amino-Acid Betaine Transport (GO:0015838)
Cellular Biogenic Amine Catabolic Process (GO:0042402)
Ethanol Oxidation (GO:0006069)
Very-Low-Density Lipoprotein Particle Remodeling (GO:0034372)
Histone H2A K63-linked Deubiquitination (GO:0070537)
Negative Regulation Of Metabolic Process (GO:0009892)
Endoderm Formation (GO:0001706)
Positive Regulation Of Mitotic Nuclear Division (GO:0045840)
Regulation Of Lipid Metabolic Process (GO:0019216)
Positive Regulation Of Neurogenesis (GO:0050769)
Positive Regulation Of Cold-Induced Thermogenesis (GO:0120162)
Regulation Of Mitotic Nuclear Division (GO:0007088)
Positive Regulation Of Cell Motility (GO:2000147)
Regulation Of Cold-Induced Thermogenesis (GO:0120161)
Regulation Of Lipid Storage (GO:0010883)

Lipid Catabolic Process (GO:0016042)
Cdc42 Protein Signal Transduction (GO:0032488)
Angiotensin Maturation (GO:0002003)
Regulation Of Mitotic Centrosome Separation (GO:0046602)
Cellular Response To Sterol Depletion (GO:0071501)
Cyclooxygenase Pathway (GO:0019371)
Synapse Pruning (GO:0098883)
Triglyceride-Rich Lipoprotein Particle Remodeling (GO:0034370)
Negative Regulation Of Chromosome Organization (GO:2001251)
Negative Regulation Of Lipase Activity (GO:0060192)
Positive Regulation Of Monocyte Chemotactic Protein-1 Production (GO:0071639)
Positive Regulation Of Respiratory Burst (GO:0060267)
Positive Regulation Of Sequestering Of Triglyceride (GO:0010890)
Regulation Of Angiotensin Levels In Blood (GO:0002002)
Chemokine-Mediated Signaling Pathway (GO:0070098)
Antimicrobial Humoral Response (GO:0019730)
Response To cAMP (GO:0051591)
Long-Chain Fatty Acid Metabolic Process (GO:0001676)
Positive Regulation Of Cytokinesis (GO:0032467)
NAD Metabolic Process (GO:0019674)
Cellular Response To Chemokine (GO:1990869)
Glucose Metabolic Process (GO:0006006)
Cellular Response To Organic Cyclic Compound (GO:0071407)
Inorganic Cation Transmembrane Transport (GO:0098662)
Kinetochore Assembly (GO:0051382)
Positive Regulation Of Platelet Activation (GO:0010572)
Regulation Of Attachment Of Spindle Microtubules To Kinetochore (GO:0051988)
Cholesterol Efflux (GO:0033344)
Cellular Response To Organic Substance (GO:0071310)
Visual System Development (GO:0150063)
Acylglycerol Catabolic Process (GO:0046464)
Regulation Of Neuroblast Proliferation (GO:1902692)
Dicarboxylic Acid Transport (GO:0006835)
Iron Ion Transport (GO:0006826)
Collagen Fibril Organization (GO:0030199)
Organic Hydroxy Compound Biosynthetic Process (GO:1901617)
Response To Tumor Necrosis Factor (GO:0034612)
Inflammatory Response (GO:0006954)
Regulation Of Osteoblast Differentiation (GO:0045667)
Epidermis Development (GO:0008544)
Positive Regulation Of Cell Division (GO:0051781)
Regulation Of Low-Density Lipoprotein Particle Clearance (GO:0010988)
Ammonium Transmembrane Transport (GO:0072488)
Regulation Of Respiratory Burst (GO:0060263)
Thyroid Hormone Generation (GO:0006590)
Glycine Transport (GO:0015816)
Negative Regulation Of Cholesterol Transport (GO:0032375)
Positive Regulation Of Lipid Localization (GO:1905954)

Regulation Of Biosynthetic Process (GO:0009889)
Regulation Of Monocyte Chemotaxis (GO:0090025)
Bile Acid Biosynthetic Process (GO:0006699)
Regulation Of Platelet Aggregation (GO:0090330)
Negative Regulation Of Lipid Biosynthetic Process (GO:0051055)
Adenylate Cyclase-Activating G Protein-Coupled Receptor Signaling Pathway (GO:0007189)
Regulation Of Cell-Substrate Adhesion (GO:0010810)
Fatty Acid Biosynthetic Process (GO:0006633)
Regulation Of GTPase Activity (GO:0043087)
Acute Inflammatory Response (GO:0002526)
Regulation Of Reactive Oxygen Species Biosynthetic Process (GO:1903426)
Regulation Of Fatty Acid Metabolic Process (GO:0019217)
Regulation Of Monocyte Chemotactic Protein-1 Production (GO:0071637)
Aspartate Family Amino Acid Catabolic Process (GO:0009068)
Cellular Response To Growth Hormone Stimulus (GO:0071378)
Intestinal Absorption (GO:0050892)
Negative Regulation Of Leukocyte Chemotaxis (GO:0002689)
Negative Regulation Of Vascular Permeability (GO:0043116)
Phospholipid Efflux (GO:0033700)
Positive Regulation Of Metaphase/Anaphase Transition Of Cell Cycle (GO:1902101)
Positive Regulation Of Mitotic Metaphase/Anaphase Transition (GO:0045842)
Positive Regulation Of Platelet Aggregation (GO:1901731)
Positive Regulation Of Protein Maturation (GO:1903319)
Protein Localization To Kinetochore (GO:0034501)
Intracellular Monoatomic Cation Homeostasis (GO:0030003)
Cholesterol Metabolic Process (GO:0008203)
Regulation Of Interleukin-10 Production (GO:0032653)
Mitotic G2/M Transition Checkpoint (GO:0044818)
Amino Acid Import Across Plasma Membrane (GO:0089718)
Positive Regulation Of Response To Wounding (GO:1903036)
Secondary Alcohol Metabolic Process (GO:1902652)
Negative Regulation Of Tumor Necrosis Factor Production (GO:0032720)
Regulation Of Monoatomic Ion Transmembrane Transporter Activity (GO:0032412)
Phospholipid Metabolic Process (GO:0006644)
Regulation Of Lipid Biosynthetic Process (GO:0046890)
Regulation Of Monooxygenase Activity (GO:0032768)
Steroid Catabolic Process (GO:0006706)
Negative Regulation Of Mononuclear Cell Migration (GO:0071676)
Negative Regulation Of Substrate Adhesion-Dependent Cell Spreading (GO:1900025)
Organophosphate Catabolic Process (GO:0046434)
Phosphate Ion Homeostasis (GO:0055062)
Phospholipid Homeostasis (GO:0055091)
Positive Regulation Of Alcohol Biosynthetic Process (GO:1902932)
Positive Regulation Of Sodium Ion Transmembrane Transporter Activity (GO:2000651)
Lipid Droplet Organization (GO:0034389)
Phosphatidic Acid Biosynthetic Process (GO:0006654)
Positive Regulation Of Neural Precursor Cell Proliferation (GO:2000179)
Regulation Of Mitotic Metaphase/Anaphase Transition (GO:0030071)

Negative Regulation Of Tumor Necrosis Factor Superfamily Cytokine Production (GO:1903556)
Cellular Response To Hypoxia (GO:0071456)
Steroid Biosynthetic Process (GO:0006694)
Positive Regulation Of Transport (GO:0051050)
Mitotic G2 DNA Damage Checkpoint Signaling (GO:0007095)
Prostaglandin Metabolic Process (GO:0006693)
acyl-CoA Biosynthetic Process (GO:0071616)
Inhibitory Synapse Assembly (GO:1904862)
Positive Regulation Of T-helper 17 Type Immune Response (GO:2000318)
Positive Regulation Of Chromosome Separation (GO:1905820)
Regulation Of T-helper 17 Type Immune Response (GO:2000316)
Regulation Of Cellular Ketone Metabolic Process (GO:0010565)
Positive Regulation Of Cellular Biosynthetic Process (GO:0031328)
Cellular Response To Decreased Oxygen Levels (GO:0036294)
Monocarboxylic Acid Transport (GO:0015718)
Positive Regulation Of Hydrolase Activity (GO:0051345)
acyl-CoA Metabolic Process (GO:0006637)
Bile Acid Metabolic Process (GO:0008206)
Cellular Response To cAMP (GO:0071320)
Triglyceride Homeostasis (GO:0070328)
Organic Cation Transport (GO:0015695)
Cellular Response To Insulin Stimulus (GO:0032869)
Regulation Of Mitotic Cell Cycle (GO:0007346)
2-Oxoglutarate Metabolic Process (GO:0006103)
Bone Morphogenesis (GO:0060349)
Regulation Of Sequestering Of Triglyceride (GO:0010889)
Centromere Complex Assembly (GO:0034508)
Ethanol Metabolic Process (GO:0006067)
Positive Regulation Of Adaptive Immune Response Based On Somatic Recombination Of Immune Receptors Bu
Positive Regulation Of Transporter Activity (GO:0032411)
Prostanoid Biosynthetic Process (GO:0046457)
Regulation Of MHC Class II Biosynthetic Process (GO:0045346)
Very Long-Chain Fatty Acid Metabolic Process (GO:0000038)
Regulation Of Cyclin-Dependent Protein Kinase Activity (GO:1904029)
Regulation Of T Cell Proliferation (GO:0042129)
Carboxylic Acid Metabolic Process (GO:0019752)
Phosphatidic Acid Metabolic Process (GO:0046473)
Regulation Of Lipoprotein Lipase Activity (GO:0051004)
Adenylate Cyclase-Activating Adrenergic Receptor Signaling Pathway (GO:0071880)
Regulation Of Systemic Arterial Blood Pressure (GO:0003073)
Defense Response To Protozoan (GO:0042832)
Digestive Tract Morphogenesis (GO:0048546)
Epiboly Involved In Wound Healing (GO:0090505)
Vasodilation (GO:0042311)
Negative Regulation Of DNA Replication (GO:0008156)
Positive Regulation Of Cell Activation (GO:0050867)
Protein Localization To Microtubule Organizing Center (GO:1905508)
Protein Homooligomerization (GO:0051260)

Superoxide Metabolic Process (GO:0006801)
Negative Regulation Of Cell Cycle Process (GO:0010948)
Purine Nucleotide Metabolic Process (GO:0006163)
Regulation Of calcineurin-NFAT Signaling Cascade (GO:0070884)
Cellular Response To Organonitrogen Compound (GO:0071417)
Positive Regulation Of Cell Migration (GO:0030335)
Regulation Of Fatty Acid Oxidation (GO:0046320)
acetyl-CoA Metabolic Process (GO:0006084)
Activation Of Cysteine-Type Endopeptidase Activity (GO:0097202)
Antifungal Innate Immune Response (GO:0061760)
Apoptotic Nuclear Changes (GO:0030262)
Regulation Of Osteoblast Proliferation (GO:0033688)
Dicarboxylic Acid Catabolic Process (GO:0043649)
Mesonephric Tubule Development (GO:0072164)
Negative Regulation Of Interleukin-10 Production (GO:0032693)
Nuclear Chromosome Segregation (GO:0098813)
Positive Regulation Of Cardiac Muscle Tissue Growth (GO:0055023)
Activation Of Protein Kinase Activity (GO:0032147)
Regulation Of ERK1 And ERK2 Cascade (GO:0070372)
Renal System Development (GO:0072001)
Inorganic Anion Transport (GO:0015698)
Negative Regulation Of T Cell Proliferation (GO:0042130)
Microtubule Cytoskeleton Organization Involved In Mitosis (GO:1902850)
Mitotic Cytokinesis (GO:0000281)
Negative Regulation Of Cytokine-Mediated Signaling Pathway (GO:0001960)
Adenylate Cyclase-Modulating G Protein-Coupled Receptor Signaling Pathway (GO:0007188)
DNA Replication Checkpoint Signaling (GO:0000076)
Transepithelial Transport (GO:0070633)
Triglyceride Biosynthetic Process (GO:0019432)
Growth Hormone Receptor Signaling Pathway (GO:0060396)
Positive Regulation Of Macrophage Derived Foam Cell Differentiation (GO:0010744)
Prostaglandin Biosynthetic Process (GO:0001516)
Protein Localization To Centrosome (GO:0071539)
Regulation Of Sodium Ion Transmembrane Transporter Activity (GO:2000649)
Mitotic Cell Cycle Phase Transition (GO:0044772)
Monoatomic Cation Transmembrane Transport (GO:0098655)
Import Into Cell (GO:0098657)
Positive Regulation Of Axonogenesis (GO:0050772)
BMP Signaling Pathway (GO:0030509)
Regulation Of Pathway-Restricted SMAD Protein Phosphorylation (GO:0060393)
Small GTPase Mediated Signal Transduction (GO:0007264)
Adrenergic Receptor Signaling Pathway (GO:0071875)
Energy Reserve Metabolic Process (GO:0006112)
fatty-acyl-CoA Metabolic Process (GO:0035337)
Ureteric Bud Development (GO:0001657)
Intracellular Lipid Transport (GO:0032365)
Melanosome Assembly (GO:1903232)
Glycerophospholipid Metabolic Process (GO:0006650)

Regulation Of T Cell Activation (GO:0050863)
Regulation Of Receptor-Mediated Endocytosis (GO:0048259)
Negative Regulation Of Lymphocyte Proliferation (GO:0050672)
Positive Regulation Of Protein Phosphorylation (GO:0001934)
Inorganic Anion Transmembrane Transport (GO:0098661)
Regulation Of Cellular Response To Growth Factor Stimulus (GO:0090287)
DNA Catabolic Process (GO:0006308)
Bicarbonate Transport (GO:0015701)
Regulation Of Smooth Muscle Cell Migration (GO:0014910)
Cellular Response To Lectin (GO:1990858)
Stimulatory C-type Lectin Receptor Signaling Pathway (GO:0002223)
Superoxide Anion Generation (GO:0042554)
Glycogen Metabolic Process (GO:0005977)
Mesoderm Development (GO:0007498)
Negative Regulation Of Peptidyl-Serine Phosphorylation (GO:0033137)
Positive Regulation Of Hormone Secretion (GO:0046887)
Receptor Clustering (GO:0043113)
Retinoid Metabolic Process (GO:0001523)
Positive Regulation Of Wound Healing (GO:0090303)
Positive Regulation Of Cytokine Production (GO:0001819)
Regulation Of Peptidyl-Tyrosine Phosphorylation (GO:0050730)
Acylglycerol Metabolic Process (GO:0006639)
Positive Regulation Of Binding (GO:0051099)
Activation Of Adenylate Cyclase Activity (GO:0007190)
Regulation Of Membrane Depolarization (GO:0003254)
Anterior/Posterior Axis Specification (GO:0009948)
Xenobiotic Transport (GO:0042908)
Hydrogen Peroxide Catabolic Process (GO:0042744)
Positive Regulation Of Protein Processing (GO:0010954)
Cellular Response To BMP Stimulus (GO:0071773)
Response To Reactive Oxygen Species (GO:0000302)
Cellular Response To Tumor Necrosis Factor (GO:0071356)
Monocyte Chemotaxis (GO:0002548)
Potassium Ion Import Across Plasma Membrane (GO:1990573)
Regulation Of Protein Phosphorylation (GO:0001932)
Regulation Of Viral Genome Replication (GO:0045069)
Negative Regulation Of Cellular Catabolic Process (GO:0031330)
Positive Regulation Of Signal Transduction (GO:0009967)
Anaphase-Promoting Complex-Dependent Catabolic Process (GO:0031145)
Negative Regulation Of Double-Strand Break Repair Via Homologous Recombination (GO:2000042)
Negative Regulation Of Reactive Oxygen Species Metabolic Process (GO:2000378)
Peptide Catabolic Process (GO:0043171)
Regulation Of Filopodium Assembly (GO:0051489)
Positive Regulation Of Receptor-Mediated Endocytosis (GO:0048260)
Negative Regulation Of Cytokine Production (GO:0001818)
Positive Regulation Of ERK1 And ERK2 Cascade (GO:0070374)
Positive Regulation Of Transmembrane Receptor Protein Serine/Threonine Kinase Signaling Pathway (GO:00901[illegible]
Bone Development (GO:0060348)

Cellular Response To Ketone (GO:1901655)
Extracellular Matrix Disassembly (GO:0022617)
Lymphocyte Chemotaxis (GO:0048247)
Mitotic Spindle Assembly (GO:0090307)
Positive Regulation Of Osteoblast Differentiation (GO:0045669)
Acylglycerol Biosynthetic Process (GO:0046463)
Regulation Of Mononuclear Cell Migration (GO:0071675)
Glucose Transmembrane Transport (GO:1904659)
Hexose Transmembrane Transport (GO:0008645)
Negative Regulation Of Cell Development (GO:0010721)
Placenta Development (GO:0001890)
Positive Regulation Of Receptor Internalization (GO:0002092)
Neutrophil Chemotaxis (GO:0030593)
Regulation Of Actin Filament Polymerization (GO:0030833)
Regulation Of Cellular Biosynthetic Process (GO:0031326)
Hexose Metabolic Process (GO:0019318)
Organic Hydroxy Compound Transport (GO:0015850)
Monocarboxylic Acid Metabolic Process (GO:0032787)
Kidney Development (GO:0001822)
Negative Regulation Of Protein Binding (GO:0032091)
Positive Regulation Of Cell-Substrate Adhesion (GO:0010811)
Bile Acid And Bile Salt Transport (GO:0015721)
Cellular Lipid Catabolic Process (GO:0044242)
Cellular Response To Interleukin-6 (GO:0071354)
Positive Regulation Of Epithelial Cell Apoptotic Process (GO:1904037)
Regulation Of Cardiac Muscle Hypertrophy (GO:0010611)
Amino Acid Transport (GO:0006865)
Cellular Component Disassembly (GO:0022411)
Response To Hydrogen Peroxide (GO:0042542)
Positive Regulation Of Blood Vessel Endothelial Cell Migration (GO:0043536)
B Cell Chemotaxis (GO:0035754)
ISG15-protein Conjugation (GO:0032020)
L-aspartate Import Across Plasma Membrane (GO:0140009)
L-aspartate Transmembrane Transport (GO:0070778)
L-phenylalanine Catabolic Process (GO:0006559)
L-phenylalanine Metabolic Process (GO:0006558)
Regulation Of Growth Hormone Receptor Signaling Pathway (GO:0060398)
RNA Decapping (GO:0110154)
Regulation Of Keratinocyte Apoptotic Process (GO:1902172)
Activation Of Cysteine-Type Endopeptidase Activity Involved In Apoptotic Process By Cytochrome C (GO:00086
Activation Of Transmembrane Receptor Protein Tyrosine Kinase Activity (GO:0007171)
Aminophospholipid Transport (GO:0015917)
Autocrine Signaling (GO:0035425)
Axonemal Central Apparatus Assembly (GO:1904158)
Basic Amino Acid Transport (GO:0015802)
Bradykinin Catabolic Process (GO:0010815)
Bundle Of His cell-Purkinje Myocyte Adhesion Involved In Cell Communication (GO:0086073)
Regulation Of Protein Targeting (GO:1903533)

Cell Junction Disassembly (GO:0150146)
Regulation Of Retinal Ganglion Cell Axon Guidance (GO:0090259)
Regulation Of Ribonuclease Activity (GO:0060700)
Cellular Response To Iron Ion (GO:0071281)
Cellular Response To Magnesium Ion (GO:0071286)
Choline Metabolic Process (GO:0019695)
Resolution Of Recombination Intermediates (GO:0071139)
Response To Bile Acid (GO:1903412)
Response To cGMP (GO:0070305)
Response To Heparin (GO:0071503)
Response To Lithium Ion (GO:0010226)
Response To Manganese Ion (GO:0010042)
Response To Testosterone (GO:0033574)
Retinal Ganglion Cell Axon Guidance (GO:0031290)
Epithelial Tube Branching Involved In Lung Morphogenesis (GO:0060441)
Epithelial Tube Formation (GO:0072175)
Erythrose 4-Phosphate/Phosphoenolpyruvate Family Amino Acid Catabolic Process (GO:1902222)
Succinate Transport (GO:0015744)
Gastrulation With Mouth Forming Second (GO:0001702)
Valine Metabolic Process (GO:0006573)
Hepatocyte Differentiation (GO:0070365)
Hexose Import Across Plasma Membrane (GO:0140271)
Humoral Immune Response Mediated By Circulating Immunoglobulin (GO:0002455)
Hydrogen Peroxide Biosynthetic Process (GO:0050665)
Immunoglobulin V(D)J Recombination (GO:0033152)
Inclusion Body Assembly (GO:0070841)
Interleukin-5-Mediated Signaling Pathway (GO:0038043)
Intracellular Phosphate Ion Homeostasis (GO:0030643)
Intracellular Water Homeostasis (GO:0009992)
Ketone Body Biosynthetic Process (GO:0046951)
Leukocyte Apoptotic Process (GO:0071887)
Lipid Droplet Disassembly (GO:1905691)
Maintenance Of DNA Repeat Elements (GO:0043570)
Mesenchymal Cell Differentiation Involved In Kidney Development (GO:0072161)
Mesenchymal To Epithelial Transition Involved In Metanephros Morphogenesis (GO:0003337)
Mesodermal Cell Fate Commitment (GO:0001710)
Monoterpenoid Metabolic Process (GO:0016098)
Negative Regulation Of Chemokine-Mediated Signaling Pathway (GO:0070100)
Negative Regulation Of Cytokinesis (GO:0032466)
Negative Regulation Of Heterochromatin Formation (GO:0031452)
Negative Regulation Of Lamellipodium Organization (GO:1902744)
Negative Regulation Of Nervous System Process (GO:0031645)
Negative Regulation Of Nuclear-Transcribed mRNA Catabolic Process, Nonsense-Mediated Decay (GO:2000623
Notochord Development (GO:0030903)
Osmosensory Signaling Pathway (GO:0007231)
Phagosome Acidification (GO:0090383)
Positive Regulation Of NK T Cell Proliferation (GO:0051142)
Positive Regulation Of T Cell Differentiation In Thymus (GO:0033089)

Positive Regulation Of Cholesterol Biosynthetic Process (GO:0045542)
Positive Regulation Of Fever Generation (GO:0031622)
Positive Regulation Of Microglial Cell Activation (GO:1903980)
Positive Regulation Of Mitotic Cytokinesis (GO:1903490)
Positive Regulation Of Monoatomic Ion Transmembrane Transport (GO:0034767)
Positive Regulation Of Osteoblast Proliferation (GO:0033690)
Positive Regulation Of Peroxisome Proliferator Activated Receptor Signaling Pathway (GO:0035360)
Positive Regulation Of Protein Catabolic Process In The Vacuole (GO:1904352)
Positive Regulation Of Sterol Biosynthetic Process (GO:0106120)
Protein Modification By Small Protein Conjugation Or Removal (GO:0070647)
Regulation Of NK T Cell Activation (GO:0051133)
Regulation Of Apoptotic DNA Fragmentation (GO:1902510)
Negative Regulation Of Catabolic Process (GO:0009895)
Cellular Response To Cytokine Stimulus (GO:0071345)
Regulation Of Interleukin-2 Production (GO:0032663)
Regulation Of Wound Healing (GO:0061041)
Granulocyte Chemotaxis (GO:0071621)
ERK1 And ERK2 Cascade (GO:0070371)
Regulation Of Execution Phase Of Apoptosis (GO:1900117)
Acylglycerol Homeostasis (GO:0055090)
Response To Hexose (GO:0009746)
Secondary Alcohol Biosynthetic Process (GO:1902653)
Ear Morphogenesis (GO:0042471)
Eosinophil Chemotaxis (GO:0048245)
Eosinophil Migration (GO:0072677)
Gliogenesis (GO:0042063)
Neuromuscular Junction Development (GO:0007528)
Positive Regulation Of Interleukin-17 Production (GO:0032740)
Cellular Response To Oxygen-Containing Compound (GO:1901701)
Cellular Response To Radiation (GO:0071478)
Myelination (GO:0042552)
Post-Translational Protein Modification (GO:0043687)
Regulation Of DNA Binding (GO:0051101)
Transmembrane Receptor Protein Serine/Threonine Kinase Signaling Pathway (GO:0007178)
Endocytosis (GO:0006897)
Regulation Of BMP Signaling Pathway (GO:0030510)
Regulation Of DNA Replication (GO:0006275)
Regulation Of Glycogen Biosynthetic Process (GO:0005979)
Regulation Of Postsynapse Organization (GO:0099175)
Regulation Of Protein Processing (GO:0070613)
Cholesterol Biosynthetic Process (GO:0006695)
Ribonucleoside Bisphosphate Metabolic Process (GO:0033875)
Sterol Transport (GO:0015918)
Wound Healing, Spreading Of Cells (GO:0044319)
Inner Ear Morphogenesis (GO:0042472)
Long-Chain Fatty Acid Biosynthetic Process (GO:0042759)
Negative Regulation Of Intracellular Protein Transport (GO:0090317)
Positive Regulation Of Interleukin-2 Production (GO:0032743)

Purine Nucleoside Bisphosphate Metabolic Process (GO:0034032)
Antibacterial Humoral Response (GO:0019731)
Erythrocyte Differentiation (GO:0030218)
Positive Regulation Of Pathway-Restricted SMAD Protein Phosphorylation (GO:0010862)
Transmembrane Receptor Protein Tyrosine Kinase Signaling Pathway (GO:0007169)
Positive Regulation Of Protein Secretion (GO:0050714)
Regulation Of Cellular Response To Hypoxia (GO:1900037)
CENP-A Containing Chromatin Assembly (GO:0034080)
DNA Strand Resection Involved In Replication Fork Processing (GO:0110025)
Regulation Of Entry Of Bacterium Into Host Cell (GO:2000535)
Regulation Of Glial Cell Differentiation (GO:0045685)
RIG-I Signaling Pathway (GO:0039529)
SA Node Cell Action Potential (GO:0086015)
Regulation Of Inositol Phosphate Biosynthetic Process (GO:0010919)
Regulation Of Intestinal Cholesterol Absorption (GO:0030300)
Actin Filament Reorganization (GO:0090527)
Anterograde Dendritic Transport (GO:0098937)
Regulation Of Mitotic Cytokinesis (GO:1902412)
Axon Extension Involved In Axon Guidance (GO:0048846)
Regulation Of Nuclease Activity (GO:0032069)
Regulation Of Prostaglandin Biosynthetic Process (GO:0031392)
Cardiac Muscle Cell-Cardiac Muscle Cell Adhesion (GO:0086042)
Catecholamine Uptake (GO:0090493)
Regulation Of Skeletal Muscle Satellite Cell Proliferation (GO:0014842)
Regulation Of Smooth Muscle Cell Differentiation (GO:0051150)
Regulation Of Striated Muscle Tissue Development (GO:0016202)
Regulation Of Systemic Arterial Blood Pressure By Renin-Angiotensin (GO:0003081)
Regulation Of Vascular Associated Smooth Muscle Cell Apoptotic Process (GO:1905459)
Chromatin Remodeling At Centromere (GO:0031055)
Citrulline Metabolic Process (GO:0000052)
Detection Of Stimulus Involved In Sensory Perception Of Pain (GO:0062149)
Epithelial Cell Differentiation Involved In Kidney Development (GO:0035850)
Sulfur Amino Acid Catabolic Process (GO:0000098)
Exocrine System Development (GO:0035272)
Synaptic Transmission, Dopaminergic (GO:0001963)
Fatty Acid Elongation, Monounsaturated Fatty Acid (GO:0034625)
Fatty Acid Elongation, Polyunsaturated Fatty Acid (GO:0034626)
Fatty Acid Elongation, Saturated Fatty Acid (GO:0019367)
Fatty Acid Elongation, Unsaturated Fatty Acid (GO:0019368)
Forebrain Neuron Development (GO:0021884)
Free Ubiquitin Chain Polymerization (GO:0010994)
Galactose Metabolic Process (GO:0006012)
Gamma-Aminobutyric Acid Transport (GO:0015812)
Gap Junction Assembly (GO:0016264)
Gland Morphogenesis (GO:0022612)
Glycogen Catabolic Process (GO:0005980)
Very Long-Chain Fatty Acid Catabolic Process (GO:0042760)
Histidine Catabolic Process (GO:0006548)

Histidine Metabolic Process (GO:0006547)
Hyaluronan Biosynthetic Process (GO:0030213)
Interleukin-3-Mediated Signaling Pathway (GO:0038156)
Intracellular Triglyceride Homeostasis (GO:0035356)
Ketone Body Metabolic Process (GO:1902224)
Leukotriene Transport (GO:0071716)
Lipid Hydroxylation (GO:0002933)
Long-Chain Fatty Acid Catabolic Process (GO:0042758)
Maintenance Of Protein Location In Extracellular Region (GO:0071694)
Membrane Biogenesis (GO:0044091)
Monoacylglycerol Biosynthetic Process (GO:0006640)
Negative Regulation Of Dendritic Spine Development (GO:0061000)
Negative Regulation Of Lipoprotein Particle Clearance (GO:0010985)
Negative Regulation Of Membrane Protein Ectodomain Proteolysis (GO:0051045)
Negative Regulation Of Response To Type II Interferon (GO:0060331)
Negative Regulation Of Smooth Muscle Cell Apoptotic Process (GO:0034392)
Negative Regulation Of Type II Interferon-Mediated Signaling Pathway (GO:0060336)
Neuron Projection Extension Involved In Neuron Projection Guidance (GO:1902284)
Neurotransmitter Receptor Transport, Postsynaptic Endosome To Lysosome (GO:0098943)
Norepinephrine Transport (GO:0015874)
Polyamine Biosynthetic Process (GO:0006596)
Positive Regulation Of cAMP-mediated Signaling (GO:0043950)
Positive Regulation Of Cysteine-Type Endopeptidase Activity Involved In Apoptotic Signaling Pathway (GO:2001
Positive Regulation Of Spindle Assembly (GO:1905832)
Regulation Of MHC Class I Biosynthetic Process (GO:0045343)
Regulation Of NK T Cell Proliferation (GO:0051140)
Regulation Of SA Node Cell Action Potential (GO:0098907)
Regulation Of Adiponectin Secretion (GO:0070163)
Regulation Of Bile Acid Metabolic Process (GO:1904251)
G Protein-Coupled Receptor Signaling Pathway, Coupled To Cyclic Nucleotide Second Messenger (GO:000718
Blood Circulation (GO:0008015)
Dicarboxylic Acid Metabolic Process (GO:0043648)
Pyruvate Metabolic Process (GO:0006090)
Chloride Transport (GO:0006821)
Embryonic Organ Development (GO:0048568)
Neutrophil Migration (GO:1990266)
L-amino Acid Transport (GO:0015807)
Spindle Assembly Checkpoint Signaling (GO:0071173)
Mitotic Spindle Assembly Checkpoint Signaling (GO:0007094)
Mitotic Spindle Checkpoint Signaling (GO:0071174)
Negative Regulation Of Receptor-Mediated Endocytosis (GO:0048261)
Organelle Fusion (GO:0048284)
Positive Regulation Of Adenylate Cyclase Activity (GO:0045762)
Positive Regulation Of Monooxygenase Activity (GO:0032770)
Positive Regulation Of Nuclear Division (GO:0051785)
Regulation Of Cardiac Muscle Cell Contraction (GO:0086004)
Positive Regulation Of Protein Modification Process (GO:0031401)
RNA Modification (GO:0009451)

Monoatomic Ion Transport (GO:0006811)
Regulation Of Insulin Secretion Involved In Cellular Response To Glucose Stimulus (GO:0061178)
In Utero Embryonic Development (GO:0001701)
Myeloid Cell Development (GO:0061515)
Positive Regulation Of Immune System Process (GO:0002684)
Regulation Of Cyclin-Dependent Protein Serine/Threonine Kinase Activity (GO:0000079)
Response To Insulin (GO:0032868)
Positive Regulation Of Endocytosis (GO:0045807)
Arachidonic Acid Metabolic Process (GO:0019369)
Cartilage Development (GO:0051216)
Cell Cycle G1/S Phase Transition (GO:0044843)
Fatty Acid Oxidation (GO:0019395)
Glycerophospholipid Biosynthetic Process (GO:0046474)
Lipid Transport (GO:0006869)
Regulation Of Cytokinesis (GO:0032465)
Carboxylic Acid Transport (GO:0046942)
Negative Regulation Of Viral Genome Replication (GO:0045071)
Protein Homotetramerization (GO:0051289)
Regulation Of Chemokine (C-C Motif) Ligand 5 Production (GO:0071649)
Regulation Of Chemokine-Mediated Signaling Pathway (GO:0070099)
Regulation Of Chromosome Condensation (GO:0060623)
Regulation Of Establishment Of Protein Localization To Mitochondrion (GO:1903747)
L-alanine Transport (GO:0015808)
NADH Metabolic Process (GO:0006734)
Regulation Of Leukocyte Proliferation (GO:0070663)
Amine Transport (GO:0015837)
Arginine Catabolic Process (GO:0006527)
Aromatic Compound Catabolic Process (GO:0019439)
Regulation Of Nitric Oxide Metabolic Process (GO:0080164)
Benzene-Containing Compound Metabolic Process (GO:0042537)
Blood Coagulation, Intrinsic Pathway (GO:0007597)
Catecholamine Transport (GO:0051937)
Regulation Of Stem Cell Division (GO:2000035)
Regulation Of Triglyceride Metabolic Process (GO:0090207)
Diacylglycerol Biosynthetic Process (GO:0006651)
Dopamine Uptake (GO:0090494)
Sinoatrial Node Development (GO:0003163)
Somatic Hypermutation Of Immunoglobulin Genes (GO:0016446)
Folic Acid Transport (GO:0015884)
Fructose Transmembrane Transport (GO:0015755)
Terpenoid Metabolic Process (GO:0006721)
Glycine Metabolic Process (GO:0006544)
Venous Blood Vessel Development (GO:0060841)
Intermediate Filament Bundle Assembly (GO:0045110)
Kidney Mesenchyme Development (GO:0072074)
Lymphocyte Apoptotic Process (GO:0070227)
Melanin Biosynthetic Process (GO:0042438)
Methionine Metabolic Process (GO:0006555)

Negative Regulation Of DNA-templated DNA Replication (GO:2000104)
Negative Regulation Of RNA Catabolic Process (GO:1902369)
Negative Regulation Of Intracellular Transport (GO:0032387)
Negative Regulation Of Keratinocyte Proliferation (GO:0010839)
Negative Regulation Of Muscle Hypertrophy (GO:0014741)
Negative Regulation Of Nitric Oxide Biosynthetic Process (GO:0045019)
Negative Regulation Of Nitric Oxide Metabolic Process (GO:1904406)
Neurotransmitter Receptor Diffusion Trapping (GO:0099628)
Nonribosomal Peptide Biosynthetic Process (GO:0019184)
Peptidyl-Proline Hydroxylation To 4-hydroxy-L-proline (GO:0018401)
Phosphatidylserine Acyl-Chain Remodeling (GO:0036150)
Positive Regulation Of DNA Demethylation (GO:1901537)
Positive Regulation Of Apoptotic Cell Clearance (GO:2000427)
Positive Regulation Of Attachment Of Mitotic Spindle Microtubules To Kinetochore (GO:1902425)
Positive Regulation Of Chromosome Condensation (GO:1905821)
Positive Regulation Of Execution Phase Of Apoptosis (GO:1900119)
Positive Regulation Of Fibroblast Migration (GO:0010763)
Positive Regulation Of Insulin-Like Growth Factor Receptor Signaling Pathway (GO:0043568)
Positive Regulation Of Myeloid Cell Apoptotic Process (GO:0033034)
Positive Regulation Of Natural Killer Cell Proliferation (GO:0032819)
Positive Regulation Of Plasminogen Activation (GO:0010756)
Positive Regulation Of Vascular Endothelial Growth Factor Signaling Pathway (GO:1900748)
Post-Translational Protein Targeting To Membrane, Translocation (GO:0031204)
Postsynaptic Neurotransmitter Receptor Diffusion Trapping (GO:0098970)
Protein Localization To Ciliary Transition Zone (GO:1904491)
Purine Nucleotide Catabolic Process (GO:0006195)
Purine-Containing Compound Metabolic Process (GO:0072521)
Regulation Of Cdc42 Protein Signal Transduction (GO:0032489)
Regulation Of Apoptotic Cell Clearance (GO:2000425)
Regulation Of Attachment Of Mitotic Spindle Microtubules To Kinetochore (GO:1902423)
Regulation Of Cardiac Muscle Cell Action Potential Involved In Regulation Of Contraction (GO:0098909)
Regulation Of Nitric-Oxide Synthase Activity (GO:0050999)
Regulation Of Platelet Activation (GO:0010543)
Regulation Of Sodium Ion Transmembrane Transport (GO:1902305)
Diterpenoid Metabolic Process (GO:0016101)
Sterol Biosynthetic Process (GO:0016126)
Fatty Acid Derivative Biosynthetic Process (GO:1901570)
Limb Development (GO:0060173)
Negative Regulation Of Mitotic Metaphase/Anaphase Transition (GO:0045841)
Phospholipid Catabolic Process (GO:0009395)
Regulation Of Cardiac Muscle Cell Action Potential (GO:0098901)
Positive Regulation Of Phosphorylation (GO:0042327)
Cellular Response To Interleukin-1 (GO:0071347)
Cytoskeleton-Dependent Cytokinesis (GO:0061640)
Response To Lipid (GO:0033993)
Sodium Ion Transmembrane Transport (GO:0035725)
Alpha-Amino Acid Metabolic Process (GO:1901605)
Regulation Of Monoatomic Ion Transmembrane Transport (GO:0034765)

Response To Alcohol (GO:0097305)
Defense Response To Fungus (GO:0050832)
Glycolytic Process (GO:0006096)
Heterotypic Cell-Cell Adhesion (GO:0034113)
Morphogenesis Of An Epithelium (GO:0002009)
Negative Regulation Of Plasma Membrane Bounded Cell Projection Assembly (GO:0120033)
Positive Regulation Of Calcium Ion Transport (GO:0051928)
Positive Regulation Of Viral Genome Replication (GO:0045070)
Positive Regulation Of GTPase Activity (GO:0043547)
Phosphatidylcholine Metabolic Process (GO:0046470)
Regulation Of MAPK Cascade (GO:0043408)
Positive Regulation Of Macromolecule Metabolic Process (GO:0010604)
Regulation Of Interleukin-1 Beta Production (GO:0032651)
Mitotic DNA Damage Checkpoint Signaling (GO:0044773)
Peptide Metabolic Process (GO:0006518)
Regulation Of Dendritic Spine Morphogenesis (GO:0061001)
Regulation Of Macrophage Derived Foam Cell Differentiation (GO:0010743)
Embryonic Skeletal System Morphogenesis (GO:0048704)
Innate Immune Response Activating Cell Surface Receptor Signaling Pathway (GO:0002220)
Positive Regulation Of Cell Communication (GO:0010647)
Positive Regulation Of Interleukin-10 Production (GO:0032733)
Positive Regulation Of Locomotion (GO:0040017)
DNA Topological Change (GO:0006265)
Regulation Of Glucan Biosynthetic Process (GO:0010962)
Regulation Of Mitotic Sister Chromatid Segregation (GO:0033047)
Regulation Of Morphogenesis Of An Epithelium (GO:1905330)
Apoptotic DNA Fragmentation (GO:0006309)
Regulation Of Organ Growth (GO:0046620)
Brown Fat Cell Differentiation (GO:0050873)
Bundle Of His Cell To Purkinje Myocyte Communication (GO:0086069)
Regulation Of Sister Chromatid Cohesion (GO:0007063)
Cellular Response To Platelet-Derived Growth Factor Stimulus (GO:0036120)
Regulation Of Urine Volume (GO:0035809)
Collagen-Activated Signaling Pathway (GO:0038065)
Response To Magnesium Ion (GO:0032026)
Response To Vitamin (GO:0033273)
Response To Vitamin D (GO:0033280)
Embryonic Digestive Tract Morphogenesis (GO:0048557)
Embryonic Placenta Development (GO:0001892)
Endosome To Melanosome Transport (GO:0035646)
Endosome To Pigment Granule Transport (GO:0043485)
Somatic Recombination Of Immunoglobulin Gene Segments (GO:0016447)
Glucan Metabolic Process (GO:0044042)
Tricarboxylic Acid Metabolic Process (GO:0072350)
Glycerol Transmembrane Transport (GO:0015793)
Tyrosine Phosphorylation Of STAT Protein (GO:0007260)
Interleukin-12-Mediated Signaling Pathway (GO:0035722)
Intermembrane Lipid Transfer (GO:0120009)

Membrane Lipid Catabolic Process (GO:0046466)
Negative Regulation Of Cytoplasmic Translation (GO:2000766)
Negative Regulation Of Systemic Arterial Blood Pressure (GO:0003085)
Negative Regulation Of Triglyceride Metabolic Process (GO:0090209)
Nicotinamide Nucleotide Biosynthetic Process (GO:0019359)
Positive Regulation Of NK T Cell Activation (GO:0051135)
Positive Regulation Of Chromatin Binding (GO:0035563)
Positive Regulation Of Dendritic Cell Cytokine Production (GO:0002732)
Positive Regulation Of Fatty Acid Beta-Oxidation (GO:0032000)
Positive Regulation Of Inositol Phosphate Biosynthetic Process (GO:0060732)
Positive Regulation Of Ligase Activity (GO:0051351)
Positive Regulation Of Membrane Invagination (GO:1905155)
Positive Regulation Of Peptide Secretion (GO:0002793)
Positive Regulation Of Ubiquitin Protein Ligase Activity (GO:1904668)
Protein Hexamerization (GO:0034214)
Regulation Of DNA Demethylation (GO:1901535)
Regulation Of Cell Killing (GO:0031341)
Response To Interleukin-1 (GO:0070555)
Integrin-Mediated Signaling Pathway (GO:0007229)
Positive Regulation Of Canonical Wnt Signaling Pathway (GO:0090263)
RNA Catabolic Process (GO:0006401)
Monoatomic Anion Transport (GO:0006820)
Positive Regulation Of Interleukin-1 Beta Production (GO:0032731)
Regulation Of Calcium Ion Transport (GO:0051924)
Glutamine Family Amino Acid Metabolic Process (GO:0009064)
Positive Regulation Of Cholesterol Transport (GO:0032376)
Positive Regulation Of Glucose Transmembrane Transport (GO:0010828)
Response To Organic Cyclic Compound (GO:0014070)
Eye Development (GO:0001654)
Glycosaminoglycan Biosynthetic Process (GO:0006024)
Positive Regulation Of Type II Interferon Production (GO:0032729)
Regulation Of Type II Interferon Production (GO:0032649)
Protein Maturation (GO:0051604)
Chordate Embryonic Development (GO:0043009)
Phospholipid Transport (GO:0015914)
Positive Regulation Of Proteolysis (GO:0045862)
Amide Transport (GO:0042886)
Regulation Of Metaphase/Anaphase Transition Of Cell Cycle (GO:1902099)
Chromosome Condensation (GO:0030261)
Transition Metal Ion Transport (GO:0000041)
Gluconeogenesis (GO:0006094)
Intracellular pH Reduction (GO:0051452)
Proteoglycan Biosynthetic Process (GO:0030166)
Regulation Of Small GTPase Mediated Signal Transduction (GO:0051056)
Regulation Of Complement Activation (GO:0030449)
Regulation Of Complement-Dependent Cytotoxicity (GO:1903659)
[2Fe-2S] Cluster Assembly (GO:0044571)
Regulation Of Natural Killer Cell Proliferation (GO:0032817)

Aspartate Transmembrane Transport (GO:0015810)
Axon Regeneration (GO:0031103)
cAMP Biosynthetic Process (GO:0006171)
Carbohydrate Phosphorylation (GO:0046835)
Cell Differentiation Involved In Metanephros Development (GO:0072202)
Regulation Of Store-Operated Calcium Entry (GO:2001256)
Response To Platelet-Derived Growth Factor (GO:0036119)
Sequestering Of Extracellular Ligand From Receptor (GO:0035581)
Serine Family Amino Acid Catabolic Process (GO:0009071)
Fructose 6-Phosphate Metabolic Process (GO:0006002)
Toxin Transport (GO:1901998)
Glucan Biosynthetic Process (GO:0009250)
Transferrin Transport (GO:0033572)
Glycerolipid Metabolic Process (GO:0046486)
Type II Interferon-Mediated Signaling Pathway (GO:0060333)
Glycogen Biosynthetic Process (GO:0005978)
Vasoconstriction (GO:0042310)
Homocysteine Metabolic Process (GO:0050667)
Homologous Recombination (GO:0035825)
Iron Ion Transmembrane Transport (GO:0034755)
Lipid Import Into Cell (GO:0140354)
Long-Chain Fatty Acid Import Into Cell (GO:0044539)
Mesenchymal To Epithelial Transition (GO:0060231)
Mitotic DNA Replication Checkpoint Signaling (GO:0033314)
Motor Neuron Axon Guidance (GO:0008045)
Myelin Maintenance (GO:0043217)
Myeloid Dendritic Cell Differentiation (GO:0043011)
Negative Regulation Of Leukocyte Migration (GO:0002686)
Negative Regulation Of Muscle Cell Apoptotic Process (GO:0010656)
Negative Regulation Of Protein Exit From Endoplasmic Reticulum (GO:0070862)
Negative Regulation Of Protein Maturation (GO:1903318)
Neuron Remodeling (GO:0016322)
Nucleoside Metabolic Process (GO:0009116)
Phosphatidylcholine Catabolic Process (GO:0034638)
Positive Regulation Of RNA Biosynthetic Process (GO:1902680)
Positive Regulation Of Epidermis Development (GO:0045684)
Positive Regulation Of Leukocyte Apoptotic Process (GO:2000108)
Positive Regulation Of Lipase Activity (GO:0060193)
Positive Regulation Of Mesenchymal Cell Proliferation (GO:0002053)
Positive Regulation Of Phagocytosis, Engulfment (GO:0060100)
Positive Regulation Of Sodium Ion Transmembrane Transport (GO:1902307)
Protein K6-linked Ubiquitination (GO:0085020)
Purine Nucleobase Metabolic Process (GO:0006144)
Regulation Of Animal Organ Morphogenesis (GO:2000027)
Regulation Of Bile Acid Biosynthetic Process (GO:0070857)
Positive Regulation Of MAPK Cascade (GO:0043410)
Cellular Response To Peptide Hormone Stimulus (GO:0071375)
Sodium Ion Transport (GO:0006814)

Actin Filament Bundle Assembly (GO:0051017)
Actin Filament Bundle Organization (GO:0061572)
Regulation Of Lamellipodium Assembly (GO:0010591)
Inositol Lipid-Mediated Signaling (GO:0048017)
Negative Regulation Of Double-Strand Break Repair (GO:2000780)
Negative Regulation Of Endocytosis (GO:0045806)
Positive Regulation Of DNA Binding (GO:0043388)
Positive Regulation Of Chromosome Organization (GO:2001252)
Protein K63-linked Deubiquitination (GO:0070536)
Fatty Acid Catabolic Process (GO:0009062)
Negative Regulation Of Viral Process (GO:0048525)
Positive Regulation Of Gene Expression (GO:0010628)
Regulation Of Interleukin-17 Production (GO:0032660)
Cellular Response To Amyloid-Beta (GO:1904646)
Cellular Response To Hydrogen Peroxide (GO:0070301)
Glial Cell Differentiation (GO:0010001)
Histone Deubiquitination (GO:0016578)
Positive Regulation Of Carbohydrate Metabolic Process (GO:0045913)
Negative Regulation Of Programmed Cell Death (GO:0043069)
Regulation Of Cellular Component Organization (GO:0051128)
Epithelium Development (GO:0060429)
Regulation Of Chromatin Binding (GO:0035561)
Regulation Of Glycoprotein Biosynthetic Process (GO:0010559)
SRP-dependent Cotranslational Protein Targeting To Membrane (GO:0006614)
Acidic Amino Acid Transport (GO:0015800)
Alpha-Amino Acid Biosynthetic Process (GO:1901607)
Regulation Of Mitotic Sister Chromatid Separation (GO:0010965)
Arginine Metabolic Process (GO:0006525)
Aromatic Amino Acid Family Catabolic Process (GO:0009074)
Regulation Of Nuclear-Transcribed mRNA Catabolic Process, Nonsense-Mediated Decay (GO:2000622)
Regulation Of Peroxisome Proliferator Activated Receptor Signaling Pathway (GO:0035358)
Regulation Of Phagocytosis, Engulfment (GO:0060099)
Regulation Of Phospholipase Activity (GO:0010517)
Cellular Response To UV-A (GO:0071492)
Regulation Of Smooth Muscle Cell Apoptotic Process (GO:0034391)
Cellular Response To Forskolin (GO:1904322)
Cellular Response To Interleukin-12 (GO:0071349)
Regulation Of Triglyceride Catabolic Process (GO:0010896)
Removal Of Superoxide Radicals (GO:0019430)
Coenzyme A Biosynthetic Process (GO:0015937)
Response To Forskolin (GO:1904321)
Response To Sterol (GO:0036314)
Ribonucleoside Bisphosphate Biosynthetic Process (GO:0034030)
Signal Release (GO:0023061)
Hematopoietic Stem Cell Proliferation (GO:0071425)
Insulin-Like Growth Factor Receptor Signaling Pathway (GO:0048009)
Kinetochore Organization (GO:0051383)
Megakaryocyte Development (GO:0035855)

Mitotic Spindle Elongation (GO:0000022)
Mitotic Spindle Midzone Assembly (GO:0051256)
Monoamine Transport (GO:0015844)
Negative Regulation Of B Cell Proliferation (GO:0030889)
Negative Regulation Of Centrosome Duplication (GO:0010826)
Negative Regulation Of Oxidoreductase Activity (GO:0051354)
Negative Regulation Of Pathway-Restricted SMAD Protein Phosphorylation (GO:0060394)
Neuron Projection Maintenance (GO:1990535)
Peptidyl-Proline Hydroxylation (GO:0019511)
Phosphate Ion Transmembrane Transport (GO:0035435)
Positive Regulation Of DNA-templated DNA Replication (GO:2000105)
Positive Regulation Of MHC Class II Biosynthetic Process (GO:0045348)
Positive Regulation Of Attachment Of Spindle Microtubules To Kinetochore (GO:0051987)
Positive Regulation Of Bone Resorption (GO:0045780)
Positive Regulation Of Cholesterol Metabolic Process (GO:0090205)
Positive Regulation Of Macrophage Differentiation (GO:0045651)
Positive Regulation Of Nitric-Oxide Synthase Biosynthetic Process (GO:0051770)
Positive Regulation Of Transforming Growth Factor Beta Production (GO:0071636)
Protein Localization To Condensed Chromosome (GO:1903083)
Purine Nucleoside Bisphosphate Biosynthetic Process (GO:0034033)
Positive Regulation Of Mitotic Cell Cycle Phase Transition (GO:1901992)
Response To Nutrient Levels (GO:0031667)
Hexose Biosynthetic Process (GO:0019319)
One-Carbon Compound Transport (GO:0019755)
Positive Regulation Of Cell Development (GO:0010720)
Regulation Of G2/M Transition Of Mitotic Cell Cycle (GO:0010389)
Regulation Of Axon Extension (GO:0030516)
Positive Regulation Of Cysteine-Type Endopeptidase Activity (GO:2001056)
Positive Regulation Of Interleukin-1 Production (GO:0032732)
Cellular Response To Hormone Stimulus (GO:0032870)
Response To Cytokine (GO:0034097)
Positive Regulation Of Multicellular Organismal Process (GO:0051240)
Base-Excision Repair (GO:0006284)
Regulation Of Peptidyl-Threonine Phosphorylation (GO:0010799)
Regulation Of Receptor Internalization (GO:0002090)
Cellular Response To Starvation (GO:0009267)
Antimicrobial Humoral Immune Response Mediated By Antimicrobial Peptide (GO:0061844)
Positive Regulation Of T Cell Proliferation (GO:0042102)
Regulation Of Glycogen Metabolic Process (GO:0070873)
Regulation Of Mesenchymal Cell Proliferation (GO:0010464)
Base-Excision Repair, AP Site Formation (GO:0006285)
Biogenic Amine Biosynthetic Process (GO:0042401)
Regulation Of Protein Exit From Endoplasmic Reticulum (GO:0070861)
Cellular Response To UV-B (GO:0071493)
Cellular Response To Fluid Shear Stress (GO:0071498)
Regulation Of Type II Interferon-Mediated Signaling Pathway (GO:0060334)
Cellular Response To Superoxide (GO:0071451)
Regulation Of Ventricular Cardiac Muscle Cell Action Potential (GO:0098911)

Cytidine To Uridine Editing (GO:0016554)
Desensitization Of G Protein-Coupled Receptor Signaling Pathway (GO:0002029)
Digestive System Development (GO:0055123)
Erythrocyte Development (GO:0048821)
Exit From Mitosis (GO:0010458)
Tyrosine Metabolic Process (GO:0006570)
Histone H2A Ubiquitination (GO:0033522)
Maintenance Of Protein Localization In Endoplasmic Reticulum (GO:0035437)
Methylguanosine-Cap Decapping (GO:0110156)
Negative Regulation Of calcineurin-NFAT Signaling Cascade (GO:0070885)
Negative Regulation Of Calcineurin-Mediated Signaling (GO:0106057)
Negative Regulation Of Cell Division (GO:0051782)
Negative Regulation Of Reactive Oxygen Species Biosynthetic Process (GO:1903427)
Neurotransmitter Uptake (GO:0001504)
Organic Cyclic Compound Catabolic Process (GO:1901361)
Polyamine Metabolic Process (GO:0006595)
Positive Regulation Of Coagulation (GO:0050820)
Positive Regulation Of Hemostasis (GO:1900048)
Positive Regulation Of Mitotic Cell Cycle Spindle Assembly Checkpoint (GO:0090267)
Positive Regulation Of Spindle Checkpoint (GO:0090232)
Positive Regulation Of Triglyceride Biosynthetic Process (GO:0010867)
Regulation Of Cell Cycle Checkpoint (GO:1901976)
Response To Lipopolysaccharide (GO:0032496)
Melanosome Organization (GO:0032438)
Negative Regulation Of Endothelial Cell Migration (GO:0010596)
Positive Regulation Of Signaling (GO:0023056)
Cellular Response To Type II Interferon (GO:0071346)
Activation Of Cysteine-Type Endopeptidase Activity Involved In Apoptotic Process (GO:0006919)
Cellular Component Assembly (GO:0022607)
MAPK Cascade (GO:0000165)
Phosphorylation (GO:0016310)
G2/M Transition Of Mitotic Cell Cycle (GO:0000086)
Regulation Of Intracellular Transport (GO:0032386)
Fatty Acid Transport (GO:0015908)
Protein Autophosphorylation (GO:0046777)
Positive Regulation Of Peptidyl-Tyrosine Phosphorylation (GO:0050731)
Regulation Of Cell Cycle Process (GO:0010564)
Intermediate Filament Organization (GO:0045109)
Regulation Of Chemokine (C-X-C Motif) Ligand 2 Production (GO:2000341)
Regulation Of Chromosome Separation (GO:1905818)
C4-dicarboxylate Transport (GO:0015740)
DNA Conformation Change (GO:0071103)
G Protein-Coupled Glutamate Receptor Signaling Pathway (GO:0007216)
V(D)J Recombination (GO:0033151)
Regulation Of Interleukin-5 Production (GO:0032674)
acetyl-CoA Biosynthetic Process (GO:0006085)
Alanine Transport (GO:0032328)
Atrial Cardiac Muscle Tissue Development (GO:0003228)

cAMP Metabolic Process (GO:0046058)
Response To Interleukin-6 (GO:0070741)
Response To Iron Ion (GO:0010039)
Sodium Ion Export Across Plasma Membrane (GO:0036376)
Sphingomyelin Metabolic Process (GO:0006684)
Spinal Cord Development (GO:0021510)
Fatty Acid Elongation (GO:0030497)
Fatty Acid Transmembrane Transport (GO:1902001)
Folic Acid Metabolic Process (GO:0046655)
Granulocyte Activation (GO:0036230)
Very Long-Chain Fatty Acid Biosynthetic Process (GO:0042761)
Histone Monoubiquitination (GO:0010390)
Mitochondrial Electron Transport, Ubiquinol To Cytochrome C (GO:0006122)
Mitochondrial Transcription (GO:0006390)
Mitotic DNA Integrity Checkpoint Signaling (GO:0044774)
Multicellular Organismal-Level Iron Ion Homeostasis (GO:0060586)
Negative Regulation Of Cardiac Muscle Hypertrophy (GO:0010614)
Negative Regulation Of Hippo Signaling (GO:0035331)
Negative Regulation Of Locomotion (GO:0040013)
Polysaccharide Biosynthetic Process (GO:0000271)
Positive Regulation Of Calcium-Mediated Signaling (GO:0050850)
Positive Regulation Of Cell Morphogenesis Involved In Differentiation (GO:0010770)
Positive Regulation Of Fatty Acid Oxidation (GO:0046321)
Positive Regulation Of Histone H3-K4 Methylation (GO:0051571)
Positive Regulation Of Integrin-Mediated Signaling Pathway (GO:2001046)
Positive Regulation Of Neuroinflammatory Response (GO:0150078)
Positive Regulation Of Reactive Oxygen Species Biosynthetic Process (GO:1903428)
Positive Regulation Of Sodium Ion Transport (GO:0010765)
Positive Regulation Of Vascular Endothelial Cell Proliferation (GO:1905564)
Pyrimidine Deoxyribonucleotide Catabolic Process (GO:0009223)
Quaternary Ammonium Group Transport (GO:0015697)
Regulation Of DNA-templated DNA Replication Initiation (GO:0030174)
Regulation Of T Cell Differentiation In Thymus (GO:0033081)
Synapse Organization (GO:0050808)
Regulation Of Monoatomic Cation Transmembrane Transport (GO:1904062)
Regulation Of Protein Targeting To Mitochondrion (GO:1903214)
Retina Homeostasis (GO:0001895)
Digestive Tract Development (GO:0048565)
Vitamin Transport (GO:0051180)
Negative Regulation Of Cell-Substrate Adhesion (GO:0010812)
Positive Regulation Of Lipid Biosynthetic Process (GO:0046889)
Protein Complex Oligomerization (GO:0051259)
Response To Molecule Of Bacterial Origin (GO:0002237)
Positive Regulation Of Phosphatidylinositol 3-Kinase Signaling (GO:0014068)
Visual Perception (GO:0007601)
Sensory Organ Development (GO:0007423)
Sulfur Compound Biosynthetic Process (GO:0044272)
Negative Regulation Of Extrinsic Apoptotic Signaling Pathway (GO:2001237)

Positive Regulation Of Protein Transport (GO:0051222)
Regulation Of G Protein-Coupled Receptor Signaling Pathway (GO:0008277)
Regulation Of Actin Filament-Based Process (GO:0032970)
L-alpha-amino Acid Transmembrane Transport (GO:1902475)
Cell Cycle G2/M Phase Transition (GO:0044839)
Regulation Of Small Molecule Metabolic Process (GO:0062012)
Water-Soluble Vitamin Metabolic Process (GO:0006767)
Positive Regulation Of Monoatomic Ion Transport (GO:0043270)
Positive Regulation Of Myeloid Leukocyte Differentiation (GO:0002763)
Protein Localization To Cilium (GO:0061512)
Sensory Perception Of Light Stimulus (GO:0050953)
Regulation Of Cytokine Production (GO:0001817)
Regulation Of Fatty Acid Beta-Oxidation (GO:0031998)
Regulation Of Humoral Immune Response (GO:0002920)
Regulation Of Immune Effector Process (GO:0002697)
Regulation Of Lamellipodium Organization (GO:1902743)
Acute-Phase Response (GO:0006953)
Regulation Of Nitric-Oxide Synthase Biosynthetic Process (GO:0051769)
Regulation Of Oxidoreductase Activity (GO:0051341)
Regulation Of Short-Term Neuronal Synaptic Plasticity (GO:0048172)
Chondroitin Sulfate Biosynthetic Process (GO:0030206)
Response To UV-A (GO:0070141)
Endonucleolytic Cleavage Of Tricistronic rRNA Transcript (SSU-rRNA, 5.8S rRNA, LSU-rRNA) (GO:0000479)
Glomerular Filtration (GO:0003094)
Interleukin-6-Mediated Signaling Pathway (GO:0070102)
Intracellular Potassium Ion Homeostasis (GO:0030007)
Killing By Host Of Symbiont Cells (GO:0051873)
Membrane Depolarization During Action Potential (GO:0086010)
Monoacylglycerol Metabolic Process (GO:0046462)
Myeloid Dendritic Cell Activation (GO:0001773)
Negative Regulation Of Bone Mineralization (GO:0030502)
Negative Regulation Of Neuroinflammatory Response (GO:0150079)
Negative Regulation Of Toll-Like Receptor Signaling Pathway (GO:0034122)
Positive Regulation Of Brown Fat Cell Differentiation (GO:0090336)
Positive Regulation Of Cardiac Muscle Cell Proliferation (GO:0060045)
Positive Regulation Of Fatty Acid Biosynthetic Process (GO:0045723)
Positive Regulation Of Glycogen Biosynthetic Process (GO:0045725)
Positive Regulation Of Glycoprotein Biosynthetic Process (GO:0010560)
Positive Regulation Of Macrophage Chemotaxis (GO:0010759)
Positive Regulation Of Potassium Ion Transmembrane Transporter Activity (GO:1901018)
Positive Regulation Of Superoxide Anion Generation (GO:0032930)
Protein Localization To Synapse (GO:0035418)
Synapse Assembly (GO:0007416)
Negative Regulation Of Cellular Response To Growth Factor Stimulus (GO:0090288)
Cellular Response To Metal Ion (GO:0071248)
Organic Substance Transport (GO:0071702)
Double-Strand Break Repair (GO:0006302)
Regulation Of Heart Rate By Cardiac Conduction (GO:0086091)

Axoneme Assembly (GO:0035082)
Carbohydrate Catabolic Process (GO:0016052)
Double-Strand Break Repair Via Nonhomologous End Joining (GO:0006303)
Stem Cell Differentiation (GO:0048863)
Lymphocyte Migration (GO:0072676)
Negative Regulation Of DNA Recombination (GO:0045910)
Negative Regulation Of mRNA Catabolic Process (GO:1902373)
Negative Regulation Of Proteolysis (GO:0045861)
Positive Regulation Of Actin Filament Polymerization (GO:0030838)
Protein Localization To Lysosome (GO:0061462)
Response To Ionizing Radiation (GO:0010212)
Second-Messenger-Mediated Signaling (GO:0019932)
Negative Regulation Of Cell Adhesion (GO:0007162)
Positive Regulation Of Apoptotic Process (GO:0043065)
Potassium Ion Transmembrane Transport (GO:0071805)
DNA Integrity Checkpoint Signaling (GO:0031570)
DNA Recombination (GO:0006310)
Amino Acid Transmembrane Transport (GO:0003333)
Regulation Of Axonogenesis (GO:0050770)
Cellular Response To Reactive Oxygen Species (GO:0034614)
Protein Tetramerization (GO:0051262)
Intrinsic Apoptotic Signaling Pathway (GO:0097193)
Regulation Of Cholesterol Storage (GO:0010885)
L-glutamate Transmembrane Transport (GO:0015813)
RNA Phosphodiester Bond Hydrolysis, Endonucleolytic (GO:0090502)
Regulation Of Histone H3-K4 Methylation (GO:0051569)
Regulation Of Integrin Activation (GO:0033623)
Regulation Of Macrophage Differentiation (GO:0045649)
Regulation Of Microglial Cell Activation (GO:1903978)
Regulation Of Non-Canonical Wnt Signaling Pathway (GO:2000050)
Regulation Of Superoxide Anion Generation (GO:0032928)
Resolution Of Meiotic Recombination Intermediates (GO:0000712)
Detection Of Mechanical Stimulus Involved In Sensory Perception (GO:0050974)
Electron Transport Chain (GO:0022900)
Embryonic Cranial Skeleton Morphogenesis (GO:0048701)
Establishment Of Chromosome Localization (GO:0051303)
Establishment Of Protein Localization To Chromosome (GO:0070199)
Establishment Of Spindle Localization (GO:0051293)
Synaptic Transmission, GABAergic (GO:0051932)
Histone H2A Monoubiquitination (GO:0035518)
Hyaluronan Metabolic Process (GO:0030212)
Icosanoid Transport (GO:0071715)
Mammary Gland Epithelium Development (GO:0061180)
Myotube Cell Development (GO:0014904)
Negative Regulation Of Peptidyl-Threonine Phosphorylation (GO:0010801)
Negative Regulation Of Smooth Muscle Cell Migration (GO:0014912)
Pancreas Development (GO:0031016)
Pathway-Restricted SMAD Protein Phosphorylation (GO:0060389)

Positive Regulation Of Acute Inflammatory Response (GO:0002675)
Positive Regulation Of Exosomal Secretion (GO:1903543)
Positive Regulation Of Glycogen Metabolic Process (GO:0070875)
Positive Regulation Of Histone Acetylation (GO:0035066)
Positive Regulation Of Response To Cytokine Stimulus (GO:0060760)
Positive Regulation Of Transcription From RNA Polymerase II Promoter Involved In Cellular Response To Chemi
Post-Translational Protein Targeting To Endoplasmic Reticulum Membrane (GO:0006620)
Primary Alcohol Metabolic Process (GO:0034308)
Protein Homotrimerization (GO:0070207)
Protein Localization To Vacuole (GO:0072665)
Protein Maturation By Iron-Sulfur Cluster Transfer (GO:0097428)
Protein Trimerization (GO:0070206)
Regulation Of Branching Involved In Ureteric Bud Morphogenesis (GO:0090189)
Regulation Of Carbohydrate Catabolic Process (GO:0043470)
Generation Of Neurons (GO:0048699)
Regulation Of Endothelial Cell Apoptotic Process (GO:2000351)
Response To Amyloid-Beta (GO:1904645)
Skeletal System Morphogenesis (GO:0048705)
Energy Derivation By Oxidation Of Organic Compounds (GO:0015980)
Ephrin Receptor Signaling Pathway (GO:0048013)
mRNA Catabolic Process (GO:0006402)
Negative Regulation Of BMP Signaling Pathway (GO:0030514)
Positive Regulation Of miRNA Transcription (GO:1902895)
Negative Regulation Of Apoptotic Process (GO:0043066)
Response To Unfolded Protein (GO:0006986)
Embryonic Organ Morphogenesis (GO:0048562)
Negative Regulation Of Immune System Process (GO:0002683)
Phosphatidylinositol Phosphate Biosynthetic Process (GO:0046854)
Positive Regulation Of I-kappaB kinase/NF-kappaB Signaling (GO:0043123)
Regulation Of Cholesterol Biosynthetic Process (GO:0045540)
DNA Alkylation (GO:0006305)
L-glutamate Import (GO:0051938)
Regulation Of Interleukin-13 Production (GO:0032656)
Amyloid-Beta Metabolic Process (GO:0050435)
Branched-Chain Amino Acid Metabolic Process (GO:0009081)
Regulation Of Systemic Arterial Blood Pressure By Hormone (GO:0001990)
Cellular Response To Sterol (GO:0036315)
Renal Filtration (GO:0097205)
Coenzyme A Metabolic Process (GO:0015936)
Dendritic Cell Chemotaxis (GO:0002407)
Dendritic Spine Morphogenesis (GO:0060997)
Endoplasmic Reticulum To Cytosol Transport (GO:1903513)
Sphingolipid Catabolic Process (GO:0030149)
Folic Acid-Containing Compound Metabolic Process (GO:0006760)
Glutamine Family Amino Acid Catabolic Process (GO:0009065)
Membrane Repolarization During Action Potential (GO:0086011)
Mitotic Chromosome Condensation (GO:0007076)
Negative Regulation Of Cell-Substrate Junction Organization (GO:0150118)

Negative Regulation Of Focal Adhesion Assembly (GO:0051895)
Negative Regulation Of Protein Processing (GO:0010955)
Negative Regulation Of Type I Interferon-Mediated Signaling Pathway (GO:0060339)
Nucleotide-Sugar Biosynthetic Process (GO:0009226)
Phagosome Maturation (GO:0090382)
Positive Regulation Of Endothelial Cell Apoptotic Process (GO:2000353)
Positive Regulation Of Secretion (GO:0051047)
Positive Regulation Of Sprouting Angiogenesis (GO:1903672)
Positive Regulation Of Transmembrane Transport (GO:0034764)
Regulation Of Rac Protein Signal Transduction (GO:0035020)
Regulation Of Carbohydrate Metabolic Process (GO:0006109)
Wnt Signaling Pathway (GO:0016055)
Positive Regulation Of Wnt Signaling Pathway (GO:0030177)
Positive Regulation Of Cysteine-Type Endopeptidase Activity Involved In Apoptotic Process (GO:0043280)
Chromosome Organization (GO:0051276)
Zymogen Activation (GO:0031638)
Regulation Of Apoptotic Signaling Pathway (GO:2001233)
Positive Regulation Of Programmed Cell Death (GO:0043068)
Cell Morphogenesis Involved In Neuron Differentiation (GO:0048667)
Positive Regulation Of JNK Cascade (GO:0046330)
Positive Regulation Of Endothelial Cell Proliferation (GO:0001938)
Positive Regulation Of Intracellular Signal Transduction (GO:1902533)
Supramolecular Fiber Organization (GO:0097435)
Cytoskeleton Organization (GO:0007010)
Negative Regulation Of Inflammatory Response (GO:0050728)
Negative Regulation Of Transmembrane Receptor Protein Serine/Threonine Kinase Signaling Pathway (GO:00901
Cellular Response To Nutrient Levels (GO:0031669)
Regulation Of Cytokine Production Involved In Inflammatory Response (GO:1900015)
T Cell Differentiation (GO:0030217)
Cardiac Conduction (GO:0061337)
Negative Regulation Of Cellular Metabolic Process (GO:0031324)
Negative Regulation Of Cold-Induced Thermogenesis (GO:0120163)
Regulation Of Interleukin-1 Production (GO:0032652)
Regulation Of Plasminogen Activation (GO:0010755)
Cell Surface Toll-Like Receptor Signaling Pathway (GO:0140895)
Regulation Of Stem Cell Proliferation (GO:0072091)
Regulation Of Steroid Biosynthetic Process (GO:0050810)
Regulation Of Transforming Growth Factor Beta Production (GO:0071634)
Cellular Response To Nitrogen Compound (GO:1901699)
Regulation Of Triglyceride Biosynthetic Process (GO:0010866)
Chondroitin Sulfate Metabolic Process (GO:0030204)
Response To UV-B (GO:0010224)
Heart Process (GO:0003015)
Male Meiosis I (GO:0007141)
Membrane Depolarization During Cardiac Muscle Cell Action Potential (GO:0086012)
Mitochondrial Electron Transport, Cytochrome C To Oxygen (GO:0006123)
Negative Regulation Of Amyloid-Beta Formation (GO:1902430)
Negative Regulation Of Anoikis (GO:2000811)

Negative Regulation Of Biosynthetic Process (GO:0009890)
Negative Regulation Of Chemokine Production (GO:0032682)
Negative Regulation Of Release Of Cytochrome C From Mitochondria (GO:0090201)
Neuron Projection Organization (GO:0106027)
Phosphatidylinositol Dephosphorylation (GO:0046856)
Positive Regulation Of Cell Migration Involved In Sprouting Angiogenesis (GO:0090050)
Positive Regulation Of Muscle Hypertrophy (GO:0014742)
Positive Regulation Of Smooth Muscle Cell Migration (GO:0014911)
Positive Regulation Of Telomere Capping (GO:1904355)
Prostanoid Metabolic Process (GO:0006692)
Protein O-linked Mannosylation (GO:0035269)
Regulation Of Apoptotic Process (GO:0042981)
Regulation Of Protein Serine/Threonine Kinase Activity (GO:0071900)
Negative Regulation Of Cellular Component Organization (GO:0051129)
Positive Regulation Of Cytoskeleton Organization (GO:0051495)
Neuron Projection Morphogenesis (GO:0048812)
Insulin Receptor Signaling Pathway (GO:0008286)
Response To Type II Interferon (GO:0034341)
DNA Damage Response, Signal Transduction By P53 Class Mediator Resulting In Cell Cycle Arrest (GO:000697[illegible]
DNA Modification (GO:0006304)
G Protein-Coupled Acetylcholine Receptor Signaling Pathway (GO:0007213)
Regulation Of Glucose Transmembrane Transport (GO:0010827)
Regulation Of Leukocyte Chemotaxis (GO:0002688)
Androgen Receptor Signaling Pathway (GO:0030521)
Regulation Of Mitotic Cell Cycle Spindle Assembly Checkpoint (GO:0090266)
Base Conversion Or Substitution Editing (GO:0016553)
Carboxylic Acid Biosynthetic Process (GO:0046394)
Carboxylic Acid Catabolic Process (GO:0046395)
Regulation Of Receptor Signaling Pathway Via STAT (GO:1904892)
Cell Migration Involved In Sprouting Angiogenesis (GO:0002042)
Regulation Of Single Stranded Viral RNA Replication Via Double Stranded DNA Intermediate (GO:0045091)
Regulation Of Ubiquitin Protein Ligase Activity (GO:1904666)
Retrograde Protein Transport, ER To Cytosol (GO:0030970)
Embryo Development Ending In Birth Or Egg Hatching (GO:0009792)
Epoxygenase P450 Pathway (GO:0019373)
Sulfur Amino Acid Metabolic Process (GO:0000096)
Extracellular Matrix Assembly (GO:0085029)
Toll-Like Receptor 4 Signaling Pathway (GO:0034142)
Leukocyte Tethering Or Rolling (GO:0050901)
Megakaryocyte Differentiation (GO:0030219)
Membrane Lipid Metabolic Process (GO:0006643)
Mesenchyme Development (GO:0060485)
Neuron Maturation (GO:0042551)
Peptidyl-Threonine Dephosphorylation (GO:0035970)
Peroxisomal Membrane Transport (GO:0015919)
Polyol Metabolic Process (GO:0019751)
Positive Chemotaxis (GO:0050918)
Positive Regulation By Host Of Viral Process (GO:0044794)

Positive Regulation Of SMAD Protein Signal Transduction (GO:0060391)
Positive Regulation Of Alpha-Beta T Cell Proliferation (GO:0046641)
Positive Regulation Of Cardiac Muscle Hypertrophy (GO:0010613)
Positive Regulation Of Dendrite Morphogenesis (GO:0050775)
Positive Regulation Of Heart Rate (GO:0010460)
Positive Regulation Of Leukocyte Proliferation (GO:0070665)
Positive Regulation Of Macrophage Activation (GO:0043032)
Positive Regulation Of Nitric-Oxide Synthase Activity (GO:0051000)
Positive Regulation Of Peptidyl-Lysine Acetylation (GO:2000758)
Positive Regulation Of Small GTPase Mediated Signal Transduction (GO:0051057)
Postsynaptic Membrane Organization (GO:0001941)
rRNA Modification (GO:0000154)
Regulation Of DNA Methylation-Dependent Heterochromatin Formation (GO:0090308)
Regulation Of DNA-templated DNA Replication (GO:0090329)
Regulation Of Brown Fat Cell Differentiation (GO:0090335)
Chloride Transmembrane Transport (GO:1902476)
Positive Regulation Of Reactive Oxygen Species Metabolic Process (GO:2000379)
Spindle Assembly (GO:0051225)
Negative Regulation Of Protein Phosphorylation (GO:0001933)
Protein Phosphorylation (GO:0006468)
Phosphate-Containing Compound Metabolic Process (GO:0006796)
Fatty Acid Beta-Oxidation (GO:0006635)
Negative Regulation Of DNA Metabolic Process (GO:0051053)
Positive Regulation Of miRNA Metabolic Process (GO:2000630)
Positive Regulation Of Nervous System Development (GO:0051962)
Regulation Of Cell Communication (GO:0010646)
Negative Regulation Of Macromolecule Metabolic Process (GO:0010605)
Regulation Of Epidermal Cell Differentiation (GO:0045604)
Regulation Of Exosomal Secretion (GO:1903541)
Actin Filament Capping (GO:0051693)
Regulation Of Lymphocyte Activation (GO:0051249)
Regulation Of Oligodendrocyte Differentiation (GO:0048713)
Barbed-End Actin Filament Capping (GO:0051016)
Regulation Of Oxidative Stress-Induced Intrinsic Apoptotic Signaling Pathway (GO:1902175)
Regulation Of Vascular Endothelial Cell Proliferation (GO:1905562)
Renal Absorption (GO:0070293)
Response To Ethanol (GO:0045471)
Dendritic Cell Migration (GO:0036336)
Response To Organophosphorus (GO:0046683)
Endothelial Cell Proliferation (GO:0001935)
Glucose 6-Phosphate Metabolic Process (GO:0051156)
Vesicle Transport Along Actin Filament (GO:0030050)
Intracellular Sodium Ion Homeostasis (GO:0006883)
Membrane Repolarization (GO:0086009)
Mitochondrial Fusion (GO:0008053)
Negative Regulation Of ERBB Signaling Pathway (GO:1901185)
Negative Regulation Of Amyloid Precursor Protein Catabolic Process (GO:1902992)
Negative Regulation Of Nervous System Development (GO:0051961)

Neutrophil Activation (GO:0042119)
Nitric Oxide Mediated Signal Transduction (GO:0007263)
Positive Regulation Of G Protein-Coupled Receptor Signaling Pathway (GO:0045745)
Positive Regulation Of Cytokine Production Involved In Inflammatory Response (GO:1900017)
Positive Regulation Of Epidermal Cell Differentiation (GO:0045606)
Positive Regulation Of Focal Adhesion Assembly (GO:0051894)
Positive Regulation Of Glycolytic Process (GO:0045821)
Positive Regulation Of Macrophage Migration (GO:1905523)
Positive Regulation Of Monocyte Chemotaxis (GO:0090026)
Positive Regulation Of Natural Killer Cell Activation (GO:0032816)
Positive Regulation Of Triglyceride Metabolic Process (GO:0090208)
Protein Localization To Chromosome, Centromeric Region (GO:0071459)
Pulmonary Valve Morphogenesis (GO:0003184)
Regulation Of DNA Damage Checkpoint (GO:2000001)
Regulation Of cAMP-mediated Signaling (GO:0043949)
Regulation Of Cell Cycle G2/M Phase Transition (GO:1902749)
Cellular Response To Molecule Of Bacterial Origin (GO:0071219)
Protein Processing (GO:0016485)
Vascular Transport (GO:0010232)
Plasma Membrane Bounded Cell Projection Morphogenesis (GO:0120039)
Positive Regulation Of Developmental Growth (GO:0048639)
Positive Regulation Of Fat Cell Differentiation (GO:0045600)
Regulation Of Cell Migration (GO:0030334)
Cytokine-Mediated Signaling Pathway (GO:0019221)
Positive Regulation Of Angiogenesis (GO:0045766)
Regulation Of Smoothened Signaling Pathway (GO:0008589)
Regulation Of Substrate Adhesion-Dependent Cell Spreading (GO:1900024)
Regulation Of Synapse Assembly (GO:0051963)
Metaphase Plate Congression (GO:0051310)
Positive Regulation Of Ras Protein Signal Transduction (GO:0046579)
Calcium-Mediated Signaling (GO:0019722)
Transport Across Blood-Brain Barrier (GO:0150104)
mRNA Metabolic Process (GO:0016071)
Mitotic Spindle Organization (GO:0007052)
Negative Regulation Of Cell Cycle (GO:0045786)
Regulation Of Centriole Replication (GO:0046599)
Regulation Of Granulocyte Chemotaxis (GO:0071622)
Regulation Of Insulin-Like Growth Factor Receptor Signaling Pathway (GO:0043567)
Regulation Of Integrin-Mediated Signaling Pathway (GO:2001044)
Regulation Of Megakaryocyte Differentiation (GO:0045652)
Cardiac Epithelial To Mesenchymal Transition (GO:0060317)
Regulation Of Tissue Remodeling (GO:0034103)
Regulation Of Transcription Involved In G1/S Transition Of Mitotic Cell Cycle (GO:0000083)
Regulation Of Vascular Endothelial Growth Factor Signaling Pathway (GO:1900746)
Chondroitin Sulfate Proteoglycan Biosynthetic Process (GO:0050650)
Cyclic Nucleotide Biosynthetic Process (GO:0009190)
Dopaminergic Neuron Differentiation (GO:0071542)
tRNA Metabolic Process (GO:0006399)

Fluid Transport (GO:0042044)
Gastrulation (GO:0007369)
Water Transport (GO:0006833)
Leukocyte Adhesion To Vascular Endothelial Cell (GO:0061756)
Mitotic Sister Chromatid Cohesion (GO:0007064)
Negative Regulation Of Execution Phase Of Apoptosis (GO:1900118)
Neuron Apoptotic Process (GO:0051402)
Phosphate Ion Transport (GO:0006817)
Phosphatidylcholine Biosynthetic Process (GO:0006656)
piRNA Processing (GO:0034587)
Positive Regulation Of Activated T Cell Proliferation (GO:0042104)
Positive Regulation Of Alpha-Beta T Cell Activation (GO:0046635)
Positive Regulation Of Axon Extension (GO:0045773)
Positive Regulation Of calcineurin-NFAT Signaling Cascade (GO:0070886)
Positive Regulation Of Calcineurin-Mediated Signaling (GO:0106058)
Positive Regulation Of Histone Methylation (GO:0031062)
Protein Heterooligomerization (GO:0051291)
rRNA Catabolic Process (GO:0016075)
Regulation Of Autophagy Of Mitochondrion (GO:1903146)
Exocytosis (GO:0006887)
Negative Regulation Of Binding (GO:0051100)
Rho Protein Signal Transduction (GO:0007266)
Cellular Response To Ionizing Radiation (GO:0071479)
Negative Regulation Of Response To Stimulus (GO:0048585)
Neurotransmitter Transport (GO:0006836)
Glucose Homeostasis (GO:0042593)
Positive Regulation Of Macromolecule Biosynthetic Process (GO:0010557)
Potassium Ion Transport (GO:0006813)
Apoptotic Process (GO:0006915)
Blood Vessel Morphogenesis (GO:0048514)
Cellular Response To Alcohol (GO:0097306)
Spermatid Development (GO:0007286)
Positive Regulation Of Chemokine Production (GO:0032722)
DNA Methylation (GO:0006306)
Regulation Of Fibroblast Migration (GO:0010762)
Actin Filament-Based Transport (GO:0099515)
Regulation Of Macrophage Chemotaxis (GO:0010758)
Ameboidal-Type Cell Migration (GO:0001667)
Regulation Of Meiotic Cell Cycle (GO:0051445)
Calcium-Mediated Signaling Using Intracellular Calcium Source (GO:0035584)
Regulation Of Spindle Organization (GO:0090224)
Regulation Of Toll-Like Receptor Signaling Pathway (GO:0034121)
Response To Ketone (GO:1901654)
Sensory Perception Of Pain (GO:0019233)
Glutamate Metabolic Process (GO:0006536)
Intracellular Monoatomic Ion Homeostasis (GO:0006873)
Microglial Cell Activation (GO:0001774)
Mitochondrial RNA Metabolic Process (GO:0000959)

Myoblast Differentiation (GO:0045445)
Negative Regulation Of B Cell Activation (GO:0050869)
Negative Regulation Of Cytokine Production Involved In Inflammatory Response (GO:1900016)
Negative Regulation Of Microtubule Depolymerization (GO:0007026)
Negative Regulation Of Reproductive Process (GO:2000242)
Negative Regulation Of Small GTPase Mediated Signal Transduction (GO:0051058)
Nuclear-Transcribed mRNA poly(A) Tail Shortening (GO:0000289)
Phosphatidylserine Metabolic Process (GO:0006658)
Positive Regulation Of Epithelial Cell Differentiation (GO:0030858)
Positive Regulation Of Leukocyte Activation (GO:0002696)
Positive Regulation Of Lipid Catabolic Process (GO:0050996)
Positive Regulation Of Phospholipase Activity (GO:0010518)
Positive Regulation Of Vascular Associated Smooth Muscle Cell Proliferation (GO:1904707)
Pulmonary Valve Development (GO:0003177)
Regulation Of Calcium Ion Import (GO:0090279)
Regulation Of Cardiac Muscle Cell Proliferation (GO:0060043)
Negative Regulation Of Gene Expression (GO:0010629)
Regulation Of Endothelial Cell Migration (GO:0010594)
Positive Regulation Of Cell Adhesion (GO:0045785)
Positive Regulation Of Epithelial Cell Proliferation (GO:0050679)
Regulation Of Focal Adhesion Assembly (GO:0051893)
Regulation Of Smooth Muscle Cell Proliferation (GO:0048660)
Mesenchymal Cell Differentiation (GO:0048762)
Monoatomic Anion Transmembrane Transport (GO:0098656)
Organelle Disassembly (GO:1903008)
Peptidyl-Tyrosine Modification (GO:0018212)
Positive Regulation Of Leukocyte Chemotaxis (GO:0002690)
Regulation Of Blood Vessel Endothelial Cell Migration (GO:0043535)
Cellular Response To Lipopolysaccharide (GO:0071222)
Enzyme-Linked Receptor Protein Signaling Pathway (GO:0007167)
Regulation Of DNA Metabolic Process (GO:0051052)
Sensory Perception Of Mechanical Stimulus (GO:0050954)
Organic Anion Transport (GO:0015711)
Negative Regulation Of Multicellular Organismal Process (GO:0051241)
Regulation Of Membrane Protein Ectodomain Proteolysis (GO:0051043)
Aminoglycan Biosynthetic Process (GO:0006023)
Regulation Of Steroid Metabolic Process (GO:0019218)
Response To Cadmium Ion (GO:0046686)
Sodium Ion Import Across Plasma Membrane (GO:0098719)
Modulation By Symbiont Of Entry Into Host (GO:0052372)
Negative Regulation Of GTPase Activity (GO:0034260)
Negative Regulation Of Biomineral Tissue Development (GO:0070168)
Negative Regulation Of Interleukin-2 Production (GO:0032703)
Negative Regulation Of Tumor Necrosis Factor-Mediated Signaling Pathway (GO:0010804)
Positive Regulation Of G2/M Transition Of Mitotic Cell Cycle (GO:0010971)
Positive Regulation Of Blood Coagulation (GO:0030194)
Positive Regulation Of Transcription Regulatory Region DNA Binding (GO:2000679)
Protein Mannosylation (GO:0035268)

Purine Nucleotide Biosynthetic Process (GO:0006164)
Regulation Of Anoikis (GO:2000209)
Regulation Of I-kappaB kinase/NF-kappaB Signaling (GO:0043122)
Neuron Migration (GO:0001764)
Positive Regulation Of Tyrosine Phosphorylation Of STAT Protein (GO:0042531)
Purine Ribonucleotide Biosynthetic Process (GO:0009152)
Regulation Of Phosphatidylinositol 3-Kinase Signaling (GO:0014066)
Regulation Of Epithelial Cell Proliferation (GO:0050678)
Regulation Of Actin Cytoskeleton Organization (GO:0032956)
Regulation Of Reactive Oxygen Species Metabolic Process (GO:2000377)
Cellular Response To Peptide (GO:1901653)
Positive Regulation Of Cellular Metabolic Process (GO:0031325)
Regulation Of Anatomical Structure Morphogenesis (GO:0022603)
Regulation Of Histone Acetylation (GO:0035065)
Acetylcholine Receptor Signaling Pathway (GO:0095500)
Blood Vessel Endothelial Cell Migration (GO:0043534)
Cellular Response To Cadmium Ion (GO:0071276)
Regulation Of Telomere Capping (GO:1904353)
Cellular Response To Low-Density Lipoprotein Particle Stimulus (GO:0071404)
Dendritic Cell Differentiation (GO:0097028)
Inositol Phosphate Metabolic Process (GO:0043647)
Mammary Gland Development (GO:0030879)
Modulation By Host Of Viral Process (GO:0044788)
Negative Regulation Of Extrinsic Apoptotic Signaling Pathway In Absence Of Ligand (GO:2001240)
Negative Regulation Of Macroautophagy (GO:0016242)
Negative Regulation Of Myeloid Cell Differentiation (GO:0045638)
Negative Regulation Of Signal Transduction In Absence Of Ligand (GO:1901099)
Platelet Dense Granule Organization (GO:0060155)
Positive Regulation Of Cholesterol Efflux (GO:0010875)
Positive Regulation Of Protein Autophosphorylation (GO:0031954)
Positive Regulation Of Synaptic Transmission, Glutamatergic (GO:0051968)
rRNA Methylation (GO:0031167)
Regulation Of Catabolic Process (GO:0009894)
Sensory Perception Of Sound (GO:0007605)
Myeloid Cell Differentiation (GO:0030099)
Regulation Of Endothelial Cell Proliferation (GO:0001936)
Positive Regulation Of Protein Kinase B Signaling (GO:0051897)
Peptidyl-Threonine Phosphorylation (GO:0018107)
Regulation Of Bone Mineralization (GO:0030500)
Regulation Of Cholesterol Metabolic Process (GO:0090181)
Regulation Of Cholesterol Transport (GO:0032374)
Regulation Of Extracellular Matrix Organization (GO:1903053)
Axon Extension (GO:0048675)
Cell-Cell Signaling By Wnt (GO:0198738)
Regulation Of Toll-Like Receptor 4 Signaling Pathway (GO:0034143)
Response To BMP (GO:0071772)
Cytosolic Pattern Recognition Receptor Signaling Pathway (GO:0002753)
Diacylglycerol Metabolic Process (GO:0046339)

Heparan Sulfate Proteoglycan Biosynthetic Process (GO:0015012)
Immune Response-Activating Cell Surface Receptor Signaling Pathway (GO:0002429)
Microtubule Nucleation (GO:0007020)
Negative Regulation Of Actin Filament Polymerization (GO:0030837)
Negative Regulation Of Epidermal Growth Factor Receptor Signaling Pathway (GO:0042059)
Negative Regulation Of Extrinsic Apoptotic Signaling Pathway Via Death Domain Receptors (GO:1902042)
Negative Regulation Of Protein Depolymerization (GO:1901880)
Positive Regulation Of Cell-Substrate Junction Organization (GO:0150117)
Positive Regulation Of Oxidoreductase Activity (GO:0051353)
Potassium Ion Homeostasis (GO:0055075)
Protein Localization To Cell Junction (GO:1902414)
Regulation Of Inflammatory Response (GO:0050727)
Defense Response To Bacterium (GO:0042742)
T Cell Receptor Signaling Pathway (GO:0050852)
Regulation Of miRNA Transcription (GO:1902893)
Anterior/Posterior Pattern Specification (GO:0009952)
Epithelial Cell Differentiation (GO:0030855)
Regulation Of Cytoplasmic Translation (GO:2000765)
Regulation Of Microtubule Depolymerization (GO:0031114)
Regulation Of Muscle Cell Differentiation (GO:0051147)
Response To Purine-Containing Compound (GO:0014074)
Fat-Soluble Vitamin Metabolic Process (GO:0006775)
Lipid Translocation (GO:0034204)
Negative Regulation Of Cell Junction Assembly (GO:1901889)
Negative Regulation Of Ossification (GO:0030279)
Outflow Tract Septum Morphogenesis (GO:0003148)
Peroxisome Organization (GO:0007031)
Positive Regulation Of BMP Signaling Pathway (GO:0030513)
Positive Regulation Of Cell Cycle G2/M Phase Transition (GO:1902751)
Positive Regulation Of Peptidyl-Threonine Phosphorylation (GO:0010800)
Protein Exit From Endoplasmic Reticulum (GO:0032527)
Protein Kinase B Signaling (GO:0043491)
Regulation Of AMPA Receptor Activity (GO:2000311)
Regulation Of Macroautophagy (GO:0016241)
Cellular Response To Transforming Growth Factor Beta Stimulus (GO:0071560)
Response To Light Stimulus (GO:0009416)
Response To Retinoic Acid (GO:0032526)
Negative Regulation Of Peptidase Activity (GO:0010466)
Positive Regulation Of Phagocytosis (GO:0050766)
Regulation Of Defense Response (GO:0031347)
Positive Regulation Of Cell Junction Assembly (GO:1901890)
Positive Regulation Of Interleukin-8 Production (GO:0032757)
Regulation Of Hippo Signaling (GO:0035330)
Regulation Of Keratinocyte Proliferation (GO:0010837)
Activation Of Protein Kinase B Activity (GO:0032148)
Regulation Of Myelination (GO:0031641)
Regulation Of Neutrophil Chemotaxis (GO:0090022)
Cardiac Conduction System Development (GO:0003161)

Regulation Of Synapse Organization (GO:0050807)
Cellular Response To Hexose Stimulus (GO:0071331)
Cellular Response To Steroid Hormone Stimulus (GO:0071383)
Secretory Granule Organization (GO:0033363)
Sodium Ion Homeostasis (GO:0055078)
Ventricular Cardiac Muscle Tissue Development (GO:0003229)
Lamellipodium Assembly (GO:0030032)
Mitotic Cell Cycle Checkpoint Signaling (GO:0007093)
Myotube Differentiation (GO:0014902)
Negative Regulation Of Protein Localization To Membrane (GO:1905476)
Negative Regulation Of Protein Transport (GO:0051224)
Negative Regulation Of Smoothened Signaling Pathway (GO:0045879)
Osteoclast Differentiation (GO:0030316)
Positive Regulation Of Glucose Import (GO:0046326)
Positive Regulation Of Peptidase Activity (GO:0010952)
Positive Regulation Of Striated Muscle Cell Differentiation (GO:0051155)
Proteoglycan Metabolic Process (GO:0006029)
Regulation Of Cell Cycle Phase Transition (GO:1901987)
Phosphatidylinositol Biosynthetic Process (GO:0006661)
Fat Cell Differentiation (GO:0045444)
Phosphatidylinositol-Mediated Signaling (GO:0048015)
Positive Regulation Of Apoptotic Signaling Pathway (GO:2001235)
Axon Development (GO:0061564)
Neuron Differentiation (GO:0030182)
Negative Regulation Of Cellular Biosynthetic Process (GO:0031327)
Response To Glucocorticoid (GO:0051384)
Dendritic Spine Organization (GO:0097061)
Meiosis I (GO:0007127)
Mitotic G1 DNA Damage Checkpoint Signaling (GO:0031571)
Negative Regulation Of Catalytic Activity (GO:0043086)
Negative Regulation Of Interleukin-1 Beta Production (GO:0032691)
Negative Regulation Of Protein Polymerization (GO:0032272)
Nucleic Acid Phosphodiester Bond Hydrolysis (GO:0090305)
Protein Export From Nucleus (GO:0006611)
G1/S Transition Of Mitotic Cell Cycle (GO:0000082)
Protein Polymerization (GO:0051258)
Hemopoiesis (GO:0030097)
Negative Regulation Of Cell Death (GO:0060548)
Positive Regulation Of Type I Interferon Production (GO:0032481)
Regulation Of Chromatin Organization (GO:1902275)
Regulation Of Extrinsic Apoptotic Signaling Pathway In Absence Of Ligand (GO:2001239)
Regulation Of Peptidase Activity (GO:0052547)
Cell Surface Receptor Signaling Pathway Involved In Cell-Cell Signaling (GO:1905114)
Regulation Of Transcription Regulatory Region DNA Binding (GO:2000677)
Respiratory Tube Development (GO:0030323)
Dorsal/Ventral Pattern Formation (GO:0009953)
Establishment Of Protein Localization To Vacuole (GO:0072666)
Glycerophospholipid Catabolic Process (GO:0046475)

Ventricular Septum Morphogenesis (GO:0060412)
Iron-Sulfur Cluster Assembly (GO:0016226)
mRNA Modification (GO:0016556)
Metallo-Sulfur Cluster Assembly (GO:0031163)
Negative Regulation Of Notch Signaling Pathway (GO:0045746)
Negative Regulation Of Neurogenesis (GO:0050768)
Peptide Cross-Linking (GO:0018149)
Positive Regulation Of TORC1 Signaling (GO:1904263)
Positive Regulation Of Cytosolic Calcium Ion Concentration Involved In Phospholipase C-activating G Protein-C
Positive Regulation Of Filopodium Assembly (GO:0051491)
Positive Regulation Of Ubiquitin-Protein Transferase Activity (GO:0051443)
Regulation Of SMAD Protein Signal Transduction (GO:0060390)
Regulation Of Actin Polymerization Or Depolymerization (GO:0008064)
Calcium Ion Homeostasis (GO:0055074)
Regulation Of Protein Secretion (GO:0050708)
Canonical Wnt Signaling Pathway (GO:0060070)
Regulation Of Tyrosine Phosphorylation Of STAT Protein (GO:0042509)
Inorganic Cation Import Across Plasma Membrane (GO:0098659)
Regulation Of Chemotaxis (GO:0050920)
Regulation Of Glucose Metabolic Process (GO:0010906)
RNA Phosphodiester Bond Hydrolysis (GO:0090501)
Regulation Of Monoatomic Ion Transport (GO:0043269)
Regulation Of Protein Targeting To Membrane (GO:0090313)
Cellular Response To Osmotic Stress (GO:0071470)
Glycosaminoglycan Metabolic Process (GO:0030203)
Negative Regulation Of G2/M Transition Of Mitotic Cell Cycle (GO:0010972)
Negative Regulation Of Cytoskeleton Organization (GO:0051494)
Negative Regulation Of Type II Interferon Production (GO:0032689)
Positive Regulation Of Fibroblast Proliferation (GO:0048146)
Regulation Of Bone Resorption (GO:0045124)
Regulation Of Endocytosis (GO:0030100)
Negative Regulation Of Endopeptidase Activity (GO:0010951)
Peptidyl-Threonine Modification (GO:0018210)
Positive Regulation Of Kinase Activity (GO:0033674)
Positive Regulation Of Vasculature Development (GO:1904018)
Proteolysis (GO:0006508)
Positive Regulation Of Stress-Activated MAPK Cascade (GO:0032874)
Regulation Of Neurogenesis (GO:0050767)
Extrinsic Apoptotic Signaling Pathway (GO:0097191)
Peptidyl-Tyrosine Phosphorylation (GO:0018108)
Regulation Of Developmental Growth (GO:0048638)
JNK Cascade (GO:0007254)
Alpha-Amino Acid Catabolic Process (GO:1901606)
Cardiac Muscle Cell Action Potential (GO:0086001)
Regulation Of Spindle Assembly (GO:0090169)
Regulation Of Striated Muscle Contraction (GO:0006942)
Chondrocyte Differentiation (GO:0002062)
Response To Steroid Hormone (GO:0048545)

Vacuolar Acidification (GO:0007035)
Integrated Stress Response Signaling (GO:0140467)
Maintenance Of Blood-Brain Barrier (GO:0035633)
Modified Amino Acid Transport (GO:0072337)
Negative Regulation Of DNA Binding (GO:0043392)
Peptidyl-Tyrosine Dephosphorylation (GO:0035335)
Positive Regulation Of Dephosphorylation (GO:0035306)
Protein K11-linked Ubiquitination (GO:0070979)
Protein Acetylation (GO:0006473)
Regulation Of Blood Coagulation (GO:0030193)
Ras Protein Signal Transduction (GO:0007265)
Actin Filament Organization (GO:0007015)
Positive Regulation Of Cell Growth (GO:0030307)
B Cell Differentiation (GO:0030183)
Positive Regulation Of Protein Kinase Activity (GO:0045860)
Regulation Of Endopeptidase Activity (GO:0052548)
Regulation Of Heart Contraction (GO:0008016)
Phagocytosis (GO:0006909)
Regulation Of Vasculature Development (GO:1901342)
Extrinsic Apoptotic Signaling Pathway Via Death Domain Receptors (GO:0008625)
Intrinsic Apoptotic Signaling Pathway In Response To Endoplasmic Reticulum Stress (GO:0070059)
Lamellipodium Organization (GO:0097581)
Negative Regulation Of Interleukin-1 Production (GO:0032692)
Negative Regulation Of Peptidyl-Tyrosine Phosphorylation (GO:0050732)
Phospholipid Dephosphorylation (GO:0046839)
Positive Regulation Of Phosphate Metabolic Process (GO:0045937)
Positive Regulation Of Protein Targeting To Mitochondrion (GO:1903955)
Regulation Of Calcium-Mediated Signaling (GO:0050848)
Regulation Of Cell Migration Involved In Sprouting Angiogenesis (GO:0090049)
Positive Regulation Of Protein Polymerization (GO:0032273)
Regulation Of Wnt Signaling Pathway (GO:0030111)
Regulation Of Centrosome Duplication (GO:0010824)
Regulation Of Dendrite Morphogenesis (GO:0048814)
Regulation Of Extrinsic Apoptotic Signaling Pathway Via Death Domain Receptors (GO:1902041)
Actin Filament Polymerization (GO:0030041)
Regulation Of Macrophage Activation (GO:0043030)
Regulation Of Protein Autophosphorylation (GO:0031952)
Cardiac Muscle Tissue Morphogenesis (GO:0055008)
Regulation Of Transmembrane Receptor Protein Serine/Threonine Kinase Signaling Pathway (GO:0090092)
Response To Estradiol (GO:0032355)
Leukocyte Cell-Cell Adhesion (GO:0007159)
Negative Chemotaxis (GO:0050919)
Negative Regulation Of Cell-Matrix Adhesion (GO:0001953)
Negative Regulation Of Chemotaxis (GO:0050922)
Negative Regulation Of Developmental Growth (GO:0048640)
Negative Regulation Of Intracellular Steroid Hormone Receptor Signaling Pathway (GO:0033144)
Negative Regulation Of Microtubule Polymerization Or Depolymerization (GO:0031111)
Non-Motile Cilium Assembly (GO:1905515)

Phospholipid Translocation (GO:0045332)
Positive Regulation Of G1/S Transition Of Mitotic Cell Cycle (GO:1900087)
Positive Regulation Of Rho Protein Signal Transduction (GO:0035025)
Positive Regulation Of Mononuclear Cell Migration (GO:0071677)
Protein Localization To Chromatin (GO:0071168)
Receptor Signaling Pathway Via STAT (GO:0097696)
Regulation Of JNK Cascade (GO:0046328)
Regulation Of Double-Strand Break Repair Via Homologous Recombination (GO:0010569)
Regulation Of Hydrolase Activity (GO:0051336)
Cell-Cell Junction Assembly (GO:0007043)
Myeloid Leukocyte Differentiation (GO:0002573)
Negative Regulation Of Supramolecular Fiber Organization (GO:1902904)
Negative Regulation Of Cell Population Proliferation (GO:0008285)
Axonogenesis (GO:0007409)
Regulation Of Cell Growth (GO:0001558)
T Cell Activation (GO:0042110)
Transforming Growth Factor Beta Receptor Signaling Pathway (GO:0007179)
Plasma Membrane Organization (GO:0007009)
Regulation Of Cholesterol Efflux (GO:0010874)
Cellular Nitrogen Compound Biosynthetic Process (GO:0044271)
Regulation Of Transmembrane Transport (GO:0034762)
Respiratory System Development (GO:0060541)
Muscle Cell Development (GO:0055001)
Negative Regulation Of G1/S Transition Of Mitotic Cell Cycle (GO:2000134)
Negative Regulation Of Fat Cell Differentiation (GO:0045599)
Negative Regulation Of Osteoblast Differentiation (GO:0045668)
Negative Regulation Of Proteasomal Ubiquitin-Dependent Protein Catabolic Process (GO:0032435)
Phosphatidylinositol 3-Kinase Signaling (GO:0014065)
Positive Regulation Of Synapse Assembly (GO:0051965)
Positive Regulation Of Telomere Maintenance Via Telomerase (GO:0032212)
Negative Regulation Of Defense Response (GO:0031348)
Positive Regulation Of Secretion By Cell (GO:1903532)
Regulation Of Rho Protein Signal Transduction (GO:0035023)
Positive Regulation Of Cellular Component Organization (GO:0051130)
Negative Regulation Of Cellular Process (GO:0048523)
Regulation Of Protein Polymerization (GO:0032271)
Regulation Of Sprouting Angiogenesis (GO:1903670)
Regulation Of Type I Interferon-Mediated Signaling Pathway (GO:0060338)
Epithelial Tube Morphogenesis (GO:0060562)
Vasculogenesis (GO:0001570)
Ventricular Cardiac Muscle Tissue Morphogenesis (GO:0055010)
Lung Development (GO:0030324)
ncRNA Catabolic Process (GO:0034661)
Negative Regulation Of Blood Vessel Endothelial Cell Migration (GO:0043537)
Negative Regulation Of Epithelial Cell Migration (GO:0010633)
Positive Regulation Of Cytokine Production Involved In Immune Response (GO:0002720)
Positive Regulation Of Establishment Of Protein Localization To Mitochondrion (GO:1903749)
Positive Regulation Of Interleukin-12 Production (GO:0032735)

Positive Regulation Of Ion Transmembrane Transporter Activity (GO:0032414)
Positive Regulation Of Protein Dephosphorylation (GO:0035307)
Positive Regulation Of Small Molecule Metabolic Process (GO:0062013)
Positive Regulation Of Telomerase Activity (GO:0051973)
Regulation Of Activated T Cell Proliferation (GO:0046006)
Regulation Of Amyloid-Beta Formation (GO:1902003)
Modification-Dependent Protein Catabolic Process (GO:0019941)
Positive Regulation Of Lymphocyte Proliferation (GO:0050671)
Regulation Of Cytoskeleton Organization (GO:0051493)
Regulation Of Interleukin-6 Production (GO:0032675)
Regulation Of Fat Cell Differentiation (GO:0045598)
Regulation Of Plasma Membrane Bounded Cell Projection Assembly (GO:0120032)
Defense Response To Gram-negative Bacterium (GO:0050829)
Negative Regulation Of Phosphorylation (GO:0042326)
DNA Damage Checkpoint Signaling (GO:0000077)
Regulation Of Neuronal Synaptic Plasticity (GO:0048168)
Response To Estrogen (GO:0043627)
Establishment Of Mitotic Spindle Localization (GO:0040001)
Transcription Initiation-Coupled Chromatin Remodeling (GO:0045815)
Microtubule Polymerization (GO:0046785)
Negative Regulation Of Ras Protein Signal Transduction (GO:0046580)
Negative Regulation Of Interleukin-6 Production (GO:0032715)
Negative Regulation Of Mitotic Cell Cycle (GO:0045930)
Regulation Of Blood Circulation (GO:1903522)
Cellular Response To Growth Factor Stimulus (GO:0071363)
Positive Regulation Of Interleukin-6 Production (GO:0032755)
Cellular Response To Oxidative Stress (GO:0034599)
Action Potential (GO:0001508)
Regulation Of Sodium Ion Transport (GO:0002028)
Regulatory ncRNA Processing (GO:0070918)
Dendrite Morphogenesis (GO:0048813)
Glutamate Receptor Signaling Pathway (GO:0007215)
Liver Development (GO:0001889)
Macrophage Activation (GO:0042116)
Negative Regulation Of Cell Cycle G1/S Phase Transition (GO:1902807)
Neutral Amino Acid Transport (GO:0015804)
Positive Regulation Of Substrate Adhesion-Dependent Cell Spreading (GO:1900026)
Positive Regulation Of Telomere Maintenance Via Telomere Lengthening (GO:1904358)
Regulation Of Cardiac Muscle Contraction (GO:0055117)
Negative Regulation Of Blood Vessel Morphogenesis (GO:2000181)
Negative Regulation Of Transforming Growth Factor Beta Receptor Signaling Pathway (GO:0030512)
Positive Regulation Of Response To Stimulus (GO:0048584)
Positive Regulation Of Synaptic Transmission (GO:0050806)
Regulation Of Protein Binding (GO:0043393)
Positive Regulation Of Cell Projection Organization (GO:0031346)
Negative Regulation Of Protein Modification Process (GO:0031400)
Organonitrogen Compound Catabolic Process (GO:1901565)
DNA Damage Response, Signal Transduction By P53 Class Mediator (GO:0030330)

Regulation Of Glucose Import (GO:0046324)
Blood Vessel Diameter Maintenance (GO:0097746)
Regulation Of Trans-Synaptic Signaling (GO:0099177)
Substrate Adhesion-Dependent Cell Spreading (GO:0034446)
Interstrand Cross-Link Repair (GO:0036297)
Negative Regulation Of Organelle Assembly (GO:1902116)
Negative Regulation Of Sequestering Of Calcium Ion (GO:0051283)
Positive Regulation Of Interferon-Beta Production (GO:0032728)
Positive Regulation Of Signaling Receptor Activity (GO:2000273)
Protein Destabilization (GO:0031648)
Receptor Signaling Pathway Via JAK-STAT (GO:0007259)
Positive Regulation Of Protein Modification By Small Protein Conjugation Or Removal (GO:1903322)
Aortic Valve Morphogenesis (GO:0003180)
Regulation Of Vascular Associated Smooth Muscle Cell Proliferation (GO:1904705)
Ventricular Septum Development (GO:0003281)
Mitochondrion Disassembly (GO:0061726)
Negative Regulation Of Smooth Muscle Cell Proliferation (GO:0048662)
Positive Regulation Of Exocytosis (GO:0045921)
Positive Regulation Of Receptor Signaling Pathway Via JAK-STAT (GO:0046427)
Positive Regulation Of Receptor Signaling Pathway Via STAT (GO:1904894)
Receptor-Mediated Endocytosis (GO:0006898)
Positive Regulation Of Cell Differentiation (GO:0045597)
Protein Modification Process (GO:0036211)
Amino Acid Catabolic Process (GO:0009063)
Connective Tissue Development (GO:0061448)
Response To Transforming Growth Factor Beta (GO:0071559)
Tumor Necrosis Factor-Mediated Signaling Pathway (GO:0033209)
Membrane Assembly (GO:0071709)
Membrane Fission (GO:0090148)
Positive Regulation Of Bone Mineralization (GO:0030501)
Positive Regulation Of Protein Tyrosine Kinase Activity (GO:0061098)
Regulation Of Interleukin-8 Production (GO:0032677)
Macromolecule Modification (GO:0043412)
Organonitrogen Compound Biosynthetic Process (GO:1901566)
Regulation Of Cell Motility (GO:2000145)
Regulation Of Angiogenesis (GO:0045765)
Protein Dephosphorylation (GO:0006470)
Regulation Of Mitotic Spindle Organization (GO:0060236)
Carboxylic Acid Transmembrane Transport (GO:1905039)
Regulation Of Protein Tyrosine Kinase Activity (GO:0061097)
Release Of Sequestered Calcium Ion Into Cytosol (GO:0051209)
Fibroblast Growth Factor Receptor Signaling Pathway (GO:0008543)
Vesicle Cytoskeletal Trafficking (GO:0099518)
Negative Regulation Of Mitochondrion Organization (GO:0010823)
Negative Regulation Of Protein Secretion (GO:0050709)
Negative Regulation Of Transport (GO:0051051)
Neuron Projection Extension (GO:1990138)
Nuclear-Transcribed mRNA Catabolic Process, Deadenylation-Dependent Decay (GO:0000288)

Odontogenesis (GO:0042476)
Positive Regulation Of ATP-dependent Activity (GO:0032781)
Positive Regulation Of Intracellular Transport (GO:0032388)
Positive Regulation Of Intrinsic Apoptotic Signaling Pathway (GO:2001244)
Positive Regulation Of Pattern Recognition Receptor Signaling Pathway (GO:0062208)
Phospholipase C-activating G Protein-Coupled Receptor Signaling Pathway (GO:0007200)
Regulation Of Cell Division (GO:0051302)
DNA Metabolic Process (GO:0006259)
Protein-Containing Complex Assembly (GO:0065003)
Protein-Containing Complex Organization (GO:0043933)
Regulation Of Cytokine-Mediated Signaling Pathway (GO:0001959)
Regulation Of Glycolytic Process (GO:0006110)
Aortic Valve Development (GO:0003176)
Regulation Of Protein Dephosphorylation (GO:0035304)
Negative Regulation Of Cell-Cell Adhesion (GO:0022408)
Negative Regulation Of Mitotic Cell Cycle Phase Transition (GO:1901991)
Positive Regulation Of Catalytic Activity (GO:0043085)
Positive Regulation Of Stem Cell Population Maintenance (GO:1902459)
Regulation Of Autophagosome Assembly (GO:2000785)
Regulation Of Cysteine-Type Endopeptidase Activity Involved In Apoptotic Process (GO:0043281)
Positive Regulation Of Protein Ubiquitination (GO:0031398)
Purine Ribonucleotide Metabolic Process (GO:0009150)
Regulation Of Response To External Stimulus (GO:0032101)
Regulation Of Kinase Activity (GO:0043549)
Defense Response To Gram-positive Bacterium (GO:0050830)
Positive Regulation Of Amide Metabolic Process (GO:0034250)
Positive Regulation Of Endothelial Cell Migration (GO:0010595)
Regulation Of Dendrite Development (GO:0050773)
Regulation Of Release Of Cytochrome C From Mitochondria (GO:0090199)
Cellular Response To Retinoic Acid (GO:0071300)
Non-Canonical Wnt Signaling Pathway (GO:0035567)
Positive Regulation Of Ossification (GO:0045778)
Positive Regulation Of Viral Process (GO:0048524)
Regulation Of Amide Metabolic Process (GO:0034248)
DNA Repair (GO:0006281)
Negative Regulation Of Angiogenesis (GO:0016525)
Metal Ion Transport (GO:0030001)
Regulation Of Neurotransmitter Receptor Activity (GO:0099601)
Regulation Of Osteoclast Differentiation (GO:0045670)
Branching Morphogenesis Of An Epithelial Tube (GO:0048754)
Cardiac Ventricle Morphogenesis (GO:0003208)
Cellular Response To Glucose Starvation (GO:0042149)
Stress-Activated MAPK Cascade (GO:0051403)
Substantia Nigra Development (GO:0021762)
Keratinocyte Differentiation (GO:0030216)
Lipid Modification (GO:0030258)
Monoatomic Cation Transport (GO:0006812)
Negative Regulation Of I-kappaB kinase/NF-kappaB Signaling (GO:0043124)

Positive Regulation Of Neuron Differentiation (GO:0045666)
Regulation Of Insulin Secretion (GO:0050796)
Positive Regulation Of Plasma Membrane Bounded Cell Projection Assembly (GO:0120034)
Regulation Of Chromosome Organization (GO:0033044)
SCF-dependent Proteasomal Ubiquitin-Dependent Protein Catabolic Process (GO:0031146)
Intracellular Glucose Homeostasis (GO:0001678)
Intracellular Steroid Hormone Receptor Signaling Pathway (GO:0030518)
Positive Regulation Of Biomineral Tissue Development (GO:0070169)
Positive Regulation Of Cell-Matrix Adhesion (GO:0001954)
Regulation Of Intracellular Signal Transduction (GO:1902531)
Regulation Of Neuron Projection Development (GO:0010975)
Regulation Of Transport (GO:0051049)
Nucleobase-Containing Compound Catabolic Process (GO:0034655)
Positive Regulation Of Biosynthetic Process (GO:0009891)
Positive Regulation Of Chemotaxis (GO:0050921)
Positive Regulation Of Cytokine-Mediated Signaling Pathway (GO:0001961)
Positive Regulation Of Leukocyte Cell-Cell Adhesion (GO:1903039)
Regulation Of B Cell Proliferation (GO:0030888)
Antigen Receptor-Mediated Signaling Pathway (GO:0050851)
Regulation Of Cellular Metabolic Process (GO:0031323)
I-kappaB kinase/NF-kappaB Signaling (GO:0007249)
Regulation Of Protein Modification Process (GO:0031399)
Regulation Of Stem Cell Differentiation (GO:2000736)
Regulation Of Telomerase Activity (GO:0051972)
Lymphocyte Differentiation (GO:0030098)
Regulation Of RNA Metabolic Process (GO:0051252)
B Cell Activation (GO:0042113)
Regulation Of Protein Kinase B Signaling (GO:0051896)
Regulation Of Epidermal Growth Factor Receptor Signaling Pathway (GO:0042058)
Regulation Of Fibroblast Proliferation (GO:0048145)
Regulation Of mRNA Catabolic Process (GO:0061013)
Replication Fork Processing (GO:0031297)
Response To Glucose (GO:0009749)
Epithelial Cell Migration (GO:0010631)
Vacuolar Transport (GO:0007034)
Homotypic Cell-Cell Adhesion (GO:0034109)
Intracellular Protein Transmembrane Transport (GO:0065002)
Positive Regulation Of Peptide Hormone Secretion (GO:0090277)
Regulation Of Peptidyl-Serine Phosphorylation (GO:0033135)
Positive Regulation Of Cellular Component Biogenesis (GO:0044089)
Golgi To Plasma Membrane Transport (GO:0006893)
Response To Organonitrogen Compound (GO:0010243)
Epithelial To Mesenchymal Transition (GO:0001837)
Negative Regulation Of Hydrolase Activity (GO:0051346)
Positive Regulation Of TOR Signaling (GO:0032008)
Positive Regulation Of Lymphocyte Activation (GO:0051251)
Positive Regulation Of Smooth Muscle Cell Proliferation (GO:0048661)
Sphingolipid Metabolic Process (GO:0006665)

Intracellular Calcium Ion Homeostasis (GO:0006874)
Cell Junction Assembly (GO:0034329)
Positive Regulation Of Epithelial Cell Migration (GO:0010634)
Recombinational Repair (GO:0000725)
Cellular Response To Mechanical Stimulus (GO:0071260)
Regulation Of Tumor Necrosis Factor-Mediated Signaling Pathway (GO:0010803)
Cytoplasmic Microtubule Organization (GO:0031122)
Negative Regulation Of ERK1 And ERK2 Cascade (GO:0070373)
Positive Regulation Of Supramolecular Fiber Organization (GO:1902905)
Autophagy Of Mitochondrion (GO:0000422)
Interferon-Mediated Signaling Pathway (GO:0140888)
Intrinsic Apoptotic Signaling Pathway In Response To DNA Damage (GO:0008630)
Neurogenesis (GO:0022008)
Nuclear Export (GO:0051168)
Regulation Of Interferon-Beta Production (GO:0032648)
Regulation Of Interleukin-12 Production (GO:0032655)
Regulation Of Mitochondrion Organization (GO:0010821)
tRNA Processing (GO:0008033)
Mitotic Metaphase Plate Congression (GO:0007080)
Positive Regulation Of Cell Death (GO:0010942)
Regulation Of TORC1 Signaling (GO:1903432)
Positive Regulation Of Transferase Activity (GO:0051347)
Regulation Of Cell Adhesion (GO:0030155)
Amide Biosynthetic Process (GO:0043604)
Regulation Of Telomere Maintenance Via Telomerase (GO:0032210)
Sensory Perception (GO:0007600)
Microtubule Bundle Formation (GO:0001578)
Negative Regulation Of MAP Kinase Activity (GO:0043407)
Positive Regulation Of Protein Localization To Cell Periphery (GO:1904377)
Positive Regulation Of Protein Localization To Plasma Membrane (GO:1903078)
Negative Regulation Of Response To External Stimulus (GO:0032102)
Adenylate Cyclase-Inhibiting G Protein-Coupled Receptor Signaling Pathway (GO:0007193)
Regulation Of Peptide Hormone Secretion (GO:0090276)
Regulation Of Protein Localization To Nucleus (GO:1900180)
Regulation Of Proteolysis (GO:0030162)
Icosanoid Metabolic Process (GO:0006690)
Negative Regulation Of Innate Immune Response (GO:0045824)
Positive Regulation Of Telomere Maintenance (GO:0032206)
Positive Regulation Of Cell Population Proliferation (GO:0008284)
Dephosphorylation (GO:0016311)
Protein Localization To Membrane (GO:0072657)
NADH Dehydrogenase Complex Assembly (GO:0010257)
Actin Cytoskeleton Reorganization (GO:0031532)
Actin Polymerization Or Depolymerization (GO:0008154)
Regulation Of Receptor Signaling Pathway Via JAK-STAT (GO:0046425)
Endosome Organization (GO:0007032)
Mitochondrial Respiratory Chain Complex I Assembly (GO:0032981)
Positive Regulation Of NIK/NF-kappaB Signaling (GO:1901224)

Regulation Of Signal Transduction (GO:0009966)
Epidermal Cell Differentiation (GO:0009913)
Negative Regulation Of Neuron Projection Development (GO:0010977)
Negative Regulation Of Protein Catabolic Process (GO:0042177)
Cellular Response To Fibroblast Growth Factor Stimulus (GO:0044344)
Regulation Of Synaptic Transmission, Glutamatergic (GO:0051966)
Negative Regulation Of MAPK Cascade (GO:0043409)
Central Nervous System Development (GO:0007417)
Regulation Of Stem Cell Population Maintenance (GO:2000036)
Pattern Recognition Receptor Signaling Pathway (GO:0002221)
post-Golgi Vesicle-Mediated Transport (GO:0006892)
Regulation Of Cation Channel Activity (GO:2001257)
Positive Regulation Of T Cell Activation (GO:0050870)
Signal Transduction In Response To DNA Damage (GO:0042770)
Endosome To Lysosome Transport (GO:0008333)
Membrane Lipid Biosynthetic Process (GO:0046467)
Positive Regulation Of DNA Metabolic Process (GO:0051054)
Regulation Of Neuron Death (GO:1901214)
Regulation Of Phagocytosis (GO:0050764)
Memory (GO:0007613)
Muscle Organ Development (GO:0007517)
Cell-Matrix Adhesion (GO:0007160)
Cellular Response To Salt (GO:1902075)
Organelle Organization (GO:0006996)
DNA-templated Transcription Initiation (GO:0006352)
Regulation Of Multicellular Organismal Development (GO:2000026)
Regulation Of Type I Interferon Production (GO:0032479)
Cilium Movement (GO:0003341)
Negative Regulation Of Protein-Containing Complex Assembly (GO:0031333)
Positive Regulation Of Protein Binding (GO:0032092)
Regulation Of Cell Death (GO:0010941)
Regulation Of Transforming Growth Factor Beta Receptor Signaling Pathway (GO:0017015)
Double-Strand Break Repair Via Homologous Recombination (GO:0000724)
Regulation Of Extrinsic Apoptotic Signaling Pathway (GO:2001236)
Homophilic Cell Adhesion Via Plasma Membrane Adhesion Molecules (GO:0007156)
Calcium Ion Transport (GO:0006816)
Protein Deubiquitination (GO:0016579)
Positive Regulation Of Organelle Assembly (GO:1902117)
Regulation Of Protein Ubiquitination (GO:0031396)
Regulation Of Canonical Wnt Signaling Pathway (GO:0060828)
Regulation Of Embryonic Development (GO:0045995)
RNA Methylation (GO:0001510)
Cell Junction Organization (GO:0034330)
Protein Modification By Small Protein Removal (GO:0070646)
Phosphatidylinositol Metabolic Process (GO:0046488)
Cellular Response To Virus (GO:0098586)
Response To Metal Ion (GO:0010038)
Positive Regulation Of T Cell Differentiation (GO:0045582)

Regulation Of Cell Population Proliferation (GO:0042127)
Nervous System Development (GO:0007399)
Cellular Response To Light Stimulus (GO:0071482)
Positive Regulation Of DNA Biosynthetic Process (GO:2000573)
Positive Regulation Of Cell Cycle (GO:0045787)
Membrane Organization (GO:0061024)
Regulation Of Protein-Containing Complex Assembly (GO:0043254)
Brain Development (GO:0007420)
tRNA Modification (GO:0006400)
Protein Secretion (GO:0009306)
Aerobic Electron Transport Chain (GO:0019646)
Cell-Cell Junction Organization (GO:0045216)
Skin Development (GO:0043588)
Modulation Of Chemical Synaptic Transmission (GO:0050804)
Cellular Response To Calcium Ion (GO:0071277)
Negative Regulation Of Growth (GO:0045926)
Neuron Projection Guidance (GO:0097485)
Mitochondrial ATP Synthesis Coupled Electron Transport (GO:0042775)
Negative Regulation Of Epithelial Cell Proliferation (GO:0050680)
Protein K48-linked Ubiquitination (GO:0070936)
Regulation Of Notch Signaling Pathway (GO:0008593)
Negative Regulation Of Cell Growth (GO:0030308)
Regulation Of Cilium Assembly (GO:1902017)
Protein Autoubiquitination (GO:0051865)
Regulation Of Cellular Localization (GO:0060341)
Positive Regulation Of Protein Localization To Nucleus (GO:1900182)
Protein Polyubiquitination (GO:0000209)
DNA Replication (GO:0006260)
Ubiquitin-Dependent ERAD Pathway (GO:0030433)
Gland Development (GO:0048732)
Regulation Of Ras Protein Signal Transduction (GO:0046578)
Negative Regulation Of Cell Motility (GO:2000146)
Regulation Of Immune Response (GO:0050776)
Sphingolipid Biosynthetic Process (GO:0030148)
Monoatomic Cation Homeostasis (GO:0055080)
Regulation Of Innate Immune Response (GO:0045088)
Proteolysis Involved In Protein Catabolic Process (GO:0051603)
Non-Membrane-Bounded Organelle Assembly (GO:0140694)
Regulation Of Neuron Differentiation (GO:0045664)
Cellular Response To UV (GO:0034644)
Positive Regulation Of Protein Localization To Membrane (GO:1905477)
Protein Localization To Plasma Membrane (GO:0072659)
Regulation Of Organelle Assembly (GO:1902115)
Regulation Of Protein Localization To Plasma Membrane (GO:1903076)
Glycoprotein Biosynthetic Process (GO:0009101)
Wound Healing (GO:0042060)
Negative Regulation Of Protein Serine/Threonine Kinase Activity (GO:0071901)
Positive Regulation Of Immune Response (GO:0050778)

Negative Regulation Of NF-kappaB Transcription Factor Activity (GO:0032088)
Regulation Of Autophagy (GO:0010506)
Regulation Of Phosphorylation (GO:0042325)
Negative Regulation Of Apoptotic Signaling Pathway (GO:2001234)
Neuron Projection Development (GO:0031175)
Regulation Of Cell Differentiation (GO:0045595)
Cilium Assembly (GO:0060271)
Calcium Ion Transmembrane Import Into Cytosol (GO:0097553)
Epigenetic Regulation Of Gene Expression (GO:0040029)
Vesicle-Mediated Transport To The Plasma Membrane (GO:0098876)
Regulation Of NIK/NF-kappaB Signaling (GO:1901222)
DNA Damage Response (GO:0006974)
Protein Localization To Cell Periphery (GO:1990778)
Ribosomal Small Subunit Biogenesis (GO:0042274)
Positive Regulation Of Protein Metabolic Process (GO:0051247)
Cellular Respiration (GO:0045333)
Macromolecule Catabolic Process (GO:0009057)
Nuclear-Transcribed mRNA Catabolic Process (GO:0000956)
Positive Regulation Of Protein Localization (GO:1903829)
Anterograde Trans-Synaptic Signaling (GO:0098916)
Positive Regulation Of Cellular Catabolic Process (GO:0031331)
Regulation Of Cell Cycle (GO:0051726)
Negative Regulation Of Neuron Death (GO:1901215)
Defense Response To Symbiont (GO:0140546)
ERAD Pathway (GO:0036503)
Cellular Response To Chemical Stress (GO:0062197)
Positive Regulation Of DNA Repair (GO:0045739)
Axon Guidance (GO:0007411)
Negative Regulation Of DNA-binding Transcription Factor Activity (GO:0043433)
Mitochondrial Respiratory Chain Complex Assembly (GO:0033108)
Neuron Development (GO:0048666)
rRNA Metabolic Process (GO:0016072)
Positive Regulation Of Neuron Projection Development (GO:0010976)
Positive Regulation Of Protein Catabolic Process (GO:0045732)
Negative Regulation Of Cell Differentiation (GO:0045596)
Cytoplasmic Translation (GO:0002181)
Negative Regulation Of Amide Metabolic Process (GO:0034249)
Protein O-linked Glycosylation (GO:0006493)
Vesicle Organization (GO:0016050)
Muscle Contraction (GO:0006936)
Protein Transport (GO:0015031)
Peptidyl-Serine Phosphorylation (GO:0018105)
Positive Regulation Of Cellular Process (GO:0048522)
Vesicle-Mediated Transport (GO:0016192)
Protein Modification By Small Protein Conjugation (GO:0032446)
Negative Regulation Of Macromolecule Biosynthetic Process (GO:0010558)
Positive Regulation Of Protein-Containing Complex Assembly (GO:0031334)
Negative Regulation Of Signal Transduction (GO:0009968)

Ubiquitin-Dependent Protein Catabolic Process (GO:0006511)
Proteasome-Mediated Ubiquitin-Dependent Protein Catabolic Process (GO:0043161)
Regulation Of Macromolecule Metabolic Process (GO:0060255)
Mitochondrion Organization (GO:0007005)
Response To UV (GO:0009411)
ncRNA Processing (GO:0034470)
Negative Regulation Of Cell Migration (GO:0030336)
rRNA Processing (GO:0006364)
Organelle Assembly (GO:0070925)
Regulation Of Gene Expression (GO:0010468)
Activation Of GTPase Activity (GO:0090630)
Protein Catabolic Process (GO:0030163)
Regulation Of RNA Splicing (GO:0043484)
Chemical Synaptic Transmission (GO:0007268)
Peptidyl-Serine Modification (GO:0018209)
Plasma Membrane Bounded Cell Projection Assembly (GO:0120031)
Positive Regulation Of Inflammatory Response (GO:0050729)
Response To Calcium Ion (GO:0051592)
Regulation Of G1/S Transition Of Mitotic Cell Cycle (GO:2000045)
Regulation Of Cellular Response To Stress (GO:0080135)
Negative Regulation Of Translation (GO:0017148)
Cilium Organization (GO:0044782)
Positive Regulation Of Translation (GO:0045727)
Protein Ubiquitination (GO:0016567)
Negative Regulation Of Intracellular Signal Transduction (GO:1902532)
RNA Metabolic Process (GO:0016070)
Response To Endoplasmic Reticulum Stress (GO:0034976)
Negative Regulation Of Canonical Wnt Signaling Pathway (GO:0090090)
Regulation Of MAP Kinase Activity (GO:0043405)
Ribonucleoprotein Complex Biogenesis (GO:0022613)
Regulation Of Protein Kinase Activity (GO:0045859)
Defense Response To Virus (GO:0051607)
Positive Regulation Of Nucleic Acid-Templated Transcription (GO:1903508)
Positive Regulation Of Intracellular Protein Transport (GO:0090316)
Regulation Of Programmed Cell Death (GO:0043067)
Endomembrane System Organization (GO:0010256)
Regulation Of DNA Repair (GO:0006282)
Golgi Organization (GO:0007030)
Plasma Membrane Bounded Cell Projection Organization (GO:0120036)
Protein Localization To Organelle (GO:0033365)
Intracellular Protein Transport (GO:0006886)
Protein Stabilization (GO:0050821)
Negative Regulation Of Wnt Signaling Pathway (GO:0030178)
Positive Regulation Of NF-kappaB Transcription Factor Activity (GO:0051092)
Ribosome Biogenesis (GO:0042254)
Positive Regulation Of Response To External Stimulus (GO:0032103)
Cell-Cell Adhesion Via Plasma-Membrane Adhesion Molecules (GO:0098742)
Negative Regulation Of Nucleic Acid-Templated Transcription (GO:1903507)

Transcription By RNA Polymerase II (GO:0006366)
Positive Regulation Of Transcription By RNA Polymerase II (GO:0045944)
DNA-templated Transcription (GO:0006351)
Regulation Of Translation (GO:0006417)
Protein Localization (GO:0008104)
Proteasomal Protein Catabolic Process (GO:0010498)
Chromatin Remodeling (GO:0006338)
Positive Regulation Of Developmental Process (GO:0051094)
Regulation Of Nucleic Acid-Templated Transcription (GO:1903506)
Translation (GO:0006412)
Positive Regulation Of DNA-binding Transcription Factor Activity (GO:0051091)
Chromatin Organization (GO:0006325)
Gene Expression (GO:0010467)
Positive Regulation Of DNA-templated Transcription (GO:0045893)
Negative Regulation Of DNA-templated Transcription (GO:0045892)
Negative Regulation Of Transcription By RNA Polymerase II (GO:0000122)
Regulation Of DNA-templated Transcription (GO:0006355)
Regulation Of Transcription By RNA Polymerase II (GO:0006357)

Overlap	P.value	Adjusted.P.value	Old.P.value	Old.Adjusted.P.value
10/72	2.29617E-05	0.01884651	0	0
16/176	2.45707E-05	0.01884651	0	0
10/73	2.59713E-05	0.01884651	0	0
6/26	5.53021E-05	0.030098182	0	0
5/23	0.000319376	0.139056507	0	0
5/25	0.000482262	0.158329573	0	0
4/15	0.000569906	0.158329573	0	0
6/39	0.000581827	0.158329573	0	0
5/28	0.000834403	0.188385441	0	0
4/17	0.000951879	0.188385441	0	0
4/17	0.000951879	0.188385441	0	0
7/60	0.001138711	0.190180859	0	0
4/18	0.00119786	0.190180859	0	0
6/45	0.001266828	0.190180859	0	0
3/9	0.001463821	0.190180859	0	0
4/19	0.001485105	0.190180859	0	0
4/19	0.001485105	0.190180859	0	0
12/163	0.00163688	0.195706167	0	0
4/20	0.001817042	0.195706167	0	0
7/65	0.001828384	0.195706167	0	0
6/49	0.001985891	0.195706167	0	0
3/10	0.002049322	0.195706167	0	0
5/34	0.002067635	0.195706167	0	0
6/50	0.002206039	0.20010616	0	0
10/127	0.002391637	0.204645444	0	0
6/51	0.002444089	0.204645444	0	0
9/108	0.002651587	0.211892361	0	0
9/109	0.002822767	0.211892361	0	0
9/110	0.003002565	0.211892361	0	0
4/23	0.003114633	0.211892361	0	0
4/23	0.003114633	0.211892361	0	0
4/23	0.003114633	0.211892361	0	0
3/12	0.003608519	0.22756855	0	0
3/12	0.003608519	0.22756855	0	0
4/24	0.003658658	0.22756855	0	0
9/118	0.00478777	0.282594056	0	0
4/26	0.00493274	0.282594056	0	0
4/26	0.00493274	0.282594056	0	0
7/78	0.005142064	0.283752733	0	0
4/27	0.005668725	0.283752733	0	0
3/14	0.005735012	0.283752733	0	0
3/14	0.005735012	0.283752733	0	0
3/14	0.005735012	0.283752733	0	0
3/14	0.005735012	0.283752733	0	0
9/122	0.005944641	0.287090063	0	0
4/28	0.006474448	0.287090063	0	0
7/82	0.006750436	0.287090063	0	0

2/5	0.006917948	0.287090063	0	0
2/5	0.006917948	0.287090063	0	0
2/5	0.006917948	0.287090063	0	0
2/5	0.006917948	0.287090063	0	0
2/5	0.006917948	0.287090063	0	0
3/15	0.007026292	0.287090063	0	0
5/45	0.007121205	0.287090063	0	0
10/149	0.007402262	0.292994998	0	0
3/16	0.008476123	0.318146908	0	0
3/16	0.008476123	0.318146908	0	0
3/16	0.008476123	0.318146908	0	0
7/86	0.008705357	0.321212923	0	0
4/31	0.009336289	0.336173158	0	0
13/228	0.009438233	0.336173158	0	0
3/17	0.010088466	0.336173158	0	0
5/49	0.010180289	0.336173158	0	0
2/6	0.010191745	0.336173158	0	0
2/6	0.010191745	0.336173158	0	0
2/6	0.010191745	0.336173158	0	0
8/111	0.010577133	0.343677888	0	0
3/18	0.011866593	0.353192502	0	0
3/18	0.011866593	0.353192502	0	0
5/51	0.01200352	0.353192502	0	0
7/92	0.012375028	0.353192502	0	0
6/71	0.012381008	0.353192502	0	0
5/52	0.012993158	0.353192502	0	0
3/19	0.013813127	0.353192502	0	0
3/19	0.013813127	0.353192502	0	0
3/19	0.013813127	0.353192502	0	0
3/19	0.013813127	0.353192502	0	0
3/19	0.013813127	0.353192502	0	0
2/7	0.014014399	0.353192502	0	0
2/7	0.014014399	0.353192502	0	0
2/7	0.014014399	0.353192502	0	0
2/7	0.014014399	0.353192502	0	0
2/7	0.014014399	0.353192502	0	0
2/7	0.014014399	0.353192502	0	0
2/7	0.014014399	0.353192502	0	0
5/53	0.01403646	0.353192502	0	0
4/35	0.014276959	0.353192502	0	0
4/35	0.014276959	0.353192502	0	0
5/54	0.015134606	0.36579631	0	0
6/75	0.015916409	0.36579631	0	0
7/98	0.01704604	0.36579631	0	0
5/56	0.017499931	0.36579631	0	0
12/221	0.01752582	0.36579631	0	0
9/146	0.017951902	0.36579631	0	0
3/21	0.018218931	0.36579631	0	0

3/21	0.018218931	0.36579631	0	0
2/8	0.018353933	0.36579631	0	0
2/8	0.018353933	0.36579631	0	0
2/8	0.018353933	0.36579631	0	0
2/8	0.018353933	0.36579631	0	0
2/8	0.018353933	0.36579631	0	0
2/8	0.018353933	0.36579631	0	0
2/8	0.018353933	0.36579631	0	0
2/8	0.018353933	0.36579631	0	0
2/8	0.018353933	0.36579631	0	0
2/8	0.018353933	0.36579631	0	0
2/8	0.018353933	0.36579631	0	0
2/8	0.018353933	0.36579631	0	0
2/8	0.018353933	0.36579631	0	0
5/57	0.018769244	0.36579631	0	0
7/100	0.018847182	0.36579631	0	0
4/38	0.018895087	0.36579631	0	0
6/78	0.01898713	0.36579631	0	0
4/39	0.020616788	0.391492443	0	0
3/22	0.020680584	0.391492443	0	0
5/59	0.021486137	0.399789053	0	0
5/59	0.021486137	0.399789053	0	0
9/152	0.022661268	0.412665074	0	0
14/284	0.023079469	0.412665074	0	0
2/9	0.023179692	0.412665074	0	0
2/9	0.023179692	0.412665074	0	0
2/9	0.023179692	0.412665074	0	0
3/23	0.023315482	0.412665074	0	0
6/82	0.023677786	0.415697908	0	0
4/41	0.024341159	0.42392563	0	0
3/24	0.026123605	0.428111584	0	0
3/24	0.026123605	0.428111584	0	0
3/24	0.026123605	0.428111584	0	0
3/24	0.026123605	0.428111584	0	0
4/42	0.026345844	0.428111584	0	0
4/42	0.026345844	0.428111584	0	0
7/108	0.027385185	0.428111584	0	0
12/236	0.027508324	0.428111584	0	0
6/85	0.027665893	0.428111584	0	0
6/85	0.027665893	0.428111584	0	0
4/43	0.028446647	0.428111584	0	0
2/10	0.028462297	0.428111584	0	0
2/10	0.028462297	0.428111584	0	0
2/10	0.028462297	0.428111584	0	0
2/10	0.028462297	0.428111584	0	0
2/10	0.028462297	0.428111584	0	0
2/10	0.028462297	0.428111584	0	0
2/10	0.028462297	0.428111584	0	0

2/10	0.028462297	0.428111584	0	0
3/25	0.029104508	0.428111584	0	0
3/25	0.029104508	0.428111584	0	0
3/25	0.029104508	0.428111584	0	0
3/25	0.029104508	0.428111584	0	0
7/110	0.029872097	0.436453391	0	0
4/44	0.030644244	0.444750125	0	0
5/65	0.031124404	0.444927511	0	0
11/214	0.031682651	0.444927511	0	0
3/26	0.032257353	0.444927511	0	0
3/26	0.032257353	0.444927511	0	0
2/11	0.034173597	0.444927511	0	0
2/11	0.034173597	0.444927511	0	0
2/11	0.034173597	0.444927511	0	0
2/11	0.034173597	0.444927511	0	0
2/11	0.034173597	0.444927511	0	0
2/11	0.034173597	0.444927511	0	0
2/11	0.034173597	0.444927511	0	0
2/11	0.034173597	0.444927511	0	0
2/11	0.034173597	0.444927511	0	0
2/11	0.034173597	0.444927511	0	0
2/11	0.034173597	0.444927511	0	0
2/11	0.034173597	0.444927511	0	0
2/11	0.034173597	0.444927511	0	0
10/190	0.034335242	0.444927511	0	0
5/67	0.034851916	0.447743929	0	0
4/46	0.035331867	0.447743929	0	0
4/46	0.035331867	0.447743929	0	0
3/27	0.035580937	0.447743929	0	0
3/27	0.035580937	0.447743929	0	0
4/47	0.037822604	0.469005243	0	0
4/47	0.037822604	0.469005243	0	0
5/69	0.038844101	0.469005243	0	0
5/69	0.038844101	0.469005243	0	0
3/28	0.039073721	0.469005243	0	0
2/12	0.040286624	0.469005243	0	0
2/12	0.040286624	0.469005243	0	0
2/12	0.040286624	0.469005243	0	0
2/12	0.040286624	0.469005243	0	0
2/12	0.040286624	0.469005243	0	0
2/12	0.040286624	0.469005243	0	0
2/12	0.040286624	0.469005243	0	0
2/12	0.040286624	0.469005243	0	0
2/12	0.040286624	0.469005243	0	0
3/29	0.042733856	0.488677393	0	0
3/29	0.042733856	0.488677393	0	0
3/29	0.042733856	0.488677393	0	0
4/49	0.043098787	0.488677393	0	0

4/49	0.043098787	0.488677393	0	0
5/72	0.045335139	0.501627472	0	0
4/50	0.045884222	0.501627472	0	0
4/50	0.045884222	0.501627472	0	0
3/30	0.046559209	0.501627472	0	0
3/30	0.046559209	0.501627472	0	0
2/13	0.046775552	0.501627472	0	0
2/13	0.046775552	0.501627472	0	0
2/13	0.046775552	0.501627472	0	0
2/13	0.046775552	0.501627472	0	0
2/13	0.046775552	0.501627472	0	0
2/13	0.046775552	0.501627472	0	0
9/174	0.04743247	0.503393649	0	0
5/73	0.047633942	0.503393649	0	0
5/73	0.047633942	0.503393649	0	0
9/176	0.050328444	0.519064414	0	0
3/31	0.050547384	0.519064414	0	0
3/31	0.050547384	0.519064414	0	0
3/31	0.050547384	0.519064414	0	0
3/31	0.050547384	0.519064414	0	0
3/31	0.050547384	0.519064414	0	0
6/99	0.052021897	0.523413796	0	0
7/125	0.053421435	0.523413796	0	0
2/14	0.053615653	0.523413796	0	0
2/14	0.053615653	0.523413796	0	0
2/14	0.053615653	0.523413796	0	0
2/14	0.053615653	0.523413796	0	0
2/14	0.053615653	0.523413796	0	0
2/14	0.053615653	0.523413796	0	0
2/14	0.053615653	0.523413796	0	0
2/14	0.053615653	0.523413796	0	0
2/14	0.053615653	0.523413796	0	0
3/32	0.054695748	0.530486271	0	0
4/53	0.054827474	0.530486271	0	0
5/77	0.057508274	0.553962441	0	0
3/33	0.059001449	0.55598803	0	0
3/33	0.059001449	0.55598803	0	0
2/15	0.060783257	0.55598803	0	0
2/15	0.060783257	0.55598803	0	0
2/15	0.060783257	0.55598803	0	0
2/15	0.060783257	0.55598803	0	0
2/15	0.060783257	0.55598803	0	0
2/15	0.060783257	0.55598803	0	0
2/15	0.060783257	0.55598803	0	0
2/15	0.060783257	0.55598803	0	0
2/15	0.060783257	0.55598803	0	0
2/15	0.060783257	0.55598803	0	0
7/129	0.061231285	0.557742707	0	0

3/34	0.063461438	0.568541357	0	0
3/34	0.063461438	0.568541357	0	0
3/34	0.063461438	0.568541357	0	0
3/34	0.063461438	0.568541357	0	0
6/105	0.065501426	0.572760646	0	0
12/272	0.067115824	0.572760646	0	0
2/16	0.068255715	0.572760646	0	0
2/16	0.068255715	0.572760646	0	0
2/16	0.068255715	0.572760646	0	0
2/16	0.068255715	0.572760646	0	0
2/16	0.068255715	0.572760646	0	0
2/16	0.068255715	0.572760646	0	0
2/16	0.068255715	0.572760646	0	0
2/16	0.068255715	0.572760646	0	0
2/16	0.068255715	0.572760646	0	0
2/16	0.068255715	0.572760646	0	0
2/16	0.068255715	0.572760646	0	0
5/81	0.068468099	0.572760646	0	0
11/244	0.068629863	0.572760646	0	0
4/58	0.071660013	0.572760646	0	0
4/58	0.071660013	0.572760646	0	0
3/36	0.07283121	0.572760646	0	0
4/59	0.075309025	0.572760646	0	0
4/59	0.075309025	0.572760646	0	0
4/59	0.075309025	0.572760646	0	0
8/163	0.075689013	0.572760646	0	0
2/17	0.076011357	0.572760646	0	0
2/17	0.076011357	0.572760646	0	0
2/17	0.076011357	0.572760646	0	0
2/17	0.076011357	0.572760646	0	0
2/17	0.076011357	0.572760646	0	0
2/17	0.076011357	0.572760646	0	0
2/17	0.076011357	0.572760646	0	0
3/37	0.077734074	0.572760646	0	0
6/111	0.080839526	0.572760646	0	0
12/281	0.08097557	0.572760646	0	0
3/38	0.08277742	0.572760646	0	0
3/38	0.08277742	0.572760646	0	0
4/61	0.082881799	0.572760646	0	0
4/61	0.082881799	0.572760646	0	0
6/112	0.083575339	0.572760646	0	0
2/18	0.084029462	0.572760646	0	0
2/18	0.084029462	0.572760646	0	0
2/18	0.084029462	0.572760646	0	0
2/18	0.084029462	0.572760646	0	0
2/18	0.084029462	0.572760646	0	0
2/18	0.084029462	0.572760646	0	0
4/62	0.086803268	0.572760646	0	0

4/62	0.086803268	0.572760646	0	0
3/39	0.087957476	0.572760646	0	0
3/39	0.087957476	0.572760646	0	0
15/377	0.089217122	0.572760646	0	0
4/63	0.090813145	0.572760646	0	0
2/19	0.092290215	0.572760646	0	0
2/19	0.092290215	0.572760646	0	0
2/19	0.092290215	0.572760646	0	0
2/19	0.092290215	0.572760646	0	0
2/19	0.092290215	0.572760646	0	0
2/19	0.092290215	0.572760646	0	0
2/19	0.092290215	0.572760646	0	0
2/19	0.092290215	0.572760646	0	0
2/19	0.092290215	0.572760646	0	0
2/19	0.092290215	0.572760646	0	0
2/19	0.092290215	0.572760646	0	0
2/19	0.092290215	0.572760646	0	0
3/40	0.093270374	0.572760646	0	0
3/40	0.093270374	0.572760646	0	0
13/320	0.095963185	0.572760646	0	0
5/90	0.097017115	0.572760646	0	0
3/41	0.098712158	0.572760646	0	0
5/91	0.100511379	0.572760646	0	0
2/20	0.100774681	0.572760646	0	0
2/20	0.100774681	0.572760646	0	0
2/20	0.100774681	0.572760646	0	0
2/20	0.100774681	0.572760646	0	0
2/20	0.100774681	0.572760646	0	0
2/20	0.100774681	0.572760646	0	0
4/66	0.103359719	0.572760646	0	0
4/66	0.103359719	0.572760646	0	0
6/119	0.10413383	0.572760646	0	0
3/42	0.104278802	0.572760646	0	0
3/42	0.104278802	0.572760646	0	0
11/265	0.106490872	0.572760646	0	0
4/67	0.107709423	0.572760646	0	0
4/67	0.107709423	0.572760646	0	0
11/266	0.108548285	0.572760646	0	0
2/21	0.109464767	0.572760646	0	0
2/21	0.109464767	0.572760646	0	0
2/21	0.109464767	0.572760646	0	0
2/21	0.109464767	0.572760646	0	0
3/43	0.109966217	0.572760646	0	0
3/43	0.109966217	0.572760646	0	0
8/178	0.110913749	0.572760646	0	0
8/179	0.113534901	0.572760646	0	0
5/95	0.115103792	0.572760646	0	0
3/44	0.115770267	0.572760646	0	0

3/44	0.115770267	0.572760646	0	0
3/44	0.115770267	0.572760646	0	0
3/44	0.115770267	0.572760646	0	0
3/44	0.115770267	0.572760646	0	0
3/44	0.115770267	0.572760646	0	0
2/22	0.118343192	0.572760646	0	0
2/22	0.118343192	0.572760646	0	0
2/22	0.118343192	0.572760646	0	0
2/22	0.118343192	0.572760646	0	0
2/22	0.118343192	0.572760646	0	0
2/22	0.118343192	0.572760646	0	0
2/22	0.118343192	0.572760646	0	0
4/70	0.121239399	0.572760646	0	0
4/70	0.121239399	0.572760646	0	0
4/70	0.121239399	0.572760646	0	0
3/45	0.121686777	0.572760646	0	0
3/45	0.121686777	0.572760646	0	0
5/97	0.122758548	0.572760646	0	0
4/71	0.125904199	0.572760646	0	0
4/71	0.125904199	0.572760646	0	0
4/71	0.125904199	0.572760646	0	0
2/23	0.127393457	0.572760646	0	0
2/23	0.127393457	0.572760646	0	0
2/23	0.127393457	0.572760646	0	0
2/23	0.127393457	0.572760646	0	0
2/23	0.127393457	0.572760646	0	0
3/46	0.127711541	0.572760646	0	0
3/46	0.127711541	0.572760646	0	0
3/46	0.127711541	0.572760646	0	0
3/46	0.127711541	0.572760646	0	0
1/5	0.128140015	0.572760646	0	0
1/5	0.128140015	0.572760646	0	0
1/5	0.128140015	0.572760646	0	0
1/5	0.128140015	0.572760646	0	0
1/5	0.128140015	0.572760646	0	0
1/5	0.128140015	0.572760646	0	0
1/5	0.128140015	0.572760646	0	0
1/5	0.128140015	0.572760646	0	0
1/5	0.128140015	0.572760646	0	0
1/5	0.128140015	0.572760646	0	0
1/5	0.128140015	0.572760646	0	0
1/5	0.128140015	0.572760646	0	0
1/5	0.128140015	0.572760646	0	0
1/5	0.128140015	0.572760646	0	0
1/5	0.128140015	0.572760646	0	0
1/5	0.128140015	0.572760646	0	0
1/5	0.128140015	0.572760646	0	0
1/5	0.128140015	0.572760646	0	0

1/5	0.128140015	0.572760646	0	0
1/5	0.128140015	0.572760646	0	0
1/5	0.128140015	0.572760646	0	0
1/5	0.128140015	0.572760646	0	0
1/5	0.128140015	0.572760646	0	0
1/5	0.128140015	0.572760646	0	0
1/5	0.128140015	0.572760646	0	0
1/5	0.128140015	0.572760646	0	0
1/5	0.128140015	0.572760646	0	0
1/5	0.128140015	0.572760646	0	0
1/5	0.128140015	0.572760646	0	0
1/5	0.128140015	0.572760646	0	0
1/5	0.128140015	0.572760646	0	0
1/5	0.128140015	0.572760646	0	0
1/5	0.128140015	0.572760646	0	0
1/5	0.128140015	0.572760646	0	0
1/5	0.128140015	0.572760646	0	0
1/5	0.128140015	0.572760646	0	0
1/5	0.128140015	0.572760646	0	0
1/5	0.128140015	0.572760646	0	0
1/5	0.128140015	0.572760646	0	0
1/5	0.128140015	0.572760646	0	0
1/5	0.128140015	0.572760646	0	0
1/5	0.128140015	0.572760646	0	0
1/5	0.128140015	0.572760646	0	0
1/5	0.128140015	0.572760646	0	0
1/5	0.128140015	0.572760646	0	0
1/5	0.128140015	0.572760646	0	0
1/5	0.128140015	0.572760646	0	0
1/5	0.128140015	0.572760646	0	0
1/5	0.128140015	0.572760646	0	0
1/5	0.128140015	0.572760646	0	0
1/5	0.128140015	0.572760646	0	0
1/5	0.128140015	0.572760646	0	0
1/5	0.128140015	0.572760646	0	0
1/5	0.128140015	0.572760646	0	0
1/5	0.128140015	0.572760646	0	0
1/5	0.128140015	0.572760646	0	0
1/5	0.128140015	0.572760646	0	0
1/5	0.128140015	0.572760646	0	0
1/5	0.128140015	0.572760646	0	0
1/5	0.128140015	0.572760646	0	0
1/5	0.128140015	0.572760646	0	0
1/5	0.128140015	0.572760646	0	0
1/5	0.128140015	0.572760646	0	0
1/5	0.128140015	0.572760646	0	0
1/5	0.128140015	0.572760646	0	0
1/5	0.128140015	0.572760646	0	0

1/5	0.128140015	0.572760646	0	0
1/5	0.128140015	0.572760646	0	0
1/5	0.128140015	0.572760646	0	0
1/5	0.128140015	0.572760646	0	0
1/5	0.128140015	0.572760646	0	0
1/5	0.128140015	0.572760646	0	0
1/5	0.128140015	0.572760646	0	0
1/5	0.128140015	0.572760646	0	0
1/5	0.128140015	0.572760646	0	0
1/5	0.128140015	0.572760646	0	0
1/5	0.128140015	0.572760646	0	0
1/5	0.128140015	0.572760646	0	0
4/72	0.130643557	0.572760646	0	0
12/308	0.132648091	0.572760646	0	0
3/47	0.133840335	0.572760646	0	0
3/47	0.133840335	0.572760646	0	0
4/73	0.135455711	0.572760646	0	0
2/24	0.136599813	0.572760646	0	0
2/24	0.136599813	0.572760646	0	0
2/24	0.136599813	0.572760646	0	0
2/24	0.136599813	0.572760646	0	0
2/24	0.136599813	0.572760646	0	0
2/24	0.136599813	0.572760646	0	0
2/24	0.136599813	0.572760646	0	0
2/24	0.136599813	0.572760646	0	0
2/24	0.136599813	0.572760646	0	0
2/24	0.136599813	0.572760646	0	0
2/24	0.136599813	0.572760646	0	0
15/406	0.139401554	0.572760646	0	0
3/48	0.140068923	0.572760646	0	0
3/48	0.140068923	0.572760646	0	0
3/48	0.140068923	0.572760646	0	0
3/48	0.140068923	0.572760646	0	0
6/130	0.141189498	0.572760646	0	0
8/189	0.141544707	0.572760646	0	0
4/75	0.145291185	0.572760646	0	0
4/75	0.145291185	0.572760646	0	0
2/25	0.145947236	0.572760646	0	0
2/25	0.145947236	0.572760646	0	0
2/25	0.145947236	0.572760646	0	0
2/25	0.145947236	0.572760646	0	0
2/25	0.145947236	0.572760646	0	0
2/25	0.145947236	0.572760646	0	0
2/25	0.145947236	0.572760646	0	0
2/25	0.145947236	0.572760646	0	0
2/25	0.145947236	0.572760646	0	0
2/25	0.145947236	0.572760646	0	0
2/25	0.145947236	0.572760646	0	0

2/25	0.145947236	0.572760646	0	0
3/49	0.146393065	0.572760646	0	0
3/49	0.146393065	0.572760646	0	0
3/49	0.146393065	0.572760646	0	0
11/284	0.149434572	0.572760646	0	0
4/76	0.150310822	0.572760646	0	0
1/6	0.151729691	0.572760646	0	0
1/6	0.151729691	0.572760646	0	0
1/6	0.151729691	0.572760646	0	0
1/6	0.151729691	0.572760646	0	0
1/6	0.151729691	0.572760646	0	0
1/6	0.151729691	0.572760646	0	0
1/6	0.151729691	0.572760646	0	0
1/6	0.151729691	0.572760646	0	0
1/6	0.151729691	0.572760646	0	0
1/6	0.151729691	0.572760646	0	0
1/6	0.151729691	0.572760646	0	0
1/6	0.151729691	0.572760646	0	0
1/6	0.151729691	0.572760646	0	0
1/6	0.151729691	0.572760646	0	0
1/6	0.151729691	0.572760646	0	0
1/6	0.151729691	0.572760646	0	0
1/6	0.151729691	0.572760646	0	0
1/6	0.151729691	0.572760646	0	0
1/6	0.151729691	0.572760646	0	0
1/6	0.151729691	0.572760646	0	0
1/6	0.151729691	0.572760646	0	0
1/6	0.151729691	0.572760646	0	0
1/6	0.151729691	0.572760646	0	0
1/6	0.151729691	0.572760646	0	0
1/6	0.151729691	0.572760646	0	0
1/6	0.151729691	0.572760646	0	0
1/6	0.151729691	0.572760646	0	0
1/6	0.151729691	0.572760646	0	0
1/6	0.151729691	0.572760646	0	0
1/6	0.151729691	0.572760646	0	0
1/6	0.151729691	0.572760646	0	0
1/6	0.151729691	0.572760646	0	0
1/6	0.151729691	0.572760646	0	0
1/6	0.151729691	0.572760646	0	0
1/6	0.151729691	0.572760646	0	0
1/6	0.151729691	0.572760646	0	0
1/6	0.151729691	0.572760646	0	0
1/6	0.151729691	0.572760646	0	0
1/6	0.151729691	0.572760646	0	0
1/6	0.151729691	0.572760646	0	0
1/6	0.151729691	0.572760646	0	0
1/6	0.151729691	0.572760646	0	0

1/6	0.151729691	0.572760646	0	0
1/6	0.151729691	0.572760646	0	0
1/6	0.151729691	0.572760646	0	0
1/6	0.151729691	0.572760646	0	0
1/6	0.151729691	0.572760646	0	0
1/6	0.151729691	0.572760646	0	0
1/6	0.151729691	0.572760646	0	0
1/6	0.151729691	0.572760646	0	0
1/6	0.151729691	0.572760646	0	0
1/6	0.151729691	0.572760646	0	0
1/6	0.151729691	0.572760646	0	0
1/6	0.151729691	0.572760646	0	0
1/6	0.151729691	0.572760646	0	0
1/6	0.151729691	0.572760646	0	0
1/6	0.151729691	0.572760646	0	0
1/6	0.151729691	0.572760646	0	0
1/6	0.151729691	0.572760646	0	0
1/6	0.151729691	0.572760646	0	0
1/6	0.151729691	0.572760646	0	0
1/6	0.151729691	0.572760646	0	0
1/6	0.151729691	0.572760646	0	0
1/6	0.151729691	0.572760646	0	0
1/6	0.151729691	0.572760646	0	0
1/6	0.151729691	0.572760646	0	0
1/6	0.151729691	0.572760646	0	0
1/6	0.151729691	0.572760646	0	0
1/6	0.151729691	0.572760646	0	0
1/6	0.151729691	0.572760646	0	0
1/6	0.151729691	0.572760646	0	0
3/50	0.152808526	0.572760646	0	0
3/50	0.152808526	0.572760646	0	0
3/50	0.152808526	0.572760646	0	0
3/50	0.152808526	0.572760646	0	0
4/77	0.155395895	0.572760646	0	0
4/77	0.155395895	0.572760646	0	0
4/77	0.155395895	0.572760646	0	0
2/26	0.155421397	0.572760646	0	0
2/26	0.155421397	0.572760646	0	0
2/26	0.155421397	0.572760646	0	0
2/26	0.155421397	0.572760646	0	0
2/26	0.155421397	0.572760646	0	0
2/26	0.155421397	0.572760646	0	0
2/26	0.155421397	0.572760646	0	0
2/26	0.155421397	0.572760646	0	0
2/26	0.155421397	0.572760646	0	0
2/26	0.155421397	0.572760646	0	0
9/225	0.157820168	0.572760646	0	0
3/51	0.159311081	0.572760646	0	0

5/107	0.16433447	0.572760646	0	0
2/27	0.165008638	0.572760646	0	0
2/27	0.165008638	0.572760646	0	0
2/27	0.165008638	0.572760646	0	0
2/27	0.165008638	0.572760646	0	0
4/79	0.165754719	0.572760646	0	0
4/79	0.165754719	0.572760646	0	0
4/79	0.165754719	0.572760646	0	0
3/52	0.165896522	0.572760646	0	0
3/52	0.165896522	0.572760646	0	0
3/52	0.165896522	0.572760646	0	0
3/52	0.165896522	0.572760646	0	0
5/108	0.16877115	0.572760646	0	0
5/108	0.16877115	0.572760646	0	0
4/80	0.17102461	0.572760646	0	0
3/53	0.172560667	0.572760646	0	0
3/53	0.172560667	0.572760646	0	0
3/53	0.172560667	0.572760646	0	0
1/7	0.174682256	0.572760646	0	0
1/7	0.174682256	0.572760646	0	0
1/7	0.174682256	0.572760646	0	0
1/7	0.174682256	0.572760646	0	0
1/7	0.174682256	0.572760646	0	0
1/7	0.174682256	0.572760646	0	0
1/7	0.174682256	0.572760646	0	0
1/7	0.174682256	0.572760646	0	0
1/7	0.174682256	0.572760646	0	0
1/7	0.174682256	0.572760646	0	0
1/7	0.174682256	0.572760646	0	0
1/7	0.174682256	0.572760646	0	0
1/7	0.174682256	0.572760646	0	0
1/7	0.174682256	0.572760646	0	0
1/7	0.174682256	0.572760646	0	0
1/7	0.174682256	0.572760646	0	0
1/7	0.174682256	0.572760646	0	0
1/7	0.174682256	0.572760646	0	0
1/7	0.174682256	0.572760646	0	0
1/7	0.174682256	0.572760646	0	0
1/7	0.174682256	0.572760646	0	0
1/7	0.174682256	0.572760646	0	0
1/7	0.174682256	0.572760646	0	0
1/7	0.174682256	0.572760646	0	0
1/7	0.174682256	0.572760646	0	0
1/7	0.174682256	0.572760646	0	0
1/7	0.174682256	0.572760646	0	0
1/7	0.174682256	0.572760646	0	0
1/7	0.174682256	0.572760646	0	0
1/7	0.174682256	0.572760646	0	0

1/7	0.174682256	0.572760646	0	0
1/7	0.174682256	0.572760646	0	0
1/7	0.174682256	0.572760646	0	0
1/7	0.174682256	0.572760646	0	0
1/7	0.174682256	0.572760646	0	0
1/7	0.174682256	0.572760646	0	0
1/7	0.174682256	0.572760646	0	0
1/7	0.174682256	0.572760646	0	0
1/7	0.174682256	0.572760646	0	0
1/7	0.174682256	0.572760646	0	0
1/7	0.174682256	0.572760646	0	0
1/7	0.174682256	0.572760646	0	0
1/7	0.174682256	0.572760646	0	0
1/7	0.174682256	0.572760646	0	0
1/7	0.174682256	0.572760646	0	0
1/7	0.174682256	0.572760646	0	0
1/7	0.174682256	0.572760646	0	0
1/7	0.174682256	0.572760646	0	0
1/7	0.174682256	0.572760646	0	0
1/7	0.174682256	0.572760646	0	0
1/7	0.174682256	0.572760646	0	0
1/7	0.174682256	0.572760646	0	0
1/7	0.174682256	0.572760646	0	0
1/7	0.174682256	0.572760646	0	0
1/7	0.174682256	0.572760646	0	0
1/7	0.174682256	0.572760646	0	0
1/7	0.174682256	0.572760646	0	0
1/7	0.174682256	0.572760646	0	0
1/7	0.174682256	0.572760646	0	0
1/7	0.174682256	0.572760646	0	0
1/7	0.174682256	0.572760646	0	0
2/28	0.174695943	0.572760646	0	0
2/28	0.174695943	0.572760646	0	0
2/28	0.174695943	0.572760646	0	0
2/28	0.174695943	0.572760646	0	0
2/28	0.174695943	0.572760646	0	0
2/28	0.174695943	0.572760646	0	0
2/28	0.174695943	0.572760646	0	0
2/28	0.174695943	0.572760646	0	0
2/28	0.174695943	0.572760646	0	0
2/28	0.174695943	0.572760646	0	0
9/231	0.175604513	0.574873721	0	0
4/81	0.176352222	0.575590387	0	0
4/81	0.176352222	0.575590387	0	0
5/110	0.177782878	0.579391205	0	0
4/82	0.181735591	0.582746477	0	0
2/29	0.184470916	0.582746477	0	0
2/29	0.184470916	0.582746477	0	0

2/29	0.184470916	0.582746477	0	0
2/29	0.184470916	0.582746477	0	0
2/29	0.184470916	0.582746477	0	0
2/29	0.184470916	0.582746477	0	0
2/29	0.184470916	0.582746477	0	0
2/29	0.184470916	0.582746477	0	0
2/29	0.184470916	0.582746477	0	0
2/29	0.184470916	0.582746477	0	0
9/234	0.184827094	0.582746477	0	0
3/55	0.186108483	0.582746477	0	0
8/204	0.189257082	0.582746477	0	0
13/364	0.189512003	0.582746477	0	0
4/84	0.192661691	0.582746477	0	0
3/56	0.192983951	0.582746477	0	0
3/56	0.192983951	0.582746477	0	0
2/30	0.194321756	0.582746477	0	0
2/30	0.194321756	0.582746477	0	0
2/30	0.194321756	0.582746477	0	0
2/30	0.194321756	0.582746477	0	0
2/30	0.194321756	0.582746477	0	0
2/30	0.194321756	0.582746477	0	0
2/30	0.194321756	0.582746477	0	0
1/8	0.197014886	0.582746477	0	0
1/8	0.197014886	0.582746477	0	0
1/8	0.197014886	0.582746477	0	0
1/8	0.197014886	0.582746477	0	0
1/8	0.197014886	0.582746477	0	0
1/8	0.197014886	0.582746477	0	0
1/8	0.197014886	0.582746477	0	0
1/8	0.197014886	0.582746477	0	0
1/8	0.197014886	0.582746477	0	0
1/8	0.197014886	0.582746477	0	0
1/8	0.197014886	0.582746477	0	0
1/8	0.197014886	0.582746477	0	0
1/8	0.197014886	0.582746477	0	0
1/8	0.197014886	0.582746477	0	0
1/8	0.197014886	0.582746477	0	0
1/8	0.197014886	0.582746477	0	0
1/8	0.197014886	0.582746477	0	0
1/8	0.197014886	0.582746477	0	0
1/8	0.197014886	0.582746477	0	0
1/8	0.197014886	0.582746477	0	0
1/8	0.197014886	0.582746477	0	0
1/8	0.197014886	0.582746477	0	0
1/8	0.197014886	0.582746477	0	0
1/8	0.197014886	0.582746477	0	0
1/8	0.197014886	0.582746477	0	0
1/8	0.197014886	0.582746477	0	0

1/8	0.197014886	0.582746477	0	0
1/8	0.197014886	0.582746477	0	0
1/8	0.197014886	0.582746477	0	0
1/8	0.197014886	0.582746477	0	0
1/8	0.197014886	0.582746477	0	0
1/8	0.197014886	0.582746477	0	0
1/8	0.197014886	0.582746477	0	0
1/8	0.197014886	0.582746477	0	0
1/8	0.197014886	0.582746477	0	0
1/8	0.197014886	0.582746477	0	0
1/8	0.197014886	0.582746477	0	0
1/8	0.197014886	0.582746477	0	0
1/8	0.197014886	0.582746477	0	0
1/8	0.197014886	0.582746477	0	0
1/8	0.197014886	0.582746477	0	0
1/8	0.197014886	0.582746477	0	0
1/8	0.197014886	0.582746477	0	0
4/85	0.198200454	0.583873327	0	0
4/85	0.198200454	0.583873327	0	0
4/85	0.198200454	0.583873327	0	0
3/57	0.199921729	0.585773356	0	0
3/57	0.199921729	0.585773356	0	0
3/57	0.199921729	0.585773356	0	0
3/57	0.199921729	0.585773356	0	0
2/31	0.204237232	0.586460998	0	0
2/31	0.204237232	0.586460998	0	0
2/31	0.204237232	0.586460998	0	0
3/58	0.206917824	0.586460998	0	0
3/58	0.206917824	0.586460998	0	0
3/58	0.206917824	0.586460998	0	0
3/58	0.206917824	0.586460998	0	0
4/87	0.20941946	0.586460998	0	0
5/117	0.2106569	0.586460998	0	0
3/59	0.213968296	0.586460998	0	0
3/59	0.213968296	0.586460998	0	0
3/59	0.213968296	0.586460998	0	0
2/32	0.214206667	0.586460998	0	0
2/32	0.214206667	0.586460998	0	0
2/32	0.214206667	0.586460998	0	0
2/32	0.214206667	0.586460998	0	0
2/32	0.214206667	0.586460998	0	0
2/32	0.214206667	0.586460998	0	0
2/32	0.214206667	0.586460998	0	0
5/118	0.215506846	0.586460998	0	0
1/9	0.218744295	0.586460998	0	0
1/9	0.218744295	0.586460998	0	0
1/9	0.218744295	0.586460998	0	0
1/9	0.218744295	0.586460998	0	0

1/9	0.218744295	0.586460998	0	0
1/9	0.218744295	0.586460998	0	0
1/9	0.218744295	0.586460998	0	0
1/9	0.218744295	0.586460998	0	0
1/9	0.218744295	0.586460998	0	0
1/9	0.218744295	0.586460998	0	0
1/9	0.218744295	0.586460998	0	0
1/9	0.218744295	0.586460998	0	0
1/9	0.218744295	0.586460998	0	0
1/9	0.218744295	0.586460998	0	0
1/9	0.218744295	0.586460998	0	0
1/9	0.218744295	0.586460998	0	0
1/9	0.218744295	0.586460998	0	0
1/9	0.218744295	0.586460998	0	0
1/9	0.218744295	0.586460998	0	0
1/9	0.218744295	0.586460998	0	0
1/9	0.218744295	0.586460998	0	0
1/9	0.218744295	0.586460998	0	0
1/9	0.218744295	0.586460998	0	0
1/9	0.218744295	0.586460998	0	0
1/9	0.218744295	0.586460998	0	0
1/9	0.218744295	0.586460998	0	0
1/9	0.218744295	0.586460998	0	0
1/9	0.218744295	0.586460998	0	0
1/9	0.218744295	0.586460998	0	0
1/9	0.218744295	0.586460998	0	0
1/9	0.218744295	0.586460998	0	0
1/9	0.218744295	0.586460998	0	0
1/9	0.218744295	0.586460998	0	0
1/9	0.218744295	0.586460998	0	0
1/9	0.218744295	0.586460998	0	0
1/9	0.218744295	0.586460998	0	0
1/9	0.218744295	0.586460998	0	0
1/9	0.218744295	0.586460998	0	0
1/9	0.218744295	0.586460998	0	0
1/9	0.218744295	0.586460998	0	0
1/9	0.218744295	0.586460998	0	0
1/9	0.218744295	0.586460998	0	0
1/9	0.218744295	0.586460998	0	0
1/9	0.218744295	0.586460998	0	0
1/9	0.218744295	0.586460998	0	0
1/9	0.218744295	0.586460998	0	0
1/9	0.218744295	0.586460998	0	0
1/9	0.218744295	0.586460998	0	0
1/9	0.218744295	0.586460998	0	0
11/310	0.220313544	0.589830333	0	0
4/89	0.220813836	0.589830333	0	0
4/89	0.220813836	0.589830333	0	0

2/33	0.224219909	0.591430856	0	0
2/33	0.224219909	0.591430856	0	0
2/33	0.224219909	0.591430856	0	0
2/33	0.224219909	0.591430856	0	0
2/33	0.224219909	0.591430856	0	0
2/33	0.224219909	0.591430856	0	0
2/33	0.224219909	0.591430856	0	0
2/33	0.224219909	0.591430856	0	0
2/33	0.224219909	0.591430856	0	0
3/61	0.228216889	0.591430856	0	0
3/61	0.228216889	0.591430856	0	0
16/480	0.230299343	0.591430856	0	0
2/34	0.234267315	0.591430856	0	0
2/34	0.234267315	0.591430856	0	0
2/34	0.234267315	0.591430856	0	0
2/34	0.234267315	0.591430856	0	0
2/34	0.234267315	0.591430856	0	0
2/34	0.234267315	0.591430856	0	0
13/381	0.234272305	0.591430856	0	0
6/153	0.23441084	0.591430856	0	0
6/154	0.238847011	0.591430856	0	0
1/10	0.239886746	0.591430856	0	0
1/10	0.239886746	0.591430856	0	0
1/10	0.239886746	0.591430856	0	0
1/10	0.239886746	0.591430856	0	0
1/10	0.239886746	0.591430856	0	0
1/10	0.239886746	0.591430856	0	0
1/10	0.239886746	0.591430856	0	0
1/10	0.239886746	0.591430856	0	0
1/10	0.239886746	0.591430856	0	0
1/10	0.239886746	0.591430856	0	0
1/10	0.239886746	0.591430856	0	0
1/10	0.239886746	0.591430856	0	0
1/10	0.239886746	0.591430856	0	0
1/10	0.239886746	0.591430856	0	0
1/10	0.239886746	0.591430856	0	0
1/10	0.239886746	0.591430856	0	0
1/10	0.239886746	0.591430856	0	0
1/10	0.239886746	0.591430856	0	0
1/10	0.239886746	0.591430856	0	0
1/10	0.239886746	0.591430856	0	0
1/10	0.239886746	0.591430856	0	0
1/10	0.239886746	0.591430856	0	0
1/10	0.239886746	0.591430856	0	0
1/10	0.239886746	0.591430856	0	0
1/10	0.239886746	0.591430856	0	0
1/10	0.239886746	0.591430856	0	0
1/10	0.239886746	0.591430856	0	0

1/10	0.239886746	0.591430856	0	0
1/10	0.239886746	0.591430856	0	0
1/10	0.239886746	0.591430856	0	0
1/10	0.239886746	0.591430856	0	0
1/10	0.239886746	0.591430856	0	0
1/10	0.239886746	0.591430856	0	0
1/10	0.239886746	0.591430856	0	0
1/10	0.239886746	0.591430856	0	0
1/10	0.239886746	0.591430856	0	0
1/10	0.239886746	0.591430856	0	0
1/10	0.239886746	0.591430856	0	0
1/10	0.239886746	0.591430856	0	0
1/10	0.239886746	0.591430856	0	0
1/10	0.239886746	0.591430856	0	0
1/10	0.239886746	0.591430856	0	0
1/10	0.239886746	0.591430856	0	0
1/10	0.239886746	0.591430856	0	0
1/10	0.239886746	0.591430856	0	0
1/10	0.239886746	0.591430856	0	0
1/10	0.239886746	0.591430856	0	0
3/63	0.242637126	0.597535095	0	0
2/35	0.24433973	0.597671451	0	0
2/35	0.24433973	0.597671451	0	0
2/35	0.24433973	0.597671451	0	0
2/35	0.24433973	0.597671451	0	0
2/35	0.24433973	0.597671451	0	0
2/35	0.24433973	0.597671451	0	0
3/64	0.24990239	0.607735486	0	0
3/64	0.24990239	0.607735486	0	0
4/94	0.249963091	0.607735486	0	0
5/125	0.250364091	0.607735486	0	0
13/387	0.251008758	0.607735486	0	0
2/36	0.254428467	0.607735486	0	0
2/36	0.254428467	0.607735486	0	0
2/36	0.254428467	0.607735486	0	0
5/126	0.255458795	0.607735486	0	0
3/65	0.257199629	0.607735486	0	0
3/65	0.257199629	0.607735486	0	0
1/11	0.260458066	0.607735486	0	0
1/11	0.260458066	0.607735486	0	0
1/11	0.260458066	0.607735486	0	0
1/11	0.260458066	0.607735486	0	0
1/11	0.260458066	0.607735486	0	0
1/11	0.260458066	0.607735486	0	0
1/11	0.260458066	0.607735486	0	0
1/11	0.260458066	0.607735486	0	0
1/11	0.260458066	0.607735486	0	0
1/11	0.260458066	0.607735486	0	0

1/11	0.260458066	0.607735486	0	0
1/11	0.260458066	0.607735486	0	0
1/11	0.260458066	0.607735486	0	0
1/11	0.260458066	0.607735486	0	0
1/11	0.260458066	0.607735486	0	0
1/11	0.260458066	0.607735486	0	0
1/11	0.260458066	0.607735486	0	0
1/11	0.260458066	0.607735486	0	0
1/11	0.260458066	0.607735486	0	0
1/11	0.260458066	0.607735486	0	0
1/11	0.260458066	0.607735486	0	0
1/11	0.260458066	0.607735486	0	0
1/11	0.260458066	0.607735486	0	0
1/11	0.260458066	0.607735486	0	0
1/11	0.260458066	0.607735486	0	0
1/11	0.260458066	0.607735486	0	0
1/11	0.260458066	0.607735486	0	0
1/11	0.260458066	0.607735486	0	0
1/11	0.260458066	0.607735486	0	0
1/11	0.260458066	0.607735486	0	0
1/11	0.260458066	0.607735486	0	0
1/11	0.260458066	0.607735486	0	0
6/159	0.261390107	0.60925724	0	0
2/37	0.26452529	0.613935675	0	0
2/37	0.26452529	0.613935675	0	0
2/37	0.26452529	0.613935675	0	0
3/66	0.264525339	0.613935675	0	0
3/67	0.271876085	0.615515267	0	0
9/260	0.272598217	0.615515267	0	0
4/98	0.273835981	0.615515267	0	0
14/429	0.273932492	0.615515267	0	0
2/38	0.274622394	0.615515267	0	0
2/38	0.274622394	0.615515267	0	0
2/38	0.274622394	0.615515267	0	0
6/162	0.275175578	0.615515267	0	0
5/130	0.276076827	0.615515267	0	0
5/130	0.276076827	0.615515267	0	0
3/68	0.279248504	0.615515267	0	0
1/12	0.280473654	0.615515267	0	0
1/12	0.280473654	0.615515267	0	0
1/12	0.280473654	0.615515267	0	0
1/12	0.280473654	0.615515267	0	0
1/12	0.280473654	0.615515267	0	0
1/12	0.280473654	0.615515267	0	0
1/12	0.280473654	0.615515267	0	0
1/12	0.280473654	0.615515267	0	0
1/12	0.280473654	0.615515267	0	0
1/12	0.280473654	0.615515267	0	0

1/12	0.280473654	0.615515267	0	0
1/12	0.280473654	0.615515267	0	0
1/12	0.280473654	0.615515267	0	0
1/12	0.280473654	0.615515267	0	0
1/12	0.280473654	0.615515267	0	0
1/12	0.280473654	0.615515267	0	0
1/12	0.280473654	0.615515267	0	0
1/12	0.280473654	0.615515267	0	0
1/12	0.280473654	0.615515267	0	0
1/12	0.280473654	0.615515267	0	0
1/12	0.280473654	0.615515267	0	0
1/12	0.280473654	0.615515267	0	0
1/12	0.280473654	0.615515267	0	0
1/12	0.280473654	0.615515267	0	0
1/12	0.280473654	0.615515267	0	0
1/12	0.280473654	0.615515267	0	0
1/12	0.280473654	0.615515267	0	0
1/12	0.280473654	0.615515267	0	0
1/12	0.280473654	0.615515267	0	0
1/12	0.280473654	0.615515267	0	0
1/12	0.280473654	0.615515267	0	0
1/12	0.280473654	0.615515267	0	0
1/12	0.280473654	0.615515267	0	0
1/12	0.280473654	0.615515267	0	0
1/12	0.280473654	0.615515267	0	0
1/12	0.280473654	0.615515267	0	0
1/12	0.280473654	0.615515267	0	0
1/12	0.280473654	0.615515267	0	0
1/12	0.280473654	0.615515267	0	0
1/12	0.280473654	0.615515267	0	0
1/12	0.280473654	0.615515267	0	0
1/12	0.280473654	0.615515267	0	0
1/12	0.280473654	0.615515267	0	0
5/131	0.281285108	0.616674401	0	0
2/39	0.28471239	0.619818873	0	0
2/39	0.28471239	0.619818873	0	0
2/39	0.28471239	0.619818873	0	0
2/39	0.28471239	0.619818873	0	0
2/39	0.28471239	0.619818873	0	0
2/39	0.28471239	0.619818873	0	0
2/39	0.28471239	0.619818873	0	0
4/100	0.285914937	0.621521179	0	0
3/69	0.286639304	0.621521179	0	0
3/69	0.286639304	0.621521179	0	0
4/101	0.291983539	0.621521179	0	0
3/70	0.29404527	0.621521179	0	0
3/70	0.29404527	0.621521179	0	0
3/70	0.29404527	0.621521179	0	0

3/70	0.29404527	0.621521179	0	0
3/70	0.29404527	0.621521179	0	0
3/70	0.29404527	0.621521179	0	0
2/40	0.294788285	0.621521179	0	0
2/40	0.294788285	0.621521179	0	0
2/40	0.294788285	0.621521179	0	0
2/40	0.294788285	0.621521179	0	0
2/40	0.294788285	0.621521179	0	0
2/40	0.294788285	0.621521179	0	0
2/40	0.294788285	0.621521179	0	0
4/102	0.298069132	0.621521179	0	0
6/167	0.298511197	0.621521179	0	0
1/13	0.299948497	0.621521179	0	0
1/13	0.299948497	0.621521179	0	0
1/13	0.299948497	0.621521179	0	0
1/13	0.299948497	0.621521179	0	0
1/13	0.299948497	0.621521179	0	0
1/13	0.299948497	0.621521179	0	0
1/13	0.299948497	0.621521179	0	0
1/13	0.299948497	0.621521179	0	0
1/13	0.299948497	0.621521179	0	0
1/13	0.299948497	0.621521179	0	0
1/13	0.299948497	0.621521179	0	0
1/13	0.299948497	0.621521179	0	0
1/13	0.299948497	0.621521179	0	0
1/13	0.299948497	0.621521179	0	0
1/13	0.299948497	0.621521179	0	0
1/13	0.299948497	0.621521179	0	0
1/13	0.299948497	0.621521179	0	0
1/13	0.299948497	0.621521179	0	0
1/13	0.299948497	0.621521179	0	0
1/13	0.299948497	0.621521179	0	0
1/13	0.299948497	0.621521179	0	0
1/13	0.299948497	0.621521179	0	0
1/13	0.299948497	0.621521179	0	0
1/13	0.299948497	0.621521179	0	0
1/13	0.299948497	0.621521179	0	0
1/13	0.299948497	0.621521179	0	0
1/13	0.299948497	0.621521179	0	0
1/13	0.299948497	0.621521179	0	0
1/13	0.299948497	0.621521179	0	0
1/13	0.299948497	0.621521179	0	0
1/13	0.299948497	0.621521179	0	0
1/13	0.299948497	0.621521179	0	0
3/71	0.301463258	0.621521179	0	0
3/71	0.301463258	0.621521179	0	0
5/135	0.302297509	0.621521179	0	0
7/201	0.30240441	0.621521179	0	0
6/168	0.30322475	0.621521179	0	0
2/41	0.304843471	0.621521179	0	0

2/41	0.304843471	0.621521179	0	0
2/41	0.304843471	0.621521179	0	0
2/41	0.304843471	0.621521179	0	0
2/41	0.304843471	0.621521179	0	0
2/41	0.304843471	0.621521179	0	0
2/41	0.304843471	0.621521179	0	0
2/41	0.304843471	0.621521179	0	0
2/41	0.304843471	0.621521179	0	0
2/41	0.304843471	0.621521179	0	0
2/41	0.304843471	0.621521179	0	0
3/72	0.308890201	0.621521179	0	0
3/72	0.308890201	0.621521179	0	0
3/72	0.308890201	0.621521179	0	0
9/270	0.309262004	0.621521179	0	0
5/137	0.312894801	0.621521179	0	0
2/42	0.314871703	0.621521179	0	0
2/42	0.314871703	0.621521179	0	0
2/42	0.314871703	0.621521179	0	0
2/42	0.314871703	0.621521179	0	0
3/73	0.316323106	0.621521179	0	0
3/73	0.316323106	0.621521179	0	0
4/105	0.316410055	0.621521179	0	0
1/14	0.318897178	0.621521179	0	0
1/14	0.318897178	0.621521179	0	0
1/14	0.318897178	0.621521179	0	0
1/14	0.318897178	0.621521179	0	0
1/14	0.318897178	0.621521179	0	0
1/14	0.318897178	0.621521179	0	0
1/14	0.318897178	0.621521179	0	0
1/14	0.318897178	0.621521179	0	0
1/14	0.318897178	0.621521179	0	0
1/14	0.318897178	0.621521179	0	0
1/14	0.318897178	0.621521179	0	0
1/14	0.318897178	0.621521179	0	0
1/14	0.318897178	0.621521179	0	0
1/14	0.318897178	0.621521179	0	0
1/14	0.318897178	0.621521179	0	0
1/14	0.318897178	0.621521179	0	0
1/14	0.318897178	0.621521179	0	0
1/14	0.318897178	0.621521179	0	0
1/14	0.318897178	0.621521179	0	0
1/14	0.318897178	0.621521179	0	0
1/14	0.318897178	0.621521179	0	0
1/14	0.318897178	0.621521179	0	0
1/14	0.318897178	0.621521179	0	0
1/14	0.318897178	0.621521179	0	0
1/14	0.318897178	0.621521179	0	0
1/14	0.318897178	0.621521179	0	0

1/14	0.318897178	0.621521179	0	0
1/14	0.318897178	0.621521179	0	0
1/14	0.318897178	0.621521179	0	0
1/14	0.318897178	0.621521179	0	0
1/14	0.318897178	0.621521179	0	0
1/14	0.318897178	0.621521179	0	0
1/14	0.318897178	0.621521179	0	0
1/14	0.318897178	0.621521179	0	0
1/14	0.318897178	0.621521179	0	0
1/14	0.318897178	0.621521179	0	0
1/14	0.318897178	0.621521179	0	0
1/14	0.318897178	0.621521179	0	0
1/14	0.318897178	0.621521179	0	0
1/14	0.318897178	0.621521179	0	0
6/172	0.322206014	0.627408311	0	0
2/43	0.324867092	0.628095612	0	0
2/43	0.324867092	0.628095612	0	0
2/43	0.324867092	0.628095612	0	0
2/43	0.324867092	0.628095612	0	0
2/43	0.324867092	0.628095612	0	0
2/43	0.324867092	0.628095612	0	0
2/43	0.324867092	0.628095612	0	0
2/43	0.324867092	0.628095612	0	0
15/482	0.326134101	0.629985747	0	0
2/44	0.334824082	0.631993003	0	0
2/44	0.334824082	0.631993003	0	0
2/44	0.334824082	0.631993003	0	0
2/44	0.334824082	0.631993003	0	0
6/175	0.336552388	0.631993003	0	0
1/15	0.337333886	0.631993003	0	0
1/15	0.337333886	0.631993003	0	0
1/15	0.337333886	0.631993003	0	0
1/15	0.337333886	0.631993003	0	0
1/15	0.337333886	0.631993003	0	0
1/15	0.337333886	0.631993003	0	0
1/15	0.337333886	0.631993003	0	0
1/15	0.337333886	0.631993003	0	0
1/15	0.337333886	0.631993003	0	0
1/15	0.337333886	0.631993003	0	0
1/15	0.337333886	0.631993003	0	0
1/15	0.337333886	0.631993003	0	0
1/15	0.337333886	0.631993003	0	0
1/15	0.337333886	0.631993003	0	0
1/15	0.337333886	0.631993003	0	0
1/15	0.337333886	0.631993003	0	0
1/15	0.337333886	0.631993003	0	0
1/15	0.337333886	0.631993003	0	0
1/15	0.337333886	0.631993003	0	0

1/15	0.337333886	0.631993003	0	0
1/15	0.337333886	0.631993003	0	0
1/15	0.337333886	0.631993003	0	0
1/15	0.337333886	0.631993003	0	0
1/15	0.337333886	0.631993003	0	0
1/15	0.337333886	0.631993003	0	0
1/15	0.337333886	0.631993003	0	0
1/15	0.337333886	0.631993003	0	0
1/15	0.337333886	0.631993003	0	0
1/15	0.337333886	0.631993003	0	0
1/15	0.337333886	0.631993003	0	0
3/76	0.338628824	0.63387356	0	0
4/109	0.340996456	0.637209687	0	0
4/109	0.340996456	0.637209687	0	0
2/45	0.344737444	0.639196758	0	0
2/45	0.344737444	0.639196758	0	0
2/45	0.344737444	0.639196758	0	0
8/245	0.344792621	0.639196758	0	0
3/77	0.346057185	0.639196758	0	0
3/77	0.346057185	0.639196758	0	0
3/77	0.346057185	0.639196758	0	0
16/525	0.348441501	0.639196758	0	0
10/316	0.351911594	0.639196758	0	0
4/111	0.353316196	0.639196758	0	0
4/111	0.353316196	0.639196758	0	0
4/111	0.353316196	0.639196758	0	0
3/78	0.353477691	0.639196758	0	0
2/46	0.354602256	0.639196758	0	0
2/46	0.354602256	0.639196758	0	0
2/46	0.354602256	0.639196758	0	0
2/46	0.354602256	0.639196758	0	0
2/46	0.354602256	0.639196758	0	0
1/16	0.355272429	0.639196758	0	0
1/16	0.355272429	0.639196758	0	0
1/16	0.355272429	0.639196758	0	0
1/16	0.355272429	0.639196758	0	0
1/16	0.355272429	0.639196758	0	0
1/16	0.355272429	0.639196758	0	0
1/16	0.355272429	0.639196758	0	0
1/16	0.355272429	0.639196758	0	0
1/16	0.355272429	0.639196758	0	0
1/16	0.355272429	0.639196758	0	0
1/16	0.355272429	0.639196758	0	0
1/16	0.355272429	0.639196758	0	0
1/16	0.355272429	0.639196758	0	0
1/16	0.355272429	0.639196758	0	0
1/16	0.355272429	0.639196758	0	0
1/16	0.355272429	0.639196758	0	0

1/16	0.355272429	0.639196758	0	0
1/16	0.355272429	0.639196758	0	0
1/16	0.355272429	0.639196758	0	0
1/16	0.355272429	0.639196758	0	0
1/16	0.355272429	0.639196758	0	0
1/16	0.355272429	0.639196758	0	0
1/16	0.355272429	0.639196758	0	0
1/16	0.355272429	0.639196758	0	0
1/16	0.355272429	0.639196758	0	0
1/16	0.355272429	0.639196758	0	0
1/16	0.355272429	0.639196758	0	0
21/705	0.356262973	0.640449621	0	0
4/112	0.359477107	0.642967536	0	0
3/79	0.360887807	0.642967536	0	0
3/79	0.360887807	0.642967536	0	0
5/146	0.361051472	0.642967536	0	0
2/47	0.364413895	0.642967536	0	0
3/80	0.368285073	0.642967536	0	0
1/17	0.372726243	0.642967536	0	0
1/17	0.372726243	0.642967536	0	0
1/17	0.372726243	0.642967536	0	0
1/17	0.372726243	0.642967536	0	0
1/17	0.372726243	0.642967536	0	0
1/17	0.372726243	0.642967536	0	0
1/17	0.372726243	0.642967536	0	0
1/17	0.372726243	0.642967536	0	0
1/17	0.372726243	0.642967536	0	0
1/17	0.372726243	0.642967536	0	0
1/17	0.372726243	0.642967536	0	0
1/17	0.372726243	0.642967536	0	0
1/17	0.372726243	0.642967536	0	0
1/17	0.372726243	0.642967536	0	0
1/17	0.372726243	0.642967536	0	0
1/17	0.372726243	0.642967536	0	0
1/17	0.372726243	0.642967536	0	0
1/17	0.372726243	0.642967536	0	0
1/17	0.372726243	0.642967536	0	0
1/17	0.372726243	0.642967536	0	0
1/17	0.372726243	0.642967536	0	0
1/17	0.372726243	0.642967536	0	0
1/17	0.372726243	0.642967536	0	0
1/17	0.372726243	0.642967536	0	0
1/17	0.372726243	0.642967536	0	0
1/17	0.372726243	0.642967536	0	0
1/17	0.372726243	0.642967536	0	0
1/17	0.372726243	0.642967536	0	0
1/17	0.372726243	0.642967536	0	0
1/17	0.372726243	0.642967536	0	0

1/17	0.372726243	0.642967536	0	0
1/17	0.372726243	0.642967536	0	0
1/17	0.372726243	0.642967536	0	0
1/17	0.372726243	0.642967536	0	0
1/17	0.372726243	0.642967536	0	0
1/17	0.372726243	0.642967536	0	0
1/17	0.372726243	0.642967536	0	0
1/17	0.372726243	0.642967536	0	0
1/17	0.372726243	0.642967536	0	0
1/17	0.372726243	0.642967536	0	0
1/17	0.372726243	0.642967536	0	0
1/17	0.372726243	0.642967536	0	0
1/17	0.372726243	0.642967536	0	0
1/17	0.372726243	0.642967536	0	0
1/17	0.372726243	0.642967536	0	0
2/48	0.374168022	0.644433374	0	0
2/48	0.374168022	0.644433374	0	0
3/81	0.375667104	0.646503782	0	0
5/149	0.37718223	0.646764987	0	0
15/500	0.377971243	0.646764987	0	0
6/184	0.379928975	0.646764987	0	0
2/49	0.383860571	0.646764987	0	0
2/49	0.383860571	0.646764987	0	0
2/49	0.383860571	0.646764987	0	0
2/49	0.383860571	0.646764987	0	0
2/49	0.383860571	0.646764987	0	0
6/186	0.389597254	0.646764987	0	0
1/18	0.389708403	0.646764987	0	0
1/18	0.389708403	0.646764987	0	0
1/18	0.389708403	0.646764987	0	0
1/18	0.389708403	0.646764987	0	0
1/18	0.389708403	0.646764987	0	0
1/18	0.389708403	0.646764987	0	0
1/18	0.389708403	0.646764987	0	0
1/18	0.389708403	0.646764987	0	0
1/18	0.389708403	0.646764987	0	0
1/18	0.389708403	0.646764987	0	0
1/18	0.389708403	0.646764987	0	0
1/18	0.389708403	0.646764987	0	0
1/18	0.389708403	0.646764987	0	0
1/18	0.389708403	0.646764987	0	0
1/18	0.389708403	0.646764987	0	0
1/18	0.389708403	0.646764987	0	0
1/18	0.389708403	0.646764987	0	0
1/18	0.389708403	0.646764987	0	0
1/18	0.389708403	0.646764987	0	0
1/18	0.389708403	0.646764987	0	0
1/18	0.389708403	0.646764987	0	0

1/18	0.389708403	0.646764987	0	0
1/18	0.389708403	0.646764987	0	0
1/18	0.389708403	0.646764987	0	0
1/18	0.389708403	0.646764987	0	0
1/18	0.389708403	0.646764987	0	0
1/18	0.389708403	0.646764987	0	0
1/18	0.389708403	0.646764987	0	0
1/18	0.389708403	0.646764987	0	0
1/18	0.389708403	0.646764987	0	0
1/18	0.389708403	0.646764987	0	0
1/18	0.389708403	0.646764987	0	0
1/18	0.389708403	0.646764987	0	0
1/18	0.389708403	0.646764987	0	0
1/18	0.389708403	0.646764987	0	0
1/18	0.389708403	0.646764987	0	0
1/18	0.389708403	0.646764987	0	0
4/117	0.390231472	0.646764987	0	0
4/117	0.390231472	0.646764987	0	0
3/83	0.390376295	0.646764987	0	0
2/50	0.393487737	0.649314436	0	0
2/50	0.393487737	0.649314436	0	0
2/50	0.393487737	0.649314436	0	0
13/434	0.393503556	0.649314436	0	0
8/257	0.393926568	0.649314436	0	0
4/119	0.402482872	0.649314436	0	0
2/51	0.403045966	0.649314436	0	0
2/51	0.403045966	0.649314436	0	0
2/51	0.403045966	0.649314436	0	0
2/51	0.403045966	0.649314436	0	0
2/51	0.403045966	0.649314436	0	0
3/85	0.404997783	0.649314436	0	0
3/85	0.404997783	0.649314436	0	0
3/85	0.404997783	0.649314436	0	0
3/85	0.404997783	0.649314436	0	0
3/85	0.404997783	0.649314436	0	0
1/19	0.406231632	0.649314436	0	0
1/19	0.406231632	0.649314436	0	0
1/19	0.406231632	0.649314436	0	0
1/19	0.406231632	0.649314436	0	0
1/19	0.406231632	0.649314436	0	0
1/19	0.406231632	0.649314436	0	0
1/19	0.406231632	0.649314436	0	0
1/19	0.406231632	0.649314436	0	0
1/19	0.406231632	0.649314436	0	0
1/19	0.406231632	0.649314436	0	0
1/19	0.406231632	0.649314436	0	0
1/19	0.406231632	0.649314436	0	0
1/19	0.406231632	0.649314436	0	0

1/19	0.406231632	0.649314436	0	0
1/19	0.406231632	0.649314436	0	0
1/19	0.406231632	0.649314436	0	0
1/19	0.406231632	0.649314436	0	0
1/19	0.406231632	0.649314436	0	0
1/19	0.406231632	0.649314436	0	0
1/19	0.406231632	0.649314436	0	0
1/19	0.406231632	0.649314436	0	0
1/19	0.406231632	0.649314436	0	0
1/19	0.406231632	0.649314436	0	0
1/19	0.406231632	0.649314436	0	0
1/19	0.406231632	0.649314436	0	0
1/19	0.406231632	0.649314436	0	0
1/19	0.406231632	0.649314436	0	0
1/19	0.406231632	0.649314436	0	0
1/19	0.406231632	0.649314436	0	0
1/19	0.406231632	0.649314436	0	0
1/19	0.406231632	0.649314436	0	0
1/19	0.406231632	0.649314436	0	0
3/86	0.412270458	0.653422309	0	0
3/86	0.412270458	0.653422309	0	0
2/52	0.41253194	0.653422309	0	0
2/52	0.41253194	0.653422309	0	0
2/52	0.41253194	0.653422309	0	0
2/52	0.41253194	0.653422309	0	0
3/87	0.419515134	0.653422309	0	0
4/122	0.420771996	0.653422309	0	0
4/122	0.420771996	0.653422309	0	0
7/228	0.421053567	0.653422309	0	0
2/53	0.421942576	0.653422309	0	0
2/53	0.421942576	0.653422309	0	0
2/53	0.421942576	0.653422309	0	0
2/53	0.421942576	0.653422309	0	0
1/20	0.422308309	0.653422309	0	0
1/20	0.422308309	0.653422309	0	0
1/20	0.422308309	0.653422309	0	0
1/20	0.422308309	0.653422309	0	0
1/20	0.422308309	0.653422309	0	0
1/20	0.422308309	0.653422309	0	0
1/20	0.422308309	0.653422309	0	0
1/20	0.422308309	0.653422309	0	0
1/20	0.422308309	0.653422309	0	0
1/20	0.422308309	0.653422309	0	0
1/20	0.422308309	0.653422309	0	0
1/20	0.422308309	0.653422309	0	0
1/20	0.422308309	0.653422309	0	0
1/20	0.422308309	0.653422309	0	0
1/20	0.422308309	0.653422309	0	0

1/20	0.422308309	0.653422309	0	0
1/20	0.422308309	0.653422309	0	0
1/20	0.422308309	0.653422309	0	0
1/20	0.422308309	0.653422309	0	0
1/20	0.422308309	0.653422309	0	0
1/20	0.422308309	0.653422309	0	0
1/20	0.422308309	0.653422309	0	0
1/20	0.422308309	0.653422309	0	0
1/20	0.422308309	0.653422309	0	0
1/20	0.422308309	0.653422309	0	0
1/20	0.422308309	0.653422309	0	0
1/20	0.422308309	0.653422309	0	0
1/20	0.422308309	0.653422309	0	0
1/20	0.422308309	0.653422309	0	0
1/20	0.422308309	0.653422309	0	0
1/20	0.422308309	0.653422309	0	0
10/336	0.424508818	0.65636058	0	0
3/88	0.426729935	0.658561469	0	0
3/88	0.426729935	0.658561469	0	0
4/123	0.426839794	0.658561469	0	0
2/54	0.431275004	0.6616365	0	0
2/54	0.431275004	0.6616365	0	0
2/54	0.431275004	0.6616365	0	0
2/54	0.431275004	0.6616365	0	0
2/54	0.431275004	0.6616365	0	0
2/54	0.431275004	0.6616365	0	0
2/54	0.431275004	0.6616365	0	0
2/54	0.431275004	0.6616365	0	0
4/124	0.432891261	0.6616365	0	0
4/124	0.432891261	0.6616365	0	0
4/124	0.432891261	0.6616365	0	0
3/89	0.433913052	0.6616365	0	0
3/89	0.433913052	0.6616365	0	0
7/231	0.434297856	0.6616365	0	0
1/21	0.437950481	0.6616365	0	0
1/21	0.437950481	0.6616365	0	0
1/21	0.437950481	0.6616365	0	0
1/21	0.437950481	0.6616365	0	0
1/21	0.437950481	0.6616365	0	0
1/21	0.437950481	0.6616365	0	0
1/21	0.437950481	0.6616365	0	0
1/21	0.437950481	0.6616365	0	0
1/21	0.437950481	0.6616365	0	0
1/21	0.437950481	0.6616365	0	0
1/21	0.437950481	0.6616365	0	0
1/21	0.437950481	0.6616365	0	0
1/21	0.437950481	0.6616365	0	0
1/21	0.437950481	0.6616365	0	0

1/21	0.437950481	0.6616365	0	0
1/21	0.437950481	0.6616365	0	0
7/232	0.438703905	0.662315118	0	0
2/55	0.440526567	0.663686047	0	0
2/55	0.440526567	0.663686047	0	0
2/55	0.440526567	0.663686047	0	0
3/90	0.441062747	0.664034302	0	0
3/91	0.448177348	0.670211146	0	0
3/91	0.448177348	0.670211146	0	0
2/56	0.449694809	0.670211146	0	0
2/56	0.449694809	0.670211146	0	0
2/56	0.449694809	0.670211146	0	0
4/127	0.450936012	0.670211146	0	0
1/22	0.45316987	0.670211146	0	0
1/22	0.45316987	0.670211146	0	0
1/22	0.45316987	0.670211146	0	0
1/22	0.45316987	0.670211146	0	0
1/22	0.45316987	0.670211146	0	0
1/22	0.45316987	0.670211146	0	0
1/22	0.45316987	0.670211146	0	0
1/22	0.45316987	0.670211146	0	0
1/22	0.45316987	0.670211146	0	0
1/22	0.45316987	0.670211146	0	0
1/22	0.45316987	0.670211146	0	0
1/22	0.45316987	0.670211146	0	0
1/22	0.45316987	0.670211146	0	0
1/22	0.45316987	0.670211146	0	0
1/22	0.45316987	0.670211146	0	0
1/22	0.45316987	0.670211146	0	0
1/22	0.45316987	0.670211146	0	0
1/22	0.45316987	0.670211146	0	0
1/22	0.45316987	0.670211146	0	0
1/22	0.45316987	0.670211146	0	0
3/92	0.45525525	0.672838207	0	0
2/57	0.458777463	0.677583811	0	0
3/93	0.462294912	0.6800987	0	0
3/93	0.462294912	0.6800987	0	0
2/58	0.467772446	0.6800987	0	0
2/58	0.467772446	0.6800987	0	0
1/23	0.467977883	0.6800987	0	0
1/23	0.467977883	0.6800987	0	0
1/23	0.467977883	0.6800987	0	0
1/23	0.467977883	0.6800987	0	0
1/23	0.467977883	0.6800987	0	0
1/23	0.467977883	0.6800987	0	0
1/23	0.467977883	0.6800987	0	0
1/23	0.467977883	0.6800987	0	0
1/23	0.467977883	0.6800987	0	0

1/23	0.467977883	0.6800987	0	0
1/23	0.467977883	0.6800987	0	0
1/23	0.467977883	0.6800987	0	0
1/23	0.467977883	0.6800987	0	0
1/23	0.467977883	0.6800987	0	0
1/23	0.467977883	0.6800987	0	0
1/23	0.467977883	0.6800987	0	0
1/23	0.467977883	0.6800987	0	0
1/23	0.467977883	0.6800987	0	0
1/23	0.467977883	0.6800987	0	0
1/23	0.467977883	0.6800987	0	0
7/240	0.473723568	0.687989464	0	0
6/204	0.47584732	0.69043758	0	0
3/95	0.476253679	0.69043758	0	0
2/59	0.476677851	0.69043758	0	0
2/59	0.476677851	0.69043758	0	0
4/132	0.480579646	0.690890456	0	0
1/24	0.482385619	0.690890456	0	0
1/24	0.482385619	0.690890456	0	0
1/24	0.482385619	0.690890456	0	0
1/24	0.482385619	0.690890456	0	0
1/24	0.482385619	0.690890456	0	0
1/24	0.482385619	0.690890456	0	0
1/24	0.482385619	0.690890456	0	0
1/24	0.482385619	0.690890456	0	0
1/24	0.482385619	0.690890456	0	0
1/24	0.482385619	0.690890456	0	0
1/24	0.482385619	0.690890456	0	0
1/24	0.482385619	0.690890456	0	0
1/24	0.482385619	0.690890456	0	0
1/24	0.482385619	0.690890456	0	0
1/24	0.482385619	0.690890456	0	0
1/24	0.482385619	0.690890456	0	0
3/96	0.48317002	0.691104556	0	0
3/96	0.48317002	0.691104556	0	0
2/60	0.485491937	0.692605469	0	0
2/60	0.485491937	0.692605469	0	0
2/60	0.485491937	0.692605469	0	0
2/60	0.485491937	0.692605469	0	0
3/97	0.490042593	0.695860428	0	0
2/61	0.494213121	0.695860428	0	0
2/61	0.494213121	0.695860428	0	0
1/25	0.496403879	0.695860428	0	0
1/25	0.496403879	0.695860428	0	0
1/25	0.496403879	0.695860428	0	0
1/25	0.496403879	0.695860428	0	0
1/25	0.496403879	0.695860428	0	0
1/25	0.496403879	0.695860428	0	0

1/25	0.496403879	0.695860428	0	0
1/25	0.496403879	0.695860428	0	0
1/25	0.496403879	0.695860428	0	0
1/25	0.496403879	0.695860428	0	0
1/25	0.496403879	0.695860428	0	0
1/25	0.496403879	0.695860428	0	0
1/25	0.496403879	0.695860428	0	0
1/25	0.496403879	0.695860428	0	0
1/25	0.496403879	0.695860428	0	0
1/25	0.496403879	0.695860428	0	0
1/25	0.496403879	0.695860428	0	0
1/25	0.496403879	0.695860428	0	0
1/25	0.496403879	0.695860428	0	0
1/25	0.496403879	0.695860428	0	0
1/25	0.496403879	0.695860428	0	0
1/25	0.496403879	0.695860428	0	0
1/25	0.496403879	0.695860428	0	0
1/25	0.496403879	0.695860428	0	0
3/98	0.496870167	0.696065864	0	0
2/62	0.502839973	0.703071691	0	0
2/62	0.502839973	0.703071691	0	0
2/62	0.502839973	0.703071691	0	0
3/99	0.503651571	0.703626297	0	0
5/173	0.503883049	0.703626297	0	0
4/137	0.509577677	0.707688964	0	0
1/26	0.510043171	0.707688964	0	0
1/26	0.510043171	0.707688964	0	0
1/26	0.510043171	0.707688964	0	0
1/26	0.510043171	0.707688964	0	0
1/26	0.510043171	0.707688964	0	0
1/26	0.510043171	0.707688964	0	0
1/26	0.510043171	0.707688964	0	0
1/26	0.510043171	0.707688964	0	0
1/26	0.510043171	0.707688964	0	0
2/63	0.51137121	0.708628341	0	0
2/63	0.51137121	0.708628341	0	0
3/101	0.517071467	0.713461886	0	0
2/64	0.519805684	0.713461886	0	0
2/64	0.519805684	0.713461886	0	0
1/27	0.523313723	0.713461886	0	0
1/27	0.523313723	0.713461886	0	0
1/27	0.523313723	0.713461886	0	0
1/27	0.523313723	0.713461886	0	0
1/27	0.523313723	0.713461886	0	0
1/27	0.523313723	0.713461886	0	0
1/27	0.523313723	0.713461886	0	0
1/27	0.523313723	0.713461886	0	0
1/27	0.523313723	0.713461886	0	0

1/27	0.523313723	0.713461886	0	0
1/27	0.523313723	0.713461886	0	0
1/27	0.523313723	0.713461886	0	0
1/27	0.523313723	0.713461886	0	0
1/27	0.523313723	0.713461886	0	0
1/27	0.523313723	0.713461886	0	0
1/27	0.523313723	0.713461886	0	0
1/27	0.523313723	0.713461886	0	0
1/27	0.523313723	0.713461886	0	0
1/27	0.523313723	0.713461886	0	0
1/27	0.523313723	0.713461886	0	0
1/27	0.523313723	0.713461886	0	0
1/27	0.523313723	0.713461886	0	0
3/102	0.523707898	0.713461886	0	0
3/102	0.523707898	0.713461886	0	0
2/65	0.528142379	0.718603725	0	0
2/65	0.528142379	0.718603725	0	0
3/103	0.530294033	0.721080644	0	0
1/28	0.536225486	0.722297085	0	0
1/28	0.536225486	0.722297085	0	0
1/28	0.536225486	0.722297085	0	0
1/28	0.536225486	0.722297085	0	0
1/28	0.536225486	0.722297085	0	0
1/28	0.536225486	0.722297085	0	0
1/28	0.536225486	0.722297085	0	0
1/28	0.536225486	0.722297085	0	0
1/28	0.536225486	0.722297085	0	0
1/28	0.536225486	0.722297085	0	0
1/28	0.536225486	0.722297085	0	0
1/28	0.536225486	0.722297085	0	0
2/66	0.536380407	0.722297085	0	0
2/66	0.536380407	0.722297085	0	0
2/66	0.536380407	0.722297085	0	0
3/104	0.536828977	0.722297085	0	0
3/104	0.536828977	0.722297085	0	0
9/330	0.537320965	0.722512502	0	0
3/105	0.543311883	0.727287337	0	0
2/67	0.544518997	0.727287337	0	0
2/67	0.544518997	0.727287337	0	0
2/67	0.544518997	0.727287337	0	0
1/29	0.548788142	0.727287337	0	0
1/29	0.548788142	0.727287337	0	0
1/29	0.548788142	0.727287337	0	0
1/29	0.548788142	0.727287337	0	0
1/29	0.548788142	0.727287337	0	0
1/29	0.548788142	0.727287337	0	0
1/29	0.548788142	0.727287337	0	0
1/29	0.548788142	0.727287337	0	0

1/29	0.548788142	0.727287337	0	0
1/29	0.548788142	0.727287337	0	0
1/29	0.548788142	0.727287337	0	0
1/29	0.548788142	0.727287337	0	0
1/29	0.548788142	0.727287337	0	0
1/29	0.548788142	0.727287337	0	0
1/29	0.548788142	0.727287337	0	0
1/29	0.548788142	0.727287337	0	0
1/29	0.548788142	0.727287337	0	0
1/29	0.548788142	0.727287337	0	0
4/144	0.5488898	0.727287337	0	0
4/144	0.5488898	0.727287337	0	0
3/106	0.549741956	0.727973381	0	0
2/68	0.552557492	0.731256936	0	0
4/145	0.554370994	0.733211212	0	0
2/69	0.560495343	0.735735658	0	0
2/69	0.560495343	0.735735658	0	0
2/69	0.560495343	0.735735658	0	0
1/30	0.561011113	0.735735658	0	0
1/30	0.561011113	0.735735658	0	0
1/30	0.561011113	0.735735658	0	0
1/30	0.561011113	0.735735658	0	0
1/30	0.561011113	0.735735658	0	0
1/30	0.561011113	0.735735658	0	0
1/30	0.561011113	0.735735658	0	0
1/30	0.561011113	0.735735658	0	0
1/30	0.561011113	0.735735658	0	0
1/30	0.561011113	0.735735658	0	0
1/30	0.561011113	0.735735658	0	0
2/70	0.568332105	0.74018461	0	0
3/109	0.568707949	0.74018461	0	0
1/31	0.572903568	0.74018461	0	0
1/31	0.572903568	0.74018461	0	0
1/31	0.572903568	0.74018461	0	0
1/31	0.572903568	0.74018461	0	0
1/31	0.572903568	0.74018461	0	0
1/31	0.572903568	0.74018461	0	0
1/31	0.572903568	0.74018461	0	0
1/31	0.572903568	0.74018461	0	0
1/31	0.572903568	0.74018461	0	0
1/31	0.572903568	0.74018461	0	0
1/31	0.572903568	0.74018461	0	0
1/31	0.572903568	0.74018461	0	0
1/31	0.572903568	0.74018461	0	0
1/31	0.572903568	0.74018461	0	0
1/31	0.572903568	0.74018461	0	0
1/31	0.572903568	0.74018461	0	0
1/31	0.572903568	0.74018461	0	0

1/31	0.572903568	0.74018461	0	0
1/31	0.572903568	0.74018461	0	0
1/31	0.572903568	0.74018461	0	0
1/31	0.572903568	0.74018461	0	0
1/31	0.572903568	0.74018461	0	0
1/31	0.572903568	0.74018461	0	0
3/110	0.574919698	0.741631457	0	0
2/71	0.576067429	0.741631457	0	0
2/71	0.576067429	0.741631457	0	0
2/71	0.576067429	0.741631457	0	0
2/71	0.576067429	0.741631457	0	0
2/71	0.576067429	0.741631457	0	0
10/379	0.576707166	0.742016254	0	0
5/188	0.577803187	0.742987323	0	0
6/227	0.580054721	0.744529449	0	0
3/111	0.581075346	0.744529449	0	0
2/72	0.583701059	0.744529449	0	0
2/72	0.583701059	0.744529449	0	0
1/32	0.584474428	0.744529449	0	0
1/32	0.584474428	0.744529449	0	0
1/32	0.584474428	0.744529449	0	0
1/32	0.584474428	0.744529449	0	0
1/32	0.584474428	0.744529449	0	0
1/32	0.584474428	0.744529449	0	0
1/32	0.584474428	0.744529449	0	0
1/32	0.584474428	0.744529449	0	0
1/32	0.584474428	0.744529449	0	0
1/32	0.584474428	0.744529449	0	0
1/32	0.584474428	0.744529449	0	0
1/32	0.584474428	0.744529449	0	0
3/112	0.587174376	0.747531355	0	0
2/73	0.591232827	0.748360864	0	0
2/73	0.591232827	0.748360864	0	0
3/113	0.59321631	0.748360864	0	0
14/537	0.594307994	0.748360864	0	0
1/33	0.595732373	0.748360864	0	0
1/33	0.595732373	0.748360864	0	0
1/33	0.595732373	0.748360864	0	0
1/33	0.595732373	0.748360864	0	0
1/33	0.595732373	0.748360864	0	0
1/33	0.595732373	0.748360864	0	0
1/33	0.595732373	0.748360864	0	0
1/33	0.595732373	0.748360864	0	0
1/33	0.595732373	0.748360864	0	0
1/33	0.595732373	0.748360864	0	0
1/33	0.595732373	0.748360864	0	0
1/33	0.595732373	0.748360864	0	0
1/33	0.595732373	0.748360864	0	0

1/33	0.595732373	0.748360864	0	0
1/33	0.595732373	0.748360864	0	0
1/33	0.595732373	0.748360864	0	0
1/33	0.595732373	0.748360864	0	0
1/33	0.595732373	0.748360864	0	0
1/33	0.595732373	0.748360864	0	0
5/192	0.596512746	0.748909024	0	0
2/74	0.598662649	0.750984426	0	0
3/114	0.599200711	0.750984426	0	0
3/114	0.599200711	0.750984426	0	0
2/75	0.605990518	0.754286179	0	0
2/75	0.605990518	0.754286179	0	0
2/75	0.605990518	0.754286179	0	0
2/75	0.605990518	0.754286179	0	0
1/34	0.606685852	0.754286179	0	0
1/34	0.606685852	0.754286179	0	0
1/34	0.606685852	0.754286179	0	0
1/34	0.606685852	0.754286179	0	0
1/34	0.606685852	0.754286179	0	0
1/34	0.606685852	0.754286179	0	0
1/34	0.606685852	0.754286179	0	0
1/34	0.606685852	0.754286179	0	0
1/34	0.606685852	0.754286179	0	0
1/34	0.606685852	0.754286179	0	0
4/155	0.607141948	0.754422387	0	0
2/76	0.613216504	0.761016926	0	0
3/117	0.616804936	0.761016926	0	0
1/35	0.617343082	0.761016926	0	0
1/35	0.617343082	0.761016926	0	0
1/35	0.617343082	0.761016926	0	0
1/35	0.617343082	0.761016926	0	0
1/35	0.617343082	0.761016926	0	0
1/35	0.617343082	0.761016926	0	0
1/35	0.617343082	0.761016926	0	0
1/35	0.617343082	0.761016926	0	0
1/35	0.617343082	0.761016926	0	0
1/35	0.617343082	0.761016926	0	0
1/35	0.617343082	0.761016926	0	0
1/35	0.617343082	0.761016926	0	0
2/77	0.620340747	0.762984072	0	0
2/77	0.620340747	0.762984072	0	0
2/77	0.620340747	0.762984072	0	0
2/77	0.620340747	0.762984072	0	0
3/118	0.622555612	0.764844	0	0
3/118	0.622555612	0.764844	0	0
2/78	0.627363454	0.765133909	0	0
2/78	0.627363454	0.765133909	0	0
1/36	0.627712063	0.765133909	0	0

1/36	0.627712063	0.765133909	0	0
1/36	0.627712063	0.765133909	0	0
1/36	0.627712063	0.765133909	0	0
1/36	0.627712063	0.765133909	0	0
1/36	0.627712063	0.765133909	0	0
1/36	0.627712063	0.765133909	0	0
1/36	0.627712063	0.765133909	0	0
1/36	0.627712063	0.765133909	0	0
1/36	0.627712063	0.765133909	0	0
1/36	0.627712063	0.765133909	0	0
1/36	0.627712063	0.765133909	0	0
2/79	0.634284893	0.772713046	0	0
1/37	0.637800576	0.773533066	0	0
1/37	0.637800576	0.773533066	0	0
1/37	0.637800576	0.773533066	0	0
1/37	0.637800576	0.773533066	0	0
1/37	0.637800576	0.773533066	0	0
1/37	0.637800576	0.773533066	0	0
1/37	0.637800576	0.773533066	0	0
1/37	0.637800576	0.773533066	0	0
3/122	0.64496488	0.780041914	0	0
7/283	0.647285384	0.780041914	0	0
18/711	0.647454544	0.780041914	0	0
1/38	0.647616194	0.780041914	0	0
1/38	0.647616194	0.780041914	0	0
1/38	0.647616194	0.780041914	0	0
1/38	0.647616194	0.780041914	0	0
1/38	0.647616194	0.780041914	0	0
1/38	0.647616194	0.780041914	0	0
1/38	0.647616194	0.780041914	0	0
1/38	0.647616194	0.780041914	0	0
2/81	0.647825347	0.780041914	0	0
2/81	0.647825347	0.780041914	0	0
3/123	0.650418003	0.782298338	0	0
3/123	0.650418003	0.782298338	0	0
5/205	0.653969737	0.78263184	0	0
3/124	0.655811223	0.78263184	0	0
1/39	0.657166285	0.78263184	0	0
1/39	0.657166285	0.78263184	0	0
1/39	0.657166285	0.78263184	0	0
1/39	0.657166285	0.78263184	0	0
1/39	0.657166285	0.78263184	0	0
1/39	0.657166285	0.78263184	0	0
1/39	0.657166285	0.78263184	0	0
1/39	0.657166285	0.78263184	0	0
1/39	0.657166285	0.78263184	0	0
1/39	0.657166285	0.78263184	0	0
1/39	0.657166285	0.78263184	0	0

1/39	0.657166285	0.78263184	0	0
1/39	0.657166285	0.78263184	0	0
1/39	0.657166285	0.78263184	0	0
1/39	0.657166285	0.78263184	0	0
1/39	0.657166285	0.78263184	0	0
2/83	0.660965399	0.786295996	0	0
2/83	0.660965399	0.786295996	0	0
7/288	0.665179834	0.787479924	0	0
8/328	0.665902662	0.787479924	0	0
3/126	0.666417744	0.787479924	0	0
1/40	0.66645802	0.787479924	0	0
1/40	0.66645802	0.787479924	0	0
1/40	0.66645802	0.787479924	0	0
1/40	0.66645802	0.787479924	0	0
1/40	0.66645802	0.787479924	0	0
1/40	0.66645802	0.787479924	0	0
1/40	0.66645802	0.787479924	0	0
1/40	0.66645802	0.787479924	0	0
1/40	0.66645802	0.787479924	0	0
2/84	0.667386523	0.787479924	0	0
2/84	0.667386523	0.787479924	0	0
2/84	0.667386523	0.787479924	0	0
3/127	0.671631007	0.79164833	0	0
2/85	0.673709137	0.79164833	0	0
2/85	0.673709137	0.79164833	0	0
2/85	0.673709137	0.79164833	0	0
2/85	0.673709137	0.79164833	0	0
1/41	0.675498375	0.79164833	0	0
1/41	0.675498375	0.79164833	0	0
1/41	0.675498375	0.79164833	0	0
1/41	0.675498375	0.79164833	0	0
1/41	0.675498375	0.79164833	0	0
1/41	0.675498375	0.79164833	0	0
1/41	0.675498375	0.79164833	0	0
7/291	0.675646576	0.79164833	0	0
2/86	0.679933862	0.795784369	0	0
4/171	0.683164409	0.795784369	0	0
1/42	0.684294138	0.795784369	0	0
1/42	0.684294138	0.795784369	0	0
1/42	0.684294138	0.795784369	0	0
1/42	0.684294138	0.795784369	0	0
1/42	0.684294138	0.795784369	0	0
1/42	0.684294138	0.795784369	0	0
1/42	0.684294138	0.795784369	0	0
1/42	0.684294138	0.795784369	0	0
1/42	0.684294138	0.795784369	0	0
1/42	0.684294138	0.795784369	0	0
1/42	0.684294138	0.795784369	0	0

1/42	0.684294138	0.795784369	0	0
2/87	0.686061355	0.797413545	0	0
2/88	0.692092312	0.802307777	0	0
1/43	0.692851916	0.802307777	0	0
1/43	0.692851916	0.802307777	0	0
1/43	0.692851916	0.802307777	0	0
1/43	0.692851916	0.802307777	0	0
1/43	0.692851916	0.802307777	0	0
1/43	0.692851916	0.802307777	0	0
7/297	0.69595883	0.805335168	0	0
4/174	0.696206149	0.805335168	0	0
1/44	0.701178136	0.80808089	0	0
1/44	0.701178136	0.80808089	0	0
1/44	0.701178136	0.80808089	0	0
1/44	0.701178136	0.80808089	0	0
1/44	0.701178136	0.80808089	0	0
1/44	0.701178136	0.80808089	0	0
1/44	0.701178136	0.80808089	0	0
3/134	0.706448682	0.813724223	0	0
1/45	0.709279051	0.814353389	0	0
1/45	0.709279051	0.814353389	0	0
1/45	0.709279051	0.814353389	0	0
1/45	0.709279051	0.814353389	0	0
1/45	0.709279051	0.814353389	0	0
2/91	0.709613403	0.814353389	0	0
2/91	0.709613403	0.814353389	0	0
2/92	0.715265801	0.817841252	0	0
3/136	0.715861765	0.817841252	0	0
1/46	0.717160749	0.817841252	0	0
1/46	0.717160749	0.817841252	0	0
1/46	0.717160749	0.817841252	0	0
1/46	0.717160749	0.817841252	0	0
1/46	0.717160749	0.817841252	0	0
1/46	0.717160749	0.817841252	0	0
1/46	0.717160749	0.817841252	0	0
1/46	0.717160749	0.817841252	0	0
1/46	0.717160749	0.817841252	0	0
1/46	0.717160749	0.817841252	0	0
2/93	0.720825594	0.821160292	0	0
2/93	0.720825594	0.821160292	0	0
1/47	0.724829149	0.822707538	0	0
1/47	0.724829149	0.822707538	0	0
1/47	0.724829149	0.822707538	0	0
1/47	0.724829149	0.822707538	0	0
1/47	0.724829149	0.822707538	0	0
1/47	0.724829149	0.822707538	0	0
1/47	0.724829149	0.822707538	0	0
2/94	0.726293647	0.823940213	0	0

3/139	0.729541334	0.827193481	0	0
2/95	0.731670844	0.827293909	0	0
2/95	0.731670844	0.827293909	0	0
2/95	0.731670844	0.827293909	0	0
1/48	0.732290015	0.827293909	0	0
1/48	0.732290015	0.827293909	0	0
1/48	0.732290015	0.827293909	0	0
1/48	0.732290015	0.827293909	0	0
2/96	0.736958089	0.832135768	0	0
1/49	0.739548952	0.832901226	0	0
1/49	0.739548952	0.832901226	0	0
1/49	0.739548952	0.832901226	0	0
1/49	0.739548952	0.832901226	0	0
1/49	0.739548952	0.832901226	0	0
1/50	0.746611417	0.837791951	0	0
1/50	0.746611417	0.837791951	0	0
1/50	0.746611417	0.837791951	0	0
1/50	0.746611417	0.837791951	0	0
1/50	0.746611417	0.837791951	0	0
1/50	0.746611417	0.837791951	0	0
1/50	0.746611417	0.837791951	0	0
3/143	0.746970224	0.837791951	0	0
3/144	0.751184696	0.841627436	0	0
1/51	0.753482716	0.841627436	0	0
1/51	0.753482716	0.841627436	0	0
1/51	0.753482716	0.841627436	0	0
1/51	0.753482716	0.841627436	0	0
1/51	0.753482716	0.841627436	0	0
1/51	0.753482716	0.841627436	0	0
1/51	0.753482716	0.841627436	0	0
3/146	0.759444668	0.845623797	0	0
1/52	0.760168016	0.845623797	0	0
1/52	0.760168016	0.845623797	0	0
1/52	0.760168016	0.845623797	0	0
1/52	0.760168016	0.845623797	0	0
1/52	0.760168016	0.845623797	0	0
1/52	0.760168016	0.845623797	0	0
1/52	0.760168016	0.845623797	0	0
11/483	0.760586623	0.845657343	0	0
3/147	0.763490883	0.848453115	0	0
4/192	0.766359335	0.848523482	0	0
1/53	0.766672342	0.848523482	0	0
1/53	0.766672342	0.848523482	0	0
1/53	0.766672342	0.848523482	0	0
1/53	0.766672342	0.848523482	0	0
1/53	0.766672342	0.848523482	0	0
1/53	0.766672342	0.848523482	0	0
1/53	0.766672342	0.848523482	0	0

4/193	0.769856516	0.851614652	0	0
1/54	0.773000584	0.853791107	0	0
1/54	0.773000584	0.853791107	0	0
1/54	0.773000584	0.853791107	0	0
1/55	0.779157501	0.859719148	0	0
1/55	0.779157501	0.859719148	0	0
2/105	0.780652101	0.860679224	0	0
6/283	0.780818313	0.860679224	0	0
1/56	0.785147722	0.863702168	0	0
1/56	0.785147722	0.863702168	0	0
1/56	0.785147722	0.863702168	0	0
1/56	0.785147722	0.863702168	0	0
2/107	0.789454081	0.868000775	0	0
1/57	0.790975752	0.868358151	0	0
1/57	0.790975752	0.868358151	0	0
1/57	0.790975752	0.868358151	0	0
2/108	0.793737346	0.870950707	0	0
1/58	0.796645976	0.872383446	0	0
1/58	0.796645976	0.872383446	0	0
1/58	0.796645976	0.872383446	0	0
1/58	0.796645976	0.872383446	0	0
2/109	0.797943496	0.872926126	0	0
2/109	0.797943496	0.872926126	0	0
9/418	0.800642813	0.874028081	0	0
1/59	0.802162658	0.874028081	0	0
1/59	0.802162658	0.874028081	0	0
1/59	0.802162658	0.874028081	0	0
1/59	0.802162658	0.874028081	0	0
1/59	0.802162658	0.874028081	0	0
1/59	0.802162658	0.874028081	0	0
1/59	0.802162658	0.874028081	0	0
2/111	0.806128566	0.877470944	0	0
2/111	0.806128566	0.877470944	0	0
1/60	0.807529949	0.878118231	0	0
1/60	0.807529949	0.878118231	0	0
2/112	0.810109544	0.880044151	0	0
2/112	0.810109544	0.880044151	0	0
1/61	0.812751886	0.882474242	0	0
2/113	0.814017524	0.883407851	0	0
4/207	0.814602354	0.883602055	0	0
1/62	0.817832399	0.885364066	0	0
1/62	0.817832399	0.885364066	0	0
1/62	0.817832399	0.885364066	0	0
2/114	0.817853531	0.885364066	0	0
1/63	0.822775312	0.890249431	0	0
1/64	0.827584344	0.894119661	0	0
1/64	0.827584344	0.894119661	0	0
1/64	0.827584344	0.894119661	0	0

17/766	0.830731806	0.896948911	0	0
9/433	0.831778026	0.896948911	0	0
1/65	0.832263115	0.896948911	0	0
1/65	0.832263115	0.896948911	0	0
1/65	0.832263115	0.896948911	0	0
3/167	0.833352139	0.897678182	0	0
2/119	0.835989773	0.900074053	0	0
3/169	0.839255995	0.903143995	0	0
1/67	0.841243866	0.904389085	0	0
1/67	0.841243866	0.904389085	0	0
1/68	0.845552608	0.907676543	0	0
1/68	0.845552608	0.907676543	0	0
1/68	0.845552608	0.907676543	0	0
2/123	0.849306895	0.911257324	0	0
1/69	0.849744617	0.911277848	0	0
2/124	0.852478948	0.912953231	0	0
2/124	0.852478948	0.912953231	0	0
1/70	0.853823049	0.912953231	0	0
1/70	0.853823049	0.912953231	0	0
1/70	0.853823049	0.912953231	0	0
1/70	0.853823049	0.912953231	0	0
2/125	0.85559007	0.914393512	0	0
1/71	0.857790977	0.915846472	0	0
1/71	0.857790977	0.915846472	0	0
1/72	0.861651388	0.919066669	0	0
1/72	0.861651388	0.919066669	0	0
4/226	0.863670176	0.92076884	0	0
1/73	0.865407192	0.921717934	0	0
1/73	0.865407192	0.921717934	0	0
1/74	0.869061217	0.9251571	0	0
1/76	0.876074869	0.93216764	0	0
2/133	0.878399988	0.933922149	0	0
1/77	0.879439782	0.933922149	0	0
1/77	0.879439782	0.933922149	0	0
1/77	0.879439782	0.933922149	0	0
1/78	0.882713491	0.936485024	0	0
1/78	0.882713491	0.936485024	0	0
2/135	0.88355899	0.936925437	0	0
1/79	0.885898463	0.938035483	0	0
1/79	0.885898463	0.938035483	0	0
1/79	0.885898463	0.938035483	0	0
2/137	0.888514984	0.938122485	0	0
1/80	0.8889971	0.938122485	0	0
1/80	0.8889971	0.938122485	0	0
1/80	0.8889971	0.938122485	0	0
1/80	0.8889971	0.938122485	0	0
1/80	0.8889971	0.938122485	0	0
1/80	0.8889971	0.938122485	0	0

1/81	0.892011737	0.940847651	0	0
4/241	0.894141464	0.942037014	0	0
1/82	0.894944647	0.942037014	0	0
1/82	0.894944647	0.942037014	0	0
3/192	0.895118092	0.942037014	0	0
3/193	0.897095901	0.942037014	0	0
4/243	0.897717407	0.942037014	0	0
1/83	0.897798043	0.942037014	0	0
1/83	0.897798043	0.942037014	0	0
1/83	0.897798043	0.942037014	0	0
1/83	0.897798043	0.942037014	0	0
7/384	0.897899313	0.942037014	0	0
2/142	0.90006186	0.943850996	0	0
1/84	0.900574076	0.943933444	0	0
3/196	0.902829735	0.94449055	0	0
1/85	0.903274839	0.94449055	0	0
1/85	0.903274839	0.94449055	0	0
1/85	0.903274839	0.94449055	0	0
1/85	0.903274839	0.94449055	0	0
3/199	0.908273825	0.948611926	0	0
2/146	0.90848663	0.948611926	0	0
5/298	0.908523595	0.948611926	0	0
1/88	0.910945611	0.950684849	0	0
2/148	0.912445528	0.951385588	0	0
1/89	0.913365126	0.951385588	0	0
1/89	0.913365126	0.951385588	0	0
1/89	0.913365126	0.951385588	0	0
2/149	0.914364267	0.951515779	0	0
2/149	0.914364267	0.951515779	0	0
1/90	0.915719021	0.952470286	0	0
2/150	0.916243521	0.952560719	0	0
1/91	0.918009074	0.953940694	0	0
1/92	0.920237012	0.955343813	0	0
1/92	0.920237012	0.955343813	0	0
3/207	0.921466456	0.955770888	0	0
1/93	0.922404518	0.955770888	0	0
1/93	0.922404518	0.955770888	0	0
1/93	0.922404518	0.955770888	0	0
1/94	0.924513228	0.957044839	0	0
1/94	0.924513228	0.957044839	0	0
5/313	0.928421406	0.960633746	0	0
2/158	0.929941144	0.961728116	0	0
11/594	0.93057103	0.961728116	0	0
7/411	0.930804382	0.961728116	0	0
6/364	0.931318379	0.961802709	0	0
1/98	0.932391305	0.961998043	0	0
1/98	0.932391305	0.961998043	0	0
4/267	0.933003063	0.96217322	0	0

6/367	0.934474511	0.962545555	0	0
5/319	0.93523762	0.962545555	0	0
2/162	0.935969034	0.962545555	0	0
2/162	0.935969034	0.962545555	0	0
1/100	0.936016968	0.962545555	0	0
1/100	0.936016968	0.962545555	0	0
2/163	0.937397406	0.962890513	0	0
1/101	0.93775635	0.962890513	0	0
5/322	0.938423174	0.962890513	0	0
23/1127	0.939348673	0.962890513	0	0
1/102	0.93944853	0.962890513	0	0
1/102	0.93944853	0.962890513	0	0
1/102	0.93944853	0.962890513	0	0
4/273	0.939902554	0.962902522	0	0
2/166	0.941503456	0.96408891	0	0
4/275	0.94205479	0.964199942	0	0
1/104	0.942696365	0.964403189	0	0
1/106	0.945770291	0.966639401	0	0
1/106	0.945770291	0.966639401	0	0
1/107	0.947244963	0.967238407	0	0
1/107	0.947244963	0.967238407	0	0
3/228	0.948266081	0.967795454	0	0
1/108	0.948679604	0.967795454	0	0
7/434	0.951106215	0.96957332	0	0
3/231	0.951313097	0.96957332	0	0
1/111	0.952754047	0.970587534	0	0
1/114	0.956505544	0.973043256	0	0
1/114	0.956505544	0.973043256	0	0
1/114	0.956505544	0.973043256	0	0
1/118	0.961049054	0.977208683	0	0
1/119	0.96210887	0.977829603	0	0
2/189	0.965478741	0.980796649	0	0
9/557	0.967417293	0.982307578	0	0
1/126	0.968763816	0.983216237	0	0
2/195	0.96997402	0.98398576	0	0
2/196	0.970666223	0.984229328	0	0
1/129	0.971245833	0.984358556	0	0
1/130	0.972028619	0.984693487	0	0
1/132	0.973530948	0.985756685	0	0
1/137	0.976943707	0.988752417	0	0
4/325	0.97753089	0.988761258	0	0
2/208	0.977860813	0.988761258	0	0
1/140	0.978776684	0.989227874	0	0
1/152	0.984764479	0.994817759	0	0
1/155	0.985976521	0.995118631	0	0
1/155	0.985976521	0.995118631	0	0
1/172	0.991234927	0.999962204	0	0
5/456	0.994830554	0.999994366	0	0

1/192	0.994959746	0.999994366	0	0
14/938	0.995840671	0.999994366	0	0
1/199	0.99584755	0.999994366	0	0
1/202	0.996178488	0.999994366	0	0
3/351	0.99635803	0.999994366	0	0
1/220	0.997678542	0.999994366	0	0
1/228	0.998139937	0.999994366	0	0
1/233	0.998380507	0.999994366	0	0
4/452	0.998395053	0.999994366	0	0
1/234	0.99842475	0.999994366	0	0
1/246	0.998870198	0.999994366	0	0
1/268	0.999385495	0.999994366	0	0
1/296	0.999716312	0.999994366	0	0
16/1243	0.999836048	0.999994366	0	0
11/1025	0.999931979	0.999994366	0	0
6/763	0.999966531	0.999994366	0	0
23/1922	0.999993796	0.999994366	0	0
22/2028	0.999994366	0.999994366	0	0

Odds.Ratio	Combined.Score
5.891804872	62.93439844
3.676	39.01690172
5.797985233	61.2181332
10.90037383	106.8530859
10.07514511	81.09625398
9.066697761	69.24258634
13.16945996	98.37637328
6.601869159	49.17955501
7.882868267	55.88002692
11.14224323	77.51739939
11.14224323	77.51739939
4.799731468	32.53189886
10.34583666	69.59870455
5.584471603	37.25534676
18.07899628	117.9962813
9.655617629	62.87998405
9.655617629	62.87998405
2.900587138	18.60716049
9.051675978	57.12101017
4.384831461	27.64339195
5.063942621	31.50626829
15.49548593	95.92087139
6.25	38.63343585
4.948598131	30.26837999
3.113300175	18.7911858
4.838380062	29.0984186
3.308270677	19.62663636
3.275018797	19.22448372
3.24242537	18.83294182
7.621287857	43.9873605
7.621287857	43.9873605
7.621287857	43.9873605
12.05080545	67.77924567
12.05080545	67.77924567
7.239851024	40.62033361
3.00320756	16.04220539
6.581005587	34.95738523
6.581005587	34.95738523
3.579574827	18.86543562
6.294551048	32.56039718
9.858736059	50.88256848
9.858736059	50.88256848
9.858736059	50.88256848
9.858736059	50.88256848
2.896300486	14.8443078
6.031967722	30.4004655
3.387965044	16.93355148

24.06431664	119.6871535
24.06431664	119.6871535
24.06431664	119.6871535
24.06431664	119.6871535
24.06431664	119.6871535
9.036710037	44.80487684
4.528684701	22.39288906
2.617566963	12.84170399
8.341149557	39.79147131
8.341149557	39.79147131
8.341149557	39.79147131
3.215758783	15.25497016
5.360921443	25.05612351
2.203770261	10.27615092
7.744954859	35.59861965
4.11613806	18.88196782
18.04730983	82.76816124
18.04730983	82.76816124
18.04730983	82.76816124
2.820597825	12.83107121
7.228252788	32.0502764
7.228252788	32.0502764
3.936769955	17.41058309
2.987838731	13.12281083
3.346196981	14.69513043
3.852810416	16.73403609
6.776138476	29.01634599
6.776138476	29.01634599
6.776138476	29.01634599
6.776138476	29.01634599
6.776138476	29.01634599
14.43710575	61.61280305
14.43710575	61.61280305
14.43710575	61.61280305
14.43710575	61.61280305
14.43710575	61.61280305
14.43710575	61.61280305
14.43710575	61.61280305
3.772349192	16.09320783
4.66822851	19.83580844
4.66822851	19.83580844
3.695172099	15.48562155
3.151564405	13.048752
2.789974071	11.36032069
3.549897571	14.36131774
2.089344344	8.449475797
2.385955765	9.591683468
6.022614622	24.12234247

6.022614622	24.12234247
12.03030303	48.09608584
12.03030303	48.09608584
12.03030303	48.09608584
12.03030303	48.09608584
12.03030303	48.09608584
12.03030303	48.09608584
12.03030303	48.09608584
12.03030303	48.09608584
12.03030303	48.09608584
12.03030303	48.09608584
12.03030303	48.09608584
12.03030303	48.09608584
12.03030303	48.09608584
3.481450918	13.84063251
2.729692723	10.84067953
4.255668748	16.89012515
3.019781931	11.97039707
4.13386539	16.04621683
5.70534142	22.12850901
3.352162797	12.87346956
3.352162797	12.87346956
2.285135917	8.654033789
1.888017429	7.115582451
10.31115823	38.81613589
10.31115823	38.81613589
10.31115823	38.81613589
5.419795539	20.37104773
2.86025578	10.70656081
3.910010569	14.52798273
5.161444503	18.81303148
5.161444503	18.81303148
5.161444503	18.81303148
5.161444503	18.81303148
3.806919533	13.84365251
3.806919533	13.84365251
2.512441132	9.039142921
1.947913854	6.999373828
2.751212587	9.870126298
2.751212587	9.870126298
3.709115217	13.20343005
9.021799629	32.11016355
9.021799629	32.11016355
9.021799629	32.11016355
9.021799629	32.11016355
9.021799629	32.11016355
9.021799629	32.11016355
9.021799629	32.11016355

9.021799629	32.11016355
4.926579926	17.42463429
4.926579926	17.42463429
4.926579926	17.42463429
4.926579926	17.42463429
2.463401331	8.648584386
3.616201117	12.60358351
3.016013682	10.46485285
1.968733154	6.796039363
4.712138355	16.18152674
4.712138355	16.18152674
8.018965162	27.07444779
8.018965162	27.07444779
8.018965162	27.07444779
8.018965162	27.07444779
8.018965162	27.07444779
8.018965162	27.07444779
8.018965162	27.07444779
8.018965162	27.07444779
8.018965162	27.07444779
8.018965162	27.07444779
8.018965162	27.07444779
8.018965162	27.07444779
8.018965162	27.07444779
2.017053777	6.800664202
2.918422003	9.796112936
3.44364636	11.51200641
3.44364636	11.51200641
4.515566914	15.06368406
4.515566914	15.06368406
3.363388333	11.01458682
3.363388333	11.01458682
2.826929804	9.18243069
2.826929804	9.18243069
4.33472119	14.05448877
7.216697588	23.17812583
7.216697588	23.17812583
7.216697588	23.17812583
7.216697588	23.17812583
7.216697588	23.17812583
7.216697588	23.17812583
7.216697588	23.17812583
7.216697588	23.17812583
7.216697588	23.17812583
4.167786674	13.14004688
4.167786674	13.14004688
4.167786674	13.14004688
3.21357335	10.10431151

3.21357335	10.10431151
2.69993317	8.352709949
3.143551129	9.687273917
3.143551129	9.687273917
4.013217679	12.30866087
4.013217679	12.30866087
6.560296846	20.09021766
6.560296846	20.09021766
6.560296846	20.09021766
6.560296846	20.09021766
6.560296846	20.09021766
6.560296846	20.09021766
1.978195489	6.03042662
2.660091089	8.097875109
2.660091089	8.097875109
1.954301922	5.841769763
3.869689326	11.55041927
3.869689326	11.55041927
3.869689326	11.55041927
3.869689326	11.55041927
3.869689326	11.55041927
2.33536328	6.90354535
2.148590745	6.294389442
6.013296228	17.59438894
6.013296228	17.59438894
6.013296228	17.59438894
6.013296228	17.59438894
6.013296228	17.59438894
6.013296228	17.59438894
6.013296228	17.59438894
6.013296228	17.59438894
6.013296228	17.59438894
3.73605948	10.85687417
2.950632767	8.567350668
2.511790008	7.173236348
3.61133829	10.22078536
3.61133829	10.22078536
5.55044955	15.54370594
5.55044955	15.54370594
5.55044955	15.54370594
5.55044955	15.54370594
5.55044955	15.54370594
5.55044955	15.54370594
5.55044955	15.54370594
5.55044955	15.54370594
5.55044955	15.54370594
5.55044955	15.54370594
2.077715356	5.80326059

3.494663629	9.635915836
3.494663629	9.635915836
3.494663629	9.635915836
3.494663629	9.635915836
2.193146417	5.977822708
1.6750618	4.524903808
5.153723827	13.83514128
5.153723827	13.83514128
5.153723827	13.83514128
5.153723827	13.83514128
5.153723827	13.83514128
5.153723827	13.83514128
5.153723827	13.83514128
5.153723827	13.83514128
5.153723827	13.83514128
5.153723827	13.83514128
5.153723827	13.83514128
2.379099568	6.379287472
1.712575917	4.58803801
2.676736327	7.055401544
2.676736327	7.055401544
3.282527881	8.598945195
2.627932961	6.796242757
2.627932961	6.796242757
2.627932961	6.796242757
1.869297343	4.824884998
4.809894867	12.39448587
4.809894867	12.39448587
4.809894867	12.39448587
4.809894867	12.39448587
4.809894867	12.39448587
4.809894867	12.39448587
4.809894867	12.39448587
3.185818937	8.138052111
2.067182911	5.199562961
1.618259886	4.067670621
3.094636219	7.710595494
3.094636219	7.710595494
2.535463426	6.31416548
2.535463426	6.31416548
2.047575384	5.082096002
4.509044527	11.16704471
4.509044527	11.16704471
4.509044527	11.16704471
4.509044527	11.16704471
4.509044527	11.16704471
4.509044527	11.16704471
2.491620112	6.089796147

2.491620112	6.089796147
3.008519207	7.313414766
3.008519207	7.313414766
1.504395731	3.635646552
2.449263012	5.87566253
4.243588344	10.11169511
4.243588344	10.11169511
4.243588344	10.11169511
4.243588344	10.11169511
4.243588344	10.11169511
4.243588344	10.11169511
4.243588344	10.11169511
4.243588344	10.11169511
4.243588344	10.11169511
4.243588344	10.11169511
4.243588344	10.11169511
4.243588344	10.11169511
2.927057169	6.943719428
2.927057169	6.943719428
1.535978679	3.600012465
2.126207199	4.960160474
2.849882606	6.599037562
2.10137539	4.827877043
4.007627293	9.196976163
4.007627293	9.196976163
4.007627293	9.196976163
4.007627293	9.196976163
4.007627293	9.196976163
4.007627293	9.196976163
2.33038986	5.288912908
2.33038986	5.288912908
1.920039699	4.343280296
2.776665713	6.277172585
2.776665713	6.277172585
1.569269054	3.514685639
2.29328131	5.110160493
2.29328131	5.110160493
1.563033666	3.47081032
3.796504248	8.398446516
3.796504248	8.398446516
3.796504248	8.398446516
3.796504248	8.398446516
2.707109665	5.976166785
2.707109665	5.976166785
1.703034985	3.744978051
1.692987942	3.683340742
2.007566335	4.340199856
2.640946595	5.694270418

2.640946595	5.694270418
2.640946595	5.694270418
2.640946595	5.694270418
2.640946595	5.694270418
2.640946595	5.694270418
3.606493506	7.696857499
3.606493506	7.696857499
3.606493506	7.696857499
3.606493506	7.696857499
3.606493506	7.696857499
3.606493506	7.696857499
3.606493506	7.696857499
2.188702669	4.618136769
2.188702669	4.618136769
2.188702669	4.618136769
2.577934148	5.429915426
2.577934148	5.429915426
1.963720798	4.118974834
2.155924289	4.467579588
2.155924289	4.467579588
2.155924289	4.467579588
3.434579026	7.076863867
3.434579026	7.076863867
3.434579026	7.076863867
3.434579026	7.076863867
3.434579026	7.076863867
2.517852511	5.181692988
2.517852511	5.181692988
2.517852511	5.181692988
2.517852511	5.181692988
9.006944444	18.50595396
9.006944444	18.50595396
9.006944444	18.50595396
9.006944444	18.50595396
9.006944444	18.50595396
9.006944444	18.50595396
9.006944444	18.50595396
9.006944444	18.50595396
9.006944444	18.50595396
9.006944444	18.50595396
9.006944444	18.50595396
9.006944444	18.50595396
9.006944444	18.50595396
9.006944444	18.50595396
9.006944444	18.50595396
9.006944444	18.50595396
9.006944444	18.50595396
9.006944444	18.50595396

9.006944444	18.50595396
9.006944444	18.50595396
9.006944444	18.50595396
9.006944444	18.50595396
9.006944444	18.50595396
9.006944444	18.50595396
9.006944444	18.50595396
9.006944444	18.50595396
9.006944444	18.50595396
9.006944444	18.50595396
9.006944444	18.50595396
9.006944444	18.50595396
9.006944444	18.50595396
9.006944444	18.50595396
9.006944444	18.50595396
9.006944444	18.50595396
9.006944444	18.50595396
9.006944444	18.50595396
9.006944444	18.50595396
9.006944444	18.50595396
9.006944444	18.50595396
9.006944444	18.50595396
9.006944444	18.50595396
9.006944444	18.50595396
9.006944444	18.50595396
9.006944444	18.50595396
9.006944444	18.50595396
9.006944444	18.50595396
9.006944444	18.50595396
9.006944444	18.50595396
9.006944444	18.50595396
9.006944444	18.50595396
9.006944444	18.50595396
9.006944444	18.50595396
9.006944444	18.50595396
9.006944444	18.50595396
9.006944444	18.50595396
9.006944444	18.50595396
9.006944444	18.50595396
9.006944444	18.50595396
9.006944444	18.50595396
9.006944444	18.50595396
9.006944444	18.50595396
9.006944444	18.50595396
9.006944444	18.50595396
9.006944444	18.50595396
9.006944444	18.50595396
9.006944444	18.50595396

9.006944444	18.50595396
9.006944444	18.50595396
9.006944444	18.50595396
9.006944444	18.50595396
9.006944444	18.50595396
9.006944444	18.50595396
9.006944444	18.50595396
9.006944444	18.50595396
9.006944444	18.50595396
9.006944444	18.50595396
9.006944444	18.50595396
9.006944444	18.50595396
2.124109979	4.323164083
1.468579165	2.966611549
2.460501859	4.948334274
2.460501859	4.948334274
2.093217823	4.184573832
3.278293135	6.526097174
3.278293135	6.526097174
3.278293135	6.526097174
3.278293135	6.526097174
3.278293135	6.526097174
3.278293135	6.526097174
3.278293135	6.526097174
3.278293135	6.526097174
3.278293135	6.526097174
3.278293135	6.526097174
3.278293135	6.526097174
1.390701429	2.740233413
2.405700124	4.728693885
2.405700124	4.728693885
2.405700124	4.728693885
2.405700124	4.728693885
1.748718722	3.423383287
1.598623449	3.125532103
2.034044116	3.923702385
2.034044116	3.923702385
3.135597322	6.034488788
3.135597322	6.034488788
3.135597322	6.034488788
3.135597322	6.034488788
3.135597322	6.034488788
3.135597322	6.034488788
3.135597322	6.034488788
3.135597322	6.034488788
3.135597322	6.034488788
3.135597322	6.034488788
3.135597322	6.034488788

3.135597322	6.034488788
2.353281073	4.521735561
2.353281073	4.521735561
2.353281073	4.521735561
1.458608059	2.772663139
2.005690048	3.80088289
7.205185185	13.58649124
7.205185185	13.58649124
7.205185185	13.58649124
7.205185185	13.58649124
7.205185185	13.58649124
7.205185185	13.58649124
7.205185185	13.58649124
7.205185185	13.58649124
7.205185185	13.58649124
7.205185185	13.58649124
7.205185185	13.58649124
7.205185185	13.58649124
7.205185185	13.58649124
7.205185185	13.58649124
7.205185185	13.58649124
7.205185185	13.58649124
7.205185185	13.58649124
7.205185185	13.58649124
7.205185185	13.58649124
7.205185185	13.58649124
7.205185185	13.58649124
7.205185185	13.58649124
7.205185185	13.58649124
7.205185185	13.58649124
7.205185185	13.58649124
7.205185185	13.58649124
7.205185185	13.58649124
7.205185185	13.58649124
7.205185185	13.58649124
7.205185185	13.58649124
7.205185185	13.58649124
7.205185185	13.58649124
7.205185185	13.58649124
7.205185185	13.58649124
7.205185185	13.58649124
7.205185185	13.58649124
7.205185185	13.58649124
7.205185185	13.58649124
7.205185185	13.58649124
7.205185185	13.58649124
7.205185185	13.58649124
7.205185185	13.58649124

7.205185185	13.58649124
7.205185185	13.58649124
7.205185185	13.58649124
7.205185185	13.58649124
7.205185185	13.58649124
7.205185185	13.58649124
7.205185185	13.58649124
7.205185185	13.58649124
7.205185185	13.58649124
7.205185185	13.58649124
7.205185185	13.58649124
7.205185185	13.58649124
7.205185185	13.58649124
7.205185185	13.58649124
7.205185185	13.58649124
7.205185185	13.58649124
7.205185185	13.58649124
7.205185185	13.58649124
7.205185185	13.58649124
7.205185185	13.58649124
7.205185185	13.58649124
7.205185185	13.58649124
7.205185185	13.58649124
7.205185185	13.58649124
7.205185185	13.58649124
7.205185185	13.58649124
7.205185185	13.58649124
7.205185185	13.58649124
7.205185185	13.58649124
2.30309262	4.326519797
2.30309262	4.326519797
2.30309262	4.326519797
2.30309262	4.326519797
1.978112803	3.68280939
1.978112803	3.68280939
1.978112803	3.68280939
3.004792826	5.593767877
3.004792826	5.593767877
3.004792826	5.593767877
3.004792826	5.593767877
3.004792826	5.593767877
3.004792826	5.593767877
3.004792826	5.593767877
3.004792826	5.593767877
3.004792826	5.593767877
3.004792826	5.593767877
1.507127193	2.782607541
2.254995353	4.142193087

1.770284606	3.196871075
2.88445269	5.197084133
2.88445269	5.197084133
2.88445269	5.197084133
2.88445269	5.197084133
1.925164494	3.459994536
1.925164494	3.459994536
1.925164494	3.459994536
2.20886124	3.967978552
2.20886124	3.967978552
2.20886124	3.967978552
2.20886124	3.967978552
1.753006811	3.118970098
1.753006811	3.118970098
1.899735372	3.354833527
2.164572491	3.803167744
2.164572491	3.803167744
2.164572491	3.803167744
6.004012346	10.4757205
6.004012346	10.4757205
6.004012346	10.4757205
6.004012346	10.4757205
6.004012346	10.4757205
6.004012346	10.4757205
6.004012346	10.4757205
6.004012346	10.4757205
6.004012346	10.4757205
6.004012346	10.4757205
6.004012346	10.4757205
6.004012346	10.4757205
6.004012346	10.4757205
6.004012346	10.4757205
6.004012346	10.4757205
6.004012346	10.4757205
6.004012346	10.4757205
6.004012346	10.4757205
6.004012346	10.4757205
6.004012346	10.4757205
6.004012346	10.4757205
6.004012346	10.4757205
6.004012346	10.4757205
6.004012346	10.4757205
6.004012346	10.4757205
6.004012346	10.4757205
6.004012346	10.4757205
6.004012346	10.4757205
6.004012346	10.4757205
6.004012346	10.4757205

6.004012346	10.4757205
6.004012346	10.4757205
6.004012346	10.4757205
6.004012346	10.4757205
6.004012346	10.4757205
6.004012346	10.4757205
6.004012346	10.4757205
6.004012346	10.4757205
6.004012346	10.4757205
6.004012346	10.4757205
6.004012346	10.4757205
6.004012346	10.4757205
6.004012346	10.4757205
6.004012346	10.4757205
6.004012346	10.4757205
6.004012346	10.4757205
6.004012346	10.4757205
6.004012346	10.4757205
6.004012346	10.4757205
6.004012346	10.4757205
6.004012346	10.4757205
6.004012346	10.4757205
6.004012346	10.4757205
6.004012346	10.4757205
6.004012346	10.4757205
6.004012346	10.4757205
6.004012346	10.4757205
6.004012346	10.4757205
6.004012346	10.4757205
6.004012346	10.4757205
6.004012346	10.4757205
2.773369488	4.838720714
2.773369488	4.838720714
2.773369488	4.838720714
2.773369488	4.838720714
2.773369488	4.838720714
2.773369488	4.838720714
2.773369488	4.838720714
2.773369488	4.838720714
2.773369488	4.838720714
2.773369488	4.838720714
1.465936801	2.550027701
1.874966746	3.253577335
1.874966746	3.253577335
1.719438522	2.969800908
1.850833214	3.156045326
2.670514671	4.513873375
2.670514671	4.513873375

2.670514671	4.513873375
2.670514671	4.513873375
2.670514671	4.513873375
2.670514671	4.513873375
2.670514671	4.513873375
2.670514671	4.513873375
2.670514671	4.513873375
2.670514671	4.513873375
1.446165414	2.441610988
2.081105233	3.49922348
1.475131141	2.455575528
1.340347924	2.22940461
1.804376164	2.971481898
2.041733885	3.358954922
2.041733885	3.358954922
2.575006626	4.218478751
2.575006626	4.218478751
2.575006626	4.218478751
2.575006626	4.218478751
2.575006626	4.218478751
2.575006626	4.218478751
2.575006626	4.218478751
5.146031746	8.359605006
5.146031746	8.359605006
5.146031746	8.359605006
5.146031746	8.359605006
5.146031746	8.359605006
5.146031746	8.359605006
5.146031746	8.359605006
5.146031746	8.359605006
5.146031746	8.359605006
5.146031746	8.359605006
5.146031746	8.359605006
5.146031746	8.359605006
5.146031746	8.359605006
5.146031746	8.359605006
5.146031746	8.359605006
5.146031746	8.359605006
5.146031746	8.359605006
5.146031746	8.359605006
5.146031746	8.359605006
5.146031746	8.359605006
5.146031746	8.359605006
5.146031746	8.359605006
5.146031746	8.359605006
5.146031746	8.359605006
5.146031746	8.359605006
5.146031746	8.359605006

5.146031746	8.359605006
5.146031746	8.359605006
5.146031746	8.359605006
5.146031746	8.359605006
5.146031746	8.359605006
5.146031746	8.359605006
5.146031746	8.359605006
5.146031746	8.359605006
5.146031746	8.359605006
5.146031746	8.359605006
5.146031746	8.359605006
5.146031746	8.359605006
5.146031746	8.359605006
5.146031746	8.359605006
5.146031746	8.359605006
5.146031746	8.359605006
5.146031746	8.359605006
1.782007955	2.884137757
1.782007955	2.884137757
1.782007955	2.884137757
2.003820735	3.225809423
2.003820735	3.225809423
2.003820735	3.225809423
2.003820735	3.225809423
2.486085343	3.949079588
2.486085343	3.949079588
2.486085343	3.949079588
1.967286245	3.099328758
1.967286245	3.099328758
1.967286245	3.099328758
1.967286245	3.099328758
1.738888515	2.718606221
1.611390592	2.509780382
1.932056559	2.979090993
1.932056559	2.979090993
1.932056559	2.979090993
2.403092146	3.702718016
2.403092146	3.702718016
2.403092146	3.702718016
2.403092146	3.702718016
2.403092146	3.702718016
2.403092146	3.702718016
2.403092146	3.702718016
1.597047946	2.451089462
4.502546296	6.843203248
4.502546296	6.843203248
4.502546296	6.843203248
4.502546296	6.843203248

4.502546296	6.843203248
4.502546296	6.843203248
4.502546296	6.843203248
4.502546296	6.843203248
4.502546296	6.843203248
4.502546296	6.843203248
4.502546296	6.843203248
4.502546296	6.843203248
4.502546296	6.843203248
4.502546296	6.843203248
4.502546296	6.843203248
4.502546296	6.843203248
4.502546296	6.843203248
4.502546296	6.843203248
4.502546296	6.843203248
4.502546296	6.843203248
4.502546296	6.843203248
4.502546296	6.843203248
4.502546296	6.843203248
4.502546296	6.843203248
4.502546296	6.843203248
4.502546296	6.843203248
4.502546296	6.843203248
4.502546296	6.843203248
4.502546296	6.843203248
4.502546296	6.843203248
4.502546296	6.843203248
4.502546296	6.843203248
4.502546296	6.843203248
4.502546296	6.843203248
4.502546296	6.843203248
4.502546296	6.843203248
4.502546296	6.843203248
4.502546296	6.843203248
4.502546296	6.843203248
4.502546296	6.843203248
4.502546296	6.843203248
4.502546296	6.843203248
4.502546296	6.843203248
4.502546296	6.843203248
4.502546296	6.843203248
4.502546296	6.843203248
4.502546296	6.843203248
4.502546296	6.843203248
4.502546296	6.843203248
1.329967817	2.011847037
1.697798225	2.564414376
1.697798225	2.564414376

2.325453348	3.476850354
2.325453348	3.476850354
2.325453348	3.476850354
2.325453348	3.476850354
2.325453348	3.476850354
2.325453348	3.476850354
2.325453348	3.476850354
2.325453348	3.476850354
2.325453348	3.476850354
1.865241636	2.755817736
1.865241636	2.755817736
1.247619048	1.831973025
2.252666976	3.269278565
2.252666976	3.269278565
2.252666976	3.269278565
2.252666976	3.269278565
2.252666976	3.269278565
2.252666976	3.269278565
1.277292284	1.853697432
1.473354949	2.137366521
1.463324072	2.095380645
4.002057613	5.71329086
4.002057613	5.71329086
4.002057613	5.71329086
4.002057613	5.71329086
4.002057613	5.71329086
4.002057613	5.71329086
4.002057613	5.71329086
4.002057613	5.71329086
4.002057613	5.71329086
4.002057613	5.71329086
4.002057613	5.71329086
4.002057613	5.71329086
4.002057613	5.71329086
4.002057613	5.71329086
4.002057613	5.71329086
4.002057613	5.71329086
4.002057613	5.71329086
4.002057613	5.71329086
4.002057613	5.71329086
4.002057613	5.71329086
4.002057613	5.71329086
4.002057613	5.71329086
4.002057613	5.71329086
4.002057613	5.71329086
4.002057613	5.71329086
4.002057613	5.71329086
4.002057613	5.71329086

4.002057613	5.71329086
4.002057613	5.71329086
4.002057613	5.71329086
4.002057613	5.71329086
4.002057613	5.71329086
4.002057613	5.71329086
4.002057613	5.71329086
4.002057613	5.71329086
4.002057613	5.71329086
4.002057613	5.71329086
4.002057613	5.71329086
4.002057613	5.71329086
4.002057613	5.71329086
4.002057613	5.71329086
4.002057613	5.71329086
4.002057613	5.71329086
4.002057613	5.71329086
4.002057613	5.71329086
4.002057613	5.71329086
4.002057613	5.71329086
1.802881041	2.553218963
2.184291899	3.078094724
2.184291899	3.078094724
2.184291899	3.078094724
2.184291899	3.078094724
2.184291899	3.078094724
2.184291899	3.078094724
1.773234201	2.458917052
1.773234201	2.458917052
1.60306228	2.222552886
1.503342662	2.081887635
1.256405971	1.736689074
2.119938885	2.901635727
2.119938885	2.901635727
2.119938885	2.901635727
1.490841248	2.034542334
1.74454371	2.368920665
1.74454371	2.368920665
3.601666667	4.845370456
3.601666667	4.845370456
3.601666667	4.845370456
3.601666667	4.845370456
3.601666667	4.845370456
3.601666667	4.845370456
3.601666667	4.845370456
3.601666667	4.845370456
3.601666667	4.845370456
3.601666667	4.845370456

3.601666667	4.845370456
3.601666667	4.845370456
3.601666667	4.845370456
3.601666667	4.845370456
3.601666667	4.845370456
3.601666667	4.845370456
3.601666667	4.845370456
3.601666667	4.845370456
3.601666667	4.845370456
3.601666667	4.845370456
3.601666667	4.845370456
3.601666667	4.845370456
3.601666667	4.845370456
3.601666667	4.845370456
3.601666667	4.845370456
3.601666667	4.845370456
3.601666667	4.845370456
3.601666667	4.845370456
3.601666667	4.845370456
3.601666667	4.845370456
3.601666667	4.845370456
3.601666667	4.845370456
1.415136522	1.898747154
2.059263186	2.738446112
2.059263186	2.738446112
2.059263186	2.738446112
1.716764029	2.282984106
1.689852463	2.200878863
1.29461103	1.682678844
1.534529894	1.987562957
1.219066779	1.578537368
2.001958359	2.587247375
2.001958359	2.587247375
2.001958359	2.587247375
1.387706686	1.790621657
1.442835821	1.857039494
1.442835821	1.857039494
1.663768945	2.122392176
3.274074074	4.1622501
3.274074074	4.1622501
3.274074074	4.1622501
3.274074074	4.1622501
3.274074074	4.1622501
3.274074074	4.1622501
3.274074074	4.1622501
3.274074074	4.1622501
3.274074074	4.1622501
3.274074074	4.1622501

3.274074074	4.1622501
3.274074074	4.1622501
3.274074074	4.1622501
3.274074074	4.1622501
3.274074074	4.1622501
3.274074074	4.1622501
3.274074074	4.1622501
3.274074074	4.1622501
3.274074074	4.1622501
3.274074074	4.1622501
3.274074074	4.1622501
3.274074074	4.1622501
3.274074074	4.1622501
3.274074074	4.1622501
3.274074074	4.1622501
3.274074074	4.1622501
3.274074074	4.1622501
3.274074074	4.1622501
3.274074074	4.1622501
3.274074074	4.1622501
3.274074074	4.1622501
3.274074074	4.1622501
3.274074074	4.1622501
3.274074074	4.1622501
3.274074074	4.1622501
3.274074074	4.1622501
3.274074074	4.1622501
3.274074074	4.1622501
3.274074074	4.1622501
3.274074074	4.1622501
3.274074074	4.1622501
3.274074074	4.1622501
3.274074074	4.1622501
1.431310708	1.815455186
1.947751091	2.446912495
1.947751091	2.446912495
1.947751091	2.446912495
1.947751091	2.446912495
1.947751091	2.446912495
1.947751091	2.446912495
1.947751091	2.446912495
1.502405338	1.881103034
1.638475836	2.047325749
1.638475836	2.047325749
1.486839832	1.830385849
1.613937746	1.975494573
1.613937746	1.975494573
1.613937746	1.975494573

1.613937746	1.975494573
1.613937746	1.975494573
1.613937746	1.975494573
1.896396836	2.316444673
1.896396836	2.316444673
1.896396836	2.316444673
1.896396836	2.316444673
1.896396836	2.316444673
1.896396836	2.316444673
1.896396836	2.316444673
1.471591989	1.781258843
1.344261914	1.625142533
3.001080247	3.613734256
3.001080247	3.613734256
3.001080247	3.613734256
3.001080247	3.613734256
3.001080247	3.613734256
3.001080247	3.613734256
3.001080247	3.613734256
3.001080247	3.613734256
3.001080247	3.613734256
3.001080247	3.613734256
3.001080247	3.613734256
3.001080247	3.613734256
3.001080247	3.613734256
3.001080247	3.613734256
3.001080247	3.613734256
3.001080247	3.613734256
3.001080247	3.613734256
3.001080247	3.613734256
3.001080247	3.613734256
3.001080247	3.613734256
3.001080247	3.613734256
3.001080247	3.613734256
3.001080247	3.613734256
3.001080247	3.613734256
3.001080247	3.613734256
3.001080247	3.613734256
3.001080247	3.613734256
3.001080247	3.613734256
3.001080247	3.613734256
3.001080247	3.613734256
1.590121365	1.906725874
1.590121365	1.906725874
1.386983352	1.659308683
1.301739449	1.556867431
1.335894773	1.594097849
1.847676133	2.19495951

1.847676133	2.19495951
1.847676133	2.19495951
1.847676133	2.19495951
1.847676133	2.19495951
1.847676133	2.19495951
1.847676133	2.19495951
1.847676133	2.19495951
1.847676133	2.19495951
1.847676133	2.19495951
1.847676133	2.19495951
1.566995313	1.840858149
1.566995313	1.840858149
1.566995313	1.840858149
1.244360902	1.460340209
1.365827114	1.586938466
1.801391466	2.081669989
1.801391466	2.081669989
1.801391466	2.081669989
1.801391466	2.081669989
1.544530005	1.777740288
1.544530005	1.777740288
1.427660084	1.642831681
2.77008547	3.165893438
2.77008547	3.165893438
2.77008547	3.165893438
2.77008547	3.165893438
2.77008547	3.165893438
2.77008547	3.165893438
2.77008547	3.165893438
2.77008547	3.165893438
2.77008547	3.165893438
2.77008547	3.165893438
2.77008547	3.165893438
2.77008547	3.165893438
2.77008547	3.165893438
2.77008547	3.165893438
2.77008547	3.165893438
2.77008547	3.165893438
2.77008547	3.165893438
2.77008547	3.165893438
2.77008547	3.165893438
2.77008547	3.165893438
2.77008547	3.165893438
2.77008547	3.165893438
2.77008547	3.165893438
2.77008547	3.165893438
2.77008547	3.165893438
2.77008547	3.165893438

2.77008547	3.165893438
2.77008547	3.165893438
2.77008547	3.165893438
2.77008547	3.165893438
2.77008547	3.165893438
2.77008547	3.165893438
2.77008547	3.165893438
2.77008547	3.165893438
2.77008547	3.165893438
2.77008547	3.165893438
2.77008547	3.165893438
2.77008547	3.165893438
2.77008547	3.165893438
2.77008547	3.165893438
1.303434298	1.476222949
1.757364587	1.975873768
1.757364587	1.975873768
1.757364587	1.975873768
1.757364587	1.975873768
1.757364587	1.975873768
1.757364587	1.975873768
1.757364587	1.975873768
1.757364587	1.975873768
1.159736527	1.299422884
1.715434226	1.876942379
1.715434226	1.876942379
1.715434226	1.876942379
1.715434226	1.876942379
1.280097329	1.394027855
2.572089947	2.795044055
2.572089947	2.795044055
2.572089947	2.795044055
2.572089947	2.795044055
2.572089947	2.795044055
2.572089947	2.795044055
2.572089947	2.795044055
2.572089947	2.795044055
2.572089947	2.795044055
2.572089947	2.795044055
2.572089947	2.795044055
2.572089947	2.795044055
2.572089947	2.795044055
2.572089947	2.795044055
2.572089947	2.795044055
2.572089947	2.795044055
2.572089947	2.795044055
2.572089947	2.795044055
2.572089947	2.795044055

2.572089947	2.795044055
2.572089947	2.795044055
2.572089947	2.795044055
2.572089947	2.795044055
2.572089947	2.795044055
2.572089947	2.795044055
2.572089947	2.795044055
2.572089947	2.795044055
2.572089947	2.795044055
2.572089947	2.795044055
2.572089947	2.795044055
1.48082701	1.603514543
1.37298927	1.477176082
1.37298927	1.477176082
1.675454114	1.784312027
1.675454114	1.784312027
1.675454114	1.784312027
1.217343118	1.296241732
1.46074048	1.550066577
1.46074048	1.550066577
1.46074048	1.550066577
1.13462438	1.196217376
1.178747707	1.231054977
1.34718669	1.401602096
1.34718669	1.401602096
1.34718669	1.401602096
1.441189591	1.49874336
1.63729128	1.69747569
1.63729128	1.69747569
1.63729128	1.69747569
1.63729128	1.69747569
1.63729128	1.69747569
2.400493827	2.484199958
2.400493827	2.484199958
2.400493827	2.484199958
2.400493827	2.484199958
2.400493827	2.484199958
2.400493827	2.484199958
2.400493827	2.484199958
2.400493827	2.484199958
2.400493827	2.484199958
2.400493827	2.484199958
2.400493827	2.484199958
2.400493827	2.484199958
2.400493827	2.484199958
2.400493827	2.484199958
2.400493827	2.484199958
2.400493827	2.484199958

2.400493827	2.484199958
2.400493827	2.484199958
2.400493827	2.484199958
2.400493827	2.484199958
2.400493827	2.484199958
2.400493827	2.484199958
2.400493827	2.484199958
2.400493827	2.484199958
2.400493827	2.484199958
2.400493827	2.484199958
2.400493827	2.484199958
1.108510459	1.144078274
1.334643769	1.365480426
1.422153199	1.449441693
1.422153199	1.449441693
1.278051233	1.301995201
1.600824572	1.61597635
1.403611259	1.402064461
2.250347222	2.220892567
2.250347222	2.220892567
2.250347222	2.220892567
2.250347222	2.220892567
2.250347222	2.220892567
2.250347222	2.220892567
2.250347222	2.220892567
2.250347222	2.220892567
2.250347222	2.220892567
2.250347222	2.220892567
2.250347222	2.220892567
2.250347222	2.220892567
2.250347222	2.220892567
2.250347222	2.220892567
2.250347222	2.220892567
2.250347222	2.220892567
2.250347222	2.220892567
2.250347222	2.220892567
2.250347222	2.220892567
2.250347222	2.220892567
2.250347222	2.220892567
2.250347222	2.220892567
2.250347222	2.220892567
2.250347222	2.220892567
2.250347222	2.220892567
2.250347222	2.220892567
2.250347222	2.220892567
2.250347222	2.220892567
2.250347222	2.220892567
2.250347222	2.220892567

2.250347222	2.220892567
2.250347222	2.220892567
2.250347222	2.220892567
2.250347222	2.220892567
2.250347222	2.220892567
2.250347222	2.220892567
2.250347222	2.220892567
2.250347222	2.220892567
2.250347222	2.220892567
2.250347222	2.220892567
2.250347222	2.220892567
2.250347222	2.220892567
2.250347222	2.220892567
2.250347222	2.220892567
2.250347222	2.220892567
1.565943373	1.539401143
1.565943373	1.539401143
1.385544753	1.356520209
1.251230825	1.219983637
1.115636392	1.085444106
1.214806259	1.175654209
1.532546481	1.467376303
1.532546481	1.467376303
1.532546481	1.467376303
1.532546481	1.467376303
1.532546481	1.467376303
1.201183801	1.132286008
2.117864924	1.995783785
2.117864924	1.995783785
2.117864924	1.995783785
2.117864924	1.995783785
2.117864924	1.995783785
2.117864924	1.995783785
2.117864924	1.995783785
2.117864924	1.995783785
2.117864924	1.995783785
2.117864924	1.995783785
2.117864924	1.995783785
2.117864924	1.995783785
2.117864924	1.995783785
2.117864924	1.995783785
2.117864924	1.995783785
2.117864924	1.995783785
2.117864924	1.995783785
2.117864924	1.995783785
2.117864924	1.995783785
2.117864924	1.995783785

2.117864924	1.995783785
2.117864924	1.995783785
2.117864924	1.995783785
2.117864924	1.995783785
2.117864924	1.995783785
2.117864924	1.995783785
2.117864924	1.995783785
2.117864924	1.995783785
2.117864924	1.995783785
2.117864924	1.995783785
2.117864924	1.995783785
2.117864924	1.995783785
2.117864924	1.995783785
2.117864924	1.995783785
2.117864924	1.995783785
2.117864924	1.995783785
1.275259142	1.200038233
1.275259142	1.200038233
1.350766729	1.270590817
1.500541126	1.399562773
1.500541126	1.399562773
1.500541126	1.399562773
1.113393436	1.038423283
1.157952636	1.078737979
1.252951178	1.140314297
1.469842111	1.335652383
1.469842111	1.335652383
1.469842111	1.335652383
1.469842111	1.335652383
1.469842111	1.335652383
1.317685194	1.191020973
1.317685194	1.191020973
1.317685194	1.191020973
1.317685194	1.191020973
1.317685194	1.191020973
2.000102881	1.801756199
2.000102881	1.801756199
2.000102881	1.801756199
2.000102881	1.801756199
2.000102881	1.801756199
2.000102881	1.801756199
2.000102881	1.801756199
2.000102881	1.801756199
2.000102881	1.801756199
2.000102881	1.801756199
2.000102881	1.801756199
2.000102881	1.801756199
2.000102881	1.801756199

2.000102881	1.801756199
2.000102881	1.801756199
2.000102881	1.801756199
2.000102881	1.801756199
2.000102881	1.801756199
2.000102881	1.801756199
2.000102881	1.801756199
2.000102881	1.801756199
2.000102881	1.801756199
2.000102881	1.801756199
2.000102881	1.801756199
2.000102881	1.801756199
2.000102881	1.801756199
2.000102881	1.801756199
2.000102881	1.801756199
2.000102881	1.801756199
2.000102881	1.801756199
2.000102881	1.801756199
2.000102881	1.801756199
1.301742285	1.153442197
1.301742285	1.153442197
1.440371058	1.275364518
1.440371058	1.275364518
1.440371058	1.275364518
1.440371058	1.275364518
1.28617897	1.117246663
1.220907111	1.05689554
1.220907111	1.05689554
1.141101903	0.987047688
1.412055731	1.218443194
1.412055731	1.218443194
1.412055731	1.218443194
1.412055731	1.218443194
1.894736842	1.633300374
1.894736842	1.633300374
1.894736842	1.633300374
1.894736842	1.633300374
1.894736842	1.633300374
1.894736842	1.633300374
1.894736842	1.633300374
1.894736842	1.633300374
1.894736842	1.633300374
1.894736842	1.633300374
1.894736842	1.633300374
1.894736842	1.633300374
1.894736842	1.633300374
1.894736842	1.633300374
1.894736842	1.633300374

1.894736842	1.633300374
1.894736842	1.633300374
1.894736842	1.633300374
1.894736842	1.633300374
1.894736842	1.633300374
1.894736842	1.633300374
1.894736842	1.633300374
1.894736842	1.633300374
1.894736842	1.633300374
1.894736842	1.633300374
1.894736842	1.633300374
1.894736842	1.633300374
1.894736842	1.633300374
1.894736842	1.633300374
1.894736842	1.633300374
1.894736842	1.633300374
1.105276536	0.947025806
1.27098185	1.082373147
1.27098185	1.082373147
1.210584793	1.030627156
1.384829456	1.164654496
1.384829456	1.164654496
1.384829456	1.164654496
1.384829456	1.164654496
1.384829456	1.164654496
1.384829456	1.164654496
1.384829456	1.164654496
1.384829456	1.164654496
1.200434513	1.005086257
1.200434513	1.005086257
1.200434513	1.005086257
1.256138152	1.048763693
1.256138152	1.048763693
1.125643727	0.938814644
1.799907407	1.486092529
1.799907407	1.486092529
1.799907407	1.486092529
1.799907407	1.486092529
1.799907407	1.486092529
1.799907407	1.486092529
1.799907407	1.486092529
1.799907407	1.486092529
1.799907407	1.486092529
1.799907407	1.486092529
1.799907407	1.486092529
1.799907407	1.486092529
1.799907407	1.486092529
1.799907407	1.486092529

1.799907407	1.486092529
1.799907407	1.486092529
1.120582605	0.923282264
1.358630588	1.113784329
1.358630588	1.113784329
1.358630588	1.113784329
1.241635688	1.016363403
1.227462825	0.985120247
1.227462825	0.985120247
1.333402048	1.06563642
1.333402048	1.06563642
1.333402048	1.06563642
1.170973944	0.93259858
1.714109347	1.356697381
1.714109347	1.356697381
1.714109347	1.356697381
1.714109347	1.356697381
1.714109347	1.356697381
1.714109347	1.356697381
1.714109347	1.356697381
1.714109347	1.356697381
1.714109347	1.356697381
1.714109347	1.356697381
1.714109347	1.356697381
1.714109347	1.356697381
1.714109347	1.356697381
1.714109347	1.356697381
1.714109347	1.356697381
1.714109347	1.356697381
1.714109347	1.356697381
1.714109347	1.356697381
1.714109347	1.356697381
1.714109347	1.356697381
1.213608454	0.954984886
1.309090909	1.020030567
1.200061958	0.925910507
1.200061958	0.925910507
1.285648025	0.976801078
1.285648025	0.976801078
1.636111111	1.242355191
1.636111111	1.242355191
1.636111111	1.242355191
1.636111111	1.242355191
1.636111111	1.242355191
1.636111111	1.242355191
1.636111111	1.242355191
1.636111111	1.242355191
1.636111111	1.242355191

1.636111111	1.242355191
1.636111111	1.242355191
1.636111111	1.242355191
1.636111111	1.242355191
1.636111111	1.242355191
1.636111111	1.242355191
1.636111111	1.242355191
1.636111111	1.242355191
1.636111111	1.242355191
1.636111111	1.242355191
1.636111111	1.242355191
1.081657585	0.808140257
1.090965732	0.810214682
1.173852433	0.870769166
1.263027699	0.935795385
1.263027699	0.935795385
1.124941806	0.824314955
1.56489533	1.140826605
1.56489533	1.140826605
1.56489533	1.140826605
1.56489533	1.140826605
1.56489533	1.140826605
1.56489533	1.140826605
1.56489533	1.140826605
1.56489533	1.140826605
1.56489533	1.140826605
1.56489533	1.140826605
1.56489533	1.140826605
1.56489533	1.140826605
1.56489533	1.140826605
1.56489533	1.140826605
1.56489533	1.140826605
1.56489533	1.140826605
1.161170404	0.844619884
1.161170404	0.844619884
1.241187384	0.896872818
1.241187384	0.896872818
1.241187384	0.896872818
1.241187384	0.896872818
1.148758206	0.819366687
1.220087419	0.859903504
1.220087419	0.859903504
1.499614198	1.050277915
1.499614198	1.050277915
1.499614198	1.050277915
1.499614198	1.050277915
1.499614198	1.050277915
1.499614198	1.050277915

1.499614198	1.050277915
1.499614198	1.050277915
1.499614198	1.050277915
1.499614198	1.050277915
1.499614198	1.050277915
1.499614198	1.050277915
1.499614198	1.050277915
1.499614198	1.050277915
1.499614198	1.050277915
1.499614198	1.050277915
1.499614198	1.050277915
1.499614198	1.050277915
1.499614198	1.050277915
1.499614198	1.050277915
1.499614198	1.050277915
1.499614198	1.050277915
1.499614198	1.050277915
1.499614198	1.050277915
1.136607318	0.794973302
1.199690785	0.824767385
1.199690785	0.824767385
1.199690785	0.824767385
1.124709572	0.771405204
1.071150942	0.734178729
1.082370731	0.729705102
1.439555556	0.969195039
1.439555556	0.969195039
1.439555556	0.969195039
1.439555556	0.969195039
1.439555556	0.969195039
1.439555556	0.969195039
1.439555556	0.969195039
1.439555556	0.969195039
1.439555556	0.969195039
1.179962894	0.791353341
1.179962894	0.791353341
1.101642516	0.726614959
1.160871387	0.759558406
1.160871387	0.759558406
1.384116809	0.896318254
1.384116809	0.896318254
1.384116809	0.896318254
1.384116809	0.896318254
1.384116809	0.896318254
1.384116809	0.896318254
1.384116809	0.896318254
1.384116809	0.896318254
1.384116809	0.896318254

1.384116809	0.896318254
1.384116809	0.896318254
1.384116809	0.896318254
1.384116809	0.896318254
1.384116809	0.896318254
1.384116809	0.896318254
1.384116809	0.896318254
1.384116809	0.896318254
1.384116809	0.896318254
1.384116809	0.896318254
1.384116809	0.896318254
1.384116809	0.896318254
1.384116809	0.896318254
1.090458488	0.705331665
1.090458488	0.705331665
1.142385959	0.729287057
1.142385959	0.729287057
1.079498141	0.684751197
1.332784636	0.830592084
1.332784636	0.830592084
1.332784636	0.830592084
1.332784636	0.830592084
1.332784636	0.830592084
1.332784636	0.830592084
1.332784636	0.830592084
1.332784636	0.830592084
1.332784636	0.830592084
1.332784636	0.830592084
1.332784636	0.830592084
1.332784636	0.830592084
1.1244782	0.700450576
1.1244782	0.700450576
1.1244782	0.700450576
1.068754831	0.664846424
1.068754831	0.664846424
1.008607969	0.626506586
1.058222174	0.645591458
1.10712145	0.672966484
1.10712145	0.672966484
1.10712145	0.672966484
1.285119048	0.771126445
1.285119048	0.771126445
1.285119048	0.771126445
1.285119048	0.771126445
1.285119048	0.771126445
1.285119048	0.771126445
1.285119048	0.771126445
1.285119048	0.771126445

1.285119048	0.771126445
1.285119048	0.771126445
1.285119048	0.771126445
1.285119048	0.771126445
1.285119048	0.771126445
1.285119048	0.771126445
1.285119048	0.771126445
1.285119048	0.771126445
1.285119048	0.771126445
1.285119048	0.771126445
1.027879755	0.616581468
1.027879755	0.616581468
1.047894034	0.626961584
1.090290662	0.646758014
1.020537	0.602036362
1.073962285	0.621753651
1.073962285	0.621753651
1.073962285	0.621753651
1.240740741	0.717166219
1.240740741	0.717166219
1.240740741	0.717166219
1.240740741	0.717166219
1.240740741	0.717166219
1.240740741	0.717166219
1.240740741	0.717166219
1.240740741	0.717166219
1.240740741	0.717166219
1.240740741	0.717166219
1.240740741	0.717166219
1.058114155	0.597886703
1.018078838	0.574591731
1.199320988	0.668067208
1.199320988	0.668067208
1.199320988	0.668067208
1.199320988	0.668067208
1.199320988	0.668067208
1.199320988	0.668067208
1.199320988	0.668067208
1.199320988	0.668067208
1.199320988	0.668067208
1.199320988	0.668067208
1.199320988	0.668067208
1.199320988	0.668067208
1.199320988	0.668067208
1.199320988	0.668067208
1.199320988	0.668067208
1.199320988	0.668067208
1.199320988	0.668067208

1.199320988	0.668067208
1.199320988	0.668067208
1.199320988	0.668067208
1.199320988	0.668067208
1.199320988	0.668067208
1.199320988	0.668067208
1.008511969	0.558236491
1.042725391	0.575094919
1.042725391	0.575094919
1.042725391	0.575094919
1.042725391	0.575094919
1.042725391	0.575094919
0.974282813	0.536265381
0.982587065	0.538970598
0.976259145	0.531702784
0.999122264	0.542398346
1.027776305	0.553320139
1.027776305	0.553320139
1.160573477	0.623276991
1.160573477	0.623276991
1.160573477	0.623276991
1.160573477	0.623276991
1.160573477	0.623276991
1.160573477	0.623276991
1.160573477	0.623276991
1.160573477	0.623276991
1.160573477	0.623276991
1.160573477	0.623276991
1.160573477	0.623276991
1.160573477	0.623276991
0.989904846	0.527058442
1.013248321	0.532507979
1.013248321	0.532507979
0.980855019	0.512198738
0.96184253	0.500502057
1.124247685	0.582319548
1.124247685	0.582319548
1.124247685	0.582319548
1.124247685	0.582319548
1.124247685	0.582319548
1.124247685	0.582319548
1.124247685	0.582319548
1.124247685	0.582319548
1.124247685	0.582319548
1.124247685	0.582319548
1.124247685	0.582319548
1.124247685	0.582319548
1.124247685	0.582319548

1.124247685	0.582319548
1.124247685	0.582319548
1.124247685	0.582319548
1.124247685	0.582319548
1.124247685	0.582319548
1.124247685	0.582319548
0.961369622	0.496696105
0.999123892	0.512607537
0.971968251	0.497801957
0.971968251	0.497801957
0.985386434	0.493571137
0.985386434	0.493571137
0.985386434	0.493571137
0.985386434	0.493571137
1.090123457	0.544782836
1.090123457	0.544782836
1.090123457	0.544782836
1.090123457	0.544782836
1.090123457	0.544782836
1.090123457	0.544782836
1.090123457	0.544782836
1.090123457	0.544782836
1.090123457	0.544782836
1.090123457	0.544782836
0.952458471	0.475269789
0.972020258	0.475354082
0.946243397	0.457227132
1.058006536	0.510308674
1.058006536	0.510308674
1.058006536	0.510308674
1.058006536	0.510308674
1.058006536	0.510308674
1.058006536	0.510308674
1.058006536	0.510308674
1.058006536	0.510308674
1.058006536	0.510308674
1.058006536	0.510308674
1.058006536	0.510308674
1.058006536	0.510308674
0.959010513	0.457914439
0.959010513	0.457914439
0.959010513	0.457914439
0.959010513	0.457914439
0.937966704	0.444523354
0.937966704	0.444523354
0.946343131	0.441212834
0.946343131	0.441212834
1.027724868	0.478584458

1.027724868	0.478584458
1.027724868	0.478584458
1.027724868	0.478584458
1.027724868	0.478584458
1.027724868	0.478584458
1.027724868	0.478584458
1.027724868	0.478584458
1.027724868	0.478584458
1.027724868	0.478584458
1.027724868	0.478584458
1.027724868	0.478584458
0.934004771	0.425212272
0.999125514	0.449336339
0.999125514	0.449336339
0.999125514	0.449336339
0.999125514	0.449336339
0.999125514	0.449336339
0.999125514	0.449336339
0.999125514	0.449336339
0.999125514	0.449336339
0.906250976	0.397444896
0.911096184	0.396297679
0.931985796	0.405140461
0.972072072	0.422323566
0.972072072	0.422323566
0.972072072	0.422323566
0.972072072	0.422323566
0.972072072	0.422323566
0.972072072	0.422323566
0.972072072	0.422323566
0.972072072	0.422323566
0.910265142	0.395177179
0.910265142	0.395177179
0.898652416	0.386546388
0.898652416	0.386546388
0.898274254	0.381491868
0.891179453	0.375972838
0.946442495	0.39733378
0.946442495	0.39733378
0.946442495	0.39733378
0.946442495	0.39733378
0.946442495	0.39733378
0.946442495	0.39733378
0.946442495	0.39733378
0.946442495	0.39733378
0.946442495	0.39733378
0.946442495	0.39733378
0.946442495	0.39733378

0.946442495	0.39733378
0.946442495	0.39733378
0.946442495	0.39733378
0.946442495	0.39733378
0.946442495	0.39733378
0.88769784	0.367554653
0.88769784	0.367554653
0.894651259	0.364747392
0.897701689	0.365016074
0.87659806	0.355757296
0.922127255	0.374179071
0.922127255	0.374179071
0.922127255	0.374179071
0.922127255	0.374179071
0.922127255	0.374179071
0.922127255	0.374179071
0.922127255	0.374179071
0.922127255	0.374179071
0.922127255	0.374179071
0.876827006	0.354576483
0.876827006	0.354576483
0.876827006	0.354576483
0.869483751	0.346094691
0.866218119	0.342118744
0.866218119	0.342118744
0.866218119	0.342118744
0.866218119	0.342118744
0.899027778	0.352692668
0.899027778	0.352692668
0.899027778	0.352692668
0.899027778	0.352692668
0.899027778	0.352692668
0.899027778	0.352692668
0.899027778	0.352692668
0.885062246	0.347019769
0.855861825	0.330157042
0.860491308	0.327864168
0.877055104	0.332726138
0.877055104	0.332726138
0.877055104	0.332726138
0.877055104	0.332726138
0.877055104	0.332726138
0.877055104	0.332726138
0.877055104	0.332726138
0.877055104	0.332726138
0.877055104	0.332726138
0.877055104	0.332726138
0.877055104	0.332726138

0.877055104	0.332726138
0.845749209	0.318668336
0.835871769	0.307630847
0.856128748	0.314147016
0.856128748	0.314147016
0.856128748	0.314147016
0.856128748	0.314147016
0.856128748	0.314147016
0.856128748	0.314147016
0.866479401	0.314068259
0.845174718	0.306045771
0.836175711	0.296836781
0.836175711	0.296836781
0.836175711	0.296836781
0.836175711	0.296836781
0.836175711	0.296836781
0.836175711	0.296836781
0.836175711	0.296836781
0.822724822	0.285900756
0.81712963	0.280689131
0.81712963	0.280689131
0.81712963	0.280689131
0.81712963	0.280689131
0.81712963	0.280689131
0.807571241	0.277025168
0.807571241	0.277025168
0.798556999	0.267597294
0.810269167	0.270847213
0.798930041	0.2656085
0.798930041	0.2656085
0.798930041	0.2656085
0.798930041	0.2656085
0.798930041	0.2656085
0.798930041	0.2656085
0.798930041	0.2656085
0.798930041	0.2656085
0.798930041	0.2656085
0.798930041	0.2656085
0.789740871	0.258528044
0.789740871	0.258528044
0.781521739	0.251508785
0.781521739	0.251508785
0.781521739	0.251508785
0.781521739	0.251508785
0.781521739	0.251508785
0.781521739	0.251508785
0.781521739	0.251508785
0.781116399	0.249801707

0.792272578	0.249834642
0.772677399	0.241403375
0.772677399	0.241403375
0.772677399	0.241403375
0.764854216	0.238312243
0.764854216	0.238312243
0.764854216	0.238312243
0.764854216	0.238312243
0.764417953	0.2333189
0.748881173	0.225948535
0.748881173	0.225948535
0.748881173	0.225948535
0.748881173	0.225948535
0.748881173	0.225948535
0.733560091	0.214353903
0.733560091	0.214353903
0.733560091	0.214353903
0.733560091	0.214353903
0.733560091	0.214353903
0.733560091	0.214353903
0.733560091	0.214353903
0.769476899	0.224479461
0.763980068	0.218577543
0.718851852	0.203470441
0.718851852	0.203470441
0.718851852	0.203470441
0.718851852	0.203470441
0.718851852	0.203470441
0.718851852	0.203470441
0.718851852	0.203470441
0.753217043	0.207261086
0.704720407	0.193245468
0.704720407	0.193245468
0.704720407	0.193245468
0.704720407	0.193245468
0.704720407	0.193245468
0.704720407	0.193245468
0.704720407	0.193245468
0.834893668	0.228481402
0.747947646	0.201836735
0.763540552	0.203181281
0.691132479	0.183630971
0.691132479	0.183630971
0.691132479	0.183630971
0.691132479	0.183630971
0.691132479	0.183630971
0.691132479	0.183630971
0.691132479	0.183630971

0.759461244	0.198637942
0.678057303	0.174583126
0.678057303	0.174583126
0.678057303	0.174583126
0.665466392	0.166061861
0.665466392	0.166061861
0.697299926	0.172669369
0.776625392	0.192147056
0.653333333	0.158030487
0.653333333	0.158030487
0.653333333	0.158030487
0.653333333	0.158030487
0.683947345	0.16169446
0.641633598	0.150455357
0.641633598	0.150455357
0.641633598	0.150455357
0.677460006	0.156495071
0.630344379	0.143305577
0.630344379	0.143305577
0.630344379	0.143305577
0.630344379	0.143305577
0.671093926	0.151477637
0.671093926	0.151477637
0.787957056	0.175194653
0.619444444	0.136552734
0.619444444	0.136552734
0.619444444	0.136552734
0.619444444	0.136552734
0.619444444	0.136552734
0.619444444	0.136552734
0.619444444	0.136552734
0.658712192	0.141960407
0.658712192	0.141960407
0.608913999	0.130170673
0.608913999	0.130170673
0.652690167	0.137447281
0.652690167	0.137447281
0.598734568	0.124135278
0.646776647	0.13308942
0.706570897	0.144886032
0.588888889	0.118424292
0.588888889	0.118424292
0.588888889	0.118424292
0.640968725	0.128880873
0.579360812	0.113017146
0.570135215	0.107894812
0.570135215	0.107894812
0.570135215	0.107894812

0.810418981	0.1502908
0.759482728	0.139888873
0.561197917	0.103039666
0.561197917	0.103039666
0.561197917	0.103039666
0.656054493	0.119598072
0.613418328	0.109887084
0.648083038	0.113569748
0.544135802	0.094066764
0.544135802	0.094066764
0.535986733	0.089919756
0.535986733	0.089919756
0.535986733	0.089919756
0.593017372	0.096860303
0.528077342	0.085981249
0.58812616	0.093868914
0.58812616	0.093868914
0.520397209	0.082239052
0.520397209	0.082239052
0.520397209	0.082239052
0.520397209	0.082239052
0.583314479	0.090976005
0.512936508	0.078681806
0.512936508	0.078681806
0.505685968	0.075298922
0.505685968	0.075298922
0.645461104	0.09460157
0.498636831	0.072080517
0.498636831	0.072080517
0.491780822	0.069017362
0.478617284	0.063322849
0.547465621	0.070980681
0.472295322	0.060675868
0.472295322	0.060675868
0.472295322	0.060675868
0.466137566	0.058152807
0.466137566	0.058152807
0.539177257	0.066748646
0.460137702	0.055747034
0.460137702	0.055747034
0.460137702	0.055747034
0.531134474	0.062782095
0.454289733	0.053452323
0.454289733	0.053452323
0.454289733	0.053452323
0.454289733	0.053452323
0.454289733	0.053452323
0.454289733	0.053452323

0.448587963	0.051262833
0.604137693	0.067597739
0.443026978	0.049173075
0.443026978	0.049173075
0.568537204	0.062993708
0.565515555	0.061410754
0.599019814	0.064634209
0.437601626	0.047177889
0.437601626	0.047177889
0.437601626	0.047177889
0.437601626	0.047177889
0.663497551	0.071456922
0.512032865	0.053912854
0.432307006	0.045272425
0.556638481	0.056900308
0.427138448	0.043452115
0.427138448	0.043452115
0.427138448	0.043452115
0.427138448	0.043452115
0.548033154	0.052725928
0.497706658	0.04776745
0.610195609	0.058538762
0.412345679	0.038460342
0.490837929	0.044973953
0.407638889	0.036940057
0.407638889	0.036940057
0.407638889	0.036940057
0.487473654	0.043641686
0.487473654	0.043641686
0.403037869	0.035485754
0.484154841	0.042350523
0.398539095	0.034094224
0.394139194	0.032762434
0.394139194	0.032762434
0.526322983	0.04304738
0.389834944	0.031487519
0.389834944	0.031487519
0.389834944	0.031487519
0.385623258	0.030266768
0.385623258	0.030266768
0.580023987	0.043078119
0.459136102	0.033348883
0.671982912	0.048353788
0.618278822	0.044334387
0.598371012	0.042576542
0.369644903	0.02587614
0.369644903	0.02587614
0.543676672	0.037702235

0.593305201	0.040208844
0.568762477	0.038081289
0.447564935	0.029616663
0.447564935	0.029616663
0.362139918	0.023945298
0.362139918	0.023945298
0.444761982	0.028752955
0.3585	0.023039045
0.563291586	0.035799595
0.738215894	0.046189094
0.35493216	0.02216986
0.35493216	0.02216986
0.35493216	0.02216986
0.53138391	0.032934683
0.436558215	0.026314533
0.527407285	0.031481913
0.348004315	0.020536095
0.341340388	0.019031625
0.341340388	0.019031625
0.338102725	0.018324338
0.338102725	0.018324338
0.476679058	0.025321258
0.33492558	0.01764527
0.584269663	0.029289167
0.470333594	0.02347531
0.325740741	0.015765361
0.317043592	0.014098515
0.317043592	0.014098515
0.317043592	0.014098515
0.306141184	0.012162936
0.303531073	0.011724696
0.382407508	0.013434433
0.583800971	0.019338607
0.28642963	0.009089683
0.370403837	0.011292128
0.368475413	0.010970477
0.279673032	0.008159648
0.277490669	0.007872419
0.273225898	0.007329466
0.263112745	0.006137433
0.44409637	0.010092262
0.346794676	0.007764018
0.257394085	0.005521558
0.236791759	0.00363541
0.232142857	0.003278492
0.232142857	0.003278492
0.208880225	0.001838921
0.39315617	0.002037671

0.186814039	0.000943971
0.532890576	0.00222109
0.180144033	0.000749597
0.177427676	0.000679341
0.306226766	0.001117305
0.162692373	0.000378123
0.156893457	0.000292103
0.153472222	0.000248749
0.316091381	0.000507717
0.152805595	0.000240897
0.145230537	0.000164175
0.133111389	8.18228E-05
0.120301318	3.41329E-05
0.452845888	7.42511E-05
0.377535261	2.56814E-05
0.277070087	9.27355E-06
0.410579842	2.5473E-06
0.368803032	2.07794E-06

Genes
CYP39A1;FABP3;LIPC;ACSM3;INSIG1;LCAT;APOC3;CYP7B1;APOB;ABCG4
VIT;POSTN;COL16A1;COL24A1;LUM;COL22A1;COL12A1;MMP8;CCDC80;COL2A1;CTSK;SMOC1;SERPINH1;
CYP39A1;FABP3;LIPC;ACSM3;INSIG1;LCAT;APOC3;CYP7B1;APOB;ABCG4
HCAR2;SREBF1;APOC1;CIDEA;APOC3;CCDC3
HCAR2;RARRES2;APOC1;CIDEA;APOC3
SLC25A1;ACSL1;ELOVL6;HSD17B12;ACSL3
LIPC;APOC1;APOC3;LCAT
PTGIS;DDAH1;CLEC7A;PTGS2;JAK2;MMP8
RMI2;CDCA2;NCAPG;CDC6;MKI67
SLC5A8;PTGIS;QPRT;NUDT12
ACSL1;ELOVL6;ACSL3;HSD17B12
CYP39A1;APOF;LCAT;MSMO1;CYP7B1;APOB;CYP8B1
HCAR2;APOC1;CIDEA;APOC3
LIPC;MOGAT2;APOC1;PNPLA3;APOC3;APOB
SLC5A8;PTGIS;NUDT12
GLDC;NR1H4;UCP1;PDK4
GPNMB;ILDR2;CTSG;VTCN1
SLC22A4;SLC5A8;CENPF;SLC36A2;AQP9;SLC6A12;SLC3A1;RAB38;SLC13A5;SLC6A2;RHBG;RHD
C3;HILPDA;CIDEA;PLIN2
CYP39A1;LIPC;ACSM3;ALDH1A2;ACSM5;CYP7B1;CYP8B1
SLC46A1;SLC36A2;UCP3;FXYD2;UCP1;OTOP1
APOC1;APOC3;APOB
TOP2A;SPAG5;ESPL1;CENPI;KNTC1
SLC46A1;GLRX3;SCARA5;FRRS1;TFR2;CP
ORM1;CLEC7A;OAS3;CIDEA;NR1H4;CD14;IGF1;JAK2;MMP8;PF4
LIPC;APOC1;LCAT;APOC3;APOB;ABCG4
CNKSR2;ADRB3;ACSL1;CLEC7A;UCP1;ADRB1;ELOVL6;JAK2;JCHAIN
VIT;POSTN;COL16A1;CCDC80;COL24A1;COL22A1;SMOC1;MMP8;MATN4
VIT;POSTN;COL16A1;CCDC80;COL24A1;COL22A1;SMOC1;MMP8;MATN4
ABCD2;FABP3;ACSL1;ACSL3
ACSL1;ELOVL6;ACSL3;HSD17B12
GPNMB;ILDR2;CTSG;VTCN1
LIPC;PNPLA3;APOC3
LIPC;APOB;MPO
C3;CCBE1;BRCA1;PTGS2
OPN1MW;SPAG5;CD28;CDCA8;NCAPG;CDC6;IGF1;CDC14A;E2F8
APOC1;PDK4;APOC3;BRCA1
C3;CCBE1;BRCA1;PTGS2
ORM1;CLEC7A;OAS3;CD14;JAK2;MMP8;PF4
DDAH1;CLEC7A;PTGS2;MMP8
UBE2C;KNTC1;CDC14A
F13A1;FBLN1;GP5
APOC1;APOC3;BRCA1
F13A1;FBLN1;GP5
ABCD2;LIPC;ACSM3;ACSL1;PNPLA3;ACSM5;MSMO1;ACSL3;CYP2E1
DDAH1;CLEC7A;PTGS2;MMP8
ORM1;CLEC7A;OAS3;CD14;JAK2;MMP8;PF4

RHBG;RHD
SIX2;FGFR2
NR1H4;APOC3
APOC1;APOC3
SLIT2;NBL1
ESPL1;UBE2C;CDCA8
SPAG5;ESPL1;UBE2C;CENPI;KNTC1
COMP;GDF10;BMP3;GDF11;COL2A1;OGN;EN1;IGF1;PTH1R;FGFR2
NR1H4;UCP1;PDK4
LIPC;APOC3;LCAT
DMTN;CTSG;JAK2
ANLN;CENPF;UBE2C;UBD;KNTC1;CDC6;CDC14A
ITGA4;LAMB3;COL12A1;MMP8
WNT10B;INSIG1;MGST1;UCP1;NR1H4;PPBP;ADCY5;PDK4;SPP1;CTSG;CD14;ADTRP;PF4
KRT17;IDH1;QPRT
GPNMB;ILDR2;CTSG;VTCN1;TNFRSF21
MGAM;PYGL
FABP3;NR1H4
ABCD2;PTGS2
TPX2;ESPL1;SPAG5;CENPI;CDCA8;NCAPG;KNTC1;KIF11
CDK1;NCAPG;CDC6
DCT;NCAN;HAPLN4
GDF10;GDF11;BMP3;FOS;RUNX2
CYP39A1;HSD11B1;BDH1;MSMO1;CYP2E1;CYP7B1;CYP8B1
SREBF1;LIPC;ACSS2;ACSM3;ACSL1;ACSM5
CCBE1;ANGPT1;ADTRP;SLIT2;E2F8
KRT17;IDH1;NUDT12
IGF1;MMP8;PTGS2
CDCA2;NEK2;MKI67
RARRES2;SIX2;FGFR2
APOC1;APOC3;BRCA1
TOP2A;NHEJ1
SREBF1;INSIG1
SLC22A4;SLC6A12
SMOX;CHDH
ACSS2;ADH7
LIPC;LCAT
BRCA1;MPND
WNT10B;APOC1;CIDEA;APOC3;CCDC3
ITGA4;LAMB3;COL12A1;MMP8
ESPL1;UBE2C;CD28;IGF1
RARRES2;APOC1;APOC3;THRSP;CCDC3
CDKL5;MME;DCT;NCAN;SLIT2;HAPLN4
CNKSR2;ADRB3;ACSL1;UCP1;ADRB1;ELOVL6;JAK2
CDCA2;CD28;NEK2;IGF1;MKI67
CCR1;CCNA2;DUOX1;CCL24;CCL11;CD151;GPNMB;F10;CLEC7A;HAS2;LAMC2;IGF1
CNKSR2;WNT10B;ADRB3;ACSL1;CIDEA;UCP1;ADRB1;ELOVL6;JAK2
C3;HILPDA;APOB

HSD11B1;LIPC;PLA1A
RHOU;RHOBTB1
ENPEP;CTSG
NEK2;KIF11
SREBF1;INSIG1
PTGIS;PTGS2
C1QB;C3
LIPC;LCAT
TOP2A;ESPL1
APOC1;APOC3
CLEC7A;OAS3
CLEC7A;JCHAIN
CIDEA;PLIN2
ENPEP;CTSG
CCR1;CCL24;CCL11;PPBP;PF4
SLPI;RARRES2;CTSG;PPBP;CXCL14;JCHAIN;PF4
HCN4;DUOX1;DMTN;AQP9
ACSL1;PNPLA3;ELOVL6;ACSL3;ADTRP;CYP2E1
OPN1MW;CDCA8;CDC6;CDC14A
KRT17;QPRT;NUDT12
CCR1;CCL24;CCL11;PPBP;PF4
SLC25A1;HK3;KRT17;GALM;GDPGP1
HCN4;DMTN;INSIG1;AQP9;SPP1;NR1H4;ADTRP;BRCA1;ADCY5
HCN4;SLC46A1;SLC36A2;SCARA5;KCNH7;UCP1;SLC6A12;SLC1A3;OTOP1;SLC6A2;RHD;FXYD2;UCP3;RHB
CENPF;KNTC1
JAK2;GP5
SPAG5;NEK2
APOC1;APOC3;ABCG4
SOCS2;CLEC3B;MME;UCP1;NR1H4;SLIT2
C3;SMOC1;CHRDL1;FREM2
LIPC;PNPLA3;APOC3
DCT;NCAN;HAPLN4
SLC46A1;SLC1A3;SLC13A5
SCARA5;TFR2;CP
COL2A1;LUM;COL12A1;SERPINH1
CYP39A1;MSMO1;CYP7B1;CYP8B1
CCL24;CCL11;UBD;HAS2;BRCA1;JAK2;TNFRSF21
CCR1;CCL24;ORM1;VNN1;CCL11;C3AR1;HP;NOX4;CD14;PPBP;FOS;PF4
CCNA2;GDF10;SMOC1;IGF1;RUNX2;FGFR2
EDAR;SCEL;DCT;LAMB3;LAMC2;FGFR2
OPN1MW;CDC6;CDC14A;FGFR2
NR1H4;APOC3
RHBG;RHD
CLEC7A;JCHAIN
DUOX1;CTSK
SLC36A2;SLC6A2
APOC1;APOC3
C3;HILPDA

UCP1;THRSP
CCR1;SLIT2;NBL1
CYP39A1;CYP7B1;CYP8B1
DMTN;CTSG;JAK2
APOC1;APOC3;BRCA1
ADGRE1;ADGRF3;ADRB3;ADRB1;PTH1R;ADCY5;PF4
VIT;DMTN;CCDC80;JAK2
LIPC;ACSM3;ACSM5;ELOVL6;CYP2E1
ARHGAP44;CCL24;CCL11;CDKL5;RGS10;RGL3;ADRB1;CPEB2;SLC6A2;NGEF;RGS7
ITIH4;VNN1;HP
ABCD2;UCP1;MMP8
FABP3;PDK4
CLEC7A;OAS3
MAT1A;ASPA
SOCS2;JAK2
SLC46A1;SLC2A5
SLIT2;NBL1
DDAH1;SLIT2
APOC1;APOC3
ESPL1;UBE2C
ESPL1;UBE2C
DMTN;JAK2
CCBE1;TFR2
CDK1;KNTC1
CCR1;SLC46A1;CCL11;SCARA5;GLRX3;FRRS1;TFR2;FXYD2;PTH1R;CP
INSIG1;APOF;LCAT;MSMO1;APOB
CLEC7A;CD28;MMP8;TNFRSF21
CDK1;BRCA1;CLSPN;CDC6
SLC22A4;SLC1A3;SLC6A2
DUOX1;DMTN;CLEC7A
IDH1;APOF;LCAT;APOB
ORM1;CIDEA;NR1H4;IGF1
HCN4;CACNG7;FXYD2;WNK3;PTPN3
SMPD3;PNPLA3;PLBD1;LCAT;AGPAT2
C3;PDK4;THRSP
POR;DDAH1
HSD11B1;SPP1
SLIT2;NBL1
DMTN;FBLN1
PON1;PLBD1
SFRP4;XPR1
FABP3;TLCD1
SREBF1;PTH1R
FXYD2;WNK3
CIDEA;PNPLA3;PLIN2
LIPC;PNPLA3;AGPAT2
DCT;NCAN;HAPLN4
ANAPC15;ESPL1;UBE2C;CDC6

ORM1;CIDEA;NR1H4;IGF1
ANGPT4;PTGIS;HILPDA;PTGS2;CPEB2
CYP39A1;MSMO1;CYP7B1;CYP8B1
CD151;SPAG5;TFR2;PON1
CDK1;BRCA1;CLSPN
HPGDS;PTGIS;PTGS2
SLC25A1;ACSS2
LGI2;HAPLN4
CLEC7A;JAK2
CDCA8;NCAPG
CLEC7A;JAK2
NR1H4;PDK4
SREBF1;DDAH1;CLEC7A;CD28;NR1H4;IGF1;PTH1R;MMP8;PTGS2
ANGPT4;PTGIS;HILPDA;PTGS2;CPEB2
ABCC3;SLC5A8;ACSL1;SLC10A1;SLC6A12
CCL24;CDKL5;CCL11;CTSK;RGS10;ADRB1;RGL3;SLC6A2;RGS7
ACSM3;ACSM5;HMGCS2
CYP39A1;CYP7B1;CYP8B1
HCN4;DMTN;AQP9
LIPC;NR1H4;APOC3
SLC22A4;SLC6A12;SLC6A2
PKLR;INSIG1;RARRES2;PDK4;OTOP1;CPEB2
ANAPC15;CDCA2;UBD;GMNN;NEK2;MKI67;PTPN3
IDH1;TAT
SFRP4;FGFR2
CIDEA;PLIN2
CENPF;CENPI
ACSS2;ADH7
CLEC7A;JAK2
PON1;WNK3
PTGIS;PTGS2
JAK2;PF4
ABCD2;ACSL1;ELOVL6
CCNA2;CCNE2;CCNF;CDC6
GPNMB;CD28;VTCN1;JAK2;TNFRSF21
AS3MT;PTGIS;PON1
LIPC;PNPLA3;AGPAT2
APOC1;APOC3
ADRB3;ADRB1
DDAH1;SLC2A5
CLEC7A;PF4
SIX2;FGFR2
CCNA2;CD151
ADRB3;ADRB1
GMNN;CDC6
JAK2;GP5
SPAG5;SNX10
COMP;POLQ;SCARA5;TMEM120A;ALDH1A2;MAT1A;THG1L

DUOX1;NOX4;SOD3
ESPL1;NEK2;E2F8
KRT17;IDH1;QPRT
CLEC7A;HOMER2;IGF1
HCN4;DMTN;AQP9;NR1H4;BRCA1;SLIT2
CCR1;CCNA2;CCL24;CCL11;DMTN;CD151;GPNMB;F10;CLEC7A;HAS2;LAMC2;IGF1
FABP3;PDK4
ACSS2;HMGCS2
PERP;CYCS
CLEC7A;RARRES2
TOP2A;DNASE2B
CCNA2;FGFR2
QPRT;ASPA
SLIT2;FGFR2
MMP8;TNFRSF21
TOP2A;CENPF
IGF1;FGFR2
TPX2;ANGPT1;CLSPN;IGF1;JAK2
CCR1;CCNA2;CCL24;CCL11;MUSK;GPNMB;ANGPT1;STYX;FBLN1;IGF1;FGFR2
CENPF;MME;SIX2;HAS2
SLC5A8;SLC4A10;SLC4A1;XPR1
GPNMB;VTCN1;TNFRSF21
ESPL1;CDK1;CDCA8;KIF11
ANLN;ESPL1;EXOC7;CDCA8
OAS3;SLIT2;OTOP1;CCDC3
ADGRE1;ADGRF3;GNAL;ADRB3;SCTR;PTH1R;ADCY5;PF4
CDC6;CLSPN
SLC1A3;RHBG
MOGAT2;PNPLA3
SOCS2;JAK2
APOB;PF4
PTGIS;PTGS2
SPAG5;SNX10
FXYD2;WNK3;PTPN3
CCNA2;CCNE2;UBE2C;CCNF;CDK1;BCAT1
HCN4;SLC46A1;SLC36A2;SCARA5;KCNH7;FXYD2;UCP3;UCP1;SLC6A12;SLC1A3;OTOP1;SLC6A2
SLC22A4;SLC46A1;SLC1A3
CDKL5;ISLR2;SLIT2
GDF10;BMP3;AMHR2;RUNX2
GDF10;GDF11;BMP3;PMEPA1
CCNA2;CNKSR2;RRAD;RAB38;RGL3;IGF1
ADRB3;ADRB1
ADRB3;PYGL
SLC25A1;ACSL3
SLIT2;FGFR2
ABCD2;FABP3
AP1S2;RAB38
PON1;PNPLA3;LCAT;PLA1A

GPNMB;ILDR2;CTSG;VTCN1
C3;APOC1;APOC3
GPNMB;VTCN1;TNFRSF21
GDF10;GDF11;ANGPT4;MUSK;ANGPT1;RARRES2;CDCA8;IGF1;C3;CCNA2;BMP3;GPNMB;WNK3;ADTRP;JAK2
SLC5A8;SLC1A3;SLC4A1;XPR1
SFRP4;SLIT2
ISG20;DNASE2B
SLC4A10;SLC4A1
IGF1;SLIT2
CLEC7A;PAK3
CLEC7A;PAK3
DUOX1;NOX4
GYS2;PYGL
GDF11;JAK2
DMTN;INPP5J
HCAR2;TFR2
ITGA4;MUSK
RARRES2;ALDH1A2;ADH7
DUOX1;DMTN;CLEC7A
CCBE1;ORM1;HILPDA;NR1H4;BRCA1;PTGS2;MMP8;AGPAT2;C3;CLEC7A;CD28;CD14;JAK2
ANGPT4;DMTN;ANGPT1;IGF1;JAK2
APOC1;PNPLA3;APOC3
GMNN;PON1;IGF1;MMP8;ADD2
ADRB3;ADRB1
HCN4;PTPN3
SIX2;WLS
ABCC3;SLC22A4
CAT;MPO
CCBE1;CLEC3B
GDF10;BMP3;AMHR2;RUNX2
CAT;HP;UCP1;FOS
CCL24;CCL11;HAS2;BRCA1;JAK2;TNFRSF21
CCL24;CCL11;CTSG
HCN4;FXYD2;KCNJ15
C3;CCNA2;MUSK;GPNMB;ANGPT1;RARRES2;CDCA8;FBLN1;IGF1;ADTRP;SLIT2
ISG20;SLPI;OAS3;CD28
APOC1;HP;APOC3;PTPN3
C3;CCBE1;DMTN;PTGIS;CRADD;CTSK;IGF1;JAK2;AGPAT2;FGFR2;WLS
ANAPC15;UBE2C
POLQ;RMI2
ABCD2;BRCA1
ENPEP;MME
ARHGAP44;GPM6A;DMTN
C3;SFRP4;ANGPT1
ABCD2;ORM1;GPNMB;HOMER2;CIDEA;NR1H4;MMP8;TNFRSF21
CCR1;CCL24;CCL11;MUSK;GPNMB;ANGPT1;IGF1;FGFR2
CCNA2;GDF10;GDF11;BMP3;JAK2
SFRP4;OGN;FGFR2

POSTN;SPP1;ADCY5
CTSK;CTSG;MMP8
CCL24;CCL11;CYP7B1
TPX2;CDCA8;KIF11
CCNA2;IGF1;RUNX2
MOGAT2;PNPLA3
RARRES2;SLIT2
PPBP;SLC2A5
PPBP;SLC2A5
CCL11;IGF1
ESRRB;E2F8
SFRP4;ANGPT1
CCL24;CCL11;PPBP;PF4
CCL24;CCL11;SLIT2;PAK3
C3;PDK4;NR1H4;JAK2
HK3;GALM;GDPGP1
ABCC3;SLC5A8;SLC10A1
VNN1;KRT17;MSMO1;ACSL3;IGF1
CENPF;MME;SIX2;HAS2
ITGA4;ADRB3;SLPI;STYX
VIT;CCDC80;DMTN;JAK2
ABCC3;SLC10A1
PLBD1;SMPDL3B
PTGIS;JAK2
SFRP4;ITGA4
GLRX3;IGF1
SLC36A2;SLC3A1;SLC6A12
CTSK;CTSG;MMP8
CAT;HP;SLC6A2
ANGPT4;ANGPT1;PTGS2
CYP7B1
UBE2L6
SLC1A3
SLC1A3
TAT
TAT
JAK2
NUDT12
SFRP4
CYCS
ANGPT1
ATP8A1
HILPDA
HYDIN
SLC3A1
MME
DSC2
SREBF1

C1QB
SLIT2
OAS3
TFR2
SLFN14
CHDH
RMI2
NR1H4
HCN4
SLIT2
SLC13A5
SLFN14
SPP1
SLIT2
FGFR2
FGFR2
TAT
SLC13A5
UGDH
BCAT1
E2F8
SLC2A5
C1QB
DUOX1
NHEJ1
UBD
JAK2
XPR1
SCTR
HMGCS2
HCAR2
PLIN2
RMI2
SIX2
SIX2
SIX2
CYP2E1
SLIT2
E2F8
APOBEC1
SLIT2
TNFRSF21
APOBEC1
COL2A1
WNK3
RAB38
JAK2
VNN1

SREBF1
PTGS2
MMP8
CDCA8
WNK3
CCNA2
PTGIS
RNF128
SREBF1
UBD
HSPH1
CIDEA
HCAR2;APOC1;CIDEA;APOC3
CCR1;DUOX1;CCL24;CCL11;PTGIS;MME;HAS2;CTSG;BRCA1;JAK2;TNFRSF21;PF4
CLEC7A;HOMER2;CD28
DUOX1;DMTN;CLEC7A
CCL24;CCL11;PPBP;PF4
CDK1;IGF1
PTGIS;CIDEA
LIPC;APOC3
GYS2;SLC2A5
INSIG1;MSMO1
OTOP1;FGFR2
CCL24;CCL11
CCL24;CCL11
NCAN;HAPLN4
MUSK;TNC
NR1H4;JAK2
HCN4;WNT10B;DMTN;INSIG1;AQP9;UCP1;NR1H4;FOS;PPBP;PDK4;CTSG;CD14;SLIT2;SLC13A5;PF4
OPN1MW;BRCA1;SLC6A2
ABCD2;MAL2;TNFRSF21
CCNA2;ESCO2;JAK2
IGF1;JAK2;MMP8
GDF10;BMP3;GDF11;AMHR2;FOS;RUNX2
SCARA5;ADRB3;TFR2;MRC1;RHOU;CD14;CD302;RHOBTB1
CCNA2;SFRP4;CHRDL1;NBL1
CCNA2;GMNN;ESCO2;CDC6
PPP1R3B;IGF1
ARHGAP44;CDKL5
CCBE1;CHAC1
INSIG1;MSMO1
ACSM3;ACSM5
LCAT;APOB
CCNA2;CD151
OTOP1;FGFR2
ELOVL6;CYP2E1
DMTN;INSIG1
CLEC7A;CD28

ACSM3;ACSM5
SLPI;CTSG;JCHAIN
DMTN;JAK2;KLF1
GDF10;GDF11;BMP3
SOCS2;SHC2;MUSK;ANGPT1;RARRES2;PDK4;IGF1;JAK2;PAK3;NGEF;FGFR2
HCAR2;EXOC7;IGF1;WLS
DDAH1
CENPI
BRCA1
EXOC7
TNFRSF21
OAS3
HCN4
PTH1R
CYP8B1
DMTN
STAU2
CDCA8
SLIT2
OAS3
PTGS2
DSC2
SLC6A2
ANGPT1
FGFR2
CENPF
ENPEP
IGF1
CENPI
DDAH1
TMEM120A
SIX2
MAT1A
FGFR2
SLC6A2
ELOVL6
ELOVL6
ELOVL6
ELOVL6
FGFR2
UBE2C
GALM
SLC6A12
GJB1
FGFR2
PYGL
ABCD2
HAL

HAL
HAS2
JAK2
NR1H4
HMGCS2
ABCC3
CYP2E1
ADTRP
NBL1
TLCD1
MOGAT2
NGEF
APOC3
PTPN3
OTOP1
IGF1
OTOP1
SLIT2
CACNG7
SLC6A2
SMOX
SCTR
JAK2
SPAG5
HSPH1
JAK2
HCN4
HCAR2
NR1H4
CCR1;LPAR3;PTH1R
HCN4;C3AR1;F5
SLC46A1;TAT;IDH1
HK3;PKLR;KRT17
SLC5A8;SLC4A10;SLC1A3;SLC4A1
COL2A1;RARRES2;ESRRB;FGFR2
CCL24;CCL11;PPBP;PF4
SLC36A2;SLC1A3
CENPF;KNTC1
CENPF;KNTC1
CENPF;KNTC1
APOC1;APOC3
CIDEA;THG1L
ADRB3;ADRB1
POR;S100A1
CD28;IGF1
HCN4;DSC2
C3;CCNA2;CDCA2;MUSK;GPNMB;ANGPT1;RARRES2;CDCA8;ADTRP
APOBEC1;TFB1M;THG1L

HCN4;SLC5A8;SLC36A2;SLC1A3;SLC4A1
NR1H4;ADCY5
ESRRB;FGFR2
DMTN;GP5
HCAR2;C7
CCNA2;CCNE2;CCNF;CDC6
PKLR;INSIG1;OTOP1;CPEB2
C3;CD151;TFR2;CLEC7A
PTGIS;CYP2E1;PTGS2
WNT10B;COL2A1;OGN
CCNA2;CCNE2;BCAT1
ABCD2;ADH7;ACAT2
LIPC;INPP5J;PNPLA3;LCAT;AGPAT2
ABCC3;FABP3;ACSL1;SLC10A1;TTPA
OPN1MW;CDC6;CDC14A;E2F8
SLC36A2;SLC6A12;SLC3A1
ISG20;SLPI;OAS3
ALDH1A2;MAT1A;THG1L
OAS3
SLIT2
NCAPG
SREBF1
SLC36A2
NUDT12
JAK2
AQP9
DDAH1
PON1
JAK2
CYP2E1
GP5
SLC6A2
ESRRB
SREBF1
MOGAT2
SLC6A2
HCN4
POLQ
SLC46A1
SLC2A5
CYP2E1
GLDC
CCBE1
PKP1
SIX2
TNFRSF21
DCT
MAT1A

GMNN
PKP1
INSIG1
FGFR2
GLRX3
PTGIS
PTGIS
CACNG7
CHAC1
P4HA1
PLA1A
APOBEC1
C3
CDCA8
NCAPG
PTGIS
DMTN
IGF1
HCAR2
JAK2
CLEC3B
CCBE1
SEC61B
CACNG7
TMEM107
NUDT12
TTR
APOC3
C3
CDCA8
HCN4
DDAH1;S100A1
JAK2;GP5
FXYD2;PTPN3
RARRES2;ADH7
INSIG1;MSMO1
SLC25A1;HMGCS2
COMP;SMOC1
CENPF;KNTC1
SMPD3;PLBD1
HCN4;DSC2
C3;CCNA2;MUSK;GPNMB;ANGPT1;RARRES2;CDCA8;ADTRP;FGFR2
CCL24;PTGIS;CCL11;HAS2
ANLN;ESPL1;EXOC7;CDCA8
SOCS2;SLPI;ALDH1A2;SPP1;JAK2
HCN4;FXYD2;SLC6A12;SLC6A2
DDAH1;NOX4
WNK3;KCNJ15

SLIT2;ADH7
CLEC7A;RARRES2
HK3;PKLR
ITGA4;DSC2
TNC;FGFR2
ARHGAP44;SLIT2
CCR1;WNK3
TOP2A;CD28
CCL24;CCL11;CDKL5;RGS10;RGL3;ADRB1;SLC6A2;NGEF;RGS7
LIPC;PON1;LCAT
CCNA2;ADRB3;WWC1;STYX;IGF1;PAK3;MMP8;FGFR2
MUSK;ANGPT1;TNC;IGF1;BRCA1;MMP8;CTCFL;SFRP4;CLEC7A;CD28;PKP1;ADTRP;PF4
ORM1;CLEC7A;IGF1;JAK2
CDK1;BRCA1;CLSPN
ENPEP;MME;CPXM2
ARHGAP44;NGEF
APOB;PF4
COL2A1;FGFR2
CLEC7A;PAK3
CLSTN3;WLS
CLEC7A;CD28
DUOX1;RARRES2
TOP2A
PPP1R3B
CDK1
SIX2
DNASE2B
WWC1
UCP1
DSC2
ESPL1
HAS2
HAS2
JAK2
SLFN14
SPP1
SPP1
SIX2
ESRRB
RAB38
RAB38
NHEJ1
PYGL
IDH1
AQP9
JAK2
JAK2
TTPA

SMPDL3B
CPEB2
ADRB3
SREBF1
QPRT
JAK2
GMNN
CLEC7A
ABCD2
PTH1R
NHEJ1
C3
TFR2
UBE2C
UGDH
APOBEC1
CFH
CCL24;PTGIS;CCL11;HAS2
COL16A1;ITGA4;ITGBL1;FBLN1
SFRP4;SCEL;FGFR2;WLS
ISG20;SLFN14;NUDT12
SLC5A8;SLC4A10;SLC4A1
ORM1;CLEC7A;JAK2
CCR1;HOMER2;WNK3
DDAH1;TAT
PON1;CYP8B1
C3;IGF1
SOCS2;GLDC;SPP1
SMOC1;CHRDL1;FREM2
UGDH;ANGPT1;HAS2
CLEC7A;CD14;JAK2
CLEC7A;NR1H4;CD14;JAK2
KEL;GLRX3;MME;CPXM2;CTSG
ALDH1A2;BRCA1;FGFR2
ATP8A1;APOC1;APOC3
CCBE1;CTSK;PERP
SLC46A1;SLC1A3
UBE2C;CDC6
TOP2A;NCAPG
TFR2;CP
SLC25A1;KRT17
RAB38;ATP6V0D2
UGDH;IGF1
ARHGAP44;RHOU;SLIT2;SLC6A2;NGEF
CFH
CFH
GLRX3
JAK2

SLC1A3
TNC
ADCY5
HK3
SIX2
HOMER2
HAS2
NBL1
GLDC
HK3
RAB40B
GYS2
TFR2
PNPLA3
JAK2
GYS2
KEL
NOX4
BRCA1
SCARA5
ACSL3
ACSL3
SIX2
CLSPN
SLIT2
ABCD2
UBD
SLIT2
IGF1
INSIG1
CHAC1
C3
UPP2
LIPC
TOP2A
SFRP4
HCAR2
FGFR2
FGFR2
C3
WNK3
BRCA1
TTR
SIX2
NR1H4
CCR1;CCL24;CCL11;MUSK;ADRB3;GPNMB;ANGPT1;WWC1;IGF1;MMP8;FGFR2
PKLR;INSIG1;OTOP1;CPEB2
HCN4;SLC5A8;SLC6A12;SLC6A2

DMTN;ADD2
DMTN;ADD2
DMTN;SLIT2
EXOC7;IGF1
POLQ;RMI2
APOC1;APOC3
IGF1;MMP8
CDK1;NCAPG
BRCA1;MPND
ABCD2;ADTRP;ACAT2
ISG20;SLPI;OAS3
ANGPT1;MUSK;HILPDA;TNC;IGF1;BRCA1;MMP8;AGPAT2;CTCFL;SFRP4;TFR2;CLEC7A;CD28;PKP1;ADTRP;PF
NR1H4;JAK2
ITGA4;IGF1
CAT;SLC6A2
NCAN;HAPLN4
BRCA1;MPND
PTH1R;IGF1
ANGPT4;ANGPT1;NR1H4;IGF1;VTCN1;MPO;COMP;SOCS2;WNK3;ALB;CAT;CD28;CDK1
SOCS2;CISH;ANGPTL7;TNC;MKI67;SLC6A2
KRT17;ALDH1A2;TNC;CDK1;JAK2;FGFR2
GMNN
IGF1
SEC61B
SLC1A3
BCAT1
CDCA8
DDAH1
TAT
APOBEC1
PTGIS
C3
FGFR2
MME
IGF1
ADCY5
JAK2
APOC3
SOD3
PANK3
ADCY5
INSIG1
PANK3
WLS
WNT10B
IGF1
CENPF
GP5

CDCA8
CDCA8
SLC6A2
TNFRSF21
CCNF
HP
PMEPA1
ABCD2
P4HA1
XPR1
E2F8
JAK2
CDCA8
SPP1
SREBF1
PF4
JAK2
PTGS2
CDK1
PANK3
ESPL1;UBE2C;CDK1
UCP1;SCTR
SLC25A1;KRT17
SLC4A10;SLC4A1
MME;CLEC7A
CENPF;CDK1
CDKL5;ISLR2
CLEC7A;PERP;CYCS
ORM1;CLEC7A;JAK2
SOCS2;UCP1;ADTRP;SLIT2
ITIH4;MME;UBD;ALDH1A2;JAK2
ACSL1;ESRRB;HILPDA;UCP1;ELOVL6;ADRB1;AGPAT2;CNKSR2;HCAR2;ADRB3;SPP1;CD28;JAK2
POLQ;NEIL3
DMTN;WNK3
SFRP4;ANGPT1
SREBF1;ALB;PDK4;PLIN2;XPR1
PPBP;CXCL14;PF4
CD28;IGF1;JAK2
PPP1R3B
FGFR2
NEIL3
SMOX
INSIG1
MME
HAS2
OTOP1
SOD3
DSC2

APOBEC1
ADRB3
FGFR2
DMTN
UBE2C
TAT
BRCA1
INSIG1
NUDT12
HOMER2
HOMER2
E2F8
ABCD2
SLC1A3
HSD11B1
SMOX
DMTN
DMTN
CDCA8
CDCA8
SREBF1
BRCA1
SLPI;CTSG;CD14;PPBP;JAK2;PF4
AP1S2;RAB38
ANGPT4;SLIT2
CLSTN3;WLS
CCL24;CCL11;JAK2
CRADD;CYCS;JAK2
RSPH1;DMTN;HYDIN;HAS2;PKP1;KNTC1;TLCD1;JCHAIN;ADD2
SBK2;CDK1;IGF1;PTPN3
CDK19;CCL11;CDKL5;PANK3;AMHR2;CDCA8;HK3;WNK3;CDK1;PDK4;NEK2;CTSG;JAK2;PAK3
CCNA2;CDK1
SPAG5;WWC1
FABP3;ACSL1
CDKL5;MUSK;WNK3;NEK2;JAK2;FGFR2
ANGPT4;ANGPT1;NOX4;IGF1;JAK2
CDCA2;SPAG5;NEK2;KIF11;MKI67
KRT17;KRT7;PKP1
OAS3
NCAPG
SLC13A5
TOP2A
HOMER2
NHEJ1
TNFRSF21
ACSS2
SLC36A2
HCN4

ADCY5
PTGIS
TFR2
FXYD2
SMPD3
VIT
ELOVL6
ABCD2
SLC46A1
CTSG
ELOVL6
BRCA1
CYCS
TFB1M
CLSPN
TFR2
GLRX3
WWC1
FBLN1
HAS2
CLEC7A
CDKL5
ABCD2
BRCA1
DMTN
MMP8
MMP8
WNK3
ITGA4
UPP2
SLC22A4
GMNN
VNN1
C1QB;GPM6A;MUSK;TNC;PAK3
HCN4;WNK3
SREBF1;RHOU
ALB;JCHAIN
RARRES2;FGFR2
SLC46A1;TTPA
DMTN;FBLN1
SREBF1;FABP3
COMP;UGDH;POLQ;TMEM120A
SLPI;CD14;JAK2
MUSK;IGF1;JAK2
COL2A1;LUM;CRYBB1;SLC4A10
SMOC1;CHRDL1;FREM2
UGDH;ANGPT1;MAT1A
BRCA1;IGF1;PF4

EXOC7;ACSL3;IGF1
C3;RGS7;KCTD16
ARHGAP44;DMTN;PAK3
SLC1A3;SLC6A2
CCNA2;CDK1
PDK4;NR1H4
SLC46A1;VNN1
CCR1;WNK3
CCR1;PF4
TMEM107;SNX10
COL2A1;LUM;CRYBB1;SLC4A10
GPNMB;HILPDA;CIDEA;CD28;VTCN1;AGPAT2
ABCD2
CFH
CFH
DMTN
ITIH4
JAK2
HP
SLC4A10
UGDH
MME
RCL1
JCHAIN
JAK2
FXYD2
PF4
HCN4
MOGAT2
UBD
CCR1
IGF1
SMPDL3B
PTGS2
FGFR2
ABCD2
IGF1
IGF1
RARRES2
FXYD2
CLEC7A
STAU2
GPM6A;LGI2;HAPLN4
SLIT2;CHRDL1;NBL1
DMTN;TFR2;SLFN14;FOS;SLC13A5
SLC22A4;CCR1;CACNG7;CENPF;AQP9;RAB38;SLC6A2
POLQ;RMI2;ANKLE1;BRCA1;MPND;NHEJ1
HCN4;DSC2

RSPH1;HYDIN
HK3;PKLR
POLQ;NHEJ1
GPM6A;FGFR2
CCL24;CCL11
POLQ;RMI2
APOBEC1;PKP1
CHAC1;PTPN3
CCL24;CCL11
CACNG7;RNF128
BRCA1;SLC6A2;NHEJ1
CNKSR2;DDAH1;RCAN2
DMTN;ANGPT1;FBLN1
TOP2A;SFRP4;WNT10B;PTGIS;CRADD;CTSK;UBD;SLIT2;SLC6A2
HCN4;KCNH7;FXYD2;KCNJ15;SLC1A3
CLSPN;CDC6
RMI2;BRCA1
SLC22A4;SLC36A2
SLIT2;PAK3
UCP1;FOS;SLC6A2
ALDH1A2;MAT1A;THG1L
LGALS12;CYCS;CHAC1;BRCA1
APOB
SLC1A3
SLFN14
BRCA1
FBLIM1
PF4
MMP8
SFRP4
CLEC7A
TOP2A
TMEM120A
POR
FGFR2
CENPF
CTCFL
ESPL1
HAPLN4
BRCA1
HAS2
ABCC3
JAK2
IGF1
DMTN
SLIT2
ILDR2
AMHR2

PTGS2
SMPD3
IGF1
BRCA1
AGPAT2
RUNX2
SEC61B
IGF1
SCARA5
RNF128
GLRX3
SCARA5
SIX2
PPP1R3B
GPM6A;WNT10B;CDKL5;EN2;EN1;RUNX2
ANGPT1;ITGA4
ITGA4;IGF1
SFRP4;FGFR2
ADRB3;CYCS
PAK3;NGEF
SLFN14;NUDT12
CHRDL1;NBL1
SREBF1;FOS
ANGPT4;ANGPT1;NR1H4;CIDEA;VTCN1;IGF1;MPO;SOCS2;COMP;ALB;WNK3;CAT;CDK1;PDK4;CD28
HSPH1;SERPINH1
SIX2;FGFR2
ADTRP;SMPDL3B
SOCS2;CISH
EDAR;CLEC7A;UBD;TNFRSF19;S100B;WLS
SREBF1
FOS
SLC1A3
TNFRSF21
MME
BCAT1
ENPEP
INSIG1
JCHAIN
PANK3
CCR1
PAK3
SEC61B
SMPD3
SLC46A1
DDAH1
KCNH7
NCAPG
DMTN

DMTN
CHAC1
OAS3
UGDH
RAB38
ITGA4
ACSL3
S100A1
C3
ARHGAP44
PDK4
SFRP4;WNT10B;WLS
SFRP4;SCEL;FGFR2;WLS
CRADD;CLEC7A;CYCS;JAK2
TOP2A;CDCA8
PERP;CYCS
CRADD;CTSK
TOP2A;SFRP4;WNT10B;CRADD;CLEC7A;UBD;SLIT2;SLC6A2
PAK3;S100B;FGFR2
EDAR;TNFRSF19;MMP8
CCL24;CCL11;ITGA4
F10;ANGPT1;MUSK;WWC1;TNFRSF19;SCTR;IGF1;MMP8;S100B;EDAR;ADRB3;CLEC7A;UBD;JAK2;FGFR2;WLS
SPTA1;COL2A1;KRT17;KATNAL2;LUM;COL12A1;SERPINH1;KRT7;XIRP2;MYO1F
CCL24;CCL11;DMTN;RHOU
PTGIS;NR1H4;IGF1;SMPDL3B
CIDEA;PMEPA1;NBL1;CHRDL1
SREBF1;ALB;PDK4
ABCD2;CLEC7A
CD28;NHEJ1
HCN4;DSC2
SREBF1;BRCA1
WNT10B;CIDEA
ORM1
CLEC3B
NR1H4
FBLN1
NR1H4
PTGS2
NR1H4
SREBF1
UGDH
MME
NOX4
SYCP3
HCN4
CYCS
IGF1
PDK4

ABCD2
OAS3
IGF1
ABCD2
INPP5J
PTGS2
IGF1
IGF1
NEK2
HPGDS
TMTC4
TOP2A;WNT10B;ANGPT4;ANGPT1;CRADD;NR1H4;CIDEA;VTCN1;IGF1;SLC6A2;MPO;SOCS2;COMP;SFRP4;U[illegible]
CCNA2;CCNE2;CCNF;CDC6
PON1;NR1H4;APOC3
CCL24;CCL11;SPAG5
GPM6A;PAK3;S100B;NBL1;FGFR2
RARRES2;PDK4
CCL24;CCL11;UBD
CRADD
NEIL3
RGS10
C3
SLIT2
PMEPA1
CDCA8
APOBEC1
UGDH
PON1
JAK2
SLIT2
TOP2A
UBE2C
SEC61B
SCEL
CYP2E1
NOX4
HAS2
NR1H4
ITGA4
GP5
SMPDL3B
FGFR2
C3
DUSP18
ABCD2
INPP5J
GPNMB
KRT17

JAK2
JAK2
IGF1
CDKL5
ADRB1
JAK2
MMP8
S100A1
BRCA1
IGF1
MUSK
TFB1M
APOBEC1
GMNN
PTGS2
SLC1A3;SLC4A1
CLEC7A;MMP8
TPX2;NEK2;KIF11
DMTN;INPP5J;PMEPA1;FBLN1;SLIT2
CDK19;CCL11;CDKL5;MUSK;AMHR2;CDCA8;WNK3;CDK1;PDK4;NEK2;CTSG;CLSPN;JAK2;PAK3;FGFR2
HK3;PANK3;INPP5J;LCAT;DUSP18;AGPAT2
ABCD2;ACAT2
GMNN;CDC6
SREBF1;FOS
MME;CLSTN3
HCN4;SOCS2
CCR1;KRT17;CD28;IGF1;CCDC3;MMP8
SFRP4
SMPD3
ADD2
CLEC7A
TNFRSF21
ADD2
VNN1
ITGA4
HAS2
ADH7
CCR1
DUOX1
ANGPT4
HK3
MYO1F
FXYD2
KCNH7
THG1L
PTPN3
IGF1
CCL11

CTSG
DDAH1
C3
CLEC7A
SFRP4
COL16A1
IGF1
RARRES2
CCR1
JAK2
SREBF1
CDK1
SLIT2
BRCA1
SCTR
CENPF
CTSG;CD14;PPBP;PF4
KEL;MME;CPXM2;CTSG
ABCC3;ATP8A1;SLC1A3
GPM6A;NBL1
CDKL5;ISLR2
RARRES2;PTGS2
CCR1;CCL24;CCL11;CD151;F10;TNC;LAMC2;IGF1;CCNA2;GPNMB;CLEC7A;HAS2;SLIT2
CCR1;DUOX1;CCL24;CCL11;CTSG;PPBP;JAK2;PF4
ANGPT4;PTGIS;S100A1;BRCA1
KCTD21;FGFR2
DMTN;FBLN1
MUSK;CLSTN3
CENPF;CDCA8
IGF1;SLC6A2
CNKSR2;DMTN;RCAN2
ABCC3;ATP8A1;SLC1A3
APOBEC1;SLFN14;NUDT12
TPX2;CDCA8;KIF11
GMNN;BRCA1;PTPN3
BRCA1
RARRES2
IGF1
DMTN
PF4
HAS2
GPNMB
ESRRB
CCBE1
UGDH
ADCY5
EN1
THG1L

AQP9
UGDH
AQP9
ITGA4
ESCO2
CIDEA
TNFRSF21
XPR1
LCAT
PNLDC1
IGF1
HSPH1
ISLR2
IGF1
IGF1
BRCA1
TMEM120A
SLFN14
SREBF1
CCR1;SCRN1;EXOC7
SLPI;STYX;JAK2
RHOU;RHOBTB1
BRCA1;SLC6A2
DDAH1;SLIT2
SLC1A3;SLC6A2
HK3;PDK4;PYGL
HSPH1;CD28;IGF1;JAK2
HCN4;KCNH7;KCNJ15;SLC1A3
CASP12;ESPL1;PRUNE2;TNFRSF19;CD14;JAK2;TNFRSF21
CCBE1;HAS2
BRCA1;ADCY5
RSPH1;SYCP3
CLEC7A;OAS3
FOS
DMTN
MYO1F
RARRES2
ITGA4
ANAPC15
DMTN
TPX2
SMPDL3B
SLIT2
MME
TAT
SLC4A1
JAK2
TFB1M

IGF1
TNFRSF21
ABCD2
TPX2
IGF1
SLIT2
PNLDC1
PLA1A
SFRP4
CLEC7A
ABCD2
FGFR2
IGF1
SLIT2
WNK3
FGFR2
CCR1;GPNMB;KRT17;SLFN14;CIDEA;CD28;IGF1;CCDC3;MMP8;NUDT12
ANGPT4;ANGPT1;SLIT2
VIT;CCDC80;JAK2
CCL24;CCL11;IGF1;FGFR2
COL16A1;DMTN
OGN;IGF1
IGF1;FGFR2
SLC1A3;SLC4A1
CDK1;PLIN2
JAK2;FGFR2
CCR1;RARRES2
ANGPT4;ANGPT1
CTSG;CD14;PPBP;PF4
SHC2;MUSK;JAK2;FGFR2
CCNA2;GMNN;ESCO2;BRCA1
COL2A1;HOMER2;CDC14A
MGST1;SLC4A10;SLC4A1
WNT10B;GPNMB;WWC1;PON1;CIDEA;NR1H4;APOC3
PTPN3
UGDH
NR1H4
FOS
HCN4
EXOC7
CPEB2
CCR1
HOMER2
CCDC3
CDK1
DMTN
IGF1
TMTC4

QPRT
PDK4
EDAR;CLEC7A;UBD;TNFRSF19;NR1H4;S100B;WLS
GPM6A;CDKL5
IGF1;JAK2
PANK3;ADCY5
MUSK;IGF1;JAK2
ANGPT1;IGF1;FGFR2
ARHGAP44;DMTN;PAK3
UCP1;BRCA1
ITGA4;IGF1
MUSK;CDK1
ARHGAP44;GPNMB;PAK3;FGFR2
BRCA1
RGS10
SLIT2
FOS
NEK2
ADTRP
UBD
INPP5J
JAK2
KRT17
PF4
FEZ1
PF4
PF4
AP1S2
PON1
GPNMB
CACNG7
TFB1M
RARRES2
COL2A1;HOMER2;CDC14A
JAK2;KLF1
CCL24;CCL11;ANGPT1
ANGPT1;F10;IGF1
WNK3;CDK1
CCNA2;CCR1
NR1H4
APOC1
ANGPTL7
SLIT2
WLS
CD14
RUNX2
OAS3
MOGAT2

UGDH
C3AR1
TPX2
SLIT2
PTPN3
BRCA1
TPX2
COL16A1
POR
FXYD2
STAU2
PTGIS;NR1H4;TNC;JAK2;PTGS2;MMP8;SMPDL3B
SLPI;RARRES2;OAS3;CTSG;MPO;JCHAIN
CD28;VTCN1;TNFRSF21
SREBF1;FOS
SIX2;WLS
KRT17;CDK1;FGFR2;E2F8
CPEB2
TPX2
FGFR2
DUOX1
ALDH1A2
ATP8A1
DMTN
CCR1
FGFR2
ABCD2
CCNA2
CDK1
WNK3
SEC61B
IGF1
CACNG7
UCHL1;EXOC7;ATP6V0D2
GDF10;CLEC3B;FOS
DCT;OPN1MW
WNT10B;ALDH1A2
SERPINH1;FETUB
C3;CLEC7A
TNC;JAK2;PTGS2
COL16A1;CLSTN3
CLEC7A;CD14
WWC1
FGFR2
IGF1
TNFRSF21
SLIT2
HCN4

CDKL5
SLC2A5
ADTRP
AP1S2
FXYD2
FGFR2
DMTN
BRCA1
IGF1
DMTN
ADTRP
KCTD21
SNX10
IGF1
CTSK
IGF1
IGF1
UBD
SOCS2;CISH;INPP5J
TMEM120A;UCP1
EXOC7;IGF1
CRADD;CTSK
PAK3;S100B;FGFR2
WNT10B;UGDH;EN2;EN1;RUNX2
PTGIS;APOC1;APOC3;BRCA1
SLIT2
PAK3
SYCP3
CRADD
HP
IGF1
SLIT2
ANKLE1
STYX
CCNE2;BCAT1
UBE2C;JAK2
WNT10B;RUNX2;FGFR2
GPNMB;ALB
OAS3;CD14
MKI67
PF4
CTSK
WLS
IGF1
FGFR2
NBL1
CACNG7
LIPC

SLIT2
GLRX3
APOBEC1
GLRX3
CHAC1
CCL11
F13A1
GPR137C
C3AR1
GPM6A
UBE2C
JAK2
PAK3
CCR1;CCL11;PTH1R
EXOC7;ADTRP;IGF1
SFRP4;WNT10B
IGF1;JAK2
HCN4;FXYD2;KCNJ15
RARRES2
PDK4
SLFN14
KCNJ15
DMTN
WNK3
UGDH
BRCA1
SLIT2
NR1H4
IGF1
SPP1
CD151;TFR2
SERPINH1;FETUB
WNK3;CDK1
CCNA2;MUSK;FGFR2
ANGPT4;PTGIS;BRCA1
ENPEP;KEL;CFH;MME;CPXM2;CTSK;UBD;CTSG;MMP8
EDAR;TNFRSF19;MMP8
CCL11;MME
CRADD;JAK2
JAK2;FGFR2
MUSK
TNFRSF19
TAT
HCN4
SPAG5
HCN4
WNT10B
ADTRP

ATP6V0D2
FOS
WNK3
SLC46A1
JAK2
DUSP18
CDCA2
UBE2C
ESCO2
DMTN
CCNA2;CNKSR2;RGL3;IGF1
SPTA1;DMTN;XIRP2;MYO1F
CDKL5;ISLR2;IGF1
ITGA4;NHEJ1
CCNA2;TPX2;NOX4;CLSPN
SERPINH1;FETUB
HCN4;S100A1
CD302;CD14
GPNMB
CRADD
CHAC1
DMTN
IGF1
DMTN
INPP5J
PTH1R
RHOU
CLEC7A
PTGS2
CCL24;CCL11
SFRP4;FGFR2;WLS
CCNF
CDKL5
BRCA1
JAK2
MMP8
GPNMB
FGFR2
SFRP4
SOCS2
ITGA4
SLIT2
DMTN
SLIT2
WWC1
BRCA1
TPX2
TMEM107

ATP8A1
CDC6
SLC6A2
CCR1
CTCFL
JAK2
EDAR;TNFRSF19;MMP8
POLQ;RMI2
ARHGAP44;NGEF
GJB1;PKP1
UBD;SNX10
TPX2;SLIT2
SFRP4;FABP3;GPNMB;DDAH1;ALDH1A2;OGN;NOX4;FBLN1;CDC6;JAK2
FEZ1;SLIT2;PAK3;S100B;FGFR2
SOCS2;CISH;TNC;BRCA1;SLIT2;SLC6A2
CLEC7A;CD28;NHEJ1
GDF10;FOS
ABCD2;TLCD1
PON1
PM20D1
KCNJ15
FGFR2
IGF1
GPNMB
WNT10B
GDF10
STYX
IGF1
CLSTN3
NEK2
PTGIS;NR1H4;SMPDL3B
EXOC7;IGF1
APOC3;SLC6A2
CD151;TFR2;CLEC7A
ANGPT1;GMNN;FBLN1;CDC6;BRCA1;SFRP4;FABP3;GPNMB;DDAH1;ALDH1A2;NOX4;SLIT2;JAK2;TNFRSF21
PAK3
S100A1
OAS3
SLIT2
HAS2
FGFR2
FGFR2
SLFN14
ANGPT4
SLIT2
CLEC7A
RHOU
CLEC7A

WNK3
CDCA2
IGF1
NEK2
IGF1
IGF1
RNF128;UBE2C;KCTD21;UBD;UBE2L6
CD28;JAK2
ARHGAP44;DMTN;PAK3
ORM1;CLEC7A;MMP8
WNT10B;RARRES2
CDKL5;DMTN
RARRES2;CTSG
FBLN1;SLIT2
CLSPN
SLC4A10
BRCA1
ESPL1
CTCFL
TPX2
ARHGAP44
ORM1
PTPN3
S100A1
CLEC3B;AMHR2;HAS2;RUNX2
CLEC7A;MMP8
UCP1;FOS;CPEB2
KCNH7
WNK3
PNLDC1
PAK3
HOMER2
E2F8
JAK2
GPNMB
SLC36A2
DMTN
NEK2
HCN4
ANGPT4;PF4
CIDEA;PMEPA1
C7;BRCA1
CACNG7;CLSTN3
SLPI;STYX;ADD2
CDKL5;MUSK;SLIT2
FBLN1;SLIT2
ENPEP;LIPC
CRADD

IGF1
KEL
CDKL5
ITGA4
NEIL3
FEZ1
GP5
OAS3
JAK2
SERF2
JAK2
ANGPT1;BRCA1
SLIT2
IGF1
SLIT2
CTSK
OGN
SMPD3
JAK2
JAK2
ADRB3;TFR2;MRC1
CCNA2;RARRES2;IGF1;APOB;NBL1;RUNX2;PF4
CDK19;CCL11;CDKL5;AMHR2;CDCA8;F13A1;DAPP1;UBE2L6;CCNA2;QPCT;WNK3;CDK1;PDK4;NEK2;CTSG
HAL
COL2A1
CLEC3B
JAK2
TLCD1
EXOC7
CCNA2
NOX4
CLEC7A;CD14
QPCT;UBE2L6
LCAT;ELOVL6;BCAT1
DUOX1;TNC;FBLN1
ANGPT4;PTGIS;GPNMB;BRCA1;PF4
DAPP1;DUSP18;PTPN3
TPX2
SLC46A1
NOX4
GP5
FGFR2
MYO1F
IGF1
ADTRP
SFRP4
S100B
PNLDC1

FGFR2
FXYD2
SPAG5
VNN1
CD14
C3AR1;PTH1R
ESRRB;FGFR2
TOP2A;ISG20;POLQ;NEIL3;RMI2;CDK1;BRCA1
ANLN;CENPF;DMTN;COL16A1;ANGPT1;KNTC1;JCHAIN;ADD2
DMTN;KNTC1;JCHAIN
AGPAT2
IGF1
SLIT2
CDCA2
ADTRP
GPNMB
NHEJ1
ESRRB
FEZ1
CLEC7A;CYCS
ANGPT1;BRCA1
ACSM3;ACSM5
TNC;JAK2;PTGS2
MUSK;FGFR2
RARRES2;CTSG
NR1H4;CD28
ANGPT4;ANGPT1
CDKL5
IGF1
WNT10B
SFRP4
CCNA2
CD28
NR1H4
POLQ;NEIL3;RMI2;CDK1;BRCA1;MPND;NHEJ1
ANGPT4;PF4
CCR1;CACNG7;SLC5A8;KCNJ15
CACNG7
CCR1
SLIT2
FGFR2
PLIN2
TNFRSF19
S100A1
SCEL
CYP2E1
HCN4
NR1H4

NBL1
NR1H4;ADCY5
GPM6A;ANLN
RMI2
CCNF
HK3
PMEPA1
CCNA2
COL16A1
ARHGAP44;CLEC7A;WWC1;RHOU;PAK3;SLC6A2;NGEF
ARHGAP44;CDKL5;MUSK;PAK3
WWC1
ISG20
MMP8
RARRES2
AGPAT2
HAS2
TNFRSF21
CD28;VTCN1;TNFRSF21
PDK4
IRAK1BP1
IGF1
ESRRB
NEK2
ITGA4;NHEJ1
OAS3;ILDR2
ITGA4;NHEJ1
ANGPT1;F10;IGF1
PTPN3
IGF1
PKP1
BRCA1
GYS2
ANLN
ATP6V0D2
DSC2
SEC61B
TFR2
DMTN;INPP5J
SMPD3;CLEC7A
EXOC7
NR1H4
IGF1
CPEB2
GPR137C
CLEC7A
IGF1
SMPD3;ELOVL6

CCR1;CCL11;PTH1R
GPM6A;PKP1
ANGPT4;ANGPT1
RMI2;BRCA1
CRADD
CCDC3
KATNAL2
FBLN1
CCL24;CCL11
CTSK
JAK2
BRCA1
CHAC1
ANKLE1
OAS3
CLEC7A
SREBF1
THG1L
CDCA8
HP
GPR137C
MUSK;NEK2;FGFR2
ANGPT1;TNC;FBLN1
PM20D1
NEK2
MME
RSPH1
UCHL1
WNK3
WNK3
PTGIS;NR1H4;SMPDL3B
ADCY5
TFR2
CDK1
PERP
PTGIS
OAS3
NEK2
CCNA2;CLEC7A;SIX2;CLEC11A;HILPDA;HAS2;LAMC2;IGF1;JAK2;S100B;FGFR2
DAPP1;DUSP18;PTPN3
RAB40B;ITGA4;WNK3;RAB38
LYRM2
DMTN
JAK2
JAK2
SNX10
LYRM2
MMP8

SOCS2;FGFR2;KCTD16;RGS7
SCEL
NGEF
PTPN3
FGFR2
CACNG7
UCHL1;FBLN1
VIT;C3;SCTR;S100B;NHEJ1;AFF2
ESRRB
CD14
EXOC7
HCN4
CD28;JAK2
CLSPN
CACNG7
ELOVL6
APOBEC1;BRCA1
GPNMB
CLEC7A
MUSK
IGF1
ITGA4;ITGBL1
DMTN;SLC13A5
CCL24;ABCD2;CCL11;DMTN;PNPLA3;CDCA8;SNX10;RAB38;RHOU
TFB1M
CDK1
CD14
HYDIN
PMEPA1
ADD2
HP
CIDEA;PMEPA1
RMI2;BRCA1
IGF1
MPZL2
CCR1;CACNG7
UCHL1;CDK1
SPAG5
ANGPT1;BRCA1
SFRP4;SCEL;FGFR2;WLS
CDK1
TFB1M
PAK3
UCHL1;CDK1
INPP5J
JAK2
TFR2
VNN1

CLEC11A;HILPDA;LAMC2;CDC6;IGF1;S100B;SFRP4;FABP3;GPNMB;DDAH1;CLEC7A;ALDH1A2;NOX4;HAS2;
GPM6A;GDF11;GJB1;FEZ1;ALDH1A2;CHAC1;S100B;NBL1;NHEJ1
OPN1MW
NEK2
FGFR2
EXOC7;TTPA;TLCD1
FBLIM1;CLEC7A
CENPF;SCTR;AFF2
THG1L
WLS
CYCS
PKP1
SCEL
CDKL5;CLSTN3
DMTN
BRCA1;SLIT2
FEZ1;SLIT2
CYCS
FGFR2
UBE2C
CHAC1
BRCA1;SLIT2
CDKL5
BRCA1
WWC1
CDK1
CBFB;UBE2C;UBE2L6;BRCA1
CDK1
SEC61B
FGFR2
IGF1
FBLN1;SLIT2
C7
ELOVL6
TFR2
CFH
CTSK
CENPF;NEK2
NBL1
MME
WNK3
RAB40B;WNK3
CDKL5
WNK3
IGF1
IGF1
UCHL1
C7

PTGIS
SREBF1;UCHL1;EXOC7;ATP6V0D2
IGF1
IGF1
GPM6A;PAK3;NBL1
SMOC1;RUNX2;FGFR2
RSPH1;TMEM107;SNX10;CDC14A
GP5
CTCFL
EXOC7
MMP8
TOP2A;POLQ;RMI2;ANKLE1;CRADD;CDK1;BRCA1
RAB40B;WNK3
RCL1
TFR2;PERP;CD28
CYCS
ISG20
PNLDC1
CDK1
SLC1A3;LPAR3;SLC6A2
ABCD2;IGF1
ANAPC15;CCNF;GMNN;BRCA1;FGFR2
GPNMB
ISG20;OAS3
SEC61B
CPEB2
BRCA1
FEZ1;SLIT2
SFRP4;PTGIS
LYRM2
GPM6A;UGDH
SLFN14
MUSK
RNF128
GDF10;WNT10B;ESRRB
RPL22L1
IGF1
TMTC4
SNX10
HCN4
CENPF;TFR2;RAB38;APOB;WLS
CDK1;CLSPN
DUOX1;SPAG5;CLEC7A;CLEC11A;HILPDA;HP;HAS2;LAMC2;IGF1;S100B;FGFR2
CCR1;SCRN1;SCARA5;EXOC7;AP1S2;RHOU;RHOBTB1
UBE2C;UBD;CCNF;UBE2L6;BRCA1;TRIM10
PF4
CLEC7A
SFRP4;KCTD21;CHAC1;SMPDL3B

UCHL1;RNF128;UBE2C;KCTD21;UBD;UBE2L6
UCHL1;ANAPC15;UBE2C;CCNF;SEC61B
IGF1;AFF2
RAB38;THG1L
CAT
THG1L
ADTRP;SLIT2
TFB1M
TMEM107;AP1S2;SNX10;RAB38;CDC14A
CCR1;BNC2;ANGPT1;MUSK;WWC1;ESRRB;TNC;ILDR2;VTCN1;IGF1;BRCA1;MMP8;CTCFL;KLF1;AFF2;SFRP4;[illegible]
NGEF
CTSK
ILDR2
SLC1A3;LPAR3;SLC6A2;HAPLN4
CDK1;CLSPN
DMTN;TMEM107;SNX10;CDC14A
MMP8
DMTN
GPNMB
BRCA1
CPEB2
TMEM107;SNX10;CDC14A
CD28
CBFB;UBE2C;UBD;CCNF;UBE2L6;BRCA1;TRIM10
WWC1;NR1H4;SLIT2
ISG20
CHAC1
SFRP4
UCHL1
RCL1
CCNA2
ISG20;OAS3
ESRRB;SPP1;NR1H4;FOS;IGF1;BRCA1;RUNX2;CTCFL;KLF1
RHOU
ALB;CIDEA
SNX10;TLCD1
BRCA1
CDK1
GPM6A
SNX10
CACNG7;ANKLE1;RAB38;WLS
WNT10B;IGF1
SFRP4
CLEC7A
RCL1
RARRES2
MPZL2
CENPF;GMNN;KCTD1;BRCA1;RUNX2

CBFB
SREBF1;SATB2;ESRRB;NR1H4;FOS;IGF1;BRCA1;RUNX2;CTCFL;CCNA2;TFR2;FGFR2;PF4;E2F8
CBFB
CD28
ANGPT1;RAB38;WLS
UCHL1
SATB2
ESRRB
BNC2;WWC1;ESRRB;KLF1
RPL22L1
CLEC7A
SATB2
NOX4
SREBF1;SATB2;ESRRB;NR1H4;FOS;IGF1;BRCA1;RUNX2;CTCFL;KLF1;CCNA2;TFR2;SPP1;FGFR2;PF4;E2F8
CENPF;CBFB;WWC1;GMNN;NR1H4;EN1;KCTD1;BRCA1;RUNX2;FGFR2;E2F8
CBFB;WWC1;NR1H4;EN1;FGFR2;E2F8
SREBF1;MGAM;CBFB;BNC2;SATB2;WWC1;GMNN;ESRRB;EN2;NR1H4;EN1;KCTD1;HR;FOS;IGF1;BRCA1;RUN
SREBF1;MGAM;CBFB;SATB2;WWC1;ESRRB;EN2;NR1H4;EN1;HR;FOS;IGF1;BRCA1;RUNX2;CTCFL;KLF1;CCN

HAS2;CTSG;MATN4

G

4

BD;ALB;WNK3;CAT;CDK1;CD28;SLIT2

;JAK2;PAK3;PTPN3

JAK2;FGFR2;PF4

KRT17;CLEC7A;CD28;PKP1;CCDC3;ADTRP;PF4

X2;CTCFL;KLF1;CENPF;SIX2;SPP1;E2F8
A2;TFR2;SIX2;FGFR2;PF4;E2F8

Term	Overlap	P.value	Adjusted.P.value	Old.P.value
SUZ12 CHEA	134/1684	2.84324E-12	2.95697E-10	0
SMC3 ENCODE	90/1181	1.20117E-07	6.24607E-06	0
ZC3H11A ENCODE	18/129	1.18079E-05	0.000409342	0
CTCF ENCODE	113/1790	2.94873E-05	0.00076667	0
GATA1 CHEA	59/807	6.44829E-05	0.00126407	0
SOX2 CHEA	57/775	7.29271E-05	0.00126407	0
ZBTB7A ENCODE	129/2184	0.000157981	0.002347146	0
RAD21 ENCODE	81/1265	0.000275063	0.003575817	0
TCF3 CHEA	67/1006	0.000324301	0.00374748	0
TP53 CHEA	27/319	0.00075995	0.007903481	0
SMAD4 CHEA	42/584	0.000983711	0.009300536	0
RUNX1 CHEA	78/1294	0.001978603	0.017147894	0
GATA2 CHEA	49/772	0.005106306	0.040850452	0
TP63 CHEA	78/1399	0.013305175	0.098838441	0
EZH2 CHEA	18/237	0.015514385	0.107566405	0
RCOR1 ENCODE	42/702	0.022479148	0.146114462	0
SALL4 CHEA	23/355	0.036967356	0.214828198	0
AR CHEA	60/1095	0.037181804	0.214828198	0
FOXP2 ENCODE	12/178	0.08747211	0.478794706	0
SP1 ENCODE	38/707	0.102642291	0.533739914	0
KLF4 CHEA	51/987	0.111529058	0.552334382	0
UBTF ENCODE	80/1631	0.135211594	0.639182083	0
PBX3 ENCODE	63/1269	0.145585845	0.648058065	0
EZH2 ENCODE	19/338	0.151169756	0.648058065	0
HNF4A ENCODE	63/1276	0.155916589	0.648058065	0
REST CHEA	63/1280	0.162014516	0.648058065	0
ZEB1 ENCODE	7/106	0.17734579	0.674474547	0
FOXA1 ENCODE	12/205	0.181589301	0.674474547	0
NELFE ENCODE	13/234	0.218269351	0.782759051	0
POU5F1 CHEA	14/261	0.245086548	0.849633367	0
ERG CHEA	8/144	0.288193578	0.959963473	0
BCL3 ENCODE	14/273	0.298962195	0.959963473	0
TRIM28 CHEA	11/210	0.304603794	0.959963473	0
NANOG CHEA	28/595	0.356351968	0.999994574	0
EGR1 CHEA	15/315	0.393617433	0.999994574	0
E2F1 CHEA	39/859	0.408484205	0.999994574	0
MYOD1 ENCODE	8/166	0.432469861	0.999994574	0
PPARD CHEA	13/285	0.467350382	0.999994574	0
HDAC2 ENCODE	8/175	0.49150824	0.999994574	0
ESR1 CHEA	7/154	0.504905222	0.999994574	0
IRF3 ENCODE	29/663	0.51017363	0.999994574	0
ETS1 ENCODE	7/156	0.518620611	0.999994574	0
FOXM1 ENCODE	4/95	0.595401254	0.999994574	0
FOXA2 ENCODE	13/316	0.618171621	0.999994574	0
EGR1 ENCODE	22/537	0.640915664	0.999994574	0
VDR CHEA	3/76	0.646531716	0.999994574	0
STAT3 CHEA	7/177	0.652139413	0.999994574	0

CEBPD ENCODE	30/734	0.660311766	0.999994574	0
SUZ12 ENCODE	4/105	0.673620338	0.999994574	0
CREB1 CHEA	59/1444	0.707788602	0.999994574	0
FOS ENCODE	25/637	0.727134911	0.999994574	0
FOSL2 ENCODE	7/196	0.751720496	0.999994574	0
SRF ENCODE	11/299	0.753936251	0.999994574	0
NFIC ENCODE	10/279	0.774670375	0.999994574	0
IRF8 CHEA	4/121	0.775658412	0.999994574	0
REST ENCODE	14/383	0.781404277	0.999994574	0
RFX5 ENCODE	21/559	0.78250241	0.999994574	0
TAF7 ENCODE	23/640	0.851772874	0.999994574	0
SP2 ENCODE	37/994	0.856316115	0.999994574	0
NFYA ENCODE	88/2250	0.868283238	0.999994574	0
CBX3 ENCODE	2/81	0.872012346	0.999994574	0
SPI1 CHEA	39/1056	0.873889365	0.999994574	0
CEBPB ENCODE	4/144	0.876223803	0.999994574	0
CHD1 ENCODE	23/655	0.878450891	0.999994574	0
E2F4 ENCODE	25/710	0.884379814	0.999994574	0
ZMIZ1 ENCODE	33/914	0.885415964	0.999994574	0
TCF7L2 ENCODE	20/583	0.887395258	0.999994574	0
TCF3 ENCODE	30/840	0.888067959	0.999994574	0
BHLHE40 ENCODE	11/348	0.893781754	0.999994574	0
ZKSCAN1 ENCODE	3/120	0.897713684	0.999994574	0
KAT2A ENCODE	2/90	0.906771148	0.999994574	0
MYC CHEA	19/573	0.911822823	0.999994574	0
ZNF384 ENCODE	25/730	0.911928485	0.999994574	0
USF2 ENCODE	34/965	0.91603106	0.999994574	0
NFE2L2 CHEA	36/1022	0.922033482	0.999994574	0
NR2C2 ENCODE	10/350	0.941688418	0.999994574	0
STAT5A ENCODE	6/233	0.942042812	0.999994574	0
NFYB ENCODE	143/3715	0.954273421	0.999994574	0
RELA ENCODE	14/484	0.961730876	0.999994574	0
TRIM28 ENCODE	2/120	0.968948727	0.999994574	0
IRF1 ENCODE	6/263	0.973945414	0.999994574	0
ZBTB33 ENCODE	21/702	0.975056459	0.999994574	0
PPARG CHEA	15/535	0.9757147	0.999994574	0
YY1 CHEA	8/332	0.977863567	0.999994574	0
SPI1 ENCODE	51/1540	0.985871202	0.999994574	0
SIN3A ENCODE	34/1131	0.992840189	0.999994574	0
ATF3 ENCODE	1/114	0.993733002	0.999994574	0
STAT3 ENCODE	19/725	0.994836678	0.999994574	0
USF1 ENCODE	44/1423	0.994898627	0.999994574	0
E2F6 ENCODE	113/3245	0.996852417	0.999994574	0
PML ENCODE	48/1596	0.998233725	0.999994574	0
FLI1 ENCODE	12/650	0.999864881	0.999994574	0
BCLAF1 ENCODE	17/851	0.99994371	0.999994574	0
MAX ENCODE	52/2073	0.99999227	0.999994574	0
BRCA1 ENCODE	90/3218	0.999992654	0.999994574	0

TAF1 ENCODE	72/3346	0.999992898	0.999994574	0
ATF2 ENCODE	74/2852	0.999993078	0.999994574	0
MYC ENCODE	33/1515	0.999993139	0.999994574	0
YY1 ENCODE	64/2753	0.999993184	0.999994574	0
ELF1 ENCODE	35/2483	0.999993339	0.999994574	0
CREB1 ENCODE	45/2238	0.999993496	0.999994574	0
GABPA ENCODE	39/2082	0.999993606	0.999994574	0
NRF1 ENCODE	37/1882	0.999993758	0.999994574	0
SIX5 ENCODE	15/1094	0.999994574	0.999994574	0

Old.Adjusted.P.value	Odds.Ratio	Combined.Score
0	2.070834139	55.05535587
0	1.912928636	30.48213832
0	3.628807631	41.17512853
0	1.557823613	16.25051646
0	1.792428759	17.29534408
0	1.802510055	17.17080068
0	1.450591152	12.69707618
0	1.56017978	12.79115104
0	1.620619327	13.01979329
0	2.071408793	14.87739206
0	1.744011508	12.0758476
0	1.454705197	9.056069563
0	1.52336537	8.03922399
0	1.331229697	5.750382784
0	1.828815471	7.618822578
0	1.423112481	5.40094974
0	1.541313182	5.082819332
0	1.298392883	4.274226
0	1.601677739	3.902384155
0	1.263518649	2.87640682
0	1.213523523	2.661827578
0	1.150788586	2.302629409
0	1.163270604	2.241610084
0	1.319815678	2.493596206
0	1.156105954	2.148546728
0	1.152048873	2.096808833
0	1.563028661	2.703498518
0	1.375647668	2.346865555
0	1.301066391	1.980256128
0	1.253401473	1.762462802
0	1.299316005	1.61650877
0	1.194569277	1.442368521
0	1.221175891	1.451664759
0	1.091416814	1.126163554
0	1.103868699	1.029220486
0	1.050586955	0.940592636
0	1.117103326	0.936403642
0	1.054265566	0.801954535
0	1.05639883	0.750335342
0	1.049997235	0.717551884
0	1.008489903	0.6787179
0	1.035794183	0.680084503
0	0.968711844	0.502296198
0	0.944847818	0.454461554
0	0.940058298	0.41819189
0	0.90548737	0.394912945
0	0.906838833	0.387670805

	0	0.937093187	0.388934138
	0	0.872341401	0.344652163
	0	0.934498623	0.322971895
	0	0.897432915	0.285960937
	0	0.814857831	0.232552851
	0	0.839832102	0.237208446
	0	0.817280613	0.208666176
	0	0.752413739	0.191145478
	0	0.83357768	0.205612457
	0	0.856892685	0.210160023
	0	0.816788622	0.131041783
	0	0.84559651	0.131165275
	0	0.885552314	0.125073023
	0	0.556991259	0.076280898
	0	0.837968053	0.112959347
	0	0.628042328	0.082985581
	0	0.796756797	0.103255915
	0	0.79863366	0.098127044
	0	0.818725319	0.099637012
	0	0.777882972	0.092929621
	0	0.809776363	0.09612613
	0	0.71585373	0.080385833
	0	0.563657922	0.060821
	0	0.499790048	0.048912042
	0	0.750473056	0.069275853
	0	0.775136081	0.071462669
	0	0.797000209	0.069900909
	0	0.796311827	0.064639611
	0	0.64417935	0.038702827
	0	0.579688666	0.034610055
	0	0.859203769	0.04021507
	0	0.650924311	0.025399472
	0	0.372137629	0.011738554
	0	0.511208212	0.013495907
	0	0.671751556	0.016968379
	0	0.629407521	0.015474016
	0	0.539994258	0.012087837
	0	0.739649309	0.010524884
	0	0.670227719	0.004815965
	0	0.194128875	0.001220433
	0	0.584079921	0.003023606
	0	0.687437076	0.003515848
	0	0.76459195	0.002410412
	0	0.664927207	0.001175483
	0	0.406366273	5.49116E-05
	0	0.438285329	2.46717E-05
	0	0.539538279	4.17074E-06
	0	0.591867797	4.34779E-06

	0	0.438116054	3.11162E-06
	0	0.548659583	3.79805E-06
	0	0.470678077	3.22931E-06
	0	0.486759212	3.31774E-06
	0	0.286359642	1.90738E-06
	0	0.42233961	2.74691E-06
	0	0.393512343	2.51629E-06
	0	0.417180463	2.60421E-06
	0	0.294218628	1.59629E-06

Genes
CHRM3;PHLDB1;FAAH;THSD7B;PLXND1;HHIP;GALNT18;CLSTN1;HS6ST2;NPPC;BAIAP2L1;DACH1;DPYSL4;[illegible]
EPHB6;ANKRD39;ACHE;MIDN;PHLDB1;MEGF10;TMEM51;PEAR1;GLI1;PTPRF;NPPC;SMPD4;KCNT1;RASSF3;[illegible]
ANKRD39;ACHE;GADD45B;SFSWAP;TANGO2;MTTP;PEAR1;PTPRF;VAV2;CTU2;POLA2;ABLIM3;TRAF5;ANKRD[illegible]
EPHB6;ANKRD39;MIDN;PHLDB1;TRRAP;IGF1R;NPPC;BAIAP2L1;EME2;ARHGEF40;LMO1;COL27A1;COL23A1[illegible]
ACHE;BTG2;TFRC;ITGB5;LDB1;THRA;ICAM2;ATP2A3;SLC2A1;SLC40A1;PTPRF;IGF1R;AQP1;NDST1;FTH1;CE[illegible]
TOP2A;HHIP;ATP2A2;NRARP;SLC6A1;ETS1;HS6ST2;CTGF;SHF;BAIAP2L1;DPYSL4;HEY1;HIST1H1A;BCAS1;H[illegible]
MIDN;TMEM200B;TRRAP;CLSTN1;IGF1R;RPS6KA4;NPPC;BAIAP2L1;RASSF3;EME2;FTH1;CEP250;FBRSL1;PI[illegible]
HRK;EPHB6;ANKRD39;ACHE;MEGF10;KIF14;TMEM51;PEAR1;GLI1;PTPRF;ALAD;NPPC;BAIAP2L1;SIX5;SNAP[illegible]
CSRNP3;ITGB5;TMEM51;CHD3;HS6ST2;CTGF;SHF;SHE;SPRED2;BAIAP2L1;RASSF3;CIC;HIST1H1A;EPHB4;[illegible]
BTG2;NOTCH1;ANKRD11;COL11A2;HS6ST2;LMNB1;ALAD;PRX;ABL1;TNRC18;ATP6V0A4;IER5;NTNG1;JAG1;[illegible]
BTG2;TENM2;THSD7B;MEGF10;GALNT18;FMN2;SLC7A1;NDRG1;HS6ST2;SH3RF1;SHE;SOX17;RPS6KC1;KC[illegible]
GPSM1;MIDN;BTG2;ZC3H4;PANK1;PRR3;ICAM2;ATP2A3;SLC2A1;CHD3;GLI1;ARHGAP6;GBA2;SLC2A6;PTP[illegible]
ACHE;BTG2;ICAM2;KIF14;CHD3;SLC2A6;PTPRF;IGF1R;SHE;FADS2;IER5;EPHB4;IER2;BCAS1;MAGI1;EGFL7;[illegible]
ITGB4;GALNT18;CLSTN1;TNC;STAB2;SLC4A3;ETS1;MEOX1;PHEX;SHF;AKAP12;NYNRIN;SPRED2;INSC;BDKR[illegible]
HRK;CHST8;COL26A1;PRKCH;TESC;SPHK1;PDGFB;ADAM11;NRXN2;NDNF;POU3F1;MAFA;COL2A1;CAMK4;[illegible]
CUL7;MIDN;NLRX1;LDB1;MAZ;TMEM51;NRXN2;NRARP;GBA2;RBM15B;RAB11FIP1;SIX5;CASKIN2;MICALL2;[illegible]
FOXC1;TCF15;LDB1;UNC5B;LAMA1;NRXN2;CDC42BPB;HS6ST2;CTGF;NELL2;PM20D2;FADS2;MYCN;SOX17[illegible]
DGKG;IRS2;PTPRF;HS6ST2;RYR3;MYLK;IGF1R;AKAP12;RAC3;HMCN1;PRKG1;RBM33;MAGI1;GUCY1A3;GAB1[illegible]
SPRED2;TSC22D4;MNT;JUND;CTDSP2;NAB2;MAZ;IRS2;TNRC18;NDRG1;SCAF1;IER2
ANKRD39;TOP2A;TOMM40;ANKRD11;CHD8;KIF14;GLI1;APOLD1;PNN;SPRED2;ARVCF;MNT;PABPN1;RAB40[illegible]
MIDN;LDB1;ATP2A3;SHF;AKAP12;DPYSL4;CCHCR1;HIST1H1A;IER2;HIST1H1C;CHST8;FOXD3;PLEKHG2;PH[illegible]
PHLDB1;LDB1;PLXND1;PANK1;FAM89A;NAB2;ATP2A3;SLC2A1;ATP2A2;NRARP;IRS2;SH3RF1;FGFRL1;IGF1R[illegible]
TOP2A;CHD8;PHF1;GALNT18;IRS2;ZC3H6;PNN;HEY1;SNRNP70;SIX5;KHSRP;PIM3;MAP3K9;IER5;DLGAP5;H[illegible]
HRK;COL26A1;CXADR;CADM4;CTTNBP2;ITGB4;PAPLN;APLP1;LAMA3;KLHL23;DUSP26;ERBB4;KIF5A;CASKI[illegible]
MIDN;PANK1;PHF1;TMEM51;AATK;ZC3H6;CTGF;LMNB1;SHF;ZFYVE27;WDR90;MAN2C1;RBM33;LZTS3;COL2[illegible]
DGKG;CHRM3;TENM2;GRIK5;GALNT18;CELF3;EFCAB6;ENDOV;KCNT1;DPYSL4;RASSF3;PIM3;MAP3K9;GIT1;[illegible]
FAM83H;DLL4;LSR;EPHA1;HIST1H4C;NFATC4;TJP2
ITGB5;MAZ;ZBTB42;MBP;LSR;SOX12;ZSWIM4;SH3RF1;RAPGEF3;ETV5;ZBTB2;EXPH5
RRM2;TFRC;GADD45B;FUS;MEPCE;CIRBP;SNRNP40;NASP;RPS2;HIST1H4C;IER5;RBMX;HIST1H1C
ATF7IP;NOTCH1;HIST1H4K;PTCH1;NRARP;FGD1;ETV5;MLLT6;AKAP12;MYCN;ADNP;TCF3;PATZ1;EPHB4
RGP1;TMEM200B;PHF12;PITPNM3;FAM181B;TBKBP1;NDRG1;TBX3
JUND;DHRS11;AMIGO3;PHF1;MAZ;SCRIB;UFSP1;SIX5;NAPRT;DVL2;ADNP;AMH;RGL2;HIST1H1C
EFNB2;FOXD3;JAG1;SOX17;HEY1;UNC5B;PTCH1;NRXN2;ADCY1;KCNK1;RAMP1
SHC4;LDB1;LEF1;SLC6A1;HS6ST2;CTGF;MLLT6;SHF;AKAP12;LDHB;RAI1;SPRED2;DPYSL4;KHSRP;SPP1;MG[illegible]
EGR2;PLEKHG2;GADD45B;TANGO2;TNK2;SEMA3B;GPR37L1;GBA2;ETV5;ALAD;ERBB4;KHSRP;TRAF5;ABL1;[illegible]
NUMA1;ANKRD11;ZBTB48;DTX3;SLC7A1;LMNB1;PNN;MNT;SMPD4;TMEM88;SNRNP70;SLC22A17;SFN;RPS[illegible]
FMNL3;NWD1;TNC;MUM1;CHD3;MAFA;NES;RYR3
SMURF1;SPNS3;TBC1D8;ITPR3;SH3RF1;INF2;FTH1;SH3BP5;BIRC5;MBP;HIST1H4C;ATP9A;HIST1H1C
HNRNPUL1;ZC3H4;SPHK1;ID1;TNIK;CPLX2;APOLD1;LMNB1
MAGI1;AFAP1L2;CASP7;SH3BP5;ID3;RYR3;THSD4
ANKRD39;TOP2A;TOMM40;ANKRD11;CHD8;PPM1K;EFNB2;PNN;ARVCF;PABPN1;KHSRP;ZSWIM4;IP6K1;HIS[illegible]
GUCD1;BCL7C;MEPCE;ARID5A;ADNP;GTPBP2;HIST1H1C
ATF7IP;KIF14;APOLD1;LMNB1
ATF7IP;ITGB5;HIST1H2AK;LSR;SOX12;NR2F2;ETV5;ZBTB2;VTN;CTDSP2;PLXNB1;HIST1H4C;EXPH5
SMIM4;TFRC;GADD45B;FUS;ZC3H4;NAB2;CEP131;SOX12;PLEKHA4;CACNA1H;ZBTB2;COL1A1;POLA2;SMP[illegible]
ARHGEF2;N4BP2;CAMP
LDHB;MYCN;NAPRT;CCDC64;ETV5;SMAD7;MLLT6

BTG2;MIDN;TFRC;MEPCE;MAZ;SLC40A1;ATP2A2;ZC3H6;APOLD1;LMNB1;PNN;SPRED2;ELMSAN1;FTH1;IER5[illegible]
HRK;SOX17;KCNA1;SLIT2
MIDN;BTG2;TENM2;TRRAP;PRR3;FADS2;SPRED2;ENDOV;ZFYVE27;SMPD4;KHSRP;WDR90;FTH1;UBTF;CIC;[illegible]
TOP2A;TOMM40;GALNT18;EFNB2;PABPN1;RAB40C;KHSRP;ABL1;DVL2;RHPN1;ZSWIM4;MPZL1;ANKZF1;KD[illegible]
AGBL5;SHC4;MNT;MICALL2;SCAF1;SH2D5;BCAR1
CTU2;EGR3;PLEKHG2;CTNNBIP1;MAPKAPK2;FOSB;ZSWIM4;PALB2;AP4B1;IER2;MAP3K11
TSC22D4;SPRED2;GUCD1;GADD45B;SNRNP70;MTTP;TWF2;PPM1K;GTPBP2;HIST1H1C
MNT;OSBPL3;TAP2;PPM1K
TFRC;CELF3;LHFPL4;SLC35F1;CRIP2;SYT14;CPLX2;MAPK8IP1;DPYSL4;CAMK2N2;CASKIN2;CCDC64;BSN;S[illegible]
MEG3;MIDN;CBX6;TOMM40;FAHD1;NUMA1;PANK1;PHF1;GRIK5;NRARP;SOX12;SNRNP40;ARVCF;NASP;RAB[illegible]
MYH7B;JUND;TFRC;ITGB5;HIST1H4K;FUS;OSBPL3;HIST1H2AK;DDX55;MEPCE;PCIF1;ZC3H6;CPLX2;LMNB1;[illegible]
ANKRD39;TOP2A;TOMM40;CHD8;ANKRD11;PPM1K;ZC3H6;UBE2J2;EFNB2;PNN;ARVCF;PABPN1;RAB40C;K[illegible]
ANKRD39;TOP2A;LDB1;PANK1;CHD8;PHF1;GALNT18;KIF14;EFCAB6;ZC3H6;SLC2A6;PNN;SIX5;KHSRP;PPF[illegible]
AGBL5;ADNP
CUL7;LDB1;ZC3H4;ATP2A3;TWF2;ARHGAP6;PRAM1;SPRED2;MNT;WDR90;SH3BP5;DVL2;SAFB2;TBC1D10C[illegible]
MNT;PLEKHG2;ELMSAN1;GTPBP2
SEPT11;JUND;NOTCH1;RRM2;FUS;NAB2;MAZ;MTTP;ATP2A2;DENND3;ZC3H6;SLC7A1;SH3RF1;SMAD7;MNT;[illegible]
TOP2A;RTEL1;MEPCE;MAZ;KIF14;LMNB1;SMPD4;SNRNP70;WDR90;HIST1H3C;IER2;DLGAP5;HNRNPA0;HIS[illegible]
BTG2;CUL7;PANK1;NDUFA4L2;SLC2A1;ATP2A2;IRS2;NDRG1;UFSP1;GBA2;TBC1D10C;PIM3;RPS2;PATZ1;N[illegible]
PANK1;TMEM51;PPM1K;AP4B1;TBX3;TTC38;NFATC4;MLLT6;VTN;SPRED2;MYO1B;HNRNPUL1;ID1;SNAPC4;[illegible]
MIDN;NOTCH1;ZC3H4;ANKRD11;NAB2;ATP2A2;ZC3H6;ZBTB2;RASSF3;BCL7C;N4BP2;HNRNPA0;TSC22D4;[illegible]
RPS6KA4;EME2;ZC3H4;SPHK1;MEPCE;DVL2;MAGED2;SOX12;ADCK3;ZC3H6;PATZ1
SMIM4;MKNK1;LMNB1
RPS2;SCAF1
CBX7;MIDN;KLHDC4;TFRC;LDB1;MAP1S;SOX12;MLLT6;ALAD;AKAP12;MNT;RASSF3;HNRNPUL1;NASP;RPS2;[illegible]
MIDN;ZC3H6;PPM1K;CHD3;CLCN2;SPRED2;CASP7;UBTF;PATZ1;N4BP2;MAP4K3;ATF7IP;GADD45B;PHF12;[illegible]
SMIM4;ZC3H4;MEPCE;MAZ;UFSP1;ZBTB2;LMNB1;MNT;RBM15B;RAB11FIP1;RAB40C;KHSRP;BCL7C;MKNK1;[illegible]
CHRM3;ZBTB48;CLSTN1;TNC;SLC40A1;IRS2;ADCY1;AK5;ETS1;EFNB2;FTH1;SH3BP5;TSPAN2;MAP3K9;PRK[illegible]
FAM83H;SART1;CCDC142;RPS6KC1;PHF12;FBXL19;FAM193B;PALB2;AP4B1;MAP3K11
PPP1R16B;FTH1;PCIF1;HIST1H4C;N4BP2;HIST1H1C
ANKRD39;MIDN;PANK1;GALNT18;MUM1;IRS2;PNN;KHSRP;FTH1;PPFIA1;PIM3;DIP2A;MAP3K9;HMCN1;IER5[illegible]
GADD45B;FUS;ZC3H4;MEPCE;ICAM2;ARID5A;PBX2;NRXN2;DENND3;LSR;GTPBP2;SCAF1;FSCN1;IER5
AGBL5;HIST1H4C
ATF7IP;CASP7;JUND;HNRNPUL1;CHD8;TNRC18
TFRC;HIST1H4K;FUS;PHF12;MEPCE;ZBTB42;SCRIB;FANCC;KAZALD1;ETV5;ZBTB2;RNF167;PNN;EXOSC10;S[illegible]
BRD2;CARD10;FAM69B;PHF12;SLC16A11;IRS2;PALB2;ALAD;EFNB2;FADS2;POLA2;CEP250;PIM3;B4GALNT[illegible]
ATF7IP;CCDC142;THRA;SFSWAP;FBXL19;TAOK2;PCIF1;CEP250
EPHB6;ATP2A3;DIXDC1;SLC2A6;PHEX;SPRED2;TBC1D10C;ANKRD2;MAN2C1;ARHGEF40;IER5;GRASP;CAM[illegible]
TFRC;ZBTB49;SLC2A1;CPLX2;NDRG1;LMNB1;SART1;SPRED2;ENDOV;MNT;SIX5;CASKIN2;DVL2;RPS2;MAN2[illegible]
DVL2
BTG2;RRM2;HIST1H2AK;LSR;GTPBP2;AP4B1;LMNB1;SHF;RASSF3;SNRNP70;MAFF;PEX5;HIST1H4C;N4BP2;[illegible]
SMIM4;ZC3H4;MEPCE;NAB2;IRS2;CLN6;LMNB1;VTN;MNT;RBM15B;RAB11FIP1;RASSF3;BCL7A;PABPN1;KHS[illegible]
ANKRD39;MIDN;TFRC;TMEM200B;PLXND1;NAB2;IRS2;FGFRL1;ZFYVE27;EME2;KHSRP;DIP2A;IER5;IER2;CEP[illegible]
MIDN;CUL7;RTEL1;TFRC;MEPCE;MAZ;ATP2A2;ZC3H6;UFSP1;ZBTB2;LMNB1;SART1;GUCD1;PABPN1;SIX5;FT[illegible]
ZBTB9;XAB2;DHRS11;SFSWAP;MPZ;SCRIB;UBE2J2;PALB2;UFSP1;CLCN2;IER2;CD276
JUND;TFRC;HIST1H4K;CPSF1;SPHK1;DDX55;HIST1H2AK;MEPCE;SCRIB;GTPBP2;AP4B1;LMNB1;EME2;TNIK;[illegible]
TFRC;PANK1;UFSP1;LMNB1;ZFYVE27;XPO4;BCL7C;PIM3;MAN2C1;SH2D5;GIT1;SPHK1;DDX55;CIRBP;SOX1[illegible]
ANKRD39;ACHE;MIDN;TFRC;NUMA1;ZC3H4;PHF1;ZC3H6;CTGF;LMNB1;PNN;SNRNP70;SIX5;KHSRP;BCL7C;[illegible]

BTG2;TFRC;ZC3H4;PANK1;SLC2A1;ATP2A2;ZC3H6;LMNB1;SART1;PNN;SPRED2;XPO4;SNRNP70;EME2;FTH1
BTG2;TFRC;ZC3H4;CHD8;PHF1;ZC3H6;GLI1;LMNB1;SHF;SART1;PNN;SPRED2;ZFYVE27;EME2;SIX5;MAN2C1
TFRC;ZBTB49;MEPCE;MAZ;SLC2A1;ZC3H6;SLC7A1;CLCN2;UFSP1;CLN6;LMNB1;MNT;XPO4;SNRNP70;EME2
ANKRD39;NUMA1;ZC3H4;CLSTN1;ZC3H6;LMNB1;PNN;SPRED2;SMPD4;XPO4;EME2;WDR90;FTH1;NKIRAS2
TOMM40;NUMA1;ANKRD11;MTTP;KIF14;ALAD;SART1;SPRED2;GUCD1;ZFYVE27;SNRNP70;EME2;MKNK1;ER
TOMM40;TFRC;NUMA1;ZC3H4;ANKRD11;ZBTB48;MTTP;KIF14;APOLD1;PNN;ENDOV;MNT;RBM15B;SMPD4;P
RTEL1;CCDC142;SFSWAP;TRRAP;ZBTB49;MTTP;UBE2J2;CLCN2;GBA2;FAM83H;SART1;ENDOV;GUCD1;RAB4
SMIM4;ZBTB49;MEPCE;CLCN2;SART1;PNN;ENDOV;ZFYVE27;EXOSC10;SNRNP70;SIX5;MKNK1;SAFB2;CEP2
LRRC45;RTEL1;NUMA1;PHF12;FBXL19;MEPCE;MTTP;ARID5A;SCRIB;GTPBP2;SCAF1;ABL1;BIRC5;PPP1R12C

SOX17;KDR;IER5;KIRREL3;LMO1;CARD10;PRKCH;COL27A1;UNC5B;TAP2;VASH1;POU3F1;HIC1;DAAM2;M
EME2;SIX5;RAC3;ANKRD2;MAN2C1;ARHGEF40;HGFAC;LMO1;FOXD2;COL27A1;PLEKHG2;PHYKPL;SPHK1
2;PLCD3;LGR6;RAMP1;TELO2
;SRCIN1;MAPK8IP3;HIC1;ZPBP;EID2;MYCN;HNRNPUL1;ADNP;AJUBA;TRIB2;TELO2;NLRX1;CCDC142;COL
P250;IER5;ERMAP;COL27A1;HIST1H2AK;COL23A1;GAB1;GPR37L1;HSPG2;VAV2;GFOD1;CDC42EP4;MAP
IST1H1C;FOXD2;FBXW10;PROX1;ETV5;MYCN;DGKQ;FSCN1;CASQ2;EPHA1;TRIB2;SHC4;NOTCH1;LAMA1;
M3;DIP2A;FAM65A;ARHGEF40;EPHB4;IER2;CARD10;COL27A1;PHYKPL;MAP1S;ARID5A;SOX12;GTPBP2;SR
C4;RAC3;ANKRD2;MAN2C1;ARHGEF40;HGFAC;LMO1;FOXD2;COL27A1;PLEKHG2;TBC1D8;VPS37D;SORC
MAGI1;LRRC45;TCF15;UNC5B;SOX12;SRCIN1;ETV5;MYCN;CDC42EP4;CTDSP2;FSCN1;CASQ2;ADNP;EPH
RRM2;UNC5B;OSBPL3;SORCS2;PTPN14;TBX2;COL1A1;NELL2;COL2A1;HNRNPUL1;ASTN2;CRLF1;EPHA2
NMB1;MYO6;CDH23;CTLA4;SRGAP3;B4GALNT3;BCAS1;PPP1R16B;MAGI1;KL;AFAP1L2;FOXD2;JAG1;MAGI
RF;ALAD;NYNRIN;HEY1;SOX17;SNRNP70;BCL7C;FTH1;FAM65A;IER5;IER2;HIST1H1C;PPP1R16B;CARD10;
TRPC3;SH2D3C;COL23A1;ADAM11;ARAP3;HSPG2;SRCIN1;PALD1;VAV2;MYL4;INF2;B3GNT8;GFOD1;MAP
RB2;FBRSL1;FAM65A;ADAMTS8;SH2D5;FAM81A;GIT1;CAMP;MAGI1;CARD10;LRRC45;PHYKPL;CTNNBIP1;R
TBX21;CDH22;TNRC18;KIF1A
DVL2;IP6K1;N4BP3;PATZ1;SH2D5;GIT1;MAP4K3;KDM4B;FAHD1;FZD5;KHNYN;MAP1S;MAGI3;ARID5A;VPS3
;HEY1;MYO6;FSCN1;CASQ2;ID3;TNRC18;N4BP2;FAM81A;AJUBA
;TMC6;PALD1;SYTL2;ANKFN1;CRISPLD1;PITPNM3;COL4A5;TNIK;EPHA1;SLC25A13;AKNA;LAMA3;PLA2G5

C;KHSRP;CASKIN2;ABL1;RPS2;ZSWIM4;EXPH5;HIST1H1C;KDM4B;HIST1H2AK;CEP131;ARID5A;LSR;SCAF1
YKPL;TMC6;SOX12;SORCS2;RNF167;BACE2;PYGO2;FSCN1;BIRC5;AJUBA;RTEL1;NOTCH1;ITPR3;FAM109A
;LMNB1;RPS6KA4;RASSF3;BCL7A;XPO4;EME2;SIX5;BCL7C;UBTF;PIM3;CIC;IER5;FOXD2;KHNYN;SPHK1;C
IST1H1C;PLEKHG2;TANGO2;CEP131;SCRIB;MAPK8IP3;ETV5;ADNP;AJUBA;KANK3;NOTCH1;ANKRD11;ME
N1;FSCN1;N4BP2;IL17C;SHANK3;PRPH
7A1;PLEKHG2;GAB1;SOX13;PROX1;LIME1;TTC38;ETV5;INF2;ERF;CLDN19;ITGA6;KANK4;SLC25A13;LAMA
PRKG1;CHST8;LMO1;TRPC3;SPNS3;APLP1;ARID5A;ARAP3;SORCS1;GRIP2;MAPK8IP1;EID2;GPRC5B;MYC

AT3;GPC4;HIST1H3C;CBX7;PTCH1;TBX3;ETV5;MYCN;CDC42EP4;ID1;CASQ2;TCF3;TRIB2
TGA5
2;N4BP2;FAM81A;DLGAP5;JAM3;ATF7IP;RRM2;FZD5;FUS;MAP1S;TPM3;DDX55;HIST1H2AK;PLET1;TBX3;KL

T1H3C;DLGAP5;ANKZF1;LRRC45;KDM4B;RRM2;HIST1H2AK;CEP131;ETV5;SPSB3;XAB2;HNRNPUL1;ADCK

D4;NKIRAS2;ERF;PPFIA1;FAM64A;SFXN2;RPS2;IER2;BOK

;;EPHB4;HIST1H1C;GADD45B;FUS;PCIF1;CIRBP;SCRIB;PALB2;HNRNPUL1;ID1;FSCN1;ADNP;HIST1H4C;R

ER2;BRD2;CEP131;CIRBP;SOX12;GTPBP2;HIC1;MAPK8IP1;CTU2;HNRNPUL1;ADNP;ARHGEF7;SLC25A13;T
14B;RRM2;HIST1H2AK;ETV5;ABTB2;SPSB3;XAB2;HNRNPUL1;ADCK3;PRR12;MXD1;MAP3K11

LC25A13
40C;TAOK2;FOSB;DIP2A;MXD1;N4BP2;ANKZF1
SART1;SNRNP70;NKIRAS2;FSCN1;RPS2;MAN2C1;HIST1H4C;IER5;HIST1H1C
HSRP;NKIRAS2;RHPN1;ABL1;MAP3K9;ZSWIM4;IP6K1;DLGAP5;MPZL1;HNRNPA0;LRRC47;LRRC45;KDM4B;N
A1;DIP2A;MAP3K9;IER5;DLGAP5;HIST1H1C;APC2;LRRC45;SZT2;MAGI3;TANGO2;HIST1H2AK;CEP131;GTP

;FLOT1;PIM3;PATZ1;N4BP2;IER2;PCK2;PHF12;DDX55;PBX2;ARAP3;PLEKHA4;PARVB;FANCE;DGKZ;PALB2;

RASSF3;UBTF;PIM3;TCF3;IP6K1;IER5;N4BP2;RGL2
T1H1C;RRM2;FUS;HIST1H2AK;FANCC;PALB2;POLA2;HNRNPUL1;NASP;TAOK2;BIRC5;FAM64A
4BP2;IER5;IER2;HIST1H1C;FAHD1;JUND;KHNYN;GADD45B;HIST1H4K;HIST1H2AK;PCIF1;ARID5A;SCAF1;T
TNRC18;MAGED2;HMCN1;TCF3;PHLDB3;N4BP2
EGR2;GADD45B;FUS;OSBPL3;PCIF1;CIRBP;TBC1D8;ARID5A;SCRIB;MAFA;ETV5;SMAD7;MYO1D;CTDSP2;EF

;TCF3;PATZ1;RBMX;IER2
DDX55;ETV5;SMAD7;ITPKB;HNRNPUL1;NASP;PSPC1;CLCF1;ID3;SFXN2;PRR12
DVL2;ZSWIM4;IP6K1;IER5;MAP4K3;CBX6;KDM4B;FAHD1;DDX55;TNK2;PTCH1;GTPBP2;SMAD7;HNRNPUL
G1;MAP4K3;HIST1H1C;JAG1;UNC5B;MAGI3;ABCA4;SOX13;KREMEN1;SORCS1;NR2F2;TMC6;GRIP2;BFSP2

;SZT2;MAGI3;TANGO2;HIST1H2AK;CTNNBIP1;CEP131;CIRBP;ARID5A;SOX12;POU3F1;GTPBP2;HNRNPUL1

NRNP70;MAFF;RPS2;TCF3;HIST1H4C;HNRNPA0;HIST1H1C
3;N4BP2

P;PPP1R16B;SZT2;SDS;KHNYN;SH2D3C;ARID5A;PROX1;PALD1;FSCN1;ERF;ARHGEF2;PPP1R12C;CUL7;T
C1;DLGAP5;HIST1H1C;RRM2;FAHD1;DDX55;HIST1H2AK;SCRIB;PALB2;CTU2;NASP;ID1;BIRC5;FOSB;CCD

ZCCHC3;MAP3K11;MPZL1;MAP4K3;HIST1H1C
SRP;DVL2;ZSWIM4;IP6K1;HOXC9;SH2D5;IER5;ATF7IP;FOXD3;CBX6;KDM4B;FAHD1;PLEKHG2;GADD45B;P
131;APLP1;CIRBP;SOX12;GTPBP2;HSPG2;SYTL2;HNRNPUL1;SPATA13;FAM64A;ADNP;TELO2;CCDC142;N
H1;NAPRT;DVL2;CEP250;FLOT2;RPS2;MAN2C1;IER5;ANKZF1;JUND;KHNYN;GADD45B;CPSF1;FUS;DDX55;

MAN2C1;HIST1H4C;RGL2
2;GTPBP2;CTU2;HNRNPUL1;GFOD1;BIRC5;ADNP;RAPGEF3;TELO2;SLC25A13;SMIM4;ZBTB49;MEPCE;MAZ
FTH1;NKIRAS2;SNAPC4;CEP250;PIM3;DIP2A;MAP3K9;MAN2C1;IER2;HIST1H1C;PLEKHG2;KHNYN;DDX55

1;NKIRAS2;SNAPC4;MAN2C1;IER5;IER2;RBM33;LRRC47;MAP1S;DDX55;HIST1H2AK;CIRBP;ETV5;PYGO2;H
1;IER5;HIST1H1C;PLEKHG2;DDX55;HIST1H2AK;CIRBP;SCRIB;MAPK8IP3;ZPBP;RNF167;HNRNPUL1;FSCN1
2;BCL7C;SAFB2;PIM3;RPS2;PPARGC1B;RRM2;FUS;DDX55;HIST1H2AK;SOX12;GTPBP2;AP4B1;CTU2;BIRC5
;CEP250;MAN2C1;ERMAP;IER2;RBM33;HIST1H1C;LRRC47;MAP1S;ACTR5;DDX55;CIRBP;HNRNPUL1;PSP(
BB2;MAN2C1;IER2;LRRC47;SZT2;KLHDC4;PRKCH;PLEKHG2;DDX55;TANGO2;TBC1D8;SCRIB;AP4B1;FBXL
ABPN1;XPO4;KHSRP;RPS2;ANKZF1;HNRNPA0;KLHDC4;EGR3;JUND;FBXL19;HIST1H2AK;CEP131;SOX12;G
40C;EME2;RPS6KC1;NKIRAS2;SNAPC4;ERBB2;MAN2C1;IER2;LRRC47;SZT2;KLHDC4;CPSF1;DDX55;SCAF1
50;DIP2A;IP6K1;BSN;MAGI1;MYH7B;CPSF1;PHF12;PCIF1;CIRBP;PALB2;HINFP;EID2;POLA2;PSPC1;CDC4
;MAP3K12

AL;ASTN2;TRIB2;KANK4;SHC4;COL13A1;DHH;CTTNBP2;CHRD;AK5;CPLX2;NDRG1;PCDH17;SCUBE2;EFNE
;COL23A1;VPS37D;LSR;SRCIN1;SORCS2;HIC1;ZPBP;VAV2;EID2;GPRC5B;HNRNPUL1;CDC42EP4;PEX5;AD

13A1;MAZ;GLIS2;EFNB2;RAI1;EFNB1;CASKIN2;SFN;PATZ1;N4BP2;PRPH;EGR2;JAG1;FZD5;GADD45B;TMEM
KAPK2;KCNQ2;TNIK;ERG;ASTN2;EPHA2;HEMGN;NOTCH1;SLC7A1;PRAM1;ABL1;CDH23;MBP;VILL;ACSS1;
SEMA3A;LEF1;CRIP2;MLLT6;DLL4;LDHB;SPP1;DVL2;MBP;GPC4;SRGAP3;IP6K1;HIST1H3C;ATP9A;NTNG1;
CIN1;PYGO2;BCAM;ERF;FLYWCH1;ADNP;PHLDB3;AJUBA;EPHA2;NOTCH3;CUL7;ANKRD11;MEPCE;MAZ;PD
S2;HIC1;ZPBP;EID2;GPRC5B;MYCN;HNRNPUL1;PEX5;ADNP;TRIB2;CCDC142;COL13A1;NDUFA4L2;NRXN2
A1;TRIB2;SLC25A13;LAMA5;LEF1;NRXN2;MLLT6;DLL4;LDHB;SPP1;MGAT3;GPC4;SRGAP3;PATZ1;N4BP2;JA

3;COL23A1;SMAD7;ITPKB;GNAO1;FMNL3;NFASC;MYO1B;ANKFN1;ID1;ID3;ITGA6;TNIK;LGR6;SLC25A13;TJ
RD2;EGFL7;SH2D3C;KREMEN1;ARID5A;SCRIB;DGKZ;TTC38;PALD1;VAV2;PTP4A3;HNRNPUL1;RARA;ITGA5
KAPK2;FAM181B;PHLDB3;SHANK3;EPHA2;DHH;CTTNBP2;RAB11FIP1;ELMSAN1;MICALL2;SH3BP5;PCK2;K
HBDF2;SHROOM2;ARAP3;ITPKB;GFOD1;TNNT2;TET3;ITGA6;ITGA5;ERG;AJUBA;CHST3;NOTCH1;AKNA;LEF1

37D;SCRIB;USB1;MAPK8IP3;ABTB2;RGP1;GFOD1;CLCF1;CTDSP2;FSCN1;ERF;MAFK;FLYWCH1;TCF3

;PPM1K;AK5;NDRG1;THSD4;NCALD;ZBTB2;SCUBE2;ABLIM3;ERBB4;RPS6KC1;MPZL1;NTNG1;COL26A1;AF

l;AP4B1;ETV5;NFATC4;SPSB3;XAB2;HNRNPUL1;FLYWCH1;TCF3;HIST1H4C;MXD1;MAP3K11
;NDRG1;MLLT6;EFNB2;DLL4;MNT;SPP1;ABL1;RPS2;PRPH;CBX7;JUND;HIST1H4K;FBXL19;IGF2;CDC42BPB;
TNNBIP1;TBC1D8;VPS37D;SRCIN1;VAV2;CTU2;HNRNPUL1;GFOD1;MAPKAPK2;PRR12;TRIB2;NLRX1;CCDC
PCE;MAZ;MTTP;SLC41A1;NRXN2;PPM1K;APOLD1;EFNB2;ARVCF;PABPN1;RAB40C;CASKIN2;RHPN1;TNRC1

5;CCDC142;TOMM40;CHRNA4;MEPCE;PDGFB;CHRD;TWF2;ZBTB42;FAM109A;CLCN2;FAM83H;VTN;PABP
N;MAPKAPK2;ERG;ZBTB49;CHRD;NRXN2;CRIP2;CPLX2;PCDH17;CACNA1H;NCALD;NWD1;TBX21;CDH22;

F2;ABTB2;TNRC6C;POLA2;SMTN;HNRNPUL1;NASP;TAOK2;ID3;HIST1H4C;RBMX

3;TCF3;MXD1;MAP3K11

GL2;BCL2L2

TOMM40;ANKRD11;MEPCE;KMT2B;SEMA3B;MTTP;SLC41A1;MNT;RAB40C;SPOCK2;DVL2;SAFB2;TMEM86A

MAGI3;TANGO2;CEP131;ETV5;SPSB3;FMNL3;HNRNPUL1;TCF3;MXD1;MAP3K11;KANK3
PBP2;ETV5;HNRNPUL1;CTDSP2;FAM64A;PRR12;RNF122;KANK3;SMIM4;TOMM40;ANKRD11;SLC41A1;NRX

FBXL12;ITPKB;AGBL5;HNRNPUL1;MAPKAPK2;BIRC5;ID3;ARHGEF2;ADNP;RGL2

TC38;RGP1;CTU2;PYGO2;BIRC5;HIST1H4C

RF;ADNP;IL17C

1;SFXN2;ADNP;ADCK3;RGL2;OTUD1;RAPGEF3
;INF2;PSPC1;DAAM2;MAFF;FSCN1;FAT4;ADCK3;CDS2

.;FAM64A;FLYWCH1;PRR12;RNF122;KANK3;CUL7;SMIM4;ANKRD11;MEPCE;MAZ;MTTP;TWF2;SLC41A1;FA

OMM40;MAZ;ASAP2;PPM1F;MNT;RAB11FIP1;RAB40C;RPS6KC1;ERBB2;PCED1B;PATZ1;HNRNPA0;PCK2;KD
C64;FAM64A;SFXN2;MXD1;HIST1H4C;RGL2

TCH1;PCIF1;MTSS1L;PALB2;HINFP;SMAD7;LRFN4;HNRNPUL1;TRIP6;MAFF;SFXN2;ADNP;RGL2;OTUD1;RAP
IOTCH1;MEPCE;MAZ;ITPR3;FAM109A;EFNB2;LDHB;GUCD1;RAB11FIP1;IP6K1;N4BP3;PATZ1;N4BP2;PPAR
PCIF1;CIRBP;SCRIB;FANCC;LSR;MAPK8IP3;PYGO2;TAOK2;TRIP6;FSCN1;ADNP;ADCK3;MXD1;PRR12;HIST

Z;SLC7A1;NDRG1;CLN6;MNT;RBM15B;DVL2;RPS2;IP6K1;N4BP2;PPARGC1B;ATF7IP;SEPT11;FAHD1;CPSF1
;MAGI3;HIST1H2AK;LSR;SOX12;GTPBP2;MAPK8IP3;ETV5;CTU2;RNF167;PYGO2;HNRNPUL1;MAPKAPK2;P

INRNPUL1;PSPC1;FSCN1;ADNP;PRR12;CUL7;CCDC142;SMIM4;SFSWAP;ZBTB49;ANKRD11;MEPCE;MAZ;C
;ARHGEF2;ADNP;CUL7;NLRX1;CCDC142;SMIM4;TOMM40;AMIGO3;MEPCE;MAZ;MTTP;DENND3;PPM1K;AI
;ADNP;ADCK3;RBMX;TELO2
C1;BIRC5;FAM193B;ADNP;PRR12;CCDC142;SFSWAP;MEPCE;MAZ;UBE2J2;APOLD1;GUCD1;MNT;RBM15B;
12;XAB2;HNRNPUL1;ID3;MAFK;HIST1H4C;PPP1R12C;BCL2L2;MAP3K11
TPBP2;SCAF1;MAPK8IP3;ESR2;SPSB3;CTU2;XAB2;HNRNPUL1;NASP;ID1;ERF;FOSB;TCF3;ADNP;PPP1R12
;PALB2;MAPK8IP3;FBXL12;SPSB3;RGP1;RNF167;XAB2;PYGO2;HNRNPUL1;PPP1R12C;RGL2;MAP3K11
2EP4;TAOK2;FOSB;MAGED2;SFXN2;FLYWCH1;ADCK3;MAP3K11;CDS2

32;SCUBE1;CDH22;MBP;TSPAN2;TNRC18;HOXC9;PROM1;ZBTB7C;PRPH;NTNG1;KL;EGR2;EGR3;IGF2;SLC
DNP;AJUBA;TRIB2;RAPGEF3;TELO2;NLRX1;CCDC142;COL13A1;NDUFA4L2;NRXN2;ASAP2;PPM1F;RASIP1;

I74B;CPSF1;SNAI3;MARCH9;USB1;MAFA;SYT8;NFATC4;COL1A1;ABTB2;GIPC3;ITGA10;ESAM;COL9A2;HIS
PPARGC1B;RAP1GAP2;TNK2;IDH2;PARVB;PLET1;MAPK13;KLF1;FBXL12;MYO1D;STK24;KIF26A;COL9A1;MA
ATF7IP;CBX7;JAG1;EGR3;GADD45B;FBXL19;TBX3;KLF2;ID1;ESAM;TCF3;LGR6;KCNK1;HIST1H4C
DGFB;NDRG1;FAM83H;RAI1;RAB11FIP1;PCBP4;CASKIN2;MYO6;PRR29;TNRC18;CHST14;PATZ1;N4BP2;RAE
2;ASAP2;PPM1F;GLIS2;EFNB2;RAI1;EFNB1;RBM15B;CHRNE;CASKIN2;CASKIN1;SPP1;N4BP2;MYH7B;COL2
M3;CBX7;JAG1;FZD5;HIST1H4K;PHF12;TNK2;PTCH1;NR2F2;MAFA;TBX3;KLF2;ARHGAP24;HINFP;SMAD7;A

P2
5;ARHGEF2;TNIK;EPHA1;EMID1;RTEL1;MTTP;LEF1;PDGFB;FAM109A;MLLT6;NEURL1B;LDHB;SCUBE1;RAB4
DM4B;PHF12;PARVB;KLF2;DLK1;MAPK13;PLXNB3;TCF3
;NRXN2;DENND3;ASAP2;CPLX2;NDRG1;EFNB2;RAI1;EFNB1;RAB40C;ABLIM3;RPS6KC1;MYO6;CASKIN1;M

AP1L2;JAG1;GADD45B;PHF12;PTCH1;SYT14;NR2F2;TBX3;KLF2;TBX2;ABTB2;MYO1D;SBK1;NBEA;SPRY1;RG

;TBX3;KLF2;TBX2;NFATC4;HINFP;GNAO1;PLXNB1;TCF3;RBMX
C142;MAZ;ZBTB48;ZBTB42;CLCN2;RASIP1;RAI1;ZDHHC18;CASKIN2;MYO6;N4BP3;PATZ1;N4BP2;ANKRD50
18;ZSWIM4;IP6K1;MPZL1;ANKZF1;HNRNPA0;KDM4B;FAHD1;JUND;GADD45B;FUS;OSBPL3;MAFA;ESR2;HIN

N1;CASKIN2;TRIM47;PATZ1;MAP4K3;PCK2;MYH7B;NPM2;FAHD1;JUND;FZD5;PHF12;KAZALD1;PALB2;FBXL
MGAT3;TNRC18;N4BP3;PPARGC1B;CPSF1;PHF12;OSBPL3;PCIF1;LHFPL4;SYT14;SEMA4F;NR2F2;PLET1;KA

;KLHDC4;KDM4B;JUND;PHF12;KLF2;HINFP;AGBL5;FMNL3;XAB2;TAOK2;ID1;MAFF;FOSB;MAFK;MXD1;RBM

N2;FAM109A;PPM1K;APOLD1;EFNB2;MNT;ARVCF;PABPN1;RAB40C;RHPN1;ABL1;RPS2;ZSWIM4;IP6K1;N4

M109A;EFNB2;LDHB;EFNB1;GUCD1;RHPN1;ABL1;TNRC18;IP6K1;HOXC9;N4BP2;HIST1H3C;ANKZF1;EXPH

M4B;NPM2;CARD9;IDH2;PBX2;NR2F2;PALB2;FBXL12;ID3;CCDC64;ADCK3;RGL2

GEF3;SLC25A13
C1B;EXPH5;PCK2;RAB11FIP5;CBX7;CADM4;FZD5;CPSF1;FUS;OSBPL3;IDH2;CYBRD1;FANCC;MAFA;FANC
1H4C;RGL2;ZCCHC3;RBMX

;NCKAP5L;AP4B1;PALB2;HINFP;FMNL3;TAOK2;SFXN2;ADCK3;MXD1;OTUD1
EX5;ADNP;RTEL1;CCDC142;SMIM4;TOMM40;ANKRD11;MEPCE;ZBTB42;NRXN2;DENND3;PPM1K;CLCN2;

:LCN2;APOLD1;ZBTB2;GUCD1;EXOSC10;ERBB2;SAFB2;ABL1;RPS2;ANKZF1;HNRNPA0;MYH7B;SEPT11;KLH
'OLD1;LDHB;MNT;RBM15B;PABPN1;RPS2;IP6K1;HNRNPA0;ATF7IP;JUND;GADD45B;HIST1H4K;FUS;PHF12

;EXOSC10;PABPN1;SAFB2;ABL1;RPS2;IP6K1;MAP4K3;ATF7IP;SEPT11;KLHDC4;FAHD1;FUS;PHF12;FBXL1

C;BCL2L2;MAP3K11;MAP3K12

16A11;BTBD11;NR2F2;MAFA;ESR2;ABTB2;GNAO1;GJB2;GIPC3;ID3;ESAM;FAT4;NES;FBXL7;HRK;GPR27;AC
GLIS2;DLL4;RAI1;EFNB1;RBM15B;ABLIM3;CASKIN2;PATZ1;COL26A1;EGR2;CBX6;FZD5;GADD45B;OSBPL3;

T1H4C;RGL2;NES;CDS2;ACHE;MEGF10;TMEM51;NRARP;PEAR1;GLI1;PTPRF;ALAD;CASP7;SMPD4;SIX5;BC
AFK;SPRY1;KCNK1;RAMP1

311FIP5;JUND;FZD5;GADD45B;TMEM74B;CPSF1;SMURF1;SNAI3;PCIF1;IDH2;PBX2;MARCH9;KLHL23;USB1
6A1;EGR2;CBX6;JAG1;GADD45B;CPSF1;USB1;NR2F2;MAFA;KAZALD1;AP4B1;SYT8;COL1A1;ABTB2;MYO1D
RHGAP33;SBK1;PTK7;ID1;ID3;AQP11;SPRY1;RGS12;TCF3;NES

0C;ELMSAN1;TBX21;CASKIN2;SH3BP5;TSPAN2;CD276;HNRNPA0;HIST1H4K;FUS;PHF12;SNAI3;TNK2;OSB

YL10;SFN;TSPAN2;CHST14;SRGAP3;BEND5;MPZL1;MAP4K3;KLHDC4;JAG1;GALNT3;ABCA4;APLN;NFATC4

GS12;LGR6;KCNK1;FBXL7

;PPARGC1B;MAP4K3;EGR2;CBX6;KDM4B;JUND;FZD5;TNK2;OSBPL3;PTCH1;IDH2;MTSS1L;KLHL23;USB1;D
IFP;FMNL3;XAB2;SPRY1;TCF3;HIST1H4C;RGL2;RBMX;MAP3K11

.12;ID1;AQP11;PLXNB1;FAT4;HIST1H4C;FGFR4;MAP3K11
AZALD1;NELL2;GNAO1;NFASC;SBK1;PPP2R2C;FOSB;FXYD6;TBKBP1

⟨;BCL2L2;MAP3K11

-BP2;MPZL1;ANKZF1;EXPH5;HNRNPA0;PCK2;KDM4B;RRM2;FAHD1;OSBPL3;SEMA4F;AP4B1;PALB2;ESR2;[illegible]

[illegible]I5;PCK2;TMEM86A;JUND;FZD5;GADD45B;PHF12;SNAI3;OSBPL3;PCIF1;CYBRD1;FANCC;MAFA;AP4B1;ESR[illegible]

[illegible]E;PALB2;ESR2;PM20D2;POLA2;NASP;SFXN2;ADCK3;MXD1;ZCCHC3;PHF1;NRARP;EFCAB6;VSIG10L;UFSF[illegible]

[illegible]POLD1;ZBTB2;GUCD1;MNT;RBM15B;EXOSC10;MKNK1;RPS2;PATZ1;N4BP2;MAP4K3;ANKZF1;HNRNPA0;[illegible]

HDC4;JUND;GADD45B;CPSF1;FUS;PCIF1;SCAF1;PALB2;POLA2;SNRNP40;NASP;ID3;SFXN2;ADCK3;MXD1;
;PCIF1;KAZALD1;AP4B1;MYO1D;AGBL5;NASP;ID1;TRIP6;MAFF;FOSB;SPRY1;TCF3;MXD1;PLCD3;HIST1H4C

;PCIF1;SCAF1;PALB2;FBXL12;XAB2;SNRNP40;NASP;TAOK2;ZCCHC3;RBMX

[illegible]HE;SLC35F1;PEAR1;CHRDL2;MEOX2;ARHGAP6;SHE;KCNT1;EMILIN3;KIF1A;ADAMTS8;PRKG1;CHST8;FOX
;MARCH9;USB1;MAFA;KAZALD1;SYT8;COL1A1;ABTB2;MYO1D;FMNL3;SLC4A8;GIPC3;KIF26A;BCL6B;ITGA1

[illegible]L7C;RAC3;ANKRD2;MAN2C1;HGFAC;FOXD2;PLEKHG2;TRPC3;SPHK1;TBC1D8;VPS37D;SORCS2;VAV2;GP

1;FANCC;MAFA;SYT8;NFATC4;PTK7;ID1;TCF3;FGFR4;RGL2;ZCCHC3;DOCK6;ACHE;THRA;ZC3H4;PHF1;FAM
[illegible];FMNL3;GIPC3;KIF26A;BCL6B;ITGA10;PLXNB3;ESAM;COL9A2;LGR6;PLCD3;RAMP1;NES;CDS2

[illegible]PL3;FANCC;PTPN14;NFATC4;NBEA;ID3;MAFK;TBKBP1;RGS12;RAMP1

;SMAD7;TNRC6C;TMIE;NFASC;PTK7;COL7A1;CCDC64;FXYD6;MXD1

[illegible]CBLD1;SMAD7;FA2H;LRFN4;STK24;CLCF1;MAFF;ID3;CCDC64;OTUD1;CDS2

HINFP;ABTB2;SPSB3;AGBL5;ARHGAP33;FMNL3;XAB2;SNRNP40;SMTN;NASP;ID1;FOSB;SFXN2;TCF3;ADCK3

2;NFATC4;COL1A1;ABTB2;ARHGAP33;FA2H;SMTN;LRFN4;NASP;ID1;PLXNB3;SFXN2;FAT4;TCF3;ADCK3;MX

P1;LMNB1;SMPD4;BCL7A;XPO4;SNRNP70;SIX5;BCL7C;SNAPC4;GIT1;BOK;SPHK1;ACTR5;DDX55;PALD1;B

IYH7B;JUND;GADD45B;CPSF1;FUS;PHF12;OSBPL3;FBXL19;IDH2;FANCC;KAZALD1;AP4B1;FBXL12;COL1A1

RGL2;ZCCHC3;RBMX
:;RGL2;RBMX;XKR6;MAP3K12

D3;FOXD2;COL25A1;SPHK1;TESC;GAB1;PROX1;DUSP26;SORCS2;ITPKB;BACE2;GPRC5B;COL2A1;PITPNM
0;ESAM;SPRY1;COL9A2;LGR6;MXD1;PLCD3;RAMP1;NES;CDS2

RC5B;CDC42EP4;PEX5;RAPGEF3;LAMA5;AMIGO3;NDUFA4L2;SEMA3G;NRXN2;ASAP2;CLCN2;PPM1F;RAS

69B;KIF14;ATP2A2;ZC3H6;GLI1;LMNB1;SPRED2;SMPD4;SIX5;NKIRAS2;UNKL;HGFAC;RBM33;TSC22D4;PL

3;KCNK1;MXD1;RBMX;MAP3K11;MAP3K12;CDS2

.D1;HIST1H4C;MAP3K11;MAP3K12;CDS2;TOP2A;NUMA1;LDB1;CHD8;PHF1;KIF14;ATP2A2;EFCAB6;ZC3H(

IRC5;RAPGEF3;SLC25A13;PPM1K;CLCN2;CLN6;APOLD1;RASIP1;MLLT6;DDN;DLL4;NEURL1B;ABLIM3;RPS

.;AGBL5;MAFF;ID3;FOSB;SFXN2;TCF3;PLCD3;RGL2;ZCCHC3;MAP3K12

13;TRAF5;ITGA7;FAM181B;CHST2;OPRD1;FOXC1;GRP;KCNA1;LEF1;NRXN2;NDNF;ADCY1;DLL4;SPOCK2;TE

IP1;DLL4;ZDHHC18;RBM15B;ABLIM3;SPP1;TRIM47;COL26A1;KAZALD1;KLF1;MYO1D;FMNL3;SLC4A8;KIF2

LEKHG2;SPHK1;TESC;VPS37D;ETV5;VAV2;CDC42EP4;CTDSP2;ARHGEF2;LAMA5;SEMA3B;ZBTB42;DENND

[illegible];SLC2A6;LMNB1;HEY1;BCL7A;SIX5;CIC;DLGAP5;HIST1H1C;LRRC45;PLEKHG2;SHROOM2;ETV5;CTDSP2;[illegible]

6KC1;CAMK2N2;TRIM47;HNRNPA0;SEPT11;RRM2;FAHD1;CDC42BPB;KAZALD1;HINFP;AGBL5;FMNL3;BCL

3X21;TRIM47;SLIT2;JAM3;KCNJ3;COL26A1;KCNJ8;GALNT3;KAZALD1;DLK1;TBX2;APLN;NELL2;HEYL;PPP2R[illegible]

.6A;BCL6B;LGR6;PLCD3;RAMP1

3;CLCN2;MLLT6;MICALL2;TRIM47;ZSWIM4;ANKRD50;MAP4K3;HNRNPA0;RRM2;NCKAP5L;FBXL19;MTSS1[illegible]

TOMM40;AMIGO3;ZBTB48;NRXN2;PPM1K;SLC7A1;APOLD1;MNT;ARVCF;PABPN1;RAB40C;ERBB2;MICALL2

6B;TAOK2;AQP11;SPRY1;OTUD1

2C;BCL6B;CLCF1;CAMK4;TMEM229A;CRLF1;SNTB1

L;KAZALD1;BAIAP3;FBXL12;TAOK2;TBKBP1;AGRN;PLCD3;MSRB1

;RPS2;ZSWIM4;MPZL1;HNRNPA0;ATF7IP;KDM4B;RRM2;FAHD1;NCKAP5L;SEMA4F;HINFP;SPSB3;AGBL5;F[illegible]

[illegible]NL3;XAB2;SNRNP40;TSPAN13;FOSB;KCNK1;RBMX;MSRB1

Term
Regulation Of Cell Migration (GO:0030334)
Cell-Cell Adhesion Via Plasma-Membrane Adhesion Molecules (GO:0098742)
Extracellular Matrix Organization (GO:0030198)
Transmembrane Receptor Protein Tyrosine Kinase Signaling Pathway (GO:0007169)
Axon Guidance (GO:0007411)
Apical Protein Localization (GO:0045176)
Extracellular Structure Organization (GO:0043062)
External Encapsulating Structure Organization (GO:0045229)
Positive Regulation Of Cell Migration (GO:0030335)
Protein Autophosphorylation (GO:0046777)
Muscle Contraction (GO:0006936)
Aorta Morphogenesis (GO:0035909)
Positive Regulation Of Cell Population Proliferation (GO:0008284)
Cellular Response To Vascular Endothelial Growth Factor Stimulus (GO:0035924)
Semaphorin-Plexin Signaling Pathway (GO:0071526)
Neuron Projection Guidance (GO:0097485)
Semaphorin-Plexin Signaling Pathway Involved In Neuron Projection Guidance (GO:1902285)
Positive Regulation Of Cell Motility (GO:2000147)
Regulation Of Cytoskeleton Organization (GO:0051493)
Endothelial Cell Differentiation (GO:0045446)
Nervous System Development (GO:0007399)
Positive Regulation Of Peptidyl-Tyrosine Phosphorylation (GO:0050731)
Synapse Organization (GO:0050808)
Negative Regulation Of DNA-templated Transcription (GO:0045892)
Axonogenesis (GO:0007409)
Cell Junction Assembly (GO:0034329)
Blood Vessel Endothelial Cell Migration (GO:0043534)
Central Nervous System Development (GO:0007417)
Negative Regulation Of Transcription By RNA Polymerase II (GO:0000122)
Endodermal Cell Differentiation (GO:0035987)
Negative Regulation Of Chemotaxis (GO:0050922)
Cardiac Ventricle Morphogenesis (GO:0003208)
Stress-Activated MAPK Cascade (GO:0051403)
Positive Regulation Of Cell Adhesion (GO:0045785)
Actin Cytoskeleton Reorganization (GO:0031532)
Blood Vessel Morphogenesis (GO:0048514)
Cell Surface Receptor Signaling Pathway Involved In Heart Development (GO:0061311)
Negative Regulation Of Blood Vessel Endothelial Cell Migration (GO:0043537)
Positive Regulation Of Endothelial Cell Proliferation (GO:0001938)
Regulation Of Cell Adhesion (GO:0030155)
Notch Signaling Involved In Heart Development (GO:0061314)
Cell Migration Involved In Sprouting Angiogenesis (GO:0002042)
Filopodium Assembly (GO:0046847)
Endoderm Formation (GO:0001706)
Regulation Of Endothelial Cell Proliferation (GO:0001936)
Protein Localization To Membrane (GO:0072657)
Cellular Response To Magnesium Ion (GO:0071286)

Substrate Adhesion-Dependent Cell Spreading (GO:0034446)
Pulmonary Valve Morphogenesis (GO:0003184)
Regulation Of Actin Filament Polymerization (GO:0030833)
Cell-Matrix Adhesion (GO:0007160)
Ventricular Septum Morphogenesis (GO:0060412)
Regulation Of Cell Motility (GO:2000145)
Aortic Valve Morphogenesis (GO:0003180)
Negative Regulation Of Cellular Response To Growth Factor Stimulus (GO:0090288)
Cardiac Epithelial To Mesenchymal Transition (GO:0060317)
Regulation Of Kinase Activity (GO:0043549)
Protein Phosphorylation (GO:0006468)
Regulation Of Anatomical Structure Morphogenesis (GO:0022603)
JNK Cascade (GO:0007254)
Regulation Of Neuron Migration (GO:2001222)
Pulmonary Valve Development (GO:0003177)
Axon Extension Involved In Axon Guidance (GO:0048846)
Neuron Projection Extension Involved In Neuron Projection Guidance (GO:1902284)
Notch Signaling Pathway (GO:0007219)
Aortic Valve Development (GO:0003176)
Regulation Of Neuron Projection Development (GO:0010975)
Regulation Of Vasculature Development (GO:1901342)
Regulation Of Cell Migration Involved In Sprouting Angiogenesis (GO:0090049)
Negative Regulation Of Nucleic Acid-Templated Transcription (GO:1903507)
Embryonic Organ Development (GO:0048568)
Positive Regulation Of JNK Cascade (GO:0046330)
Positive Regulation Of Cell Projection Organization (GO:0031346)
Negative Regulation Of Cell Migration Involved In Sprouting Angiogenesis (GO:0090051)
Positive Regulation Of Cardiac Muscle Cell Proliferation (GO:0060045)
Negative Chemotaxis (GO:0050919)
Branching Morphogenesis Of An Epithelial Tube (GO:0048754)
Collagen Fibril Organization (GO:0030199)
Mesenchymal Cell Migration (GO:0090497)
Ephrin Receptor Signaling Pathway (GO:0048013)
Positive Regulation Of Cellular Process (GO:0048522)
Lymphatic Endothelial Cell Differentiation (GO:0060836)
Postsynaptic Density Organization (GO:0097106)
Protein Localization To Plasma Membrane (GO:0072659)
Cell-Cell Junction Organization (GO:0045216)
Vasculogenesis (GO:0001570)
Negative Regulation Of Developmental Process (GO:0051093)
Enzyme-Linked Receptor Protein Signaling Pathway (GO:0007167)
Regulation Of Actin Filament-Based Process (GO:0032970)
Regulation Of Hydrolase Activity (GO:0051336)
Synapse Assembly (GO:0007416)
Protein Localization To Cell Periphery (GO:1990778)
Integrin-Mediated Signaling Pathway (GO:0007229)
Regulation Of Glial Cell Proliferation (GO:0060251)
Response To Magnesium Ion (GO:0032026)

Semaphorin-Plexin Signaling Pathway Involved In Axon Guidance (GO:1902287)
Endochondral Ossification (GO:0001958)
Skin Morphogenesis (GO:0043589)
Lamellipodium Assembly (GO:0030032)
Phosphorylation (GO:0016310)
Neural Crest Cell Migration (GO:0001755)
Positive Regulation Of Cardiac Muscle Tissue Growth (GO:0055023)
Regulation Of Endothelial Cell Migration (GO:0010594)
Ventricular Septum Development (GO:0003281)
Negative Regulation Of Endothelial Cell Migration (GO:0010596)
Heterophilic Cell-Cell Adhesion Via Plasma Membrane Cell Adhesion Molecules (GO:0007157)
Positive Regulation Of DNA-templated Transcription (GO:0045893)
Sensory Perception Of Mechanical Stimulus (GO:0050954)
Negative Regulation Of Cell Motility (GO:2000146)
Postsynaptic Membrane Organization (GO:0001941)
Positive Regulation Of Axonogenesis (GO:0050772)
Negative Regulation Of Notch Signaling Pathway (GO:0045746)
Regulation Of Peptidyl-Tyrosine Phosphorylation (GO:0050730)
Regulation Of Phosphatidylinositol 3-Kinase Signaling (GO:0014066)
Regulation Of Granulocyte Differentiation (GO:0030852)
Regulation Of Receptor Binding (GO:1900120)
Smooth Muscle Tissue Development (GO:0048745)
Glycerolipid Metabolic Process (GO:0046486)
Wound Healing, Spreading Of Epidermal Cells (GO:0035313)
Lipid Phosphorylation (GO:0046834)
Motor Neuron Axon Guidance (GO:0008045)
Negative Regulation Of Receptor Binding (GO:1900121)
Peptidyl-Cysteine Modification (GO:0018198)
Regulation Of GTPase Activity (GO:0043087)
Positive Regulation Of Phosphorylation (GO:0042327)
Positive Regulation Of Stress-Activated MAPK Cascade (GO:0032874)
Regulation Of Cytokine Production (GO:0001817)
Response To Calcium Ion (GO:0051592)
Cell-Substrate Junction Assembly (GO:0007044)
Vascular Endothelial Growth Factor Receptor Signaling Pathway (GO:0048010)
Negative Regulation Of Cytoskeleton Organization (GO:0051494)
Sensory Perception Of Sound (GO:0007605)
Mesenchyme Morphogenesis (GO:0072132)
Positive Regulation Of Epidermal Cell Differentiation (GO:0045606)
Positive Regulation Of GTPase Activity (GO:0043547)
Negative Regulation Of Apoptotic Process (GO:0043066)
Sprouting Angiogenesis (GO:0002040)
Positive Regulation Of Nucleic Acid-Templated Transcription (GO:1903508)
Engulfment Of Apoptotic Cell (GO:0043652)
Protein Kinase C Signaling (GO:0070528)
Cardiac Ventricle Development (GO:0003231)
Peptidyl-Tyrosine Phosphorylation (GO:0018108)
Regulation Of JNK Cascade (GO:0046328)

Peptidyl-Tyrosine Modification (GO:0018212)
Negative Regulation Of Axon Extension (GO:0030517)
Negative Regulation Of Cell Differentiation (GO:0045596)
Response To Fibroblast Growth Factor (GO:0071774)
Peripheral Nervous System Development (GO:0007422)
Regulation Of Cardiac Muscle Cell Proliferation (GO:0060043)
Regulation Of Heart Contraction (GO:0008016)
Regulation Of DNA-templated Transcription (GO:0006355)
Regulation Of Cell Population Proliferation (GO:0042127)
Positive Regulation Of DNA-binding Transcription Factor Activity (GO:0051091)
Neural Crest Cell Development (GO:0014032)
Cell-Cell Junction Assembly (GO:0007043)
Negative Regulation Of Supramolecular Fiber Organization (GO:1902904)
Negative Regulation Of Cell Migration (GO:0030336)
Endocardial Cushion Morphogenesis (GO:0003203)
Negative Regulation Of Signal Transduction (GO:0009968)
Activation Of GTPase Activity (GO:0090630)
Regulation Of Lamellipodium Assembly (GO:0010591)
Regulation Of Protein Polymerization (GO:0032271)
Ventricular Cardiac Muscle Tissue Morphogenesis (GO:0055010)
Endochondral Bone Morphogenesis (GO:0060350)
Endothelium Development (GO:0003158)
Synaptic Membrane Adhesion (GO:0099560)
Icosanoid Secretion (GO:0032309)
Negative Regulation Of Stem Cell Differentiation (GO:2000737)
Regulation Of miRNA Transcription (GO:1902893)
Heart Morphogenesis (GO:0003007)
Regulation Of Small GTPase Mediated Signal Transduction (GO:0051056)
Regulation Of Vascular Endothelial Growth Factor Receptor Signaling Pathway (GO:0030947)
Homophilic Cell Adhesion Via Plasma Membrane Adhesion Molecules (GO:0007156)
Positive Regulation Of Kinase Activity (GO:0033674)
Positive Regulation Of NF-kappaB Transcription Factor Activity (GO:0051092)
Epithelial To Mesenchymal Transition (GO:0001837)
Insulin Receptor Signaling Pathway (GO:0008286)
Brain Development (GO:0007420)
Negative Regulation Of Macromolecule Metabolic Process (GO:0010605)
Positive Regulation Of Cell Junction Assembly (GO:1901890)
Regulation Of Extracellular Matrix Organization (GO:1903053)
Endocardial Cushion Development (GO:0003197)
Negative Regulation Of Actin Filament Polymerization (GO:0030837)
Protein Localization To Cell Junction (GO:1902414)
Regulation Of Axon Extension Involved In Axon Guidance (GO:0048841)
Adherens Junction Organization (GO:0034332)
Regulation Of Axon Extension (GO:0030516)
Branching Involved In Blood Vessel Morphogenesis (GO:0001569)
Peptidyl-Tyrosine Autophosphorylation (GO:0038083)
Positive Regulation Of Keratinocyte Differentiation (GO:0045618)
Regulation Of Ras Protein Signal Transduction (GO:0046578)

Regulation Of Actin Cytoskeleton Organization (GO:0032956)
DNA Methylation Involved In Embryo Development (GO:0043045)
T-helper 1 Cell Differentiation (GO:0045063)
Venous Blood Vessel Morphogenesis (GO:0048845)
Hepatocyte Differentiation (GO:0070365)
Vocal Learning (GO:0042297)
Imitative Learning (GO:0098596)
Intracellular Water Homeostasis (GO:0009992)
Negative Regulation Of Lamellipodium Organization (GO:1902744)
Negative Regulation Of Lens Fiber Cell Differentiation (GO:1902747)
Negative Regulation Of Morphogenesis Of An Epithelium (GO:1905331)
Negative Regulation Of Neuron Migration (GO:2001223)
Neurotransmitter-Gated Ion Channel Clustering (GO:0072578)
Notochord Development (GO:0030903)
Pericardium Morphogenesis (GO:0003344)
Plasma Membrane Raft Organization (GO:0044857)
Positive Regulation Of Granulocyte Differentiation (GO:0030854)
Positive Regulation Of Podosome Assembly (GO:0071803)
Postsynaptic Density Assembly (GO:0097107)
Cellular Response To Growth Factor Stimulus (GO:0071363)
Positive Regulation Of Epithelial Cell Proliferation (GO:0050679)
Positive Regulation Of Neuron Projection Development (GO:0010976)
Iron Ion Transport (GO:0006826)
Regulation Of Peptidyl-Threonine Phosphorylation (GO:0010799)
Cell Chemotaxis (GO:0060326)
Regulation Of Integrin Activation (GO:0033623)
Cardiac Cell Development (GO:0055006)
Smooth Muscle Contraction (GO:0006939)
Glucosamine-Containing Compound Metabolic Process (GO:1901071)
Vascular Endothelial Growth Factor Signaling Pathway (GO:0038084)
Mammary Gland Epithelium Development (GO:0061180)
Negative Regulation Of Blood Pressure (GO:0045776)
Negative Regulation Of mRNA Splicing, Via Spliceosome (GO:0048025)
Negative Regulation Of Peptidyl-Threonine Phosphorylation (GO:0010801)
Neuron Cell-Cell Adhesion (GO:0007158)
Regulation Of Postsynapse Organization (GO:0099175)
Negative Regulation Of Smoothened Signaling Pathway (GO:0045879)
Positive Regulation Of Transferase Activity (GO:0051347)
Cytoskeleton Organization (GO:0007010)
Regulation Of Gene Expression (GO:0010468)
Regulation Of Transcription By RNA Polymerase II (GO:0006357)
Negative Regulation Of Response To Stimulus (GO:0048585)
Negative Regulation Of Protein Polymerization (GO:0032272)
Protein Export From Nucleus (GO:0006611)
N-acetylglucosamine Metabolic Process (GO:0006044)
Cellular Response To Estrogen Stimulus (GO:0071391)
Negative Regulation Of mRNA Processing (GO:0050686)
Protein Localization To Cell-Cell Junction (GO:0150105)

Regulation Of Bicellular Tight Junction Assembly (GO:2000810)
AV Node Cell To Bundle Of His Cell Communication (GO:0086067)
Regulation Of Extracellular Matrix Assembly (GO:1901201)
Regulation Of Ketone Biosynthetic Process (GO:0010566)
Regulation Of Lens Fiber Cell Differentiation (GO:1902746)
Atrioventricular Valve Formation (GO:0003190)
Cellular Hyperosmotic Response (GO:0071474)
Cellular Response To Caffeine (GO:0071313)
Response To Caffeine (GO:0031000)
Dendrite Arborization (GO:0140059)
Dorsal Aorta Development (GO:0035907)
Dorsal Aorta Morphogenesis (GO:0035912)
Enteric Nervous System Development (GO:0048484)
Spinal Cord Dorsal/Ventral Patterning (GO:0021513)
Vascular Wound Healing (GO:0061042)
Negative Regulation Of T-helper 17 Type Immune Response (GO:2000317)
Neurotransmitter Receptor Transport, Endosome To Postsynaptic Membrane (GO:0098887)
Neurotrophin TRK Receptor Signaling Pathway (GO:0048011)
Parallel Actin Filament Bundle Assembly (GO:0030046)
Positive Regulation Of Gliogenesis (GO:0014015)
Regulation Of Adiponectin Secretion (GO:0070163)
Positive Regulation Of Cellular Component Organization (GO:0051130)
Positive Regulation Of Developmental Process (GO:0051094)
MAPK Cascade (GO:0000165)
Actin Polymerization Or Depolymerization (GO:0008154)
Vascular Transport (GO:0010232)
In Utero Embryonic Development (GO:0001701)
Regulation Of Actin Polymerization Or Depolymerization (GO:0008064)
Endothelial Cell Migration (GO:0043542)
Regulation Of Blood Vessel Endothelial Cell Migration (GO:0043535)
Skeletal System Development (GO:0001501)
Positive Regulation Of Phosphatidylinositol 3-Kinase Signaling (GO:0014068)
Autonomic Nervous System Development (GO:0048483)
Regulation Of Osteoblast Proliferation (GO:0033688)
Synaptic Transmission, Cholinergic (GO:0007271)
Regulation Of Blood Vessel Endothelial Cell Proliferation Involved In Sprouting Angiogenesis (GO:1903587)
Transport Across Blood-Brain Barrier (GO:0150104)
Positive Regulation Of Protein Localization (GO:1903829)
Cellular Response To Fibroblast Growth Factor Stimulus (GO:0044344)
Cellular Response To Retinoic Acid (GO:0071300)
Regulation Of Chemotaxis (GO:0050920)
Apoptotic Cell Clearance (GO:0043277)
Establishment Of Endothelial Barrier (GO:0061028)
Negative Regulation Of Actin Filament Bundle Assembly (GO:0032232)
Positive Regulation Of Fibroblast Proliferation (GO:0048146)
Positive Regulation Of Cell-Substrate Adhesion (GO:0010811)
Regulation Of Microvillus Organization (GO:0032530)
Anterograde Axonal Protein Transport (GO:0099641)

Axo-Dendritic Protein Transport (GO:0099640)
Cardiac Atrium Development (GO:0003230)
Regulation Of Response To Wounding (GO:1903034)
Ventricular Cardiac Muscle Cell Membrane Repolarization (GO:0099625)
Ventricular Trabecula Myocardium Morphogenesis (GO:0003222)
Lens Fiber Cell Development (GO:0070307)
Leukocyte Aggregation (GO:0070486)
Negative Regulation Of T-helper 17 Cell Differentiation (GO:2000320)
Negative Regulation Of Osteoblast Proliferation (GO:0033689)
Neurotransmitter Receptor Transport, Endosome To Plasma Membrane (GO:0099639)
Pericardium Development (GO:0060039)
Positive Regulation Of Astrocyte Differentiation (GO:0048711)
Positive Regulation Of Cardiac Epithelial To Mesenchymal Transition (GO:0062043)
Postsynaptic Membrane Assembly (GO:0097104)
Protein Localization To Presynapse (GO:1905383)
Regulation Of Alpha-Beta T Cell Proliferation (GO:0046640)
Positive Regulation Of Neuron Differentiation (GO:0045666)
Cellular Response To Alkaloid (GO:0071312)
Embryonic Axis Specification (GO:0000578)
Muscle Tissue Morphogenesis (GO:0060415)
Positive Regulation Of Glial Cell Differentiation (GO:0045687)
Positive Regulation Of Leukocyte Adhesion To Vascular Endothelial Cell (GO:1904996)
Defense Response To Fungus (GO:0050832)
Heterotypic Cell-Cell Adhesion (GO:0034113)
Maintenance Of Blood-Brain Barrier (GO:0035633)
Supramolecular Fiber Organization (GO:0097435)
Metal Ion Transport (GO:0030001)
Negative Regulation Of Cell Adhesion (GO:0007162)
Regulation Of Filopodium Assembly (GO:0051489)
Skeletal System Morphogenesis (GO:0048705)
Negative Regulation Of BMP Signaling Pathway (GO:0030514)
Positive Regulation Of miRNA Transcription (GO:1902895)
Regulation Of JUN Kinase Activity (GO:0043506)
Regulation Of Cell-Cell Adhesion (GO:0022407)
Positive Regulation Of Response To External Stimulus (GO:0032103)
Regulation Of Dendritic Spine Morphogenesis (GO:0061001)
Artery Morphogenesis (GO:0048844)
Lamellipodium Organization (GO:0097581)
Positive Regulation Of JUN Kinase Activity (GO:0043507)
Positive Regulation Of Protein Phosphorylation (GO:0001934)
Cardiac Atrium Morphogenesis (GO:0003209)
Heart Trabecula Morphogenesis (GO:0061384)
Vesicle Transport Along Actin Filament (GO:0030050)
Positive Regulation Of Nervous System Process (GO:0031646)
Regulation Of Cell-Cell Adhesion Mediated By Cadherin (GO:2000047)
Regulation Of B Cell Proliferation (GO:0030888)
Negative Regulation Of Neuron Apoptotic Process (GO:0043524)
Positive Regulation Of Transcription By RNA Polymerase II (GO:0045944)

Chordate Embryonic Development (GO:0043009)
Regulation Of Cell Differentiation (GO:0045595)
Actin Filament Polymerization (GO:0030041)
Cardiac Muscle Tissue Morphogenesis (GO:0055008)
Regulation Of Plasma Membrane Bounded Cell Projection Assembly (GO:0120032)
Regulation Of Stress Fiber Assembly (GO:0051492)
Auditory Behavior (GO:0031223)
Regulation Of Podosome Assembly (GO:0071801)
Regulation Of Toll-Like Receptor 3 Signaling Pathway (GO:0034139)
Regulation Of Tumor Necrosis Factor Superfamily Cytokine Production (GO:1903555)
Dehydroascorbic Acid Transport (GO:0070837)
Embryonic Placenta Development (GO:0001892)
Somite Development (GO:0061053)
Epithelial To Mesenchymal Transition Involved In Endocardial Cushion Formation (GO:0003198)
Sympathetic Ganglion Development (GO:0061549)
Learned Vocalization Behavior Or Vocal Learning (GO:0098598)
Negative Regulation Of T Cell Cytokine Production (GO:0002725)
Neuronal Action Potential (GO:0019228)
Positive Regulation Of Peptide Secretion (GO:0002793)
Regulation Of CD8-positive, Alpha-Beta T Cell Proliferation (GO:2000564)
Atrioventricular Valve Morphogenesis (GO:0003181)
Regulation Of Smooth Muscle Cell Migration (GO:0014910)
Establishment Of Skin Barrier (GO:0061436)
Mesoderm Development (GO:0007498)
Negative Regulation Of RNA Splicing (GO:0033119)
Negative Regulation Of Axon Extension Involved In Axon Guidance (GO:0048843)
Regulation Of Fibroblast Proliferation (GO:0048145)
Response To Hydrogen Peroxide (GO:0042542)
Positive Regulation Of Cell-Cell Adhesion (GO:0022409)
Negative Regulation Of Osteoblast Differentiation (GO:0045668)
Actin Filament Organization (GO:0007015)
Positive Regulation Of Synaptic Transmission (GO:0050806)
Regulation Of Apoptotic Process (GO:0042981)
Cell Junction Organization (GO:0034330)
Negative Regulation Of Kinase Activity (GO:0033673)
Positive Regulation Of Epithelial To Mesenchymal Transition (GO:0010718)
Anterograde Trans-Synaptic Signaling (GO:0098916)
Actin Filament Bundle Assembly (GO:0051017)
Actin Filament Bundle Organization (GO:0061572)
Regulation Of Sprouting Angiogenesis (GO:1903670)
Epithelial Tube Morphogenesis (GO:0060562)
Regulation Of Cell Adhesion Mediated By Integrin (GO:0033628)
Neuron Projection Morphogenesis (GO:0048812)
Actin Filament-Based Transport (GO:0099515)
Response To ATP (GO:0033198)
Endothelial Cell Development (GO:0001885)
Stress-Activated Protein Kinase Signaling Cascade (GO:0031098)
Myoblast Differentiation (GO:0045445)

Negative Regulation Of miRNA Transcription (GO:1902894)
Adherens Junction Assembly (GO:0034333)
Arachidonate Transport (GO:1903963)
Arachidonic Acid Secretion (GO:0050482)
Axon Regeneration (GO:0031103)
Camera-Type Eye Morphogenesis (GO:0048593)
Cellular Response To Purine-Containing Compound (GO:0071415)
Response To Laminar Fluid Shear Stress (GO:0034616)
Ectoderm Development (GO:0007398)
Excitatory Synapse Assembly (GO:1904861)
Granulocyte Migration (GO:0097530)
Mitral Valve Morphogenesis (GO:0003183)
Morphogenesis Of An Epithelial Sheet (GO:0002011)
Negative Regulation Of Cell-Cell Adhesion Mediated By Cadherin (GO:2000048)
Negative Regulation Of Extracellular Matrix Organization (GO:1903054)
Negative Regulation Of Hormone Secretion (GO:0046888)
Negative Regulation Of Potassium Ion Transmembrane Transporter Activity (GO:1901017)
Negative Regulation Of Transcription By Competitive Promoter Binding (GO:0010944)
Neurotransmitter Receptor Transport To Postsynaptic Membrane (GO:0098969)
Positive Regulation Of Vascular Endothelial Growth Factor Receptor Signaling Pathway (GO:0030949)
Postsynapse Assembly (GO:0099068)
Regulation Of CD8-positive, Alpha-Beta T Cell Activation (GO:2001185)
Regulation Of Bile Acid Biosynthetic Process (GO:0070857)
Myelination (GO:0042552)
Smoothened Signaling Pathway (GO:0007224)
Regulation Of Blood Circulation (GO:1903522)
Regulation Of Nucleic Acid-Templated Transcription (GO:1903506)
Intrinsic Apoptotic Signaling Pathway In Response To DNA Damage (GO:0008630)
Positive Regulation Of miRNA Metabolic Process (GO:2000630)
Skin Epidermis Development (GO:0098773)
Focal Adhesion Assembly (GO:0048041)
Innate Immune Response In Mucosa (GO:0002227)
Negative Regulation Of miRNA Metabolic Process (GO:2000629)
Circulatory System Development (GO:0072359)
Regulation Of Angiogenesis (GO:0045765)
Action Potential (GO:0001508)
Liver Development (GO:0001889)
Intracellular Iron Ion Homeostasis (GO:0006879)
Plasma Membrane Bounded Cell Projection Morphogenesis (GO:0120039)
Positive Regulation Of Transport (GO:0051050)
Regulation Of Endocytosis (GO:0030100)
Regulation Of ERK1 And ERK2 Cascade (GO:0070372)
Angiogenesis Involved In Wound Healing (GO:0060055)
Sarcoplasmic Reticulum Calcium Ion Transport (GO:0070296)
Heparan Sulfate Proteoglycan Biosynthetic Process, Enzymatic Modification (GO:0015015)
Inositol Phosphate Biosynthetic Process (GO:0032958)
Insulin-Like Growth Factor Receptor Signaling Pathway (GO:0048009)
Negative Regulation Of T-helper Cell Differentiation (GO:0045623)

Negative Regulation Of Lipopolysaccharide-Mediated Signaling Pathway (GO:0031665)
Nuclear RNA Surveillance (GO:0071027)
Regulation Of Axon Regeneration (GO:0048679)
Regulation Of Canonical Wnt Signaling Pathway (GO:0060828)
Regulation Of Neural Precursor Cell Proliferation (GO:2000177)
Regulation Of Protein Export From Nucleus (GO:0046825)
Placenta Development (GO:0001890)
Regulation Of Epithelial To Mesenchymal Transition (GO:0010717)
Regulation Of Smoothened Signaling Pathway (GO:0008589)
Regulation Of Stress-Activated MAPK Cascade (GO:0032872)
Regulation Of Synapse Assembly (GO:0051963)
Heart Development (GO:0007507)
Positive Regulation Of Angiogenesis (GO:0045766)
Positive Regulation Of Macromolecule Metabolic Process (GO:0010604)
Negative Regulation Of Endothelial Cell Proliferation (GO:0001937)
Axon Extension (GO:0048675)
Diacylglycerol Metabolic Process (GO:0046339)
Hepaticobiliary System Development (GO:0061008)
Regulation Of Cell Junction Assembly (GO:1901888)
Regulation Of Osteoblast Differentiation (GO:0045667)
Negative Regulation Of Cell Cycle (GO:0045786)
Positive Regulation Of Canonical Wnt Signaling Pathway (GO:0090263)
Positive Regulation Of Endothelial Cell Migration (GO:0010595)
Response To Molecule Of Bacterial Origin (GO:0002237)
Negative Regulation Of Cysteine-Type Endopeptidase Activity (GO:2000117)
Retinoic Acid Receptor Signaling Pathway (GO:0048384)
Ganglion Development (GO:0061548)
Podocyte Differentiation (GO:0072112)
Positive Regulation Of Myelination (GO:0031643)
Regulation Of T-helper 17 Cell Differentiation (GO:2000319)
Regulation Of Alcohol Biosynthetic Process (GO:1902930)
Regulation Of Astrocyte Differentiation (GO:0048710)
Calcium-Dependent Cell-Cell Adhesion Via Plasma Membrane Cell Adhesion Molecules (GO:0016339)
Heart Looping (GO:0001947)
Regulation Of Notch Signaling Pathway (GO:0008593)
Positive Regulation Of Macromolecule Biosynthetic Process (GO:0010557)
Regulation Of Heterotypic Cell-Cell Adhesion (GO:0034114)
Regulation Of Microtubule Depolymerization (GO:0031114)
Regulation Of Muscle Cell Differentiation (GO:0051147)
Protein Kinase B Signaling (GO:0043491)
Negative Regulation Of Wnt Signaling Pathway (GO:0030178)
Mesenchymal Cell Differentiation (GO:0048762)
Regulation Of Neuron Apoptotic Process (GO:0043523)
Intrinsic Apoptotic Signaling Pathway (GO:0097193)
Modulation Of Chemical Synaptic Transmission (GO:0050804)
Negative Regulation Of Protein Binding (GO:0032091)
Regulation Of Protein Tyrosine Kinase Activity (GO:0061097)
Vesicle Cytoskeletal Trafficking (GO:0099518)

Negative Regulation Of Protein Secretion (GO:0050709)
Odontogenesis (GO:0042476)
Negative Regulation Of Neuron Death (GO:1901215)
Negative Regulation Of Protein Kinase Activity (GO:0006469)
Regulation Of Synaptic Transmission, Glutamatergic (GO:0051966)
Neuron Migration (GO:0001764)
Purine Ribonucleotide Biosynthetic Process (GO:0009152)
Positive Regulation Of Defense Response (GO:0031349)
Cellular Response To Hypoxia (GO:0071456)
Regulation Of Myelination (GO:0031641)
Ventricular Cardiac Muscle Tissue Development (GO:0003229)
Wound Healing, Spreading Of Cells (GO:0044319)
Negative Regulation Of Stress Fiber Assembly (GO:0051497)
Regulation Of Hematopoietic Stem Cell Differentiation (GO:1902036)
Regulation Of Interleukin-5 Production (GO:0032674)
acetyl-CoA Biosynthetic Process (GO:0006085)
Cell Projection Assembly (GO:0030031)
Regulation Of Vascular Associated Smooth Muscle Cell Migration (GO:1904752)
Dendrite Self-Avoidance (GO:0070593)
Detection Of Calcium Ion (GO:0005513)
Gamma-Delta T Cell Activation (GO:0046629)
Low-Density Lipoprotein Particle Remodeling (GO:0034374)
Mesoderm Morphogenesis (GO:0048332)
Morphogenesis Of A Polarized Epithelium (GO:0001738)
Multicellular Organismal-Level Iron Ion Homeostasis (GO:0060586)
Positive Regulation Of Nuclear-Transcribed mRNA poly(A) Tail Shortening (GO:0060213)
Positive Regulation Of Protein Export From Nucleus (GO:0046827)
Positive Regulation Of Vascular Endothelial Cell Proliferation (GO:1905564)
Negative Regulation Of Cell-Cell Adhesion (GO:0022408)
Positive Regulation Of Insulin Secretion (GO:0032024)
Positive Regulation Of Myeloid Leukocyte Differentiation (GO:0002763)
Cellular Response To Decreased Oxygen Levels (GO:0036294)
Regulation Of Intracellular Signal Transduction (GO:1902531)
Positive Regulation Of MAP Kinase Activity (GO:0043406)
Striated Muscle Contraction (GO:0006941)
Non-Canonical Wnt Signaling Pathway (GO:0035567)
Wnt Signaling Pathway, Planar Cell Polarity Pathway (GO:0060071)
Regulation Of Keratinocyte Differentiation (GO:0045616)
Acute Inflammatory Response (GO:0002526)
Cell-Cell Adhesion Mediated By Cadherin (GO:0044331)
Cellular Response To Xenobiotic Stimulus (GO:0071466)
Dendritic Spine Organization (GO:0097061)
Positive Regulation Of Nuclear Division (GO:0051785)
Protein Localization To Cell Surface (GO:0034394)
Cellular Response To Salt (GO:1902075)
Regulation Of mRNA Stability (GO:0043488)
Positive Regulation Of Lymphocyte Proliferation (GO:0050671)
Response To Lipid (GO:0033993)

Memory (GO:0007613)
Muscle Organ Development (GO:0007517)
Positive Regulation Of Neurogenesis (GO:0050769)
Regulation Of BMP Signaling Pathway (GO:0030510)
Regulation Of Gastrulation (GO:0010470)
Regulation Of Lamellipodium Organization (GO:1902743)
acyl-CoA Biosynthetic Process (GO:0071616)
Atrioventricular Valve Development (GO:0003171)
Regulation Of Nitrogen Compound Metabolic Process (GO:0051171)
Regulation Of Vasculogenesis (GO:2001212)
Heparan Sulfate Proteoglycan Metabolic Process (GO:0030201)
Inhibitory Synapse Assembly (GO:1904862)
Microvillus Organization (GO:0032528)
Negative Regulation Of Myoblast Differentiation (GO:0045662)
Positive Regulation Of Chromosome Separation (GO:1905820)
Positive Regulation Of Glycogen Biosynthetic Process (GO:0045725)
Positive Regulation Of Insulin Secretion Involved In Cellular Response To Glucose Stimulus (GO:0035774)
Protein Localization To Synapse (GO:0035418)
Regulation Of T-helper 17 Type Immune Response (GO:2000316)
Determination Of Heart Left/Right Asymmetry (GO:0061371)
Embryonic Heart Tube Morphogenesis (GO:0003143)
Regulation Of Axonogenesis (GO:0050770)
Regulation Of MAPK Cascade (GO:0043408)
Peptidyl-Serine Modification (GO:0018209)
Regulation Of Peptidyl-Serine Phosphorylation (GO:0033135)
Negative Regulation Of Transmembrane Receptor Protein Serine/Threonine Kinase Signaling Pathway (GO:00901
Regulation Of Insulin Secretion Involved In Cellular Response To Glucose Stimulus (GO:0061178)
Cortical Cytoskeleton Organization (GO:0030865)
Positive Regulation Of Response To Wounding (GO:1903036)
Regulation Of Catecholamine Secretion (GO:0050433)
Wnt Signaling Pathway (GO:0016055)
Negative Regulation Of Protein-Containing Complex Assembly (GO:0031333)
Inorganic Cation Transmembrane Transport (GO:0098662)
Calcium Ion Transport (GO:0006816)
Regulation Of mRNA Splicing, Via Spliceosome (GO:0048024)
Negative Regulation Of DNA-binding Transcription Factor Activity (GO:0043433)
Negative Regulation Of Protein Phosphorylation (GO:0001933)
Positive Regulation Of Programmed Cell Death (GO:0043068)
Positive Regulation Of Stress Fiber Assembly (GO:0051496)
Response To Retinoic Acid (GO:0032526)
Positive Regulation Of Epithelial Cell Migration (GO:0010634)
Regulation Of Protein Transport (GO:0051223)
Mucosal Immune Response (GO:0002385)
Negative Regulation Of Wound Healing (GO:0061045)
Positive Regulation Of mRNA Processing (GO:0050685)
Primary Neural Tube Formation (GO:0014020)
Protein Depolymerization (GO:0051261)
Regulation Of Calcium Ion-Dependent Exocytosis (GO:0017158)

Regulation Of Extracellular Matrix Disassembly (GO:0010715)
RNA Phosphodiester Bond Hydrolysis, Endonucleolytic (GO:0090502)
Aorta Development (GO:0035904)
Regulation Of Neuron Projection Arborization (GO:0150011)
Regulation Of Nuclear-Transcribed mRNA poly(A) Tail Shortening (GO:0060211)
Cellular Response To Exogenous dsRNA (GO:0071360)
Centromere Complex Assembly (GO:0034508)
Renal Water Homeostasis (GO:0003091)
Resolution Of Meiotic Recombination Intermediates (GO:0000712)
Detection Of Mechanical Stimulus Involved In Sensory Perception (GO:0050974)
Establishment Of Apical/Basal Cell Polarity (GO:0035089)
Membrane Raft Organization (GO:0031579)
Membrane Repolarization During Cardiac Muscle Cell Action Potential (GO:0086013)
Microvillus Assembly (GO:0030033)
Negative Regulation Of Smooth Muscle Cell Migration (GO:0014912)
Odontogenesis Of Dentin-Containing Tooth (GO:0042475)
Pathway-Restricted SMAD Protein Phosphorylation (GO:0060389)
Positive Regulation Of Glycogen Metabolic Process (GO:0070875)
Positive Regulation Of Heterotypic Cell-Cell Adhesion (GO:0034116)
Positive Regulation Of Protein-Containing Complex Disassembly (GO:0043243)
Cellular Response To Ketone (GO:1901655)
Synaptic Vesicle Recycling (GO:0036465)
Regulation Of Cell-Substrate Adhesion (GO:0010810)
Organonitrogen Compound Catabolic Process (GO:1901565)
Negative Regulation Of Canonical Wnt Signaling Pathway (GO:0090090)
Regulation Of Cellular Component Organization (GO:0051128)
Regulation Of Establishment Of Planar Polarity (GO:0090175)
Establishment Of Mitotic Spindle Orientation (GO:0000132)
Tube Closure (GO:0060606)
Negative Regulation Of Plasma Membrane Bounded Cell Projection Assembly (GO:0120033)
Phosphatidic Acid Biosynthetic Process (GO:0006654)
Positive Regulation Of B Cell Proliferation (GO:0030890)
Positive Regulation Of Neural Precursor Cell Proliferation (GO:2000179)
Regulation Of Stem Cell Differentiation (GO:2000736)
Positive Regulation Of Apoptotic Signaling Pathway (GO:2001235)
Neuron Projection Development (GO:0031175)
Cellular Response To Metal Ion (GO:0071248)
3'-UTR-mediated mRNA Destabilization (GO:0061158)
Regulation Of Cellular Response To Heat (GO:1900034)
Regulation Of Interleukin-13 Production (GO:0032656)
Amyloid-Beta Metabolic Process (GO:0050435)
Astrocyte Development (GO:0014002)
Cardiac Myofibril Assembly (GO:0055003)
Regulation Of Telomere Maintenance In Response To DNA Damage (GO:1904505)
Dendritic Spine Morphogenesis (GO:0060997)
Retrograde Axonal Transport (GO:0008090)
Digestive Tract Morphogenesis (GO:0048546)
Sphingolipid Catabolic Process (GO:0030149)

Sympathetic Nervous System Development (GO:0048485)
Vasodilation (GO:0042311)
Negative Regulation Of Epithelial Cell Differentiation (GO:0030857)
Negative Regulation Of Platelet Activation (GO:0010544)
Neurotrophin Signaling Pathway (GO:0038179)
Platelet-Derived Growth Factor Receptor Signaling Pathway (GO:0048008)
Polyol Biosynthetic Process (GO:0046173)
Positive Regulation Of DNA Damage Response, Signal Transduction By P53 Class Mediator (GO:0043517)
Positive Regulation Of Cyclin-Dependent Protein Serine/Threonine Kinase Activity (GO:0045737)
Positive Regulation Of Isotype Switching (GO:0045830)
Positive Regulation Of Mitotic Sister Chromatid Separation (GO:1901970)
Positive Regulation Of Sprouting Angiogenesis (GO:1903672)
Positive Regulation Of Syncytium Formation By Plasma Membrane Fusion (GO:0060143)
Protein Localization To Microtubule Organizing Center (GO:1905508)
Regulation Of Rac Protein Signal Transduction (GO:0035020)
Protein Polymerization (GO:0051258)
Regulation Of mRNA Catabolic Process (GO:0061013)
Regulation Of Nervous System Development (GO:0051960)
Epithelial Cell Migration (GO:0010631)
Positive Regulation Of Peptide Hormone Secretion (GO:0090277)
Chemical Synaptic Transmission (GO:0007268)
Cellular Response To Insulin Stimulus (GO:0032869)
Negative Regulation Of Epithelial To Mesenchymal Transition (GO:0010719)
Phagocytosis, Engulfment (GO:0006911)
Positive Regulation Of Locomotion (GO:0040017)
Positive Regulation Of Protein Targeting To Mitochondrion (GO:1903955)
Response To Metal Ion (GO:0010038)
Plasma Membrane Bounded Cell Projection Assembly (GO:0120031)
Positive Regulation Of Gene Expression (GO:0010628)
Cellular Response To Organic Substance (GO:0071310)
Regulation Of Wound Healing (GO:0061041)
Secondary Alcohol Metabolic Process (GO:1902652)
Negative Regulation Of Cell-Matrix Adhesion (GO:0001953)
Negative Regulation Of Developmental Growth (GO:0048640)
Positive Regulation Of Lamellipodium Organization (GO:1902745)
Presynaptic Endocytosis (GO:0140238)
Positive Regulation Of Cell Cycle (GO:0045787)
acetyl-CoA Metabolic Process (GO:0006084)
Apoptotic Nuclear Changes (GO:0030262)
Regulation Of Presynapse Assembly (GO:1905606)
Regulation Of Presynapse Organization (GO:0099174)
Regulation Of Protein Kinase A Signaling (GO:0010738)
Regulation Of Steroid Biosynthetic Process (GO:0050810)
Regulation Of Synaptic Transmission, GABAergic (GO:0032228)
Chondroitin Sulfate Metabolic Process (GO:0030204)
Extrinsic Apoptotic Signaling Pathway In Absence Of Ligand (GO:0097192)
Mesonephric Tubule Development (GO:0072164)
Negative Regulation Of ATP-dependent Activity (GO:0032780)

Negative Regulation Of Anoikis (GO:2000811)
Negative Regulation Of Chemokine Production (GO:0032682)
Negative Regulation Of Cytokine Production Involved In Immune Response (GO:0002719)
Nuclear Chromosome Segregation (GO:0098813)
Positive Regulation Of Myeloid Leukocyte Cytokine Production Involved In Immune Response (GO:0061081)
Positive Regulation Of Smooth Muscle Cell Migration (GO:0014911)
Regulation Of Intrinsic Apoptotic Signaling Pathway (GO:2001242)
Negative Regulation Of ERK1 And ERK2 Cascade (GO:0070373)
Negative Regulation Of Programmed Cell Death (GO:0043069)
Regulation Of Cysteine-Type Endopeptidase Activity Involved In Apoptotic Process (GO:0043281)
Transition Metal Ion Transport (GO:0000041)
Gluconeogenesis (GO:0006094)
Neural Tube Closure (GO:0001843)
Cellular Component Assembly (GO:0022607)
Negative Regulation Of Cysteine-Type Endopeptidase Activity Involved In Apoptotic Process (GO:0043154)
Neurogenesis (GO:0022008)
Nuclear Export (GO:0051168)
Macromolecule Catabolic Process (GO:0009057)
DNA Damage Response (GO:0006974)
Activation Of Cysteine-Type Endopeptidase Activity Involved In Apoptotic Process (GO:0006919)
Regulation Of Neurogenesis (GO:0050767)
Positive Regulation Of Inflammatory Response (GO:0050729)
Positive Regulation Of Vasculature Development (GO:1904018)
Cellular Response To dsRNA (GO:0071359)
Cellular Response To Glucocorticoid Stimulus (GO:0071385)
Response To Acidic pH (GO:0010447)
Embryonic Digit Morphogenesis (GO:0042733)
Embryonic Heart Tube Development (GO:0035050)
Sensory Perception Of Taste (GO:0050909)
Hippo Signaling (GO:0035329)
Intracellular Estrogen Receptor Signaling Pathway (GO:0030520)
mRNA Cis Splicing, Via Spliceosome (GO:0045292)
Microtubule Depolymerization (GO:0007019)
ncRNA Metabolic Process (GO:0034660)
Negative Regulation Of Cellular Senescence (GO:2000773)
Negative Regulation Of Insulin Secretion (GO:0046676)
Negative Regulation Of Vascular Associated Smooth Muscle Cell Proliferation (GO:1904706)
Neural Tube Development (GO:0021915)
Neuron Maturation (GO:0042551)
Peptidyl-Threonine Dephosphorylation (GO:0035970)
Positive Chemotaxis (GO:0050918)
Positive Regulation Of Macrophage Derived Foam Cell Differentiation (GO:0010744)
Positive Regulation Of Stem Cell Differentiation (GO:2000738)
Protein Localization To Centrosome (GO:0071539)
Positive Regulation Of Developmental Growth (GO:0048639)
Regulation Of TORC1 Signaling (GO:1903432)
Cardiac Muscle Contraction (GO:0060048)
Signal Transduction By P53 Class Mediator (GO:0072331)

Negative Regulation Of Epithelial Cell Migration (GO:0010633)
Phosphatidic Acid Metabolic Process (GO:0046473)
Positive Regulation Of Establishment Of Protein Localization To Mitochondrion (GO:1903749)
Positive Regulation Of Nitrogen Compound Metabolic Process (GO:0051173)
Skin Development (GO:0043588)
Positive Regulation Of Multicellular Organismal Process (GO:0051240)
Positive Regulation Of Cell Growth (GO:0030307)
Positive Regulation Of Protein Kinase Activity (GO:0045860)
Microtubule Bundle Formation (GO:0001578)
Positive Regulation Of Actin Filament Bundle Assembly (GO:0032233)
Positive Regulation Of Protein Localization To Cell Periphery (GO:1904377)
Regulated Exocytosis (GO:0045055)
Cellular Response To Calcium Ion (GO:0071277)
Establishment Of Protein Localization To Membrane (GO:0090150)
Positive Regulation Of Protein Modification Process (GO:0031401)
Apoptotic Mitochondrial Changes (GO:0008637)
Cellular Response To Glucose Stimulus (GO:0071333)
Cellular Response To Hydrogen Peroxide (GO:0070301)
Response To Estrogen (GO:0043627)
Embryonic Limb Morphogenesis (GO:0030326)
Establishment Of Mitotic Spindle Localization (GO:0040001)
Negative Regulation Of Ras Protein Signal Transduction (GO:0046580)
Negative Regulation Of Mitotic Cell Cycle (GO:0045930)
Regulation Of DNA Biosynthetic Process (GO:2000278)
3'-UTR-mediated mRNA Stabilization (GO:0070935)
Regulation Of Epidermal Cell Differentiation (GO:0045604)
Regulation Of Epithelial Cell Differentiation (GO:0030856)
Actin Filament Capping (GO:0051693)
Regulation Of Oligodendrocyte Differentiation (GO:0048713)
Barbed-End Actin Filament Capping (GO:0051016)
Cell Junction Maintenance (GO:0034331)
Regulation Of Vascular Endothelial Cell Proliferation (GO:1905562)
Renal Absorption (GO:0070293)
Response To Organophosphorus (GO:0046683)
Ureteric Bud Development (GO:0001657)
Myoblast Fusion (GO:0007520)
ncRNA-mediated Post-Transcriptional Gene Silencing (GO:0035194)
Negative Regulation Of Nervous System Development (GO:0051961)
Nitric Oxide Mediated Signal Transduction (GO:0007263)
Positive Regulation Of CREB Transcription Factor Activity (GO:0032793)
Positive Regulation Of DNA Replication (GO:0045740)
Positive Regulation Of Cyclin-Dependent Protein Kinase Activity (GO:1904031)
Positive Regulation Of Cytokine Production Involved In Inflammatory Response (GO:1900017)
Positive Regulation Of Establishment Of Protein Localization (GO:1904951)
Positive Regulation Of Focal Adhesion Assembly (GO:0051894)
Positive Regulation Of Stress-Activated Protein Kinase Signaling Cascade (GO:0070304)
Regulation Of T Cell Cytokine Production (GO:0002724)
Positive Regulation Of Plasma Membrane Bounded Cell Projection Assembly (GO:0120034)

Negative Regulation Of Extrinsic Apoptotic Signaling Pathway (GO:2001237)
Regulation Of Peptide Hormone Secretion (GO:0090276)
Regulation Of Cell Growth (GO:0001558)
Protein Localization (GO:0008104)
Positive Regulation Of MAPK Cascade (GO:0043410)
Regulation Of Cellular Senescence (GO:2000772)
Dendrite Morphogenesis (GO:0048813)
Establishment Of Spindle Orientation (GO:0051294)
Hexose Biosynthetic Process (GO:0019319)
One-Carbon Compound Transport (GO:0019755)
Positive Regulation Of Mitotic Nuclear Division (GO:0045840)
Positive Regulation Of Wnt Signaling Pathway (GO:0030177)
Positive Regulation Of Cysteine-Type Endopeptidase Activity Involved In Apoptotic Process (GO:0043280)
Tight Junction Assembly (GO:0120192)
mRNA Stabilization (GO:0048255)
Positive Regulation Of NIK/NF-kappaB Signaling (GO:1901224)
Regulation Of Alternative mRNA Splicing, Via Spliceosome (GO:0000381)
Protein Homooligomerization (GO:0051260)
Regulation Of Integrin-Mediated Signaling Pathway (GO:2001044)
Regulation Of Neuroinflammatory Response (GO:0150077)
Bicarbonate Transport (GO:0015701)
Regulation Of Protein-Containing Complex Disassembly (GO:0043244)
Release Of Cytochrome C From Mitochondria (GO:0001836)
Cortical Actin Cytoskeleton Organization (GO:0030866)
Epithelial Structure Maintenance (GO:0010669)
Fluid Transport (GO:0042044)
Water Transport (GO:0006833)
Multicellular Organismal-Level Water Homeostasis (GO:0050891)
Negative Regulation Of Peptidyl-Serine Phosphorylation (GO:0033137)
Oligodendrocyte Differentiation (GO:0048709)
Positive Regulation Of Axon Extension (GO:0045773)
Positive Regulation Of Hormone Secretion (GO:0046887)
C-terminal Protein Deglutamylation (GO:0035609)
DNA Replication-Dependent Chromatin Assembly (GO:0006335)
ERBB2-EGFR Signaling Pathway (GO:0038134)
Regulation Of Establishment Of T Cell Polarity (GO:1903903)
Regulation Of Excitatory Synapse Assembly (GO:1904889)
L-ascorbic Acid Metabolic Process (GO:0019852)
L-ornithine Transmembrane Transport (GO:1903352)
Regulation Of Glomerular Filtration (GO:0003093)
Regulation Of Glutamate Receptor Signaling Pathway (GO:1900449)
Regulation Of Glycogen (Starch) Synthase Activity (GO:2000465)
Regulation Of Guanylate Cyclase Activity (GO:0031282)
Regulation Of Hormone Biosynthetic Process (GO:0046885)
Regulation Of Hyaluronan Biosynthetic Process (GO:1900125)
Activation Of Cysteine-Type Endopeptidase Activity Involved In Apoptotic Process By Cytochrome C (GO:00086
Regulation Of Long-Chain Fatty Acid Import Across Plasma Membrane (GO:0010746)
Amylin Receptor Signaling Pathway (GO:0097647)

Anterior/Posterior Axis Specification, Embryo (GO:0008595)
Astrocyte Differentiation (GO:0048708)
Autocrine Signaling (GO:0035425)
Basic Amino Acid Transport (GO:0015802)
Blood Vessel Endothelial Cell Proliferation Involved In Sprouting Angiogenesis (GO:0002043)
Regulation Of pro-B Cell Differentiation (GO:2000973)
Regulation Of Protein Depolymerization (GO:1901879)
Cardiolipin Acyl-Chain Remodeling (GO:0035965)
Cell-Cell Signaling Involved In Cardiac Conduction (GO:0086019)
Regulation Of Resting Membrane Potential (GO:0060075)
Regulation Of Retinal Ganglion Cell Axon Guidance (GO:0090259)
Regulation Of Retrograde Transport, Endosome To Golgi (GO:1905279)
Regulation Of Sarcomere Organization (GO:0060297)
Regulation Of T-Circle Formation (GO:1904429)
Regulation Of Ventricular Cardiac Muscle Cell Membrane Depolarization (GO:0060373)
Regulation Of Vitamin D Biosynthetic Process (GO:0060556)
Regulation Of Voltage-Gated Sodium Channel Activity (GO:1905150)
Renal Filtration Cell Differentiation (GO:0061318)
Reproductive System Development (GO:0061458)
Response To Bile Acid (GO:1903412)
Response To Heparin (GO:0071503)
Dermatan Sulfate Metabolic Process (GO:0030205)
Dermatan Sulfate Proteoglycan Metabolic Process (GO:0050655)
Response To Testosterone (GO:0033574)
Detection Of Virus (GO:0009597)
Retinal Ganglion Cell Axon Guidance (GO:0031290)
Ear Development (GO:0043583)
Short-Chain Fatty Acid Catabolic Process (GO:0019626)
snRNA Modification (GO:0040031)
Epithelial Tube Branching Involved In Lung Morphogenesis (GO:0060441)
Establishment Of Lymphocyte Polarity (GO:0001767)
Establishment Of Mitochondrion Localization, Microtubule-Mediated (GO:0034643)
Female Gonad Development (GO:0008585)
Forebrain Neuron Differentiation (GO:0021879)
Galactolipid Biosynthetic Process (GO:0019375)
Galactosylceramide Biosynthetic Process (GO:0006682)
Glomerular Epithelial Cell Differentiation (GO:0072311)
Transmembrane Receptor Protein Tyrosine Phosphatase Signaling Pathway (GO:0007185)
Tripartite Regional Subdivision (GO:0007351)
Glycoside Metabolic Process (GO:0016137)
Glycosyl Compound Catabolic Process (GO:1901658)
Glycosylceramide Catabolic Process (GO:0046477)
Vascular Endothelial Growth Factor Receptor-2 Signaling Pathway (GO:0036324)
Ventricular Cardiac Muscle Cell Development (GO:0055015)
Immunoglobulin V(D)J Recombination (GO:0033152)
Inflammatory Response To Wounding (GO:0090594)
Inner Ear Auditory Receptor Cell Differentiation (GO:0042491)
Iron Coordination Entity Transport (GO:1901678)

Isoleucine Metabolic Process (GO:0006549)
Lipoprotein Metabolic Process (GO:0042157)
mRNA 5'-Splice Site Recognition (GO:0000395)
Metanephric Collecting Duct Development (GO:0072205)
Negative Regulation Of Amyloid Precursor Protein Biosynthetic Process (GO:0042985)
Negative Regulation Of Chemokine-Mediated Signaling Pathway (GO:0070100)
Negative Regulation Of Erythrocyte Differentiation (GO:0045647)
Negative Regulation Of Glial Cell Proliferation (GO:0060253)
Negative Regulation Of Heterochromatin Formation (GO:0031452)
Negative Regulation Of Meiotic Cell Cycle (GO:0051447)
Negative Regulation Of Monocyte Chemotaxis (GO:0090027)
Negative Regulation Of Protein Kinase Activity By Regulation Of Protein Phosphorylation (GO:0044387)
Nucleoside Triphosphate Biosynthetic Process (GO:0009142)
Ornithine Transport (GO:0015822)
Pexophagy (GO:0000425)
Plasma Membrane Raft Assembly (GO:0044854)
Podocyte Cell Migration (GO:0090521)
Positive Regulation Of I-kappaB Phosphorylation (GO:1903721)
Positive Regulation Of T-helper 1 Cell Cytokine Production (GO:2000556)
Positive Regulation Of T-helper 2 Cell Differentiation (GO:0045630)
Positive Regulation Of Wnt Signaling Pathway, Planar Cell Polarity Pathway (GO:2000096)
Positive Regulation Of Cardiac Muscle Cell Differentiation (GO:2000727)
Positive Regulation Of Epithelial Cell Differentiation Involved In Kidney Development (GO:2000698)
Positive Regulation Of Fever Generation (GO:0031622)
Positive Regulation Of Hemoglobin Biosynthetic Process (GO:0046985)
Positive Regulation Of Killing Of Cells Of Another Organism (GO:0051712)
Positive Regulation Of Mitotic Cytokinesis (GO:1903490)
Positive Regulation Of Osteoblast Proliferation (GO:0033690)
Positive Regulation Of Protein Localization To Cilium (GO:1903566)
Positive Regulation Of Tau-Protein Kinase Activity (GO:1902949)
Positive Regulation Of Transcription Of Notch Receptor Target (GO:0007221)
Protein Localization To Membrane Raft (GO:1903044)
Protein Repair (GO:0030091)
Protein Targeting To Vacuole Involved In Autophagy (GO:0071211)
Regulation Of T-helper 1 Cell Cytokine Production (GO:2000554)
Regulation Of T-helper 17 Cell Lineage Commitment (GO:2000328)
Regulation Of Calcidiol 1-Monooxygenase Activity (GO:0060558)
Second-Messenger-Mediated Signaling (GO:0019932)
Protein Targeting To Membrane (GO:0006612)
DNA Damage Response, Signal Transduction By P53 Class Mediator (GO:0030330)
Regulation Of Trans-Synaptic Signaling (GO:0099177)
Interstrand Cross-Link Repair (GO:0036297)
Regulation Of Focal Adhesion Assembly (GO:0051893)
Regulation Of Myoblast Differentiation (GO:0045661)
Negative Regulation Of Protein Catabolic Process (GO:0042177)
Negative Regulation Of Gene Expression (GO:0010629)
Anterior/Posterior Axis Specification (GO:0009948)
Cell Projection Organization (GO:0030030)

Regulation Of Smooth Muscle Contraction (GO:0006940)
Leukotriene Metabolic Process (GO:0006691)
Limb Morphogenesis (GO:0035108)
Long-Term Memory (GO:0007616)
Mesoderm Formation (GO:0001707)
Negative Regulation Of Microtubule Depolymerization (GO:0007026)
Negative Regulation Of Reproductive Process (GO:2000242)
Negative Regulation Of Small GTPase Mediated Signal Transduction (GO:0051058)
Regulation Of Cardiac Muscle Contraction By Calcium Ion Signaling (GO:0010882)
Epithelial Cell Differentiation (GO:0030855)
Positive Regulation Of Tyrosine Phosphorylation Of STAT Protein (GO:0042531)
Regulation Of Vascular Associated Smooth Muscle Cell Proliferation (GO:1904705)
Positive Regulation Of Receptor Signaling Pathway Via JAK-STAT (GO:0046427)
Positive Regulation Of Receptor Signaling Pathway Via STAT (GO:1904894)
Positive Regulation Of Signaling (GO:0023056)
Cellular Response To Hormone Stimulus (GO:0032870)
Defense Response To Gram-negative Bacterium (GO:0050829)
Regulation Of MAP Kinase Activity (GO:0043405)
Response To cAMP (GO:0051591)
Connective Tissue Development (GO:0061448)
Positive Regulation Of Protein Tyrosine Kinase Activity (GO:0061098)
Regulation Of Dendritic Spine Development (GO:0060998)
Regulation Of Lipopolysaccharide-Mediated Signaling Pathway (GO:0031664)
Regulation Of Neurotransmitter Transport (GO:0051588)
Negative Regulation Of GTPase Activity (GO:0034260)
Negative Regulation Of Biomineral Tissue Development (GO:0070168)
Positive Regulation Of T Cell Cytokine Production (GO:0002726)
Positive Regulation Of Heart Contraction (GO:0045823)
Positive Regulation Of Signal Transduction By P53 Class Mediator (GO:1901798)
Positive Regulation Of Smoothened Signaling Pathway (GO:0045880)
Regulation Of Anoikis (GO:2000209)
Regulation Of Cardiac Conduction (GO:1903779)
CENP-A Containing Chromatin Assembly (GO:0034080)
Regulation Of Collagen Fibril Organization (GO:1904026)
Regulation Of Cortisol Biosynthetic Process (GO:2000064)
Regulation Of Glial Cell Apoptotic Process (GO:0034350)
Regulation Of Hemoglobin Biosynthetic Process (GO:0046984)
Anterograde Dendritic Transport (GO:0098937)
Regulation Of Mitotic Cytokinesis (GO:1902412)
Antigen Processing And Presentation Of Endogenous Peptide Antigen Via MHC Class I (GO:0019885)
Regulation Of Myosin-Light-Chain-Phosphatase Activity (GO:0035507)
Autophagosome Membrane Docking (GO:0016240)
Regulation Of Osteoclast Development (GO:2001204)
Bleb Assembly (GO:0032060)
Calcitonin Family Receptor Signaling Pathway (GO:0097646)
Cardiac Muscle Cell Myoblast Differentiation (GO:0060379)
Cardiac Muscle Cell-Cardiac Muscle Cell Adhesion (GO:0086042)
Cardiac Pacemaker Cell Development (GO:0060926)

Regulation Of Respiratory System Process (GO:0044065)
Cellular Hypotonic Response (GO:0071476)
Cellular Response To Gonadotropin Stimulus (GO:0071371)
Cellular Response To Hepatocyte Growth Factor Stimulus (GO:0035729)
Cellular Response To Laminar Fluid Shear Stress (GO:0071499)
Regulation Of Vascular Associated Smooth Muscle Cell Differentiation (GO:1905063)
Cholesterol Catabolic Process (GO:0006707)
Release Of Sequestered Calcium Ion Into Cytosol By Sarcoplasmic Reticulum (GO:0014808)
Chromatin Remodeling At Centromere (GO:0031055)
Response To Hepatocyte Growth Factor (GO:0035728)
Delamination (GO:0060232)
Dense Core Granule Cytoskeletal Transport (GO:0099519)
Detection Of Stimulus Involved In Sensory Perception Of Pain (GO:0062149)
Development Of Primary Female Sexual Characteristics (GO:0046545)
Serine Transport (GO:0032329)
Skeletal Muscle Cell Differentiation (GO:0035914)
Skeletal Muscle Thin Filament Assembly (GO:0030240)
Sterol Catabolic Process (GO:0016127)
Gamma-Aminobutyric Acid Transport (GO:0015812)
Gap Junction Assembly (GO:0016264)
Glomerular Epithelial Cell Development (GO:0072310)
Ureter Development (GO:0072189)
Vascular Associated Smooth Muscle Cell Development (GO:0097084)
Glycosylceramide Biosynthetic Process (GO:0046476)
Heart Trabecula Formation (GO:0060347)
Hypotonic Response (GO:0006971)
Inner Ear Receptor Cell Differentiation (GO:0060113)
Insulin Secretion Involved In Cellular Response To Glucose Stimulus (GO:0035773)
Intracellular Sequestering Of Iron Ion (GO:0006880)
Macropinocytosis (GO:0044351)
Maintenance Of Lens Transparency (GO:0036438)
Meiotic DNA Double-Strand Break Formation (GO:0042138)
Membrane Invagination (GO:0010324)
Mitochondrion-Endoplasmic Reticulum Membrane Tethering (GO:1990456)
Monoacylglycerol Catabolic Process (GO:0052651)
Negative Regulation Of Blood Vessel Endothelial Cell Proliferation Involved In Sprouting Angiogenesis (GO:190
Negative Regulation Of Hematopoietic Progenitor Cell Differentiation (GO:1901533)
Negative Regulation Of Lipoprotein Particle Clearance (GO:0010985)
Negative Regulation Of Oligodendrocyte Differentiation (GO:0048715)
Negative Regulation Of Response To Reactive Oxygen Species (GO:1901032)
Negative Regulation Of Ruffle Assembly (GO:1900028)
Negative Regulation Of Type 2 Immune Response (GO:0002829)
Nose Development (GO:0043584)
Nucleobase Catabolic Process (GO:0046113)
Peptide Transport (GO:0015833)
Placenta Blood Vessel Development (GO:0060674)
Podocyte Development (GO:0072015)
Positive Regulation Of B Cell Receptor Signaling Pathway (GO:0050861)

Positive Regulation Of Blood Circulation (GO:1903524)
Positive Regulation Of Cell Killing (GO:0031343)
Positive Regulation Of Chemokine (C-X-C Motif) Ligand 2 Production (GO:2000343)
Positive Regulation Of Endoplasmic Reticulum Stress-Induced Intrinsic Apoptotic Signaling Pathway (GO:19022
Positive Regulation Of Gastrulation (GO:2000543)
Positive Regulation Of Interleukin-5 Production (GO:0032754)
Positive Regulation Of Membrane Depolarization (GO:1904181)
Positive Regulation Of Neurotransmitter Secretion (GO:0001956)
Positive Regulation Of Protein Localization To Centrosome (GO:1904781)
Positive Regulation Of Spindle Assembly (GO:1905832)
Positive Regulation Of Synaptic Plasticity (GO:0031915)
Positive Regulation Of Toll-Like Receptor 3 Signaling Pathway (GO:0034141)
Positive Regulation Of Trophoblast Cell Migration (GO:1901165)
Protein Deglutamylation (GO:0035608)
Protein Import Into Peroxisome Matrix, Receptor Recycling (GO:0016562)
Regulation Of I-kappaB Phosphorylation (GO:1903719)
Regulation Of Rab Protein Signal Transduction (GO:0032483)
Regulation Of TORC2 Signaling (GO:1903939)
Regulation Of Aldosterone Biosynthetic Process (GO:0032347)
Regulation Of Bile Acid Metabolic Process (GO:1904251)
Regulation Of Blood-Brain Barrier Permeability (GO:1905603)
Regulation Of Cardiac Muscle Hypertrophy In Response To Stress (GO:1903242)
Regulation Of Cell Adhesion Molecule Production (GO:0060353)
Positive Regulation Of Interleukin-6 Production (GO:0032755)
Regulation Of Mitochondrial Membrane Potential (GO:0051881)
Anterograde Axonal Transport (GO:0008089)
Regulation Of Nitric Oxide Biosynthetic Process (GO:0045428)
Regulation Of Protein Targeting To Mitochondrion (GO:1903214)
Regulation Of Receptor-Mediated Endocytosis (GO:0048259)
Digestive Tract Development (GO:0048565)
Fibroblast Growth Factor Receptor Signaling Pathway (GO:0008543)
Negative Regulation Of Cell-Substrate Adhesion (GO:0010812)
Neuron Projection Extension (GO:1990138)
Positive Regulation Of Intrinsic Apoptotic Signaling Pathway (GO:2001244)
Positive Regulation Of Pattern Recognition Receptor Signaling Pathway (GO:0062208)
Negative Regulation Of Transforming Growth Factor Beta Receptor Signaling Pathway (GO:0030512)
Renal System Development (GO:0072001)
Positive Regulation Of Protein Serine/Threonine Kinase Activity (GO:0071902)
Regulation Of Dopamine Secretion (GO:0014059)
Fc-epsilon Receptor Signaling Pathway (GO:0038095)
Regulation Of Mononuclear Cell Migration (GO:0071675)
Regulation Of Regulatory T Cell Differentiation (GO:0045589)
Glucose Transmembrane Transport (GO:1904659)
Vesicle Transport Along Microtubule (GO:0047496)
Hexose Transmembrane Transport (GO:0008645)
Inositol Phosphate Metabolic Process (GO:0043647)
Mammary Gland Development (GO:0030879)
Membrane Protein Ectodomain Proteolysis (GO:0006509)

Negative Regulation Of Axonogenesis (GO:0050771)
Negative Regulation Of Cell Communication (GO:0010648)
Negative Regulation Of Cell Development (GO:0010721)
Negative Regulation Of Extrinsic Apoptotic Signaling Pathway In Absence Of Ligand (GO:2001240)
Negative Regulation Of Signal Transduction In Absence Of Ligand (GO:1901099)
Positive Regulation Of Excitatory Postsynaptic Potential (GO:2000463)
Positive Regulation Of Protein Autophosphorylation (GO:0031954)
Positive Regulation Of Synaptic Transmission, Glutamatergic (GO:0051968)
Peptidyl-Serine Phosphorylation (GO:0018105)
Regulation Of Cytokine-Mediated Signaling Pathway (GO:0001959)
Cyclic-Nucleotide-Mediated Signaling (GO:0019935)
Synaptic Vesicle Endocytosis (GO:0048488)
Positive Regulation Of ERK1 And ERK2 Cascade (GO:0070374)
RNA Splicing (GO:0008380)
Positive Regulation Of Protein-Containing Complex Assembly (GO:0031334)
Regulation Of Multicellular Organismal Development (GO:2000026)
Glucose Metabolic Process (GO:0006006)
Phospholipid Transport (GO:0015914)
Organic Substance Transport (GO:0071702)
Regulation Of Neuron Differentiation (GO:0045664)
Negative Regulation Of Cellular Component Organization (GO:0051129)
Positive Regulation Of Cytoskeleton Organization (GO:0051495)
Positive Regulation Of Endocytosis (GO:0045807)
Regulation Of Protein-Containing Complex Assembly (GO:0043254)
Regulation Of Chemokine Production (GO:0032642)
Acylglycerol Metabolic Process (GO:0006639)
Heart Contraction (GO:0060047)
Regulation Of Extrinsic Apoptotic Signaling Pathway (GO:2001236)
Response To Light Stimulus (GO:0009416)
Regulation Of Cellular Response To Transforming Growth Factor Beta Stimulus (GO:1903844)
Regulation Of Interleukin-4 Production (GO:0032673)
Cell-Cell Signaling By Wnt (GO:0198738)
Regulation Of Toll-Like Receptor 4 Signaling Pathway (GO:0034143)
Glial Cell Development (GO:0021782)
Heparan Sulfate Proteoglycan Biosynthetic Process (GO:0015012)
Negative Regulation Of Protein Depolymerization (GO:1901880)
Negative Regulation Of Protein Tyrosine Kinase Activity (GO:0061099)
Positive Regulation Of Cell-Substrate Junction Organization (GO:0150117)
Positive Regulation Of Lamellipodium Assembly (GO:0010592)
Positive Regulation Of mRNA Splicing, Via Spliceosome (GO:0048026)
Regulation Of Chaperone-Mediated Autophagy (GO:1904714)
Regulation Of Chemokine-Mediated Signaling Pathway (GO:0070099)
DNA Ligation (GO:0006266)
Regulation Of Extracellular Matrix Constituent Secretion (GO:0003330)
Regulation Of Hydrogen Peroxide-Induced Cell Death (GO:1903205)
Adenylate Cyclase-Inhibiting G Protein-Coupled Acetylcholine Receptor Signaling Pathway (GO:0007197)
Regulation Of Meiotic Nuclear Division (GO:0040020)
Regulation Of Myeloid Leukocyte Differentiation (GO:0002761)

Arginine Catabolic Process (GO:0006527)
Axon Ensheathment In Central Nervous System (GO:0032291)
Regulation Of Positive Chemotaxis (GO:0050926)
Regulation Of Protein Glycosylation (GO:0060049)
Regulation Of Protein Localization To Cell Cortex (GO:1904776)
Regulation Of Protein Localization To Cilium (GO:1903564)
Cell Migration Involved In Heart Development (GO:0060973)
Regulation Of Vascular Wound Healing (GO:0061043)
Release Of Sequestered Calcium Ion Into Cytosol By Endoplasmic Reticulum (GO:1903514)
Collecting Duct Development (GO:0072044)
Coronary Vasculature Development (GO:0060976)
Coronary Vasculature Morphogenesis (GO:0060977)
Dendritic Spine Maintenance (GO:0097062)
Dermatan Sulfate Proteoglycan Biosynthetic Process (GO:0050651)
snRNA Transcription By RNA Polymerase III (GO:0042796)
Establishment Of Planar Polarity (GO:0001736)
Fructose Metabolic Process (GO:0006000)
Termination Of RNA Polymerase II Transcription (GO:0006369)
Fructose Transmembrane Transport (GO:0015755)
Galactosylceramide Metabolic Process (GO:0006681)
Tooth Mineralization (GO:0034505)
Transcription Initiation At RNA Polymerase I Promoter (GO:0006361)
Glycoside Catabolic Process (GO:0016139)
Hair Cell Differentiation (GO:0035315)
Venous Blood Vessel Development (GO:0060841)
Heparan Sulfate Proteoglycan Biosynthetic Process, Polysaccharide Chain Biosynthetic Process (GO:0015014)
Hyperosmotic Response (GO:0006972)
Lactate Metabolic Process (GO:0006089)
Lymphocyte Apoptotic Process (GO:0070227)
Mammary Gland Epithelial Cell Differentiation (GO:0060644)
Monosaccharide Biosynthetic Process (GO:0046364)
Myeloid Leukocyte Mediated Immunity (GO:0002444)
Myoblast Migration (GO:0051451)
Negative Regulation Of B Cell Apoptotic Process (GO:0002903)
Negative Regulation Of Amyloid-Beta Clearance (GO:1900222)
Negative Regulation Of Glial Cell Apoptotic Process (GO:0034351)
Negative Regulation Of Inflammatory Response To Wounding (GO:0106015)
Negative Regulation Of Mitochondrial Fusion (GO:0010637)
Negative Regulation Of Mitochondrial Membrane Potential (GO:0010917)
Negative Regulation Of Muscle Cell Differentiation (GO:0051148)
Negative Regulation Of Muscle Hypertrophy (GO:0014741)
Negative Regulation Of Nitric Oxide Biosynthetic Process (GO:0045019)
Negative Regulation Of Nitric Oxide Metabolic Process (GO:1904406)
Negative Regulation Of Vascular Associated Smooth Muscle Cell Differentiation (GO:1905064)
Nucleobase-Containing Small Molecule Interconversion (GO:0015949)
Olefinic Compound Biosynthetic Process (GO:0120255)
Osteoblast Development (GO:0002076)
Peptidyl-Lysine Hydroxylation (GO:0017185)

Phosphatidylserine Acyl-Chain Remodeling (GO:0036150)
Positive Regulation Of Attachment Of Mitotic Spindle Microtubules To Kinetochore (GO:1902425)
Positive Regulation Of Calcium Ion Import (GO:0090280)
Positive Regulation Of Cell Migration By Vascular Endothelial Growth Factor Signaling Pathway (GO:0038089)
Positive Regulation Of Epithelial Tube Formation (GO:1905278)
Positive Regulation Of Extracellular Matrix Disassembly (GO:0090091)
Positive Regulation Of Meiotic Nuclear Division (GO:0045836)
Positive Regulation Of Microtubule Nucleation (GO:0090063)
Positive Regulation Of Neuron Projection Arborization (GO:0150012)
Positive Regulation Of Steroid Biosynthetic Process (GO:0010893)
Positive Regulation Of Synaptic Transmission, GABAergic (GO:0032230)
Positive Regulation Of Vascular Associated Smooth Muscle Cell Migration (GO:1904754)
Proximal/Distal Pattern Formation (GO:0009954)
Pyrimidine Nucleobase Catabolic Process (GO:0006208)
Regulation Of CD4-positive, Alpha-Beta T Cell Activation (GO:2000514)
Regulation Of Cdc42 Protein Signal Transduction (GO:0032489)
Regulation Of T-helper 2 Cell Differentiation (GO:0045628)
Regulation Of Alpha-Beta T Cell Differentiation (GO:0046637)
Regulation Of Attachment Of Mitotic Spindle Microtubules To Kinetochore (GO:1902423)
Regulation Of Cardiac Muscle Cell Action Potential Involved In Regulation Of Contraction (GO:0098909)
Regulation Of Organelle Organization (GO:0033043)
Protein Complex Oligomerization (GO:0051259)
Positive Regulation Of Interleukin-8 Production (GO:0032757)
Positive Regulation Of Organelle Assembly (GO:1902117)
RNA Processing (GO:0006396)
Amino Acid Transmembrane Transport (GO:0003333)
Keratinocyte Differentiation (GO:0030216)
Lipid Modification (GO:0030258)
Positive Regulation Of Growth (GO:0045927)
Potassium Ion Transport (GO:0006813)
RNA Splicing, Via Transesterification Reactions (GO:0000375)
Amyloid Fibril Formation (GO:1990000)
Cell Morphogenesis Involved In Differentiation (GO:0000904)
Regulation Of Supramolecular Fiber Organization (GO:1902903)
Response To Purine-Containing Compound (GO:0014074)
Ear Morphogenesis (GO:0042471)
Lipid Translocation (GO:0034204)
Negative Regulation Of Cell Junction Assembly (GO:1901889)
Outflow Tract Septum Morphogenesis (GO:0003148)
Positive Regulation Of BMP Signaling Pathway (GO:0030513)
Positive Regulation Of p38MAPK Cascade (GO:1900745)
Regulation Of AMPA Receptor Activity (GO:2000311)
Regulation Of Embryonic Development (GO:0045995)
Negative Regulation Of Apoptotic Signaling Pathway (GO:2001234)
Positive Regulation Of Apoptotic Process (GO:0043065)
Regulation Of Endothelial Cell Apoptotic Process (GO:2000351)
SCF-dependent Proteasomal Ubiquitin-Dependent Protein Catabolic Process (GO:0031146)
Regulation Of Primary Metabolic Process (GO:0080090)

Learning (GO:0007612)
Positive Regulation Of Cell-Matrix Adhesion (GO:0001954)
Negative Regulation Of Cell Population Proliferation (GO:0008285)
Regulation Of NIK/NF-kappaB Signaling (GO:1901222)
Regulation Of Glycogen Biosynthetic Process (GO:0005979)
Regulation Of Neutrophil Chemotaxis (GO:0090022)
Regulation Of Synapse Organization (GO:0050807)
Cellular Response To Hexose Stimulus (GO:0071331)
Inner Ear Morphogenesis (GO:0042472)
Metanephros Development (GO:0001656)
Myotube Differentiation (GO:0014902)
Negative Regulation Of TORC1 Signaling (GO:1904262)
Negative Regulation Of Protein Transport (GO:0051224)
Negative Regulation Of Response To Wounding (GO:1903035)
Organophosphate Ester Transport (GO:0015748)
Positive Regulation Of Striated Muscle Cell Differentiation (GO:0051155)
Proteoglycan Metabolic Process (GO:0006029)
Regulation Of Actin Cytoskeleton Reorganization (GO:2000249)
Regulation Of Biomineral Tissue Development (GO:0070167)
Regulation Of Cell Size (GO:0008361)
Positive Regulation Of Chemotaxis (GO:0050921)
Positive Regulation Of Leukocyte Cell-Cell Adhesion (GO:1903039)
CD40 Signaling Pathway (GO:0023035)
DNA Damage Response, Signal Transduction By P53 Class Mediator Resulting In Transcription Of P21 Class Me[illegible]
Regulation Of Defense Response To Bacterium (GO:1900424)
DNA Topological Change (GO:0006265)
Regulation Of Endothelial Cell Development (GO:1901550)
Regulation Of Epidermis Development (GO:0045682)
L-arginine Transmembrane Transport (GO:1903826)
Regulation Of Generation Of Precursor Metabolites And Energy (GO:0043467)
Regulation Of Lysosomal Protein Catabolic Process (GO:1905165)
Adult Heart Development (GO:0007512)
Alcohol Catabolic Process (GO:0046164)
Regulation Of Morphogenesis Of An Epithelium (GO:1905330)
Astral Microtubule Organization (GO:0030953)
Regulation Of Nephron Tubule Epithelial Cell Differentiation (GO:0072182)
Regulation Of Neutrophil Migration (GO:1902622)
Basic Amino Acid Transmembrane Transport (GO:1990822)
Regulation Of Pattern Recognition Receptor Signaling Pathway (GO:0062207)
Regulation Of Protein Localization To Early Endosome (GO:1902965)
Cardioblast Differentiation (GO:0010002)
Cellular Response To Platelet-Derived Growth Factor Stimulus (GO:0036120)
Regulation Of Vesicle Fusion (GO:0031338)
Collagen-Activated Signaling Pathway (GO:0038065)
Detection Of Mechanical Stimulus Involved In Sensory Perception Of Sound (GO:0050910)
Response To Vitamin (GO:0033273)
Response To Vitamin D (GO:0033280)
Embryonic Forelimb Morphogenesis (GO:0035115)

Sensory Perception Of Sour Taste (GO:0050915)
Sequestering Of Actin Monomers (GO:0042989)
Skeletal Myofibril Assembly (GO:0014866)
Endothelial Cell Chemotaxis (GO:0035767)
Somatic Recombination Of Immunoglobulin Gene Segments (GO:0016447)
Exonucleolytic Trimming Involved In rRNA Processing (GO:0000459)
Exonucleolytic Trimming To Generate Mature 3'-End Of 5.8S rRNA From Tricistronic rRNA Transcript (SSU-rRNA, 5
Fructose 1,6-Bisphosphate Metabolic Process (GO:0030388)
Transcription-Coupled Nucleotide-Excision Repair (GO:0006283)
Tricarboxylic Acid Metabolic Process (GO:0072350)
Glycerol Transmembrane Transport (GO:0015793)
Lactation (GO:0007595)
Mesodermal Cell Differentiation (GO:0048333)
Mitochondrial Fragmentation Involved In Apoptotic Process (GO:0043653)
Negative Regulation Of Chromosome Organization (GO:2001251)
Negative Regulation Of Glial Cell Differentiation (GO:0045686)
Negative Regulation Of Glycoprotein Biosynthetic Process (GO:0010561)
Negative Regulation Of Membrane Potential (GO:0045837)
Negative Regulation Of Platelet Aggregation (GO:0090331)
Negative Regulation Of Production Of Molecular Mediator Of Immune Response (GO:0002701)
Negative Regulation Of Protein-Containing Complex Disassembly (GO:0043242)
Negative Regulation Of Smooth Muscle Cell Differentiation (GO:0051151)
Negative Regulation Of Systemic Arterial Blood Pressure (GO:0003085)
Nucleoside Diphosphate Metabolic Process (GO:0009132)
Positive Regulation Of Actin Cytoskeleton Reorganization (GO:2000251)
Positive Regulation Of Blood Vessel Endothelial Cell Proliferation Involved In Sprouting Angiogenesis (GO:1903
Positive Regulation Of Calcium Ion Transport Into Cytosol (GO:0010524)
Positive Regulation Of Cell-Cell Adhesion Mediated By Cadherin (GO:2000049)
Positive Regulation Of Extracellular Matrix Assembly (GO:1901203)
Positive Regulation Of Fatty Acid Beta-Oxidation (GO:0032000)
Positive Regulation Of Macrophage Cytokine Production (GO:0060907)
Positive Regulation Of Megakaryocyte Differentiation (GO:0045654)
Positive Regulation Of Opsonization (GO:1903028)
Positive Regulation Of Protein Localization To Early Endosome (GO:1902966)
Positive Regulation Of Protein Localization To Endosome (GO:1905668)
Presynaptic Membrane Assembly (GO:0097105)
Protein K29-linked Ubiquitination (GO:0035519)
Pyrimidine-Containing Compound Catabolic Process (GO:0072529)
Regulation Of DNA-templated Transcription Initiation (GO:2000142)
Regulation Of Wnt Signaling Pathway, Planar Cell Polarity Pathway (GO:2000095)
Regulation Of Mitotic Cell Cycle (GO:0007346)
Positive Regulation Of Double-Strand Break Repair (GO:2000781)
Negative Regulation Of Multicellular Organismal Process (GO:0051241)
Positive Regulation Of Intracellular Protein Transport (GO:0090316)
Calcium-Mediated Signaling (GO:0019722)
Epidermis Development (GO:0008544)
Regulation Of Protein Modification Process (GO:0031399)
Epidermal Growth Factor Receptor Signaling Pathway (GO:0007173)

Negative Regulation Of Response To Biotic Stimulus (GO:0002832)
Positive Regulation Of mRNA Catabolic Process (GO:0061014)
Negative Regulation Of Cellular Process (GO:0048523)
Intracellular Monoatomic Cation Homeostasis (GO:0030003)
Canonical Wnt Signaling Pathway (GO:0060070)
Regulation Of Tyrosine Phosphorylation Of STAT Protein (GO:0042509)
Positive Regulation Of T Cell Proliferation (GO:0042102)
Regulation Of Cell-Matrix Adhesion (GO:0001952)
Fc Receptor Signaling Pathway (GO:0038093)
Regulation Of Nucleotide-Excision Repair (GO:2000819)
cAMP-mediated Signaling (GO:0019933)
Calcium-Ion Regulated Exocytosis (GO:0017156)
Camera-Type Eye Development (GO:0043010)
Cardiac Muscle Cell Development (GO:0055013)
Regulation Of Transcription By RNA Polymerase III (GO:0006359)
Regulation Of Vasoconstriction (GO:0019229)
Response To Glucocorticoid (GO:0051384)
Establishment Or Maintenance Of Apical/Basal Cell Polarity (GO:0035088)
Telomere Maintenance Via Telomere Lengthening (GO:0010833)
miRNA-mediated Gene Silencing (GO:0035195)
Negative Regulation Of Catalytic Activity (GO:0043086)
Negative Regulation Of Endothelial Cell Apoptotic Process (GO:2000352)
Positive Regulation Of Cytokine Production (GO:0001819)
Negative Regulation Of Angiogenesis (GO:0016525)
B Cell Receptor Signaling Pathway (GO:0050853)
Regulation Of Cytokine Production Involved In Inflammatory Response (GO:1900015)
Response To Glucose (GO:0009749)
Homotypic Cell-Cell Adhesion (GO:0034109)
Negative Regulation Of Cellular Metabolic Process (GO:0031324)
Negative Regulation Of Cold-Induced Thermogenesis (GO:0120163)
Positive Regulation Of Blood Vessel Endothelial Cell Migration (GO:0043536)
Regulation Of Insulin Secretion (GO:0050796)
Protein Targeting (GO:0006605)
Positive Regulation Of DNA Metabolic Process (GO:0051054)
Regulation Of Extrinsic Apoptotic Signaling Pathway In Absence Of Ligand (GO:2001239)
Regulation Of Microtubule Polymerization Or Depolymerization (GO:0031110)
Regulation Of Mitochondrial Fission (GO:0090140)
Cell Surface Receptor Signaling Pathway Involved In Cell-Cell Signaling (GO:1905114)
Cellular Response To Epidermal Growth Factor Stimulus (GO:0071364)
Dorsal/Ventral Pattern Formation (GO:0009953)
Synaptic Transmission, Glutamatergic (GO:0035249)
Glycerophospholipid Catabolic Process (GO:0046475)
Microtubule Polymerization Or Depolymerization (GO:0031109)
Negative Regulation Of Neurogenesis (GO:0050768)
Positive Regulation Of TORC1 Signaling (GO:1904263)
Positive Regulation Of Filopodium Assembly (GO:0051491)
Positive Regulation Of Nitric Oxide Biosynthetic Process (GO:0045429)
Regulation Of Androgen Receptor Signaling Pathway (GO:0060765)

Inflammatory Response (GO:0006954)
Regulation Of Signal Transduction (GO:0009966)
Regulation Of Ceramide Biosynthetic Process (GO:2000303)
DNA Damage Response, Signal Transduction Resulting In Transcription (GO:0042772)
Regulation Of Inflammatory Response To Wounding (GO:0106014)
T-helper 1 Type Immune Response (GO:0042088)
Regulation Of Leukocyte Mediated Cytotoxicity (GO:0001910)
Regulation Of Long-Term Synaptic Depression (GO:1900452)
Aminoglycan Metabolic Process (GO:0006022)
Aspartate Transmembrane Transport (GO:0015810)
Autophagy Of Peroxisome (GO:0030242)
Biomineral Tissue Development (GO:0031214)
cGMP Biosynthetic Process (GO:0006182)
Regulation Of Protein Localization To Centrosome (GO:1904779)
Cell Differentiation In Spinal Cord (GO:0021515)
Regulation Of Sequestering Of Calcium Ion (GO:0051282)
Regulation Of Tau-Protein Kinase Activity (GO:1902947)
Regulation Of Transmembrane Transporter Activity (GO:0022898)
Regulation Of Viral-Induced Cytoplasmic Pattern Recognition Receptor Signaling Pathway (GO:0039531)
Response To Interleukin-4 (GO:0070670)
Response To Platelet-Derived Growth Factor (GO:0036119)
Dosage Compensation (GO:0007549)
Embryonic Eye Morphogenesis (GO:0048048)
Serine Family Amino Acid Catabolic Process (GO:0009071)
Endodermal Cell Fate Commitment (GO:0001711)
Forelimb Morphogenesis (GO:0035136)
Fructose 6-Phosphate Metabolic Process (GO:0006002)
Telomeric Loop Disassembly (GO:0090657)
Transferrin Transport (GO:0033572)
Hematopoietic Stem Cell Differentiation (GO:0060218)
Iron Ion Transmembrane Transport (GO:0034755)
Ketone Biosynthetic Process (GO:0042181)
Kinetochore Assembly (GO:0051382)
Long-Chain Fatty Acid Import Into Cell (GO:0044539)
Mitochondrion Morphogenesis (GO:0070584)
Muscle Cell Fate Commitment (GO:0042693)
Muscle Cell Migration (GO:0014812)
Myelin Assembly (GO:0032288)
Myeloid Dendritic Cell Differentiation (GO:0043011)
Negative Regulation Of Cell Adhesion Mediated By Integrin (GO:0033629)
Negative Regulation Of Establishment Of Protein Localization (GO:1904950)
Negative Regulation Of Leukocyte Migration (GO:0002686)
Negative Regulation Of Lipid Localization (GO:1905953)
Negative Regulation Of Lymphocyte Apoptotic Process (GO:0070229)
Negative Regulation Of Necroptotic Process (GO:0060546)
Neuron Projection Regeneration (GO:0031102)
Neuron Remodeling (GO:0016322)
Nuclear mRNA Surveillance (GO:0071028)

Oligosaccharide Catabolic Process (GO:0009313)
Peptidyl-Serine Dephosphorylation (GO:0070262)
Phosphatidylcholine Catabolic Process (GO:0034638)
Pinocytosis (GO:0006907)
Positive Regulation Of RIG-I Signaling Pathway (GO:1900246)
Positive Regulation Of RNA Biosynthetic Process (GO:1902680)
Positive Regulation Of Cardiocyte Differentiation (GO:1905209)
Positive Regulation Of Epidermis Development (GO:0045684)
Positive Regulation Of Lipase Activity (GO:0060193)
Positive Regulation Of Mitochondrial Membrane Permeability (GO:0035794)
Positive Regulation Of Monocyte Differentiation (GO:0045657)
Positive Regulation Of Myoblast Fusion (GO:1901741)
Positive Regulation Of Protein Acetylation (GO:1901985)
Positive Regulation Of Protein Kinase A Signaling (GO:0010739)
Positive Regulation Of Receptor Recycling (GO:0001921)
Positive Regulation Of Skeletal Muscle Tissue Development (GO:0048643)
Positive Regulation Of Smooth Muscle Contraction (GO:0045987)
Positive Regulation Of Vasculogenesis (GO:2001214)
Presynaptic Membrane Organization (GO:0097090)
Protein O-linked Glycosylation Via Serine (GO:0018242)
Protein O-linked Glycosylation Via Threonine (GO:0018243)
Protein Localization To Nuclear Envelope (GO:0090435)
rRNA 3'-End Processing (GO:0031125)
Receptor Guanylyl Cyclase Signaling Pathway (GO:0007168)
Regulation Of PERK-mediated Unfolded Protein Response (GO:1903897)
Regulation Of Cardiac Muscle Cell Differentiation (GO:2000725)
Regulation Of Cellular Extravasation (GO:0002691)
Generation Of Neurons (GO:0048699)
Cholesterol Metabolic Process (GO:0008203)
Bicellular Tight Junction Assembly (GO:0070830)
Cognition (GO:0050890)
Positive Regulation Of Smooth Muscle Cell Proliferation (GO:0048661)
Transmembrane Receptor Protein Serine/Threonine Kinase Signaling Pathway (GO:0007178)
Regulation Of Double-Strand Break Repair (GO:2000779)
Regulation Of Wnt Signaling Pathway (GO:0030111)
Monoatomic Cation Transmembrane Transport (GO:0098655)
Cellular Response To Organic Cyclic Compound (GO:0071407)
Neuropeptide Signaling Pathway (GO:0007218)
Positive Regulation Of Cellular Biosynthetic Process (GO:0031328)
Regulation Of Vesicle-Mediated Transport (GO:0060627)
Positive Regulation Of Peptidyl-Serine Phosphorylation (GO:0033138)
Regulation Of Glucose Metabolic Process (GO:0010906)
RNA Phosphodiester Bond Hydrolysis (GO:0090501)
Regulation Of Monoatomic Ion Transport (GO:0043269)
Regulation Of Plasma Membrane Bounded Cell Projection Organization (GO:0120035)
Cellular Response To Osmotic Stress (GO:0071470)
Diterpenoid Metabolic Process (GO:0016101)
Limb Development (GO:0060173)

Negative Regulation Of Cation Channel Activity (GO:2001258)
Negative Regulation Of Signaling (GO:0023057)
Positive Regulation Of Nitric Oxide Metabolic Process (GO:1904407)
Regulation Of DNA Damage Response, Signal Transduction By P53 Class Mediator (GO:0043516)
Regulation Of Cardiac Muscle Cell Action Potential (GO:0098901)
Positive Regulation Of Intracellular Signal Transduction (GO:1902533)
Positive Regulation Of Protein Metabolic Process (GO:0051247)
Positive Regulation Of Cell Differentiation (GO:0045597)
Epithelium Development (GO:0060429)
Positive Regulation Of Hydrolase Activity (GO:0051345)
Regulation Of Mitotic Metaphase/Anaphase Transition (GO:0030071)
Regulation Of Cell Communication (GO:0010646)
L-serine Metabolic Process (GO:0006563)
Regulation Of Gene Expression By Genomic Imprinting (GO:0006349)
Rap Protein Signal Transduction (GO:0032486)
T-helper Cell Lineage Commitment (GO:0002295)
Acidic Amino Acid Transport (GO:0015800)
Actin Filament Depolymerization (GO:0030042)
Regulation Of Long-Term Neuronal Synaptic Plasticity (GO:0048169)
Regulation Of Low-Density Lipoprotein Particle Clearance (GO:0010988)
Alpha-Amino Acid Biosynthetic Process (GO:1901607)
Regulation Of Microtubule Nucleation (GO:0010968)
Regulation Of Mitochondrial Depolarization (GO:0051900)
Regulation Of Mitotic Sister Chromatid Separation (GO:0010965)
Arginine Metabolic Process (GO:0006525)
Regulation Of Opsonization (GO:1903027)
Basement Membrane Organization (GO:0071711)
Regulation Of Phospholipase Activity (GO:0010517)
calcineurin-NFAT Signaling Cascade (GO:0033173)
Regulation Of Protein Localization To Cajal Body (GO:1904869)
Cardiac Muscle Cell Membrane Repolarization (GO:0099622)
Cell Proliferation In Forebrain (GO:0021846)
Cellular Response To Cholesterol (GO:0071397)
Regulation Of Synapse Maturation (GO:0090128)
Coenzyme A Biosynthetic Process (GO:0015937)
Response To Corticosteroid (GO:0031960)
Ribonucleoside Bisphosphate Biosynthetic Process (GO:0034030)
Diol Metabolic Process (GO:0034311)
Embryonic Appendage Morphogenesis (GO:0035113)
Signal Release (GO:0023061)
snRNA Transcription By RNA Polymerase II (GO:0042795)
Establishment Of Endothelial Intestinal Barrier (GO:0090557)
Surfactant Homeostasis (GO:0043129)
Synaptic Vesicle Transport (GO:0048489)
Very-Low-Density Lipoprotein Particle Assembly (GO:0034379)
Kinetochore Organization (GO:0051383)
Mast Cell Activation Involved In Immune Response (GO:0002279)
Mast Cell Degranulation (GO:0043303)

Microtubule Anchoring At Centrosome (GO:0034454)
Mitotic Spindle Elongation (GO:0000022)
Mitotic Spindle Midzone Assembly (GO:0051256)
Muscle Filament Sliding (GO:0030049)
Negative Regulation Of B Cell Proliferation (GO:0030889)
Negative Regulation Of Activated T Cell Proliferation (GO:0046007)
Negative Regulation Of Dephosphorylation (GO:0035305)
Negative Regulation Of Exocytosis (GO:0045920)
Negative Regulation Of Glycolytic Process (GO:0045820)
Negative Regulation Of Hemostasis (GO:1900047)
Negative Regulation Of Homotypic Cell-Cell Adhesion (GO:0034111)
Negative Regulation Of Hydrogen Peroxide-Induced Cell Death (GO:1903206)
Negative Regulation Of Pathway-Restricted SMAD Protein Phosphorylation (GO:0060394)
Negative Regulation Of Programmed Necrotic Cell Death (GO:0062099)
Negative Regulation Of Response To Cytokine Stimulus (GO:0060761)
Negative Regulation Of Sodium Ion Transmembrane Transporter Activity (GO:2000650)
Negative Regulation Of Sprouting Angiogenesis (GO:1903671)
Negative Regulation Of Steroid Biosynthetic Process (GO:0010894)
Negative Regulation Of Transmembrane Transport (GO:0034763)
Negative Regulation Of Vascular Endothelial Growth Factor Receptor Signaling Pathway (GO:0030948)
Nucleoside Triphosphate Metabolic Process (GO:0009141)
Positive Regulation Of Attachment Of Spindle Microtubules To Kinetochore (GO:0051987)
Positive Regulation Of Bone Resorption (GO:0045780)
Positive Regulation Of Defense Response To Bacterium (GO:1900426)
Positive Regulation Of Dendrite Development (GO:1900006)
Positive Regulation Of Integrin Activation (GO:0033625)
Positive Regulation Of Interleukin-13 Production (GO:0032736)
Positive Regulation Of Mitochondrial Outer Membrane Permeabilization Involved In Apoptotic Signaling Pathway
Positive Regulation Of Nitric-Oxide Synthase Biosynthetic Process (GO:0051770)
Positive Regulation Of Non-Canonical Wnt Signaling Pathway (GO:2000052)
Positive Regulation Of Protein Depolymerization (GO:1901881)
Positive Regulation Of Protein Localization To Cajal Body (GO:1904871)
Positive Regulation Of Toll-Like Receptor 4 Signaling Pathway (GO:0034145)
Prostaglandin Transport (GO:0015732)
Purine Nucleoside Bisphosphate Biosynthetic Process (GO:0034033)
Purine Ribonucleoside Triphosphate Biosynthetic Process (GO:0009206)
Regulation Of B Cell Apoptotic Process (GO:0002902)
Regulation Of T-helper 2 Cell Cytokine Production (GO:2000551)
Regulation Of Amyloid Precursor Protein Biosynthetic Process (GO:0042984)
Regulation Of Monoatomic Ion Transmembrane Transport (GO:0034765)
Vacuolar Acidification (GO:0007035)
mRNA Splice Site Recognition (GO:0006376)
Morphogenesis Of An Epithelium (GO:0002009)
Negative Regulation Of DNA Binding (GO:0043392)
Positive Regulation Of Calcium Ion Transport (GO:0051928)
Positive Regulation Of Microtubule Polymerization (GO:0031116)
Positive Regulation Of Binding (GO:0051099)
Positive Regulation Of Protein Transport (GO:0051222)

Antigen Receptor-Mediated Signaling Pathway (GO:0050851)
Regulation Of Protein Phosphorylation (GO:0001932)
Blood Circulation (GO:0008015)
Negative Regulation Of Cell Projection Organization (GO:0031345)
Pyruvate Metabolic Process (GO:0006090)
Positive Regulation Of Signal Transduction (GO:0009967)
Kidney Development (GO:0001822)
Regulation Of Interleukin-6 Production (GO:0032675)
Regulation Of Macrophage Derived Foam Cell Differentiation (GO:0010743)
Response To Epidermal Growth Factor (GO:0070849)
Embryonic Skeletal System Morphogenesis (GO:0048704)
Immunoglobulin Mediated Immune Response (GO:0016064)
Negative Regulation Of Blood Coagulation (GO:0030195)
Negative Regulation Of Carbohydrate Metabolic Process (GO:0045912)
Negative Regulation Of Peptidyl-Tyrosine Phosphorylation (GO:0050732)
Positive Regulation Of Cell Communication (GO:0010647)
Positive Regulation Of Lipid Kinase Activity (GO:0090218)
Positive Regulation Of Phosphate Metabolic Process (GO:0045937)
Positive Regulation Of Cellular Component Biogenesis (GO:0044089)
Transforming Growth Factor Beta Receptor Signaling Pathway (GO:0007179)
Negative Regulation Of Catabolic Process (GO:0009895)
Sensory Perception (GO:0007600)
Positive Regulation Of DNA Recombination (GO:0045911)
Positive Regulation Of Protein Localization To Plasma Membrane (GO:1903078)
Negative Regulation Of Cellular Biosynthetic Process (GO:0031327)
Response To Lipopolysaccharide (GO:0032496)
Regulation Of Endocytic Recycling (GO:2001135)
Regulation Of Epithelial Cell Apoptotic Process (GO:1904035)
Actin Crosslink Formation (GO:0051764)
Regulation Of Leukocyte Tethering Or Rolling (GO:1903236)
Regulation Of Mesenchymal Cell Proliferation (GO:0010464)
Regulation Of Mitochondrial Fusion (GO:0010635)
Regulation Of Muscle System Process (GO:0090257)
Aspartate Family Amino Acid Catabolic Process (GO:0009068)
Brain Morphogenesis (GO:0048854)
cGMP Metabolic Process (GO:0046068)
Calcineurin-Mediated Signaling (GO:0097720)
Regulation Of Pseudopodium Assembly (GO:0031272)
Cell Differentiation In Hindbrain (GO:0021533)
Cell Part Morphogenesis (GO:0032990)
Cellular Response To Acetylcholine (GO:1905145)
Cellular Response To Fluid Shear Stress (GO:0071498)
Central Nervous System Myelination (GO:0022010)
Central Nervous System Projection Neuron Axonogenesis (GO:0021952)
Detection Of Light Stimulus Involved In Sensory Perception (GO:0050962)
Detection Of Light Stimulus Involved In Visual Perception (GO:0050908)
Dosage Compensation By Inactivation Of X Chromosome (GO:0009048)
Self Proteolysis (GO:0097264)

Smooth Muscle Cell Differentiation (GO:0051145)
sno(s)RNA 3'-End Processing (GO:0031126)
Sphingoid Metabolic Process (GO:0046519)
Epithelial Cell-Cell Adhesion (GO:0090136)
Telomere Maintenance In Response To DNA Damage (GO:0043247)
Intestinal Absorption (GO:0050892)
Intrinsic Apoptotic Signaling Pathway In Response To Oxidative Stress (GO:0008631)
Magnesium Ion Transport (GO:0015693)
Mast Cell Mediated Immunity (GO:0002448)
Mitochondrion Distribution (GO:0048311)
Negative Regulation Of Cellular Response To Vascular Endothelial Growth Factor Stimulus (GO:1902548)
Negative Regulation Of Fibrinolysis (GO:0051918)
Negative Regulation Of Leukocyte Chemotaxis (GO:0002689)
Negative Regulation Of Potassium Ion Transmembrane Transport (GO:1901380)
Negative Regulation Of Release Of Sequestered Calcium Ion Into Cytosol (GO:0051280)
Negative Regulation Of Ryanodine-Sensitive Calcium-Release Channel Activity (GO:0060315)
Negative Regulation Of Vascular Permeability (GO:0043116)
Negative Regulation Of Voltage-Gated Potassium Channel Activity (GO:1903817)
Nucleoside Phosphate Biosynthetic Process (GO:1901293)
Phagosome-Lysosome Fusion (GO:0090385)
Positive Regulation Of Behavior (GO:0048520)
Positive Regulation Of Endoplasmic Reticulum Unfolded Protein Response (GO:1900103)
Positive Regulation Of Metaphase/Anaphase Transition Of Cell Cycle (GO:1902101)
Positive Regulation Of Mitotic Cell Cycle Spindle Assembly Checkpoint (GO:0090267)
Positive Regulation Of Mitotic Metaphase/Anaphase Transition (GO:0045842)
Positive Regulation Of Muscle Contraction (GO:0045933)
Positive Regulation Of Pseudopodium Assembly (GO:0031274)
Positive Regulation Of Spindle Checkpoint (GO:0090232)
Positive Regulation Of Vascular Permeability (GO:0043117)
Presynapse Organization (GO:0099172)
Pyrimidine Nucleobase Metabolic Process (GO:0006206)
Regulation Of CD4-positive, Alpha-Beta T Cell Differentiation (GO:0043370)
Regulation Of Amyloid Precursor Protein Catabolic Process (GO:1902991)
Cellular Response To Reactive Oxygen Species (GO:0034614)
Regulation Of Rho Protein Signal Transduction (GO:0035023)
Regulation Of Dendrite Morphogenesis (GO:0048814)
Regulation Of Gluconeogenesis (GO:0006111)
Regulation Of Long-Term Synaptic Potentiation (GO:1900271)
Bile Acid Metabolic Process (GO:0008206)
Regulation Of Protein Autophosphorylation (GO:0031952)
Regulation Of Protein Localization To Cell Surface (GO:2000008)
Cellular Response To cAMP (GO:0071320)
Regulation Of Transmembrane Receptor Protein Serine/Threonine Kinase Signaling Pathway (GO:0090092)
Central Nervous System Neuron Differentiation (GO:0021953)
Modulation Of Excitatory Postsynaptic Potential (GO:0098815)
Negative Regulation Of DNA Biosynthetic Process (GO:2000279)
Negative Regulation Of Microtubule Polymerization Or Depolymerization (GO:0031111)
Phospholipid Translocation (GO:0045332)

Positive Regulation Of Cholesterol Transport (GO:0032376)
Positive Regulation Of Extrinsic Apoptotic Signaling Pathway (GO:2001238)
Positive Regulation Of Leukocyte Migration (GO:0002687)
Adenylate Cyclase-Inhibiting G Protein-Coupled Receptor Signaling Pathway (GO:0007193)
Cardiac Muscle Tissue Development (GO:0048738)
Cartilage Development (GO:0051216)
Positive Regulation Of B Cell Activation (GO:0050871)
Regulation Of Cell Development (GO:0060284)
T Cell Receptor Signaling Pathway (GO:0050852)
Cellular Response To Oxidative Stress (GO:0034599)
Protein Maturation (GO:0051604)
Gland Development (GO:0048732)
Regulation Of Cell Cycle G1/S Phase Transition (GO:1902806)
Positive Regulation Of Cell Cycle Process (GO:0090068)
Regulation Of Receptor Signaling Pathway Via JAK-STAT (GO:0046425)
Negative Regulation Of Metabolic Process (GO:0009892)
Negative Regulation Of Viral Genome Replication (GO:0045071)
Amide Transport (GO:0042886)
Regulation Of Metaphase/Anaphase Transition Of Cell Cycle (GO:1902099)
Cellular Nitrogen Compound Biosynthetic Process (GO:0044271)
Regulation Of Transcription By RNA Polymerase I (GO:0006356)
Regulation Of Transmembrane Transport (GO:0034762)
Regulation Of Ubiquitin-Protein Transferase Activity (GO:0051438)
Female Gamete Generation (GO:0007292)
Glycosphingolipid Metabolic Process (GO:0006687)
Muscle Cell Development (GO:0055001)
Negative Regulation Of Fat Cell Differentiation (GO:0045599)
Phosphatidylinositol 3-Kinase Signaling (GO:0014065)
Positive Regulation Of Synapse Assembly (GO:0051965)
Protein Transmembrane Import Into Intracellular Organelle (GO:0044743)
Adenylate Cyclase-Modulating G Protein-Coupled Receptor Signaling Pathway (GO:0007188)
Regulation Of Chemokine (C-X-C Motif) Ligand 2 Production (GO:2000341)
C-terminal Protein Amino Acid Modification (GO:0018410)
Regulation Of Chromosome Separation (GO:1905818)
C4-dicarboxylate Transport (GO:0015740)
DNA Conformation Change (GO:0071103)
V(D)J Recombination (GO:0033151)
Actin-Myosin Filament Sliding (GO:0033275)
Regulation Of Leukocyte Adhesion To Vascular Endothelial Cell (GO:1904994)
Aspartate Family Amino Acid Metabolic Process (GO:0009066)
Astrocyte Activation (GO:0048143)
Atrial Cardiac Muscle Cell Action Potential (GO:0086014)
Atrial Cardiac Muscle Tissue Development (GO:0003228)
cAMP Metabolic Process (GO:0046058)
Calcium Ion Import (GO:0070509)
Cardiac Right Ventricle Morphogenesis (GO:0003215)
Cardiolipin Metabolic Process (GO:0032048)
Cell-Cell Junction Maintenance (GO:0045217)

Regulation Of Retinoic Acid Receptor Signaling Pathway (GO:0048385)
Cellular Component Maintenance (GO:0043954)
Cellular Response To Acidic pH (GO:0071468)
Cellular Response To Leucine Starvation (GO:1990253)
Regulation Of Transcription From RNA Polymerase II Promoter In Response To Stress (GO:0043618)
snRNA Transcription (GO:0009301)
Sphingomyelin Metabolic Process (GO:0006684)
Spinal Cord Development (GO:0021510)
Steroid Catabolic Process (GO:0006706)
Eye Photoreceptor Cell Differentiation (GO:0001754)
Fatty Acid Transmembrane Transport (GO:1902001)
Folic Acid Metabolic Process (GO:0046655)
Glucose Import (GO:0046323)
Granulocyte Activation (GO:0036230)
Granulocyte Differentiation (GO:0030851)
Very Long-Chain Fatty Acid Biosynthetic Process (GO:0042761)
Lipid Droplet Formation (GO:0140042)
Microtubule Anchoring At Microtubule Organizing Center (GO:0072393)
Myelination In Peripheral Nervous System (GO:0022011)
Negative Regulation By Host Of Viral Process (GO:0044793)
Negative Regulation Of JUN Kinase Activity (GO:0043508)
Negative Regulation Of Cardiac Muscle Hypertrophy (GO:0010614)
Negative Regulation Of Coagulation (GO:0050819)
Negative Regulation Of Locomotion (GO:0040013)
Negative Regulation Of Mononuclear Cell Migration (GO:0071676)
Negative Regulation Of Potassium Ion Transport (GO:0043267)
Neutrophil Mediated Immunity (GO:0002446)
Nuclear Body Organization (GO:0030575)
Phosphate Ion Homeostasis (GO:0055062)
Polysaccharide Biosynthetic Process (GO:0000271)
Positive Regulation Of T-helper 1 Type Immune Response (GO:0002827)
Positive Regulation Of T-helper Cell Differentiation (GO:0045624)
Positive Regulation Of Cell Morphogenesis Involved In Differentiation (GO:0010770)
Positive Regulation Of Cellular Extravasation (GO:0002693)
Positive Regulation Of Fatty Acid Oxidation (GO:0046321)
Positive Regulation Of Insulin Receptor Signaling Pathway (GO:0046628)
Positive Regulation Of Integrin-Mediated Signaling Pathway (GO:2001046)
Positive Regulation Of mRNA Metabolic Process (GO:1903313)
Positive Regulation Of Myeloid Leukocyte Mediated Immunity (GO:0002888)
Positive Regulation Of Neuroinflammatory Response (GO:0150078)
Positive Regulation Of Neuron Migration (GO:2001224)
Positive Regulation Of Protein Localization To Cell Surface (GO:2000010)
Positive Regulation Of Purine Nucleotide Biosynthetic Process (GO:1900373)
Positive Regulation Of Regulated Secretory Pathway (GO:1903307)
Positive Regulation Of Sodium Ion Transmembrane Transporter Activity (GO:2000651)
Regulation Of T Cell Differentiation In Thymus (GO:0033081)
Regulation Of Cell Communication By Electrical Coupling (GO:0010649)
Regulation Of Protein Kinase Activity (GO:0045859)

ERBB Signaling Pathway (GO:0038127)
Apical Junction Assembly (GO:0043297)
Regulation Of Smooth Muscle Cell Proliferation (GO:0048660)
Epidermal Cell Differentiation (GO:0009913)
Negative Regulation Of Neuron Projection Development (GO:0010977)
Positive Regulation Of Protein Secretion (GO:0050714)
Vesicle-Mediated Transport (GO:0016192)
Carboxylic Acid Metabolic Process (GO:0019752)
ncRNA Catabolic Process (GO:0034661)
Negative Regulation Of Small Molecule Metabolic Process (GO:0062014)
Positive Regulation Of Notch Signaling Pathway (GO:0045747)
Positive Regulation Of Reproductive Process (GO:2000243)
Regulation Of G0 To G1 Transition (GO:0070316)
Regulation Of Activated T Cell Proliferation (GO:0046006)
Ceramide Metabolic Process (GO:0006672)
Axon Development (GO:0061564)
Actomyosin Structure Organization (GO:0031032)
Cell Morphogenesis Involved In Neuron Differentiation (GO:0048667)
Chloride Transport (GO:0006821)
Monoatomic Cation Homeostasis (GO:0055080)
Neutrophil Migration (GO:1990266)
Regulation Of T Cell Proliferation (GO:0042129)
Ras Protein Signal Transduction (GO:0007265)
Embryonic Skeletal System Development (GO:0048706)
Glial Cell Differentiation (GO:0010001)
Membrane Protein Proteolysis (GO:0033619)
Organophosphate Biosynthetic Process (GO:0090407)
Positive Regulation Of Carbohydrate Metabolic Process (GO:0045913)
Positive Regulation Of Immunoglobulin Production (GO:0002639)
Positive Regulation Of Neuron Death (GO:1901216)
Positive Regulation Of Transcription By RNA Polymerase I (GO:0045943)
Protein Targeting To Vacuole (GO:0006623)
ERBB2 Signaling Pathway (GO:0038128)
Regulation Of Fatty Acid Beta-Oxidation (GO:0031998)
Acute-Phase Response (GO:0006953)
Regulation Of Macrophage Cytokine Production (GO:0010935)
Regulation Of Myoblast Fusion (GO:1901739)
Regulation Of Nitric-Oxide Synthase Biosynthetic Process (GO:0051769)
Regulation Of Oxidoreductase Activity (GO:0051341)
Body Fluid Secretion (GO:0007589)
Cardiac Left Ventricle Morphogenesis (GO:0003214)
Regulation Of Response To Endoplasmic Reticulum Stress (GO:1905897)
Cellular Response To Arsenic-Containing Substance (GO:0071243)
Cellular Response To Interleukin-4 (GO:0071353)
Regulation Of Trophoblast Cell Migration (GO:1901163)
Chondroitin Sulfate Biosynthetic Process (GO:0030206)
Clathrin Coat Assembly (GO:0048268)
Response To Chemokine (GO:1990868)

Secondary Palate Development (GO:0062009)
Embryonic Hemopoiesis (GO:0035162)
Sphingoid Biosynthetic Process (GO:0046520)
Sphingosine Biosynthetic Process (GO:0046512)
Synaptic Vesicle Localization (GO:0097479)
Killing By Host Of Symbiont Cells (GO:0051873)
Membrane Depolarization (GO:0051899)
Monoacylglycerol Metabolic Process (GO:0046462)
Myeloid Dendritic Cell Activation (GO:0001773)
Negative Regulation Of T Cell Differentiation (GO:0045581)
Negative Regulation Of Cartilage Development (GO:0061037)
Negative Regulation Of Cell Activation (GO:0050866)
Negative Regulation Of Fibroblast Growth Factor Receptor Signaling Pathway (GO:0040037)
Outer Mitochondrial Membrane Organization (GO:0007008)
Plasma Membrane Tubulation (GO:0097320)
Positive Regulation Of Brown Fat Cell Differentiation (GO:0090336)
Positive Regulation Of Cellular Response To Insulin Stimulus (GO:1900078)
Positive Regulation Of Cyclase Activity (GO:0031281)
Positive Regulation Of Glycoprotein Biosynthetic Process (GO:0010560)
Positive Regulation Of Leukocyte Degranulation (GO:0043302)
Positive Regulation Of Type 2 Immune Response (GO:0002830)
Protein Hydroxylation (GO:0018126)
Protein Import Into Peroxisome Matrix (GO:0016558)
Protein Insertion Into Mitochondrial Outer Membrane (GO:0045040)
Regulation Of ERBB Signaling Pathway (GO:1901184)
Regulation Of Cell Fate Commitment (GO:0010453)
Regulation Of Cell Projection Organization (GO:0031344)
Regulation Of Cellular Ketone Metabolic Process (GO:0010565)
Response To UV (GO:0009411)
Regulation Of Mitotic Nuclear Division (GO:0007088)
Nucleotide-Excision Repair (GO:0006289)
Peptide Metabolic Process (GO:0006518)
post-Golgi Vesicle-Mediated Transport (GO:0006892)
Protein Modification Process (GO:0036211)
Visual Perception (GO:0007601)
Regulation Of Cold-Induced Thermogenesis (GO:0120161)
Positive Regulation Of Protein Localization To Membrane (GO:1905477)
Signal Transduction In Response To DNA Damage (GO:0042770)
Regulation Of Regulated Secretory Pathway (GO:1903305)
Response To Exogenous dsRNA (GO:0043330)
Positive Regulation Of Substrate Adhesion-Dependent Cell Spreading (GO:1900026)
Regulation Of Cardiac Muscle Contraction (GO:0055117)
Calcium Ion Homeostasis (GO:0055074)
Regulation Of Protein Secretion (GO:0050708)
Sensory Perception Of Light Stimulus (GO:0050953)
Calcium Ion Transmembrane Transport (GO:0070588)
Regulation Of Protein Localization To Plasma Membrane (GO:1903076)
2-Oxoglutarate Metabolic Process (GO:0006103)

Regulation Of Endothelial Cell Chemotaxis (GO:2001026)
Regulation Of Establishment Of Protein Localization (GO:0070201)
Regulation Of Exit From Mitosis (GO:0007096)
L-glutamate Transmembrane Transport (GO:0015813)
MHC Class II Protein Complex Assembly (GO:0002399)
Regulation Of Microglial Cell Activation (GO:1903978)
Regulation Of Non-Canonical Wnt Signaling Pathway (GO:2000050)
Bone Morphogenesis (GO:0060349)
Calcium Ion-Regulated Exocytosis Of Neurotransmitter (GO:0048791)
Cellular Response To ATP (GO:0071318)
Regulation Of Superoxide Anion Generation (GO:0032928)
Ceramide Transport (GO:0035627)
Respiratory Electron Transport Chain (GO:0022904)
Contractile Actin Filament Bundle Assembly (GO:0030038)
Endoderm Development (GO:0007492)
Establishment Of Chromosome Localization (GO:0051303)
Stress Fiber Assembly (GO:0043149)
Striated Muscle Tissue Development (GO:0014706)
Synaptonemal Complex Assembly (GO:0007130)
Synaptonemal Complex Organization (GO:0070193)
Genitalia Development (GO:0048806)
Histone mRNA Catabolic Process (GO:0071044)
miRNA-mediated Gene Silencing By Inhibition Of Translation (GO:0035278)
Mitochondrion Transport Along Microtubule (GO:0047497)
Mitotic intra-S DNA Damage Checkpoint Signaling (GO:0031573)
Monosaccharide Transmembrane Transport (GO:0015749)
Negative Regulation Of T Cell Mediated Immunity (GO:0002710)
Negative Regulation Of Androgen Receptor Signaling Pathway (GO:0060766)
Negative Regulation Of Chondrocyte Differentiation (GO:0032331)
Negative Regulation Of Transcription Regulatory Region DNA Binding (GO:2000678)
Pancreas Development (GO:0031016)
Peptide Antigen Assembly With MHC Class II Protein Complex (GO:0002503)
Phagolysosome Assembly (GO:0001845)
Polarized Epithelial Cell Differentiation (GO:0030859)
Positive Regulation Of Acute Inflammatory Response (GO:0002675)
Positive Regulation Of Endothelial Cell Chemotaxis (GO:2001028)
Positive Regulation Of Long-Term Synaptic Potentiation (GO:1900273)
Positive Regulation Of Lyase Activity (GO:0051349)
Positive Regulation Of Membrane Protein Ectodomain Proteolysis (GO:0051044)
Positive Regulation Of Nuclear-Transcribed mRNA Catabolic Process, Deadenylation-Dependent Decay (GO:190
Positive Regulation Of Telomere Maintenance In Response To DNA Damage (GO:1904507)
Protein Homotrimerization (GO:0070207)
Protein Localization To Vacuole (GO:0072665)
Protein Transport To Vacuole Involved In Ubiquitin-Dependent Protein Catabolic Process Via The Multivesicular
Protein Trimerization (GO:0070206)
Regulation Of Carbohydrate Catabolic Process (GO:0043470)
Regulation Of Phagocytosis (GO:0050764)
Response To Organic Cyclic Compound (GO:0014070)

Glycosaminoglycan Biosynthetic Process (GO:0006024)
Positive Regulation Of Mitochondrion Organization (GO:0010822)
Positive Regulation Of Type II Interferon Production (GO:0032729)
Proteolysis (GO:0006508)
Regulation Of Erythrocyte Differentiation (GO:0045646)
Regulation Of Intracellular Protein Transport (GO:0033157)
Blood Vessel Diameter Maintenance (GO:0097746)
Histone H3 Acetylation (GO:0043966)
Negative Regulation Of T Cell Proliferation (GO:0042130)
Positive Regulation Of RNA Splicing (GO:0033120)
Positive Regulation Of Double-Strand Break Repair Via Homologous Recombination (GO:1905168)
Positive Regulation Of Myeloid Cell Differentiation (GO:0045639)
Regulation Of Interleukin-8 Production (GO:0032677)
Activation Of Protein Kinase Activity (GO:0032147)
Regulation Of Inflammatory Response (GO:0050727)
Anterior/Posterior Pattern Specification (GO:0009952)
Microtubule Cytoskeleton Organization Involved In Mitosis (GO:1902850)
Positive Regulation Of Proteolysis (GO:0045862)
Regulation Of Tumor Necrosis Factor Production (GO:0032680)
Regulation Of Phosphorylation (GO:0042325)
Sodium Ion Transmembrane Transport (GO:0035725)
Cellular Response To Organonitrogen Compound (GO:0071417)
Negative Regulation Of MAPK Cascade (GO:0043409)
RNA Stabilization (GO:0043489)
Regulation Of p38MAPK Cascade (GO:1900744)
Regulation Of Sodium Ion Transmembrane Transporter Activity (GO:2000649)
Synaptic Vesicle Cycle (GO:0099504)
Melanosome Organization (GO:0032438)
Muscle Tissue Development (GO:0060537)
Negative Regulation Of Smooth Muscle Cell Proliferation (GO:0048662)
Negative Regulation Of Transferase Activity (GO:0051348)
Protein Acylation (GO:0043543)
Regulation Of Cellular Component Biogenesis (GO:0044087)
Regulation Of Clathrin-Dependent Endocytosis (GO:2000369)
Regulation Of Dephosphorylation (GO:0035303)
L-glutamate Import (GO:0051938)
Regulation Of Isotype Switching (GO:0045191)
Regulation Of mRNA Metabolic Process (GO:1903311)
Regulation Of Monocyte Differentiation (GO:0045655)
Regulation Of Multicellular Organism Growth (GO:0040014)
Regulation Of Systemic Arterial Blood Pressure (GO:0003073)
Regulation Of Systemic Arterial Blood Pressure By Hormone (GO:0001990)
Cellular Response To Sterol (GO:0036315)
Coenzyme A Metabolic Process (GO:0015936)
tRNA Wobble Base Modification (GO:0002097)
tRNA Wobble Uridine Modification (GO:0002098)
Glutamine Family Amino Acid Catabolic Process (GO:0009065)
Keratan Sulfate Biosynthetic Process (GO:0018146)

mRNA Methylation (GO:0080009)
miRNA Metabolic Process (GO:0010586)
Negative Regulation Of Cell-Substrate Junction Organization (GO:0150118)
Negative Regulation Of Focal Adhesion Assembly (GO:0051895)
Negative Regulation Of Gluconeogenesis (GO:0045721)
Negative Regulation Of Telomere Maintenance (GO:0032205)
Nitric Oxide Biosynthetic Process (GO:0006809)
Positive Regulation Of Collagen Biosynthetic Process (GO:0032967)
Positive Regulation Of Glucose Metabolic Process (GO:0010907)
Positive Regulation Of Immunoglobulin Mediated Immune Response (GO:0002891)
Positive Regulation Of Muscle Cell Differentiation (GO:0051149)
Positive Regulation Of Secretion (GO:0051047)
Positive Regulation Of Transcription By RNA Polymerase III (GO:0045945)
Presynapse Assembly (GO:0099054)
Receptor Catabolic Process (GO:0032801)
Regulation Of Activin Receptor Signaling Pathway (GO:0032925)
Regulation Of Amyloid-Beta Clearance (GO:1900221)
Sterol Metabolic Process (GO:0016125)
Positive Regulation Of Ubiquitin-Dependent Protein Catabolic Process (GO:2000060)
G2/M Transition Of Mitotic Cell Cycle (GO:0000086)
Regulation Of Intracellular Transport (GO:0032386)
Signal Release From Synapse (GO:0099643)
Tumor Necrosis Factor-Mediated Signaling Pathway (GO:0033209)
Import Into Cell (GO:0098657)
Membrane Assembly (GO:0071709)
Negative Regulation Of Epithelial Cell Apoptotic Process (GO:1904036)
Positive Regulation Of Bone Mineralization (GO:0030501)
BMP Signaling Pathway (GO:0030509)
Negative Regulation Of Viral Process (GO:0048525)
Cellular Response To Oxygen-Containing Compound (GO:1901701)
Positive Regulation Of T Cell Activation (GO:0050870)
Glycerophospholipid Biosynthetic Process (GO:0046474)
Positive Regulation Of Metabolic Process (GO:0009893)
Positive Regulation Of Amide Metabolic Process (GO:0034250)
RNA Methylation (GO:0001510)
DNA Repair-Dependent Chromatin Remodeling (GO:0140861)
DNA-templated Transcription Termination (GO:0006353)
Regulation Of Establishment Of Endothelial Barrier (GO:1903140)
Regulation Of Fibrinolysis (GO:0051917)
Antifungal Innate Immune Response (GO:0061760)
Regulation Of Nucleocytoplasmic Transport (GO:0046822)
Regulation Of Protein Kinase C Signaling (GO:0090036)
Cell Communication By Electrical Coupling Involved In Cardiac Conduction (GO:0086064)
Regulation Of Secretion (GO:0051046)
Dicarboxylic Acid Catabolic Process (GO:0043649)
Endoplasmic Reticulum Tubular Network Organization (GO:0071786)
Stabilization Of Membrane Potential (GO:0030322)
Establishment Of Melanosome Localization (GO:0032401)

Lymphocyte Mediated Immunity (GO:0002449)
Maturation Of 5.8S rRNA (GO:0000460)
Negative Regulation Of Inflammatory Response To Antigenic Stimulus (GO:0002862)
Negative Regulation Of Interleukin-10 Production (GO:0032693)
Nitric Oxide Metabolic Process (GO:0046209)
Nuclear Migration (GO:0007097)
Oligodendrocyte Development (GO:0014003)
Pharyngeal System Development (GO:0060037)
Positive Regulation By Host Of Viral Transcription (GO:0043923)
Positive Regulation Of Cell Migration Involved In Sprouting Angiogenesis (GO:0090050)
Positive Regulation Of Collagen Metabolic Process (GO:0010714)
Positive Regulation Of Positive Chemotaxis (GO:0050927)
Positive Regulation Of Telomere Capping (GO:1904355)
Protein Targeting To Peroxisome (GO:0006625)
Regulation Of B Cell Receptor Signaling Pathway (GO:0050855)
Regulation Of DNA Strand Elongation (GO:0060382)
Regulation Of Cardiac Muscle Contraction By Regulation Of The Release Of Sequestered Calcium Ion (GO:0010[illegible]
Vitamin Transport (GO:0051180)
Negative Regulation Of Lymphocyte Proliferation (GO:0050672)
Positive Regulation Of ATP-dependent Activity (GO:0032781)
Positive Regulation Of Cytokinesis (GO:0032467)
Positive Regulation Of Intracellular Transport (GO:0032388)
Negative Regulation Of Cytokine Production (GO:0001818)
Regulation Of Mitotic Cell Cycle Phase Transition (GO:1901990)
Negative Regulation Of Binding (GO:0051100)
Regulation Of Cellular Catabolic Process (GO:0031329)
Adenylate Cyclase-Activating G Protein-Coupled Receptor Signaling Pathway (GO:0007189)
Regulation Of Cytosolic Calcium Ion Concentration (GO:0051480)
L-alpha-amino Acid Transmembrane Transport (GO:1902475)
Regulation Of Glycolytic Process (GO:0006110)
Regulation Of Microtubule Polymerization (GO:0031113)
Cell Cycle G2/M Phase Transition (GO:0044839)
Regulation Of Small Molecule Metabolic Process (GO:0062012)
Retinoid Metabolic Process (GO:0001523)
Negative Regulation Of Secretion By Cell (GO:1903531)
Nucleic Acid-Templated Transcription (GO:0097659)
Positive Regulation Of Catalytic Activity (GO:0043085)
Positive Regulation Of Cell Cycle G1/S Phase Transition (GO:1902808)
Positive Regulation Of Stem Cell Population Maintenance (GO:1902459)
Positive Regulation Of Wound Healing (GO:0090303)
Regulation Of Type II Interferon Production (GO:0032649)
Defense Response To Bacterium (GO:0042742)
Positive Regulation Of T Cell Differentiation (GO:0045582)
Positive Regulation Of Cysteine-Type Endopeptidase Activity (GO:2001056)
Regulation Of Cellular Respiration (GO:0043457)
G Protein-Coupled Acetylcholine Receptor Signaling Pathway (GO:0007213)
NAD Biosynthetic Process (GO:0009435)
Regulation Of Hematopoietic Progenitor Cell Differentiation (GO:1901532)

Rac Protein Signal Transduction (GO:0016601)
Regulation Of Inflammatory Response To Antigenic Stimulus (GO:0002861)
Regulation Of Leukocyte Chemotaxis (GO:0002688)
Amyloid Precursor Protein Metabolic Process (GO:0042982)
Regulation Of Mitotic Cell Cycle Spindle Assembly Checkpoint (GO:0090266)
cGMP-mediated Signaling (GO:0019934)
Regulation Of Receptor Recycling (GO:0001919)
Regulation Of Single Stranded Viral RNA Replication Via Double Stranded DNA Intermediate (GO:0045091)
Regulation Of Telomerase RNA Localization To Cajal Body (GO:1904872)
Regulation Of Telomere Maintenance Via Telomere Lengthening (GO:1904356)
Regulation Of Vacuole Organization (GO:0044088)
Central Nervous System Neuron Development (GO:0021954)
Detection Of Visible Light (GO:0009584)
Diol Biosynthetic Process (GO:0034312)
Transcription By RNA Polymerase I (GO:0006360)
Transepithelial Transport (GO:0070633)
Triglyceride Biosynthetic Process (GO:0019432)
Ventricular Cardiac Muscle Cell Action Potential (GO:0086005)
Keratan Sulfate Metabolic Process (GO:0042339)
Leukocyte Tethering Or Rolling (GO:0050901)
Mesenchyme Development (GO:0060485)
Negative Regulation Of Rho Protein Signal Transduction (GO:0035024)
Negative Regulation Of Fibroblast Proliferation (GO:0048147)
Negative Regulation Of Interleukin-8 Production (GO:0032717)
Negative Regulation Of Ubiquitin-Protein Transferase Activity (GO:0051444)
Nicotinamide Nucleotide Metabolic Process (GO:0046496)
Nuclear Envelope Organization (GO:0006998)
Peroxisomal Membrane Transport (GO:0015919)
Polyol Metabolic Process (GO:0019751)
Positive Regulation Of Amyloid-Beta Formation (GO:1902004)
Positive Regulation Of Cardiac Muscle Hypertrophy (GO:0010613)
Positive Regulation Of Cartilage Development (GO:0061036)
Positive Regulation Of Cell Adhesion Mediated By Integrin (GO:0033630)
Positive Regulation Of Cell Cycle Phase Transition (GO:1901989)
Positive Regulation Of Dendrite Extension (GO:1903861)
Positive Regulation Of Dendrite Morphogenesis (GO:0050775)
Positive Regulation Of Heart Rate (GO:0010460)
Positive Regulation Of Nitric-Oxide Synthase Activity (GO:0051000)
Positive Regulation Of Peptidyl-Lysine Acetylation (GO:2000758)
Positive Regulation Of Regulatory T Cell Differentiation (GO:0045591)
Positive Regulation Of Small GTPase Mediated Signal Transduction (GO:0051057)
Positive Regulation Of Transcription From RNA Polymerase II Promoter In Response To Stress (GO:0036003)
Prostaglandin Biosynthetic Process (GO:0001516)
Receptor Recycling (GO:0001881)
Regulation Of DNA Methylation-Dependent Heterochromatin Formation (GO:0090308)
Regulation Of Brown Fat Cell Differentiation (GO:0090335)
Regulation Of Cartilage Development (GO:0061035)
Regulation Of Transforming Growth Factor Beta Receptor Signaling Pathway (GO:0017015)

Cellular Response To Lipid (GO:0071396)
Regulation Of Cell Cycle (GO:0051726)
Regulation Of Dendrite Development (GO:0050773)
Regulation Of Microtubule-Based Process (GO:0032886)
Synaptic Vesicle Exocytosis (GO:0016079)
Negative Regulation Of mRNA Catabolic Process (GO:1902373)
Positive Regulation Of Actin Filament Polymerization (GO:0030838)
Small GTPase Mediated Signal Transduction (GO:0007264)
Antimicrobial Humoral Immune Response Mediated By Antimicrobial Peptide (GO:0061844)
Monocarboxylic Acid Biosynthetic Process (GO:0072330)
Cellular Response To Chemical Stress (GO:0062197)
Sodium Ion Transport (GO:0006814)
Organic Anion Transport (GO:0015711)
Positive Regulation Of DNA Repair (GO:0045739)
Regulation Of Osteoclast Differentiation (GO:0045670)
Cellular Response To Amino Acid Starvation (GO:0034198)
Ceramide Biosynthetic Process (GO:0046513)
Skeletal Muscle Tissue Development (GO:0007519)
Translational Elongation (GO:0006414)
Lysosome Localization (GO:0032418)
Monoatomic Cation Transport (GO:0006812)
Potassium Ion Import Across Plasma Membrane (GO:1990573)
Regulation Of Collagen Biosynthetic Process (GO:0032965)
Regulation Of Dendrite Extension (GO:1903859)
Regulation Of Exosomal Secretion (GO:1903541)
T-helper Cell Differentiation (GO:0042093)
Regulation Of Mitochondrial Outer Membrane Permeabilization Involved In Apoptotic Signaling Pathway (GO:19[illegible]
Antigen Processing And Presentation Of Peptide Antigen Via MHC Class I (GO:0002474)
Associative Learning (GO:0008306)
Regulation Of Protein Deacetylation (GO:0090311)
Regulation Of Synaptic Vesicle Exocytosis (GO:2000300)
Cellular Response To Inorganic Substance (GO:0071241)
Response To Axon Injury (GO:0048678)
Response To Cholesterol (GO:0070723)
Endothelial Cell Proliferation (GO:0001935)
Epithelial Cell Development (GO:0002064)
Establishment Of Vesicle Localization (GO:0051650)
Glycerolipid Catabolic Process (GO:0046503)
Vesicle-Mediated Transport In Synapse (GO:0099003)
Icosanoid Biosynthetic Process (GO:0046456)
Lysosomal Lumen Acidification (GO:0007042)
Melanosome Localization (GO:0032400)
Negative Regulation Of DNA-templated Transcription, Elongation (GO:0032785)
Negative Regulation Of T Cell Receptor Signaling Pathway (GO:0050860)
Negative Regulation Of Amyloid Precursor Protein Catabolic Process (GO:1902992)
Negative Regulation Of Lipid Catabolic Process (GO:0050995)
Negative Regulation Of Lipid Storage (GO:0010888)
Negative Regulation Of Monoatomic Ion Transport (GO:0043271)

Negative Regulation Of Peptide Hormone Secretion (GO:0090278)
Neuromuscular Process (GO:0050905)
Neuron Fate Commitment (GO:0048663)
Neutrophil Activation (GO:0042119)
Peptide Antigen Assembly With MHC Protein Complex (GO:0002501)
Positive Regulation Of G Protein-Coupled Receptor Signaling Pathway (GO:0045745)
Positive Regulation Of Chromosome Segregation (GO:0051984)
Positive Regulation Of Extracellular Matrix Organization (GO:1903055)
Positive Regulation Of Interleukin-4 Production (GO:0032753)
Positive Regulation Of Osteoclast Differentiation (GO:0045672)
Positive Regulation Of Response To Endoplasmic Reticulum Stress (GO:1905898)
Protein Import Into Mitochondrial Matrix (GO:0030150)
Regulation Of DNA Damage Checkpoint (GO:2000001)
Regulation Of RIG-I Signaling Pathway (GO:0039535)
Regulation Of Cell Cycle G2/M Phase Transition (GO:1902749)
Response To Reactive Oxygen Species (GO:0000302)
Negative Regulation Of Endopeptidase Activity (GO:0010951)
Peptidyl-Threonine Modification (GO:0018210)
Protein Stabilization (GO:0050821)
Regulation Of Viral Genome Replication (GO:0045069)
Extrinsic Apoptotic Signaling Pathway (GO:0097191)
DNA-templated DNA Replication Maintenance Of Fidelity (GO:0045005)
Intracellular Glucose Homeostasis (GO:0001678)
Intracellular Steroid Hormone Receptor Signaling Pathway (GO:0030518)
mRNA Destabilization (GO:0061157)
Neurotransmitter Secretion (GO:0007269)
Regulation Of Cellular Component Size (GO:0032535)
Intracellular Calcium Ion Homeostasis (GO:0006874)
Regulation Of Cellular Response To Growth Factor Stimulus (GO:0090287)
DNA Catabolic Process (GO:0006308)
Regulation Of Fibroblast Growth Factor Receptor Signaling Pathway (GO:0040036)
NADP Metabolic Process (GO:0006739)
Regulation Of Granulocyte Chemotaxis (GO:0071622)
RNA-templated DNA Biosynthetic Process (GO:0006278)
Regulation Of Lymphocyte Differentiation (GO:0045619)
Regulation Of Megakaryocyte Differentiation (GO:0045652)
Regulation Of Nuclear Division (GO:0051783)
Calcium-Independent Cell-Cell Adhesion Via Plasma Membrane Cell-Adhesion Molecules (GO:0016338)
Cellular Response To Catecholamine Stimulus (GO:0071870)
Regulation Of Transcription Involved In G1/S Transition Of Mitotic Cell Cycle (GO:0000083)
Regulation Of Vascular Endothelial Growth Factor Signaling Pathway (GO:1900746)
Chondroitin Sulfate Proteoglycan Biosynthetic Process (GO:0050650)
Cyclic Nucleotide Biosynthetic Process (GO:0009190)
Embryonic Digestive Tract Development (GO:0048566)
Skeletal Muscle Contraction (GO:0003009)
snRNA Processing (GO:0016180)
Sphingosine Metabolic Process (GO:0006670)
Eye Morphogenesis (GO:0048592)

Telomere Maintenance Via Telomerase (GO:0007004)
Ganglioside Metabolic Process (GO:0001573)
Heme Biosynthetic Process (GO:0006783)
Immunoglobulin Production Involved In Immunoglobulin-Mediated Immune Response (GO:0002381)
Inositol Phosphate-Mediated Signaling (GO:0048016)
Insulin Secretion (GO:0030073)
Interleukin-1-Mediated Signaling Pathway (GO:0070498)
Leukocyte Adhesion To Vascular Endothelial Cell (GO:0061756)
Leukotriene Biosynthetic Process (GO:0019370)
Negative Regulation Of Cyclin-Dependent Protein Serine/Threonine Kinase Activity (GO:0045736)
Negative Regulation Of Protein Dephosphorylation (GO:0035308)
Negative Regulation Of Telomere Maintenance Via Telomerase (GO:0032211)
Nucleotide Biosynthetic Process (GO:0009165)
Positive Regulation Of Activated T Cell Proliferation (GO:0042104)
Positive Regulation Of calcineurin-NFAT Signaling Cascade (GO:0070886)
Positive Regulation Of Calcineurin-Mediated Signaling (GO:0106058)
Positive Regulation Of Mitochondrial Fission (GO:0090141)
rRNA Catabolic Process (GO:0016075)
Receptor Clustering (GO:0043113)
Regulation Of Autophagy Of Mitochondrion (GO:1903146)
Telomere Maintenance (GO:0000723)
Regulation Of Transport (GO:0051049)
Embryonic Organ Morphogenesis (GO:0048562)
Extracellular Matrix Disassembly (GO:0022617)
Nucleus Organization (GO:0006997)
Phosphatidylinositol Phosphate Biosynthetic Process (GO:0046854)
Positive Regulation Of Nucleocytoplasmic Transport (GO:0046824)
Positive Regulation Of Osteoblast Differentiation (GO:0045669)
Regulation Of DNA-templated Transcription Elongation (GO:0032784)
Protein Processing (GO:0016485)
Negative Regulation Of Amide Metabolic Process (GO:0034249)
Positive Regulation Of Protein Kinase B Signaling (GO:0051897)
Endocytosis (GO:0006897)
Regulation Of Monoatomic Ion Transmembrane Transporter Activity (GO:0032412)
Phagocytosis (GO:0006909)
Regulation Of Protein Binding (GO:0043393)
Regulation Of Cellular Metabolic Process (GO:0031323)
I-kappaB kinase/NF-kappaB Signaling (GO:0007249)
Chromosome Organization (GO:0051276)
Establishment Of Protein Localization To Extracellular Region (GO:0035592)
Hexose Metabolic Process (GO:0019318)
Regulation Of Apoptotic Signaling Pathway (GO:2001233)
DNA Methylation (GO:0006306)
Fc-gamma Receptor Signaling Pathway Involved In Phagocytosis (GO:0038096)
Regulation Of Fibroblast Migration (GO:0010762)
T Cell Mediated Immunity (GO:0002456)
Regulation Of Macrophage Chemotaxis (GO:0010758)
Ameboidal-Type Cell Migration (GO:0001667)

Regulation Of Meiotic Cell Cycle (GO:0051445)
Regulation Of Membrane Depolarization (GO:0003254)
Regulation Of Necroptotic Process (GO:0060544)
Calcium-Mediated Signaling Using Intracellular Calcium Source (GO:0035584)
Regulation Of Spindle Organization (GO:0090224)
Cellular Response To Copper Ion (GO:0071280)
Cellular Response To pH (GO:0071467)
Response To Ketone (GO:1901654)
Sensory Perception Of Pain (GO:0019233)
Endoplasmic Reticulum Calcium Ion Homeostasis (GO:0032469)
Epithelial Cilium Movement Involved In Extracellular Fluid Movement (GO:0003351)
Hydrogen Peroxide Catabolic Process (GO:0042744)
Lipopolysaccharide-Mediated Signaling Pathway (GO:0031663)
Long-Term Synaptic Potentiation (GO:0060291)
Microglial Cell Activation (GO:0001774)
Monocarboxylic Acid Catabolic Process (GO:0072329)
Negative Regulation Of B Cell Activation (GO:0050869)
Negative Regulation Of Cyclin-Dependent Protein Kinase Activity (GO:1904030)
Negative Regulation Of Phosphoprotein Phosphatase Activity (GO:0032515)
Negative Regulation Of Protein Localization To Cell Periphery (GO:1904376)
Negative Regulation Of Protein Localization To Plasma Membrane (GO:1903077)
Phosphatidylglycerol Metabolic Process (GO:0046471)
Phosphatidylserine Metabolic Process (GO:0006658)
Positive Regulation Of Antigen Receptor-Mediated Signaling Pathway (GO:0050857)
Positive Regulation Of Embryonic Development (GO:0040019)
Positive Regulation Of Epithelial Cell Differentiation (GO:0030858)
Positive Regulation Of Lipid Catabolic Process (GO:0050996)
Positive Regulation Of Phospholipase Activity (GO:0010518)
Positive Regulation Of Vascular Associated Smooth Muscle Cell Proliferation (GO:1904707)
Receptor Metabolic Process (GO:0043112)
Regulation Of Calcium Ion Import (GO:0090279)
Regulation Of Calcium Ion Transmembrane Transport (GO:1903169)
Sulfur Compound Biosynthetic Process (GO:0044272)
Negative Regulation Of Epithelial Cell Proliferation (GO:0050680)
Neutrophil Chemotaxis (GO:0030593)
Positive Regulation Of Protein Polymerization (GO:0032273)
Regulation Of G Protein-Coupled Receptor Signaling Pathway (GO:0008277)
T Cell Differentiation (GO:0030217)
Cardiac Conduction (GO:0061337)
Cellular Component Disassembly (GO:0022411)
Replication Fork Processing (GO:0031297)
Intracellular Protein Transmembrane Transport (GO:0065002)
Myofibril Assembly (GO:0030239)
Regulation Of Cilium Assembly (GO:1902017)
Regulation Of Double-Strand Break Repair Via Homologous Recombination (GO:0010569)
Positive Regulation Of Supramolecular Fiber Organization (GO:1902905)
DNA Replication Initiation (GO:0006270)
Regulation Of Lipid Storage (GO:0010883)

Regulation Of Membrane Protein Ectodomain Proteolysis (GO:0051043)
Antigen Processing And Presentation Of Endogenous Peptide Antigen (GO:0002483)
Regulation Of Steroid Metabolic Process (GO:0019218)
Regulation Of Transferase Activity (GO:0051338)
snRNA Metabolic Process (GO:0016073)
Sodium Ion Import Across Plasma Membrane (GO:0098719)
Histone H2A Acetylation (GO:0043968)
Maturation Of 5.8S rRNA From Tricistronic rRNA Transcript (SSU-rRNA, 5.8S rRNA, LSU-rRNA) (GO:0000466)
Negative Regulation Of Interleukin-2 Production (GO:0032703)
Negative Regulation Of Osteoclast Differentiation (GO:0045671)
poly(A)+ mRNA Export From Nucleus (GO:0016973)
Porphyrin-Containing Compound Biosynthetic Process (GO:0006779)
Positive Regulation Of Amyloid Precursor Protein Catabolic Process (GO:1902993)
Positive Regulation Of Blood Coagulation (GO:0030194)
Positive Regulation Of Dendritic Spine Development (GO:0060999)
Positive Regulation Of Transcription Regulatory Region DNA Binding (GO:2000679)
Regulation Of Cell-Substrate Junction Assembly (GO:0090109)
Plasma Membrane Organization (GO:0007009)
Positive Regulation Of Protein Localization To Nucleus (GO:1900182)
Negative Regulation Of Hydrolase Activity (GO:0051346)
Positive Regulation Of TOR Signaling (GO:0032008)
Protein Monoubiquitination (GO:0006513)
Regulation Of T Cell Differentiation (GO:0045580)
Receptor-Mediated Endocytosis (GO:0006898)
Chromatin Organization (GO:0006325)
Granulocyte Chemotaxis (GO:0071621)
Positive Regulation Of Secretion By Cell (GO:1903532)
Positive Regulation Of Cold-Induced Thermogenesis (GO:0120162)
Regulation Of Tumor Necrosis Factor-Mediated Signaling Pathway (GO:0010803)
Chloride Transmembrane Transport (GO:1902476)
Positive Regulation Of Reactive Oxygen Species Metabolic Process (GO:2000379)
Post-Translational Protein Modification (GO:0043687)
Regulation Of DNA Binding (GO:0051101)
Fc Receptor Mediated Stimulatory Signaling Pathway (GO:0002431)
Regulation Of Intracellular Steroid Hormone Receptor Signaling Pathway (GO:0033143)
Acetylcholine Receptor Signaling Pathway (GO:0095500)
Regulation Of Release Of Sequestered Calcium Ion Into Cytosol By Sarcoplasmic Reticulum (GO:0010880)
Regulation Of Telomere Capping (GO:1904353)
Cytoskeleton-Dependent Intracellular Transport (GO:0030705)
Dendritic Cell Differentiation (GO:0097028)
snRNA 3'-End Processing (GO:0034472)
Homologous Chromosome Segregation (GO:0045143)
Modulation By Host Of Viral Process (GO:0044788)
Negative Regulation Of G Protein-Coupled Receptor Signaling Pathway (GO:0045744)
Negative Regulation Of Myeloid Cell Differentiation (GO:0045638)
Oxoacid Metabolic Process (GO:0043436)
peptidyl-L-cysteine S-palmitoylation (GO:0018230)
peptidyl-S-diacylglycerol-L-cysteine Biosynthetic Process From Peptidyl-Cysteine (GO:0018231)

Peptidyl-Glutamic Acid Modification (GO:0018200)
Positive Regulation Of Cholesterol Efflux (GO:0010875)
Positive Regulation Of Cilium Assembly (GO:0045724)
Positive Regulation Of Receptor Internalization (GO:0002092)
Protein Dephosphorylation (GO:0006470)
Antibacterial Humoral Response (GO:0019731)
Negative Regulation Of DNA Metabolic Process (GO:0051053)
Negative Regulation Of Tumor Necrosis Factor Superfamily Cytokine Production (GO:1903556)
Positive Regulation Of Nervous System Development (GO:0051962)
Response To Cytokine (GO:0034097)
Negative Regulation Of Cell Growth (GO:0030308)
Negative Regulation Of Phosphorylation (GO:0042326)
Regulation Of DNA Replication (GO:0006275)
Neuron Development (GO:0048666)
Regulation Of Centrosome Cycle (GO:0046605)
Regulation Of Cholesterol Metabolic Process (GO:0090181)
Regulation Of Lipid Catabolic Process (GO:0050994)
Adaptive Immune Response Based On Somatic Recombination Of Immune Receptors Built From Immunoglobu
Artery Development (GO:0060840)
Regulation Of Nuclear-Transcribed mRNA Catabolic Process, Deadenylation-Dependent Decay (GO:1900151)
Cellular Lipid Catabolic Process (GO:0044242)
Rescue Of Stalled Ribosome (GO:0072344)
Histone mRNA Metabolic Process (GO:0008334)
Immune Response-Activating Cell Surface Receptor Signaling Pathway (GO:0002429)
Negative Regulation Of Neuron Differentiation (GO:0045665)
Negative Regulation Of Oxidative Stress-Induced Cell Death (GO:1903202)
Positive Regulation Of Morphogenesis Of An Epithelium (GO:1905332)
Positive Regulation Of Neuron Apoptotic Process (GO:0043525)
Positive Regulation Of Oxidoreductase Activity (GO:0051353)
Positive Regulation Of Protein Targeting To Membrane (GO:0090314)
Protein N-linked Glycosylation Via Asparagine (GO:0018279)
Regulation Of Cardiac Muscle Hypertrophy (GO:0010611)
Positive Regulation Of I-kappaB kinase/NF-kappaB Signaling (GO:0043123)
Hemopoiesis (GO:0030097)
G Protein-Coupled Receptor Signaling Pathway, Coupled To Cyclic Nucleotide Second Messenger (GO:000718
Regulation Of Mitochondrion Organization (GO:0010821)
Steroid Biosynthetic Process (GO:0006694)
Positive Regulation Of Cell Death (GO:0010942)
Protein Catabolic Process (GO:0030163)
Sphingolipid Biosynthetic Process (GO:0030148)
Negative Regulation Of Blood Vessel Morphogenesis (GO:2000181)
Regulation Of Cysteine-Type Endopeptidase Activity (GO:2000116)
Regulation Of Cytokine Production Involved In Immune Response (GO:0002718)
ERK1 And ERK2 Cascade (GO:0070371)
Regulation Of Endoplasmic Reticulum Stress-Induced Intrinsic Apoptotic Signaling Pathway (GO:1902235)
Fc-gamma Receptor Signaling Pathway (GO:0038094)
Regulation Of Execution Phase Of Apoptosis (GO:1900117)
Acylglycerol Catabolic Process (GO:0046464)

Regulation Of Ruffle Assembly (GO:1900027)
Regulation Of Ryanodine-Sensitive Calcium-Release Channel Activity (GO:0060314)
Response To Copper Ion (GO:0046688)
Response To Hexose (GO:0009746)
Stress Granule Assembly (GO:0034063)
mRNA Polyadenylation (GO:0006378)
Multivesicular Body Sorting Pathway (GO:0071985)
Negative Regulation Of Antigen Receptor-Mediated Signaling Pathway (GO:0050858)
Negative Regulation Of Ion Transmembrane Transporter Activity (GO:0032413)
Negative Regulation Of Ossification (GO:0030279)
Neuromuscular Junction Development (GO:0007528)
Positive Regulation Of Interleukin-17 Production (GO:0032740)
Positive Regulation Of Peptidyl-Threonine Phosphorylation (GO:0010800)
RNA Modification (GO:0009451)
Amide Biosynthetic Process (GO:0043604)
Regulation Of Release Of Sequestered Calcium Ion Into Cytosol (GO:0051279)
Regulation Of Substrate Adhesion-Dependent Cell Spreading (GO:1900024)
Metaphase Plate Congression (GO:0051310)
Negative Regulation Of MAP Kinase Activity (GO:0043407)
Positive Regulation Of Ras Protein Signal Transduction (GO:0046579)
Receptor Internalization (GO:0031623)
Inorganic Cation Import Across Plasma Membrane (GO:0098659)
Chromatin Remodeling (GO:0006338)
Negative Regulation Of Protein Modification Process (GO:0031400)
Positive Regulation Of Tumor Necrosis Factor Production (GO:0032760)
Rho Protein Signal Transduction (GO:0007266)
Regulation Of Protein Localization To Nucleus (GO:1900180)
Regulation Of Proteolysis (GO:0030162)
Cell Cycle G1/S Phase Transition (GO:0044843)
Icosanoid Metabolic Process (GO:0006690)
Positive Regulation Of Telomere Maintenance (GO:0032206)
ATP Biosynthetic Process (GO:0006754)
Regulation Of Chondrocyte Differentiation (GO:0032330)
RNA Polyadenylation (GO:0043631)
Activation Of Protein Kinase B Activity (GO:0032148)
Regulation Of Lysosomal Lumen pH (GO:0035751)
Regulation Of Monocyte Chemotaxis (GO:0090025)
Bile Acid Biosynthetic Process (GO:0006699)
Regulation Of Platelet Aggregation (GO:0090330)
Cardiac Conduction System Development (GO:0003161)
Chemical Synaptic Transmission, Postsynaptic (GO:0099565)
Sterol Transport (GO:0015918)
Vesicle-Mediated Transport Between Endosomal Compartments (GO:0098927)
Late Endosome To Vacuole Transport Via Multivesicular Body Sorting Pathway (GO:0032511)
Negative Regulation Of Intrinsic Apoptotic Signaling Pathway In Response To DNA Damage (GO:1902230)
Negative Regulation Of Protein Localization To Membrane (GO:1905476)
Negative Regulation Of Response To Endoplasmic Reticulum Stress (GO:1903573)
Negative Regulation Of Signaling Receptor Activity (GO:2000272)

Negative Regulation Of Telomere Maintenance Via Telomere Lengthening (GO:1904357)
Osteoclast Differentiation (GO:0030316)
Positive Regulation Of Glucose Import (GO:0046326)
Positive Regulation Of Release Of Cytochrome C From Mitochondria (GO:0090200)
Regulation Of Cell Cycle Process (GO:0010564)
Regulation Of Cyclin-Dependent Protein Serine/Threonine Kinase Activity (GO:0000079)
Response To Insulin (GO:0032868)
Positive Regulation Of Catabolic Process (GO:0009896)
Regulation Of Macromolecule Biosynthetic Process (GO:0010556)
Carboxylic Acid Transport (GO:0046942)
Cellular Response To Alcohol (GO:0097306)
Mitochondrial Transport (GO:0006839)
Protein Homotetramerization (GO:0051289)
Negative Regulation Of Intracellular Signal Transduction (GO:1902532)
Regulation Of Organelle Assembly (GO:1902115)
Negative Regulation Of Protein Serine/Threonine Kinase Activity (GO:0071901)
Regulation Of G1/S Transition Of Mitotic Cell Cycle (GO:2000045)
Regulation Of I-kappaB kinase/NF-kappaB Signaling (GO:0043122)
L-amino Acid Transport (GO:0015807)
Antigen Processing And Presentation Of Exogenous Peptide Antigen Via MHC Class II (GO:0019886)
Regulation Of Potassium Ion Transmembrane Transporter Activity (GO:1901016)
Histone H3-K4 Methylation (GO:0051568)
Negative Regulation Of Interleukin-1 Beta Production (GO:0032691)
Negative Regulation Of Lipid Metabolic Process (GO:0045833)
Nucleic Acid Phosphodiester Bond Hydrolysis (GO:0090305)
Positive Regulation Of Erythrocyte Differentiation (GO:0045648)
Positive Regulation Of Microtubule Polymerization Or Depolymerization (GO:0031112)
Positive Regulation Of Monooxygenase Activity (GO:0032770)
Positive Regulation Of Phosphatidylinositol 3-Kinase Activity (GO:0043552)
Regulation Of Actin Filament Bundle Assembly (GO:0032231)
Regulation Of Cardiac Muscle Cell Contraction (GO:0086004)
Regulation Of Exocytosis (GO:0017157)
Regulation Of Lipid Metabolic Process (GO:0019216)
Monoatomic Anion Transmembrane Transport (GO:0098656)
Negative Regulation Of TOR Signaling (GO:0032007)
Nucleosome Assembly (GO:0006334)
Protein K63-linked Ubiquitination (GO:0070534)
Regulation Of Cellular Response To Stress (GO:0080135)
Cellular Response To Interleukin-1 (GO:0071347)
Cytoskeleton-Dependent Cytokinesis (GO:0061640)
Phosphate-Containing Compound Metabolic Process (GO:0006796)
Response To Tumor Necrosis Factor (GO:0034612)
Lipid Transport (GO:0006869)
Positive Regulation Of Tumor Necrosis Factor Superfamily Cytokine Production (GO:1903557)
Regulation Of Immunoglobulin Production (GO:0002637)
Activation Of Phospholipase C Activity (GO:0007202)
Amino Acid Import Across Plasma Membrane (GO:0089718)
Cardiac Muscle Cell Differentiation (GO:0055007)

Cellular Response To Acid Chemical (GO:0071229)
Regulation Of Transcription Regulatory Region DNA Binding (GO:2000677)
Respiratory Tube Development (GO:0030323)
Establishment Of Protein Localization To Vacuole (GO:0072666)
mRNA Modification (GO:0016556)
Monoubiquitinated Histone H2A Deubiquitination (GO:0035522)
Monoubiquitinated Histone Deubiquitination (GO:0035521)
Peptide Cross-Linking (GO:0018149)
Positive Regulation Of Cytosolic Calcium Ion Concentration Involved In Phospholipase C-activating G Protein-C
Positive Regulation Of Mitotic Cell Cycle (GO:0045931)
Positive Regulation Of Ubiquitin-Protein Transferase Activity (GO:0051443)
Protein Quality Control For Misfolded Or Incompletely Synthesized Proteins (GO:0006515)
Epigenetic Regulation Of Gene Expression (GO:0040029)
Vesicle-Mediated Transport To The Plasma Membrane (GO:0098876)
Phospholipase C-activating G Protein-Coupled Receptor Signaling Pathway (GO:0007200)
Positive Regulation Of Proteasomal Ubiquitin-Dependent Protein Catabolic Process (GO:0032436)
Regulation Of Cell Division (GO:0051302)
Regulation Of Stem Cell Population Maintenance (GO:2000036)
Cellular Response To Peptide (GO:1901653)
Mitotic DNA Damage Checkpoint Signaling (GO:0044773)
Regulation Of Protein Kinase B Signaling (GO:0051896)
DNA Metabolic Process (GO:0006259)
Regulation Of Chromosome Segregation (GO:0051983)
Antigen Processing And Presentation Of Peptide Antigen Via MHC Class II (GO:0002495)
Regulation Of Nitric-Oxide Synthase Activity (GO:0050999)
Regulation Of Platelet Activation (GO:0010543)
Regulation Of Protein Targeting To Membrane (GO:0090313)
Regulation Of Sodium Ion Transmembrane Transport (GO:1902305)
Determination Of Bilateral Symmetry (GO:0009855)
Transcription By RNA Polymerase III (GO:0006383)
Glycosaminoglycan Metabolic Process (GO:0030203)
Hematopoietic Progenitor Cell Differentiation (GO:0002244)
Negative Regulation Of Type II Interferon Production (GO:0032689)
Phospholipid Catabolic Process (GO:0009395)
Protein Insertion Into Mitochondrial Membrane (GO:0051204)
Pyroptosis (GO:0070269)
Regulation Of Bone Resorption (GO:0045124)
Regulation Of Interleukin-1 Beta Production (GO:0032651)
Positive Regulation Of Protein Ubiquitination (GO:0031398)
Potassium Ion Transmembrane Transport (GO:0071805)
Protein Localization To Organelle (GO:0033365)
Membrane Lipid Biosynthetic Process (GO:0046467)
Monoatomic Anion Transport (GO:0006820)
Positive Regulation Of Interleukin-1 Beta Production (GO:0032731)
RNA Metabolic Process (GO:0016070)
T Cell Activation (GO:0042110)
Establishment Of Protein Localization To Organelle (GO:0072594)
Mitotic Spindle Organization (GO:0007052)

Regulation Of Protein Serine/Threonine Kinase Activity (GO:0071900)
Regulation Of Neuron Death (GO:1901214)
Selective Autophagy (GO:0061912)
Eye Development (GO:0001654)
Inorganic Anion Transport (GO:0015698)
Peptidyl-Threonine Phosphorylation (GO:0018107)
RNA Biosynthetic Process (GO:0032774)
Alpha-Amino Acid Metabolic Process (GO:1901605)
Regulation Of Muscle Contraction (GO:0006937)
Carbohydrate Derivative Biosynthetic Process (GO:1901137)
Cardiac Muscle Cell Action Potential (GO:0086001)
Regulation Of Spindle Assembly (GO:0090169)
Regulation Of Striated Muscle Contraction (GO:0006942)
Chondrocyte Differentiation (GO:0002062)
Response To Alcohol (GO:0097305)
Sarcomere Organization (GO:0045214)
Lipid Droplet Organization (GO:0034389)
Modified Amino Acid Transport (GO:0072337)
Multivesicular Body Assembly (GO:0036258)
Organic Cyclic Compound Biosynthetic Process (GO:1901362)
Peptidyl-Tyrosine Dephosphorylation (GO:0035335)
Positive Regulation Of Dephosphorylation (GO:0035306)
Positive Regulation Of Viral Genome Replication (GO:0045070)
Protein Acetylation (GO:0006473)
Regulation Of Blood Coagulation (GO:0030193)
Exocytosis (GO:0006887)
DNA-templated Transcription Initiation (GO:0006352)
Mitotic Cytokinesis (GO:0000281)
Negative Regulation Of Cytokine-Mediated Signaling Pathway (GO:0001960)
Positive Regulation Of Protein Binding (GO:0032092)
protein-DNA Complex Assembly (GO:0065004)
Organelle Organization (GO:0006996)
DNA Geometric Change (GO:0032392)
Regulation Of Potassium Ion Transport (GO:0043266)
Extrinsic Apoptotic Signaling Pathway Via Death Domain Receptors (GO:0008625)
Homologous Chromosome Pairing At Meiosis (GO:0007129)
Intrinsic Apoptotic Signaling Pathway In Response To Endoplasmic Reticulum Stress (GO:0070059)
Mitotic G2 DNA Damage Checkpoint Signaling (GO:0007095)
Multivesicular Body Organization (GO:0036257)
Negative Regulation Of Calcium Ion Transmembrane Transporter Activity (GO:1901020)
Negative Regulation Of Interleukin-1 Production (GO:0032692)
Negative Regulation Of Myeloid Leukocyte Differentiation (GO:0002762)
Prostaglandin Metabolic Process (GO:0006693)
Protein Palmitoylation (GO:0018345)
Negative Regulation Of Peptidase Activity (GO:0010466)
Positive Regulation Of Phagocytosis (GO:0050766)
Regulation Of Centrosome Duplication (GO:0010824)
Regulation Of Macrophage Activation (GO:0043030)

Antigen Processing And Presentation Of Exogenous Peptide Antigen (GO:0002478)
Toll-Like Receptor Signaling Pathway (GO:0002224)
Leukocyte Cell-Cell Adhesion (GO:0007159)
Negative Regulation Of Intracellular Steroid Hormone Receptor Signaling Pathway (GO:0033144)
Non-Motile Cilium Assembly (GO:1905515)
Positive Regulation Of G1/S Transition Of Mitotic Cell Cycle (GO:1900087)
Positive Regulation Of Rho Protein Signal Transduction (GO:0035025)
Positive Regulation Of Glucose Transmembrane Transport (GO:0010828)
Positive Regulation Of Mononuclear Cell Migration (GO:0071677)
Positive Regulation Of Proteolysis Involved In Protein Catabolic Process (GO:1903052)
Protein Localization To Chromatin (GO:0071168)
Receptor Signaling Pathway Via STAT (GO:0097696)
Regulation Of Cell Morphogenesis (GO:0022604)
Negative Regulation Of Autophagy (GO:0010507)
Negative Regulation Of Intrinsic Apoptotic Signaling Pathway (GO:2001243)
Cellular Response To Molecule Of Bacterial Origin (GO:0071219)
Positive Regulation Of Proteasomal Protein Catabolic Process (GO:1901800)
mRNA Export From Nucleus (GO:0006406)
Phosphatidylinositol-Mediated Signaling (GO:0048015)
Regulation Of T Cell Activation (GO:0050863)
Regulation Of Cholesterol Efflux (GO:0010874)
NIK/NF-kappaB Signaling (GO:0038061)
Chromosome Condensation (GO:0030261)
Respiratory System Development (GO:0060541)
Very Long-Chain Fatty Acid Metabolic Process (GO:0000038)
Heterochromatin Formation (GO:0031507)
Histone Lysine Methylation (GO:0034968)
Intracellular pH Reduction (GO:0051452)
Left/Right Pattern Formation (GO:0060972)
Proteoglycan Biosynthetic Process (GO:0030166)
Regulation Of Epithelial Cell Proliferation (GO:0050678)
Lymphocyte Differentiation (GO:0030098)
Regulation Of RNA Metabolic Process (GO:0051252)
Cellular Response To Tumor Necrosis Factor (GO:0071356)
Negative Regulation Of Response To External Stimulus (GO:0032102)
Telomere Organization (GO:0032200)
Inorganic Anion Transmembrane Transport (GO:0098661)
Positive Regulation Of Mitotic Cell Cycle Phase Transition (GO:1901992)
Neuron Differentiation (GO:0030182)
B Cell Activation (GO:0042113)
Steroid Metabolic Process (GO:0008202)
Glycolipid Metabolic Process (GO:0006664)
Ubiquitin-Dependent Protein Catabolic Process Via The Multivesicular Body Sorting Pathway (GO:0043162)
Inositol Lipid-Mediated Signaling (GO:0048017)
Lung Development (GO:0030324)
Monoubiquitinated Protein Deubiquitination (GO:0035520)
Positive Regulation Of DNA Binding (GO:0043388)
Positive Regulation Of Chromosome Organization (GO:2001252)

Positive Regulation Of Cytokine Production Involved In Immune Response (GO:0002720)
Positive Regulation Of Epidermal Growth Factor Receptor Signaling Pathway (GO:0045742)
Positive Regulation Of Interleukin-12 Production (GO:0032735)
Positive Regulation Of Ion Transmembrane Transporter Activity (GO:0032414)
Positive Regulation Of Protein Dephosphorylation (GO:0035307)
Positive Regulation Of Small Molecule Metabolic Process (GO:0062013)
Protein K63-linked Deubiquitination (GO:0070536)
Regulation Of Amyloid-Beta Formation (GO:1902003)
Negative Regulation Of Protein Metabolic Process (GO:0051248)
Protein Glycosylation (GO:0006486)
Regulation Of Secretion By Cell (GO:1903530)
Endocytic Recycling (GO:0032456)
Positive Regulation Of Interleukin-1 Production (GO:0032732)
Positive Regulation Of Macroautophagy (GO:0016239)
Positive Regulation Of Type I Interferon Production (GO:0032481)
Protein N-linked Glycosylation (GO:0006487)
Protein O-linked Glycosylation (GO:0006493)
ATP Metabolic Process (GO:0046034)
DNA Damage Checkpoint Signaling (GO:0000077)
Regulation Of Interleukin-17 Production (GO:0032660)
Regulation Of Phosphatidylinositol 3-Kinase Activity (GO:0043551)
Cellular Response To Amyloid-Beta (GO:1904646)
Sister Chromatid Segregation (GO:0000819)
Superoxide Metabolic Process (GO:0006801)
Transcription Initiation-Coupled Chromatin Remodeling (GO:0045815)
Histone Deubiquitination (GO:0016578)
Negative Regulation Of JNK Cascade (GO:0046329)
Negative Regulation Of Interleukin-6 Production (GO:0032715)
Positive Regulation Of Organelle Organization (GO:0010638)
Purine Nucleotide Metabolic Process (GO:0006163)
Regulation Of T Cell Receptor Signaling Pathway (GO:0050856)
Regulation Of calcineurin-NFAT Signaling Cascade (GO:0070884)
Autophagosome Assembly (GO:0000045)
Positive Regulation Of DNA Biosynthetic Process (GO:2000573)
Retrograde Transport, Endosome To Golgi (GO:0042147)
Sphingolipid Metabolic Process (GO:0006665)
Regulation Of Protein Catabolic Process (GO:0042176)
Cellular Response To BMP Stimulus (GO:0071773)
Negative Regulation Of Immune Response (GO:0050777)
Regulation Of mRNA Processing (GO:0050684)
Regulation Of Postsynaptic Membrane Potential (GO:0060078)
Skeletal Muscle Organ Development (GO:0060538)
Intrinsic Apoptotic Signaling Pathway By P53 Class Mediator (GO:0072332)
Macrophage Activation (GO:0042116)
Positive Regulation Of Telomere Maintenance Via Telomere Lengthening (GO:1904358)
Regulation Of G2/M Transition Of Mitotic Cell Cycle (GO:0010389)
Cellular Response To Lipopolysaccharide (GO:0071222)
Regulation Of Protein Metabolic Process (GO:0051246)

Cellular Response To Transforming Growth Factor Beta Stimulus (GO:0071560)
Protein Secretion (GO:0009306)
Regulation Of Glucose Import (GO:0046324)
Regulation Of Receptor Internalization (GO:0002090)
Histone Deacetylation (GO:0016575)
Negative Regulation Of Protein Kinase B Signaling (GO:0051898)
Positive Regulation Of Interferon-Beta Production (GO:0032728)
Positive Regulation Of Phospholipase C Activity (GO:0010863)
Positive Regulation Of Signaling Receptor Activity (GO:2000273)
Protein Destabilization (GO:0031648)
Receptor Signaling Pathway Via JAK-STAT (GO:0007259)
Regulation Of Protein Localization (GO:0032880)
Monocarboxylic Acid Metabolic Process (GO:0032787)
RNA Splicing, Via Transesterification Reactions With Bulged Adenosine As Nucleophile (GO:0000377)
Endosomal Transport (GO:0016197)
B Cell Differentiation (GO:0030183)
Protein-Containing Complex Organization (GO:0043933)
Negative Regulation Of Macromolecule Biosynthetic Process (GO:0010558)
Phosphatidylinositol Biosynthetic Process (GO:0006661)
Regulation Of Ossification (GO:0030278)
Calcium Ion Import Across Plasma Membrane (GO:0098703)
Spliceosomal snRNP Assembly (GO:0000387)
Type I Interferon-Mediated Signaling Pathway (GO:0060337)
Muscle Cell Differentiation (GO:0042692)
Positive Regulation Of Exocytosis (GO:0045921)
Positive Regulation Of Immune Effector Process (GO:0002699)
Regulation Of Response To External Stimulus (GO:0032101)
Cytosolic Transport (GO:0016482)
Regulation Of Endopeptidase Activity (GO:0052548)
Sensory Organ Development (GO:0007423)
Regulation Of Cellular Biosynthetic Process (GO:0031326)
Antimicrobial Humoral Response (GO:0019730)
RNA 3'-End Processing (GO:0031123)
Regulation Of Neurotransmitter Secretion (GO:0046928)
Regulation Of Telomere Maintenance (GO:0032204)
Cellular Response To Type I Interferon (GO:0071357)
Response To Transforming Growth Factor Beta (GO:0071559)
mRNA Transcription By RNA Polymerase II (GO:0042789)
Membrane Fission (GO:0090148)
Positive Regulation Of Production Of Molecular Mediator Of Immune Response (GO:0002702)
DNA Repair (GO:0006281)
Regulation Of DNA Repair (GO:0006282)
Myeloid Leukocyte Differentiation (GO:0002573)
Protein Autoubiquitination (GO:0051865)
Golgi Organization (GO:0007030)
Proteasome-Mediated Ubiquitin-Dependent Protein Catabolic Process (GO:0043161)
RNA Transport (GO:0050658)
Regulation Of Mitotic Spindle Organization (GO:0060236)

Axonal Transport (GO:0098930)
Retina Homeostasis (GO:0001895)
Heterochromatin Organization (GO:0070828)
Histone H4 Acetylation (GO:0043967)
Internal Peptidyl-Lysine Acetylation (GO:0018393)
Negative Regulation Of Mitochondrion Organization (GO:0010823)
Negative Regulation Of Proteasomal Protein Catabolic Process (GO:1901799)
Negative Regulation Of Transport (GO:0051051)
Nuclear Transport (GO:0051169)
Organelle Transport Along Microtubule (GO:0072384)
Regulation Of RNA Splicing (GO:0043484)
mRNA Processing (GO:0006397)
Regulation Of Cellular Localization (GO:0060341)
Cholesterol Homeostasis (GO:0042632)
Plasma Membrane Bounded Cell Projection Organization (GO:0120036)
Regulation Of Microtubule Cytoskeleton Organization (GO:0070507)
Regulation Of Protein Dephosphorylation (GO:0035304)
Cellular Response To Heat (GO:0034605)
Establishment Or Maintenance Of Epithelial Cell Apical/Basal Polarity (GO:0045197)
Water-Soluble Vitamin Metabolic Process (GO:0006767)
mRNA 3'-End Processing (GO:0031124)
Positive Regulation Of Monoatomic Ion Transport (GO:0043270)
Protein Deacetylation (GO:0006476)
Protein Localization To Chromosome (GO:0034502)
Regulation Of Autophagosome Assembly (GO:2000785)
Sterol Homeostasis (GO:0055092)
Ubiquitin-Dependent ERAD Pathway (GO:0030433)
Protein Tetramerization (GO:0051262)
Regulation Of Macromolecule Metabolic Process (GO:0060255)
DNA Duplex Unwinding (GO:0032508)
O-glycan Processing (GO:0016266)
Regulation Of Heart Rate By Cardiac Conduction (GO:0086091)
Carbohydrate Catabolic Process (GO:0016052)
Regulation Of Release Of Cytochrome C From Mitochondria (GO:0090199)
Response To Amino Acid Starvation (GO:1990928)
Stem Cell Differentiation (GO:0048863)
Visual System Development (GO:0150063)
Lymphocyte Migration (GO:0072676)
Negative Regulation Of DNA Recombination (GO:0045910)
Positive Regulation Of Ossification (GO:0045778)
Positive Regulation Of Response To Biotic Stimulus (GO:0002833)
Protein Localization To Lysosome (GO:0061462)
Regulation Of Amide Metabolic Process (GO:0034248)
Regulation Of Fat Cell Differentiation (GO:0045598)
Nucleosome Organization (GO:0034728)
Transcription By RNA Polymerase II (GO:0006366)
DNA Integrity Checkpoint Signaling (GO:0031570)
DNA Recombination (GO:0006310)

Regulation Of Neurotransmitter Receptor Activity (GO:0099601)
Cellular Response To Glucose Starvation (GO:0042149)
Regulation Of Ubiquitin-Dependent Protein Catabolic Process (GO:2000058)
Modulation By Host Of Symbiont Process (GO:0051851)
Monocyte Chemotaxis (GO:0002548)
Negative Regulation Of I-kappaB kinase/NF-kappaB Signaling (GO:0043124)
Organic Hydroxy Compound Biosynthetic Process (GO:1901617)
Positive Regulation Of Calcium Ion Transmembrane Transport (GO:1904427)
Proteasomal Protein Catabolic Process (GO:0010498)
Negative Regulation Of Translation (GO:0017148)
Regulation Of Chromosome Organization (GO:0033044)
Response To Amyloid-Beta (GO:1904645)
Energy Derivation By Oxidation Of Organic Compounds (GO:0015980)
mRNA Catabolic Process (GO:0006402)
mRNA Transcription (GO:0009299)
Positive Regulation Of T Cell Mediated Immunity (GO:0002711)
Positive Regulation Of Biomineral Tissue Development (GO:0070169)
Positive Regulation Of Cell Division (GO:0051781)
Positive Regulation Of Receptor-Mediated Endocytosis (GO:0048260)
Positive Regulation Of Response To Stimulus (GO:0048584)
Positive Regulation Of Translation (GO:0045727)
Regulation Of Programmed Cell Death (GO:0043067)
Regulation Of Proteasomal Ubiquitin-Dependent Protein Catabolic Process (GO:0032434)
Endomembrane System Organization (GO:0010256)
Cellular Response To Nutrient Levels (GO:0031669)
Regulation Of TOR Signaling (GO:0032006)
Regulation Of Insulin Receptor Signaling Pathway (GO:0046626)
Autophagosome Maturation (GO:0097352)
Bone Development (GO:0060348)
Establishment Of Protein Localization To Mitochondrion (GO:0072655)
Mitotic Spindle Assembly (GO:0090307)
Nucleobase-Containing Compound Catabolic Process (GO:0034655)
Positive Regulation Of Biosynthetic Process (GO:0009891)
Regulation Of DNA Recombination (GO:0000018)
Cellular Response To UV (GO:0034644)
Positive Regulation Of Protein Modification By Small Protein Conjugation Or Removal (GO:1903322)
Determination Of Left/Right Symmetry (GO:0007368)
Zymogen Activation (GO:0031638)
Positive Regulation Of Innate Immune Response (GO:0045089)
Cellular Response To Cytokine Stimulus (GO:0071345)
Mitotic Cell Cycle Phase Transition (GO:0044772)
Negative Regulation Of Inflammatory Response (GO:0050728)
DNA-templated Transcription (GO:0006351)
Regulation Of Cytokinesis (GO:0032465)
Glycoprotein Biosynthetic Process (GO:0009101)
Apoptotic Process (GO:0006915)
Regulation Of Epidermal Growth Factor Receptor Signaling Pathway (GO:0042058)
Regulation Of Interleukin-10 Production (GO:0032653)

Amino Acid Transport (GO:0006865)
Antiviral Innate Immune Response (GO:0140374)
Vacuolar Transport (GO:0007034)
Mitotic G2/M Transition Checkpoint (GO:0044818)
Protein Sumoylation (GO:0016925)
Histone Modification (GO:0016570)
Macromolecule Modification (GO:0043412)
Golgi To Plasma Membrane Transport (GO:0006893)
Regulation Of Interleukin-2 Production (GO:0032663)
Negative Regulation Of Tumor Necrosis Factor Production (GO:0032720)
Positive Regulation Of Lymphocyte Activation (GO:0051251)
Ubiquitin-Dependent Protein Catabolic Process (GO:0006511)
Response To Endoplasmic Reticulum Stress (GO:0034976)
Cellular Response To Mechanical Stimulus (GO:0071260)
Cytoplasmic Microtubule Organization (GO:0031122)
Retrograde Vesicle-Mediated Transport, Golgi To Endoplasmic Reticulum (GO:0006890)
Oligosaccharide Metabolic Process (GO:0009311)
Dephosphorylation (GO:0016311)
Erythrocyte Differentiation (GO:0030218)
Unsaturated Fatty Acid Metabolic Process (GO:0033559)
Interferon-Mediated Signaling Pathway (GO:0140888)
Negative Regulation Of T Cell Activation (GO:0050868)
Proton Transmembrane Transport (GO:1902600)
Response To Interleukin-1 (GO:0070555)
Defense Response To Gram-positive Bacterium (GO:0050830)
Regulation Of Epithelial Cell Migration (GO:0010632)
Regulation Of Interferon-Beta Production (GO:0032648)
Regulation Of Interleukin-12 Production (GO:0032655)
Dicarboxylic Acid Metabolic Process (GO:0043648)
Mitotic Metaphase Plate Congression (GO:0007080)
Nucleocytoplasmic Transport (GO:0006913)
Positive Regulation Of Fat Cell Differentiation (GO:0045600)
SMAD Protein Signal Transduction (GO:0060395)
Regulation Of Telomere Maintenance Via Telomerase (GO:0032210)
Cholesterol Transport (GO:0030301)
Negative Regulation Of Gene Expression, Epigenetic (GO:0045814)
Positive Regulation Of Leukocyte Mediated Cytotoxicity (GO:0001912)
Glucose Homeostasis (GO:0042593)
mRNA Splicing, Via Spliceosome (GO:0000398)
Arachidonic Acid Metabolic Process (GO:0019369)
Negative Regulation Of Innate Immune Response (GO:0045824)
Neurotransmitter Transport (GO:0006836)
ERAD Pathway (GO:0036503)
Cellular Response To Peptide Hormone Stimulus (GO:0071375)
Gonad Development (GO:0008406)
Positive Regulation Of Chemokine Production (GO:0032722)
Endoplasmic Reticulum Organization (GO:0007029)
Negative Regulation Of Protein Modification By Small Protein Conjugation Or Removal (GO:1903321)

Negative Regulation Of Protein Ubiquitination (GO:0031397)
Negative Regulation Of Growth (GO:0045926)
Response To Peptide Hormone (GO:0043434)
Phosphatidylcholine Metabolic Process (GO:0046470)
Positive Regulation Of Protein Catabolic Process (GO:0045732)
Cellular Response To Starvation (GO:0009267)
Regulation Of Reactive Oxygen Species Metabolic Process (GO:2000377)
Pattern Recognition Receptor Signaling Pathway (GO:0002221)
Positive Regulation Of Cellular Metabolic Process (GO:0031325)
Regulation Of Cation Channel Activity (GO:2001257)
Peptidyl-Lysine Modification (GO:0018205)
RNA Catabolic Process (GO:0006401)
Chemokine-Mediated Signaling Pathway (GO:0070098)
Endosome To Lysosome Transport (GO:0008333)
Myeloid Cell Differentiation (GO:0030099)
Post-Transcriptional Regulation Of Gene Expression (GO:0010608)
Regulation Of Calcium Ion Transport (GO:0051924)
Positive Regulation Of Transmembrane Receptor Protein Serine/Threonine Kinase Signaling Pathway (GO:00901
Recombinational Repair (GO:0000725)
Modification-Dependent Protein Catabolic Process (GO:0019941)
mRNA Transport (GO:0051028)
Regulation Of Bone Mineralization (GO:0030500)
Regulation Of Macroautophagy (GO:0016241)
Mitochondrion Organization (GO:0007005)
Cellular Response To Chemokine (GO:1990869)
Regulation Of Type I Interferon Production (GO:0032479)
Protein Targeting To Mitochondrion (GO:0006626)
Regulation Of Defense Response (GO:0031347)
Histone Acetylation (GO:0016573)
protein-DNA Complex Organization (GO:0071824)
Protein Polyubiquitination (GO:0000209)
Cytokine-Mediated Signaling Pathway (GO:0019221)
Autophagosome Organization (GO:1905037)
Regulation Of Pathway-Restricted SMAD Protein Phosphorylation (GO:0060393)
Fatty Acid Catabolic Process (GO:0009062)
Non-Membrane-Bounded Organelle Assembly (GO:0140694)
Spliceosomal Complex Assembly (GO:0000245)
Fat Cell Differentiation (GO:0045444)
Glycerophospholipid Metabolic Process (GO:0006650)
Protein Lipidation (GO:0006497)
Protein-Containing Complex Disassembly (GO:0032984)
Double-Strand Break Repair (GO:0006302)
rRNA Processing (GO:0006364)
G1/S Transition Of Mitotic Cell Cycle (GO:0000082)
Phosphatidylinositol Metabolic Process (GO:0046488)
Protein Ubiquitination (GO:0016567)
Macroautophagy (GO:0016236)
RNA Export From Nucleus (GO:0006405)

Cellular Response To Light Stimulus (GO:0071482)
Fatty Acid Biosynthetic Process (GO:0006633)
Gene Expression (GO:0010467)
Cellular Response To Type II Interferon (GO:0071346)
tRNA Modification (GO:0006400)
Negative Regulation Of Cellular Catabolic Process (GO:0031330)
Monoatomic Ion Transport (GO:0006811)
Positive Regulation Of Autophagy (GO:0010508)
Protein-Containing Complex Assembly (GO:0065003)
Intermediate Filament Organization (GO:0045109)
DNA-templated DNA Replication (GO:0006261)
Phospholipid Metabolic Process (GO:0006644)
Positive Regulation Of Cellular Catabolic Process (GO:0031331)
Protein K48-linked Ubiquitination (GO:0070936)
Double-Strand Break Repair Via Homologous Recombination (GO:0000724)
Mitotic Sister Chromatid Segregation (GO:0000070)
Lipid Biosynthetic Process (GO:0008610)
Negative Regulation Of Defense Response (GO:0031348)
Defense Response To Symbiont (GO:0140546)
Regulation Of Protein Ubiquitination (GO:0031396)
protein-RNA Complex Assembly (GO:0022618)
DNA Replication (GO:0006260)
Monocarboxylic Acid Transport (GO:0015718)
Transcription Initiation At RNA Polymerase II Promoter (GO:0006367)
Defense Response To Virus (GO:0051607)
Regulation Of Immune Response (GO:0050776)
Regulation Of Transcription Elongation By RNA Polymerase II (GO:0034243)
Regulation Of Innate Immune Response (GO:0045088)
Long-Chain Fatty Acid Metabolic Process (GO:0001676)
Proteolysis Involved In Protein Catabolic Process (GO:0051603)
Vesicle Fusion (GO:0006906)
Fatty Acid Metabolic Process (GO:0006631)
Protein Localization To Nucleus (GO:0034504)
Response To Type II Interferon (GO:0034341)
Wound Healing (GO:0042060)
Positive Regulation Of Immune Response (GO:0050778)
Cilium Organization (GO:0044782)
Organonitrogen Compound Biosynthetic Process (GO:1901566)
Spindle Assembly (GO:0051225)
Negative Regulation Of NF-kappaB Transcription Factor Activity (GO:0032088)
Regulation Of DNA Metabolic Process (GO:0051052)
Intracellular Protein Transport (GO:0006886)
Nitrogen Compound Transport (GO:0071705)
Calcium Ion Transmembrane Import Into Cytosol (GO:0097553)
Ribosomal Small Subunit Biogenesis (GO:0042274)
Purine Ribonucleotide Metabolic Process (GO:0009150)
Cellular Respiration (GO:0045333)
mRNA Metabolic Process (GO:0016071)

Nuclear-Transcribed mRNA Catabolic Process (GO:0000956)
Regulation Of Translation (GO:0006417)
Protein Modification By Small Protein Conjugation (GO:0032446)
Cilium Assembly (GO:0060271)
rRNA Metabolic Process (GO:0016072)
Cytoplasmic Translation (GO:0002181)
Protein Transport (GO:0015031)
Organelle Assembly (GO:0070925)
ncRNA Processing (GO:0034470)
Lysosomal Transport (GO:0007041)
Golgi Vesicle Transport (GO:0048193)
Sensory Perception Of Chemical Stimulus (GO:0007606)
Protein Deubiquitination (GO:0016579)
Regulation Of Autophagy (GO:0010506)
Ribonucleoprotein Complex Biogenesis (GO:0022613)
Membrane Organization (GO:0061024)
Macromolecule Biosynthetic Process (GO:0009059)
Ribosome Biogenesis (GO:0042254)
Peptide Biosynthetic Process (GO:0043043)
Translation (GO:0006412)

Overlap	P.value	Adjusted.P.value	Old.P.value	Old.Adjusted.P.value
47/434	8.22284E-09	2.42327E-05	0	0
25/172	1.13199E-07	0.000166799	0	0
24/176	6.69767E-07	0.000615044	0	0
32/284	8.34806E-07	0.000615044	0	0
21/149	1.92312E-06	0.001133488	0	0
5/9	1.65755E-05	0.007193948	0	0
16/109	1.87455E-05	0.007193948	0	0
16/110	2.10669E-05	0.007193948	0	0
28/272	2.197E-05	0.007193948	0	0
20/162	2.5064E-05	0.007386367	0	0
14/94	5.21986E-05	0.013222931	0	0
6/17	5.38429E-05	0.013222931	0	0
40/483	7.48808E-05	0.015893591	0	0
8/34	8.08971E-05	0.015893591	0	0
8/34	8.08971E-05	0.015893591	0	0
16/124	9.31778E-05	0.016061284	0	0
5/12	9.34018E-05	0.016061284	0	0
23/221	9.81008E-05	0.016061284	0	0
15/114	0.000122348	0.01897688	0	0
5/13	0.000146359	0.021566071	0	0
36/433	0.000156199	0.021914825	0	0
16/130	0.000163599	0.021914825	0	0
16/131	0.000179004	0.022935916	0	0
69/1025	0.000194822	0.023922555	0	0
20/188	0.000203412	0.023978176	0	0
13/95	0.000229593	0.026023518	0	0
6/22	0.00026944	0.028362812	0	0
26/283	0.000273355	0.028362812	0	0
54/763	0.000293688	0.028362812	0	0
7/31	0.000298353	0.028362812	0	0
7/31	0.000298353	0.028362812	0	0
8/42	0.000386807	0.034093803	0	0
8/42	0.000386807	0.034093803	0	0
12/88	0.000408635	0.034093803	0	0
9/53	0.000416484	0.034093803	0	0
9/53	0.000416484	0.034093803	0	0
5/16	0.000445519	0.034853132	0	0
7/33	0.000449413	0.034853132	0	0
11/77	0.000470083	0.035521381	0	0
16/144	0.000527423	0.038857878	0	0
4/10	0.000599743	0.041718426	0	0
5/17	0.000608718	0.041718426	0	0
5/17	0.000608718	0.041718426	0	0
7/35	0.000655966	0.043934814	0	0
12/93	0.000680307	0.04455252	0	0
19/192	0.000717109	0.04594175	0	0
3/5	0.000762793	0.047828719	0	0

7/36	0.000784168	0.048144651	0	0
5/18	0.000812916	0.048891103	0	0
10/70	0.000841505	0.049598301	0	0
13/109	0.000882138	0.050324644	0	0
6/27	0.000887982	0.050324644	0	0
14/123	0.00090712	0.050439319	0	0
7/37	0.000931376	0.050477487	0	0
10/71	0.000942064	0.050477487	0	0
5/19	0.001064118	0.055999218	0	0
11/85	0.00109669	0.056700779	0	0
37/500	0.001164557	0.059171552	0	0
14/127	0.001241799	0.062026815	0	0
6/29	0.001323399	0.063935366	0	0
6/29	0.001323399	0.063935366	0	0
5/20	0.001368565	0.065050976	0	0
3/6	0.001476316	0.067251843	0	0
3/6	0.001476316	0.067251843	0	0
7/40	0.001506561	0.067251843	0	0
7/40	0.001506561	0.067251843	0	0
17/174	0.001536274	0.067251843	0	0
6/30	0.001594277	0.067251843	0	0
6/30	0.001594277	0.067251843	0	0
34/456	0.001597431	0.067251843	0	0
10/77	0.00177265	0.072281254	0	0
10/77	0.00177265	0.072281254	0	0
13/118	0.001838089	0.072281254	0	0
4/13	0.00183953	0.072281254	0	0
4/13	0.00183953	0.072281254	0	0
6/31	0.0019053	0.073880508	0	0
7/42	0.002022339	0.076408114	0	0
7/42	0.002022339	0.076408114	0	0
6/32	0.002260127	0.08431133	0	0
7/43	0.002326641	0.085707622	0	0
41/594	0.002399628	0.087304992	0	0
3/7	0.0025003	0.088775709	0	0
3/7	0.0025003	0.088775709	0	0
14/137	0.002551943	0.089530683	0	0
9/68	0.00259751	0.090057206	0	0
6/33	0.00266251	0.091237395	0	0
12/109	0.002726165	0.092344925	0	0
13/124	0.002860468	0.095793164	0	0
9/70	0.00317462	0.105119163	0	0
9/71	0.003498702	0.11330411	0	0
9/71	0.003498702	0.11330411	0	0
14/142	0.003545505	0.113571761	0	0
10/85	0.003709184	0.116432259	0	0
3/8	0.003871857	0.116432259	0	0
3/8	0.003871857	0.116432259	0	0

3/8	0.003871857	0.116432259	0	0
3/8	0.003871857	0.116432259	0	0
3/8	0.003871857	0.116432259	0	0
5/25	0.003918543	0.116645927	0	0
31/429	0.004000105	0.1178831	0	0
6/36	0.004193532	0.121933186	0	0
4/16	0.004220287	0.121933186	0	0
10/88	0.004764911	0.134686932	0	0
6/37	0.004824919	0.134686932	0	0
6/37	0.004824919	0.134686932	0	0
7/49	0.004955302	0.134686932	0	0
73/1243	0.005148748	0.134686932	0	0
10/89	0.005164876	0.134686932	0	0
13/133	0.005222788	0.134686932	0	0
4/17	0.005331542	0.134686932	0	0
6/38	0.005523438	0.134686932	0	0
5/27	0.005542915	0.134686932	0	0
10/90	0.005590663	0.134686932	0	0
10/90	0.005590663	0.134686932	0	0
3/9	0.005621477	0.134686932	0	0
3/9	0.005621477	0.134686932	0	0
3/9	0.005621477	0.134686932	0	0
3/9	0.005621477	0.134686932	0	0
3/9	0.005621477	0.134686932	0	0
3/9	0.005621477	0.134686932	0	0
3/9	0.005621477	0.134686932	0	0
3/9	0.005621477	0.134686932	0	0
3/9	0.005621477	0.134686932	0	0
18/214	0.005747872	0.136604664	0	0
19/231	0.005869554	0.137301859	0	0
11/105	0.005870388	0.137301859	0	0
15/167	0.006207463	0.144042472	0	0
11/106	0.006300709	0.145063991	0	0
5/28	0.006511135	0.145647395	0	0
5/28	0.006511135	0.145647395	0	0
5/28	0.006511135	0.145647395	0	0
10/92	0.006524119	0.145647395	0	0
4/18	0.006622583	0.145647395	0	0
4/18	0.006622583	0.145647395	0	0
19/234	0.006717059	0.146630914	0	0
33/482	0.006824286	0.147876255	0	0
7/52	0.006901129	0.148449834	0	0
37/557	0.006993562	0.149348034	0	0
3/10	0.007773656	0.163635468	0	0
3/10	0.007773656	0.163635468	0	0
4/19	0.008104844	0.16939699	0	0
8/67	0.008265006	0.170528497	0	0
11/110	0.008274712	0.170528497	0	0

7/54	0.008480634	0.173558528	0	0
5/30	0.008791447	0.178678588	0	0
17/207	0.008978342	0.181227217	0	0
4/20	0.009788919	0.193062133	0	0
4/20	0.009788919	0.193062133	0	0
4/20	0.009788919	0.193062133	0	0
8/69	0.009826712	0.193062133	0	0
104/1922	0.010573977	0.206367617	0	0
47/766	0.010864259	0.210637963	0	0
19/246	0.011156339	0.214887127	0	0
6/44	0.011344677	0.217095859	0	0
8/71	0.011597601	0.217938249	0	0
8/71	0.011597601	0.217938249	0	0
14/163	0.011661067	0.217938249	0	0
4/21	0.011684507	0.217938249	0	0
20/267	0.0128424	0.23430618	0	0
10/102	0.013125833	0.23430618	0	0
5/33	0.013161157	0.23430618	0	0
5/33	0.013161157	0.23430618	0	0
5/33	0.013161157	0.23430618	0	0
3/12	0.013357122	0.23430618	0	0
3/12	0.013357122	0.23430618	0	0
3/12	0.013357122	0.23430618	0	0
3/12	0.013357122	0.23430618	0	0
3/12	0.013357122	0.23430618	0	0
7/59	0.013571579	0.234902698	0	0
7/59	0.013571579	0.234902698	0	0
11/118	0.013630255	0.234902698	0	0
4/22	0.013800391	0.236452046	0	0
7/60	0.014806195	0.241898423	0	0
10/104	0.014898247	0.241898423	0	0
13/152	0.015220967	0.241898423	0	0
6/47	0.015473931	0.241898423	0	0
6/47	0.015473931	0.241898423	0	0
14/169	0.015608617	0.241898423	0	0
15/186	0.015794146	0.241898423	0	0
7/61	0.016118758	0.241898423	0	0
4/23	0.016144407	0.241898423	0	0
4/23	0.016144407	0.241898423	0	0
4/23	0.016144407	0.241898423	0	0
4/23	0.016144407	0.241898423	0	0
4/23	0.016144407	0.241898423	0	0
5/35	0.016770997	0.241898423	0	0
5/35	0.016770997	0.241898423	0	0
3/13	0.016812408	0.241898423	0	0
3/13	0.016812408	0.241898423	0	0
3/13	0.016812408	0.241898423	0	0
8/76	0.01703969	0.241898423	0	0

9/91	0.017185628	0.241898423	0	0
2/5	0.017237417	0.241898423	0	0
2/5	0.017237417	0.241898423	0	0
2/5	0.017237417	0.241898423	0	0
2/5	0.017237417	0.241898423	0	0
2/5	0.017237417	0.241898423	0	0
2/5	0.017237417	0.241898423	0	0
2/5	0.017237417	0.241898423	0	0
2/5	0.017237417	0.241898423	0	0
2/5	0.017237417	0.241898423	0	0
2/5	0.017237417	0.241898423	0	0
2/5	0.017237417	0.241898423	0	0
2/5	0.017237417	0.241898423	0	0
2/5	0.017237417	0.241898423	0	0
2/5	0.017237417	0.241898423	0	0
2/5	0.017237417	0.241898423	0	0
2/5	0.017237417	0.241898423	0	0
2/5	0.017237417	0.241898423	0	0
2/5	0.017237417	0.241898423	0	0
13/155	0.017641954	0.246402082	0	0
11/123	0.018105914	0.251689285	0	0
9/92	0.018341697	0.253769862	0	0
4/24	0.018723442	0.257686002	0	0
5/36	0.018799624	0.257686002	0	0
6/50	0.020541827	0.270174755	0	0
3/14	0.020719204	0.270174755	0	0
3/14	0.020719204	0.270174755	0	0
3/14	0.020719204	0.270174755	0	0
3/14	0.020719204	0.270174755	0	0
3/14	0.020719204	0.270174755	0	0
3/14	0.020719204	0.270174755	0	0
3/14	0.020719204	0.270174755	0	0
3/14	0.020719204	0.270174755	0	0
3/14	0.020719204	0.270174755	0	0
3/14	0.020719204	0.270174755	0	0
4/25	0.021543428	0.278458258	0	0
4/25	0.021543428	0.278458258	0	0
12/143	0.02196839	0.28271111	0	0
10/111	0.022519399	0.283844899	0	0
63/1127	0.023401101	0.283844899	0	0
106/2028	0.024326414	0.283844899	0	0
6/52	0.024482914	0.283844899	0	0
4/26	0.02460935	0.283844899	0	0
4/26	0.02460935	0.283844899	0	0
3/15	0.025079866	0.283844899	0	0
3/15	0.025079866	0.283844899	0	0
3/15	0.025079866	0.283844899	0	0
3/15	0.025079866	0.283844899	0	0

3/15	0.025079866	0.283844899	0	0
2/6	0.02511799	0.283844899	0	0
2/6	0.02511799	0.283844899	0	0
2/6	0.02511799	0.283844899	0	0
2/6	0.02511799	0.283844899	0	0
2/6	0.02511799	0.283844899	0	0
2/6	0.02511799	0.283844899	0	0
2/6	0.02511799	0.283844899	0	0
2/6	0.02511799	0.283844899	0	0
2/6	0.02511799	0.283844899	0	0
2/6	0.02511799	0.283844899	0	0
2/6	0.02511799	0.283844899	0	0
2/6	0.02511799	0.283844899	0	0
2/6	0.02511799	0.283844899	0	0
2/6	0.02511799	0.283844899	0	0
2/6	0.02511799	0.283844899	0	0
2/6	0.02511799	0.283844899	0	0
2/6	0.02511799	0.283844899	0	0
2/6	0.02511799	0.283844899	0	0
2/6	0.02511799	0.283844899	0	0
2/6	0.02511799	0.283844899	0	0
10/113	0.025138622	0.283844899	0	0
17/233	0.025853978	0.290807918	0	0
9/98	0.026489608	0.296824616	0	0
6/53	0.026630411	0.297272046	0	0
8/83	0.027446625	0.305227185	0	0
4/27	0.027925257	0.30822372	0	0
4/27	0.027925257	0.30822372	0	0
5/40	0.028500723	0.313401611	0	0
6/54	0.028899136	0.316601312	0	0
12/149	0.029101455	0.317636997	0	0
7/69	0.029696093	0.32035095	0	0
3/16	0.029893624	0.32035095	0	0
3/16	0.029893624	0.32035095	0	0
3/16	0.029893624	0.32035095	0	0
3/16	0.029893624	0.32035095	0	0
8/85	0.031086483	0.326808635	0	0
8/85	0.031086483	0.326808635	0	0
6/55	0.031291312	0.326808635	0	0
5/41	0.031339523	0.326808635	0	0
4/28	0.031494283	0.326808635	0	0
4/28	0.031494283	0.326808635	0	0
4/28	0.031494283	0.326808635	0	0
4/28	0.031494283	0.326808635	0	0
4/28	0.031494283	0.326808635	0	0
7/71	0.034018995	0.332292124	0	0
2/7	0.034165088	0.332292124	0	0
2/7	0.034165088	0.332292124	0	0

2/7	0.034165088	0.332292124	0	0
2/7	0.034165088	0.332292124	0	0
2/7	0.034165088	0.332292124	0	0
2/7	0.034165088	0.332292124	0	0
2/7	0.034165088	0.332292124	0	0
2/7	0.034165088	0.332292124	0	0
2/7	0.034165088	0.332292124	0	0
2/7	0.034165088	0.332292124	0	0
2/7	0.034165088	0.332292124	0	0
2/7	0.034165088	0.332292124	0	0
2/7	0.034165088	0.332292124	0	0
2/7	0.034165088	0.332292124	0	0
2/7	0.034165088	0.332292124	0	0
2/7	0.034165088	0.332292124	0	0
2/7	0.034165088	0.332292124	0	0
2/7	0.034165088	0.332292124	0	0
5/42	0.034349205	0.332983903	0	0
3/17	0.035156937	0.333602925	0	0
3/17	0.035156937	0.333602925	0	0
3/17	0.035156937	0.333602925	0	0
3/17	0.035156937	0.333602925	0	0
3/17	0.035156937	0.333602925	0	0
4/29	0.035318667	0.333602925	0	0
4/29	0.035318667	0.333602925	0	0
4/29	0.035318667	0.333602925	0	0
21/316	0.035586258	0.335056553	0	0
13/171	0.035720854	0.335252729	0	0
7/72	0.03632929	0.339880695	0	0
5/43	0.037531856	0.344568159	0	0
5/43	0.037531856	0.344568159	0	0
5/43	0.037531856	0.344568159	0	0
5/43	0.037531856	0.344568159	0	0
5/43	0.037531856	0.344568159	0	0
5/43	0.037531856	0.344568159	0	0
12/155	0.037774761	0.345721187	0	0
4/30	0.039399784	0.356169209	0	0
4/30	0.039399784	0.356169209	0	0
4/30	0.039399784	0.356169209	0	0
4/30	0.039399784	0.356169209	0	0
24/377	0.039971425	0.360231774	0	0
3/18	0.040863817	0.361863195	0	0
3/18	0.040863817	0.361863195	0	0
3/18	0.040863817	0.361863195	0	0
3/18	0.040863817	0.361863195	0	0
3/18	0.040863817	0.361863195	0	0
5/44	0.04088919	0.361863195	0	0
7/74	0.041254577	0.364003712	0	0
52/938	0.04191936	0.367444006	0	0

6/59	0.042132528	0.367444006	0	0
14/193	0.042144991	0.367444006	0	0
4/31	0.04373817	0.367444006	0	0
4/31	0.04373817	0.367444006	0	0
7/75	0.043872038	0.367444006	0	0
7/75	0.043872038	0.367444006	0	0
2/8	0.044262851	0.367444006	0	0
2/8	0.044262851	0.367444006	0	0
2/8	0.044262851	0.367444006	0	0
2/8	0.044262851	0.367444006	0	0
2/8	0.044262851	0.367444006	0	0
2/8	0.044262851	0.367444006	0	0
2/8	0.044262851	0.367444006	0	0
2/8	0.044262851	0.367444006	0	0
2/8	0.044262851	0.367444006	0	0
2/8	0.044262851	0.367444006	0	0
2/8	0.044262851	0.367444006	0	0
2/8	0.044262851	0.367444006	0	0
2/8	0.044262851	0.367444006	0	0
2/8	0.044262851	0.367444006	0	0
3/19	0.047006133	0.383731503	0	0
3/19	0.047006133	0.383731503	0	0
3/19	0.047006133	0.383731503	0	0
3/19	0.047006133	0.383731503	0	0
3/19	0.047006133	0.383731503	0	0
3/19	0.047006133	0.383731503	0	0
5/46	0.048132916	0.38969149	0	0
5/46	0.048132916	0.38969149	0	0
5/46	0.048132916	0.38969149	0	0
4/32	0.048333564	0.390243875	0	0
11/144	0.04918163	0.396006185	0	0
7/77	0.049421895	0.396856468	0	0
40/705	0.05112622	0.401425401	0	0
6/62	0.051640701	0.401425401	0	0
6/62	0.051640701	0.401425401	0	0
5/47	0.052020894	0.401425401	0	0
14/199	0.052042617	0.401425401	0	0
4/33	0.053184936	0.401425401	0	0
4/33	0.053184936	0.401425401	0	0
4/33	0.053184936	0.401425401	0	0
4/33	0.053184936	0.401425401	0	0
4/33	0.053184936	0.401425401	0	0
11/146	0.05334254	0.401425401	0	0
3/20	0.053573885	0.401425401	0	0
3/20	0.053573885	0.401425401	0	0
3/20	0.053573885	0.401425401	0	0
3/20	0.053573885	0.401425401	0	0
3/20	0.053573885	0.401425401	0	0

3/20	0.053573885	0.401425401	0	0
2/9	0.055303262	0.401425401	0	0
2/9	0.055303262	0.401425401	0	0
2/9	0.055303262	0.401425401	0	0
2/9	0.055303262	0.401425401	0	0
2/9	0.055303262	0.401425401	0	0
2/9	0.055303262	0.401425401	0	0
2/9	0.055303262	0.401425401	0	0
2/9	0.055303262	0.401425401	0	0
2/9	0.055303262	0.401425401	0	0
2/9	0.055303262	0.401425401	0	0
2/9	0.055303262	0.401425401	0	0
2/9	0.055303262	0.401425401	0	0
2/9	0.055303262	0.401425401	0	0
2/9	0.055303262	0.401425401	0	0
2/9	0.055303262	0.401425401	0	0
2/9	0.055303262	0.401425401	0	0
2/9	0.055303262	0.401425401	0	0
2/9	0.055303262	0.401425401	0	0
2/9	0.055303262	0.401425401	0	0
2/9	0.055303262	0.401425401	0	0
2/9	0.055303262	0.401425401	0	0
2/9	0.055303262	0.401425401	0	0
5/48	0.056086741	0.406112101	0	0
4/34	0.058290528	0.420005344	0	0
4/34	0.058290528	0.420005344	0	0
27/452	0.059071376	0.424593523	0	0
5/49	0.06033036	0.428983	0	0
5/49	0.06033036	0.428983	0	0
3/21	0.060555456	0.428983	0	0
3/21	0.060555456	0.428983	0	0
3/21	0.060555456	0.428983	0	0
3/21	0.060555456	0.428983	0	0
10/132	0.061584334	0.435225496	0	0
14/205	0.063436106	0.446596041	0	0
4/35	0.063647892	0.446596041	0	0
4/35	0.063647892	0.446596041	0	0
5/50	0.064751311	0.451116111	0	0
5/50	0.064751311	0.451116111	0	0
5/50	0.064751311	0.451116111	0	0
6/66	0.066199867	0.456212439	0	0
16/244	0.06670468	0.456212439	0	0
2/10	0.06718568	0.456212439	0	0
2/10	0.06718568	0.456212439	0	0
2/10	0.06718568	0.456212439	0	0
2/10	0.06718568	0.456212439	0	0
2/10	0.06718568	0.456212439	0	0
2/10	0.06718568	0.456212439	0	0

2/10	0.06718568	0.456212439	0	0
2/10	0.06718568	0.456212439	0	0
2/10	0.06718568	0.456212439	0	0
14/207	0.067578248	0.457106897	0	0
3/22	0.067937842	0.457106897	0	0
3/22	0.067937842	0.457106897	0	0
3/22	0.067937842	0.457106897	0	0
7/83	0.068643762	0.460804482	0	0
5/51	0.069348822	0.46176685	0	0
5/51	0.069348822	0.46176685	0	0
5/51	0.069348822	0.46176685	0	0
12/171	0.069413884	0.46176685	0	0
9/119	0.074258437	0.49280566	0	0
22/364	0.074417744	0.49280566	0	0
4/37	0.075104936	0.49280566	0	0
3/23	0.075706865	0.49280566	0	0
3/23	0.075706865	0.49280566	0	0
3/23	0.075706865	0.49280566	0	0
3/23	0.075706865	0.49280566	0	0
7/85	0.075919162	0.49280566	0	0
7/85	0.075919162	0.49280566	0	0
7/85	0.075919162	0.49280566	0	0
7/85	0.075919162	0.49280566	0	0
6/69	0.078537317	0.508032297	0	0
5/53	0.079068818	0.508032297	0	0
2/11	0.079816408	0.508032297	0	0
2/11	0.079816408	0.508032297	0	0
2/11	0.079816408	0.508032297	0	0
2/11	0.079816408	0.508032297	0	0
2/11	0.079816408	0.508032297	0	0
2/11	0.079816408	0.508032297	0	0
2/11	0.079816408	0.508032297	0	0
4/38	0.081196632	0.514594566	0	0
4/38	0.081196632	0.514594566	0	0
6/70	0.082918152	0.523761035	0	0
9/122	0.083792166	0.523761035	0	0
3/24	0.083847361	0.523761035	0	0
3/24	0.083847361	0.523761035	0	0
3/24	0.083847361	0.523761035	0	0
3/24	0.083847361	0.523761035	0	0
10/140	0.083887074	0.523761035	0	0
5/54	0.084188176	0.524529716	0	0
8/105	0.086191493	0.534750169	0	0
8/105	0.086191493	0.534750169	0	0
9/123	0.087125917	0.534769497	0	0
6/71	0.087431923	0.534769497	0	0
4/39	0.087524211	0.534769497	0	0
4/39	0.087524211	0.534769497	0	0

4/39	0.087524211	0.534769497	0	0
4/39	0.087524211	0.534769497	0	0
7/88	0.087646307	0.534769497	0	0
7/88	0.087646307	0.534769497	0	0
5/55	0.089477866	0.541203346	0	0
5/55	0.089477866	0.541203346	0	0
5/55	0.089477866	0.541203346	0	0
9/124	0.090537489	0.541203346	0	0
6/72	0.092077619	0.541203346	0	0
3/25	0.092343359	0.541203346	0	0
3/25	0.092343359	0.541203346	0	0
3/25	0.092343359	0.541203346	0	0
3/25	0.092343359	0.541203346	0	0
2/12	0.093108278	0.541203346	0	0
2/12	0.093108278	0.541203346	0	0
2/12	0.093108278	0.541203346	0	0
2/12	0.093108278	0.541203346	0	0
2/12	0.093108278	0.541203346	0	0
2/12	0.093108278	0.541203346	0	0
2/12	0.093108278	0.541203346	0	0
2/12	0.093108278	0.541203346	0	0
2/12	0.093108278	0.541203346	0	0
2/12	0.093108278	0.541203346	0	0
2/12	0.093108278	0.541203346	0	0
2/12	0.093108278	0.541203346	0	0
2/12	0.093108278	0.541203346	0	0
2/12	0.093108278	0.541203346	0	0
2/12	0.093108278	0.541203346	0	0
4/40	0.094082381	0.543648583	0	0
4/40	0.094082381	0.543648583	0	0
4/40	0.094082381	0.543648583	0	0
6/73	0.096854061	0.558569308	0	0
18/297	0.097112583	0.558966372	0	0
7/91	0.10034051	0.570119046	0	0
5/57	0.100558837	0.570119046	0	0
4/41	0.100865397	0.570119046	0	0
3/26	0.101178236	0.570119046	0	0
3/26	0.101178236	0.570119046	0	0
3/26	0.101178236	0.570119046	0	0
3/26	0.101178236	0.570119046	0	0
3/26	0.101178236	0.570119046	0	0
3/26	0.101178236	0.570119046	0	0
3/26	0.101178236	0.570119046	0	0
3/26	0.101178236	0.570119046	0	0
8/109	0.101511154	0.570126282	0	0
6/74	0.1017599	0.570126282	0	0
6/74	0.1017599	0.570126282	0	0
8/110	0.105564534	0.577391264	0	0

5/58	0.106344766	0.577391264	0	0
5/58	0.106344766	0.577391264	0	0
6/75	0.106793629	0.577391264	0	0
6/75	0.106793629	0.577391264	0	0
2/13	0.106980267	0.577391264	0	0
2/13	0.106980267	0.577391264	0	0
2/13	0.106980267	0.577391264	0	0
2/13	0.106980267	0.577391264	0	0
2/13	0.106980267	0.577391264	0	0
2/13	0.106980267	0.577391264	0	0
2/13	0.106980267	0.577391264	0	0
2/13	0.106980267	0.577391264	0	0
2/13	0.106980267	0.577391264	0	0
2/13	0.106980267	0.577391264	0	0
2/13	0.106980267	0.577391264	0	0
2/13	0.106980267	0.577391264	0	0
2/13	0.106980267	0.577391264	0	0
2/13	0.106980267	0.577391264	0	0
2/13	0.106980267	0.577391264	0	0
4/42	0.107867105	0.577391264	0	0
4/42	0.107867105	0.577391264	0	0
4/42	0.107867105	0.577391264	0	0
13/204	0.107871576	0.577391264	0	0
11/166	0.107954729	0.577391264	0	0
7/93	0.109331323	0.583694582	0	0
8/111	0.109706217	0.583764533	0	0
3/27	0.110334864	0.583764533	0	0
3/27	0.110334864	0.583764533	0	0
3/27	0.110334864	0.583764533	0	0
3/27	0.110334864	0.583764533	0	0
6/76	0.111953586	0.591267414	0	0
5/59	0.112290347	0.591985067	0	0
17/284	0.113424319	0.59649134	0	0
8/112	0.113935528	0.59649134	0	0
7/94	0.113982315	0.59649134	0	0
10/149	0.114172234	0.59649134	0	0
10/149	0.114172234	0.59649134	0	0
15/245	0.114359555	0.59649134	0	0
4/43	0.115080981	0.599193729	0	0
5/60	0.118392309	0.601074685	0	0
7/95	0.118735533	0.601074685	0	0
3/28	0.119795739	0.601074685	0	0
3/28	0.119795739	0.601074685	0	0
3/28	0.119795739	0.601074685	0	0
3/28	0.119795739	0.601074685	0	0
3/28	0.119795739	0.601074685	0	0
3/28	0.119795739	0.601074685	0	0
3/28	0.119795739	0.601074685	0	0

2/14	0.121357122	0.601074685	0	0
2/14	0.121357122	0.601074685	0	0
2/14	0.121357122	0.601074685	0	0
2/14	0.121357122	0.601074685	0	0
2/14	0.121357122	0.601074685	0	0
2/14	0.121357122	0.601074685	0	0
2/14	0.121357122	0.601074685	0	0
2/14	0.121357122	0.601074685	0	0
2/14	0.121357122	0.601074685	0	0
2/14	0.121357122	0.601074685	0	0
2/14	0.121357122	0.601074685	0	0
2/14	0.121357122	0.601074685	0	0
2/14	0.121357122	0.601074685	0	0
2/14	0.121357122	0.601074685	0	0
2/14	0.121357122	0.601074685	0	0
2/14	0.121357122	0.601074685	0	0
2/14	0.121357122	0.601074685	0	0
2/14	0.121357122	0.601074685	0	0
2/14	0.121357122	0.601074685	0	0
2/14	0.121357122	0.601074685	0	0
4/44	0.122500164	0.602435358	0	0
4/44	0.122500164	0.602435358	0	0
4/44	0.122500164	0.602435358	0	0
6/78	0.122644783	0.602435358	0	0
8/114	0.122653958	0.602435358	0	0
10/153	0.12937142	0.627900514	0	0
3/29	0.129543099	0.627900514	0	0
3/29	0.129543099	0.627900514	0	0
3/29	0.129543099	0.627900514	0	0
3/29	0.129543099	0.627900514	0	0
3/29	0.129543099	0.627900514	0	0
3/29	0.129543099	0.627900514	0	0
3/29	0.129543099	0.627900514	0	0
4/45	0.130117495	0.628981335	0	0
5/62	0.13105125	0.628981335	0	0
12/192	0.13154912	0.628981335	0	0
9/135	0.133116973	0.628981335	0	0
2/15	0.136169017	0.628981335	0	0
2/15	0.136169017	0.628981335	0	0
2/15	0.136169017	0.628981335	0	0
2/15	0.136169017	0.628981335	0	0
2/15	0.136169017	0.628981335	0	0
2/15	0.136169017	0.628981335	0	0
2/15	0.136169017	0.628981335	0	0
2/15	0.136169017	0.628981335	0	0
2/15	0.136169017	0.628981335	0	0
2/15	0.136169017	0.628981335	0	0
2/15	0.136169017	0.628981335	0	0

2/15	0.136169017	0.628981335	0	0
2/15	0.136169017	0.628981335	0	0
2/15	0.136169017	0.628981335	0	0
2/15	0.136169017	0.628981335	0	0
2/15	0.136169017	0.628981335	0	0
2/15	0.136169017	0.628981335	0	0
2/15	0.136169017	0.628981335	0	0
2/15	0.136169017	0.628981335	0	0
2/15	0.136169017	0.628981335	0	0
2/15	0.136169017	0.628981335	0	0
2/15	0.136169017	0.628981335	0	0
2/15	0.136169017	0.628981335	0	0
2/15	0.136169017	0.628981335	0	0
2/15	0.136169017	0.628981335	0	0
2/15	0.136169017	0.628981335	0	0
5/63	0.137600677	0.63214091	0	0
4/46	0.137925553	0.63214091	0	0
4/46	0.137925553	0.63214091	0	0
4/46	0.137925553	0.63214091	0	0
4/46	0.137925553	0.63214091	0	0
16/273	0.138384025	0.633257333	0	0
7/99	0.138744275	0.633714105	0	0
3/30	0.139559028	0.633714105	0	0
3/30	0.139559028	0.633714105	0	0
3/30	0.139559028	0.633714105	0	0
3/30	0.139559028	0.633714105	0	0
5/64	0.144291413	0.651555668	0	0
16/275	0.144512072	0.651555668	0	0
26/480	0.145371819	0.651555668	0	0
6/82	0.145452792	0.651555668	0	0
4/47	0.145916687	0.651555668	0	0
4/47	0.145916687	0.651555668	0	0
3/31	0.149825552	0.651555668	0	0
3/31	0.149825552	0.651555668	0	0
3/31	0.149825552	0.651555668	0	0
3/31	0.149825552	0.651555668	0	0
5/65	0.151119257	0.651555668	0	0
2/16	0.151351224	0.651555668	0	0
2/16	0.151351224	0.651555668	0	0
2/16	0.151351224	0.651555668	0	0
2/16	0.151351224	0.651555668	0	0
2/16	0.151351224	0.651555668	0	0
2/16	0.151351224	0.651555668	0	0
2/16	0.151351224	0.651555668	0	0
2/16	0.151351224	0.651555668	0	0
2/16	0.151351224	0.651555668	0	0
2/16	0.151351224	0.651555668	0	0
2/16	0.151351224	0.651555668	0	0

2/16	0.151351224	0.651555668	0	0
2/16	0.151351224	0.651555668	0	0
2/16	0.151351224	0.651555668	0	0
2/16	0.151351224	0.651555668	0	0
2/16	0.151351224	0.651555668	0	0
2/16	0.151351224	0.651555668	0	0
4/48	0.154083049	0.651555668	0	0
4/48	0.154083049	0.651555668	0	0
21/381	0.156714545	0.651555668	0	0
6/84	0.157531057	0.651555668	0	0
3/32	0.160324727	0.651555668	0	0
3/32	0.160324727	0.651555668	0	0
3/32	0.160324727	0.651555668	0	0
15/260	0.161233512	0.651555668	0	0
4/49	0.162416623	0.651555668	0	0
4/49	0.162416623	0.651555668	0	0
4/49	0.162416623	0.651555668	0	0
6/85	0.16372943	0.651555668	0	0
21/384	0.165114952	0.651555668	0	0
5/67	0.165168758	0.651555668	0	0
5/67	0.165168758	0.651555668	0	0
7/104	0.165878259	0.651555668	0	0
7/104	0.165878259	0.651555668	0	0
2/17	0.166843802	0.651555668	0	0
2/17	0.166843802	0.651555668	0	0
2/17	0.166843802	0.651555668	0	0
2/17	0.166843802	0.651555668	0	0
2/17	0.166843802	0.651555668	0	0
2/17	0.166843802	0.651555668	0	0
2/17	0.166843802	0.651555668	0	0
2/17	0.166843802	0.651555668	0	0
2/17	0.166843802	0.651555668	0	0
2/17	0.166843802	0.651555668	0	0
2/17	0.166843802	0.651555668	0	0
2/17	0.166843802	0.651555668	0	0
2/17	0.166843802	0.651555668	0	0
2/17	0.166843802	0.651555668	0	0
2/17	0.166843802	0.651555668	0	0
2/17	0.166843802	0.651555668	0	0
2/17	0.166843802	0.651555668	0	0
2/17	0.166843802	0.651555668	0	0
2/17	0.166843802	0.651555668	0	0
2/17	0.166843802	0.651555668	0	0
2/17	0.166843802	0.651555668	0	0
4/50	0.170909261	0.651555668	0	0
4/50	0.170909261	0.651555668	0	0
3/33	0.17103871	0.651555668	0	0
3/33	0.17103871	0.651555668	0	0

3/33	0.17103871	0.651555668	0	0
3/33	0.17103871	0.651555668	0	0
3/33	0.17103871	0.651555668	0	0
3/33	0.17103871	0.651555668	0	0
5/68	0.17238134	0.651555668	0	0
21/387	0.173754309	0.651555668	0	0
7/106	0.17734579	0.651555668	0	0
9/145	0.179393535	0.651555668	0	0
4/51	0.179552705	0.651555668	0	0
4/51	0.179552705	0.651555668	0	0
4/51	0.179552705	0.651555668	0	0
4/51	0.179552705	0.651555668	0	0
5/69	0.179712905	0.651555668	0	0
5/69	0.179712905	0.651555668	0	0
13/225	0.181023381	0.651555668	0	0
3/34	0.181949827	0.651555668	0	0
3/34	0.181949827	0.651555668	0	0
3/34	0.181949827	0.651555668	0	0
3/34	0.181949827	0.651555668	0	0
3/34	0.181949827	0.651555668	0	0
3/34	0.181949827	0.651555668	0	0
3/34	0.181949827	0.651555668	0	0
3/34	0.181949827	0.651555668	0	0
3/34	0.181949827	0.651555668	0	0
2/18	0.182591306	0.651555668	0	0
2/18	0.182591306	0.651555668	0	0
2/18	0.182591306	0.651555668	0	0
2/18	0.182591306	0.651555668	0	0
2/18	0.182591306	0.651555668	0	0
2/18	0.182591306	0.651555668	0	0
2/18	0.182591306	0.651555668	0	0
2/18	0.182591306	0.651555668	0	0
2/18	0.182591306	0.651555668	0	0
2/18	0.182591306	0.651555668	0	0
2/18	0.182591306	0.651555668	0	0
2/18	0.182591306	0.651555668	0	0
2/18	0.182591306	0.651555668	0	0
2/18	0.182591306	0.651555668	0	0
2/18	0.182591306	0.651555668	0	0
2/18	0.182591306	0.651555668	0	0
2/18	0.182591306	0.651555668	0	0
2/18	0.182591306	0.651555668	0	0
2/18	0.182591306	0.651555668	0	0
2/18	0.182591306	0.651555668	0	0
2/18	0.182591306	0.651555668	0	0
2/18	0.182591306	0.651555668	0	0
2/18	0.182591306	0.651555668	0	0
6/88	0.182926606	0.651555668	0	0

5/70	0.187158657	0.651555668	0	0
4/52	0.188338616	0.651555668	0	0
13/227	0.189021629	0.651555668	0	0
19/351	0.190958206	0.651555668	0	0
17/310	0.192675143	0.651555668	0	0
3/35	0.193040636	0.651555668	0	0
3/35	0.193040636	0.651555668	0	0
3/35	0.193040636	0.651555668	0	0
3/35	0.193040636	0.651555668	0	0
3/35	0.193040636	0.651555668	0	0
3/35	0.193040636	0.651555668	0	0
7/109	0.195153274	0.651555668	0	0
7/109	0.195153274	0.651555668	0	0
4/53	0.197258601	0.651555668	0	0
4/53	0.197258601	0.651555668	0	0
4/53	0.197258601	0.651555668	0	0
4/53	0.197258601	0.651555668	0	0
8/129	0.198251849	0.651555668	0	0
2/19	0.198542508	0.651555668	0	0
2/19	0.198542508	0.651555668	0	0
2/19	0.198542508	0.651555668	0	0
2/19	0.198542508	0.651555668	0	0
2/19	0.198542508	0.651555668	0	0
2/19	0.198542508	0.651555668	0	0
2/19	0.198542508	0.651555668	0	0
2/19	0.198542508	0.651555668	0	0
2/19	0.198542508	0.651555668	0	0
2/19	0.198542508	0.651555668	0	0
2/19	0.198542508	0.651555668	0	0
2/19	0.198542508	0.651555668	0	0
2/19	0.198542508	0.651555668	0	0
2/19	0.198542508	0.651555668	0	0
1/5	0.19898205	0.651555668	0	0
1/5	0.19898205	0.651555668	0	0
1/5	0.19898205	0.651555668	0	0
1/5	0.19898205	0.651555668	0	0
1/5	0.19898205	0.651555668	0	0
1/5	0.19898205	0.651555668	0	0
1/5	0.19898205	0.651555668	0	0
1/5	0.19898205	0.651555668	0	0
1/5	0.19898205	0.651555668	0	0
1/5	0.19898205	0.651555668	0	0
1/5	0.19898205	0.651555668	0	0
1/5	0.19898205	0.651555668	0	0
1/5	0.19898205	0.651555668	0	0
1/5	0.19898205	0.651555668	0	0
1/5	0.19898205	0.651555668	0	0
1/5	0.19898205	0.651555668	0	0

1/5	0.19898205	0.651555668	0	0
1/5	0.19898205	0.651555668	0	0
1/5	0.19898205	0.651555668	0	0
1/5	0.19898205	0.651555668	0	0
1/5	0.19898205	0.651555668	0	0
1/5	0.19898205	0.651555668	0	0
1/5	0.19898205	0.651555668	0	0
1/5	0.19898205	0.651555668	0	0
1/5	0.19898205	0.651555668	0	0
1/5	0.19898205	0.651555668	0	0
1/5	0.19898205	0.651555668	0	0
1/5	0.19898205	0.651555668	0	0
1/5	0.19898205	0.651555668	0	0
1/5	0.19898205	0.651555668	0	0
1/5	0.19898205	0.651555668	0	0
1/5	0.19898205	0.651555668	0	0
1/5	0.19898205	0.651555668	0	0
1/5	0.19898205	0.651555668	0	0
1/5	0.19898205	0.651555668	0	0
1/5	0.19898205	0.651555668	0	0
1/5	0.19898205	0.651555668	0	0
1/5	0.19898205	0.651555668	0	0
1/5	0.19898205	0.651555668	0	0
1/5	0.19898205	0.651555668	0	0
1/5	0.19898205	0.651555668	0	0
1/5	0.19898205	0.651555668	0	0
1/5	0.19898205	0.651555668	0	0
1/5	0.19898205	0.651555668	0	0
1/5	0.19898205	0.651555668	0	0
1/5	0.19898205	0.651555668	0	0
1/5	0.19898205	0.651555668	0	0
1/5	0.19898205	0.651555668	0	0
1/5	0.19898205	0.651555668	0	0
1/5	0.19898205	0.651555668	0	0
1/5	0.19898205	0.651555668	0	0
1/5	0.19898205	0.651555668	0	0
1/5	0.19898205	0.651555668	0	0
1/5	0.19898205	0.651555668	0	0
1/5	0.19898205	0.651555668	0	0
1/5	0.19898205	0.651555668	0	0
1/5	0.19898205	0.651555668	0	0
1/5	0.19898205	0.651555668	0	0
1/5	0.19898205	0.651555668	0	0
1/5	0.19898205	0.651555668	0	0
1/5	0.19898205	0.651555668	0	0
1/5	0.19898205	0.651555668	0	0
1/5	0.19898205	0.651555668	0	0
1/5	0.19898205	0.651555668	0	0

1/5	0.19898205	0.651555668	0	0
1/5	0.19898205	0.651555668	0	0
1/5	0.19898205	0.651555668	0	0
1/5	0.19898205	0.651555668	0	0
1/5	0.19898205	0.651555668	0	0
1/5	0.19898205	0.651555668	0	0
1/5	0.19898205	0.651555668	0	0
1/5	0.19898205	0.651555668	0	0
1/5	0.19898205	0.651555668	0	0
1/5	0.19898205	0.651555668	0	0
1/5	0.19898205	0.651555668	0	0
1/5	0.19898205	0.651555668	0	0
1/5	0.19898205	0.651555668	0	0
1/5	0.19898205	0.651555668	0	0
1/5	0.19898205	0.651555668	0	0
1/5	0.19898205	0.651555668	0	0
1/5	0.19898205	0.651555668	0	0
1/5	0.19898205	0.651555668	0	0
1/5	0.19898205	0.651555668	0	0
1/5	0.19898205	0.651555668	0	0
1/5	0.19898205	0.651555668	0	0
1/5	0.19898205	0.651555668	0	0
1/5	0.19898205	0.651555668	0	0
1/5	0.19898205	0.651555668	0	0
1/5	0.19898205	0.651555668	0	0
1/5	0.19898205	0.651555668	0	0
1/5	0.19898205	0.651555668	0	0
1/5	0.19898205	0.651555668	0	0
1/5	0.19898205	0.651555668	0	0
1/5	0.19898205	0.651555668	0	0
1/5	0.19898205	0.651555668	0	0
1/5	0.19898205	0.651555668	0	0
1/5	0.19898205	0.651555668	0	0
1/5	0.19898205	0.651555668	0	0
1/5	0.19898205	0.651555668	0	0
1/5	0.19898205	0.651555668	0	0
1/5	0.19898205	0.651555668	0	0
5/72	0.202373125	0.661190242	0	0
5/72	0.202373125	0.661190242	0	0
3/36	0.204293975	0.665253419	0	0
3/36	0.204293975	0.665253419	0	0
3/36	0.204293975	0.665253419	0	0
4/54	0.206304236	0.66873023	0	0
4/54	0.206304236	0.66873023	0	0
4/54	0.206304236	0.66873023	0	0
18/336	0.210799505	0.66873023	0	0
2/20	0.214650136	0.66873023	0	0
2/20	0.214650136	0.66873023	0	0

2/20	0.214650136	0.66873023	0	0
2/20	0.214650136	0.66873023	0	0
2/20	0.214650136	0.66873023	0	0
2/20	0.214650136	0.66873023	0	0
2/20	0.214650136	0.66873023	0	0
2/20	0.214650136	0.66873023	0	0
2/20	0.214650136	0.66873023	0	0
2/20	0.214650136	0.66873023	0	0
2/20	0.214650136	0.66873023	0	0
8/132	0.215282018	0.66873023	0	0
4/55	0.21546709	0.66873023	0	0
3/37	0.21569301	0.66873023	0	0
3/37	0.21569301	0.66873023	0	0
3/37	0.21569301	0.66873023	0	0
3/37	0.21569301	0.66873023	0	0
6/94	0.223747566	0.66873023	0	0
5/75	0.225927159	0.66873023	0	0
7/114	0.226296913	0.66873023	0	0
3/38	0.227221272	0.66873023	0	0
3/38	0.227221272	0.66873023	0	0
3/38	0.227221272	0.66873023	0	0
2/21	0.230870632	0.66873023	0	0
2/21	0.230870632	0.66873023	0	0
2/21	0.230870632	0.66873023	0	0
2/21	0.230870632	0.66873023	0	0
2/21	0.230870632	0.66873023	0	0
2/21	0.230870632	0.66873023	0	0
2/21	0.230870632	0.66873023	0	0
2/21	0.230870632	0.66873023	0	0
2/21	0.230870632	0.66873023	0	0
2/21	0.230870632	0.66873023	0	0
2/21	0.230870632	0.66873023	0	0
1/6	0.233754874	0.66873023	0	0
1/6	0.233754874	0.66873023	0	0
1/6	0.233754874	0.66873023	0	0
1/6	0.233754874	0.66873023	0	0
1/6	0.233754874	0.66873023	0	0
1/6	0.233754874	0.66873023	0	0
1/6	0.233754874	0.66873023	0	0
1/6	0.233754874	0.66873023	0	0
1/6	0.233754874	0.66873023	0	0
1/6	0.233754874	0.66873023	0	0
1/6	0.233754874	0.66873023	0	0
1/6	0.233754874	0.66873023	0	0
1/6	0.233754874	0.66873023	0	0
1/6	0.233754874	0.66873023	0	0
1/6	0.233754874	0.66873023	0	0
1/6	0.233754874	0.66873023	0	0

1/6	0.233754874	0.66873023	0	0
1/6	0.233754874	0.66873023	0	0
1/6	0.233754874	0.66873023	0	0
1/6	0.233754874	0.66873023	0	0
1/6	0.233754874	0.66873023	0	0
1/6	0.233754874	0.66873023	0	0
1/6	0.233754874	0.66873023	0	0
1/6	0.233754874	0.66873023	0	0
1/6	0.233754874	0.66873023	0	0
1/6	0.233754874	0.66873023	0	0
1/6	0.233754874	0.66873023	0	0
1/6	0.233754874	0.66873023	0	0
1/6	0.233754874	0.66873023	0	0
1/6	0.233754874	0.66873023	0	0
1/6	0.233754874	0.66873023	0	0
1/6	0.233754874	0.66873023	0	0
1/6	0.233754874	0.66873023	0	0
1/6	0.233754874	0.66873023	0	0
1/6	0.233754874	0.66873023	0	0
1/6	0.233754874	0.66873023	0	0
1/6	0.233754874	0.66873023	0	0
1/6	0.233754874	0.66873023	0	0
1/6	0.233754874	0.66873023	0	0
1/6	0.233754874	0.66873023	0	0
1/6	0.233754874	0.66873023	0	0
1/6	0.233754874	0.66873023	0	0
1/6	0.233754874	0.66873023	0	0
1/6	0.233754874	0.66873023	0	0
1/6	0.233754874	0.66873023	0	0
1/6	0.233754874	0.66873023	0	0
1/6	0.233754874	0.66873023	0	0
1/6	0.233754874	0.66873023	0	0
1/6	0.233754874	0.66873023	0	0
1/6	0.233754874	0.66873023	0	0
1/6	0.233754874	0.66873023	0	0
1/6	0.233754874	0.66873023	0	0
1/6	0.233754874	0.66873023	0	0
1/6	0.233754874	0.66873023	0	0
1/6	0.233754874	0.66873023	0	0
1/6	0.233754874	0.66873023	0	0
1/6	0.233754874	0.66873023	0	0
1/6	0.233754874	0.66873023	0	0
1/6	0.233754874	0.66873023	0	0
1/6	0.233754874	0.66873023	0	0
1/6	0.233754874	0.66873023	0	0
1/6	0.233754874	0.66873023	0	0
1/6	0.233754874	0.66873023	0	0
1/6	0.233754874	0.66873023	0	0

1/6	0.233754874	0.66873023	0	0
1/6	0.233754874	0.66873023	0	0
1/6	0.233754874	0.66873023	0	0
1/6	0.233754874	0.66873023	0	0
1/6	0.233754874	0.66873023	0	0
1/6	0.233754874	0.66873023	0	0
1/6	0.233754874	0.66873023	0	0
1/6	0.233754874	0.66873023	0	0
1/6	0.233754874	0.66873023	0	0
1/6	0.233754874	0.66873023	0	0
1/6	0.233754874	0.66873023	0	0
1/6	0.233754874	0.66873023	0	0
1/6	0.233754874	0.66873023	0	0
1/6	0.233754874	0.66873023	0	0
1/6	0.233754874	0.66873023	0	0
1/6	0.233754874	0.66873023	0	0
1/6	0.233754874	0.66873023	0	0
1/6	0.233754874	0.66873023	0	0
1/6	0.233754874	0.66873023	0	0
1/6	0.233754874	0.66873023	0	0
1/6	0.233754874	0.66873023	0	0
1/6	0.233754874	0.66873023	0	0
1/6	0.233754874	0.66873023	0	0
5/76	0.233953467	0.66873023	0	0
3/39	0.238862695	0.671301239	0	0
3/39	0.238862695	0.671301239	0	0
3/39	0.238862695	0.671301239	0	0
3/39	0.238862695	0.671301239	0	0
3/39	0.238862695	0.671301239	0	0
3/39	0.238862695	0.671301239	0	0
3/39	0.238862695	0.671301239	0	0
3/39	0.238862695	0.671301239	0	0
3/39	0.238862695	0.671301239	0	0
3/39	0.238862695	0.671301239	0	0
3/39	0.238862695	0.671301239	0	0
5/77	0.242058711	0.671301239	0	0
4/58	0.243574964	0.671301239	0	0
7/117	0.245755133	0.671301239	0	0
2/22	0.247163913	0.671301239	0	0
2/22	0.247163913	0.671301239	0	0
2/22	0.247163913	0.671301239	0	0
2/22	0.247163913	0.671301239	0	0
2/22	0.247163913	0.671301239	0	0
2/22	0.247163913	0.671301239	0	0
2/22	0.247163913	0.671301239	0	0
2/22	0.247163913	0.671301239	0	0
2/22	0.247163913	0.671301239	0	0
2/22	0.247163913	0.671301239	0	0

2/22	0.247163913	0.671301239	0	0
2/22	0.247163913	0.671301239	0	0
2/22	0.247163913	0.671301239	0	0
2/22	0.247163913	0.671301239	0	0
2/22	0.247163913	0.671301239	0	0
2/22	0.247163913	0.671301239	0	0
2/22	0.247163913	0.671301239	0	0
2/22	0.247163913	0.671301239	0	0
9/158	0.248589697	0.671301239	0	0
3/40	0.25060164	0.671301239	0	0
3/40	0.25060164	0.671301239	0	0
3/40	0.25060164	0.671301239	0	0
10/179	0.251075666	0.671301239	0	0
6/98	0.252469516	0.671301239	0	0
6/98	0.252469516	0.671301239	0	0
4/59	0.253122937	0.671301239	0	0
4/59	0.253122937	0.671301239	0	0
4/59	0.253122937	0.671301239	0	0
11/201	0.257690854	0.671301239	0	0
5/79	0.258485435	0.671301239	0	0
5/79	0.258485435	0.671301239	0	0
5/79	0.258485435	0.671301239	0	0
5/79	0.258485435	0.671301239	0	0
7/119	0.259006744	0.671301239	0	0
3/41	0.262422922	0.671301239	0	0
3/41	0.262422922	0.671301239	0	0
3/41	0.262422922	0.671301239	0	0
4/60	0.262746553	0.671301239	0	0
4/60	0.262746553	0.671301239	0	0
2/23	0.263493156	0.671301239	0	0
2/23	0.263493156	0.671301239	0	0
2/23	0.263493156	0.671301239	0	0
2/23	0.263493156	0.671301239	0	0
2/23	0.263493156	0.671301239	0	0
2/23	0.263493156	0.671301239	0	0
2/23	0.263493156	0.671301239	0	0
2/23	0.263493156	0.671301239	0	0
2/23	0.263493156	0.671301239	0	0
2/23	0.263493156	0.671301239	0	0
2/23	0.263493156	0.671301239	0	0
1/7	0.267019846	0.671301239	0	0
1/7	0.267019846	0.671301239	0	0
1/7	0.267019846	0.671301239	0	0
1/7	0.267019846	0.671301239	0	0
1/7	0.267019846	0.671301239	0	0
1/7	0.267019846	0.671301239	0	0
1/7	0.267019846	0.671301239	0	0
1/7	0.267019846	0.671301239	0	0

1/7	0.267019846	0.671301239	0	0
1/7	0.267019846	0.671301239	0	0
1/7	0.267019846	0.671301239	0	0
1/7	0.267019846	0.671301239	0	0
1/7	0.267019846	0.671301239	0	0
1/7	0.267019846	0.671301239	0	0
1/7	0.267019846	0.671301239	0	0
1/7	0.267019846	0.671301239	0	0
1/7	0.267019846	0.671301239	0	0
1/7	0.267019846	0.671301239	0	0
1/7	0.267019846	0.671301239	0	0
1/7	0.267019846	0.671301239	0	0
1/7	0.267019846	0.671301239	0	0
1/7	0.267019846	0.671301239	0	0
1/7	0.267019846	0.671301239	0	0
1/7	0.267019846	0.671301239	0	0
1/7	0.267019846	0.671301239	0	0
1/7	0.267019846	0.671301239	0	0
1/7	0.267019846	0.671301239	0	0
1/7	0.267019846	0.671301239	0	0
1/7	0.267019846	0.671301239	0	0
1/7	0.267019846	0.671301239	0	0
1/7	0.267019846	0.671301239	0	0
1/7	0.267019846	0.671301239	0	0
1/7	0.267019846	0.671301239	0	0
1/7	0.267019846	0.671301239	0	0
1/7	0.267019846	0.671301239	0	0
1/7	0.267019846	0.671301239	0	0
1/7	0.267019846	0.671301239	0	0
1/7	0.267019846	0.671301239	0	0
1/7	0.267019846	0.671301239	0	0
1/7	0.267019846	0.671301239	0	0
1/7	0.267019846	0.671301239	0	0
1/7	0.267019846	0.671301239	0	0
1/7	0.267019846	0.671301239	0	0
1/7	0.267019846	0.671301239	0	0
1/7	0.267019846	0.671301239	0	0
1/7	0.267019846	0.671301239	0	0
1/7	0.267019846	0.671301239	0	0
1/7	0.267019846	0.671301239	0	0
1/7	0.267019846	0.671301239	0	0
1/7	0.267019846	0.671301239	0	0
1/7	0.267019846	0.671301239	0	0
1/7	0.267019846	0.671301239	0	0
1/7	0.267019846	0.671301239	0	0
1/7	0.267019846	0.671301239	0	0
1/7	0.267019846	0.671301239	0	0
1/7	0.267019846	0.671301239	0	0

1/7	0.267019846	0.671301239	0	0
1/7	0.267019846	0.671301239	0	0
1/7	0.267019846	0.671301239	0	0
1/7	0.267019846	0.671301239	0	0
1/7	0.267019846	0.671301239	0	0
1/7	0.267019846	0.671301239	0	0
1/7	0.267019846	0.671301239	0	0
1/7	0.267019846	0.671301239	0	0
1/7	0.267019846	0.671301239	0	0
1/7	0.267019846	0.671301239	0	0
1/7	0.267019846	0.671301239	0	0
1/7	0.267019846	0.671301239	0	0
1/7	0.267019846	0.671301239	0	0
1/7	0.267019846	0.671301239	0	0
1/7	0.267019846	0.671301239	0	0
1/7	0.267019846	0.671301239	0	0
1/7	0.267019846	0.671301239	0	0
1/7	0.267019846	0.671301239	0	0
1/7	0.267019846	0.671301239	0	0
1/7	0.267019846	0.671301239	0	0
6/100	0.267199306	0.671301239	0	0
6/100	0.267199306	0.671301239	0	0
4/61	0.272437737	0.683252118	0	0
4/61	0.272437737	0.683252118	0	0
10/183	0.272651677	0.683252118	0	0
3/42	0.274311831	0.684295406	0	0
3/42	0.274311831	0.684295406	0	0
3/42	0.274311831	0.684295406	0	0
5/81	0.275166266	0.684295406	0	0
7/122	0.279251246	0.684295406	0	0
2/24	0.279824592	0.684295406	0	0
2/24	0.279824592	0.684295406	0	0
2/24	0.279824592	0.684295406	0	0
2/24	0.279824592	0.684295406	0	0
2/24	0.279824592	0.684295406	0	0
2/24	0.279824592	0.684295406	0	0
2/24	0.279824592	0.684295406	0	0
2/24	0.279824592	0.684295406	0	0
2/24	0.279824592	0.684295406	0	0
2/24	0.279824592	0.684295406	0	0
2/24	0.279824592	0.684295406	0	0
2/24	0.279824592	0.684295406	0	0
4/62	0.282188529	0.684295406	0	0
5/82	0.283589218	0.684295406	0	0
14/270	0.285159335	0.684295406	0	0
3/43	0.286254144	0.684295406	0	0
3/43	0.286254144	0.684295406	0	0
3/43	0.286254144	0.684295406	0	0

3/43	0.286254144	0.684295406	0	0
3/43	0.286254144	0.684295406	0	0
19/379	0.291452526	0.684295406	0	0
5/83	0.292060455	0.684295406	0	0
2/25	0.296127308	0.684295406	0	0
2/25	0.296127308	0.684295406	0	0
2/25	0.296127308	0.684295406	0	0
2/25	0.296127308	0.684295406	0	0
2/25	0.296127308	0.684295406	0	0
2/25	0.296127308	0.684295406	0	0
2/25	0.296127308	0.684295406	0	0
2/25	0.296127308	0.684295406	0	0
2/25	0.296127308	0.684295406	0	0
2/25	0.296127308	0.684295406	0	0
2/25	0.296127308	0.684295406	0	0
2/25	0.296127308	0.684295406	0	0
2/25	0.296127308	0.684295406	0	0
2/25	0.296127308	0.684295406	0	0
2/25	0.296127308	0.684295406	0	0
2/25	0.296127308	0.684295406	0	0
3/44	0.298236142	0.684295406	0	0
3/44	0.298236142	0.684295406	0	0
1/8	0.298842276	0.684295406	0	0
1/8	0.298842276	0.684295406	0	0
1/8	0.298842276	0.684295406	0	0
1/8	0.298842276	0.684295406	0	0
1/8	0.298842276	0.684295406	0	0
1/8	0.298842276	0.684295406	0	0
1/8	0.298842276	0.684295406	0	0
1/8	0.298842276	0.684295406	0	0
1/8	0.298842276	0.684295406	0	0
1/8	0.298842276	0.684295406	0	0
1/8	0.298842276	0.684295406	0	0
1/8	0.298842276	0.684295406	0	0
1/8	0.298842276	0.684295406	0	0
1/8	0.298842276	0.684295406	0	0
1/8	0.298842276	0.684295406	0	0
1/8	0.298842276	0.684295406	0	0
1/8	0.298842276	0.684295406	0	0
1/8	0.298842276	0.684295406	0	0
1/8	0.298842276	0.684295406	0	0
1/8	0.298842276	0.684295406	0	0
1/8	0.298842276	0.684295406	0	0
1/8	0.298842276	0.684295406	0	0
1/8	0.298842276	0.684295406	0	0
1/8	0.298842276	0.684295406	0	0
1/8	0.298842276	0.684295406	0	0
1/8	0.298842276	0.684295406	0	0

1/8	0.298842276	0.684295406	0	0
1/8	0.298842276	0.684295406	0	0
1/8	0.298842276	0.684295406	0	0
1/8	0.298842276	0.684295406	0	0
1/8	0.298842276	0.684295406	0	0
1/8	0.298842276	0.684295406	0	0
1/8	0.298842276	0.684295406	0	0
1/8	0.298842276	0.684295406	0	0
1/8	0.298842276	0.684295406	0	0
1/8	0.298842276	0.684295406	0	0
1/8	0.298842276	0.684295406	0	0
1/8	0.298842276	0.684295406	0	0
1/8	0.298842276	0.684295406	0	0
1/8	0.298842276	0.684295406	0	0
1/8	0.298842276	0.684295406	0	0
1/8	0.298842276	0.684295406	0	0
1/8	0.298842276	0.684295406	0	0
1/8	0.298842276	0.684295406	0	0
1/8	0.298842276	0.684295406	0	0
1/8	0.298842276	0.684295406	0	0
1/8	0.298842276	0.684295406	0	0
1/8	0.298842276	0.684295406	0	0
1/8	0.298842276	0.684295406	0	0
1/8	0.298842276	0.684295406	0	0
1/8	0.298842276	0.684295406	0	0
1/8	0.298842276	0.684295406	0	0
1/8	0.298842276	0.684295406	0	0
1/8	0.298842276	0.684295406	0	0
1/8	0.298842276	0.684295406	0	0
1/8	0.298842276	0.684295406	0	0
1/8	0.298842276	0.684295406	0	0
1/8	0.298842276	0.684295406	0	0
1/8	0.298842276	0.684295406	0	0
1/8	0.298842276	0.684295406	0	0
1/8	0.298842276	0.684295406	0	0
1/8	0.298842276	0.684295406	0	0
1/8	0.298842276	0.684295406	0	0
1/8	0.298842276	0.684295406	0	0
1/8	0.298842276	0.684295406	0	0
1/8	0.298842276	0.684295406	0	0
7/125	0.299872949	0.684345465	0	0
5/84	0.300574973	0.684345465	0	0
12/231	0.30291392	0.684345465	0	0
7/126	0.30681832	0.684345465	0	0
5/85	0.309127819	0.684345465	0	0
5/85	0.309127819	0.684345465	0	0
3/45	0.310244616	0.684345465	0	0
3/45	0.310244616	0.684345465	0	0

3/45	0.310244616	0.684345465	0	0
3/45	0.310244616	0.684345465	0	0
26/537	0.310297194	0.684345465	0	0
10/190	0.311654724	0.684345465	0	0
4/65	0.311720917	0.684345465	0	0
4/65	0.311720917	0.684345465	0	0
4/65	0.311720917	0.684345465	0	0
4/65	0.311720917	0.684345465	0	0
2/26	0.312373063	0.684345465	0	0
2/26	0.312373063	0.684345465	0	0
2/26	0.312373063	0.684345465	0	0
2/26	0.312373063	0.684345465	0	0
2/26	0.312373063	0.684345465	0	0
2/26	0.312373063	0.684345465	0	0
2/26	0.312373063	0.684345465	0	0
2/26	0.312373063	0.684345465	0	0
2/26	0.312373063	0.684345465	0	0
2/26	0.312373063	0.684345465	0	0
2/26	0.312373063	0.684345465	0	0
2/26	0.312373063	0.684345465	0	0
2/26	0.312373063	0.684345465	0	0
2/26	0.312373063	0.684345465	0	0
16/320	0.316206164	0.684345465	0	0
5/86	0.317714094	0.684345465	0	0
3/46	0.322266874	0.684345465	0	0
3/46	0.322266874	0.684345465	0	0
3/46	0.322266874	0.684345465	0	0
3/46	0.322266874	0.684345465	0	0
3/46	0.322266874	0.684345465	0	0
3/46	0.322266874	0.684345465	0	0
3/46	0.322266874	0.684345465	0	0
5/87	0.326328963	0.684345465	0	0
7/129	0.327831895	0.684345465	0	0
6/108	0.327915544	0.684345465	0	0
2/27	0.328536117	0.684345465	0	0
2/27	0.328536117	0.684345465	0	0
2/27	0.328536117	0.684345465	0	0
2/27	0.328536117	0.684345465	0	0
2/27	0.328536117	0.684345465	0	0
2/27	0.328536117	0.684345465	0	0
2/27	0.328536117	0.684345465	0	0
2/27	0.328536117	0.684345465	0	0
2/27	0.328536117	0.684345465	0	0
2/27	0.328536117	0.684345465	0	0
2/27	0.328536117	0.684345465	0	0
2/27	0.328536117	0.684345465	0	0
2/27	0.328536117	0.684345465	0	0
2/27	0.328536117	0.684345465	0	0

12/236	0.328539377	0.684345465	0	0
10/193	0.32875927	0.684345465	0	0
1/9	0.329284652	0.684345465	0	0
1/9	0.329284652	0.684345465	0	0
1/9	0.329284652	0.684345465	0	0
1/9	0.329284652	0.684345465	0	0
1/9	0.329284652	0.684345465	0	0
1/9	0.329284652	0.684345465	0	0
1/9	0.329284652	0.684345465	0	0
1/9	0.329284652	0.684345465	0	0
1/9	0.329284652	0.684345465	0	0
1/9	0.329284652	0.684345465	0	0
1/9	0.329284652	0.684345465	0	0
1/9	0.329284652	0.684345465	0	0
1/9	0.329284652	0.684345465	0	0
1/9	0.329284652	0.684345465	0	0
1/9	0.329284652	0.684345465	0	0
1/9	0.329284652	0.684345465	0	0
1/9	0.329284652	0.684345465	0	0
1/9	0.329284652	0.684345465	0	0
1/9	0.329284652	0.684345465	0	0
1/9	0.329284652	0.684345465	0	0
1/9	0.329284652	0.684345465	0	0
1/9	0.329284652	0.684345465	0	0
1/9	0.329284652	0.684345465	0	0
1/9	0.329284652	0.684345465	0	0
1/9	0.329284652	0.684345465	0	0
1/9	0.329284652	0.684345465	0	0
1/9	0.329284652	0.684345465	0	0
1/9	0.329284652	0.684345465	0	0
1/9	0.329284652	0.684345465	0	0
1/9	0.329284652	0.684345465	0	0
1/9	0.329284652	0.684345465	0	0
1/9	0.329284652	0.684345465	0	0
1/9	0.329284652	0.684345465	0	0
1/9	0.329284652	0.684345465	0	0
1/9	0.329284652	0.684345465	0	0
1/9	0.329284652	0.684345465	0	0
1/9	0.329284652	0.684345465	0	0
1/9	0.329284652	0.684345465	0	0
1/9	0.329284652	0.684345465	0	0
1/9	0.329284652	0.684345465	0	0
1/9	0.329284652	0.684345465	0	0
1/9	0.329284652	0.684345465	0	0
1/9	0.329284652	0.684345465	0	0
1/9	0.329284652	0.684345465	0	0
1/9	0.329284652	0.684345465	0	0
1/9	0.329284652	0.684345465	0	0
1/9	0.329284652	0.684345465	0	0
1/9	0.329284652	0.684345465	0	0

1/9	0.329284652	0.684345465	0	0
1/9	0.329284652	0.684345465	0	0
1/9	0.329284652	0.684345465	0	0
1/9	0.329284652	0.684345465	0	0
1/9	0.329284652	0.684345465	0	0
1/9	0.329284652	0.684345465	0	0
1/9	0.329284652	0.684345465	0	0
1/9	0.329284652	0.684345465	0	0
1/9	0.329284652	0.684345465	0	0
1/9	0.329284652	0.684345465	0	0
1/9	0.329284652	0.684345465	0	0
1/9	0.329284652	0.684345465	0	0
1/9	0.329284652	0.684345465	0	0
1/9	0.329284652	0.684345465	0	0
1/9	0.329284652	0.684345465	0	0
1/9	0.329284652	0.684345465	0	0
1/9	0.329284652	0.684345465	0	0
1/9	0.329284652	0.684345465	0	0
1/9	0.329284652	0.684345465	0	0
1/9	0.329284652	0.684345465	0	0
1/9	0.329284652	0.684345465	0	0
1/9	0.329284652	0.684345465	0	0
1/9	0.329284652	0.684345465	0	0
1/9	0.329284652	0.684345465	0	0
1/9	0.329284652	0.684345465	0	0
1/9	0.329284652	0.684345465	0	0
1/9	0.329284652	0.684345465	0	0
9/172	0.331184124	0.687808042	0	0
4/67	0.331567493	0.688119297	0	0
3/47	0.334290749	0.692152498	0	0
3/47	0.334290749	0.692152498	0	0
3/47	0.334290749	0.692152498	0	0
7/130	0.334886836	0.692152498	0	0
5/88	0.334967658	0.692152498	0	0
6/109	0.335649268	0.692152498	0	0
14/281	0.336637454	0.692152498	0	0
8/152	0.339831373	0.692152498	0	0
4/68	0.341516626	0.692152498	0	0
9/174	0.343416137	0.692152498	0	0
5/89	0.343625483	0.692152498	0	0
5/89	0.343625483	0.692152498	0	0
2/28	0.344593066	0.692152498	0	0
2/28	0.344593066	0.692152498	0	0
2/28	0.344593066	0.692152498	0	0
2/28	0.344593066	0.692152498	0	0
2/28	0.344593066	0.692152498	0	0
2/28	0.344593066	0.692152498	0	0
2/28	0.344593066	0.692152498	0	0

2/28	0.344593066	0.692152498	0	0
2/28	0.344593066	0.692152498	0	0
2/28	0.344593066	0.692152498	0	0
2/28	0.344593066	0.692152498	0	0
2/28	0.344593066	0.692152498	0	0
25/525	0.344915696	0.692152498	0	0
10/196	0.346042877	0.692152498	0	0
14/283	0.346219736	0.692152498	0	0
8/154	0.352968743	0.692152498	0	0
9/176	0.355716216	0.692152498	0	0
3/49	0.358297285	0.692152498	0	0
3/49	0.358297285	0.692152498	0	0
1/10	0.358406756	0.692152498	0	0
1/10	0.358406756	0.692152498	0	0
1/10	0.358406756	0.692152498	0	0
1/10	0.358406756	0.692152498	0	0
1/10	0.358406756	0.692152498	0	0
1/10	0.358406756	0.692152498	0	0
1/10	0.358406756	0.692152498	0	0
1/10	0.358406756	0.692152498	0	0
1/10	0.358406756	0.692152498	0	0
1/10	0.358406756	0.692152498	0	0
1/10	0.358406756	0.692152498	0	0
1/10	0.358406756	0.692152498	0	0
1/10	0.358406756	0.692152498	0	0
1/10	0.358406756	0.692152498	0	0
1/10	0.358406756	0.692152498	0	0
1/10	0.358406756	0.692152498	0	0
1/10	0.358406756	0.692152498	0	0
1/10	0.358406756	0.692152498	0	0
1/10	0.358406756	0.692152498	0	0
1/10	0.358406756	0.692152498	0	0
1/10	0.358406756	0.692152498	0	0
1/10	0.358406756	0.692152498	0	0
1/10	0.358406756	0.692152498	0	0
1/10	0.358406756	0.692152498	0	0
1/10	0.358406756	0.692152498	0	0
1/10	0.358406756	0.692152498	0	0
1/10	0.358406756	0.692152498	0	0
1/10	0.358406756	0.692152498	0	0
1/10	0.358406756	0.692152498	0	0
1/10	0.358406756	0.692152498	0	0
1/10	0.358406756	0.692152498	0	0
1/10	0.358406756	0.692152498	0	0
1/10	0.358406756	0.692152498	0	0
1/10	0.358406756	0.692152498	0	0
1/10	0.358406756	0.692152498	0	0
1/10	0.358406756	0.692152498	0	0

1/10	0.358406756	0.692152498	0	0
1/10	0.358406756	0.692152498	0	0
1/10	0.358406756	0.692152498	0	0
1/10	0.358406756	0.692152498	0	0
1/10	0.358406756	0.692152498	0	0
1/10	0.358406756	0.692152498	0	0
1/10	0.358406756	0.692152498	0	0
1/10	0.358406756	0.692152498	0	0
1/10	0.358406756	0.692152498	0	0
1/10	0.358406756	0.692152498	0	0
1/10	0.358406756	0.692152498	0	0
1/10	0.358406756	0.692152498	0	0
1/10	0.358406756	0.692152498	0	0
1/10	0.358406756	0.692152498	0	0
1/10	0.358406756	0.692152498	0	0
1/10	0.358406756	0.692152498	0	0
1/10	0.358406756	0.692152498	0	0
1/10	0.358406756	0.692152498	0	0
1/10	0.358406756	0.692152498	0	0
1/10	0.358406756	0.692152498	0	0
1/10	0.358406756	0.692152498	0	0
1/10	0.358406756	0.692152498	0	0
1/10	0.358406756	0.692152498	0	0
1/10	0.358406756	0.692152498	0	0
1/10	0.358406756	0.692152498	0	0
1/10	0.358406756	0.692152498	0	0
1/10	0.358406756	0.692152498	0	0
1/10	0.358406756	0.692152498	0	0
1/10	0.358406756	0.692152498	0	0
1/10	0.358406756	0.692152498	0	0
1/10	0.358406756	0.692152498	0	0
1/10	0.358406756	0.692152498	0	0
1/10	0.358406756	0.692152498	0	0
1/10	0.358406756	0.692152498	0	0
1/10	0.358406756	0.692152498	0	0
1/10	0.358406756	0.692152498	0	0
1/10	0.358406756	0.692152498	0	0
1/10	0.358406756	0.692152498	0	0
1/10	0.358406756	0.692152498	0	0
2/29	0.360522691	0.693059602	0	0
2/29	0.360522691	0.693059602	0	0
2/29	0.360522691	0.693059602	0	0
2/29	0.360522691	0.693059602	0	0
2/29	0.360522691	0.693059602	0	0
2/29	0.360522691	0.693059602	0	0
2/29	0.360522691	0.693059602	0	0
5/91	0.360980138	0.693486614	0	0
4/70	0.361432266	0.693902859	0	0

7/134	0.363289697	0.697014802	0	0
13/265	0.365457733	0.700718243	0	0
3/50	0.37025822	0.704409202	0	0
3/50	0.37025822	0.704409202	0	0
3/50	0.37025822	0.704409202	0	0
13/266	0.370512467	0.704409202	0	0
4/71	0.37138561	0.704409202	0	0
6/114	0.37455957	0.704409202	0	0
2/30	0.376305806	0.704409202	0	0
2/30	0.376305806	0.704409202	0	0
2/30	0.376305806	0.704409202	0	0
2/30	0.376305806	0.704409202	0	0
2/30	0.376305806	0.704409202	0	0
2/30	0.376305806	0.704409202	0	0
2/30	0.376305806	0.704409202	0	0
2/30	0.376305806	0.704409202	0	0
2/30	0.376305806	0.704409202	0	0
2/30	0.376305806	0.704409202	0	0
5/93	0.378356998	0.704409202	0	0
4/72	0.381327519	0.704409202	0	0
4/72	0.381327519	0.704409202	0	0
3/51	0.382177312	0.704409202	0	0
3/51	0.382177312	0.704409202	0	0
3/51	0.382177312	0.704409202	0	0
7/137	0.384713259	0.704409202	0	0
8/159	0.386032967	0.704409202	0	0
1/11	0.386265786	0.704409202	0	0
1/11	0.386265786	0.704409202	0	0
1/11	0.386265786	0.704409202	0	0
1/11	0.386265786	0.704409202	0	0
1/11	0.386265786	0.704409202	0	0
1/11	0.386265786	0.704409202	0	0
1/11	0.386265786	0.704409202	0	0
1/11	0.386265786	0.704409202	0	0
1/11	0.386265786	0.704409202	0	0
1/11	0.386265786	0.704409202	0	0
1/11	0.386265786	0.704409202	0	0
1/11	0.386265786	0.704409202	0	0
1/11	0.386265786	0.704409202	0	0
1/11	0.386265786	0.704409202	0	0
1/11	0.386265786	0.704409202	0	0
1/11	0.386265786	0.704409202	0	0
1/11	0.386265786	0.704409202	0	0
1/11	0.386265786	0.704409202	0	0
1/11	0.386265786	0.704409202	0	0
1/11	0.386265786	0.704409202	0	0
1/11	0.386265786	0.704409202	0	0
1/11	0.386265786	0.704409202	0	0

1/11	0.386265786	0.704409202	0	0
1/11	0.386265786	0.704409202	0	0
1/11	0.386265786	0.704409202	0	0
1/11	0.386265786	0.704409202	0	0
1/11	0.386265786	0.704409202	0	0
1/11	0.386265786	0.704409202	0	0
1/11	0.386265786	0.704409202	0	0
1/11	0.386265786	0.704409202	0	0
1/11	0.386265786	0.704409202	0	0
1/11	0.386265786	0.704409202	0	0
1/11	0.386265786	0.704409202	0	0
1/11	0.386265786	0.704409202	0	0
1/11	0.386265786	0.704409202	0	0
1/11	0.386265786	0.704409202	0	0
1/11	0.386265786	0.704409202	0	0
1/11	0.386265786	0.704409202	0	0
1/11	0.386265786	0.704409202	0	0
1/11	0.386265786	0.704409202	0	0
1/11	0.386265786	0.704409202	0	0
1/11	0.386265786	0.704409202	0	0
1/11	0.386265786	0.704409202	0	0
1/11	0.386265786	0.704409202	0	0
1/11	0.386265786	0.704409202	0	0
1/11	0.386265786	0.704409202	0	0
1/11	0.386265786	0.704409202	0	0
1/11	0.386265786	0.704409202	0	0
1/11	0.386265786	0.704409202	0	0
1/11	0.386265786	0.704409202	0	0
1/11	0.386265786	0.704409202	0	0
1/11	0.386265786	0.704409202	0	0
1/11	0.386265786	0.704409202	0	0
1/11	0.386265786	0.704409202	0	0
1/11	0.386265786	0.704409202	0	0
4/73	0.391251901	0.705020169	0	0
4/73	0.391251901	0.705020169	0	0
2/31	0.39192513	0.705020169	0	0
2/31	0.39192513	0.705020169	0	0
2/31	0.39192513	0.705020169	0	0
2/31	0.39192513	0.705020169	0	0
2/31	0.39192513	0.705020169	0	0
2/31	0.39192513	0.705020169	0	0
2/31	0.39192513	0.705020169	0	0
2/31	0.39192513	0.705020169	0	0
2/31	0.39192513	0.705020169	0	0
2/31	0.39192513	0.705020169	0	0
2/31	0.39192513	0.705020169	0	0
2/31	0.39192513	0.705020169	0	0
2/31	0.39192513	0.705020169	0	0

2/31	0.39192513	0.705020169	0	0
2/31	0.39192513	0.705020169	0	0
2/31	0.39192513	0.705020169	0	0
3/52	0.394044984	0.705020169	0	0
3/52	0.394044984	0.705020169	0	0
3/52	0.394044984	0.705020169	0	0
3/52	0.394044984	0.705020169	0	0
3/52	0.394044984	0.705020169	0	0
5/95	0.395721578	0.705020169	0	0
6/117	0.39798209	0.705020169	0	0
6/117	0.39798209	0.705020169	0	0
4/74	0.401152868	0.705020169	0	0
4/74	0.401152868	0.705020169	0	0
6/118	0.405783554	0.705020169	0	0
3/53	0.405852163	0.705020169	0	0
3/53	0.405852163	0.705020169	0	0
3/53	0.405852163	0.705020169	0	0
2/32	0.407365152	0.705020169	0	0
2/32	0.407365152	0.705020169	0	0
2/32	0.407365152	0.705020169	0	0
2/32	0.407365152	0.705020169	0	0
2/32	0.407365152	0.705020169	0	0
2/32	0.407365152	0.705020169	0	0
2/32	0.407365152	0.705020169	0	0
2/32	0.407365152	0.705020169	0	0
2/32	0.407365152	0.705020169	0	0
2/32	0.407365152	0.705020169	0	0
2/32	0.407365152	0.705020169	0	0
2/32	0.407365152	0.705020169	0	0
2/32	0.407365152	0.705020169	0	0
8/163	0.412581505	0.705020169	0	0
1/12	0.412916461	0.705020169	0	0
1/12	0.412916461	0.705020169	0	0
1/12	0.412916461	0.705020169	0	0
1/12	0.412916461	0.705020169	0	0
1/12	0.412916461	0.705020169	0	0
1/12	0.412916461	0.705020169	0	0
1/12	0.412916461	0.705020169	0	0
1/12	0.412916461	0.705020169	0	0
1/12	0.412916461	0.705020169	0	0
1/12	0.412916461	0.705020169	0	0
1/12	0.412916461	0.705020169	0	0
1/12	0.412916461	0.705020169	0	0
1/12	0.412916461	0.705020169	0	0
1/12	0.412916461	0.705020169	0	0
1/12	0.412916461	0.705020169	0	0
1/12	0.412916461	0.705020169	0	0
1/12	0.412916461	0.705020169	0	0

1/12	0.412916461	0.705020169	0	0
1/12	0.412916461	0.705020169	0	0
1/12	0.412916461	0.705020169	0	0
1/12	0.412916461	0.705020169	0	0
1/12	0.412916461	0.705020169	0	0
1/12	0.412916461	0.705020169	0	0
1/12	0.412916461	0.705020169	0	0
1/12	0.412916461	0.705020169	0	0
1/12	0.412916461	0.705020169	0	0
1/12	0.412916461	0.705020169	0	0
1/12	0.412916461	0.705020169	0	0
1/12	0.412916461	0.705020169	0	0
1/12	0.412916461	0.705020169	0	0
1/12	0.412916461	0.705020169	0	0
1/12	0.412916461	0.705020169	0	0
1/12	0.412916461	0.705020169	0	0
1/12	0.412916461	0.705020169	0	0
1/12	0.412916461	0.705020169	0	0
1/12	0.412916461	0.705020169	0	0
1/12	0.412916461	0.705020169	0	0
1/12	0.412916461	0.705020169	0	0
1/12	0.412916461	0.705020169	0	0
1/12	0.412916461	0.705020169	0	0
1/12	0.412916461	0.705020169	0	0
1/12	0.412916461	0.705020169	0	0
1/12	0.412916461	0.705020169	0	0
1/12	0.412916461	0.705020169	0	0
1/12	0.412916461	0.705020169	0	0
1/12	0.412916461	0.705020169	0	0
1/12	0.412916461	0.705020169	0	0
1/12	0.412916461	0.705020169	0	0
1/12	0.412916461	0.705020169	0	0
1/12	0.412916461	0.705020169	0	0
1/12	0.412916461	0.705020169	0	0
1/12	0.412916461	0.705020169	0	0
1/12	0.412916461	0.705020169	0	0
1/12	0.412916461	0.705020169	0	0
1/12	0.412916461	0.705020169	0	0
1/12	0.412916461	0.705020169	0	0
1/12	0.412916461	0.705020169	0	0
1/12	0.412916461	0.705020169	0	0
1/12	0.412916461	0.705020169	0	0
1/12	0.412916461	0.705020169	0	0
1/12	0.412916461	0.705020169	0	0
1/12	0.412916461	0.705020169	0	0
1/12	0.412916461	0.705020169	0	0
1/12	0.412916461	0.705020169	0	0
6/119	0.413577011	0.705739115	0	0

3/54	0.417590273	0.710530332	0	0
3/54	0.417590273	0.710530332	0	0
3/54	0.417590273	0.710530332	0	0
3/54	0.417590273	0.710530332	0	0
3/54	0.417590273	0.710530332	0	0
4/76	0.420862026	0.715357614	0	0
19/411	0.421972587	0.715357614	0	0
2/33	0.422612014	0.715357614	0	0
2/33	0.422612014	0.715357614	0	0
2/33	0.422612014	0.715357614	0	0
2/33	0.422612014	0.715357614	0	0
2/33	0.422612014	0.715357614	0	0
2/33	0.422612014	0.715357614	0	0
2/33	0.422612014	0.715357614	0	0
3/55	0.429251222	0.716582151	0	0
5/99	0.430283756	0.716582151	0	0
4/77	0.430659465	0.716582151	0	0
4/77	0.430659465	0.716582151	0	0
4/77	0.430659465	0.716582151	0	0
4/77	0.430659465	0.716582151	0	0
4/77	0.430659465	0.716582151	0	0
4/77	0.430659465	0.716582151	0	0
7/144	0.434710335	0.716582151	0	0
2/34	0.437653396	0.716582151	0	0
2/34	0.437653396	0.716582151	0	0
2/34	0.437653396	0.716582151	0	0
2/34	0.437653396	0.716582151	0	0
2/34	0.437653396	0.716582151	0	0
2/34	0.437653396	0.716582151	0	0
2/34	0.437653396	0.716582151	0	0
2/34	0.437653396	0.716582151	0	0
2/34	0.437653396	0.716582151	0	0
1/13	0.438411136	0.716582151	0	0
1/13	0.438411136	0.716582151	0	0
1/13	0.438411136	0.716582151	0	0
1/13	0.438411136	0.716582151	0	0
1/13	0.438411136	0.716582151	0	0
1/13	0.438411136	0.716582151	0	0
1/13	0.438411136	0.716582151	0	0
1/13	0.438411136	0.716582151	0	0
1/13	0.438411136	0.716582151	0	0
1/13	0.438411136	0.716582151	0	0
1/13	0.438411136	0.716582151	0	0
1/13	0.438411136	0.716582151	0	0
1/13	0.438411136	0.716582151	0	0
1/13	0.438411136	0.716582151	0	0
1/13	0.438411136	0.716582151	0	0
1/13	0.438411136	0.716582151	0	0

1/13	0.438411136	0.716582151	0	0
1/13	0.438411136	0.716582151	0	0
1/13	0.438411136	0.716582151	0	0
1/13	0.438411136	0.716582151	0	0
1/13	0.438411136	0.716582151	0	0
1/13	0.438411136	0.716582151	0	0
1/13	0.438411136	0.716582151	0	0
1/13	0.438411136	0.716582151	0	0
1/13	0.438411136	0.716582151	0	0
1/13	0.438411136	0.716582151	0	0
1/13	0.438411136	0.716582151	0	0
1/13	0.438411136	0.716582151	0	0
1/13	0.438411136	0.716582151	0	0
1/13	0.438411136	0.716582151	0	0
1/13	0.438411136	0.716582151	0	0
1/13	0.438411136	0.716582151	0	0
1/13	0.438411136	0.716582151	0	0
1/13	0.438411136	0.716582151	0	0
1/13	0.438411136	0.716582151	0	0
1/13	0.438411136	0.716582151	0	0
1/13	0.438411136	0.716582151	0	0
1/13	0.438411136	0.716582151	0	0
1/13	0.438411136	0.716582151	0	0
1/13	0.438411136	0.716582151	0	0
1/13	0.438411136	0.716582151	0	0
1/13	0.438411136	0.716582151	0	0
1/13	0.438411136	0.716582151	0	0
1/13	0.438411136	0.716582151	0	0
5/100	0.438867146	0.716929866	0	0
3/56	0.440827396	0.718538902	0	0
3/56	0.440827396	0.718538902	0	0
3/56	0.440827396	0.718538902	0	0
3/56	0.440827396	0.718538902	0	0
32/711	0.441741195	0.719630349	0	0
5/101	0.447420432	0.728479565	0	0
7/146	0.44891509	0.729197375	0	0
4/79	0.450114732	0.729197375	0	0
3/57	0.452311646	0.729197375	0	0
2/35	0.452478406	0.729197375	0	0
2/35	0.452478406	0.729197375	0	0
2/35	0.452478406	0.729197375	0	0
2/35	0.452478406	0.729197375	0	0
5/102	0.455940187	0.729197375	0	0
5/102	0.455940187	0.729197375	0	0
5/102	0.455940187	0.729197375	0	0
4/80	0.459763049	0.729197375	0	0
4/80	0.459763049	0.729197375	0	0
1/14	0.462799897	0.729197375	0	0

1/14	0.462799897	0.729197375	0	0
1/14	0.462799897	0.729197375	0	0
1/14	0.462799897	0.729197375	0	0
1/14	0.462799897	0.729197375	0	0
1/14	0.462799897	0.729197375	0	0
1/14	0.462799897	0.729197375	0	0
1/14	0.462799897	0.729197375	0	0
1/14	0.462799897	0.729197375	0	0
1/14	0.462799897	0.729197375	0	0
1/14	0.462799897	0.729197375	0	0
1/14	0.462799897	0.729197375	0	0
1/14	0.462799897	0.729197375	0	0
1/14	0.462799897	0.729197375	0	0
1/14	0.462799897	0.729197375	0	0
1/14	0.462799897	0.729197375	0	0
1/14	0.462799897	0.729197375	0	0
1/14	0.462799897	0.729197375	0	0
1/14	0.462799897	0.729197375	0	0
1/14	0.462799897	0.729197375	0	0
1/14	0.462799897	0.729197375	0	0
1/14	0.462799897	0.729197375	0	0
1/14	0.462799897	0.729197375	0	0
1/14	0.462799897	0.729197375	0	0
1/14	0.462799897	0.729197375	0	0
1/14	0.462799897	0.729197375	0	0
1/14	0.462799897	0.729197375	0	0
1/14	0.462799897	0.729197375	0	0
1/14	0.462799897	0.729197375	0	0
1/14	0.462799897	0.729197375	0	0
1/14	0.462799897	0.729197375	0	0
1/14	0.462799897	0.729197375	0	0
1/14	0.462799897	0.729197375	0	0
1/14	0.462799897	0.729197375	0	0
1/14	0.462799897	0.729197375	0	0
1/14	0.462799897	0.729197375	0	0
1/14	0.462799897	0.729197375	0	0
1/14	0.462799897	0.729197375	0	0
1/14	0.462799897	0.729197375	0	0
1/14	0.462799897	0.729197375	0	0
1/14	0.462799897	0.729197375	0	0
1/14	0.462799897	0.729197375	0	0
1/14	0.462799897	0.729197375	0	0
1/14	0.462799897	0.729197375	0	0
1/14	0.462799897	0.729197375	0	0
1/14	0.462799897	0.729197375	0	0
1/14	0.462799897	0.729197375	0	0
1/14	0.462799897	0.729197375	0	0
1/14	0.462799897	0.729197375	0	0
3/58	0.463697279	0.729197375	0	0
3/58	0.463697279	0.729197375	0	0

3/58	0.463697279	0.729197375	0	0
3/58	0.463697279	0.729197375	0	0
3/58	0.463697279	0.729197375	0	0
15/330	0.464643186	0.730295183	0	0
2/36	0.467077484	0.731002308	0	0
2/36	0.467077484	0.731002308	0	0
2/36	0.467077484	0.731002308	0	0
2/36	0.467077484	0.731002308	0	0
2/36	0.467077484	0.731002308	0	0
2/36	0.467077484	0.731002308	0	0
2/36	0.467077484	0.731002308	0	0
2/36	0.467077484	0.731002308	0	0
4/81	0.469352489	0.73378344	0	0
4/81	0.469352489	0.73378344	0	0
11/240	0.470280359	0.73484423	0	0
3/59	0.474978042	0.73963784	0	0
3/59	0.474978042	0.73963784	0	0
3/59	0.474978042	0.73963784	0	0
6/127	0.475301253	0.73963784	0	0
4/82	0.478878806	0.73963784	0	0
4/82	0.478878806	0.73963784	0	0
5/105	0.481265647	0.73963784	0	0
5/105	0.481265647	0.73963784	0	0
2/37	0.481442305	0.73963784	0	0
2/37	0.481442305	0.73963784	0	0
2/37	0.481442305	0.73963784	0	0
2/37	0.481442305	0.73963784	0	0
2/37	0.481442305	0.73963784	0	0
2/37	0.481442305	0.73963784	0	0
2/37	0.481442305	0.73963784	0	0
2/37	0.481442305	0.73963784	0	0
2/37	0.481442305	0.73963784	0	0
2/37	0.481442305	0.73963784	0	0
1/15	0.486130664	0.73963784	0	0
1/15	0.486130664	0.73963784	0	0
1/15	0.486130664	0.73963784	0	0
1/15	0.486130664	0.73963784	0	0
1/15	0.486130664	0.73963784	0	0
1/15	0.486130664	0.73963784	0	0
1/15	0.486130664	0.73963784	0	0
1/15	0.486130664	0.73963784	0	0
1/15	0.486130664	0.73963784	0	0
1/15	0.486130664	0.73963784	0	0
1/15	0.486130664	0.73963784	0	0
1/15	0.486130664	0.73963784	0	0
1/15	0.486130664	0.73963784	0	0
1/15	0.486130664	0.73963784	0	0
1/15	0.486130664	0.73963784	0	0

1/15	0.486130664	0.73963784	0	0
1/15	0.486130664	0.73963784	0	0
1/15	0.486130664	0.73963784	0	0
1/15	0.486130664	0.73963784	0	0
1/15	0.486130664	0.73963784	0	0
1/15	0.486130664	0.73963784	0	0
1/15	0.486130664	0.73963784	0	0
1/15	0.486130664	0.73963784	0	0
1/15	0.486130664	0.73963784	0	0
1/15	0.486130664	0.73963784	0	0
1/15	0.486130664	0.73963784	0	0
1/15	0.486130664	0.73963784	0	0
1/15	0.486130664	0.73963784	0	0
1/15	0.486130664	0.73963784	0	0
1/15	0.486130664	0.73963784	0	0
1/15	0.486130664	0.73963784	0	0
1/15	0.486130664	0.73963784	0	0
3/60	0.486148116	0.73963784	0	0
4/83	0.488337959	0.742586153	0	0
2/38	0.495565689	0.750478975	0	0
2/38	0.495565689	0.750478975	0	0
2/38	0.495565689	0.750478975	0	0
2/38	0.495565689	0.750478975	0	0
2/38	0.495565689	0.750478975	0	0
2/38	0.495565689	0.750478975	0	0
2/38	0.495565689	0.750478975	0	0
2/38	0.495565689	0.750478975	0	0
3/61	0.497202096	0.752059834	0	0
3/61	0.497202096	0.752059834	0	0
18/406	0.497375167	0.752059834	0	0
5/107	0.497923798	0.752503299	0	0
5/108	0.506176622	0.754813553	0	0
5/108	0.506176622	0.754813553	0	0
4/85	0.507039595	0.754813553	0	0
3/62	0.508134984	0.754813553	0	0
1/16	0.508449282	0.754813553	0	0
1/16	0.508449282	0.754813553	0	0
1/16	0.508449282	0.754813553	0	0
1/16	0.508449282	0.754813553	0	0
1/16	0.508449282	0.754813553	0	0
1/16	0.508449282	0.754813553	0	0
1/16	0.508449282	0.754813553	0	0
1/16	0.508449282	0.754813553	0	0
1/16	0.508449282	0.754813553	0	0
1/16	0.508449282	0.754813553	0	0
1/16	0.508449282	0.754813553	0	0
1/16	0.508449282	0.754813553	0	0
1/16	0.508449282	0.754813553	0	0

1/16	0.508449282	0.754813553	0	0
1/16	0.508449282	0.754813553	0	0
1/16	0.508449282	0.754813553	0	0
1/16	0.508449282	0.754813553	0	0
1/16	0.508449282	0.754813553	0	0
1/16	0.508449282	0.754813553	0	0
1/16	0.508449282	0.754813553	0	0
1/16	0.508449282	0.754813553	0	0
1/16	0.508449282	0.754813553	0	0
1/16	0.508449282	0.754813553	0	0
1/16	0.508449282	0.754813553	0	0
1/16	0.508449282	0.754813553	0	0
1/16	0.508449282	0.754813553	0	0
1/16	0.508449282	0.754813553	0	0
1/16	0.508449282	0.754813553	0	0
1/16	0.508449282	0.754813553	0	0
1/16	0.508449282	0.754813553	0	0
2/39	0.509441519	0.754813553	0	0
2/39	0.509441519	0.754813553	0	0
2/39	0.509441519	0.754813553	0	0
2/39	0.509441519	0.754813553	0	0
2/39	0.509441519	0.754813553	0	0
8/178	0.510832757	0.75649454	0	0
4/86	0.516274981	0.757186935	0	0
4/86	0.516274981	0.757186935	0	0
3/63	0.518942172	0.757186935	0	0
5/110	0.522516546	0.757186935	0	0
2/40	0.523064664	0.757186935	0	0
2/40	0.523064664	0.757186935	0	0
2/40	0.523064664	0.757186935	0	0
2/40	0.523064664	0.757186935	0	0
2/40	0.523064664	0.757186935	0	0
2/40	0.523064664	0.757186935	0	0
2/40	0.523064664	0.757186935	0	0
2/40	0.523064664	0.757186935	0	0
2/40	0.523064664	0.757186935	0	0
2/40	0.523064664	0.757186935	0	0
2/40	0.523064664	0.757186935	0	0
2/40	0.523064664	0.757186935	0	0
2/40	0.523064664	0.757186935	0	0
4/87	0.525429001	0.757186935	0	0
9/204	0.52801287	0.757186935	0	0
3/64	0.529619434	0.757186935	0	0
3/64	0.529619434	0.757186935	0	0
1/17	0.529799613	0.757186935	0	0
1/17	0.529799613	0.757186935	0	0
1/17	0.529799613	0.757186935	0	0
1/17	0.529799613	0.757186935	0	0

1/17	0.529799613	0.757186935	0	0
1/17	0.529799613	0.757186935	0	0
1/17	0.529799613	0.757186935	0	0
1/17	0.529799613	0.757186935	0	0
1/17	0.529799613	0.757186935	0	0
1/17	0.529799613	0.757186935	0	0
1/17	0.529799613	0.757186935	0	0
1/17	0.529799613	0.757186935	0	0
1/17	0.529799613	0.757186935	0	0
1/17	0.529799613	0.757186935	0	0
1/17	0.529799613	0.757186935	0	0
1/17	0.529799613	0.757186935	0	0
1/17	0.529799613	0.757186935	0	0
1/17	0.529799613	0.757186935	0	0
1/17	0.529799613	0.757186935	0	0
1/17	0.529799613	0.757186935	0	0
1/17	0.529799613	0.757186935	0	0
1/17	0.529799613	0.757186935	0	0
1/17	0.529799613	0.757186935	0	0
1/17	0.529799613	0.757186935	0	0
1/17	0.529799613	0.757186935	0	0
1/17	0.529799613	0.757186935	0	0
1/17	0.529799613	0.757186935	0	0
1/17	0.529799613	0.757186935	0	0
1/17	0.529799613	0.757186935	0	0
1/17	0.529799613	0.757186935	0	0
1/17	0.529799613	0.757186935	0	0
1/17	0.529799613	0.757186935	0	0
1/17	0.529799613	0.757186935	0	0
1/17	0.529799613	0.757186935	0	0
1/17	0.529799613	0.757186935	0	0
1/17	0.529799613	0.757186935	0	0
1/17	0.529799613	0.757186935	0	0
1/17	0.529799613	0.757186935	0	0
1/17	0.529799613	0.757186935	0	0
1/17	0.529799613	0.757186935	0	0
1/17	0.529799613	0.757186935	0	0
1/17	0.529799613	0.757186935	0	0
1/17	0.529799613	0.757186935	0	0
1/17	0.529799613	0.757186935	0	0
1/17	0.529799613	0.757186935	0	0
1/17	0.529799613	0.757186935	0	0
1/17	0.529799613	0.757186935	0	0
1/17	0.529799613	0.757186935	0	0
1/17	0.529799613	0.757186935	0	0
1/17	0.529799613	0.757186935	0	0
1/17	0.529799613	0.757186935	0	0
5/111	0.53059869	0.757961385	0	0

10/228	0.532037317	0.759648242	0	0
13/298	0.532579977	0.760054814	0	0
2/41	0.536430899	0.76210759	0	0
2/41	0.536430899	0.76210759	0	0
2/41	0.536430899	0.76210759	0	0
2/41	0.536430899	0.76210759	0	0
2/41	0.536430899	0.76210759	0	0
5/112	0.538619169	0.76210759	0	0
3/65	0.540162905	0.76210759	0	0
3/65	0.540162905	0.76210759	0	0
4/89	0.543480843	0.76210759	0	0
4/89	0.543480843	0.76210759	0	0
4/89	0.543480843	0.76210759	0	0
4/89	0.543480843	0.76210759	0	0
2/42	0.549536845	0.76210759	0	0
2/42	0.549536845	0.76210759	0	0
2/42	0.549536845	0.76210759	0	0
2/42	0.549536845	0.76210759	0	0
2/42	0.549536845	0.76210759	0	0
2/42	0.549536845	0.76210759	0	0
2/42	0.549536845	0.76210759	0	0
2/42	0.549536845	0.76210759	0	0
1/18	0.550223619	0.76210759	0	0
1/18	0.550223619	0.76210759	0	0
1/18	0.550223619	0.76210759	0	0
1/18	0.550223619	0.76210759	0	0
1/18	0.550223619	0.76210759	0	0
1/18	0.550223619	0.76210759	0	0
1/18	0.550223619	0.76210759	0	0
1/18	0.550223619	0.76210759	0	0
1/18	0.550223619	0.76210759	0	0
1/18	0.550223619	0.76210759	0	0
1/18	0.550223619	0.76210759	0	0
1/18	0.550223619	0.76210759	0	0
1/18	0.550223619	0.76210759	0	0
1/18	0.550223619	0.76210759	0	0
1/18	0.550223619	0.76210759	0	0
1/18	0.550223619	0.76210759	0	0
1/18	0.550223619	0.76210759	0	0
1/18	0.550223619	0.76210759	0	0
1/18	0.550223619	0.76210759	0	0
1/18	0.550223619	0.76210759	0	0
1/18	0.550223619	0.76210759	0	0
1/18	0.550223619	0.76210759	0	0
1/18	0.550223619	0.76210759	0	0
1/18	0.550223619	0.76210759	0	0
1/18	0.550223619	0.76210759	0	0
1/18	0.550223619	0.76210759	0	0

1/18	0.550223619	0.76210759	0	0
1/18	0.550223619	0.76210759	0	0
1/18	0.550223619	0.76210759	0	0
1/18	0.550223619	0.76210759	0	0
1/18	0.550223619	0.76210759	0	0
1/18	0.550223619	0.76210759	0	0
1/18	0.550223619	0.76210759	0	0
1/18	0.550223619	0.76210759	0	0
1/18	0.550223619	0.76210759	0	0
1/18	0.550223619	0.76210759	0	0
1/18	0.550223619	0.76210759	0	0
1/18	0.550223619	0.76210759	0	0
1/18	0.550223619	0.76210759	0	0
1/18	0.550223619	0.76210759	0	0
1/18	0.550223619	0.76210759	0	0
3/66	0.550569073	0.76210759	0	0
3/66	0.550569073	0.76210759	0	0
3/66	0.550569073	0.76210759	0	0
9/208	0.551617495	0.763200356	0	0
3/67	0.560834766	0.770576861	0	0
3/67	0.560834766	0.770576861	0	0
2/43	0.562379895	0.770576861	0	0
2/43	0.562379895	0.770576861	0	0
2/43	0.562379895	0.770576861	0	0
2/43	0.562379895	0.770576861	0	0
2/43	0.562379895	0.770576861	0	0
2/43	0.562379895	0.770576861	0	0
6/139	0.563773254	0.770576861	0	0
1/19	0.569761446	0.770576861	0	0
1/19	0.569761446	0.770576861	0	0
1/19	0.569761446	0.770576861	0	0
1/19	0.569761446	0.770576861	0	0
1/19	0.569761446	0.770576861	0	0
1/19	0.569761446	0.770576861	0	0
1/19	0.569761446	0.770576861	0	0
1/19	0.569761446	0.770576861	0	0
1/19	0.569761446	0.770576861	0	0
1/19	0.569761446	0.770576861	0	0
1/19	0.569761446	0.770576861	0	0
1/19	0.569761446	0.770576861	0	0
1/19	0.569761446	0.770576861	0	0
1/19	0.569761446	0.770576861	0	0
1/19	0.569761446	0.770576861	0	0
1/19	0.569761446	0.770576861	0	0
1/19	0.569761446	0.770576861	0	0
1/19	0.569761446	0.770576861	0	0
1/19	0.569761446	0.770576861	0	0
1/19	0.569761446	0.770576861	0	0

1/19	0.569761446	0.770576861	0	0
1/19	0.569761446	0.770576861	0	0
1/19	0.569761446	0.770576861	0	0
1/19	0.569761446	0.770576861	0	0
1/19	0.569761446	0.770576861	0	0
1/19	0.569761446	0.770576861	0	0
1/19	0.569761446	0.770576861	0	0
1/19	0.569761446	0.770576861	0	0
1/19	0.569761446	0.770576861	0	0
1/19	0.569761446	0.770576861	0	0
1/19	0.569761446	0.770576861	0	0
1/19	0.569761446	0.770576861	0	0
1/19	0.569761446	0.770576861	0	0
1/19	0.569761446	0.770576861	0	0
1/19	0.569761446	0.770576861	0	0
1/19	0.569761446	0.770576861	0	0
1/19	0.569761446	0.770576861	0	0
1/19	0.569761446	0.770576861	0	0
1/19	0.569761446	0.770576861	0	0
1/19	0.569761446	0.770576861	0	0
3/68	0.570957135	0.771839761	0	0
2/44	0.574958161	0.774406627	0	0
2/44	0.574958161	0.774406627	0	0
2/44	0.574958161	0.774406627	0	0
2/44	0.574958161	0.774406627	0	0
2/44	0.574958161	0.774406627	0	0
2/44	0.574958161	0.774406627	0	0
2/44	0.574958161	0.774406627	0	0
2/44	0.574958161	0.774406627	0	0
5/117	0.577723221	0.774527278	0	0
4/93	0.578485155	0.774527278	0	0
4/93	0.578485155	0.774527278	0	0
8/189	0.579341467	0.774527278	0	0
3/69	0.580933645	0.774527278	0	0
3/69	0.580933645	0.774527278	0	0
5/118	0.585331166	0.774527278	0	0
2/45	0.587270411	0.774527278	0	0
2/45	0.587270411	0.774527278	0	0
2/45	0.587270411	0.774527278	0	0
2/45	0.587270411	0.774527278	0	0
2/45	0.587270411	0.774527278	0	0
2/45	0.587270411	0.774527278	0	0
1/20	0.588451502	0.774527278	0	0
1/20	0.588451502	0.774527278	0	0
1/20	0.588451502	0.774527278	0	0
1/20	0.588451502	0.774527278	0	0
1/20	0.588451502	0.774527278	0	0
1/20	0.588451502	0.774527278	0	0

1/20	0.588451502	0.774527278	0	0
1/20	0.588451502	0.774527278	0	0
1/20	0.588451502	0.774527278	0	0
1/20	0.588451502	0.774527278	0	0
1/20	0.588451502	0.774527278	0	0
1/20	0.588451502	0.774527278	0	0
1/20	0.588451502	0.774527278	0	0
1/20	0.588451502	0.774527278	0	0
1/20	0.588451502	0.774527278	0	0
1/20	0.588451502	0.774527278	0	0
1/20	0.588451502	0.774527278	0	0
1/20	0.588451502	0.774527278	0	0
1/20	0.588451502	0.774527278	0	0
1/20	0.588451502	0.774527278	0	0
1/20	0.588451502	0.774527278	0	0
1/20	0.588451502	0.774527278	0	0
1/20	0.588451502	0.774527278	0	0
1/20	0.588451502	0.774527278	0	0
1/20	0.588451502	0.774527278	0	0
1/20	0.588451502	0.774527278	0	0
1/20	0.588451502	0.774527278	0	0
1/20	0.588451502	0.774527278	0	0
1/20	0.588451502	0.774527278	0	0
1/20	0.588451502	0.774527278	0	0
1/20	0.588451502	0.774527278	0	0
1/20	0.588451502	0.774527278	0	0
1/20	0.588451502	0.774527278	0	0
1/20	0.588451502	0.774527278	0	0
1/20	0.588451502	0.774527278	0	0
1/20	0.588451502	0.774527278	0	0
1/20	0.588451502	0.774527278	0	0
1/20	0.588451502	0.774527278	0	0
3/70	0.590762059	0.775835912	0	0
3/70	0.590762059	0.775835912	0	0
3/70	0.590762059	0.775835912	0	0
3/70	0.590762059	0.775835912	0	0
3/70	0.590762059	0.775835912	0	0
2/46	0.599316017	0.784970801	0	0
2/46	0.599316017	0.784970801	0	0
2/46	0.599316017	0.784970801	0	0
2/46	0.599316017	0.784970801	0	0
2/46	0.599316017	0.784970801	0	0
2/46	0.599316017	0.784970801	0	0
3/71	0.600440425	0.785745085	0	0
3/71	0.600440425	0.785745085	0	0
4/96	0.603706133	0.786468344	0	0
1/21	0.606330532	0.786468344	0	0
1/21	0.606330532	0.786468344	0	0

1/21	0.606330532	0.786468344	0	0
1/21	0.606330532	0.786468344	0	0
1/21	0.606330532	0.786468344	0	0
1/21	0.606330532	0.786468344	0	0
1/21	0.606330532	0.786468344	0	0
1/21	0.606330532	0.786468344	0	0
1/21	0.606330532	0.786468344	0	0
1/21	0.606330532	0.786468344	0	0
1/21	0.606330532	0.786468344	0	0
1/21	0.606330532	0.786468344	0	0
1/21	0.606330532	0.786468344	0	0
1/21	0.606330532	0.786468344	0	0
1/21	0.606330532	0.786468344	0	0
1/21	0.606330532	0.786468344	0	0
1/21	0.606330532	0.786468344	0	0
1/21	0.606330532	0.786468344	0	0
1/21	0.606330532	0.786468344	0	0
3/72	0.609967066	0.790489421	0	0
3/72	0.609967066	0.790489421	0	0
2/47	0.611094909	0.790560447	0	0
2/47	0.611094909	0.790560447	0	0
2/47	0.611094909	0.790560447	0	0
2/47	0.611094909	0.790560447	0	0
5/122	0.614999912	0.795263159	0	0
11/268	0.618201928	0.796384519	0	0
3/73	0.619340566	0.796384519	0	0
3/73	0.619340566	0.796384519	0	0
4/98	0.620001796	0.796384519	0	0
2/48	0.622607523	0.796384519	0	0
2/48	0.622607523	0.796384519	0	0
2/48	0.622607523	0.796384519	0	0
2/48	0.622607523	0.796384519	0	0
2/48	0.622607523	0.796384519	0	0
1/22	0.62343369	0.796384519	0	0
1/22	0.62343369	0.796384519	0	0
1/22	0.62343369	0.796384519	0	0
1/22	0.62343369	0.796384519	0	0
1/22	0.62343369	0.796384519	0	0
1/22	0.62343369	0.796384519	0	0
1/22	0.62343369	0.796384519	0	0
1/22	0.62343369	0.796384519	0	0
1/22	0.62343369	0.796384519	0	0
1/22	0.62343369	0.796384519	0	0
1/22	0.62343369	0.796384519	0	0
1/22	0.62343369	0.796384519	0	0
1/22	0.62343369	0.796384519	0	0
1/22	0.62343369	0.796384519	0	0
1/22	0.62343369	0.796384519	0	0

1/22	0.62343369	0.796384519	0	0
1/22	0.62343369	0.796384519	0	0
1/22	0.62343369	0.796384519	0	0
1/22	0.62343369	0.796384519	0	0
5/124	0.629358101	0.803604127	0	0
2/49	0.633854757	0.807483818	0	0
2/49	0.633854757	0.807483818	0	0
2/49	0.633854757	0.807483818	0	0
2/49	0.633854757	0.807483818	0	0
5/125	0.636414209	0.807483818	0	0
5/125	0.636414209	0.807483818	0	0
3/75	0.637623709	0.807483818	0	0
3/75	0.637623709	0.807483818	0	0
6/150	0.638097468	0.807483818	0	0
1/23	0.63979461	0.807483818	0	0
1/23	0.63979461	0.807483818	0	0
1/23	0.63979461	0.807483818	0	0
1/23	0.63979461	0.807483818	0	0
1/23	0.63979461	0.807483818	0	0
1/23	0.63979461	0.807483818	0	0
1/23	0.63979461	0.807483818	0	0
1/23	0.63979461	0.807483818	0	0
1/23	0.63979461	0.807483818	0	0
1/23	0.63979461	0.807483818	0	0
1/23	0.63979461	0.807483818	0	0
1/23	0.63979461	0.807483818	0	0
1/23	0.63979461	0.807483818	0	0
1/23	0.63979461	0.807483818	0	0
1/23	0.63979461	0.807483818	0	0
1/23	0.63979461	0.807483818	0	0
1/23	0.63979461	0.807483818	0	0
1/23	0.63979461	0.807483818	0	0
7/175	0.640398072	0.807899451	0	0
4/101	0.643641138	0.811643318	0	0
2/50	0.644837937	0.811763093	0	0
2/50	0.644837937	0.811763093	0	0
2/50	0.644837937	0.811763093	0	0
2/50	0.644837937	0.811763093	0	0
4/102	0.651302395	0.814473733	0	0
3/77	0.655283287	0.814473733	0	0
3/77	0.655283287	0.814473733	0	0
1/24	0.655445466	0.814473733	0	0
1/24	0.655445466	0.814473733	0	0
1/24	0.655445466	0.814473733	0	0
1/24	0.655445466	0.814473733	0	0
1/24	0.655445466	0.814473733	0	0
1/24	0.655445466	0.814473733	0	0
1/24	0.655445466	0.814473733	0	0

1/24	0.655445466	0.814473733	0	0
1/24	0.655445466	0.814473733	0	0
1/24	0.655445466	0.814473733	0	0
1/24	0.655445466	0.814473733	0	0
1/24	0.655445466	0.814473733	0	0
1/24	0.655445466	0.814473733	0	0
1/24	0.655445466	0.814473733	0	0
1/24	0.655445466	0.814473733	0	0
1/24	0.655445466	0.814473733	0	0
1/24	0.655445466	0.814473733	0	0
1/24	0.655445466	0.814473733	0	0
1/24	0.655445466	0.814473733	0	0
1/24	0.655445466	0.814473733	0	0
2/51	0.655558769	0.814473733	0	0
2/51	0.655558769	0.814473733	0	0
2/51	0.655558769	0.814473733	0	0
2/51	0.655558769	0.814473733	0	0
2/51	0.655558769	0.814473733	0	0
2/51	0.655558769	0.814473733	0	0
2/51	0.655558769	0.814473733	0	0
2/51	0.655558769	0.814473733	0	0
4/103	0.658853068	0.818221657	0	0
9/228	0.661346627	0.820972414	0	0
3/78	0.663878131	0.821862818	0	0
3/78	0.663878131	0.821862818	0	0
2/52	0.666019311	0.821862818	0	0
2/52	0.666019311	0.821862818	0	0
2/52	0.666019311	0.821862818	0	0
2/52	0.666019311	0.821862818	0	0
2/52	0.666019311	0.821862818	0	0
2/52	0.666019311	0.821862818	0	0
1/25	0.670417041	0.821862818	0	0
1/25	0.670417041	0.821862818	0	0
1/25	0.670417041	0.821862818	0	0
1/25	0.670417041	0.821862818	0	0
1/25	0.670417041	0.821862818	0	0
1/25	0.670417041	0.821862818	0	0
1/25	0.670417041	0.821862818	0	0
1/25	0.670417041	0.821862818	0	0
1/25	0.670417041	0.821862818	0	0
1/25	0.670417041	0.821862818	0	0
1/25	0.670417041	0.821862818	0	0
1/25	0.670417041	0.821862818	0	0
1/25	0.670417041	0.821862818	0	0
1/25	0.670417041	0.821862818	0	0
1/25	0.670417041	0.821862818	0	0
1/25	0.670417041	0.821862818	0	0
1/25	0.670417041	0.821862818	0	0

1/25	0.670417041	0.821862818	0	0
1/25	0.670417041	0.821862818	0	0
1/25	0.670417041	0.821862818	0	0
1/25	0.670417041	0.821862818	0	0
5/130	0.670430341	0.821862818	0	0
3/79	0.672316152	0.823147362	0	0
3/79	0.672316152	0.823147362	0	0
3/79	0.672316152	0.823147362	0	0
2/53	0.676221935	0.826079177	0	0
2/53	0.676221935	0.826079177	0	0
2/53	0.676221935	0.826079177	0	0
2/53	0.676221935	0.826079177	0	0
2/53	0.676221935	0.826079177	0	0
9/231	0.676392621	0.826079177	0	0
3/80	0.680597434	0.830107108	0	0
3/80	0.680597434	0.830107108	0	0
4/106	0.680836062	0.830107108	0	0
9/232	0.681317638	0.830107108	0	0
1/26	0.684738786	0.830107108	0	0
1/26	0.684738786	0.830107108	0	0
1/26	0.684738786	0.830107108	0	0
1/26	0.684738786	0.830107108	0	0
1/26	0.684738786	0.830107108	0	0
1/26	0.684738786	0.830107108	0	0
1/26	0.684738786	0.830107108	0	0
1/26	0.684738786	0.830107108	0	0
1/26	0.684738786	0.830107108	0	0
1/26	0.684738786	0.830107108	0	0
1/26	0.684738786	0.830107108	0	0
1/26	0.684738786	0.830107108	0	0
1/26	0.684738786	0.830107108	0	0
2/54	0.686169296	0.830107108	0	0
2/54	0.686169296	0.830107108	0	0
2/54	0.686169296	0.830107108	0	0
2/54	0.686169296	0.830107108	0	0
2/54	0.686169296	0.830107108	0	0
2/54	0.686169296	0.830107108	0	0
4/107	0.687939444	0.831907075	0	0
3/81	0.688722232	0.832170733	0	0
3/81	0.688722232	0.832170733	0	0
7/184	0.691390574	0.835052468	0	0
4/108	0.694930316	0.83704732	0	0
4/108	0.694930316	0.83704732	0	0
3/82	0.696690962	0.83704732	0	0
1/27	0.698438874	0.83704732	0	0
1/27	0.698438874	0.83704732	0	0
1/27	0.698438874	0.83704732	0	0
1/27	0.698438874	0.83704732	0	0

1/27	0.698438874	0.83704732	0	0
1/27	0.698438874	0.83704732	0	0
1/27	0.698438874	0.83704732	0	0
1/27	0.698438874	0.83704732	0	0
1/27	0.698438874	0.83704732	0	0
1/27	0.698438874	0.83704732	0	0
1/27	0.698438874	0.83704732	0	0
1/27	0.698438874	0.83704732	0	0
1/27	0.698438874	0.83704732	0	0
1/27	0.698438874	0.83704732	0	0
1/27	0.698438874	0.83704732	0	0
1/27	0.698438874	0.83704732	0	0
3/83	0.704504191	0.842541099	0	0
3/83	0.704504191	0.842541099	0	0
3/83	0.704504191	0.842541099	0	0
3/83	0.704504191	0.842541099	0	0
3/83	0.704504191	0.842541099	0	0
2/56	0.705310109	0.842541099	0	0
2/56	0.705310109	0.842541099	0	0
2/56	0.705310109	0.842541099	0	0
5/136	0.708390939	0.844171067	0	0
11/288	0.709894466	0.844171067	0	0
1/28	0.711544259	0.844171067	0	0
1/28	0.711544259	0.844171067	0	0
1/28	0.711544259	0.844171067	0	0
1/28	0.711544259	0.844171067	0	0
1/28	0.711544259	0.844171067	0	0
1/28	0.711544259	0.844171067	0	0
1/28	0.711544259	0.844171067	0	0
1/28	0.711544259	0.844171067	0	0
1/28	0.711544259	0.844171067	0	0
1/28	0.711544259	0.844171067	0	0
1/28	0.711544259	0.844171067	0	0
1/28	0.711544259	0.844171067	0	0
1/28	0.711544259	0.844171067	0	0
1/28	0.711544259	0.844171067	0	0
1/28	0.711544259	0.844171067	0	0
3/84	0.712162631	0.844224969	0	0
3/84	0.712162631	0.844224969	0	0
5/137	0.714409666	0.845307546	0	0
5/137	0.714409666	0.845307546	0	0
2/57	0.714510043	0.845307546	0	0
2/57	0.714510043	0.845307546	0	0
2/57	0.714510043	0.845307546	0	0
4/111	0.715227625	0.845477661	0	0
4/111	0.715227625	0.845477661	0	0
3/85	0.719667124	0.846772185	0	0
3/85	0.719667124	0.846772185	0	0

4/112	0.721768672	0.846772185	0	0
2/58	0.723467632	0.846772185	0	0
2/58	0.723467632	0.846772185	0	0
2/58	0.723467632	0.846772185	0	0
2/58	0.723467632	0.846772185	0	0
2/58	0.723467632	0.846772185	0	0
1/29	0.724080728	0.846772185	0	0
1/29	0.724080728	0.846772185	0	0
1/29	0.724080728	0.846772185	0	0
1/29	0.724080728	0.846772185	0	0
1/29	0.724080728	0.846772185	0	0
1/29	0.724080728	0.846772185	0	0
1/29	0.724080728	0.846772185	0	0
1/29	0.724080728	0.846772185	0	0
1/29	0.724080728	0.846772185	0	0
1/29	0.724080728	0.846772185	0	0
1/29	0.724080728	0.846772185	0	0
1/29	0.724080728	0.846772185	0	0
1/29	0.724080728	0.846772185	0	0
1/29	0.724080728	0.846772185	0	0
1/29	0.724080728	0.846772185	0	0
1/29	0.724080728	0.846772185	0	0
1/29	0.724080728	0.846772185	0	0
1/29	0.724080728	0.846772185	0	0
1/29	0.724080728	0.846772185	0	0
3/86	0.727018639	0.849870658	0	0
2/59	0.732186554	0.853912066	0	0
2/59	0.732186554	0.853912066	0	0
2/59	0.732186554	0.853912066	0	0
2/59	0.732186554	0.853912066	0	0
2/59	0.732186554	0.853912066	0	0
16/418	0.732214384	0.853912066	0	0
1/30	0.736072952	0.85435486	0	0
1/30	0.736072952	0.85435486	0	0
1/30	0.736072952	0.85435486	0	0
1/30	0.736072952	0.85435486	0	0
1/30	0.736072952	0.85435486	0	0
1/30	0.736072952	0.85435486	0	0
1/30	0.736072952	0.85435486	0	0
1/30	0.736072952	0.85435486	0	0
1/30	0.736072952	0.85435486	0	0
1/30	0.736072952	0.85435486	0	0
1/30	0.736072952	0.85435486	0	0
1/30	0.736072952	0.85435486	0	0
2/60	0.740670627	0.859014695	0	0
2/60	0.740670627	0.859014695	0	0
1/31	0.747544531	0.861898956	0	0
1/31	0.747544531	0.861898956	0	0

1/31	0.747544531	0.861898956	0	0
1/31	0.747544531	0.861898956	0	0
1/31	0.747544531	0.861898956	0	0
1/31	0.747544531	0.861898956	0	0
1/31	0.747544531	0.861898956	0	0
1/31	0.747544531	0.861898956	0	0
1/31	0.747544531	0.861898956	0	0
1/31	0.747544531	0.861898956	0	0
1/31	0.747544531	0.861898956	0	0
1/31	0.747544531	0.861898956	0	0
1/31	0.747544531	0.861898956	0	0
1/31	0.747544531	0.861898956	0	0
1/31	0.747544531	0.861898956	0	0
2/61	0.748923787	0.862814074	0	0
2/61	0.748923787	0.862814074	0	0
4/117	0.752802915	0.866944194	0	0
3/90	0.754918193	0.868772901	0	0
2/62	0.75695007	0.868772901	0	0
2/62	0.75695007	0.868772901	0	0
2/62	0.75695007	0.868772901	0	0
1/32	0.758518044	0.868772901	0	0
1/32	0.758518044	0.868772901	0	0
1/32	0.758518044	0.868772901	0	0
1/32	0.758518044	0.868772901	0	0
1/32	0.758518044	0.868772901	0	0
1/32	0.758518044	0.868772901	0	0
1/32	0.758518044	0.868772901	0	0
1/32	0.758518044	0.868772901	0	0
1/32	0.758518044	0.868772901	0	0
1/32	0.758518044	0.868772901	0	0
3/91	0.761523143	0.87119903	0	0
3/91	0.761523143	0.87119903	0	0
3/91	0.761523143	0.87119903	0	0
4/119	0.764446576	0.871984407	0	0
5/146	0.764615104	0.871984407	0	0
2/63	0.764753597	0.871984407	0	0
2/63	0.764753597	0.871984407	0	0
2/63	0.764753597	0.871984407	0	0
6/173	0.766697711	0.871984407	0	0
3/92	0.7679831	0.871984407	0	0
3/92	0.7679831	0.871984407	0	0
1/33	0.769015091	0.871984407	0	0
1/33	0.769015091	0.871984407	0	0
1/33	0.769015091	0.871984407	0	0
1/33	0.769015091	0.871984407	0	0
1/33	0.769015091	0.871984407	0	0
1/33	0.769015091	0.871984407	0	0
1/33	0.769015091	0.871984407	0	0

1/33	0.769015091	0.871984407	0	0
1/33	0.769015091	0.871984407	0	0
1/33	0.769015091	0.871984407	0	0
1/33	0.769015091	0.871984407	0	0
1/33	0.769015091	0.871984407	0	0
1/33	0.769015091	0.871984407	0	0
1/33	0.769015091	0.871984407	0	0
1/33	0.769015091	0.871984407	0	0
5/147	0.769757171	0.872154703	0	0
5/147	0.769757171	0.872154703	0	0
2/64	0.772338559	0.87306549	0	0
2/64	0.772338559	0.87306549	0	0
2/64	0.772338559	0.87306549	0	0
2/64	0.772338559	0.87306549	0	0
2/64	0.772338559	0.87306549	0	0
2/64	0.772338559	0.87306549	0	0
3/93	0.774299689	0.874946773	0	0
1/34	0.779056339	0.875287469	0	0
1/34	0.779056339	0.875287469	0	0
1/34	0.779056339	0.875287469	0	0
1/34	0.779056339	0.875287469	0	0
1/34	0.779056339	0.875287469	0	0
1/34	0.779056339	0.875287469	0	0
1/34	0.779056339	0.875287469	0	0
1/34	0.779056339	0.875287469	0	0
1/34	0.779056339	0.875287469	0	0
1/34	0.779056339	0.875287469	0	0
1/34	0.779056339	0.875287469	0	0
1/34	0.779056339	0.875287469	0	0
1/34	0.779056339	0.875287469	0	0
1/34	0.779056339	0.875287469	0	0
1/34	0.779056339	0.875287469	0	0
2/65	0.779709204	0.875353533	0	0
2/65	0.779709204	0.875353533	0	0
3/94	0.780474604	0.875545739	0	0
3/94	0.780474604	0.875545739	0	0
4/122	0.781104456	0.875918885	0	0
2/66	0.786869822	0.881374899	0	0
2/66	0.786869822	0.881374899	0	0
1/35	0.788661557	0.881374899	0	0
1/35	0.788661557	0.881374899	0	0
1/35	0.788661557	0.881374899	0	0
1/35	0.788661557	0.881374899	0	0
1/35	0.788661557	0.881374899	0	0
1/35	0.788661557	0.881374899	0	0
1/35	0.788661557	0.881374899	0	0
4/124	0.79168134	0.884414294	0	0
3/96	0.792406472	0.88455374	0	0

3/96	0.792406472	0.88455374	0	0
2/67	0.793824736	0.885801399	0	0
1/36	0.797849658	0.886395705	0	0
1/36	0.797849658	0.886395705	0	0
1/36	0.797849658	0.886395705	0	0
1/36	0.797849658	0.886395705	0	0
1/36	0.797849658	0.886395705	0	0
1/36	0.797849658	0.886395705	0	0
1/36	0.797849658	0.886395705	0	0
1/36	0.797849658	0.886395705	0	0
1/36	0.797849658	0.886395705	0	0
3/97	0.798167092	0.886395705	0	0
3/97	0.798167092	0.886395705	0	0
6/180	0.798267459	0.886395705	0	0
6/180	0.798267459	0.886395705	0	0
2/68	0.800578288	0.888626823	0	0
4/126	0.801844518	0.889697212	0	0
3/98	0.803793356	0.891188495	0	0
3/98	0.803793356	0.891188495	0	0
1/37	0.806638735	0.891474212	0	0
1/37	0.806638735	0.891474212	0	0
1/37	0.806638735	0.891474212	0	0
1/37	0.806638735	0.891474212	0	0
1/37	0.806638735	0.891474212	0	0
1/37	0.806638735	0.891474212	0	0
1/37	0.806638735	0.891474212	0	0
4/127	0.806773574	0.891474212	0	0
4/127	0.806773574	0.891474212	0	0
2/69	0.807134833	0.891539113	0	0
2/70	0.813498727	0.896581132	0	0
2/70	0.813498727	0.896581132	0	0
3/100	0.814650613	0.896581132	0	0
1/38	0.815046099	0.896581132	0	0
1/38	0.815046099	0.896581132	0	0
1/38	0.815046099	0.896581132	0	0
1/38	0.815046099	0.896581132	0	0
1/38	0.815046099	0.896581132	0	0
1/38	0.815046099	0.896581132	0	0
1/38	0.815046099	0.896581132	0	0
1/38	0.815046099	0.896581132	0	0
10/291	0.816154979	0.897325919	0	0
4/129	0.816332131	0.897325919	0	0
2/71	0.819674319	0.899384963	0	0
2/71	0.819674319	0.899384963	0	0
4/130	0.820963926	0.899384963	0	0
11/319	0.821795548	0.899384963	0	0
1/39	0.823088308	0.899384963	0	0
1/39	0.823088308	0.899384963	0	0

1/39	0.823088308	0.899384963	0	0
1/39	0.823088308	0.899384963	0	0
1/39	0.823088308	0.899384963	0	0
1/39	0.823088308	0.899384963	0	0
1/39	0.823088308	0.899384963	0	0
1/39	0.823088308	0.899384963	0	0
1/39	0.823088308	0.899384963	0	0
1/39	0.823088308	0.899384963	0	0
1/39	0.823088308	0.899384963	0	0
1/39	0.823088308	0.899384963	0	0
3/102	0.824994161	0.900865433	0	0
7/214	0.825607135	0.900865433	0	0
2/72	0.825665943	0.900865433	0	0
2/72	0.825665943	0.900865433	0	0
4/132	0.829938483	0.902528698	0	0
1/40	0.830781206	0.902528698	0	0
1/40	0.830781206	0.902528698	0	0
1/40	0.830781206	0.902528698	0	0
1/40	0.830781206	0.902528698	0	0
1/40	0.830781206	0.902528698	0	0
1/40	0.830781206	0.902528698	0	0
1/40	0.830781206	0.902528698	0	0
1/40	0.830781206	0.902528698	0	0
1/40	0.830781206	0.902528698	0	0
1/40	0.830781206	0.902528698	0	0
2/73	0.831477915	0.902528698	0	0
2/73	0.831477915	0.902528698	0	0
2/73	0.831477915	0.902528698	0	0
5/162	0.836906346	0.904761329	0	0
1/41	0.838139948	0.904761329	0	0
1/41	0.838139948	0.904761329	0	0
1/41	0.838139948	0.904761329	0	0
1/41	0.838139948	0.904761329	0	0
1/41	0.838139948	0.904761329	0	0
1/41	0.838139948	0.904761329	0	0
1/41	0.838139948	0.904761329	0	0
1/41	0.838139948	0.904761329	0	0
1/41	0.838139948	0.904761329	0	0
1/41	0.838139948	0.904761329	0	0
1/41	0.838139948	0.904761329	0	0
1/41	0.838139948	0.904761329	0	0
1/41	0.838139948	0.904761329	0	0
1/41	0.838139948	0.904761329	0	0
2/75	0.842580009	0.908035949	0	0
2/75	0.842580009	0.908035949	0	0
6/192	0.844573264	0.908035949	0	0
1/42	0.845179032	0.908035949	0	0
1/42	0.845179032	0.908035949	0	0

1/42	0.845179032	0.908035949	0	0
1/42	0.845179032	0.908035949	0	0
1/42	0.845179032	0.908035949	0	0
1/42	0.845179032	0.908035949	0	0
1/42	0.845179032	0.908035949	0	0
1/42	0.845179032	0.908035949	0	0
1/42	0.845179032	0.908035949	0	0
1/42	0.845179032	0.908035949	0	0
7/220	0.846009947	0.908597418	0	0
3/107	0.848713421	0.911168834	0	0
1/43	0.851912329	0.911614246	0	0
1/43	0.851912329	0.911614246	0	0
1/43	0.851912329	0.911614246	0	0
1/43	0.851912329	0.911614246	0	0
1/43	0.851912329	0.911614246	0	0
1/43	0.851912329	0.911614246	0	0
1/43	0.851912329	0.911614246	0	0
1/43	0.851912329	0.911614246	0	0
1/43	0.851912329	0.911614246	0	0
2/77	0.853014464	0.912230392	0	0
3/108	0.853107214	0.912230392	0	0
6/195	0.854696331	0.913530736	0	0
3/109	0.857389241	0.913530736	0	0
6/196	0.857948194	0.913530736	0	0
2/78	0.857991724	0.913530736	0	0
2/78	0.857991724	0.913530736	0	0
1/44	0.858353108	0.913530736	0	0
1/44	0.858353108	0.913530736	0	0
1/44	0.858353108	0.913530736	0	0
1/44	0.858353108	0.913530736	0	0
1/44	0.858353108	0.913530736	0	0
1/44	0.858353108	0.913530736	0	0
1/44	0.858353108	0.913530736	0	0
1/44	0.858353108	0.913530736	0	0
2/79	0.862814455	0.917616095	0	0
2/79	0.862814455	0.917616095	0	0
1/45	0.864514062	0.918307321	0	0
1/45	0.864514062	0.918307321	0	0
1/45	0.864514062	0.918307321	0	0
10/308	0.86471083	0.918307321	0	0
3/111	0.865626437	0.918617612	0	0
3/111	0.865626437	0.918617612	0	0
6/199	0.867345817	0.919598284	0	0
2/80	0.867486675	0.919598284	0	0
2/80	0.867486675	0.919598284	0	0
7/228	0.870182261	0.920046779	0	0
1/46	0.870407336	0.920046779	0	0
1/46	0.870407336	0.920046779	0	0

1/46	0.870407336	0.920046779	0	0
1/46	0.870407336	0.920046779	0	0
1/46	0.870407336	0.920046779	0	0
1/46	0.870407336	0.920046779	0	0
1/46	0.870407336	0.920046779	0	0
2/81	0.872012346	0.921082574	0	0
2/81	0.872012346	0.921082574	0	0
1/47	0.876044546	0.924016921	0	0
1/47	0.876044546	0.924016921	0	0
1/47	0.876044546	0.924016921	0	0
1/47	0.876044546	0.924016921	0	0
12/367	0.877156884	0.924570244	0	0
3/114	0.877196608	0.924570244	0	0
1/48	0.881436808	0.92771224	0	0
1/48	0.881436808	0.92771224	0	0
1/48	0.881436808	0.92771224	0	0
1/48	0.881436808	0.92771224	0	0
4/147	0.885936937	0.931145663	0	0
1/49	0.886594751	0.931145663	0	0
1/49	0.886594751	0.931145663	0	0
1/49	0.886594751	0.931145663	0	0
1/49	0.886594751	0.931145663	0	0
1/49	0.886594751	0.931145663	0	0
2/85	0.888726626	0.93271986	0	0
2/85	0.888726626	0.93271986	0	0
1/50	0.891528548	0.933333794	0	0
1/50	0.891528548	0.933333794	0	0
1/50	0.891528548	0.933333794	0	0
1/50	0.891528548	0.933333794	0	0
1/50	0.891528548	0.933333794	0	0
1/50	0.891528548	0.933333794	0	0
1/50	0.891528548	0.933333794	0	0
1/51	0.896247927	0.936336256	0	0
1/51	0.896247927	0.936336256	0	0
1/51	0.896247927	0.936336256	0	0
1/51	0.896247927	0.936336256	0	0
1/51	0.896247927	0.936336256	0	0
2/87	0.896302877	0.936336256	0	0
6/211	0.899907288	0.939662371	0	0
1/52	0.900762198	0.939662371	0	0
1/52	0.900762198	0.939662371	0	0
1/52	0.900762198	0.939662371	0	0
2/89	0.903396649	0.941743871	0	0
2/89	0.903396649	0.941743871	0	0
1/53	0.905080264	0.942831933	0	0
1/53	0.905080264	0.942831933	0	0
1/54	0.909210644	0.946131274	0	0
1/54	0.909210644	0.946131274	0	0

1/54	0.909210644	0.946131274	0	0
3/124	0.90962287	0.94622612	0	0
1/55	0.913161487	0.948933831	0	0
1/55	0.913161487	0.948933831	0	0
2/92	0.913191837	0.948933831	0	0
3/126	0.915093235	0.950574467	0	0
1/56	0.916940587	0.951152379	0	0
1/56	0.916940587	0.951152379	0	0
1/56	0.916940587	0.951152379	0	0
1/56	0.916940587	0.951152379	0	0
2/94	0.919195907	0.952555046	0	0
1/57	0.920555402	0.952555046	0	0
1/57	0.920555402	0.952555046	0	0
1/57	0.920555402	0.952555046	0	0
1/57	0.920555402	0.952555046	0	0
1/57	0.920555402	0.952555046	0	0
1/57	0.920555402	0.952555046	0	0
2/95	0.922049365	0.953431396	0	0
2/95	0.922049365	0.953431396	0	0
5/192	0.923444266	0.954457241	0	0
1/58	0.924013067	0.954457241	0	0
1/58	0.924013067	0.954457241	0	0
2/96	0.924807713	0.954943353	0	0
4/162	0.925137562	0.95494935	0	0
1/59	0.927320405	0.956021458	0	0
1/59	0.927320405	0.956021458	0	0
1/59	0.927320405	0.956021458	0	0
2/97	0.92747382	0.956021458	0	0
1/60	0.930483946	0.958407282	0	0
1/60	0.930483946	0.958407282	0	0
6/226	0.930764045	0.958407282	0	0
7/257	0.933019134	0.959893152	0	0
1/61	0.933509933	0.959893152	0	0
1/61	0.933509933	0.959893152	0	0
1/61	0.933509933	0.959893152	0	0
3/135	0.936161644	0.960857798	0	0
1/62	0.936404342	0.960857798	0	0
1/62	0.936404342	0.960857798	0	0
1/62	0.936404342	0.960857798	0	0
1/62	0.936404342	0.960857798	0	0
1/62	0.936404342	0.960857798	0	0
4/168	0.937082438	0.9610776	0	0
2/101	0.937270791	0.9610776	0	0
1/63	0.939172887	0.962358309	0	0
1/63	0.939172887	0.962358309	0	0
13/434	0.941117548	0.964015785	0	0
2/104	0.94378036	0.965987953	0	0
1/65	0.944354018	0.965987953	0	0

1/65	0.944354018	0.965987953	0	0
1/65	0.944354018	0.965987953	0	0
8/296	0.946586206	0.967794429	0	0
1/66	0.946776837	0.967794429	0	0
1/67	0.949094279	0.969228018	0	0
1/67	0.949094279	0.969228018	0	0
2/107	0.949643952	0.969228018	0	0
2/107	0.949643952	0.969228018	0	0
9/328	0.949823725	0.969228018	0	0
1/68	0.95131092	0.970409582	0	0
1/69	0.953431142	0.971899542	0	0
1/69	0.953431142	0.971899542	0	0
3/146	0.955336814	0.97329349	0	0
1/70	0.955459134	0.97329349	0	0
2/111	0.956559676	0.973741404	0	0
2/111	0.956559676	0.973741404	0	0
1/71	0.957398902	0.974259173	0	0
2/112	0.958141008	0.974385724	0	0
3/148	0.958184536	0.974385724	0	0
2/113	0.959667109	0.975556733	0	0
3/150	0.960861363	0.97593118	0	0
1/73	0.961028939	0.97593118	0	0
1/73	0.961028939	0.97593118	0	0
1/76	0.965902906	0.98054284	0	0
4/189	0.966479894	0.980790718	0	0
1/77	0.967388267	0.981036897	0	0
1/77	0.967388267	0.981036897	0	0
1/78	0.968808988	0.98146445	0	0
1/78	0.968808988	0.98146445	0	0
1/78	0.968808988	0.98146445	0	0
1/79	0.970167878	0.982041364	0	0
2/122	0.9711981	0.982041364	0	0
2/122	0.9711981	0.982041364	0	0
1/80	0.971467627	0.982041364	0	0
1/80	0.971467627	0.982041364	0	0
1/80	0.971467627	0.982041364	0	0
5/228	0.971894113	0.982041364	0	0
2/123	0.972263027	0.982041364	0	0
1/81	0.972710804	0.982041364	0	0
1/81	0.972710804	0.982041364	0	0
2/124	0.973289905	0.982242089	0	0
8/325	0.973576227	0.982242089	0	0
3/163	0.974699255	0.983038571	0	0
1/83	0.975037178	0.983042957	0	0
1/84	0.976124977	0.983466772	0	0
1/84	0.976124977	0.983466772	0	0
1/85	0.97716542	0.983506316	0	0
1/85	0.97716542	0.983506316	0	0

1/85	0.97716542	0.983506316	0	0
4/202	0.977633084	0.983641071	0	0
9/364	0.978554379	0.984231998	0	0
5/243	0.981939754	0.987300053	0	0
1/91	0.982522885	0.987549435	0	0
1/93	0.98401344	0.988438821	0	0
7/313	0.984078555	0.988438821	0	0
7/322	0.987566728	0.99160448	0	0
1/100	0.988298886	0.992001641	0	0
1/105	0.990637372	0.994010329	0	0
3/197	0.992262566	0.995210449	0	0
1/110	0.992508826	0.995210449	0	0
1/112	0.993148179	0.995512818	0	0
4/241	0.993714027	0.995741325	0	0
1/118	0.994757245	0.996280679	0	0
2/167	0.994928414	0.996280679	0	0
2/183	0.997308853	0.998325132	0	0
1/155	0.998993994	0.999459084	0	0
1/158	0.999119939	0.999459084	0	0
2/234	0.999654896	0.999654896	0	0

Odds.Ratio	Combined.Score
2.772867902	51.62068156
3.830061087	61.25844405
3.550760788	50.47880587
2.867775499	40.1375752
3.681043388	48.44827754
27.70567787	304.9726707
3.844515119	41.84584676
3.803416242	40.95445608
2.580327869	27.67616737
3.154065905	33.41441762
3.904098361	38.49618846
12.09934613	118.9297859
2.038036663	19.36055954
6.835778175	64.40897218
6.835778175	64.40897218
3.307946444	30.70105341
15.82933289	146.8740456
2.602845018	24.02299678
3.38075953	30.45603841
13.84994206	122.2872974
2.041937125	17.89631823
3.132855613	27.31252488
3.105450092	26.79413384
1.641884992	14.02731751
2.662286613	22.63017723
3.532306376	29.59790532
8.31612529	68.35160274
2.267854007	18.60714923
1.723786487	14.01954494
6.472899729	52.54203017
6.472899729	52.54203017
5.225170999	41.05722714
5.225170999	41.05722714
3.515002459	27.42646906
4.545242883	35.37864202
4.545242883	35.37864202
10.07110502	77.71137476
5.974358974	46.04778461
3.70789576	28.41212877
2.78814554	21.04355068
14.75771605	109.4876309
9.231363461	68.35044826
9.231363461	68.35044826
5.547038328	40.65647156
3.297161648	24.04609129
2.452535114	17.75704727
33.1734104	238.1361354

5.355480796	38.29643916
8.520812907	60.6245829
3.704739705	26.23073775
3.014961014	21.20470837
6.334438184	44.50930772
2.861031734	20.04220059
5.176693767	36.12735751
3.643815201	25.38805517
7.911769575	54.16087977
3.305654546	22.52955318
1.795315436	12.12809968
2.759175976	18.46218182
5.783012206	38.3272126
5.783012206	38.3272126
7.38393202	48.6895936
22.11445087	144.1465375
22.11445087	144.1465375
4.705346144	30.57499112
4.705346144	30.57499112
2.414357032	15.64115908
5.541763341	35.69635387
5.541763341	35.69635387
1.807482924	11.63903069
3.31645966	21.01069889
3.31645966	21.01069889
2.755232526	17.35528974
9.836934156	61.95542462
9.836934156	61.95542462
5.319814385	33.31861401
4.436004646	27.51875724
4.436004646	27.51875724
5.114938426	31.16191406
4.312556459	26.14845236
1.665618556	10.04774644
16.5849711	99.36627688
16.5849711	99.36627688
2.533519925	15.1273943
3.387008938	20.1635481
4.925238464	29.19920786
2.750987571	16.24419479
2.605479163	15.25969252
3.275615947	18.84320117
3.222614443	18.2250555
3.222614443	18.2250555
2.433913934	13.73232447
2.961460761	16.57512838
13.26728324	73.68677166
13.26728324	73.68677166

13.26728324	73.68677166
13.26728324	73.68677166
13.26728324	73.68677166
5.536500579	30.68348182
1.743346361	9.625772959
4.432018561	24.26180849
7.37654321	40.33384808
2.847110155	15.22200734
4.288825687	22.87643007
4.288825687	22.87643007
3.695315525	19.61213793
1.40969306	7.427675172
2.810923254	14.80196811
2.408927875	12.65825114
6.808760684	35.63783437
4.154582367	21.59865513
5.032655641	26.14582778
2.775641026	14.39629882
2.775641026	14.39629882
11.05549133	57.28027882
11.05549133	57.28027882
11.05549133	57.28027882
11.05549133	57.28027882
11.05549133	57.28027882
11.05549133	57.28027882
11.05549133	57.28027882
11.05549133	57.28027882
11.05549133	57.28027882
2.045906363	10.55467873
1.997244261	10.26179419
2.59959284	13.35627779
2.195810452	11.15911525
2.572093595	13.03303755
4.813592624	24.23278753
4.813592624	24.23278753
4.813592624	24.23278753
2.707658195	13.62561121
6.322089947	31.71963139
6.322089947	31.71963139
1.969063467	9.851430968
1.644476748	8.20144555
3.448419151	17.15957596
1.593640655	7.908864383
9.475639967	46.02332206
9.475639967	46.02332206
5.900308642	28.41171701
3.007173827	14.42157799
2.467652016	11.83128397

3.301331949	15.74725462
4.428041715	20.96224287
1.991551735	9.386063936
5.53125	25.59035189
5.53125	25.59035189
5.53125	25.59035189
2.90827297	13.44393066
1.296413411	5.897850404
1.466055338	6.629908187
1.863788961	8.379124456
3.497496642	15.66531075
2.815651532	12.54923776
2.815651532	12.54923776
2.088568599	9.29726234
5.205610022	23.16231737
1.803242686	7.853127433
2.412080673	10.45196277
3.952987916	17.11835651
3.952987916	17.11835651
3.952987916	17.11835651
7.369171484	31.80317434
7.369171484	31.80317434
7.369171484	31.80317434
7.369171484	31.80317434
7.369171484	31.80317434
2.983114447	12.82672818
2.983114447	12.82672818
2.282195008	9.803084962
4.916152263	21.05616707
2.926675871	12.32923548
2.360511829	9.929520657
2.077571627	8.694806295
3.241072944	13.51073195
3.241072944	13.51073195
2.007086198	8.349342367
1.949877625	8.088318388
2.872327612	11.85631234
4.657163743	19.21630335
4.657163743	19.21630335
4.657163743	19.21630335
4.657163743	19.21630335
4.657163743	19.21630335
3.689069139	15.08129932
3.689069139	15.08129932
6.631907514	27.0955738
6.631907514	27.0955738
6.631907514	27.0955738
2.607934337	10.62005611

2.434055481	9.891227021
14.72594303	59.7972368
14.72594303	59.7972368
14.72594303	59.7972368
14.72594303	59.7972368
14.72594303	59.7972368
14.72594303	59.7972368
14.72594303	59.7972368
14.72594303	59.7972368
14.72594303	59.7972368
14.72594303	59.7972368
14.72594303	59.7972368
14.72594303	59.7972368
14.72594303	59.7972368
14.72594303	59.7972368
14.72594303	59.7972368
14.72594303	59.7972368
14.72594303	59.7972368
14.72594303	59.7972368
2.033358043	8.209633173
2.17973829	8.744056459
2.404603279	9.614994484
4.424074074	17.59887356
3.569880013	14.186412
3.019616115	11.73209066
6.02869154	23.37139396
6.02869154	23.37139396
6.02869154	23.37139396
6.02869154	23.37139396
6.02869154	23.37139396
6.02869154	23.37139396
6.02869154	23.37139396
6.02869154	23.37139396
6.02869154	23.37139396
6.02869154	23.37139396
4.213183422	16.16886857
4.213183422	16.16886857
2.033352358	7.763645718
2.196104226	8.330653785
1.328963714	4.990221822
1.245599373	4.628887066
2.888025825	10.71393989
4.021464646	14.89803386
4.021464646	14.89803386
5.526011561	20.36716497
5.526011561	20.36716497
5.526011561	20.36716497
5.526011561	20.36716497

5.526011561	20.36716497
11.04387991	40.68754141
11.04387991	40.68754141
11.04387991	40.68754141
11.04387991	40.68754141
11.04387991	40.68754141
11.04387991	40.68754141
11.04387991	40.68754141
11.04387991	40.68754141
11.04387991	40.68754141
11.04387991	40.68754141
11.04387991	40.68754141
11.04387991	40.68754141
11.04387991	40.68754141
11.04387991	40.68754141
11.04387991	40.68754141
11.04387991	40.68754141
11.04387991	40.68754141
11.04387991	40.68754141
11.04387991	40.68754141
11.04387991	40.68754141
2.153235114	7.931118335
1.749423336	6.394650997
2.241788858	8.139941584
2.82643037	10.24779272
2.363658915	8.498564149
3.846417069	13.7633408
3.846417069	13.7633408
3.161231584	11.24711133
2.767401392	9.80751443
1.943686472	6.87475509
2.500655652	8.794155272
5.100666963	17.90390236
5.100666963	17.90390236
5.100666963	17.90390236
5.100666963	17.90390236
2.302023558	7.990282761
2.302023558	7.990282761
2.710781761	9.391272408
3.073258658	10.64231139
3.68595679	12.74585147
3.68595679	12.74585147
3.68595679	12.74585147
3.68595679	12.74585147
3.68595679	12.74585147
2.422256098	8.189251152
8.834642032	29.83061924
8.834642032	29.83061924

8.834642032	29.83061924
8.834642032	29.83061924
8.834642032	29.83061924
8.834642032	29.83061924
8.834642032	29.83061924
8.834642032	29.83061924
8.834642032	29.83061924
8.834642032	29.83061924
8.834642032	29.83061924
8.834642032	29.83061924
8.834642032	29.83061924
8.834642032	29.83061924
8.834642032	29.83061924
8.834642032	29.83061924
8.834642032	29.83061924
8.834642032	29.83061924
2.990041026	10.07995578
4.736085879	15.85609978
4.736085879	15.85609978
4.736085879	15.85609978
4.736085879	15.85609978
4.736085879	15.85609978
3.538333333	11.82986423
3.538333333	11.82986423
3.538333333	11.82986423
1.583162908	5.281108082
1.825908653	6.08396527
2.384865541	7.906141601
2.911203269	9.556214595
2.911203269	9.556214595
2.911203269	9.556214595
2.911203269	9.556214595
2.911203269	9.556214595
2.911203269	9.556214595
1.861544997	6.098633785
3.402065527	11.00226275
3.402065527	11.00226275
3.402065527	11.00226275
3.402065527	11.00226275
1.512747875	4.870428611
4.420115607	14.13336513
4.420115607	14.13336513
4.420115607	14.13336513
4.420115607	14.13336513
4.420115607	14.13336513
2.836408474	9.067684596
2.313432836	7.375208158
1.312342318	4.162759697

2.505669133	7.935291803
1.735781665	5.496594697
3.275891632	10.25201455
3.275891632	10.25201455
2.279292205	7.126157206
2.279292205	7.126157206
7.361816782	22.95127016
7.361816782	22.95127016
7.361816782	22.95127016
7.361816782	22.95127016
7.361816782	22.95127016
7.361816782	22.95127016
7.361816782	22.95127016
7.361816782	22.95127016
7.361816782	22.95127016
7.361816782	22.95127016
7.361816782	22.95127016
7.361816782	22.95127016
7.361816782	22.95127016
7.361816782	22.95127016
4.143641618	12.6690898
4.143641618	12.6690898
4.143641618	12.6690898
4.143641618	12.6690898
4.143641618	12.6690898
4.143641618	12.6690898
2.697764463	8.184448204
2.697764463	8.184448204
2.697764463	8.184448204
3.158730159	9.569780635
1.833542433	5.523060869
2.213937282	6.658110275
1.341542261	3.989019299
2.371063971	7.026517977
2.371063971	7.026517977
2.63339403	7.784601994
1.678954364	4.962472552
3.049648787	8.947608783
3.049648787	8.947608783
3.049648787	8.947608783
3.049648787	8.947608783
3.049648787	8.947608783
1.806188686	5.293977228
3.899693982	11.41320925
3.899693982	11.41320925
3.899693982	11.41320925
3.899693982	11.41320925
3.899693982	11.41320925

3.899693982	11.41320925
6.309798746	18.26638396
6.309798746	18.26638396
6.309798746	18.26638396
6.309798746	18.26638396
6.309798746	18.26638396
6.309798746	18.26638396
6.309798746	18.26638396
6.309798746	18.26638396
6.309798746	18.26638396
6.309798746	18.26638396
6.309798746	18.26638396
6.309798746	18.26638396
6.309798746	18.26638396
6.309798746	18.26638396
6.309798746	18.26638396
6.309798746	18.26638396
6.309798746	18.26638396
6.309798746	18.26638396
6.309798746	18.26638396
6.309798746	18.26638396
6.309798746	18.26638396
6.309798746	18.26638396
2.57201757	7.40961185
2.947839506	8.378690423
2.947839506	8.378690423
1.413132825	3.997765209
2.513430949	7.057512587
2.513430949	7.057512587
3.682851638	10.32743677
3.682851638	10.32743677
3.682851638	10.32743677
3.682851638	10.32743677
1.816080095	5.062046787
1.625697365	4.483221517
2.852598566	7.857166326
2.852598566	7.857166326
2.457448178	6.726530408
2.457448178	6.726530408
2.457448178	6.726530408
2.212529002	6.007186206
1.557038135	4.215649872
5.520785219	14.90774956
5.520785219	14.90774956
5.520785219	14.90774956
5.520785219	14.90774956
5.520785219	14.90774956
5.520785219	14.90774956

5.520785219	14.90774956
5.520785219	14.90774956
5.520785219	14.90774956
1.60868088	4.334540966
3.488834804	9.382042246
3.488834804	9.382042246
3.488834804	9.382042246
2.038510911	5.460814018
2.403899441	6.41506074
2.403899441	6.41506074
2.403899441	6.41506074
1.672809028	4.462499752
1.811810774	4.711077404
1.428739303	3.711951675
2.679433221	6.936701597
3.314219653	8.553624547
3.314219653	8.553624547
3.314219653	8.553624547
3.314219653	8.553624547
1.986032937	5.120164041
1.986032937	5.120164041
1.986032937	5.120164041
1.986032937	5.120164041
2.106839023	5.360180648
2.303495558	5.844974158
4.907108032	12.4052976
4.907108032	12.4052976
4.907108032	12.4052976
4.907108032	12.4052976
4.907108032	12.4052976
4.907108032	12.4052976
4.907108032	12.4052976
2.600490196	6.529522766
2.600490196	6.529522766
2.073810905	5.16358442
1.763431444	4.372279718
3.156234517	7.82353923
3.156234517	7.82353923
3.156234517	7.82353923
3.156234517	7.82353923
1.703604088	4.222014304
2.256367205	5.583833707
1.82546152	4.474541692
1.82546152	4.474541692
1.747870841	4.265505546
2.041799036	4.975649477
2.526058201	6.153073157
2.526058201	6.153073157

2.526058201	6.153073157
2.526058201	6.153073157
1.912175048	4.655086515
1.912175048	4.655086515
2.211123986	5.337131458
2.211123986	5.337131458
2.211123986	5.337131458
1.732580857	4.161644101
2.010757224	4.795904044
3.012611666	7.176768499
3.012611666	7.176768499
3.012611666	7.176768499
3.012611666	7.176768499
4.416166282	10.48394421
4.416166282	10.48394421
4.416166282	10.48394421
4.416166282	10.48394421
4.416166282	10.48394421
4.416166282	10.48394421
4.416166282	10.48394421
4.416166282	10.48394421
4.416166282	10.48394421
4.416166282	10.48394421
4.416166282	10.48394421
4.416166282	10.48394421
4.416166282	10.48394421
4.416166282	10.48394421
4.416166282	10.48394421
2.455761317	5.804399348
2.455761317	5.804399348
2.455761317	5.804399348
1.980642033	4.623907787
1.430967742	3.336851245
1.843592722	4.238762168
2.12585792	4.883121737
2.389264264	5.480896611
2.881477758	6.601095579
2.881477758	6.601095579
2.881477758	6.601095579
2.881477758	6.601095579
2.881477758	6.601095579
2.881477758	6.601095579
2.881477758	6.601095579
2.881477758	6.601095579
1.752797605	4.009676308
1.951412584	4.459249322
1.951412584	4.459249322
1.735522116	3.902204874

2.085638077	4.674058733
2.085638077	4.674058733
1.923030364	4.301543944
1.923030364	4.301543944
4.014486668	8.972822838
4.014486668	8.972822838
4.014486668	8.972822838
4.014486668	8.972822838
4.014486668	8.972822838
4.014486668	8.972822838
4.014486668	8.972822838
4.014486668	8.972822838
4.014486668	8.972822838
4.014486668	8.972822838
4.014486668	8.972822838
4.014486668	8.972822838
4.014486668	8.972822838
4.014486668	8.972822838
4.014486668	8.972822838
2.326267057	5.180260159
2.326267057	5.180260159
2.326267057	5.180260159
1.507810539	3.35761342
1.571475891	3.498173403
1.800529401	3.985241983
1.718582073	3.797979141
2.761271676	6.086492551
2.761271676	6.086492551
2.761271676	6.086492551
2.761271676	6.086492551
1.895459065	4.150431571
2.046907858	4.47590664
1.411448087	3.072185371
1.7019678	3.696882615
1.779740211	3.865083132
1.592544147	3.455895884
1.592544147	3.455895884
1.445180692	3.133741095
2.266500475	4.900444231
2.009586011	4.287957191
1.759423503	3.749079311
2.650682081	5.624660322
2.650682081	5.624660322
2.650682081	5.624660322
2.650682081	5.624660322
2.650682081	5.624660322
2.650682081	5.624660322
2.650682081	5.624660322

3.679753657	7.760665454
3.679753657	7.760665454
3.679753657	7.760665454
3.679753657	7.760665454
3.679753657	7.760665454
3.679753657	7.760665454
3.679753657	7.760665454
3.679753657	7.760665454
3.679753657	7.760665454
3.679753657	7.760665454
3.679753657	7.760665454
3.679753657	7.760665454
3.679753657	7.760665454
3.679753657	7.760665454
3.679753657	7.760665454
3.679753657	7.760665454
3.679753657	7.760665454
3.679753657	7.760665454
3.679753657	7.760665454
3.679753657	7.760665454
2.209722222	4.639627606
2.209722222	4.639627606
2.209722222	4.639627606
1.842614076	3.866657546
1.669679684	3.503636209
1.547671443	3.16509301
2.548599378	5.208678678
2.548599378	5.208678678
2.548599378	5.208678678
2.548599378	5.208678678
2.548599378	5.208678678
2.548599378	5.208678678
2.548599378	5.208678678
2.15571364	4.396184402
1.938870932	3.940109161
1.476012461	2.993906718
1.580409114	3.186937708
3.396518032	6.772175996
3.396518032	6.772175996
3.396518032	6.772175996
3.396518032	6.772175996
3.396518032	6.772175996
3.396518032	6.772175996
3.396518032	6.772175996
3.396518032	6.772175996
3.396518032	6.772175996
3.396518032	6.772175996
3.396518032	6.772175996

3.396518032	6.772175996
3.396518032	6.772175996
3.396518032	6.772175996
3.396518032	6.772175996
3.396518032	6.772175996
3.396518032	6.772175996
3.396518032	6.772175996
3.396518032	6.772175996
3.396518032	6.772175996
3.396518032	6.772175996
3.396518032	6.772175996
3.396518032	6.772175996
3.396518032	6.772175996
3.396518032	6.772175996
3.396518032	6.772175996
1.90534223	3.779054696
2.104276896	4.168659247
2.104276896	4.168659247
2.104276896	4.168659247
2.104276896	4.168659247
1.379222155	2.727718919
1.682573347	3.323288956
2.454078356	4.832737068
2.454078356	4.832737068
2.454078356	4.832737068
2.454078356	4.832737068
1.872950095	3.625882153
1.368426777	2.647074128
1.270386221	2.449889714
1.745268042	3.364708717
2.055232558	3.955746092
2.055232558	3.955746092
2.366308836	4.491925366
2.366308836	4.491925366
2.366308836	4.491925366
2.366308836	4.491925366
1.841637698	3.48011692
3.153744639	5.954749749
3.153744639	5.954749749
3.153744639	5.954749749
3.153744639	5.954749749
3.153744639	5.954749749
3.153744639	5.954749749
3.153744639	5.954749749
3.153744639	5.954749749
3.153744639	5.954749749
3.153744639	5.954749749
3.153744639	5.954749749

3.153744639	5.954749749
3.153744639	5.954749749
3.153744639	5.954749749
3.153744639	5.954749749
3.153744639	5.954749749
3.153744639	5.954749749
2.008417508	3.756270051
2.008417508	3.756270051
1.292837466	2.396053571
1.700339104	3.142452221
2.284592386	4.182069674
2.284592386	4.182069674
2.284592386	4.182069674
1.355623609	2.473879664
1.963683128	3.56917179
1.963683128	3.56917179
1.963683128	3.56917179
1.678727716	3.037725003
1.281947954	2.308933599
1.782043135	3.209081094
1.782043135	3.209081094
1.595423686	2.86618047
1.595423686	2.86618047
2.943341032	5.270632606
2.943341032	5.270632606
2.943341032	5.270632606
2.943341032	5.270632606
2.943341032	5.270632606
2.943341032	5.270632606
2.943341032	5.270632606
2.943341032	5.270632606
2.943341032	5.270632606
2.943341032	5.270632606
2.943341032	5.270632606
2.943341032	5.270632606
2.943341032	5.270632606
2.943341032	5.270632606
2.943341032	5.270632606
2.943341032	5.270632606
2.943341032	5.270632606
2.943341032	5.270632606
2.943341032	5.270632606
2.943341032	5.270632606
2.943341032	5.270632606
1.92089372	3.393494064
1.92089372	3.393494064
2.208323699	3.899602358
2.208323699	3.899602358

2.208323699	3.899602358
2.208323699	3.899602358
2.208323699	3.899602358
2.208323699	3.899602358
1.753664772	3.083023628
1.27123696	2.224808325
1.563028661	2.703498518
1.463432171	2.514430178
1.879925138	3.228370048
1.879925138	3.228370048
1.879925138	3.228370048
1.879925138	3.228370048
1.726173233	2.962794539
1.726173233	2.962794539
1.356945824	2.319195569
2.136975573	3.641458317
2.136975573	3.641458317
2.136975573	3.641458317
2.136975573	3.641458317
2.136975573	3.641458317
2.136975573	3.641458317
2.136975573	3.641458317
2.136975573	3.641458317
2.136975573	3.641458317
2.759237875	4.692097593
2.759237875	4.692097593
2.759237875	4.692097593
2.759237875	4.692097593
2.759237875	4.692097593
2.759237875	4.692097593
2.759237875	4.692097593
2.759237875	4.692097593
2.759237875	4.692097593
2.759237875	4.692097593
2.759237875	4.692097593
2.759237875	4.692097593
2.759237875	4.692097593
2.759237875	4.692097593
2.759237875	4.692097593
2.759237875	4.692097593
2.759237875	4.692097593
2.759237875	4.692097593
2.759237875	4.692097593
2.759237875	4.692097593
2.759237875	4.692097593
2.759237875	4.692097593
2.759237875	4.692097593
1.617056194	2.746845273

1.699527587	2.848065933
1.84066358	3.073013227
1.344121987	2.239164526
1.267259852	2.098203016
1.284427475	2.115130569
2.070086705	3.404991562
2.070086705	3.404991562
2.070086705	3.404991562
2.070086705	3.404991562
2.070086705	3.404991562
2.070086705	3.404991562
1.516818109	2.478435299
1.516818109	2.478435299
1.803004535	2.926708571
1.803004535	2.926708571
1.803004535	2.926708571
1.803004535	2.926708571
1.461541418	2.365091307
2.596793914	4.198371902
2.596793914	4.198371902
2.596793914	4.198371902
2.596793914	4.198371902
2.596793914	4.198371902
2.596793914	4.198371902
2.596793914	4.198371902
2.596793914	4.198371902
2.596793914	4.198371902
2.596793914	4.198371902
2.596793914	4.198371902
2.596793914	4.198371902
2.596793914	4.198371902
2.596793914	4.198371902
5.515570934	8.905113537
5.515570934	8.905113537
5.515570934	8.905113537
5.515570934	8.905113537
5.515570934	8.905113537
5.515570934	8.905113537
5.515570934	8.905113537
5.515570934	8.905113537
5.515570934	8.905113537
5.515570934	8.905113537
5.515570934	8.905113537
5.515570934	8.905113537
5.515570934	8.905113537
5.515570934	8.905113537
5.515570934	8.905113537
5.515570934	8.905113537

5.515570934	8.905113537
5.515570934	8.905113537
5.515570934	8.905113537
5.515570934	8.905113537
5.515570934	8.905113537
5.515570934	8.905113537
5.515570934	8.905113537
5.515570934	8.905113537
5.515570934	8.905113537
5.515570934	8.905113537
5.515570934	8.905113537
5.515570934	8.905113537
5.515570934	8.905113537
5.515570934	8.905113537
5.515570934	8.905113537
5.515570934	8.905113537
5.515570934	8.905113537
5.515570934	8.905113537
5.515570934	8.905113537
5.515570934	8.905113537
5.515570934	8.905113537
5.515570934	8.905113537
5.515570934	8.905113537
5.515570934	8.905113537
5.515570934	8.905113537
5.515570934	8.905113537
5.515570934	8.905113537
5.515570934	8.905113537
5.515570934	8.905113537
5.515570934	8.905113537
5.515570934	8.905113537
5.515570934	8.905113537
5.515570934	8.905113537
5.515570934	8.905113537
5.515570934	8.905113537
5.515570934	8.905113537
5.515570934	8.905113537
5.515570934	8.905113537
5.515570934	8.905113537
5.515570934	8.905113537
5.515570934	8.905113537
5.515570934	8.905113537
5.515570934	8.905113537
5.515570934	8.905113537
5.515570934	8.905113537
5.515570934	8.905113537
5.515570934	8.905113537
5.515570934	8.905113537

5.515570934	8.905113537
5.515570934	8.905113537
5.515570934	8.905113537
5.515570934	8.905113537
5.515570934	8.905113537
5.515570934	8.905113537
5.515570934	8.905113537
5.515570934	8.905113537
5.515570934	8.905113537
5.515570934	8.905113537
5.515570934	8.905113537
5.515570934	8.905113537
5.515570934	8.905113537
5.515570934	8.905113537
5.515570934	8.905113537
5.515570934	8.905113537
5.515570934	8.905113537
5.515570934	8.905113537
5.515570934	8.905113537
5.515570934	8.905113537
5.515570934	8.905113537
5.515570934	8.905113537
5.515570934	8.905113537
5.515570934	8.905113537
5.515570934	8.905113537
5.515570934	8.905113537
5.515570934	8.905113537
5.515570934	8.905113537
5.515570934	8.905113537
5.515570934	8.905113537
5.515570934	8.905113537
5.515570934	8.905113537
5.515570934	8.905113537
5.515570934	8.905113537
5.515570934	8.905113537
5.515570934	8.905113537
5.515570934	8.905113537
1.648622473	2.633908725
1.648622473	2.633908725
2.007251708	3.187907664
2.007251708	3.187907664
2.007251708	3.187907664
1.766851852	2.788804839
1.766851852	2.788804839
1.766851852	2.788804839
1.252874584	1.950535051
2.452399281	3.773619216
2.452399281	3.773619216

2.452399281	3.773619216
2.452399281	3.773619216
2.452399281	3.773619216
2.452399281	3.773619216
2.452399281	3.773619216
2.452399281	3.773619216
2.452399281	3.773619216
2.452399281	3.773619216
2.452399281	3.773619216
1.425956489	2.189993097
1.732116921	2.65870784
1.948112887	2.988208669
1.948112887	2.988208669
1.948112887	2.988208669
1.948112887	2.988208669
1.506327779	2.255329384
1.577718921	2.346924166
1.445558848	2.147966544
1.892353427	2.804147914
1.892353427	2.804147914
1.892353427	2.804147914
2.323204084	3.405579666
2.323204084	3.405579666
2.323204084	3.405579666
2.323204084	3.405579666
2.323204084	3.405579666
2.323204084	3.405579666
2.323204084	3.405579666
2.323204084	3.405579666
2.323204084	3.405579666
2.323204084	3.405579666
2.323204084	3.405579666
4.412226067	6.413092306
4.412226067	6.413092306
4.412226067	6.413092306
4.412226067	6.413092306
4.412226067	6.413092306
4.412226067	6.413092306
4.412226067	6.413092306
4.412226067	6.413092306
4.412226067	6.413092306
4.412226067	6.413092306
4.412226067	6.413092306
4.412226067	6.413092306
4.412226067	6.413092306
4.412226067	6.413092306
4.412226067	6.413092306
4.412226067	6.413092306

4.412226067	6.413092306
4.412226067	6.413092306
4.412226067	6.413092306
4.412226067	6.413092306
4.412226067	6.413092306
4.412226067	6.413092306
4.412226067	6.413092306
4.412226067	6.413092306
4.412226067	6.413092306
4.412226067	6.413092306
4.412226067	6.413092306
4.412226067	6.413092306
4.412226067	6.413092306
4.412226067	6.413092306
4.412226067	6.413092306
4.412226067	6.413092306
4.412226067	6.413092306
4.412226067	6.413092306
4.412226067	6.413092306
4.412226067	6.413092306
4.412226067	6.413092306
4.412226067	6.413092306
4.412226067	6.413092306
4.412226067	6.413092306
4.412226067	6.413092306
4.412226067	6.413092306
4.412226067	6.413092306
4.412226067	6.413092306
4.412226067	6.413092306
4.412226067	6.413092306
4.412226067	6.413092306
4.412226067	6.413092306
4.412226067	6.413092306
4.412226067	6.413092306
4.412226067	6.413092306
4.412226067	6.413092306
4.412226067	6.413092306
4.412226067	6.413092306
4.412226067	6.413092306
4.412226067	6.413092306
4.412226067	6.413092306
4.412226067	6.413092306
4.412226067	6.413092306
4.412226067	6.413092306
4.412226067	6.413092306
4.412226067	6.413092306
4.412226067	6.413092306
4.412226067	6.413092306

4.412226067	6.413092306
4.412226067	6.413092306
4.412226067	6.413092306
4.412226067	6.413092306
4.412226067	6.413092306
4.412226067	6.413092306
4.412226067	6.413092306
4.412226067	6.413092306
4.412226067	6.413092306
4.412226067	6.413092306
4.412226067	6.413092306
4.412226067	6.413092306
4.412226067	6.413092306
4.412226067	6.413092306
4.412226067	6.413092306
4.412226067	6.413092306
4.412226067	6.413092306
4.412226067	6.413092306
4.412226067	6.413092306
4.412226067	6.413092306
4.412226067	6.413092306
4.412226067	6.413092306
4.412226067	6.413092306
1.555415925	2.259448568
1.839691715	2.634192737
1.839691715	2.634192737
1.839691715	2.634192737
1.839691715	2.634192737
1.839691715	2.634192737
1.839691715	2.634192737
1.839691715	2.634192737
1.839691715	2.634192737
1.839691715	2.634192737
1.839691715	2.634192737
1.839691715	2.634192737
1.533732458	2.175714482
1.635631001	2.310051587
1.405912786	1.973085607
2.206928406	3.084631666
2.206928406	3.084631666
2.206928406	3.084631666
2.206928406	3.084631666
2.206928406	3.084631666
2.206928406	3.084631666
2.206928406	3.084631666
2.206928406	3.084631666
2.206928406	3.084631666
2.206928406	3.084631666

2.206928406	3.084631666
2.206928406	3.084631666
2.206928406	3.084631666
2.206928406	3.084631666
2.206928406	3.084631666
2.206928406	3.084631666
2.206928406	3.084631666
2.206928406	3.084631666
1.334836043	1.85802709
1.789876582	2.476993545
1.789876582	2.476993545
1.789876582	2.476993545
1.307775065	1.80734635
1.440532634	1.982842414
1.440532634	1.982842414
1.605808081	2.206187595
1.605808081	2.206187595
1.605808081	2.206187595
1.279629061	1.735170165
1.492123642	2.018717846
1.492123642	2.018717846
1.492123642	2.018717846
1.492123642	2.018717846
1.380662021	1.865137952
1.742683298	2.331358004
1.742683298	2.331358004
1.742683298	2.331358004
1.577050265	2.107830801
1.577050265	2.107830801
2.101726603	2.803131375
2.101726603	2.803131375
2.101726603	2.803131375
2.101726603	2.803131375
2.101726603	2.803131375
2.101726603	2.803131375
2.101726603	2.803131375
2.101726603	2.803131375
2.101726603	2.803131375
2.101726603	2.803131375
2.101726603	2.803131375
3.676662822	4.854784328
3.676662822	4.854784328
3.676662822	4.854784328
3.676662822	4.854784328
3.676662822	4.854784328
3.676662822	4.854784328
3.676662822	4.854784328
3.676662822	4.854784328

3.676662822	4.854784328
3.676662822	4.854784328
3.676662822	4.854784328
3.676662822	4.854784328
3.676662822	4.854784328
3.676662822	4.854784328
3.676662822	4.854784328
3.676662822	4.854784328
3.676662822	4.854784328
3.676662822	4.854784328
3.676662822	4.854784328
3.676662822	4.854784328
3.676662822	4.854784328
3.676662822	4.854784328
3.676662822	4.854784328
3.676662822	4.854784328
3.676662822	4.854784328
3.676662822	4.854784328
3.676662822	4.854784328
3.676662822	4.854784328
3.676662822	4.854784328
3.676662822	4.854784328
3.676662822	4.854784328
3.676662822	4.854784328
3.676662822	4.854784328
3.676662822	4.854784328
3.676662822	4.854784328
3.676662822	4.854784328
3.676662822	4.854784328
3.676662822	4.854784328
3.676662822	4.854784328
3.676662822	4.854784328
3.676662822	4.854784328
3.676662822	4.854784328
3.676662822	4.854784328
3.676662822	4.854784328
3.676662822	4.854784328
3.676662822	4.854784328
3.676662822	4.854784328
3.676662822	4.854784328
3.676662822	4.854784328
3.676662822	4.854784328
3.676662822	4.854784328
3.676662822	4.854784328
3.676662822	4.854784328
3.676662822	4.854784328
3.676662822	4.854784328
3.676662822	4.854784328

3.676662822	4.854784328
3.676662822	4.854784328
3.676662822	4.854784328
3.676662822	4.854784328
3.676662822	4.854784328
3.676662822	4.854784328
3.676662822	4.854784328
3.676662822	4.854784328
3.676662822	4.854784328
3.676662822	4.854784328
3.676662822	4.854784328
3.676662822	4.854784328
3.676662822	4.854784328
3.676662822	4.854784328
3.676662822	4.854784328
3.676662822	4.854784328
3.676662822	4.854784328
3.676662822	4.854784328
3.676662822	4.854784328
3.676662822	4.854784328
1.409734906	1.860512355
1.409734906	1.860512355
1.549301494	2.014626728
1.549301494	2.014626728
1.277268011	1.659886683
1.697910182	2.196229418
1.697910182	2.196229418
1.697910182	2.196229418
1.452704763	1.874540824
1.344432662	1.715016623
2.0060886	2.55493905
2.0060886	2.55493905
2.0060886	2.55493905
2.0060886	2.55493905
2.0060886	2.55493905
2.0060886	2.55493905
2.0060886	2.55493905
2.0060886	2.55493905
2.0060886	2.55493905
2.0060886	2.55493905
2.0060886	2.55493905
2.0060886	2.55493905
1.522509579	1.9262485
1.433763224	1.80686928
1.208760246	1.516640166
1.655375723	2.070668515
1.655375723	2.070668515
1.655375723	2.070668515

1.655375723	2.070668515
1.655375723	2.070668515
1.166954587	1.43871281
1.415307365	1.741952464
1.918766944	2.335073794
1.918766944	2.335073794
1.918766944	2.335073794
1.918766944	2.335073794
1.918766944	2.335073794
1.918766944	2.335073794
1.918766944	2.335073794
1.918766944	2.335073794
1.918766944	2.335073794
1.918766944	2.335073794
1.918766944	2.335073794
1.918766944	2.335073794
1.918766944	2.335073794
1.918766944	2.335073794
1.918766944	2.335073794
1.918766944	2.335073794
1.614916114	1.953838048
1.614916114	1.953838048
3.151260504	3.806216439
3.151260504	3.806216439
3.151260504	3.806216439
3.151260504	3.806216439
3.151260504	3.806216439
3.151260504	3.806216439
3.151260504	3.806216439
3.151260504	3.806216439
3.151260504	3.806216439
3.151260504	3.806216439
3.151260504	3.806216439
3.151260504	3.806216439
3.151260504	3.806216439
3.151260504	3.806216439
3.151260504	3.806216439
3.151260504	3.806216439
3.151260504	3.806216439
3.151260504	3.806216439
3.151260504	3.806216439
3.151260504	3.806216439
3.151260504	3.806216439
3.151260504	3.806216439
3.151260504	3.806216439
3.151260504	3.806216439
3.151260504	3.806216439
3.151260504	3.806216439

3.151260504	3.806216439
3.151260504	3.806216439
3.151260504	3.806216439
3.151260504	3.806216439
3.151260504	3.806216439
3.151260504	3.806216439
3.151260504	3.806216439
3.151260504	3.806216439
3.151260504	3.806216439
3.151260504	3.806216439
3.151260504	3.806216439
3.151260504	3.806216439
3.151260504	3.806216439
3.151260504	3.806216439
3.151260504	3.806216439
3.151260504	3.806216439
3.151260504	3.806216439
3.151260504	3.806216439
3.151260504	3.806216439
3.151260504	3.806216439
3.151260504	3.806216439
3.151260504	3.806216439
3.151260504	3.806216439
3.151260504	3.806216439
3.151260504	3.806216439
3.151260504	3.806216439
3.151260504	3.806216439
3.151260504	3.806216439
3.151260504	3.806216439
3.151260504	3.806216439
3.151260504	3.806216439
3.151260504	3.806216439
3.151260504	3.806216439
3.151260504	3.806216439
3.151260504	3.806216439
3.151260504	3.806216439
3.151260504	3.806216439
3.151260504	3.806216439
3.151260504	3.806216439
3.151260504	3.806216439
1.310045473	1.577814049
1.397318744	1.67965826
1.210664448	1.445904546
1.298968368	1.534730474
1.379779838	1.619862129
1.379779838	1.619862129
1.576383154	1.844989717
1.576383154	1.844989717

1.576383154	1.844989717
1.576383154	1.844989717
1.125235322	1.316778222
1.227143227	1.430676415
1.447404372	1.687162547
1.447404372	1.687162547
1.447404372	1.687162547
1.447404372	1.687162547
1.838722094	2.139458132
1.838722094	2.139458132
1.838722094	2.139458132
1.838722094	2.139458132
1.838722094	2.139458132
1.838722094	2.139458132
1.838722094	2.139458132
1.838722094	2.139458132
1.838722094	2.139458132
1.838722094	2.139458132
1.838722094	2.139458132
1.838722094	2.139458132
1.838722094	2.139458132
1.838722094	2.139458132
1.163083766	1.339129127
1.362673991	1.562446599
1.539642425	1.743453014
1.539642425	1.743453014
1.539642425	1.743453014
1.539642425	1.743453014
1.539642425	1.743453014
1.539642425	1.743453014
1.539642425	1.743453014
1.34598536	1.507300787
1.266826603	1.412833837
1.298621537	1.447961962
1.765080831	1.964726481
1.765080831	1.964726481
1.765080831	1.964726481
1.765080831	1.964726481
1.765080831	1.964726481
1.765080831	1.964726481
1.765080831	1.964726481
1.765080831	1.964726481
1.765080831	1.964726481
1.765080831	1.964726481
1.765080831	1.964726481
1.765080831	1.964726481
1.765080831	1.964726481
1.765080831	1.964726481

1.18332777	1.317160461
1.206835059	1.342518919
2.757208766	3.062797655
2.757208766	3.062797655
2.757208766	3.062797655
2.757208766	3.062797655
2.757208766	3.062797655
2.757208766	3.062797655
2.757208766	3.062797655
2.757208766	3.062797655
2.757208766	3.062797655
2.757208766	3.062797655
2.757208766	3.062797655
2.757208766	3.062797655
2.757208766	3.062797655
2.757208766	3.062797655
2.757208766	3.062797655
2.757208766	3.062797655
2.757208766	3.062797655
2.757208766	3.062797655
2.757208766	3.062797655
2.757208766	3.062797655
2.757208766	3.062797655
2.757208766	3.062797655
2.757208766	3.062797655
2.757208766	3.062797655
2.757208766	3.062797655
2.757208766	3.062797655
2.757208766	3.062797655
2.757208766	3.062797655
2.757208766	3.062797655
2.757208766	3.062797655
2.757208766	3.062797655
2.757208766	3.062797655
2.757208766	3.062797655
2.757208766	3.062797655
2.757208766	3.062797655
2.757208766	3.062797655
2.757208766	3.062797655
2.757208766	3.062797655
2.757208766	3.062797655
2.757208766	3.062797655
2.757208766	3.062797655
2.757208766	3.062797655
2.757208766	3.062797655
2.757208766	3.062797655
2.757208766	3.062797655
2.757208766	3.062797655

2.757208766	3.062797655
2.757208766	3.062797655
2.757208766	3.062797655
2.757208766	3.062797655
2.757208766	3.062797655
2.757208766	3.062797655
2.757208766	3.062797655
2.757208766	3.062797655
2.757208766	3.062797655
2.757208766	3.062797655
2.757208766	3.062797655
2.757208766	3.062797655
2.757208766	3.062797655
2.757208766	3.062797655
2.757208766	3.062797655
2.757208766	3.062797655
2.757208766	3.062797655
2.757208766	3.062797655
2.757208766	3.062797655
2.757208766	3.062797655
2.757208766	3.062797655
2.757208766	3.062797655
2.757208766	3.062797655
2.757208766	3.062797655
2.757208766	3.062797655
2.757208766	3.062797655
2.757208766	3.062797655
1.219287658	1.347411373
1.401308054	1.54693744
1.504571729	1.648625684
1.504571729	1.648625684
1.504571729	1.648625684
1.256461101	1.374521461
1.329698865	1.454319965
1.285945982	1.403852455
1.158285749	1.26108214
1.226614987	1.323892603
1.379340278	1.481906526
1.204381416	1.287257719
1.313800143	1.403405158
1.313800143	1.403405158
1.697104281	1.808079756
1.697104281	1.808079756
1.697104281	1.808079756
1.697104281	1.808079756
1.697104281	1.808079756
1.697104281	1.808079756
1.697104281	1.808079756

1.697104281	1.808079756
1.697104281	1.808079756
1.697104281	1.808079756
1.697104281	1.808079756
1.697104281	1.808079756
1.10510083	1.176330383
1.187181994	1.259828735
1.149552075	1.219308769
1.209684613	1.259736247
1.18983221	1.229836761
1.439004775	1.476983324
1.439004775	1.476983324
2.45072408	2.514655498
2.45072408	2.514655498
2.45072408	2.514655498
2.45072408	2.514655498
2.45072408	2.514655498
2.45072408	2.514655498
2.45072408	2.514655498
2.45072408	2.514655498
2.45072408	2.514655498
2.45072408	2.514655498
2.45072408	2.514655498
2.45072408	2.514655498
2.45072408	2.514655498
2.45072408	2.514655498
2.45072408	2.514655498
2.45072408	2.514655498
2.45072408	2.514655498
2.45072408	2.514655498
2.45072408	2.514655498
2.45072408	2.514655498
2.45072408	2.514655498
2.45072408	2.514655498
2.45072408	2.514655498
2.45072408	2.514655498
2.45072408	2.514655498
2.45072408	2.514655498
2.45072408	2.514655498
2.45072408	2.514655498
2.45072408	2.514655498
2.45072408	2.514655498
2.45072408	2.514655498
2.45072408	2.514655498
2.45072408	2.514655498
2.45072408	2.514655498
2.45072408	2.514655498
2.45072408	2.514655498

2.45072408	2.514655498
2.45072408	2.514655498
2.45072408	2.514655498
2.45072408	2.514655498
2.45072408	2.514655498
2.45072408	2.514655498
2.45072408	2.514655498
2.45072408	2.514655498
2.45072408	2.514655498
2.45072408	2.514655498
2.45072408	2.514655498
2.45072408	2.514655498
2.45072408	2.514655498
2.45072408	2.514655498
2.45072408	2.514655498
2.45072408	2.514655498
2.45072408	2.514655498
2.45072408	2.514655498
2.45072408	2.514655498
2.45072408	2.514655498
2.45072408	2.514655498
2.45072408	2.514655498
2.45072408	2.514655498
2.45072408	2.514655498
2.45072408	2.514655498
2.45072408	2.514655498
2.45072408	2.514655498
2.45072408	2.514655498
2.45072408	2.514655498
2.45072408	2.514655498
2.45072408	2.514655498
2.45072408	2.514655498
2.45072408	2.514655498
2.45072408	2.514655498
2.45072408	2.514655498
2.45072408	2.514655498
2.45072408	2.514655498
2.45072408	2.514655498
2.45072408	2.514655498
1.634163031	1.667173749
1.634163031	1.667173749
1.634163031	1.667173749
1.634163031	1.667173749
1.634163031	1.667173749
1.634163031	1.667173749
1.634163031	1.667173749
1.283111914	1.307404228
1.337401796	1.361047894

1.216631458	1.231905901
1.139144157	1.146667803
1.408313861	1.39923675
1.408313861	1.39923675
1.408313861	1.39923675
1.134581513	1.126489887
1.317371476	1.304875385
1.226089198	1.204025016
1.575717585	1.540032549
1.575717585	1.540032549
1.575717585	1.540032549
1.575717585	1.540032549
1.575717585	1.540032549
1.575717585	1.540032549
1.575717585	1.540032549
1.575717585	1.540032549
1.575717585	1.540032549
1.575717585	1.540032549
1.253818603	1.218607727
1.297930283	1.25133023
1.297930283	1.25133023
1.378901734	1.326325054
1.378901734	1.326325054
1.378901734	1.326325054
1.18836773	1.135196596
1.169320807	1.112997555
2.205536332	2.097971403
2.205536332	2.097971403
2.205536332	2.097971403
2.205536332	2.097971403
2.205536332	2.097971403
2.205536332	2.097971403
2.205536332	2.097971403
2.205536332	2.097971403
2.205536332	2.097971403
2.205536332	2.097971403
2.205536332	2.097971403
2.205536332	2.097971403
2.205536332	2.097971403
2.205536332	2.097971403
2.205536332	2.097971403
2.205536332	2.097971403
2.205536332	2.097971403
2.205536332	2.097971403
2.205536332	2.097971403
2.205536332	2.097971403
2.205536332	2.097971403
2.205536332	2.097971403

2.205536332	2.097971403
2.205536332	2.097971403
2.205536332	2.097971403
2.205536332	2.097971403
2.205536332	2.097971403
2.205536332	2.097971403
2.205536332	2.097971403
2.205536332	2.097971403
2.205536332	2.097971403
2.205536332	2.097971403
2.205536332	2.097971403
2.205536332	2.097971403
2.205536332	2.097971403
2.205536332	2.097971403
2.205536332	2.097971403
2.205536332	2.097971403
2.205536332	2.097971403
2.205536332	2.097971403
2.205536332	2.097971403
2.205536332	2.097971403
2.205536332	2.097971403
2.205536332	2.097971403
2.205536332	2.097971403
2.205536332	2.097971403
2.205536332	2.097971403
2.205536332	2.097971403
2.205536332	2.097971403
2.205536332	2.097971403
2.205536332	2.097971403
2.205536332	2.097971403
2.205536332	2.097971403
2.205536332	2.097971403
2.205536332	2.097971403
1.279052603	1.200267668
1.279052603	1.200267668
1.521302859	1.424980736
1.521302859	1.424980736
1.521302859	1.424980736
1.521302859	1.424980736
1.521302859	1.424980736
1.521302859	1.424980736
1.521302859	1.424980736
1.521302859	1.424980736
1.521302859	1.424980736
1.521302859	1.424980736
1.521302859	1.424980736
1.521302859	1.424980736
1.521302859	1.424980736

1.521302859	1.424980736
1.521302859	1.424980736
1.521302859	1.424980736
1.350690103	1.257884462
1.350690103	1.257884462
1.350690103	1.257884462
1.350690103	1.257884462
1.350690103	1.257884462
1.225827218	1.13639626
1.192763529	1.098950619
1.192763529	1.098950619
1.260714286	1.151552448
1.260714286	1.151552448
1.182051707	1.066134254
1.323606936	1.19358415
1.323606936	1.19358415
1.323606936	1.19358415
1.470515781	1.32058981
1.470515781	1.32058981
1.470515781	1.32058981
1.470515781	1.32058981
1.470515781	1.32058981
1.470515781	1.32058981
1.470515781	1.32058981
1.470515781	1.32058981
1.470515781	1.32058981
1.470515781	1.32058981
1.470515781	1.32058981
1.470515781	1.32058981
1.470515781	1.32058981
1.138904726	1.008296847
2.004928174	1.773378978
2.004928174	1.773378978
2.004928174	1.773378978
2.004928174	1.773378978
2.004928174	1.773378978
2.004928174	1.773378978
2.004928174	1.773378978
2.004928174	1.773378978
2.004928174	1.773378978
2.004928174	1.773378978
2.004928174	1.773378978
2.004928174	1.773378978
2.004928174	1.773378978
2.004928174	1.773378978
2.004928174	1.773378978
2.004928174	1.773378978
2.004928174	1.773378978

2.004928174	1.773378978
2.004928174	1.773378978
2.004928174	1.773378978
2.004928174	1.773378978
2.004928174	1.773378978
2.004928174	1.773378978
2.004928174	1.773378978
2.004928174	1.773378978
2.004928174	1.773378978
2.004928174	1.773378978
2.004928174	1.773378978
2.004928174	1.773378978
2.004928174	1.773378978
2.004928174	1.773378978
2.004928174	1.773378978
2.004928174	1.773378978
2.004928174	1.773378978
2.004928174	1.773378978
2.004928174	1.773378978
2.004928174	1.773378978
2.004928174	1.773378978
2.004928174	1.773378978
2.004928174	1.773378978
2.004928174	1.773378978
2.004928174	1.773378978
2.004928174	1.773378978
2.004928174	1.773378978
2.004928174	1.773378978
2.004928174	1.773378978
2.004928174	1.773378978
2.004928174	1.773378978
2.004928174	1.773378978
2.004928174	1.773378978
2.004928174	1.773378978
2.004928174	1.773378978
2.004928174	1.773378978
2.004928174	1.773378978
2.004928174	1.773378978
2.004928174	1.773378978
2.004928174	1.773378978
2.004928174	1.773378978
2.004928174	1.773378978
2.004928174	1.773378978
2.004928174	1.773378978
2.004928174	1.773378978
2.004928174	1.773378978
2.004928174	1.773378978
1.171529475	1.034356892

1.297585855	1.133122733
1.297585855	1.133122733
1.297585855	1.133122733
1.297585855	1.133122733
1.297585855	1.133122733
1.225565844	1.060666239
1.069866109	0.923096447
1.423005289	1.225635515
1.423005289	1.225635515
1.423005289	1.225635515
1.423005289	1.225635515
1.423005289	1.225635515
1.423005289	1.225635515
1.423005289	1.225635515
1.272565585	1.076225172
1.173417815	0.989555434
1.208713851	1.018266003
1.208713851	1.018266003
1.208713851	1.018266003
1.208713851	1.018266003
1.208713851	1.018266003
1.208713851	1.018266003
1.127232805	0.939069882
1.378464203	1.139063589
1.378464203	1.139063589
1.378464203	1.139063589
1.378464203	1.139063589
1.378464203	1.139063589
1.378464203	1.139063589
1.378464203	1.139063589
1.378464203	1.139063589
1.378464203	1.139063589
1.83775471	1.51540912
1.83775471	1.51540912
1.83775471	1.51540912
1.83775471	1.51540912
1.83775471	1.51540912
1.83775471	1.51540912
1.83775471	1.51540912
1.83775471	1.51540912
1.83775471	1.51540912
1.83775471	1.51540912
1.83775471	1.51540912
1.83775471	1.51540912
1.83775471	1.51540912
1.83775471	1.51540912
1.83775471	1.51540912
1.83775471	1.51540912

1.83775471	1.51540912
1.83775471	1.51540912
1.83775471	1.51540912
1.83775471	1.51540912
1.83775471	1.51540912
1.83775471	1.51540912
1.83775471	1.51540912
1.83775471	1.51540912
1.83775471	1.51540912
1.83775471	1.51540912
1.83775471	1.51540912
1.83775471	1.51540912
1.83775471	1.51540912
1.83775471	1.51540912
1.83775471	1.51540912
1.83775471	1.51540912
1.83775471	1.51540912
1.83775471	1.51540912
1.83775471	1.51540912
1.83775471	1.51540912
1.83775471	1.51540912
1.83775471	1.51540912
1.83775471	1.51540912
1.83775471	1.51540912
1.83775471	1.51540912
1.83775471	1.51540912
1.83775471	1.51540912
1.83775471	1.51540912
1.161005062	0.956155635
1.248489475	1.022640068
1.248489475	1.022640068
1.248489475	1.022640068
1.248489475	1.022640068
1.040257626	0.849922833
1.148850908	0.923970882
1.110896649	0.889741029
1.176358025	0.939031049
1.225305074	0.972137262
1.336622577	1.059962072
1.336622577	1.059962072
1.336622577	1.059962072
1.336622577	1.059962072
1.136947355	0.89295123
1.136947355	0.89295123
1.136947355	0.89295123
1.160818713	0.902007255
1.160818713	0.902007255
1.69630024	1.306932342

1.69630024	1.306932342
1.69630024	1.306932342
1.69630024	1.306932342
1.69630024	1.306932342
1.69630024	1.306932342
1.69630024	1.306932342
1.69630024	1.306932342
1.69630024	1.306932342
1.69630024	1.306932342
1.69630024	1.306932342
1.69630024	1.306932342
1.69630024	1.306932342
1.69630024	1.306932342
1.69630024	1.306932342
1.69630024	1.306932342
1.69630024	1.306932342
1.69630024	1.306932342
1.69630024	1.306932342
1.69630024	1.306932342
1.69630024	1.306932342
1.69630024	1.306932342
1.69630024	1.306932342
1.69630024	1.306932342
1.69630024	1.306932342
1.69630024	1.306932342
1.69630024	1.306932342
1.69630024	1.306932342
1.69630024	1.306932342
1.69630024	1.306932342
1.69630024	1.306932342
1.69630024	1.306932342
1.69630024	1.306932342
1.69630024	1.306932342
1.69630024	1.306932342
1.69630024	1.306932342
1.69630024	1.306932342
1.69630024	1.306932342
1.69630024	1.306932342
1.69630024	1.306932342
1.69630024	1.306932342
1.69630024	1.306932342
1.69630024	1.306932342
1.69630024	1.306932342
1.69630024	1.306932342
1.69630024	1.306932342
1.69630024	1.306932342
1.202963741	0.924505732
1.202963741	0.924505732

1.202963741	0.924505732
1.202963741	0.924505732
1.202963741	0.924505732
1.050466142	0.805167076
1.297242223	0.987538765
1.297242223	0.987538765
1.297242223	0.987538765
1.297242223	0.987538765
1.297242223	0.987538765
1.297242223	0.987538765
1.297242223	0.987538765
1.297242223	0.987538765
1.145683021	0.866596032
1.145683021	0.866596032
1.059515014	0.799325943
1.181420314	0.879551714
1.181420314	0.879551714
1.181420314	0.879551714
1.093612778	0.813436249
1.130935423	0.832716492
1.130935423	0.832716492
1.102665122	0.806418569
1.102665122	0.806418569
1.260112174	0.921102783
1.260112174	0.921102783
1.260112174	0.921102783
1.260112174	0.921102783
1.260112174	0.921102783
1.260112174	0.921102783
1.260112174	0.921102783
1.260112174	0.921102783
1.260112174	0.921102783
1.260112174	0.921102783
1.575053551	1.136051216
1.575053551	1.136051216
1.575053551	1.136051216
1.575053551	1.136051216
1.575053551	1.136051216
1.575053551	1.136051216
1.575053551	1.136051216
1.575053551	1.136051216
1.575053551	1.136051216
1.575053551	1.136051216
1.575053551	1.136051216
1.575053551	1.136051216
1.575053551	1.136051216
1.575053551	1.136051216
1.575053551	1.136051216

1.575053551	1.136051216
1.575053551	1.136051216
1.575053551	1.136051216
1.575053551	1.136051216
1.575053551	1.136051216
1.575053551	1.136051216
1.575053551	1.136051216
1.575053551	1.136051216
1.575053551	1.136051216
1.575053551	1.136051216
1.575053551	1.136051216
1.575053551	1.136051216
1.575053551	1.136051216
1.575053551	1.136051216
1.575053551	1.136051216
1.575053551	1.136051216
1.575053551	1.136051216
1.160632796	0.837097045
1.116561181	0.800292518
1.225044906	0.860049347
1.225044906	0.860049347
1.225044906	0.860049347
1.225044906	0.860049347
1.225044906	0.860049347
1.225044906	0.860049347
1.225044906	0.860049347
1.225044906	0.860049347
1.140562089	0.796977688
1.140562089	0.796977688
1.023020012	0.714488097
1.080930634	0.753741826
1.070379912	0.728789158
1.070379912	0.728789158
1.088877458	0.739528745
1.121171745	0.75904241
1.469973087	0.994274815
1.469973087	0.994274815
1.469973087	0.994274815
1.469973087	0.994274815
1.469973087	0.994274815
1.469973087	0.994274815
1.469973087	0.994274815
1.469973087	0.994274815
1.469973087	0.994274815
1.469973087	0.994274815
1.469973087	0.994274815
1.469973087	0.994274815
1.469973087	0.994274815

1.469973087	0.994274815
1.469973087	0.994274815
1.469973087	0.994274815
1.469973087	0.994274815
1.469973087	0.994274815
1.469973087	0.994274815
1.469973087	0.994274815
1.469973087	0.994274815
1.469973087	0.994274815
1.469973087	0.994274815
1.469973087	0.994274815
1.469973087	0.994274815
1.469973087	0.994274815
1.469973087	0.994274815
1.469973087	0.994274815
1.469973087	0.994274815
1.469973087	0.994274815
1.191873166	0.803847193
1.191873166	0.803847193
1.191873166	0.803847193
1.191873166	0.803847193
1.191873166	0.803847193
1.037592339	0.696964292
1.075542005	0.711057756
1.075542005	0.711057756
1.102427746	0.723151616
1.049881366	0.681476556
1.160447308	0.75202809
1.160447308	0.75202809
1.160447308	0.75202809
1.160447308	0.75202809
1.160447308	0.75202809
1.160447308	0.75202809
1.160447308	0.75202809
1.160447308	0.75202809
1.160447308	0.75202809
1.160447308	0.75202809
1.160447308	0.75202809
1.160447308	0.75202809
1.160447308	0.75202809
1.062527889	0.683779416
1.017480075	0.649798002
1.084298304	0.689176293
1.084298304	0.689176293
1.378027682	0.875400949
1.378027682	0.875400949
1.378027682	0.875400949
1.378027682	0.875400949

1.378027682	0.875400949
1.378027682	0.875400949
1.378027682	0.875400949
1.378027682	0.875400949
1.378027682	0.875400949
1.378027682	0.875400949
1.378027682	0.875400949
1.378027682	0.875400949
1.378027682	0.875400949
1.378027682	0.875400949
1.378027682	0.875400949
1.378027682	0.875400949
1.378027682	0.875400949
1.378027682	0.875400949
1.378027682	0.875400949
1.378027682	0.875400949
1.378027682	0.875400949
1.378027682	0.875400949
1.378027682	0.875400949
1.378027682	0.875400949
1.378027682	0.875400949
1.378027682	0.875400949
1.378027682	0.875400949
1.378027682	0.875400949
1.378027682	0.875400949
1.378027682	0.875400949
1.378027682	0.875400949
1.378027682	0.875400949
1.378027682	0.875400949
1.378027682	0.875400949
1.378027682	0.875400949
1.378027682	0.875400949
1.378027682	0.875400949
1.378027682	0.875400949
1.378027682	0.875400949
1.378027682	0.875400949
1.378027682	0.875400949
1.378027682	0.875400949
1.378027682	0.875400949
1.378027682	0.875400949
1.378027682	0.875400949
1.378027682	0.875400949
1.378027682	0.875400949
1.378027682	0.875400949
1.378027682	0.875400949
1.378027682	0.875400949
1.378027682	0.875400949
1.039922167	0.659049953

1.011205919	0.63811305
1.005482713	0.633476432
1.130633031	0.704178065
1.130633031	0.704178065
1.130633031	0.704178065
1.130633031	0.704178065
1.130633031	0.704178065
1.030149121	0.637401172
1.066753683	0.656997069
1.066753683	0.656997069
1.037418301	0.632577034
1.037418301	0.632577034
1.037418301	0.632577034
1.037418301	0.632577034
1.102309469	0.659930033
1.102309469	0.659930033
1.102309469	0.659930033
1.102309469	0.659930033
1.102309469	0.659930033
1.102309469	0.659930033
1.102309469	0.659930033
1.102309469	0.659930033
1.296899383	0.774807251
1.296899383	0.774807251
1.296899383	0.774807251
1.296899383	0.774807251
1.296899383	0.774807251
1.296899383	0.774807251
1.296899383	0.774807251
1.296899383	0.774807251
1.296899383	0.774807251
1.296899383	0.774807251
1.296899383	0.774807251
1.296899383	0.774807251
1.296899383	0.774807251
1.296899383	0.774807251
1.296899383	0.774807251
1.296899383	0.774807251
1.296899383	0.774807251
1.296899383	0.774807251
1.296899383	0.774807251
1.296899383	0.774807251
1.296899383	0.774807251
1.296899383	0.774807251
1.296899383	0.774807251
1.296899383	0.774807251
1.296899383	0.774807251
1.296899383	0.774807251

1.296899383	0.774807251
1.296899383	0.774807251
1.296899383	0.774807251
1.296899383	0.774807251
1.296899383	0.774807251
1.296899383	0.774807251
1.296899383	0.774807251
1.296899383	0.774807251
1.296899383	0.774807251
1.296899383	0.774807251
1.296899383	0.774807251
1.296899383	0.774807251
1.296899383	0.774807251
1.296899383	0.774807251
1.296899383	0.774807251
1.049766034	0.626503368
1.049766034	0.626503368
1.049766034	0.626503368
0.996817615	0.593007215
1.033309249	0.597592654
1.033309249	0.597592654
1.075367544	0.618957563
1.075367544	0.618957563
1.075367544	0.618957563
1.075367544	0.618957563
1.075367544	0.618957563
1.075367544	0.618957563
0.994312928	0.569843862
1.224785339	0.68898771
1.224785339	0.68898771
1.224785339	0.68898771
1.224785339	0.68898771
1.224785339	0.68898771
1.224785339	0.68898771
1.224785339	0.68898771
1.224785339	0.68898771
1.224785339	0.68898771
1.224785339	0.68898771
1.224785339	0.68898771
1.224785339	0.68898771
1.224785339	0.68898771
1.224785339	0.68898771
1.224785339	0.68898771
1.224785339	0.68898771
1.224785339	0.68898771
1.224785339	0.68898771
1.224785339	0.68898771
1.224785339	0.68898771

1.224785339	0.68898771
1.224785339	0.68898771
1.224785339	0.68898771
1.224785339	0.68898771
1.224785339	0.68898771
1.224785339	0.68898771
1.224785339	0.68898771
1.224785339	0.68898771
1.224785339	0.68898771
1.224785339	0.68898771
1.224785339	0.68898771
1.224785339	0.68898771
1.224785339	0.68898771
1.224785339	0.68898771
1.224785339	0.68898771
1.224785339	0.68898771
1.224785339	0.68898771
1.224785339	0.68898771
1.224785339	0.68898771
1.224785339	0.68898771
1.017358826	0.570169742
1.049708567	0.580969608
1.049708567	0.580969608
1.049708567	0.580969608
1.049708567	0.580969608
1.049708567	0.580969608
1.049708567	0.580969608
1.049708567	0.580969608
1.049708567	0.580969608
0.983901672	0.539827867
0.990584686	0.542188992
0.990584686	0.542188992
0.973968907	0.531653807
1.00189175	0.544146181
1.00189175	0.544146181
0.975143305	0.52226481
1.025243031	0.545706006
1.025243031	0.545706006
1.025243031	0.545706006
1.025243031	0.545706006
1.025243031	0.545706006
1.025243031	0.545706006
1.160262247	0.615241548
1.160262247	0.615241548
1.160262247	0.615241548
1.160262247	0.615241548
1.160262247	0.615241548
1.160262247	0.615241548

1.160262247	0.615241548
1.160262247	0.615241548
1.160262247	0.615241548
1.160262247	0.615241548
1.160262247	0.615241548
1.160262247	0.615241548
1.160262247	0.615241548
1.160262247	0.615241548
1.160262247	0.615241548
1.160262247	0.615241548
1.160262247	0.615241548
1.160262247	0.615241548
1.160262247	0.615241548
1.160262247	0.615241548
1.160262247	0.615241548
1.160262247	0.615241548
1.160262247	0.615241548
1.160262247	0.615241548
1.160262247	0.615241548
1.160262247	0.615241548
1.160262247	0.615241548
1.160262247	0.615241548
1.160262247	0.615241548
1.160262247	0.615241548
1.160262247	0.615241548
1.160262247	0.615241548
1.160262247	0.615241548
1.160262247	0.615241548
1.160262247	0.615241548
1.160262247	0.615241548
1.160262247	0.615241548
1.160262247	0.615241548
0.986886377	0.519439701
0.986886377	0.519439701
0.986886377	0.519439701
0.986886377	0.519439701
0.986886377	0.519439701
1.001889565	0.512933639
1.001889565	0.512933639
1.001889565	0.512933639
1.001889565	0.512933639
1.001889565	0.512933639
1.001889565	0.512933639
0.972322339	0.495973703
0.972322339	0.495973703
0.958132045	0.483538328
1.102191465	0.551459466
1.102191465	0.551459466

1.102191465	0.551459466
1.102191465	0.551459466
1.102191465	0.551459466
1.102191465	0.551459466
1.102191465	0.551459466
1.102191465	0.551459466
1.102191465	0.551459466
1.102191465	0.551459466
1.102191465	0.551459466
1.102191465	0.551459466
1.102191465	0.551459466
1.102191465	0.551459466
1.102191465	0.551459466
1.102191465	0.551459466
1.102191465	0.551459466
1.102191465	0.551459466
1.102191465	0.551459466
0.958180447	0.473676805
0.958180447	0.473676805
0.979574031	0.482443147
0.979574031	0.482443147
0.979574031	0.482443147
0.979574031	0.482443147
0.941606996	0.457746379
0.942683054	0.453374111
0.944442609	0.452482426
0.944442609	0.452482426
0.937647754	0.44822648
0.958228738	0.454046088
0.958228738	0.454046088
0.958228738	0.454046088
0.958228738	0.454046088
0.958228738	0.454046088
1.049651233	0.495973717
1.049651233	0.495973717
1.049651233	0.495973717
1.049651233	0.495973717
1.049651233	0.495973717
1.049651233	0.495973717
1.049651233	0.495973717
1.049651233	0.495973717
1.049651233	0.495973717
1.049651233	0.495973717
1.049651233	0.495973717
1.049651233	0.495973717
1.049651233	0.495973717
1.049651233	0.495973717
1.049651233	0.495973717

1.049651233	0.495973717
1.049651233	0.495973717
1.049651233	0.495973717
1.049651233	0.495973717
0.925684295	0.428642617
0.937791755	0.427572496
0.937791755	0.427572496
0.937791755	0.427572496
0.937791755	0.427572496
0.917921978	0.414814133
0.917921978	0.414814133
0.918111753	0.413156686
0.918111753	0.413156686
0.917826759	0.412346738
1.001887386	0.447450998
1.001887386	0.447450998
1.001887386	0.447450998
1.001887386	0.447450998
1.001887386	0.447450998
1.001887386	0.447450998
1.001887386	0.447450998
1.001887386	0.447450998
1.001887386	0.447450998
1.001887386	0.447450998
1.001887386	0.447450998
1.001887386	0.447450998
1.001887386	0.447450998
1.001887386	0.447450998
1.001887386	0.447450998
1.001887386	0.447450998
1.001887386	0.447450998
1.001887386	0.447450998
0.91773132	0.409001012
0.908505155	0.400300042
0.918206313	0.402868762
0.918206313	0.402868762
0.918206313	0.402868762
0.918206313	0.402868762
0.899187453	0.385554707
0.893204187	0.377546368
0.893204187	0.377546368
0.958276917	0.404814668
0.958276917	0.404814668
0.958276917	0.404814668
0.958276917	0.404814668
0.958276917	0.404814668
0.958276917	0.404814668
0.958276917	0.404814668

0.958276917	0.404814668
0.958276917	0.404814668
0.958276917	0.404814668
0.958276917	0.404814668
0.958276917	0.404814668
0.958276917	0.404814668
0.958276917	0.404814668
0.958276917	0.404814668
0.958276917	0.404814668
0.958276917	0.404814668
0.958276917	0.404814668
0.958276917	0.404814668
0.958276917	0.404814668
0.899420276	0.379795793
0.899420276	0.379795793
0.899420276	0.379795793
0.899420276	0.379795793
0.899420276	0.379795793
0.899420276	0.379795793
0.899420276	0.379795793
0.899420276	0.379795793
0.890057987	0.371380906
0.904827212	0.374125403
0.881248555	0.361009361
0.881248555	0.361009361
0.881385681	0.358227411
0.881385681	0.358227411
0.881385681	0.358227411
0.881385681	0.358227411
0.881385681	0.358227411
0.881385681	0.358227411
0.918300654	0.367187393
0.918300654	0.367187393
0.918300654	0.367187393
0.918300654	0.367187393
0.918300654	0.367187393
0.918300654	0.367187393
0.918300654	0.367187393
0.918300654	0.367187393
0.918300654	0.367187393
0.918300654	0.367187393
0.918300654	0.367187393
0.918300654	0.367187393
0.918300654	0.367187393
0.918300654	0.367187393
0.918300654	0.367187393
0.918300654	0.367187393
0.918300654	0.367187393

0.918300654	0.367187393
0.918300654	0.367187393
0.918300654	0.367187393
0.918300654	0.367187393
0.880973349	0.352244395
0.869607545	0.345257313
0.869607545	0.345257313
0.869607545	0.345257313
0.864058325	0.338048953
0.864058325	0.338048953
0.864058325	0.338048953
0.864058325	0.338048953
0.864058325	0.338048953
0.892458232	0.348934722
0.858268899	0.330248386
0.858268899	0.330248386
0.863743646	0.332052194
0.888409194	0.340906286
0.881522491	0.333848301
0.881522491	0.333848301
0.881522491	0.333848301
0.881522491	0.333848301
0.881522491	0.333848301
0.881522491	0.333848301
0.881522491	0.333848301
0.881522491	0.333848301
0.881522491	0.333848301
0.881522491	0.333848301
0.881522491	0.333848301
0.881522491	0.333848301
0.881522491	0.333848301
0.847397406	0.319156043
0.847397406	0.319156043
0.847397406	0.319156043
0.847397406	0.319156043
0.847397406	0.319156043
0.847397406	0.319156043
0.855312837	0.319933583
0.847220987	0.315943309
0.847220987	0.315943309
0.870653622	0.321315054
0.84704416	0.308276389
0.84704416	0.308276389
0.836452769	0.302305197
0.847573419	0.304200554
0.847573419	0.304200554
0.847573419	0.304200554
0.847573419	0.304200554

0.847573419	0.304200554
0.847573419	0.304200554
0.847573419	0.304200554
0.847573419	0.304200554
0.847573419	0.304200554
0.847573419	0.304200554
0.847573419	0.304200554
0.847573419	0.304200554
0.847573419	0.304200554
0.847573419	0.304200554
0.847573419	0.304200554
0.847573419	0.304200554
0.825953757	0.289299387
0.825953757	0.289299387
0.825953757	0.289299387
0.825953757	0.289299387
0.825953757	0.289299387
0.815926781	0.284854483
0.815926781	0.284854483
0.815926781	0.284854483
0.840358062	0.289721143
0.873692547	0.299361105
0.816139092	0.277746545
0.816139092	0.277746545
0.816139092	0.277746545
0.816139092	0.277746545
0.816139092	0.277746545
0.816139092	0.277746545
0.816139092	0.277746545
0.816139092	0.277746545
0.816139092	0.277746545
0.816139092	0.277746545
0.816139092	0.277746545
0.816139092	0.277746545
0.816139092	0.277746545
0.816139092	0.277746545
0.816139092	0.277746545
0.81571398	0.276893278
0.81571398	0.276893278
0.833947821	0.280455584
0.833947821	0.280455584
0.801049759	0.269279465
0.801049759	0.269279465
0.801049759	0.269279465
0.823165455	0.27588755
0.823165455	0.27588755
0.805723953	0.26505619
0.805723953	0.26505619

0.815500686	0.26589448
0.786704058	0.254655689
0.786704058	0.254655689
0.786704058	0.254655689
0.786704058	0.254655689
0.786704058	0.254655689
0.786950074	0.254068712
0.786950074	0.254068712
0.786950074	0.254068712
0.786950074	0.254068712
0.786950074	0.254068712
0.786950074	0.254068712
0.786950074	0.254068712
0.786950074	0.254068712
0.786950074	0.254068712
0.786950074	0.254068712
0.786950074	0.254068712
0.786950074	0.254068712
0.786950074	0.254068712
0.786950074	0.254068712
0.786950074	0.254068712
0.786950074	0.254068712
0.786950074	0.254068712
0.786950074	0.254068712
0.786950074	0.254068712
0.79597465	0.253759236
0.772861715	0.240916409
0.772861715	0.240916409
0.772861715	0.240916409
0.772861715	0.240916409
0.772861715	0.240916409
0.874967884	0.272711682
0.759774092	0.232814571
0.759774092	0.232814571
0.759774092	0.232814571
0.759774092	0.232814571
0.759774092	0.232814571
0.759774092	0.232814571
0.759774092	0.232814571
0.759774092	0.232814571
0.759774092	0.232814571
0.759774092	0.232814571
0.759774092	0.232814571
0.759774092	0.232814571
0.759496695	0.228000338
0.759496695	0.228000338
0.734409842	0.213684918
0.734409842	0.213684918

0.734409842	0.213684918
0.734409842	0.213684918
0.734409842	0.213684918
0.734409842	0.213684918
0.734409842	0.213684918
0.734409842	0.213684918
0.734409842	0.213684918
0.734409842	0.213684918
0.734409842	0.213684918
0.734409842	0.213684918
0.734409842	0.213684918
0.734409842	0.213684918
0.734409842	0.213684918
0.746584726	0.215851123
0.746584726	0.215851123
0.779211734	0.221258589
0.759218657	0.213451204
0.734103156	0.204416886
0.734103156	0.204416886
0.734103156	0.204416886
0.710681996	0.196424467
0.710681996	0.196424467
0.710681996	0.196424467
0.710681996	0.196424467
0.710681996	0.196424467
0.710681996	0.196424467
0.710681996	0.196424467
0.710681996	0.196424467
0.710681996	0.196424467
0.710681996	0.196424467
0.75055176	0.204476356
0.75055176	0.204476356
0.75055176	0.204476356
0.76557971	0.205637111
0.780347296	0.209431717
0.722030818	0.193649815
0.722030818	0.193649815
0.722030818	0.193649815
0.790460842	0.209995941
0.742079626	0.195899783
0.742079626	0.195899783
0.68843714	0.180814356
0.68843714	0.180814356
0.68843714	0.180814356
0.68843714	0.180814356
0.68843714	0.180814356
0.68843714	0.180814356
0.68843714	0.180814356

0.68843714	0.180814356
0.68843714	0.180814356
0.68843714	0.180814356
0.68843714	0.180814356
0.68843714	0.180814356
0.68843714	0.180814356
0.68843714	0.180814356
0.68843714	0.180814356
0.774811091	0.202752703
0.774811091	0.202752703
0.71034791	0.183505793
0.71034791	0.183505793
0.71034791	0.183505793
0.71034791	0.183505793
0.71034791	0.183505793
0.71034791	0.183505793
0.733795761	0.187702229
0.667540456	0.166666103
0.667540456	0.166666103
0.667540456	0.166666103
0.667540456	0.166666103
0.667540456	0.166666103
0.667540456	0.166666103
0.667540456	0.166666103
0.667540456	0.166666103
0.667540456	0.166666103
0.667540456	0.166666103
0.667540456	0.166666103
0.667540456	0.166666103
0.667540456	0.166666103
0.667540456	0.166666103
0.667540456	0.166666103
0.699035888	0.173944067
0.699035888	0.173944067
0.725693959	0.179865482
0.725693959	0.179865482
0.745998117	0.184296143
0.688077367	0.164926953
0.688077367	0.164926953
0.64787299	0.153816711
0.64787299	0.153816711
0.64787299	0.153816711
0.64787299	0.153816711
0.64787299	0.153816711
0.64787299	0.153816711
0.64787299	0.153816711
0.733487654	0.171340014
0.710013052	0.165206402

0.710013052	0.165206402
0.677456031	0.15641957
0.629329379	0.142124662
0.629329379	0.142124662
0.629329379	0.142124662
0.629329379	0.142124662
0.629329379	0.142124662
0.629329379	0.142124662
0.629329379	0.142124662
0.629329379	0.142124662
0.629329379	0.142124662
0.702422826	0.158352316
0.702422826	0.158352316
0.75838067	0.170871944
0.75838067	0.170871944
0.667156554	0.148389596
0.721387371	0.159311589
0.694992394	0.151795418
0.694992394	0.151795418
0.611815968	0.131466633
0.611815968	0.131466633
0.611815968	0.131466633
0.611815968	0.131466633
0.611815968	0.131466633
0.611815968	0.131466633
0.611815968	0.131466633
0.715484794	0.153623334
0.715484794	0.153623334
0.657164524	0.140807058
0.647466377	0.133644128
0.647466377	0.133644128
0.680591145	0.13951843
0.595249228	0.12173478
0.595249228	0.12173478
0.595249228	0.12173478
0.595249228	0.12173478
0.595249228	0.12173478
0.595249228	0.12173478
0.595249228	0.12173478
0.595249228	0.12173478
0.781881227	0.158839966
0.703962963	0.142858009
0.638049336	0.126874955
0.638049336	0.126874955
0.698339212	0.137765643
0.784464077	0.153961775
0.579554422	0.112834484
0.579554422	0.112834484

0.579554422	0.112834484
0.579554422	0.112834484
0.579554422	0.112834484
0.579554422	0.112834484
0.579554422	0.112834484
0.579554422	0.112834484
0.579554422	0.112834484
0.579554422	0.112834484
0.579554422	0.112834484
0.579554422	0.112834484
0.666771764	0.128272866
0.743293665	0.142442005
0.628901353	0.120475496
0.628901353	0.120475496
0.687355324	0.128125574
0.564664478	0.104682475
0.564664478	0.104682475
0.564664478	0.104682475
0.564664478	0.104682475
0.564664478	0.104682475
0.564664478	0.104682475
0.564664478	0.104682475
0.564664478	0.104682475
0.564664478	0.104682475
0.564664478	0.104682475
0.620011059	0.114423377
0.620011059	0.114423377
0.620011059	0.114423377
0.700231012	0.124671305
0.550519031	0.09720525
0.550519031	0.09720525
0.550519031	0.09720525
0.550519031	0.09720525
0.550519031	0.09720525
0.550519031	0.09720525
0.550519031	0.09720525
0.550519031	0.09720525
0.550519031	0.09720525
0.550519031	0.09720525
0.550519031	0.09720525
0.550519031	0.09720525
0.550519031	0.09720525
0.550519031	0.09720525
0.602961182	0.103279204
0.602961182	0.103279204
0.709003817	0.119767613
0.537063606	0.090337751
0.537063606	0.090337751

0.537063606	0.090337751
0.537063606	0.090337751
0.537063606	0.090337751
0.537063606	0.090337751
0.537063606	0.090337751
0.537063606	0.090337751
0.537063606	0.090337751
0.537063606	0.090337751
0.722126799	0.120757049
0.634548688	0.104087369
0.524248915	0.084022243
0.524248915	0.084022243
0.524248915	0.084022243
0.524248915	0.084022243
0.524248915	0.084022243
0.524248915	0.084022243
0.524248915	0.084022243
0.524248915	0.084022243
0.524248915	0.084022243
0.586820631	0.093292025
0.628472337	0.099845431
0.697639303	0.109535678
0.622510634	0.095781523
0.693930883	0.106318234
0.579068919	0.088690673
0.579068919	0.088690673
0.512030257	0.078207356
0.512030257	0.078207356
0.512030257	0.078207356
0.512030257	0.078207356
0.512030257	0.078207356
0.512030257	0.078207356
0.512030257	0.078207356
0.512030257	0.078207356
0.571518551	0.084330769
0.571518551	0.084330769
0.500366992	0.072847283
0.500366992	0.072847283
0.500366992	0.072847283
0.736612381	0.10707407
0.610918433	0.088156648
0.610918433	0.088156648
0.683036197	0.097208015
0.564161781	0.08019849
0.564161781	0.08019849
0.695692161	0.0967378
0.489222094	0.067901079
0.489222094	0.067901079

0.489222094	0.067901079
0.489222094	0.067901079
0.489222094	0.067901079
0.489222094	0.067901079
0.489222094	0.067901079
0.556991259	0.076280898
0.556991259	0.076280898
0.478561757	0.063332067
0.478561757	0.063332067
0.478561757	0.063332067
0.478561757	0.063332067
0.741490062	0.097186669
0.594313389	0.077869395
0.468355052	0.059107329
0.468355052	0.059107329
0.468355052	0.059107329
0.468355052	0.059107329
0.61476949	0.074454431
0.458573626	0.055197259
0.458573626	0.055197259
0.458573626	0.055197259
0.458573626	0.055197259
0.458573626	0.055197259
0.530037007	0.062526133
0.530037007	0.062526133
0.449191441	0.051575182
0.449191441	0.051575182
0.449191441	0.051575182
0.449191441	0.051575182
0.449191441	0.051575182
0.449191441	0.051575182
0.449191441	0.051575182
0.440184544	0.048217023
0.440184544	0.048217023
0.440184544	0.048217023
0.440184544	0.048217023
0.440184544	0.048217023
0.517511208	0.056655518
0.642646143	0.067775734
0.431530859	0.045101011
0.431530859	0.045101011
0.431530859	0.045101011
0.505561307	0.051361776
0.505561307	0.051361776
0.423210008	0.042207432
0.423210008	0.042207432
0.415203151	0.039518405
0.415203151	0.039518405

0.415203151	0.039518405
0.544909951	0.051616701
0.407492845	0.037017685
0.407492845	0.037017685
0.488632281	0.044372357
0.535993233	0.047558317
0.400062913	0.034690495
0.400062913	0.034690495
0.400062913	0.034690495
0.400062913	0.034690495
0.477959635	0.04027097
0.392898336	0.032523375
0.392898336	0.032523375
0.392898336	0.032523375
0.392898336	0.032523375
0.392898336	0.032523375
0.392898336	0.032523375
0.472795451	0.038370432
0.472795451	0.038370432
0.586965008	0.046748729
0.385985147	0.030504045
0.385985147	0.030504045
0.467741143	0.036563064
0.555965776	0.043261274
0.379310345	0.028621293
0.379310345	0.028621293
0.379310345	0.028621293
0.462793242	0.034844032
0.372861807	0.026864863
0.372861807	0.026864863
0.598354777	0.042931642
0.61404878	0.042571738
0.36662822	0.025225369
0.36662822	0.025225369
0.36662822	0.025225369
0.499211771	0.032931563
0.360599013	0.023694206
0.360599013	0.023694206
0.360599013	0.023694206
0.360599013	0.023694206
0.360599013	0.023694206
0.535456188	0.034796096
0.444001213	0.028763748
0.354764297	0.022263481
0.354764297	0.022263481
0.67575947	0.04100997
0.430874428	0.024931174
0.343641869	0.019674928

0.343641869	0.019674928
0.343641869	0.019674928
0.608656331	0.033411114
0.338337326	0.0185043
0.333193527	0.017408409
0.333193527	0.017408409
0.418497746	0.021623005
0.418497746	0.021623005
0.617897898	0.031808682
0.328203274	0.016382046
0.323359794	0.015420405
0.323359794	0.015420405
0.46054408	0.021042865
0.318656704	0.014519046
0.403055279	0.017900532
0.403055279	0.017900532
0.314087988	0.013673867
0.399370145	0.017077196
0.454143911	0.019398708
0.39575141	0.016292617
0.447917895	0.017883186
0.305331283	0.012137149
0.305331283	0.012137149
0.293071895	0.010167239
0.474149149	0.016166013
0.28920051	0.009588543
0.28920051	0.009588543
0.28542968	0.009044641
0.28542968	0.009044641
0.28542968	0.009044641
0.281755538	0.008533291
0.365896844	0.010693267
0.365896844	0.010693267
0.278174412	0.008052408
0.278174412	0.008052408
0.278174412	0.008052408
0.491273013	0.014005416
0.362853816	0.010206681
0.274682814	0.007600051
0.274682814	0.007600051
0.359860675	0.009742613
0.552123835	0.01478541
0.411242775	0.010538636
0.267955102	0.006773819
0.264712831	0.006396693
0.264712831	0.006396693
0.261547756	0.006041577
0.261547756	0.006041577

0.261547756	0.006041577
0.442714179	0.010014571
0.554175343	0.012013923
0.459945276	0.008382651
0.244034346	0.004302726
0.238704177	0.003846891
0.500185982	0.008027762
0.485662666	0.006076228
0.22174455	0.002609958
0.211028303	0.001985087
0.338561468	0.002629784
0.201295197	0.001513614
0.197647474	0.001358906
0.369100641	0.00232748
0.187452558	0.000985353
0.26547694	0.001349815
0.241805214	0.000651611
0.142138139	0.000143064
0.139400084	0.000122735
0.188142072	6.49398E-05

Genes
ROBO4;NOTCH1;PLXND1;LDB1;MAZ;SEMA3B;LEF1;PDGFB;TNC;CHRD;SEMA3G;KIF14;PPM1F;MYLK;IGF1R;F
ROBO4;TENM2;AMIGO3;FGFRL1;PTPRF;MPZ;CDH22;CDH23;HMCN1;GPC4;SCARF1;CD177;KIRREL3;NTNG1
COL27A1;COL13A1;COL11A1;APLP1;COL23A1;COL11A2;SCX;P3H4;NDNF;THSD4;COLQ;COL1A1;COL2A1;S
EPHB6;SHC4;FOXC1;PDGFB;IRS2;SOGA1;IGF1R;EFNB2;EFNB1;ZFYVE27;ERBB4;ERBB2;KDR;BDKRB2;EPHB4
EPHB6;NOTCH3;ROBO4;NOTCH1;PLXND1;UNC5B;SEMA3A;SEMA3B;SEMA3G;SEMA4F;EFNB2;NELL2;EFNB1
INSC;SHROOM2;MAL;CELSR1;JAM3
COL13A1;COL11A1;APLP1;COL23A1;NDNF;THSD4;COLQ;COL1A1;SPOCK2;COL7A1;COL4A6;COL9A1;COL4
COL13A1;COL11A1;APLP1;COL23A1;NDNF;THSD4;COLQ;COL1A1;SPOCK2;COL7A1;COL4A6;COL9A1;COL4
NOTCH1;SEMA3A;MAZ;LEF1;SEMA3B;PDGFB;SEMA3G;PPM1F;MYLK;IGF1R;FAM83H;SYDE1;VTN;KDR;SPHK1
AATK;IGF1R;STK24;ERBB4;STK26;MKNK1;CAMK4;MAPKAPK2;ERBB2;TAOK2;KDR;ABL1;PIM3;MAP3K9;TNIK;E
TPM3;RYR3;CACNA1H;MYLK;GNAO1;SMTN;PABPN1;TNNT2;CHRNE;BDKRB2;CASQ2;MYH13;ANKRD2;SNTB1
DLL4;JAG1;HEY1;PROX1;MYLK;TBX2
NOTCH1;MEGF10;MAZ;LEF1;PDGFB;CHRD;KIF14;SCX;IRS2;GLI1;TNF;AQP1;IGF1R;EFNB2;DLL4;ESM1;BCL7A
DLL4;FOXC1;NOTCH1;EGR3;SPHK1;MAPKAPK2;KDR;GAB1
NELL2;PLXND1;SEMA3A;SEMA3B;SEMA3G;PLXNB3;SEMA4F;PLXNB1
EPHB6;NOTCH3;ROBO4;NOTCH1;SEMA3A;SEMA3B;SEMA3G;SEMA4F;EFNB2;NELL2;EFNB1;NFASC;KIF5A;SI
NELL2;PLXND1;SEMA3A;PLXNB3;PLXNB1
NOTCH1;SPHK1;MAZ;LEF1;SEMA3B;PDGFB;SEMA3G;SEMA4F;PLET1;PPM1F;MYLK;IGF1R;COL1A1;FAM83H;I
PHLDB1;TWF2;CELSR1;SYDE1;BAIAP2L1;CAPN6;DAAM2;TAOK2;FSCN1;RAC3;ESAM;PLXNB1;S100A9;S100A
JAG1;SCUBE1;TIE1;KDR;PROX1
CHRM3;NLGN2;PLXND1;LDB1;ADAM22;SLC2A1;NRXN2;CELSR1;GBA2;GLIS2;RPS6KA6;DPYSL4;ERBB4;SPC
TNK2;PDGFB;IGF2;TNF;SRCIN1;VTN;GPRC5B;ERBB4;DGKQ;CLCF1;ABL1;CSPG4;ARHGEF2;ITGA5;AMH;CRLF
KIRREL3;NTNG1;NLGN2;PLXND1;FZD5;TNC;NRXN2;SLC6A1;PTPRF;LRFN4;PTK7;SPOCK2;PPFIA1;GPC4;AGR
TENM2;LDB1;THRA;ZC3H4;CHD8;NAB2;CHD3;TNF;DACH1;HEY1;BCL7A;ANKRD2;CIC;FOXD3;SOX13;ARID5A
EPHB6;NTNG1;NOTCH3;ROBO4;NOTCH1;MAP1S;SEMA3A;APLP1;SEMA3B;SEMA3G;SEMA4F;EFNB2;NELL2;I
KIRREL3;NLGN2;PLXND1;FZD5;NRXN2;ARVCF;SPOCK2;CDH22;CDHR3;FSCN1;ITGA6;DCHS1;SHANK3
EFNB2;EGR3;ID1;KDR;SLIT2;EPHB4
AMIGO3;CHD8;ADAM22;SLC2A1;CELSR1;GBA2;GLIS2;RPS6KA6;CIC;GIT1;CHST8;EGR2;SZT2;KDM4B;MAP1
TENM2;LDB1;THRA;CHD8;CHD3;TNF;DACH1;HEY1;ANKRD2;FOXD3;SOX13;ARID5A;SOX12;PROX1;DUSP26;
VTN;ITGB5;COL11A1;COL7A1;LAMA3;ITGA7;ITGA5
NOTCH1;SEMA3A;SEMA3B;SEMA3G;SEMA4F;SLIT2;ELANE
DLL4;HEYL;NOTCH1;JAG1;HEY1;TNNT2;PROX1;SMAD7
MAPK10;SH2D3C;TAOK2;MAP4K3;MAP3K11;MAPK13;MAPK8IP1;MAP3K12
MAGI1;MEGF10;LAMA1;ERBB2;CHRD;PDGFB;RAC3;NDNF;DUSP26;TNF;PPM1F;SMAD7
SYDE1;CXADR;THSD7B;PTK7;MICALL2;TNIK;CDC42BPB;PARVB;BCAR1
EFNB2;EGFL7;CUL7;SOX17;ID1;KDR;PROX1;RASIP1;SMAD7
DLL4;HEYL;JAG1;NOTCH1;HEY1
DLL4;CARD10;NOTCH1;MMRN2;VASH1;MEOX2;TNF
PPP1R16B;NELL2;EGFL7;EGR3;KDR;PDGFB;IGF2;PLXNB3;NRARP;PROX1;APLN
PLXND1;LAMA1;TESC;TNC;CHRD;KIF14;ADAM22;GPR4;DUSP26;TNF;VTN;TNN;ERBB2;PLXNB3;PLXNB1;EPH
HEYL;JAG1;NOTCH1;HEY1
EFNB2;EGR3;KDR;SLIT2;EPHB4
FMNL3;FGD5;SPATA13;SRGAP3;FGD1
VTN;ITGB5;SOX17;COL7A1;LAMA3;ITGA7;ITGA5
PPP1R16B;NELL2;EGFL7;EGR3;TIE1;KDR;PDGFB;PLXNB3;NRARP;PROX1;VASH1;NR2F2
NLGN2;SMURF1;PTCH1;TESC;NRXN2;TNF;PRAM1;ANO1;ZFYVE27;RAB40C;TMEM88;FLOT1;FLOT2;TNIK;AGR
KCNA1;SLC41A1;RYR3

LAMA5;KIF14;MICALL2;SRGAP3;PARVB;EPHA1;SRCIN1
HEYL;JAG1;NOTCH1;HEY1;SLIT2
BAIAP2L1;PLEKHG2;DAAM2;CDC42EP4;TWF2;ESAM;SLIT2;GBA2;KANK4;KANK3
COL13A1;ITGB5;ITGB4;VTN;TNN;ITGA10;TRIP6;TAOK2;PPFIA1;ITGA7;ITGA6;ITGA5;ARHGEF7
HEYL;NOTCH1;HEY1;SLIT2;PROX1;SMAD7
ROBO4;CADM4;PLXND1;LDB1;TNC;KIF14;SRCIN1;TNN;ERBB4;SPOCK2;SPATA13;ABL1;MGAT3;PLXNB1
DLL4;HEYL;NOTCH1;JAG1;HEY1;TIE1;SLIT2
NOTCH1;CADM4;SMURF1;MMRN2;CHRD;SPRY1;SLIT2;CHRDL2;APLN;SMAD7
HEYL;NOTCH1;HEY1;SPRY1;TBX3
LDB1;ERBB4;TIE1;ERBB2;KDR;IRS2;EPHA1;FGFR4;EPHB4;IGF1R;EPHA2
EPHB6;PDGFB;IGF1R;RPS6KA4;ERBB4;MKNK1;ERBB2;ABL1;KDR;PIM3;MAP3K9;AMH;PRKG1;EPHB4;MAP4K3
PHLDB1;PLXND1;VASH1;ETS1;SRCIN1;INF2;ADGRB3;ERBB2;TNIK;KIF1A;SHANK3;RAPGEF3;LZTS3;EPHA2
MAPK10;SH2D3C;MAP4K3;MAP3K11;MAPK8IP1;MAP3K12
NTNG1;TNN;KIF26A;SEMA3A;SRGAP3;ARHGEF2
HEYL;JAG1;NOTCH1;HEY1;SLIT2
NELL2;SEMA3A;SLIT2
NELL2;SEMA3A;SLIT2
DLL4;NOTCH3;HEYL;NOTCH1;JAG1;HEY1;NRARP
DLL4;HEYL;NOTCH1;JAG1;HEY1;TIE1;SLIT2
NTNG1;SEMA3A;TWF2;NDNF;SRCIN1;EFNB2;MAG;TNN;PTK7;KIF26A;PLXNB3;ITGA6;KIF1A;S100A9;SCARF1;
PLXND1;ERBB2;VASH1;ETS1;RAPGEF3;EPHA2
DLL4;CARD10;NOTCH1;MMRN2;KDR;MEOX2
NOTCH1;LDB1;THRA;CHD8;LEF1;PDGFB;CHD3;TNF;PPM1F;GLIS2;RBM15B;DACH1;HEY1;BCL7A;TBX21;CIC
COL27A1;COL2A1;HEY1;SOX17;KDR;PDGFB;IGF2;TNF;NES;TBX2
GADD45B;SEMA3A;TRAF5;CARD9;TAOK2;TNIK;MAPK8IP3;SH3RF1;MAP3K11;MAPK8IP1
CUL7;PLXND1;TWF2;NDNF;TNN;PTK7;DVL2;PLXNB3;PLXNB1;ITGA6;SLIT2;S100A9;SCARF1
DLL4;NOTCH1;MMRN2;MEOX2
NOTCH1;ERBB4;GLI1;TBX2
SEMA3A;SEMA3B;SEMA3G;SEMA4F;SLIT2;LGR6
DLL4;TIE1;KDR;NRARP;SLIT2;RASIP1;EPHA2
COL1A1;COL27A1;COL2A1;COL11A1;COL11A2;SCX;P3H4
ERBB4;SEMA3A;SEMA3B;SEMA3G;SEMA4F;SOX10
EPHB6;EFNB2;EFNB1;ARHGEF7;EPHA1;EPHB4;EPHA2
NOTCH1;NUMA1;LAMA1;MAZ;LEF1;PDGFB;CHRD;KIF14;SCX;IRS2;GLI1;TNF;NCMAP;IGF1R;EFNB2;ESM1;BC
TIE1;NR2F2;PROX1
NLGN2;NRXN2;SHANK3
SMURF1;PTCH1;TESC;TNF;PRAM1;ZFYVE27;RAB40C;TMEM88;FLOT1;FLOT2;TNIK;RAMP1;EPHA2;PACSIN1
ARVCF;CXADR;NLGN2;CDH22;CDHR3;FSCN1;DCHS1;CD177;TJP2
CUL7;EGFL7;NOTCH1;SOX17;KDR;RASIP1
RPS6KA6;FOXC1;NOTCH1;TFRC;BCL7A;HEY1;ARHGEF15;BCL7C;VASH1;AMH;RAPGEF3;TBX2
SHC4;TIE1;PTPRF;NPPC;DOK4;ERBB4;ERBB2;KDR;BDKRB2;FGFR4;MPZL1;BCAR1;EPHA2
BAIAP2L1;DAAM2;TAOK2;FSCN1;TWF2;RAC3;ESAM;CELSR1;RAPGEF3
FGD5;PLXND1;PLXNB3;PLXNB1;EPHA1;PRKG1;FGD1;RASIP1;VAV2
KIRREL3;NLGN2;PLXND1;FZD5;SPOCK2;NRXN2;PLXNB1;SRGAP3;SHANK3
SMURF1;PTCH1;TESC;TNF;PRAM1;ZFYVE27;RAB40C;TMEM88;FLOT1;FLOT2;TNIK;RAMP1;EPHA2;PACSIN1
LAMA5;ITGB5;ITGB4;ITGA10;ADAM11;ITGA7;ITGA6;ITGA5;PRAM1;BCAR1
NOTCH1;PRKCH;TNF
KCNA1;SLC41A1;RYR3

PLXND1;PLXNB3;PLXNB1
FOXC1;COL13A1;SCX
COL1A1;ITGB4;ITGA6
ABLIM3;SPATA13;SRGAP3;PARVB;ARHGEF7
DGKG;EPHB6;PDGFB;RPS6KA4;MKNK1;ERBB2;ABL1;PIM3;MAP3K9;PRKG1;MAP4K3;BRD2;PRKCH;SPHK1;TN
ERBB4;SEMA3A;SEMA3B;SEMA3G;SEMA4F;SOX10
NOTCH1;ERBB4;GLI1;TBX2
DLL4;NELL2;KDR;ABL1;PROX1;SLIT2;NR2F2;ETS1;BCAR1;EPHA2
HEYL;NOTCH1;HEY1;SLIT2;PROX1;SMAD7
DLL4;NOTCH1;SLIT2;NR2F2;VASH1;TNF
TENM2;CXADR;AMIGO3;DCHS1;HMCN1;FAT4;SCARF1
CSRNP3;LDB1;THRA;TRRAP;CHD8;SCX;CHD3;GLI1;MEOX2;ETS1;TNF;RPS6KA4;HEY1;SOX17;UBTF;IER5;IER
COL1A1;MYH7B;TMIE;COL2A1;COL11A1;COL11A2;CDH23;BIRC5;LHFPL4;ATP6V0A4
JAG1;CHRD;SMAD7;DACH1;STK24;STK26;MMRN2;PLXNB3;CLDN19;SRGAP3;SLIT2;EPHA1;AMH
NLGN2;LHFPL4;NRXN2;SHANK3
ZFYVE27;PLXND1;TWF2;PLXNB3;PLXNB1;SLIT2
DLL4;EGFL7;HEY1;NRARP;DLK1
VTN;CADM4;DGKQ;TNK2;PDGFB;IGF2;CSPG4;ARHGEF2;ITGA5;AMH
PPP1R16B;UNC5B;MAZ;KDR;PDGFB;PLXNB1;TNF;LIME1;RAPGEF3;IGF1R
TESC;LEF1;RARA
ATP2A3;ATP2A2;PTPRF
PTCH1;PROX1;MYLK
DGKG;DGKQ;DGKZ
ITGA5;PLET1;ARHGAP24
DGKG;DGKQ;DGKZ
SEMA3A;RAC3;SLIT2
ATP2A3;ATP2A2;PTPRF
NOS2;S100A9;S100A8
DOCK6;GPSM1;PLXND1;FGD1;RASIP1;VAV2;RGP1;FGD5;ERBB2;PLXNB3;PLXNB1;ITGA6;SRGAP3;EPHA1;AR
TFRC;TIE1;IGF2;TNF;IGF1R;ERBB4;ERBB2;KDR;CLDN19;DVL2;FLOT1;BIRC5;ITGA6;TNIK;EPHA1;FGFR4;AMH;
GADD45B;SPHK1;SEMA3A;TRAF5;CARD9;TAOK2;TNIK;MAPK8IP3;MAP3K11;SH3RF1;MAPK8IP1
AFAP1L2;NOS2;ZBTB49;CARD9;TNF;ZBTB2;RPS6KA4;BCL6B;MAPKAPK2;FLOT1;PATZ1;AMH;ERMAP;CD276;L
ITPKB;TRPC3;KCNMB1;TNNT2;CASQ2;SYT15;ITPR3;ADCY1;SYT8;RYR3;SLC25A13
ITGB4;TAOK2;TRIP6;ITGA6;ARHGEF7
MAPKAPK2;KDR;CLEC14A;BCAR1;VAV2
TWF2;SLIT2;SHANK3;KANK4;KANK3
COL1A1;MYH7B;TMIE;COL2A1;COL11A1;COL11A2;CDH23;BIRC5;LHFPL4;ATP6V0A4
HEYL;NOTCH1;HEY1;TBX2
FOXC1;PRKCH;NUMA1;SFN
APC2;RAP1GAP2;DOCK6;ARHGEF15;TBC1D8;SCRIB;ARHGAP24;RGP1;SYDE1;ERBB2;TBC1D10C;PLXNB1;IT
NOTCH1;TFRC;MAZ;LEF1;KIF14;SCX;NDNF;FMN2;TNF;MPO;SH3RF1;AQP1;IGF1R;ERBB4;ERBB2;KDR;SFN;PI
EFNB2;ESM1;EGR3;KDR;NRARP;SLIT2;EPHB4
FOXC1;NOTCH1;TRRAP;CHD8;KMT2B;MAZ;ZBTB48;LEF1;PDGFB;SCX;CHD3;GLI1;TNF;ETS1;GLIS2;SOX17;EI
MEGF10;RAC3;XKR6
DGKQ;FLOT1;PDGFB
DLL4;HEYL;NOTCH1;HEY1
ERBB4;ERBB2;KDR;PDGFB;ABL1;EPHA1;FGFR4;IGF1R
GADD45B;SEMA3A;TRAF5;CARD9;TAOK2;TNIK;MAPK8IP3;MAP3K11;SH3RF1;IGF1R;MAPK8IP1

ERBB4;ERBB2;KDR;PDGFB;ABL1;EPHA1;FGFR4
MAG;SEMA3A;SEMA3B;SEMA3G;SEMA4F
JAG1;NOTCH1;PTCH1;IGF2;CHRD;TNF;TBX3;SMAD7;SPRED2;HEY1;BCL7A;TNN;CHADL;BCL7C;SPRY1;ANKR
DLL4;EGR3;NDNF;IER2
EGR2;NFASC;ADGRB2;SOX10
NOTCH1;ERBB4;GLI1;TBX2
TNNT2;ATP2A3;ATP2A2;SLC4A3;CACNA1H;APLN;SMAD7;TBX2
TRRAP;NAB2;SCX;ETS1;TNF;RPS6KA4;DACH1;SOX17;SOX13;ARID5A;ATP1B4;POU3F1;SOX10;HIC1;EID2;M
BTG2;NOTCH1;TFRC;ZBTB49;MAZ;PDGFB;KIF14;SCX;IRS2;GLI1;ETS1;IGF1R;EFNB2;DLL4;ESM1;BCL7A;ERBB
OPRD1;PRKCH;TFRC;SPHK1;CARD9;ADCY1;TNF;ESR2;RPS6KA4;TRAF5;DVL2;FLOT1;FLOT2;ARHGEF2;TCF3;S
ERBB4;SEMA3A;SEMA3B;SEMA3G;SEMA4F;SOX10
ARVCF;GJB2;CDH22;CDHR3;FSCN1;DCHS1;JAM3;SMAD7
APC2;CHADL;TWF2;ARHGEF2;SLIT2;SHANK3;KANK4;KANK3
JAG1;CHRD;SMAD7;DACH1;STK24;TNN;STK26;MMRN2;PLXNB3;CLDN19;SRGAP3;SLIT2;EPHA1;AMH
HEYL;NOTCH1;HEY1;TBX2
EGFL7;CADM4;HHIP;PTCH1;CTNNBIP1;MAZ;NRARP;TNF;HIC1;DLK1;GLIS2;APLN;SIGIRR;DLL4;HEY1;WIF1;K
RAP1GAP2;APC2;SYDE1;ARHGEF15;TBC1D10C;TBC1D8;SCRIB;EPHA1;ARHGAP24;EPHA2
TWF2;FSCN1;PLXNB3;SLIT2;EPHA2
PLEKHG2;DAAM2;ABL1;ESAM;GBA2
DLL4;NOTCH1;TNNT2;PROX1;SMAD7
FOXC1;COL13A1;SCX
JAG1;SCUBE1;KDR
NTNG1;LRFN4;PTPRF
NOS2;BDKRB2;PLA2G5
JAG1;NOTCH1;TCF15
MYCN;RARA;PDGFB;ETS1;TNF;APLN;NFATC4
HEYL;NOTCH1;HEY1;SOX17;EPHB4;TBX3;TBX2
SYDE1;FGD5;ARHGEF15;SPATA13;SRGAP3;ARHGEF2;SLIT2;ARHGEF40;ARHGAP6;FGD1;VAV2
VTN;CADM4;MMRN2;ITGA5
KIRREL3;ROBO4;CADM4;PTK7;PLXNB3;ESAM;HMCN1
ERBB4;TIE1;ERBB2;KDR;EPHA1;FGFR4;EPHB4;IGF1R;EPHA2;MAP3K12
PRKCH;TFRC;SPHK1;CARD9;TNF;RPS6KA4;TRAF5;FLOT1;FLOT2;ARHGEF2;S100A9;S100A8;LTF
HEYL;CUL7;NOTCH1;HEY1;LEF1;TBX3
GAB1;IGF2;IRS2;SOGA1;BCAR1;IGF1R
EGR2;KDM4B;AMIGO3;MAP1S;PTCH1;CHD8;SHROOM2;PROX1;HSPG2;NFASC;CIC;NES;SHANK3;GIT1
OPRD1;NOTCH1;NOS2;PDGFB;TNF;APLN;HINFP;DLL4;MYCN;DGKQ;KDR;CLDN19;FLOT2;DIP2A;AMH
NLGN2;CLSTN1;FLOT1;KDR;CLDN19;PPM1F;EPHA2
NOTCH1;LAMA1;TIE1;FGFR4
HEYL;NOTCH1;HEY1;TBX2
TWF2;SLIT2;KANK4;KANK3
NLGN2;BSN;JAM3;TJP2
SEMA3A;SEMA3B;SEMA3G;SEMA4F
CDH22;CDHR3;DCHS1;JAM3;SMAD7
MAG;ZFYVE27;SEMA3A;SEMA3G;TWF2
DLL4;KDR;NRARP
ABL1;KDR;IGF1R
FOXC1;PRKCH;NUMA1
SYDE1;NOTCH1;CADM4;KIF14;DENND3;SPRY1;ARHGEF2;GIT1

BAIAP2L1;DAAM2;TAOK2;FSCN1;TWF2;RAC3;ESAM;CELSR1;RAPGEF3
ZDBF2;TET3
TBX21;LEF1
NOTCH1;PROX1
PROX1;PCK2
NRXN2;SHANK3
NRXN2;SHANK3
AQP11;AQP4
PLXNB3;SLIT2
SPRED2;SPRY1
TNF;TBX2
TNN;SRGAP3
LHFPL4;SHANK3
COL27A1;COL2A1
DLL4;NOTCH1
FA2H;FLOT1
TESC;LEF1
FSCN1;TNF
NRXN2;SHANK3
FOXC1;NOTCH1;EGR3;SPHK1;PDGFB;TWF2;NDNF;SMAD7;DLL4;MAPKAPK2;ERBB2;KDR;BCAR1
PPP1R16B;NELL2;EGFL7;EGR3;ERBB2;KDR;PDGFB;PLXNB3;NRARP;PROX1;TNF
TNN;PTK7;TWF2;PLXNB3;ITGA6;NDNF;SCARF1;S100A9;PACSIN1
TFRC;FTH1;SLC40A1;LTF
SPRED2;CADM4;SPHK1;DGKQ;SMAD7
EGR3;LEF1;PDGFB;PLXNB3;BCAR1;EPHA2
KIF14;RASIP1;JAM3
CXADR;JAG1;NOTCH1
SMTN;BDKRB2;MYLK
MGAT3;CHST2;CHST3
FOXC1;GAB1;KDR
ERBB4;SCRIB;EPHA2
NOS2;GPR37L1;APLN
SFSWAP;PCBP4;RBMX
SPRED2;DGKQ;SMAD7
NLGN2;NRXN2;ASTN2
KIF1A;SRCIN1;SHANK3;LZTS3
HHIP;PTCH1;GLI1;GLIS2
PABPN1;ERBB4;TIE1;ERBB2;KDR;IGF2;RPS2;EPHA1;FGFR4;EPHB4;IGF1R;EPHA2
FMNL3;CECR2;FGD5;RAC3;ARAP3;TNIK;CDC42BPB;AJUBA;FGD1;PACSIN1
TFRC;TRRAP;PHF1;NAB2;TNC;SCX;CHD3;ETS1;TNF;RPS6KA4;SOX17;SNRNP70;KHSRP;KDR;FLOT2;DIP2A;A[illegible]
TRRAP;SCX;ETS1;TNF;RPS6KA4;DACH1;SOX17;IER5;IER2;LMO1;SOX13;ARID5A;SOX12;POU3F1;SOX10;HI[illegible]
RTEL1;KIF26A;HHIP;AQP11;PIM3;SLIT2
TWF2;SLIT2;KANK4;KANK3
EGR2;XPO4;SMURF1;CCHCR1
MGAT3;CHST2;CHST3
ARID5A;RARA;ESR2
SFSWAP;PCBP4;RBMX
LSR;SCRIB;TJP2

PRKCH;TNF;EPHA2
GJC3;CXADR
NOTCH1;TIE1
DGKQ;CLCN2
SPRED2;SPRY1
NOTCH1;HEY1
ARHGEF2;AQP1
CASQ2;RYR3
CASQ2;RYR3
SEMA3A;TAOK2
DLL4;HEY1
DLL4;HEY1
KIF26A;SOX10
DLL4;CHRD
KDR;NDNF
TBX21;SMAD7
SCRIB;GRIP2
ZFYVE27;BCAR1
FSCN1;FMN2
PRKCH;TNF
RAB11FIP1;RAB11FIP5
HRK;TFRC;CALY;FLOT1;CLDN19;TNF;AJUBA;SHANK3;RAPGEF3;IGF1R
PHLDB1;TFRC;NUMA1;LEF1;PDGFB;SCX;TNF;SYDE1;BCL7A;BCL7C;MMRN2;KDR;DVL2;ARHGEF7;SHANK3;R[illegible]
PABPN1;MAPKAPK2;TAOK2;KDR;TNIK;TNF;SHANK3;MAP3K11;MAPK13
INF2;TWF2;MICALL2;VILL;ARHGAP6;MSRB1
SLC4A8;TFRC;KCNJ8;ATP2A3;SLC2A1;SLC4A3;SLC6A1;SLC7A1
FOXD3;PDGFB;IGF2;HINFP
PLEKHG2;DAAM2;ESAM;GBA2
PTP4A3;EGR3;PLXND1;ID1;S100A9
KDR;PDGFB;VASH1;ETS1;TNF;EPHA2
COL1A1;LAMA5;COL27A1;COL2A1;COL13A1;HHIP;ANKRD11;COL11A2;CHRD;COL9A2;PHEX;TBX3
UNC5B;MAZ;KDR;PDGFB;PLXNB1;TNF;IGF1R
KIF26A;SEMA3A;SOX10
TNN;PLXNB1;LTF
CHRNA4;CHRNE;COLQ
PPP1R16B;DLL4;MMRN2
SLC4A8;TFRC;KCNJ8;ATP2A3;SLC2A1;SLC4A3;SLC6A1;SLC7A1
CARD10;PTP4A3;NLGN2;NUMA1;TFRC;CROCC;CEP250;TNF
DLL4;KL;EGR3;GALNT3;NDNF;FGFR4
PTK7;TESC;TWF2;RARA;AQP1
EFNB2;PDGFB;PPM1F;ELANE
MEGF10;RARA;RAC3;XKR6
PPP1R16B;ROBO4;RAPGEF3;TJP2
PPFIA1;RHPN1;ARHGAP6;SHANK3
SPHK1;PDGFB;AMH;AQP1
PDGFB;ABL1;RAC3;NDNF;ARHGEF7;EPHA1;PPM1F
FSCN1;TWF2
KIF5A;MAPK8IP3

KIF5A;MAPK8IP3
DLL4;NOTCH1
CADM4;STK24
KCNJ8;KCNJ3
DLL4;NOTCH1
PROX1;EPHA2
S100A9;S100A8
TBX21;SMAD7
TNN;PLXNB1
SCRIB;GRIP2
DLL4;NOTCH1
NOTCH1;CLCF1
JAG1;NOTCH1
NLGN2;NRXN2
KIF5A;MAPK8IP3
SH3RF1;MAPK8IP1
HEYL;GPRC5B;PROX1;ARHGEF2;TCF3
CASP7;CASQ2;RYR3
FZD5;PTCH1;TBX3
NOTCH1;PROX1;MYLK
EGR2;NOTCH1;CLCF1
ETS1;TNF;CHST2
CARD9;S100A9;S100A8;LTF
CXADR;ITGA7;ITGA5;JAM3
LSR;ESAM;JAM3;TJP2
COL27A1;TPM3;FUS;COL11A1;NCKAP5L;COL11A2;KIF14;SHROOM2;SCX;P3H4;THSD4;COL1A1;MYO1D;BF
TRPC3;KCNJ8;CHRNA4;KCNA1;ATP2A3;SLC41A1;RYR3;AQP1;KCNMB1;CDH23;KCNK1;RAMP1;KCNJ3
NOTCH1;JAG1;PLXND1;ADAM22;PLXNB3;PLXNB1;JAM3
PPP1R16B;TENM2;DAAM2;FSCN1;AGRN
LAMA5;ANKRD11;HHIP;HOXC9;LTF
NOTCH1;SMURF1;CHRD;CHRDL2;SMAD7
MYCN;PDGFB;ETS1;TNF;APLN
DVL2;TNF;MAP3K11;MAPK8IP1;MAP3K12
MAGI1;JAG1;MEGF10;FLOT2;SMAD7
VTN;GPRC5B;MMRN2;PDGFB;GPR4;ETS1;TNF;S100A9;SCARF1;S100A8;PPM1F;MAPK13
KIF1A;SRCIN1;SHANK3;LZTS3
NOTCH1;JAG1;SMAD7;TBX2
ABLIM3;SPATA13;PARVB;ARHGEF7
DVL2;TNF;MAP3K11;MAP3K12
OPRD1;TFRC;SPHK1;TNK2;SH2D3C;PDGFB;IGF2;TNF;VTN;ERBB4;DGKQ;TAOK2;ERBB2;KDR;CLDN19;DVL2;
DLL4;NOTCH1;PROX1
DLL4;NOTCH1;HEY1
MYO1D;MYO1B;MYO6
EGR2;NLGN2;NCMAP
NOTCH1;FLOT1;PPM1F
TFRC;CLCF1;CTLA4;IRS2;AMH
MAG;UNC5B;CLCF1;KIF14;NDNF;CRLF1;NFATC4
CSRNP3;LDB1;THRA;CHD8;SCX;GLI1;MEOX2;ETS1;TNF;RPS6KA4;HEY1;SOX17;IER5;IER2;LMO1;FOXD3;FO

COL1A1;FOXD3;PTCH1;IGF2;PROX1;HINFP
APC2;HEMGN;PHLDB1;MEGF10;LEF1;IGF2;CHD3;TNF;SMAD7;HEY1;BCL7A;BCL7C;SPOCK2;FLOT2
INF2;MICALL2;ARHGAP6;MSRB1
NOTCH1;TNNT2;PROX1;SMAD7
PPP1R16B;DAAM2;TBC1D10C;TBC1D8;FSCN1;TWF2;EPHA2
ARHGEF15;PPFIA1;RHPN1;EPHA1;ARHGAP6;RAPGEF3;PPM1F
NRXN2;SHANK3
FSCN1;TNF
FLOT1;FLOT2
MAPKAPK2;LTF
SLC2A1;SLC2A6
IGF2;PDGFB
PTCH1;MEOX1
HEYL;NOTCH1
NELL2;SEMA3A
NRXN2;SHANK3
TBX21;SMAD7
MYH7B;KCNA1
GRP;RASL10B
SH3RF1;MAPK8IP1
HEYL;NOTCH1;HEY1
VTN;PDGFB;SLIT2
FA2H;LSR;SFN
TCF15;TIE1;TBX3
SFSWAP;PCBP4;RBMX
SEMA3B;SEMA3G;SEMA4F
FTH1;SPHK1;PDGFB;AMH;AQP1
STK24;STK26;ANKZF1;AQP1;MAPK13
MAGI1;MEGF10;FLOT1;TNF;SMAD7
NOTCH1;TNN;PTCH1;CHRD
MYO1D;MYO1B;BAIAP2L1;TPM3;MYO6;SHROOM2;RAC3;VILL;PROX1;ARHGEF2;BCAR1
NLGN2;CLSTN1;FLOT1;TNF;SHANK3;BAIAP3;NFATC4
HRK;TOP2A;CSRNP3;TFRC;TRRAP;LEF1;KIF14;SCX;FMN2;TNF;MPO;SH3RF1;AQP1;IGF1R;ERBB4;ERBB2;ABL[illegible]
CXADR;NLGN2;PTK7;PPFIA1;SLC6A1;TJP2
CAMK2N2;IRS2;SFN;EPHA1;TRIB2;PPM1F
COL1A1;FOXC1;NOTCH1;JAG1;LEF1
CHRM3;NLGN2;CHRNA4;KCNA1;NRXN2;SLC6A1;GABRR2;KCNMB1;CHRNE;MPZ;KCNQ2;KIF5A;AMPH;BSN
MYO1B;BAIAP2L1;SHROOM2;FSCN1
MYO1B;BAIAP2L1;SHROOM2;FSCN1
PPP1R16B;TNN;ITGA5;KLF2
SOX17;SCRIB;SLIT2;RASIP1
TESC;RAC3;JAM3;EPHA2
NTNG1;TNN;MAP1S;APLP1;TAOK2;DIP2A;PLXNB1;TNIK;SRGAP3;SHANK3;PACSIN1
MYO1D;MYO1B;MYO6
TRPC3;DGKQ;RYR3
PPP1R16B;ROBO4;RAPGEF3
STK26;TAOK2;MAPK13
JAG1;NOTCH1;HINFP

PDGFB;RARA;NFATC4
JAM3;SMAD7
BDKRB2;PLA2G5
BDKRB2;PLA2G5
TNC;MAPK8IP3
SHROOM2;TBX2
CASQ2;RYR3
KLF2;SMAD7
SMURF1;ITGA6
SRGAP3;SHANK3
CD177;JAM3
NOTCH1;DCHS1
DLL4;NOTCH1
NOTCH1;PPM1F
NOTCH1;CHADL
RAB11FIP1;RAB11FIP5
GRP;CASQ2
DACH1;SMAD7
SCRIB;GRIP2
VTN;ITGA5
NLGN2;NRXN2
SH3RF1;MAPK8IP1
PROX1;FGFR4
NFASC;MPZ;MAL;SOX10;BCAS1
EVC2;DHH;PTCH1;GLI1
TNNT2;BDKRB2;CACNA1H;TBX2
FOXC1;NOTCH1;TRRAP;PHF1;NAB2;CHD3;MLLT6;RPS6KA4;SOX17;DVL2;PATZ1;PPARGC1B;ATF7IP;FUS;SN[illegible]
SFN;HIC1;BCL2L2;BOK;EPHA2
MYCN;PDGFB;ETS1;TNF;APLN
FA2H;LSR;SFN
TAOK2;TRIP6;ARHGEF7
NOS2;CAMP;LTF
PDGFB;RARA;NFATC4
HEYL;FOXC1;CXADR;NOTCH1;SOX17;HEY1;ERBB4;PDGFB;DVL2;HSPG2
PPP1R16B;FOXC1;PLXND1;SPHK1;GAB1;VASH1;HSPG2;ETS1;AQP1;ERBB2;KDR;EPHA1;RAPGEF3;EPHA2
MYH7B;CHRNA4;KCNA1;SLC4A3
PHF1;LSR;PROX1;PCK2
TFRC;SLC22A17;FTH1;SLC40A1;CYBRD1
MAP1S;PLXNB1;TNIK;SRGAP3;PACSIN1
NUMA1;CALY;FLOT1;TNF;BOK
CALY;SPHK1;FLOT1;ABL1;CD177;PACSIN1
NOTCH1;CARD9;PDGFB;PLA2G5;DUSP26;TNF;SPRED2;RPS6KA6;ERBB4;ERBB2;KDR;SPRY1;FGFR4;AMH;EP[illegible]
KDR;NDNF
ATP2A2;RYR3
NDST1;HS6ST2
ITPKB;IP6K1
GIGYF1;IGF1R
TBX21;SMAD7

SIGIRR;LTF
EXOSC10;ZC3H4
STK24;SCARF1
APC2;CHD8;SOX13;KREMEN1;NRARP;PLEKHA4;GLI1;COL1A1;GPRC5B;SOX17;TMEM88;DAAM2;PTK7;LGR6
DLL4;PROX1;NES
XPO4;PTPN14;RAPGEF3
CUL7;IGF2;PDGFB
COL1A1;SPRED2;PHLDB1;FOXC1;LEF1;SPRY1;SMAD7
DHH;HHIP;PTCH1;GLI1;GLIS2
CARD9;TAOK2;MAPK8IP3;IGF1R;MAPK8IP1
NLGN2;LRFN4;CLSTN1;LHFPL4;GPC4
FOXC1;CXADR;NOTCH1;SCUBE1;SOX17;ERBB4;PDGFB;DVL2;EPHB4;TBX3;APLN;TBX2
TNN;SPHK1;KDR;GAB1;ITGA5;EPHA1;ETS1;RAPGEF3;AQP1
NOTCH1;TFRC;TESC;MAZ;LEF1;PDGFB;TNC;SCX;SCRIB;POU3F1;ETS1;TNF;PPM1F;KLF2;HINFP;DLL4;NWD1;
DLL4;MMRN2;NR2F2;VASH1
NELL2;SEMA3A;SLIT2
DGKG;DGKQ;DGKZ
PHF1;LSR;PROX1
PRKCH;CLDN19;FLOT1
HEMGN;NOTCH1;TNN;PTCH1;CTNNBIP1;CHRD;LTF
NPPC;RNF167;BTG2;FOXC1;SCRIB;NR2F2;HOXC9
COL1A1;GPRC5B;DAAM2;PTK7;NRARP;PLEKHA4;LGR6
NELL2;KDR;PDGFB;ABL1;PROX1;ETS1;BCAR1
FZD5;NOS2;MAPKAPK2;S100A9;S100A8;HNRNPA0
SFN;TNF;SH3RF1;AQP1;LTF
THRA;RARA
NELL2;SEMA3A
JAG1;PROM1
EGR2;NCMAP
TBX21;SMAD7
DGKQ;CLCN2
NOTCH1;CLCF1
CDH22;CDH23;CDHR3;DCHS1
NOTCH1;SOX17;DVL2;TBX2
DLL4;EGFL7;JAG1;HEY1;NRARP;DLK1
LDB1;PCIF1;ERBB2;KDR;CIRBP;PDGFB;IGF2;IRS2;FGFR4
FLOT1;FLOT2;TNF
APC2;MAP1S;ARHGEF2
MEGF10;LAMA1;IGF2
KDR;TNF;EPHA2
APC2;SOX17;TMEM88;WIF1;CHD8;CTNNBIP1;SOX13;KREMEN1;GLI1;HIC1
HEYL;CUL7;FOXC1;NOTCH1;LEF1
MAG;UNC5B;CLCF1;KIF14;NDNF;TNF;CRLF1;NFATC4
STK24;ATP2A3;SFN;HIC1;BCL2L2;PPM1F;EPHA2;BOK
RNF167;SLC4A8;NLGN2;GRIK5;CLSTN1;CPLX2;TNF;DGKZ;BAIAP3
CTNNBIP1;ATP2A3;PDGFB;ATP2A2;NES;PTPRF
GPRC5B;PDGFB;SH3BP5;SRCIN1
MYO1D;MYO1B;MYO6;KIF1A

MIDN;RAB11FIP1;RHBDF2;RAB11FIP5
COL1A1;FOXC1;ANKRD11;AQP1
MAG;UNC5B;CLCF1;KIF14;NDNF;CRLF1;NFATC4
CAMK2N2;SH3BP5;SFN;EPHA1;SRCIN1;TRIB2;PPM1F
RNF167;NLGN2;TNF;DGKZ;SHANK3
SEMA3A;NDNF;CELSR1;ASTN2;SH3RF1
NPPC;PANK1;ADCY1;AK5;SLC25A13
GPRC5B;GRP;MMRN2;GPR4;ETS1;TNF;S100A9;S100A8;MAPK13
OPRD1;NOTCH1;NDNF;FMN2;NDRG1;AQP1
EGR2;KIF14;NCMAP
TNNT2;PROX1;SMAD7
ITGA5;PLET1;ARHGAP24
PPFIA1;RHPN1;ARHGAP6
FOXC1;TCF15
LEF1;RARA
DIP2A;ACSS1
RAC3;PARVB
PDGFB;PRKG1
ROBO4;TNN
KCNMB1;CASQ2
CXADR;SOX13
PLA2G5;MPO
SCX;TBX3
SCRIB;CELSR1
SLC40A1;CYBRD1
TNRC6C;BTG2
XPO4;RAPGEF3
IGF2;APLN
AKNA;NOTCH1;JAG1;PPM1F
ANO1;NLGN2;IRS2;BAIAP3
TESC;LEF1;CTNNBIP1;TNF
OPRD1;NOTCH1;NDNF;FMN2;NDRG1;AQP1
PPP1R16B;ARHGEF15;ARHGAP6;LIME1;TNF;FGD1;VAV2;AKAP12;FGD5;SPATA13;TRAF5;KDR;ARHGEF2;TNIK
ERBB2;PDGFB;DVL2;TNF;ELANE;MAP3K11;MAP3K12
MYL4;MYH7B;TNNT2;CASQ2;RYR3
FZD5;SMURF1;DVL2;CELSR1
SMURF1;DVL2;CELSR1
FOXC1;PRKCH;NUMA1
SIGIRR;CTNNBIP1;ELANE
CDH22;CDHR3;DCHS1
TFRC;PPM1F;VAV2
DIP2A;SHANK3;IGF1R
NPM2;PDGFB;IGF2
SMURF1;CD177;JAM3
ITPKB;PDGFB;SYT15;ADCY1;SYT8;CACNA1H;RYR3;MAPK13
PCBP4;FUS;CSDC2;KHSRP;MAPKAPK2;CIRBP
TFRC;CLCF1;IRS2;AMH;SLC7A1;CD276
NOS2;MAPKAPK2;RARA;SPP1;FGFR4;S100A9;S100A8;HNRNPA0

CAMK4;SLC6A1;CIC;SHANK3;NFATC4
SMTN;EGR3;ITGA7;ZBTB42;CACNA1H
CUL7;PLXND1;PLXNB3;PLXNB1;SLIT2;SHANK3
NOTCH1;NUMA1;SMURF1;CHRD;CHRDL2;SMAD7
PHLDB1;SCX
TWF2;EPHA2
DIP2A;ACSS1
HEYL;NOTCH1
PM20D2;TNF
HEY1;KDR
NDST1;HS6ST2
PLXNB1;SRGAP3
STK26;TNIK
NOTCH1;TBX3
NUMA1;BIRC5
IGF2;IRS2
ANO1;BAIAP3
NLGN2;NRXN2
CARD9;SMAD7
NOTCH1;SOX17;DVL2;TBX2
NOTCH1;SOX17;DVL2;TBX2
PLXND1;PLXNB3;PLXNB1;SLIT2
PDGFB;IGF2;GPR37L1;DUSP26;TNF;IGF1R;SPRED2;DOK4;TAOK2;ERBB2;KDR;TNIK;EPHA2
PRKCH;SBK1;GALNT3;DGKQ;MKNK1;CAMK4;MAPKAPK2;PDGFB;NDNF;MAP3K12;MAPK13
OPRD1;TFRC;SH2D3C;AMH;TNF;PPM1F;SMAD7
SPRED2;EID2;NOTCH1;SMURF1;CHRD;SPRY1;CHRDL2;SMAD7
ANO1;PIM3;BAIAP3
FMNL3;RAC3;TNF
VTN;SCARF1;MYLK
CHRNA4;SYT15;SYT8
FZD5;LEF1;DIXDC1;DVL2;RAC3;GPC4
OPRD1;THRA;PEX5;KIF14;JAM3
TRPC3;KCNJ8;KCNA1;ATP2A3;SLC40A1;ATP1B4;SLC41A1;ATP2A2;SLC6A1;RYR3;CACNA1H;AQP1;SLC4A8;
TRPC3;CHRNA4;CDH23;ATP2A3;ATP2A2;RAMP1;CACNA1H;RYR3
RBM15B;SFSWAP;PCBP4;CELF3;CIRBP;RBMX;HNRNPA0
SIGIRR;HEYL;NWD1;PTCH1;CTNNBIP1;ID1;ID3;PROX1;GLIS2;SMAD7
SPRED2;CADM4;DGKQ;CAMK2N2;SFN;SLIT2;EPHA1;TRIB2;PPM1F;SMAD7
HRK;TOP2A;CSRNP3;GADD45B;NOS2;SCRIB;TNF;CTLA4;ITGA6;PLEKHN1;SLIT2;ARHGEF7;RNF122;MAP3K1
ARHGEF15;EPHA1;PPM1F;RAPGEF3
PTK7;TESC;RARA;TWF2;AQP1
NELL2;KDR;ABL1;PROX1;ETS1;PPM1F;BCAR1
RHBDF2;TNF;PPM1F
NOS2;CAMP;LTF
VTN;CLDN19;TNF
CELF3;CIRBP;RBMX
DVL2;SCRIB;CELSR1
NCKAP5L;KIF14;TWF2
SYT15;SYT8;BAIAP3

FSCN1;FGFR4
NYNRIN;KHNYN
JAG1;TBX2
NTNG1;DVL2
TNRC6C;BTG2
FLOT1;ZCCHC3
NASP;DLGAP5
AQP4;AQP1
TOP2A;EME2
COL11A1;KCNA1
FSCN1;SCRIB
FA2H;FLOT1
KCNJ8;KCNJ3
STK26;TNIK
SLIT2;PRKG1
FOXC1;ANKRD11
AMH;SMAD7
IGF2;IRS2
FLOT1;TNF
TNF;IGF1R
SPP1;ADCY1;AQP1;PCK2
NLGN2;SYT8;GIT1;PACSIN1
NOTCH1;PDGFB;NDNF;PPM1F
ACHE;CASP7;DENND3;PLA2G5;CLN6;ELANE
APC2;SOX17;TMEM88;TNN;CHD8;SOX13;KREMEN1;GLI1
TFRC;CTTNBP2;SPHK1;TAOK2;TNC;KIF14;TNF;BCAR1;CD177;PACSIN1
SMURF1;DVL2;CELSR1
GPSM1;NUMA1;INSC
DVL2;SCRIB;CELSR1
PLXNB3;SLIT2;ARHGAP24
DGKG;DGKQ;DGKZ
TFRC;CLCF1;IRS2
DLL4;PROX1;NES
NOTCH1;JAG1;TCF15;CHD3
LTB;TNF;S100A9;S100A8;BOK
BTG2;ATCAY;ZFYVE27;MAP1S;MICALL2;DIP2A;PLXNB1;TNIK;SRGAP3;MAPK8IP3;SHANK3;PACSIN1
ITPKB;KCNA1;SLC41A1;SYT15;ADCY1;SYT8;CACNA1H;RYR3;AQP1
KHSRP;PLEKHN1
MAPKAPK2;IER5
LEF1;RARA
BACE2;MGAT3
TNF;S100A9
ADPRHL1;PROX1
RTEL1;ACTR5
DIP2A;SHANK3
KIF5A;KIF1A
SOX17;GLI1
SMPD4;SPHK1

NELL2;SEMA3A
BDKRB2;KLF2
SPRED2;SPRY1
PDGFB;PRKG1
ZFYVE27;BCAR1
PDGFB;BCAR1
ITPKB;IP6K1
SPRED2;HIC1
PDGFB;PROX1
TFRC;CLCF1
BIRC5;DLGAP5
TNN;ITGA5
FLOT1;RAPGEF3
CEP131;CEP250
CADM4;ARHGAP24
INF2;CASQ2;MICALL2;ARHGAP6;MSRB1
PCBP4;CSDC2;KHSRP;MAPKAPK2
HEYL;HEY1;LEF1;KIF14
PTP4A3;PLXND1;DAAM2;S100A9
NLGN2;GRP;RASL10B;IRS2
CHRM3;NLGN2;CHRNA4;GRIK5;KCNA1;NRXN2;SLC6A1;GABRR2;KCNMB1;CHRNE;MPZ;KCNQ2;KIF5A;CDHF
IGF2;GAB1;IRS2;SOGA1;BCAR1;IGF1R;PCK2
SPRED2;SPRY1;SMAD7
MEGF10;RAC3;XKR6
SPOCK2;PDGFB;PPM1F
FZD5;MICALL2;UBE2J2
TRPC3;TNNT2;ITPR3;NDRG1;SLC25A13
NOTCH1;CEP131;PARVB;FGD1;MYLK;FMNL3;FGD5;ABLIM3;WDR90;STK26;SPATA13;FSCN1;CEP250;TNIK;S
NOTCH1;TFRC;PHF1;MAZ;LEF1;PDGFB;TNC;SCX;TNF;ETS1;PPM1F;DLL4;NWD1;ERBB2;FLOT1;AMH;TESC;CA
KHSRP;LEF1;SLIT2;TNF;RAMP1;CACNA1H
VTN;CADM4;CLDN19;MYLK
OSBPL5;IDH2;GBA2;CLN6
JAG1;PLET1;ARHGAP6
MAG;PTCH1;SEMA3G
TWF2;FSCN1;ARHGEF7
NLGN2;SYT8;PACSIN1
SPHK1;RARA;PDGFB;PROX1;TBX3
DIP2A;ACSS1
TOP2A;CECR2
LRFN4;GPC4
LRFN4;GPC4
AKAP12;AMH
DGKQ;FGFR4
NLGN2;BAIAP3
NDNF;CHST3
BCL2L2;BOK
FOXC1;SLIT2
TNNT2;LTF

NOTCH1;ITGA5
SIGIRR;ELANE
TNF;SMAD7
TOP2A;MAP1S
GPRC5B;CAMK4
VTN;PDGFB
S100A9;BCL2L2;S100A8;BOK
SPRED2;RPS6KA6;SPRY1;DUSP26
EGR3;TFRC;SPHK1;LEF1;KIF14;SCX;FMN2;MAPK8IP3;MPO;SH3RF1;TBX3;AQP1;IGF1R;ERBB4;ERBB2;KDR;B[illegible]
CARD9;SFN;TNF;SH3RF1;PPM1F;AQP1
TFRC;FTH1;LTF
KHSRP;PCK2;SLC25A13
DVL2;SCRIB;CELSR1
MAGI1;CARD10;NLGN2;SLC2A1;SHROOM2;P3H4;FANCC;PARVB;THSD4;MYO1B;BAIAP2L1;ZFYVE27;FSCN1;[illegible]
SFN;TNF;SH3RF1;AQP1
ERBB4;ERBB2;DCHS1;FAT4
EGR2;XPO4;SMURF1;CCHCR1
CASP7;EXOSC10;SMURF1;DENND3;CLN6;ELANE
TOP2A;RTEL1;BTG2;TRRAP;CHRNA4;PHF1;FANCC;FMN2;HIC1;HINFP;ENDOV;TAOK2;MAPKAPK2;FSCN1;ABL[illegible]
ATP2A3;TNF;S100A9;S100A8;BOK
HEYL;NOTCH1;HEY1;LEF1;TNF
GPRC5B;GPR4;ETS1;TNF;S100A9;S100A8;MAPK13
SPHK1;KDR;GAB1;EPHA1;ETS1;RAPGEF3;AQP1
FLOT1;ZCCHC3
AQP1;PCK2
GPR4;RAB11FIP5
TBX3;TBX2
SOX17;TBX2
ITPR3;ASIC3
DCHS1;FAT4
ESR2;PPARGC1B
SART1;SFSWAP
NCKAP5L;KIF14
KHSRP;MEPCE
AMH;TBX2
MIDN;PIM3
PRKG1;APLN
PTCH1;PROX1
ADGRB3;SCARF1
PPM1K;PPM1F
PLXNB3;SCRIB
PRKCH;PLA2G5
FOXC1;TCF15
CEP131;CEP250
ZFYVE27;SMURF1;TWF2;AGRN
RNF167;SZT2;DGKQ;TELO2
MYL4;TNNT2;CASQ2
MYO6;NDRG1;BOK

DLL4;SLIT2;NR2F2
DGKG;DGKQ;DGKZ
FZD5;MICALL2;UBE2J2
PDGFB;TNF;KLF2
COL1A1;JAG1;ITGB4;ITGA6;EPHA2
ACHE;FOXC1;PRKCH;GRP;NUMA1;THRA;CARD9;LEF1;PDGFB;SCX;TNF;IGF1R;COL1A1;SYDE1;BCL7A;BCL7C
ZFYVE27;SMURF1;SPHK1;ERBB2;TWF2;S100A9;S100A8
GPRC5B;NAB2;TAOK2;PDGFB;KIF14;SRCIN1;TELO2;MAP3K12;LTF
NUMA1;CAPN6;MAP1S;NCKAP5L
ARHGEF15;EPHA1;RAPGEF3;PPM1F
PRKCH;NUMA1;TNF;EPHA2
RAB11FIP1;SYT15;CPLX2;RAB11FIP5
ITPKB;SYT15;ADCY1;SYT8;RYR3
ZDHHC18;TAOK2;VPS37D;AQP11;NACAD
PPP1R16B;TFRC;IGF2;TNF;ERBB4;ERBB2;KDR;CLDN19;DVL2;FLOT1;BIRC5;TNIK;RAMP1
ERBB4;SFN;BOK
IGF1R;PCK2;RAB11FIP5
ANKZF1;AQP1;MAPK13
ARID5A;RARA;ESR2
PTCH1;TBX3;TBX2
GPSM1;NUMA1;INSC
SPRY1;RASIP1;ARHGAP24
BTG2;FOXC1;SCRIB
DACH1;PDGFB;FGFR4
MAPKAPK2;HNRNPA0
MAFF;SFN
MAFF;APOLD1
TWF2;VILL
NOTCH1;DAAM2
TWF2;VILL
NLGN2;CD177
IGF2;APLN
KLHL3;MAGED2
TRPC3;DGKQ
FOXC1;SLIT2
ADGRB3;CACNA1H
TNRC6C;AJUBA
NOTCH1;TNF
NOS2;NDNF
OPRD1;RPS6KA4
NPM2;GLI1
PDGFB;PROX1
CARD9;TNF
PTP4A3;TNF
KDR;PPM1F
CARD9;TAOK2
FZD5;SMAD7
TENM2;CDC42EP4;CROCC;FSCN1;TWF2;AGRN

UNC5B;ITGA6;ARHGEF2;TNF;SH3RF1
GRP;NOS2;RASL10B;TNF
TFRC;SPHK1;KIF14;TNC;ESR2;SOX17;KIF26A;TAOK2;ERBB2;SLIT2;S100A9;S100A8;BCAR1
SMURF1;SHROOM2;CELSR1;AP4B1;ANO1;NBEA;CROCC;INSC;MYO6;ID1;PEX5;MGAT3;CEP250;MAL;ESAM;
NOTCH1;CARD9;PDGFB;IGF2;GPR37L1;PLA2G5;TNF;IGF1R;DOK4;ERBB4;TAOK2;ERBB2;KDR;FGFR4;AMH;EL
VASH1;AMH;TBX2
TAOK2;DIP2A;SHANK3
GPSM1;NUMA1;INSC
KHSRP;PCK2;SLC25A13
SLC4A8;AQP11;SLC4A3
PDGFB;IGF2;DLGAP5
COL1A1;GPRC5B;DAAM2;PTK7;NRARP;PLEKHA4;LGR6
CARD9;ATP2A3;TNF;S100A9;S100A8;PPM1F;BOK
CLDN19;MICALL2;LSR;ESAM
FUS;MAPKAPK2;CIRBP;HNRNPA0
PTP4A3;SPHK1;TRIP6;TNF
RBM15B;CELF3;RBMX;HNRNPA0
ALAD;FUS;CARD9;AQP11;AQP4;AMH;RBMX;RYR3
LAMA1;CD177
SPHK1;TNF
SLC4A8;SLC4A3
TNF;IGF1R
SFN;BOK
FMNL3;TNF
CXADR;LSR
AQP11;AQP4
AQP11;AQP4
AQP4;AQP1
PPM1F;SMAD7
SOX13;SOX10
ZFYVE27;TWF2
GRP;RASL10B
AGBL5
NASP
ERBB2
FLOT2
LRFN4
SLC2A1
SLC7A1
PDGFB
SHANK3
IGF2
NOS2
DGKQ
PDGFB
BOK
IRS2
RAMP1

TBX3
S100A9
S100A9
SLC7A1
NRARP
NOTCH1
MAP1S
PLA2G5
CASQ2
KCNK1
SLIT2
ATP9A
PROX1
RTEL1
SMAD7
TNF
GRP
PROM1
TBX3
FGFR4
SLIT2
CHST14
CHST14
SPP1
ZCCHC3
SLIT2
SHROOM2
PCK2
MEPCE
CELSR1
SCRIB
MAP1S
NR2F2
SHANK3
FA2H
FA2H
PROM1
PTPRF
TBX3
GBA2
GBA2
GBA2
KDR
PROX1
TCF3
TNF
MYO6
SLC22A17

SDS
MTTP
SFSWAP
PTCH1
BACE2
SLIT2
LDB1
NOTCH1
PHF1
NPPC
SLIT2
PPM1F
AK5
SLC7A1
PEX5
FLOT1
DAAM2
TNF
TBX21
RARA
PLEKHA4
EFNB2
PROM1
TNF
LDB1
NOS2
BIRC5
LTF
CROCC
NAB2
NOTCH1
FLOT2
MSRB1
SMURF1
TBX21
TBX21
TNF
CHRM3;NOS2;SPHK1;NDNF;NCALD
ZDHHC18;PEX5;TAOK2;VPS37D;NACAD
RPS6KA6;MYO6;NDRG1
SLC4A8;NLGN2;GRIK5
FSCN1;FANCC;FANCE
LDB1;KDR;ARHGAP6;PPM1F
NOTCH1;FLOT2;ANKRD2;TBX3
ALAD;RGP1;SNRNP70;FMN2
OPRD1;BTG2;NOTCH1;NOS2;PCIF1;PDGFB;TNF;HINFP;APLN;RPS6KA4;DLL4;MYCN;DGKQ;KDR;CLDN19;FLO
FZD5;TBX3
RAC3;PARVB

CHRM3;SPHK1
MAPKAPK2;PLA2G5
PTCH1;TBX3
CAMK4;NFATC4
ITGB4;SCX
APC2;ARHGEF2
NPPC;AMH
SPRY1;SLIT2
CASQ2;ATP2A2
SCUBE1;JAG1;ERBB4;KDR;SCRIB;PROX1;GLI1;PCK2
ERBB4;CLCF1;TNF;CRLF1
PDGFB;PRKG1;APLN
NOTCH1;ERBB4;TNF
NOTCH1;ERBB4;TNF
CLSTN1;SCRIB;TNF
ARID5A;RARA;SLIT2;RAMP1;CACNA1H;ESR2
NOS2;CAMP;ELANE;AQP1;LTF
ERBB2;PDGFB;SPRY1;TNF;TRIB2;LIME1;ELANE
DGKQ;RAPGEF3;AQP1
COL2A1;COL11A2;SCX
GPRC5B;PDGFB;SRCIN1
KIF1A;SHANK3
SIGIRR;LTF
FLOT1;CPLX2
GPSM1;PLXNB3
NOTCH1;HEY1
FZD5;TBX21
ATP2A2;APLN
SPRED2;HIC1
DHH;GLI1
NOTCH1;ITGA5
ATP2A3;ATP2A2
NASP
CHADL
DGKQ
PRKCH
LDB1
KIF5A
BIRC5
TAP2
TNF
ATP2A2
LTF
MYLK
RAMP1
NOTCH1
CXADR
TBX3

NLGN2
MYLK
NOTCH1
BCAR1
KLF2
PDGFB
SCARF1
RYR3
NASP
BCAR1
AKNA
KIF5A
KCNA1
NR2F2
SFXN2
MEGF10
PROX1
SCARF1
SLC6A1
GJB2
JAG1
SOX17
NOTCH1
FA2H
HEY1
MYLK
MYO6
RAB11FIP5
FTH1
MAPKAPK2
FOXC1
SPO11
SMURF1
ATP2A2
FAAH
MMRN2
TCF15
KHSRP
NOTCH1
GPR37L1
ARHGAP24
TBX21
PROX1
DPYSL4
TAP2
HEY1
JAG1
PRKCH

APLN
NOS2
TNF
BOK
SCX
RARA
KDR
SLC4A8
CEP250
NUMA1
CPLX2
FLOT1
SYDE1
AGBL5
PEX5
TNF
DENND3
TELO2
CLCN2
FGFR4
TJP2
TRPC3
NOTCH1
NOS2;CARD9;ARHGEF2;TNF;MAPK13
OPRD1;ABL1;RNF122
KIF5A;KIF1A;MAPK8IP3
KHSRP;TNF;KLF2
FZD5;MICALL2;UBE2J2
VTN;TNK2;FLOT1
CHD8;GLI1;TNF
KL;GALNT3;FGFR4
NOTCH1;JAG1;PLET1
NELL2;TNN;SEMA3A
S100A9;S100A8;BOK
FLOT1;ZCCHC3;LTF
SPRED2;EID2;SMURF1;SPRY1;SMAD7
PYGO2;FOXC1;SOX17;PROX1
ERBB2;PDGFB;PROX1;TNF;TELO2;ELANE;LTF
SYT15;SYT8
MAPK10;VAV2
SLIT2;TNF
CTLA4;SOX12
SLC2A1;SLC2A6
KIF5A;KIF1A
SLC2A1;SLC2A6
ITPKB;IP6K1
ERBB4;TBX3
BACE2;RBMX

MAG;SEMA3G
KIF26A;HHIP
NOTCH1;TNF
UNC5B;TNF
UNC5B;TNF
NLGN2;SHANK3
TAOK2;PDGFB
NLGN2;SHANK3
PRKCH;SBK1;DGKQ;MKNK1;CAMK4;MAPKAPK2;PDGFB;MAP3K12;MAPK13
SIGIRR;SPHK1;MAPKAPK2
DGKQ;RAPGEF3;AQP1
NLGN2;SYT8;PACSIN1
NOTCH1;ERBB4;CARD9;KDR;PDGFB;PLA2G5;FGFR4;AMH;TNF;MAP3K12
ARVCF;SNRNP40;FUS;KHSRP;CELF3;USB1
HRK;TFRC;FSCN1;TNF;AJUBA;RASIP1
SOX17;TNN;ACTR5;SCX
KHSRP;IRS2;SLC25A13;PCK2
OSBPL5;MTTP;ABCA4;ATP9A
TRPC3;CHRNA4;ABCA4;CDH23;ATP2A3;AQP11;AQP4;ABCA13;RAMP1;RYR3;AQP1
HEYL;GPRC5B;HEY1;ARHGEF2;TCF3
OPRD1;RTEL1;PEX5;TNF;RAPGEF3
BAIAP2L1;NUMA1;CDC42EP4;PROX1;NES
VTN;CALY;FLOT1;PLA2G5;TNF
HRK;OPRD1;ATF7IP;TFRC;PEX5;TNF;AJUBA
SIGIRR;TNF;ELANE
DGKG;DGKQ;DGKZ
MYL4;TNNT2;CASQ2
ITGA6;LTB;TNF;SH3RF1
MAPK10;CIRBP;ELANE;MAP4K3
EID2;SMAD7
LEF1;RARA
RAC3;GPC4
FLOT1;LTF
SOX10;S100A9
NDST1;HS6ST2
APC2;ARHGEF2
SH3BP5;SRCIN1
KDR;PPM1F
TWF2;FSCN1
CIRBP;RBMX
SNRNP70
SLIT2
TOP2A
NOTCH1
GPR37L1
CHRM3
NPM2
CAMK4

NOS2
SOX10
KDR
RAMP1
NUMA1
CROCC
NOTCH1
TNF
RYR3
PTCH1
APLN
NOTCH1
IGF1R
CHST14
SNAPC4
CELSR1
KHSRP
MAZ
SLC2A6
FA2H
COL1A1
UBTF
GBA2
MYO6
PROX1
NDST1
ARHGEF2
LDHB
CASP7
ERBB4
CHST14
CARD9
MEGF10
IRS2
TNF
PRKCH
GIT1
TFRC
RNF122
IGF2
NOTCH1
KHSRP
KHSRP
PDGFB
AK5
CACNA1H
TNN
P3H4

OSBPL5
BIRC5
PDGFB
KDR
MMRN2
FSCN1
NPM2
GIT1
DVL2
TNF
NLGN2
PDGFB
HOXC9
DPYSL4
SH3RF1
ABL1
RARA
SH3RF1
BIRC5
ATP2A2
SYDE1;CAPN6;ACTR5;PLXNB1;S100A9;S100A8
ALAD;FUS;CARD9;AQP11;RBMX;BOK
AFAP1L2;NOS2;TNF;ELANE
NUMA1;CROCC;FSCN1;TNF
ARVCF;SNRNP40;EXOSC10;HNRNPUL1;PABPN1;FUS;KHSRP;CELF3;USB1;HNRNPA0
SFXN2;SLC7A1;SLC25A13
JAG1;EXPH5;EPHA2
DGKG;DGKQ;DGKZ
SPHK1;ERBB2;S100A9;S100A8;PPM1F
KCNJ8;KCNMB1;KCNA1;KCNQ2;KCNK1;AQP1;KCNJ3
SNRNP40;KHSRP
FUS;CAMP
PROX1;EPHA2
MAP1S;GBA2
TRPC3;DGKQ
TMIE;MYO6
ABCA4;ATP9A
ARHGAP6;TNF
NELL2;TBX2
NOTCH1;NUMA1
GADD45B;SPHK1
NLGN2;SHANK3
PHLDB1;RPS6KA6;SOX17;ACTR5
MAZ;ITGA6;TNF;SH3RF1;BCL2L2
HRK;TOP2A;CSRNP3;GADD45B;SCRIB;TNF;CTLA4;ITGA6;PLEKHN1;SLIT2;ARHGEF7;RNF122;MAP3K11;BOK
KDR;NDNF;TNF
KIF14;FBXL7;FBXL12
PM20D2;IRS2;FGFR4

SLC6A1;CIC;SHANK3
KDR;EPHA1;PPM1F
BTG2;NOTCH1;TESC;ZBTB49;CTNNBIP1;PROX1;ETS1;PTPN14;TBX3;DLL4;TNN;ERBB4;FTH1;MMRN2;CLDN1
PTP4A3;SPHK1;TRIP6;TNF;LIME1
IGF2;IRS2
SLIT2;JAM3
CTTNBP2;TNF
IGF1R;PCK2
TMIE;MYO6
SOX17;PTCH1
ADGRB3;CACNA1H
RNF167;SZT2
RHBDF2;PPM1F
CLDN19;GIT1
OSBPL5;MTTP
EFNB2;PROX1
CHST8;CHST14
BAIAP2L1;RAPGEF3
NOTCH1;HEY1
RAP1GAP2;VAV2
PDGFB;KDR;PPM1F
GNAO1;ETS1;TNF
ITGA5
RPS6KA6
MMRN2
TOP2A
TNF
MAFF
SLC7A1
NOS2
MGAT3
SCUBE1
SCARF1
MMRN2
NUMA1
PROM1
JAM3
SLC7A1
FLOT1
MGAT3
NOTCH1
PDGFB
SPHK1
COL4A6
COL11A1
SPP1
SPP1
TBX3

ASIC3
TWF2
PROX1
EGR3
TCF3
EXOSC10
EXOSC10
KHSRP
XAB2
IDH2
AQP11
ERBB4
ITGB4
ERBB4
TOP2A
NOTCH1
BACE2
RNF122
PRKG1
TNF
TNF
PDGFB
GPR37L1
RRM2
BAIAP2L1
PPP1R16B
TRPC3
FLOT1
PHLDB1
IRS2
GPRC5B
TESC
PLA2G5
MGAT3
MGAT3
NLGN2
RNF167
DPYSL4
THRA
PLEKHA4
CUL7;BTG2;FOXC1;SPHK1;PIM3;SCRIB;DLGAP5
BCL7A;TRRAP;FUS;BCL7C;FMN2
RPS6KA4;SPRED2;RPS6KA6;RAB11FIP1;NOTCH1;PTCH1;ID1;SPRY1;IP6K1;TNF;RAB11FIP5;SMAD7
FZD5;XPO4;CEP131;ERBB2;MICALL2;UBE2J2;RAPGEF3
CHRM3;SPHK1;KDR;ATP2A2;NCALD
COL7A1;LAMA3;ATP2A2;FLOT2;GLI1
PPP1R16B;SPRED2;CADM4
ERBB2;GAB1;BCAR1

SIGIRR;ZDHHC18;LTF
TNRC6C;BTG2;KHSRP
BTG2;NOTCH1;PLXND1;ZBTB49;ADAM22;ETS1;DLL4;SOX17;BCL7A;ERBB4;FTH1;BCL7C;SLIT2;HOXC9;ADAI
TFRC;SLC22A17;FTH1;ATP2A3;SLC40A1;CASQ2;SLC41A1;CYBRD1;ATP2A2;ELANE
FZD5;LEF1;DIXDC1;DVL2
ERBB4;CLCF1;TNF;CRLF1
TFRC;IGF2;SLC7A1;CD276
JAG1;LDB1;EPHA1;PLET1
MAPK10;VAV2
BCL7A;BCL7C
DGKQ;RAPGEF3
SYT15;SYT8
SHROOM2;NES
CXADR;ADPRHL1
CHD8;NAB2
CHRM3;BDKRB2
SLIT2;TNF
FSCN1;SCRIB
ZBTB48;TELO2
TNRC6C;AJUBA
NOTCH1;NES
KDR;NDNF
AFAP1L2;FZD5;NOS2;SPHK1;CARD9;SCRIB;TNF;MAPK13;RARA;FLOT1;ARHGEF2;LTB;AMH;ZCCHC3;ELANE;C
FOXC1;VASH1;TNF;HSPG2;KLF2
CTLA4;LIME1;BCAR1
NOS2;CARD9;TNF
IRS2;IGF1R;PCK2
CXADR;MEGF10;TJP2
AMH;GIT1;TBX2
NOTCH1;ID1;IP6K1
PDGFB;KDR;ETS1
MIDN;NLGN2;NOS2;IRS2;TNF
ZDHHC18;TOMM40;TAOK2;VPS37D;AQP11;NACAD;AP4B1
RTEL1;NPM2;ACTR5;PDGFB;FGFR4;GLI1
UNC5B;TNF
MAP1S;GBA2
INF2;KDR
RAC3;GPC4
FOXC1;ERBB2
PTCH1;CHRD
GRIK5;CDHR3
SMPD4;PLA2G5
NCKAP5L;KIF14
NOTCH1;TNF
RNF167;TELO2
TENM2;FSCN1
TNF;KLF2
HEYL;SAFB2

AFAP1L2;SCUBE1;NOS2;MAPKAPK2;BDKRB2;ADCY1;HSPG2;TNF;S100A9;S100A8;NFATC4;HNRNPA0
KIF26A;HHIP;DGKQ;PTCH1;UBTF;RGS12;GLI1;KAZALD1;GIT1;CD177
SPHK1
RPS6KA6
GIT1
LEF1
NOS2
SHANK3
CLN6
SLC25A13
PEX5
COL1A1
NPPC
CEP250
SOX13
LIME1
NAB2
CHRM3
FLOT2
LEF1
PDGFB
RBM15B
TBX2
SDS
SOX17
TBX3
KHSRP
RTEL1
TFRC
MEOX1
SLC40A1
CACNA1H
DLGAP5
SLC2A1
MYH7B
TBX2
MEGF10
NCMAP
CAMK4
JAM3
PPM1F
SLIT2
TNF
IRS2
ARHGEF2
MAPK8IP3
SCARF1
EXOSC10

MAN2C1
PPM1F
PLA2G5
MAPKAPK2
ZCCHC3
TOP2A
EFNB2
SFN
PLA2G5
BOK
CTNNBIP1
FLOT1
DIP2A
AMH
SCRIB
FLOT1
SPHK1
KDR
NLGN2
GALNT3
GALNT3
LMNB1
EXOSC10
NPPC
BOK
EFNB2
JAM3
FZD5;LDB1;SEMA3A;ERBB2;NDNF;CELSR1;ASTN2;IER2;SH3RF1
OSBPL5;GBA2;SCARF1;CLN6
MICALL2;CLDN19;ESAM
CHRNA4;MGAT3;TNF
PDGFB;TNF;ELANE
EID2;ITGB5;SMURF1;ID1;SCX;AMH;SMAD7
RTEL1;BCL7A;TRRAP;BCL7C;FMN2
DAAM2;WIF1;CTNNBIP1;KREMEN1;LGR6;HIC1
TRPC3;KCNJ8;KCNA1;ATP2A3;SLC40A1;SLC41A1;ATP2A2;SLC6A1;RYR3;SLC4A8;KCNQ2;ATP6V0A4;KCNK1[illegible]
CASP7;PDGFB;SPP1;ADCY1;TNF;RYR3;RAPGEF3;AQP1
OPRD1;GRP;SORCS1;SORCS2
PCIF1;ERBB2;CIRBP;PDGFB;IGF2;IRS2;FGFR4;TNF;KLF2
SPHK1;CPLX2;ATP9A;CD177;PACSIN1
OPRD1;TFRC;SH2D3C;AMH;TNF
DGKQ;IRS2
NYNRIN;KHNYN
KCNJ8;KCNJ3
NTNG1;KIF26A
ARHGEF2;MYLK
RDH12;RDH5
PTCH1;KREMEN1

GRP;CASQ2
KIF26A;HHIP
TNF;KLF2
SPRED2;HIC1
CXADR;ATP2A2
TFRC;MAZ;PDGFB;TNF;IGF1R;ERBB2;KDR;AMH;BOK;UNC5B;SPHK1;CARD9;IGF2;GPR37L1;GPRC5B;PTP4A3;
APC2;LDB1;CARD9;PCIF1;ERBB2;CIRBP;FGFR4;TNF;CLN6;KLF2
FOXC1;PRKCH;TCF15;LAMA1;CTNNBIP1;LEF1;PLA2G5;COL1A1;HEYL;GPRC5B;KDR;ARHGEF2;TCF3;LTF
SOX17;PTCH1;TNC;KDR;PROX1;SOX10;MEOX1;TBX2
DOCK6;RGP1;ERBB2;PLXNB1;ITGA6;SRGAP3;ARHGEF7;AGRN;RAPGEF3
BCL7A;BCL7C;DLGAP5
CXADR;CASQ2;KAZALD1
SDS
IGF2
RAPGEF3
TBX21
SLC6A1
TWF2
SHANK3
KHSRP
SDS
GIT1
KDR
BIRC5
NOS2
PLA2G5
HMCN1
PLA2G5
NFATC4
MEPCE
KCNJ3
KIF14
PTCH1
ARHGEF15
PANK1
TNF
PANK1
SPHK1
PTCH1
NOS2
SNAPC4
TJP2
NKIRAS2
KIF5A
MTTP
DLGAP5
GRP
GRP

MAP1S
BIRC5
BIRC5
TNNT2
CTLA4
SCRIB
PPP1R16B
ATP9A
GIT1
VTN
PRKG1
GPR37L1
SMAD7
ARHGEF2
SIGIRR
GRP
KLF2
PROX1
IRS2
MMRN2
AK5
BIRC5
SPP1
MMRN2
PACSIN1
RASIP1
RARA
BOK
KDR
PLEKHA4
NES
MEPCE
LTF
NOS2
PANK1
SLC25A13
IRS2
TBX21
BACE2
KCNJ8;KCNJ3
ATP6V0A4;CLN6
SFSWAP;CELF3
TNC;SOX10
HEY1;LEF1
PDGFB;MYLK
NUMA1;GIT1
GPSM1;VTN;FOXC1;FLOT1;RARA
ACHE;CEP131;TNF;EXPH5

GNAO1;CTLA4;ERMAP;LIME1;PRAM1;BCAR1;CD276
CADM4;TFRC;IGF2;TNF;ERBB4;ERBB2;KDR;CLDN19;DVL2;FLOT1;BIRC5;TNIK;SLIT2
DLL4;BDKRB2;TBC1D8
EFNB2;MAG;SEMA3A
LDHB;SDS;PCK2
JAG1;NLGN2;DHH;LAMA1;IGF2;GLI1;KLF2;DLL4;VTN;ESM1;ITGA5;LGR6;SHANK3
PYGO2;FOXC1;SOX17;PROX1
NOS2;MAPKAPK2;CARD9;ARHGEF2;TNF;MAPK13
PRKCH;PLA2G5
FOXC1;ERBB2
COL2A1;HOXC9
GNAO1;CARD9
VTN;PDGFB
SOGA1;GIT1
SH3BP5;SRCIN1
CLSTN1;TNF
PDGFB;DGKZ
ITGA6;AMH
HRK;TFRC;FLOT1;CLDN19;TNF
EID2;ITGB5;ID1;SMAD7
RGP1;FMN2;TNF;GIT1
CHRNA4;CDH23;ASIC3
TFRC;TRRAP;FUS
PRKCH;TNF;EPHA2
BTG2;DACH1;KHSRP;PCIF1;PDGFB;PROX1;SOGA1
CASP7;PABPN1;NOS2;MAPKAPK2;S100A9;S100A8;GIT1;HNRNPA0
ATP9A
TNF
BAIAP2L1
CHST2
CTNNBIP1
TFRC
KCNA1
SDS
SHANK3
NPPC
NFATC4
CDC42EP4
PROX1
MYH7B
CHRM3
KLF2
SOX10
SZT2
CEP250
CEP250
RBM15B
DHH

HEY1
EXOSC10
SPHK1
ITGB5
RTEL1
TJP2
STK24
SLC41A1
GRP
ATCAY
CADM4
VTN
SLIT2
CASQ2
CASQ2
CASQ2
SLIT2
GRP
AK5
PLA2G5
GRP
BOK
DLGAP5
BIRC5
DLGAP5
SPHK1
CDC42EP4
BIRC5
TJP2
NLGN2
DPYSL4
SH3RF1
FLOT2
PEX5;ANKZF1;AQP1;MAPK13
ABL1;GPR4;ARHGEF2;RASIP1
CUL7;TNIK
DGKQ;SOGA1
ADCY1;SHANK3
OSBPL3;GBA2
TAOK2;PDGFB
TNF;RAB11FIP5
RAPGEF3;AQP1
EID2;SMAD7
PROX1;GBA2
NLGN2;SHANK3
DACH1;EXOSC10
APC2;ARHGEF2
ABCA4;ATP9A

PTCH1;ABCA13
LTB;TNF
TNF;JAM3
AKAP12;CHRM3;GPR37L1
NOTCH1;HEY1;TBX2
COL2A1;COL11A2;SCX
TFRC;CLCF1;IRS2
HEYL;HEY1;LEF1
GNAO1;CTLA4;ERMAP;PRAM1;CD276
PEX5;ABL1;MGAT3;FANCC;ANKRD2;ETV5
CPM;BACE2;CASP7;PTCH1;TESC;PHEX
PHF1;LSR;PROX1;TBX3
BCL7A;BCL7C;KIF14;GLI1
NPM2;NUMA1;PDGFB;IGF2;KIF14;BIRC5
NOTCH1;ERBB4;TNF
NOTCH1;ID1;IP6K1
PROX1;TNF;LTF
AQP11;ABCA13
BCL7A;BCL7C
NOS2;SPHK1
ERBB2;UBTF
KCNJ8;KCNJ3
RPS2;SMAD7
SPO11;FMN2
GBA2;CLN6
CXADR;MEGF10
TNF;TRIB2
ERBB2;IGF1R
NLGN2;CLSTN1
TOMM40;PEX5
GNAO1;AKAP12;ADGRB2;ADGRB3;GPR4;GPR37L1;ADCY1;RAMP1
TNF
AGBL5
NUMA1
SLC25A13
TOP2A
TCF3
TNNT2
TNF
SLC25A13
TNF
KCNJ3
PROX1
EPHA2
CACNA1H
JAG1
PLA2G5
NLGN2

KLF2
IGF1R
RAB11FIP5
RNF167
KLF2
SNAPC4
SMPD4
SOX12
SPP1
PROM1
SLC2A1
PM20D2
SLC2A1
CAMP
LEF1
TECRL
CDS2
MAP1S
NCMAP
LTF
MAPK8IP1
NOTCH1
VTN
ELANE
SLIT2
CASQ2
CARD9
ETS1
FGFR4
NDST1
TBX21
RARA
CUL7
JAM3
IRS2
IGF2
LAMA1
KHSRP
CD177
TNF
SEMA3A
TNF
NOS2
SLC4A8
TESC
CAMK4
CASQ2
CAMK2N2;SFN;EPHA1;TRIB2;PPM1F;MAP3K12

ERBB4;GAB1;BCAR1
CLDN19;MICALL2;ESAM
PDGFB;TNF;ELANE
JAG1;GLI1;EPHA2
EFNB2;MAG;SEMA3A
ACHE;NLGN2;IRS2;EXPH5
STAB2;ARAP3;SCRIB;DENND3;FMN2;MAPK8IP3;AP4B1;NCALD;SYTL2;MAPK8IP1;CECR2;ZFYVE27;CALY;MY(
SLC2A1;AMH
EXOSC10;ZC3H4
SOGA1;GIT1
DLL4;JAG1
SYDE1;CACNA1H
BCL7A;BCL7C
IGF2;SCRIB
FA2H;SMPD4;CLN6
NTNG1;MAP1S;APLP1;TAOK2;MAPK8IP3
MYH7B;ITGB5;TNNT2;CDC42BPB
NTNG1;MAP1S;APLP1;TAOK2
SLC4A8;CLIC3;SLC6A1;CLCN2
KCNA1;SLC40A1;CYBRD1;ITPR3
CXADR;S100A9;S100A8;CD177
TFRC;PLA2G5;SLC7A1;CD276
DOK4;NKIRAS2;RAC3;CELSR1;RGL2;ARHGAP6;RAPGEF3
COL1A1;HOXC9
EGR2;SOX10
BACE2;RBMX
ITPKB;IP6K1
PDGFB;IRS2
TFRC;CLCF1
EFNB2;TNF
ERBB2;UBTF
SMURF1;VPS37D
ERBB2
IRS2
SIGIRR
GPRC5B
FLOT1
KDR
SZT2
ERBB4
NOTCH1
AQP11
MAPK13
LEF1
SYDE1
CHST3
CALY
FOXC1

LEF1
KDR
SPHK1
SPHK1
KIF5A
CAMP
CHRNA4
FAAH
CAMK4
CTLA4
CHADL
PDGFB
SPRY1
TOMM40
PACSIN1
SOX13
IGF2
NOS2
RAMP1
CD177
RARA
P3H4
PEX5
TOMM40
RHBDF2
CHD3
NTNG1
FGFR4
CIRBP;ELANE;MAP4K3;AQP1;MAPK13
CUL7;PDGFB;IGF2
XAB2;FSCN1;FANCC
CPM;BACE2;MGAT3
MYO1B;OSBPL5;SORCS1
EPHB6;CHRM3;PDGFB;PHEX;PPM1F;RPS6KA4;MKNK1;ERBB2;ABL1;PIM3;MAP3K9;PRKG1;MAP4K3;BRD2;P[illegible]
COL1A1;COL2A1;RDH12;COL11A1;CEP250
ACHE;NOTCH1;THRA;ID1;IP6K1;PPARGC1B;IGF1R
PRKCH;ERBB2;TNF;EPHA2
MYO6;NDRG1;HINFP
SYT15;SYT8
FLOT1;ZCCHC3
RAC3;ARHGEF7
ATP2A2;SMAD7
ATP2A3;CASQ2;ATP2A2;ITPR3;ELANE
ACHE;NOS2;RHBDF2;TNF;EXPH5
COL1A1;COL2A1;RDH12;COL11A1;CDH23
TRPC3;ATP2A3;ATP2A2;RYR3
PRKCH;PPFIA1;TNF;EPHA2
IDH2

NOTCH1
PTP4A3
NPM2
SLC25A13
GNAO1
SPHK1
DAAM2
LTF
SYT8
RYR3
CD177
ABCA13
SLC25A13
ITGB5
SOX17
MAP1S
ITGB5
TBX2
P3H4
P3H4
TBX3
EXOSC10
TNRC6C
MAP1S
EME2
SLC2A6
SMAD7
HEYL
CHADL
HEY1
PROX1
GNAO1
PLA2G5
SCRIB
TNF
KDR
SHANK3
NOS2
TNF
TNRC6C
ACTR5
AMH
SZT2
VPS37D
AMH
SLC2A6
SPHK1;PLA2G5;TNF
SPP1;FGFR4;TNF

CHST14;CHST2;CHST3
HRK;KDR;BOK
FZD5;SCRIB;CD276
CPM;CUL7;DHH;PTCH1;ADAM22;ADAM11;VASH1;PHEX;PM20D2;BACE2;CASP7;CAPN6;ADAMTS8;ELANE;[illegible]
LDB1;ETS1
CEP131;PTPN14
BDKRB2;KLF2
LDB1;TRRAP
SCRIB;PLA2G5
CIRBP;RBMX
TRRAP;FUS
TESC;ETS1
AFAP1L2;NOS2;TNF;ELANE
CARD10;TAOK2;KIF14;PDGFB
GPRC5B;AKNA;SPHK1;TNC;GPR4;ETS1;TNF;S100A9;ELANE;S100A8;MAPK13
HEYL;HEY1;HOXC9
MAP1S;BIRC5;DLGAP5
FGFR4;TNF;CLN6
FZD5;MAPKAPK2;RARA;ARHGEF2;AMH;LTF
CADM4;LDB1;ITGA6;AMH
SLC4A8;SLC6A1;KCNK1;ASIC3
SLIT2;RYR3;RAPGEF3;AQP1;MAPK13
SPRED2;RPS6KA6;SPRY1;DUSP26;IGF1R
FUS;CIRBP
GADD45B;SPHK1
FXYD6;FGF11
CPLX2;GIT1
BACE2;SHROOM2
MYH7B;PTCH1
PRKG1;APLN
IRS2;SMAD7
ZDHHC18;SPHK1
NOTCH1;TIE1
TNK2
PPP1R16B
SLC25A13
TFRC
SAFB2
CTNNBIP1
PTCH1
GPR37L1
RASL10B
PTCH1
PANK1
CTU2
CTU2
NOS2
CHST2

PCIF1
KHSRP
ARHGAP6
ARHGAP6
SOGA1
RTEL1
NOS2
SCX
IRS2
TFRC
LAMA1
AQP1
CHD8
NLGN2
SMURF1
SMAD7
TNF
OSBPL5;GBA2;CLN6
SMURF1;TRIB2;SH3RF1;SMAD7
NES;FBXL7
NUMA1;ATP9A
NRXN2;CPLX2
TRAF5;TNF
NEURL1B;SLC7A1
NLGN2;NRXN2
KDR;NDNF
KL;LTF
SMURF1;CHRD;SCX
PROX1;TNF;LTF
TESC;PDGFB;TWF2;SYT15;ADCY1;SYT8;RYR3;CACNA1H;AQP1;ITPKB;CASP7;PABPN1;PTK7;PEX5;RARA;SLIT[illegible]
GNAO1;TFRC;FLOT2;SLC7A1;CD276
DGKG;DGKQ;PITPNM3;DGKZ;CDS2
ACHE;THRA;TNF;PPARGC1B;IGF1R
ERBB2;PCIF1;CIRBP;TNF
RBM15B;MEPCE;PCIF1
PHF1
MAZ
TNF
VTN
CARD9
PTPN14
DGKG
GJC3
MYO6
PM20D2
ZFYVE27
KCNK1
SHROOM2

JAG1
EXOSC10
RHBDF2
TRIB2
NOS2
LMNB1
SOX10
PTCH1
LEF1
KDR
SCX
KDR
RTEL1
PEX5
PRKCH
ACTR5
CASQ2
SLC2A1;SLC2A6
CTLA4;PLA2G5
MYL4;TNNT2
KIF14;BIRC5
NUMA1;CEP131
RPS6KA4;SIGIRR;TBX21;LEF1;RARA;TNF;TRIB2;ELANE
NPM2;BCL7A;BCL7C;KIF14
CTNNBIP1;LEF1;PDGFB;NES
ABL1;SOGA1;BOK
ADGRB2;ADGRB3;GPR4;ADCY1;RAMP1
TRPC3;CDH23
SLC7A1;SLC25A13
SLC2A6;GIT1
NUMA1;GBA2
NES;FBXL7
DGKQ;SLC2A6
RDH12;RDH5
NOTCH1;RHBDF2
XAB2;SCX
IGF2;FGFR4
RRM2;GLI1
BCL7A;BCL7C
VTN;MYLK
FZD5;RARA;SCRIB;CD276
NOS2;STAB2;MPO;S100A9;ELANE;CAMP;S100A8;AQP1;LTF
SART1;LEF1;SOX12
CARD9;TNF;PPM1F
NOS2
CHRM3
NAPRT
FOXC1

RAC3
RHBDF2
SLIT2
ACHE
BIRC5
AQP1
SCRIB
TOP2A
EXOSC10
RTEL1
EFNB1
GBA2
CEP250
SPHK1
UBTF
CXADR
PCK2
KCNJ8
CHST2
TNF
MEOX1
RASIP1
FTH1
ELANE
SMAD7
IDH2
LMNB1
PEX5
IP6K1
TNF
TRPC3
SCX
RAC3
GLI1
SMURF1
CUL7
ATP2A2
TNF
DIP2A
SOX12
NOTCH1
KLF2
AMH
PEX5
ATF7IP
SOX13
SCX
SPRED2;EID2;SMURF1;SPRY1;SMAD7

CASP7;PABPN1;PTK7;TESC;TWF2;RARA;SPP1;ADCY1;GIT1;AQP1
AFAP1L2;JUND;GADD45B;TRRAP;ACTR5;ZBTB49;PROX1;TBX3;RNF167;RARA;PIM3;HOXC9;DLGAP5
TNIK;PACSIN1
PHLDB1;ERBB2
CPLX2;SYT8
FUS;CIRBP
BAIAP2L1;CDC42EP4
ARHGAP33;DOK4;TNK2;RGL2;RAPGEF3
S100A9;CAMP;LTF
SDS;OSBPL3;CHST14
ABL1;MGAT3;FANCC;ETV5
SLC4A8;SLC6A1;KCNK1;ASIC3
SLC4A8;SLC2A1;SLC4A3;SLC2A6
BCL7A;BCL7C;ACTR5;FMN2
CAMK4;TNF
RNF167;SZT2
FA2H;SMPD4
MYH7B;EGR2
GTPBP2;ANKZF1
RNF167;GRP
ANO1;SLC41A1
KCNJ8;KCNJ3
SCX
SMURF1
ATP9A
LEF1
BOK
TAP2
SLC6A1
SPRED2
SLC4A8
AQP1
MAPK8IP3
PTCH1
NRARP
EXPH5
KIF5A
SMPD4
CPLX2
PLA2G5
CLN6
SHROOM2
ZC3H4
DGKZ
FLOT2
TNF
TNF
CASQ2

MIDN
KCNA1
DLL4
CAMP
GNAO1
GRP
NUMA1
FSCN1
RARA
TNF
BOK
TOMM40
TELO2
ZCCHC3
KIF14
STK24;STK26;PEX5
VTN;SPOCK2;LTF
SBK1;GALNT3;CDC42BPB
ATF7IP;SOX17;TMEM88;TESC;FLOT1;FLOT2;MAPK8IP3;TELO2;SMAD7
PROX1;TNF;LTF
TNF;BCL2L2;BOK
RTEL1;EME2
IGF1R;PCK2
PPARGC1B;ESR2
KHSRP;PLEKHN1
NRXN2;CPLX2
RAP1GAP2;VAV2
TRPC3;CDH23;ATP2A3;CASQ2;ATP2A2;ELANE
SLIT2
CECR2
SPRY1
IDH2
JAM3
TELO2
TBX21
TESC
CUL7
CLDN19
APLP1
HINFP
CADM4
CHST3
NPPC
TNF
MYH7B
USB1
SPHK1
SHROOM2

TELO2
CLN6
ALAD
GNAO1
NFATC4
RAB11FIP5
RPS6KA4
TNF
PLA2G5
NR2F2
PPP1R16B
EXOSC10
RRM2
IGF2
TNF
TNF
KDR
EXOSC10
NRXN2
FZD5
RTEL1;ACTR5;ZBTB48
MYO6;CLDN19
SOX17;HOXC9
FLOT1;ELANE
NUMA1;LMNB1
ITPKB;IP6K1
XPO4;RAPGEF3
CTNNBIP1;LTF
LDB1;ZC3H4
CPM;BACE2;PTCH1;PHEX;HGFAC
BTG2;ATCAY;SPHK1;PCIF1
MAZ;IGF2;TNF;IGF1R
NEURL1B;MYO6;STAB2;AMPH;HSPG2;SCARF1;ELANE;ATP9A
CHRM3;NLGN2;FXYD6
MEGF10;RARA;ELANE
VTN;CTNNBIP1;PDGFB;FLOT1;NES
VASH1;SLC2A6
NKIRAS2;TBKBP1
TOP2A;LDB1
TANGO2;MTTP
KHSRP;IRS2
MAZ;TNF
ZDBF2
VAV2
ARHGEF7
JAG1
AMH
SRGAP3

NPPC
SMAD7
ARHGEF2
KDR
NUMA1
AQP1
RAB11FIP5
SLIT2
CHRNA4
ATP2A2
AQP4
MPO
TNF
NFATC4
TNF
FAAH
CTLA4
NR2F2
TNF
PPFIA1
PPFIA1
CDS2
OSBPL5
PRKCH
SCX
SFN
IRS2
PLA2G5
PDGFB
SMURF1
PDGFB
LIME1
CHST14;CHST2;CHST3
AQP11;VASH1;NR2F2
CXADR;S100A9;S100A8
BAIAP2L1;NUMA1;CDC42EP4
DGKQ;RGS12;GIT1
DLL4;SOX13
SLC4A3;KCNJ3
FLOT1;ELANE
RTEL1;EME2
TOMM40;PEX5
ADPRHL1;TNNT2
CROCC;TBC1D10C;TBC1D8
RTEL1;TRRAP;FUS
BAIAP2L1;NUMA1;CDC42EP4;PROX1
POLA2
TNF

TNF
TAP2
DGKQ
LDB1
MEPCE
SLC6A1
TRRAP
EXOSC10
TBX21
LTF
PABPN1
ALAD
TNF
VTN
SHANK3
FOXC1
LDB1
FA2H;FLOT1;PACSIN1
CARD10;TFRC;MEPCE
GPSM1;PLXNB3
RNF167;TELO2
FANCC;FANCE
TBX21;CAMK4
TFRC;STAB2;HSPG2;SCARF1;RAMP1
BRD2;CECR2;KDM4B;NPM2;NASP;BCL7A;PHF1;CHD8;BCL7C;ACTR5;CHD3
CXADR;S100A9;S100A8
ACHE;EXPH5;BAIAP3
ACHE;THRA;PPARGC1B;IGF1R
SPHK1;MAPKAPK2
SLC4A8;SLC6A1
PDGFB;CD177
RPS6KA4;MAP3K12
FOXC1;LEF1
VAV2
SAFB2
CHRM3
CASQ2
RTEL1
KIF1A
CAMK4
USB1
FMN2
LTF
APLP1
LDB1
AMH
ZDHHC18
ZDHHC18

AGBL5
PTCH1
CROCC
APLN
CTDSP2;PPM1K;DUSP26;PTPRF;PPM1F
CAMP;LTF
RTEL1;DACH1
RARA;LTF
NLGN2;CLSTN1
KHSRP;SPHK1;MAPKAPK2;LEF1;MAP4K3
MAG;SOX17;SEMA3G;SLIT2;ESR2
CADM4;IRS2;SLIT2
NPM2;ACTR5;GLI1
BTG2;TENM2;ATCAY;ZFYVE27;MICALL2;GBA2
CEP131
DGKQ
TNF
JAG1
SMAD7
TNRC6C
FAAH
ANKZF1
EXOSC10
BCAR1
HEY1
GPR37L1
MMRN2
TNF
TNF
ERBB2
NUDT14
NOTCH1
GPRC5B;TFRC;TRAF5;CARD9;LTB;TNF;LTF
KIRREL3;JAG1;KDR;PDGFB
OPRD1;CHRM3
INF2;FZD5
DHRS11;OSBPL3
EFNB2;TNF
CASP7;DENND3;ELANE;CLN6
FA2H;SMPD4;SPHK1
FOXC1;VASH1;HSPG2
LTF
TNF
KDR
BOK
VAV2
BOK
FAAH

ARHGAP24
CASQ2
AQP1
IRS2
CIRBP
CPSF1
EXPH5
DGKZ
CASQ2
NOTCH1
TNC
SPHK1
SPHK1
RBM15B;MEPCE
FA2H;SMPD4
CASQ2;LIME1
RAC3;ARHGEF7
MAP1S;KIF14
SPRY1;MAPK8IP1
NOTCH1;GPR4
TFRC;RAMP1
KCNJ8;SLC6A1;CACNA1H;KCNJ3
CECR2;KDM4B;NPM2;BCL7A;PHF1;CHD8;BCL7C;ACTR5;CHD3
PPP1R16B;CADM4;SLIT2
FZD5;ARHGEF2;AMH
CELSR1;ARHGAP6
CARD10;TFRC
FGFR4;CLN6
MAP3K11;HINFP
FAAH;MAPKAPK2
RTEL1;ACTR5
SLC25A13
CHADL
CPSF1
PDGFB
CLN6
SLIT2
OSBPL3
PRKG1
HEY1
CHRNA4
OSBPL5
MYO1D
VPS37D
AMH
PPFIA1
AQP11
TNF

EXOSC10
EPHA2
IRS2
HRK
CUL7;BCL7A;BCL7C;CEP250;GIT1
PDGFB;PROX1;NR2F2
SLC2A1;IRS2;PCK2
APC2;SMURF1;TNF
PCIF1;S100A9
SLC16A11;SLC7A1
PTCH1;ADCY1
TOMM40;SLC25A13
AQP4;RYR3
DGKG;SPRED2;NLRX1;SLIT2;DUSP26;AJUBA;BCL2L2;EPHA2;IGF1R
EFNB1;TBC1D10C;TBC1D8
SPRY1;NR2F2;RASIP1
RRM2;BCL7A;BCL7C;KIF14
GPRC5B;NLRX1;TFRC;CARD9;TRAF5;LTB;LIME1;TNF;LTF
SLC7A1
GNAO1
CASQ2
KMT2B
GIT1
TNF
SPO11
ETS1
NUMA1
TNF
PDGFB
SHANK3
ATP2A2
CPLX2;EXPH5
IRS2;FGFR4
SLC4A8;SLC6A1
RNF167;SZT2
BRD2;NASP
RNF167;TRAF5
TRRAP;ACTR5;MAPKAPK2;AJUBA
RPS6KA4;RBMX;MAPK13
CECR2;CUL7;BIRC5
KHSRP;TNK2;SPHK1;PLA2G5;IP6K1;DUSP15;NUDT14
SMPD4;SPHK1;ARHGEF2;MAP4K3
OSBPL5;MTTP;ABCA4;ABCA13
FZD5;ARHGEF2;AMH
CLCF1
ARHGAP6
SLC7A1
CXADR

KLF2
HEY1
PROX1
VPS37D
PCIF1
TRRAP
TRRAP
NDNF
GPR4
SPHK1
RPS2
ANKZF1
ZDBF2;PHF1;IGF2
OSBPL5;MICALL2;ANKRD50
OPRD1;ANO1;GPR4
TRIB2;SH3RF1;SMAD7
INSC;SFN;GIT1
BCL7A;BCL7C
ARHGEF2;IGF1R
EME2;TAOK2
MAZ;IGF2;TNF;IGF1R;EPHA2
TOP2A;ENDOV;RTEL1;POLA2;BTG2;TRRAP;CHRNA4;ACTR5;FSCN1;FANCC;HINFP
NUMA1
GNAO1
TNF
PDGFB
ERBB2
FXYD6
NOTCH1
SNAPC4
CLN6
MEOX1
RARA
SMPD4
TOMM40
ELANE
SPP1
FZD5;TNF;GIT1
SPHK1;RPS2;SMAD7
KCNJ8;KCNA1;KCNQ2;KCNK1;KCNJ3
FAM83H;COL1A1;CROCC;CEP250;TSPAN10
FA2H;SPHK1
CLIC3;CLCN2
FZD5;TNF
SNRNP40;EXOSC10;HNRNPUL1;PABPN1
DLL4;CXADR;SCRIB;CD276
NEURL1B;SCRIB;TSPAN10
MAP1S;BIRC5;DLGAP5

TRIB2;LIME1;TELO2;LTF
EFNB2;KIF14
PEX5;KLHL3
FOXC1;SHROOM2
CLIC3;CLCN2
SBK1;CDC42BPB
POLA2
SLC25A13
KCNA1
RRM2
SLC4A3
NUMA1
SMAD7
SCX
SLIT2
TNNT2
CDS2
SLC7A1
VPS37D
DHRS11
PTPRF
PPP1R16B
TOP2A
SPHK1
VTN
RAB11FIP1;SYTL2;RAB11FIP5
MAZ;UBTF
CUL7;BIRC5
SIGIRR;SLIT2
VTN;FLOT1
BRD2;NASP
CUL7;CXADR;NUMA1;TANGO2;ARAP3;CDC42BPB;FGD1;FMNL3;CECR2;FGD5;SMPD4;FAM174B;TNIK;ARHG
RTEL1
CASQ2
TNF
P3H4
ATP2A3
TAOK2
VPS37D
CASQ2
GIT1
RARA
AMH
ZDHHC18
VTN;SPOCK2
PLA2G5;TNF
CEP131
SPHK1

GNAO1
MAPKAPK2
CD177
HEYL
CEP250
RRM2
GPR4
IRS2
TNF
SMURF1
LEF1
CLCF1
NTNG1
SNRNP70;RASIP1
ARHGEF2;BCL2L2
CASP7;FZD5;PABPN1;GIT1
TRIB2;SH3RF1;SMAD7
RBM15B;PABPN1
ERBB2;IGF1R
GNAO1;TBX21
PTCH1
CARD10
TOP2A
PROX1
TECRL
LMNB1
KMT2B
ATP6V0A4
NOTCH1
CHST8
TIE1;ERBB2;AQP11
DLL4;CLCF1;TCF3
TRRAP;SNRNP70;FUS
SMPD4;TRAF5;ARHGEF2;TNF
SIGIRR;ZDHHC18;CLDN19;ELANE;LTF
RTEL1;ACTR5
SLC4A8;SLC6A1
RRM2;DLGAP5
TENM2;FZD5;LDB1;ERBB4;ERBB2;IER2
CHRNA4;CLCF1;TCF3
DHRS11;RDH5;GBA2
GBA2
VPS37D
IGF1R
PROX1
TRRAP
FOXC1
RTEL1

FZD5
AFAP1L2
LTB
SHANK3
PPP1R16B
IRS2
OTUD1
TNF
RGP1;BTG2;PCIF1;FLOT2;FMN2
GALNT3;GALNT18;TET3;MGAT3;AQP11
RHBDF2;CPLX2
MICALL2;ANKRD50
FZD5;TNF
GPSM1;KDR
FLOT1;ZCCHC3
MGAT3;NUDT14
GALNT3;GALNT18;TET3
SLC25A13
HINFP
SPHK1
PDGFB
IGF1R
TOP2A
NOS2
PHF1
TRRAP
MAPK8IP1
TNF
PLA2G5
IDH2
DGKZ
TNF
SMURF1;ATP2A2
PDGFB;FGFR4
RGP1;TBC1D10C;BAIAP3
FA2H;SPHK1;GBA2
APC2;RGP1;FMN2;TNF
SMURF1;SCX
ZDHHC18;RHBDF2
SAFB2
CHRNA4
MYH7B
BOK
TNF
RTEL1
KIF14
CASP7;PABPN1;TNF;GIT1
PM20D2;PCIF1;KLF2

ITGB5;ID1;SMAD7
TANGO2;MTTP
IRS2
FLOT1
CHD3
EPHA2
FLOT1
ARHGAP6
SHANK3
SOX17
CLCF1
ATCAY;PPM1F;RAB11FIP5
LDHB;FA2H;GBA2
PNN;SART1;XAB2;SNRNP40;SNRNP70;RBMX
RGP1;CALY;TBC1D10C;MICALL2;ANKRD50;BAIAP3
CLCF1;TCF3
MAGI1;CARD10;SLC2A1;FANCC
BTG2;DACH1;PCIF1
ITPKB;PITPNM3;IP6K1
NOTCH1
CACNA1H
SART1
TBKBP1
MEGF10
EXPH5
PLA2G5
EFNB2;AKNA;STK24;TNC
MYO1D;RGP1;TBC1D10C;BAIAP3
VTN;SPOCK2
FOXC1;SHROOM2
PCIF1;FGFR4
S100A9;CAMP;LTF
USB1
CPLX2
RTEL1
TBKBP1
SMAD7
RARA
VPS37D
CLCF1
ENDOV;BTG2;TRRAP;EME2;CHRNA4;ACTR5;FSCN1;FANCC;FANCE;HINFP
BCL7A;TRRAP;BCL7C;ACTR5
CAMK4;EPHA2
SH3RF1;RNF122
CUL7;FAM174B;TANGO2;ARHGEF7
APC2;SPSB3;SMURF1;TBX21;FBXL19;KIF14;UBE2J2;RNF122;FBXL7;ANKZF1;FBXL12
FLOT1
NUMA1

KIF1A
LTF
LMNB1
TRRAP
TRRAP
TFRC
AQP11
PPM1F
RBM15B
MAP1S
TRRAP;FUS;SNRNP70
PNN;XAB2;SART1;SNRNP40;SNRNP70;RBMX;HNRNPA0
ATCAY;PPM1F
MTTP;FGFR4
BTG2;ATCAY;ZFYVE27;MICALL2
PHLDB1
PPP1R16B
IER5
SCRIB
SLC2A1
CPSF1
MYLK
CHD3
LEF1
EFNB1
MTTP;FGFR4
UBE2J2;ANKZF1
AQP4;RYR3
PM20D2;DHH;KHSRP;PROX1;HINFP
RTEL1
GALNT3
KCNJ3
MAN2C1
HRK
SZT2
MEOX1
FOXC1
TBX21
RTEL1
KL
MMRN2
SZT2
PCIF1
TNF;TRIB2
BRD2;NASP
MNT;THRA;MAZ;SNAPC4;RARA;RBMX
HINFP
RTEL1

NLGN2
SZT2
SMURF1
LEF1
PDGFB
NLRX1
OSBPL3
TRPC3
APC2;SPSB3;SMURF1;TBX21;FBXL19;KIF14;RNF122
TNRC6C;BTG2;PCIF1
ACTR5
IGF1R
SLC25A13
EXOSC10
RARA
FZD5
KL
KIF14
VTN
ACTR5;IER5
PCIF1;ERBB2;CIRBP
TRRAP;STK26;FBXO10;TNF;BCL2L2;BCAR1
TRIB2;SH3RF1;SMAD7
CUL7;SMPD4;FAM174B;TANGO2;ARHGEF7;LMNB1
STK24;STK26
DGKQ;TELO2
IGF2
ATP2A2
LTF
TOMM40
BIRC5
EXOSC10
SCX
RTEL1
AQP1;MAPK13
SPHK1;SMAD7
NOTCH1
HGFAC
PLA2G5
FOXC1;SMPD4;CLCF1;KHSRP;LEF1;AQP4;ARHGEF2;RBMX;CRLF1;MAPK13
NES;FBXL7;HINFP
RHBDF2;ELANE;GIT1
XAB2;MNT;THRA;UBTF;SCX;RBMX
KIF14;GIT1
CHST8;AQP11
CASP7;ATCAY;MAGI3;TAOK2;KREMEN1;MAL;BOK
RHBDF2
TRIB2

SLC7A1
TNF
VPS37D
TAOK2
EGR2
TRRAP;CHD3
CHRM3;PHEX
OSBPL5
TBX21
RARA
GNAO1
APC2;SPSB3;RNF167;SMURF1;TBX21;FBXL19;KIF14;VPS37D;KLHL3;FBXO10;RNF122;FBXL7
ATP2A3;FLOT1;ATP2A2
AQP1
NUMA1
ATP9A
MAN2C1
CTDSP2;DUSP15;DUSP26;PPM1F
KLF1
FAAH
TBKBP1
PLA2G5
ATP6V0A4
RBMX;MAPK13
STAB2;CAMP
PPM1F
FLOT1
LTB
IDH2
KIF14
RBM15B
SOX13
SMAD7
EXOSC10
OSBPL5
LMNB1
NOS2
BACE2;FGFR4
PNN;XAB2;SART1;SNRNP40;SNRNP70;RBMX
FAAH
ZDHHC18
NRXN2
UBE2J2;ANKZF1
IRS2;PCK2
NR2F2
TNF
SMPD4
SMAD7

SMAD7
SOX17;SLIT2;ESR2
SLC2A1
PLA2G5
APC2;TNF
SZT2;STK24;STK26
PDGFB
MAPKAPK2
PDGFB
FGF11
EGR2;P3H4
EXOSC10
FOXC1
DENND3
KLF1
PCIF1
MYLK
NOTCH1;NUMA1
FSCN1;PALB2
RNF167;SMURF1;KLHL3;FBXO10;FBXL7
RBM15B
KL
GPSM1;KDR
MYH7B;ATCAY;CXADR;TOMM40
FOXC1
ZCCHC3
TOMM40
AKNA;TNC
TRRAP
PHF1
RNF167;SMURF1;TRAF5;KLHL3;UBE2J2;FBXL7
RPS6KA4;FOXC1;CLCF1;TRAF5;PDGFB;TNF;CRLF1
ATP2A2
SMAD7
FAAH
CIRBP;DLGAP5;CDS2
CELF3
EGR2
SMPD4
ZDHHC18
PEX5
EME2;ACTR5;FSCN1;PALB2
SART1;EXOSC10
HINFP
PITPNM3
CUL7;SMURF1;FANCC;KLHL3;FBXO10;FANCE;UBE2J2;SH3RF1;RNF167;RNF225;TRIM47;RNF122;FBXL7
VPS37D;KLHL3
RBM15B

MAPK13
TECRL
RBM15B;CASP7;SNRNP40;EXOSC10;HNRNPUL1;PABPN1;TESC;RPS2
AQP4
CTU2
RASIP1
CHRNA4;AQP11
GPSM1;KDR
MAGI1;CARD10;EID2;CALY;SLC2A1;CASQ2;FANCC;DLGAP5;BOK
BFSP2
POLA2
PLA2G5
KHSRP;IRS2;TNF
KLHL3
FSCN1;PALB2
KIF14;DLGAP5
DHRS11
ZDHHC18;ELANE
AGBL5;TBKBP1;ZCCHC3
SPHK1;SMAD7
SART1;XAB2;CELF3
POLA2
SLC16A11
MAZ
AGBL5;TBKBP1;TNF;ZCCHC3
CD276
LDB1
ZDHHC18
FAAH
ANKZF1
PLA2G5
FA2H;FAAH
COL1A1;LMNB1
AQP4
ERBB2
CD177
NOTCH1;ABLIM3;WDR90;CEP131;CEP250
FA2H;SPHK1
NUMA1
NWD1
TRRAP;ACTR5
EGR2;XPO4;SMURF1;MYO6;VPS37D;ARHGEF2;CCHCR1;AP4B1
SLC7A1;RAMP1;AQP1
CACNA1H
EXOSC10
EPHA2
SLC25A13
HNRNPA0

EXOSC10
BTG2;PCIF1;ERBB2;CIRBP
EGR2;RNF225;CUL7;SMURF1;TRIM47;KLHL3;FBXO10;FBXL7;SH3RF1
NOTCH1;ABLIM3;WDR90;CEP131;CEP250
EXOSC10
RPS2
NEURL1B;TFRC;TANGO2;MTTP;MYO6;ARHGEF2;RAMP1
NOTCH1;ABLIM3;WDR90;CEP131;CEP250;ATP2A2;VPS37D
EXOSC10
DENND3
MYO1B;SORCS1;ATP9A
ITPR3
OTUD1
FZD5;SOGA1;RASIP1;BOK
EXOSC10
VPS37D;LMNB1
RPS2;GTPBP2
EXOSC10
RPS2
RPS2;GTPBP2

AM83H;DACH1;TNN;ERBB4;KDR;MGAT3;SRGAP3;SLIT2;AMH;NTNG1;JAG1;SPHK1;SEMA4F;PLET1;SRCIN1;
;CXADR;CADM4;MAG;LRFN4;PTK7;PLXNB3;CDHR3;CLDN19;DCHS1;ESAM;FAT4
SPOCK2;COL7A1;COL4A6;COL9A1;FLOT1;COL4A5;COL9A3;HMCN1;COL9A2;ADAMTS8;ELANE
I;MPZL1;KL;GALNT3;TIE1;IGF2;GAB1;NFATC4;VAV2;GIGYF1;DOK4;MAPKAPK2;COL4A6;EPHA1;ARHGEF7;F
L;NFASC;KIF5A;PLXNB3;RAC3;PLXNB1;SLIT2;ARHGEF40;LGR6

4A5;COL9A3;COL9A2;ADAMTS8
4A5;COL9A3;COL9A2;ADAMTS8
;GAB1;SEMA4F;PLET1;COL1A1;DAAM2;TRIP6;ITGA6;ARHGEF2;EPHA1;ARHGEF7;LGR6;BCAR1;EPHA2
EPHA1;FGFR4;EPHB4;MAP3K11;MAP3K12

A;ERBB4;BCL7C;ERBB2;KDR;AMH;ELANE;PRKCH;SPHK1;CEP131;IGF2;TBC1D8;PROX1;TBX3;TBX2;CLCF1;

LIT2;ARHGEF40;LGR6

DAAM2;SPOCK2;TRIP6;KDR;ITGA6;EPHA1;LGR6;BCAR1;EPHA2
A8;RAPGEF3

CK2;ERBB2;APC2;CHST8;KIRREL3;EGR2;SZT2;JAG1;FZD5;MAP1S;APLP1;SEMA4F;PROX1;SOX10;NFASC;A
1
N;SHANK3
A;SOX12;PROX1;DUSP26;SOX10;ETV5;HIC1;EID2;PSPC1;RARA;ERF;BIRC5;FLYWCH1;FOXC1;NOTCH1;ZBT
EFNB1;NFASC;KIF5A;TAOK2;SLIT2;ARHGEF40;LGR6

S;PTCH1;APLP1;SHROOM2;LSR;SOX12;PROX1;HSPG2;SOX10;NFASC;MAL;NES
;SOX10;HIC1;ETV5;RARA;ERF;FLYWCH1;FOXC1;NOTCH1;ZBTB49;MAZ;LEF1;GLIS2;ZBTB2;DLL4;MNT;TBX2

A2

N;SHANK3;RAMP1;EPHA2;PACSIN1

3;BRD2;PRKCH;CDC42BPB;SMAD7;MAPK13;MAPK10;SBK1;STK24;DGKQ;STK26;CAMK4;TAOK2;MAPKAPK

;SHANK3;LZTS3

;PATZ1;BEND5;ATF7IP;PHF12;SOX13;ARID5A;NR2F2;PROX1;TBX3;HINFP;TBX2;HEYL;EID2;PSPC1;ID1;RAF

L7A;ERBB4;BCL7C;SPOCK2;ERBB2;KDR;AMH;IER5;EGR2;SPHK1;CEP131;IGF2;TBC1D8;PROX1;DUSP26;T

NK2;CDC42BPB;DGKZ;MAPK10;STK24;DGKQ;STK26;CAMK4;TAOK2;MAPKAPK2;RARA;BIRC5;ERG;TNIK;TRI

2;LMO1;FOXD3;FOXD2;TCF15;ACTR5;TESC;SOX12;PROX1;POU3F1;SOX10;ETV5;MYCN;TET3;RARA;ITGA6;

HGEF7;AGRN;RAPGEF3;PRKG1
EPHB4;EPHA2

TF

GA6;SRGAP3;EPHA1;ARHGEF7;AGRN;RAPGEF3;EPHA2
M3;AMH;PRKCH;EGR3;UNC5B;SPHK1;MAPK8IP3;TBX3;NFATC4;MAG;CLCF1;BIRC5;ITGA5;CRLF1;BCL2L2;

RBB4;TBX21;SPP1;ATF7IP;AFAP1L2;EGR2;TESC;ACTR5;NR2F2;POU3F1;SOX10;KLF2;ESR2;HINFP;KLF1;CO

D2;TRIB2

YCN;PSPC1;DGKQ;ERF;ERG;ADNP;AJUBA;NOTCH1;KMT2B;MAZ;PDGFB;GLIS2;DVL2;SAFB2;ABL1;HOXC9;P
34;FTH1;BCL7C;ERBB2;KDR;ADAMTS8;AMH;IER5;ZBTB7C;BOK;CADM4;JAG1;JUND;TESC;CEP131;IGF2;TBC
S100A9;PPARGC1B;S100A8;LTF

IF26A;MMRN2;SPRY1;LTF

MH;ERMAP;ACTR5;TESC;SOX13;ATP1B4;PROX1;POU3F1;HIC1;PTP4A3;MYCN;PSPC1;DGKQ;CLDN19;ADN
C1;MYCN;DGKQ;ERF;FLYWCH1;ERG;ADNP;NOTCH1;AKNA;MAZ;GLIS2;ZBTB2;DVL2;SAFB2;ABL1;HOXC9;PA

APGEF3;PACSIN1

SP2;MYO1B;COL2A1;MYO6;RAC3;ARHGEF2;CAMP;BCAR1

FLOT1;BIRC5;CSPG4;ARHGEF2;TNIK;ITGA5;AMH;MAP3K12

KD2;TCF15;SOX12;PROX1;ETV5;MYCN;TET3;RARA;ITGA6;ARHGEF2;ERG;FOXC1;NOTCH1;AKNA;ZBTB49;MB

1;KDR;CTLA4;PIM3;PLEKHN1;SLIT2;AMH;BOK;EGR3;GADD45B;SPHK1;SCRIB;FBXO10;MAPK8IP3;TBX3;STK

AI3;ACTR5;SOX13;IGF2;ATP1B4;HIC1;ESR2;HINFP;KLF1;PTP4A3;PSPC1;TCF3;AJUBA

HA2;MAP3K12

MYCN;DGKQ;CLDN19;FGFR4;AMH

K;SRGAP3;ARHGEF40;TELO2;RAPGEF3

KCNQ2;ATP6V0A4;KCNK1;ASIC3

1;BOK

₹3;AMPH;BSN

RGAP3;ARHGEF7
RD9;PCIF1;CIRBP;POU3F1;HINFP;MYCN;DGKQ;CLDN19;FGFR4

IRC5;PIM3;AMH;BCL2L2;LTF

RAC3;BSN

.1;CTLA4;SFN;FLYWCH1;BCL2L2;EPHA2;BOK

;MMRN2;SPP1;FLOT1;AMH;PPARGC1B

ARHGEF2;JAM3;CD177;PACSIN1
ANE;MAP3K12

T2;DIP2A;AMH

9;AQP11;PLXNB1;ADAMTS8;ZBTB7C

MTS8;ZBTB7C;TESC;PROX1;PTPN14;ESR2;RNF167;MMRN2;PLXNB3;CLDN19;AQP11;PLXNB1

CD276

l;ASIC3

DOK4;TRIP6;TAOK2;TRAF5;PLXNB1;LTB;S100A9;S100A8;LTF

)6;KIF5A;AMPH;KIF1A;ATP9A;EXPH5

RKCH;CDC42BPB;DUSP26;MAPK10;STK24;STK26;CTDSP2;CAMK4;TAOK2;MAPKAPK2;RARA;BIRC5;AQP11

HGFAC

2;RAPGEF3;GIT1

EF7;AJUBA;PACSIN1

;SMAD7;COL1A1;STK24;DAAM2;KIF26A;STK26;TRIP6;SPATA13;MMRN2;PLXNB3;CLDN19;PLXNB1;ITGA6;L

GFR4;BCAR1;EPHA2

RARA;BIRC5;AQP11;EPHA1;FGFR4;NES;CRLF1;LTF

ADGRB2;KCNQ2;MAL;DCHS1;FAT4;NES;SHANK3;FGF11

TB49;MAZ;LEF1;PDGFB;PPM1F;GLIS2;ZBTB2;DLL4;MNT;RBM15B;TBX21;PATZ1;BEND5;ELANE;ATF7IP;CBX7

1;PATZ1;ELANE;CBX7;CBX6;JUND;PHF12;PTCH1;IGF2;NR2F2;KLF2;TBX3;ESR2;TBX2;SMAD7;BCL6B;MAFF;

2;RARA;BIRC5;ERG;TNIK;EPHA1;FGFR4;TRIB2;MAP3K11;MAP3K12

ɾA;BIRC5;ID3

BX3;CLCF1;RARA;BIRC5;AQP11;EPHA1;FGFR4;S100A9;CRLF1;S100A8

B2;MAP3K11;MAP3K12

;ARHGEF2;ERG;FOXC1;NOTCH1;AKNA;ZBTB49;KMT2B;MAZ;ZBTB48;LEF1;PDGFB;GLIS2;ERBB4;TBX21;ERB

_TF

L1A1;MYCN;CAMK4;RARA;TCF3;MAP3K12

ATZ1;PPARGC1B;ZBTB7C;EGR2;AFAP1L2;EGR3;JUND;FUS;PHF12;SNAI3;IGF2;NR2F2;ESR2;NFATC4;SMAD
C1D8;PROX1;PTPN14;TBX3;EID2;CLCF1;MMRN2;RARA;CLDN19;BIRC5;AQP11;ARHGEF2;EPHA1;FGFR4;CR

P;AJUBA;LTF;OPRD1;FOXC1;NOTCH1;DHH;ZBTB49;MAZ;LEF1;PDGFB;PPM1F;MLLT6;ZBTB2;DLL4;NWD1;DV
TZ1;PPARGC1B;ELANE;ZBTB7C;CBX7;EGR2;CBX6;JAG1;EGR3;JUND;FZD5;FUS;PHF12;PTCH1;IGF2;NR2F2;

EPCE;LEF1;GLIS2;TBX21;DVL2;ABL1;PPARGC1B;EGR2;JAG1;JUND;FZD5;IGF2;NR2F2;TBX3;KLF2;HINFP;HE[illegible]

‹26;BIRC5;ITGA6;ARHGEF7;RNF122;BCL2L2;MAP3K11;BCAR1;LTF

;ERG;TNIK;TRIB2;MAP3K11;MAP3K12

.GR6;EPHA1;BCAR1;EPHA2

';CBX6;JUND;PHF12;PTCH1;IGF2;NR2F2;TBX3;KLF2;ESR2;TBX2;NFATC4;HINFP;SMAD7;HEYL;BCL6B;ID1;M

ID1;FOSB;ID3;MAFK;MAGED2;TCF3;MXD1

B2;SPP1;DVL2;ABL1;PPARGC1B;ATF7IP;EGR2;AFAP1L2;JAG1;JUND;FZD5;IGF2;NR2F2;TBX3;KLF2;ESR2;AP

7;COL1A1;ZBTB9;ID1;MAFF;ID3;MAFK;TCF3;MXD1;MAP3K12;CSRNP3;LDB1;THRA;CHD8;PHF1;CHD3;GLI1
LF1

'L2;ABL1;PATZ1;PPARGC1B;CD276;ATF7IP;NOS2;FUS;SNAI3;IGF2;ESR2;APLN;HINFP;KLF1;BCL6B;TCF3;F[illegible]

ESR2;NFATC4;SMAD7;ZBTB9;ID1;MAFF;ID3;MAFK;MAGED2;TCF3;MXD1;TENM2;CSRNP3;LDB1;THRA;CHD[illegible]

YL;MAFF;TCF3;AGRN;RBMX

1AFF;ID3;FOSB;MAFK;MAGED2;TCF3;MXD1

LN;HINFP;KLF1;COL1A1;HEYL;CAMK4;MAFF;TCF3;AGRN;RBMX;MAP3K12

.;MEOX2;MEOX1;HEY1;BCL7A;SIX5;BCL7C;ANKRD2;CIC;FOXD3;BRD2;FOXD2;TCF15;ACTR5;TESC;PROX1;

GFR4
8;PHF1;CHD3;GLI1;MEOX2;MEOX1;HEY1;BCL7A;SIX5;BCL7C;ANKRD2;CIC;FOXD3;BRD2;FOXD2;TCF15;PI

LIME1;ETV5;PTP4A3;RARA;BIRC5;FOXC1;ZBTB48;LEF1;ZBTB42;PPM1F;MLLT6;MNT;RBM15B;ERBB4;TBX21

ROX1;DUSP26;LIME1;ETV5;TET3;RARA;ITGA6;ARHGEF2;FOXC1;ZBTB49;ZBTB48;LEF1;ZBTB42;MLLT6;DLL4;

.;SPP1;BEND5;ATF7IP;FBXL19;TBX3;KLF2;TBX2;HINFP;KLF1;HEYL;CAMK4;FOSB

;MNT;TBX21;FBXL19;TBX3;KLF2;TBX2;HINFP;KLF1;HEYL;BCL6B;FOSB;AGRN;RBMX

Term	Overlap	P.value	Adjusted.P.value	Old.P.value
TAF1 ENCODE	251/3346	7.65643E-24	7.96268E-22	0
BRCA1 ENCODE	221/3218	4.89335E-16	2.54454E-14	0
YY1 ENCODE	187/2753	5.82539E-13	2.01947E-11	0
UBTF ENCODE	126/1631	2.06276E-12	5.36316E-11	0
ATF2 ENCODE	182/2852	2.7456E-10	5.12237E-09	0
CHD1 ENCODE	63/655	2.95522E-10	5.12237E-09	0
MAX ENCODE	142/2073	4.20921E-10	6.25369E-09	0
CREB1 ENCODE	147/2238	3.47853E-09	4.52209E-08	0
TCF3 ENCODE	70/840	1.34838E-08	1.55812E-07	0
MYC ENCODE	104/1515	1.2064E-07	1.24368E-06	0
PML ENCODE	108/1596	1.37795E-07	1.24368E-06	0
CREB1 CHEA	100/1444	1.43502E-07	1.24368E-06	0
NFE2L2 CHEA	76/1022	3.51726E-07	2.81381E-06	0
ZNF384 ENCODE	59/730	5.43145E-07	4.03479E-06	0
NRF1 ENCODE	120/1882	6.04796E-07	4.18415E-06	0
USF2 ENCODE	72/965	6.43716E-07	4.18415E-06	0
MYC CHEA	49/573	1.03413E-06	6.32646E-06	0
USF1 ENCODE	91/1423	1.4489E-05	8.37145E-05	0
ZMIZ1 ENCODE	64/914	2.08408E-05	0.000114076	0
ZBTB33 ENCODE	52/702	2.92572E-05	0.000150996	0
ELF1 ENCODE	141/2483	3.04897E-05	0.000150996	0
BHLHE40 ENCODE	29/348	0.000252805	0.001195079	0
CEBPD ENCODE	50/734	0.000319076	0.001385611	0
TAF7 ENCODE	45/640	0.000319756	0.001385611	0
PBX3 ENCODE	77/1269	0.00035244	0.00146615	0
RCOR1 ENCODE	48/702	0.000384554	0.001538214	0
KAT2A ENCODE	11/90	0.001117495	0.004304425	0
E2F1 CHEA	54/859	0.00123239	0.004577448	0
SIN3A ENCODE	67/1131	0.00156232	0.005602804	0
PPARG CHEA	36/535	0.00258609	0.008965114	0
EGR1 ENCODE	36/537	0.002749848	0.009225296	0
FOS ENCODE	41/637	0.003033799	0.009859847	0
PPARD CHEA	22/285	0.003480241	0.010968033	0
E2F6 ENCODE	161/3245	0.004653242	0.014233447	0
RELA ENCODE	32/484	0.005616839	0.016137272	0
KLF4 CHEA	57/987	0.005789966	0.016137272	0
TCF7L2 ENCODE	37/583	0.005891955	0.016137272	0
GABPA ENCODE	108/2082	0.005979701	0.016137272	0
SIX5 ENCODE	62/1094	0.00613812	0.016137272	0
SALL4 CHEA	25/355	0.006292576	0.016137272	0
ERG CHEA	13/144	0.006361809	0.016137272	0
SPI1 CHEA	60/1056	0.006606152	0.01635809	0
RFX5 ENCODE	35/559	0.008864342	0.021439339	0
SPI1 ENCODE	81/1540	0.012071412	0.027920606	0
ETS1 ENCODE	13/156	0.012081032	0.027920606	0
BCLAF1 ENCODE	48/851	0.016015337	0.036208587	0
NR2C2 ENCODE	23/350	0.018201738	0.040276187	0

STAT3 ENCODE	41/725	0.023765501	0.051491918	0
FLI1 ENCODE	37/650	0.028078587	0.059595369	0
NELFE ENCODE	16/234	0.032081338	0.066729183	0
TP53 CHEA	20/319	0.040133776	0.081524157	0
NFYB ENCODE	172/3715	0.040762079	0.081524157	0
IRF1 ENCODE	17/263	0.043681585	0.085714807	0
TCF3 CHEA	52/1006	0.05075861	0.097380503	0
AR CHEA	56/1095	0.05164428	0.097380503	0
ESR1 CHEA	11/154	0.052435655	0.097380503	0
NFYA ENCODE	107/2250	0.056063018	0.102290419	0
SP1 ENCODE	37/707	0.07709061	0.138231438	0
SP2 ENCODE	50/994	0.078660563	0.138655908	0
ATF3 ENCODE	8/114	0.096590566	0.167423648	0
SOX2 CHEA	39/775	0.109212236	0.185312446	0
RUNX1 CHEA	62/1294	0.11199504	0.185312446	0
STAT3 CHEA	11/177	0.112256578	0.185312446	0
CEBPB ENCODE	9/144	0.137819555	0.223956777	0
ZBTB7A ENCODE	99/2184	0.153000842	0.244801347	0
HDAC2 ENCODE	10/175	0.182697843	0.287887509	0
YY1 CHEA	17/332	0.205694136	0.31928642	0
FOXA2 ENCODE	16/316	0.227228491	0.347525927	0
IRF3 ENCODE	31/663	0.249259802	0.375695933	0
HNF4A ENCODE	57/1276	0.266745024	0.393080174	0
ZEB1 ENCODE	6/106	0.268352811	0.393080174	0
FOSL2 ENCODE	10/196	0.284662549	0.411179237	0
EGR1 CHEA	15/315	0.312949842	0.445846351	0
FOXA1 ENCODE	10/205	0.332646844	0.467503673	0
NFIC ENCODE	13/279	0.358312912	0.496860572	0
NANOG CHEA	26/595	0.397264612	0.543625258	0
CTCF ENCODE	75/1790	0.438790526	0.592471818	0
BCL3 ENCODE	12/273	0.444353864	0.592471818	0
SRF ENCODE	13/299	0.454425457	0.596960449	0
E2F4 ENCODE	30/710	0.459200345	0.596960449	0
STAT5A ENCODE	10/233	0.486947182	0.625216135	0
TRIM28 CHEA	9/210	0.494127765	0.626698629	0
ZKSCAN1 ENCODE	5/120	0.549277313	0.688251091	0
TP63 CHEA	56/1399	0.595838444	0.732120161	0
FOXP2 ENCODE	7/178	0.598367439	0.732120161	0
SMAD4 CHEA	23/584	0.609384556	0.736930161	0
EZH2 ENCODE	13/338	0.632930049	0.756606035	0
CBX3 ENCODE	3/81	0.65080823	0.769136999	0
IRF8 CHEA	4/121	0.736141015	0.860209725	0
FOXM1 ENCODE	3/95	0.752895269	0.870012311	0
SUZ12 ENCODE	3/105	0.810020493	0.917722292	0
MYOD1 ENCODE	5/166	0.815493564	0.917722292	0
VDR CHEA	2/76	0.824136386	0.917722292	0
GATA2 CHEA	27/772	0.829479764	0.917722292	0
POU5F1 CHEA	8/261	0.843683873	0.923611819	0

ZC3H11A ENCODE	3/129	0.903195277	0.97846155	0
REST CHEA	43/1280	0.930371325	0.997511524	0
SMC3 ENCODE	38/1181	0.954721998	0.999950517	0
TRIM28 ENCODE	2/120	0.960065399	0.999950517	0
REST ENCODE	7/383	0.9960026	0.999950517	0
SUZ12 CHEA	47/1684	0.998803702	0.999950517	0
RAD21 ENCODE	32/1265	0.999243136	0.999950517	0
GATA1 CHEA	17/807	0.999470193	0.999950517	0
EZH2 CHEA	1/237	0.999950517	0.999950517	0

Old.Adjusted.P.value	Odds.Ratio	Combined.Score
0	2.292566104	122.0252627
0	1.99222205	70.23277022
0	1.912737844	53.88446458
0	2.132229743	57.37186012
0	1.763951017	38.83488435
0	2.613097576	57.33731693
0	1.870855732	40.38910982
0	1.785109608	34.76796446
0	2.231515152	40.43902737
0	1.829179353	29.13966494
0	1.803506705	28.49090007
0	1.843171296	29.04269697
0	1.968935416	29.25919251
0	2.138591272	30.85108072
0	1.694632723	24.26438512
0	1.971160122	28.10087492
0	2.26271027	31.18455394
0	1.67262736	18.6366108
0	1.825583567	19.67722738
0	1.930208333	20.15018516
0	1.492976435	15.52415209
0	2.167681876	17.95467411
0	1.755904914	14.13517549
0	1.813662239	14.5962657
0	1.563898398	12.43397815
0	1.761277749	13.8496798
0	3.287556133	22.34441991
0	1.609149733	10.7793722
0	1.514957713	9.789025284
0	1.719040121	10.24136719
0	1.711994379	10.0942779
0	1.641116213	9.515092766
0	1.982970735	11.22491061
0	1.275099441	6.847527615
0	1.682583891	8.719126541
0	1.465987401	7.552223058
0	1.612699348	8.279868583
0	1.322137222	6.768529078
0	1.438373116	7.325974766
0	1.796264532	9.104159743
0	2.342452018	11.84681631
0	1.441344325	7.235193953
0	1.587397287	7.501592781
0	1.331292588	5.880206589
0	2.14453081	9.470502606
0	1.422928268	5.882682116
0	1.663804251	6.665596093

	0	1.423207141	5.322111996
	0	1.431268868	5.113563004
	0	1.730978137	5.953666042
	0	1.57867893	5.07630051
	0	1.171482283	3.748746908
	0	1.629448511	5.101524118
	0	1.293555643	3.855667697
	0	1.279794809	3.792513021
	0	1.810116954	5.33652974
	0	1.193068008	3.43756167
	0	1.305484074	3.345660381
	0	1.254402377	3.189460236
	0	1.772841342	4.143616337
	0	1.251384791	2.771144286
	0	1.191592708	2.608754744
	0	1.55743369	3.40605788
	0	1.565556926	3.102636406
	0	1.125804488	2.113486113
	0	1.422745978	2.418556654
	0	1.267884323	2.004987878
	0	1.252404643	1.855812199
	0	1.153093966	1.6019468
	0	1.100720678	1.454560597
	0	1.406388206	1.850037169
	0	1.260719501	1.584032075
	0	1.172670807	1.362306159
	0	1.201962646	1.32296889
	0	1.145437944	1.175618651
	0	1.071051338	0.988743926
	0	1.025202027	0.844492887
	0	1.076533515	0.873212983
	0	1.064210882	0.839365885
	0	1.033134773	0.804056438
	0	1.04949344	0.755215078
	0	1.047849532	0.738693223
	0	1.017071219	0.609380094
	0	0.973510114	0.504069631
	0	0.957129396	0.491534056
	0	0.957773916	0.474390933
	0	0.934572491	0.427469131
	0	0.899256191	0.386266736
	0	0.798684431	0.244663863
	0	0.761854079	0.216236392
	0	0.686802506	0.144706357
	0	0.724726594	0.147816504
	0	0.631269411	0.122099653
	0	0.84251462	0.15751364
	0	0.737047061	0.125281352

	0	0.555283558	0.056537026
	0	0.8027361	0.057934667
	0	0.766822625	0.035530789
	0	0.394969127	0.016096521
	0	0.430595902	0.001724714
	0	0.651588706	0.000779961
	0	0.59108855	0.000447543
	0	0.492819648	0.000261168
	0	0.098011217	4.84999E-06

Genes
ATF1;EIF4A2;RPL4;RPL5;VPS29;TMEM41B;RPL32;GDE1;THUMPD3;EPRS;CYB5D2;UBE2L3;IKZF5;LSM10;PSI
EIF4A2;RPL4;SMARCAL1;VPS29;TMEM41B;GDE1;JMJD1C;THUMPD3;CYB5D2;IKZF5;TFG;RPL36AL;GPBP1;RF
ATF1;EIF4A2;RPL4;ANKRD13C;RPL5;VPS29;UHRF1BP1L;POGLUT1;RPL32;GDE1;CYB5D2;UBE2L3;IKZF5;PSI
NCKAP1;KDELR1;UBE2D3;SPPL2A;COL4A3BP;RPL36AL;GPBP1;KPNA4;EIF2A;AGFG1;MEF2A;RALGAPA1;DIC
EIF4A2;RPL4;ANKRD13C;SMARCAL1;RPL5;VPS29;TMEM41B;RPL32;UBE2D3;THUMPD3;PARK7;EPRS;BZW1;
ARF1;UHRF1BP1L;BMPR2;USPL1;ATP8A1;KDELR1;CLTC;PTEN;THUMPD3;HMGB1;EPRS;BZW1;LAPTM4A;MEI
ATF1;RPL4;RPL5;VPS29;POGLUT1;RPL32;GOLT1B;RFESD;UBE2D3;THUMPD3;SAYSD1;PARK7;EPRS;IPO7;IKZ
ANKRD13C;SMARCAL1;RPL5;GDE1;PARK7;EPRS;CYB5D2;LSM10;PSMD8;LAPTM4A;COL4A3BP;GPBP1;NAMI
ATF1;EIF4A2;WDR47;UBE2D3;PTEN;AFF4;RAB22A;TUBB6;ZMYM2;LAMP1;RPL36AL;GPBP1;NAMPT;RPL35;IT
ATF1;EIF4A2;RPL4;RPL5;POGLUT1;RPL32;RFESD;THUMPD3;PARK7;EPRS;IPO7;NAMPT;PAPOLA;RPS11;RPL3
ATF1;EIF4A2;SPPL2A;BZW1;HDDC3;CYB5D2;IKZF5;PSMD8;RNF115;RPL36AL;PAPOLA;RPL35;NBN;EIF2A;RP
EIF4A2;ANKRD13C;EPRS;EFR3A;COL4A3BP;GPBP1;PAPOLA;MAP3K7;CHID1;RALGAPA1;ATG12;RALGAPB;TI
ATF1;RPL5;USPL1;EIF4E3;CLTC;SNX13;ZBTB20;SRA1;RORB;SLC4A4;CDH9;AACS;EFR3A;RNF115;SENP5;SU
VPS29;WDR47;CAB39L;CLTC;SPPL2A;AFF4;CDC73;TFG;AP1G1;AKT2;LAMP2;UBXN4;ARL5A;RPS10;TNPO3;I
RPL5;TMEM41B;SAYSD1;PARK7;TMEM141;LAPTM4A;TFG;AKT2;CHORDC1;NAMPT;PAPOLA;VPS35;ATG3;CO
ATF1;POGLUT1;USP31;PHF20;RFESD;HEXA;CLTC;PTEN;THUMPD3;AFF4;IPO7;LAPTM4A;RNF115;MED13;LAI
RPL4;RPL5;RPL32;THUMPD3;IPO7;ARHGAP35;RPL7A;LAPTM4A;RPL36AL;PPP6R3;RPL35;RRP12;RPS11;MAI
ATF1;NCKAP1;USPL1;POGLUT1;USP31;PHF20;RFESD;HEXA;UBE2D3;CLTC;PTEN;SNX13;BMI1;AFF4;IPO7;R
ARF1;BMPR2;CAB39L;SNX13;SYNCRIP;UBL5;EFR3A;SENP5;TFG;RPL36AL;KPNA4;SNAP29;JAK1;MEF2A;CCT
RPL4;ANKRD13C;EIF4A2;VPS29;SMARCAL1;TMEM41B;CAB39L;BZW1;IKZF5;RPL7A;SEPT7;LAMP1;GPBP1;NI
RPL4;TMEM41B;RPL32;GOLT1B;SPPL2A;GDE1;BZW1;CYB5D2;UBE2L3;THG1L;PSMD8;RNF115;IPO8;TFG;LAI
CAMK2D;CLTC;HEXA;UBE2D3;VPS26A;GABARAP;ARHGAP12;SOCS2;SYNCRIP;FAM83G;HSPH1;LAMP1;NAM
EIF4A2;PTEN;HNMT;AFF4;SYNCRIP;KIF21A;TMED5;CAST;ARHGEF12;ANXA4;ARRDC3;ASH1L;MFSD11;RAD2
RPL4;RPL5;DDX6;RPL32;RPL10;CLTC;SAV1;UBE2L3;PSMD8;SEP15;SEPT7;MAT2A;PRDX1;PSMB1;RPL35;GF
EIF4A2;RPL4;GSK3B;USPL1;PHF20;CLTC;SPPL2A;PTEN;CHD6;ZBTB20;JMJD1C;AFF4;ELAVL1;AACS;LAPTM4
INO80C;NCKAP1;ARF1;USPL1;CBFB;USP31;PTEN;TFB1M;TM9SF3;EFR3A;SENP5;SCRN3;WDR7;JAK2;YAF2;S
RPL4;RPL5;GOLT1B;RPL32;TMEM258;RPS8;CLTC;RPL24;CALM1;TROVE2;CALM2
VPS29;USPL1;PHF20;SPPL2A;TUBB6;GPBP1;PPP6R3;LAMP2;PAPOLA;GNPNAT1;NBN;SNAP29;RPL39;LRRC
RPL4;USPL1;GOLT1B;HEXA;CLTC;RSF1;HMGB1;BZW1;CYB5D2;IKZF5;SYNCRIP;UBL5;RNF115;LAMP1;CHMP
INO80C;GTF2A1;UHRF1BP1L;ABCD2;CAMK2D;ROCK2;PVT1;HSPB8;PON2;PTEN;MGST1;SNX10;HSPA13;PPI
DCUN1D3;CLTC;ZCCHC9;VPS26A;UBFD1;BZW1;CDC73;IPO8;LAMP1;WDR7;MAPK6;RMRP;B3GALNT2;CD16
SCARNA13;GSK3B;VCP;RPN2;GOLT1B;DCTN4;HEXA;CHD6;ZBTB20;HMGB1;NLK;AFF4;TTBK2;LAPTM4A;ZMYI
MEF2A;SLMO2;CAMK2D;SMOX;EPT1;OSGIN1;TUBD1;ACSL3;THY1;EPRS;SLC4A4;CYB5D2;EFR3A;ZEB1;QPC
ANKRD13C;VPS29;GOLT1B;RFESD;UBE2D3;THUMPD3;SAYSD1;IPO7;TMEM141;THG1L;RNF115;COL4A3BP;
INO80C;EIF4A2;RPL5;GSK3B;CAMK2D;POGLUT1;CLTC;SPPL2A;PTEN;PARK7;LMAN1;RPL7A;AP1G1;GPBP1;
EIF4A2;RPL5;TMEM41B;UBE2D3;CLTC;SNX10;AACS;RPL7A;LAPTM4A;SENP5;SGCB;SUMO2;METTL16;RPS1
ATF1;INO80C;ITGB1;IDI1;SEMA3C;TMEM41B;ROCK2;SPPL2A;PSMD8;SOCS2;PCNP;LMAN1;AKT2;PSD3;MET
RPL4;RPL32;SPPL2A;THUMPD3;IPO7;THG1L;PTAR1;TFG;RPL36AL;RPL35;MAP3K7;EIF2A;PIH1D1;MFSD11;A
SMARCAL1;RPL32;USP31;GDE1;IPO7;LSM10;ARHGAP44;IPO8;COL4A3BP;NUDCD1;WAPAL;CEP170;POLK;
ITGB1;SH2D4B;CLTC;PRSS23;ADIPOR2;ARHGAP12;CST3;SYNCRIP;FGD4;GNPNAT1;JAK2;DRAM2;SLC38A2;
RANBP2;IDI1;CD164;TTC37;ELL2;SOD1;MIB1;EFR3A;TTLL4;LAMP1;PSMA2;NAMPT;POLR2J
GSK3B;USPL1;SPPL2A;JMJD1C;THUMPD3;SRA1;SNX10;SYNCRIP;IPO8;TFG;LAMP1;CHMP1B;TLK1;KPNA4;IT
DDX6;IDI1;GSK3B;VCP;CCDC186;DERL2;RAB40B;PPP6R3;EMC2;BPNT1;RAB6A;XBP1;DUSP3;SORT1;ARRD
RPL4;SMARCAL1;ACSM3;PHF20;CPQ;HEXA;SPPL2A;GDE1;SRA1;SNX10;HMGB1;TFB1M;SYNCRIP;LAPTM4A;
CLTC;HEXA;NFATC3;AFF4;LAMP1;PSMB1;RPL35;ARIH1;RPS11;UBXN4;CALM2;CRK;SNX5
VPS29;USPL1;RPL10;RFESD;CLTC;THUMPD3;TROVE2;CDC73;SAV1;APOO;NUDCD1;PRDX1;PSMB1;ZKSCAN
TIMM8B;RWDD1;NEK9;TTC37;TMED10;ZFR;KNOP1;EPT1;TTC9C;CWC15;IKZF2;RPS26;RPS25;UBL5;PTAR1;T

ANKRD13C;USPL1;RPN2;DCUN1D3;HEATR1;GDE1;COX7A2;HSPA13;AFF4;TROVE2;CYB5D2;PCNP;LAPTM4
SCARNA13;NCKAP1;TMEM87A;UBA6;CLTC;ZCCHC9;THUMPD3;SRA1;COX7A2;THG1L;PSMD8;IPO8;PSMB1;
EIF4A2;HSPA8;RPL10;NUFIP2;UAP1;MFSD11;TUBB4B;EEF2;MAT2A;PRDX1;MALAT1;CALR;STT3B;CALM1;RP
CCBE1;CAMK2D;ARHGEF12;TMEM30A;BNC2;RRAD;DCUN1D3;RPS27L;RORA;TUBB4B;EDA2R;TUBB6;FER;P
VPS29;ACSM3;GOLT1B;RFESD;UBE2D3;GDE1;ZBTB20;THUMPD3;THG1L;LAPTM4A;DEPTOR;TFG;RPL36AL;R
B3GALNT2;SMARCAL1;DCTN4;CLTC;UBFD1;DNAJA1;DYNC1LI1;PSMA3;FER;PSMA2;NAMPT;PSMB1;G3BP2;
EIF4A2;WDR47;HSPB8;SNX13;SNX10;BMI1;SYNCRIP;KIF21A;METTL15;DST;GPX4;RPS8;ZFR;CPED1;TWSG1
WDR47;ATP8A1;BNC2;GOLT1B;CPQ;CHD6;F13A1;RORA;IKZF2;GHR;PTER;PPP6R3;TMEM38B;WLS;NUDT12;
XBP1;DEPTOR;SEMA3C;GNPNAT1;TOP1;MPDZ;ESR1;CTSD;EBAG9;FREM2;CAP2
VPS29;ACSM3;GOLT1B;GDE1;ZBTB20;LAPTM4A;RPL36AL;KPNA4;VPS35;PIH1D1;SACM1L;ARRDC3;DICER1;
SCARNA13;INO80C;GSK3B;VCP;RPN2;GOLT1B;DCTN4;PTEN;CHD6;ZBTB20;NLK;TTBK2;ZMYM2;PPP6R3;VP
PHF20;CHD6;ZBTB20;THG1L;LAPTM4A;RNF115;ZMYM2;RPL36AL;PPP6R3;VPS35;WDR7;CCT3;ATP6AP1;TFB
ATG3;GNPTG;LAMP1;CNOT1;SYNRG;DPH3;EMC3;SNAP29
SMARCAL1;NUFIP2;SH2D4B;NDUFB4;UBE2D3;PTEN;SNX10;RSF1;ADIPOR2;ARHGAP12;SOCS2;LAPTM4A;C
SMARCAL1;SCOC;POGLUT1;PHF20;CLTC;PTEN;THUMPD3;EPRS;AFF4;BZW1;LAMP1;GPBP1;KPNA4;CTSD;Z
SOCS2;ITGB1;GTF2A2;NAMPT;ZFR;ALG14;KPNA4;NUP153;IL6ST;AGFG1;NUDT12
CAST;SYNCRIP;GPX4;BUD31;ETF1;LSM14A;OGT;RAD23B;RC3H2
NCKAP1;TMEM41B;KDELR1;TMEM141;IPO8;DEPTOR;AKT2;MEF2A;S100A1;RPS8;CASD1;TMEM170B;ATG12
USPL1;TTLL4;P4HA1;RAB14;NDUFAF4;UBFD1;ESYT2;CALR;ZHX1;EBAG9
RPL4;MTMR10;TMED10;EXOC7;DCTN4;ACTR3B;COL4A3BP;DEPTOR;ZC3H11A;CAPRIN1;NMT1;PCGF1;CCD
UHRF1BP1L;NAA20;PIH1D1;DCUN1D1;NR1H3;CASD1;SGPP1;PIK3CA;PSME4;MDM2;RICTOR;ATF6;EPS15;
SCARNA13;GSK3B;VCP;RPN2;GOLT1B;PHF20;DCTN4;CHD6;ZBTB20;NLK;TTBK2;ARNTL;LAPTM4A;ZMYM2;R
MTMR10;UHRF1BP1L;SLC46A1;USP31;SPPL2A;PTEN;HNMT;SYNCRIP;LAPTM4A;NUDCD1;AKT2;KIF21A;ARL
GOLPH3;AP1G1;SCRN3;MPZL3;TMEM141;SAV1
FAM83G;TUBB6;GPX4;KDELC2;PSME4;RNASE4;ANG;JAK2;CAP2;THOC6
SMAD3;CLTC;ERLIN1;ZBTB20;HOOK3;ESR1;SOD1;ZMYM2;RAB40B;NDUFAF4;EMC2;GNPNAT1;RICTOR;TPT1;
PCNP;UHRF1BP1L;AKIRIN1;PIH1D1;RPL7L1;NINJ1;PSME4;NBR1;MDM2;PCMTD1
GPX4;CLTC;SLC39A10;NUP153;CDC73;THOC6;LYRM2;LAMP1;NBR1;WDFY1;MDM2;PKN2;NOP10
EIF4A2;DCUN1D3;SNX10;ADH7;BMI1;CST3;FGD4;SYNCRIP;DST;ZFR;OTOP1;TUBB4B;TDRD3;TWSG1;UFM1;
ATF1;NCKAP1;RPL5;RPL32;CELF1;KDELR1;RSF1;PARK7;TMEM141;HDDC3;CDC73;GHR;MED13;DEPTOR;ZM
GSK3B;LAMP1;GPX4;SYNRG;NDUFAF4;MTERF4;SRA1;MALAT1;CALM1;GABARAP;CDC73;TSPAN31
RWDD1;RPL32;RPL10;NUP153;MAPK14;DDAH1;WDR60;CNOT1;CHMP1B;EMC3;RPL24;TCEA1;KDM7A
EIF4A2;VPS29;GTF2A1;GOLT1B;DERL2;HMGB1;TROVE2;SUMO2;METTL4;EMC3;VPS35;MAP3K7;SNX5;TMED
SOCS2;LAPTM4A;GDE1;EPT1;GPCPD1;SEC22B;MAPK14;EPS15;UBE2L3;THG1L
GSK3B;ARL4A;BMPR2;HTRA1;GGH;ITGAV;ACSL3;TOP1;SOD1
SYNRG;CBFA2T2;SEC22B;ELAVL1;UBE2L3
SLC46A1;POGLUT1;USP31;CPQ;SPPL2A;AACS;RAB22A;ARHGAP35;MEDAG;LAMP1;PSD3;TLK1;RRP12;METT
UBE2W;UBA6;PIH1D1;TCEA1;ARIH1;TROVE2;PCMTD1
ZHX2;BNC2;DST;SLC35F3;CPQ;CELF1;SMOX;ANXA4;SACM1L;HSPB8;LGI2;SNX13;TDRD3;FGD4;TWSG1;PP
GSK3B;SHC2;PCOLCE2;SLC30A4;SLC4A4;HDDC3;CNOT1;CDC27;CHMP4C;RGL3;SVEP1;MPND;KPNB1
LAMP1;ZFR;NOP10
TMEM87A;USO1;RNASE4;VPS26A
SCARNA13;TUBB4B;CALM2
EYA4;OTOP1;BMI1
MUSK;USO1;CACNG1;LSM14A;PRKAG3
ABCD2;SERINC2
INO80C;SLC35F3;HSPA13;ARHGAP12;MAT2A;PCGF1;MFSD1;MEF2C;ZHX2;COG6;SYNRG;C1GALT1;MITF;SC
SOCS2;DST;NUFIP2;CLTC;BMI1;IL6ST;ADIPOR2;OGT

SOCS2;DUSP3;ARRDC3
SMARCAL1;TPH2;PTEN;CHD6;RORA;THY1;ADRA1A;ARHGAP35;TM9SF3;ARNTL;EFR3A;PSD3;PAPOLA;SC5D;
TPH2;SEMA3C;KDELR1;RRAD;ZCCHC9;CREM;PTEN;VPS26A;CDC73;SOCS2;APOO;MED13;DEPTOR;NBN;JAK
SACM1L;AURKAIP1
TPH2;TAOK1;TMED2;NMBR;CASD1;GDAP1;JAK1
POPDC3;GABRB2;CAMK2D;EIF4E3;SEMA3C;RRAD;PON2;HTRA1;HR;RORA;THY1;RORB;AEBP1;BMI1;CXCL14
ATF1;TPH2;ZCCHC9;MRGPRG;HDDC3;CDC73;SOCS2;MED13;DEPTOR;ZMYM2;NBN;CD164;ARHGEF12;SCA
ATF1;INO80C;SMAD3;AMHR2;ATP8A1;SH2D4B;NUP153;MITF;ELAVL1;RCL1;DAB2;RAP1A;STIM2;CAPZA2;ZM
CXCL14

MD8;RNF115;RPL36AL;GPBP1;CHORDC1;NAMPT;RPL35;KIF21A;RPS11;RPL39;RPS10;EIF2A;MEF2A;PIH1D
PL35;KIF21A;RPS11;EIF2A;MEF2A;PIH1D1;RNASE4;MFSD11;TMEM170B;RC3H2;ATG12;NINJ1;CRYZL1;RPL
MD8;PTAR1;IPO8;COL4A3BP;DEPTOR;GPBP1;PAPOLA;KIF21A;CEP97;RPS11;EIF2A;RPS8;RPGRIP1L;CASD
ER1;ACSL3;CASD1;MFSD11;ATG12;TWSG1;STIM2;TBL1XR1;IL6ST;EIF1B;KPNB1;DDX6;VCP;CBFB;DCUN1
HDDC3;CYB5D2;THG1L;PSMD8;LAMP1;GPBP1;RPL35;NBN;RPS11;MAP3K7;EIF2A;MEF2A;RALGAPA1;RPS
D13;RPL36AL;AKT2;NAMPT;SUMO2;ARIH1;RPL39;PBRM1;GPX4;RPS8;MFSD11;GPCPD1;ATG12;MORF4L2;
ZF5;THG1L;LAMP1;RPL36AL;GPBP1;NAMPT;PAPOLA;RPL35;NBN;RPS11;RPL39;RPS10;ATG3;AGTPBP1;RPS
PT;PAPOLA;RPS11;MAP3K7;EIF2A;ATG3;ACOT9;RALGAPA1;RPS8;SACM1L;RPGRIP1L;MFSD11;TMEM170B;
GAV;JAK2;YAF2;RPS10;NCK1;MEF2A;ZHX2;GPX4;SLC39A10;ACSL3;CASD1;MFSD11;RAD23B;ARL4A;ZEB1;
9;RPS10;EIF2A;RPS8;MFSD11;ATG12;THOC6;PPA1;TBL1XR1;MORF4L2;RPL7L1;UBE2V2;NHP2;RPL24;NU
S8;PIH1D1;ARRDC3;ACSL3;MFSD11;THOC6;RPL7L1;RPL24;AURKAIP1;PPIA;INO80C;DDX6;IDI1;ZCCHC9;
MEM258;MORF4L2;RFX5;ULK2;RPL24;NUP98;PPIA;KPNB1;IDI1;VCP;DCUN1D3;CREM;NLK;ACTR3B;TTBK2;
MO2;CHORDC1;WAPAL;CEP170;YAF2;METTL15;BUD31;GPT2;SLC30A4;SLC39A10;GPCPD1;CGRRF1;MAF
PBRM1;ARRDC3;AHSA2;DNAJC3;AFTPH;PSMA3;RRM2B;PSMA2;STIM2;TBL1XR1;MORF4L2;NHP2;RPL24;BIF
G6;PIH1D1;ARRDC3;MFSD11;ZFYVE16;TMX3;RALGAPB;PPA1;TMX1;STIM2;RPL7L1;PPIA;INO80C;PFKFB4;C
MP1;NPTN;MFSD1;VPS35;UBXN4;YAF2;SNAP29;CTSD;ATG3;ATP6V0B;DUSP3;GAA;ARRDC3;MFSD11;RC3H
P3K7;EIF2A;CHID1;RPS8;RAD23B;TBL1XR1;NHP2;YME1L1;ETF1;EEF1E1;PPIA;KPNB1;TMED10;RPL10;RPS
AB22A;RNF115;MED13;LAMP1;NPTN;MFSD1;VPS35;YAF2;SNAP29;CTSD;ATG3;TTC37;ATP6V0B;DUSP3;TM
3;ACOT9;PBRM1;ATP6AP1;GPX4;S100A1;RPS8;ARRDC3;DNAJC3;PPIA;CRK;MPND;INO80C;TMEM87A;DDX
PTN;TMED2;METTL16;RPS11;MAP3K7;PCMTD1;MKL2;HSBP1;PIH1D1;SACM1L;RPGRIP1L;RC3H2;RPL24;N
MP1;RPL36AL;CHORDC1;RPS11;MAP3K7;RPS10;EIF2A;MEF2A;ATG3;PIH1D1;SACM1L;ALG14;ATG12;ARM
PT;NPTN;MAPK6;SLC38A2;ATG3;CD164;ARFGEF2;AGTPBP1;SYNRG;ANXA4;BNIP3;ERLIN1;ZC3H11A;RFX5
3B;RC3H2;TMBIM4;PIK3CA;TAX1BP1;RPL24;MALAT1;ETF1;NUP98;GTF2A1;DDX6;PFKFB4;RPL10;CBFB;NU
PBP1L1;MAPK6;SLC38A2;ARL5A;RPS10;TNPO3;SNX5;HSPA8;RPS8;SYNRG;PTGES3;ARRDC3;SLC39A10;AS
A;ZMYM2;AP1G1;RPL36AL;GPBP1;PPP6R3;VPS35;RPS11;NCK1;MEF2A;CCT3;CAST;ARHGEF12;ATP6AP1;P
ERF2;TNPO3;NEK9;AKIRIN1;ZFHX2;ARHGEF12;TMEM30A;PDPK1;GAA;BNIP3;MTERF4;OSGIN1;TRAPPC8;U

C1;ACOT9;ANXA4;ZDHHC13;RNASE4;CASD1;CBFA2T2;RAD23B;PSMA1;TMEM258;CRYZL1;NUP98;EXOC5;
1B;TMED2;MFSD1;VPS35;RRP12;METTL15;ATG3;PBRM1;BUD31;ARRDC3;TFB2M;MFSD11;ZHX1;THOC6;SL
P6R3;POLR2J;RANBP2;MKL2;XBP1;EPT1;SLC39A10;RNASE4;DNAJC15;SOD1;VMP1;RCL1;DYNC1LI1;TMX3
4;HSPA8;GPX4;ERCC6L2;HSPA4;DICER1;CASD1;TMEM170B;DNAJC15;MARCH5;RAD23B;ITFG1;THOC6;D
M2;LAMP1;KDELC2;PPP6R3;NPTN;CCT3;GPX4;EPT1;VWA5A;TUBB4B;THOC6;PNRC2;ARMC1;FER;CNOT1;N
T;MFSD1;SVEP1;MALAT1;CEPT1;STT3B;SLC38A4;FKTN
TFG;LAMP1;RPL36AL;NAMPT;PAPOLA;KPNA4;NBN;RPS11;MEF2A;ATG3;AGTPBP1;PIH1D1;GPT2;SACM1L;F
GPBP1L1;NBN;RMRP;CD164;NUP153;EEF2;PNRC2;P4HA1;CNOT1;RPL7L1;RFX5;NINJ1;YME1L1;MALAT1;IL
1;PCMTD1;AGFG1;ACOT9;DST;IGFBP5;RPS8;SACM1L;EN2;ARL4A;TBL1XR1;CRYZL1;SMCR8;ETF1;IL6ST;PP
TL16;MAP3K7;SLC38A2;YAF2;AGTPBP1;SMAD3;ARHGEF12;SMURF2;HSPA4;IDH1;TMEM192;GGH;SGPP1;
TG12;TMX3;TMEM258;YME1L1;WDPCP;AURKAIP1;INO80C;TMED10;NPRL2;ZCCHC9;UBFD1;COX7A2;VCP
CEP97;RPS11;UBXN4;TMED5;EIF2A;CCT3;TTC37;ATP6V0B;HSBP1;GPT2;ZFR;ALG14;MITF;RPGRIP1L;MFSD
PTGES3;SMAD5;OXR1;PNRC2;TOX4;DDAH1;TBL1XR1;CPD;IL6ST;CPEB2;OGT;MBTPS2

GAV;RRP12;RPS11;SNAP29;TNPO3;JAK1;EXOC7;SLC25A16;TOX4;TMEM33;TMX1;TAX1BP1;FAM96B;SMCF
C3;TTC9C;GPCPD1;PNRC2;ARMC1;TM9SF2;RCL1;COPS3;AIMP2;CNOT1;MORF4L2;CPD;RFX5;NHP2;UBE2
NUDCD1;TFG;LAMP1;RPL36AL;CHMP1B;KPNA4;ITGAV;RRP12;RPS11;ARL5A;RNF111;TMED5;ATP6V0B;ARI

N8;CEP170;GPBP1L1;NBN;SLC38A2;ARL5A;UGGT1;TNPO3;NUS1;TMEM30A;API5;ERCC6L2;RPS8;MTERF4
TMBIM4;WDR60;RPL35;YME1L1;TCEA1;WDR7;EBAG9;TAF2

A;TFG;CHMP1B;NAMPT;DNAJB9;TMED2;BPNT1;TNPO3;CD164;RALGAPA1;ALG14;RNASE4;ACSL4;TTC9C;AI
CHORDC1;DPH3;DNAJB9;RRP12;RAB6A;RPS11;UGGT1;CHID1;BTF3;HSPA4;TFB2M;MAPK14;RAB11A;ARCI
L39;CALM2
2RY6;RRM2B;SGCB;MDM2;CRELD1;ASCC3;CPEB2
PL35;KPNA4;VPS35;CEP97;MAP3K7;ACOT9;S100A1;PIH1D1;SACM1L;ARRDC3;DICER1;ACSL3;MFSD11;T
NBN;TMED7;SEC22B;LRRCC1
;DDAH1;TBL1XR1;ZC3H11A;CRYZL1;UBE2V2;LCOR;SMCR8;RHOU;IL6ST;RAI14;PCOLCE2;NUFIP2;DCUN1I
MKL2;MEF2C;SCARA5;ACSL4;ARMC1;TOM1L1;AR;ARL4A;MAF;PSMA1;KIF2A;STIM2;TBL1XR1;RHOU;ATF6;S

;RC3H2;ARMC1;MORF4L2;RFX5;CRYZL1;NHP2;UPRT;WDPCP;NUP98;IL6ST;PPIA;KPNB1;IDI1;VCP;TMED10
S35;BPNT1;EPS15;GPX4;ATP6AP1;TUBB4B;TM9SF2;DRAP1;ARL4A;CNOT1;MORF4L2;CNEP1R1;CRYZL1;NB
32M;TOX4;ARL4A;RPL7L1;CRYZL1;UPRT;NUP98;IL6ST;PPIA;MPND;SCARNA13;VCP;RPN2;RPL10;DCTN4;RP

HORDC1;ITGAV;METTL16;SLC38A2;METTL15;PCMTD1;SLC38A4;DST;PLEKHA1;GPT2;ARRDC3;VWA5A;NF
HX2;ATP6V0B;ARHGEF12;PIH1D1;AHSA2;RNASE4;ACSL3;ZHX1;PSMA3;AFTPH;PSMA4;TMBIM4;NINJ1;TAX1

;THOC6;DYNC1LI1;TWSG1;ZFYVE16;STIM2;TBL1XR1;WDFY1;UBE2V2;KPNB1;XPR1;RAI14;DDX6;TPH2;SH

C43;SMCR8;NAA15;EEF1E1;METTL16
UBE2K;CRK;PCMTD1
AB40B;VPS35;RWDD1;SORT1;TUBB4B;RCL1;AIMP2;ARL4A;FER;CNOT1;NBR1;NMT1;INTS4;HNRNPC;CALR;I
5A;RMRP;TMEM150A;SCARA5;ZFR;AHSA2;GPX7;ACSL3;IL17RE;GPCPD1;SLC25A16;MAF;NINJ1;UBE2V2;FA

;NUDT12

DDAH1;TBL1XR1;NBR1;LCOR;TCEA1;SMCR8;IL6ST;OGT;KPNB1;MBTPS2;RAI14
IYM2;SEPT7;NBN;AGFG1;CCBE1;ZHX2;ARHGEF12;SCARA5;S100A1;GAA;ARRDC3;TOX4;CPED1;ARL4A;AF

05;LRRCC1;HSPA8;PIH1D1;PTGES3;NUP153;TTC9C;MFSD11;NET1;COPS3;TMEM258;WNK1;TMX1;YME1L

TL16;WLS;DST;MUSK;COG6;SACM1L;SLC39A10;TGFBR1;ZEB1;SLC7A8;RNF128;TBL1XR1;FEZ1;LCOR;ETF1

P6R3;ULK2;RAB6A;SEC22B;MAP3K7;SLC38A2;CALM2;DDR2

ORBS2;MAPK14;ELL2;RAB11A;CNOT7;PIK3CA;PPA1;TBL1XR1;TMEM56;QPCT;NINJ1;CAPZA2;ATF6;CALM2

CACNG1;MAP3K7;DLGAP4;SLMO2;AGTPBP1;API5;HSPA4;ANXA4;TRAPPC8;SYT9;GPCPD1;RAD23B;GDAP1
K2;AGFG1;XBP1;ARHGEF12;SCARA5;SORT1;ARRDC3;EPT1;NR1H3;EEF2;MAPK14;ELL2;MARCH5;CPED1;A

;SLC4A4;PRSS23;ADRA1A;DPP4;DUOXA1;SVEP1;DUOX2;WLS;CCBE1;ZHX2;IGFBP5;SLC30A4;EYA4;LGI2;
RA5;IGFBP5;SORT1;IDH1;ARRDC3;EEF2;MAPK14;MARCH5;CPED1;ARL4A;AFTPH;MAF;WNK1;NDUFAF4;MC
AT5;LCOR;TLK1

)1;ARRDC3;ACSL3;MFSD11;ATG12;ARMC1;ZFYVE16;PSME4;RPL24;YME1L1;WDPCP;AURKAIP1;TMEM87A;
24;YME1L1;WDPCP;NUP98;AURKAIP1;EIF1B;RPN2;NPRL2;DCUN1D3;MTMR6;SEP15;HSPH1;PRDX1;DPH3
1;MFSD11;ATG12;DYNC1LI1;ZC3H11A;RPL7L1;WDFY1;UBE2V2;RPL24;YME1L1;MALAT1;EIF1B;PPIA;XPR1;
D1;UAP1;SAV1;ARHGAP12;RAB6A;SNX5;XBP1;NDFIP2;SMAD3;SMURF2;SYNRG;IDH1;NFATC3;NUP153;TR
8;PIH1D1;SACM1L;ARRDC3;MFSD11;RC3H2;ATG12;ARMC1;STIM2;ZC3H11A;MORF4L2;RPL7L1;RFX5;RPL
YME1L1;CHMP3;BIRC6;PPIA;GTF2A1;DDX6;CD151;RPN2;RPL10;DCUN1D3;DCUN1D1;RPS27L;CDC42;MA
8;ATG12;THOC6;TMEM258;PPA1;ZC3H11A;MORF4L2;RPL7L1;UBE2V2;NHP2;NUP98;ATF6;PPIA;KPNB1;IN
ATG12;THOC6;MORF4L2;RPL7L1;RFX5;UBE2V2;NHP2;RPL24;YME1L1;WDPCP;NUP98;AURKAIP1;ATF6;IDI
TBL1XR1;BIRC6;ETF1;MALAT1;RHOU;EXOC5;IL6ST;PPIA;KPNB1;GTF2A1;CAMK2D;USO1;MRPL57;TROVE2;
P98;PPIA;KPNB1;INO80C;UBA6;UBFD1;G3BP2;SNX5;B3GALNT2;BTF3;CD164;NUS1;HSPA8;ERLIN1;NUP1
CREM;VPS26A;GABARAP;SAV1;PRDX1;DPH3;SLC38A2;B3GALNT2;BTF3;HSPA8;XBP1;HSPA4;MTERF4;STY
HSPH1;RPRD1A;RAB6A;SERF2;EDEM3;NUS1;HSPA8;XBP1;AKIRIN1;NUP153;DNAJC13;EEF2;ELL2;RCL1;C
;PIK3CA;KIF2A;STIM2;TBL1XR1;ZC3H11A;PAPD5;TAX1BP1;UBE2V2;PFDN1;RHOU;CRELD1;DDR2;RAI14;N
RC6;NUP98;PRKAA1;RPL10;CBFB;NUFIP2;NAA20;NLK;HSPA13;TSPAN31;RNFT1;SOCS2;NAA25;MAPK6;SL
CBFB;NPRL2;CREM;ADIPOR2;TTBK2;VCPIP1;ZMAT5;G3BP2;ZC3H14;ATP6V1F;B3GALNT2;BTF3;HSPA8;SLM
H2;THOC6;KIF2A;TMEM258;PPA1;ZC3H11A;SMCR8;EIF1B;KPNB1;GTF2A1;TMED10;DCTN4;USO1;UBFD1;V
27L;MRPL57;MAT2A;G3BP2;NAA25;ATP6V1F;HSPA8;ST13;PTGES3;TUBB4B;EEF2;SOD1;RCL1;AIMP2;RPS2
MEM150A;ATP6AP1;GAA;PIH1D1;ARRDC3;SLC39A10;ACSL3;MFSD11;RC3H2;THOC6;ARMC1;TMEM258;PI
X6;GTF2A1;NUFIP2;ROCK2;UBA6;RPS27L;UAP1;MRPL57;RNFT1;TM9SF3;MAT2A;PRDX1;ATAD1;DYNC1H1;I
IAA35;ETF1;AURKAIP1;ATF6;GTF2A1;CAMK2D;MRPL57;HSPH1;PRDX1;RICTOR;SNX5;EDEM3;ERCC6L2;HS
C1;ZFYVE16;TMEM258;TMX1;RPL7L1;NHP2;YME1L1;WDPCP;AURKAIP1;ATF6;EIF1B;INO80C;TMEM87A;RI
;NBR1;CPEB2;PPIA
FIP2;STAU2;DCUN1D3;SAV1;MAT2A;PSMB1;GPBP1L1;ASCC3;SLC38A2;SYNRG;IDH1;EPT1;TUBB4B;EEF2;
H1L;ACSL3;TUBB4B;EEF2;MFSD11;GPCPD1;RAD23B;ITFG1;PNRC2;RPS26;ARL4A;RPL7L1;CAPRIN1;EIF3E
DPK1;PIH1D1;ZDHHC13;ARL4A;CRYZL1;WDFY1;BIRC6;UPRT;MALAT1;WDPCP;NUP98;IL6ST;PPIA;MPND;K
BE2A;GPCPD1;CLK4;ITFG1;ACVR2A;RPS26;AIMP2;RPS25;RAB14;CAPRIN1;NHP2;MDM2;TCEA1;BIRC6;CI

EEF1E1;GTF2A1;MGST1;RPS27L;ADIPOR2;MAT2A;KDELC2;EMC3;UGGT1;EDEM3;TIMM8B;RANBP2;DYNC1
LC25A16;ARMC1;TAX1BP1;RPL24;YME1L1;WDPCP;ATF6;KDM7A;TMEM87A;GTF2A1;HEATR1;RPS27L;MRPI
;FMNL2;TMBIM4;TBL1XR1;NBR1;CAPZA2;TAX1BP1;ULK2;TMCC1;KPNB1
DRAP1;DNAJA1;AGO3;YME1L1;POLR3G;INTS4;MALAT1;EIF1B;TPT1;KDM7A
MORF4L2;CNEP1R1;NBR1;NMT1;TPP1;HNRNPC;CALR;IL6ST;MPND;KBTBD8;PPIA;KPNB1

RNASE4;ACSL4;DICER1;MFSD11;ATG12;TWSG1;TMX3;TMEM258;PPA1;ZC3H11A;RFX5;UBE2V2;YME1L1;N
L6ST;CALM2;PLA1A;DDR2
IA;GTF2A1;RPL10;DCTN4;ADIPOR2;GABARAP;ARHGAP12;SOCS2;CST3;VCPIP1;PRDX1;METTL4;DRAM2;SL
FMNL2;KIF2A;TMEM33;CNOT1;NMT1;CHMP4C;BIRC6;NUP98;EXOC5;CALM2
IP1;ZKSCAN8;DPH3;EDEM3;BTF3;NEK9;MTERF4;FAM103A1;KNOP1;NUP153;CWC15;RPS26;GNPTG;COP
)11;THOC6;TMEM258;PSMA2;TMEM33;ZC3H11A;CRYZL1;WDPCP;AURKAIP1;EIF1B;CRK;NAPB;TMED10;CL

R8;ETF1;AURKAIP1;CLDND1;RPL10;CBFB;ROCK2;DCTN4;GABARAP;MTMR6;CDC42;PCNP;RAB6A;UGGT1;TI
N;TPP2;TPP1;NUP98;CRELD1;EBAG9;KPNB1
HGEF12;EXOC7;SACM1L;ACSL4;TFB2M;ATG12;CPED1;AFTPH;ZEB1;RRM2B;PIK3CA;TMEM258;TMEM33;TN

;ARRDC3;NFATC3;NR1H3;NUP153;THOC6;PNRC2;ERP44;RPS25;AGO3;COPS2;RPL7L1;INTS4;RPL22L1;BI

RMC1;PSMA3;MORF4L2;COPS2;DMXL1;PSME4;NBR1;RPL24;INTS4;ANG;EIF3E;NUP98;IL6ST;ATF6;RSL24D
N1;GNPTG;UBLCP1;TMEM258;TMEM33;TBCE;TAX1BP1;NAA35;AURKAIP1;RSL24D1;ITPA

MEM170B;CGRRF1;RC3H2;ARMC1;MORF4L2;RFX5;CRYZL1;NHP2;UPRT;WDPCP;NUP98;IL6ST;PPIA;KPNB1

D3;SLC1A3;AEBP1;ADIPOR2;TM9SF3;SOCS2;CST3;ZMAT5;CEPT1;DRAM2;SLC38A4;PLEKHA1;NFATC3;SNX
T6GALNAC5;GAS7;MAOA;TM9SF3;SC5D;BRD7;RAB6A;MAPK6;PLEKHH1;SNX5;CAP2;MIR99AHG;BTBD7;SC

;RPN2;UBFD1;NLK;TTBK2;SEP15;HSPH1;DPH3;EDEM3;HSPA8;XBP1;TMEM184C;EPT1;TMEM192;TDRD3;
R1;TPP2;INTS4;MALAT1;NUP98;HNRNPC;CALR;EEF1E1;MPND;KPNB1
S27L;COX7A2;NLK;TTBK2;PCNP;RAB40B;PSMB1;ZKSCAN8;UGGT1;SORT1;EPT1;TUBB4B;RCL1;COPS3;FEI

ATC3;RNASE4;ELL2;RCL1;RPS26;UFM1;TWSG1;TBL1XR1;CAPRIN1;NBR1;TCEA1;PKN2;RHOU;IL6ST;OGT
BP1;FAM96B;IL6ST;ATF6;EIF1B;KPNB1;GAS7;TMEM87A;GTF2A1;NUFIP2;ZCCHC9;CREM;DERL2;AEBP1;AD

C2;CD151;FAM83G;ZKSCAN8;SLC38A2;IDH1;KLHDC1;OSGIN1;SMAD5;FER;NDUFAF4;MDM2;UBE2N;CALR

MPND;KPNB1
AM96B;NAA35;CCDC43;CRK;MPND;ROCK2;UBA6;PON2;CST3;VCPIP1;PERP;PSMB1;EPS15;RWDD1;IDH1;

PH;MAF;RALGAPB;MORF4L2;CNEP1R1;CRELD1;AURKAIP1;EIF1B;SMPDL3A;CRK;KDM7A;TPH2;PRKAA1;ST

1;TCEA1;PPIA;KPNB1

;TOP1;PPIA;PFKFB4;CCL11;HTRA1;SLC1A3;ADH7;TTBK2;CDC42;DUOX1;GPNMB;PERP;ASCC3;ZC3H14;SI

;DYNC1LI1;WNK1;TBL1XR1;COPS2;RPL7L1;NAA35;TPP1;TOP1;SEC22B;CALM1;CALM2;CPEB2;PCNXL4;K
FTPH;GGCX;WNK1;TBL1XR1;NDUFAF4;MORF4L2;CNEP1R1;MPND;NOP10;KDM7A

EN2;NMBR;OTOP1;ZHX1;TGFBR1;SGPP1;CPED1;AR;GREM2;CPEB1;PLCXD3;MAF;SLC7A8;TMEM56;RGL3;
RF4L2;CNEP1R1;LCOR;NOP10;KDM7A

;SEP15;PRDX1;SNX5;BTF3;NUS1;XBP1;SYNRG;MTERF4;EPT1;PRDX6;RPS26;AIMP2;GNPTG;RPS25;TADA2/
3;SNX5;EDEM3;B3GALNT2;BTF3;NUS1;XBP1;ST13;ERLIN1;NFATC3;PRDX6;ELL2;RPS26;AIMP2;GNPTG;RPS
TMEM87A;DDX6;TMED10;RPN2;UBFD1;COX7A2;ACTR3B;SEP15;VCPIP1;ZMAT5;ABL2;RPRD1A;ZBED6;RAI
APPC8;UBE2A;ELL2;MARCH5;SMAD5;TDRD3;DRAP1;WNK1;DMXL1;UBE2N;STT3B;INTS8;UBE2K;LSM14A;
24;YME1L1;WDPCP;NUP98;AURKAIP1;EIF1B;PPIA;INO80C;TMEM87A;MAOA;ZCCHC9;UBFD1;VPS26A;CO›
AT2A;MAPK6;BTF3;CD164;HSPA8;AKIRIN1;YTHDF3;TTC9C;UBE2A;EEF2;SOD1;RPS26;UBE2W;GOLPH3;P4I
IO80C;UBA6;UBFD1;SOCS2;HSPH1;PRDX1;RAB6A;SNX5;SERF2;B3GALNT2;CD164;NUS1;XBP1;SMAD3;ST
1;VCP;UBA6;MAOA;NPRL2;DCUN1D3;ZCCHC9;CREM;UAP1;UBFD1;ADIPOR2;HSPH1;DPH3;G3BP2;RAB6A
;SAV1;CDC42;VCPIP1;DNAJB9;RAB6A;MAPK6;SNX5;RANBP2;NUS1;XBP1;SMAD3;SYNRG;STYX;C1GALT1;E
53;EEF2;RCL1;RPS26;AIMP2;RPS25;TBCK;NDUFAF4;CAPRIN1;NMT1;STT3B;OGT;CALM2;RSL24D1;NOP10
X;EEF2;AIMP2;UBE2W;RPS25;FER;GGCX;P4HA1;CAPRIN1;STT3B;DYNLRB1;CALM1;CALM2;LSM14A;CPEB
NOT1;CPD;NMT1;UBE2N;TPP2;TPP1;CALR;HNRNPC;CALM2;GSK3B;ARF1;USPL1;PHF20;CLTC;PTEN;SRA1
IAPB;PRKAA1;ROCK2;MAOA;CREM;ADH7;MRPL57;CCL8;PRDX1;EMC2;RICTOR;POLR2J;CAP2;B3GALNT2;C
C38A2;CD164;XBP1;ST13;PNPLA8;HSPA4;C1GALT1;TTC9C;MARCH5;TDRD3;SOD1;RPS26;P4HA1;NBR1;E
O2;NDFIP2;EPT1;BTBD7;DNAJC13;EEF2;SMAD5;GNPTG;CNIH1;P4HA1;WDR60;CNOT1;NDUFAF4;OSTC;SE
/PS26A;GABARAP;ARHGAP12;SOCS2;FAM83G;SC5D;DNAJB9;SLC38A2;ATP6V1F;RANBP2;ARFGEF2;CD16
25;CAPRIN1;OSTC;EIF3E;HNRNPC;CALR;ABCE1;NOP10
PA1;ZC3H11A;WDFY1;SMCR8;ETF1;TOP1;EIF1B;RAI14;GTF2A1;CAMK2D;TMED10;DCTN4;HEATR1;USO1;U
NDFIP2;SMURF2;STYX;SPRYD7;EPT1;UBE2A;TUBB4B;SMAD5;TDRD3;SOD1;DNAJA1;UBE2W;ITCH;OSTM1;F
PA4;PTGES3;FAM103A1;NFATC3;GGH;MAPK14;VMP1;COPS3;TAOK1;DMXL1;UBE2N;RSL24D1;LSM14A
N2;NPRL2;NLK;ADIPOR2;GABARAP;ARHGAP12;VCPIP1;HSPH1;DPH3;RAB6A;EDEM3;HSPA8;AKIRIN1;TME

MIB1;CNOT7;RGL3;CALM1;RSL24D1;OGT;CALM2;LSM14A;TPT1
E;OGT;CALM2;LSM14A
PNB1;KDM7A;SCARNA13;PFKFB4;VCP;GTF2A2;RPN2;NUFIP2;DCTN4;UBFD1;NLK;GABARAP;TSPAN31;TTB
RELD1;IL6ST;CALM2;MPND;LSM14A;MBTPS2

1H1;HUWE1;NUP153;EEF2;PUM2;TM9SF2;COPS3;WNK1;TPP2;PKN2;EIF3E;HNRNPC;OGT;CALM2;MBTPS2
57;GABARAP;HSPH1;PRDX1;PSMB1;EMC3;DNAJB9;SLC38A2;UGGT1;POLR2K;RWDD1;CD164;ERCC6L2;I

IUP98;AURKAIP1;PPIA;KPNB1;RAI14;INO80C;NPRL2;CREM;UBFD1;ACTR3B;FAM83G;RAP1A;G3BP2;SLC3

LC38A2;RWDD1;DYNC1H1;CD164;XBP1;BNIP3;NFATC3;TUBB4B;RPS25;CNOT7;NMT2;TCEA1;SERINC1;C

S3;RPS25;AGO3;UBLCP1;COPS2;UBE2N;INTS4;HNRNPC;NOL12;RSL24D1;NOP10;ITPA;GSK3B;USPL1;CL
LDND1;NPRL2;HSPA13;VCPIP1;HSPH1;KCTD21;ZMAT5;GPBP1L1;EDEM3;TBC1D8B;PLEKHA1;NR1H3;NUP

IMM8B;RANBP2;TMEM184C;YTHDF3;KLHDC1;OSGIN1;MIB1;VMP1;RPS25;LYRM2;UFM1;NBR1;CAPZA2;TP

MX1;ZC3H11A;NHP2;SMCR8;CCDC69;PON2;VPS26A;GABARAP;ARHGAP12;CDC42;PCNP;VCPIP1;HSPH1;

RC6;WDPCP;SEC22B;AURKAIP1;OGT;EIF1B;RSL24D1;MBTPS2

)1

1;DDX6;IDI1;VCP;RPN2;CREM;UAP1;UBFD1;VPS26A;NLK;ACTR3B;ADIPOR2;TTBK2;SEP15;HSPH1;DPH3;G

K24;ACVR2A;TDRD3;ARCN1;RPS26;UFM1;DMXL1;PKN2;OGT;UBE2K;KBTBD8;MBTPS2
ORBS2;ELL2;ACVR2A;WARS2;RCL1;TM9SF2;PLCXD3;FER;TADA2A;P4HA1;SLC25A32;CALM1;KBTBD8

NET1;RCL1;AIMP2;COPS3;FER;CNOT1;DMXL1;CPD;NMT1;INTS4;TPP2;CALR;STT3B;HNRNPC;DYNLRB1;CA

R;CNOT1;NBR1;NMT1;INTS4;CALR;HNRNPC;DYNLRB1

DIPOR2;PTS;SOCS2;PRDX1;SC5D;RICTOR;SLC38A2;UGGT1;CD164;HSPA8;SMAD3;API5;SYNRG;C1GALT1;

R;DYNLRB1;ITPA;BMPR2;HEXA;CLTC;CHD6;HMGB1;BMI1;AFF4;SYNCRIP;UBE2Q2;WAPAL;CEP170;UBXN4;

C1GALT1;NFATC3;NR1H3;TUBB4B;DNAJC15;ACVR2A;VMP1;ERP44;GOLPH3;GGCX;CNOT1;CALM1;CALM2

AU2;NAA20;ZCCHC9;UBFD1;VPS26A;MTMR6;SOCS2;APOO;MAT2A;PCGF1;POLR2J;SERF2;CD164;NUS1;A

LC38A4;ARHGEF37;YTHDF3;SYNRG;PUM2;ITCH;UFM1;TADA2A;FMNL2;QPCT;MDM2;TPP2;POLR3G;SERINC

PNB1;ST6GALNAC5;KDM7A

CPEB2;SERINC2;ST6GALNAC5

\;AGO3;WNK1;NMT1;INTS4;CALR;HNRNPC;CALM1;CALM2;INTS8;ARF1;WDR47;USPL1;HEXA;CLTC;SNX13
S25;AGO3;WNK1;WDR60;NMT1;INTS4;CALR;HNRNPC;CALM1;CALM2;ARF1;USPL1;CAB39L;CLTC;TFB1M;S
36A;SNX5;BTF3;CD164;NUS1;HSPA8;AKIRIN1;HSPA4;SYNRG;IDH1;MTERF4;KNOP1;BTBD7;TRAPPC8;DNA.
CPEB2;GSK3B;BMPR2;CPQ;CLTC;SETD7;PTEN;SNX13;HMGB1;BMI1;AFF4;ELAVL1;CDC73;MED13;ZMYM2;
K7A2;GABARAP;SEP15;HSPH1;PRDX1;G3BP2;RAB6A;SNX5;B3GALNT2;CD164;NUS1;HSPA8;AKIRIN1;API5
HA1;CAPRIN1;MDM2;UBE2N;CALR;CALM1;CPEB2;OGT;LSM14A
T13;HSPA4;SYNRG;FAM103A1;KNOP1;TMEM192;NUP153;BTBD7;TRAPPC8;EEF2;PRDX6;RCL1;AIMP2;RPS.
\;SLC38A2;SNX5;EDEM3;B3GALNT2;CD164;NUS1;AKIRIN1;API5;ST13;HSPA4;EPT1;NUP153;EEF2;ELL2;M
RLIN1;EEF2;TUBB4B;ELL2;TDRD3;PNRC2;P4HA1;DMXL1;CAPRIN1;CAPZA2;CALM1;LSM14A;TPT1
;USPL1;CLTC;PTEN;CDC73;SYNCRIP;RPL7A;NUDCD1;RRP12;TMED7;RMRP;CCT3;PBRM1;SLC39A10;TFB2
2;PCNXL4;GSK3B;ARF1;USPL1;HEXA;SRA1;CDC73;SYNCRIP;UBL5;RPL7A;MED13;TMED2;ITGAV;ARIH1;PC
;HMGB1;AACS;SEPT7;AP1G1;CHMP1B;PPP6R3;ARIH1;RRP12;UBXN4;CCT3;ZHX2;DUSP3;ZFR;SLC25A16;/
D164;SMAD3;IDH1;DENND4C;EYA4;C1GALT1;OSGIN1;SORBS2;NETO2;PRDX6;OXR1;ESR1;VMP1;GOLPH3
ESYT2;CALM2;MBTPS2
RINC1;HNRNPC;TMCC1;DYNLRB1;RSL24D1;ARF1;RSF1;TFB1M;CEP170;SNAP29;METTL15;TMED5;CCT3;N
4;ST13;SYNRG;NFATC3;TMEM192;BTBD7;TRAPPC8;DNAJC13;EEF2;CLK4;AIMP2;GNPTG;OSTM1;TPP2;PKN

JBFD1;VPS26A;NPL;GABARAP;ARHGAP12;SOCS2;FAM83G;EMC2;SC5D;DNAJB9;SLC38A2;ARFGEF2;CD16
P4HA1;TAOK1;ESYT2;CALR;STT3B;CALM1;CALM2;LSM14A

M184C;SYNRG;FAM103A1;KNOP1;TMEM192;NUP153;TRAPPC8;RPS26;GNPTG;RPS25;TBCK;COPS2;NMT

K2;ARNTL;RAB40B;SORT1;SYNRG;EPT1;TUBB4B;DRAP1;RPS26;COPS3;CNOT1;NBR1;NMT1;MDM2;CNTN1;

2;TPT1
KNOP1;TTC9C;EEF2;VMP1;AIMP2;GNPTG;WDR60;COPS2;MDM2;POLR3G;SLC25A32;ABCE1;OGT;CALM2

3A2;SNX5;SERF2;B3GALNT2;SLMO2;SMURF2;FAM103A1;ERLIN1;NFATC3;NUP153;BTBD7;TRAPPC8;NETO2

ALR;OGT;TPT1

TC;SRA1;TFB1M;ELAVL1;UBL5;SENP5;NUDCD1;SCRN3;RRP12;TMED7;TNPO3;PDPK1;HSBP1;BUD31;TFB2
153;MARCH5;GDAP1;RPS26;ERP44;TTLL4;WNK1;CNOT1;NMT1;KBTBD8

P2;NOL12;EBAG9;CPEB2;PCNXL4;RSL24D1

GPNMB;EMC3;DNAJB9;ZBED6;MPZL3;UGGT1;TIMM8B;HSPA8;AKIRIN1;YTHDF3;NUP153;TRAPPC8;DNAJC1

;3BP2;RAB6A;EDEM3;NUS1;HSPA8;XBP1;TMEM184C;SMAD3;STYX;EPT1;NFATC3;TMEM192;TRAPPC8;CW

LM2;GSK3B;PHF20;CAB39L;CHD6;HMGB1;TFB1M;MED13;ZMYM2;PPP6R3;WAPAL;TMED7;METTL15;NCK1

;NUP153;TUBB4B;OXR1;WNK1;CD28;STT3B;CALM1

YAF2;JAK1;RMRP;LRRCC1;DUSP3;ARHGEF12;GPX4;EXOC7;GAA;ANXA4;ZHX1;TGFBR1;TMEM37;AFTPH;PSN

;UBE2K;LSM14A;TPT1

API5;SORT1;IDH1;SPRYD7;C1GALT1;NR1H3;EEF2;MAPK14;ELL2;MARCH5;OXR1;RPS26;TTLL4;CNIH1;P4HA

:2

;SRA1;RSF1;TFB1M;SYNCRIP;UBL5;ZMYM2;CHMP1B;TMED2;ARIH1;JAK2;PCMTD1;PBRM1;EXOC7;SLC39A1
YNCRIP;CHMP1B;TMED2;ARIH1;UBXN4;TMED5;PCMTD1;ZYG11A;DUSP3;HSBP1;GAA;TFB2M;ASH1L;ZHX1
JC13;CWC15;EEF2;CLK4;DRAP1;RPS26;UBE2W;GNPTG;COPS3;RPS25;GGCX;WNK1;CNOT1;COPS2;CAPR
;UBE2Q2;WAPAL;TMED2;ITGAV;ARIH1;POLK;JAK2;TMED7;YAF2;PCMTD1;RMRP;NCK1;PBRM1;GPX4;GPCPD
;ST13;HSPA4;MTERF4;EPT1;NUP153;UBE2A;EEF2;ELL2;MARCH5;DRAP1;RPS26;COPS3;RPS25;TBCK;FER

25;TADA2A;NDUFAF4;CAPRIN1;TPP2;STT3B;NOL12;RSL24D1;NOP10;USPL1;USP31;PHF20;HEXA;CLTC;PT
IARCH5;DRAP1;RPS26;UBE2W;TBCK;CNOT7;GGCX;CNOT1;UBE2N;TPP2;SERINC1;TPP1;HNRNPC;CALM2;

2M;RAD23B;KIF2A;BIRC6;ETF1;TOP1;EEF1E1;SCARNA13;GTF2A1;RPL10;PVT1;HEATR1;USO1;TROVE2;MAT
MTD1;NCK1;BUD31;SLC39A10;TFB2M;SLC25A16;SIKE1;TMBIM4;FAM96B;NAA35;BIRC6;CRELD1;GTF2A1
AFTPH;TAX1BP1;ETF1;TOP1;CRK;CLDND1;NUFIP2;DCTN4;DERL2;HSPA13;CDC42;PCNP;PSMB1;EMC2;DN
3;FMNL2;P4HA1;QPCT;TBCE;MDM2;SLC25A30;CNTN1

MKL2;TTC37;GAA;ZFR;SLC39A10;TFB2M;ASH1L;RAD23B;DNAJC3;PSMA3;AFTPH;SIKE1;PSMA4;KIF2A;PSM
2;SLC25A32;TPP1;CALR;STT3B

4;NUS1;TMEM30A;ST13;SYNRG;NFATC3;TMEM192;BTBD7;TRAPPC8;DNAJC13;EEF2;CLK4;PRDX6;GNPTG;

1;INTS4;TPP2;NOL12;NOP10;ITPA;ITGB1;USPL1;CLTC;SRA1;UBL5;NUDCD1;SCRN3;CHMP1B;TMED2;MFSI

;INTS4;CALR;HNRNPC;DYNLRB1;CALM1;OGT;CALM2

2;TDRD3;DRAP1;RCL1;AIMP2;COPS3;CPEB1;TADA2A;P4HA1;WNK1;CNOT1;CPD;MDM2;CALR;STT3B;CALN

M;TOX4;AFTPH;TMEM33;TAX1BP1;FAM96B;SMCR8;BIRC6;ETF1;EXOC5;SEC22B;CRK;SCARNA13;GTF2A2;

13;NETO2;HOOK3;TTC9C;RPS25;UBLCP1;CNOT1;NBR1;INTS4;SLC25A32;SERINC1;HNRNPC;ABCE1;CPEB

C15;PRDX6;ELL2;TDRD3;NET1;RCL1;AIMP2;UBE2W;COPS3;FER;CNOT1;DMXL1;CPD;TBCE;NMT1;MDM2;I

;CCT3;ATP6AP1;GPX4;ZDHHC13;TFB2M;TOX4;DNAJC3;PSMA3;ARL4A;CNEP1R1;ETF1;CRELD1;EEF1E1;M

MA4;PSMA1;DDAH1;BIRC6;CCDC43;TOP1;CRELD1;NAPB;GTF2A1;PRKAA1;STAU2;ROCK2;ZBTB41;TSPAN3

A1;WNK1;NDUFAF4;MDM2;NOP10;TPT1

10;TFB2M;ASH1L;ZHX1;ITFG1;PSMA3;SIKE1;PSMA4;PSMA2;TAX1BP1;EXOC5;EEF1E1;RPL10;USO1;RPS27
;PSMA3;ZEB1;TAX1BP1;CRELD1;EEF1E1;RPL10;USO1;MRPL57;HSPA13;METTL4;EMC3;SC5D;RICTOR;GPI
IN1;NMT1;UBE2N;INTS4;STT3B;HNRNPC;OGT;INTS8;UBE2K;LSM14A;PCNXL4;GSK3B;ARF1;USP31;CELF1
D1;CBFA2T2;TGFBR1;DNAJC3;PSMA4;ZEB1;TOP1;CRK;KDM7A;SCARNA13;GTF2A1;GTF2A2;STAU2;ROCK2;
;UBLCP1;GGCX;WNK1;CNOT1;CAPRIN1;MDM2;STT3B;HNRNPC;CALM1;NOL12;OGT;CALM2;LSM14A;RSL2

TEN;TFB1M;AFF4;CDC73;SYNCRIP;RPL7A;NUDCD1;GNPNAT1;MFSD1;RRP12;TMED7;SNAP29;TMED5;RMR
CPEB2;ARF1;USPL1;PHF20;CLTC;SRA1;HMGB1;SENP5;NUDCD1;AP1G1;CHMP1B;WAPAL;TMED2;ARIH1;F

2A;NAA25;DNAJB9;GPBP1L1;RANBP2;RWDD1;PTGES3;TTC9C;TUBB4B;WARS2;VMP1;DNAJA1;POLR3G;RP
;RPL10;NUFIP2;RPS27L;MRPL57;TROVE2;MAT2A;DNAJB9;MAPK6;UGGT1;RANBP2;DYNC1H1;YTHDF3;ERC
IAJB9;MPZL3;POLR2K;RANBP2;TMEM30A;PLEKHA1;YTHDF3;PTGES3;OXR1;RAB11A;VMP1;GOLPH3;ESYT2;

A2;PFDN1;CHMP3;BIRC6;TOP1;CRK;KDM7A;NAPB;GTF2A1;RPL10;NDUFB4;DCTN4;USO1;TUBD1;ZBTB41;F

FER;TTLL4;OSTM1;TPP2;PKN2;SLC25A32;TPP1;CALR;STT3B;NOP10

D1;ARIH1;UBXN4;TNPO3;LRRCC1;PDPK1;HSBP1;BUD31;ANXA4;ZDHHC13;TFB2M;ITFG1;SLC25A16;TOX4;

M2;LSM14A;RSL24D1;ITGB1;ARF1;USPL1;SLC35F3;PHF20;CPQ;HEXA;PTEN;AFF4;CDC73;PTER;UBE2Q2;S

NAA20;DERL2;TM9SF3;PCNP;PSMB1;EMC3;DNAJB9;GPBP1L1;MPZL3;UGGT1;POLR2K;TIMM8B;TMEM30A;

52;RSL24D1;KBTBD8

NTS4;TPP2;CALR;STT3B;HNRNPC;DYNLRB1;NOL12;CALM2;GSK3B;WDR47;PHF20;CAB39L;CHD6;SNX13;

PND;SCARNA13;CCDC186;NUFIP2;DCTN4;DERL2;HSPA13;TSPAN31;ARNTL;RAB40B;SC5D;DNAJB9;POLR

1;TM9SF3;CDC42;MAT2A;KDELC2;PCGF1;CEPT1;DRAM2;TMEM30A;SMOX;C1GALT1;TUBB4B;RAB11A;ITC

L;DERL2;MRPL57;PCNP;LMAN1;PSMB1;EMC2;SC5D;RICTOR;GPBP1L1;CEPT1;RANBP2;DYNC1H1;YTHDF3
3P1L1;RANBP2;YTHDF3;ERCC6L2;PTGES3;TTC9C;RAB11A;VMP1;LYRM2;TAOK1;RPL22L1;PKN2;ESYT2;ANI
;CAB39L;CLTC;PTEN;SNX13;SRA1;RSF1;HMGB1;BMI1;ELAVL1;CDC73;RAB22A;SYNCRIP;RPL7A;MED13;ZN
CCDC69;PON2;ZDHHC21;MRPL57;TM9SF3;HECTD2;NAA25;RICTOR;CEPT1;DRAM2;EPS15;RWDD1;ARFGE
4D1;NOP10;GSK3B;USPL1;PHF20;CAB39L;HEXA;CLTC;PTEN;RSF1;AFF4;ELAVL1;CDC73;SYNCRIP;RPL7A;

P;NCK1;CCT3;TTC37;PBRM1;ATP6V0B;BUD31;ZFR;TFB2M;ARL4A;KIF2A;TMEM33;CHMP3;SMCR8;BIRC6;E
RP12;POLK;UBXN4;METTL15;NCK1;PBRM1;ATP6V0B;DUSP3;TMEM150A;DYNLT3;GPCPD1;CBFA2T2;SLC2

L22L1;SLC25A32;EIF3E;NAA15;ABCE1;EBAG9;MBTPS2
C6L2;PTGES3;TUBB4B;RAB11A;PNRC2;DNAJA1;LYRM2;GOLPH3;TAOK1;TCEA1;RPL22L1;PKN2;ESYT2
TAF2;TPT1

PTS;CDC42;NAA25;PCGF1;DNAJB9;GPBP1L1;BPNT1;ATAD1;TIMM8B;RWDD1;BNIP3;PTGES3;SPRYD7;TTC9

;PSMA4;TMBIM4;PIK3CA;KIF2A;TMEM33;FAM96B;NAA35;CHMP3;SMCR8;BIRC6;ETF1;EXOC5;SEC22B;GTI

UMO2;PSD3;NPTN;WAPAL;GNPNAT1;MFSD1;POLK;TMED5;RMRP;NCK1;LRRCC1;TTC37;ZYG11A;GAA;ANXA

YTHDF3;ERCC6L2;NR1H3;TTC9C;GDAP1;ACVR2A;RAB11A;VMP1;TM9SF2;DNAJA1;ERP44;SLC25A32;TAF2

RSF1;HMGB1;TFB1M;ELAVL1;MED13;ZMYM2;SUMO2;PPP6R3;NPTN;WAPAL;TMED2;WDR7;TMED7;YAF2;MI

2K;RWDD1;PNPLA8;SORT1;TTC9C;TUBB4B;SOD1;PNRC2;ERP44;NBR1;EBAG9

H;GOLPH3;CAPZA2;TCEA1;ESYT2

;ERCC6L2;PTGES3;C1GALT1;HUWE1;NR1H3;TTC9C;TUBB4B;RAB11A;MIB1;VMP1;LYRM2;ITCH;TAOK1;CA
KRD13C;ATP8A1;UBE2D3;ZBTB20;PARK7;BZW1;IPO7;TMEM141;HDDC3;LAMP1;PAPOLA;NBN;MAP3K7;AG
MYM2;SEPT7;AP1G1;SUMO2;PPP6R3;WAPAL;WDR7;POLK;METTL16;UBXN4;TNPO3;PCMTD1;RMRP;DUSP3
EF2;SMOX;SPRYD7;TTC9C;TUBB4B;ACVR2A;MIB1;PNRC2;VMP1;ERP44;ITCH;CAPZA2;PKN2;NAA15;KBTBD
MED13;SEPT7;AP1G1;TRPS1;PPP6R3;TMED2;ARIH1;UBXN4;TNPO3;RMRP;PBRM1;ATP6V0B;ARHGEF12;BU

TF1;TOP1;CRELD1;EEF1E1;SCARNA13;GTF2A1;RPL10;PVT1;HEATR1;USO1;MRPL57;MAT2A;EMC2;NAA25
25A16;TOX4;AFTPH;SIKE1;TAX1BP1;PFDN1;ETF1;CRELD1;SEC22B;CRK;GTF2A1;CLDND1;RPL10;DCTN4;D

9C;TUBB4B;MIB1;WARS2;TM9SF2;ITCH;TTLL4;RAB14;RPS6KB1;CAPZA2;EIF3E

F2A1;GTF2A2;NAA20;HEATR1;RPS27L;ZDHHC21;TROVE2;CDC42;PCNP;PSMB1;METTL4;EMC3;DNAJB9;MF

A4;ZFR;AHSA2;EN2;GPCPD1;CBFA2T2;DNAJC3;ARL4A;PSMA1;KIF2A;DDAH1;CHMP3;SMCR8;ANG;TOP1;CF

;MBTPS2

ETTL15;TMED5;NCK1;CCT3;ATP6AP1;GPX4;ZDHHC13;TFB2M;GPCPD1;RAD23B;ZHX1;TGFBR1;TOX4;DNAJ

PZA2;TCEA1;RPL22L1;PKN2;EBAG9;ANKRD13C;UHRF1BP1L;POGLUT1;ATP8A1;PARK7;BZW1;IPO7;LAPTM
TPBP1;RPS8;CSGALNACT2;RPGRIP1L;CGRRF1;THOC6;NHP2;MALAT1;PPIA;KPNB1;INO80C;IDI1;PFKFB4;Z
;ATP6AP1;PDPK1;EXOC7;ZFR;ASH1L;RAD23B;ZHX1;ITFG1;AFTPH;PSMA4;TMBIM4;PSMA2;PFDN1;SMCR8;
8;MBTPS2;TPT1
JD31;AHSA2;SLC39A10;TFB2M;GPCPD1;RAD23B;PSMA4;NAA35;CHMP3;ETF1;CRELD1;EXOC5;CRK;MPN

;SC5D;DNAJB9;MAPK6;RANBP2;RWDD1;PTGES3;TTC9C;TUBB4B;WARS2;VMP1;OSTM1;POLR3G;RPL22L1;
ERL2;TROVE2;HSPA13;TSPAN31;CDC42;PCNP;RAB40B;PSMB1;METTL4;EMC3;MAPK6;POLR2K;RANBP2;Y

ZL3;GPR155;UGGT1;POLR2K;TIMM8B;PLEKHA1;ERCC6L2;TTC9C;GDAP1;VMP1;TM9SF2;DNAJA1;ERP44;L

RELD1;GTF2A1;RPL10;NAA20;PVT1;DCTN4;PON2;DERL2;NPL;MRPL57;TROVE2;KDELC2;EMC2;NAA25;EM

C3;PSMA3;ARL4A;PSMA4;TMEM33;CNEP1R1;ETF1;CRELD1;EEF1E1;MPND;SCARNA13;GTF2A1;CCDC186

4A;PTAR1;COL4A3BP;PAPOLA;KPNA4;RPS8;TMEM258;TMX1;MORF4L2;RPL7L1;UBE2V2;MALAT1;IL6ST;PP
CCHC9;CREM;SAV1;FAM83G;ZMAT5;G3BP2;RPRD1A;RAB6A;SLC38A2;ATP6V1F;CD164;HSPA8;AKIRIN1;Z
BIRC6;ETF1;CCDC43;EEF1E1;CRK;NAPB;GTF2A1;PRKAA1;CCDC186;NUFIP2;STAU2;DCTN4;TUBD1;PCNP

D;RBMS2;GTF2A1;CAMK2D;RPL10;NUFIP2;DCTN4;HEATR1;USO1;MRPL57;TROVE2;ZBTB41;TSPAN31;CDC

SLC25A32;EIF3E;ABCE1;EBAG9
THDF3;PTGES3;WARS2;VMP1;GOLPH3;NBR1;RPL22L1;PKN2;SLC25A32;ESYT2;ABCE1;TAF2;TPT1

YRM2;ITCH;UFM1;OSTM1;NBR1;RPL22L1;SLC25A32;TAF2

C3;DNAJB9;ASCC3;MAPK6;UGGT1;RANBP2;PNPLA8;ERCC6L2;BNIP3;PTGES3;SNX24;TTC9C;ACVR2A;RAB

;RPL10;NUFIP2;DCTN4;USO1;DERL2;HSPA13;PTS;TSPAN31;ARNTL;LMAN1;RAB40B;PSMB1;EMC2;PCGF1

IA;KPNB1;XPR1;INO80C;DDX6;IDI1;VCP;CREM;UBFD1;COX7A2;ZKSCAN8;ZMAT5;G3BP2;SLC38A2;CD
FHX2;SMAD3;SMURF2;HSPA4;FAM103A1;GGH;NUP153;MARCH5;TDRD3;UBE2W;COPS3;FER;CNOT7;P4H
;LMAN1;MAT2A;HECTD2;PSMB1;EMC2;NAA25;METTL4;PCGF1;RICTOR;GPBP1L1;TAF9B;MPZL3;MAPK6

42;PCNP;MAT2A;KCTD21;PSMB1;METTL4;EMC3;DNAJB9;GPBP1L1;MPZL3;UGGT1;TIMM8B;RANBP2;RWDI

11A;TTLL7;OSTM1;CAPZA2;SLC25A30;TCEA1;POLR3G;ESYT2;ABCE1;EBAG9;TPT1

;SC5D;DNAJB9;GPBP1L1;BRD7;POLR2K;RWDD1;TMEM30A;SORT1;C1GALT1;TTC9C;TUBB4B;SOD1;WARS2

A

)1;PLEKH

:;PNRC2;ERP

Term
Cytoplasmic Translation (GO:0002181)
Intracellular Protein Transport (GO:0006886)
Ubiquitin-Dependent Protein Catabolic Process (GO:0006511)
snRNA Modification (GO:0040031)
Negative Regulation Of Translation (GO:0017148)
Golgi To Lysosome Transport (GO:0090160)
N-terminal Peptidyl-Methionine Acetylation (GO:0017196)
Regulation Of Translation (GO:0006417)
Protein Phosphorylation (GO:0006468)
Negative Regulation Of Programmed Cell Death (GO:0043069)
Peptidyl-Methionine Modification (GO:0018206)
Regulation Of Apoptotic Process (GO:0042981)
Negative Regulation Of Gene Expression (GO:0010629)
Positive Regulation Of Macromolecule Biosynthetic Process (GO:0010557)
Cytosolic Transport (GO:0016482)
Protein Transport (GO:0015031)
Regulation Of Ubiquitin-Protein Transferase Activity (GO:0051438)
Translation (GO:0006412)
Organelle Organization (GO:0006996)
Endosome Organization (GO:0007032)
Proteasomal Protein Catabolic Process (GO:0010498)
Myelin Maintenance (GO:0043217)
Proteasome-Mediated Ubiquitin-Dependent Protein Catabolic Process (GO:0043161)
Response To Insulin (GO:0032868)
Autophagosome Maturation (GO:0097352)
Negative Regulation Of Apoptotic Process (GO:0043066)
Golgi To Vacuole Transport (GO:0006896)
Positive Regulation Of Toll-Like Receptor 4 Signaling Pathway (GO:0034145)
Hydrogen Peroxide Biosynthetic Process (GO:0050665)
Positive Regulation Of Ubiquitin-Protein Transferase Activity (GO:0051443)
Cellular Response To Insulin Stimulus (GO:0032869)
Protein-Containing Complex Disassembly (GO:0032984)
Transmembrane Receptor Protein Serine/Threonine Kinase Signaling Pathway (GO:0007178)
Regulation Of Mitotic Spindle Organization (GO:0060236)
Autophagosome Membrane Docking (GO:0016240)
Regulation Of GTPase Activity (GO:0043087)
Peptidyl-Serine Modification (GO:0018209)
N-terminal Protein Amino Acid Modification (GO:0031365)
Eye Photoreceptor Cell Development (GO:0042462)
Interleukin-6-Mediated Signaling Pathway (GO:0070102)
Peptidyl-Glutamic Acid Modification (GO:0018200)
Positive Regulation Of Protein Autophosphorylation (GO:0031954)
mRNA Destabilization (GO:0061157)
Regulation Of Macroautophagy (GO:0016241)
Response To Unfolded Protein (GO:0006986)
Regulation Of Lamellipodium Assembly (GO:0010591)
Positive Regulation Of Lamellipodium Assembly (GO:0010592)

Folic Acid Transport (GO:0015884)
Peptidyl-Serine Phosphorylation (GO:0018105)
Positive Regulation Of Cellular Biosynthetic Process (GO:0031328)
Positive Regulation Of Amide Metabolic Process (GO:0034250)
Endosome Transport Via Multivesicular Body Sorting Pathway (GO:0032509)
Response To Purine-Containing Compound (GO:0014074)
Stress Granule Assembly (GO:0034063)
N-terminal Protein Amino Acid Acetylation (GO:0006474)
Endosome To Lysosome Transport Via Multivesicular Body Sorting Pathway (GO:0032510)
Regulation Of Cholesterol Biosynthetic Process (GO:0045540)
Endoplasmic Reticulum To Golgi Vesicle-Mediated Transport (GO:0006888)
Positive Regulation Of Cellular Catabolic Process (GO:0031331)
Protein Localization (GO:0008104)
Positive Regulation Of Protein Metabolic Process (GO:0051247)
Endosomal Transport (GO:0016197)
Golgi Vesicle Transport (GO:0048193)
Positive Regulation Of Muscle Hypertrophy (GO:0014742)
ERAD Pathway (GO:0036503)
Macromolecule Biosynthetic Process (GO:0009059)
Negative Regulation Of Smooth Muscle Cell Proliferation (GO:0048662)
Lysosomal Transport (GO:0007041)
Membrane Fission (GO:0090148)
Positive Regulation Of Myoblast Differentiation (GO:0045663)
Positive Regulation Of SMAD Protein Signal Transduction (GO:0060391)
Positive Regulation Of Cardiac Muscle Hypertrophy (GO:0010613)
rRNA Modification (GO:0000154)
Protein Modification By Small Protein Conjugation (GO:0032446)
Regulation Of TOR Signaling (GO:0032006)
Positive Regulation Of Pattern Recognition Receptor Signaling Pathway (GO:0062208)
Multivesicular Body-Lysosome Fusion (GO:0061763)
Peptidyl-Cysteine Modification (GO:0018198)
Regulation Of Protein Acetylation (GO:1901983)
Vesicle Fusion With Vacuole (GO:0051469)
Regulation Of Translational Initiation (GO:0006446)
Retrograde Transport, Endosome To Golgi (GO:0042147)
3'-UTR-mediated mRNA Stabilization (GO:0070935)
Regulation Of Protein Neddylation (GO:2000434)
Gene Expression (GO:0010467)
Peptide Biosynthetic Process (GO:0043043)
Modification-Dependent Protein Catabolic Process (GO:0019941)
Transcription By RNA Polymerase II (GO:0006366)
Activation Of Protein Kinase Activity (GO:0032147)
Protein K11-linked Ubiquitination (GO:0070979)
Regulation Of Protein Modification By Small Protein Conjugation Or Removal (GO:1903320)
Regulation Of Spindle Assembly (GO:0090169)
Phosphorylation (GO:0016310)
Positive Regulation Of Viral Process (GO:0048524)
Vesicle-Mediated Transport (GO:0016192)

Regulation Of Myoblast Differentiation (GO:0045661)
Positive Regulation Of MHC Class II Biosynthetic Process (GO:0045348)
Thyroid Hormone Generation (GO:0006590)
Vacuole Fusion (GO:0097576)
Negative Regulation Of Macromolecule Biosynthetic Process (GO:0010558)
Response To Endoplasmic Reticulum Stress (GO:0034976)
Golgi Organization (GO:0007030)
Protein Modification Process (GO:0036211)
Positive Regulation Of Protein Ubiquitination (GO:0031398)
Establishment Of Protein Localization To Organelle (GO:0072594)
Positive Regulation Of Lamellipodium Organization (GO:1902745)
Regulation Of Protein Autophosphorylation (GO:0031952)
Regulation Of Mitotic Spindle Assembly (GO:1901673)
P-body Assembly (GO:0033962)
Cellular Response To Growth Hormone Stimulus (GO:0071378)
Lysosomal Membrane Organization (GO:0097212)
Positive Regulation Of Endoplasmic Reticulum Unfolded Protein Response (GO:1900103)
Positive Regulation Of Protein Polyubiquitination (GO:1902916)
'De Novo' Post-Translational Protein Folding (GO:0051084)
Negative Regulation Of G1/S Transition Of Mitotic Cell Cycle (GO:2000134)
Peptidyl-Threonine Phosphorylation (GO:0018107)
Positive Regulation Of mRNA Catabolic Process (GO:0061014)
Macroautophagy (GO:0016236)
Positive Regulation Of Establishment Of Protein Localization To Mitochondrion (GO:1903749)
Positive Regulation Of Release Of Sequestered Calcium Ion Into Cytosol (GO:0051281)
Ubiquitin-Dependent Protein Catabolic Process Via The Multivesicular Body Sorting Pathway (GO:0043162)
Cellular Response To Peptide Hormone Stimulus (GO:0071375)
Regulation Of mRNA Catabolic Process (GO:0061013)
Endocytosis (GO:0006897)
Negative Regulation Of Macroautophagy (GO:0016242)
Postreplication Repair (GO:0006301)
Protein Neddylation (GO:0045116)
rRNA Methylation (GO:0031167)
Early Endosome To Golgi Transport (GO:0034498)
Mitochondrial Transcription (GO:0006390)
Myelination In Peripheral Nervous System (GO:0022011)
Regulation Of Cell Communication By Electrical Coupling (GO:0010649)
Cellular Response To Growth Factor Stimulus (GO:0071363)
Superoxide Metabolic Process (GO:0006801)
Insulin Receptor Signaling Pathway (GO:0008286)
Protein Polyubiquitination (GO:0000209)
Regulation Of Actin Cytoskeleton Organization (GO:0032956)
Positive Regulation Of Autophagy (GO:0010508)
Positive Regulation Of Protein Secretion (GO:0050714)
Positive Regulation Of Cell Differentiation (GO:0045597)
Negative Regulation Of Kinase Activity (GO:0033673)
Positive Regulation Of Translation (GO:0045727)
Cellular Response To Interleukin-6 (GO:0071354)

Protein N-linked Glycosylation Via Asparagine (GO:0018279)
Regulation Of Cardiac Muscle Hypertrophy (GO:0010611)
Regulation Of Toll-Like Receptor 4 Signaling Pathway (GO:0034143)
Negative Regulation Of Cell Cycle G1/S Phase Transition (GO:1902807)
Response To Nutrient Levels (GO:0031667)
Positive Regulation Of Endothelial Cell Proliferation (GO:0001938)
Regulation Of Proteasomal Ubiquitin-Dependent Protein Catabolic Process (GO:0032434)
Regulation Of Trophoblast Cell Migration (GO:1901163)
Positive Regulation Of Pathway-Restricted SMAD Protein Phosphorylation (GO:0010862)
Vesicle Organization (GO:0016050)
Endomembrane System Organization (GO:0010256)
COPI Coating Of Golgi Vesicle (GO:0048205)
COPI-coated Vesicle Budding (GO:0035964)
Golgi Transport Vesicle Coating (GO:0048200)
Aminophospholipid Transport (GO:0015917)
Cellular Response To Lipoteichoic Acid (GO:0071223)
Mitochondrial Protein Catabolic Process (GO:0035694)
Negative Regulation Of Cell Size (GO:0045792)
Nucleotide-Binding Domain, Leucine Rich Repeat Containing Receptor Signaling Pathway (GO:0035872)
Positive Regulation Of Mitochondrial Membrane Potential (GO:0010918)
Protein Targeting To Vacuole Involved In Autophagy (GO:0071211)
Response To Lipoteichoic Acid (GO:0070391)
Dicarboxylic Acid Transport (GO:0006835)
Multivesicular Body Sorting Pathway (GO:0071985)
Positive Regulation Of Peptidyl-Threonine Phosphorylation (GO:0010800)
Regulation Of Supramolecular Fiber Organization (GO:1902903)
Protein Import Into Nucleus (GO:0006606)
Negative Regulation Of Cell Death (GO:0060548)
Positive Regulation Of Transport (GO:0051050)
Regulation Of Cytokinesis (GO:0032465)
Muscle Cell Differentiation (GO:0042692)
DNA-templated Transcription (GO:0006351)
Positive Regulation Of Cellular Component Organization (GO:0051130)
Regulation Of Protein Ubiquitination (GO:0031396)
Negative Regulation Of Nucleocytoplasmic Transport (GO:0046823)
Positive Regulation Of Telomerase RNA Localization To Cajal Body (GO:1904874)
Regulation Of MHC Class II Biosynthetic Process (GO:0045346)
Regulation Of Extracellular Matrix Disassembly (GO:0010715)
Activation Of Protein Kinase B Activity (GO:0032148)
Osteoclast Differentiation (GO:0030316)
Cellular Response To Type II Interferon (GO:0071346)
Peptidyl-Threonine Modification (GO:0018210)
Response To Transforming Growth Factor Beta (GO:0071559)
Regulation Of Cytoskeleton Organization (GO:0051493)
Extrinsic Apoptotic Signaling Pathway (GO:0097191)
Peptidyl-Tyrosine Phosphorylation (GO:0018108)
Positive Regulation Of Cell Motility (GO:2000147)
Positive Regulation Of Ubiquitin-Dependent Protein Catabolic Process (GO:2000060)

Vesicle-Mediated Transport To The Plasma Membrane (GO:0098876)
miRNA-mediated Gene Silencing (GO:0035195)
Protein Targeting To Lysosome (GO:0006622)
Regulation Of Protein Targeting To Mitochondrion (GO:1903214)
Negative Regulation Of Macromolecule Metabolic Process (GO:0010605)
Protein Ubiquitination (GO:0016567)
DNA Double-Strand Break Processing (GO:0000729)
Golgi To Endosome Transport (GO:0006895)
Folic Acid-Containing Compound Metabolic Process (GO:0006760)
Midbody Abscission (GO:0061952)
Positive Regulation Of Sprouting Angiogenesis (GO:1903672)
Regulation Of Rac Protein Signal Transduction (GO:0035020)
Retrograde Axonal Transport (GO:0008090)
ATF6-mediated Unfolded Protein Response (GO:0036500)
Histidine Catabolic Process (GO:0006548)
Histidine Metabolic Process (GO:0006547)
Mitochondrion-Endoplasmic Reticulum Membrane Tethering (GO:1990456)
Negative Regulation Of Establishment Of Protein Localization To Mitochondrion (GO:1903748)
Negative Regulation Of Response To Type II Interferon (GO:0060331)
Negative Regulation Of Type II Interferon-Mediated Signaling Pathway (GO:0060336)
Polyamine Biosynthetic Process (GO:0006596)
Positive Regulation Of Chemokine (C-X-C Motif) Ligand 2 Production (GO:2000343)
Positive Regulation Of Protein K63-linked Ubiquitination (GO:1902523)
Positive Regulation Of Toll-Like Receptor 3 Signaling Pathway (GO:0034141)
protein-DNA Covalent Cross-Linking Repair (GO:0106300)
Regulation Of Cellular Response To Hypoxia (GO:1900037)
Regulation Of Entry Of Bacterium Into Host Cell (GO:2000535)
Retinal Rod Cell Differentiation (GO:0060221)
Positive Regulation Of Protein Phosphorylation (GO:0001934)
Mesenchymal Cell Differentiation (GO:0048762)
Peptidyl-Tyrosine Modification (GO:0018212)
Phagocytosis (GO:0006909)
Cellular Response To Organic Cyclic Compound (GO:0071407)
Negative Regulation Of Mitotic Cell Cycle Phase Transition (GO:1901991)
Negative Regulation Of Protein Localization (GO:1903828)
Regulation Of Protein Dephosphorylation (GO:0035304)
Mitotic Spindle Organization (GO:0007052)
Amino Acid Import Across Plasma Membrane (GO:0089718)
Chaperone Cofactor-Dependent Protein Refolding (GO:0051085)
Glycerophospholipid Catabolic Process (GO:0046475)
Negative Regulation Of Neurogenesis (GO:0050768)
Positive Regulation Of Calcium Ion Transmembrane Transporter Activity (GO:1901021)
Positive Regulation Of Mitotic Cell Cycle (GO:0045931)
Protein Quality Control For Misfolded Or Incompletely Synthesized Proteins (GO:0006515)
Regulation Of SMAD Protein Signal Transduction (GO:0060390)
Regulation Of Chromatin Organization (GO:1902275)
Regulation Of Cellular Component Organization (GO:0051128)
Protein Catabolic Process (GO:0030163)

Positive Regulation Of Protein Transport (GO:0051222)
Regulation Of Actin Filament-Based Process (GO:0032970)
Positive Regulation Of Actin Filament Polymerization (GO:0030838)
Protein Localization To Organelle (GO:0033365)
Activin Receptor Signaling Pathway (GO:0032924)
Late Endosome To Lysosome Transport (GO:1902774)
Neuron Projection Organization (GO:0106027)
Phosphatidylinositol Dephosphorylation (GO:0046856)
Positive Regulation Of Homotypic Cell-Cell Adhesion (GO:0034112)
Regulation Of Cardiac Muscle Contraction By Regulation Of The Release Of Sequestered Calcium Ion (GO:0010[illegible]
Cellular Response To Lipid (GO:0071396)
Phospholipid Catabolic Process (GO:0009395)
Protein Localization To Golgi Apparatus (GO:0034067)
Positive Regulation Of Signal Transduction (GO:0009967)
post-Golgi Vesicle-Mediated Transport (GO:0006892)
Protein Localization To Membrane (GO:0072657)
Protein Localization To Nucleus (GO:0034504)
Cellular Response To Organonitrogen Compound (GO:0071417)
Cellular Response To Oxygen-Containing Compound (GO:1901701)
RNA-mediated Gene Silencing (GO:0031047)
Multivesicular Body Assembly (GO:0036258)
Positive Regulation Of Dephosphorylation (GO:0035306)
Positive Regulation Of Viral Genome Replication (GO:0045070)
Regulation Of Developmental Growth (GO:0048638)
Positive Regulation Of Hydrolase Activity (GO:0051345)
Ubiquitin-Dependent ERAD Pathway (GO:0030433)
Regulation Of Cell Motility (GO:2000145)
NLS-bearing Protein Import Into Nucleus (GO:0006607)
Growth Hormone Receptor Signaling Pathway (GO:0060396)
Mesenchyme Development (GO:0060485)
Regulation Of Telomerase RNA Localization To Cajal Body (GO:1904872)
NADH Metabolic Process (GO:0006734)
Cellular Biogenic Amine Catabolic Process (GO:0042402)
Negative Regulation Of Mitochondrial Fusion (GO:0010637)
Neurotransmitter Receptor Transport, Endosome To Plasma Membrane (GO:0099639)
Plasma Membrane To Endosome Transport (GO:0048227)
Positive Regulation Of Apoptotic Cell Clearance (GO:2000427)
Positive Regulation Of Fibroblast Migration (GO:0010763)
Positive Regulation Of Membrane Potential (GO:0045838)
Positive Regulation Of Ryanodine-Sensitive Calcium-Release Channel Activity (GO:0060316)
Protein Desumoylation (GO:0016926)
rRNA Pseudouridine Synthesis (GO:0031118)
Regulation Of Apoptotic Cell Clearance (GO:2000425)
Venous Blood Vessel Development (GO:0060841)
Positive Regulation Of Proteasomal Protein Catabolic Process (GO:1901800)
Positive Regulation Of T Cell Activation (GO:0050870)
Regulation Of Cellular Response To Stress (GO:0080135)
Import Into Nucleus (GO:0051170)

Regulation Of mRNA Stability (GO:0043488)
Positive Regulation Of Cell Migration (GO:0030335)
Multivesicular Body Organization (GO:0036257)
Phospholipid Dephosphorylation (GO:0046839)
Positive Regulation Of Locomotion (GO:0040017)
Positive Regulation Of Protein Targeting To Mitochondrion (GO:1903955)
Mitotic Spindle Assembly (GO:0090307)
Positive Regulation Of Osteoblast Differentiation (GO:0045669)
Positive Regulation Of Transferase Activity (GO:0051347)
Positive Regulation Of Neuron Projection Development (GO:0010976)
Glycerolipid Catabolic Process (GO:0046503)
Intracellular Lipid Transport (GO:0032365)
Mitotic Cytokinetic Process (GO:1902410)
ncRNA-mediated Post-Transcriptional Gene Silencing (GO:0035194)
Positive Regulation Of Extracellular Matrix Organization (GO:1903055)
Positive Regulation Of Glycolytic Process (GO:0045821)
Positive Regulation Of Response To Endoplasmic Reticulum Stress (GO:1905898)
Regulation Of Oxidative Stress-Induced Intrinsic Apoptotic Signaling Pathway (GO:1902175)
Regulation Of Vascular Endothelial Cell Proliferation (GO:1905562)
Response To Organophosphorus (GO:0046683)
Triglyceride Homeostasis (GO:0070328)
BMP Signaling Pathway (GO:0030509)
Autophagosome Organization (GO:1905037)
Negative Regulation Of Intrinsic Apoptotic Signaling Pathway (GO:2001243)
Regulation Of Pathway-Restricted SMAD Protein Phosphorylation (GO:0060393)
Cytoskeleton Organization (GO:0007010)
DNA Topological Change (GO:0006265)
Golgi Ribbon Formation (GO:0090161)
RISC Complex Assembly (GO:0070922)
UDP-N-acetylglucosamine Biosynthetic Process (GO:0006048)
Anoikis (GO:0043276)
Astral Microtubule Organization (GO:0030953)
Collagen-Activated Signaling Pathway (GO:0038065)
Glucan Metabolic Process (GO:0044042)
Mitochondrial Fragmentation Involved In Apoptotic Process (GO:0043653)
p38MAPK Cascade (GO:0038066)
Positive Regulation Of Fatty Acid Beta-Oxidation (GO:0032000)
Protein Hexamerization (GO:0034214)
Regulation Of Endothelial Cell Development (GO:1901550)
Regulation Of Endothelial Cell Differentiation (GO:0045601)
Regulation Of miRNA Metabolic Process (GO:2000628)
Regulation Of Organ Growth (GO:0046620)
Regulation Of Pinocytosis (GO:0048548)
Regulation Of Rhodopsin Mediated Signaling Pathway (GO:0022400)
Regulation Of Sterol Biosynthetic Process (GO:0106118)
Regulation Of Toll-Like Receptor 3 Signaling Pathway (GO:0034139)
Tyrosine Phosphorylation Of STAT Protein (GO:0007260)
Regulation Of Autophagy (GO:0010506)

Amide Transport (GO:0042886)
miRNA Processing (GO:0035196)
Cellular Response To Nutrient Levels (GO:0031669)
COPII Vesicle Coating (GO:0048208)
RNA Destabilization (GO:0050779)
RNA-templated DNA Biosynthetic Process (GO:0006278)
Cellular Response To Misfolded Protein (GO:0071218)
Cortical Actin Cytoskeleton Organization (GO:0030866)
Glycogen Metabolic Process (GO:0005977)
Negative Regulation Of Leukocyte Cell-Cell Adhesion (GO:1903038)
Regulation Of Tissue Remodeling (GO:0034103)
Superoxide Anion Generation (GO:0042554)
tRNA Metabolic Process (GO:0006399)
Telomere Maintenance Via Telomerase (GO:0007004)
Vesicle Targeting, Rough ER To cis-Golgi (GO:0048207)
Phosphatidylinositol-Mediated Signaling (GO:0048015)
Positive Regulation Of Transmembrane Receptor Protein Serine/Threonine Kinase Signaling Pathway (GO:00901[illegible]
Regulation Of Cell Cycle Process (GO:0010564)
Positive Regulation Of TOR Signaling (GO:0032008)
Protein Monoubiquitination (GO:0006513)
Positive Regulation Of Intracellular Signal Transduction (GO:1902533)
Negative Regulation Of Protein Phosphorylation (GO:0001933)
Cellular Response To Transforming Growth Factor Beta Stimulus (GO:0071560)
Protein Polymerization (GO:0051258)
Positive Regulation Of Protein Dephosphorylation (GO:0035307)
Positive Regulation Of Small Molecule Metabolic Process (GO:0062013)
Retrograde Vesicle-Mediated Transport, Golgi To Endoplasmic Reticulum (GO:0006890)
Late Endosome To Vacuole Transport (GO:0045324)
Mitochondrial RNA Metabolic Process (GO:0000959)
Regulation Of Cardiac Muscle Contraction By Calcium Ion Signaling (GO:0010882)
Regulation Of Spindle Organization (GO:0090224)
Striated Muscle Cell Differentiation (GO:0051146)
Endocytic Recycling (GO:0032456)
Protein N-linked Glycosylation (GO:0006487)
Protein Stabilization (GO:0050821)
Macromolecule Modification (GO:0043412)
Negative Regulation Of JNK Cascade (GO:0046329)
Positive Regulation Of Carbohydrate Metabolic Process (GO:0045913)
Positive Regulation Of Organelle Organization (GO:0010638)
Autophagy Of Mitochondrion (GO:0000422)
Proton Transmembrane Transport (GO:1902600)
Intracellular Monoatomic Cation Homeostasis (GO:0030003)
Autophagosome Assembly (GO:0000045)
ER-associated Misfolded Protein Catabolic Process (GO:0071712)
Golgi Lumen Acidification (GO:0061795)
I-kappaB Phosphorylation (GO:0007252)
Actin-Mediated Cell Contraction (GO:0070252)
Amino Sugar Biosynthetic Process (GO:0046349)

Histone Ubiquitination (GO:0016574)
Lipid Import Into Cell (GO:0140354)
Long-Chain Fatty Acid Import Into Cell (GO:0044539)
Muscle Cell Fate Commitment (GO:0042693)
Negative Regulation Of Establishment Of Protein Localization (GO:1904950)
Negative Regulation Of Extracellular Matrix Organization (GO:1903054)
Negative Regulation Of Lipid Localization (GO:1905953)
Peptidyl-Amino Acid Modification (GO:0018193)
Positive Regulation Of Protein Acetylation (GO:1901985)
Positive Regulation Of Skeletal Muscle Tissue Development (GO:0048643)
Positive Regulation Of Sodium Ion Transmembrane Transport (GO:1902307)
Positive Regulation Of Vasculogenesis (GO:2001214)
Protein Insertion Into ER Membrane By Stop-Transfer Membrane-Anchor Sequence (GO:0045050)
Quinone Metabolic Process (GO:1901661)
Regulation Of PERK-mediated Unfolded Protein Response (GO:1903897)
Regulation Of Cell-Cell Adhesion Mediated By Integrin (GO:0033632)
Regulation Of Protein Localization To Chromatin (GO:1905634)
Type II Interferon-Mediated Signaling Pathway (GO:0060333)
Negative Regulation Of GTPase Activity (GO:0034260)
Plasma Membrane Repair (GO:0001778)
snRNA Metabolic Process (GO:0016073)
Regulation Of Organelle Organization (GO:0033043)
Cellular Response To BMP Stimulus (GO:0071773)
Mitotic Metaphase Plate Congression (GO:0007080)
Positive Regulation Of Proteasomal Ubiquitin-Dependent Protein Catabolic Process (GO:0032436)
Negative Regulation Of Cellular Biosynthetic Process (GO:0031327)
Regulation Of Protein Kinase Activity (GO:0045859)
Metaphase Plate Congression (GO:0051310)
Regulation Of Release Of Sequestered Calcium Ion Into Cytosol (GO:0051279)
Negative Regulation Of Organelle Assembly (GO:1902116)
Positive Regulation Of Signaling Receptor Activity (GO:2000273)
Receptor Signaling Pathway Via JAK-STAT (GO:0007259)
Regulation Of Peptidyl-Threonine Phosphorylation (GO:0010799)
Positive Regulation Of GTPase Activity (GO:0043547)
NAD Metabolic Process (GO:0019674)
Negative Regulation Of Axonogenesis (GO:0050771)
Negative Regulation Of Cell Development (GO:0010721)
Positive Regulation Of Phosphatase Activity (GO:0010922)
Regulation Of Catabolic Process (GO:0009894)
Regulation Of Mononuclear Cell Migration (GO:0071675)
Regulation Of Release Of Sequestered Calcium Ion Into Cytosol By Sarcoplasmic Reticulum (GO:0010880)
Response To Gamma Radiation (GO:0010332)
Regulation Of Osteoblast Differentiation (GO:0045667)
UDP-N-acetylglucosamine Metabolic Process (GO:0006047)
Fatty Acid Homeostasis (GO:0055089)
Formation Of Extrachromosomal Circular DNA (GO:0001325)
Negative Regulation Of Autophagosome Assembly (GO:1902902)
Nuclear Pore Complex Assembly (GO:0051292)

Nucleoside Triphosphate Metabolic Process (GO:0009141)
Positive Regulation Of Cellular Respiration (GO:1901857)
Positive Regulation Of Mitochondrial Outer Membrane Permeabilization Involved In Apoptotic Signaling Pathway
Positive Regulation Of Nitric-Oxide Synthase Biosynthetic Process (GO:0051770)
Positive Regulation Of Transcription From RNA Polymerase II Promoter In Response To Endoplasmic Reticulum
Protein Localization To Endosome (GO:0036010)
rRNA Base Methylation (GO:0070475)
Receptor-Mediated Endocytosis Of Virus By Host Cell (GO:0019065)
Regulation Of Calcium Ion Transmembrane Transport Via High Voltage-Gated Calcium Channel (GO:1902514)
Regulation Of Cysteine-Type Endopeptidase Activity Involved In Apoptotic Signaling Pathway (GO:2001267)
Regulation Of Glycoprotein Biosynthetic Process (GO:0010559)
Regulation Of Lipid Transport (GO:0032368)
Regulation Of Programmed Necrotic Cell Death (GO:0062098)
Removal Of Superoxide Radicals (GO:0019430)
Response To Sterol (GO:0036314)
Signal Release (GO:0023061)
T-Circle Formation (GO:0090656)
Telomere Maintenance Via Telomere Trimming (GO:0090737)
Regulation Of Proteolysis (GO:0030162)
Mitochondrion Disassembly (GO:0061726)
Muscle Tissue Development (GO:0060537)
Negative Regulation Of Stress-Activated MAPK Cascade (GO:0032873)
Negative Regulation Of Transferase Activity (GO:0051348)
Regulation Of Ossification (GO:0030278)
Regulation Of Phosphoprotein Phosphatase Activity (GO:0043666)
Regulation Of Vascular Associated Smooth Muscle Cell Proliferation (GO:1904705)
mRNA Stabilization (GO:0048255)
Mitochondrial Transport (GO:0006839)
Long-Chain Fatty Acid Transport (GO:0015909)
Negative Regulation Of Protein Depolymerization (GO:1901880)
Nuclear Membrane Reassembly (GO:0031468)
Regulation Of Cell Junction Assembly (GO:1901888)
Regulation Of Centrosome Cycle (GO:0046605)
Regulation Of Nuclear-Transcribed mRNA Catabolic Process, Deadenylation-Dependent Decay (GO:1900151)
Response To BMP (GO:0071772)
Negative Regulation Of Extrinsic Apoptotic Signaling Pathway (GO:2001237)
Protein K48-linked Ubiquitination (GO:0070936)
Membrane Assembly (GO:0071709)
Response To cAMP (GO:0051591)
Negative Regulation Of Protein Kinase Activity (GO:0006469)
Positive Regulation Of Plasma Membrane Bounded Cell Projection Assembly (GO:0120034)
Negative Regulation Of Protein Modification By Small Protein Conjugation Or Removal (GO:1903321)
Organelle Disassembly (GO:1903008)
Positive Regulation Of Leukocyte Chemotaxis (GO:0002690)
Protein Autoubiquitination (GO:0051865)
Biogenic Amine Biosynthetic Process (GO:0042401)
Cellular Response To Superoxide (GO:0071451)
Endosomal Lumen Acidification (GO:0048388)

Inner Ear Receptor Cell Stereocilium Organization (GO:0060122)
Melanocyte Differentiation (GO:0030318)
Methylguanosine-Cap Decapping (GO:0110156)
Mitochondrion Distribution (GO:0048311)
Negative Regulation Of CD4-positive, Alpha-Beta T Cell Activation (GO:2000515)
Negative Regulation Of Reactive Oxygen Species Biosynthetic Process (GO:1903427)
Nucleoside Phosphate Catabolic Process (GO:1901292)
Nucleotide-Binding Oligomerization Domain Containing Signaling Pathway (GO:0070423)
Polyamine Metabolic Process (GO:0006595)
Positive Regulation Of Platelet Aggregation (GO:1901731)
Post-Transcriptional Gene Silencing (GO:0016441)
Protein Polyglutamylation (GO:0018095)
Regulation Of Alcohol Biosynthetic Process (GO:1902930)
Regulation Of Amyloid Precursor Protein Catabolic Process (GO:1902991)
Regulation Of Cell Communication By Electrical Coupling Involved In Cardiac Conduction (GO:1901844)
Regulation Of Cytoplasmic Transport (GO:1903649)
Regulation Of mRNA 3'-End Processing (GO:0031440)
Regulation Of Mitochondrial Fusion (GO:0010635)
Regulation Of Phospholipase C Activity (GO:1900274)
Regulation Of Protein K63-linked Ubiquitination (GO:1900044)
Regulation Of Stress-Activated Protein Kinase Signaling Cascade (GO:0070302)
Regulation Of Type II Interferon-Mediated Signaling Pathway (GO:0060334)
Positive Regulation Of DNA Repair (GO:0045739)
Acylglycerol Homeostasis (GO:0055090)
Eosinophil Chemotaxis (GO:0048245)
Eosinophil Migration (GO:0072677)
Positive Regulation Of Vascular Endothelial Growth Factor Production (GO:0010575)
Protein Exit From Endoplasmic Reticulum (GO:0032527)
Regulation Of Cytoplasmic Translation (GO:2000765)
Regulation Of Endoplasmic Reticulum Stress-Induced Intrinsic Apoptotic Signaling Pathway (GO:1902235)
Regulation Of Ryanodine-Sensitive Calcium-Release Channel Activity (GO:0060314)
Response To Peptide Hormone (GO:0043434)
Axonal Transport (GO:0098930)
Carboxylic Acid Transmembrane Transport (GO:1905039)
Negative Regulation Of Transport (GO:0051051)
Regulation Of Mitochondrial Membrane Potential (GO:0051881)
Regulation Of Nitric Oxide Biosynthetic Process (GO:0045428)
Vitamin Transport (GO:0051180)
Positive Regulation Of Intracellular Protein Transport (GO:0090316)
Transforming Growth Factor Beta Receptor Signaling Pathway (GO:0007179)
Positive Regulation Of Secretion By Cell (GO:1903532)
Positive Regulation Of DNA-templated Transcription Initiation (GO:2000144)
mRNA 3'-End Processing (GO:0031124)
Positive Regulation Of Wound Healing (GO:0090303)
Regulation Of Glycolytic Process (GO:0006110)
Regulation Of Microtubule Cytoskeleton Organization (GO:0070507)
Negative Regulation Of Protein Metabolic Process (GO:0051248)
Cellular Response To Hexose Stimulus (GO:0071331)

Myotube Differentiation (GO:0014902)
Negative Regulation Of Intracellular Protein Transport (GO:0090317)
Negative Regulation Of Response To Endoplasmic Reticulum Stress (GO:1903573)
Positive Regulation Of Striated Muscle Cell Differentiation (GO:0051155)
Regulation Of Biomineral Tissue Development (GO:0070167)
Regulation Of Cell Size (GO:0008361)
Regulation Of Chondrocyte Differentiation (GO:0032330)
Regulation Of Glycogen Biosynthetic Process (GO:0005979)
Wound Healing, Spreading Of Cells (GO:0044319)
Positive Regulation Of Lymphocyte Proliferation (GO:0050671)
Membrane Organization (GO:0061024)
C-terminal Protein Amino Acid Modification (GO:0018410)
DNA Conformation Change (GO:0071103)
MyD88-dependent Toll-Like Receptor Signaling Pathway (GO:0002755)
Adipose Tissue Development (GO:0060612)
Biogenic Amine Metabolic Process (GO:0006576)
Endochondral Bone Morphogenesis (GO:0060350)
Folic Acid Metabolic Process (GO:0046655)
Histone H4-K16 Acetylation (GO:0043984)
Negative Regulation Of Alcohol Biosynthetic Process (GO:1902931)
Negative Regulation Of Complement Activation (GO:0045916)
Negative Regulation Of Locomotion (GO:0040013)
Negative Regulation Of Macrophage Derived Foam Cell Differentiation (GO:0010745)
Positive Regulation Of Fatty Acid Oxidation (GO:0046321)
Positive Regulation Of Histone H3-K4 Methylation (GO:0051571)
Positive Regulation Of Nuclear-Transcribed mRNA poly(A) Tail Shortening (GO:0060213)
Positive Regulation Of Vascular Endothelial Cell Proliferation (GO:1905564)
pre-miRNA Processing (GO:0031054)
Pseudouridine Synthesis (GO:0001522)
Regulation Of Actomyosin Structure Organization (GO:0110020)
Regulation Of Chemokine (C-X-C Motif) Ligand 2 Production (GO:2000341)
Regulation Of Dosage Compensation By Inactivation Of X Chromosome (GO:1900095)
Regulation Of Monooxygenase Activity (GO:0032768)
Regulation Of Retinoic Acid Receptor Signaling Pathway (GO:0048385)
Regulation Of Sensory Perception (GO:0051931)
Regulation Of Vascular Associated Smooth Muscle Cell Migration (GO:1904752)
Relaxation Of Cardiac Muscle (GO:0055119)
snRNA Transcription (GO:0009301)
Sodium Ion Export Across Plasma Membrane (GO:0036376)
Negative Regulation Of Cell Differentiation (GO:0045596)
Regulation Of Cell Growth (GO:0001558)
Regulation Of Cell Migration (GO:0030334)
Mitotic Sister Chromatid Segregation (GO:0000070)
Protein Localization To Lysosome (GO:0061462)
Regulation Of Microtubule-Based Process (GO:0032886)
Negative Regulation Of Amide Metabolic Process (GO:0034249)
Regulation Of Bone Mineralization (GO:0030500)
L-amino Acid Transport (GO:0015807)

Nuclear Membrane Organization (GO:0071763)
Regulation Of Vascular Endothelial Growth Factor Production (GO:0010574)
Telomere Maintenance Via Telomere Lengthening (GO:0010833)
protein-RNA Complex Assembly (GO:0022618)
Cellular Response To Hormone Stimulus (GO:0032870)
Positive Regulation Of Gene Expression (GO:0010628)
Monocyte Chemotaxis (GO:0002548)
Regulation Of Axonogenesis (GO:0050770)
Substantia Nigra Development (GO:0021762)
DNA-templated Transcription Initiation (GO:0006352)
Negative Regulation Of Protein-Containing Complex Assembly (GO:0031333)
Regulation Of Cell Death (GO:0010941)
T Cell Receptor Signaling Pathway (GO:0050852)
Regulation Of T Cell Proliferation (GO:0042129)
Cortical Cytoskeleton Organization (GO:0030865)
Positive Regulation Of Response To Wounding (GO:1903036)
Cellular Response To Interleukin-4 (GO:0071353)
Clathrin Coat Assembly (GO:0048268)
Endoplasmic Reticulum Mannose Trimming (GO:1904380)
Inhibitory Synapse Assembly (GO:1904862)
Membrane Protein Intracellular Domain Proteolysis (GO:0031293)
Negative Regulation Of Cartilage Development (GO:0061037)
Negative Regulation Of Endoplasmic Reticulum Unfolded Protein Response (GO:1900102)
Neuromuscular Process Controlling Balance (GO:0050885)
Positive Regulation Of Fatty Acid Biosynthetic Process (GO:0045723)
Positive Regulation Of Macrophage Chemotaxis (GO:0010759)
Regulation Of IRE1-mediated Unfolded Protein Response (GO:1903894)
Regulation Of Fatty Acid Beta-Oxidation (GO:0031998)
Regulation Of Lamellipodium Organization (GO:1902743)
Regulation Of Nitric-Oxide Synthase Biosynthetic Process (GO:0051769)
Regulation Of Nitrogen Compound Metabolic Process (GO:0051171)
Regulation Of Response To Endoplasmic Reticulum Stress (GO:1905897)
Regulation Of Vasculogenesis (GO:2001212)
Relaxation Of Muscle (GO:0090075)
Thymus Development (GO:0048538)
Negative Regulation Of BMP Signaling Pathway (GO:0030514)
Regulation Of Extrinsic Apoptotic Signaling Pathway (GO:2001236)
Antigen Receptor-Mediated Signaling Pathway (GO:0050851)
Regulation Of Neuron Projection Development (GO:0010975)
Positive Regulation Of Interleukin-8 Production (GO:0032757)
Positive Regulation Of Organelle Assembly (GO:1902117)
Nucleus Organization (GO:0006997)
Phosphatidylinositol Phosphate Biosynthetic Process (GO:0046854)
Regulation Of Insulin Receptor Signaling Pathway (GO:0046626)
Positive Regulation Of Cytoskeleton Organization (GO:0051495)
Positive Regulation Of Protein Modification By Small Protein Conjugation Or Removal (GO:1903322)
Apoptotic Cell Clearance (GO:0043277)
Membrane Fusion (GO:0061025)

Regulation Of DNA Damage Response, Signal Transduction By P53 Class Mediator (GO:0043516)
Regulation Of Glucose Metabolic Process (GO:0010906)
Regulation Of Lipid Biosynthetic Process (GO:0046890)
Regulation Of Nitric-Oxide Synthase Activity (GO:0050999)
Regulation Of Plasma Membrane Bounded Cell Projection Organization (GO:0120035)
Regulation Of Protein Transport (GO:0051223)
Vesicle Coating (GO:0006901)
Regulation Of Protein Kinase B Signaling (GO:0051896)
Phosphatidylinositol Biosynthetic Process (GO:0006661)
Protein Maturation (GO:0051604)
RNA Methylation (GO:0001510)
Glycerophospholipid Metabolic Process (GO:0006650)
Regulation Of Embryonic Development (GO:0045995)
Wound Healing (GO:0042060)
2-Oxoglutarate Metabolic Process (GO:0006103)
Establishment Of Spindle Localization (GO:0051293)
Histone H2A Monoubiquitination (GO:0035518)
Mammary Gland Epithelium Development (GO:0061180)
Maturation Of LSU-rRNA From Tricistronic rRNA Transcript (SSU-rRNA, 5.8S rRNA, LSU-rRNA) (GO:0000463)
miRNA-mediated Gene Silencing By Inhibition Of Translation (GO:0035278)
Negative Regulation Of Chondrocyte Differentiation (GO:0032331)
Negative Regulation Of Peptidyl-Threonine Phosphorylation (GO:0010801)
Negative Regulation Of Smooth Muscle Cell Migration (GO:0014912)
Nuclear Pore Organization (GO:0006999)
Pathway-Restricted SMAD Protein Phosphorylation (GO:0060389)
Positive Regulation Of Acute Inflammatory Response (GO:0002675)
Positive Regulation Of Nuclear-Transcribed mRNA Catabolic Process, Deadenylation-Dependent Decay (GO:190
Positive Regulation Of Protein-Containing Complex Disassembly (GO:0043243)
Positive Regulation Of Transcription From RNA Polymerase II Promoter Involved In Cellular Response To Chemi
Positive Regulation Of Transporter Activity (GO:0032411)
Prostanoid Biosynthetic Process (GO:0046457)
Protein Localization To Vacuole (GO:0072665)
Regulation Of Establishment Of Protein Localization (GO:0070201)
Regulation Of Histone H3-K4 Methylation (GO:0051569)
Regulation Of Nuclear-Transcribed mRNA poly(A) Tail Shortening (GO:0060211)
Regulation Of Sensory Perception Of Pain (GO:0051930)
Protein Localization To Plasma Membrane (GO:0072659)
I-kappaB kinase/NF-kappaB Signaling (GO:0007249)
Positive Regulation Of Cell Projection Organization (GO:0031346)
Carbohydrate Derivative Biosynthetic Process (GO:1901137)
Heterotypic Cell-Cell Adhesion (GO:0034113)
Modified Amino Acid Transport (GO:0072337)
Protein Acetylation (GO:0006473)
Response To Alcohol (GO:0097305)
Response To Steroid Hormone (GO:0048545)
Vacuolar Acidification (GO:0007035)
Regulation Of Interleukin-8 Production (GO:0032677)
Negative Regulation Of Cytokine Production (GO:0001818)

ncRNA Processing (GO:0034470)
Antiviral Innate Immune Response (GO:0140374)
Protein Sumoylation (GO:0016925)
Positive Regulation Of Macroautophagy (GO:0016239)
DNA Geometric Change (GO:0032392)
Regulation Of Dendritic Spine Morphogenesis (GO:0061001)
Activation Of NF-kappaB-inducing Kinase Activity (GO:0007250)
Endoplasmic Reticulum To Cytosol Transport (GO:1903513)
Histone H4-K5 Acetylation (GO:0043981)
Histone H4-K8 Acetylation (GO:0043982)
mRNA Methylation (GO:0080009)
Negative Regulation Of Endoplasmic Reticulum Stress-Induced Intrinsic Apoptotic Signaling Pathway (GO:1902
Negative Regulation Of Synaptic Transmission (GO:0050805)
Negative Regulation Of Type I Interferon-Mediated Signaling Pathway (GO:0060339)
Neurotrophin Signaling Pathway (GO:0038179)
Nucleotide-Sugar Biosynthetic Process (GO:0009226)
Positive Regulation Of Secretion (GO:0051047)
Protein Localization To Microtubule Organizing Center (GO:1905508)
Regulation Of Cellular Response To Heat (GO:1900034)
Regulation Of Lipoprotein Lipase Activity (GO:0051004)
Regulation Of miRNA-mediated Gene Silencing (GO:0060964)
Regulation Of Multicellular Organism Growth (GO:0040014)
Regulation Of Systemic Arterial Blood Pressure (GO:0003073)
Transmembrane Receptor Protein Tyrosine Kinase Signaling Pathway (GO:0007169)
Vascular Transport (GO:0010232)
Regulation Of RNA Splicing (GO:0043484)
Regulation Of Protein Secretion (GO:0050708)
Golgi To Plasma Membrane Transport (GO:0006893)
Epithelial To Mesenchymal Transition (GO:0001837)
Negative Regulation Of Hydrolase Activity (GO:0051346)
Regulation Of T Cell Differentiation (GO:0045580)
Regulation Of Wound Healing (GO:0061041)
Response To Organonitrogen Compound (GO:0010243)
Positive Regulation Of T Cell Proliferation (GO:0042102)
Positive Regulation Of Cell Cycle (GO:0045787)
Fatty Acid Metabolic Process (GO:0006631)
Receptor-Mediated Endocytosis (GO:0006898)
Protein-Containing Complex Assembly (GO:0065003)
Protein Localization To Cell Periphery (GO:1990778)
Protein Autophosphorylation (GO:0046777)
Ribosomal Small Subunit Biogenesis (GO:0042274)
Cellular Response To Cytokine Stimulus (GO:0071345)
Actin Filament Polymerization (GO:0030041)
Negative Regulation Of Developmental Growth (GO:0048640)
Negative Regulation Of Intracellular Steroid Hormone Receptor Signaling Pathway (GO:0033144)
Receptor Signaling Pathway Via STAT (GO:0097696)
Regulation Of Centrosome Duplication (GO:0010824)
Regulation Of Transmembrane Receptor Protein Serine/Threonine Kinase Signaling Pathway (GO:0090092)

Response To Estradiol (GO:0032355)
Toll-Like Receptor Signaling Pathway (GO:0002224)
Cytoplasmic Microtubule Organization (GO:0031122)
Regulation Of Endocytosis (GO:0030100)
Positive Regulation Of Protein Modification Process (GO:0031401)
Integrin-Mediated Signaling Pathway (GO:0007229)
Positive Regulation Of Endothelial Cell Migration (GO:0010595)
Transport Across Blood-Brain Barrier (GO:0150104)
Positive Regulation Of MAPK Cascade (GO:0043410)
Extrinsic Apoptotic Signaling Pathway In Absence Of Ligand (GO:0097192)
Heart Process (GO:0003015)
Negative Regulation Of Anoikis (GO:2000811)
Negative Regulation Of Biosynthetic Process (GO:0009890)
Negative Regulation Of Intrinsic Apoptotic Signaling Pathway By P53 Class Mediator (GO:1902254)
Nuclear Migration (GO:0007097)
Pharyngeal System Development (GO:0060037)
Protein Alpha-1,2-Demannosylation (GO:0036508)
Regulation Of Autophagosome Maturation (GO:1901096)
Regulation Of Establishment Of Endothelial Barrier (GO:1903140)
Regulation Of Interleukin-1 Production (GO:0032652)
Regulation Of Transforming Growth Factor Beta Production (GO:0071634)
Regulation Of Triglyceride Biosynthetic Process (GO:0010866)
Reticulophagy (GO:0061709)
Tail-Anchored Membrane Protein Insertion Into ER Membrane (GO:0071816)
Vasculature Development (GO:0001944)
Activation Of Cysteine-Type Endopeptidase Activity Involved In Apoptotic Process (GO:0006919)
Nuclear Export (GO:0051168)
Regulation Of Cell Communication (GO:0010646)
Exocytosis (GO:0006887)
Negative Regulation Of Binding (GO:0051100)
Regulation Of Mitotic Cell Cycle (GO:0007346)
NIK/NF-kappaB Signaling (GO:0038061)
Intracellular pH Reduction (GO:0051452)
Negative Regulation Of Proteasomal Ubiquitin-Dependent Protein Catabolic Process (GO:0032435)
Phosphatidylinositol 3-Kinase Signaling (GO:0014065)
Regulation Of Calcium Ion Transmembrane Transporter Activity (GO:1901019)
Vesicle Budding From Membrane (GO:0006900)
Regulation Of G1/S Transition Of Mitotic Cell Cycle (GO:2000045)
Response To Calcium Ion (GO:0051592)
Chromatin Remodeling (GO:0006338)
Cellular Response To Starvation (GO:0009267)
Dicarboxylic Acid Metabolic Process (GO:0043648)
Intracellular Iron Ion Homeostasis (GO:0006879)
Regulation Of TORC1 Signaling (GO:1903432)
Ribosome Assembly (GO:0042255)
Cardiac Muscle Contraction (GO:0060048)
Inositol Lipid-Mediated Signaling (GO:0048017)
Positive Regulation Of Interleukin-12 Production (GO:0032735)

Regulation Of G0 To G1 Transition (GO:0070316)
Regulation Of Sprouting Angiogenesis (GO:1903670)
Regulation Of Type I Interferon-Mediated Signaling Pathway (GO:0060338)
NAD Biosynthetic Process (GO:0009435)
Central Nervous System Neuron Development (GO:0021954)
Embryo Development Ending In Birth Or Egg Hatching (GO:0009792)
Leukocyte Tethering Or Rolling (GO:0050901)
Long-Chain fatty-acyl-CoA Biosynthetic Process (GO:0035338)
mRNA Cis Splicing, Via Spliceosome (GO:0045292)
Negative Regulation Of Oxidative Stress-Induced Intrinsic Apoptotic Signaling Pathway (GO:1902176)
Negative Regulation Of Ubiquitin-Protein Transferase Activity (GO:0051444)
Negative Regulation Of Vascular Associated Smooth Muscle Cell Proliferation (GO:1904706)
Positive Regulation Of Leukocyte Proliferation (GO:0070665)
Positive Regulation Of Transcription From RNA Polymerase II Promoter In Response To Stress (GO:0036003)
Prostaglandin Biosynthetic Process (GO:0001516)
Protein Localization To Centrosome (GO:0071539)
Regulation Of DNA-templated DNA Replication (GO:0090329)
Regulation Of Aerobic Respiration (GO:1903715)
Regulation Of Cartilage Development (GO:0061035)
Regulation Of Oxidative Phosphorylation (GO:0002082)
Regulation Of Tubulin Deacetylation (GO:0090043)
Regulation Of Ubiquitin Protein Ligase Activity (GO:1904666)
Retrograde Protein Transport, ER To Cytosol (GO:0030970)
Transepithelial Transport (GO:0070633)
Negative Regulation Of Neuron Death (GO:1901215)
Cellular Response To Calcium Ion (GO:0071277)
Regulation Of Heart Contraction (GO:0008016)
Positive Regulation Of Phosphorylation (GO:0042327)
Regulation Of Smoothened Signaling Pathway (GO:0008589)
Regulation Of Stress-Activated MAPK Cascade (GO:0032872)
Cellular Response To Chemical Stress (GO:0062197)
Positive Regulation Of Protein Polymerization (GO:0032273)
Regulation Of G Protein-Coupled Receptor Signaling Pathway (GO:0008277)
Regulation Of Actin Filament Polymerization (GO:0030833)
Regulation Of Cellular Biosynthetic Process (GO:0031326)
Positive Regulation Of Cysteine-Type Endopeptidase Activity Involved In Apoptotic Process (GO:0043280)
Regulation Of DNA Repair (GO:0006282)
Apoptotic Mitochondrial Changes (GO:0008637)
Cellular Response To Glucose Stimulus (GO:0071333)
Negative Regulation Of Cell Cycle Process (GO:0010948)
Rho Protein Signal Transduction (GO:0007266)
Negative Regulation Of Innate Immune Response (GO:0045824)
Negative Regulation Of Response To Stimulus (GO:0048585)
Positive Regulation Of Transcription Initiation By RNA Polymerase II (GO:0060261)
Regulation Of JNK Cascade (GO:0046328)
Negative Regulation Of Protein Binding (GO:0032091)
Negative Regulation Of Supramolecular Fiber Organization (GO:1902904)
Positive Regulation Of Cell-Substrate Adhesion (GO:0010811)

Regulation Of Hydrolase Activity (GO:0051336)
Neuron Projection Development (GO:0031175)
Autophagy Of Nucleus (GO:0044804)
Cardiac Muscle Cell Contraction (GO:0086003)
Energy Reserve Metabolic Process (GO:0006112)
Excitatory Postsynaptic Potential (GO:0060079)
fatty-acyl-CoA Metabolic Process (GO:0035337)
Lysosomal Lumen Acidification (GO:0007042)
Mitochondrial Fusion (GO:0008053)
Negative Regulation Of ERBB Signaling Pathway (GO:1901185)
Negative Regulation Of T Cell Receptor Signaling Pathway (GO:0050860)
Negative Regulation Of Lipid Storage (GO:0010888)
Negative Regulation Of Nervous System Development (GO:0051961)
Neural Crest Cell Differentiation (GO:0014033)
Positive Regulation Of Macrophage Migration (GO:1905523)
Positive Regulation Of Natural Killer Cell Activation (GO:0032816)
Positive Regulation Of Phosphoprotein Phosphatase Activity (GO:0032516)
Positive Regulation Of Triglyceride Metabolic Process (GO:0090208)
Regulation Of Epidermal Cell Differentiation (GO:0045604)
Regulation Of Histone Modification (GO:0031056)
Regulation Of Nervous System Process (GO:0031644)
Response To Ethanol (GO:0045471)
Positive Regulation Of Macromolecule Metabolic Process (GO:0010604)
Regulation Of RNA Metabolic Process (GO:0051252)
Glycoprotein Metabolic Process (GO:0009100)
Dendrite Morphogenesis (GO:0048813)
Neutral Amino Acid Transport (GO:0015804)
Positive Regulation Of Substrate Adhesion-Dependent Cell Spreading (GO:1900026)
Regulatory ncRNA Processing (GO:0070918)
Skeletal Muscle Organ Development (GO:0060538)
Cellular Response To Hypoxia (GO:0071456)
Organelle Assembly (GO:0070925)
Cellular Response To Decreased Oxygen Levels (GO:0036294)
Protein K63-linked Ubiquitination (GO:0070534)
Regulation Of Blood Vessel Endothelial Cell Migration (GO:0043535)
Regulation Of Focal Adhesion Assembly (GO:0051893)
Regulation Of Smooth Muscle Cell Proliferation (GO:0048660)
NADP Metabolic Process (GO:0006739)
Bicarbonate Transport (GO:0015701)
Establishment Of Epithelial Cell Polarity (GO:0090162)
Ganglioside Metabolic Process (GO:0001573)
Glycosphingolipid Biosynthetic Process (GO:0006688)
Leukocyte Adhesion To Vascular Endothelial Cell (GO:0061756)
Negative Regulation Of Cyclin-Dependent Protein Serine/Threonine Kinase Activity (GO:0045736)
Negative Regulation Of Peptidyl-Serine Phosphorylation (GO:0033137)
Pore Complex Assembly (GO:0046931)
Positive Regulation Of Histone Methylation (GO:0031062)
Positive Regulation Of Mitochondrial Fission (GO:0090141)

Receptor Clustering (GO:0043113)
Regulation Of Early Endosome To Late Endosome Transport (GO:2000641)
Regulation Of Granulocyte Chemotaxis (GO:0071622)
Regulation Of Protein-Containing Complex Disassembly (GO:0043244)
snRNA Processing (GO:0016180)
Positive Regulation Of Protein Kinase B Signaling (GO:0051897)
Regulation Of Endothelial Cell Proliferation (GO:0001936)
COPII-coated Vesicle Budding (GO:0090114)
DNA Damage Response, Signal Transduction By P53 Class Mediator (GO:0030330)
Histone H3 Acetylation (GO:0043966)
Lytic Vacuole Organization (GO:0080171)
Negative Regulation Of Protein Kinase B Signaling (GO:0051898)
Positive Regulation Of RNA Splicing (GO:0033120)
Positive Regulation Of Phospholipase C Activity (GO:0010863)
Protein Destabilization (GO:0031648)
Ribosome Biogenesis (GO:0042254)
Peptidyl-Lysine Modification (GO:0018205)
C-terminal Protein Deglutamylation (GO:0035609)
CD4-positive, Alpha-Beta T Cell Activation (GO:0035710)
L-aspartate Import Across Plasma Membrane (GO:0140009)
L-aspartate Transmembrane Transport (GO:0070778)
RNA Decapping (GO:0110154)
TORC2 Signaling (GO:0038203)
Adiponectin-Activated Signaling Pathway (GO:0033211)
Axo-Dendritic Transport (GO:0008088)
Chondrocyte Development (GO:0002063)
Dermatan Sulfate Proteoglycan Metabolic Process (GO:0050655)
Embryonic Brain Development (GO:1990403)
Equilibrioception (GO:0050957)
Histamine Metabolic Process (GO:0001692)
Inner Ear Auditory Receptor Cell Differentiation (GO:0042491)
interleukin-17A-mediated Signaling Pathway (GO:0038173)
Interleukin-4-Mediated Signaling Pathway (GO:0035771)
Interleukin-5-Mediated Signaling Pathway (GO:0038043)
Intracellular Phosphate Ion Homeostasis (GO:0030643)
Intracellular Water Homeostasis (GO:0009992)
Leukocyte Chemotaxis Involved In Inflammatory Response (GO:0002232)
Lipid Droplet Disassembly (GO:1905691)
Lipoprotein Metabolic Process (GO:0042157)
mRNA 3'-End Processing By Stem-Loop Binding And Cleavage (GO:0006398)
Negative Regulation Of PERK-mediated Unfolded Protein Response (GO:1903898)
Negative Regulation Of Actin Filament Depolymerization (GO:0030835)
Negative Regulation Of Cytokinesis (GO:0032466)
Negative Regulation Of Excitatory Postsynaptic Potential (GO:0090394)
Negative Regulation Of Glycogen Biosynthetic Process (GO:0045719)
Negative Regulation Of Insulin-Like Growth Factor Receptor Signaling Pathway (GO:0043569)
Negative Regulation Of Lipoprotein Lipase Activity (GO:0051005)
Negative Regulation Of Mast Cell Activation (GO:0033004)

Negative Regulation Of Protein Localization To Chromatin (GO:0120186)
Negative Regulation Of Protein Polyubiquitination (GO:1902915)
Negative Regulation Of Transcription From RNA Polymerase II Promoter In Response To Stress (GO:0097201)
Negative Regulation Of Trophoblast Cell Migration (GO:1901164)
Nephron Tubule Development (GO:0072080)
Osmosensory Signaling Pathway (GO:0007231)
Peptidyl-Diphthamide Biosynthetic Process From Peptidyl-Histidine (GO:0017183)
Peptidyl-Diphthamide Metabolic Process (GO:0017182)
Positive Regulation Of NK T Cell Proliferation (GO:0051142)
Positive Regulation Of Activin Receptor Signaling Pathway (GO:0032927)
Positive Regulation Of Autophagy Of Mitochondrion In Response To Mitochondrial Depolarization (GO:1904925
Positive Regulation Of Cardiac Muscle Cell Differentiation (GO:2000727)
Positive Regulation Of Cholesterol Biosynthetic Process (GO:0045542)
Positive Regulation Of Extrinsic Apoptotic Signaling Pathway Via Death Domain Receptors (GO:1902043)
Positive Regulation Of Focal Adhesion Disassembly (GO:0120183)
Positive Regulation Of Monoatomic Ion Transmembrane Transport (GO:0034767)
Positive Regulation Of Protein Catabolic Process In The Vacuole (GO:1904352)
Positive Regulation Of Protein Localization To Cilium (GO:1903566)
Positive Regulation Of Skeletal Muscle Fiber Development (GO:0048743)
Positive Regulation Of Sterol Biosynthetic Process (GO:0106120)
Positive Regulation Of Sterol Transport (GO:0032373)
Positive Regulation Of Toll-Like Receptor 2 Signaling Pathway (GO:0034137)
Positive Regulation Of Translation In Response To Stress (GO:0032056)
Postsynaptic Density Assembly (GO:0097107)
Prepulse Inhibition (GO:0060134)
Presynaptic Active Zone Organization (GO:1990709)
Protein Import (GO:0017038)
Protein Modification By Small Protein Conjugation Or Removal (GO:0070647)
Protein Ufmylation (GO:0071569)
Purine Ribonucleoside Catabolic Process (GO:0046130)
Regulation Of 3'-UTR-mediated mRNA Stabilization (GO:1905868)
Regulation Of NK T Cell Activation (GO:0051133)
Regulation Of Blood Vessel Remodeling (GO:0060312)
Regulation Of Endoplasmic Reticulum Tubular Network Organization (GO:1903371)
Regulation Of Establishment Of T Cell Polarity (GO:1903903)
Regulation Of Focal Adhesion Disassembly (GO:0120182)
Regulation Of Glycogen (Starch) Synthase Activity (GO:2000465)
Regulation Of Growth Hormone Receptor Signaling Pathway (GO:0060398)
Regulation Of Hepatocyte Proliferation (GO:2000345)
Regulation Of Histone Deacetylase Activity (GO:1901725)
Regulation Of Hyaluronan Biosynthetic Process (GO:1900125)
Regulation Of Long-Chain Fatty Acid Import Across Plasma Membrane (GO:0010746)
Regulation Of mRNA Binding (GO:1902415)
Regulation Of Macrophage Apoptotic Process (GO:2000109)
Regulation Of Post-Transcriptional Gene Silencing By RNA (GO:1900368)
Regulation Of Stress Granule Assembly (GO:0062028)
Regulation Of System Process (GO:0044057)
Regulation Of Translation In Response To Endoplasmic Reticulum Stress (GO:0036490)

Response To Angiotensin (GO:1990776)
Response To cGMP (GO:0070305)
Response To Testosterone (GO:0033574)
Signal Complex Assembly (GO:0007172)
snoRNA Localization (GO:0048254)
tRNA Catabolic Process (GO:0016078)
Tight Junction Organization (GO:0120193)
Tongue Development (GO:0043586)
Ventricular Cardiac Muscle Cell Development (GO:0055015)
Ventricular Compact Myocardium Morphogenesis (GO:0003223)
Zymogen Inhibition (GO:0097341)
Regulation Of Cellular Component Biogenesis (GO:0044087)
Regulation Of BMP Signaling Pathway (GO:0030510)
Regulation Of Fat Cell Differentiation (GO:0045598)
RNA Polymerase II Preinitiation Complex Assembly (GO:0051123)
Peptide Metabolic Process (GO:0006518)
Regulation Of Cation Channel Activity (GO:2001257)
Cell Projection Organization (GO:0030030)
Cellular Response To Estradiol Stimulus (GO:0071392)
Maturation Of LSU-rRNA (GO:0000470)
Mesoderm Formation (GO:0001707)
Negative Regulation Of Cyclin-Dependent Protein Kinase Activity (GO:1904030)
Negative Regulation Of Cytokine Production Involved In Inflammatory Response (GO:1900016)
Negative Regulation Of miRNA Transcription (GO:1902894)
Negative Regulation Of Microtubule Depolymerization (GO:0007026)
Negative Regulation Of Phosphoprotein Phosphatase Activity (GO:0032515)
Negative Regulation Of Protein Localization To Cell Periphery (GO:1904376)
Negative Regulation Of Protein Localization To Plasma Membrane (GO:1903077)
Phosphatidylserine Metabolic Process (GO:0006658)
Positive Regulation Of Lipid Catabolic Process (GO:0050996)
Positive Regulation Of Phospholipase Activity (GO:0010518)
Positive Regulation Of Protein Processing (GO:0010954)
Positive Regulation Of Vascular Associated Smooth Muscle Cell Proliferation (GO:1904707)
Regulation Of Calcium Ion Transmembrane Transport (GO:1903169)
Regulation Of Cardiac Muscle Cell Proliferation (GO:0060043)
Regulation Of Fibroblast Migration (GO:0010762)
Regulation Of Macrophage Chemotaxis (GO:0010758)
Regulation Of Necroptotic Process (GO:0060544)
Stress-Activated Protein Kinase Signaling Cascade (GO:0031098)
Positive Regulation Of ERK1 And ERK2 Cascade (GO:0070374)
G2/M Transition Of Mitotic Cell Cycle (GO:0000086)
RNA 3'-End Processing (GO:0031123)
Import Into Cell (GO:0098657)
Negative Regulation Of Epithelial Cell Apoptotic Process (GO:1904036)
Positive Regulation Of Bone Mineralization (GO:0030501)
Positive Regulation Of Cation Channel Activity (GO:2001259)
Regulation Of ERK1 And ERK2 Cascade (GO:0070372)
Regulation Of Gene Expression (GO:0010468)

Cellular Response To Molecule Of Bacterial Origin (GO:0071219)
Cellular Response To Oxidative Stress (GO:0034599)
Chemokine-Mediated Signaling Pathway (GO:0070098)
Endosome To Lysosome Transport (GO:0008333)
Post-Transcriptional Regulation Of Gene Expression (GO:0010608)
Regulation Of Transcription Initiation By RNA Polymerase II (GO:0060260)
Response To Lipopolysaccharide (GO:0032496)
Regulation Of Protein Localization (GO:0032880)
Regulation Of Small GTPase Mediated Signal Transduction (GO:0051056)
Ribonucleoprotein Complex Biogenesis (GO:0022613)
Positive Regulation Of DNA-binding Transcription Factor Activity (GO:0051091)
Chaperone-Mediated Protein Complex Assembly (GO:0051131)
Negative Regulation Of miRNA Metabolic Process (GO:2000629)
Negative Regulation Of Reactive Oxygen Species Metabolic Process (GO:2000378)
Peptide Catabolic Process (GO:0043171)
Positive Regulation Of G2/M Transition Of Mitotic Cell Cycle (GO:0010971)
Positive Regulation Of Interferon-Alpha Production (GO:0032727)
Positive Regulation Of Translational Initiation (GO:0045948)
Regulation Of Anoikis (GO:2000209)
Regulation Of Cell-Substrate Junction Assembly (GO:0090109)
Regulation Of Histone Deacetylation (GO:0031063)
Zinc Ion Transmembrane Transport (GO:0071577)
Negative Regulation Of Transcription By RNA Polymerase II (GO:0000122)
Muscle Organ Development (GO:0007517)
Positive Regulation Of Mitochondrion Organization (GO:0010822)
Regulation Of Neuron Death (GO:1901214)
Response To Organic Cyclic Compound (GO:0014070)
Selective Autophagy (GO:0061912)
RNA Transport (GO:0050658)
Histone H4 Acetylation (GO:0043967)
Negative Regulation Of Ubiquitin-Dependent Protein Catabolic Process (GO:2000059)
Nuclear Transport (GO:0051169)
Positive Regulation Of Lipid Biosynthetic Process (GO:0046889)
Positive Regulation Of Lymphocyte Differentiation (GO:0045621)
Retina Homeostasis (GO:0001895)
Negative Regulation Of Cell Population Proliferation (GO:0008285)
Negative Regulation Of Protein Modification Process (GO:0031400)
Proteolysis Involved In Protein Catabolic Process (GO:0051603)
Positive Regulation Of Angiogenesis (GO:0045766)
DNA Repair (GO:0006281)
Mitochondrion Organization (GO:0007005)
DNA Strand Resection Involved In Replication Fork Processing (GO:0110025)
IRE1-mediated Unfolded Protein Response (GO:0036498)
N-acetylneuraminate Catabolic Process (GO:0019262)
SMAD Protein Complex Assembly (GO:0007183)
Adenosine Metabolic Process (GO:0046085)
Aldehyde Biosynthetic Process (GO:0046184)
Anterograde Dendritic Transport (GO:0098937)

Auditory Receptor Cell Morphogenesis (GO:0002093)
Auditory Receptor Cell Stereocilium Organization (GO:0060088)
Cellular Response To Bacterial Lipopeptide (GO:0071221)
Cellular Response To Histamine (GO:0071420)
Cellular Response To Interleukin-9 (GO:0071355)
Citrulline Metabolic Process (GO:0000052)
Clathrin Coat Disassembly (GO:0072318)
Epithelial Cell Differentiation Involved In Kidney Development (GO:0035850)
Free Ubiquitin Chain Polymerization (GO:0010994)
Ganglioside Catabolic Process (GO:0006689)
Glucan Catabolic Process (GO:0009251)
Glycogen Catabolic Process (GO:0005980)
Histone H2A-K119 Monoubiquitination (GO:0036353)
Inner Ear Receptor Cell Differentiation (GO:0060113)
Interleukin-3-Mediated Signaling Pathway (GO:0038156)
Interleukin-9-Mediated Signaling Pathway (GO:0038113)
Intracellular Triglyceride Homeostasis (GO:0035356)
Locomotor Rhythm (GO:0045475)
Lymphocyte Migration Into Lymphoid Organs (GO:0097021)
Membrane Biogenesis (GO:0044091)
Negative Regulation Of CD4-positive, Alpha-Beta T Cell Proliferation (GO:2000562)
Negative Regulation Of T-helper 17 Type Immune Response (GO:2000317)
Negative Regulation Of Dendritic Spine Development (GO:0061000)
Negative Regulation Of Digestive System Process (GO:0060457)
Negative Regulation Of Glycogen Metabolic Process (GO:0070874)
Negative Regulation Of Low-Density Lipoprotein Receptor Activity (GO:1905598)
Negative Regulation Of Neuron Projection Regeneration (GO:0070571)
Negative Regulation Of Protein Sumoylation (GO:0033234)
Negative Regulation Of Response To Reactive Oxygen Species (GO:1901032)
Negative Regulation Of Retinoic Acid Receptor Signaling Pathway (GO:0048387)
Negative Regulation Of Stress-Activated Protein Kinase Signaling Cascade (GO:0070303)
Negative Regulation Of Telomere Capping (GO:1904354)
Neurotransmitter Receptor Transport, Endosome To Postsynaptic Membrane (GO:0098887)
Neurotransmitter Receptor Transport, Postsynaptic Endosome To Lysosome (GO:0098943)
Neurotrophin TRK Receptor Signaling Pathway (GO:0048011)
Nuclear-Transcribed mRNA Catabolic Process, Deadenylation-Independent Decay (GO:0031086)
Nucleotide Catabolic Process (GO:0009166)
Parallel Actin Filament Bundle Assembly (GO:0030046)
Peptidyl-Arginine Modification (GO:0018195)
Pharyngeal Arch Artery Morphogenesis (GO:0061626)
Positive Regulation Of B Cell Receptor Signaling Pathway (GO:0050861)
Positive Regulation Of IRE1-mediated Unfolded Protein Response (GO:1903896)
Positive Regulation Of RNA Binding (GO:1905216)
Positive Regulation Of Aspartic-Type Endopeptidase Activity Involved In Amyloid Precursor Protein Catabolic Pro
Positive Regulation Of Autophagosome Maturation (GO:1901098)
Positive Regulation Of cAMP-mediated Signaling (GO:0043950)
Positive Regulation Of Cysteine-Type Endopeptidase Activity Involved In Apoptotic Signaling Pathway (GO:2001
Positive Regulation Of Endoplasmic Reticulum Stress-Induced Intrinsic Apoptotic Signaling Pathway (GO:19022

Positive Regulation Of Lipoprotein Lipase Activity (GO:0051006)
Positive Regulation Of mRNA Binding (GO:1902416)
Positive Regulation Of Mitophagy In Response To Mitochondrial Depolarization (GO:0098779)
Positive Regulation Of Multicellular Organism Growth (GO:0040018)
Positive Regulation Of Pinocytosis (GO:0048549)
Positive Regulation Of Protein Localization To Centrosome (GO:1904781)
Positive Regulation Of Receptor Catabolic Process (GO:2000646)
Positive Regulation Of Spindle Assembly (GO:1905832)
Positive Regulation Of Trophoblast Cell Migration (GO:1901165)
Positive Regulation Of Unsaturated Fatty Acid Biosynthetic Process (GO:2001280)
Protein Catabolic Process In The Vacuole (GO:0007039)
Protein Deglutamylation (GO:0035608)
Receptor-Mediated Virion Attachment To Host Cell (GO:0046813)
Regulation Of MHC Class I Biosynthetic Process (GO:0045343)
Regulation Of NAD(P)H Oxidase Activity (GO:0033860)
Regulation Of NK T Cell Proliferation (GO:0051140)
Regulation Of Rab Protein Signal Transduction (GO:0032483)
Regulation Of T Cell Mediated Immune Response To Tumor Cell (GO:0002840)
Regulation Of TORC2 Signaling (GO:1903939)
Regulation Of Extracellular Matrix Assembly (GO:1901201)
Regulation Of Microtubule Binding (GO:1904526)
Regulation Of Mitotic Cytokinesis (GO:1902412)
Regulation Of Myosin-Light-Chain-Phosphatase Activity (GO:0035507)
Regulation Of Oxidative Stress-Induced Neuron Intrinsic Apoptotic Signaling Pathway (GO:1903376)
Regulation Of Respiratory Gaseous Exchange (GO:0043576)
Regulation Of Skeletal Muscle Satellite Cell Proliferation (GO:0014842)
Regulation Of Transforming Growth Factor Beta2 Production (GO:0032909)
Regulation Of Vascular Associated Smooth Muscle Cell Differentiation (GO:1905063)
Ribosome-Associated Ubiquitin-Dependent Protein Catabolic Process (GO:1990116)
Serine Transport (GO:0032329)
Transcription-Dependent Tethering Of RNA Polymerase II Gene DNA At Nuclear Periphery (GO:0000972)
Transepithelial Chloride Transport (GO:0030321)
Tubulin Complex Assembly (GO:0007021)
Very Long-Chain Fatty Acid Catabolic Process (GO:0042760)
Vesicle Fusion With Golgi Apparatus (GO:0048280)
Cellular Response To Chemokine (GO:1990869)
Microtubule Cytoskeleton Organization Involved In Mitosis (GO:1902850)
Negative Regulation Of Cytokine-Mediated Signaling Pathway (GO:0001960)
Positive Regulation Of Proteolysis (GO:0045862)
Protein Targeting To Mitochondrion (GO:0006626)
Transcription Preinitiation Complex Assembly (GO:0070897)
Positive Regulation Of Catabolic Process (GO:0009896)
Positive Regulation Of Protein Localization To Membrane (GO:1905477)
Phosphate-Containing Compound Metabolic Process (GO:0006796)
L-alpha-amino Acid Transmembrane Transport (GO:1902475)
Cell Cycle G2/M Phase Transition (GO:0044839)
Positive Regulation Of Catalytic Activity (GO:0043085)
Regulation Of Autophagosome Assembly (GO:2000785)

Fc-epsilon Receptor Signaling Pathway (GO:0038095)
Cellular Response To Interferon-Beta (GO:0035458)
Cellular Response To Topologically Incorrect Protein (GO:0035967)
Mammary Gland Development (GO:0030879)
Modulation By Host Of Viral Process (GO:0044788)
Negative Regulation Of Protein Localization To Nucleus (GO:1900181)
Organelle Fission (GO:0048285)
Placenta Development (GO:0001890)
Positive Regulation Of Cilium Assembly (GO:0045724)
Regulation Of Intracellular Steroid Hormone Receptor Signaling Pathway (GO:0033143)
Regulation Of Protein Export From Nucleus (GO:0046825)
Ribosomal Large Subunit Assembly (GO:0000027)
Glycoprotein Biosynthetic Process (GO:0009101)
Positive Regulation Of Immune Response (GO:0050778)
Regulation Of Protein Localization To Plasma Membrane (GO:1903076)
Response To Type II Interferon (GO:0034341)
Histone Acetylation (GO:0016573)
rRNA Processing (GO:0006364)
Heart Contraction (GO:0060047)
Lymphocyte Migration (GO:0072676)
Positive Regulation Of Ossification (GO:0045778)
Histone Modification (GO:0016570)
Positive Regulation Of Growth (GO:0045927)
Spindle Assembly (GO:0051225)
Ras Protein Signal Transduction (GO:0007265)
Regulation Of Intracellular Signal Transduction (GO:1902531)
Negative Regulation Of Intracellular Signal Transduction (GO:1902532)
Positive Regulation Of Epithelial Cell Proliferation (GO:0050679)
Calcium Ion Homeostasis (GO:0055074)
Sensory Perception Of Light Stimulus (GO:0050953)
Positive Regulation Of Cytokine Production (GO:0001819)
Artery Development (GO:0060840)
Cell-Cell Signaling By Wnt (GO:0198738)
Cytosolic Pattern Recognition Receptor Signaling Pathway (GO:0002753)
Endocardial Cushion Development (GO:0003197)
Long-Chain fatty-acyl-CoA Metabolic Process (GO:0035336)
Negative Regulation Of Epidermal Growth Factor Receptor Signaling Pathway (GO:0042059)
Negative Regulation Of Extrinsic Apoptotic Signaling Pathway Via Death Domain Receptors (GO:1902042)
Negative Regulation Of Lymphocyte Activation (GO:0051250)
Negative Regulation Of Neuron Differentiation (GO:0045665)
Negative Regulation Of Oxidative Stress-Induced Cell Death (GO:1903202)
Positive Regulation Of Cell-Substrate Junction Organization (GO:0150117)
Positive Regulation Of mRNA Splicing, Via Spliceosome (GO:0048026)
Positive Regulation Of Oxidoreductase Activity (GO:0051353)
Protein Localization To Cell Junction (GO:1902414)
Regulation Of Cellular Response To Transforming Growth Factor Beta Stimulus (GO:1903844)
Regulation Of Cholesterol Metabolic Process (GO:0090181)
Regulation Of Lipid Catabolic Process (GO:0050994)

Regulation Of Myotube Differentiation (GO:0010830)
Zinc Ion Transport (GO:0006829)
Positive Regulation Of Protein Kinase Activity (GO:0045860)
Regulation Of I-kappaB kinase/NF-kappaB Signaling (GO:0043122)
Cellular Response To Organic Substance (GO:0071310)
Negative Regulation Of Apoptotic Signaling Pathway (GO:2001234)
Enzyme-Linked Receptor Protein Signaling Pathway (GO:0007167)
Amino Acid Transmembrane Transport (GO:0003333)
Collagen Fibril Organization (GO:0030199)
Regulation Of Neurotransmitter Receptor Activity (GO:0099601)
Regulation Of Ubiquitin-Dependent Protein Catabolic Process (GO:2000058)
Skeletal Muscle Tissue Development (GO:0007519)
Negative Regulation Of Response To External Stimulus (GO:0032102)
Regulation Of Cell Division (GO:0051302)
DNA Dealkylation (GO:0035510)
DNA Dealkylation Involved In DNA Repair (GO:0006307)
ER Overload Response (GO:0006983)
SREBP Signaling Pathway (GO:0032933)
T-helper 17 Cell Lineage Commitment (GO:0072540)
Amino Sugar Catabolic Process (GO:0046348)
Arginine Catabolic Process (GO:0006527)
Aromatic Amino Acid Transport (GO:0015801)
Aromatic Compound Catabolic Process (GO:0019439)
Catecholamine Catabolic Process (GO:0042424)
Cellular Response To Interleukin-2 (GO:0071352)
Coronary Vasculature Morphogenesis (GO:0060977)
Cytoplasmic Sequestering Of NF-kappaB (GO:0007253)
Dermatan Sulfate Proteoglycan Biosynthetic Process (GO:0050651)
Dopamine Catabolic Process (GO:0042420)
Establishment Of Golgi Localization (GO:0051683)
Establishment Of Planar Polarity (GO:0001736)
Ethanol Oxidation (GO:0006069)
Hair Cell Differentiation (GO:0035315)
Histone H2A K63-linked Deubiquitination (GO:0070537)
Histone Lysine Demethylation (GO:0070076)
Inhibition Of Cysteine-Type Endopeptidase Activity (GO:0097340)
Interleukin-2-Mediated Signaling Pathway (GO:0038110)
Lactate Metabolic Process (GO:0006089)
Late Nucleophagy (GO:0044805)
Lipoxygenase Pathway (GO:0019372)
Lymphatic Endothelial Cell Differentiation (GO:0060836)
Mammary Gland Epithelial Cell Differentiation (GO:0060644)
Mitochondrial Outer Membrane Permeabilization (GO:0097345)
Mitral Valve Development (GO:0003174)
Myoblast Migration (GO:0051451)
Negative Regulation Of T-helper 17 Cell Differentiation (GO:2000320)
Negative Regulation Of Complement Activation, Classical Pathway (GO:0045959)
Negative Regulation Of miRNA-mediated Gene Silencing (GO:0060965)

Negative Regulation Of Muscle Cell Differentiation (GO:0051148)
Negative Regulation Of Nitric Oxide Biosynthetic Process (GO:0045019)
Negative Regulation Of Nitric Oxide Metabolic Process (GO:1904406)
Negative Regulation Of Vascular Associated Smooth Muscle Cell Differentiation (GO:1905064)
Neurotransmitter Receptor Diffusion Trapping (GO:0099628)
Peptidyl-Proline Hydroxylation To 4-hydroxy-L-proline (GO:0018401)
Peptidyl-Tyrosine Dephosphorylation Involved In Inactivation Of Protein Kinase Activity (GO:1990264)
Phosphatidylserine Acyl-Chain Remodeling (GO:0036150)
Positive Regulation Of Arp2/3 Complex-Mediated Actin Nucleation (GO:2000601)
Positive Regulation Of Adipose Tissue Development (GO:1904179)
Positive Regulation Of Aspartic-Type Peptidase Activity (GO:1905247)
Positive Regulation Of Cardiac Epithelial To Mesenchymal Transition (GO:0062043)
Positive Regulation Of Dendritic Cell Chemotaxis (GO:2000510)
Positive Regulation Of Extracellular Matrix Disassembly (GO:0090091)
Positive Regulation Of Leukocyte Differentiation (GO:1902107)
Positive Regulation Of Myeloid Cell Apoptotic Process (GO:0033034)
Positive Regulation Of Myotube Differentiation (GO:0010831)
Positive Regulation Of Natural Killer Cell Proliferation (GO:0032819)
Positive Regulation Of Plasminogen Activation (GO:0010756)
Positive Regulation Of Triglyceride Lipase Activity (GO:0061365)
Positive Regulation Of Vascular Associated Smooth Muscle Cell Migration (GO:1904754)
Positive Regulation Of Vascular Endothelial Growth Factor Signaling Pathway (GO:1900748)
Postsynaptic Density Organization (GO:0097106)
Postsynaptic Neurotransmitter Receptor Diffusion Trapping (GO:0098970)
Protein Deglycosylation (GO:0006517)
Protein Retention In ER Lumen (GO:0006621)
protein-RNA Complex Disassembly (GO:0032988)
Purine Nucleotide Catabolic Process (GO:0006195)
Regulation Of ATPase-coupled Calcium Transmembrane Transporter Activity (GO:1901894)
Regulation Of CD4-positive, Alpha-Beta T Cell Activation (GO:2000514)
Regulation Of Carbohydrate Biosynthetic Process (GO:0043255)
Regulation Of Cardiac Muscle Cell Action Potential Involved In Regulation Of Contraction (GO:0098909)
Regulation Of Collateral Sprouting (GO:0048670)
Regulation Of Cyclic-Nucleotide Phosphodiesterase Activity (GO:0051342)
Regulation Of Cytoplasmic Translational Elongation (GO:1900247)
Regulation Of Dendritic Cell Chemotaxis (GO:2000508)
Regulation Of Dopamine Receptor Signaling Pathway (GO:0060159)
Regulation Of Endodeoxyribonuclease Activity (GO:0032071)
Regulation Of Epithelial To Mesenchymal Transition Involved In Endocardial Cushion Formation (GO:1905005)
Regulation Of Establishment Of Protein Localization To Mitochondrion (GO:1903747)
Regulation Of Extent Of Cell Growth (GO:0061387)
Regulation Of Extracellular Matrix Constituent Secretion (GO:0003330)
Regulation Of Hydrogen Peroxide-Induced Cell Death (GO:1903205)
Regulation Of Leukocyte Degranulation (GO:0043300)
Regulation Of Leukocyte Proliferation (GO:0070663)
Regulation Of Lipoprotein Metabolic Process (GO:0050746)
Regulation Of Microvillus Organization (GO:0032530)
Regulation Of Nitric Oxide Metabolic Process (GO:0080164)

Regulation Of Oxidative Stress-Induced Cell Death (GO:1903201)
Regulation Of Positive Chemotaxis (GO:0050926)
Regulation Of Protein Glycosylation (GO:0060049)
Regulation Of Protein Localization To Cilium (GO:1903564)
Regulation Of Skeletal Muscle Fiber Development (GO:0048742)
Regulation Of Transforming Growth Factor Beta Activation (GO:1901388)
Regulation Of Vascular Wound Healing (GO:0061043)
Regulation Of Wound Healing, Spreading Of Epidermal Cells (GO:1903689)
Response To UV-C (GO:0010225)
siRNA Processing (GO:0030422)
Spermidine Metabolic Process (GO:0008216)
Store-Operated Calcium Entry (GO:0002115)
Stress-Induced Premature Senescence (GO:0090400)
tRNA Aminoacylation For Mitochondrial Protein Translation (GO:0070127)
Taurine Metabolic Process (GO:0019530)
Thyroid Hormone Transport (GO:0070327)
Translocation Of Molecules Into Host (GO:0044417)
Ventricular Trabecula Myocardium Morphogenesis (GO:0003222)
Viral mRNA Export From Host Cell Nucleus (GO:0046784)
Protein Glycosylation (GO:0006486)
Cytokine-Mediated Signaling Pathway (GO:0019221)
Intracellular Glucose Homeostasis (GO:0001678)
Lysosome Organization (GO:0007040)
mRNA Catabolic Process (GO:0006402)
Positive Regulation Of Biomineral Tissue Development (GO:0070169)
Positive Regulation Of Stress Fiber Assembly (GO:0051496)
Regulation Of Cellular Component Size (GO:0032535)
Ear Morphogenesis (GO:0042471)
Iron Ion Transport (GO:0006826)
Lipid Translocation (GO:0034204)
mRNA Polyadenylation (GO:0006378)
Negative Regulation Of Antigen Receptor-Mediated Signaling Pathway (GO:0050858)
Negative Regulation Of Cell Junction Assembly (GO:1901889)
Negative Regulation Of Ion Transmembrane Transporter Activity (GO:0032413)
Outflow Tract Septum Morphogenesis (GO:0003148)
Positive Regulation Of Cell Cycle G2/M Phase Transition (GO:1902751)
Positive Regulation Of Cellular Response To Transforming Growth Factor Beta Stimulus (GO:1903846)
Positive Regulation Of Lipid Metabolic Process (GO:0045834)
Positive Regulation Of Transforming Growth Factor Beta Receptor Signaling Pathway (GO:0030511)
Regulation Of AMPA Receptor Activity (GO:2000311)
Regulation Of Heterotypic Cell-Cell Adhesion (GO:0034114)
Regulation Of Microtubule Depolymerization (GO:0031114)
Regulation Of Muscle Cell Differentiation (GO:0051147)
Regulation Of Cellular Catabolic Process (GO:0031329)
Positive Regulation Of Double-Strand Break Repair (GO:2000781)
Regulation Of Anatomical Structure Morphogenesis (GO:0022603)
Establishment Of Protein Localization To Mitochondrion (GO:0072655)
Lymphocyte Chemotaxis (GO:0048247)

mRNA Metabolic Process (GO:0016071)
Nuclear-Transcribed mRNA Catabolic Process (GO:0000956)
Positive Regulation Of Canonical Wnt Signaling Pathway (GO:0090263)
Regulation Of Kinase Activity (GO:0043549)
RNA Polyadenylation (GO:0043631)
Cellular Response To Steroid Hormone Stimulus (GO:0071383)
Chemical Synaptic Transmission, Postsynaptic (GO:0099565)
fatty-acyl-CoA Biosynthetic Process (GO:0046949)
Inner Ear Morphogenesis (GO:0042472)
Lamellipodium Assembly (GO:0030032)
Mitotic Cell Cycle Checkpoint Signaling (GO:0007093)
Negative Regulation Of TORC1 Signaling (GO:1904262)
Negative Regulation Of Intrinsic Apoptotic Signaling Pathway In Response To DNA Damage (GO:1902230)
Negative Regulation Of Protein Localization To Membrane (GO:1905476)
Negative Regulation Of Protein Transport (GO:0051224)
Negative Regulation Of Smoothened Signaling Pathway (GO:0045879)
Phosphatidylethanolamine Metabolic Process (GO:0046337)
Positive Regulation Of T Cell Migration (GO:2000406)
Positive Regulation Of Interleukin-2 Production (GO:0032743)
Proteoglycan Metabolic Process (GO:0006029)
Purine Nucleoside Bisphosphate Metabolic Process (GO:0034032)
Regulation Of Hippo Signaling (GO:0035330)
Regulation Of Intracellular Estrogen Receptor Signaling Pathway (GO:0033146)
Regulation Of Lysosomal Lumen pH (GO:0035751)
Regulation Of Neutrophil Chemotaxis (GO:0090022)
Regulation Of Platelet Aggregation (GO:0090330)
Regulation Of Postsynapse Organization (GO:0099175)
Ribonucleoside Bisphosphate Metabolic Process (GO:0033875)
Vesicle Cargo Loading (GO:0035459)
Neuron Development (GO:0048666)
Regulation Of Cell-Matrix Adhesion (GO:0001952)
Regulation Of Tyrosine Phosphorylation Of STAT Protein (GO:0042509)
Epidermal Growth Factor Receptor Signaling Pathway (GO:0007173)
Positive Regulation Of Innate Immune Response (GO:0045089)
Regulation Of Apoptotic Signaling Pathway (GO:2001233)
Negative Regulation Of Cellular Process (GO:0048523)
CD40 Signaling Pathway (GO:0023035)
Cdc42 Protein Signal Transduction (GO:0032488)
DNA Damage Response, Signal Transduction By P53 Class Mediator Resulting In Transcription Of P21 Class Me
RNA Secondary Structure Unwinding (GO:0010501)
T-helper 17 Cell Differentiation (GO:0072539)
Angiotensin Maturation (GO:0002003)
Apoptotic DNA Fragmentation (GO:0006309)
Bone Resorption (GO:0045453)
Box C/D snoRNP Assembly (GO:0000492)
Cellular Response To Sterol Depletion (GO:0071501)
Cyclooxygenase Pathway (GO:0019371)
Endochondral Ossification (GO:0001958)

Glutamine Transport (GO:0006868)
Inner Ear Receptor Cell Development (GO:0060119)
Interleukin-12-Mediated Signaling Pathway (GO:0035722)
Intestinal Epithelial Cell Differentiation (GO:0060575)
Isoprenoid Biosynthetic Process (GO:0008299)
Mesodermal Cell Differentiation (GO:0048333)
Negative Regulation Of Alpha-Beta T Cell Differentiation (GO:0046639)
Negative Regulation Of Cholesterol Storage (GO:0010887)
Negative Regulation Of Cytoplasmic Translation (GO:2000766)
Negative Regulation Of Lipase Activity (GO:0060192)
Negative Regulation Of Membrane Potential (GO:0045837)
Negative Regulation Of Mitotic Nuclear Division (GO:0045839)
Negative Regulation Of Oxidative Stress-Induced Neuron Death (GO:1903204)
Negative Regulation Of Protein Import Into Nucleus (GO:0042308)
Negative Regulation Of Regulated Secretory Pathway (GO:1903306)
Negative Regulation Of Smooth Muscle Cell Differentiation (GO:0051151)
Negative Regulation Of Systemic Arterial Blood Pressure (GO:0003085)
Negative Regulation Of Transporter Activity (GO:0032410)
Negative Regulation Of Ubiquitin Protein Ligase Activity (GO:1904667)
Nicotinamide Nucleotide Biosynthetic Process (GO:0019359)
Nucleoside Diphosphate Metabolic Process (GO:0009132)
Peptidyl-Lysine Monomethylation (GO:0018026)
Positive Regulation Of ATP Biosynthetic Process (GO:2001171)
Positive Regulation Of NK T Cell Activation (GO:0051135)
Positive Regulation Of Adaptive Immune Response (GO:0002821)
Positive Regulation Of Dendritic Spine Morphogenesis (GO:0061003)
Positive Regulation Of Extracellular Matrix Assembly (GO:1901203)
Positive Regulation Of Inositol Phosphate Biosynthetic Process (GO:0060732)
Positive Regulation Of Mitochondrial Membrane Permeability Involved In Apoptotic Process (GO:1902110)
Positive Regulation Of Protein Localization To Early Endosome (GO:1902966)
Positive Regulation Of Protein Localization To Endosome (GO:1905668)
Positive Regulation Of Tumor Necrosis Factor-Mediated Signaling Pathway (GO:1903265)
Positive Regulation Of Ubiquitin Protein Ligase Activity (GO:1904668)
Presynaptic Membrane Assembly (GO:0097105)
Proline Transport (GO:0015824)
Protein Localization To Perinuclear Region Of Cytoplasm (GO:1905719)
Regulation Of DNA-templated Transcription Initiation (GO:2000142)
Regulation Of RNA Biosynthetic Process (GO:2001141)
Regulation Of Angiotensin Levels In Blood (GO:0002002)
Regulation Of Aspartic-Type Endopeptidase Activity Involved In Amyloid Precursor Protein Catabolic Process (G
Regulation Of Complement Activation, Classical Pathway (GO:0030450)
Regulation Of Dendritic Spine Maintenance (GO:1902950)
Regulation Of Epidermis Development (GO:0045682)
Regulation Of Fibroblast Apoptotic Process (GO:2000269)
Regulation Of Glucan Biosynthetic Process (GO:0010962)
Regulation Of Intrinsic Apoptotic Signaling Pathway By P53 Class Mediator (GO:1902253)
Regulation Of Mitochondrial Gene Expression (GO:0062125)
Regulation Of Mitotic Centrosome Separation (GO:0046602)

Regulation Of Morphogenesis Of An Epithelium (GO:1905330)
Regulation Of Protein Localization To Early Endosome (GO:1902965)
Regulation Of Skeletal Muscle Cell Differentiation (GO:2001014)
Regulation Of Tumor Necrosis Factor Superfamily Cytokine Production (GO:1903555)
Regulation Of Type B Pancreatic Cell Development (GO:2000074)
Response To Misfolded Protein (GO:0051788)
Retrograde Transport, Vesicle Recycling Within Golgi (GO:0000301)
Sensory Organ Morphogenesis (GO:0090596)
Translational Termination (GO:0006415)
Tricarboxylic Acid Metabolic Process (GO:0072350)
Ubiquitin-Dependent Protein Catabolic Process Via The C-end Degron Rule Pathway (GO:0140627)
Vitamin K Metabolic Process (GO:0042373)
Negative Regulation Of Immune Response (GO:0050777)
DNA-templated Transcription Elongation (GO:0006354)
Fc Receptor Signaling Pathway (GO:0038093)
Wnt Signaling Pathway, Planar Cell Polarity Pathway (GO:0060071)
Cell-Cell Adhesion Mediated By Cadherin (GO:0044331)
Dendritic Spine Organization (GO:0097061)
Mitotic G1 DNA Damage Checkpoint Signaling (GO:0031571)
Negative Regulation Of Insulin Receptor Signaling Pathway (GO:0046627)
Nucleic Acid Phosphodiester Bond Hydrolysis (GO:0090305)
Organelle Fusion (GO:0048284)
Plasma Membrane Invagination (GO:0099024)
Positive Regulation Of Erythrocyte Differentiation (GO:0045648)
Positive Regulation Of Monooxygenase Activity (GO:0032770)
Protein Export From Nucleus (GO:0006611)
Regulation Of Actin Filament Bundle Assembly (GO:0032231)
Regulation Of Fatty Acid Biosynthetic Process (GO:0042304)
Regulation Of Interferon-Alpha Production (GO:0032647)
Regulation Of Keratinocyte Differentiation (GO:0045616)
Regulation Of Nucleotide-Excision Repair (GO:2000819)
Regulation Of Potassium Ion Transmembrane Transporter Activity (GO:1901016)
Regulation Of Reactive Oxygen Species Biosynthetic Process (GO:1903426)
Regulation Of Vasoconstriction (GO:0019229)
Cellular Response To Salt (GO:1902075)
Negative Regulation Of Developmental Process (GO:0051093)
Positive Regulation Of Wnt Signaling Pathway (GO:0030177)
Positive Regulation Of I-kappaB kinase/NF-kappaB Signaling (GO:0043123)
Amino Acid Transport (GO:0006865)
Epithelial Cell Migration (GO:0010631)
Positive Regulation Of Blood Vessel Endothelial Cell Migration (GO:0043536)
Regulation Of Cytokine Production Involved In Inflammatory Response (GO:1900015)
Regulation Of Epidermal Growth Factor Receptor Signaling Pathway (GO:0042058)
Regulation Of Fibroblast Proliferation (GO:0048145)
Response To Glucose (GO:0009749)
Vacuolar Transport (GO:0007034)
Regulation Of Protein Phosphorylation (GO:0001932)
Regulation Of Double-Strand Break Repair (GO:2000779)

Protein Secretion (GO:0009306)
Regulation Of Viral Genome Replication (GO:0045069)
Epithelial Cell Differentiation (GO:0030855)
Plasma Membrane Bounded Cell Projection Organization (GO:0120036)
Regulation Of Vesicle-Mediated Transport (GO:0060627)
Positive Regulation Of Epithelial To Mesenchymal Transition (GO:0010718)
Regulation Of Interleukin-2 Production (GO:0032663)
RNA Metabolic Process (GO:0016070)
Mitotic Cell Cycle Phase Transition (GO:0044772)
Regulation Of Transforming Growth Factor Beta Receptor Signaling Pathway (GO:0017015)
Telomere Maintenance (GO:0000723)
DNA Synthesis Involved In DNA Repair (GO:0000731)
Activation Of Phospholipase C Activity (GO:0007202)
Cell Surface Receptor Signaling Pathway Involved In Cell-Cell Signaling (GO:1905114)
Cellular Response To Acid Chemical (GO:0071229)
Cellular Response To Epidermal Growth Factor Stimulus (GO:0071364)
mRNA Modification (GO:0016556)
Negative Regulation Of Cellular Response To Insulin Stimulus (GO:1900077)
Positive Regulation Of Nitric Oxide Biosynthetic Process (GO:0045429)
Regulation Of Immunoglobulin Production (GO:0002637)
Regulation Of Mitochondrial Fission (GO:0090140)
Regulation Of Peptidase Activity (GO:0052547)
Ventricular Septum Morphogenesis (GO:0060412)
Negative Regulation Of Cell Motility (GO:2000146)
Chromatin Organization (GO:0006325)
Regulation Of Nucleic Acid-Templated Transcription (GO:1903506)
Calcium Ion Transport (GO:0006816)
Response To Molecule Of Bacterial Origin (GO:0002237)
B Cell Homeostasis (GO:0001782)
DNA Damage Response, Signal Transduction Resulting In Transcription (GO:0042772)
ESCRT III Complex Disassembly (GO:1904903)
ESCRT Complex Disassembly (GO:1904896)
Golgi Inheritance (GO:0048313)
NAD Biosynthesis Via Nicotinamide Riboside Salvage Pathway (GO:0034356)
T-helper 1 Type Immune Response (GO:0042088)
Amine Metabolic Process (GO:0009308)
Aminoglycan Metabolic Process (GO:0006022)
Arachidonate Transport (GO:1903963)
Arachidonic Acid Secretion (GO:0050482)
Aspartate Transmembrane Transport (GO:0015810)
Biomineral Tissue Development (GO:0031214)
Carbohydrate Phosphorylation (GO:0046835)
Cellular Response To Purine-Containing Compound (GO:0071415)
Chondroitin Sulfate Proteoglycan Metabolic Process (GO:0050654)
Ectoderm Development (GO:0007398)
Embryonic Eye Morphogenesis (GO:0048048)
Excitatory Synapse Assembly (GO:1904861)
Glucan Biosynthetic Process (GO:0009250)

Glycogen Biosynthetic Process (GO:0005978)
Glycosphingolipid Catabolic Process (GO:0046479)
Homocysteine Metabolic Process (GO:0050667)
Homologous Recombination (GO:0035825)
Hormone Transport (GO:0009914)
Iron Ion Transmembrane Transport (GO:0034755)
Leucine Transport (GO:0015820)
Mitochondrial DNA Replication (GO:0006264)
Mitochondrial Ribosome Assembly (GO:0061668)
Mitral Valve Morphogenesis (GO:0003183)
Myelin Assembly (GO:0032288)
Negative Regulation Of Alpha-Beta T Cell Proliferation (GO:0046642)
Negative Regulation Of Cell Adhesion Mediated By Integrin (GO:0033629)
Negative Regulation Of Heterotypic Cell-Cell Adhesion (GO:0034115)
Negative Regulation Of Humoral Immune Response Mediated By Circulating Immunoglobulin (GO:0002924)
Negative Regulation Of Phosphatidylinositol 3-Kinase Signaling (GO:0014067)
Nerve Growth Factor Signaling Pathway (GO:0038180)
Neurotransmitter Receptor Transport To Postsynaptic Membrane (GO:0098969)
Peptidyl-Lysine Dimethylation (GO:0018027)
Peptidyl-Serine Dephosphorylation (GO:0070262)
Phosphatidylcholine Catabolic Process (GO:0034638)
Phosphatidylethanolamine Biosynthetic Process (GO:0006646)
Piecemeal Microautophagy Of The Nucleus (GO:0034727)
Pinocytosis (GO:0006907)
Positive Regulation Of ATP Metabolic Process (GO:1903580)
Positive Regulation Of RIG-I Signaling Pathway (GO:1900246)
Positive Regulation Of Cardiocyte Differentiation (GO:1905209)
Positive Regulation Of Establishment Of Protein Localization To Telomere (GO:1904851)
Positive Regulation Of Leukocyte Apoptotic Process (GO:2000108)
Positive Regulation Of Lipase Activity (GO:0060193)
Positive Regulation Of mRNA 3'-End Processing (GO:0031442)
Positive Regulation Of miRNA-mediated Gene Silencing (GO:2000637)
Positive Regulation Of Mitochondrial Membrane Permeability (GO:0035794)
Positive Regulation Of Myoblast Fusion (GO:1901741)
Positive Regulation Of Platelet Activation (GO:0010572)
Positive Regulation Of rRNA Processing (GO:2000234)
Positive Regulation Of Tissue Remodeling (GO:0034105)
Positive Regulation Of Transcription Of Nucleolar Large rRNA By RNA Polymerase I (GO:1901838)
Presynaptic Membrane Organization (GO:0097090)
Protein K11-linked Deubiquitination (GO:0035871)
Protein O-linked Glycosylation Via Serine (GO:0018242)
Purine Nucleobase Metabolic Process (GO:0006144)
Regulation Of CD4-positive, Alpha-Beta T Cell Proliferation (GO:2000561)
Regulation Of Animal Organ Morphogenesis (GO:2000027)
Regulation Of Attachment Of Spindle Microtubules To Kinetochore (GO:0051988)
Regulation Of Cardiac Muscle Cell Differentiation (GO:2000725)
Regulation Of Cell-Cell Adhesion Involved In Gastrulation (GO:0070587)
Regulation Of Cellular Extravasation (GO:0002691)

Regulation Of Endoplasmic Reticulum Unfolded Protein Response (GO:1900101)
Regulation Of High Voltage-Gated Calcium Channel Activity (GO:1901841)
Regulation Of Mast Cell Activation Involved In Immune Response (GO:0033006)
Regulation Of Myeloid Leukocyte Mediated Immunity (GO:0002886)
Regulation Of Natural Killer Cell Proliferation (GO:0032817)
Regulation Of Postsynaptic Neurotransmitter Receptor Activity (GO:0098962)
Regulation Of Protein Localization To Centrosome (GO:1904779)
Regulation Of Receptor Binding (GO:1900120)
Response To Histamine (GO:0034776)
Response To Interleukin-4 (GO:0070670)
Response To Platelet-Derived Growth Factor (GO:0036119)
Sequestering Of Extracellular Ligand From Receptor (GO:0035581)
Skeletal Muscle Tissue Regeneration (GO:0043403)
Synaptic Vesicle Fusion To Presynaptic Active Zone Membrane (GO:0031629)
Telomere Maintenance Via Recombination (GO:0000722)
Toxin Transport (GO:1901998)
Transferrin Transport (GO:0033572)
Wound Healing, Spreading Of Epidermal Cells (GO:0035313)
Regulation Of DNA Binding (GO:0051101)
Positive Regulation Of Cellular Process (GO:0048522)
RNA Phosphodiester Bond Hydrolysis (GO:0090501)
Cell-Substrate Junction Assembly (GO:0007044)
Diterpenoid Metabolic Process (GO:0016101)
Negative Regulation Of G2/M Transition Of Mitotic Cell Cycle (GO:0010972)
Negative Regulation Of Wound Healing (GO:0061045)
Nuclear-Transcribed mRNA Catabolic Process, Nonsense-Mediated Decay (GO:0000184)
Positive Regulation Of Fibroblast Proliferation (GO:0048146)
Positive Regulation Of mRNA Processing (GO:0050685)
Positive Regulation Of Nitric Oxide Metabolic Process (GO:1904407)
Protein Insertion Into Mitochondrial Membrane (GO:0051204)
Regulation Of Cardiac Muscle Cell Action Potential (GO:0098901)
Regulation Of Chemotaxis (GO:0050920)
Transcription By RNA Polymerase III (GO:0006383)
Transcription Elongation By RNA Polymerase II (GO:0006368)
Neutrophil Chemotaxis (GO:0030593)
Positive Regulation Of Protein Catabolic Process (GO:0045732)
Protein Modification By Small Protein Removal (GO:0070646)
Negative Regulation Of T Cell Activation (GO:0050868)
Myeloid Leukocyte Differentiation (GO:0002573)
Regulation Of Cilium Assembly (GO:1902017)
Positive Regulation Of Cellular Component Biogenesis (GO:0044089)
TOR Signaling (GO:0031929)
Alpha-Amino Acid Metabolic Process (GO:1901605)
Chondrocyte Differentiation (GO:0002062)
Lipid Droplet Organization (GO:0034389)
Maintenance Of Blood-Brain Barrier (GO:0035633)
Negative Regulation Of DNA Binding (GO:0043392)
Negative Regulation Of Plasma Membrane Bounded Cell Projection Assembly (GO:0120033)

Positive Regulation Of B Cell Proliferation (GO:0030890)
Positive Regulation Of Calcium Ion Transport (GO:0051928)
Protein Insertion Into ER Membrane (GO:0045048)
Regulation Of Establishment Of Planar Polarity (GO:0090175)
Regulation Of Monoatomic Ion Transmembrane Transport (GO:0034765)
Regulation Of Striated Muscle Contraction (GO:0006942)
Response To Interferon-Beta (GO:0035456)
Blood Circulation (GO:0008015)
Nucleocytoplasmic Transport (GO:0006913)
Plasma Membrane Bounded Cell Projection Morphogenesis (GO:0120039)
Positive Regulation Of Fat Cell Differentiation (GO:0045600)
Regulation Of Interferon-Beta Production (GO:0032648)
Regulation Of Interleukin-12 Production (GO:0032655)
Regulation Of Mitochondrion Organization (GO:0010821)
Negative Regulation Of DNA-templated Transcription (GO:0045892)
Cholesterol Homeostasis (GO:0042632)
Plasma Membrane Organization (GO:0007009)
Positive Regulation Of Protein Localization To Nucleus (GO:1900182)
CRD-mediated mRNA Stabilization (GO:0070934)
N-acetylneuraminate Metabolic Process (GO:0006054)
Rap Protein Signal Transduction (GO:0032486)
T-helper Cell Lineage Commitment (GO:0002295)
Acidic Amino Acid Transport (GO:0015800)
Arginine Metabolic Process (GO:0006525)
Atrial Septum Morphogenesis (GO:0060413)
Branched-Chain Amino Acid Transport (GO:0015803)
calcineurin-NFAT Signaling Cascade (GO:0033173)
Cell Proliferation In Forebrain (GO:0021846)
Cellular Response To Increased Oxygen Levels (GO:0036295)
Cellular Response To Interleukin-12 (GO:0071349)
Cellular Response To Interleukin-15 (GO:0071350)
Cellular Response To Type III Interferon (GO:0071358)
Coenzyme A Biosynthetic Process (GO:0015937)
Diol Metabolic Process (GO:0034311)
Epithelial Cell Apoptotic Process (GO:1904019)
Establishment Of Epithelial Cell Apical/Basal Polarity (GO:0045198)
Glycine Transport (GO:0015816)
Histone H3-K14 Acetylation (GO:0044154)
Insulin-Like Growth Factor Receptor Signaling Pathway (GO:0048009)
Interleukin-15-Mediated Signaling Pathway (GO:0035723)
Lens Fiber Cell Differentiation (GO:0070306)
Lipoprotein Catabolic Process (GO:0042159)
Microtubule Anchoring At Centrosome (GO:0034454)
Mitochondrial Protein Processing (GO:0034982)
Mitochondrial Respirasome Assembly (GO:0097250)
Mitotic G1/S Transition Checkpoint Signaling (GO:0044819)
Negative Regulation Of T-helper Cell Differentiation (GO:0045623)
Negative Regulation Of Alpha-Beta T Cell Activation (GO:0046636)

Negative Regulation Of Exocytosis (GO:0045920)
Negative Regulation Of Hydrogen Peroxide-Induced Cell Death (GO:1903206)
Negative Regulation Of Intracellular Estrogen Receptor Signaling Pathway (GO:0033147)
Negative Regulation Of Nuclear Division (GO:0051784)
Negative Regulation Of Sodium Ion Transmembrane Transporter Activity (GO:2000650)
Negative Regulation Of Transmembrane Transport (GO:0034763)
Neuron Projection Fasciculation (GO:0106030)
Neuron Projection Maintenance (GO:1990535)
Peptidyl-Proline Hydroxylation (GO:0019511)
Phosphate Ion Transmembrane Transport (GO:0035435)
Positive Regulation Of B Cell Differentiation (GO:0045579)
Positive Regulation Of Actin Nucleation (GO:0051127)
Positive Regulation Of Autophagy Of Mitochondrion (GO:1903599)
Positive Regulation Of Cellular Senescence (GO:2000774)
Positive Regulation Of Cholesterol Metabolic Process (GO:0090205)
Positive Regulation Of Non-Canonical Wnt Signaling Pathway (GO:2000052)
Positive Regulation Of Phospholipid Transport (GO:2001140)
Positive Regulation Of Post-Transcriptional Gene Silencing By RNA (GO:1900370)
Positive Regulation Of Protein Localization To Cajal Body (GO:1904871)
Protein Deneddylation (GO:0000338)
Protein Oxidation (GO:0018158)
Protein Prenylation (GO:0018342)
Purine Nucleoside Bisphosphate Biosynthetic Process (GO:0034033)
Regulation Of ERAD Pathway (GO:1904292)
Regulation Of Biosynthetic Process (GO:0009889)
Regulation Of Cardiac Muscle Cell Apoptotic Process (GO:0010665)
Regulation Of Dendritic Cell Differentiation (GO:2001198)
Regulation Of Establishment Of Protein Localization To Telomere (GO:0070203)
Regulation Of Long-Term Neuronal Synaptic Plasticity (GO:0048169)
Regulation Of mRNA Polyadenylation (GO:1900363)
Regulation Of Peptidyl-Lysine Acetylation (GO:2000756)
Regulation Of Phospholipase Activity (GO:0010517)
Regulation Of Protein Localization To Cajal Body (GO:1904869)
Regulation Of Toll-Like Receptor 2 Signaling Pathway (GO:0034135)
Regulation Of Toll-Like Receptor 9 Signaling Pathway (GO:0034163)
Ribonucleoside Bisphosphate Biosynthetic Process (GO:0034030)
Small Nucleolar Ribonucleoprotein Complex Assembly (GO:0000491)
snRNA Transcription By RNA Polymerase II (GO:0042795)
Steroid Hormone Mediated Signaling Pathway (GO:0043401)
Type III Interferon-Mediated Signaling Pathway (GO:0038196)
Intracellular Calcium Ion Homeostasis (GO:0006874)
RNA Modification (GO:0009451)
SMAD Protein Signal Transduction (GO:0060395)
Positive Regulation Of Actin Filament Bundle Assembly (GO:0032233)
Positive Regulation Of Protein Localization To Cell Periphery (GO:1904377)
Positive Regulation Of Protein Localization To Plasma Membrane (GO:1903078)
Receptor Internalization (GO:0031623)
Regulation Of Substrate Adhesion-Dependent Cell Spreading (GO:1900024)

Regulation Of Synapse Assembly (GO:0051963)
Negative Regulation Of Multicellular Organismal Process (GO:0051241)
Artery Morphogenesis (GO:0048844)
Cytoplasmic Translational Initiation (GO:0002183)
Extrinsic Apoptotic Signaling Pathway Via Death Domain Receptors (GO:0008625)
Lamellipodium Organization (GO:0097581)
Maturation Of SSU-rRNA From Tricistronic rRNA Transcript (SSU-rRNA, 5.8S rRNA, LSU-rRNA) (GO:0000462)
Mitotic G2 DNA Damage Checkpoint Signaling (GO:0007095)
Negative Regulation Of Calcium Ion Transmembrane Transporter Activity (GO:1901020)
Phagocytosis, Engulfment (GO:0006911)
Positive Regulation Of Interleukin-10 Production (GO:0032733)
Positive Regulation Of Phosphate Metabolic Process (GO:0045937)
Prostaglandin Metabolic Process (GO:0006693)
Protein Palmitoylation (GO:0018345)
Regulation Of Arp2/3 Complex-Mediated Actin Nucleation (GO:0034315)
Regulation Of Calcium-Mediated Signaling (GO:0050848)
Regulation Of Macrophage Derived Foam Cell Differentiation (GO:0010743)
Response To Amino Acid (GO:0043200)
Response To Epidermal Growth Factor (GO:0070849)
Granulocyte Chemotaxis (GO:0071621)
Sterol Homeostasis (GO:0055092)
Negative Regulation Of Cell Migration (GO:0030336)
Nitrogen Compound Transport (GO:0071705)
Positive Regulation Of Cell Cycle Process (GO:0090068)
Positive Regulation Of Supramolecular Fiber Organization (GO:1902905)
Regulation Of Protein Metabolic Process (GO:0051246)
Arachidonic Acid Metabolic Process (GO:0019369)
Fatty Acid Oxidation (GO:0019395)
Positive Regulation Of B Cell Activation (GO:0050871)
Regulation Of Protein Localization To Nucleus (GO:1900180)
Gland Development (GO:0048732)
Negative Regulation Of Neuron Apoptotic Process (GO:0043524)
Regulation Of Cell Cycle G1/S Phase Transition (GO:1902806)
Regulation Of Protein-Containing Complex Assembly (GO:0043254)
acyl-CoA Metabolic Process (GO:0006637)
Cellular Response To cAMP (GO:0071320)
Negative Chemotaxis (GO:0050919)
Negative Regulation Of DNA Biosynthetic Process (GO:2000279)
Negative Regulation Of Chemotaxis (GO:0050922)
Negative Regulation Of Microtubule Polymerization Or Depolymerization (GO:0031111)
Phospholipid Translocation (GO:0045332)
Positive Regulation Of Mononuclear Cell Migration (GO:0071677)
Regulation Of Dendrite Morphogenesis (GO:0048814)
Aspartate Family Amino Acid Catabolic Process (GO:0009068)
Brain Morphogenesis (GO:0048854)
Calcineurin-Mediated Signaling (GO:0097720)
Cellular Response To UV-B (GO:0071493)
Cellular Response To Acetylcholine (GO:1905145)

Cellular Response To Fluid Shear Stress (GO:0071498)
Cellular Response To Nitrogen Levels (GO:0043562)
Cellular Response To Nitrogen Starvation (GO:0006995)
Central Nervous System Myelination (GO:0022010)
Central Nervous System Neuron Axonogenesis (GO:0021955)
Endoplasmic Reticulum Membrane Organization (GO:0090158)
Exit From Mitosis (GO:0010458)
Histone H2A Ubiquitination (GO:0033522)
Intestinal Absorption (GO:0050892)
Intracellular Copper Ion Homeostasis (GO:0006878)
Lipoprotein Transport (GO:0042953)
Lysosomal Microautophagy (GO:0016237)
Maintenance Of Protein Localization In Endoplasmic Reticulum (GO:0035437)
Mitochondrial Genome Maintenance (GO:0000002)
Negative Regulation Of RNA Metabolic Process (GO:0051253)
Negative Regulation Of Amyloid Fibril Formation (GO:1905907)
Negative Regulation Of calcineurin-NFAT Signaling Cascade (GO:0070885)
Negative Regulation Of Calcineurin-Mediated Signaling (GO:0106057)
Negative Regulation Of Cell Division (GO:0051782)
Negative Regulation Of Myotube Differentiation (GO:0010832)
Negative Regulation Of Nitrogen Compound Metabolic Process (GO:0051172)
Negative Regulation Of Nuclear-Transcribed mRNA Catabolic Process, Deadenylation-Dependent Decay (GO:19[illegible]
Negative Regulation Of Release Of Sequestered Calcium Ion Into Cytosol (GO:0051280)
Negative Regulation Of Ryanodine-Sensitive Calcium-Release Channel Activity (GO:0060315)
Negative Regulation Of Vascular Permeability (GO:0043116)
Neurotransmitter Uptake (GO:0001504)
Phosphatidylinositol-3-Phosphate Biosynthetic Process (GO:0036092)
Positive Regulation Of Behavior (GO:0048520)
Positive Regulation Of Circadian Rhythm (GO:0042753)
Positive Regulation Of Mitotic Cell Cycle Spindle Assembly Checkpoint (GO:0090267)
Positive Regulation Of Muscle Contraction (GO:0045933)
Positive Regulation Of Myelination (GO:0031643)
Positive Regulation Of Protein Exit From Endoplasmic Reticulum (GO:0070863)
Positive Regulation Of Protein Localization To Chromosome, Telomeric Region (GO:1904816)
Positive Regulation Of Protein Maturation (GO:1903319)
Positive Regulation Of Pseudopodium Assembly (GO:0031274)
Positive Regulation Of Spindle Checkpoint (GO:0090232)
Positive Regulation Of Triglyceride Biosynthetic Process (GO:0010867)
Regulation Of CD4-positive, Alpha-Beta T Cell Differentiation (GO:0043370)
Regulation Of RNA Export From Nucleus (GO:0046831)
Regulation Of T Cell Mediated Immunity (GO:0002709)
Regulation Of T-helper 17 Cell Differentiation (GO:2000319)
Regulation Of Actin Filament Depolymerization (GO:0030834)
Regulation Of Adipose Tissue Development (GO:1904177)
Regulation Of Endosome Size (GO:0051036)
Regulation Of Glycogen Metabolic Process (GO:0070873)
Regulation Of Homotypic Cell-Cell Adhesion (GO:0034110)
Regulation Of Keratinocyte Migration (GO:0051547)

Regulation Of Leukocyte Cell-Cell Adhesion (GO:1903037)
Regulation Of Microtubule-Based Movement (GO:0060632)
Regulation Of Protein Exit From Endoplasmic Reticulum (GO:0070861)
Regulation Of Pseudopodium Assembly (GO:0031272)
Smooth Muscle Cell Differentiation (GO:0051145)
Sphingoid Metabolic Process (GO:0046519)
Negative Regulation Of Phosphorylation (GO:0042326)
Regulation Of Plasma Membrane Bounded Cell Projection Assembly (GO:0120032)
NADH Dehydrogenase Complex Assembly (GO:0010257)
Actin Polymerization Or Depolymerization (GO:0008154)
Carboxylic Acid Transport (GO:0046942)
Mitochondrial Respiratory Chain Complex I Assembly (GO:0032981)
Positive Regulation Of Chemokine Production (GO:0032722)
Regulation Of Macromolecule Biosynthetic Process (GO:0010556)
Regulation Of Receptor Signaling Pathway Via JAK-STAT (GO:0046425)
Defense Response To Virus (GO:0051607)
MAPK Cascade (GO:0000165)
Positive Regulation Of Interleukin-6 Production (GO:0032755)
Transcription Initiation At RNA Polymerase II Promoter (GO:0006367)
Glycosphingolipid Metabolic Process (GO:0006687)
Negative Regulation Of Fat Cell Differentiation (GO:0045599)
Negative Regulation Of Osteoblast Differentiation (GO:0045668)
Proteoglycan Biosynthetic Process (GO:0030166)
Regulation Of Metaphase/Anaphase Transition Of Cell Cycle (GO:1902099)
Regulation Of Transcription By RNA Polymerase I (GO:0006356)
ERBB Signaling Pathway (GO:0038127)
Endoplasmic Reticulum Organization (GO:0007029)
Negative Regulation Of TOR Signaling (GO:0032007)
Negative Regulation Of Neuron Projection Development (GO:0010977)
Negative Regulation Of Protein Ubiquitination (GO:0031397)
Regulation Of Lipid Metabolic Process (GO:0019216)
Ribosomal Large Subunit Biogenesis (GO:0042273)
Chloride Transport (GO:0006821)
Neutrophil Migration (GO:1990266)
Positive Regulation Of JNK Cascade (GO:0046330)
Positive Regulation Of Response To Stimulus (GO:0048584)
Regulation Of Immune Response (GO:0050776)
Regulation Of Transcription Elongation By RNA Polymerase II (GO:0034243)
Protein Complex Oligomerization (GO:0051259)
Neuron Projection Morphogenesis (GO:0048812)
Positive Regulation Of Tyrosine Phosphorylation Of STAT Protein (GO:0042531)
Regulation Of Synaptic Transmission, Glutamatergic (GO:0051966)
intra-Golgi Vesicle-Mediated Transport (GO:0006891)
Negative Regulation Of Blood Vessel Endothelial Cell Migration (GO:0043537)
Positive Regulation Of DNA Binding (GO:0043388)
Positive Regulation Of Notch Signaling Pathway (GO:0045747)
Positive Regulation Of RNA Metabolic Process (GO:0051254)
Positive Regulation Of Ion Transmembrane Transporter Activity (GO:0032414)

Regulation Of Amyloid-Beta Formation (GO:1902003)
DNA Strand Elongation (GO:0022616)
NADPH Regeneration (GO:0006740)
V(D)J Recombination (GO:0033151)
Alanine Transport (GO:0032328)
Axonal Fasciculation (GO:0007413)
Axonal Transport Of Mitochondrion (GO:0019896)
Cardiolipin Metabolic Process (GO:0032048)
Cell Projection Assembly (GO:0030031)
Detection Of Calcium Ion (GO:0005513)
Exonucleolytic Catabolism Of Deadenylated mRNA (GO:0043928)
Eye Photoreceptor Cell Differentiation (GO:0001754)
Fatty Acid Transmembrane Transport (GO:1902001)
Glucose Import (GO:0046323)
Icosanoid Secretion (GO:0032309)
Lipoprotein Localization (GO:0044872)
Mesoderm Morphogenesis (GO:0048332)
Microtubule Anchoring At Microtubule Organizing Center (GO:0072393)
Morphogenesis Of A Polarized Epithelium (GO:0001738)
Negative Regulation By Host Of Viral Process (GO:0044793)
Negative Regulation Of JUN Kinase Activity (GO:0043508)
Negative Regulation Of Cell Cycle Phase Transition (GO:1901988)
Negative Regulation Of Hippo Signaling (GO:0035331)
Negative Regulation Of Intrinsic Apoptotic Signaling Pathway In Response To DNA Damage By P53 Class Mediat[illegible]
Negative Regulation Of Stem Cell Differentiation (GO:2000737)
Nuclear Body Organization (GO:0030575)
Nucleotide Metabolic Process (GO:0009117)
Oligosaccharide-Lipid Intermediate Biosynthetic Process (GO:0006490)
Phosphate Ion Homeostasis (GO:0055062)
Phosphatidylcholine Acyl-Chain Remodeling (GO:0036151)
Polysaccharide Biosynthetic Process (GO:0000271)
Positive Regulation By Symbiont Of Entry Into Host (GO:0075294)
Positive Regulation Of ERAD Pathway (GO:1904294)
Positive Regulation Of T Cell Chemotaxis (GO:0010820)
Positive Regulation Of Alcohol Biosynthetic Process (GO:1902932)
Positive Regulation Of Cellular Extravasation (GO:0002693)
Positive Regulation Of Insulin Receptor Signaling Pathway (GO:0046628)
Positive Regulation Of mRNA Metabolic Process (GO:1903313)
Positive Regulation Of Protein Export From Nucleus (GO:0046827)
Positive Regulation Of Purine Nucleotide Biosynthetic Process (GO:1900373)
Positive Regulation Of Reactive Oxygen Species Biosynthetic Process (GO:1903428)
Positive Regulation Of Sodium Ion Transmembrane Transporter Activity (GO:2000651)
Positive Regulation Of Sodium Ion Transport (GO:0010765)
Positive Regulation Of Viral Entry Into Host Cell (GO:0046598)
Primary miRNA Processing (GO:0031053)
Protein Insertion Into Mitochondrial Inner Membrane (GO:0045039)
Regulation Of DNA-templated DNA Replication Initiation (GO:0030174)
Regulation Of T Cell Differentiation In Thymus (GO:0033081)

Regulation Of Actin Filament Organization (GO:0110053)
Regulation Of Hematopoietic Stem Cell Differentiation (GO:1902036)
Regulation Of Leukocyte Adhesion To Vascular Endothelial Cell (GO:1904994)
Regulation Of Platelet-Derived Growth Factor Receptor Signaling Pathway (GO:0010640)
Regulation Of Retrograde Protein Transport, ER To Cytosol (GO:1904152)
Regulation Of Tube Diameter (GO:0035296)
Ribosome Disassembly (GO:0032790)
Sphingomyelin Metabolic Process (GO:0006684)
Vascular Process In Circulatory System (GO:0003018)
Long-Chain Fatty Acid Metabolic Process (GO:0001676)
Organonitrogen Compound Catabolic Process (GO:1901565)
Positive Regulation Of Tumor Necrosis Factor Production (GO:0032760)
Cellular Response To Lipopolysaccharide (GO:0071222)
Visual Perception (GO:0007601)
DNA Metabolic Process (GO:0006259)
Cellular Response To Peptide (GO:1901653)
Mitotic DNA Damage Checkpoint Signaling (GO:0044773)
Positive Regulation Of Cellular Metabolic Process (GO:0031325)
Regulation Of Mitotic Nuclear Division (GO:0007088)
Regulation Of Stem Cell Population Maintenance (GO:2000036)
Negative Regulation Of Cell Growth (GO:0030308)
Positive Regulation Of Endocytosis (GO:0045807)
Regulation Of Neuron Differentiation (GO:0045664)
Vesicle Fusion (GO:0006906)
Cellular Response To Vascular Endothelial Growth Factor Stimulus (GO:0035924)
Establishment Of Mitotic Spindle Localization (GO:0040001)
Histone Deubiquitination (GO:0016578)
Inner Mitochondrial Membrane Organization (GO:0007007)
Membrane Protein Proteolysis (GO:0033619)
Negative Regulation Of Ras Protein Signal Transduction (GO:0046580)
Negative Regulation Of Mitotic Cell Cycle (GO:0045930)
Positive Regulation Of Immunoglobulin Production (GO:0002639)
Positive Regulation Of Transcription By RNA Polymerase I (GO:0045943)
Regulation Of DNA Biosynthetic Process (GO:2000278)
Regulation Of T Cell Receptor Signaling Pathway (GO:0050856)
Regulation Of Blood Circulation (GO:1903522)
Myeloid Cell Differentiation (GO:0030099)
Atrial Septum Development (GO:0003283)
Atrioventricular Valve Development (GO:0003171)
Cellular Response To Arsenic-Containing Substance (GO:0071243)
Chondroitin Sulfate Biosynthetic Process (GO:0030206)
Cytoplasmic Sequestering Of Transcription Factor (GO:0042994)
Endolysosomal Toll-Like Receptor Signaling Pathway (GO:0140894)
Endonucleolytic Cleavage Of Tricistronic rRNA Transcript (SSU-rRNA, 5.8S rRNA, LSU-rRNA) (GO:0000479)
Endothelial Cell Differentiation (GO:0045446)
Glomerular Filtration (GO:0003094)
Intracellular Potassium Ion Homeostasis (GO:0030007)
Lipid Oxidation (GO:0034440)

Microvillus Organization (GO:0032528)
Mitochondrial DNA Metabolic Process (GO:0032042)
Mitochondrial Fission (GO:0000266)
Myeloid Dendritic Cell Activation (GO:0001773)
ncRNA Transcription (GO:0098781)
Negative Regulation Of DNA Damage Response, Signal Transduction By P53 Class Mediator (GO:0043518)
Negative Regulation Of NLRP3 Inflammasome Complex Assembly (GO:1900226)
Negative Regulation Of Myoblast Differentiation (GO:0045662)
Negative Regulation Of Secretion (GO:0051048)
Negative Regulation Of Translational Initiation (GO:0045947)
Outer Mitochondrial Membrane Organization (GO:0007008)
Positive Regulation Of T-helper 17 Type Immune Response (GO:2000318)
Positive Regulation Of Cardiac Muscle Cell Proliferation (GO:0060045)
Positive Regulation Of Cellular Response To Insulin Stimulus (GO:1900078)
Positive Regulation Of Cyclase Activity (GO:0031281)
Positive Regulation Of Glycogen Biosynthetic Process (GO:0045725)
Positive Regulation Of Leukocyte Degranulation (GO:0043302)
Positive Regulation Of Potassium Ion Transmembrane Transporter Activity (GO:1901018)
Positive Regulation Of Superoxide Anion Generation (GO:0032930)
Protein Insertion Into Mitochondrial Outer Membrane (GO:0045040)
Protein Localization To Synapse (GO:0035418)
Purine Ribonucleoside Bisphosphate Metabolic Process (GO:0034035)
Regulation Of ERBB Signaling Pathway (GO:1901184)
Regulation Of T Cell Chemotaxis (GO:0010819)
Regulation Of T-helper 17 Type Immune Response (GO:2000316)
Regulation Of Gastrulation (GO:0010470)
Regulation Of Intrinsic Apoptotic Signaling Pathway In Response To DNA Damage By P53 Class Mediator (GO:19[illegible]
Regulation Of Myoblast Fusion (GO:1901739)
Regulation Of Oxidoreductase Activity (GO:0051341)
Regulation Of Response To Stress (GO:0080134)
Regulation Of Short-Term Neuronal Synaptic Plasticity (GO:0048172)
Regulation Of Transcription Of Nucleolar Large rRNA By RNA Polymerase I (GO:1901836)
Response To Chemokine (GO:1990868)
Response To Nerve Growth Factor (GO:1990089)
Response To Progesterone (GO:0032570)
Regulation Of Response To External Stimulus (GO:0032101)
Positive Regulation Of Programmed Cell Death (GO:0043068)
Cellular Response To Amino Acid Stimulus (GO:0071230)
One-Carbon Compound Transport (GO:0019755)
Protein Methylation (GO:0006479)
Regulation Of G2/M Transition Of Mitotic Cell Cycle (GO:0010389)
Regulation Of Cardiac Muscle Contraction (GO:0055117)
Regulation Of Cellular Senescence (GO:2000772)
Regulation Of mRNA Processing (GO:0050684)
Regulation Of Postsynaptic Membrane Potential (GO:0060078)
Regulation Of Sodium Ion Transport (GO:0002028)
Positive Regulation Of Vasculature Development (GO:1904018)
Cellular Response To Interleukin-1 (GO:0071347)

Negative Regulation Of NF-kappaB Transcription Factor Activity (GO:0032088)
Eye Development (GO:0001654)
Positive Regulation Of Apoptotic Process (GO:0043065)
Positive Regulation Of Nucleic Acid-Templated Transcription (GO:1903508)
Positive Regulation Of Tumor Necrosis Factor Superfamily Cytokine Production (GO:1903557)
Protein Targeting (GO:0006605)
Histone Deacetylation (GO:0016575)
Interstrand Cross-Link Repair (GO:0036297)
Negative Regulation Of T Cell Proliferation (GO:0042130)
Positive Regulation Of Interferon-Beta Production (GO:0032728)
Positive Regulation Of Myeloid Cell Differentiation (GO:0045639)
Regulation Of Erythrocyte Differentiation (GO:0045646)
Regulation Of Receptor Internalization (GO:0002090)
Substrate Adhesion-Dependent Cell Spreading (GO:0034446)
Anterior/Posterior Pattern Specification (GO:0009952)
Mitotic Cytokinesis (GO:0000281)
Regulation Of miRNA Transcription (GO:1902893)
Regulation Of Multicellular Organismal Development (GO:2000026)
Regulation Of Type I Interferon Production (GO:0032479)
Positive Regulation Of Cell Growth (GO:0030307)
L-glutamate Transmembrane Transport (GO:0015813)
Blood Coagulation, Fibrin Clot Formation (GO:0072378)
Bone Morphogenesis (GO:0060349)
Ceramide Transport (GO:0035627)
Contractile Actin Filament Bundle Assembly (GO:0030038)
Copper Ion Homeostasis (GO:0055070)
Dolichol Metabolic Process (GO:0019348)
Embryonic Cranial Skeleton Morphogenesis (GO:0048701)
Establishment Of Apical/Basal Cell Polarity (GO:0035089)
Ethanol Metabolic Process (GO:0006067)
Formation Of Cytoplasmic Translation Initiation Complex (GO:0001732)
Lipoprotein Biosynthetic Process (GO:0042158)
Microvillus Assembly (GO:0030033)
Mitochondrion Transport Along Microtubule (GO:0047497)
Mitotic Recombination (GO:0006312)
Negative Regulation Of Blood Pressure (GO:0045776)
Negative Regulation Of Cilium Assembly (GO:1902018)
Negative Regulation Of Defense Response To Virus (GO:0050687)
Negative Regulation Of Fatty Acid Biosynthetic Process (GO:0045717)
Phagolysosome Assembly (GO:0001845)
Photoreceptor Cell Development (GO:0042461)
Polarized Epithelial Cell Differentiation (GO:0030859)
Positive Regulation Of Adaptive Immune Response Based On Somatic Recombination Of Immune Receptors Bu
Positive Regulation Of Cytoplasmic Translation (GO:2000767)
Positive Regulation Of Glycogen Metabolic Process (GO:0070875)
Positive Regulation Of Heterotypic Cell-Cell Adhesion (GO:0034116)
Positive Regulation Of Lyase Activity (GO:0051349)
Positive Regulation Of Telomere Maintenance In Response To DNA Damage (GO:1904507)

Positive Regulation Of Type I Interferon-Mediated Signaling Pathway (GO:0060340)
Primary Alcohol Metabolic Process (GO:0034308)
Protein Activation Cascade (GO:0072376)
Protein Homotrimerization (GO:0070207)
Protein Trimerization (GO:0070206)
Regulation Of ATP Biosynthetic Process (GO:2001169)
Regulation Of Carbohydrate Catabolic Process (GO:0043470)
Regulation Of Cholesterol Storage (GO:0010885)
Regulation Of Mast Cell Degranulation (GO:0043304)
Regulation Of Superoxide Anion Generation (GO:0032928)
Regulation Of Vesicle Size (GO:0097494)
Response To Muscle Stretch (GO:0035994)
Smooth Muscle Contraction (GO:0006939)
Stress Fiber Assembly (GO:0043149)
Striated Muscle Tissue Development (GO:0014706)
Synaptic Transmission, GABAergic (GO:0051932)
Synaptic Vesicle Membrane Organization (GO:0048499)
Telomere Capping (GO:0016233)
Vascular Endothelial Growth Factor Signaling Pathway (GO:0038084)
Phospholipase C-activating G Protein-Coupled Receptor Signaling Pathway (GO:0007200)
Regulation Of Epithelial To Mesenchymal Transition (GO:0010717)
Positive Regulation Of Phagocytosis (GO:0050766)
Regulation Of Cell Cycle (GO:0051726)
Negative Regulation Of Endothelial Cell Migration (GO:0010596)
Positive Regulation Of Receptor Signaling Pathway Via JAK-STAT (GO:0046427)
Positive Regulation Of Receptor Signaling Pathway Via STAT (GO:1904894)
Protein Acylation (GO:0043543)
Regulation Of Sodium Ion Transmembrane Transporter Activity (GO:2000649)
Ventricular Septum Development (GO:0003281)
Purine Ribonucleotide Metabolic Process (GO:0009150)
Positive Regulation Of DNA Metabolic Process (GO:0051054)
Circulatory System Development (GO:0072359)
Regulation Of MAPK Cascade (GO:0043408)
Defense Response To Gram-positive Bacterium (GO:0050830)
Positive Regulation Of Protein Localization (GO:1903829)
Amino Acid Catabolic Process (GO:0009063)
Connective Tissue Development (GO:0061448)
Establishment Of Protein Localization To Plasma Membrane (GO:0061951)
mRNA Transcription By RNA Polymerase II (GO:0042789)
Maturation Of SSU-rRNA (GO:0030490)
Negative Regulation Of Type I Interferon Production (GO:0032480)
Positive Regulation Of Production Of Molecular Mediator Of Immune Response (GO:0002702)
Regulation Of Intracellular Transport (GO:0032386)
Regulation Of Neurotransmitter Secretion (GO:0046928)
Regulation Of Protein Import Into Nucleus (GO:0042306)
3'-Phosphoadenosine 5'-Phosphosulfate Metabolic Process (GO:0050427)
L-glutamate Import (GO:0051938)
Cardiac Myofibril Assembly (GO:0055003)

Cellular Response To Corticosteroid Stimulus (GO:0071384)
Cellular Response To Estrogen Stimulus (GO:0071391)
Cellular Response To Sterol (GO:0036315)
Ceramide Catabolic Process (GO:0046514)
Coenzyme A Metabolic Process (GO:0015936)
Dendritic Cell Chemotaxis (GO:0002407)
Dendritic Spine Morphogenesis (GO:0060997)
Dolichol-Linked Oligosaccharide Biosynthetic Process (GO:0006488)
Epiboly Involved In Wound Healing (GO:0090505)
Ganglioside Biosynthetic Process (GO:0001574)
Glutamine Family Amino Acid Catabolic Process (GO:0009065)
Melanosome Transport (GO:0032402)
miRNA Metabolic Process (GO:0010586)
Negative Regulation Of Cation Transmembrane Transport (GO:1904063)
Negative Regulation Of Cell-Substrate Junction Organization (GO:0150118)
Negative Regulation Of Epithelial Cell Differentiation (GO:0030857)
Negative Regulation Of Focal Adhesion Assembly (GO:0051895)
Negative Regulation Of Immune Effector Process (GO:0002698)
Negative Regulation Of Macrophage Activation (GO:0043031)
Negative Regulation Of Phagocytosis (GO:0050765)
Negative Regulation Of Telomere Maintenance (GO:0032205)
Phagosome Maturation (GO:0090382)
Pigment Granule Transport (GO:0051904)
Platelet-Derived Growth Factor Receptor Signaling Pathway (GO:0048008)
Positive Regulation Of DNA Damage Response, Signal Transduction By P53 Class Mediator (GO:0043517)
Positive Regulation Of Cell Activation (GO:0050867)
Positive Regulation Of Glucose Metabolic Process (GO:0010907)
Positive Regulation Of Muscle Cell Differentiation (GO:0051149)
Positive Regulation Of Syncytium Formation By Plasma Membrane Fusion (GO:0060143)
Presynapse Assembly (GO:0099054)
Protein Localization To Cell-Cell Junction (GO:0150105)
Protein Localization To Chromosome, Telomeric Region (GO:0070198)
Regulation Of Activin Receptor Signaling Pathway (GO:0032925)
Regulation Of Amyloid Fibril Formation (GO:1905906)
Regulation Of Bicellular Tight Junction Assembly (GO:2000810)
Regulation Of Carbohydrate Metabolic Process (GO:0006109)
Regulation Of Clathrin-Dependent Endocytosis (GO:2000369)
Regulation Of Intrinsic Apoptotic Signaling Pathway In Response To DNA Damage (GO:1902229)
Regulation Of rRNA Processing (GO:2000232)
Regulation Of Telomere Maintenance In Response To DNA Damage (GO:1904505)
Renal Filtration (GO:0097205)
Sphingolipid Catabolic Process (GO:0030149)
Stem Cell Development (GO:0048864)
tRNA Wobble Uridine Modification (GO:0002098)
Vasodilation (GO:0042311)
Cell Junction Organization (GO:0034330)
Protein Lipidation (GO:0006497)
Regulation Of T Cell Activation (GO:0050863)

Response To Lipid (GO:0033993)
Heterochromatin Organization (GO:0070828)
Internal Peptidyl-Lysine Acetylation (GO:0018393)
Negative Regulation Of Lymphocyte Proliferation (GO:0050672)
Negative Regulation Of Mitochondrion Organization (GO:0010823)
Negative Regulation Of Proteasomal Protein Catabolic Process (GO:1901799)
Negative Regulation Of Protein Secretion (GO:0050709)
Nuclear-Transcribed mRNA Catabolic Process, Deadenylation-Dependent Decay (GO:0000288)
Organelle Transport Along Microtubule (GO:0072384)
Positive Regulation Of ATP-dependent Activity (GO:0032781)
Positive Regulation Of Cytokinesis (GO:0032467)
Positive Regulation Of Intrinsic Apoptotic Signaling Pathway (GO:2001244)
Regulation Of Monoatomic Cation Transmembrane Transport (GO:1904062)
Regulation Of Protein Tyrosine Kinase Activity (GO:0061097)
Cellular Response To Metal Ion (GO:0071248)
Non-Membrane-Bounded Organelle Assembly (GO:0140694)
Negative Regulation Of Transmembrane Receptor Protein Serine/Threonine Kinase Signaling Pathway (GO:00901
Inorganic Anion Transmembrane Transport (GO:0098661)
Phosphatidylinositol Metabolic Process (GO:0046488)
Positive Regulation Of Mitotic Cell Cycle Phase Transition (GO:1901992)
Telomere Organization (GO:0032200)
Regulation Of Cell Population Proliferation (GO:0042127)
Monoatomic Cation Transmembrane Transport (GO:0098655)
Positive Regulation Of Developmental Process (GO:0051094)
Negative Regulation Of Defense Response (GO:0031348)
Activation Of Cysteine-Type Endopeptidase Activity (GO:0097202)
Antifungal Innate Immune Response (GO:0061760)
Apoptotic Nuclear Changes (GO:0030262)
Cell Communication By Electrical Coupling Involved In Cardiac Conduction (GO:0086064)
Cell Surface Toll-Like Receptor Signaling Pathway (GO:0140895)
Cellular Response To Gamma Radiation (GO:0071480)
Chondroitin Sulfate Metabolic Process (GO:0030204)
Dicarboxylic Acid Catabolic Process (GO:0043649)
Endoplasmic Reticulum Tubular Network Organization (GO:0071786)
Establishment Of Melanosome Localization (GO:0032401)
Glycoprotein Catabolic Process (GO:0006516)
Male Meiosis I (GO:0007141)
Mitochondrial Electron Transport, Cytochrome C To Oxygen (GO:0006123)
Mitophagy (GO:0000423)
Negative Regulation Of Inflammatory Response To Antigenic Stimulus (GO:0002862)
Negative Regulation Of Phosphatase Activity (GO:0010923)
Positive Regulation Of Cardiac Muscle Tissue Growth (GO:0055023)
Positive Regulation Of Lymphocyte Migration (GO:2000403)
Positive Regulation Of Positive Chemotaxis (GO:0050927)
Positive Regulation Of Smooth Muscle Cell Migration (GO:0014911)
Protein O-linked Mannosylation (GO:0035269)
Protein Localization To Mitochondrion (GO:0070585)
Regulation Of B Cell Receptor Signaling Pathway (GO:0050855)

Regulation Of DNA Strand Elongation (GO:0060382)
Regulation Of Golgi Organization (GO:1903358)
Regulation Of NMDA Receptor Activity (GO:2000310)
Regulation Of Nucleocytoplasmic Transport (GO:0046822)
Regulation Of Plasminogen Activation (GO:0010755)
Regulation Of Presynapse Assembly (GO:1905606)
Regulation Of Presynapse Organization (GO:0099174)
Regulation Of Stem Cell Proliferation (GO:0072091)
Response To UV-B (GO:0010224)
Ruffle Organization (GO:0031529)
Cellular Response To Virus (GO:0098586)
Positive Regulation Of T Cell Differentiation (GO:0045582)
Positive Regulation Of Cysteine-Type Endopeptidase Activity (GO:2001056)
Positive Regulation Of Interleukin-1 Production (GO:0032732)
Positive Regulation Of Type I Interferon Production (GO:0032481)
Response To Metal Ion (GO:0010038)
Notch Signaling Pathway (GO:0007219)
Aortic Valve Development (GO:0003176)
Cellular Response To Heat (GO:0034605)
Endothelial Cell Migration (GO:0043542)
Negative Regulation Of Secretion By Cell (GO:1903531)
Nucleic Acid-Templated Transcription (GO:0097659)
Positive Regulation Of Cell Cycle G1/S Phase Transition (GO:1902808)
Positive Regulation Of Monoatomic Ion Transport (GO:0043270)
Protein Deacetylation (GO:0006476)
Protein Localization To Chromosome (GO:0034502)
Regulation Of Small Molecule Metabolic Process (GO:0062012)
Retinoid Metabolic Process (GO:0001523)
Water-Soluble Vitamin Metabolic Process (GO:0006767)
Regulation Of Macromolecule Metabolic Process (GO:0060255)
Inorganic Cation Transmembrane Transport (GO:0098662)
RNA Export From Nucleus (GO:0006405)
Fatty Acid Biosynthetic Process (GO:0006633)
Monocarboxylic Acid Biosynthetic Process (GO:0072330)
Inflammatory Response (GO:0006954)
Regulation Of Interleukin-6 Production (GO:0032675)
DNA Duplex Unwinding (GO:0032508)
Cellular Response To Retinoic Acid (GO:0071300)
Double-Strand Break Repair Via Nonhomologous End Joining (GO:0006303)
Negative Regulation Of mRNA Catabolic Process (GO:1902373)
Non-Canonical Wnt Signaling Pathway (GO:0035567)
Positive Regulation Of Response To Biotic Stimulus (GO:0002833)
Regulation Of Amide Metabolic Process (GO:0034248)
Visual System Development (GO:0150063)
Regulation Of Phosphatidylinositol 3-Kinase Signaling (GO:0014066)
DNA Biosynthetic Process (GO:0071897)
DNA Damage Response, Signal Transduction By P53 Class Mediator Resulting In Cell Cycle Arrest (GO:000697
Rac Protein Signal Transduction (GO:0016601)

Carbohydrate Derivative Catabolic Process (GO:1901136)
Carboxylic Acid Catabolic Process (GO:0046395)
Cellular Response To Glucocorticoid Stimulus (GO:0071385)
Cellular Response To Unfolded Protein (GO:0034620)
Embryonic Digit Morphogenesis (GO:0042733)
Filopodium Assembly (GO:0046847)
Hippo Signaling (GO:0035329)
Intracellular Estrogen Receptor Signaling Pathway (GO:0030520)
Megakaryocyte Differentiation (GO:0030219)
Membrane Lipid Metabolic Process (GO:0006643)
Microtubule Depolymerization (GO:0007019)
ncRNA Metabolic Process (GO:0034660)
Negative Regulation Of Rho Protein Signal Transduction (GO:0035024)
Negative Regulation Of Cellular Senescence (GO:2000773)
Negative Regulation Of Fibroblast Proliferation (GO:0048147)
Negative Regulation Of Interleukin-8 Production (GO:0032717)
Negative Regulation Of Leukocyte Activation (GO:0002695)
Negative Regulation Of Striated Muscle Cell Differentiation (GO:0051154)
Neural Tube Development (GO:0021915)
Nicotinamide Nucleotide Metabolic Process (GO:0046496)
Nuclear-Transcribed mRNA Catabolic Process, Exonucleolytic (GO:0000291)
Peroxisomal Membrane Transport (GO:0015919)
Positive Chemotaxis (GO:0050918)
Positive Regulation Of Alpha-Beta T Cell Proliferation (GO:0046641)
Positive Regulation Of Amyloid-Beta Formation (GO:1902004)
Positive Regulation Of Cartilage Development (GO:0061036)
Positive Regulation Of Cell Cycle Phase Transition (GO:1901989)
Positive Regulation Of Dendrite Morphogenesis (GO:0050775)
Positive Regulation Of Fatty Acid Metabolic Process (GO:0045923)
Positive Regulation Of Glial Cell Differentiation (GO:0045687)
Positive Regulation Of Lymphocyte Chemotaxis (GO:0140131)
Positive Regulation Of Nitric-Oxide Synthase Activity (GO:0051000)
Positive Regulation Of Peptidyl-Lysine Acetylation (GO:2000758)
Positive Regulation Of Potassium Ion Transmembrane Transport (GO:1901381)
Positive Regulation Of Small GTPase Mediated Signal Transduction (GO:0051057)
Postsynaptic Membrane Organization (GO:0001941)
Regulation Of Cellular Respiration (GO:0043457)
Regulation Of Establishment Of Cell Polarity (GO:2000114)
Regulation Of Hematopoietic Progenitor Cell Differentiation (GO:1901532)
Regulation Of Inflammatory Response To Antigenic Stimulus (GO:0002861)
Regulation Of Mitotic Cell Cycle Spindle Assembly Checkpoint (GO:0090266)
Regulation Of Protein Sumoylation (GO:0033233)
Regulation Of Receptor Signaling Pathway Via STAT (GO:1904892)
Regulation Of Viral Transcription (GO:0046782)
Ribosomal Small Subunit Assembly (GO:0000028)
Sulfur Amino Acid Metabolic Process (GO:0000096)
Toll-Like Receptor 4 Signaling Pathway (GO:0034142)
Response To Reactive Oxygen Species (GO:0000302)

Positive Regulation Of Cell Population Proliferation (GO:0008284)
rRNA Metabolic Process (GO:0016072)
DNA Recombination (GO:0006310)
Cellular Response To Glucose Starvation (GO:0042149)
Modulation By Host Of Symbiont Process (GO:0051851)
Negative Regulation Of I-kappaB kinase/NF-kappaB Signaling (GO:0043124)
Positive Regulation Of Calcium Ion Transmembrane Transport (GO:1904427)
Positive Regulation Of Neuron Differentiation (GO:0045666)
Stress-Activated MAPK Cascade (GO:0051403)
Translational Elongation (GO:0006414)
Positive Regulation Of Multicellular Organismal Process (GO:0051240)
Positive Regulation Of Protein Serine/Threonine Kinase Activity (GO:0071902)
Protein Processing (GO:0016485)
T-helper Cell Differentiation (GO:0042093)
Actin Filament Capping (GO:0051693)
Adrenergic Receptor Signaling Pathway (GO:0071875)
Antigen Processing And Presentation Of Peptide Antigen Via MHC Class I (GO:0002474)
Barbed-End Actin Filament Capping (GO:0051016)
Cardiac Atrium Morphogenesis (GO:0003209)
Dendritic Cell Migration (GO:0036336)
Endothelial Cell Proliferation (GO:0001935)
Establishment Of Vesicle Localization (GO:0051650)
Heart Trabecula Morphogenesis (GO:0061384)
Intracellular Sodium Ion Homeostasis (GO:0006883)
Maintenance Of Protein Location In Nucleus (GO:0051457)
Melanosome Assembly (GO:1903232)
Melanosome Localization (GO:0032400)
Membrane Repolarization (GO:0086009)
Myoblast Fusion (GO:0007520)
Negative Regulation Of Lipid Catabolic Process (GO:0050995)
Negative Regulation Of Signal Transduction By P53 Class Mediator (GO:1901797)
Neuromuscular Process (GO:0050905)
Neuron Fate Commitment (GO:0048663)
Nitric Oxide Mediated Signal Transduction (GO:0007263)
Peptide Antigen Assembly With MHC Protein Complex (GO:0002501)
Positive Regulation Of Establishment Of Protein Localization (GO:1904951)
Positive Regulation Of Focal Adhesion Assembly (GO:0051894)
Positive Regulation Of Monocyte Chemotaxis (GO:0090026)
Positive Regulation Of Nervous System Process (GO:0031646)
Positive Regulation Of Neutrophil Chemotaxis (GO:0090023)
Positive Regulation Of Stress-Activated Protein Kinase Signaling Cascade (GO:0070304)
Regulation Of RIG-I Signaling Pathway (GO:0039535)
Regulation Of T Cell Cytokine Production (GO:0002724)
Regulation Of Adaptive Immune Response (GO:0002819)
Regulation Of cAMP-mediated Signaling (GO:0043949)
Regulation Of Defense Response To Virus (GO:0050688)
Regulation Of Establishment Or Maintenance Of Cell Polarity (GO:0032878)
Regulation Of Exosomal Secretion (GO:1903541)

Regulation Of Mitochondrial Membrane Permeability (GO:0046902)
Regulation Of Mitochondrial Outer Membrane Permeabilization Involved In Apoptotic Signaling Pathway (GO:19[illegible]
Regulation Of Protein Deacetylation (GO:0090311)
Regulation Of Synaptic Vesicle Exocytosis (GO:2000300)
Response To Axon Injury (GO:0048678)
Response To Cholesterol (GO:0070723)
Regulation Of Cytokine Production (GO:0001817)
Glycolipid Biosynthetic Process (GO:0009247)
mRNA Transcription (GO:0009299)
Positive Regulation Of Cell Division (GO:0051781)
Positive Regulation Of Cell-Matrix Adhesion (GO:0001954)
Positive Regulation Of Receptor-Mediated Endocytosis (GO:0048260)
Positive Regulation Of Transcription Elongation By RNA Polymerase II (GO:0032968)
Regulation Of JUN Kinase Activity (GO:0043506)
Regulation Of Filopodium Assembly (GO:0051489)
Regulation Of Primary Metabolic Process (GO:0080090)
Protein O-linked Glycosylation (GO:0006493)
Regulation Of Protein Binding (GO:0043393)
Double-Strand Break Repair (GO:0006302)
Regulation Of Signal Transduction (GO:0009966)
Regulation Of mRNA Splicing, Via Spliceosome (GO:0048024)
Establishment Of Protein Localization To Membrane (GO:0090150)
Positive Regulation Of Phosphatidylinositol 3-Kinase Signaling (GO:0014068)
Regulation Of Monoatomic Ion Transmembrane Transporter Activity (GO:0032412)
Bone Development (GO:0060348)
Cellular Response To Ketone (GO:1901655)
Neural Crest Cell Development (GO:0014032)
Positive Regulation Of Biosynthetic Process (GO:0009891)
Positive Regulation Of Chemotaxis (GO:0050921)
Positive Regulation Of Nucleocytoplasmic Transport (GO:0046824)
Regulation Of B Cell Proliferation (GO:0030888)
Regulation Of DNA-templated Transcription Elongation (GO:0032784)
Regulation Of Cell-Substrate Adhesion (GO:0010810)
Regulation Of Transport (GO:0051049)
DNA Catabolic Process (GO:0006308)
N-glycan Processing (GO:0006491)
Atrioventricular Valve Morphogenesis (GO:0003181)
Cardiac Epithelial To Mesenchymal Transition (GO:0060317)
Cellular Response To Lectin (GO:1990858)
Chondroitin Sulfate Proteoglycan Biosynthetic Process (GO:0050650)
Dopamine Metabolic Process (GO:0042417)
Embryonic Digestive Tract Development (GO:0048566)
Eye Morphogenesis (GO:0048592)
Inositol Phosphate-Mediated Signaling (GO:0048016)
Insulin Secretion (GO:0030073)
Interleukin-1-Mediated Signaling Pathway (GO:0070498)
Intrinsic Apoptotic Signaling Pathway In Response To DNA Damage By P53 Class Mediator (GO:0042771)
Linoleic Acid Metabolic Process (GO:0043651)

Mesoderm Development (GO:0007498)
Negative Regulation Of RNA Splicing (GO:0033119)
Negative Regulation Of Axon Extension Involved In Axon Guidance (GO:0048843)
Negative Regulation Of Calcium Ion Transmembrane Transport (GO:1903170)
Negative Regulation Of Fatty Acid Metabolic Process (GO:0045922)
Negative Regulation Of Protein Dephosphorylation (GO:0035308)
Negative Regulation Of Telomere Maintenance Via Telomerase (GO:0032211)
Neuron Apoptotic Process (GO:0051402)
Nucleotide Biosynthetic Process (GO:0009165)
Phosphate Ion Transport (GO:0006817)
Positive Regulation Of Activated T Cell Proliferation (GO:0042104)
Positive Regulation Of Alpha-Beta T Cell Activation (GO:0046635)
Protein Heterooligomerization (GO:0051291)
Regulation Of B Cell Differentiation (GO:0045577)
Regulation Of Acute Inflammatory Response (GO:0002673)
Regulation Of Autophagy Of Mitochondrion (GO:1903146)
Regulation Of Cellular Response To Growth Factor Stimulus (GO:0090287)
Regulation Of Fibroblast Growth Factor Receptor Signaling Pathway (GO:0040036)
Regulation Of Insulin-Like Growth Factor Receptor Signaling Pathway (GO:0043567)
Regulation Of Megakaryocyte Differentiation (GO:0045652)
Regulation Of Smooth Muscle Cell Migration (GO:0014910)
Regulation Of Vascular Endothelial Growth Factor Signaling Pathway (GO:1900746)
Sphingosine Metabolic Process (GO:0006670)
Stimulatory C-type Lectin Receptor Signaling Pathway (GO:0002223)
Vesicle Docking (GO:0048278)
Cell Junction Assembly (GO:0034329)
Positive Regulation Of Epithelial Cell Migration (GO:0010634)
Sulfur Compound Biosynthetic Process (GO:0044272)
Heart Development (GO:0007507)
Negative Regulation Of Response To Biotic Stimulus (GO:0002832)
Regulation Of Protein Modification Process (GO:0031399)
Regulation Of Cold-Induced Thermogenesis (GO:0120161)
Cell-Cell Junction Assembly (GO:0007043)
Lipid Biosynthetic Process (GO:0008610)
Negative Regulation Of Cellular Response To Growth Factor Stimulus (GO:0090288)
Synapse Assembly (GO:0007416)
Regulation Of Protein Catabolic Process (GO:0042176)
Regulation Of Defense Response (GO:0031347)
DNA Methylation (GO:0006306)
Ameboidal-Type Cell Migration (GO:0001667)
Anterior/Posterior Axis Specification (GO:0009948)
Endoplasmic Reticulum Calcium Ion Homeostasis (GO:0032469)
Endothelial Cell Development (GO:0001885)
Gamma-Aminobutyric Acid Signaling Pathway (GO:0007214)
Hydrogen Peroxide Catabolic Process (GO:0042744)
Intracellular Monoatomic Ion Homeostasis (GO:0006873)
Long-Term Synaptic Potentiation (GO:0060291)
Microglial Cell Activation (GO:0001774)

Myoblast Differentiation (GO:0045445)
Negative Regulation Of Reproductive Process (GO:2000242)
Negative Regulation Of Stem Cell Population Maintenance (GO:1902455)
Negative Regulation Of Viral Life Cycle (GO:1903901)
Nuclear-Transcribed mRNA poly(A) Tail Shortening (GO:0000289)
Phosphatidylglycerol Metabolic Process (GO:0046471)
Positive Regulation Of Antigen Receptor-Mediated Signaling Pathway (GO:0050857)
Positive Regulation Of Epithelial Cell Differentiation (GO:0030858)
Pulmonary Valve Development (GO:0003177)
Regulation Of Calcium Ion Import (GO:0090279)
Regulation Of Meiotic Cell Cycle (GO:0051445)
Regulation Of Membrane Depolarization (GO:0003254)
Regulation Of Phosphatase Activity (GO:0010921)
Regulation Of Smooth Muscle Contraction (GO:0006940)
Response To Ketone (GO:1901654)
Transport Along Microtubule (GO:0010970)
B Cell Receptor Signaling Pathway (GO:0050853)
Cardiac Conduction (GO:0061337)
Intracellular Protein Transmembrane Transport (GO:0065002)
Mitotic G2/M Transition Checkpoint (GO:0044818)
Negative Regulation Of Cellular Metabolic Process (GO:0031324)
Negative Regulation Of Cold-Induced Thermogenesis (GO:0120163)
Positive Regulation Of Cell-Cell Adhesion (GO:0022409)
Regulation Of Interleukin-10 Production (GO:0032653)
Replication Fork Processing (GO:0031297)
Response To Hydrogen Peroxide (GO:0042542)
Neuron Differentiation (GO:0030182)
Negative Regulation Of Catabolic Process (GO:0009895)
Regulation Of Cellular Localization (GO:0060341)
Response To Ionizing Radiation (GO:0010212)
Modulation Of Chemical Synaptic Transmission (GO:0050804)
Positive Regulation Of Cold-Induced Thermogenesis (GO:0120162)
Positive Regulation Of Protein-Containing Complex Assembly (GO:0031334)
Negative Regulation Of Growth (GO:0045926)
Positive Regulation Of Defense Response (GO:0031349)
Protein Dephosphorylation (GO:0006470)
Regulation Of DNA Metabolic Process (GO:0051052)
Positive Regulation Of Smooth Muscle Cell Proliferation (GO:0048661)
Regulation Of Signal Transduction By P53 Class Mediator (GO:1901796)
Cellular Response To Reactive Oxygen Species (GO:0034614)
Aminoglycan Biosynthetic Process (GO:0006023)
Anaphase-Promoting Complex-Dependent Catabolic Process (GO:0031145)
Cellular Response To Nerve Growth Factor Stimulus (GO:1990090)
Focal Adhesion Assembly (GO:0048041)
Histone H2A Acetylation (GO:0043968)
Innate Immune Response In Mucosa (GO:0002227)
Lipid Catabolic Process (GO:0016042)
Macrophage Differentiation (GO:0030225)

Modulation By Host Of Viral Genome Replication (GO:0044827)
Modulation By Symbiont Of Entry Into Host (GO:0052372)
Negative Regulation Of Biomineral Tissue Development (GO:0070168)
Negative Regulation Of Interleukin-2 Production (GO:0032703)
poly(A)+ mRNA Export From Nucleus (GO:0016973)
Positive Regulation Of T Cell Cytokine Production (GO:0002726)
Positive Regulation Of Amyloid Precursor Protein Catabolic Process (GO:1902993)
Positive Regulation Of Blood Coagulation (GO:0030194)
Positive Regulation Of Dendritic Spine Development (GO:0060999)
Positive Regulation Of Granulocyte Chemotaxis (GO:0071624)
Positive Regulation Of Natural Killer Cell Mediated Cytotoxicity (GO:0045954)
Positive Regulation Of Signal Transduction By P53 Class Mediator (GO:1901798)
Positive Regulation Of Smoothened Signaling Pathway (GO:0045880)
Positive Regulation Of Transcription Regulatory Region DNA Binding (GO:2000679)
Protein Mannosylation (GO:0035268)
Purine Nucleotide Biosynthetic Process (GO:0006164)
Regulation Of Dendritic Spine Development (GO:0060998)
Regulation Of Lipid Storage (GO:0010883)
Regulation Of Neurotransmitter Transport (GO:0051588)
Regulation Of Steroid Metabolic Process (GO:0019218)
Sulfur Compound Transport (GO:0072348)
Cellular Response To Mechanical Stimulus (GO:0071260)
Chloride Transmembrane Transport (GO:1902476)
Myelination (GO:0042552)
Negative Regulation Of ERK1 And ERK2 Cascade (GO:0070373)
Positive Regulation Of Reactive Oxygen Species Metabolic Process (GO:2000379)
Post-Translational Protein Modification (GO:0043687)
Regulation Of Tumor Necrosis Factor-Mediated Signaling Pathway (GO:0010803)
Protein-Containing Complex Organization (GO:0043933)
Apoptotic Process (GO:0006915)
Positive Regulation Of NF-kappaB Transcription Factor Activity (GO:0051092)
Hemopoiesis (GO:0030097)
Regulation Of Stress Fiber Assembly (GO:0051492)
Regulation Of Tumor Necrosis Factor Production (GO:0032680)
Acylglycerol Biosynthetic Process (GO:0046463)
Cellular Response To Low-Density Lipoprotein Particle Stimulus (GO:0071404)
Cytoskeleton-Dependent Intracellular Transport (GO:0030705)
Glucose Transmembrane Transport (GO:1904659)
Membrane Protein Ectodomain Proteolysis (GO:0006509)
Natural Killer Cell Mediated Immunity (GO:0002228)
Negative Regulation Of G Protein-Coupled Receptor Signaling Pathway (GO:0045744)
Negative Regulation Of Extrinsic Apoptotic Signaling Pathway In Absence Of Ligand (GO:2001240)
Negative Regulation Of Signal Transduction In Absence Of Ligand (GO:1901099)
Oligosaccharide Biosynthetic Process (GO:0009312)
Oxoacid Metabolic Process (GO:0043436)
peptidyl-L-cysteine S-palmitoylation (GO:0018230)
peptidyl-S-diacylglycerol-L-cysteine Biosynthetic Process From Peptidyl-Cysteine (GO:0018231)
Platelet Dense Granule Organization (GO:0060155)

Positive Regulation Of Cholesterol Efflux (GO:0010875)
Positive Regulation Of Excitatory Postsynaptic Potential (GO:2000463)
Positive Regulation Of Receptor Internalization (GO:0002092)
Positive Regulation Of Synaptic Transmission, Glutamatergic (GO:0051968)
Positive Regulation Of Viral Life Cycle (GO:1903902)
Protein Peptidyl-Prolyl Isomerization (GO:0000413)
Regulation Of Dopamine Secretion (GO:0014059)
Regulation Of Telomere Capping (GO:1904353)
snRNA 3'-End Processing (GO:0034472)
tRNA Aminoacylation (GO:0043039)
Interferon-Mediated Signaling Pathway (GO:0140888)
Negative Regulation Of Cysteine-Type Endopeptidase Activity Involved In Apoptotic Process (GO:0043154)
Regulation Of Mitotic Metaphase/Anaphase Transition (GO:0030071)
Unsaturated Fatty Acid Metabolic Process (GO:0033559)
Activation Of GTPase Activity (GO:0090630)
Wnt Signaling Pathway (GO:0016055)
Regulation Of Ras Protein Signal Transduction (GO:0046578)
Epithelium Development (GO:0060429)
Negative Regulation Of Cell Projection Organization (GO:0031345)
Regulation Of Epithelial Cell Migration (GO:0010632)
Protein Homooligomerization (GO:0051260)
Negative Regulation Of Transforming Growth Factor Beta Receptor Signaling Pathway (GO:0030512)
Sphingolipid Biosynthetic Process (GO:0030148)
Regulation Of Canonical Wnt Signaling Pathway (GO:0060828)
Axon Extension (GO:0048675)
Endocrine System Development (GO:0035270)
Hepaticobiliary System Development (GO:0061008)
Histone mRNA Metabolic Process (GO:0008334)
Immune Response-Activating Cell Surface Receptor Signaling Pathway (GO:0002429)
Negative Regulation Of Protein Tyrosine Kinase Activity (GO:0061099)
Positive Regulation Of Neutrophil Migration (GO:1902624)
Potassium Ion Homeostasis (GO:0055075)
Protein Alkylation (GO:0008213)
Regulation Of T Cell Migration (GO:2000404)
Regulation Of Axon Extension Involved In Axon Guidance (GO:0048841)
Regulation Of Cholesterol Transport (GO:0032374)
Regulation Of Extracellular Matrix Organization (GO:1903053)
Rescue Of Stalled Ribosome (GO:0072344)
Translesion Synthesis (GO:0019985)
Positive Regulation Of Kinase Activity (GO:0033674)
Negative Regulation Of Gene Expression, Epigenetic (GO:0045814)
Regulated Exocytosis (GO:0045055)
Regulation Of Telomere Maintenance Via Telomerase (GO:0032210)
Regulation Of Innate Immune Response (GO:0045088)
Positive Regulation Of Stress-Activated MAPK Cascade (GO:0032874)
Regulation Of Neuron Apoptotic Process (GO:0043523)
Negative Regulation Of Cellular Component Organization (GO:0051129)
ERK1 And ERK2 Cascade (GO:0070371)

Amyloid Fibril Formation (GO:1990000)
Cell Morphogenesis Involved In Differentiation (GO:0000904)
Early Endosome To Late Endosome Transport (GO:0045022)
Fat-Soluble Vitamin Metabolic Process (GO:0006775)
Natural Killer Cell Mediated Cytotoxicity (GO:0042267)
Negative Regulation Of Ossification (GO:0030279)
Neuromuscular Junction Development (GO:0007528)
Peroxisome Organization (GO:0007031)
Positive Regulation Of BMP Signaling Pathway (GO:0030513)
Positive Regulation Of Interleukin-17 Production (GO:0032740)
Positive Regulation Of Natural Killer Cell Mediated Immunity (GO:0002717)
Protein K48-linked Deubiquitination (GO:0071108)
Protein Kinase B Signaling (GO:0043491)
Regulation Of Cysteine-Type Endopeptidase Activity (GO:2000116)
Regulation Of Viral Life Cycle (GO:1903900)
Cardiac Muscle Tissue Development (GO:0048738)
Cartilage Development (GO:0051216)
Cellular Response To Ionizing Radiation (GO:0071479)
Icosanoid Metabolic Process (GO:0006690)
Neurotransmitter Transport (GO:0006836)
Positive Regulation Of Telomere Maintenance (GO:0032206)
Actin Cytoskeleton Reorganization (GO:0031532)
Blood Vessel Morphogenesis (GO:0048514)
Negative Regulation Of Cysteine-Type Endopeptidase Activity (GO:2000117)
Negative Regulation Of Metabolic Process (GO:0009892)
Carbohydrate Biosynthetic Process (GO:0016051)
Cardiac Conduction System Development (GO:0003161)
Cholesterol Biosynthetic Process (GO:0006695)
Late Endosome To Vacuole Transport Via Multivesicular Body Sorting Pathway (GO:0032511)
Long-Chain Fatty Acid Biosynthetic Process (GO:0042759)
Negative Regulation Of Lipid Biosynthetic Process (GO:0051055)
Negative Regulation Of Proteolysis Involved In Protein Catabolic Process (GO:1903051)
Negative Regulation Of Response To Wounding (GO:1903035)
Negative Regulation Of Telomere Maintenance Via Telomere Lengthening (GO:1904357)
Peptide Hormone Secretion (GO:0030072)
Positive Regulation Of Glucose Import (GO:0046326)
Positive Regulation Of Peptidase Activity (GO:0010952)
Positive Regulation Of Release Of Cytochrome C From Mitochondria (GO:0090200)
Regulation Of Actin Cytoskeleton Reorganization (GO:2000249)
Regulation Of Monocyte Chemotaxis (GO:0090025)
Regulation Of Myelination (GO:0031641)
Regulation Of Protein Processing (GO:0070613)
Regulation Of Synapse Organization (GO:0050807)
Response To Zinc Ion (GO:0010043)
Secretory Granule Organization (GO:0033363)
Sodium Ion Homeostasis (GO:0055078)
Vesicle-Mediated Transport Between Endosomal Compartments (GO:0098927)
Regulation Of DNA-templated Transcription (GO:0006355)

Cytoskeleton-Dependent Cytokinesis (GO:0061640)
Glycerophospholipid Biosynthetic Process (GO:0046474)
Positive Regulation Of Metabolic Process (GO:0009893)
Response To Tumor Necrosis Factor (GO:0034612)
mRNA Processing (GO:0006397)
Monoatomic Anion Transmembrane Transport (GO:0098656)
Regulation Of Exocytosis (GO:0017157)
Regulation Of Phosphorylation (GO:0042325)
Acute Inflammatory Response (GO:0002526)
Antigen Processing And Presentation Of Exogenous Peptide Antigen Via MHC Class II (GO:0019886)
cAMP-mediated Signaling (GO:0019933)
Calcium-Ion Regulated Exocytosis (GO:0017156)
Camera-Type Eye Development (GO:0043010)
Cardiac Muscle Cell Development (GO:0055013)
Cellular Response To Xenobiotic Stimulus (GO:0071466)
Meiosis I (GO:0007127)
Negative Regulation Of Endothelial Cell Apoptotic Process (GO:2000352)
Negative Regulation Of Lipid Metabolic Process (GO:0045833)
Negative Regulation Of Receptor-Mediated Endocytosis (GO:0048261)
Organelle Membrane Fusion (GO:0090174)
Positive Regulation Of Nuclear Division (GO:0051785)
Regulation Of Cardiac Muscle Cell Contraction (GO:0086004)
Regulation Of Myeloid Cell Differentiation (GO:0045637)
Regulation Of Transcription By RNA Polymerase III (GO:0006359)
Response To Glucocorticoid (GO:0051384)
Phosphatidylcholine Metabolic Process (GO:0046470)
Negative Regulation Of Inflammatory Response (GO:0050728)
Nucleotide-Excision Repair (GO:0006289)
Pattern Recognition Receptor Signaling Pathway (GO:0002221)
Positive Regulation Of DNA-templated Transcription, Elongation (GO:0032786)
Cardiac Muscle Cell Differentiation (GO:0055007)
Clathrin-Dependent Endocytosis (GO:0072583)
Establishment Of Protein Localization To Vacuole (GO:0072666)
In Utero Embryonic Development (GO:0001701)
Microtubule Polymerization Or Depolymerization (GO:0031109)
Monoubiquitinated Histone H2A Deubiquitination (GO:0035522)
Monoubiquitinated Histone Deubiquitination (GO:0035521)
Myeloid Cell Development (GO:0061515)
Peptide Cross-Linking (GO:0018149)
Peptidyl-Lysine Methylation (GO:0018022)
Peptidyl-Proline Modification (GO:0018208)
Phospholipid Biosynthetic Process (GO:0008654)
Positive Regulation Of TORC1 Signaling (GO:1904263)
Positive Regulation Of Cytosolic Calcium Ion Concentration Involved In Phospholipase C-activating G Protein-C
Positive Regulation Of Filopodium Assembly (GO:0051491)
Positive Regulation Of Immune System Process (GO:0002684)
Regulation Of Actin Polymerization Or Depolymerization (GO:0008064)
Regulation Of Androgen Receptor Signaling Pathway (GO:0060765)

Regulation Of Catecholamine Secretion (GO:0050433)
Regulation Of Extrinsic Apoptotic Signaling Pathway In Absence Of Ligand (GO:2001239)
Regulation Of Transcription Regulatory Region DNA Binding (GO:2000677)
Regulation Of Protein Serine/Threonine Kinase Activity (GO:0071900)
Small GTPase Mediated Signal Transduction (GO:0007264)
Response To Interleukin-1 (GO:0070555)
RNA Catabolic Process (GO:0006401)
Regulation Of Calcium Ion Transport (GO:0051924)
Signal Transduction In Response To DNA Damage (GO:0042770)
Striated Muscle Contraction (GO:0006941)
Golgi To Plasma Membrane Protein Transport (GO:0043001)
Antigen Processing And Presentation Of Peptide Antigen Via MHC Class II (GO:0002495)
Cellular Response To Osmotic Stress (GO:0071470)
Establishment Of Endothelial Barrier (GO:0061028)
Glycosaminoglycan Metabolic Process (GO:0030203)
Mucosal Immune Response (GO:0002385)
Negative Regulation Of Cation Channel Activity (GO:2001258)
Negative Regulation Of Cytoskeleton Organization (GO:0051494)
Negative Regulation Of Type II Interferon Production (GO:0032689)
Positive Regulation Of Protein Import Into Nucleus (GO:0042307)
Protein Depolymerization (GO:0051261)
Regulation Of Calcium Ion-Dependent Exocytosis (GO:0017158)
Regulation Of Chromosome Segregation (GO:0051983)
Regulation Of Platelet Activation (GO:0010543)
Regulation Of Sodium Ion Transmembrane Transport (GO:1902305)
Vascular Endothelial Growth Factor Receptor Signaling Pathway (GO:0048010)
Regulation Of Programmed Cell Death (GO:0043067)
Glycosaminoglycan Biosynthetic Process (GO:0006024)
Inorganic Anion Transport (GO:0015698)
Memory (GO:0007613)
Positive Regulation Of Type II Interferon Production (GO:0032729)
Regulation Of Phagocytosis (GO:0050764)
Renal System Development (GO:0072001)
Brain Development (GO:0007420)
Glucose Homeostasis (GO:0042593)
Regulation Of Type II Interferon Production (GO:0032649)
Chordate Embryonic Development (GO:0043009)
Phospholipid Transport (GO:0015914)
JNK Cascade (GO:0007254)
RNA Biosynthetic Process (GO:0032774)
Defense Response To Fungus (GO:0050832)
Establishment Of Mitotic Spindle Orientation (GO:0000132)
Integrated Stress Response Signaling (GO:0140467)
mRNA Splice Site Recognition (GO:0006376)
Morphogenesis Of An Epithelium (GO:0002009)
Peptidyl-Tyrosine Dephosphorylation (GO:0035335)
Positive Regulation Of Endopeptidase Activity (GO:0010950)
Regulation Of Muscle Contraction (GO:0006937)

Proteolysis (GO:0006508)
Regulation Of Endothelial Cell Migration (GO:0010594)
Homophilic Cell Adhesion Via Plasma Membrane Adhesion Molecules (GO:0007156)
Negative Regulation Of Peptidase Activity (GO:0010466)
Response To Light Stimulus (GO:0009416)
Response To Retinoic Acid (GO:0032526)
Organic Anion Transport (GO:0015711)
Sensory Perception Of Mechanical Stimulus (GO:0050954)
Embryonic Skeletal System Morphogenesis (GO:0048704)
Innate Immune Response Activating Cell Surface Receptor Signaling Pathway (GO:0002220)
Negative Regulation Of Axon Extension (GO:0030517)
Negative Regulation Of Carbohydrate Metabolic Process (GO:0045912)
Negative Regulation Of Epithelial To Mesenchymal Transition (GO:0010719)
Negative Regulation Of Peptidyl-Tyrosine Phosphorylation (GO:0050732)
Positive Regulation Of JUN Kinase Activity (GO:0043507)
Positive Regulation Of Cell Communication (GO:0010647)
Regulation Of Vasculature Development (GO:1901342)
Mitochondrial Respiratory Chain Complex Assembly (GO:0033108)
Regulation Of Peptidyl-Tyrosine Phosphorylation (GO:0050730)
Negative Regulation Of Autophagy (GO:0010507)
Cellular Response To Tumor Necrosis Factor (GO:0071356)
Dephosphorylation (GO:0016311)
Positive Regulation Of MAP Kinase Activity (GO:0043406)
Positive Regulation Of Binding (GO:0051099)
B Cell Proliferation (GO:0042100)
Antigen Processing And Presentation Of Exogenous Peptide Antigen (GO:0002478)
Central Nervous System Neuron Differentiation (GO:0021953)
Endodermal Cell Differentiation (GO:0035987)
Glutamine Family Amino Acid Metabolic Process (GO:0009064)
Leukocyte Cell-Cell Adhesion (GO:0007159)
Modulation Of Excitatory Postsynaptic Potential (GO:0098815)
Negative Regulation Of Cell-Matrix Adhesion (GO:0001953)
Non-Motile Cilium Assembly (GO:1905515)
Organic Cation Transport (GO:0015695)
Positive Regulation Of G1/S Transition Of Mitotic Cell Cycle (GO:1900087)
Positive Regulation Of Cholesterol Transport (GO:0032376)
Positive Regulation Of Glucose Transmembrane Transport (GO:0010828)
Positive Regulation Of Leukocyte Migration (GO:0002687)
Positive Regulation Of Proteolysis Involved In Protein Catabolic Process (GO:1903052)
Protein Localization To Chromatin (GO:0071168)
Regulation Of Cell Morphogenesis (GO:0022604)
Regulation Of Extrinsic Apoptotic Signaling Pathway Via Death Domain Receptors (GO:1902041)
Regulation Of Gluconeogenesis (GO:0006111)
Regulation Of Long-Term Synaptic Potentiation (GO:1900271)
Regulation Of Macrophage Activation (GO:0043030)
Regulation Of Response To Biotic Stimulus (GO:0002831)
mRNA Export From Nucleus (GO:0006406)
Central Nervous System Development (GO:0007417)

B Cell Activation (GO:0042113)
Sensory Perception Of Sound (GO:0007605)
Heterochromatin Formation (GO:0031507)
Mesenchymal Cell Migration (GO:0090497)
Mitochondrial Membrane Organization (GO:0007006)
Positive Regulation Of Telomere Maintenance Via Telomerase (GO:0032212)
Protein Transmembrane Import Into Intracellular Organelle (GO:0044743)
Purine Ribonucleoside Triphosphate Metabolic Process (GO:0009205)
Regulation Of Cholesterol Efflux (GO:0010874)
Regulation Of Natural Killer Cell Mediated Cytotoxicity (GO:0042269)
Very Long-Chain Fatty Acid Metabolic Process (GO:0000038)
Regulation Of Peptidyl-Serine Phosphorylation (GO:0033135)
Regulation Of Angiogenesis (GO:0045765)
Cellular Component Assembly (GO:0022607)
Sphingolipid Metabolic Process (GO:0006665)
Actin Filament Bundle Assembly (GO:0051017)
Actin Filament Bundle Organization (GO:0061572)
Epithelial Tube Morphogenesis (GO:0060562)
Monoubiquitinated Protein Deubiquitination (GO:0035520)
ncRNA Catabolic Process (GO:0034661)
Negative Regulation Of Endocytosis (GO:0045806)
Negative Regulation Of Epithelial Cell Migration (GO:0010633)
Positive Regulation Of Chromosome Organization (GO:2001252)
Positive Regulation Of Cytokine Production Involved In Immune Response (GO:0002720)
Positive Regulation Of Nitrogen Compound Metabolic Process (GO:0051173)
Positive Regulation Of Reproductive Process (GO:2000243)
Positive Regulation Of Telomerase Activity (GO:0051973)
Protein K63-linked Deubiquitination (GO:0070536)
Regulation Of NLRP3 Inflammasome Complex Assembly (GO:1900225)
Regulation Of Activated T Cell Proliferation (GO:0046006)
Regulation Of Cell Adhesion Mediated By Integrin (GO:0033628)
Signal Transduction By P53 Class Mediator (GO:0072331)
Ventricular Cardiac Muscle Tissue Morphogenesis (GO:0055010)
Antimicrobial Humoral Immune Response Mediated By Antimicrobial Peptide (GO:0061844)
Canonical Wnt Signaling Pathway (GO:0060070)
Positive Regulation Of DNA Biosynthetic Process (GO:2000573)
Supramolecular Fiber Organization (GO:0097435)
Neuron Projection Guidance (GO:0097485)
ATP Metabolic Process (GO:0046034)
DNA Damage Checkpoint Signaling (GO:0000077)
Cellular Response To Amyloid-Beta (GO:1904646)
Cellular Response To Hydrogen Peroxide (GO:0070301)
Embryonic Limb Morphogenesis (GO:0030326)
Mitochondrial Electron Transport, NADH To Ubiquinone (GO:0006120)
Negative Regulation Of Interleukin-6 Production (GO:0032715)
Organophosphate Biosynthetic Process (GO:0090407)
Protein Targeting To Vacuole (GO:0006623)
Purine Nucleotide Metabolic Process (GO:0006163)

Regulation Of calcineurin-NFAT Signaling Cascade (GO:0070884)
Regulation Of Interleukin-17 Production (GO:0032660)
Regulation Of Metal Ion Transport (GO:0010959)
Regulation Of Neuronal Synaptic Plasticity (GO:0048168)
Response To Estrogen (GO:0043627)
Semaphorin-Plexin Signaling Pathway (GO:0071526)
Smoothened Signaling Pathway (GO:0007224)
Transcription Initiation-Coupled Chromatin Remodeling (GO:0045815)
Monocarboxylic Acid Metabolic Process (GO:0032787)
Positive Regulation Of Response To External Stimulus (GO:0032103)
Regulation Of Neurogenesis (GO:0050767)
tRNA Modification (GO:0006400)
Adherens Junction Organization (GO:0034332)
Endoderm Formation (GO:0001706)
Establishment Of Spindle Orientation (GO:0051294)
Intrinsic Apoptotic Signaling Pathway By P53 Class Mediator (GO:0072332)
Liver Development (GO:0001889)
Macrophage Activation (GO:0042116)
Platelet Aggregation (GO:0070527)
Positive Regulation Of Cell Development (GO:0010720)
Positive Regulation Of Mitotic Nuclear Division (GO:0045840)
Positive Regulation Of Telomere Maintenance Via Telomere Lengthening (GO:1904358)
Regulation Of Axon Extension (GO:0030516)
Regulation Of Regulated Secretory Pathway (GO:1903305)
tRNA Aminoacylation For Protein Translation (GO:0006418)
Negative Regulation Of Signal Transduction (GO:0009968)
RNA Splicing (GO:0008380)
Regulation Of Inflammatory Response (GO:0050727)
Negative Regulation Of Nucleic Acid-Templated Transcription (GO:1903507)
B Cell Differentiation (GO:0030183)
Aerobic Electron Transport Chain (GO:0019646)
Cell-Cell Junction Organization (GO:0045216)
Base-Excision Repair (GO:0006284)
Blood Vessel Diameter Maintenance (GO:0097746)
Neural Crest Cell Migration (GO:0001755)
Positive Regulation Of Double-Strand Break Repair Via Homologous Recombination (GO:1905168)
Regulation Of Glucose Import (GO:0046324)
Regulation Of Intracellular Protein Transport (GO:0033157)
tRNA Methylation (GO:0030488)
Phospholipid Metabolic Process (GO:0006644)
Regulation Of Transcription By RNA Polymerase II (GO:0006357)
Antimicrobial Humoral Response (GO:0019730)
Positive Regulation Of Peptidyl-Tyrosine Phosphorylation (GO:0050731)
Mitochondrial ATP Synthesis Coupled Electron Transport (GO:0042775)
Regulation Of Notch Signaling Pathway (GO:0008593)
Sensory Organ Development (GO:0007423)
Axonogenesis (GO:0007409)
RNA Stabilization (GO:0043489)

Aortic Valve Morphogenesis (GO:0003180)
Melanosome Organization (GO:0032438)
Negative Regulation Of Endothelial Cell Proliferation (GO:0001937)
Positive Regulation Of Immune Effector Process (GO:0002699)
Positive Regulation Of Signaling (GO:0023056)
Spliceosomal snRNP Assembly (GO:0000387)
Type I Interferon-Mediated Signaling Pathway (GO:0060337)
Synapse Organization (GO:0050808)
Kidney Development (GO:0001822)
Calcium-Dependent Cell-Cell Adhesion Via Plasma Membrane Cell Adhesion Molecules (GO:0016339)
Cellular Response To Type I Interferon (GO:0071357)
Heart Looping (GO:0001947)
Lymphocyte Proliferation (GO:0046651)
Positive Regulation Of Protein Tyrosine Kinase Activity (GO:0061098)
Regulation Of Telomere Maintenance (GO:0032204)
Tumor Necrosis Factor-Mediated Signaling Pathway (GO:0033209)
Mitochondrial Gene Expression (GO:0140053)
Protein Targeting To Membrane (GO:0006612)
Second-Messenger-Mediated Signaling (GO:0019932)
Positive Regulation Of Inflammatory Response (GO:0050729)
Anterograde Axonal Transport (GO:0008089)
Digestive Tract Development (GO:0048565)
Fibroblast Growth Factor Receptor Signaling Pathway (GO:0008543)
Neuron Projection Extension (GO:1990138)
Positive Regulation Of Intracellular Transport (GO:0032388)
Regulation Of Receptor-Mediated Endocytosis (GO:0048259)
Vesicle Cytoskeletal Trafficking (GO:0099518)
Regulation Of Rho Protein Signal Transduction (GO:0035023)
Regulation Of Cell Differentiation (GO:0045595)
Negative Regulation Of MAPK Cascade (GO:0043409)
Cyclic-Nucleotide-Mediated Signaling (GO:0019935)
Establishment Or Maintenance Of Epithelial Cell Apical/Basal Polarity (GO:0045197)
Negative Regulation Of Cell-Cell Adhesion (GO:0022408)
Positive Regulation Of Insulin Secretion (GO:0032024)
Positive Regulation Of Stem Cell Population Maintenance (GO:1902459)
Protein Localization To Cilium (GO:0061512)
Regulation Of Cytokine-Mediated Signaling Pathway (GO:0001959)
Positive Regulation Of Neurogenesis (GO:0050769)
O-glycan Processing (GO:0016266)
Negative Regulation Of Proteolysis (GO:0045861)
Regulation Of Chemokine Production (GO:0032642)
Regulation Of Dendrite Development (GO:0050773)
Regulation Of Heart Rate By Cardiac Conduction (GO:0086091)
Regulation Of Release Of Cytochrome C From Mitochondria (GO:0090199)
Response To Amino Acid Starvation (GO:1990928)
Stem Cell Differentiation (GO:0048863)
Synaptic Vesicle Exocytosis (GO:0016079)
DNA Integrity Checkpoint Signaling (GO:0031570)

Cardiac Ventricle Morphogenesis (GO:0003208)
Cellular Response To Amino Acid Starvation (GO:0034198)
Ceramide Biosynthetic Process (GO:0046513)
Determination Of Heart Left/Right Asymmetry (GO:0061371)
Embryonic Heart Tube Morphogenesis (GO:0003143)
Lipid Modification (GO:0030258)
Monoatomic Cation Transport (GO:0006812)
Organic Hydroxy Compound Biosynthetic Process (GO:1901617)
Potassium Ion Import Across Plasma Membrane (GO:1990573)
Cell-Matrix Adhesion (GO:0007160)
Actomyosin Structure Organization (GO:0031032)
Cell Morphogenesis Involved In Neuron Differentiation (GO:0048667)
Embryonic Organ Development (GO:0048568)
Monoatomic Cation Homeostasis (GO:0055080)
Positive Regulation Of Synaptic Transmission (GO:0050806)
Positive Regulation Of DNA-templated Transcription (GO:0045893)
Metal Ion Transport (GO:0030001)
DNA-templated DNA Replication Maintenance Of Fidelity (GO:0045005)
Energy Derivation By Oxidation Of Organic Compounds (GO:0015980)
Ephrin Receptor Signaling Pathway (GO:0048013)
Intracellular Steroid Hormone Receptor Signaling Pathway (GO:0030518)
Learning (GO:0007612)
Positive Regulation Of T Cell Mediated Immunity (GO:0002711)
Positive Regulation Of miRNA Transcription (GO:1902895)
Regulation Of Cell-Cell Adhesion (GO:0022407)
Regulation Of Chromosome Organization (GO:0033044)
Regulation Of Endothelial Cell Apoptotic Process (GO:2000351)
Response To Amyloid-Beta (GO:1904645)
Skeletal System Morphogenesis (GO:0048705)
Organic Substance Transport (GO:0071702)
T Cell Activation (GO:0042110)
Regulation Of Cyclin-Dependent Protein Serine/Threonine Kinase Activity (GO:0000079)
Embryonic Organ Morphogenesis (GO:0048562)
Negative Regulation Of Immune System Process (GO:0002683)
Positive Regulation Of Cytokine-Mediated Signaling Pathway (GO:0001961)
Positive Regulation Of Leukocyte Cell-Cell Adhesion (GO:1903039)
Regulation Of Viral Entry Into Host Cell (GO:0046596)
Positive Regulation Of Transcription By RNA Polymerase II (GO:0045944)
Calcium Ion Transmembrane Transport (GO:0070588)
Negative Regulation Of Protein Serine/Threonine Kinase Activity (GO:0071901)
Regulation Of Organelle Assembly (GO:1902115)
Regulation Of Cell Adhesion (GO:0030155)
Establishment Of Protein Localization To Extracellular Region (GO:0035592)
Hexose Metabolic Process (GO:0019318)
Mitotic Nuclear Division (GO:0140014)
Natural Killer Cell Activation (GO:0030101)
Regulation Of Cellular Metabolic Process (GO:0031323)
Regulation Of Stem Cell Differentiation (GO:2000736)

Regulation Of Telomerase Activity (GO:0051972)
Zymogen Activation (GO:0031638)
Negative Regulation Of Canonical Wnt Signaling Pathway (GO:0090090)
T Cell Differentiation (GO:0030217)
Homotypic Cell-Cell Adhesion (GO:0034109)
Positive Regulation Of Peptide Hormone Secretion (GO:0090277)
Sodium Ion Transmembrane Transport (GO:0035725)
Epigenetic Regulation Of Gene Expression (GO:0040029)
Cognition (GO:0050890)
Negative Regulation Of Tumor Necrosis Factor Production (GO:0032720)
Positive Regulation Of Lymphocyte Activation (GO:0051251)
Secondary Alcohol Metabolic Process (GO:1902652)
Defense Response To Symbiont (GO:0140546)
Regulation Of Cysteine-Type Endopeptidase Activity Involved In Apoptotic Process (GO:0043281)
Axon Guidance (GO:0007411)
Negative Regulation Of DNA-binding Transcription Factor Activity (GO:0043433)
Skeletal System Development (GO:0001501)
RNA Splicing, Via Transesterification Reactions With Bulged Adenosine As Nucleophile (GO:0000377)
Cellular Defense Response (GO:0006968)
Cellular Response To Radiation (GO:0071478)
Oligosaccharide Metabolic Process (GO:0009311)
Calcium-Mediated Signaling (GO:0019722)
Cellular Respiration (GO:0045333)
Epidermis Development (GO:0008544)
Negative Regulation Of Cell Cycle (GO:0045786)
Antibacterial Humoral Response (GO:0019731)
Erythrocyte Differentiation (GO:0030218)
Fatty Acid Beta-Oxidation (GO:0006635)
Heterophilic Cell-Cell Adhesion Via Plasma Membrane Cell Adhesion Molecules (GO:0007157)
Intrinsic Apoptotic Signaling Pathway In Response To DNA Damage (GO:0008630)
Negative Regulation Of DNA Metabolic Process (GO:0051053)
Negative Regulation Of Tumor Necrosis Factor Superfamily Cytokine Production (GO:1903556)
Positive Regulation Of miRNA Metabolic Process (GO:2000630)
Regulation Of Mitotic Cell Cycle Phase Transition (GO:1901990)
RNA Processing (GO:0006396)
Positive Regulation Of Cell Death (GO:0010942)
Positive Regulation Of Developmental Growth (GO:0048639)
tRNA Processing (GO:0008033)
Plasma Membrane Bounded Cell Projection Assembly (GO:0120031)
Positive Regulation Of Cell Adhesion (GO:0045785)
Negative Regulation Of MAP Kinase Activity (GO:0043407)
Positive Regulation Of DNA Recombination (GO:0045911)
Positive Regulation Of Leukocyte Mediated Cytotoxicity (GO:0001912)
Sensory Perception (GO:0007600)
Cell Cycle G1/S Phase Transition (GO:0044843)
Sprouting Angiogenesis (GO:0002040)
Cellular Response To Alcohol (GO:0097306)
Positive Regulation Of NIK/NF-kappaB Signaling (GO:1901224)

Protein Homotetramerization (GO:0051289)
Proton Motive Force-Driven Mitochondrial ATP Synthesis (GO:0042776)
Regulation Of Proteasomal Protein Catabolic Process (GO:0061136)
Spermatid Development (GO:0007286)
Tight Junction Assembly (GO:0120192)
Lymphocyte Differentiation (GO:0030098)
Apical Junction Assembly (GO:0043297)
Negative Regulation Of Protein Catabolic Process (GO:0042177)
Nucleosome Assembly (GO:0006334)
Cellular Response To Fibroblast Growth Factor Stimulus (GO:0044344)
Ceramide Metabolic Process (GO:0006672)
Neuron Migration (GO:0001764)
Purine Ribonucleotide Biosynthetic Process (GO:0009152)
Regulation Of Reactive Oxygen Species Metabolic Process (GO:2000377)
Recombinational Repair (GO:0000725)
Nervous System Development (GO:0007399)
Membrane Lipid Biosynthetic Process (GO:0046467)
Monoatomic Anion Transport (GO:0006820)
Positive Regulation Of Interleukin-1 Beta Production (GO:0032731)
mRNA Transport (GO:0051028)
Aerobic Respiration (GO:0009060)
Glucose Metabolic Process (GO:0006006)
Heart Morphogenesis (GO:0003007)
Positive Regulation Of Protein Binding (GO:0032092)
protein-DNA Complex Assembly (GO:0065004)
protein-DNA Complex Organization (GO:0071824)
Proton Motive Force-Driven ATP Synthesis (GO:0015986)
DNA Damage Response (GO:0006974)
Response To UV (GO:0009411)
Fatty Acid Catabolic Process (GO:0009062)
Negative Regulation Of Viral Process (GO:0048525)
Positive Regulation Of Cell Junction Assembly (GO:1901890)
Fat Cell Differentiation (GO:0045444)
Positive Regulation Of Apoptotic Signaling Pathway (GO:2001235)
Spliceosomal Complex Assembly (GO:0000245)
Cell-Cell Adhesion Via Plasma-Membrane Adhesion Molecules (GO:0098742)
G1/S Transition Of Mitotic Cell Cycle (GO:0000082)
Oxidative Phosphorylation (GO:0006119)
Negative Regulation Of Wnt Signaling Pathway (GO:0030178)
Regulation Of Secretion By Cell (GO:1903530)
mRNA Splicing, Via Spliceosome (GO:0000398)
Negative Regulation Of Endopeptidase Activity (GO:0010951)
Monoatomic Ion Transport (GO:0006811)
Regulation Of Wnt Signaling Pathway (GO:0030111)
Neuropeptide Signaling Pathway (GO:0007218)
DNA-templated DNA Replication (GO:0006261)
Regulation Of Endopeptidase Activity (GO:0052548)
Double-Strand Break Repair Via Homologous Recombination (GO:0000724)

Negative Regulation Of Epithelial Cell Proliferation (GO:0050680)
Protein Deubiquitination (GO:0016579)
Regulation Of Double-Strand Break Repair Via Homologous Recombination (GO:0010569)
Regulation Of MAP Kinase Activity (GO:0043405)
Negative Regulation Of Cell Adhesion (GO:0007162)
DNA Replication (GO:0006260)
Protein Tetramerization (GO:0051262)
Defense Response To Gram-negative Bacterium (GO:0050829)
Nucleosome Organization (GO:0034728)
Regulation Of DNA Replication (GO:0006275)
Negative Regulation Of Blood Vessel Morphogenesis (GO:2000181)
Cellular Response To UV (GO:0034644)
Organonitrogen Compound Biosynthetic Process (GO:1901566)
Response To Cytokine (GO:0034097)
Regulation Of NIK/NF-kappaB Signaling (GO:1901222)
Defense Response To Bacterium (GO:0042742)
Regulation Of Interleukin-1 Beta Production (GO:0032651)
Macromolecule Catabolic Process (GO:0009057)
Negative Regulation Of Angiogenesis (GO:0016525)
Cilium Assembly (GO:0060271)
Regulation Of Insulin Secretion (GO:0050796)
Generation Of Neurons (GO:0048699)
Positive Regulation Of Peptidyl-Serine Phosphorylation (GO:0033138)
Sodium Ion Transport (GO:0006814)
Extracellular Matrix Organization (GO:0030198)
Regulation Of Epithelial Cell Proliferation (GO:0050678)
Potassium Ion Transmembrane Transport (GO:0071805)
Muscle Contraction (GO:0006936)
Actin Filament Organization (GO:0007015)
Mitochondrial Translation (GO:0032543)
Axon Development (GO:0061564)
Cilium Organization (GO:0044782)
Inorganic Cation Import Across Plasma Membrane (GO:0098659)
Intrinsic Apoptotic Signaling Pathway (GO:0097193)
Lipid Transport (GO:0006869)
Anterograde Trans-Synaptic Signaling (GO:0098916)
Extracellular Structure Organization (GO:0043062)
Adenylate Cyclase-Activating G Protein-Coupled Receptor Signaling Pathway (GO:0007189)
Adenylate Cyclase-Modulating G Protein-Coupled Receptor Signaling Pathway (GO:0007188)
Potassium Ion Transport (GO:0006813)
Chemical Synaptic Transmission (GO:0007268)

Overlap	P.value	Adjusted.P.value	Old.P.value	Old.Adjusted.P.value
18/93	3.58818E-08	0.000111664	0	0
35/325	2.00265E-07	0.000311613	0	0
34/367	8.80806E-06	0.009136895	0	0
4/5	1.3571E-05	0.01055825	0	0
15/107	3.06676E-05	0.01908753	0	0
5/11	4.30155E-05	0.022310731	0	0
4/7	8.89096E-05	0.031512571	0	0
21/202	8.97775E-05	0.031512571	0	0
39/500	9.87792E-05	0.031512571	0	0
32/381	0.000107077	0.031512571	0	0
5/13	0.000111878	0.031512571	0	0
50/705	0.000122813	0.031512571	0	0
29/336	0.00013918	0.031512571	0	0
15/122	0.000141766	0.031512571	0	0
15/127	0.000222338	0.042382256	0	0
27/313	0.000235431	0.042382256	0	0
7/32	0.000260172	0.042382256	0	0
22/234	0.000265856	0.042382256	0	0
33/418	0.00026626	0.042382256	0	0
9/53	0.00027433	0.042382256	0	0
21/220	0.000295839	0.042382256	0	0
4/9	0.000299617	0.042382256	0	0
27/319	0.000318785	0.043132941	0	0
11/79	0.000364696	0.045858749	0	0
8/44	0.000368403	0.045858749	0	0
36/482	0.000413251	0.049462952	0	0
5/17	0.000469101	0.05370141	0	0
4/10	0.000483175	0.05370141	0	0
3/5	0.000645374	0.068357329	0	0
6/27	0.000658972	0.068357329	0	0
12/99	0.000728845	0.073166661	0	0
9/62	0.000904391	0.084809516	0	0
14/130	0.000907458	0.084809516	0	0
7/39	0.000926582	0.084809516	0	0
3/6	0.001251366	0.111264305	0	0
19/214	0.001345619	0.113217033	0	0
16/166	0.001346089	0.113217033	0	0
4/13	0.001490663	0.115973566	0	0
4/13	0.001490663	0.115973566	0	0
4/13	0.001490663	0.115973566	0	0
5/22	0.001683745	0.12190531	0	0
5/22	0.001683745	0.12190531	0	0
7/43	0.001684424	0.12190531	0	0
11/96	0.001898384	0.133705503	0	0
7/44	0.001933402	0.133705503	0	0
6/33	0.002000253	0.13532148	0	0
5/23	0.002079646	0.136316734	0	0

3/7	0.002123213	0.136316734	0	0
15/158	0.002177905	0.136316734	0	0
16/174	0.002190179	0.136316734	0	0
10/85	0.002469504	0.144062389	0	0
5/24	0.002539342	0.144062389	0	0
5/24	0.002539342	0.144062389	0	0
5/24	0.002539342	0.144062389	0	0
4/15	0.002665448	0.144062389	0	0
4/15	0.002665448	0.144062389	0	0
4/15	0.002665448	0.144062389	0	0
12/115	0.002684967	0.144062389	0	0
14/146	0.002747869	0.144300476	0	0
26/351	0.002782143	0.144300476	0	0
17/196	0.003002211	0.153161973	0	0
16/180	0.003079997	0.154595954	0	0
17/197	0.003164061	0.156294587	0	0
4/16	0.003439721	0.16602979	0	0
10/89	0.003467846	0.16602979	0	0
16/183	0.00362612	0.169732425	0	0
6/37	0.003654265	0.169732425	0	0
11/105	0.003851747	0.176274088	0	0
6/38	0.004191733	0.185601433	0	0
6/38	0.004191733	0.185601433	0	0
4/17	0.004353761	0.185601433	0	0
4/17	0.004353761	0.185601433	0	0
4/17	0.004353761	0.185601433	0	0
26/364	0.004502132	0.186373145	0	0
9/78	0.004552252	0.186373145	0	0
6/39	0.004785398	0.186373145	0	0
3/9	0.004791083	0.186373145	0	0
3/9	0.004791083	0.186373145	0	0
3/9	0.004791083	0.186373145	0	0
3/9	0.004791083	0.186373145	0	0
8/65	0.004935074	0.189604329	0	0
10/94	0.005139211	0.195039333	0	0
4/18	0.005418346	0.198939616	0	0
4/18	0.005418346	0.198939616	0	0
22/296	0.005433762	0.198939616	0	0
14/158	0.005581355	0.20196717	0	0
16/192	0.005759714	0.202144528	0	0
16/192	0.005759714	0.202144528	0	0
9/81	0.00584461	0.202144528	0	0
5/29	0.005991948	0.202144528	0	0
5/29	0.005991948	0.202144528	0	0
5/29	0.005991948	0.202144528	0	0
29/429	0.006040952	0.202144528	0	0
6/41	0.006154669	0.203563943	0	0
28/411	0.006229948	0.203563943	0	0

7/54	0.006279608	0.203563943	0	0
3/10	0.006637337	0.208640335	0	0
3/10	0.006637337	0.208640335	0	0
3/10	0.006637337	0.208640335	0	0
10/98	0.006883803	0.214223951	0	0
11/114	0.007153224	0.219696681	0	0
12/130	0.007213814	0.219696681	0	0
43/711	0.007271452	0.219696681	0	0
9/84	0.007402978	0.221519887	0	0
9/85	0.007987207	0.231652072	0	0
5/31	0.008015083	0.231652072	0	0
5/31	0.008015083	0.231652072	0	0
4/20	0.008039339	0.231652072	0	0
3/11	0.008850835	0.243750435	0	0
3/11	0.008850835	0.243750435	0	0
3/11	0.008850835	0.243750435	0	0
3/11	0.008850835	0.243750435	0	0
3/11	0.008850835	0.243750435	0	0
5/32	0.009185564	0.248569345	0	0
5/32	0.009185564	0.248569345	0	0
7/58	0.009265453	0.248569732	0	0
6/45	0.009713721	0.258368369	0	0
10/104	0.010320698	0.2688542	0	0
5/33	0.010468658	0.2688542	0	0
5/33	0.010468658	0.2688542	0	0
5/33	0.010468658	0.2688542	0	0
9/89	0.010680868	0.2688542	0	0
6/46	0.010793375	0.2688542	0	0
15/189	0.011269706	0.2688542	0	0
4/22	0.011376571	0.2688542	0	0
4/22	0.011376571	0.2688542	0	0
4/22	0.011376571	0.2688542	0	0
4/22	0.011376571	0.2688542	0	0
3/12	0.011445648	0.2688542	0	0
3/12	0.011445648	0.2688542	0	0
3/12	0.011445648	0.2688542	0	0
3/12	0.011445648	0.2688542	0	0
13/155	0.011490234	0.2688542	0	0
5/34	0.011869135	0.27559137	0	0
6/47	0.011955281	0.27559137	0	0
17/226	0.012055088	0.275848786	0	0
9/91	0.012259112	0.278469747	0	0
10/107	0.01246506	0.279159478	0	0
8/76	0.012468884	0.279159478	0	0
20/283	0.013085213	0.280507996	0	0
7/62	0.013176794	0.280507996	0	0
10/108	0.013249506	0.280507996	0	0
4/23	0.013333823	0.280507996	0	0

4/23	0.013333823	0.280507996	0	0
4/23	0.013333823	0.280507996	0	0
4/23	0.013333823	0.280507996	0	0
5/35	0.013391554	0.280507996	0	0
5/35	0.013391554	0.280507996	0	0
8/77	0.013430492	0.280507996	0	0
10/109	0.014070419	0.282496465	0	0
3/13	0.014432152	0.282496465	0	0
6/49	0.014537873	0.282496465	0	0
9/94	0.014943531	0.282496465	0	0
15/196	0.015306774	0.282496465	0	0
2/5	0.015458241	0.282496465	0	0
2/5	0.015458241	0.282496465	0	0
2/5	0.015458241	0.282496465	0	0
2/5	0.015458241	0.282496465	0	0
2/5	0.015458241	0.282496465	0	0
2/5	0.015458241	0.282496465	0	0
2/5	0.015458241	0.282496465	0	0
2/5	0.015458241	0.282496465	0	0
2/5	0.015458241	0.282496465	0	0
2/5	0.015458241	0.282496465	0	0
2/5	0.015458241	0.282496465	0	0
4/24	0.015492723	0.282496465	0	0
4/24	0.015492723	0.282496465	0	0
4/24	0.015492723	0.282496465	0	0
4/24	0.015492723	0.282496465	0	0
8/79	0.01551441	0.282496465	0	0
7/64	0.015522781	0.282496465	0	0
6/50	0.015964355	0.288843449	0	0
8/80	0.016639806	0.299324139	0	0
5/37	0.016819312	0.300814357	0	0
15/199	0.017344831	0.303704879	0	0
10/113	0.017738206	0.303704879	0	0
10/113	0.017738206	0.303704879	0	0
3/14	0.017817385	0.303704879	0	0
3/14	0.017817385	0.303704879	0	0
3/14	0.017817385	0.303704879	0	0
3/14	0.017817385	0.303704879	0	0
4/25	0.017859252	0.303704879	0	0
4/25	0.017859252	0.303704879	0	0
7/66	0.018150975	0.305098564	0	0
7/66	0.018150975	0.305098564	0	0
5/38	0.018732585	0.305098564	0	0
10/114	0.018755994	0.305098564	0	0
7/67	0.019575796	0.305098564	0	0
7/67	0.019575796	0.305098564	0	0
16/221	0.020178973	0.305098564	0	0
8/83	0.020367727	0.305098564	0	0

8/83	0.020367727	0.305098564	0	0
4/26	0.020438632	0.305098564	0	0
4/26	0.020438632	0.305098564	0	0
5/39	0.020783645	0.305098564	0	0
14/186	0.021275762	0.305098564	0	0
27/434	0.021279228	0.305098564	0	0
3/15	0.021605377	0.305098564	0	0
3/15	0.021605377	0.305098564	0	0
3/15	0.021605377	0.305098564	0	0
3/15	0.021605377	0.305098564	0	0
3/15	0.021605377	0.305098564	0	0
3/15	0.021605377	0.305098564	0	0
3/15	0.021605377	0.305098564	0	0
2/6	0.0225617	0.305098564	0	0
2/6	0.0225617	0.305098564	0	0
2/6	0.0225617	0.305098564	0	0
2/6	0.0225617	0.305098564	0	0
2/6	0.0225617	0.305098564	0	0
2/6	0.0225617	0.305098564	0	0
2/6	0.0225617	0.305098564	0	0
2/6	0.0225617	0.305098564	0	0
2/6	0.0225617	0.305098564	0	0
2/6	0.0225617	0.305098564	0	0
2/6	0.0225617	0.305098564	0	0
2/6	0.0225617	0.305098564	0	0
2/6	0.0225617	0.305098564	0	0
2/6	0.0225617	0.305098564	0	0
2/6	0.0225617	0.305098564	0	0
24/377	0.022614908	0.305098564	0	0
6/54	0.022634447	0.305098564	0	0
6/54	0.022634447	0.305098564	0	0
7/69	0.022656187	0.305098564	0	0
12/152	0.022737756	0.305098564	0	0
5/40	0.022975798	0.305098564	0	0
5/40	0.022975798	0.305098564	0	0
5/40	0.022975798	0.305098564	0	0
8/85	0.023160517	0.305098564	0	0
4/27	0.023235334	0.305098564	0	0
4/27	0.023235334	0.305098564	0	0
4/27	0.023235334	0.305098564	0	0
4/27	0.023235334	0.305098564	0	0
4/27	0.023235334	0.305098564	0	0
4/27	0.023235334	0.305098564	0	0
4/27	0.023235334	0.305098564	0	0
4/27	0.023235334	0.305098564	0	0
4/27	0.023235334	0.305098564	0	0
12/153	0.023789824	0.311066942	0	0
9/102	0.024190399	0.313980043	0	0

7/70	0.024315293	0.313980043	0	0
7/70	0.024315293	0.313980043	0	0
5/41	0.02531207	0.322416454	0	0
11/137	0.025492922	0.322416454	0	0
3/16	0.02579746	0.322416454	0	0
3/16	0.02579746	0.322416454	0	0
3/16	0.02579746	0.322416454	0	0
3/16	0.02579746	0.322416454	0	0
3/16	0.02579746	0.322416454	0	0
3/16	0.02579746	0.322416454	0	0
16/228	0.026075028	0.324030828	0	0
4/28	0.026253082	0.324030828	0	0
4/28	0.026253082	0.324030828	0	0
18/266	0.026343123	0.324030828	0	0
6/56	0.026582119	0.325683285	0	0
14/192	0.027035023	0.329933305	0	0
10/122	0.028494708	0.337999658	0	0
9/105	0.028528388	0.337999658	0	0
25/406	0.028885222	0.337999658	0	0
4/29	0.029494869	0.337999658	0	0
4/29	0.029494869	0.337999658	0	0
4/29	0.029494869	0.337999658	0	0
4/29	0.029494869	0.337999658	0	0
4/29	0.029494869	0.337999658	0	0
13/176	0.02952614	0.337999658	0	0
7/73	0.029788008	0.337999658	0	0
10/123	0.029924734	0.337999658	0	0
3/17	0.030392547	0.337999658	0	0
3/17	0.030392547	0.337999658	0	0
3/17	0.030392547	0.337999658	0	0
3/17	0.030392547	0.337999658	0	0
2/7	0.030737115	0.337999658	0	0
2/7	0.030737115	0.337999658	0	0
2/7	0.030737115	0.337999658	0	0
2/7	0.030737115	0.337999658	0	0
2/7	0.030737115	0.337999658	0	0
2/7	0.030737115	0.337999658	0	0
2/7	0.030737115	0.337999658	0	0
2/7	0.030737115	0.337999658	0	0
2/7	0.030737115	0.337999658	0	0
2/7	0.030737115	0.337999658	0	0
2/7	0.030737115	0.337999658	0	0
2/7	0.030737115	0.337999658	0	0
2/7	0.030737115	0.337999658	0	0
8/90	0.031296916	0.342943672	0	0
9/107	0.031706755	0.343303768	0	0
9/107	0.031706755	0.343303768	0	0
7/74	0.031782436	0.343303768	0	0

7/74	0.031782436	0.343303768	0	0
18/272	0.031881359	0.343303768	0	0
4/30	0.032962969	0.350104977	0	0
4/30	0.032962969	0.350104977	0	0
4/30	0.032962969	0.350104977	0	0
4/30	0.032962969	0.350104977	0	0
5/44	0.033211542	0.350353627	0	0
5/44	0.033211542	0.350353627	0	0
11/143	0.033480482	0.3519975	0	0
8/92	0.035038696	0.358715236	0	0
3/18	0.035387396	0.358715236	0	0
3/18	0.035387396	0.358715236	0	0
3/18	0.035387396	0.358715236	0	0
3/18	0.035387396	0.358715236	0	0
3/18	0.035387396	0.358715236	0	0
3/18	0.035387396	0.358715236	0	0
3/18	0.035387396	0.358715236	0	0
3/18	0.035387396	0.358715236	0	0
3/18	0.035387396	0.358715236	0	0
3/18	0.035387396	0.358715236	0	0
4/31	0.036658957	0.362564464	0	0
6/61	0.038369357	0.362564464	0	0
6/61	0.038369357	0.362564464	0	0
6/61	0.038369357	0.362564464	0	0
6/61	0.038369357	0.362564464	0	0
9/111	0.038783952	0.362564464	0	0
2/8	0.039884865	0.362564464	0	0
2/8	0.039884865	0.362564464	0	0
2/8	0.039884865	0.362564464	0	0
2/8	0.039884865	0.362564464	0	0
2/8	0.039884865	0.362564464	0	0
2/8	0.039884865	0.362564464	0	0
2/8	0.039884865	0.362564464	0	0
2/8	0.039884865	0.362564464	0	0
2/8	0.039884865	0.362564464	0	0
2/8	0.039884865	0.362564464	0	0
2/8	0.039884865	0.362564464	0	0
2/8	0.039884865	0.362564464	0	0
2/8	0.039884865	0.362564464	0	0
2/8	0.039884865	0.362564464	0	0
2/8	0.039884865	0.362564464	0	0
2/8	0.039884865	0.362564464	0	0
2/8	0.039884865	0.362564464	0	0
2/8	0.039884865	0.362564464	0	0
2/8	0.039884865	0.362564464	0	0
2/8	0.039884865	0.362564464	0	0
2/8	0.039884865	0.362564464	0	0
16/241	0.040306915	0.362564464	0	0

4/32	0.040583732	0.362564464	0	0
4/32	0.040583732	0.362564464	0	0
7/78	0.040646698	0.362564464	0	0
3/19	0.040776852	0.362564464	0	0
3/19	0.040776852	0.362564464	0	0
3/19	0.040776852	0.362564464	0	0
3/19	0.040776852	0.362564464	0	0
3/19	0.040776852	0.362564464	0	0
3/19	0.040776852	0.362564464	0	0
3/19	0.040776852	0.362564464	0	0
3/19	0.040776852	0.362564464	0	0
3/19	0.040776852	0.362564464	0	0
3/19	0.040776852	0.362564464	0	0
3/19	0.040776852	0.362564464	0	0
3/19	0.040776852	0.362564464	0	0
6/62	0.041068563	0.364117854	0	0
8/95	0.041200023	0.364245655	0	0
10/130	0.041377112	0.36477499	0	0
5/47	0.042489191	0.372468625	0	0
5/47	0.042489191	0.372468625	0	0
30/525	0.042684753	0.373131887	0	0
11/149	0.04309371	0.375651611	0	0
8/96	0.04340378	0.377297664	0	0
6/63	0.043884676	0.380415349	0	0
4/33	0.044737541	0.385659908	0	0
4/33	0.044737541	0.385659908	0	0
5/48	0.045894628	0.387344489	0	0
3/20	0.046554068	0.387344489	0	0
3/20	0.046554068	0.387344489	0	0
3/20	0.046554068	0.387344489	0	0
3/20	0.046554068	0.387344489	0	0
3/20	0.046554068	0.387344489	0	0
6/64	0.046818667	0.387344489	0	0
6/64	0.046818667	0.387344489	0	0
14/208	0.047905279	0.387344489	0	0
7/81	0.048256525	0.387344489	0	0
4/34	0.04912	0.387344489	0	0
4/34	0.04912	0.387344489	0	0
4/34	0.04912	0.387344489	0	0
5/49	0.049457899	0.387344489	0	0
5/49	0.049457899	0.387344489	0	0
13/190	0.049609454	0.387344489	0	0
6/65	0.049871354	0.387344489	0	0
2/9	0.049911677	0.387344489	0	0
2/9	0.049911677	0.387344489	0	0
2/9	0.049911677	0.387344489	0	0
2/9	0.049911677	0.387344489	0	0
2/9	0.049911677	0.387344489	0	0

2/9	0.049911677	0.387344489	0	0
2/9	0.049911677	0.387344489	0	0
2/9	0.049911677	0.387344489	0	0
2/9	0.049911677	0.387344489	0	0
2/9	0.049911677	0.387344489	0	0
2/9	0.049911677	0.387344489	0	0
2/9	0.049911677	0.387344489	0	0
2/9	0.049911677	0.387344489	0	0
2/9	0.049911677	0.387344489	0	0
2/9	0.049911677	0.387344489	0	0
2/9	0.049911677	0.387344489	0	0
2/9	0.049911677	0.387344489	0	0
2/9	0.049911677	0.387344489	0	0
2/9	0.049911677	0.387344489	0	0
2/9	0.049911677	0.387344489	0	0
2/9	0.049911677	0.387344489	0	0
2/9	0.049911677	0.387344489	0	0
2/9	0.049911677	0.387344489	0	0
3/21	0.052710715	0.40602907	0	0
3/21	0.052710715	0.40602907	0	0
3/21	0.052710715	0.40602907	0	0
8/100	0.052989339	0.406578927	0	0
6/66	0.053043395	0.406578927	0	0
5/50	0.053179407	0.406619941	0	0
7/83	0.053800721	0.410362365	0	0
10/137	0.05551477	0.420918993	0	0
9/119	0.05599268	0.420918993	0	0
5/51	0.057059274	0.420918993	0	0
5/51	0.057059274	0.420918993	0	0
4/36	0.058566367	0.420918993	0	0
4/36	0.058566367	0.420918993	0	0
4/36	0.058566367	0.420918993	0	0
4/36	0.058566367	0.420918993	0	0
15/234	0.058903964	0.420918993	0	0
3/22	0.059237166	0.420918993	0	0
3/22	0.059237166	0.420918993	0	0
3/22	0.059237166	0.420918993	0	0
3/22	0.059237166	0.420918993	0	0
3/22	0.059237166	0.420918993	0	0
3/22	0.059237166	0.420918993	0	0
3/22	0.059237166	0.420918993	0	0
3/22	0.059237166	0.420918993	0	0
7/85	0.059727416	0.420918993	0	0
2/10	0.060730279	0.420918993	0	0
2/10	0.060730279	0.420918993	0	0
2/10	0.060730279	0.420918993	0	0
2/10	0.060730279	0.420918993	0	0
2/10	0.060730279	0.420918993	0	0

2/10	0.060730279	0.420918993	0	0
2/10	0.060730279	0.420918993	0	0
2/10	0.060730279	0.420918993	0	0
2/10	0.060730279	0.420918993	0	0
2/10	0.060730279	0.420918993	0	0
2/10	0.060730279	0.420918993	0	0
2/10	0.060730279	0.420918993	0	0
2/10	0.060730279	0.420918993	0	0
2/10	0.060730279	0.420918993	0	0
2/10	0.060730279	0.420918993	0	0
2/10	0.060730279	0.420918993	0	0
2/10	0.060730279	0.420918993	0	0
2/10	0.060730279	0.420918993	0	0
2/10	0.060730279	0.420918993	0	0
2/10	0.060730279	0.420918993	0	0
2/10	0.060730279	0.420918993	0	0
2/10	0.060730279	0.420918993	0	0
2/10	0.060730279	0.420918993	0	0
5/52	0.061097344	0.422522077	0	0
4/37	0.063626621	0.433273618	0	0
4/37	0.063626621	0.433273618	0	0
4/37	0.063626621	0.433273618	0	0
4/37	0.063626621	0.433273618	0	0
4/37	0.063626621	0.433273618	0	0
4/37	0.063626621	0.433273618	0	0
4/37	0.063626621	0.433273618	0	0
5/53	0.065293192	0.441574604	0	0
5/53	0.065293192	0.441574604	0	0
3/23	0.066122675	0.441574604	0	0
3/23	0.066122675	0.441574604	0	0
3/23	0.066122675	0.441574604	0	0
3/23	0.066122675	0.441574604	0	0
3/23	0.066122675	0.441574604	0	0
3/23	0.066122675	0.441574604	0	0
3/23	0.066122675	0.441574604	0	0
6/70	0.066932827	0.445074698	0	0
6/70	0.066932827	0.445074698	0	0
4/38	0.068908277	0.447308916	0	0
4/38	0.068908277	0.447308916	0	0
7/88	0.069341155	0.447308916	0	0
7/88	0.069341155	0.447308916	0	0
5/54	0.069646128	0.447308916	0	0
5/54	0.069646128	0.447308916	0	0
5/54	0.069646128	0.447308916	0	0
6/71	0.070706083	0.447308916	0	0
2/11	0.072259059	0.447308916	0	0
2/11	0.072259059	0.447308916	0	0
2/11	0.072259059	0.447308916	0	0

2/11	0.072259059	0.447308916	0	0
2/11	0.072259059	0.447308916	0	0
2/11	0.072259059	0.447308916	0	0
2/11	0.072259059	0.447308916	0	0
2/11	0.072259059	0.447308916	0	0
2/11	0.072259059	0.447308916	0	0
2/11	0.072259059	0.447308916	0	0
2/11	0.072259059	0.447308916	0	0
2/11	0.072259059	0.447308916	0	0
2/11	0.072259059	0.447308916	0	0
2/11	0.072259059	0.447308916	0	0
2/11	0.072259059	0.447308916	0	0
2/11	0.072259059	0.447308916	0	0
2/11	0.072259059	0.447308916	0	0
2/11	0.072259059	0.447308916	0	0
2/11	0.072259059	0.447308916	0	0
2/11	0.072259059	0.447308916	0	0
2/11	0.072259059	0.447308916	0	0
2/11	0.072259059	0.447308916	0	0
2/11	0.072259059	0.447308916	0	0
2/11	0.072259059	0.447308916	0	0
2/11	0.072259059	0.447308916	0	0
7/89	0.072739438	0.447308916	0	0
3/24	0.073355533	0.447308916	0	0
3/24	0.073355533	0.447308916	0	0
3/24	0.073355533	0.447308916	0	0
3/24	0.073355533	0.447308916	0	0
3/24	0.073355533	0.447308916	0	0
3/24	0.073355533	0.447308916	0	0
3/24	0.073355533	0.447308916	0	0
3/24	0.073355533	0.447308916	0	0
5/55	0.074155209	0.447308916	0	0
4/39	0.074408239	0.447308916	0	0
4/39	0.074408239	0.447308916	0	0
4/39	0.074408239	0.447308916	0	0
4/39	0.074408239	0.447308916	0	0
4/39	0.074408239	0.447308916	0	0
4/39	0.074408239	0.447308916	0	0
9/126	0.074576146	0.447308916	0	0
6/72	0.074599398	0.447308916	0	0
6/73	0.078612361	0.464170498	0	0
5/56	0.078819241	0.464170498	0	0
4/40	0.080122959	0.464170498	0	0
4/40	0.080122959	0.464170498	0	0
4/40	0.080122959	0.464170498	0	0
4/40	0.080122959	0.464170498	0	0
10/147	0.08069357	0.464170498	0	0
3/25	0.080923214	0.464170498	0	0

3/25	0.080923214	0.464170498	0	0
3/25	0.080923214	0.464170498	0	0
3/25	0.080923214	0.464170498	0	0
3/25	0.080923214	0.464170498	0	0
3/25	0.080923214	0.464170498	0	0
3/25	0.080923214	0.464170498	0	0
3/25	0.080923214	0.464170498	0	0
3/25	0.080923214	0.464170498	0	0
3/25	0.080923214	0.464170498	0	0
6/74	0.082744416	0.464170498	0	0
11/167	0.082770415	0.464170498	0	0
2/12	0.084421755	0.464170498	0	0
2/12	0.084421755	0.464170498	0	0
2/12	0.084421755	0.464170498	0	0
2/12	0.084421755	0.464170498	0	0
2/12	0.084421755	0.464170498	0	0
2/12	0.084421755	0.464170498	0	0
2/12	0.084421755	0.464170498	0	0
2/12	0.084421755	0.464170498	0	0
2/12	0.084421755	0.464170498	0	0
2/12	0.084421755	0.464170498	0	0
2/12	0.084421755	0.464170498	0	0
2/12	0.084421755	0.464170498	0	0
2/12	0.084421755	0.464170498	0	0
2/12	0.084421755	0.464170498	0	0
2/12	0.084421755	0.464170498	0	0
2/12	0.084421755	0.464170498	0	0
2/12	0.084421755	0.464170498	0	0
2/12	0.084421755	0.464170498	0	0
2/12	0.084421755	0.464170498	0	0
2/12	0.084421755	0.464170498	0	0
2/12	0.084421755	0.464170498	0	0
2/12	0.084421755	0.464170498	0	0
2/12	0.084421755	0.464170498	0	0
2/12	0.084421755	0.464170498	0	0
2/12	0.084421755	0.464170498	0	0
2/12	0.084421755	0.464170498	0	0
2/12	0.084421755	0.464170498	0	0
2/12	0.084421755	0.464170498	0	0
13/207	0.084627954	0.46436029	0	0
14/227	0.084754706	0.46436029	0	0
24/434	0.085527337	0.467769899	0	0
8/111	0.085916916	0.468151789	0	0
4/41	0.086048465	0.468151789	0	0
4/41	0.086048465	0.468151789	0	0
7/93	0.0872995	0.47412922	0	0
5/58	0.088606195	0.478173951	0	0
3/26	0.088812514	0.478173951	0	0

3/26	0.088812514	0.478173951	0	0
3/26	0.088812514	0.478173951	0	0
3/26	0.088812514	0.478173951	0	0
10/150	0.089423607	0.480632584	0	0
7/94	0.091179889	0.489044769	0	0
26/480	0.091303024	0.489044769	0	0
4/42	0.092180392	0.491207841	0	0
4/42	0.092180392	0.491207841	0	0
4/42	0.092180392	0.491207841	0	0
5/59	0.093725567	0.495609721	0	0
5/59	0.093725567	0.495609721	0	0
5/59	0.093725567	0.495609721	0	0
7/95	0.095155562	0.495609721	0	0
6/77	0.095847457	0.495609721	0	0
3/27	0.097009668	0.495609721	0	0
3/27	0.097009668	0.495609721	0	0
2/13	0.09714715	0.495609721	0	0
2/13	0.09714715	0.495609721	0	0
2/13	0.09714715	0.495609721	0	0
2/13	0.09714715	0.495609721	0	0
2/13	0.09714715	0.495609721	0	0
2/13	0.09714715	0.495609721	0	0
2/13	0.09714715	0.495609721	0	0
2/13	0.09714715	0.495609721	0	0
2/13	0.09714715	0.495609721	0	0
2/13	0.09714715	0.495609721	0	0
2/13	0.09714715	0.495609721	0	0
2/13	0.09714715	0.495609721	0	0
2/13	0.09714715	0.495609721	0	0
2/13	0.09714715	0.495609721	0	0
2/13	0.09714715	0.495609721	0	0
2/13	0.09714715	0.495609721	0	0
2/13	0.09714715	0.495609721	0	0
2/13	0.09714715	0.495609721	0	0
2/13	0.09714715	0.495609721	0	0
4/43	0.098514012	0.501760399	0	0
5/60	0.09899281	0.503375204	0	0
9/134	0.09996169	0.507472723	0	0
11/174	0.102847173	0.521138799	0	0
5/61	0.104405621	0.521138799	0	0
5/61	0.104405621	0.521138799	0	0
4/44	0.105044262	0.521138799	0	0
4/44	0.105044262	0.521138799	0	0
4/44	0.105044262	0.521138799	0	0
6/79	0.105161831	0.521138799	0	0
6/79	0.105161831	0.521138799	0	0
3/28	0.105500464	0.521138799	0	0
3/28	0.105500464	0.521138799	0	0

3/28	0.105500464	0.521138799	0	0
3/28	0.105500464	0.521138799	0	0
3/28	0.105500464	0.521138799	0	0
3/28	0.105500464	0.521138799	0	0
3/28	0.105500464	0.521138799	0	0
3/28	0.105500464	0.521138799	0	0
3/28	0.105500464	0.521138799	0	0
9/136	0.106997334	0.52119525	0	0
7/98	0.107647946	0.52119525	0	0
8/117	0.107981451	0.52119525	0	0
5/62	0.109961506	0.52119525	0	0
5/62	0.109961506	0.52119525	0	0
5/62	0.109961506	0.52119525	0	0
6/80	0.109989027	0.52119525	0	0
2/14	0.110368788	0.52119525	0	0
2/14	0.110368788	0.52119525	0	0
2/14	0.110368788	0.52119525	0	0
2/14	0.110368788	0.52119525	0	0
2/14	0.110368788	0.52119525	0	0
2/14	0.110368788	0.52119525	0	0
2/14	0.110368788	0.52119525	0	0
2/14	0.110368788	0.52119525	0	0
2/14	0.110368788	0.52119525	0	0
2/14	0.110368788	0.52119525	0	0
2/14	0.110368788	0.52119525	0	0
2/14	0.110368788	0.52119525	0	0
2/14	0.110368788	0.52119525	0	0
2/14	0.110368788	0.52119525	0	0
2/14	0.110368788	0.52119525	0	0
2/14	0.110368788	0.52119525	0	0
2/14	0.110368788	0.52119525	0	0
2/14	0.110368788	0.52119525	0	0
2/14	0.110368788	0.52119525	0	0
2/14	0.110368788	0.52119525	0	0
2/14	0.110368788	0.52119525	0	0
2/14	0.110368788	0.52119525	0	0
9/137	0.110617203	0.521576871	0	0
4/45	0.111765777	0.526195307	0	0
8/118	0.111935694	0.526199213	0	0
3/29	0.114270346	0.531553536	0	0
3/29	0.114270346	0.531553536	0	0
3/29	0.114270346	0.531553536	0	0
3/29	0.114270346	0.531553536	0	0
3/29	0.114270346	0.531553536	0	0
3/29	0.114270346	0.531553536	0	0
3/29	0.114270346	0.531553536	0	0
6/81	0.114927629	0.533813105	0	0
11/178	0.115499375	0.535669233	0	0

7/100	0.116439497	0.539225765	0	0
4/46	0.118672912	0.547937835	0	0
4/46	0.118672912	0.547937835	0	0
5/64	0.121491614	0.555915358	0	0
3/30	0.123304504	0.555915358	0	0
3/30	0.123304504	0.555915358	0	0
2/15	0.124024708	0.555915358	0	0
2/15	0.124024708	0.555915358	0	0
2/15	0.124024708	0.555915358	0	0
2/15	0.124024708	0.555915358	0	0
2/15	0.124024708	0.555915358	0	0
2/15	0.124024708	0.555915358	0	0
2/15	0.124024708	0.555915358	0	0
2/15	0.124024708	0.555915358	0	0
2/15	0.124024708	0.555915358	0	0
2/15	0.124024708	0.555915358	0	0
2/15	0.124024708	0.555915358	0	0
2/15	0.124024708	0.555915358	0	0
2/15	0.124024708	0.555915358	0	0
2/15	0.124024708	0.555915358	0	0
2/15	0.124024708	0.555915358	0	0
2/15	0.124024708	0.555915358	0	0
2/15	0.124024708	0.555915358	0	0
16/284	0.124750742	0.555915358	0	0
6/83	0.125132564	0.555915358	0	0
7/102	0.125592826	0.555915358	0	0
7/102	0.125592826	0.555915358	0	0
4/47	0.125759773	0.555915358	0	0
4/47	0.125759773	0.555915358	0	0
4/47	0.125759773	0.555915358	0	0
4/47	0.125759773	0.555915358	0	0
4/47	0.125759773	0.555915358	0	0
4/47	0.125759773	0.555915358	0	0
5/65	0.127459971	0.561834886	0	0
5/65	0.127459971	0.561834886	0	0
8/122	0.128522655	0.564918787	0	0
8/122	0.128522655	0.564918787	0	0
18/328	0.129337153	0.567697065	0	0
9/142	0.129720049	0.56793805	0	0
10/162	0.129757054	0.56793805	0	0
6/84	0.130395377	0.569930354	0	0
17/308	0.132558592	0.572279793	0	0
3/31	0.13258796	0.572279793	0	0
3/31	0.13258796	0.572279793	0	0
3/31	0.13258796	0.572279793	0	0
3/31	0.13258796	0.572279793	0	0
3/31	0.13258796	0.572279793	0	0
3/31	0.13258796	0.572279793	0	0

3/31	0.13258796	0.572279793	0	0
3/31	0.13258796	0.572279793	0	0
4/48	0.133020243	0.573350412	0	0
5/66	0.133559693	0.574879341	0	0
13/225	0.135398058	0.57746498	0	0
6/85	0.135762565	0.57746498	0	0
6/85	0.135762565	0.57746498	0	0
6/85	0.135762565	0.57746498	0	0
17/310	0.137993617	0.57746498	0	0
2/16	0.13805718	0.57746498	0	0
2/16	0.13805718	0.57746498	0	0
2/16	0.13805718	0.57746498	0	0
2/16	0.13805718	0.57746498	0	0
2/16	0.13805718	0.57746498	0	0
2/16	0.13805718	0.57746498	0	0
2/16	0.13805718	0.57746498	0	0
2/16	0.13805718	0.57746498	0	0
2/16	0.13805718	0.57746498	0	0
2/16	0.13805718	0.57746498	0	0
2/16	0.13805718	0.57746498	0	0
2/16	0.13805718	0.57746498	0	0
2/16	0.13805718	0.57746498	0	0
2/16	0.13805718	0.57746498	0	0
2/16	0.13805718	0.57746498	0	0
2/16	0.13805718	0.57746498	0	0
5/67	0.139787469	0.583917588	0	0
4/49	0.14044801	0.584963969	0	0
4/49	0.14044801	0.584963969	0	0
6/86	0.141232115	0.584963969	0	0
6/86	0.141232115	0.584963969	0	0
8/125	0.141749743	0.584963969	0	0
3/32	0.142105643	0.584963969	0	0
3/32	0.142105643	0.584963969	0	0
3/32	0.142105643	0.584963969	0	0
3/32	0.142105643	0.584963969	0	0
3/32	0.142105643	0.584963969	0	0
3/32	0.142105643	0.584963969	0	0
7/106	0.144947641	0.595088467	0	0
7/106	0.144947641	0.595088467	0	0
13/228	0.145215966	0.595404593	0	0
8/126	0.146303887	0.598151854	0	0
4/50	0.148036587	0.598151854	0	0
4/50	0.148036587	0.598151854	0	0
4/50	0.148036587	0.598151854	0	0
4/50	0.148036587	0.598151854	0	0
3/33	0.151842457	0.598151854	0	0
3/33	0.151842457	0.598151854	0	0
3/33	0.151842457	0.598151854	0	0

3/33	0.151842457	0.598151854	0	0
3/33	0.151842457	0.598151854	0	0
3/33	0.151842457	0.598151854	0	0
2/17	0.152412472	0.598151854	0	0
2/17	0.152412472	0.598151854	0	0
2/17	0.152412472	0.598151854	0	0
2/17	0.152412472	0.598151854	0	0
2/17	0.152412472	0.598151854	0	0
2/17	0.152412472	0.598151854	0	0
2/17	0.152412472	0.598151854	0	0
2/17	0.152412472	0.598151854	0	0
2/17	0.152412472	0.598151854	0	0
2/17	0.152412472	0.598151854	0	0
2/17	0.152412472	0.598151854	0	0
2/17	0.152412472	0.598151854	0	0
2/17	0.152412472	0.598151854	0	0
2/17	0.152412472	0.598151854	0	0
2/17	0.152412472	0.598151854	0	0
2/17	0.152412472	0.598151854	0	0
2/17	0.152412472	0.598151854	0	0
2/17	0.152412472	0.598151854	0	0
2/17	0.152412472	0.598151854	0	0
2/17	0.152412472	0.598151854	0	0
2/17	0.152412472	0.598151854	0	0
6/88	0.152469782	0.598151854	0	0
5/69	0.152613294	0.598151854	0	0
5/69	0.152613294	0.598151854	0	0
13/231	0.155405828	0.600073229	0	0
4/51	0.155779339	0.600073229	0	0
4/51	0.155779339	0.600073229	0	0
6/89	0.158233423	0.600073229	0	0
5/70	0.159204101	0.600073229	0	0
5/70	0.159204101	0.600073229	0	0
5/70	0.159204101	0.600073229	0	0
5/70	0.159204101	0.600073229	0	0
7/109	0.160338145	0.600073229	0	0
8/129	0.160387195	0.600073229	0	0
3/34	0.161783343	0.600073229	0	0
3/34	0.161783343	0.600073229	0	0
3/34	0.161783343	0.600073229	0	0
4/52	0.163669508	0.600073229	0	0
4/52	0.163669508	0.600073229	0	0
4/52	0.163669508	0.600073229	0	0
4/52	0.163669508	0.600073229	0	0
7/110	0.165626762	0.600073229	0	0
5/71	0.165908498	0.600073229	0	0
5/71	0.165908498	0.600073229	0	0
5/71	0.165908498	0.600073229	0	0

5/71	0.165908498	0.600073229	0	0
11/192	0.166287915	0.600073229	0	0
2/18	0.167040613	0.600073229	0	0
2/18	0.167040613	0.600073229	0	0
2/18	0.167040613	0.600073229	0	0
2/18	0.167040613	0.600073229	0	0
2/18	0.167040613	0.600073229	0	0
2/18	0.167040613	0.600073229	0	0
2/18	0.167040613	0.600073229	0	0
2/18	0.167040613	0.600073229	0	0
2/18	0.167040613	0.600073229	0	0
2/18	0.167040613	0.600073229	0	0
2/18	0.167040613	0.600073229	0	0
2/18	0.167040613	0.600073229	0	0
2/18	0.167040613	0.600073229	0	0
2/18	0.167040613	0.600073229	0	0
2/18	0.167040613	0.600073229	0	0
2/18	0.167040613	0.600073229	0	0
2/18	0.167040613	0.600073229	0	0
2/18	0.167040613	0.600073229	0	0
2/18	0.167040613	0.600073229	0	0
2/18	0.167040613	0.600073229	0	0
19/364	0.168690637	0.600073229	0	0
6/91	0.170038531	0.600073229	0	0
4/53	0.17170023	0.600073229	0	0
3/35	0.171913336	0.600073229	0	0
3/35	0.171913336	0.600073229	0	0
3/35	0.171913336	0.600073229	0	0
3/35	0.171913336	0.600073229	0	0
3/35	0.171913336	0.600073229	0	0
5/72	0.172722612	0.600073229	0	0
17/322	0.173180175	0.600073229	0	0
5/73	0.179642484	0.600073229	0	0
4/54	0.17986456	0.600073229	0	0
4/54	0.17986456	0.600073229	0	0
4/54	0.17986456	0.600073229	0	0
4/54	0.17986456	0.600073229	0	0
2/19	0.181895179	0.600073229	0	0
2/19	0.181895179	0.600073229	0	0
2/19	0.181895179	0.600073229	0	0
2/19	0.181895179	0.600073229	0	0
2/19	0.181895179	0.600073229	0	0
2/19	0.181895179	0.600073229	0	0
2/19	0.181895179	0.600073229	0	0
2/19	0.181895179	0.600073229	0	0
2/19	0.181895179	0.600073229	0	0
2/19	0.181895179	0.600073229	0	0
2/19	0.181895179	0.600073229	0	0

2/19	0.181895179	0.600073229	0	0
2/19	0.181895179	0.600073229	0	0
2/19	0.181895179	0.600073229	0	0
2/19	0.181895179	0.600073229	0	0
2/19	0.181895179	0.600073229	0	0
6/93	0.182197519	0.600073229	0	0
6/93	0.182197519	0.600073229	0	0
3/36	0.182217608	0.600073229	0	0
3/36	0.182217608	0.600073229	0	0
3/36	0.182217608	0.600073229	0	0
3/36	0.182217608	0.600073229	0	0
3/36	0.182217608	0.600073229	0	0
3/36	0.182217608	0.600073229	0	0
3/36	0.182217608	0.600073229	0	0
3/36	0.182217608	0.600073229	0	0
9/155	0.186738955	0.600073229	0	0
6/94	0.188403267	0.600073229	0	0
1/5	0.188882142	0.600073229	0	0
1/5	0.188882142	0.600073229	0	0
1/5	0.188882142	0.600073229	0	0
1/5	0.188882142	0.600073229	0	0
1/5	0.188882142	0.600073229	0	0
1/5	0.188882142	0.600073229	0	0
1/5	0.188882142	0.600073229	0	0
1/5	0.188882142	0.600073229	0	0
1/5	0.188882142	0.600073229	0	0
1/5	0.188882142	0.600073229	0	0
1/5	0.188882142	0.600073229	0	0
1/5	0.188882142	0.600073229	0	0
1/5	0.188882142	0.600073229	0	0
1/5	0.188882142	0.600073229	0	0
1/5	0.188882142	0.600073229	0	0
1/5	0.188882142	0.600073229	0	0
1/5	0.188882142	0.600073229	0	0
1/5	0.188882142	0.600073229	0	0
1/5	0.188882142	0.600073229	0	0
1/5	0.188882142	0.600073229	0	0
1/5	0.188882142	0.600073229	0	0
1/5	0.188882142	0.600073229	0	0
1/5	0.188882142	0.600073229	0	0
1/5	0.188882142	0.600073229	0	0
1/5	0.188882142	0.600073229	0	0
1/5	0.188882142	0.600073229	0	0
1/5	0.188882142	0.600073229	0	0
1/5	0.188882142	0.600073229	0	0
1/5	0.188882142	0.600073229	0	0
1/5	0.188882142	0.600073229	0	0
1/5	0.188882142	0.600073229	0	0

1/5	0.188882142	0.600073229	0	0
1/5	0.188882142	0.600073229	0	0
1/5	0.188882142	0.600073229	0	0
1/5	0.188882142	0.600073229	0	0
1/5	0.188882142	0.600073229	0	0
1/5	0.188882142	0.600073229	0	0
1/5	0.188882142	0.600073229	0	0
1/5	0.188882142	0.600073229	0	0
1/5	0.188882142	0.600073229	0	0
1/5	0.188882142	0.600073229	0	0
1/5	0.188882142	0.600073229	0	0
1/5	0.188882142	0.600073229	0	0
1/5	0.188882142	0.600073229	0	0
1/5	0.188882142	0.600073229	0	0
1/5	0.188882142	0.600073229	0	0
1/5	0.188882142	0.600073229	0	0
1/5	0.188882142	0.600073229	0	0
1/5	0.188882142	0.600073229	0	0
1/5	0.188882142	0.600073229	0	0
1/5	0.188882142	0.600073229	0	0
1/5	0.188882142	0.600073229	0	0
1/5	0.188882142	0.600073229	0	0
1/5	0.188882142	0.600073229	0	0
1/5	0.188882142	0.600073229	0	0
1/5	0.188882142	0.600073229	0	0
1/5	0.188882142	0.600073229	0	0
1/5	0.188882142	0.600073229	0	0
1/5	0.188882142	0.600073229	0	0
1/5	0.188882142	0.600073229	0	0
1/5	0.188882142	0.600073229	0	0
1/5	0.188882142	0.600073229	0	0
1/5	0.188882142	0.600073229	0	0
1/5	0.188882142	0.600073229	0	0
1/5	0.188882142	0.600073229	0	0
1/5	0.188882142	0.600073229	0	0
1/5	0.188882142	0.600073229	0	0
1/5	0.188882142	0.600073229	0	0
1/5	0.188882142	0.600073229	0	0
1/5	0.188882142	0.600073229	0	0
1/5	0.188882142	0.600073229	0	0
1/5	0.188882142	0.600073229	0	0
1/5	0.188882142	0.600073229	0	0
1/5	0.188882142	0.600073229	0	0
1/5	0.188882142	0.600073229	0	0
1/5	0.188882142	0.600073229	0	0
1/5	0.188882142	0.600073229	0	0
1/5	0.188882142	0.600073229	0	0
1/5	0.188882142	0.600073229	0	0

1/5	0.188882142	0.600073229	0	0
1/5	0.188882142	0.600073229	0	0
1/5	0.188882142	0.600073229	0	0
1/5	0.188882142	0.600073229	0	0
1/5	0.188882142	0.600073229	0	0
1/5	0.188882142	0.600073229	0	0
1/5	0.188882142	0.600073229	0	0
1/5	0.188882142	0.600073229	0	0
1/5	0.188882142	0.600073229	0	0
1/5	0.188882142	0.600073229	0	0
1/5	0.188882142	0.600073229	0	0
3/37	0.192681521	0.600073229	0	0
5/75	0.193783316	0.600073229	0	0
5/75	0.193783316	0.600073229	0	0
4/56	0.196565973	0.600073229	0	0
4/56	0.196565973	0.600073229	0	0
4/56	0.196565973	0.600073229	0	0
2/20	0.196933085	0.600073229	0	0
2/20	0.196933085	0.600073229	0	0
2/20	0.196933085	0.600073229	0	0
2/20	0.196933085	0.600073229	0	0
2/20	0.196933085	0.600073229	0	0
2/20	0.196933085	0.600073229	0	0
2/20	0.196933085	0.600073229	0	0
2/20	0.196933085	0.600073229	0	0
2/20	0.196933085	0.600073229	0	0
2/20	0.196933085	0.600073229	0	0
2/20	0.196933085	0.600073229	0	0
2/20	0.196933085	0.600073229	0	0
2/20	0.196933085	0.600073229	0	0
2/20	0.196933085	0.600073229	0	0
2/20	0.196933085	0.600073229	0	0
2/20	0.196933085	0.600073229	0	0
2/20	0.196933085	0.600073229	0	0
2/20	0.196933085	0.600073229	0	0
2/20	0.196933085	0.600073229	0	0
2/20	0.196933085	0.600073229	0	0
2/20	0.196933085	0.600073229	0	0
2/20	0.196933085	0.600073229	0	0
2/20	0.196933085	0.600073229	0	0
10/179	0.200725007	0.600073229	0	0
3/38	0.203290659	0.600073229	0	0
3/38	0.203290659	0.600073229	0	0
3/38	0.203290659	0.600073229	0	0
3/38	0.203290659	0.600073229	0	0
3/38	0.203290659	0.600073229	0	0
3/38	0.203290659	0.600073229	0	0
13/244	0.203615202	0.600073229	0	0
52/1127	0.204488706	0.600073229	0	0

7/117	0.204687825	0.600073229	0	0
7/117	0.204687825	0.600073229	0	0
4/57	0.205088932	0.600073229	0	0
4/57	0.205088932	0.600073229	0	0
4/57	0.205088932	0.600073229	0	0
4/57	0.205088932	0.600073229	0	0
9/159	0.206206036	0.600073229	0	0
6/97	0.207493234	0.600073229	0	0
7/118	0.210537059	0.600073229	0	0
7/118	0.210537059	0.600073229	0	0
13/246	0.211576009	0.600073229	0	0
2/21	0.212114391	0.600073229	0	0
2/21	0.212114391	0.600073229	0	0
2/21	0.212114391	0.600073229	0	0
2/21	0.212114391	0.600073229	0	0
2/21	0.212114391	0.600073229	0	0
2/21	0.212114391	0.600073229	0	0
2/21	0.212114391	0.600073229	0	0
2/21	0.212114391	0.600073229	0	0
2/21	0.212114391	0.600073229	0	0
2/21	0.212114391	0.600073229	0	0
2/21	0.212114391	0.600073229	0	0
36/763	0.213181735	0.600073229	0	0
4/58	0.213717283	0.600073229	0	0
4/58	0.213717283	0.600073229	0	0
4/58	0.213717283	0.600073229	0	0
4/58	0.213717283	0.600073229	0	0
4/58	0.213717283	0.600073229	0	0
3/39	0.214030862	0.600073229	0	0
3/39	0.214030862	0.600073229	0	0
3/39	0.214030862	0.600073229	0	0
3/39	0.214030862	0.600073229	0	0
3/39	0.214030862	0.600073229	0	0
3/39	0.214030862	0.600073229	0	0
3/39	0.214030862	0.600073229	0	0
19/379	0.214624923	0.600073229	0	0
5/78	0.215685029	0.600073229	0	0
5/78	0.215685029	0.600073229	0	0
7/119	0.216447649	0.600073229	0	0
15/291	0.21644874	0.600073229	0	0
9/162	0.221326147	0.600073229	0	0
1/6	0.222146245	0.600073229	0	0
1/6	0.222146245	0.600073229	0	0
1/6	0.222146245	0.600073229	0	0
1/6	0.222146245	0.600073229	0	0
1/6	0.222146245	0.600073229	0	0
1/6	0.222146245	0.600073229	0	0
1/6	0.222146245	0.600073229	0	0

1/6	0.222146245	0.600073229	0	0
1/6	0.222146245	0.600073229	0	0
1/6	0.222146245	0.600073229	0	0
1/6	0.222146245	0.600073229	0	0
1/6	0.222146245	0.600073229	0	0
1/6	0.222146245	0.600073229	0	0
1/6	0.222146245	0.600073229	0	0
1/6	0.222146245	0.600073229	0	0
1/6	0.222146245	0.600073229	0	0
1/6	0.222146245	0.600073229	0	0
1/6	0.222146245	0.600073229	0	0
1/6	0.222146245	0.600073229	0	0
1/6	0.222146245	0.600073229	0	0
1/6	0.222146245	0.600073229	0	0
1/6	0.222146245	0.600073229	0	0
1/6	0.222146245	0.600073229	0	0
1/6	0.222146245	0.600073229	0	0
1/6	0.222146245	0.600073229	0	0
1/6	0.222146245	0.600073229	0	0
1/6	0.222146245	0.600073229	0	0
1/6	0.222146245	0.600073229	0	0
1/6	0.222146245	0.600073229	0	0
1/6	0.222146245	0.600073229	0	0
1/6	0.222146245	0.600073229	0	0
1/6	0.222146245	0.600073229	0	0
1/6	0.222146245	0.600073229	0	0
1/6	0.222146245	0.600073229	0	0
1/6	0.222146245	0.600073229	0	0
1/6	0.222146245	0.600073229	0	0
1/6	0.222146245	0.600073229	0	0
1/6	0.222146245	0.600073229	0	0
1/6	0.222146245	0.600073229	0	0
1/6	0.222146245	0.600073229	0	0
1/6	0.222146245	0.600073229	0	0
1/6	0.222146245	0.600073229	0	0
1/6	0.222146245	0.600073229	0	0
1/6	0.222146245	0.600073229	0	0
1/6	0.222146245	0.600073229	0	0
1/6	0.222146245	0.600073229	0	0
1/6	0.222146245	0.600073229	0	0
1/6	0.222146245	0.600073229	0	0
1/6	0.222146245	0.600073229	0	0
1/6	0.222146245	0.600073229	0	0
1/6	0.222146245	0.600073229	0	0
1/6	0.222146245	0.600073229	0	0
1/6	0.222146245	0.600073229	0	0
1/6	0.222146245	0.600073229	0	0
1/6	0.222146245	0.600073229	0	0

1/6	0.222146245	0.600073229	0	0
1/6	0.222146245	0.600073229	0	0
1/6	0.222146245	0.600073229	0	0
1/6	0.222146245	0.600073229	0	0
1/6	0.222146245	0.600073229	0	0
1/6	0.222146245	0.600073229	0	0
1/6	0.222146245	0.600073229	0	0
1/6	0.222146245	0.600073229	0	0
1/6	0.222146245	0.600073229	0	0
1/6	0.222146245	0.600073229	0	0
1/6	0.222146245	0.600073229	0	0
1/6	0.222146245	0.600073229	0	0
1/6	0.222146245	0.600073229	0	0
1/6	0.222146245	0.600073229	0	0
1/6	0.222146245	0.600073229	0	0
1/6	0.222146245	0.600073229	0	0
1/6	0.222146245	0.600073229	0	0
1/6	0.222146245	0.600073229	0	0
1/6	0.222146245	0.600073229	0	0
1/6	0.222146245	0.600073229	0	0
1/6	0.222146245	0.600073229	0	0
1/6	0.222146245	0.600073229	0	0
1/6	0.222146245	0.600073229	0	0
1/6	0.222146245	0.600073229	0	0
1/6	0.222146245	0.600073229	0	0
1/6	0.222146245	0.600073229	0	0
1/6	0.222146245	0.600073229	0	0
1/6	0.222146245	0.600073229	0	0
1/6	0.222146245	0.600073229	0	0
1/6	0.222146245	0.600073229	0	0
1/6	0.222146245	0.600073229	0	0
1/6	0.222146245	0.600073229	0	0
1/6	0.222146245	0.600073229	0	0
1/6	0.222146245	0.600073229	0	0
1/6	0.222146245	0.600073229	0	0
4/59	0.222443955	0.600073229	0	0
4/59	0.222443955	0.600073229	0	0
4/59	0.222443955	0.600073229	0	0
4/59	0.222443955	0.600073229	0	0
4/59	0.222443955	0.600073229	0	0
4/59	0.222443955	0.600073229	0	0
5/79	0.223152813	0.600073229	0	0
5/79	0.223152813	0.600073229	0	0
10/184	0.224272065	0.600073229	0	0
3/40	0.224888261	0.600073229	0	0
3/40	0.224888261	0.600073229	0	0
3/40	0.224888261	0.600073229	0	0
3/40	0.224888261	0.600073229	0	0

2/22	0.227402116	0.600073229	0	0
2/22	0.227402116	0.600073229	0	0
2/22	0.227402116	0.600073229	0	0
2/22	0.227402116	0.600073229	0	0
2/22	0.227402116	0.600073229	0	0
2/22	0.227402116	0.600073229	0	0
2/22	0.227402116	0.600073229	0	0
2/22	0.227402116	0.600073229	0	0
2/22	0.227402116	0.600073229	0	0
2/22	0.227402116	0.600073229	0	0
2/22	0.227402116	0.600073229	0	0
2/22	0.227402116	0.600073229	0	0
5/80	0.230697066	0.600073229	0	0
5/80	0.230697066	0.600073229	0	0
5/80	0.230697066	0.600073229	0	0
5/80	0.230697066	0.600073229	0	0
4/60	0.231261894	0.600073229	0	0
6/101	0.233944917	0.600073229	0	0
3/41	0.235849298	0.600073229	0	0
3/41	0.235849298	0.600073229	0	0
3/41	0.235849298	0.600073229	0	0
5/81	0.238313477	0.600073229	0	0
5/81	0.238313477	0.600073229	0	0
5/81	0.238313477	0.600073229	0	0
8/144	0.239189672	0.600073229	0	0
15/297	0.23930697	0.600073229	0	0
12/231	0.24049492	0.600073229	0	0
7/123	0.24066203	0.600073229	0	0
6/102	0.240716922	0.600073229	0	0
6/102	0.240716922	0.600073229	0	0
16/320	0.242098291	0.600073229	0	0
2/23	0.242762065	0.600073229	0	0
2/23	0.242762065	0.600073229	0	0
2/23	0.242762065	0.600073229	0	0
2/23	0.242762065	0.600073229	0	0
2/23	0.242762065	0.600073229	0	0
2/23	0.242762065	0.600073229	0	0
2/23	0.242762065	0.600073229	0	0
2/23	0.242762065	0.600073229	0	0
2/23	0.242762065	0.600073229	0	0
2/23	0.242762065	0.600073229	0	0
2/23	0.242762065	0.600073229	0	0
2/23	0.242762065	0.600073229	0	0
2/23	0.242762065	0.600073229	0	0
2/23	0.242762065	0.600073229	0	0
2/23	0.242762065	0.600073229	0	0
2/23	0.242762065	0.600073229	0	0
2/23	0.242762065	0.600073229	0	0

2/23	0.242762065	0.600073229	0	0
2/23	0.242762065	0.600073229	0	0
8/145	0.244867023	0.600073229	0	0
12/232	0.244946447	0.600073229	0	0
5/82	0.245997726	0.600073229	0	0
5/82	0.245997726	0.600073229	0	0
7/124	0.246848027	0.600073229	0	0
3/42	0.246900753	0.600073229	0	0
3/42	0.246900753	0.600073229	0	0
3/42	0.246900753	0.600073229	0	0
3/42	0.246900753	0.600073229	0	0
3/42	0.246900753	0.600073229	0	0
8/146	0.250588395	0.600073229	0	0
5/83	0.253745493	0.600073229	0	0
1/7	0.254047775	0.600073229	0	0
1/7	0.254047775	0.600073229	0	0
1/7	0.254047775	0.600073229	0	0
1/7	0.254047775	0.600073229	0	0
1/7	0.254047775	0.600073229	0	0
1/7	0.254047775	0.600073229	0	0
1/7	0.254047775	0.600073229	0	0
1/7	0.254047775	0.600073229	0	0
1/7	0.254047775	0.600073229	0	0
1/7	0.254047775	0.600073229	0	0
1/7	0.254047775	0.600073229	0	0
1/7	0.254047775	0.600073229	0	0
1/7	0.254047775	0.600073229	0	0
1/7	0.254047775	0.600073229	0	0
1/7	0.254047775	0.600073229	0	0
1/7	0.254047775	0.600073229	0	0
1/7	0.254047775	0.600073229	0	0
1/7	0.254047775	0.600073229	0	0
1/7	0.254047775	0.600073229	0	0
1/7	0.254047775	0.600073229	0	0
1/7	0.254047775	0.600073229	0	0
1/7	0.254047775	0.600073229	0	0
1/7	0.254047775	0.600073229	0	0
1/7	0.254047775	0.600073229	0	0
1/7	0.254047775	0.600073229	0	0
1/7	0.254047775	0.600073229	0	0
1/7	0.254047775	0.600073229	0	0
1/7	0.254047775	0.600073229	0	0
1/7	0.254047775	0.600073229	0	0
1/7	0.254047775	0.600073229	0	0
1/7	0.254047775	0.600073229	0	0
1/7	0.254047775	0.600073229	0	0
1/7	0.254047775	0.600073229	0	0
1/7	0.254047775	0.600073229	0	0

1/7	0.254047775	0.600073229	0	0
1/7	0.254047775	0.600073229	0	0
1/7	0.254047775	0.600073229	0	0
1/7	0.254047775	0.600073229	0	0
1/7	0.254047775	0.600073229	0	0
1/7	0.254047775	0.600073229	0	0
1/7	0.254047775	0.600073229	0	0
1/7	0.254047775	0.600073229	0	0
1/7	0.254047775	0.600073229	0	0
1/7	0.254047775	0.600073229	0	0
1/7	0.254047775	0.600073229	0	0
1/7	0.254047775	0.600073229	0	0
1/7	0.254047775	0.600073229	0	0
1/7	0.254047775	0.600073229	0	0
1/7	0.254047775	0.600073229	0	0
1/7	0.254047775	0.600073229	0	0
1/7	0.254047775	0.600073229	0	0
1/7	0.254047775	0.600073229	0	0
1/7	0.254047775	0.600073229	0	0
1/7	0.254047775	0.600073229	0	0
1/7	0.254047775	0.600073229	0	0
1/7	0.254047775	0.600073229	0	0
1/7	0.254047775	0.600073229	0	0
1/7	0.254047775	0.600073229	0	0
1/7	0.254047775	0.600073229	0	0
1/7	0.254047775	0.600073229	0	0
1/7	0.254047775	0.600073229	0	0
1/7	0.254047775	0.600073229	0	0
1/7	0.254047775	0.600073229	0	0
1/7	0.254047775	0.600073229	0	0
1/7	0.254047775	0.600073229	0	0
1/7	0.254047775	0.600073229	0	0
1/7	0.254047775	0.600073229	0	0
1/7	0.254047775	0.600073229	0	0
1/7	0.254047775	0.600073229	0	0
1/7	0.254047775	0.600073229	0	0
1/7	0.254047775	0.600073229	0	0
1/7	0.254047775	0.600073229	0	0
1/7	0.254047775	0.600073229	0	0
1/7	0.254047775	0.600073229	0	0
1/7	0.254047775	0.600073229	0	0
1/7	0.254047775	0.600073229	0	0
1/7	0.254047775	0.600073229	0	0
1/7	0.254047775	0.600073229	0	0
1/7	0.254047775	0.600073229	0	0
1/7	0.254047775	0.600073229	0	0
1/7	0.254047775	0.600073229	0	0
1/7	0.254047775	0.600073229	0	0

1/7	0.254047775	0.600073229	0	0
1/7	0.254047775	0.600073229	0	0
1/7	0.254047775	0.600073229	0	0
1/7	0.254047775	0.600073229	0	0
1/7	0.254047775	0.600073229	0	0
1/7	0.254047775	0.600073229	0	0
1/7	0.254047775	0.600073229	0	0
1/7	0.254047775	0.600073229	0	0
1/7	0.254047775	0.600073229	0	0
1/7	0.254047775	0.600073229	0	0
1/7	0.254047775	0.600073229	0	0
1/7	0.254047775	0.600073229	0	0
1/7	0.254047775	0.600073229	0	0
1/7	0.254047775	0.600073229	0	0
1/7	0.254047775	0.600073229	0	0
1/7	0.254047775	0.600073229	0	0
1/7	0.254047775	0.600073229	0	0
1/7	0.254047775	0.600073229	0	0
1/7	0.254047775	0.600073229	0	0
8/147	0.256352114	0.600073229	0	0
13/257	0.257613837	0.600073229	0	0
3/43	0.258029759	0.600073229	0	0
3/43	0.258029759	0.600073229	0	0
3/43	0.258029759	0.600073229	0	0
3/43	0.258029759	0.600073229	0	0
3/43	0.258029759	0.600073229	0	0
3/43	0.258029759	0.600073229	0	0
2/24	0.25816266	0.600073229	0	0
2/24	0.25816266	0.600073229	0	0
2/24	0.25816266	0.600073229	0	0
2/24	0.25816266	0.600073229	0	0
2/24	0.25816266	0.600073229	0	0
2/24	0.25816266	0.600073229	0	0
2/24	0.25816266	0.600073229	0	0
2/24	0.25816266	0.600073229	0	0
2/24	0.25816266	0.600073229	0	0
2/24	0.25816266	0.600073229	0	0
2/24	0.25816266	0.600073229	0	0
2/24	0.25816266	0.600073229	0	0
2/24	0.25816266	0.600073229	0	0
2/24	0.25816266	0.600073229	0	0
2/24	0.25816266	0.600073229	0	0
2/24	0.25816266	0.600073229	0	0
4/63	0.258193462	0.600073229	0	0
5/84	0.261552462	0.607426315	0	0
7/127	0.265689143	0.610317104	0	0
3/44	0.26922382	0.610317104	0	0
3/44	0.26922382	0.610317104	0	0

5/85	0.26941433	0.610317104	0	0
5/85	0.26941433	0.610317104	0	0
5/85	0.26941433	0.610317104	0	0
5/85	0.26941433	0.610317104	0	0
2/25	0.273574786	0.610317104	0	0
2/25	0.273574786	0.610317104	0	0
2/25	0.273574786	0.610317104	0	0
2/25	0.273574786	0.610317104	0	0
2/25	0.273574786	0.610317104	0	0
2/25	0.273574786	0.610317104	0	0
2/25	0.273574786	0.610317104	0	0
2/25	0.273574786	0.610317104	0	0
2/25	0.273574786	0.610317104	0	0
2/25	0.273574786	0.610317104	0	0
2/25	0.273574786	0.610317104	0	0
2/25	0.273574786	0.610317104	0	0
2/25	0.273574786	0.610317104	0	0
2/25	0.273574786	0.610317104	0	0
2/25	0.273574786	0.610317104	0	0
2/25	0.273574786	0.610317104	0	0
2/25	0.273574786	0.610317104	0	0
2/25	0.273574786	0.610317104	0	0
2/25	0.273574786	0.610317104	0	0
2/25	0.273574786	0.610317104	0	0
2/25	0.273574786	0.610317104	0	0
2/25	0.273574786	0.610317104	0	0
2/25	0.273574786	0.610317104	0	0
2/25	0.273574786	0.610317104	0	0
2/25	0.273574786	0.610317104	0	0
8/150	0.273880418	0.610317104	0	0
4/65	0.27647721	0.610317104	0	0
4/65	0.27647721	0.610317104	0	0
3/45	0.280470823	0.610317104	0	0
3/45	0.280470823	0.610317104	0	0
3/45	0.280470823	0.610317104	0	0
25/537	0.284317532	0.610317104	0	0
1/8	0.28464248	0.610317104	0	0
1/8	0.28464248	0.610317104	0	0
1/8	0.28464248	0.610317104	0	0
1/8	0.28464248	0.610317104	0	0
1/8	0.28464248	0.610317104	0	0
1/8	0.28464248	0.610317104	0	0
1/8	0.28464248	0.610317104	0	0
1/8	0.28464248	0.610317104	0	0
1/8	0.28464248	0.610317104	0	0
1/8	0.28464248	0.610317104	0	0
1/8	0.28464248	0.610317104	0	0
1/8	0.28464248	0.610317104	0	0

1/8	0.28464248	0.610317104	0	0
1/8	0.28464248	0.610317104	0	0
1/8	0.28464248	0.610317104	0	0
1/8	0.28464248	0.610317104	0	0
1/8	0.28464248	0.610317104	0	0
1/8	0.28464248	0.610317104	0	0
1/8	0.28464248	0.610317104	0	0
1/8	0.28464248	0.610317104	0	0
1/8	0.28464248	0.610317104	0	0
1/8	0.28464248	0.610317104	0	0
1/8	0.28464248	0.610317104	0	0
1/8	0.28464248	0.610317104	0	0
1/8	0.28464248	0.610317104	0	0
1/8	0.28464248	0.610317104	0	0
1/8	0.28464248	0.610317104	0	0
1/8	0.28464248	0.610317104	0	0
1/8	0.28464248	0.610317104	0	0
1/8	0.28464248	0.610317104	0	0
1/8	0.28464248	0.610317104	0	0
1/8	0.28464248	0.610317104	0	0
1/8	0.28464248	0.610317104	0	0
1/8	0.28464248	0.610317104	0	0
1/8	0.28464248	0.610317104	0	0
1/8	0.28464248	0.610317104	0	0
1/8	0.28464248	0.610317104	0	0
1/8	0.28464248	0.610317104	0	0
1/8	0.28464248	0.610317104	0	0
1/8	0.28464248	0.610317104	0	0
1/8	0.28464248	0.610317104	0	0
1/8	0.28464248	0.610317104	0	0
1/8	0.28464248	0.610317104	0	0
1/8	0.28464248	0.610317104	0	0
1/8	0.28464248	0.610317104	0	0
1/8	0.28464248	0.610317104	0	0
1/8	0.28464248	0.610317104	0	0
1/8	0.28464248	0.610317104	0	0
1/8	0.28464248	0.610317104	0	0
1/8	0.28464248	0.610317104	0	0
1/8	0.28464248	0.610317104	0	0
1/8	0.28464248	0.610317104	0	0
1/8	0.28464248	0.610317104	0	0
1/8	0.28464248	0.610317104	0	0
1/8	0.28464248	0.610317104	0	0
1/8	0.28464248	0.610317104	0	0
1/8	0.28464248	0.610317104	0	0
1/8	0.28464248	0.610317104	0	0
1/8	0.28464248	0.610317104	0	0
1/8	0.28464248	0.610317104	0	0

1/8	0.28464248	0.610317104	0	0
1/8	0.28464248	0.610317104	0	0
1/8	0.28464248	0.610317104	0	0
1/8	0.28464248	0.610317104	0	0
1/8	0.28464248	0.610317104	0	0
1/8	0.28464248	0.610317104	0	0
1/8	0.28464248	0.610317104	0	0
1/8	0.28464248	0.610317104	0	0
1/8	0.28464248	0.610317104	0	0
1/8	0.28464248	0.610317104	0	0
1/8	0.28464248	0.610317104	0	0
1/8	0.28464248	0.610317104	0	0
4/66	0.285697694	0.610317104	0	0
2/26	0.288971642	0.610317104	0	0
2/26	0.288971642	0.610317104	0	0
2/26	0.288971642	0.610317104	0	0
2/26	0.288971642	0.610317104	0	0
2/26	0.288971642	0.610317104	0	0
2/26	0.288971642	0.610317104	0	0
2/26	0.288971642	0.610317104	0	0
2/26	0.288971642	0.610317104	0	0
2/26	0.288971642	0.610317104	0	0
2/26	0.288971642	0.610317104	0	0
2/26	0.288971642	0.610317104	0	0
2/26	0.288971642	0.610317104	0	0
2/26	0.288971642	0.610317104	0	0
2/26	0.288971642	0.610317104	0	0
2/26	0.288971642	0.610317104	0	0
2/26	0.288971642	0.610317104	0	0
2/26	0.288971642	0.610317104	0	0
2/26	0.288971642	0.610317104	0	0
2/26	0.288971642	0.610317104	0	0
2/26	0.288971642	0.610317104	0	0
6/109	0.289571504	0.610317104	0	0
6/109	0.289571504	0.610317104	0	0
6/109	0.289571504	0.610317104	0	0
9/175	0.291126845	0.610317104	0	0
3/46	0.291759049	0.610317104	0	0
3/46	0.291759049	0.610317104	0	0
3/46	0.291759049	0.610317104	0	0
3/46	0.291759049	0.610317104	0	0
3/46	0.291759049	0.610317104	0	0
3/46	0.291759049	0.610317104	0	0
3/46	0.291759049	0.610317104	0	0
3/46	0.291759049	0.610317104	0	0
13/265	0.293150709	0.610317104	0	0
5/88	0.293286602	0.610317104	0	0

4/67	0.294961835	0.610317104	0	0
4/67	0.294961835	0.610317104	0	0
7/132	0.297902393	0.610317104	0	0
7/132	0.297902393	0.610317104	0	0
5/89	0.301325481	0.610317104	0	0
3/47	0.30307718	0.610317104	0	0
3/47	0.30307718	0.610317104	0	0
6/111	0.30390675	0.610317104	0	0
6/111	0.30390675	0.610317104	0	0
6/111	0.30390675	0.610317104	0	0
4/68	0.304263207	0.610317104	0	0
2/27	0.304328599	0.610317104	0	0
2/27	0.304328599	0.610317104	0	0
2/27	0.304328599	0.610317104	0	0
2/27	0.304328599	0.610317104	0	0
2/27	0.304328599	0.610317104	0	0
2/27	0.304328599	0.610317104	0	0
2/27	0.304328599	0.610317104	0	0
2/27	0.304328599	0.610317104	0	0
2/27	0.304328599	0.610317104	0	0
2/27	0.304328599	0.610317104	0	0
2/27	0.304328599	0.610317104	0	0
2/27	0.304328599	0.610317104	0	0
7/133	0.304446765	0.610317104	0	0
13/268	0.306839863	0.610317104	0	0
21/452	0.308786128	0.610317104	0	0
6/112	0.31112232	0.610317104	0	0
4/69	0.313595509	0.610317104	0	0
1/9	0.313983831	0.610317104	0	0
1/9	0.313983831	0.610317104	0	0
1/9	0.313983831	0.610317104	0	0
1/9	0.313983831	0.610317104	0	0
1/9	0.313983831	0.610317104	0	0
1/9	0.313983831	0.610317104	0	0
1/9	0.313983831	0.610317104	0	0
1/9	0.313983831	0.610317104	0	0
1/9	0.313983831	0.610317104	0	0
1/9	0.313983831	0.610317104	0	0
1/9	0.313983831	0.610317104	0	0
1/9	0.313983831	0.610317104	0	0
1/9	0.313983831	0.610317104	0	0
1/9	0.313983831	0.610317104	0	0
1/9	0.313983831	0.610317104	0	0
1/9	0.313983831	0.610317104	0	0
1/9	0.313983831	0.610317104	0	0
1/9	0.313983831	0.610317104	0	0
1/9	0.313983831	0.610317104	0	0
1/9	0.313983831	0.610317104	0	0

1/9	0.313983831	0.610317104	0	0
1/9	0.313983831	0.610317104	0	0
1/9	0.313983831	0.610317104	0	0
1/9	0.313983831	0.610317104	0	0
1/9	0.313983831	0.610317104	0	0
1/9	0.313983831	0.610317104	0	0
1/9	0.313983831	0.610317104	0	0
1/9	0.313983831	0.610317104	0	0
1/9	0.313983831	0.610317104	0	0
1/9	0.313983831	0.610317104	0	0
1/9	0.313983831	0.610317104	0	0
1/9	0.313983831	0.610317104	0	0
1/9	0.313983831	0.610317104	0	0
1/9	0.313983831	0.610317104	0	0
1/9	0.313983831	0.610317104	0	0
1/9	0.313983831	0.610317104	0	0
1/9	0.313983831	0.610317104	0	0
1/9	0.313983831	0.610317104	0	0
1/9	0.313983831	0.610317104	0	0
1/9	0.313983831	0.610317104	0	0
1/9	0.313983831	0.610317104	0	0
1/9	0.313983831	0.610317104	0	0
1/9	0.313983831	0.610317104	0	0
1/9	0.313983831	0.610317104	0	0
1/9	0.313983831	0.610317104	0	0
1/9	0.313983831	0.610317104	0	0
1/9	0.313983831	0.610317104	0	0
1/9	0.313983831	0.610317104	0	0
1/9	0.313983831	0.610317104	0	0
1/9	0.313983831	0.610317104	0	0
1/9	0.313983831	0.610317104	0	0
1/9	0.313983831	0.610317104	0	0
1/9	0.313983831	0.610317104	0	0
1/9	0.313983831	0.610317104	0	0
1/9	0.313983831	0.610317104	0	0
1/9	0.313983831	0.610317104	0	0
1/9	0.313983831	0.610317104	0	0
1/9	0.313983831	0.610317104	0	0
1/9	0.313983831	0.610317104	0	0
1/9	0.313983831	0.610317104	0	0
1/9	0.313983831	0.610317104	0	0
1/9	0.313983831	0.610317104	0	0
1/9	0.313983831	0.610317104	0	0
1/9	0.313983831	0.610317104	0	0
1/9	0.313983831	0.610317104	0	0
1/9	0.313983831	0.610317104	0	0
1/9	0.313983831	0.610317104	0	0
1/9	0.313983831	0.610317104	0	0

1/9	0.313983831	0.610317104	0	0
1/9	0.313983831	0.610317104	0	0
1/9	0.313983831	0.610317104	0	0
1/9	0.313983831	0.610317104	0	0
1/9	0.313983831	0.610317104	0	0
1/9	0.313983831	0.610317104	0	0
1/9	0.313983831	0.610317104	0	0
1/9	0.313983831	0.610317104	0	0
1/9	0.313983831	0.610317104	0	0
1/9	0.313983831	0.610317104	0	0
1/9	0.313983831	0.610317104	0	0
1/9	0.313983831	0.610317104	0	0
1/9	0.313983831	0.610317104	0	0
1/9	0.313983831	0.610317104	0	0
1/9	0.313983831	0.610317104	0	0
1/9	0.313983831	0.610317104	0	0
1/9	0.313983831	0.610317104	0	0
1/9	0.313983831	0.610317104	0	0
3/48	0.314414308	0.610772364	0	0
27/594	0.31748092	0.615131102	0	0
2/28	0.319623069	0.615131102	0	0
2/28	0.319623069	0.615131102	0	0
2/28	0.319623069	0.615131102	0	0
2/28	0.319623069	0.615131102	0	0
2/28	0.319623069	0.615131102	0	0
2/28	0.319623069	0.615131102	0	0
2/28	0.319623069	0.615131102	0	0
2/28	0.319623069	0.615131102	0	0
2/28	0.319623069	0.615131102	0	0
2/28	0.319623069	0.615131102	0	0
2/28	0.319623069	0.615131102	0	0
2/28	0.319623069	0.615131102	0	0
2/28	0.319623069	0.615131102	0	0
2/28	0.319623069	0.615131102	0	0
4/70	0.322952562	0.61936424	0	0
5/92	0.325628321	0.61936424	0	0
6/114	0.325634692	0.61936424	0	0
3/49	0.325759935	0.61936424	0	0
4/71	0.332328326	0.61936424	0	0
4/71	0.332328326	0.61936424	0	0
5/93	0.333777797	0.61936424	0	0
2/29	0.33483438	0.61936424	0	0
2/29	0.33483438	0.61936424	0	0
2/29	0.33483438	0.61936424	0	0
2/29	0.33483438	0.61936424	0	0
2/29	0.33483438	0.61936424	0	0
2/29	0.33483438	0.61936424	0	0
2/29	0.33483438	0.61936424	0	0

2/29	0.33483438	0.61936424	0	0
2/29	0.33483438	0.61936424	0	0
2/29	0.33483438	0.61936424	0	0
2/29	0.33483438	0.61936424	0	0
2/29	0.33483438	0.61936424	0	0
2/29	0.33483438	0.61936424	0	0
2/29	0.33483438	0.61936424	0	0
3/50	0.337103977	0.61936424	0	0
3/50	0.337103977	0.61936424	0	0
3/50	0.337103977	0.61936424	0	0
3/50	0.337103977	0.61936424	0	0
3/50	0.337103977	0.61936424	0	0
3/50	0.337103977	0.61936424	0	0
3/50	0.337103977	0.61936424	0	0
45/1025	0.337680299	0.61936424	0	0
4/72	0.341716896	0.61936424	0	0
4/72	0.341716896	0.61936424	0	0
4/72	0.341716896	0.61936424	0	0
1/10	0.342123113	0.61936424	0	0
1/10	0.342123113	0.61936424	0	0
1/10	0.342123113	0.61936424	0	0
1/10	0.342123113	0.61936424	0	0
1/10	0.342123113	0.61936424	0	0
1/10	0.342123113	0.61936424	0	0
1/10	0.342123113	0.61936424	0	0
1/10	0.342123113	0.61936424	0	0
1/10	0.342123113	0.61936424	0	0
1/10	0.342123113	0.61936424	0	0
1/10	0.342123113	0.61936424	0	0
1/10	0.342123113	0.61936424	0	0
1/10	0.342123113	0.61936424	0	0
1/10	0.342123113	0.61936424	0	0
1/10	0.342123113	0.61936424	0	0
1/10	0.342123113	0.61936424	0	0
1/10	0.342123113	0.61936424	0	0
1/10	0.342123113	0.61936424	0	0
1/10	0.342123113	0.61936424	0	0
1/10	0.342123113	0.61936424	0	0
1/10	0.342123113	0.61936424	0	0
1/10	0.342123113	0.61936424	0	0
1/10	0.342123113	0.61936424	0	0
1/10	0.342123113	0.61936424	0	0
1/10	0.342123113	0.61936424	0	0
1/10	0.342123113	0.61936424	0	0
1/10	0.342123113	0.61936424	0	0
1/10	0.342123113	0.61936424	0	0
1/10	0.342123113	0.61936424	0	0
1/10	0.342123113	0.61936424	0	0

1/10	0.342123113	0.61936424	0	0
1/10	0.342123113	0.61936424	0	0
1/10	0.342123113	0.61936424	0	0
1/10	0.342123113	0.61936424	0	0
1/10	0.342123113	0.61936424	0	0
1/10	0.342123113	0.61936424	0	0
1/10	0.342123113	0.61936424	0	0
1/10	0.342123113	0.61936424	0	0
1/10	0.342123113	0.61936424	0	0
1/10	0.342123113	0.61936424	0	0
1/10	0.342123113	0.61936424	0	0
1/10	0.342123113	0.61936424	0	0
1/10	0.342123113	0.61936424	0	0
1/10	0.342123113	0.61936424	0	0
1/10	0.342123113	0.61936424	0	0
1/10	0.342123113	0.61936424	0	0
1/10	0.342123113	0.61936424	0	0
1/10	0.342123113	0.61936424	0	0
1/10	0.342123113	0.61936424	0	0
1/10	0.342123113	0.61936424	0	0
1/10	0.342123113	0.61936424	0	0
1/10	0.342123113	0.61936424	0	0
1/10	0.342123113	0.61936424	0	0
1/10	0.342123113	0.61936424	0	0
1/10	0.342123113	0.61936424	0	0
1/10	0.342123113	0.61936424	0	0
1/10	0.342123113	0.61936424	0	0
1/10	0.342123113	0.61936424	0	0
1/10	0.342123113	0.61936424	0	0
1/10	0.342123113	0.61936424	0	0
1/10	0.342123113	0.61936424	0	0
1/10	0.342123113	0.61936424	0	0
1/10	0.342123113	0.61936424	0	0
1/10	0.342123113	0.61936424	0	0
1/10	0.342123113	0.61936424	0	0
1/10	0.342123113	0.61936424	0	0
1/10	0.342123113	0.61936424	0	0
1/10	0.342123113	0.61936424	0	0
1/10	0.342123113	0.61936424	0	0
1/10	0.342123113	0.61936424	0	0
7/139	0.344238923	0.622832285	0	0
3/51	0.348436764	0.62372546	0	0
3/51	0.348436764	0.62372546	0	0
3/51	0.348436764	0.62372546	0	0
3/51	0.348436764	0.62372546	0	0
3/51	0.348436764	0.62372546	0	0
3/51	0.348436764	0.62372546	0	0
3/51	0.348436764	0.62372546	0	0

3/51	0.348436764	0.62372546	0	0
11/231	0.349274007	0.62372546	0	0
2/30	0.349943655	0.62372546	0	0
2/30	0.349943655	0.62372546	0	0
2/30	0.349943655	0.62372546	0	0
2/30	0.349943655	0.62372546	0	0
2/30	0.349943655	0.62372546	0	0
2/30	0.349943655	0.62372546	0	0
2/30	0.349943655	0.62372546	0	0
2/30	0.349943655	0.62372546	0	0
2/30	0.349943655	0.62372546	0	0
2/30	0.349943655	0.62372546	0	0
2/30	0.349943655	0.62372546	0	0
2/30	0.349943655	0.62372546	0	0
2/30	0.349943655	0.62372546	0	0
2/30	0.349943655	0.62372546	0	0
2/30	0.349943655	0.62372546	0	0
2/30	0.349943655	0.62372546	0	0
2/30	0.349943655	0.62372546	0	0
4/73	0.351112512	0.625092756	0	0
4/73	0.351112512	0.625092756	0	0
8/163	0.352929771	0.62760997	0	0
8/163	0.352929771	0.62760997	0	0
6/118	0.354903682	0.62803106	0	0
5/96	0.358316512	0.62803106	0	0
5/96	0.358316512	0.62803106	0	0
3/52	0.359749043	0.62803106	0	0
3/52	0.359749043	0.62803106	0	0
3/52	0.359749043	0.62803106	0	0
3/52	0.359749043	0.62803106	0	0
4/74	0.360509563	0.62803106	0	0
4/74	0.360509563	0.62803106	0	0
4/74	0.360509563	0.62803106	0	0
6/119	0.362255328	0.62803106	0	0
2/31	0.3649337	0.62803106	0	0
2/31	0.3649337	0.62803106	0	0
2/31	0.3649337	0.62803106	0	0
2/31	0.3649337	0.62803106	0	0
2/31	0.3649337	0.62803106	0	0
2/31	0.3649337	0.62803106	0	0
2/31	0.3649337	0.62803106	0	0
2/31	0.3649337	0.62803106	0	0
2/31	0.3649337	0.62803106	0	0
1/11	0.369109515	0.62803106	0	0
1/11	0.369109515	0.62803106	0	0
1/11	0.369109515	0.62803106	0	0
1/11	0.369109515	0.62803106	0	0
1/11	0.369109515	0.62803106	0	0

1/11	0.369109515	0.62803106	0	0
1/11	0.369109515	0.62803106	0	0
1/11	0.369109515	0.62803106	0	0
1/11	0.369109515	0.62803106	0	0
1/11	0.369109515	0.62803106	0	0
1/11	0.369109515	0.62803106	0	0
1/11	0.369109515	0.62803106	0	0
1/11	0.369109515	0.62803106	0	0
1/11	0.369109515	0.62803106	0	0
1/11	0.369109515	0.62803106	0	0
1/11	0.369109515	0.62803106	0	0
1/11	0.369109515	0.62803106	0	0
1/11	0.369109515	0.62803106	0	0
1/11	0.369109515	0.62803106	0	0
1/11	0.369109515	0.62803106	0	0
1/11	0.369109515	0.62803106	0	0
1/11	0.369109515	0.62803106	0	0
1/11	0.369109515	0.62803106	0	0
1/11	0.369109515	0.62803106	0	0
1/11	0.369109515	0.62803106	0	0
1/11	0.369109515	0.62803106	0	0
1/11	0.369109515	0.62803106	0	0
1/11	0.369109515	0.62803106	0	0
1/11	0.369109515	0.62803106	0	0
1/11	0.369109515	0.62803106	0	0
1/11	0.369109515	0.62803106	0	0
1/11	0.369109515	0.62803106	0	0
1/11	0.369109515	0.62803106	0	0
1/11	0.369109515	0.62803106	0	0
1/11	0.369109515	0.62803106	0	0
1/11	0.369109515	0.62803106	0	0
1/11	0.369109515	0.62803106	0	0
1/11	0.369109515	0.62803106	0	0
1/11	0.369109515	0.62803106	0	0
1/11	0.369109515	0.62803106	0	0
1/11	0.369109515	0.62803106	0	0
1/11	0.369109515	0.62803106	0	0
1/11	0.369109515	0.62803106	0	0
1/11	0.369109515	0.62803106	0	0
1/11	0.369109515	0.62803106	0	0
1/11	0.369109515	0.62803106	0	0
1/11	0.369109515	0.62803106	0	0
1/11	0.369109515	0.62803106	0	0
1/11	0.369109515	0.62803106	0	0
1/11	0.369109515	0.62803106	0	0
1/11	0.369109515	0.62803106	0	0
1/11	0.369109515	0.62803106	0	0
1/11	0.369109515	0.62803106	0	0

1/11	0.369109515	0.62803106	0	0
1/11	0.369109515	0.62803106	0	0
1/11	0.369109515	0.62803106	0	0
1/11	0.369109515	0.62803106	0	0
1/11	0.369109515	0.62803106	0	0
1/11	0.369109515	0.62803106	0	0
4/75	0.369902589	0.628210821	0	0
4/75	0.369902589	0.628210821	0	0
3/53	0.37103197	0.628210821	0	0
3/53	0.37103197	0.628210821	0	0
3/53	0.37103197	0.628210821	0	0
3/53	0.37103197	0.628210821	0	0
3/53	0.37103197	0.628210821	0	0
3/53	0.37103197	0.628210821	0	0
3/53	0.37103197	0.628210821	0	0
9/189	0.371504477	0.628668805	0	0
5/98	0.37471281	0.633753404	0	0
4/76	0.379286285	0.637556819	0	0
4/76	0.379286285	0.637556819	0	0
2/32	0.3797889	0.637556819	0	0
2/32	0.3797889	0.637556819	0	0
2/32	0.3797889	0.637556819	0	0
2/32	0.3797889	0.637556819	0	0
2/32	0.3797889	0.637556819	0	0
2/32	0.3797889	0.637556819	0	0
3/54	0.382277109	0.637556819	0	0
3/54	0.382277109	0.637556819	0	0
3/54	0.382277109	0.637556819	0	0
3/54	0.382277109	0.637556819	0	0
3/54	0.382277109	0.637556819	0	0
3/54	0.382277109	0.637556819	0	0
3/54	0.382277109	0.637556819	0	0
4/77	0.388655503	0.637556819	0	0
4/77	0.388655503	0.637556819	0	0
4/77	0.388655503	0.637556819	0	0
4/77	0.388655503	0.637556819	0	0
4/77	0.388655503	0.637556819	0	0
4/77	0.388655503	0.637556819	0	0
5/100	0.39110459	0.637556819	0	0
7/146	0.391342325	0.637556819	0	0
3/55	0.39347643	0.637556819	0	0
3/55	0.39347643	0.637556819	0	0
2/33	0.394495119	0.637556819	0	0
2/33	0.394495119	0.637556819	0	0
2/33	0.394495119	0.637556819	0	0
2/33	0.394495119	0.637556819	0	0
2/33	0.394495119	0.637556819	0	0
2/33	0.394495119	0.637556819	0	0

2/33	0.394495119	0.637556819	0	0
1/12	0.394990214	0.637556819	0	0
1/12	0.394990214	0.637556819	0	0
1/12	0.394990214	0.637556819	0	0
1/12	0.394990214	0.637556819	0	0
1/12	0.394990214	0.637556819	0	0
1/12	0.394990214	0.637556819	0	0
1/12	0.394990214	0.637556819	0	0
1/12	0.394990214	0.637556819	0	0
1/12	0.394990214	0.637556819	0	0
1/12	0.394990214	0.637556819	0	0
1/12	0.394990214	0.637556819	0	0
1/12	0.394990214	0.637556819	0	0
1/12	0.394990214	0.637556819	0	0
1/12	0.394990214	0.637556819	0	0
1/12	0.394990214	0.637556819	0	0
1/12	0.394990214	0.637556819	0	0
1/12	0.394990214	0.637556819	0	0
1/12	0.394990214	0.637556819	0	0
1/12	0.394990214	0.637556819	0	0
1/12	0.394990214	0.637556819	0	0
1/12	0.394990214	0.637556819	0	0
1/12	0.394990214	0.637556819	0	0
1/12	0.394990214	0.637556819	0	0
1/12	0.394990214	0.637556819	0	0
1/12	0.394990214	0.637556819	0	0
1/12	0.394990214	0.637556819	0	0
1/12	0.394990214	0.637556819	0	0
1/12	0.394990214	0.637556819	0	0
1/12	0.394990214	0.637556819	0	0
1/12	0.394990214	0.637556819	0	0
1/12	0.394990214	0.637556819	0	0
1/12	0.394990214	0.637556819	0	0
1/12	0.394990214	0.637556819	0	0
1/12	0.394990214	0.637556819	0	0
1/12	0.394990214	0.637556819	0	0
1/12	0.394990214	0.637556819	0	0
1/12	0.394990214	0.637556819	0	0
1/12	0.394990214	0.637556819	0	0
1/12	0.394990214	0.637556819	0	0
1/12	0.394990214	0.637556819	0	0
1/12	0.394990214	0.637556819	0	0
1/12	0.394990214	0.637556819	0	0
1/12	0.394990214	0.637556819	0	0
1/12	0.394990214	0.637556819	0	0
1/12	0.394990214	0.637556819	0	0
1/12	0.394990214	0.637556819	0	0
1/12	0.394990214	0.637556819	0	0

1/12	0.394990214	0.637556819	0	0
1/12	0.394990214	0.637556819	0	0
1/12	0.394990214	0.637556819	0	0
1/12	0.394990214	0.637556819	0	0
1/12	0.394990214	0.637556819	0	0
1/12	0.394990214	0.637556819	0	0
1/12	0.394990214	0.637556819	0	0
1/12	0.394990214	0.637556819	0	0
1/12	0.394990214	0.637556819	0	0
4/78	0.398005256	0.641425353	0	0
4/78	0.398005256	0.641425353	0	0
4/78	0.398005256	0.641425353	0	0
6/124	0.399083725	0.642830026	0	0
5/101	0.399289987	0.642830026	0	0
13/288	0.40140549	0.645901698	0	0
3/56	0.404622301	0.649398969	0	0
3/56	0.404622301	0.649398969	0	0
3/56	0.404622301	0.649398969	0	0
3/56	0.404622301	0.649398969	0	0
3/56	0.404622301	0.649398969	0	0
6/125	0.40644512	0.651115722	0	0
4/79	0.407330717	0.651115722	0	0
4/79	0.407330717	0.651115722	0	0
4/79	0.407330717	0.651115722	0	0
2/34	0.4090396	0.651115722	0	0
2/34	0.4090396	0.651115722	0	0
2/34	0.4090396	0.651115722	0	0
2/34	0.4090396	0.651115722	0	0
2/34	0.4090396	0.651115722	0	0
2/34	0.4090396	0.651115722	0	0
2/34	0.4090396	0.651115722	0	0
2/34	0.4090396	0.651115722	0	0
2/34	0.4090396	0.651115722	0	0
2/34	0.4090396	0.651115722	0	0
2/34	0.4090396	0.651115722	0	0
2/34	0.4090396	0.651115722	0	0
3/57	0.415707485	0.652572508	0	0
1/13	0.419810463	0.652572508	0	0
1/13	0.419810463	0.652572508	0	0
1/13	0.419810463	0.652572508	0	0
1/13	0.419810463	0.652572508	0	0
1/13	0.419810463	0.652572508	0	0
1/13	0.419810463	0.652572508	0	0
1/13	0.419810463	0.652572508	0	0
1/13	0.419810463	0.652572508	0	0
1/13	0.419810463	0.652572508	0	0
1/13	0.419810463	0.652572508	0	0
1/13	0.419810463	0.652572508	0	0

1/13	0.419810463	0.652572508	0	0
1/13	0.419810463	0.652572508	0	0
1/13	0.419810463	0.652572508	0	0
1/13	0.419810463	0.652572508	0	0
1/13	0.419810463	0.652572508	0	0
1/13	0.419810463	0.652572508	0	0
1/13	0.419810463	0.652572508	0	0
1/13	0.419810463	0.652572508	0	0
1/13	0.419810463	0.652572508	0	0
1/13	0.419810463	0.652572508	0	0
1/13	0.419810463	0.652572508	0	0
1/13	0.419810463	0.652572508	0	0
1/13	0.419810463	0.652572508	0	0
1/13	0.419810463	0.652572508	0	0
1/13	0.419810463	0.652572508	0	0
1/13	0.419810463	0.652572508	0	0
1/13	0.419810463	0.652572508	0	0
1/13	0.419810463	0.652572508	0	0
1/13	0.419810463	0.652572508	0	0
1/13	0.419810463	0.652572508	0	0
1/13	0.419810463	0.652572508	0	0
1/13	0.419810463	0.652572508	0	0
1/13	0.419810463	0.652572508	0	0
1/13	0.419810463	0.652572508	0	0
1/13	0.419810463	0.652572508	0	0
1/13	0.419810463	0.652572508	0	0
1/13	0.419810463	0.652572508	0	0
1/13	0.419810463	0.652572508	0	0
1/13	0.419810463	0.652572508	0	0
1/13	0.419810463	0.652572508	0	0
1/13	0.419810463	0.652572508	0	0
1/13	0.419810463	0.652572508	0	0
1/13	0.419810463	0.652572508	0	0
1/13	0.419810463	0.652572508	0	0
1/13	0.419810463	0.652572508	0	0
6/127	0.421143483	0.654317783	0	0
11/245	0.422690968	0.654572614	0	0
2/35	0.423410884	0.654572614	0	0
2/35	0.423410884	0.654572614	0	0
2/35	0.423410884	0.654572614	0	0
2/35	0.423410884	0.654572614	0	0
2/35	0.423410884	0.654572614	0	0
2/35	0.423410884	0.654572614	0	0
2/35	0.423410884	0.654572614	0	0
2/35	0.423410884	0.654572614	0	0
2/35	0.423410884	0.654572614	0	0
5/104	0.423763881	0.654793047	0	0
4/81	0.42589027	0.657425854	0	0

4/81	0.42589027	0.657425854	0	0
3/58	0.426725129	0.658388002	0	0
12/270	0.4292692	0.66198501	0	0
24/557	0.430792069	0.663076711	0	0
4/82	0.435115526	0.663076711	0	0
6/129	0.435793271	0.663076711	0	0
2/36	0.437598719	0.663076711	0	0
2/36	0.437598719	0.663076711	0	0
2/36	0.437598719	0.663076711	0	0
2/36	0.437598719	0.663076711	0	0
2/36	0.437598719	0.663076711	0	0
2/36	0.437598719	0.663076711	0	0
2/36	0.437598719	0.663076711	0	0
2/36	0.437598719	0.663076711	0	0
3/59	0.437668759	0.663076711	0	0
3/59	0.437668759	0.663076711	0	0
3/59	0.437668759	0.663076711	0	0
3/59	0.437668759	0.663076711	0	0
3/59	0.437668759	0.663076711	0	0
5/106	0.439979277	0.663076711	0	0
1/14	0.443613661	0.663076711	0	0
1/14	0.443613661	0.663076711	0	0
1/14	0.443613661	0.663076711	0	0
1/14	0.443613661	0.663076711	0	0
1/14	0.443613661	0.663076711	0	0
1/14	0.443613661	0.663076711	0	0
1/14	0.443613661	0.663076711	0	0
1/14	0.443613661	0.663076711	0	0
1/14	0.443613661	0.663076711	0	0
1/14	0.443613661	0.663076711	0	0
1/14	0.443613661	0.663076711	0	0
1/14	0.443613661	0.663076711	0	0
1/14	0.443613661	0.663076711	0	0
1/14	0.443613661	0.663076711	0	0
1/14	0.443613661	0.663076711	0	0
1/14	0.443613661	0.663076711	0	0
1/14	0.443613661	0.663076711	0	0
1/14	0.443613661	0.663076711	0	0
1/14	0.443613661	0.663076711	0	0
1/14	0.443613661	0.663076711	0	0
1/14	0.443613661	0.663076711	0	0
1/14	0.443613661	0.663076711	0	0
1/14	0.443613661	0.663076711	0	0
1/14	0.443613661	0.663076711	0	0
1/14	0.443613661	0.663076711	0	0
1/14	0.443613661	0.663076711	0	0
1/14	0.443613661	0.663076711	0	0
1/14	0.443613661	0.663076711	0	0

1/14	0.443613661	0.663076711	0	0
1/14	0.443613661	0.663076711	0	0
1/14	0.443613661	0.663076711	0	0
1/14	0.443613661	0.663076711	0	0
1/14	0.443613661	0.663076711	0	0
1/14	0.443613661	0.663076711	0	0
1/14	0.443613661	0.663076711	0	0
1/14	0.443613661	0.663076711	0	0
1/14	0.443613661	0.663076711	0	0
1/14	0.443613661	0.663076711	0	0
1/14	0.443613661	0.663076711	0	0
1/14	0.443613661	0.663076711	0	0
1/14	0.443613661	0.663076711	0	0
1/14	0.443613661	0.663076711	0	0
1/14	0.443613661	0.663076711	0	0
1/14	0.443613661	0.663076711	0	0
1/14	0.443613661	0.663076711	0	0
1/14	0.443613661	0.663076711	0	0
1/14	0.443613661	0.663076711	0	0
4/83	0.444298816	0.66346349	0	0
4/83	0.444298816	0.66346349	0	0
3/60	0.448532275	0.669463999	0	0
13/298	0.449612322	0.670754336	0	0
2/37	0.451593982	0.671778429	0	0
2/37	0.451593982	0.671778429	0	0
2/37	0.451593982	0.671778429	0	0
2/37	0.451593982	0.671778429	0	0
2/37	0.451593982	0.671778429	0	0
2/37	0.451593982	0.671778429	0	0
4/84	0.45343613	0.67326797	0	0
5/108	0.456086021	0.67326797	0	0
6/132	0.457636625	0.67326797	0	0
9/204	0.459403988	0.67326797	0	0
4/85	0.462523623	0.67326797	0	0
4/85	0.462523623	0.67326797	0	0
2/38	0.465388606	0.67326797	0	0
2/38	0.465388606	0.67326797	0	0
2/38	0.465388606	0.67326797	0	0
2/38	0.465388606	0.67326797	0	0
2/38	0.465388606	0.67326797	0	0
2/38	0.465388606	0.67326797	0	0
2/38	0.465388606	0.67326797	0	0
2/38	0.465388606	0.67326797	0	0
2/38	0.465388606	0.67326797	0	0
2/38	0.465388606	0.67326797	0	0
1/15	0.466441434	0.67326797	0	0
1/15	0.466441434	0.67326797	0	0
1/15	0.466441434	0.67326797	0	0

1/15	0.466441434	0.67326797	0	0
1/15	0.466441434	0.67326797	0	0
1/15	0.466441434	0.67326797	0	0
1/15	0.466441434	0.67326797	0	0
1/15	0.466441434	0.67326797	0	0
1/15	0.466441434	0.67326797	0	0
1/15	0.466441434	0.67326797	0	0
1/15	0.466441434	0.67326797	0	0
1/15	0.466441434	0.67326797	0	0
1/15	0.466441434	0.67326797	0	0
1/15	0.466441434	0.67326797	0	0
1/15	0.466441434	0.67326797	0	0
1/15	0.466441434	0.67326797	0	0
1/15	0.466441434	0.67326797	0	0
1/15	0.466441434	0.67326797	0	0
1/15	0.466441434	0.67326797	0	0
1/15	0.466441434	0.67326797	0	0
1/15	0.466441434	0.67326797	0	0
1/15	0.466441434	0.67326797	0	0
1/15	0.466441434	0.67326797	0	0
1/15	0.466441434	0.67326797	0	0
1/15	0.466441434	0.67326797	0	0
1/15	0.466441434	0.67326797	0	0
1/15	0.466441434	0.67326797	0	0
1/15	0.466441434	0.67326797	0	0
1/15	0.466441434	0.67326797	0	0
1/15	0.466441434	0.67326797	0	0
1/15	0.466441434	0.67326797	0	0
1/15	0.466441434	0.67326797	0	0
1/15	0.466441434	0.67326797	0	0
1/15	0.466441434	0.67326797	0	0
1/15	0.466441434	0.67326797	0	0
1/15	0.466441434	0.67326797	0	0
1/15	0.466441434	0.67326797	0	0
1/15	0.466441434	0.67326797	0	0
1/15	0.466441434	0.67326797	0	0
1/15	0.466441434	0.67326797	0	0
1/15	0.466441434	0.67326797	0	0
1/15	0.466441434	0.67326797	0	0
1/15	0.466441434	0.67326797	0	0
1/15	0.466441434	0.67326797	0	0
1/15	0.466441434	0.67326797	0	0
1/15	0.466441434	0.67326797	0	0
1/15	0.466441434	0.67326797	0	0
1/15	0.466441434	0.67326797	0	0
1/15	0.466441434	0.67326797	0	0
1/15	0.466441434	0.67326797	0	0
1/15	0.466441434	0.67326797	0	0
3/62	0.469996356	0.677456535	0	0
3/62	0.469996356	0.677456535	0	0
3/62	0.469996356	0.677456535	0	0

5/110	0.47206149	0.68011822	0	0
2/39	0.478975505	0.68523639	0	0
2/39	0.478975505	0.68523639	0	0
2/39	0.478975505	0.68523639	0	0
2/39	0.478975505	0.68523639	0	0
2/39	0.478975505	0.68523639	0	0
2/39	0.478975505	0.68523639	0	0
2/39	0.478975505	0.68523639	0	0
2/39	0.478975505	0.68523639	0	0
2/39	0.478975505	0.68523639	0	0
2/39	0.478975505	0.68523639	0	0
2/39	0.478975505	0.68523639	0	0
2/39	0.478975505	0.68523639	0	0
2/39	0.478975505	0.68523639	0	0
6/135	0.479270326	0.68523639	0	0
6/135	0.479270326	0.68523639	0	0
5/111	0.479993314	0.68523639	0	0
3/63	0.480586496	0.68523639	0	0
3/63	0.480586496	0.68523639	0	0
3/63	0.480586496	0.68523639	0	0
3/63	0.480586496	0.68523639	0	0
32/766	0.482530151	0.68523639	0	0
12/281	0.484370258	0.68523639	0	0
10/233	0.486947182	0.68523639	0	0
5/112	0.487884478	0.68523639	0	0
1/16	0.488333708	0.68523639	0	0
1/16	0.488333708	0.68523639	0	0
1/16	0.488333708	0.68523639	0	0
1/16	0.488333708	0.68523639	0	0
1/16	0.488333708	0.68523639	0	0
1/16	0.488333708	0.68523639	0	0
1/16	0.488333708	0.68523639	0	0
1/16	0.488333708	0.68523639	0	0
1/16	0.488333708	0.68523639	0	0
1/16	0.488333708	0.68523639	0	0
1/16	0.488333708	0.68523639	0	0
1/16	0.488333708	0.68523639	0	0
1/16	0.488333708	0.68523639	0	0
1/16	0.488333708	0.68523639	0	0
1/16	0.488333708	0.68523639	0	0
1/16	0.488333708	0.68523639	0	0
1/16	0.488333708	0.68523639	0	0
1/16	0.488333708	0.68523639	0	0
1/16	0.488333708	0.68523639	0	0
1/16	0.488333708	0.68523639	0	0
1/16	0.488333708	0.68523639	0	0
1/16	0.488333708	0.68523639	0	0
1/16	0.488333708	0.68523639	0	0

1/16	0.488333708	0.68523639	0	0
1/16	0.488333708	0.68523639	0	0
1/16	0.488333708	0.68523639	0	0
1/16	0.488333708	0.68523639	0	0
1/16	0.488333708	0.68523639	0	0
1/16	0.488333708	0.68523639	0	0
1/16	0.488333708	0.68523639	0	0
1/16	0.488333708	0.68523639	0	0
1/16	0.488333708	0.68523639	0	0
1/16	0.488333708	0.68523639	0	0
3/64	0.49107565	0.68523639	0	0
3/64	0.49107565	0.68523639	0	0
3/64	0.49107565	0.68523639	0	0
3/64	0.49107565	0.68523639	0	0
3/64	0.49107565	0.68523639	0	0
3/64	0.49107565	0.68523639	0	0
2/40	0.492348511	0.68523639	0	0
2/40	0.492348511	0.68523639	0	0
2/40	0.492348511	0.68523639	0	0
2/40	0.492348511	0.68523639	0	0
2/40	0.492348511	0.68523639	0	0
2/40	0.492348511	0.68523639	0	0
2/40	0.492348511	0.68523639	0	0
2/40	0.492348511	0.68523639	0	0
2/40	0.492348511	0.68523639	0	0
2/40	0.492348511	0.68523639	0	0
2/40	0.492348511	0.68523639	0	0
2/40	0.492348511	0.68523639	0	0
2/40	0.492348511	0.68523639	0	0
7/162	0.498183557	0.688545242	0	0
12/284	0.499240253	0.688545242	0	0
3/65	0.501459441	0.688545242	0	0
3/65	0.501459441	0.688545242	0	0
3/65	0.501459441	0.688545242	0	0
10/236	0.503250843	0.688545242	0	0
5/114	0.503535206	0.688545242	0	0
2/41	0.505502311	0.688545242	0	0
2/41	0.505502311	0.688545242	0	0
2/41	0.505502311	0.688545242	0	0
2/41	0.505502311	0.688545242	0	0
2/41	0.505502311	0.688545242	0	0
2/41	0.505502311	0.688545242	0	0
2/41	0.505502311	0.688545242	0	0
2/41	0.505502311	0.688545242	0	0
4/90	0.507090422	0.688545242	0	0
1/17	0.509328775	0.688545242	0	0
1/17	0.509328775	0.688545242	0	0
1/17	0.509328775	0.688545242	0	0

1/17	0.509328775	0.688545242	0	0
1/17	0.509328775	0.688545242	0	0
1/17	0.509328775	0.688545242	0	0
1/17	0.509328775	0.688545242	0	0
1/17	0.509328775	0.688545242	0	0
1/17	0.509328775	0.688545242	0	0
1/17	0.509328775	0.688545242	0	0
1/17	0.509328775	0.688545242	0	0
1/17	0.509328775	0.688545242	0	0
1/17	0.509328775	0.688545242	0	0
1/17	0.509328775	0.688545242	0	0
1/17	0.509328775	0.688545242	0	0
1/17	0.509328775	0.688545242	0	0
1/17	0.509328775	0.688545242	0	0
1/17	0.509328775	0.688545242	0	0
1/17	0.509328775	0.688545242	0	0
1/17	0.509328775	0.688545242	0	0
1/17	0.509328775	0.688545242	0	0
1/17	0.509328775	0.688545242	0	0
1/17	0.509328775	0.688545242	0	0
1/17	0.509328775	0.688545242	0	0
1/17	0.509328775	0.688545242	0	0
1/17	0.509328775	0.688545242	0	0
1/17	0.509328775	0.688545242	0	0
1/17	0.509328775	0.688545242	0	0
1/17	0.509328775	0.688545242	0	0
1/17	0.509328775	0.688545242	0	0
1/17	0.509328775	0.688545242	0	0
1/17	0.509328775	0.688545242	0	0
1/17	0.509328775	0.688545242	0	0
1/17	0.509328775	0.688545242	0	0
1/17	0.509328775	0.688545242	0	0
1/17	0.509328775	0.688545242	0	0
1/17	0.509328775	0.688545242	0	0
1/17	0.509328775	0.688545242	0	0
1/17	0.509328775	0.688545242	0	0
1/17	0.509328775	0.688545242	0	0
1/17	0.509328775	0.688545242	0	0
1/17	0.509328775	0.688545242	0	0
1/17	0.509328775	0.688545242	0	0
1/17	0.509328775	0.688545242	0	0
1/17	0.509328775	0.688545242	0	0
1/17	0.509328775	0.688545242	0	0
1/17	0.509328775	0.688545242	0	0
1/17	0.509328775	0.688545242	0	0
1/17	0.509328775	0.688545242	0	0
1/17	0.509328775	0.688545242	0	0
3/66	0.511733805	0.691496136	0	0

20/483	0.514521692	0.69496159	0	0
4/91	0.515807199	0.696395663	0	0
2/42	0.518432382	0.697519054	0	0
2/42	0.518432382	0.697519054	0	0
2/42	0.518432382	0.697519054	0	0
2/42	0.518432382	0.697519054	0	0
2/42	0.518432382	0.697519054	0	0
2/42	0.518432382	0.697519054	0	0
2/42	0.518432382	0.697519054	0	0
2/42	0.518432382	0.697519054	0	0
16/387	0.522648229	0.698306691	0	0
5/117	0.526648541	0.698306691	0	0
5/117	0.526648541	0.698306691	0	0
1/18	0.529463361	0.698306691	0	0
1/18	0.529463361	0.698306691	0	0
1/18	0.529463361	0.698306691	0	0
1/18	0.529463361	0.698306691	0	0
1/18	0.529463361	0.698306691	0	0
1/18	0.529463361	0.698306691	0	0
1/18	0.529463361	0.698306691	0	0
1/18	0.529463361	0.698306691	0	0
1/18	0.529463361	0.698306691	0	0
1/18	0.529463361	0.698306691	0	0
1/18	0.529463361	0.698306691	0	0
1/18	0.529463361	0.698306691	0	0
1/18	0.529463361	0.698306691	0	0
1/18	0.529463361	0.698306691	0	0
1/18	0.529463361	0.698306691	0	0
1/18	0.529463361	0.698306691	0	0
1/18	0.529463361	0.698306691	0	0
1/18	0.529463361	0.698306691	0	0
1/18	0.529463361	0.698306691	0	0
1/18	0.529463361	0.698306691	0	0
1/18	0.529463361	0.698306691	0	0
1/18	0.529463361	0.698306691	0	0
1/18	0.529463361	0.698306691	0	0
1/18	0.529463361	0.698306691	0	0
1/18	0.529463361	0.698306691	0	0
1/18	0.529463361	0.698306691	0	0
1/18	0.529463361	0.698306691	0	0
1/18	0.529463361	0.698306691	0	0
1/18	0.529463361	0.698306691	0	0
1/18	0.529463361	0.698306691	0	0
1/18	0.529463361	0.698306691	0	0
1/18	0.529463361	0.698306691	0	0
1/18	0.529463361	0.698306691	0	0
1/18	0.529463361	0.698306691	0	0
1/18	0.529463361	0.698306691	0	0
1/18	0.529463361	0.698306691	0	0
1/18	0.529463361	0.698306691	0	0

1/18	0.529463361	0.698306691	0	0
1/18	0.529463361	0.698306691	0	0
1/18	0.529463361	0.698306691	0	0
1/18	0.529463361	0.698306691	0	0
1/18	0.529463361	0.698306691	0	0
1/18	0.529463361	0.698306691	0	0
7/167	0.530476295	0.698306691	0	0
2/43	0.531134942	0.698306691	0	0
2/43	0.531134942	0.698306691	0	0
2/43	0.531134942	0.698306691	0	0
2/43	0.531134942	0.698306691	0	0
2/43	0.531134942	0.698306691	0	0
2/43	0.531134942	0.698306691	0	0
2/43	0.531134942	0.698306691	0	0
2/43	0.531134942	0.698306691	0	0
2/43	0.531134942	0.698306691	0	0
4/93	0.533021298	0.700490827	0	0
5/118	0.534247867	0.7018064	0	0
7/168	0.536839279	0.704529959	0	0
8/193	0.538140098	0.704529959	0	0
4/94	0.541513577	0.704529959	0	0
3/69	0.541864246	0.704529959	0	0
3/69	0.541864246	0.704529959	0	0
3/69	0.541864246	0.704529959	0	0
2/44	0.543606893	0.704529959	0	0
2/44	0.543606893	0.704529959	0	0
2/44	0.543606893	0.704529959	0	0
2/44	0.543606893	0.704529959	0	0
2/44	0.543606893	0.704529959	0	0
2/44	0.543606893	0.704529959	0	0
2/44	0.543606893	0.704529959	0	0
2/44	0.543606893	0.704529959	0	0
2/44	0.543606893	0.704529959	0	0
2/44	0.543606893	0.704529959	0	0
1/19	0.548772693	0.704529959	0	0
1/19	0.548772693	0.704529959	0	0
1/19	0.548772693	0.704529959	0	0
1/19	0.548772693	0.704529959	0	0
1/19	0.548772693	0.704529959	0	0
1/19	0.548772693	0.704529959	0	0
1/19	0.548772693	0.704529959	0	0
1/19	0.548772693	0.704529959	0	0
1/19	0.548772693	0.704529959	0	0
1/19	0.548772693	0.704529959	0	0
1/19	0.548772693	0.704529959	0	0
1/19	0.548772693	0.704529959	0	0
1/19	0.548772693	0.704529959	0	0
1/19	0.548772693	0.704529959	0	0

1/19	0.548772693	0.704529959	0	0
1/19	0.548772693	0.704529959	0	0
1/19	0.548772693	0.704529959	0	0
1/19	0.548772693	0.704529959	0	0
1/19	0.548772693	0.704529959	0	0
1/19	0.548772693	0.704529959	0	0
1/19	0.548772693	0.704529959	0	0
1/19	0.548772693	0.704529959	0	0
1/19	0.548772693	0.704529959	0	0
1/19	0.548772693	0.704529959	0	0
1/19	0.548772693	0.704529959	0	0
1/19	0.548772693	0.704529959	0	0
1/19	0.548772693	0.704529959	0	0
1/19	0.548772693	0.704529959	0	0
1/19	0.548772693	0.704529959	0	0
1/19	0.548772693	0.704529959	0	0
1/19	0.548772693	0.704529959	0	0
1/19	0.548772693	0.704529959	0	0
1/19	0.548772693	0.704529959	0	0
1/19	0.548772693	0.704529959	0	0
1/19	0.548772693	0.704529959	0	0
1/19	0.548772693	0.704529959	0	0
1/19	0.548772693	0.704529959	0	0
1/19	0.548772693	0.704529959	0	0
1/19	0.548772693	0.704529959	0	0
4/95	0.549926224	0.705428858	0	0
4/95	0.549926224	0.705428858	0	0
3/70	0.551666256	0.707369341	0	0
7/171	0.555714413	0.711711463	0	0
2/45	0.555845772	0.711711463	0	0
2/45	0.555845772	0.711711463	0	0
6/146	0.555967406	0.711711463	0	0
3/71	0.561342924	0.714576743	0	0
3/71	0.561342924	0.714576743	0	0
3/71	0.561342924	0.714576743	0	0
3/71	0.561342924	0.714576743	0	0
5/122	0.564069482	0.714576743	0	0
4/97	0.56650411	0.714576743	0	0
1/20	0.567290556	0.714576743	0	0
1/20	0.567290556	0.714576743	0	0
1/20	0.567290556	0.714576743	0	0
1/20	0.567290556	0.714576743	0	0
1/20	0.567290556	0.714576743	0	0
1/20	0.567290556	0.714576743	0	0
1/20	0.567290556	0.714576743	0	0
1/20	0.567290556	0.714576743	0	0
1/20	0.567290556	0.714576743	0	0
1/20	0.567290556	0.714576743	0	0

1/20	0.567290556	0.714576743	0	0
1/20	0.567290556	0.714576743	0	0
1/20	0.567290556	0.714576743	0	0
1/20	0.567290556	0.714576743	0	0
1/20	0.567290556	0.714576743	0	0
1/20	0.567290556	0.714576743	0	0
1/20	0.567290556	0.714576743	0	0
1/20	0.567290556	0.714576743	0	0
1/20	0.567290556	0.714576743	0	0
1/20	0.567290556	0.714576743	0	0
1/20	0.567290556	0.714576743	0	0
1/20	0.567290556	0.714576743	0	0
1/20	0.567290556	0.714576743	0	0
1/20	0.567290556	0.714576743	0	0
1/20	0.567290556	0.714576743	0	0
1/20	0.567290556	0.714576743	0	0
2/46	0.567849706	0.714576743	0	0
2/46	0.567849706	0.714576743	0	0
2/46	0.567849706	0.714576743	0	0
2/46	0.567849706	0.714576743	0	0
2/46	0.567849706	0.714576743	0	0
2/46	0.567849706	0.714576743	0	0
2/46	0.567849706	0.714576743	0	0
2/46	0.567849706	0.714576743	0	0
2/46	0.567849706	0.714576743	0	0
2/46	0.567849706	0.714576743	0	0
7/173	0.568108128	0.714612972	0	0
3/72	0.570891876	0.717244859	0	0
3/72	0.570891876	0.717244859	0	0
3/72	0.570891876	0.717244859	0	0
5/123	0.571372529	0.717559044	0	0
4/98	0.574665437	0.721112436	0	0
4/98	0.574665437	0.721112436	0	0
5/124	0.57861155	0.723638149	0	0
5/124	0.57861155	0.723638149	0	0
5/124	0.57861155	0.723638149	0	0
5/124	0.57861155	0.723638149	0	0
2/47	0.579617366	0.723638149	0	0
2/47	0.579617366	0.723638149	0	0
3/73	0.580310985	0.723638149	0	0
1/21	0.585049352	0.723638149	0	0
1/21	0.585049352	0.723638149	0	0
1/21	0.585049352	0.723638149	0	0
1/21	0.585049352	0.723638149	0	0
1/21	0.585049352	0.723638149	0	0
1/21	0.585049352	0.723638149	0	0
1/21	0.585049352	0.723638149	0	0
1/21	0.585049352	0.723638149	0	0

1/21	0.585049352	0.723638149	0	0
1/21	0.585049352	0.723638149	0	0
1/21	0.585049352	0.723638149	0	0
1/21	0.585049352	0.723638149	0	0
1/21	0.585049352	0.723638149	0	0
1/21	0.585049352	0.723638149	0	0
1/21	0.585049352	0.723638149	0	0
1/21	0.585049352	0.723638149	0	0
1/21	0.585049352	0.723638149	0	0
1/21	0.585049352	0.723638149	0	0
1/21	0.585049352	0.723638149	0	0
1/21	0.585049352	0.723638149	0	0
1/21	0.585049352	0.723638149	0	0
1/21	0.585049352	0.723638149	0	0
1/21	0.585049352	0.723638149	0	0
1/21	0.585049352	0.723638149	0	0
1/21	0.585049352	0.723638149	0	0
1/21	0.585049352	0.723638149	0	0
1/21	0.585049352	0.723638149	0	0
1/21	0.585049352	0.723638149	0	0
1/21	0.585049352	0.723638149	0	0
2/48	0.591147926	0.729152733	0	0
2/48	0.591147926	0.729152733	0	0
2/48	0.591147926	0.729152733	0	0
2/48	0.591147926	0.729152733	0	0
2/48	0.591147926	0.729152733	0	0
2/48	0.591147926	0.729152733	0	0
2/48	0.591147926	0.729152733	0	0
5/126	0.592892	0.731014226	0	0
9/228	0.593766945	0.731803063	0	0
6/152	0.595523138	0.73320159	0	0
4/101	0.59861826	0.73320159	0	0
3/75	0.598752347	0.73320159	0	0
5/127	0.59993087	0.73320159	0	0
1/22	0.60208016	0.73320159	0	0
1/22	0.60208016	0.73320159	0	0
1/22	0.60208016	0.73320159	0	0
1/22	0.60208016	0.73320159	0	0
1/22	0.60208016	0.73320159	0	0
1/22	0.60208016	0.73320159	0	0
1/22	0.60208016	0.73320159	0	0
1/22	0.60208016	0.73320159	0	0
1/22	0.60208016	0.73320159	0	0
1/22	0.60208016	0.73320159	0	0
1/22	0.60208016	0.73320159	0	0
1/22	0.60208016	0.73320159	0	0
1/22	0.60208016	0.73320159	0	0
1/22	0.60208016	0.73320159	0	0

1/22	0.60208016	0.73320159	0	0
1/22	0.60208016	0.73320159	0	0
1/22	0.60208016	0.73320159	0	0
1/22	0.60208016	0.73320159	0	0
1/22	0.60208016	0.73320159	0	0
1/22	0.60208016	0.73320159	0	0
1/22	0.60208016	0.73320159	0	0
1/22	0.60208016	0.73320159	0	0
1/22	0.60208016	0.73320159	0	0
1/22	0.60208016	0.73320159	0	0
2/49	0.602441024	0.73320159	0	0
2/49	0.602441024	0.73320159	0	0
2/49	0.602441024	0.73320159	0	0
2/49	0.602441024	0.73320159	0	0
4/102	0.606420475	0.737756262	0	0
3/76	0.607771494	0.738822222	0	0
3/76	0.607771494	0.738822222	0	0
6/154	0.608285994	0.739158927	0	0
2/50	0.613496726	0.744909017	0	0
2/50	0.613496726	0.744909017	0	0
5/129	0.613800074	0.744986674	0	0
3/77	0.616654568	0.74535267	0	0
3/77	0.616654568	0.74535267	0	0
8/207	0.617762787	0.74535267	0	0
1/23	0.618412788	0.74535267	0	0
1/23	0.618412788	0.74535267	0	0
1/23	0.618412788	0.74535267	0	0
1/23	0.618412788	0.74535267	0	0
1/23	0.618412788	0.74535267	0	0
1/23	0.618412788	0.74535267	0	0
1/23	0.618412788	0.74535267	0	0
1/23	0.618412788	0.74535267	0	0
1/23	0.618412788	0.74535267	0	0
1/23	0.618412788	0.74535267	0	0
1/23	0.618412788	0.74535267	0	0
1/23	0.618412788	0.74535267	0	0
1/23	0.618412788	0.74535267	0	0
1/23	0.618412788	0.74535267	0	0
1/23	0.618412788	0.74535267	0	0
4/104	0.621744081	0.749077654	0	0
2/51	0.624315491	0.751303097	0	0
2/51	0.624315491	0.751303097	0	0
2/51	0.624315491	0.751303097	0	0
3/78	0.625400534	0.752317921	0	0
4/105	0.629263299	0.756379832	0	0
4/105	0.629263299	0.756379832	0	0
3/79	0.634008546	0.756433006	0	0
1/24	0.634075824	0.756433006	0	0

1/24	0.634075824	0.756433006	0	0
1/24	0.634075824	0.756433006	0	0
1/24	0.634075824	0.756433006	0	0
1/24	0.634075824	0.756433006	0	0
1/24	0.634075824	0.756433006	0	0
1/24	0.634075824	0.756433006	0	0
1/24	0.634075824	0.756433006	0	0
1/24	0.634075824	0.756433006	0	0
1/24	0.634075824	0.756433006	0	0
1/24	0.634075824	0.756433006	0	0
1/24	0.634075824	0.756433006	0	0
1/24	0.634075824	0.756433006	0	0
1/24	0.634075824	0.756433006	0	0
1/24	0.634075824	0.756433006	0	0
1/24	0.634075824	0.756433006	0	0
2/52	0.63489814	0.756433006	0	0
2/52	0.63489814	0.756433006	0	0
2/52	0.63489814	0.756433006	0	0
2/52	0.63489814	0.756433006	0	0
2/52	0.63489814	0.756433006	0	0
2/52	0.63489814	0.756433006	0	0
2/53	0.645245824	0.765727403	0	0
2/53	0.645245824	0.765727403	0	0
2/53	0.645245824	0.765727403	0	0
2/53	0.645245824	0.765727403	0	0
1/25	0.649096687	0.765727403	0	0
1/25	0.649096687	0.765727403	0	0
1/25	0.649096687	0.765727403	0	0
1/25	0.649096687	0.765727403	0	0
1/25	0.649096687	0.765727403	0	0
1/25	0.649096687	0.765727403	0	0
1/25	0.649096687	0.765727403	0	0
1/25	0.649096687	0.765727403	0	0
1/25	0.649096687	0.765727403	0	0
1/25	0.649096687	0.765727403	0	0
1/25	0.649096687	0.765727403	0	0
1/25	0.649096687	0.765727403	0	0
1/25	0.649096687	0.765727403	0	0
1/25	0.649096687	0.765727403	0	0
1/25	0.649096687	0.765727403	0	0
1/25	0.649096687	0.765727403	0	0
1/25	0.649096687	0.765727403	0	0
1/25	0.649096687	0.765727403	0	0
1/25	0.649096687	0.765727403	0	0
1/25	0.649096687	0.765727403	0	0
1/25	0.649096687	0.765727403	0	0
1/25	0.649096687	0.765727403	0	0
76/1922	0.650555011	0.766802302	0	0

3/81	0.65080823	0.766802302	0	0
4/108	0.651239873	0.766802302	0	0
4/108	0.651239873	0.766802302	0	0
4/108	0.651239873	0.766802302	0	0
8/214	0.654719688	0.770608044	0	0
2/54	0.655359997	0.770778651	0	0
2/54	0.655359997	0.770778651	0	0
3/82	0.658999085	0.77476583	0	0
1/26	0.663501675	0.775081536	0	0
1/26	0.663501675	0.775081536	0	0
1/26	0.663501675	0.775081536	0	0
1/26	0.663501675	0.775081536	0	0
1/26	0.663501675	0.775081536	0	0
1/26	0.663501675	0.775081536	0	0
1/26	0.663501675	0.775081536	0	0
1/26	0.663501675	0.775081536	0	0
1/26	0.663501675	0.775081536	0	0
1/26	0.663501675	0.775081536	0	0
1/26	0.663501675	0.775081536	0	0
1/26	0.663501675	0.775081536	0	0
1/26	0.663501675	0.775081536	0	0
1/26	0.663501675	0.775081536	0	0
1/26	0.663501675	0.775081536	0	0
1/26	0.663501675	0.775081536	0	0
1/26	0.663501675	0.775081536	0	0
2/55	0.665242387	0.77682338	0	0
4/111	0.672330884	0.783571533	0	0
2/56	0.674894973	0.783571533	0	0
2/56	0.674894973	0.783571533	0	0
2/56	0.674894973	0.783571533	0	0
1/27	0.677316011	0.783571533	0	0
1/27	0.677316011	0.783571533	0	0
1/27	0.677316011	0.783571533	0	0
1/27	0.677316011	0.783571533	0	0
1/27	0.677316011	0.783571533	0	0
1/27	0.677316011	0.783571533	0	0
1/27	0.677316011	0.783571533	0	0
1/27	0.677316011	0.783571533	0	0
1/27	0.677316011	0.783571533	0	0
1/27	0.677316011	0.783571533	0	0
1/27	0.677316011	0.783571533	0	0
1/27	0.677316011	0.783571533	0	0
1/27	0.677316011	0.783571533	0	0
1/27	0.677316011	0.783571533	0	0
1/27	0.677316011	0.783571533	0	0
1/27	0.677316011	0.783571533	0	0
1/27	0.677316011	0.783571533	0	0
1/27	0.677316011	0.783571533	0	0

1/27	0.677316011	0.783571533	0	0
1/27	0.677316011	0.783571533	0	0
1/27	0.677316011	0.783571533	0	0
4/112	0.679162226	0.785123643	0	0
4/112	0.679162226	0.785123643	0	0
3/85	0.682734095	0.788959712	0	0
2/57	0.684319963	0.789619476	0	0
2/57	0.684319963	0.789619476	0	0
2/57	0.684319963	0.789619476	0	0
2/57	0.684319963	0.789619476	0	0
1/28	0.690563883	0.792124882	0	0
1/28	0.690563883	0.792124882	0	0
1/28	0.690563883	0.792124882	0	0
1/28	0.690563883	0.792124882	0	0
1/28	0.690563883	0.792124882	0	0
1/28	0.690563883	0.792124882	0	0
1/28	0.690563883	0.792124882	0	0
1/28	0.690563883	0.792124882	0	0
1/28	0.690563883	0.792124882	0	0
1/28	0.690563883	0.792124882	0	0
1/28	0.690563883	0.792124882	0	0
1/28	0.690563883	0.792124882	0	0
1/28	0.690563883	0.792124882	0	0
1/28	0.690563883	0.792124882	0	0
1/28	0.690563883	0.792124882	0	0
1/28	0.690563883	0.792124882	0	0
7/195	0.692317902	0.793468207	0	0
2/58	0.693519769	0.793468207	0	0
2/58	0.693519769	0.793468207	0	0
2/58	0.693519769	0.793468207	0	0
2/58	0.693519769	0.793468207	0	0
2/58	0.693519769	0.793468207	0	0
2/58	0.693519769	0.793468207	0	0
6/169	0.696458177	0.796537247	0	0
3/87	0.69786098	0.797555405	0	0
3/87	0.69786098	0.797555405	0	0
2/59	0.702496988	0.800208978	0	0
2/59	0.702496988	0.800208978	0	0
1/29	0.703268494	0.800208978	0	0
1/29	0.703268494	0.800208978	0	0
1/29	0.703268494	0.800208978	0	0
1/29	0.703268494	0.800208978	0	0
1/29	0.703268494	0.800208978	0	0
1/29	0.703268494	0.800208978	0	0
1/29	0.703268494	0.800208978	0	0
1/29	0.703268494	0.800208978	0	0
1/29	0.703268494	0.800208978	0	0
1/29	0.703268494	0.800208978	0	0

12/330	0.705206096	0.801839277	0	0
3/88	0.705216614	0.801839277	0	0
2/60	0.711254382	0.807524129	0	0
2/60	0.711254382	0.807524129	0	0
2/60	0.711254382	0.807524129	0	0
2/60	0.711254382	0.807524129	0	0
3/89	0.712434484	0.808274195	0	0
3/89	0.712434484	0.808274195	0	0
1/30	0.715452096	0.809043213	0	0
1/30	0.715452096	0.809043213	0	0
1/30	0.715452096	0.809043213	0	0
1/30	0.715452096	0.809043213	0	0
1/30	0.715452096	0.809043213	0	0
1/30	0.715452096	0.809043213	0	0
1/30	0.715452096	0.809043213	0	0
1/30	0.715452096	0.809043213	0	0
1/30	0.715452096	0.809043213	0	0
3/90	0.719515307	0.813047071	0	0
3/90	0.719515307	0.813047071	0	0
2/61	0.719794862	0.813067736	0	0
4/119	0.724178158	0.813681164	0	0
5/147	0.725151543	0.813681164	0	0
3/91	0.726459893	0.813681164	0	0
3/91	0.726459893	0.813681164	0	0
1/31	0.727136028	0.813681164	0	0
1/31	0.727136028	0.813681164	0	0
1/31	0.727136028	0.813681164	0	0
1/31	0.727136028	0.813681164	0	0
1/31	0.727136028	0.813681164	0	0
1/31	0.727136028	0.813681164	0	0
1/31	0.727136028	0.813681164	0	0
1/31	0.727136028	0.813681164	0	0
1/31	0.727136028	0.813681164	0	0
1/31	0.727136028	0.813681164	0	0
1/31	0.727136028	0.813681164	0	0
1/31	0.727136028	0.813681164	0	0
1/31	0.727136028	0.813681164	0	0
1/31	0.727136028	0.813681164	0	0
1/31	0.727136028	0.813681164	0	0
1/31	0.727136028	0.813681164	0	0
1/31	0.727136028	0.813681164	0	0
1/31	0.727136028	0.813681164	0	0
1/31	0.727136028	0.813681164	0	0
1/31	0.727136028	0.813681164	0	0
1/31	0.727136028	0.813681164	0	0
1/31	0.727136028	0.813681164	0	0
2/62	0.728121469	0.814307769	0	0
10/283	0.728219319	0.814307769	0	0

3/92	0.733269151	0.819365744	0	0
3/92	0.733269151	0.819365744	0	0
1/32	0.738340759	0.822375248	0	0
1/32	0.738340759	0.822375248	0	0
1/32	0.738340759	0.822375248	0	0
1/32	0.738340759	0.822375248	0	0
1/32	0.738340759	0.822375248	0	0
1/32	0.738340759	0.822375248	0	0
1/32	0.738340759	0.822375248	0	0
1/32	0.738340759	0.822375248	0	0
1/32	0.738340759	0.822375248	0	0
3/93	0.739944071	0.823866172	0	0
7/205	0.740420394	0.824101669	0	0
9/260	0.743510548	0.8272452	0	0
3/94	0.746485728	0.827825066	0	0
1/33	0.749085921	0.827825066	0	0
1/33	0.749085921	0.827825066	0	0
1/33	0.749085921	0.827825066	0	0
1/33	0.749085921	0.827825066	0	0
1/33	0.749085921	0.827825066	0	0
1/33	0.749085921	0.827825066	0	0
1/33	0.749085921	0.827825066	0	0
1/33	0.749085921	0.827825066	0	0
1/33	0.749085921	0.827825066	0	0
1/33	0.749085921	0.827825066	0	0
1/33	0.749085921	0.827825066	0	0
1/33	0.749085921	0.827825066	0	0
1/33	0.749085921	0.827825066	0	0
1/33	0.749085921	0.827825066	0	0
1/33	0.749085921	0.827825066	0	0
1/33	0.749085921	0.827825066	0	0
1/33	0.749085921	0.827825066	0	0
1/33	0.749085921	0.827825066	0	0
2/65	0.751850111	0.829785266	0	0
2/65	0.751850111	0.829785266	0	0
2/65	0.751850111	0.829785266	0	0
11/316	0.751926237	0.829785266	0	0
4/124	0.753344992	0.831056227	0	0
1/34	0.759390341	0.832413787	0	0
1/34	0.759390341	0.832413787	0	0
1/34	0.759390341	0.832413787	0	0
1/34	0.759390341	0.832413787	0	0
1/34	0.759390341	0.832413787	0	0
1/34	0.759390341	0.832413787	0	0
1/34	0.759390341	0.832413787	0	0
1/34	0.759390341	0.832413787	0	0
1/34	0.759390341	0.832413787	0	0
1/34	0.759390341	0.832413787	0	0

1/34	0.759390341	0.832413787	0	0
1/34	0.759390341	0.832413787	0	0
1/34	0.759390341	0.832413787	0	0
1/34	0.759390341	0.832413787	0	0
1/34	0.759390341	0.832413787	0	0
1/34	0.759390341	0.832413787	0	0
1/34	0.759390341	0.832413787	0	0
1/34	0.759390341	0.832413787	0	0
3/97	0.765322941	0.838226434	0	0
5/155	0.766542315	0.838226434	0	0
2/67	0.766660148	0.838226434	0	0
2/67	0.766660148	0.838226434	0	0
1/35	0.769272075	0.838226434	0	0
1/35	0.769272075	0.838226434	0	0
1/35	0.769272075	0.838226434	0	0
1/35	0.769272075	0.838226434	0	0
1/35	0.769272075	0.838226434	0	0
1/35	0.769272075	0.838226434	0	0
1/35	0.769272075	0.838226434	0	0
1/35	0.769272075	0.838226434	0	0
1/35	0.769272075	0.838226434	0	0
1/35	0.769272075	0.838226434	0	0
1/35	0.769272075	0.838226434	0	0
1/35	0.769272075	0.838226434	0	0
1/35	0.769272075	0.838226434	0	0
9/267	0.770601742	0.839381387	0	0
3/98	0.771343696	0.839895585	0	0
8/240	0.77223552	0.840572556	0	0
16/456	0.773099898	0.841069234	0	0
2/68	0.773772884	0.841069234	0	0
2/68	0.773772884	0.841069234	0	0
2/68	0.773772884	0.841069234	0	0
1/36	0.778748442	0.844412944	0	0
1/36	0.778748442	0.844412944	0	0
1/36	0.778748442	0.844412944	0	0
1/36	0.778748442	0.844412944	0	0
1/36	0.778748442	0.844412944	0	0
1/36	0.778748442	0.844412944	0	0
1/36	0.778748442	0.844412944	0	0
2/69	0.78069552	0.846229348	0	0
77/2028	0.782263695	0.84763392	0	0
3/100	0.783006025	0.848142969	0	0
4/130	0.785141283	0.849530766	0	0
2/70	0.787431664	0.849530766	0	0
2/70	0.787431664	0.849530766	0	0
2/70	0.787431664	0.849530766	0	0
6/188	0.78757879	0.849530766	0	0
1/37	0.787836051	0.849530766	0	0

1/37	0.787836051	0.849530766	0	0
1/37	0.787836051	0.849530766	0	0
1/37	0.787836051	0.849530766	0	0
1/37	0.787836051	0.849530766	0	0
1/37	0.787836051	0.849530766	0	0
1/37	0.787836051	0.849530766	0	0
1/37	0.787836051	0.849530766	0	0
4/131	0.790109999	0.851687674	0	0
2/71	0.793984951	0.855568271	0	0
1/38	0.796550832	0.856257751	0	0
1/38	0.796550832	0.856257751	0	0
1/38	0.796550832	0.856257751	0	0
1/38	0.796550832	0.856257751	0	0
1/38	0.796550832	0.856257751	0	0
1/38	0.796550832	0.856257751	0	0
1/38	0.796550832	0.856257751	0	0
3/103	0.799573943	0.859210673	0	0
2/72	0.800359028	0.859460764	0	0
2/72	0.800359028	0.859460764	0	0
3/104	0.804856009	0.861966238	0	0
1/39	0.804908062	0.861966238	0	0
1/39	0.804908062	0.861966238	0	0
1/39	0.804908062	0.861966238	0	0
1/39	0.804908062	0.861966238	0	0
1/39	0.804908062	0.861966238	0	0
1/39	0.804908062	0.861966238	0	0
1/39	0.804908062	0.861966238	0	0
2/73	0.806557554	0.863435538	0	0
6/193	0.807733752	0.86439733	0	0
3/105	0.810020493	0.866546501	0	0
1/40	0.812922395	0.867563269	0	0
1/40	0.812922395	0.867563269	0	0
1/40	0.812922395	0.867563269	0	0
1/40	0.812922395	0.867563269	0	0
1/40	0.812922395	0.867563269	0	0
1/40	0.812922395	0.867563269	0	0
1/40	0.812922395	0.867563269	0	0
2/75	0.818442583	0.872772292	0	0
1/41	0.820607882	0.872772292	0	0
1/41	0.820607882	0.872772292	0	0
1/41	0.820607882	0.872772292	0	0
1/41	0.820607882	0.872772292	0	0
1/41	0.820607882	0.872772292	0	0
1/41	0.820607882	0.872772292	0	0
1/41	0.820607882	0.872772292	0	0
1/41	0.820607882	0.872772292	0	0
1/41	0.820607882	0.872772292	0	0
1/42	0.827978001	0.877430453	0	0

1/42	0.827978001	0.877430453	0	0
1/42	0.827978001	0.877430453	0	0
1/42	0.827978001	0.877430453	0	0
1/42	0.827978001	0.877430453	0	0
1/42	0.827978001	0.877430453	0	0
1/42	0.827978001	0.877430453	0	0
1/42	0.827978001	0.877430453	0	0
1/42	0.827978001	0.877430453	0	0
1/42	0.827978001	0.877430453	0	0
3/109	0.829536199	0.877430453	0	0
2/77	0.829669224	0.877430453	0	0
2/77	0.829669224	0.877430453	0	0
2/77	0.829669224	0.877430453	0	0
2/77	0.829669224	0.877430453	0	0
2/77	0.829669224	0.877430453	0	0
45/1243	0.829780792	0.877430453	0	0
5/171	0.834854947	0.879114394	0	0
1/43	0.835045678	0.879114394	0	0
1/43	0.835045678	0.879114394	0	0
1/43	0.835045678	0.879114394	0	0
1/43	0.835045678	0.879114394	0	0
1/43	0.835045678	0.879114394	0	0
1/43	0.835045678	0.879114394	0	0
1/43	0.835045678	0.879114394	0	0
1/43	0.835045678	0.879114394	0	0
1/43	0.835045678	0.879114394	0	0
1/43	0.835045678	0.879114394	0	0
1/43	0.835045678	0.879114394	0	0
1/43	0.835045678	0.879114394	0	0
6/201	0.836837897	0.880703259	0	0
3/111	0.838632783	0.882293855	0	0
2/79	0.840266413	0.88371378	0	0
1/44	0.841823309	0.883857671	0	0
1/44	0.841823309	0.883857671	0	0
1/44	0.841823309	0.883857671	0	0
1/44	0.841823309	0.883857671	0	0
1/44	0.841823309	0.883857671	0	0
33/938	0.842784459	0.884568376	0	0
2/80	0.845337901	0.8863516	0	0
2/80	0.845337901	0.8863516	0	0
2/80	0.845337901	0.8863516	0	0
4/144	0.846633538	0.886792241	0	0
1/45	0.848322783	0.886792241	0	0
1/45	0.848322783	0.886792241	0	0
1/45	0.848322783	0.886792241	0	0
1/45	0.848322783	0.886792241	0	0
1/45	0.848322783	0.886792241	0	0
1/45	0.848322783	0.886792241	0	0

1/45	0.848322783	0.886792241	0	0
1/45	0.848322783	0.886792241	0	0
3/114	0.85149048	0.889804693	0	0
1/46	0.854555503	0.892108932	0	0
1/46	0.854555503	0.892108932	0	0
1/46	0.854555503	0.892108932	0	0
2/82	0.855044272	0.892319844	0	0
2/83	0.859686086	0.896543971	0	0
1/47	0.860532404	0.896543971	0	0
1/47	0.860532404	0.896543971	0	0
1/47	0.860532404	0.896543971	0	0
1/47	0.860532404	0.896543971	0	0
4/148	0.861215792	0.896955671	0	0
2/84	0.864191542	0.899350214	0	0
4/149	0.8646709	0.899350214	0	0
4/149	0.8646709	0.899350214	0	0
4/149	0.8646709	0.899350214	0	0
5/180	0.865502884	0.899804234	0	0
1/48	0.866263973	0.899804234	0	0
1/48	0.866263973	0.899804234	0	0
1/48	0.866263973	0.899804234	0	0
2/85	0.868564	0.900990389	0	0
2/85	0.868564	0.900990389	0	0
2/85	0.868564	0.900990389	0	0
2/85	0.868564	0.900990389	0	0
1/49	0.87176027	0.901900917	0	0
1/49	0.87176027	0.901900917	0	0
1/49	0.87176027	0.901900917	0	0
1/49	0.87176027	0.901900917	0	0
1/49	0.87176027	0.901900917	0	0
1/49	0.87176027	0.901900917	0	0
1/49	0.87176027	0.901900917	0	0
1/49	0.87176027	0.901900917	0	0
2/86	0.872806775	0.902683511	0	0
5/183	0.874594715	0.904232144	0	0
1/50	0.877030939	0.905848086	0	0
1/50	0.877030939	0.905848086	0	0
1/50	0.877030939	0.905848086	0	0
8/275	0.880558772	0.909190079	0	0
2/88	0.8809163	0.909257555	0	0
1/51	0.882085233	0.909257783	0	0
1/51	0.882085233	0.909257783	0	0
1/51	0.882085233	0.909257783	0	0
1/51	0.882085233	0.909257783	0	0
1/52	0.886932024	0.913648612	0	0
1/52	0.886932024	0.913648612	0	0
1/53	0.89157982	0.91631321	0	0
1/53	0.89157982	0.91631321	0	0

1/53	0.89157982	0.91631321	0	0
1/53	0.89157982	0.91631321	0	0
1/53	0.89157982	0.91631321	0	0
1/53	0.89157982	0.91631321	0	0
1/53	0.89157982	0.91631321	0	0
2/91	0.89218802	0.916635562	0	0
1/54	0.896036783	0.919678915	0	0
1/54	0.896036783	0.919678915	0	0
1/54	0.896036783	0.919678915	0	0
1/55	0.900310739	0.922848162	0	0
1/55	0.900310739	0.922848162	0	0
1/55	0.900310739	0.922848162	0	0
1/55	0.900310739	0.922848162	0	0
1/56	0.904409192	0.92674396	0	0
2/95	0.905678269	0.927738898	0	0
13/433	0.906061598	0.927826158	0	0
1/57	0.908339342	0.929241299	0	0
1/57	0.908339342	0.929241299	0	0
1/57	0.908339342	0.929241299	0	0
1/58	0.912108092	0.932790135	0	0
1/59	0.915722062	0.934949821	0	0
1/59	0.915722062	0.934949821	0	0
1/59	0.915722062	0.934949821	0	0
1/59	0.915722062	0.934949821	0	0
1/59	0.915722062	0.934949821	0	0
1/60	0.919187601	0.937872726	0	0
1/60	0.919187601	0.937872726	0	0
11/384	0.919914785	0.938307051	0	0
2/100	0.92032381	0.938416676	0	0
1/61	0.922510798	0.939722947	0	0
1/61	0.922510798	0.939722947	0	0
1/61	0.922510798	0.939722947	0	0
1/62	0.925697494	0.94202577	0	0
1/62	0.925697494	0.94202577	0	0
1/62	0.925697494	0.94202577	0	0
4/172	0.925982272	0.94202577	0	0
1/63	0.928753288	0.944227453	0	0
1/63	0.928753288	0.944227453	0	0
3/140	0.9301167	0.945304758	0	0
1/64	0.931683551	0.946588055	0	0
5/211	0.936929616	0.951327386	0	0
1/66	0.937187877	0.951327386	0	0
2/107	0.937265349	0.951327386	0	0
2/109	0.941440124	0.955253233	0	0
1/68	0.942249195	0.955762547	0	0
1/69	0.94462497	0.957548178	0	0
1/69	0.94462497	0.957548178	0	0
2/111	0.94535001	0.957971095	0	0

1/70	0.94690312	0.959231732	0	0
2/112	0.947210512	0.959231732	0	0
1/71	0.949087651	0.96082003	0	0
2/114	0.950751981	0.962191924	0	0
1/72	0.951182407	0.962314581	0	0
1/73	0.953191072	0.963720148	0	0
1/73	0.953191072	0.963720148	0	0
1/75	0.95696412	0.966592776	0	0
1/75	0.95696412	0.966592776	0	0
1/75	0.95696412	0.966592776	0	0
1/77	0.960433365	0.969782165	0	0
1/79	0.963623242	0.972687489	0	0
2/123	0.96406412	0.972816971	0	0
2/125	0.96651312	0.974972068	0	0
1/83	0.969252969	0.977419067	0	0
4/204	0.970001437	0.977856972	0	0
1/84	0.970518756	0.978061648	0	0
1/85	0.971732491	0.978967793	0	0
1/86	0.97289631	0.979656219	0	0
5/243	0.973045428	0.979656219	0	0
1/87	0.974012265	0.980138182	0	0
3/172	0.974154048	0.980138182	0	0
1/89	0.976108364	0.981469864	0	0
1/89	0.976108364	0.981469864	0	0
3/176	0.977270298	0.982320791	0	0
1/91	0.97803557	0.982578318	0	0
2/137	0.978157978	0.982578318	0	0
1/94	0.980639138	0.984752823	0	0
2/144	0.983024164	0.986829419	0	0
1/98	0.983637384	0.987126584	0	0
1/99	0.984311453	0.987484604	0	0
4/228	0.985343894	0.988201804	0	0
1/103	0.986741492	0.989284641	0	0
1/105	0.987811622	0.990038573	0	0
1/108	0.98925721	0.990893507	0	0
3/199	0.989301454	0.990893507	0	0
1/109	0.98969995	0.990973695	0	0
1/110	0.99012446	0.991079872	0	0
2/163	0.991510972	0.992148599	0	0
1/122	0.994041102	0.994360627	0	0
4/273	0.996431822	0.996431822	0	0

Odds.Ratio	Combined.Score
5.717206983	98.0102867
2.904238963	44.79388366
2.448242135	28.4971547
94.01470588	1053.676797
3.86605455	40.17721032
19.60531697	197.1108556
31.33496732	292.2891416
2.75882837	25.70724975
2.027660739	18.70035238
2.191148023	20.03139391
14.70245399	133.7644179
1.836522256	16.53759959
2.253845172	20.01356291
3.321472108	29.43266808
3.172360248	26.68371073
2.249305549	18.7909044
6.59704797	54.45313687
2.466638294	20.30673627
2.047013977	16.84904537
4.826364757	39.58188502
2.506908762	20.37037293
18.79901961	152.5165201
2.202387327	17.73140925
3.821566204	30.25322134
5.239189929	41.42278726
1.928777341	15.02798318
9.799591002	75.11085952
15.66503268	119.6045985
35.21052632	258.6452873
6.724815725	49.25813197
3.259303517	23.54536863
4.004908917	28.0673969
2.854624797	19.99625587
5.152060271	35.98202957
23.47246022	156.8786481
2.309388905	15.26714119
2.524709784	16.68972437
10.44172113	67.96030143
10.44172113	67.96030143
10.44172113	67.96030143
6.915553952	44.16780871
6.915553952	44.16780871
4.578652453	29.24079219
3.054533556	19.1420056
4.454672385	27.83490473
5.228774229	32.49412065
6.531015678	40.33266454

17.60342717	108.3460074
2.48060635	15.20460874
2.395868757	14.67175415
3.144855967	18.88089162
6.186955118	36.97231674
6.186955118	36.97231674
6.186955118	36.97231674
8.542335116	50.63369243
8.542335116	50.63369243
8.542335116	50.63369243
2.750696914	16.2843642
2.506504248	14.780679
1.899748111	11.17913205
2.247278015	13.05310376
2.307486955	13.34379744
2.234675522	12.86256623
7.830065359	44.4149885
2.984997656	16.90768839
2.2656776	12.73218384
4.553142585	25.5516002
2.760776372	15.3477863
4.410626536	24.14659724
4.410626536	24.14659724
7.227375566	39.29318246
7.227375566	39.29318246
7.227375566	39.29318246
1.82542143	9.863124721
3.073661073	16.57358997
4.276747822	22.84718254
11.73439412	62.67338385
11.73439412	62.67338385
11.73439412	62.67338385
11.73439412	62.67338385
3.305332296	17.55590094
2.806584362	14.7931011
6.710784314	35.01663609
6.710784314	35.01663609
1.902255639	9.920498213
2.296181417	11.91333249
2.148801447	11.08108431
2.148801447	11.08108431
2.94512947	15.14454896
4.896728016	25.05821597
4.896728016	25.05821597
4.896728016	25.05821597
1.721302149	8.794466171
4.031941032	20.52477469
1.735092439	8.811471445

3.505037816	17.77211128
10.05752754	50.43894747
10.05752754	50.43894747
10.05752754	50.43894747
2.678451178	13.33489421
2.518355395	12.44115944
2.399144152	11.83199698
1.533643138	7.551351019
2.826880395	13.86831591
2.789538581	13.47323199
4.51958471	21.81345958
4.51958471	21.81345958
5.871323529	28.31979112
8.799877601	41.59916376
8.799877601	41.59916376
8.799877601	41.59916376
8.799877601	41.59916376
8.799877601	41.59916376
4.351965462	20.41124972
4.351965462	20.41124972
3.229457589	15.11858478
3.617652618	16.7649832
2.506698188	11.46464463
4.196319018	19.13256869
4.196319018	19.13256869
4.196319018	19.13256869
2.649506782	12.02690913
3.527027027	15.97328016
2.035339473	9.129794208
5.218409586	23.35864109
5.218409586	23.35864109
5.218409586	23.35864109
5.218409586	23.35864109
7.821705426	34.96416278
7.821705426	34.96416278
7.821705426	34.96416278
7.821705426	34.96416278
2.159746584	9.645985052
4.051406812	17.96318404
3.440822197	15.23108216
1.92166338	8.490424663
2.584613997	11.37614184
2.428789614	10.64981919
2.769052449	12.14096309
1.798193916	7.797458824
2.993961758	12.96175269
2.403880071	10.39388463
4.943498452	21.34331443

4.943498452	21.34331443
4.943498452	21.34331443
4.943498452	21.34331443
3.916155419	16.89089162
3.916155419	16.89089162
2.728778468	11.76165634
2.37947375	10.14531613
7.039167687	29.83408174
3.280441118	13.87954021
2.493000653	10.47927036
1.955904053	8.174622379
15.62917685	65.16761884
15.62917685	65.16761884
15.62917685	65.16761884
15.62917685	65.16761884
15.62917685	65.16761884
15.62917685	65.16761884
15.62917685	65.16761884
15.62917685	65.16761884
15.62917685	65.16761884
15.62917685	65.16761884
15.62917685	65.16761884
4.696078431	19.570366
4.696078431	19.570366
4.696078431	19.570366
4.696078431	19.570366
2.651633942	11.04666982
2.88860836	12.03234378
3.205718115	13.26332801
2.614668856	10.70957253
3.67101227	14.99692045
1.923710505	7.799608719
2.286587558	9.219599798
2.286587558	9.219599798
6.398909536	25.77212409
6.398909536	25.77212409
6.398909536	25.77212409
6.398909536	25.77212409
4.472222222	18.00173904
4.472222222	18.00173904
2.790397565	11.18679032
2.790397565	11.18679032
3.559583566	14.1582108
2.264482431	9.004129943
2.743747437	10.79242463
2.743747437	10.79242463
1.842009465	7.189573179
2.509688013	9.772232319

2.509688013	9.772232319
4.268716578	16.60670944
4.268716578	16.60670944
3.454709491	13.38212433
1.919556812	7.390652314
1.570470734	6.046349816
5.865361077	22.49256317
5.865361077	22.49256317
5.865361077	22.49256317
5.865361077	22.49256317
5.865361077	22.49256317
5.865361077	22.49256317
5.865361077	22.49256317
11.72127139	44.44121786
11.72127139	44.44121786
11.72127139	44.44121786
11.72127139	44.44121786
11.72127139	44.44121786
11.72127139	44.44121786
11.72127139	44.44121786
11.72127139	44.44121786
11.72127139	44.44121786
11.72127139	44.44121786
11.72127139	44.44121786
11.72127139	44.44121786
11.72127139	44.44121786
11.72127139	44.44121786
11.72127139	44.44121786
1.608068672	6.093206854
2.937960688	11.12982458
2.937960688	11.12982458
2.654961711	10.05519572
2.01980198	7.64238223
3.355828221	12.66259318
3.355828221	12.66259318
3.355828221	12.66259318
2.44424541	9.203332633
4.082907076	15.3602277
4.082907076	15.3602277
4.082907076	15.3602277
4.082907076	15.3602277
4.082907076	15.3602277
4.082907076	15.3602277
4.082907076	15.3602277
4.082907076	15.3602277
4.082907076	15.3602277
2.005371814	7.497077218
2.277594368	8.476749448

2.612682793	9.710426994
2.612682793	9.710426994
3.262440354	11.99427692
2.056173603	7.54482969
5.413896997	19.80121578
5.413896997	19.80121578
5.413896997	19.80121578
5.413896997	19.80121578
5.413896997	19.80121578
5.413896997	19.80121578
1.780531306	6.493200981
3.912581699	14.24168734
3.912581699	14.24168734
1.713337624	6.230634536
2.82014742	10.23013132
1.854267154	6.695057949
2.101851852	7.478466442
2.20607275	7.846682241
1.551610294	5.499566581
3.755882353	13.23399781
3.755882353	13.23399781
3.755882353	13.23399781
3.755882353	13.23399781
3.755882353	13.23399781
1.879421625	6.6202238
2.493533117	8.761401057
2.083142139	7.309891383
5.026927785	17.56186302
5.026927785	17.56186302
5.026927785	17.56186302
5.026927785	17.56186302
9.376528117	32.65173743
9.376528117	32.65173743
9.376528117	32.65173743
9.376528117	32.65173743
9.376528117	32.65173743
9.376528117	32.65173743
9.376528117	32.65173743
9.376528117	32.65173743
9.376528117	32.65173743
9.376528117	32.65173743
9.376528117	32.65173743
9.376528117	32.65173743
9.376528117	32.65173743
2.294605311	7.949053666
2.160824379	7.457492292
2.160824379	7.457492292
2.456187696	8.471001992

2.456187696	8.471001992
1.672334911	5.762420925
3.611236802	12.32287798
3.611236802	12.32287798
3.611236802	12.32287798
3.611236802	12.32287798
3.011011483	10.25206593
3.011011483	10.25206593
1.962093119	6.664823461
2.239737274	7.506036541
4.691554468	15.67635803
4.691554468	15.67635803
4.691554468	15.67635803
4.691554468	15.67635803
4.691554468	15.67635803
4.691554468	15.67635803
4.691554468	15.67635803
4.691554468	15.67635803
4.691554468	15.67635803
4.691554468	15.67635803
3.477305737	11.49631179
2.56310029	8.356978612
2.56310029	8.356978612
2.56310029	8.356978612
2.56310029	8.356978612
2.075650976	6.745344123
7.813365933	25.17277695
7.813365933	25.17277695
7.813365933	25.17277695
7.813365933	25.17277695
7.813365933	25.17277695
7.813365933	25.17277695
7.813365933	25.17277695
7.813365933	25.17277695
7.813365933	25.17277695
7.813365933	25.17277695
7.813365933	25.17277695
7.813365933	25.17277695
7.813365933	25.17277695
7.813365933	25.17277695
7.813365933	25.17277695
7.813365933	25.17277695
7.813365933	25.17277695
7.813365933	25.17277695
7.813365933	25.17277695
7.813365933	25.17277695
7.813365933	25.17277695
1.676506357	5.383651278

3.352941176	10.74412439
3.352941176	10.74412439
2.317325849	7.422018515
4.398102815	14.07234885
4.398102815	14.07234885
4.398102815	14.07234885
4.398102815	14.07234885
4.398102815	14.07234885
4.398102815	14.07234885
4.398102815	14.07234885
4.398102815	14.07234885
4.398102815	14.07234885
4.398102815	14.07234885
4.398102815	14.07234885
4.398102815	14.07234885
2.517199017	8.036188954
2.162165223	6.89582916
1.96090535	6.245537289
2.795501022	8.829605546
2.795501022	8.829605546
1.433448408	4.520972275
1.876193547	5.899462157
2.137483206	6.705731002
2.472908315	7.730781485
3.237153482	10.05764906
3.237153482	10.05764906
2.730346697	8.413310005
4.139174887	12.69543255
4.139174887	12.69543255
4.139174887	12.69543255
4.139174887	12.69543255
4.139174887	12.69543255
2.430144878	7.439823611
2.430144878	7.439823611
1.699905349	5.165212682
2.223031149	6.738505897
3.129084967	9.429463117
3.129084967	9.429463117
3.129084967	9.429463117
2.668153932	8.022160989
2.668153932	8.022160989
1.729492646	5.194658913
2.38883105	7.162452463
6.696821516	20.07372442
6.696821516	20.07372442
6.696821516	20.07372442
6.696821516	20.07372442
6.696821516	20.07372442

6.696821516	20.07372442
6.696821516	20.07372442
6.696821516	20.07372442
6.696821516	20.07372442
6.696821516	20.07372442
6.696821516	20.07372442
6.696821516	20.07372442
6.696821516	20.07372442
6.696821516	20.07372442
6.696821516	20.07372442
6.696821516	20.07372442
6.696821516	20.07372442
6.696821516	20.07372442
6.696821516	20.07372442
6.696821516	20.07372442
6.696821516	20.07372442
6.696821516	20.07372442
6.696821516	20.07372442
3.909016728	11.50398812
3.909016728	11.50398812
3.909016728	11.50398812
2.044120797	6.004941183
2.348894349	6.897868686
2.60872529	7.654219227
2.164303748	6.325109321
1.852143482	5.354743431
1.923887457	5.545671605
2.551880501	7.307729991
2.551880501	7.307729991
2.933210784	8.323263357
2.933210784	8.323263357
2.933210784	8.323263357
2.933210784	8.323263357
1.613290224	4.568590904
3.703085744	10.46568361
3.703085744	10.46568361
3.703085744	10.46568361
3.703085744	10.46568361
3.703085744	10.46568361
3.703085744	10.46568361
3.703085744	10.46568361
3.703085744	10.46568361
2.108588009	5.941925393
5.859413203	16.41404966
5.859413203	16.41404966
5.859413203	16.41404966
5.859413203	16.41404966
5.859413203	16.41404966

5.859413203	16.41404966
5.859413203	16.41404966
5.859413203	16.41404966
5.859413203	16.41404966
5.859413203	16.41404966
5.859413203	16.41404966
5.859413203	16.41404966
5.859413203	16.41404966
5.859413203	16.41404966
5.859413203	16.41404966
5.859413203	16.41404966
5.859413203	16.41404966
5.859413203	16.41404966
5.859413203	16.41404966
5.859413203	16.41404966
5.859413203	16.41404966
5.859413203	16.41404966
5.859413203	16.41404966
2.49745464	6.981102194
2.844177065	7.834920923
2.844177065	7.834920923
2.844177065	7.834920923
2.844177065	7.834920923
2.844177065	7.834920923
2.844177065	7.834920923
2.844177065	7.834920923
2.445296524	6.672890234
2.445296524	6.672890234
3.517747858	9.555059912
3.517747858	9.555059912
3.517747858	9.555059912
3.517747858	9.555059912
3.517747858	9.555059912
3.517747858	9.555059912
3.517747858	9.555059912
2.201627764	5.95334621
2.201627764	5.95334621
2.760380623	7.383960151
2.760380623	7.383960151
2.030173265	5.417957263
2.030173265	5.417957263
2.395267309	6.381778163
2.395267309	6.381778163
2.395267309	6.381778163
2.167643168	5.742571577
5.208095626	13.68425862
5.208095626	13.68425862
5.208095626	13.68425862

5.208095626	13.68425862
5.208095626	13.68425862
5.208095626	13.68425862
5.208095626	13.68425862
5.208095626	13.68425862
5.208095626	13.68425862
5.208095626	13.68425862
5.208095626	13.68425862
5.208095626	13.68425862
5.208095626	13.68425862
5.208095626	13.68425862
5.208095626	13.68425862
5.208095626	13.68425862
5.208095626	13.68425862
5.208095626	13.68425862
5.208095626	13.68425862
5.208095626	13.68425862
5.208095626	13.68425862
5.208095626	13.68425862
5.208095626	13.68425862
5.208095626	13.68425862
5.208095626	13.68425862
2.005310053	5.255660086
3.3500612	8.751824993
3.3500612	8.751824993
3.3500612	8.751824993
3.3500612	8.751824993
3.3500612	8.751824993
3.3500612	8.751824993
3.3500612	8.751824993
3.3500612	8.751824993
2.347239264	6.106565849
2.681372549	6.966711597
2.681372549	6.966711597
2.681372549	6.966711597
2.681372549	6.966711597
2.681372549	6.966711597
2.681372549	6.966711597
1.808119131	4.693758991
2.134688407	5.540845982
2.102717371	5.347686174
2.301094671	5.846156845
2.606753813	6.579949295
2.606753813	6.579949295
2.606753813	6.579949295
2.606753813	6.579949295
1.716049383	4.319461687
3.197618783	8.03962756

3.197618783	8.03962756
3.197618783	8.03962756
3.197618783	8.03962756
3.197618783	8.03962756
3.197618783	8.03962756
3.197618783	8.03962756
3.197618783	8.03962756
3.197618783	8.03962756
3.197618783	8.03962756
2.07168666	5.162640565
1.658140788	4.131563855
4.687041565	11.58603936
4.687041565	11.58603936
4.687041565	11.58603936
4.687041565	11.58603936
4.687041565	11.58603936
4.687041565	11.58603936
4.687041565	11.58603936
4.687041565	11.58603936
4.687041565	11.58603936
4.687041565	11.58603936
4.687041565	11.58603936
4.687041565	11.58603936
4.687041565	11.58603936
4.687041565	11.58603936
4.687041565	11.58603936
4.687041565	11.58603936
4.687041565	11.58603936
4.687041565	11.58603936
4.687041565	11.58603936
4.687041565	11.58603936
4.687041565	11.58603936
4.687041565	11.58603936
4.687041565	11.58603936
4.687041565	11.58603936
4.687041565	11.58603936
4.687041565	11.58603936
4.687041565	11.58603936
4.687041565	11.58603936
1.576527549	3.89322003
1.546721187	3.817298622
1.380316215	3.394086077
1.824764455	4.478655422
2.536168521	6.220827248
2.536168521	6.220827248
1.911639349	4.661361549
2.214029402	5.365818717
3.058432228	7.405160873

3.058432228	7.405160873
3.058432228	7.405160873
3.058432228	7.405160873
1.679012346	4.05375799
1.889567516	4.525364779
1.350647477	3.232871133
2.469298246	5.886826379
2.469298246	5.886826379
2.469298246	5.886826379
2.172915247	5.144125357
2.172915247	5.144125357
2.172915247	5.144125357
1.867997316	4.393982176
1.983839153	4.652097548
2.930844553	6.837498084
2.930844553	6.837498084
4.260724605	9.934000601
4.260724605	9.934000601
4.260724605	9.934000601
4.260724605	9.934000601
4.260724605	9.934000601
4.260724605	9.934000601
4.260724605	9.934000601
4.260724605	9.934000601
4.260724605	9.934000601
4.260724605	9.934000601
4.260724605	9.934000601
4.260724605	9.934000601
4.260724605	9.934000601
4.260724605	9.934000601
4.260724605	9.934000601
4.260724605	9.934000601
4.260724605	9.934000601
4.260724605	9.934000601
4.260724605	9.934000601
2.405857215	5.575710006
2.133296152	4.933691198
1.691689273	3.895906711
1.586348366	3.608167045
2.095092025	4.733801267
2.095092025	4.733801267
2.345588235	5.285486307
2.345588235	5.285486307
2.345588235	5.285486307
1.92928545	4.345242538
1.92928545	4.345242538
2.813463892	6.327592634
2.813463892	6.327592634

2.813463892	6.327592634
2.813463892	6.327592634
2.813463892	6.327592634
2.813463892	6.327592634
2.813463892	6.327592634
2.813463892	6.327592634
2.813463892	6.327592634
1.664873734	3.720911822
1.806131138	4.025666077
1.723776382	3.836774263
2.058228393	4.543796286
2.058228393	4.543796286
2.058228393	4.543796286
1.903114417	4.200886571
3.905460473	8.607353292
3.905460473	8.607353292
3.905460473	8.607353292
3.905460473	8.607353292
3.905460473	8.607353292
3.905460473	8.607353292
3.905460473	8.607353292
3.905460473	8.607353292
3.905460473	8.607353292
3.905460473	8.607353292
3.905460473	8.607353292
3.905460473	8.607353292
3.905460473	8.607353292
3.905460473	8.607353292
3.905460473	8.607353292
3.905460473	8.607353292
3.905460473	8.607353292
3.905460473	8.607353292
3.905460473	8.607353292
3.905460473	8.607353292
3.905460473	8.607353292
3.905460473	8.607353292
1.65178021	3.636690894
2.288259206	5.014376525
1.708016122	3.740266202
2.705112513	5.867898103
2.705112513	5.867898103
2.705112513	5.867898103
2.705112513	5.867898103
2.705112513	5.867898103
2.705112513	5.867898103
2.705112513	5.867898103
1.877641278	4.062188024
1.548026321	3.34139958

1.767104445	3.799952209
2.233660131	4.760787939
2.233660131	4.760787939
1.988249974	4.191052087
2.604787162	5.452075693
2.604787162	5.452075693
3.60485236	7.524316326
3.60485236	7.524316326
3.60485236	7.524316326
3.60485236	7.524316326
3.60485236	7.524316326
3.60485236	7.524316326
3.60485236	7.524316326
3.60485236	7.524316326
3.60485236	7.524316326
3.60485236	7.524316326
3.60485236	7.524316326
3.60485236	7.524316326
3.60485236	7.524316326
3.60485236	7.524316326
3.60485236	7.524316326
3.60485236	7.524316326
3.60485236	7.524316326
1.404321675	2.923007937
1.82867992	3.800694685
1.729720981	3.588669661
1.729720981	3.588669661
2.181600547	4.52329078
2.181600547	4.52329078
2.181600547	4.52329078
2.181600547	4.52329078
2.181600547	4.52329078
2.181600547	4.52329078
1.955010225	4.027229016
1.955010225	4.027229016
1.64774004	3.380586001
1.64774004	3.380586001
1.366181321	2.794295326
1.589266013	3.245879746
1.545484081	3.156019733
1.805140805	3.677404121
1.374198132	2.776884114
2.511627907	5.074766806
2.511627907	5.074766806
2.511627907	5.074766806
2.511627907	5.074766806
2.511627907	5.074766806
2.511627907	5.074766806

2.511627907	5.074766806
2.511627907	5.074766806
2.131907308	4.300598453
1.922860304	3.871115373
1.441303687	2.881938991
1.782197618	3.558777326
1.782197618	3.558777326
1.782197618	3.558777326
1.36467343	2.702801024
3.347188264	6.62772507
3.347188264	6.62772507
3.347188264	6.62772507
3.347188264	6.62772507
3.347188264	6.62772507
3.347188264	6.62772507
3.347188264	6.62772507
3.347188264	6.62772507
3.347188264	6.62772507
3.347188264	6.62772507
3.347188264	6.62772507
3.347188264	6.62772507
3.347188264	6.62772507
3.347188264	6.62772507
3.347188264	6.62772507
3.347188264	6.62772507
1.891747477	3.72226304
2.084422658	4.091550532
2.084422658	4.091550532
1.75982801	3.444600294
1.75982801	3.444600294
1.605237674	3.136140237
2.424893428	4.731414555
2.424893428	4.731414555
2.424893428	4.731414555
2.424893428	4.731414555
2.424893428	4.731414555
2.424893428	4.731414555
1.659485383	3.20510136
1.659485383	3.20510136
1.420967695	2.74180438
1.591550472	3.059070467
2.039002558	3.895098076
2.039002558	3.895098076
2.039002558	3.895098076
2.039002558	3.895098076
2.343941248	4.418122431
2.343941248	4.418122431
2.343941248	4.418122431

2.343941248	4.418122431
2.343941248	4.418122431
2.343941248	4.418122431
3.123879381	5.876531928
3.123879381	5.876531928
3.123879381	5.876531928
3.123879381	5.876531928
3.123879381	5.876531928
3.123879381	5.876531928
3.123879381	5.876531928
3.123879381	5.876531928
3.123879381	5.876531928
3.123879381	5.876531928
3.123879381	5.876531928
3.123879381	5.876531928
3.123879381	5.876531928
3.123879381	5.876531928
3.123879381	5.876531928
3.123879381	5.876531928
3.123879381	5.876531928
3.123879381	5.876531928
3.123879381	5.876531928
3.123879381	5.876531928
3.123879381	5.876531928
1.716725595	3.228798362
1.83243865	3.444706215
1.83243865	3.444706215
1.40119141	2.608619541
1.995515227	3.710290923
1.995515227	3.710290923
1.695953347	3.126802011
1.804152902	3.315254081
1.804152902	3.315254081
1.804152902	3.315254081
1.804152902	3.315254081
1.610423751	2.947832827
1.551846273	2.840133828
2.26821179	4.131541481
2.26821179	4.131541481
2.26821179	4.131541481
1.953839869	3.536266658
1.953839869	3.536266658
1.953839869	3.536266658
1.953839869	3.536266658
1.594704976	2.867308963
1.776724298	3.191563363
1.776724298	3.191563363
1.776724298	3.191563363

1.776724298	3.191563363
1.427237774	2.560513896
2.928484108	5.240575908
2.928484108	5.240575908
2.928484108	5.240575908
2.928484108	5.240575908
2.928484108	5.240575908
2.928484108	5.240575908
2.928484108	5.240575908
2.928484108	5.240575908
2.928484108	5.240575908
2.928484108	5.240575908
2.928484108	5.240575908
2.928484108	5.240575908
2.928484108	5.240575908
2.928484108	5.240575908
2.928484108	5.240575908
2.928484108	5.240575908
2.928484108	5.240575908
2.928484108	5.240575908
2.928484108	5.240575908
2.928484108	5.240575908
1.294993577	2.304685551
1.655875126	2.933763991
1.913865546	3.372240993
2.197215422	3.868779556
2.197215422	3.868779556
2.197215422	3.868779556
2.197215422	3.868779556
2.197215422	3.868779556
1.750114458	3.073320649
1.310148419	2.297244043
1.724287261	2.960233267
1.875490196	3.217499376
1.875490196	3.217499376
1.875490196	3.217499376
1.875490196	3.217499376
2.756076514	4.697249268
2.756076514	4.697249268
2.756076514	4.697249268
2.756076514	4.697249268
2.756076514	4.697249268
2.756076514	4.697249268
2.756076514	4.697249268
2.756076514	4.697249268
2.756076514	4.697249268
2.756076514	4.697249268
2.756076514	4.697249268

2.756076514	4.697249268
2.756076514	4.697249268
2.756076514	4.697249268
2.756076514	4.697249268
2.756076514	4.697249268
1.617639583	2.754296541
1.617639583	2.754296541
2.130521865	3.62732779
2.130521865	3.62732779
2.130521865	3.62732779
2.130521865	3.62732779
2.130521865	3.62732779
2.130521865	3.62732779
2.130521865	3.62732779
2.130521865	3.62732779
1.4467679	2.427739614
1.599173554	2.66929344
5.853479853	9.755597092
5.853479853	9.755597092
5.853479853	9.755597092
5.853479853	9.755597092
5.853479853	9.755597092
5.853479853	9.755597092
5.853479853	9.755597092
5.853479853	9.755597092
5.853479853	9.755597092
5.853479853	9.755597092
5.853479853	9.755597092
5.853479853	9.755597092
5.853479853	9.755597092
5.853479853	9.755597092
5.853479853	9.755597092
5.853479853	9.755597092
5.853479853	9.755597092
5.853479853	9.755597092
5.853479853	9.755597092
5.853479853	9.755597092
5.853479853	9.755597092
5.853479853	9.755597092
5.853479853	9.755597092
5.853479853	9.755597092
5.853479853	9.755597092
5.853479853	9.755597092
5.853479853	9.755597092
5.853479853	9.755597092
5.853479853	9.755597092
5.853479853	9.755597092
5.853479853	9.755597092

5.853479853	9.755597092
5.853479853	9.755597092
5.853479853	9.755597092
5.853479853	9.755597092
5.853479853	9.755597092
5.853479853	9.755597092
5.853479853	9.755597092
5.853479853	9.755597092
5.853479853	9.755597092
5.853479853	9.755597092
5.853479853	9.755597092
5.853479853	9.755597092
5.853479853	9.755597092
5.853479853	9.755597092
5.853479853	9.755597092
5.853479853	9.755597092
5.853479853	9.755597092
5.853479853	9.755597092
5.853479853	9.755597092
5.853479853	9.755597092
5.853479853	9.755597092
5.853479853	9.755597092
5.853479853	9.755597092
5.853479853	9.755597092
5.853479853	9.755597092
5.853479853	9.755597092
5.853479853	9.755597092
5.853479853	9.755597092
5.853479853	9.755597092
5.853479853	9.755597092
5.853479853	9.755597092
5.853479853	9.755597092
5.853479853	9.755597092
5.853479853	9.755597092
5.853479853	9.755597092
5.853479853	9.755597092
5.853479853	9.755597092
5.853479853	9.755597092
5.853479853	9.755597092
5.853479853	9.755597092
5.853479853	9.755597092
5.853479853	9.755597092
5.853479853	9.755597092
5.853479853	9.755597092
5.853479853	9.755597092
5.853479853	9.755597092
5.853479853	9.755597092
5.853479853	9.755597092

5.853479853	9.755597092
5.853479853	9.755597092
5.853479853	9.755597092
5.853479853	9.755597092
5.853479853	9.755597092
5.853479853	9.755597092
5.853479853	9.755597092
5.853479853	9.755597092
5.853479853	9.755597092
5.853479853	9.755597092
5.853479853	9.755597092
2.067751458	3.405000653
1.674846626	2.748447888
1.674846626	2.748447888
1.803167421	2.933315518
1.803167421	2.933315518
1.803167421	2.933315518
2.602825319	4.22930816
2.602825319	4.22930816
2.602825319	4.22930816
2.602825319	4.22930816
2.602825319	4.22930816
2.602825319	4.22930816
2.602825319	4.22930816
2.602825319	4.22930816
2.602825319	4.22930816
2.602825319	4.22930816
2.602825319	4.22930816
2.602825319	4.22930816
2.602825319	4.22930816
2.602825319	4.22930816
2.602825319	4.22930816
2.602825319	4.22930816
2.602825319	4.22930816
2.602825319	4.22930816
2.602825319	4.22930816
2.602825319	4.22930816
2.602825319	4.22930816
1.388779312	2.230128806
2.008567931	3.199886745
2.008567931	3.199886745
2.008567931	3.199886745
2.008567931	3.199886745
2.008567931	3.199886745
2.008567931	3.199886745
1.321429913	2.103086533
1.140334302	1.809987104

1.492675836	2.367785801
1.492675836	2.367785801
1.769052904	2.802731004
1.769052904	2.802731004
1.769052904	2.802731004
1.769052904	2.802731004
1.407891492	2.222890926
1.546210546	2.431658136
1.479150737	2.304655281
1.479150737	2.304655281
1.309948891	2.034574585
2.465705829	3.823396372
2.465705829	3.823396372
2.465705829	3.823396372
2.465705829	3.823396372
2.465705829	3.823396372
2.465705829	3.823396372
2.465705829	3.823396372
2.465705829	3.823396372
2.465705829	3.823396372
2.465705829	3.823396372
2.465705829	3.823396372
1.165518064	1.801436678
1.736201888	2.679135291
1.736201888	2.679135291
1.736201888	2.679135291
1.736201888	2.679135291
1.736201888	2.679135291
1.952672379	3.010308198
1.952672379	3.010308198
1.952672379	3.010308198
1.952672379	3.010308198
1.952672379	3.010308198
1.952672379	3.010308198
1.952672379	3.010308198
1.240047163	1.908263096
1.60576519	2.463141247
1.60576519	2.463141247
1.465867159	2.243372725
1.27626249	1.95319406
1.38006818	2.081305505
4.682539683	7.04450332
4.682539683	7.04450332
4.682539683	7.04450332
4.682539683	7.04450332
4.682539683	7.04450332
4.682539683	7.04450332
4.682539683	7.04450332

4.682539683	7.04450332
4.682539683	7.04450332
4.682539683	7.04450332
4.682539683	7.04450332
4.682539683	7.04450332
4.682539683	7.04450332
4.682539683	7.04450332
4.682539683	7.04450332
4.682539683	7.04450332
4.682539683	7.04450332
4.682539683	7.04450332
4.682539683	7.04450332
4.682539683	7.04450332
4.682539683	7.04450332
4.682539683	7.04450332
4.682539683	7.04450332
4.682539683	7.04450332
4.682539683	7.04450332
4.682539683	7.04450332
4.682539683	7.04450332
4.682539683	7.04450332
4.682539683	7.04450332
4.682539683	7.04450332
4.682539683	7.04450332
4.682539683	7.04450332
4.682539683	7.04450332
4.682539683	7.04450332
4.682539683	7.04450332
4.682539683	7.04450332
4.682539683	7.04450332
4.682539683	7.04450332
4.682539683	7.04450332
4.682539683	7.04450332
4.682539683	7.04450332
4.682539683	7.04450332
4.682539683	7.04450332
4.682539683	7.04450332
4.682539683	7.04450332
4.682539683	7.04450332
4.682539683	7.04450332
4.682539683	7.04450332
4.682539683	7.04450332
4.682539683	7.04450332
4.682539683	7.04450332
4.682539683	7.04450332
4.682539683	7.04450332
4.682539683	7.04450332
4.682539683	7.04450332

4.682539683	7.04450332
4.682539683	7.04450332
4.682539683	7.04450332
4.682539683	7.04450332
4.682539683	7.04450332
4.682539683	7.04450332
4.682539683	7.04450332
4.682539683	7.04450332
4.682539683	7.04450332
4.682539683	7.04450332
4.682539683	7.04450332
4.682539683	7.04450332
4.682539683	7.04450332
4.682539683	7.04450332
4.682539683	7.04450332
4.682539683	7.04450332
4.682539683	7.04450332
4.682539683	7.04450332
4.682539683	7.04450332
4.682539683	7.04450332
4.682539683	7.04450332
4.682539683	7.04450332
4.682539683	7.04450332
4.682539683	7.04450332
4.682539683	7.04450332
4.682539683	7.04450332
4.682539683	7.04450332
4.682539683	7.04450332
4.682539683	7.04450332
4.682539683	7.04450332
4.682539683	7.04450332
4.682539683	7.04450332
4.682539683	7.04450332
4.682539683	7.04450332
4.682539683	7.04450332
1.704545455	2.56206835
1.704545455	2.56206835
1.704545455	2.56206835
1.704545455	2.56206835
1.704545455	2.56206835
1.704545455	2.56206835
1.583982756	2.375813328
1.583982756	2.375813328
1.348517099	2.015891992
1.899798207	2.834786969
1.899798207	2.834786969
1.899798207	2.834786969
1.899798207	2.834786969

2.342298289	3.469026666
2.342298289	3.469026666
2.342298289	3.469026666
2.342298289	3.469026666
2.342298289	3.469026666
2.342298289	3.469026666
2.342298289	3.469026666
2.342298289	3.469026666
2.342298289	3.469026666
2.342298289	3.469026666
2.342298289	3.469026666
2.342298289	3.469026666
1.562781186	2.292052761
1.562781186	2.292052761
1.562781186	2.292052761
1.562781186	2.292052761
1.674019608	2.451106993
1.480796586	2.15110817
1.849706887	2.67201673
1.849706887	2.67201673
1.849706887	2.67201673
1.542137552	2.211684857
1.542137552	2.211684857
1.542137552	2.211684857
1.379600116	1.973515808
1.248711511	1.785667648
1.285840228	1.832394735
1.415023116	2.015504724
1.46529484	2.086775662
1.46529484	2.086775662
1.235663786	1.752679693
2.230643847	3.157863322
2.230643847	3.157863322
2.230643847	3.157863322
2.230643847	3.157863322
2.230643847	3.157863322
2.230643847	3.157863322
2.230643847	3.157863322
2.230643847	3.157863322
2.230643847	3.157863322
2.230643847	3.157863322
2.230643847	3.157863322
2.230643847	3.157863322
2.230643847	3.157863322
2.230643847	3.157863322
2.230643847	3.157863322
2.230643847	3.157863322
2.230643847	3.157863322

2.230643847	3.157863322
2.230643847	3.157863322
1.369458128	1.926882334
1.279927993	1.800494774
1.522030117	2.134545243
1.522030117	2.134545243
1.402855311	1.9625699
1.802184352	2.520839299
1.802184352	2.520839299
1.802184352	2.520839299
1.802184352	2.520839299
1.802184352	2.520839299
1.359463126	1.881420217
1.502438257	2.060479149
3.901912902	5.346529585
3.901912902	5.346529585
3.901912902	5.346529585
3.901912902	5.346529585
3.901912902	5.346529585
3.901912902	5.346529585
3.901912902	5.346529585
3.901912902	5.346529585
3.901912902	5.346529585
3.901912902	5.346529585
3.901912902	5.346529585
3.901912902	5.346529585
3.901912902	5.346529585
3.901912902	5.346529585
3.901912902	5.346529585
3.901912902	5.346529585
3.901912902	5.346529585
3.901912902	5.346529585
3.901912902	5.346529585
3.901912902	5.346529585
3.901912902	5.346529585
3.901912902	5.346529585
3.901912902	5.346529585
3.901912902	5.346529585
3.901912902	5.346529585
3.901912902	5.346529585
3.901912902	5.346529585
3.901912902	5.346529585
3.901912902	5.346529585
3.901912902	5.346529585
3.901912902	5.346529585
3.901912902	5.346529585
3.901912902	5.346529585
3.901912902	5.346529585

3.901912902	5.346529585
3.901912902	5.346529585
3.901912902	5.346529585
3.901912902	5.346529585
3.901912902	5.346529585
3.901912902	5.346529585
3.901912902	5.346529585
3.901912902	5.346529585
3.901912902	5.346529585
3.901912902	5.346529585
3.901912902	5.346529585
3.901912902	5.346529585
3.901912902	5.346529585
3.901912902	5.346529585
3.901912902	5.346529585
3.901912902	5.346529585
3.901912902	5.346529585
3.901912902	5.346529585
3.901912902	5.346529585
3.901912902	5.346529585
3.901912902	5.346529585
3.901912902	5.346529585
3.901912902	5.346529585
3.901912902	5.346529585
3.901912902	5.346529585
3.901912902	5.346529585
3.901912902	5.346529585
3.901912902	5.346529585
3.901912902	5.346529585
3.901912902	5.346529585
3.901912902	5.346529585
3.901912902	5.346529585
3.901912902	5.346529585
3.901912902	5.346529585
3.901912902	5.346529585
3.901912902	5.346529585
3.901912902	5.346529585
3.901912902	5.346529585
3.901912902	5.346529585
3.901912902	5.346529585
3.901912902	5.346529585
3.901912902	5.346529585
3.901912902	5.346529585
3.901912902	5.346529585
3.901912902	5.346529585
3.901912902	5.346529585
3.901912902	5.346529585
3.901912902	5.346529585

3.901912902	5.346529585
3.901912902	5.346529585
3.901912902	5.346529585
3.901912902	5.346529585
3.901912902	5.346529585
3.901912902	5.346529585
3.901912902	5.346529585
3.901912902	5.346529585
3.901912902	5.346529585
3.901912902	5.346529585
3.901912902	5.346529585
3.901912902	5.346529585
3.901912902	5.346529585
3.901912902	5.346529585
3.901912902	5.346529585
3.901912902	5.346529585
3.901912902	5.346529585
3.901912902	5.346529585
3.901912902	5.346529585
1.349611936	1.837096265
1.250167591	1.695594266
1.757037944	2.380224784
1.757037944	2.380224784
1.757037944	2.380224784
1.757037944	2.380224784
1.757037944	2.380224784
1.757037944	2.380224784
2.129139809	2.883207522
2.129139809	2.883207522
2.129139809	2.883207522
2.129139809	2.883207522
2.129139809	2.883207522
2.129139809	2.883207522
2.129139809	2.883207522
2.129139809	2.883207522
2.129139809	2.883207522
2.129139809	2.883207522
2.129139809	2.883207522
2.129139809	2.883207522
2.129139809	2.883207522
2.129139809	2.883207522
2.129139809	2.883207522
2.129139809	2.883207522
1.588650715	2.15110634
1.483342393	1.989340738
1.367568676	1.812614212
1.714093799	2.249254795
1.714093799	2.249254795

1.464723926	1.920992493
1.464723926	1.920992493
1.464723926	1.920992493
1.464723926	1.920992493
2.036462209	2.639622104
2.036462209	2.639622104
2.036462209	2.639622104
2.036462209	2.639622104
2.036462209	2.639622104
2.036462209	2.639622104
2.036462209	2.639622104
2.036462209	2.639622104
2.036462209	2.639622104
2.036462209	2.639622104
2.036462209	2.639622104
2.036462209	2.639622104
2.036462209	2.639622104
2.036462209	2.639622104
2.036462209	2.639622104
2.036462209	2.639622104
2.036462209	2.639622104
2.036462209	2.639622104
2.036462209	2.639622104
2.036462209	2.639622104
2.036462209	2.639622104
2.036462209	2.639622104
2.036462209	2.639622104
2.036462209	2.639622104
2.036462209	2.639622104
1.320890862	1.710637807
1.536403086	1.975241115
1.536403086	1.975241115
1.673194614	2.127108181
1.673194614	2.127108181
1.673194614	2.127108181
1.146570362	1.441999804
3.344322344	4.202212397
3.344322344	4.202212397
3.344322344	4.202212397
3.344322344	4.202212397
3.344322344	4.202212397
3.344322344	4.202212397
3.344322344	4.202212397
3.344322344	4.202212397
3.344322344	4.202212397
3.344322344	4.202212397
3.344322344	4.202212397
3.344322344	4.202212397

3.344322344	4.202212397
3.344322344	4.202212397
3.344322344	4.202212397
3.344322344	4.202212397
3.344322344	4.202212397
3.344322344	4.202212397
3.344322344	4.202212397
3.344322344	4.202212397
3.344322344	4.202212397
3.344322344	4.202212397
3.344322344	4.202212397
3.344322344	4.202212397
3.344322344	4.202212397
3.344322344	4.202212397
3.344322344	4.202212397
3.344322344	4.202212397
3.344322344	4.202212397
3.344322344	4.202212397
3.344322344	4.202212397
3.344322344	4.202212397
3.344322344	4.202212397
3.344322344	4.202212397
3.344322344	4.202212397
3.344322344	4.202212397
3.344322344	4.202212397
3.344322344	4.202212397
3.344322344	4.202212397
3.344322344	4.202212397
3.344322344	4.202212397
3.344322344	4.202212397
3.344322344	4.202212397
3.344322344	4.202212397
3.344322344	4.202212397
3.344322344	4.202212397
3.344322344	4.202212397
3.344322344	4.202212397
3.344322344	4.202212397
3.344322344	4.202212397
3.344322344	4.202212397
3.344322344	4.202212397
3.344322344	4.202212397
3.344322344	4.202212397
3.344322344	4.202212397
3.344322344	4.202212397
3.344322344	4.202212397
3.344322344	4.202212397
3.344322344	4.202212397
3.344322344	4.202212397

3.344322344	4.202212397
3.344322344	4.202212397
3.344322344	4.202212397
3.344322344	4.202212397
3.344322344	4.202212397
3.344322344	4.202212397
3.344322344	4.202212397
3.344322344	4.202212397
3.344322344	4.202212397
3.344322344	4.202212397
3.344322344	4.202212397
3.344322344	4.202212397
1.511543327	1.893693284
1.951507742	2.422653858
1.951507742	2.422653858
1.951507742	2.422653858
1.951507742	2.422653858
1.951507742	2.422653858
1.951507742	2.422653858
1.951507742	2.422653858
1.951507742	2.422653858
1.951507742	2.422653858
1.951507742	2.422653858
1.951507742	2.422653858
1.951507742	2.422653858
1.951507742	2.422653858
1.951507742	2.422653858
1.951507742	2.422653858
1.951507742	2.422653858
1.951507742	2.422653858
1.951507742	2.422653858
1.951507742	2.422653858
1.951507742	2.422653858
1.951507742	2.422653858
1.365210754	1.691978071
1.365210754	1.691978071
1.365210754	1.691978071
1.271121477	1.568559091
1.634197717	2.01304886
1.634197717	2.01304886
1.634197717	2.01304886
1.634197717	2.01304886
1.634197717	2.01304886
1.634197717	2.01304886
1.634197717	2.01304886
1.634197717	2.01304886
1.209968333	1.484713953
1.411560352	1.731426963

1.487472767	1.816069342
1.487472767	1.816069342
1.312521525	1.589449639
1.312521525	1.589449639
1.394683027	1.673011922
1.596973406	1.906415406
1.596973406	1.906415406
1.339066339	1.594874029
1.339066339	1.594874029
1.339066339	1.594874029
1.464154412	1.7421419
1.873349633	2.228625228
1.873349633	2.228625228
1.873349633	2.228625228
1.873349633	2.228625228
1.873349633	2.228625228
1.873349633	2.228625228
1.873349633	2.228625228
1.873349633	2.228625228
1.873349633	2.228625228
1.873349633	2.228625228
1.873349633	2.228625228
1.873349633	2.228625228
1.302036354	1.548458497
1.195543893	1.412450568
1.143334621	1.343539811
1.3263641	1.548621781
1.441553544	1.67169946
2.926129426	3.389668669
2.926129426	3.389668669
2.926129426	3.389668669
2.926129426	3.389668669
2.926129426	3.389668669
2.926129426	3.389668669
2.926129426	3.389668669
2.926129426	3.389668669
2.926129426	3.389668669
2.926129426	3.389668669
2.926129426	3.389668669
2.926129426	3.389668669
2.926129426	3.389668669
2.926129426	3.389668669
2.926129426	3.389668669
2.926129426	3.389668669
2.926129426	3.389668669
2.926129426	3.389668669
2.926129426	3.389668669
2.926129426	3.389668669

2.926129426	3.389668669
2.926129426	3.389668669
2.926129426	3.389668669
2.926129426	3.389668669
2.926129426	3.389668669
2.926129426	3.389668669
2.926129426	3.389668669
2.926129426	3.389668669
2.926129426	3.389668669
2.926129426	3.389668669
2.926129426	3.389668669
2.926129426	3.389668669
2.926129426	3.389668669
2.926129426	3.389668669
2.926129426	3.389668669
2.926129426	3.389668669
2.926129426	3.389668669
2.926129426	3.389668669
2.926129426	3.389668669
2.926129426	3.389668669
2.926129426	3.389668669
2.926129426	3.389668669
2.926129426	3.389668669
2.926129426	3.389668669
2.926129426	3.389668669
2.926129426	3.389668669
2.926129426	3.389668669
2.926129426	3.389668669
2.926129426	3.389668669
2.926129426	3.389668669
2.926129426	3.389668669
2.926129426	3.389668669
2.926129426	3.389668669
2.926129426	3.389668669
2.926129426	3.389668669
2.926129426	3.389668669
2.926129426	3.389668669
2.926129426	3.389668669
2.926129426	3.389668669
2.926129426	3.389668669
2.926129426	3.389668669
2.926129426	3.389668669
2.926129426	3.389668669
2.926129426	3.389668669
2.926129426	3.389668669
2.926129426	3.389668669
2.926129426	3.389668669
2.926129426	3.389668669

2.926129426	3.389668669
2.926129426	3.389668669
2.926129426	3.389668669
2.926129426	3.389668669
2.926129426	3.389668669
2.926129426	3.389668669
2.926129426	3.389668669
2.926129426	3.389668669
2.926129426	3.389668669
2.926129426	3.389668669
2.926129426	3.389668669
2.926129426	3.389668669
2.926129426	3.389668669
2.926129426	3.389668669
2.926129426	3.389668669
2.926129426	3.389668669
2.926129426	3.389668669
2.926129426	3.389668669
1.561403509	1.806612108
1.117696511	1.282375185
1.801203686	2.054476134
1.801203686	2.054476134
1.801203686	2.054476134
1.801203686	2.054476134
1.801203686	2.054476134
1.801203686	2.054476134
1.801203686	2.054476134
1.801203686	2.054476134
1.801203686	2.054476134
1.801203686	2.054476134
1.801203686	2.054476134
1.801203686	2.054476134
1.801203686	2.054476134
1.801203686	2.054476134
1.419637552	1.604545105
1.346378958	1.510635396
1.301665302	1.460441267
1.527380129	1.713101251
1.398375768	1.540495306
1.398375768	1.540495306
1.331009481	1.460489798
1.734401884	1.897642499
1.734401884	1.897642499
1.734401884	1.897642499
1.734401884	1.897642499
1.734401884	1.897642499
1.734401884	1.897642499
1.734401884	1.897642499

1.734401884	1.897642499
1.734401884	1.897642499
1.734401884	1.897642499
1.734401884	1.897642499
1.734401884	1.897642499
1.734401884	1.897642499
1.734401884	1.897642499
1.494804552	1.625396448
1.494804552	1.625396448
1.494804552	1.625396448
1.494804552	1.625396448
1.494804552	1.625396448
1.494804552	1.625396448
1.494804552	1.625396448
1.078341014	1.170707059
1.377739331	1.479378847
1.377739331	1.479378847
1.377739331	1.479378847
2.600868268	2.78965132
2.600868268	2.78965132
2.600868268	2.78965132
2.600868268	2.78965132
2.600868268	2.78965132
2.600868268	2.78965132
2.600868268	2.78965132
2.600868268	2.78965132
2.600868268	2.78965132
2.600868268	2.78965132
2.600868268	2.78965132
2.600868268	2.78965132
2.600868268	2.78965132
2.600868268	2.78965132
2.600868268	2.78965132
2.600868268	2.78965132
2.600868268	2.78965132
2.600868268	2.78965132
2.600868268	2.78965132
2.600868268	2.78965132
2.600868268	2.78965132
2.600868268	2.78965132
2.600868268	2.78965132
2.600868268	2.78965132
2.600868268	2.78965132
2.600868268	2.78965132
2.600868268	2.78965132
2.600868268	2.78965132
2.600868268	2.78965132
2.600868268	2.78965132

2.600868268	2.78965132
2.600868268	2.78965132
2.600868268	2.78965132
2.600868268	2.78965132
2.600868268	2.78965132
2.600868268	2.78965132
2.600868268	2.78965132
2.600868268	2.78965132
2.600868268	2.78965132
2.600868268	2.78965132
2.600868268	2.78965132
2.600868268	2.78965132
2.600868268	2.78965132
2.600868268	2.78965132
2.600868268	2.78965132
2.600868268	2.78965132
2.600868268	2.78965132
2.600868268	2.78965132
2.600868268	2.78965132
2.600868268	2.78965132
2.600868268	2.78965132
2.600868268	2.78965132
2.600868268	2.78965132
2.600868268	2.78965132
2.600868268	2.78965132
2.600868268	2.78965132
2.600868268	2.78965132
2.600868268	2.78965132
2.600868268	2.78965132
2.600868268	2.78965132
2.600868268	2.78965132
2.600868268	2.78965132
2.600868268	2.78965132
2.600868268	2.78965132
2.600868268	2.78965132
2.600868268	2.78965132
2.600868268	2.78965132
2.600868268	2.78965132
2.600868268	2.78965132
2.600868268	2.78965132
1.242461516	1.324984962
1.463586291	1.543056855
1.463586291	1.543056855
1.463586291	1.543056855
1.463586291	1.543056855
1.463586291	1.543056855
1.463586291	1.543056855
1.463586291	1.543056855

1.463586291	1.543056855
1.171817058	1.232632659
1.672371638	1.755961996
1.672371638	1.755961996
1.672371638	1.755961996
1.672371638	1.755961996
1.672371638	1.755961996
1.672371638	1.755961996
1.672371638	1.755961996
1.672371638	1.755961996
1.672371638	1.755961996
1.672371638	1.755961996
1.672371638	1.755961996
1.672371638	1.755961996
1.672371638	1.755961996
1.672371638	1.755961996
1.672371638	1.755961996
1.672371638	1.755961996
1.672371638	1.755961996
1.357701051	1.42103585
1.357701051	1.42103585
1.209280153	1.259448579
1.209280153	1.259448579
1.254914005	1.299976519
1.286927796	1.320823636
1.286927796	1.320823636
1.433642245	1.465682132
1.433642245	1.465682132
1.433642245	1.465682132
1.433642245	1.465682132
1.338235294	1.365316888
1.338235294	1.365316888
1.338235294	1.365316888
1.243743341	1.262904439
1.614619341	1.627600212
1.614619341	1.627600212
1.614619341	1.627600212
1.614619341	1.627600212
1.614619341	1.627600212
1.614619341	1.627600212
1.614619341	1.627600212
1.614619341	1.627600212
1.614619341	1.627600212
2.340659341	2.332845966
2.340659341	2.332845966
2.340659341	2.332845966
2.340659341	2.332845966
2.340659341	2.332845966

2.340659341	2.332845966
2.340659341	2.332845966
2.340659341	2.332845966
2.340659341	2.332845966
2.340659341	2.332845966
2.340659341	2.332845966
2.340659341	2.332845966
2.340659341	2.332845966
2.340659341	2.332845966
2.340659341	2.332845966
2.340659341	2.332845966
2.340659341	2.332845966
2.340659341	2.332845966
2.340659341	2.332845966
2.340659341	2.332845966
2.340659341	2.332845966
2.340659341	2.332845966
2.340659341	2.332845966
2.340659341	2.332845966
2.340659341	2.332845966
2.340659341	2.332845966
2.340659341	2.332845966
2.340659341	2.332845966
2.340659341	2.332845966
2.340659341	2.332845966
2.340659341	2.332845966
2.340659341	2.332845966
2.340659341	2.332845966
2.340659341	2.332845966
2.340659341	2.332845966
2.340659341	2.332845966
2.340659341	2.332845966
2.340659341	2.332845966
2.340659341	2.332845966
2.340659341	2.332845966
2.340659341	2.332845966
2.340659341	2.332845966
2.340659341	2.332845966
2.340659341	2.332845966
2.340659341	2.332845966
2.340659341	2.332845966
2.340659341	2.332845966
2.340659341	2.332845966
2.340659341	2.332845966
2.340659341	2.332845966
2.340659341	2.332845966
2.340659341	2.332845966
2.340659341	2.332845966

2.340659341	2.332845966
2.340659341	2.332845966
2.340659341	2.332845966
2.340659341	2.332845966
2.340659341	2.332845966
2.340659341	2.332845966
1.319317868	1.312082176
1.319317868	1.312082176
1.404895961	1.392908052
1.404895961	1.392908052
1.404895961	1.392908052
1.404895961	1.392908052
1.404895961	1.392908052
1.404895961	1.392908052
1.404895961	1.392908052
1.171393342	1.159907085
1.259119995	1.235946379
1.300925926	1.261200839
1.300925926	1.261200839
1.560717196	1.510992288
1.560717196	1.510992288
1.560717196	1.510992288
1.560717196	1.510992288
1.560717196	1.510992288
1.560717196	1.510992288
1.377276982	1.324402655
1.377276982	1.324402655
1.377276982	1.324402655
1.377276982	1.324402655
1.377276982	1.324402655
1.377276982	1.324402655
1.377276982	1.324402655
1.283037873	1.212550241
1.283037873	1.212550241
1.283037873	1.212550241
1.283037873	1.212550241
1.283037873	1.212550241
1.283037873	1.212550241
1.232483048	1.157030758
1.17945791	1.106535083
1.350720271	1.259862873
1.350720271	1.259862873
1.51029261	1.404796425
1.51029261	1.404796425
1.51029261	1.404796425
1.51029261	1.404796425
1.51029261	1.404796425
1.51029261	1.404796425

1.51029261	1.404796425
2.127761128	1.976465158
2.127761128	1.976465158
2.127761128	1.976465158
2.127761128	1.976465158
2.127761128	1.976465158
2.127761128	1.976465158
2.127761128	1.976465158
2.127761128	1.976465158
2.127761128	1.976465158
2.127761128	1.976465158
2.127761128	1.976465158
2.127761128	1.976465158
2.127761128	1.976465158
2.127761128	1.976465158
2.127761128	1.976465158
2.127761128	1.976465158
2.127761128	1.976465158
2.127761128	1.976465158
2.127761128	1.976465158
2.127761128	1.976465158
2.127761128	1.976465158
2.127761128	1.976465158
2.127761128	1.976465158
2.127761128	1.976465158
2.127761128	1.976465158
2.127761128	1.976465158
2.127761128	1.976465158
2.127761128	1.976465158
2.127761128	1.976465158
2.127761128	1.976465158
2.127761128	1.976465158
2.127761128	1.976465158
2.127761128	1.976465158
2.127761128	1.976465158
2.127761128	1.976465158
2.127761128	1.976465158
2.127761128	1.976465158
2.127761128	1.976465158
2.127761128	1.976465158
2.127761128	1.976465158
2.127761128	1.976465158
2.127761128	1.976465158
2.127761128	1.976465158
2.127761128	1.976465158
2.127761128	1.976465158
2.127761128	1.976465158
2.127761128	1.976465158
2.127761128	1.976465158

2.127761128	1.976465158
2.127761128	1.976465158
2.127761128	1.976465158
2.127761128	1.976465158
2.127761128	1.976465158
2.127761128	1.976465158
2.127761128	1.976465158
2.127761128	1.976465158
2.127761128	1.976465158
1.26563328	1.166015371
1.26563328	1.166015371
1.26563328	1.166015371
1.190730021	1.093785603
1.219580777	1.119657281
1.107423679	1.010837692
1.325165701	1.199011564
1.325165701	1.199011564
1.325165701	1.199011564
1.325165701	1.199011564
1.325165701	1.199011564
1.180661945	1.062957464
1.24869281	1.121488289
1.24869281	1.121488289
1.24869281	1.121488289
1.46301956	1.307856541
1.46301956	1.307856541
1.46301956	1.307856541
1.46301956	1.307856541
1.46301956	1.307856541
1.46301956	1.307856541
1.46301956	1.307856541
1.46301956	1.307856541
1.46301956	1.307856541
1.46301956	1.307856541
1.46301956	1.307856541
1.46301956	1.307856541
1.300557596	1.141594897
1.95034595	1.692806568
1.95034595	1.692806568
1.95034595	1.692806568
1.95034595	1.692806568
1.95034595	1.692806568
1.95034595	1.692806568
1.95034595	1.692806568
1.95034595	1.692806568
1.95034595	1.692806568
1.95034595	1.692806568
1.95034595	1.692806568

1.95034595	1.692806568
1.95034595	1.692806568
1.95034595	1.692806568
1.95034595	1.692806568
1.95034595	1.692806568
1.95034595	1.692806568
1.95034595	1.692806568
1.95034595	1.692806568
1.95034595	1.692806568
1.95034595	1.692806568
1.95034595	1.692806568
1.95034595	1.692806568
1.95034595	1.692806568
1.95034595	1.692806568
1.95034595	1.692806568
1.95034595	1.692806568
1.95034595	1.692806568
1.95034595	1.692806568
1.95034595	1.692806568
1.95034595	1.692806568
1.95034595	1.692806568
1.95034595	1.692806568
1.95034595	1.692806568
1.95034595	1.692806568
1.95034595	1.692806568
1.95034595	1.692806568
1.95034595	1.692806568
1.95034595	1.692806568
1.95034595	1.692806568
1.95034595	1.692806568
1.95034595	1.692806568
1.95034595	1.692806568
1.95034595	1.692806568
1.95034595	1.692806568
1.95034595	1.692806568
1.161025037	1.004033193
1.100894847	0.947995898
1.418611543	1.219172087
1.418611543	1.219172087
1.418611543	1.219172087
1.418611543	1.219172087
1.418611543	1.219172087
1.418611543	1.219172087
1.418611543	1.219172087
1.418611543	1.219172087
1.418611543	1.219172087
1.182437876	1.015216168
1.216131907	1.038058026

1.216131907	1.038058026
1.276844331	1.087380039
1.089224039	0.921125238
1.054823838	0.888298528
1.200477627	0.998969902
1.142026727	0.948552894
1.376815763	1.137873456
1.376815763	1.137873456
1.376815763	1.137873456
1.376815763	1.137873456
1.376815763	1.137873456
1.376815763	1.137873456
1.376815763	1.137873456
1.376815763	1.137873456
1.253977968	1.036153107
1.253977968	1.036153107
1.253977968	1.036153107
1.253977968	1.036153107
1.253977968	1.036153107
1.15890178	0.951490406
1.800225416	1.463225431
1.800225416	1.463225431
1.800225416	1.463225431
1.800225416	1.463225431
1.800225416	1.463225431
1.800225416	1.463225431
1.800225416	1.463225431
1.800225416	1.463225431
1.800225416	1.463225431
1.800225416	1.463225431
1.800225416	1.463225431
1.800225416	1.463225431
1.800225416	1.463225431
1.800225416	1.463225431
1.800225416	1.463225431
1.800225416	1.463225431
1.800225416	1.463225431
1.800225416	1.463225431
1.800225416	1.463225431
1.800225416	1.463225431
1.800225416	1.463225431
1.800225416	1.463225431
1.800225416	1.463225431
1.800225416	1.463225431
1.800225416	1.463225431
1.800225416	1.463225431
1.800225416	1.463225431
1.800225416	1.463225431

1.800225416	1.463225431
1.800225416	1.463225431
1.800225416	1.463225431
1.800225416	1.463225431
1.800225416	1.463225431
1.800225416	1.463225431
1.800225416	1.463225431
1.800225416	1.463225431
1.800225416	1.463225431
1.800225416	1.463225431
1.800225416	1.463225431
1.800225416	1.463225431
1.800225416	1.463225431
1.800225416	1.463225431
1.800225416	1.463225431
1.800225416	1.463225431
1.800225416	1.463225431
1.800225416	1.463225431
1.800225416	1.463225431
1.185219657	0.961518852
1.185219657	0.961518852
1.231913934	0.98771735
1.068001478	0.853727888
1.337408313	1.063201857
1.337408313	1.063201857
1.337408313	1.063201857
1.337408313	1.063201857
1.337408313	1.063201857
1.337408313	1.063201857
1.170343137	0.925625391
1.136279707	0.892063479
1.114660115	0.871307301
1.080432514	0.840387754
1.155833939	0.891214598
1.155833939	0.891214598
1.300190166	0.994492719
1.300190166	0.994492719
1.300190166	0.994492719
1.300190166	0.994492719
1.300190166	0.994492719
1.300190166	0.994492719
1.300190166	0.994492719
1.300190166	0.994492719
1.300190166	0.994492719
1.300190166	0.994492719
1.671550672	1.27476267
1.671550672	1.27476267
1.671550672	1.27476267

1.671550672	1.27476267
1.671550672	1.27476267
1.671550672	1.27476267
1.671550672	1.27476267
1.671550672	1.27476267
1.671550672	1.27476267
1.671550672	1.27476267
1.671550672	1.27476267
1.671550672	1.27476267
1.671550672	1.27476267
1.671550672	1.27476267
1.671550672	1.27476267
1.671550672	1.27476267
1.671550672	1.27476267
1.671550672	1.27476267
1.671550672	1.27476267
1.671550672	1.27476267
1.671550672	1.27476267
1.671550672	1.27476267
1.671550672	1.27476267
1.671550672	1.27476267
1.671550672	1.27476267
1.671550672	1.27476267
1.671550672	1.27476267
1.671550672	1.27476267
1.671550672	1.27476267
1.671550672	1.27476267
1.671550672	1.27476267
1.671550672	1.27476267
1.671550672	1.27476267
1.671550672	1.27476267
1.671550672	1.27476267
1.671550672	1.27476267
1.671550672	1.27476267
1.671550672	1.27476267
1.671550672	1.27476267
1.671550672	1.27476267
1.671550672	1.27476267
1.671550672	1.27476267
1.671550672	1.27476267
1.671550672	1.27476267
1.671550672	1.27476267
1.671550672	1.27476267
1.671550672	1.27476267
1.671550672	1.27476267
1.190029666	0.898508502
1.190029666	0.898508502
1.190029666	0.898508502

1.114519427	0.83660958
1.26498381	0.931161946
1.26498381	0.931161946
1.26498381	0.931161946
1.26498381	0.931161946
1.26498381	0.931161946
1.26498381	0.931161946
1.26498381	0.931161946
1.26498381	0.931161946
1.26498381	0.931161946
1.26498381	0.931161946
1.26498381	0.931161946
1.26498381	0.931161946
1.26498381	0.931161946
1.088566368	0.800630207
1.088566368	0.800630207
1.103947216	0.810278605
1.170134639	0.857413881
1.170134639	0.857413881
1.170134639	0.857413881
1.170134639	0.857413881
1.020539703	0.743679397
1.044075969	0.756856589
1.04949344	0.755215078
1.093572616	0.784831505
1.56003256	1.118163134
1.56003256	1.118163134
1.56003256	1.118163134
1.56003256	1.118163134
1.56003256	1.118163134
1.56003256	1.118163134
1.56003256	1.118163134
1.56003256	1.118163134
1.56003256	1.118163134
1.56003256	1.118163134
1.56003256	1.118163134
1.56003256	1.118163134
1.56003256	1.118163134
1.56003256	1.118163134
1.56003256	1.118163134
1.56003256	1.118163134
1.56003256	1.118163134
1.56003256	1.118163134
1.56003256	1.118163134
1.56003256	1.118163134
1.56003256	1.118163134
1.56003256	1.118163134
1.56003256	1.118163134

1.56003256	1.118163134
1.56003256	1.118163134
1.56003256	1.118163134
1.56003256	1.118163134
1.56003256	1.118163134
1.56003256	1.118163134
1.56003256	1.118163134
1.56003256	1.118163134
1.56003256	1.118163134
1.56003256	1.118163134
1.150891908	0.818464939
1.150891908	0.818464939
1.150891908	0.818464939
1.150891908	0.818464939
1.150891908	0.818464939
1.150891908	0.818464939
1.231630421	0.872694467
1.231630421	0.872694467
1.231630421	0.872694467
1.231630421	0.872694467
1.231630421	0.872694467
1.231630421	0.872694467
1.231630421	0.872694467
1.231630421	0.872694467
1.231630421	0.872694467
1.231630421	0.872694467
1.231630421	0.872694467
1.231630421	0.872694467
1.231630421	0.872694467
1.056818633	0.736377148
1.032396622	0.717172722
1.13226991	0.781529547
1.13226991	0.781529547
1.13226991	0.781529547
1.03539823	0.710973319
1.073394495	0.73645773
1.199987462	0.81863465
1.199987462	0.81863465
1.199987462	0.81863465
1.199987462	0.81863465
1.199987462	0.81863465
1.199987462	0.81863465
1.199987462	0.81863465
1.199987462	0.81863465
1.088349293	0.73906094
1.462454212	0.986661624
1.462454212	0.986661624
1.462454212	0.986661624

1.462454212	0.986661624
1.462454212	0.986661624
1.462454212	0.986661624
1.462454212	0.986661624
1.462454212	0.986661624
1.462454212	0.986661624
1.462454212	0.986661624
1.462454212	0.986661624
1.462454212	0.986661624
1.462454212	0.986661624
1.462454212	0.986661624
1.462454212	0.986661624
1.462454212	0.986661624
1.462454212	0.986661624
1.462454212	0.986661624
1.462454212	0.986661624
1.462454212	0.986661624
1.462454212	0.986661624
1.462454212	0.986661624
1.462454212	0.986661624
1.462454212	0.986661624
1.462454212	0.986661624
1.462454212	0.986661624
1.462454212	0.986661624
1.462454212	0.986661624
1.462454212	0.986661624
1.462454212	0.986661624
1.462454212	0.986661624
1.462454212	0.986661624
1.462454212	0.986661624
1.462454212	0.986661624
1.462454212	0.986661624
1.462454212	0.986661624
1.462454212	0.986661624
1.462454212	0.986661624
1.462454212	0.986661624
1.462454212	0.986661624
1.462454212	0.986661624
1.462454212	0.986661624
1.462454212	0.986661624
1.462454212	0.986661624
1.462454212	0.986661624
1.462454212	0.986661624
1.462454212	0.986661624
1.462454212	0.986661624
1.462454212	0.986661624
1.462454212	0.986661624
1.462454212	0.986661624
1.462454212	0.986661624
1.462454212	0.986661624
1.114239086	0.746485258

1.010637149	0.671586137
1.075783187	0.712192383
1.16992665	0.768578248
1.16992665	0.768578248
1.16992665	0.768578248
1.16992665	0.768578248
1.16992665	0.768578248
1.16992665	0.768578248
1.16992665	0.768578248
1.16992665	0.768578248
1.008917676	0.654632847
1.044478528	0.669742462
1.044478528	0.669742462
1.37635567	0.875212612
1.37635567	0.875212612
1.37635567	0.875212612
1.37635567	0.875212612
1.37635567	0.875212612
1.37635567	0.875212612
1.37635567	0.875212612
1.37635567	0.875212612
1.37635567	0.875212612
1.37635567	0.875212612
1.37635567	0.875212612
1.37635567	0.875212612
1.37635567	0.875212612
1.37635567	0.875212612
1.37635567	0.875212612
1.37635567	0.875212612
1.37635567	0.875212612
1.37635567	0.875212612
1.37635567	0.875212612
1.37635567	0.875212612
1.37635567	0.875212612
1.37635567	0.875212612
1.37635567	0.875212612
1.37635567	0.875212612
1.37635567	0.875212612
1.37635567	0.875212612
1.37635567	0.875212612
1.37635567	0.875212612
1.37635567	0.875212612
1.37635567	0.875212612
1.37635567	0.875212612
1.37635567	0.875212612
1.37635567	0.875212612
1.37635567	0.875212612
1.37635567	0.875212612

1.37635567	0.875212612
1.37635567	0.875212612
1.37635567	0.875212612
1.37635567	0.875212612
1.37635567	0.875212612
1.37635567	0.875212612
1.023523985	0.648893742
1.14133222	0.722165593
1.14133222	0.722165593
1.14133222	0.722165593
1.14133222	0.722165593
1.14133222	0.722165593
1.14133222	0.722165593
1.14133222	0.722165593
1.14133222	0.722165593
1.14133222	0.722165593
1.051498127	0.661596205
1.035181063	0.648950223
1.017113215	0.632701911
1.011583012	0.626813602
1.039760349	0.637775626
1.063424947	0.651602765
1.063424947	0.651602765
1.063424947	0.651602765
1.11409943	0.679075819
1.11409943	0.679075819
1.11409943	0.679075819
1.11409943	0.679075819
1.11409943	0.679075819
1.11409943	0.679075819
1.11409943	0.679075819
1.11409943	0.679075819
1.11409943	0.679075819
1.11409943	0.679075819
1.299823633	0.779986417
1.299823633	0.779986417
1.299823633	0.779986417
1.299823633	0.779986417
1.299823633	0.779986417
1.299823633	0.779986417
1.299823633	0.779986417
1.299823633	0.779986417
1.299823633	0.779986417
1.299823633	0.779986417
1.299823633	0.779986417
1.299823633	0.779986417
1.299823633	0.779986417
1.299823633	0.779986417

1.299823633	0.779986417
1.299823633	0.779986417
1.299823633	0.779986417
1.299823633	0.779986417
1.299823633	0.779986417
1.299823633	0.779986417
1.299823633	0.779986417
1.299823633	0.779986417
1.299823633	0.779986417
1.299823633	0.779986417
1.299823633	0.779986417
1.299823633	0.779986417
1.299823633	0.779986417
1.299823633	0.779986417
1.299823633	0.779986417
1.299823633	0.779986417
1.299823633	0.779986417
1.299823633	0.779986417
1.299823633	0.779986417
1.299823633	0.779986417
1.299823633	0.779986417
1.299823633	0.779986417
1.299823633	0.779986417
1.299823633	0.779986417
1.299823633	0.779986417
1.028280543	0.614882096
1.028280543	0.614882096
1.047498127	0.623064481
0.998349983	0.586531376
1.08813328	0.63902195
1.08813328	0.63902195
1.002457002	0.588487981
1.032039744	0.595923782
1.032039744	0.595923782
1.032039744	0.595923782
1.032039744	0.595923782
0.999580515	0.572337651
1.006061564	0.571715554
1.2313476	0.698030838
1.2313476	0.698030838
1.2313476	0.698030838
1.2313476	0.698030838
1.2313476	0.698030838
1.2313476	0.698030838
1.2313476	0.698030838
1.2313476	0.698030838
1.2313476	0.698030838
1.2313476	0.698030838

1.2313476	0.698030838
1.2313476	0.698030838
1.2313476	0.698030838
1.2313476	0.698030838
1.2313476	0.698030838
1.2313476	0.698030838
1.2313476	0.698030838
1.2313476	0.698030838
1.2313476	0.698030838
1.2313476	0.698030838
1.2313476	0.698030838
1.2313476	0.698030838
1.2313476	0.698030838
1.2313476	0.698030838
1.2313476	0.698030838
1.2313476	0.698030838
1.063347411	0.601746701
1.063347411	0.601746701
1.063347411	0.601746701
1.063347411	0.601746701
1.063347411	0.601746701
1.063347411	0.601746701
1.063347411	0.601746701
1.063347411	0.601746701
1.063347411	0.601746701
1.063347411	0.601746701
0.986217934	0.557650533
1.017029429	0.570101386
1.017029429	0.570101386
1.017029429	0.570101386
0.991057502	0.554708627
0.995306633	0.551367284
0.995306633	0.551367284
0.982677734	0.537646498
0.982677734	0.537646498
0.982677734	0.537646498
0.982677734	0.537646498
1.039663135	0.567018869
1.039663135	0.567018869
1.00244798	0.545523307
1.16971917	0.627038574
1.16971917	0.627038574
1.16971917	0.627038574
1.16971917	0.627038574
1.16971917	0.627038574
1.16971917	0.627038574
1.16971917	0.627038574
1.16971917	0.627038574

1.16971917	0.627038574
1.16971917	0.627038574
1.16971917	0.627038574
1.16971917	0.627038574
1.16971917	0.627038574
1.16971917	0.627038574
1.16971917	0.627038574
1.16971917	0.627038574
1.16971917	0.627038574
1.16971917	0.627038574
1.16971917	0.627038574
1.16971917	0.627038574
1.16971917	0.627038574
1.16971917	0.627038574
1.16971917	0.627038574
1.16971917	0.627038574
1.16971917	0.627038574
1.16971917	0.627038574
1.16971917	0.627038574
1.16971917	0.627038574
1.16971917	0.627038574
1.017008611	0.534630234
1.017008611	0.534630234
1.017008611	0.534630234
1.017008611	0.534630234
1.017008611	0.534630234
1.017008611	0.534630234
1.017008611	0.534630234
0.966333722	0.505144209
0.960812797	0.500841334
0.960957221	0.498078577
0.964372347	0.49484952
0.974500204	0.499828181
0.958362667	0.489666634
1.113960114	0.565184024
1.113960114	0.565184024
1.113960114	0.565184024
1.113960114	0.565184024
1.113960114	0.565184024
1.113960114	0.565184024
1.113960114	0.565184024
1.113960114	0.565184024
1.113960114	0.565184024
1.113960114	0.565184024
1.113960114	0.565184024
1.113960114	0.565184024
1.113960114	0.565184024
1.113960114	0.565184024

1.113960114	0.565184024
1.113960114	0.565184024
1.113960114	0.565184024
1.113960114	0.565184024
1.113960114	0.565184024
1.113960114	0.565184024
1.113960114	0.565184024
1.113960114	0.565184024
1.113960114	0.565184024
1.113960114	0.565184024
0.995318109	0.504392883
0.995318109	0.504392883
0.995318109	0.504392883
0.995318109	0.504392883
0.954481793	0.477414307
0.961100585	0.478586091
0.961100585	0.478586091
0.947871705	0.471196619
0.974531377	0.476136883
0.974531377	0.476136883
0.942806254	0.460170548
0.948063118	0.458337577
0.948063118	0.458337577
0.939723247	0.452618392
1.063270063	0.511006639
1.063270063	0.511006639
1.063270063	0.511006639
1.063270063	0.511006639
1.063270063	0.511006639
1.063270063	0.511006639
1.063270063	0.511006639
1.063270063	0.511006639
1.063270063	0.511006639
1.063270063	0.511006639
1.063270063	0.511006639
1.063270063	0.511006639
1.063270063	0.511006639
1.063270063	0.511006639
1.063270063	0.511006639
0.935294118	0.444476753
0.954593084	0.449708271
0.954593084	0.449708271
0.954593084	0.449708271
0.935373317	0.439029608
0.925985246	0.428921469
0.925985246	0.428921469
0.923017458	0.420612451
1.016987843	0.463326172

1.016987843	0.463326172
1.016987843	0.463326172
1.016987843	0.463326172
1.016987843	0.463326172
1.016987843	0.463326172
1.016987843	0.463326172
1.016987843	0.463326172
1.016987843	0.463326172
1.016987843	0.463326172
1.016987843	0.463326172
1.016987843	0.463326172
1.016987843	0.463326172
1.016987843	0.463326172
1.016987843	0.463326172
1.016987843	0.463326172
0.935452323	0.424967292
0.935452323	0.424967292
0.935452323	0.424967292
0.935452323	0.424967292
0.935452323	0.424967292
0.935452323	0.424967292
0.917062179	0.401786869
0.917062179	0.401786869
0.917062179	0.401786869
0.917062179	0.401786869
0.974562475	0.421180168
0.974562475	0.421180168
0.974562475	0.421180168
0.974562475	0.421180168
0.974562475	0.421180168
0.974562475	0.421180168
0.974562475	0.421180168
0.974562475	0.421180168
0.974562475	0.421180168
0.974562475	0.421180168
0.974562475	0.421180168
0.974562475	0.421180168
0.974562475	0.421180168
0.974562475	0.421180168
0.974562475	0.421180168
0.974562475	0.421180168
0.974562475	0.421180168
0.974562475	0.421180168
0.974562475	0.421180168
0.974562475	0.421180168
0.974562475	0.421180168
0.974562475	0.421180168
0.959196869	0.412386951

0.899256191	0.386266736
0.89913273	0.385617561
0.89913273	0.385617561
0.89913273	0.385617561
0.907456119	0.384351308
0.899379349	0.380051254
0.899379349	0.380051254
0.88782672	0.370253159
0.935531136	0.383777231
0.935531136	0.383777231
0.935531136	0.383777231
0.935531136	0.383777231
0.935531136	0.383777231
0.935531136	0.383777231
0.935531136	0.383777231
0.935531136	0.383777231
0.935531136	0.383777231
0.935531136	0.383777231
0.935531136	0.383777231
0.935531136	0.383777231
0.935531136	0.383777231
0.935531136	0.383777231
0.935531136	0.383777231
0.935531136	0.383777231
0.935531136	0.383777231
0.882363796	0.359654848
0.873785963	0.34689711
0.865978448	0.340501163
0.865978448	0.340501163
0.865978448	0.340501163
0.899502207	0.350461652
0.899502207	0.350461652
0.899502207	0.350461652
0.899502207	0.350461652
0.899502207	0.350461652
0.899502207	0.350461652
0.899502207	0.350461652
0.899502207	0.350461652
0.899502207	0.350461652
0.899502207	0.350461652
0.899502207	0.350461652
0.899502207	0.350461652
0.899502207	0.350461652
0.899502207	0.350461652
0.899502207	0.350461652
0.899502207	0.350461652
0.899502207	0.350461652
0.899502207	0.350461652

0.899502207	0.350461652
0.899502207	0.350461652
0.899502207	0.350461652
0.865649964	0.334915868
0.865649964	0.334915868
0.855210914	0.326391087
0.850188931	0.322501902
0.850188931	0.322501902
0.850188931	0.322501902
0.850188931	0.322501902
0.866142088	0.32068633
0.866142088	0.32068633
0.866142088	0.32068633
0.866142088	0.32068633
0.866142088	0.32068633
0.866142088	0.32068633
0.866142088	0.32068633
0.866142088	0.32068633
0.866142088	0.32068633
0.866142088	0.32068633
0.866142088	0.32068633
0.866142088	0.32068633
0.866142088	0.32068633
0.866142088	0.32068633
0.866142088	0.32068633
0.866142088	0.32068633
0.86980189	0.319834881
0.834963325	0.305576148
0.834963325	0.305576148
0.834963325	0.305576148
0.834963325	0.305576148
0.834963325	0.305576148
0.834963325	0.305576148
0.859965934	0.311090556
0.834761322	0.300293169
0.834761322	0.300293169
0.820271951	0.289649645
0.820271951	0.289649645
0.835164835	0.29399183
0.835164835	0.29399183
0.835164835	0.29399183
0.835164835	0.29399183
0.835164835	0.29399183
0.835164835	0.29399183
0.835164835	0.29399183
0.835164835	0.29399183
0.835164835	0.29399183
0.835164835	0.29399183

0.880907902	0.307670461
0.824897401	0.28809564
0.806087176	0.27465416
0.806087176	0.27465416
0.806087176	0.27465416
0.806087176	0.27465416
0.815262873	0.276429
0.815262873	0.276429
0.806323944	0.269990021
0.806323944	0.269990021
0.806323944	0.269990021
0.806323944	0.269990021
0.806323944	0.269990021
0.806323944	0.269990021
0.806323944	0.269990021
0.806323944	0.269990021
0.806323944	0.269990021
0.805849829	0.265267615
0.805849829	0.265267615
0.792383241	0.26052691
0.812659847	0.262259832
0.82251793	0.264336388
0.796650718	0.254587265
0.796650718	0.254587265
0.779405779	0.248351191
0.779405779	0.248351191
0.779405779	0.248351191
0.779405779	0.248351191
0.779405779	0.248351191
0.779405779	0.248351191
0.779405779	0.248351191
0.779405779	0.248351191
0.779405779	0.248351191
0.779405779	0.248351191
0.779405779	0.248351191
0.779405779	0.248351191
0.779405779	0.248351191
0.779405779	0.248351191
0.779405779	0.248351191
0.779405779	0.248351191
0.779405779	0.248351191
0.779405779	0.248351191
0.779405779	0.248351191
0.779405779	0.248351191
0.779405779	0.248351191
0.779405779	0.248351191
0.779136104	0.247210062
0.855017411	0.271171349

0.787658328	0.244365052
0.787658328	0.244365052
0.75422427	0.228793802
0.75422427	0.228793802
0.75422427	0.228793802
0.75422427	0.228793802
0.75422427	0.228793802
0.75422427	0.228793802
0.75422427	0.228793802
0.75422427	0.228793802
0.75422427	0.228793802
0.778865769	0.234579318
0.825437648	0.248074682
0.836903926	0.248035163
0.770266453	0.225209566
0.730616606	0.211076297
0.730616606	0.211076297
0.730616606	0.211076297
0.730616606	0.211076297
0.730616606	0.211076297
0.730616606	0.211076297
0.730616606	0.211076297
0.730616606	0.211076297
0.730616606	0.211076297
0.730616606	0.211076297
0.730616606	0.211076297
0.730616606	0.211076297
0.730616606	0.211076297
0.730616606	0.211076297
0.730616606	0.211076297
0.730616606	0.211076297
0.730616606	0.211076297
0.730616606	0.211076297
0.741917957	0.211608575
0.741917957	0.211608575
0.741917957	0.211608575
0.841455754	0.239913381
0.778594771	0.220522954
0.708439708	0.194990486
0.708439708	0.194990486
0.708439708	0.194990486
0.708439708	0.194990486
0.708439708	0.194990486
0.708439708	0.194990486
0.708439708	0.194990486
0.708439708	0.194990486
0.708439708	0.194990486
0.708439708	0.194990486

0.708439708	0.194990486
0.708439708	0.194990486
0.708439708	0.194990486
0.708439708	0.194990486
0.708439708	0.194990486
0.708439708	0.194990486
0.708439708	0.194990486
0.708439708	0.194990486
0.745566291	0.199407214
0.778323108	0.206929167
0.719014482	0.191050537
0.719014482	0.191050537
0.687567335	0.180356179
0.687567335	0.180356179
0.687567335	0.180356179
0.687567335	0.180356179
0.687567335	0.180356179
0.687567335	0.180356179
0.687567335	0.180356179
0.687567335	0.180356179
0.687567335	0.180356179
0.687567335	0.180356179
0.687567335	0.180356179
0.687567335	0.180356179
0.687567335	0.180356179
0.813896137	0.212087974
0.737679572	0.191517275
0.804654323	0.207975541
0.84758028	0.218122247
0.708083278	0.18160699
0.708083278	0.18160699
0.708083278	0.18160699
0.667887668	0.167016806
0.667887668	0.167016806
0.667887668	0.167016806
0.667887668	0.167016806
0.667887668	0.167016806
0.667887668	0.167016806
0.667887668	0.167016806
0.697478378	0.172674767
0.915176191	0.224733768
0.722393973	0.176708321
0.741285403	0.179310711
0.687185388	0.164222662
0.687185388	0.164222662
0.687185388	0.164222662
0.769419769	0.18373118
0.649301316	0.154835813

0.649301316	0.154835813
0.649301316	0.154835813
0.649301316	0.154835813
0.649301316	0.154835813
0.649301316	0.154835813
0.649301316	0.154835813
0.649301316	0.154835813
0.735409912	0.17325015
0.677190744	0.156221655
0.631719632	0.143693685
0.631719632	0.143693685
0.631719632	0.143693685
0.631719632	0.143693685
0.631719632	0.143693685
0.631719632	0.143693685
0.631719632	0.143693685
0.700611995	0.156710274
0.667481663	0.14864474
0.667481663	0.14864474
0.693638887	0.150583375
0.615063299	0.133485476
0.615063299	0.133485476
0.615063299	0.133485476
0.615063299	0.133485476
0.615063299	0.133485476
0.615063299	0.133485476
0.615063299	0.133485476
0.658046076	0.14146676
0.748649963	0.159853829
0.686802506	0.144706357
0.599261138	0.124118745
0.599261138	0.124118745
0.599261138	0.124118745
0.599261138	0.124118745
0.599261138	0.124118745
0.599261138	0.124118745
0.599261138	0.124118745
0.63995043	0.12821537
0.584249084	0.115511825
0.584249084	0.115511825
0.584249084	0.115511825
0.584249084	0.115511825
0.584249084	0.115511825
0.584249084	0.115511825
0.584249084	0.115511825
0.584249084	0.115511825
0.584249084	0.115511825
0.569969326	0.107592365

0.569969326	0.107592365
0.569969326	0.107592365
0.569969326	0.107592365
0.569969326	0.107592365
0.569969326	0.107592365
0.569969326	0.107592365
0.569969326	0.107592365
0.569969326	0.107592365
0.569969326	0.107592365
0.660746865	0.12348601
0.622819886	0.116298026
0.622819886	0.116298026
0.622819886	0.116298026
0.622819886	0.116298026
0.622819886	0.116298026
0.871549356	0.162625636
0.702712691	0.126837733
0.556369556	0.100296101
0.556369556	0.100296101
0.556369556	0.100296101
0.556369556	0.100296101
0.556369556	0.100296101
0.556369556	0.100296101
0.556369556	0.100296101
0.556369556	0.100296101
0.556369556	0.100296101
0.556369556	0.100296101
0.556369556	0.100296101
0.556369556	0.100296101
0.717633718	0.127828433
0.648442812	0.114114491
0.60657924	0.105566794
0.543402334	0.093565804
0.543402334	0.093565804
0.543402334	0.093565804
0.543402334	0.093565804
0.543402334	0.093565804
0.846735979	0.14482914
0.598771237	0.100604854
0.598771237	0.100604854
0.598771237	0.100604854
0.666666667	0.110991558
0.531024531	0.087350389
0.531024531	0.087350389
0.531024531	0.087350389
0.531024531	0.087350389
0.531024531	0.087350389
0.531024531	0.087350389

0.531024531	0.087350389
0.531024531	0.087350389
0.630818089	0.101414706
0.519196853	0.081604155
0.519196853	0.081604155
0.519196853	0.081604155
0.583740831	0.091415
0.576503969	0.087160466
0.507883421	0.076286125
0.507883421	0.076286125
0.507883421	0.076286125
0.507883421	0.076286125
0.648011983	0.096819585
0.569443616	0.08311647
0.643509128	0.093570286
0.643509128	0.093570286
0.643509128	0.093570286
0.666257669	0.096237304
0.497051412	0.071359483
0.497051412	0.071359483
0.497051412	0.071359483
0.562553392	0.079271652
0.562553392	0.079271652
0.562553392	0.079271652
0.562553392	0.079271652
0.486670737	0.066791087
0.486670737	0.066791087
0.486670737	0.066791087
0.486670737	0.066791087
0.486670737	0.066791087
0.486670737	0.066791087
0.486670737	0.066791087
0.486670737	0.066791087
0.555827221	0.075615336
0.654925209	0.087756495
0.476713762	0.062551047
0.476713762	0.062551047
0.476713762	0.062551047
0.697883803	0.088769846
0.542844146	0.068828655
0.467155067	0.058612354
0.467155067	0.058612354
0.467155067	0.058612354
0.467155067	0.058612354
0.457971223	0.054950564
0.457971223	0.054950564
0.449140603	0.051543515
0.449140603	0.051543515

0.449140603	0.051543515
0.449140603	0.051543515
0.449140603	0.051543515
0.449140603	0.051543515
0.449140603	0.051543515
0.524463614	0.059829961
0.440643214	0.048371086
0.440643214	0.048371086
0.440643214	0.048371086
0.432460544	0.045414978
0.432460544	0.045414978
0.432460544	0.045414978
0.432460544	0.045414978
0.424575425	0.042658526
0.501800878	0.049713989
0.719537381	0.070980913
0.416971917	0.040086532
0.416971917	0.040086532
0.416971917	0.040086532
0.409635199	0.037685117
0.402551472	0.035441592
0.402551472	0.035441592
0.402551472	0.035441592
0.402551472	0.035441592
0.402551472	0.035441592
0.39570787	0.03334434
0.39570787	0.03334434
0.685574817	0.057227835
0.476074048	0.039528287
0.389092389	0.031382713
0.389092389	0.031382713
0.389092389	0.031382713
0.382693809	0.029546939
0.382693809	0.029546939
0.382693809	0.029546939
0.554738562	0.0426595
0.376501635	0.027828043
0.376501635	0.027828043
0.510403917	0.036976323
0.370506037	0.02621777
0.565072369	0.036812835
0.359068282	0.023293301
0.444172779	0.028777442
0.435824783	0.026299642
0.348313378	0.020719596
0.343173167	0.019549644
0.343173167	0.019549644
0.427783136	0.024041429

0.338181947	0.018450697
0.423871972	0.022988238
0.333333333	0.017418041
0.416259169	0.021021941
0.3286213	0.016447309
0.324040157	0.015534453
0.324040157	0.015534453
0.315249315	0.013867622
0.315249315	0.013867622
0.315249315	0.013867622
0.306921149	0.012390614
0.299020068	0.011080155
0.385115884	0.014094268
0.378814082	0.012902561
0.284374162	0.008880903
0.465196078	0.014168815
0.280933257	0.008406801
0.277574278	0.007959367
0.274294333	0.007536997
0.488271382	0.013341776
0.271090666	0.007138192
0.413064104	0.010816425
0.264901765	0.006405767
0.264901765	0.006405767
0.403428588	0.009275632
0.258987926	0.005751925
0.344924386	0.007617341
0.250594089	0.004899299
0.327800544	0.005612462
0.240209962	0.003962975
0.237746381	0.003759463
0.414828431	0.006124762
0.228375111	0.003048165
0.223959801	0.002746478
0.21764632	0.002350778
0.355656583	0.003825509
0.215619771	0.002232411
0.213630406	0.002120202
0.288827469	0.002462331
0.192322829	0.00114946
0.34461331	0.001231841

Genes
EIF4A2;RPL4;RWDD1;RPL5;RPL32;RPL10;RPS8;RPS26;RPL7A;RPS25;RPL36AL;RPL35;RPL24;ETF1;RPL22L1
VPS29;ARF1;TMED10;CLTC;USO1;SNX13;VPS26A;IPO7;GABARAP;RAB22A;LMAN1;CACNG7;IPO8;AKT2;LAM
GSK3B;VCP;UBA6;UBE2D3;UBE2L3;PSMD8;PCNP;RNF115;KCTD21;CHMP1B;PSMB1;ARIH1;RNF111;NDFIP
METTL4;NHP2;METTL16;NOP10
DDX6;IGFBP5;YTHDF3;CELF1;ROCK2;SYNCRIP;CPEB1;AGO3;CNOT1;CAPRIN1;EIF3E;CALR;METTL16;CPEB2
LAMP1;AP1G1;SORT1;RBSN;ASB15
NAA20;NAA25;NAA35;NAA15
EIF4A2;RPL5;DDX6;RPL10;YTHDF3;IGFBP5;RPS27L;EEF2;BZW1;ELAVL1;SYNCRIP;RPS6KB1;CNOT1;CAPRIN
GSK3B;PRKAA1;CAMK2D;CCL11;BMPR2;AMHR2;ROCK2;PRKAG3;TTBK2;EFEMP1;AKT2;ABL2;TLK1;RICTOR;J
GSK3B;PRKAA1;RPL10;SOCS2;AKT2;RICTOR;TAF9B;XBP1;GPX4;API5;ANXA4;BNIP3;SMAD5;THOC6;DNAJC3
NAA20;NAA25;NAA35;PFDN1;NAA15
ANKRD13C;ITGB1;GSK3B;PARK7;HMGB1;IER3IP1;AKT2;CTSD;MEF2C;ANXA4;THOC6;DNAJC3;TMBIM4;MOR
DDX6;GSK3B;PRKAA1;CELF1;ROCK2;PARK7;BMI1;SYNCRIP;GPNMB;NPTN;METTL16;NUDT12;MEF2C;NDFIP
RPL5;XBP1;YTHDF3;RPS27L;EEF2;ELAVL1;HSPH1;RPS6KB1;AKT2;NAMPT;RFX5;CD28;EIF3E;JAK2;OGT
VPS29;TMEM87A;SORT1;CLTC;VPS26A;HOOK3;RBSN;ASB15;LAMP1;RAB14;AP1G1;VPS35;RAB6A;EPS15;S
VPS29;ARF1;TMED10;CLTC;USO1;SNX13;VPS26A;RAB22A;CHMP1B;LAMP2;TMED2;TLK1;VPS35;TMED7;RA
DCUN1D3;ARRDC3;DCUN1D1;UBE2N;PARK7;BMI1;UBE2L3
RPL4;RWDD1;RPL5;RPL32;RPL10;RPS8;EEF2;MRPL57;WARS2;RPS26;RPS25;RPL7A;RPL36AL;RPL35;RPL24
ABCD2;CCL11;TMED10;SNX10;THY1;PARK7;CDC42;FGD4;LMAN1;CHMP1B;TMED2;RICTOR;TMED7;EPS15;
ARFGEF2;ATP6V0B;ATP6AP1;SNX10;DNAJC13;HOOK3;TMCC1;RAB22A;ATP6V1F
GSK3B;XBP1;VCP;SMURF2;UBE2D3;UBE2A;RAD23B;PSMD8;PCNP;UBE2W;PSMA3;ITCH;PSMA4;PSMA1;PS
ABCD2;PTEN;PRDX6;SOD1
GSK3B;VCP;UBE2D3;DERL2;PSMD8;PCNP;CDC27;PSMB1;DNAJB9;ASCC3;UBXN4;SMURF2;KLHDC1;ERLIN
XBP1;CPEB1;FER;RPS6KB1;PDPK1;SORT1;AKT2;DENND4C;OTOP1;CPEB2;OGT
VMP1;VCP;CHMP1B;LAMP2;CHMP4C;CHMP3;CALM1;SNAP29
ITGB1;GSK3B;PRKAA1;RPL10;PARK7;SOCS2;AKT2;RICTOR;TAF9B;MEF2C;XBP1;API5;ANXA4;BNIP3;SMAD5;
LAMP1;SORT1;AP1G1;RBSN;ASB15
NINJ1;WDFY1;NR1H3;TIRAP
DUOX1;DUOX2;SOD1
DCUN1D3;DCUN1D1;PTEN;UBE2N;BMI1;UBE2L3
GSK3B;XBP1;CPEB1;FER;RPS6KB1;PDPK1;AKT2;RARRES2;NAMPT;DENND4C;OTOP1;CPEB2
GSK3B;HSPA8;VCP;CHMP1B;LAMP2;CHMP4C;CHMP3;ETF1;SNAP29
ITGB1;SMAD3;BMPR2;AMHR2;SMURF2;MSTN;SMAD5;TGFBR1;ACVR2A;TMEM100;FAM83G;TWSG1;MAP3K7
DYNC1H1;CHMP1B;CLTC;CHMP4C;CHMP3;CEP97;MAP9
VMP1;CALM1;SNAP29
ITGB1;CCL24;GSK3B;CCL11;SNX13;THY1;ARHGAP12;SOD1;ARHGAP44;FGD4;RAP1A;CCL8;WNK1;TMED2;
GSK3B;CAMK2D;POGLUT1;PDPK1;ROCK2;MAPK14;TGFBR1;TTBK2;RPS6KB1;WNK1;AKT2;ULK2;TLK1;RICTO
NAA20;NAA35;PFDN1;NAA15
USH1C;RORB;RPGRIP1L;THY1
FER;IL6ST;JAK2;JAK1
TTLL7;AGTPBP1;TTLL4;GGCX;NAA20
TOM1L1;GPNMB;NBN;CALM1;CALM2
YTHDF3;AGO3;CELF1;ROCK2;CNOT1;METTL16;RC3H2
SCOC;ATP6V0B;PIK3CA;EXOC7;BNIP3;VPS35;SMCR8;VPS26A;MAP3K7;ATG12;SNX5
DNAJA1;HSPA8;ERP44;HSPH1;HSPA4;HSPB8;STT3B
CDC42;NCKAP1;AKIRIN1;FER;MSTN;PIK3CA
CDC42;NCKAP1;AKIRIN1;MSTN;PIK3CA

SLC46A1;SLC25A32;FOLR2
GSK3B;CAMK2D;PDPK1;ROCK2;MAPK14;TGFBR1;TTBK2;RPS6KB1;WNK1;AKT2;ULK2;TLK1;RICTOR;PKN2;TC
RPL5;SMAD3;YTHDF3;RPS27L;NR1H3;PARK7;EEF2;ELAVL1;DDAH1;RPS6KB1;AKT2;DUOXA1;CNEP1R1;CD2
RPL5;YTHDF3;RPS6KB1;ROCK2;CD28;RPS27L;EIF3E;EEF2;ELAVL1;OGT
VCP;SORT1;CHMP1B;CHMP4C;CHMP3
ATF1;DUOX1;P2RY6;TLR7;DUOX2
DDX6;DYNC1H1;YTHDF3;G3BP2;LSM14A
NAA20;NAA25;NAA35;NAA15
VCP;CHMP1B;CHMP4C;CHMP3
PRKAA1;ERLIN1;MBTPS2;SOD1
LMAN1;TMED10;TFG;KDELR1;USO1;TMED2;TRAPPC8;IER3IP1;TMED7;SEC22B;TMED5;ARCN1
GSK3B;ABCD2;PRKAA1;NPRL2;BNIP3;ZBTB20;HMGB1;SLC4A4;ITCH;CNOT7;CNOT1;AKT2;ULK2;ATF6
VPS29;ARF1;TMED10;CLTC;USO1;USH1C;SNX13;VPS26A;RAB22A;TM9SF3;DUOXA1;TMED2;TLK1;VPS35;T
RPL5;GSK3B;VCP;YTHDF3;ROCK2;RPS27L;NR1H3;EEF2;ELAVL1;RPS6KB1;PERP;CD28;VPS35;EIF3E;AURKA
VPS29;TMEM87A;SORT1;CLTC;VPS26A;RBSN;ASB15;ZFYVE16;RAB14;CHMP1B;CHMP3;VPS35;TMCC1;RAB
TMED10;COG6;KDELR1;USO1;TRAPPC8;IER3IP1;RBSN;ARCN1;LMAN1;GOLPH3;TFG;TMED2;EXOC5;TMED7;
MEF2A;CAMK2D;ROCK2;IL6ST
VCP;XBP1;ERLIN1;DERL2;DNAJB9;CALR;STT3B;UBXN4;ATF6;UGGT1
RPL4;RPL5;RPL32;RPL10;RPS8;EEF2;RPS26;RPS25;RPL7A;GOLPH3;RPL36AL;RPL35;RPL24;RPS11;RPL39;R
MEF2C;BMPR2;IGFBP5;OGN;PTEN;ANG
ZFYVE16;LAMP1;AP1G1;SORT1;CHMP1B;CHMP4C;CHMP3;VPS35;HOOK3;RBSN;ASB15
EXOC7;CHMP1B;CHMP4C;CHMP3;EXOC5;TMCC1
AKIRIN1;MEF2C;PBRM1;MYF6;BRD7;MAPK14
DAB2;BMPR2;JAK2;TGFBR1
MEF2A;CAMK2D;ROCK2;IL6ST
NHP2;TFB2M;TFB1M;NOP10
VCP;UBA6;DCUN1D3;UBE2D3;DCUN1D1;UBE2L3;PCNP;VCPIP1;RNF115;SENP5;SUMO2;ARIH1;RNF111;P
TBCK;PRKAA1;GOLPH3;DEPTOR;PIK3CA;PIH1D1;NPRL2;SMCR8;RICTOR
NINJ1;WDFY1;NR1H3;HMGB1;TIRAP;PUM2
CHMP1B;CHMP4C;CHMP3
TMX3;PARK7;RAB6A
GSK3B;TAOK1;PARK7
CHMP1B;CHMP4C;CHMP3
EIF4A2;DNAJC3;YTHDF3;RPS6KB1;EIF3E;BZW1;EIF1B;NCK1
VPS29;TMEM87A;LMAN1;CLTC;VPS35;VPS26A;RAB6A;RBSN;ASB15;SNX5
HNRNPC;MAPK14;ELAVL1;TIRAP
RPL5;COPS3;DCUN1D3;DCUN1D1
RPL4;RPL5;RPL32;RPL10;RPS8;THOC6;RPS26;RPS25;SYNCRIP;RPL7A;GOLPH3;RPL36AL;RPL35;RPL24;NOX
RPL4;RPL5;RPL32;RPL10;RPS8;RPS26;RPS25;RPL7A;RPL36AL;RPL35;RPL24;RPS11;RPL39;RPS10
NDFIP2;XBP1;SMURF2;UBA6;UBE2D3;UBE2A;RC3H2;UBE2L3;MIB1;COPS3;ITCH;RNF128;KCTD21;MDM2;A
GTF2A1;XBP1;PBRM1;GTF2A2;CBFB;NR1H3;ELL2;CDC73;DRAP1;MAF;COPS2;TCEA1;RPRD1A;POLR2J;POLR
TOM1L1;CAB39L;PDPK1;ANG;PARK7;JAK2;MAP3K7;PPIA;TIRAP
UBE2W;UBE2D3;CDC27;UBE2A;UBE2L3
COPS3;ITCH;DCUN1D3;DCUN1D1;TGFBR1
DYNC1H1;CHMP1B;CHMP4C;CHMP3;CEP97
GSK3B;PRKAA1;CAMK2D;CCL11;BMPR2;AMHR2;ROCK2;PRKAG3;TLK1;RICTOR;JAK2;MAPK6;JAK1;CDKL2;PD
CNOT7;CD28;RSF1;PKN2;PPIA;ATG12
ARF1;TMED10;EQTN;RAB22A;CDC42;CCL8;AP1G1;CHMP1B;TMED2;ARL5A;SNAP29;EPS15;TBC1D8B;SCAR

MEF2C;PBRM1;AKIRIN1;MSTN;MYF6;BRD7;MAPK14
XBP1;RFX5;JAK2
DUOX1;CPQ;DUOX2
CHMP1B;CHMP4C;CHMP3
DDX6;GSK3B;SYNCRIP;BMPR2;IGFBP5;CNOT1;CAPRIN1;CLTC;CALR;LSM14A
GSK3B;ERP44;VCP;XBP1;UFM1;TMX1;TMEM33;DNAJB9;ATF6;MBTPS2;NCK1
CDC42;VMP1;VCPIP1;LMAN1;GOLPH3;TMED10;COG6;TMED2;TRAPPC8;TMED7;SEC22B;TMED5
GSK3B;PRKAA1;CAMK2D;CCL11;BMPR2;RPN2;AMHR2;ROCK2;UBE2D3;PTEN;F13A1;PARK7;MTMR6;PRKAG[illegible]
GSK3B;NDFIP2;DCUN1D3;ARRDC3;DCUN1D1;HUWE1;UBE2N;BMI1;UBE2L3
IPO8;LAMP1;KPNA4;NUP153;NUP98;IPO7;TNPO3;KPNB1;RAB11A
CDC42;NCKAP1;AKIRIN1;MSTN;PIK3CA
TOM1L1;GPNMB;NBN;CALM1;CALM2
CHMP1B;CHMP4C;CHMP3;CEP97
DDX6;DYNC1H1;LSM14A
GHR;SOCS2;JAK2
CHMP1B;CHMP4C;CHMP3
XBP1;TMEM33;ATF6
UBE2N;UBE2V2;GABARAP
HSPA8;ST13;PTGES3;HSPA13;UGGT1
GPNMB;DCUN1D3;PTEN;BRD7;CDC73
GSK3B;CAMK2D;PDPK1;ROCK2;WNK1;WNK3;TGFBR1
CNOT7;YTHDF3;CELF1;ROCK2;CNOT1;METTL16
VCP;CHMP1B;LAMP2;NBR1;CHMP4C;CHMP3;CALR;SNAP29;ATG12;GABARAP
UBE2D3;HUWE1;NMT1;RHOU;UBE2L3
P2RY6;PDPK1;THY1;CALM1;CALM2
TOM1L1;RNF115;CHMP1B;CHMP4C;CHMP3
XBP1;CPEB1;FER;RPS6KB1;PDPK1;AKT2;DENND4C;OTOP1;CPEB2
CNOT7;YTHDF3;CNOT1;CAPRIN1;ZC3H14;PUM2
ITGB1;SCARA5;SORT1;EQTN;CLTC;DNAJC13;RAB22A;MIB1;CDC42;DAB2;P2RY6;MRC1;RHOU;EPS15;SNX5
PIK3CA;FEZ1;SMCR8;SEC22B
VCP;UBE2N;UBE2V2;UBE2A
COPS3;DCUN1D3;COPS2;DCUN1D1
TFB2M;TFB1M;METTL16;METTL15
LMAN1;RAB6A;RBSN
MTERF4;TFB2M;TFB1M
DICER1;PRDX6;SOD1
CAMK2D;CALM1;CALM2
MEF2C;SMAD3;BMPR2;DUSP3;AMHR2;PDPK1;MAPK14;TGFBR1;ACVR2A;TMEM100;CLEC3B;RAP1A;RPS6KB[illegible]
DUOX1;PRDX1;NOX4;DUOX2;SOD1
GSK3B;FER;PIK3CA;AKT2;RARRES2;NAMPT
CBFB;UBE2D3;HUWE1;UBE2A;RC3H2;UBE2L3;UBE2W;RNF115;ITCH;UBE2Q2;CDC27;UBE2V2;MDM2;UBE[illegible]
ARHGAP44;CDC42;PIK3CA;ROCK2;TAOK1;ABL2;RICTOR;CRK;ARHGAP35
GSK3B;SCOC;PRKAA1;NPRL2;BNIP3;ULK2;PARK7;HMGB1;MAP3K7;ATF6
GOLPH3;TMED10;EXOC7;ACSL4;ANG;IER3IP1;PPIA;WLS
GSK3B;MEF2C;XBP1;PBRM1;AKIRIN1;SMAD3;BMPR2;RARRES2;MAPK14;TGFBR1;ACVR2A;MEDAG;TMEM10[illegible]
DEPTOR;PDPK1;NPRL2;SMCR8;PARK7;THY1;PPIA
RPL5;SYNCRIP;YTHDF3;RPS6KB1;CD28;RPS27L;EIF3E;EEF2;OGT;ELAVL1
FER;IL6ST;JAK2;JAK1

RPN2;OSTC;STT3B;UGGT1
MEF2A;CAMK2D;ROCK2;IL6ST
NINJ1;WDFY1;NR1H3;TIRAP
GPNMB;DCUN1D3;PTEN;BRD7;CDC73
PRKAA1;RPS6KB1;SCTR;RICTOR;PRKAG3
CCL24;BMPR2;CCL11;PDPK1;CNTN1;ANG;HMGB1;TGFBR1
GSK3B;VCP;DAB2;SUMO2;MDM2;PARK7;ARIH1;OGT;RAD23B;GABARAP
SMURF2;YTHDF3;CALR
TWSG1;DAB2;BMPR2;MSTN;TGFBR1;ACVR2A
ARFGEF2;ZFYVE16;SORT1;CHMP4C;SNX10;DNAJC13;HOOK3;EPS15;RAB22A
ARFGEF2;TMED10;COG6;SNX10;DNAJC13;TRAPPC8;HOOK3;RAB22A;VMP1;CDC42;LMAN1;GOLPH3;TMED2
TMED10;TMED2
TMED10;TMED2
TMED10;TMED2
TMEM30A;ATP8A1
MAPK14;TIRAP
BNIP3;YME1L1
DEPTOR;PTEN
ITCH;MAP3K7
VCP;AKT2
HSPA8;LAMP2
MAPK14;TIRAP
SLC46A1;SLC1A3;SLC25A32;FOLR2
SORT1;CHMP1B;CHMP4C;CHMP3
WNK3;CALM1;CALM2;UBE2K
HSPA8;PIK3CA;AEBP1;PARK7
RANBP2;IPO8;KPNA4;NUP153;NUP98;IPO7;TNPO3;KPNB1
GPNMB;CHMP1B;BNIP3;CHMP4C;CHMP3;VPS35;PARK7
GSK3B;DYNC1H1;DAB2;CD151;AKT2;NR1H3
CDC42;CHMP4C;BIRC6;PKN2;CALM1;CALM2;MAP9;RAB11A
POPDC3;MEF2C;NINJ1;MYF6;RORA
MEF2A;GTF2A1;XBP1;GTF2A2;CBFB;MTERF4;RSF1;TFB2M;DRAP1;MAF;COPS2;TCEA1;POLR3G;POLR2J;POLR
GSK3B;LMAN1;VCP;DAB2;CD151;TMEM30A;BNIP3;PARK7;TGFBR1;TIRAP
DNAJA1;GSK3B;NDFIP2;DCUN1D1;UBE2N;HUWE1;PARK7;OGT;TGFBR1;UBE2L3
UFM1;NUP153;PARK7
CCT3;NHP2;NOP10
XBP1;RFX5;JAK2
CST3;DPP4;DDR2
PDPK1;ANG;PARK7;PPIA
ATP6AP1;OSTM1;SNX10;MAPK14
SYNCRIP;CCL24;CCL11;CCL8;JAK2;CALM1;JAK1
GSK3B;CAMK2D;PDPK1;ROCK2;WNK1;WNK3;TGFBR1
CLEC3B;MEF2C;SMAD3;ROCK2;TGFBR1
ARHGAP44;CDC42;GSK3B;PRKAA1;ROCK2;TAOK1;RICTOR;MPDZ;CRK;ARHGAP35
GSK3B;SMAD3;PDPK1;SORT1;ITGAV;JAK2;GABARAP
FER;EFEMP1;ABL2;JAK2;CLK4;JAK1;DDR2
ITGB1;CCL24;XBP1;CCL11;CD151;SEMA3C;ROCK2;TGFBR1;CDC42;DUOX1;DAB2;FER;GPNMB;AKT2;CNTN
GSK3B;VCP;DAB2;SUMO2;PTEN;MDM2;ARIH1;GABARAP

VPS29;ARFGEF2;RAB14;EXOC7;VPS35;VPS26A;EXOC5;EPS15
DDX6;CNOT7;AGO3;CNOT1
HSPA8;GNPTG;ZFYVE16;LAMP2
LMAN1;UBE2D3;HUWE1;RHOU;UBE2L3
GSK3B;MEF2C;NDFIP2;PRKAA1;SMAD3;CELF1;ROCK2;PARK7;DICER1;ESR1;SMAD5;CNOT7;CD28;SMCR8
VCP;CBFB;UBA6;UBE2D3;RNFT1;UBE2L3;PCNP;VCPIP1;RNF115;ARIH1;RNF111;PCMTD1;ATG3;SMURF2;HU
UBE2N;UBE2V2;NBN
RAB14;SORT1;EPS15
SLC46A1;GGH;SLC25A32
CHMP1B;CHMP4C;CHMP3
PDPK1;S100A1;JAK1
ARHGAP44;OGT;GABARAP
DYNC1H1;DST;SOD1
XBP1;MBTPS2
HAL;HNMT
HAL;HNMT
VMP1;CALM1
DNAJA1;LMAN1
NR1H3;OTOP1
NR1H3;OTOP1
SMOX;SLC38A2
HMGB1;TIRAP
UBE2N;UBE2V2
WDFY1;TIRAP
VCPIP1;VCP
DDAH1;ROCK2
EXOC7;ITGAV
RORB;RPGRIP1L
BMPR2;MUSK;MSTN;ROCK2;RARRES2;PARK7;TGFBR1;ACVR2A;TOM1L1;GHR;TWSG1;DAB2;RAP1A;GPNMB;A
GSK3B;MEF2C;SEMA3C;ROCK2;SLC39A10;TGFBR1
FER;EFEMP1;ABL2;JAK2;CLK4;DDR2
ITGB1;CDC42;P2RY6;NR1H3;ITGAV;HMGB1;ARHGAP12
HSPA8;GABRB2;MEF2C;P2RY6;GOLPH3;RAP1A;IGFBP5;ROCK2;LCOR;RORA;ESR1;UBE2L3
GPNMB;DCUN1D3;PTEN;BRD7;CDC73
BTF3;GSK3B;VCP;ROCK2;TTBK2
CNEP1R1;PPP6R3;CALM1;CALM2;PPIA
DYNC1H1;VCP;KIF2A;TBCE;LSM14A;KPNB1;MAP9;RAB11A
SLC7A8;SLC1A3;SLC38A2;SLC38A4
HSPA8;ST13;PTGES3;HSPA13
PNPLA8;GDE1;GPCPD1;PRDX6
CCL11;PTEN;THY1;HOOK3
VMP1;P2RY6;CALM1;CALM2
DUSP3;RPS6KB1;MDM2;PKN2
UBE2W;VCP;YME1L1;UGGT1
DAB2;BMPR2;JAK2;TGFBR1
PHF20;METTL4;TLK1;OGT
SOCS2;HSPA8;CAMK2D;ITCH;ZFYVE16;RAP1A;ROCK2;METTL4;ABL2;TLK1;PARK7;EBAG9
HSPA8;VCP;BNIP3;LAMP2;MDM2;YME1L1;TPP1;STT3B;CTSD

GOLPH3;TMED10;EXOC7;ANG;ACSL3;IER3IP1;PPIA
ARHGAP44;CDC42;ROCK2;TAOK1;RICTOR;CRK;ARHGAP35
NCKAP1;CCL24;FER;CCL11;NCK1
DNAJA1;PRKAA1;TMEM30A;LAMP1;CNEP1R1;MDM2;VPS35;SNX10;CALR;RAB6A;RAB11A
SMAD3;ACVR2A;TGFBR1
CHMP1B;CHMP4C;CHMP3
GSK3B;ABCD2;ARF1
SACM1L;PTEN;MTMR6
IL6ST;JAK2;JAK1
CAMK2D;CALM1;CALM2
HSPA8;GSK3B;MEF2C;XBP1;ROCK2;MGST1;NR1H3;RORA;RORB;HMGB1;MAPK14;ESR1;CDC73;UBE2L3;ABL
GDE1;GPCPD1;PRDX6;SMPDL3A
GOLPH3;TMED10;RAB6A;ARL5A
CCBE1;XBP1;POGLUT1;PIH1D1;PTEN;TUBD1;CDC73;TGFBR1;P2RY6;CNTN1;UBE2N;NPTN;VPS35;ITGAV;JAI
ARFGEF2;RAB14;EXOC7;SORT1;EXOC5;EPS15
TMEM150A;ROCK2;DENND4C;THY1;RAB11A;TM9SF3;TM9SF2;EFR3A;RAP1A;RAB40B;WNK3;TMED2;RAB6A;
IPO8;CNEP1R1;MDM2;KPNA4;NUP153;NUP98;CALR;IPO7;KPNB1;TNPO3
GABRB2;MEF2C;XBP1;CPEB1;P2RY6;GOLPH3;RAP1A;IGFBP5;ROCK2
GSK3B;PRKAA1;VCP;CAMK2D;ROCK2;RORA;RORB;HMGB1;CDC73;P2RY6;RAP1A;ABL2;MEF2A;MEF2C;XBP1
CNOT7;AGO3;CELF1;DICER1
CHMP1B;CHMP4C;CHMP3;RAB11A
CNEP1R1;CALM1;CALM2;PPIA
CNOT7;CD28;PKN2;PPIA
GHR;GSK3B;MUSK;SOD1
ITGB1;GSK3B;CCL24;CCL11;SNX13;THY1;RAP1A;CCL8;RGL3;SMCR8;CALM1;CALM2;RGS7
VCP;ERLIN1;DERL2;DNAJB9;CALR;STT3B;UBXN4
DUOX1;KIF2A;ROCK2;AKT2;OSGIN1;ABL2;PKN2;CRK;DUOX2;TTBK2
RANBP2;KPNA4;KPNB1
GHR;SOCS2;JAK2
BMPR2;CRELD1;TGFBR1
CCT3;NHP2;NOP10
VCP;NUDT12
SMOX;HNMT
BNIP3;HUWE1
VPS35;RAB11A
SORT1;RAB11A
C4B;C2
ITGB1;DDR2
VCP;AKT2
CALM1;CALM2
SENP5;USPL1
NHP2;NOP10
C4B;HMGB1
CCBE1;BMPR2
GSK3B;VCP;DAB2;SUMO2;MDM2;ARIH1;GABARAP;RNFT1
XBP1;PBRM1;CD28;ABL2;THY1;IL6ST;JAK2;BRD7;NCK1
INO80C;GSK3B;ROCK2;CHORDC1;UBE2V2;UBE2N;SLC38A2;MBTPS2;TAF2
IPO8;KPNA4;NUP153;NUP98;IPO7;TNPO3;KPNB1

YTHDF3;CELF1;ROCK2;METTL16;ELAVL1;ZC3H14;PUM2
ITGB1;CCL24;XBP1;CCL11;CD151;BMPR2;SMURF2;SEMA3C;ROCK2;RAB11A;TGFBR1;CDC42;DAB2;FER;GF
CHMP1B;CHMP4C;CHMP3;RAB11A
SACM1L;PTEN;MTMR6;SGPP1
DUOX1;AKT2;RARRES2;DUOX2
UBE2D3;HUWE1;RHOU;UBE2L3
KIF2A;LSM14A;MAP9;RAB11A;KPNB1
MEF2C;BMPR2;IL6ST;ACVR2A;DDR2
MUSK;DCUN1D3;PTGES3;ARRDC3;DCUN1D1;UBE2N;SERINC1;NBN;BMI1;UBE2L3;DDR2
RAP1A;MUSK;CAPRIN1;NPTN;ATP1B2;CBFA2T2;ARHGAP35;DDR2
GDE1;GPCPD1;PRDX6
ABCD2;TMEM41B;SGPP1
CHMP1B;CHMP4C;CHMP3
CNOT7;CELF1;CNOT1
SMAD3;IER3IP1;DDR2
PRKAA1;ZBTB20;SLC4A4
XBP1;RNFT1;NCK1
PARK7;PPIA;SOD1
MEF2C;PDPK1;HMGB1
DUOX1;TLR7;DUOX2
XBP1;PNPLA8;NR1H3;RORA
TMEM100;FAM83G;BMPR2;AMHR2;SMAD5;ACVR2A
VMP1;ATG3;TMEM41B;ULK2;ATG12;GABARAP
DNAJA1;XBP1;MDM2;PARK7;PPIA;TPT1
TWSG1;DAB2;BMPR2;MSTN;TGFBR1;ACVR2A
FGD4;CCL24;CCL11;FMNL2;DST;WDPCP;RHOU;RICTOR;THY1
HMGB1;TOP1
GOLPH3;TMED5
AGO3;DICER1
GNPNAT1;UAP1
PIK3CA;MAP3K7
KPNB1;RAB11A
JAK2;DDR2
GSK3B;PPP1R3C
BNIP3;VPS35
MAPK14;MAP3K7
ABCD2;AKT2
MAT2A;YME1L1
ROCK2;ZDHHC21
TMEM100;ZEB1
SMAD3;RC3H2
PTEN;SOD1
CDC42;NR1H3
NMT1;NMT2
ERLIN1;MBTPS2
WDFY1;TIRAP
JAK2;JAK1
VPS29;GSK3B;ATP6V0B;PRKAA1;EXOC7;NPRL2;BNIP3;VPS26A;HMGB1;ULK2;ABL2;VPS35;SMCR8;DRAM2;A

SLC46A1;SLC1A3;SLC25A32;FOLR2
SMAD3;AGO3;DICER1;PUM2
HSPA8;PRKAA1;XBP1;BMPR2;LAMP2;RICTOR;PRKAG3
TMED10;TMED2;TRAPPC8
YTHDF3;ROCK2;METTL16
PTGES3;NHP2;NOP10
UBE2W;VCP;UGGT1
NCKAP1;FMNL2;ROCK2
GSK3B;PPP1R3C;GAA
DUSP3;GPNMB;WNK1
CST3;GPNMB;DDR2
DUOX1;NOX4;DUOX2
ANG;DICER1;THG1L
PTGES3;NHP2;NOP10
TMED10;TMED2;TRAPPC8
PIK3CA;PLEKHA1;RPS6KB1;EXOC7;PTEN;OGT
TWSG1;DAB2;BMPR2;MSTN;JAK2;RNF111;TGFBR1;ACVR2A
CDC42;PBRM1;DCUN1D3;BIRC6;CALM1;BRD7;CALM2;MAP9;RAB11A;PUM2
GOLPH3;PIK3CA;PIH1D1;RICTOR;SMCR8
UBE2W;ITCH;UBE2D3;HUWE1;KBTBD8
ITGB1;BMPR2;GOLT1B;ROCK2;PARK7;HMGB1;EDA2R;TFG;RICTOR;ITGAV;JAK2;MAP3K7;WLS;NDFIP2;MUSK;
DNAJC3;DEPTOR;PDPK1;PTEN;SMCR8;PARK7;THY1;CALM1;PPIA;CALM2;NCK1
ITGB1;MEF2C;TWSG1;CLEC3B;SMAD3;MAP3K7;SMAD5;TGFBR1
GPX4;CHMP3;ANG;JAK2;UBE2K;GAS7
CNEP1R1;CALM1;CALM2;PPIA
PRKAA1;AKT2;ZBTB20;SLC4A4
GOLPH3;TMED10;KDELR1;RAB6A;SEC22B
CHMP1B;CHMP4C;CHMP3
MTERF4;TFB2M;TFB1M
CAMK2D;CALM1;CALM2
DYNC1H1;CLTC;MAP9
POPDC3;MEF2C;SORT1
VPS29;ATP6AP1;RAB14;VPS35;VPS26A;EPS15
TMEM258;RPN2;OSTC;ALG14;STT3B;UGGT1
CCT3;RPL5;PIH1D1;PTGES3;PTEN;PARK7;ATP1B2;LAMP1;LAMP2;MFSD1;PFDN1;CALR;NAA15;TAF9B
RPN2;GGCX;AKT2;QPCT;UBE2D3;ABL2;UBE2L3
DNAJA1;ITCH;DUSP3;FKTN
PRKAA1;AKT2;ZBTB20;SLC4A4
GSK3B;LMAN1;AKT2;SMCR8
ATG3;BNIP3;OGT;ATG12;GABARAP
SLC46A1;ATP6V0B;ATP6AP1;OTOP1;ATP6V1F
SLC46A1;ARF1;CCL11;SCARA5;ATP6AP1;SLC39A10;ATP1B2;SOD1;CCL8;STIM2;NPTN;CALR;TPT1
VMP1;ATG3;TMEM41B;ULK2;ATG12;GABARAP
VCP;UGGT1
ATP6V0B;ATP6V1F
TLR7;MAP3K7
CAMK2D;PIK3CA
GNPNAT1;UAP1

UBE2N;UBE2A
RPS6KB1;ACSL3
RPS6KB1;ACSL3
MEF2C;MYF6
DNAJA1;NDFIP2
CST3;DPP4
NR1H3;ITGAV
TTLL4;PFDN1
PRKAA1;TAOK1
MEF2C;MYF6
WNK3;ATP1B2
TMEM100;RAP1A
EMC2;EMC3
GGCX;CRYZL1
TMEM33;NCK1
DPP4;WNK1
VCPIP1;VCP
JAK2;JAK1
WNK1;TMED2;CPEB2
CHMP1B;CHMP4C;CHMP3
USPL1;INTS4;INTS8
INO80C;LMAN1;GOLPH3;TMEM33;TAOK1;VPS35;RBSN;ATG12
TMEM100;FAM83G;BMPR2;AMHR2;SMAD5;ACVR2A
CHMP1B;CHMP4C;CHMP3;KPNB1;RAB11A
GSK3B;VCP;DAB2;SUMO2;MDM2;ARIH1;GABARAP
DDX6;SYNCRIP;BMPR2;IGFBP5;CNOT1;ROCK2;CAPRIN1;ERLIN1;CALR;LSM14A
DEPTOR;RAP1A;PDPK1;SMCR8;PARK7;THY1;PPIA;DDR2;SOD1
CHMP1B;CHMP4C;CHMP3;KPNB1;RAB11A
CAMK2D;PDPK1;THY1;CALM1;CALM2
FEZ1;SMCR8;CEP97;SEC22B
ITGB1;BUD31;PARK7;JAK2
GHR;FER;JAK2;JAK1
WNK3;CALM1;CALM2;UBE2K
TBC1D8B;ITGB1;CCL24;GSK3B;CCL11;RALGAPA1;SNX13;THY1;TBCK;RALGAPB;RAP1A;CCL8;RGL3;SMCR8;
VCP;NAMPT;NUDT12
PTEN;ULK2;THY1
GSK3B;CCL11;HOOK3
MEF2C;CALM1;CALM2
ITGB1;RARRES2;MDM2
AKIRIN1;MSTN;RARRES2
CAMK2D;CALM1;CALM2
PRKAA1;DCUN1D3;MDM2
MEF2C;BMPR2;NBR1;RORB;IL6ST;ACVR2A;DDR2
GNPNAT1;UAP1
PRKAA1;XBP1
SMARCAL1;NBN
SMCR8;SEC22B
NUP153;NUP98

SMPDL3A;ITPA
VCP;PARK7
GSK3B;NMT1
NAMPT;JAK2
ATF6;MBTPS2
TMEM30A;VPS35
METTL16;METTL15
DPP4;EPS15
CAMK2D;CACNG1
PIH1D1;PARK7
TM9SF2;FKTN
NR1H3;ITGAV
ITCH;OGT
PRDX1;SOD1
RORA;TGFBR1
KPNA4;WLS
SMARCAL1;NBN
SMARCAL1;NBN
CST3;ITCH;PERP;AURKAIP1;OGT
ATG3;BNIP3;GABARAP;ATG12
POPDC3;MEF2C;POGLUT1;MYF6
ITCH;DUSP3;PPIA;FKTN
EIF4A2;GSK3B;NPRL2;PARK7
MEF2C;BMPR2;NBR1;DDR2
GSK3B;PPP6R3;CALM1;CALM2
P2RY6;MEF2C;IGFBP5;PTEN
SYNCRIP;HNRNPC;MAPK14;ELAVL1;TIRAP
SLC25A16;TIMM8B;BNIP3;VPS35;GDAP1
ABCD2;RPS6KB1;ACSL3
PIK3CA;TAOK1;TTBK2
CHMP1B;CHMP4C;CHMP3
MEF2C;RAP1A;ROCK2
CHMP1B;CHMP4C;CHMP3
SYNCRIP;CNOT7;CNOT1
TMEM100;BMPR2;ACVR2A
GSK3B;EYA4;ITGAV;BIRC6;PARK7;TGFBR1
RNF115;ITCH;UBE2Q2;UBE2D3;UBE2A;UBE2K
CHMP1B;PTEN;CHMP4C;CHMP3
DUOX1;RAP1A;IGFBP5;DUOX2
DEPTOR;DUSP3;PDPK1;SMCR8;PARK7;THY1;PPIA
CDC42;GSK3B;NCKAP1;AKIRIN1;MSTN;PIK3CA;ARHGAP35
DNAJA1;RPL5;DCUN1D3;PARK7;OGT
PRKAA1;VCP;UFM1;ULK2;ASCC3
AKIRIN1;MSTN;RARRES2;HMGB1;CALR
RNF115;ITCH;UBE2D3;MDM2;UBE2A;RNFT1
SMOX;SLC38A2
PRDX1;SOD1
ATP6V0B;ATP6V1F

USH1C;SOD1
MEF2C;MITF
NUDT12;PNRC2
ARMC1;MEF2A
TWSG1;HMGB1
ABCD2;PARK7
SMPDL3A;ITPA
ITCH;MAP3K7
SMOX;SLC38A2
IL6ST;JAK2
CELF1;DICER1
TTLL7;TTLL4
ERLIN1;MBTPS2
TMED10;ROCK2
CAMK2D;CALM2
CHMP3;DNAJC13
PAPOLA;CDC73
BNIP3;HUWE1
ABL2;ESR1
UBE2N;UBE2V2
NBR1;TIRAP
NR1H3;OTOP1
INO80C;PBRM1;EYA4;UBE2V2;UBE2N;HMGB1;BRD7
PNPLA8;NR1H3;RORA
CCL24;CCL11;CCL8
CCL24;CCL11;CCL8
CCBE1;RORA;IL6ST
LMAN1;VCP;DERL2
SYNCRIP;CPEB1;CPEB2
XBP1;PARK7;NCK1
CAMK2D;CALM1;CALM2
XBP1;IGFBP5;ROCK2;SORT1;OGT
DYNC1H1;DST;HSBP1;SOD1
SLC46A1;SLC7A8;SLC38A2;SLC38A4
NDFIP2;PTEN;NR1H3;ITGAV
VCP;AKT2;PARK7;SOD1
SMAD3;DDAH1;ROCK2;JAK2
SLC46A1;SLC35F3;SLC25A32;FOLR2
GSK3B;SMAD3;TMEM30A;AKT2;UBE2D3;NMT1;HUWE1;RHOU;UBE2L3
ITGB1;TWSG1;SMAD3;MAP3K7;SMAD5;TGFBR1
GOLPH3;TMED10;EXOC7;ANG;IER3IP1;PPIA
GTF2A1;GTF2A2;MED13;MITF;TAF2
LSM10;PAPOLA;RPRD1A;CDC73
ITGB1;DUOX1;XBP1;DUOX2
PRKAA1;ZBTB20;SLC4A4;OGT
GSK3B;PRKAA1;TAOK1;MPDZ
CST3;DDX6;SYNCRIP;IGFBP5;CNOT1;CAPRIN1;PFDN1;ITGAV;CALR;LSM14A
PRKAA1;XBP1;ZBTB20

ITGB1;MEF2C;SORT1
LMAN1;UFM1;PARK7
XBP1;NR1H3;PARK7
MEF2C;MYF6;MAPK14
ROCK2;NBR1;DDR2
DEPTOR;PTEN;ARHGAP35
EFEMP1;TRPS1;TGFBR1
GSK3B;PPP1R3C;AKT2
ITGB1;CD151;ARHGAP35
MEF2C;CD28;JAK2;IL6ST;TIRAP;NCK1
DPP4;HSPA8;EXOC7;TMEM33;CHMP1B;USO1;CHMP4C;CHMP3;TMCC1;EXOC5;SNAP29
AGTPBP1;ATG12
HMGB1;TOP1
MAP3K7;TIRAP
XBP1;NAMPT
MAOA;HNMT
MEF2C;BMPR2
SLC46A1;SLC25A32
PHF20;OGT
ERLIN1;SOD1
SVEP1;VSIG4
DUSP3;CRK
NR1H3;ITGAV
ABCD2;AKT2
PHF20;OGT
CNOT7;CNOT1
PDPK1;HMGB1
AGO3;DICER1
NHP2;NOP10
CDC42;ROCK2
HMGB1;TIRAP
PHF20;OGT
DDAH1;ZDHHC21
CNOT1;CALR
TMEM100;ZFHX2
MEF2C;IGFBP5
CAMK2D;PIK3CA
USPL1;ELL2
ATP1B2;SLC4A4
SMAD3;MSTN;PTEN;NR1H3;RORA;RORB;CDC73;TGFBR1;EFEMP1;NBR1;ITGAV;CALR;ZBED6
CAMK2D;SMAD3;BMPR2;DCUN1D3;OSGIN1;DERL2;CGRRF1;TGFBR1;SOCS2;CDC42;ITCH;RICTOR;TAF9B;E
ITGB1;CCL24;XBP1;DUSP3;CCL11;CD151;YTHDF3;IGFBP5;SEMA3C;ROCK2;PTEN;OSGIN1;MITF;THY1;TGFB
KIF2A;CHMP1B;CHMP4C;CHMP3;LSM14A;KPNB1;MAP9;RAB11A
CACNG7;ZFYVE16;RNF128;MFSD1
GSK3B;PRKAA1;TAOK1;MPDZ
SYNCRIP;DDX6;IGFBP5;CNOT1;CAPRIN1;CALR;LSM14A
MEF2C;BMPR2;NBR1;ACVR2A;DDR2
SLC7A8;SLC1A3;SLC38A2

CHMP1B;CHMP4C;CHMP3
CCBE1;RORA;IL6ST
PTGES3;NHP2;NOP10
RPL5;AGO3;RPL10;CELF1;PTGES3;RPS27L;EIF3E;DICER1;HNRNPC;CDC73
GHR;SOCS2;HSPA8;MEF2C;XBP1;ESR1;UBE2L3
RPL5;GSK3B;PRKAA1;BMPR2;CELF1;ROCK2;RPS27L;PARK7;ELAVL1;VPS35;BRD7;SLC38A2;MEF2C;SMAD3;
CCL24;CCL11;CCL8;FOLR2
GSK3B;PTEN;THY1;ARHGAP35
CDC42;S100A1;CALM1;CALM2
GTF2A2;RSF1;TFB2M;TFB1M;TAF2
CDC42;GSK3B;HSPA8;HMGB1;PARK7
CHMP1B;CHMP4C;CHMP3;VPS35;PARK7
PIK3CA;WNK1;CD28;UBE2N;THY1;MAP3K7;RC3H2
GPNMB;CD28;VSIG4;JAK2;IL6ST;NCK1
NCKAP1;FMNL2;ROCK2
ITGB1;DUOX1;DUOX2
XBP1;JAK1
CLTC;EPS15
EDEM3;UGGT1
GABRB2;LGI2
SPPL2A;MBTPS2
EFEMP1;TGFBR1
DNAJB9;NCK1
USH1C;TPP1
ABCD2;NR1H3
MSTN;RARRES2
TMEM33;DNAJB9
ABCD2;AKT2
CDC42;FER
NAMPT;JAK2
ROCK2;VPS35
NR1H3;MBTPS2
TMEM100;RAP1A
CAMK2D;PIK3CA
TGFBR1;SOD1
GREM2;SMURF2;UBE2D3;GPR155
DEPTOR;ITGAV;BIRC6;PARK7;TGFBR1
MEF2C;PIK3CA;PLEKHA1;WNK1;CD28;UBE2N;THY1;MAP3K7;RC3H2
ARHGAP44;GSK3B;RAP1A;MUSK;PTEN;NPTN;ABL2;ATP1B2;CBFA2T2;ARHGAP35;DDR2
CNTN1;TLR7;HMGB1;PARK7;TIRAP
GSK3B;DYNC1H1;CNOT1;CAPRIN1;G3BP2
CHMP1B;CHMP4C;CHMP3;NUP98
SOCS2;EFR3A;TMEM150A;PIK3CA
RPS6KB1;OGT;SNX5;NCK1
NCKAP1;CCL24;DYNC1H1;FER;CCL11;NCK1
GSK3B;NDFIP2;DCUN1D3;DCUN1D1;HUWE1;UBE2L3
NR1H3;ITGAV;HMGB1
DPP4;USO1;SNAP29

COPS3;MDM2;EEF1E1
AKT2;RORA;OGT
ZBTB20;NR1H3;THRSP
DDAH1;S100A1;ZDHHC21
GSK3B;PTEN;VPS35
DNAJA1;HSPA8;NDFIP2
TMED10;TMED2;TRAPPC8
ITGB1;MSTN;PIK3CA;PLEKHA1;CNTN1;PTEN;RICTOR;PARK7;TGFBR1
SOCS2;EFR3A;TMEM150A;PIK3CA;SACM1L;PTEN;MTMR6
NAA20;CPD;CNTN1;NAA35;AEBP1;NAA15;OGT;MBTPS2
METTL4;THUMPD3;TFB2M;TFB1M;METTL16
GDE1;SERINC1;GPCPD1;PRDX6;PLA1A
INO80C;POGLUT1;TADA2A;RAB14;WDPCP
SMAD3;DST;CHMP1B;CHMP4C;CHMP3;TGFBR1
GPT2;IDH1
DYNC1H1;KPNB1
PCGF1;BMI1
AKT2;JAK2
RPL7L1;RPL35
DDX6;AGO3
EFEMP1;TGFBR1
CALM1;CALM2
MEF2C;IGFBP5
NUP153;NUP98
AMHR2;TGFBR1
PARK7;IL6ST
CNOT7;CNOT1
BNIP3;SMCR8
XBP1;SMAD5
WNK3;NR1H3
PNPLA8;PTGES3
RNF128;MFSD1
DNAJA1;CTSD
PHF20;OGT
CNOT7;CNOT1
TMEM100;ZFHX2
EFR3A;RAP1A;TMEM150A;RAB40B;ROCK2;DENND4C;WNK3;TMED2;RAB11A
ROCK2;TLR7;MAP3K7;TIRAP
RAP1A;MUSK;CAPRIN1;NPTN;ATP1B2;CBFA2T2;ARHGAP35;DDR2
GOLPH3;RRM2B;DDR2
ITGB1;NINJ1;ITGAV
SLC46A1;SLC25A32;FOLR2
NAA20;NAA35;NAA15
ADH7;TGFBR1;SOD1
HSPA8;NR1H3;UBE2L3
ATP6V0B;DMXL1;ATP6V1F
ANXA4;CNTN1;TLR7;HMGB1;PARK7;TIRAP
CHID1;TWSG1;ABCD2;ITCH;GPNMB;ANXA4;NPTN;VSIG4;NMBR;HMGB1;ATG12

RPL5;RPS25;PIH1D1;INTS4;RRP12;INTS8;THG1L
UBE2W;UBE2N;NMBR;NCK1
RANBP2;SENP5;SUMO2;MDM2
SCOC;HSPB8;BNIP3;SMCR8;MAP3K7
HMGB1;ASCC3;NBN
ARHGAP44;CAPRIN1;PTEN
MAP3K7;TIRAP
VCP;DERL2
PHF20;OGT
PHF20;OGT
METTL4;METTL16
XBP1;PARK7
ATAD1;ARF1
CNOT7;YTHDF3
RAP1A;SORT1
GNPNAT1;UAP1
XBP1;ACSL3
SNX10;HOOK3
GSK3B;CHORDC1
SORT1;NR1H3
ELAVL1;PUM2
GHR;SOD1
BMPR2;DDAH1
GSK3B;SHC2;MUSK;SORT1;RARRES2;MAPK14;SOCS2;GHR;FER;PIK3CA;RAB14;AKT2;NAMPT;JAK2;CRK;DDF
ATP8A1;SLC7A8;SLC1A3;ATP1B2;SLC4A4;SLC38A2
RPS26;CELF1;METTL4;METTL16;SLC38A2;CLK4;PRDX6
GOLPH3;TMED10;EXOC7;DPH3;ANG;IER3IP1;PPIA
ARFGEF2;GOLPH3;EXOC7;EXOC5
GSK3B;ROCK2;SLC39A10;TGFBR1
CST3;WNK1;TMED2;CPEB2
PBRM1;XBP1;BRD7;SOD1
ITGB1;DUOX1;CRK;DUOX2
ATF1;VCP;XBP1;ATF6
CD28;HMGB1;JAK2;IL6ST;NCK1
DUSP3;RPS6KB1;MDM2;PKN2;CALR
XBP1;ABCD2;ACSM3;GPX4;PNPLA8;ACSL4;ACSL3;ADIPOR2
DPP4;ITGB1;DAB2;ITCH;CLTC;MRC1;DNAJC13;EPS15
TMED10;SMAD3;GPX4;HSPA4;PIH1D1;PTGES3;CLTC;USH1C;MITF;COX7A2;TRAPPC8;CAPZA2;TBCE;MDM2;
EFR3A;RAP1A;TMEM150A;RAB40B;ROCK2;DENND4C;WNK3;TMED2;RAB11A
GSK3B;FER;CAMK2D;MUSK;PDPK1;TAOK1;WNK3;ULK2;JAK2;DDR2
RCL1;RPS25;RPS8;HEATR1;RPS27L;RPS11
CCL24;GSK3B;XBP1;CCL11;GHR;SYNCRIP;DUOX1;GREM2;FER;CCL8;WNK1;CALM1;JAK2;IL6ST;UBE2K;DUC
ANG;JAK2;GAS7
MSTN;PTEN;ULK2
DAB2;CNOT1;CALR
GHR;JAK2;JAK1
CHMP1B;CHMP4C;CHMP3
TWSG1;SMAD3;SMURF2

SOCS2;LCOR;ESR1
TLR7;MAP3K7;TIRAP
DYNC1H1;HOOK3;RAB11A;KPNB1
DAB2;ZFYVE16;CD151;ABL2;ITGAV
MUSK;ROCK2;RARRES2;ACVR2A;DAB2;GPNMB;AKT2;CNEP1R1;TAOK1;NPTN;CALM1;CALM2;PPIA
ITGB1;CDC42;DAB2;DST;ITGAV;THY1
BMPR2;PDPK1;ROCK2;HMGB1;CALR;MAPK14
ATP8A1;SLC7A8;SLC1A3;ATP1B2;SLC4A4;SLC38A2
CCL24;MEF2C;CCL11;MUSK;ROCK2;HMGB1;TGFBR1;TIRAP;SOD1;GHR;P2RY6;RAP1A;CCL8;GPNMB;TAOK1[illegible]
GSK3B;ITGAV
NOX4;SOD1
ITGB1;PIK3CA
ABCD2;PARK7
MDM2;TAF9B
DYNC1H1;HOOK3
BMPR2;TGFBR1
EDEM3;UGGT1
SMCR8;ATG12
ROCK2;ZDHHC21
TMED10;HMGB1
SMAD3;ITGAV
CNEP1R1;NR1H3
UFM1;ULK2
EMC2;EMC3
BMPR2;PIK3CA
VCP;SMAD3;CNTN1;RPS27L;JAK2
RANBP2;NUP153;NUP98;CALR
SOCS2;CAMK2D;ITGAV;CRK
TMED10;CCL8;EXOC7;EXOC5;SNAP29;RAB11A
GSK3B;DAB2;STYX;RSF1;PARK7;JAK2
NEK9;DUSP3;RPS6KB1;DYNLT3;CDC27;MDM2;PKN2;BRD7
DICER1;MAP3K7;TIRAP
ATP6V0B;DMXL1;ATP6V1F
STYX;PARK7;OGT
PLEKHA1;PIK3CA;PTEN
CAMK2D;CALM1;CALM2
GOLPH3;TMED10;CHMP4C
LSM10;PBRM1;GPNMB;DCUN1D3;PTEN;BRD7;CDC73
MEF2A;MEF2C;CAMK2D;PRKAA1;SYT9;CALM1;CALM2
INO80C;PBRM1;PIH1D1;SETD7;CHD6;RSF1;BMI1;ESR1;TADA2A;PCGF1;HNRNPC;TOP1;BRD7
HSPA8;XBP1;PRKAA1;BMPR2;NPRL2;LAMP2;XPR1;GABARAP
SLC46A1;GPT2;IDH1;SLC25A32
SLC46A1;ATP6AP1;SCARA5;SOD1
DEPTOR;PIH1D1;NPRL2;SMCR8
RPL5;RPL10;MTERF4;RPS27L
CAMK2D;PIK3CA;GAA
RPS6KB1;EXOC7;OGT
HMGB1;MAPK14;TIRAP

PBRM1;RRM2B;BRD7
PDPK1;S100A1;JAK1
CNOT7;YTHDF3;UBE2K
NAMPT;NUDT12
TAOK1;PTEN
CELF1;RICTOR
ITGB1;GOLPH3
ACSL4;ACSL3
CWC15;HNRNPC
PARK7;PPIA
RPL5;PARK7
MEF2C;PTEN
NMBR;JAK2
ATF6;MBTPS2
PNPLA8;PTGES3
SNX10;HOOK3
METTL4;NBN
VCP;COX7A2
BMPR2;TRPS1
VCP;COX7A2
PRKAA1;TADA2A
RPL5;PTEN
VCP;DERL2
OSTM1;SLC1A3
MEF2C;GPNMB;VPS35;PARK7;IL6ST;SOD1
MEF2A;MEF2C;CAMK2D;PRKAA1;SYT9
MEF2A;CAMK2D;S100A1;CALM1;CALM2
MUSK;ROCK2;PTGES3;RARRES2;ACVR2A;DAB2;GPNMB;AKT2;NPTN;ANG;NBN;PPIA;DDR2
VCP;KCTD21;TUBD1;RORA
TAOK1;NBR1;PPIA;TIRAP
PRKAA1;PIK3CA;GPX7;PARK7;PPIA;CPEB2
NCKAP1;CCL24;FER;CCL11;NCK1
ROCK2;NMT1;NMT2;RPGRIP1L;RGS7
NCKAP1;CCL24;FER;CCL11;NCK1
TOM1L1;TM9SF2;RPL10;MITF;JAK2
VCP;SMAD3;CNTN1;RPS27L;HMGB1;JAK2;CTSD
INO80C;PBRM1;MORF4L2;EYA4;UBE2V2;UBE2N;BRD7;TAF2
ATG3;BNIP3;VPS35
PRKAA1;XBP1;ZBTB20
TOM1L1;CHMP4C;AURKAIP1
CDC42;ROCK2;RHOU;ARHGAP35
CNOT7;YTHDF3;VSIG4;ATG12
DDAH1;NR1H3;SMCR8;THY1
GTF2A1;GTF2A2;MED13;TAF2
ITCH;DUSP3;TAOK1;HMGB1;EDA2R;TIRAP;FKTN
GSK3B;DAB2;STYX;PARK7;TTBK2
HSPA8;PIK3CA;TAOK1;PFDN1;TTBK2
CDC42;GSK3B;CALR;JAK2;CRK

ARHGAP44;FGD4;CRK;ARHGAP12;SOD1
CDC42;NCKAP1;GSK3B;PTEN;USH1C;DICER1;HMGB1;RAB6A;ARHGAP35;RAB11A;GAS7
ATG3;ULK2
CAMK2D;PIK3CA
GSK3B;PPP1R3C
GSK3B;MEF2C
ACSL4;ACSL3
ATP6V0B;ATP6V1F
GDAP1;THG1L
RNF115;DUSP3
DUSP3;THY1
NR1H3;ITGAV
CCL11;HOOK3
MEF2C;KBTBD8
MSTN;RARRES2
LAMP1;JAK2
CALM1;CALM2
CNEP1R1;NR1H3
ROCK2;SGPP1
TADA2A;UBE2N
ROCK2;DLGAP4
ADH7;SOD1
RPL5;GSK3B;MEF2C;PRKAA1;SMAD3;BMPR2;MUSK;CELF1;ROCK2;NR1H3;MITF;PARK7;MAPK14;TGFBR1;ITG
CELF1;METTL4;MITF;CLK4;PRDX6;RC3H2
ERP44;GOLPH3;PARK7;STT3B
CDC42;MEF2A;PTEN
SLC7A8;SLC38A2;SLC38A4
CDC42;CALR;CRK
AGO3;DICER1;PUM2
POPDC3;MEF2C;MYF6
CPEB1;BNIP3;MDM2;RORA;CPEB2
ATG3;TMEM41B;YTHDF3;SNX10;ATG12;GABARAP;RAB11A;TTBK2;VMP1;AP1G1;CHMP1B;CAPRIN1;CHMP4C
CPEB1;BNIP3;MDM2;RORA;CPEB2
RNF115;ITCH;UBE2V2;UBE2N
MEF2C;PDPK1;HMGB1;MAPK14
DUSP3;ROCK2;PTEN;THY1
BMPR2;IGFBP5;OGN;ANG
IDH1;NUDT12
SLC4A10;SLC4A4
CDC42;GOLPH3
HEXA;ST6GALNAC5
TM9SF2;ST6GALNAC5
ITGB1;GOLPH3
PTEN;IPO7
PTEN;NCK1
NUP153;NUP98
PHF20;OGT
BNIP3;VPS35

MUSK;THY1
DAB2;DNAJC13
MSTN;RARRES2
BNIP3;ETF1
INTS4;INTS8
ITGB1;PIK3CA;CNTN1;RICTOR;PARK7;TGFBR1
CCL24;BMPR2;CCL11;CNTN1;ANG;TGFBR1
TMED10;TMED2;TRAPPC8
MDM2;RPS27L;NBN
TADA2A;MAP3K7;TAF2
GAA;TPP1;HOOK3
MSTN;PLEKHA1;PTEN
NUP98;SLC38A2;PRDX6
P2RY6;ANG;ESR1
MDM2;CDC73;SERF2
RCL1;RPL5;RPS25;RPS8;PIH1D1;HEATR1;RRP12;RPS11;RSL24D1
RANBP2;USPL1;SENP5;SUMO2;MDM2;PARK7
AGTPBP1
HMGB1
SLC1A3
SLC1A3
NUDT12
RICTOR
ADIPOR2
HSPA8
BMPR2
CSGALNACT2
RPL10
USH1C
HNMT
USH1C
MAP3K7
JAK1
JAK2
XPR1
SCTR
NINJ1
PRKAA1
CTSD
LSM10
NCK1
PIK3CA
CHMP4C
PTEN
GSK3B
IGFBP5
SORT1
FER

VCP
PPIA
NCK1
CALR
MEF2C
WNK3
DPH3
DPH3
JAK2
ACVR2A
HUWE1
MEF2C
PRKAA1
PTEN
DUSP3
WNK3
RNF128
GSK3B
MYF6
PRKAA1
NR1H3
TIRAP
NCK1
PTEN
PTEN
CAST
LAMP2
UBA6
UFM1
TADA2A
METTL16
HSPH1
CST3
TMEM33
ABL2
DUSP3
GSK3B
JAK2
XBP1
CAMK2D
CLTC
AKT2
EIF3E
MEF2C
PUM2
G3BP2
ROCK2
NCK1

ROCK2
TLR7
ROCK2
NCK1
PIH1D1
DICER1
MPDZ
WDPCP
MEF2A
TGFBR1
CAST
HSPA8;RAP1A;ROCK2
TWSG1;BMPR2;SMURF2;UBE2D3;GPR155
XBP1;SMAD3;RARRES2;RORA;MEDAG
GTF2A1;GTF2A2;ESR1;TAF2
CPQ;CPD;TPP1;AEBP1
ITGB1;CAMK2D;CALM1;CALM2
ITGB1;NCKAP1
LCOR;ESR1
RPL7L1;RPL35
ITGB1;BMPR2
PTEN;IPO7
CHID1;ABCD2
NFATC3;ESR1
TAOK1;TTBK2
GSK3B;ROCK2
DAB2;CLTC
DAB2;CLTC
SERINC1;PLA1A
ABCD2;AKT2
PDPK1;ESR1
CCBE1;CLEC3B
P2RY6;IGFBP5
CAMK2D;CACNG1
MEF2C;TGFBR1
ITGB1;DDR2
MSTN;RARRES2
ITCH;OGT
MAPK14;MAP3K7
CCL24;P2RY6;RAP1A;CCL11;CCL8;MUSK;GPNMB;HMGB1;TIRAP;DDR2
USH1C;UBE2A;TAF2
PAPOLA;RPRD1A;INTS8
SLC46A1;SLC7A8;SLC1A3
CAST;PRKAA1;PDPK1
MEF2C;BMPR2;ACVR2A
P2RY6;CALM1;CALM2
CCL24;DUSP3;CCL11;MUSK;STYX;PTEN;HMGB1;TIRAP;P2RY6;RAP1A;CCL8;GPNMB;DDR2
GSK3B;RPL5;BMPR2;BNC2;CELF1;PHF20;CHD6;ZBTB20;JMJD1C;RORA;RSF1;RORB;PARK7;BMI1;AFF4;UBE2[illegible]

MEF2C;XBP1;NR1H3;HMGB1;MAPK14;CDC73;TIRAP
FER;PRKAA1;ERCC6L2;GPX7;PARK7;PPIA;CPEB2
CCL24;CCL11;CCL8;WNK1
CACNG7;VCP;SORT1;HOOK3
RPL10;CELF1;METTL16;PUM2
GTF2A1;GTF2A2;MED13;TAF2
MEF2C;XBP1;FER;NR1H3;HMGB1;JAK2;MAPK14;CDC73;TIRAP
VCPIP1;VCP;PRKAA1;RAB14;WDPCP;CTSD
ARHGAP44;FGD4;ARHGEF12;ITGAV;RHOU;ARHGAP35;ARHGAP12
RCL1;RPL5;RPS25;RPS8;HEATR1;RPS11;RSL24D1
SMAD3;BUD31;PTEN;PARK7;ESR1;EDA2R;TIRAP;FER;UBE2N;MAP3K7;PPIA;MBTPS2;DDR2
HSPA4;PTGES3
NFATC3;ESR1
ABCD2;PARK7
CPQ;TPP1
RRM2B;RAB11A
TLR7;NMBR
YTHDF3;RPS6KB1
ITGB1;PIK3CA
DUSP3;ROCK2
CAMK2D;TADA2A
SLC30A4;SLC39A10
RPL10;CBFB;UBE2D3;CREM;ZBTB20;AEBP1;HMGB1;BMI1;CDC73;IKZF5;TRPS1;PCGF1;TAF9B;ZBED6;MEF2/
MEF2A;MEF2C;MSTN;FKTN
GSK3B;BNIP3;VPS35;PARK7
MEF2C;GPNMB;VPS35;PARK7
ATF1;SOCS2;IGFBP5;ESR1
UFM1;ULK2;CALR;OGT
NUP153;NUP98;KPNB1
PHF20;MORF4L2;OGT
RPL5;PARK7;OGT
RANBP2;NUP153;NUP98
CNEP1R1;ZBTB20;NR1H3
PBRM1;XBP1;BRD7
PRDX1;ATP1B2;SOD1
PBRM1;SMAD3;BMPR2;MSTN;IGFBP5;CELF1;PTEN;CGRRF1;CDC73;SAV1;CNOT7;GPNMB;DDAH1;OGN;NOX
GSK3B;PRKAA1;PTEN;PARK7;PPIA
UBE2W;VCP;CHMP4C;MDM2;YME1L1
ITGB1;XBP1;S100A1;PDPK1;NINJ1;CNTN1;JAK1
SMARCAL1;VCP;ERCC6L2;HUWE1;PARK7;UBE2A;RAD23B;VCPIP1;RRM2B;PSME4;UBE2V2;UBE2N;NBN;AS(
MEF2A;TIMM8B;AGTPBP1;BNIP3;YME1L1;COX7A2;PARK7;GDAP1;THG1L
NBN
XBP1
NPL
SMAD3
TADA2A
DDR2
STAU2

SOD1
SOD1
TIRAP
GABRB2
JAK1
DDAH1
HSPA8
MEF2C
UBE2K
HEXA
GAA
GAA
PCGF1
USH1C
JAK2
JAK1
XBP1
PTEN
WNK1
SERINC1
TWSG1
RC3H2
PTEN
NR1H3
GSK3B
ITGAV
THY1
PARK7
PARK7
CNOT1
PPIA
NBN
RAB11A
CACNG7
SORT1
PNRC2
VCP
USH1C
PARK7
BMPR2
SLC39A10
TMEM33
EIF3E
ROCK2
SMCR8
SCTR
JAK2
NCK1

NR1H3
EIF3E
HUWE1
GHR
CDC42
GSK3B
ITCH
DYNC1H1
SMURF2
ABCD2
LAMP2
AGTPBP1
DPP4
HSPH1
PARK7
JAK2
DENND4C
HMGB1
PIH1D1
SMAD3
TTBK2
MAP9
ROCK2
PARK7
NMBR
MSTN
SMAD3
NFATC3
ASCC3
SLC38A2
NUP98
OSTM1
TBCE
ABCD2
SEC22B
CCL24;CCL11;CCL8;WNK1
DYNC1H1;KIF2A;TBCE;KPNB1
CNOT7;YTHDF3;NR1H3;OTOP1
CCBE1;PERP;AURKAIP1;OGT
TIMM8B;HSPA4;DNAJC15;GDAP1
GTF2A1;GTF2A2;ESR1;TAF2
GSK3B;VCP;PTEN;VPS35;SEC22B
ITGB1;PDPK1;AKT2;WNK3;NMT1
PFKFB4;DUSP3;GPX4;PIK3CA;PPA1;TAOK1;GDE1;RICTOR;TOP1;HNMT
SLC7A8;SLC1A3;SLC38A2
USH1C;UBE2A;TAF2
ABL2;MAPK14;SOD1
FEZ1;SMCR8;SEC22B

FER;MAP3K7
CALM1;UBE2K
UBE2W;HSPB8
JAK2;ARHGAP35
CDC42;ROCK2
GSK3B;UFM1
TMCC1;GDAP1
ANG;SOD1
GSK3B;ARHGAP35
UFM1;PARK7
GSK3B;PARK7
RPL5;RPL10
B3GALNT2;GOLPH3;BMPR2;CSGALNACT2;FKTN
XBP1;C7;POLR3G;IL6ST;TIRAP
ITGB1;DAB2;PDPK1;CLTC;WNK3
SYNCRIP;CCL24;CCL11;CCL8;CALM1
TADA2A;MORF4L2;MAP3K7;TAF2
RPL5;RPS25;PIH1D1;NHP2;RRP12;TFB1M
PIK3CA;GAA;SOD1
CCL24;CCL11;CCL8
MEF2C;BMPR2;ACVR2A
TADA2A;TBL1XR1;MORF4L2;UBE2N;UBE2A
CDC42;DERL2;RICTOR;TAF9B;TGFBR1
KIF2A;LSM14A;KPNB1;MAP9;RAB11A
NCKAP1;RAP1A;ROCK2;G3BP2;RGL3;PARK7;ARHGAP35;KPNB1
RPL5;ARHGEF12;PDPK1;PTEN;ARHGAP35;ARHGAP44;FGD4;TBCK;TMBIM4;UBE2N;RHOU;RICTOR;ITGAV;CA[illegible]
PRKAA1;DUSP3;DEPTOR;MSTN;PLEKHA1;NPRL2;MDM2;PTEN;RORA;MAPK14;ESR1;ARHGAP12
CCL24;XBP1;BMPR2;CCL11;CNTN1;ANG;TGFBR1
CCL11;CCL8;STIM2;NPTN;CALR;TPT1
EFEMP1;EYA4;SLC4A10;USH1C;DRAM2;ATF6
CCBE1;XBP1;TMED10;ROCK2;RORA;PARK7;HMGB1;MAPK14;TIRAP;SOD1;CD28;CNTN1;TLR7;JAK2;IL6ST;M[illegible]
BMPR2;TGFBR1
VPS35;WLS
ITCH;MAP3K7
BMPR2;CRELD1
ACSL4;ACSL3
RNF115;DUSP3
GSK3B;PARK7
DUSP3;GPNMB
GSK3B;CALR
PARK7;PPIA
DUSP3;THY1
NUP98;PRDX6
ABL2;PARK7
STAU2;JAK1
SMAD3;SMURF2
ERLIN1;MBTPS2
PRKAA1;RARRES2

XBP1;MAPK14
SLC30A4;SLC39A10
TOM1L1;RAP1A;CAB39L;PIH1D1;NOX4;CALM1;CALM2;DDR2
NDFIP2;TFG;GOLT1B;PDPK1;UBE2N;ZDHHC13;RORA;MAP3K7;ESR1;EDA2R;TIRAP;WLS
GHR;SOCS2;CLEC3B;IGFBP5;PARK7
PIH1D1;ITGAV;BIRC6;PARK7;TGFBR1
SHC2;BMPR2;MUSK;IL6ST;JAK2;ACVR2A;DDR2
SLC7A8;SLC38A2;SLC38A4
DPT;TGFBR1;DDR2
CACNG7;MEF2C;DLGAP4
RPL5;PTEN;RAD23B
POPDC3;MEF2C;MYF6
ITCH;DUSP3;PTEN;NR1H3;RORA;VPS35;CRK;ATG12
TADA2A;BIRC6;CALM1;CALM2;RAB11A
ASCC3
ASCC3
GSK3B
ERLIN1
JAK1
NPL
DDAH1
SLC7A8
PON2
MAOA
JAK1
TGFBR1
G3BP2
CSGALNACT2
MAOA
CDC42
WDPCP
ADH7
USH1C
MPND
JMJD1C
CAST
JAK1
PARK7
ULK2
GPX4
BMPR2
AKT2
BNIP3
BMPR2
MSTN
RC3H2
SVEP1
ELAVL1

ZBED6
ROCK2
ROCK2
NFATC3
CACNG7
P4HA1
DUSP3
PLA1A
NCKAP1
PRKAA1
ROCK2
TGFBR1
CALR
DDR2
HMGB1
MEF2C
MAPK14
JAK2
CLEC3B
NR1H3
IGFBP5
CCBE1
PTEN
CACNG7
PARK7
KDELR1
VCP
NUDT12
VMP1
TWSG1
OGT
CAMK2D
ULK2
CALM2
DPH3
CALR
VPS35
HMGB1
TGFBR1
DNAJA1
GSK3B
IER3IP1
PARK7
FER
JAK2
ITGAV
USH1C
JAK2

PARK7
CNTN1
FKTN
GSK3B
MYF6
ITGAV
XBP1
PTEN
DCUN1D3
DICER1
SLC38A2
STIM2
MAPK14
WARS2
GHR
SLC7A8
THOC6
TGFBR1
THOC6
B3GALNT2;POGLUT1;TMEM258;RPN2;OSTC;STT3B;OGT;FKTN
CCL24;CCL11;ADIPOR2;GHR;DUOX1;GREM2;FER;CCL8;JAK2;IL6ST;MAP3K7;DUOX2;JAK1
PRKAA1;XBP1;ZBTB20
GAA;TPP1;HOOK3
AGO3;METTL16;NUDT12
MEF2C;BMPR2;ACVR2A
CDC42;ROCK2;TGFBR1
PTEN;ARHGAP35;RAB22A
USH1C;OTOP1
SCARA5;CLTC
TMEM30A;ATP8A1
PAPOLA;CDC73
DUSP3;THY1
ROCK2;PTEN
CAMK2D;CALM2
BMPR2;SEMA3C
RRM2B;RAB11A
DAB2;RNF111
NR1H3;ZBTB20
DAB2;RNF111
CACNG7;MEF2C
WNK1;THY1
TAOK1;TTBK2
MAPK14;ZBED6
VPS29;ABL2;SMCR8;DRAM2
PBRM1;MORF4L2;UBE2V2;UBE2N;BRD7
ARHGAP44;GSK3B;MEF2C;POGLUT1;FMNL2;GPNMB;ARHGAP35
TIMM8B;DNAJC15;GDAP1
CCL24;CCL11;CCL8

AGO3;CELF1;ANG;METTL16;NUDT12
CNOT7;CNOT1;EIF3E;RC3H2;PNRC2
VCP;SMURF2;TBL1XR1;WNK1;WLS
MUSK;NPRL2;NBN;DDR2;SOD1
PAPOLA;CDC73
HSPA8;UBE2L3
GSK3B;MEF2C
ACSL4;ACSL3
USH1C;OTOP1
ITGB1;GOLPH3
CHMP4C;NBN
DEPTOR;NPRL2
TAF9B;TPT1
DAB2;CLTC
NDFIP2;DPH3
VCP;KCTD21
PNPLA8;CEPT1
WNK1;ABL2
CD28;MAP3K7
BMPR2;CSGALNACT2
ACOT9;ACSM3
ARRDC3;MAPK14
UFM1;CNOT1
ATP6V0B;ATP6V1F
DPP4;TIRAP
JAK2;IL6ST
ARHGAP44;VPS35
ACOT9;ACSM3
TMED10;TMED2
GSK3B;MEF2C;XBP1;WNK1;TAOK1;HMGB1;RAB6A;RAB11A
GSK3B;DUSP3;ROCK2;THY1
GHR;CNOT7;JAK2;IL6ST
EFEMP1;PIK3CA;PDPK1
POLR3G;UBE2K;TIRAP
DEPTOR;PPIA;TGFBR1
BMPR2;CELF1;DCUN1D3;PTEN;PARK7;CDC73;SAV1;GPNMB;CHMP1B;VPS35;JAK2;FKTN;CD164;PBRM1;S[illegible]
ITGB1
RHOU
RPS27L
AGO3
JAK1
CPA3
DICER1
TPP1
PIH1D1
ERLIN1
PTGES3
MEF2C

SLC38A2
USH1C
JAK2
IL6ST
IDI1
ITGB1
HMGB1
NR1H3
CPEB2
SORT1
BNIP3
TOM1L1
PARK7
UFM1
RAP1A
NFATC3
BMPR2
NDFIP2
RPL5
NAMPT
RRM2B
SETD7
VCP
JAK2
IL6ST
CAPRIN1
SMAD3
P2RY6
BNIP3
ROCK2
ROCK2
UBE2K
PTEN
PTEN
SLC38A2
HOOK3
MITF
MITF
CPA3
ROCK2
SVEP1
VPS35
SGPP1
IER3IP1
PPP1R3C
MDM2
METTL4
MAP9

BTBD7
ROCK2
MEF2C
PARK7
GSK3B
UBE2W
COG6
WDPCP
ETF1
IDH1
KLHDC1
GGCX
FER;SMCR8;MAPK14;ATG12
PBRM1;CDC73
FER;MAP3K7
CDC42;SMURF2
FER;CDH9
CDC42;PTEN
MDM2;RPS27L
RPS6KB1;NCK1
DICER1;PNRC2
GDAP1;THG1L
CDC42;ARHGAP12
MAPK14;ACVR2A
S100A1;PARK7
STYX;CALR
CDC42;ROCK2
ERLIN1;NR1H3
TLR7;NMBR
ROCK2;SGPP1
PBRM1;BRD7
ITGB1;ATP1B2
ABCD2;PARK7
BMPR2;ZDHHC21
MEF2A;MEF2C;CAMK2D;PRKAA1;ROCK2;SYT9
CST3;ROCK2;BNIP3;HUWE1;THY1;OGT
VCP;SMURF2;TBL1XR1;WNK1;CDC73;WLS
NDFIP2;GOLT1B;TFG;UBE2N;ZDHHC13;MAP3K7;EDA2R;TIRAP;WLS
SLC7A8;SLC38A2;SLC38A4
DPP4;PIK3CA;PKN2
PDPK1;HMGB1;MAPK14
CHID1;ABCD2;MAPK14
RNF115;FER;DUSP3
BMI1;CDC73;DDR2
PRKAA1;XBP1;ZBTB20
AP1G1;CHMP1B;CHMP3
MUSK;ROCK2;RARRES2;PTEN;PARK7;ACVR2A;SOD1;DAB2;FER;GPNMB;AKT2;NPTN;PPIA
PBRM1;MORF4L2;UBE2V2;UBE2N;BRD7

GOLPH3;AP1G1;PARK7;WLS
CNOT7;CD28;PKN2;PPIA
DAB2;MAF;PTER;AKT2;PIH1D1;ANXA4;TPP1
GSK3B;PLEKHA1;WDPCP;ATP1B2;HMGB1;RAB6A;RAB11A
ZFYVE16;CHMP3;ABL2;DNAJC13;RAB11A
DAB2;SMAD3;TGFBR1
CD28;VSIG4;MAP3K7
SYNCRIP;AGO3;PAPOLA;ANG;DICER1;RBMS2
RPS6KB1;CDC27;PPP2R2D;USH1C;UBE2A;TAF2
DAB2;SMAD3;SMURF2;PDPK1;OGT;RNF111
INO80C;PTGES3;PTEN;NBN
VCP;RRM2B
P2RY6;ANG
VPS35;WLS
XBP1;CPEB1
DUSP3;PDPK1
METTL4;METTL16
RPS6KB1;NCK1
SMAD3;DDAH1
XBP1;DNAJB9
BNIP3;VPS35
CST3;PSMA3
BMPR2;TGFBR1
DUSP3;IGFBP5;PTEN;MITF;THY1;OGT;CRK
INO80C;PBRM1;PIH1D1;SETD7;CHD6;RSF1;BMI1;ESR1;TADA2A;PCGF1;HNRNPC;TOP1;BRD7
MEF2C;SMAD3;BNC2;CREM;JMJD1C;RORA;MITF;RSF1;ASH1L;RORB;ESR1;TGFBR1;UBE2L3;SBNO1;TADA2A;[illegible]
CACNG7;CCL8;STIM2;ITGAV;CACNG1;TPT1
C4B;FER;JAK2;TIRAP
MEF2C
RPS27L
CHMP1B
CHMP1B
VCPIP1
NUDT12
HMGB1
MAOA
HEXA
PNPLA8
PNPLA8
SLC1A3
DDR2
GNPTG
P2RY6
CSGALNACT2
EDA2R
EFEMP1
PTEN
PPP1R3C

PPP1R3C
HEXA
NOX4
NBN
SLC7A8
SCARA5
SLC7A8
RRM2B
MTERF4
BMPR2
DICER1
TWSG1
WNK1
WNK1
SVEP1
PTEN
RAP1A
RAB11A
SETD7
NCK1
PNPLA8
CEPT1
ULK2
SNX5
VCP
PUM2
MEF2C
CCT3
MEF2C
PDPK1
CPEB1
PUM2
GSK3B
MAPK14
JAK2
HEATR1
ROCK2
PIH1D1
PTEN
VCPIP1
POGLUT1
SLC25A16
TWSG1
BTBD7
CDC42
MEF2C
WNK1
THY1

XBP1
CALM1
FER
FER
JAK2
DLGAP4
GSK3B
GREM2
GABRB2
XBP1
FER
GREM2
MSTN
SNAP29
PTEN
RAB40B
CLTC
ITGB1
RSF1;HMGB1;JAK2
GSK3B;PRKAA1;PTEN;DERL2;TSPAN31;CDC42;DPP4;DUOX1;AKT2;NAMPT;RICTOR;ITGAV;BRD7;TAF9B;DUO[illegible]
NHP2;DICER1
DST;THY1
RARRES2;ADH7
CHMP4C;NBN
PTEN;CRK
EIF3E;PNRC2
BMI1;DDR2
NUP98;PRDX6
SMAD3;DDAH1
TIMM8B;HSPA4
CAMK2D;CALM1
DUSP3;RARRES2
POLR3G;POLR2K
PBRM1;CDC73
CCL24;CCL11;CCL8;PPIA
GSK3B;VCP;RNF128;VPS35;SEC22B
VCPIP1;COPS3;USPL1;SENP5;USP31;COPS2
DUSP3;GPNMB;VSIG4
CDC42;OSTM1;SNX10;MAPK14
GSK3B;ATG3;WDPCP;CEP97
GSK3B;VCP;SMAD3;PARK7;TIRAP
RPS6KB1;RICTOR
DDAH1;NOX4
MEF2C;BMPR2
PRKAA1;PPIA
ITGB1;WNK3
RSF1;JAK2
ARHGAP44;CEP97

MEF2C;TIRAP
STIM2;WNK3
EMC2;EMC3
CDC42;SMURF2
WNK1;WNK3
CALM1;CALM2
CALM1;UBE2K
ROCK2;C3AR1;SOD1
RANBP2;NUP153;NUP98
NCKAP1;DICER1;GAS7
XBP1;RARRES2;MEDAG
POLR3G;TLR7;TIRAP
HMGB1;MAPK14;TIRAP
GSK3B;GOLPH3;VPS35
RPL10;CBFB;UBE2D3;CREM;ZBTB20;RSF1;RORB;HMGB1;AEBP1;BMI1;CDC73;IKZF5;ZMYM2;TRPS1;PCGF1;Z
XBP1;ACSM3;NR1H3;RORA
ABCD2;CHMP1B;CHMP4C;CHMP3
CCT3;SMAD3;PARK7;JAK1
SYNCRIP
NPL
RAP1A
JAK1
SLC1A3
DDAH1
BMPR2
SLC7A8
NFATC3
HOOK3
ATP6AP1
JAK2
JAK1
JAK1
SLC25A16
SGPP1
BMPR2
CDC42
SLC7A8
TADA2A
GHR
JAK1
MAF
CTSD
HOOK3
YME1L1
COX7A2
RPS27L
RC3H2
TWSG1

SMCR8
PARK7
CNOT1
TOM1L1
CAMK2D
AKT2
ARHGAP35
ABCD2
P4HA1
XPR1
XBP1
NCKAP1
PARK7
EEF1E1
PRKAA1
DAB2
TMEM30A
PUM2
CCT3
COPS3
TMX3
PTAR1
SLC25A16
RNFT1
THRSP
CAMK2D
HMGB1
CCT3
NPTN
ZC3H14
PRKAA1
PDPK1
CCT3
TIRAP
HMGB1
SLC25A16
PIH1D1
ELL2
ESR1
JAK1
CAMK2D;CCL11;CCL8;STIM2;NPTN;CALR;TPT1
TFB1M;NOP10;THG1L
SMAD3;MSTN;SMAD5
CDC42;ROCK2;TGFBR1
ITGB1;PDPK1;WNK3
ITGB1;PDPK1;WNK3
ITGB1;ITCH;CLTC
CDC42;CALR;CRK

MEF2C;MUSK;VPS35
GSK3B;MEF2C;TWSG1;BMPR2;GPNMB;ARRDC3;PTEN;NPTN;NR1H3;CALR;OGT
BMPR2;TGFBR1
EIF4A2;EIF3E
SORT1;GABARAP
ITGB1;GOLPH3
RPS8;HEATR1
TAOK1;NBN
CALM1;CALM2
CDC42;ARHGAP12
CD28;HMGB1
PTGES3;ANG
PNPLA8;PTGES3
ZDHHC13;ZDHHC21
NCKAP1;ARF1
TMBIM4;CALM2
NR1H3;ITGAV
XBP1;CPEB1
DUSP3;PDPK1
CCL24;CCL11;CCL8;PPIA
XBP1;ACSM3;NR1H3;RORA
DUSP3;IGFBP5;PTEN;MITF;ATP1B2;THY1;CALR;OGT
TMEM30A;SLC35F3;SLC7A8;CHMP1B;ARRDC3;CHMP3;SLC38A2;SLC38A4
CDC42;DYNC1LI1;DYNC1H1;RRM2B;CD28;PKN2
NCKAP1;CCL24;FER;CCL11;NCK1
RPL10;ROCK2;MDM2;NR1H3;VPS35
GPX4;PNPLA8;PTGES3
ABCD2;ADH7;ADIPOR2
MEF2C;XBP1;TIRAP
GSK3B;PARK7;JAK1
XBP1;ARHGAP35;TGFBR1;SOD1
MEF2C;PARK7;IL6ST;SOD1
PBRM1;PTEN;BRD7;CDC73
CDC42;HSPA8;GSK3B;VCP;PARK7;TIRAP
ACOT9;ACSM3
RAP1A;IGFBP5
SEMA3C;ITGAV
BMPR2;HNRNPC
DUSP3;SEMA3C
TAOK1;TTBK2
TMEM30A;ATP8A1
HMGB1;CALR
GSK3B;CAPRIN1
ASPA
PTEN
NFATC3
MAPK14
ROCK2

MEF2C
GABARAP
GABARAP
PTEN
PTEN
VCPIP1
PPP2R2D
UBE2A
SLC46A1
ARF1
VMP1
ULK2
KDELR1
MEF2A
RPS26
PFDN1
GSK3B
GSK3B
CHMP4C
XBP1
CLTC
SYNCRIP
CALM2
CALM2
DDAH1
SLC1A3
PIK3CA
MEF2C
RORA
DYNC1LI1
CACNG1
DICER1
TMEM30A
CCT3
CCBE1
CDC42
DYNC1LI1
NR1H3
HMGB1
NUP153
HMGB1
RC3H2
PIK3CA
PRKAA1
RAB11A
PPP1R3C
JAK1
PTEN

WNK1
LAMP1
TMEM30A
CDC42
MEF2C
SGPP1
NPRL2;PTEN;PARK7;PPIA
CDC42;ATG3;FER;WDPCP
LYRM2;NDUFB4;NDUFAF4
ANG;JAK2;GAS7
SLC7A8;SLC38A2;SLC38A4
LYRM2;NDUFB4;NDUFAF4
TLR7;HMGB1;TIRAP
TOM1L1;RPL10;MITF
GHR;JAK2;CALM1
UBE2W;CNOT7;NDUFAF4;BNIP3;UBE2N;CHMP3;TLR7;NMBR;NCK1
MEF2A;MEF2C;ITGAV;MAPK14;MAP3K7
XBP1;TLR7;HMGB1;TIRAP
GTF2A1;GTF2A2;ESR1;TAF2
TM9SF2;ST6GALNAC5
SMAD3;RORA
NBR1;RORB
BMPR2;CSGALNACT2
PBRM1;BRD7
HEATR1;POLR2K
EFEMP1;PIK3CA;PDPK1
VCPIP1;TMEM33;TMCC1
PRKAA1;DEPTOR;NPRL2
PTEN;THY1;CBFA2T2
DNAJA1;PARK7;OGT
RARRES2;RORA;THRSP
RPL5;RPL10;RSL24D1
GABRB2;OSTM1;SLC4A10;SLC1A3
CCL24;CCL11;CCL8;PPIA
TAOK1;HMGB1;EDA2R;TIRAP
INO80C;C7;EYA4;UBE2N
C7;SPPL2A;SMCR8;TIRAP
INTS4;ELL2;INTS8;CDC73
MAT2A;NINJ1;G3BP2;YME1L1;ELAVL1
MEF2A;CDC42;NCKAP1;PTEN;DICER1;ARHGAP35;GAS7
GHR;JAK2;IL6ST
CACNG7;MEF2C;SLC38A2
COG6;RAB6A
MEF2C;HMGB1
HMGB1;PARK7
POGLUT1;IL6ST
HEATR1;SLC38A2
WNK3;ATP1B2

TMED10;ROCK2
NBN
DDR2
HMGB1
SLC38A2
ARHGAP35
HSBP1
PNPLA8
NCKAP1
CALM2
CNOT7
RORB
ABCD2
SORT1
PNPLA8
VMP1
BMPR2
HOOK3
WDPCP
CCL8
DNAJA1
PTEN
MAPK14
TAF9B
GSK3B
USPL1
PARK7
ALG14
XPR1
NR1H3
CSGALNACT2
HMGB1
RNFT1
WNK1
PRKAA1
THY1
SNX5
CNOT7
GSK3B
VCP
DUOXA1
WNK3
WNK3
HMGB1
SMAD3
TIMM8B
NBN
SOD1

PIK3CA
ITCH
ZDHHC21
CNTN1
DERL2
ROCK2
ASCC3
SMPDL3A
ROCK2
GPX4;PNPLA8;ACSL4;ACSL3
CPQ;PNPLA8;TPP1;NPL
ARFGEF2;HMGB1;JAK2;TIRAP
MEF2C;XBP1;NR1H3;HMGB1;MAPK14;CDC73
EFEMP1;EYA4;SLC4A10;DRAM2;ATF6
INO80C;SMARCAL1;VCP;PTGES3;PARK7;UBE2A;HMGB1;RRM2B;PSME4;UBE2V2;UBE2N;NBN;TOP1
GSK3B;XBP1;MDM2
TAOK1;RPS27L;NBN
MUSK;EEF1E1;MAPK14
TOM1L1;CD28;AURKAIP1
CNOT1;ELAVL1;OGT
SMAD3;BMPR2;DCUN1D3;OSGIN1;ULK2;CGRRF1
CDC42;DAB2;CD151;CALR
MEF2C;ZEB1;ZFHX2;CALR
CHMP1B;CHMP4C;CHMP3;SEC22B
PIK3CA;MAPK14
ITGB1;KPNB1
MPND;TAF2
TIMM8B;APOO
SPPL2A;MBTPS2
ARHGAP44;ARHGAP35
TOM1L1;AURKAIP1
XBP1;DNAJB9
PIH1D1;HEATR1
TOM1L1;BMPR2
DUSP3;THY1
CAMK2D;S100A1
MAF;JAK2;TIRAP
BMPR2
BMPR2
VCP
CSGALNACT2
G3BP2
TLR7
RCL1
BMPR2
TBC1D8B
ATP1B2
ADIPOR2

RAP1A
RRM2B
GDAP1
HMGB1
USPL1
MDM2
HSPA8
MSTN
NR1H3
EIF3E
HSPA4
JAK2
MEF2C
SNX5
MAPK14
AKT2
LAMP1
ATP1B2
SOD1
HSPA4
STAU2
BPNT1
FER
WNK1
JAK2
POGLUT1
TAF9B
MAPK14
ABL2
SLC38A2
SLC4A10
PIH1D1
WNK1
RAP1A
NR1H3
CELF1;NMT1;NMT2;PARK7;JAK2;TIRAP
ITGB1;DNAJA1;DCUN1D3;BNIP3;HTRA1;HMGB1;EEF1E1;ATF6;CTSD;SAV1;SOD1
XBP1;CPEB1
SLC4A10;SLC4A4
ETF1;RAB6A
RRM2B;RAB11A
CALM1;CALM2
PTEN;EEF1E1
PAPOLA;METTL16
GSK3B;MEF2C
WNK1;WNK3
ITGB1;XBP1;PDPK1;NINJ1;CNTN1
CCL24;CCL11;CCL8;MAP3K7

ITCH;ANXA4;TAX1BP1;G3BP2
EFEMP1;ATF6;FREM2
ITGB1;DNAJA1;DCUN1D3;BNIP3;HTRA1;HMGB1;EEF1E1;ATF6;CTSD;TGFBR1;SAV1;SOD1
INO80C;MEF2C;PRKAA1;SMAD3;MSTN;PHF20;NR1H3;RORA;MITF;RSF1;RORB;ESR1;SMAD5;TGFBR1;ZXDA;M[illegible]
ARFGEF2;HMGB1;JAK2;TIRAP
TIMM8B;ATG3;LAMP2;ZDHHC21;GDAP1;GABARAP
TBL1XR1;MORF4L2
VCP;ERCC6L2
GPNMB;VSIG4
POLR3G;TLR7
MAPK14;ACVR2A
MAPK14;ACVR2A
ATAD1;ARF1
FER;ITGAV
BMPR2;TGFBR1;WLS
EXOC7;ROCK2;EXOC5
SMAD3;NFATC3;ESR1
INO80C;TADA2A;RAB14
ITCH;ATG12;TIRAP
CDC42;DERL2;RICTOR;TAF9B;TGFBR1
SLC1A3
F13A1
BMPR2
SGPP1
ITGB1
ARF1
ALG14
TGFBR1
CDC42
ADH7
EIF3E
ATG12
RAP1A
HSBP1
PTEN
BMPR2
CEP97
ITCH
ERLIN1
RAB14
RORB
CDC42
JAK2
SYNCRIP
AKT2
THY1
PARK7
INO80C

UBE2K
GDE1
F13A1
SCARA5
SCARA5
VCP
OGT
NR1H3
FER
SOD1
RAB22A
PIK3CA
ROCK2
ITGB1
BMPR2
GABRB2
SNAP29
NBN
PIK3CA
P2RY6;C3AR1;NMBR;ESR1
DAB2;SMAD3;PTEN;TGFBR1
C4B;CALR;C2
INO80C;NEK9;DCUN1D3;DYNLT3;TGFBR1;TADA2A;AKT2;MORF4L2;CDC27;MDM2;CALR;NBN;BRD7
MEF2C;HMGB1
GHR;JAK2
GHR;JAK2
ZDHHC13;ZDHHC21
CAMK2D;WNK3
BMPR2;TGFBR1
ACOT9;HSPA8;ACSM3;BPNT1
INO80C;EYA4;UBE2N;NBN;HMGB1
MEF2A;POPDC3;MEF2C;PIK3CA;PTEN;TGFBR1
DUSP3;ROCK2;NBR1;STYX;HMGB1;PPIA;TGFBR1;TIRAP;SOD1
RARRES2;ANG;RPL39;TIRAP
GSK3B;PRKAA1;PARK7;JAK1
HAL;HNMT
XBP1;NAMPT
GOLPH3;ATP6AP1
NR1H3;TAF2
RPS8;HEATR1
ITCH;ATG12
XBP1;DNAJB9
DYNC1H1;LAMP1
MEF2C;SLC38A2
UFM1;SMAD3
BPNT1
SLC1A3
MEF2A

UBE2L3
ESR1
RORA
HEXA
SLC25A16
HMGB1
PTEN
ALG14
CD151
ST6GALNAC5
DDAH1
RAB11A
DICER1
CAMK2D
PTEN
ZEB1
PTEN
FER
NR1H3
HMGB1
NBN
RAB14
RAB11A
FER
EEF1E1
JAK2
AKT2
MAPK14
MAPK14
PTEN
JAK1
TPP1
ACVR2A
PFDN1
ROCK2
RORA
DAB2
TPT1
HEATR1
INO80C
TBC1D8B
SMPDL3A
KBTBD8
DPH3
SOD1
CDC42;VMP1;SMAD3
ZDHHC13;ZDHHC21;ATG12
DUSP3;GPNMB;THY1

SOCS2;FER;JAK2;ESR1;TIRAP
SETD7;HMGB1
TADA2A;MORF4L2
GPNMB;VSIG4
BNIP3;HUWE1
PARK7;OGT
DPH3;NR1H3
CNOT7;RC3H2
CDC42;RAB6A
VMP1;ATP1B2
CDC42;PKN2
NCK1;SOD1
WNK1;WNK3
NOX4;THY1
MEF2A;MEF2C;CAMK2D;PRKAA1;BNIP3;SYT9
DDX6;DYNC1H1;YTHDF3;CAPRIN1;G3BP2;LSM14A
SMURF2;PDPK1;UBE2D3;OGT;GPR155
GABRB2;SLC1A3;XPR1
SACM1L;PTEN;MTMR6
LSM10;RRM2B;RAB11A
INO80C;PTGES3;NBN
BMPR2;CELF1;PTEN;DERL2;CDC73;SAV1;TSPAN31;DPP4;GPNMB;AKT2;NAMPT;ITGAV;JAK2;FKTN;XBP1;PBR
SLC46A1;ATP6V0B;SCARA5;ATP6AP1;SLC30A4;SLC39A10;SLC1A3;ITGAV;OTOP1;CACNG1;SLC4A4;ATP6V[illegible]
GSK3B;XBP1;PBRM1;PRKAA1;BMPR2;SMURF2;BNIP3;VPS35;BRD7;OGT
ITCH;NR1H3;RORA;VPS35;ATG12
PERP
RARRES2
DICER1
ATP1B2
TIRAP
MDM2
CSGALNACT2
ASPA
TMEM33
RAB11A
STT3B
SYCP3
COX7A2
OGT
MAPK14
GSK3B
MEF2C
ABL2
CNTN1
IGFBP5
FKTN
DNAJA1
SLC39A10

INO80C
RBSN
MEF2C
GSK3B
CLEC3B
VPS35
VPS35
AGO3
MAPK14
PLEKHA1
JAK2;MAPK14;JAK1
PBRM1;XBP1;BRD7
PERP;HMGB1;CTSD
TMED10;HMGB1;JAK2
POLR3G;TLR7;NMBR
SLC30A4;CALM1;CALM2
SORBS2;MIB1
BMPR2;ROCK2
VCP;HSBP1
DPP4;PIK3CA
DPH3;SMCR8
MEF2A;TCEA1
LSM10;CDC73
STIM2;WNK3
TBL1XR1;MORF4L2
TPP1;ESR1
RORA;OGT
RARRES2;ADH7
SLC46A1;SLC25A32
ROCK2;SORT1;MDM2;CHD6;VPS35;BMI1;AFF4
SLC46A1;ATP6V0B;SCARA5;ATP6AP1;SLC30A4;SLC39A10;SLC1A3;ITGAV;OTOP1;CACNG1;SLC4A4;ATP6V[illegible]
NUP153;NUP98;THOC6
XBP1;ACSM3;GPX4
XBP1;ACSM3;PARK7
CCL24;ITCH;CCL11;CCL8;C3AR1;NFATC3;NOX4;HMGB1;FOLR2;MAP3K7
XBP1;TLR7;NMBR;HMGB1;TIRAP
ASCC3;NBN
GSK3B;RORB
SMARCAL1;HMGB1
SYNCRIP;ELAVL1
CDC42;SMURF2
POLR3G;TIRAP
TMED10;RPL10
ATF6;FREM2
MUSK;PTEN;JAK2;DDR2
RRM2B
MDM2
NCKAP1

STT3B
NPL
UBE2L3
HSPB8
WDPCP
FGD4
SAV1
ESR1
MAF
SERINC1
KIF2A
DICER1
ARHGAP35
PTEN
CDC73
ANXA4
NR1H3
XBP1
WDPCP
IDH1
CNOT7
ABCD2
GPNMB
JAK2
ROCK2
BMPR2
CDC73
CAPRIN1
NR1H3
DICER1
WNK1
S100A1
PRKAA1
ATP1B2
ITGAV
MUSK
VCP
ROCK2
ITCH
MAPK14
DYNC1LI1
PARK7
JAK2
RSF1
RPS27L
NOX4
TIRAP
FER;ERCC6L2;SOD1

MEF2C;XBP1;PTEN;DERL2;BMI1;TGFBR1;TSPAN31;DPP4;FER;CNOT7;AKT2;NAMPT;MDM2;PFDN1;ITGAV;CA[illegible]
RPL5;RPS25;PIH1D1;RRP12
HMGB1;NBN
PRKAA1;XBP1
CDC42;ROCK2
RORA;ESR1
PDPK1;THY1
MEF2C;ZEB1
MAPK14;MAP3K7
ASCC3;EEF2
DYNC1H1;PRKAA1;XBP1;BMPR2;SMAD3;SMURF2;NMBR;ADIPOR2;TGFBR1;SOD1;GHR;DAB2;CD28;CACNG1[illegible]
GHR;MEF2C;PIH1D1;CALM1;CALM2
CPD;CNTN1;YME1L1;AEBP1;OGT
HMGB1
CAPZA2
ZDHHC21
CALR
CAPZA2
BMPR2
HMGB1
BMPR2
RAB11A
TGFBR1
ATP1B2
PARK7
AP1G1
RAB11A
ATP1B2
ITGB1
PRKAA1
MDM2
TPP1
TGFBR1
DDAH1
CALR
CCT3
THY1
HMGB1
DICER1
TIRAP
TAOK1
PUM2
MAP3K7
IL6ST
SCTR
ITCH
ROCK2
CHMP3

BNIP3
GSK3B
TADA2A
RAP1A
SOD1
TGFBR1
TWSG1;GPNMB;CD28;NPTN;ZBTB20;MAPK14;SOD1
TM9SF2;ST6GALNAC5
NR1H3;TAF2
CDC42;PKN2
GSK3B;THY1
ATAD1;DAB2
ELL2;CDC73
DNAJA1;MAP3K7
ARHGAP44;CDC42
ROCK2;VPS35
B3GALNT2;POGLUT1;OGT;FKTN
GSK3B;DAB2;STYX;PARK7;CRK
SMARCAL1;VCP;UBE2V2;UBE2N;NBN;HMGB1;MPND
SOCS2;IGFBP5;RORA;PPIA;OGT;CRK;RGS7;NCK1
CELF1;NUP98;METTL16;PRDX6
ATG3;ZDHHC21;RAB11A
MUSK;JAK2;DDR2
CACNG7;MEF2C;WNK3
OGN;MBTPS2
GOLPH3;ROCK2
SEMA3C;KBTBD8
ZBTB20;NR1H3
RARRES2;CNTN1
GSK3B;SMAD3
MEF2C;TIRAP
INTS4;INTS8
THY1;JAK2
DNAJA1;RAB11A
DICER1
GNPTG
BMPR2
TGFBR1
MAP3K7
CSGALNACT2
MAOA
RARRES2
EFEMP1
NFATC3
PARK7
MAP3K7
RPS27L
PNPLA8

JAK2
RPS26
SEMA3C
CALM2
ERLIN1
GSK3B
HNRNPC
BNIP3
RRM2B
XPR1
HMGB1
HSPH1
MAT2A
XBP1
IL6ST
PARK7
TWSG1
NPTN
IGFBP5
MEF2C
IGFBP5
CCBE1
SGPP1
MAP3K7
USO1
CDC42;VMP1;PTEN;CDH9
BMPR2;ROCK2;CALR;RAB11A
MAT2A;CSGALNACT2;METTL16
MEF2A;POPDC3;MEF2C;BMPR2;PTEN;CRELD1;TGFBR1
ITCH;ATG12
FER;FKTN
DYNC1H1;ARRDC3;NR1H3;JAK2;ADIPOR2;OGT
PKN2;CDH9;MPDZ
PRKAA1;XBP1;ACSM3
SMURF2;UBE2D3;GPR155
GABRB2;LGI2;PTEN
GSK3B;VCP;MDM2;VPS35;SEC22B
CELF1;PARK7;JAK2;TIRAP
METTL4
PKN2
WLS
CAMK2D
RAP1A
GABRB2
PRDX1
SLC39A10
NPTN
JAK2

ITGB1
CALR
OGT
PPIA
CNOT7
PNPLA8
SLC39A10
TMEM100
BMPR2
WNK3
CDC27
CAMK2D
PPP6R3
ZDHHC21
NR1H3
CDC42
MEF2C;PLEKHA1
MEF2A;CAMK2D
AKT2;DNAJC15
TAOK1;NBN
PTEN;ITGAV
ARRDC3;NR1H3
THY1;JAK1
CD28;HMGB1
SMARCAL1;NBN
PLEKHA1;SOD1
MEF2C;XBP1;WNK1;EN2;ACSL4;RORB;TGFBR1
CST3;RPL5;PRKAA1
CAMK2D;RAB14;WDPCP
PRKAA1;DCUN1D3;MAPK14
ITGB1;GSK3B;MEF2C;PTEN;DLGAP4
DYNC1H1;JAK2;ADIPOR2;OGT
GSK3B;VCP;PARK7;TIRAP
SMAD3;BMPR2;DCUN1D3;OSGIN1;CGRRF1
MEF2C;NINJ1;POLR3G;TLR7;TIRAP
DUSP3;UBLCP1;PTEN;MTMR6;NCK1
TOM1L1;INO80C;UBE2V2;UBE2N;TAF2
P2RY6;IGFBP5
RPL5;MDM2
FER;PLEKHA1;ERCC6L2
HEXA
CDC27
RAP1A
THY1
MORF4L2
RPL39
PLA1A
CDC42

TMEM41B
EXOC7
ROCK2
VSIG4
ZC3H11A
MAP3K7
ROCK2
CNTN1
CAPRIN1
TIRAP
LAMP1
EEF1E1
TUBD1
PARK7
FKTN
NAMPT
MEF2C
ITGAV
MEF2C
RORA
SLC35F3
BNIP3;TLR7
GABRB2;SLC1A3
ABCD2;MAL2
DUSP3;PTEN
MAPK14;SOD1
STT3B;JAK2
SPPL2A;UBE2K
CAPZA2;MDM2;USH1C;MITF;NUP98
NCKAP1;CASP12;PIK3CA;BNIP3;MDM2;JAK2;MAP3K7;PPIA;OGT
FER;UBE2N;MAP3K7;PPIA;EDA2R;TIRAP
CD164;BMI1;OGT;TIRAP
CDC42;ROCK2;TGFBR1
ARFGEF2;DICER1;HMGB1;JAK2;TIRAP
ANG
ITGB1
HOOK3
SORT1
SPPL2A
TUBB4B
RPGRIP1L
EYA4
EYA4
ST6GALNAC5
BPNT1
ZDHHC21
ZDHHC21
AP1G1

NR1H3
PTEN
ATAD1
CACNG7
HMGB1
PPIA
SYT9
NBN
INTS8
WARS2
JAK2;JAK1
PIH1D1;PARK7
PBRM1;BRD7
GPX4;PNPLA8
TBC1D8B;TBCK;RALGAPB;RALGAPA1
PTEN;VPS35;WLS
DENND4C;OGT;GABARAP
PTER;PIH1D1;ANXA4;WDPCP;TPP1;JAK2
PTEN;CBFA2T2
BMPR2;RAB11A
SCARA5;NINJ1;G3BP2;ELAVL1;THG1L
SMURF2;PDPK1;OGT
TM9SF2;ST6GALNAC5;SGPP1
GSK3B;VCP;DAB2;SMURF2;WNK1;TBL1XR1;TMEM170B;WLS
ULK2
TGFBR1
XBP1
LSM10
C3AR1
THY1
TIRAP
ATP1B2
ETF1
ABL2
SEMA3C
NR1H3
DDR2
ASCC3
VCP
RAP1A;MUSK;NBN;DDR2
HMGB1;BMI1
TMED10;SYT9
CCT3;HNRNPC
POLR3G;ATG12;TIRAP
TAOK1;HMGB1;EDA2R;TIRAP
MEF2C;PARK7;IL6ST;SOD1
CDC42;GSK3B;HSPA8
ITGAV

MDM2
MEF2C
HOOK3
GGCX
TUBB4B
MEF2C
MUSK
ABCD2
BMPR2
JAK2
LAMP1
VCPIP1
PTEN
CST3
PPIA
BMPR2;SMAD5
MEF2C;OGN
MDM2;MAPK14
GPX4;PNPLA8
SLC1A3;SLC38A2
INO80C;NBN
FER;RICTOR
CCBE1;TGFBR1
CAST;CST3
ARRDC3;NR1H3
ST6GALNAC5
SMAD5
SC5D
CHMP4C
GPX4
ERLIN1
RPL5
CRK
HNRNPC
PARK7
AKT2
VCP
BNIP3
ABL2
HMGB1
DICER1
CCBE1
MEF2C
SLC30A4
AP1G1
ATP1B2
HOOK3
ATF1;SMARCAL1;MSTN;BNC2;PHF20;JMJD1C;HR;RORA;RSF1;RORB;HMGB1;IKZF2;UBE2L3;IKZF5;ZXDA;MED[illegible]

EXOC7;ROCK2;EXOC5
SACM1L;PTEN;CEPT1;MTMR6
DYNC1H1;JAK2;ADIPOR2;OGT
CCL24;CCL11;CCL8;JAK2
SYNCRIP;HSPA8;CELF1;BUD31;PAPOLA;HNRNPC;RPRD1A;CWC15
GABRB2;SLC1A3
CHMP3;SMCR8
FER;PTGES3;ANG
CNTN1
CTSD
ATF1
SYT9
EFEMP1
SORBS2
MEF2C
SYCP3
PDPK1
PRKAA1
ITGAV
VCPIP1
CD28
CAMK2D
MEF2C
POLR3G
UBE2L3
PNPLA8;NR1H3
NR1H3;RORA;VPS35;MAPK14
RAD23B;RNF111
TLR7;MAP3K7
ELL2;CDC73
MEF2C
CLTC
CACNG7
TGFBR1
KIF2A
TAF2
TAF2
ATP6AP1
F13A1
SETD7
PPIA
IDI1
PIH1D1
C3AR1
CDC42
C7
ARHGAP35
PARK7

SYT9
EYA4
PARK7
PIH1D1;THY1;CALM1;CALM2
RRAD;G3BP2;RGL3;PARK7
CCL24;CCL11;CCL8
METTL16;NUDT12
STIM2;WNK3
NBN;MAPK14
PIK3CA;GAA
GOLPH3
CTSD
WNK3
RAP1A
HEXA
RPL39
CALM2
PIK3CA
HMGB1
SMAD3
KIF2A
SYT9
PUM2
JAK2
CAMK2D
MAPK14
VCP;MORF4L2;BNIP3;OSGIN1;ABL2;CALR;TPT1
HEXA;CSGALNACT2
SLC4A10;XPR1
ATAD1;MUSK
TLR7;JAK2
ITGAV;CALR
C1GALT1;TGFBR1
UFM1;ZFHX2;CNTN1;PTEN;SCTR;IER3IP1
PRKAA1;PARK7;ADIPOR2
TLR7;HMGB1;JAK2
WDPCP;TGFBR1
TMEM30A;ATP8A1
MAP3K7
RSF1
RARRES2
ITGB1
MAF
HNRNPC
ARHGAP12
DUSP3
ROCK2
CACNG1

CPA3;DPP4;CPQ;CPD;SPPL2A;HTRA1;CNTN1;MDM2;TPP1;AEBP1;CTSD;OGT
BMPR2;ROCK2;CALR
ITGB1;NPTN
CST3;PARK7
COPS3;UBE2A
GSK3B;RORB
MGST1;SLC4A10;SLC4A4
TIMM8B;USH1C;SOD1
TGFBR1
MAP3K7
SEMA3C
CLTC
PTEN
THY1
MAP3K7
WLS
GPNMB
LYRM2;NDUFB4;NDUFAF4
GHR;CNOT7;JAK2
PIK3CA;SMCR8
CCL24;CCL11;CCL8;JAK2
DUSP3;UBLCP1;PTEN;MTMR6;SGPP1
GHR;MEF2C;MAP3K7
GSK3B;DPH3;HMGB1
MEF2C
CTSD
TAOK1
ITGAV
DDAH1
ITGB1
PTEN
PTEN
RPGRIP1L
SLC35F3
LSM10
NR1H3
AKT2
THY1
PTEN
ESR1
FMNL2
GSK3B
OGT
GSK3B
NR1H3
TIRAP
ZC3H11A;THOC6
NCKAP1;UFM1;ZFHX2;SCTR;CNTN1;PTEN;TPP1;IER3IP1;PTS;THOC6

ITGB1;MEF2C;DNAJB9
TIMM8B;USH1C;SOD1
HMGB1
SEMA3C
BNIP3
CCT3
DNAJC15
HSPA8
NR1H3
LAMP1
ABCD2
PTEN;PARK7;NCK1
ITGB1;XBP1;GPNMB;PDPK1;ROCK2;NINJ1;CNTN1
VMP1;CDC42;NCKAP1;VCPIP1;YTHDF3;CAPZA2;MDM2;USH1C;MITF
TM9SF2;SERINC1;SGPP1
USH1C
USH1C
MEF2C
TAF2
DICER1
NR1H3
PTEN
NBN
MAP3K7
NR1H3
SMURF2
PTGES3
MPND
HSPA8
HMGB1
DPP4
NBN
TGFBR1
CXCL14;RPL39
GSK3B;PTEN
CCT3;PTGES3
CST3;CDC42;DYNC1H1;KIF2A;MDM2;DPT;HOOK3;TGFBR1;AGFG1;DDR2;ARHGAP12
SEMA3C;FEZ1;CNTN1;ARHGAP35
HSPA8
NBN
GSK3B
PLEKHA1
WDPCP
NDUFB4
NMBR
DDR2
ZFYVE16
IDH1

GSK3B
JAK2
WNK1
SLC4A10
ESR1
SEMA3C
TTBK2
BRD7
PNPLA8;ACSL4;ACSL3
RARRES2;NINJ1;POLR3G;TLR7;TIRAP
CCL11;HOOK3
THUMPD3;THG1L
CDH9
ITGAV
ITGB1
RPS27L
XBP1
JAK2
PPIA
MYF6
CD28
CCT3
GSK3B
SYT9
WARS2
VCP;IGFBP5;RPS6KB1;CNOT1;KCTD21;CALR;RPGRIP1L;SMAD5;NCK1
SYNCRIP;ZMAT5;HNRNPC
CELF1;NINJ1;NR1H3;VPS35;TLR7;RORA;PARK7;JAK2
EIF4A2;HSPA8;ZHX2;SMURF2;RSF1;RORB;CBFA2T2;ZHX1;ZEB1;CNOT7;ZMYM2;MDM2;CALR;BRD7;ZBED6;[illegible]
ITGB1;DNAJB9
NDUFB4;COX7A2
SMAD3;CDH9
HUWE1
ROCK2
SEMA3C
MORF4L2
AKT2
GSK3B
THUMPD3
GPX4;GDE1
ATF1;SMARCAL1;BMPR2;PHF20;UBE2D3;CHD6;ZBTB20;JMJD1C;HR;RORA;RORB;HMGB1;PARK7;IKZF2;BMI1;[illegible]
RARRES2;CXCL14;RPL39
GHR;NOX4;IL6ST;JAK2
NDUFB4;COX7A2
POGLUT1;IL6ST
ATF6;FREM2
SEMA3C;FEZ1;CNTN1;PTEN;ULK2;ARHGAP35
ELAVL1

ROCK2
AP1G1
MEF2C
XBP1
WLS
HNRNPC
JAK1
CAST;CDC42;MUSK;PTEN
C1GALT1;TGFBR1
CDH9
JAK1
MEF2C
MEF2C
NOX4
NBN
JAK2
MTERF4;TFB2M;MRPL57
ATG3;ZDHHC21
DDAH1;PDPK1
NINJ1;TLR7;IL6ST
SOD1
RARRES2
RAB14
ULK2
DYNC1H1
ARF1
CCDC186
ABL2;ARHGAP35
GSK3B;PBRM1;ZFHX2;TRPS1;BRD7;TGFBR1
DUSP3;PTEN;PPIA
ATF1
CDC42
WNK1
ACSL4
OGT
SNX10
SPPL2A
CAPRIN1;NPTN
C1GALT1
CST3
TLR7
GSK3B
CAMK2D
BNIP3
NPRL2
MEF2C
SNAP29
NBN

MEF2C
NPRL2
ST6GALNAC5
MEF2C
MEF2C
SGPP1
NDFIP2
PARK7
ATP1B2
ITGB1;ITGAV;THY1
ITGB1;ROCK2
MEF2A;MEF2C
RPL10;RARRES2
WNK1;SLC39A10
CACNG7;NPTN
ATF1;INO80C;GTF2A1;PRKAA1;GTF2A2;BMPR2;MSTN;PHF20;HEATR1;CHD6;RORA;RSF1;RORB;HMGB1;PARK
CACNG7;NDFIP2;CCL8;SLC4A4;TPT1
SMARCAL1
COX7A2
CRK
ESR1
ATAD1
MAP3K7
SMAD3
DPP4
INO80C
PDPK1
GSK3B
TGFBR1
CACNG7;CCL8;CHMP1B;ARRDC3;CHMP3;TPT1
DPP4;CD28;NCK1
PTEN;IPO7
EFEMP1
SMCR8
UBE2K
THY1
HMGB1
ATF1;GTF2A1;GTF2A2;BMPR2;CHD6;RORA;RORB;HMGB1;PARK7;CDC73;MED13;NAMPT;NCK1;MEF2A;MEF2
ITGAV;CACNG1
PTEN;IPO7
ATG3;WDPCP
CD164;ROCK2;ABL2;ITGAV
GOLPH3
GAA
PPP2R2D
PRDX1
OGT
ITCH

PTGES3
PERP
GSK3B;DAB2;TMEM170B
CD28
PPIA
ACSL4
ATP1B2;SLC4A4
BMI1;BRD7
GPR155
DICER1
THY1
IDH1
CNOT7;NDUFAF4;BNIP3;TLR7
HMGB1;CTSD
SEMA3C;FEZ1;CNTN1;ARHGAP35
ITCH;ANXA4;TAX1BP1;ESR1
PRDX1;TRPS1;OGN;TGFBR1
HSPA8;SYNCRIP;BUD31;CWC15;HNRNPC
ITGB1
MAPK14
ST6GALNAC5
PRKAA1;PDPK1
NDUFB4;COX7A2
SVEP1;EDA2R
PTEN;IPO7
RPL39
JAK2
ABCD2
CD164
RPS27L
BMPR2
DICER1
SMAD3
PBRM1;BRD7
SYNCRIP;CELF1;ZMAT5;HNRNPC;RBMS2
BNIP3
GHR
THG1L
ITGB1;FGD4;GOLPH3;RAP1A;WDPCP;SNX10;SNAP29;TTBK2
ITGAV;JAK2
DNAJA1
MORF4L2
LAMP1
USH1C
RPS6KB1
CCBE1
GOLPH3
TLR7

THG1L
NDUFB4
RAD23B
SYCP3
MPDZ
ITGB1;DNAJB9
PKN2
CST3
RSF1
RAB14
TM9SF2
MEF2C
SLC25A16
MAPK14
UBE2N;NBN
NCKAP1;MEF2C;PTEN;PTS;TGFBR1;THOC6;SLC25A16;FEZ1;NINJ1;TPP1;WDPCP;BPNT1;FKTN
SGPP1
SLC4A10
JAK2
THOC6
NDUFB4
GAA
TGFBR1
GSK3B
RSF1
SETD7
NDUFB4
SMARCAL1;VCPIP1;VCP;RRM2B;TAOK1;PSME4;SETD7;SUSD6;PARK7;UBE2A;MAPK14
DCUN1D3;UBE2A
ABCD2
PPIA
THY1
AKT2
TGFBR1
CELF1
ITGB1;CD164;NPTN;CDH9
RPS6KB1
NDUFB4
GSK3B;DAB2;TMEM170B
MEF2C
SYNCRIP;HSPA8;BUD31;HNRNPC;CWC15
CST3
WNK1;SLC1A3
CDC73;WLS
SORT1
RRM2B
PSMA3
UBE2N;NBN

CDC73
VCPIP1;USP31
MORF4L2
GHR;MEF2C
CD164
TOP1
THG1L
RARRES2
RSF1
INO80C
ROCK2
MAPK14
GOLPH3;SGPP1
IL6ST;JAK2
TLR7
RARRES2;ANG;RPL39;TIRAP
JAK2
TPP1
ROCK2
WDPCP;SNX10;RPGRIP1L;SNAP29;TTBK2
ACSL4
MEF2C;EN2;ACSL4
PARK7
SLC4A4
DPT;TGFBR1;DDR2
CDC73
SLC1A3;ATP1B2
ROCK2
CDC42;ARHGAP12
MRPL57
ARHGAP35
WDPCP;SNX10;SNAP29;TTBK2
ATP1B2
BNIP3
TMEM41B
GABRB2;SLC1A3;GABARAP
TGFBR1
ADGRE1
ADGRE1;SCTR
SLC1A3
GABRB2;PTEN;SLC1A3;GABARAP

;RPS11;RPL39;RPS10
IP2;TMED2;TLK1;VPS35;KPNA4;TMED7;RAB6A;ARL5A;TMED5;TNPO3;WLS;ATP6AP1;SYNRG;NUP153;DNAJ
2;XBP1;SMURF2;UBE2A;RAD23B;RC3H2;MIB1;TOM1L1;COPS3;UBE2W;PSMA3;ITCH;PSMA4;RNF128;PSM

;LSM14A

1;CD28;ETF1;EIF3E;CALR;EIF1B;LSM14A;OGT
AK2;MAPK6;MAP3K7;JAK1;CDKL2;MUSK;PDPK1;MAPK14;CLK4;TGFBR1;ACVR2A;TBCK;FER;RPS6KB1;WNK
;DNAJA1;ITCH;DAB2;GOLPH3;UFM1;TMBIM4;RPS6KB1;WNK3;CD28;TAX1BP1;MDM2;YME1L1;NAA35;BIRC

F4L2;TAX1BP1;YME1L1;NAA35;BIRC6;IL6ST;EEF1E1;ATF6;PPIA;PRKAA1;VCP;RPL10;DCUN1D3;HTRA1;SA
2;SMAD3;IGFBP5;DICER1;ESR1;SMAD5;RPS26;TWSG1;CNOT7;AGO3;CNOT1;CAPRIN1;CD28;SMCR8;CAL

NX5
B6A;ARL5A;TMED5;WLS;SYNRG;ARRDC3;DNAJC15;ARCN1;VMP1;ARL4A;GOLPH3;RAB14;CHMP3

;RPL22L1;ETF1;RPS11;AURKAIP1;ABCE1;RPL39;RPS10
CCL24;AGTPBP1;YTHDF3;DST;COG6;SORT1;TRAPPC8;VMP1;GOLPH3;ZFYVE16;FMNL2;RAB14;CHMP4C;Y

MA2;TBL1XR1;PSME4;PSMB1;MDM2;ATF6;UBE2K

1;UBE2A;RAD23B;UBE2W;PSMA3;ITCH;PSMA4;PSMA1;PSMA2;TBL1XR1;MDM2;CALR;STT3B;UBE2K

THOC6;SOD1;DNAJC3;DNAJA1;DAB2;ITCH;GOLPH3;UFM1;PIK3CA;TMBIM4;RPS6KB1;WNK3;MDM2;CD28;

;RNF111

RGL3;SMCR8;CPEB2;CRK;RGS7
R;PKN2;TOP1

)P1
8;EIF3E;OGT

MED7;RAB6A;ARL5A;TMED5;WLS;SYNRG;HOOK3;DNAJC15;ARCN1;TM9SF2;ARL4A;RAB14
IP1;SEC22B;OGT
6A;EPS15;SNX5
;RAB6A;SEC22B;TMED5

PS10

CMTD1;RANBP2;ATG3;SMURF2;MIB1;COPS3;ITCH;UFM1;COPS2;UBE2V2;MDM2;UBE2N;BIRC6

K4;KPNA4;RPS11;RPL39;RPS10;RBMS2;MBTPS2;AGFG1

RIH1;UBE2K
2K;TAF2

)PK1;TGFBR1;ACVR2A;GNPTG;TBCK;FER;PIK3CA;WNK1;TAOK1;COPS2;WNK3;PKN2;BIRC6;TOP1;TAF2

A5;EXOC7;SORT1;HOOK3;RBSN;RAB11A;MIB1;ARL4A;ZFYVE16;RAB14;CHMP4C;CHMP3;RHOU;EXOC5;TM

3;UBE2L3;PTAR1;AKT2;ABL2;TLK1;JAK2;MAPK6;JAK1;FKTN;B3GALNT2;CDKL2;PDPK1;ATG12;TGFBR1;ACVF

31

2N;TPP2;ARIH1;UBE2K

);DAB2;ZEB1;RAP1A;MYF6;BRD7;IL6ST;DDR2

2;TMCC1;TMED7

2K

1;DUOX2

JWE1;UBE2A;RC3H2;MIB1;UBE2W;ITCH;MDM2;UBE2V2;UBE2N;TPP2;BIRC6;KBTBD8;UBE2K

AKT2;WNK3;NPTN;NBN;CALM1;JAK2;CALM2;PPIA;UBE2K;DDR2

_2;LCOR

‹2;IL6ST;SNX5;WLS

ARL5A

;IGFBP5;ERCC6L2;NR1H3;SYT9;MAPK14;ESR1;TIRAP;CPEB1;FER;LCOR

PNMB;AKT2;CNTN1;DDR2

ATF6;SNX5

ARRDC3;SCTR;ZDHHC13;TIRAP;TGFBR1;SOD1;DAB2;GOLPH3;PIK3CA;CNTN1;UBE2N;SMCR8;TLR7;PPIA;[illegible]

RGS7

BAG9
R1;TTBK2;CDC42;DAB2;FER;GPNMB;KIF2A;AKT2;CNTN1;OGT

YTHDF3;MUSK;MITF;EEF2;MAPK14;TGFBR1;SOD1;RPS6KB1;CD28;CNTN1;EIF3E;OGT

R2

;TMED2;CHMP3;EPS15;NCK1

DX2;JAK1

;PPIA;DDR2

CH;CD28;CNTN1;VPS35;SLC38A2

;ULK2;CHMP3;WDPCP;SNAP29

L3;EFEMP1;GPBP1;VPS35;METTL16;YAF2;MEF2C;MUSK;MITF;DICER1;ASH1L;TGFBR1;SMCR8;PRKAA1;RO

A;MEF2C;ZHX2;SMAD3;SMURF2;HSBP1;NR1H3;MITF;DICER1;ZHX1;ESR1;DRAP1;DAB2;ZEB1;MAF;CNOT1;[illegible]

4;ANG;JAK2;EEF1E1;FKTN

CC3;MPND

LM2;CRK

AP3K7

IAD3;OSGIN1;NR1H3;CGRRF1;CNOT7;DDAH1;CHMP4C;NOX4;CHMP3;EEF1E1;CRK

EFEMP1;GPBP1;PCGF1;GPBP1L1;CALR;YAF2

X2;DYNC1H1;XBP1;PBRM1;DICER1;TGFBR1;FER;CNOT7;MDM2;PFDN1;CALR;IL6ST;FOLR2

ZBED6;BRD7;TAF9B;YAF2;MEF2A;HSPA8;MEF2C;ZHX2;SMAD3;SMURF2;HSBP1;NFATC3;NR1H3;MITF;DICER

MED13;TBL1XR1;MORF4L2;NAA15;OGT;YAF2;RNF111;SNX5;TAF2

M1;SMAD3;OSGIN1;CGRRF1;TGFBR1;FER;CNOT7;AGO3;DDAH1;MDM2;NOX4;PFDN1;BIRC6;CALR;IL6ST;F
1F

1F

LR;JAK2;IL6ST;FOLR2;DDR2

.;JAK2;OGT

13;EFEMP1;ZMYM2;TRPS1;GPBP1;YAF2;RNF111;MEF2C;PBRM1;ZHX2;ANXA4;EN2;MITF;ASH1L;CBFA2T2;Z

AF2

;CDC73;IKZF5;ZXDA;MED13;TRPS1;NAMPT;NCK1;MEF2A;MEF2C;PBRM1;ZHX2;HSBP1;BUD31;ANXA4;EN2

(7;CDC73;ZXDA;MED13;NAMPT;YAF2;SNX5;RNF111;NCK1;MEF2A;MEF2C;XBP1;AKIRIN1;SMAD3;NFATC3;

2C;XBP1;AKIRIN1;SMAD3;NFATC3;NR1H3;MITF;ELL2;ESR1;SMAD5;ACVR2A;DAB2;TBL1XR1;RFX5;MYF6;TC

C15;ARCN1;ARL4A;RAB14;NUP98;CALR;KPNB1
A1;PSMA2;TBL1XR1;CHMP4C;MDM2;CHMP3;UBE2K

1;TAOK1;COPS2;WNK3;ULK2;PKN2;BIRC6;TLR7;TOP1;TAF2;DDR2
6;NAA15;IL6ST;TPT1

V1;SOCS2;ABL2;RICTOR;TAF9B;XBP1;API5;BNIP3;OSGIN1;SMAD5;SOD1;DNAJA1;DAB2;ITCH;GOLPH3;UF
R;LSM14A

ME1L1;CHMP3;WDPCP;RHOU;TMCC1;PPIA

TAX1BP1;YME1L1;NAA35;BIRC6;NAA15;IL6ST;TPT1

R2A;TBCK;FER;GGCX;UBLCP1;WNK1;TAOK1;COPS2;QPCT;WNK3;PFDN1;PKN2;BIRC6;STT3B;TAF2

DDR2

CK2;CREM;SBNO1;METTL4;PCGF1;GPBP1L1;SLC38A2;NDFIP2;SMAD3;SORT1;MAPK14;CLK4;PRDX6;ESR1

TBL1XR1;COPS2;MDM2;LCOR;CALR;OGT

R1;CBFA2T2;ZHX1;ESR1;DRAP1;DAB2;MAF;ZEB1;CNOT7;CNOT1;TBL1XR1;COPS2;MDM2;LCOR;CALR;OGT

OLR2;EEF1E1

:HX1;TGFBR1;TOX4;MAF;ZEB1;TBL1XR1;MORF4L2;RFX5;LCOR;ATF6;CRK;KDM7A;INO80C;PRKAA1;CAMK2D

;MITF;DICER1;ZHX1;TOX4;MAF;ZEB1;TBL1XR1;RFX5;LCOR;ATF6;CRK;KDM7A;GTF2A1;GTF2A2;CAMK2D;RF

NR1H3;MITF;ESR1;SMAD5;ACVR2A;TGFBR1;DAB2;TBL1XR1;MORF4L2;RFX5;MYF6;TCEA1;NAA15;ATF6;OG

EA1;ATF6;OGT;TAF2

11;RPS6KB1;WNK3;MDM2;CD28;CALR;NAA15;TPT1

.;SMAD5;PUM2;TADA2A;CNOT7;MDM2;CD28;CNTN1;CALR;OGT

);CBFB;CREM;ZBTB41;SBNO1;ZKSCAN8;METTL4;PCGF1;GPBP1L1;ZBED6;BRD7;SNX5;POLR2K;HSPA8;XBP

PL10;CBFB;CREM;AEBP1;ZBTB41;ZKSCAN8;PCGF1;ZBED6;BRD7;TAF9B;XBP1;AKIRIN1;ZFHX2;SMAD3;SMU

T;TAF2

1;ZFHX2;SMAD3;SMURF2;NFATC3;NR1H3;MAPK14;ESR1;SMAD5;TADA2A;CNOT7;MDM2;MYF6;POLR3G;C

RF2;NFATC3;NR1H3;MAPK14;ESR1;SMAD5;ACVR2A;DRAP1;DAB2;TADA2A;CNOT1;COPS2;MDM2;MYF6;TC

ALR;NAA15;OGT;TAF2

:EA1;CALR;OGT;TAF2

baseMean	log2FoldChange	lfcSE	stat	pvalue	padj
148.7106929	2.313483056	0.21990599	10.52032761	6.96311E-26	1.09209E-21
151.7433681	1.68926698	0.209941002	8.046389046	8.52726E-16	1.91059E-12
189.7408942	1.029074958	0.135443223	7.597832771	3.01131E-14	3.93578E-11
692.8194092	0.887263666	0.11136223	7.967366179	1.62092E-15	3.17781E-12
45.19373102	4.116083155	0.502815636	8.186068327	2.69899E-16	8.46618E-13
46.3951178	0.695257338	0.222162237	3.129502772	0.001751024	0.024368291
21.14927995	2.177807273	0.653899139	3.330494171	0.00086692	0.014884084
6.154760337	2.245936407	0.525939318	4.270333726	1.95181E-05	0.000977672
185.9529859	0.684276386	0.186696536	3.665179865	0.000247165	0.006418101
10.85596508	0.903917064	0.319509745	2.829075103	0.004668274	0.04547653
18.95743152	1.7978627	0.504693955	3.56228301	0.000367644	0.008462615
3.772706408	3.066910391	1.000086304	3.066645728	0.002164753	0.027736582
91.63929267	1.469093558	0.232921894	6.307236884	2.84061E-10	1.33088E-07
141.8612637	0.751827549	0.149728797	5.021262196	5.1333E-07	6.00826E-05
31.16032048	1.02145948	0.311026158	3.284159403	0.001022871	0.016829906
144.5129523	0.60509513	0.170851737	3.541638733	0.00039765	0.008916368
5.345990696	3.091226311	0.623945861	4.954318165	7.25844E-07	7.69198E-05
208.2472469	0.949037228	0.273070004	3.475435649	0.000510024	0.010623136
33.00218447	0.985132058	0.230030586	4.28261335	1.84711E-05	0.000935305
55.95410661	1.09265915	0.387386876	2.820588969	0.004793558	0.046209073
25.93573853	0.966179646	0.279355023	3.458608462	0.000542974	0.011146594
16.49112818	0.94960743	0.27632205	3.436596648	0.000589072	0.011665044
1086.332233	0.699319268	0.176136467	3.970326417	7.17742E-05	0.002564253
35.27616777	1.063984953	0.357482569	2.97632681	0.002917238	0.0337917
301.8836301	0.676886552	0.214018945	3.162741275	0.001562912	0.022675954
5.75177219	1.935492803	0.595581948	3.249750616	0.001155063	0.018317493
7.501980405	1.201203666	0.379226629	3.167508749	0.001537511	0.022431926
21.9801907	1.49066585	0.306443676	4.864404027	1.14802E-06	0.00010979
6.115875418	2.407050929	0.839515812	2.86718951	0.004141349	0.042232071
219.5372823	0.825422824	0.159054031	5.189575	2.10775E-07	2.89981E-05
176.3937992	0.611895849	0.199761692	3.06312909	0.002190356	0.027918062
6.557612023	3.434906932	0.964309382	3.562038281	0.000367987	0.008462615
87.40233729	0.884859049	0.185710398	4.7647254	1.89111E-06	0.000164779
335.6556954	2.076758891	0.325428237	6.381618594	1.75226E-10	8.58826E-08
16.66713366	1.379321652	0.306841765	4.495221352	6.94976E-06	0.000434263
9956.242537	0.628497362	0.182802729	3.438118049	0.000585772	0.011665044
333.8336767	0.78825116	0.213491184	3.692195362	0.000222327	0.0059344
68.50742139	1.271079646	0.338216681	3.758181421	0.000171153	0.004889545
603.1226115	0.716207924	0.190034162	3.768837759	0.000164009	0.004773708
3.812330921	2.1714823	0.656415752	3.308089871	0.000939347	0.015790688
44.66975461	0.961908562	0.22029864	4.366384481	1.2632E-05	0.000687775
51.80066553	0.989573129	0.272224437	3.635137016	0.000277833	0.006983224
304.5525137	1.059157989	0.299704408	3.534008711	0.000409308	0.009041662
44.29178754	0.777319713	0.205690139	3.779081085	0.000157408	0.00461456
20.50561438	0.733865459	0.261489861	2.806477678	0.005008638	0.047470074
195.6915504	0.768694138	0.21645474	3.551292695	0.000383344	0.008700962
33.17202626	1.520754545	0.419634843	3.623994931	0.000290087	0.007189519

215.3896899	0.653746131	0.155554009	4.20269549	2.63755E-05	0.001223887
141.8921763	0.977887222	0.230705113	4.238688981	2.24829E-05	0.001076665
40.61222406	1.074279398	0.314973188	3.410701096	0.000647961	0.01239914
13.72388784	0.961816494	0.297429931	3.233758252	0.001221728	0.019118304
17.17348879	1.330121295	0.288235062	4.614710246	3.93644E-06	0.000278104
15.70183431	5.497716221	1.375871751	3.995805726	6.44746E-05	0.002395444
385.4475237	0.598378679	0.179385474	3.335714235	0.000850806	0.014679908
74.72252845	0.68013064	0.189882066	3.58185823	0.000341159	0.008012353
45.12755448	0.728024772	0.184326777	3.949641957	7.82682E-05	0.002752373
11.5319071	1.083306752	0.376877869	2.874423892	0.004047653	0.041710507
243.4502411	1.018896176	0.216246972	4.711724584	2.45629E-06	0.000190715
339.689726	1.360771736	0.239030745	5.692873247	1.24919E-08	2.88122E-06
9.540046755	1.51847006	0.391961894	3.874024699	0.000107053	0.003505245
28.76002049	0.956351112	0.268015969	3.568261681	0.000359358	0.008312925
65.74061231	0.716996418	0.210871268	3.400161741	0.00067346	0.012725961
272.7858215	1.134281607	0.210181667	5.396672417	6.78882E-08	1.16123E-05
14.20334177	1.067675153	0.3680209	2.901126412	0.003718238	0.039376673
82.14831238	0.607553565	0.21570229	2.816630104	0.004853038	0.046468286
13.29526844	3.321564449	0.719716394	4.615101834	3.92903E-06	0.000278104
415.550923	0.653835923	0.190771079	3.427332525	0.000609542	0.011971582
24.01586182	1.551932852	0.381419781	4.068831585	4.72495E-05	0.00193995
28.77744739	0.882489252	0.220574086	4.000874569	6.31088E-05	0.002373756
30.98211831	1.138029056	0.292315933	3.893147542	9.89519E-05	0.003309148
54.78699696	0.59237362	0.201965553	2.933042846	0.003356576	0.036840121
84.88636358	0.851877366	0.243530859	3.498026367	0.000468715	0.009974658
30.19452259	0.590887432	0.211525977	2.793450909	0.005214894	0.04894698
50.27595876	0.762081605	0.20768247	3.669455604	0.000243068	0.006396428
51.5197704	0.676057126	0.202370693	3.34068692	0.000835714	0.014467262
101.6670631	2.046126689	0.31411058	6.514033011	7.31594E-11	4.4132E-08
13.83193242	4.912692928	1.601488844	3.067578613	0.002158007	0.027734345
11.62913851	1.202506036	0.314210507	3.827071377	0.000129677	0.004043445
18.70852271	1.080697621	0.322145482	3.354688118	0.000794545	0.013970454
8.4981764	1.316834938	0.448744888	2.934484542	0.003341022	0.036824025
29.15885858	0.99506881	0.208246994	4.778310548	1.76774E-06	0.00015753
93.9565527	1.672429273	0.235018569	7.116158012	1.10977E-12	1.16038E-09
65.16885519	0.60925453	0.185853467	3.278144548	0.001044919	0.017074327
20.59609825	0.747636345	0.263364578	2.838788532	0.004528516	0.044613845
11.84319751	1.457964537	0.36851737	3.95629801	7.61202E-05	0.002682852
50.57327914	2.27614336	0.38290454	5.944414658	2.77446E-09	7.77048E-07
239.1645339	0.599762774	0.149796627	4.003846991	6.23207E-05	0.00236096
75.1615243	0.821018526	0.256173253	3.204934615	0.001350932	0.020534482
128.3764019	0.689959751	0.219623787	3.141552924	0.001680544	0.023767049
10.76196194	1.387183193	0.385102705	3.602112304	0.000315642	0.007601441
151.3998264	1.560534909	0.290597726	5.370086447	7.86989E-08	1.24678E-05
185.3009931	0.799151101	0.282125283	2.83261072	0.004616957	0.045088642
33.16299018	0.907961807	0.241392387	3.761352294	0.000168997	0.004845616
4.049011104	1.67761275	0.575543502	2.914832229	0.003558798	0.03817454
4.707172926	2.293827822	0.786005475	2.918335681	0.003519053	0.037855162

142.7416441	0.994710795	0.251185187	3.960069492	7.4928E-05	0.002646779
15.93676547	1.674942888	0.396582109	4.223445414	2.40596E-05	0.001136597
403.1852708	1.11899193	0.347991514	3.215572468	0.001301846	0.019959097
278.2002228	0.81780699	0.176164155	4.642300766	3.44551E-06	0.000252076
209.7161597	0.600561889	0.136671954	4.394185276	1.11189E-05	0.000629562
222.5527667	0.714288642	0.210943708	3.386157613	0.000708787	0.013120652
56.07165824	0.692513666	0.157097122	4.408188102	1.04239E-05	0.000592349
195.9011872	0.586729445	0.202002215	2.904569371	0.003677587	0.039104593
8.223722579	1.824052171	0.622775708	2.928907066	0.003401561	0.037074416
79.84441369	1.244938285	0.201904384	6.16597946	7.00482E-10	2.6796E-07
53.70215713	5.495484695	1.463535966	3.754936553	0.000173385	0.004913071
3.145573729	2.420048692	0.750925166	3.222756144	0.001269636	0.019541678
3.683157989	2.995332983	0.801081955	3.739109294	0.000184673	0.005135493
3249.324212	0.974692617	0.260063837	3.747897552	0.000178323	0.005019031
16.00665712	1.29196138	0.38156899	3.385918178	0.000709405	0.013120652
182.9288884	0.956523097	0.230722084	4.145780408	3.38658E-05	0.001498918
87.37167123	0.585977173	0.20876401	2.806887895	0.005002265	0.047462504
29.45592784	2.345384964	0.83571327	2.806446959	0.005009116	0.047470074
31.2290792	0.640661384	0.212692115	3.012153893	0.002594011	0.031392331
22.48348433	0.950393014	0.276277629	3.439992656	0.00058173	0.011633224
122.1911592	1.012219674	0.333239226	3.037516579	0.002385363	0.029598125
50.95385674	0.959077997	0.178405623	5.375828292	7.62314E-08	1.22794E-05
59.31568222	0.612312539	0.195907442	3.125519545	0.001774913	0.0245599
247.5799083	0.624389462	0.196785455	3.172945176	0.00150901	0.022160408
20.81310407	1.208749752	0.316415343	3.820136345	0.000133378	0.004134188
346.3687171	0.861093786	0.198169814	4.34523185	1.39129E-05	0.000744742
23.85117412	1.741680731	0.602662688	2.889976042	0.003852712	0.040418684
105.842135	1.160324179	0.344534683	3.367800792	0.000757703	0.013627275
5.234514426	2.403689621	0.860294304	2.794031776	0.005205536	0.048888402
172.9774228	1.257043119	0.19937779	6.304830251	2.8851E-10	1.33088E-07
2.67697516	2.247241437	0.751526707	2.990234966	0.002787629	0.032799082
14.9718303	0.701461521	0.244794583	2.865510801	0.004163371	0.042360859
28.87192488	1.095000795	0.317194933	3.452138359	0.000556162	0.011278092
14.02694777	1.324001043	0.428660775	3.088691854	0.002010398	0.026653498
21.34888433	1.675340419	0.600954178	2.787800601	0.00530672	0.049535953
17.20395859	1.349608483	0.388988949	3.469529118	0.000521372	0.010792045
15.23014904	1.172010848	0.311248058	3.765520196	0.000166203	0.004808696
192.1214924	0.594339733	0.180719114	3.288748595	0.001006339	0.016666754
24.35293551	1.844651079	0.48018885	3.841511691	0.000122279	0.003851049
10.15450118	1.281124565	0.458697161	2.792963796	0.005222754	0.048991429
342.4109879	0.716196047	0.143993274	4.973815989	6.56476E-07	7.20012E-05
56.69713077	1.443547041	0.464846391	3.10542809	0.001900038	0.025756438
148.8103039	0.882626108	0.165538934	5.331833961	9.72258E-08	1.49499E-05
6.287496472	1.49293244	0.439623577	3.395933515	0.00068395	0.012827455
72.148209	2.933004955	0.554789087	5.286702682	1.24541E-07	1.82551E-05
346.5749398	1.554897148	0.23148256	6.717124392	1.85346E-11	1.36047E-08
18.71098557	1.33656077	0.300827904	4.442941469	8.87373E-06	0.000515465
174.1639701	1.49819115	0.279680663	5.356792035	8.47125E-08	1.32863E-05

3.274295117	2.836875471	0.80013644	3.545489653	0.000391884	0.008865217
10.30359596	1.646432855	0.41024839	4.013258544	5.98863E-05	0.00229647
24.77522068	1.098742758	0.352545826	3.116595568	0.001829524	0.025133238
6.858146694	2.506672099	0.845364035	2.965198417	0.003024878	0.034528516
17.3850267	1.100623697	0.376245465	2.925280963	0.003441454	0.037353465
10.68582897	1.224768874	0.344111779	3.559218104	0.000371961	0.00852899
21.17600576	2.548711459	0.860347139	2.962422192	0.003052289	0.034689933
32.03749428	3.730967786	1.069501748	3.488510226	0.00048572	0.010275041
397.1103279	0.634954581	0.227753412	2.787903703	0.005305031	0.049535953
1840.310253	0.84113545	0.24955131	3.3705912	0.000750071	0.013537525
20.92821636	2.577085875	0.839216788	3.070822596	0.002134699	0.02764709
25.22982055	2.965983915	0.800822786	3.703670733	0.000212502	0.005736458
69.14803611	0.936967443	0.246095653	3.80733032	0.000140475	0.004269788
171.1957583	0.765736257	0.161990544	4.727042938	2.27813E-06	0.000179832
30.60866535	0.80619554	0.264702719	3.045663991	0.002321671	0.029014417
77.04839682	0.748796269	0.231613061	3.232962185	0.001225138	0.019119464
31.34781831	1.116188913	0.22270364	5.011992238	5.38694E-07	6.16706E-05
272.7958354	0.62066131	0.126702	4.898591246	9.65262E-07	9.6753E-05
2.801724172	2.845319642	0.967153416	2.941952741	0.003261497	0.03630879
17.08362192	1.843621323	0.431811849	4.269501472	1.9591E-05	0.000977672
201.021836	0.661395061	0.219662074	3.010966111	0.002604179	0.031427701
140.8125925	0.90044889	0.21702693	4.149019159	3.33903E-05	0.00148355
35.87509926	0.63142733	0.218358232	2.891703806	0.00383159	0.040250941
4.291031099	3.840322625	1.342271213	2.861063092	0.00422223	0.042613551
64.22276916	0.714586019	0.249569998	2.863268924	0.004192945	0.042468884
112.7924139	0.791052059	0.247553246	3.195482475	0.001395973	0.021052317
82.49637604	0.643042144	0.227751392	2.82343892	0.004751148	0.045968644
272.6901969	0.652487192	0.11784153	5.536988441	3.07717E-08	6.10915E-06
4.139560504	1.780528883	0.621963767	2.862753392	0.004199773	0.042468884
168.3071978	0.844800125	0.240394334	3.51422644	0.000441037	0.009540992
8.32651305	1.417874765	0.468420155	3.026929456	0.002470516	0.030318915
11.09564939	1.495838208	0.526051153	2.843522344	0.004461788	0.04420637
18.45031111	1.900040244	0.512933946	3.704259113	0.000212009	0.005733028
11.09321973	1.160828489	0.408392527	2.842433229	0.004477061	0.044301717
60.84955897	0.759744408	0.178684861	4.2518678	2.11995E-05	0.00103904
47.92754695	0.63711549	0.185385809	3.436700426	0.000588847	0.011665044
58.08058083	0.612667889	0.158849678	3.856903569	0.000114832	0.003675575
322.9020181	0.830362521	0.284517623	2.918492404	0.003517284	0.037855162
101.0229927	0.932496122	0.266019371	3.50536925	0.000455974	0.009769812
3.061261408	2.702378769	0.785288675	3.44125524	0.000579022	0.011598189
798.6068401	1.064600211	0.258031243	4.125857783	3.69355E-05	0.001600268
41.25726198	0.648445611	0.192622405	3.366408025	0.00076154	0.013645843
82.95090919	0.777192629	0.205621355	3.779727196	0.000157	0.004611222
29.39568205	1.285225995	0.315094211	4.078862603	4.52566E-05	0.001877789
71.84673618	0.748328293	0.240301246	3.114125731	0.001844909	0.025249167
435.8341444	0.734415663	0.120027494	6.118728648	9.43249E-10	3.14764E-07
34.59694492	1.017613186	0.314393526	3.236749815	0.001208994	0.018942912
26.97534357	0.822085595	0.274344264	2.996547417	0.002730558	0.032370421

11.77449417	0.952246258	0.317708285	2.997234575	0.00272441	0.032370421
15.18578058	1.814893963	0.505547315	3.589958666	0.00033073	0.007812011
55.31509983	1.407407149	0.414750381	3.393383618	0.000690349	0.012905163
3.336696976	2.577616418	0.681064728	3.784686406	0.000153903	0.004562962
45.56323596	0.890365005	0.300012179	2.967762863	0.002999757	0.034366825
298.723377	0.63226092	0.153034267	4.131498987	3.60405E-05	0.001578938
77.31538156	4.9854339	1.340443333	3.719242566	0.000199821	0.00545992
14.90763031	2.133551616	0.444909874	4.79546924	1.62294E-06	0.00014799
18.53996533	1.116499097	0.39502795	2.826380002	0.004707738	0.045730975
12.05291489	1.047528531	0.375105008	2.792627424	0.005228187	0.049013082
211.4834195	0.974359068	0.14514791	6.712870111	1.90833E-11	1.36047E-08
72.81245402	0.679614678	0.173249927	3.92274149	8.75471E-05	0.002991478
36.28023349	0.839382306	0.297904354	2.817623491	0.004838051	0.046409777
12.87404989	1.797364528	0.518264565	3.46804441	0.000524261	0.010833338
19.7764115	0.948219556	0.292758458	3.238914295	0.001199856	0.018837382
83.35481602	0.717520324	0.136735623	5.247501051	1.54176E-07	2.17847E-05
237.3269359	0.599865471	0.205053391	2.925411128	0.003440014	0.037353465
20.39855987	1.712547128	0.578477938	2.960436373	0.003072036	0.03482693
1805.680117	1.024759485	0.167321322	6.124500284	9.09687E-10	3.10164E-07
37.06399983	0.815086424	0.225183314	3.619657284	0.000294993	0.007279945
129.3584475	0.601025955	0.152441236	3.942673067	8.05785E-05	0.002820966
28.72697353	0.803858797	0.272122862	2.95402889	0.003136546	0.035340226
11.61011	1.204453543	0.350709107	3.4343378	0.000594003	0.011733438
918.1264872	1.799363692	0.281150074	6.400011452	1.55365E-10	8.1225E-08
363.8975977	0.697220689	0.193607523	3.601206606	0.000316744	0.007601441
19.18572999	1.22830669	0.290496233	4.228305046	2.35458E-05	0.00111907
49.47414788	0.899390072	0.241781366	3.719848581	0.000199342	0.005456342
56.0570425	0.982363152	0.333110718	2.949058977	0.003187431	0.035794049
250.195302	0.647500343	0.204097918	3.172498525	0.001511333	0.022173761
6.547175667	2.932872688	0.867502545	3.38082315	0.00072269	0.013288014
6.091691156	2.212843793	0.618082439	3.580175806	0.000343363	0.008049786
933.0789832	0.787185269	0.115501288	6.815380853	9.40144E-12	8.67365E-09
90.60379961	0.899862138	0.26550991	3.389184745	0.000701008	0.013042235
236.4868786	1.151942298	0.409172088	2.81530029	0.004873167	0.046632556
18.40802959	2.244894873	0.664899613	3.376291442	0.0007347	0.013398887
258.6702784	0.778400133	0.240324779	3.238950795	0.001199703	0.018837382
28.37115389	4.598986306	0.841629428	5.464383909	4.64517E-08	8.61777E-06
6.34658423	1.765686925	0.531560603	3.321703893	0.000894696	0.01525262
58.42009473	2.210667352	0.571105565	3.870855907	0.000108454	0.003536363
11.52056183	2.07882275	0.725426942	2.865654182	0.004161486	0.042360859
8.106387991	1.986585619	0.635380623	3.126607179	0.00176836	0.024544215
283.373054	1.070322068	0.371998427	2.877222028	0.004011932	0.041522935
33.40564857	0.973868138	0.329221961	2.958089839	0.003095519	0.035028946
49.28400497	0.718473068	0.164433532	4.369382926	1.24598E-05	0.000683286
83.79078131	1.97244042	0.307120726	6.422361804	1.34176E-10	7.25661E-08
197.5564247	0.614017195	0.162147382	3.78678451	0.000152609	0.004533191
140.8344435	0.887993452	0.144348698	6.15172473	7.66448E-10	2.73204E-07
3.209148656	1.958210754	0.681042061	2.875315442	0.00403624	0.041647627

9.436597368	1.214044822	0.385305288	3.15086468	0.001627879	0.023275629
996.2384449	0.632212103	0.198053359	3.192130169	0.001412277	0.021135637
16.33980882	1.696557285	0.550387968	3.082475245	0.002052868	0.026943249
344.9741595	0.606884077	0.133131866	4.558518531	5.15157E-06	0.000343818
177.0583818	0.937430161	0.259687199	3.609843554	0.000306382	0.007496554
31.95171109	1.282506533	0.321932846	3.983770374	6.78304E-05	0.002485636
7.942465321	1.328182115	0.421404936	3.151795343	0.0016227	0.023255949
34.04202121	0.757721448	0.242374517	3.126242224	0.001770557	0.024552969
438.9018661	1.219943459	0.274043294	4.451645004	8.5215E-06	0.000496844
75.52013306	1.106842862	0.362696783	3.051703005	0.002275471	0.028596547
14.2133014	1.872372341	0.454623333	4.118513514	3.81324E-05	0.001640543
138.1275045	0.977418444	0.271264451	3.603194004	0.000314331	0.007601441
36.05069633	0.700557426	0.200135133	3.500422017	0.000464522	0.009912333
42.37299432	0.694296203	0.23422953	2.964170246	0.003035003	0.03459077
10.37699956	2.424646716	0.721636584	3.359927654	0.000779629	0.013816609
69.6328431	0.879751472	0.18506161	4.753830212	1.99599E-06	0.000166249
903.9819283	0.684712774	0.194698331	3.516788099	0.000436802	0.009466112
345.1762007	0.585189396	0.190403454	3.073417961	0.002116219	0.02754421
989.0062355	1.0883066	0.381529799	2.852481252	0.004337937	0.043390437
116.4844243	0.869507857	0.285082268	3.050024343	0.002288228	0.028710857
72.68770447	0.697800929	0.189516479	3.682006606	0.000231405	0.006141053
14.70719338	1.545501433	0.449306497	3.439748683	0.000582255	0.011633224
57.62371798	0.583344733	0.174603471	3.34096871	0.000834866	0.014467262
14.21999683	0.947916037	0.290642199	3.261453566	0.001108426	0.017775614
350.4548759	1.033543944	0.321088408	3.218876538	0.001286939	0.019769195
35.23968778	0.608750212	0.198488115	3.066935326	0.002162656	0.027734345
125.7038676	1.024077539	0.214085067	4.783507581	1.72262E-06	0.000154459
14.76183201	1.296135504	0.307422325	4.216139817	2.4852E-05	0.001160055
5.568003095	1.823362903	0.54037605	3.374248179	0.000740176	0.01345182
10.80179471	1.864128348	0.526962566	3.537496718	0.000403939	0.008988385
73.65318773	0.669402243	0.173114486	3.866818198	0.000110265	0.003565751
39.77702969	0.847402851	0.298729421	2.836690302	0.00455838	0.044795513
593.2811349	0.671859421	0.135028281	4.975694106	6.50142E-07	7.18087E-05
15.58779111	1.187093095	0.400808078	2.961749424	0.003058966	0.034740641
105.7434689	0.583177152	0.186048804	3.134538569	0.001721246	0.024103595
183.0194782	0.751046755	0.222559465	3.37458915	0.00073926	0.013450753
44.92832534	2.259497149	0.419558713	5.385413482	7.22783E-08	1.18085E-05
2.958427882	2.379611637	0.823705768	2.888909765	0.0038658	0.04045803
17.12632164	1.906080818	0.581276019	3.279132041	0.001041269	0.017065434
224.7236652	0.740850231	0.220445212	3.360700039	0.000777452	0.013793618
8.046704167	1.41690035	0.489719592	2.893289085	0.003812302	0.04012896
515.2348592	0.599250672	0.154671471	3.8743452	0.000106912	0.003505245
95.50906277	0.655248056	0.159100451	4.11845505	3.81421E-05	0.001640543
108.9763032	1.534098152	0.262494216	5.844312216	5.08665E-09	1.35219E-06
284.7669919	0.945675078	0.1881343	5.026595775	4.99263E-07	5.88755E-05
69.29524386	1.269287861	0.253101341	5.014939301	5.30502E-07	6.11794E-05
6.631363339	1.173701358	0.399006025	2.941562996	0.003265604	0.03630879
210.397686	0.911999227	0.160473259	5.683185065	1.32209E-08	2.96224E-06

34.88994794	0.72340064	0.256317167	2.822287124	0.004768247	0.046049988
32.47602569	1.129687852	0.283925818	3.978813402	6.92601E-05	0.002501018
32.46203837	1.131232818	0.314211447	3.600227905	0.000317938	0.007601441
503.2248564	0.646730998	0.156594418	4.129974792	3.62803E-05	0.001580612
14.70677738	0.937376028	0.314253265	2.982868063	0.002855611	0.033373619
134.3171541	1.152319741	0.202693012	5.685049174	1.30775E-08	2.96224E-06
32.02757112	1.408562854	0.253476892	5.556967519	2.74502E-08	5.5196E-06
28.95537637	0.990337272	0.306364444	3.232546367	0.001226922	0.019128279
161.1184802	1.098176413	0.225987019	4.85946678	1.17702E-06	0.000111881
20.18278334	1.444263225	0.430104086	3.357938863	0.00078526	0.013869386
174.5933474	1.486336693	0.494211645	3.007490225	0.002634146	0.031633955
3.101666192	1.810121172	0.608798127	2.973269943	0.002946452	0.034029567
117.5555297	0.706602242	0.179424399	3.938161408	8.21084E-05	0.002849087
8.991737528	1.301745923	0.463117325	2.810833997	0.004941327	0.047112327
16.28192796	2.112268887	0.391586611	5.394129492	6.88565E-08	1.16123E-05
75.38772534	0.612427378	0.19084197	3.209081196	0.001331599	0.020355552
14.70327417	1.600340742	0.47696451	3.355261677	0.000792899	0.013957166
7.556300851	2.075143154	0.469615844	4.41880993	9.92459E-06	0.000568092
11.75619775	3.086004398	0.717971266	4.298228276	1.72169E-05	0.000882449
14.49908992	1.33680086	0.468367969	2.854167977	0.004314971	0.043243453
3.43633939	3.176501325	0.860370349	3.692016266	0.000222483	0.0059344
357.5442071	1.402051039	0.384685279	3.644670375	0.000267735	0.006805756
91.82329988	0.982519239	0.231816163	4.238355198	2.25163E-05	0.001076665
242.2168145	0.79467097	0.166371526	4.776484239	1.78387E-06	0.000158069
561.8584988	0.633463717	0.200315856	3.162324395	0.001565151	0.022687457
324.4943775	0.588767159	0.188918484	3.116514318	0.001830028	0.025133238
238.0924674	0.619174358	0.169504928	3.652839858	0.000259356	0.00663579
491.290016	0.828321294	0.182999773	4.526351484	6.00107E-06	0.000390001
2695.512373	1.906420729	0.552704582	3.449258051	0.000562129	0.011376045
76.10373575	3.3881128	1.15620198	2.93038142	0.003385462	0.036969932
104.8413217	4.278416644	1.20991183	3.536139195	0.000406021	0.009007113
3.885490824	1.459511552	0.519828844	2.807677122	0.004990023	0.047425411
10.76026472	1.95882079	0.482205516	4.062211495	4.861E-05	0.001980258
25.46059524	1.663533215	0.501782474	3.315247747	0.000915619	0.015511549
362.759718	1.639539357	0.309813764	5.292015869	1.20975E-07	1.78998E-05
85.27722059	0.697393542	0.161217283	4.325798881	1.5198E-05	0.000799884
23.17822175	0.76332185	0.245738391	3.106237682	0.001894843	0.025708239
89.98312991	3.342512874	0.892705584	3.744249988	0.000180933	0.005061126
38.65737331	1.719654373	0.457661508	3.757480895	0.000171632	0.004894334
12.71806387	1.57039812	0.553845939	2.835442148	0.00457623	0.044858497
13.12880372	2.469557026	0.701407334	3.520860002	0.00043015	0.009370093
47.15565274	0.670326868	0.198029499	3.384984915	0.000711822	0.013134366
15.08201115	0.861310436	0.27516654	3.130142335	0.001747216	0.024336891
31.67910536	1.880536383	0.435645749	4.316664139	1.58405E-05	0.000822656
1417.07707	0.632990287	0.161274177	3.924932679	8.6754E-05	0.002970851
8.710464633	2.186938914	0.47436892	4.610206994	4.02268E-06	0.000282923
78.33374583	0.641028014	0.227417901	2.818722758	0.004821514	0.046305965
9.947738414	3.766472012	1.196817305	3.147073489	0.001649135	0.023390902

807.2613422	0.585037707	0.196595505	2.975844774	0.002921827	0.03381988
11.3409639	1.347507798	0.374198768	3.601048196	0.000316937	0.007601441
61.27775574	1.067970406	0.382083496	2.795123097	0.005187996	0.04875286
169.42115	1.293021915	0.448369063	2.883833929	0.003928659	0.040851128
179.753216	2.018925235	0.707456897	2.853778433	0.004320265	0.043268862
24.88336609	1.231443898	0.365568467	3.368572538	0.000755585	0.013605737
29.30491634	0.945420489	0.238087849	3.970889296	7.16048E-05	0.002564213
38.08411591	0.863448781	0.280813387	3.074813461	0.002106342	0.0274901
45.02110795	0.765223861	0.24730754	3.094219693	0.001973313	0.026272871
53.9089057	1.605909317	0.302076343	5.316236619	1.05935E-07	1.58237E-05
15.70634114	1.53105954	0.448352062	3.414860042	0.000638148	0.012280628
9.978468325	1.524921213	0.33238091	4.587872436	4.47786E-06	0.000309387
6.658638785	1.680767107	0.439458943	3.824628297	0.00013097	0.004067576
10.82838683	2.379380908	0.717180905	3.317685806	0.000907665	0.015406733
191.7349714	0.67365004	0.20855229	3.230125348	0.00123736	0.019252726
187.5112669	0.668141323	0.142062065	4.703164933	2.56159E-06	0.000196941
18.89654354	3.148129275	1.009445553	3.118671697	0.001816683	0.025015672
31.34428931	1.734924516	0.389672201	4.45226658	8.49686E-06	0.000496844
14.7203812	3.438527597	0.762944366	4.506917869	6.57761E-06	0.000415981
14.68928737	1.501647794	0.348787128	4.305341784	1.66728E-05	0.000857365
103.6543948	0.861771315	0.188378694	4.574675059	4.7696E-06	0.000326665
18.44703145	1.297936886	0.335624121	3.867233627	0.000110077	0.003565751
37.60716347	0.798962956	0.253738713	3.148762549	0.001639634	0.02331468
4.023438618	1.949704409	0.682821723	2.855363769	0.004298756	0.043136078
11.07055608	1.180198348	0.397122854	2.971872141	0.002959899	0.034077886
16.97406128	1.368270736	0.379859383	3.602045377	0.000315723	0.007601441
7.517502643	2.490573923	0.721080632	3.453946499	0.000552447	0.011269949
80.95026231	1.407393398	0.450502605	3.124051635	0.001783792	0.024629485
66.99083273	0.650315445	0.154155534	4.218566965	2.4586E-05	0.001151065
82.70043725	1.225672594	0.195503668	6.269307397	3.62658E-10	1.53728E-07
231.0942796	0.843308706	0.226503101	3.723166265	0.00019674	0.005394525
15.14190516	0.99521895	0.301842584	3.297145607	0.000976729	0.0162795
469.807134	0.637389781	0.179959267	3.541855844	0.000397323	0.008916368
63.94082363	0.582657492	0.189967601	3.067141389	0.002161166	0.027734345
73.08680997	0.781191983	0.255864843	3.053143109	0.002264579	0.028551173
60.08810536	1.010586601	0.237859775	4.248665417	2.15048E-05	0.001047457
98.37663857	0.851878951	0.248328481	3.430452063	0.000602576	0.011872875
18.3025134	0.927315125	0.301717531	3.073454574	0.002115959	0.02754421
129.6870201	0.691383544	0.22271956	3.104278507	0.001907437	0.025813573
5.82532275	1.334858951	0.442706068	3.015226236	0.002567876	0.031196416
61.09059541	1.38702385	0.255782327	5.422672722	5.87144E-08	1.05471E-05
13.99965983	1.460684831	0.295254864	4.947199897	7.52887E-07	7.87218E-05
93.07555788	0.841546892	0.213671844	3.938501559	8.19921E-05	0.002849087
38.74696976	2.161287936	0.61097564	3.537437164	0.00040403	0.008988385
803.3562388	0.649309053	0.152041187	4.270612888	1.94937E-05	0.000977672
42.26482519	1.524618239	0.488765129	3.119326952	0.001812647	0.024982035
20.73893818	1.841773371	0.462937588	3.978448536	6.93664E-05	0.002501018
18.24439212	1.373414256	0.352677252	3.894252457	9.8502E-05	0.003308149

40.1130419	1.827248734	0.645044246	2.832749449	0.004614954	0.045088642
551.99086	0.643161552	0.169333921	3.798184961	0.00014576	0.004404803
22.57960005	1.260978158	0.366950359	3.4363726	0.00058956	0.011665044
24.4095571	0.792426018	0.275871492	2.872446194	0.004073074	0.041917387
13.98431098	1.433716173	0.381825251	3.754901409	0.00017341	0.004913071
13.48607155	1.281839945	0.330057247	3.883689744	0.000102883	0.003411458
5.42952342	1.686492409	0.52922922	3.186695564	0.001439082	0.021397627
6444.14198	1.73694469	0.530964107	3.271303402	0.00107053	0.017309476
9.087979393	1.19648528	0.406763218	2.941478548	0.003266495	0.03630879
14.65680013	2.140205275	0.45640837	4.689233191	2.74231E-06	0.000206781
7.608632813	1.684847079	0.419608533	4.015283163	5.93745E-05	0.002282424
502.2743032	0.943969618	0.2477479	3.810202292	0.000138853	0.004236911
159.0119924	0.890011288	0.224134895	3.970873373	7.16096E-05	0.002564213
14.79563994	1.151532734	0.314460829	3.661927431	0.000250325	0.006478703
9.909160933	1.388038148	0.396185674	3.503504137	0.00045918	0.009825064
31.14439126	1.406824669	0.43142207	3.260901026	0.001110588	0.017792096
153.7689	1.176978278	0.2374632	4.956466009	7.17869E-07	7.65922E-05
22.99950915	0.925791665	0.298602991	3.100409887	0.00193253	0.025994319
26.94074388	0.599551811	0.193371937	3.100510962	0.001931871	0.025994319
134.4907717	0.810987219	0.253837679	3.194904798	0.00139877	0.021052317
38.90329532	0.895877494	0.196882173	4.550323063	5.35636E-06	0.000355971
33.44321108	1.217786473	0.2020014	6.028604109	1.65382E-09	5.08598E-07
9.971134678	1.406729465	0.408431767	3.444221481	0.000572706	0.011505047
6.372492792	1.833530837	0.554019729	3.309504589	0.000934612	0.015740712
29.7797928	1.449849422	0.325684046	4.45170539	8.5191E-06	0.000496844
44.14699	0.776158842	0.218243093	3.556395904	0.000375977	0.008583448
88.63023985	0.790145946	0.266016998	2.97028367	0.002975249	0.034185935
3462.03614	0.604209622	0.151160091	3.997150432	6.41096E-05	0.002394034
18.3618313	0.693797296	0.242060323	2.866216521	0.0041541	0.042334572
29.09306219	0.637019014	0.199044394	3.200386607	0.001372434	0.020757231
11.5909009	1.581301829	0.513505993	3.079422344	0.002074024	0.027175436
6.920376615	2.368987561	0.752010641	3.150204838	0.00163156	0.02328425
182.7376989	1.334709816	0.406149918	3.286249137	0.001015312	0.016744635
12.75444316	1.036556421	0.323430037	3.204886068	0.00135116	0.020534482
100.9405037	0.590476246	0.174518715	3.383455151	0.000715799	0.013192234
17.997275	1.519311194	0.318941864	4.76359916	1.9017E-06	0.000164786
71.60535733	0.936198678	0.249815736	3.747556866	0.000178565	0.005019031
74.15269909	0.942694115	0.328374311	2.870791298	0.004094458	0.042027141
28.30276471	0.656084384	0.205489943	3.192780999	0.001409098	0.021129476
6.281612247	1.386170147	0.457007757	3.033143583	0.002420204	0.029920446
30.0206609	1.527169409	0.368404102	4.145364837	3.39273E-05	0.001498918
478.3621632	0.636805247	0.146846263	4.336543761	1.44741E-05	0.00076693
15.47259783	0.76482567	0.257194818	2.973721149	0.002942123	0.034004612
78.4557829	0.693546337	0.236441299	2.93327071	0.003354113	0.036840121
59.25027122	1.238895039	0.400014795	3.097123045	0.001954087	0.026083326
1772.802442	0.763712024	0.273529121	2.792068431	0.005237228	0.049068508
196.9885272	0.60223557	0.178022199	3.38292401	0.000717185	0.01320226
5.990132219	1.321959481	0.458461293	2.883470214	0.003933198	0.040853168

17.97686735	1.248630043	0.327693635	3.810357941	0.000138766	0.004236911
59.22080572	0.634595243	0.155709935	4.075496163	4.59163E-05	0.001900137
12.8215368	1.848143094	0.378668588	4.88063482	1.05745E-06	0.000102379
530.1752241	1.067317144	0.162526129	6.567049573	5.1322E-11	3.35389E-08
56.84531093	1.090970186	0.256748523	4.249178039	2.14556E-05	0.001047457
14.01649101	0.899126791	0.31064659	2.894371994	0.003799178	0.040072496
22.9877917	4.845739958	1.529285365	3.168630308	0.001531591	0.022387203
84.3833611	0.850838616	0.154612739	5.50303047	3.73318E-08	7.14039E-06
45.60440613	0.737744444	0.160654519	4.592117586	4.38771E-06	0.000304499
220.217175	1.950806209	0.333571543	5.848239308	4.96803E-09	1.34343E-06
178.5280635	0.690320229	0.218666741	3.156951197	0.00159428	0.022961149
10.12520745	1.043792137	0.32824443	3.179923376	0.00147314	0.021755864
19.75273627	1.61696594	0.531787376	3.040624906	0.002360877	0.029387303
10.3971317	1.79676475	0.551941761	3.255352062	0.001132519	0.018106447
2.673727526	3.096227939	0.999866571	3.096641121	0.001957267	0.026103547
10.34464424	1.820646993	0.613072573	2.969708762	0.002980822	0.034224899
22.46430381	1.312139218	0.233458771	5.620432304	1.9048E-08	3.98332E-06
57.65476235	0.724079468	0.221585567	3.267719462	0.001084178	0.017465721
777.7486845	0.941485981	0.232067542	4.056948122	4.97181E-05	0.002014933
75.79714958	1.20697513	0.25371595	4.757190595	1.96306E-06	0.00016553
289.4547088	0.647268191	0.120072744	5.390633796	7.02096E-08	1.16438E-05
82.16824861	0.638536663	0.201089857	3.17537977	0.001496405	0.022037201
865.7118764	0.656320653	0.144762535	4.533774254	5.7939E-06	0.000381813
97.72691693	2.088357318	0.351298485	5.944680676	2.76996E-09	7.77048E-07
3454.95098	0.950844224	0.168538809	5.641693024	1.68386E-08	3.61776E-06
68.53214437	0.772225353	0.260598788	2.963273	0.003043865	0.034619271
19.10563427	0.786333162	0.276847428	2.840312322	0.004506938	0.044429178
11.41852312	0.978827623	0.31920831	3.066422746	0.002166368	0.027736582
12.08654858	1.360766547	0.403152174	3.375317395	0.000737306	0.013430787
31.93917683	0.888490567	0.246324159	3.606997261	0.000309761	0.007543929
244.6860041	0.591679585	0.206132236	2.870388436	0.004099678	0.042053208
24.90137257	0.748122402	0.203288837	3.680095829	0.000233146	0.006166386
23.40962918	1.03960713	0.31825968	3.26653735	0.001088714	0.017513228
23.38244865	1.054132778	0.339581524	3.104211225	0.001907871	0.025813573
67.1560328	0.700914746	0.204348617	3.42999506	0.000603592	0.011877968
7.333738668	2.216036978	0.604567797	3.66548961	0.000246866	0.006418101
645.4926713	0.733974284	0.212615724	3.452116673	0.000556207	0.011278092
17.49904022	1.069576956	0.365349729	2.927542762	0.003416521	0.037185782
229.7925272	0.743652302	0.257499935	2.887970828	0.003877358	0.040493118
113.0624325	0.590590774	0.124454491	4.745435627	2.08058E-06	0.000169957
72.14004223	0.624405322	0.205248633	3.042189923	0.002348637	0.029304706
6.179675381	1.782829628	0.537206208	3.318706304	0.000904355	0.015383842
28.02577321	1.813545015	0.585151657	3.099273483	0.001939959	0.026005393
33.15489648	0.771998782	0.251354448	3.071355169	0.002130895	0.02764709
3.680188674	2.544998899	0.749478967	3.395690889	0.000684556	0.012827455
38.69304291	0.689206581	0.172304473	3.999934345	6.33601E-05	0.002377366
5.007009422	2.128639652	0.700771023	3.037568024	0.002384956	0.029598125
3703.074518	1.086629206	0.285179609	3.810332762	0.00013878	0.004236911

600.4542837		1.548970608	0.249040208	6.219761131	4.97912E-10	2.05507E-07
3.347417529		2.182904445	0.702605081	3.106872556	0.001890779	0.025697553
124.3794443		0.713443449	0.169056357	4.220151569	2.44138E-05	0.001149869
14.79155593		1.384508678	0.468303899	2.956432095	0.003112208	0.03514173
659.2311052		0.623502416	0.17460199	3.570992608	0.000355631	0.008274669
17.36264261		0.796686835	0.26962573	2.954787861	0.003128841	0.035278752
287.467799		0.594386373	0.211194282	2.814405612	0.004886753	0.046707884
35.43172905		2.073661872	0.441476798	4.697102732	2.63878E-06	0.000200906
486.6987325		1.434314779	0.199321977	7.195969064	6.20187E-13	6.94787E-10
109.6106647		0.597396685	0.178677832	3.343429222	0.000827498	0.014372623
36.79452203		0.580753416	0.172386898	3.368895335	0.000754701	0.013605434
60.61000265		0.806392366	0.218065665	3.697933674	0.000217362	0.005847513
7.370600965		2.722131703	0.867233216	3.138869283	0.001696011	0.023835335
328.3775203		0.923661382	0.188582002	4.89792966	9.68517E-07	9.6753E-05
14.88186441		0.969173019	0.344589969	2.81253985	0.004915193	0.046920202
138.9930194		0.675480531	0.199214671	3.390716805	0.000697101	0.013000394
45.05817534		0.932158609	0.236054414	3.948914122	7.85065E-05	0.002754577
643.4502012		0.641793655	0.104478662	6.142820364	8.10689E-10	2.82552E-07
116.8108978		0.891549217	0.2483157	3.590386012	0.000330189	0.007810978
9.903658339		2.032578146	0.683679616	2.972998023	0.002949064	0.034034667
100.6266118		0.796678887	0.127074711	6.269373989	3.62503E-10	1.53728E-07
9.931019333		1.736920781	0.402916003	4.310875641	1.62609E-05	0.000841705
2.614400751		2.38309787	0.838238157	2.84298424	0.004469328	0.044253119
156.1844373		1.068875186	0.312323005	3.422338955	0.000620849	0.012111182
8.745307356		1.12258968	0.382431348	2.93540183	0.00333116	0.036753938
8.791848691		1.229201151	0.437198508	2.811540133	0.004930494	0.047037632
31.77254237		0.881360292	0.284132664	3.101932313	0.001922619	0.025905806
613.1296879		0.622972822	0.110923598	5.616233466	1.95165E-08	4.02759E-06
61.42957891		2.322823531	0.583674398	3.979656365	6.9015E-05	0.00249984
423.6228219		0.724865594	0.197605135	3.668252825	0.000244214	0.006405095
23.61023171		0.735691854	0.222947703	3.299840476	0.000967398	0.016141139
1012.846673		1.510035297	0.3526121	4.282426206	1.84866E-05	0.000935305
14.00400933		1.8586876	0.39857954	4.663279001	3.1121E-06	0.000230237
79.73533167		0.626864941	0.172639468	3.631063899	0.000282255	0.007071712
132.6490595		1.288103841	0.239662333	5.374661201	7.67268E-08	1.22794E-05
155.0442201		0.789311881	0.231915703	3.403443019	0.000665423	0.012650294
52.80000235		0.615831797	0.206126788	2.987635926	0.002811442	0.032980302
7.784542407		1.268913578	0.423303046	2.997648118	0.002720716	0.032370421
41.63598689		1.109647286	0.335140709	3.310989252	0.000929668	0.015678397
6.51859313		1.979411591	0.519786246	3.80812614	0.000140024	0.004264338
3.797493135		2.444140135	0.611749978	3.995325249	6.46055E-05	0.002395444
105.9466914		0.83381184	0.164775663	5.060285148	4.1863E-07	5.13481E-05
18.28997738		1.948948342	0.628711984	3.099906463	0.001935818	0.025994319
187.8622888		0.951659636	0.194589098	4.890611273	1.00523E-06	9.97853E-05
66.17736887		1.243800889	0.246586094	5.044083656	4.557E-07	5.42292E-05
30.64804634		4.832609607	1.281601909	3.770757185	0.000162753	0.004753478
105.4917117		5.076147828	1.342878757	3.780049241	0.000156797	0.004611222
17.09675933		1.286868835	0.293204103	4.388986443	1.1388E-05	0.000638955

9.585919956	2.000423882	0.514115435	3.891001408	9.98314E-05	0.00332432
86.67303359	5.018314054	1.33361857	3.762930546	0.000167934	0.004832797
30.40037283	1.129677169	0.257988171	4.37879444	1.19338E-05	0.000659046
50.72728309	0.605493716	0.159829847	3.788364493	0.000151642	0.004530204
5.753511259	1.427408208	0.486928117	2.931455707	0.003373775	0.036945324
25.43486124	1.475086421	0.351223694	4.199848832	2.67093E-05	0.001232085
35.61285648	0.786380563	0.228704176	3.438418035	0.000585124	0.011665044
92.59307562	1.121310621	0.293063947	3.826163652	0.000130156	0.004050324
21.87761698	0.936749964	0.255675629	3.663821885	0.00024848	0.006441577
32.52324798	1.11576788	0.234520476	4.757656548	1.95853E-06	0.00016553
141.1534148	0.654162277	0.14100929	4.639143132	3.49857E-06	0.000254035
7.156933049	1.962968982	0.435661053	4.505725187	6.61466E-06	0.000416644
16.14546175	1.058821778	0.332395612	3.185426462	0.001445408	0.021447289
177.4305028	1.552299873	0.291257952	5.329639454	9.84079E-08	1.49848E-05
57.99540334	0.601814196	0.200680394	2.998868927	0.002709839	0.032295675
11.56770316	1.065191109	0.362045207	2.942149453	0.003259426	0.03630879
58.63913624	0.680311824	0.23697394	2.8708297	0.00409396	0.042027141
5.559336595	2.137938077	0.563115834	3.796622203	0.000146681	0.004424127
30.02187526	1.19412652	0.246878541	4.836898797	1.31881E-06	0.00012312
104.5917053	0.657718185	0.216969662	3.031383186	0.002434361	0.03001613
50.4286252	0.81482792	0.251347268	3.241841167	0.001187602	0.018738781
45.93831069	0.789923689	0.221303739	3.569409591	0.000357787	0.008297741
80.00685811	0.810185454	0.134628828	6.017919522	1.76673E-09	5.32873E-07
36.98046091	0.894803574	0.306006137	2.924136038	0.003454138	0.037423737
8.850350593	0.907441361	0.453361152	2.001586056	0.04532927	0.188729565
25.61399584	1.115689963	0.609067112	1.831801357	0.066981021	0.239519
140.037652	0.568784723	0.182860885	3.110477797	0.001867849	0.025474217
755.2262616	-0.249782168	0.122129548	-2.045223063	0.040832872	0.177157057
872.691782	-0.406577323	0.165723261	-2.453350978	0.014153217	0.09353526
328.5078269	-0.239430792	0.076268134	-3.13932937	0.00169335	0.023835335
264.387456	-0.179205775	0.135165757	-1.325822299	0.184898565	0.430769788
1634.940347	-0.271532419	0.112010459	-2.424170215	0.015343412	0.098787387
1045.863005	-0.289017438	0.102168164	-2.82884049	0.004671698	0.04548163
238.9118613	0.251140382	0.104097456	2.412550618	0.015841338	0.100216166
466.9675715	-0.015414381	0.120311065	-0.128121058	0.898053171	0.957387882
25.78360007	-0.103636343	0.240135515	-0.43157441	0.66605076	0.837650559
13.03538926	0.060984435	0.390467341	0.156183191	0.875888626	0.948628307
11.48331436	0.465841021	0.319838661	1.456487526	0.145257895	0.37526352
545.2664202	-0.254893717	0.056279877	-4.529038267	5.92528E-06	0.000388837
129.3688075	-0.133575253	0.179768478	-0.743040461	0.457457184	0.694284175
2.910374933	0.639989864	0.570280788	1.122236409	0.261761936	0.51962191
29.86143494	-0.333481033	0.195025824	-1.709932696	0.08727832	0.279207439
4.581999578	-0.143197609	0.630663857	-0.227058531	0.820378229	0.921144748
1019.997766	-0.095977972	0.134730026	-0.712372552	0.476234107	0.70906168
104.4970949	-0.088717829	0.173582701	-0.511098335	0.609282202	0.802570863
2410.004461	-0.27962573	0.124861827	-2.239481336	0.025124614	0.131516192
605.8025468	-0.011378355	0.092696092	-0.122749025	0.902305841	0.958662553
290.5275212	-0.052030769	0.113031565	-0.460320701	0.645286044	0.82563765

1026.464629	-0.4373503	0.215596254	-2.028561681	0.042502955	0.181194983
774.1997745	-0.022920883	0.070582189	-0.32474033	0.745377608	0.882102347
339.8136145	-0.136879704	0.212832736	-0.643132755	0.520137962	0.743254883
272.380561	-0.175291183	0.108307473	-1.618458805	0.105563758	0.310829552
346.2104971	-0.297830584	0.152731182	-1.950031289	0.05117239	0.202418099
22.1885535	0.4736917	0.298271712	1.588121437	0.112258882	0.322584884
4.314317796	0.270366295	0.512268557	0.527782335	0.597650419	0.794878848
58.5250543	0.165100535	0.145263615	1.136558081	0.255723072	0.512836005
22.24668712	0.663429834	0.325348092	2.039138541	0.041436203	0.178687215
2636.504964	-0.17319527	0.114347732	-1.514636684	0.129864437	0.351838629
73.45831727	0.193011633	0.145559789	1.325995555	0.18484117	0.430769788
178.1693423	-0.473530082	0.143702941	-3.295201058	0.000983513	0.016375176
445.3826524	-0.168150109	0.133135414	-1.263000616	0.206588958	0.456725202
645.6052413	-0.005330094	0.157289575	-0.033887141	0.972967147	0.986800481
7.266447433	-0.735103112	0.679145235	-1.082394567	0.279077241	0.53751602
8565.587848	-0.180869873	0.176554645	-1.024441319	0.305626874	0.56446678
48.01557757	0.103185916	0.374549876	0.275493126	0.782937406	0.900791541
4.25058342	-0.453192798	0.503484861	-0.900112065	0.368060616	0.623287987
4.082211383	-0.349372101	0.708743	-0.492946104	0.62205067	0.810641951
24.27876753	0.906964784	0.53444241	1.697029964	0.089690996	0.283955102
68.21419079	0.451226221	0.249638816	1.807516265	0.070681821	0.24687097
26.79764173	0.620637377	0.257763497	2.407778385	0.01604992	0.100811755
9.106042884	-0.011339208	0.412537046	-0.02748652	0.978071692	0.988880488
56.28541934	0.249201644	0.257771058	0.966755718	0.333666166	0.591993229
16.2300543	-0.116078385	0.282898444	-0.410318217	0.68157253	0.84736731
30.38778546	-0.193342254	0.489225406	-0.395200764	0.692694729	0.853431717
784.5313957	-0.499511046	0.279867655	-1.784811633	0.074291876	0.254187125
4.515984695	-0.012840302	0.472525729	-0.027173762	0.978321143	0.988913947
49.9118149	-0.269419253	0.579742168	-0.46472254	0.642130165	0.824312819
2.738391729	0.08706004	0.566683909	0.153630691	0.877900936	0.949450031
10.859319	-0.02711902	0.387081029	-0.070060319	0.94414565	0.975492778
152.9239415	-0.275721315	0.196188439	-1.405390227	0.159905271	0.397563536
13.315543	-0.064938411	0.358114782	-0.181334071	0.856105365	0.938518178
176.4745009	0.054989726	0.124466721	0.441802639	0.658632028	0.833668367
151.6745379	0.008190415	0.205124765	0.039928943	0.968149776	0.984725103
464.3080622	0.007974865	0.131788482	0.060512609	0.951747374	0.978862788
119.4980358	-0.052426614	0.369128777	-0.142027979	0.887057905	0.95312914
722.5656081	-0.177947339	0.069318568	-2.56709486	0.010255455	0.075936734
881.8700541	-0.1249184	0.057723124	-2.164096321	0.030456958	0.148534492
85.02593292	-0.365964971	0.251885315	-1.452903164	0.146250642	0.3765876
30.81379165	0.398634077	0.333973276	1.193610704	0.232630285	0.487972902
102.964526	0.133485882	0.115953022	1.151206583	0.249647257	0.505482518
152.5523927	0.044554234	0.173889036	0.256222215	0.797779256	0.907614235
253.5659829	-0.025536109	0.101611881	-0.251310265	0.801574239	0.909885675
3.929167633	-0.437509162	0.722864319	-0.60524382	0.545017004	0.759994702
26581.48174	-0.281142636	0.095382562	-2.947526573	0.003203272	0.03593714
94.1706005	-0.322338192	0.172133646	-1.872604225	0.061123058	0.226311151
122.3454331	-0.087380418	0.120379232	-0.725876188	0.467914675	0.703082369

45.03806965	-0.078664292	0.413290128	-0.190336732	0.849045272	0.934802172
30.16031593	0.653076038	0.25541867	2.556884496	0.010561428	0.077296051
754.6109324	0.217229875	0.091239033	2.380887506	0.017270985	0.105400052
1103.54682	-0.105504341	0.118078203	-0.893512417	0.371582825	0.625604127
558.0520322	0.19587161	0.09592	2.042030964	0.04114846	0.178050523
1570.252262	-0.224693512	0.077831389	-2.886926646	0.003890249	0.040595255
31.58629634	1.163918503	0.519749483	2.239383666	0.025130963	0.131516192
944.3693109	-0.016260727	0.071655578	-0.226928979	0.820478968	0.921144748
60.30964447	-0.328215297	0.223913913	-1.465810199	0.142700002	0.371531678
219.993073	-0.147643177	0.143269613	-1.03052681	0.302762773	0.561492585
241.1702345	0.419150443	0.194944304	2.150103558	0.031547024	0.151309947
385.7893129	0.325099729	0.157006448	2.070613875	0.038394894	0.170445945
25415.58275	-0.040984785	0.139921556	-0.292912587	0.769588968	0.893892718
133.6807018	0.391529186	0.135431447	2.89097691	0.003840463	0.040317154
209.2052061	0.266735222	0.132230454	2.017199618	0.043674686	0.184437888
2426.436244	0.036722109	0.142728744	0.257286009	0.796957992	0.907074684
1183.192173	0.054002555	0.130558983	0.413625736	0.679148222	0.845914923
4.53361138	0.618269386	0.50242312	1.230575108	0.21848182	0.4711493
6.506929627	0.390457502	0.390961431	0.998711051	0.317934686	0.576478535
1126.228361	0.096749182	0.084748484	1.141603688	0.25361879	0.510230517
12.01117046	0.23307078	0.354839871	0.656833685	0.511287862	0.73623199
689.7690006	0.036623786	0.090183525	0.406102841	0.684667039	0.848677613
1917.661187	-0.257458529	0.134551074	-1.913463195	0.055688781	0.213974712
154.4331141	-0.129315018	0.118667674	-1.089724046	0.275834721	0.534543001
231.0974124	0.195727515	0.131743377	1.485672523	0.137365762	0.363251493
26.05170499	0.407127611	0.282047215	1.443473251	0.148887202	0.380813256
20.13312736	-0.756573092	0.310043342	-2.440217185	0.014678434	0.095684357
77.49054096	-0.024177197	0.171338961	-0.141107411	0.887785089	0.953373594
69.94626002	-0.287130717	0.175112947	-1.63968868	0.101069914	0.304491074
19.48576545	-0.124410172	0.379311981	-0.327989039	0.742919951	0.880589216
754.3072329	-0.243669755	0.095328425	-2.556108057	0.010585024	0.077396513
309.8200359	-0.178543833	0.067524331	-2.644140713	0.00818986	0.065602537
1279.317637	-0.107894048	0.094244707	-1.144828727	0.252280113	0.508385108
413.3577288	-0.021692345	0.084598812	-0.256414294	0.797630952	0.907511342
137.6914696	-0.330845041	0.275610623	-1.200407434	0.229981143	0.48527166
19.60771807	0.470226859	0.300297142	1.56587191	0.117378607	0.33099428
14.10634387	0.321931197	0.509393939	0.631988668	0.527394282	0.748701296
335.0925419	0.122152732	0.131430305	0.9294107	0.352676284	0.609517889
14.4305999	-0.886918473	0.663750095	-1.336223496	0.181476234	0.426968393
1283.838974	-0.363345956	0.152306406	-2.385624901	0.017050132	0.104499517
373.2460319	0.450050608	0.119011264	3.78157992	0.000155836	0.00460289
371.8246658	-0.02921967	0.102212994	-0.285870409	0.774977379	0.896822533
4.627579676	-0.334390838	0.464963604	-0.719176373	0.472032255	0.706224734
138.9155859	0.208517774	0.118243222	1.763464923	0.077822108	0.260784867
3.284274721	0.942917641	0.868011417	1.086296359	0.277347897	0.535639013
633.1255317	0.286224826	0.234051314	1.222914845	0.221361861	0.474540521
12.18290788	0.058511679	0.285005622	0.205300086	0.837337677	0.930780365
19.77467035	-0.944463767	0.486239268	-1.942384807	0.052090533	0.204861565

163.7708234	-0.281648757	0.305605642	-0.9216085	0.356732823	0.612881761
34.5237953	-0.364626978	0.307780267	-1.184699011	0.236136474	0.492010908
51.39245277	-0.112852677	0.333737767	-0.338147757	0.735251843	0.876856177
9.673519201	1.356290462	0.764609287	1.77383467	0.076090502	0.2579099
9.66277732	0.980837726	0.902175573	1.087191624	0.276952132	0.535402099
23.68633026	0.819130148	0.324114538	2.527286045	0.011494784	0.081245692
483.6321854	-0.163139915	0.194012583	-0.840872856	0.400419166	0.649919715
19.16045582	-0.180136352	0.303523171	-0.593484681	0.552856832	0.765246364
1445.387919	-0.155355394	0.160042077	-0.970715933	0.331689755	0.590423575
289.0256302	0.054312441	0.089423711	0.607360623	0.543611614	0.759149191
437.1029986	-0.081811379	0.095764991	-0.854293187	0.392942561	0.643530057
316.6638501	-0.294406362	0.123250811	-2.388676879	0.016909167	0.104041052
32.04461997	0.488846432	0.38715982	1.262647635	0.206715841	0.45676687
231.220224	-0.052770302	0.086044073	-0.613293855	0.539682056	0.757302797
9.408764197	1.58372461	0.794206688	1.994096289	0.046141533	0.19074428
120.3156458	-0.331719865	0.137656491	-2.409765517	0.015962776	0.100627082
836.7168343	-0.303839289	0.067181998	-4.522629531	6.10761E-06	0.000392589
315.2400246	0.221908542	0.284705578	0.779431662	0.435725479	0.679451025
27.6523941	-0.264550452	0.193598972	-1.36648686	0.171786202	0.413687851
68.59525821	0.118140251	0.141022088	0.837742885	0.402175122	0.651758072
123.6810374	-0.264887603	0.122969111	-2.154098712	0.031232432	0.150398973
263.6074908	-0.007877792	0.087768237	-0.089756752	0.928480516	0.970107815
438.4617355	-0.306056395	0.380137422	-0.805120406	0.420750202	0.665360573
76.59603201	-0.742172368	0.298706578	-2.484620104	0.012968975	0.088320666
2.675426435	-0.360732546	0.846110151	-0.426342297	0.669858441	0.840552028
4776.357499	-0.340271009	0.510304876	-0.666799448	0.504900246	0.731061249
633.8934626	-0.005862663	0.358964982	-0.016332129	0.986969425	0.992541292
195.8363312	-0.1941492	0.088942419	-2.182863949	0.029045831	0.144157627
593.6101422	0.326697945	0.112240991	2.910682999	0.003606397	0.038556736
8.394934965	0.315677279	0.377543788	0.836134214	0.403079403	0.652549272
139.3286434	-0.378817985	0.123590835	-3.065097702	0.002175989	0.027814353
67.31834164	-0.412684997	0.342872036	-1.20361229	0.228739467	0.483823304
884.9340136	0.150140654	0.181787218	0.82591425	0.408852737	0.656743786
177.9458476	-0.709857876	0.256979634	-2.7623118	0.005739364	0.051942403
366.2107059	0.571190066	0.123303213	4.632402124	3.61447E-06	0.000259459
5.636111119	0.318332089	0.652753191	0.487676036	0.625779328	0.812948148
409.137636	0.465788428	0.223790014	2.081363772	0.037400622	0.16804498
1261.543458	-0.228905493	0.112402732	-2.036476246	0.041702556	0.179293555
269.0733782	-0.089270251	0.092996905	-0.959927115	0.337091899	0.595725198
26.22549328	0.312186401	0.264053484	1.182284728	0.237092743	0.492684668
143.1703518	0.048583964	0.183006706	0.265476414	0.790642477	0.904476207
85.22909989	0.32545614	0.162038236	2.008514455	0.044588652	0.186887867
369.9653087	-0.20862973	0.126705091	-1.646577322	0.099644955	0.301938075
13.75912424	-0.367785383	0.400361564	-0.918633095	0.358287515	0.614347157
717.864887	-0.539749848	0.155928527	-3.461520847	0.000537133	0.011041136
59.31013819	0.253187369	0.15498168	1.633659988	0.102330264	0.306521747
96.64564966	-0.244472672	0.120824716	-2.02336642	0.043035384	0.18262093
308.079259	-0.24068258	0.158723316	-1.516365627	0.129426926	0.35120799

1111.144464	-0.058575963	0.114953608	-0.509561763	0.610358518	0.803167633
165.895201	-0.084711534	0.371161113	-0.228233862	0.819464438	0.920862667
234.820579	0.186901816	0.1867162	1.00099411	0.316829656	0.575733312
6.722369336	-0.534116489	0.423638896	-1.260782458	0.207387235	0.457548517
227.5386126	-0.044583546	0.086924567	-0.512899256	0.608021794	0.801631962
158.065918	-0.11877505	0.092794202	-1.279983518	0.200550932	0.449208007
211.4499094	0.430926085	0.333176621	1.293386323	0.195877478	0.444015373
1904.253091	-0.070076613	0.056098643	-1.249167714	0.211603738	0.462972839
223.5828772	-0.033865252	0.139390998	-0.242951499	0.80804297	0.913987158
1519.521947	-0.139672691	0.122633603	-1.13894306	0.254726904	0.511720904
368.7241144	-0.089886964	0.131796488	-0.682013346	0.495230512	0.722933298
410.0079994	-0.334720344	0.144413638	-2.317789022	0.02046079	0.116312806
49.12005276	0.205303001	0.238185472	0.861945943	0.388717266	0.639780065
136.0262107	0.024039757	0.138722503	0.173293853	0.862420456	0.941673797
613.2157324	-0.210390747	0.132519016	-1.587626841	0.112370745	0.322788052
12.74794822	0.652118437	0.629259148	1.036327305	0.300049472	0.558241509
14.59963514	0.063453201	0.381163811	0.166472259	0.867785313	0.944769182
10.95241142	0.618177188	0.410998534	1.504086116	0.132559195	0.355698616
38.71325842	-0.196821107	0.173656115	-1.133395777	0.257048087	0.513998503
31.6701449	-0.261929559	0.202342863	-1.294483805	0.19549836	0.443604785
166.0186681	0.010314337	0.114411551	0.090151182	0.928167077	0.969855368
48.15159588	0.410822383	0.239887009	1.712566199	0.086792376	0.278431505
66.24657075	-0.024851309	0.15621211	-0.159086955	0.873600366	0.947352611
587.312488	-0.009616373	0.15999193	-0.060105362	0.95207172	0.979038346
50.07397618	-0.984090898	0.477747673	-2.059854927	0.039412412	0.173538536
796.8908811	-0.336767385	0.079139357	-4.255371786	2.08702E-05	0.001026107
722.2962633	-0.08159556	0.10302922	-0.791965232	0.428380949	0.673125134
100.5580214	0.049529111	0.134863349	0.367254047	0.713429528	0.864200403
102.2011668	-0.221066126	0.114089789	-1.937650411	0.052665888	0.206348185
212.3551895	0.011857852	0.085671719	0.138410347	0.88991612	0.953820886
422.5993754	-0.260720977	0.105759716	-2.465220102	0.013692911	0.091816852
651.2777493	0.243762084	0.194282392	1.254679239	0.209595225	0.461244775
10.44068794	-0.183584819	0.407288748	-0.450748566	0.652170775	0.829708504
4.253220852	0.067120815	0.625956179	0.107229255	0.914607107	0.96370157
713.671592	0.014711783	0.073923383	0.19901393	0.842251848	0.93182686
75.99327936	-0.073957885	0.377036061	-0.196155999	0.844488067	0.932309255
2209.586686	-0.126534134	0.126171456	-1.002874491	0.315921422	0.575044176
843.067975	-0.219588768	0.126322216	-1.738322642	0.082153979	0.269674132
48.4693254	-0.17552264	0.182505067	-0.961741189	0.336179627	0.594703504
4.4193255	0.309119774	0.501783914	0.616041617	0.537867053	0.755431795
481.094839	-0.098696758	0.092871148	-1.062727875	0.287905376	0.547328265
3.389703045	0.767013564	0.512375163	1.496976473	0.13439938	0.358453483
1683.917525	0.069667526	0.150420181	0.463152787	0.643254855	0.824451185
21.31761894	-0.078541605	0.23231179	-0.338087037	0.735297599	0.876856177
293.1879662	-0.174413777	0.106841855	-1.632448037	0.102585137	0.306759453
165.5572502	-0.176192125	0.116726474	-1.509444414	0.13118525	0.353645491
2999.993231	-0.328079515	0.115026165	-2.852216423	0.004341553	0.043398928
348.6684595	-0.047750917	0.131380565	-0.363454951	0.716265059	0.865878001

15.22172391	-0.263371286	0.449377914	-0.586079727	0.557821939	0.768771196
180.8976319	-0.188464142	0.113697819	-1.657588021	0.097400649	0.297814105
114.3841665	-0.06258983	0.109893144	-0.569551725	0.568981779	0.775924721
1517.678256	-0.09183588	0.087774018	-1.046276355	0.295433459	0.554602454
98.55406797	-0.023198218	0.116077497	-0.199851124	0.841597017	0.93182686
317.9300093	0.325587333	0.184646465	1.763301198	0.077849703	0.260784867
762.8928357	-0.028795523	0.122805469	-0.234480786	0.814611748	0.918370519
353.7652999	0.260702649	0.139610175	1.867361385	0.061851138	0.227866016
439.7797682	0.447911727	0.216574401	2.068165601	0.038624454	0.171227159
13.43700207	0.316757414	0.354796929	0.892785105	0.371972264	0.626024556
990.8804107	-0.006745981	0.057965684	-0.116378873	0.907352278	0.960444971
52.06130411	0.205494186	0.13129201	1.565169021	0.117543283	0.331275626
4476.341806	0.024338037	0.160058133	0.152057486	0.879141595	0.95014173
1054.321444	-0.073884274	0.096878374	-0.762649812	0.445672269	0.685757272
3.267917961	-0.505926365	0.736615727	-0.686825364	0.492192784	0.720914422
12.08643218	-0.596115254	0.460072617	-1.295698184	0.195079487	0.443103067
61.28811119	-0.636953059	0.470557231	-1.353614432	0.17585942	0.419176162
284.4482357	-0.08876818	0.127753505	-0.694839493	0.487155895	0.717692379
6.705250893	-0.333578616	0.405744425	-0.822139739	0.410997371	0.658233714
3.375638032	0.146739526	0.564477275	0.25995648	0.794897343	0.905927111
100.3670903	0.294439437	0.151534115	1.943057086	0.052009261	0.204733671
437.4038689	-0.125430206	0.068917684	-1.820000321	0.068758956	0.242831473
116.8679401	0.00715613	0.118329207	0.060476447	0.951776174	0.978862788
260.1233383	-0.356338194	0.156853317	-2.271792536	0.023099044	0.125445085
304.3238009	0.071827363	0.084440179	0.850630161	0.394974829	0.645455477
381.1879396	-0.270318327	0.181612656	-1.488433311	0.136636657	0.362188015
38.16663249	-0.119161867	0.215219753	-0.553675326	0.579801064	0.782986042
6.312859465	0.484139187	0.499692585	0.968874067	0.332608026	0.591386949
145.9992136	-0.265269164	0.1383433	-1.917470264	0.055178215	0.212624484
547.6388917	-0.262765734	0.116825652	-2.249212635	0.024498971	0.129461543
2876.381197	0.144165393	0.121197378	1.189509178	0.234239362	0.489929172
57784.07069	-0.329072042	0.164400767	-2.001645421	0.04532288	0.188729565
427.0332079	-0.174026083	0.123921293	-1.404327528	0.160221341	0.397864393
17.49751033	0.857089529	0.9015496	0.95068483	0.341764391	0.59937747
406.3342443	-0.365753403	0.126175511	-2.898766975	0.003746332	0.039647418
9.384956227	0.617415418	0.709722851	0.869938762	0.384333871	0.635384466
375.0308564	-0.065645938	0.078231192	-0.839127417	0.401397812	0.650901912
3266.771862	-0.835448672	0.388460576	-2.150665278	0.031502629	0.151258267
945.9439685	0.215005811	0.155134352	1.385932952	0.165767378	0.405536664
144.9630615	0.199665137	0.17088033	1.168450095	0.242625258	0.49834135
389.0967257	-0.301011481	0.169086724	-1.780219484	0.075040048	0.25564492
7.633260656	-0.887746697	0.429992104	-2.064565113	0.038964167	0.172047859
376.6680202	-0.25027619	0.082648637	-3.028195014	0.002460193	0.030215867
1922.50323	0.047249249	0.230844577	0.204679915	0.837822213	0.930810213
3.564605225	-0.755750246	1.013688999	-0.745544488	0.455942627	0.693666133
11059.05323	0.234896038	0.134461005	1.746945437	0.080646803	0.266287255
6.905513996	0.505352575	0.710690225	0.711072923	0.477039048	0.709683822
7.244886646	1.908017341	1.176249652	1.622119367	0.104777792	0.309829354

784.7018654	0.053067998	0.102393107	0.518277057	0.604264978	0.798962394
857.4519266	0.17502853	0.132602449	1.319949456	0.186851894	0.433198094
287.9879672	0.246300637	0.117539172	2.095477039	0.036128607	0.16433906
594.0952536	-0.015439113	0.079298202	-0.194696889	0.845630247	0.93321593
3.047040557	-0.095853053	0.582399716	-0.164582933	0.869272266	0.945403663
64.63138024	-0.497241822	0.490371038	-1.014011398	0.310577313	0.570118748
1205.486752	0.119855199	0.10681125	1.122121487	0.261810789	0.51962191
3.732774314	0.683574561	0.569882118	1.199501685	0.23033293	0.48564897
298.4175209	-0.180403217	0.082999448	-2.173547195	0.029739158	0.146353608
273.3943904	0.099532732	0.080520719	1.236113296	0.216416453	0.469066467
1070.284628	-0.366092222	0.195991784	-1.867895752	0.061776603	0.227847604
313.7038639	-0.137922267	0.109686979	-1.257416957	0.208602692	0.459572597
171.8069159	-0.217140738	0.136660781	-1.5889031	0.112082271	0.322313594
128.0846446	0.023971719	0.088953362	0.269486377	0.787555424	0.903200814
11.74153574	-0.307606831	0.377721008	-0.814375755	0.415429723	0.661679388
248.2648073	-0.423816012	0.08683634	-4.880629608	1.05748E-06	0.000102379
659.7116032	-0.192186771	0.116130518	-1.654920466	0.097940632	0.298612622
14.70105998	0.76811128	0.361614711	2.12411513	0.033660518	0.157075739
4.595290247	1.577691064	0.775127638	2.035395188	0.041811125	0.179514286
32.88912365	-0.530431906	0.221870556	-2.390726897	0.016815056	0.103790035
4.207285255	-0.573248492	0.452233316	-1.267594562	0.204942775	0.454803778
273.5818488	0.236225599	0.113983198	2.07245982	0.03822258	0.17001785
237.5959622	0.213333623	0.213646551	0.998535297	0.318019857	0.576493309
101.1873943	-0.0837132	0.133864157	-0.625359332	0.531735247	0.751414479
372.8668057	0.092046783	0.109212038	0.84282635	0.399325569	0.649354145
1806.591563	-0.006301286	0.136401632	-0.04619656	0.963153584	0.98274378
110.5622359	0.380323253	0.121260603	3.136412359	0.001710285	0.023971508
40.19768259	0.19192714	0.308385275	0.622361557	0.533704153	0.752550205
206.4809255	-0.038561972	0.111315464	-0.346420614	0.729026635	0.873448484
7310.780449	-0.006623519	0.105527388	-0.062765877	0.949952939	0.977692886
639.7847632	0.016009101	0.138227485	0.115817061	0.907797529	0.960645318
7.651788245	0.314745984	0.413800233	0.760623024	0.446882264	0.686849577
399.1861324	-0.012330808	0.075979666	-0.162290894	0.871076785	0.946199847
38864.01652	0.215258589	0.140105571	1.536402783	0.124439625	0.343189051
111.1425724	-0.246001552	0.198988003	-1.236263234	0.216360732	0.469025809
12186.17297	0.105868571	0.136714623	0.774376356	0.438708262	0.681324391
272.7227688	-0.058857327	0.101900782	-0.577594461	0.563537947	0.772666244
95.66521679	0.214826194	0.183645568	1.169786982	0.242086704	0.49765417
11.29621951	0.027341602	0.38354808	0.071285984	0.94317015	0.974993168
1546.767308	0.241381301	0.249523221	0.967370089	0.333359057	0.591900483
40.68098379	0.582465054	0.21005927	2.772860503	0.005556593	0.050964685
238.55559	0.181773095	0.088328662	2.057917449	0.039598058	0.173782322
84.97023555	0.327610588	0.166046194	1.973008712	0.04849457	0.196225524
8.665904804	-0.888698838	0.7460469	-1.191210416	0.233570991	0.489137368
12.76493051	-0.152433371	0.368241901	-0.413949011	0.678911449	0.845876523
505.9336958	-0.201497238	0.097747913	-2.061396821	0.039265198	0.173036067
7.191984866	-0.956376845	0.61152817	-1.563912985	0.117838005	0.331986935
64.01589914	-0.505345146	0.276350816	-1.828636343	0.067454104	0.240348628

18.7410896	0.157082178	0.326778939	0.480698599	0.630730727	0.816068366
3.309022598	2.282746863	1.153053833	1.979740058	0.047732745	0.194553111
375.2654468	-0.399315198	0.114768894	-3.479298114	0.000502729	0.010527105
279.7799938	0.044392693	0.15839568	0.280264544	0.779274551	0.898614978
932.454383	-0.029504457	0.100854529	-0.292544696	0.769870191	0.894019225
14.40602604	-0.753798305	0.394999283	-1.908353602	0.056345529	0.215489705
3048.095744	-0.302776177	0.122800418	-2.465595647	0.013678565	0.091759886
207.0990484	0.212241448	0.385472423	0.55060086	0.581907319	0.784201399
2314.368048	-0.348320132	0.17880298	-1.948066706	0.051406982	0.203038808
161.4408334	-0.052200232	0.096094887	-0.5432155	0.586981436	0.787731397
88.91609306	0.27263792	0.233073033	1.169753172	0.242100314	0.49765417
86.79247907	0.530881488	0.210510736	2.521873699	0.011673161	0.082136321
1070.21541	-0.205229911	0.103734897	-1.97840762	0.047882741	0.194961814
4.61833906	-0.120202695	0.58312192	-0.206136472	0.83668431	0.930545789
467.4628995	-0.233218431	0.075804275	-3.076586787	0.002093853	0.027370411
2.748959192	-0.091031139	0.715928102	-0.127151231	0.898820704	0.957506447
5.752331843	0.629156885	0.462501627	1.360334425	0.173724119	0.416746572
123.7176866	-0.109404657	0.220960957	-0.495131168	0.620507534	0.809425942
3547.865657	-0.193515169	0.069573545	-2.781447587	0.005411707	0.050139147
20.35031514	-0.44089184	0.252115923	-1.748766341	0.080331417	0.265525384
8.670361681	0.336563749	0.705723008	0.476906301	0.633428845	0.817960211
4.461222068	0.071855278	0.653554669	0.109945322	0.912452739	0.962595598
12.78606856	-0.959242319	0.639479281	-1.500036588	0.133604925	0.357038617
47.91012385	0.557421203	0.530441868	1.050862002	0.293321974	0.55260803
44.63453742	0.428642829	0.470405381	0.91122008	0.362179426	0.617840126
184.5249198	0.305675167	0.292476588	1.045126959	0.295964296	0.554787142
19.10740047	0.818618696	0.592150485	1.382450434	0.16683344	0.406609306
2722.990191	-0.256107656	0.101438091	-2.524768093	0.011577466	0.08157373
492.8279798	-0.124037461	0.148042738	-0.837849006	0.402115512	0.651728809
4.517473542	0.804864555	0.54188962	1.485292438	0.137466374	0.363395013
2180.181978	-0.147605713	0.103604949	-1.424697508	0.15424466	0.38887588
52.31762602	-0.225904677	0.141338478	-1.598323963	0.109970897	0.318242753
53.88662463	0.32094869	0.176504805	1.818356675	0.069009636	0.243278743
1976.938735	-0.098807141	0.073498383	-1.344344423	0.178837037	0.423578692
2.932240469	-0.796673625	0.668483278	-1.191762983	0.233354194	0.48890291
3.659983187	-0.60438825	0.624773976	-0.967371039	0.333358583	0.591900483
20.56699969	0.31340442	0.417744217	0.750230421	0.45311594	0.691607974
545.0264302	0.296425276	0.272758531	1.086768119	0.277139301	0.535573218
222.9505855	0.008959051	0.144186039	0.062135357	0.950455041	0.978017115
147.4620127	-0.05996332	0.100561608	-0.596284416	0.55098524	0.764274809
272.2263133	0.132090822	0.109262385	1.208932264	0.22668887	0.481042922
32.95219333	0.951438554	0.45408435	2.095290347	0.03614519	0.164366819
3.042334633	-1.066888231	0.955387151	-1.116707745	0.264119305	0.521850238
609.7944976	0.026504235	0.094749455	0.279729688	0.779684899	0.898747855
62.11218907	-0.350678764	0.297972377	-1.176883464	0.239242023	0.494631148
776.1634464	-0.104050472	0.058342657	-1.783437303	0.074515145	0.254503138
28.75843646	0.185680371	0.230114116	0.806905609	0.419720867	0.664585052
5.060962979	0.532011834	0.658078145	0.808432613	0.418841584	0.663816586

115.6403535	-0.198043082	0.278126817	-0.712060362	0.476427398	0.709176698
134.5546729	0.771007166	0.392183994	1.96593226	0.049306436	0.198287729
366.6368429	0.004880777	0.065525627	0.074486544	0.940623248	0.974550053
405.8360985	-0.18507581	0.234250603	-0.790076131	0.429483309	0.674275897
4.400176105	1.124051635	0.49647055	2.264085222	0.023568874	0.126898117
994.0061164	0.128746131	0.074566159	1.726602686	0.084239043	0.274108953
400.0341996	-0.054859303	0.082105096	-0.668159532	0.504031764	0.730167397
10.34931322	0.155509673	0.540430817	0.287751305	0.773537115	0.896155718
6.274206759	0.735061664	0.664282871	1.106549177	0.268488891	0.527493394
8.435480128	0.437201901	0.425369658	1.027816376	0.304036212	0.562389898
2.907415826	0.079986104	1.119504935	0.071447746	0.943041411	0.974993168
3.30887386	0.438540168	0.708281718	0.619160649	0.535810533	0.754028927
29.10342744	0.214878211	0.204531669	1.050586507	0.293448541	0.552647324
43.19393552	0.254010549	0.207623685	1.223417981	0.221171865	0.474471281
29.40530918	-0.193200495	0.465193519	-0.415312095	0.677913448	0.845854775
88.72703914	0.230867589	0.138864988	1.662532743	0.096406004	0.295780861
702.2460306	0.246982384	0.10208985	2.419264841	0.015551913	0.099354867
38.32782636	0.089355678	0.183952981	0.485752814	0.627142433	0.813801939
3.502635108	0.364248807	0.52603597	0.692440874	0.488660505	0.718626475
293.4967103	0.182759256	0.19695574	0.927920437	0.353448842	0.610315055
234.6653979	-0.266342498	0.164661049	-1.617519747	0.105766137	0.310992892
732.8257856	-0.187386782	0.10415933	-1.79903982	0.072012382	0.249234728
1714.347298	-0.159209893	0.21300522	-0.747445971	0.454794405	0.692659256
14.10184329	-0.343753391	0.438293787	-0.784299028	0.43286467	0.677482235
1944.237533	0.11483111	0.130527816	0.879744356	0.378997815	0.631220317
316.1473319	-0.213267919	0.087198429	-2.445777066	0.014454034	0.09473342
134.6155496	0.037957379	0.10921574	0.347544949	0.728181956	0.873123696
915.470443	0.053715688	0.137228083	0.391433641	0.69547673	0.854503374
333.3680156	0.145553562	0.10798509	1.347904253	0.17768918	0.421963374
75.30885612	0.34148781	0.1570367	2.174573269	0.029662109	0.146203809
615.5197402	0.136961603	0.266498116	0.513930847	0.60730034	0.801442647
94.74322959	0.118012175	0.123432268	0.956088532	0.339027508	0.597335157
327.895832	0.025547008	0.121412178	0.210415528	0.833343374	0.929163639
21.05736455	-0.605123744	0.486313104	-1.244308943	0.213385881	0.465277931
941.5649442	-0.221571386	0.109477193	-2.023904529	0.042979976	0.182465501
2287.166045	0.006272958	0.128008062	0.049004401	0.960915788	0.98222115
470.3441571	-0.092944606	0.220850946	-0.420847667	0.673866322	0.843351295
5244.930841	0.195033674	0.131158769	1.487004456	0.137013634	0.362809697
249.9459177	-0.026354559	0.156264649	-0.168653365	0.866069302	0.943555914
991.5596064	0.129027771	0.071401391	1.807076418	0.070750365	0.246952274
3.840433007	0.312384145	0.522508313	0.597854881	0.549936768	0.763630657
82.33107157	0.172695603	0.121550615	1.420771117	0.15538331	0.390299781
8.868568689	0.714538175	0.648552143	1.101743603	0.270573155	0.529531989
291.5680937	-0.227884772	0.163904312	-1.390352507	0.164421861	0.403782513
142.912668	-0.278923938	0.095543396	-2.919342931	0.003507701	0.037855162
11.52177893	0.03213409	0.434923225	0.07388451	0.941102281	0.974550053
291.270797	-0.157571249	0.089587278	-1.758857409	0.078601731	0.262352803
6568.853891	-0.110538122	0.112679632	-0.980994706	0.326595352	0.585151858

1037.88364	-0.168129745	0.071145748	-2.363173466	0.018119186	0.108099837
236.221675	0.02165845	0.123689904	0.175102816	0.860998846	0.940710965
678.560363	-0.051162186	0.066443009	-0.770016078	0.441290355	0.682742007
375.5055338	-0.043064375	0.14520892	-0.296568387	0.766796049	0.892250595
263.8258768	0.024682033	0.079271557	0.311360521	0.755526563	0.887493231
116.5013436	0.126524513	0.13435289	0.941732722	0.346329497	0.604006654
145.1328163	0.229980172	0.15961635	1.440830919	0.149632456	0.382283017
177.7632078	0.000696243	0.175346139	0.003970676	0.996831867	0.998423335
4.702224765	-0.55802812	0.492367918	-1.133355971	0.257064796	0.513998503
596.5342963	0.02805982	0.19067927	0.147157162	0.883007972	0.95143563
118.4179372	-0.174595169	0.323778913	-0.539241938	0.589719926	0.789919491
268.7697668	-0.096900271	0.151126743	-0.641185466	0.521402191	0.744065636
126.3539576	0.252331124	0.177205464	1.423946635	0.154461921	0.389005157
41.8078741	-0.195768826	0.172645107	-1.13393788	0.256820606	0.513818445
835.472398	0.145777739	0.132514929	1.100085398	0.271294915	0.530481168
7.402905961	0.699677251	0.375188889	1.864866665	0.062200096	0.228700076
35.31110813	0.318962944	0.240745305	1.32489788	0.185205024	0.431116715
248.0591973	-0.137940599	0.074726421	-1.845941464	0.064900705	0.234918685
310.3215598	0.050540165	0.090140239	0.56068372	0.575013167	0.780215115
63.69183979	-0.179934687	0.378542348	-0.475335686	0.634547727	0.818575962
391.7624113	0.085960488	0.129869414	0.661899405	0.508035693	0.733544682
437.4554312	0.208323695	0.092378563	2.255108624	0.024126511	0.128620056
162.8093093	0.135156327	0.284480711	0.475098389	0.634716847	0.818618539
223.3673446	-0.331095625	0.187430213	-1.766500822	0.077311864	0.26028339
308.8572465	-0.01752093	0.098172327	-0.17847117	0.858352961	0.93958737
2.94230377	1.344070711	1.04858867	1.281790229	0.199916243	0.448439123
35.20162972	-1.035476346	0.403327312	-2.56733505	0.010248354	0.075936734
24.87819594	0.370107779	0.311055107	1.189846335	0.234106794	0.489760032
39.34081424	0.059148487	0.231368983	0.255645706	0.798224422	0.907769715
114.2019618	-0.393706939	0.182269804	-2.160022836	0.030770902	0.14918418
499.286566	-0.095949498	0.093272836	-1.028697122	0.303622023	0.562210657
166.1137968	-0.128379999	0.091356225	-1.405268217	0.159941536	0.397563536
1722.761621	0.018328627	0.081640219	0.224504879	0.822364471	0.922469201
12.60853104	-0.798425594	0.386813598	-2.064109424	0.039007343	0.172093154
648.1573444	0.131899616	0.213409702	0.618058201	0.536536974	0.75450963
50.20978967	0.420585108	0.259264207	1.622225885	0.104754991	0.309829354
92.90235145	0.260439351	0.33537824	0.776554113	0.437421883	0.680596258
180.2302276	0.178438104	0.177559083	1.004950578	0.314920648	0.573869373
81.62688519	-0.180670333	0.140284229	-1.287887701	0.197785057	0.44611812
94.04351042	-0.043170691	0.150171303	-0.2874763	0.773747646	0.896159371
541.9765869	-0.317486378	0.143509594	-2.212300717	0.026945894	0.137571418
134.1645554	0.043427098	0.144567858	0.300392486	0.763877794	0.891351784
724.249666	-0.186252475	0.178246186	-1.044917031	0.296061318	0.554857408
18.91093194	-0.091393619	0.236979276	-0.385660808	0.699747892	0.857008117
449.4019404	-0.144490785	0.12102901	-1.193852492	0.232535674	0.487913329
62.17764541	0.177785929	0.554822575	0.320437446	0.748636743	0.884690979
669.6763523	-0.167143652	0.122987706	-1.359027317	0.174137933	0.417483848
311.5366852	-0.056071704	0.079231653	-0.707693224	0.479135786	0.711221434

3.427635115	-0.508487911	0.61251307	-0.830166629	0.40644458	0.655022276
241.1485427	0.041211913	0.096335796	0.427794387	0.668800824	0.839560759
4.862812577	-2.020694857	1.029953444	-1.961928347	0.049770827	0.199609773
626.0714511	0.109270919	0.203547392	0.536832813	0.591383104	0.79088016
278.5774238	-0.32192588	0.116981708	-2.751933483	0.005924455	0.053140675
37.97939331	-0.457499601	0.282820218	-1.617634001	0.105741497	0.310992892
53.88894136	0.447909559	0.332469461	1.347220156	0.177909338	0.422329357
5.980987459	0.470823574	0.436698412	1.078143544	0.280969702	0.539411971
3.687321308	0.369701202	0.643650082	0.57438228	0.565709142	0.774060678
595.8317519	0.072009163	0.099323325	0.724997506	0.468453561	0.703622452
7.945122849	0.680347591	0.397862742	1.710005786	0.087264803	0.279207439
641.1861677	-0.129281285	0.052367275	-2.468741875	0.013558898	0.091230272
151.0172752	0.076836775	0.133298323	0.576427169	0.564326485	0.773206656
1180.449013	-0.215781219	0.116097213	-1.858625313	0.063080267	0.230617926
821.1618683	-0.306815031	0.091415822	-3.356257408	0.00079005	0.013922634
22.25961301	0.750741901	0.610557072	1.229601515	0.218846362	0.471611205
611.8567379	-0.110203542	0.132122788	-0.834099431	0.404224956	0.653396496
47.59493497	0.601567013	0.380194602	1.582260795	0.113590047	0.324370417
902.1993506	-0.2302946	0.154351785	-1.492011253	0.135696195	0.360905397
413.1198758	0.00991993	0.11579655	0.085666884	0.931731228	0.971304259
117.3812957	-0.005193985	0.122849707	-0.04227918	0.966276142	0.983959158
7.267805028	0.257589371	0.537470493	0.479262348	0.631752004	0.81678327
6.354424988	1.870780582	0.696216959	2.687065515	0.007208281	0.061011702
4.630918568	0.568684915	0.979314534	0.580696901	0.561444751	0.771009498
3354.211469	0.475070102	0.124522689	3.815128825	0.000136112	0.004194065
312.7233969	-0.023950409	0.099322995	-0.241136595	0.809449251	0.914983931
58.57774487	0.723510205	0.506290652	1.429041206	0.15299239	0.386958982
34.54124036	0.517791448	0.620837612	0.834020746	0.404269293	0.653396496
401.9523545	-0.095063523	0.169469966	-0.56094614	0.574834255	0.780163053
5.738380154	0.357937698	0.511303743	0.700049047	0.483896675	0.715310442
32.9677609	0.900929864	0.839477841	1.073202674	0.283180191	0.541854464
58.54960424	-0.202151378	0.232325099	-0.870122853	0.38423327	0.635352094
27.6791811	0.192275327	0.260663735	0.737637427	0.460734805	0.697145057
56.3708943	-0.01151844	0.155985607	-0.073842967	0.941135337	0.974550053
2143.835517	-0.120700862	0.071815717	-1.680702598	0.092820696	0.289193443
98.48588537	-0.367849842	0.203358202	-1.808876344	0.070470216	0.246268911
1040.075265	-0.008481397	0.091895119	-0.092294314	0.926464206	0.968986559
692.2662571	0.102404814	0.092168733	1.111058061	0.266543359	0.525184175
113.8141559	-0.081458327	0.203704886	-0.399884012	0.689241948	0.851119654
7.988446542	-1.241886937	0.49869116	-2.490292666	0.012763794	0.087517934
28.66562609	0.278936166	0.442999008	0.629654156	0.528920883	0.749308566
770.8742891	0.391167525	0.214758304	1.821431431	0.068541301	0.242335835
436.2280014	0.030242524	0.064790932	0.466770944	0.640663766	0.823232665
298.0078116	-0.182481768	0.073448953	-2.48447064	0.012974421	0.088320666
27.94911417	-0.0640195	0.20227492	-0.31649747	0.751624946	0.88558395
11.4918659	0.349298032	0.388064127	0.900103894	0.368064964	0.623287987
16.59526158	0.764011355	0.331254475	2.306418211	0.021087275	0.118287849
995.5733301	-0.005749466	0.084342014	-0.068168472	0.945651525	0.976005209

908.5364536	0.108320723	0.090212929	1.200722816	0.229858741	0.485078645
679.0599504	0.071264917	0.143822844	0.495504852	0.620243798	0.80924247
409.2153676	0.357989537	0.203121739	1.762438318	0.077995269	0.260963931
23.22613243	0.730091007	0.263267089	2.773195117	0.005550883	0.050942098
6.537154198	0.60046064	0.59181038	1.014616608	0.31028862	0.569788866
193.9762341	0.566144834	0.146247479	3.87114251	0.000108326	0.003536363
113.0635419	-0.041798545	0.178373919	-0.234331032	0.814727996	0.918435556
1599.95294	-0.235833425	0.058618795	-4.023170823	5.74198E-05	0.00224023
1436.076394	-0.238682425	0.069296969	-3.444341485	0.000572452	0.011505047
4.385182708	0.394841612	0.762456219	0.517854799	0.604559581	0.799020152
663.1260018	0.302742183	0.232550209	1.301835778	0.19297253	0.440314141
1099.677394	-0.234924311	0.35640218	-0.659155089	0.509796183	0.73483854
710.1256872	1.218419682	0.693137898	1.757831574	0.078776171	0.262639264
21.86386819	1.547237372	0.601680776	2.571525357	0.01012516	0.075302523
2187.448476	-0.674839475	0.505692986	-1.334484546	0.182045108	0.427527932
162.3549737	-0.687388083	0.46367631	-1.482474022	0.138214211	0.364794717
87.48005226	-0.455039338	0.352894262	-1.289449524	0.197241854	0.445691001
38.48095464	0.405157063	0.188032429	2.154719082	0.031183824	0.150211023
211.7587691	-0.04659472	0.12294327	-0.378993659	0.704692573	0.860203048
645.9359663	0.035335483	0.120709507	0.292731567	0.769727339	0.893921036
3372.657202	-0.001916283	0.088138314	-0.021741767	0.982653947	0.991070527
382.4244438	0.025266796	0.128301075	0.196933629	0.843879477	0.932271104
120.0178306	-0.142629346	0.128550468	-1.109520244	0.267205816	0.525910227
480.8324812	0.079171499	0.08642389	0.916083492	0.359623106	0.61570305
3175.124951	-0.005789796	0.131868594	-0.043905797	0.964979494	0.983605536
34.19188958	-0.211279969	0.221120948	-0.955495041	0.339327412	0.597508828
2.678143382	1.787874532	1.056901557	1.691618789	0.090718682	0.285395303
25.39738287	1.466017527	0.84564056	1.7336178	0.08298592	0.271382646
261.8544741	0.22988503	0.120169696	1.913003341	0.055747625	0.213985743
7.741570831	0.637513783	0.476020782	1.3392562	0.180487282	0.425746014
236.1594315	-0.189566313	0.121656387	-1.558210931	0.119183255	0.334192367
955.5954197	-0.035087362	0.112324138	-0.312375974	0.754754807	0.887201677
121.1330454	0.052373459	0.242058157	0.216367254	0.828701489	0.926595434
1170.374788	0.035890096	0.136717696	0.26251244	0.792926396	0.905326977
761.8601324	-0.035133148	0.066549271	-0.527926869	0.597550095	0.794878848
718.1534303	-0.079870986	0.22942423	-0.348136663	0.727737551	0.87295111
325.8421092	0.418772024	0.175531627	2.385735437	0.017045008	0.104499517
1983.69848	0.067375177	0.169419218	0.397683201	0.690863722	0.852317047
1153.919142	1.860228037	0.845078647	2.201248422	0.027718442	0.140182155
3.6420004	1.37722665	0.8604521	1.600584913	0.109468886	0.317334055
7.676796789	0.66889618	0.862004611	0.775977497	0.437762273	0.680661785
1511.783117	0.28014191	0.527118205	0.531459372	0.595100488	0.792928048
132.9759867	0.030909191	0.264267339	0.116961829	0.906890302	0.960313075
20226.85404	-0.067122465	0.163638052	-0.410188609	0.681667597	0.84736731
90.92194547	0.687736757	0.487130817	1.41181123	0.158005551	0.394674162
495.2083471	-0.068032154	0.132230096	-0.514498258	0.606903678	0.801235462
6.125152446	0.455984955	0.425728169	1.07107067	0.284137655	0.543001703
115.5038141	0.02907702	0.114233416	0.254540406	0.799078092	0.908235437

293.6981818	-0.359702616	0.123603218	-2.910139567	0.003612674	0.038597532
2.957061439	-0.941050508	0.600368237	-1.567455521	0.117008255	0.330301921
6.331550958	0.653409922	0.507146194	1.288405453	0.197604862	0.44611812
146.3946625	0.07501873	0.652562092	0.114960294	0.908476594	0.960992992
2229.168866	-0.168221865	0.125608844	-1.339251751	0.18048873	0.425746014
80.213662	0.054054848	0.173350105	0.31182472	0.755173736	0.887389343
853.8222588	-0.197880905	0.116455363	-1.699199583	0.089281585	0.283287958
11.93763039	0.269814227	0.308013399	0.875982108	0.38103976	0.632873833
458.1486149	-0.326648153	0.097922965	-3.335766566	0.000850646	0.014679908
11.19819568	2.08844349	0.947310378	2.204603198	0.027481951	0.139400686
600.799614	-0.929135973	0.420012254	-2.212163966	0.026955338	0.137574851
26.22534526	-0.278427252	0.297603265	-0.935565177	0.349497111	0.606632725
531.8220697	-0.226011159	0.077544768	-2.91458941	0.003561568	0.03817454
208.9580044	-0.388779083	0.297471281	-1.306946613	0.191230844	0.438494359
289.6973654	-0.264221057	0.129727805	-2.036734203	0.041676685	0.179231458
247.0006974	-0.295833141	0.096408875	-3.06852602	0.002151176	0.02767764
181.6045729	-0.03151876	0.142584701	-0.22105289	0.825051251	0.92429313
109.1288147	0.630959243	0.295658027	2.134084606	0.032835856	0.154857295
2.831930656	0.86690935	1.049246009	0.826221251	0.408678595	0.656531301
19.35293958	-0.048460822	0.70909675	-0.068341622	0.945513692	0.976005209
40.36667893	-0.162840867	0.362202275	-0.449585434	0.653009393	0.830317856
8.291114756	-0.178916265	0.539516471	-0.33162336	0.740173678	0.879355136
330.5285399	-0.236771688	0.118439224	-1.999098619	0.045597685	0.189494988
2358.151221	0.022858258	0.04960815	0.460776262	0.644959134	0.825444522
192.1779711	-0.968650452	0.617001553	-1.569931952	0.116430943	0.329345551
2172.445456	-0.052742238	0.082521094	-0.639136429	0.522734183	0.744849907
132.0438562	0.024865211	0.103952627	0.239197527	0.81095242	0.915758284
221.1766507	-0.102621697	0.097308081	-1.054606116	0.291605506	0.550962625
196.6240652	-0.229883191	0.143553617	-1.601375118	0.10929386	0.317176115
249.5740072	0.343011722	0.173545027	1.976499858	0.048098192	0.19544517
797.0897447	-0.499215451	0.064834117	-7.699888178	1.36185E-14	2.13593E-11
1655.079661	0.047959205	0.16036847	0.29905632	0.764897074	0.891746525
87.81606653	0.03193899	0.153164101	0.208527913	0.83481679	0.929483538
88.71302083	0.121378391	0.135576589	0.895275448	0.370639861	0.625026387
405.1389516	-0.512648996	0.321988196	-1.592135992	0.111354152	0.320866896
280.3914213	-0.08201351	0.31581499	-0.259688465	0.795104091	0.905927111
47.69387828	-0.257553673	0.500752902	-0.514332863	0.607019289	0.801320641
1035.041025	-0.225258468	0.086531243	-2.603203893	0.009235701	0.071235615
8.545388646	0.10745758	0.441448954	0.243420172	0.807679919	0.913774208
35.78977586	-0.452440051	0.29288815	-1.544753693	0.122405852	0.339546521
2699.563709	-0.065835857	0.112891177	-0.583179827	0.55977225	0.770157539
712.9504518	-0.080127869	0.118172674	-0.67805751	0.49773523	0.72436479
11.26967775	0.630067554	0.397972647	1.583193115	0.113377455	0.324077445
108.3402081	-0.193165267	0.574063807	-0.336487451	0.736503313	0.877682597
5.533730758	-0.146729521	0.442864791	-0.331319003	0.740403539	0.879363902
2.723336752	1.168320535	0.637358533	1.833066436	0.066792692	0.239172735
4.632039589	0.883948803	0.546149028	1.618512087	0.105552284	0.310829552
6356.423134	-0.39327164	0.292148603	-1.346135616	0.178258784	0.422902855

7.228312215	-0.294059118	0.42792153	-0.687180002	0.491969305	0.72084769
223.2042184	-0.020124416	0.134422558	-0.149710108	0.880993333	0.950743683
167.5234276	0.258183096	0.142973427	1.805811762	0.070947748	0.247029455
20.37969132	0.552603525	0.515079178	1.072851609	0.283337701	0.541869101
290.6486543	0.145480701	0.124723144	1.166429072	0.24344101	0.499015555
243.8078404	0.009879386	0.144038871	0.068588335	0.945317304	0.976005209
2060.77252	-0.111887261	0.060077099	-1.862394544	0.062547498	0.229651199
2087.353011	-0.204085123	0.09729822	-2.097521653	0.035947422	0.163918072
952.4550182	-0.059524875	0.051633474	-1.152834968	0.248978129	0.504648872
8.031758508	-0.797213567	0.577480631	-1.380502693	0.167431921	0.407187604
91.97077564	-0.403739897	0.444244644	-0.908823331	0.363443389	0.618784858
922.2717436	-0.179900312	0.063304481	-2.841825861	0.004485599	0.044330263
125.8451544	0.074283535	0.134953071	0.550439751	0.58201779	0.784223971
705.8385737	-0.10538805	0.082461655	-1.278024923	0.201240639	0.450058203
60.0304836	0.965794532	0.411470494	2.347178096	0.018916211	0.110992087
99.22736574	-0.451970526	0.229011762	-1.97356905	0.048430766	0.196225299
391.3521869	-0.192838477	0.135869667	-1.419290127	0.155814449	0.39088193
44.74220761	-0.058173394	0.199162407	-0.292090232	0.770217635	0.894291781
20.23275796	-0.061162292	0.4367893	-0.140026992	0.888638664	0.953660331
72.53423744	-0.020220939	0.263211767	-0.076823841	0.938763684	0.974295153
4.435595258	1.223734609	0.712569973	1.7173536	0.085914584	0.277180856
17.23849417	0.470528947	0.494889973	0.950774864	0.341718675	0.599364314
733.7484263	-0.208716963	0.103819113	-2.010390542	0.044389871	0.186551643
78.41231335	0.369774071	0.309426917	1.19502878	0.232075786	0.487396441
289.4901286	-0.052055682	0.135462921	-0.38427993	0.700770982	0.857636942
428.8761658	-0.257543488	0.13287338	-1.938262481	0.052591207	0.206107071
299.9034999	0.194300288	0.152892353	1.270830652	0.203788902	0.453300969
10.43248525	-0.061827876	0.380877816	-0.162329948	0.871046033	0.946199847
1310.703764	-0.153227467	0.091792213	-1.669286114	0.095060691	0.292971483
9.241675088	0.639367267	0.669539165	0.954936321	0.339609901	0.597673737
133.3094476	-0.212562527	0.112608563	-1.887623117	0.059076564	0.221792559
116.0953043	0.050737965	0.187954579	0.269948012	0.787200249	0.903046278
236.1686418	-0.039274036	0.132164506	-0.297160238	0.766344177	0.892250595
10.3735523	0.43647543	0.355646241	1.227274128	0.219719576	0.472454323
219.0215209	-0.073477213	0.085888251	-0.855497834	0.392275603	0.64295648
1069.663999	-0.202356967	0.07032746	-2.877353579	0.00401026	0.041522935
323.9982263	0.115162406	0.126445229	0.910769092	0.362417051	0.618026146
159.0912535	-0.109044194	0.103688913	-1.051647577	0.292961271	0.552335575
181.2928677	0.262176207	0.191649509	1.367998322	0.171312601	0.413173434
8.129169018	0.057393989	0.485707282	0.118165799	0.905936288	0.960133527
115.0969645	0.034513644	0.31115081	0.11092256	0.911677756	0.962232976
166.5663812	0.240680951	0.158154354	1.521810466	0.128056578	0.348808506
208.1425351	0.055884636	0.107796224	0.518428508	0.604159328	0.798890052
576.0081979	-0.022081911	0.324960877	-0.067952521	0.94582343	0.976005209
1942.893611	0.06243626	0.086575566	0.721176458	0.470800954	0.705535576
3.53786465	0.044171809	0.674592921	0.065479207	0.947792462	0.976593642
385.8477745	-0.096956883	0.238356347	-0.406772818	0.684174852	0.848557456
466.2488919	0.052546833	0.222497111	0.236168608	0.813301838	0.917677859

1213.678587	-0.21081089	0.138637208	-1.520593882	0.128361783	0.349275886
53.50661014	-0.053078517	0.190968214	-0.277944248	0.781055156	0.899351668
243.7116039	-0.055420167	0.213898592	-0.25909552	0.795561541	0.906077061
567.1377893	-0.152673497	0.115287388	-1.324286201	0.185408012	0.431359424
149.9185835	0.15875187	0.135325189	1.17311397	0.240750105	0.496110133
10.57914415	0.677873872	0.483623994	1.401654758	0.161018367	0.399211518
28.40666453	-0.42722291	0.190511234	-2.242507703	0.024928578	0.130893813
5.759196266	1.403592746	0.899351524	1.560672004	0.11860116	0.333301505
4.177091351	-1.053308325	0.640403725	-1.644756713	0.100019992	0.30249008
33.00269143	0.017125536	0.400016687	0.042812053	0.965851356	0.983885343
19.72320883	0.117892124	0.656452636	0.179589689	0.8574747	0.939073885
120.3111303	0.25386734	0.172537874	1.471371673	0.14119063	0.369534051
94.04819537	-0.171493101	0.162227634	-1.057113988	0.290459568	0.549857317
290.0744574	-0.130326102	0.111123977	-1.172799113	0.240876374	0.496110133
28.11079948	-0.179052546	0.233934615	-0.765395688	0.44403596	0.685056997
2714.923052	0.629198038	0.254137146	2.475820829	0.013293027	0.089788042
33.33598159	-0.188289498	0.314305246	-0.599065719	0.549129061	0.763184775
619.6089876	-0.04034247	0.09830814	-0.410367543	0.681536352	0.84736731
228.8633713	-0.106949392	0.100975598	-1.05916076	0.289526573	0.549072905
28.89760251	0.253828137	0.206591114	1.228649829	0.219203124	0.472055719
3082.926544	-0.035757069	0.128569523	-0.278114663	0.78092434	0.899311917
1970.376188	0.041414054	0.182411854	0.227035978	0.820395766	0.921144748
3.357523749	-0.321868279	0.626922849	-0.513409711	0.607664754	0.801498108
1601.253219	-0.022970266	0.109824238	-0.209154794	0.834327401	0.929483538
585.2834436	-0.261760207	0.097776551	-2.677126608	0.007425657	0.062081025
20.42233049	0.865496729	0.394091112	2.196184339	0.028078752	0.141088009
412.8966651	0.04745766	0.077596902	0.611592196	0.540807602	0.757639765
1244.448667	0.242210217	0.134071195	1.806579087	0.070827933	0.246968718
110.7772816	-0.203128211	0.184589838	-1.100430087	0.271144776	0.530319823
106.2468407	-0.052328909	0.140061286	-0.373614369	0.708691242	0.862039895
427.0016577	-0.234794462	0.088417196	-2.655529375	0.007918404	0.064515577
40.45761382	-0.212958807	0.2308614	-0.922453071	0.356292298	0.612326146
117.9103228	0.179696592	0.164773988	1.090564073	0.275464745	0.534437044
237.3527961	-0.21686376	0.088273492	-2.45672575	0.014020968	0.092902392
189.8338153	0.072402962	0.216713728	0.33409495	0.738307911	0.878437596
1031.134256	-0.095960022	0.125518429	-0.764509426	0.444563722	0.685329016
38.85334356	0.057449603	0.442063202	0.129957896	0.896599738	0.956747655
790.2619909	-0.172062514	0.182102377	-0.944866934	0.344726814	0.60239957
7.196933304	0.347594768	0.366384633	0.948715467	0.342765345	0.600522632
612.2087858	-0.03344507	0.089176576	-0.375043215	0.707628327	0.861889372
3.568333328	0.378459059	1.41587207	0.267297496	0.789240114	0.903718901
1251.981672	0.023608558	0.165389881	0.142744875	0.886491674	0.95296336
96.08120619	-0.131866276	0.241710136	-0.545555427	0.585371571	0.786559328
246.1773037	0.312133072	0.207950073	1.501000058	0.133355547	0.356941063
794.9778545	-0.574971365	0.181777804	-3.163044942	0.001561282	0.022673288
563.4715376	-0.328054228	0.068946666	-4.758086873	1.95436E-06	0.00016553
235.1093794	-0.150264983	0.097923528	-1.534513573	0.124903357	0.343758904
18.32128484	0.811910019	0.411735372	1.971921956	0.048618516	0.196417802

21.26968129	0.431029993	0.452971931	0.951560048	0.341320151	0.59908004
84.72829248	-0.07316726	0.356638439	-0.205158086	0.837448615	0.930780365
570.8087719	-0.228534921	0.07095051	-3.221046923	0.001277232	0.019639326
73.93328634	-0.068730241	0.173411258	-0.396342441	0.691852422	0.85293695
44.09366765	-0.430569106	0.398813505	-1.079625191	0.280309119	0.53883665
2.869807081	1.636104186	1.13132405	1.446185278	0.148125242	0.379731332
60.55037994	-1.379771445	0.620165706	-2.224843187	0.026091767	0.134865403
6.180136704	-0.525192643	0.415690497	-1.263422299	0.206437454	0.456642941
2903.047376	0.151323934	0.172570644	0.876881092	0.380551227	0.632388957
179.7776301	0.750505685	0.445241616	1.685614412	0.091870091	0.287373457
6.894616378	0.467461513	0.444134879	1.052521508	0.292560348	0.552234505
305.1792526	0.149549131	0.158972567	0.940722874	0.346846894	0.604501728
15.52391588	0.540507677	0.80627789	0.670373929	0.50261945	0.728907743
40.52665507	-0.191353946	0.599595131	-0.319138592	0.74962142	0.88518749
5402.726284	-0.08328219	0.134200447	-0.620580573	0.534875625	0.753066663
7.633116209	-0.203723401	0.655387223	-0.310844329	0.755918968	0.8875741
126.2513221	0.067050674	0.160488019	0.417792393	0.676098913	0.84484991
142.3049408	0.409653256	0.246434206	1.662323031	0.096448022	0.295785247
209.1726163	-0.042307383	0.110733301	-0.382065584	0.702412719	0.858728471
457.8828237	-0.221293931	0.129243257	-1.712228056	0.086854649	0.278460408
424.2982555	-0.061110165	0.092316938	-0.661960482	0.507996548	0.733544682
183.5001125	-0.18414058	0.091931091	-2.00302832	0.045174249	0.188484415
201.2397525	0.089520398	0.145291688	0.616142599	0.537800409	0.755405841
50.98672995	-0.481557901	0.181286409	-2.656337585	0.007899449	0.064495035
5.198621119	0.14743179	0.603248418	0.244396481	0.806923767	0.91335864
82.0723485	0.231036942	0.565122964	0.408825968	0.682667382	0.847675388
199.477901	0.170965568	0.110674714	1.544757255	0.12240499	0.339546521
5.884321798	0.178823273	0.550999745	0.324543295	0.745526749	0.882212278
115.4911326	0.457956551	0.187591112	2.441248665	0.014636573	0.095548023
14.64914201	0.552287427	0.28551211	1.934374784	0.053067065	0.207557067
7.727356658	2.39969774	0.871817719	2.752522331	0.005913811	0.05312269
17.03538151	0.610375037	0.855078924	0.713823039	0.475336611	0.708263292
4.25735688	0.795323377	0.858218195	0.926714653	0.354074708	0.61085774
21.95125975	-0.856299772	0.689157105	-1.242532024	0.214040333	0.46592315
5.865530206	2.101144678	1.137835457	1.846615576	0.064802875	0.23461872
31.07231949	0.66311681	0.567434392	1.168622872	0.242555609	0.49834135
11.96632708	0.485723971	0.369854907	1.313282482	0.189087779	0.436736716
413.3124887	0.002667847	0.074213483	0.035948278	0.971323601	0.986149125
1748.132187	-0.085837406	0.05545283	-1.547935519	0.121637822	0.338467634
1114.339441	0.11484301	0.094196621	1.219183966	0.222774376	0.476215527
3702.047507	0.067468913	0.113568352	0.594081995	0.552457273	0.76490866
536.6175709	0.150996789	0.138750387	1.088262116	0.27647941	0.5350158
17256.99871	0.096401952	0.207887102	0.463722623	0.642846487	0.824387858
29.7112659	0.514008369	0.333159472	1.542829824	0.122872071	0.340481549
17383.37509	0.159509219	0.159654065	0.999092753	0.317749761	0.5764038
123.8567915	0.45568926	0.201323708	2.263465459	0.023607012	0.127059839
5.855610799	1.384764461	0.852799496	1.623786679	0.104421344	0.309651042
215.2942008	0.036492016	0.108807136	0.335382567	0.737336517	0.878218859

13.26290931	0.061262026	0.421678847	0.145281241	0.884488825	0.9521814
4.478016601	0.548640953	0.714925319	0.76741016	0.44283769	0.683810803
242.9730768	0.241562619	0.256191628	0.942898178	0.345732987	0.603475283
2.730332823	-0.917680461	0.812358605	-1.129649462	0.25862396	0.515570067
246.2371551	0.086408764	0.175320128	0.492862772	0.622109553	0.810641951
8.683650596	-1.441837434	0.629270059	-2.291285615	0.0219469	0.121159866
45.09360904	0.256008067	0.315250786	0.81207749	0.416747169	0.662355886
221.2213182	-0.298157295	0.198600127	-1.501294586	0.133279385	0.356837465
17.47251104	0.559023807	0.551545899	1.013558089	0.310793665	0.570315647
6.891105901	-7.59976E-05	1.006906157	-7.54763E-05	0.999939779	0.999939779
8.201670084	0.34485835	0.569817941	0.605207953	0.545040832	0.759994702
17.56572245	-0.283340749	0.464602769	-0.609855919	0.541957255	0.758121441
39.4750546	-0.73262033	0.351573107	-2.083834956	0.037175182	0.16733144
132.5975681	0.103712168	0.292930551	0.354050365	0.723301139	0.870759523
152.4007314	-1.119689342	0.449103106	-2.493167666	0.012660904	0.087044994
54.85174241	-1.305257535	0.530559592	-2.460152552	0.013887797	0.092490959
83.88365755	0.66440436	0.824891713	0.805444338	0.420563315	0.665360573
2653.374303	-0.014786388	0.142755539	-0.103578383	0.917503943	0.965132921
10.01897151	-0.014434701	0.598907546	-0.024101719	0.980771472	0.99019174
26.94848526	-0.317581597	0.307109019	-1.034100523	0.301089172	0.559711102
71.06839689	-0.087082496	0.164741348	-0.528601335	0.597082034	0.794488387
106.8438436	-0.567624227	0.214440267	-2.647003924	0.008120841	0.065428258
396.1045226	-0.403731039	0.133441708	-3.025523627	0.00248203	0.030412625
137.3569312	-0.235234536	0.098041329	-2.399340536	0.016424632	0.102164672
251.419815	0.353260693	0.137912697	2.561480563	0.010422707	0.076795237
1470.110128	-0.08316417	0.129710784	-0.641150778	0.521424726	0.744065636
227.1639622	-0.140562505	0.119082358	-1.180380602	0.237848874	0.493412267
764.0978515	-0.215303533	0.125950945	-1.709423729	0.087372489	0.279207439
175.2104991	-0.032527444	0.080668621	-0.403223009	0.686784175	0.849863221
2314.918297	0.127441892	0.05275333	2.415807532	0.015700358	0.099912932
119.3826583	-0.235991546	0.167073523	-1.412501165	0.157802448	0.394355257
97.35680872	0.142175598	0.226106236	0.628799986	0.529480009	0.74980955
117.8590399	0.305140918	0.152033313	2.007066164	0.044742618	0.187281352
387.0830296	-0.443660489	0.124846222	-3.553655705	0.000379916	0.008654984
1162.559512	-0.100527591	0.120756526	-0.832481647	0.405137133	0.654067923
379.3430776	0.010722619	0.14603523	0.07342488	0.94146802	0.974594762
1119.722175	-0.140277099	0.102656113	-1.366475848	0.171789656	0.413687851
848.5577029	0.082832899	0.086457137	0.958080527	0.338022154	0.596415734
155.1923738	0.061710697	0.164787934	0.374485534	0.708043117	0.861889372
5.430326199	0.746780189	0.441963326	1.689688137	0.091087634	0.285931737
966.9128934	0.120467919	0.096671808	1.246153572	0.21270802	0.464445578
53.02945954	-0.300939755	0.153517835	-1.96029181	0.049961693	0.19985689
308.7519999	0.05526334	0.068301084	0.80911366	0.418449772	0.663730402
1216.084836	-0.265268464	0.107923106	-2.457939488	0.013973672	0.092865706
198.1940337	0.156850529	0.127952048	1.225853995	0.220253624	0.473019011
130.595022	0.092678128	0.119560716	0.77515535	0.438247868	0.681014522
11.88665344	-0.044455926	0.296793626	-0.149787334	0.880932403	0.950743683
371.2902891	0.109762141	0.074645253	1.470450392	0.141439812	0.36984695

300.1521071	0.168864096	0.085073835	1.984912231	0.047154237	0.193048044
273.4065683	0.169238008	0.092856585	1.82257411	0.06836792	0.242084363
342.4020519	-0.152017983	0.113822858	-1.335566386	0.181691043	0.427200576
2791.752702	-0.081783722	0.047706902	-1.714295396	0.086474488	0.277752586
560.940048	0.306230949	0.207755265	1.473998505	0.140481994	0.368245065
913.2863327	0.183792904	0.168668985	1.08966627	0.275860179	0.534543001
390.5994692	-0.050583578	0.115615158	-0.437516833	0.661736571	0.835398185
244.1714522	-0.033873534	0.150537195	-0.225017708	0.821965499	0.922285512
380.4286606	-0.180113365	0.079841053	-2.255899175	0.024076946	0.128530573
258.5748688	0.025762082	0.115389355	0.223262203	0.823331442	0.923257803
1082.979533	-0.139022916	0.076298819	-1.822084779	0.068442123	0.242103013
6.079936527	1.068270453	0.435745237	2.451594102	0.014222499	0.093803902
32.98927452	-0.664118423	0.319244728	-2.080279999	0.037499858	0.168138301
82.14108469	0.199905983	0.189642969	1.054117557	0.291829099	0.551252269
375.9133195	-0.145854166	0.083125785	-1.754620007	0.079324327	0.263529494
317.5831551	-0.068928069	0.094054756	-0.73285044	0.463649654	0.699296945
1908.092864	-0.12696696	0.066120113	-1.920247153	0.054826688	0.211695407
79.07887212	0.07419046	0.166000385	0.446929443	0.654926004	0.831662169
7.724627464	0.145270503	0.41792204	0.347601918	0.728139165	0.873123696
378.0444856	0.296249275	0.212966825	1.391058327	0.16420774	0.403483346
137.7569028	0.225061192	0.176243511	1.276989952	0.201605795	0.450536698
20.94109869	-0.206433105	0.464207426	-0.444700136	0.656536471	0.832281264
554.6700713	0.071207392	0.130599727	0.545233851	0.585592693	0.786664905
89.57243321	0.608484088	0.273151128	2.227646258	0.025904113	0.134218736
678.5206578	-0.06965368	0.055709175	-1.250308948	0.211186711	0.462669699
1027.559007	-0.015934054	0.076202571	-0.209101265	0.834369187	0.929483538
3.857133655	0.093523902	0.549790685	0.170108197	0.864925047	0.942899493
4.683516521	0.169476803	0.435607946	0.389058108	0.697233162	0.855397756
171.5261424	-0.427571944	0.111719843	-3.827179966	0.00012962	0.004043445
3446.07126	-0.24891329	0.083074185	-2.99627724	0.002732978	0.032374647
7.502507849	-0.28190506	0.451239897	-0.624734343	0.532145429	0.751568565
35.442924	-0.500265268	0.353359855	-1.415738832	0.156851984	0.392730927
528.6650076	0.055371147	0.154442907	0.358521784	0.719952869	0.868595446
89.35072742	-0.064745301	0.14734816	-0.439403527	0.660369172	0.834476254
264.4887444	-0.138230131	0.081191605	-1.702517533	0.088658395	0.281863212
334.2250902	0.262609032	0.204779541	1.282398772	0.199702795	0.448315412
12.18720116	0.284857189	0.343842026	0.828453673	0.407413621	0.655304608
273.7153707	0.113885867	0.108596933	1.048702421	0.2943151	0.553481777
65.4816487	0.14386421	0.203518034	0.70688679	0.479636834	0.711575578
11.29903048	2.086871333	0.980987596	2.127316738	0.033393775	0.156446473
1050.093738	0.36150345	0.123853123	2.918807712	0.003513729	0.037855162
2397.832359	-0.153859618	0.142132965	-1.082504809	0.279028279	0.53751602
435.4410646	-0.093043604	0.065336974	-1.424057435	0.154429847	0.389005157
37.71896224	0.384076243	0.281321841	1.365255687	0.172172701	0.414200348
269.2379879	0.404693949	0.207033946	1.954722675	0.050615812	0.201129566
93.00117749	-0.04715439	0.156649863	-0.301017755	0.763400955	0.891126867
6.523604989	0.38039599	0.682647531	0.557236308	0.577365986	0.781716861
3.365087096	0.72154949	0.867421912	0.831832214	0.405503658	0.654312692

42.08154387	0.697337028	0.392635183	1.776043152	0.075725805	0.257074356
331.4496235	-0.109153736	0.167399234	-0.652056364	0.514364812	0.738695881
57.22192548	-0.161379131	0.208180752	-0.775187567	0.438228834	0.681014522
1258.457136	0.042286671	0.1248576	0.338679193	0.734851417	0.876677323
7.822788119	0.360570307	0.987333818	0.365195946	0.714965145	0.864906543
130.8863153	0.073597995	0.181070759	0.406459857	0.684404747	0.848557456
1408.325923	0.0682649	0.27269617	0.250333182	0.802329698	0.910590181
7.485471845	0.10200252	0.706185027	0.144441635	0.885151742	0.952291996
3.735554255	0.4626119	0.519973588	0.889683458	0.373635879	0.627431154
233.8084567	0.071575113	0.105927419	0.675699587	0.499231398	0.725734105
457.4229558	-0.201316958	0.120243731	-1.674240782	0.094083287	0.291474017
48.13381979	0.272191246	0.199387219	1.365138885	0.172209403	0.414200348
7.545403578	0.054944296	0.786469729	0.069861933	0.944303552	0.975591654
30.11119929	0.502611643	0.193324051	2.599840223	0.009326717	0.07188218
47.70441917	0.419433247	0.212600176	1.972873474	0.048509979	0.196225524
3.721501674	1.426902807	0.616827762	2.31329213	0.020706583	0.116904985
179.5660641	-0.003752493	0.086347395	-0.043458086	0.965336375	0.983619889
18.2662784	0.485501602	0.289841949	1.675056369	0.093923172	0.291182257
73.4714092	0.041453879	0.146415145	0.283125623	0.777080541	0.897873228
18.1496047	1.202759025	0.524612541	2.292661595	0.021867495	0.120934342
124.4120571	-1.016586205	0.397025587	-2.560505518	0.010452	0.076853809
361.6094257	-0.316755796	0.337845373	-0.937576244	0.348462224	0.605772725
16.25655674	0.28217891	0.361918914	0.779674394	0.435582555	0.679363244
605.6402825	0.220665449	0.165435908	1.333842522	0.182255472	0.427598328
623.7879668	-0.292585913	0.171693974	-1.704112884	0.08836	0.281331352
51.74439397	0.0271072	0.166336354	0.162966182	0.870545062	0.946199847
33.59178117	0.077145766	0.204469635	0.377296931	0.705952953	0.860776344
338.5769272	-0.133404433	0.088314491	-1.510561085	0.130900315	0.353093825
12.97248346	0.46182795	0.408241274	1.13126227	0.25794472	0.515109686
574.1565745	-0.560037566	0.239560218	-2.337773655	0.019398993	0.112494686
112.9840017	0.217757084	0.215146689	1.012133096	0.31147442	0.570697037
62.19909868	0.801763822	0.357256962	2.244221684	0.024818142	0.130663891
493.6189061	0.481136894	0.153945078	3.125380163	0.001775754	0.0245599
125.2495813	0.353361014	0.097163718	3.63675889	0.00027609	0.006961733
3.59117418	-0.187791698	0.511699738	-0.366995884	0.713622088	0.864250676
186.2364452	-0.332296858	0.123306415	-2.694887024	0.00704125	0.05985635
12.00339264	0.341245005	0.342054141	0.997634479	0.318456636	0.576818787
906.8232297	-0.566015694	0.1892191	-2.991324302	0.002777703	0.032731404
2653.144513	0.215864285	0.159412532	1.354123688	0.17569692	0.418979853
57.6053406	0.218202289	0.174195346	1.252629845	0.210340448	0.461824914
13.42358199	0.316704581	0.479409925	0.660613317	0.508860326	0.734084378
89.52263534	0.022668111	0.125643441	0.180416188	0.856825847	0.938857569
3.78502624	-0.909343534	0.601182065	-1.512592586	0.130383179	0.352520887
3.277910265	-1.124441063	0.700537528	-1.605111815	0.1084692	0.315276302
171.1750653	-0.208513683	0.203124901	-1.026529404	0.304642111	0.563303271
7.979171234	0.972965841	0.668075991	1.456370014	0.14529036	0.375285575
58.14945578	-0.332232574	0.182336231	-1.822087539	0.068441704	0.242103013
1112.611863	-0.035178396	0.128077917	-0.274664024	0.783574373	0.901193845

12.02553526	0.126090339	0.378063909	0.333515938	0.738744861	0.878561905
48.14731127	0.070721904	0.134640657	0.525264101	0.59939961	0.795772707
62.17057694	0.131442034	0.184507412	0.712394331	0.476220625	0.70906168
577.6258748	0.028890998	0.064732643	0.446312657	0.655371414	0.831896116
147.8388736	-0.039543698	0.110218396	-0.358775846	0.719762784	0.868512899
774.1927095	0.084375296	0.114058748	0.739752957	0.459449908	0.695896896
208.5899651	-0.083834889	0.114628378	-0.731362429	0.464557808	0.700184956
11.57515381	2.023762576	0.755156593	2.679924396	0.007363879	0.061796753
2109.377032	-0.177744336	0.111002761	-1.601260488	0.109319236	0.317176115
86.49137716	-0.164558528	0.237787437	-0.692040462	0.48891192	0.718842604
650.5335091	-0.04252212	0.210327174	-0.20217131	0.839782802	0.931173342
25.68313356	0.622896062	0.245783558	2.534327634	0.011266334	0.080163497
456.9992769	-0.220562489	0.103543985	-2.130133285	0.033160611	0.155720751
3.226294964	0.114008071	0.716773467	0.159057325	0.873623709	0.947352611
56.07574283	-0.296832429	0.447728226	-0.662974572	0.507346839	0.73318233
465.1857625	-0.048315533	0.07568869	-0.63834548	0.523248813	0.745244677
366.2581095	-0.043102474	0.115850482	-0.372052613	0.709853677	0.862515113
456.0110847	-0.089548873	0.166940298	-0.536412561	0.591673452	0.79088016
2107.968169	0.309720202	0.130188856	2.379007017	0.017359346	0.105651523
6.965750431	1.378082841	0.779876549	1.767052546	0.077219429	0.260173903
12.02306681	0.840536535	0.698412083	1.203496553	0.228784224	0.483852721
199.8332344	-0.03980643	0.106439493	-0.373981767	0.708417882	0.861905675
103.6295073	0.447239136	0.37369626	1.196798534	0.23138509	0.486759558
6.439389728	0.62838672	0.467292905	1.344738413	0.178709725	0.423524226
190.4785447	-0.171814453	0.104258872	-1.64796002	0.099360875	0.301543338
707.9523659	0.132186919	0.116503238	1.134620132	0.256534514	0.513818445
158.453243	-0.25430082	0.141513142	-1.797012045	0.072333708	0.249940927
117.1499133	-0.164303181	0.126306912	-1.300824939	0.193318383	0.440650447
49.88416861	-0.083361224	0.143446546	-0.581130922	0.56115222	0.770873642
38.60571086	-0.240413093	0.244470475	-0.983403387	0.325408944	0.584078637
2234.730182	-0.350475426	0.135612697	-2.584385042	0.009755282	0.073842588
71.85048605	0.187216466	0.222469736	0.841536782	0.400047288	0.649641838
182.032156	-0.131225751	0.071222525	-1.842475415	0.065405639	0.236146881
5.55294513	-0.947566028	0.597583023	-1.585664238	0.112815496	0.323444764
604.5884893	0.056155614	0.202181493	0.27774854	0.781205397	0.899392604
6687.234864	-0.114669641	0.14632787	-0.783648673	0.433246288	0.677730654
539.5798016	0.030497925	0.102875386	0.296455027	0.766882608	0.892250595
21.6468769	-0.008235943	0.477379373	-0.017252406	0.986235255	0.992541292
316.4288694	0.278126218	0.112890896	2.463672691	0.013752163	0.092056733
62.16817479	0.033826027	0.171047805	0.197757737	0.843234614	0.932221964
239.9397387	0.160787774	0.110357169	1.456976243	0.145122938	0.375211299
8.390721493	-0.335853188	0.475390023	-0.706479251	0.479890152	0.711667657
6.375145932	0.986526829	0.42054066	2.345853616	0.018983561	0.111097787
128.0557921	-0.093590305	0.205016919	-0.456500399	0.648030184	0.827595913
483.5770178	-0.353704208	0.239233665	-1.478488437	0.13927709	0.366555585
2.961872822	2.498588377	1.340152402	1.864406148	0.06226469	0.22886323
234.4407881	-0.294348291	0.131229213	-2.243008883	0.024896242	0.130840064
352.5367761	-0.176027527	0.147200789	-1.195832764	0.231761827	0.486997923

138.2208877	0.208498632	0.165054775	1.263208722	0.206514179	0.456642941
5.708900885	-0.227856347	0.483769787	-0.471001607	0.637639587	0.820943957
1052.212278	-0.02999243	0.091146109	-0.329058808	0.742111238	0.880313054
678.6059664	-0.030437743	0.173270586	-0.175665955	0.860556383	0.940613387
47.52829395	0.232766277	0.237297785	0.980903707	0.326640229	0.585151858
15.6250306	1.458080479	0.632667066	2.304656835	0.0211858	0.118658889
656.4745256	-0.15820257	0.111083856	-1.424172474	0.154396551	0.3890049
9.522577659	0.341797097	0.316681391	1.079309067	0.280449972	0.538872897
3.240772449	0.682215548	0.904062784	0.754610807	0.450482537	0.69003832
12.12834214	-0.866342195	0.488287433	-1.774246349	0.076022411	0.257802268
2448.467721	-0.203728546	0.094545485	-2.154820475	0.031175885	0.150211023
5.982979901	-0.651887275	0.450605972	-1.44669027	0.147983691	0.379505869
341.9224531	0.299105151	0.3161299	0.946146351	0.344073945	0.602015651
6.745165031	1.007140698	0.665641927	1.51303675	0.130270324	0.352451227
9.160997318	0.48215569	0.403109345	1.19609157	0.231660827	0.486916163
242.488621	0.00383738	0.192300691	0.019955103	0.984079188	0.991458113
230.1684892	-0.187864883	0.104623963	-1.795620023	0.072554969	0.250539881
3.121121363	0.250671739	0.651297984	0.384880263	0.70032613	0.857427527
1102.0952	-0.269655309	0.211371384	-1.275741796	0.202046809	0.450798249
4.918969958	-0.795122075	0.490949887	-1.619558525	0.105327145	0.310575473
89.83269953	-0.179710549	0.202010072	-0.889611826	0.373674354	0.627431154
5.622895492	-1.435423123	0.773466067	-1.855832059	0.0634775	0.231530489
13.14429054	-1.644303733	0.644726193	-2.55039077	0.010760224	0.078022814
275.0538565	-0.048888674	0.101992173	-0.479337508	0.631698543	0.81678327
14.06587756	0.611346041	0.334185067	1.82936373	0.067345137	0.240218588
81.60889535	0.613293047	0.239072902	2.565297203	0.010308746	0.076229315
53.25489059	0.10838408	0.18496877	0.585958807	0.557903197	0.768771196
265.7008963	0.023423744	0.066498882	0.352242681	0.724656271	0.871803199
8.382669429	-0.009916155	0.482393902	-0.020556137	0.983599731	0.991149398
169.1611778	0.147478045	0.114213014	1.291254291	0.196615515	0.445162804
859.238594	0.102780294	0.180269265	0.57014874	0.56857682	0.775709712
121.1983488	-0.06069293	0.122827305	-0.494132227	0.621212799	0.810102398
171.5163643	-0.055282342	0.327614699	-0.168741948	0.865999622	0.943555914
1859.343359	-0.218697282	0.229018466	-0.954932959	0.339611601	0.597673737
11.45854328	-0.327044054	0.406943978	-0.803658665	0.421594133	0.666224924
154.2491374	0.064866594	0.12644777	0.512991201	0.607957476	0.801631962
9.698682599	-0.050700531	0.637092082	-0.079581166	0.936570375	0.973575955
699.8703083	-0.294240665	0.217189422	-1.354765176	0.175492385	0.41887423
75.8090887	-0.198732044	0.152570621	-1.302557744	0.192725792	0.440233029
505.158128	-0.142547403	0.081126268	-1.757105395	0.078899846	0.262898914
19.57885606	1.02049551	0.513704915	1.986540288	0.046973364	0.192551907
9.878503305	0.424190234	0.372080522	1.140049555	0.254265656	0.511138497
56.83228082	-0.173985153	0.160743508	-1.082377479	0.279084831	0.53751602
436.1521589	-0.297540419	0.161972027	-1.836986446	0.066211896	0.238169444
309.5304337	-0.163393863	0.15388833	-1.061769033	0.288340551	0.547763227
80.13608135	0.213838985	0.345340751	0.619211559	0.535776998	0.754028927
11.0875738	0.403692046	0.406493917	0.993107224	0.320657704	0.579213006
69.34392312	0.170982595	0.183541235	0.931575922	0.351555731	0.608637468

145.9187525	-0.172238745	0.220198829	-0.78219646	0.434099125	0.678391604
3.939590849	-0.76012634	0.522557992	-1.454625806	0.145772883	0.375878512
364.8401541	0.253758878	0.140456393	1.806673748	0.070813163	0.246968718
9.326252447	0.773459718	0.326038532	2.372295426	0.01767795	0.106720924
10782.72432	-0.31279841	0.164955098	-1.896263971	0.057925148	0.219112681
151.7917919	-0.187835598	0.220386626	-0.852300349	0.394047412	0.644513465
105.6496178	0.165775916	0.114259346	1.450874014	0.146814945	0.377359161
149.0700454	-0.268795503	0.197771914	-1.359118687	0.174108982	0.417478257
460.622168	-0.297401152	0.095417266	-3.116848402	0.001827955	0.025133238
21.57544854	-0.279326804	0.323705058	-0.862905282	0.388189546	0.639466951
3.079031669	0.317257824	0.914044018	0.347092501	0.728521827	0.873219977
202.5983317	-0.72898414	0.327510057	-2.225837415	0.026025074	0.134667521
1206.039453	0.013776964	0.134026306	0.102792986	0.918127272	0.965350236
66.04168368	0.21164501	0.189253879	1.118312669	0.263433478	0.521277253
123.2637601	-0.048645006	0.098618779	-0.493263113	0.621826688	0.810633345
61.74988135	0.171565131	0.200498505	0.855692816	0.392167715	0.642846827
22.24551422	0.036120868	0.212863179	0.169690542	0.865253513	0.943060187
130.5687893	0.189797904	0.231338361	0.820434206	0.411968622	0.658847341
593.3374717	0.053840661	0.110095613	0.489035482	0.624816577	0.81216834
899.8090242	-0.129123229	0.199960233	-0.645744543	0.518444809	0.741851962
837.9242731	-0.069309461	0.179257062	-0.386648428	0.699016501	0.8567814
15.45049541	-0.256833085	0.269918542	-0.951520717	0.341340107	0.59908004
17.7573115	0.426632113	0.2808144	1.51926722	0.128695247	0.349943297
226.0947104	0.245545932	0.203622149	1.205890088	0.227859873	0.482548507
140.8085236	0.055668745	0.169534466	0.328362407	0.742637663	0.880449218
59.30722562	-0.031135567	0.142838848	-0.217976882	0.827447124	0.925836979
1417.160872	-0.172401323	0.089954821	-1.916532335	0.055297372	0.212882664
29.07425209	0.09867375	0.247098656	0.399329365	0.689650532	0.851289072
20.28876046	-0.558420082	0.266675912	-2.094002713	0.036259739	0.164702132
15.55574471	-0.035836185	0.303925751	-0.117910985	0.906138189	0.960133527
52.09667165	0.083873027	0.200898258	0.417490066	0.676319989	0.845004996
889.5352886	-0.178035658	0.138323317	-1.287097948	0.19806015	0.446254188
99.61614281	-0.160090975	0.124721688	-1.283585701	0.199286956	0.447851546
113.762836	-0.060588858	0.133671233	-0.453267745	0.650355956	0.828805376
392.2814421	-0.86805472	0.313749294	-2.766714502	0.005662432	0.051453988
323.5462057	-0.033700976	0.123601922	-0.27265738	0.785116599	0.902044615
15.78526985	0.279287989	0.295190941	0.946126558	0.344084039	0.602015651
168.4159365	0.106802673	0.095778868	1.11509642	0.264809106	0.522834476
5.762611945	-0.050296964	0.450155527	-0.111732414	0.911035577	0.962059189
204.6577686	-0.018485135	0.119222542	-0.155047316	0.876784016	0.948920022
94.41044642	0.064830877	0.116487362	0.556548591	0.577835887	0.781880601
124.1769596	0.584581394	0.250082955	2.337549926	0.019410608	0.112494686
107.2685056	0.214577821	0.114429755	1.875192518	0.060766243	0.225309163
1718.261008	-0.554715066	0.109475091	-5.067043661	4.04042E-07	5.02936E-05
8.312695903	-0.263458243	0.375940965	-0.700796846	0.483429807	0.715091304
42.14955904	0.045821826	0.150047349	0.305382443	0.760074892	0.889367077
440.8482228	-0.005610938	0.138577313	-0.040489588	0.967702808	0.984398161
4.484698992	-0.484540959	0.473329568	-1.023686225	0.305983504	0.565058904

556.5832469	0.307611475	0.197867104	1.554636767	0.120032602	0.335757327
173.194779	-0.173672408	0.160814979	-1.079951686	0.280163695	0.53868915
901.8322917	-0.226031166	0.09032092	-2.502533924	0.012330781	0.085389239
411.2150919	-0.432700209	0.130641738	-3.312113084	0.000925941	0.015632357
599.2905748	-0.136006138	0.0720114	-1.888675108	0.058935376	0.221505497
11.3942298	0.297915112	0.312311063	0.953905087	0.340131688	0.598370657
818.1986428	-0.212434637	0.145641797	-1.458610375	0.144672376	0.374855543
25.91870909	0.523272454	0.274235284	1.908114982	0.056376357	0.215502505
249.3815231	-0.08616036	0.157024165	-0.548707644	0.5832061	0.785021177
158.2167409	-0.007858679	0.109487303	-0.07177708	0.942779313	0.974993168
282.1228033	0.037184696	0.130405251	0.285147227	0.775531349	0.897140705
2535.926178	-0.174448908	0.151276648	-1.153178037	0.248837316	0.504493856
27.64482028	-0.157439504	0.388186944	-0.405576506	0.685053795	0.848888656
188.1081226	0.041883018	0.138879223	0.301578717	0.762973235	0.891042078
381.9404195	0.020238616	0.178408955	0.113439465	0.909682153	0.9616745
122.6261157	0.179648744	0.146264996	1.228241546	0.219356307	0.472320746
171.8324135	-0.172676879	0.077908687	-2.216400829	0.026664063	0.136577127
22.38503155	0.742329905	0.388969261	1.908453902	0.056332576	0.215489705
383.610101	-0.006815843	0.105134817	-0.064829552	0.948309712	0.976899148
377.45753	-0.479876345	0.115376186	-4.159232161	3.19319E-05	0.001430915
559.6801815	-0.074277403	0.155030109	-0.479115981	0.631856122	0.81678327
111.977378	0.256538453	0.196598466	1.304885322	0.1919319	0.439197537
8.954863339	0.44400627	0.315704061	1.406400249	0.159605306	0.39715209
499.1779549	-0.432399567	0.073611566	-5.874071039	4.25221E-09	1.17003E-06
25.63960685	0.520676317	0.597569047	0.871324108	0.383577211	0.634974705
45.99299588	-0.246152951	0.611982443	-0.402222243	0.687520474	0.850122158
54.61439243	-0.197569108	0.68183035	-0.289762854	0.771997667	0.895430514
606.890015	0.454994162	0.294801073	1.543393849	0.122735245	0.340282762
10.68062827	1.345954067	0.505908872	2.660467414	0.007803228	0.064091503
257.2421116	0.13177135	0.094673677	1.391847813	0.163968488	0.403299571
3.019457774	0.489882793	0.617504497	0.793326681	0.427587516	0.672443858
488.8295096	-0.308848699	0.07723032	-3.999060223	6.35945E-05	0.002380468
2.592513405	-0.565907572	0.673285583	-0.840516397	0.400618912	0.650042108
1522.443512	0.379478359	0.277992288	1.365067936	0.172231699	0.414200348
44.25388405	0.22381455	0.420856055	0.531807841	0.594859092	0.792808463
272.1827979	0.299891298	0.272357738	1.101093367	0.270856024	0.529953328
13.6578306	0.012367671	0.584667222	0.02115335	0.983123327	0.991149398
49.72446102	0.085282386	0.265257499	0.321507918	0.747825513	0.884198669
197.6433373	-0.094481712	0.108028019	-0.874603758	0.381789542	0.633716497
13.67782061	0.639021599	0.348299277	1.834691148	0.066551465	0.238799627
543.0207089	-0.362714567	0.258094257	-1.405356985	0.159915151	0.397563536
114.8568948	-0.055700386	0.128662124	-0.432919839	0.665073007	0.836844909
19.84957284	0.557392985	0.267448503	2.084113314	0.037149861	0.16733144
327.3045234	-0.334078437	0.306098989	-1.091406537	0.275094037	0.534091809
325.9080637	-0.120409344	0.1234933	-0.975027346	0.329546696	0.587876521
2198.150241	-0.363526371	0.078317482	-4.641701444	3.45552E-06	0.000252076
5.245585128	-0.05462843	0.988537953	-0.055261844	0.95592986	0.980690995
696.767238	-0.342653225	0.107579978	-3.185102201	0.001447029	0.021451042

500.294284	-0.055012435	0.100276364	-0.548608197	0.583274359	0.785040337
844.4778924	0.066923541	0.17776376	0.376474603	0.706564093	0.86122222
2854.44299	-0.258575933	0.050672687	-5.102866013	3.34548E-07	4.26589E-05
5.372760188	0.402757026	0.421448493	0.955649463	0.339249363	0.59750556
5.173179391	-0.893474668	0.57017265	-1.567024775	0.117108901	0.330526543
28.82618825	0.177317251	0.219973507	0.806084578	0.420194083	0.665067412
90.5773328	0.126121573	0.162023788	0.778413926	0.436325031	0.680183063
90.69223973	-0.278103382	0.343130742	-0.810488095	0.417659704	0.662879458
571.6017303	0.239817361	0.104124629	2.30317615	0.021268934	0.118841684
719.3421802	0.166429512	0.093919001	1.77205368	0.076385647	0.258307996
649.3346406	-0.148217881	0.084191219	-1.760490958	0.078324599	0.261814005
373.7290764	-0.954147529	0.478562062	-1.99378013	0.046176088	0.190786555
348.591367	-0.219837024	0.086113068	-2.552888076	0.010683382	0.077717145
154.2626007	-0.134657954	0.094973089	-1.417853783	0.156233458	0.391682474
9350.577927	-0.057116609	0.07353697	-0.776706045	0.43733222	0.680596258
371.0153683	0.387729266	0.360336233	1.076020756	0.281917974	0.540466121
110.8833344	-0.643083657	0.284359074	-2.261519729	0.023727092	0.127400109
6.608753104	0.416659678	0.558191138	0.746446244	0.455397893	0.693264981
300.7441158	-0.461689257	0.255905799	-1.804137536	0.071209749	0.247529632
3.150600712	-1.240905539	0.705599995	-1.758652986	0.078636467	0.262411564
29.91628678	0.564248553	0.266961102	2.113598382	0.034549584	0.159616584
4.049557682	1.408669127	0.763246096	1.845628998	0.064946093	0.234974515
233.6429613	0.379484733	0.102389904	3.706271021	0.000210333	0.005707381
3.041291545	0.272260759	0.571554316	0.476351506	0.633823977	0.818118933
1415.564192	0.143635505	0.070890402	2.026162924	0.042748092	0.18199269
1967.604418	-0.160410838	0.132511417	-1.210543532	0.226070397	0.480315376
341.7327234	-0.283042805	0.099624404	-2.841099102	0.004495834	0.044347584
1578.783356	-0.081114374	0.076749987	-1.056864983	0.290573212	0.549939695
277.0521993	0.303371262	0.132444473	2.290554336	0.021989203	0.121308003
287.4249292	-0.010320182	0.19655257	-0.052505961	0.958125546	0.981531095
16.2368758	0.322780788	0.342132745	0.943437286	0.345457279	0.603089043
19.13795095	0.008489518	0.255599978	0.033214082	0.973503869	0.986906773
135.1221122	0.352934848	0.137965277	2.55814256	0.010523294	0.077171427
231.1542965	0.692244196	0.336243736	2.058757151	0.039517508	0.173782322
240.1909354	-0.01091537	0.109955356	-0.099270923	0.920923165	0.966719692
4.686208215	-0.081943854	0.544419365	-0.150516053	0.880357486	0.950735166
546.335069	-0.311632535	0.092980466	-3.351591458	0.000803485	0.014096038
113.9465858	0.130117297	0.39184436	0.332063722	0.739841143	0.879332259
412.9410028	-0.136181962	0.06929967	-1.965117031	0.049400694	0.198498377
749.1169114	-0.117383664	0.069880775	-1.679770491	0.093001979	0.289643176
557.5757195	-0.016796749	0.079209434	-0.212054901	0.832064203	0.928693708
6.382868284	0.604065387	0.483691197	1.248865786	0.211714168	0.462972839
421.2300912	-0.115803789	0.092816411	-1.247665025	0.212153755	0.463622612
83.03982475	0.155544453	0.19228679	0.808919077	0.418561695	0.663815578
427.7370264	-0.433039167	0.102445683	-4.227012355	2.36815E-05	0.001122115
29.34151697	1.054745715	0.508253665	2.075234845	0.037964775	0.169592573
224.4252388	0.134143528	0.113820846	1.178549738	0.238577516	0.494147253
73.31756394	0.510624462	0.186417493	2.739144566	0.006159928	0.054575001

16.69213863	-0.052899318	0.301383186	-0.175521797	0.860669645	0.940613387
3.870281617	0.515386214	0.883119165	0.583597587	0.559491084	0.770079698
7515.624591	-0.103113065	0.134657335	-0.765744139	0.443828558	0.68493625
470.909791	-0.099270982	0.076620988	-1.295610826	0.195109597	0.443107287
227.4450538	-0.108779479	0.087974213	-1.23649278	0.216275448	0.468970568
145.1290729	0.177550192	0.180574405	0.983252264	0.325483298	0.584078637
511.0889258	0.161256008	0.209880515	0.768322909	0.442295368	0.683444389
98.95612219	0.288178002	0.240143057	1.200026378	0.230129096	0.485322676
10.67292683	0.290647926	0.297643604	0.97649646	0.328818501	0.587245431
388.8232889	-0.199647013	0.157181926	-1.270165205	0.204025789	0.453634884
1041.854653	-0.255853479	0.083749343	-3.054990868	0.002250674	0.028467393
141.0184772	0.021245259	0.104391333	0.203515541	0.8387321	0.930862799
678.3701615	-0.034310687	0.11163694	-0.307341702	0.758583297	0.88881073
9.966797787	0.160223818	0.36966714	0.433427267	0.664704395	0.83676248
15.0552356	1.029685147	0.643425742	1.60031699	0.10952828	0.317354801
340.8489352	0.223399755	0.141968632	1.573585317	0.11558335	0.327813609
17.85103836	-0.56881219	0.570587412	-0.996888782	0.318818497	0.577074358
522.8595535	-0.313193889	0.239191618	-1.309384887	0.190404008	0.43813035
8.417415947	0.467554533	1.053243891	0.443918581	0.65710145	0.832739103
2798.19413	0.019051749	0.138169803	0.137886486	0.890330131	0.953820886
2305.727121	-0.192840197	0.100629802	-1.91633287	0.05532274	0.21292806
278.8937488	-0.142017473	0.247021967	-0.574918395	0.565346488	0.77379303
106.8824262	-0.610152467	0.252694991	-2.414580776	0.01575333	0.100045497
118.9300873	0.19020102	0.171988298	1.105895123	0.268771916	0.527663112
205.776156	-0.269361217	0.302117346	-0.891578126	0.372619099	0.626450632
534.2555889	0.134629957	0.154048168	0.87394715	0.382147036	0.633976208
1552.953947	-0.125972263	0.167989002	-0.749883991	0.453324577	0.691759407
1482.170402	-0.037376374	0.170512631	-0.219200029	0.826494233	0.925380893
447.3825033	-0.192041478	0.08075323	-2.378127512	0.017400807	0.10571922
467.3558864	-0.236284937	0.087134281	-2.711733359	0.006693241	0.057584636
14.80009491	1.038524861	0.522613382	1.987176175	0.046902877	0.192343583
10.36377314	0.499169476	0.425603826	1.172850068	0.240855936	0.496110133
253.8520002	0.244254914	0.154944304	1.576404601	0.114932589	0.326434042
1261.368237	-0.127141442	0.066928644	-1.899656615	0.057478197	0.218064314
32.54757306	0.593965392	0.326943919	1.816719496	0.069260075	0.243608574
3.819412732	0.288148017	0.562756533	0.512029626	0.608630275	0.802097071
846.360573	0.40695207	0.118146987	3.444455758	0.00057221	0.011505047
394.806975	0.471374806	0.18269968	2.580052722	0.009878523	0.07441631
4.915100471	0.857658653	0.716050583	1.197762664	0.231009426	0.486525022
136.082269	-0.213327439	0.340934057	-0.625714665	0.531502113	0.751380545
61.49956369	0.303467517	0.258737367	1.172878586	0.240844498	0.496110133
475.960488	0.218586365	0.168163466	1.299844549	0.193654254	0.44095141
118.0856822	-0.183614769	0.113112918	-1.623287353	0.104527992	0.309733048
50.84769452	0.129301592	0.211016351	0.612756273	0.540037508	0.757466143
120.3724661	-0.225674033	0.209315501	-1.078152509	0.280965702	0.539411971
86.75246926	0.005110282	0.19743157	0.025883815	0.97935001	0.989273349
325.6145345	-0.306064535	0.157735966	-1.940359848	0.052335973	0.205414764
9.362822461	0.594029309	0.389018357	1.526995573	0.126762112	0.347308746

201.3319109	0.066706476	0.097685718	0.68286826	0.494690092	0.722813893
50.44496696	0.338314828	0.328440413	1.030064555	0.302979703	0.561618659
4598.867875	0.000420419	0.137585807	0.003055683	0.997561922	0.998771859
13.67907259	0.919954672	0.487452827	1.887269129	0.059124136	0.221798083
280.416778	-0.321564338	0.095555311	-3.365216803	0.000764835	0.01364696
65.1471423	-0.130119978	0.175343017	-0.742088168	0.458033918	0.694415566
28.59913356	0.568891705	0.28092351	2.025076882	0.04285947	0.182219554
288.2709921	0.352732248	0.118548686	2.975420984	0.002925867	0.033841667
2481.262772	0.181814534	0.142609548	1.274911369	0.202340615	0.451231367
13.14311289	-0.096736836	0.276067913	-0.35040956	0.726031353	0.872122772
5.838030321	1.257874826	0.614869167	2.045760129	0.040779977	0.177123554
101.6618041	-0.062884555	0.163822116	-0.383858763	0.701083132	0.857806901
622.7790169	0.133251405	0.073156988	1.821444647	0.068539294	0.242335835
106.247174	0.023923406	0.153400062	0.155954347	0.876069007	0.948628307
3.79170297	0.262207656	0.953233004	0.275071945	0.783260965	0.901097703
892.8345851	0.128127056	0.176811135	0.724654903	0.468663768	0.703870778
1036.725405	0.298864679	0.178835878	1.671167346	0.094688628	0.292687514
41.01139834	0.466060309	0.215881301	2.158872981	0.030860023	0.149339276
81.06664308	-0.030734614	0.142468277	-0.21572953	0.829198582	0.927019072
318.2117277	0.467663692	0.301975902	1.548678845	0.121458942	0.338118928
278.7136884	0.061784804	0.068335136	0.904144019	0.36591904	0.621852229
297.0836568	0.351733499	0.167121325	2.104659583	0.035320951	0.161791413
11.86308213	0.339743265	0.31544117	1.07704161	0.281461677	0.540059327
116.8251752	0.059312489	0.118012992	0.502592873	0.615250541	0.806889329
100.8637266	0.112740867	0.602306797	0.187181794	0.851518099	0.93609097
498.9106271	0.186216844	0.164240664	1.133804741	0.256876462	0.513818445
474.8942203	0.009086116	0.076385865	0.118950221	0.905314794	0.959974986
855.0047339	-0.125662964	0.125032875	-1.005039383	0.314877886	0.573869373
21.99074899	0.165831142	0.356328637	0.465388196	0.641653486	0.824014843
15.27536035	1.6597235	0.819746156	2.02467982	0.042900252	0.182294108
67.03265641	0.017842283	0.157804852	0.113065491	0.909978634	0.9616745
231.3098178	-0.07142225	0.211669699	-0.337423118	0.73579796	0.877119039
59.45918943	0.349564428	0.334939985	1.043662878	0.29664139	0.555344561
2069.77077	0.172342429	0.175686891	0.980963508	0.326610737	0.585151858
263.2020948	-0.015765522	0.095151563	-0.165688524	0.868402078	0.945025059
1114.499353	-0.089676444	0.050619149	-1.77159129	0.076462427	0.258511899
1395.735549	0.138203161	0.080925874	1.707774717	0.087678152	0.279614505
685.6910244	0.146704353	0.075302928	1.948189214	0.051392327	0.203032054
58.69446357	-0.469274852	0.268859521	-1.745427689	0.080910449	0.266824178
332.0262225	-0.060312774	0.152920808	-0.394405277	0.693281848	0.85351767
127.4589018	0.286493253	0.11294469	2.536580112	0.011194113	0.079949209
161.6331149	0.527457276	0.139128355	3.791155847	0.000149948	0.004505329
325.921989	-0.064309016	0.105048093	-0.612186427	0.540414421	0.757542051
20.37984448	-0.100486167	0.355214823	-0.282888439	0.777262357	0.898017149
230.5851432	0.170136491	0.115989713	1.466823972	0.142423947	0.371222252
7.727320232	-0.524039211	0.414690499	-1.263687526	0.206342204	0.456584527
160.4266315	-0.292423709	0.130945456	-2.233171865	0.025537613	0.132714354
2224.813111	-0.217657346	0.152804401	-1.424418043	0.154325494	0.388950836

58.73440474	0.012177771	0.180942748	0.067301792	0.94634145	0.976282267
2.689792891	0.080648222	0.700247328	0.115171052	0.908309543	0.960978931
4973.830968	-0.057268194	0.124637478	-0.459478122	0.645890859	0.826141921
29.93355694	0.295246415	0.199875675	1.477150306	0.139635351	0.366893988
523.4989769	0.169384108	0.11080495	1.528669145	0.126346485	0.346497337
491.630642	0.036308306	0.103742883	0.34998358	0.72635102	0.872288622
13.92067493	0.131026786	0.333269097	0.393156123	0.694204175	0.853551135
283.3144176	0.197781787	0.196955825	1.004193638	0.315285288	0.57422143
66.33536993	0.339845331	0.154052032	2.206042507	0.027381024	0.138978633
318.3312802	0.144651906	0.273012558	0.529836088	0.596225582	0.793955003
56.78826924	0.234401289	0.326799634	0.71726301	0.473211827	0.706757551
13.82837417	1.276793981	1.070749784	1.192429828	0.23309275	0.488616239
2507.703764	-0.860957872	0.560372983	-1.536401467	0.124439947	0.343189051
477.0749607	0.218463298	0.082206218	2.657503314	0.007872182	0.064372939
1220.926424	0.251418764	0.12238475	2.054330819	0.039943684	0.174944635
12.49803407	0.587764922	0.305763919	1.922283452	0.054570101	0.211119256
112.2932124	0.689760204	0.263859655	2.614117734	0.00894582	0.069596353
355.7292156	-0.071331185	0.093963551	-0.759136755	0.447770754	0.68770432
45.3069062	0.058652018	0.169123192	0.346800562	0.728741155	0.873219977
1016.299813	0.164717587	0.086509934	1.904030895	0.056906161	0.216525042
111.6166185	0.580392448	0.369106055	1.572427329	0.11585148	0.328233994
482.7871251	0.217322408	0.18789775	1.156599307	0.247436105	0.502785584
93.87798891	-0.881254148	0.379066531	-2.324800731	0.020082618	0.114912725
266.2264301	0.04428767	0.078278682	0.565769234	0.571550663	0.778277531
1740.918562	-0.40192899	0.088118545	-4.561230416	5.08547E-06	0.000340857
279.0166616	0.311503672	0.312997327	0.9952279	0.319625435	0.577868049
1537.159104	0.102937588	0.120109304	0.857032592	0.391426872	0.642169359
31.63130711	0.482202634	0.235565113	2.047003595	0.040657732	0.176837455
3178.424029	0.159032824	0.145414248	1.093653655	0.274106908	0.533044162
571.9949045	0.502324036	0.124820122	4.024383481	5.71248E-05	0.002234276
11.15078197	0.316073795	0.392696182	0.804881253	0.420888207	0.665511709
326.1245421	0.230775299	0.066237442	3.484061151	0.000493867	0.010369216
3461.002297	0.231217915	0.172367482	1.341424219	0.179782759	0.424747406
127.0364362	-0.258454586	0.15977956	-1.617569764	0.10575535	0.310992892
427.9987226	0.184041921	0.087801965	2.096102535	0.036073096	0.164181788
1841.937628	-0.074720339	0.086353049	-0.865288949	0.386880215	0.638114343
105.056237	0.277926187	0.122616705	2.26662579	0.023413095	0.126363037
28.92844616	0.294426453	0.182983874	1.609029508	0.10760989	0.314000652
48.34513057	-0.002695019	0.16585583	-0.016249166	0.987035612	0.992541292
600.0457723	0.381219413	0.156588109	2.43453615	0.01491089	0.096797353
9.405452172	0.447219331	0.430332342	1.039241738	0.298692323	0.5571706
122.386871	0.330059647	0.137337873	2.403267494	0.016249296	0.10167835
771.1925678	-0.13824988	0.104670024	-1.320816369	0.186562601	0.432655305
1465.60776	-0.058443874	0.141221199	-0.413846326	0.678986654	0.845876523
312.879012	0.505915456	0.179113239	2.824556453	0.004734611	0.045838046
373.2925521	0.209973959	0.098712632	2.127123496	0.033409824	0.156464518
61.42146878	0.662972437	0.263789804	2.51326028	0.011962104	0.083606794
724.509042	0.213244039	0.152276912	1.400370127	0.161402511	0.399906178

17.93499118	0.455244247	0.229467493	1.983916069	0.047265197	0.193300484
175.4346143	-0.265214254	0.104731155	-2.532333901	0.011330604	0.080484234
1229.670864	0.197771157	0.117952802	1.67669741	0.093601667	0.290634968
542.7712543	0.218273617	0.097499062	2.238725311	0.025173795	0.131696396
3992.523507	-0.10795189	0.119362681	-0.90440236	0.365782087	0.621686851
101.5828027	0.307283185	0.317697211	0.967220281	0.333433926	0.591900483
36.38686384	0.354449756	0.253141088	1.40020634	0.161451538	0.399906178
827.7351538	-0.090386689	0.088176143	-1.02506967	0.305330315	0.564118349
728.1741245	0.201312606	0.098548256	2.042782031	0.04107402	0.177809807
168.6894386	-0.033960574	0.12058602	-0.281629443	0.778227661	0.898338312
286.2193131	0.128137666	0.076693198	1.670782665	0.094764614	0.292749301
2916.762578	0.201172722	0.211924419	0.949266361	0.342485158	0.600305902
156.4847935	0.342225363	0.167875241	2.038569597	0.041493002	0.178833813
61.23002855	0.136312282	0.216747854	0.628897956	0.529415864	0.74980955
236.5252313	0.379047085	0.159398531	2.377983557	0.017407602	0.10571922
9.224264336	0.51863422	0.371219245	1.397110273	0.162380413	0.401193194
515.9759302	0.087635903	0.174979569	0.500835058	0.61648721	0.80702296
76.32895096	0.331523356	0.239401376	1.384801377	0.16611321	0.405813019
209.6183081	0.365210779	0.127248671	2.870055749	0.004103994	0.042064683
52.31941077	0.106467677	0.177165218	0.600951348	0.547872391	0.762398361
311.8729876	-0.017197659	0.104279725	-0.164918531	0.869008107	0.945247461
1043.131934	0.018993171	0.093418411	0.203312933	0.838890447	0.930862799
297.0837905	-0.061546604	0.109046446	-0.56440724	0.572477013	0.778738936
2.912745797	0.030784651	0.67867525	0.045359913	0.963820433	0.982935149
1716.290634	0.06649712	0.132780573	0.500804588	0.616508656	0.80702296
821.3524903	0.103514095	0.111506731	0.928321494	0.353240828	0.610139776
231.8904919	0.189754079	0.113686615	1.669097807	0.095097998	0.292986838
20332.78541	-0.178248633	0.04398076	-4.052877475	5.05915E-05	0.002030194
4.625799574	0.798089689	0.607310189	1.314138479	0.188799607	0.436423439
296.6293958	-0.113505904	0.087017175	-1.304408054	0.19209449	0.439441363
1512.301879	-0.114311479	0.232675061	-0.491292356	0.623219686	0.810950402
58.780248	0.386380559	0.14809458	2.609012149	0.009080402	0.070368451
17.08122907	0.785048721	0.333589044	2.353340841	0.018605571	0.109702923
271.554652	-0.310151405	0.122005049	-2.54211943	0.011018252	0.079361966
692.6588608	-0.025911069	0.1213544	-0.213515694	0.830924749	0.928287183
8.503358039	0.204196813	0.476631595	0.428416444	0.668347955	0.839214838
595.3522905	0.191589572	0.097764623	1.959702461	0.050030577	0.199969311
193.7140449	0.311592554	0.120643343	2.582757946	0.009801406	0.073977505
11.91057134	1.055668411	0.433224041	2.436772459	0.014819001	0.096320436
176.0778866	0.023035324	0.12203531	0.188759496	0.850281319	0.935523059
65.35944474	0.39544175	0.284184013	1.391498931	0.164074184	0.403299571
51.52712782	0.349666221	0.25319399	1.381021017	0.167272499	0.406933747
308.5963714	0.389478244	0.174232849	2.235389288	0.025391803	0.132452965
420.5520631	0.164531766	0.16448741	1.000269661	0.317180025	0.576008142
11.43619593	0.388706079	0.306175122	1.269554746	0.204243278	0.453989735
6244.726705	-0.046501961	0.12527204	-0.371207824	0.710482746	0.862677974
7.456781769	-0.316841677	0.347152433	-0.912687475	0.361406933	0.617320505
6.686020813	0.857668551	0.444668315	1.928782693	0.053757845	0.209243639

617.6648389	0.125983349	0.113347545	1.111478409	0.266362479	0.525013602
30.55654856	1.108388517	0.544945266	2.033944667	0.041957175	0.179762383
16.78909691	0.079190819	0.572718206	0.138271873	0.890025554	0.953820886
611.0938444	0.002116023	0.058262858	0.036318552	0.971028357	0.985937912
6849.534448	0.165193655	0.140418103	1.176441294	0.239418579	0.494646201
112.618883	0.133272648	0.121521803	1.096697422	0.272773684	0.531715444
44.63723537	-0.289976889	0.162099426	-1.788882894	0.073633677	0.252928295
42.11284329	0.034511267	0.304361191	0.113389183	0.909722015	0.9616745
75.41627085	0.290339133	0.139496735	2.08133282	0.037403453	0.16804498
108.4950038	-0.063903314	0.128158341	-0.498627817	0.618041605	0.808044267
9.209152245	-0.011543559	0.375071738	-0.030776935	0.975447435	0.988030139
17.17550233	-0.519996527	0.273381847	-1.902088719	0.057159557	0.217112899
2.98756579	-0.216485577	0.697563167	-0.310345481	0.756298248	0.887592168
26.62330435	-0.124943594	0.256567721	-0.486980957	0.626271826	0.813271002
26.1948247	0.336693487	0.22279109	1.511252028	0.130724251	0.352919666
80.995974	-0.065846329	0.144744965	-0.454912745	0.649172014	0.828042767
50.01194313	0.121833704	0.160927103	0.757073865	0.44900561	0.688654515
37.94423974	0.218288425	0.246791873	0.884504106	0.376424147	0.629675376
294.1745446	-0.181622401	0.130234604	-1.394578666	0.163142932	0.402126944
465.6833214	0.07619865	0.132904653	0.573333198	0.566419111	0.774448801
1496.804862	-0.310485194	0.113383916	-2.738353068	0.006174775	0.054600664
46.42923169	0.29737875	0.153855495	1.932844514	0.053255355	0.208085945
76.08570096	-0.272975317	0.391234744	-0.697727697	0.485347504	0.716441435
45.65492643	-0.813490111	0.472200301	-1.722764915	0.084931046	0.275332477
137.471454	0.004341527	0.101113197	0.04293729	0.965751523	0.983880928
77.61964049	0.278553556	0.215304189	1.29376747	0.195745752	0.443909251
405.9744866	-0.194376804	0.090188878	-2.155219229	0.031144683	0.15019591
379.0763579	0.163334377	0.099902094	1.634944484	0.102060686	0.3060095
5.948537284	0.222723867	0.444564021	0.500993911	0.616375408	0.80702296
526.4483067	0.381003464	0.153750127	2.47806926	0.01320955	0.089455347
120.0190961	0.054834977	0.258623774	0.212026049	0.832086712	0.928693708
455.217802	0.027611174	0.113685519	0.242873274	0.80810357	0.913988445
295.6450671	-0.102878219	0.072207017	-1.424767618	0.154224386	0.38887588
695.8123234	0.027491824	0.104992566	0.261845438	0.793440606	0.905368772
5.306203509	0.164344307	0.473475235	0.347102225	0.728514522	0.873219977
214.7121001	-0.092144651	0.120059252	-0.767493134	0.442788374	0.683810803
112.6318139	-0.118516805	0.166918055	-0.710029873	0.477685612	0.709875036
229.5820804	-0.147702922	0.124577848	-1.185627495	0.235769439	0.49179517
287.4332433	0.035773564	0.075555793	0.473472161	0.635876358	0.819624255
323.6001522	-0.170992045	0.076979591	-2.221264661	0.026333041	0.13550112
447.718176	0.089618689	0.075809355	1.182158709	0.237142733	0.492684668
153.0193011	0.175573248	0.127700825	1.374879517	0.169168801	0.409957273
79.59651448	0.080514944	0.161858625	0.497439937	0.618878849	0.808403087
2450.403689	0.048532975	0.062995871	0.770415177	0.441053653	0.682669052
1576.700231	-0.173758108	0.082359924	-2.109740995	0.034880671	0.160713411
38.12433511	0.041909594	0.197266556	0.212451594	0.831754738	0.928693708
2043.042295	0.013454085	0.12955894	0.103845287	0.917292127	0.965104295
20.04548297	0.326456887	0.252878188	1.290964988	0.196715819	0.445162804

3.166727402	0.768036879	1.170612075	0.656098545	0.511760721	0.736709972
506.3756583	-0.483952605	0.230764243	-2.097173278	0.035978239	0.163940355
3.323636809	0.129439622	1.158457784	0.111734432	0.911033978	0.962059189
53.05639715	-0.158540831	0.157526162	-1.006441268	0.314203351	0.573019227
116.3497639	-0.25405815	0.103646377	-2.451201455	0.014238024	0.093866823
144.7508966	-0.426226825	0.40647015	-1.048605477	0.294359734	0.553499349
438.5435625	-0.265969822	0.122556625	-2.17017907	0.029993283	0.147004578
1122.038884	-0.313602078	0.122419595	-2.56169838	0.010416173	0.076795237
25086.41209	0.494324099	0.303259629	1.630035954	0.103093898	0.307458586
5.079592641	0.783476074	1.04609713	0.748951556	0.453886404	0.692078503
3.881253288	0.260084943	0.599146101	0.434092691	0.664221133	0.836652459
498.127779	-0.170174875	0.125414795	-1.356896326	0.174814154	0.417988969
474.965516	-0.087669392	0.085094925	-1.030254061	0.302890758	0.561529391
8.671393848	0.738732904	0.463867697	1.592550871	0.111260982	0.320866896
56.83556147	0.389668501	0.363966286	1.070617022	0.284341666	0.543156866
4.294297243	-0.645240879	0.687931426	-0.9379436	0.348273395	0.605634643
252.4269521	-0.161899927	0.075354226	-2.148518217	0.031672611	0.151651309
10.5037185	0.104626891	0.310581997	0.336873651	0.736212149	0.877546082
52.47878326	-0.525984702	0.213882843	-2.459218771	0.013923974	0.092628441
615.5214867	-0.571960102	0.12003443	-4.764967051	1.88885E-06	0.000164779
5.834914225	0.553275413	0.400321642	1.382077198	0.166947999	0.406609306
126.7866947	0.285162501	0.343767597	0.829521174	0.406809561	0.655071986
114.8170636	-0.243810862	0.141247025	-1.726130953	0.084323856	0.274295993
8632.551993	0.175358197	0.131971633	1.328756741	0.183928238	0.429498408
1120.538819	0.01923319	0.136570722	0.140829527	0.888004616	0.953471838
161.8643743	-0.07782878	0.089730877	-0.867357848	0.38574597	0.637114553
167.7901091	0.255767765	0.167971471	1.522685752	0.127837344	0.348467226
247.829823	0.453039744	0.189960824	2.384911446	0.017083233	0.104620627
2.69828753	0.804069222	0.573026621	1.403196978	0.160558109	0.398467573
273.8241468	0.177102457	0.187437175	0.94486303	0.344728808	0.60239957
3.226660578	0.175828667	0.519957234	0.338159863	0.735242721	0.876856177
6.030715785	-0.395130462	0.449057969	-0.879909698	0.37890823	0.631138136
199.058038	0.084827702	0.149846817	0.56609612	0.571328441	0.778130154
4.460110683	0.504217167	0.526113416	0.958381124	0.33787061	0.596282508
5.832177135	-0.500238269	0.592840507	-0.843799071	0.398781695	0.648806234
1851.385797	0.009405616	0.093117407	0.101008134	0.919544003	0.966087273
562.19393	-0.207319325	0.078871377	-2.628574945	0.008574346	0.067815264
160.3629599	0.068307576	0.142291991	0.480052144	0.631190316	0.816460941
175.6650639	-0.072955736	0.122636236	-0.59489543	0.551913372	0.76481793
1085.599435	-0.181022361	0.190436332	-0.950566305	0.34182458	0.599416001
624.7094373	-0.126848339	0.085859626	-1.477392163	0.139570546	0.366887151
2.672665806	-0.496144518	0.59641014	-0.83188478	0.405473984	0.654312692
61.31375022	0.343706503	0.137243524	2.504354982	0.012267488	0.085209605
231.3391822	-0.087105054	0.107117381	-0.81317386	0.416118384	0.661839645
80.03014098	0.414924262	0.5079183	0.816911426	0.413979048	0.660580668
82.17354316	-0.425656131	0.240585659	-1.769249811	0.076852195	0.25938236
5.888523839	-2.659861022	0.9881797	-2.691677457	0.007109366	0.060337286
1090.595406	-0.284883041	0.187038497	-1.523125163	0.127727394	0.348395905

424.4955608	-0.433679299	0.133239979	-3.254873667	0.001134428	0.018118504
5728.363148	0.026608922	0.196667045	0.135299342	0.892375205	0.95479462
803.3352672	-0.171292882	0.072856064	-2.351113571	0.01871732	0.110320348
364.1159233	0.18183016	0.303618505	0.598877067	0.549254865	0.763291982
4276.405902	-0.611358563	0.298535773	-2.047856966	0.040574017	0.176718377
405.6954102	-0.320047723	0.181804306	-1.760396832	0.078340546	0.261814005
27.45721872	0.717991535	0.667426772	1.075760765	0.282034264	0.540466121
1609.777269	-0.044544624	0.075974722	-0.586308475	0.557668237	0.768771196
72.55727899	0.148506378	0.132838463	1.117947123	0.263589578	0.521277253
83.14780638	0.094766903	0.168292337	0.563108844	0.57336077	0.779186406
223.9240573	0.222164985	0.201178095	1.104319958	0.269454368	0.528147648
3.639196391	-0.02552704	0.562734975	-0.045362456	0.963818406	0.982935149
495.7852727	0.08599874	0.086013888	0.999823892	0.317395741	0.576094758
215.9644791	-0.173283454	0.0798646	-2.16971543	0.03002841	0.147038898
148.5441409	0.112821827	0.108454935	1.040264573	0.298216998	0.556589137
60.78237899	-0.190357019	0.148257738	-1.28396009	0.19915592	0.447694059
29.83608136	-0.529905774	0.265177823	-1.998303513	0.045683765	0.189601527
4.783050951	-0.446068465	0.50343325	-0.886052847	0.375589051	0.628747857
55.03595562	0.216656763	0.179049864	1.210035899	0.226265118	0.480468739
3.715349871	1.107398821	0.530690263	2.086714038	0.036913992	0.166702863
62.52762012	0.29892941	0.173650996	1.721437926	0.085171387	0.275940516
88.52045701	0.125870116	0.120329251	1.046047528	0.295539089	0.554602454
11.92133469	-0.120582929	0.42236304	-0.28549593	0.775264222	0.896964006
113.1717378	-0.401406727	0.285708788	-1.404950581	0.160035974	0.397563536
34.40219414	0.11839351	0.220381869	0.537219828	0.591115777	0.79088016
12.32099803	0.365338652	0.283700616	1.287761221	0.197829095	0.44611812
715.7027608	-0.378686887	0.111328097	-3.40153921	0.000670075	0.012687447
29.65269311	0.349241858	0.360576665	0.968564779	0.332762383	0.591532966
714.516483	0.206851199	0.101251955	2.042935354	0.041058838	0.177793157
31.03791978	0.106779487	0.293590745	0.363701816	0.716080687	0.865788583
122.5758232	0.437670307	0.161212928	2.714858621	0.006630407	0.057231261
19.4888883	1.222415803	0.491379254	2.487723676	0.012856358	0.087772159
30.17396838	0.245146029	0.217144287	1.12895454	0.258917011	0.515926109
397.308137	-0.10288924	0.085197026	-1.207662335	0.227177171	0.481753212
623.6659123	-0.113679203	0.106039894	-1.072041844	0.283701238	0.542432063
32.58528573	0.498714622	0.196250065	2.541220167	0.011046633	0.079380341
739.5045911	-0.159405052	0.072364861	-2.202796346	0.027609106	0.13977444
3.755221878	-0.069607281	0.49269353	-0.141279065	0.887649488	0.953293246
67.12522504	-0.088903129	0.216285056	-0.411046102	0.68103873	0.847181914
302.9117308	-0.092600233	0.092455547	-1.001564925	0.316553768	0.575699131
3.628195735	0.939922934	0.935940836	1.004254648	0.315255888	0.57422143
19.94213154	-0.319209647	0.249547625	-1.279153217	0.200843107	0.449553772
362.5549543	0.239873564	0.107865727	2.223816308	0.026160806	0.134982713
29.83012658	-0.263855989	0.256612586	-1.028226996	0.303843062	0.562231547
459.9688689	-0.150864913	0.085591233	-1.762621093	0.077964417	0.260963931
808.3909598	-0.120303222	0.090412709	-1.330600783	0.183320405	0.428941851
292.4960598	-0.074186867	0.120018278	-0.61812974	0.53648982	0.75450963
118.7388244	-0.360969077	0.146785372	-2.459162464	0.013926158	0.092628441

610.6575476	0.076524111	0.114074803	0.670823957	0.502332684	0.728860202
752.5400107	-0.505654103	0.213394629	-2.369572776	0.01780865	0.107224073
5.323607035	-0.175461575	0.517342121	-0.339159655	0.73448946	0.876558568
2064.821325	0.328161813	0.128283644	2.558095502	0.010524718	0.077171427
374.8472162	-0.204558038	0.098674046	-2.073068323	0.038165922	0.169872686
267.708211	-0.252941595	0.134847768	-1.875756631	0.060688706	0.225206985
5.990921082	-0.118764521	0.595445044	-0.199455049	0.841906802	0.93182686
360.212976	-0.629404794	0.303739236	-2.07218798	0.038247914	0.170044074
1008.050993	-0.112354667	0.074874609	-1.500571013	0.133466554	0.356954457
439.5659952	0.019708477	0.067115526	0.293650044	0.769025331	0.893581791
115.4069957	-0.102682814	0.129518689	-0.792803064	0.427892571	0.672578929
1142.745359	-0.445139841	0.629769654	-0.706829613	0.479672369	0.711575578
29.7101306	-0.670864431	0.631174443	-1.062882755	0.287835124	0.54728446
664.6233216	0.017490091	0.065687927	0.266260362	0.790038698	0.904145475
652.5178544	-0.303621793	0.07622779	-3.983085319	6.80263E-05	0.002487004
88.24603582	-0.096086035	0.12027782	-0.798867447	0.424367276	0.669258558
127.3547951	0.250334042	0.165693774	1.510823469	0.130833434	0.353061179
485.4679539	-0.165994992	0.225196258	-0.737112566	0.461053898	0.697145057
38.96128673	0.352122201	0.275398974	1.278589371	0.201041695	0.449869874
598.9744666	-0.450538794	0.177264911	-2.541612949	0.011034229	0.079380341
534.8745833	-0.091708109	0.071597826	-1.280878406	0.200236379	0.44877213
2.886068024	0.968363893	0.869166543	1.114129278	0.26522373	0.523307206
148.0231421	0.104657854	0.12619778	0.829316129	0.406925546	0.655147881
118.5644079	-0.189776213	0.251946996	-0.75323864	0.451306524	0.690422789
475.9959434	-0.330917318	0.108878933	-3.03931449	0.002371172	0.029492038
96.22158765	0.016174438	0.140495884	0.115123931	0.908346892	0.960978931
6.560893859	-0.685630756	0.537466081	-1.275672607	0.202071276	0.450798249
23.339888	-0.237059446	0.225354864	-1.05193845	0.29282779	0.552281525
2774.25649	-0.274346987	0.136499985	-2.009868258	0.044445134	0.186583471
203.7448985	0.021041393	0.143639131	0.146487889	0.88353625	0.951745378
386.4905326	0.102674735	0.120743401	0.850354838	0.395127837	0.645455477
15.13510446	0.182688348	0.325713916	0.560885915	0.574875312	0.780163053
916.9951587	-0.263641129	0.175845872	-1.499273914	0.133802587	0.357207219
232.3920798	0.162476536	0.12140389	1.338314087	0.180794072	0.426017761
620.4871131	-0.130043979	0.059899982	-2.171018659	0.029929762	0.146876842
40.56892018	-0.014347321	0.225402801	-0.063651921	0.949247389	0.977223239
64.26843663	-0.890436866	0.368017642	-2.419549405	0.01553975	0.099354867
5.656823769	-0.283740983	0.473082893	-0.599770119	0.54865945	0.762847578
303.8091017	0.028176831	0.13291728	0.211987716	0.832116617	0.928693708
17.95144613	0.430411897	0.485469729	0.886588537	0.375300467	0.628533105
86.24207479	0.071404134	0.316266054	0.225772363	0.821378473	0.921865923
3.460515917	1.351465696	0.746692116	1.809937011	0.070305556	0.24607729
1380.727654	-0.080911197	0.072566153	-1.114999122	0.264850798	0.522834476
2581.792723	-0.140884674	0.094518147	-1.490556884	0.136077871	0.361369003
41.19749481	-0.543920146	0.378318702	-1.437729996	0.150510679	0.38383894
124.3245344	0.26650057	0.127741998	2.086240814	0.036956815	0.166800197
26.55551361	0.185323012	0.187357253	0.989142449	0.322593451	0.581267519
2329.00642	0.275620179	0.08927584	3.08728745	0.002019922	0.026667047

110.9923893	0.3723736	0.228033901	1.632974741	0.102474309	0.306660383
183.1574429	0.130635242	0.170762185	0.76501271	0.444263976	0.685136303
73.71417162	0.131032956	0.176204707	0.743640498	0.457093996	0.694071278
265.9595999	0.018780424	0.155750894	0.120579877	0.904023808	0.959382191
241.2039293	-0.285804796	0.076119015	-3.754709608	0.000173543	0.004913071
3.662573042	-0.024279855	0.530310923	-0.045784188	0.963482262	0.982781984
98.57381748	0.840576592	0.306141331	2.745714174	0.006037934	0.053775673
399.7445465	0.213527414	0.169824611	1.257340808	0.208630253	0.459572597
20.92691876	-0.69112897	0.365990385	-1.888380126	0.058974937	0.221548004
40.95195161	0.203459299	0.218677508	0.930407982	0.352159887	0.609028082
164.1767561	0.156070393	0.114064393	1.36826566	0.171228936	0.413035162
248.247289	0.289791257	0.085283196	3.397987759	0.000678835	0.012765997
14.88241619	0.216265493	0.312231724	0.6926442	0.488532865	0.718626475
223.1455514	0.260425073	0.097086604	2.682399656	0.007309608	0.061558565
869.0061159	0.088386517	0.056022337	1.577701355	0.114634235	0.325769766
345.0262362	0.286867234	0.131026988	2.189375165	0.028569583	0.142709289
121.8663896	0.13100857	0.18584616	0.704930199	0.480853676	0.712692218
961.823813	0.020929772	0.131380661	0.159306335	0.87342753	0.947352611
44.95494901	0.93026799	0.74327948	1.251572275	0.210725762	0.462176319
1820.438897	-0.045700168	0.122169026	-0.37407328	0.708349797	0.861905675
1780.426105	-0.198031063	0.181711884	-1.08980799	0.275797734	0.534543001
272.2903779	0.206488377	0.10123271	2.039739702	0.041376258	0.178526888
3.040214065	1.245140756	0.61951142	2.009875389	0.044444379	0.186583471
6210.237486	-0.238572225	0.224002149	-1.06504436	0.286855856	0.546332392
424.4633094	0.017600528	0.15781472	0.111526528	0.91119883	0.962059189
5.717135971	1.36390859	0.512488875	2.661342827	0.007782967	0.064044098
74.13255401	0.51604186	0.25370672	2.034009423	0.041950645	0.179762383
261.7257079	-0.147113511	0.070437428	-2.088570179	0.036746432	0.166233354
32.52654305	0.075328018	0.269497926	0.279512424	0.779851605	0.898757629
7.137992858	0.045822784	0.417159289	0.109844813	0.912532451	0.962614942
19.80024171	0.090084787	0.345504287	0.26073421	0.794297483	0.905623853
3.205089432	-0.202148995	0.660532994	-0.306039209	0.759574793	0.889224856
19.98016567	0.39360931	0.502364692	0.783513084	0.433325873	0.677730654
7544.674346	-0.049810356	0.10761189	-0.462870372	0.643457286	0.824643248
678.31189	-0.302546431	0.092264354	-3.279125873	0.001041292	0.017065434
30176.5662	0.073465281	0.131263777	0.559676727	0.57569996	0.780736608
6.760226271	0.55781064	0.493262894	1.13085871	0.258114564	0.515243582
927.9920959	0.153453602	0.096907027	1.583513664	0.113304435	0.324077445
1097.019015	-0.215325256	0.076801726	-2.803651257	0.005052753	0.047734421
4.70065893	0.262181527	0.598802042	0.437843408	0.661499802	0.835344221
254.9355802	0.15763766	0.125757639	1.253503651	0.210022471	0.461731489
35.90095637	0.342299138	0.335577099	1.020031282	0.307713625	0.566586111
164.8533616	0.299745303	0.326302973	0.918610395	0.358299393	0.614347157
231.0904302	0.368067794	0.136522069	2.696031476	0.007017104	0.059765103
218.8298221	-0.207493878	0.116976715	-1.77380497	0.076095416	0.2579099
9266.628327	0.046976185	0.119916674	0.391740227	0.695250164	0.854463425
2771.486668	0.150158717	0.192512347	0.779995253	0.435393669	0.679203731
1553.274747	0.0716863	0.135392164	0.529471553	0.596478373	0.794021966

14.47273972	-0.05049585	0.358583841	-0.140820206	0.88801198	0.953471838
277.2244751	-0.1676474	0.108374776	-1.546922692	0.121881889	0.338875295
362.496661	0.101307286	0.063513505	1.595051092	0.110700811	0.31963025
1248.558999	0.089860942	0.067801426	1.325354748	0.185053518	0.430939775
728.1034674	-0.014062755	0.118197208	-0.118977049	0.905293539	0.959974986
108.3961242	0.481795793	0.316388746	1.522796874	0.127809532	0.348467226
316.433924	-0.026579474	0.151682653	-0.17523081	0.860898276	0.940666613
812.5765573	0.012904891	0.181609628	0.071058408	0.94335127	0.974993168
97.73612305	0.047565914	0.143350676	0.331815068	0.740028906	0.879355136
2850.078502	-0.250690483	0.142071057	-1.764542959	0.077640609	0.260752744
427.4413304	-0.071222806	0.07519037	-0.947233087	0.343520019	0.601215055
92.78635072	0.293131071	0.216699182	1.352709634	0.176148412	0.419609916
1139.778076	-0.335063641	0.11253247	-2.977484098	0.002906247	0.033739142
6.543442565	1.207597426	0.491510554	2.45691047	0.014013761	0.092902392
466.5741152	-0.296176016	0.121786389	-2.431930364	0.015018594	0.097352624
1397.639435	-0.016874141	0.089547932	-0.188436974	0.850534118	0.935533846
72.64949284	0.586415974	0.30367053	1.931092801	0.053471577	0.208618959
7.437455295	0.323077266	0.444774153	0.726384984	0.467602794	0.702883096
211.4167919	0.395559594	0.194078924	2.038137813	0.041536153	0.178921455
114.4931512	0.069360464	0.13193735	0.525707569	0.599091404	0.795609617
58.37400211	-0.960265036	0.347002988	-2.767310571	0.005652088	0.051389769
43.02817715	0.094081471	0.18124923	0.51907239	0.603710262	0.798632907
365.5972052	0.015715931	0.067179423	0.233939658	0.815031825	0.918646004
17.05243749	-0.146490775	0.290744881	-0.503846445	0.614369287	0.80620548
96.29109269	-0.174806283	0.125864274	-1.388847499	0.164879128	0.404562617
36.67482568	-0.259027196	0.344793283	-0.751253604	0.452500046	0.690975632
853.0018259	-0.248862138	0.162825097	-1.528401593	0.12641286	0.346604975
384.8133573	-0.13452345	0.125204611	-1.074428881	0.282630504	0.541176514
1432.507935	0.462224129	0.145767991	3.170957667	0.001519373	0.022246589
6.60306087	0.186560566	0.497734722	0.374819272	0.707794881	0.861889372
3.351272937	-0.738872789	0.562631784	-1.313243955	0.189100757	0.436736716
3.939788278	0.104152815	0.599078176	0.173855131	0.861979316	0.94137167
514.3366787	-0.076423508	0.165606582	-0.461476269	0.644456943	0.825182684
1512.763137	-0.041102975	0.123061677	-0.334003048	0.738377259	0.878437596
539.8247319	0.031901816	0.053912076	0.591737844	0.554026151	0.766061319
3.525981651	0.199653896	0.51113917	0.390605745	0.696088678	0.854510951
371.784681	-0.187386298	0.080455801	-2.329058884	0.019855946	0.114157135
297.2052054	0.408333464	0.100834164	4.049554719	5.13152E-05	0.002047907
31.71858502	0.130441105	0.228971099	0.56968371	0.568892241	0.775924721
553.1761023	0.100833462	0.168998334	0.596653584	0.550738688	0.764270535
318.0135846	0.183957818	0.084373188	2.180287632	0.029236147	0.144846762
289.1997992	-0.112117504	0.077498954	-1.446696999	0.147981805	0.379505869
33.81992421	0.224573237	0.204787126	1.09661794	0.272808442	0.531717112
38.14763274	0.026713686	0.421194467	0.063423639	0.949429164	0.977282077
93.00967707	0.020615281	0.290602325	0.070939834	0.94344564	0.975026451
1077.138387	0.345926974	0.113118362	3.05809744	0.002227471	0.028265098
441.8655706	-0.256484581	0.115425374	-2.222081446	0.026277802	0.135350097
564.0455313	-0.058569713	0.050504512	-1.159692687	0.246173949	0.501557834

32.71355794	0.466398083	0.271918669	1.715211699	0.086306421	0.277496905
4.29605303	1.133085048	0.504888744	2.244227193	0.024817787	0.130663891
183.216193	0.203704985	0.319740038	0.637095642	0.524062547	0.745794119
407.3649989	-0.479193229	0.234842494	-2.04048774	0.041301771	0.178500131
312.9881239	-0.525847745	0.259013327	-2.030195713	0.042336649	0.180617323
394.6594173	-0.118170604	0.061787009	-1.912547735	0.055805976	0.214104924
5.061832199	1.627929062	0.655627704	2.48300835	0.013027803	0.088530359
3.139196872	0.202199616	0.753194618	0.268456002	0.788348337	0.903504225
431.6816224	0.022100619	0.179678378	0.123000994	0.902106311	0.958662553
16.78738396	1.273658111	0.593023106	2.14773775	0.031734595	0.151884462
91.03782962	0.126984113	0.125859018	1.008939327	0.313003733	0.571976009
146.948512	0.190465417	0.104487563	1.82285252	0.068325731	0.242084363
393.9227699	0.065240216	0.075433994	0.864864928	0.38711293	0.638296803
74.76619887	0.208507165	0.202241995	1.030978582	0.302550864	0.561386357
37.55564693	0.134817615	0.222245181	0.606616594	0.544105385	0.759365444
3.151441018	1.200063834	1.042992228	1.150597101	0.249898026	0.505794379
7.888227889	0.199799471	0.405512765	0.492708217	0.622218771	0.810641951
1726.396006	0.060998419	0.071983984	0.847388755	0.39677847	0.647292857
228.7905836	-0.018225349	0.074239551	-0.245493792	0.806074114	0.913010135
2282.930409	0.034148275	0.16115238	0.211900529	0.832184638	0.928702424
73.40796069	-0.36595376	0.122238269	-2.993774076	0.002755499	0.032543107
1595.430664	-0.013186188	0.09267831	-0.142279121	0.886859538	0.953108598
21.8027898	0.267647753	0.355397915	0.753093199	0.451393911	0.690422789
214.5018439	-0.020232572	0.086677328	-0.233424041	0.815432146	0.918701084
3.659855436	-0.087073736	0.475343582	-0.183180629	0.854656293	0.937700546
5.990295438	1.507669111	0.562580829	2.679915547	0.007364074	0.061796753
886.1658032	0.047280951	0.079997332	0.591031599	0.554499249	0.766234909
132.5099548	0.253602168	0.255730872	0.991675999	0.321355602	0.579926507
3.508303318	1.317394774	0.765807768	1.72026823	0.085383696	0.27617197
1240.466196	0.199919757	0.075184418	2.659058376	0.007835938	0.06421048
273.8122317	0.258474978	0.14974608	1.726088447	0.084331502	0.274295993
1435.047735	0.416826721	0.10965551	3.801238266	0.000143975	0.004359267
240.2020592	0.052312683	0.086473341	0.604957351	0.545207335	0.76009172
812.1343469	-0.3658732	0.125418272	-2.917224046	0.00353162	0.037964309
433.0921078	-0.148525506	0.167551063	-0.886449202	0.375375516	0.628591671
500.1097717	-0.093255595	0.135972689	-0.685840628	0.492813614	0.721493215
942.1953285	0.018133894	0.122825663	0.147639295	0.882627442	0.951417787
409.3555181	-0.053706451	0.117930572	-0.455407366	0.648816197	0.827875514
236.5101635	0.226567799	0.178271306	1.270915683	0.203758646	0.453300969
13806.5358	-0.022485349	0.191589386	-0.117362186	0.906573048	0.960203382
51.86948515	0.156530148	0.232240717	0.673999587	0.500311576	0.726893893
270.0869758	0.167158522	0.102683651	1.627898124	0.103546489	0.308336615
96.83720795	-0.70433575	0.501316865	-1.404971185	0.160029847	0.397563536
564.659624	-0.097934192	0.085859294	-1.140635894	0.254021474	0.510716932
2808.219254	-0.085475213	0.166536119	-0.513253305	0.607774143	0.80150758
1550.800682	0.066287981	0.109722168	0.60414392	0.545747962	0.760687675
11.34257022	0.017339592	0.304323065	0.056977582	0.954563053	0.979993907
26.62155991	0.076579831	0.243986226	0.313869485	0.753620168	0.8866675

314.790512	0.130987661	0.098592433	1.328577233	0.183987487	0.429517084
567.0943586	-0.03526696	0.094035674	-0.375038099	0.707632132	0.861889372
13685.09089	0.019196446	0.140467126	0.136661488	0.891298372	0.954379567
817.7391316	-0.031689757	0.073104821	-0.433483813	0.664663323	0.83676248
385.1503978	0.08073757	0.196276438	0.411346214	0.680818687	0.847121006
1121.786345	-0.132209893	0.056355686	-2.345990299	0.018976601	0.111097787
28.24754424	-0.454814755	0.325954501	-1.395332028	0.162915738	0.401756358
12.92543231	0.226930457	0.312857291	0.725348149	0.468238474	0.70336675
251.5593269	0.219063166	0.104709898	2.092096066	0.036429923	0.165203628
591.8659238	0.123512817	0.104328393	1.183884974	0.236458599	0.492010908
29.06867919	0.208962138	0.297146529	0.703229276	0.481912877	0.713857345
6.05309151	0.423553778	0.566168225	0.748105879	0.454396296	0.69248182
323.6709716	0.48475794	0.304953929	1.589610407	0.11192265	0.322028806
568.996941	0.172904974	0.149538085	1.156260453	0.247574639	0.502785584
169.5914058	0.000718923	0.110041705	0.006533187	0.994787308	0.997203384
6.837210854	-0.324252032	0.644303223	-0.503259987	0.614781495	0.80643696
54.05583328	-0.885659377	0.356954878	-2.481152187	0.013095845	0.088915683
8.441541654	0.510473925	0.605722229	0.842752504	0.399366876	0.649354145
6.395852359	0.861217389	0.514634277	1.673455166	0.094237726	0.291686543
818.9946614	-0.10991321	0.154624351	-0.710840234	0.477183245	0.709683822
650.8868566	0.099074721	0.167329832	0.592092398	0.553788717	0.765862233
287.9049434	-0.386253498	0.142686394	-2.707010017	0.006789221	0.058186962
546.62747	-0.547588198	0.2871567	-1.906931645	0.05652944	0.215772142
5.638352895	1.129086189	0.586649261	1.924635832	0.054274933	0.210469729
81.43303352	-0.028111479	0.424617357	-0.066204263	0.947215203	0.976502838
34.44737698	-0.840467026	0.326162753	-2.576833251	0.009971004	0.074825466
1875.725504	-0.189776232	0.119728471	-1.585055173	0.112953799	0.323573952
356.9376442	-0.333898773	0.131939891	-2.530688573	0.011383887	0.080716496
90.83694847	-0.106574757	0.144327975	-0.738420649	0.46025887	0.696718475
260.6746468	-0.27136093	0.138331913	-1.961665415	0.049801451	0.199664099
59.7970639	0.343515283	0.17848808	1.924583887	0.054281437	0.210469729
121.6558048	-0.086207431	0.181941684	-0.473819022	0.635628969	0.819431592
156.6276579	0.575395674	0.101052726	5.694014364	1.24087E-08	2.88122E-06
926.1843292	-0.127733232	0.106365024	-1.200895065	0.229791909	0.485070119
1268.163513	-0.228594974	0.071909335	-3.178933222	0.001478181	0.021809781
912.1782149	0.08974491	0.112712707	0.796227084	0.425900058	0.670866377
21.50302358	0.267449368	0.273643141	0.977365508	0.328388233	0.586744252
383.3631315	0.106450622	0.090140172	1.180945405	0.237624412	0.493172063
461.6464999	-0.19599243	0.06471661	-3.028471797	0.00245794	0.03021186
1083.706343	-0.165246329	0.127016779	-1.300980314	0.193265193	0.440641269
235.2776783	0.155021428	0.107994573	1.435455723	0.151157277	0.384829693
494.8284391	-0.061935349	0.07222936	-0.857481625	0.391178766	0.641829455
6943.950713	-0.207918046	0.156221157	-1.330921175	0.183214949	0.428880565
16.4371803	0.658476014	0.422521788	1.558442743	0.119128331	0.334138972
4691.88779	-0.032934437	0.193609734	-0.17010734	0.864925721	0.942899493
49.02508033	-0.5789261	0.1716364	-3.372979742	0.000743594	0.013467127
421.4977893	-0.067877554	0.093967879	-0.722348481	0.470080253	0.705255279
32.40392215	-0.41418044	0.244243307	-1.695769862	0.089929472	0.284251076

94.74388083	-0.037680839	0.383693847	-0.098205482	0.921769129	0.966973977
303.1905694	-0.149930242	0.166791917	-0.898905923	0.368702773	0.623914531
337.9878214	-0.00335579	0.100696264	-0.033325869	0.973414725	0.986906773
1032.932144	-0.060060841	0.087106213	-0.689512716	0.490500674	0.720006182
54.74728029	0.318928819	0.210948298	1.511881449	0.130564024	0.352576817
658.0670684	-0.096240492	0.067618016	-1.423296595	0.154650194	0.389268759
2330.166584	-0.155240863	0.073070469	-2.12453629	0.033625325	0.156958213
134.6233436	-0.358441526	0.113301845	-3.163598329	0.001558317	0.022651199
318.075538	-0.321521283	0.101615388	-3.164100322	0.001555632	0.022633141
456.3182352	-0.003505653	0.155700233	-0.022515401	0.982036827	0.990754251
259.6029623	-0.082671473	0.108096223	-0.764795211	0.4443935	0.685268671
87.42097207	0.048125989	0.151628573	0.317393929	0.750944713	0.885437575
123.8328299	-0.126229193	0.116635222	-1.082256207	0.2791387	0.53751602
2.611035036	0.063378075	0.685411558	0.092467182	0.926326865	0.968954952
41.97002015	-0.006613381	0.185939021	-0.035567472	0.971627245	0.986184096
65.5955708	0.161233186	0.153913848	1.047554772	0.294843784	0.55408305
114.3507874	0.801285877	0.353486903	2.266804995	0.023402141	0.126363037
601.9630921	0.282233443	0.18604057	1.517053208	0.129253251	0.350910158
206.4584304	-0.031536755	0.087078474	-0.36216477	0.717228905	0.866709156
534.24499	-0.011362005	0.071386821	-0.159161103	0.873541949	0.947352611
111.2672593	-0.276453263	0.132444531	-2.087313545	0.036859801	0.166554054
140.2341635	0.108277613	0.249512326	0.433956969	0.664319689	0.836652459
119.4160021	-0.171593414	0.124082518	-1.382897583	0.166696272	0.406604094
327.7188241	-0.079986784	0.116379437	-0.687293102	0.491898045	0.720819297
828.9784366	0.416808026	0.156428445	2.664528346	0.007709637	0.063711356
6641.401981	-0.199448048	0.15404887	-1.294706333	0.195421554	0.443604785
56.86659725	0.150252432	0.125418166	1.19801171	0.230912458	0.486386113
14.57431201	0.378360045	0.350949033	1.078105392	0.280986725	0.539411971
6446.751943	0.136454752	0.142125425	0.960100924	0.337004424	0.595725198
491.8659836	-0.463212121	0.203025551	-2.281545941	0.022516161	0.123347355
5668.506957	-0.347587473	0.12068015	-2.880237343	0.003973759	0.041219865
8.51473677	0.033598268	0.34628978	0.097023561	0.922707684	0.967233479
384.6304938	-0.044990479	0.077963212	-0.57707319	0.563890015	0.772932515
143.3863056	-0.169090232	0.118660457	-1.42499225	0.154159441	0.38887588
122.8978131	-0.171383653	0.138821282	-1.234563252	0.216993092	0.469594725
95.33907847	-0.569164643	0.334882151	-1.699596832	0.089206787	0.2831079
1025.80402	0.089501314	0.154561515	0.579065974	0.562544658	0.771716083
3027.976538	-0.174875452	0.129061227	-1.354980544	0.175423756	0.418774153
65.89892809	-0.005814085	0.205310527	-0.028318493	0.977408131	0.988491024
375.0443305	0.197869003	0.100643894	1.966030876	0.049295044	0.198287729
47.32621279	0.031928242	0.212832447	0.150015858	0.880752104	0.950743683
232.4736748	-0.044964241	0.093284399	-0.482012439	0.62979711	0.815331232
4.616418527	1.704308737	1.371231415	1.242903801	0.213903285	0.46582326
190.1753276	-0.268497749	0.348069905	-0.771390301	0.440475622	0.682478371
163.0140449	-0.180409779	0.12372306	-1.458174239	0.144792523	0.374863969
7.440615551	-0.26603503	0.363805427	-0.731256354	0.464622585	0.700215299
204.3879402	0.678308665	0.316075305	2.146034991	0.031870187	0.152115647
899.4867601	-0.148664532	0.071950224	-2.066213602	0.038808315	0.171634538

16.23237476	0.459145525	0.311110425	1.475828157	0.13999003	0.367588084
845.8580848	-0.053678045	0.104495361	-0.513688306	0.607469929	0.801442647
8.844562415	0.863412922	0.409728481	2.107280706	0.035093255	0.161313777
19.87358654	-0.453399357	0.29315862	-1.546600801	0.121959536	0.338989742
194.0638147	0.530922919	0.636706787	0.833857798	0.404361121	0.653410243
50.744345	-0.031027059	0.487150677	-0.063690888	0.94921636	0.977223239
3.242290144	1.137066427	0.828079905	1.37313612	0.169710034	0.410824537
598.9673839	0.071860138	0.191456481	0.375334058	0.70741204	0.861818427
1077.382231	-0.521496961	0.228907492	-2.27819962	0.022714687	0.124128345
420.3857736	-0.062959424	0.089607101	-0.702616467	0.482294797	0.714002102
4.093796895	-0.215081241	0.643194268	-0.334395457	0.738081167	0.878437596
776.9902289	-0.062546403	0.1194948	-0.523423637	0.600679479	0.796908894
4.805110938	1.127134667	1.162485284	0.969590482	0.332250659	0.590885513
110.9260732	-0.04938808	0.105831698	-0.466666233	0.640738692	0.823232665
10576.09627	-0.245267101	0.145228966	-1.688830452	0.091251926	0.286181806
5.093586007	-1.281991759	0.564245452	-2.272046241	0.023083718	0.125445085
9.117436747	-0.200920653	0.528889143	-0.379891809	0.704025728	0.85974352
418.3001328	-0.026525181	0.084091482	-0.315432435	0.752433344	0.886266194
20.3443289	0.089866131	0.281967281	0.3187112	0.749945521	0.88518749
2.883290689	1.336161718	0.769234273	1.737002321	0.082386761	0.270091838
278.5280346	0.127214033	0.093996771	1.353387261	0.175931945	0.41928531
483.0994897	-0.183429508	0.113237213	-1.619869496	0.105260314	0.310500845
9.824647841	-0.374970346	0.357490657	-1.048895511	0.294226212	0.553447338
129.3286976	-0.269180341	0.091425955	-2.94424423	0.003237444	0.036165295
334.8600063	0.36031842	0.21230435	1.697178696	0.089662881	0.283955102
428.9471704	-0.10106577	0.221123724	-0.457055299	0.647631299	0.82742337
8.195743823	-0.410342417	0.414155381	-0.990793398	0.321786474	0.580503687
796.7837883	-0.193345315	0.073806558	-2.619622444	0.008802716	0.068961941
76.89467532	0.054412214	0.125669687	0.432978036	0.665030726	0.836844909
202.7238859	-0.079876258	0.365183749	-0.218728949	0.826861197	0.925638145
365.2694286	0.466060512	0.426532749	1.092672281	0.274537714	0.533628642
9.770968369	0.790681334	0.765575527	1.032793377	0.301700604	0.560316432
5.080114289	0.870000645	0.570066849	1.526137936	0.126975516	0.347432657
125.415869	-0.120651463	0.093688648	-1.287791702	0.197818482	0.44611812
2.905661486	0.809253072	0.854074693	0.947520256	0.34337374	0.601109766
39.04654216	-0.389285929	0.542888785	-0.717063863	0.473334692	0.706757551
769.7303963	-0.244758658	0.116084659	-2.108449647	0.034992114	0.160990412
2991.617915	-0.044585722	0.05283525	-0.843863181	0.398745865	0.648806234
8.321503435	0.108423908	0.405945398	0.267089881	0.789399958	0.903718901
3.354134031	-0.290882397	0.529740125	-0.549103955	0.582934111	0.784884699
368.4051787	0.131242254	0.096815311	1.355593992	0.175228384	0.418435137
240.2543791	-0.110063253	0.178226894	-0.617545705	0.536874844	0.7548494
210.5442369	0.123118637	0.180828093	0.680860121	0.495960004	0.723257713
10.77399789	-1.175512289	0.518316538	-2.267942857	0.02333269	0.126227021
14.81096371	-0.644732129	0.391334814	-1.647520499	0.099451106	0.301642072
60.68048135	-0.007662686	0.198116895	-0.038677598	0.969147435	0.985192723
29.97915513	0.222280486	0.282147657	0.787816167	0.430804243	0.675808537
6.848715011	0.099561781	0.524917201	0.1896714	0.849566633	0.935119121

32.77719495	1.325049554	0.887210846	1.493500174	0.135306309	0.360234959
32.36175677	1.474870026	0.789614491	1.867835562	0.061784995	0.227847604
28.78114567	0.583076762	0.654977051	0.890224719	0.373345234	0.627379314
48.5403163	0.095116075	0.307309517	0.3095123	0.756931857	0.887988809
335.3674651	-0.05751969	0.079954223	-0.71940778	0.471889705	0.706078814
550.7570463	0.132133546	0.067120082	1.968614206	0.04899741	0.197441962
188.989876	0.423097153	0.206918411	2.044753539	0.040879162	0.17721083
350.1645276	-0.032147115	0.107144386	-0.300035458	0.76415011	0.891536883
205.7930815	-0.274753092	0.190141896	-1.444989752	0.148460764	0.380094454
871.6417682	-0.188473247	0.093306596	-2.019934879	0.043390143	0.183579984
8.484072476	0.248902416	0.537042992	0.463468325	0.643028714	0.824387858
635.2297419	-0.178747327	0.09209058	-1.940994689	0.052258922	0.205301071
777.1111566	0.060606011	0.07219187	0.839512968	0.401181515	0.650685718
64.1582317	-0.01292806	0.154875746	-0.083473756	0.933474845	0.972410963
5184.491753	-0.052496084	0.105484819	-0.497664826	0.618720304	0.808403087
535.7327111	0.185803992	0.105701285	1.757821504	0.078777885	0.262639264
45.27779136	-1.128659525	0.518328126	-2.17750006	0.029443277	0.145474083
301.1048324	0.209802281	0.123676136	1.696384513	0.089813085	0.28416287
1720.536648	-0.182471229	0.089130584	-2.047234742	0.040635042	0.176829885
1084.686375	-0.129407219	0.083915264	-1.542117751	0.123044982	0.340780063
383.0610248	-0.039894595	0.105388453	-0.378548061	0.705023498	0.860203048
454.1521965	-0.502637206	0.394907456	-1.272797459	0.203089923	0.452130923
99.24755335	-0.30751205	0.109733229	-2.802360349	0.005073017	0.047844381
46.42602614	0.047875806	0.215236495	0.222433494	0.823976441	0.923645097
83.76507287	-0.101709125	0.157755721	-0.644725432	0.519105133	0.742376666
23.78247475	-0.370492693	0.242416243	-1.52833279	0.126429933	0.346604975
62.43415047	-0.337107254	0.157033724	-2.146718845	0.031815672	0.152090717
466.8492632	-0.216543163	0.076953217	-2.81395855	0.004893554	0.046742081
253.7597497	0.232653569	0.077631401	2.996900313	0.002727399	0.032370421
1198.926348	0.026447923	0.094805681	0.278969813	0.780267992	0.898986384
144.0790589	-0.437230932	0.122070705	-3.581784268	0.000341256	0.008012353
163.3404031	0.069077276	0.129249225	0.534450214	0.593030086	0.791576954
19.82171679	0.242960535	0.298139341	0.814922762	0.415116523	0.661601456
164.1199632	-0.53265272	0.147737352	-3.6054032	0.000311669	0.007578622
1018.368553	-0.20943838	0.131641906	-1.590970436	0.111616229	0.321326898
3904.343571	-0.416499444	0.172303355	-2.41724512	0.015638482	0.099704858
343.5311727	0.157106611	0.189065162	0.830965419	0.405993165	0.654824855
295.6450192	-0.233989308	0.109104525	-2.144634307	0.031982097	0.152480917
3797.883139	-0.435611725	0.159012702	-2.739477533	0.006153692	0.054558794
46.91136738	0.216323647	0.197374337	1.096006957	0.27307573	0.531877602
1402.962269	-0.104416439	0.07781392	-1.341873521	0.179637009	0.424747406
7.963632165	0.152329549	0.367914397	0.414035302	0.678848253	0.845876523
5.890987883	0.56609439	0.465074613	1.217211981	0.223523573	0.477296626
1926.858925	-0.24203313	0.131451578	-1.841234115	0.065587258	0.236600527
16.66572903	0.087389561	0.325814325	0.268218905	0.788530824	0.903520748
14700.21746	-0.021235753	0.139945453	-0.151743074	0.879389583	0.95027878
805.7386394	-0.026532728	0.068592576	-0.386816323	0.698892192	0.856695986
1517.927322	-0.238249069	0.077788816	-3.062767653	0.002193003	0.027918062

48.47205048	-0.151349677	0.161325433	-0.938163772	0.348160253	0.605516235
2054.620032	-0.272521634	0.057798483	-4.715030924	2.41674E-06	0.000188578
6.781549326	0.002117411	0.451328747	0.004691505	0.996256734	0.998186672
2678.660577	-0.219952942	0.13018571	-1.689532146	0.091117497	0.285931737
3.462773994	-1.030620931	0.565041107	-1.823975139	0.068155831	0.241954742
14.27541964	-0.200695333	0.310807736	-0.645721808	0.518459535	0.741851962
6.524829865	0.758224327	0.742971559	1.020529411	0.307477448	0.56641723
370.82663	0.026549794	0.237975145	0.111565406	0.911168003	0.962059189
124.3890917	-0.262031686	0.102266167	-2.562251967	0.010399584	0.076720167
200.7141162	0.089563999	0.09542474	0.938582579	0.347945101	0.605319822
202.6354861	0.443994887	0.280909201	1.5805637	0.113977831	0.325141561
18.09873512	0.543810235	0.299294662	1.816972716	0.069221291	0.243608574
205.979207	0.045303986	0.083132931	0.544958364	0.585782154	0.786664905
20.22228475	0.48821558	0.267137404	1.827582262	0.06761227	0.240788111
59.83598057	0.17230313	0.317075434	0.543413684	0.586845006	0.787659342
59.63125911	0.268108965	0.159771377	1.678078829	0.093331711	0.290097884
304.4402327	-0.348805691	0.17916348	-1.946857093	0.05155187	0.203355012
809.504987	-0.089220958	0.124075168	-0.71908795	0.472086731	0.706238868
1687.060495	0.045065534	0.14892455	0.30260648	0.762189775	0.890906576
426.2645915	0.166443777	0.164392305	1.012479124	0.311309024	0.570697037
356.7710166	-0.234504579	0.112139339	-2.091189233	0.036511104	0.165407324
71.26604242	-0.116548277	0.163058913	-0.714761772	0.47475626	0.707869302
738.7369591	-0.112571899	0.105734231	-1.064668445	0.287025995	0.546523698
2674.030023	-0.326233363	0.176992567	-1.843203746	0.065299268	0.235871424
391.5088038	0.156087133	0.085898038	1.81712106	0.069198579	0.243608574
2764.291125	0.038779674	0.06496243	0.596955408	0.550537153	0.764058464
322.7223451	-0.114330967	0.172962993	-0.661014039	0.50860331	0.733916121
90.31291544	-0.862522864	0.415916297	-2.073789536	0.038098861	0.169824595
170.1662906	-0.070337601	0.220553826	-0.318913538	0.749792078	0.88518749
2.65089681	-0.983213893	0.607714826	-1.617886961	0.105686961	0.310992892
3188.138141	0.396491545	0.352943625	1.123384915	0.261274054	0.519040185
146.3530438	-0.279549628	0.120101289	-2.327615545	0.019932527	0.114425677
1385.843533	-0.204136734	0.07382091	-2.765296912	0.005687101	0.051588482
14.3487881	-0.190902042	0.341185235	-0.559526095	0.575802728	0.780746086
3.46805123	0.205528562	0.575328206	0.357237069	0.720914337	0.869287343
596.5629748	0.018191743	0.121950881	0.149172705	0.881417357	0.950743683
217.774046	-0.028978894	0.092479923	-0.313353356	0.754012218	0.886841482
106.0653708	-0.498586981	0.261508182	-1.906582717	0.056574645	0.215820017
12.28728156	-0.095356349	0.279358873	-0.341339967	0.732847663	0.875531897
425.6971192	0.210105016	0.1555653	1.350590494	0.176826648	0.420714372
3074.180597	-0.147302264	0.089905412	-1.63841377	0.101335409	0.304822506
362.6591656	-0.087109824	0.099172822	-0.878363875	0.379746282	0.631863005
347.138902	-0.097190093	0.089339875	-1.087869136	0.276652883	0.535219417
135.4537186	-0.252464757	0.144962133	-1.741591075	0.081580024	0.268408034
15.0391166	1.354790867	0.644291342	2.10276125	0.035486645	0.162364724
158.7268403	0.230998925	0.304450724	0.758739942	0.448008138	0.687884956
290.1743093	-0.317478381	0.280950333	-1.130016034	0.258469468	0.515361701
104.1742684	0.440650723	0.160936775	2.738036253	0.006180726	0.054600664

374.3084752	-0.108941281	0.113270634	-0.961778679	0.33616079	0.594703504
416.9716422	0.255308248	0.161007265	1.585693957	0.112808751	0.323444764
39.31387657	-0.18875219	0.197094876	-0.957671727	0.338228318	0.596670665
99.32630136	-0.304090805	0.141825577	-2.144118222	0.032023415	0.152568422
1189.048998	-0.08026571	0.088885539	-0.903023266	0.366513547	0.622358722
5.662207084	0.688905125	1.305610086	0.527649972	0.597742302	0.794878848
6.125701533	0.2325089	0.549577958	0.423068096	0.672245579	0.842065303
165.6571299	-0.244522297	0.221904608	-1.101925278	0.270494158	0.529443451
15.75365203	0.057998811	0.30421033	0.190653653	0.848796952	0.934780308
214.6363174	0.14344279	0.104889632	1.367559284	0.171450069	0.413377844
101864.6296	0.106284977	0.160783001	0.661046111	0.508582743	0.733916121
148.4297429	0.025619933	0.182741699	0.140197523	0.888503929	0.953660331
5.87798073	-0.001160393	0.481411434	-0.002410397	0.998076783	0.998977228
254.0893845	-0.158232501	0.12382308	-1.277891815	0.201287575	0.45009899
192.5036855	-0.178466177	0.110809878	-1.61056198	0.107275226	0.313490711
311.9098736	-0.047997123	0.132890758	-0.361177281	0.717966924	0.867400496
51.00987726	0.49797013	0.179697659	2.77115536	0.005585777	0.051082988
2412.799807	-0.201064425	0.051360336	-3.914780185	9.04866E-05	0.003071844
199.6015212	-0.115023389	0.147280065	-0.780984099	0.434811847	0.678647416
353.4117364	0.186274346	0.12835757	1.451214338	0.146720186	0.377301097
304.7739968	-0.041987413	0.11100335	-0.378253569	0.705242234	0.860311053
123.5322677	-0.05031528	0.141190956	-0.356363338	0.721568482	0.869608119
373.6559846	0.093861365	0.078643019	1.19351173	0.232669021	0.487988891
24.67951136	0.142860897	0.208653192	0.684681102	0.493545177	0.721816725
7.560533325	-0.26364163	0.68390177	-0.385496341	0.699869717	0.857032569
1954.003093	0.162294395	0.064281017	2.524763941	0.011577603	0.08157373
6.21573783	-0.08950528	0.441952603	-0.202522352	0.839508386	0.931066348
957.4138089	0.201511949	0.12303637	1.63782424	0.101458363	0.305133839
19.54899949	-0.420281623	0.271270917	-1.549305868	0.12130821	0.337939248
553.5526932	-0.347220917	0.087229566	-3.980541622	6.87584E-05	0.002496313
156.8987006	0.007867524	0.113073927	0.069578584	0.944529083	0.975750616
2.59831144	-0.890616908	0.604421805	-1.47350228	0.140615649	0.368367437
208.3225562	-0.028347747	0.096203224	-0.294665246	0.768249615	0.892934503
77.3251721	-0.44293633	0.509136843	-0.86997501	0.384314061	0.635384466
19.48736153	0.11603393	0.323175209	0.359043415	0.719562611	0.868512899
707.8009975	0.362173572	0.106579955	3.398139675	0.000678458	0.012765997
933.391561	0.016225377	0.186284365	0.087100047	0.930591988	0.970891022
2075.827421	0.161012056	0.126351241	1.274321128	0.202549632	0.45153974
35.26008361	0.043429349	0.204423288	0.212448145	0.831757428	0.928693708
269.1811937	-0.162820789	0.095009138	-1.713738198	0.086576818	0.277910523
1283.743187	-0.175219483	0.152276522	-1.150666436	0.24986949	0.505794379
240.0240429	-0.008747611	0.075899035	-0.115253253	0.90824439	0.960978931
5.596282472	0.532037008	0.454622565	1.170282887	0.241887146	0.497476791
4221.821606	-0.108535169	0.130691442	-0.830468829	0.406273765	0.654881575
330.0643086	0.057007596	0.090879318	0.627288994	0.530469816	0.750628072
1595.524949	0.027878553	0.065076993	0.428393388	0.668364738	0.839214838
1229.857647	0.18765198	0.211135496	0.888775138	0.37412394	0.627635028
116.9348352	0.065412428	0.115237062	0.567633596	0.57028379	0.777227229

808.3257908	-0.29805385	0.07686424	-3.877665983	0.000105463	0.00348229
129.3160217	-0.062001343	0.229491066	-0.270168875	0.787030337	0.903033152
224.8543047	-0.261313989	0.116245702	-2.247945388	0.024579672	0.129844252
413.5759263	0.094982931	0.10279337	0.924018064	0.355476911	0.612138993
160.0918323	0.002210387	0.119991531	0.018421189	0.985302849	0.992070995
54.53176974	0.040439431	0.237618129	0.17018664	0.864863358	0.942899493
1519.027572	-0.017234307	0.165539053	-0.10411022	0.91708188	0.96494782
1491.37912	0.177401461	0.171907732	1.031957425	0.302092063	0.560711469
4.723003525	0.786404503	0.948002449	0.829538472	0.406799777	0.655071986
496.0886864	0.074827317	0.064702635	1.156480209	0.24748479	0.502785584
5.155174889	0.113950401	0.614312497	0.185492565	0.852842711	0.936643758
222.0201485	-0.062398689	0.120710308	-0.51692925	0.605205548	0.799666707
251.2082331	0.227619242	0.197618235	1.151812949	0.249397946	0.505173367
32.35559728	0.430435382	0.320310891	1.343805015	0.179011448	0.423878692
77.54160397	0.397570375	0.229395454	1.733122289	0.083073935	0.271557233
27.90368201	0.171029254	0.1738155	0.983970099	0.325130215	0.583983313
587.6216404	-0.037888373	0.086074773	-0.440179758	0.659806919	0.834290513
54.46451574	0.488625742	0.238683532	2.047169903	0.040641405	0.176829885
3.648155387	0.208505398	0.536256458	0.388816573	0.69741184	0.855416228
88.57009678	-0.003419117	0.173705303	-0.019683433	0.984295906	0.991566382
1969.727958	-0.445073851	0.112947247	-3.940546258	8.12963E-05	0.002839757
289.1011822	-0.129764649	0.083859816	-1.547399635	0.121766909	0.338615639
73.23372191	0.254932183	0.356454636	0.715188294	0.474492702	0.707610871
19.27567467	-0.430946261	0.349082451	-1.234511389	0.217012405	0.469594725
215.6075351	-0.217981641	0.133936681	-1.627497712	0.103631434	0.308358075
737.7017949	-0.274238331	0.140586058	-1.950679425	0.051095192	0.202163723
3412.022596	-0.1177481	0.0745887	-1.578631873	0.11442052	0.325525707
65.97054471	0.105298749	0.161294246	0.652836362	0.513861779	0.738414866
96.67570152	-0.046194409	0.139013453	-0.332301719	0.739661442	0.879251937
646.909294	-0.195677367	0.132772783	-1.473776197	0.140541859	0.368270499
54.4272471	-0.034468379	0.227423461	-0.151560348	0.87953371	0.950338711
44.28223887	0.38059852	0.364906366	1.043003235	0.296946795	0.555412298
70.15043958	-0.233231835	0.281375781	-0.828898047	0.407162101	0.655228755
3.300165688	-0.307993389	0.657084536	-0.468727192	0.639264648	0.821941899
4.226503513	-0.048113463	0.518459155	-0.092800874	0.926061757	0.968928443
66.93495793	-0.11538771	0.167479249	-0.688967206	0.490843905	0.720068699
28.10070171	-0.464396502	0.21848308	-2.125549043	0.033540826	0.15677578
40.37296855	0.190950103	0.234483732	0.814342647	0.415448685	0.661679388
14.91421956	-0.079903847	0.337138698	-0.23700586	0.812652244	0.917150306
644.1418186	-0.012031181	0.157564376	-0.076357241	0.939134887	0.974295153
70.72611346	-0.135387729	0.128039596	-1.057389531	0.290333848	0.549818409
466.0533508	0.249257507	0.225408122	1.105805349	0.268810779	0.527663112
23.37239934	0.33801711	0.418115149	0.808430671	0.418842702	0.663816586
517.668395	0.27125638	0.147719685	1.836291351	0.066314578	0.238331312
12.94387125	0.354001321	0.252286666	1.403170953	0.160565867	0.398467573
19.45006783	0.647329413	0.350403633	1.847382136	0.064691776	0.23432467
10.59366181	0.718902143	0.36305476	1.980147963	0.047686905	0.194517924
352.7674831	-0.0638669	0.099017731	-0.645004689	0.518924147	0.742203467

15.57324787	0.842194071	0.318861632	2.641252466	0.008260014	0.065995956
1059.398788	-0.235579147	0.113572627	-2.074259904	0.03805518	0.169824595
5.217186773	0.86858304	0.575652158	1.50886786	0.131332555	0.353920926
48.52762376	0.190700232	0.152287783	1.252235924	0.21048391	0.461904245
3.940917908	-0.050133867	0.587314252	-0.08536123	0.931974215	0.971363874
3.026529351	0.135289636	0.699141205	0.193508314	0.846560895	0.933717375
22.8650067	0.120663944	0.224424759	0.537658789	0.590812636	0.790746649
61.96985537	-0.189425578	0.149414981	-1.267781694	0.204875921	0.454803778
123.7766095	-0.035709981	0.098985411	-0.360760038	0.718278839	0.867576843
176.1549901	0.663946078	0.446750677	1.486166919	0.137234975	0.36307079
1423.998207	-0.163536853	0.25769208	-0.634621182	0.52567551	0.747140435
30247.01461	0.415471102	0.373814231	1.11143736	0.266380139	0.525013602
101733.2545	0.687129427	0.29343518	2.341673641	0.019197494	0.111869291
626447.7277	-0.249857869	0.17134849	-1.458185416	0.144789443	0.374863969
719.0764417	0.036602613	0.156702843	0.233579763	0.81531124	0.918696852
6.028194922	1.176118569	0.494798063	2.376966803	0.017455659	0.105949906
2007.568336	0.350432137	0.193540513	1.810639702	0.070196642	0.245766866
7.659860331	0.553656171	0.367804557	1.50529992	0.132246986	0.355061731
172.3057077	-0.051082061	0.120329531	-0.42451808	0.671188022	0.841252436
386.3572219	0.268724181	0.215185974	1.248799701	0.211738343	0.462972839
16.21352818	-0.217858596	0.27706772	-0.786300894	0.431691229	0.676575305
67.76982756	0.149754469	0.270741001	0.55312815	0.580175662	0.783198895
6.501621745	0.679778317	0.514444968	1.321381994	0.186374027	0.432345842
683.4049242	0.349250876	0.200942716	1.738061889	0.082199909	0.269712004
787.9847468	-0.069075178	0.10086695	-0.68481478	0.493460807	0.721760636
4.574326188	1.361512548	0.621500866	2.190684875	0.028474604	0.142405574
61.86303125	-0.098371695	0.243503868	-0.403984119	0.686224397	0.849533779
115.0531831	-0.23602582	0.232859922	-1.013595717	0.310775702	0.570315647
328.7780782	0.195336358	0.230846795	0.846173142	0.397456162	0.647859328
216.0507865	0.157349699	0.1373548	1.145571173	0.251972631	0.508197352
6.123626517	0.569499178	1.060759575	0.536878659	0.591351433	0.79088016
249.945481	0.210920244	0.157638464	1.33799987	0.180896479	0.426147734
15.62693742	-0.311898915	0.26825215	-1.162707976	0.244948004	0.500229753
515.4659668	0.042294113	0.102099128	0.414245586	0.67869426	0.845876523
1739.300485	0.299794368	0.276063046	1.085963415	0.277495179	0.535780415
48.63468195	0.45458976	0.248904023	1.826365661	0.067795201	0.241165781
149.7808402	-0.209394027	0.083524726	-2.506970526	0.012177087	0.084788555
2.950209676	-0.541413636	0.600039995	-0.902295914	0.366899695	0.622563077
3.340062775	-0.689968316	1.011053209	-0.682425327	0.494970046	0.722843347
291.078766	0.537964635	0.180979333	2.972519716	0.002953662	0.03403765
67.3989342	-0.239134299	0.139137596	-1.71868931	0.08567096	0.276758667
112.8464773	-0.21062821	0.153993571	-1.367772753	0.171383219	0.413280198
67.4435662	0.399676599	0.133707532	2.989185373	0.002797224	0.032873837
966.187783	-0.016071973	0.095829956	-0.167713454	0.866808711	0.943902508
8.762625131	0.53306441	0.426287728	1.250480311	0.211124143	0.462597243
14.53054718	0.831921456	0.342887076	2.426225762	0.015256776	0.09847213
61.88606317	0.093365425	0.17706613	0.52729127	0.597991335	0.794956442
185.7634489	0.072222183	0.152199438	0.474523323	0.63512677	0.818918798

28.3940215	0.057899211	0.215058701	0.26922515	0.787756428	0.903200814
1762.198484	0.293781891	0.154093571	1.906516216	0.056583264	0.215820017
3.447061809	0.268322392	0.619920988	0.432833211	0.665135944	0.836844909
93.70623702	-0.230976391	0.197106836	-1.171833489	0.241263914	0.49659462
91.61741499	-1.079902479	0.498929495	-2.164439043	0.03043067	0.148503155
938.5578729	-0.134684038	0.117137025	-1.149799025	0.250226657	0.50626353
398.5617366	-0.028154747	0.057249633	-0.491789122	0.622868427	0.810950402
5.759796069	1.061362986	0.413471074	2.566958255	0.010259496	0.075936734
39.34750803	0.257340575	0.191049663	1.346982619	0.177985831	0.422383079
1503.681153	-0.063903943	0.058493512	-1.092496249	0.274615038	0.533712796
1138.096072	0.199171406	0.108129339	1.841973768	0.065478987	0.236351047
388.3505515	-0.107051	0.156798122	-0.682731391	0.49477659	0.722813893
257.5457614	0.020882121	0.176900215	0.118044632	0.906032294	0.960133527
126.6181922	0.046748535	0.162576684	0.2875476	0.77369306	0.896159371
5.423076016	0.345874891	0.447465991	0.772963528	0.439543966	0.681475638
45.77239818	0.031179092	0.249662964	0.12488473	0.900614807	0.95813193
12.29412604	-0.095130198	0.381127841	-0.249601806	0.802895302	0.910923749
6.82985209	0.015902856	0.453925917	0.035034035	0.972052602	0.986391887
727.8343428	0.093064269	0.09016688	1.032133626	0.302009523	0.560690976
294.9967704	0.055978398	0.100705461	0.555862579	0.578304801	0.782042809
2782.239465	-0.132285147	0.073019341	-1.811645308	0.070041019	0.245589839
161.4710369	0.491264683	0.20862474	2.354776728	0.018533838	0.109444546
5753.765595	0.177918288	0.159150822	1.117922514	0.263600088	0.521277253
2.88431888	0.123327884	0.601365771	0.205079653	0.837509893	0.930780365
209.5917471	-0.192070202	0.077152985	-2.489472078	0.012793297	0.087543659
1453.842866	0.112352581	0.131842002	0.852175928	0.394116455	0.644559174
1007.327493	-0.280345016	0.093789686	-2.989081508	0.002798175	0.032873837
195.9314908	0.070883715	0.104480214	0.678441512	0.497491796	0.72427934
127.4574869	0.483195791	0.121760176	3.96842226	7.23501E-05	0.00257895
3943.509104	0.205622457	0.157937943	1.301919304	0.192943973	0.440314141
3.287094995	0.819174662	0.627037068	1.30642143	0.191409282	0.438769831
94.1231582	-0.184639704	0.116160386	-1.589523851	0.111942174	0.322028806
2.735850492	-0.680328028	0.812977383	-0.836835123	0.402685252	0.652313807
96.96739804	-0.73798603	0.413938457	-1.782839978	0.074612356	0.25468527
20.07990075	0.646027693	0.284693357	2.269205363	0.02325584	0.126040739
151.3479371	0.298171222	0.134125427	2.223077521	0.026210573	0.13504817
347.7770187	-0.077769452	0.094658824	-0.821576363	0.411318047	0.65849534
72.47310543	0.976369361	0.373809809	2.611941523	0.009002965	0.069922782
3.841521346	0.622351653	0.866913047	0.717893974	0.472822664	0.706665776
431.8225895	0.236345541	0.085732068	2.756792725	0.005837134	0.052682793
110.0718619	-0.037999883	0.31540684	-0.120478943	0.904103759	0.959402122
35.93713941	-0.494231815	0.275058834	-1.796822187	0.072363853	0.249990015
23.60694816	-0.008288842	0.378439486	-0.021902689	0.98252558	0.991056093
6.100093531	1.363950132	0.544455576	2.505163307	0.012239487	0.085090474
1087.73307	0.061658892	0.15012196	0.410725337	0.681273946	0.847216981
222.9725938	-0.093076473	0.08594652	-1.082958019	0.278827058	0.5373708
645.8899077	0.076851793	0.062807696	1.223604712	0.221101381	0.474384961
89.80994995	0.074607486	0.199100063	0.374723568	0.707866063	0.861889372

3127.955591	0.126048173	0.179532101	0.702092677	0.482621369	0.714303947
358.7718975	0.207935354	0.072890929	2.852691793	0.004335064	0.043389375
1638.102365	0.039347577	0.104733139	0.375693664	0.707144649	0.861693471
53464.05618	0.02466543	0.200548511	0.122989846	0.902115139	0.958662553
495.7007336	-0.103619619	0.126117153	-0.821614005	0.411296616	0.65849534
868.8222213	-0.077823765	0.069305259	-1.122912836	0.261474516	0.519306864
42.01788753	-0.005624022	0.166297874	-0.033818962	0.973021515	0.986800481
14765.35731	0.275025559	0.165597919	1.660803231	0.096752972	0.296400619
187.3339438	0.093009401	0.134390678	0.692082236	0.488885687	0.718842604
847.9964255	-0.159144181	0.092707521	-1.716626426	0.086047451	0.277180856
462.630185	0.034145961	0.073237359	0.46623693	0.641045918	0.82343687
61.8137102	-0.019419051	0.175278462	-0.110789715	0.911783101	0.962232976
641.3867817	-0.053809296	0.098771019	-0.544788302	0.585899125	0.78675016
236.9462659	-0.054007508	0.087514747	-0.617124657	0.537152507	0.754901426
172.8296268	-0.280296197	0.098349036	-2.850014687	0.004371721	0.043534014
81.86862799	0.076711833	0.171306059	0.447805718	0.654293416	0.830926149
7.595413904	0.409991274	0.774941822	0.529060715	0.596763332	0.794156453
43.27005571	0.451269023	0.20769485	2.172750182	0.029799125	0.146590087
63.22590661	0.222861905	0.142623144	1.562592848	0.118148392	0.332622397
174.2473267	-0.194768679	0.102664737	-1.89713318	0.057810363	0.218915758
50.13166054	-0.14167362	0.167751214	-0.84454602	0.398364359	0.648613091
12.03883354	0.034366003	0.326332541	0.105309764	0.916130016	0.964528641
39.65612964	0.389971105	0.296533362	1.315100271	0.188476206	0.435868595
25.53060748	-0.28568988	0.237816626	-1.201303228	0.229633599	0.485060385
10.26787907	-0.77855928	0.344806831	-2.257957817	0.023948289	0.128061702
3904.659974	-0.120742416	0.162267079	-0.744096814	0.456817906	0.693937939
7.563089652	-0.610698116	0.557073681	-1.096260938	0.272964599	0.531855456
1361.922317	0.126891465	0.089565957	1.416737665	0.156559641	0.392186776
841.2064713	0.289671854	0.165883683	1.746234769	0.080770166	0.266470189
1534.245157	-0.26777943	0.12636467	-2.119100459	0.034081978	0.158148444
472.6723466	-0.043146061	0.157832016	-0.273366978	0.784571134	0.901845062
154.6844639	-0.489830661	0.172396438	-2.841303834	0.004492948	0.044347584
1010.024443	0.058996124	0.049029378	1.203281084	0.228867567	0.483898479
16.51205113	0.220066907	0.600794885	0.36629291	0.714146519	0.864524493
20.06011996	0.431411526	0.356347526	1.210648296	0.226030226	0.480315376
198.0645007	-0.010917508	0.116442771	-0.093758574	0.92530094	0.96872171
11.02957061	1.405351491	0.704505337	1.994806026	0.04606404	0.190624908
8.925502941	1.767702581	0.811972126	2.177048356	0.02947696	0.145474083
104.0934488	0.019426957	0.171406613	0.113338433	0.909762249	0.9616745
566.2673928	-0.112563784	0.137764337	-0.817074911	0.41388562	0.660498785
41.67424617	0.208623184	0.273085126	0.763949274	0.444897472	0.685379023
113.5704114	-0.014656953	0.178716728	-0.082012208	0.934637006	0.972907108
33.76451634	-0.018031324	0.260254013	-0.06928356	0.944763912	0.975797539
14.5659983	0.02665272	0.350211595	0.07610462	0.939335864	0.974295153
7.880756564	-0.089291961	0.595951456	-0.14983093	0.880898006	0.950743683
3609.58352	0.274437079	0.114383964	2.399261841	0.016428163	0.102164672
3024.853295	-0.209780521	0.102408807	-2.048461711	0.04051478	0.176509392
175.9243035	-0.514594447	0.075780308	-6.790609061	1.11661E-11	9.21733E-09

312.9064843	0.359299305	0.095335975	3.768769416	0.000164054	0.004773708
108.6944643	0.100680718	0.108783707	0.925512842	0.354699207	0.611365136
290.9026167	-0.210704164	0.108340181	-1.944838586	0.051794408	0.204157703
3.841365285	-0.864943172	0.67725876	-1.277123637	0.201558601	0.450512341
115.4678481	-0.363336147	0.163068351	-2.228121791	0.025872395	0.13409869
600.9270727	0.091442715	0.068427002	1.33635424	0.181433516	0.426968393
55.64002179	-0.103286233	0.13356507	-0.773302726	0.439343243	0.681440998
74.23382848	-0.010870864	0.123909789	-0.087732084	0.930089618	0.970625163
78.79408469	-0.250845787	0.362662979	-0.691677402	0.489139941	0.719061847
906.6184984	0.00262364	0.137586491	0.019069025	0.984786041	0.991899255
111.5783506	0.065491273	0.130470262	0.501963221	0.615693393	0.80702296
543.8928084	-0.270267009	0.292797433	-0.923051159	0.355980546	0.612269826
4.245065207	0.438825328	1.010469084	0.434278826	0.664085978	0.836652459
324.073922	0.126953138	0.146120158	0.868827001	0.384941762	0.636019781
287.5060974	-0.285599789	0.120542933	-2.369278582	0.017822823	0.107224073
20.20083805	0.063844931	0.2459983	0.259534035	0.795223225	0.905927111
3685.038138	-0.121845229	0.05438447	-2.240441578	0.02506227	0.131416517
364.9007971	0.030159684	0.137000945	0.220142163	0.825760439	0.924823388
130.7551824	0.423330567	0.118908335	3.560142068	0.000370654	0.00851148
479.821973	-0.082791632	0.19548862	-0.423511262	0.671922284	0.841900389
1632.907721	0.06993817	0.146056396	0.478843597	0.632049899	0.816899103
244.9589136	0.175911832	0.14727864	1.194415105	0.232315631	0.487769526
418.010656	-0.042043278	0.093534084	-0.449496872	0.653073264	0.830317856
1329.296073	0.066251015	0.147629927	0.448764124	0.653601822	0.8307205
1176.158168	-0.424332159	0.051504764	-8.238697291	1.74096E-16	6.8263E-13
4475.372081	-0.052020173	0.125537484	-0.414379608	0.678596121	0.845876523
32.78841978	-0.313505652	0.373826607	-0.838639215	0.401671798	0.651211544
135.7650763	-0.070242857	0.166563187	-0.421718978	0.673230151	0.842875465
352.4617726	-0.033002141	0.079001958	-0.417738268	0.676138489	0.84484991
11.62719559	0.192168675	0.293089063	0.655666483	0.512038739	0.736907284
84.91674095	0.074266586	0.130250766	0.570181573	0.568554553	0.775709712
256.765855	-0.056294963	0.109574359	-0.513760366	0.607419541	0.801442647
131.1998258	0.155939407	0.178835075	0.871973279	0.383222955	0.634886324
622.276395	0.04094602	0.111678213	0.366642866	0.713885429	0.864388407
266.4023641	-0.218724399	0.150467127	-1.453635773	0.146047313	0.376249353
289.948312	0.491195061	0.183325876	2.679354778	0.007376419	0.061834184
360.8374988	-0.50943087	0.630527876	-0.807943454	0.419123135	0.664126818
353.6408632	-0.161169936	0.073674741	-2.187587409	0.02869967	0.143124204
1744.574503	-0.401491429	0.112992621	-3.553253516	0.000380498	0.008654984
294.6361551	-0.007087217	0.100275117	-0.070677727	0.943654248	0.975177784
27.06115709	-0.279626022	0.435714432	-0.641764426	0.521026151	0.743835259
116.4625581	0.078795076	0.107526897	0.732794099	0.463684021	0.699296945
8.883566935	0.271984861	0.353477903	0.769453646	0.441624052	0.682858945
1903.232875	-0.14073208	0.115919904	-1.214045865	0.224730211	0.479025363
1362.80959	0.209727643	0.165293381	1.26882057	0.204505067	0.454378449
237.312286	0.323510875	0.155953302	2.074408629	0.038041377	0.169824595
747.4592637	0.324299086	0.074156471	4.373173127	1.22454E-05	0.000673881
84.22932194	0.502212937	0.198276623	2.532890302	0.011312635	0.08042945

64.58774798	-0.325882969	0.426611089	-0.763887712	0.444934161	0.685379023
3018.041548	-0.206753731	0.067244063	-3.074676359	0.002107311	0.0274901
1209.030987	-0.235244183	0.068770824	-3.420697456	0.000624608	0.012124193
8.27500407	0.028486162	0.347045082	0.082082021	0.93458149	0.972907108
4315.669447	0.039036666	0.095534614	0.4086128	0.682823836	0.847799955
6.933688921	0.358079762	0.372016679	0.962536851	0.335780001	0.59439882
448.0684165	0.254088261	0.087953997	2.888876789	0.003866205	0.04045803
25.58744852	0.445338567	0.262849062	1.694274894	0.090213059	0.284688454
1048.337523	0.433678136	0.084685062	5.121070084	3.03807E-07	3.93794E-05
296.8519962	0.23349653	0.114912082	2.031958054	0.042157903	0.180164726
170.115207	0.056824711	0.122651899	0.4633007	0.643148844	0.824387858
83.6236329	0.230826016	0.184557007	1.250703072	0.211042829	0.462548314
21.97393496	0.799228971	0.408428172	1.956840947	0.050366171	0.200645931
35.57809448	0.108869743	0.26332707	0.413439236	0.679284833	0.845954666
1275.786675	-0.119750252	0.07159921	-1.672507995	0.094424193	0.29198522
3.017979531	0.073858111	0.54032586	0.136691794	0.891274416	0.954379567
100.3171528	-0.239458925	0.234105707	-1.02286667	0.30637089	0.565441403
26.65031648	0.051900466	0.27604568	0.188014049	0.850865638	0.935636028
1251.717503	-0.109606983	0.053598	-2.044982718	0.040856562	0.177161824
314.5422686	-0.145708959	0.11785768	-1.236312803	0.216342314	0.469025809
8084.071344	-0.171110637	0.109942044	-1.556371249	0.119619838	0.334841609
13.10533367	0.546668101	0.297054103	1.840298102	0.065724484	0.236916296
30.66646186	-0.454424814	0.383916895	-1.183654117	0.236550009	0.492049116
4.645462601	-0.113460029	0.627531308	-0.180803774	0.856521601	0.938762039
205.501555	-0.090890094	0.088298105	-1.029354982	0.303312896	0.561847107
56.49169503	0.340852232	0.355928224	0.957643168	0.338242724	0.596670665
7.395578053	0.192337635	0.369764896	0.520161964	0.602950693	0.798626949
166.3393457	0.554266528	0.135668214	4.085456075	4.39903E-05	0.001832455
98.78454567	-0.125178477	0.109045923	-1.147942746	0.2509922	0.507158164
1095.890819	0.111516372	0.135088166	0.825508081	0.409083196	0.656912138
988.3817049	0.088239896	0.088408542	0.998092422	0.318234544	0.576493309
90.68869715	-0.132733326	0.126437185	-1.049796586	0.293811645	0.553105021
28.07096224	0.21950277	0.284702505	0.770989951	0.440712888	0.682478371
226.4196679	-0.236352364	0.084153018	-2.808602344	0.004975706	0.047325033
115.9536412	-0.101461925	0.120083745	-0.844926386	0.398151941	0.648386984
231.8511073	-0.058283364	0.140693542	-0.414257566	0.678685487	0.845876523
119.9011968	0.190755984	0.290854267	0.655847296	0.511922382	0.736875059
1996.197829	0.16218698	0.112637173	1.439906352	0.149893896	0.382866618
110.8703653	0.214509351	0.149522088	1.434633194	0.15139165	0.385021347
170.8974247	0.193659923	0.265067334	0.730606521	0.465019528	0.700611554
1147.932697	-0.112393923	0.082195558	-1.367396559	0.17150104	0.413437184
17.96698529	0.121619258	0.233100445	0.521746145	0.601847091	0.797917987
27.06253868	0.124505215	0.182767176	0.681223064	0.495730357	0.723170992
45.53570595	-0.554850197	0.30672327	-1.808960229	0.070457182	0.246268911
28.81727295	0.920699883	0.523605767	1.75838377	0.078682233	0.262452603
3.999245696	0.664793913	0.588537126	1.129570053	0.258657436	0.515570067
14.5223975	-0.263627107	0.415767975	-0.634072663	0.526033402	0.747513625
567.5870631	-0.196880077	0.09833082	-2.002221447	0.04526092	0.188634008

415.3832833	0.011458485	0.078871409	0.145280588	0.884489341	0.9521814
114.548442	0.466113056	0.287195674	1.622980767	0.104593516	0.30982542
71.96057318	0.601130345	0.279267126	2.152528133	0.031355782	0.150854014
138.3714609	0.01367998	0.169887855	0.080523589	0.935820836	0.973367862
90.0420836	-0.335077874	0.345389914	-0.970143772	0.331974833	0.590796923
35.07293933	0.162873852	0.204766044	0.795414361	0.426372509	0.671340873
770.0505236	-0.157853543	0.092000826	-1.715783973	0.086201589	0.277313494
149.0666745	-0.079492278	0.18706167	-0.424952251	0.670871483	0.841015774
999.3286554	-0.172100879	0.136547316	-1.260375405	0.20753397	0.457800672
836.8812816	-0.143653142	0.091265916	-1.574006474	0.115485953	0.327655876
1159.394459	-0.141970757	0.099324849	-1.429357897	0.152901393	0.386853597
16.72121619	0.360257242	0.373395226	0.964814806	0.334637578	0.592978848
6.302393225	0.793567379	0.484996447	1.636233386	0.101790752	0.305570205
28.33591775	-0.344611966	0.495243642	-0.695843291	0.486526974	0.717237435
4.861076161	-0.516395221	0.723758364	-0.713491196	0.475541859	0.708388598
8.178334674	-0.0266585	0.574599932	-0.046394889	0.96299551	0.98274378
10.08966505	-0.500836479	0.698275514	-0.717247662	0.473221295	0.706757551
2.969916559	0.707453648	0.950842416	0.744028281	0.456859366	0.693937939
3.965948911	0.232232723	1.072445257	0.216545061	0.828562905	0.926506531
6.125323619	0.465986854	0.430889038	1.081454417	0.279495027	0.537667116
152.771609	0.028042734	0.113592936	0.246870406	0.80500852	0.912199526
9.286087346	0.06282648	0.384211585	0.163520525	0.870108614	0.945862776
496.9312221	0.01831834	0.096273264	0.190274427	0.849094092	0.934802172
209.6066621	0.188371329	0.159629086	1.180056427	0.237977773	0.493559008
141.9989834	0.046974587	0.155756202	0.301590474	0.762964271	0.891042078
4.849404203	0.930628311	0.531068399	1.752369965	0.079710213	0.264363496
83.53990967	0.058532458	0.127850154	0.457820784	0.647081203	0.826964966
3026.515356	-0.206295957	0.159497984	-1.293407932	0.195870008	0.444015373
7.255253257	-0.170226668	0.389258488	-0.4373101	0.661886472	0.835495165
652.5667961	-0.344328128	0.249038056	-1.382632571	0.166777557	0.406609306
11.51277057	0.598115682	0.439347643	1.361372231	0.173396087	0.416286449
41.76983738	-0.137059162	0.457499857	-0.299582961	0.764495283	0.891638188
7.517178571	0.705630158	0.55738967	1.265954855	0.205529245	0.455428184
73.80011991	0.182299095	0.634323792	0.28739123	0.773812776	0.896159371
7.884264846	2.02051659	0.864266566	2.337839585	0.019395571	0.112494686
23.21561762	2.030283747	0.853581681	2.378546533	0.017381043	0.105701543
21.78511102	0.511850376	0.330149094	1.55036129	0.121054824	0.337297995
17.57877631	-0.402300343	0.748303129	-0.537616812	0.590841621	0.790746649
3.289882894	4.977829108	2.980177111	1.670313181	0.094857417	0.292781203
12.46365291	0.461875603	0.326704073	1.413743022	0.157437369	0.393838828
13.85647236	0.02652508	0.341989163	0.077561171	0.93817713	0.974203529
135.233964	-0.26935648	0.102478888	-2.628409475	0.008578518	0.067815264
952.4753976	-0.074059869	0.071412698	-1.037068638	0.29970387	0.558061914
26.40708364	0.608555419	0.308629028	1.971802274	0.048632183	0.196417802
63.15089088	0.128174548	0.25551006	0.501641884	0.615919451	0.80702296
227.8146851	0.035141436	0.089289363	0.393567999	0.693900012	0.85351767
4.539892534	-0.194209322	0.494455371	-0.39277422	0.694486247	0.853764093
58.3188135	-0.612323201	0.362533143	-1.689013026	0.091216934	0.286129277

112.970685	-0.576283727	0.461007803	-1.250052002	0.211280552	0.462721491
16.95663767	-0.758671562	0.579611918	-1.30893023	0.190557986	0.438182526
14.16122248	2.278558066	0.869587524	2.620274558	0.0087859	0.068864595
325.2633507	-0.070264254	0.068807631	-1.021169497	0.307174141	0.566057952
230.8816925	-0.152031231	0.096215162	-1.580117193	0.114080031	0.325255626
1312.615245	-0.278150864	0.088308718	-3.149755421	0.001634072	0.023298895
55.45537556	-1.056286483	0.393175381	-2.686552955	0.00721935	0.061072433
193.6067108	-0.471886326	0.121736873	-3.876280991	0.000106065	0.003489529
727.6600941	-0.606539303	0.218999515	-2.769591994	0.005612655	0.051207287
136.3003174	0.186491987	0.099998514	1.864947592	0.062188751	0.228700076
98.14752487	0.398327039	0.180944305	2.201379247	0.027709187	0.140182155
348.1030764	0.464561786	0.150055959	3.095923603	0.001962009	0.026144562
7.671724452	0.154222616	0.336776111	0.457938109	0.646996907	0.826964966
678.2272752	0.134776795	0.056120639	2.401554895	0.016325559	0.101910875
19.67119911	0.582998963	0.241162067	2.417457152	0.015629374	0.099687313
66.38306618	-0.300466007	0.132228844	-2.272318183	0.023067299	0.125429377
72.98479506	-0.098470251	0.158006471	-0.623203912	0.533150532	0.752219683
85.24766851	-0.153743689	0.153106891	-1.004159173	0.315301897	0.57422143
62.78544282	-0.00838551	0.275204583	-0.030470094	0.975692144	0.988070826
507.8610496	-0.036603737	0.156578313	-0.233772711	0.815161436	0.918660053
220.5053756	0.101721008	0.089364134	1.138275535	0.255005446	0.512035005
4.687289343	-0.703599365	0.45181512	-1.55727273	0.119405747	0.33448111
125.1765412	0.315379677	0.136811161	2.305218921	0.021154316	0.118602989
559.7238774	-0.119898391	0.092386995	-1.297784285	0.194361471	0.442176576
48.01795407	-0.021072672	0.14676677	-0.14357931	0.885832679	0.952712045
8.666278087	-0.179416376	0.343814047	-0.521841319	0.601780818	0.797917646
15.82824809	0.360851033	0.310573053	1.161887771	0.245281055	0.500388666
15.65130031	0.274644957	0.386612633	0.710387954	0.477463591	0.709767761
5.447423518	0.230766579	0.750641435	0.307425847	0.758519257	0.888802094
139.6447785	0.049368351	0.244794311	0.201672789	0.840172537	0.931255553
132.3168688	-0.11680315	0.129993866	-0.898528164	0.368904037	0.624001322
1794.025913	-0.33570168	0.059584016	-5.634089491	1.75986E-08	3.72994E-06
359.1397565	-0.513207468	0.118714307	-4.323046476	1.53889E-05	0.000807224
621.2849777	-0.300898725	0.08832447	-3.406742502	0.000657431	0.012528737
101.6684428	-0.577728732	0.161937842	-3.567595592	0.000360272	0.008321805
728.6265541	-0.263524022	0.081074765	-3.25038281	0.001152498	0.018295316
6.589766022	-0.02039187	0.41827073	-0.048752802	0.961116296	0.98222115
166.6260734	0.145890476	0.12964825	1.125279174	0.260470752	0.517881447
3845.547265	-0.273225389	0.073032089	-3.741169045	0.000183166	0.005102628
273.6910845	-0.157313655	0.096267218	-1.634135258	0.102230453	0.306383729
229.2386598	-0.237237861	0.168498967	-1.407948461	0.159146332	0.396513275
70.1884621	-0.200831959	0.154918817	-1.296369051	0.194848369	0.442963013
179.0137892	0.055787047	0.104693374	0.532861293	0.594129601	0.792172802
7.282943026	0.967749081	0.579723393	1.669329015	0.095052193	0.292971483
32.99385623	-0.136099937	0.23834946	-0.571010051	0.56799283	0.775517738
172.1927974	-0.194285743	0.1128864	-1.721073062	0.085237567	0.276040883
37.78232042	0.551736946	0.258427707	2.134975975	0.032762973	0.154790608
6.148685726	-0.78002339	0.497322387	-1.568446162	0.116777045	0.329886739

3.130751115	-0.072731989	0.686572743	-0.105934862	0.915634033	0.96420063
124.4364467	0.256302315	0.199393914	1.285406906	0.198650129	0.447326436
373.9750599	-0.006525224	0.079944741	-0.081621685	0.934947557	0.973012718
30.62532915	0.632139643	0.25896404	2.441032523	0.014645336	0.095548023
3.259462658	0.745514522	0.555673295	1.341641804	0.179712165	0.424747406
179.3983855	-0.128532585	0.127019212	-1.011914522	0.311578925	0.570697037
4.319679087	1.886592654	1.057511	1.783993409	0.074424736	0.254495706
46.90072387	0.074070863	0.168003791	0.440888044	0.659294049	0.834044622
2479.200307	-0.017360167	0.168201544	-0.103210511	0.917795898	0.965181096
1173.736726	0.150151564	0.090681626	1.655810231	0.097760255	0.298370621
2160.766602	-0.23868672	0.138977371	-1.717450244	0.085896938	0.277180856
41.72996503	0.193038444	0.323708181	0.59633477	0.550951607	0.764274809
2546.264302	0.155660403	0.225298399	0.690907718	0.489623535	0.7193682
563.2835301	-0.026293545	0.139687546	-0.18823113	0.85069547	0.935636028
2969.829638	-0.023487334	0.157261315	-0.149352265	0.881275676	0.950743683
13.02319378	-0.124660814	0.306647721	-0.406527768	0.684354858	0.848557456
22.91860507	-0.219266508	0.25357789	-0.864690958	0.387208433	0.638304143
17.77593853	0.416164006	0.282901882	1.471054215	0.141276456	0.369534051
1032.590067	0.057905686	0.130060909	0.445219752	0.656160954	0.832064366
13.621943	0.177387834	0.277003859	0.64038037	0.521925342	0.744256234
147.5398385	-0.233913429	0.25663309	-0.911470258	0.36204765	0.617682512
74.44432112	-0.087052888	0.132855738	-0.65524372	0.51231085	0.737095988
257.8952336	0.182119393	0.163486107	1.113974736	0.265290025	0.523372171
156.8452839	0.146641464	0.141232771	1.038296303	0.299132129	0.557460589
90.74599376	0.574136286	0.284265179	2.019720768	0.04341236	0.183624447
68.14966244	-0.097546845	0.135389568	-0.720490114	0.471223285	0.705620202
743.6972485	-0.355044812	0.398209452	-0.891603176	0.372605667	0.626450632
355.8715238	-0.071078628	0.158030377	-0.44977826	0.652870335	0.830317856
661.3971263	-0.331248206	0.157289828	-2.105973477	0.035206656	0.161613987
271.8798468	-0.169308788	0.219507904	-0.77131067	0.44052281	0.682478371
163.2825453	-0.364199086	0.099982608	-3.642624373	0.000269873	0.006837933
144.7291169	-0.022570909	0.224906108	-0.10035703	0.920060883	0.966373094
212.3044269	-0.01657899	0.101799675	-0.162858968	0.870629478	0.946199847
2173.384162	0.004640298	0.139063354	0.033368232	0.973380942	0.986906773
104.157817	0.04208735	0.167630258	0.251072512	0.801758048	0.910028457
5004.167861	-0.171197075	0.11151544	-1.535187198	0.124737852	0.343425257
3377.079445	-0.185214656	0.188848052	-0.980760215	0.326711002	0.585151858
167.5110893	0.045737095	0.14730699	0.310488287	0.756189666	0.887592168
540.9186273	0.093316492	0.080519224	1.158934316	0.24648296	0.501861449
2747.966379	-0.128972795	0.124196718	-1.038455746	0.299057928	0.557454783
38.78466359	-0.203276921	0.438168007	-0.463924608	0.642701761	0.824387858
59.2714461	0.907485946	0.341779391	2.655180416	0.0079266	0.064527019
117.3465966	-0.066368389	0.131783517	-0.503616769	0.614530707	0.806333036
67.89287603	0.082509905	0.211004392	0.391034066	0.695772056	0.854510951
71.47796623	1.548930594	0.584950011	2.647970876	0.00809765	0.065331041
538.8636833	-0.211284202	0.109073972	-1.937072588	0.052736471	0.206469997
5.031059273	1.515797248	0.698433112	2.170282627	0.029985442	0.147004578
163.4210722	-0.173939001	0.167980446	-1.035471714	0.30044867	0.558785359

143.0327407	0.231199493	0.13250555	1.744828745	0.081014684	0.266939979
1541.856176	-0.061713272	0.097565132	-0.632534088	0.527037941	0.748534191
39.39485632	0.128064788	0.256145331	0.499969247	0.617096731	0.807352781
775.7819376	0.404355934	0.192062926	2.105330489	0.03526255	0.161646902
87.26788868	0.812652277	0.328069462	2.477073821	0.01324645	0.08955057
5986.947912	0.270757743	0.119122087	2.272943232	0.023029601	0.125328336
175.7352204	-0.108046452	0.165531412	-0.652724766	0.513933733	0.738414866
6.22073855	0.42356869	0.407234771	1.040109344	0.298289103	0.556617049
517.2459432	0.063110186	0.064863651	0.972966901	0.330569753	0.589031584
79.7025019	0.181033245	0.310440934	0.58314876	0.559793162	0.770157539
348.4338467	-0.189531275	0.08777991	-2.159164594	0.0308374	0.149321945
2462.749078	0.010891181	0.121927111	0.08932534	0.928823356	0.970144322
8.916187161	1.052193059	0.553903101	1.899597704	0.057485934	0.218064314
12.18101732	1.024814158	0.410671682	2.495458546	0.012579445	0.086761662
1121.337929	-0.08064713	0.151631859	-0.531861385	0.594822004	0.792808463
14.98212977	0.470879581	0.327622616	1.437262136	0.150643523	0.383982231
31.095391	-0.2237142	0.180407451	-1.240049666	0.214957025	0.466957774
1271.441757	-0.028846037	0.108978501	-0.264694748	0.791244624	0.904476207
203.3301359	0.285910762	0.180595225	1.583157927	0.113385473	0.324077445
64.58893209	0.578662758	0.268411998	2.155875151	0.031093415	0.150112302
15.19718088	0.408889905	0.439123333	0.931150485	0.351775727	0.608637468
7.137375786	0.787651491	0.507593794	1.551735858	0.120725439	0.336914197
124.0114771	0.165437608	0.418691226	0.395130343	0.692746697	0.853431717
171.2954472	-0.038595148	0.130122476	-0.296606316	0.766767088	0.892250595
437.5870632	0.038233833	0.061148025	0.625266852	0.531795932	0.751414479
3594.108752	0.163069617	0.139965124	1.16507321	0.24398936	0.499495198
9.731299482	-0.948278263	0.401613563	-2.361170911	0.018217333	0.108474049
3.911917154	0.080824395	0.583445129	0.138529556	0.889821913	0.953820886
4.886133542	-0.078060929	0.494368563	-0.157900269	0.874535386	0.947863958
8.098758427	0.317368433	0.372880812	0.851125675	0.394699545	0.645109177
4.572651235	-0.044073852	0.562096944	-0.078409698	0.937502159	0.973825013
16.40684709	0.110717573	0.356588406	0.310491231	0.756187427	0.887592168
12.94095445	-0.184450416	0.363108875	-0.507975509	0.611470519	0.804218333
18.96346886	0.354490577	0.318261209	1.113835324	0.26534984	0.523424335
25.62664838	0.08806352	0.273516863	0.321967425	0.747477372	0.883853672
11.47321926	-0.049872989	0.318726301	-0.156475914	0.875657904	0.948599155
157.5845689	0.059377069	0.119621383	0.496375045	0.619629829	0.809092462
220.0740347	0.093520311	0.126496452	0.739311734	0.459717726	0.696168081
26.10913576	0.16374859	0.214231267	0.7643543	0.444656134	0.685379023
8217.607352	-0.11123845	0.150839393	-0.737462859	0.460840922	0.697145057
208.6838545	-0.109178479	0.109053436	-1.00114662	0.316755929	0.575732992
283.8250565	0.106098918	0.115260629	0.920513086	0.357304698	0.613393923
6.354490291	-0.308825531	0.475535827	-0.649426424	0.516062789	0.740130678
147.0944116	0.216802539	0.179544794	1.207512259	0.227234927	0.481810544
35.54074141	0.003196957	0.194257928	0.016457281	0.986869582	0.992541292
205.0132314	0.143720054	0.104578382	1.374280717	0.16935455	0.410294394
7.283592967	1.111670826	0.429488648	2.588359045	0.009643441	0.073342536
118.9572314	0.108372105	0.235495745	0.460187106	0.645381925	0.82569297

2.654561542	0.286054414	0.624783881	0.457845381	0.647063529	0.826964966
420.0099195	-0.181973122	0.111268896	-1.635435674	0.101957749	0.305814752
114.1665165	0.175162152	0.149291819	1.173287006	0.240680731	0.496110133
1629.132129	-0.146381857	0.041395944	-3.53614008	0.000406019	0.009007113
1208.154514	-0.102979795	0.094302784	-1.09201225	0.274827717	0.533865621
511.6151765	-0.160111394	0.139945893	-1.144094982	0.252584249	0.508867226
26.19502669	-0.576138771	0.471419076	-1.222137161	0.221655762	0.474989613
1611.529903	-0.185129602	0.150129401	-1.233133559	0.217525939	0.470186993
344.9728984	0.476141475	0.215021192	2.214393249	0.026801739	0.137045706
10.92264207	0.772196839	0.400779641	1.926736689	0.054012453	0.209841212
17.71263298	0.561263228	0.355486994	1.578857278	0.114368797	0.325525707
1895.030832	0.207774764	0.07251388	2.865310259	0.004166008	0.042360859
148.4854826	-0.139013716	0.17293695	-0.803840451	0.421489125	0.666126102
67.85765548	0.401063881	0.190274723	2.107814823	0.03504701	0.161148434
636.8811052	-0.172909724	0.073068451	-2.366407397	0.017961667	0.107605342
1548.576074	0.09847842	0.101072229	0.97433707	0.329889204	0.588286785
167.8394016	0.000379383	0.088948937	0.004265178	0.996596891	0.998379256
3.498631329	0.466788138	0.534161461	0.873870865	0.382188583	0.633976208
1115.388568	-0.029262764	0.081241518	-0.360194705	0.718701534	0.867702973
33.52104185	0.376137384	0.290339642	1.29550819	0.195144978	0.443123474
422.6057907	0.314586413	0.270153328	1.164473579	0.244232144	0.499749345
5.606473361	-0.137971185	0.454230242	-0.303747246	0.761320461	0.890421335
49.72625317	0.60582164	0.243273704	2.490288227	0.012763954	0.087517934
8.708688015	1.565964804	0.648220663	2.415789705	0.015701127	0.099912932
1929.976926	-0.211995688	0.138093557	-1.53515988	0.124744561	0.343425257
1427.375229	-0.221790585	0.095050278	-2.333402814	0.019627014	0.113159006
4.922729489	0.522457244	0.609996053	0.856492828	0.391725238	0.64232291
694.6723922	0.192169537	0.073946864	2.598751686	0.009356343	0.071988004
842.9209458	-0.091204804	0.08547067	-1.067088912	0.285931688	0.544879542
612.7673877	0.010007334	0.138239347	0.072391355	0.942290464	0.974860399
4.998303426	-0.05745052	0.501918463	-0.114461858	0.908871681	0.961209942
2.73025454	2.192609118	0.983980634	2.228305154	0.025860173	0.13409869
200.4585177	0.293707921	0.107393366	2.734879566	0.00624031	0.054800127
19550.10147	0.375593843	0.249567171	1.504980969	0.13232897	0.355202389
10.07377195	-0.807779091	0.421439471	-1.916714373	0.055274228	0.212882664
36.21448003	0.283174011	0.173068369	1.636197373	0.101798286	0.305570205
117.7021762	0.561425611	0.12552554	4.472600655	7.7274E-06	0.000466141
129.8802863	0.067671757	0.170388757	0.39716093	0.691248791	0.852590912
5.586240598	1.205296994	0.516416945	2.333960971	0.019597766	0.113087328
10.02698658	1.43959407	0.824002645	1.747074574	0.080624403	0.266269349
463.7044228	-0.092084505	0.165335975	-0.556953832	0.577558973	0.781775691
1384.857897	0.222097816	0.104613242	2.123037313	0.033750725	0.157289799
651.3084824	0.024678506	0.091148884	0.27074941	0.786583772	0.903033152
948.7837295	0.331175297	0.10274758	3.223193172	0.0012677	0.019531053
1514.37243	-0.113068992	0.279653807	-0.404317728	0.68597909	0.849431238
777.5820741	0.182751528	0.071002883	2.573860677	0.010057076	0.075147777
14.81867756	0.193525439	0.247812461	0.780935058	0.434840691	0.678647416
416.6609945	0.086482442	0.075245867	1.149331446	0.250419336	0.506457365

5.867704393	0.366133384	0.492644058	0.743200649	0.45736021	0.694273861
295.8810602	0.189794979	0.077672036	2.443543233	0.014543827	0.095083527
11.76620944	0.154361191	0.734237272	0.210233391	0.83348552	0.929163639
267.3018302	0.454086553	0.127124381	3.571986347	0.000354284	0.008267672
1305.740103	0.048393135	0.267493841	0.180913083	0.856435799	0.9387336
3.845457955	-0.613984501	0.655912114	-0.936077393	0.349233342	0.606632725
3.191093385	0.241667081	0.676314389	0.357329498	0.720845149	0.869287343
297.1984187	0.053002235	0.076963896	0.68866362	0.491034975	0.720179947
97.67844681	0.008753301	0.256893645	0.034073638	0.97281843	0.986785944
24.55284409	0.382701365	0.205651009	1.860926269	0.06275459	0.230124616
3.200755324	0.292454142	0.693213912	0.421881525	0.673111496	0.842875465
10.45841971	0.197850099	0.439830969	0.449832124	0.652831493	0.830317856
4129.610034	-0.163023919	0.102912333	-1.584104781	0.113169877	0.324012101
5.738690069	0.574223162	0.752232362	0.763358759	0.445249468	0.685510224
1202.623638	0.049826273	0.094655795	0.526394322	0.59861426	0.795178764
111.2246944	0.506884003	0.121814345	4.161119128	3.16692E-05	0.001426711
77.90942349	0.15433491	0.355241795	0.434450316	0.663961467	0.836652459
80.84570471	-0.126086444	0.16349185	-0.771209354	0.440582851	0.682478371
11.99984328	0.300636893	0.287457147	1.045849428	0.295630556	0.554625556
118.7002836	0.297084636	0.116001651	2.561037994	0.010435994	0.076814497
510.5815715	-0.326104945	0.274731853	-1.186993579	0.235230153	0.491127492
4.25625901	-0.108050904	0.619485764	-0.174420318	0.861535148	0.941100241
140.8339121	0.483363736	0.197773024	2.444032695	0.01452411	0.095073516
872.2963188	0.187846413	0.076797165	2.446007122	0.014444815	0.094712573
98.32950536	0.37584686	0.132943202	2.827123564	0.00469682	0.045697845
9.66176223	0.561636719	0.376749445	1.490743322	0.136028897	0.361369003
19.53762755	0.400283736	0.406954713	0.983607569	0.325308502	0.584078637
196.2939803	0.063478389	0.097754081	0.649368174	0.51610043	0.740130678
3.507880169	0.472232686	0.598551837	0.788958711	0.430136139	0.675030538
519.9360006	-0.107781171	0.14506243	-0.742998518	0.457482578	0.694284175
74.16924682	-0.137911301	0.136245034	-1.012229923	0.311428132	0.570697037
3321.132647	-0.041807679	0.112478591	-0.371694549	0.710120284	0.862535761
288.5792424	0.125588372	0.099111073	1.267147738	0.205102469	0.454803778
923.2423292	0.070179436	0.170044275	0.412712726	0.6798171	0.846546359
3790.526222	0.385567615	0.149249053	2.583383993	0.009783636	0.073977505
1003.192792	-0.173695903	0.094959298	-1.829161607	0.067375402	0.240256908
119.4837627	0.116307947	0.156605777	0.742679802	0.457675561	0.694284175
132.0003008	-0.019906275	0.105595469	-0.188514479	0.850473367	0.935532633
120.62918	-0.043188574	0.117480345	-0.367623825	0.713153747	0.864136406
15.70373439	0.404327424	0.277967099	1.454587344	0.145783537	0.375878512
575.7834484	-0.022306029	0.074752041	-0.298400265	0.765397687	0.891997126
270.2037184	-0.219309502	0.09897252	-2.215862564	0.026700916	0.136713497
26.54485412	0.415388641	0.289688827	1.433913228	0.151597026	0.385356201
714.7935326	0.230420256	0.160874639	1.432296958	0.152058853	0.386175827
14.88199283	0.141584906	0.276757874	0.511584019	0.608942172	0.802440685
57.1044	0.196623574	0.210574405	0.933748686	0.350433545	0.607449128
665.357769	0.151715606	0.083758781	1.811339716	0.070088281	0.245688304
155.3752853	-0.081927445	0.135771612	-0.603421027	0.546228638	0.76117725

275.7504689	-0.046366092	0.066230189	-0.700074886	0.483880539	0.715310442
17.3342035	0.768953736	0.332147503	2.315097141	0.020607616	0.116808766
295.6802083	-0.144971602	0.118274255	-1.225724073	0.220302528	0.473059262
14.83982278	-0.482486431	0.25298909	-1.907143235	0.056502042	0.215720064
570.7312334	-0.055979363	0.088758729	-0.630691357	0.528242351	0.748978364
735.882015	-0.056084549	0.078200845	-0.717185973	0.473259353	0.706757551
17.70076049	0.769469465	0.446500545	1.723333765	0.084828185	0.275283521
996.7054749	-0.333002715	0.509723706	-0.653300427	0.513562617	0.738142613
242.0155342	0.079538418	0.156968222	0.506716689	0.612353619	0.804570174
196.9177804	-0.138779807	0.233774746	-0.593647559	0.552747864	0.765163063
130.1859486	-0.091395593	0.12588264	-0.726038106	0.467815411	0.703067928
762.5400056	-0.358928801	0.135299198	-2.652852388	0.007981477	0.064714842
51.69610982	-0.034087145	0.302845021	-0.112556401	0.910382253	0.961925804
452.4905317	-0.287197164	0.284463892	-1.009608502	0.312682895	0.57164221
18.73301632	0.171757142	0.256125512	0.670597557	0.502476939	0.728907743
9.510696931	0.768535764	0.80285068	0.957258658	0.338436716	0.596811498
1570.73058	-0.200300662	0.066630426	-3.006144106	0.002645836	0.031725755
80.94681584	-0.985424261	0.382646746	-2.57528457	0.010015764	0.074991831
231.4075172	-0.223013502	0.140454653	-1.58779718	0.112332209	0.322736467
64.14595045	-0.166080272	0.202624911	-0.819643901	0.412419139	0.65909739
333.5758046	-0.017517555	0.062693544	-0.279415616	0.779925888	0.898777196
26.84762448	0.545013155	0.334010997	1.631722188	0.102738024	0.307097992
2472.93243	-0.326640798	0.106953919	-3.054033016	0.002257872	0.028512455
249.3478606	0.025823508	0.170770852	0.151217304	0.8798043	0.950464985
391.6027491	0.337158435	0.149663619	2.252774833	0.02427335	0.128870448
61.32975946	0.603254328	0.302127425	1.996688407	0.045859042	0.190077489
111.8075299	0.28421186	0.166224584	1.709806414	0.087301677	0.279207439
654.3133903	-0.203933162	0.105147879	-1.939489062	0.052441815	0.205778691
397.1182555	-0.139231957	0.120952878	-1.151125624	0.249680558	0.505484686
1235.955595	0.165255985	0.081506276	2.027524679	0.042608783	0.181547448
931.1578337	0.271261989	0.18384814	1.475467685	0.14008685	0.367657657
196.4343817	-0.080952119	0.103693682	-0.780685165	0.434987688	0.678705422
303.8317917	0.146300771	0.143203056	1.021631628	0.306955282	0.566053692
868.08435	0.278773485	0.094314593	2.955783149	0.003118763	0.035190414
141.9712261	2.475243866	0.96165216	2.57394926	0.010054502	0.075147777
13.46508552	0.721909244	0.323861574	2.229067296	0.025809428	0.133905081
472.0212455	-0.071164919	0.151844445	-0.46866989	0.639305612	0.821941899
503.5519643	0.099072083	0.196872482	0.503229715	0.614802775	0.80643696
38.42793975	0.508429953	0.242468852	2.0968877	0.036003518	0.163995446
42.5043949	0.596358186	0.49993128	1.19288032	0.232916248	0.488376796
965.8406112	-0.049117248	0.076279852	-0.643908532	0.519634749	0.742880209
76.15081776	0.557060483	0.232700229	2.393897445	0.016670409	0.103220963
451.4256547	-0.421413948	0.147469411	-2.857636341	0.004268092	0.04288959
78.17901917	0.327053072	0.197832785	1.653179339	0.09829437	0.299464827
16.30109254	0.057110676	0.286097607	0.19961955	0.841778137	0.93182686
423.1695667	0.08843875	0.08353165	1.058745401	0.289715745	0.549179267
2.910949379	0.234557233	0.656228958	0.357432007	0.720768419	0.869287343
5.281414039	0.808223272	1.321582037	0.611557398	0.540830632	0.757639765

90.83412944	0.147961798	0.12586463	1.175562963	0.239769563	0.494938908
560.353696	-0.252999918	0.058158858	-4.350152771	1.36043E-05	0.000730717
143.6576739	0.560036733	0.185919376	3.01225587	0.002593139	0.031392331
284.0096661	0.136755985	0.125856201	1.086605064	0.277211386	0.535573218
493.5628862	0.118812278	0.110250575	1.077656761	0.281186961	0.539664256
40.88187667	-0.169677512	0.18867928	-0.899290647	0.368497868	0.623801464
194.8642304	0.960400808	0.388365043	2.472933201	0.013400919	0.090400005
152.0578158	0.337051142	0.195497473	1.724069048	0.084695379	0.275080208
143.6442778	0.706812788	0.281462836	2.511211777	0.012031749	0.083906605
22.1674453	0.608575511	0.374845142	1.623538478	0.104474345	0.309691103
525.7298598	-0.012109109	0.122876665	-0.098546855	0.921498068	0.966898349
18.62155779	0.20531321	0.25948036	0.791247593	0.428799524	0.673472034
771.7989278	-0.074578831	0.072264732	-1.032022523	0.302061567	0.560711469
68.55821998	0.228836762	0.163414704	1.400343769	0.1614104	0.399906178
3.777886598	0.418884019	0.672201834	0.623152152	0.533184541	0.752219683
311.5154242	-0.155592437	0.09357831	-1.662697668	0.096372969	0.295780861
234.4297807	-0.239784589	0.077715611	-3.085410837	0.002032712	0.026723427
836.6252429	-0.213313176	0.114355108	-1.865357663	0.062131287	0.228587172
954.6769946	0.117737621	0.052265932	2.252664724	0.024280297	0.128870448
608.7478928	-0.289365183	0.085612995	-3.37992129	0.000725066	0.013300507
5.400114814	-0.621707618	0.409410009	-1.518545235	0.128877005	0.350373886
90.80962229	0.375708689	0.441366925	0.851238884	0.394636667	0.64507363
91.36786246	0.119259721	0.105443112	1.131033778	0.258040875	0.515162052
768.0973425	0.083193889	0.083948064	0.991016167	0.321677686	0.580374189
67.59663085	0.139013486	0.163684436	0.84927736	0.395726976	0.646182393
339.9183008	0.141374272	0.208985109	0.676480122	0.498735862	0.725282639
347.4392397	-0.13375919	0.132628854	-1.008522549	0.313203668	0.572127455
70.849768	-0.298314728	0.251454284	-1.186357709	0.235481066	0.491585922
931.0419334	-0.656248352	0.320088632	-2.050208243	0.040344114	0.175961369
876.2625416	0.026272762	0.075230519	0.349230099	0.726916571	0.87245941
481.6895309	-0.130283762	0.173030227	-0.752953775	0.451477692	0.690422789
512.3018796	0.028738216	0.095888399	0.299704825	0.764402318	0.891632155
132.8174081	0.780530436	0.339696806	2.297726743	0.021577346	0.119794372
177.9161813	0.04596069	0.102110973	0.450105297	0.652634517	0.830163809
1201.257644	-0.248135219	0.112826676	-2.199260199	0.027859426	0.140467798
671.8802091	0.023998873	0.059303301	0.404680216	0.685712586	0.849339439
377.1093445	0.115163308	0.084333308	1.365573242	0.17207295	0.414120097
973.1394743	-0.091711368	0.119715437	-0.766078048	0.443629864	0.684802994
268.2492787	0.089915676	0.170587141	0.527095275	0.598127428	0.795026486
9.542626422	1.659128495	0.781709881	2.122435106	0.033801217	0.157404479
10920.13143	-0.079853952	0.137348274	-0.58139757	0.560972535	0.770873642
347.6484948	0.211105785	0.115998624	1.819899036	0.068774382	0.242831473
14.5503775	0.003862386	0.285279763	0.013538943	0.989197817	0.993496587
1301.729397	-0.092423266	0.122382757	-0.755198432	0.450129929	0.68971096
862.1155521	0.372202364	0.169679725	2.19355827	0.028267181	0.141552513
15.32152282	0.859621033	0.425485809	2.02032833	0.043349342	0.183506365
48.62432072	0.253464589	0.2094866	1.209932226	0.2263049	0.480488162
4304.551184	-0.015889191	0.057068024	-0.278425472	0.780685769	0.899190394

54.81561827	-0.288343719	0.174066513	-1.656514595	0.097617652	0.298205055
100.0145609	0.133765472	0.11042274	1.211394248	0.225744343	0.480208093
593.093319	-0.335942875	0.066390709	-5.060088702	4.19061E-07	5.13481E-05
128.6522426	-0.227443579	0.255494187	-0.890210385	0.373352929	0.627379314
16.43645856	0.029246595	0.264203971	0.110697029	0.911856602	0.962232976
1263.412684	-0.010404835	0.088915226	-0.117019719	0.906844427	0.960313075
217.7679401	-0.100103084	0.093452337	-1.071167257	0.284094231	0.54298488
1367.886014	0.3432463	0.164338626	2.088652607	0.036739006	0.166233354
826.4636903	-0.121588238	0.090996013	-1.336193029	0.181486189	0.426968393
692.6286166	-0.030147845	0.058095592	-0.518935146	0.603805968	0.798692148
2406.144921	-0.546675532	0.144250711	-3.789759711	0.000150793	0.004522062
1253.412429	-0.119628742	0.124998199	-0.957043723	0.338545187	0.596866551
8.18530507	0.253899683	0.60347293	0.420730856	0.673951627	0.843351295
37.71575905	0.083887526	0.191952904	0.437021398	0.66209583	0.835562337
229.489802	0.219648675	0.135980128	1.615299808	0.106245785	0.311646729
6.784178497	0.796022801	0.428404608	1.858109804	0.063153423	0.230724036
15.21003682	0.968870614	0.352707606	2.746951289	0.006015207	0.053664677
835.9190937	0.005374957	0.088755874	0.06055889	0.951710514	0.978862788
610.3524367	-0.14807976	0.093537715	-1.583102176	0.113398178	0.324077445
95.96288206	0.2441529	0.247689143	0.985723059	0.324269031	0.582970596
55.44766173	0.071225711	0.224936372	0.31664826	0.751510513	0.885551531
562.1646043	-0.022658594	0.127165524	-0.178181892	0.858580131	0.939770449
2309.602512	0.321293926	0.130479926	2.462401189	0.01380102	0.092187048
46.37791343	0.14454076	0.196464846	0.735708005	0.461908417	0.698002853
981.6509975	-0.21481359	0.148624143	-1.445347882	0.148360195	0.3799287
1088.621191	-0.200070784	0.156771616	-1.276192648	0.201887427	0.450669366
726.1040123	-0.435514815	0.21027314	-2.071186152	0.038341403	0.170304891
229.0173963	-0.08631657	0.181053843	-0.476745308	0.633543496	0.817960211
477.1790308	-0.054973144	0.14917529	-0.368513739	0.712490203	0.863744352
465.4709635	0.103861706	0.280535939	0.370226026	0.711214086	0.863140543
1476.073661	-0.004919152	0.190236449	-0.025858096	0.979370523	0.989273349
31.90335469	0.68218082	0.27003843	2.526236068	0.011529198	0.081342305
5.071350283	0.283540947	0.463819977	0.611316805	0.540989868	0.757639765
53.60447782	0.221133835	0.244816136	0.90326495	0.366385294	0.622307446
333.364609	0.037253015	0.062473923	0.59629703	0.550976814	0.764274809
466.3978064	0.001709258	0.089812582	0.019031387	0.984816067	0.991899255
61.29726664	-0.039124638	0.150861733	-0.259341037	0.795372119	0.905927111
7.81788644	0.508448509	0.405502982	1.253871198	0.209888825	0.461683841
15.72138954	1.400946432	0.5204594	2.691749697	0.007107827	0.060337286
2.880551536	-0.156426022	0.573091549	-0.272951194	0.784890733	0.902022871
840.8993756	0.072733147	0.154306516	0.471354996	0.637387248	0.820756024
336.2381643	-0.112861257	0.117879312	-0.957430571	0.338349974	0.596790851
3.62712978	-0.030076037	0.525505107	-0.057232625	0.954359889	0.979940437
563.9329733	-0.420186961	0.162775926	-2.581382706	0.009840542	0.074165819
49.28338735	0.139064836	0.468707709	0.296698418	0.766696765	0.892250595
14.21560539	-0.51936534	0.596611152	-0.870525699	0.384013181	0.635122086
14.7388564	-0.036010792	0.655420573	-0.05494303	0.956183851	0.980759108
8.144371963	-0.782334125	0.772248211	-1.013060455	0.311031285	0.570618163

5.953227523	-0.615097277	0.548589929	-1.121233265	0.262188584	0.519869247
13.73669244	1.467604686	0.547720139	2.67947914	0.00737368	0.061834184
5.909376948	0.50174365	1.006902395	0.498304158	0.618269677	0.808078468
64.22343508	0.667969304	0.399868661	1.670471756	0.094826063	0.292781203
94.60812038	0.223105133	0.144272891	1.546410631	0.122005428	0.338989742
183.8923466	-0.227375207	0.129876934	-1.750697371	0.079998053	0.2650122
4.462085713	0.338230423	0.541520903	0.624593475	0.532237903	0.75157465
61.24104303	0.381215178	0.147575671	2.583184452	0.009789297	0.073977505
59.20630286	-0.122830083	0.274214173	-0.447934847	0.654200219	0.830926149
6.314597052	0.294572153	0.554875366	0.530879854	0.595502039	0.793328292
44.18459711	0.539248775	0.252963037	2.131729532	0.033029088	0.155377388
68.38143602	0.062102521	0.312015633	0.199036568	0.84223414	0.93182686
292.1407531	-0.000405853	0.120021738	-0.003381494	0.997301964	0.998575332
2455.805415	0.144737687	0.217516789	0.665409266	0.50578876	0.731718404
220.9775722	0.161338685	0.124702418	1.293789547	0.195738124	0.443909251
5.060227769	0.355164515	0.525192373	0.676256042	0.498878097	0.725354971
24.36705327	0.742581202	0.371217384	2.000394473	0.045457685	0.189163791
54.51830009	-0.142176835	0.295453162	-0.481216158	0.630362878	0.815727012
354.005264	-0.224252206	0.165248537	-1.357060157	0.174762097	0.417988969
313.3099763	-0.350393023	0.096807853	-3.619469005	0.000295208	0.007279945
272.4974098	0.092803292	0.107815807	0.860757757	0.389371475	0.640338461
281.4207738	0.064907204	0.090071523	0.720618476	0.471144283	0.705571694
598.8063424	-0.095764584	0.115550977	-0.828764816	0.407237501	0.655228755
300.685546	-0.044609809	0.089086828	-0.500745282	0.6165504	0.80702296
345.5202168	-0.12404679	0.129123131	-0.960686046	0.33671005	0.59550749
673.1168124	0.072860442	0.072770316	1.0012385	0.316711518	0.575732992
2044.376899	0.240928865	0.141518807	1.702451214	0.088670816	0.281863212
507.6155374	0.335250277	0.097103981	3.452487472	0.000555443	0.011278092
99.22622557	0.152679483	0.120561196	1.266406503	0.205367584	0.455198585
180.9892747	0.244104275	0.111706544	2.185228071	0.028872128	0.143618922
163.2868866	0.049828394	0.100354124	0.496525623	0.619523614	0.809092462
22.12390535	1.063238189	0.400448466	2.655118645	0.007928052	0.064527019
9.513789023	0.500526554	0.436955782	1.145485597	0.252008058	0.508197352
154.4031213	0.042993819	0.103991749	0.413434907	0.679288004	0.845954666
58.96267029	0.300320136	0.231751214	1.295872982	0.195019249	0.443078525
736.2597455	0.248090192	0.167996981	1.476753868	0.139741627	0.367058729
206.7652795	0.251164925	0.115347168	2.177469371	0.029445565	0.145474083
21.49779482	1.315434239	0.566265822	2.322997765	0.020179272	0.115171656
773.9550588	-0.061363983	0.114137955	-0.537629949	0.59083255	0.790746649
182.0515054	0.570895357	0.19163427	2.979088013	0.002891077	0.033662699
84.21446371	-0.049290608	0.1809173	-0.272448284	0.785277351	0.902071932
470.6880703	0.024435921	0.124092989	0.19691621	0.843893108	0.932271104
192.540268	0.188284563	0.126492574	1.488502904	0.136618317	0.362188015
2254.513468	-0.276077924	0.117858736	-2.342447688	0.019157719	0.111698763
70.80580905	-0.098237757	0.177875835	-0.552282758	0.580754642	0.783396904
3.119715687	0.789326932	0.653960164	1.206995433	0.227433903	0.482000506
10.52494172	0.476034832	0.325716772	1.461499296	0.143878469	0.373590478
1113.663449	-0.0208329	0.072661102	-0.28671324	0.774331899	0.896493493

12.12960162	-0.607723253	0.428876249	-1.417013078	0.156479104	0.392110284
244.0142709	0.077617834	0.144157845	0.538422546	0.590285365	0.790528908
3.281750537	0.598162334	0.572295613	1.045198181	0.295931384	0.554787142
168.689594	0.18537832	0.109612843	1.691209854	0.09079673	0.285542151
946.4840318	0.241551182	0.138069814	1.749485816	0.080207079	0.265338076
9.866747163	-0.059554544	0.297287575	-0.200326382	0.841225331	0.93176399
413.8781817	0.358483508	0.08043399	4.456865904	8.31666E-06	0.000494085
377.1666413	-0.189226131	0.194327737	-0.973747411	0.330181971	0.588541201
4.711176193	0.81529418	0.437657767	1.862857788	0.062482277	0.229555408
42.92972602	0.220065771	0.189867347	1.159050117	0.246435757	0.501830487
61.07329985	0.263613726	0.154352231	1.707871176	0.087660249	0.279614505
85.79222438	-0.074983207	0.224461061	-0.334058862	0.738335142	0.878437596
263.4105865	0.028500258	0.073063128	0.39007717	0.696479484	0.854807437
250.8961361	-0.151144896	0.149553879	-1.01063842	0.31218952	0.571273149
1183.482995	0.154743637	0.082243519	1.881529858	0.059899878	0.223523597
240.771526	-0.344871875	0.101484236	-3.398280242	0.000678109	0.012765997
54.25444546	0.590789365	0.229938544	2.569335933	0.010189363	0.075613821
133.1867055	0.524199548	0.323555148	1.62012427	0.105205585	0.310500845
1118.733479	0.237745496	0.086867649	2.736870386	0.006202673	0.054622525
269.114585	0.54006543	0.260349981	2.074382441	0.038043807	0.169824595
8.039209873	-0.307144436	0.333587638	-0.92073087	0.357190955	0.613393923
1180.477427	0.229391663	0.080498286	2.849646561	0.004376784	0.043555104
25.86490461	0.973072378	0.501135003	1.941737001	0.052168947	0.205067109
3.401750523	2.08431809	1.008857703	2.066017917	0.038826788	0.171634538
1687.508975	-0.065917496	0.099103326	-0.665139084	0.505961538	0.731729311
106.1396243	0.536628481	0.220745132	2.430986704	0.015057767	0.097468436
57.20561286	0.265409552	0.199429124	1.330846506	0.183239522	0.428880565
77.34371787	0.11738065	0.163092005	0.719720445	0.471697135	0.705992735
68.45616504	0.463320061	0.333578261	1.388939614	0.164851113	0.404562617
289.8090271	0.12691145	0.088247685	1.438127803	0.150397795	0.383675833
39.25488464	0.36400026	0.244454751	1.489029188	0.136479683	0.362006992
690.1010434	0.263420587	0.099791457	2.639710806	0.00829768	0.066195734
493.7463967	0.043490653	0.088136641	0.493445771	0.621697648	0.810532495
1940.575883	-0.537277648	0.247772504	-2.168431278	0.030125888	0.147378175
37.57986323	0.238617011	0.272450523	0.875817777	0.381129104	0.632955196
243.9440491	-0.350827241	0.306286251	-1.145422755	0.252034077	0.508197352
260.7851682	0.133936743	0.130591119	1.025619074	0.305071173	0.563838826
209.8700036	-0.683287132	0.255193774	-2.677522738	0.007416882	0.062040733
2.917327083	-0.765454055	0.642528535	-1.191315269	0.233529842	0.489137368
127.8771507	-0.04504204	0.134537003	-0.33479295	0.737781279	0.87841506
25.46849227	0.62626249	0.334602537	1.871660913	0.06125353	0.226633727
28.38265615	0.142920768	0.230288827	0.620615291	0.534852776	0.753066663
88.19740654	0.378720588	0.347018571	1.091355389	0.275116534	0.534091809
1027.24869	0.024420486	0.185504986	0.131643284	0.895266448	0.955795033
3147.468297	-0.338327658	0.116807584	-2.896452833	0.003774074	0.039914074
327.9826046	0.066117686	0.079187659	0.83494937	0.403746216	0.653161684
15.91917579	-0.132460058	0.357379259	-0.370642825	0.710903581	0.863055326
93.33361732	-0.072181398	0.209861508	-0.343947771	0.730885565	0.874577971

66.27534755	-0.094228832	0.138674162	-0.679498117	0.496822301	0.723707714
5.454542466	0.559209167	0.50906983	1.098492061	0.271989683	0.531045211
115.8658156	0.365682367	0.116120109	3.149173469	0.00163733	0.02331468
984.6330081	0.148959458	0.070733204	2.105933995	0.035210086	0.161613987
17.47226948	-0.050276774	0.282348745	-0.17806622	0.85867097	0.939804292
11.6007575	-0.431379236	0.345137281	-1.249877251	0.211344391	0.462721491
254.6958816	0.104434292	0.114220351	0.914322982	0.360547158	0.616530923
84.80495754	-0.14122629	0.293370065	-0.481392982	0.630237224	0.815631704
143.3768068	0.537400372	0.138037662	3.893143095	9.89537E-05	0.003309148
88.33256591	0.046586749	0.150223592	0.31011606	0.7564727	0.88766406
125.9798623	0.068388645	0.191652481	0.356836731	0.721214037	0.869515024
846.8963494	0.218566288	0.091485706	2.389075822	0.016890816	0.10397
51.78504848	0.453636862	0.150218387	3.019849115	0.002529006	0.030819686
392.0158749	0.140160866	0.113215078	1.238005297	0.215714097	0.468076909
1214.945841	0.176309109	0.149708383	1.177683611	0.238922763	0.49442766
11.0617665	-0.250219618	0.4106278	-0.609358688	0.542286714	0.758244168
194.4868536	0.401313687	0.162625223	2.467721051	0.013597624	0.091334102
31.46377482	0.614778727	0.331925419	1.852159226	0.064002962	0.232635563
298.5796714	-0.029089317	0.147358208	-0.197405476	0.843510245	0.932221964
1563.312344	-0.024784102	0.075195731	-0.329594531	0.741706356	0.879971526
7.16716938	0.430676267	0.374642437	1.149566158	0.250322604	0.50632702
95.45530625	0.289391629	0.197146284	1.467903038	0.142130563	0.370937242
71.68512254	0.155845484	0.131399926	1.18603936	0.235606756	0.491612622
43.69459698	-0.025537064	0.192739733	-0.132495065	0.894592726	0.955539338
408.5061232	0.020056973	0.106339406	0.188612798	0.850396301	0.935523059
929.5218838	-0.019971593	0.138325184	-0.14438147	0.885199248	0.952291996
195.7719423	-0.29720563	0.315815697	-0.941073017	0.346667442	0.604461608
3.017536619	-0.548857445	0.833148811	-0.658774805	0.510040389	0.734975511
214.9624598	0.354308472	0.146148235	2.424308932	0.015337552	0.098787387
574.9673486	-0.238320748	0.194650704	-1.224350814	0.220819916	0.473910722
2191.728848	-0.222444167	0.098736754	-2.252901361	0.024265369	0.128870448
971.06967	0.027097172	0.115905879	0.233786003	0.815151116	0.918660053
262.2543036	0.047141699	0.105894019	0.445178108	0.656191046	0.832064366
262.8111124	0.045122912	0.147298113	0.306337342	0.75934781	0.889224856
3.982041203	-0.536608638	0.59681244	-0.899124418	0.368586393	0.62381666
308.801738	0.117896476	0.098716448	1.194294149	0.232362926	0.487802942
91.22721842	0.465468214	0.276659906	1.682456341	0.092480384	0.288476998
388.0103153	-0.120283046	0.109875983	-1.09471645	0.273640879	0.53261151
32.73637494	0.131344922	0.191661624	0.685295884	0.493157229	0.721618408
979.210643	-0.005321967	0.149798945	-0.035527402	0.971659197	0.986184096
27.12960283	0.432351839	0.247094213	1.749744901	0.080162342	0.265301999
285.3523899	0.082050803	0.087819319	0.934313807	0.350142044	0.607212276
113.3709742	-0.170229495	0.124790765	-1.364119331	0.172530012	0.414706621
98.80684992	0.452770092	0.139208538	3.252459208	0.00114411	0.018217486
92.12670582	0.290033638	0.163166298	1.777533975	0.075480426	0.256528242
323.1079399	-0.873107257	0.396405853	-2.202558944	0.02762585	0.139814081
188.5865281	0.157633115	0.102463277	1.538435228	0.123942234	0.342057013
454.0213527	-0.046699206	0.098299699	-0.475069671	0.634737315	0.818618539

348.3248671	0.077858654	0.114040859	0.682725952	0.494780028	0.722813893
3108.736582	-0.808348404	0.491863999	-1.643438848	0.100292168	0.302904364
138.2059126	0.109991212	0.157070838	0.70026501	0.483761819	0.715310442
3589.023677	-0.065364854	0.187352542	-0.348886935	0.727174194	0.872609033
1020.042199	0.124430489	0.087574139	1.42085884	0.155357801	0.390299781
23.83361936	0.174026054	0.271645614	0.640636348	0.521758978	0.744256234
85.81600585	-0.141031126	0.151223699	-0.932599364	0.351026862	0.608090959
80.76297854	0.075076355	0.126923061	0.591510752	0.554178253	0.766061319
74.04563011	0.051946679	0.272740764	0.190461736	0.848947325	0.934802172
1954.563841	0.006123494	0.163100793	0.037544233	0.970051072	0.985572392
793.5876172	-0.172082446	0.0726265	-2.369416759	0.017816165	0.107224073
266.9577569	-0.287935186	0.125639823	-2.291750977	0.021920017	0.121054065
119.0189758	0.123447763	0.168468172	0.732766083	0.463701111	0.699296945
188.9867312	0.470747197	0.193959061	2.427044116	0.015222405	0.098331217
333.2335329	0.008869354	0.127594022	0.069512302	0.944581841	0.975750616
24.61877014	0.331628997	0.308260064	1.075809147	0.282012621	0.540466121
64.92668025	0.078271727	0.133984674	0.584184181	0.559096404	0.769671552
87.44687963	-0.176254334	0.26897149	-0.655290023	0.512281043	0.737095988
81.39494986	0.089320343	0.148930431	0.599745412	0.548675918	0.762847578
4883.700834	-0.206724342	0.126771505	-1.630684614	0.102956885	0.307225389
721.9625762	-0.189417988	0.14026062	-1.350471627	0.176864749	0.420741198
31.4268917	-0.236458018	0.175158883	-1.349963038	0.177027839	0.420937634
760.9365711	-0.095176319	0.063679737	-1.494609175	0.13501647	0.359707545
42.52116108	0.528236303	0.342952868	1.54025918	0.12349719	0.341461865
1626.906223	0.135931591	0.125237876	1.085387223	0.277750189	0.535953982
17.20618016	0.180761056	0.3267261	0.553249513	0.580092567	0.783198895
206.8905418	0.721443649	0.294674811	2.448270508	0.014354384	0.094297412
1039.506303	0.139313098	0.131683239	1.057941004	0.29008234	0.549474809
87.4240655	-0.064951034	0.243356267	-0.266896903	0.789548541	0.90381326
42.83923084	-0.029187262	0.23975064	-0.121740081	0.903104869	0.958861141
49.60733896	-0.171810193	0.149866447	-1.146422007	0.251620581	0.507898255
889.2604276	-0.157474021	0.082004268	-1.920314939	0.05481813	0.211695407
573.3122369	-0.041105693	0.10114056	-0.406421451	0.684432961	0.848557456
6.407046301	-0.604441798	0.473654494	-1.276123854	0.20191174	0.450669366
17.60973392	0.15697732	0.241776394	0.64926653	0.516166115	0.740130678
8.20839957	0.409281068	0.4550668	0.899386788	0.368446674	0.623782128
3.516022785	1.537005322	0.589118632	2.608991192	0.009080958	0.070368451
129.1772755	-0.225181257	0.130955005	-1.719531505	0.085517636	0.276377211
6.68970935	-0.260148976	0.665082957	-0.391152672	0.695684389	0.854510951
84.45176345	-0.037475491	0.146620307	-0.255595501	0.798263193	0.907769715
30.75124088	0.416904793	0.37742992	1.104588618	0.269337885	0.528147648
2023.451229	-0.228555841	0.155764233	-1.467319138	0.14228926	0.371078276
506.6237998	-0.044878359	0.081233065	-0.552464187	0.580630364	0.783396904
169.8585723	0.077187931	0.123167186	0.626692333	0.530860931	0.75083622
6.091697476	-0.272803206	0.472783504	-0.577015068	0.563929276	0.772932515
44.34339283	0.210855228	0.935865879	0.225304963	0.821742039	0.922100747
162.5001025	-0.202895073	0.120765844	-1.680070015	0.092943694	0.289519146
3.643487558	0.990374711	0.632363556	1.566147672	0.11731405	0.33099428

113.9252785	-0.032633883	0.102775885	-0.317524706	0.750845496	0.885437575
763.558452	0.083941246	0.218402985	0.384341113	0.70072564	0.857636942
40.82503056	0.433906784	0.357748347	1.212882708	0.225174668	0.479516564
2048.584866	-0.290488098	0.058119633	-4.9981062	5.78961E-07	6.58002E-05
72.68312756	0.645114353	0.248169129	2.599494767	0.00933611	0.071883924
2804.843258	-0.280079918	0.099431091	-2.816824342	0.004850104	0.046468286
227.011946	-0.061643412	0.113822578	-0.541574554	0.588111625	0.788573372
100.5556127	-0.164282199	0.186241068	-0.882094378	0.377725772	0.630440674
67.10988874	-0.670237528	0.401927441	-1.667558521	0.095403398	0.293566195
392.0729202	-0.077566372	0.142069192	-0.545976023	0.585082419	0.786366419
33.50609642	-0.019807052	0.465341778	-0.042564526	0.966048674	0.983885343
27.61248571	0.182266255	0.335438921	0.543366447	0.586877523	0.787659342
603.6506829	0.088534076	0.073710725	1.201101687	0.229711759	0.485070119
114.0143399	-0.105695449	0.256220508	-0.41251752	0.679960142	0.846657262
6.443456668	0.274576235	0.399371137	0.687521478	0.491754171	0.720810506
1380.052913	-0.11103188	0.117389551	-0.945841249	0.344229563	0.602017893
777.8756305	-0.186829825	0.07128442	-2.620906862	0.008769622	0.068854114
12.39073016	-0.262998546	0.28498293	-0.922857187	0.356081634	0.612269826
378.3157174	0.481586646	0.124493969	3.868353233	0.000109573	0.003558055
3.976090441	0.57681687	0.529615825	1.089123178	0.27609957	0.534794106
5204.67294	-0.080366238	0.10831542	-0.741964888	0.458108609	0.694415566
27.84407331	0.171206033	0.247790908	0.69092944	0.489609884	0.7193682
22.89591857	-1.167227587	0.574010705	-2.033459614	0.04200611	0.179882722
1437.588062	-0.12803901	0.142911888	-0.895929735	0.370290292	0.62480271
3516.368238	-0.332666262	0.082055158	-4.054178552	5.03108E-05	0.002030194
97.19946528	-0.185732506	0.219818424	-0.844936027	0.398146558	0.648386984
42.96506956	-0.491754039	0.508854828	-0.96639358	0.333847276	0.59218136
516.6572634	-0.164546837	0.091037328	-1.807465576	0.070689717	0.24687097
5.698930197	-0.6607529	0.505812162	-1.306320704	0.191443519	0.438775498
168.764156	-0.058264075	0.086714514	-0.671906834	0.501643009	0.72809263
503.156438	0.068140943	0.205894217	0.330951223	0.74068133	0.879520987
71.18200506	0.358589664	0.281529921	1.273717773	0.202763455	0.451724721
9.94174074	1.008499131	0.438720269	2.298729285	0.021520316	0.119707191
218.4363898	0.488280303	0.138501635	3.525447935	0.000422767	0.009273681
299.7428125	-0.277511533	0.102616863	-2.704346284	0.006843893	0.058559526
209.9658976	-0.10196233	0.110372147	-0.923804902	0.355587903	0.612188877
34.00417601	-0.093757611	0.324514996	-0.288916113	0.772645573	0.89579137
314.0156091	-0.051169755	0.094670497	-0.54050371	0.588849703	0.789276572
204.7495236	-0.127324378	0.185698767	-0.685650099	0.492933781	0.721493215
90.5334642	-0.148160977	0.119263835	-1.242295934	0.214127395	0.46592315
21.17648882	0.293927944	0.280486142	1.047923231	0.294673978	0.553891021
166.3016357	0.299065282	0.206030168	1.451560635	0.146623811	0.377176948
43.95517855	0.378445024	0.250862812	1.508573637	0.131407775	0.35401991
15.89470597	0.1576045	0.257513001	0.612025408	0.540520947	0.757542051
1310.936141	-0.121887794	0.065579537	-1.858625425	0.063080251	0.230617926
40.44385873	0.181597572	0.422200833	0.430121303	0.667107405	0.838599606
156.9239398	0.124018526	0.302722953	0.409676651	0.68204316	0.84736731
6.472129732	0.238214684	0.456470303	0.521862391	0.601766145	0.797917646

130.443603	0.897612975	0.757482109	1.184995611	0.236019182	0.492010908
58.21857375	0.544981437	0.301851982	1.805459195	0.071002855	0.247029455
7.75473882	-1.014112512	0.531852923	-1.90675367	0.056552493	0.215807617
6.9628266	-0.105237503	0.416329646	-0.252774463	0.800442503	0.909061565
1015.209876	-0.124069405	0.063932022	-1.940645723	0.052301264	0.205329919
59.35909436	-0.039911479	0.153396852	-0.260184471	0.794721483	0.905927111
608.21648	-0.135560063	0.145607802	-0.9309945	0.351856409	0.608637468
383.5029457	-0.024502143	0.070202995	-0.349018483	0.727075433	0.87255728
64.76436039	0.124809675	0.146489094	0.852006601	0.394210428	0.644645642
472.977874	0.133864312	0.21390339	0.625816691	0.531435183	0.751377392
7.295859891	0.136100759	0.452302497	0.300906495	0.763485798	0.891135578
19.7682437	0.829852693	0.331506393	2.5032781	0.012304881	0.085389239
120.9209382	-0.017476491	0.108318287	-0.161343862	0.871822579	0.946470917
11.61519838	-0.072068042	0.374498497	-0.192438801	0.847398501	0.934372901
201.152351	-0.068492158	0.11211918	-0.61088708	0.541274339	0.757650127
14.43645456	0.143889883	0.353407104	0.40715051	0.683897447	0.848557456
6.394979434	0.84936445	0.81008475	1.048488383	0.294413652	0.55353437
903.9602202	0.023493065	0.188913179	0.12435906	0.901030987	0.958216029
2512.468243	-0.143898906	0.145718018	-0.987516218	0.323389636	0.581789752
189.3254384	-0.174991132	0.122638745	-1.42688293	0.153613642	0.387842299
309.989069	-0.170939329	0.138455916	-1.234611955	0.216974957	0.469594725
36.77591624	-0.129703308	0.159858893	-0.811361231	0.417158256	0.662355886
228.4737641	-0.099942392	0.071763818	-1.392657105	0.163723506	0.402987989
28.38212764	-0.176714756	0.286244117	-0.617356815	0.536999399	0.754870587
1014.147849	-0.12841384	0.092660968	-1.385846083	0.165793908	0.40553831
462.6350403	-0.477136916	0.563603647	-0.846582377	0.39722794	0.64762111
87.69581204	0.24212178	0.59821598	0.40473974	0.685668827	0.849339439
232.5512139	-0.234094415	0.129171016	-1.812282825	0.069942507	0.245299257
1657.640574	-0.325376354	0.110185844	-2.952977822	0.003147246	0.035409901
180.7399754	-0.061692497	0.152749948	-0.403879008	0.686301694	0.849562413
14.73556076	0.180788064	0.36394141	0.496750462	0.619365033	0.808970784
1869.99738	-0.476145574	0.131362559	-3.624667315	0.000289334	0.007189519
8.682715099	-0.816100753	0.487733769	-1.673250458	0.094278001	0.291698919
249.8220249	0.00158295	0.113859394	0.01390267	0.988907631	0.993404681
12.75073244	-0.535614418	0.293091779	-1.827463124	0.067630166	0.240797167
141.7093717	-0.224204894	0.09287064	-2.414163328	0.015771391	0.100064116
48.84399959	-0.20421033	0.278105529	-0.734290797	0.462771525	0.698835799
281.9803669	0.08352847	0.141635643	0.589741881	0.555363713	0.766909874
1127.795489	-0.390585498	0.096386217	-4.052296158	5.07174E-05	0.002030194
98.09630039	-0.151781545	0.209862312	-0.723243461	0.469530322	0.704600003
254.517707	0.554989481	0.118929618	4.666537138	3.06318E-06	0.000227692
5.974896322	-0.486519275	0.414855767	-1.172743189	0.240898806	0.496110133
1029.76928	0.12932235	0.130543779	0.990643532	0.321859673	0.58056896
90.71858517	-0.19255278	0.121244984	-1.588129868	0.112256976	0.322584884
4.397666966	1.157662643	0.530336768	2.18288211	0.029044493	0.144157627
52.44426502	-0.077493239	0.200871028	-0.385786045	0.699655132	0.856961428
204.7037862	0.366757249	0.111682399	3.283930611	0.001023701	0.016829906
73.33983363	-0.510504283	0.232238651	-2.19818829	0.027935691	0.140610839

21.37149359	0.247276598	0.276716826	0.893608828	0.371531221	0.625604127
71.80787888	-0.043344094	0.245668955	-0.176432932	0.859953835	0.940452149
410.7470571	-0.531221515	0.159569179	-3.329098502	0.000871276	0.014901952
65.94740801	-0.226262189	0.214838535	-1.053173207	0.292261616	0.551805815
286.6232723	0.092501016	0.209840358	0.440816139	0.659346109	0.834044622
568.0103677	-0.087044896	0.096134497	-0.905449127	0.3652275	0.621215498
2.765349749	-0.534849024	0.825275565	-0.648085375	0.516929733	0.740751569
100.7407112	-0.26534996	0.11273219	-2.353808256	0.018582194	0.109647526
716.9150758	0.1016319	0.124909564	0.81364386	0.415849004	0.661828708
70.27449954	-0.38268753	0.141310283	-2.708136456	0.00676622	0.058053276
42.25803015	-0.108354497	0.159776103	-0.678164601	0.497667335	0.72436479
369.7260941	-0.463478516	0.147206284	-3.148496814	0.001641125	0.02331468
1813.810243	-0.408377843	0.110338081	-3.701150498	0.000214624	0.005783788
20.30935221	-0.278793896	0.4956911	-0.562434742	0.573819854	0.779540112
122.5152212	0.122319488	0.138805855	0.881227152	0.378194886	0.630630602
191.8631985	-0.87618155	0.338701812	-2.586881789	0.009684881	0.073451487
144.9283555	0.43448316	0.180371421	2.408824836	0.016003977	0.10072487
114.9244485	0.085751287	0.162903412	0.52639344	0.598614873	0.795178764
215.1163907	-0.157639147	0.110129104	-1.431403149	0.152314706	0.386376675
21.80092091	0.323379428	0.272602039	1.186269293	0.235515969	0.491593354
2714.264341	-0.312865258	0.118435823	-2.6416438	0.008250477	0.065953358
477.3654215	-0.062670648	0.143359719	-0.437156607	0.661997777	0.835562337
4956.326225	-0.038737405	0.075405027	-0.513724431	0.607444669	0.801442647
920.0469493	-0.104239884	0.085453204	-1.21984758	0.222522661	0.476160216
88.51166609	0.157862348	0.159731423	0.988298642	0.32300641	0.581433782
94.509714	-0.160521808	0.148859927	-1.0783413	0.280881473	0.539407985
1154.962508	-0.180798324	0.12929702	-1.39831779	0.162017658	0.400738834
2403.348326	-0.095138867	0.161076864	-0.590642659	0.554759876	0.766392486
4.230231016	0.574752533	0.58289914	0.986023986	0.324121343	0.582873043
516.0578236	-0.43418931	0.35402903	-1.2264229	0.220039573	0.472883072
192.4292299	0.026591422	0.102931608	0.258340685	0.79614399	0.906411319
147.4218943	-0.193508476	0.104377741	-1.853924748	0.063749927	0.232200151
317.1273053	-0.245239552	0.120368153	-2.03741227	0.041608745	0.179037466
3008.009155	-0.100393611	0.139307513	-0.720661856	0.471117586	0.705571694
149.3485366	0.684211423	0.412955341	1.656865416	0.097546688	0.298060108
67.70236635	0.031161989	0.199698828	0.156044925	0.87599761	0.948628307
10.26092599	0.28085828	0.312496354	0.898756984	0.368782117	0.623945925
12.25778269	-0.188904352	0.458868367	-0.411674384	0.680578103	0.847023248
144.0092946	-0.322178894	0.130798095	-2.46317726	0.013771181	0.092075497
124.0860028	-0.131481918	0.18372339	-0.715651492	0.47420657	0.707453233
882.2349769	-0.110250877	0.084610712	-1.303036854	0.19256218	0.440060737
379.525866	-0.19515305	0.181413556	-1.075735763	0.282045448	0.540466121
4.80639173	-0.175052137	0.448166626	-0.390596101	0.696095808	0.854510951
1492.715528	-0.25549584	0.084182406	-3.035026577	0.002405145	0.02979644
206.5842915	-0.278985582	0.114727837	-2.431716576	0.015027461	0.097352624
82.93520671	0.096292253	0.153710117	0.626453579	0.531017476	0.750982442
3.298418451	-0.172320037	0.592511321	-0.290829949	0.771181379	0.894957138
2.888952287	0.44351983	0.976357623	0.454259607	0.649641985	0.828305414

449.9864462	-0.439407228	0.092488188	-4.750955091	2.02458E-06	0.000166249
37.29671429	-0.417737254	0.19125152	-2.184229722	0.028945372	0.143846392
141.7569991	0.492486592	0.140592187	3.502944245	0.000460146	0.009832325
1497.993654	-0.320515087	0.144438732	-2.219038356	0.026484115	0.135913723
50.44670707	-0.071414176	0.136656175	-0.522582869	0.601264564	0.797617646
291.2592431	-0.113828661	0.09271703	-1.227699601	0.219559756	0.472408814
358.6089533	0.264578515	0.078454663	3.372374618	0.00074523	0.01346783
852.0780029	-0.10895203	0.099553994	-1.094401391	0.273778974	0.532681978
77.18536017	-0.071070477	0.235636778	-0.301610292	0.762949161	0.891042078
5.796631878	0.799555375	0.776418752	1.029799156	0.303104297	0.561659711
21.87918436	-0.185425384	0.24766898	-0.748682313	0.454048707	0.692194782
185.5248558	-0.161245684	0.114443827	-1.408950465	0.158849816	0.39593927
537.3084761	-0.208767888	0.129195779	-1.615903316	0.106115219	0.311483045
339.1177139	-0.059021702	0.099044487	-0.595911025	0.551234667	0.764350148
590.5321609	-0.218652038	0.15388515	-1.420878087	0.155352205	0.390299781
82.01595797	0.171850893	0.172358038	0.997057608	0.318736548	0.577074358
5.89052946	0.14695359	0.947931094	0.155025604	0.876801132	0.948920022
4.386500865	-0.405770018	0.530616252	-0.764714644	0.444441484	0.685275288
1603.083612	-0.349447229	0.128074843	-2.728461109	0.006363059	0.055474276
479.0923081	-0.331085319	0.105092532	-3.150417178	0.001630375	0.02328425
189.3682802	-0.074068715	0.184334177	-0.401817591	0.687818275	0.850202389
35.89608693	0.220614878	0.221022351	0.99815642	0.318203515	0.576493309
34.58870073	-0.102839098	0.227860709	-0.4513244	0.651755762	0.8296516
12.34339828	0.798768841	0.421627364	1.894490041	0.058159995	0.219643959
1359.211222	-0.182920566	0.167508534	-1.092007447	0.274829828	0.533865621
468.4205318	-0.150172517	0.070185929	-2.139638504	0.032383992	0.153679436
427.2112703	0.128280785	0.14839912	0.8644309	0.387351225	0.638304143
61.068463	0.276008142	0.192221347	1.435887046	0.151034485	0.384674386
1339.838711	-0.187722996	0.103335943	-1.816628265	0.069274052	0.243608574
662.2229368	-0.12293054	0.166667374	-0.737580113	0.460769644	0.697145057
101.0616244	-0.336452627	0.16546268	-2.033404923	0.04201163	0.179882722
13.61029111	1.290491266	0.499223664	2.584996186	0.009738007	0.07377243
16.93375799	0.163106484	0.297811519	0.547683598	0.583909176	0.785490309
627.776229	0.079540491	0.062015095	1.282598871	0.199632646	0.448251742
32.09747641	0.560964102	0.192925582	2.90767091	0.003641313	0.038834298
42.67261084	0.153143053	0.186455921	0.821336496	0.411454625	0.65849534
8.77231802	-0.139607692	0.383445049	-0.364087872	0.715792393	0.86551272
621.2104002	-0.553981957	0.113427425	-4.884021257	1.03944E-06	0.000102342
979.7050781	0.164618595	0.135008062	1.219324179	0.222721175	0.476210832
199.0780586	0.124558781	0.428924182	0.290398131	0.771511674	0.895065396
232.3835613	-0.348529547	0.106526035	-3.271778091	0.001068734	0.017309476
536.7436677	0.094180954	0.076499111	1.231137891	0.218271296	0.470983132
505.0175442	-0.103868457	0.097871116	-1.061277951	0.288563603	0.547987837
878.6621855	-0.313064259	0.101910873	-3.071941683	0.002126713	0.02764709
37.96606839	0.104535	0.187541621	0.55739627	0.577256714	0.781636389
324.5059631	0.25374855	0.105564991	2.403718772	0.016229253	0.101671801
122.8197052	0.166039651	0.130184236	1.275420562	0.202160425	0.450893643
168.3728105	0.005796356	0.092799836	0.062460839	0.950195847	0.977878718

128.941613	0.097349056	0.154192741	0.631346555	0.527813953	0.748800234
367.0404275	-0.049891226	0.126590356	-0.394115539	0.693495739	0.85351767
4.827460359	-0.07107768	0.453608654	-0.156693835	0.875486146	0.948507163
33.16052664	-0.660461544	0.334687095	-1.973370216	0.048453398	0.196225524
94.8054838	-0.144149077	0.11079343	-1.3010616	0.193237371	0.440641269
66.00878076	0.064773547	0.188858778	0.342973452	0.731618433	0.874922414
5.552228573	-0.272518023	0.495986088	-0.549446908	0.58269879	0.784669685
137.0622413	-0.38403309	0.114651932	-3.349556214	0.000809411	0.014136756
174.5454877	0.032066131	0.132501249	0.242006254	0.808775317	0.914485767
2488.259783	-0.108654277	0.124527095	-0.872535227	0.382916459	0.634646702
1114.897442	-0.193607811	0.1269385	-1.525209545	0.12720684	0.347641067
558.786623	-0.312383127	0.13673189	-2.28463986	0.022333954	0.122864163
221.4668645	-0.042081103	0.151704754	-0.27738816	0.781482072	0.899579069
77.98909643	-0.211504363	0.121882838	-1.735308812	0.082686122	0.270740946
60.24796963	-0.47164197	0.240887212	-1.957936933	0.050237413	0.200439665
8.312128436	0.682192845	0.510078211	1.337427928	0.181082993	0.426506332
10.61925761	0.611463214	0.495188009	1.234810221	0.216901143	0.469594725
500.9724017	-0.205946695	0.124230807	-1.657774748	0.09736294	0.29778478
61.28816901	0.262350079	0.173112914	1.515485315	0.129649546	0.351544296
218.4733043	-0.057833199	0.239699267	-0.241273992	0.809342767	0.914936815
722.5713179	-0.063474425	0.083147637	-0.763394213	0.44522833	0.685510224
16.31447078	-0.076892185	0.24008802	-0.320266646	0.748766205	0.884777305
364.3965934	-0.423596402	0.132257719	-3.202810431	0.001360935	0.020643046
1372.127985	0.184502382	0.100649951	1.833109512	0.066786287	0.239172735
641.3387294	0.032020347	0.111377115	0.287494854	0.773733442	0.896159371
34.34396875	0.357968074	0.256176465	1.397349573	0.162308475	0.401141842
80.2782789	0.141586561	0.124622466	1.13612389	0.255904718	0.512852678
310.1055362	-0.148344282	0.077716768	-1.908780897	0.056290362	0.21543632
90.13607133	0.20849769	0.185168207	1.125990759	0.260169431	0.517571496
263.1026909	0.324519158	0.178104797	1.822068597	0.068444578	0.242103013
8.835622736	0.455497734	0.360666431	1.262933544	0.206613064	0.456725202
10391.17413	0.145315546	0.113341454	1.282104136	0.199806118	0.448315412
268.5414531	-0.181413701	0.161507156	-1.123254877	0.261329262	0.519084112
52.31466383	0.112256119	0.333673691	0.336424841	0.736550519	0.877682597
198.9633719	-0.137962886	0.103049457	-1.338802657	0.180634926	0.425962739
324.466342	-0.061614228	0.118674785	-0.519185501	0.603631389	0.798632907
424.9969252	-0.170296986	0.090737914	-1.876800768	0.060545405	0.224862452
599.9726075	-0.201304656	0.10224371	-1.968870821	0.048967927	0.197381899
2.606375946	-0.703931226	0.720182185	-0.977434932	0.328353876	0.586744252
751.4430141	0.109167437	0.13071437	0.835160185	0.403627524	0.653161684
637.4467235	-0.146741147	0.117094296	-1.253187831	0.210137357	0.461824914
365.8629528	-0.251701736	0.095732394	-2.629222214	0.008558042	0.067722339
5.047606295	0.670618145	0.551678098	1.215596826	0.224138544	0.478092671
70.21446273	0.173705683	0.356561263	0.487169252	0.626138394	0.813212534
14.06151801	0.249659272	0.299506519	0.83356874	0.404524047	0.653606177
5.40773308	0.521806092	0.616955964	0.845775261	0.397678129	0.648090702
465.4293956	-0.101592539	0.128055293	-0.793348998	0.427574517	0.672443858
47.02930433	-0.0134453	0.247994329	-0.054216158	0.956762947	0.9810323

533.2296116	-0.008478344	0.062973518	-0.134633488	0.892901663	0.954945086
7.304292297	0.178755871	0.571834006	0.312600982	0.754583832	0.887201677
591.2191436	0.186375255	0.168637951	1.105179784	0.269081695	0.52805889
275.8207795	0.690792246	0.297360049	2.323083577	0.020174663	0.115171656
1091.08352	-0.160588334	0.094246818	-1.703912519	0.088397432	0.281339109
499.8073383	-0.145294737	0.155418896	-0.934858893	0.349861024	0.606859136
3.984417431	-0.389857743	0.539967385	-0.722002391	0.470293008	0.705306146
142.040501	-0.566204743	0.356265491	-1.589277541	0.111997747	0.322129592
154.758912	-0.190111031	0.133078321	-1.428565	0.1531293	0.387180387
248.4058551	-0.175220934	0.112657736	-1.555338685	0.119865429	0.335349516
3.91998534	0.308060871	0.818363042	0.376435463	0.706593186	0.86122222
1223.75747	-0.232790132	0.080726348	-2.883694591	0.003930397	0.040851128
1278.442473	0.233182648	0.12044368	1.936030584	0.052863955	0.206917463
41.83053815	0.347900261	0.180108086	1.931619336	0.053406507	0.208416935
181.9793655	-0.78650841	0.457288695	-1.71993845	0.08544363	0.276308845
1644.36773	0.065722899	0.231747413	0.283597121	0.776719144	0.897654045
79.2849271	-0.245244188	0.172933806	-1.418139081	0.156150163	0.39156431
40.83007331	-0.421113285	0.19341892	-2.177208332	0.029465027	0.145474083
59.51271714	-0.295108834	0.130317418	-2.264538685	0.023541003	0.126857304
659.9974441	-0.254846392	0.098469153	-2.588083524	0.009651158	0.073342536
4.719999751	-0.269240074	0.4330922	-0.621669183	0.534159419	0.752718449
68.78323979	-0.009753459	0.12647035	-0.077120519	0.93852767	0.974295153
63.85571154	0.163152683	0.385343249	0.423395723	0.672006565	0.841900389
2359.889881	-0.02988444	0.100890539	-0.296206564	0.767072336	0.892250595
511.7480118	0.284415155	0.168987343	1.68305596	0.092364259	0.288229414
9.49119963	-0.000378504	0.384278689	-0.000984974	0.999214105	0.999596506
230.9364733	0.068688789	0.156524362	0.438837687	0.660779151	0.834769247
254.0290257	-0.102473608	0.10987354	-0.932650462	0.35100047	0.608090959
594.7528176	0.169200649	0.103410815	1.63619878	0.101797992	0.305570205
746.7666055	0.104118794	0.053350833	1.951587042	0.050987253	0.201968642
386.6205728	-0.235470014	0.118453381	-1.987870777	0.046825983	0.192238027
69.53930881	0.495086661	0.151028749	3.278095486	0.0010451	0.017074327
294.3162165	-0.040945769	0.095235366	-0.429942902	0.667237177	0.838604686
64.90457106	0.536466532	0.189054309	2.837631866	0.004544957	0.044696298
277.7396	-0.162128503	0.126453214	-1.282122438	0.199799699	0.448315412
14.54285215	-0.407700084	0.502446944	-0.811429125	0.417119279	0.662355886
11.31583219	-0.210530972	0.548750972	-0.383654851	0.70123428	0.857806901
45.87726978	-0.151868748	0.168175947	-0.903034888	0.366507379	0.622358722
145.0686919	-0.089494618	0.115638389	-0.773917892	0.438979348	0.681391372
8.731925611	0.409810241	0.409714755	1.000233057	0.317197735	0.576008142
511.340311	0.361776883	0.108630449	3.33034509	0.000867384	0.014884084
866.2451793	-0.179570948	0.118196092	-1.519262994	0.128696311	0.349943297
230.8058692	-0.106384824	0.076298788	-1.39431866	0.163221398	0.402228979
26.65771542	-0.150429656	0.229730095	-0.654810404	0.512589832	0.737294472
226.1195231	0.071093772	0.118288471	0.601020293	0.54782647	0.762398361
307.3875763	-0.149074771	0.118072959	-1.262564884	0.206745595	0.456768264
33.49152863	-0.188375894	0.18442887	-1.021401334	0.307064334	0.566057952
92.73228518	-0.015033205	0.113737996	-0.132173994	0.89484667	0.955539338

2180.459607	-0.186003456	0.104895269	-1.773230164	0.076190576	0.257978772
138.4388226	0.291158001	0.137105714	2.123602236	0.033703418	0.157150167
99.26706453	-0.257128029	0.109031472	-2.358291821	0.018359254	0.108864477
515.61282	-0.217104894	0.203464747	-1.067039362	0.285954062	0.544879542
211.1845586	-0.179855945	0.121243911	-1.483422493	0.137962195	0.36428847
57.02021017	0.021648333	0.644543982	0.033587054	0.973206446	0.986860406
698.826396	-0.224742231	0.082064568	-2.738602483	0.006170093	0.054600664
99.04438515	-0.342680526	0.128850917	-2.659511725	0.007825401	0.064191205
681.9146988	-0.00716896	0.069867	-0.102608671	0.918273561	0.965359778
41.63575726	0.10801115	0.16330215	0.66141903	0.508343625	0.733676398
3376.246241	0.005020118	0.128508006	0.039064629	0.968838861	0.985106561
19.04093793	-0.171700605	0.26918852	-0.637845198	0.523574454	0.745455256
19.84508786	0.417771708	0.303333742	1.377267509	0.168429557	0.40892402
341.6587283	0.041302665	0.119412566	0.345882063	0.72943135	0.873451767
411.8137055	-0.175176164	0.114744627	-1.526661152	0.126845292	0.347308746
107.9362578	-0.001016093	0.2114814	-0.004804644	0.996166464	0.998186672
44.87827326	0.022133398	0.190534106	0.116165018	0.907521761	0.960494723
3166.57996	-0.314816073	0.09295385	-3.386799709	0.00070713	0.013120652
1137.126294	-0.220629636	0.057577444	-3.831876186	0.00012717	0.003981098
83.73379803	0.537492071	0.372792447	1.44179979	0.149358862	0.381708391
86.79624436	0.212443773	0.232635889	0.913202919	0.361135829	0.616999384
3.840451326	-0.228757691	0.991651156	-0.230683633	0.81756059	0.920041637
13.85901932	0.596685147	0.365869886	1.630867062	0.102918373	0.307225389
36.75892157	-0.277571514	0.207402185	-1.338324926	0.18079054	0.426017761
181.3720465	0.019748138	0.120251752	0.164223285	0.869555371	0.945629354
572.4128074	-0.094629178	0.13285907	-0.712252293	0.47630856	0.709105217
54.7041183	-0.126616832	0.160389184	-0.789434978	0.429857819	0.674661266
413.2374306	0.088605112	0.061863509	1.432267794	0.152067196	0.386175827
103.7361096	0.260544	0.270848554	0.961954553	0.336072433	0.594703504
129.6142191	0.023444645	0.144252918	0.162524582	0.870892772	0.946199847
716.9644694	0.105454663	0.115037469	0.916698386	0.359300712	0.615271576
768.6075201	-0.209591555	0.107319741	-1.952963671	0.0508239	0.201649899
2.703315526	0.418265257	0.713522606	0.586197625	0.557742717	0.768771196
3.193567271	0.510847837	0.726516495	0.703146921	0.481964194	0.713865937
171.3309784	-0.080856763	0.091233749	-0.886259348	0.37547779	0.628628699
538.8744468	-0.310308118	0.236408881	-1.312590782	0.189320877	0.437177362
144.2706864	0.191335618	0.176229249	1.085719986	0.277602896	0.535801602
15.84690806	-0.755716406	0.285640339	-2.645692165	0.008152397	0.065442486
180.2104334	0.068985487	0.100681165	0.685187615	0.493225538	0.721618408
719.3323217	-0.123164228	0.103978921	-1.184511497	0.236210648	0.492010908
3.29621001	0.052854864	0.593262754	0.089091829	0.929008931	0.970164118
162.074273	-0.090792031	0.2557818	-0.354958918	0.722620368	0.870207145
61.89238791	0.0328167	0.138700783	0.236600687	0.812966587	0.917439052
2.836565982	-0.460787812	0.679351939	-0.678275553	0.497596996	0.72436479
10.12764747	0.727535986	0.353165319	2.06004369	0.039394364	0.173507781
148.6906271	-0.55040771	0.266259628	-2.067184254	0.038716795	0.171486647
599.2651516	0.005416277	0.090497631	0.059849931	0.952275158	0.979060167
287.6954015	0.022866472	0.12533244	0.182446559	0.85523229	0.938018632

21.74994616	-0.152579607	0.229777359	-0.664032383	0.506669586	0.732473572
45.82303396	0.720496604	0.277854874	2.593068073	0.009512394	0.072670428
62.45457686	0.978945055	0.449147412	2.179562942	0.029289874	0.145052854
96.78485072	-0.507984487	0.572890537	-0.886704272	0.375238138	0.628533105
492.3614052	-0.40719209	0.084468658	-4.82062931	1.43106E-06	0.000132028
2180.195605	-0.317119498	0.162315179	-1.953726688	0.050733548	0.201476699
1206.749197	-0.069769659	0.046449444	-1.502055852	0.133082685	0.356371663
223.2655825	-0.191585531	0.247748548	-0.773306373	0.439341085	0.681440998
113.269817	-0.249773028	0.210159563	-1.188492329	0.2346395	0.490432513
7.812401189	0.61148171	0.734962474	0.831990383	0.405414373	0.654303254
55.09115409	0.04770417	0.131937289	0.361567002	0.717675627	0.867182168
1071.641346	-0.085319184	0.103344767	-0.825578171	0.409043422	0.656912138
620.7449516	-0.256660646	0.064333637	-3.989524873	6.62058E-05	0.002437492
235.9272518	0.128599832	0.104272115	1.233309899	0.217460166	0.470109614
64.34523547	0.021893219	0.139178999	0.157302608	0.875006365	0.948155301
1878.949709	0.038505186	0.127984181	0.30085895	0.763522054	0.891135578
56.49003836	0.25818896	0.163647288	1.577716098	0.114630846	0.325769766
14.105232	-1.075813454	0.567687894	-1.895079084	0.058081925	0.21945481
956.9556649	-0.067183493	0.093714251	-0.716897297	0.47343747	0.70677644
247.240898	0.143990187	0.113012344	1.274110262	0.202624342	0.451543077
2.713682515	0.378105051	0.618575478	0.611251277	0.541033242	0.757639765
5.98537961	0.022759149	0.496304356	0.045857243	0.963424034	0.982781984
1559.329765	0.067126996	0.111370668	0.602734966	0.546685017	0.761677872
211.7969945	0.000836095	0.16437715	0.005086441	0.995941625	0.998105332
195.5839148	0.010894525	0.197424486	0.05518325	0.955992473	0.980691082
25.51604308	0.536768244	0.355922306	1.508105096	0.131527631	0.354192865
1015.341437	0.22225518	0.137469756	1.616756928	0.10593076	0.311292877
366.8546366	-0.104422838	0.080213625	-1.301809248	0.192981601	0.440314141
20.17210552	0.149050114	0.263950392	0.564689874	0.572284723	0.778668655
9.706699964	-0.12447453	0.446404313	-0.278838099	0.780369075	0.899023694
326.5360094	-0.027688407	0.249834669	-0.110826922	0.911753596	0.962232976
1156.814782	-0.111458347	0.087292198	-1.276842033	0.201658022	0.450536698
208.719854	-0.125832693	0.137076142	-0.917976613	0.35863111	0.61459466
366.031133	0.014966114	0.070830621	0.211294412	0.832657543	0.928833635
202.5127068	0.582245818	0.324476242	1.794417412	0.07274657	0.250840427
517.4857654	-0.464266722	0.35484976	-1.308347289	0.190755542	0.438241275
426.0203539	-0.131853132	0.077643842	-1.698178877	0.089474006	0.283554316
137.5357664	-0.14833599	0.158482432	-0.935977499	0.349284773	0.606632725
55.66430651	0.044816071	0.127780423	0.350727206	0.725793013	0.872122772
325.4404294	0.013468781	0.076685358	0.175636938	0.860579181	0.940613387
452.9772687	-0.232992969	0.067257874	-3.464173883	0.000531863	0.010961541
8.082558541	0.373429894	0.878413522	0.425118562	0.670750247	0.840998231
109.4561019	-0.83588679	0.498978482	-1.675196067	0.093895769	0.291154852
3.279676145	-0.329964282	0.58537246	-0.563682621	0.572970147	0.779023105
7717.957535	-0.156063689	0.141668296	-1.101613371	0.270629793	0.529576753
219.3443336	-0.403349336	0.216011806	-1.867255972	0.061865851	0.227866016
1128.84498	0.04557063	0.075117049	0.606661609	0.544075505	0.759365444
444.498016	-1.109707333	0.539456382	-2.057084448	0.039678103	0.173878562

365.536544	-0.357308057	0.198348846	-1.80141233	0.071637914	0.248412347
10.01069354	0.159692757	0.380766948	0.419397635	0.674925553	0.843921157
29.26598765	0.229440211	0.60750667	0.377675213	0.705671883	0.860575204
2.656329913	-0.595794807	0.928232361	-0.641859551	0.520964379	0.743835259
6.0404556	0.136834139	0.914388102	0.149645581	0.881044244	0.950743683
2.717969964	0.088388959	1.09219088	0.080928124	0.935499114	0.973226857
99.15870259	-0.590758195	0.372594413	-1.585526177	0.112846834	0.323444764
17.2331139	1.422873348	0.754507545	1.88583051	0.059317798	0.222303547
5.331307744	0.583851064	1.051043299	0.555496681	0.57855498	0.782148794
14.70287894	-0.440632857	0.682456923	-0.645656659	0.518501735	0.741851962
2.643009265	-1.122856041	1.021800295	-1.098899703	0.271811816	0.530854517
5.382694093	-1.8910329	1.175417179	-1.608818497	0.107656035	0.314053154
4.249601204	-1.926768931	0.805120847	-2.393142518	0.016704751	0.103351998
197.853976	0.141971195	0.434627768	0.326650079	0.743932562	0.881323235
1298.589632	-0.27391703	0.06916539	-3.96031933	7.48496E-05	0.002646779
166.8478831	0.161994987	0.254014743	0.637738523	0.523643904	0.745468911
280.6180968	0.419567608	0.175379771	2.392337533	0.016741438	0.103497326
10.17346478	0.432453862	0.315116075	1.372363698	0.169950245	0.41115219
588.1556387	0.19808376	0.148943787	1.329922943	0.183543661	0.429117213
24.94420624	0.432857998	0.565273437	0.765749759	0.443825213	0.68493625
915.7655642	-0.103036745	0.062393883	-1.651391779	0.098658602	0.300167123
104.6200853	-0.32214305	0.137447256	-2.343757599	0.019090574	0.111473032
914.9475016	-0.294152747	0.100333227	-2.931758066	0.003370492	0.036941158
111.5090785	0.442782098	0.177401899	2.49592649	0.012562863	0.086685415
4.761604727	0.332711373	0.719859282	0.46218946	0.643945461	0.824864474
1672.210644	-0.080846054	0.110831407	-0.729450756	0.46572598	0.701518184
1454.189642	-0.302229537	0.156548324	-1.930583032	0.053534638	0.208761129
390.0093091	0.356478735	0.185342246	1.923353916	0.054435618	0.210827676
64.17409678	0.107310535	0.246662521	0.435050022	0.663526119	0.836542958
5.486314061	0.625021575	0.517826943	1.207008602	0.227428831	0.482000506
1304.811978	0.032806366	0.137215162	0.239087032	0.811038097	0.915758284
13.4951535	0.103952376	0.296531783	0.350560653	0.725917979	0.872122772
59.28464183	0.829090661	0.322275646	2.572613445	0.010093387	0.075217161
11.90842473	0.805093441	0.45469033	1.770641219	0.076620383	0.2589343
464.1212141	-0.030362623	0.069401946	-0.437489514	0.661756379	0.835398185
164.8868331	0.137710477	0.140991599	0.976728245	0.328703708	0.5871511
4.257749688	0.423006197	0.549679475	0.76955065	0.441566489	0.682856321
20.9954375	0.445796967	0.23560014	1.892176154	0.058467514	0.220433773
530.9695101	0.101046667	0.1390572	0.726655415	0.467437072	0.702812011
51.5043276	-0.064009887	0.173146048	-0.369687255	0.711615529	0.863318221
7.946040359	0.396962269	0.335271039	1.184004052	0.236411459	0.492010908
4.043785466	0.542072151	0.577448464	0.938736848	0.34786587	0.605291756
5.578638404	-0.408350026	0.55542212	-0.735206632	0.462213661	0.698307949
3.211353027	-1.444257846	0.850177917	-1.698771301	0.089362283	0.283429332
1291.289101	-0.077233942	0.081009912	-0.953388785	0.340393122	0.598586551
10.05003628	0.225455046	0.330980735	0.681172715	0.495762211	0.723170992
6739.277795	-0.212240404	0.040966447	-5.180835072	2.20895E-07	2.98665E-05
27.68513915	-0.095766397	0.220451035	-0.434411192	0.663989872	0.836652459

421.4156984	-0.306232874	0.143190904	-2.138633578	0.032465356	0.153832822
175.6806361	0.04764149	0.090053758	0.52903389	0.596781941	0.794156453
13.8726903	0.347076496	0.26575107	1.306021066	0.191545394	0.438775498
217.4525434	0.265310535	0.107936637	2.45802114	0.013970495	0.092865706
158.8313249	0.087328914	0.095945166	0.910196087	0.362719107	0.61811082
680.7893618	-0.577587039	0.229017239	-2.522024286	0.011668165	0.082136321
1787.293149	-0.258784176	0.246008975	-1.05192982	0.292831749	0.552281525
163.0264116	-0.001765298	0.142279348	-0.012407266	0.990100688	0.994028575
3.804733897	0.308348529	0.558239691	0.552358663	0.580702646	0.783396904
400.7620701	0.089786978	0.122506838	0.73291401	0.463610878	0.699296945
946.4291121	0.336410145	0.08276369	4.064706944	4.80929E-05	0.001964292
20.50345716	0.054143554	0.34180486	0.158404867	0.874137779	0.947672422
498.1561305	-0.263601478	0.237445491	-1.110155752	0.266931916	0.525619607
2.654189031	0.162260802	0.800496665	0.20270016	0.839369398	0.931066348
2440.356973	-0.085499559	0.056044983	-1.525552406	0.127121373	0.347589193
34.0294966	-0.348860495	0.180938045	-1.928066017	0.053846915	0.209406154
2.666313847	0.155493865	0.625577051	0.248560693	0.803700617	0.91131004
890.439047	-0.090399609	0.061560892	-1.468458405	0.141979747	0.370937242
126.891315	0.341170788	0.265374691	1.285619161	0.198576006	0.447223734
47.47868287	-0.015709753	0.19686956	-0.079797776	0.936398093	0.973575955
213.0973057	0.020986462	0.108205122	0.193950727	0.846214464	0.933532225
211.0311575	-0.227470933	0.099758895	-2.280207033	0.022595412	0.123651936
926.8124612	0.043910522	0.09028781	0.486339424	0.626726531	0.813572167
113.5846022	0.167242354	0.114730686	1.457695056	0.144924615	0.374955566
171.7855057	0.306897191	0.160670821	1.910099104	0.056120454	0.21489092
231.5362455	0.260815058	0.104845404	2.487615557	0.012860267	0.087772159
1280.935343	-0.001258764	0.175730757	-0.007163027	0.99428478	0.996827058
4.11845666	0.215304113	0.662549537	0.324963042	0.745209043	0.881969411
5.374669515	0.68770459	0.57053492	1.205368097	0.228061232	0.482779371
203.0871948	0.562629872	0.119282083	4.716801193	2.39582E-06	0.00018788
104.4399838	0.078753464	0.399057619	0.197348604	0.843554746	0.932221964
22.48347471	0.421974056	0.495042857	0.85239904	0.393992653	0.644491111
382.6817236	0.444343791	0.143980016	3.086149054	0.002027671	0.026712588
9551.18245	0.252435319	0.182255681	1.385061454	0.166033677	0.405696555
122.3217617	0.447169355	0.191626727	2.333543769	0.019619624	0.113159006
644.1954477	0.107955893	0.184845423	0.584033355	0.559197871	0.769743673
352.1012582	0.16291588	0.091917708	1.772410161	0.076326496	0.25821932
131.884965	-0.822248049	0.54209974	-1.516783698	0.129321304	0.351034153
10.90218279	0.366699748	0.552696075	0.663474492	0.507026711	0.732922298
63.3073164	0.601762811	0.30320794	1.98465387	0.047182994	0.193115367
5.88317913	1.895597096	0.928412363	2.041762013	0.041175144	0.178076271
742.1876986	0.330674812	0.145060621	2.279562921	0.022633624	0.123817845
62.0281733	0.506113039	0.17955644	2.818684971	0.004822082	0.046305965
375.8216975	0.222237341	0.084329048	2.635359294	0.008404826	0.066744961
216.950273	0.236203567	0.090105245	2.621418628	0.008756466	0.068831417
976.3163817	-0.001064224	0.064478275	-0.016505158	0.986831387	0.992541292
2.628117555	0.26757718	0.627727895	0.426263007	0.66991621	0.840557267
644.2834224	0.035538255	0.081556259	0.435751416	0.663017097	0.836213705

263.982096	0.428614333	0.117314625	3.653545602	0.000258644	0.006628382
290.0209202	-0.213907949	0.224403393	-0.953229563	0.340473771	0.598586551
391.3064819	-0.277777266	0.117779002	-2.358461715	0.018350853	0.108855814
149.1973525	0.110688776	0.302686977	0.365687275	0.714598442	0.864729725
233.7979029	-0.515558131	0.129969266	-3.966769595	7.28534E-05	0.002591002
1052.298392	-0.217344393	0.052790917	-4.117079321	3.83704E-05	0.001644266
16.39621799	0.525377346	0.484554134	1.084249021	0.278254402	0.536596832
54.79865777	0.076922308	0.297686046	0.258400784	0.796097612	0.906411319
4.143286591	0.574168365	0.593668779	0.967152704	0.333467702	0.591900483
128.3655544	0.334376084	0.193062872	1.731954363	0.083281688	0.271896336
10.23751285	0.580294224	0.751227888	0.772460971	0.439841454	0.681734694
343.8324442	-0.142986372	0.114871097	-1.244755004	0.21322182	0.465114191
23.74551548	0.798435007	0.327168828	2.44043729	0.014669493	0.095665831
1449.20564	-0.179699488	0.09501567	-1.891261592	0.058589433	0.220681236
466.7450293	-0.024593973	0.079590382	-0.309006845	0.75731632	0.888321678
233.9481894	-0.187517372	0.111295693	-1.684857403	0.092016087	0.287543396
718.6875714	-0.514750844	0.169713395	-3.033059618	0.002420878	0.029920446
25.08827372	0.163982589	0.229012491	0.716042118	0.473965342	0.707227897
7.118661005	-0.883213281	0.621010513	-1.422219532	0.15496253	0.389804704
3.46845666	0.713270274	0.62868033	1.134551599	0.256563243	0.513818445
890.1336601	0.163532564	0.13729275	1.19112308	0.23360527	0.489137368
519.0829089	-0.136074743	0.053420462	-2.547240127	0.010857869	0.07854927
129.691983	0.005399943	0.249504802	0.021642641	0.982733019	0.991070527
616.3735378	-0.147324944	0.088051544	-1.673167077	0.09429441	0.291698919
42.4917552	-0.550239977	0.177003961	-3.108630867	0.001879564	0.025589479
3.250301164	-0.865301098	0.599320185	-1.443804363	0.148794014	0.380636979
450.6119123	-0.120399142	0.082596062	-1.45768623	0.144927049	0.374955566
1512.466337	0.004122453	0.092635387	0.044501927	0.964504316	0.983440755
764.1038856	0.133552105	0.095296468	1.401438146	0.161083092	0.399308869
1318.929057	-0.132847997	0.090312593	-1.470979766	0.141296589	0.369534051
23.98123901	0.063199593	0.35953048	0.175783687	0.860463886	0.940613387
193.8862497	-0.232615092	0.108919468	-2.135661301	0.032707031	0.154737487
549.4504766	-0.048434559	0.087426466	-0.55400339	0.579576526	0.782818409
14088.7631	0.619793544	0.247668322	2.50251441	0.012331461	0.085389239
341.5751492	-0.24912745	0.07911974	-3.148739481	0.001639763	0.02331468
386.1154995	-0.120109729	0.067923922	-1.76829791	0.077011113	0.259862801
3.604862624	1.120029855	0.945970302	1.184001076	0.236412637	0.492010908
52.44627963	-0.252190691	0.209725078	-1.202482287	0.229176725	0.484346971
77.56035521	0.163741861	0.285509478	0.573507621	0.566301041	0.774448801
52.58092081	0.376547309	0.282175142	1.334445358	0.182057943	0.427527932
2644.135859	-0.233110848	0.279333972	-0.834523799	0.403985882	0.653167263
571.1843884	0.281363999	0.301704682	0.932580818	0.351036441	0.608090959
3.293609996	0.165579406	0.614722448	0.269356369	0.787655459	0.903200814
1175.941153	-0.551182172	0.189833982	-2.903495817	0.003690219	0.039185778
173.2901629	-0.278375502	0.093889442	-2.96492871	0.003027531	0.034533668
121.0378032	-0.361967849	0.136559822	-2.650617456	0.008034479	0.064955032
399.389424	-0.046752025	0.090384105	-0.517259367	0.604975115	0.799429581
156.6451337	-0.209343698	0.192783732	-1.085899187	0.277523597	0.535780415

587.85726	-0.278372652	0.067427045	-4.128501438	3.65135E-05	0.001586366
153.2078836	-0.105558518	0.132941741	-0.794020879	0.427183277	0.672077692
82.00694564	0.063050736	0.218787155	0.288182987	0.773206671	0.895996959
17.93761091	1.042243691	0.40106947	2.598661254	0.009358808	0.071988004
14.06594691	-0.133015659	0.273424856	-0.486479764	0.626627049	0.8135129
84.84164718	0.239754447	0.113668785	2.109237347	0.0349241	0.160771817
820.2798811	0.157871199	0.071165925	2.218353779	0.02653072	0.136071881
393.7222102	-0.189912989	0.106074284	-1.790377286	0.073393279	0.252292273
144.9588569	-0.28960436	0.125304758	-2.311200021	0.020821809	0.117386505
607.9154837	-0.211776008	0.091490194	-2.314739968	0.020627167	0.116846413
72.87394675	-0.053734236	0.177407312	-0.302886255	0.761976546	0.890832903
1527.373	0.268995575	0.22216136	1.210811702	0.225967579	0.480315376
110.7189267	-0.839246706	0.337222779	-2.488701113	0.01282107	0.087695448
8.29477172	0.020319342	0.554218892	0.036663027	0.970753689	0.985839217
4.582973416	0.811476949	0.86212047	0.941257026	0.34657316	0.604364403
158.8571415	0.483109674	0.324444105	1.489038224	0.136477304	0.362006992
21.63345143	-0.205854094	0.585821516	-0.351393878	0.725292875	0.871978571
247.0273569	0.083702355	0.107914597	0.77563515	0.43796444	0.680708976
113.5809541	0.011511385	0.129358234	0.088988418	0.929091115	0.970164118
246.7665549	-0.115432369	0.078699837	-1.466742162	0.142446209	0.371222252
20.45574073	0.023110649	0.278160036	0.083084002	0.933784747	0.972604593
403.5323618	-0.157630911	0.123404061	-1.27735594	0.201476612	0.450393271
4.123459085	-0.416991	0.50902697	-0.819192351	0.412676679	0.659240276
12.20992487	-0.024067694	0.278016994	-0.086569145	0.931013992	0.970997058
330.9283782	-0.262211277	0.083809185	-3.128669934	0.001755994	0.024415166
14.24369838	2.178572863	0.932252679	2.336890964	0.019444854	0.112494686
5.097577499	2.577849148	1.16973152	2.203795575	0.027538724	0.139553262
5.886612406	2.56065471	1.152467994	2.221887915	0.026290881	0.135373008
8.309708434	3.403348559	1.290748639	2.636724499	0.008371079	0.066544349
16.66849442	2.227601905	0.830834987	2.681160447	0.007336733	0.061699367
17.24582899	2.915248365	1.07375433	2.71500499	0.006627477	0.057231261
30.52379773	1.991355848	0.733387094	2.715286189	0.006621852	0.057221557
30.81685297	0.257917546	0.603681389	0.427241175	0.669203673	0.839864801
2.594792956	0.324839725	1.322258211	0.245670416	0.805937375	0.912922273
24.09766586	0.137254922	0.698944448	0.196374579	0.844316992	0.932309255
5.842007206	-0.121197394	0.558127534	-0.217150001	0.828091449	0.926287434
6.577830567	2.822332384	1.039654462	2.714683086	0.006633922	0.057231261
88.87545466	-0.482324694	0.218115589	-2.211326099	0.027013263	0.137601826
6.985462976	2.526821442	1.232695166	2.049834795	0.040380555	0.176071344
13.73879029	1.199398736	0.647326687	1.852849204	0.063903976	0.232571274
164.0319623	-0.094089543	0.108054274	-0.870761888	0.383884179	0.634975686
127.9821237	-0.058545271	0.125618973	-0.466054364	0.641176588	0.823469833
4.342237001	-0.507333747	0.528625627	-0.959722195	0.33719505	0.595725198
608.1397047	0.71016512	0.279233226	2.543268687	0.010982074	0.079192117
12.86084757	1.622208244	0.864962788	1.875465936	0.060728651	0.225267401
9.240240922	1.402875419	0.927731822	1.512156192	0.130494132	0.352570194
18.6669456	0.471520891	0.752760959	0.626388611	0.531060078	0.750982442
73.73081431	0.346019999	0.427291744	0.809797998	0.418056283	0.663307511

77.23780432	0.683970494	0.577785275	1.183779725	0.23650027	0.492010908
111.7006493	0.46634184	0.434414542	1.073495001	0.28304908	0.541779568
143.8231266	0.52589568	0.265487066	1.98087119	0.047605721	0.194338397
8.350282777	-0.049510037	0.923622852	-0.05360417	0.957250534	0.98117193
15.33662261	0.295870669	0.810873143	0.364879108	0.71520165	0.865059203
7.088328989	2.563697728	1.011635864	2.534210005	0.011270117	0.080163497
25.53099896	0.222408829	0.409186799	0.543538621	0.586759007	0.787659342
457.3943235	0.953608854	0.404677887	2.356463954	0.018449858	0.109154121
169.7363113	0.385470617	0.360853069	1.06822042	0.285421096	0.544260725
466.7049311	0.231883173	0.142031883	1.632613523	0.102550305	0.306759453
7.742589849	0.059783434	0.546407145	0.109411882	0.91287581	0.962912382
298.8121253	-0.16526103	0.200966743	-0.82233024	0.41088897	0.658207516
185.7735221	-0.324469514	0.198973845	-1.6307144	0.102950597	0.307225389
18.73989383	1.202876501	0.652464084	1.843590368	0.065242861	0.235840507
6.456996773	2.718005783	1.078994385	2.51901754	0.011768279	0.082583308
4.956549978	3.261548225	1.23321272	2.644757203	0.008174955	0.065518964
5.738447935	1.82283412	0.968262171	1.882583224	0.059756872	0.223096114
111.6469475	1.422152174	0.562940414	2.526292549	0.011527345	0.081342305
8.993003205	1.998044803	1.013256576	1.971904106	0.048620555	0.196417802
64.33269574	-0.113423783	0.30120621	-0.376565219	0.706496739	0.86122222
129.2105301	-0.462869432	0.492154997	-0.940495241	0.34696359	0.604507547
151.6636868	0.095914629	0.475074788	0.201893747	0.83999979	0.931195696
2.702553173	0.525893532	1.286043185	0.408923696	0.682595659	0.847675388
28.61831546	-0.10728857	0.387310435	-0.277009242	0.781773009	0.899847929
1148.735507	0.073792696	0.063275201	1.166218268	0.243526209	0.499015555
483.4694004	-0.045736175	0.144535724	-0.31643509	0.751672287	0.88558395
103.8010613	0.053242506	0.118896111	0.447806964	0.654292517	0.830926149
197.8610749	0.114522134	0.138535022	0.826665574	0.408426639	0.656395472
5.984173658	0.171934795	0.48238845	0.356423947	0.721523098	0.869608119
378.4449173	-0.234629137	0.121807895	-1.926222731	0.054076569	0.209882926
455.1478511	-0.154208689	0.12779605	-1.206678057	0.227556153	0.482035481
17.88746176	0.431330257	0.311187523	1.386078251	0.165723011	0.405491373
71.73915981	0.317165007	0.262820381	1.20677478	0.227518891	0.482023803
2.843485036	-1.002608528	0.619972158	-1.61718315	0.105838752	0.311148077
443.861428	-0.261322818	0.130107745	-2.00851086	0.044589033	0.186887867
264.5315548	-0.224622907	0.108640654	-2.067576906	0.038679826	0.171371295
33.99175961	-0.065334051	0.293451997	-0.222639655	0.823815971	0.923568955
52.04138109	0.505393471	0.196230783	2.575505547	0.010009367	0.074991831
4514.389464	0.10607889	0.18270264	0.58060951	0.561503661	0.771022888
448.8372951	-0.158696405	0.122427329	-1.296249837	0.194889425	0.442992008
64.67729918	-0.441588873	0.342672946	-1.288659868	0.19751636	0.445999903
398.2891268	-0.196399683	0.070755462	-2.775752944	0.005507404	0.050661657
8.285062681	0.587658364	0.382043097	1.538199143	0.12399993	0.342156036
23.86099234	1.234021978	0.476371744	2.590460062	0.009584774	0.072974563
5.131262518	0.038099593	0.577988576	0.065917553	0.947443465	0.976502838
39.89479418	-0.169603753	0.266496359	-0.636420525	0.524502366	0.746081545
21586.89634	-0.57839847	0.225811425	-2.561422519	0.010424449	0.076795237
142.367204	0.07445037	0.097685375	0.762144485	0.445973775	0.686007186

85.6857603	-0.01396012	0.472815746	-0.029525497	0.976445484	0.988130653
55.32928152	0.094866511	0.339030247	0.27981725	0.779617717	0.898747855
14.16912944	0.279696939	0.380458789	0.735156994	0.462243887	0.698307949
64.06652426	-0.066584122	0.21143499	-0.314915344	0.752825931	0.886369034
4.96708228	0.264879357	0.455660589	0.581308464	0.561032577	0.770873642
13.2322077	-0.514005056	0.370778381	-1.38628648	0.165659444	0.405435688
318.5829146	-0.203954881	0.12992936	-1.569736675	0.116476385	0.329393369
135.1888074	-0.123608877	0.277998558	-0.444638556	0.65658098	0.832281264
203.8880098	-0.064436655	0.129005717	-0.499486816	0.617436472	0.807595166
88.51255938	0.121770407	0.281346535	0.432812889	0.665150709	0.836844909
17.80549946	0.405711121	0.289172685	1.403006374	0.160614938	0.398526292
47.26985112	0.080781477	0.222819824	0.362541697	0.71694727	0.866502349
349.0151444	0.099084267	0.158770039	0.624074088	0.53257893	0.751842299
121.8810788	0.512864428	0.22347938	2.294907156	0.021738446	0.120347964
3.758603258	1.239883073	0.523425097	2.36878797	0.017846481	0.107238214
571.5719265	-0.014387228	0.07112377	-0.202284384	0.839694408	0.931173342
4.210997457	-1.471308023	0.799355425	-1.840618049	0.065677551	0.236801542
627.8388883	-0.017942187	0.098336129	-0.182457733	0.855223522	0.938018632
166.5236223	0.014950657	0.126810589	0.117897546	0.906148838	0.960133527
56.0507358	-0.425900935	0.161571796	-2.635985647	0.008389328	0.066655633
67.00662125	-0.029124707	0.148668492	-0.195903697	0.844685543	0.932309255
316.6543726	0.075761773	0.097825842	0.774455621	0.438661402	0.681319611
19.55952577	0.358868815	0.351476477	1.021032242	0.307239164	0.566111259
25.38285669	-1.303123844	0.738493685	-1.764570059	0.077636051	0.260752744
1146.746362	-0.027773839	0.098024622	-0.28333533	0.776919797	0.897819783
904.9054328	-0.006608499	0.065506408	-0.100883252	0.919643138	0.966087273
7.298656401	0.423716182	0.345143783	1.227651207	0.21957793	0.472408814
128.8994501	0.231180817	0.112315485	2.058316511	0.03955976	0.173782322
3.891141648	0.72243852	0.83927055	0.860793364	0.38935186	0.640338461
94.8888928	-0.117791792	0.107163201	-1.099181355	0.27168897	0.530722357
210.1774227	0.421256205	0.163100065	2.582808321	0.009799975	0.073977505
4.783424586	0.550774448	0.544084561	1.012295675	0.311396702	0.570697037
713.0753859	0.185984333	0.110644719	1.680914683	0.092779487	0.289122488
130.7684953	-0.096230593	0.113844463	-0.845281279	0.397953811	0.648266262
108.0219682	-0.475204763	0.135437655	-3.508660595	0.000450369	0.009676152
49.75082268	-0.006361956	0.230174692	-0.027639687	0.977949528	0.988880488
179.0351949	0.119067663	0.198017861	0.601297593	0.547641793	0.762302191
295.2586984	0.029561546	0.093282933	0.316901976	0.751317983	0.885524254
331.6332753	0.10984894	0.122509912	0.896653494	0.369903844	0.62442922
1066.638544	-0.237706628	0.136909788	-1.736228153	0.082523501	0.270377814
207.3796985	0.866615958	0.342678147	2.528950167	0.011440427	0.080970964
994.0475443	-0.305060151	0.082885026	-3.680521867	0.000232757	0.006166386
3.646431709	-1.045602066	0.655425572	-1.59530252	0.110644602	0.31952678
3.688988855	-0.146920532	0.565065762	-0.260006077	0.794859086	0.905927111
630.7534724	0.05015876	0.111871816	0.448359219	0.653893969	0.830755205
19.55709464	-0.100977194	0.261655696	-0.385916285	0.69955867	0.856925408
53.52456022	0.5585118	0.209732186	2.662976099	0.007745292	0.063834552
100.1422416	-0.035319321	0.144407333	-0.244581219	0.806780708	0.91335864

170.6209284	0.185038815	0.193007218	0.958714478	0.337702603	0.59618726
486.0066535	0.015129048	0.123223249	0.122777544	0.902283257	0.958662553
610.1187302	-0.113309383	0.094773458	-1.195581403	0.231859952	0.487138847
578.5146771	-0.033716787	0.088254403	-0.382040847	0.702431067	0.858728471
20.65730037	-0.442636094	0.55254936	-0.801079732	0.423085489	0.667572717
695.1901158	0.03700287	0.084307676	0.438902739	0.660732012	0.834769247
420.5297615	0.051155311	0.08079824	0.63312407	0.526652625	0.748122432
336.5870814	0.016996146	0.208370988	0.081566761	0.934991234	0.973012718
1602.213408	0.259163658	0.182355975	1.421196418	0.155259666	0.390299781
2053.426617	-0.234942056	0.158982932	-1.477781625	0.139466238	0.366826846
2538.302682	-0.129446409	0.134109047	-0.965232489	0.334428378	0.592876079
3421.399044	-0.418335319	0.146383156	-2.857810497	0.004265751	0.04288959
488.2276804	0.006088488	0.160247949	0.03799417	0.969692331	0.98542524
1063.315823	-0.151543036	0.051877792	-2.921154318	0.00348737	0.037695326
2240.135567	-0.205089947	0.110123649	-1.86236061	0.062552278	0.229651199
130.2414226	-0.173347083	0.157615897	-1.099807104	0.271416177	0.530593591
41.26871319	-0.388617162	0.193256412	-2.010888835	0.0443372	0.186380231
237.4765223	-0.180906132	0.102031758	-1.773037483	0.076222496	0.257978772
361.2897048	0.209116661	0.145985675	1.43244644	0.152016096	0.386175827
552.1601583	-0.088899376	0.11974683	-0.742394395	0.457848415	0.694284175
13.70165234	-0.10852424	0.435746108	-0.249053837	0.803319137	0.911140971
13.17401312	0.213000091	0.369931843	0.575781986	0.564762551	0.773466281
80.68227466	-0.022478878	0.162102845	-0.138670472	0.889710553	0.953820886
8.935658428	0.225010687	0.316045921	0.711955676	0.476492224	0.709176698
6.557689163	-0.169819972	0.574093673	-0.295805337	0.767378747	0.892250595
100.2309251	0.076323968	0.143073605	0.533459458	0.593715568	0.791890039
1054.93653	0.077596494	0.066652756	1.164190329	0.244346889	0.499749345
2549.23813	-0.078509896	0.060276826	-1.302488893	0.192749312	0.440233029
115.275017	0.13795026	0.151299584	0.911768928	0.361890371	0.617682512
3373.8145	0.176241049	0.122402328	1.439850463	0.149909711	0.382866618
552.7482018	0.111521582	0.120321214	0.926865494	0.353996375	0.610806055
16.10254734	0.236044608	0.623598033	0.378520449	0.705044006	0.860203048
27.58176857	0.354002512	0.54669095	0.647536807	0.517284581	0.741192341
757.3147393	-0.075337937	0.086162966	-0.87436564	0.381919163	0.633864566
146.1282372	-0.145524151	0.105302817	-1.381958767	0.166984362	0.406611199
21.67830748	-0.145732859	0.287344903	-0.507170501	0.612035191	0.804353941
175.517393	0.137517325	0.192048751	0.71605425	0.473957852	0.707227897
201.9843451	-0.10953485	0.257853484	-0.42479492	0.670986182	0.841092333
20.7955848	0.279259928	0.416873905	0.669890642	0.502927506	0.729188199
236.0620552	-0.284753484	0.121386123	-2.345848744	0.01898381	0.111097787
1248.514212	-0.138046318	0.097666269	-1.41344929	0.157523662	0.393909616
13.37323685	0.190323735	0.342515739	0.555664203	0.578440432	0.782148794
60.2337486	-0.061217639	0.154625822	-0.395908253	0.692172712	0.853059628
4.939724119	0.973896219	0.589229516	1.652829997	0.098365467	0.299507667
4.519753015	1.037072475	0.458561719	2.261576649	0.023723572	0.127400109
531.4704594	-0.096776243	0.060612728	-1.596632364	0.11034768	0.319139409
111.7918263	0.270639456	0.230128555	1.17603596	0.239580506	0.494780688
6.763376441	1.411863471	0.556683071	2.536206947	0.011206049	0.07999803

5.799242505	0.488506887	0.7770542	0.628665139	0.529568306	0.74980955
223.1865159	0.079531553	0.094508296	0.841529863	0.400051162	0.649641838
833.8270414	0.172674025	0.124648097	1.385292113	0.165963164	0.405696555
429.9057079	-0.611266395	0.353732687	-1.728046113	0.083979957	0.273543522
8.846006303	0.534495157	0.472825406	1.130428167	0.258295851	0.515323948
110.0389298	0.32584999	0.401156531	0.812276418	0.416633039	0.662355886
8.33793834	0.029819021	0.529832454	0.056280096	0.955118675	0.980179369
750.9618731	-0.065274068	0.131641812	-0.495846013	0.620003059	0.809215665
315.8517009	-0.652322724	0.311911086	-2.091373961	0.036494555	0.165407324
18.61474474	0.593164938	0.315045081	1.882793841	0.059728312	0.223096114
237.1224686	-0.084898182	0.08466338	-1.002773356	0.315970227	0.575044176
5.921691413	0.389722417	0.562574661	0.692747903	0.488467772	0.718612563
132.8598565	-0.373008538	0.40723195	-0.915960886	0.35968741	0.615732083
13.77543092	0.836222865	0.413635859	2.021640162	0.04321354	0.183114554
177.5234915	0.189426392	0.107824729	1.756799147	0.078952051	0.263016984
98.89880264	-0.237367872	0.119346218	-1.988901507	0.046712075	0.19194805
2491.187581	-0.118368734	0.130826326	-0.904777635	0.365583202	0.621550889
50.04035043	0.368672599	0.217495403	1.695082262	0.090059817	0.284376518
137.945326	-0.114443763	0.143085319	-0.799828825	0.42380998	0.668446875
654.1220387	-0.118296605	0.116104495	-1.018880492	0.308259706	0.567325186
1174.44647	0.046439424	0.12263951	0.378666092	0.704935836	0.860203048
251.0641922	0.002691679	0.098823777	0.027237162	0.978270576	0.988913947
500.4915257	0.188285199	0.09202513	2.046019371	0.040754465	0.177063725
4.271897783	0.617824082	0.538261155	1.147814731	0.251045055	0.507194071
117.9046197	0.126418174	0.146580668	0.862447791	0.388441151	0.639618116
30.17404738	-0.22062488	0.318616024	-0.692447534	0.488656324	0.718626475
40.21571345	-0.185168624	0.511088183	-0.36230269	0.717125848	0.866651395
204.4297412	0.105733729	0.093558347	1.130136778	0.258418595	0.515361701
240.9323944	0.013489169	0.076906539	0.175396908	0.86076777	0.94065508
2657.615559	-0.06327721	0.045312276	-1.396469486	0.162573163	0.401290131
380.5658471	0.143663846	0.131132121	1.095565641	0.273268905	0.532121882
174.328673	-0.008151733	0.281480261	-0.028960229	0.97689631	0.988174249
8.814447904	1.279682879	0.85175782	1.502402266	0.132993252	0.356366734
847.6296182	-0.159896188	0.060205728	-2.655830181	0.007911344	0.064515577
20.4719144	1.376148946	0.731675734	1.880818075	0.05999667	0.223774527
337.1072263	0.363006234	0.238725612	1.520600286	0.128360175	0.349275886
6.000615543	1.451578009	0.587108563	2.472418393	0.013420235	0.090491387
104.4795042	-0.281931793	0.234285819	-1.203366872	0.228834382	0.483893547
2120.787084	-0.12701389	0.153280017	-0.828639588	0.40730838	0.655228755
39.2914409	0.230254126	0.437761485	0.525980777	0.598901563	0.795485457
143.7633996	-0.079932995	0.128287265	-0.623078176	0.53323315	0.752219683
414.4743735	0.368636102	0.110050202	3.349708551	0.000808966	0.014136756
534.256455	0.165104081	0.131894355	1.251790349	0.210646268	0.462171603
89.32302371	-0.260531825	0.120607339	-2.1601656	0.030759852	0.149176723
102.4595992	-0.114160074	0.151309982	-0.754478139	0.450562167	0.69003832
893.2288217	0.085305211	0.070119595	1.216567369	0.223768864	0.477657248
923.0468424	-0.132990529	0.061583514	-2.159515099	0.030810227	0.149236443
298.3057338	-0.264968175	0.115236008	-2.299352243	0.021484945	0.119577674

61.73907036	-0.111202794	0.141435432	-0.786244243	0.431724411	0.676575305
10.40110487	-0.58113779	0.308396855	-1.884382999	0.059513188	0.222736124
29.80845639	0.631009512	0.240769752	2.62080061	0.008772355	0.068854114
672.1629222	0.221395705	0.102544546	2.159019793	0.030848631	0.149330227
22.69616517	0.982122329	0.713206813	1.377051243	0.168496405	0.408959706
5.33832269	-0.636309751	0.663386557	-0.95918397	0.337466075	0.596029769
6.901584841	0.158490365	0.418939628	0.378313137	0.705197988	0.860311053
606.291851	-0.531724139	0.34501173	-1.541176991	0.123273716	0.341112378
838.303864	-0.255160527	0.061958746	-4.118232592	3.81789E-05	0.001640543
3.619106661	0.885229684	0.769502161	1.150392719	0.249982158	0.505899375
757.8166448	0.409835211	0.126941665	3.228531862	0.001244274	0.019321973
57.72472948	0.183041425	0.223392102	0.819372858	0.412573715	0.659142931
1076.579037	-0.155411272	0.077402472	-2.007833442	0.044660994	0.187039527
164.9745887	-0.191903368	0.097552903	-1.967172299	0.04916335	0.197827364
3.058134645	-0.342407499	0.75084862	-0.45602734	0.648370318	0.827826143
136.7469613	-0.201456206	0.126372119	-1.594150728	0.110902279	0.320094101
1842.058694	0.013743656	0.045198784	0.304071377	0.761073513	0.890265288
5.990809602	0.428592398	0.389700957	1.099798169	0.27142007	0.530593591
65.37302918	0.127974448	0.126096593	1.014892201	0.310157216	0.56961426
353.9765132	-0.419255479	0.304799192	-1.375513749	0.168972226	0.40960748
1006.362258	-0.397771362	0.1125195	-3.535132665	0.00040757	0.009015988
264.562045	-0.217793841	0.076513958	-2.846458945	0.004420843	0.043939481
236.7779277	0.018022422	0.18394706	0.097976135	0.921951244	0.967053651
2031.004152	-0.095982211	0.113836493	-0.843158534	0.399139786	0.649254138
44.44608023	-0.121171198	0.179878838	-0.673626754	0.50054864	0.726962688
758.9084378	0.153367134	0.109165473	1.404905141	0.160049488	0.397563536
422.4148923	0.399648703	0.217469383	1.837723992	0.066103087	0.237952906
3.209827855	0.216697261	0.635928356	0.340757349	0.733286262	0.875761442
287.3586072	-0.839303411	0.407841215	-2.057917101	0.039598092	0.173782322
7.64779648	0.055319644	0.514798535	0.107458823	0.914424991	0.963574413
19.33895481	0.112367909	0.285275574	0.3938925	0.693660408	0.85351767
46.69948244	0.525567276	0.247059535	2.127289996	0.033395995	0.156446473
12.41511082	0.509426704	0.361289614	1.410023108	0.15853286	0.395361643
7.16133229	0.077028912	0.447919908	0.171970279	0.863460895	0.942350614
58.16074621	0.01589825	0.171154086	0.092888519	0.925992127	0.968927915
9.081646891	0.439220735	0.311072127	1.411957858	0.15796237	0.394629152
8.442570336	-0.045144646	0.341593556	-0.13215895	0.894858569	0.955539338
9.038945773	0.432959241	0.434868568	0.995609417	0.319439957	0.577738842
709.0000391	0.633935309	0.239924684	2.642226295	0.008236301	0.065873604
12.80625031	-0.148817203	0.523555921	-0.284243186	0.776224022	0.897409958
611.7260683	0.205049069	0.151301247	1.355237132	0.175342018	0.418642747
96.25437708	0.400627142	0.167610315	2.390229628	0.016837842	0.103847702
79.32083454	0.442344462	0.148029759	2.98821308	0.002806138	0.032942721
469.7869761	-0.349233148	0.082336714	-4.241524002	2.22007E-05	0.001070643
21.2988219	-0.003953763	0.292053182	-0.013537818	0.989198714	0.993496587
470.1447408	-0.266179092	0.139012678	-1.914782853	0.055520202	0.213635636
310.3450054	-0.005963447	0.100445001	-0.05937027	0.952657193	0.97906267
251.5055056	-0.13435932	0.107214638	-1.253180746	0.210139935	0.461824914

42.71310927	-0.073747132	0.194475201	-0.379210981	0.704531198	0.860179613
285.2691346	-0.091489861	0.118268885	-0.773575074	0.439182117	0.681440998
1120.317871	-0.23385692	0.169819024	-1.377094951	0.168482894	0.408959706
36.01952597	0.009173111	0.172804685	0.053083695	0.957665222	0.981434349
234.046262	0.169928633	0.160132225	1.061176992	0.288609474	0.548008594
133.2886856	-0.235515978	0.096226384	-2.447519778	0.014384323	0.094355384
1822.044296	0.07189024	0.177183543	0.405738808	0.684934525	0.848874997
8.235514016	-0.091428357	0.345457782	-0.264658553	0.791272509	0.904476207
120.0672796	-0.348748687	0.125553169	-2.777697212	0.005474561	0.05047796
232.0859195	-0.0367959	0.089517907	-0.411045135	0.681039439	0.847181914
5.832017851	0.263054008	0.514778379	0.511004384	0.609347987	0.802570863
31.32843281	-0.162072411	0.193894168	-0.835880792	0.403221969	0.652712702
32.42067822	-0.238386609	0.218384352	-1.091591987	0.27501248	0.534022005
23.62737238	-0.593870981	0.25814339	-2.300546919	0.021417254	0.119285588
117.0392776	-0.280989229	0.167678138	-1.675765439	0.093784146	0.290981315
7.42247809	0.155116427	0.393994094	0.393702415	0.693800758	0.85351767
12.14007833	-0.11732403	0.372554984	-0.314917356	0.752824404	0.886369034
50.76892238	-0.434674837	0.188729055	-2.303168619	0.021269358	0.118841684
26.85050814	-0.202490278	0.297523377	-0.680586111	0.496133418	0.723376083
40.75277495	-0.274221963	0.176696198	-1.551940365	0.120676493	0.336837537
51.26133747	-0.155995327	0.16051233	-0.971858841	0.331120778	0.58967847
18.28616597	-0.562217762	0.25079096	-2.241778425	0.024975696	0.13105347
16.57419672	-0.389503436	0.238576374	-1.632615287	0.102549934	0.306759453
10.22218105	0.382212373	0.323061755	1.183093841	0.236771958	0.492249655
72.1824067	0.066192342	0.166880845	0.396644337	0.691629752	0.852859583
189.0464219	-0.029021321	0.159326433	-0.182150069	0.855464957	0.938193999
63.5425675	-0.223940426	0.141109253	-1.587000294	0.112512578	0.323026249
57.12871776	-0.234979341	0.179258719	-1.310839116	0.189912124	0.437805721
72.49352289	0.038160257	0.139869118	0.272828324	0.784985186	0.902022871
87.75940718	-0.136700256	0.148069644	-0.923215944	0.355894682	0.612269826
110.6695935	-0.231551231	0.174194114	-1.329271271	0.183758489	0.429262457
96.20152151	-0.18763254	0.148765655	-1.261262486	0.207214292	0.457400854
100.8631387	-0.323489552	0.147189946	-2.197769352	0.027965547	0.140715955
4.450967004	-0.227964037	0.477514804	-0.477396795	0.633079596	0.817716475
34.96309416	-0.531902384	0.218834077	-2.43061954	0.015073033	0.097527
4.66072472	-0.595329102	0.44302767	-1.343774085	0.179021452	0.423878692
19.74576137	-0.582973912	0.248155349	-2.349229679	0.018812299	0.110589239
336.6551761	-0.030320986	0.132662521	-0.228557287	0.819213026	0.92071257
102.1927978	-0.347371632	0.130728968	-2.657189425	0.007879516	0.064399334
496.8738004	0.032832986	0.156174974	0.210232056	0.833486562	0.929163639
209.0692721	0.132031927	0.09765762	1.351987967	0.176379165	0.42003201
5.056490545	-0.580430693	0.540109969	-1.074652805	0.282530201	0.54111658
665.5254188	0.053774163	0.074743544	0.719448938	0.471864353	0.706078814
49.4530382	-0.373302425	0.270033305	-1.382431047	0.166839389	0.406609306
65.56213532	0.082164247	0.174850258	0.469912069	0.638417841	0.821608716
1078.981622	0.049739049	0.088244543	0.563650141	0.572992256	0.779023105
11.26398357	-0.764417522	0.621154445	-1.230640026	0.218457528	0.4711493
4.839402759	-1.199304549	0.879428416	-1.36373186	0.172651973	0.414936185

3.077836397	-2.261628996	0.922915848	-2.450525691	0.014264778	0.093901418
3.337654123	-2.213963219	0.836663204	-2.646182131	0.008140597	0.065442486
896.4656199	0.098419702	0.138386057	0.711196665	0.476962375	0.709683822
201.4373244	-0.048322478	0.123345396	-0.391765562	0.695231443	0.854463425
67.28578197	0.441108483	0.251183171	1.756122756	0.07906745	0.263312141
382.1935924	-0.200968463	0.117878645	-1.704875913	0.08821757	0.28099195
4.641648026	0.624816202	0.507149757	1.232015187	0.217943411	0.470699572
2.926205343	-0.333220305	0.828443635	-0.402224474	0.687518832	0.850122158
282.0016033	0.139163074	0.203529672	0.683748334	0.494134097	0.722475918
34.67738595	0.272823165	0.304196846	0.896863885	0.369791553	0.624407399
5.227569093	1.801115977	0.98768232	1.82357823	0.06821586	0.242078012
154.3683637	0.198265199	0.109481553	1.810946169	0.070149185	0.245749344
257.2933354	-0.235381439	0.116012703	-2.028928143	0.04246561	0.181084998
11.25863252	0.592032875	0.400740659	1.477346662	0.139582736	0.366887151
4.629122389	0.200941695	0.535136222	0.375496343	0.707291365	0.86173835
629.6391661	-0.14462007	0.080908135	-1.787460179	0.073863143	0.253272744
151.108946	-0.055349072	0.097613653	-0.567021827	0.570699352	0.777523336
9118.080682	0.080009288	0.149117495	0.536551982	0.59157712	0.79088016
504.9546626	-0.139088758	0.113169061	-1.229035182	0.219058615	0.4718093
398.7543592	-0.042807347	0.186610827	-0.229393693	0.818562938	0.920415839
57.12854371	-0.518592842	0.190455832	-2.722903456	0.006471097	0.056135672
314.7305017	-0.134408197	0.104415075	-1.287248963	0.198007526	0.446254188
1098.483519	-0.291910783	0.066086666	-4.417090501	1.00038E-05	0.000570546
44.00393298	-0.417971815	0.32447657	-1.288141747	0.197696625	0.44611812
566.14406	-0.440549138	0.146208848	-3.013149644	0.002585514	0.031386378
336.706294	-0.181450869	0.14835507	-1.223085055	0.221297573	0.474540521
1079.891262	-0.277421598	0.144687833	-1.917380282	0.055189637	0.212624484
10.68282007	-0.274174272	0.365618677	-0.74989132	0.453320163	0.691759407
109.3849404	-0.33189996	0.114859318	-2.88962154	0.003857059	0.040437239
60.56267702	-0.351205132	0.156050894	-2.25058071	0.024412106	0.129220208
448.2492648	-0.300650175	0.068861686	-4.366000776	1.26542E-05	0.000687775
47.44655447	0.415252067	0.420603068	0.987277789	0.323506476	0.5819332
247.8840337	0.044915609	0.147827616	0.303837744	0.761251509	0.89040709
92.70690719	0.012021233	0.176284506	0.068192224	0.945632617	0.976005209
233.3222385	-0.090362975	0.100611578	-0.898136935	0.36911255	0.624168327
18.34775772	0.464730265	0.258112083	1.800497905	0.071782054	0.248692233
8.95216587	0.254169954	0.35426792	0.717451228	0.47309572	0.706757551
3.917858768	0.818541901	0.576438444	1.419998803	0.15560803	0.390541756
122.0471213	-0.243308544	0.15856006	-1.53448822	0.124909589	0.343758904
40.89544718	-0.521376795	0.175787068	-2.965956489	0.003017432	0.034468609
48.33152415	0.048649823	0.164147861	0.296378049	0.766941387	0.892250595
129.7422857	-0.014073098	0.100939177	-0.139421562	0.889117035	0.953766542
9030.92915	-0.529851306	0.186715597	-2.837745284	0.004543342	0.044696298
141.8741613	-0.280600518	0.108468492	-2.586931134	0.009683494	0.073451487
199.3859464	-0.290546168	0.114351363	-2.54081946	0.011059301	0.079384017
497.696553	-0.144779974	0.068240804	-2.121604166	0.033870992	0.157495595
2495.981236	-0.135969338	0.138912595	-0.978812161	0.3276728	0.586125954
14.86492234	0.797886305	0.601811569	1.325807522	0.184903461	0.430769788

3.329816975	2.321546649	1.225681247	1.894086782	0.058213492	0.219793069
228.1369375	-0.514752848	0.336933554	-1.527757747	0.126572697	0.346875096
62.6633357	-0.176686025	0.154690497	-1.14219056	0.253374819	0.509978266
310.2652164	-0.278743798	0.077571124	-3.593396384	0.000326395	0.00776249
1745.905002	-0.194019703	0.112915318	-1.718276201	0.085746248	0.276887823
79.32570444	-0.01750325	0.173060789	-0.101139315	0.919439869	0.966087273
3.901043314	0.643986067	0.600078952	1.073168898	0.283195342	0.541854464
200.1823557	0.087015641	0.085673963	1.015660277	0.30979119	0.569075314
11.60196714	0.303892808	0.337067461	0.901578595	0.367280766	0.622716515
91.97219599	-0.331488678	0.109717747	-3.02128585	0.002517036	0.030721553
9444.98652	0.281424197	0.221323384	1.271552028	0.20353233	0.452987239
141.4683377	0.009684461	0.151385386	0.063972234	0.948992336	0.977223239
223.0217813	-0.032848967	0.126125794	-0.260446063	0.794519716	0.905811385
35.68234369	-0.122966051	0.242936889	-0.506164591	0.61274111	0.804784705
22.94643056	-0.457750943	0.219946442	-2.081192766	0.037416265	0.168051749
251.8540319	-0.290132454	0.083616327	-3.469806264	0.000520834	0.010792045
145.4898989	0.554407655	0.294781817	1.88073898	0.060007434	0.223774527
845.5268898	0.811374768	0.330174653	2.457410833	0.013994255	0.092902392
270.5399273	0.22424193	0.100340715	2.234804984	0.025430154	0.132452965
60443.11779	-0.102664924	0.143116127	-0.717353984	0.473155705	0.706757551
78.7558696	-0.215600976	0.194712926	-1.107276133	0.26817456	0.527139967
24.99412969	0.444819962	0.296412151	1.500680592	0.133438195	0.356954457
188.6767451	-0.137696987	0.142698271	-0.964952039	0.334568835	0.592978848
1324.389256	-0.392254138	0.183628298	-2.136131212	0.03266872	0.154656266
1423.296121	0.052317764	0.097936758	0.534199464	0.59320354	0.79167838
7.428047216	1.264260433	1.016987487	1.243142565	0.213815303	0.465696321
143.2642034	0.232725622	0.263434897	0.88342746	0.37700536	0.630244305
143.6166176	-0.038697018	0.363064186	-0.10658451	0.915118608	0.964046228
381.2931219	-0.239747842	0.087347797	-2.744749714	0.006055706	0.053811727
14.70062435	-0.353480715	0.32664037	-1.082170934	0.279176581	0.53751602
495.1197519	-0.015502591	0.087884911	-0.176396507	0.859982449	0.940452149
153.6850422	-0.011145466	0.094685647	-0.11771019	0.906297292	0.960133527
372.1571441	-0.134880731	0.075655959	-1.782817023	0.074616094	0.25468527
71.42686809	0.221570406	0.169660039	1.305966965	0.191563792	0.438775498
62.76224825	0.482609026	0.18758704	2.572720518	0.010090265	0.075217161
3.577877111	-0.73179449	0.529581324	-1.381835909	0.167022091	0.406639936
340.7642641	0.006552079	0.09785714	0.066955551	0.946617089	0.976309753
10.42628205	0.582296666	0.451555288	1.289535704	0.197211912	0.445687555
110.1518199	0.226234291	0.335749073	0.673819555	0.500426041	0.726931743
113.0998197	-0.014108266	0.434589065	-0.032463462	0.974102453	0.987273492
5.755571595	-0.09591743	0.754594402	-0.12711124	0.898852355	0.957506447
5.832588198	-0.433949233	0.525088694	-0.82643035	0.408560013	0.656409064
132.5984903	0.079276879	0.109824758	0.721848882	0.470387392	0.705378704
16.48595383	-0.074280508	0.320874353	-0.231494064	0.816930996	0.919531057
1277.620263	-0.247964864	0.0807792	-3.069662267	0.00214301	0.02766339
118.0577625	0.421376936	0.188552856	2.23479477	0.025430825	0.132452965
14.87970662	-0.706561978	0.305580237	-2.312197888	0.020766781	0.117202658
320.6058229	-0.216085052	0.102389067	-2.110430907	0.034821257	0.160533977

911.6342669	-0.121068136	0.139832283	-0.86580962	0.386594573	0.637892359
159.3232293	-0.185712719	0.228872912	-0.811422887	0.41712286	0.662355886
117.4096424	0.111210712	0.349273717	0.318405613	0.750177281	0.88518749
162.0652485	-0.161638106	0.151267569	-1.06855757	0.285269076	0.544103149
150.261273	0.038412978	0.092623965	0.414719652	0.678347144	0.845876523
149.0643535	-0.108274699	0.087042228	-1.243932988	0.213524227	0.465450171
983.6501222	-0.220333583	0.076961631	-2.862901687	0.004197808	0.042468884
6.473359156	1.493459098	0.543342435	2.748651683	0.005984094	0.05344791
632.6412547	-0.098566489	0.112350403	-0.87731318	0.380316555	0.632388957
950.616366	-0.105311132	0.053259773	-1.977310951	0.048006493	0.195355198
22.04096929	0.045560508	0.264419624	0.172303808	0.863198691	0.942195579
85.21061705	0.274574054	0.164506977	1.66907239	0.095103034	0.292986838
260.2901078	-0.091054754	0.107303091	-0.84857531	0.39611765	0.64648379
269.203629	0.22445823	0.132605137	1.692681262	0.090516154	0.285147895
4.555649426	-0.332583968	0.46992717	-0.707735132	0.479109756	0.711221434
750.6862994	0.524524274	0.205611786	2.551041861	0.010740143	0.078009094
497.6234402	0.23144578	0.14137958	1.6370524	0.101619521	0.30527849
570.7223817	-0.155510473	0.081761634	-1.901998105	0.057171402	0.217112899
24.27350668	-0.613162349	0.310230453	-1.976473759	0.048101145	0.19544517
93.39004476	0.13255789	0.121014493	1.095388547	0.27334645	0.532170521
37.71549125	0.048630556	0.562171285	0.086504873	0.931065082	0.970997058
25.62579484	-0.225076064	0.662010844	-0.339988486	0.733865199	0.876280303
2.859153835	0.954237329	0.819062523	1.165036005	0.244004419	0.499495198
118.4050075	-0.110041696	0.13813878	-0.796602491	0.42568193	0.670590136
3.768518915	0.783011128	0.536146435	1.460442664	0.144168455	0.373927244
28.49786418	0.182586663	0.242794196	0.752022355	0.452037615	0.690807557
215.2793576	-0.195641752	0.091599122	-2.135847457	0.032691849	0.154719061
140.7560991	-0.460883907	0.150669356	-3.058909378	0.002221443	0.028211431
133.1637718	-0.303096624	0.14545598	-2.08376874	0.037181208	0.16733144
20.98775599	0.108387805	0.395780191	0.273858589	0.784193297	0.901707307
326.7291359	-0.123143566	0.076589295	-1.607843061	0.107869555	0.314157226
191.2569753	-0.371933947	0.145605772	-2.554390125	0.0106374	0.077496095
6.447001863	1.005730676	1.26007211	0.798153271	0.424781551	0.669777204
237.2126197	-0.015029904	0.106004951	-0.141784925	0.887249892	0.95312914
2192.226587	-0.339568362	0.291484258	-1.164962954	0.244033989	0.499495198
17.63203373	0.3772544	0.753231224	0.500848063	0.616478057	0.80702296
1874.153431	-0.316951729	0.175824951	-1.802655014	0.071442411	0.248036203
7.543228094	-0.058818574	0.403095638	-0.145917168	0.883986778	0.951901726
442.3053162	-0.073947208	0.06947698	-1.06434113	0.287174192	0.546589349
1612.724317	-0.172566891	0.110454078	-1.562340604	0.118207772	0.332724594
172.8113769	0.198647351	0.169785991	1.169986697	0.242006322	0.497591395
17.98321095	0.081042647	0.247961856	0.326835136	0.743792583	0.881319493
390.3453866	-0.265093634	0.103425556	-2.563134733	0.010373179	0.076597426
452.3383127	-0.070315809	0.138628341	-0.507225351	0.611996709	0.804353941
91.66108084	-0.239720256	0.142990403	-1.676477932	0.093644615	0.290634968
5.304656618	0.30449603	0.740962735	0.410946484	0.681111777	0.847181914
70.41254584	0.051766273	0.181902201	0.284582994	0.775963641	0.897389272
130.57616	0.123045708	0.106683656	1.153369808	0.248758628	0.504399525

128.7634052	0.03435066	0.124993514	0.274819538	0.783454887	0.901193338
85.63091397	0.358917082	0.359381923	0.998706555	0.317936864	0.576478535
10.07401478	-0.38551756	0.462750292	-0.833100632	0.404787977	0.653771166
363.2932693	-0.197146898	0.078895973	-2.498820786	0.012460731	0.086056408
69.92035271	-0.233712442	0.15465294	-1.51120594	0.130735989	0.352919666
335.7567729	-0.284250391	0.10023148	-2.835939264	0.004569113	0.044837853
12.64513747	0.169308004	0.285644498	0.592722791	0.553366686	0.765681792
80.65685059	0.20099079	0.31718397	0.633672597	0.526294512	0.747749173
147.2068351	-0.285588468	0.123432257	-2.313726381	0.020682736	0.116859938
47.00064959	-0.334360885	0.182067034	-1.836471319	0.06628798	0.238290323
58.40612163	-0.29100241	0.142272763	-2.045383834	0.040817031	0.177157057
23.4473336	-0.214595306	0.334345648	-0.641836695	0.520979221	0.743835259
3.250847043	1.1021139	1.545862899	0.712944143	0.475880322	0.708736775
5032.245102	-0.169936875	0.150879403	-1.126309307	0.26003462	0.517429964
22.77021259	0.729928805	0.40590208	1.798287915	0.072131394	0.249434089
2.67136371	0.639804827	0.920306739	0.695208238	0.48692481	0.717486727
160.7526905	0.292159365	0.263840948	1.107331398	0.268150674	0.527139967
812.5082289	-0.337153049	0.113165762	-2.979284912	0.00288922	0.033662699
218.0974124	-0.082809679	0.139846799	-0.592145685	0.553753037	0.765862233
473.8632699	0.133491354	0.194869686	0.685028837	0.493325724	0.721697664
3.648543808	-1.03474211	0.888653276	-1.164393512	0.244264576	0.499749345
2969.125274	-0.372603452	0.078427075	-4.750954347	2.02459E-06	0.000166249
293.071529	0.1697661	0.107659526	1.576879508	0.114823253	0.326247807
395.3453269	0.013674279	0.110646072	0.123585758	0.901643269	0.958518064
499.7649365	-0.218697641	0.085835733	-2.547862458	0.010838519	0.078517939
391.1622311	-0.20769534	0.105952407	-1.960270154	0.049964223	0.19985689
2.864184147	2.084817425	1.137185541	1.833313343	0.066755986	0.2391505
47.36109508	-0.325924662	0.24896444	-1.309121343	0.190493251	0.438142857
364.4767648	0.127449273	0.115250372	1.105846956	0.268792767	0.527663112
142.5192594	-0.200601192	0.171678656	-1.168469027	0.242617626	0.49834135
95.9276592	-0.204581418	0.154357943	-1.325370196	0.185048397	0.430939775
20.30616984	-0.217299951	0.275124819	-0.789823148	0.429631058	0.674440348
969.1115748	-0.069905821	0.139228175	-0.502095361	0.615600443	0.80702296
110.3806722	-0.271329548	0.330337501	-0.821370711	0.411435142	0.65849534
325.3904587	-0.025861809	0.124428887	-0.207844096	0.835350701	0.929721855
26.50408839	0.71019419	0.331419804	2.142883982	0.032122415	0.152918638
400.0378641	0.175116666	0.177534049	0.986383551	0.323944934	0.582655085
166.9910127	0.172360899	0.105479804	1.634065403	0.102245118	0.306383729
436.5999098	0.123113049	0.120384347	1.022666589	0.306465514	0.565490503
79.83899693	-0.244602638	0.556166264	-0.439801286	0.660081036	0.834290513
9.404022346	0.808272325	0.483442918	1.671908503	0.094542366	0.292293015
312.5430551	-0.00263152	0.103196259	-0.025500152	0.979656027	0.989402677
54.55052982	-0.757383122	0.371653737	-2.037873013	0.041562634	0.178937237
2204.057717	0.471121127	0.197310604	2.38771316	0.016953568	0.104111105
693.2438213	-0.205142885	0.125590967	-1.63342069	0.102380548	0.30661381
142.9385426	0.009323133	0.324369366	0.028742336	0.977070091	0.988286296
678.9798546	-0.166993506	0.16020804	-1.042354087	0.297247545	0.555665136
96.071901	0.257012756	0.199443837	1.288647268	0.197520743	0.445999903

214.0654328	-0.159198837	0.092792836	-1.715637162	0.086228472	0.277313494
40.47100613	0.126522815	0.29058599	0.435405764	0.663267927	0.836326722
2.795029873	0.987923395	1.243653819	0.794371698	0.426979076	0.671823819
9.324037364	-0.506226057	0.369591957	-1.369689052	0.170783988	0.412501531
740.205449	0.232865556	0.113646283	2.049038031	0.040458396	0.176312722
7.457252402	0.673481279	0.73344566	0.918242913	0.358491707	0.614548692
97.22048837	-0.423805193	0.19833394	-2.136826365	0.032612117	0.154470328
359.2925148	-0.182094243	0.131596528	-1.383731363	0.166440727	0.406317874
248.3065746	-0.026747674	0.139361524	-0.191930122	0.847796942	0.934372901
2366.564299	-0.091359957	0.141401432	-0.646103483	0.51821234	0.741776247
112.7174928	-0.15097344	0.103963001	-1.452184314	0.146450363	0.376916228
4.163489193	-0.367995609	0.573737214	-0.641400976	0.521262199	0.744055368
47.10618833	-0.510005415	0.438742058	-1.162426546	0.245062246	0.500306136
2.762256442	0.178875643	0.565366548	0.3163888	0.751707417	0.88558395
11.31546647	-0.307402113	0.344371125	-0.892647759	0.372045835	0.626024556
871.1846545	0.160910877	0.098954632	1.62610757	0.103926774	0.308857032
106.8549166	-0.106344606	0.142952243	-0.743917018	0.456926679	0.693951586
3.734350835	0.046147229	0.946356491	0.04876305	0.961108129	0.98222115
512.039444	-0.257706281	0.123842383	-2.080921529	0.037441089	0.168057997
736.4802297	-0.037069961	0.088931041	-0.416839395	0.676795885	0.845132696
1291.123991	-0.07441778	0.141046429	-0.52761194	0.597768704	0.794878848
38.21078977	-0.172110132	0.343624476	-0.500866917	0.616464787	0.80702296
347.0053225	0.113120854	0.131261432	0.861798108	0.388798626	0.639798306
10.02738015	1.633522324	0.663472959	2.462078224	0.013813454	0.092191577
8.413732041	0.4513117	0.345318782	1.306942232	0.191232332	0.438494359
171.09065	-0.24806874	0.131943959	-1.880106846	0.060093518	0.223820279
532.0876327	-0.186369113	0.089957388	-2.071748818	0.038288872	0.170167943
1112.129031	0.040828978	0.058343214	0.69980679	0.484047974	0.715377439
157.063595	-0.014823538	0.109590396	-0.135263109	0.892403851	0.95479462
616.7016093	-0.382302842	0.171089173	-2.234523874	0.025448623	0.132452965
2097.768641	-0.180530179	0.155052679	-1.164315124	0.24429633	0.499749345
1282.064	-0.155625527	0.099185323	-1.569037867	0.116639116	0.329794104
324.5541939	0.052053727	0.159317969	0.326728541	0.743873211	0.881319493
290.2942597	-0.172341229	0.143375223	-1.202029374	0.229352148	0.484596401
90.16413008	-0.07730863	0.138805499	-0.556956539	0.577557123	0.781775691
349.204683	0.255925062	0.19830171	1.290584243	0.196847883	0.445249811
604.3661489	-0.261439932	0.111378112	-2.34731877	0.01890907	0.110991709
92.11285397	-0.294659262	0.306066895	-0.962728303	0.335683889	0.594295757
67.87341312	-0.402310435	0.142562014	-2.822003021	0.004772473	0.046062439
34.66215108	0.38373769	0.304274719	1.261155354	0.207252881	0.457400854
240.6470168	0.01117799	0.147766137	0.075646493	0.939700346	0.974295153
475.0473446	-0.187511517	0.091726569	-2.044244311	0.040929418	0.177330659
232.7576471	-0.099885358	0.081799832	-1.221094899	0.222050091	0.475639665
168.0129383	0.055874911	0.138166891	0.404401592	0.68591743	0.849421948
237.5165651	-0.318073171	0.125163321	-2.541265037	0.011045216	0.079380341
9.101518771	-0.258910401	0.340508984	-0.760362906	0.44703769	0.686914776
854.4952589	-0.291747974	0.149677271	-1.949180208	0.051273908	0.202727643
1252.331415	-0.675157779	0.604844597	-1.116249995	0.264315139	0.522039875

3163.453438	-0.316895193	0.141309978	-2.242553555	0.024925618	0.130893813
8.702131003	-0.256836516	0.387593366	-0.662644252	0.507558421	0.733352951
258.525991	0.071835978	0.172683338	0.415998318	0.677411234	0.8455982
15489.36368	-0.191290475	0.128701783	-1.486307889	0.137197701	0.36307079
3407.649201	0.097940098	0.160157194	0.611524812	0.540852198	0.757639765
1246.969799	-0.004083046	0.052279054	-0.078100983	0.937747725	0.973951084
165.6951346	0.117372201	0.121182372	0.968558375	0.33276558	0.591532966
29.12252145	0.014837272	0.200753736	0.073907823	0.941083731	0.974550053
369.9409477	-0.394608837	0.153289291	-2.574275305	0.010045031	0.075129358
916.9371649	0.131454358	0.055007635	2.389747521	0.016859959	0.103924805
239.6775363	0.062595154	0.141439027	0.442559283	0.658084541	0.833446217
2746.817036	0.051267621	0.145614246	0.352078336	0.724779515	0.871803199
438.0376134	-0.679603725	0.301875198	-2.251273801	0.024368201	0.129074929
1437.796419	-0.00991388	0.100039834	-0.099099329	0.921059405	0.966733301
301.8423575	-0.240178726	0.148170887	-1.620957601	0.105026732	0.310271709
753.8337242	-0.004992164	0.161922563	-0.030830567	0.975404663	0.988030139
715.7862581	-0.052550679	0.086421143	-0.608076643	0.543136643	0.75862099
228.0380653	-0.574971939	0.275858872	-2.084297431	0.037133121	0.167307058
1140.655345	-0.293294756	0.136207911	-2.153287239	0.031296111	0.150613135
1509.506264	-0.391612372	0.145999604	-2.682283786	0.00731214	0.061558565
2931.491601	-0.112103423	0.104333632	-1.074470627	0.282611803	0.541176514
58.79877103	0.136931797	0.139286263	0.983096212	0.325560089	0.584086528
421.3581338	-0.157105754	0.288292019	-0.544953532	0.585785477	0.786664905
26.86586445	-0.219416733	0.19275353	-1.138327962	0.254983562	0.512035005
6.828081728	-0.086448685	0.387026865	-0.223366108	0.82325058	0.923257803
1599.706118	0.18286944	0.164445509	1.112036693	0.266122374	0.524684264
3.205179674	0.903478566	0.660070846	1.368759994	0.17107431	0.412756553
229.8264241	0.20085987	0.113905782	1.763386074	0.077835397	0.260784867
256.2110388	0.119310346	0.083748167	1.424632333	0.154263509	0.38887588
2135.136283	-0.236821509	0.124706777	-1.899026771	0.057560956	0.218222392
1270.749921	-0.068098628	0.126220259	-0.539522172	0.589526603	0.789819339
124.6001655	0.243217072	0.174634245	1.392722672	0.16370367	0.402987989
53.89529018	-0.004918459	0.187431949	-0.026241305	0.979064871	0.989273349
304.8054475	0.297509864	0.136178644	2.184702784	0.028910646	0.143764924
13.71375411	0.32489971	0.328729671	0.988349207	0.322981654	0.581433782
1767.773661	0.207877049	0.105248473	1.975107499	0.04825595	0.195822365
774.3820993	0.152548711	0.086785881	1.757759553	0.07878843	0.262639264
9.560407893	0.360994881	0.39265203	0.919376073	0.3578989	0.61387646
1134.664014	-0.179683519	0.080810336	-2.223521484	0.026180656	0.134982713
350.1877518	-0.048642911	0.082959635	-0.586344326	0.557644149	0.768771196
296.8774666	-0.248928757	0.152802897	-1.62908401	0.103295236	0.307707973
159.7805049	-0.175233307	0.091711747	-1.910696425	0.056043605	0.214701488
1149.817146	-0.031076827	0.158637565	-0.195898284	0.84468978	0.932309255
696.7522635	-0.245267611	0.110467482	-2.220269763	0.026400461	0.13558115
144.575484	0.312108269	0.113353514	2.753406214	0.005897867	0.053040218
252.5378775	-0.213656462	0.219403327	-0.973806843	0.330152455	0.588541201
425.0489569	-0.069124885	0.107444928	-0.643351776	0.519995867	0.743254883
215.2391782	-0.053705248	0.103140045	-0.520702199	0.602574242	0.798428447

188.0192973	0.731663525	0.304026199	2.406580509	0.016102654	0.101021612
167.4897831	0.105408092	0.153719901	0.685715329	0.492892638	0.721493215
21.58962231	0.319487963	0.330208793	0.967533178	0.333277564	0.591900483
571.6168832	-0.238966247	0.274244188	-0.871363034	0.383555963	0.634974705
410.6095618	-0.234899776	0.135016851	-1.739781175	0.081897449	0.269282934
382.3475358	-0.079253965	0.089134515	-0.889150122	0.373922405	0.627431154
17.95678347	-0.176976195	0.284017857	-0.623116436	0.53320801	0.752219683
68.08101415	-0.823238239	0.483857342	-1.70140694	0.088866599	0.282282414
35.49165763	-0.206922402	0.370896318	-0.557898235	0.576913877	0.781306068
250.0719593	-0.094711365	0.086569724	-1.094047214	0.273934271	0.532804472
357.8183984	0.017281247	0.338026803	0.051123895	0.959226796	0.981758066
218.6724808	-0.126428888	0.384235971	-0.329039698	0.742125682	0.880313054
106.4705847	0.05187891	0.11952694	0.434035287	0.664262817	0.836652459
408.2345277	-0.160675669	0.058621218	-2.740913158	0.00612687	0.054351712
175.7638511	0.043912308	0.104294346	0.421042073	0.67372436	0.843244183
368.4995888	-0.371720032	0.229928137	-1.616679181	0.10594755	0.311292877
14.20951423	0.31622528	0.758432924	0.416945612	0.67671819	0.845111062
7.049796993	0.102180951	0.515081743	0.198378127	0.84274923	0.931991382
123.382796	-0.312527663	0.14658253	-2.13209352	0.032999159	0.155376408
440.1029726	-0.180978037	0.104938024	-1.724618295	0.084596284	0.274986139
121.495213	-0.210247689	0.130160548	-1.615295047	0.106246816	0.311646729
254.905014	-0.195376804	0.08972062	-2.17761316	0.029434849	0.145474083
1310.61885	-0.257931701	0.051408161	-5.017329873	5.23946E-07	6.08708E-05
109.5264937	0.367599888	0.131683805	2.791534516	0.005245876	0.049120193
15.38857847	0.739461284	0.529826278	1.395667438	0.162814664	0.401570246
930.3927102	-0.435368478	0.310048691	-1.404193891	0.16026112	0.397900176
1636.102958	0.105786594	0.070799487	1.49417175	0.135130734	0.35992814
1441.340607	2.09787141	0.868883563	2.414444809	0.01575921	0.100045497
140.974878	0.002771059	0.134684318	0.020574473	0.983585104	0.991149398
781.0775287	-0.037038069	0.106524121	-0.347696551	0.728068087	0.873123696
10.02244034	-0.226619295	0.412355358	-0.549572815	0.582612409	0.784649556
727.873421	-0.216662127	0.083463122	-2.595902485	0.009434284	0.072320286
451.7525702	0.500984938	0.156138597	3.208591263	0.00133387	0.020370411
89.21073226	-0.057559214	0.107641848	-0.534728968	0.592837288	0.791458973
288.1616539	-0.076901126	0.085553447	-0.898866486	0.368723781	0.623914531
111.9657687	-0.205262065	0.142019743	-1.445306551	0.148371799	0.3799287
84.10329761	0.17265356	0.166567347	1.036539051	0.299950731	0.558237448
1443.095157	-0.030863465	0.131490737	-0.23471969	0.814426304	0.918293469
405.5301933	0.094812144	0.287127828	0.330208831	0.741242175	0.879770661
419.7022873	-0.370227032	0.115968107	-3.192490102	0.001410518	0.021129476
197.606284	-0.058742934	0.150410299	-0.390551274	0.696128948	0.854510951
365.7569159	-0.260787289	0.106646157	-2.445351017	0.014471122	0.094805798
977.2523581	-0.177446379	0.082576627	-2.148869306	0.031644762	0.151593293
440.6401466	-0.111567906	0.084687116	-1.317412994	0.187700228	0.434606279
13.49421687	0.627058102	0.472088642	1.328263478	0.18409108	0.429655432
260.1116092	0.098365571	0.067121805	1.465478639	0.142790376	0.371632936
5.92928692	0.067692254	0.441147008	0.153446023	0.878046554	0.949481671
6402.312025	0.087225098	0.162530291	0.536669798	0.591495722	0.79088016

7.255383428	-0.212980238	0.690629917	-0.30838548	0.757789029	0.888509678
31.9276477	0.537996273	0.320499303	1.678619164	0.093226289	0.290053783
92.6877411	0.454354378	0.586413515	0.774802025	0.438456653	0.681138928
6.4151756	0.61299129	0.634449138	0.966178775	0.333954732	0.59218136
13.03651061	0.39404925	0.324182399	1.215517101	0.22416893	0.478092671
526.9910222	-0.103175065	0.101871158	-1.012799575	0.311155904	0.570697037
92.07328927	-0.411087903	0.309720292	-1.327287599	0.184413564	0.430216026
41.09540295	-0.496201158	0.532880475	-0.931167835	0.351766753	0.608637468
209.7071832	0.041155218	0.153485277	0.268137884	0.788593186	0.903520748
2087.333444	0.086386099	0.073794427	1.170631753	0.241746828	0.497292024
10.2807778	-0.631817639	0.302245832	-2.090409764	0.036581007	0.165580521
439.0012701	-0.036116785	0.477263674	-0.075674698	0.939677906	0.974295153
346.3505521	0.417393615	0.129355707	3.226712037	0.001252214	0.019387687
1552.604347	-0.319866977	0.116886941	-2.736550158	0.006208713	0.054645036
17513.35354	-0.205839195	0.15764258	-1.305733482	0.191643208	0.438793003
1163.098241	-0.186496249	0.058529358	-3.186371014	0.001440697	0.021397627
1957.489395	-0.219203117	0.101852988	-2.152152042	0.031385381	0.150857592
4378.455549	-0.094736327	0.11604099	-0.816403988	0.414269116	0.660728031
229.4389163	-0.008648429	0.196160821	-0.044088465	0.964833888	0.983521038
342.1813007	0.011706435	0.147734158	0.079239869	0.936841833	0.973575955
14.28934218	1.444074851	1.07002352	1.349572999	0.17715299	0.421107532
9.268128646	0.679743254	0.65121806	1.043802831	0.296576622	0.555344561
345.7057806	0.075930663	0.120268421	0.63134331	0.527816074	0.748800234
26.98019637	-0.100901637	0.342489672	-0.294612203	0.768290139	0.892934503
118.5748475	0.073034364	0.134660032	0.542361105	0.587569769	0.788116331
852.334693	-0.29725611	0.116168368	-2.558838647	0.010502247	0.077094864
91.59311335	-0.38444993	0.133197697	-2.88631065	0.003897872	0.040620748
2352.207953	-0.078633234	0.099363078	-0.791372769	0.428726496	0.673424773
129.758181	-0.000881872	0.213402846	-0.00413243	0.996702808	0.99842159
68.89525416	-0.052085954	0.147258295	-0.353704723	0.723560183	0.870937675
8.780343791	1.152590761	0.633781228	1.818594037	0.068973388	0.243205625
401.3552324	-0.322803743	0.097277253	-3.318388773	0.000905384	0.015384657
472.9530028	-0.283830885	0.130384347	-2.176878526	0.029489632	0.145490843
261.9139746	-0.381695014	0.113401689	-3.365867086	0.000763035	0.013645879
1575.481999	0.090255602	0.122426264	0.737224179	0.460986032	0.697145057
6.107024989	0.867230112	0.442916556	1.95799886	0.050230146	0.200439665
60.08355268	0.612116086	0.388427808	1.575881217	0.115053181	0.326664389
26.75116929	0.418821229	0.356143076	1.175991497	0.239598274	0.494780688
148.2835068	-0.001123137	0.105576542	-0.010638132	0.991512158	0.994746798
421.7410255	-0.115808769	0.066001778	-1.754631038	0.079322439	0.263529494
119.2107844	0.424098536	0.384780009	1.102184432	0.270381499	0.529355065
163.6144033	0.112948847	0.152954118	0.738449207	0.460241522	0.696718475
174.4047263	0.117528562	0.092237447	1.27419574	0.202594054	0.45153974
3183.59084	-0.078593758	0.147321758	-0.533483706	0.593698787	0.791890039
315.3343915	0.038686966	0.137429238	0.281504623	0.778323383	0.898382686
19.99077678	-0.064999215	0.230417707	-0.28209297	0.777872224	0.898328042
83.8839451	-0.075599019	0.15144471	-0.499185601	0.617648637	0.80773795
533.6661341	-0.268907722	0.067840225	-3.9638389	7.3754E-05	0.0026171

173.4591144	-0.233789246	0.099241695	-2.355756288	0.018485041	0.109279827
467.3048926	-0.242145162	0.112202532	-2.15810783	0.030919449	0.149521001
5.253882969	0.561069803	0.413540429	1.35674716	0.174861562	0.417988969
86.14694888	-0.14789396	0.375528984	-0.393828349	0.693707773	0.85351767
2.670698957	-5.054540034	2.773797545	-1.822245479	0.068417747	0.242103013
4.476886011	-0.100032365	0.787327306	-0.127053087	0.898898381	0.957506447
148.3395993	0.275186917	0.115114463	2.390550325	0.016823143	0.103797868
2.631327172	2.066474864	0.940894269	2.196288076	0.028071331	0.141088009
27233.684	-0.304780021	0.248455154	-1.226700334	0.219935242	0.472767285
124.2659772	0.023028662	0.154176895	0.149365195	0.881265474	0.950743683
641.5554398	-0.088706945	0.058868018	-1.506878406	0.131841827	0.354469729
750.0028014	0.216225511	0.256030688	0.844529662	0.398373495	0.648613091
398.9826173	-0.095466085	0.137186601	-0.695884904	0.486500912	0.717237435
34.01641206	0.046707086	0.186617629	0.250282282	0.802369058	0.910590181
164.2763249	0.058700631	0.107114615	0.548017012	0.583680222	0.785317029
156.4694494	0.08366404	0.264143124	0.316737528	0.751442771	0.885551531
6.45386688	0.2985776	0.80418442	0.371280011	0.710428985	0.862677974
8.077891612	-0.664272885	0.554320008	-1.198356319	0.23077833	0.486234192
124.593691	0.053934327	0.115925798	0.4652487	0.641753367	0.824075635
8.628457871	-0.165259605	0.355533748	-0.464821148	0.642059542	0.824312819
14734.6843	0.056120579	0.128453723	0.43689336	0.662188688	0.835607642
37.40095383	-0.648009518	0.367446325	-1.76354878	0.077807978	0.260784867
30.72645651	-0.50055525	0.379907334	-1.31757196	0.187646977	0.43459173
285.5304134	0.461185381	0.153251117	3.009344346	0.002618122	0.031497308
7.429726576	0.254265449	0.344086947	0.738956972	0.459933126	0.696427027
255.9435405	0.280328207	0.08876442	3.158114557	0.001587931	0.022931461
29.6518031	0.10155595	0.199527102	0.508983233	0.610763977	0.803626025
701.4363715	-0.203070585	0.066415115	-3.057595941	0.002231202	0.028266699
6.502428237	0.592957766	0.407337441	1.455691783	0.145477842	0.375475519
406.025106	0.275145405	0.127132325	2.164244268	0.030445607	0.148525321
1093.202596	0.122666561	0.037664341	3.256835457	0.001126617	0.018030474
4.431839036	-0.276105224	0.548447579	-0.503430473	0.614661651	0.806386728
59.0713444	0.024105689	0.1685285	0.143036275	0.886261532	0.952949894
642.3066099	-0.325271163	0.085796209	-3.791206688	0.000149917	0.004505329
133.9892292	-0.082060073	0.102286935	-0.802253708	0.422406215	0.667104931
20.66698104	0.4995211	0.264117777	1.89128163	0.05858676	0.220681236
316.9823916	-0.170672629	0.076363216	-2.235011009	0.025416626	0.132452965
323.4367214	0.017855783	0.125264548	0.142544589	0.886649862	0.953002771
595.7119906	0.092641572	0.081509118	1.136579252	0.255714218	0.512836005
36.08092646	0.289218504	0.41252025	0.70110135	0.483239768	0.714959594
254.5404373	0.033383764	0.219061225	0.152394675	0.878875656	0.949985237
325.0905991	-0.720520673	0.475828641	-1.514244017	0.129963961	0.351986664
614.6872263	0.128418237	0.086512271	1.484393316	0.137704607	0.363718264
421.9285646	-0.089576857	0.095727294	-0.935750432	0.349401698	0.606632725
66.85550219	0.435071296	0.162441437	2.678327058	0.007399093	0.061958022
55.45832912	0.231454354	0.163623853	1.414551417	0.157200062	0.393414037
368.6423794	0.06288439	0.14505335	0.433525942	0.664632724	0.83676248
4.088807833	1.423587928	0.754187112	1.887579231	0.059082461	0.221792559

873.4574747	0.112497024	0.10279245	1.094409404	0.273775461	0.532681978
55.0568275	0.320036398	0.150990903	2.119574039	0.034041984	0.158015758
81.56631763	0.041216091	0.120969802	0.34071388	0.733318989	0.875761442
3.730958751	2.465466285	1.679589877	1.4678978	0.142131986	0.370937242
50.77924849	0.092878392	0.132179299	0.702669725	0.482261599	0.714002102
17.91304632	0.142319145	0.266099123	0.534835076	0.592763906	0.791428373
571.4656795	0.269175471	0.09364221	2.874510004	0.004046549	0.041710507
580.5519603	0.42687728	0.090311471	4.726722674	2.28173E-06	0.000179832
42.84874569	0.577070987	0.17041172	3.386333911	0.000708331	0.013120652
52.83246526	0.064429694	0.21422717	0.300754074	0.763602031	0.891162606
3.807936036	0.544438189	0.499493581	1.089980352	0.275721799	0.534543001
1328.760329	1.979716985	0.807033137	2.453080171	0.014163877	0.09353526
3.194933175	0.712662338	0.869714488	0.819421026	0.412546243	0.659142931
9804.118654	2.000009279	0.887268066	2.254120661	0.024188578	0.128679492
159.1065109	0.005031756	0.113704864	0.04425278	0.964702911	0.983511961
188.4453135	-0.423583999	0.11910434	-3.556411114	0.000375956	0.008583448
3.361123845	-0.616033905	0.636740932	-0.967479667	0.333304301	0.591900483
234.4251107	-0.094333588	0.12146038	-0.776661395	0.437358569	0.680596258
92.42441126	-0.093728221	0.207860715	-0.450918399	0.652048362	0.829708504
4.373201372	0.888232393	0.454206311	1.955570344	0.050515789	0.200945172
239.3369402	0.287173666	0.152421998	1.884069692	0.059555549	0.222736124
11.62257693	0.473365364	0.287703814	1.645321827	0.099903461	0.302254221
1679.074787	-0.011440964	0.131813172	-0.086796822	0.930833014	0.970948722
1614.705094	-0.085136857	0.158169384	-0.538263824	0.590394923	0.790528908
337.3291468	0.208498946	0.08298044	2.512627627	0.011983575	0.08371955
187.1149167	0.346819754	0.16285674	2.129600251	0.03320463	0.155829271
180.880841	0.121885596	0.110926758	1.098793458	0.271858166	0.530854517
330.4182119	0.217226713	0.109095115	1.991168097	0.04646241	0.191364611
118.3253491	0.341224874	0.258117486	1.32197504	0.186176463	0.432335156
3.435255993	0.534783373	0.565358522	0.945919009	0.344189897	0.602015651
852.6163381	0.071214857	0.108803844	0.654525197	0.512773499	0.737355786
285.3074096	0.10071866	0.097499103	1.033021402	0.301593883	0.560316432
4.093383202	-0.150875033	0.515012279	-0.292954245	0.769557126	0.893892718
181.0692733	-0.173962702	0.105572403	-1.6478047	0.099392754	0.301581729
395.6219886	-0.148173575	0.107858176	-1.373781583	0.1695095	0.410494459
56.70525895	0.173975514	0.214283261	0.811895029	0.416851867	0.662355886
163.7121788	0.243139638	0.15110311	1.609097511	0.107595021	0.314000652
5.924144769	0.188194976	0.773878806	0.243184041	0.807862829	0.913849305
171.7131418	0.143724111	0.10139343	1.417489397	0.156339892	0.391824044
307.602766	0.157458911	0.118290453	1.331121043	0.183149186	0.428880565
183.7419416	0.03458209	0.096338801	0.358963262	0.719622573	0.868512899
10.42758656	0.794903474	0.639094098	1.243797237	0.213574197	0.465473842
164.5604443	0.291007507	0.219887729	1.323436779	0.185690167	0.431717253
20.9517351	0.619063463	0.472995647	1.308814294	0.190597264	0.438189312
145.0132284	0.447857948	0.157060812	2.851493912	0.004351432	0.043414668
784.6907512	0.10908331	0.139288831	0.783144704	0.433542142	0.677866111
152.775083	-0.716970516	0.355782914	-2.015190968	0.043884643	0.185122848
12.46935264	1.573056808	1.041881155	1.50982365	0.131088429	0.353506004

173.0332139	-0.672916663	0.32640178	-2.061620694	0.039243862	0.172990651
8.560944249	1.740737684	2.506681646	0.694439075	0.487406896	0.717927288
58.89658257	0.067021019	0.19127554	0.350389907	0.7260461	0.872122772
627.8129173	0.072857964	0.083986364	0.867497547	0.385669455	0.637055264
150.3831207	0.029504958	0.097974753	0.301148588	0.763301192	0.891117256
22.5990421	0.038051905	0.221867665	0.171507213	0.863824959	0.94255118
217.1179075	0.282769113	0.089648751	3.154189092	0.001609448	0.023115913
415.4598415	-0.019649323	0.089999582	-0.218326827	0.827174473	0.925812063
909.9581722	-0.274766144	0.082518627	-3.329746936	0.000869249	0.014899791
75.04295797	-0.079767061	0.142718669	-0.558911187	0.576222334	0.780913931
94.09432688	-0.274344208	0.107348342	-2.555644568	0.010599132	0.077427476
24.35585367	0.836474867	0.319561979	2.617566926	0.008855912	0.069205842
161.259079	-0.249105497	0.169786518	-1.467168895	0.142330116	0.371123116
315.3808176	0.547874817	0.178199637	3.074500186	0.002108556	0.0274901
703.5712064	-0.01422448	0.088154193	-0.161359088	0.871810588	0.946470917
155.0211405	-0.0783943	0.099681666	-0.786446522	0.431605938	0.676524838
13.12302086	0.380984266	0.418718997	0.909880539	0.362885514	0.618235515
280.2997033	-0.287900214	0.093291438	-3.086030409	0.002028481	0.026712588
148.5858876	0.066277117	0.230007514	0.288151962	0.773230418	0.895996959
524.7565405	-0.171065217	0.109768591	-1.558416801	0.119134477	0.334138972
32.43213354	0.158645549	0.393967336	0.402687061	0.687178454	0.850122158
364.0534337	-0.068312855	0.091345109	-0.74785455	0.454547894	0.692484621
7.176387033	-0.41200358	0.617441652	-0.667275327	0.504596283	0.730756058
29.11183141	-0.064654225	0.333565632	-0.193827598	0.846310877	0.933572921
8.040518306	-0.188836101	0.396520058	-0.476233415	0.633908097	0.818154591
74.62623454	-0.074423615	0.154348525	-0.482178984	0.629678805	0.815331232
261.5867543	-0.385897589	0.071208699	-5.419247833	5.98503E-08	1.05471E-05
378.3278241	0.076103289	0.109633978	0.694157875	0.487583207	0.718038917
1375.938759	-0.226960137	0.132810932	-1.708896502	0.087470123	0.279348687
22.38721076	0.618587742	0.261456302	2.36593166	0.017984764	0.107661464
293.3510094	-0.167299039	0.077327077	-2.163524669	0.030500848	0.148702302
839.5516701	-0.288804985	0.092188385	-3.132769781	0.001731652	0.024227682
165.9917443	0.473262782	0.137369078	3.445191514	0.000570655	0.011505047
574.1512214	-0.133511458	0.140680925	-0.949037388	0.342601597	0.600442892
228.171516	0.202282149	0.292892377	0.690636442	0.48979404	0.719520451
253.6316686	0.051341991	0.189970275	0.270263286	0.786957708	0.903033152
1523.918424	-0.11448449	0.149173766	-0.767457264	0.442809693	0.683810803
9.150608432	0.35784157	0.679779648	0.526408183	0.598604631	0.795178764
52.63218509	-0.020201299	0.180874993	-0.111686521	0.911071967	0.962059189
2383.360533	-0.354774207	0.132196728	-2.683683725	0.007281595	0.061466385
86.15812523	-0.183504278	0.20300778	-0.903927314	0.366033945	0.62196771
83.10453802	-0.017199061	0.170433124	-0.100913843	0.919618854	0.966087273
27.67411944	-0.078257901	0.211492042	-0.37002764	0.711361896	0.863147994
4.056533334	-0.528310583	0.599575712	-0.881140734	0.378241652	0.630630602
94.20330491	-0.203526583	0.113460892	-1.793803837	0.072844484	0.251042165
97.05838735	-0.697776072	0.31655793	-2.204260281	0.027506045	0.139460691
6.392265291	-0.80870361	0.383282014	-2.109944063	0.034863174	0.160679996
264.2711335	-0.093629919	0.099651887	-0.939569957	0.347438193	0.604864093

147.1904244	0.014029649	0.111782434	0.125508526	0.900120976	0.957962773
4525.23525	-0.314184276	0.154469362	-2.033958529	0.041955777	0.179762383
826.3483412	0.240646478	0.100753832	2.388459806	0.016919159	0.104041052
7.19689519	-0.482073369	0.393953437	-1.223681085	0.221072558	0.474384961
4.145361489	0.92502508	0.84813329	1.09066003	0.275422505	0.534437044
29.06184113	-0.03175122	0.265651308	-0.119522168	0.904861679	0.959974986
231.2078902	-0.157952363	0.088768579	-1.779372437	0.075178722	0.255881745
36.22570089	-0.110820789	0.164983579	-0.671707997	0.501769609	0.72820899
100.1542773	0.300183043	0.143884122	2.08628332	0.036952967	0.166800197
5.278575759	-0.181655026	0.532489456	-0.341142954	0.732995966	0.875575684
3.665996136	0.526641475	0.730165735	0.721262926	0.470747762	0.705535576
190.8558773	-0.009397713	0.142921723	-0.065754264	0.947573469	0.976525546
530.359163	0.195439952	0.09156546	2.134428774	0.032807698	0.154852183
307.1672472	0.070392971	0.068279542	1.030952596	0.30256305	0.561386357
161.0056901	0.115715556	0.088449725	1.308263608	0.190783914	0.438241275
1344.638121	7.46782E-05	0.089465848	0.000834712	0.999333997	0.999629461
35.12810783	0.570056927	0.244088261	2.335454082	0.019519712	0.112807492
529.0000265	0.365451965	0.110263204	3.314360105	0.000918531	0.015540717
863.3369304	0.015777735	0.119328384	0.132221138	0.894809382	0.955539338
406.6125394	-0.548131092	0.187093902	-2.929711159	0.003392772	0.037004338
2.593314485	1.002824822	1.501673959	0.66780463	0.504258311	0.730334043
175.0129481	-0.222865511	0.145125115	-1.535678445	0.124617264	0.343425257
222.027373	-0.328055611	0.33727773	-0.972657195	0.330723706	0.589172036
325.9248346	0.543612555	0.232597111	2.337142333	0.019431784	0.112494686
3.224873712	1.404770245	0.734716871	1.911988551	0.055877663	0.214198364
436.1514824	-0.244243013	0.166700632	-1.465159497	0.142877407	0.371746724
8.991478151	0.512015737	0.416018611	1.230752	0.218415633	0.471136129
101.7547009	0.923192141	0.466098058	1.980682232	0.047626921	0.194361413
1935.475532	-0.004814786	0.129348756	-0.037223288	0.970306971	0.985640837
7.716546385	0.263641734	0.383550543	0.687371556	0.491848617	0.720814214
736.4069505	0.064939463	0.109668261	0.592144553	0.553753795	0.765862233
2.809031102	-0.623637392	0.751666811	-0.829672646	0.406723891	0.655071986
712.5945204	0.058209077	0.108695532	0.535524106	0.592287493	0.791183678
680.9479105	-0.173333223	0.095006066	-1.824443745	0.068085014	0.241914826
201.6497563	-0.095599425	0.166451656	-0.57433748	0.565739451	0.774060678
31.02075776	0.021331083	0.189698032	0.11244757	0.910468539	0.961925804
10.53825521	0.191696294	0.363497412	0.527366324	0.597939225	0.794954548
8.486620661	0.113182649	0.683491135	0.165594904	0.868475758	0.945025059
869.6926015	-0.093732029	0.137985698	-0.679288	0.496955398	0.723718151
9.950747196	-0.095561467	0.343967742	-0.277821013	0.78114976	0.899392604
133.4918785	0.456423019	0.286666242	1.592175682	0.111345236	0.320866896
22.38673509	0.297475396	0.566107867	0.525474761	0.599253194	0.795689731
589.8310666	-0.254480458	0.276444433	-0.920548319	0.357286295	0.613393923
70.8525117	-0.209638253	0.317488959	-0.660300924	0.509060737	0.734235432
126.3342165	-0.18002039	0.35868085	-0.501895739	0.615740863	0.80702296
153.3904257	0.101852291	0.111456175	0.913832644	0.36080479	0.616769737
572.6883077	0.033812507	0.124649845	0.271259922	0.786191129	0.902680942
46.23179648	0.145467198	0.186982861	0.777970757	0.436586253	0.680328006

36.65357581	0.207225028	0.198688635	1.04296367	0.296965119	0.555412298
675.1322337	-0.091857095	0.128118104	-0.716972013	0.473391366	0.706774886
85.9777839	0.213339295	0.256688163	0.831122451	0.405904458	0.65475733
422.8678402	0.21451585	0.077112096	2.781870309	0.005404663	0.050128172
4.922912367	0.981676915	0.633030418	1.550757891	0.120959715	0.337233141
47.26900943	0.103133586	0.316780051	0.325568435	0.744750897	0.881693318
925.9029686	0.04592921	0.153440006	0.299330084	0.764688203	0.891645393
284.7209835	0.050124911	0.10055988	0.498458341	0.618161025	0.808044267
210.7884104	-0.369261039	0.13954922	-2.646098902	0.008142601	0.065442486
39.44095828	0.248148721	0.183220193	1.354374301	0.175616992	0.41891664
194.7545438	0.047788077	0.104056172	0.459252692	0.646052716	0.826214187
4.860210084	-0.244001416	0.81590919	-0.299054624	0.764898367	0.891746525
334.803592	-0.002760728	0.187014666	-0.014762093	0.988221982	0.993288488
39.39351613	0.259904906	0.340207805	0.763959269	0.444891516	0.685379023
157.5512895	-0.514497764	0.236832493	-2.172412058	0.029824596	0.146590087
517.3402052	-0.139905867	0.098187513	-1.424884514	0.154190587	0.38887588
67.78696959	0.038056799	0.503404568	0.075598835	0.939738264	0.974295153
1044.657123	0.05251866	0.152960232	0.343348456	0.731336331	0.874922414
26.60398683	-0.257687514	0.490991484	-0.524830924	0.599700732	0.795879699
45.27775235	-0.215470593	0.43830715	-0.491597258	0.623004082	0.810950402
367.727638	-0.215876534	0.203396354	-1.061358921	0.288526818	0.547984332
2.855340268	1.754004374	1.186591847	1.478186773	0.139357793	0.366603091
237.6892012	-0.142528875	0.089966294	-1.584247493	0.113137409	0.323981583
485.1695833	-0.22510636	0.122640744	-1.835494082	0.066432515	0.238536531
75.82490546	-0.297856867	0.237784287	-1.252634774	0.210338653	0.461824914
2979.13114	-0.29892014	0.145638179	-2.052484743	0.040122576	0.175434203
144.4690672	-0.159380624	0.114837564	-1.387878825	0.165173947	0.404923456
30.69863449	-0.046601932	0.244496041	-0.190604034	0.848835829	0.934780308
4314.732342	-0.070362317	0.166308468	-0.423083188	0.672234568	0.842065303
9.047127013	0.610430706	0.39084699	1.56181504	0.118331568	0.332899069
378.4293919	-0.124012754	0.083373925	-1.487428524	0.136901668	0.362635663
172.284633	0.466301531	0.192393131	2.423691163	0.015363665	0.098877193
14.29805625	-0.021424336	0.758343633	-0.028251488	0.977461572	0.988491024
234.8301343	0.045691376	0.642409966	0.071124949	0.943298312	0.974993168
530.3225974	0.165557733	0.079194408	2.090523011	0.036570844	0.165580521
340.5260977	-0.00663654	0.072456319	-0.091593666	0.927020884	0.969422292
19.08694964	0.249489817	0.292574369	0.852739828	0.393803599	0.64435403
10705.56237	-0.516726918	0.108024729	-4.78341324	1.72343E-06	0.000154459
29.62530508	-0.383447661	0.272161488	-1.408897574	0.158865457	0.39593927
111.9000833	-0.072087974	0.099382659	-0.725357669	0.468232635	0.70336675
14.78975549	-0.239034174	0.289947943	-0.824403757	0.409710181	0.657435871
274.2601121	-0.101488097	0.098254854	-1.032906698	0.301647564	0.560316432
106.3148976	0.13100466	0.112608649	1.163362331	0.244682526	0.499851416
2305.889897	0.218134685	0.088522875	2.464161783	0.013733411	0.091970459
2.94218762	0.348747721	0.676200081	0.515746346	0.60603158	0.800353596
11.67637745	-0.21964507	0.589063051	-0.372871919	0.709243772	0.862208683
5.446828792	-0.255191945	0.638090372	-0.399930725	0.689207541	0.851119654
22.91761322	0.411119526	0.294540543	1.395799444	0.162774898	0.401570246

425.8884159	-0.407046153	0.125087442	-3.254092866	0.001137551	0.018149896
22.97485899	0.041163172	0.556107746	0.074020138	0.940994362	0.974550053
2.800233741	3.981102482	1.477202604	2.695028069	0.00703827	0.05985635
1114.395303	0.122226794	0.100874119	1.211676442	0.225636261	0.480128028
134.3540593	0.4681161	0.199510519	2.346322899	0.018959674	0.111097787
2503.657297	-0.153770283	0.119515316	-1.286615711	0.198228265	0.446504682
459.7315819	-0.271006959	0.208149644	-1.301981374	0.192922753	0.440314141
276.7477313	-0.174121247	0.101916077	-1.708476742	0.087547918	0.279426445
139.5047912	-0.119274429	0.166474715	-0.716471743	0.473700109	0.707033928
157.5259406	-0.033999067	0.1222455	-0.278121219	0.780919308	0.899311917
356.8397214	-0.024940818	0.085692325	-0.291050777	0.771012485	0.894883432
188.5311133	0.204064438	0.278671122	0.732276944	0.463999549	0.699612471
638.2835957	-0.128274117	0.129317279	-0.991933311	0.321230058	0.579766655
12.47669038	1.154102662	0.478498404	2.411925831	0.015868509	0.100216166
9.227723427	3.117682654	2.352731873	1.325133004	0.185127041	0.431046989
7.024517895	1.053821209	0.863418994	1.220521225	0.222267348	0.475845085
553.0687748	-0.288779186	0.095559825	-3.021972724	0.002511332	0.03069971
16.99597303	-0.56569943	0.31901248	-1.773283072	0.076181813	0.257978772
139.6344064	0.019675902	0.13091652	0.1502935	0.880533061	0.950743683
30.55756139	0.318386938	0.34909052	0.912046934	0.361744011	0.617500334
133.3319685	0.523652172	0.181633134	2.88302118	0.00393881	0.040884374
457.7463709	-0.256150915	0.14684085	-1.744411829	0.081087304	0.267123141
30.23483023	0.52315739	0.309890743	1.688199478	0.091372943	0.286446781
193.8786384	-0.100725232	0.073459109	-1.371174152	0.170320674	0.411798723
200.162867	0.114806535	0.128560961	0.893012421	0.371850521	0.625963676
77.84566469	0.477361725	0.223159339	2.139107095	0.032426996	0.153794039
218.9658689	0.285696006	0.214135656	1.334182318	0.182144113	0.427528924
1402.083469	-0.154771505	0.11898138	-1.300804416	0.19332541	0.440650447
53.75806311	-0.268353098	0.139265207	-1.926921323	0.053989436	0.209804338
73.01614988	-0.333027278	0.1751895	-1.900954558	0.057307965	0.217578824
1244.173094	0.18439258	0.104566096	1.763406953	0.077831878	0.260784867
580.2304259	0.052001369	0.24183862	0.215025082	0.829747763	0.927302545
158.1095236	-0.283866431	0.121025306	-2.345513022	0.019000915	0.111156415
1057.379886	-0.004255019	0.099515492	-0.042757356	0.965894958	0.983885343
4127.207644	-0.019883018	0.107484069	-0.184985722	0.853240235	0.936802229
11.37739252	-0.656304498	0.363490524	-1.805561504	0.07098686	0.247029455
3.48630532	1.68692055	0.985147551	1.712353188	0.0868316	0.278443431
224.3959779	-0.149334502	0.216303971	-0.690391866	0.48994779	0.719574974
59.62700255	-0.280139544	0.228633414	-1.225278223	0.22047041	0.473354951
46.42250947	0.19834828	0.158801598	1.249032017	0.211653364	0.462972839
152.8786328	-0.087072072	0.139929666	-0.622255986	0.533773558	0.75258041
470.6562396	-0.01210034	0.059905707	-0.201989762	0.839924728	0.931178302
179.1862439	-0.257429462	0.08896406	-2.893634369	0.003808113	0.040111784
271.0972756	0.248013543	0.124926431	1.985276782	0.047113686	0.19294297
2.898231724	0.544454111	0.57764002	0.942549153	0.345911558	0.60354621
5.496926578	-0.285682562	0.463413071	-0.616474975	0.537581083	0.755368367
2.663868938	0.828297666	0.607747812	1.362896993	0.172914976	0.41533843
21.37468841	0.197888058	0.259387056	0.762906448	0.44551919	0.685723551

137.5636271	0.004983702	0.21376737	0.023313671	0.981400067	0.990430387
75.88120208	0.201070271	0.139723634	1.439056987	0.150134378	0.383377984
395.0951656	-0.001678823	0.124630487	-0.013470402	0.989252499	0.993496587
357.5620513	-0.13626396	0.102231116	-1.33290103	0.182564285	0.428194742
3.313449067	-0.369808431	0.6495622	-0.569319506	0.569139332	0.776004632
3.768738047	-1.014308824	1.058531997	-0.958222168	0.337950741	0.596356821
55.89396971	0.078105478	0.213255969	0.366252248	0.714176858	0.864524493
628.2636646	-0.34537897	0.098044867	-3.522662423	0.000427235	0.00931955
146.082117	-0.12608468	0.119854556	-1.051980701	0.292808404	0.552281525
3735.070627	-0.340599046	0.130168728	-2.616596554	0.008881124	0.069345634
163.5995994	-0.085600729	0.147651334	-0.579749107	0.562083823	0.771414313
2824.285781	-0.06072534	0.099335123	-0.611317911	0.540989136	0.757639765
198.0513412	0.319588561	0.18756932	1.703842397	0.088410535	0.281339109
459.6015016	0.334788753	0.102339445	3.271355953	0.001070331	0.017309476
1131.405291	0.097593178	0.086578776	1.12721827	0.259650208	0.516922217
7.137153086	0.365369114	0.414019162	0.882493246	0.37751013	0.630416193
403.9593101	0.049248549	0.100745647	0.488840462	0.624954649	0.812197296
36.33774237	0.359380693	0.261331613	1.375190278	0.169072461	0.409787125
49.08366203	-0.124293263	0.264986489	-0.469055095	0.639030256	0.821857362
9.457515192	-0.26667273	0.356796502	-0.747408474	0.454817032	0.692659256
1170.981414	-0.248948279	0.052014924	-4.786093254	1.70059E-06	0.000154174
401.5578062	-0.283781462	0.142271032	-1.994653857	0.046080645	0.190643324
5.893028619	-0.64267727	0.40713464	-1.578537433	0.114442196	0.325525707
605.6479759	-0.236387795	0.091746044	-2.576544844	0.009979326	0.074852104
100.4804494	-0.030860113	0.132828139	-0.232331138	0.81628083	0.919051478
40.96094668	0.080517919	0.226009241	0.356259411	0.721646303	0.869635084
20.32749533	-0.65281226	0.370296227	-1.762946021	0.077909594	0.260892704
112.3255866	-0.711865218	0.365584321	-1.947198437	0.051510949	0.203347023
12.99101806	-0.693824647	0.404407989	-1.715655145	0.086225179	0.277313494
4371.503226	-0.470920478	0.171564279	-2.744863219	0.006053612	0.053811727
96.30747531	-0.143462622	0.214684551	-0.668248466	0.503975003	0.730167397
3500.447357	-0.363206863	0.123459785	-2.941904219	0.003262008	0.03630879
45.10426918	-0.139130839	0.253519017	-0.548798432	0.583143787	0.785021177
382.1051947	-0.090920255	0.111928537	-0.812306298	0.416615898	0.662355886
29.45812152	0.027606878	0.262628748	0.105117503	0.916282571	0.964607645
25.25847613	0.549420979	0.252154765	2.178903812	0.029338815	0.145203524
3776.163906	-0.213053422	0.20201764	-1.054627815	0.291595578	0.550962625
29.66048681	-0.300012787	0.690728352	-0.434342656	0.664039633	0.836652459
46.79047716	-0.182997424	0.324767996	-0.563471236	0.573114042	0.779066086
701.8625443	-0.129441825	0.144479918	-0.895915683	0.370297798	0.62480271
4.206735617	-0.009726058	0.578244745	-0.016819968	0.98658024	0.992541292
351.3604458	-0.336002287	0.06752541	-4.975938475	6.49323E-07	7.18087E-05
560.8484129	-0.343852748	0.147250851	-2.335149483	0.019535613	0.112807492
59.09387354	0.678163889	0.310330616	2.185294828	0.028867236	0.143618922
98.8329299	0.40867231	0.638143209	0.640408461	0.521907083	0.744256234
743.3298728	-0.332881596	0.094574184	-3.519793488	0.000431883	0.009393675
217.7201123	-0.192116197	0.287255564	-0.668798872	0.503623786	0.729954299
2718.057617	-0.120177904	0.083969024	-1.43121711	0.152368001	0.386376675

386.7029165	0.240866525	0.142328058	1.69233339	0.090582425	0.285222797
30.67054094	0.656566723	0.429295	1.529406873	0.12616361	0.346177407
183.9715058	-0.204875811	0.086823855	-2.359671898	0.018291104	0.108748174
327.5471245	-0.066754277	0.086158417	-0.774785319	0.438466526	0.681138928
310.9920356	0.079699503	0.084785329	0.940015263	0.347209731	0.604802024
1461.405342	-0.043830043	0.05849368	-0.749312449	0.453668906	0.692019366
6.489407988	0.87838841	0.434585457	2.021209858	0.043258046	0.183218792
705.1046664	0.330684282	0.141499235	2.337004029	0.019438975	0.112494686
28.62480043	0.246069684	0.326279116	0.754169275	0.450747584	0.690248498
304.319467	-0.264022374	0.115897079	-2.278076164	0.02272204	0.124128345
2.706891429	0.884414108	0.885066629	0.999262744	0.317667428	0.5764038
8.92367746	-0.411915029	0.590902541	-0.697094698	0.485743532	0.716804808
29.65696189	-0.735550837	0.428261813	-1.717526092	0.085883091	0.277180856
694.8112401	0.020926306	0.135134081	0.154855875	0.87693494	0.948961489
149.4905723	-0.161107905	0.076755892	-2.098964666	0.035820016	0.16345683
47.671377	-0.073266246	0.154518203	-0.47415932	0.6353863	0.819186085
14.64683552	-0.005675265	0.341125682	-0.016636874	0.986726307	0.992541292
8.71056903	0.212703589	0.388570598	0.547400113	0.584103878	0.785617462
198.706316	0.067887394	0.153074266	0.443493189	0.657409045	0.832948475
154.8131346	-0.285331692	0.304920298	-0.935758274	0.34939766	0.606632725
484.9887147	-0.138615043	0.068018178	-2.037911733	0.041558761	0.178937237
1229.846107	0.327304856	0.441268324	0.741736576	0.458246954	0.694544379
3.776093182	1.440474609	0.676240837	2.130120705	0.033161649	0.155720751
13.73366096	0.809872687	0.86850458	0.93249098	0.351082846	0.60810418
479.1152302	-0.387251058	0.152434135	-2.54044842	0.011071042	0.07941135
1100.236346	-0.194582645	0.06088539	-3.195884034	0.001394031	0.021043298
4.881966514	-0.556359326	0.447522663	-1.243198104	0.213794841	0.465696321
152.6882335	0.098909284	0.204371139	0.483968942	0.628407911	0.814743298
989.915873	-0.308022254	0.081340121	-3.786842827	0.000152574	0.004533191
1085.421538	0.103652642	0.13856444	0.748046483	0.45443212	0.69248182
546.523002	-0.170475093	0.084664853	-2.013528494	0.04405906	0.185708762
796.3471489	0.073164472	0.100344083	0.729135886	0.465918545	0.70158715
4.971251491	1.068453775	0.464848991	2.298496491	0.021533547	0.119707191
34.18799534	-0.211672661	0.433059194	-0.48878459	0.624994209	0.812197296
2665.028303	0.179514042	0.091981113	1.951640257	0.05098093	0.201968642
2555.859176	0.123652934	0.149335205	0.828022661	0.407657666	0.655428277
15.77299697	0.710891814	0.385605752	1.843571601	0.065245598	0.235840507
356.1419924	0.161488054	0.125365876	1.288134053	0.197699303	0.44611812
2605.885178	-0.062915946	0.128050095	-0.491338532	0.623187031	0.810950402
179.6001769	0.110832545	0.148832373	0.744680359	0.456464976	0.69386622
29.74349369	-0.084597055	0.45318471	-0.18667235	0.851917537	0.936363053
1753.29652	-0.014731721	0.084832987	-0.173655571	0.862136156	0.941428912
4.908710211	-0.260803067	0.511178268	-0.510199834	0.609911469	0.80299368
117.4948647	-0.06870881	0.298116252	-0.230476567	0.81772147	0.920090647
822.295028	0.467229984	0.197598488	2.364542305	0.018052365	0.108024914
4915.526185	-0.217444154	0.114709609	-1.895605386	0.058012245	0.219244348
572.6436711	0.295350024	0.172656839	1.710618738	0.087151518	0.279126894
1073.243152	-0.179291966	0.077465109	-2.314486729	0.020641038	0.116846413

99.07245507	-0.0366796	0.130389738	-0.281307409	0.778474628	0.89838333
31.48988797	-0.097503569	0.265416415	-0.367360735	0.713349956	0.864200403
255.0462777	0.096101626	0.287012376	0.334834433	0.737749985	0.87841506
276.8874697	-0.230331597	0.140655183	-1.637562104	0.101513074	0.305181339
152.9508979	-0.550557331	0.137767398	-3.996281682	6.43452E-05	0.002395444
3.945986157	-0.335709498	0.527981809	-0.63583535	0.524883744	0.746353277
14.69334725	0.192625801	0.283578388	0.679268271	0.496967897	0.723718151
2096.159611	-0.166039402	0.102959148	-1.612672652	0.106815645	0.312621288
350.1334787	-0.321048636	0.130642717	-2.457455298	0.013992522	0.092902392
123.9139738	0.346929268	0.157974846	2.196104482	0.028084466	0.141088009
72.60478186	-0.40850835	0.152295691	-2.682336885	0.00731098	0.061558565
50.16425672	0.058231141	0.167517196	0.347612916	0.728130905	0.873123696
6.468349822	-0.963317152	0.559939057	-1.720396424	0.085360407	0.276153593
103.2863173	-0.210668417	0.141758691	-1.486105834	0.137251129	0.36307079
48.8928413	0.47542881	0.285222968	1.666867203	0.095540814	0.293873576
1146.185265	-0.05005762	0.091088485	-0.549549377	0.582628489	0.784649556
2432.234925	0.090194776	0.150176651	0.600591209	0.548112295	0.762447293
165.7804774	-0.503870711	0.172873698	-2.914675387	0.003560587	0.03817454
34.65650487	0.04838944	0.23161773	0.20891941	0.834511151	0.929483538
409.3934915	0.037775149	0.085492448	0.441853638	0.658595122	0.833668367
337.7334408	-0.005396051	0.084117656	-0.064148857	0.9488517	0.977223239
1848.797712	0.138278173	0.269683779	0.512741899	0.608131877	0.801680474
830.814646	0.025592715	0.157713184	0.162273785	0.871090257	0.946199847
610.4334158	-0.30283896	0.058691641	-5.159831198	2.47173E-07	3.293E-05
4.176673023	0.232251396	0.566827827	0.409738875	0.68199751	0.84736731
7.133473221	0.693605526	0.415086681	1.670989598	0.094723733	0.292738329
389.5676585	-0.418934428	0.123496954	-3.392265271	0.000693173	0.012942529
3908.211586	-0.178111848	0.069792518	-2.552019249	0.01071006	0.07783901
332.8003439	-0.246593833	0.256578589	-0.961084999	0.336509432	0.595219795
540.8965354	-0.066845974	0.130780018	-0.511132931	0.609257978	0.802570863
343.0940244	-0.106628865	0.090662493	-1.176107793	0.239551804	0.494780688
119.158646	-0.094492026	0.14361853	-0.657937567	0.51057825	0.735547834
6.853543045	-0.283463302	1.027372807	-0.275910847	0.782616542	0.900554501
142.0201969	-0.24799224	0.081102641	-3.057757904	0.002229997	0.028266699
128.6743131	0.020296577	0.123005493	0.165005453	0.86893969	0.945238598
96.93796818	-0.294078967	0.125137725	-2.350042461	0.01877127	0.11043083
43.82145599	0.012048138	0.234692111	0.051335932	0.959057837	0.981758066
1011.952724	-0.349580116	0.098758262	-3.53975563	0.000400498	0.008947871
2467.634775	0.01179775	0.215477247	0.05475172	0.956336265	0.980851293
282.7576197	-0.225492566	0.157905994	-1.428017768	0.153286744	0.387328709
1167.250399	-0.293283424	0.091984424	-3.188403103	0.001430609	0.021328592
488.8113673	-0.407591461	0.132005603	-3.087683049	0.002017235	0.026667047
119.3751254	-0.386560669	0.122834129	-3.147013548	0.001649473	0.023390902
231.9939372	-0.168515851	0.100897794	-1.670163874	0.094886945	0.292781203
77.19047728	0.706089556	0.56310757	1.253915937	0.209872562	0.461683841
179.989636	-0.395560484	0.113396408	-3.48829819	0.000486106	0.010275041
30.35983987	-0.424646394	0.243544428	-1.743609566	0.081227194	0.267415473
529.1239215	-0.359353914	0.085706914	-4.192822939	2.75504E-05	0.001263454

2.810525979	1.618451552	0.653015339	2.47842808	0.013196271	0.089442661
1061.016322	-0.111901764	0.086907199	-1.287600626	0.197885021	0.446180085
50.91763347	-0.183066599	0.156862315	-1.167052767	0.243189061	0.498715642
888.5869707	0.03927875	0.07546525	0.520487903	0.602723557	0.798472529
96.07868727	-0.061927798	0.109752591	-0.564249073	0.572584635	0.778738936
265.173674	-0.007753894	0.160394719	-0.048342575	0.961443225	0.98240675
7.758628164	0.133077052	0.389269964	0.341863139	0.732453891	0.875328164
9.755547199	0.298531031	0.450699787	0.662372248	0.507732685	0.733404422
130.8418634	-0.191970635	0.212770142	-0.902244238	0.36692714	0.622563077
427.661337	-0.332677125	0.268191792	-1.240444845	0.214810903	0.466762842
282.3487628	-0.188012492	0.064239285	-2.92675254	0.003425213	0.037239186
6.471429145	0.160932903	0.435993163	0.369117952	0.712039808	0.863498984
567.5499752	-0.838984804	0.310769528	-2.699700996	0.006940182	0.059253816
352.1145352	-0.179603325	0.116181591	-1.545884531	0.122132457	0.339151108
37.04312405	0.5475086	0.223984918	2.444399404	0.014509354	0.095016578
17.01180169	0.039119161	0.476149983	0.082157225	0.934521688	0.972907108
699.6162269	-0.316049385	0.089191211	-3.543503664	0.000394848	0.008897691
913.0975278	-0.193682286	0.074052557	-2.615470613	0.008910459	0.069448507
67.70415385	-0.396441204	0.178286181	-2.223622731	0.026173838	0.134982713
11.10783106	-1.495700296	0.847398693	-1.765049095	0.077555515	0.260752744
139.2974008	-0.151388633	0.110861337	-1.365567451	0.172074769	0.414120097
655.8911861	-0.352135224	0.07907022	-4.453449392	8.45016E-06	0.000496844
65.52809164	0.067420135	0.349954595	0.192653949	0.847229991	0.934350155
33.3743595	0.314046725	0.222484059	1.411547083	0.158083363	0.39474279
6023.484576	0.350866039	0.250507632	1.400620159	0.161327689	0.399852004
897.0010863	0.532351253	0.219763871	2.422378388	0.015419286	0.098991436
335.011463	-0.102380529	0.156661564	-0.653514025	0.513424951	0.738089544
3166.359656	0.030965591	0.167100672	0.18531099	0.852985118	0.936643758
79.5521031	0.106708437	0.174329574	0.612107481	0.540466648	0.757542051
694.1067677	-0.254625887	0.105953267	-2.403190513	0.016252718	0.10167835
158.7023421	-0.376859103	0.166242364	-2.266925791	0.023394759	0.126363037
3192.61192	-0.044325394	0.152142417	-0.291341463	0.770790179	0.89469162
524.0643086	-0.111407884	0.157391558	-0.707839007	0.47904524	0.711221434
48.429215	0.038881442	0.308879508	0.125878994	0.899827711	0.9578373
514.167292	-0.233762879	0.077343306	-3.022406084	0.002507739	0.030679701
152.1660299	0.104499265	0.111720817	0.935360729	0.349602428	0.606678964
328.5338871	0.002622738	0.126942901	0.020660769	0.983516264	0.991149398
77.45115609	0.191322165	0.150251105	1.273349465	0.202894061	0.451887313
17.5604761	0.286672596	0.256520863	1.117541055	0.263763056	0.52147081
94.84441271	-0.03928716	0.288353017	-0.136246744	0.891626224	0.954427088
7.211514915	-0.679943749	0.4241492	-1.603076817	0.108917696	0.316345396
3.003487917	0.411948764	0.607819039	0.677749031	0.497930833	0.724515
478.622003	-0.025191174	0.067474485	-0.373343694	0.708892661	0.862039895
461.7201664	0.120261681	0.250344838	0.480384106	0.630954294	0.816222958
5.674728778	0.370403701	0.564372101	0.656311148	0.511623946	0.736580684
776.5380286	-0.372102593	0.064437013	-5.774671662	7.71033E-09	1.86044E-06
6.879791638	0.841102137	0.51792648	1.623979793	0.104380121	0.309587334
36.05565442	0.289500032	0.374132703	0.7737897	0.439055165	0.681391372

1353.122412	-0.178317951	0.068404484	-2.606816698	0.009138827	0.070712067
4.45043078	2.09613191	0.803306023	2.609381543	0.009070604	0.070357745
26.83269966	0.327573318	0.687680197	0.476345428	0.633828306	0.818118933
12.15254336	0.459405085	0.418594952	1.097493133	0.272425881	0.531518716
535.9985257	0.509094837	0.150623189	3.379923362	0.00072506	0.013300507
443.7410791	-0.207671878	0.233905309	-0.887845934	0.374623631	0.627949503
81.68723181	-0.049908605	0.158237808	-0.315402529	0.752456047	0.886266194
2.806491322	-0.114307965	0.603131478	-0.189524124	0.849682049	0.935121273
131.7490409	0.406381074	0.251562258	1.615429427	0.106217732	0.311646729
189.7102988	0.460614196	0.45379308	1.015031335	0.310090891	0.569559144
929.5253984	0.036630936	0.109524753	0.334453492	0.73803738	0.878437596
685.2255064	-0.221698171	0.084377446	-2.627457716	0.008602553	0.067943266
277.660518	-0.163933177	0.110957238	-1.477444642	0.139556487	0.366887151
1776.920789	0.021334492	0.095574723	0.223223161	0.823361827	0.923257803
531.4417071	-0.071808919	0.073597187	-0.975701953	0.329212184	0.587680844
499.2931739	0.232060253	0.121636147	1.907823108	0.056414083	0.215541651
5.979788743	0.544671258	0.467869627	1.164151779	0.244362508	0.499749345
8660.445576	0.411027268	0.109339295	3.759190769	0.000170464	0.004878749
3.113672356	1.085135971	0.697630761	1.555458894	0.119836817	0.335329285
13.96144354	-0.75534555	0.44554208	-1.695340538	0.090010839	0.284376518
76.50519812	0.119835888	0.138283622	0.866594947	0.386163983	0.637536412
157.5292419	0.074429827	0.152724982	0.487345462	0.626013536	0.81318505
6.295430602	-0.277680472	0.500045541	-0.555310365	0.57868239	0.782148794
1485.981395	0.360696313	0.195415135	1.845795172	0.064921952	0.234941369
665.7514434	-0.254109731	0.372730209	-0.681752443	0.4953955	0.723106843
397.1340117	-0.629301954	0.262606401	-2.396369439	0.016558391	0.102730147
432.6969604	-0.454102614	0.20414341	-2.224429452	0.026119564	0.134933875
11.13628983	1.8198836	1.068849985	1.702655775	0.088632506	0.281855681
222.5473692	0.149059887	0.228186294	0.653237688	0.513603057	0.738142613
55.30343291	0.17351192	0.160262248	1.082674943	0.27895273	0.53751602
148.358141	-0.12871216	0.133398016	-0.964873121	0.334608366	0.592978848
75.10738159	0.208033315	0.126330483	1.646738857	0.099611734	0.301938075
68.41202275	0.046307107	0.369359803	0.125371268	0.900229633	0.957994774
102.8420288	0.498598386	0.154450264	3.228213226	0.001245661	0.019324377
1000.269913	-0.022857458	0.140536536	-0.162644241	0.870798552	0.946199847
53.1056394	0.113734825	0.157919076	0.72020954	0.471395991	0.705744056
77.74673904	-0.088869795	0.139395049	-0.637539103	0.523773748	0.745586083
189.3684301	0.02844533	0.099268532	0.286549312	0.774457431	0.896493493
86.03437177	-0.242019625	0.118712319	-2.038706905	0.041479288	0.178823847
47.95426239	0.314384458	0.144011507	2.183050959	0.029032057	0.144157627
13.13588542	0.511561963	0.259310017	1.972781338	0.04852048	0.196225524
559.5777308	0.321711116	0.113548822	2.833240449	0.004607871	0.045056016
578.6037745	0.004244659	0.060560968	0.070089016	0.944122809	0.975492778
170.3219469	0.332357623	0.165081899	2.013289307	0.044084203	0.185742468
128.3541566	-0.013424784	0.3084835	-0.043518644	0.965288103	0.983619889
43938.65335	0.165684796	0.140659661	1.177912661	0.238831427	0.49442766
159.1529241	0.418302512	0.157892738	2.649282781	0.008066281	0.065144978
1525.492482	-0.128577869	0.127061386	-1.011935041	0.311569113	0.570697037

13.52086537	0.161167242	0.353891012	0.455414907	0.648810773	0.827875514
966.9275619	-0.013033889	0.156799575	-0.083124517	0.933752532	0.972604593
194.3983606	-0.076035977	0.10926909	-0.695859894	0.486516576	0.717237435
340.2095989	-0.203347238	0.096263816	-2.112395371	0.034652552	0.159991351
3613.328645	0.196220262	0.170626582	1.149998199	0.250144613	0.506162832
299.9353208	0.071579688	0.060742174	1.178418273	0.238629896	0.494147253
1117.115046	0.155117672	0.152962363	1.014090455	0.310539592	0.570116231
46.63479563	0.164912094	0.167269025	0.985909336	0.324177605	0.582873043
550.3671292	0.124643559	0.087985186	1.416642555	0.15658746	0.392193824
324.6631331	-0.013552528	0.106124435	-0.127704126	0.898383125	0.957387882
435.9721644	0.024419839	0.070927555	0.344292686	0.730626184	0.874409849
271.2669083	0.007241437	0.207263848	0.034938256	0.972128976	0.986405567
106.4829211	0.172474506	0.141050995	1.222781212	0.221412343	0.474540521
63.76680514	-0.055988798	0.145241986	-0.385486313	0.699877145	0.857032569
86.37421633	0.327509719	0.135050929	2.425083047	0.015304885	0.098633834
624.5137346	0.276099801	0.076685662	3.600409701	0.000317716	0.007601441
2.7335984	0.985180508	1.015953867	0.969709885	0.332191122	0.590846627
421.0594723	-0.141323158	0.096306594	-1.467429722	0.142259194	0.371061566
11.05770822	0.456792483	0.456163069	1.001379801	0.316643226	0.575732992
1220.858297	0.146588793	0.128855513	1.137621426	0.255278596	0.512353414
54.62520694	-0.081577936	0.157142077	-0.519134894	0.603666677	0.798632907
328.9408393	0.113912174	0.143129437	0.795868249	0.426108618	0.671127492
411.1411038	0.224832338	0.13499904	1.66543657	0.095825689	0.29451893
84.1977332	0.084213643	0.154463021	0.545202614	0.585614174	0.786664905
15.67291813	0.954406156	0.472113538	2.021560661	0.04322176	0.183114554
168.2113966	0.13561378	0.090867966	1.492426707	0.135587318	0.360788734
28.2911695	0.165973037	0.241828986	0.686324002	0.492508817	0.721175267
411.8621763	0.184501064	0.145374838	1.269140289	0.204391033	0.454253785
135.3931796	0.237635374	0.131522247	1.806807434	0.070792309	0.246968718
192.4146428	0.273265121	0.1271904	2.148472855	0.031676211	0.151651309
25.18659236	0.331287113	0.218520825	1.516043669	0.129508311	0.351298572
103.9643436	0.168302654	0.158110823	1.064460047	0.287120344	0.546589349
2909.515032	0.090496435	0.102588685	0.882128817	0.37770715	0.630440674
20.38596119	-0.085521625	0.286422551	-0.298585515	0.765256319	0.89196493
126.448929	0.235556955	0.103599962	2.273716615	0.022983031	0.125205228
17164.31639	0.438897568	0.279271499	1.571580231	0.116047934	0.328477856
593.2964755	0.314590063	0.193901542	1.622421666	0.104713093	0.309829354
2039.668123	0.172523648	0.130861974	1.318363491	0.187381996	0.43417037
39.10511424	0.23826726	0.22682398	1.050450048	0.293511246	0.552665238
31.72732577	0.788786743	0.496464619	1.588807567	0.112103844	0.322316534
15.72248634	0.665508662	0.766889115	0.867802983	0.385502197	0.636846056
3.021919456	1.070753481	0.790827084	1.353966629	0.175747024	0.419035624
4.376742355	1.045123143	1.331927072	0.784669946	0.432647108	0.677356331
2685.982463	0.647925436	0.25730881	2.518084931	0.011799487	0.082691308
1253.360157	0.083304192	0.125766916	0.662369683	0.507734329	0.733404422
54.3060064	-0.402837063	0.386192469	-1.043099219	0.296902342	0.555412298
526.3704391	-0.049873053	0.068428913	-0.728830122	0.466105583	0.701641229
80.40505678	-0.132607544	0.13542902	-0.979166381	0.327497777	0.585964825

52473.13871	-0.183941498	0.21119201	-0.87096807	0.383771588	0.634974705
16.38868581	0.759965758	0.698260109	1.088370577	0.276431545	0.5349973
101.2841219	0.476083993	0.517279436	0.920361337	0.357383966	0.613444778
135.5509172	0.401608078	0.569520271	0.705169066	0.48070503	0.712606586
166.1420401	0.221727674	0.184735671	1.200242882	0.230045026	0.485275882
506.1887181	0.189917354	0.139946679	1.357069387	0.174759164	0.417988969
133.3408152	0.270568549	0.207150792	1.30614296	0.191503946	0.438775498
10.14680595	0.513526424	0.378700065	1.356024124	0.175091492	0.418235603
4.730942714	-0.008595026	0.986687666	-0.008710989	0.993049724	0.995779801
55.97606288	0.201091987	0.385366891	0.521819574	0.60179596	0.797917646
3.237972459	1.148341639	0.628740874	1.826414803	0.067787804	0.241165781
34.79467317	-0.830177022	0.400512107	-2.072788834	0.038191936	0.169929738
115.1177873	-0.235400616	0.550874687	-0.427321534	0.66914515	0.839858558
104.846568	-0.016126703	0.327086328	-0.049304119	0.960676937	0.98222115
297.550049	0.51256818	0.163042113	3.143777825	0.00166782	0.023608385
241.4940991	0.523088061	0.23281073	2.246838283	0.024650364	0.130130027
7.080653574	0.181164206	0.402254123	0.450372526	0.652441852	0.829986049
649.2030434	0.05709206	0.057067189	1.000435827	0.317099639	0.576008142
47.85756576	0.881902728	0.488289306	1.806106987	0.07090163	0.247029455
42.69987423	-0.053758931	0.200400473	-0.268257505	0.788501114	0.903520748
1278.5572	-0.427670088	0.180691507	-2.366852183	0.017940096	0.107557137
124.8184344	-0.462211984	0.092998123	-4.970121644	6.69109E-07	7.28771E-05
490.8148634	0.219403724	0.118435628	1.852514545	0.063951971	0.232615883
8.841059616	0.417892433	0.399534807	1.045947503	0.295585271	0.554606937
1359.079963	0.168854425	0.112539141	1.500406206	0.133509213	0.356965308
114.027915	-0.066984021	0.14416097	-0.464647405	0.642183978	0.824312819
76.62170312	0.156314852	0.18301173	0.854124772	0.393035859	0.643530057
671.0237237	-0.169932694	0.073660273	-2.306978873	0.021055998	0.118230857
423.2155618	-0.036743692	0.152166016	-0.241471077	0.80919003	0.914888728
189.2288282	-0.020968473	0.126689312	-0.165510985	0.868541804	0.945025059
3.442803786	0.245234696	0.64291915	0.381439401	0.702877229	0.859163468
430.439688	0.345288884	0.151673618	2.276525658	0.022814567	0.124417129
34.65492901	0.638636178	0.25243021	2.529951463	0.011407831	0.080776711
30.29435263	0.32557388	0.349034747	0.932783579	0.350931722	0.608090959
261.5528924	-0.229025001	0.114810315	-1.994812061	0.046063381	0.190624908
352.8168157	-0.018912344	0.11109917	-0.17022939	0.864829739	0.942899493
7.379791884	0.189970683	0.396863534	0.478680117	0.632166213	0.816982109
709.4269064	0.142615804	0.103915981	1.372414542	0.169934426	0.41115219
40.22623262	-0.085508363	0.277570407	-0.308060083	0.758036614	0.888509678
2568.600521	0.078732791	0.077321953	1.018246281	0.30856093	0.56761314
19.72524256	0.23712462	0.255069226	0.929648097	0.352553316	0.609439679
88.45276412	-0.039284278	0.150314678	-0.261346919	0.793824987	0.905377878
399.5172229	0.068909673	0.110441764	0.623945788	0.532663188	0.751893569
314.284813	0.083238887	0.079552839	1.046334589	0.295406581	0.554602454
527.1276547	0.192176176	0.080949309	2.374031078	0.017595069	0.106392904
2240.530733	0.001972223	0.074385555	0.026513515	0.978847754	0.989127516
195.2544751	0.185211632	0.132697516	1.395743022	0.162791894	0.401570246
77.0083137	0.189890937	0.117233388	1.619768391	0.105282039	0.310500845

6.468052408	0.66792207	0.440166639	1.517430016	0.12915815	0.35089493
349.4255544	0.50514247	0.112398801	4.494198045	6.98326E-06	0.000434625
17.64497467	0.512724179	0.588502761	0.871234959	0.383625876	0.634974705
117.812522	0.670883753	0.443276444	1.513465834	0.130161373	0.352338795
420.4833178	0.347756226	0.116519397	2.984535073	0.002840096	0.033266671
827.5556284	0.079707988	0.275523833	0.289296162	0.772354749	0.895712207
3.867347661	0.320953873	0.470924548	0.681539908	0.495529924	0.723170992
3.192509866	0.675078596	0.67684366	0.997392214	0.318574169	0.576965043
417.8123964	0.092746261	0.161887889	0.572904261	0.566709519	0.774712115
278.3693816	0.227006654	0.106380166	2.133918965	0.032849415	0.154857295
587.6449293	-0.039691165	0.09146219	-0.433962545	0.66431564	0.836652459
1471.218927	-0.015764919	0.07504022	-0.210086254	0.833600354	0.929224446
54.3835792	-0.135304386	0.162507819	-0.832602312	0.405069054	0.654067923
38.28010882	-0.451926392	0.517891512	-0.872627532	0.382866129	0.63463646
16.09602107	0.728195454	0.657278832	1.107894272	0.26790748	0.526747011
7.46822955	-0.579520793	0.567443062	-1.021284481	0.307119677	0.566057952
3.183700137	0.409247808	0.731950013	0.559119886	0.576079904	0.780913931
3925.418051	0.1063176	0.081831293	1.299229131	0.193865307	0.441367902
1472.992031	0.129250307	0.122931397	1.05140192	0.293074034	0.552345884
230.5773934	0.051864272	0.103944015	0.498963524	0.617805081	0.807875178
569.6072182	-0.054139647	0.140684734	-0.384829575	0.700363686	0.857427527
64.49632102	0.473106305	0.309883847	1.526721413	0.1268303	0.347308746
12.91424309	-0.289816417	0.567999726	-0.510240416	0.609883041	0.80299368
148.7768437	0.025792535	0.112007671	0.230274723	0.817878301	0.920171284
814.7848317	-0.327867504	0.079380846	-4.130310009	3.62274E-05	0.001580612
108.0066351	-0.265500993	0.147311033	-1.802315739	0.071495744	0.248036203
6.134121709	-0.456563563	0.408930617	-1.116481732	0.264215986	0.521909763
720.1794775	-0.101306359	0.136474062	-0.742312188	0.457898209	0.694284175
1622.78288	-0.127398607	0.090538269	-1.407124405	0.159390502	0.396995496
146.257661	-0.01862241	0.09903534	-0.188038028	0.850846841	0.935636028
367.5972769	0.094006365	0.151027177	0.622446678	0.533648195	0.752538958
861.92399	-0.013519718	0.066512777	-0.203264974	0.838927931	0.930862799
3979.424894	0.058728818	0.138092226	0.425286925	0.670627524	0.840911584
2293.062443	-0.394245566	0.309943275	-1.271992645	0.203375732	0.45270295
309.3541472	0.071350429	0.176191981	0.404958435	0.685508064	0.849317361
16045.79613	0.113645473	0.125628426	0.904615909	0.365668904	0.62162921
115.5737048	0.026772568	0.101400216	0.264028705	0.7917578	0.90458703
149.9091895	-0.008887679	0.129773594	-0.068486037	0.945398735	0.976005209
139.9855072	0.01546574	0.115523156	0.13387567	0.893500889	0.95506051
212.4649206	-0.137066674	0.126069387	-1.087232018	0.276934284	0.535402099
198.056579	-0.213842874	0.116708008	-1.832289632	0.066908281	0.239477288
94.18447391	-0.439440287	0.172783515	-2.543299848	0.010981095	0.079192117
409.5257623	0.264324591	0.178204726	1.483263646	0.138004377	0.36432598
3.068325046	1.543849679	0.864971601	1.784855915	0.074284691	0.254187125
3.326164827	0.77287603	1.056302283	0.731680735	0.464363458	0.699959296
16.37179931	0.729063898	0.300640785	2.425033246	0.015306985	0.098633834
131.6183469	0.009791793	0.225533828	0.043416074	0.965369865	0.983619889
24.30238994	1.584140414	0.595349815	2.660856481	0.007794217	0.064090804

640.9372862	0.120422335	0.184944443	0.651127082	0.514964456	0.73923724
1426.42117	-0.132705653	0.137033943	-0.968414471	0.332837415	0.591593611
229.7105013	0.562154856	0.125599246	4.475782102	7.61321E-06	0.000461978
3.405472587	0.798566432	0.644257234	1.239514885	0.21515488	0.467251334
957.5046838	0.090618591	0.118579763	0.764199461	0.444748387	0.685379023
102.5487556	0.166016011	0.186152594	0.891827541	0.372485377	0.626427263
7.323553846	0.840815939	0.517597836	1.624457987	0.1042781	0.309401763
43.8965584	0.436504619	0.472385913	0.924042414	0.355464234	0.612138993
150.1880647	0.295580174	0.114587568	2.579513456	0.00989396	0.074454985
256.860094	-0.148691703	0.079086846	-1.880106628	0.060093547	0.223820279
383.6613184	0.126280321	0.127395688	0.991244861	0.32156603	0.580239486
216.4086987	-0.280395441	0.335977983	-0.834564926	0.403962717	0.653167263
585.4644227	0.140814088	0.159351401	0.883670221	0.37687426	0.630092303
58.94916379	-0.569405798	0.37066222	-1.536185148	0.124492978	0.34327494
127.8626289	-0.321361948	0.231823157	-1.386237474	0.165674402	0.405435688
10.50943674	-0.253645105	0.73907537	-0.343192474	0.731453666	0.874922414
761.8066375	0.174782473	0.065698119	2.66038777	0.007805073	0.064091503
59.35408855	-0.327771729	0.142342986	-2.302689705	0.021296308	0.118868199
16.50264426	-0.000126722	0.335025095	-0.000378246	0.999698204	0.9998257
262.8506353	0.106195955	0.142507642	0.74519481	0.45615396	0.693718482
322.2956344	-0.316257588	0.264924987	-1.193762778	0.232570776	0.487913329
128.9263864	-0.059812298	0.13136226	-0.455323301	0.648876666	0.827875514
128.6787975	-0.815318113	0.562049721	-1.450615636	0.146886919	0.377482294
7.106762605	-0.828834071	0.716153838	-1.157340821	0.247133142	0.502434185
2064.016835	0.143805191	0.147418753	0.975487768	0.329318366	0.587727238
611.5256292	0.403426815	0.135899783	2.968561129	0.002991976	0.034302741
21.240851	1.105519658	0.643976534	1.716707985	0.08603254	0.277180856
17.83667967	0.424954599	0.439835887	0.966166271	0.333960988	0.59218136
105.717758	-0.096715201	0.116757351	-0.828343574	0.407475952	0.655337658
50.75804538	0.029926646	0.285745787	0.104731714	0.916588696	0.964752507
5.421994012	-0.396199502	0.527951389	-0.750446936	0.452985572	0.691515058
21.25810269	0.45969271	0.230814133	1.991614226	0.046413401	0.191263212
335.4457474	-0.058900688	0.109825471	-0.536311724	0.591743129	0.79088016
1972.858543	-0.079104282	0.143408655	-0.551600473	0.581222112	0.783690475
4.62530972	0.1894694	0.530838424	0.356924804	0.7211481	0.869502368
121.9277256	0.025608518	0.113488008	0.225649555	0.821473996	0.921865923
217.6169933	-0.529395204	0.219833384	-2.40816565	0.016032904	0.100785601
354.4441884	-0.026079388	0.095650167	-0.272653872	0.785119296	0.902044615
51.55014043	0.011797999	0.231893768	0.050876741	0.95942374	0.981770858
519.295098	0.235751511	0.254358515	0.926847334	0.354005805	0.610806055
124.8896323	-0.005921037	0.14020846	-0.042230241	0.966315155	0.983959158
72.62460207	0.333483729	0.267402182	1.24712419	0.212351965	0.463797272
117.2404544	0.50455853	0.113175286	4.458204163	8.26492E-06	0.000492878
10.00622775	2.348893623	0.859795952	2.731919843	0.006296646	0.055171281
102.0344044	0.352815927	0.156073605	2.260573955	0.023785652	0.127539886
244.2384983	0.301237467	0.121928692	2.470603615	0.013488524	0.090873714
169.6305166	-0.12529388	0.324262218	-0.386396788	0.699202828	0.856875853
304.1214261	0.145238883	0.097120437	1.495451298	0.134796701	0.359253908

115.4749749	0.040940381	0.187782132	0.218020644	0.827413027	0.925836979
809.6451442	0.094353228	0.059260919	1.592166136	0.11134738	0.320866896
121.0332408	-0.355693233	0.141886221	-2.506890599	0.01217984	0.084788555
58.96591972	-0.026821381	0.141460965	-0.1896027	0.849620471	0.935119121
75.83856209	0.084863261	0.202040777	0.420030366	0.674463271	0.843697714
625.8033893	0.46528185	0.147481023	3.154859129	0.001605756	0.023084033
24.90273481	0.219300215	0.223924245	0.979350026	0.32740706	0.585964825
526.7995131	0.211039901	0.117606066	1.794464416	0.072739073	0.250840427
227.0938531	0.235586857	0.110020335	2.141302846	0.032249624	0.153180829
366.8682497	0.209161877	0.077389206	2.702726748	0.006877326	0.058813511
146.0179885	0.079191024	0.109738733	0.721632392	0.470520519	0.705443439
1417.312568	0.040794105	0.151517764	0.269236447	0.787747734	0.903200814
32.72388251	0.579028358	0.311383631	1.859533711	0.062951526	0.230617846
455.6048358	0.046017411	0.297372369	0.154746762	0.877020963	0.948961489
228.0400968	0.270727037	0.116208782	2.329660745	0.019824089	0.114015772
1487.722795	0.008780468	0.097990374	0.089605412	0.928600784	0.970140453
1445.477635	0.013110876	0.066110834	0.198316608	0.842797359	0.931991382
239.740758	0.14946013	0.126445568	1.182011616	0.237201092	0.492684668
605.3092465	0.140839594	0.1011108	1.392923346	0.163642972	0.402987989
8.234693572	0.503470371	0.320191707	1.572402907	0.11585714	0.328233994
4.692966386	0.084339465	0.463606298	0.181920447	0.855645158	0.938326015
16.73483991	0.328217698	0.281115313	1.167555388	0.242986154	0.498560288
2.66843436	0.702579444	0.664589841	1.05716248	0.29043744	0.549857317
8.750383474	1.210221899	0.458705997	2.638338953	0.008331326	0.066381962
87.38420387	-0.124896428	0.201006356	-0.621355617	0.534365668	0.752806174
707.4610419	0.547534892	0.143472772	3.816298266	0.000135469	0.004182464
9.051270883	0.604683105	0.35185325	1.718566206	0.085693389	0.276774119
21.91565166	-0.017374096	0.212941991	-0.081590748	0.934972159	0.973012718
485.4063794	-0.204721949	0.09430644	-2.170816211	0.029945068	0.146905989
89.83138987	-0.390699035	0.291255373	-1.341431168	0.179780504	0.424747406
740.15288	-0.38736552	0.121105796	-3.198571271	0.001381104	0.02086824
954.1198528	-0.557175612	0.153959204	-3.618982164	0.000295764	0.007282202
42.72495111	-0.470351663	0.304438695	-1.544979895	0.122351126	0.339546521
371.5125032	0.233926219	0.148735399	1.572767616	0.115772637	0.328159463
11.97798845	0.81632752	0.364060155	2.242287459	0.0249428	0.130924656
291.1474345	0.006776259	0.090899664	0.074546578	0.94057548	0.974550053
244.0775693	-0.254707061	0.080400784	-3.16796738	0.001535087	0.022417422
590.1427838	0.079718293	0.177775403	0.448421386	0.65384911	0.830755205
253.6351097	-0.05977934	0.078802817	-0.758593938	0.4480955	0.687884956
138.2257601	0.231110049	0.131808934	1.753371649	0.079538234	0.264016437
174.0962908	-0.931983214	0.341360287	-2.730203979	0.006329515	0.055274004
32.53918981	-0.313716203	0.192413076	-1.630430786	0.103010483	0.30726824
49.79692107	0.191783297	0.335189366	0.572164027	0.567210856	0.774924657
176.513274	-0.250063065	0.225098388	-1.110905621	0.266608976	0.525247479
217.7377297	-0.226833267	0.178925216	-1.267754608	0.204885597	0.454803778
1247.955312	-0.104590072	0.071316218	-1.466567837	0.142493655	0.371222252
107.9742659	0.318240576	0.16142932	1.971392656	0.04867898	0.196469668
670.4905019	0.001924808	0.093324885	0.020624812	0.983544948	0.991149398

1275.998317	-0.035035851	0.037106802	-0.944189449	0.345072846	0.60255205
40.48921889	0.773503886	0.599254493	1.29077695	0.196781033	0.445162804
1965.890971	-0.498576594	0.128274101	-3.88680637	0.000101572	0.003375107
53.24567686	0.097145831	0.239842297	0.405040444	0.685447782	0.849309766
178.6992385	0.132748971	0.124921639	1.062657938	0.287937102	0.547328265
15.04975305	0.413817498	0.335407299	1.233776067	0.21728636	0.469928195
577.3092352	0.200691053	0.102659746	1.954914766	0.050593131	0.201090388
456.915876	0.429573313	0.164096845	2.617803605	0.008849772	0.069192338
8.446993607	0.55032748	0.840646169	0.654648174	0.5126943	0.737355786
67.1531794	-0.035931061	0.128240215	-0.280185593	0.77933512	0.898614978
205.5756257	0.200782574	0.148310878	1.35379533	0.175801684	0.419102236
43750.57344	0.102572024	0.206933051	0.495677337	0.620122079	0.809215665
1484.358028	0.198761228	0.139069134	1.429226042	0.152939275	0.386887031
270.3565645	-0.461964131	0.144683058	-3.192938669	0.001408329	0.021129476
625.4205836	0.116828941	0.123262999	0.947802192	0.343230164	0.601007245
3.64442685	-0.862431514	0.495230871	-1.741473654	0.081600588	0.268419382
4.409443191	-0.052326276	0.44657716	-0.11717186	0.906723866	0.960298272
239.0981725	-0.167516024	0.094389978	-1.774722563	0.075943708	0.257646791
20.86317854	0.320290866	0.239279151	1.338565708	0.180712096	0.426016611
2.780042536	0.158965382	0.580862446	0.273671302	0.784337234	0.901740593
836.0795801	0.209776422	0.146731705	1.429659806	0.152814682	0.386760913
18.6922647	0.359152002	0.22368207	1.60563608	0.108353893	0.315057928
931.776993	-0.011748567	0.099806068	-0.117713954	0.90629431	0.960133527
15.86207355	0.573979935	0.335340411	1.711633659	0.086964201	0.278640763
8.683515059	-0.026247961	0.347265625	-0.075584679	0.939749526	0.974295153
381.1916339	0.036958604	0.183407916	0.201510406	0.840299493	0.931267525
20.08041617	0.151831218	0.44284542	0.342853762	0.731708478	0.874922414
448.8204702	0.065314139	0.07093577	0.920750406	0.357180753	0.613393923
5672.787557	0.068698809	0.065927615	1.042033897	0.297395964	0.555810092
18.77750071	0.186892971	0.259189535	0.721066808	0.470868411	0.705535576
86.18848676	0.404085657	0.169485605	2.384188654	0.017116825	0.104703699
302.6626651	0.039302694	0.331065359	0.118715816	0.905500506	0.959974986
363.6689173	0.032727006	0.329320134	0.099377483	0.920838561	0.966695581
4.704302717	-0.123663786	0.537856389	-0.229919711	0.818154159	0.920181415
68.73221707	0.60164333	0.435613404	1.381140534	0.167235755	0.406933747
53.88911717	-0.509079014	0.209337969	-2.431852262	0.015021833	0.097352624
438.5951486	0.186170385	0.127441144	1.460834228	0.14406094	0.373746018
56.93692555	0.091183149	0.243238811	0.374870886	0.707756493	0.861889372
342.5652957	0.216941377	0.120570928	1.799284296	0.071973721	0.249191135
2854.686358	0.120842252	0.101414226	1.19157101	0.233429498	0.488995358
47.47495474	-0.120688131	0.15168165	-0.795667313	0.42622543	0.671244066
2.717512773	-0.136672673	0.648420629	-0.2107778	0.833060663	0.929019015
2.814763717	1.839168078	0.737318699	2.494400427	0.012617012	0.086867962
16.73496845	0.596318731	0.644155656	0.92573701	0.354582669	0.611263418
16.60712699	0.150476223	0.302414275	0.497583069	0.618777939	0.808403087
156.6273633	0.354203984	0.127348619	2.781372799	0.005412954	0.050139147
7205.745056	0.099227203	0.194713036	0.509607398	0.61032654	0.803167633
667.7509004	-0.005555117	0.185397748	-0.029963237	0.976096373	0.988130653

124.3599615	0.06087638	0.247321989	0.246142205	0.805572153	0.912706324
384.9464292	-0.139260345	0.237018066	-0.587551605	0.556833303	0.768174292
1057.308942	-0.18449061	0.078999925	-2.335326413	0.019526375	0.112807492
12.90260854	0.695409749	0.362590154	1.917894742	0.055124359	0.212529608
482.40604	0.047300324	0.134630129	0.351335351	0.725336777	0.871978571
8.937561775	0.721813878	0.344136342	2.097464842	0.035952446	0.163918072
596.9759319	-0.223506868	0.079348763	-2.816765609	0.004850991	0.046468286
606.0943669	-0.370162337	0.092490672	-4.002158567	6.27672E-05	0.002372147
8.607703537	0.069071067	0.382962552	0.180359846	0.856870076	0.938857569
3.154153899	0.113265712	0.699319066	0.161965714	0.871332854	0.946283664
430.4276741	-0.120650139	0.069586055	-1.733826408	0.082948888	0.271318116
186.6192319	-0.072984368	0.107190742	-0.680883136	0.49594544	0.723257713
130.7906329	0.052092003	0.116748267	0.446190799	0.655459429	0.831914417
169.6257677	-0.134175543	0.110464097	-1.214652966	0.224498478	0.478661519
412.7237756	0.190591305	0.083044916	2.295038812	0.0217309	0.120347964
10.73578044	-0.168399411	0.325054409	-0.518065303	0.604412708	0.799020152
790.7218853	-0.048676752	0.138623838	-0.351142722	0.725481278	0.871978571
40.28910791	-0.050940631	0.287260914	-0.177332274	0.859247401	0.940172753
18.49838671	0.647510163	0.898551108	0.720615843	0.471145904	0.705571694
7.5124311	-0.816344412	0.703431311	-1.160517593	0.245838135	0.501134041
11.26662088	0.705824061	0.623083087	1.132792842	0.257301258	0.514340165
5.266306885	0.870418225	0.622834077	1.397512206	0.162259599	0.401084247
50.81575618	0.263935878	0.227787802	1.158691887	0.246581799	0.501930622
254.4618532	0.145950675	0.133005293	1.09732983	0.272497235	0.531559364
838.1447129	-0.272600833	0.183584208	-1.484881708	0.137575162	0.363560042
10.91154417	0.093948395	0.311819455	0.301290999	0.763192604	0.891117256
88.29780174	0.496186955	0.125975533	3.938756552	8.1905E-05	0.002849087
2162.863559	0.118657294	0.317249866	0.374018421	0.708390611	0.861905675
226.8743474	-0.279439729	0.12927994	-2.161508811	0.030656057	0.14894969
2.811070342	0.138308734	0.549318657	0.251782335	0.801209312	0.909603102
31.45728668	0.024550841	0.321281086	0.076415458	0.939088572	0.974295153
2.811671423	-0.659056148	0.904077277	-0.728982096	0.466012615	0.70158715
7.207272537	-0.189204042	0.699618457	-0.270438894	0.786822622	0.903033152
19.3978763	-1.083296464	0.583210241	-1.857471606	0.063244087	0.230893916
4478.120219	-0.059607705	0.093138923	-0.639987054	0.522181016	0.744467508
1241.114878	0.154255348	0.128459099	1.200812936	0.229823773	0.485070119
125.1103482	0.313278416	0.132796097	2.359093546	0.018319637	0.108800299
449.2408952	0.148085725	0.2279356	0.649682304	0.515897457	0.740016071
4.522166752	0.809301484	0.794793175	1.018254194	0.308557171	0.56761314
156.6393274	-0.332300511	0.231607669	-1.434756078	0.151356618	0.385021347
632.0070503	0.26821495	0.101359403	2.646177277	0.008140714	0.065442486
68.46504221	0.660475715	0.422171114	1.564473961	0.117706303	0.331675469
228.4888563	0.394000701	0.154850787	2.544389397	0.010946897	0.079011108
222.5068752	-0.115328872	0.086601199	-1.331723734	0.182950987	0.428838596
20.32349641	0.271975759	0.543242343	0.500652724	0.61661555	0.80702296
569.6021322	-0.110292578	0.113013293	-0.9759257	0.329101285	0.587616639
113.7490203	-0.047095724	0.115885922	-0.40639728	0.684450718	0.848557456
67.25823617	0.294393166	0.18442763	1.596253047	0.110432308	0.319274102

803.0265815	-0.014545417	0.123296538	-0.11797101	0.906090628	0.960133527
138.3079773	-0.240661726	0.17252334	-1.394951699	0.163030405	0.401912744
1611.240322	-0.165399905	0.105228721	-1.571813316	0.115993853	0.32838404
152.9285818	0.339157854	0.110699547	3.063769129	0.002185675	0.027915414
223.2303725	0.022684428	0.109291674	0.207558609	0.835573626	0.929903977
28.59829006	0.285026867	0.195938901	1.454672174	0.14576004	0.375878512
1182.67273	-0.149099118	0.091652409	-1.626788862	0.103781948	0.308630276
945.1184075	-0.115432776	0.186995058	-0.617303888	0.537034303	0.754870587
1229.941569	0.09404579	0.095465463	0.985128931	0.324560744	0.58330813
12.98462426	0.386480494	0.38701132	0.998628396	0.317974739	0.576478535
56.36658753	-0.213938468	0.236952181	-0.902876131	0.36659164	0.622360875
771.7484096	-0.038186532	0.144178686	-0.264855598	0.791120705	0.904476207
3.961084965	0.0256463	0.552297476	0.046435665	0.962963011	0.98274378
135.6925806	0.392733081	0.127882419	3.071048274	0.002133087	0.02764709
72.33272484	-0.128199253	0.2009393	-0.637999896	0.523473748	0.745429659
94.94620921	0.043560857	0.146811197	0.29671345	0.766685287	0.892250595
102.3171113	-0.020797079	0.138067449	-0.150629846	0.880267717	0.950735166
3.750686092	1.047960742	0.700543431	1.495925443	0.134673085	0.35903666
78.38536967	-0.002911557	0.167932827	-0.017337628	0.986167267	0.992541292
5.878493617	0.645989668	0.468133508	1.379926147	0.167609385	0.40750048
139.6597353	0.213937348	0.100364861	2.131596124	0.033040063	0.155382412
1136.527086	-0.059479834	0.081064462	-0.73373501	0.463110257	0.699078081
57.28313849	0.262367672	0.184509241	1.421975782	0.155033282	0.389920139
13.2490938	0.994026199	0.802452016	1.238735998	0.215443283	0.467618661
362.3535412	-0.010184719	0.083445397	-0.122052494	0.902857444	0.958728244
206.0805916	0.382742799	0.315932366	1.211470684	0.225715064	0.480208093
258.4379646	0.546207041	0.176648344	3.092058646	0.001987736	0.026397669
152.5269774	0.115348726	0.722151734	0.15972921	0.87309439	0.947195989
39.36666561	0.524783138	0.475864149	1.102800324	0.270113887	0.529029247
604.936162	0.671441055	0.248725942	2.699521607	0.006943925	0.059253816
7.301031932	1.567667322	0.575460912	2.724194277	0.006445858	0.055978312
2.822515649	-0.386046755	0.705920749	-0.546869823	0.584468169	0.785905244
4.833605082	2.173213065	1.101562151	1.972846528	0.04851305	0.196225524
167.8145939	0.190956026	0.178685267	1.068672473	0.285217279	0.544103149
87.47769673	-0.152521714	0.150750045	-1.011752361	0.311656473	0.570697037
175.7132962	-0.277982769	0.12143831	-2.289086277	0.02207434	0.121734862
475.3397387	-0.180199829	0.123626721	-1.457612303	0.144947437	0.374955566
23.00631845	0.23876015	0.223906308	1.066339542	0.286270181	0.545415636
108.0008574	0.071238782	0.111163878	0.640844705	0.521623584	0.744256234
53.59552448	0.594523793	0.315365075	1.885192246	0.059403887	0.222466707
191.1650949	0.057211733	0.130070974	0.439850114	0.660045669	0.834290513
69.40838434	-0.028976733	0.244829033	-0.118354972	0.905786402	0.960133527
1809.578795	-0.038201279	0.116750548	-0.327204279	0.743513385	0.881092854
2072.02123	-0.025900874	0.06752812	-0.383556872	0.701306911	0.857806901
1724.575342	-0.027974889	0.118684815	-0.235707401	0.813659727	0.917856025
17510.75845	0.000270228	0.148244498	0.001822855	0.998545573	0.999106169
110.6111621	0.263666215	0.203065696	1.298428147	0.194140252	0.441865581
119.6141607	0.419384956	0.13744644	3.051260944	0.002278824	0.028615757

275.5658467	-0.077092615	0.108750127	-0.708896778	0.478388534	0.710717607
301.6232855	0.311090336	0.168598312	1.845156879	0.065014721	0.235114339
217.7053044	0.253018006	0.212908827	1.188386642	0.234681117	0.490432513
1378.755529	-0.135147868	0.114762151	-1.177634493	0.238942352	0.49442766
54.91161791	0.184238311	0.202318313	0.910635861	0.362487269	0.618026146
251.1612409	0.071023997	0.10851219	0.654525522	0.51277329	0.737355786
1158.55048	0.103200397	0.103398656	0.998082581	0.318239316	0.576493309
13.15713103	0.315894999	0.281805286	1.120969034	0.262301044	0.519960764
378.0778024	-0.134053924	0.104150722	-1.287114693	0.198054315	0.446254188
1030.274093	0.185932874	0.071986206	2.582895857	0.009797489	0.073977505
16.69013271	-0.269123542	0.247147793	-1.088917438	0.276190296	0.534794106
81.66188031	0.28651671	0.143774872	1.992814917	0.046281718	0.190921216
197.7408547	0.126755566	0.100902806	1.256214477	0.209038219	0.460212722
15.44272041	0.104798708	0.294868308	0.355408519	0.722283568	0.870068777
70.81748322	0.570996069	0.209434089	2.726375981	0.006403401	0.055671255
659.1227672	-0.097871138	0.049076853	-1.994242356	0.046125575	0.19074428
851.8372835	0.041335324	0.066103257	0.62531448	0.531764678	0.751414479
222.7310071	-0.072394809	0.173508987	-0.417239537	0.676503209	0.845004996
386.0991645	-0.050962158	0.193032097	-0.264008729	0.791773193	0.90458703
119.5047936	-0.033517439	0.130059517	-0.257708465	0.796631913	0.906835166
10.9862522	-0.207059462	0.346151301	-0.598176175	0.549722387	0.763540225
136.2861492	0.068799869	0.093118711	0.738840441	0.460003892	0.696466941
100.1171663	-0.133675149	0.134604907	-0.993092687	0.320664787	0.579213006
3423.012984	-0.39056814	0.148894527	-2.623119518	0.00871287	0.06856631
641.0531708	-0.060190712	0.132442568	-0.454466512	0.64949309	0.828275395
99.383562	0.516561021	0.2430322	2.125483871	0.033546258	0.15677578
261.2435806	-0.021627486	0.133899956	-0.161519738	0.871684067	0.946451568
170.3393358	-0.011373602	0.098256978	-0.115753631	0.9078478	0.960645318
299.5956147	0.166572764	0.264932472	0.628736685	0.529521457	0.74980955
1559.012985	-0.00017974	0.071886611	-0.002500331	0.998005026	0.998977228
1258.29769	-0.144570491	0.097786564	-1.478428985	0.139292992	0.366555585
141.2370379	0.134405237	0.123609891	1.087334	0.276889228	0.535402099
52.79326189	-0.181224734	0.19743427	-0.917899077	0.358671705	0.614597074
10.27576576	0.542430192	0.372641645	1.455634922	0.145493569	0.375475519
64.53747339	0.742889971	0.450610203	1.648631047	0.099223244	0.301282309
319.5928341	0.187220104	0.14447134	1.29589788	0.19501067	0.443078525
1308.194052	-0.26054969	0.121062319	-2.152194778	0.031382017	0.150857592
20.36489733	0.548736221	0.414628354	1.32344114	0.185688718	0.431717253
21.58418097	-0.593857616	0.368096513	-1.613320404	0.106674916	0.312520396
156.8452787	-0.215740852	0.433132924	-0.498093865	0.618417884	0.808204825
114.5199315	0.402102788	0.277950273	1.446671682	0.147988899	0.379505869
189.0582979	0.048793315	0.118971327	0.410126677	0.681713024	0.84736731
349.114717	-0.053354772	0.144491681	-0.369258435	0.711935104	0.863498984
496.3685568	-0.024160751	0.083857683	-0.288116125	0.77325785	0.895996959
18.87505276	0.004966166	0.292625112	0.016971088	0.986459681	0.992541292
1312.635479	-0.156751598	0.086141754	-1.819693599	0.068805679	0.242881362
2.610918374	-0.901733738	0.861956315	-1.046147842	0.29549278	0.554602454
234.8037521	0.006624284	0.158918915	0.04168342	0.966751072	0.984141646

606.5497193	-0.022762865	0.113320196	-0.200872094	0.840798589	0.93162028
15.10607653	-0.125612505	0.288820039	-0.43491617	0.663623277	0.836542958
185.3451637	0.108261482	0.090985338	1.189878336	0.234094214	0.489760032
1030.18496	-0.334825967	0.098406901	-3.402464283	0.000667811	0.012680321
4.348220366	-0.645077918	0.691526636	-0.932831628	0.350906908	0.608090959
188.7587762	-0.38759449	0.205389108	-1.887122908	0.059143796	0.22181045
198.3966788	-0.077428236	0.094754232	-0.817148051	0.413843826	0.660498785
417.3680613	-0.148926264	0.110247219	-1.350839191	0.176746951	0.420660652
30.44622285	-0.082656223	0.214873569	-0.384673758	0.70047914	0.857501938
778.0036151	-0.182553978	0.109990986	-1.659717624	0.096971272	0.296760475
167.8229895	-0.055859015	0.121094749	-0.461283549	0.644595186	0.825292318
838.509699	-0.005889472	0.056511492	-0.104217249	0.916996946	0.964923187
19.32854686	0.018606442	0.246194383	0.075576224	0.939756253	0.974295153
995.561622	-0.065726863	0.044599082	-1.473726796	0.140555165	0.368270499
330.1699136	-0.075580569	0.085620469	-0.882739495	0.377377038	0.630416193
648.1326172	0.391363455	0.187794273	2.084001018	0.037160075	0.16733144
501.5415225	-0.197829399	0.131102914	-1.508962639	0.131308331	0.353916456
1026.453036	-0.13914726	0.075623712	-1.839995101	0.065768957	0.237022131
18.09395597	0.161413941	0.384394464	0.419917445	0.674545763	0.843727031
109.2356497	-0.278361746	0.097082792	-2.867261436	0.004140408	0.042232071
110.5652075	0.476613215	0.313380673	1.520876227	0.128290901	0.349204181
494.5642106	-0.123422304	0.069047573	-1.787496641	0.073857255	0.253272744
99.82834461	-0.010762435	0.104797282	-0.102697656	0.918202934	0.965350236
404.7884417	-0.481779304	0.327420001	-1.471441275	0.141171818	0.369534051
50.0662968	-0.040130295	0.307825341	-0.130367093	0.896276	0.956572177
115.0940233	-0.628337441	0.24814454	-2.532142921	0.011336777	0.080491631
176.2005688	0.005242773	0.139418851	0.037604476	0.970003039	0.985572392
1063.415258	-0.120698321	0.133389338	-0.904857336	0.365540972	0.621546466
255.4491713	0.162669731	0.08634141	1.884029117	0.059561037	0.222736124
358.2339162	0.226140748	0.074947361	3.01732771	0.00255014	0.031029006
1401.410357	-0.176494002	0.090158	-1.957607771	0.050276055	0.200439665
28.82825172	0.309587991	0.196035314	1.579246027	0.114279636	0.325525707
314.3102956	0.038008906	0.063129253	0.602080715	0.547120411	0.762139761
35.64988284	-0.103116684	0.286294934	-0.360176418	0.718715208	0.867702973
4.266179223	0.563196134	0.556362036	1.012283545	0.3114025	0.570697037
1095.161983	-0.299023724	0.095300223	-3.13770226	0.001702777	0.023887622
83.18212155	0.249605253	0.137357551	1.817193526	0.069187486	0.243608574
14.6408378	0.799746952	0.313377934	2.552020626	0.010710018	0.07783901
732.8764512	-0.15305743	0.082326037	-1.859161894	0.063004194	0.230617846
21.09721143	0.089622902	0.234594063	0.382033975	0.702436164	0.858728471
318.1272832	-0.352379156	0.100907708	-3.492093542	0.00047925	0.010157517
689.2163423	-0.078166817	0.077926915	-1.003078546	0.315822965	0.574988455
6.271222684	0.974050838	0.471008233	2.068012339	0.038638863	0.171238181
377.2897364	-0.135141937	0.072283159	-1.869618584	0.061536803	0.227404652
108.2439101	-0.2076269	0.116735671	-1.778607156	0.07530419	0.256142033
34.3098811	0.644583628	0.240109815	2.684536774	0.007263039	0.061375812
212.1107596	0.088715914	0.157480739	0.563344534	0.573200298	0.779066086
17.54266343	0.59999694	0.306887619	1.955103113	0.050570901	0.201052981

222.2791244	0.261663123	0.087747994	2.981984099	0.002863869	0.033420325
21.43544973	0.316856954	0.252084195	1.256948909	0.208772137	0.459755994
378.217252	0.157388703	0.114662151	1.37262995	0.169867416	0.411078623
335.604497	0.000223732	0.080181347	0.002790325	0.997773646	0.998920073
162.7389934	0.473485786	0.250455861	1.890495928	0.058691665	0.220907144
11.24152208	0.252307583	0.400121154	0.630577966	0.52831651	0.748993595
636.1378593	0.088401466	0.064543276	1.369646407	0.170797306	0.412501531
4.548671096	0.738614639	0.49579811	1.489748799	0.136290298	0.361688162
3.553524022	0.276108702	0.544318945	0.507255359	0.611975656	0.804353941
126.1291585	0.110498488	0.104639483	1.055992298	0.290971734	0.550361876
155.9414196	-0.545310293	0.330515506	-1.649878093	0.098967872	0.300932941
304.4849338	0.135005928	0.123015482	1.097471033	0.272435537	0.531518716
269.7203825	0.479543206	0.204546754	2.344418557	0.019056772	0.111317099
37.86029814	-0.200698391	0.250668565	-0.800652412	0.423332899	0.667828726
4.78858629	0.244959562	0.7257494	0.337526373	0.735720135	0.877092931
5.54585948	0.292816993	0.421893733	0.694053904	0.487648405	0.718038917
489.2145976	0.106007119	0.097278609	1.089726917	0.275833456	0.534543001
149.9300507	-0.073725615	0.12207921	-0.603916221	0.545899344	0.76078597
190.1072043	0.179440542	0.14370899	1.248638249	0.211797416	0.463037451
225.7464013	0.089851452	0.100305409	0.89577873	0.370370952	0.62480271
377.4480402	0.340034698	0.12129689	2.803325774	0.005057855	0.047734421
5.851868795	1.099387072	0.48423432	2.270361736	0.023185644	0.125784726
256.911014	0.236631805	0.083265444	2.841896864	0.0044846	0.044330263
500.2691344	-0.279012276	0.135535276	-2.058595257	0.039533028	0.173782322
217.7795794	0.079519826	0.121617234	0.653853271	0.513206342	0.737842907
34.29766433	0.253901116	0.178065232	1.425888221	0.15390061	0.388403012
250.885416	0.306499701	0.468339019	0.654439815	0.51282849	0.737367257
4.582116993	0.735705319	0.797352107	0.922685615	0.356171064	0.612269826
46.54648881	0.733563106	0.395729049	1.853700425	0.063782031	0.232243256
170.0345598	0.736542135	0.313415155	2.350052709	0.018770753	0.11043083
42.10866382	0.413108981	0.297660232	1.387854127	0.165181469	0.404923456
494.9880315	-0.04050929	0.063029473	-0.642703938	0.520416225	0.743546194
161.918984	0.212531397	0.140511582	1.512554295	0.130392911	0.352520887
132.8915807	-0.2392991	0.103002859	-2.323227742	0.020166921	0.115171656
341.457742	-0.03085064	0.08402979	-0.367139322	0.713515097	0.864218919
8.207197403	2.012105872	0.811366783	2.479896779	0.013142042	0.089152157
34.68133375	0.940949859	0.59853573	1.572086363	0.115930524	0.328264007
3613.43145	-0.1772673	0.098247057	-1.804301376	0.071184074	0.247529632
225.6478971	0.034039127	0.111201535	0.306103026	0.759526204	0.889224856
5.89221454	0.245583487	0.426031394	0.576444577	0.564314721	0.773206656
413.6649384	0.130677757	0.091054373	1.435161784	0.151241001	0.384829693
4.107538714	-1.132803764	0.792225582	-1.429900511	0.152745575	0.386708895
1191.567647	-0.247963877	0.121289645	-2.044394449	0.040914595	0.177315422
670.7655309	-0.094862873	0.088129738	-1.076400269	0.281748282	0.540432677
483.3964142	0.073644412	0.073118102	1.007198079	0.313839596	0.572488977
775.3675549	-0.056516965	0.063625358	-0.888277348	0.374391581	0.627949503
22.65189247	0.070442001	0.233515595	0.301658658	0.762912287	0.891042078
663.9524332	0.030324128	0.101771691	0.297962313	0.765731928	0.892047813

3.267022193	0.111063116	0.590586653	0.18805558	0.850833082	0.935636028
96.34134659	-0.166763002	0.174261367	-0.956970583	0.338582103	0.596866551
37.88075844	-0.130366327	0.154187816	-0.845503427	0.39782982	0.648131599
3187.271337	-0.094722636	0.095862617	-0.988108177	0.323099671	0.581534915
8833.88541	0.021880433	0.102924493	0.212587231	0.831648932	0.928693708
5.265574843	0.177364251	0.481343992	0.368477127	0.712517497	0.863744352
785.1301053	0.532850124	0.202265877	2.634404432	0.008428503	0.066899108
52.93388657	-0.081735624	0.178530576	-0.457824231	0.647078726	0.826964966
85.49587388	0.295570369	0.18382919	1.607853301	0.107867312	0.314157226
78.74578425	0.41204789	0.132392948	3.112309959	0.001856295	0.025338667
42.99105502	0.290067569	0.32798035	0.884405328	0.376477448	0.629692185
15.04991288	0.175185575	0.330834107	0.529526949	0.596439955	0.794021966
234.9705088	-0.001776708	0.09519685	-0.01866352	0.98510953	0.992028201
749.0342125	-0.215104343	0.14176628	-1.517316688	0.129186747	0.350910158
138.3409344	0.029159044	0.16868946	0.172856351	0.862764342	0.941918136
124.6657969	0.156506931	0.097920305	1.598309257	0.109974168	0.318242753
36.61842645	-0.303296906	0.208792573	-1.452623058	0.14632844	0.376726077
52.16077516	0.130211771	0.163335303	0.797205312	0.425331801	0.670375236
534.3616357	-0.240951064	0.091948616	-2.620496905	0.008780173	0.068854114
20.33926708	0.334153443	0.334445842	0.99912572	0.317733793	0.5764038
348.2869312	0.019024885	0.102693223	0.185259406	0.853025576	0.936643758
112.1602105	0.074683394	0.107425044	0.695213995	0.486921203	0.717486727
302.4536918	-0.01760285	0.099357817	-0.17716623	0.859377821	0.940184273
15.37517681	0.530493447	0.889700478	0.59626072	0.551001067	0.764274809
4.672241748	-0.725982401	0.644936882	-1.125664265	0.260307655	0.517710533
3.319323333	0.456928485	1.046574967	0.436594128	0.662405722	0.835683509
7.008893788	-0.448718276	0.721254074	-0.62213621	0.533852307	0.752623783
6.029706503	0.34247637	0.973535688	0.351786148	0.724998648	0.871933041
3040.978242	-0.440156375	0.107599795	-4.090680414	4.30109E-05	0.001802961
6.916455012	0.725731332	0.48299539	1.502563683	0.132951594	0.356324813
71.60664696	-0.477227636	0.336022725	-1.42022429	0.155542394	0.390462795
60.50475168	-0.068780027	0.163642505	-0.420306615	0.674261478	0.843579852
820.7162618	-0.920801463	0.557103383	-1.652837681	0.098363903	0.299507667
131.4602012	0.139359101	0.279524487	0.498557757	0.618090971	0.808044267
32.06995447	0.508626497	0.302423328	1.681836187	0.09260061	0.288737169
32.99817775	0.091278405	0.371927568	0.245419843	0.806131366	0.913010135
848.2177072	-0.622693529	0.230735166	-2.698736992	0.006960316	0.05936139
67.2756426	0.109081836	0.213840907	0.510107432	0.609976199	0.80299368
1486.038024	-0.313762555	0.122582777	-2.559597369	0.01047935	0.077006898
149.876594	-0.25312811	0.11793385	-2.146356712	0.03184453	0.152090717
2.93746547	0.417153888	0.742316056	0.561962637	0.574141476	0.779855959
229.7162027	-0.554089735	0.167749225	-3.303083716	0.000956278	0.015989626
75.52351818	0.703242215	0.291912121	2.40908878	0.015992407	0.100717784
64.52296982	0.737939458	0.297438314	2.480983196	0.013102055	0.088919355
54.01579745	-0.115138392	0.234325528	-0.491360858	0.623171243	0.810950402
2068.341437	0.219862393	0.412025002	0.533614204	0.593608479	0.791890039
90.12693519	-0.114962901	0.407827375	-0.281891085	0.778027026	0.898328042
2785.373187	-0.208052742	0.128024257	-1.625104078	0.104140384	0.309111914

140.4597416	-0.1490297	0.108064388	-1.379082446	0.167869336	0.407942773
538.1149323	-0.486861362	0.176661133	-2.755905352	0.005852993	0.052727366
93.24423601	-0.526236255	0.213330573	-2.466764358	0.013634005	0.091500098
2212.527282	-0.12556921	0.126119024	-0.995640513	0.319424843	0.577738842
26.91169208	0.452292172	0.332317539	1.361024074	0.173506082	0.416363571
154.7220826	0.159244818	0.160985482	0.989187452	0.322571437	0.581267519
2.687427786	0.122584795	0.883452585	0.138756508	0.889642563	0.953820886
6.637604789	-0.090682654	0.53079349	-0.170843568	0.86434677	0.942788256
4.945126073	0.97567752	0.996449059	0.97915444	0.327503676	0.585964825
1018.977835	-0.183186009	0.132588985	-1.381608049	0.167092081	0.406747199
52.10515551	0.455302161	0.176483413	2.579858091	0.009884092	0.074422517
233.9948669	0.130823451	0.104671585	1.249846851	0.211355498	0.462721491
203.0914157	0.251509841	0.241653885	1.040785423	0.297975147	0.556528111
88.97143024	0.128497719	0.147196917	0.872964744	0.382682295	0.634526812
516.8533193	0.035287298	0.12710361	0.277626242	0.781299285	0.899434674
1454.776228	-0.08567329	0.099790586	-0.858530783	0.39059944	0.641408659
629.1641996	-0.263581652	0.447049763	-0.589602487	0.555457184	0.76692073
312.3092518	0.162862707	0.470482943	0.346160704	0.729221945	0.873448484
1378.324575	-0.226507926	0.095960534	-2.360427945	0.018253864	0.108579298
2.93527475	1.447437059	1.040190736	1.391511201	0.164070466	0.403299571
18.63550088	-0.469581254	0.277439506	-1.692553669	0.090540456	0.285147895
685.7639929	0.123345407	0.156737194	0.786956841	0.431307132	0.676259227
116.5076262	-0.317674866	0.125278307	-2.535753183	0.011220579	0.080065312
385.5566473	-0.308294196	0.067506057	-4.566911644	4.94963E-06	0.00033606
8.01357602	0.226418801	0.35565991	0.636616035	0.524374978	0.746035664
584.6521039	-0.140932391	0.133817265	-1.053170463	0.292262873	0.551805815
72.44135808	-0.437625389	0.192099734	-2.278115542	0.022719694	0.124128345
120.2308739	-0.088332484	0.105386268	-0.838178306	0.401930569	0.651563726
126.2312282	0.001697359	0.447709507	0.003791206	0.996975063	0.998431589
408.0078246	-0.231619364	0.092966089	-2.491439253	0.012722672	0.087327085
59.08855409	0.081403138	0.136855125	0.594812495	0.551968815	0.764827183
557.8727409	-0.104726215	0.189350801	-0.553080392	0.580208363	0.783198895
228.7410321	-0.060141694	0.086567623	-0.694736579	0.4872204	0.717719992
722.1289694	-0.072633757	0.083959682	-0.865102819	0.386982358	0.638215699
38.62430864	-0.313649728	0.197839808	-1.585372181	0.112881798	0.323485861
2460.540672	-0.014686541	0.098927055	-0.148458286	0.881981104	0.950949027
4.139143936	0.127982625	0.482820074	0.265073123	0.790953132	0.904476207
980.7089207	0.412153045	0.11991193	3.437131274	0.000587911	0.011665044
32.5495644	0.617103811	0.256113067	2.409497564	0.015974503	0.100660547
9.268112783	0.511778612	0.360351992	1.42021863	0.155544042	0.390462795
707.9629697	-0.071247709	0.078976337	-0.902139947	0.366982531	0.622580207
1045.74875	-0.264244795	0.122619176	-2.155003843	0.031161534	0.15019591
446.0061835	-0.004907013	0.072535763	-0.067649565	0.946064598	0.976125068
50.97454721	0.171439488	0.237446901	0.722011898	0.470287162	0.705306146
1089.253438	-0.056606462	0.097390828	-0.581229906	0.561085515	0.770873642
73.5348448	-0.050366236	0.125429904	-0.401548865	0.688016069	0.850342319
507.0171141	-0.187470323	0.111169392	-1.686348374	0.091728719	0.287160324
254.1813582	-0.059803974	0.09077358	-0.658825771	0.510007656	0.734975511

9.920576119	0.141577158	0.306260438	0.462277005	0.643882687	0.824864474
689.1796912	-0.166633991	0.068225786	-2.442390191	0.014590368	0.095348052
6.286663081	0.065311383	0.420561026	0.155295853	0.876588084	0.94888588
813.8083768	-0.27387227	0.169797246	-1.612937063	0.106758182	0.312621288
1387.675885	0.378453299	0.108870735	3.476171058	0.000508628	0.010608135
120.9918839	-0.044367503	0.114482278	-0.387549088	0.698349752	0.856365716
485.995589	0.223020893	0.135622891	1.644419254	0.100089631	0.302525683
65.96570353	0.190840354	0.157423901	1.212270523	0.225408844	0.479946253
498.4534204	0.540450694	0.157794486	3.425029023	0.000614734	0.012036809
3.4206533	-0.195449661	0.548882644	-0.356086429	0.72177584	0.86972436
14.45717941	0.501405088	0.302042872	1.660046092	0.09690518	0.296696688
140.2243039	0.042932273	0.440917326	0.097370347	0.922432292	0.967074069
178.8408661	-0.662078535	0.283151649	-2.338247149	0.019374431	0.112494686
105.2515721	-0.176395439	0.09902835	-1.781262025	0.074869655	0.255272972
42.01930708	0.155944437	0.291471337	0.535024949	0.592632605	0.791387806
9.005500516	0.445495086	0.361809898	1.231296016	0.218212172	0.470969215
1074.558927	0.156553994	0.133066737	1.17650735	0.239392197	0.494646201
58.5799435	0.187548201	0.151841192	1.235160224	0.21677088	0.469460712
285.6038797	0.21997593	0.284183209	0.774063782	0.438893074	0.681391372
231.3578955	-0.034712678	0.173091807	-0.200544892	0.841054453	0.931706317
145.5134249	0.048262205	0.246131983	0.196082623	0.844545497	0.932309255
2082.45579	-0.345093747	0.135061388	-2.555088113	0.010616092	0.077466386
47.06604322	0.141531228	0.142590266	0.992572864	0.320918154	0.579431215
344.2090864	-0.053226838	0.118777433	-0.448122476	0.654064808	0.830904944
108.0827447	0.009922237	0.134955869	0.073522089	0.941390667	0.974594762
325.4325206	-0.04370815	0.403936557	-0.108205482	0.913832697	0.963484061
25.90671712	-0.626801365	0.537351378	-1.16646461	0.243426649	0.499015555
11.13937313	1.423964506	0.711961978	2.000056955	0.045494114	0.189213074
110.3327679	0.073546661	0.387673647	0.189712819	0.849534175	0.935119121
6.409748954	-0.079195317	0.404123955	-0.195967885	0.844635303	0.932309255
56.28429753	-0.129695062	0.147928447	-0.876741858	0.380626866	0.632388957
36.79470204	0.124584726	0.205570288	0.606044423	0.544485255	0.759653377
1208.75091	-0.132377523	0.080586725	-1.642671576	0.100450903	0.303208614
93.17603036	0.373768454	0.480882965	0.777254512	0.437008627	0.680390522
2894.545278	-0.19500305	0.176209457	-1.10665485	0.268443183	0.527469667
16.61124191	0.551848249	0.416527923	1.324876962	0.185211963	0.431116715
8.643979448	0.509178313	0.393099668	1.295290623	0.195219993	0.443229642
234.9609768	-0.092059883	0.121038974	-0.760580497	0.446907672	0.686849577
79.58559705	0.116184437	0.146060272	0.795455435	0.426348624	0.671340873
838.1167213	-0.435660094	0.142339795	-3.060704792	0.002208167	0.028088313
1043.110544	-0.561426044	0.28949993	-1.939295955	0.052465311	0.205819395
2656.249818	-0.356544441	0.128767003	-2.768911542	0.00562439	0.051207287
7.161599055	-0.562940927	0.538153252	-1.046060626	0.295533043	0.554602454
17.85572855	0.098019515	0.224999443	0.435643366	0.663095502	0.836213705
2114.347295	-0.193042286	0.055390185	-3.485135224	0.000491888	0.010369216
320.1814853	0.166656727	0.207241281	0.804167618	0.421300178	0.6659617
602.6685279	-0.248757031	0.165095291	-1.506748188	0.131875215	0.354469729
348.1186863	0.105478339	0.09870868	1.068582208	0.285257969	0.544103149

3.054449426	0.557064325	0.774854468	0.71892768	0.472185481	0.706251867
376.0170935	-0.093326416	0.146961178	-0.635041293	0.525401484	0.74688633
150.4902713	-0.276898047	0.094185109	-2.939934445	0.003282817	0.036438571
147.9780692	0.079152881	0.095419892	0.829521808	0.406809202	0.655071986
784.4327209	0.109365901	0.134640975	0.812277993	0.416632135	0.662355886
42.80677325	0.146228449	0.270056491	0.541473556	0.58818122	0.788599269
5.723512191	1.067596928	0.613827934	1.739244614	0.081991745	0.269367098
7.359451947	-0.596301567	0.477642829	-1.248425665	0.211875215	0.463078438
3.946979173	0.127362429	0.67529848	0.188601682	0.850405015	0.935523059
83.87872713	-0.002822646	0.136828069	-0.020629141	0.983541494	0.991149398
1113.594383	-0.313000292	0.091539912	-3.419276755	0.000627878	0.012142593
16.0468393	-0.03328727	0.232908116	-0.142920179	0.886353221	0.952949894
118.839366	-0.122845891	0.142916021	-0.859566972	0.390027788	0.640812469
29.25424842	-0.785097939	0.302144128	-2.59842196	0.009365333	0.072002884
3.226236263	0.2699491	0.725055697	0.372314983	0.709658344	0.862457036
123.0608368	-0.134945146	0.118975528	-1.134226073	0.25669973	0.513818445
63.03697498	0.296653991	0.13561778	2.187426981	0.028711368	0.143137031
44.7764414	0.211246984	0.366189548	0.576878792	0.564021338	0.772991145
14.81577037	-0.154267711	0.362530493	-0.425530303	0.670450136	0.840859135
474.656409	-0.164264314	0.115026838	-1.428052071	0.153276871	0.387328709
3801.022215	-0.387604374	0.157281186	-2.464403943	0.013724134	0.091947597
2499.200383	-0.199491612	0.119175215	-1.67393541	0.094143294	0.291576505
13.09523149	1.565284645	0.564245682	2.774118961	0.005535143	0.050874284
100.9625499	0.157711012	0.135952062	1.160048692	0.246028982	0.501392744
3.603668934	-0.215769184	0.557033577	-0.387354	0.698494153	0.856475827
194.3305004	0.358510606	0.185512133	1.932545325	0.053292233	0.208099808
106.0505209	-0.277995452	0.13040607	-2.131767735	0.033025945	0.155377388
98.31563869	-0.06724588	0.128120732	-0.524863377	0.59967817	0.795879699
10.65226141	-0.522993699	0.583775747	-0.89588117	0.370316232	0.62480271
4.003408033	-0.945295686	1.073128303	-0.880878534	0.378383567	0.630733114
2.679030427	-0.107222143	0.840436273	-0.127579147	0.898482034	0.957387882
1062.888716	-0.819442326	0.359909765	-2.27679937	0.022798209	0.12437117
112.3630189	-0.189288094	0.17063868	-1.109291832	0.267304307	0.52602268
285.0123316	-0.229666125	0.072533169	-3.166360018	0.001543596	0.022486539
12.16409032	0.466155753	0.500218169	0.93190488	0.351385685	0.608523581
180.9044891	0.260112996	0.14382111	1.808587045	0.070515183	0.246371157
5.478493501	0.722612617	0.669435207	1.079436232	0.280393307	0.538866392
6.391698726	-0.329738532	0.568804329	-0.579704681	0.562113786	0.771414313
132.4954128	-0.179317722	0.133727179	-1.340922047	0.179945763	0.424912579
244.1805108	0.250089805	0.169626741	1.474353651	0.140386397	0.368197366
30.64907688	-0.820968708	0.402480229	-2.039774005	0.04137284	0.178526888
131.0785267	-0.181599186	0.137789365	-1.317947765	0.187521135	0.434428578
73.96231472	0.172662728	0.145841841	1.183903923	0.236451097	0.492010908
14.72619252	3.019256315	1.277122164	2.364109245	0.018073482	0.108068812
63.09706557	-0.207757885	0.184636341	-1.125227481	0.260492651	0.517881447
23.17037813	0.565460161	0.518399544	1.090780591	0.275369439	0.534437044
55.85979536	0.250892041	0.140990615	1.779494616	0.075158707	0.255881745
429.8999696	0.00811409	0.243752033	0.033288297	0.973444686	0.986906773

422.7029055	-0.12454292	0.065430155	-1.903448348	0.056982067	0.216551186
19.46973679	0.492073724	0.281747176	1.746508095	0.080722702	0.266388692
679.2441978	0.017595581	0.100976398	0.174254392	0.86166554	0.941177125
51.12216766	-0.026659225	0.336100393	-0.079319233	0.936778709	0.973575955
102.1708237	0.1764589	0.242653558	0.72720508	0.467100335	0.70266657
34.89344995	0.728296842	0.323145884	2.25377106	0.024210574	0.128679492
194.8405349	-0.26832946	0.089133612	-3.010418338	0.002608881	0.031427701
1694.807379	-0.317215551	0.093681261	-3.386115291	0.000708896	0.013120652
23.67048957	0.374640715	0.191578159	1.95555024	0.05051816	0.200945172
290.1137072	-0.167367782	0.095109013	-1.759746799	0.078450747	0.262014804
365.4242752	-0.314657602	0.091235264	-3.44885943	0.00056296	0.01137817
183.6721852	-0.052238002	0.10059696	-0.519280125	0.603565411	0.798632907
242.8614176	-0.109044981	0.109916496	-0.992071115	0.321162836	0.57971204
3.899735767	0.169264211	0.623776017	0.271354151	0.786118662	0.902680942
352.0328297	0.086062555	0.209962562	0.409894768	0.681883144	0.84736731
436.6261088	-0.260201265	0.079023889	-3.292691213	0.000992334	0.016504523
11.2757919	-0.697571527	0.460032429	-1.516352942	0.129430131	0.35120799
1029.233564	-0.086137006	0.090810936	-0.948531201	0.342859097	0.600558641
159.508688	-0.375601436	0.155473616	-2.415853221	0.015698388	0.099912932
318.5165222	-0.157427474	0.089653301	-1.755958473	0.079095499	0.263325929
249.8709392	0.177146792	0.20196073	0.877134838	0.380413404	0.632388957
1426.913696	0.234050801	0.156103733	1.49932866	0.133788391	0.357207219
111.249635	0.359750955	0.215483245	1.669507786	0.095016789	0.292971483
331.0955885	-0.078274355	0.10773533	-0.726543051	0.467505925	0.702812011
1232.455977	-0.387677198	0.17186771	-2.25567209	0.024091175	0.128562771
477.4422492	0.262162155	0.185034753	1.416826575	0.156533638	0.392184277
471.4946626	-0.35258146	0.100893651	-3.494585189	0.000474799	0.010090445
126.1937882	-0.174557962	0.131791183	-1.324504092	0.185335685	0.431314428
785.7306004	-0.296678433	0.184676415	-1.606477107	0.108169121	0.314637332
33.64221688	-0.441182752	0.188297513	-2.34300891	0.019128926	0.111613869
63.96534388	-0.128278489	0.157992762	-0.811926366	0.416833884	0.662355886
81.08835783	0.138176111	0.131513517	1.050660903	0.293414359	0.552647324
6.880486158	0.045785669	0.379600163	0.120615515	0.90399558	0.959382191
295.9604637	-0.785617082	0.351139005	-2.237339263	0.025264175	0.132021097
10.86244029	0.480588307	0.508261672	0.945552919	0.344376668	0.602073755
494.9160871	-0.538848218	0.099867673	-5.395622028	6.82866E-08	1.16123E-05
320.316974	-0.371660357	0.138033118	-2.692544831	0.0070909	0.060245763
412.6646292	-0.128002603	0.091047747	-1.40588435	0.159758469	0.397470151
322.9086288	-0.21189379	0.097802876	-2.166539461	0.030269988	0.147898595
115.9293714	-1.094972906	0.457647052	-2.392614353	0.016728814	0.103460064
12.56838499	0.367280086	0.337220539	1.089139135	0.276092534	0.534794106
1021.881046	-0.081398856	0.173088174	-0.470273932	0.63815932	0.821410814
71.25153983	0.462579152	0.190681648	2.425923823	0.015269475	0.098513552
14.83496057	0.224540065	0.344152577	0.652443364	0.514115198	0.738489119
172.8173204	-0.526792798	0.098968103	-5.322854374	1.02152E-07	1.54052E-05
10.43062715	1.025135088	0.982252876	1.043656998	0.296644112	0.555344561
1091.707987	-0.604682237	0.246903582	-2.449062235	0.01432287	0.094188633
2320.770468	-0.114189617	0.075270467	-1.517057372	0.1292522	0.350910158

58.62949954	-0.563923522	0.173862005	-3.243512135	0.001180658	0.018666771
2.684596108	-0.321795245	0.727511827	-0.442323043	0.658255459	0.833483348
3.239681799	0.213543043	0.634061972	0.33678576	0.736278409	0.877558368
8.046708335	0.789608853	0.399366029	1.977155778	0.048024024	0.195355198
77.06860828	0.067802346	0.157601167	0.430214745	0.667039437	0.838599606
5.399587657	0.246163519	0.523778711	0.469976183	0.638372034	0.821608716
73.12690733	0.428933825	0.27158744	1.579358103	0.114253942	0.325514772
6.204054636	0.812083825	0.46210336	1.757364034	0.07885578	0.262807915
191.8110131	-0.222971097	0.123821677	-1.800743629	0.071743298	0.248612876
402.5549424	0.166200821	0.095765145	1.735504296	0.082651521	0.270697156
2120.072248	-0.16934351	0.169187214	-1.000923805	0.316863647	0.575733312
500.0974495	-0.116274073	0.081407859	-1.428290517	0.153208256	0.387317584
1817.59967	0.055246208	0.169764786	0.32542796	0.744857197	0.881697142
13.68535613	-0.18802071	0.299108959	-0.628602737	0.529609168	0.74980955
136.5074571	0.148885417	0.172139718	0.864910309	0.387088019	0.638296803
829.6916105	0.251921256	0.138214963	1.822677166	0.068352301	0.242084363
1038.850695	-0.213485947	0.272727636	-0.782780762	0.433755867	0.67799751
108.2138851	-0.224742092	0.138100804	-1.627377141	0.103657023	0.308374767
1043.250875	-0.12364841	0.079294755	-1.55935169	0.118913164	0.333842733
24.7188249	-0.217385642	0.18881326	-1.151326138	0.249598088	0.505482518
590.4990643	-0.232633077	0.084548497	-2.751475017	0.005932754	0.053140675
1075.684053	-0.124072563	0.095126681	-1.304287729	0.192135498	0.439471073
11.9715635	-0.238819357	0.373640598	-0.639168651	0.522713223	0.744849907
105.3416299	0.315223105	0.284949751	1.106241024	0.268622211	0.527612445
693.0695376	-0.163098008	0.068512598	-2.380555012	0.01728658	0.105454188
170.8871589	0.379193404	0.24723796	1.533718383	0.125098949	0.344098899
1092.58589	-0.263505367	0.09327295	-2.825099532	0.004726593	0.045816988
453.7331675	0.058009104	0.077766756	0.745937045	0.455705446	0.693372547
13.41976359	-0.150442324	0.305172968	-0.492973953	0.622030992	0.810641951
19.99505029	0.670647395	0.376873955	1.77950051	0.075157741	0.255881745
45.59235418	-0.992329064	0.52862344	-1.877194593	0.060491428	0.224717833
236.7957468	0.047526245	0.105844111	0.449021156	0.653416395	0.830564416
11.0813009	0.225875613	0.452027159	0.499694782	0.617290007	0.807470932
182.3532273	0.01291391	0.147505104	0.0875489	0.930235218	0.970712519
324.3869915	0.309153949	0.168956806	1.829780969	0.067282698	0.240050462
7.043481245	0.150758057	0.360164134	0.418581538	0.675521984	0.84441594
271.212636	0.061786085	0.091010515	0.678889525	0.497207863	0.723933172
4.358749159	-0.451629318	0.560934117	-0.805137902	0.420740106	0.665360573
134.2119177	0.245951133	0.35586738	0.691131435	0.489482947	0.719344203
106.8496394	-0.154793597	0.286831062	-0.539668181	0.589425888	0.78979544
6.735457841	0.242438016	0.484226505	0.500670685	0.616602907	0.80702296
324.0251196	-0.007957872	0.159917704	-0.049762295	0.960311813	0.98222115
888.1720934	-0.264010314	0.135678225	-1.94585619	0.051672017	0.2037777
215.487733	-0.246636101	0.111718531	-2.207656136	0.027268253	0.13849588
327.8814828	-0.092799395	0.150012705	-0.618610242	0.536173153	0.75433618
701.187934	0.025700351	0.074090584	0.346877422	0.728683409	0.873219977
4957.023844	-0.04832507	0.030518261	-1.583480473	0.113311994	0.324077445
50.97034309	0.4152083	0.278959194	1.488419485	0.136640301	0.362188015

23.62296142	0.248184249	0.207102639	1.198363527	0.230775525	0.486234192
112.268003	-0.319058917	0.091575154	-3.484120999	0.000493756	0.010369216
1863.857958	-0.083956342	0.087416496	-0.960417605	0.336845081	0.595611979
3757.041642	-0.00669456	0.118018929	-0.056724457	0.954764691	0.980093538
518.7719069	-0.174416752	0.295503577	-0.590235672	0.55503266	0.766699187
88.47521643	-0.416108939	0.182878269	-2.275332885	0.022885968	0.12476313
183.0380366	0.068171147	0.225955617	0.301701494	0.762879629	0.891042078
5.837418137	0.208982477	0.450641228	0.463744691	0.642830674	0.824387858
567.8870936	-0.147245428	0.155410469	-0.947461448	0.343403692	0.601109766
9.621329893	1.096568767	0.425330049	2.578159643	0.00993281	0.074681777
146.3832959	-0.450302273	0.486694871	-0.925225023	0.35484887	0.611508243
29.1577688	0.450026387	0.229994767	1.956680987	0.050384987	0.200669916
207.8567429	0.017098431	0.120577416	0.141804593	0.887234356	0.95312914
20.63877757	-0.385665669	0.248976456	-1.549004572	0.121380621	0.33801762
1143.05955	0.042935398	0.105178027	0.408216421	0.683114794	0.848026945
280.1637031	-0.076200418	0.110118147	-0.691987834	0.488944969	0.718842604
6.919065731	0.012232611	0.7237892	0.016900793	0.98651576	0.992541292
381.1820776	-0.310484519	0.232341885	-1.336326076	0.181442717	0.426968393
316.5480581	0.094836845	0.109388299	0.866974304	0.385956089	0.63738131
14.92603622	0.360678564	0.332414401	1.085026889	0.277909746	0.536129946
602.5328484	-0.254941815	0.137939814	-1.848210516	0.064571895	0.233998521
1459.409214	0.053194944	0.075095678	0.708362251	0.478720328	0.711116261
175.3448234	-0.252314328	0.131879158	-1.91322369	0.055719422	0.213974712
2343.977875	-0.058028749	0.082959267	-0.699484834	0.484249087	0.715377439
36.64572973	-0.26271128	0.646370133	-0.406440934	0.684418648	0.848557456
775.026542	0.203831651	0.101730234	2.003648703	0.045107705	0.188306959
88.38582079	-0.07185099	0.152216377	-0.472031931	0.636903997	0.820499075
1181.639847	-0.105631342	0.061366184	-1.721328191	0.085191287	0.275947984
270.7738154	0.178829662	0.119839895	1.492238135	0.135636728	0.360808421
179.6324697	-0.022709658	0.158903697	-0.142914598	0.886357629	0.952949894
18.73836516	-0.151009345	0.262259289	-0.575801703	0.564749223	0.773466281
2797.750446	-0.39559118	0.210384452	-1.880325174	0.060063774	0.223820279
6.404891791	3.408662718	2.260735485	1.507767158	0.13161413	0.354192865
12.74967833	0.165885381	0.606908164	0.273328637	0.784600604	0.901845062
7.339162458	-0.181200237	0.792802026	-0.228556728	0.819213461	0.92071257
16.0199304	0.399686446	0.313122136	1.276455416	0.201794578	0.450654443
29.59851	-0.705559295	0.660079837	-1.068899936	0.285114761	0.544007288
186.8410464	-0.225098994	0.127801487	-1.761317493	0.078184681	0.261404506
2495.363573	-0.46426965	0.166886181	-2.781953832	0.005403273	0.050128172
214.5345032	-0.077828738	0.083912188	-0.927502187	0.353665857	0.610488147
41.12557763	0.087238048	0.14919895	0.584709533	0.558743043	0.7695904
20.97206032	-0.043155267	0.216268543	-0.19954482	0.841836587	0.93182686
110.6407618	0.065770995	0.172851754	0.380505222	0.703570421	0.859407981
778.7814849	-0.074759853	0.061307829	-1.219417725	0.222685686	0.476210832
195.2730314	-0.428064069	0.192331117	-2.22566205	0.026036827	0.134683901
51.10005243	-0.443561539	0.152574644	-2.90717729	0.003647064	0.038859074
4.031335338	0.213079938	0.461383121	0.461828638	0.644204212	0.825061152
1713.182992	0.082592408	0.064266426	1.285156387	0.198737641	0.44733082

788.2744516	-0.125169756	0.195157209	-0.641379105	0.521276405	0.744055368
14.93440848	0.186791166	0.329191801	0.567423508	0.570426482	0.77728792
155.3040058	-0.018996458	0.153542937	-0.123720822	0.901536325	0.958493473
454.2855064	-0.171912429	0.086473525	-1.988035397	0.046807775	0.192231775
745.1961655	0.348417688	0.215263332	1.618564967	0.105540898	0.310829552
325.9071328	-0.237953474	0.101435306	-2.345864408	0.018983012	0.111097787
271.1247306	-0.105370483	0.133633087	-0.788505943	0.430400824	0.675378341
2144.890716	-0.293071063	0.115379768	-2.540055925	0.011083474	0.07941135
103.3725274	-0.045259132	0.116272743	-0.389249714	0.697091431	0.855290777
10.86217447	-0.043934349	0.358686124	-0.122486893	0.902513426	0.958694616
22.57922778	0.069524727	0.261556581	0.265811421	0.790384447	0.904317892
80.72928029	0.001616886	0.140778945	0.01148528	0.990836273	0.994259508
7.287987263	0.358455718	0.59608247	0.601352558	0.54760519	0.762302191
3.706445189	0.995286865	0.770020811	1.292545411	0.19616833	0.444481955
79.01179382	0.555492082	0.358596989	1.54907068	0.12136473	0.33801762
5.689541076	-0.984424378	0.581827996	-1.69195086	0.090655344	0.28533783
116.1826546	0.061103254	0.148457669	0.411587046	0.680642128	0.847035716
196.571215	-0.315712821	0.134096521	-2.354369955	0.018554135	0.109523164
192.6634812	-0.513040943	0.259033086	-1.980600051	0.047636143	0.194361413
2371.742862	0.012785599	0.165278541	0.077357886	0.938338842	0.974295153
4.755258872	-0.51661084	0.483705974	-1.068026586	0.28550852	0.544337665
1075.076077	-0.387593917	0.219614964	-1.764879359	0.077584043	0.260752744
20.15005021	0.726441906	0.396939701	1.830106449	0.067234023	0.23993138
165.1989584	-0.227987794	0.176486135	-1.291817026	0.196420518	0.444860563
306.2829769	-0.022810029	0.118417618	-0.192623607	0.847253755	0.934350155
481.0611417	-0.232885705	0.141854362	-1.64172396	0.100647224	0.303539528
49.3576616	0.116227905	0.158079224	0.735250979	0.462186657	0.698307949
77.09383016	-0.025891976	0.245032025	-0.105667724	0.915845989	0.96435908
95.18205642	-0.283144566	0.183745123	-1.540963704	0.12332562	0.341195806
9.701979488	0.008773592	0.374197552	0.023446419	0.981294179	0.990387252
4571.37108	-0.037767942	0.112939696	-0.334408035	0.738071677	0.878437596
28.59994329	0.646975062	0.267413309	2.419382429	0.015546886	0.099354867
3.2515059	0.440473137	0.687600144	0.640594887	0.521785922	0.744256234
14.0437648	0.086389687	0.267651988	0.32276871	0.746870412	0.88345435
7.402757671	-0.357826434	0.375849414	-0.952047338	0.341072975	0.59908004
431.1059103	0.088089581	0.114558334	0.768949563	0.441923253	0.683003971
7.357374318	-0.075322171	0.368825289	-0.204221817	0.838180163	0.930862799
42.19212151	0.807114764	0.308816644	2.613572743	0.008960101	0.06967289
873.1109696	0.032145282	0.076221186	0.421736841	0.673217111	0.842875465
57.02657359	-0.028967139	0.200461482	-0.14450227	0.885103864	0.952291996
8.005149477	0.544984926	0.951930935	0.572504691	0.566980109	0.774744383
97.28353479	0.282531059	0.343951715	0.821426514	0.411403366	0.65849534
4.632492196	1.428196811	0.548448236	2.604068567	0.009212432	0.071106196
2.66049764	0.298369927	0.970193568	0.307536493	0.758435051	0.888769825
2939.5457	0.114959406	0.093301631	1.232126434	0.217901858	0.470699572
118.7785375	0.019393208	0.115475566	0.167942089	0.866628838	0.943901208
25.60716933	0.171016807	0.240198244	0.71198192	0.476475972	0.709176698
51.72409491	-0.566079169	0.237893345	-2.379550251	0.017333779	0.105577863

11.09503785	-0.576482235	0.458444202	-1.257475246	0.208581597	0.459572597
649.2565305	0.056466516	0.168646924	0.334820905	0.73776019	0.87841506
1514.861309	-0.146400136	0.091744749	-1.595733133	0.110548388	0.31948423
6.96417579	-0.681903727	0.510421322	-1.335962464	0.181561542	0.427056273
88.92666311	-0.58899551	0.269433138	-2.186054452	0.028811622	0.143499993
338.2690475	-0.032161363	0.060779593	-0.52914739	0.596703209	0.794156453
5613.676625	-0.050245315	0.152937471	-0.328535018	0.742507172	0.880432636
18.28134335	0.223467098	0.345476399	0.646837522	0.517737105	0.741501167
5.722157026	-0.230436698	0.51626588	-0.446352755	0.655342454	0.831896116
8.116714678	-0.139575328	0.350302882	-0.398441849	0.690304515	0.851828168
1009.011185	0.141501693	0.116571522	1.213861592	0.224800582	0.479053017
65.90344389	-0.084904037	0.313863642	-0.270512495	0.786766006	0.903033152
1337.675604	0.184819209	0.177961051	1.038537411	0.299019928	0.557450202
12.36092757	0.064123916	0.386724049	0.165813108	0.868304031	0.945025059
757.6198633	-0.633288183	0.358917126	-1.764441253	0.077657718	0.260754367
2.664851648	0.294887233	0.698058712	0.422439013	0.672704607	0.842505714
136.8516515	-0.06179328	0.115801961	-0.533611686	0.593610222	0.791890039
376.2979049	0.058735547	0.078403365	0.749145743	0.453769367	0.692078503
107.8378513	0.060453618	0.192627378	0.313837105	0.753644762	0.8866675
17.66707574	-0.167799501	0.303053263	-0.553696402	0.579786638	0.782986042
7.473303955	-0.358751868	0.477464087	-0.751369323	0.45243042	0.690975632
68.47653113	-0.035039308	0.131664488	-0.266125731	0.790142379	0.904145475
38.30635833	-0.694857582	0.302494638	-2.297090575	0.021613603	0.119910773
6.445526251	-1.325366866	0.512108201	-2.588060226	0.00965181	0.073342536
94.57610926	0.248315168	0.205679955	1.207289096	0.227320828	0.481927531
481.2507508	0.184592334	0.112988817	1.633722164	0.102317202	0.306521747
915.6068111	-0.318849453	0.081190001	-3.927201009	8.59402E-05	0.002955889
97.89704064	0.013823896	0.115327765	0.119866161	0.904589171	0.959787347
112.1583111	0.207874749	0.168205165	1.235840467	0.216517869	0.469171907
69.04774975	0.101241687	0.169849993	0.596065299	0.551131605	0.764274809
24.14175609	0.077270198	0.3089327	0.250119842	0.802494672	0.910666842
580.6164848	0.065845053	0.062645448	1.051074826	0.293224225	0.552490238
617.8070612	0.171465358	0.13396592	1.279917741	0.200574067	0.449208007
31.08734844	-0.227857344	0.20756819	-1.097746935	0.272315009	0.531518716
23.29853386	0.365214072	0.405505526	0.900638954	0.367780316	0.623124822
230.2474831	-0.191328388	0.108755356	-1.759254854	0.078534231	0.262237785
3.879730557	-1.175645635	0.539597026	-2.178747432	0.029350436	0.145215217
1967.000363	-0.157562926	0.072302962	-2.179204298	0.029316495	0.145138859
4.434907096	0.659018533	0.497887299	1.323629935	0.185625978	0.431696003
3.362233432	0.778931505	0.534979394	1.456002819	0.14539184	0.375424041
3.548758274	-0.177757337	0.524798791	-0.338715217	0.734824276	0.876677323
70.29474386	0.54112854	0.16961427	3.190347961	0.001421016	0.021225917
10.60363074	-0.40899613	0.297683997	-1.373927163	0.169464296	0.410483091
4.791932056	1.14803601	1.267542898	0.905717678	0.365085303	0.621108352
2.60137871	-0.589386172	1.408766472	-0.418370386	0.675676335	0.844474272
1243.961668	-0.0033162	0.069373414	-0.047802176	0.961873903	0.982457049
133.7510968	0.091907808	0.105316826	0.872679247	0.382837932	0.63463646
18.06109697	-0.640087394	0.306855136	-2.085959528	0.036982289	0.166867153

117.5622438	-0.286333365	0.149181118	-1.919367335	0.054937862	0.21197341
53.61460546	-0.042647687	0.168887971	-0.252520571	0.800638716	0.909191326
11.36671837	-0.024048542	0.326279504	-0.073705341	0.941244849	0.974550053
85.0666185	-0.557330475	0.18499537	-3.01267256	0.002589582	0.031392331
45.26901561	-0.143316971	0.202334106	-0.708318403	0.478747551	0.711116261
39.22648073	0.268732996	0.189060027	1.42141626	0.155195782	0.390203695
20.0393521	-0.475335298	0.248612169	-1.911955075	0.055881957	0.214198364
52.52059716	0.040556626	0.198547499	0.204266618	0.838145155	0.930862799
95.46228089	-0.138238207	0.141537311	-0.976690926	0.328722189	0.5871511
10.20820312	-0.193242101	0.362638342	-0.532878294	0.594117832	0.792172802
10.41211216	-0.115112601	0.390287547	-0.294943055	0.768037381	0.892884018
104.7308676	-0.446536746	0.117082369	-3.813868396	0.000136808	0.004207259
185.6558519	-0.34177763	0.152784953	-2.236984883	0.025287328	0.132057
72.57464812	-0.327067258	0.210815428	-1.551438906	0.120796538	0.337052641
75.11444913	-0.257187068	0.135393276	-1.899555686	0.057491452	0.218064314
90.99907535	-0.216075432	0.131352533	-1.645003919	0.099969003	0.302394184
76.68248414	-0.043734054	0.167419413	-0.261224511	0.793919377	0.905377878
24.95188159	-0.099310375	0.205821724	-0.482506766	0.629445993	0.815213126
165.6791393	0.088046957	0.153589467	0.573261685	0.566467524	0.774448801
33.73245731	-0.037505262	0.245225981	-0.152941633	0.878444304	0.94964988
4.90339037	0.01069973	0.415839789	0.025730414	0.979472365	0.989312505
32.56847929	-0.074176714	0.223592699	-0.331749268	0.740078595	0.879355136
34.59383751	0.427913919	0.217142358	1.970660735	0.048762695	0.196605167
6.585138391	2.811373371	1.053188711	2.669391858	0.007598874	0.06329301
60.96642272	-0.465083836	0.129444595	-3.592918165	0.000326995	0.00776249
354.9657021	-0.112673995	0.125858273	-0.895245045	0.37065611	0.625026387
108.5360264	-0.351990503	0.224524078	-1.567718287	0.116946892	0.330188128
14.45916026	1.257591278	0.550725302	2.283518249	0.022399859	0.123005439
72.81530215	0.081167026	0.269983127	0.300637403	0.763691007	0.89120013
252.2722716	0.307387298	0.098716826	3.11382881	0.001846766	0.025252554
467.1168472	0.443076186	0.149487601	2.963966135	0.003037017	0.03459077
17.19136615	0.766124736	0.334788505	2.288384233	0.022115155	0.121917079
6089.706477	-0.049629997	0.076496844	-0.6487849	0.516477419	0.740509355
979.1436419	-0.015756112	0.141291764	-0.111514725	0.91120819	0.962059189
5.378263996	-0.290662962	0.601569751	-0.483174164	0.628972078	0.815070485
99.4290618	0.124939199	0.115844773	1.078505273	0.28080833	0.539333559
1210.377483	-0.311033277	0.057381106	-5.420482468	5.94384E-08	1.05471E-05
95.50062106	0.239162088	0.141241235	1.693287991	0.090400663	0.284993768
283.0222445	0.13516479	0.103265509	1.308905469	0.190566374	0.438182526
24.81536122	0.202603264	0.230334603	0.879604114	0.37907381	0.63127985
37.86312895	0.43370851	0.232357755	1.866554914	0.061963769	0.228077857
12.1985546	0.396300787	0.652093601	0.607736047	0.543362551	0.758868945
527.0490469	0.108309343	0.115258971	0.939704235	0.347369292	0.604864093
44.46450099	-0.044287477	0.224078866	-0.197642364	0.843324887	0.932221964
250.1941819	0.08653058	0.086148931	1.004430098	0.315171349	0.574183696
958.1153175	0.059786117	0.130784808	0.457133502	0.647575092	0.82742337
20.35502467	0.397362647	0.227824243	1.744163138	0.081130647	0.267153699
245.2598656	0.169320661	0.077949298	2.172189694	0.029841358	0.146626521

644.955785	0.316165141	0.187610132	1.685224232	0.091945318	0.287436787
117.4364996	-0.248447777	0.136263874	-1.823284265	0.068260348	0.242084363
271.1282692	0.02261611	0.13389643	0.168907493	0.865869406	0.943555914
4.550941905	0.572389192	0.540871904	1.058271261	0.289931791	0.549255974
1841.509252	0.233059766	0.087695355	2.65760672	0.007869767	0.064372939
1545.875597	0.008486294	0.109363008	0.077597483	0.938148245	0.974203529
29.1349971	0.201017281	0.233223466	0.861908472	0.388737887	0.639780065
388.0619378	0.185442333	0.192256086	0.964558972	0.334765758	0.593138968
579.8535082	0.061311205	0.076076593	0.805914178	0.420292336	0.665105942
443.3959261	-0.109911862	0.116383396	-0.944394693	0.344967992	0.60255205
18.1644648	-0.134186932	0.237325426	-0.565413215	0.571792738	0.7784837
232.2164621	0.138661068	0.094179005	1.472314002	0.140936104	0.369042996
24.37159109	0.668512365	0.280376686	2.384336494	0.017109949	0.104703699
9.121798303	0.40647587	0.462292016	0.879262146	0.379259155	0.631515084
1458.304526	0.14425883	0.173851062	0.829784006	0.406660915	0.655071986
5827.784247	-0.08976938	0.159995378	-0.561074834	0.574746523	0.780163053
570.2524386	0.509109877	0.149791375	3.398793001	0.000676839	0.012765997
6.161703909	-0.780063681	0.441409254	-1.76721189	0.077192749	0.26015197
395.1680985	0.161784788	0.090558859	1.786515319	0.074015859	0.253519269
30.67724691	-0.232812226	0.263772951	-0.882623578	0.377439685	0.630416193
134.0207049	-0.098300179	0.115278954	-0.852715743	0.393816958	0.64435403
309.4601685	0.234977325	0.074512285	3.153538049	0.001613042	0.023146341
96.1333903	0.49030797	0.136221719	3.599337712	0.000319029	0.007615899
483.8425061	0.072853386	0.148910474	0.489242857	0.624669772	0.812044815
86.22100259	-0.047291452	0.164728879	-0.287086589	0.774046021	0.89628186
582.0610065	0.401685499	0.086880037	4.623449914	3.7741E-06	0.000269059
99.8545599	0.085674675	0.185618245	0.461563869	0.64439411	0.825169596
418.6824115	0.208085518	0.092486598	2.249899159	0.024455347	0.129361777
161.1705641	-0.012220494	0.127652573	-0.095732455	0.923733064	0.967661594
168.3702578	0.24020611	0.103289347	2.325565195	0.020041758	0.114846524
117.1705265	0.38600634	0.110491227	3.49354741	0.000476648	0.010116039
3.016009619	0.870209934	0.880350617	0.988481087	0.322917093	0.581433782
295.4570609	0.032777264	0.097546982	0.336015149	0.736859443	0.877984008
309.7563292	0.122334448	0.177369097	0.68971681	0.490372293	0.719996166
125.9588582	0.350464168	0.112934836	3.103242368	0.001914128	0.025813573
128.2571109	-0.449137397	0.094505935	-4.7524782	2.00938E-06	0.000166249
111.6445976	-0.049453892	0.135660572	-0.364541379	0.715453781	0.865230711
416.160478	0.078490613	0.078552164	0.999216429	0.317689859	0.5764038
15.15626752	0.793950109	0.547513265	1.45010205	0.147030063	0.377788256
195.67191	-0.316260935	0.339467919	-0.931637183	0.35152406	0.608637468
3.431224453	-0.978423019	0.536815569	-1.822642776	0.068357513	0.242084363
9.63449308	1.163743902	0.467368434	2.489992512	0.012774579	0.08753014
6.209534417	0.195618106	0.480883319	0.406789127	0.684162873	0.848557456
165.2780167	0.050099885	0.10205784	0.490896973	0.623499318	0.811128343
18.29244666	1.251463687	0.553768032	2.259905979	0.023827087	0.127674762
49.85974802	-0.037836508	0.147980461	-0.255685833	0.798193435	0.907769715
11.197559	0.498748512	0.607881979	0.820469317	0.411948614	0.658847341
362.6292683	0.122296803	0.118900519	1.028564081	0.303684564	0.562210657

87.91658478	0.340488548	0.164826855	2.06573467	0.03885354	0.1717044
11.49610598	0.584494664	0.311151153	1.878491074	0.060314017	0.224428243
283.7523781	0.025721184	0.147127271	0.174822684	0.861218964	0.940885917
270.4528103	-0.106732979	0.15703661	-0.679669401	0.496713815	0.723616894
56.34393646	0.347819928	0.271275305	1.282165835	0.199784478	0.448315412
232.5100852	0.085046903	0.119751783	0.710193204	0.477584335	0.709791785
80.47453011	-0.106084273	0.163976031	-0.646949877	0.517664383	0.741465588
391.2497994	0.204993438	0.136374481	1.503165676	0.132796326	0.356058035
535.9298732	0.248794298	0.203803271	1.220757142	0.222177985	0.475718706
1395.8107	0.144862889	0.167570239	0.864490558	0.387318465	0.638304143
459.1014326	0.183985747	0.148467998	1.239228308	0.21526096	0.467416987
169.2662867	-0.172344132	0.167265405	-1.030363281	0.302839503	0.561500741
26.26205913	0.485103927	0.214360791	2.263025461	0.02363412	0.127162105
135.0716811	0.311801131	0.099807359	3.124029454	0.001783926	0.024629485
14.40485713	-0.153231554	0.316427685	-0.484254576	0.628205209	0.814615171
380.7595757	0.338652864	0.231322074	1.463988532	0.143197082	0.372454897
138.6641251	0.178475374	0.17355893	1.028327229	0.303795927	0.562210657
1871.916613	-0.05059222	0.123655735	-0.409137671	0.682438632	0.847590078
84.60813839	-0.285152397	0.15974012	-1.785101934	0.074244784	0.254136883
25.29440577	0.017867709	0.296170741	0.060329082	0.95189354	0.978919302
42.76136986	0.295707031	0.349187707	0.846842615	0.397082851	0.647587339
419.1375168	0.140031232	0.178961625	0.782465132	0.433941269	0.678219718
350.4410672	0.081521858	0.067309712	1.211145549	0.225839627	0.480281725
4.268819789	1.032621343	0.770440956	1.340299129	0.180148114	0.425198349
6.977441315	-0.186954157	0.423445334	-0.441507185	0.658845861	0.83373717
15.75865928	0.442263291	0.316692829	1.396505543	0.162562313	0.401290131
796.2922883	0.069281019	0.10902732	0.635446404	0.525137311	0.746646142
360.2927275	0.116928966	0.098192885	1.190808946	0.233728596	0.489245332
123.9559909	0.51714045	0.144490677	3.579057549	0.000344836	0.00807224
1398.495874	-0.189733032	0.246192672	-0.770668886	0.440903218	0.682560519
521.2395319	-0.305380219	0.136497588	-2.237257249	0.025269532	0.132021097
142.7031653	-0.261456674	0.192415539	-1.358812681	0.174205954	0.417583093
808.633858	-0.098403852	0.103609366	-0.949758266	0.342235096	0.600001704
142.0374729	0.037510751	0.100770625	0.372238938	0.709714957	0.862457036
13.51241066	0.245877118	0.453070422	0.542690731	0.587342757	0.787946609
257.1619491	0.415924872	0.119746838	3.47336832	0.000513969	0.01067695
382.7568384	0.162121637	0.144749854	1.120012441	0.262708461	0.520308056
63.59464734	0.006979309	0.216165682	0.032286852	0.974243294	0.987273492
78.02297461	0.504426056	0.153432171	3.287615974	0.001010396	0.016716292
185.4903422	0.417373056	0.174682558	2.38932301	0.016879455	0.103940859
196.5334264	0.126373431	0.106016717	1.192014196	0.233255679	0.488827107
846.7867877	-0.367823838	0.141958137	-2.591072589	0.009567731	0.07288018
413.3489276	-0.756911842	0.39132874	-1.934209692	0.053087351	0.207584646
388.2073024	0.396831416	0.167136571	2.374294341	0.017582528	0.106390575
4.885385429	0.207091199	0.509082571	0.406792946	0.684160068	0.848557456
7.072521239	0.687443307	0.459764035	1.495208964	0.134859915	0.359351496
26.84413903	-0.379416732	0.359760218	-1.054637821	0.291590999	0.550962625
1533.110731	0.301369343	0.136914483	2.201150209	0.027725392	0.140182155

761.6620379	-0.359085109	0.122049122	-2.942135938	0.003259568	0.03630879
2.858821941	0.879449178	0.581456635	1.512493151	0.130408454	0.352520887
4473.092917	0.07339077	0.13648399	0.53772439	0.590767338	0.790746649
201.4006305	-0.05556775	0.099945224	-0.555982042	0.578223131	0.781999792
912.7390283	-0.036825807	0.094059506	-0.391516062	0.695415819	0.854503374
109.2645032	-0.004296368	0.11834216	-0.036304624	0.971039463	0.985937912
421.7559207	0.274913303	0.113917951	2.413257085	0.015810663	0.100191693
2.899726185	0.591549546	0.82480041	0.717203264	0.473248686	0.706757551
16.12062129	0.317807265	0.307797362	1.032521082	0.301828077	0.560420452
199.8191478	0.164331549	0.076972564	2.134936674	0.032766183	0.154790608
46.59709792	1.089118372	0.435130981	2.502966739	0.012315712	0.085389239
359.5388712	-0.306509999	0.160814283	-1.905987415	0.056651838	0.215976528
11.38396582	0.193225403	0.368135075	0.524876373	0.599669135	0.795879699
143.8776381	0.468423058	0.161379603	2.902616248	0.003700598	0.039216336
1506.255712	-0.213262146	0.13057037	-1.633311959	0.102403403	0.306623706
111.287584	0.125114826	0.120884088	1.0349983	0.300669705	0.55906386
917.2942222	-0.751338125	0.365051155	-2.058172162	0.03957361	0.173782322
262.2140266	-0.165025434	0.085884156	-1.921488675	0.054670129	0.211454081
3.347557475	1.131711717	0.712931284	1.587406449	0.11242062	0.322872184
1881.909392	-0.24327056	0.079986815	-3.041383269	0.002354939	0.029354031
301.9395297	0.173971816	0.189655074	0.917306416	0.358982096	0.614927391
385.92166	0.064074819	0.098210158	0.652425575	0.51412667	0.738489119
433.2625622	-0.09755556	0.141584462	-0.689027301	0.490806087	0.720068699
554.3819938	0.195168782	0.094130194	2.073391907	0.038135822	0.169824595
6.864454502	0.079972831	0.378963851	0.211030236	0.832863677	0.928931441
198.7724727	-0.343142451	0.226649921	-1.513975606	0.130032027	0.352110205
10.57678359	1.01391101	0.634364805	1.598309053	0.109974214	0.318242753
55.29280453	0.172471129	0.191354246	0.901318541	0.367418978	0.622716515
699.019942	-0.083145242	0.095172717	-0.873624753	0.382322642	0.634064535
527.6509261	-0.442830768	0.260279874	-1.70136385	0.088874685	0.282282414
926.3991077	0.198217998	0.098625615	2.009802406	0.044452106	0.186583471
48.26583684	-0.265648677	0.572486881	-0.46402579	0.642629268	0.824387858
181.6721958	0.366880643	0.311605745	1.177387288	0.239040961	0.494476185
204.31484	0.034430522	0.108720367	0.316688792	0.751479754	0.885551531
8.316661214	0.210366548	0.314323684	0.66926725	0.503325014	0.729656116
45.59036384	0.200795359	0.24692488	0.81318399	0.416112577	0.661839645
5.872731664	0.145433775	0.414165536	0.351148905	0.72547664	0.871978571
50.82680437	0.237855653	0.200345732	1.187225958	0.235138505	0.491125597
81.34873592	0.031557505	0.137177096	0.230049377	0.818053401	0.920181415
211.0155474	0.350132382	0.27763582	1.261121071	0.20726523	0.457400854
1752.64279	-0.106757631	0.104877643	-1.01792553	0.308713348	0.56782692
253.169659	-0.157566774	0.096705942	-1.629339112	0.103241251	0.307664027
11.45571636	-0.012894367	0.315527762	-0.040866029	0.967402699	0.984352254
780.9150057	-0.145215882	0.158092133	-0.918552231	0.358329827	0.614347157
26.07230148	1.161908879	0.602547933	1.928326057	0.053814583	0.209384251
124.2535251	-0.202970298	0.584032105	-0.347532775	0.7281911	0.873123696
3.217637701	2.382258629	1.438741825	1.65579299	0.097763748	0.298370621
9.078836626	-0.056273758	0.482711494	-0.116578451	0.907194115	0.960404418

22.73671027	0.903438131	0.421627609	2.142739496	0.032134022	0.152918638
31.83655982	0.282536644	0.280465278	1.007385461	0.313749575	0.572488977
125.6907603	-0.134865437	0.100752764	-1.338578036	0.18070808	0.426016611
1030.437933	0.051093212	0.122986197	0.415438585	0.677820865	0.845854775
170.8641126	-0.104750459	0.14201342	-0.737609574	0.460751736	0.697145057
10.46325645	0.883568297	0.410600248	2.151894211	0.031405687	0.150908946
205.9175584	0.166828678	0.10739861	1.553359751	0.120337213	0.336101324
488.8872903	-0.430811851	0.295505668	-1.457880163	0.144873577	0.374950031
2.76359534	-1.79874188	1.032486776	-1.742145199	0.08148304	0.268145196
160.839649	0.040694439	0.101687095	0.400192756	0.68901455	0.851048861
6.651983717	-0.836980665	0.458744562	-1.824502643	0.068076117	0.241914826
813.3340767	-0.051017228	0.101222063	-0.504012925	0.614252295	0.806186861
18.83589586	0.230474256	0.248640349	0.926938273	0.353958584	0.610806055
717.6997765	0.096051644	0.053631875	1.79094323	0.073302405	0.252232323
433.9849682	0.078298529	0.083061005	0.942662907	0.345853352	0.60351179
335.2123065	-0.038085271	0.092949447	-0.409741768	0.681995387	0.84736731
14.19564965	1.05146851	0.734061995	1.432397423	0.152030116	0.386175827
62.07779878	-0.519276556	0.356097158	-1.458244036	0.14477329	0.374863969
45.84307308	0.010023169	0.196644604	0.050970982	0.959348644	0.981758066
35.41735263	-0.266568002	0.342392276	-0.778545605	0.436247432	0.680134315
1077.975579	-0.166111524	0.072138337	-2.302680254	0.02129684	0.118868199
188.7449897	0.237470742	0.16285215	1.458198381	0.14478587	0.374863969
194.6210099	-0.400713976	0.136918289	-2.926665095	0.003426176	0.037239186
3.177961125	0.280704187	0.928558376	0.302301066	0.762422565	0.891042078
113.601869	-0.00696136	0.10915153	-0.06377703	0.949147769	0.977223239
28.24171009	-0.090369744	0.210516868	-0.429275546	0.667722711	0.838710639
70.97367075	-0.215399538	0.112117791	-1.921189637	0.054707804	0.211495489
65.55269347	-0.07025425	0.224902017	-0.312377146	0.754753917	0.887201677
120.7703985	-0.004096163	0.16305094	-0.025121984	0.979957665	0.989611488
11.2211403	-0.003721083	0.317540014	-0.011718471	0.990650227	0.994151605
4.380485147	-1.27905744	0.528522572	-2.420062092	0.015517857	0.099324339
6.039330013	-0.969885568	0.448417742	-2.16290632	0.030548386	0.148795304
150.2578118	-0.03152486	0.147359956	-0.213930979	0.830600882	0.928123698
139.2934904	-0.138176608	0.163774315	-0.843701336	0.398836321	0.648827804
57.34423752	-0.044549211	0.168996561	-0.263610162	0.792080327	0.904862734
54.52910108	-0.132626494	0.153395577	-0.864604422	0.387255945	0.638304143
210.2390784	0.188663424	0.178686531	1.055834613	0.291043781	0.550365405
73.31529165	0.201485759	0.421353423	0.478187071	0.632517064	0.817300842
74.60536349	0.369557234	0.17803846	2.075715738	0.037920249	0.169490223
959.5210657	0.2289726	0.108891911	2.102751237	0.035487521	0.162364724
740.4342855	0.08942071	0.084362088	1.059963205	0.289161339	0.548724313
634.0709479	-0.129186474	0.09436067	-1.369071174	0.170977028	0.412744914
166.1078608	-1.081594029	0.462790728	-2.337112571	0.019433332	0.112494686
308.0859324	0.500311093	0.164946832	3.033165814	0.002420026	0.029920446
1014.948595	-0.117079252	0.121685267	-0.962148131	0.3359752	0.594610137
882.7077437	0.012345921	0.089148275	0.138487488	0.889855157	0.953820886
98.51370559	0.128656527	0.149867063	0.858470994	0.39063244	0.641408659
8.408093151	0.679497335	0.601584945	1.129511869	0.258681966	0.515570067

14.90530528	0.028839938	0.331765169	0.086928769	0.930728131	0.970903892
195.8961506	0.150685019	0.085235673	1.767863308	0.077083758	0.259996057
286.0615186	0.278456414	0.111164843	2.504896386	0.012248727	0.085116984
732.5558931	0.217973637	0.163586172	1.332469819	0.182705853	0.428462709
399.7638286	0.528756117	0.122731368	4.308239403	1.64559E-05	0.000848996
236.3528594	-0.163520862	0.14952673	-1.093589502	0.274135056	0.533044162
147.366561	-0.028797384	0.101270659	-0.284360585	0.776134061	0.897389272
24.72341111	0.694045888	0.259586718	2.673657163	0.007502911	0.062626747
468.1030106	-0.251543489	0.168021697	-1.497089325	0.134370017	0.358453483
577.7468024	-0.041207772	0.07104624	-0.580013408	0.561905576	0.771414313
7.119791207	0.146002365	0.643242918	0.226978581	0.820440397	0.921144748
419.8705695	0.887576477	0.342778648	2.589357541	0.00961552	0.073173127
169.630016	0.116027001	0.128900856	0.900125919	0.368053244	0.623287987
505.9068554	0.257170497	0.197616897	1.301358845	0.193135654	0.440538044
557.9527034	0.008263669	0.107434	0.076918565	0.938688329	0.974295153
486.5175102	0.202894867	0.127742852	1.588307004	0.112216934	0.322582551
1657.457188	0.245859027	0.063963186	3.843758296	0.000121164	0.003823625
44.8311136	0.061338779	0.307613392	0.199402172	0.841948161	0.93182686
11.18467762	1.140571176	0.70429035	1.619461599	0.105347982	0.310578526
58.16706765	0.625754214	0.392795852	1.593077448	0.111142818	0.320670338
129.7485536	1.069333275	0.444015514	2.408324126	0.016025946	0.100782251
112.5474606	0.726682378	0.371975508	1.953575877	0.050751396	0.201476699
4.656920722	1.676366031	0.866073367	1.93559356	0.0529175	0.207075366
103.2378547	-0.083179304	0.495730236	-0.167791468	0.866747335	0.943901208
172.4894633	0.305883789	0.446575628	0.684954059	0.493372912	0.721699379
170.7764052	0.149855301	0.478244301	0.313344665	0.75401882	0.886841482
3.685984152	0.25532706	0.805060888	0.317152483	0.751127903	0.885437575
418.3553701	0.504697161	0.206915665	2.439144279	0.014722089	0.095849417
41.20005983	0.883853237	0.514524119	1.717807201	0.085831788	0.27710699
201.2362625	0.35262306	0.117335074	3.005265589	0.00265349	0.031793234
6.356347381	1.494041186	0.61204303	2.441072136	0.014643729	0.095548023
151.1568048	-0.076299946	0.110803363	-0.688606768	0.491070761	0.720179947
786.5818331	0.06623817	0.12134241	0.545878151	0.585149698	0.786366419
16.60124579	0.87876764	0.322211721	2.727298803	0.006385519	0.055577399
341.8076063	-0.030471267	0.110601123	-0.275505946	0.782927558	0.900791541
5.465298676	0.331275856	0.532564094	0.622039413	0.533915952	0.752645856
39.8823058	0.057176928	0.168146243	0.340042855	0.733824255	0.876280303
159.6983206	0.180608774	0.180604586	1.000023192	0.317299285	0.576008142
3737.598592	-0.158613085	0.225235193	-0.704210934	0.481301423	0.713221043
10.63089982	-0.351614714	0.316620531	-1.110524047	0.266773272	0.525419661
39.62360487	-0.729016121	0.420605295	-1.733254739	0.083050402	0.271536898
55.42828904	0.314688672	0.174932993	1.798909782	0.072032953	0.249234728
76.69324752	0.245745767	0.155229684	1.583110663	0.113396244	0.324077445
586.6843813	0.088993639	0.080132839	1.110576384	0.266750733	0.525419661
93.23637666	0.231768315	0.123903739	1.870551424	0.061407283	0.22704192
291.4170558	-0.833182326	0.38124857	-2.185404461	0.028859204	0.143618922
83.74630478	0.326120891	0.192226489	1.69654501	0.089782714	0.284130767
779.894086	0.030624949	0.096398186	0.317692172	0.75071845	0.885437575

209.3001918	-0.048155946	0.110980175	-0.43391485	0.664350275	0.836652459
54.62035741	-0.303368851	0.265559253	-1.142377259	0.25329724	0.509966459
46.73205682	0.019063582	0.360107201	0.05293863	0.957780805	0.981434349
43.65698911	0.759054341	0.313662753	2.419969645	0.015521803	0.099324339
153.227254	0.70697019	0.260481978	2.714084854	0.006645914	0.057303198
61.64939868	0.135550026	0.138275033	0.980292846	0.326941585	0.585359797
886.7252514	0.221827415	0.116253513	1.908135151	0.056373751	0.215502505
167.7495897	0.18422875	0.159861758	1.152425396	0.24914631	0.50484376
3.694204283	0.606143451	0.784350871	0.772796301	0.439642943	0.68156172
25.5231205	1.07859158	0.523997688	2.058389959	0.039552715	0.173782322
144.8059213	0.268331862	0.329521799	0.814306862	0.41546918	0.661679388
101.1937165	0.443320783	0.219160496	2.022813371	0.043092393	0.182813385
4.320123747	1.419070705	0.757244625	1.873992444	0.060931467	0.225761663
743.8990317	-0.014143807	0.073620792	-0.192117015	0.847650547	0.934372901
4.614127303	0.821130234	0.587332239	1.398067702	0.162092738	0.400816408
694.0312113	0.044070059	0.09197814	0.47913623	0.631841717	0.81678327
73.25200037	0.211200046	0.187777214	1.124737351	0.260700349	0.518163005
168.0876189	0.228588385	0.122294084	1.869169607	0.061599221	0.22753702
9.165022269	0.038367686	0.354704842	0.108167924	0.913862489	0.963484061
2785.193203	0.119828269	0.077143687	1.553312705	0.120348446	0.336101324
3196.599236	0.125148941	0.064004367	1.955318777	0.050545456	0.201002773
7.050540836	0.48721971	0.593781136	0.820537535	0.41190974	0.658847341
9.775435598	1.51102388	0.652870394	2.314431615	0.020644058	0.116846413
9.664866518	0.153678866	0.464443488	0.330888192	0.740728941	0.879520987
95.22919822	0.397869389	0.173938215	2.287417912	0.022171442	0.122098628
12.98816197	0.255875184	0.287755925	0.88920909	0.373890718	0.627431154
962.5935902	0.116145234	0.067659353	1.71661758	0.086049068	0.277180856
604.5102998	-0.157770782	0.33322731	-0.473462942	0.635882934	0.819624255
13.94263813	0.546315637	0.387364013	1.410341745	0.158438798	0.395315639
5.406966746	-0.374865704	0.673303784	-0.556755676	0.577694371	0.781880601
21.51137752	1.292415917	0.620957838	2.081326361	0.037404044	0.16804498
6.562386571	0.366908893	0.6127692	0.598771761	0.549325096	0.763321946
18.11046772	1.383111989	0.596439158	2.318949002	0.020397802	0.116099368
240.6846232	0.219577034	0.114341038	1.920369441	0.054811251	0.211695407
112.1457911	-0.089765686	0.16868014	-0.532165113	0.594611643	0.792613379
392.7885495	-0.16994901	0.111943087	-1.518173333	0.128970708	0.350566122
6.727967983	0.894516176	0.562168156	1.591189692	0.111566892	0.321243828
82.42935474	0.250587013	0.191763029	1.306753519	0.191296436	0.438575252
685.9281592	0.166705513	0.055235904	3.018064321	0.002543949	0.030977715
861.0290119	0.260562615	0.308162909	0.845535291	0.397812037	0.648131599
169.1248738	0.228348795	0.196627918	1.161324382	0.245510008	0.500595289
27.38979524	0.536273787	0.43591462	1.230226658	0.21861224	0.471328542
173.6991977	0.467228843	0.31462741	1.485022691	0.137537813	0.363522593
280.4369778	0.13394291	0.118402343	1.131252194	0.257948959	0.515109686
444.0966092	-0.10739108	0.09300077	-1.154733232	0.248199686	0.503526566
62.40120608	0.407285659	0.229704679	1.773083857	0.076214813	0.257978772
216.7429004	-0.0661511	0.117230021	-0.564284635	0.572560437	0.778738936
484.6100537	0.336794841	0.156909488	2.146427506	0.031838887	0.152090717

31.43763015	0.443934498	0.180806179	2.455306007	0.014076471	0.093154162
4.97913494	0.702774959	0.474812946	1.480109093	0.138844135	0.365865493
1055.983514	0.024030901	0.088897056	0.270322795	0.78691193	0.903033152
390.8010734	0.27781159	0.136074307	2.041616784	0.041189559	0.178076271
20.73105699	0.388850783	0.321155081	1.210788201	0.225976588	0.480315376
126.4524866	0.561352982	0.363769732	1.543154731	0.122793238	0.340383377
242.4637286	0.519154447	0.312086824	1.66349364	0.096213663	0.295418349
3.004029907	0.428366498	0.702327612	0.609924045	0.541912124	0.758121441
46.28063837	-0.298917866	0.372573511	-0.802305737	0.422376125	0.667104931
5.676418501	0.871772568	0.586878412	1.485439829	0.137427352	0.363353099
6.9472852	0.561878717	0.489301258	1.148328781	0.250832862	0.50703217
38.70726892	0.126244862	0.279572107	0.451564583	0.651582689	0.829498612
2.995998666	0.048772328	0.565928872	0.086181022	0.931322517	0.971030824
8.597638997	0.741667976	0.461099399	1.608477429	0.107730656	0.314060893
108.4476056	-0.240995975	0.134447144	-1.792496053	0.073053541	0.251509133
156.5814256	-0.015820856	0.101654035	-0.155634312	0.876321278	0.948728026
292.9376202	-0.15675927	0.1044989	-1.5001045	0.133587335	0.357038617
4.941299968	-0.763721638	0.547205169	-1.395676945	0.1628118	0.401570246
14.00043894	0.630967542	1.061631042	0.594337879	0.552286148	0.76490866
30.38411274	0.088828119	0.333756594	0.266146409	0.790126454	0.904145475
279.7369697	-0.090976199	0.118762303	-0.766035994	0.443654885	0.684802994
69.78118434	-0.097973005	0.169161365	-0.579168923	0.562475198	0.771716083
74.1383667	-0.035325189	0.14040867	-0.251588372	0.801359247	0.909707472
5.446341348	-0.093298682	0.498354936	-0.187213319	0.851493382	0.93609097
7.857692803	-0.257739035	0.379360851	-0.679403355	0.496882325	0.723718151
165.0042421	0.510534991	0.190914345	2.674157312	0.00749173	0.062566716
33.72437852	0.422438559	0.382787455	1.10358517	0.269773127	0.528493845
548714.1793	0.510767757	0.314313723	1.625025314	0.104157165	0.309111914
5.956197212	2.284851363	1.465294043	1.559312531	0.118922427	0.333842733
3.776337682	0.393959928	0.526638243	0.748065553	0.454420618	0.69248182
29.05277025	0.196758436	0.239270837	0.822325186	0.410891846	0.658207516
1148.615592	-0.167172283	0.129674732	-1.289166212	0.197340309	0.445722264
19.82815808	0.019653638	0.529669478	0.037105476	0.970400906	0.985672418
5.832544397	-0.82708151	0.427957809	-1.932623947	0.05328254	0.208099808
10.40786164	-0.260197729	0.346211609	-0.751556916	0.452317561	0.69097325
388.6328739	0.025887882	0.12769722	0.202728625	0.839347148	0.931066348
268.8199168	-0.435424174	0.158852874	-2.741053165	0.00612426	0.054351712
1692.560175	0.213544694	0.154259123	1.384324568	0.166259095	0.406042921
429.6924239	-0.218267677	0.293130238	-0.7446099	0.456507581	0.69386622
15.46097679	-0.28322238	0.507137572	-0.558472486	0.576521788	0.781045843
4.593350494	0.415577563	0.537604636	0.773017074	0.439512276	0.681475638
133.2684666	-0.090211443	0.134674831	-0.669846344	0.502955747	0.729188199
76.23884784	-0.509290389	0.188640874	-2.699788117	0.006938365	0.059253816
634.5093417	-0.377049942	0.175890445	-2.143663588	0.032059852	0.152695632
274.8595835	-0.249711685	0.142241252	-1.755550384	0.07916521	0.263447308
908.2939412	-0.351755921	0.165262924	-2.128462408	0.033298764	0.156130884
2208.887557	-0.176626496	0.121469316	-1.454083238	0.14592323	0.376053227
35.57621928	0.371128899	0.382618767	0.969970452	0.332061221	0.590816584

94.70934763	-0.231636212	0.15837764	-1.462556287	0.143588832	0.373320792
5.773225057	-0.19127593	0.428132674	-0.446767886	0.65504266	0.831742963
471.9029379	-0.072672269	0.069644793	-1.043470233	0.29673056	0.555360633
162.1157086	-0.138328766	0.122566115	-1.128605285	0.25906438	0.516023081
8.012746073	0.109188644	0.397664231	0.274574968	0.7836428	0.901206458
261.4364327	0.04117782	0.179481234	0.229426883	0.818537143	0.920415839
34.95306382	0.545239719	0.555749969	0.981088168	0.326549265	0.585151858
238.6270591	0.160212161	0.118274256	1.354581854	0.175550818	0.41891664
11808.12085	-0.231101266	0.089321818	-2.58728799	0.00967347	0.073435968
426.2701052	-0.02061893	0.107711129	-0.191428035	0.848190257	0.934489113
945.1998859	0.152466135	0.115354621	1.321716754	0.186262487	0.432345842
676.2725018	0.29880595	0.115692099	2.582768859	0.009801096	0.073977505
3.726085816	0.07061173	0.53092901	0.132996556	0.894196105	0.955475416
2944.350623	0.350513852	0.126445675	2.772050939	0.005570432	0.050972377
372.7592258	-0.147055156	0.121858262	-1.206772144	0.227519907	0.482023803
6.507606975	0.159668418	0.41362082	0.386026067	0.699477364	0.856925408
148.2793562	-0.048403937	0.162164192	-0.298487211	0.765331336	0.891986079
653.4507212	0.319270933	0.098737015	3.233548548	0.001222626	0.019118304
242.0495698	-0.105756437	0.101700167	-1.039884604	0.298393516	0.556745647
327.6366422	0.072304077	0.098440685	0.734493842	0.462647812	0.69878354
122.3958615	-0.071015525	0.110825453	-0.640787138	0.521660991	0.744256234
411.7775852	-0.062380795	0.09592541	-0.650305221	0.515495085	0.739680406
4.523703929	-0.139213683	0.594578709	-0.234138359	0.814877568	0.918538146
425.4972763	-0.014095996	0.092293298	-0.152730444	0.878610852	0.949764463
117.6611417	0.169443403	0.131253919	1.290958812	0.19671796	0.445162804
461.9423223	-0.06197943	0.094247773	-0.657622228	0.510780908	0.735772204
9.031648848	0.33263762	0.485457411	0.685204535	0.493214863	0.721618408
3.928233049	-0.127103549	0.634061862	-0.200459224	0.841121445	0.931714722
47.60638148	-0.273778702	0.28687074	-0.95436259	0.339900136	0.598114409
712.7269755	0.161344781	0.077106241	2.092499635	0.036393844	0.165162342
1130.795608	0.207791642	0.130230286	1.595570801	0.110584651	0.319487865
206.2704194	0.178264682	0.116108544	1.535327854	0.124703315	0.343425257
673.9180933	0.229533789	0.148553605	1.545124329	0.122316193	0.339541093
104.6034066	0.050032536	0.140053098	0.357239769	0.720912316	0.869287343
1201.277116	0.478393992	0.176897866	2.704351396	0.006843787	0.058559526
158.6375306	-0.174808015	0.108485412	-1.611350429	0.107103365	0.313221924
463.3512345	0.098400279	0.062567854	1.572697035	0.115788987	0.328159463
413.4635539	0.168047955	0.184001772	0.913295307	0.36108725	0.616999384
30.24430563	0.465005501	0.362998864	1.281010899	0.200189838	0.448731945
129.7680744	0.267279544	0.147613327	1.810673537	0.070191402	0.245766866
133.1346153	0.011437773	0.145276751	0.078730922	0.937246649	0.973688577
372.4611415	-0.19727615	0.057104478	-3.454652892	0.000551002	0.011269949
425.8133447	-0.152532236	0.111172122	-1.372036745	0.170051999	0.41133491
102.0247921	0.446868572	0.161103177	2.773803599	0.005540512	0.050876688
2525.420787	0.001809389	0.099382027	0.018206404	0.985474194	0.992116134
64.90670103	0.176706237	0.2973973	0.594175659	0.552394631	0.76490866
254.2316081	0.03767933	0.162436363	0.231963638	0.816566256	0.919186476
2.606171778	-1.090888103	0.998825809	-1.09217052	0.274758158	0.53385864

10.0125002	-0.215121256	0.381129596	-0.56443073	0.57246103	0.778738936
180.3503606	0.05947081	0.107842851	0.551458062	0.581319708	0.783753517
279.6053036	0.913738241	0.339536644	2.691132923	0.007120982	0.060370527
283.5421989	-0.075864672	0.086288797	-0.879194929	0.379295594	0.631515084
200.4335324	0.055864917	0.088622624	0.63036857	0.528453469	0.749041961
178.5848409	0.128797088	0.106468688	1.209717994	0.226387123	0.480597677
275.2198735	0.105181347	0.152330219	0.690482475	0.489890827	0.719558693
13.52305536	0.204718443	0.251262065	0.814760648	0.41520933	0.661601456
7.356900619	0.095568503	0.477712842	0.200054289	0.841438122	0.93182686
63.74096857	0.373587932	0.177464623	2.10514031	0.035279096	0.161646902
4.735085162	0.614652696	0.449566178	1.367212938	0.171558571	0.413448775
16.27609636	-0.133090167	0.414177324	-0.321336198	0.747955628	0.88426827
958.4629078	-0.042852269	0.161690895	-0.265025861	0.79098954	0.904476207
7.018689964	1.347059084	0.537644206	2.50548424	0.012228385	0.085050992
147.7392515	-0.094482427	0.178071814	-0.530586089	0.595705637	0.793532123
3.397114566	2.470124821	1.619273544	1.525452466	0.127146281	0.347596701
8.322250852	2.135764178	1.121949498	1.903618819	0.056959846	0.216551186
6.012234281	1.910820802	1.074709752	1.777987777	0.075405862	0.256432252
19.01626777	-0.795489087	0.645392642	-1.232566092	0.217737694	0.470515017
133.7815257	0.23212588	0.232431406	0.998685525	0.317947055	0.576478535
110.9992467	-0.098841893	0.154554254	-0.639528774	0.522479001	0.744756944
3376.647872	0.170264869	0.159089665	1.070244688	0.284509184	0.543182233
306.733032	0.174373907	0.11600754	1.503125631	0.13280665	0.356058035
187.061335	0.211419033	0.198636419	1.064351814	0.287169354	0.546589349
947.770386	-0.136446719	0.116562839	-1.170585072	0.241765601	0.497292024
287.2833419	-0.152721717	0.092995471	-1.642248968	0.100538418	0.303356012
1305.54029	0.050930246	0.123956515	0.410871876	0.681166485	0.847181914
4.120830114	0.995005783	0.68207949	1.458782733	0.144624916	0.374844469
524.3771613	-0.108497975	0.088952279	-1.219732375	0.222566344	0.476160216
4.923844193	-0.476677925	0.507149075	-0.939916779	0.34726025	0.604822849
1326.238641	-0.134766799	0.092247174	-1.46093147	0.144034249	0.373746018
7.003167112	0.21207894	0.478182806	0.443510175	0.657396761	0.832948475
13.98157901	0.930875409	1.301229981	0.715381157	0.474373553	0.707506124
42.74507133	0.12122132	0.30114096	0.402540126	0.687286564	0.850122158
9.615535353	0.934637725	0.653300685	1.430639439	0.152533578	0.386588123
43.47365699	-0.101688179	0.195604753	-0.519865582	0.603157265	0.798632907
43.66730862	-0.16042915	0.160070452	-1.002240876	0.31622727	0.575300613
45.51773965	-0.08385431	0.172574083	-0.485903263	0.627035755	0.813771516
4.4473311	-0.308394838	0.873385566	-0.353102742	0.724011417	0.871347074
59.90678685	0.16659388	0.149026026	1.117884472	0.263616338	0.521277253
46.43356922	-0.460379807	0.225116489	-2.045073686	0.040847594	0.177161824
169.2550293	0.144050098	0.146098435	0.98597975	0.32414305	0.582873043
25.45509185	0.367971631	0.389078756	0.945751023	0.344275592	0.602031261
678.262463	-0.186195325	0.108529495	-1.715619564	0.086231695	0.277313494
392.9104714	0.176963349	0.070786577	2.499956286	0.012420863	0.085894541
8571.410951	0.068471716	0.261162144	0.262180861	0.793182008	0.905368772
258.2901949	0.060311282	0.191579942	0.31481	0.752905918	0.8863867
436.434322	-0.342662467	0.443697799	-0.772287957	0.439943896	0.681826094

141.6203067	0.333085213	0.214779555	1.550823643	0.120943953	0.337233141
17.50672821	0.494987577	0.300601765	1.646655589	0.099628857	0.301938075
128.2723321	-0.113297513	0.10797658	-1.049278588	0.294049918	0.55324843
752.3570672	-0.128429166	0.112395225	-1.142656781	0.253181121	0.509908862
7084.221566	-0.016297441	0.150858156	-0.108031555	0.913970662	0.963484061
101.2769563	0.240737146	0.175241703	1.373743477	0.169521334	0.410494459
369.8190689	-0.146309903	0.191358148	-0.76458674	0.444517668	0.685325381
11.48075518	-0.375719929	0.614461898	-0.611461719	0.540893954	0.757639765
17.50680505	1.564122711	0.633677951	2.468324343	0.013574726	0.091258465
591.8482182	0.237019945	0.064771052	3.659349955	0.000252856	0.006515031
1186.817258	0.167870051	0.120585331	1.392126636	0.163884054	0.403299571
32.30161718	0.355206674	0.175833796	2.020127424	0.043370172	0.183545002
458.5237568	0.381612656	0.111054541	3.436263396	0.000589797	0.011665044
587.8257556	0.228896072	0.115156164	1.987701427	0.046844721	0.192238027
307.4933476	-0.23255475	0.115740279	-2.009281063	0.044507335	0.186745062
1604.748323	-0.043584923	0.111777451	-0.389925896	0.696591344	0.854877828
1248.75584	0.141103053	0.141356995	0.998203539	0.318180671	0.576493309
586.4959027	0.141392011	0.073229105	1.930817123	0.053505672	0.208700065
55.27208621	0.186681515	0.171800663	1.086616965	0.277206124	0.535573218
1131.822648	-0.007660589	0.1376787	-0.055641059	0.955627754	0.980445195
44.96726118	0.371881423	0.242126048	1.535900106	0.124562882	0.343403657
286.1639005	0.132257361	0.085520864	1.546492337	0.121985709	0.338989742
4.00797078	0.252250282	0.5234751	0.481876371	0.629893772	0.815381783
83.93925421	0.598572293	0.259138641	2.309853482	0.020896267	0.117648108
3.352387835	0.236724459	0.54463741	0.434645977	0.663819417	0.836652459
352.1369784	-0.010660907	0.073483981	-0.145077968	0.884649313	0.952251634
320.3525184	0.008164463	0.124768974	0.065436647	0.947826347	0.976593642
173.1204999	0.300894666	0.217696701	1.382173757	0.166918356	0.406609306
174.6115265	0.139733934	0.11348672	1.231280056	0.218218139	0.470969215
779.6816145	0.331949831	0.106864547	3.106267126	0.001894655	0.025708239
5.60835862	0.198373368	0.435870016	0.455120472	0.649022571	0.827939679
249.2405397	-0.059137354	0.133940615	-0.441519202	0.658837163	0.83373717
36.23100703	1.236054457	0.668279545	1.849606899	0.064370228	0.233472484
14.23759073	-0.846994389	0.32688794	-2.59108485	0.00956739	0.07288018
5.541396865	0.182178886	0.47076246	0.386986858	0.698765937	0.856624099
1674.140864	-0.960593336	0.510042113	-1.883360826	0.059651485	0.22296804
311.3490674	-0.19876837	0.07914123	-2.511565345	0.012019703	0.083897206
1496.375238	-0.073978909	0.12361269	-0.598473414	0.549524093	0.763445693
21.65691845	-0.352172412	0.277257032	-1.270201911	0.204012717	0.453634884
96.31390778	-0.424159105	0.122480701	-3.463068885	0.000534052	0.010992213
48.69562676	-0.240996788	0.295574484	-0.815350447	0.41487174	0.661332287
2468.173704	-0.186447197	0.152698643	-1.221014105	0.222080679	0.475640226
49.8859778	0.008473901	0.176343652	0.048053339	0.961673734	0.98240675
227.9624965	-0.379365162	0.113767531	-3.334564438	0.000854331	0.014724536
5.526870231	-0.090440753	0.42709692	-0.211756977	0.832296635	0.928726028
2474.36259	-0.22433425	0.109029193	-2.057561323	0.039632263	0.173835375
630.6276912	-0.023259907	0.175052944	-0.132873554	0.894293382	0.955475416
59.00072173	-0.515004596	0.716331906	-0.718946889	0.472173644	0.706251867

1476.517244	-0.259856904	0.13472994	-1.928724263	0.053765102	0.209243639
165.5993591	-0.392971119	0.356564203	-1.102104799	0.270416113	0.529356755
402.2291402	-0.011758567	0.096007452	-0.122475571	0.902522392	0.958694616
598.7147458	0.001625028	0.059365281	0.027373366	0.978161941	0.988880488
666.6569078	-0.461721322	0.091542569	-5.043788141	4.56405E-07	5.42292E-05
530.4377658	-0.069703801	0.091905762	-0.758426891	0.448195465	0.687884956
3092.062749	-0.129445046	0.117665559	-1.100109904	0.271284239	0.530481168
218.2794702	0.217061337	0.076987437	2.819438423	0.004810776	0.046305965
24.6553927	0.613999916	0.363070023	1.691133604	0.090811288	0.285542151
152.0808604	0.605433856	0.271963395	2.226159351	0.02600351	0.134600345
2244.854884	-0.067300999	0.120919722	-0.556575868	0.577817245	0.781880601
1191.254656	0.163399504	0.090321406	1.809089462	0.070437106	0.246268911
2.881829043	-0.704861886	0.739419021	-0.953264476	0.340456086	0.598586551
1015.997271	-0.365235614	0.16317042	-2.238369029	0.025197	0.131729916
37.06147512	0.340699813	0.231861834	1.469408772	0.141721949	0.370461176
54.4980857	0.385023113	0.195238895	1.972061521	0.048602584	0.196417802
249.7424189	0.005249863	0.11294353	0.046482194	0.962925926	0.98274378
25.2019189	0.510595779	0.236821162	2.156039493	0.031080581	0.150112302
6.234989348	-0.206375508	0.366267898	-0.563455079	0.57312504	0.779066086
49.40422831	-0.15316712	0.301181958	-0.508553436	0.611065274	0.803955017
216.6715512	0.137544714	0.254950239	0.539496313	0.58954444	0.789819339
1889.406737	0.21076855	0.135644721	1.553827878	0.120225479	0.335997222
32091.83544	0.029289794	0.127386685	0.229928224	0.818147544	0.920181415
108.2434402	0.509740075	0.413771703	1.231935559	0.217973157	0.470699572
262.2536676	-0.34290534	0.298641308	-1.148218048	0.25087856	0.507059193
429.3646638	-0.026685134	0.13879897	-0.192257435	0.847540558	0.934372901
3.433004759	1.481936894	0.918329514	1.613731096	0.106585766	0.312455597
3.391294084	0.633484395	0.543409118	1.165759596	0.243711657	0.499297213
12.69756644	0.445689588	0.328509104	1.356703915	0.174875308	0.417988969
476.5348481	-0.122786902	0.074930536	-1.638676402	0.101280671	0.304741614
3.786963196	0.172419371	0.613283385	0.281141435	0.778601922	0.89838333
85.14553266	0.517484235	0.312200295	1.657539227	0.097410505	0.297814105
1908.944477	0.054048696	0.170355426	0.317270175	0.751038606	0.885437575
385.9595613	-0.204661709	0.097081751	-2.108137803	0.035019071	0.161067189
158.960677	0.280730611	0.378035359	0.742604109	0.4577214	0.694284175
331.4333797	-0.249215448	0.104927038	-2.375130881	0.017542729	0.106272752
155.2103682	-0.059372385	0.275954989	-0.215152424	0.829648482	0.927302545
1251.114305	-0.366943743	0.28164176	-1.302874061	0.192617761	0.440060737
3.873920588	-0.600525825	0.719466098	-0.834682588	0.403896448	0.653167263
347.8606713	-0.166754813	0.134418321	-1.240566105	0.21476608	0.466730109
149.6152568	-0.181965351	0.106641401	-1.706329326	0.087946781	0.280186332
821.986453	-0.216285877	0.140396503	-1.540536073	0.123429737	0.341423632
77.08899382	-0.327266549	0.141459008	-2.313508018	0.020694724	0.116880107
17.76730241	-0.443267123	0.620554532	-0.71430809	0.475036691	0.708152785
279.5868125	-0.052060069	0.125151703	-0.415975719	0.677427771	0.8455982
3697.137275	-0.155676187	0.049086792	-3.171447587	0.001516812	0.022233351
163.3257283	-0.059245454	0.12108045	-0.489306519	0.624624706	0.812044815
611.8335389	-0.333153749	0.152178454	-2.189230736	0.028580074	0.142709289

932.2274128	-0.056887393	0.142349079	-0.399633023	0.68942683	0.851213918
414.4030494	-0.237181758	0.117570014	-2.017366076	0.043657325	0.184437888
1795.7137	0.189079751	0.157977129	1.196880539	0.231353121	0.486759558
772.8478308	-0.167925058	0.120114366	-1.398043076	0.162100132	0.400816408
54.62446927	-0.110530641	0.291733816	-0.378874971	0.704780712	0.860203048
188.6998313	0.148209696	0.164661992	0.90008443	0.368075321	0.623287987
369.979621	-0.154955369	0.096387068	-1.60763651	0.107914811	0.314157226
167.5802067	-0.12166487	0.10805995	-1.125901596	0.260207174	0.517576324
38.80511273	0.278161203	0.26034714	1.068424269	0.285329174	0.544151601
116.4928105	0.119214318	0.16389911	0.727364038	0.467002978	0.702587502
42.01984209	-0.181241439	0.216894267	-0.83562116	0.403368061	0.652881803
385.6688968	-0.069524672	0.088987677	-0.781284271	0.434635319	0.678647416
193.1468187	0.100003151	0.121341752	0.824144613	0.409857393	0.657435871
364.5158174	-0.131637299	0.159928234	-0.823102312	0.410449814	0.657946778
454.988998	0.12131483	0.089302165	1.358475798	0.174312757	0.417775257
98.20833745	-0.157883845	0.164613606	-0.959117835	0.337499387	0.596029769
95.20987965	-0.391117069	0.561810666	-0.696172381	0.486320882	0.717172559
2963.470608	-0.391855522	0.169812521	-2.307577315	0.021022657	0.118178982
336.9445989	-0.051163932	0.066359008	-0.771017133	0.440696777	0.682478371
42.32099638	-0.466967523	0.27202539	-1.716632123	0.086046409	0.277180856
368.3949532	0.059275903	0.105616555	0.561236852	0.574636084	0.780163053
40.19746426	-0.07270702	0.190752234	-0.381159465	0.703084925	0.859283407
267.8133612	-0.017302865	0.084593742	-0.20454072	0.837930974	0.930810213
29.74203692	0.142288945	0.186883635	0.761377236	0.44643178	0.686319942
688.3006404	-0.133768304	0.078286937	-1.708692539	0.087507917	0.279394892
5.382775697	-0.45686186	0.534828449	-0.854221314	0.392982376	0.643530057
511.7956843	0.300229624	0.086535852	3.469424712	0.000521574	0.010792045
16.44510763	0.877010011	0.390509575	2.245809241	0.024716229	0.130346112
49.58382117	-0.011461251	0.21256734	-0.053918214	0.957000325	0.98108328
647.4015612	-0.406922902	0.187618502	-2.16888472	0.030091437	0.147255569
1471.201356	0.380952435	0.191974003	1.984395958	0.047211716	0.193173826
131.2909928	0.2843933	0.206193522	1.379254295	0.167816363	0.407940458
726.2244383	-0.180876703	0.085209577	-2.122727377	0.033776704	0.157337041
167.4177001	0.169660036	0.143513664	1.18218733	0.237131379	0.492684668
8.990328097	1.211759171	0.702807507	1.724169362	0.084677273	0.275078367
492.1296691	0.188624491	0.279625324	0.674561547	0.49995437	0.726660727
372.3362181	-0.095576744	0.080997146	-1.180001378	0.237999667	0.493559008
88.35522371	0.486079751	0.187091626	2.59808395	0.009374557	0.072022586
26.65318767	-0.023567988	0.210132792	-0.11215759	0.910698455	0.962059189
1712.492258	0.101240267	0.137331914	0.737194028	0.461004365	0.697145057
23.99863627	0.525017528	0.193268882	2.716513505	0.00659735	0.057072721
1710.633622	0.068851197	0.135120673	0.50955339	0.610364385	0.803167633
3.316714259	-0.059077845	0.523518312	-0.112847715	0.910151289	0.96178499
295.4743353	0.154998168	0.106213185	1.459311929	0.144479274	0.374547591
22.32123306	-0.059375976	0.240397988	-0.246990319	0.804915717	0.912160267
84.47122204	-0.261009271	0.666334149	-0.391709281	0.695273032	0.854463425
107.449719	0.188955464	0.132040281	1.431044089	0.15241758	0.386439916
585.0845852	-0.181259712	0.107258292	-1.689936591	0.091040086	0.285931737

10.43824051	-0.274921856	0.353752764	-0.777158185	0.437065449	0.680390522
3140.425007	0.2829016	0.117899652	2.399511742	0.016416953	0.102164672
2029.509215	-0.230679896	0.106720149	-2.161540235	0.030653632	0.14894969
11.17263833	-0.099240531	0.343624815	-0.2888049	0.772730682	0.89579137
233.031629	-0.118300142	0.123703716	-0.956318421	0.338911386	0.597245638
353.8016511	-0.108373533	0.105384459	-1.028363515	0.303778864	0.562210657
314.4317363	-0.237697011	0.096117397	-2.472986362	0.013398926	0.090400005
47.51066248	0.218233686	0.172826791	1.262730647	0.206685996	0.456765276
352.3254211	0.31028074	0.140479676	2.208723337	0.02719389	0.138207704
1317.376381	-0.360032792	0.085968662	-4.187953867	2.81481E-05	0.001283356
246.4904275	0.119700817	0.111794014	1.070726533	0.284292409	0.543156866
194.6062863	0.079382714	0.142042804	0.558864734	0.576254039	0.780913931
702.8764912	-0.082667358	0.079367491	-1.041577054	0.297607812	0.55613975
348.0238386	-0.015823443	0.143055085	-0.110610838	0.911924953	0.962232976
143.7691593	0.031088289	0.252610667	0.123067996	0.902053254	0.958662553
100.9354435	0.037443334	0.186595734	0.200665542	0.840960105	0.931667605
2.641843473	-0.222974157	0.652099927	-0.341932498	0.732401692	0.875328164
7.994993978	1.02339656	0.562428483	1.819603009	0.068819483	0.242881362
130.4792806	-0.355480835	0.119354349	-2.97836517	0.002897905	0.033717167
8.072943075	0.542315243	0.457676902	1.18493033	0.236044994	0.492010908
26.00382719	-0.220629703	0.203086213	-1.086384444	0.277308941	0.535629733
4.331847821	-0.095622794	0.694454453	-0.137694839	0.890481599	0.953833039
139.9046658	-0.478214341	0.139535653	-3.427183885	0.000609876	0.011971582
15.5866761	0.31610409	0.2757669	1.146272778	0.251682303	0.507898255
726.146605	-0.257625678	0.124475877	-2.069683576	0.038481986	0.170784229
24.21910191	0.778257455	0.327984244	2.372850127	0.017651425	0.106606997
59.43019101	0.099985438	0.317750457	0.314666544	0.753014849	0.8863867
13.24240949	0.725184656	0.345781114	2.097236162	0.035972675	0.163940355
253.8386997	-0.134982284	0.087231629	-1.547400706	0.121766651	0.338615639
691.485958	0.27205066	0.090581433	3.003382164	0.002669969	0.031941874
7320.892523	0.037464506	0.110699834	0.33843326	0.735036714	0.876811364
167.3341714	-0.123050277	0.139419605	-0.882589481	0.377458114	0.630416193
30.20134755	0.304403248	0.245478335	1.240041192	0.214960158	0.466957774
864.0870112	-0.240168957	0.07015836	-3.423240761	0.000618792	0.012086103
472.7766956	-0.272363136	0.075572873	-3.603980161	0.000313381	0.007601441
2404.064089	-0.140474295	0.081118521	-1.731716676	0.08332402	0.271921334
518.6097095	0.162680365	0.067391988	2.413942223	0.015780965	0.10008437
945.4874373	-0.042253967	0.106320709	-0.397419915	0.691057832	0.852422418
15.95614698	1.215846188	0.588481784	2.066072767	0.038821609	0.171634538
111.6912065	-0.038168851	0.177365561	-0.215198773	0.829612347	0.927302545
659.7254402	-0.055612423	0.066045095	-0.84203714	0.399767165	0.649600934
95.73358818	0.323888348	0.166744586	1.942421975	0.052086037	0.204861565
1422.853731	-0.216993893	0.087206664	-2.488271927	0.012836554	0.087743627
211.0458539	-0.318481859	0.2586201	-1.231466	0.218148626	0.470969215
165.9421054	-0.196000753	0.099573008	-1.968412504	0.049020594	0.197441962
545.6762296	-0.222445574	0.080092873	-2.777345413	0.00548049	0.050502943
482.7821175	0.127149005	0.129740228	0.980027605	0.327072492	0.585527334
43.59538793	0.150121011	0.166277934	0.902831826	0.366615157	0.622360875

253.4236947	-0.215958292	0.112302525	-1.923004774	0.05447945	0.210872581
58.89910333	-0.076574677	0.171779493	-0.445773101	0.655761154	0.831914417
14.92865684	-0.389482865	0.305176078	-1.276256209	0.201864965	0.450669366
31.74720016	-0.605150883	0.249405173	-2.426376628	0.015250434	0.098471723
59.86074306	-0.460235353	0.2149621	-2.141006963	0.032273477	0.153201334
25.8553622	0.175325974	0.369320769	0.474725466	0.634982664	0.818867629
50.15937098	-0.198785959	0.313817974	-0.633443509	0.52644406	0.747893898
3.557878324	-1.103042729	0.679991306	-1.622142401	0.104772861	0.309829354
82.34349271	-0.283258102	0.185901584	-1.523699238	0.12758386	0.348246651
61.44371827	-0.07290242	0.157142343	-0.463926007	0.642700759	0.824387858
1845.232225	-0.216778573	0.078446933	-2.76337855	0.005720638	0.051802819
154.8827557	0.170955066	0.112877906	1.514513083	0.129895758	0.351862706
1091.108114	-0.094858765	0.223860479	-0.42374056	0.671755032	0.841854248
23.52508669	0.16418221	0.297400957	0.552056765	0.580909463	0.783403612
115.8068866	0.483620465	0.132690822	3.644716777	0.000267686	0.006805756
39.74316604	-0.115643307	0.209421238	-0.552204294	0.580808393	0.783402032
19.04042879	0.276233489	0.262762284	1.051267651	0.29313568	0.552389764
558.5200138	0.167212392	0.075995891	2.200282006	0.027786893	0.140357369
291.8073555	-0.282321013	0.141510181	-1.995057958	0.046036559	0.19061583
509.6641841	-0.251629892	0.088267288	-2.850771773	0.004361326	0.043458093
257.4538841	-0.060606182	0.133985006	-0.452335558	0.651027264	0.829060702
94.19168386	-0.134311253	0.112181408	-1.197268386	0.231201963	0.48673444
33.18492	-0.021854738	0.263966911	-0.082793474	0.934015758	0.972716013
2.989435253	0.100666622	0.679595953	0.148127165	0.882242412	0.951068113
21.61863833	1.128791454	0.496547096	2.273281756	0.023009206	0.125304302
3.321811656	1.356147542	0.791282031	1.713861163	0.086554227	0.277894882
17.03912563	0.502253373	0.91534841	0.548701857	0.583210071	0.785021177
77.03370791	0.083788732	0.421506633	0.198783898	0.842431792	0.93182686
782.1790435	-0.208157371	0.066931951	-3.109985103	0.001870968	0.025494578
34.94674958	0.167837749	0.267727913	0.626896716	0.53072694	0.750782117
67.82277148	-0.125469469	0.171296079	-0.732471341	0.463880929	0.699500864
189.2091241	-0.113665234	0.16405397	-0.692852686	0.488402005	0.718612563
9.374554317	0.957800508	0.369175581	2.594430825	0.009474768	0.072470018
4.387267118	0.510695564	0.62282011	0.819972823	0.4122316	0.659066301
1633.029809	-0.096502924	0.143234243	-0.673741987	0.500475363	0.726936062
5.027188295	-0.455458157	0.418299127	-1.08883363	0.276227258	0.534794106
654.6415034	-0.389674406	0.162513244	-2.397800913	0.016493827	0.102451163
434.9638955	-0.071484242	0.080749842	-0.885255495	0.376018847	0.629276766
101.3836518	-0.174490732	0.154807509	-1.12714643	0.259680576	0.516922217
492.4944452	-0.211161027	0.11527129	-1.831861404	0.066972072	0.239519
340.3925971	-0.291863125	0.084502878	-3.45388385	0.000552575	0.011269949
1256.61467	-0.200083351	0.076473696	-2.616368274	0.008887065	0.069345634
29.92575801	-0.15641172	0.188547972	-0.829559281	0.406788007	0.655071986
319.1261245	-0.461175093	0.143629374	-3.210868919	0.001323343	0.020249082
67.88182508	0.010336982	0.249689186	0.041399399	0.966977492	0.984170992
365.8572927	0.012820752	0.06441811	0.199024035	0.842243943	0.93182686
119.6344294	0.018671204	0.131466683	0.142022328	0.88706237	0.95312914
1088.909458	-0.147274262	0.173931291	-0.846738161	0.397141083	0.647614967

112.7574121	-0.247895499	0.134156028	-1.847814837	0.064629134	0.234151845
128.2134604	0.006000828	0.103763213	0.057831944	0.953882493	0.979872479
11.2313558	-0.41568361	0.356735489	-1.165243219	0.243920556	0.499495198
331.2968589	-0.154683698	0.097137344	-1.592422558	0.111289791	0.320866896
750.6448062	-0.183087348	0.107482409	-1.703416858	0.088490085	0.281459845
234.7167899	0.057628055	0.139229713	0.413906295	0.678942733	0.845876523
271.1970527	0.045002562	0.086887878	0.517938324	0.604501302	0.799020152
79.84293336	-0.068142188	0.119538987	-0.570041536	0.568649527	0.775741426
880.3094209	0.001770105	0.045833706	0.038620163	0.969193227	0.985192723
89.52907207	0.106353261	0.171615952	0.619716639	0.535444359	0.753648867
275.6537948	-0.118986231	0.144700264	-0.822294498	0.410909308	0.658207516
149.2968653	-0.044277495	0.197180435	-0.224553189	0.822326885	0.922469201
22.05566242	-0.163756001	0.293401106	-0.558130144	0.576755518	0.781160065
656.6132941	-0.155133014	0.065831665	-2.356510546	0.018447544	0.109154121
486.355911	-0.168754008	0.074616052	-2.261631429	0.023720185	0.127400109
213.5712793	0.104157072	0.089682069	1.161403536	0.245477832	0.500595289
81.31338353	0.208917507	0.162944698	1.2821375	0.199794416	0.448315412
7328.275971	0.063242235	0.166497153	0.379839738	0.704064383	0.85974352
307.6437255	0.147302444	0.233847584	0.629907914	0.528754834	0.749140996
6.181465321	-0.620918753	0.53001219	-1.171517872	0.241390678	0.496716268
331.8775343	-0.087765302	0.07417622	-1.183199975	0.236729903	0.492227468
55.31926762	0.708063399	0.254680281	2.780205038	0.005432459	0.050207826
636.6579943	0.305164312	0.226983918	1.344431425	0.178808918	0.423578692
3.82098559	0.263330426	0.802462353	0.328152997	0.742795985	0.880508822
41.39346545	0.162586213	0.302979118	0.536625145	0.591526571	0.79088016
53.80102974	0.548771273	0.184672302	2.971594915	0.002962573	0.034077886
17.62944388	0.174474963	0.25315262	0.6892086	0.490692006	0.720068699
1386.515387	-0.120021774	0.065305617	-1.83784764	0.066084859	0.237952906
189.7689299	-0.195632559	0.109282259	-1.79015844	0.073428444	0.252292273
1903.101331	0.196532566	0.139374308	1.410106125	0.158508349	0.395361643
57.12257547	0.165618295	0.161196474	1.027431256	0.30421744	0.562658765
212.9273057	0.083856815	0.145456118	0.576509372	0.564270937	0.773206656
8.185399861	-0.708862368	0.39298155	-1.803805721	0.071261769	0.247655569
5.299978284	0.198909745	0.534874735	0.371880987	0.709981461	0.862535761
312.1231021	0.019037376	0.105587876	0.180298884	0.856917932	0.938857569
33.98924287	0.906212829	0.454626666	1.993312087	0.046227284	0.190847254
260.7421529	-0.012317232	0.071292346	-0.172770747	0.862831632	0.941926033
3.769467996	0.397594081	0.614472944	0.647048963	0.517600255	0.741441447
618.2312644	0.038295759	0.092124189	0.415697104	0.677631661	0.845705019
993.9319779	0.083571821	0.050645669	1.650127688	0.098916823	0.300894385
10.85806482	0.91201891	0.431228047	2.114934121	0.034435563	0.159317806
189.5542414	0.877820293	0.437339408	2.007183158	0.044730164	0.187279204
1511.04726	-0.377764844	0.073993489	-5.105379539	3.30131E-07	4.24408E-05
5371.199441	-0.071339003	0.120879643	-0.59016557	0.555079653	0.766699187
1085.201342	-0.090494262	0.074320986	-1.217613844	0.223370751	0.477184317
55.49024637	-0.085661582	0.159360729	-0.537532567	0.590899795	0.79075703
239.1284534	-0.259800046	0.095966451	-2.707196552	0.006785407	0.058186072
38.62683048	0.269447783	0.230697519	1.167970008	0.242818863	0.498465298

22.59518945	-0.564243805	0.350631254	-1.609222789	0.107567635	0.314000652
1144.268435	-0.330611681	0.237380796	-1.392748217	0.163695942	0.402987989
1091.93614	-0.059899707	0.082963501	-0.722000717	0.470294037	0.705306146
936.6571399	-0.246550986	0.096669346	-2.550456752	0.010758187	0.078022814
35.97702854	0.056724332	0.21680688	0.261635293	0.793602631	0.905368772
10.54075666	0.447233863	0.458788685	0.9748145	0.329652283	0.587931136
1351.477984	0.081259122	0.148199844	0.548307739	0.583480614	0.785173165
37.58953804	0.428046288	0.161104071	2.656955126	0.007884994	0.064410543
450.9082936	-0.20192397	0.089944511	-2.24498381	0.024769172	0.130581409
49.92732126	0.274211323	0.213478027	1.284494369	0.198969033	0.447627814
4.109418511	0.314505165	0.478580703	0.657162236	0.511076605	0.736062945
58.17161192	-0.187688126	0.410628985	-0.457074714	0.647617344	0.82742337
488.147933	-0.042476401	0.063733535	-0.666468618	0.505111616	0.731164798
387.7569126	-0.05634926	0.131403466	-0.428826285	0.668049648	0.838953534
12.03849329	0.340286599	0.509231505	0.668235558	0.503983241	0.730167397
292.9142031	0.000460767	0.114913796	0.004009679	0.996800747	0.998423335
126.5169581	-0.370717295	0.168876517	-2.195197421	0.028149439	0.141233463
658.734843	-0.063609316	0.112117537	-0.567344931	0.570479856	0.777291813
426.3097331	-0.298304976	0.095765547	-3.114950906	0.001839755	0.025205238
103.4382362	-0.347152206	0.279425655	-1.242377714	0.214097234	0.46592315
1190.202888	-0.105233226	0.064045712	-1.643095568	0.100363162	0.303060421
68.12248851	0.007774789	0.135890242	0.057213738	0.954374935	0.979940437
254.8766768	-0.356530957	0.099413194	-3.586354514	0.000335333	0.007896941
178.3802497	0.046706366	0.11286772	0.413815097	0.679009527	0.845876523
637.9771628	-0.066642964	0.066086902	-1.008414115	0.3132557	0.572155863
40.17657172	-1.238176671	0.464513098	-2.665536615	0.007686556	0.063711356
95.44904398	0.065648935	0.129680582	0.506235657	0.612691226	0.804784705
1436.366398	0.152996542	0.220803082	0.692909451	0.488366378	0.718612563
579.3104362	-0.087094749	0.065895648	-1.321707153	0.186265686	0.432345842
115.7084339	0.086662138	0.1068114	0.811356639	0.417160893	0.662355886
9.140861422	0.750086366	0.468674933	1.600440546	0.109500886	0.317334055
285.7443082	-0.004656129	0.092550767	-0.050308922	0.959876214	0.982105718
213.9366868	0.07790468	0.110080399	0.707707096	0.47912717	0.711221434
395.3751456	-0.110168946	0.106203441	-1.037338761	0.299578007	0.558061914
258.8356646	-0.728919641	0.392171106	-1.858677574	0.063072854	0.230617926
11.61553013	-1.440342319	0.636825226	-2.261754497	0.023712576	0.127400109
2.98255671	-0.429358722	0.605437936	-0.709170497	0.47821868	0.710532566
3.172654448	-0.484162546	0.54469941	-0.888861888	0.37407731	0.627623934
8.557675677	-0.361381255	0.367563367	-0.983180826	0.32551845	0.584078637
5.541563242	0.078015592	0.519589642	0.150148475	0.880647476	0.950743683
5.17378553	0.046644427	0.62619922	0.074488159	0.940621962	0.974550053
2.774192277	0.082694966	0.653470036	0.126547449	0.899298592	0.957602384
10.2284304	-0.374572886	0.543263141	-0.689487024	0.490516836	0.720006182
3.434209111	0.497988554	0.729757923	0.682402395	0.494984542	0.722843347
4.590176313	0.941303417	0.47302	1.989986505	0.046592422	0.191547981
9.92219318	0.232073177	0.344041294	0.674550355	0.499961483	0.726660727
9.221190636	0.259736771	0.327356451	0.793437154	0.427523171	0.672443858
17.36216751	-0.009065725	0.238080852	-0.038078347	0.969625215	0.98542524

7.188563939	0.339022143	0.377332093	0.898471529	0.368934217	0.624001322
31.35878873	0.221063674	0.25767167	0.857927741	0.390932365	0.641559403
31.76652409	0.301049073	0.25411777	1.184683278	0.236142697	0.492010908
19.08799223	0.355581985	0.31359486	1.133889709	0.256840814	0.513818445
18.11052656	0.589171418	0.279132974	2.110719524	0.034796427	0.160524791
15.98278865	0.188840671	0.301080994	0.627208874	0.530522327	0.750628072
6.877725162	0.260438952	0.587737039	0.443121557	0.657677814	0.83310328
15.39942505	-0.02251054	0.376915063	-0.05972311	0.952376166	0.979060167
6.482767019	-0.183649276	0.34442792	-0.533200897	0.593894521	0.791994019
125.1508456	0.165283451	0.134696718	1.227078539	0.219793073	0.472547575
30.55738588	0.065831387	0.21518465	0.305929756	0.75965813	0.889224856
55.64057643	0.394893738	0.162664712	2.427654607	0.015196808	0.09820632
65.09543351	0.205941513	0.202505755	1.016966225	0.309169497	0.568266072
44.91657817	0.256992278	0.158495468	1.621448749	0.104921433	0.310020301
62.78619151	0.232137725	0.197217133	1.177066727	0.239168874	0.494631148
104.2201808	-0.075296582	0.155622248	-0.483842014	0.628497996	0.814777754
80.07335852	0.267422475	0.145788052	1.834323667	0.066605963	0.238866395
77.27738358	0.089345243	0.161645236	0.552724259	0.580452239	0.783393247
88.53685309	0.280589485	0.153924809	1.822899683	0.068318586	0.242084363
91.7248497	0.066207256	0.136692437	0.48435201	0.628136071	0.814592867
99.87776601	0.354218647	0.137547866	2.575239138	0.01001708	0.074991831
86.90571693	0.196579558	0.14185724	1.385756255	0.165821344	0.405542174
86.94170764	0.290154515	0.130079471	2.230594217	0.02570802	0.133467259
126.2221445	0.287344892	0.15684642	1.832014347	0.066949283	0.239519
103.2105982	0.165212073	0.157442263	1.049350218	0.294016961	0.55324843
103.6482172	0.319558125	0.126285076	2.530450431	0.011391618	0.080731662
88.16430936	0.219951127	0.153350536	1.434302956	0.151485827	0.385135956
69.01617028	0.239147856	0.189309881	1.263261352	0.206495271	0.456642941
84.50280288	0.327054096	0.189184383	1.728758422	0.08385234	0.273247477
372.6736338	-0.102602395	0.128008357	-0.801528881	0.422825531	0.667381765
42.93300032	0.297178156	0.184901396	1.607225056	0.108005007	0.314276537
29.703711	0.389793812	0.198792802	1.960804452	0.049901839	0.199811191
1969.866616	-0.257257202	0.131834677	-1.951362173	0.051013978	0.201995261
603.9059028	-0.065920447	0.096445146	-0.683501965	0.494289709	0.722581985
25.01414068	0.246430343	0.319734774	0.770733633	0.440864832	0.682560519
188.2266239	0.347464013	0.14465595	2.402002911	0.016305579	0.10188713
18.3958357	0.468101163	0.240904176	1.943101073	0.052003947	0.204733671
931.3444615	0.127405093	0.106965992	1.191080367	0.233622037	0.489137368
77.51224396	0.472226119	0.171724682	2.749902423	0.005961301	0.053305046
1043.10222	-0.134167385	0.103346731	-1.298225733	0.194209778	0.441959686
116.1012798	-0.271048955	0.157184083	-1.724404596	0.084634828	0.275054424
543.5321288	-0.141991329	0.098025772	-1.44851018	0.147474421	0.378589689
137.7236512	0.577035596	0.226380612	2.548962079	0.010804405	0.078306971
512.7222916	0.190699254	0.209026574	0.912320623	0.361599961	0.617456047
13.17564222	0.274204287	0.380294569	0.721031297	0.470890259	0.705535576
298.2092882	-0.254752413	0.252165725	-1.010257891	0.31237175	0.571273149
2125.912409	-0.324748977	0.127305819	-2.550935844	0.01074341	0.078009094
180.2022651	0.029878512	0.09539821	0.313197826	0.75413037	0.886906173

6.111947518	-0.403516229	0.56880785	-0.709406928	0.47807199	0.710381913
7.316324383	-0.049955428	0.430394745	-0.116068861	0.907597968	0.960510562
803.2756547	0.370770288	0.161765613	2.29202166	0.021904393	0.121020762
948.8137611	0.011956652	0.075396085	0.158584522	0.873996224	0.947584459
6.207462889	-0.172128618	0.373305315	-0.461093404	0.644731594	0.825396185
424.4294535	-0.199537295	0.088225006	-2.261686389	0.023716786	0.127400109
492.7419643	0.012106901	0.063274042	0.19134073	0.848258652	0.934489113
6.596704372	-0.355423776	0.415645972	-0.855111802	0.392489256	0.643172239
6.500072395	-0.881829598	0.424913895	-2.075313632	0.037957477	0.169592573
4.62366188	0.036098918	0.53852934	0.067032408	0.946555903	0.976309753
309.0073126	0.039785951	0.222059527	0.179167954	0.857805826	0.939250668
5.260839182	-0.090761627	0.581349345	-0.156122352	0.87593658	0.948628307
3.654086412	0.126401511	0.593601862	0.212939883	0.831373853	0.92859048
29.82273637	-0.214285437	0.52516924	-0.408031203	0.683250767	0.848061493
13510.37235	-0.155009676	0.154670548	-1.002192584	0.316250589	0.575300613
377.4192025	-0.093719229	0.07282981	-1.286825111	0.198155252	0.446404335
202.0017079	0.075408281	0.153216385	0.492168517	0.622600219	0.810904341
268.0570323	-0.20887551	0.099432331	-2.100680016	0.035669065	0.163052641
58.15658605	0.755410269	0.288747546	2.61616169	0.008892444	0.069353101
10.74671633	0.103390576	0.391276488	0.264239173	0.791595628	0.904581845
255.7491226	-0.00656241	0.090438013	-0.072562517	0.942154256	0.974783781
8.722526828	0.319376636	0.313032462	1.020266824	0.307601933	0.566457486
5.257140972	-0.475898219	0.410747321	-1.158615516	0.246612942	0.501930622
366.7412236	-0.061694484	0.075394768	-0.818286017	0.413193889	0.659795725
22.60438573	-0.856682199	0.61876093	-1.38451243	0.166201605	0.405965733
645.9714997	-0.284181168	0.070130386	-4.05218316	5.07419E-05	0.002030194
190.2030654	-0.2717775	0.179909691	-1.510632909	0.130882005	0.353093825
441.4656483	-0.3675001	0.455614552	-0.80660308	0.419895199	0.664745765
284.8184281	-0.302637278	0.104560887	-2.894364102	0.003799273	0.040072496
121.3803344	-0.562578002	0.259053731	-2.171665315	0.029880917	0.146728959
211.7868971	-0.588120247	0.325844577	-1.804910342	0.071088713	0.247273315
80.351321	-0.265501962	0.170561846	-1.556631618	0.119557972	0.334787938
280.0470176	0.187995986	0.174196432	1.079218348	0.280490402	0.538872897
216.5628065	-0.085277511	0.137164646	-0.621716409	0.53412836	0.752718449
84.58410391	-0.019114646	0.185055665	-0.10329133	0.917731757	0.965181096
1609.333994	-0.239210674	0.07766448	-3.080052459	0.002069641	0.027140682
69.91974719	0.020091821	0.351388051	0.057178441	0.954403051	0.979940437
1160.712587	-0.027239133	0.130652375	-0.208485554	0.834849861	0.929483538
16.6338064	0.349778615	0.479138377	0.73001586	0.46538049	0.701088042
147.2294035	0.042664428	0.137566994	0.310135643	0.756457809	0.88766406
344.6609175	0.09045832	0.415099839	0.21791943	0.827491888	0.925836979
72.21595578	0.327851863	0.134627014	2.43526061	0.014881068	0.096664957
344.5145027	-0.402207634	0.216267529	-1.859768949	0.062918222	0.230616826
111.0257302	-0.408992958	0.144336242	-2.833612359	0.004602512	0.045045671
152.526537	-0.202052699	0.199185035	-1.014396982	0.310393364	0.569914484
2.742732854	0.013455472	0.655750488	0.020519195	0.9836292	0.991149398
7.840854026	0.247481337	0.355911371	0.695345405	0.486838866	0.717486727
360.7865476	-0.114997514	0.080418207	-1.429993517	0.15271888	0.386703731

238.254523	-0.051057199	0.09942451	-0.513527296	0.607582522	0.801457046
159.0102647	0.39749034	0.165528893	2.401335098	0.01633537	0.101910875
171.1710706	-0.426199472	0.18419279	-2.313877063	0.020674467	0.116859938
353.3477112	0.178522427	0.172455609	1.035179015	0.300585317	0.558973217
175.8425768	-0.199581364	0.098213953	-2.03210805	0.042142719	0.180148923
45.18921748	0.231434314	0.333655439	0.693632672	0.487912599	0.71826743
101.32015	-0.369453045	0.154686908	-2.388392463	0.01692226	0.104041052
527.7335995	-0.018775647	0.074002771	-0.253715453	0.799715395	0.908443048
622.7009094	-0.059873354	0.27418874	-0.218365472	0.827144366	0.925812063
5.471209636	0.775671083	0.540264311	1.435725195	0.151080553	0.384729241
6.342345187	0.615185768	0.401520765	1.532139361	0.125488049	0.344927191
13.5406082	0.661618077	0.359678391	1.8394713	0.065845895	0.237241748
2691.887813	-0.327935198	0.118217042	-2.774009504	0.005537006	0.050874284
7.684865957	1.53750408	0.630776987	2.437476496	0.014790176	0.096172934
10.17336979	-1.023757062	0.483506097	-2.117361224	0.034229202	0.15859699
541.8900303	-0.114367678	0.117419788	-0.974006845	0.330053141	0.588445317
3.328333773	1.475466512	0.672285693	2.194701638	0.028185007	0.141262312
13.23347673	0.684899348	0.358990824	1.907846393	0.056411073	0.215541651
19.81607586	-0.40860371	0.533939224	-0.765262583	0.4441152	0.685056997
165.6596697	0.937976487	0.719716439	1.303258389	0.192486562	0.440017379
22.4598447	0.712714597	0.500288719	1.424606572	0.154270959	0.38887588
538.8406305	-0.175472139	0.050466588	-3.476996273	0.000507065	0.010589621
225.6588733	0.171444031	0.072563711	2.362669013	0.018143866	0.108159786
3423.554419	-0.084534187	0.190619957	-0.443469762	0.657425986	0.832948475
2546.111791	0.004137096	0.138840142	0.029797548	0.976228514	0.988130653
1344.517625	0.234008381	0.070790672	3.30563863	0.000947602	0.015895393
2364.997055	-0.167075122	0.163278225	-1.023254155	0.306187693	0.565169799
565.5626063	0.455248156	0.430915437	1.056467504	0.29075468	0.550084004
475.7163932	0.169664231	0.066414403	2.554630085	0.01063007	0.077496095
28.53174753	-0.565253861	0.329634187	-1.714791376	0.086383483	0.277630851
4202.515599	-0.124401127	0.152355911	-0.816516577	0.414204746	0.660728031
10.50330351	-0.354146122	0.380367279	-0.931063586	0.351820673	0.608637468
645.392646	0.376376261	0.16429203	2.290897868	0.021969321	0.12124097
670.7497516	-1.005600901	0.44998802	-2.234728165	0.0254352	0.132452965
483.1610752	0.062280149	0.075565569	0.824186869	0.409833387	0.657435871
154.9234521	-0.195330061	0.101228139	-1.929602413	0.05365612	0.20902697
10.2548656	0.063874059	0.307281208	0.207868419	0.835331709	0.929721855
521.434245	0.416546936	0.131113315	3.176999491	0.001488073	0.021935091
76.57766308	-0.440420763	0.134697773	-3.269695959	0.001076631	0.017390202
2335.793988	-0.05431364	0.062712648	-0.866071548	0.386450927	0.637832421
48.44419472	-0.05514624	0.212389016	-0.259647327	0.795135826	0.905927111
137.6102666	-0.029508599	0.129940089	-0.22709388	0.820350743	0.921144748
9.779997038	-0.006221162	0.357312552	-0.01741098	0.98610875	0.992541292
4.517588581	0.577979153	0.617097157	0.936609651	0.348959386	0.606353912
17.10610365	0.357326747	0.32062193	1.114480057	0.265073295	0.523207848
235.4327276	-0.151884273	0.105825011	-1.435239854	0.15121876	0.384829693
37.45971928	0.164268726	0.183393565	0.895716954	0.370403953	0.62480271
621.4115343	0.094272493	0.075198551	1.253647736	0.209970072	0.461683841

444.1797396	0.5565001	0.144382928	3.8543345	0.000116045	0.003706818
51.75034005	1.108462254	0.406099212	2.729535591	0.00634236	0.055352441
410.4653302	0.226134789	0.124849354	1.811261185	0.070100431	0.245688304
440.5837781	0.018836193	0.054774935	0.343883438	0.730933948	0.874577971
198.5917763	0.099637541	0.357843566	0.278438823	0.780675521	0.899190394
336.372963	-0.061368438	0.093186636	-0.658554067	0.510182167	0.735095166
79.69759979	0.523963425	0.276352038	1.895999859	0.057960063	0.219112681
661.5365375	-0.122627929	0.098669437	-1.242815732	0.213935744	0.465829267
972.3661832	-0.258620799	0.068688979	-3.76509891	0.000166483	0.004808696
1746.603238	0.043997956	0.078051812	0.563701919	0.572957011	0.779023105
1131.34989	-0.127520825	0.094556285	-1.34862346	0.177457941	0.421541336
133.4178523	0.039191181	0.196035345	0.199918954	0.841543967	0.93182686
586.5361617	-0.018707652	0.091434565	-0.204601526	0.837883463	0.930810213
1684.637197	0.083869969	0.113233413	0.740682157	0.458886181	0.69524448
161.6251199	0.311066201	0.162922658	1.90928754	0.056225009	0.21523872
402.8401158	-0.15465954	0.132434741	-1.167816982	0.242880597	0.498465298
18.28722062	1.001493508	0.427696064	2.341600947	0.019201233	0.111869291
117.301935	0.001631741	0.13020394	0.012532192	0.990001019	0.993993725
791.59723	-0.289058205	0.071659393	-4.03377969	5.48868E-05	0.002165405
6.967165308	0.029012424	0.593787686	0.048859929	0.961030923	0.98222115
37.36284799	-0.543125363	0.371538742	-1.461826995	0.143788625	0.373559846
287.4484718	-0.382092913	0.079331193	-4.816427165	1.46151E-06	0.000134049
37.10776123	0.880213488	0.399473038	2.203436538	0.027563995	0.139591121
53.41313013	0.476825335	0.215857505	2.208981958	0.027175896	0.138161021
10.64004566	0.551824108	0.355202418	1.553548288	0.120292203	0.3360927
10.95072605	-0.858278921	0.502458391	-1.708159196	0.087606807	0.279557511
261.1838521	-0.493864119	0.171247864	-2.883914044	0.003927659	0.040851128
1365.868336	0.191720552	0.153165436	1.251722045	0.210671164	0.462171603
6.973757691	-0.244561146	0.584399606	-0.418482736	0.675594206	0.844438917
237.3928857	-0.134094154	0.180166088	-0.744280765	0.456706636	0.693937939
146.4516302	-0.12043128	0.138260518	-0.87104606	0.383729004	0.634974705
138.4502297	-0.207929435	0.106078525	-1.960146373	0.049978684	0.199863765
218.3960108	-0.154365927	0.081721774	-1.888920405	0.058902495	0.221434978
270.1496974	-0.080185066	0.089507023	-0.895852226	0.370331692	0.62480271
5.466953039	0.495140089	0.450357318	1.099438312	0.271576927	0.530661391
133.5825451	0.27874176	0.301672225	0.92398881	0.355492142	0.612138993
197.9138638	0.101568163	0.11355102	0.894471603	0.371069617	0.625300839
3477.851678	0.048165765	0.136022735	0.354100842	0.723263311	0.870759523
497.0384322	-0.169622644	0.103868592	-1.633050386	0.1024584	0.306660383
55.31617615	-0.354847233	0.291005749	-1.21938221	0.222699159	0.476210832
21.22988404	-0.151916773	0.386005233	-0.393561434	0.693904859	0.85351767
1214.091193	0.008234992	0.08858264	0.09296395	0.925932201	0.968927915
4.764697531	0.221307998	0.4480252	0.493963281	0.621332112	0.810123273
28.75400943	0.467455726	0.571654437	0.817724303	0.41351463	0.66004106
52.47249028	0.157383829	0.259703276	0.60601403	0.544505437	0.759653377
3.657884566	-0.114660764	0.605627402	-0.189325589	0.849837641	0.935217465
26.99901845	0.064233077	0.275988429	0.232738297	0.815964629	0.91890495
543.7849989	-0.073184317	0.085061702	-0.860367416	0.389586541	0.640605162

298.9219924	-0.168523608	0.088079633	-1.913309611	0.055708428	0.213974712
1190.736848	-0.003528581	0.084233422	-0.04189051	0.966585982	0.984126069
233.1241576	0.085301665	0.097774132	0.872435922	0.382970611	0.634669385
88.04989501	0.077835892	0.319548101	0.243581143	0.807555234	0.913699054
14.59586228	0.563780626	0.381007153	1.479711394	0.138950284	0.366083697
312.0296578	0.202081634	0.106975533	1.889045361	0.058885751	0.221425106
309.9452015	0.128305799	0.084666838	1.515419742	0.129666141	0.351544296
5.305831601	-0.120436408	0.418467642	-0.287803394	0.773497239	0.896155718
32.24779727	0.669756538	0.245006119	2.733631875	0.006264003	0.054969174
19.95922463	0.493647566	0.300015611	1.645406268	0.099886058	0.302254221
1768.81903	0.251775393	0.142530854	1.766462389	0.077318306	0.26028339
4.004430876	2.024625167	1.044352653	1.938641282	0.052545033	0.20597758
1035.202471	-0.313999998	0.121816613	-2.577645111	0.00994761	0.074721419
214.9133064	-0.012282389	0.12426338	-0.09884158	0.921264054	0.966754009
1098.34503	-0.129634135	0.094712013	-1.368719036	0.171087118	0.412756553
360.4110526	0.395911694	0.137632231	2.876591421	0.004019957	0.041561642
293.0884472	0.212880063	0.153699372	1.385041853	0.16603967	0.405696555
10.38258702	-0.386993099	0.331977507	-1.16572084	0.243727331	0.499297213
2.954779028	0.640279137	0.5709203	1.121486024	0.262081037	0.51973671
403.1709539	-0.147680942	0.088987099	-1.659576985	0.096999582	0.2967892
232.7548717	-0.350291125	0.104605584	-3.348684768	0.000811961	0.014165517
455.4456568	-0.10604971	0.119495719	-0.887477068	0.374822107	0.628134408
14.839939	-0.925402205	0.53810624	-1.719738846	0.085479923	0.276328231
1041.595322	-0.305242387	0.110695418	-2.757497938	0.005824558	0.052622332
438.1656016	0.159450555	0.178410873	0.893726669	0.371468153	0.625604127
12.06095445	0.877660009	0.43838659	2.002022936	0.045282265	0.188634008
617.98851	-0.105524399	0.114378334	-0.922590804	0.35622049	0.612269826
3.959005639	0.103352652	0.536999559	0.192463197	0.847379394	0.934372901
86.56903615	0.151335208	0.140507031	1.077065019	0.28145122	0.540059327
27189.41215	0.227490207	0.162901234	1.396491615	0.162566504	0.401290131
621.9228536	-0.239537169	0.12051582	-1.987599368	0.046856016	0.192238027
1308.239565	-0.071603797	0.077842261	-0.919857617	0.357647169	0.613713152
32.91005829	1.364621714	0.878014568	1.554213066	0.120133602	0.335860144
426.1079998	0.120825521	0.125322627	0.964115765	0.334987889	0.593465498
41.51038311	-0.060793324	0.238499909	-0.25489873	0.798801318	0.908109225
142.7859421	0.286068806	0.152353802	1.877661092	0.060427543	0.224637492
4.091458609	0.510954938	0.686687117	0.744086973	0.45682386	0.693937939
138.217673	0.096687899	0.107305252	0.901054681	0.367559247	0.622884844
855.6223823	0.057594465	0.082419326	0.698798052	0.484678246	0.715790359
9.49629126	-0.027074114	0.445225605	-0.060809876	0.951510625	0.978862788
214.3684801	0.406305272	0.17076055	2.379386051	0.017341504	0.105583907
9.286384241	0.797144493	0.420263768	1.896771869	0.057858054	0.219031068
217.4821977	0.032434629	0.128465881	0.252476601	0.800672698	0.909191326
4.050013015	0.357481547	0.688555727	0.519175911	0.603638076	0.798632907
998.0577203	0.055086745	0.109990896	0.500830038	0.616490744	0.80702296
581.6341462	-0.016889972	0.078884753	-0.214109461	0.830461698	0.928034291
514.4733359	-0.032429207	0.072874504	-0.445000722	0.656319232	0.83215124
687.7392307	0.007941459	0.081765155	0.097125225	0.922626949	0.967213493

33.8663011	-0.07513302	0.199542424	-0.376526549	0.706525482	0.86122222
399.175026	0.134680467	0.080012904	1.683234334	0.092329737	0.288229414
748.9035649	0.069079412	0.11989002	0.576189848	0.564486866	0.773291293
402.5306577	-0.020497362	0.074586265	-0.274814167	0.783459013	0.901193338
4.310553775	-0.903273165	0.483764609	-1.867174961	0.061877159	0.227866016
295.5535445	-0.304943536	0.17917971	-1.701886534	0.088776641	0.282085257
7.77594275	-0.736947258	0.515335131	-1.430034971	0.152706982	0.386703731
318.4737906	-0.292554993	0.243764428	-1.200154576	0.230079313	0.48528294
171.1452804	-0.054105657	0.129430629	-0.418028233	0.675926475	0.844719589
396.0682939	-0.21237762	0.123091804	-1.725359559	0.084462695	0.274665748
100.8949009	0.105357883	0.142939666	0.737079397	0.461074068	0.697145057
10.63491525	1.031137289	0.788577789	1.307591088	0.191012041	0.438371796
362.6821443	-0.235769407	0.08212504	-2.870858966	0.004093581	0.042027141
147.8514733	0.051233573	0.123737708	0.414049798	0.678837637	0.845876523
201.6319608	-0.092352559	0.091549543	-1.008771375	0.313084292	0.571976009
286.0192672	-0.007709651	0.289178553	-0.026660521	0.978730502	0.989127516
13.23871209	0.836402411	0.317892242	2.631087837	0.008511204	0.067487217
2.756102132	1.852498664	0.76757126	2.41345496	0.015802081	0.100177783
1506.534339	-0.170531614	0.053182237	-3.206552099	0.00134336	0.020455587
705.3475963	0.182444958	0.142063688	1.284247653	0.199055316	0.447660393
58.98546957	-0.298492134	0.213225638	-1.399888574	0.161546688	0.400015512
575.3367293	-0.058511441	0.068278568	-0.856951778	0.391471536	0.642175459
60.93566277	0.477360768	0.241953092	1.972947578	0.048501535	0.196225524
167.4984012	0.365776157	0.372953607	0.980755113	0.326713518	0.585151858
1111.972471	0.008189441	0.151859729	0.053927668	0.956992793	0.98108328
50.12825974	0.537950037	0.385315836	1.396127506	0.162676102	0.401481037
201.3146611	-0.314346003	0.437550627	-0.718422013	0.472497118	0.706381165
22.43457714	-0.086324294	0.62391611	-0.138358816	0.889956844	0.953820886
26.39474297	-0.119990679	0.279309213	-0.429597999	0.667488093	0.838710639
140.3807543	0.214696684	0.154451315	1.390060582	0.164510482	0.403910832
36.00765798	0.500804304	0.187709857	2.6679702	0.007631103	0.063426716
4790.226987	0.201766718	0.173596012	1.162277376	0.245122813	0.500326159
691.2431673	0.065046696	0.207845105	0.312957554	0.754312911	0.887054337
44.17145106	-0.637563195	0.275530429	-2.313948395	0.020670553	0.116859938
111.642486	-0.050004967	0.207801007	-0.240638714	0.809835142	0.915222249
148.7138401	0.503071186	0.373035949	1.348586342	0.17746987	0.421541336
523.1696686	-0.257404151	0.110704294	-2.325150558	0.020063911	0.114889515
605.2946197	0.045811658	0.076435865	0.599347673	0.548941063	0.762991106
7.050491796	0.127305284	0.399646195	0.318544968	0.75007159	0.88518749
334.3251374	-0.116232811	0.261770307	-0.444025957	0.657023818	0.832708005
13.32489295	0.731291661	0.476316761	1.535305329	0.124708846	0.343425257
241.3046191	-0.144441368	0.089426355	-1.615199105	0.106267584	0.311649363
632.3126996	0.483870787	0.18459249	2.621291841	0.008759724	0.068831417
1508.118748	0.105878845	0.07267157	1.456950022	0.145130177	0.375211299
55.58244052	0.290058783	0.220517602	1.315354334	0.188390845	0.43573544
17.38315591	0.372910357	0.226040405	1.649750876	0.0989939	0.300945218
118.8088139	0.153835321	0.142424573	1.080117831	0.280089712	0.538612929
18.56999894	0.672456299	0.309348289	2.173783799	0.029721376	0.146353608

1022.9243	0.13846827	0.101968102	1.357956728	0.174477415	0.417988969
1193.756361	0.074373325	0.063951508	1.162964376	0.244843956	0.500082382
8.421570855	-0.788896104	0.332126114	-2.375290806	0.017535129	0.10626776
38.82904486	0.003309335	0.365129865	0.009063447	0.992768515	0.995576123
3273.111405	-0.102373438	0.387171954	-0.264413363	0.791461416	0.904494378
73.68784114	0.191835986	0.181840304	1.054969564	0.291439245	0.550847568
15.53522114	0.195444667	0.269009194	0.726535271	0.467510693	0.702812011
118.9918803	0.171139629	0.127301244	1.344367289	0.178829646	0.423578692
114.6159286	0.25591864	0.110603179	2.313845237	0.020676213	0.116859938
53.415654	-0.024888043	0.175370075	-0.141917276	0.887145348	0.95312914
224.7364889	0.267326273	0.137585867	1.942977703	0.052018852	0.204733671
108.818428	0.370753709	0.138462669	2.677643816	0.007414202	0.062040733
362.8890338	-0.017278785	0.081736529	-0.211396121	0.832578183	0.928811169
74793.3889	-0.196749848	0.181047225	-1.086732191	0.277155183	0.535573218
60.75824603	-0.015738634	0.215760903	-0.072944789	0.941850052	0.97465603
85.64013125	-0.130608883	0.137029087	-0.953147141	0.340515524	0.598592858
248.2093149	0.14057161	0.118415102	1.1871088	0.235184708	0.491125597
48.73274076	-0.158182029	0.168541634	-0.93853385	0.34797013	0.605319822
305.8367748	-0.068510607	0.108689143	-0.63033533	0.528475213	0.749041961
95.39003408	0.095045047	0.155664688	0.610575514	0.541480637	0.757650127
285.7289811	0.003087793	0.105937537	0.0291473	0.976747112	0.988174249
133.9979673	0.009664047	0.09629052	0.100363435	0.920055798	0.966373094
20.48063782	0.140082662	0.356077792	0.393404659	0.694020629	0.853551135
220.5316435	-0.49274684	0.283445955	-1.738415495	0.082137628	0.269674132
291.6326812	-0.20128901	0.096413556	-2.087766697	0.036818885	0.166417116
12.15018467	0.453450659	0.282372702	1.605858697	0.10830496	0.31497404
1334.381837	0.197188074	0.116905124	1.686735933	0.091654139	0.287016553
910.4171153	-0.196781386	0.179350217	-1.09719068	0.272558047	0.531559364
272.1290672	0.415561105	0.155905778	2.665463146	0.007688236	0.063711356
1155.343294	0.152812449	0.075262771	2.030385632	0.042317356	0.180617323
83.90070856	-0.373914064	0.15908474	-2.350408118	0.018752837	0.11043083
466.6487242	0.065867383	0.153123605	0.430158259	0.667080523	0.838599606
232.3761653	-0.042750632	0.084843038	-0.503879085	0.614346348	0.80620548
355.7407562	0.349919356	0.123828709	2.825833849	0.004715772	0.045740362
14.97588373	-0.02706108	0.257833268	-0.104955733	0.916410935	0.964630141
2045.874326	-0.515973819	0.158149836	-3.262563097	0.001104096	0.017724299
264.7344508	-0.190766424	0.08369552	-2.279290744	0.022649788	0.123863066
260.5792862	0.177522007	0.213675908	0.830800293	0.406086457	0.654848858
177.5976489	-0.099211244	0.124822783	-0.794816796	0.426720079	0.671685842
12.2264989	1.009247495	0.460896398	2.189749147	0.028542435	0.142657599
99.41628116	-0.424313976	0.298008082	-1.423833786	0.154494593	0.389005157
258.2927456	-0.212202378	0.110190761	-1.925772868	0.054132741	0.209997009
610.2114256	0.159623969	0.337272129	0.473279455	0.636013818	0.819658235
109.0743466	0.104516053	0.128312724	0.814541611	0.415334745	0.661679388
14.86384002	-0.370266146	0.255698128	-1.448059669	0.147600364	0.378695257
82.05616607	-0.248742356	0.192537179	-1.291918566	0.196385347	0.444845146
62.08745716	0.610855146	0.307588069	1.98595202	0.047038652	0.192725761
108.5877165	0.212165357	0.119280485	1.778709711	0.075287366	0.256140358

203.907827	0.010525976	0.094906192	0.11090926	0.911688302	0.962232976
333.1864596	0.13952947	0.118403101	1.178427493	0.238626222	0.494147253
272.4281001	0.718024196	0.28318009	2.535574435	0.011226307	0.080069757
522.3816908	0.217330224	0.129853723	1.673654156	0.094198589	0.291632582
359.2494652	0.482637653	0.114017518	4.233013146	2.30581E-05	0.00109922
21.81920525	0.103516656	0.220866368	0.468684559	0.639295125	0.821941899
4987.862209	0.054390255	0.141782707	0.383616986	0.701262349	0.857806901
209.8781867	0.064004957	0.133998584	0.477653984	0.632896501	0.817716475
149.8487735	0.042481911	0.132438262	0.320767657	0.748386471	0.884595178
1053.025846	0.109019306	0.083358347	1.307839111	0.190927885	0.43834164
914.1057373	-0.128283499	0.078869844	-1.626521519	0.103838759	0.308740683
92646.82	-0.309693427	0.082458445	-3.755751497	0.000172822	0.004913071
20071.6179	0.000692407	0.119387664	0.005799654	0.995372572	0.9977263
175.1655493	0.315083297	0.172872961	1.822629142	0.068359579	0.242084363
27.46971057	0.056400295	0.461582968	0.122188857	0.902749451	0.958724768
5.747281839	0.545176456	0.516494604	1.055531757	0.291182193	0.550560762
602.9750786	0.01158455	0.075125375	0.154202889	0.877449764	0.949163535
236.0039736	0.236087496	0.108799884	2.169924143	0.030012593	0.147007342
137.9699271	0.489850704	0.169158499	2.89580901	0.003781825	0.039969098
1127.39909	-0.040951732	0.119117386	-0.343793072	0.731001911	0.874592568
36.47242083	0.114528362	0.180655366	0.63396048	0.526106613	0.74754993
381.1722377	0.109893465	0.07293031	1.506828429	0.13185464	0.354469729
211.0119257	-0.090329699	0.114848221	-0.786513697	0.431566598	0.676524838
1398.368694	-0.055592334	0.191912349	-0.289675649	0.772064387	0.895441681
113.5095341	0.114997295	0.131833667	0.872290796	0.383049758	0.634733481
49.77595056	-0.224165872	0.17205026	-1.302909228	0.192605753	0.440060737
275.7766394	0.145242956	0.083240186	1.744865828	0.081008227	0.266939979
93.65385464	-0.022298636	0.124004916	-0.179820583	0.857293424	0.93895182
4.41958162	1.267616551	0.58799254	2.155837811	0.031096332	0.150112302
116.4967405	0.099206051	0.210401319	0.471508694	0.637277512	0.820756024
96.76840922	-0.036769621	0.119377974	-0.308010089	0.758074655	0.888509678
358.1040363	-0.048803229	0.15625863	-0.312323412	0.754794749	0.887201677
7.952075716	0.461668151	0.686033832	0.672952455	0.500977539	0.727396012
439.3819491	-0.02084588	0.099998743	-0.208461422	0.834868701	0.929483538
645.0512605	0.122048402	0.056512043	2.159688359	0.030796803	0.149236443
580.1315185	0.005520767	0.177519708	0.031099461	0.975190219	0.98797774
357.2749452	0.075638668	0.108212873	0.698980317	0.484564331	0.715689516
133.7832155	0.124333589	0.150842094	0.824263211	0.409790017	0.657435871
272.7496851	0.599267697	0.273051345	2.194706999	0.028184622	0.141262312
1148.415456	0.048182517	0.08924215	0.539907622	0.589260741	0.789641615
93442.45429	0.012151896	0.169279296	0.071786071	0.942772158	0.974993168
171.7497098	0.728680364	0.300785577	2.422590772	0.015410275	0.098991436
3.369816805	0.589800425	0.683949832	0.862344572	0.388497932	0.639624802
244.0520411	0.250170402	0.088199649	2.836410389	0.004562378	0.044806722
32.3536868	1.203721923	0.526390389	2.286747532	0.022210564	0.12227114
1274.178552	0.186452211	0.108218906	1.722917171	0.084903505	0.275305355
324.4945616	0.123863765	0.159621385	0.77598478	0.437757973	0.680661785
1309.050581	0.38024751	0.19489091	1.951078738	0.05104768	0.202077692

5.211376289	0.816575785	0.544486728	1.499716602	0.133687828	0.35713846
6.926059156	0.519926838	0.532564813	0.976269602	0.328930879	0.587379245
88.29736115	-0.035281483	0.11966805	-0.294827929	0.76812533	0.892920077
89.69462042	-0.052186809	0.124046386	-0.420703982	0.673971253	0.843351295
353.8599089	0.224951381	0.084269412	2.669430995	0.007597988	0.06329301
509.3642814	-0.370581335	0.144219006	-2.569573486	0.010182379	0.075613821
53.68347942	0.334654232	0.203693389	1.642931238	0.100397161	0.30310473
1405.136054	0.4093182	0.147652392	2.772174529	0.005568317	0.050972377
197.4794424	0.063234776	0.108826129	0.581062442	0.56119837	0.770873642
75.55576621	0.31135976	0.157325473	1.979080396	0.047806955	0.19475436
131.9355954	0.260370973	0.142189515	1.831154521	0.067077483	0.239668244
62.38997824	0.762945344	0.446310216	1.709450776	0.087367482	0.279207439
752.7366718	-0.22285844	0.120227781	-1.853635143	0.063791376	0.232243256
2378.265864	0.151487404	0.172783288	0.87674801	0.380623523	0.632388957
268.0226503	0.218837771	0.192629126	1.136057538	0.255932485	0.512852678
476.7138146	0.089924152	0.084901264	1.059161528	0.289526223	0.549072905
354.7089947	-0.232893522	0.178406749	-1.305407578	0.191754099	0.438854705
295.5813728	0.182709995	0.103104083	1.772092728	0.076379166	0.258307996
16.54356781	0.585995746	0.412613275	1.420205749	0.15554779	0.390462795
728.0324273	0.104770836	0.077565243	1.350744637	0.176777248	0.420660652
127.2548755	0.171179881	0.116015556	1.47549076	0.140080651	0.367657657
230.5774846	0.311959181	0.138986386	2.244530496	0.024798289	0.130663891
744.5073666	-0.086497885	0.096360058	-0.897652891	0.369370632	0.624195321
4.855989872	-0.521315465	0.490024	-1.063857005	0.287393483	0.54675878
624.1018346	-0.24321859	0.194434285	-1.250903822	0.210969569	0.462452373
2016.218923	0.066509964	0.109334498	0.608316365	0.542977669	0.75862099
144.4770615	-0.762961021	0.481499556	-1.584551868	0.113068188	0.323842488
470.3745652	-0.026081141	0.16068901	-0.162308179	0.871063174	0.946199847
59.9529916	0.057067164	0.190336482	0.299822519	0.764312538	0.89162218
62.54304198	-0.233174018	0.341512366	-0.682768888	0.494752892	0.722813893
105.5683746	0.066974258	0.114869041	0.583048818	0.559860437	0.770182536
228.2846419	0.120768524	0.119797107	1.008108854	0.313402208	0.572290165
463.0333617	-0.07348974	0.122582899	-0.59951054	0.548832483	0.762907797
372.7046392	-0.128686386	0.17009179	-0.756570239	0.449307377	0.688855098
49.78073997	-0.311252731	0.209664447	-1.484527947	0.137668915	0.363718264
43.25434685	0.096454443	0.165474818	0.582894994	0.559963991	0.770257431
7.959313305	0.350289745	0.407456966	0.859697524	0.3899558	0.640761317
182.8370719	-0.143301137	0.093920751	-1.525766526	0.12706802	0.347574555
15.18942988	0.217407079	0.256143392	0.848770987	0.396008737	0.646440574
202.4443617	-0.425848414	0.077047427	-5.527094551	3.25578E-08	6.38295E-06
1287.805656	0.110888489	0.103083093	1.07571946	0.282052742	0.540466121
7.413898981	0.882746423	0.391263198	2.256144783	0.024061565	0.128492199
244.2031804	-0.248516658	0.194391378	-1.278434573	0.20109624	0.449913941
181.7688839	-0.684880223	0.274593557	-2.494159839	0.012625568	0.086888724
899.7560436	-0.06678567	0.171763694	-0.38882297	0.697407108	0.855416228
215.5246592	-0.119331766	0.160267346	-0.744579411	0.456526019	0.69386622
38.82824193	-0.024902822	0.331327956	-0.075160643	0.940086898	0.974509115
230.4725622	0.189611918	0.09611428	1.972775712	0.048521121	0.196225524

536.6233345	0.001686601	0.12068453	0.013975286	0.988849698	0.993404681
318.5181841	0.163839181	0.10443936	1.568749366	0.116706351	0.329865274
88.72692007	0.175911067	0.136247722	1.291111985	0.196664849	0.445162804
183.1835468	0.419060884	0.213988632	1.958332459	0.050191014	0.20043272
569.6862671	0.247654892	0.125266742	1.977020296	0.048039336	0.195355198
172.9499942	0.265008049	0.102031217	2.597323207	0.009395347	0.07212757
2866.043169	-0.25791943	0.093317175	-2.763900947	0.005711487	0.051749838
40346.32491	-0.058097693	0.097085256	-0.598419315	0.549560181	0.763445693
2.814188065	0.198679707	0.585431359	0.339373188	0.734328614	0.876558568
419.0758674	0.020984834	0.094631631	0.221752848	0.824506287	0.923946599
20.36274129	0.092069219	0.226437762	0.406598342	0.684303015	0.848557456
43.98837547	0.341904837	0.342097527	0.999436739	0.31758317	0.576368252
9307.607833	0.415079021	0.11505294	3.607721969	0.000308897	0.007534595
6.799515826	0.619645045	0.359162474	1.725249961	0.084482436	0.274672993
150.7430247	-0.282258759	0.440873006	-0.640226903	0.522025095	0.744312872
336.9288679	0.204253888	0.397542775	0.513790969	0.607398143	0.801442647
224.5575884	-0.349171144	0.204955253	-1.703645746	0.08844729	0.281380789
96.08212471	0.785491487	0.311470721	2.521879053	0.011672984	0.082136321
154.9693199	-0.475801133	0.139186707	-3.418438029	0.000629817	0.012164348
6.730254841	0.242805322	0.453434239	0.535480784	0.592317442	0.791183678
229.4300788	-0.323414997	0.177455768	-1.822510482	0.068377565	0.242084363
55.5861948	0.467199679	0.189165061	2.469799006	0.013518899	0.091039247
5.729254953	0.694108785	0.596224416	1.164173701	0.244353626	0.499749345
7.053676499	-0.175128294	0.384814076	-0.455098461	0.649038406	0.827939679
408.1734566	-0.0691592	0.094816234	-0.729402525	0.465755474	0.701518184
397.3603585	0.057765384	0.082402782	0.701012554	0.483295181	0.714959594
34.6732061	0.114335463	0.274054201	0.417200185	0.67653199	0.845004996
19.03593731	0.252351054	0.226004612	1.116574799	0.264176172	0.521896849
160.3016621	0.094122909	0.145621082	0.646354962	0.518049501	0.741669386
51.82290284	0.308654125	0.236316098	1.306107067	0.19151615	0.438775498
2971.575865	-0.0368993	0.044111938	-0.836492372	0.402877966	0.652321447
358.1170244	-3.18576E-05	0.111982942	-0.000284486	0.999773013	0.999836762
3.618804754	0.770813589	0.610112432	1.263395973	0.206446911	0.456642941
3.591535363	0.321089445	0.59122531	0.543091509	0.587066799	0.787778548
17.54404436	1.30018608	0.615999909	2.110691998	0.034798794	0.160524791
2926.700021	0.106493761	0.207341216	0.513615977	0.607520507	0.801442647
2081.307857	0.262513693	0.111079794	2.363289326	0.018113522	0.108099837
2.656540001	-1.56092781	0.67442566	-2.314454956	0.020642779	0.116846413
223.0625261	-0.016287949	0.130459556	-0.124850564	0.900641857	0.95813193
54.01543104	0.264163219	0.262852278	1.004987367	0.314902933	0.573869373
6.556278317	-0.355147486	0.529979384	-0.670115661	0.502784062	0.729074078
5.064587224	1.172332248	0.894802944	1.310156896	0.190142764	0.437826367
2.86546216	0.817094249	1.197721154	0.682207413	0.495107808	0.722888742
2.733261563	0.287526172	0.871053561	0.330090117	0.74133187	0.879770661
33.40460908	0.463997628	0.510112841	0.909598017	0.363034545	0.618290129
7.143149374	-0.058234907	0.707914672	-0.082262608	0.934437888	0.972907108
25.97540666	-0.608379337	0.321562482	-1.891947508	0.058497975	0.220442633
10.72921736	-0.455937803	0.436673492	-1.044116052	0.2964317	0.555265112

3.51354495	2.12704083	2.932383686	0.725362387	0.468229742	0.70336675
321.2730602	-0.032236662	0.132844435	-0.242664753	0.808265115	0.913988445
111.4076704	-0.17339451	0.140217035	-1.23661515	0.216229993	0.468936837
562.5781319	-0.070094861	0.061371517	-1.142139937	0.253395857	0.509978266
1772.276351	0.144251841	0.088892249	1.622771865	0.104638182	0.30982542
142.8302579	0.530945931	0.695431751	0.763476689	0.445179159	0.685510224
198.4200101	0.015213866	0.331932673	0.045834193	0.963442405	0.982781984
70.79298224	0.361679803	0.51546703	0.701654581	0.482894604	0.714636627
3.845841345	-0.169792111	0.706085521	-0.240469612	0.809966219	0.915275758
2184.395191	-0.265185681	0.135821982	-1.95245038	0.050884757	0.201687269
18.24182278	-0.403703234	0.679640552	-0.593995212	0.552515315	0.76490866
509.9290568	-0.167539153	0.134052627	-1.249801346	0.211372124	0.462721491
68.55600682	0.1658164	0.367513705	0.451184263	0.651856751	0.829708504
85.3874729	0.199221633	0.137781667	1.445922652	0.148198898	0.37976509
551.3857065	-0.028288891	0.116118154	-0.243621606	0.807523893	0.913699054
101.0077237	0.076835592	0.136399747	0.563311835	0.57322256	0.779066086
31.79362487	0.009485023	0.229788303	0.041277224	0.96707489	0.984206253
1395.219742	-0.238615043	0.110676783	-2.155962943	0.031086559	0.150112302
406.9400798	-1.380498144	0.735988401	-1.875706386	0.060695608	0.225206985
62.89578483	-0.140593088	0.49385417	-0.284685433	0.775885152	0.897389272
1026.126526	-0.201541154	0.125097106	-1.611077672	0.107162794	0.313278894
104.8582367	-0.298935459	0.28158711	-1.061609173	0.288413148	0.547834784
268.678655	-0.156774918	0.166467307	-0.94177602	0.346307325	0.604006654
45.39595094	-0.237816243	0.166430967	-1.428918228	0.153027738	0.386985979
1468.350194	-0.165491484	0.143474125	-1.153458739	0.248722144	0.504390755
60.95101999	0.020230957	0.180712022	0.111951362	0.910861972	0.962059189
12.79890162	0.118930009	0.307957852	0.386189239	0.699356523	0.856925408
528.0118481	0.017167694	0.379796035	0.045202403	0.963945979	0.982999267
373.9690243	-0.079003803	0.212503387	-0.371776677	0.71005913	0.862535761
38.31415142	-0.298561268	0.362952246	-0.822591047	0.410740591	0.658207516
1740.204088	0.00080492	0.224810769	0.003580435	0.997143233	0.998480142
13.66284946	-0.155655161	0.817931212	-0.190303486	0.849071322	0.934802172
3861.512355	-0.020721941	0.133538159	-0.155176177	0.876682428	0.948920022
393.3708361	0.102203955	0.237884089	0.429637624	0.667459264	0.838710639
67.09766812	-0.192285726	0.137067478	-1.402854477	0.160660238	0.398575637
277.0739659	-0.137606314	0.087772994	-1.567752305	0.11693895	0.330188128
[truncated: 523,758 more chars]
